# Supplementary material for: An interpretable machine learning model of cross-sectional U.S. county-level obesity prevalence using explainable artificial intelligence
Source: PLoS One. 2023 Oct 5;18(10):e0292341. doi: 10.1371/journal.pone.0292341 (PMC10553328; doi:10.1371/journal.pone.0292341)
Supplement: S1 Table — (PDF) [file pone.0292341.s001.pdf]

S1 Table: Data Sources

Category	Variables	Data Source	Year
HEALTH OUTCOMES			
Length of Life	Premature age adjusted mortality	National Center for Health Statistics - Mortality Files	2018-2020
Quality of Life	Poor mental health days	Behavioral Risk Factor Surveillance System	2019
	Low birthweight	National Center for Health Statistics - Natality files	2014-2020
	Diabetes prevalence	Behavioral Risk Factor Surveillance System	2019
HEALTH BEHAVIORS			
Diet and Exercise	Adult obesity	CDC Diabetes Interactive Atlas	2019
	Food environment index	USDA Food Environment Atlas, Map the Meal Gap from Feeding America	2019
	Physical inactivity	CDC Diabetes Interactive Atlas	2019
	Access to exercise opportunities	Business Analyst, Delorme map data, ESRI, & US Census TIGER/Line Files	2010 & 2021
	Food insecurity	Map the Meal Gap	2019
	Limited access to healthy foods	USDA Food Environment Atlas	2019
Alcohol and Drug Use	Excessive drinking	Behavioral Risk Factor Surveillance System	2019
	Alcohol impaired driving deaths	Fatality Analysis Reporting System	2016-2020
Tobacco Use	Adult smoking	Behavioral Risk Factor Surveillance System	2019
Sexual Activity	Sexually transmitted infections	National Center for HIV/AIDS, Viral Hepatitis, STD, and TB Prevention	2019
	Teen births	National Center for Health Statistics - Natality files	2014-2020
Sleep	Insufficient sleep	Behavioral Risk Factor Surveillance System	2018
CLINICAL CARE			
Access to Care	Primary care physicians	Area Health Resource File/American Medical Association	2019
	Dentists	Area Health Resource File/National Provider Identification file	2020
	Mental health providers	CMS, National Provider Identification	2021
	Uninsured adults	Small Area Health Insurance Estimates	2019
	Uninsured children	Small Area Health Insurance Estimates	2019

Category	Variables	Data Source	Year
	Other primary care providers	CMS, National Provider Identification	2021
Quality of Care	Preventable hospital stays	Mapping Medicare Disparities Tool	2019
	Mammography screening	Mapping Medicare Disparities Tool	2019
	Flu vaccinations	Mapping Medicare Disparities Tool	2019
SOCIAL AND ECONOMIC FACTORS			
Income	Gender pay gap		2016-2020
	Median household income	Small Area Income and Poverty Estimates	2020
	Children eligible for free or reduced price lunch	National Center for Education Statistics	2019-2020
	Children in poverty	Small Area Income and Poverty Estimates	2020
	Income inequality	American Community Survey, 5-year estimates	2016-2020
Education	High school completion	State-specific sources & EDFacts	2016-2020
	Some college	American Community Survey, 5-year estimates	2016-2020
	School segregation	National Center for Education Statistics	2020-2021
	School funding adequacy	School Finance Indicators Database	2019
Employment	Unemployment	Bureau of Labor Statistics	2020
Family and Social Support	Children in single parent households	American Community Survey, 5-year estimates	2016-2020
	Social associations	County Business Patterns	2019
Family and Social Support	Childcare cost burden	The Living Wage Calculator, Small Area Income and Poverty Estimates	2021 & 2020
	Childcare centers	Homeland Infrastructure Foundation-Level Data (HIFLD)	2021
Community Safety	Violent crime	Uniform Crime Reporting - FBI	2014 & 2016
	Injury deaths	CDC WONDER mortality data	2016-2020

Category	Variables	Data Source	Year
PHYSICAL ENVIRONMENT			
Air and Water Quality	Air pollution particulate matter	Environmental Public Health Tracking Network	2018
	Drinking water violations	Safe Drinking Water Information System	2020
Housing and Transit	Severe housing problems	Comprehensive Housing Affordability Strategy (CHAS) data	2014-2018
	Driving alone to work	American Community Survey, 5-year estimates	2016-2020
	Long commute driving alone	American Community Survey, 5-year estimates	2016-2020
	Traffic volume	EJSCREEN: Environmental Justice Screening and Mapping Tool	2016-2020
	Homeownership	American Community Survey, 5-year estimates	2019
	Severe housing cost burden	American Community Survey, 5-year estimates	2016-2020
	Broadband access	American Community Survey, 5-year estimates	2016-2020
DEMOGRAPHICS			
	Population	Census Population Estimates	2020
	% Below 18 years of age	Census Population Estimates	2020
	% 65 and older	Census Population Estimates	2020
	% Non-Hispanic Black	Census Population Estimates	2020
Category	Variables	Data Source	Year
	% American Indian Alaska Native	Census Population Estimates	2020
	% Asian	Census Population Estimates	2020
	% Native Hawaiian Other Pacific Islander	Census Population Estimates	2020
	% Hispanic	Census Population Estimates	2020
	% Non-Hispanic white	Census Population Estimates	2020
	% Not proficient in English	American Community Survey, 5-year estimates	2016-2020
	% Female	Census Population Estimates	2020
	% Rural	Census Population Estimates	2010
SEVERE HOUSING PROBLEMS			
	Homeownership	Comprehensive Housing Affordability Strategy	2014-2018

Category	Variables	Data Source	Year
	Severe housing cost burden	Comprehensive Housing Affordability Strategy	2014-2019
	Broadband access raw value	Comprehensive Housing Affordability Strategy	2014-2020
