# Supplementary material for: An interpretable machine learning model of cross-sectional U.S. county-level obesity prevalence using explainable artificial intelligence
Source: PLoS One. 2023 Oct 5;18(10):e0292341. doi: 10.1371/journal.pone.0292341 (PMC10553328; doi:10.1371/journal.pone.0292341)
Supplement: S2 Table — (PDF) [file pone.0292341.s002.pdf]

S2 Table

Predicted Obesity Prevalence	Surrogate Decision Tree Rules
0.24	Inactivity < 0.18 & Smoking < 0.15
0.29	Inactivity is 0.18 to 0.27 & Smoking < 0.15
0.31	Inactivity < 0.27 & Smoking >= 0.15 & Uninsured Adults >= 0.11
0.33	Inactivity < 0.27 & Smoking >= 0.15 & Uninsured Adults < 0.11
0.35	Inactivity is 0.27 to 0.30
0.37	Inactivity is 0.30 to 0.34
0.39	Inactivity is 0.34 to 0.38 & Diabetes < 0.16
0.4	Inactivity >= 0.38 & Diabetes < 0.16
0.44	Inactivity >= 0.34 & Diabetes >= 0.16
