# Supplementary material for: An interpretable machine learning model of cross-sectional U.S. county-level obesity prevalence using explainable artificial intelligence
Source: PLoS One. 2023 Oct 5;18(10):e0292341. doi: 10.1371/journal.pone.0292341 (PMC10553328; doi:10.1371/journal.pone.0292341)
Supplement: S2 File — (DOCX) [file pone.0292341.s004.docx]

STROBE Statement—checklist of items that should be included in reports of observational studies

|  | Item No. | Recommendation | Page  No. | Relevant text from manuscript |
| --- | --- | --- | --- | --- |
| **Title and abstract** | 1 | (*a*) Indicate the study’s design with a commonly used term in the title or the abstract | 1 | An Interpretable Machine Learning Model of Cross-Sectional  U.S. County-Level Obesity Prevalence Rates Using Explainable Artificial Intelligence |
|  |  | (*b*) Provide in the abstract an informative and balanced summary of what was done and what was found | 2 | **Methods** This study shows the application of explainable artificial intelligence methods to machine learning models of cross-sectional obesity prevalence data collected from 3,142 counties in the United States. County-level features from 7 broad categories: health outcomes, health behaviors, clinical care, social and economic factors, physical environment, demographics, and severe housing conditions. Explainable methods applied to random forest prediction models include feature importance, accumulated local effects, global surrogate decision tree models, and local interpretable model-agnostic explanations.   **Results**  The results show that machine learning models explained 79% of the variance in obesity prevalence, with physical inactivity, diabetes, and smoking prevalence being the most important factors in predicting obesity prevalence. |
| Introduction | | | |  |
| Background/rationale | 2 | Explain the scientific background and rationale for the investigation being reported | 3 | Identifying the principal factors that impact health is an important theme in obesity research.^1–3^ Multiple health behaviors and environmental conditions contribute to the obesity crisis.^4^ There is also substantial geographic heterogeneity in the prevalence of obesity across the United States.^5–7^ Machine learning may be the most powerful approach to modeling variation in obesity prevalence across the United States, but machine learning models are often opaque and difficult to interpret.^8^ To open the black box of machine learning models, the field of explainable artificial intelligence has emerged with the goal of extracting domain knowledge about the outcomes being predicted.^4,9,10^ This paper shows an application of explainable artificial intelligence methods to machine learning models of geographic variation in obesity prevalence. |
| Objectives | 3 | State specific objectives, including any prespecified hypotheses | 3 | Understanding what machine learning models discover about obesity has the potential to inform public health strategies that address the obesity crisis. Here, an explainable artificial intelligence approach applied to the County Health Rankings data from 2022 helps to better understand the most important factors contributing to the heterogeneity in county-level obesity prevalence.^11^ This paper employs four explainable artificial intelligence approaches: 1) Iterative random forest estimates of feature importance. 2) Accumulated effects plots that visualize the direction and nature of the discovered associations. 3) A surrogate decision tree model trained to mimic the predictions from the iterative random forest model that offers a visual aid in interpreting what the iterative random forest model has learned. 4) Local interpretable model-agnostic explanations offer explanations of obesity prevalence predictions for individual counties. These four explainable artificial intelligence approaches have the potential to leverage the power of machine learning models while extracting information about the important contributing factors to the obesity crisis. |
| Methods | | | |  |
| Study design | 4 | Present key elements of study design early in the paper | 4 | This paper follows the reporting guidelines for cross-sectional studies outlined by the Strengthening the Reporting of Observational Studies in Epidemiology.^12^ |
| Setting | 5 | Describe the setting, locations, and relevant dates, including periods of recruitment, exposure, follow-up, and data collection | 4 | The analyses in this paper are based on data from the 2022 County Health Rankings.^11,13^ The County Health Rankings dataset is an aggregation of statistics relevant to health for 3,142 counties across the United States. Analysis of publicly available and unidentifiable data does not require approval from the institutional review board. |
| Participants | 6 | (*a*) *Cohort study*—Give the eligibility criteria, and the sources and methods of selection of participants. Describe methods of follow-up  *Case-control study*—Give the eligibility criteria, and the sources and methods of case ascertainment and control selection. Give the rationale for the choice of cases and controls  *Cross-sectional study*—Give the eligibility criteria, and the sources and methods of selection of participants |  |  |
|  |  | (*b*) *Cohort study*—For matched studies, give matching criteria and number of exposed and unexposed  *Case-control study*—For matched studies, give matching criteria and the number of controls per case | 4 | Supplemental eTable 1 contains the sources of all analyzed variables. |
| Variables | 7 | Clearly define all outcomes, exposures, predictors, potential confounders, and effect modifiers. Give diagnostic criteria, if applicable | 4 | County-level rate of obesity based on a body mass index of ≥ 30 is the predicted outcome in all analyses. The County Health Rankings dataset calculates obesity prevalence using self-reported height and weight from the Behavioral Risk Factor Surveillance System.^14^ The predictors used from the county health rankings data include 64 variables from 7 broad categories: health outcomes, health behaviors, clinical care , social and economic factors, physical environment, demographics, and severe housing conditions. |
| Data sources/ measurement | 8* | For each variable of interest, give sources of data and details of methods of assessment (measurement). Describe comparability of assessment methods if there is more than one group | *4* | *The analyses in this paper are based on data from the 2022 County Health Rankings.^11^*  ^11.Stiff J. COUNTY HEALTH RANKINGS 2022:-ANALYTIC DATASET CODEBOOK-Non-standard measure variables. Published online 2022.^ |
| Bias | 9 | Describe any efforts to address potential sources of bias |  |  |
| Study size | 10 | Explain how the study size was arrived at | 4 | The County Health Rankings dataset is an aggregation of statistics relevant to health for 3,142 counties across the United States. |

Continued on next page

| Quantitative variables | 11 | Explain how quantitative variables were handled in the analyses. If applicable, describe which groupings were chosen and why | NA | No groupings were chosen. |
| --- | --- | --- | --- | --- |
| Statistical methods | 12 | (*a*) Describe all statistical methods, including those used to control for confounding | 5-6 | The iterative random forest R package (version 3.0.0) was used to build a prediction model of county-level obesity prevalence using a 2-fold cross-validation scheme.^9^ First, the modeling algorithm generates a forest of 1,000 decision trees separately for each data partition. The algorithm generates each decision using a subset of 8 features ($\surd$64 features), selected at random from the entire set of 64 features. The algorithm estimates the importance of each feature based on the variance explained in the outcome, averaged across all the decision trees. The algorithm then generates a second prediction model using the same process with one exception: each iteration weights the probability of selecting each feature for a decision tree based on the importance of that feature in the first prediction model. The algorithm iterates 100 times, using the importance from the previously run model, and keeps the model with the best performance based on out-of-bag error.  *2.4 Accumulated Local Effects*  The random forest algorithm estimates the importance of features in predicting obesity prevalence but does not describe the nature of the direction of the relationship. Accumulated local effects plots increase the transparency of what the machine learning model learned about the relationship between individual features and obesity prevalence by showing how the predicted obesity prevalence rate differs as the value of a feature increases.^16^ Subsets of data within specific ranges of feature values are the basis of estimating the accumulated effects. The R package *iml* (version 0.11.1) generated the accumulated local effects plots.  *2.5 Interpretable Decision Tree Surrogate for Random Forest Model*  First is a merger of predictions for the two data partitions. A grid search discovered the optimal decision tree settings for max depth, minsplit, and complexity. The R package *rpart* (version 4.1.16) implemented the decision tree algorithm. The selected decision tree parameters shared the most variance with the random forest predictions.  *2.6 Local interpretable model-agnostic explanations offer explanations*  Whereas the decision tree model described above serves as a global surrogate model, the local interpretable model-agnostic explanations approach serves as a local surrogate for individual predictions. The R package *lime* (version 0.5.3) implemented the local interpretable model-agnostic explanations algorithm. The training for each local model uses the prediction model that was not trained on the observation. Interrogation of the local model using the plot_features() function identifies the model features that increase or decrease the predicted prevalence for that county. The main results show the local models for two exemplar counties at lower and higher ends of the obesity prevalence distribution, respectfully. |
|  |  | (*b*) Describe any methods used to examine subgroups and interactions | NA | No subgroups or interactions are examined. |
|  |  | (*c*) Explain how missing data were addressed | 5 | To estimate missing values for each data partition separately, the multivariate imputation by chained equations R package (M.I.C.E. version 3.14.7) performs 10 imputations with 100 iterations.^15^ The final analysis uses the median imputed values. Trace lines of means and standard deviations across iterations showed convergence for each variable. Percentage of adults with obesity, the primary outcome, was not used to impute any variable. |
|  |  | (*d*) *Cohort study*—If applicable, explain how loss to follow-up was addressed  *Case-control study*—If applicable, explain how matching of cases and controls was addressed  *Cross-sectional study*—If applicable, describe analytical methods taking account of sampling strategy | NA |  |
|  |  | (*e*) Describe any sensitivity analyses | NA |  |
| Results | | | | |
| Participants | 13* | (a) Report numbers of individuals at each stage of study—eg numbers potentially eligible, examined for eligibility, confirmed eligible, included in the study, completing follow-up, and analysed |  | All eligible 3,142 Counties are analyzed |
|  |  | (b) Give reasons for non-participation at each stage | NA |  |
|  |  | (c) Consider use of a flow diagram | NA |  |
| Descriptive data | 14* | (a) Give characteristics of study participants (eg demographic, clinical, social) and information on exposures and potential confounders | 6 | Of the 3,142 counties in the analyses, the mean prevalence of adults with obesity was 35.7% (standard deviation = 4.3%; min = 16.4%; max = 51%; see Figure 1a). |
|  |  | (b) Indicate number of participants with missing data for each variable of interest | NA |  |
|  |  | (c) *Cohort study*—Summarise follow-up time (eg, average and total amount) | NA |  |
| Outcome data | 15* | *Cohort study*—Report numbers of outcome events or summary measures over time |  |  |
|  |  | *Case-control study—*Report numbers in each exposure category, or summary measures of exposure |  |  |
|  |  | *Cross-sectional study—*Report numbers of outcome events or summary measures | *6* | Of the 3,142 counties in the analyses, the mean prevalence of adults with obesity was 35.7% (standard deviation = 4.3%; min = 16.4%; max = 51%; see Figure 1a). |
| Main results | 16 | (*a*) Give unadjusted estimates and, if applicable, confounder-adjusted estimates and their precision (eg, 95% confidence interval). Make clear which confounders were adjusted for and why they were included | NA |  |
|  |  | (*b*) Report category boundaries when continuous variables were categorized | NA |  |
|  |  | (*c*) If relevant, consider translating estimates of relative risk into absolute risk for a meaningful time period | NA |  |

Continued on next page

| Other analyses | 17 | Report other analyses done—eg analyses of subgroups and interactions, and sensitivity analyses | NA |  |
| --- | --- | --- | --- | --- |
| Discussion | | | | |
| Key results | 18 | Summarise key results with reference to study objectives | 16-17 | This paper shows an explainable artificial intelligence approach to creating an interpretable machine learning model of county-level obesity prevalence. Using a cross-validation approach, two random forest models learned to predict obesity prevalence, and both explained 79% of the heterogeneity in county-level obesity prevalence. Physical inactivity explains most of the heterogeneity in county-level obesity, but the model also highlights the importance of diabetes and smoking.  Physical inactivity dominated the random forest models, as well as the global and local surrogate models. The accumulated local effects plot and the example local feature plots suggest a strong linear relationship, with higher levels of physical inactivity linked to higher levels of obesity. While the data analyzed in this paper did not include metrics of energy consumption, higher physical inactivity linked to obesity is consistent with the energy balance model.^17,18^ The analyzed data includes information on the food environment (i.e., food environment index), yet none of these features predicted obesity prevalence. Overall, the data suggest county-level efforts to increase the number of people engaging in monthly physical activities or exercises may be essential to reducing obesity prevalence.  Diabetes played a prominent role in differentiating medium vs. high levels of obesity prevalence in the surrogate decision tree. The accumulated local effects plot showed a linear relationship, with a higher prevalence of diabetes linked to a higher prevalence of obesity. Each diabetes node in the surrogate decision tree showed higher obesity estimates with higher diabetes prevalence. These findings are consistent with extensive research showing obesity causes insulin resistance and can lead to diabetes.^19,20^  Adult smoking played a prominent role in differentiating low vs. medium levels of obesity prevalence in the surrogate decision tree. The accumulated local effects plot showed a weak linear relationship, yet still showing higher prevalence of adult smoking linked to higher prevalence of obesity. The association between higher smoking prevalence and obesity is consistent with evidence that heavy cigarette smoking is associated with higher visceral adiposity and a greater risk of obesity compared to light smokers.^21,22^ |
| Limitations | 19 | Discuss limitations of the study, taking into account sources of potential bias or imprecision. Discuss both direction and magnitude of any potential bias | 18 | Many of the county-level estimates analyzed here are interpolations of self-reported data, sampled from each county. For example, self-reported height and weight are used to estimate obesity prevalence, which introduces error in the primary outcome of the analysis reported here. Moreover, body mass index is an imperfect metric for obesity, compared to waist circumference or skinfold measurements.^23^ Yet, the World Health Organization and Centers for Disease Control consider body mass index a reasonable obesity proxy. ^24,25^  The cross-sectional data reported here restricts causal claims between features of the model and county-level obesity. This issue is most obvious for diabetes, as obesity is a known risk factor for diabetes, while diabetes is not a risk factor for obesity. Similarly, physical inactivity is a known risk of obesity, yet obesity may reduce the probability of living an active lifestyle. Future studies may show that decreasing physical inactivity prevalence also decreases the prevalence of obesity and diabetes.  Finally, this study does not distinguish between type-1 and type-2 diabetes. Here, diabetes prevalence is based on whether a person self-reports having been told by a doctor they have diabetes. However, type-2 diabetes accounts for 90% to 95% of the diabetes diagnoses in adults, making the findings reported here biased towards resembling a pure measure of type-2 diabetes prevalence.^26^ |
| Interpretation | 20 | Give a cautious overall interpretation of results considering objectives, limitations, multiplicity of analyses, results from similar studies, and other relevant evidence | 18 | County-level physical inactivity explained most of the heterogeneity in county-level obesity prevalence. Explainable artificial intelligence approaches can make black-box models transparent by revealing what the model learned from the data. |
| Generalisability | 21 | Discuss the generalisability (external validity) of the study results | 18-19 | The local interpretable model-agnostic explanations may be useful for county health departments to identify the most important factors contributing to their obesity prevalence. |
| Other information | |  | | |
| Funding | 22 | Give the source of funding and the role of the funders for the present study and, if applicable, for the original study on which the present article is based | NA |  |

*Give information separately for cases and controls in case-control studies and, if applicable, for exposed and unexposed groups in cohort and cross-sectional studies.

**Note:** An Explanation and Elaboration article discusses each checklist item and gives methodological background and published examples of transparent reporting. The STROBE checklist is best used in conjunction with this article (freely available on the Web sites of PLoS Medicine at http://www.plosmedicine.org/, Annals of Internal Medicine at http://www.annals.org/, and Epidemiology at http://www.epidem.com/). Information on the STROBE Initiative is available at www.strobe-statement.org.
