# Supplementary material for: An interpretable machine learning model of cross-sectional U.S. county-level obesity prevalence using explainable artificial intelligence
Source: PLoS One. 2023 Oct 5;18(10):e0292341. doi: 10.1371/journal.pone.0292341 (PMC10553328; doi:10.1371/journal.pone.0292341)
Supplement: S1 Fig — (PDF) [file pone.0292341.s005.pdf]

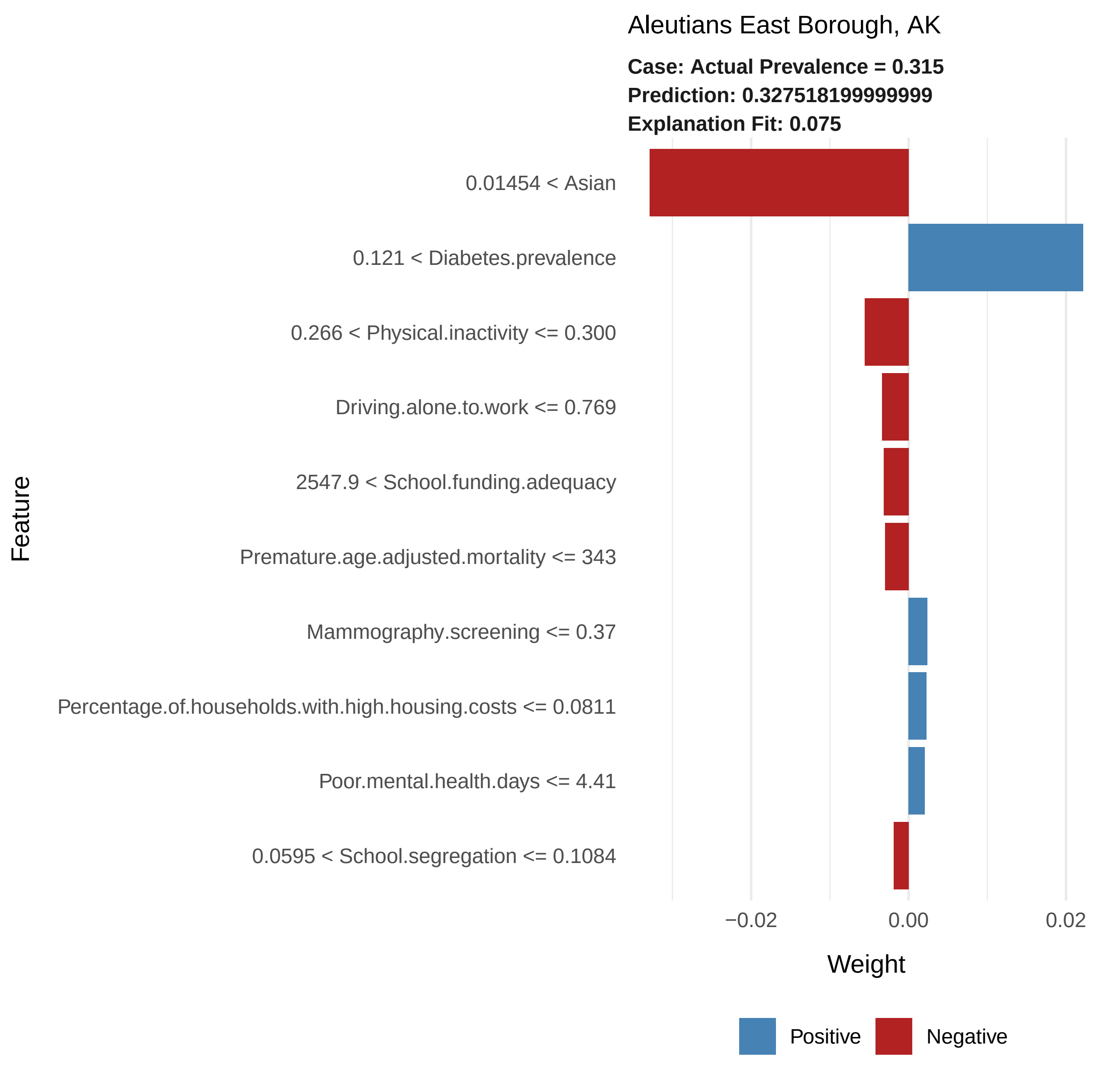

Aleutians East Borough, AK
Case: Actual Prevalence = 0.315
Prediction: 0.327518199999999
Explanation Fit: 0.075
0.01454 < Asian
0.121 < Diabetes.prevalence
0.266 < Physical.inactivity <= 0.300
Driving.alone.to.work <= 0.769
2547.9 < School.funding.adequacy
Premature.age.adjusted.mortality <= 343
Mammography.screening <= 0.37
Percentage.of.households.with.high.housing.costs <= 0.0811
Poor.mental.health.days <= 4.41
0.0595 < School.segregation <= 0.1084
Feature
−0.02
0.00
0.02
Weight
Positive
Negative

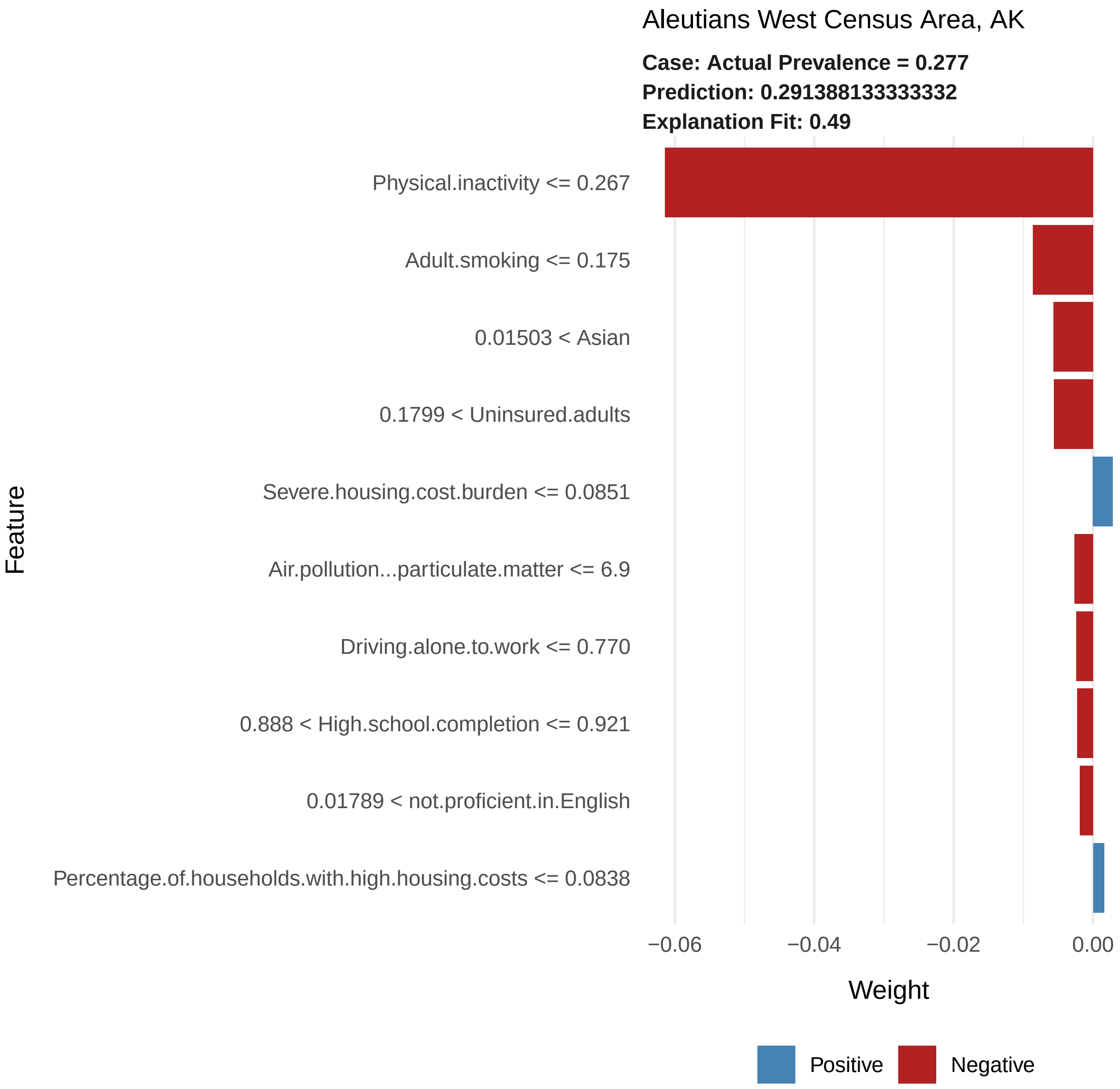
Aleutians West Census Area, AK
Case: Actual Prevalence = 0.277
Prediction: 0.291388133333332
Explanation Fit: 0.49
Physical.inactivity <= 0.267
Adult.smoking <= 0.175
0.01503 < Asian
0.1799 < Uninsured.adults
Severe.housing.cost.burden <= 0.0851
Air.pollution...particulate.matter <= 6.9
Driving.alone.to.work <= 0.770
0.888 < High.school.completion <= 0.921
0.01789 < not.proficient.in.English
Percentage.of.households.with.high.housing.costs <= 0.0838
Feature
−0.06
−0.04
−0.02
0.00
Weight
Positive
Negative

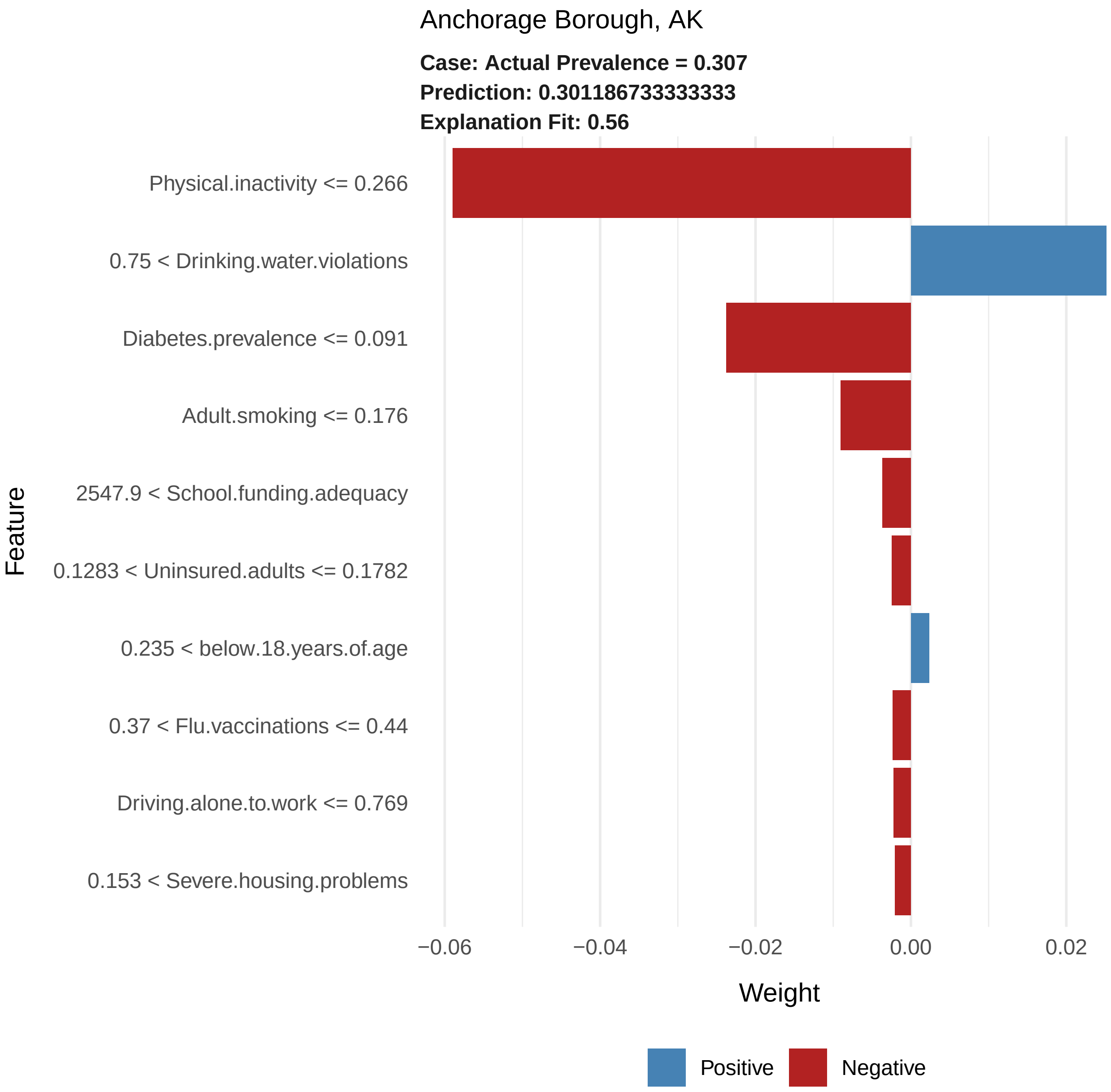
Anchorage Borough, AK
Case: Actual Prevalence = 0.307
Prediction: 0.301186733333333
Explanation Fit: 0.56
Physical.inactivity <= 0.266
0.75 < Drinking.water.violations
Diabetes.prevalence <= 0.091
Adult.smoking <= 0.176
2547.9 < School.funding.adequacy
0.1283 < Uninsured.adults <= 0.1782
0.235 < below.18.years.of.age
0.37 < Flu.vaccinations <= 0.44
Driving.alone.to.work <= 0.769
0.153 < Severe.housing.problems
Feature
−0.06
−0.04
−0.02
0.00
0.02
Weight
Positive
Negative

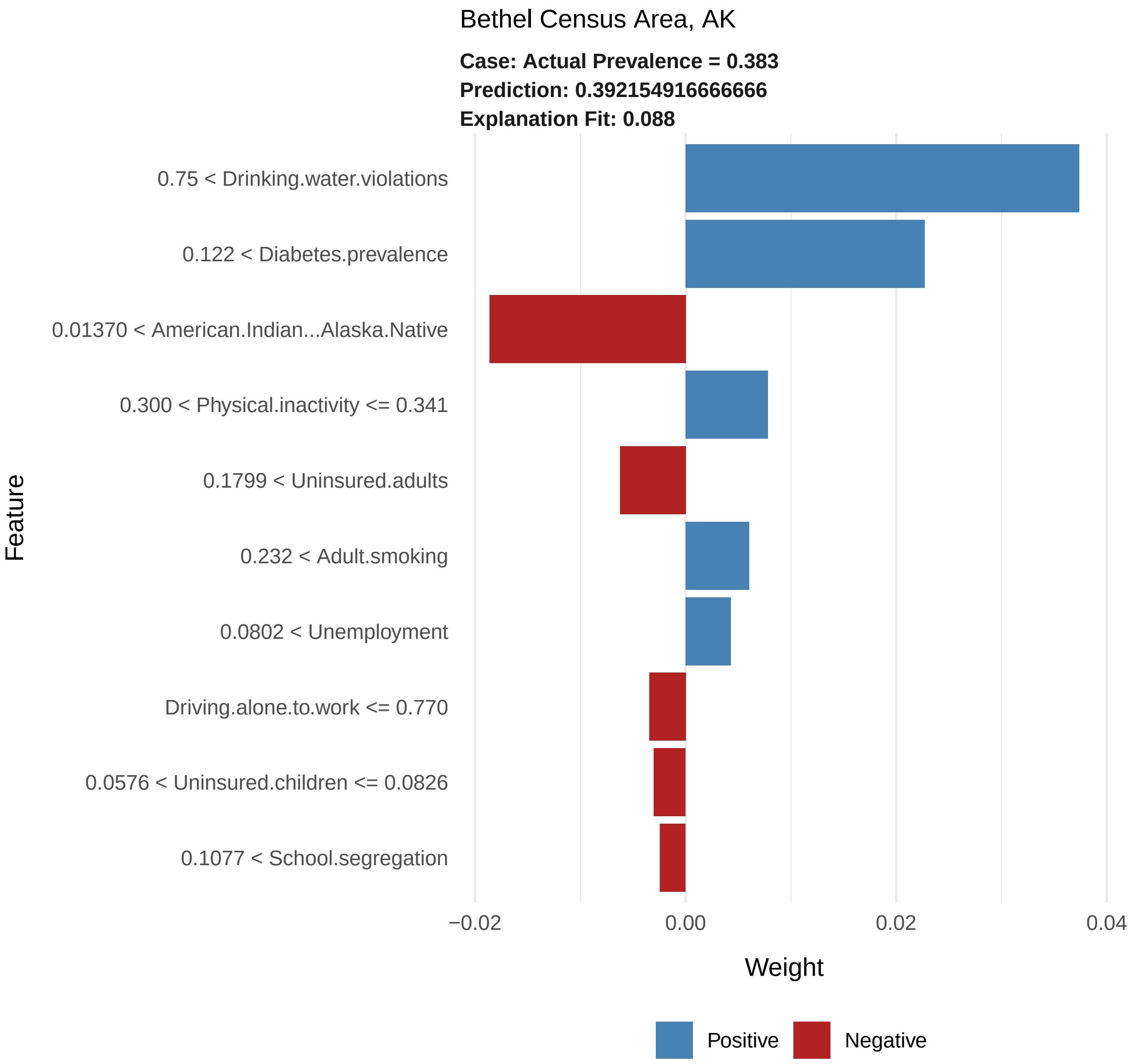

Bethel Census Area, AK
Case: Actual Prevalence = 0.383
Prediction: 0.392154916666666
Explanation Fit: 0.088
0.75 < Drinking.water.violations
0.122 < Diabetes.prevalence
0.01370 < American.Indian...Alaska.Native
0.300 < Physical.inactivity <= 0.341
0.1799 < Uninsured.adults
0.232 < Adult.smoking
0.0802 < Unemployment
Driving.alone.to.work <= 0.770
0.0576 < Uninsured.children <= 0.0826
0.1077 < School.segregation
Feature
−0.02
0.00
0.02
0.04
Weight
Positive
Negative

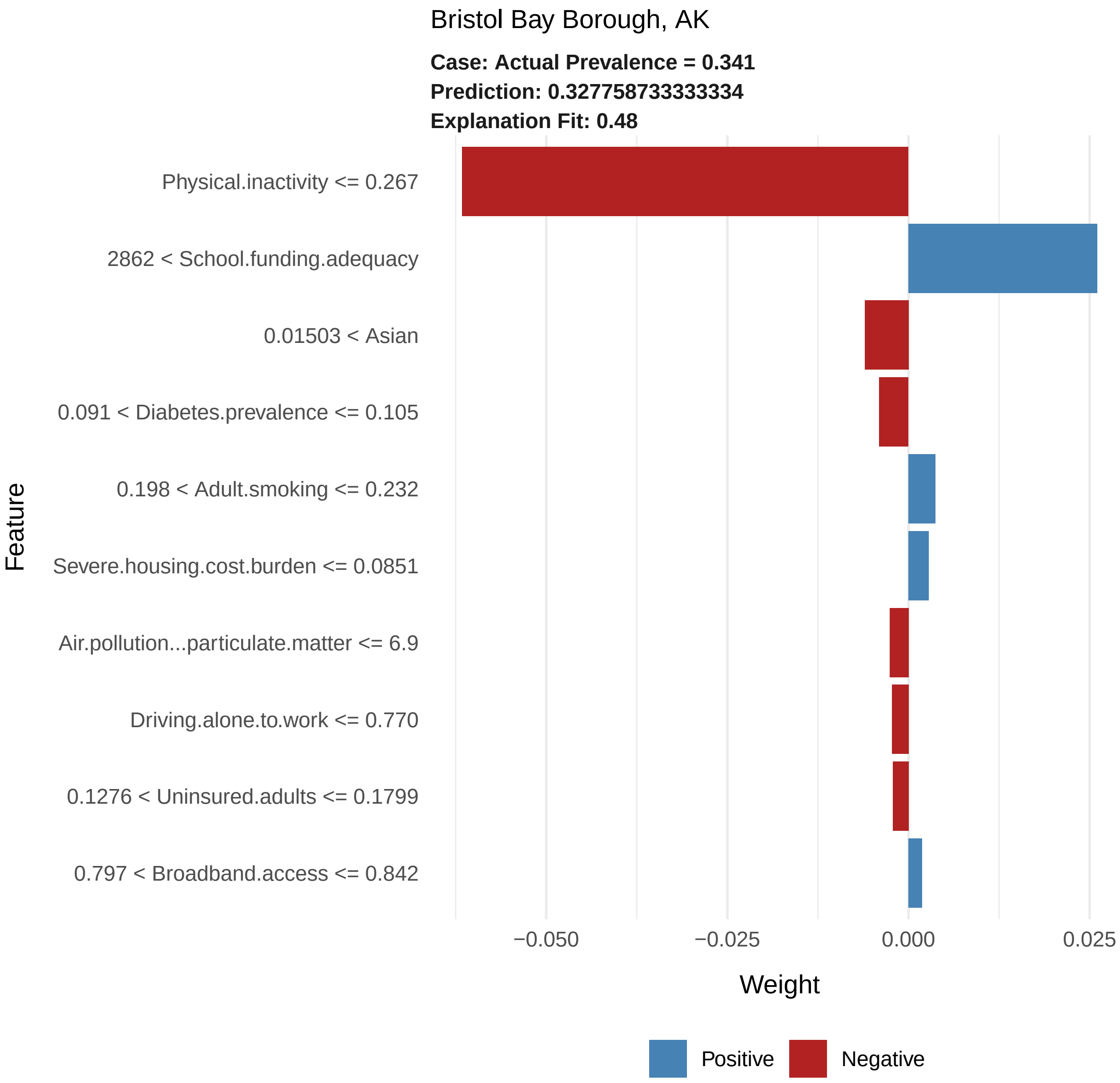

Bristol Bay Borough, AK
Case: Actual Prevalence = 0.341
Prediction: 0.327758733333334
Explanation Fit: 0.48
Physical.inactivity <= 0.267
2862 < School.funding.adequacy
0.01503 < Asian
0.091 < Diabetes.prevalence <= 0.105
0.198 < Adult.smoking <= 0.232
Severe.housing.cost.burden <= 0.0851
Air.pollution...particulate.matter <= 6.9
Driving.alone.to.work <= 0.770
0.1276 < Uninsured.adults <= 0.1799
0.797 < Broadband.access <= 0.842
Feature
−0.050
−0.025
0.000
0.025
Weight
Positive
Negative

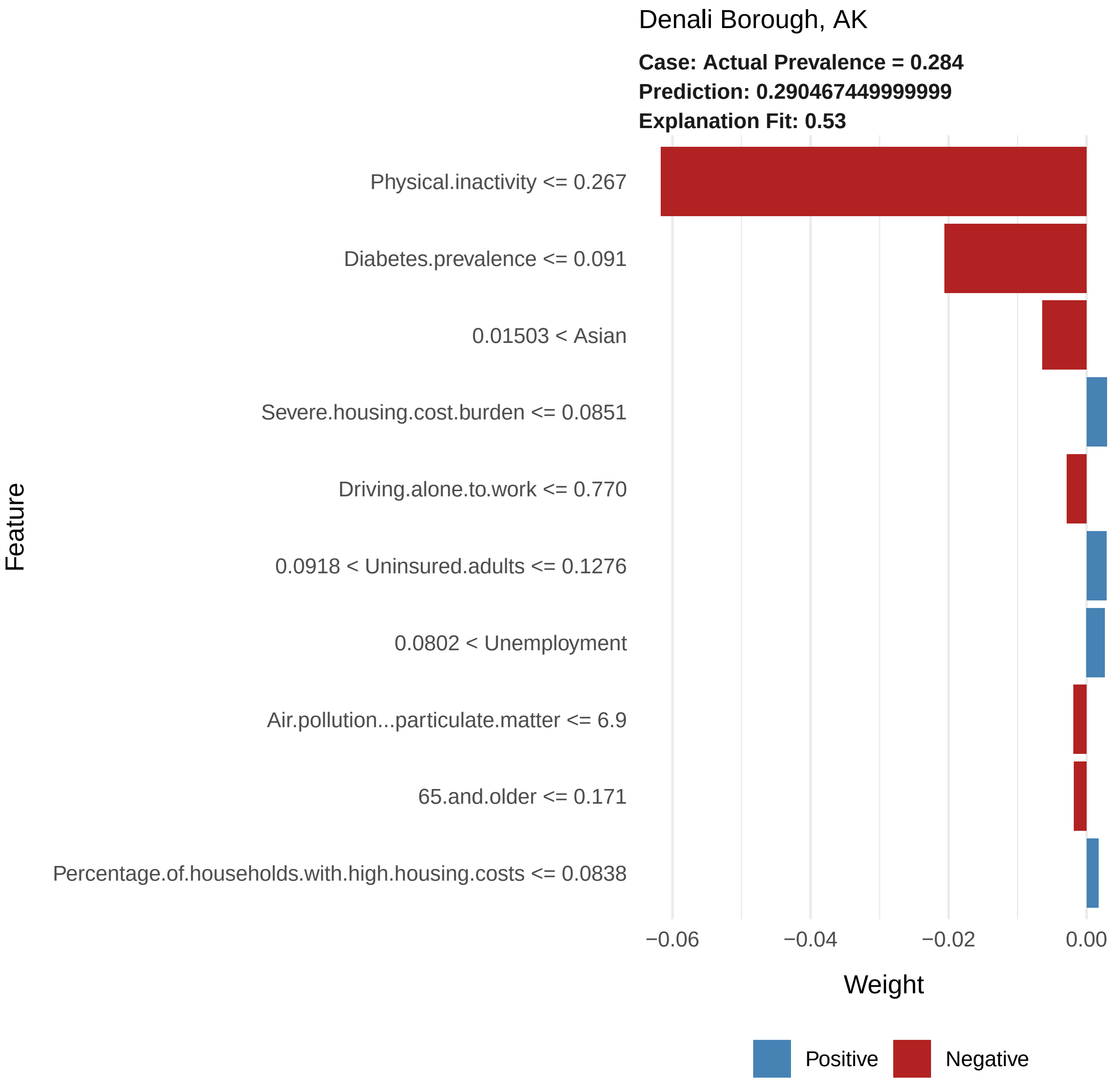

Denali Borough, AK
Case: Actual Prevalence = 0.284
Prediction: 0.290467449999999
Explanation Fit: 0.53
Physical.inactivity <= 0.267
Diabetes.prevalence <= 0.091
0.01503 < Asian
Severe.housing.cost.burden <= 0.0851
Driving.alone.to.work <= 0.770
0.0918 < Uninsured.adults <= 0.1276
0.0802 < Unemployment
Air.pollution...particulate.matter <= 6.9
65.and.older <= 0.171
Percentage.of.households.with.high.housing.costs <= 0.0838
Feature
−0.06
−0.04
−0.02
0.00
Weight
Positive
Negative

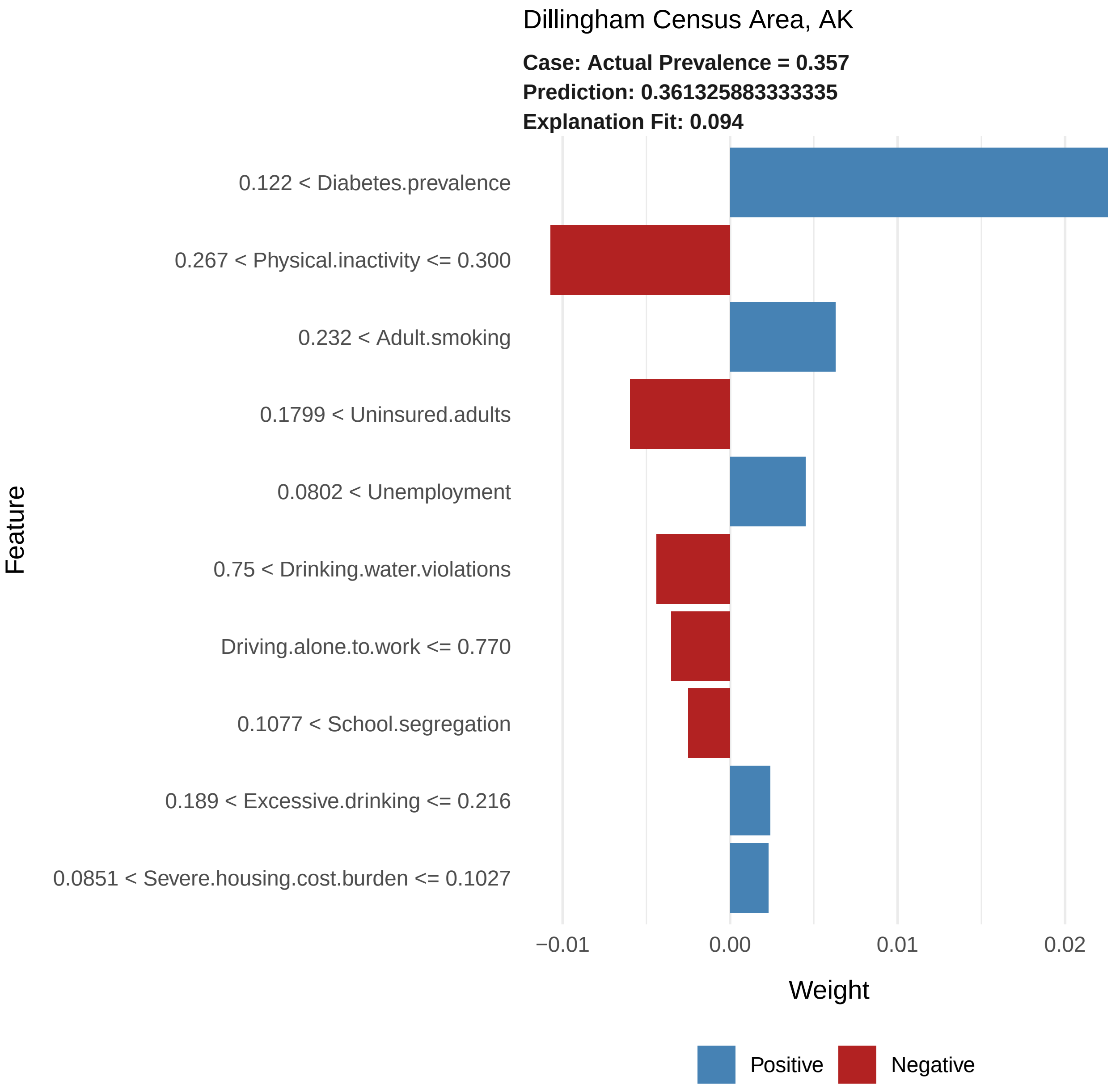

Dillingham Census Area, AK
Case: Actual Prevalence = 0.357
Prediction: 0.361325883333335
Explanation Fit: 0.094
Feature
0.122 < Diabetes.prevalence
0.267 < Physical.inactivity <= 0.300
0.232 < Adult.smoking
0.1799 < Uninsured.adults
0.0802 < Unemployment
0.75 < Drinking.water.violations
Driving.alone.to.work <= 0.770
0.1077 < School.segregation
0.189 < Excessive.drinking <= 0.216
0.0851 < Severe.housing.cost.burden <= 0.1027
−0.01
0.00
0.01
0.02
Weight
Positive
Negative

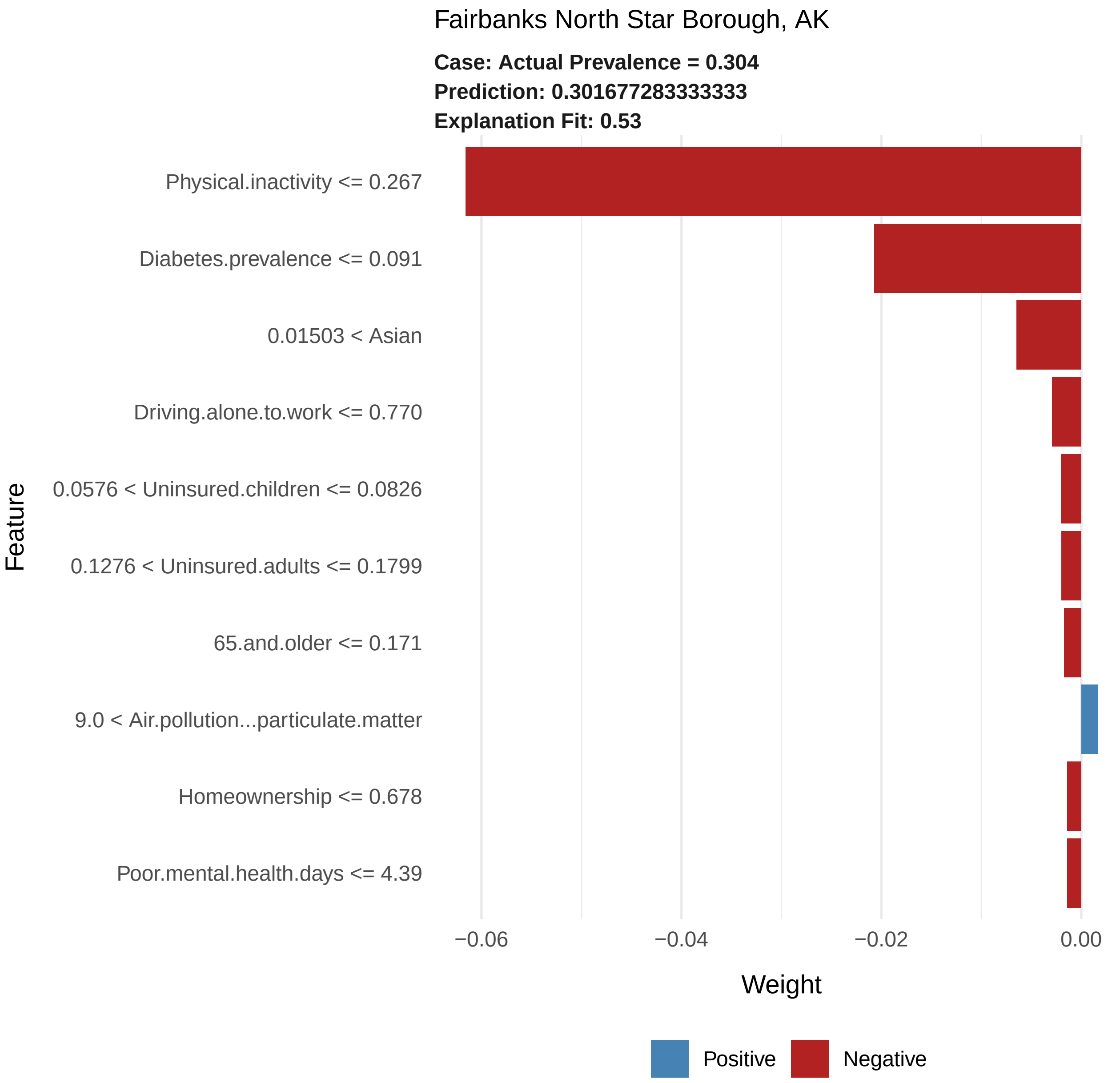
Fairbanks North Star Borough, AK
Case: Actual Prevalence = 0.304
Prediction: 0.301677283333333
Explanation Fit: 0.53
Physical.inactivity <= 0.267
Diabetes.prevalence <= 0.091
0.01503 < Asian
Driving.alone.to.work <= 0.770
0.0576 < Uninsured.children <= 0.0826
0.1276 < Uninsured.adults <= 0.1799
65.and.older <= 0.171
9.0 < Air.pollution...particulate.matter
Homeownership <= 0.678
Poor.mental.health.days <= 4.39
Feature
−0.06
−0.04
−0.02
0.00
Weight
Positive
Negative

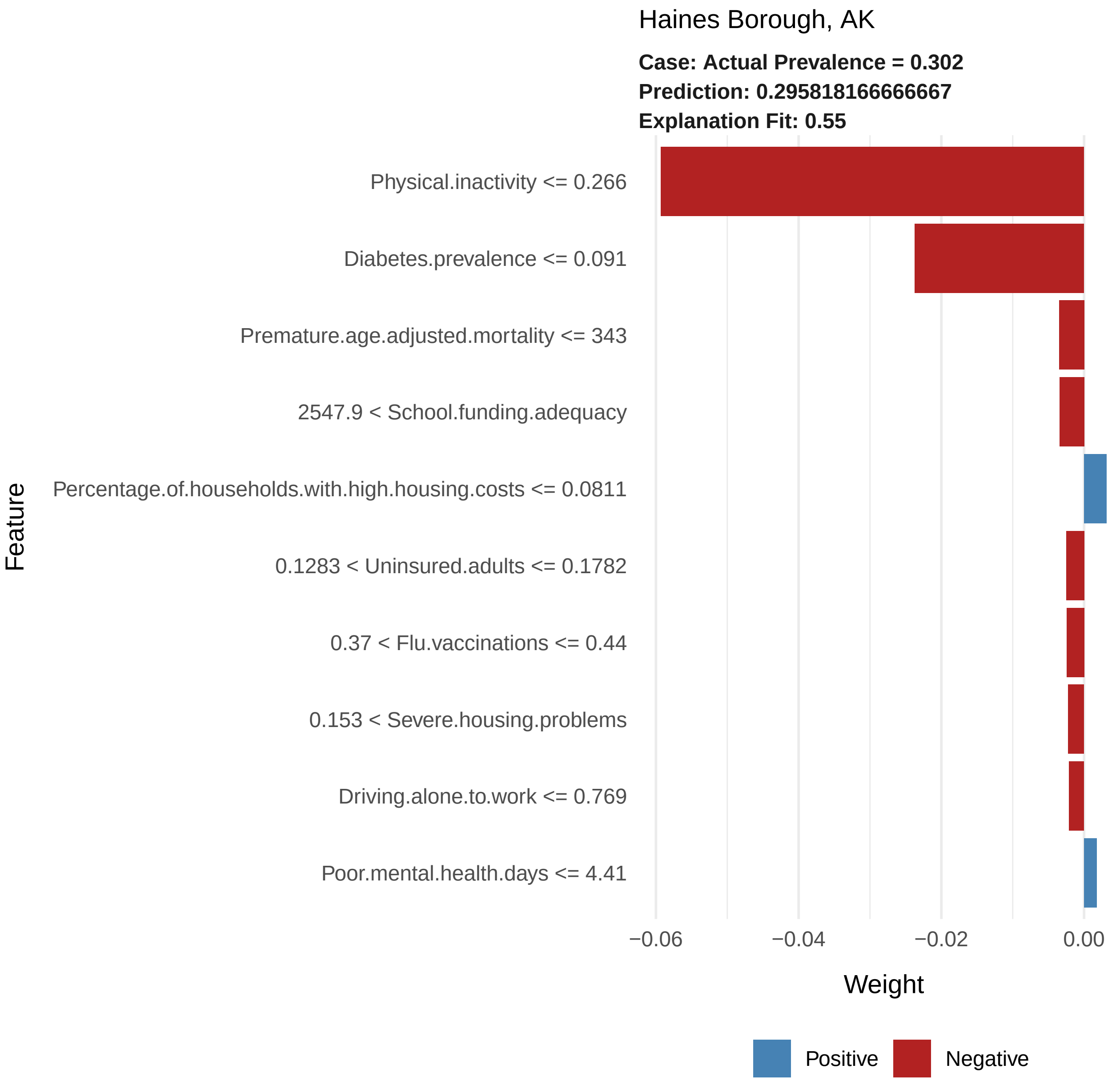
Haines Borough, AK
Case: Actual Prevalence = 0.302
Prediction: 0.295818166666667
Explanation Fit: 0.55
Physical.inactivity <= 0.266
Diabetes.prevalence <= 0.091
Premature.age.adjusted.mortality <= 343
2547.9 < School.funding.adequacy
Percentage.of.households.with.high.housing.costs <= 0.0811
0.1283 < Uninsured.adults <= 0.1782
0.37 < Flu.vaccinations <= 0.44
0.153 < Severe.housing.problems
Driving.alone.to.work <= 0.769
Poor.mental.health.days <= 4.41
Feature
−0.06
−0.04
−0.02
0.00
Weight
Positive
Negative

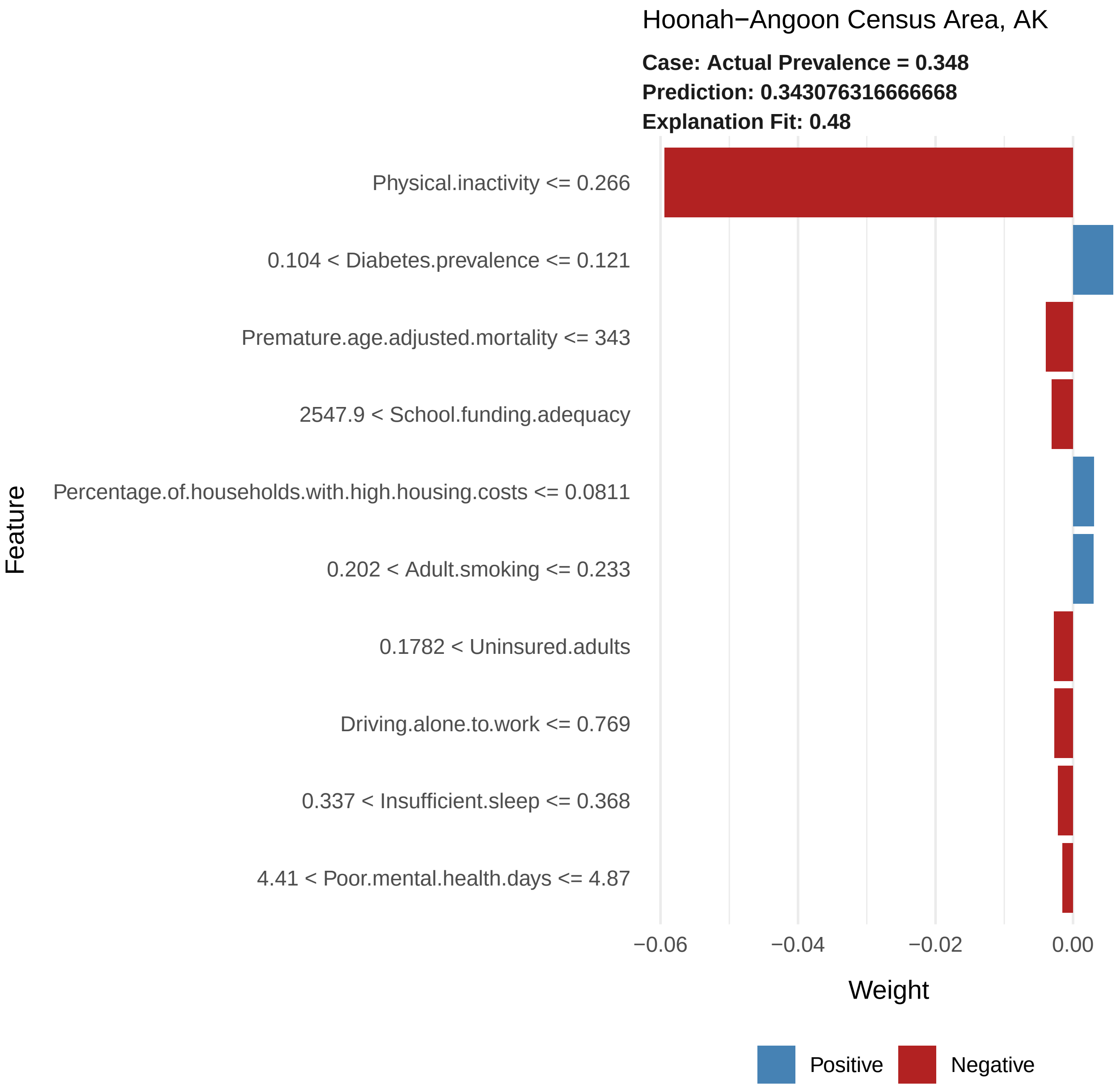

Hoonah−Angoon Census Area, AK
Case: Actual Prevalence = 0.348
Prediction: 0.343076316666668
Explanation Fit: 0.48
Feature
Physical.inactivity <= 0.266
0.104 < Diabetes.prevalence <= 0.121
Premature.age.adjusted.mortality <= 343
2547.9 < School.funding.adequacy
Percentage.of.households.with.high.housing.costs <= 0.0811
0.202 < Adult.smoking <= 0.233
0.1782 < Uninsured.adults
Driving.alone.to.work <= 0.769
0.337 < Insufficient.sleep <= 0.368
4.41 < Poor.mental.health.days <= 4.87
−0.06
−0.04
−0.02
0.00
Weight
Positive
Negative

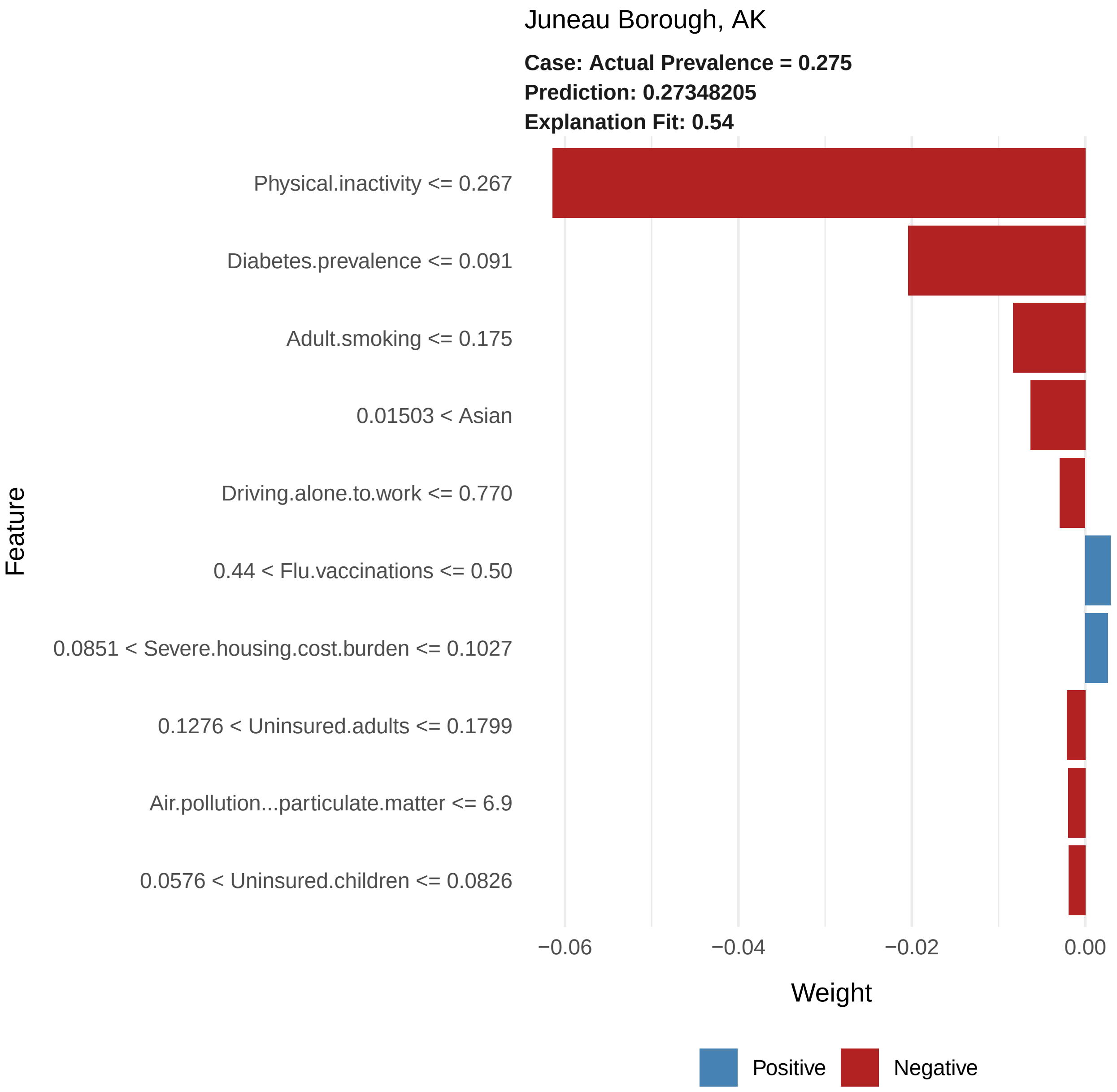
Juneau Borough, AK
Case: Actual Prevalence = 0.275
Prediction: 0.27348205
Explanation Fit: 0.54
Physical.inactivity <= 0.267
Diabetes.prevalence <= 0.091
Adult.smoking <= 0.175
0.01503 < Asian
Driving.alone.to.work <= 0.770
0.44 < Flu.vaccinations <= 0.50
0.0851 < Severe.housing.cost.burden <= 0.1027
0.1276 < Uninsured.adults <= 0.1799
Air.pollution...particulate.matter <= 6.9
0.0576 < Uninsured.children <= 0.0826
Feature
−0.06
−0.04
−0.02
0.00
Weight
Positive
Negative

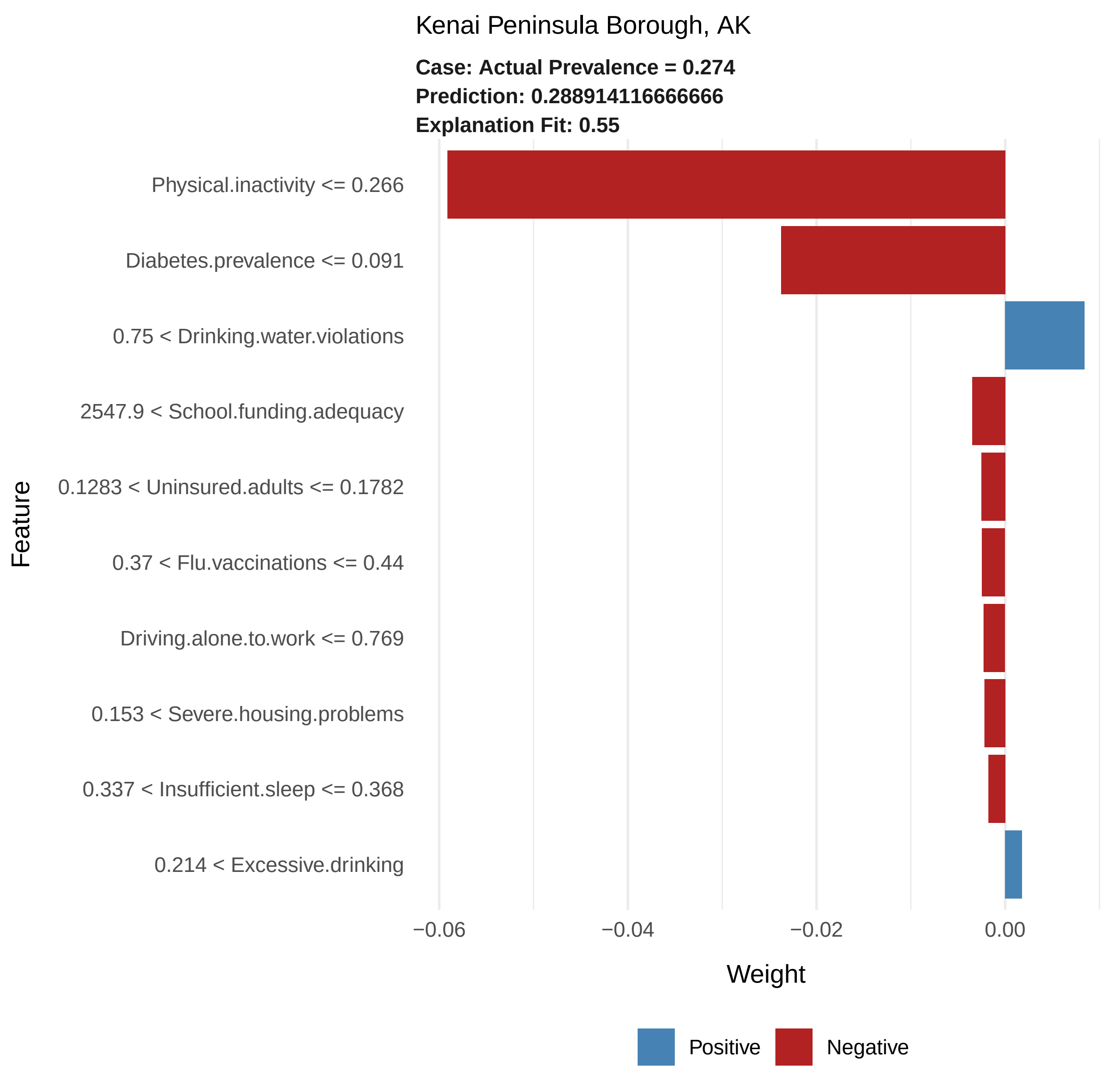
Kenai Peninsula Borough, AK
Case: Actual Prevalence = 0.274
Prediction: 0.288914116666666
Explanation Fit: 0.55
Physical.inactivity <= 0.266
Diabetes.prevalence <= 0.091
0.75 < Drinking.water.violations
2547.9 < School.funding.adequacy
0.1283 < Uninsured.adults <= 0.1782
0.37 < Flu.vaccinations <= 0.44
Driving.alone.to.work <= 0.769
0.153 < Severe.housing.problems
0.337 < Insufficient.sleep <= 0.368
0.214 < Excessive.drinking
Feature
−0.06
−0.04
−0.02
0.00
Weight
Positive
Negative

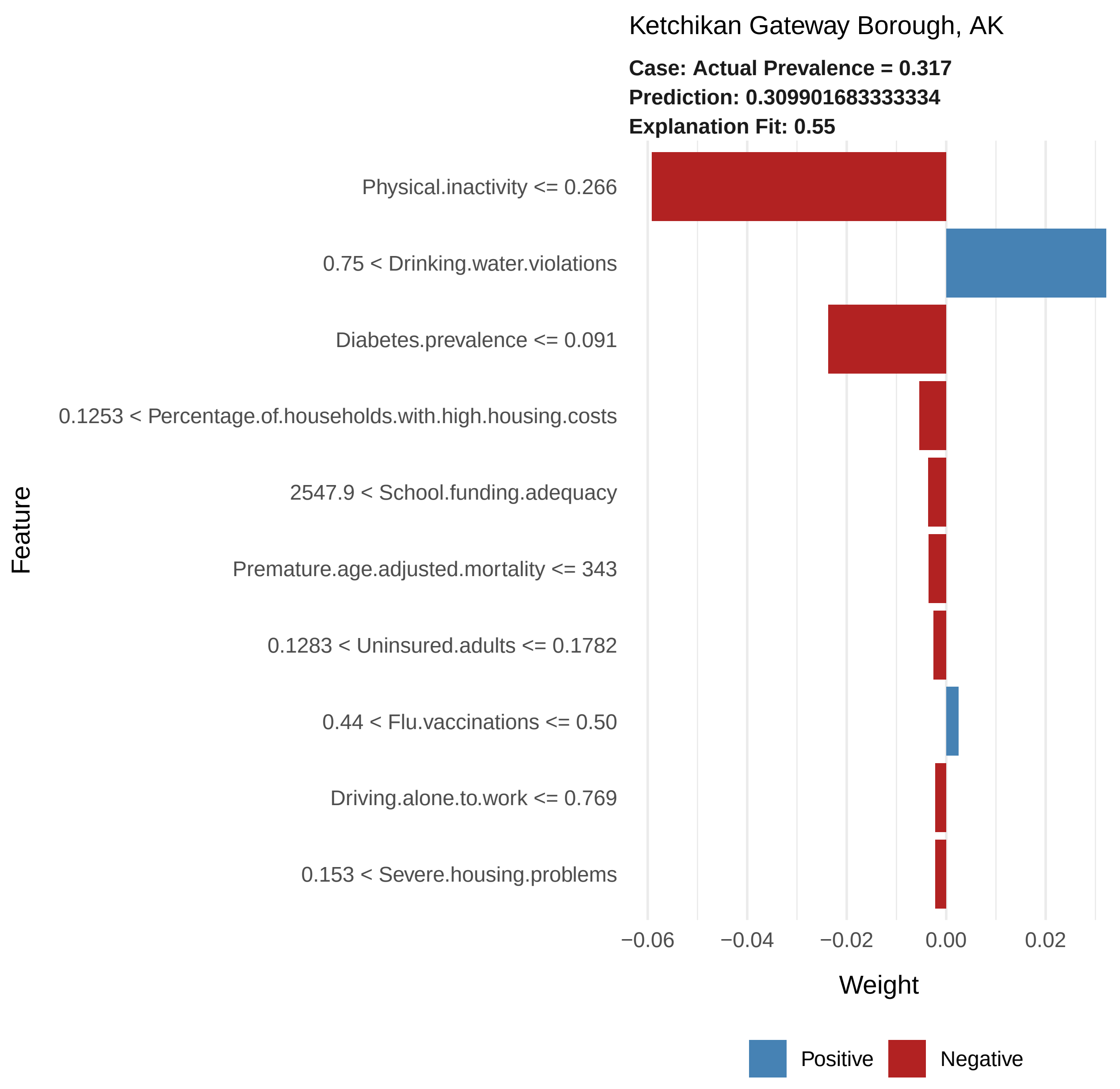

Ketchikan Gateway Borough, AK
Case: Actual Prevalence = 0.317
Prediction: 0.309901683333334
Explanation Fit: 0.55
Physical.inactivity <= 0.266
0.75 < Drinking.water.violations
Diabetes.prevalence <= 0.091
0.1253 < Percentage.of.households.with.high.housing.costs
2547.9 < School.funding.adequacy
Premature.age.adjusted.mortality <= 343
0.1283 < Uninsured.adults <= 0.1782
0.44 < Flu.vaccinations <= 0.50
Driving.alone.to.work <= 0.769
0.153 < Severe.housing.problems
Feature
−0.06
−0.04
−0.02
0.00
0.02
Weight
Positive
Negative

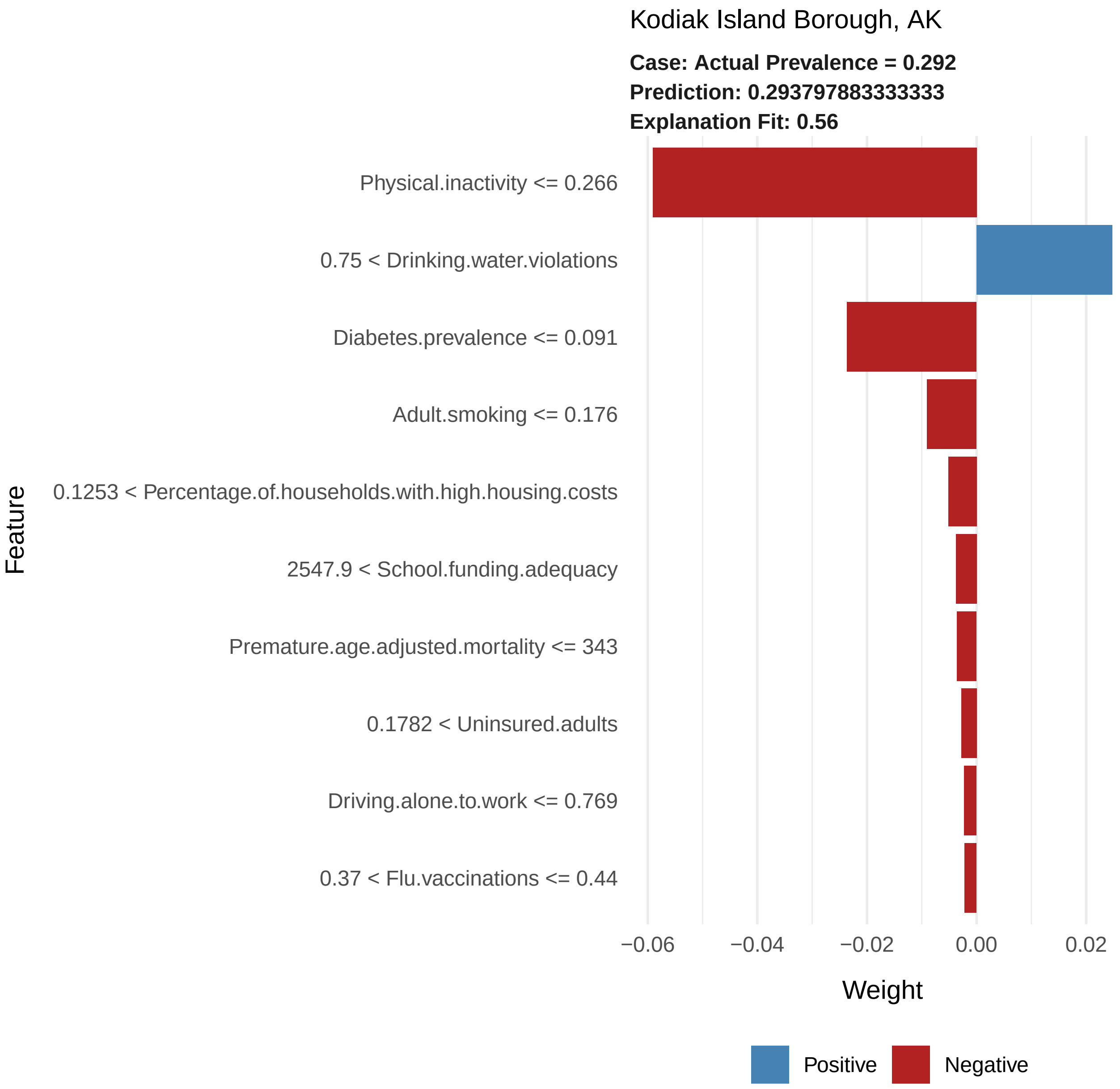
Kodiak Island Borough, AK
Case: Actual Prevalence = 0.292
Prediction: 0.293797883333333
Explanation Fit: 0.56
Physical.inactivity <= 0.266
0.75 < Drinking.water.violations
Diabetes.prevalence <= 0.091
Adult.smoking <= 0.176
0.1253 < Percentage.of.households.with.high.housing.costs
2547.9 < School.funding.adequacy
Premature.age.adjusted.mortality <= 343
0.1782 < Uninsured.adults
Driving.alone.to.work <= 0.769
0.37 < Flu.vaccinations <= 0.44
Feature
−0.06
−0.04
−0.02
0.00
0.02
Weight
Positive
Negative

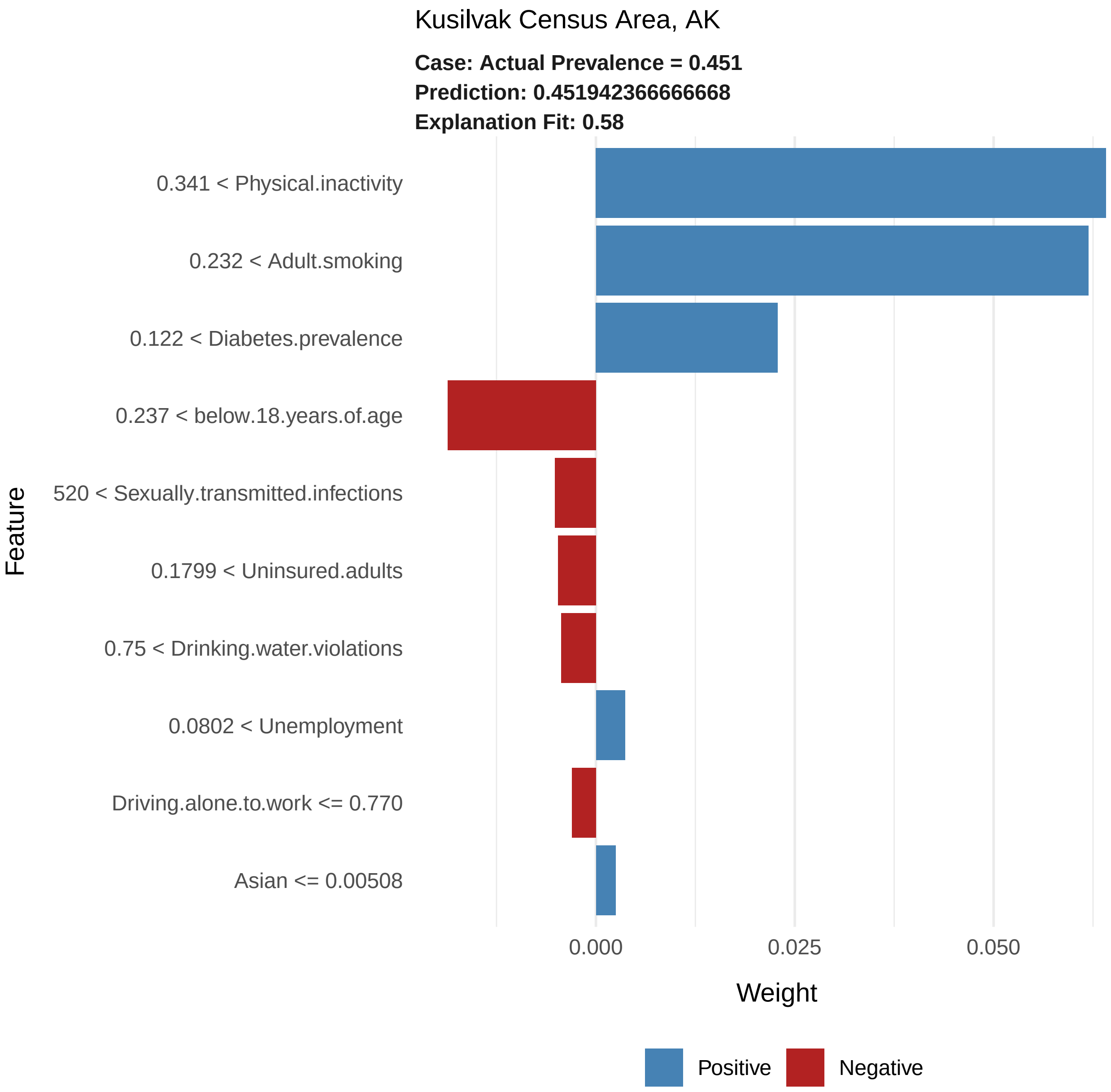
Kusilvak Census Area, AK
Case: Actual Prevalence = 0.451
Prediction: 0.451942366666668
Explanation Fit: 0.58
0.341 < Physical.inactivity
0.232 < Adult.smoking
0.122 < Diabetes.prevalence
0.237 < below.18.years.of.age
520 < Sexually.transmitted.infections
0.1799 < Uninsured.adults
0.75 < Drinking.water.violations
0.0802 < Unemployment
Driving.alone.to.work <= 0.770
Asian <= 0.00508
Feature
0.000
0.025
0.050
Weight
Positive
Negative

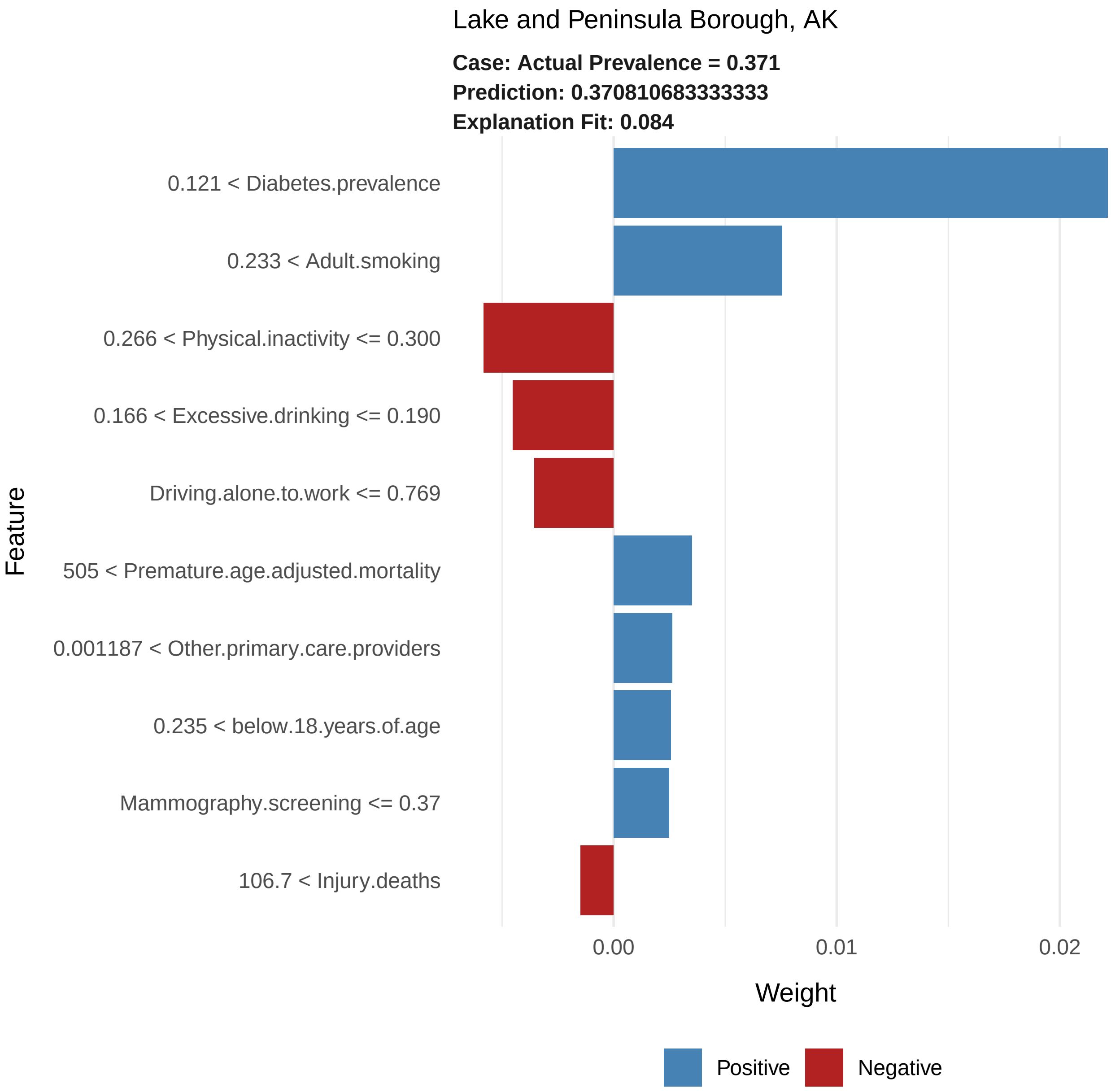

Lake and Peninsula Borough, AK
Case: Actual Prevalence = 0.371
Prediction: 0.370810683333333
Explanation Fit: 0.084
0.121 < Diabetes.prevalence
0.233 < Adult.smoking
0.266 < Physical.inactivity <= 0.300
0.166 < Excessive.drinking <= 0.190
Driving.alone.to.work <= 0.769
505 < Premature.age.adjusted.mortality
0.001187 < Other.primary.care.providers
0.235 < below.18.years.of.age
Mammography.screening <= 0.37
106.7 < Injury.deaths
Feature
0.00
0.01
0.02
Weight
Positive
Negative

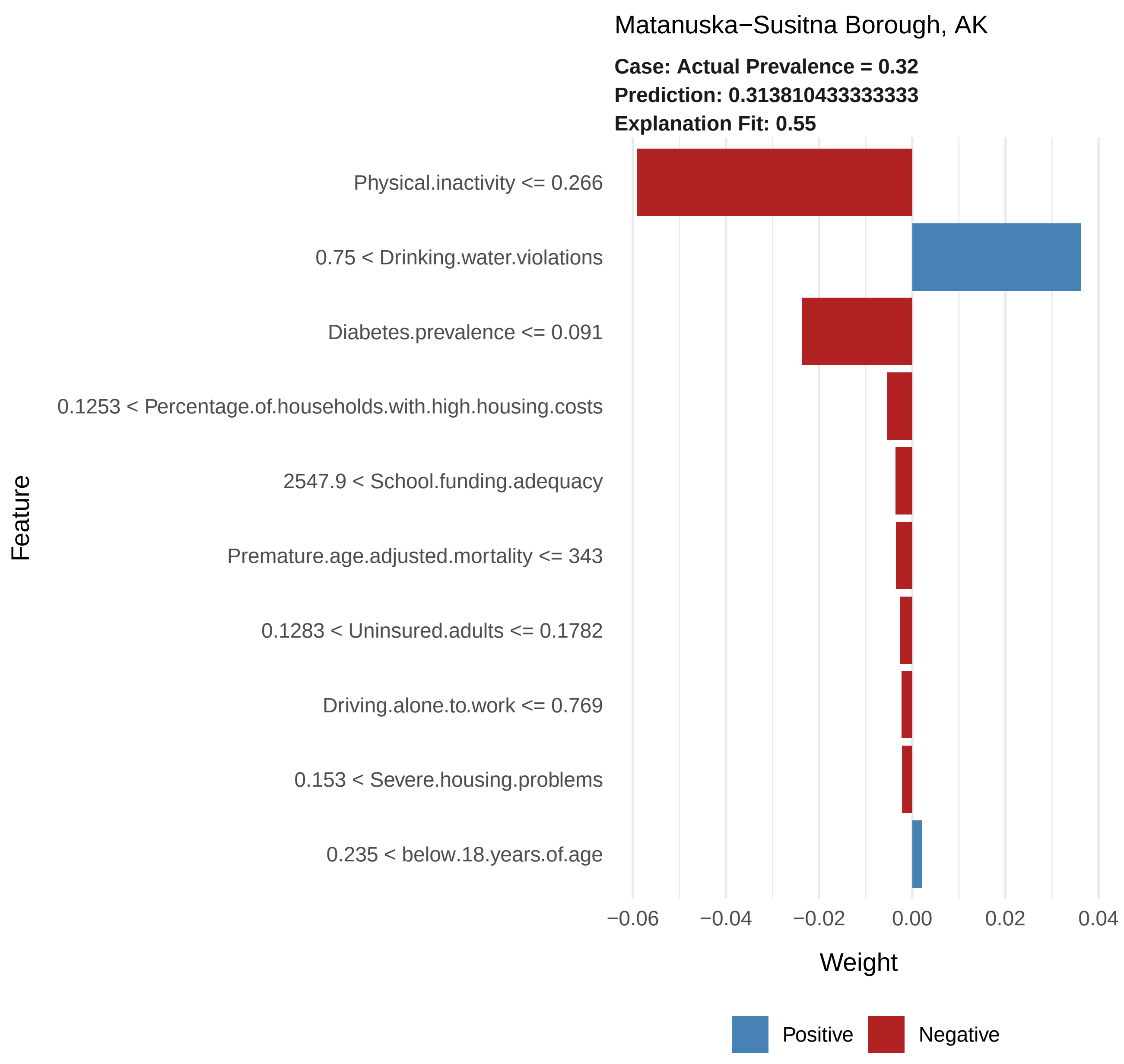
Matanuska−Susitna Borough, AK
Case: Actual Prevalence = 0.32
Prediction: 0.313810433333333
Explanation Fit: 0.55
Physical.inactivity <= 0.266
0.75 < Drinking.water.violations
Diabetes.prevalence <= 0.091
0.1253 < Percentage.of.households.with.high.housing.costs
2547.9 < School.funding.adequacy
Premature.age.adjusted.mortality <= 343
0.1283 < Uninsured.adults <= 0.1782
Driving.alone.to.work <= 0.769
0.153 < Severe.housing.problems
0.235 < below.18.years.of.age
Feature
−0.06
−0.04
−0.02
0.00
0.02
0.04
Weight
Positive
Negative

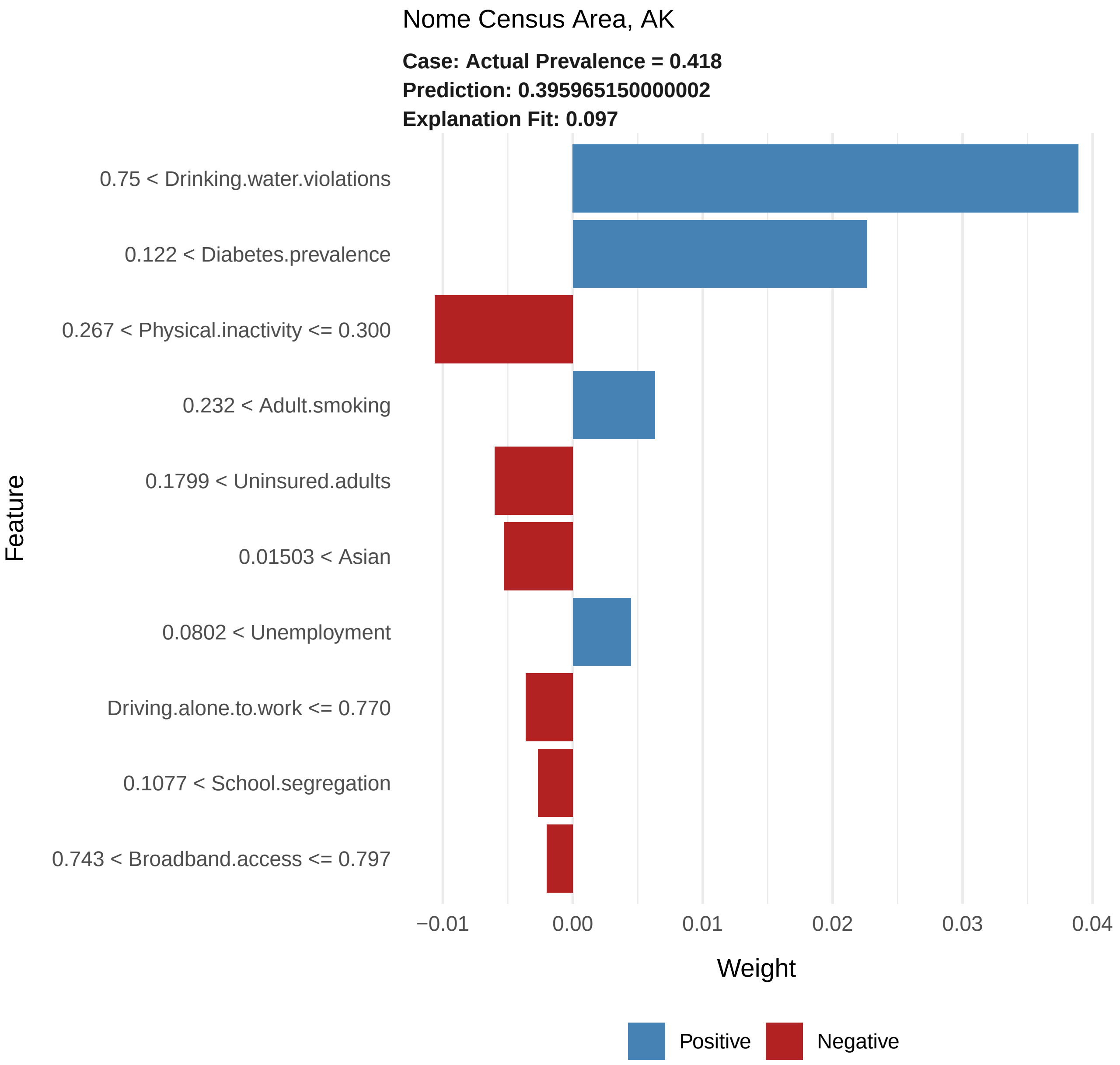
Nome Census Area, AK
Case: Actual Prevalence = 0.418
Prediction: 0.395965150000002
Explanation Fit: 0.097
0.75 < Drinking.water.violations
0.122 < Diabetes.prevalence
0.267 < Physical.inactivity <= 0.300
0.232 < Adult.smoking
0.1799 < Uninsured.adults
0.01503 < Asian
0.0802 < Unemployment
Driving.alone.to.work <= 0.770
0.1077 < School.segregation
0.743 < Broadband.access <= 0.797
Feature
−0.01
0.00
0.01
0.02
0.03
0.04
Weight
Positive
Negative

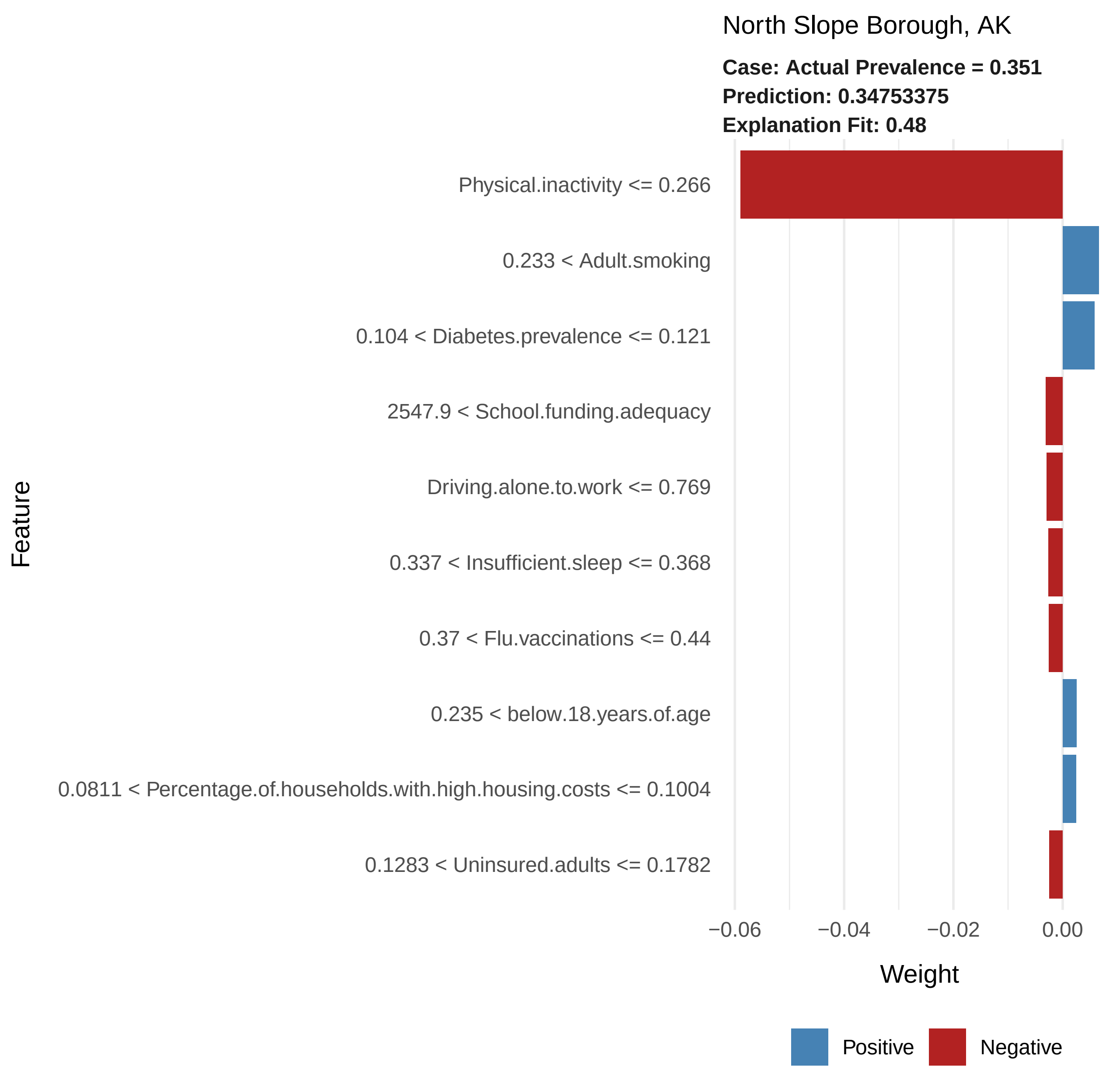
North Slope Borough, AK
Case: Actual Prevalence = 0.351
Prediction: 0.34753375
Explanation Fit: 0.48
Physical.inactivity <= 0.266
0.233 < Adult.smoking
0.104 < Diabetes.prevalence <= 0.121
2547.9 < School.funding.adequacy
Driving.alone.to.work <= 0.769
0.337 < Insufficient.sleep <= 0.368
0.37 < Flu.vaccinations <= 0.44
0.235 < below.18.years.of.age
0.0811 < Percentage.of.households.with.high.housing.costs <= 0.1004
0.1283 < Uninsured.adults <= 0.1782
Feature
−0.06
−0.04
−0.02
0.00
Weight
Positive
Negative

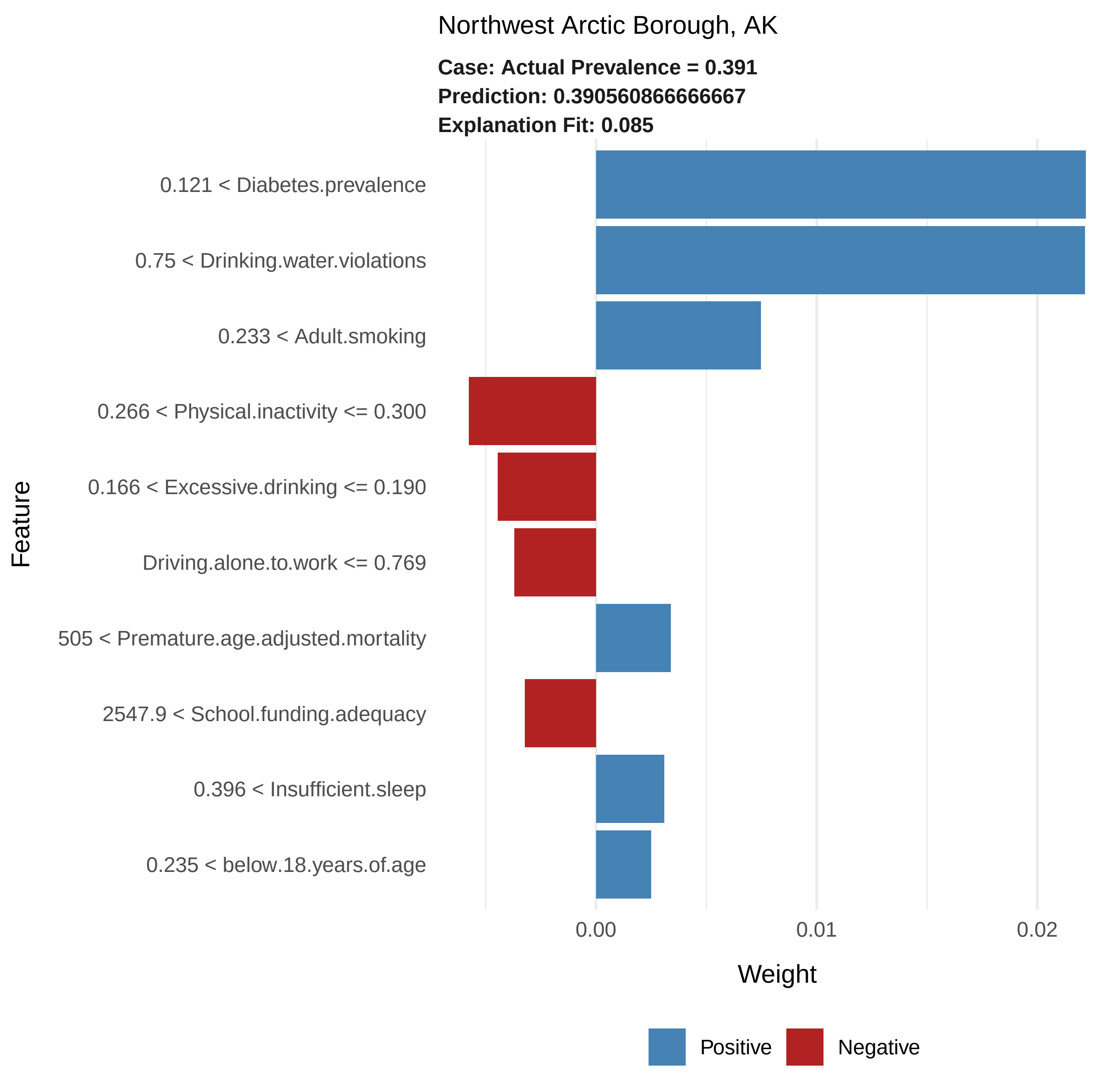

Northwest Arctic Borough, AK
Case: Actual Prevalence = 0.391
Prediction: 0.390560866666667
Explanation Fit: 0.085
0.121 < Diabetes.prevalence
0.75 < Drinking.water.violations
0.233 < Adult.smoking
0.266 < Physical.inactivity <= 0.300
0.166 < Excessive.drinking <= 0.190
Driving.alone.to.work <= 0.769
505 < Premature.age.adjusted.mortality
2547.9 < School.funding.adequacy
0.396 < Insufficient.sleep
0.235 < below.18.years.of.age
Feature
0.00
0.01
0.02
Weight
Positive
Negative

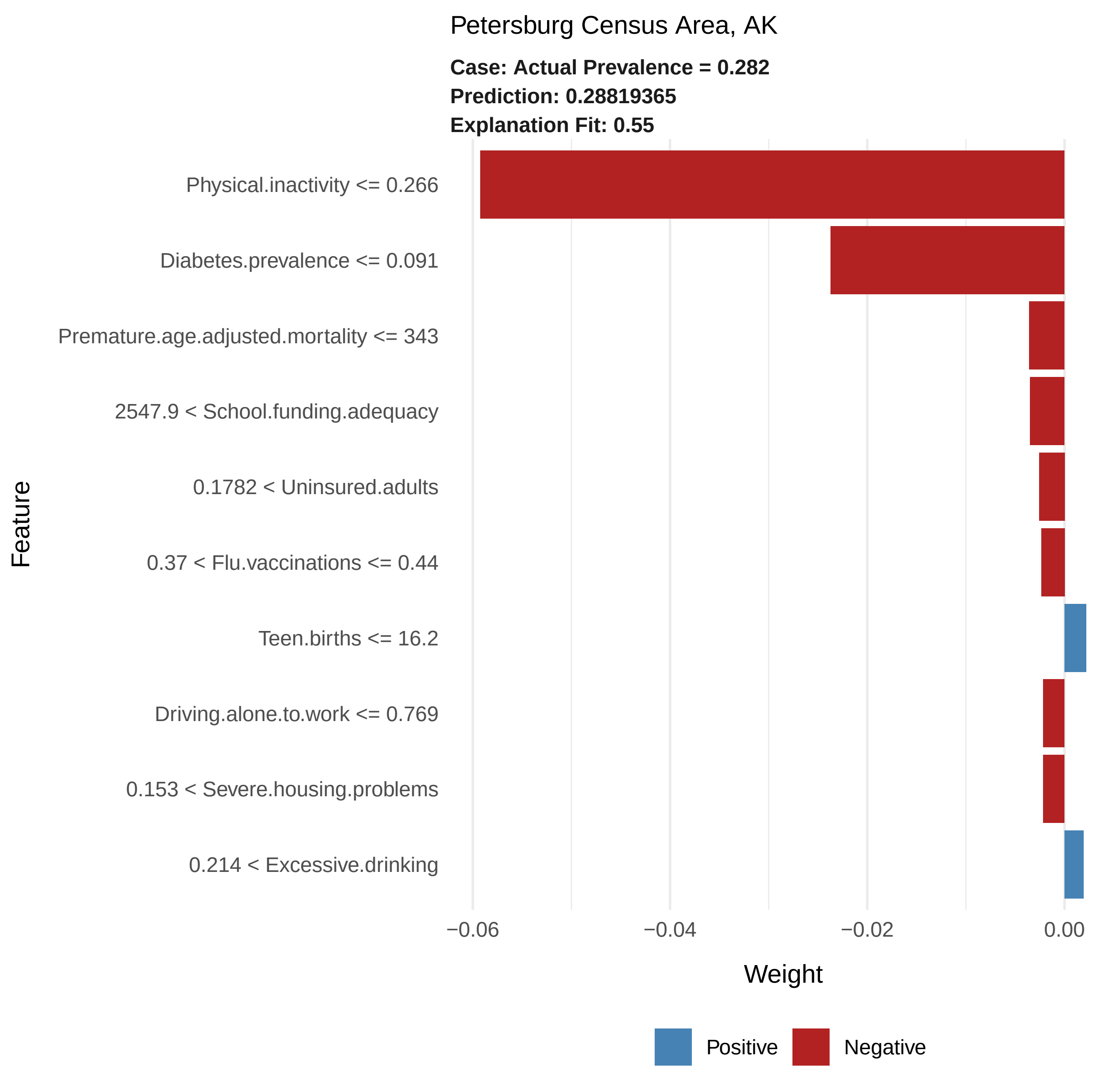

Petersburg Census Area, AK
Case: Actual Prevalence = 0.282
Prediction: 0.28819365
Explanation Fit: 0.55
Physical.inactivity <= 0.266
Diabetes.prevalence <= 0.091
Premature.age.adjusted.mortality <= 343
2547.9 < School.funding.adequacy
0.1782 < Uninsured.adults
0.37 < Flu.vaccinations <= 0.44
Teen.births <= 16.2
Driving.alone.to.work <= 0.769
0.153 < Severe.housing.problems
0.214 < Excessive.drinking
Feature
−0.06
−0.04
−0.02
0.00
Weight
Positive
Negative

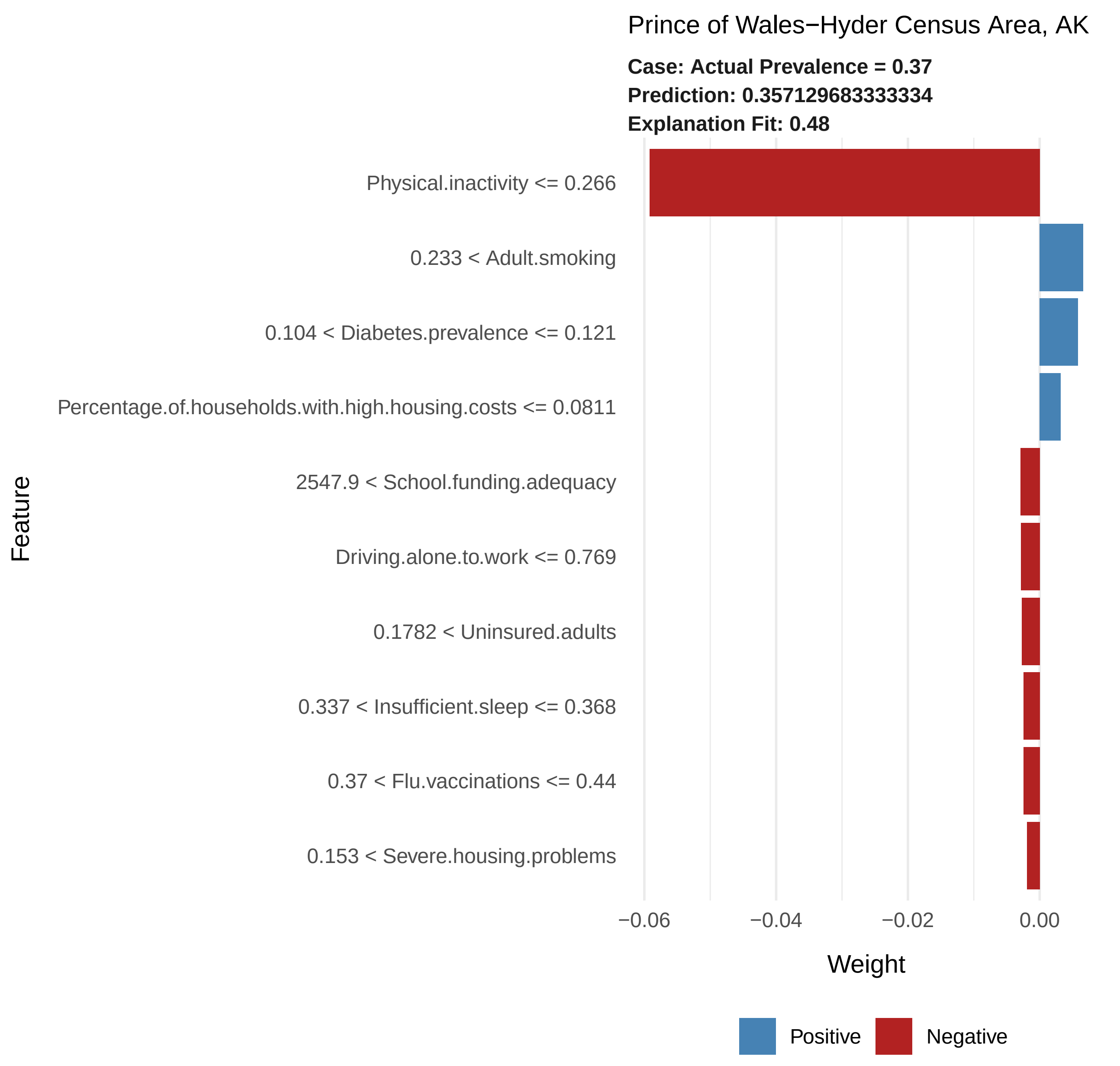

Prince of Wales−Hyder Census Area, AK
Case: Actual Prevalence = 0.37
Prediction: 0.357129683333334
Explanation Fit: 0.48
Physical.inactivity <= 0.266
0.233 < Adult.smoking
0.104 < Diabetes.prevalence <= 0.121
Percentage.of.households.with.high.housing.costs <= 0.0811
2547.9 < School.funding.adequacy
Driving.alone.to.work <= 0.769
0.1782 < Uninsured.adults
0.337 < Insufficient.sleep <= 0.368
0.37 < Flu.vaccinations <= 0.44
0.153 < Severe.housing.problems
Feature
−0.06
−0.04
−0.02
0.00
Weight
Positive
Negative

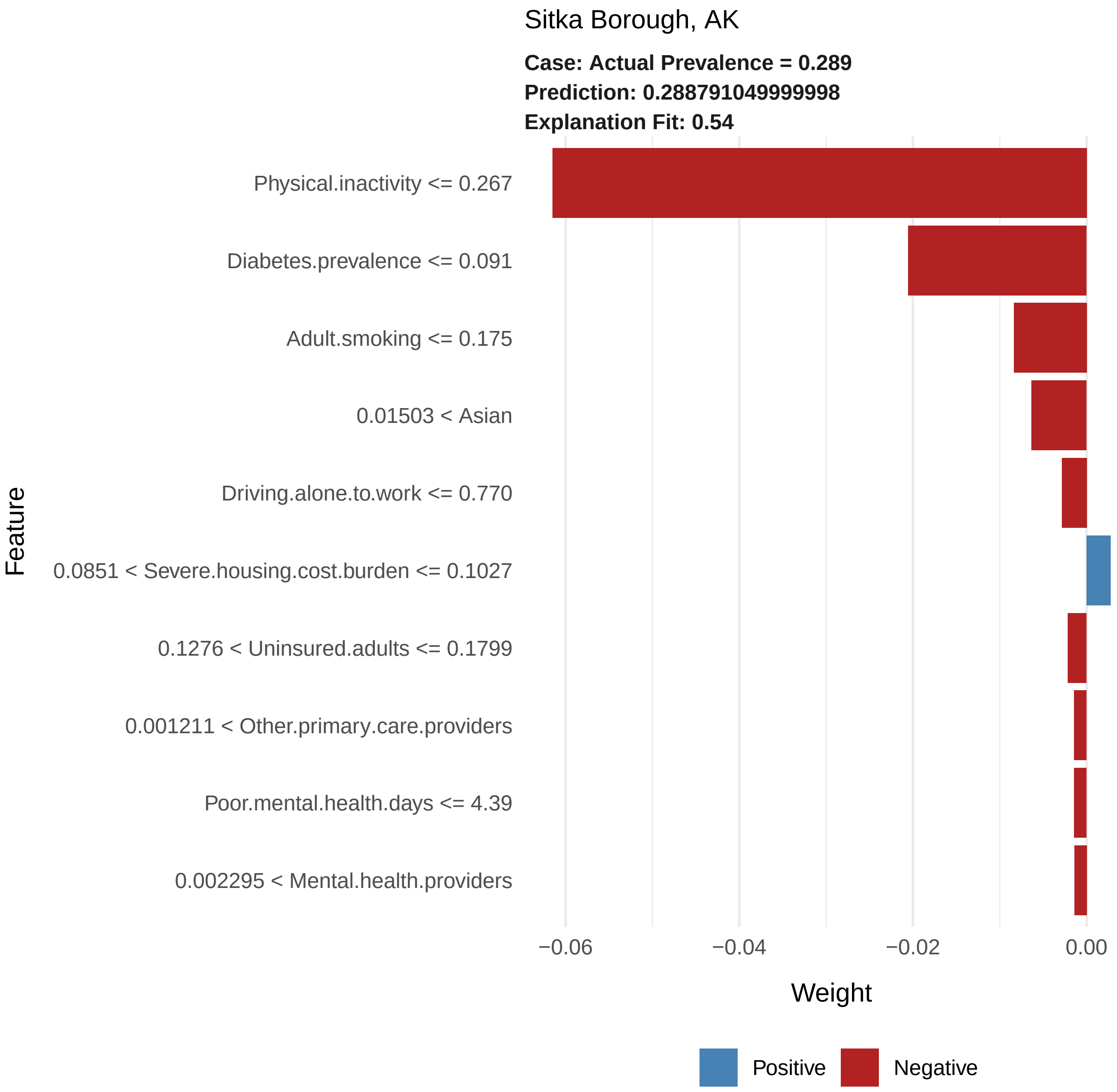
Sitka Borough, AK
Case: Actual Prevalence = 0.289
Prediction: 0.288791049999998
Explanation Fit: 0.54
Feature
Physical.inactivity <= 0.267
Diabetes.prevalence <= 0.091
Adult.smoking <= 0.175
0.01503 < Asian
Driving.alone.to.work <= 0.770
0.0851 < Severe.housing.cost.burden <= 0.1027
0.1276 < Uninsured.adults <= 0.1799
0.001211 < Other.primary.care.providers
Poor.mental.health.days <= 4.39
0.002295 < Mental.health.providers
−0.06
−0.04
−0.02
0.00
Weight
Positive
Negative

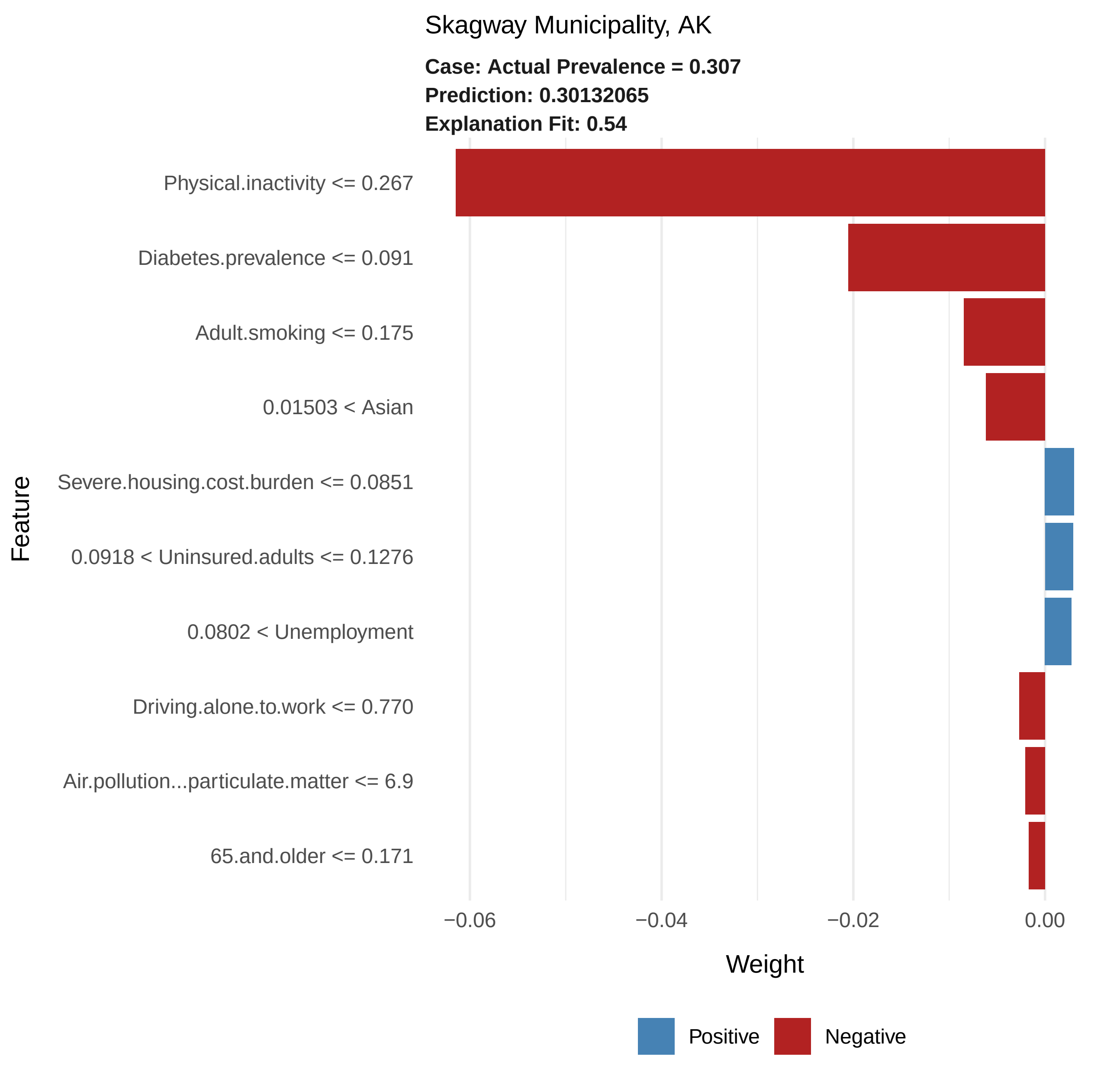
Skagway Municipality, AK
Case: Actual Prevalence = 0.307
Prediction: 0.30132065
Explanation Fit: 0.54
Physical.inactivity <= 0.267
Diabetes.prevalence <= 0.091
Adult.smoking <= 0.175
0.01503 < Asian
Severe.housing.cost.burden <= 0.0851
0.0918 < Uninsured.adults <= 0.1276
0.0802 < Unemployment
Driving.alone.to.work <= 0.770
Air.pollution...particulate.matter <= 6.9
65.and.older <= 0.171
Feature
−0.06
−0.04
−0.02
0.00
Weight
Positive
Negative

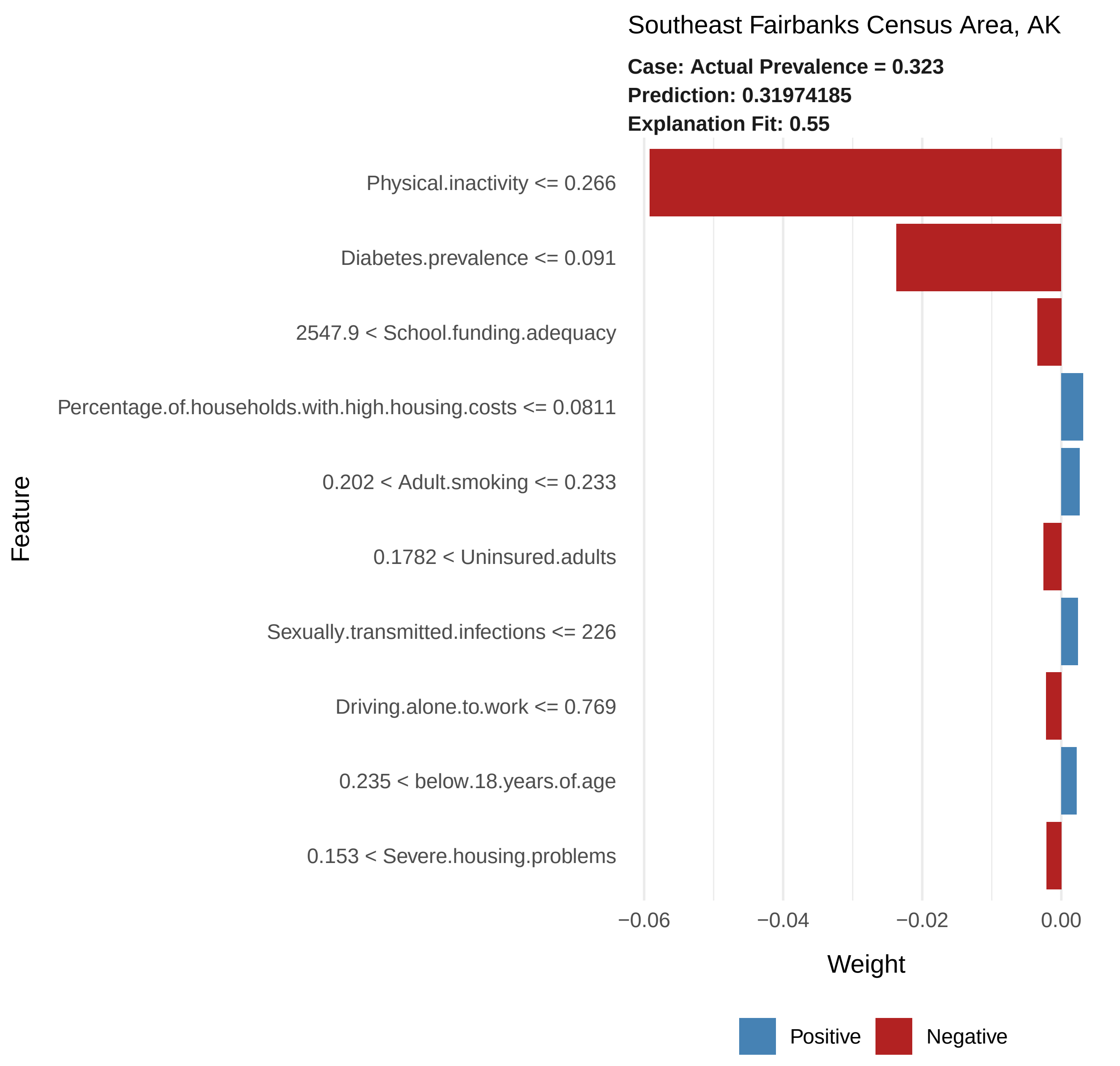

Southeast Fairbanks Census Area, AK
Case: Actual Prevalence = 0.323
Prediction: 0.31974185
Explanation Fit: 0.55
Physical.inactivity <= 0.266
Diabetes.prevalence <= 0.091
2547.9 < School.funding.adequacy
Percentage.of.households.with.high.housing.costs <= 0.0811
0.202 < Adult.smoking <= 0.233
0.1782 < Uninsured.adults
Sexually.transmitted.infections <= 226
Driving.alone.to.work <= 0.769
0.235 < below.18.years.of.age
0.153 < Severe.housing.problems
Feature
−0.06
−0.04
−0.02
0.00
Weight
Positive
Negative

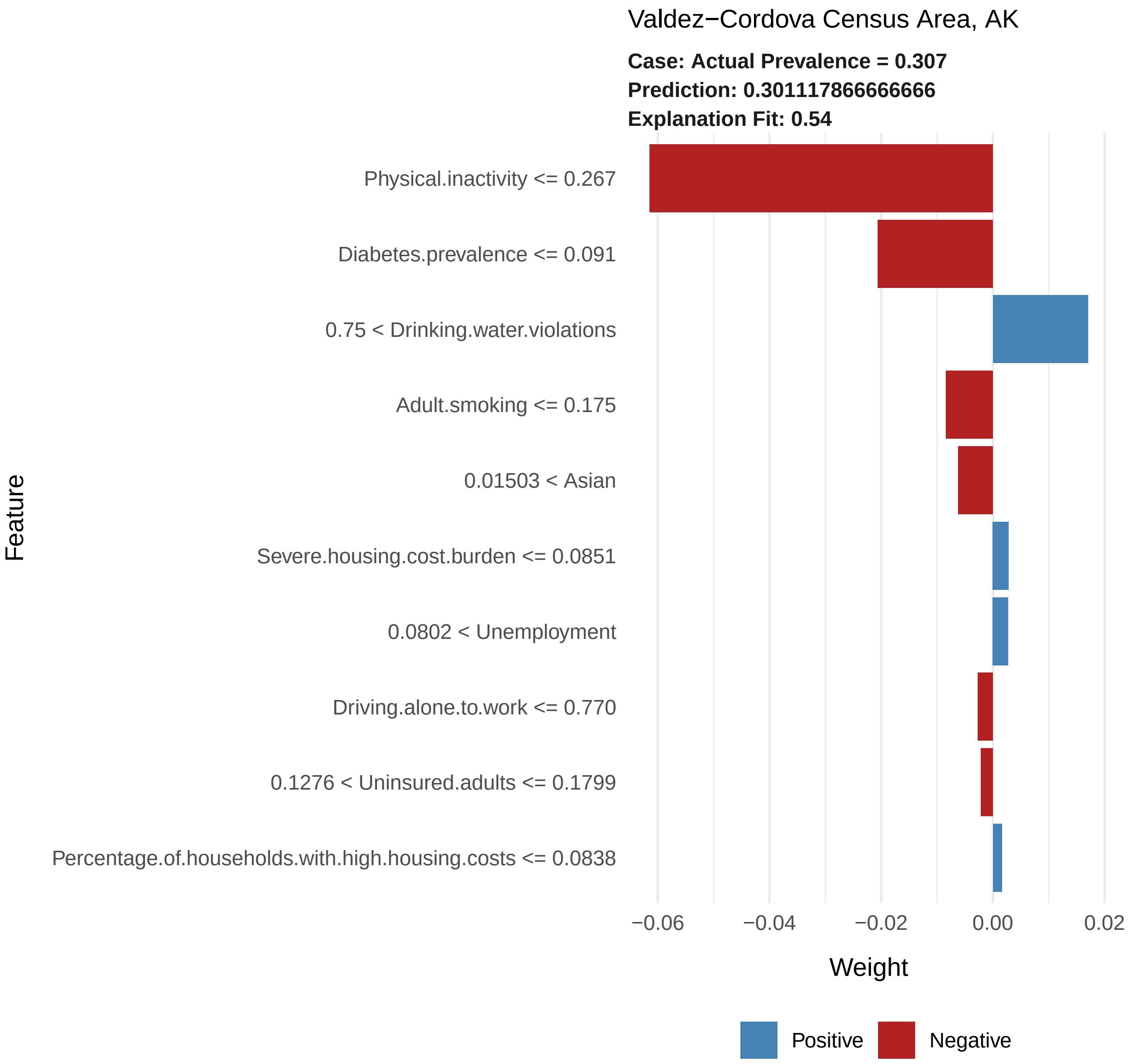

Valdez−Cordova Census Area, AK
Case: Actual Prevalence = 0.307
Prediction: 0.301117866666666
Explanation Fit: 0.54
Physical.inactivity <= 0.267
Diabetes.prevalence <= 0.091
0.75 < Drinking.water.violations
Adult.smoking <= 0.175
0.01503 < Asian
Severe.housing.cost.burden <= 0.0851
0.0802 < Unemployment
Driving.alone.to.work <= 0.770
0.1276 < Uninsured.adults <= 0.1799
Percentage.of.households.with.high.housing.costs <= 0.0838
Feature
−0.06
−0.04
−0.02
0.00
0.02
Weight
Positive
Negative

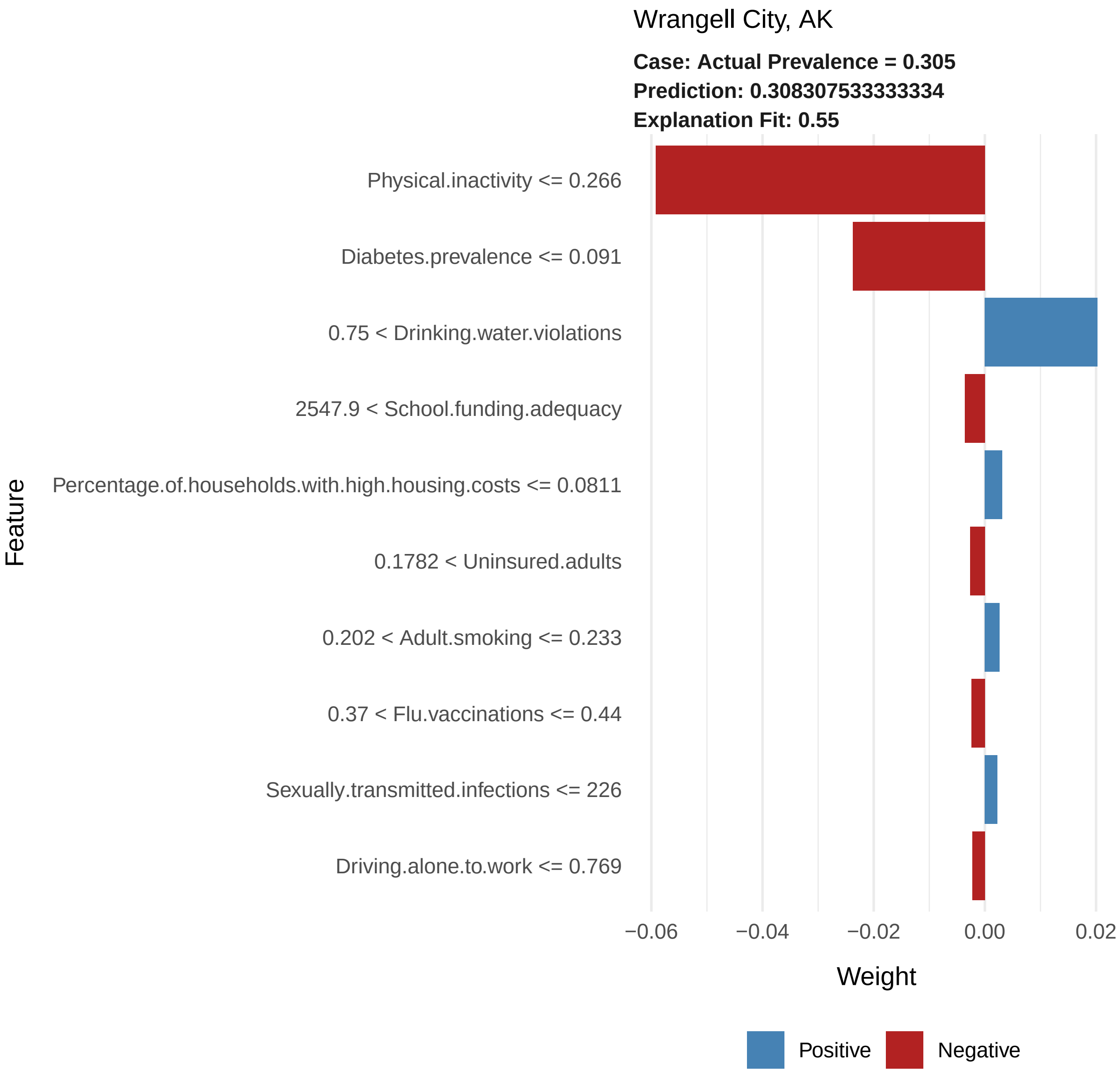
Wrangell City, AK
Case: Actual Prevalence = 0.305
Prediction: 0.308307533333334
Explanation Fit: 0.55
Physical.inactivity <= 0.266
Diabetes.prevalence <= 0.091
0.75 < Drinking.water.violations
2547.9 < School.funding.adequacy
Percentage.of.households.with.high.housing.costs <= 0.0811
0.1782 < Uninsured.adults
0.202 < Adult.smoking <= 0.233
0.37 < Flu.vaccinations <= 0.44
Sexually.transmitted.infections <= 226
Driving.alone.to.work <= 0.769
Feature
−0.06
−0.04
−0.02
0.00
0.02
Weight
Positive
Negative

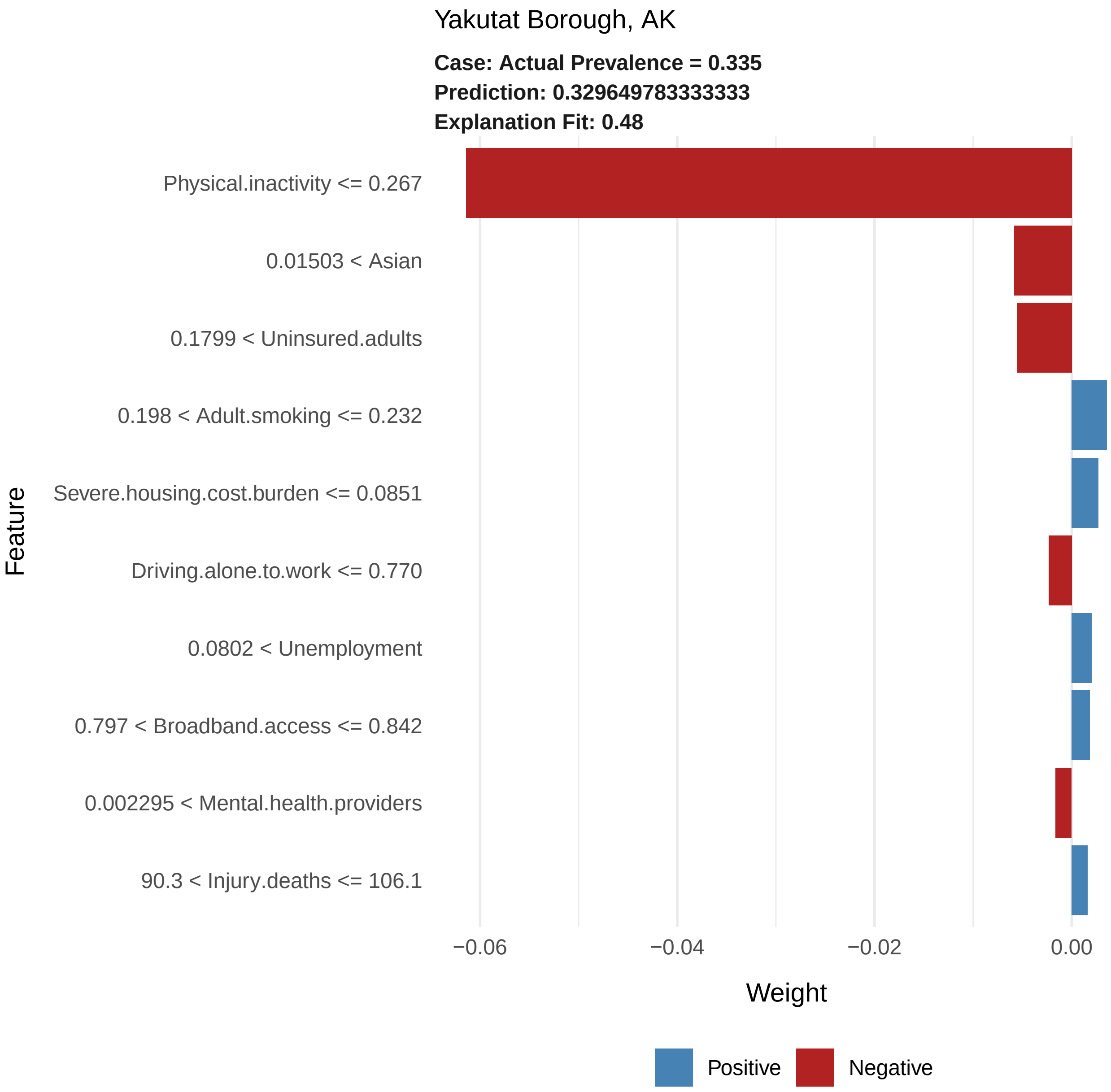
Yakutat Borough, AK
Case: Actual Prevalence = 0.335
Prediction: 0.329649783333333
Explanation Fit: 0.48
Physical.inactivity <= 0.267
0.01503 < Asian
0.1799 < Uninsured.adults
0.198 < Adult.smoking <= 0.232
Severe.housing.cost.burden <= 0.0851
Driving.alone.to.work <= 0.770
0.0802 < Unemployment
0.797 < Broadband.access <= 0.842
0.002295 < Mental.health.providers
90.3 < Injury.deaths <= 106.1
Feature
−0.06
−0.04
−0.02
0.00
Weight
Positive
Negative

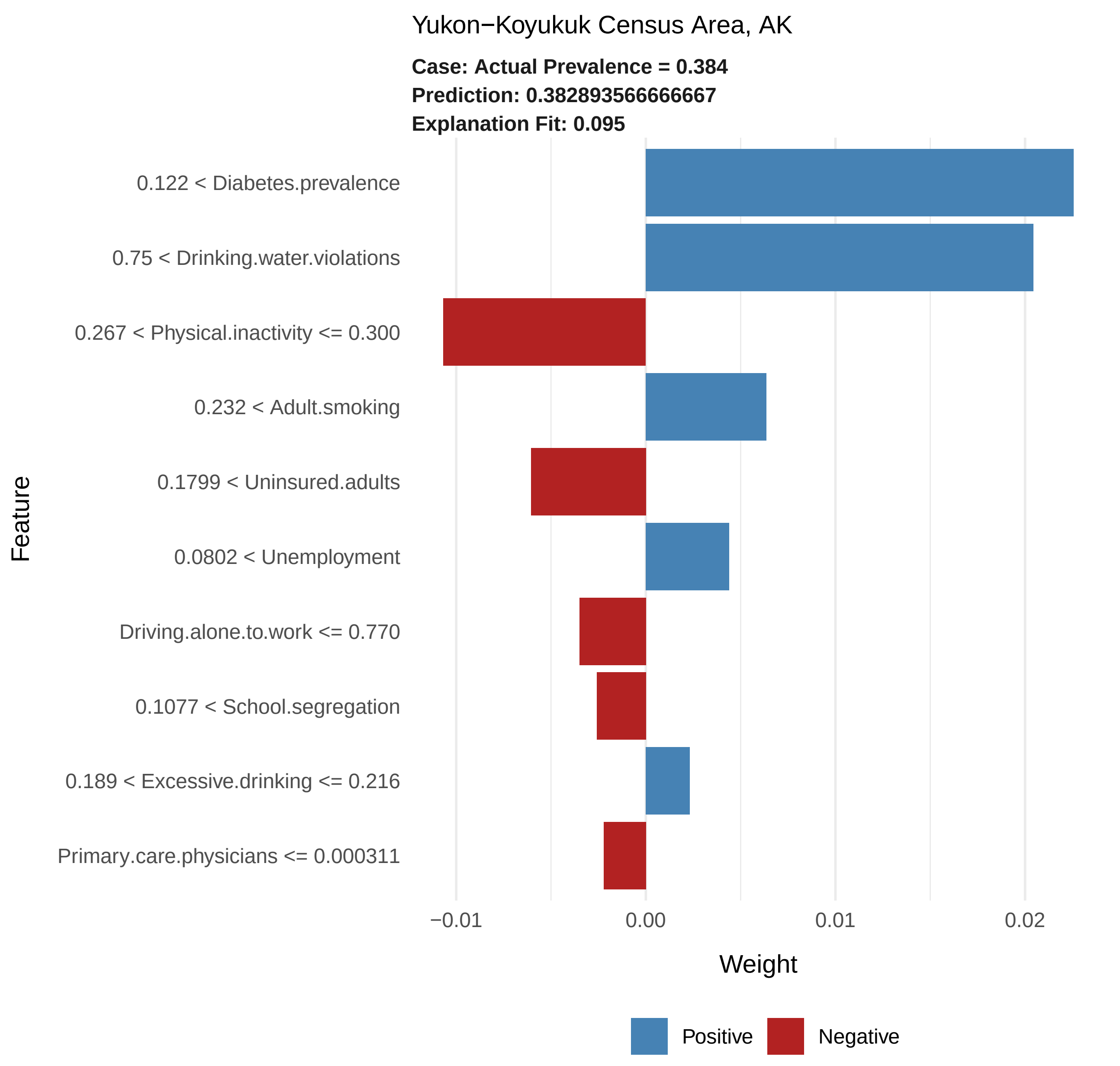
Yukon−Koyukuk Census Area, AK
Case: Actual Prevalence = 0.384
Prediction: 0.382893566666667
Explanation Fit: 0.095
0.122 < Diabetes.prevalence
0.75 < Drinking.water.violations
0.267 < Physical.inactivity <= 0.300
0.232 < Adult.smoking
0.1799 < Uninsured.adults
0.0802 < Unemployment
Driving.alone.to.work <= 0.770
0.1077 < School.segregation
0.189 < Excessive.drinking <= 0.216
Primary.care.physicians <= 0.000311
Feature
−0.01
0.00
0.01
0.02
Weight
Positive
Negative

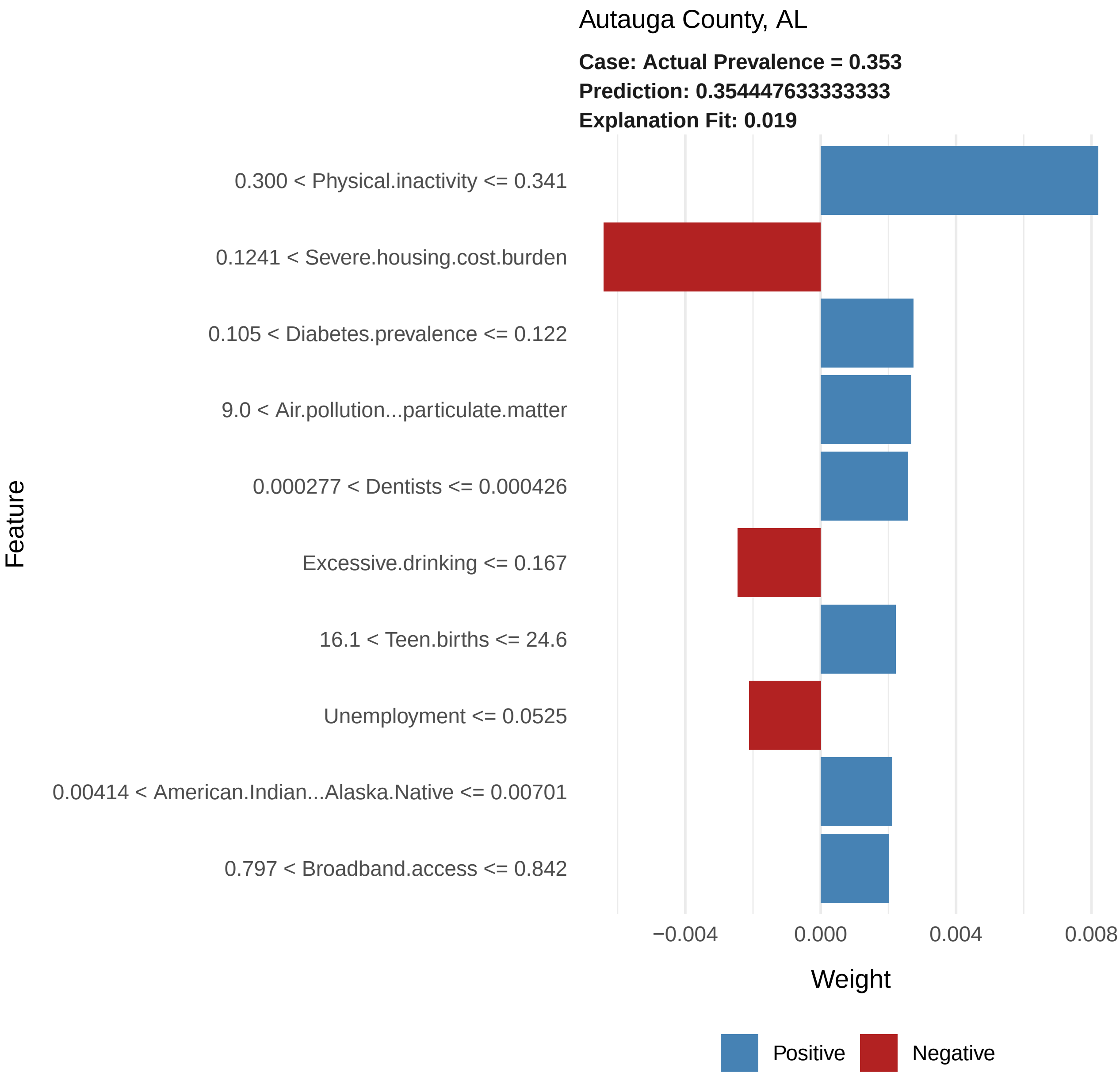

Autauga County, AL
Case: Actual Prevalence = 0.353
Prediction: 0.354447633333333
Explanation Fit: 0.019
0.300 < Physical.inactivity <= 0.341
0.1241 < Severe.housing.cost.burden
0.105 < Diabetes.prevalence <= 0.122
9.0 < Air.pollution...particulate.matter
0.000277 < Dentists <= 0.000426
Excessive.drinking <= 0.167
16.1 < Teen.births <= 24.6
Unemployment <= 0.0525
0.00414 < American.Indian...Alaska.Native <= 0.00701
0.797 < Broadband.access <= 0.842
Feature
−0.004
0.000
0.004
0.008
Weight
Positive
Negative

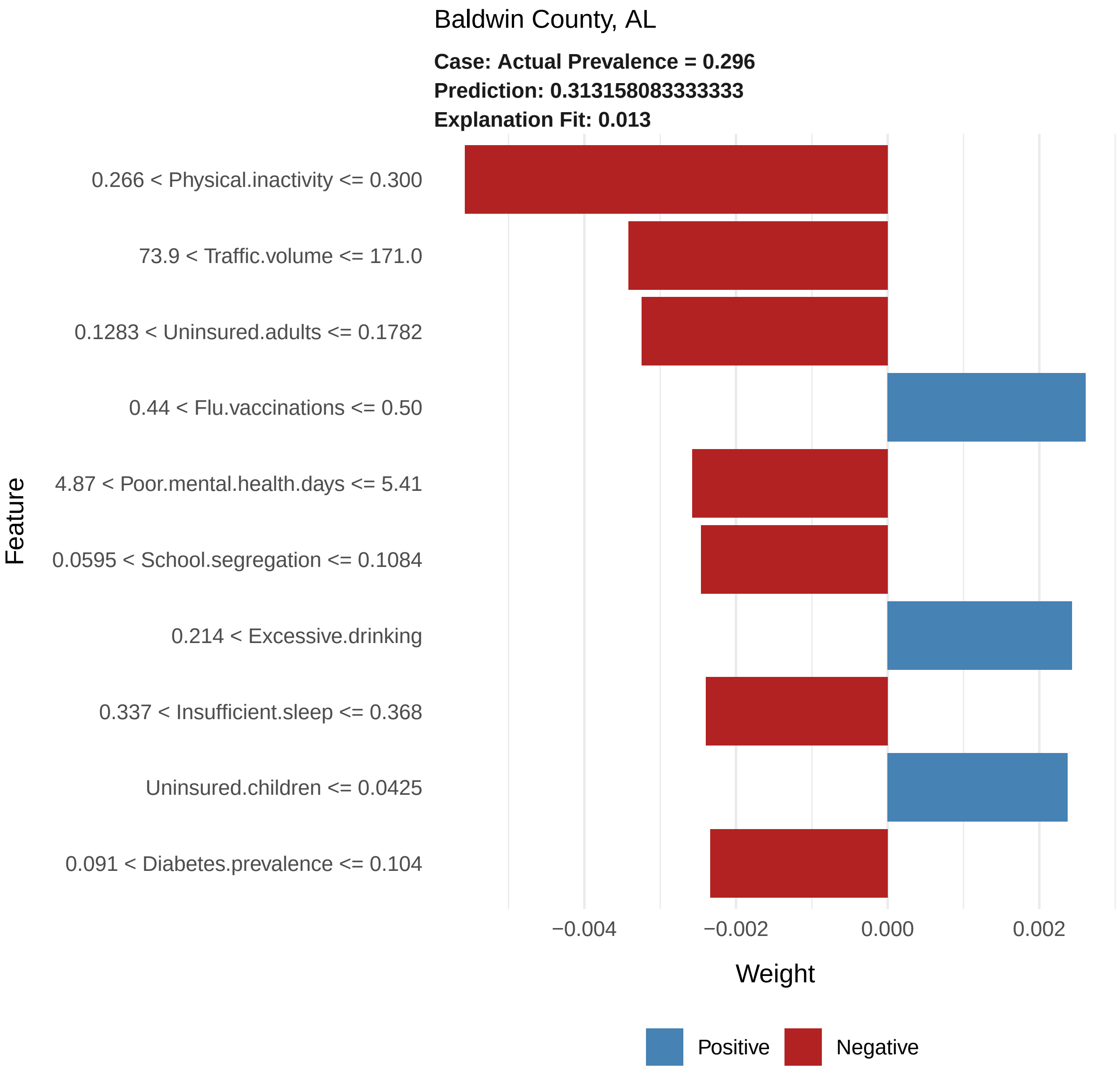
Baldwin County, AL
Case: Actual Prevalence = 0.296
Prediction: 0.313158083333333
Explanation Fit: 0.013
0.266 < Physical.inactivity <= 0.300
73.9 < Traffic.volume <= 171.0
0.1283 < Uninsured.adults <= 0.1782
0.44 < Flu.vaccinations <= 0.50
4.87 < Poor.mental.health.days <= 5.41
0.0595 < School.segregation <= 0.1084
0.214 < Excessive.drinking
0.337 < Insufficient.sleep <= 0.368
Uninsured.children <= 0.0425
0.091 < Diabetes.prevalence <= 0.104
Feature
−0.004
−0.002
0.000
0.002
Weight
Positive
Negative

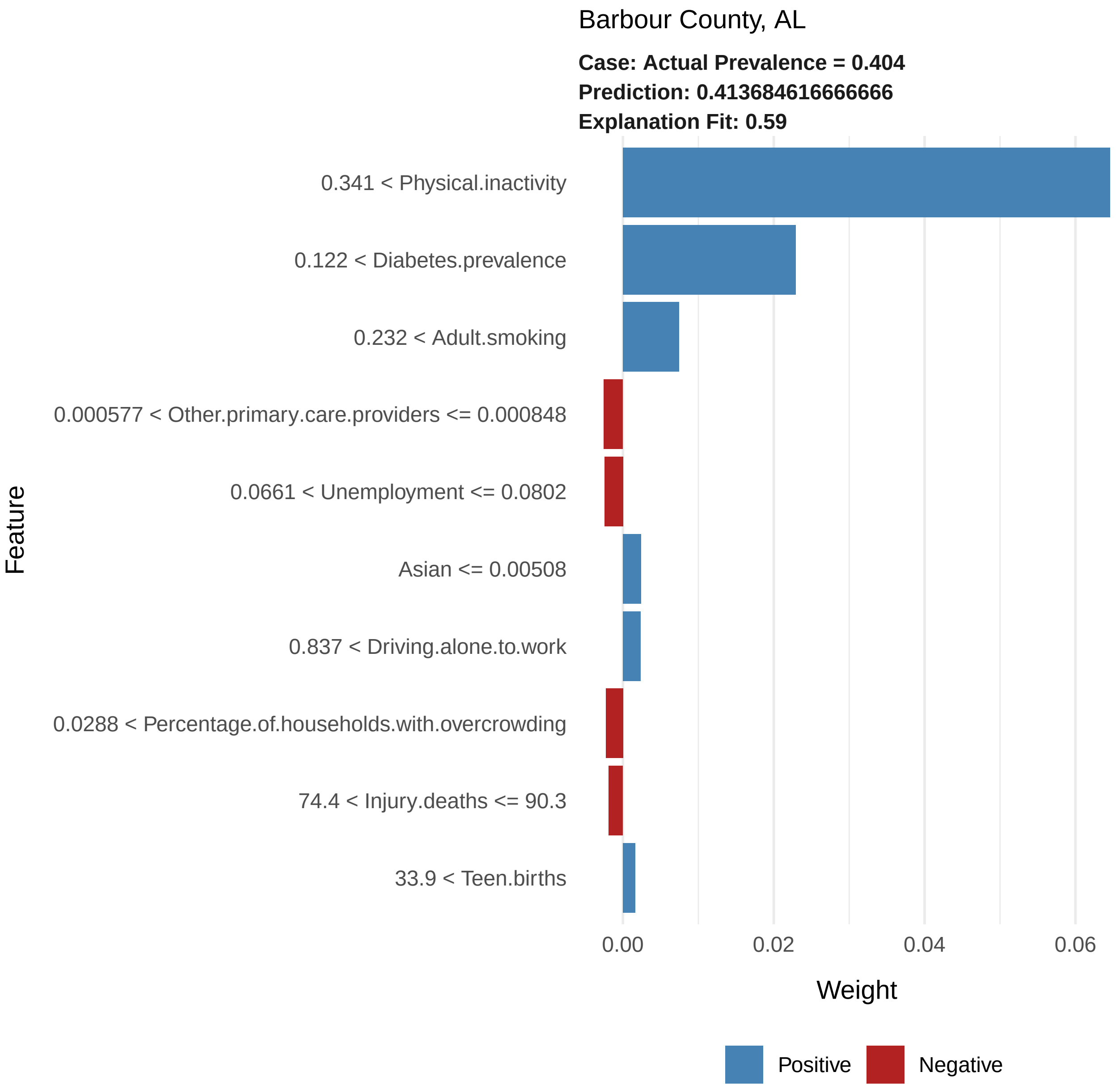

Barbour County, AL
Case: Actual Prevalence = 0.404
Prediction: 0.413684616666666
Explanation Fit: 0.59
0.341 < Physical.inactivity
0.122 < Diabetes.prevalence
0.232 < Adult.smoking
0.000577 < Other.primary.care.providers <= 0.000848
0.0661 < Unemployment <= 0.0802
Asian <= 0.00508
0.837 < Driving.alone.to.work
0.0288 < Percentage.of.households.with.overcrowding
74.4 < Injury.deaths <= 90.3
33.9 < Teen.births
Feature
0.00
0.02
0.04
0.06
Weight
Positive
Negative

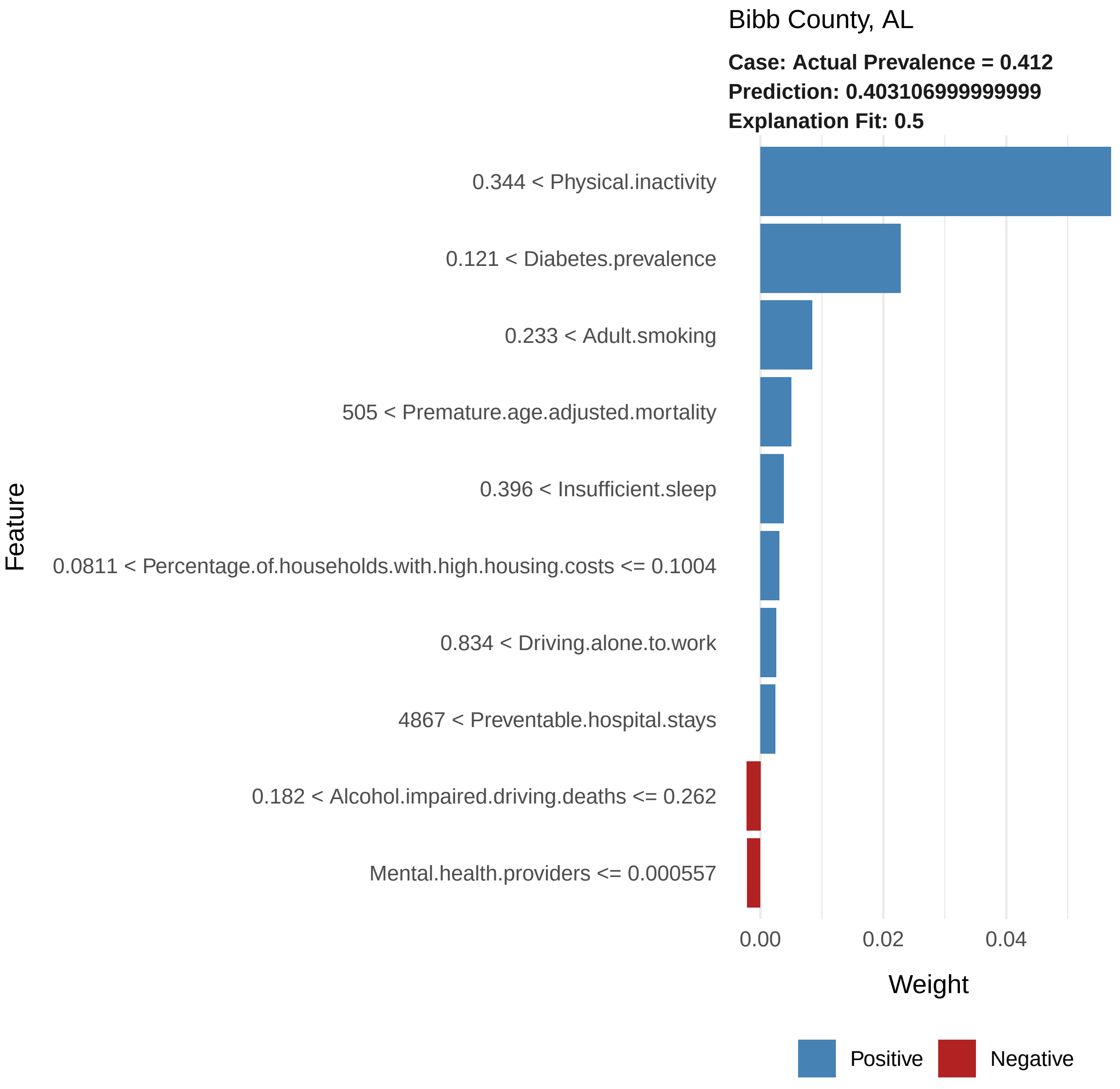
Bibb County, AL
Case: Actual Prevalence = 0.412
Prediction: 0.403106999999999
Explanation Fit: 0.5
0.344 < Physical.inactivity
0.121 < Diabetes.prevalence
0.233 < Adult.smoking
505 < Premature.age.adjusted.mortality
0.396 < Insufficient.sleep
0.0811 < Percentage.of.households.with.high.housing.costs <= 0.1004
0.834 < Driving.alone.to.work
4867 < Preventable.hospital.stays
0.182 < Alcohol.impaired.driving.deaths <= 0.262
Mental.health.providers <= 0.000557
Feature
0.00
0.02
0.04
Weight
Positive
Negative

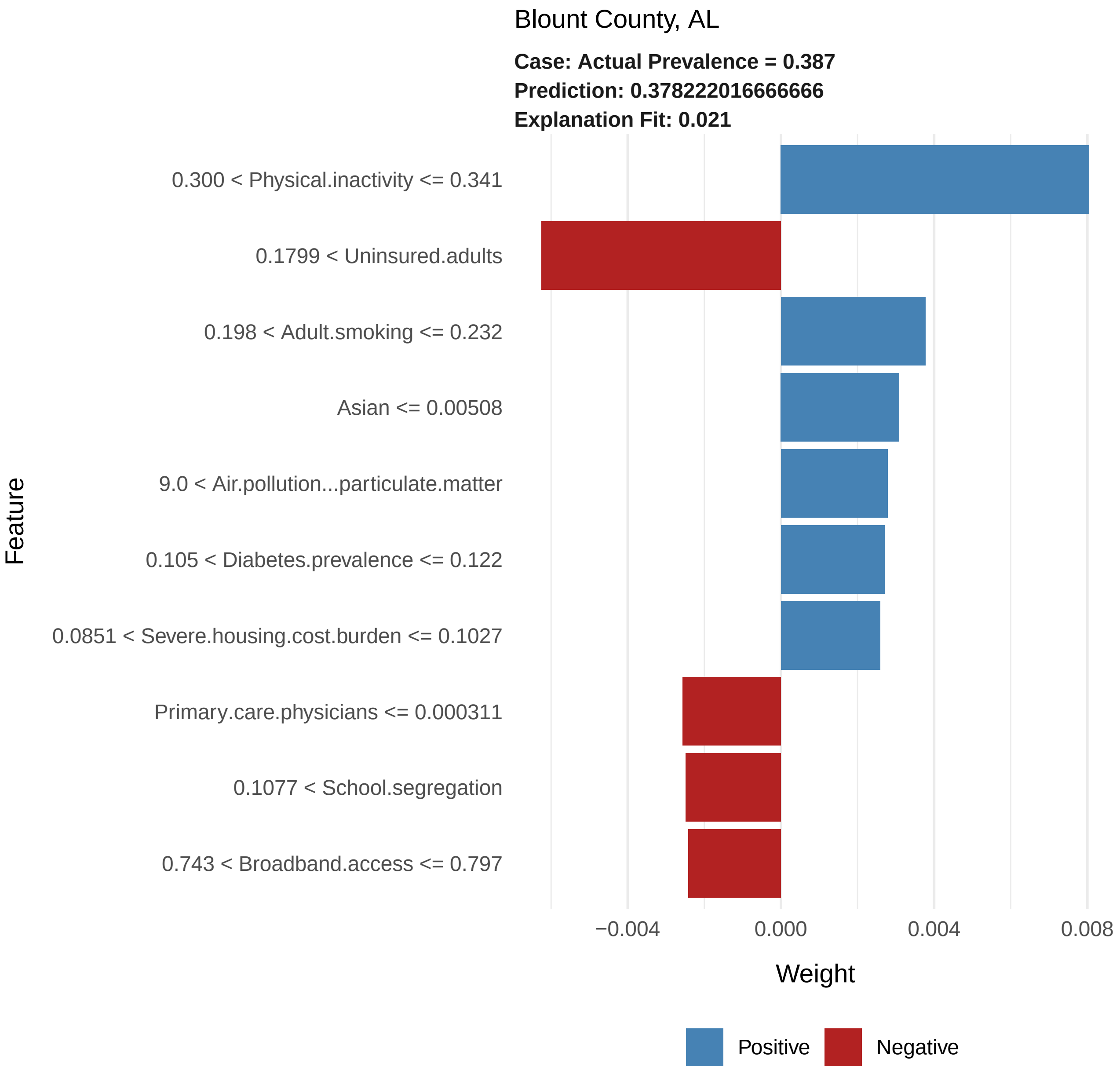
Blount County, AL
Case: Actual Prevalence = 0.387
Prediction: 0.378222016666666
Explanation Fit: 0.021
0.300 < Physical.inactivity <= 0.341
0.1799 < Uninsured.adults
0.198 < Adult.smoking <= 0.232
Asian <= 0.00508
9.0 < Air.pollution...particulate.matter
0.105 < Diabetes.prevalence <= 0.122
0.0851 < Severe.housing.cost.burden <= 0.1027
Primary.care.physicians <= 0.000311
0.1077 < School.segregation
0.743 < Broadband.access <= 0.797
Feature
−0.004
0.000
0.004
0.008
Weight
Positive
Negative

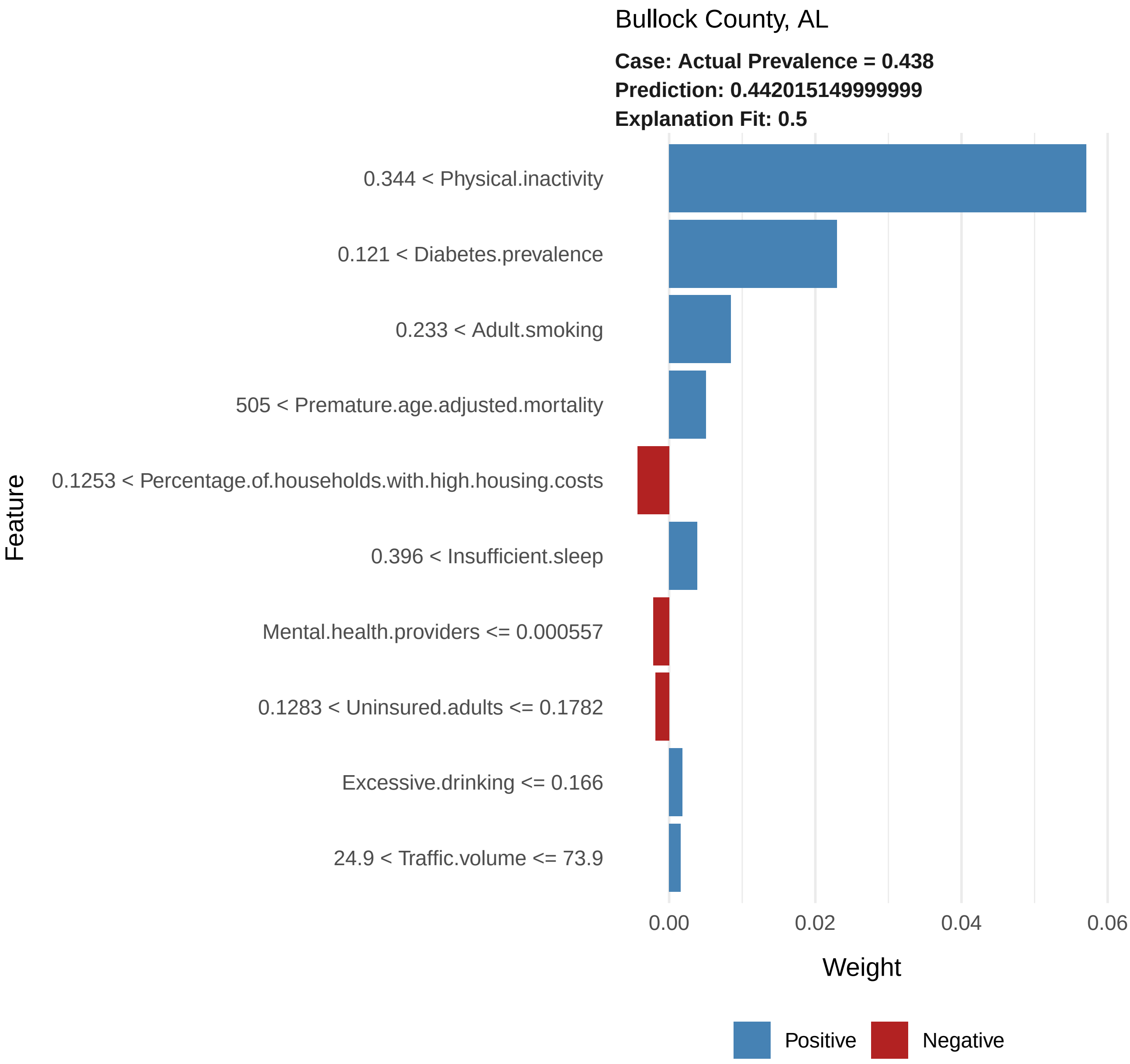

Bullock County, AL
Case: Actual Prevalence = 0.438
Prediction: 0.442015149999999
Explanation Fit: 0.5
0.344 < Physical.inactivity
0.121 < Diabetes.prevalence
0.233 < Adult.smoking
505 < Premature.age.adjusted.mortality
0.1253 < Percentage.of.households.with.high.housing.costs
0.396 < Insufficient.sleep
Mental.health.providers <= 0.000557
0.1283 < Uninsured.adults <= 0.1782
Excessive.drinking <= 0.166
24.9 < Traffic.volume <= 73.9
Feature
0.00
0.02
0.04
0.06
Weight
Positive
Negative

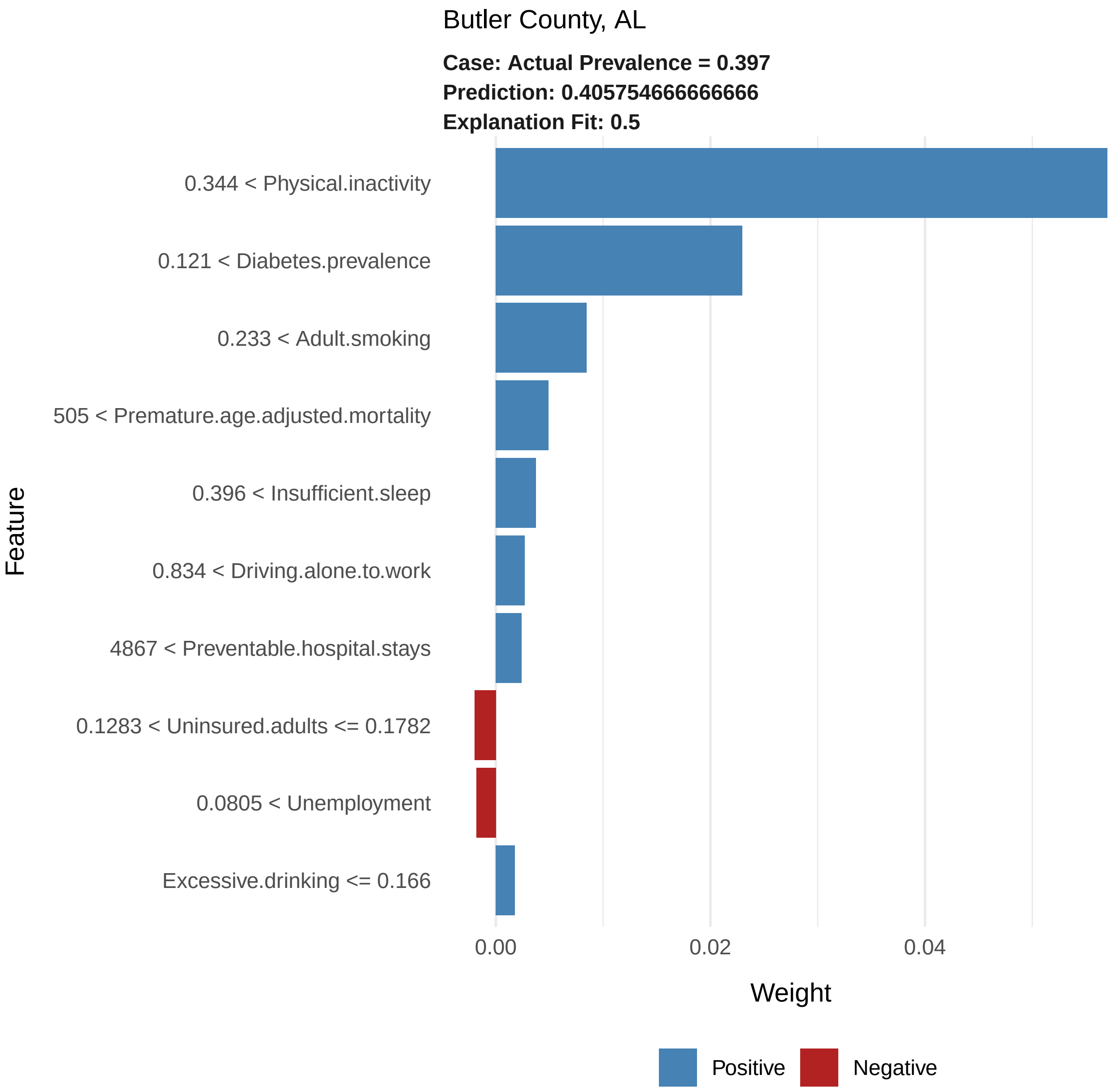

Butler County, AL
Case: Actual Prevalence = 0.397
Prediction: 0.405754666666666
Explanation Fit: 0.5
0.344 < Physical.inactivity
0.121 < Diabetes.prevalence
0.233 < Adult.smoking
505 < Premature.age.adjusted.mortality
0.396 < Insufficient.sleep
0.834 < Driving.alone.to.work
4867 < Preventable.hospital.stays
0.1283 < Uninsured.adults <= 0.1782
0.0805 < Unemployment
Excessive.drinking <= 0.166
Feature
0.00
0.02
0.04
Weight
Positive
Negative

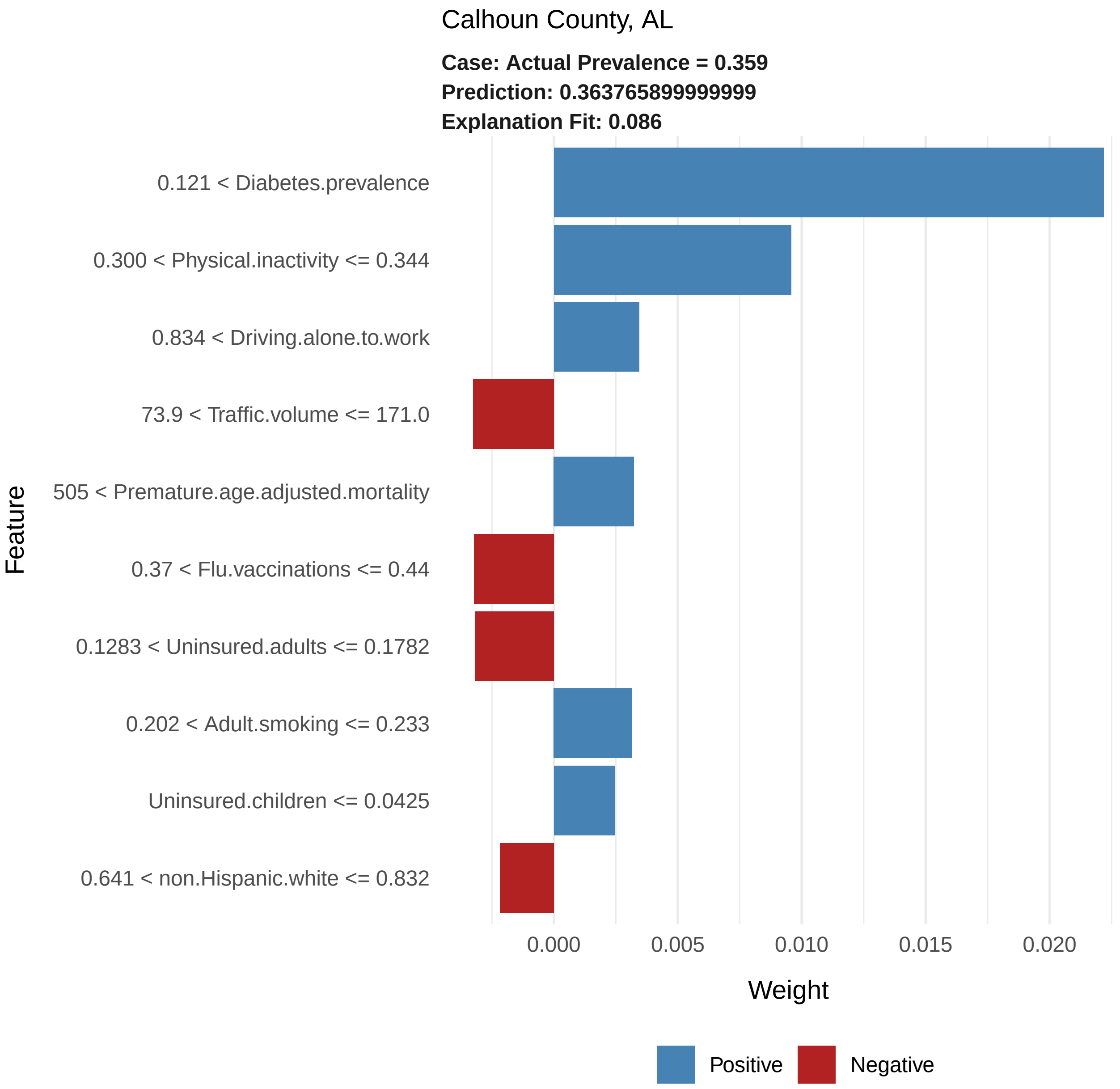
Calhoun County, AL
Case: Actual Prevalence = 0.359
Prediction: 0.363765899999999
Explanation Fit: 0.086
0.121 < Diabetes.prevalence
0.300 < Physical.inactivity <= 0.344
0.834 < Driving.alone.to.work
73.9 < Traffic.volume <= 171.0
505 < Premature.age.adjusted.mortality
0.37 < Flu.vaccinations <= 0.44
0.1283 < Uninsured.adults <= 0.1782
0.202 < Adult.smoking <= 0.233
Uninsured.children <= 0.0425
0.641 < non.Hispanic.white <= 0.832
Feature
0.000
0.005
0.010
0.015
0.020
Weight
Positive
Negative

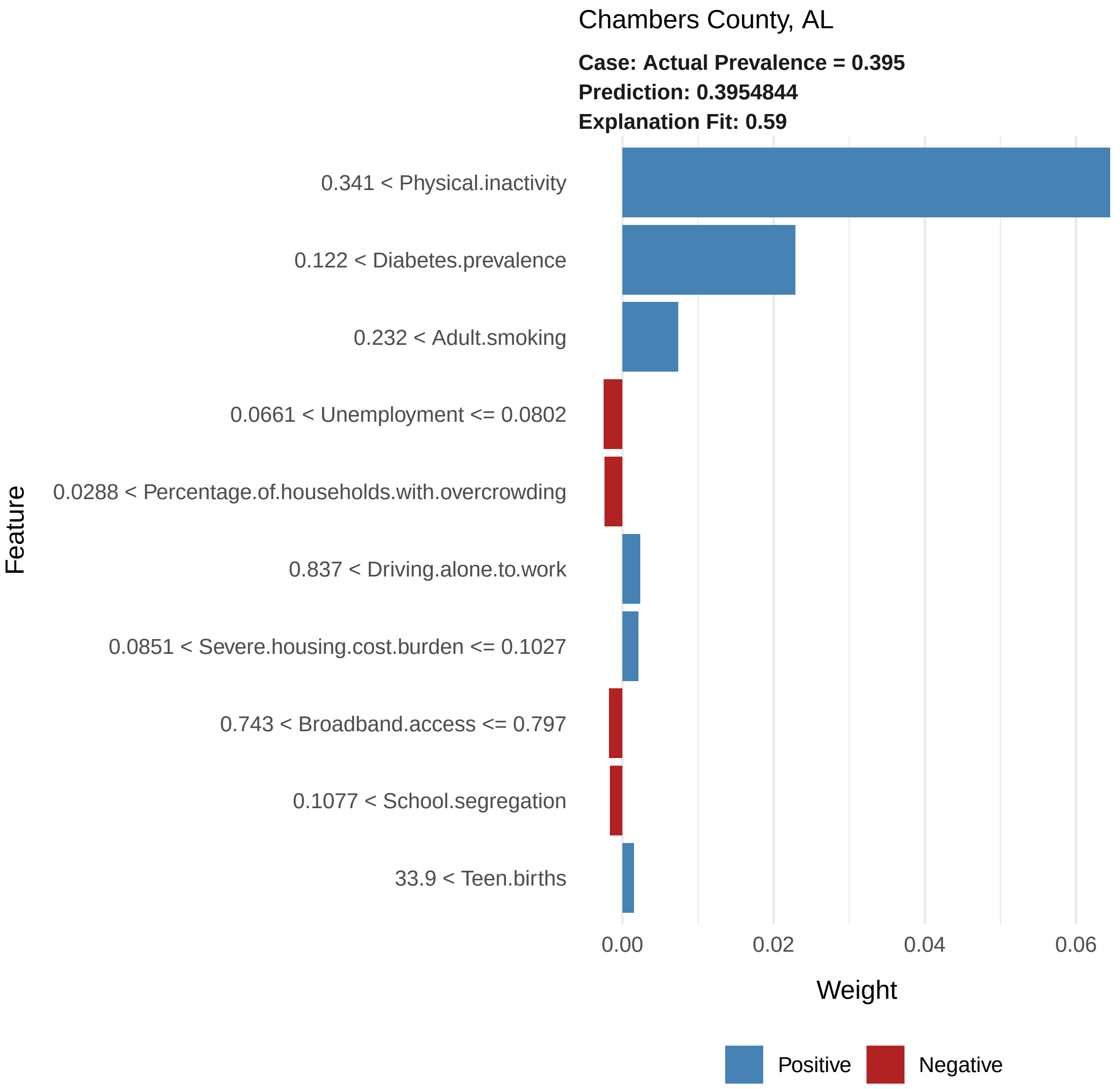

Chambers County, AL
Case: Actual Prevalence = 0.395
Prediction: 0.3954844
Explanation Fit: 0.59
Feature
0.341 < Physical.inactivity
0.122 < Diabetes.prevalence
0.232 < Adult.smoking
0.0661 < Unemployment <= 0.0802
0.0288 < Percentage.of.households.with.overcrowding
0.837 < Driving.alone.to.work
0.0851 < Severe.housing.cost.burden <= 0.1027
0.743 < Broadband.access <= 0.797
0.1077 < School.segregation
33.9 < Teen.births
0.00
0.02
0.04
0.06
Weight
Positive
Negative

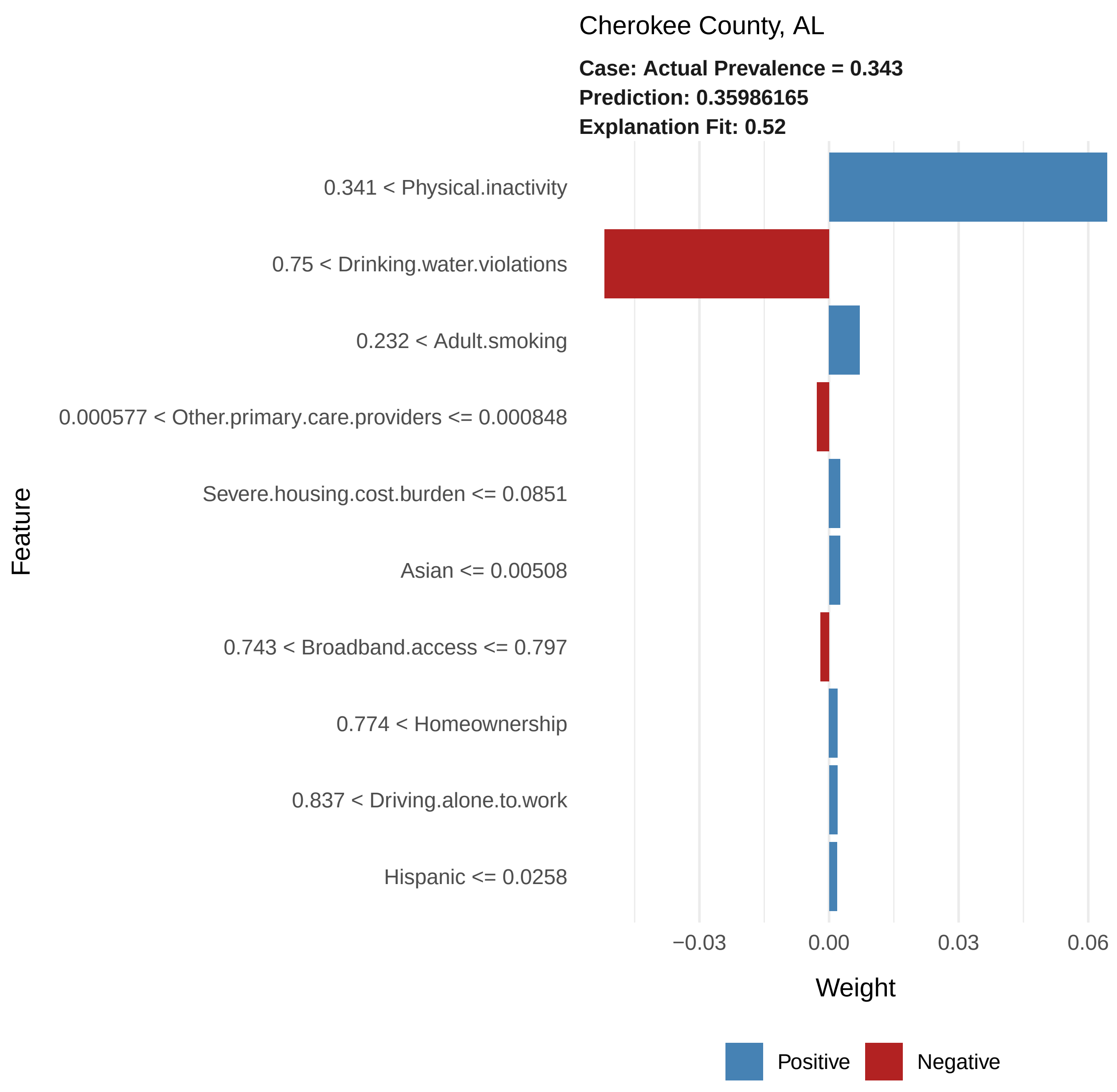

Cherokee County, AL
Case: Actual Prevalence = 0.343
Prediction: 0.35986165
Explanation Fit: 0.52
0.341 < Physical.inactivity
0.75 < Drinking.water.violations
0.232 < Adult.smoking
0.000577 < Other.primary.care.providers <= 0.000848
Severe.housing.cost.burden <= 0.0851
Asian <= 0.00508
0.743 < Broadband.access <= 0.797
0.774 < Homeownership
0.837 < Driving.alone.to.work
Hispanic <= 0.0258
Feature
−0.03
0.00
0.03
0.06
Weight
Positive
Negative

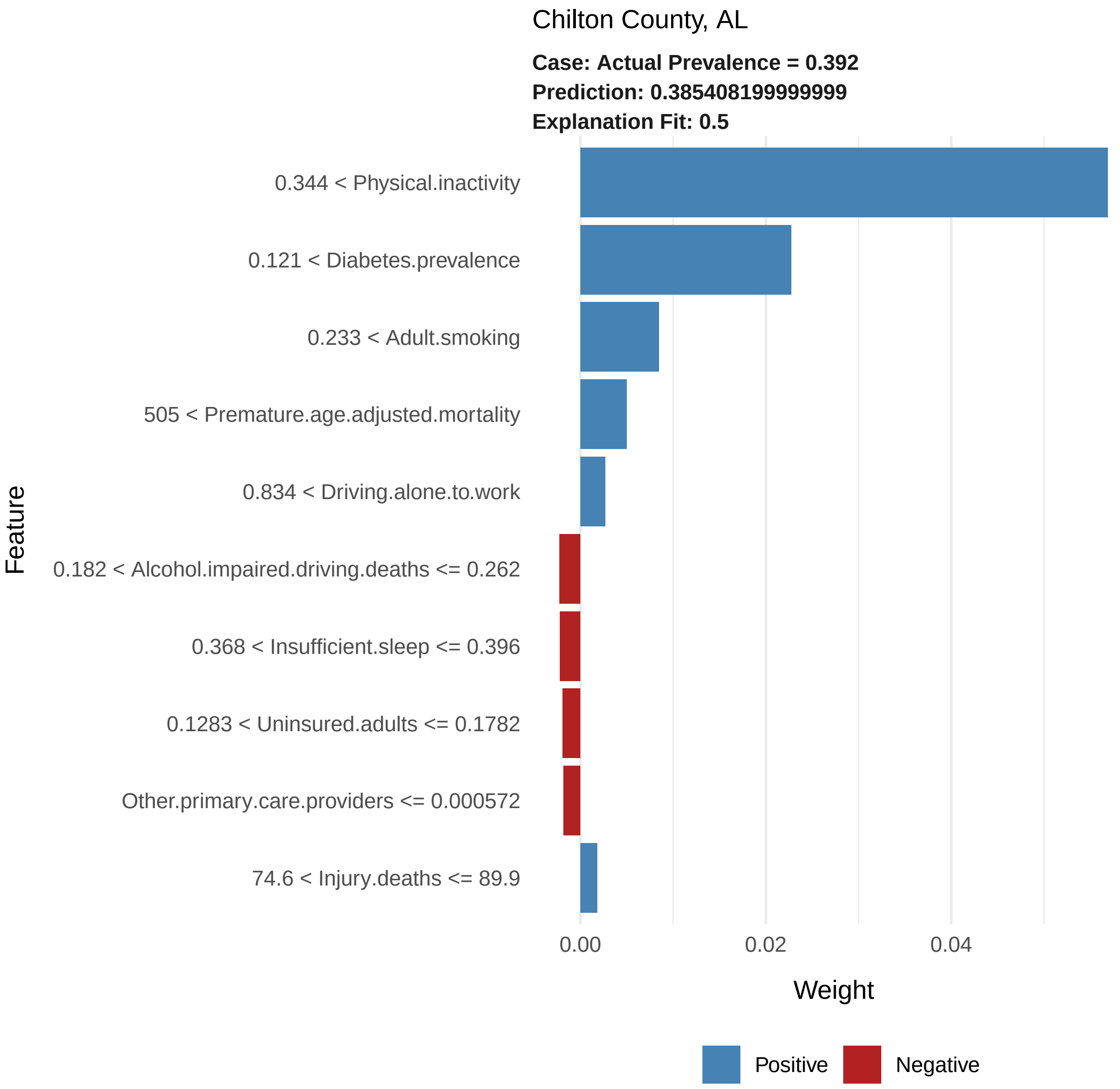

Chilton County, AL
Case: Actual Prevalence = 0.392
Prediction: 0.385408199999999
Explanation Fit: 0.5
0.344 < Physical.inactivity
0.121 < Diabetes.prevalence
0.233 < Adult.smoking
505 < Premature.age.adjusted.mortality
0.834 < Driving.alone.to.work
0.182 < Alcohol.impaired.driving.deaths <= 0.262
0.368 < Insufficient.sleep <= 0.396
0.1283 < Uninsured.adults <= 0.1782
Other.primary.care.providers <= 0.000572
74.6 < Injury.deaths <= 89.9
Feature
0.00
0.02
0.04
Weight
Positive
Negative

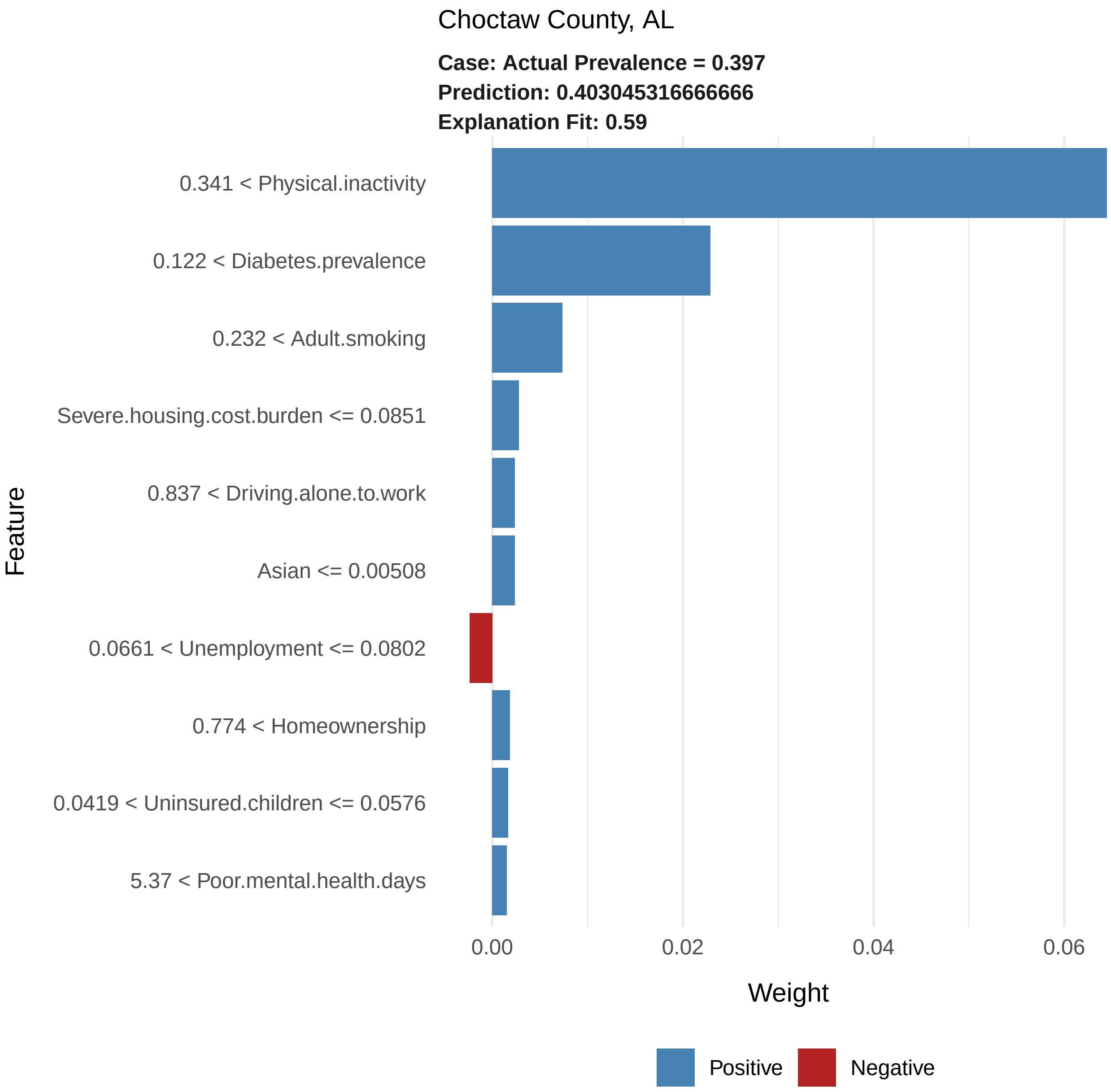
Choctaw County, AL
Case: Actual Prevalence = 0.397
Prediction: 0.403045316666666
Explanation Fit: 0.59
0.341 < Physical.inactivity
0.122 < Diabetes.prevalence
0.232 < Adult.smoking
Severe.housing.cost.burden <= 0.0851
0.837 < Driving.alone.to.work
Asian <= 0.00508
0.0661 < Unemployment <= 0.0802
0.774 < Homeownership
0.0419 < Uninsured.children <= 0.0576
5.37 < Poor.mental.health.days
Feature
0.00
0.02
0.04
0.06
Weight
Positive
Negative

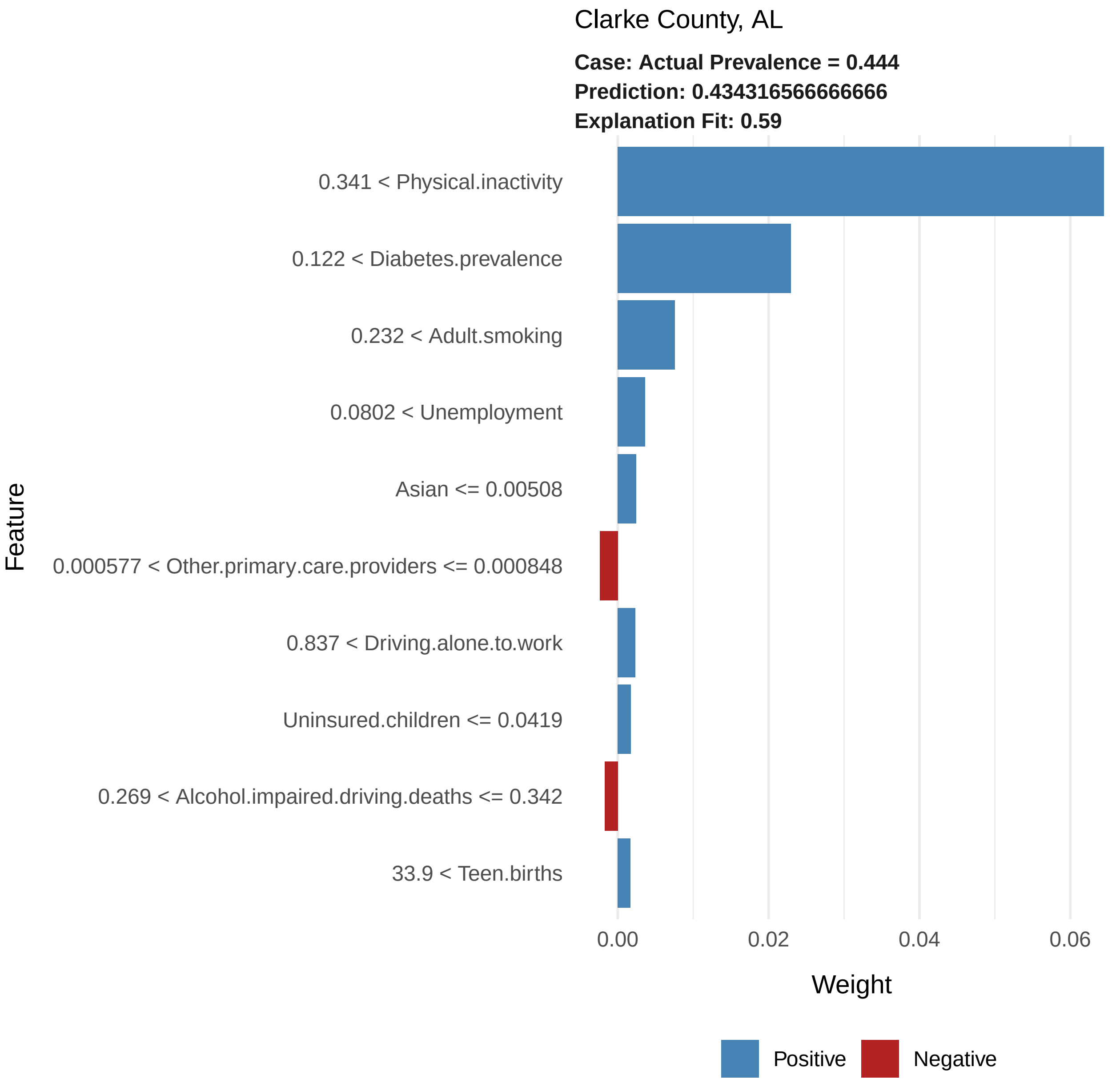

Clarke County, AL
Case: Actual Prevalence = 0.444
Prediction: 0.434316566666666
Explanation Fit: 0.59
0.341 < Physical.inactivity
0.122 < Diabetes.prevalence
0.232 < Adult.smoking
0.0802 < Unemployment
Asian <= 0.00508
0.000577 < Other.primary.care.providers <= 0.000848
0.837 < Driving.alone.to.work
Uninsured.children <= 0.0419
0.269 < Alcohol.impaired.driving.deaths <= 0.342
33.9 < Teen.births
Feature
0.00
0.02
0.04
0.06
Weight
Positive
Negative

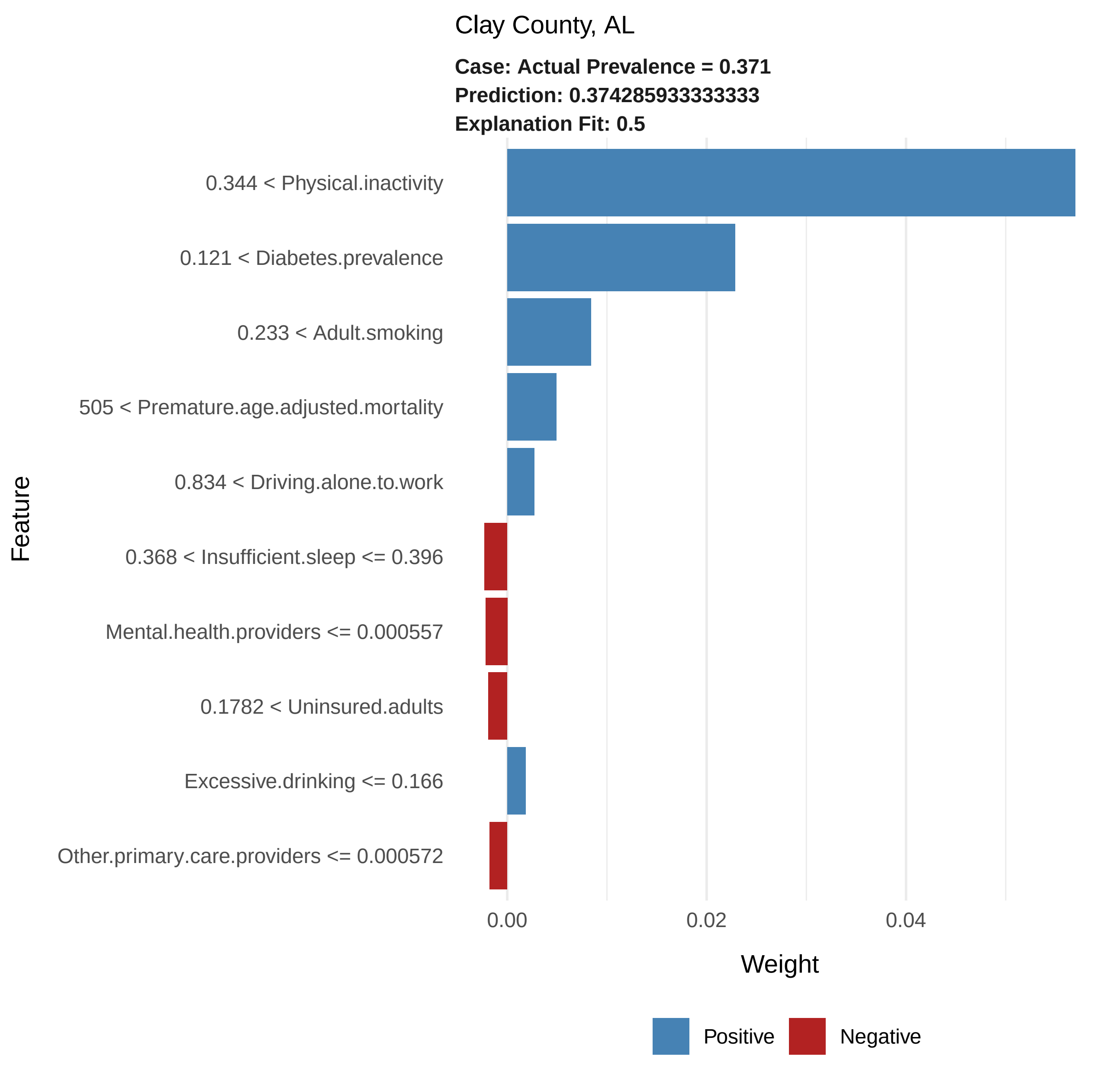
Clay County, AL
Case: Actual Prevalence = 0.371
Prediction: 0.374285933333333
Explanation Fit: 0.5
0.344 < Physical.inactivity
0.121 < Diabetes.prevalence
0.233 < Adult.smoking
505 < Premature.age.adjusted.mortality
0.834 < Driving.alone.to.work
0.368 < Insufficient.sleep <= 0.396
Mental.health.providers <= 0.000557
0.1782 < Uninsured.adults
Excessive.drinking <= 0.166
Other.primary.care.providers <= 0.000572
Feature
0.00
0.02
0.04
Weight
Positive
Negative

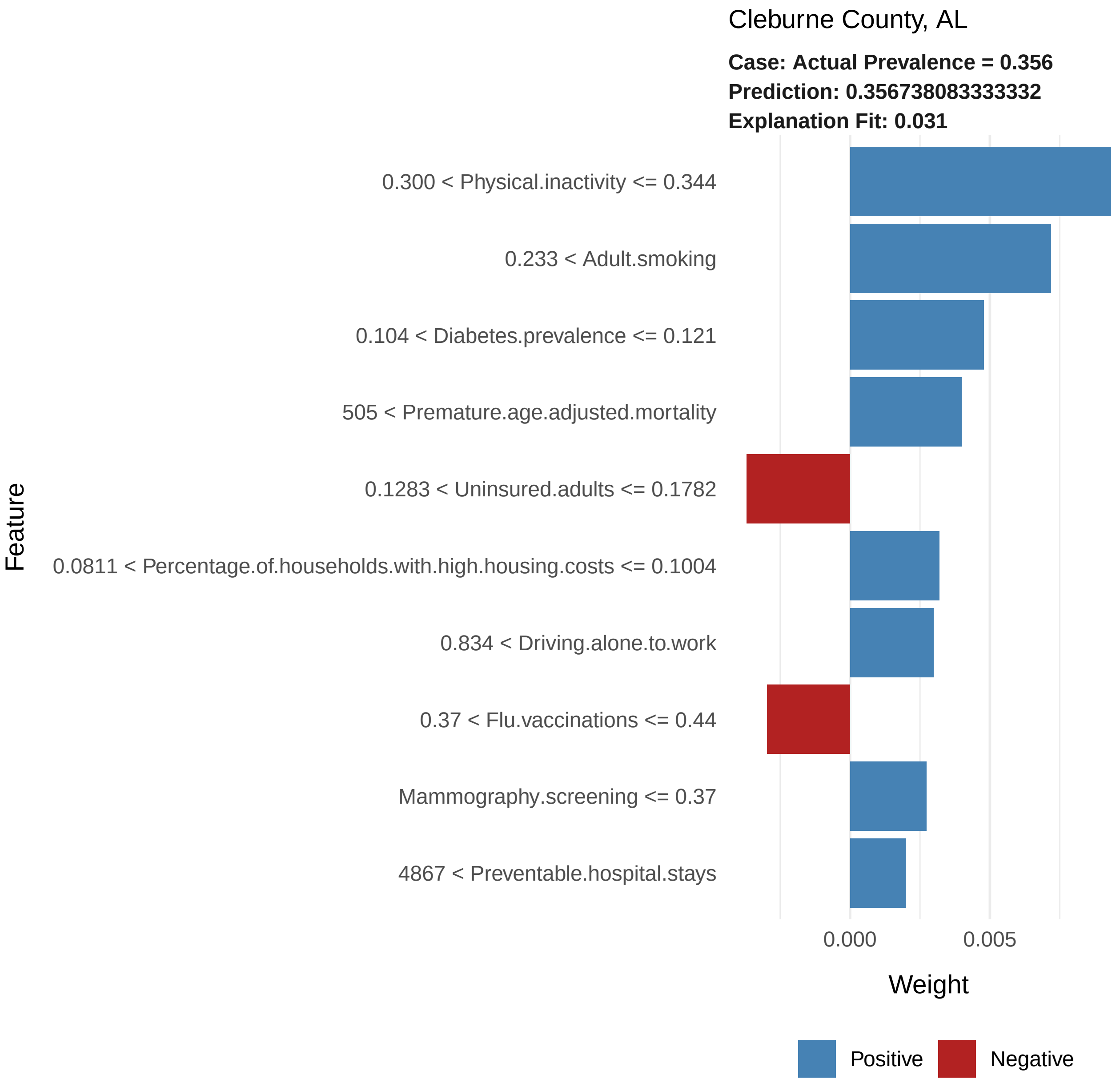

Cleburne County, AL
Case: Actual Prevalence = 0.356
Prediction: 0.356738083333332
Explanation Fit: 0.031
0.300 < Physical.inactivity <= 0.344
0.233 < Adult.smoking
0.104 < Diabetes.prevalence <= 0.121
505 < Premature.age.adjusted.mortality
0.1283 < Uninsured.adults <= 0.1782
0.0811 < Percentage.of.households.with.high.housing.costs <= 0.1004
0.834 < Driving.alone.to.work
0.37 < Flu.vaccinations <= 0.44
Mammography.screening <= 0.37
4867 < Preventable.hospital.stays
Feature
0.000
0.005
Weight
Positive
Negative

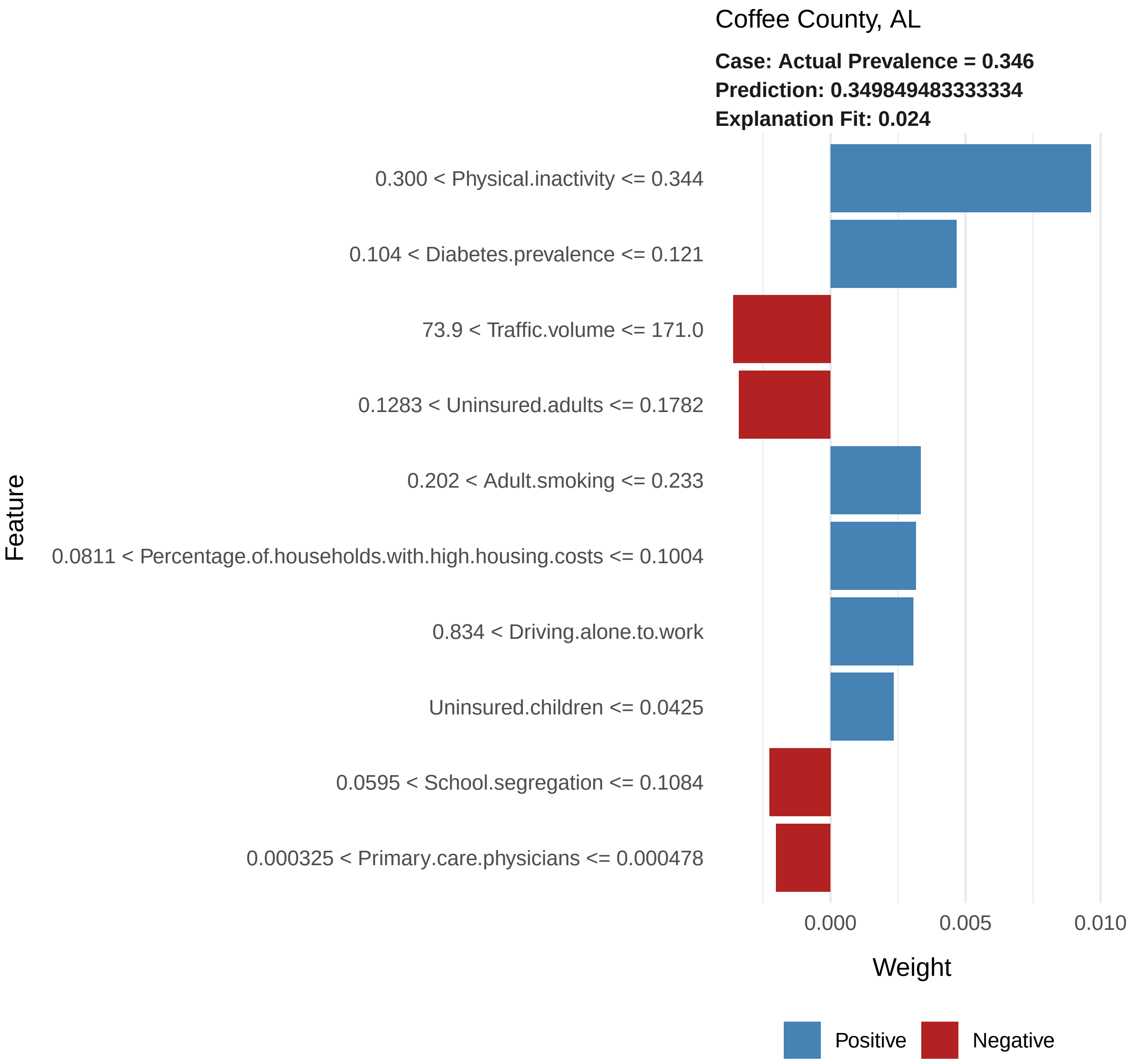
Coffee County, AL
Case: Actual Prevalence = 0.346
Prediction: 0.349849483333334
Explanation Fit: 0.024
0.300 < Physical.inactivity <= 0.344
0.104 < Diabetes.prevalence <= 0.121
73.9 < Traffic.volume <= 171.0
0.1283 < Uninsured.adults <= 0.1782
0.202 < Adult.smoking <= 0.233
0.0811 < Percentage.of.households.with.high.housing.costs <= 0.1004
0.834 < Driving.alone.to.work
Uninsured.children <= 0.0425
0.0595 < School.segregation <= 0.1084
0.000325 < Primary.care.physicians <= 0.000478
Feature
0.000
0.005
0.010
Weight
Positive
Negative

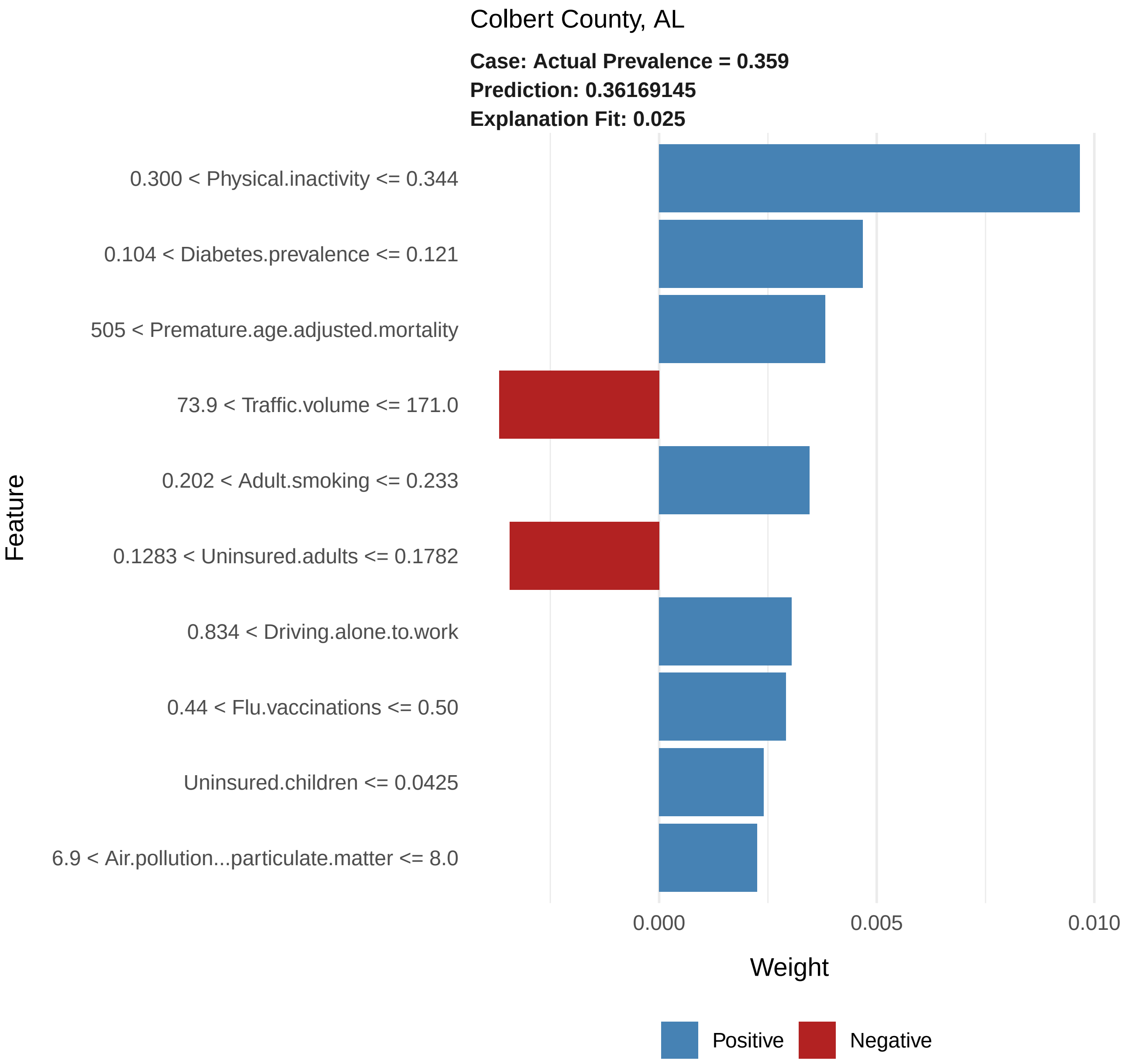

Colbert County, AL
Case: Actual Prevalence = 0.359
Prediction: 0.36169145
Explanation Fit: 0.025
0.300 < Physical.inactivity <= 0.344
0.104 < Diabetes.prevalence <= 0.121
505 < Premature.age.adjusted.mortality
73.9 < Traffic.volume <= 171.0
0.202 < Adult.smoking <= 0.233
0.1283 < Uninsured.adults <= 0.1782
0.834 < Driving.alone.to.work
0.44 < Flu.vaccinations <= 0.50
Uninsured.children <= 0.0425
6.9 < Air.pollution...particulate.matter <= 8.0
Feature
0.000
0.005
0.010
Weight
Positive
Negative

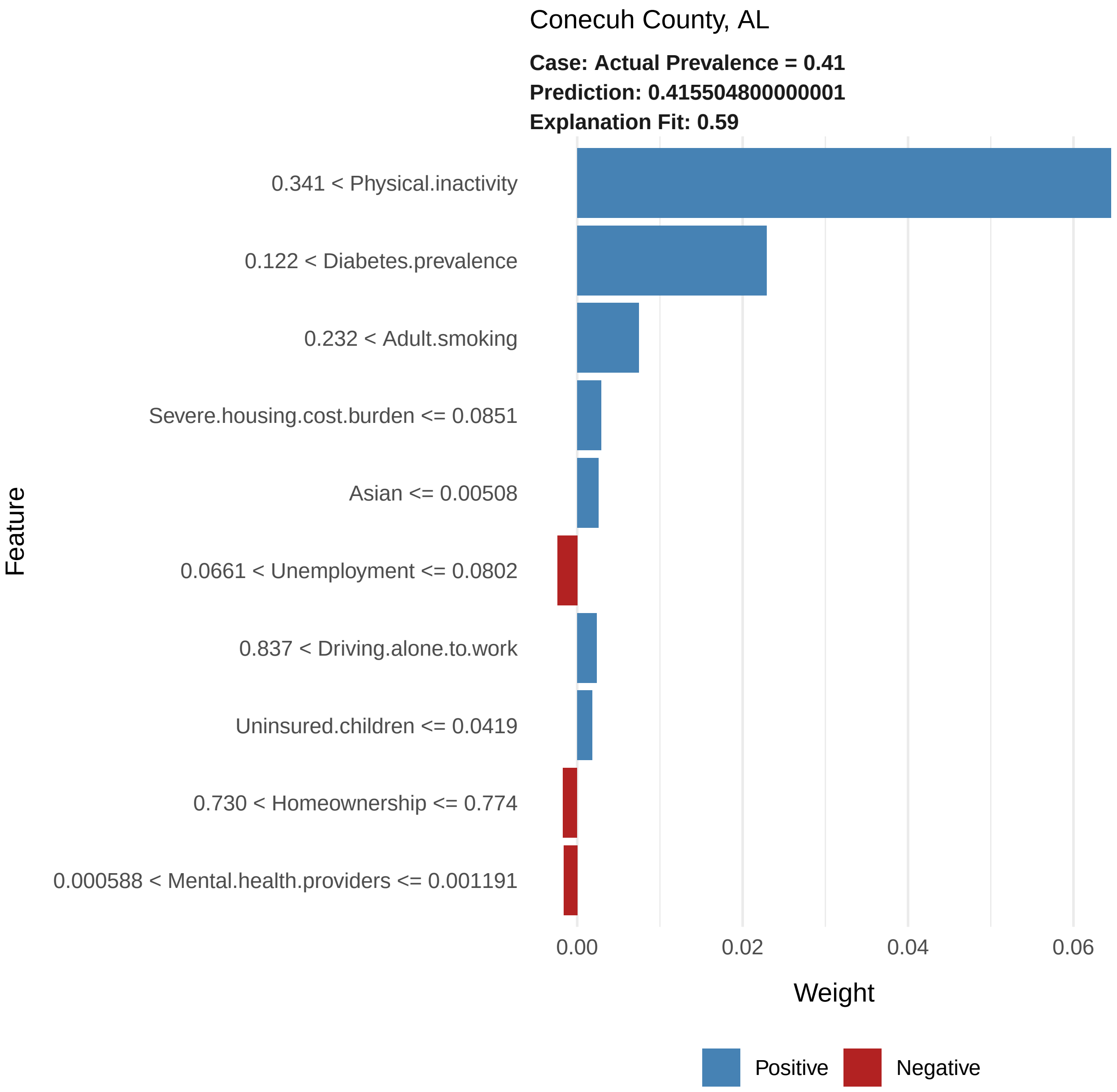

Conecuh County, AL
Case: Actual Prevalence = 0.41
Prediction: 0.415504800000001
Explanation Fit: 0.59
0.341 < Physical.inactivity
0.122 < Diabetes.prevalence
0.232 < Adult.smoking
Severe.housing.cost.burden <= 0.0851
Asian <= 0.00508
0.0661 < Unemployment <= 0.0802
0.837 < Driving.alone.to.work
Uninsured.children <= 0.0419
0.730 < Homeownership <= 0.774
0.000588 < Mental.health.providers <= 0.001191
Feature
0.00
0.02
0.04
0.06
Weight
Positive
Negative

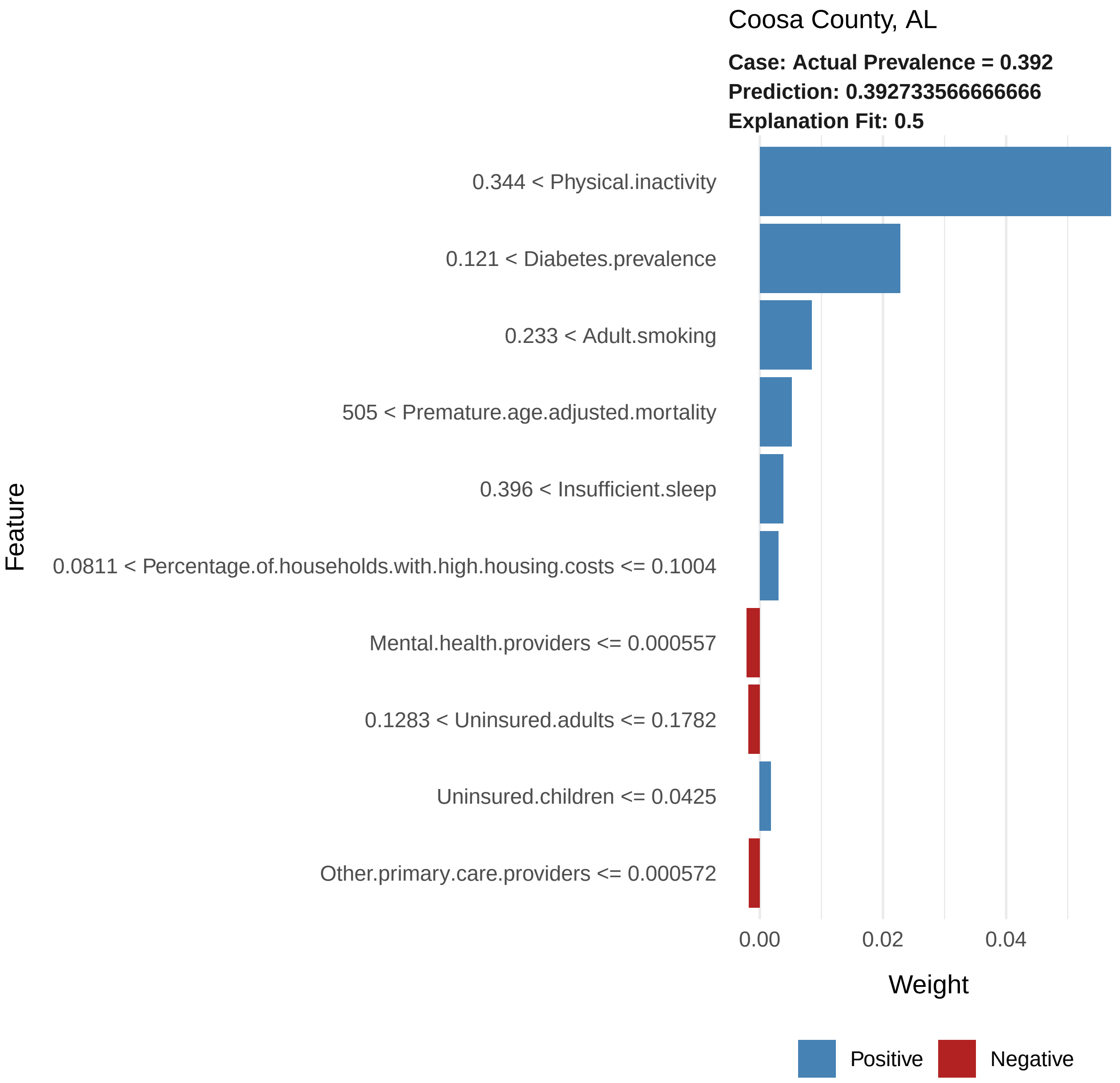

Coosa County, AL
Case: Actual Prevalence = 0.392
Prediction: 0.392733566666666
Explanation Fit: 0.5
Feature
0.344 < Physical.inactivity
0.121 < Diabetes.prevalence
0.233 < Adult.smoking
505 < Premature.age.adjusted.mortality
0.396 < Insufficient.sleep
0.0811 < Percentage.of.households.with.high.housing.costs <= 0.1004
Mental.health.providers <= 0.000557
0.1283 < Uninsured.adults <= 0.1782
Uninsured.children <= 0.0425
Other.primary.care.providers <= 0.000572
0.00
0.02
0.04
Weight
Positive
Negative

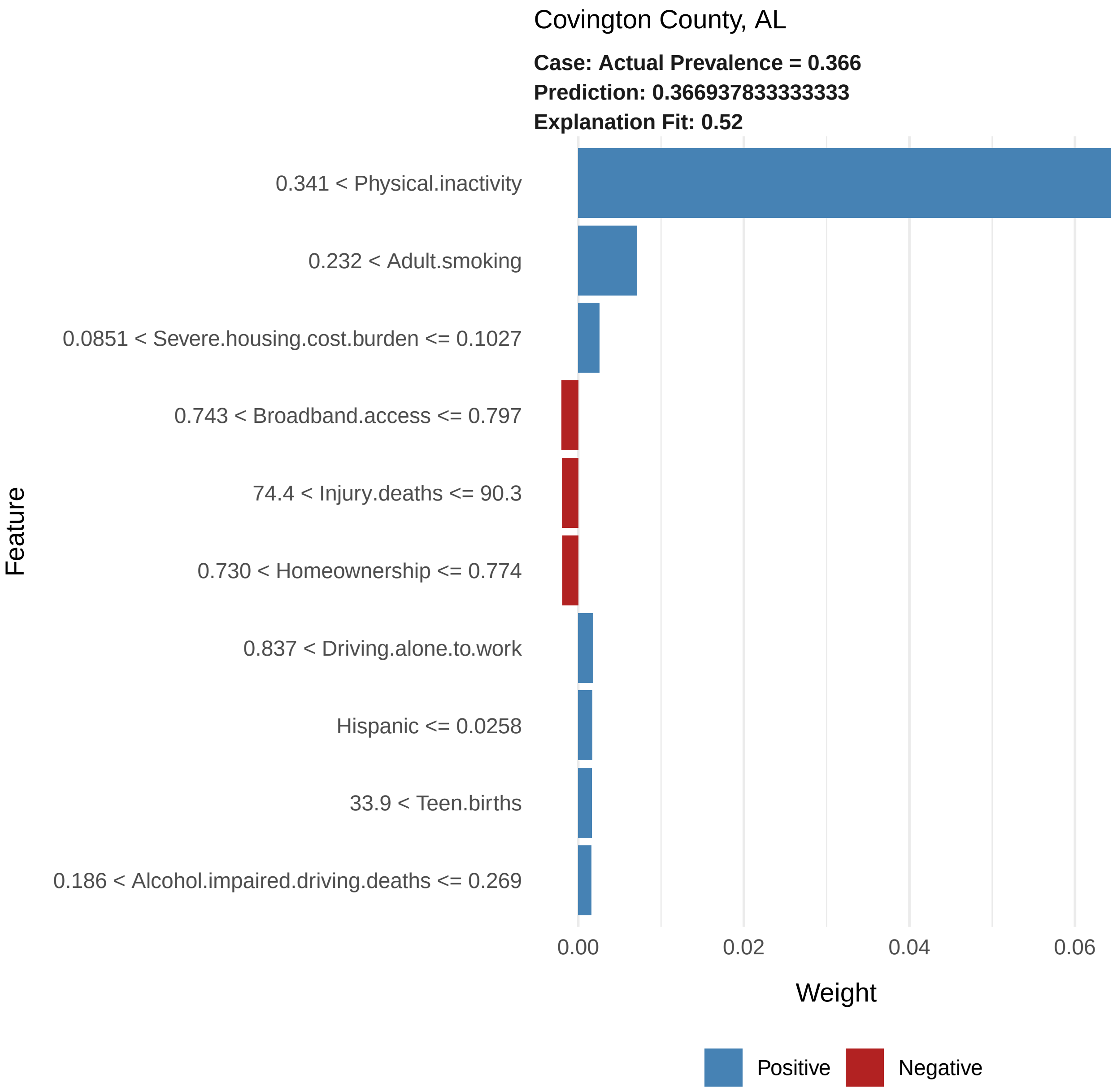
Covington County, AL
Case: Actual Prevalence = 0.366
Prediction: 0.366937833333333
Explanation Fit: 0.52
0.341 < Physical.inactivity
0.232 < Adult.smoking
0.0851 < Severe.housing.cost.burden <= 0.1027
0.743 < Broadband.access <= 0.797
74.4 < Injury.deaths <= 90.3
0.730 < Homeownership <= 0.774
0.837 < Driving.alone.to.work
Hispanic <= 0.0258
33.9 < Teen.births
0.186 < Alcohol.impaired.driving.deaths <= 0.269
Feature
0.00
0.02
0.04
0.06
Weight
Positive
Negative

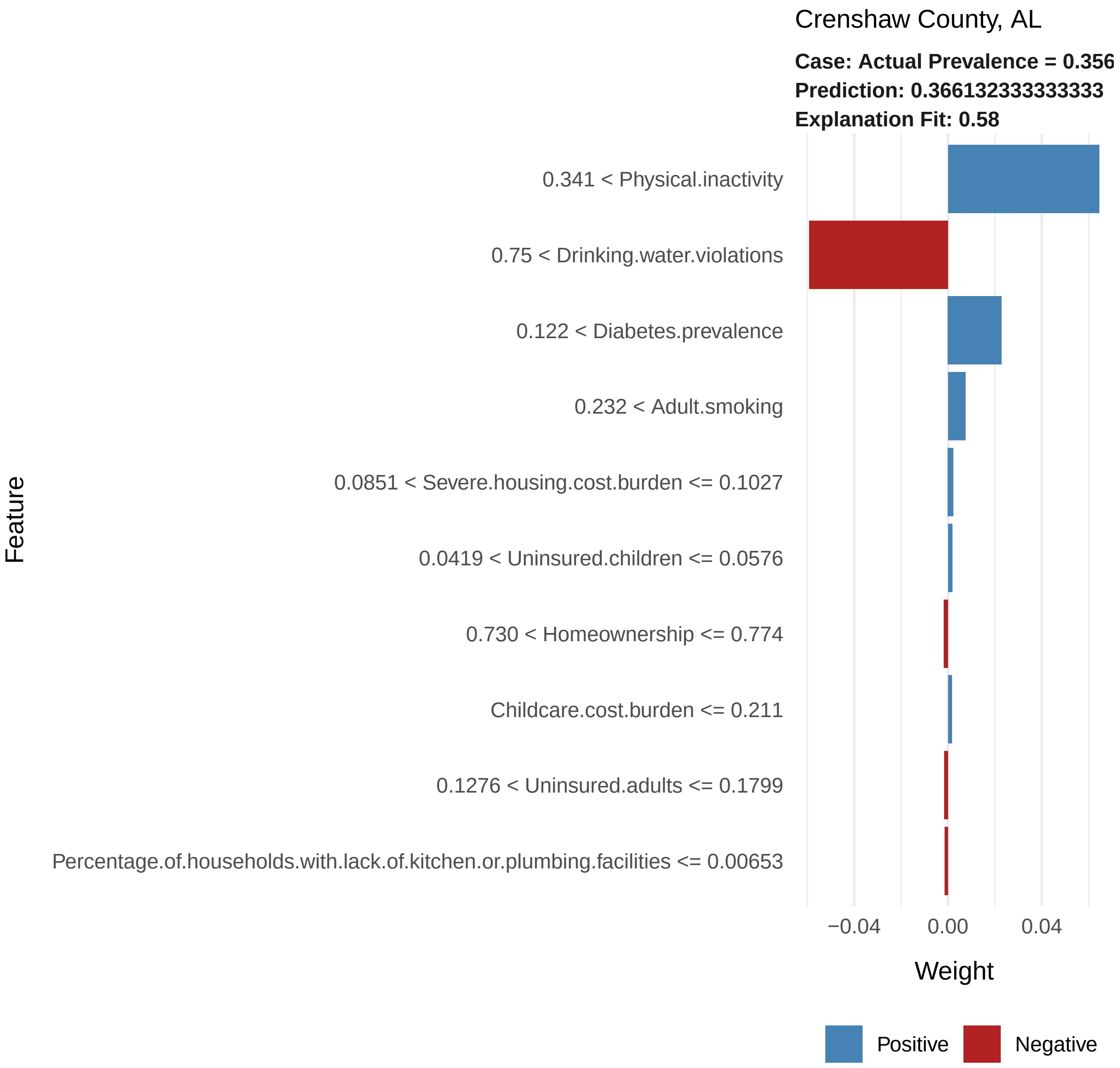

Crenshaw County, AL
Case: Actual Prevalence = 0.356
Prediction: 0.366132333333333
Explanation Fit: 0.58
0.341 < Physical.inactivity
0.75 < Drinking.water.violations
0.122 < Diabetes.prevalence
0.232 < Adult.smoking
0.0851 < Severe.housing.cost.burden <= 0.1027
0.0419 < Uninsured.children <= 0.0576
0.730 < Homeownership <= 0.774
Childcare.cost.burden <= 0.211
0.1276 < Uninsured.adults <= 0.1799
Percentage.of.households.with.lack.of.kitchen.or.plumbing.facilities <= 0.00653
Feature
−0.04
0.00
0.04
Weight
Positive
Negative

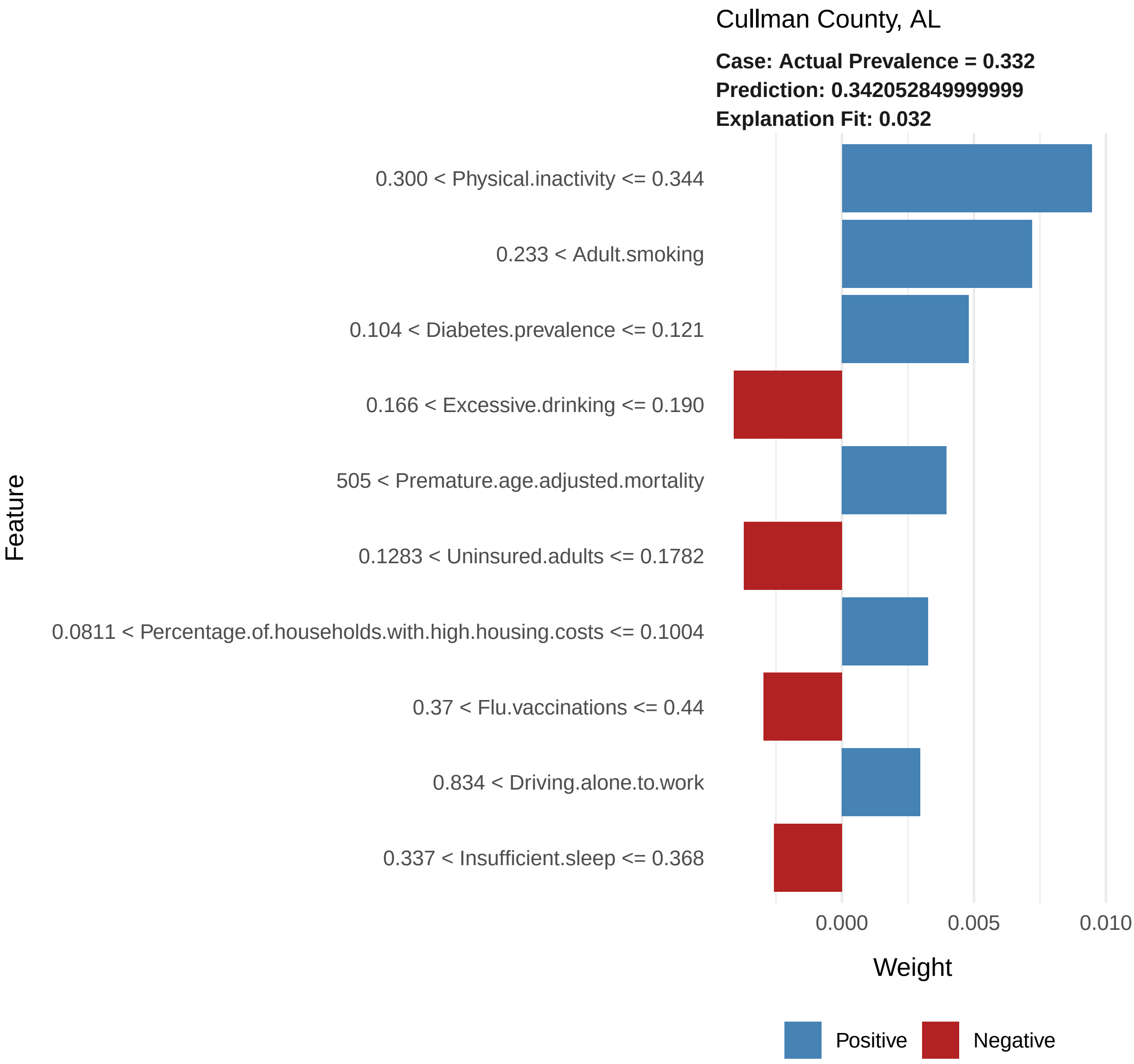

Cullman County, AL
Case: Actual Prevalence = 0.332
Prediction: 0.342052849999999
Explanation Fit: 0.032
0.300 < Physical.inactivity <= 0.344
0.233 < Adult.smoking
0.104 < Diabetes.prevalence <= 0.121
0.166 < Excessive.drinking <= 0.190
505 < Premature.age.adjusted.mortality
0.1283 < Uninsured.adults <= 0.1782
0.0811 < Percentage.of.households.with.high.housing.costs <= 0.1004
0.37 < Flu.vaccinations <= 0.44
0.834 < Driving.alone.to.work
0.337 < Insufficient.sleep <= 0.368
Feature
0.000
0.005
0.010
Weight
Positive
Negative

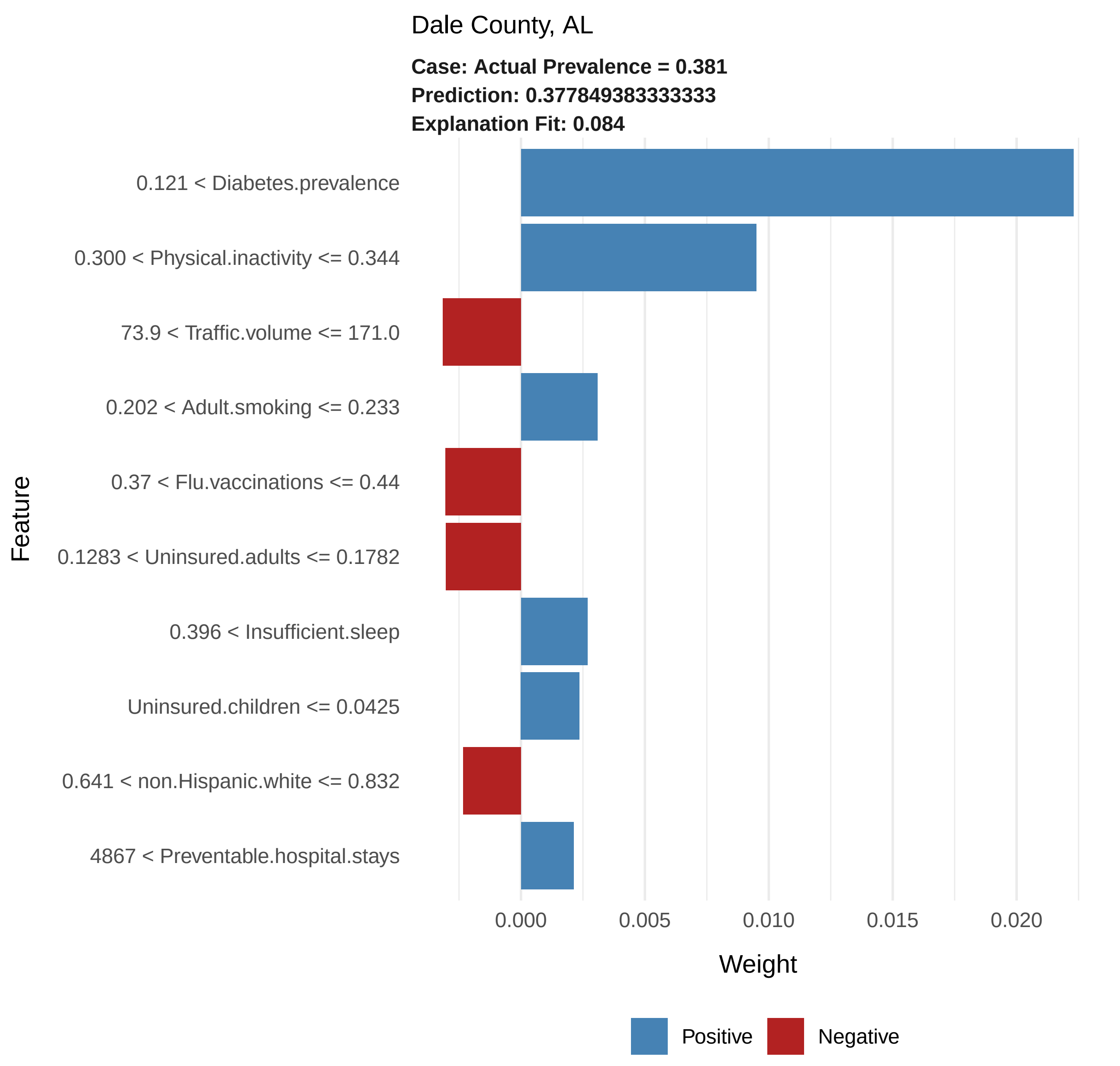
Dale County, AL
Case: Actual Prevalence = 0.381
Prediction: 0.377849383333333
Explanation Fit: 0.084
Feature
0.121 < Diabetes.prevalence
0.300 < Physical.inactivity <= 0.344
73.9 < Traffic.volume <= 171.0
0.202 < Adult.smoking <= 0.233
0.37 < Flu.vaccinations <= 0.44
0.1283 < Uninsured.adults <= 0.1782
0.396 < Insufficient.sleep
Uninsured.children <= 0.0425
0.641 < non.Hispanic.white <= 0.832
4867 < Preventable.hospital.stays
0.000
0.005
0.010
0.015
0.020
Weight
Positive
Negative

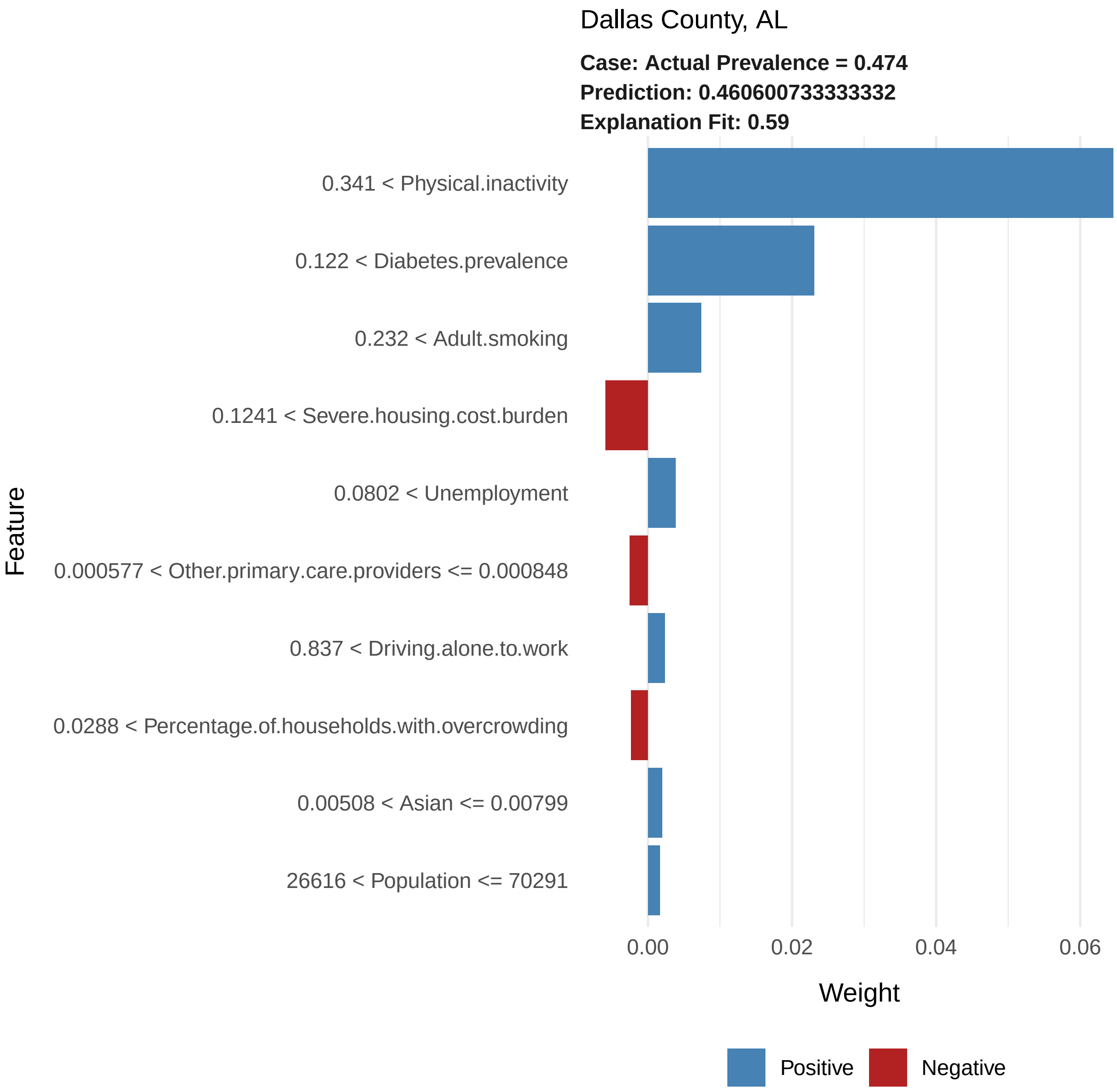

Dallas County, AL
Case: Actual Prevalence = 0.474
Prediction: 0.460600733333332
Explanation Fit: 0.59
0.341 < Physical.inactivity
0.122 < Diabetes.prevalence
0.232 < Adult.smoking
0.1241 < Severe.housing.cost.burden
0.0802 < Unemployment
0.000577 < Other.primary.care.providers <= 0.000848
0.837 < Driving.alone.to.work
0.0288 < Percentage.of.households.with.overcrowding
0.00508 < Asian <= 0.00799
26616 < Population <= 70291
Feature
0.00
0.02
0.04
0.06
Weight
Positive
Negative

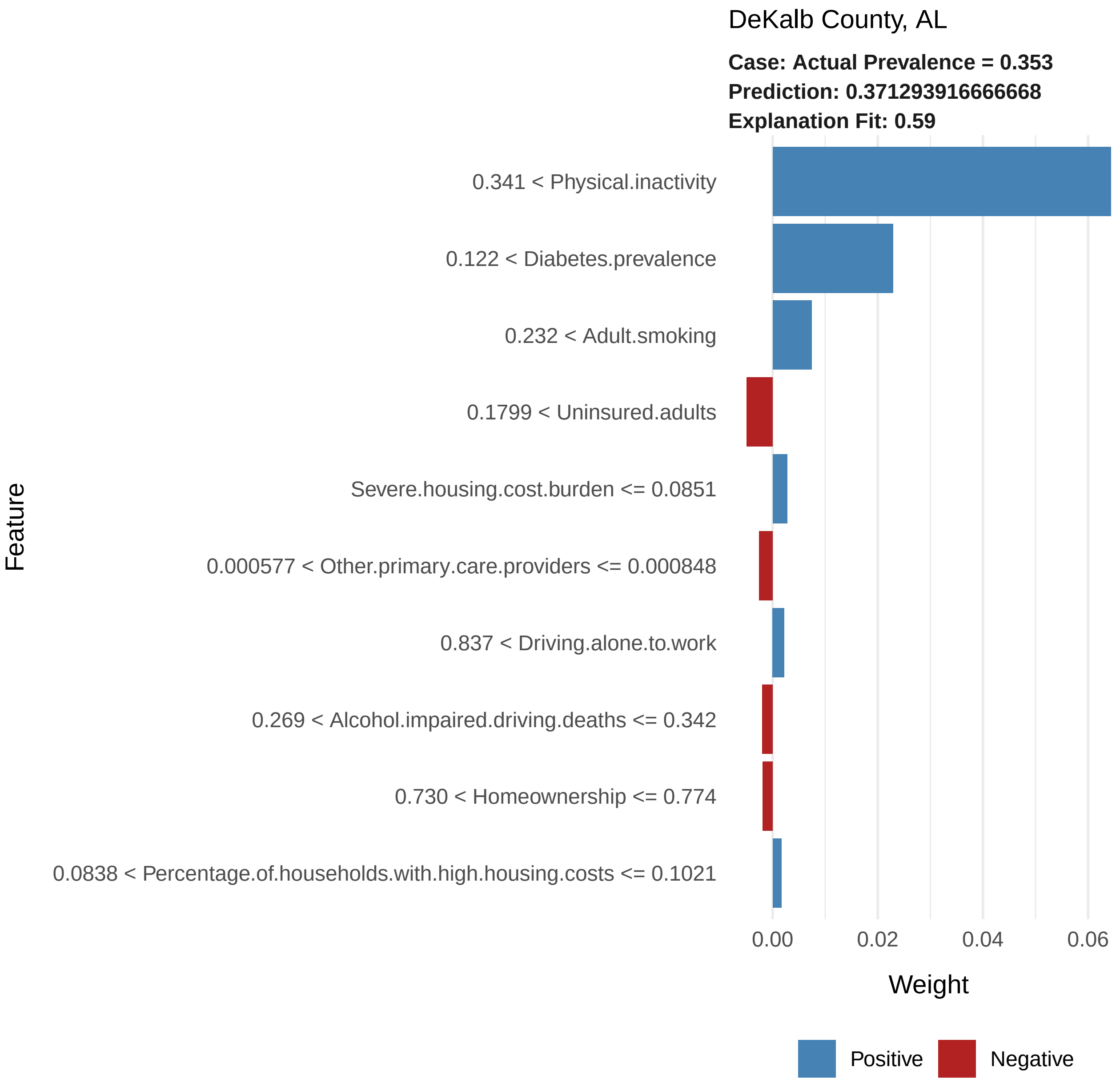
DeKalb County, AL
Case: Actual Prevalence = 0.353
Prediction: 0.371293916666668
Explanation Fit: 0.59
0.341 < Physical.inactivity
0.122 < Diabetes.prevalence
0.232 < Adult.smoking
0.1799 < Uninsured.adults
Severe.housing.cost.burden <= 0.0851
0.000577 < Other.primary.care.providers <= 0.000848
0.837 < Driving.alone.to.work
0.269 < Alcohol.impaired.driving.deaths <= 0.342
0.730 < Homeownership <= 0.774
0.0838 < Percentage.of.households.with.high.housing.costs <= 0.1021
Feature
0.00
0.02
0.04
0.06
Weight
Positive
Negative

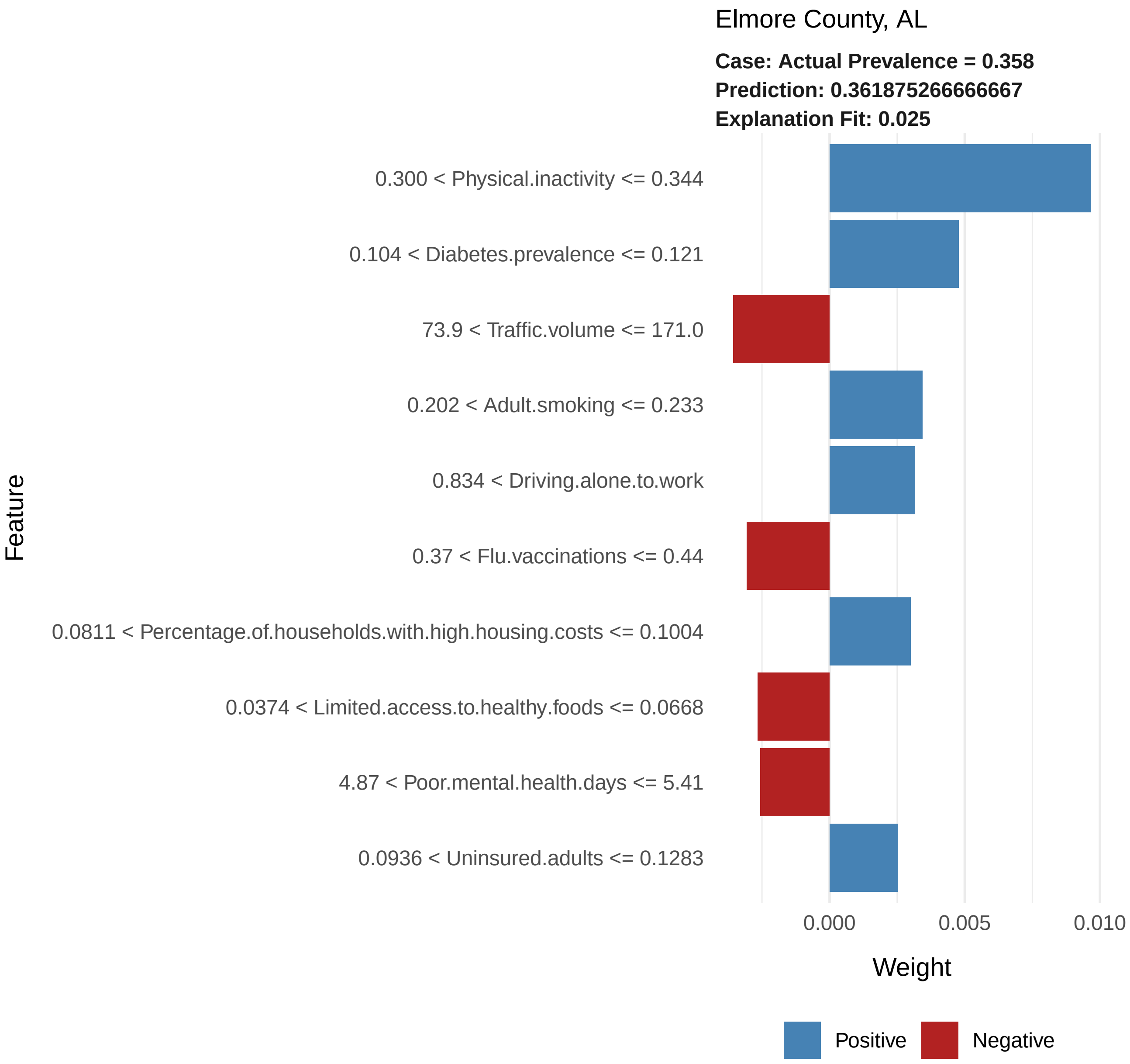
Elmore County, AL
Case: Actual Prevalence = 0.358
Prediction: 0.361875266666667
Explanation Fit: 0.025
0.300 < Physical.inactivity <= 0.344
0.104 < Diabetes.prevalence <= 0.121
73.9 < Traffic.volume <= 171.0
0.202 < Adult.smoking <= 0.233
0.834 < Driving.alone.to.work
0.37 < Flu.vaccinations <= 0.44
0.0811 < Percentage.of.households.with.high.housing.costs <= 0.1004
0.0374 < Limited.access.to.healthy.foods <= 0.0668
4.87 < Poor.mental.health.days <= 5.41
0.0936 < Uninsured.adults <= 0.1283
Feature
0.000
0.005
0.010
Weight
Positive
Negative

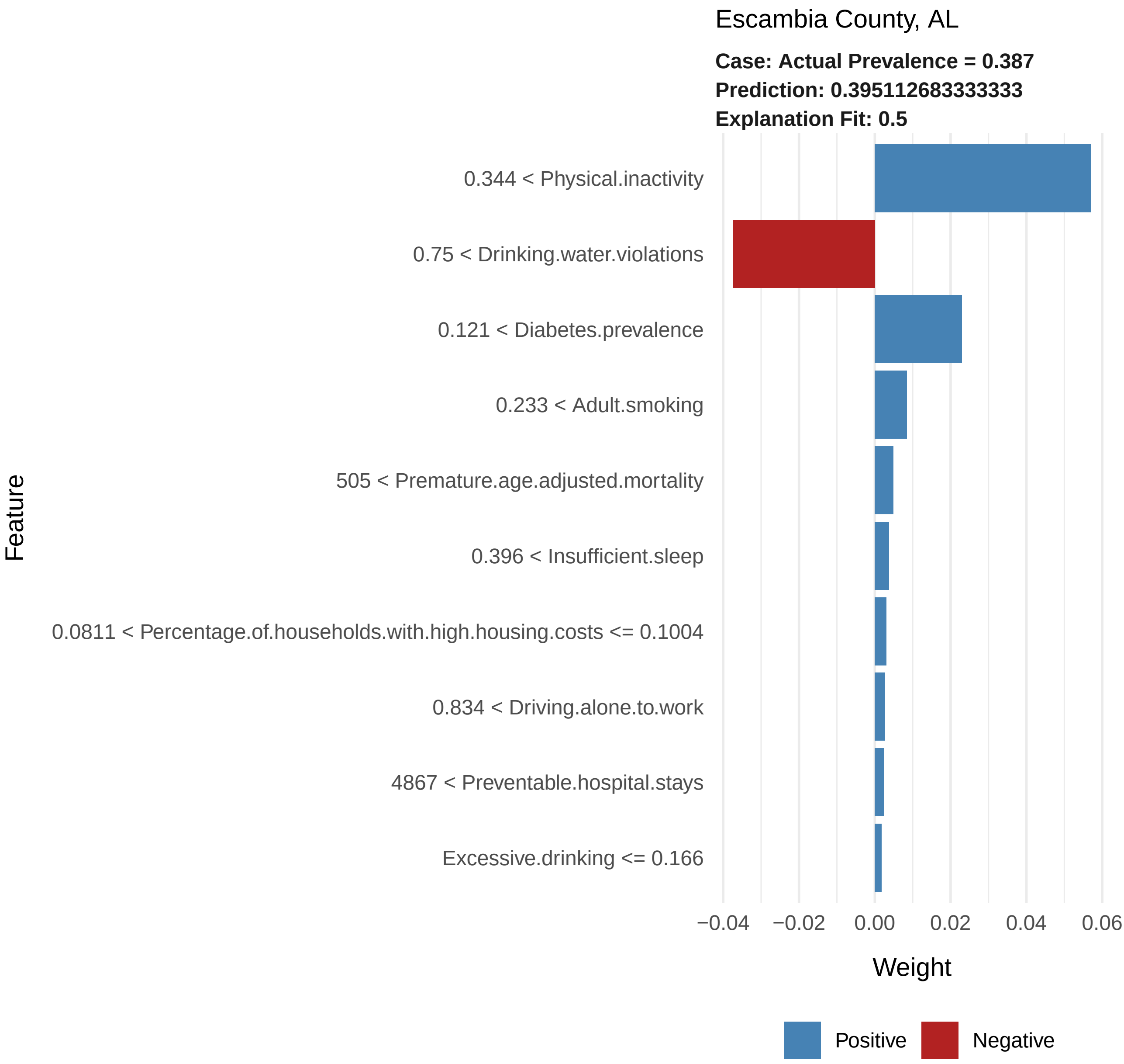

Escambia County, AL
Case: Actual Prevalence = 0.387
Prediction: 0.395112683333333
Explanation Fit: 0.5
0.344 < Physical.inactivity
0.75 < Drinking.water.violations
0.121 < Diabetes.prevalence
0.233 < Adult.smoking
505 < Premature.age.adjusted.mortality
0.396 < Insufficient.sleep
0.0811 < Percentage.of.households.with.high.housing.costs <= 0.1004
0.834 < Driving.alone.to.work
4867 < Preventable.hospital.stays
Excessive.drinking <= 0.166
Feature
−0.04
−0.02
0.00
0.02
0.04
0.06
Weight
Positive
Negative

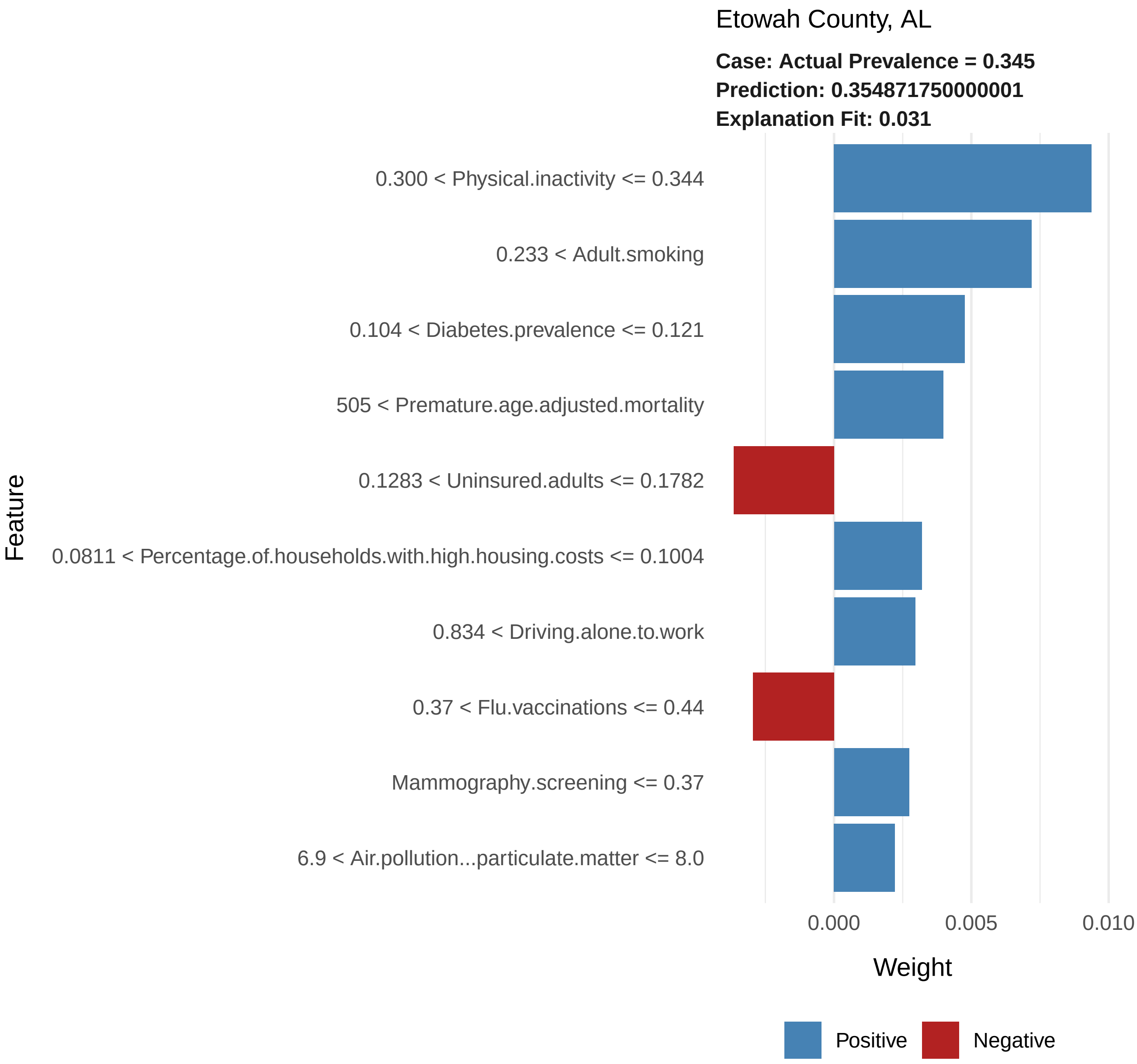

Etowah County, AL
Case: Actual Prevalence = 0.345
Prediction: 0.354871750000001
Explanation Fit: 0.031
0.300 < Physical.inactivity <= 0.344
0.233 < Adult.smoking
0.104 < Diabetes.prevalence <= 0.121
505 < Premature.age.adjusted.mortality
0.1283 < Uninsured.adults <= 0.1782
0.0811 < Percentage.of.households.with.high.housing.costs <= 0.1004
0.834 < Driving.alone.to.work
0.37 < Flu.vaccinations <= 0.44
Mammography.screening <= 0.37
6.9 < Air.pollution...particulate.matter <= 8.0
Feature
0.000
0.005
0.010
Weight
Positive
Negative

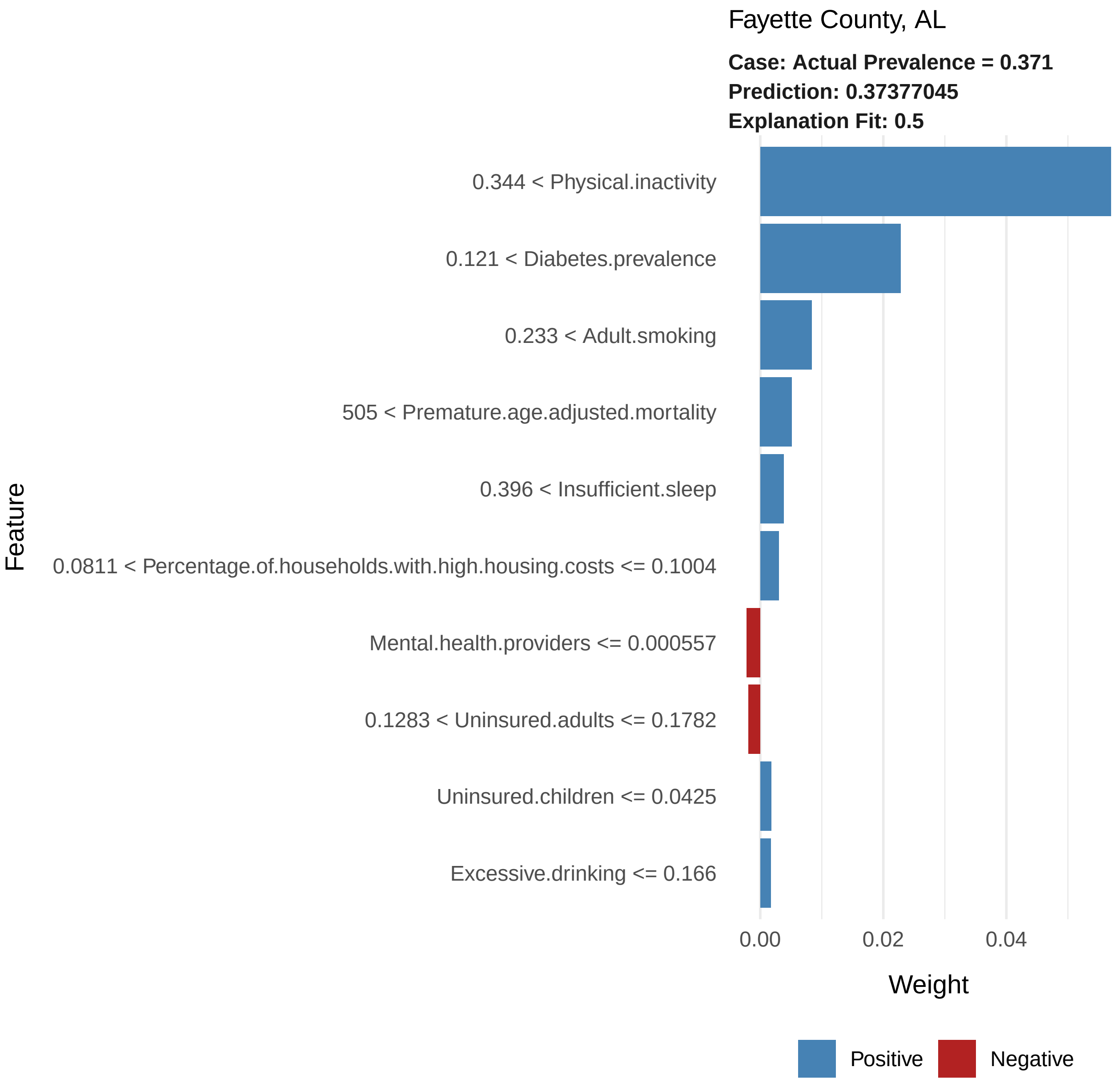

Fayette County, AL
Case: Actual Prevalence = 0.371
Prediction: 0.37377045
Explanation Fit: 0.5
0.344 < Physical.inactivity
0.121 < Diabetes.prevalence
0.233 < Adult.smoking
505 < Premature.age.adjusted.mortality
0.396 < Insufficient.sleep
0.0811 < Percentage.of.households.with.high.housing.costs <= 0.1004
Mental.health.providers <= 0.000557
0.1283 < Uninsured.adults <= 0.1782
Uninsured.children <= 0.0425
Excessive.drinking <= 0.166
Feature
0.00
0.02
0.04
Weight
Positive
Negative

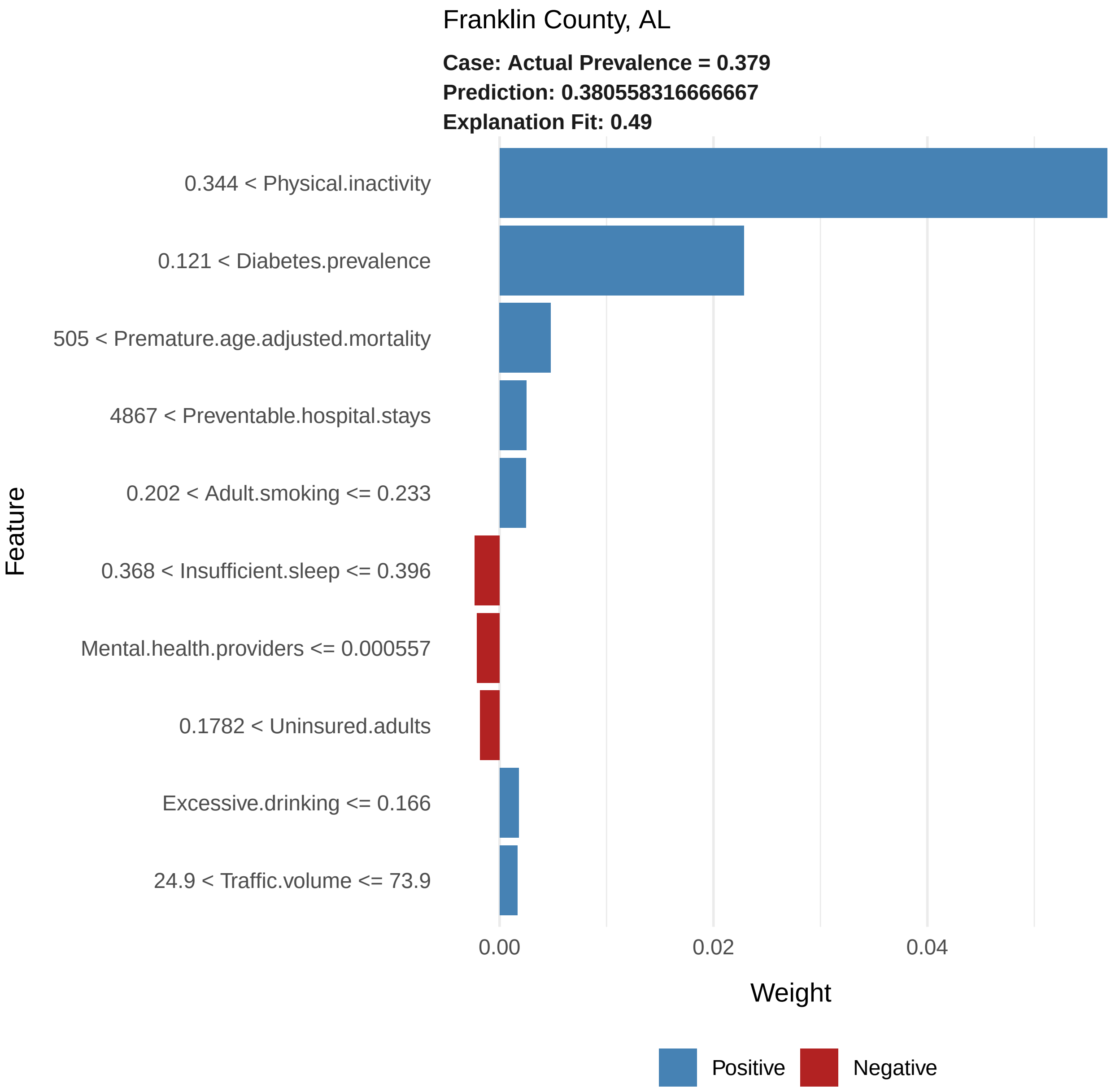

Franklin County, AL
Case: Actual Prevalence = 0.379
Prediction: 0.380558316666667
Explanation Fit: 0.49
0.344 < Physical.inactivity
0.121 < Diabetes.prevalence
505 < Premature.age.adjusted.mortality
4867 < Preventable.hospital.stays
0.202 < Adult.smoking <= 0.233
0.368 < Insufficient.sleep <= 0.396
Mental.health.providers <= 0.000557
0.1782 < Uninsured.adults
Excessive.drinking <= 0.166
24.9 < Traffic.volume <= 73.9
Feature
0.00
0.02
0.04
Weight
Positive
Negative

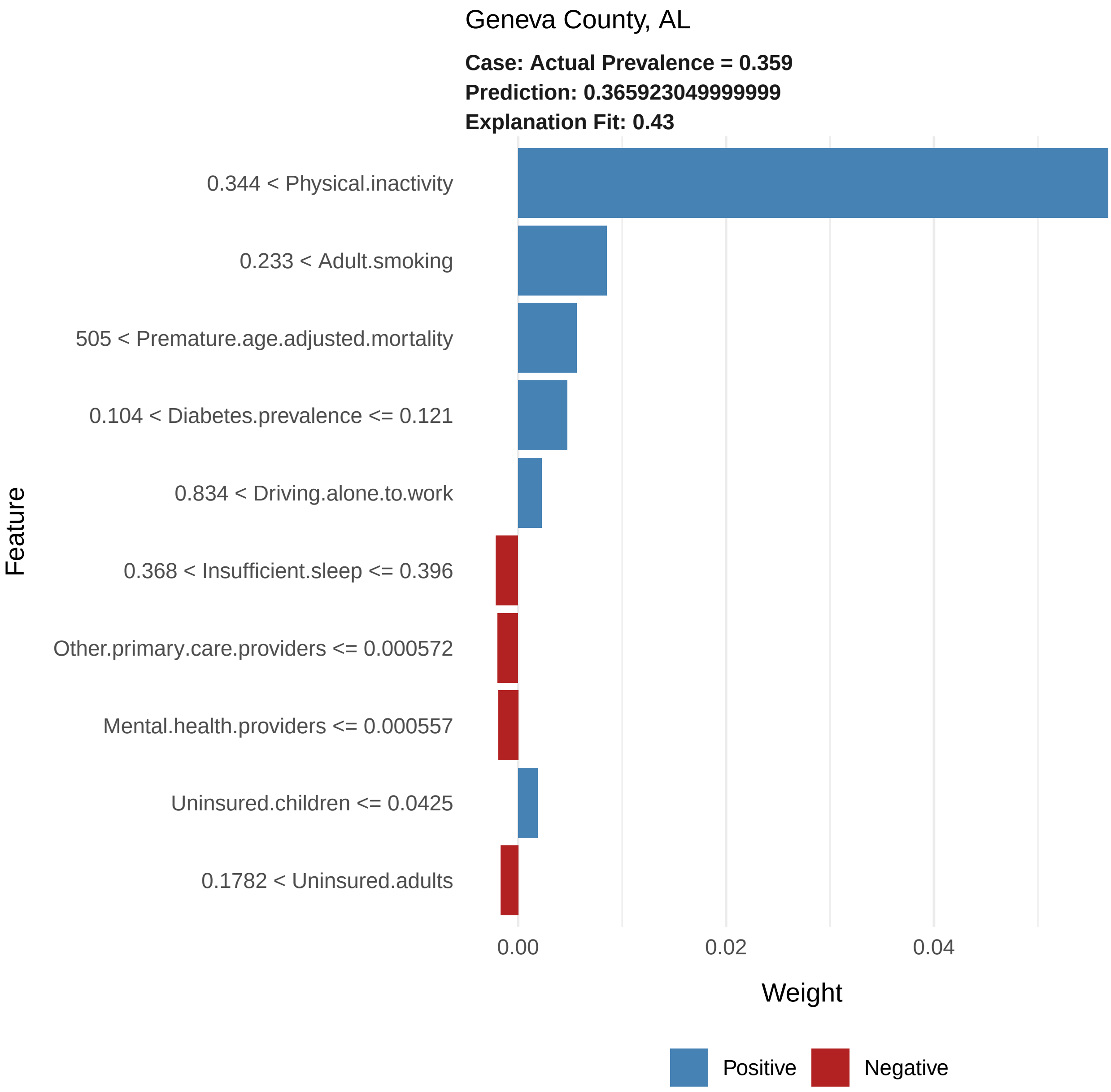
Geneva County, AL
Case: Actual Prevalence = 0.359
Prediction: 0.365923049999999
Explanation Fit: 0.43
0.344 < Physical.inactivity
0.233 < Adult.smoking
505 < Premature.age.adjusted.mortality
0.104 < Diabetes.prevalence <= 0.121
0.834 < Driving.alone.to.work
0.368 < Insufficient.sleep <= 0.396
Other.primary.care.providers <= 0.000572
Mental.health.providers <= 0.000557
Uninsured.children <= 0.0425
0.1782 < Uninsured.adults
Feature
0.00
0.02
0.04
Weight
Positive
Negative

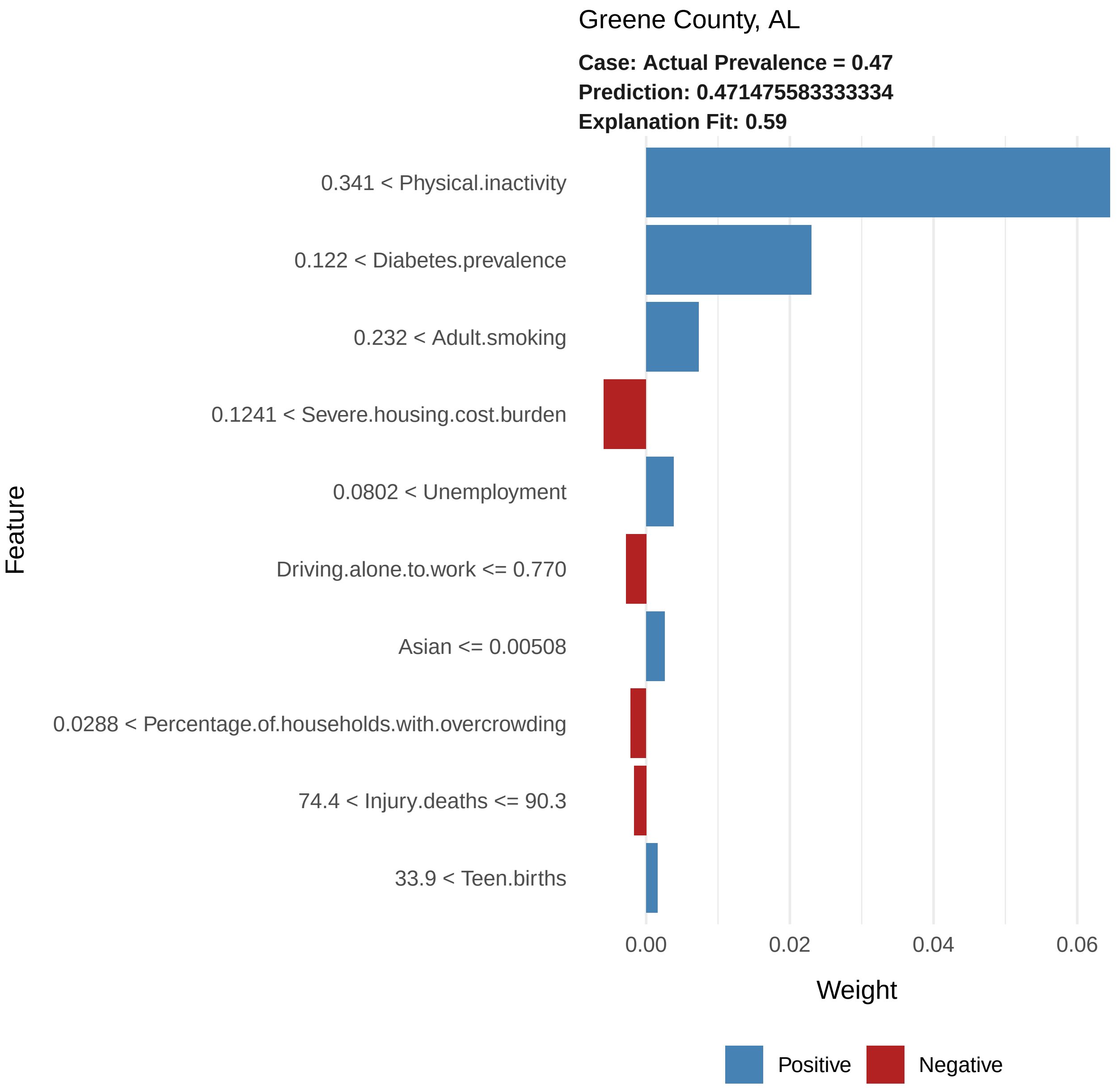

Greene County, AL
Case: Actual Prevalence = 0.47
Prediction: 0.471475583333334
Explanation Fit: 0.59
0.341 < Physical.inactivity
0.122 < Diabetes.prevalence
0.232 < Adult.smoking
0.1241 < Severe.housing.cost.burden
0.0802 < Unemployment
Driving.alone.to.work <= 0.770
Asian <= 0.00508
0.0288 < Percentage.of.households.with.overcrowding
74.4 < Injury.deaths <= 90.3
33.9 < Teen.births
Feature
0.00
0.02
0.04
0.06
Weight
Positive
Negative

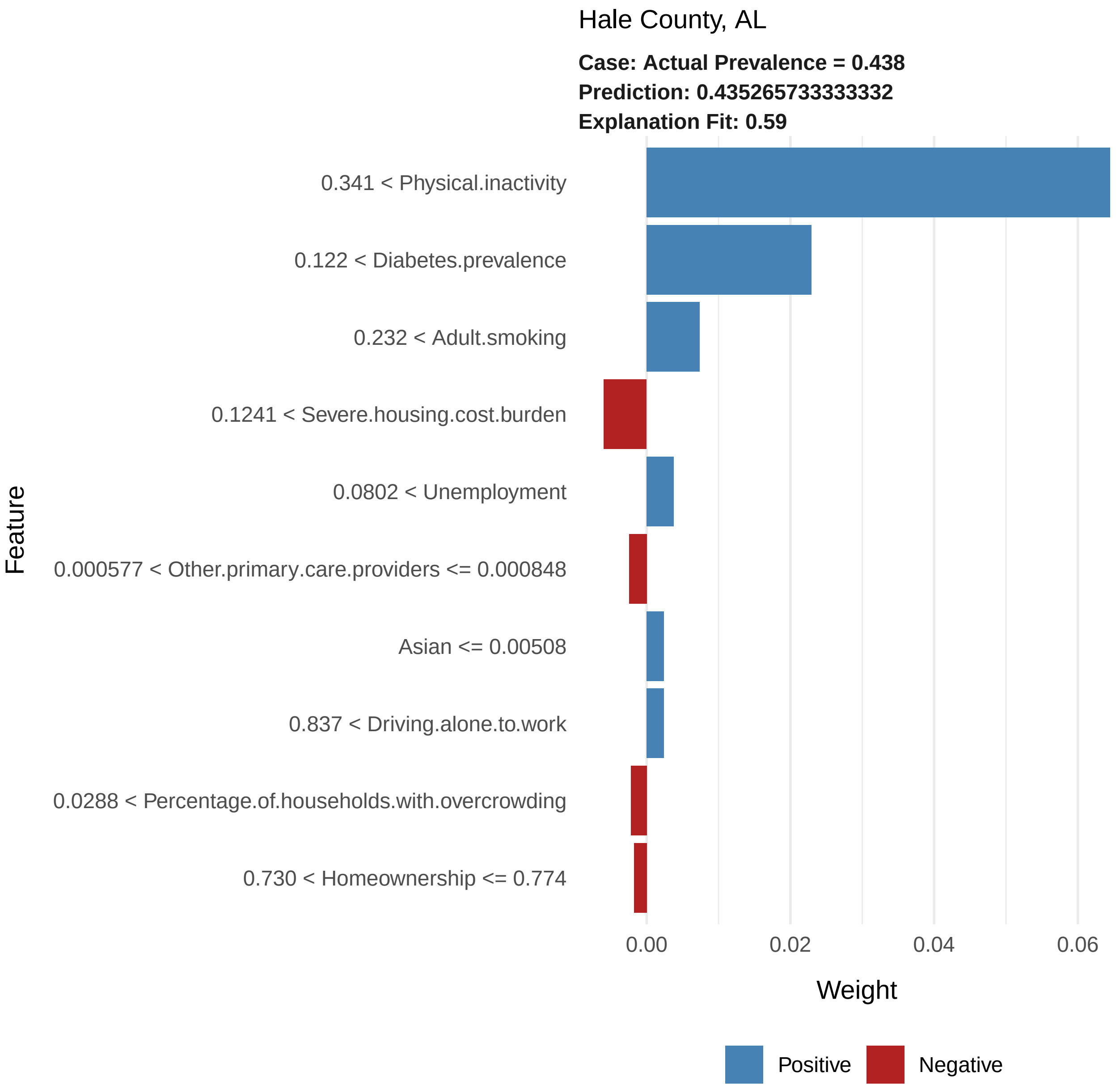

Hale County, AL
Case: Actual Prevalence = 0.438
Prediction: 0.435265733333332
Explanation Fit: 0.59
0.341 < Physical.inactivity
0.122 < Diabetes.prevalence
0.232 < Adult.smoking
0.1241 < Severe.housing.cost.burden
0.0802 < Unemployment
0.000577 < Other.primary.care.providers <= 0.000848
Asian <= 0.00508
0.837 < Driving.alone.to.work
0.0288 < Percentage.of.households.with.overcrowding
0.730 < Homeownership <= 0.774
Feature
0.00
0.02
0.04
0.06
Weight
Positive
Negative

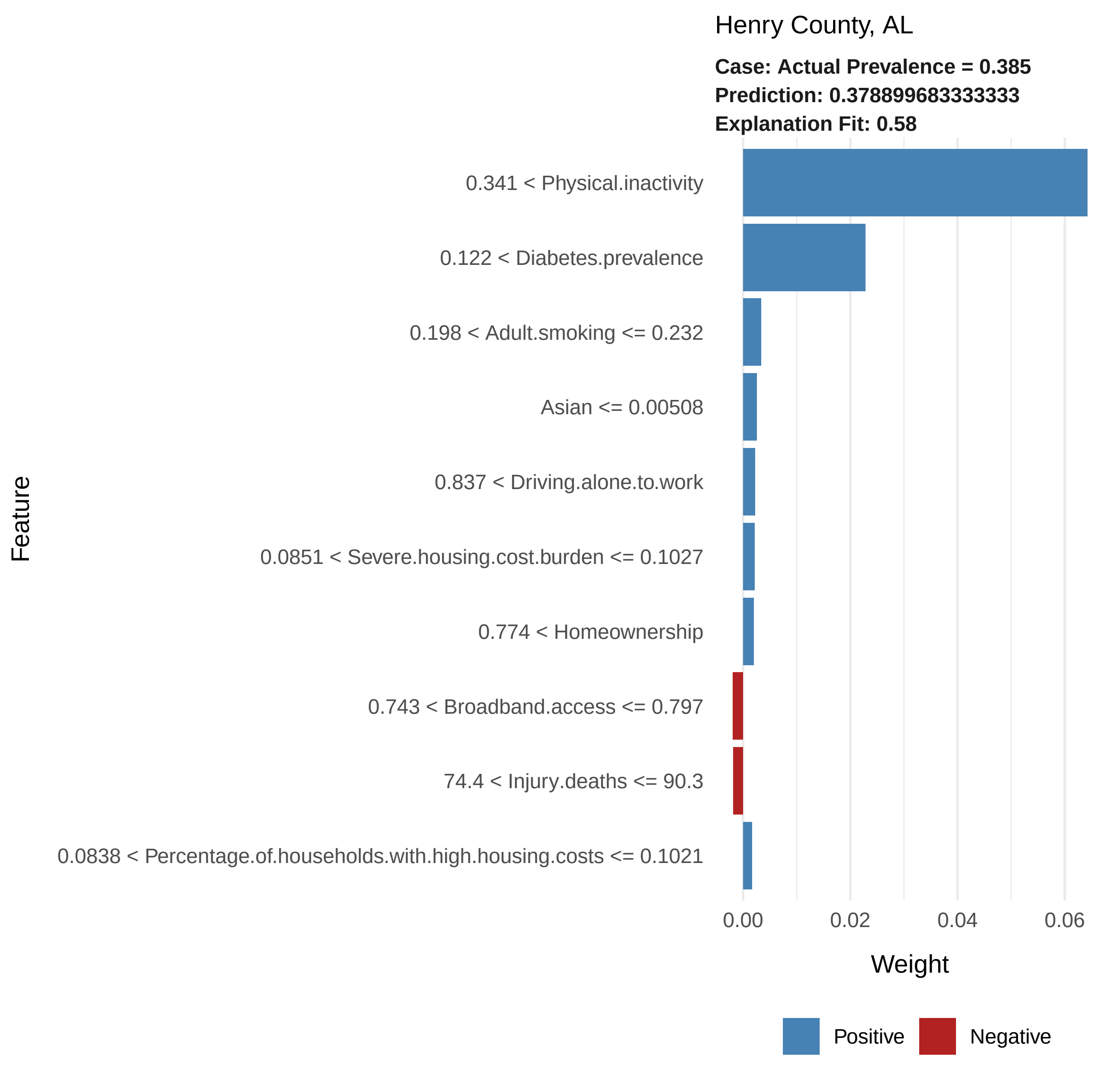

Henry County, AL
Case: Actual Prevalence = 0.385
Prediction: 0.378899683333333
Explanation Fit: 0.58
0.341 < Physical.inactivity
0.122 < Diabetes.prevalence
0.198 < Adult.smoking <= 0.232
Asian <= 0.00508
0.837 < Driving.alone.to.work
0.0851 < Severe.housing.cost.burden <= 0.1027
0.774 < Homeownership
0.743 < Broadband.access <= 0.797
74.4 < Injury.deaths <= 90.3
0.0838 < Percentage.of.households.with.high.housing.costs <= 0.1021
Feature
0.00
0.02
0.04
0.06
Weight
Positive
Negative

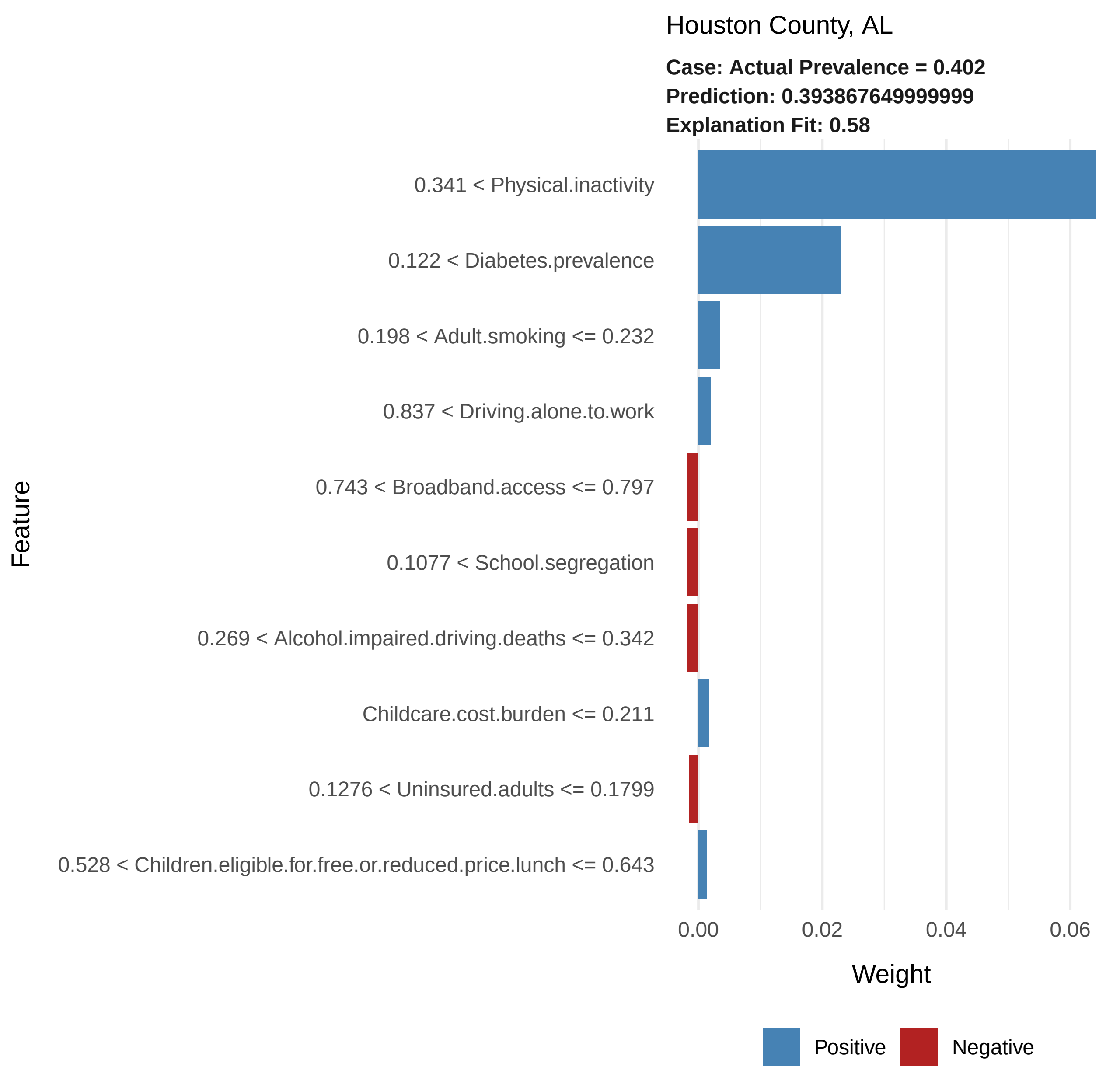

Houston County, AL
Case: Actual Prevalence = 0.402
Prediction: 0.393867649999999
Explanation Fit: 0.58
0.341 < Physical.inactivity
0.122 < Diabetes.prevalence
0.198 < Adult.smoking <= 0.232
0.837 < Driving.alone.to.work
0.743 < Broadband.access <= 0.797
0.1077 < School.segregation
0.269 < Alcohol.impaired.driving.deaths <= 0.342
Childcare.cost.burden <= 0.211
0.1276 < Uninsured.adults <= 0.1799
0.528 < Children.eligible.for.free.or.reduced.price.lunch <= 0.643
Feature
0.00
0.02
0.04
0.06
Weight
Positive
Negative

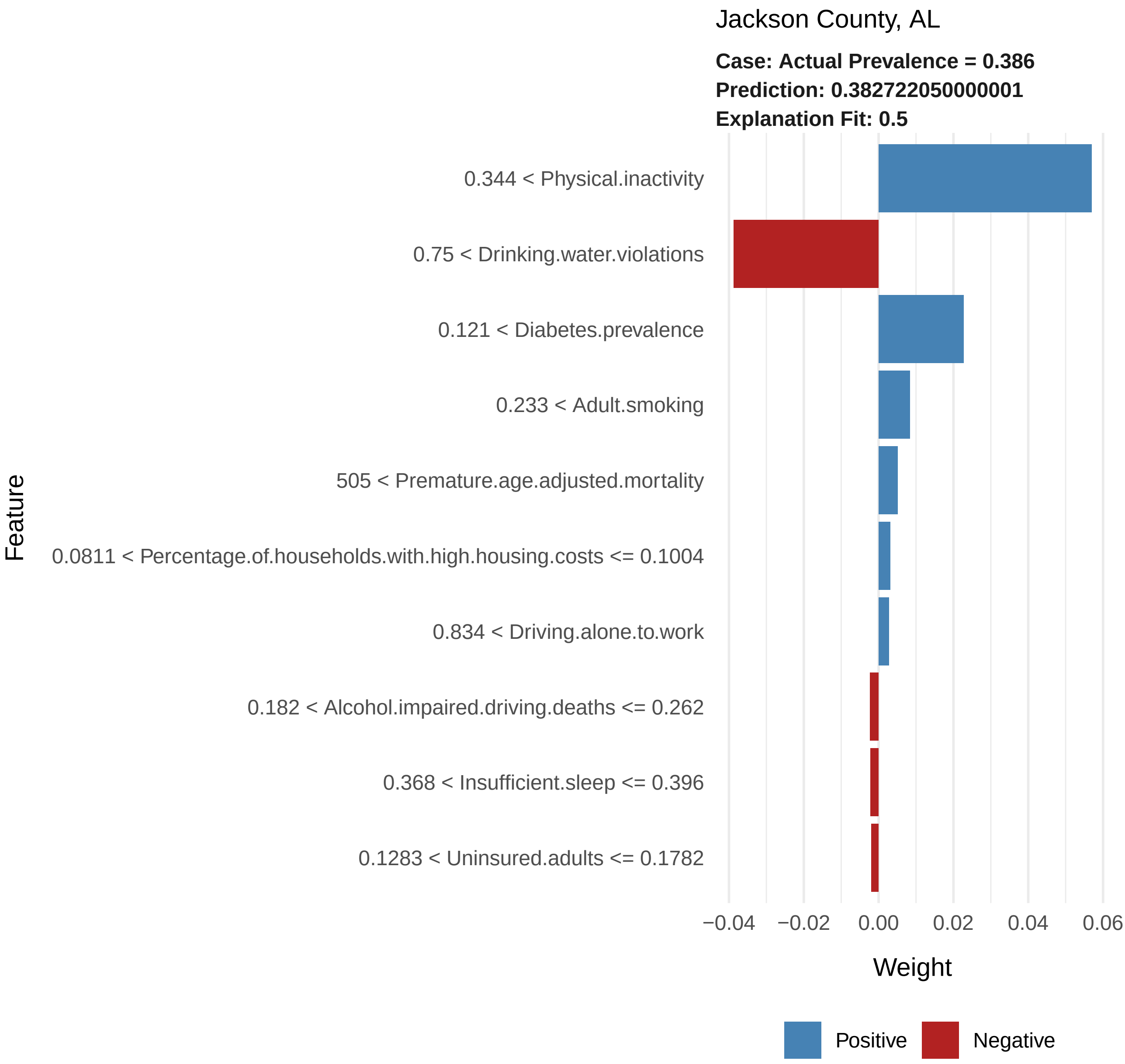

Jackson County, AL
Case: Actual Prevalence = 0.386
Prediction: 0.382722050000001
Explanation Fit: 0.5
0.344 < Physical.inactivity
0.75 < Drinking.water.violations
0.121 < Diabetes.prevalence
0.233 < Adult.smoking
505 < Premature.age.adjusted.mortality
0.0811 < Percentage.of.households.with.high.housing.costs <= 0.1004
0.834 < Driving.alone.to.work
0.182 < Alcohol.impaired.driving.deaths <= 0.262
0.368 < Insufficient.sleep <= 0.396
0.1283 < Uninsured.adults <= 0.1782
Feature
−0.04
−0.02
0.00
0.02
0.04
0.06
Weight
Positive
Negative

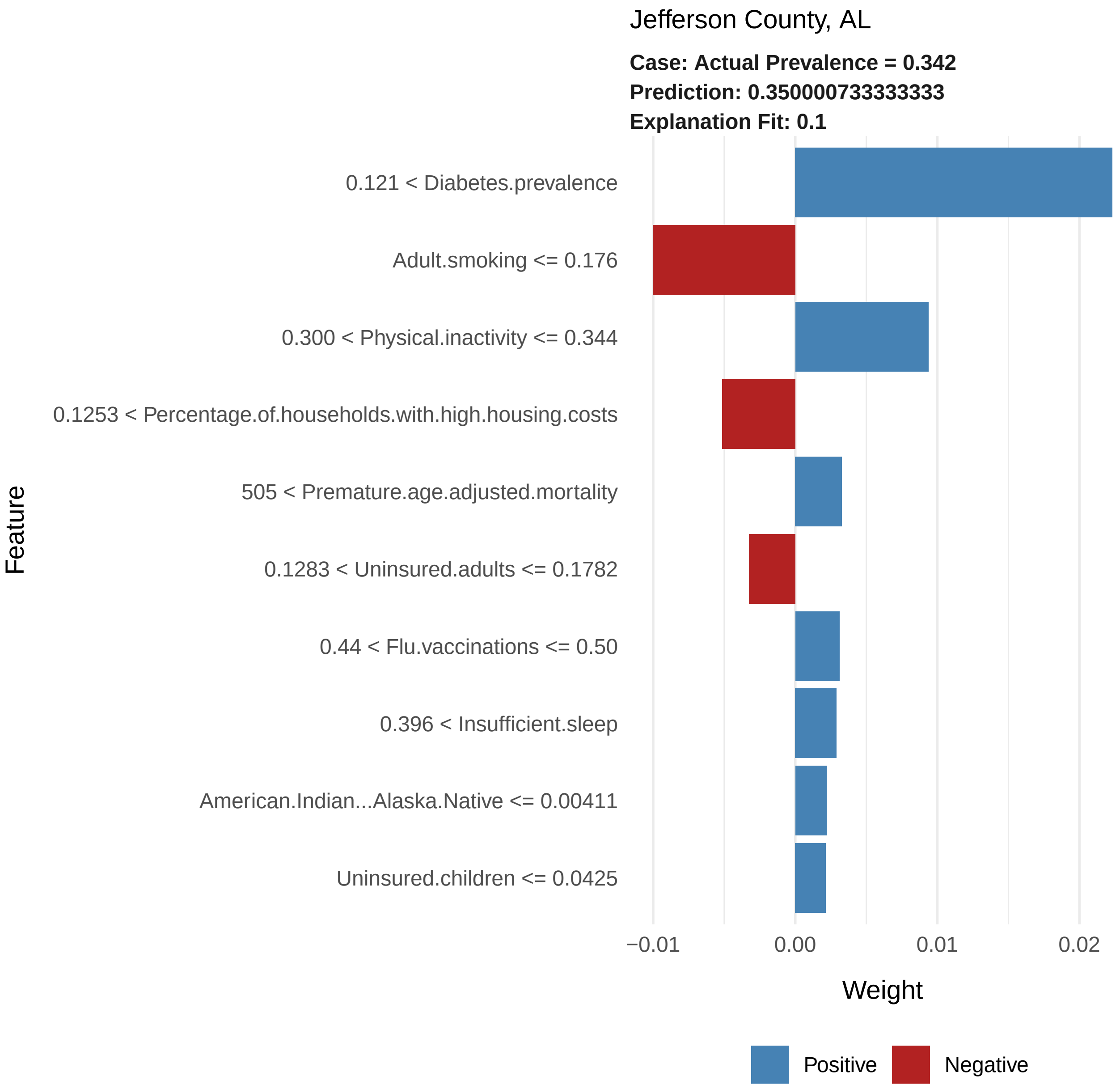
Jefferson County, AL
Case: Actual Prevalence = 0.342
Prediction: 0.350000733333333
Explanation Fit: 0.1
0.121 < Diabetes.prevalence
Adult.smoking <= 0.176
0.300 < Physical.inactivity <= 0.344
0.1253 < Percentage.of.households.with.high.housing.costs
505 < Premature.age.adjusted.mortality
0.1283 < Uninsured.adults <= 0.1782
0.44 < Flu.vaccinations <= 0.50
0.396 < Insufficient.sleep
American.Indian...Alaska.Native <= 0.00411
Uninsured.children <= 0.0425
Feature
−0.01
0.00
0.01
0.02
Weight
Positive
Negative

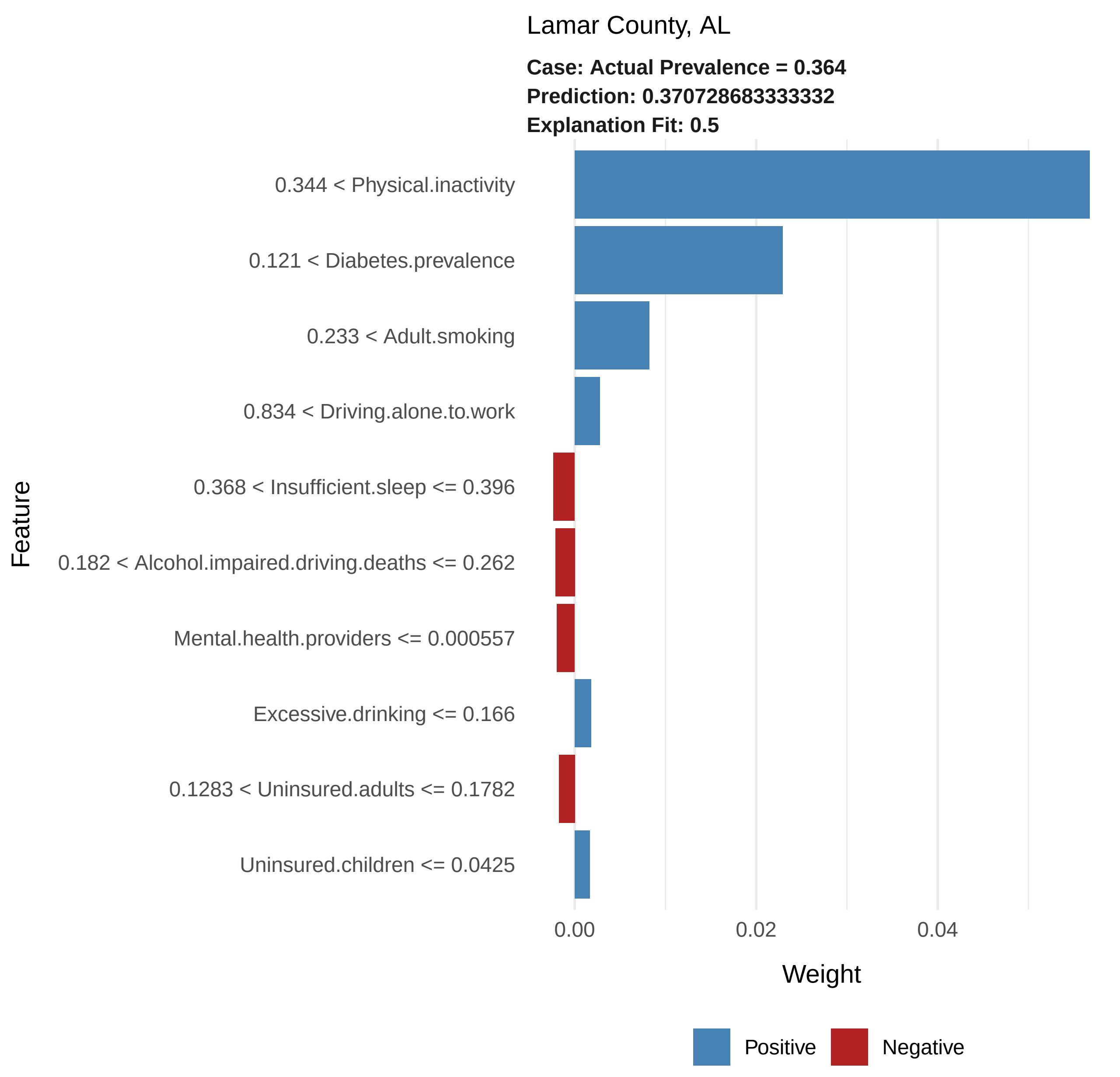

Lamar County, AL
Case: Actual Prevalence = 0.364
Prediction: 0.370728683333332
Explanation Fit: 0.5
0.344 < Physical.inactivity
0.121 < Diabetes.prevalence
0.233 < Adult.smoking
0.834 < Driving.alone.to.work
0.368 < Insufficient.sleep <= 0.396
0.182 < Alcohol.impaired.driving.deaths <= 0.262
Mental.health.providers <= 0.000557
Excessive.drinking <= 0.166
0.1283 < Uninsured.adults <= 0.1782
Uninsured.children <= 0.0425
Feature
0.00
0.02
0.04
Weight
Positive
Negative

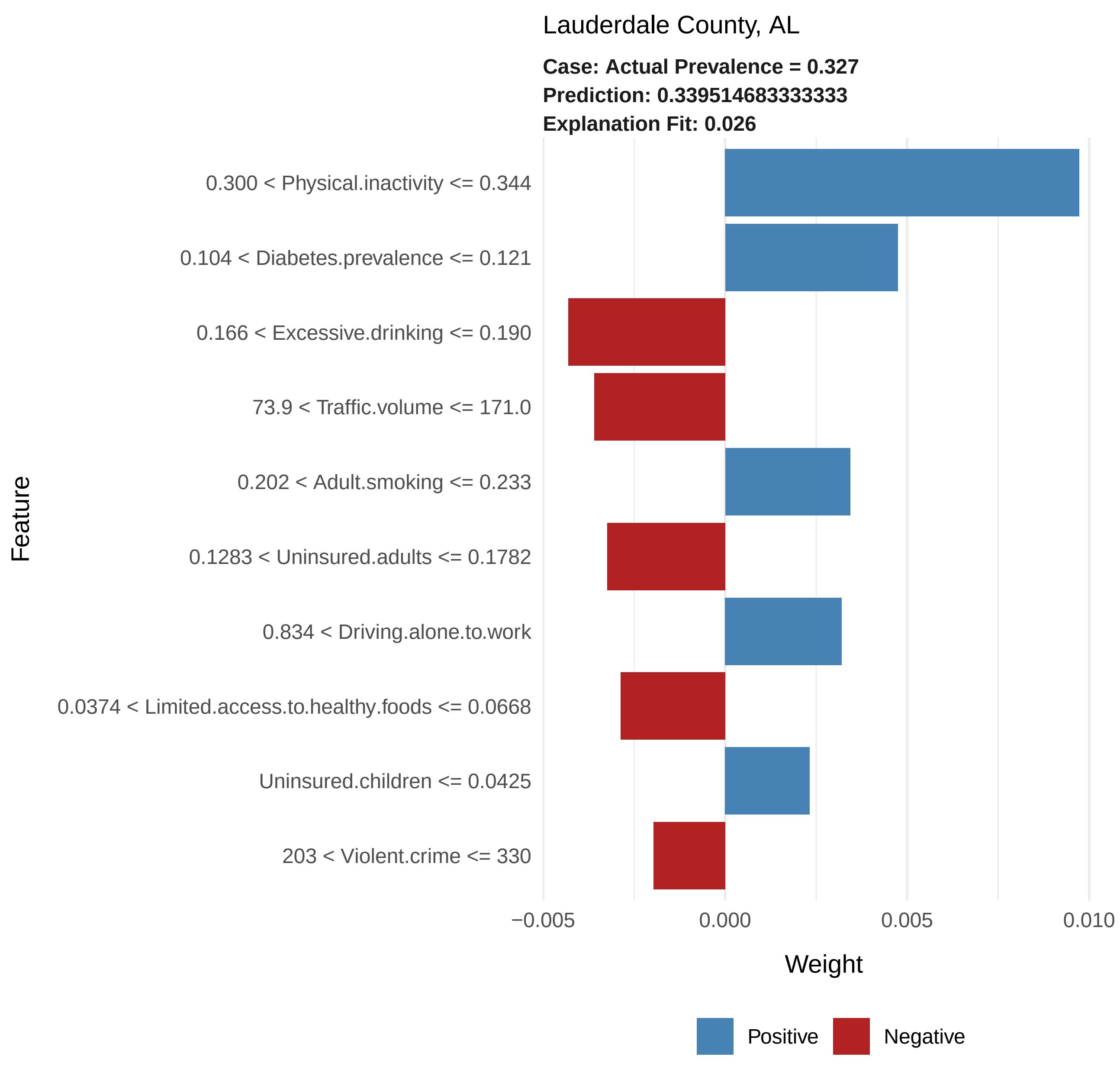
Lauderdale County, AL
Case: Actual Prevalence = 0.327
Prediction: 0.339514683333333
Explanation Fit: 0.026
Feature
0.300 < Physical.inactivity <= 0.344
0.104 < Diabetes.prevalence <= 0.121
0.166 < Excessive.drinking <= 0.190
73.9 < Traffic.volume <= 171.0
0.202 < Adult.smoking <= 0.233
0.1283 < Uninsured.adults <= 0.1782
0.834 < Driving.alone.to.work
0.0374 < Limited.access.to.healthy.foods <= 0.0668
Uninsured.children <= 0.0425
203 < Violent.crime <= 330
−0.005
0.000
0.005
0.010
Weight
Positive
Negative

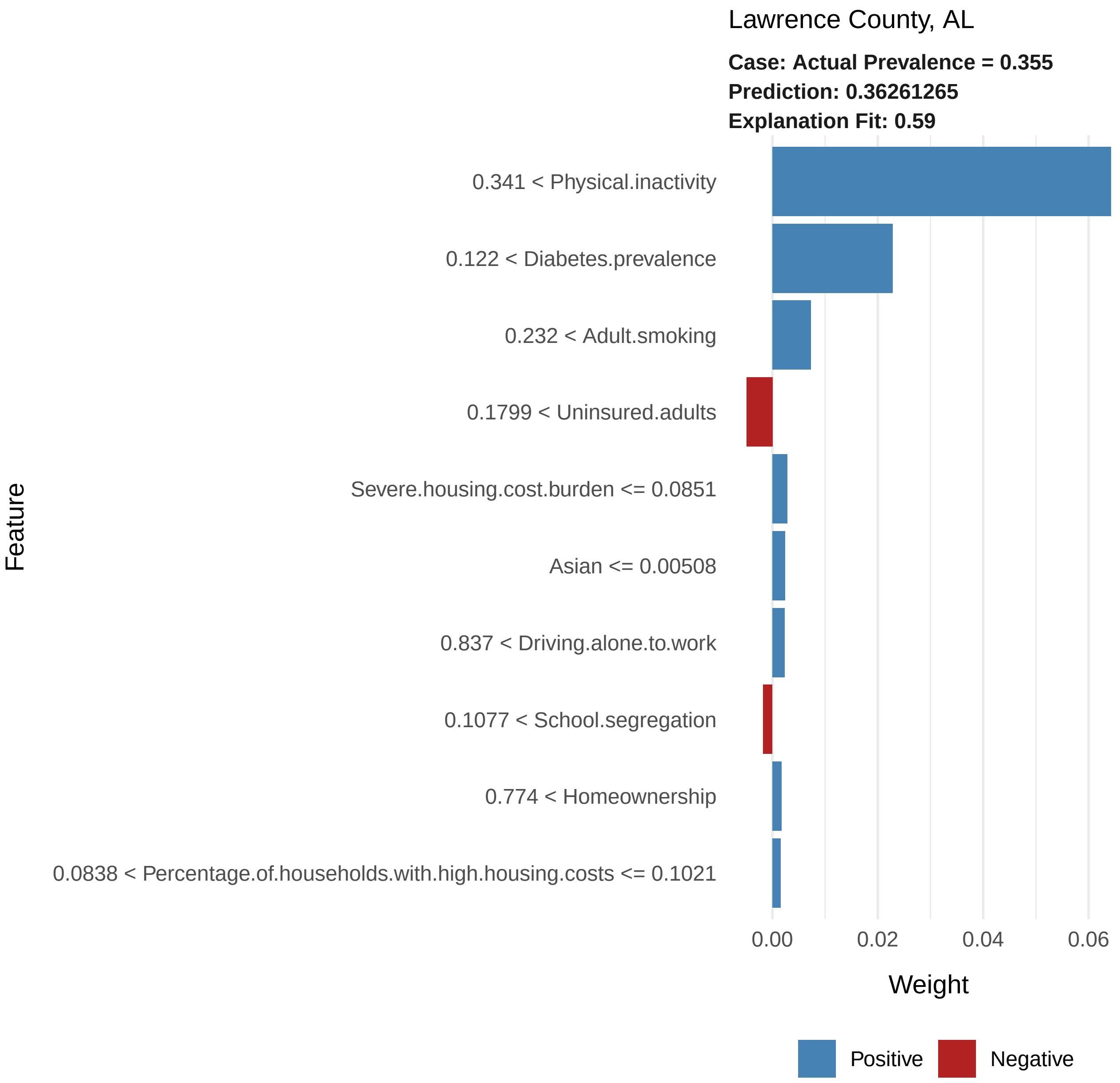
Lawrence County, AL
Case: Actual Prevalence = 0.355
Prediction: 0.36261265
Explanation Fit: 0.59
0.341 < Physical.inactivity
0.122 < Diabetes.prevalence
0.232 < Adult.smoking
0.1799 < Uninsured.adults
Severe.housing.cost.burden <= 0.0851
Asian <= 0.00508
0.837 < Driving.alone.to.work
0.1077 < School.segregation
0.774 < Homeownership
0.0838 < Percentage.of.households.with.high.housing.costs <= 0.1021
Feature
0.00
0.02
0.04
0.06
Weight
Positive
Negative

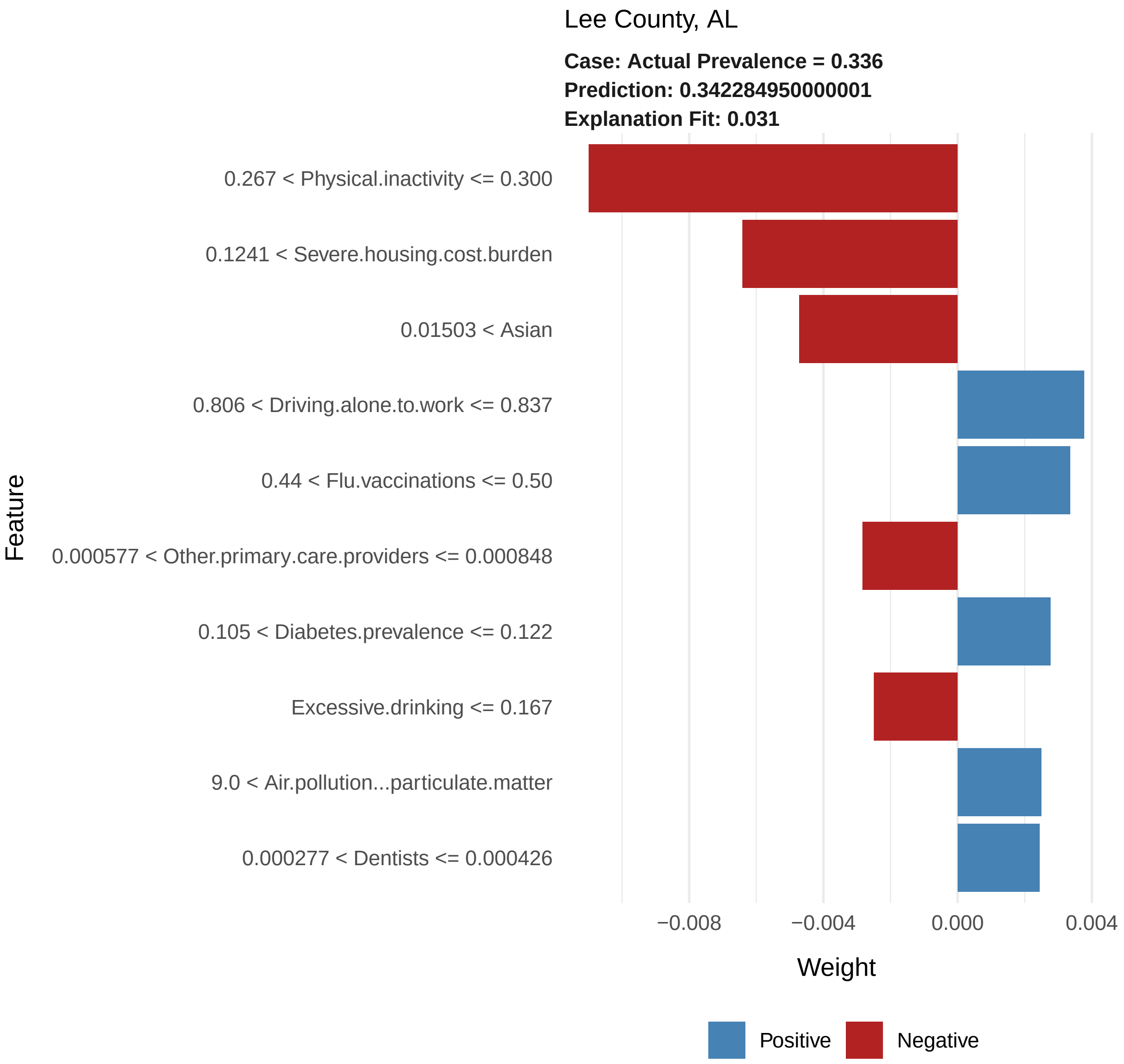
Lee County, AL
Case: Actual Prevalence = 0.336
Prediction: 0.342284950000001
Explanation Fit: 0.031
0.267 < Physical.inactivity <= 0.300
0.1241 < Severe.housing.cost.burden
0.01503 < Asian
0.806 < Driving.alone.to.work <= 0.837
0.44 < Flu.vaccinations <= 0.50
0.000577 < Other.primary.care.providers <= 0.000848
0.105 < Diabetes.prevalence <= 0.122
Excessive.drinking <= 0.167
9.0 < Air.pollution...particulate.matter
0.000277 < Dentists <= 0.000426
Feature
−0.008
−0.004
0.000
0.004
Weight
Positive
Negative

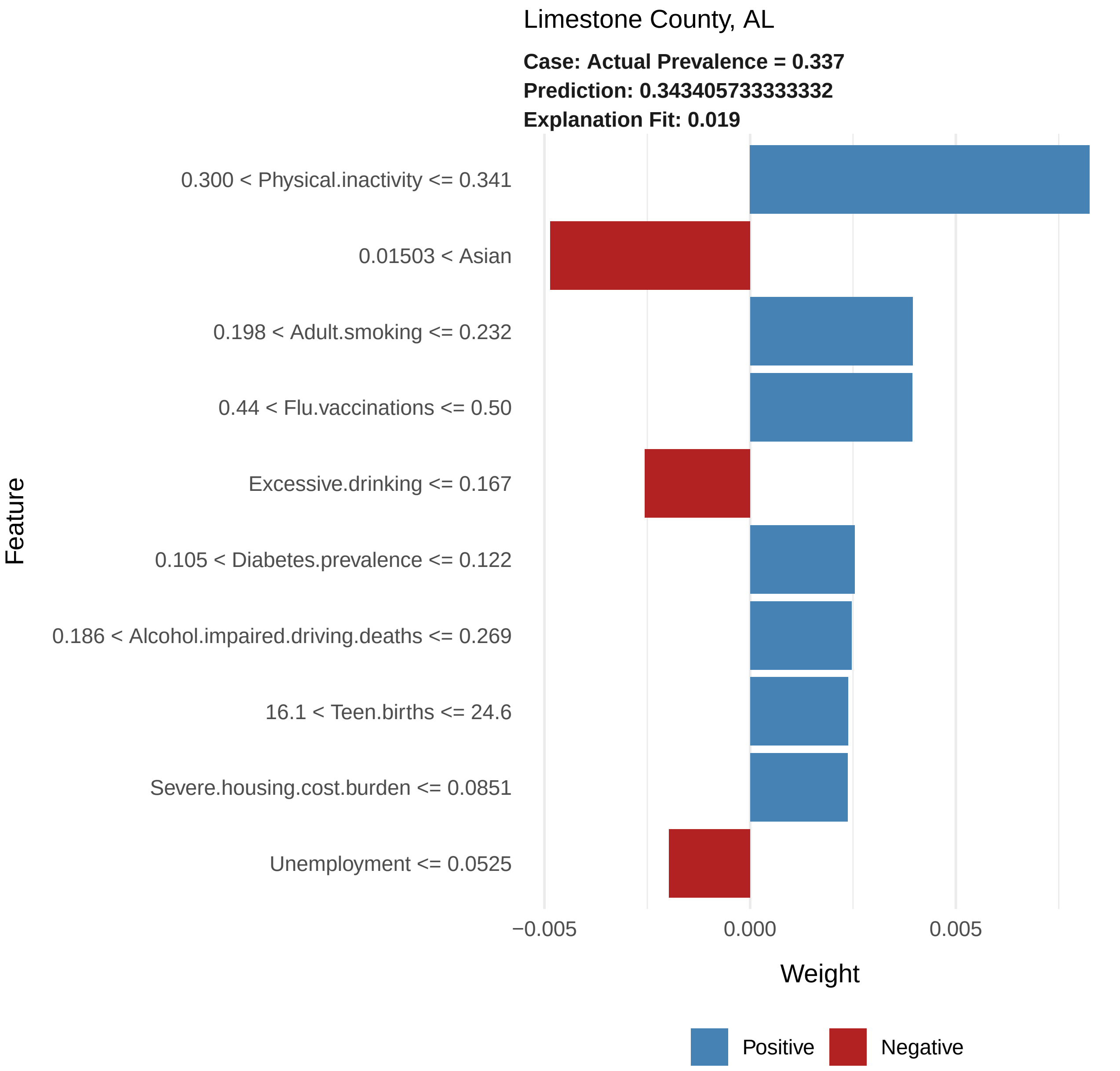

Limestone County, AL
Case: Actual Prevalence = 0.337
Prediction: 0.343405733333332
Explanation Fit: 0.019
0.300 < Physical.inactivity <= 0.341
0.01503 < Asian
0.198 < Adult.smoking <= 0.232
0.44 < Flu.vaccinations <= 0.50
Excessive.drinking <= 0.167
0.105 < Diabetes.prevalence <= 0.122
0.186 < Alcohol.impaired.driving.deaths <= 0.269
16.1 < Teen.births <= 24.6
Severe.housing.cost.burden <= 0.0851
Unemployment <= 0.0525
Feature
−0.005
0.000
0.005
Weight
Positive
Negative

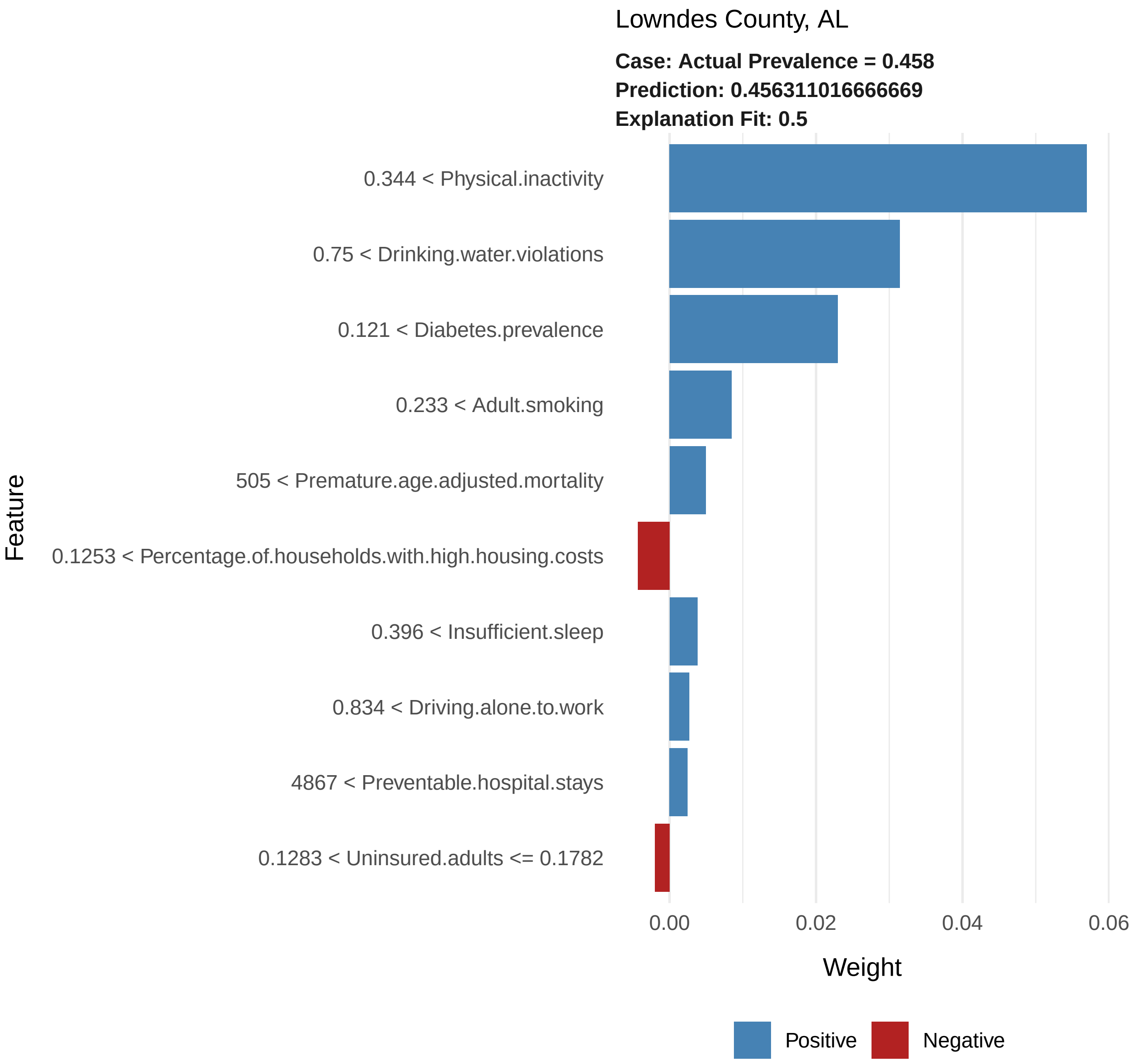

Lowndes County, AL
Case: Actual Prevalence = 0.458
Prediction: 0.456311016666669
Explanation Fit: 0.5
0.344 < Physical.inactivity
0.75 < Drinking.water.violations
0.121 < Diabetes.prevalence
0.233 < Adult.smoking
505 < Premature.age.adjusted.mortality
0.1253 < Percentage.of.households.with.high.housing.costs
0.396 < Insufficient.sleep
0.834 < Driving.alone.to.work
4867 < Preventable.hospital.stays
0.1283 < Uninsured.adults <= 0.1782
Feature
0.00
0.02
0.04
0.06
Weight
Positive
Negative

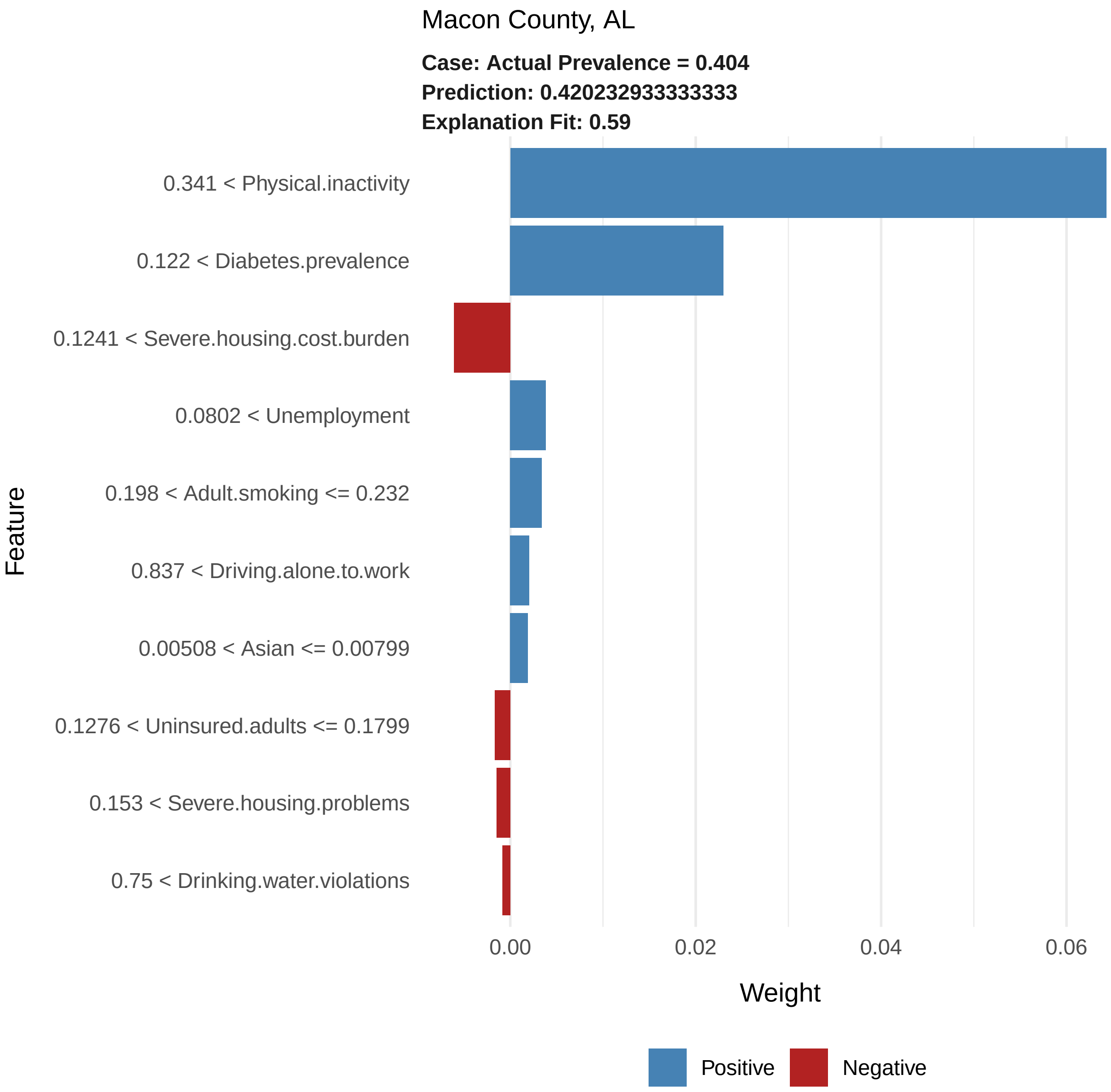

Macon County, AL
Case: Actual Prevalence = 0.404
Prediction: 0.420232933333333
Explanation Fit: 0.59
0.341 < Physical.inactivity
0.122 < Diabetes.prevalence
0.1241 < Severe.housing.cost.burden
0.0802 < Unemployment
0.198 < Adult.smoking <= 0.232
0.837 < Driving.alone.to.work
0.00508 < Asian <= 0.00799
0.1276 < Uninsured.adults <= 0.1799
0.153 < Severe.housing.problems
0.75 < Drinking.water.violations
Feature
0.00
0.02
0.04
0.06
Weight
Positive
Negative

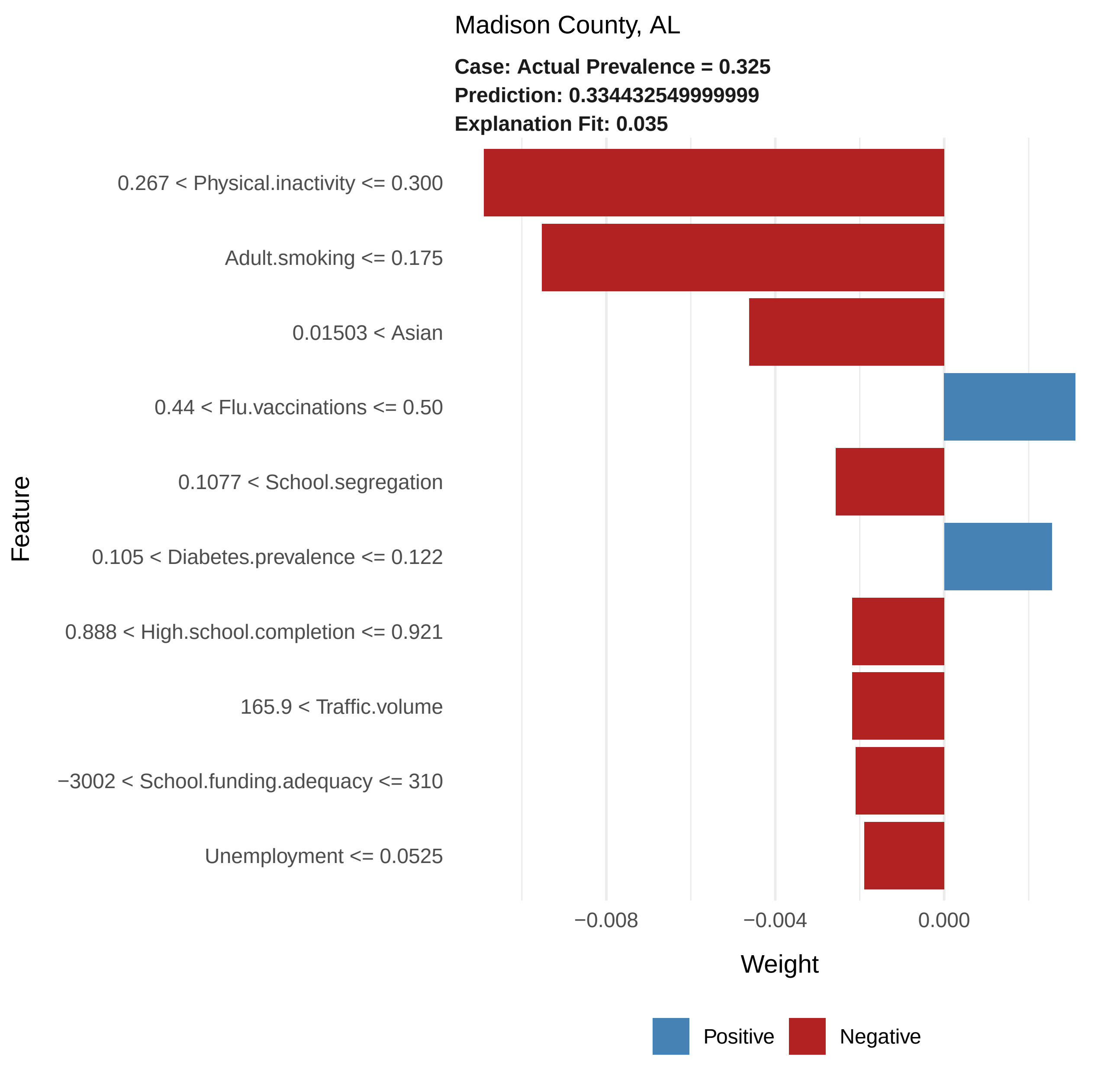

Madison County, AL
Case: Actual Prevalence = 0.325
Prediction: 0.334432549999999
Explanation Fit: 0.035
0.267 < Physical.inactivity <= 0.300
Adult.smoking <= 0.175
0.01503 < Asian
0.44 < Flu.vaccinations <= 0.50
0.1077 < School.segregation
0.105 < Diabetes.prevalence <= 0.122
0.888 < High.school.completion <= 0.921
165.9 < Traffic.volume
−3002 < School.funding.adequacy <= 310
Unemployment <= 0.0525
Feature
−0.008
−0.004
0.000
Weight
Positive
Negative

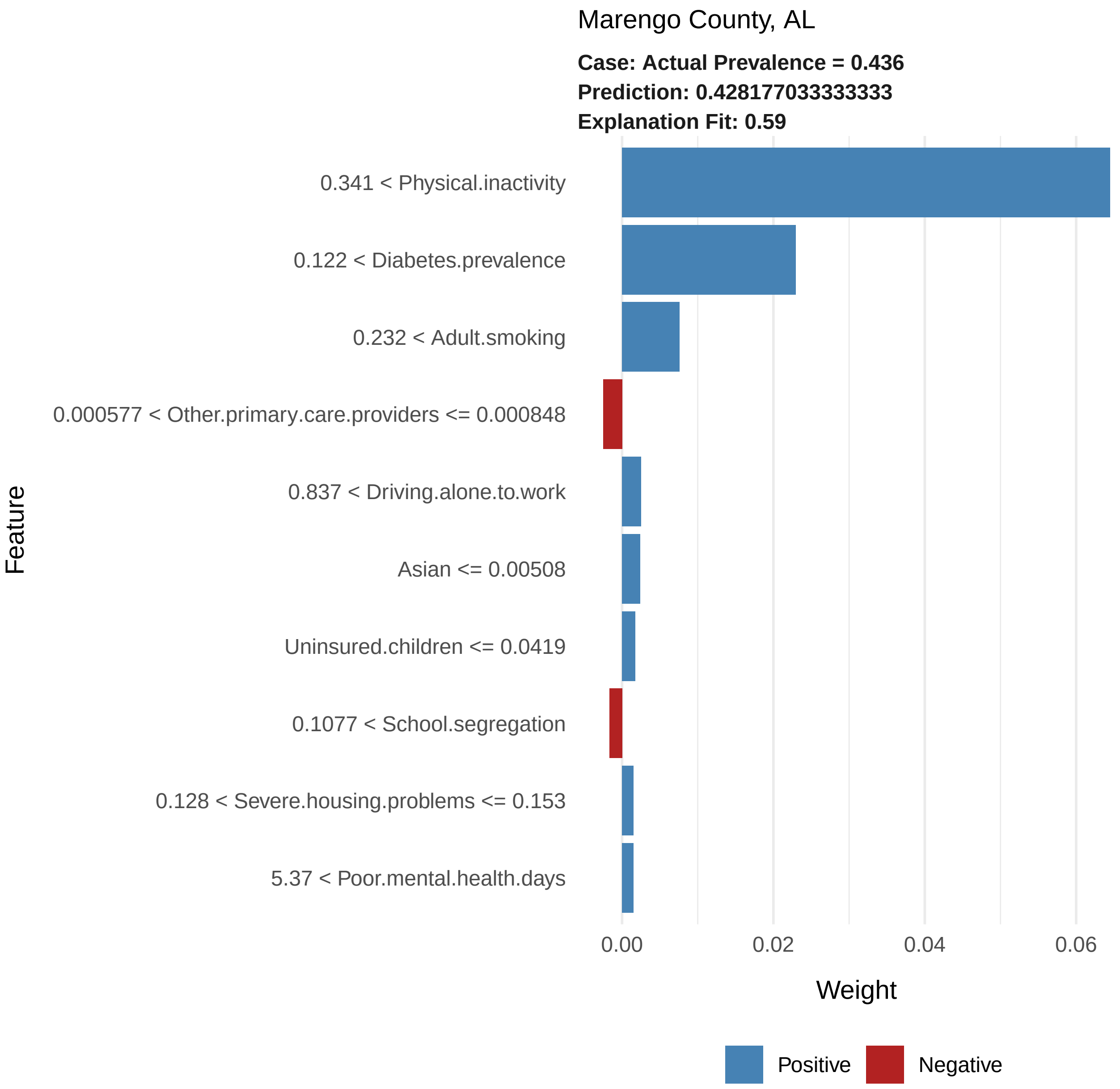
Marengo County, AL
Case: Actual Prevalence = 0.436
Prediction: 0.428177033333333
Explanation Fit: 0.59
0.341 < Physical.inactivity
0.122 < Diabetes.prevalence
0.232 < Adult.smoking
0.000577 < Other.primary.care.providers <= 0.000848
0.837 < Driving.alone.to.work
Asian <= 0.00508
Uninsured.children <= 0.0419
0.1077 < School.segregation
0.128 < Severe.housing.problems <= 0.153
5.37 < Poor.mental.health.days
Feature
0.00
0.02
0.04
0.06
Weight
Positive
Negative

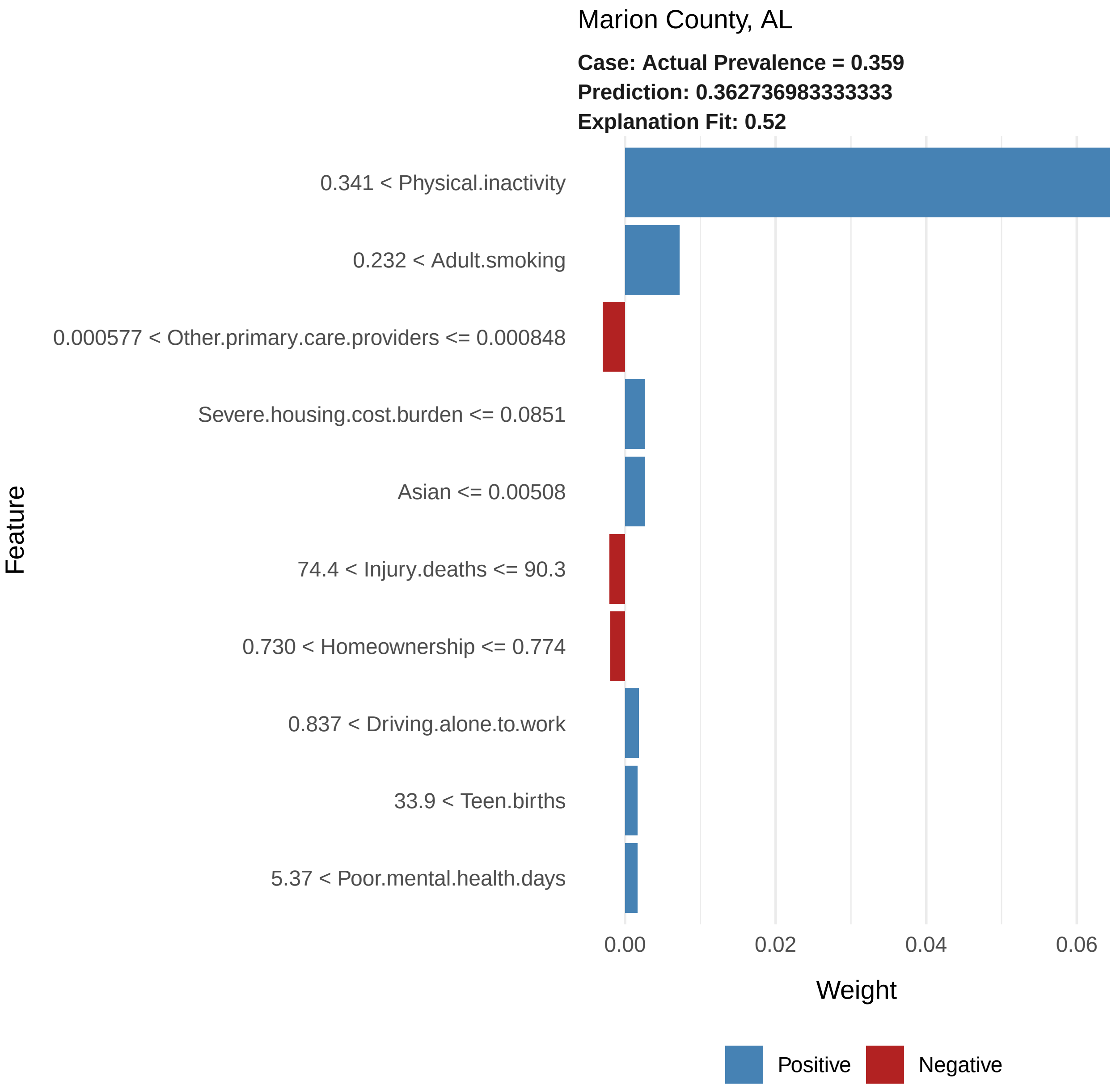
Marion County, AL
Case: Actual Prevalence = 0.359
Prediction: 0.362736983333333
Explanation Fit: 0.52
0.341 < Physical.inactivity
0.232 < Adult.smoking
0.000577 < Other.primary.care.providers <= 0.000848
Severe.housing.cost.burden <= 0.0851
Asian <= 0.00508
74.4 < Injury.deaths <= 90.3
0.730 < Homeownership <= 0.774
0.837 < Driving.alone.to.work
33.9 < Teen.births
5.37 < Poor.mental.health.days
Feature
0.00
0.02
0.04
0.06
Weight
Positive
Negative

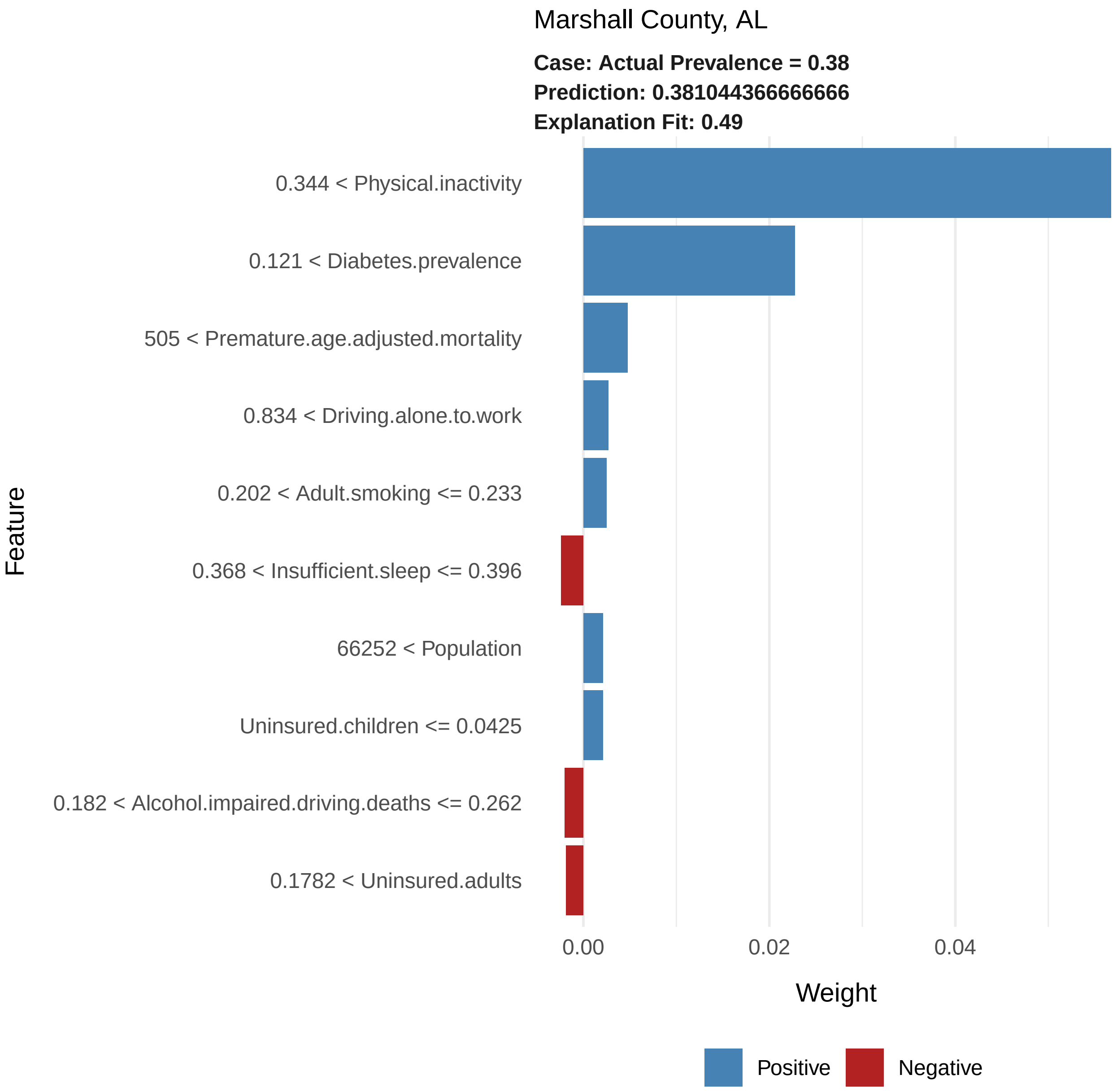

Marshall County, AL
Case: Actual Prevalence = 0.38
Prediction: 0.381044366666666
Explanation Fit: 0.49
0.344 < Physical.inactivity
0.121 < Diabetes.prevalence
505 < Premature.age.adjusted.mortality
0.834 < Driving.alone.to.work
0.202 < Adult.smoking <= 0.233
0.368 < Insufficient.sleep <= 0.396
66252 < Population
Uninsured.children <= 0.0425
0.182 < Alcohol.impaired.driving.deaths <= 0.262
0.1782 < Uninsured.adults
Feature
0.00
0.02
0.04
Weight
Positive
Negative

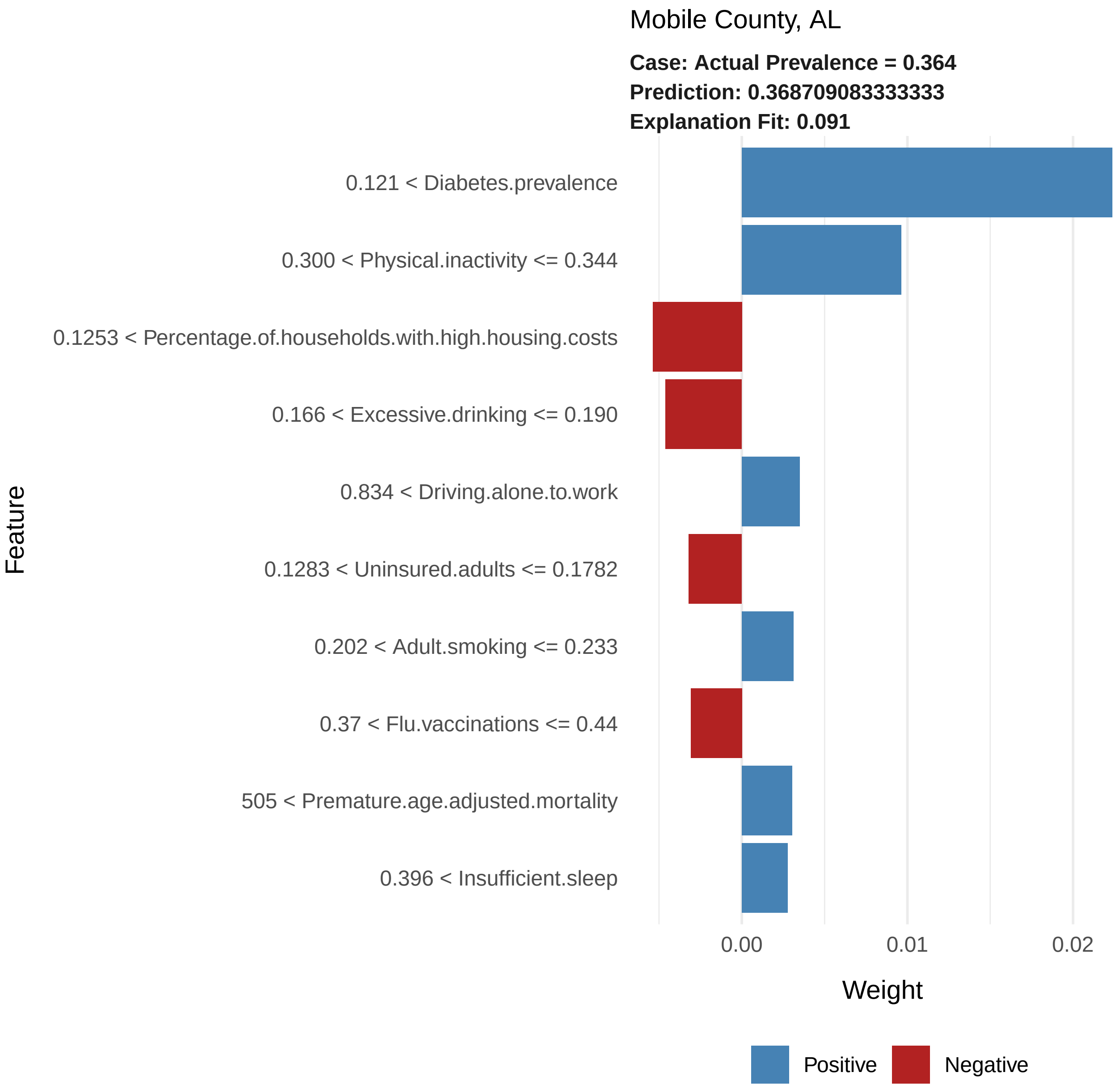

Mobile County, AL
Case: Actual Prevalence = 0.364
Prediction: 0.368709083333333
Explanation Fit: 0.091
0.121 < Diabetes.prevalence
0.300 < Physical.inactivity <= 0.344
0.1253 < Percentage.of.households.with.high.housing.costs
0.166 < Excessive.drinking <= 0.190
0.834 < Driving.alone.to.work
0.1283 < Uninsured.adults <= 0.1782
0.202 < Adult.smoking <= 0.233
0.37 < Flu.vaccinations <= 0.44
505 < Premature.age.adjusted.mortality
0.396 < Insufficient.sleep
Feature
0.00
0.01
0.02
Weight
Positive
Negative

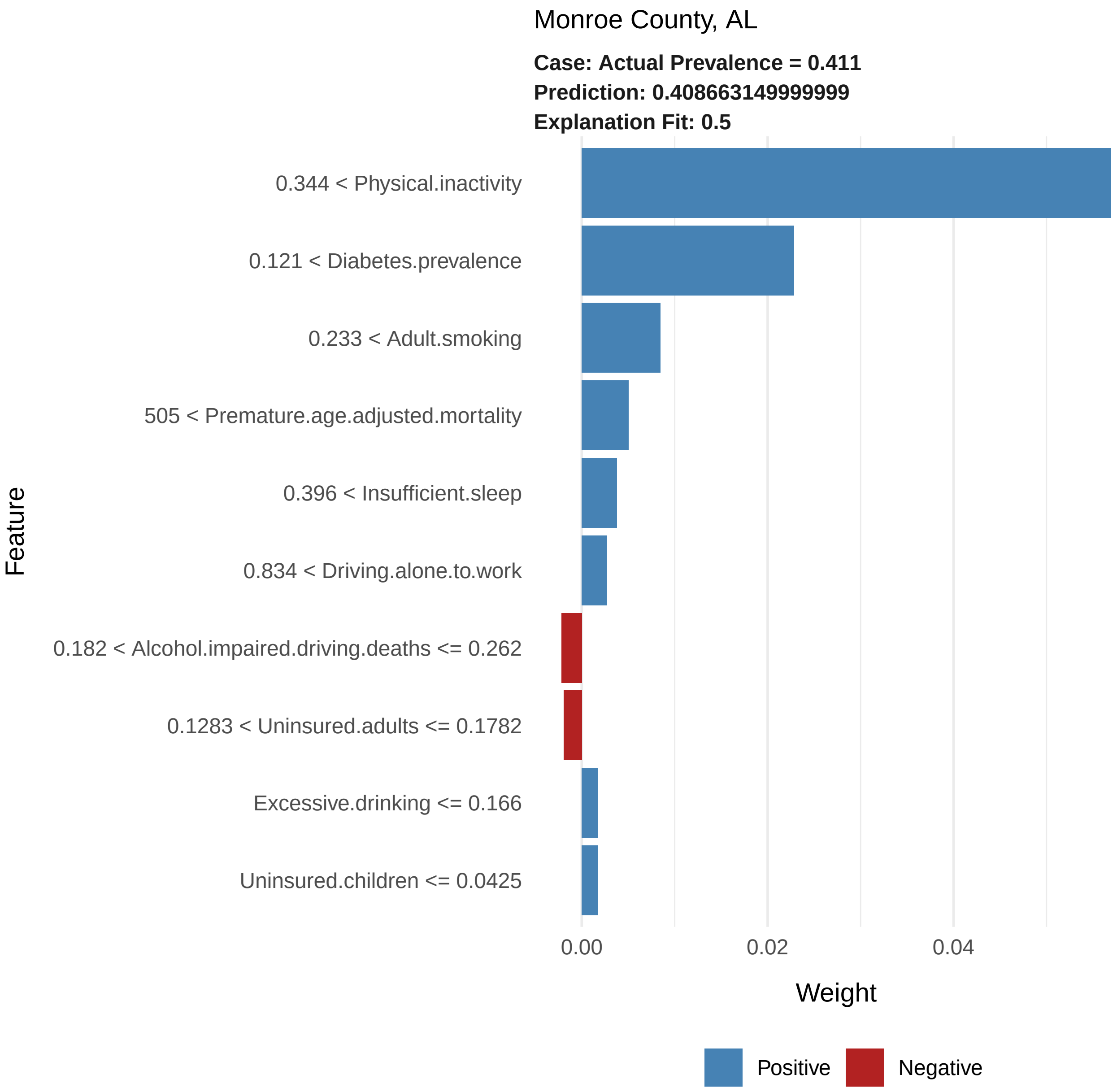
Monroe County, AL
Case: Actual Prevalence = 0.411
Prediction: 0.408663149999999
Explanation Fit: 0.5
0.344 < Physical.inactivity
0.121 < Diabetes.prevalence
0.233 < Adult.smoking
505 < Premature.age.adjusted.mortality
0.396 < Insufficient.sleep
0.834 < Driving.alone.to.work
0.182 < Alcohol.impaired.driving.deaths <= 0.262
0.1283 < Uninsured.adults <= 0.1782
Excessive.drinking <= 0.166
Uninsured.children <= 0.0425
Feature
0.00
0.02
0.04
Weight
Positive
Negative

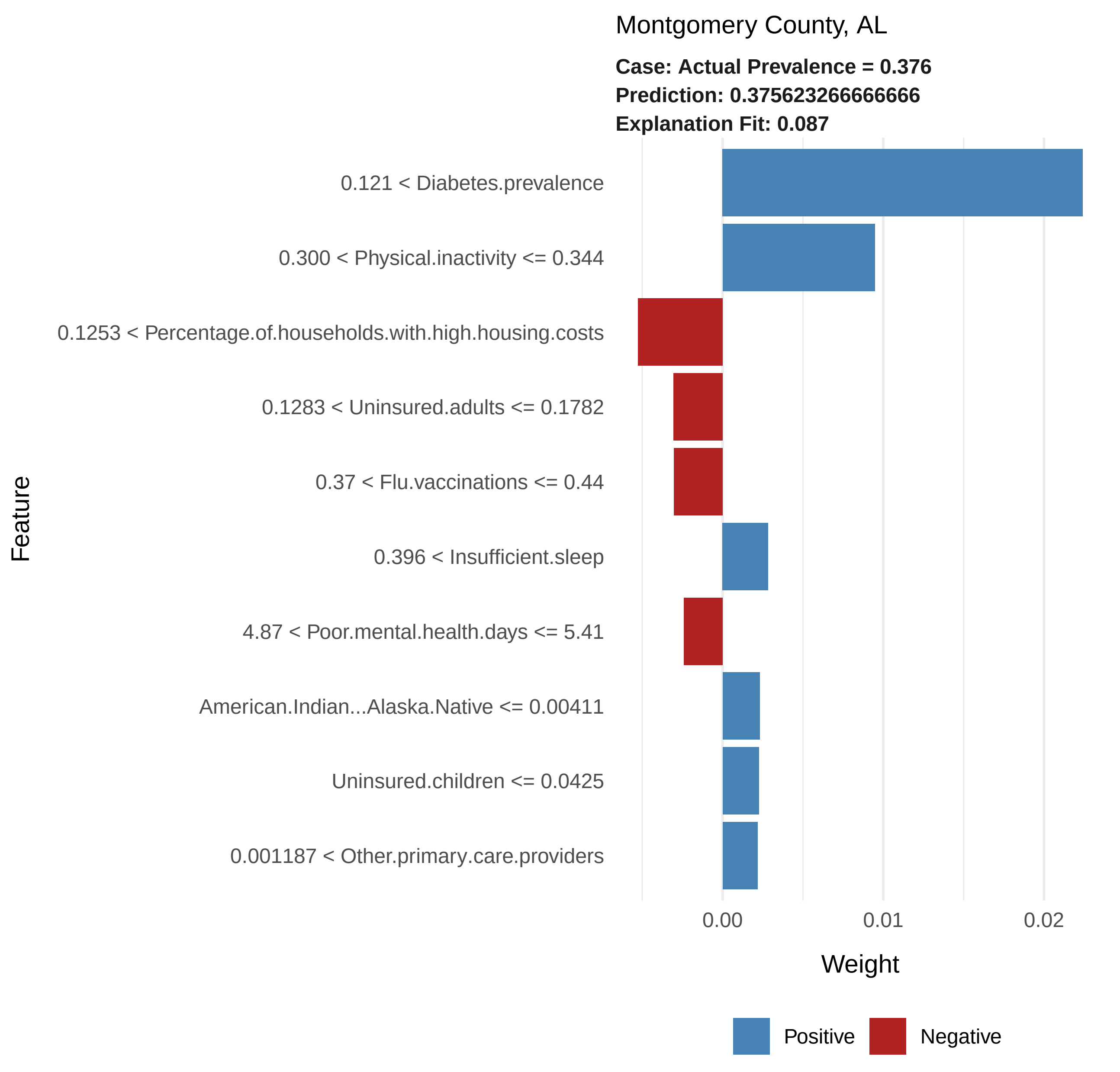

Montgomery County, AL
Case: Actual Prevalence = 0.376
Prediction: 0.375623266666666
Explanation Fit: 0.087
0.121 < Diabetes.prevalence
0.300 < Physical.inactivity <= 0.344
0.1253 < Percentage.of.households.with.high.housing.costs
0.1283 < Uninsured.adults <= 0.1782
0.37 < Flu.vaccinations <= 0.44
0.396 < Insufficient.sleep
4.87 < Poor.mental.health.days <= 5.41
American.Indian...Alaska.Native <= 0.00411
Uninsured.children <= 0.0425
0.001187 < Other.primary.care.providers
Feature
0.00
0.01
0.02
Weight
Positive
Negative

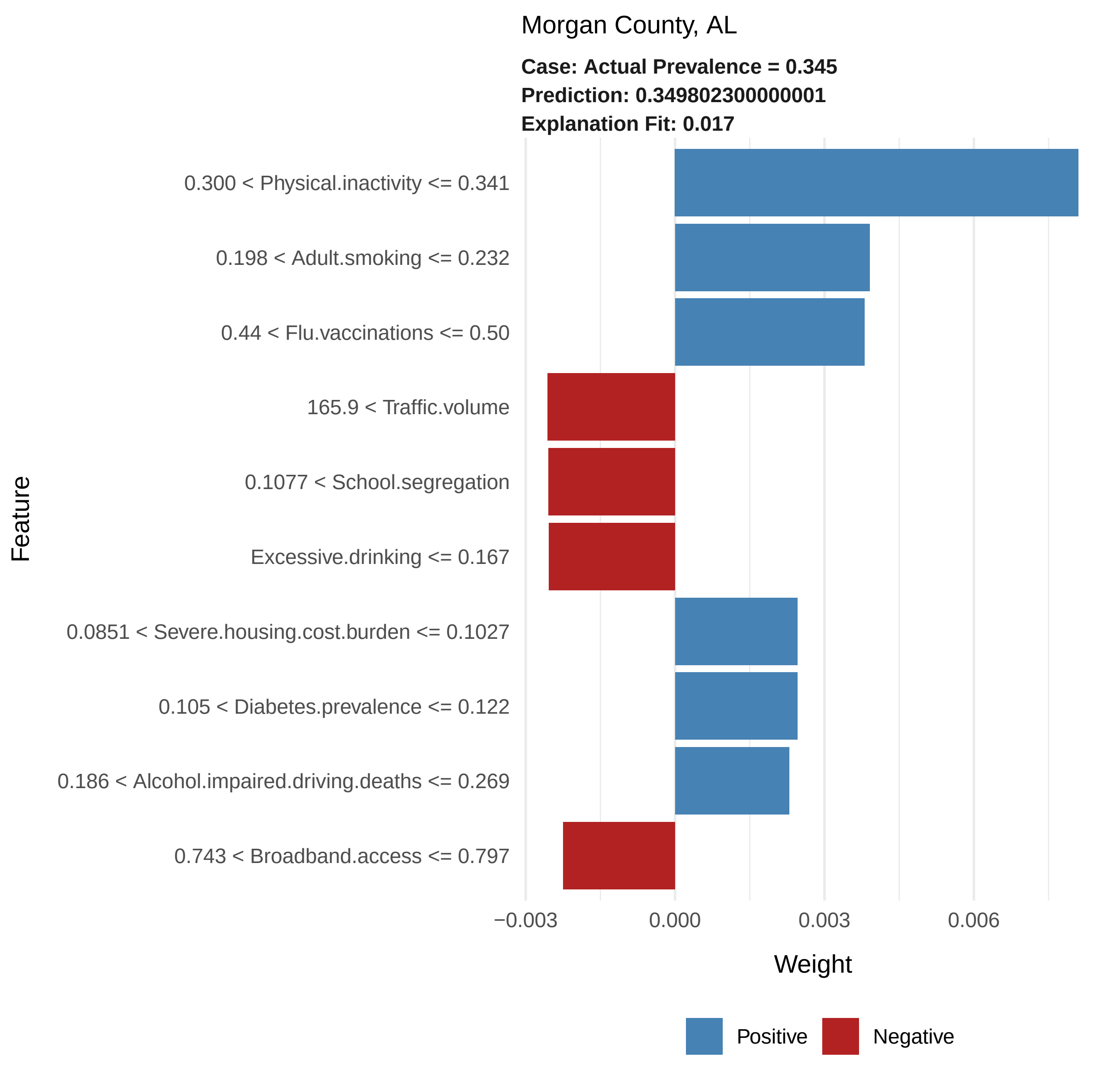

Morgan County, AL
Case: Actual Prevalence = 0.345
Prediction: 0.349802300000001
Explanation Fit: 0.017
0.300 < Physical.inactivity <= 0.341
0.198 < Adult.smoking <= 0.232
0.44 < Flu.vaccinations <= 0.50
165.9 < Traffic.volume
0.1077 < School.segregation
Excessive.drinking <= 0.167
0.0851 < Severe.housing.cost.burden <= 0.1027
0.105 < Diabetes.prevalence <= 0.122
0.186 < Alcohol.impaired.driving.deaths <= 0.269
0.743 < Broadband.access <= 0.797
Feature
−0.003
0.000
0.003
0.006
Weight
Positive
Negative

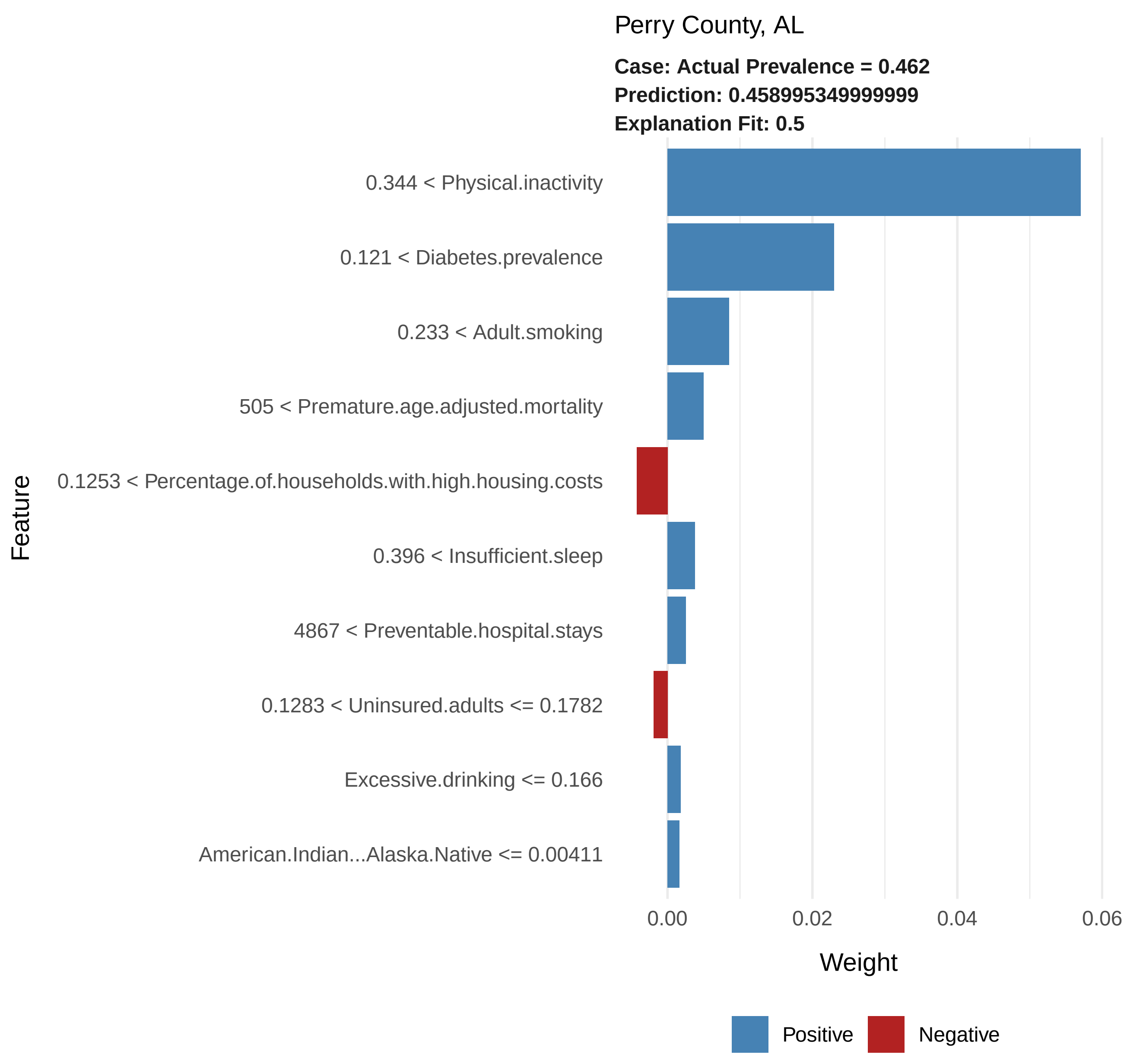

Perry County, AL
Case: Actual Prevalence = 0.462
Prediction: 0.458995349999999
Explanation Fit: 0.5
0.344 < Physical.inactivity
0.121 < Diabetes.prevalence
0.233 < Adult.smoking
505 < Premature.age.adjusted.mortality
0.1253 < Percentage.of.households.with.high.housing.costs
0.396 < Insufficient.sleep
4867 < Preventable.hospital.stays
0.1283 < Uninsured.adults <= 0.1782
Excessive.drinking <= 0.166
American.Indian...Alaska.Native <= 0.00411
Feature
0.00
0.02
0.04
0.06
Weight
Positive
Negative

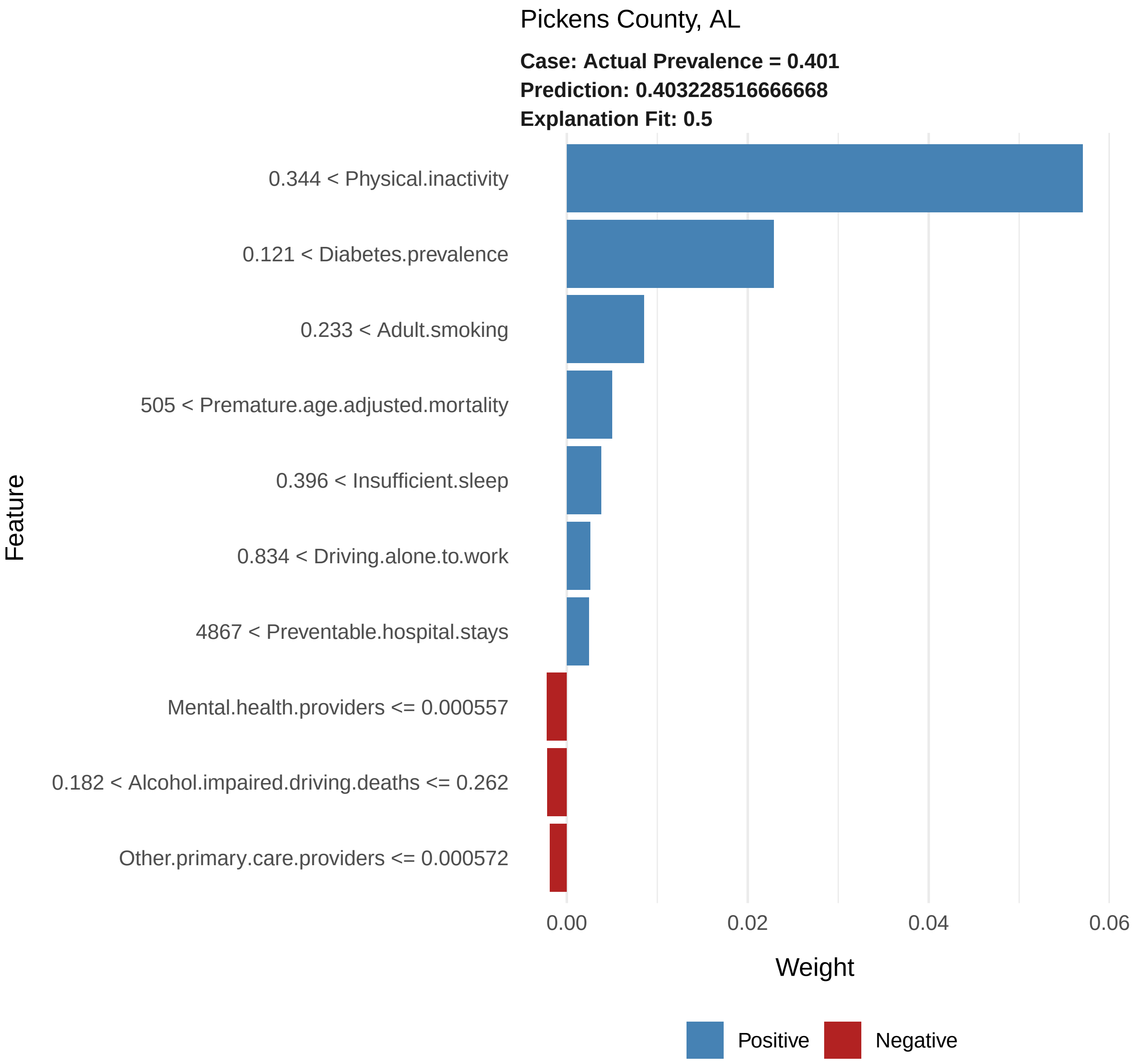

Pickens County, AL
Case: Actual Prevalence = 0.401
Prediction: 0.403228516666668
Explanation Fit: 0.5
0.344 < Physical.inactivity
0.121 < Diabetes.prevalence
0.233 < Adult.smoking
505 < Premature.age.adjusted.mortality
0.396 < Insufficient.sleep
0.834 < Driving.alone.to.work
4867 < Preventable.hospital.stays
Mental.health.providers <= 0.000557
0.182 < Alcohol.impaired.driving.deaths <= 0.262
Other.primary.care.providers <= 0.000572
Feature
0.00
0.02
0.04
0.06
Weight
Positive
Negative

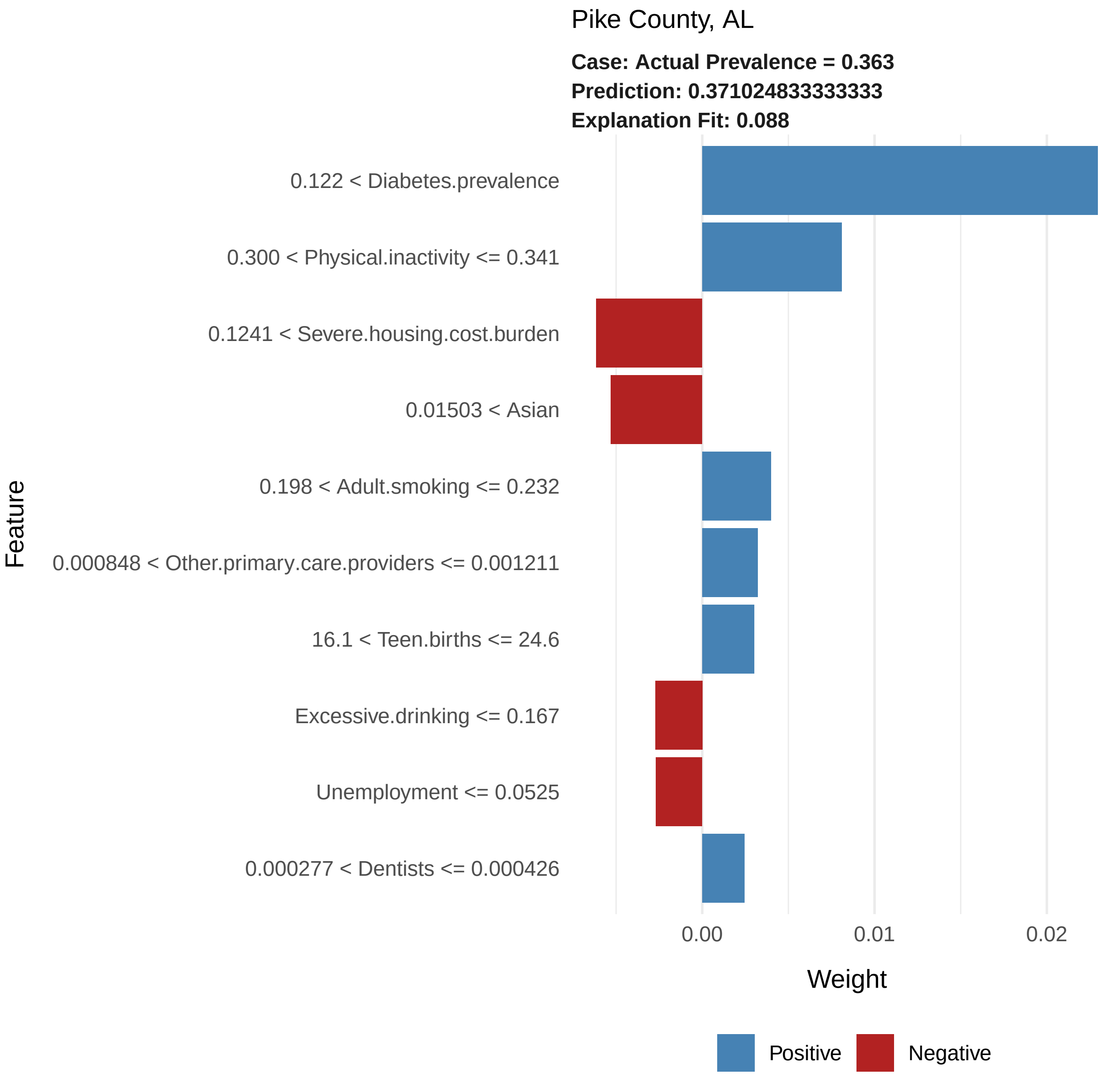

Pike County, AL
Case: Actual Prevalence = 0.363
Prediction: 0.371024833333333
Explanation Fit: 0.088
0.122 < Diabetes.prevalence
0.300 < Physical.inactivity <= 0.341
0.1241 < Severe.housing.cost.burden
0.01503 < Asian
0.198 < Adult.smoking <= 0.232
0.000848 < Other.primary.care.providers <= 0.001211
16.1 < Teen.births <= 24.6
Excessive.drinking <= 0.167
Unemployment <= 0.0525
0.000277 < Dentists <= 0.000426
Feature
0.00
0.01
0.02
Weight
Positive
Negative

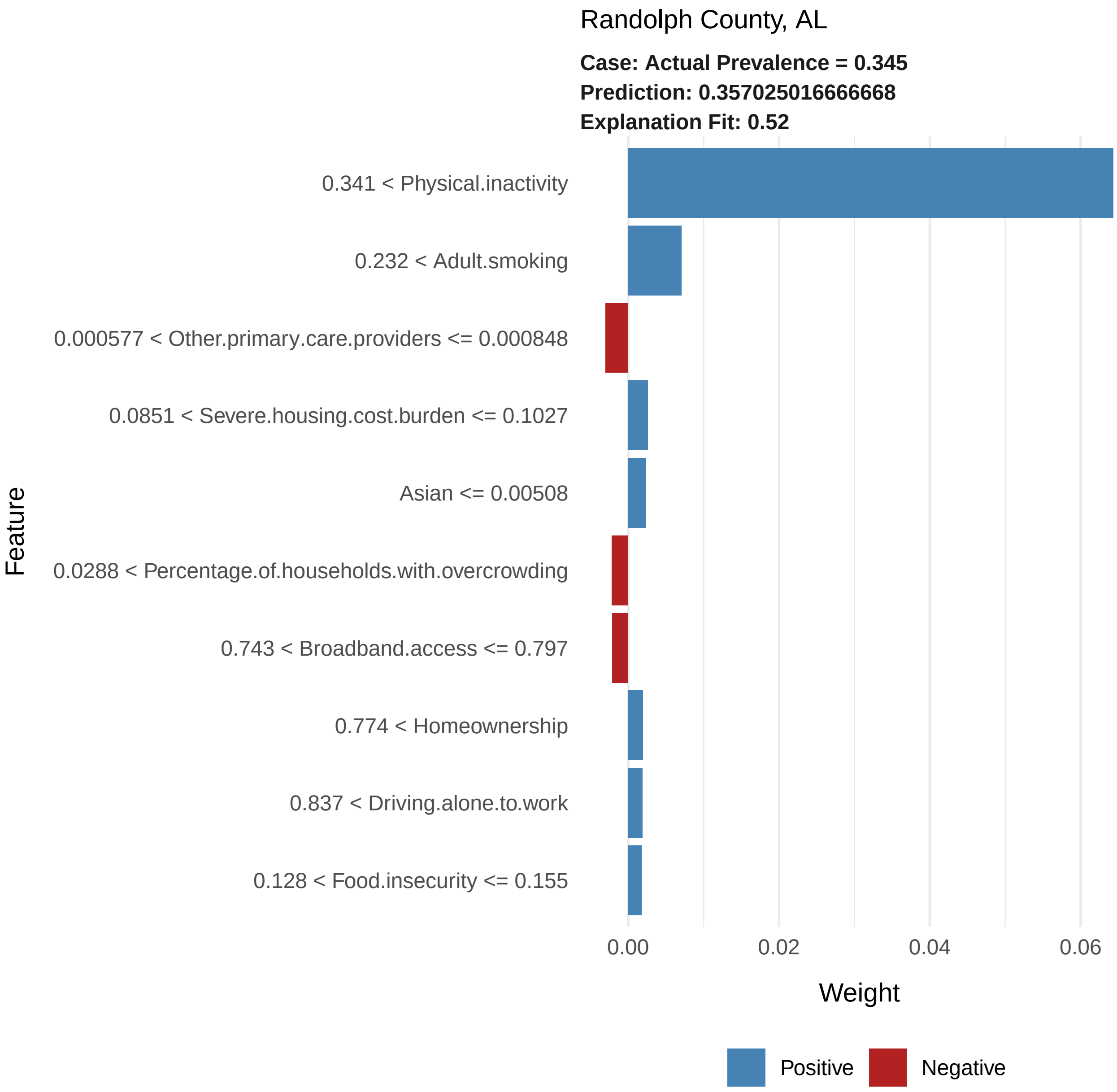
Randolph County, AL
Case: Actual Prevalence = 0.345
Prediction: 0.357025016666668
Explanation Fit: 0.52
0.341 < Physical.inactivity
0.232 < Adult.smoking
0.000577 < Other.primary.care.providers <= 0.000848
0.0851 < Severe.housing.cost.burden <= 0.1027
Asian <= 0.00508
0.0288 < Percentage.of.households.with.overcrowding
0.743 < Broadband.access <= 0.797
0.774 < Homeownership
0.837 < Driving.alone.to.work
0.128 < Food.insecurity <= 0.155
Feature
0.00
0.02
0.04
0.06
Weight
Positive
Negative

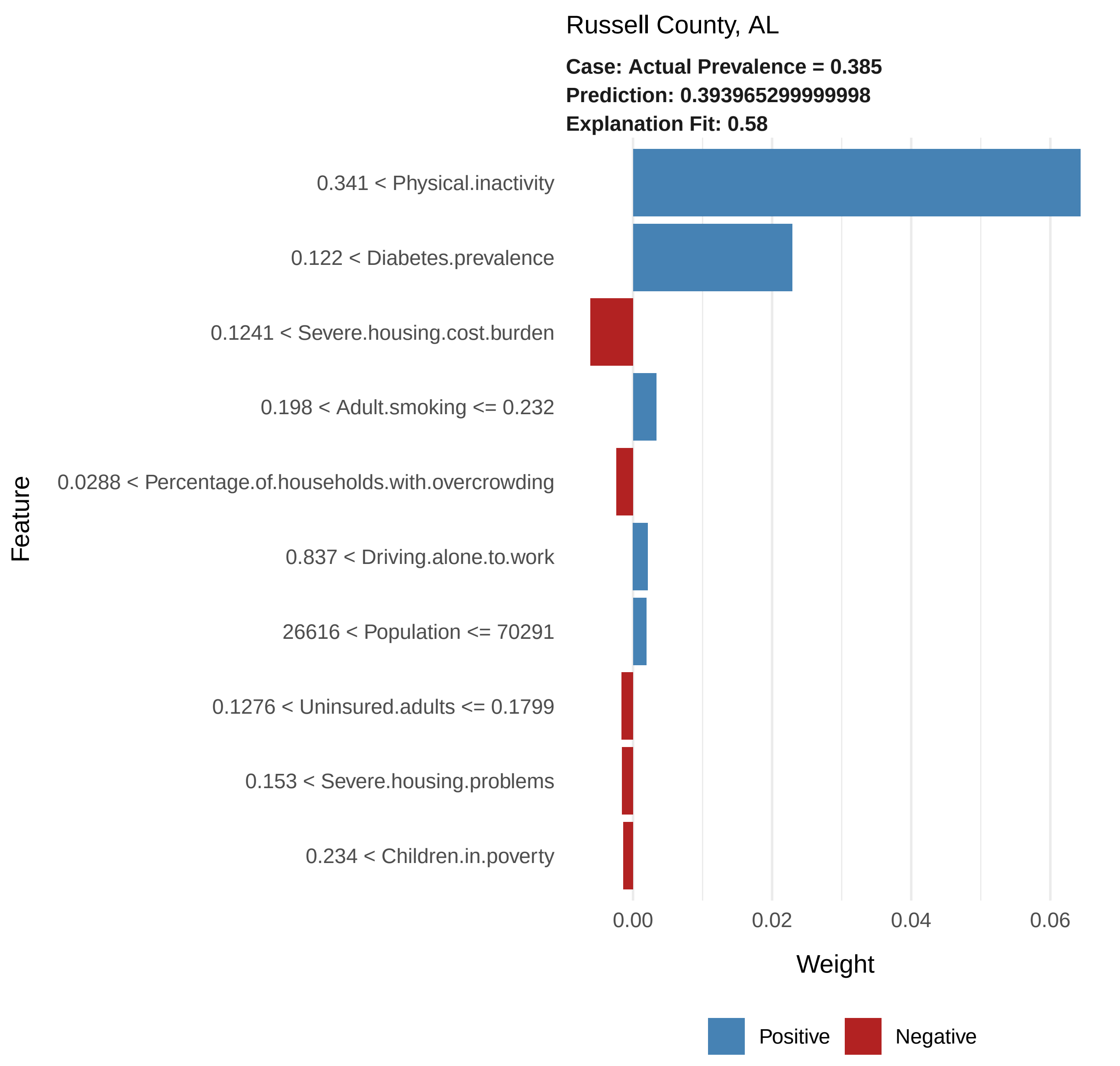

Russell County, AL
Case: Actual Prevalence = 0.385
Prediction: 0.393965299999998
Explanation Fit: 0.58
0.341 < Physical.inactivity
0.122 < Diabetes.prevalence
0.1241 < Severe.housing.cost.burden
0.198 < Adult.smoking <= 0.232
0.0288 < Percentage.of.households.with.overcrowding
0.837 < Driving.alone.to.work
26616 < Population <= 70291
0.1276 < Uninsured.adults <= 0.1799
0.153 < Severe.housing.problems
0.234 < Children.in.poverty
Feature
0.00
0.02
0.04
0.06
Weight
Positive
Negative

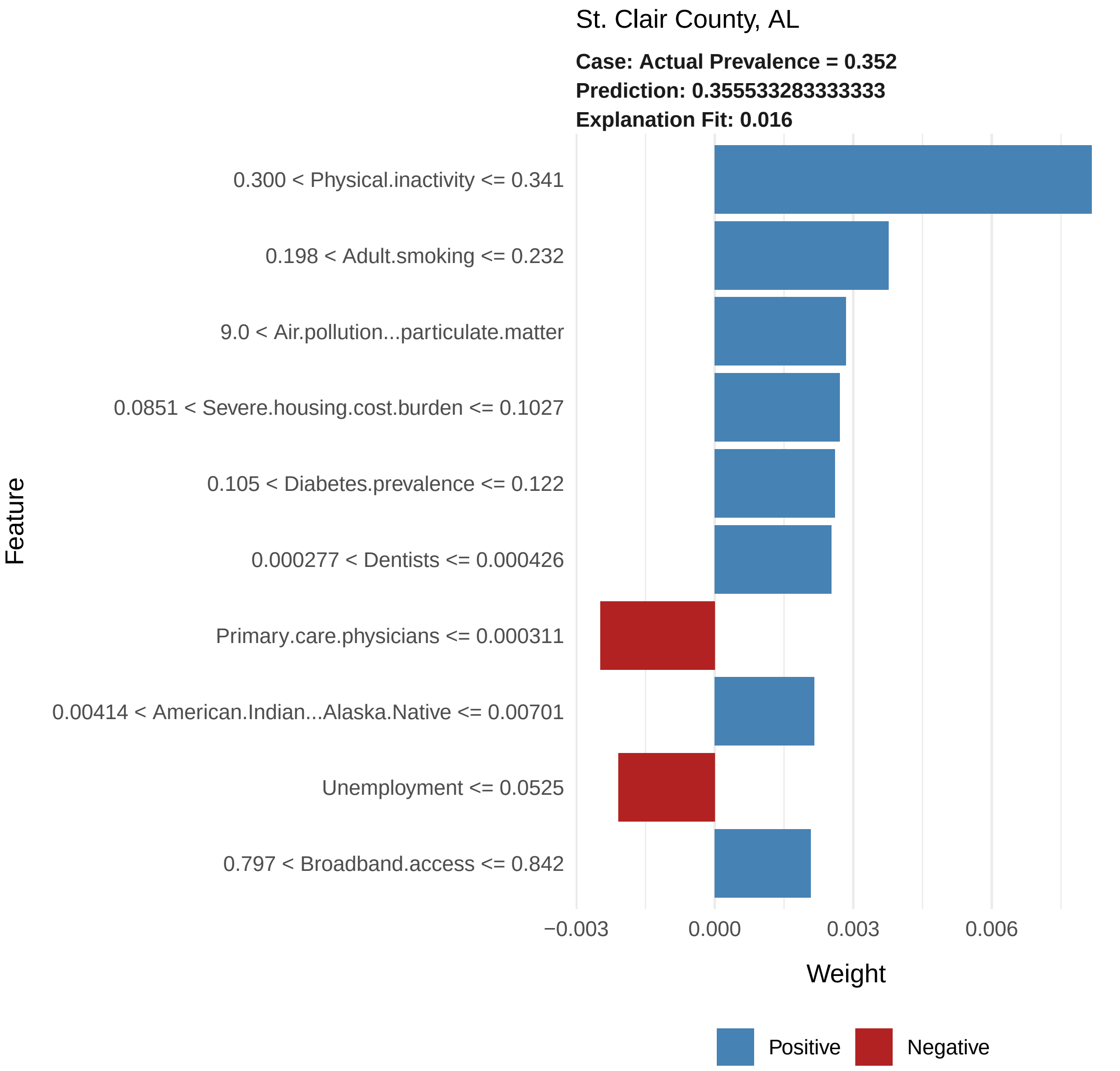

St. Clair County, AL
Case: Actual Prevalence = 0.352
Prediction: 0.355533283333333
Explanation Fit: 0.016
0.300 < Physical.inactivity <= 0.341
0.198 < Adult.smoking <= 0.232
9.0 < Air.pollution...particulate.matter
0.0851 < Severe.housing.cost.burden <= 0.1027
0.105 < Diabetes.prevalence <= 0.122
0.000277 < Dentists <= 0.000426
Primary.care.physicians <= 0.000311
0.00414 < American.Indian...Alaska.Native <= 0.00701
Unemployment <= 0.0525
0.797 < Broadband.access <= 0.842
Feature
−0.003
0.000
0.003
0.006
Weight
Positive
Negative

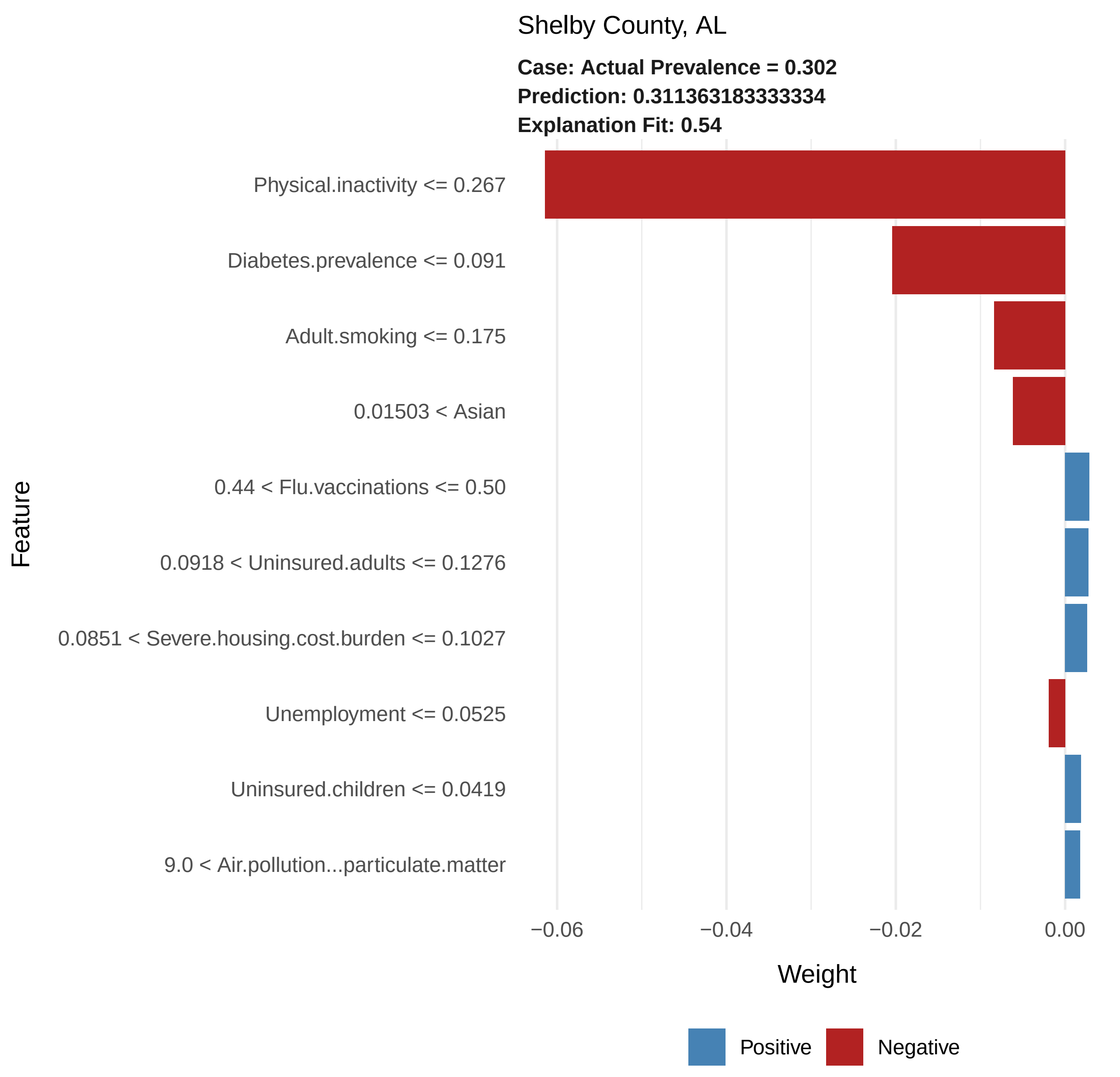

Shelby County, AL
Case: Actual Prevalence = 0.302
Prediction: 0.311363183333334
Explanation Fit: 0.54
Physical.inactivity <= 0.267
Diabetes.prevalence <= 0.091
Adult.smoking <= 0.175
0.01503 < Asian
0.44 < Flu.vaccinations <= 0.50
0.0918 < Uninsured.adults <= 0.1276
0.0851 < Severe.housing.cost.burden <= 0.1027
Unemployment <= 0.0525
Uninsured.children <= 0.0419
9.0 < Air.pollution...particulate.matter
Feature
−0.06
−0.04
−0.02
0.00
Weight
Positive
Negative

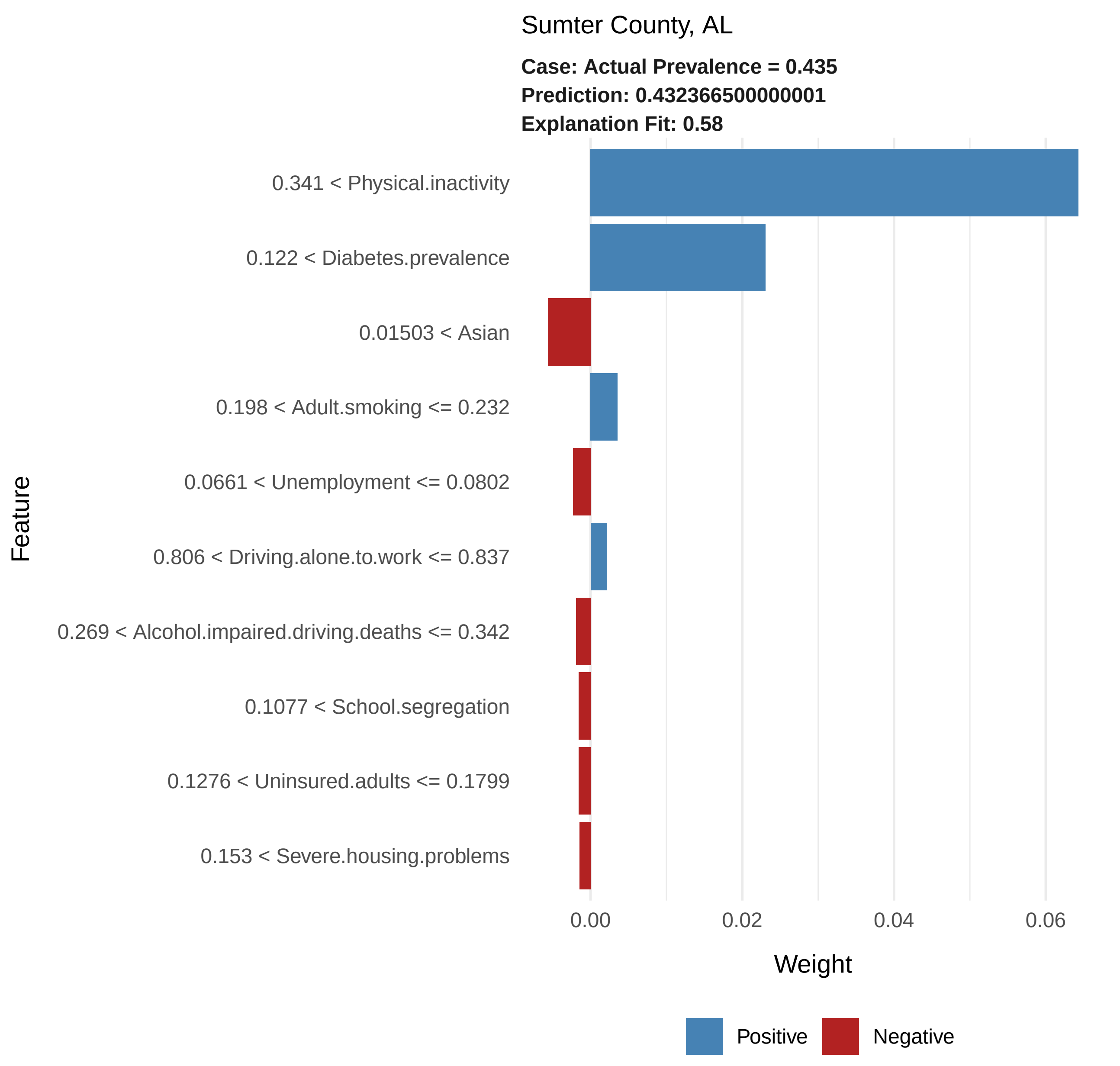

Sumter County, AL
Case: Actual Prevalence = 0.435
Prediction: 0.432366500000001
Explanation Fit: 0.58
0.341 < Physical.inactivity
0.122 < Diabetes.prevalence
0.01503 < Asian
0.198 < Adult.smoking <= 0.232
0.0661 < Unemployment <= 0.0802
0.806 < Driving.alone.to.work <= 0.837
0.269 < Alcohol.impaired.driving.deaths <= 0.342
0.1077 < School.segregation
0.1276 < Uninsured.adults <= 0.1799
0.153 < Severe.housing.problems
Feature
0.00
0.02
0.04
0.06
Weight
Positive
Negative

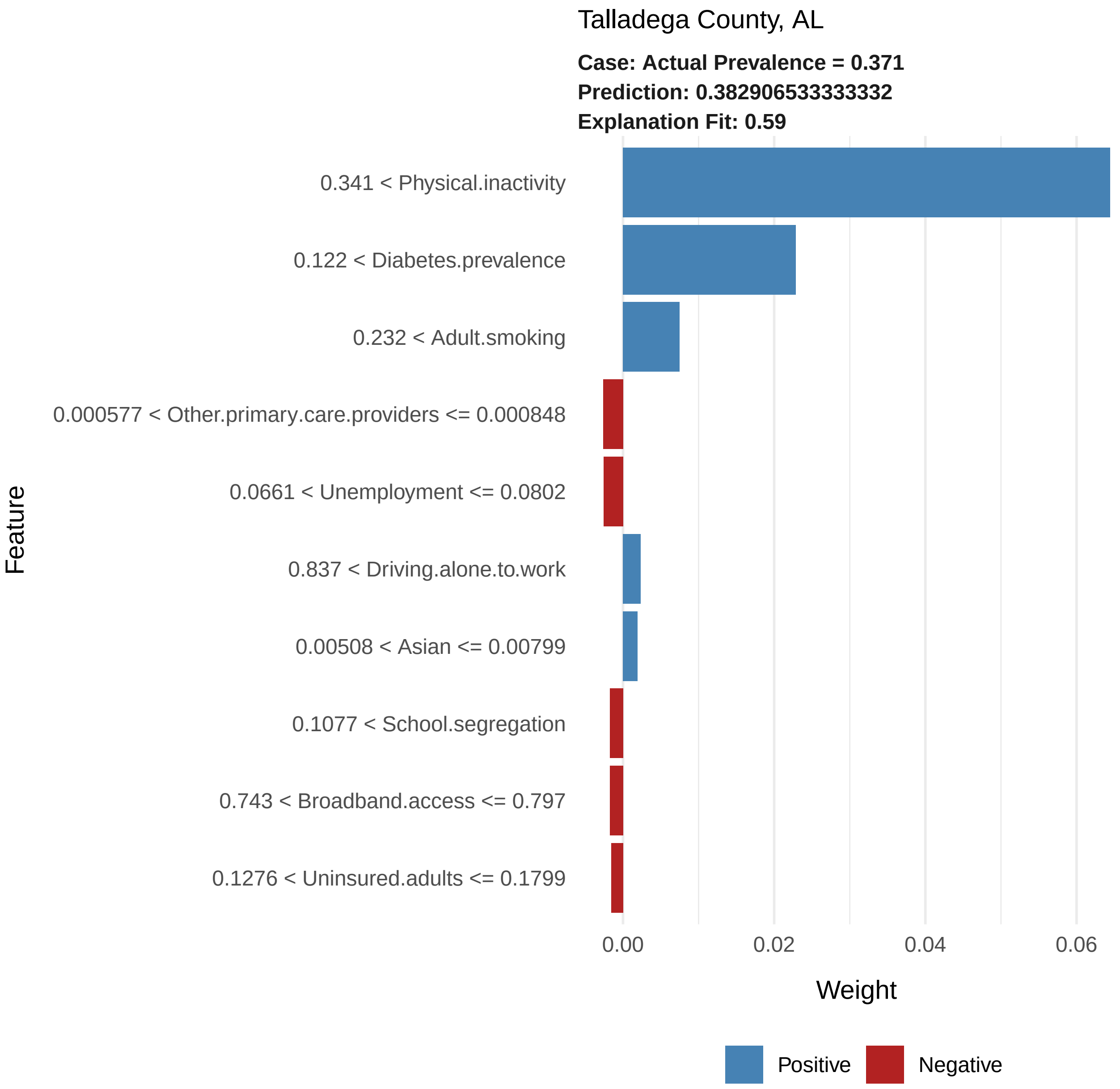
Talladega County, AL
Case: Actual Prevalence = 0.371
Prediction: 0.382906533333332
Explanation Fit: 0.59
0.341 < Physical.inactivity
0.122 < Diabetes.prevalence
0.232 < Adult.smoking
0.000577 < Other.primary.care.providers <= 0.000848
0.0661 < Unemployment <= 0.0802
0.837 < Driving.alone.to.work
0.00508 < Asian <= 0.00799
0.1077 < School.segregation
0.743 < Broadband.access <= 0.797
0.1276 < Uninsured.adults <= 0.1799
Feature
0.00
0.02
0.04
0.06
Weight
Positive
Negative

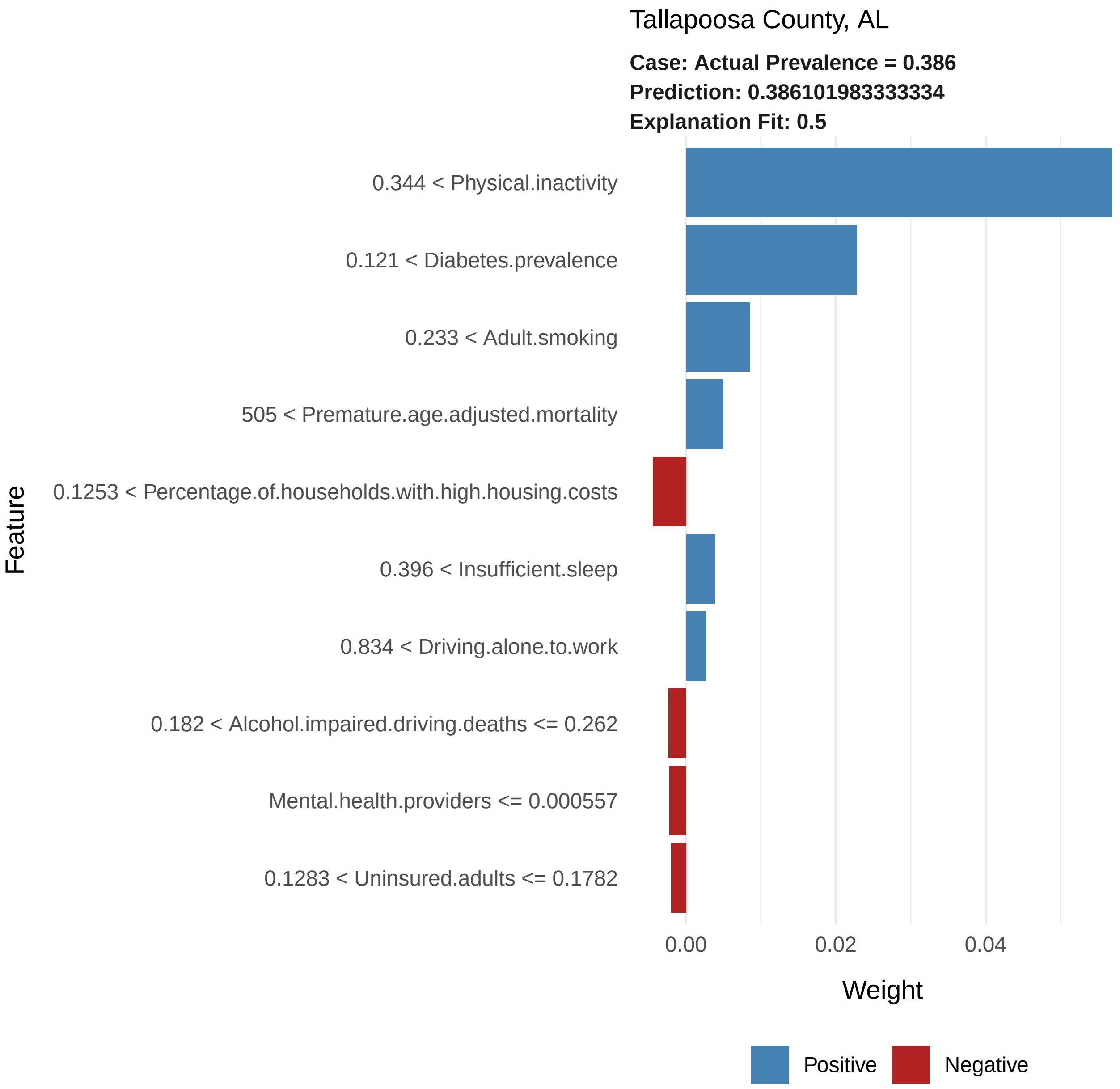

Tallapoosa County, AL
Case: Actual Prevalence = 0.386
Prediction: 0.386101983333334
Explanation Fit: 0.5
0.344 < Physical.inactivity
0.121 < Diabetes.prevalence
0.233 < Adult.smoking
505 < Premature.age.adjusted.mortality
0.1253 < Percentage.of.households.with.high.housing.costs
0.396 < Insufficient.sleep
0.834 < Driving.alone.to.work
0.182 < Alcohol.impaired.driving.deaths <= 0.262
Mental.health.providers <= 0.000557
0.1283 < Uninsured.adults <= 0.1782
Feature
0.00
0.02
0.04
Weight
Positive
Negative

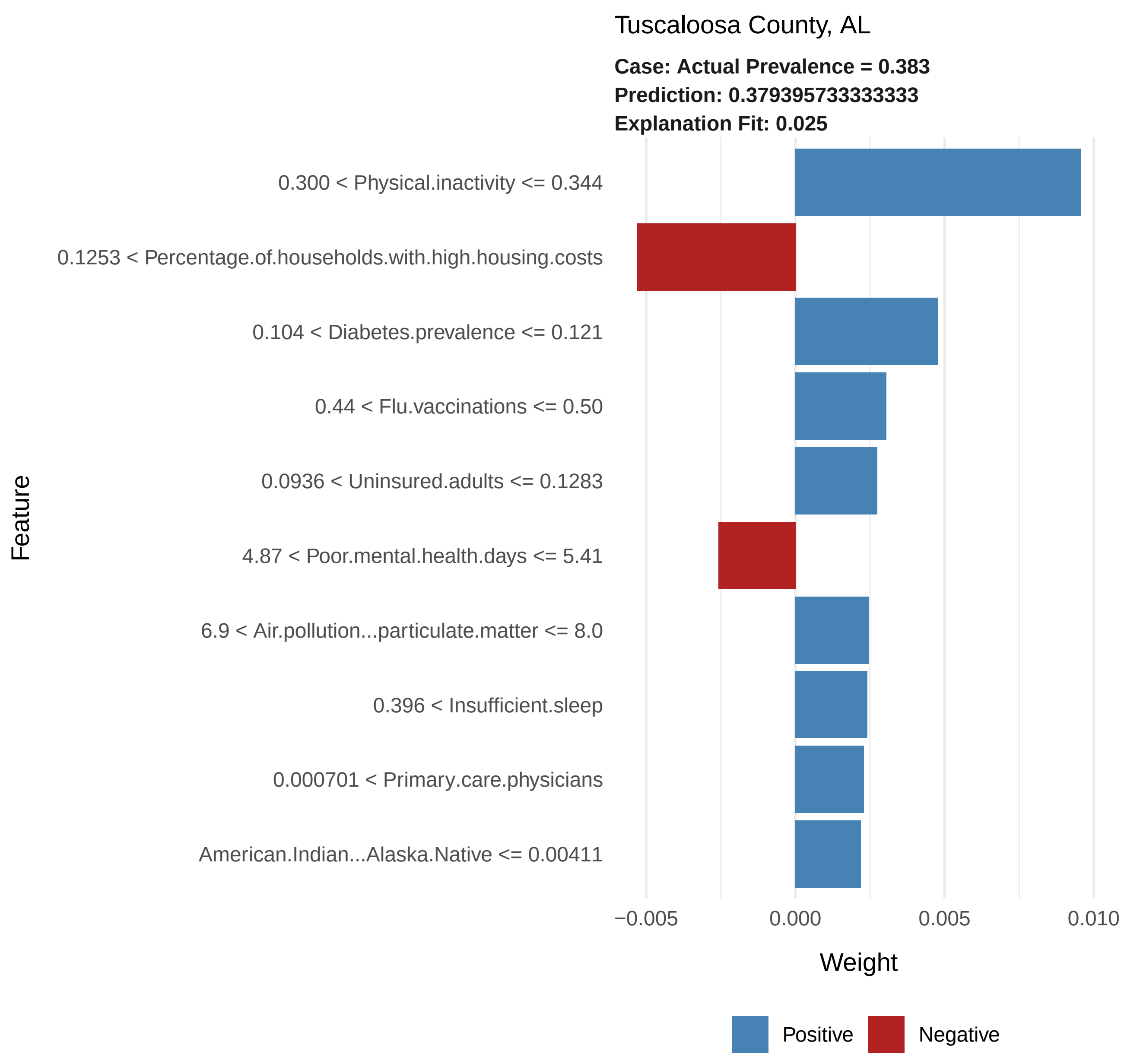
Tuscaloosa County, AL
Case: Actual Prevalence = 0.383
Prediction: 0.379395733333333
Explanation Fit: 0.025
0.300 < Physical.inactivity <= 0.344
0.1253 < Percentage.of.households.with.high.housing.costs
0.104 < Diabetes.prevalence <= 0.121
0.44 < Flu.vaccinations <= 0.50
0.0936 < Uninsured.adults <= 0.1283
4.87 < Poor.mental.health.days <= 5.41
6.9 < Air.pollution...particulate.matter <= 8.0
0.396 < Insufficient.sleep
0.000701 < Primary.care.physicians
American.Indian...Alaska.Native <= 0.00411
Feature
−0.005
0.000
0.005
0.010
Weight
Positive
Negative

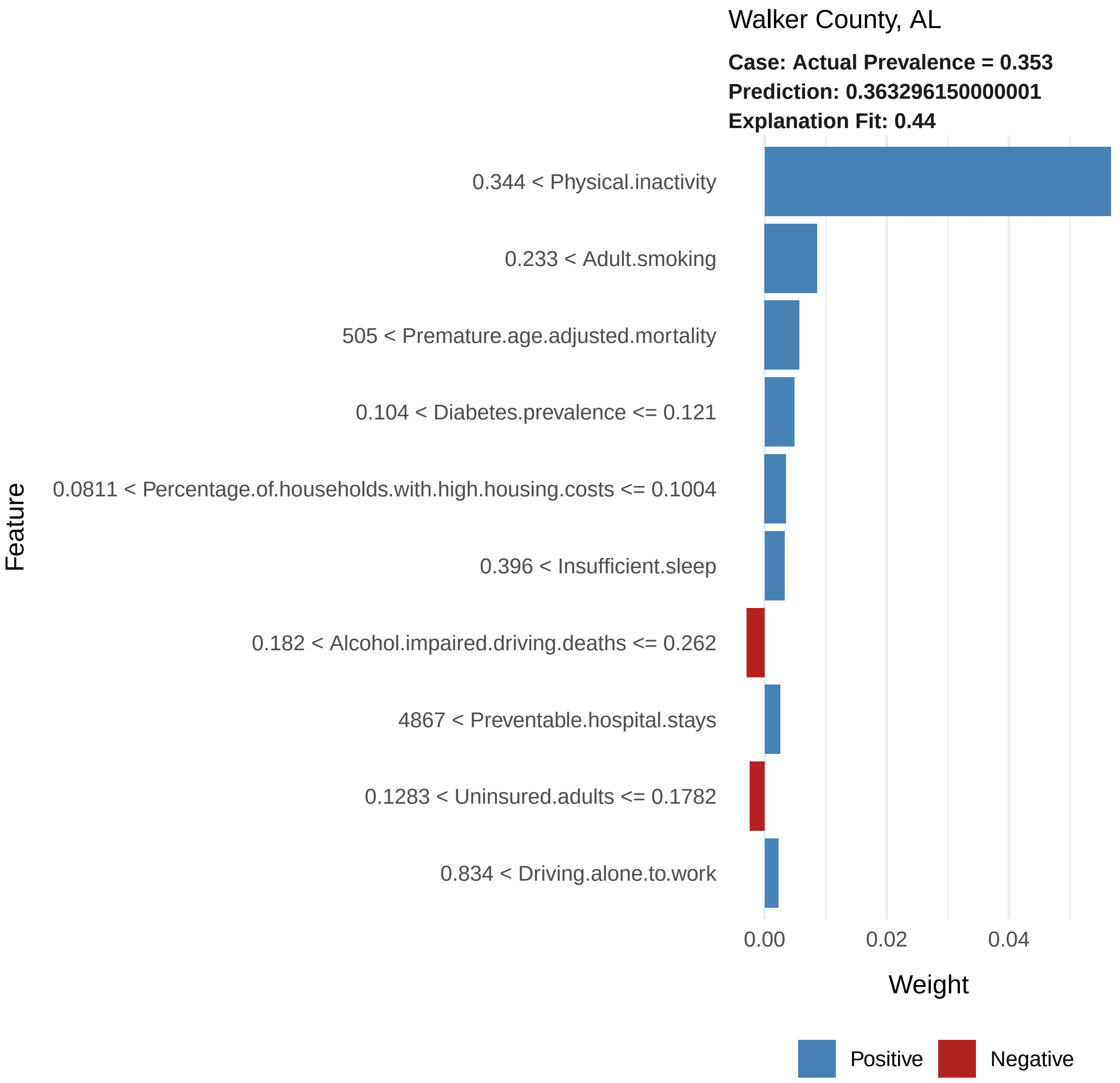

Walker County, AL
Case: Actual Prevalence = 0.353
Prediction: 0.363296150000001
Explanation Fit: 0.44
0.344 < Physical.inactivity
0.233 < Adult.smoking
505 < Premature.age.adjusted.mortality
0.104 < Diabetes.prevalence <= 0.121
0.0811 < Percentage.of.households.with.high.housing.costs <= 0.1004
0.396 < Insufficient.sleep
0.182 < Alcohol.impaired.driving.deaths <= 0.262
4867 < Preventable.hospital.stays
0.1283 < Uninsured.adults <= 0.1782
0.834 < Driving.alone.to.work
Feature
0.00
0.02
0.04
Weight
Positive
Negative

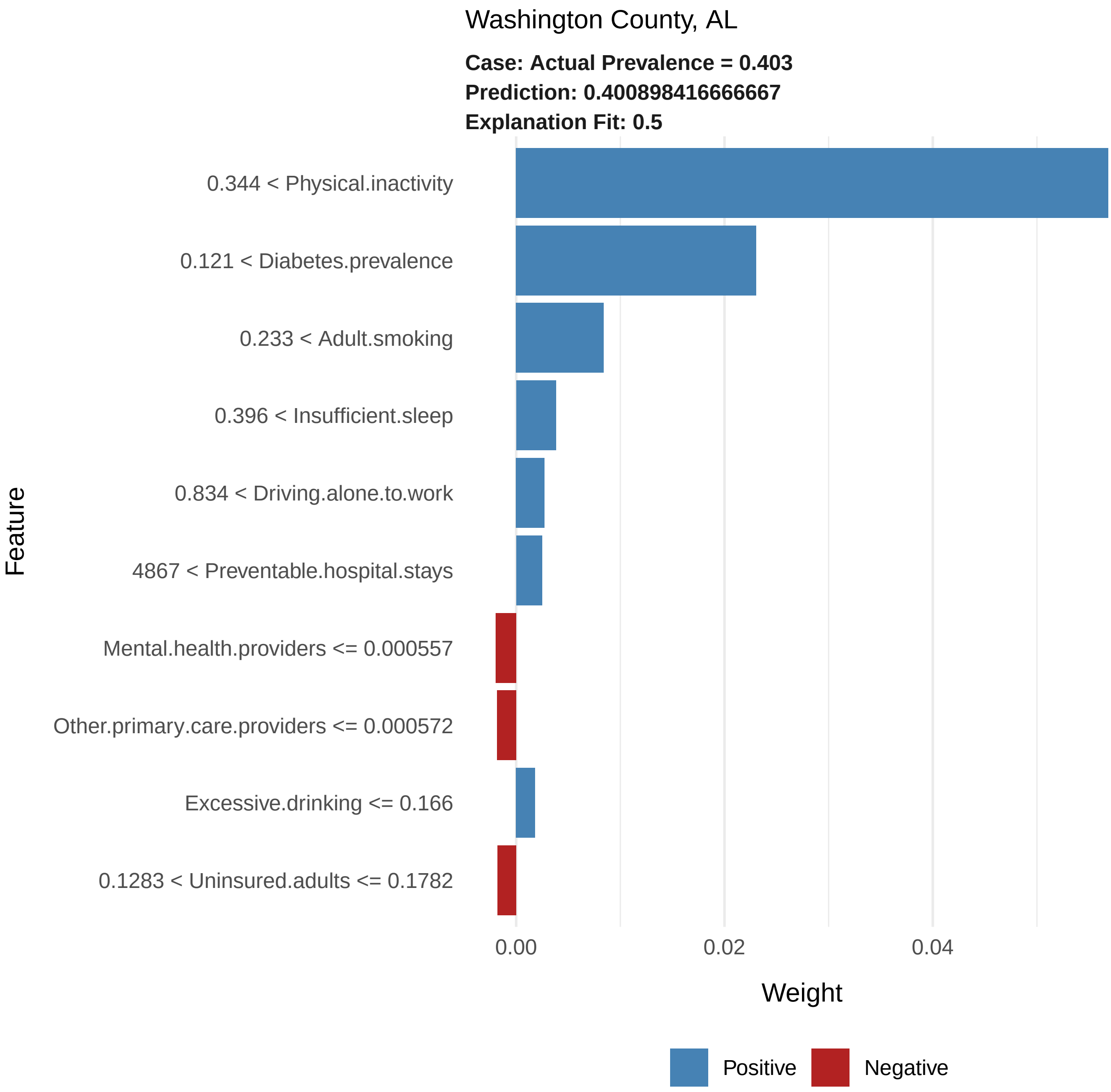
Washington County, AL
Case: Actual Prevalence = 0.403
Prediction: 0.400898416666667
Explanation Fit: 0.5
0.344 < Physical.inactivity
0.121 < Diabetes.prevalence
0.233 < Adult.smoking
0.396 < Insufficient.sleep
0.834 < Driving.alone.to.work
4867 < Preventable.hospital.stays
Mental.health.providers <= 0.000557
Other.primary.care.providers <= 0.000572
Excessive.drinking <= 0.166
0.1283 < Uninsured.adults <= 0.1782
Feature
0.00
0.02
0.04
Weight
Positive
Negative

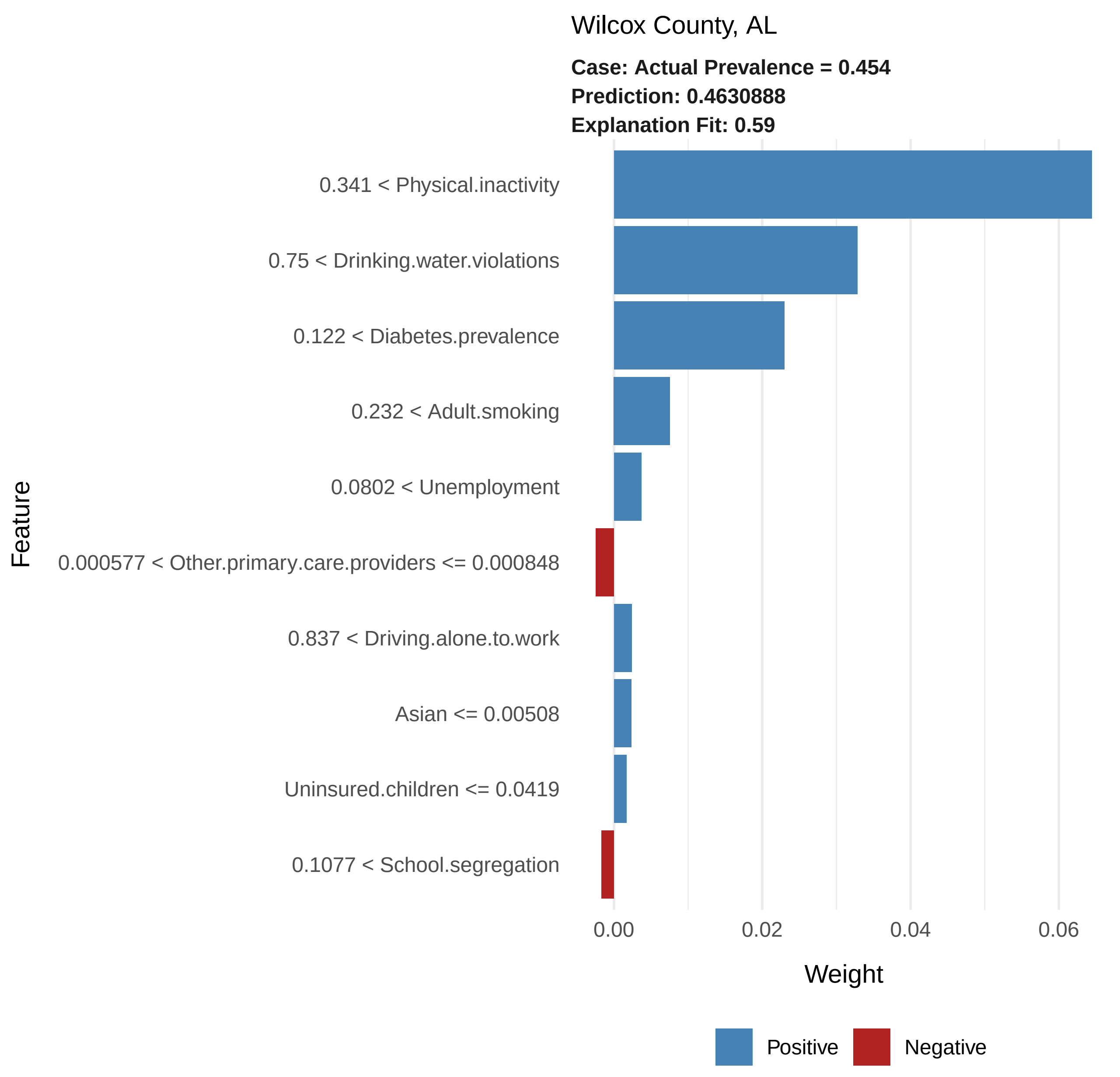

Wilcox County, AL
Case: Actual Prevalence = 0.454
Prediction: 0.4630888
Explanation Fit: 0.59
0.341 < Physical.inactivity
0.75 < Drinking.water.violations
0.122 < Diabetes.prevalence
0.232 < Adult.smoking
0.0802 < Unemployment
0.000577 < Other.primary.care.providers <= 0.000848
0.837 < Driving.alone.to.work
Asian <= 0.00508
Uninsured.children <= 0.0419
0.1077 < School.segregation
Feature
0.00
0.02
0.04
0.06
Weight
Positive
Negative

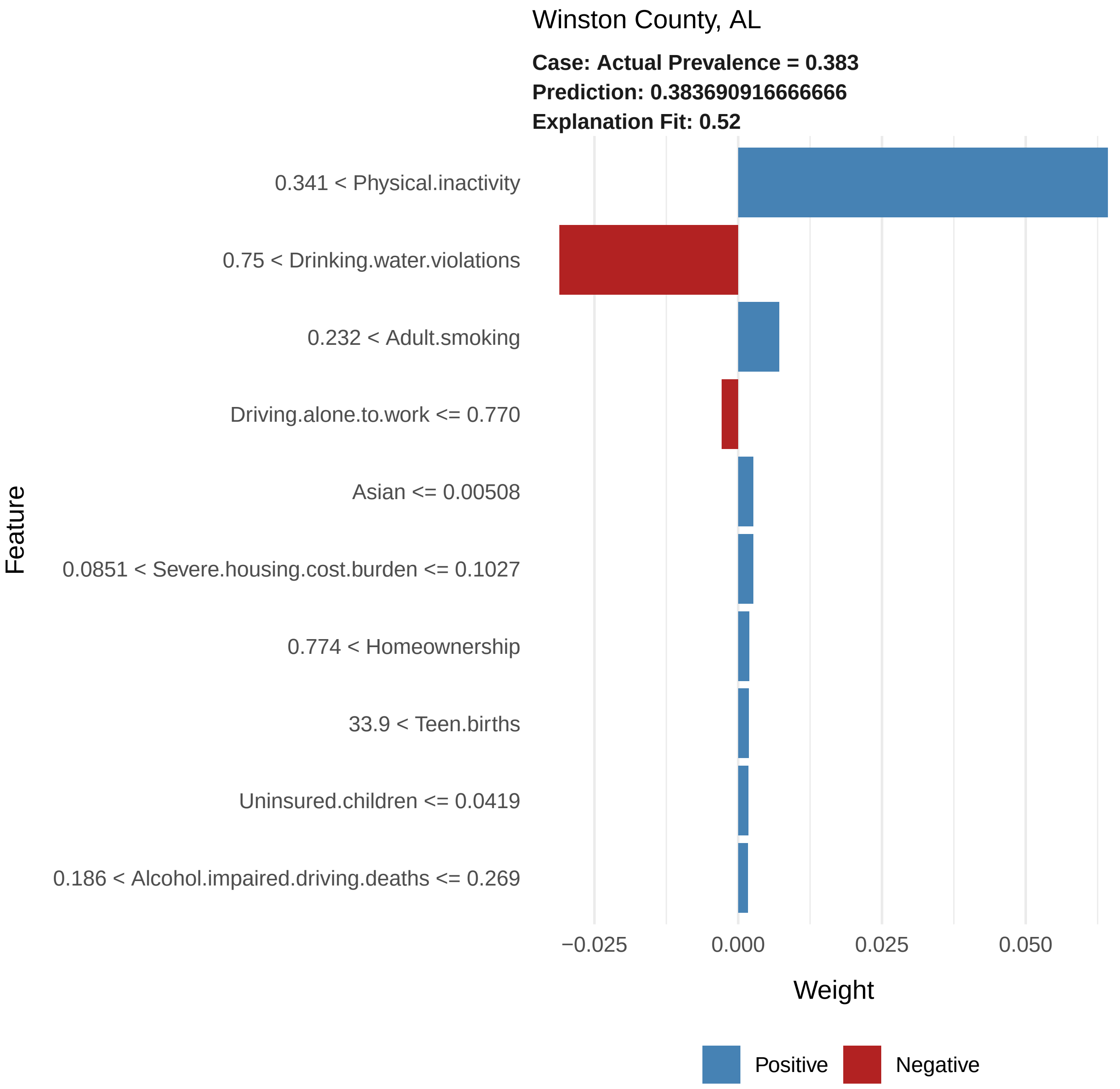

Winston County, AL
Case: Actual Prevalence = 0.383
Prediction: 0.383690916666666
Explanation Fit: 0.52
0.341 < Physical.inactivity
0.75 < Drinking.water.violations
0.232 < Adult.smoking
Driving.alone.to.work <= 0.770
Asian <= 0.00508
0.0851 < Severe.housing.cost.burden <= 0.1027
0.774 < Homeownership
33.9 < Teen.births
Uninsured.children <= 0.0419
0.186 < Alcohol.impaired.driving.deaths <= 0.269
Feature
−0.025
0.000
0.025
0.050
Weight
Positive
Negative

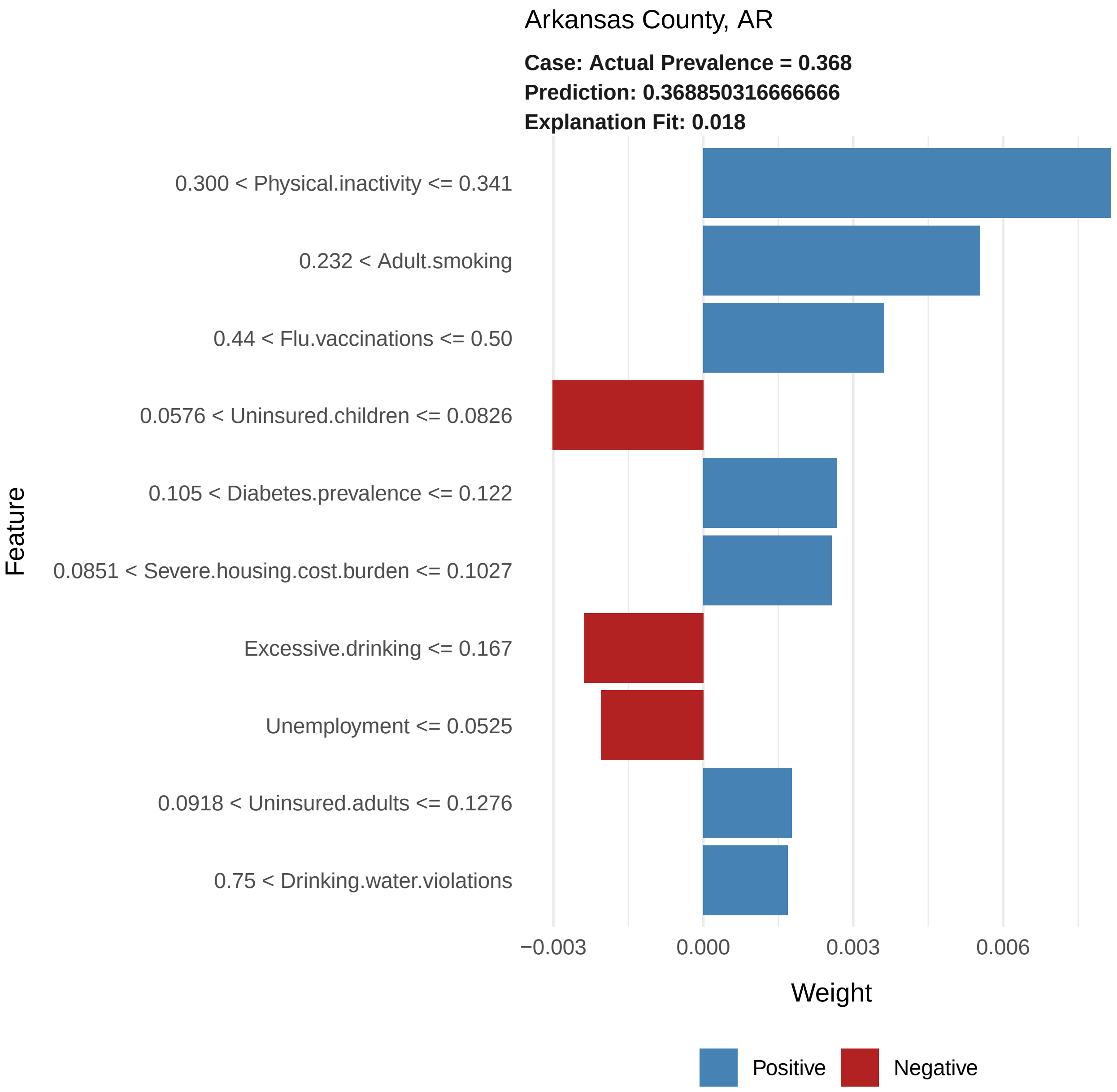

Arkansas County, AR
Case: Actual Prevalence = 0.368
Prediction: 0.368850316666666
Explanation Fit: 0.018
0.300 < Physical.inactivity <= 0.341
0.232 < Adult.smoking
0.44 < Flu.vaccinations <= 0.50
0.0576 < Uninsured.children <= 0.0826
0.105 < Diabetes.prevalence <= 0.122
0.0851 < Severe.housing.cost.burden <= 0.1027
Excessive.drinking <= 0.167
Unemployment <= 0.0525
0.0918 < Uninsured.adults <= 0.1276
0.75 < Drinking.water.violations
Feature
−0.003
0.000
0.003
0.006
Weight
Positive
Negative

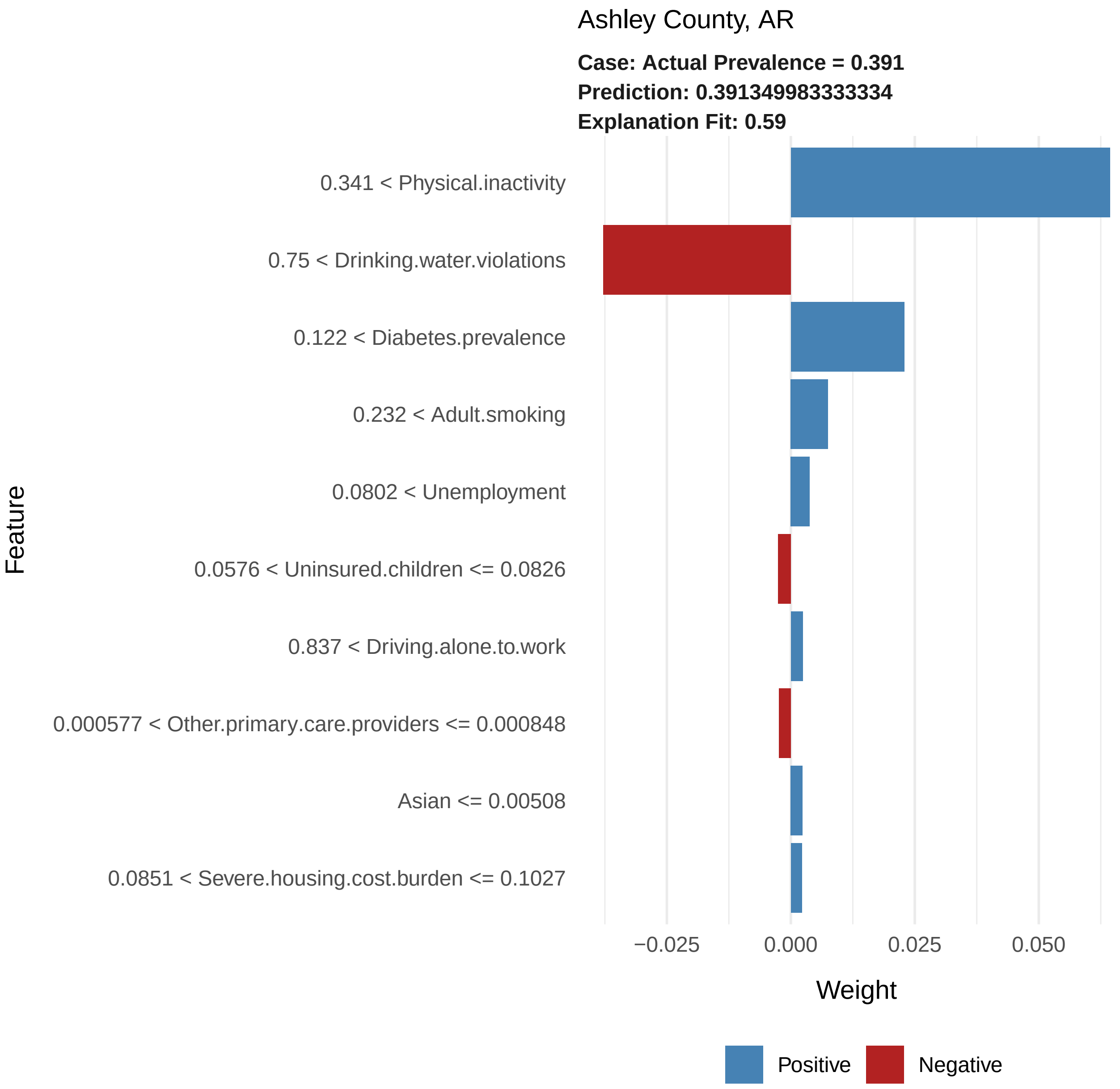
Ashley County, AR
Case: Actual Prevalence = 0.391
Prediction: 0.391349983333334
Explanation Fit: 0.59
0.341 < Physical.inactivity
0.75 < Drinking.water.violations
0.122 < Diabetes.prevalence
0.232 < Adult.smoking
0.0802 < Unemployment
0.0576 < Uninsured.children <= 0.0826
0.837 < Driving.alone.to.work
0.000577 < Other.primary.care.providers <= 0.000848
Asian <= 0.00508
0.0851 < Severe.housing.cost.burden <= 0.1027
Feature
−0.025
0.000
0.025
0.050
Weight
Positive
Negative

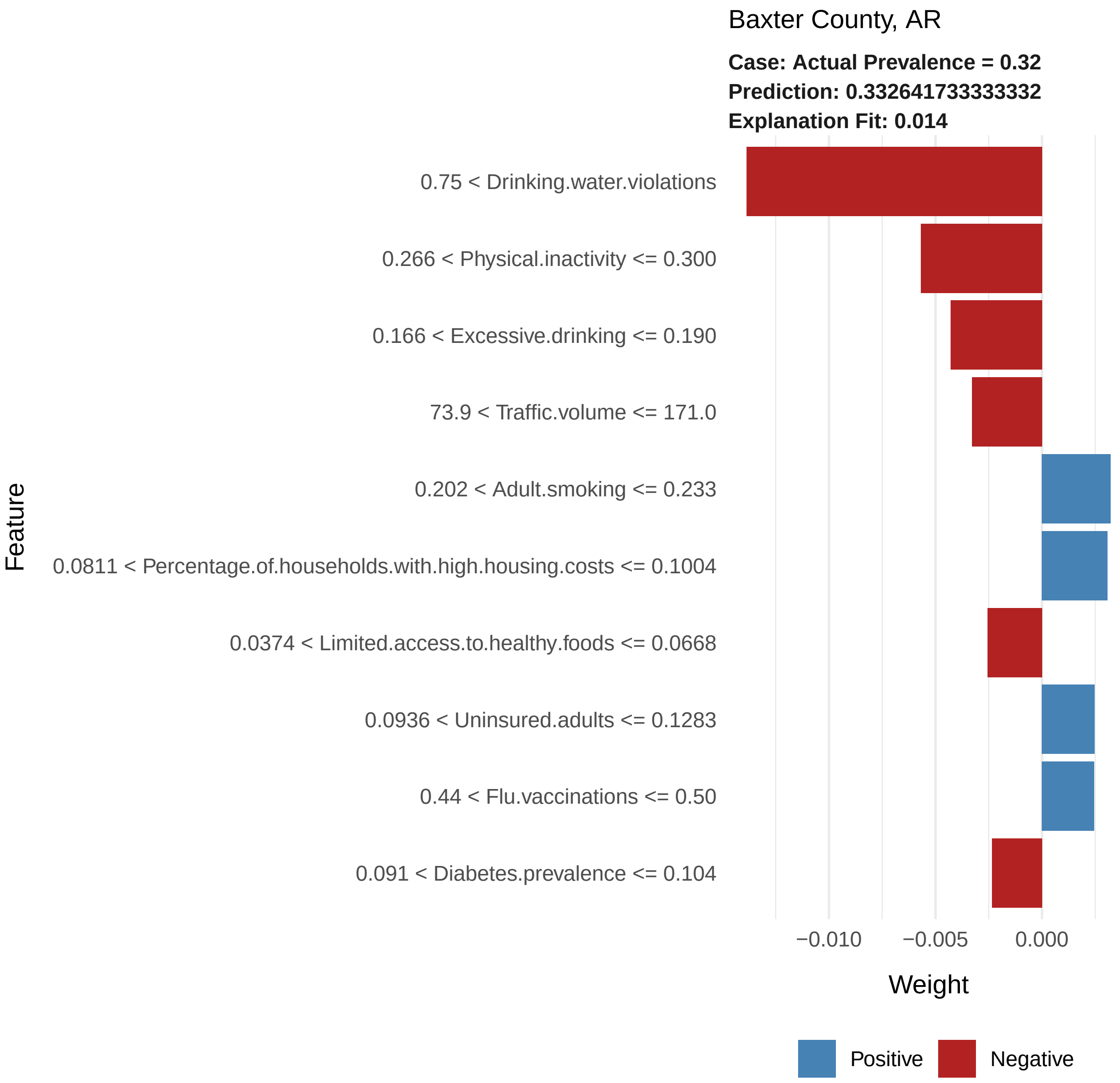

Baxter County, AR
Case: Actual Prevalence = 0.32
Prediction: 0.332641733333332
Explanation Fit: 0.014
0.75 < Drinking.water.violations
0.266 < Physical.inactivity <= 0.300
0.166 < Excessive.drinking <= 0.190
73.9 < Traffic.volume <= 171.0
0.202 < Adult.smoking <= 0.233
0.0811 < Percentage.of.households.with.high.housing.costs <= 0.1004
0.0374 < Limited.access.to.healthy.foods <= 0.0668
0.0936 < Uninsured.adults <= 0.1283
0.44 < Flu.vaccinations <= 0.50
0.091 < Diabetes.prevalence <= 0.104
Feature
−0.010
−0.005
0.000
Weight
Positive
Negative

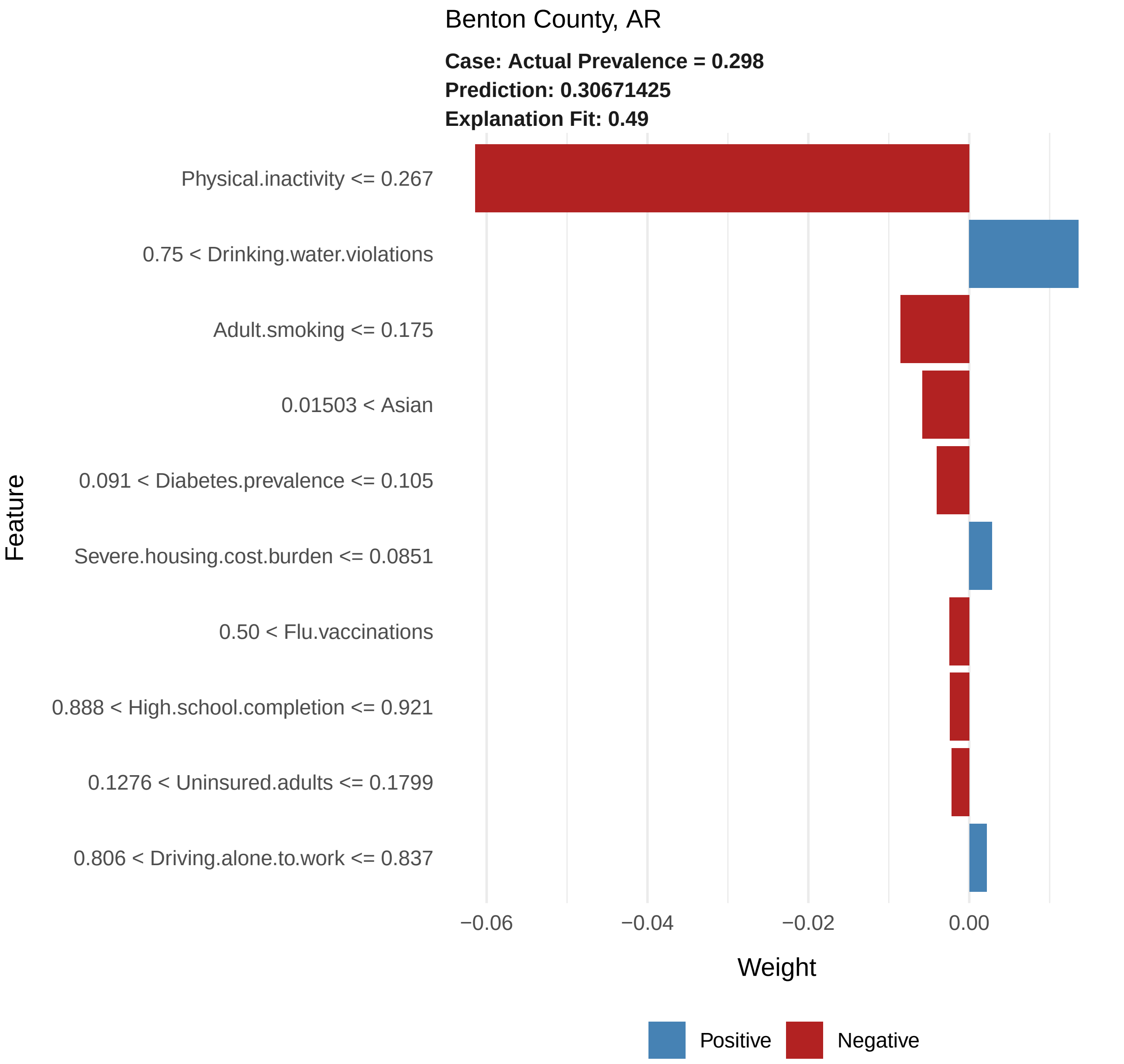

Benton County, AR
Case: Actual Prevalence = 0.298
Prediction: 0.30671425
Explanation Fit: 0.49
Physical.inactivity <= 0.267
0.75 < Drinking.water.violations
Adult.smoking <= 0.175
0.01503 < Asian
0.091 < Diabetes.prevalence <= 0.105
Severe.housing.cost.burden <= 0.0851
0.50 < Flu.vaccinations
0.888 < High.school.completion <= 0.921
0.1276 < Uninsured.adults <= 0.1799
0.806 < Driving.alone.to.work <= 0.837
Feature
−0.06
−0.04
−0.02
0.00
Weight
Positive
Negative

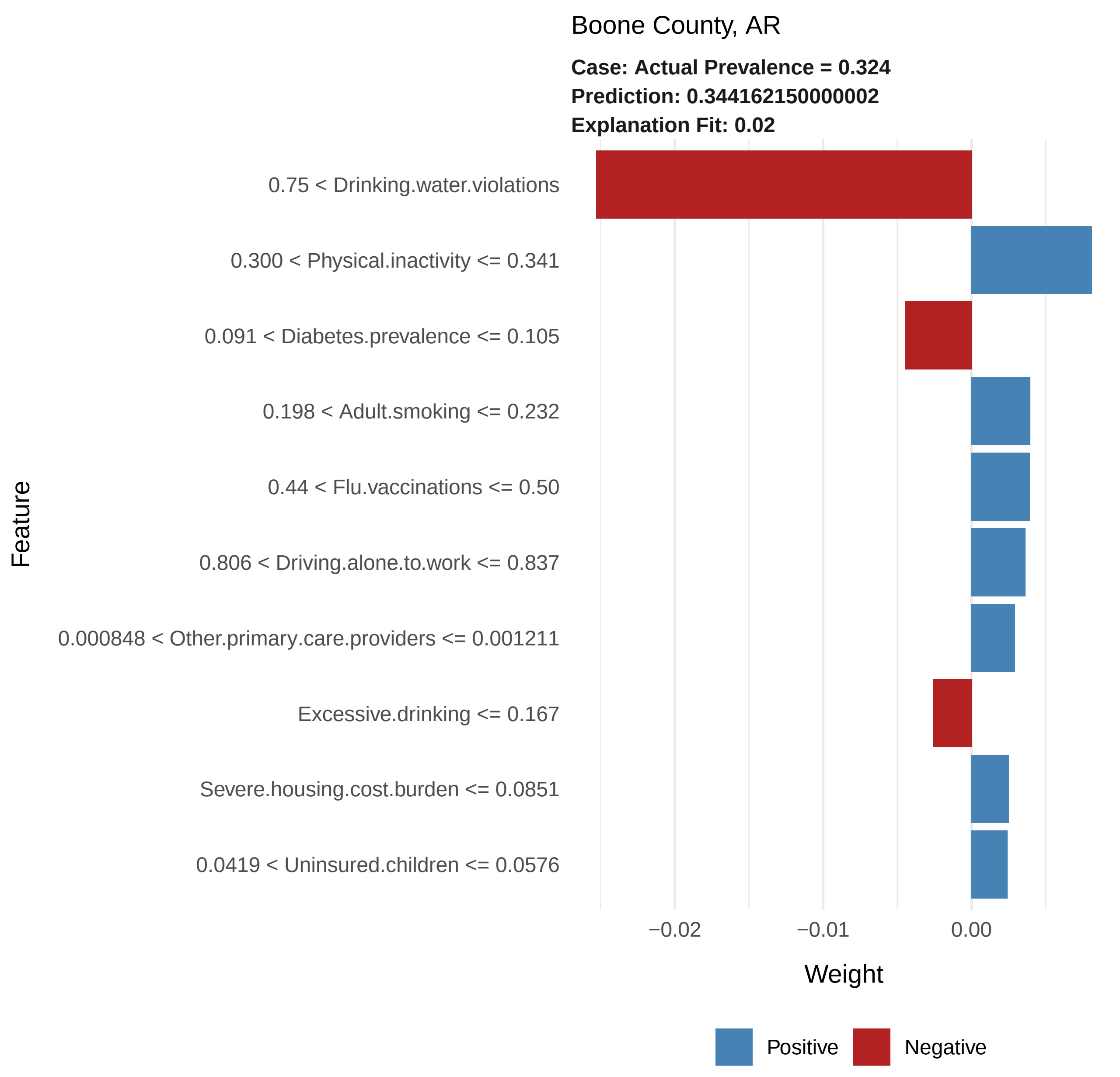

Boone County, AR
Case: Actual Prevalence = 0.324
Prediction: 0.344162150000002
Explanation Fit: 0.02
0.75 < Drinking.water.violations
0.300 < Physical.inactivity <= 0.341
0.091 < Diabetes.prevalence <= 0.105
0.198 < Adult.smoking <= 0.232
0.44 < Flu.vaccinations <= 0.50
0.806 < Driving.alone.to.work <= 0.837
0.000848 < Other.primary.care.providers <= 0.001211
Excessive.drinking <= 0.167
Severe.housing.cost.burden <= 0.0851
0.0419 < Uninsured.children <= 0.0576
Feature
−0.02
−0.01
0.00
Weight
Positive
Negative

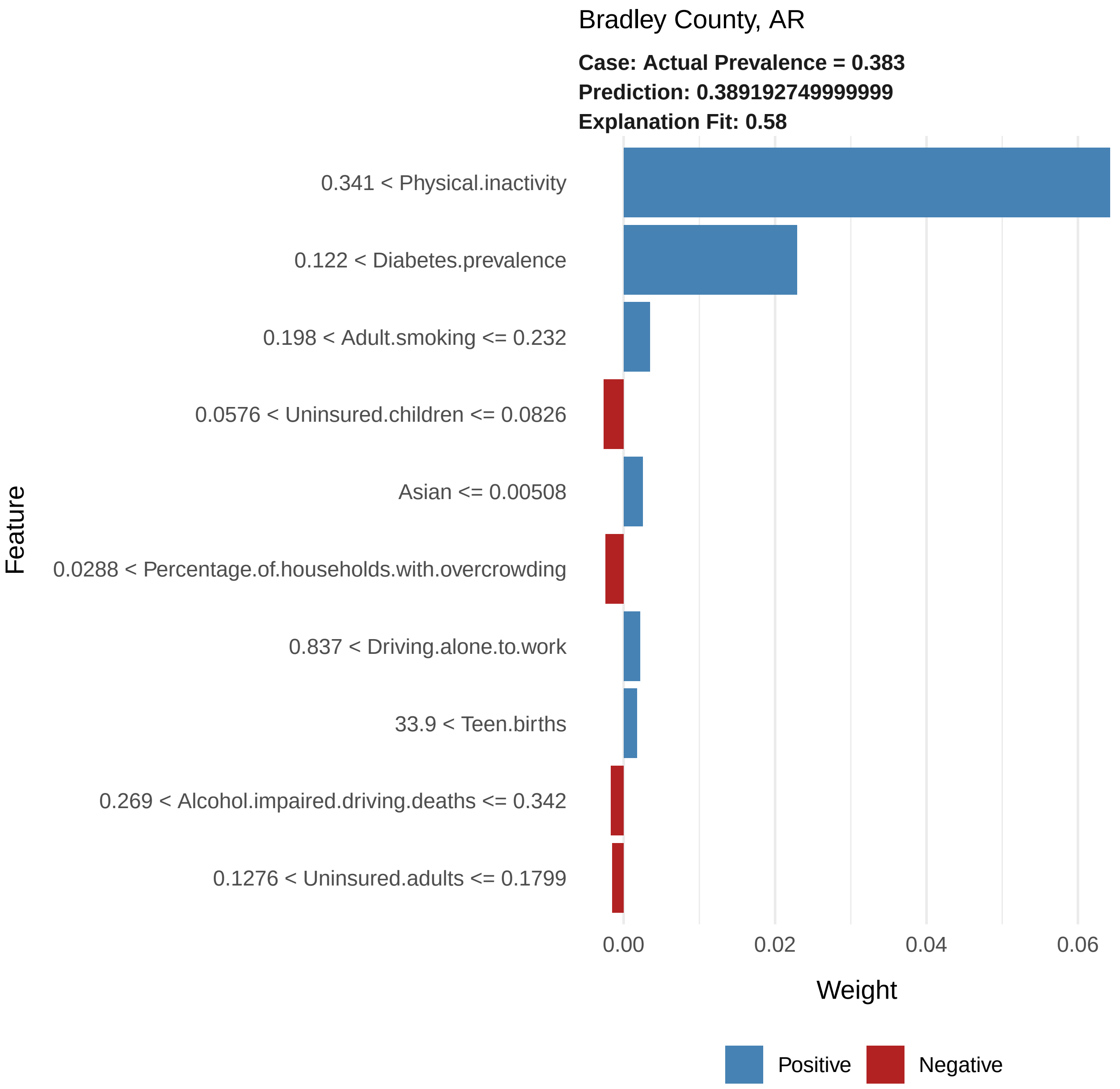

Bradley County, AR
Case: Actual Prevalence = 0.383
Prediction: 0.389192749999999
Explanation Fit: 0.58
0.341 < Physical.inactivity
0.122 < Diabetes.prevalence
0.198 < Adult.smoking <= 0.232
0.0576 < Uninsured.children <= 0.0826
Asian <= 0.00508
0.0288 < Percentage.of.households.with.overcrowding
0.837 < Driving.alone.to.work
33.9 < Teen.births
0.269 < Alcohol.impaired.driving.deaths <= 0.342
0.1276 < Uninsured.adults <= 0.1799
Feature
0.00
0.02
0.04
0.06
Weight
Positive
Negative

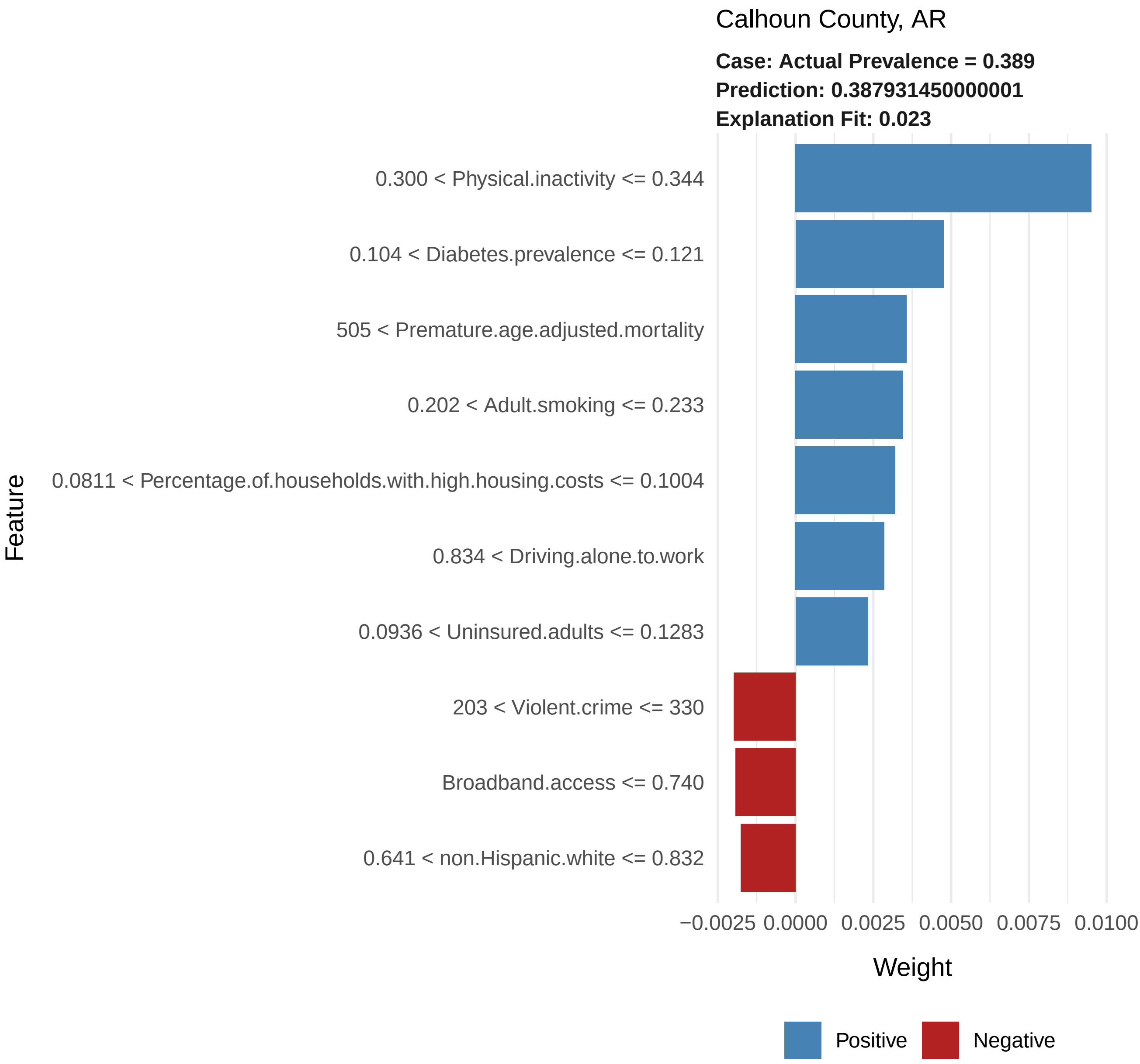
Calhoun County, AR
Case: Actual Prevalence = 0.389
Prediction: 0.387931450000001
Explanation Fit: 0.023
0.300 < Physical.inactivity <= 0.344
0.104 < Diabetes.prevalence <= 0.121
505 < Premature.age.adjusted.mortality
0.202 < Adult.smoking <= 0.233
0.0811 < Percentage.of.households.with.high.housing.costs <= 0.1004
0.834 < Driving.alone.to.work
0.0936 < Uninsured.adults <= 0.1283
203 < Violent.crime <= 330
Broadband.access <= 0.740
0.641 < non.Hispanic.white <= 0.832
Feature
−0.0025
0.0000
0.0025
0.0050
0.0075
0.0100
Weight
Positive
Negative

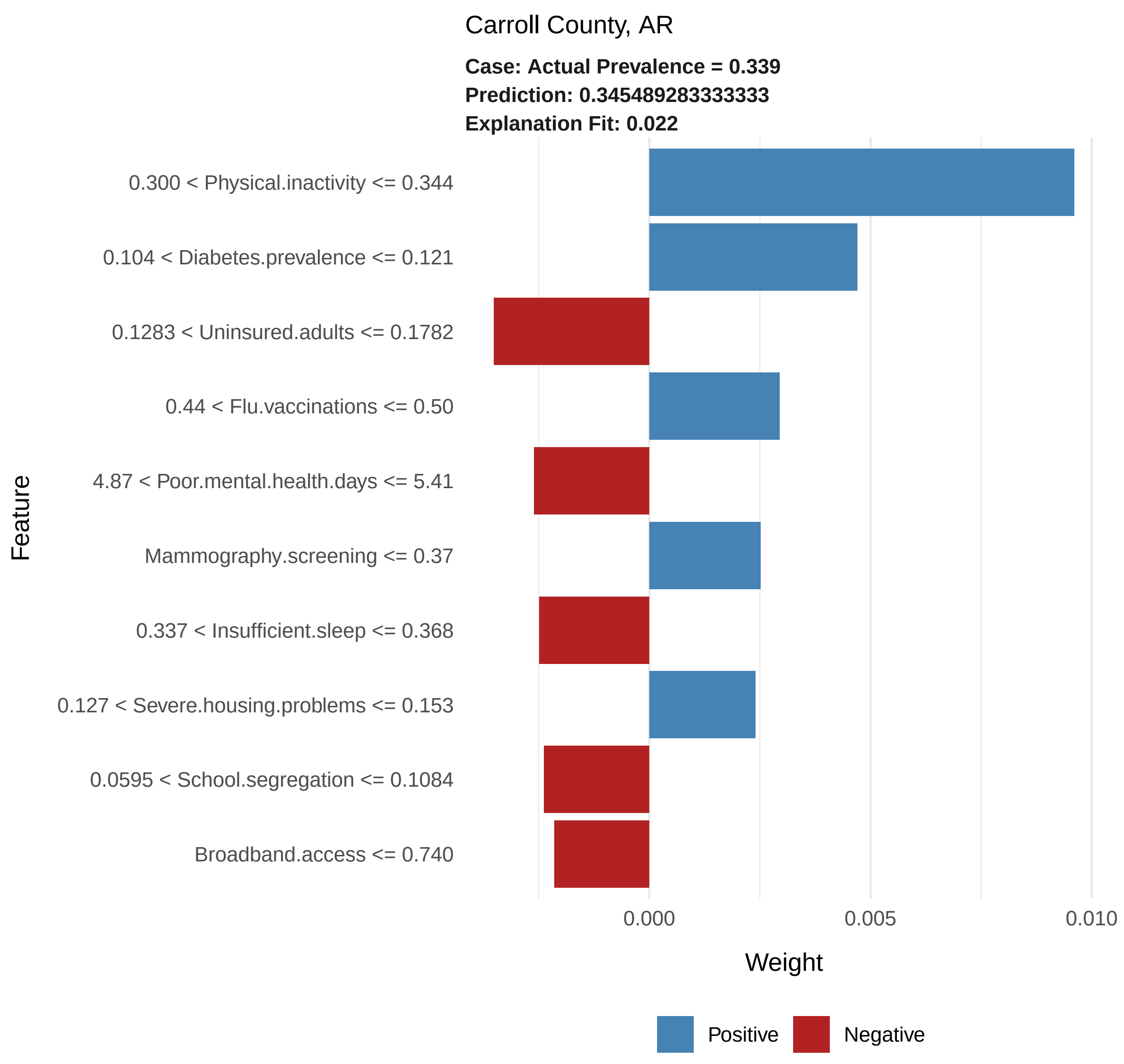
Carroll County, AR
Case: Actual Prevalence = 0.339
Prediction: 0.345489283333333
Explanation Fit: 0.022
0.300 < Physical.inactivity <= 0.344
0.104 < Diabetes.prevalence <= 0.121
0.1283 < Uninsured.adults <= 0.1782
0.44 < Flu.vaccinations <= 0.50
4.87 < Poor.mental.health.days <= 5.41
Mammography.screening <= 0.37
0.337 < Insufficient.sleep <= 0.368
0.127 < Severe.housing.problems <= 0.153
0.0595 < School.segregation <= 0.1084
Broadband.access <= 0.740
Feature
0.000
0.005
0.010
Weight
Positive
Negative

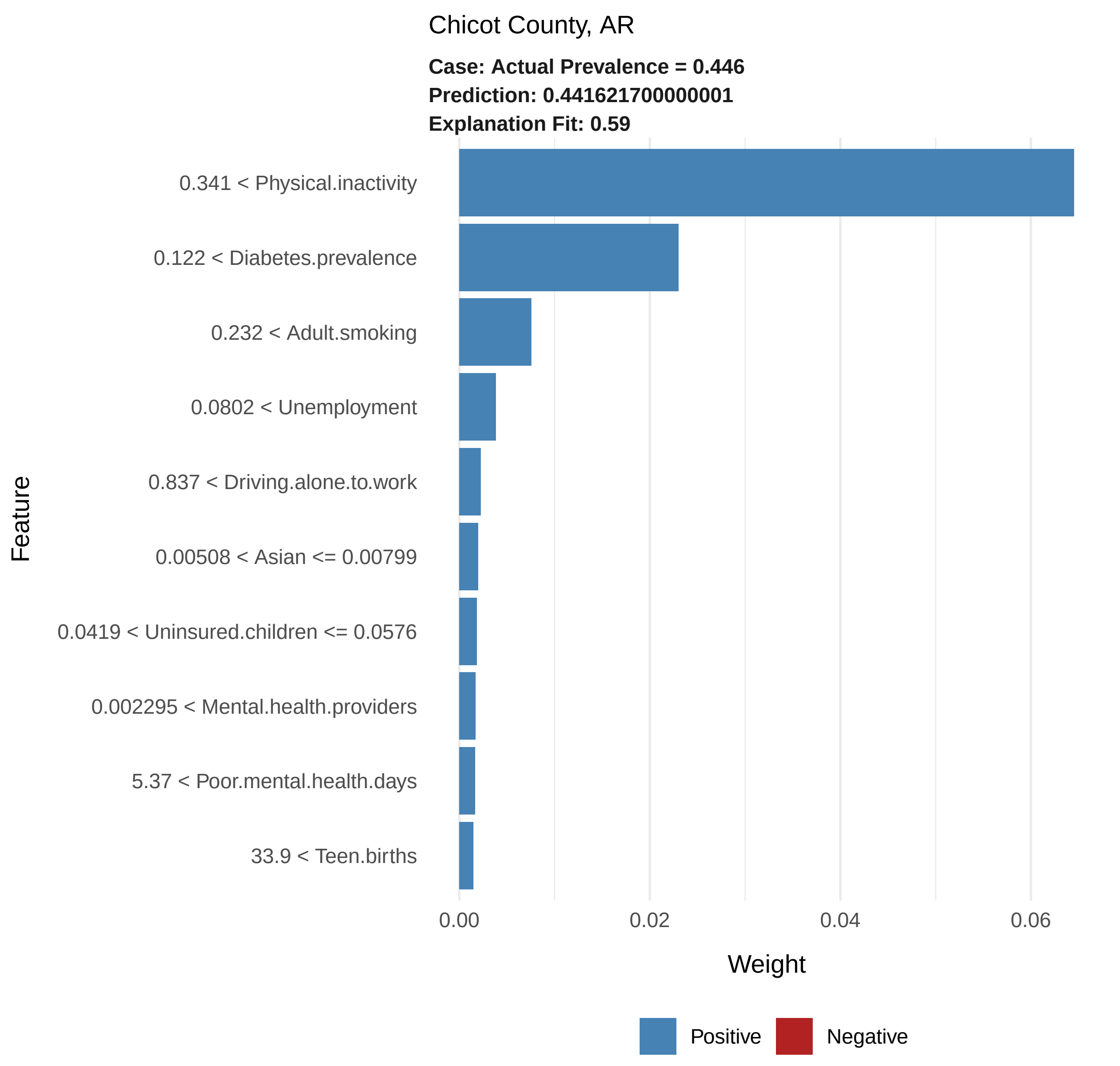
Chicot County, AR
Case: Actual Prevalence = 0.446
Prediction: 0.441621700000001
Explanation Fit: 0.59
0.341 < Physical.inactivity
0.122 < Diabetes.prevalence
0.232 < Adult.smoking
0.0802 < Unemployment
0.837 < Driving.alone.to.work
0.00508 < Asian <= 0.00799
0.0419 < Uninsured.children <= 0.0576
0.002295 < Mental.health.providers
5.37 < Poor.mental.health.days
33.9 < Teen.births
Feature
0.00
0.02
0.04
0.06
Weight
Positive
Negative

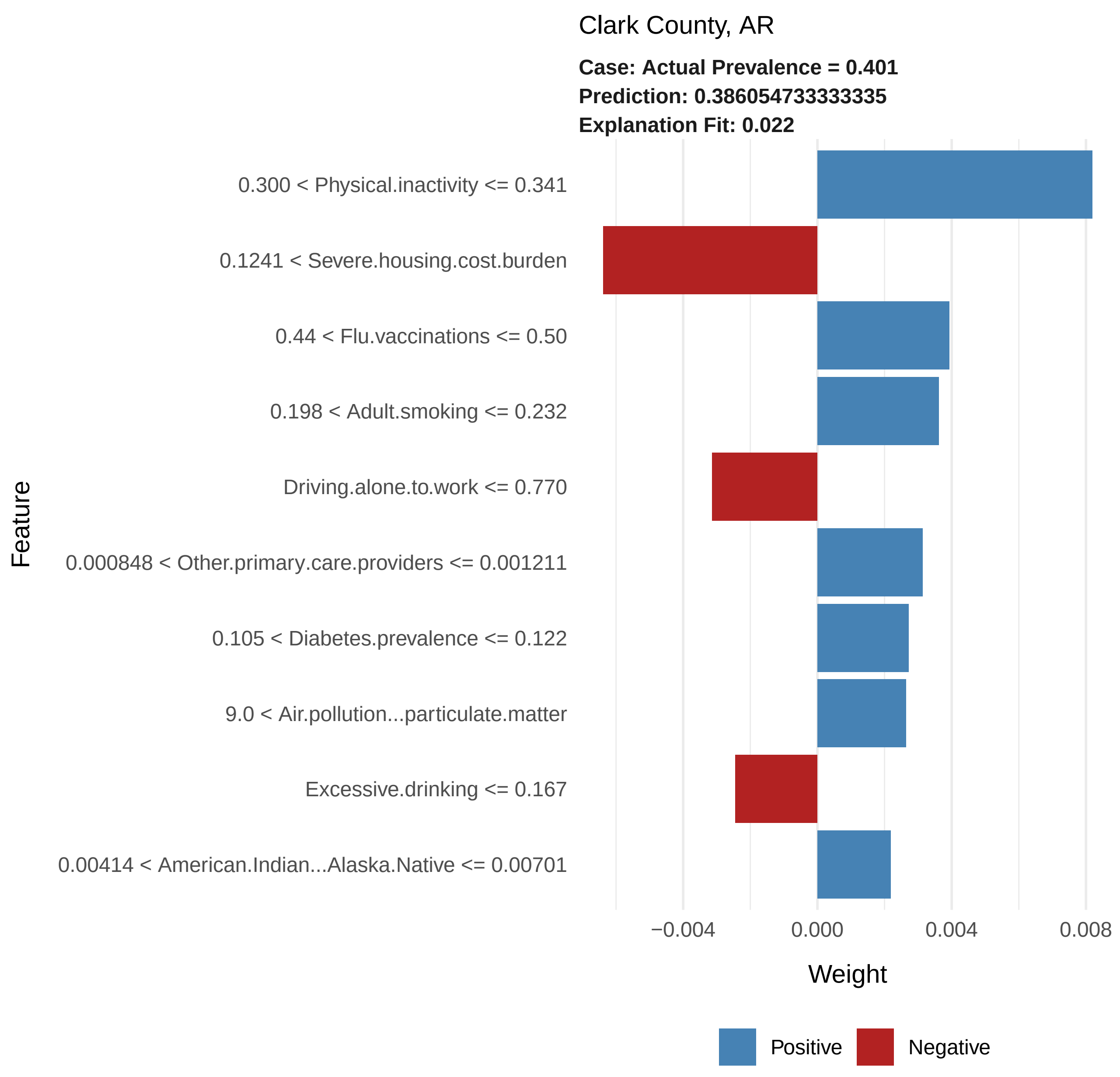

Clark County, AR
Case: Actual Prevalence = 0.401
Prediction: 0.386054733333335
Explanation Fit: 0.022
0.300 < Physical.inactivity <= 0.341
0.1241 < Severe.housing.cost.burden
0.44 < Flu.vaccinations <= 0.50
0.198 < Adult.smoking <= 0.232
Driving.alone.to.work <= 0.770
0.000848 < Other.primary.care.providers <= 0.001211
0.105 < Diabetes.prevalence <= 0.122
9.0 < Air.pollution...particulate.matter
Excessive.drinking <= 0.167
0.00414 < American.Indian...Alaska.Native <= 0.00701
Feature
−0.004
0.000
0.004
0.008
Weight
Positive
Negative

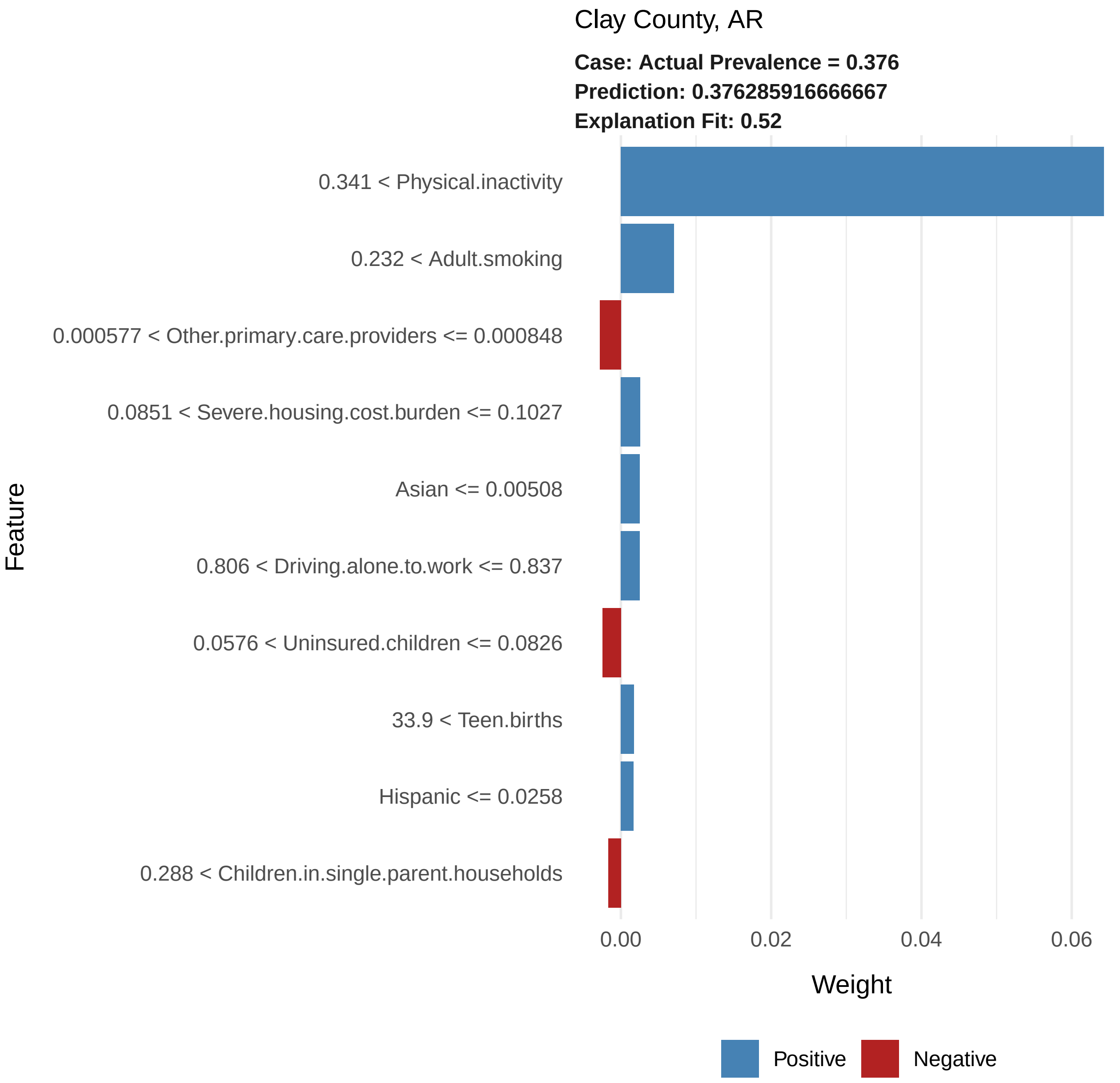
Clay County, AR
Case: Actual Prevalence = 0.376
Prediction: 0.376285916666667
Explanation Fit: 0.52
0.341 < Physical.inactivity
0.232 < Adult.smoking
0.000577 < Other.primary.care.providers <= 0.000848
0.0851 < Severe.housing.cost.burden <= 0.1027
Asian <= 0.00508
0.806 < Driving.alone.to.work <= 0.837
0.0576 < Uninsured.children <= 0.0826
33.9 < Teen.births
Hispanic <= 0.0258
0.288 < Children.in.single.parent.households
Feature
0.00
0.02
0.04
0.06
Weight
Positive
Negative

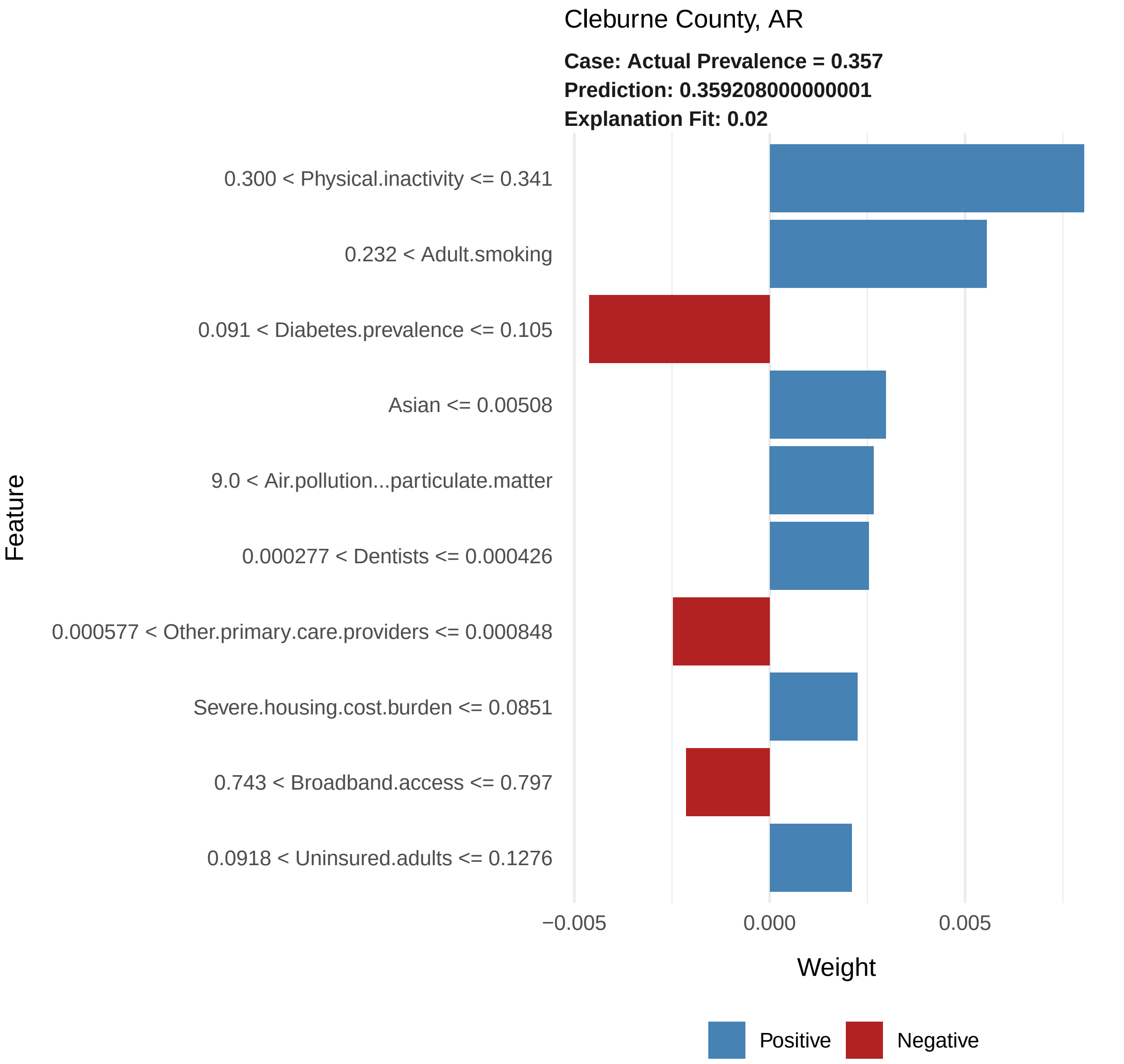
Cleburne County, AR
Case: Actual Prevalence = 0.357
Prediction: 0.359208000000001
Explanation Fit: 0.02
0.300 < Physical.inactivity <= 0.341
0.232 < Adult.smoking
0.091 < Diabetes.prevalence <= 0.105
Asian <= 0.00508
9.0 < Air.pollution...particulate.matter
0.000277 < Dentists <= 0.000426
0.000577 < Other.primary.care.providers <= 0.000848
Severe.housing.cost.burden <= 0.0851
0.743 < Broadband.access <= 0.797
0.0918 < Uninsured.adults <= 0.1276
Feature
−0.005
0.000
0.005
Weight
Positive
Negative

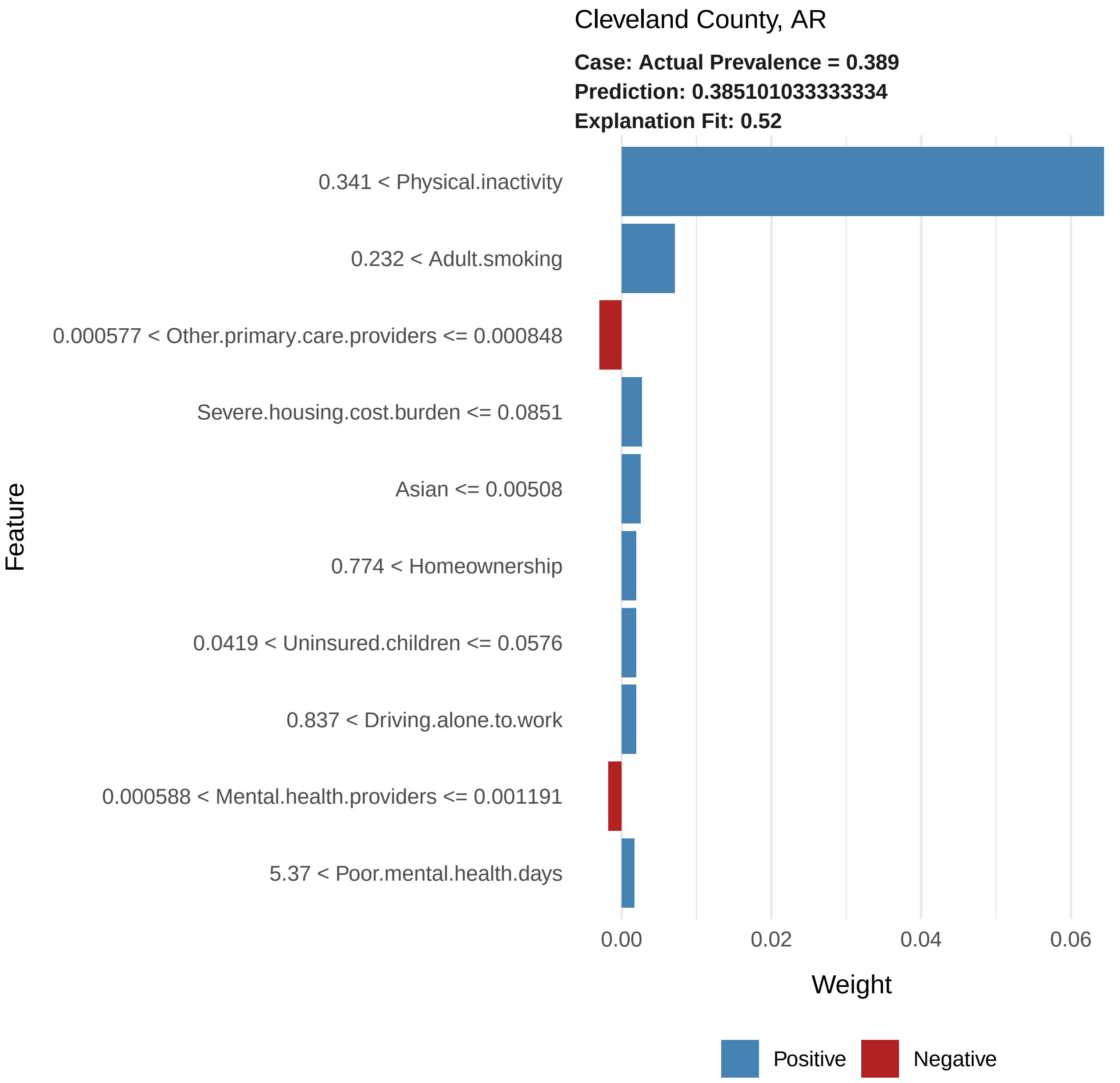

Cleveland County, AR
Case: Actual Prevalence = 0.389
Prediction: 0.385101033333334
Explanation Fit: 0.52
0.341 < Physical.inactivity
0.232 < Adult.smoking
0.000577 < Other.primary.care.providers <= 0.000848
Severe.housing.cost.burden <= 0.0851
Asian <= 0.00508
0.774 < Homeownership
0.0419 < Uninsured.children <= 0.0576
0.837 < Driving.alone.to.work
0.000588 < Mental.health.providers <= 0.001191
5.37 < Poor.mental.health.days
Feature
0.00
0.02
0.04
0.06
Weight
Positive
Negative

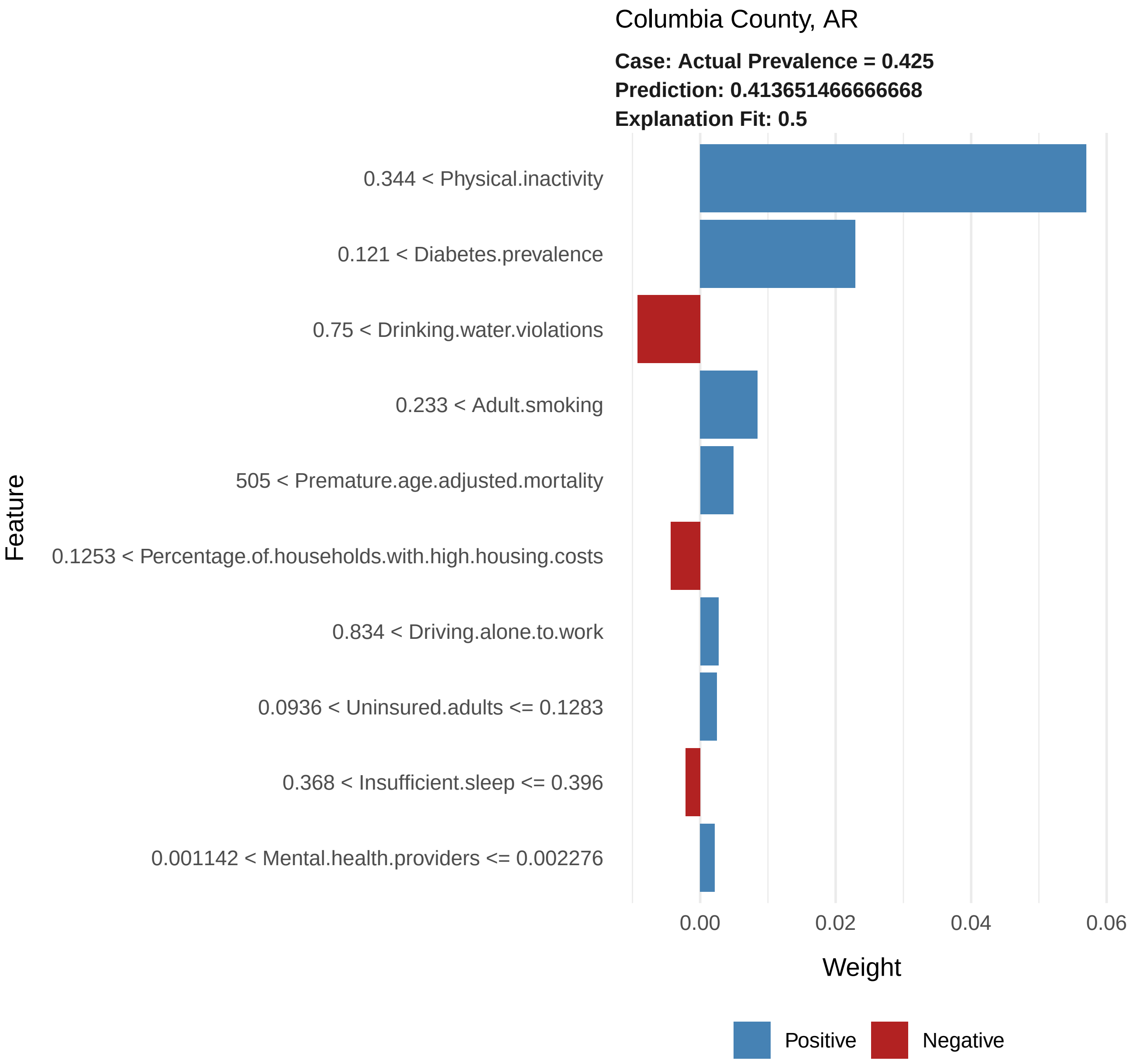
Columbia County, AR
Case: Actual Prevalence = 0.425
Prediction: 0.413651466666668
Explanation Fit: 0.5
0.344 < Physical.inactivity
0.121 < Diabetes.prevalence
0.75 < Drinking.water.violations
0.233 < Adult.smoking
505 < Premature.age.adjusted.mortality
0.1253 < Percentage.of.households.with.high.housing.costs
0.834 < Driving.alone.to.work
0.0936 < Uninsured.adults <= 0.1283
0.368 < Insufficient.sleep <= 0.396
0.001142 < Mental.health.providers <= 0.002276
Feature
0.00
0.02
0.04
0.06
Weight
Positive
Negative

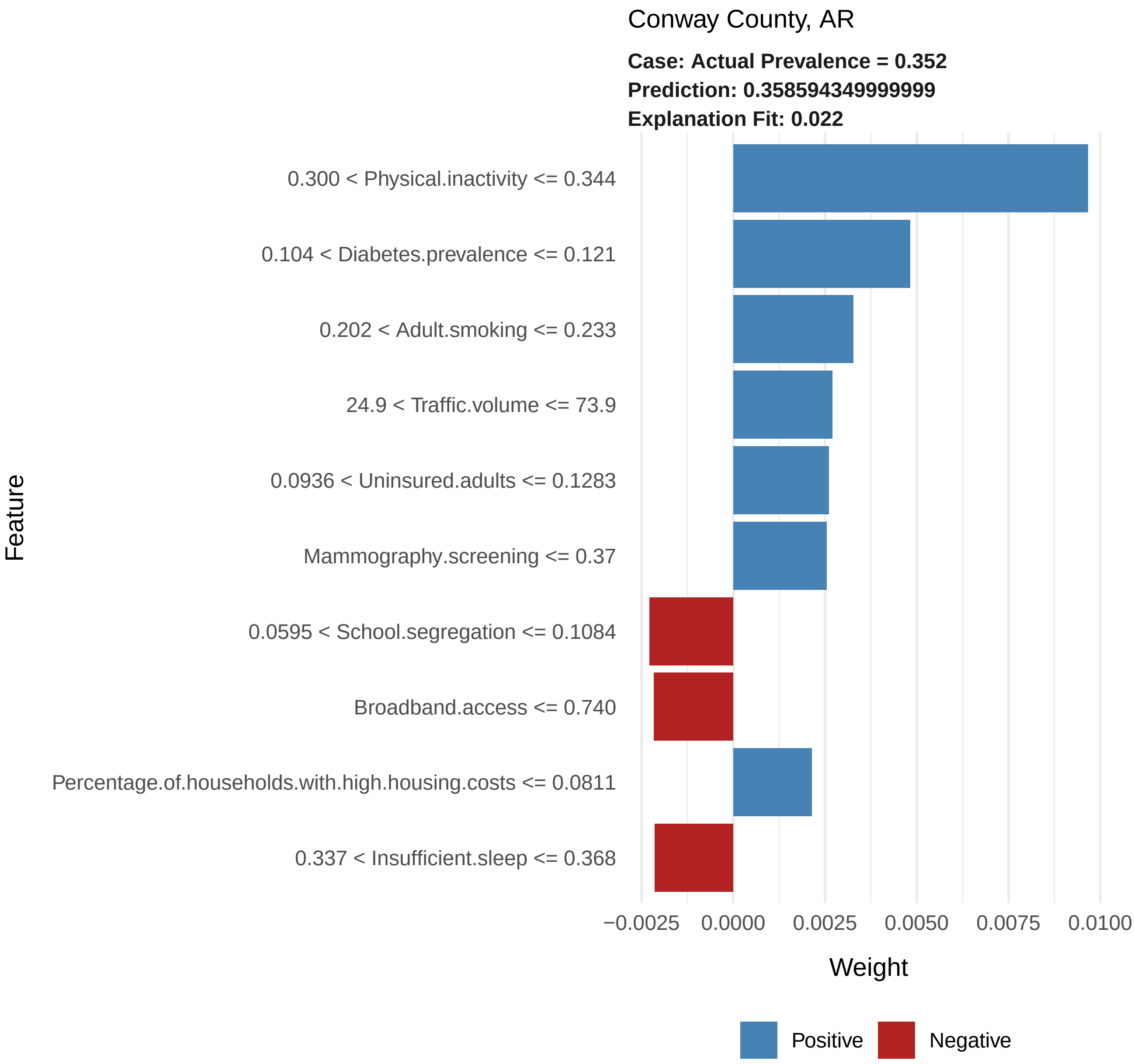
Conway County, AR
Case: Actual Prevalence = 0.352
Prediction: 0.358594349999999
Explanation Fit: 0.022
0.300 < Physical.inactivity <= 0.344
0.104 < Diabetes.prevalence <= 0.121
0.202 < Adult.smoking <= 0.233
24.9 < Traffic.volume <= 73.9
0.0936 < Uninsured.adults <= 0.1283
Mammography.screening <= 0.37
0.0595 < School.segregation <= 0.1084
Broadband.access <= 0.740
Percentage.of.households.with.high.housing.costs <= 0.0811
0.337 < Insufficient.sleep <= 0.368
Feature
−0.0025
0.0000
0.0025
0.0050
0.0075
0.0100
Weight
Positive
Negative

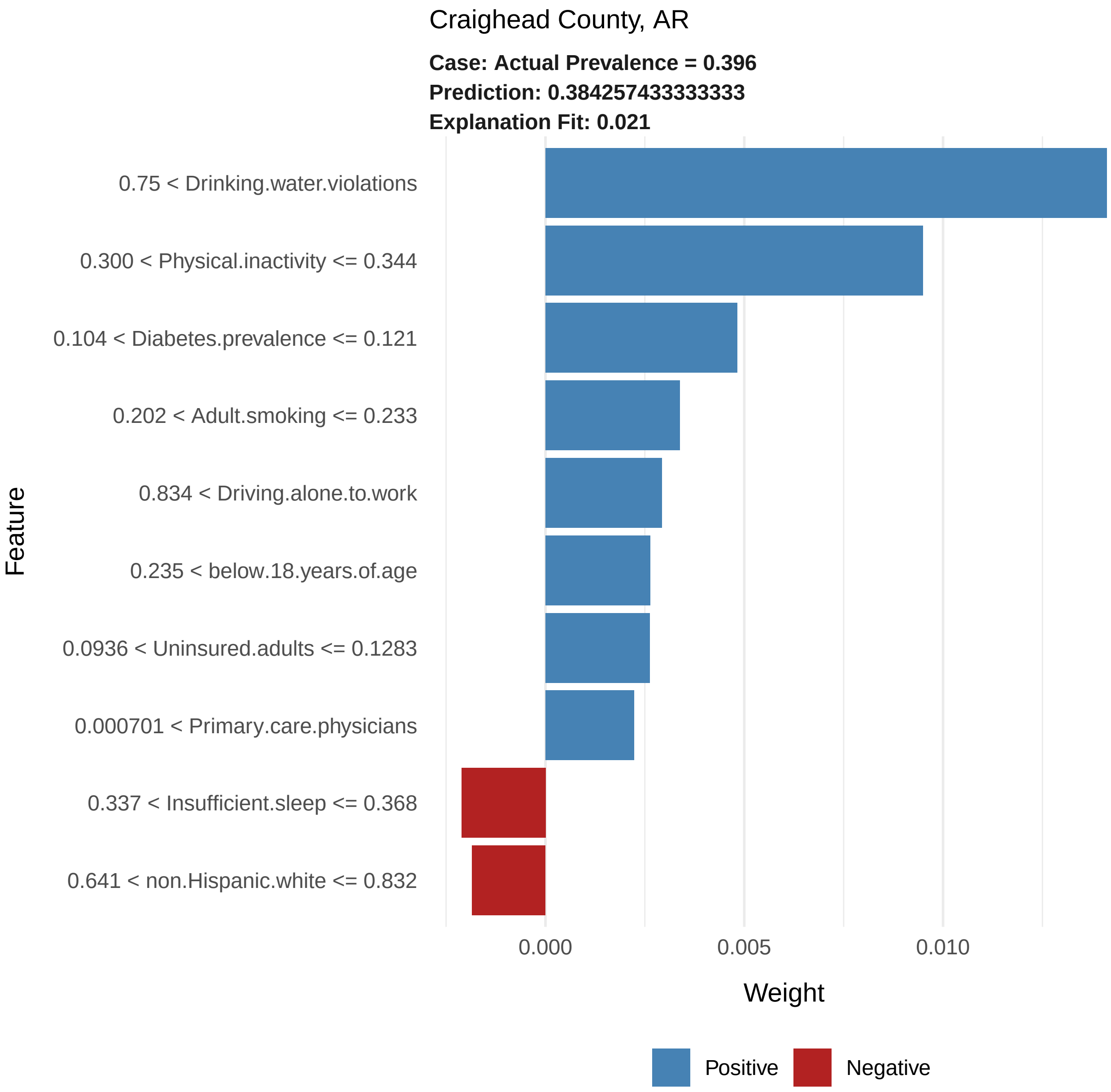

Craighead County, AR
Case: Actual Prevalence = 0.396
Prediction: 0.384257433333333
Explanation Fit: 0.021
0.75 < Drinking.water.violations
0.300 < Physical.inactivity <= 0.344
0.104 < Diabetes.prevalence <= 0.121
0.202 < Adult.smoking <= 0.233
0.834 < Driving.alone.to.work
0.235 < below.18.years.of.age
0.0936 < Uninsured.adults <= 0.1283
0.000701 < Primary.care.physicians
0.337 < Insufficient.sleep <= 0.368
0.641 < non.Hispanic.white <= 0.832
Feature
0.000
0.005
0.010
Weight
Positive
Negative

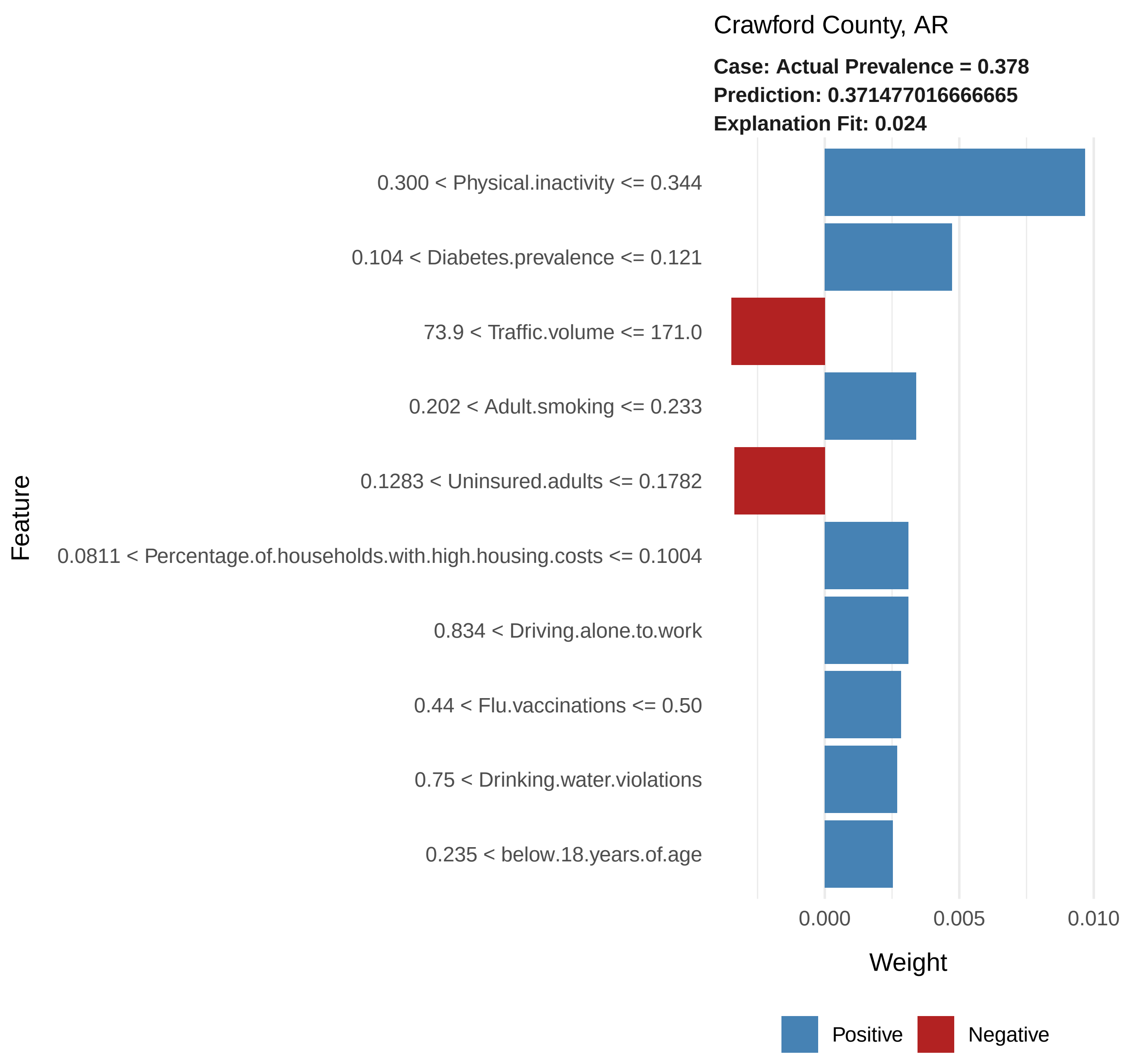
Crawford County, AR
Case: Actual Prevalence = 0.378
Prediction: 0.371477016666665
Explanation Fit: 0.024
Feature
0.300 < Physical.inactivity <= 0.344
0.104 < Diabetes.prevalence <= 0.121
73.9 < Traffic.volume <= 171.0
0.202 < Adult.smoking <= 0.233
0.1283 < Uninsured.adults <= 0.1782
0.0811 < Percentage.of.households.with.high.housing.costs <= 0.1004
0.834 < Driving.alone.to.work
0.44 < Flu.vaccinations <= 0.50
0.75 < Drinking.water.violations
0.235 < below.18.years.of.age
0.000
0.005
0.010
Weight
Positive
Negative

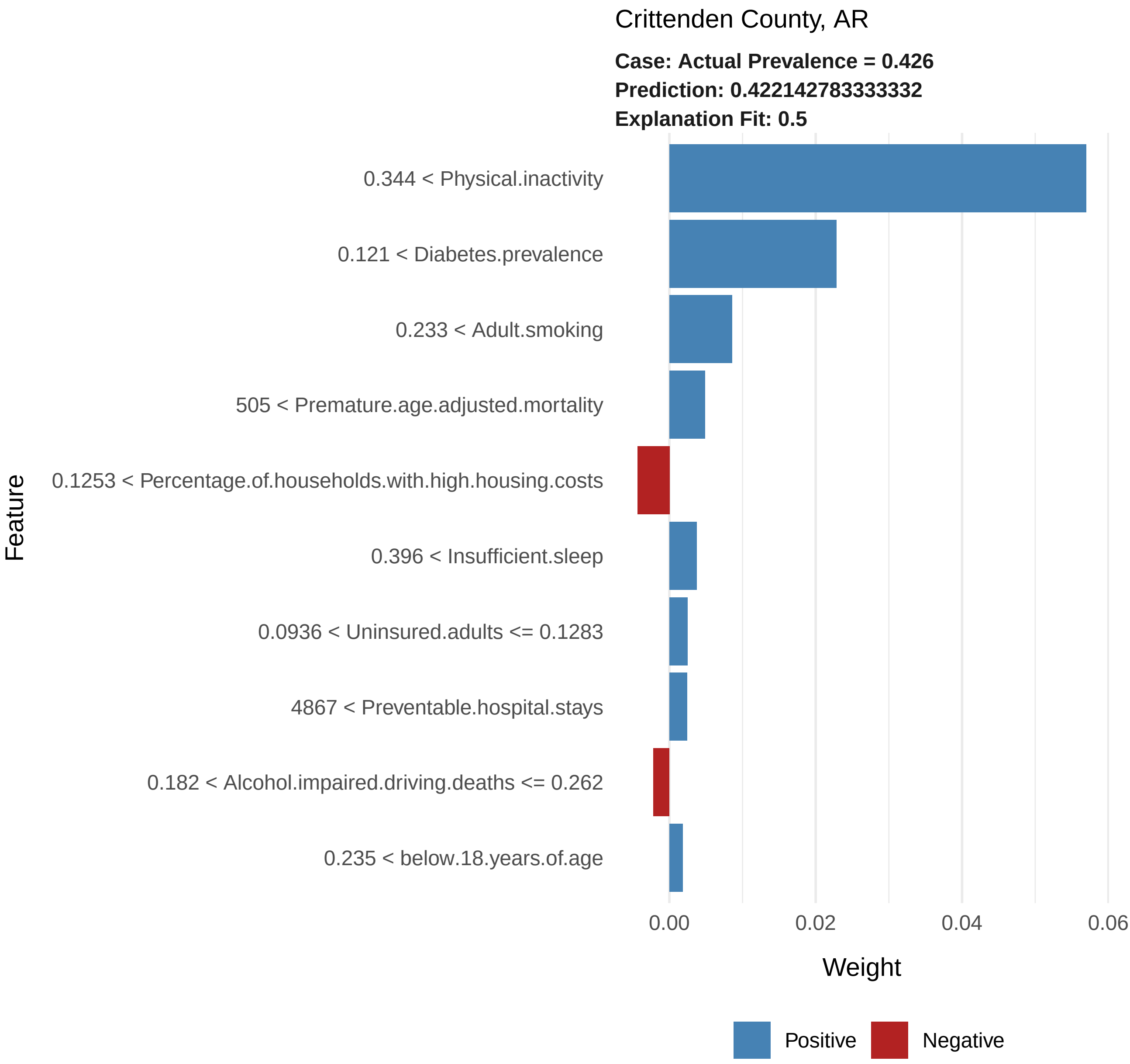
Crittenden County, AR
Case: Actual Prevalence = 0.426
Prediction: 0.422142783333332
Explanation Fit: 0.5
0.344 < Physical.inactivity
0.121 < Diabetes.prevalence
0.233 < Adult.smoking
505 < Premature.age.adjusted.mortality
0.1253 < Percentage.of.households.with.high.housing.costs
0.396 < Insufficient.sleep
0.0936 < Uninsured.adults <= 0.1283
4867 < Preventable.hospital.stays
0.182 < Alcohol.impaired.driving.deaths <= 0.262
0.235 < below.18.years.of.age
Feature
0.00
0.02
0.04
0.06
Weight
Positive
Negative

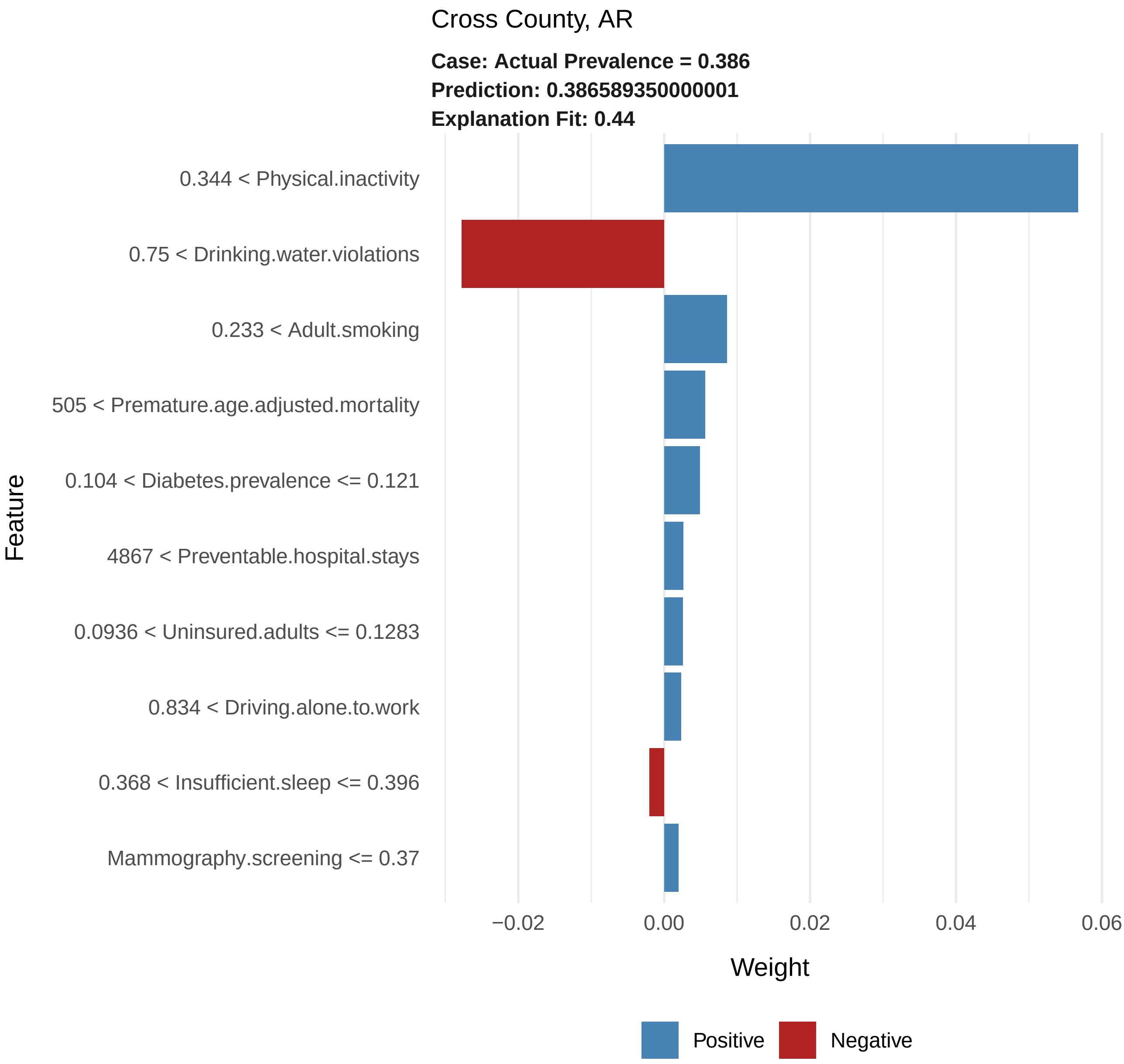

Cross County, AR
Case: Actual Prevalence = 0.386
Prediction: 0.386589350000001
Explanation Fit: 0.44
0.344 < Physical.inactivity
0.75 < Drinking.water.violations
0.233 < Adult.smoking
505 < Premature.age.adjusted.mortality
0.104 < Diabetes.prevalence <= 0.121
4867 < Preventable.hospital.stays
0.0936 < Uninsured.adults <= 0.1283
0.834 < Driving.alone.to.work
0.368 < Insufficient.sleep <= 0.396
Mammography.screening <= 0.37
Feature
−0.02
0.00
0.02
0.04
0.06
Weight
Positive
Negative

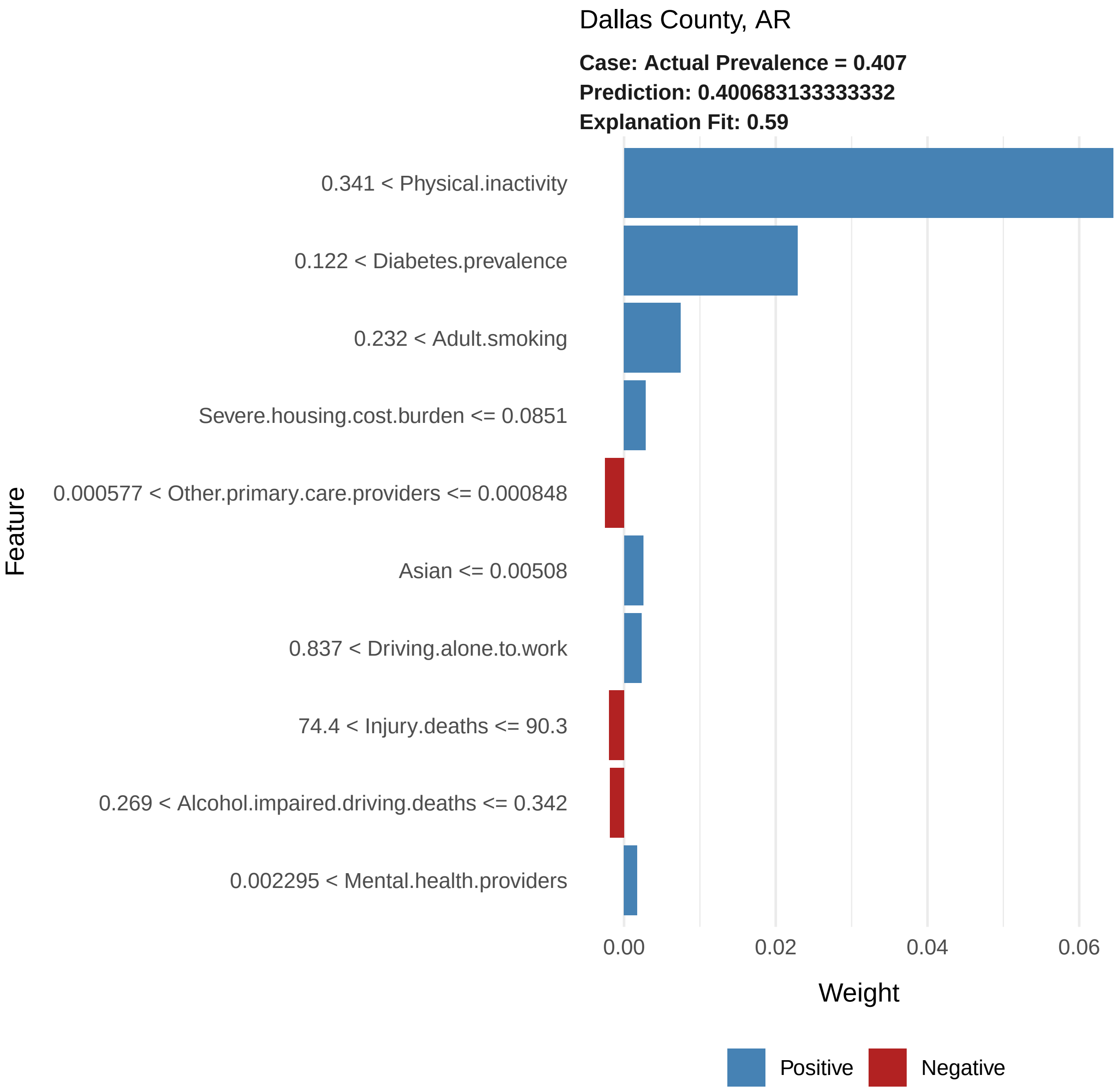

Dallas County, AR
Case: Actual Prevalence = 0.407
Prediction: 0.400683133333332
Explanation Fit: 0.59
0.341 < Physical.inactivity
0.122 < Diabetes.prevalence
0.232 < Adult.smoking
Severe.housing.cost.burden <= 0.0851
0.000577 < Other.primary.care.providers <= 0.000848
Asian <= 0.00508
0.837 < Driving.alone.to.work
74.4 < Injury.deaths <= 90.3
0.269 < Alcohol.impaired.driving.deaths <= 0.342
0.002295 < Mental.health.providers
Feature
0.00
0.02
0.04
0.06
Weight
Positive
Negative

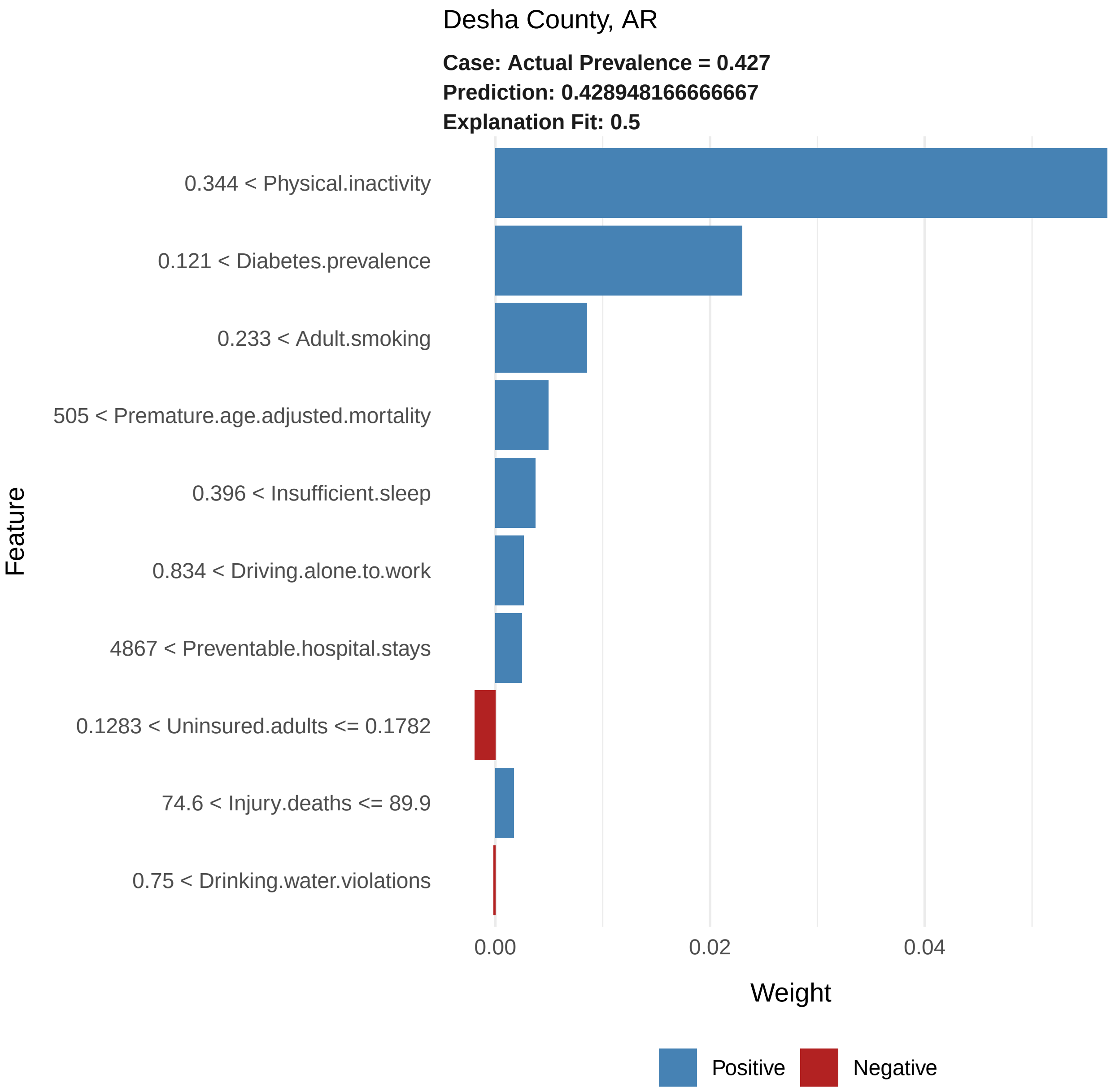

Desha County, AR
Case: Actual Prevalence = 0.427
Prediction: 0.428948166666667
Explanation Fit: 0.5
0.344 < Physical.inactivity
0.121 < Diabetes.prevalence
0.233 < Adult.smoking
505 < Premature.age.adjusted.mortality
0.396 < Insufficient.sleep
0.834 < Driving.alone.to.work
4867 < Preventable.hospital.stays
0.1283 < Uninsured.adults <= 0.1782
74.6 < Injury.deaths <= 89.9
0.75 < Drinking.water.violations
Feature
0.00
0.02
0.04
Weight
Positive
Negative

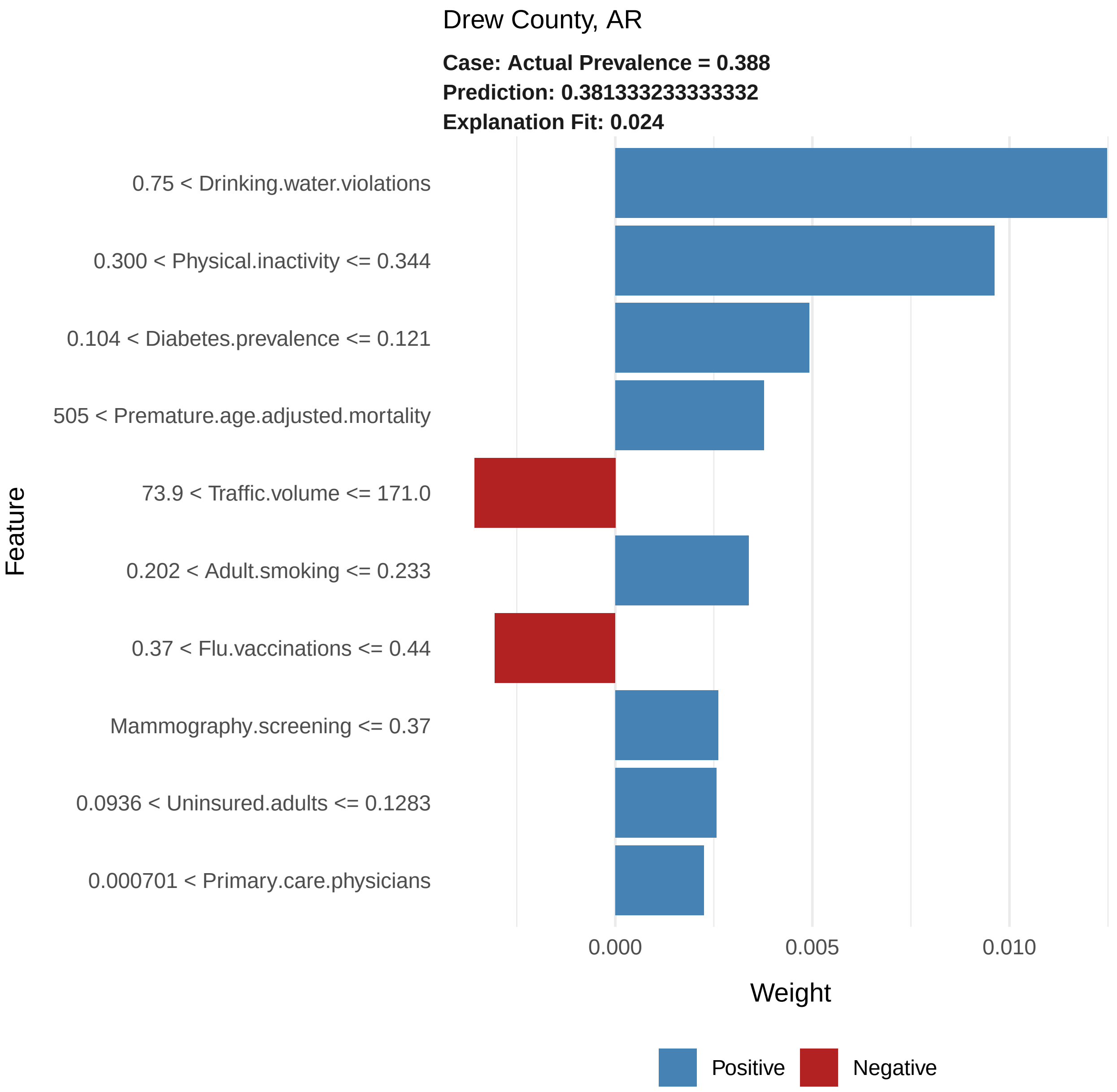

Drew County, AR
Case: Actual Prevalence = 0.388
Prediction: 0.381333233333332
Explanation Fit: 0.024
0.75 < Drinking.water.violations
0.300 < Physical.inactivity <= 0.344
0.104 < Diabetes.prevalence <= 0.121
505 < Premature.age.adjusted.mortality
73.9 < Traffic.volume <= 171.0
0.202 < Adult.smoking <= 0.233
0.37 < Flu.vaccinations <= 0.44
Mammography.screening <= 0.37
0.0936 < Uninsured.adults <= 0.1283
0.000701 < Primary.care.physicians
Feature
0.000
0.005
0.010
Weight
Positive
Negative

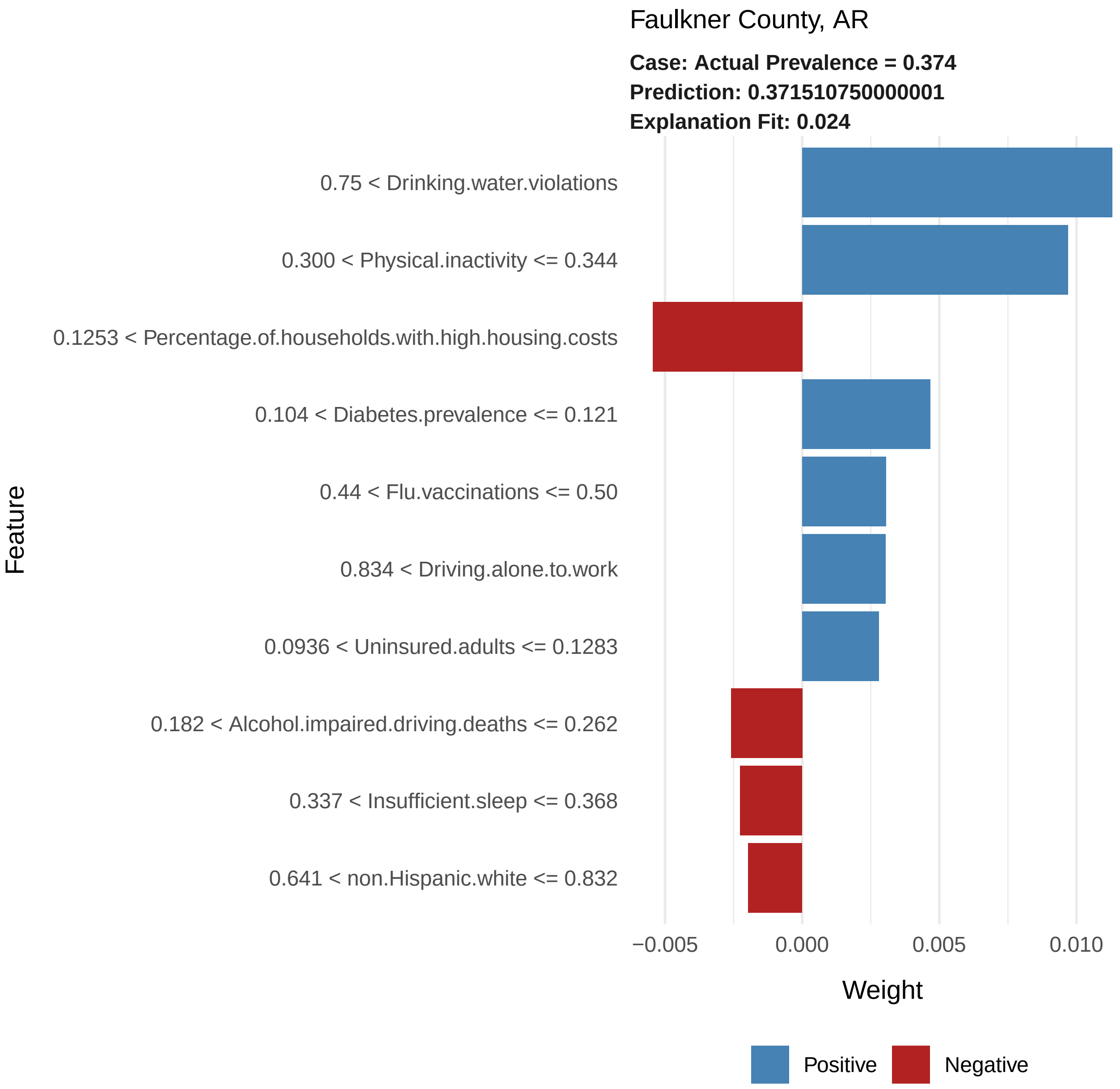

Faulkner County, AR
Case: Actual Prevalence = 0.374
Prediction: 0.371510750000001
Explanation Fit: 0.024
0.75 < Drinking.water.violations
0.300 < Physical.inactivity <= 0.344
0.1253 < Percentage.of.households.with.high.housing.costs
0.104 < Diabetes.prevalence <= 0.121
0.44 < Flu.vaccinations <= 0.50
0.834 < Driving.alone.to.work
0.0936 < Uninsured.adults <= 0.1283
0.182 < Alcohol.impaired.driving.deaths <= 0.262
0.337 < Insufficient.sleep <= 0.368
0.641 < non.Hispanic.white <= 0.832
Feature
−0.005
0.000
0.005
0.010
Weight
Positive
Negative

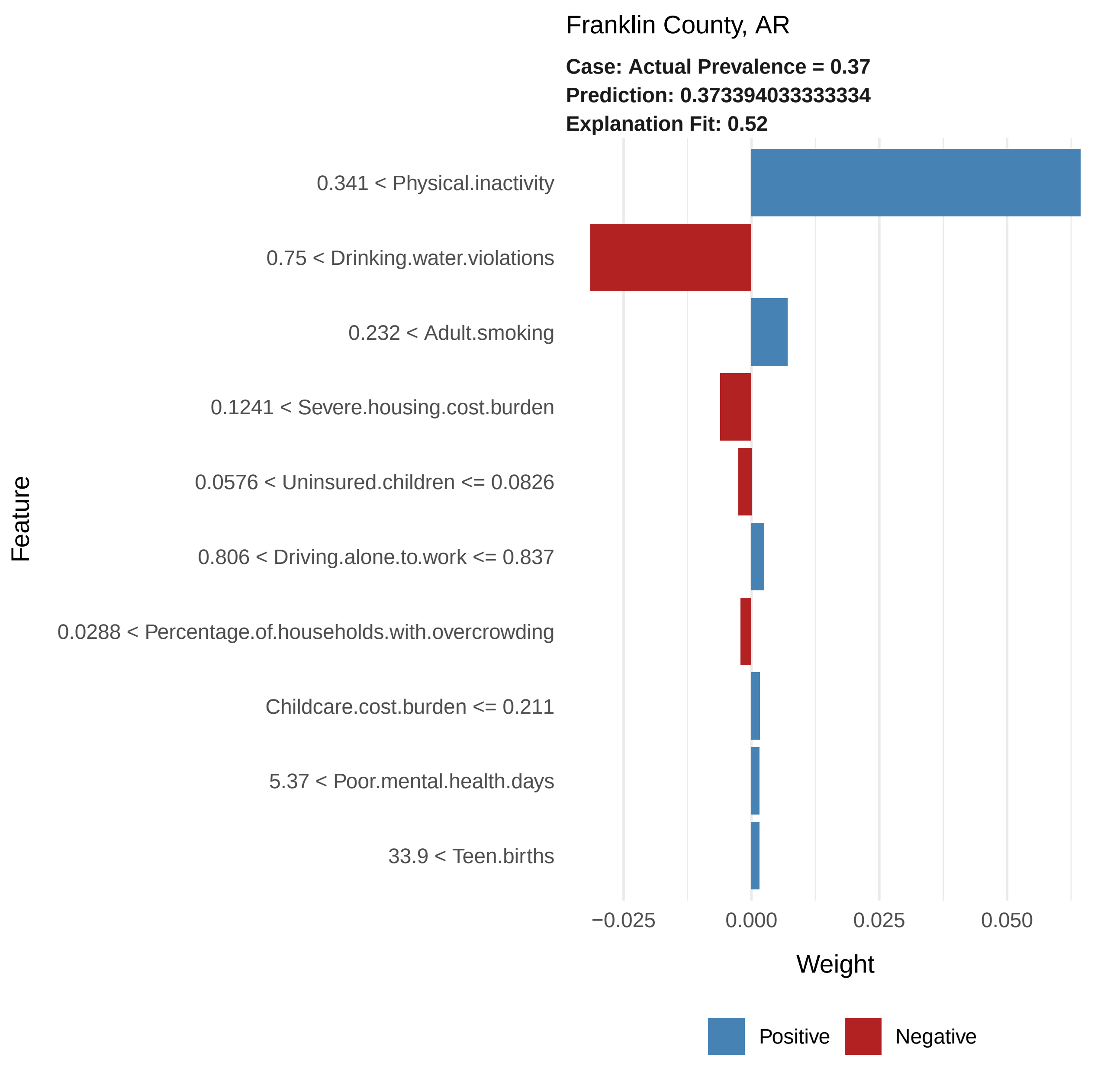

Franklin County, AR
Case: Actual Prevalence = 0.37
Prediction: 0.373394033333334
Explanation Fit: 0.52
0.341 < Physical.inactivity
0.75 < Drinking.water.violations
0.232 < Adult.smoking
0.1241 < Severe.housing.cost.burden
0.0576 < Uninsured.children <= 0.0826
0.806 < Driving.alone.to.work <= 0.837
0.0288 < Percentage.of.households.with.overcrowding
Childcare.cost.burden <= 0.211
5.37 < Poor.mental.health.days
33.9 < Teen.births
Feature
−0.025
0.000
0.025
0.050
Weight
Positive
Negative

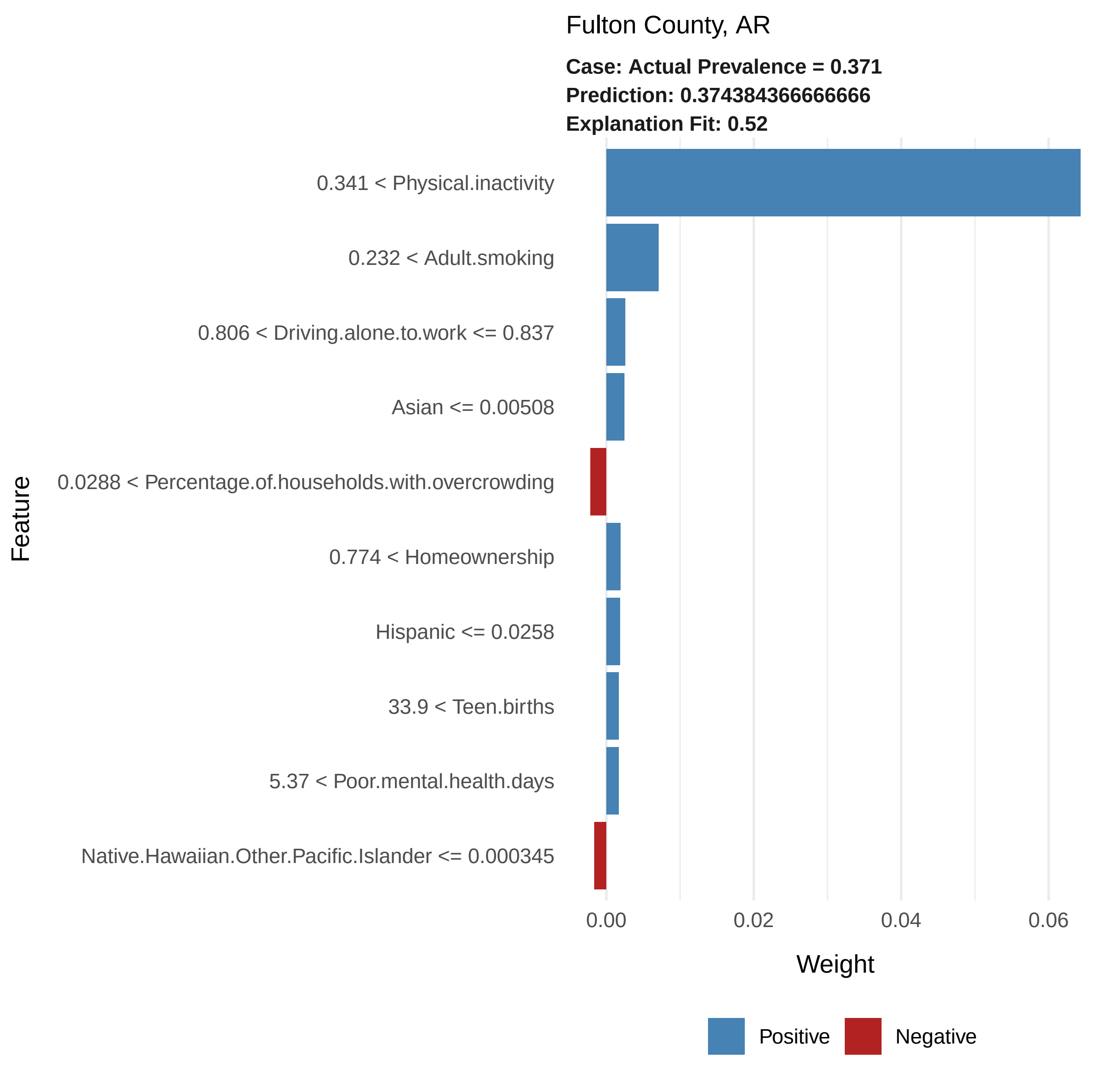
Fulton County, AR
Case: Actual Prevalence = 0.371
Prediction: 0.374384366666666
Explanation Fit: 0.52
0.341 < Physical.inactivity
0.232 < Adult.smoking
0.806 < Driving.alone.to.work <= 0.837
Asian <= 0.00508
0.0288 < Percentage.of.households.with.overcrowding
0.774 < Homeownership
Hispanic <= 0.0258
33.9 < Teen.births
5.37 < Poor.mental.health.days
Native.Hawaiian.Other.Pacific.Islander <= 0.000345
Feature
0.00
0.02
0.04
0.06
Weight
Positive
Negative

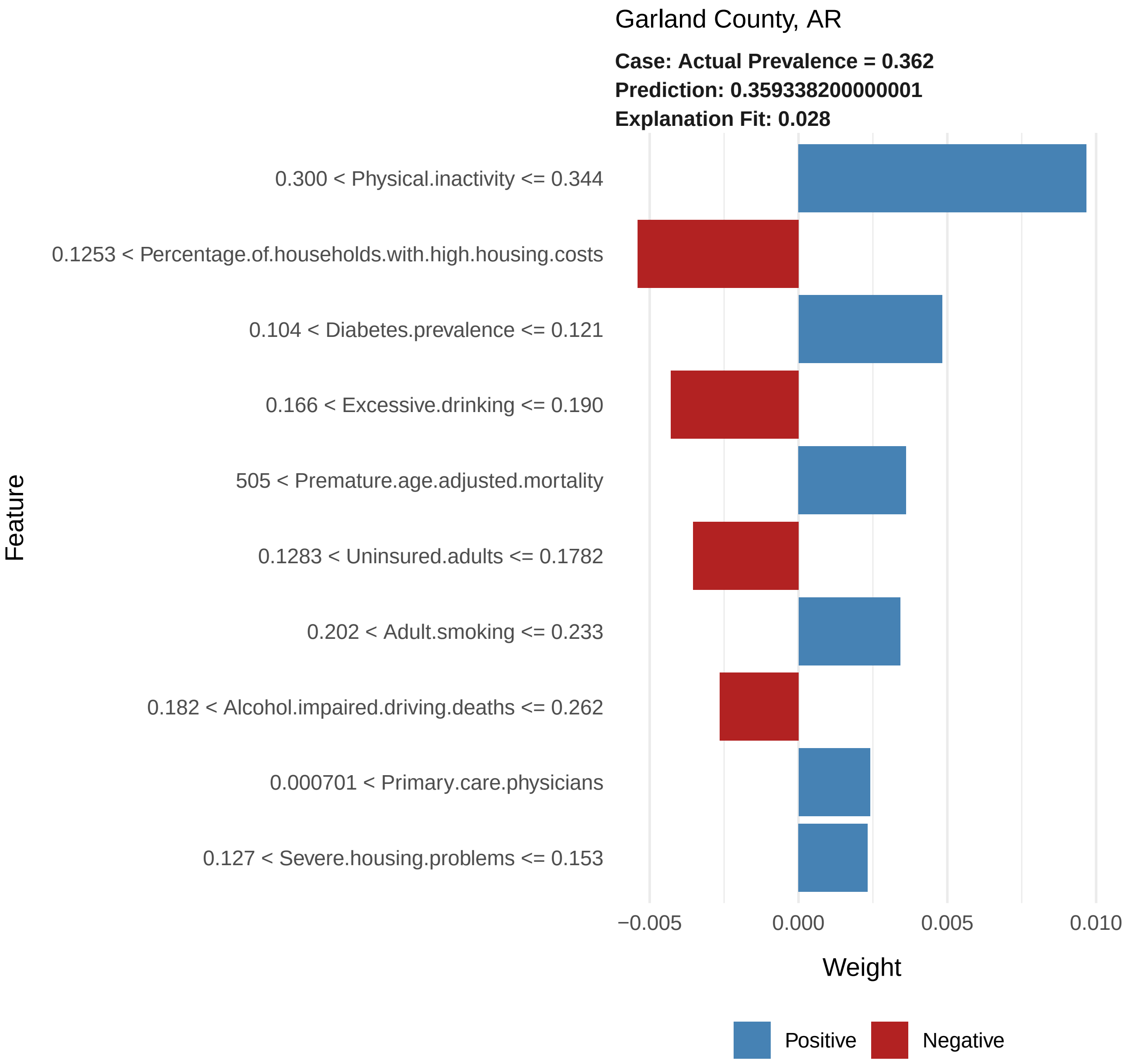
Garland County, AR
Case: Actual Prevalence = 0.362
Prediction: 0.359338200000001
Explanation Fit: 0.028
0.300 < Physical.inactivity <= 0.344
0.1253 < Percentage.of.households.with.high.housing.costs
0.104 < Diabetes.prevalence <= 0.121
0.166 < Excessive.drinking <= 0.190
505 < Premature.age.adjusted.mortality
0.1283 < Uninsured.adults <= 0.1782
0.202 < Adult.smoking <= 0.233
0.182 < Alcohol.impaired.driving.deaths <= 0.262
0.000701 < Primary.care.physicians
0.127 < Severe.housing.problems <= 0.153
Feature
−0.005
0.000
0.005
0.010
Weight
Positive
Negative

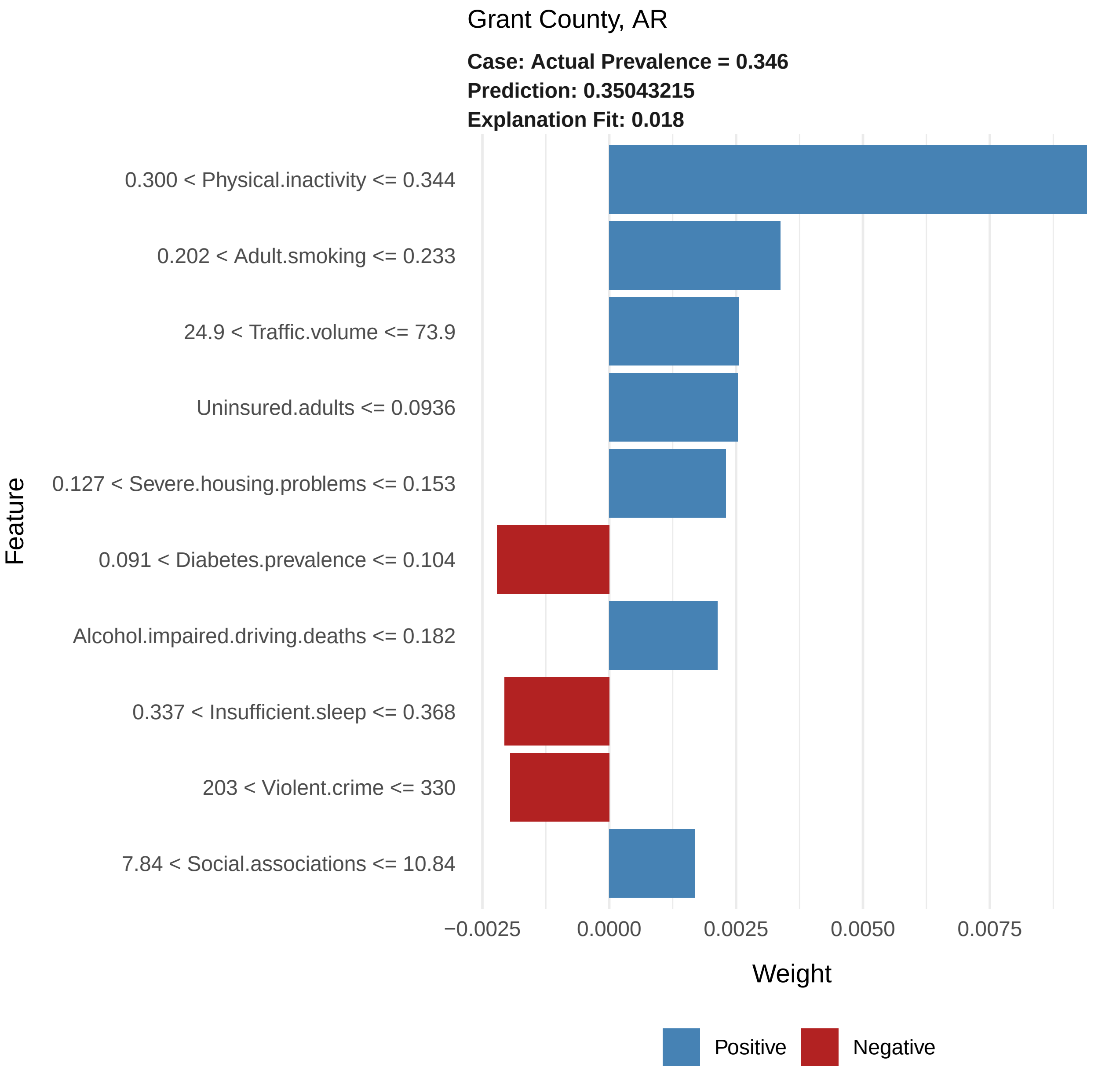

Grant County, AR
Case: Actual Prevalence = 0.346
Prediction: 0.35043215
Explanation Fit: 0.018
0.300 < Physical.inactivity <= 0.344
0.202 < Adult.smoking <= 0.233
24.9 < Traffic.volume <= 73.9
Uninsured.adults <= 0.0936
0.127 < Severe.housing.problems <= 0.153
0.091 < Diabetes.prevalence <= 0.104
Alcohol.impaired.driving.deaths <= 0.182
0.337 < Insufficient.sleep <= 0.368
203 < Violent.crime <= 330
7.84 < Social.associations <= 10.84
Feature
−0.0025
0.0000
0.0025
0.0050
0.0075
Weight
Positive
Negative

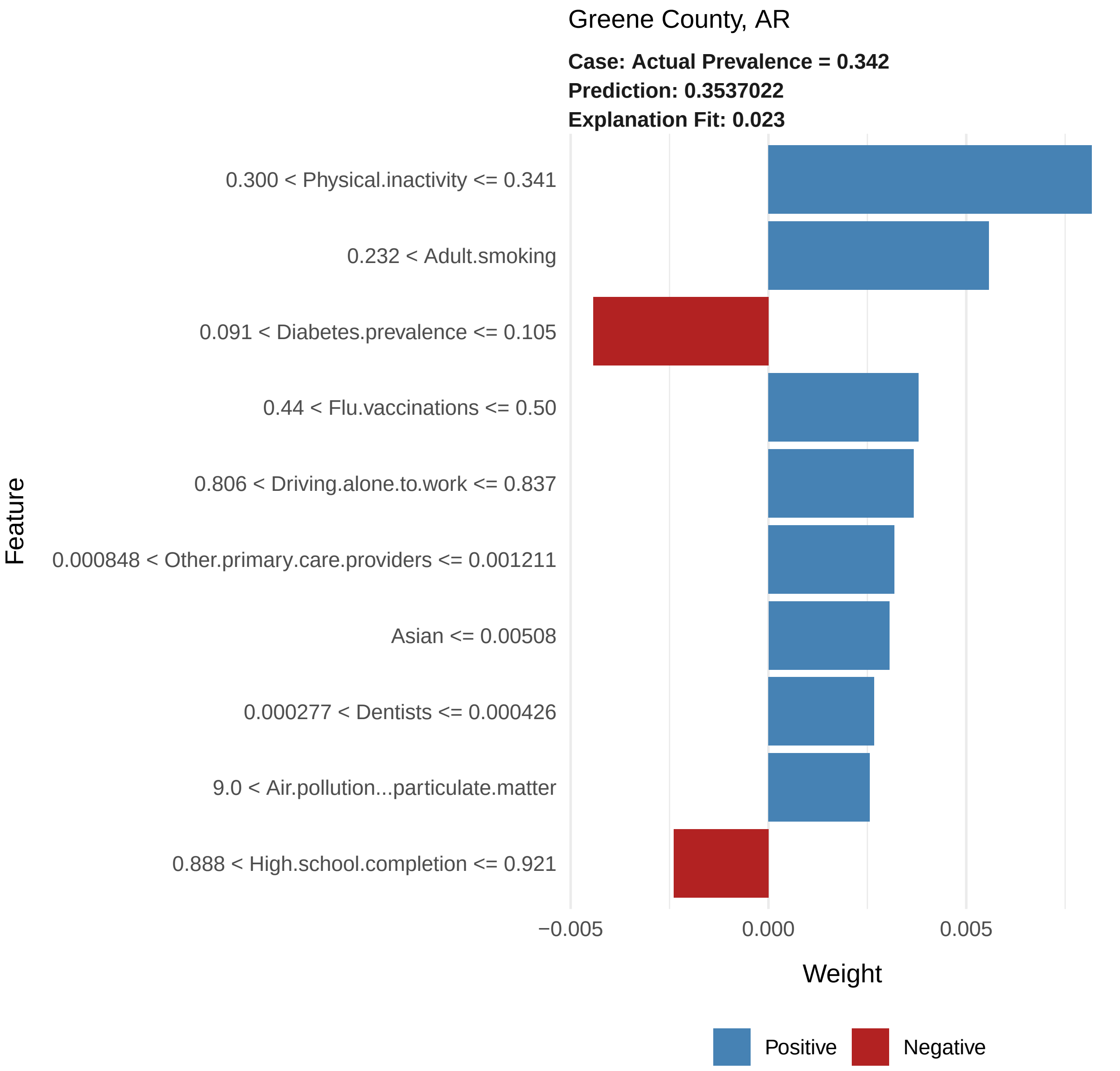
Greene County, AR
Case: Actual Prevalence = 0.342
Prediction: 0.3537022
Explanation Fit: 0.023
0.300 < Physical.inactivity <= 0.341
0.232 < Adult.smoking
0.091 < Diabetes.prevalence <= 0.105
0.44 < Flu.vaccinations <= 0.50
0.806 < Driving.alone.to.work <= 0.837
0.000848 < Other.primary.care.providers <= 0.001211
Asian <= 0.00508
0.000277 < Dentists <= 0.000426
9.0 < Air.pollution...particulate.matter
0.888 < High.school.completion <= 0.921
Feature
−0.005
0.000
0.005
Weight
Positive
Negative

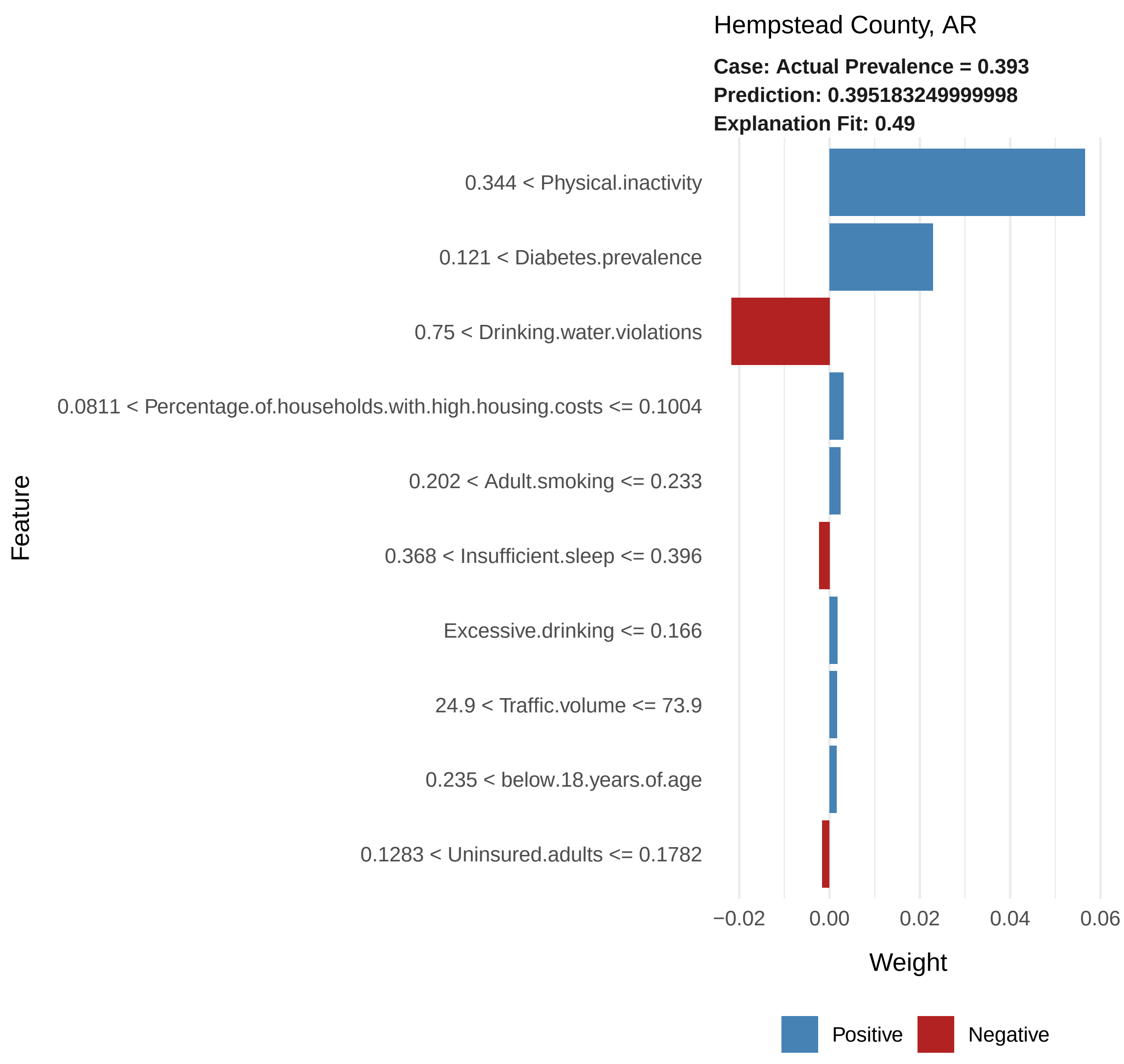

Hempstead County, AR
Case: Actual Prevalence = 0.393
Prediction: 0.395183249999998
Explanation Fit: 0.49
0.344 < Physical.inactivity
0.121 < Diabetes.prevalence
0.75 < Drinking.water.violations
0.0811 < Percentage.of.households.with.high.housing.costs <= 0.1004
0.202 < Adult.smoking <= 0.233
0.368 < Insufficient.sleep <= 0.396
Excessive.drinking <= 0.166
24.9 < Traffic.volume <= 73.9
0.235 < below.18.years.of.age
0.1283 < Uninsured.adults <= 0.1782
Feature
−0.02
0.00
0.02
0.04
0.06
Weight
Positive
Negative

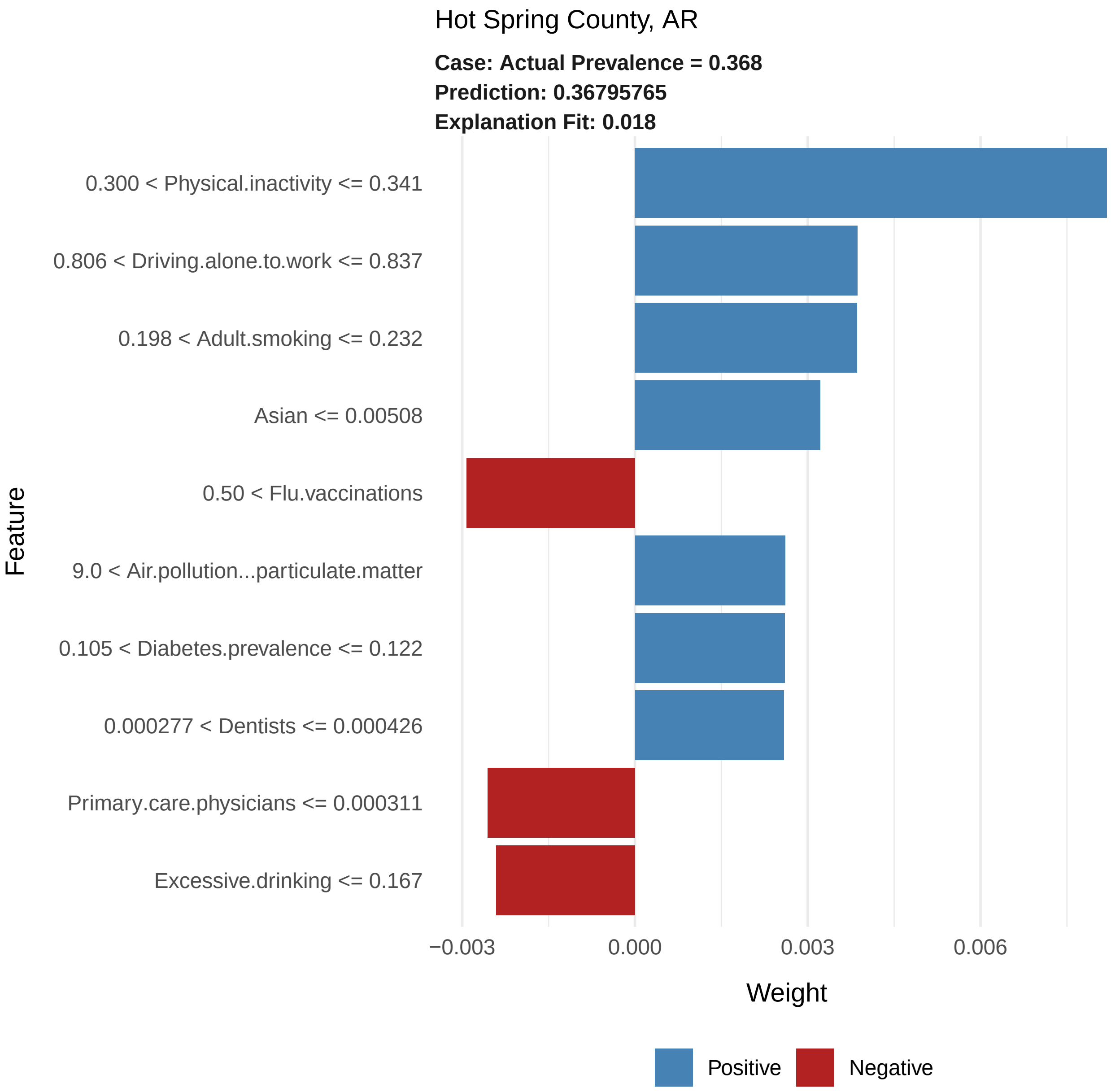
Hot Spring County, AR
Case: Actual Prevalence = 0.368
Prediction: 0.36795765
Explanation Fit: 0.018
0.300 < Physical.inactivity <= 0.341
0.806 < Driving.alone.to.work <= 0.837
0.198 < Adult.smoking <= 0.232
Asian <= 0.00508
0.50 < Flu.vaccinations
9.0 < Air.pollution...particulate.matter
0.105 < Diabetes.prevalence <= 0.122
0.000277 < Dentists <= 0.000426
Primary.care.physicians <= 0.000311
Excessive.drinking <= 0.167
Feature
−0.003
0.000
0.003
0.006
Weight
Positive
Negative

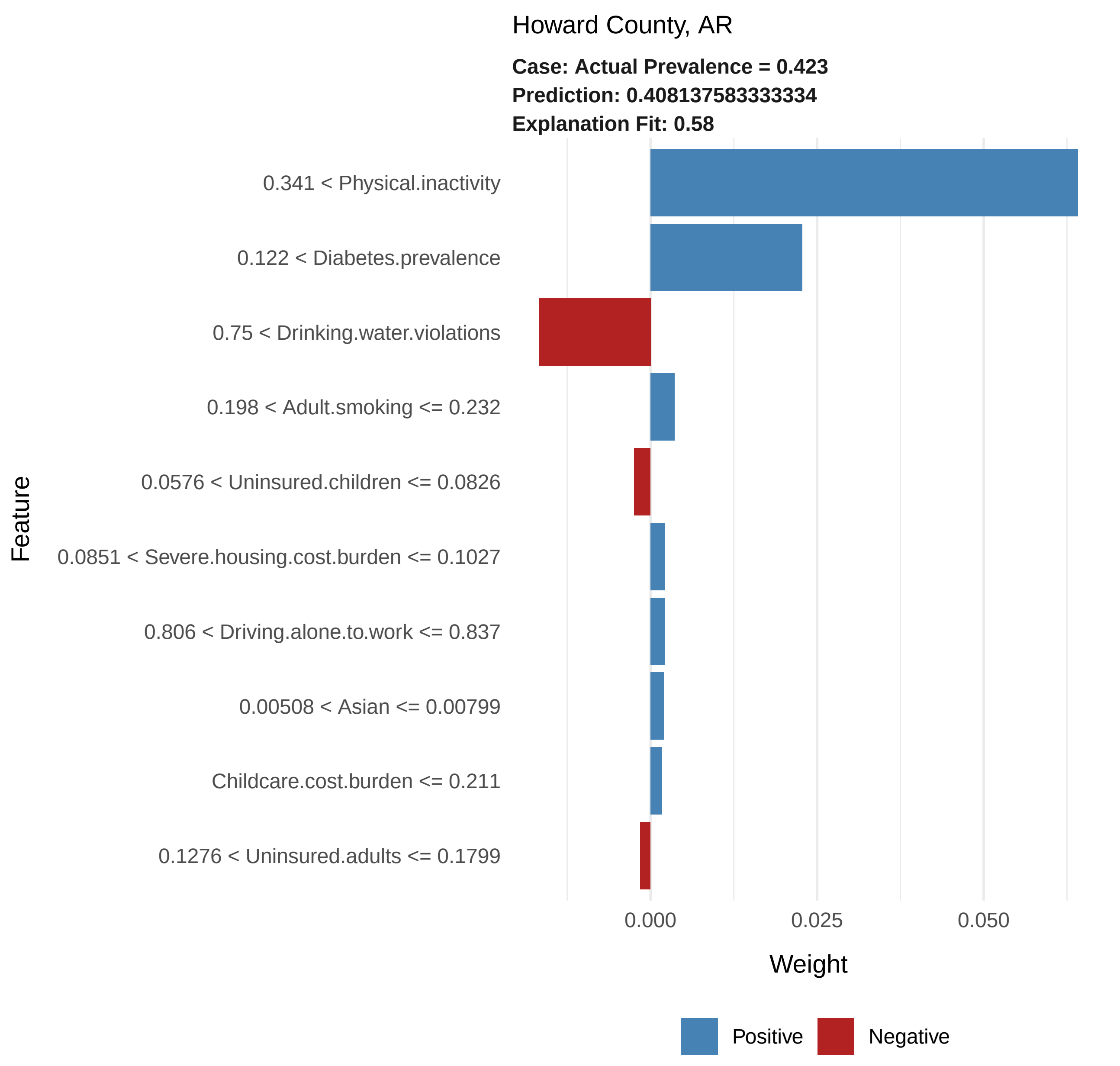

Howard County, AR
Case: Actual Prevalence = 0.423
Prediction: 0.408137583333334
Explanation Fit: 0.58
0.341 < Physical.inactivity
0.122 < Diabetes.prevalence
0.75 < Drinking.water.violations
0.198 < Adult.smoking <= 0.232
0.0576 < Uninsured.children <= 0.0826
0.0851 < Severe.housing.cost.burden <= 0.1027
0.806 < Driving.alone.to.work <= 0.837
0.00508 < Asian <= 0.00799
Childcare.cost.burden <= 0.211
0.1276 < Uninsured.adults <= 0.1799
Feature
0.000
0.025
0.050
Weight
Positive
Negative

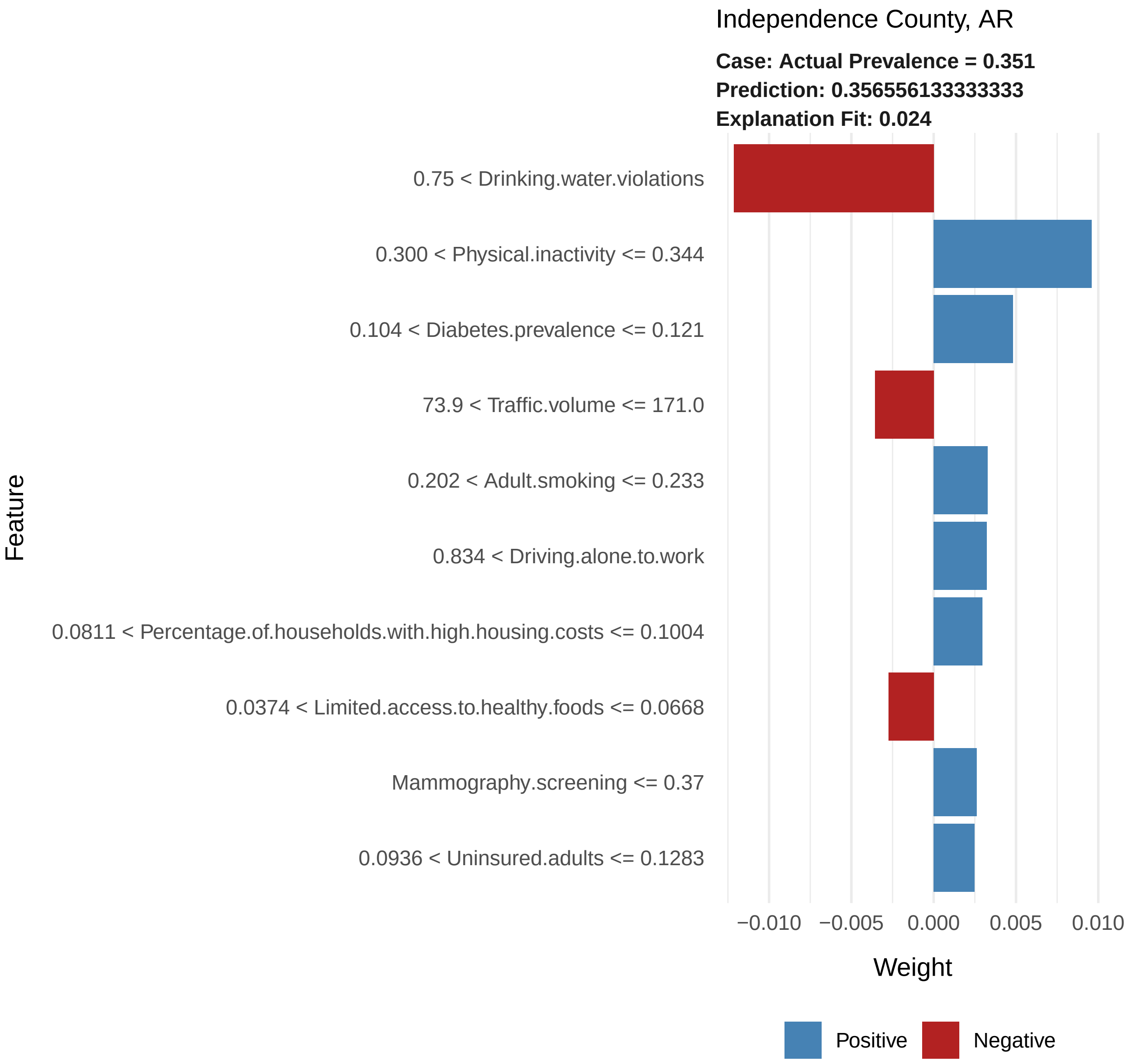

Independence County, AR
Case: Actual Prevalence = 0.351
Prediction: 0.356556133333333
Explanation Fit: 0.024
0.75 < Drinking.water.violations
0.300 < Physical.inactivity <= 0.344
0.104 < Diabetes.prevalence <= 0.121
73.9 < Traffic.volume <= 171.0
0.202 < Adult.smoking <= 0.233
0.834 < Driving.alone.to.work
0.0811 < Percentage.of.households.with.high.housing.costs <= 0.1004
0.0374 < Limited.access.to.healthy.foods <= 0.0668
Mammography.screening <= 0.37
0.0936 < Uninsured.adults <= 0.1283
Feature
−0.010
−0.005
0.000
0.005
0.010
Weight
Positive
Negative

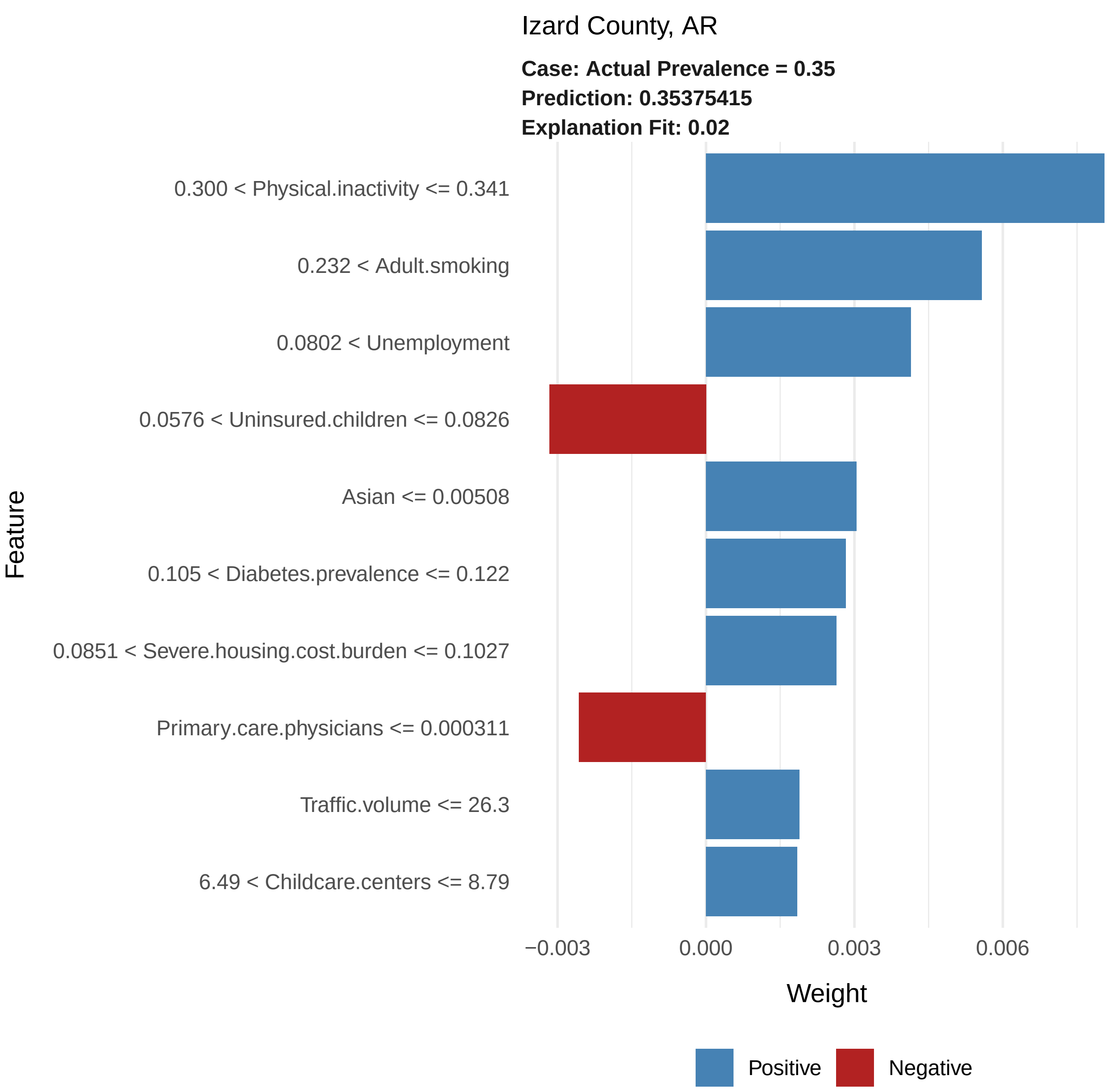

Izard County, AR
Case: Actual Prevalence = 0.35
Prediction: 0.35375415
Explanation Fit: 0.02
0.300 < Physical.inactivity <= 0.341
0.232 < Adult.smoking
0.0802 < Unemployment
0.0576 < Uninsured.children <= 0.0826
Asian <= 0.00508
0.105 < Diabetes.prevalence <= 0.122
0.0851 < Severe.housing.cost.burden <= 0.1027
Primary.care.physicians <= 0.000311
Traffic.volume <= 26.3
6.49 < Childcare.centers <= 8.79
Feature
−0.003
0.000
0.003
0.006
Weight
Positive
Negative

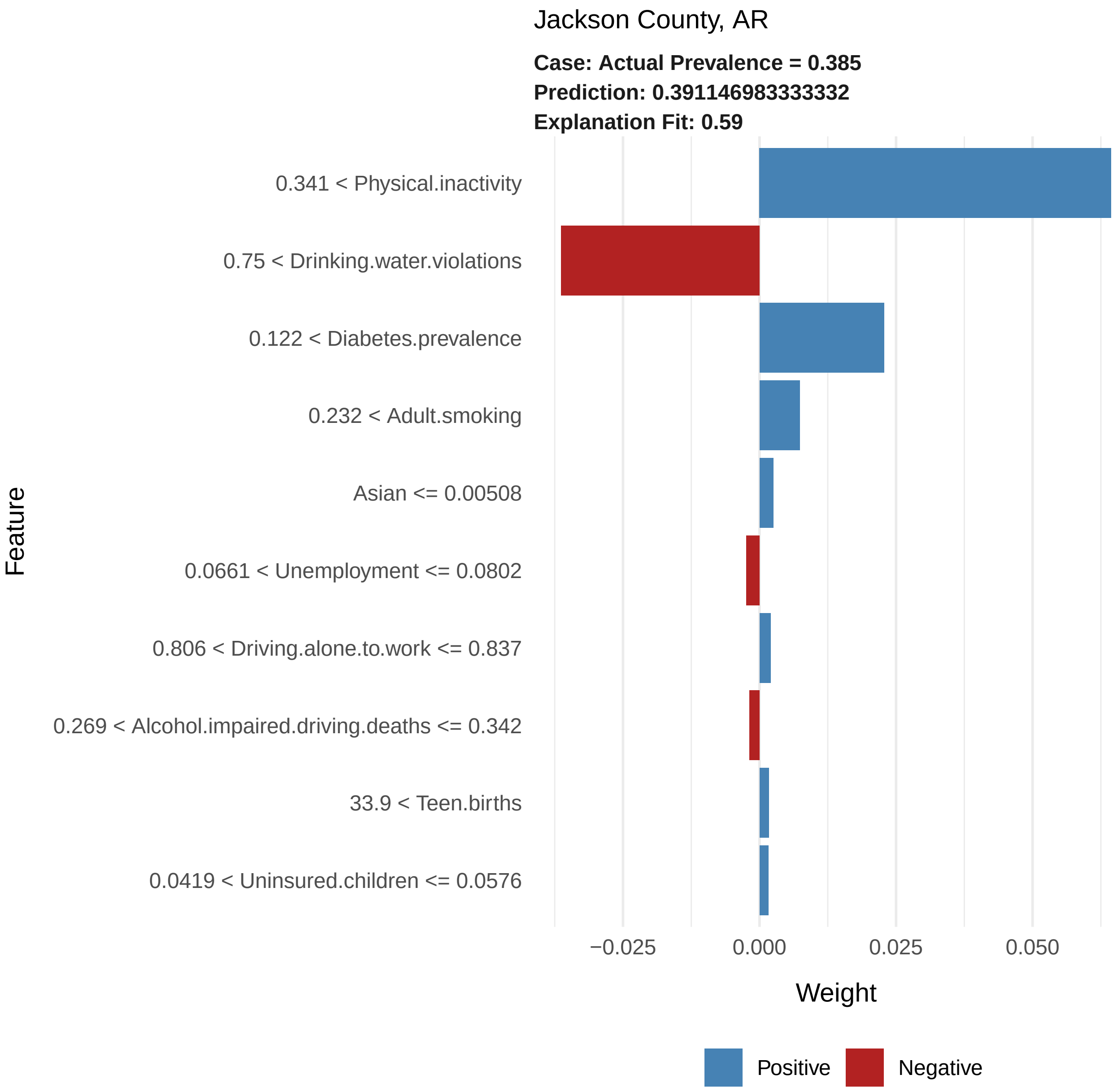
Jackson County, AR
Case: Actual Prevalence = 0.385
Prediction: 0.391146983333332
Explanation Fit: 0.59
0.341 < Physical.inactivity
0.75 < Drinking.water.violations
0.122 < Diabetes.prevalence
0.232 < Adult.smoking
Asian <= 0.00508
0.0661 < Unemployment <= 0.0802
0.806 < Driving.alone.to.work <= 0.837
0.269 < Alcohol.impaired.driving.deaths <= 0.342
33.9 < Teen.births
0.0419 < Uninsured.children <= 0.0576
Feature
−0.025
0.000
0.025
0.050
Weight
Positive
Negative

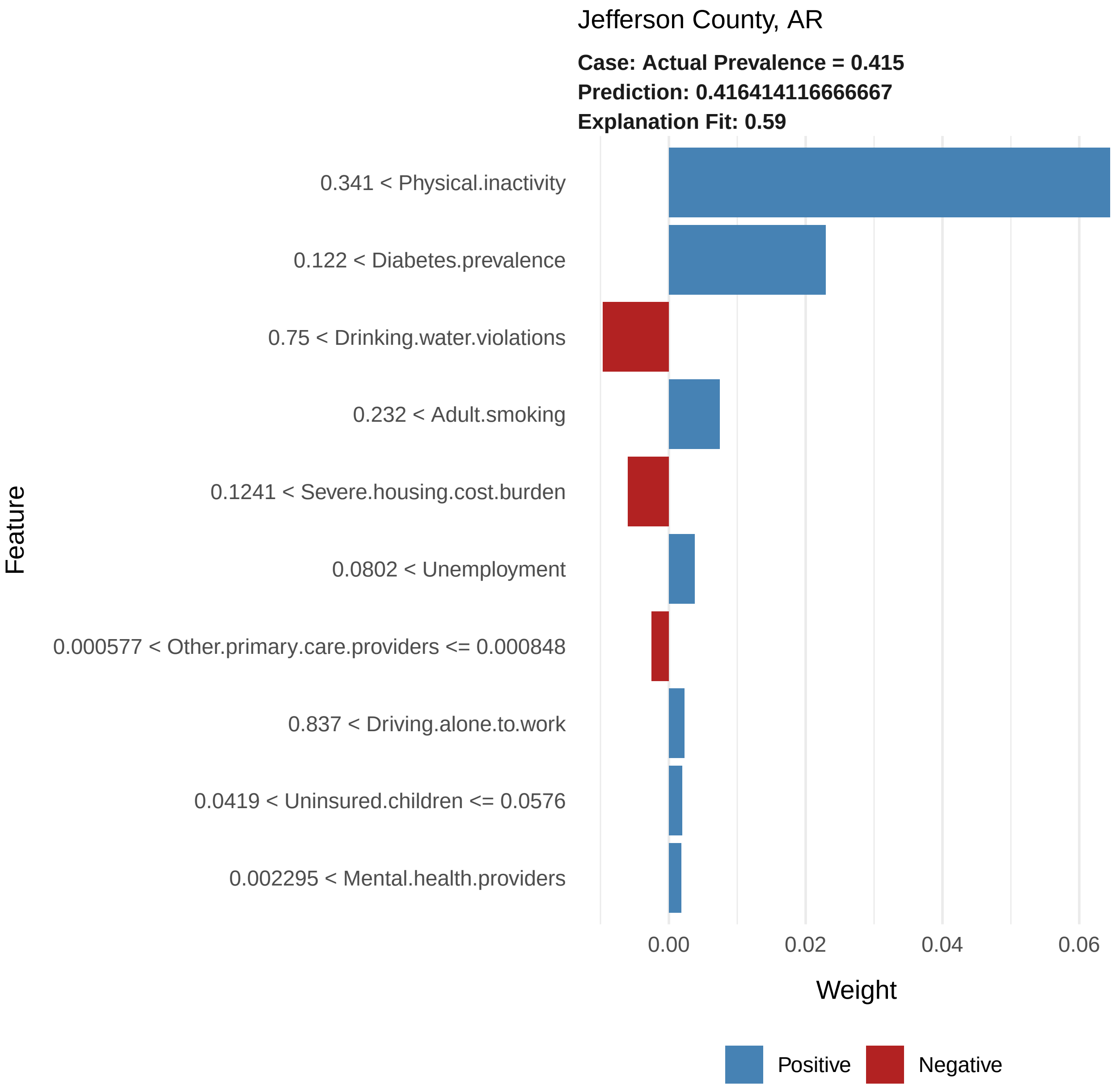

Jefferson County, AR
Case: Actual Prevalence = 0.415
Prediction: 0.416414116666667
Explanation Fit: 0.59
0.341 < Physical.inactivity
0.122 < Diabetes.prevalence
0.75 < Drinking.water.violations
0.232 < Adult.smoking
0.1241 < Severe.housing.cost.burden
0.0802 < Unemployment
0.000577 < Other.primary.care.providers <= 0.000848
0.837 < Driving.alone.to.work
0.0419 < Uninsured.children <= 0.0576
0.002295 < Mental.health.providers
Feature
0.00
0.02
0.04
0.06
Weight
Positive
Negative

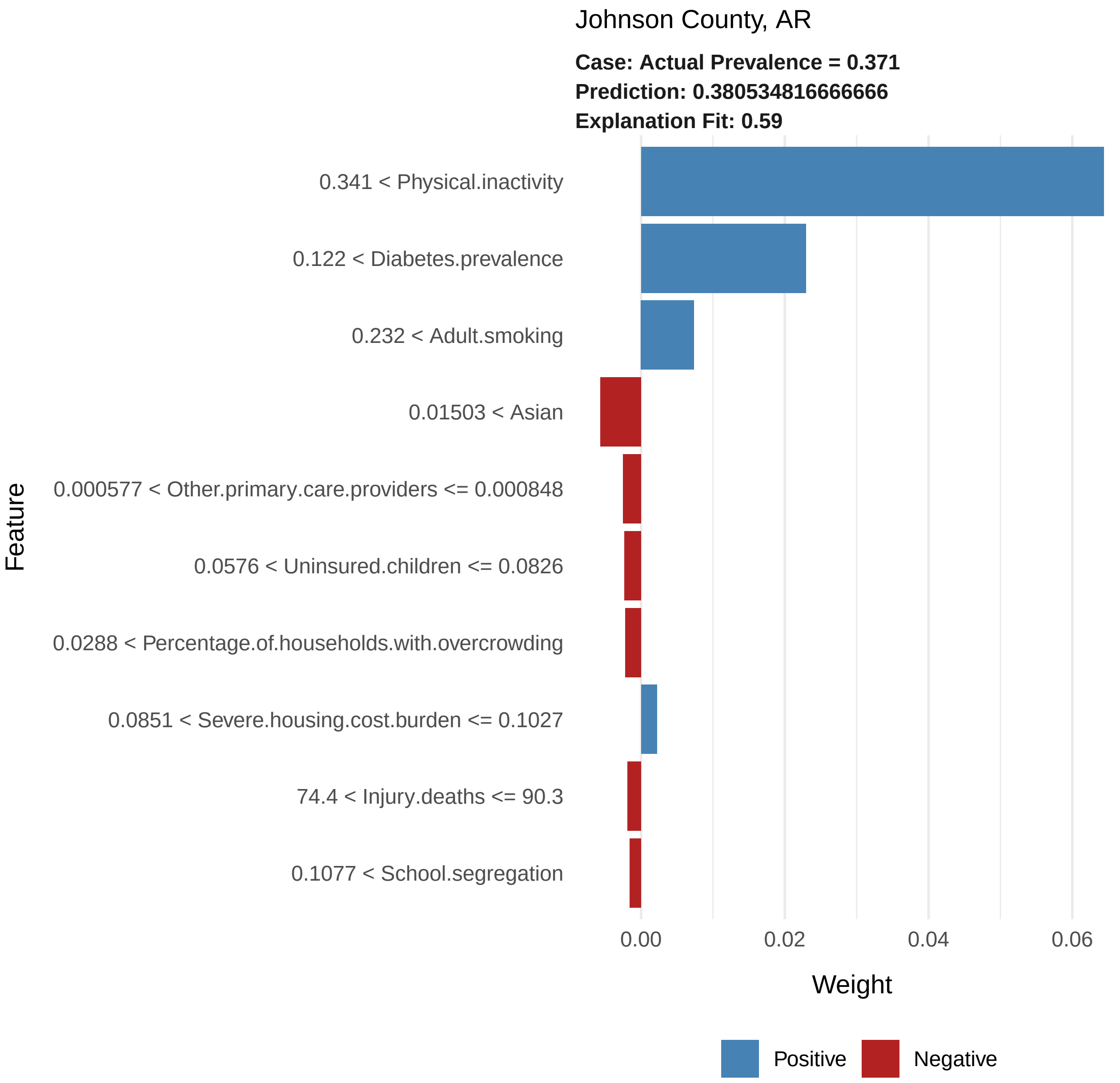

Johnson County, AR
Case: Actual Prevalence = 0.371
Prediction: 0.380534816666666
Explanation Fit: 0.59
0.341 < Physical.inactivity
0.122 < Diabetes.prevalence
0.232 < Adult.smoking
0.01503 < Asian
0.000577 < Other.primary.care.providers <= 0.000848
0.0576 < Uninsured.children <= 0.0826
0.0288 < Percentage.of.households.with.overcrowding
0.0851 < Severe.housing.cost.burden <= 0.1027
74.4 < Injury.deaths <= 90.3
0.1077 < School.segregation
Feature
0.00
0.02
0.04
0.06
Weight
Positive
Negative

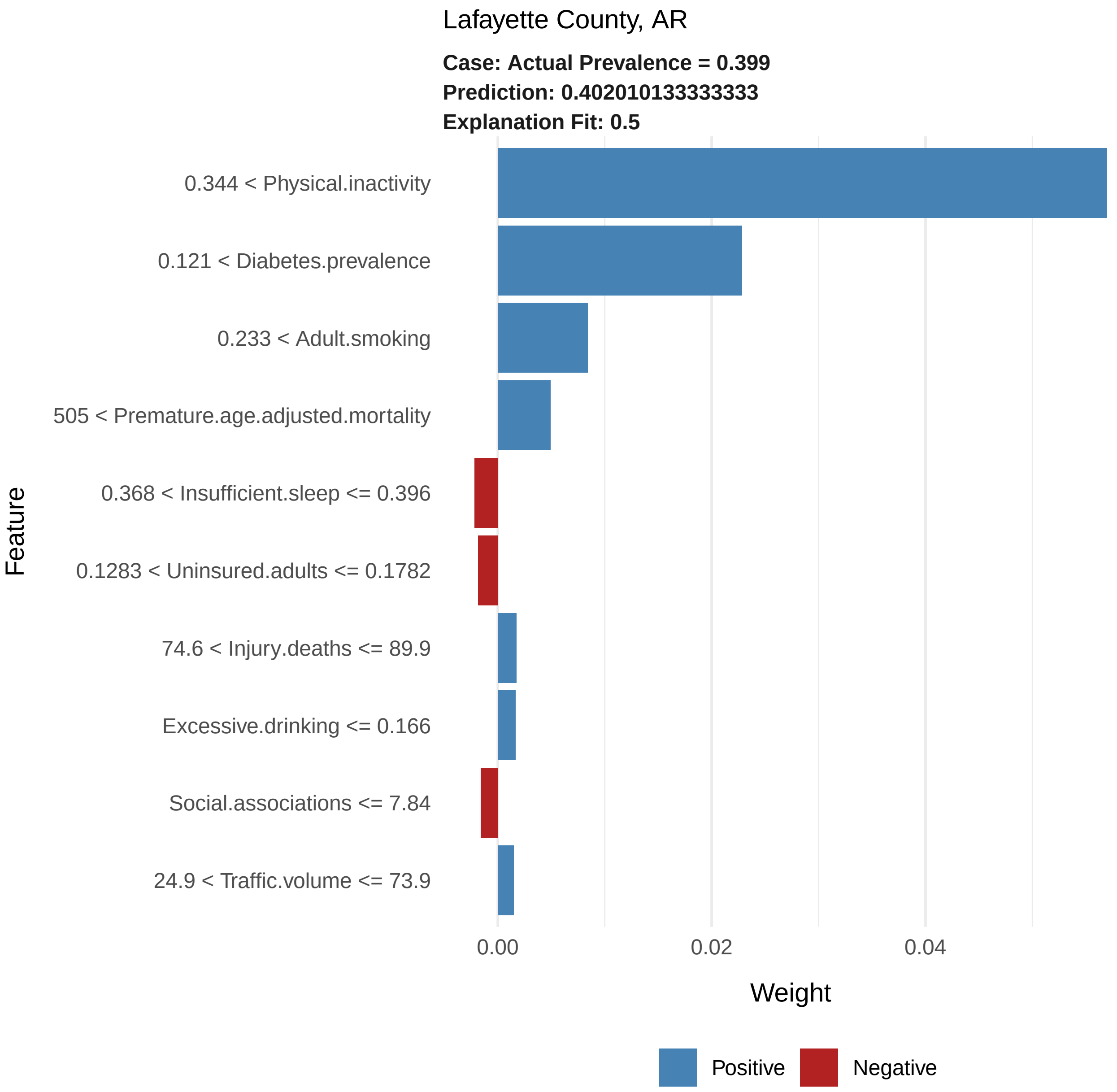

Lafayette County, AR
Case: Actual Prevalence = 0.399
Prediction: 0.402010133333333
Explanation Fit: 0.5
0.344 < Physical.inactivity
0.121 < Diabetes.prevalence
0.233 < Adult.smoking
505 < Premature.age.adjusted.mortality
0.368 < Insufficient.sleep <= 0.396
0.1283 < Uninsured.adults <= 0.1782
74.6 < Injury.deaths <= 89.9
Excessive.drinking <= 0.166
Social.associations <= 7.84
24.9 < Traffic.volume <= 73.9
Feature
0.00
0.02
0.04
Weight
Positive
Negative

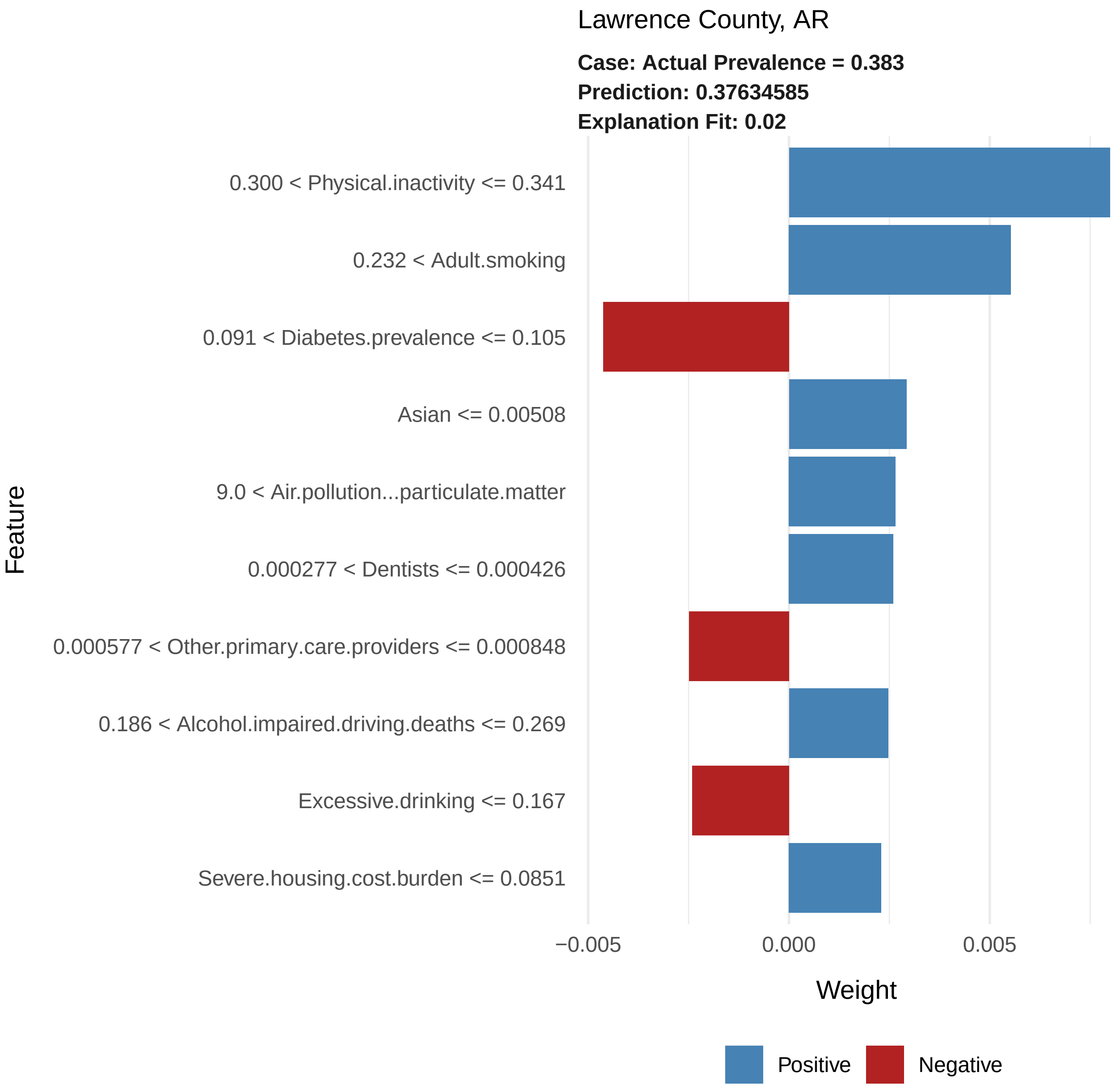
Lawrence County, AR
Case: Actual Prevalence = 0.383
Prediction: 0.37634585
Explanation Fit: 0.02
0.300 < Physical.inactivity <= 0.341
0.232 < Adult.smoking
0.091 < Diabetes.prevalence <= 0.105
Asian <= 0.00508
9.0 < Air.pollution...particulate.matter
0.000277 < Dentists <= 0.000426
0.000577 < Other.primary.care.providers <= 0.000848
0.186 < Alcohol.impaired.driving.deaths <= 0.269
Excessive.drinking <= 0.167
Severe.housing.cost.burden <= 0.0851
Feature
−0.005
0.000
0.005
Weight
Positive
Negative

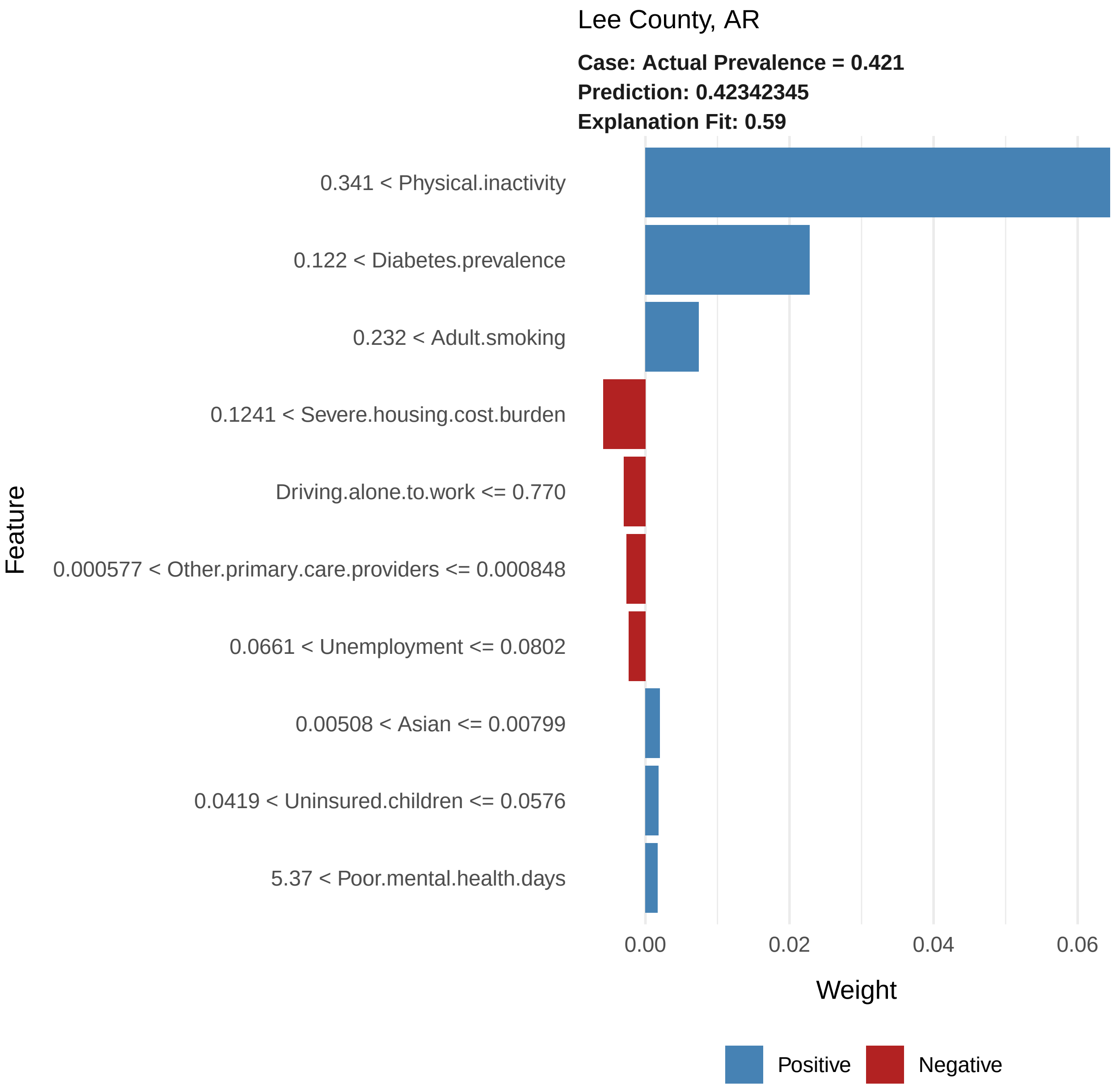
Lee County, AR
Case: Actual Prevalence = 0.421
Prediction: 0.42342345
Explanation Fit: 0.59
0.341 < Physical.inactivity
0.122 < Diabetes.prevalence
0.232 < Adult.smoking
0.1241 < Severe.housing.cost.burden
Driving.alone.to.work <= 0.770
0.000577 < Other.primary.care.providers <= 0.000848
0.0661 < Unemployment <= 0.0802
0.00508 < Asian <= 0.00799
0.0419 < Uninsured.children <= 0.0576
5.37 < Poor.mental.health.days
Feature
0.00
0.02
0.04
0.06
Weight
Positive
Negative

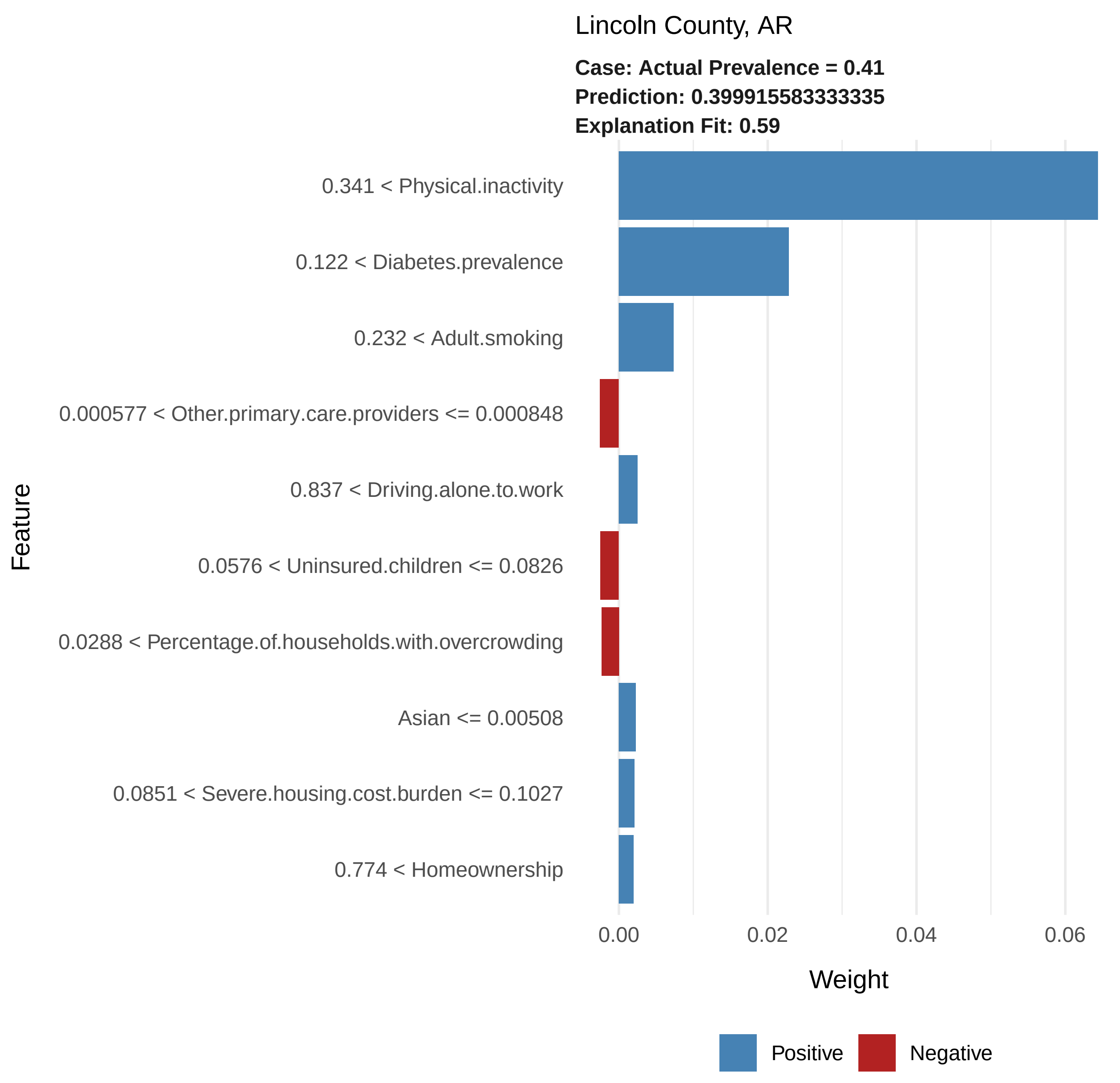

Lincoln County, AR
Case: Actual Prevalence = 0.41
Prediction: 0.399915583333335
Explanation Fit: 0.59
0.341 < Physical.inactivity
0.122 < Diabetes.prevalence
0.232 < Adult.smoking
0.000577 < Other.primary.care.providers <= 0.000848
0.837 < Driving.alone.to.work
0.0576 < Uninsured.children <= 0.0826
0.0288 < Percentage.of.households.with.overcrowding
Asian <= 0.00508
0.0851 < Severe.housing.cost.burden <= 0.1027
0.774 < Homeownership
Feature
0.00
0.02
0.04
0.06
Weight
Positive
Negative

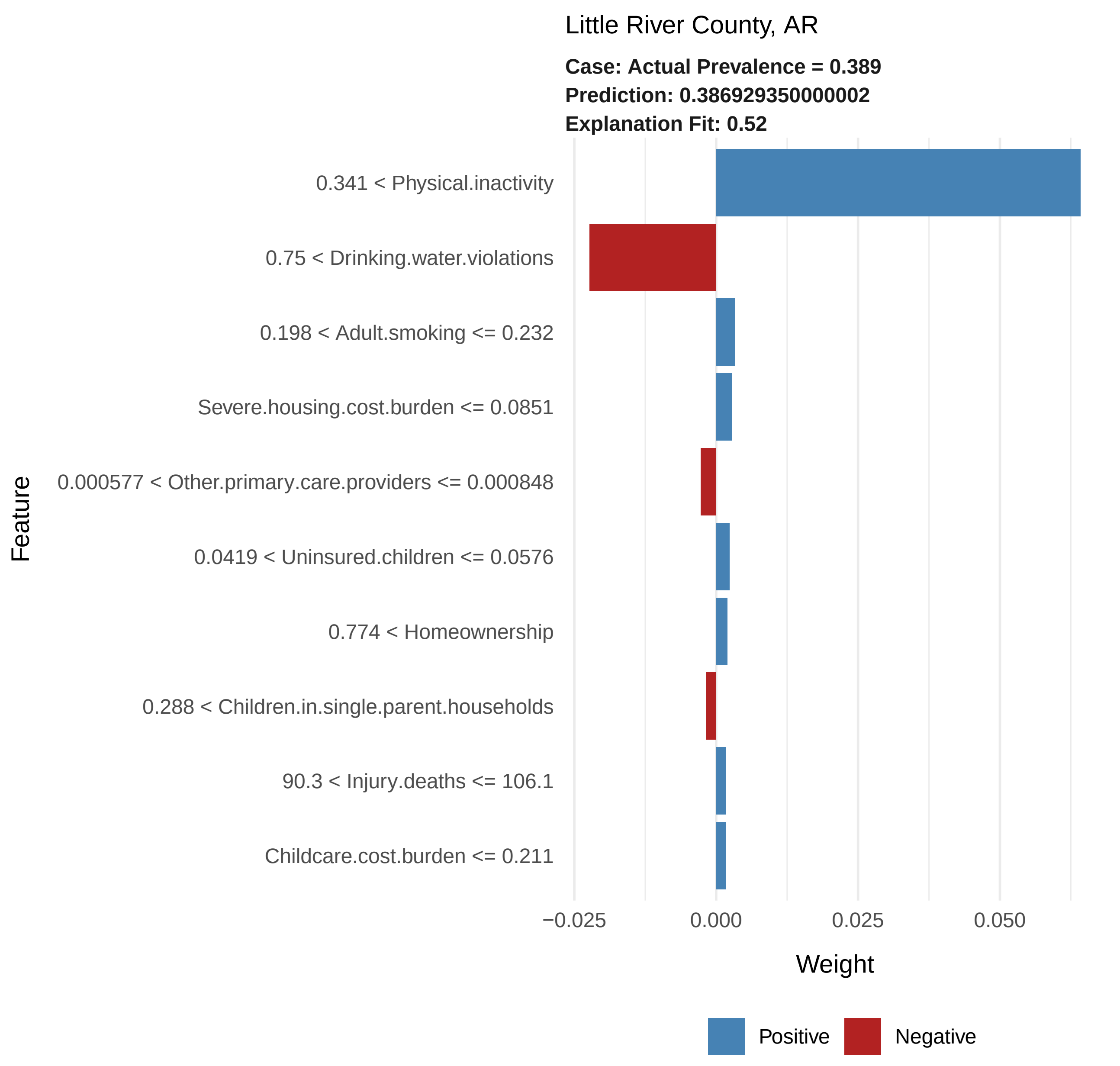

Little River County, AR
Case: Actual Prevalence = 0.389
Prediction: 0.386929350000002
Explanation Fit: 0.52
0.341 < Physical.inactivity
0.75 < Drinking.water.violations
0.198 < Adult.smoking <= 0.232
Severe.housing.cost.burden <= 0.0851
0.000577 < Other.primary.care.providers <= 0.000848
0.0419 < Uninsured.children <= 0.0576
0.774 < Homeownership
0.288 < Children.in.single.parent.households
90.3 < Injury.deaths <= 106.1
Childcare.cost.burden <= 0.211
Feature
−0.025
0.000
0.025
0.050
Weight
Positive
Negative

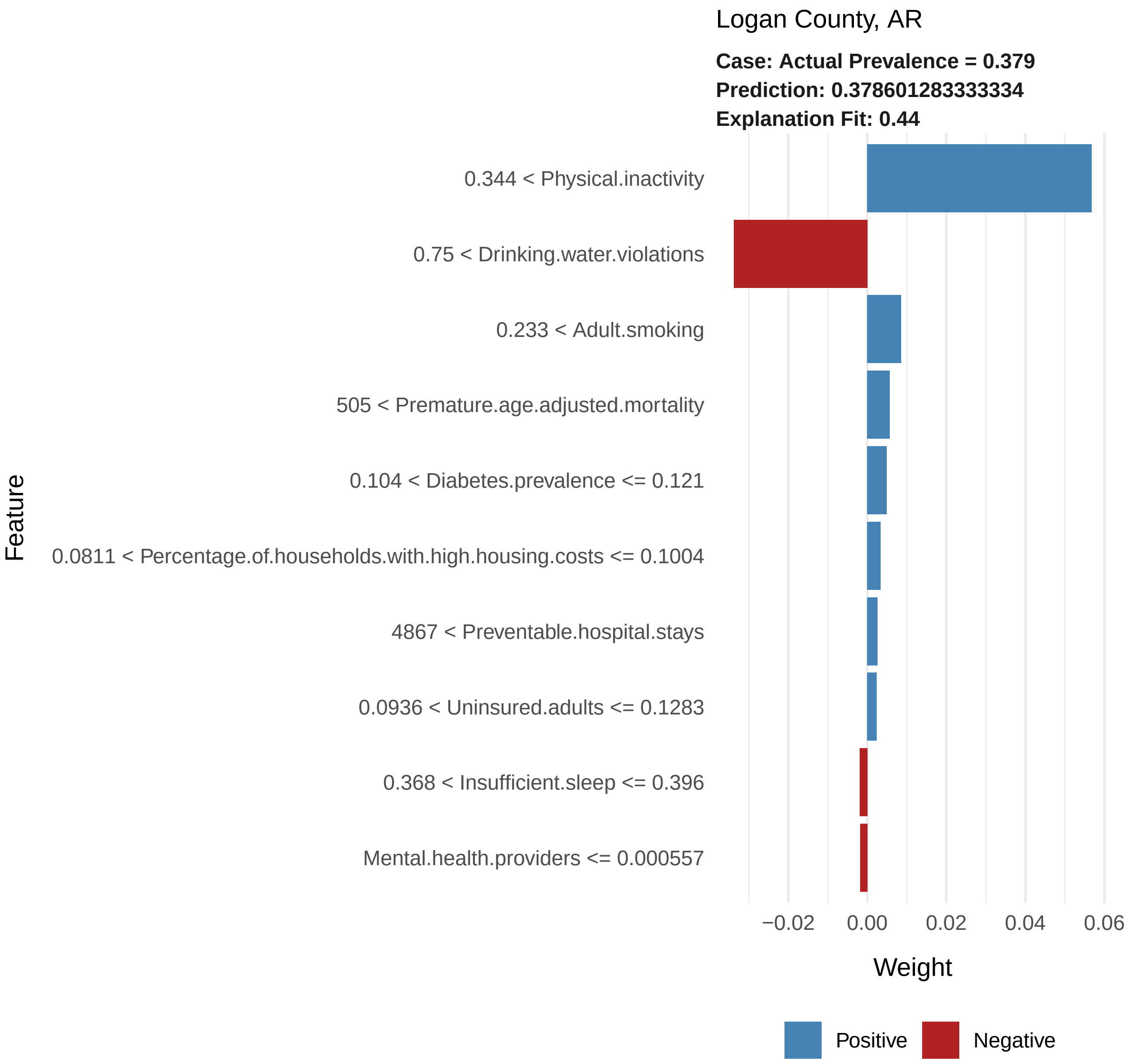
Logan County, AR
Case: Actual Prevalence = 0.379
Prediction: 0.378601283333334
Explanation Fit: 0.44
0.344 < Physical.inactivity
0.75 < Drinking.water.violations
0.233 < Adult.smoking
505 < Premature.age.adjusted.mortality
0.104 < Diabetes.prevalence <= 0.121
0.0811 < Percentage.of.households.with.high.housing.costs <= 0.1004
4867 < Preventable.hospital.stays
0.0936 < Uninsured.adults <= 0.1283
0.368 < Insufficient.sleep <= 0.396
Mental.health.providers <= 0.000557
Feature
−0.02
0.00
0.02
0.04
0.06
Weight
Positive
Negative

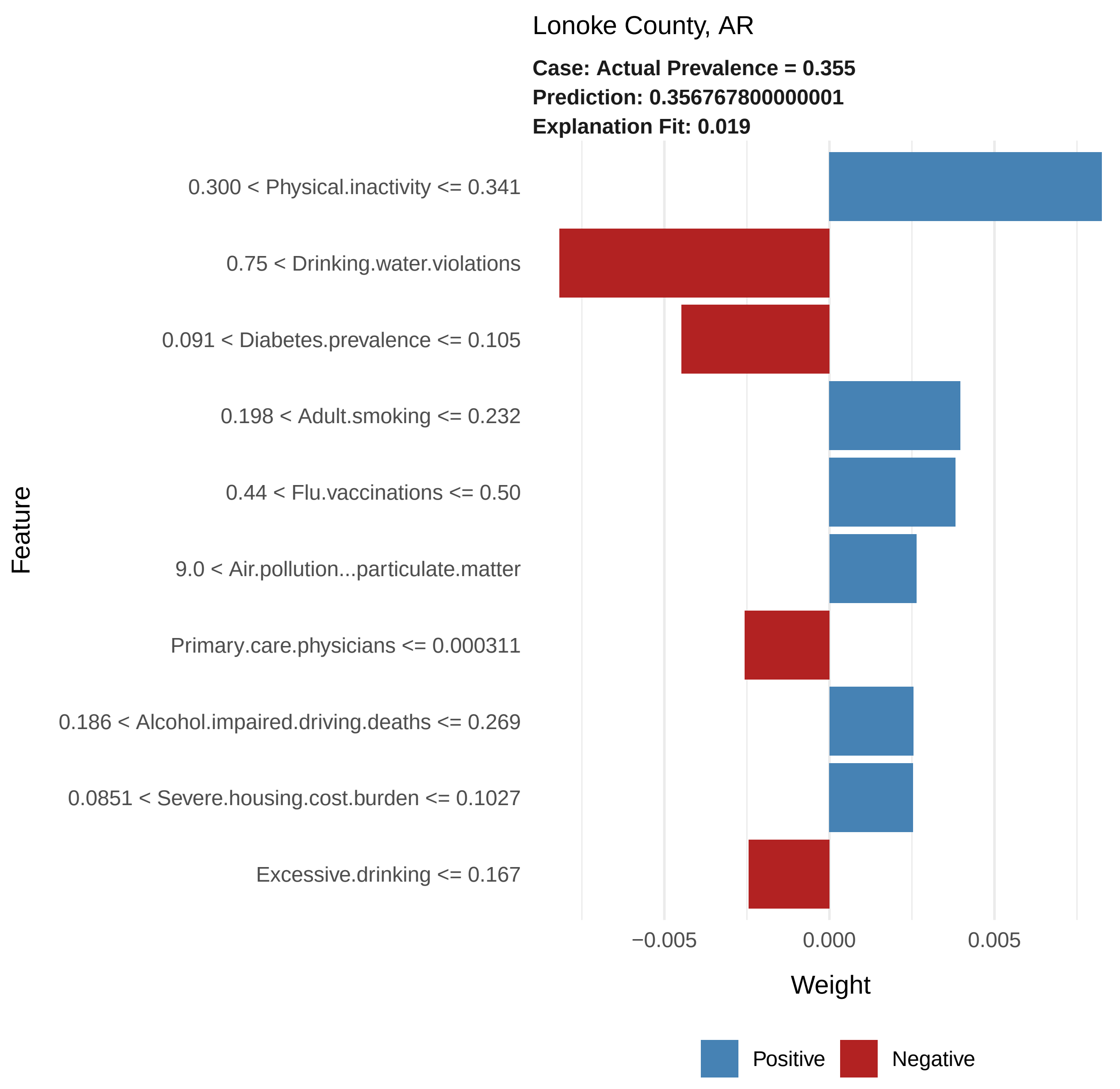

Lonoke County, AR
Case: Actual Prevalence = 0.355
Prediction: 0.356767800000001
Explanation Fit: 0.019
0.300 < Physical.inactivity <= 0.341
0.75 < Drinking.water.violations
0.091 < Diabetes.prevalence <= 0.105
0.198 < Adult.smoking <= 0.232
0.44 < Flu.vaccinations <= 0.50
9.0 < Air.pollution...particulate.matter
Primary.care.physicians <= 0.000311
0.186 < Alcohol.impaired.driving.deaths <= 0.269
0.0851 < Severe.housing.cost.burden <= 0.1027
Excessive.drinking <= 0.167
Feature
−0.005
0.000
0.005
Weight
Positive
Negative

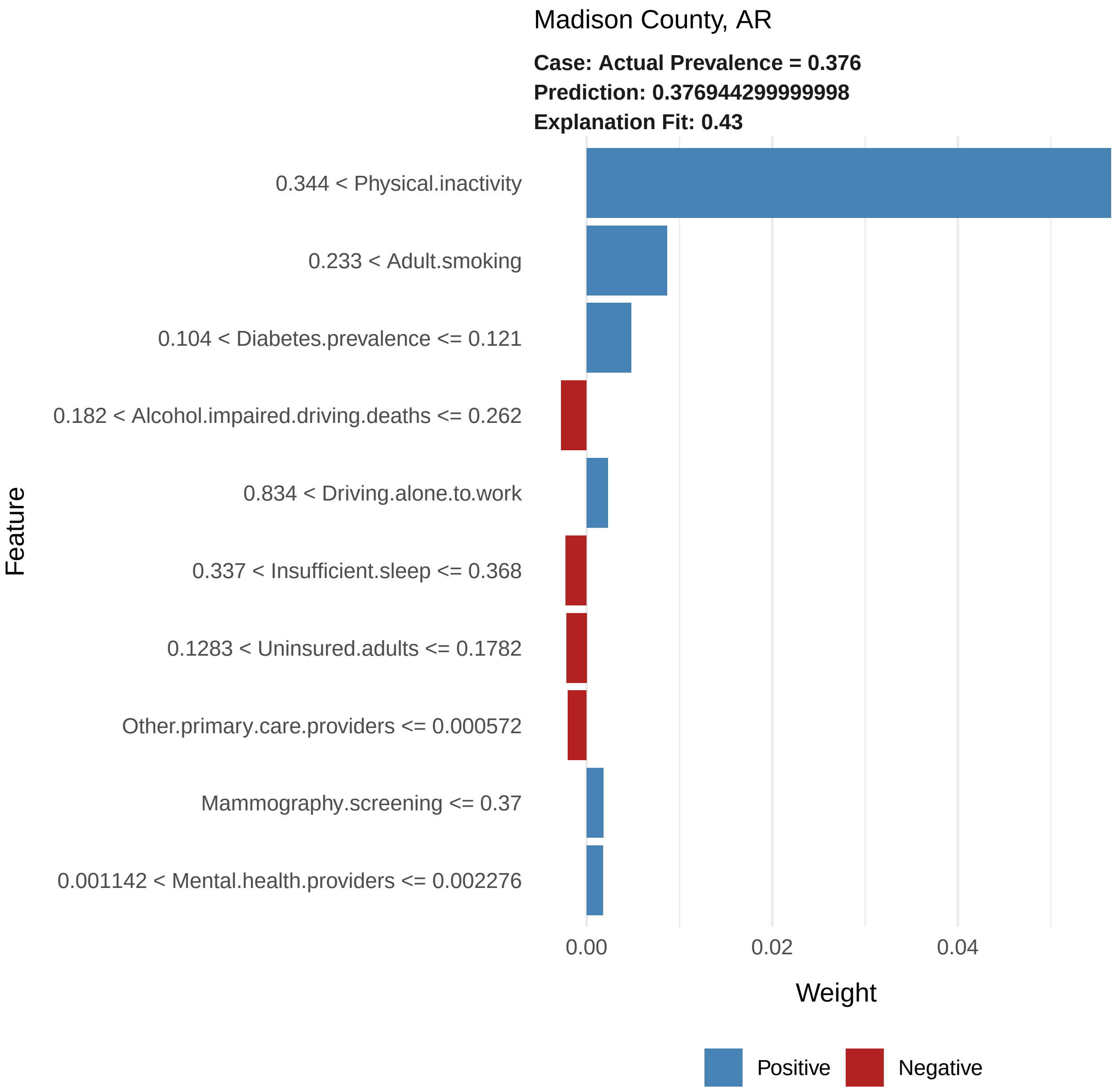

Madison County, AR
Case: Actual Prevalence = 0.376
Prediction: 0.376944299999998
Explanation Fit: 0.43
0.344 < Physical.inactivity
0.233 < Adult.smoking
0.104 < Diabetes.prevalence <= 0.121
0.182 < Alcohol.impaired.driving.deaths <= 0.262
0.834 < Driving.alone.to.work
0.337 < Insufficient.sleep <= 0.368
0.1283 < Uninsured.adults <= 0.1782
Other.primary.care.providers <= 0.000572
Mammography.screening <= 0.37
0.001142 < Mental.health.providers <= 0.002276
Feature
0.00
0.02
0.04
Weight
Positive
Negative

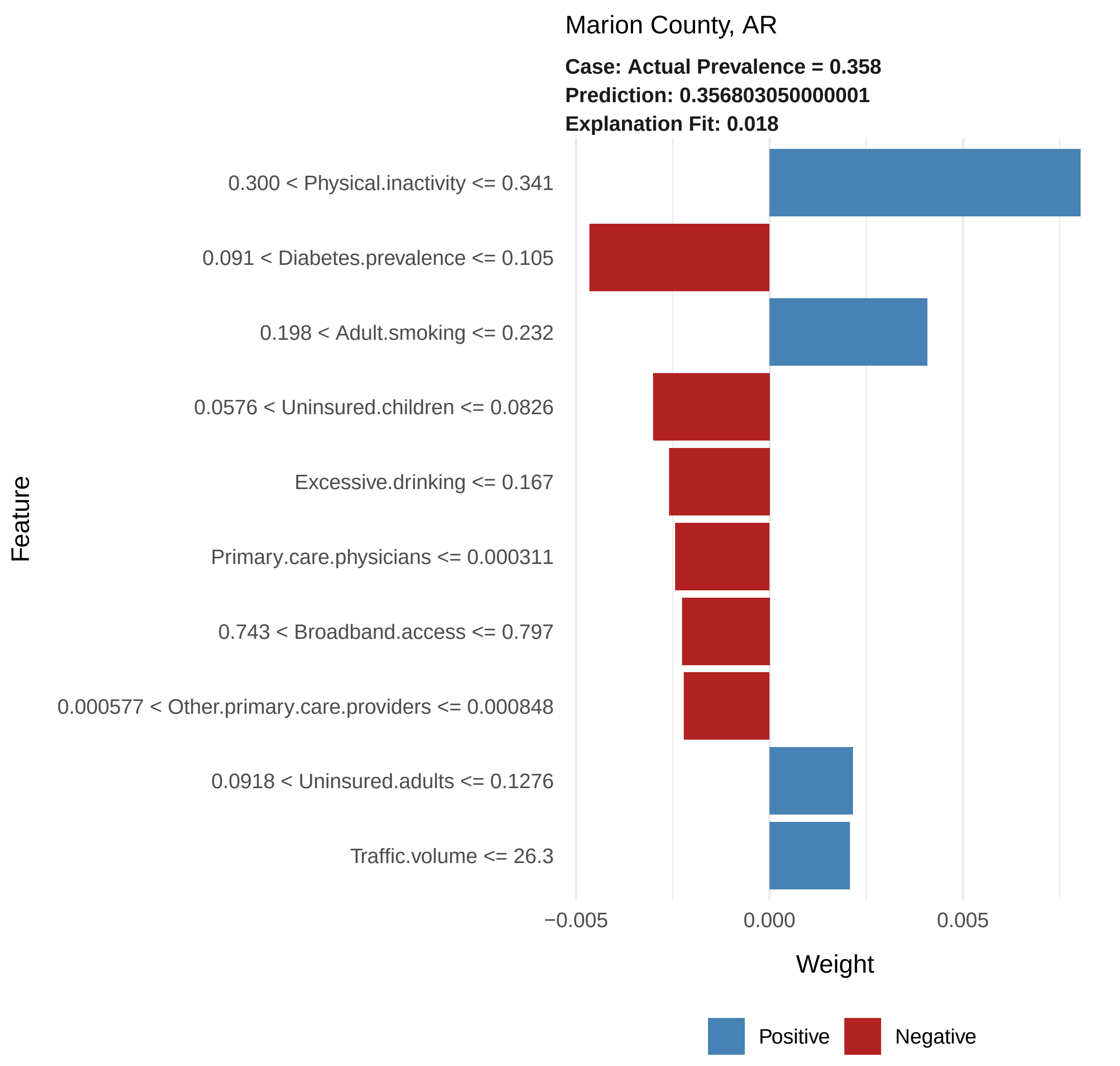

Marion County, AR
Case: Actual Prevalence = 0.358
Prediction: 0.356803050000001
Explanation Fit: 0.018
0.300 < Physical.inactivity <= 0.341
0.091 < Diabetes.prevalence <= 0.105
0.198 < Adult.smoking <= 0.232
0.0576 < Uninsured.children <= 0.0826
Excessive.drinking <= 0.167
Primary.care.physicians <= 0.000311
0.743 < Broadband.access <= 0.797
0.000577 < Other.primary.care.providers <= 0.000848
0.0918 < Uninsured.adults <= 0.1276
Traffic.volume <= 26.3
Feature
−0.005
0.000
0.005
Weight
Positive
Negative

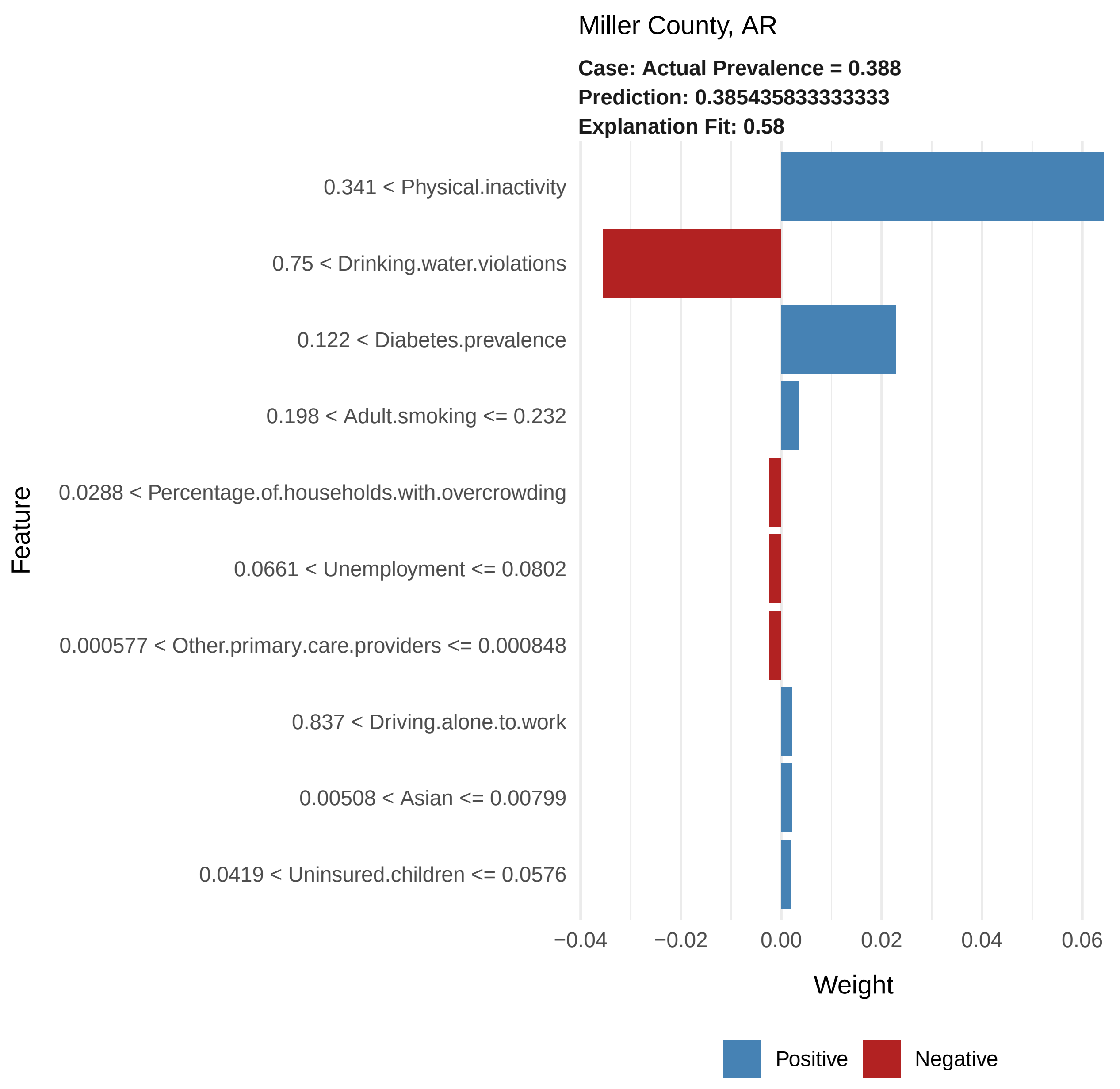
Miller County, AR
Case: Actual Prevalence = 0.388
Prediction: 0.385435833333333
Explanation Fit: 0.58
0.341 < Physical.inactivity
0.75 < Drinking.water.violations
0.122 < Diabetes.prevalence
0.198 < Adult.smoking <= 0.232
0.0288 < Percentage.of.households.with.overcrowding
0.0661 < Unemployment <= 0.0802
0.000577 < Other.primary.care.providers <= 0.000848
0.837 < Driving.alone.to.work
0.00508 < Asian <= 0.00799
0.0419 < Uninsured.children <= 0.0576
Feature
−0.04
−0.02
0.00
0.02
0.04
0.06
Weight
Positive
Negative

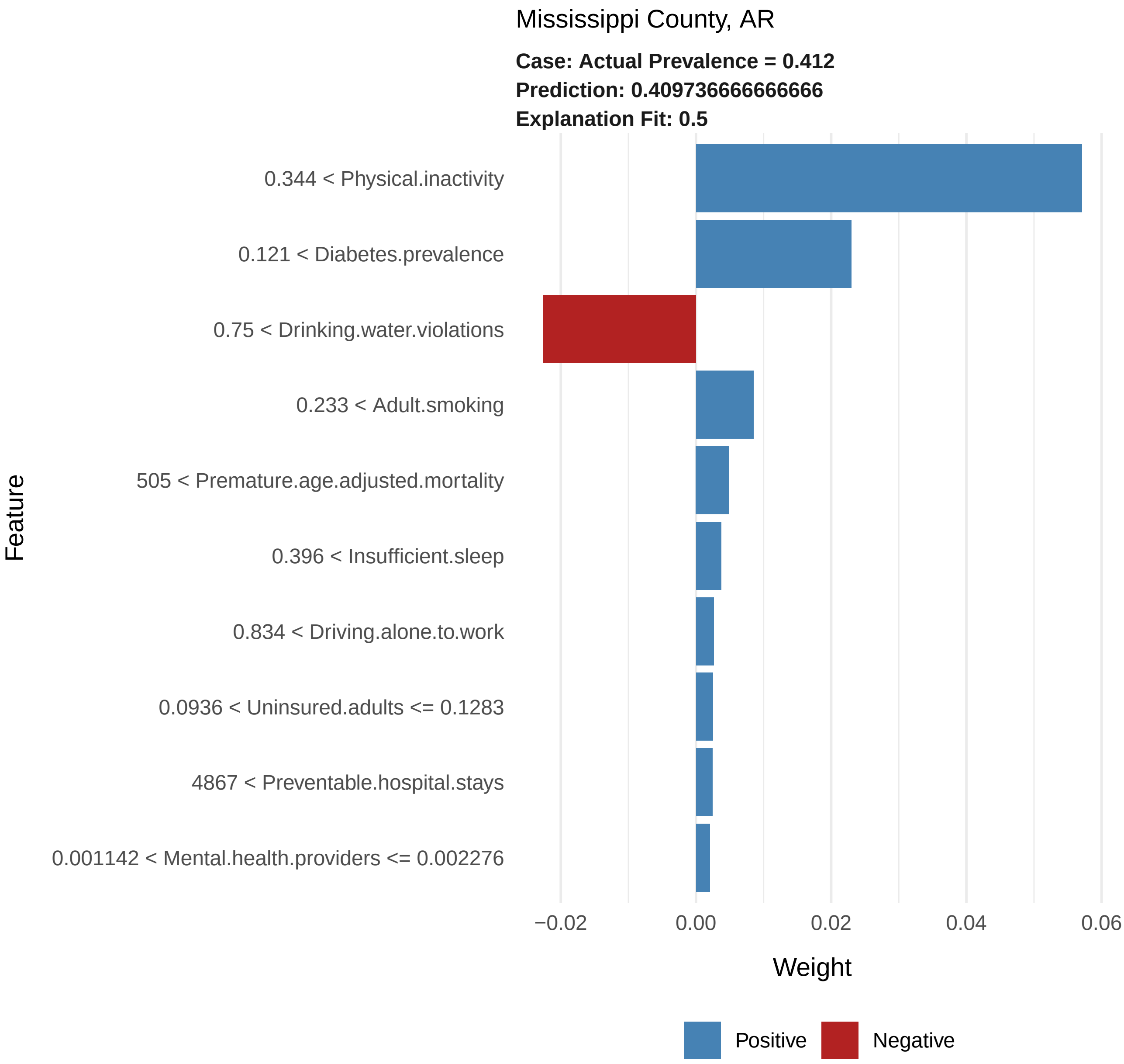
Mississippi County, AR
Case: Actual Prevalence = 0.412
Prediction: 0.409736666666666
Explanation Fit: 0.5
0.344 < Physical.inactivity
0.121 < Diabetes.prevalence
0.75 < Drinking.water.violations
0.233 < Adult.smoking
505 < Premature.age.adjusted.mortality
0.396 < Insufficient.sleep
0.834 < Driving.alone.to.work
0.0936 < Uninsured.adults <= 0.1283
4867 < Preventable.hospital.stays
0.001142 < Mental.health.providers <= 0.002276
Feature
−0.02
0.00
0.02
0.04
0.06
Weight
Positive
Negative

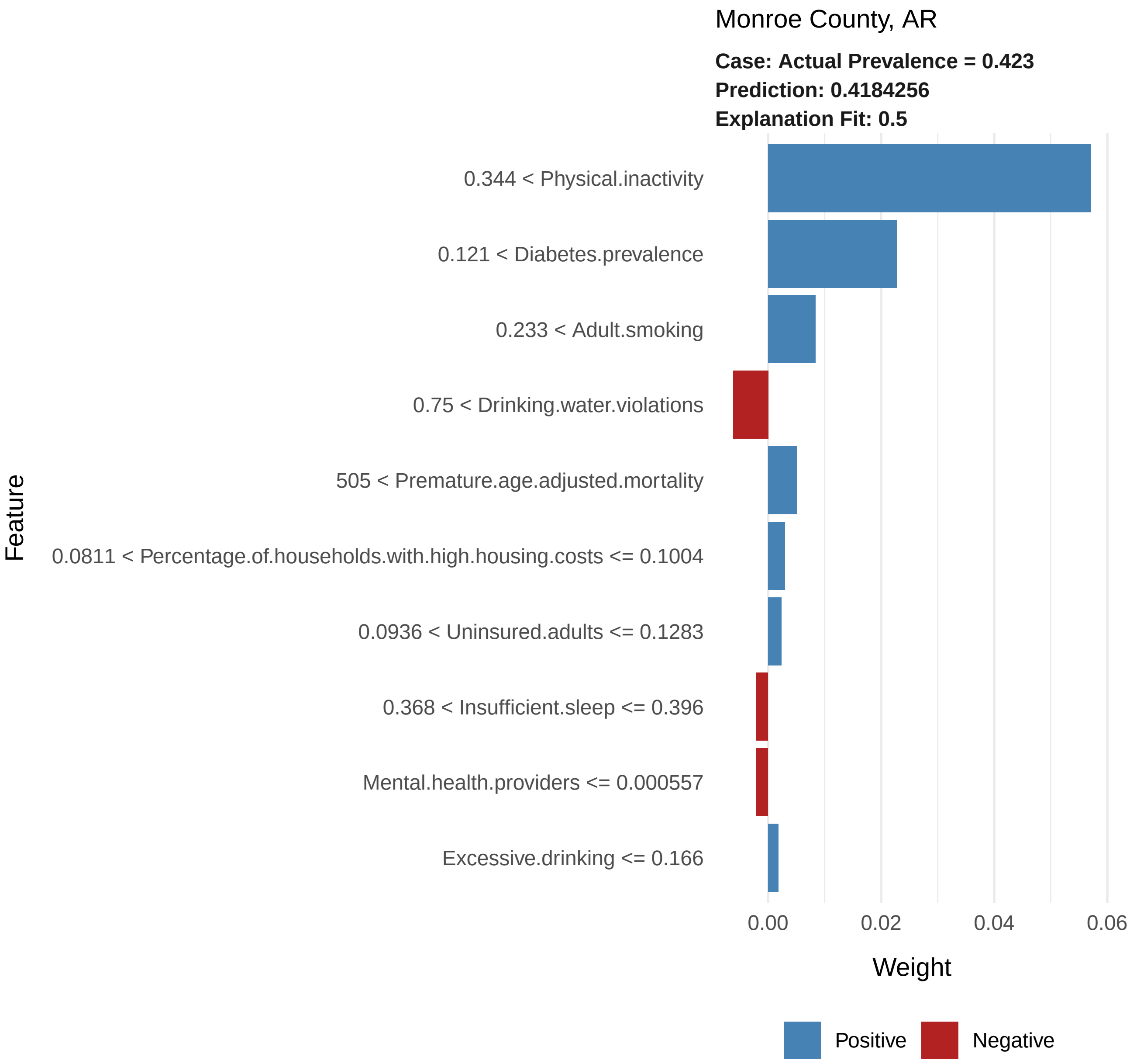
Monroe County, AR
Case: Actual Prevalence = 0.423
Prediction: 0.4184256
Explanation Fit: 0.5
0.344 < Physical.inactivity
0.121 < Diabetes.prevalence
0.233 < Adult.smoking
0.75 < Drinking.water.violations
505 < Premature.age.adjusted.mortality
0.0811 < Percentage.of.households.with.high.housing.costs <= 0.1004
0.0936 < Uninsured.adults <= 0.1283
0.368 < Insufficient.sleep <= 0.396
Mental.health.providers <= 0.000557
Excessive.drinking <= 0.166
Feature
0.00
0.02
0.04
0.06
Weight
Positive
Negative

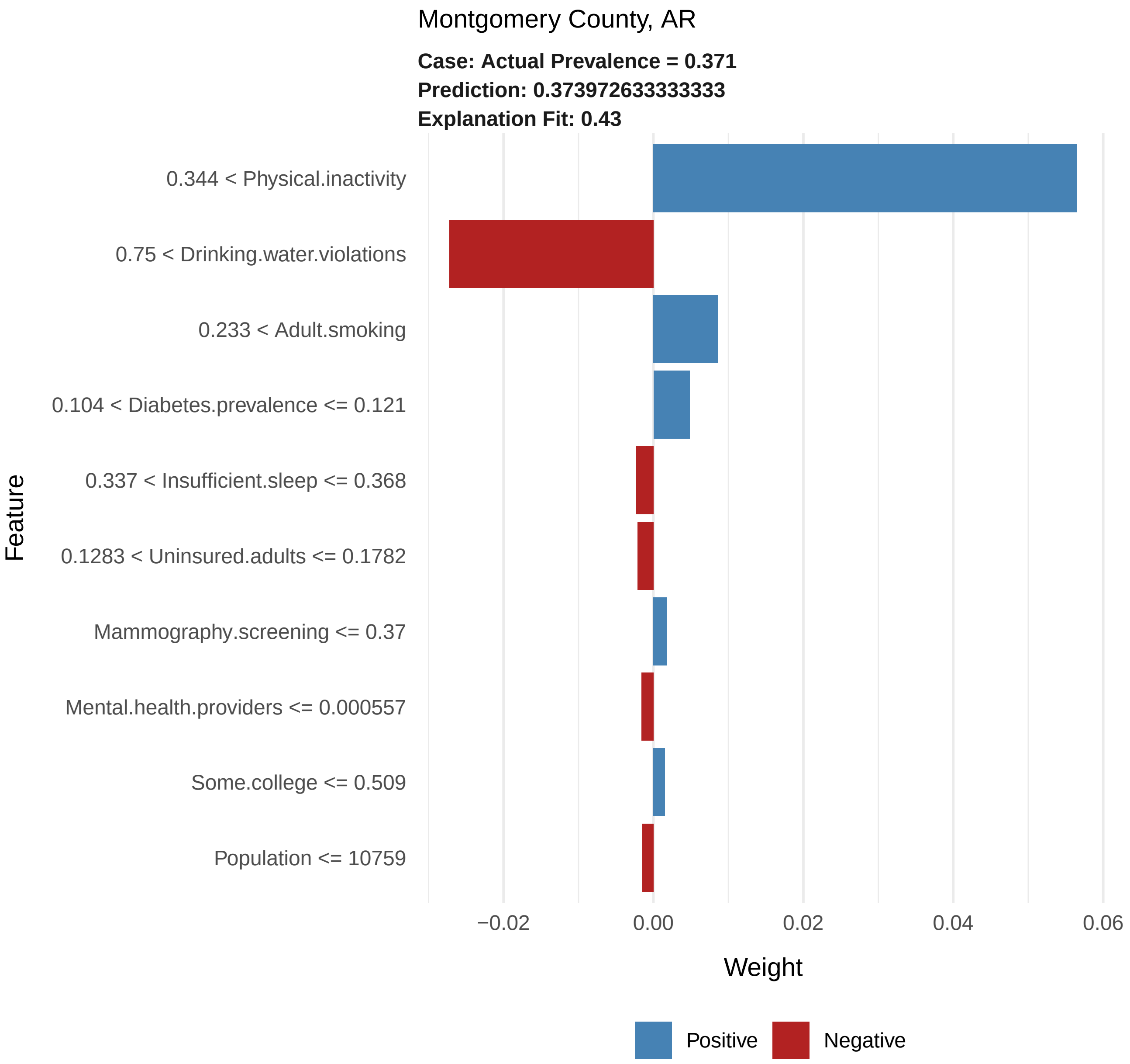

Montgomery County, AR
Case: Actual Prevalence = 0.371
Prediction: 0.373972633333333
Explanation Fit: 0.43
0.344 < Physical.inactivity
0.75 < Drinking.water.violations
0.233 < Adult.smoking
0.104 < Diabetes.prevalence <= 0.121
0.337 < Insufficient.sleep <= 0.368
0.1283 < Uninsured.adults <= 0.1782
Mammography.screening <= 0.37
Mental.health.providers <= 0.000557
Some.college <= 0.509
Population <= 10759
Feature
−0.02
0.00
0.02
0.04
0.06
Weight
Positive
Negative

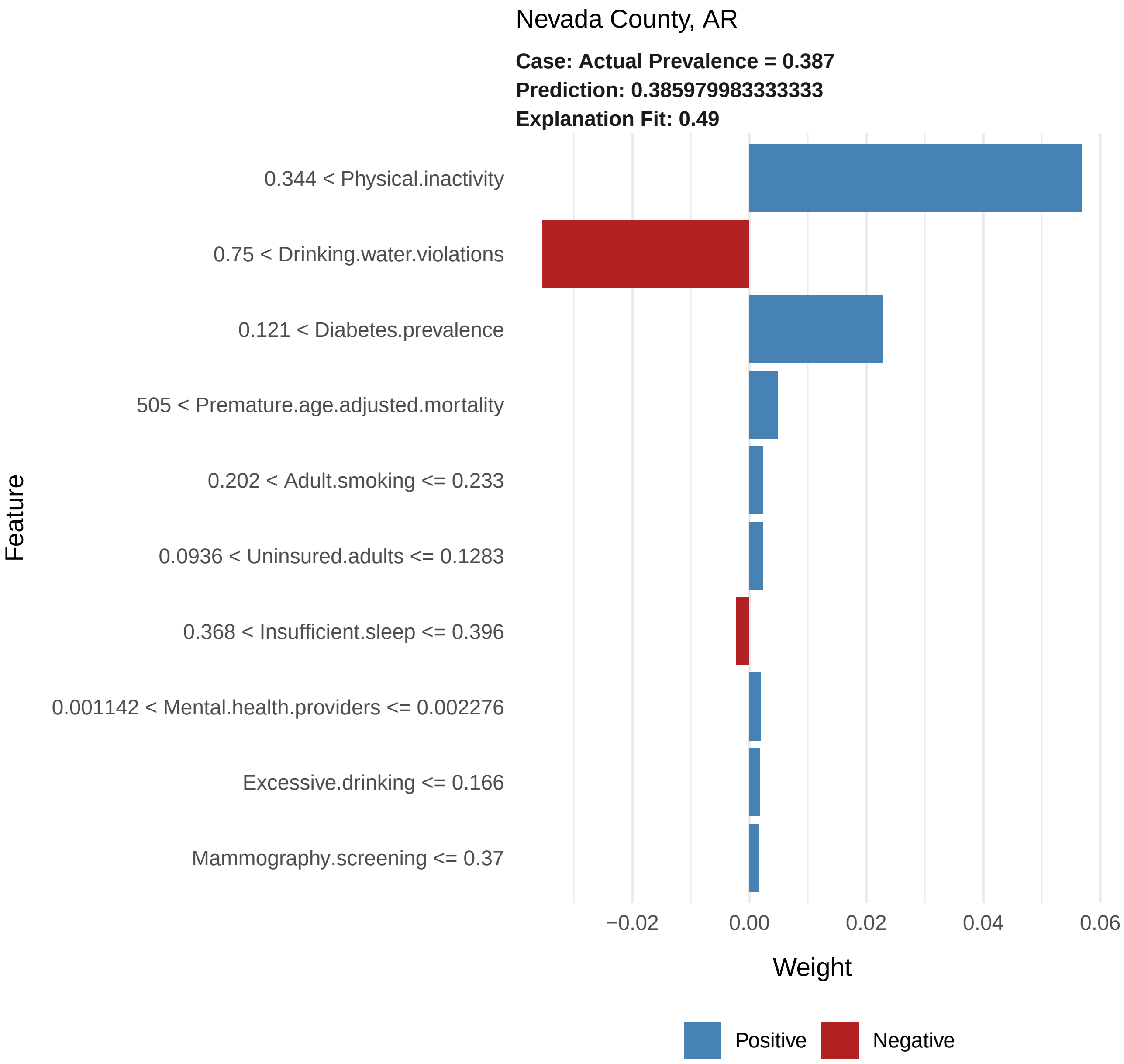

Nevada County, AR
Case: Actual Prevalence = 0.387
Prediction: 0.385979983333333
Explanation Fit: 0.49
0.344 < Physical.inactivity
0.75 < Drinking.water.violations
0.121 < Diabetes.prevalence
505 < Premature.age.adjusted.mortality
0.202 < Adult.smoking <= 0.233
0.0936 < Uninsured.adults <= 0.1283
0.368 < Insufficient.sleep <= 0.396
0.001142 < Mental.health.providers <= 0.002276
Excessive.drinking <= 0.166
Mammography.screening <= 0.37
Feature
−0.02
0.00
0.02
0.04
0.06
Weight
Positive
Negative

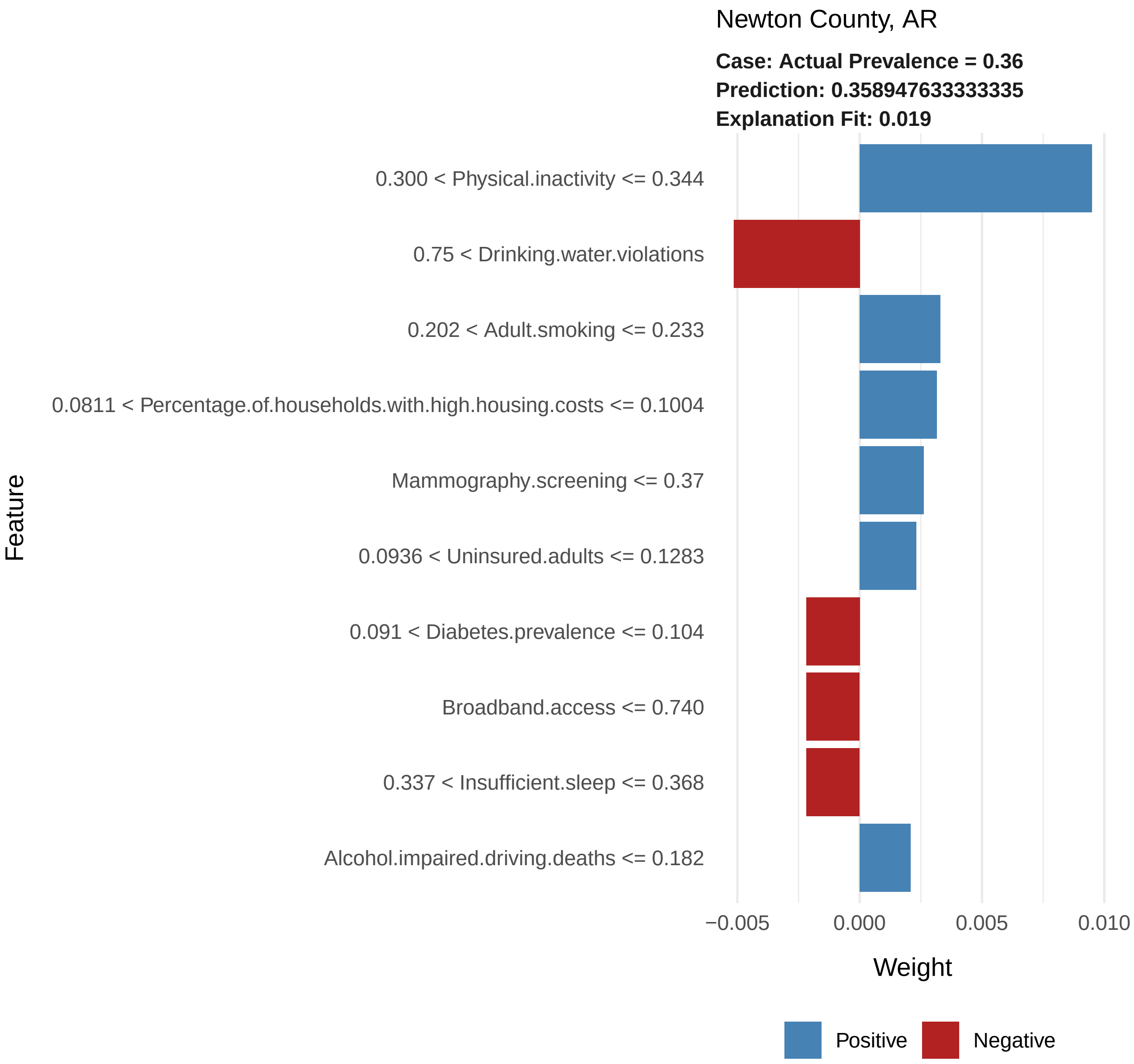

Newton County, AR
Case: Actual Prevalence = 0.36
Prediction: 0.358947633333335
Explanation Fit: 0.019
0.300 < Physical.inactivity <= 0.344
0.75 < Drinking.water.violations
0.202 < Adult.smoking <= 0.233
0.0811 < Percentage.of.households.with.high.housing.costs <= 0.1004
Mammography.screening <= 0.37
0.0936 < Uninsured.adults <= 0.1283
0.091 < Diabetes.prevalence <= 0.104
Broadband.access <= 0.740
0.337 < Insufficient.sleep <= 0.368
Alcohol.impaired.driving.deaths <= 0.182
Feature
−0.005
0.000
0.005
0.010
Weight
Positive
Negative

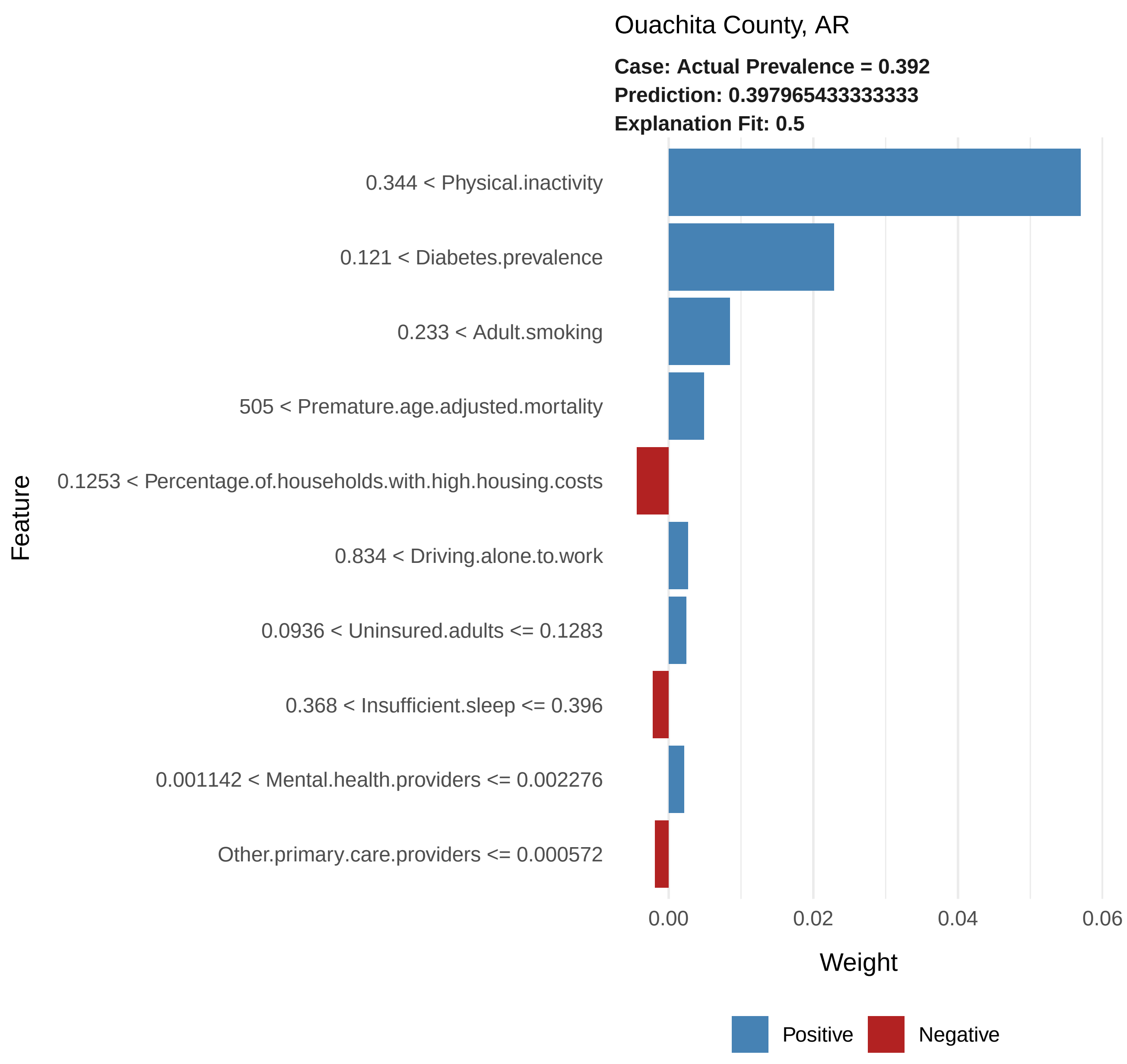

Ouachita County, AR
Case: Actual Prevalence = 0.392
Prediction: 0.397965433333333
Explanation Fit: 0.5
0.344 < Physical.inactivity
0.121 < Diabetes.prevalence
0.233 < Adult.smoking
505 < Premature.age.adjusted.mortality
0.1253 < Percentage.of.households.with.high.housing.costs
0.834 < Driving.alone.to.work
0.0936 < Uninsured.adults <= 0.1283
0.368 < Insufficient.sleep <= 0.396
0.001142 < Mental.health.providers <= 0.002276
Other.primary.care.providers <= 0.000572
Feature
0.00
0.02
0.04
0.06
Weight
Positive
Negative

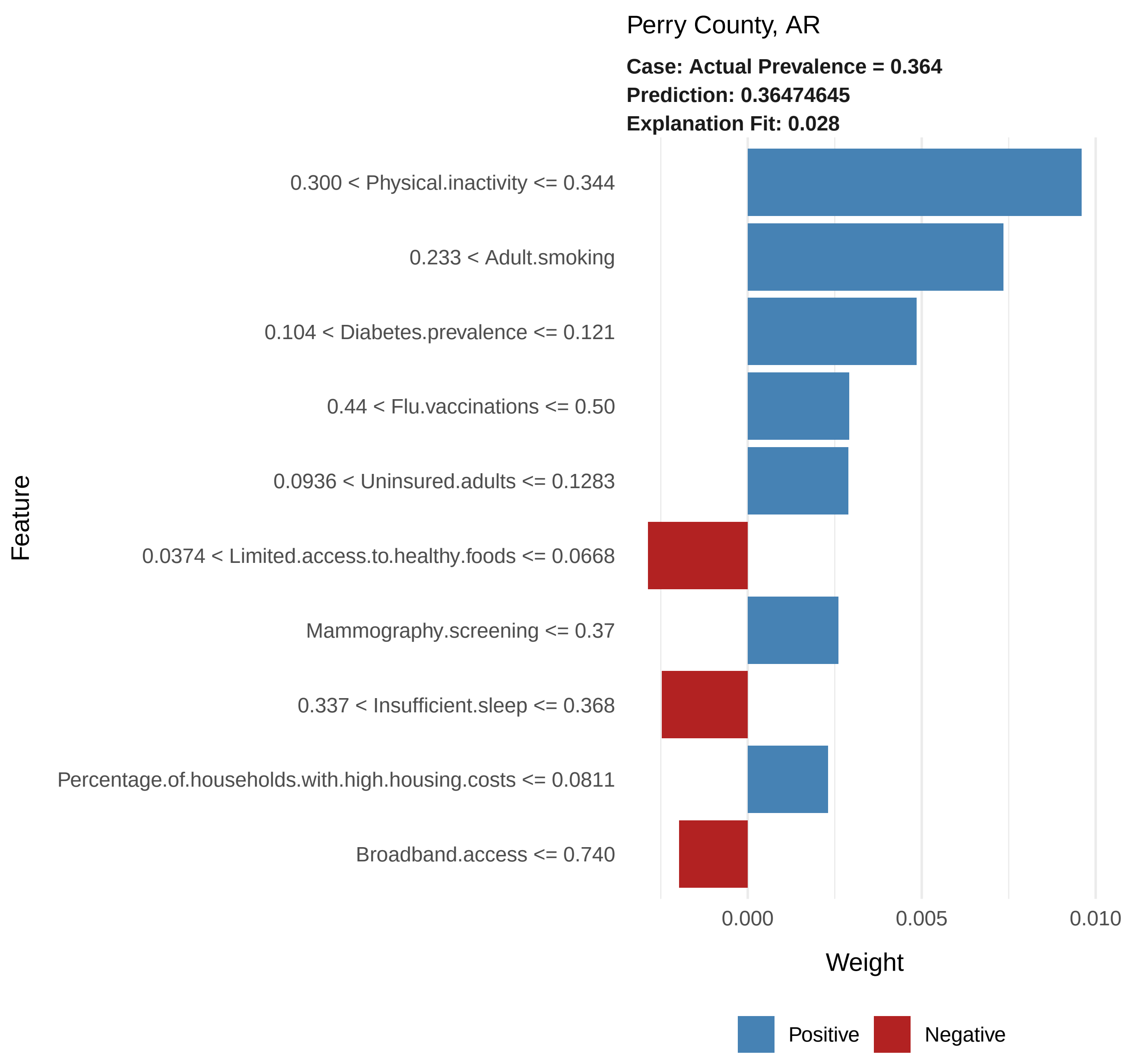

Perry County, AR
Case: Actual Prevalence = 0.364
Prediction: 0.36474645
Explanation Fit: 0.028
0.300 < Physical.inactivity <= 0.344
0.233 < Adult.smoking
0.104 < Diabetes.prevalence <= 0.121
0.44 < Flu.vaccinations <= 0.50
0.0936 < Uninsured.adults <= 0.1283
0.0374 < Limited.access.to.healthy.foods <= 0.0668
Mammography.screening <= 0.37
0.337 < Insufficient.sleep <= 0.368
Percentage.of.households.with.high.housing.costs <= 0.0811
Broadband.access <= 0.740
Feature
0.000
0.005
0.010
Weight
Positive
Negative

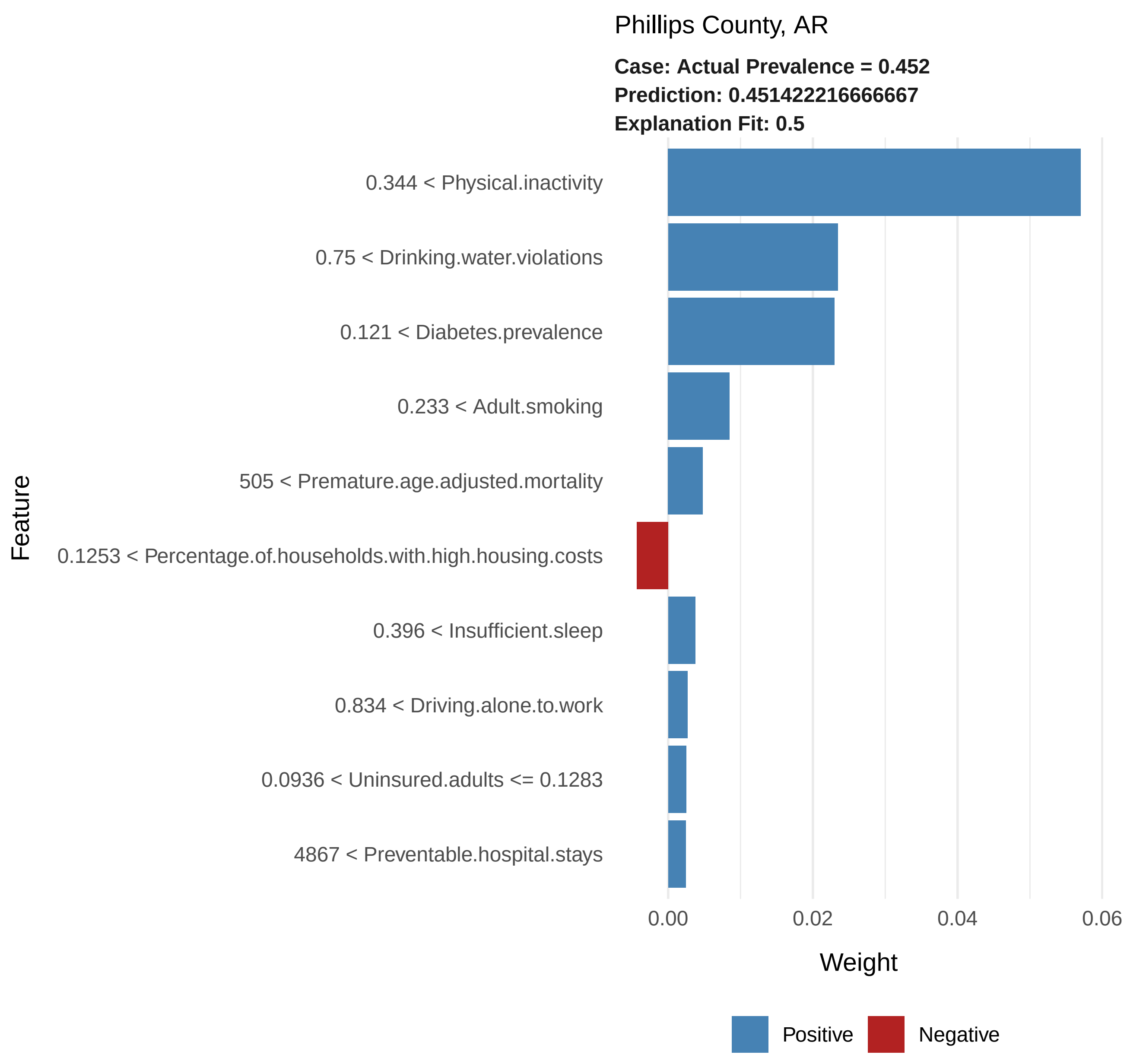

Phillips County, AR
Case: Actual Prevalence = 0.452
Prediction: 0.451422216666667
Explanation Fit: 0.5
0.344 < Physical.inactivity
0.75 < Drinking.water.violations
0.121 < Diabetes.prevalence
0.233 < Adult.smoking
505 < Premature.age.adjusted.mortality
0.1253 < Percentage.of.households.with.high.housing.costs
0.396 < Insufficient.sleep
0.834 < Driving.alone.to.work
0.0936 < Uninsured.adults <= 0.1283
4867 < Preventable.hospital.stays
Feature
0.00
0.02
0.04
0.06
Weight
Positive
Negative

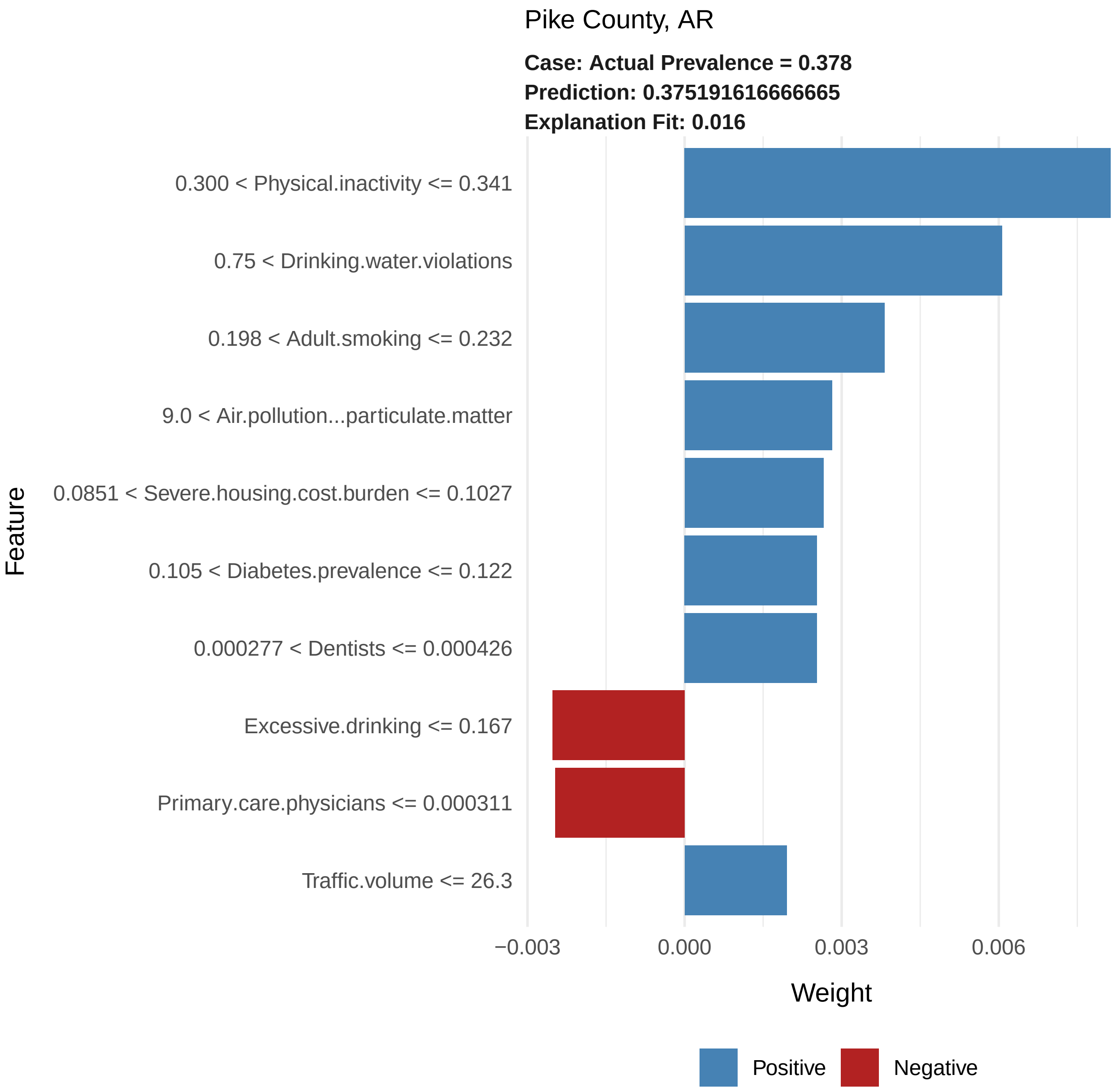

Pike County, AR
Case: Actual Prevalence = 0.378
Prediction: 0.375191616666665
Explanation Fit: 0.016
0.300 < Physical.inactivity <= 0.341
0.75 < Drinking.water.violations
0.198 < Adult.smoking <= 0.232
9.0 < Air.pollution...particulate.matter
0.0851 < Severe.housing.cost.burden <= 0.1027
0.105 < Diabetes.prevalence <= 0.122
0.000277 < Dentists <= 0.000426
Excessive.drinking <= 0.167
Primary.care.physicians <= 0.000311
Traffic.volume <= 26.3
Feature
−0.003
0.000
0.003
0.006
Weight
Positive
Negative

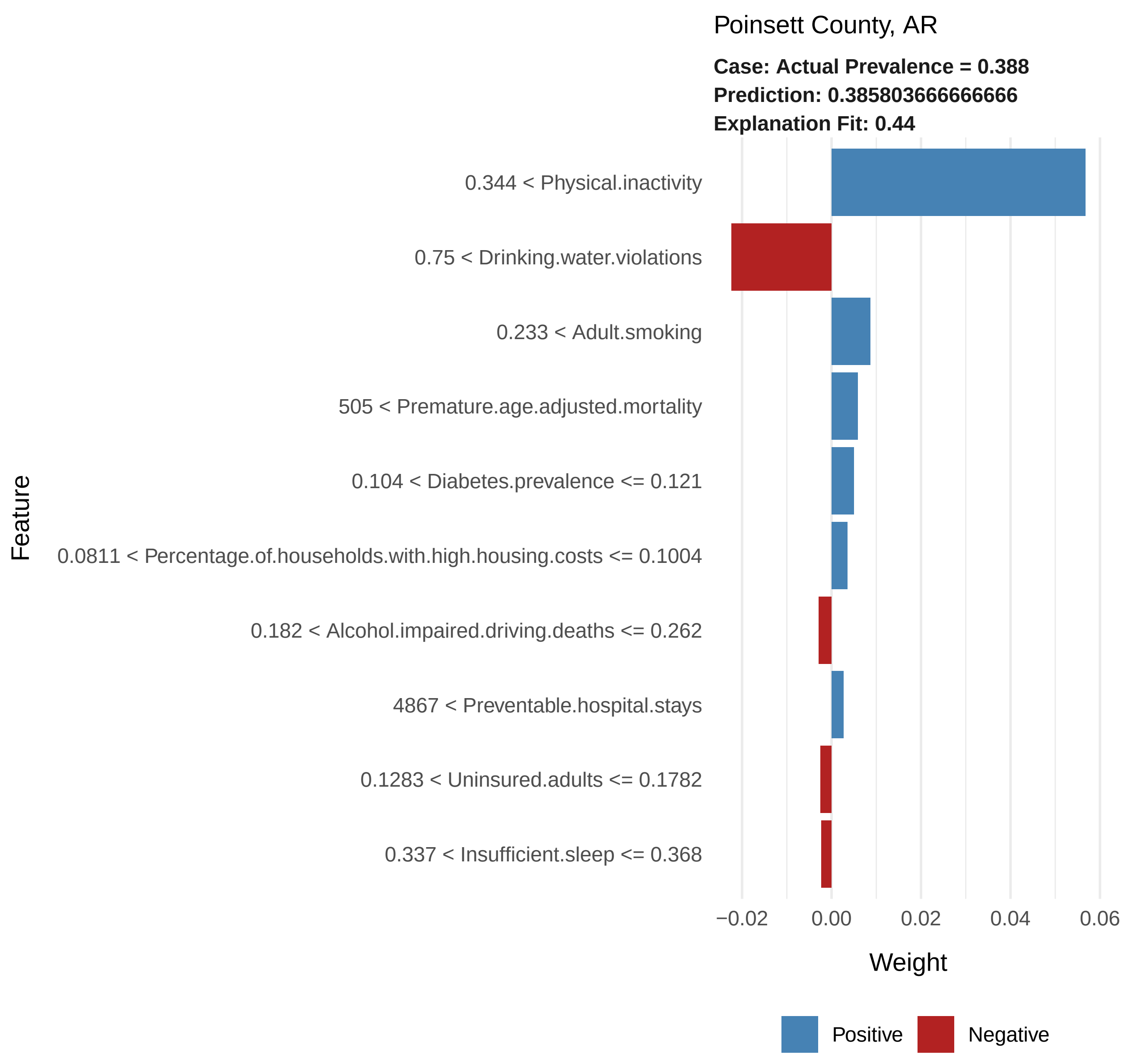
Poinsett County, AR
Case: Actual Prevalence = 0.388
Prediction: 0.385803666666666
Explanation Fit: 0.44
0.344 < Physical.inactivity
0.75 < Drinking.water.violations
0.233 < Adult.smoking
505 < Premature.age.adjusted.mortality
0.104 < Diabetes.prevalence <= 0.121
0.0811 < Percentage.of.households.with.high.housing.costs <= 0.1004
0.182 < Alcohol.impaired.driving.deaths <= 0.262
4867 < Preventable.hospital.stays
0.1283 < Uninsured.adults <= 0.1782
0.337 < Insufficient.sleep <= 0.368
Feature
−0.02
0.00
0.02
0.04
0.06
Weight
Positive
Negative

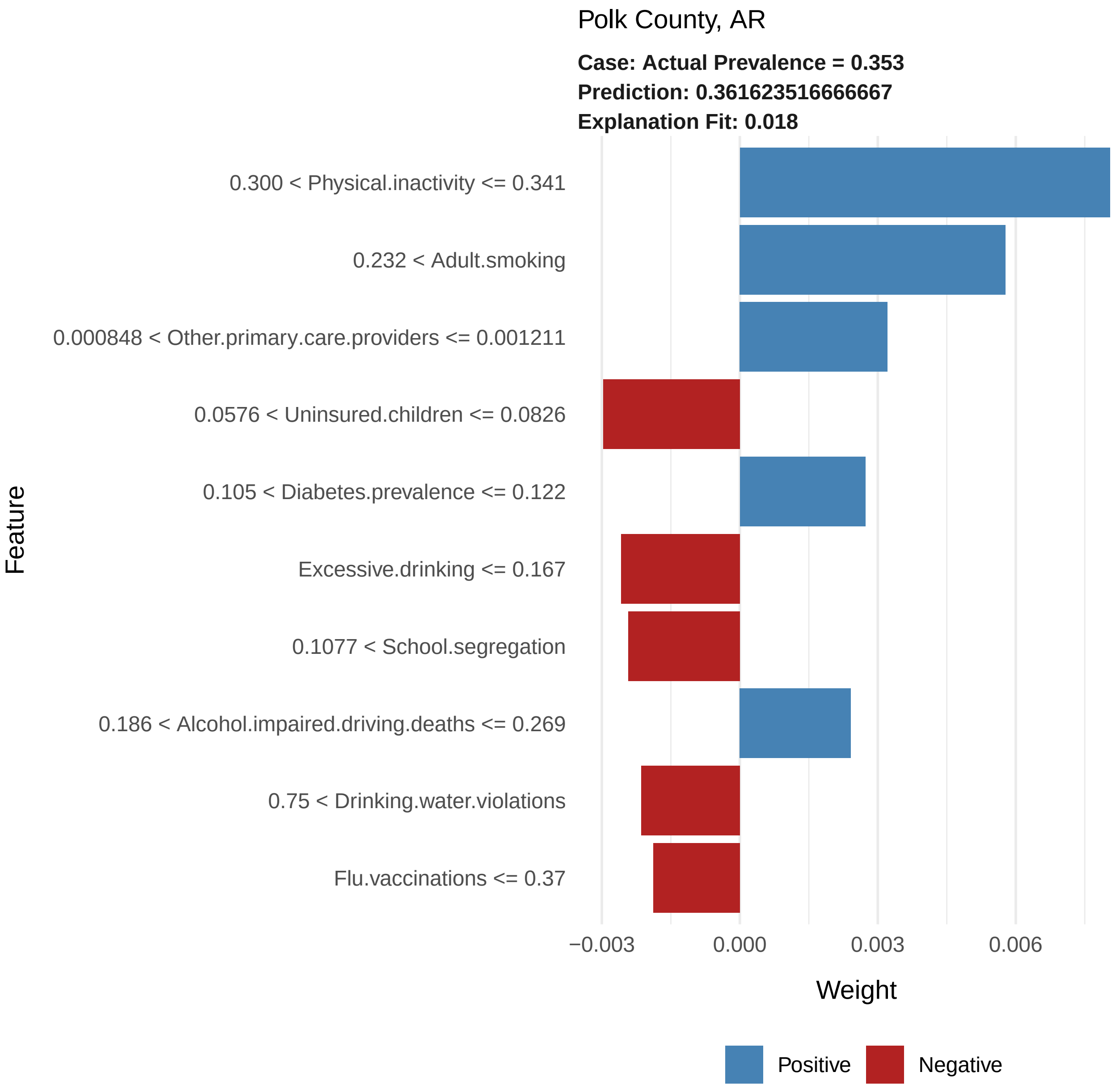

Polk County, AR
Case: Actual Prevalence = 0.353
Prediction: 0.361623516666667
Explanation Fit: 0.018
0.300 < Physical.inactivity <= 0.341
0.232 < Adult.smoking
0.000848 < Other.primary.care.providers <= 0.001211
0.0576 < Uninsured.children <= 0.0826
0.105 < Diabetes.prevalence <= 0.122
Excessive.drinking <= 0.167
0.1077 < School.segregation
0.186 < Alcohol.impaired.driving.deaths <= 0.269
0.75 < Drinking.water.violations
Flu.vaccinations <= 0.37
Feature
−0.003
0.000
0.003
0.006
Weight
Positive
Negative

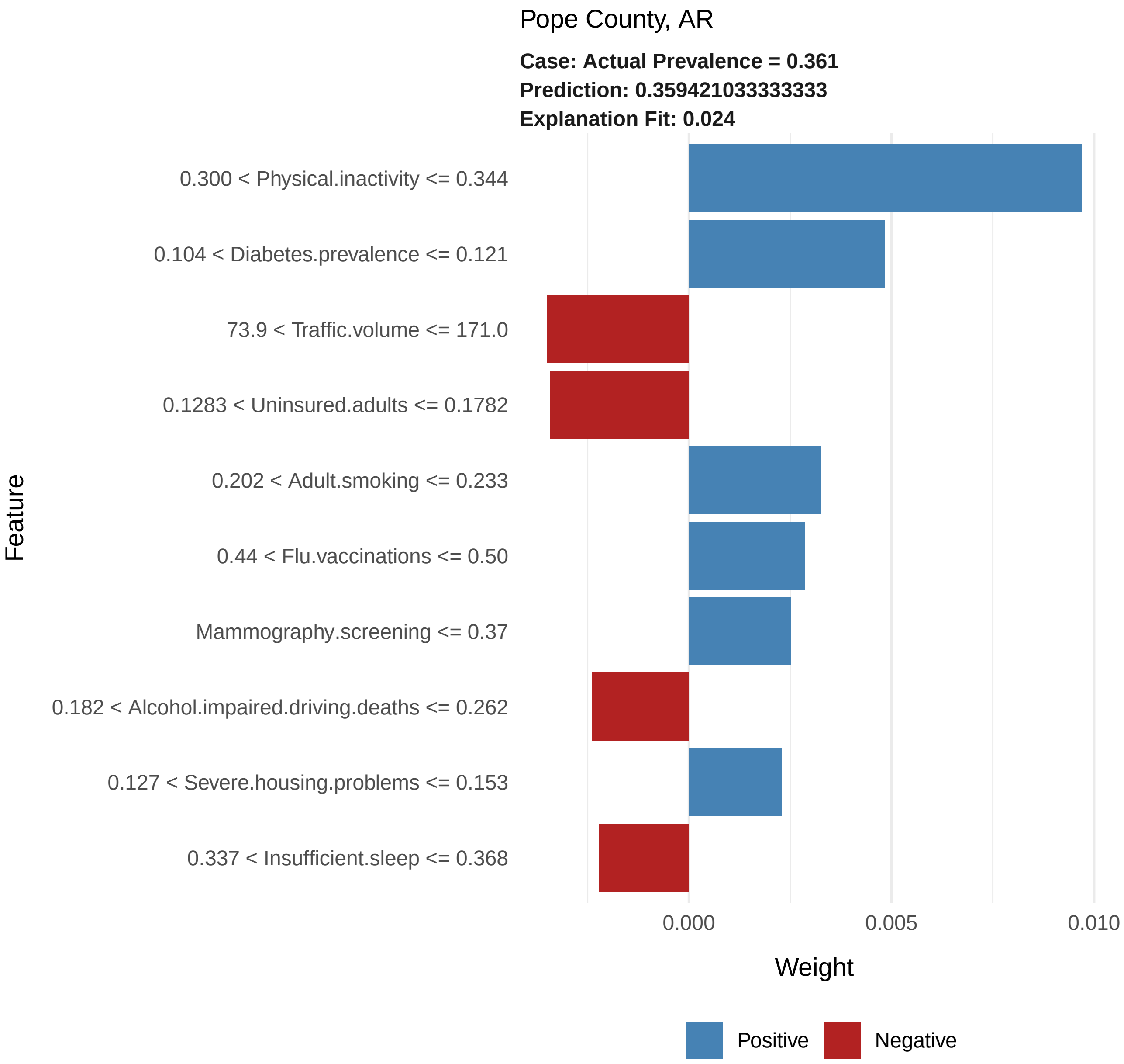

Pope County, AR
Case: Actual Prevalence = 0.361
Prediction: 0.359421033333333
Explanation Fit: 0.024
0.300 < Physical.inactivity <= 0.344
0.104 < Diabetes.prevalence <= 0.121
73.9 < Traffic.volume <= 171.0
0.1283 < Uninsured.adults <= 0.1782
0.202 < Adult.smoking <= 0.233
0.44 < Flu.vaccinations <= 0.50
Mammography.screening <= 0.37
0.182 < Alcohol.impaired.driving.deaths <= 0.262
0.127 < Severe.housing.problems <= 0.153
0.337 < Insufficient.sleep <= 0.368
Feature
0.000
0.005
0.010
Weight
Positive
Negative

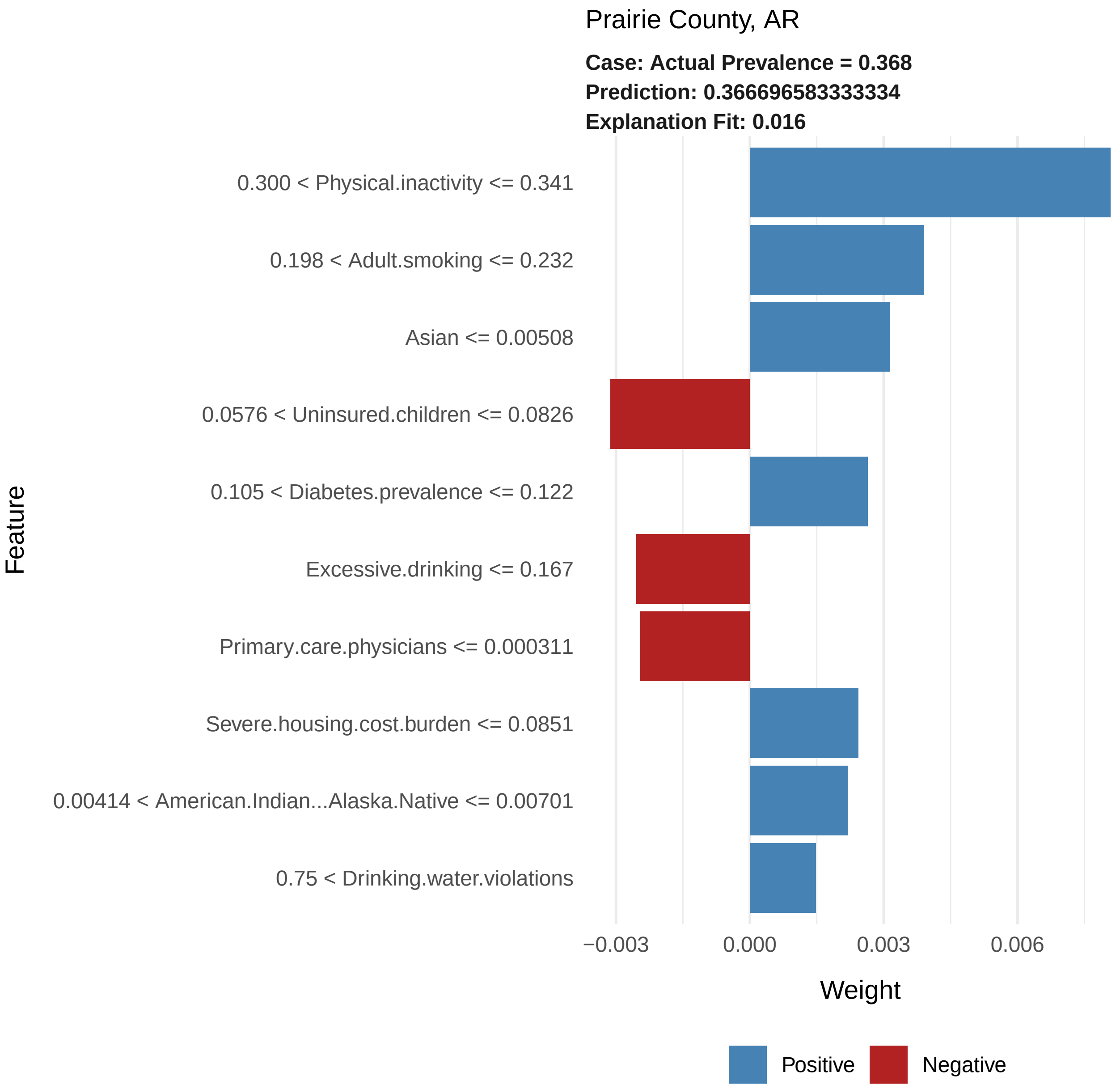

Prairie County, AR
Case: Actual Prevalence = 0.368
Prediction: 0.366696583333334
Explanation Fit: 0.016
0.300 < Physical.inactivity <= 0.341
0.198 < Adult.smoking <= 0.232
Asian <= 0.00508
0.0576 < Uninsured.children <= 0.0826
0.105 < Diabetes.prevalence <= 0.122
Excessive.drinking <= 0.167
Primary.care.physicians <= 0.000311
Severe.housing.cost.burden <= 0.0851
0.00414 < American.Indian...Alaska.Native <= 0.00701
0.75 < Drinking.water.violations
Feature
−0.003
0.000
0.003
0.006
Weight
Positive
Negative

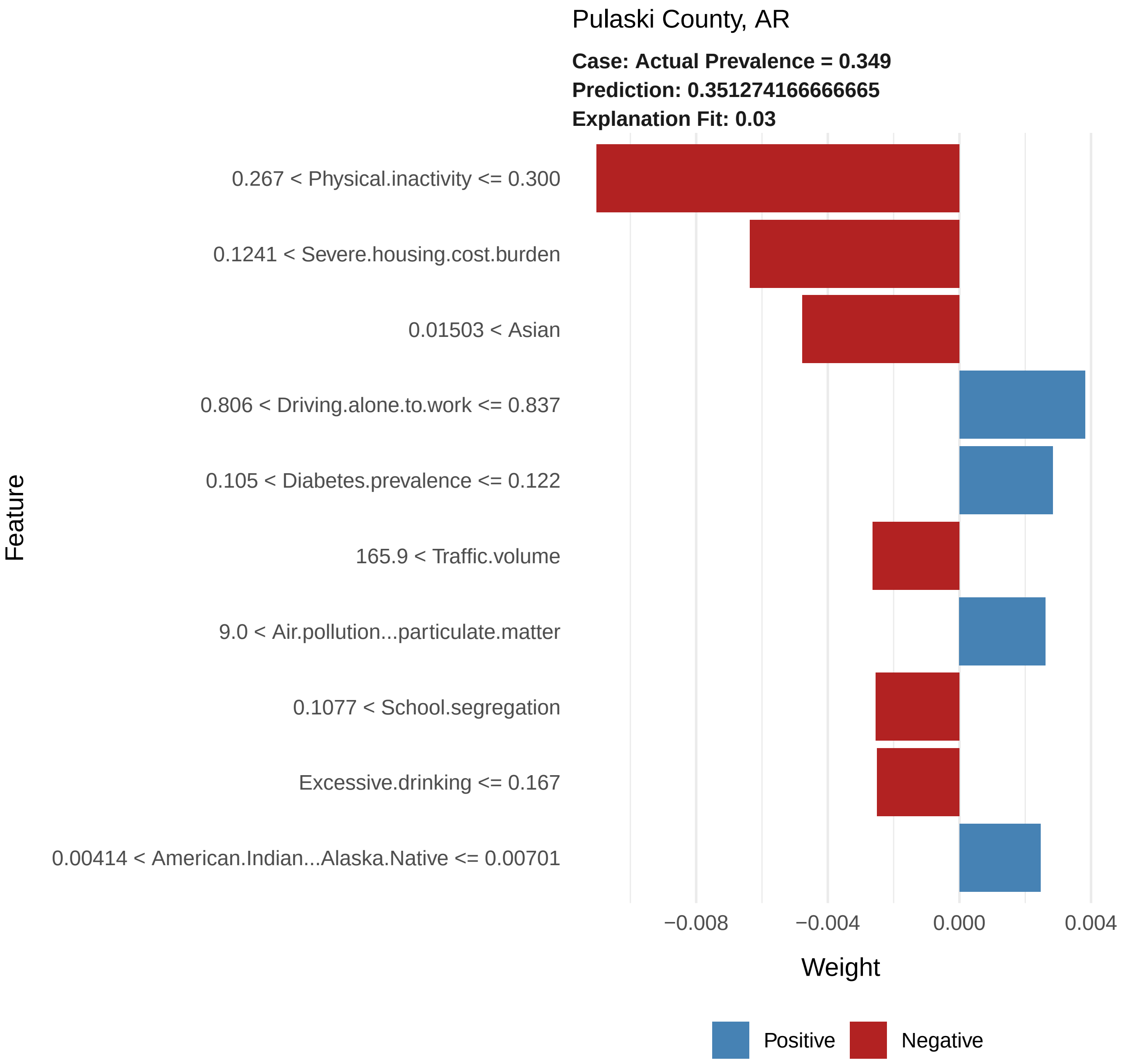

Pulaski County, AR
Case: Actual Prevalence = 0.349
Prediction: 0.351274166666665
Explanation Fit: 0.03
0.267 < Physical.inactivity <= 0.300
0.1241 < Severe.housing.cost.burden
0.01503 < Asian
0.806 < Driving.alone.to.work <= 0.837
0.105 < Diabetes.prevalence <= 0.122
165.9 < Traffic.volume
9.0 < Air.pollution...particulate.matter
0.1077 < School.segregation
Excessive.drinking <= 0.167
0.00414 < American.Indian...Alaska.Native <= 0.00701
Feature
−0.008
−0.004
0.000
0.004
Weight
Positive
Negative

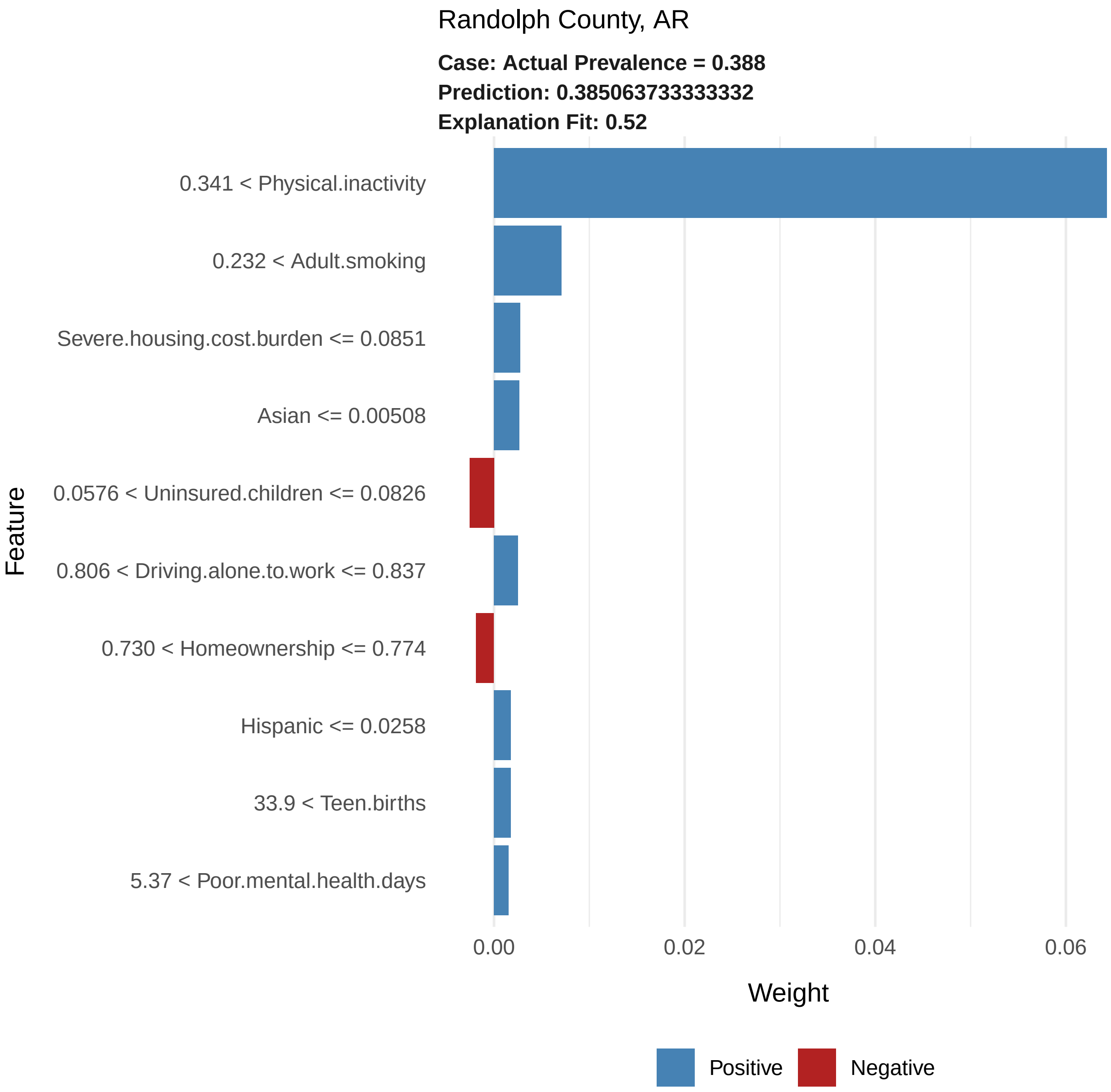

Randolph County, AR
Case: Actual Prevalence = 0.388
Prediction: 0.385063733333332
Explanation Fit: 0.52
0.341 < Physical.inactivity
0.232 < Adult.smoking
Severe.housing.cost.burden <= 0.0851
Asian <= 0.00508
0.0576 < Uninsured.children <= 0.0826
0.806 < Driving.alone.to.work <= 0.837
0.730 < Homeownership <= 0.774
Hispanic <= 0.0258
33.9 < Teen.births
5.37 < Poor.mental.health.days
Feature
0.00
0.02
0.04
0.06
Weight
Positive
Negative

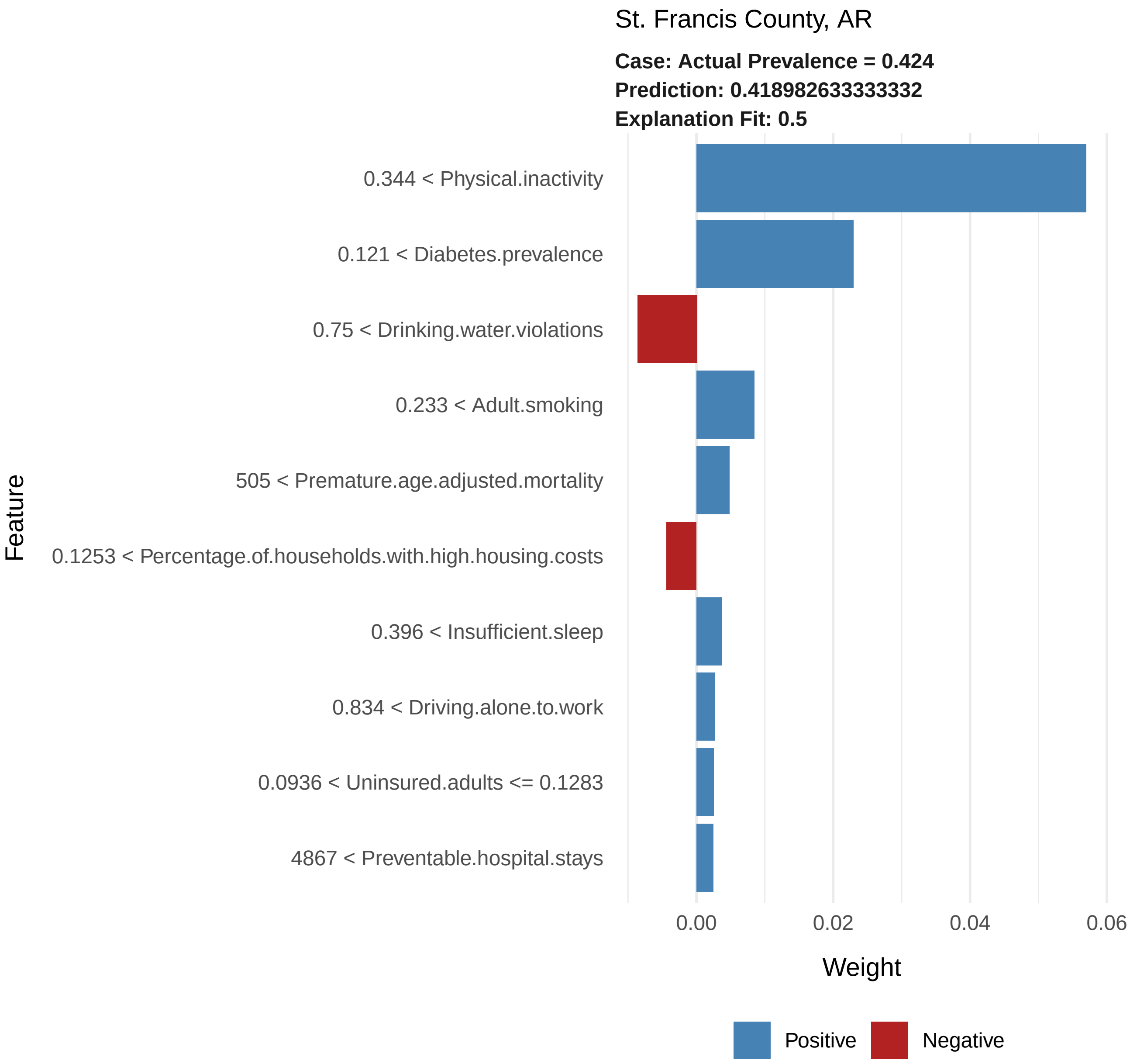
St. Francis County, AR
Case: Actual Prevalence = 0.424
Prediction: 0.418982633333332
Explanation Fit: 0.5
0.344 < Physical.inactivity
0.121 < Diabetes.prevalence
0.75 < Drinking.water.violations
0.233 < Adult.smoking
505 < Premature.age.adjusted.mortality
0.1253 < Percentage.of.households.with.high.housing.costs
0.396 < Insufficient.sleep
0.834 < Driving.alone.to.work
0.0936 < Uninsured.adults <= 0.1283
4867 < Preventable.hospital.stays
Feature
0.00
0.02
0.04
0.06
Weight
Positive
Negative

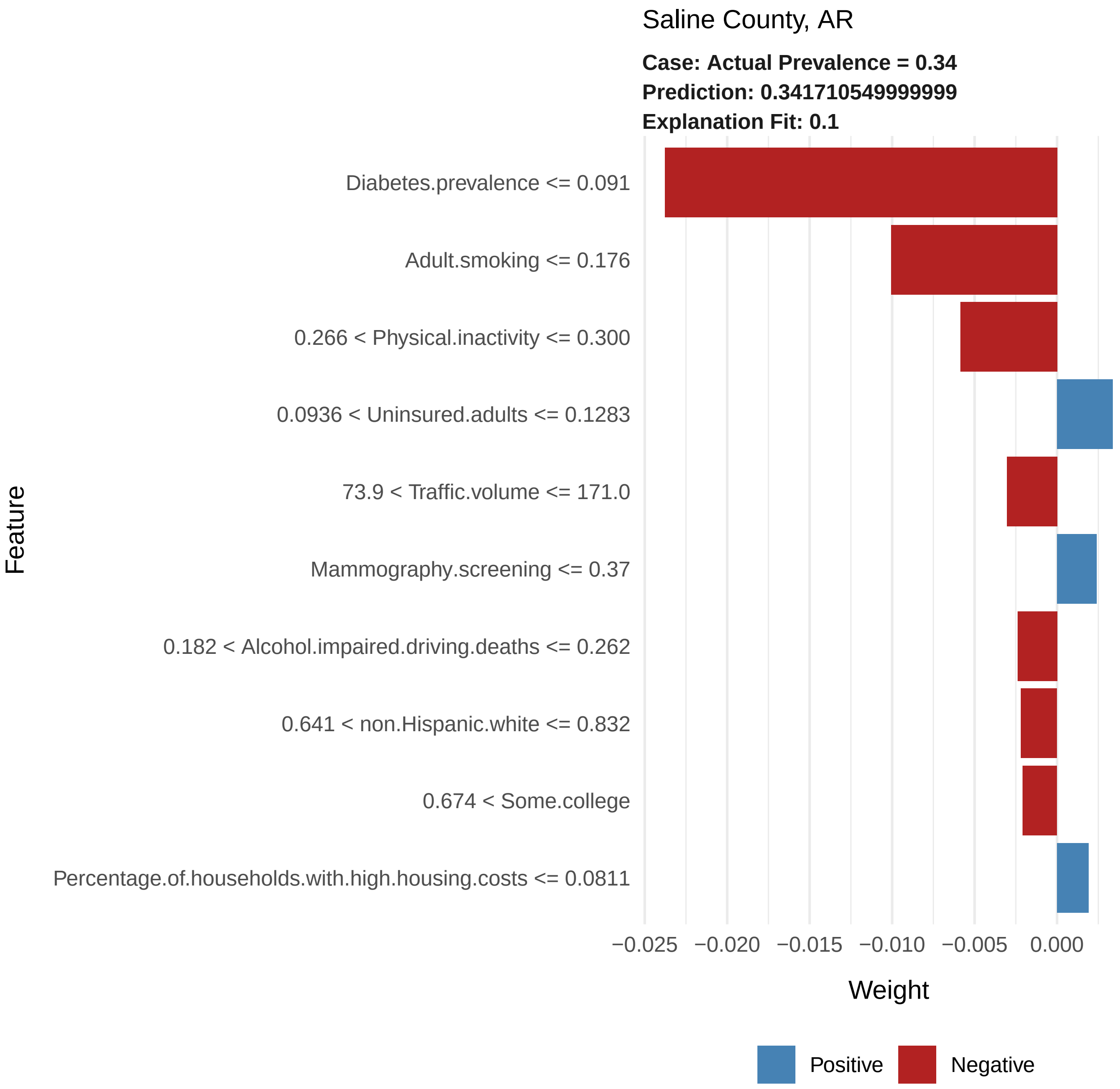
Saline County, AR
Case: Actual Prevalence = 0.34
Prediction: 0.341710549999999
Explanation Fit: 0.1
Diabetes.prevalence <= 0.091
Adult.smoking <= 0.176
0.266 < Physical.inactivity <= 0.300
0.0936 < Uninsured.adults <= 0.1283
73.9 < Traffic.volume <= 171.0
Mammography.screening <= 0.37
0.182 < Alcohol.impaired.driving.deaths <= 0.262
0.641 < non.Hispanic.white <= 0.832
0.674 < Some.college
Percentage.of.households.with.high.housing.costs <= 0.0811
Feature
−0.025
−0.020
−0.015
−0.010
−0.005
0.000
Weight
Positive
Negative

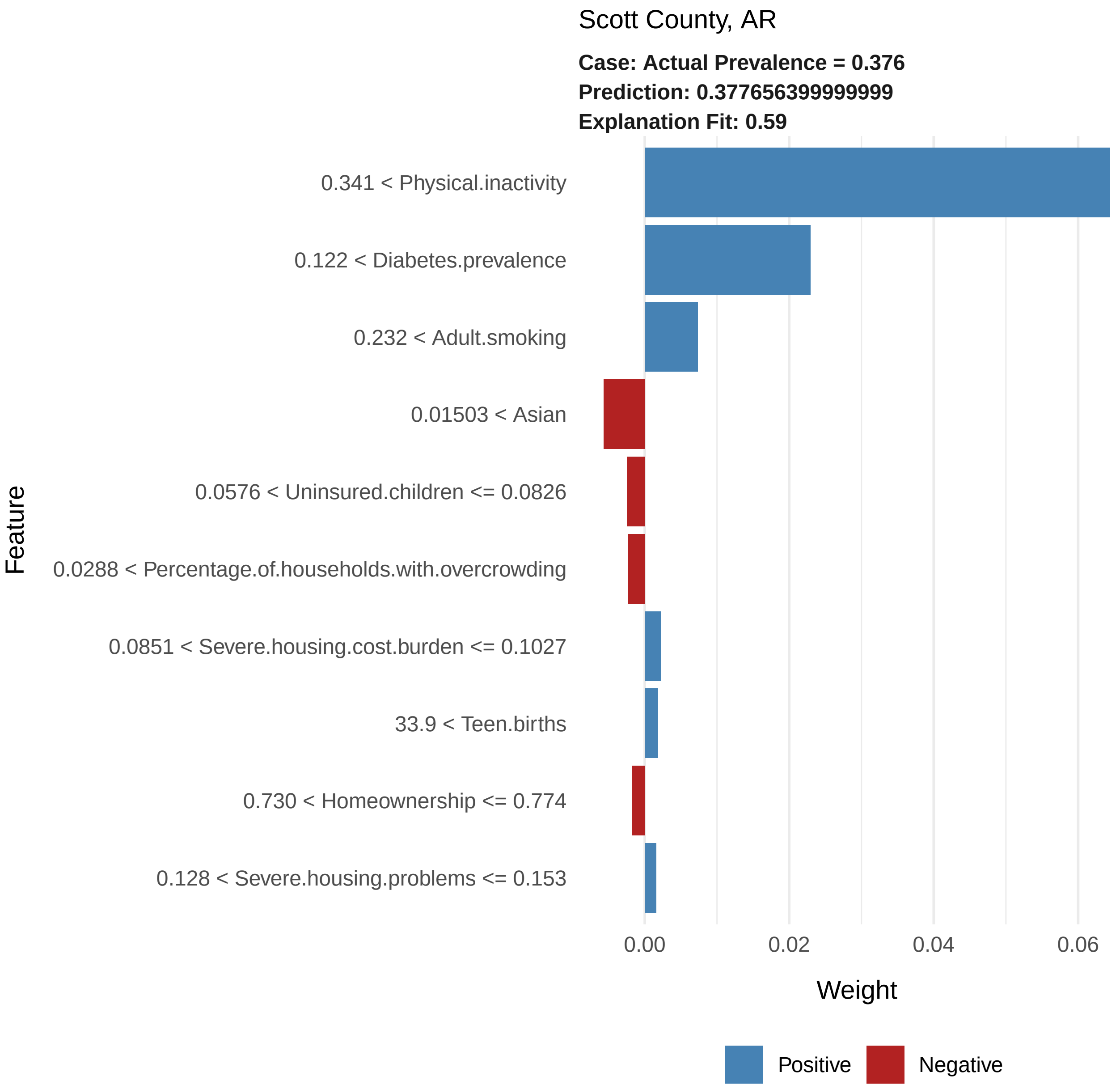
Scott County, AR
Case: Actual Prevalence = 0.376
Prediction: 0.377656399999999
Explanation Fit: 0.59
0.341 < Physical.inactivity
0.122 < Diabetes.prevalence
0.232 < Adult.smoking
0.01503 < Asian
0.0576 < Uninsured.children <= 0.0826
0.0288 < Percentage.of.households.with.overcrowding
0.0851 < Severe.housing.cost.burden <= 0.1027
33.9 < Teen.births
0.730 < Homeownership <= 0.774
0.128 < Severe.housing.problems <= 0.153
Feature
0.00
0.02
0.04
0.06
Weight
Positive
Negative

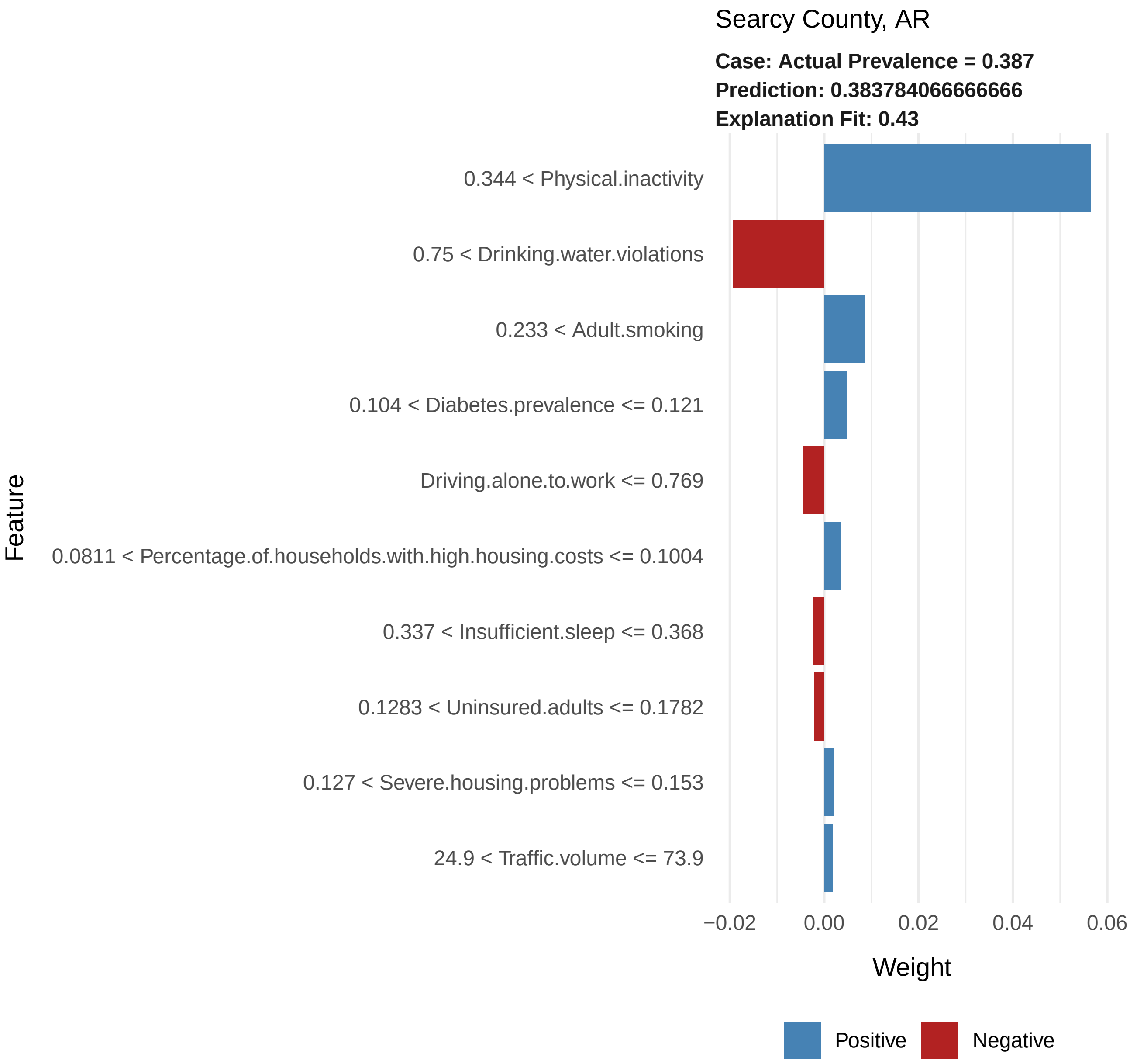
Searcy County, AR
Case: Actual Prevalence = 0.387
Prediction: 0.383784066666666
Explanation Fit: 0.43
Feature
0.344 < Physical.inactivity
0.75 < Drinking.water.violations
0.233 < Adult.smoking
0.104 < Diabetes.prevalence <= 0.121
Driving.alone.to.work <= 0.769
0.0811 < Percentage.of.households.with.high.housing.costs <= 0.1004
0.337 < Insufficient.sleep <= 0.368
0.1283 < Uninsured.adults <= 0.1782
0.127 < Severe.housing.problems <= 0.153
24.9 < Traffic.volume <= 73.9
−0.02
0.00
0.02
0.04
0.06
Weight
Positive
Negative

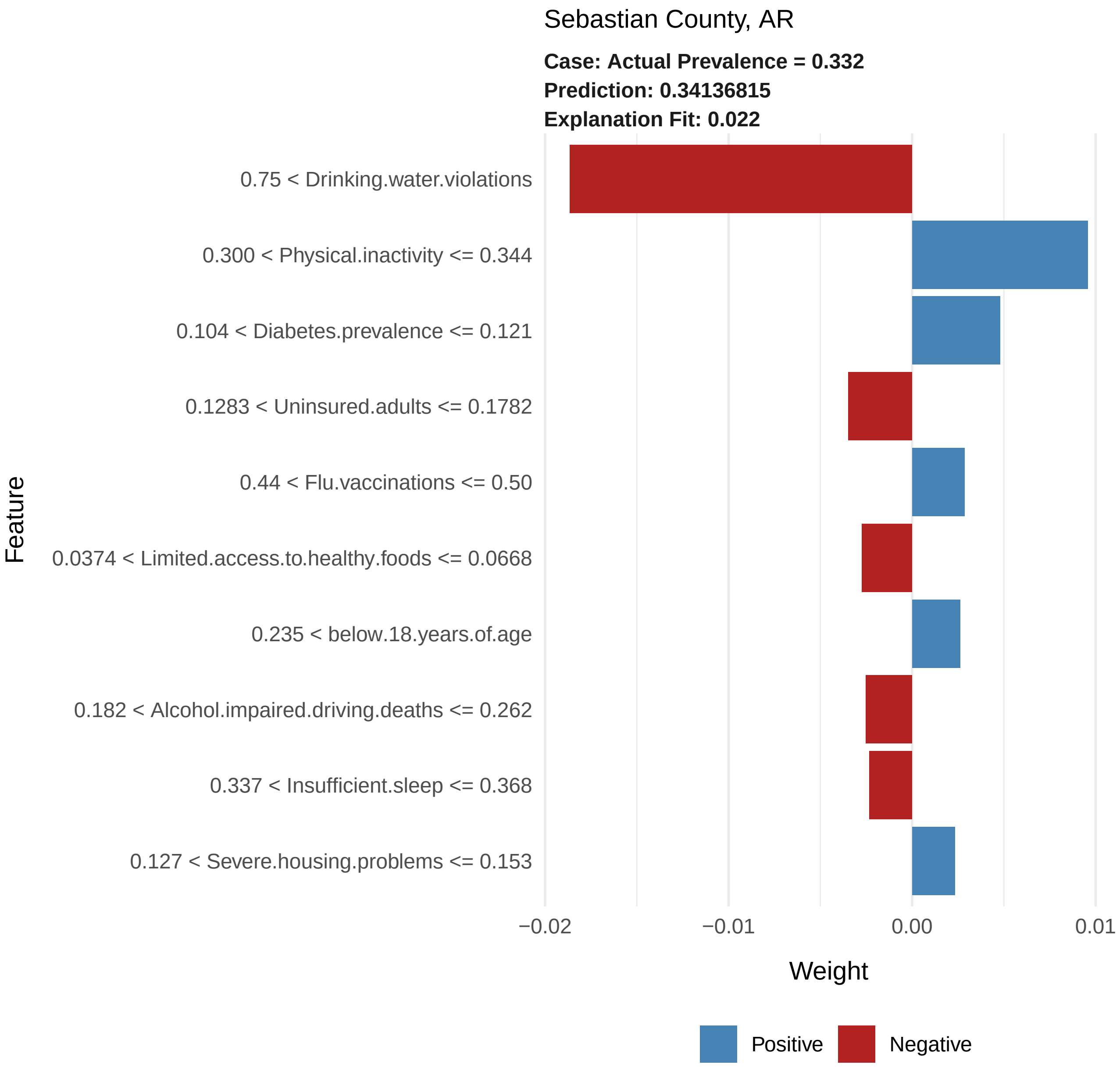

Sebastian County, AR
Case: Actual Prevalence = 0.332
Prediction: 0.34136815
Explanation Fit: 0.022
0.75 < Drinking.water.violations
0.300 < Physical.inactivity <= 0.344
0.104 < Diabetes.prevalence <= 0.121
0.1283 < Uninsured.adults <= 0.1782
0.44 < Flu.vaccinations <= 0.50
0.0374 < Limited.access.to.healthy.foods <= 0.0668
0.235 < below.18.years.of.age
0.182 < Alcohol.impaired.driving.deaths <= 0.262
0.337 < Insufficient.sleep <= 0.368
0.127 < Severe.housing.problems <= 0.153
Feature
−0.02
−0.01
0.00
0.01
Weight
Positive
Negative

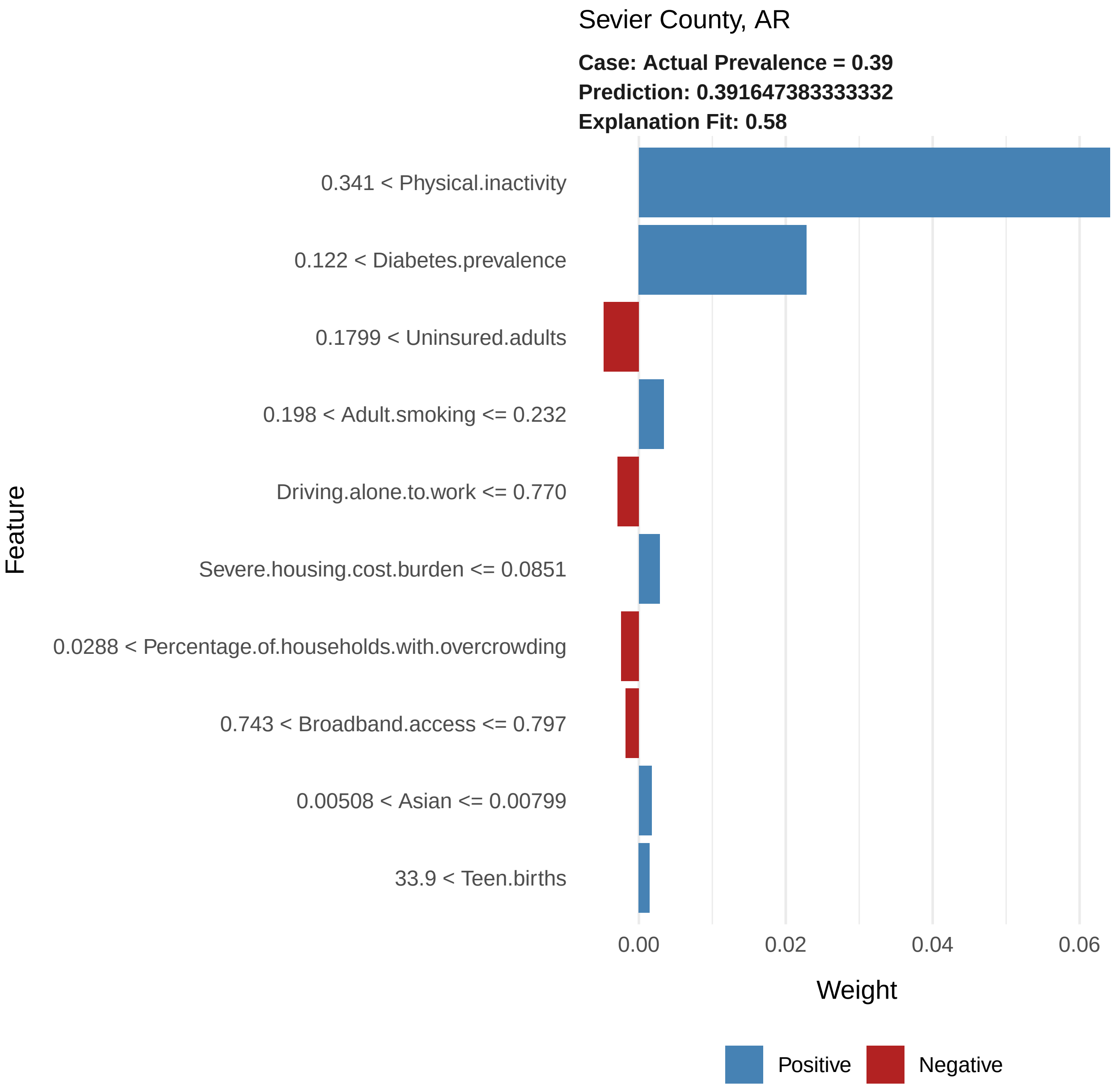
Sevier County, AR
Case: Actual Prevalence = 0.39
Prediction: 0.391647383333332
Explanation Fit: 0.58
0.341 < Physical.inactivity
0.122 < Diabetes.prevalence
0.1799 < Uninsured.adults
0.198 < Adult.smoking <= 0.232
Driving.alone.to.work <= 0.770
Severe.housing.cost.burden <= 0.0851
0.0288 < Percentage.of.households.with.overcrowding
0.743 < Broadband.access <= 0.797
0.00508 < Asian <= 0.00799
33.9 < Teen.births
Feature
0.00
0.02
0.04
0.06
Weight
Positive
Negative

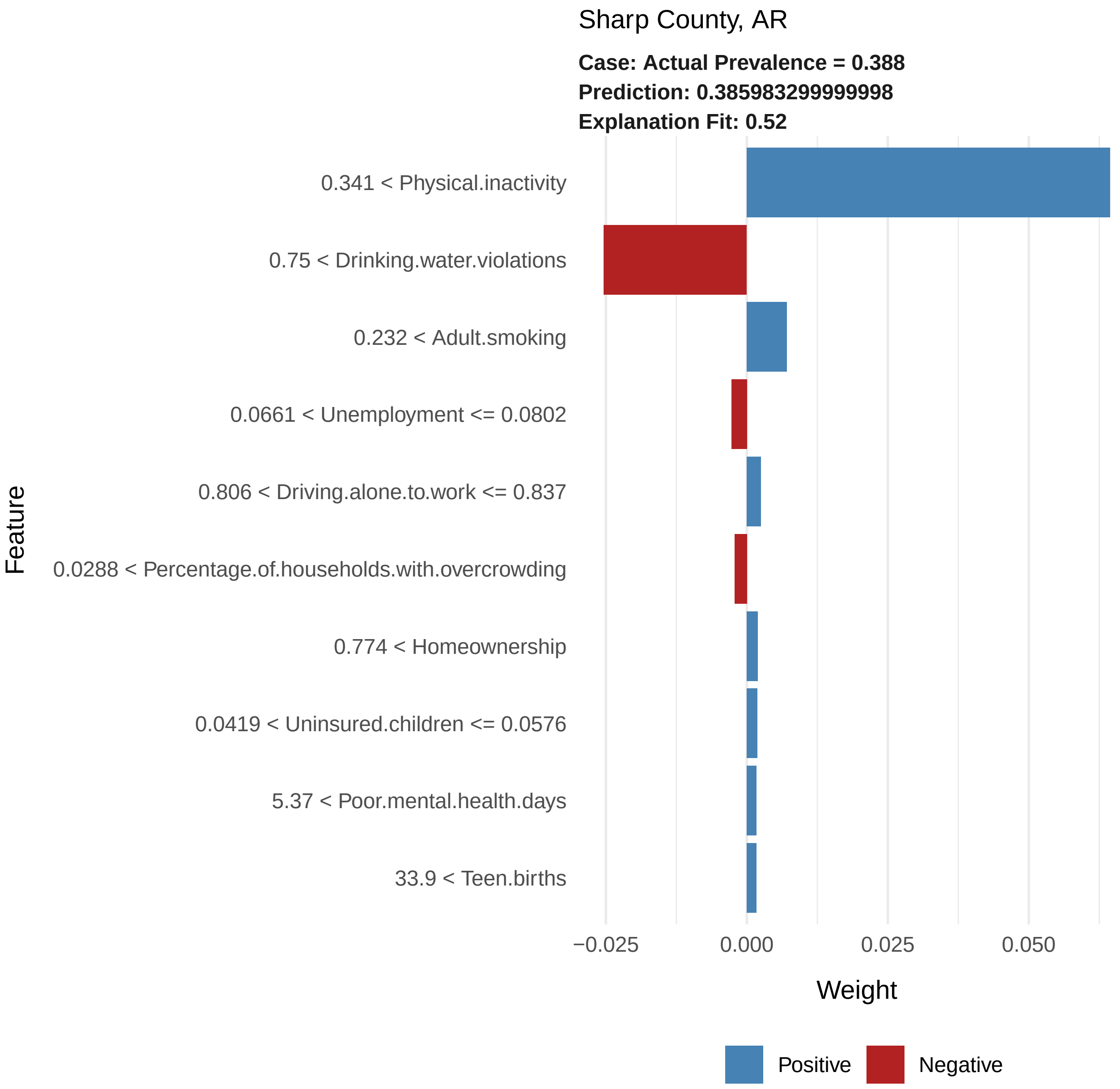

Sharp County, AR
Case: Actual Prevalence = 0.388
Prediction: 0.385983299999998
Explanation Fit: 0.52
0.341 < Physical.inactivity
0.75 < Drinking.water.violations
0.232 < Adult.smoking
0.0661 < Unemployment <= 0.0802
0.806 < Driving.alone.to.work <= 0.837
0.0288 < Percentage.of.households.with.overcrowding
0.774 < Homeownership
0.0419 < Uninsured.children <= 0.0576
5.37 < Poor.mental.health.days
33.9 < Teen.births
Feature
−0.025
0.000
0.025
0.050
Weight
Positive
Negative

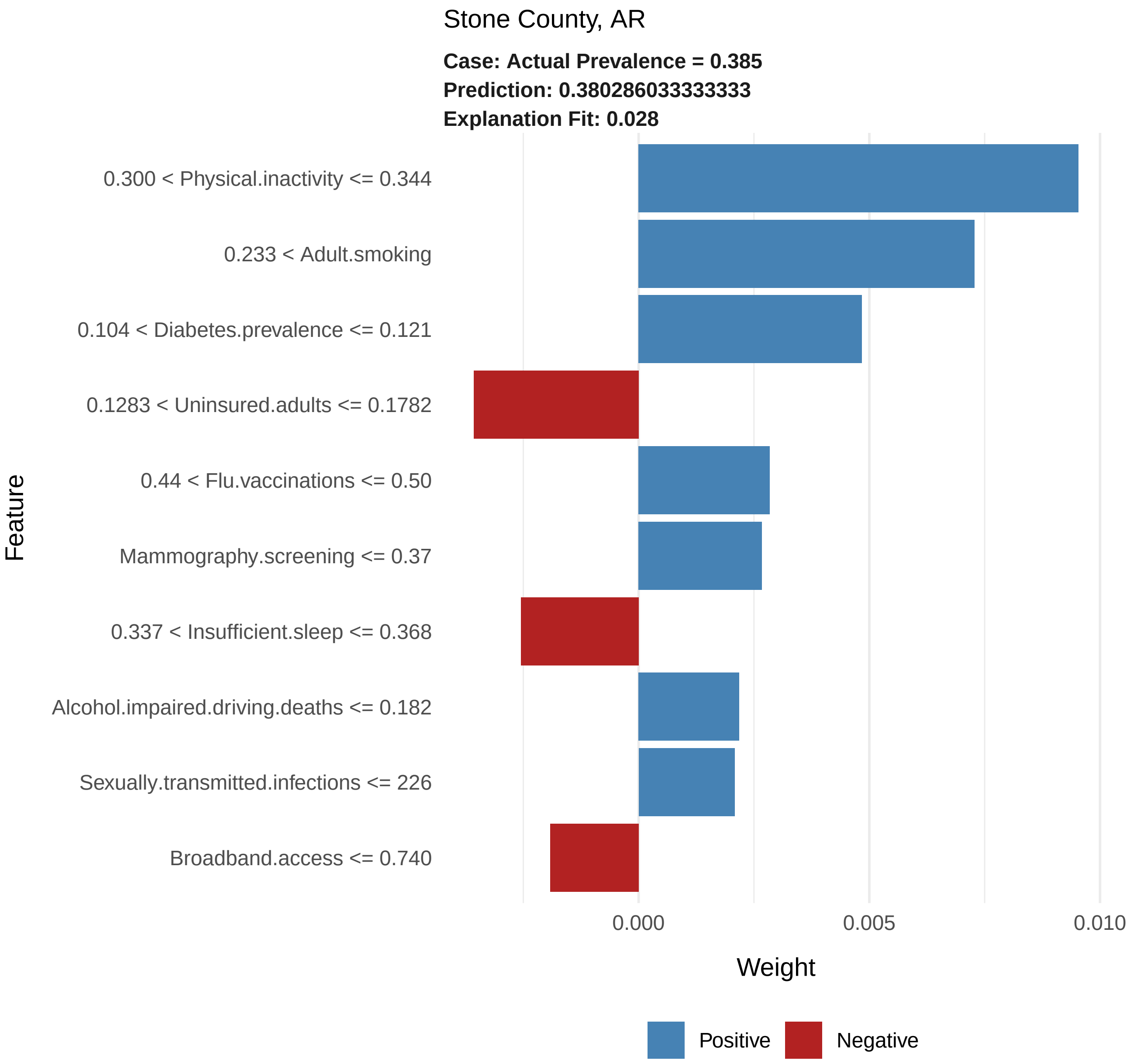

Stone County, AR
Case: Actual Prevalence = 0.385
Prediction: 0.380286033333333
Explanation Fit: 0.028
0.300 < Physical.inactivity <= 0.344
0.233 < Adult.smoking
0.104 < Diabetes.prevalence <= 0.121
0.1283 < Uninsured.adults <= 0.1782
0.44 < Flu.vaccinations <= 0.50
Mammography.screening <= 0.37
0.337 < Insufficient.sleep <= 0.368
Alcohol.impaired.driving.deaths <= 0.182
Sexually.transmitted.infections <= 226
Broadband.access <= 0.740
Feature
0.000
0.005
0.010
Weight
Positive
Negative

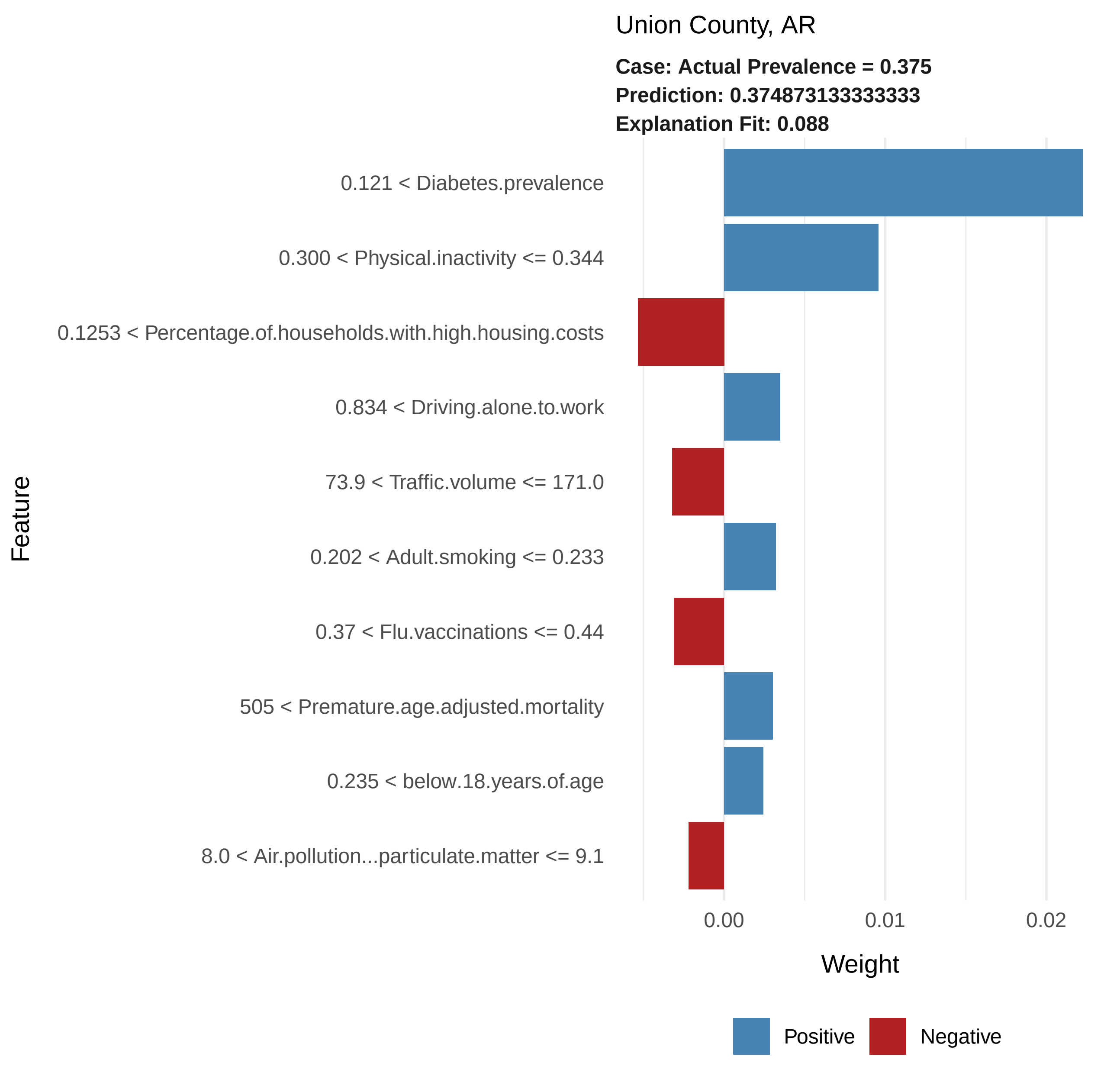
Union County, AR
Case: Actual Prevalence = 0.375
Prediction: 0.374873133333333
Explanation Fit: 0.088
0.121 < Diabetes.prevalence
0.300 < Physical.inactivity <= 0.344
0.1253 < Percentage.of.households.with.high.housing.costs
0.834 < Driving.alone.to.work
73.9 < Traffic.volume <= 171.0
0.202 < Adult.smoking <= 0.233
0.37 < Flu.vaccinations <= 0.44
505 < Premature.age.adjusted.mortality
0.235 < below.18.years.of.age
8.0 < Air.pollution...particulate.matter <= 9.1
Feature
0.00
0.01
0.02
Weight
Positive
Negative

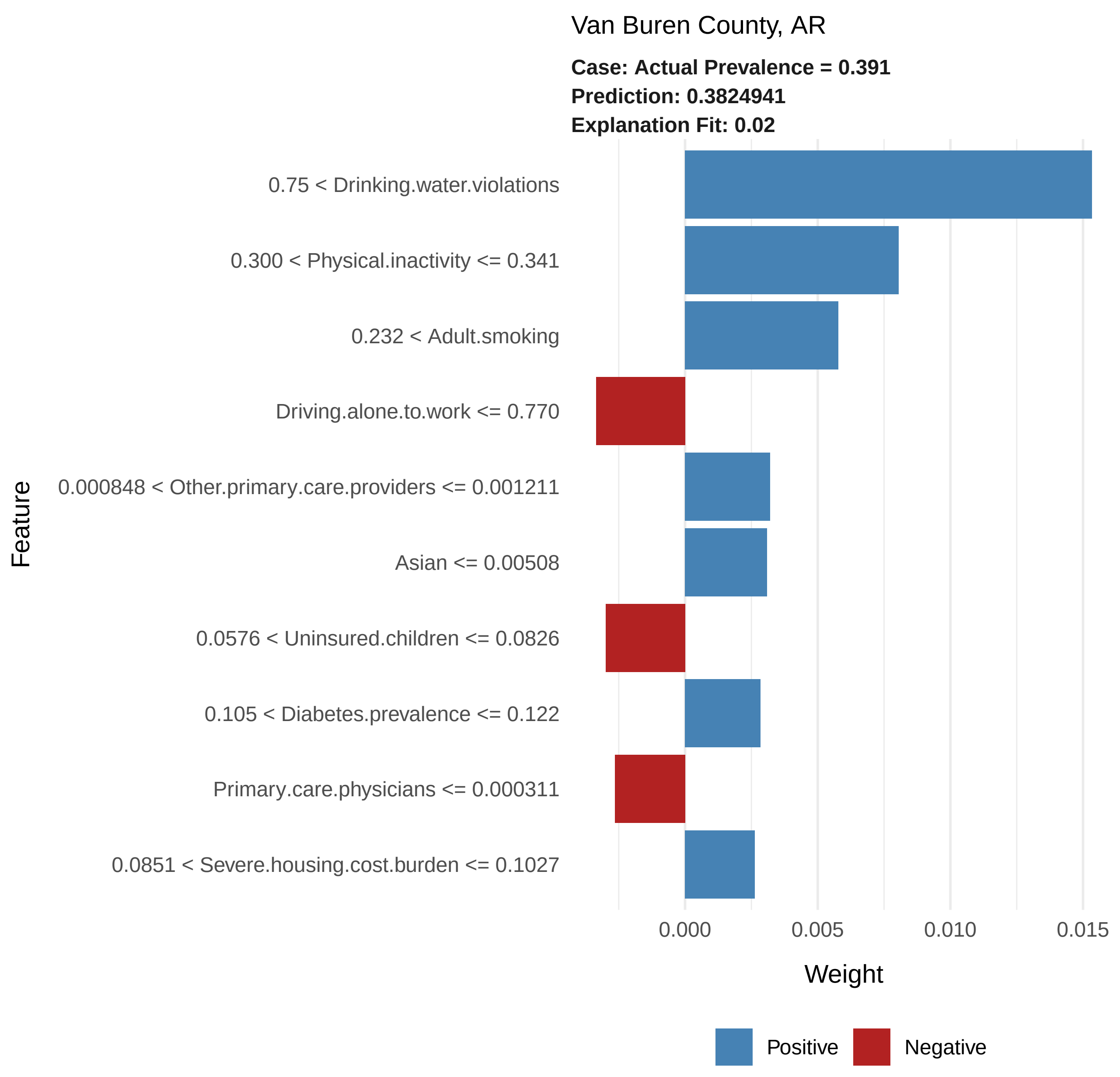

Van Buren County, AR
Case: Actual Prevalence = 0.391
Prediction: 0.3824941
Explanation Fit: 0.02
0.75 < Drinking.water.violations
0.300 < Physical.inactivity <= 0.341
0.232 < Adult.smoking
Driving.alone.to.work <= 0.770
0.000848 < Other.primary.care.providers <= 0.001211
Asian <= 0.00508
0.0576 < Uninsured.children <= 0.0826
0.105 < Diabetes.prevalence <= 0.122
Primary.care.physicians <= 0.000311
0.0851 < Severe.housing.cost.burden <= 0.1027
Feature
0.000
0.005
0.010
0.015
Weight
Positive
Negative

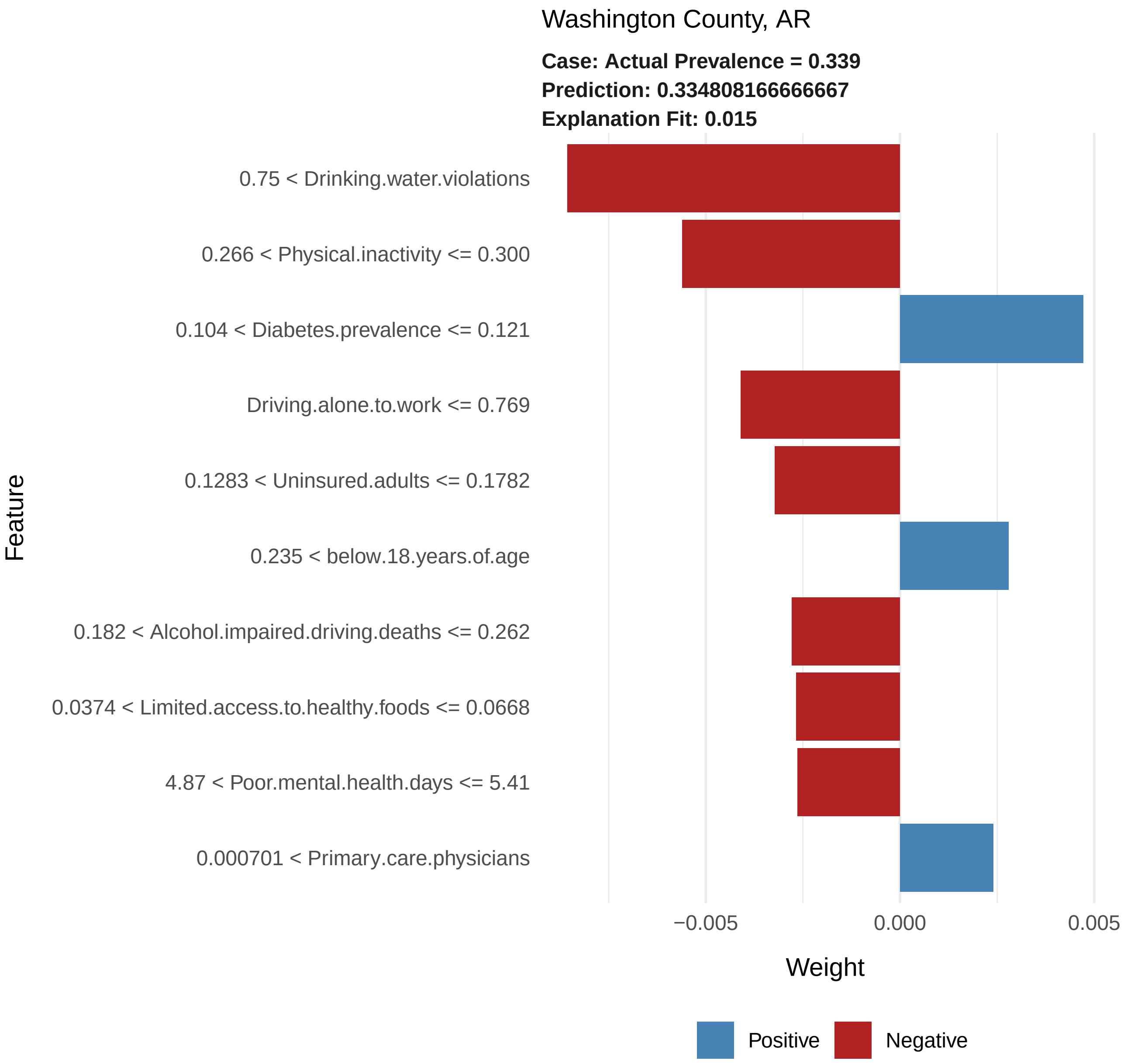
Washington County, AR
Case: Actual Prevalence = 0.339
Prediction: 0.334808166666667
Explanation Fit: 0.015
0.75 < Drinking.water.violations
0.266 < Physical.inactivity <= 0.300
0.104 < Diabetes.prevalence <= 0.121
Driving.alone.to.work <= 0.769
0.1283 < Uninsured.adults <= 0.1782
0.235 < below.18.years.of.age
0.182 < Alcohol.impaired.driving.deaths <= 0.262
0.0374 < Limited.access.to.healthy.foods <= 0.0668
4.87 < Poor.mental.health.days <= 5.41
0.000701 < Primary.care.physicians
Feature
−0.005
0.000
0.005
Weight
Positive
Negative

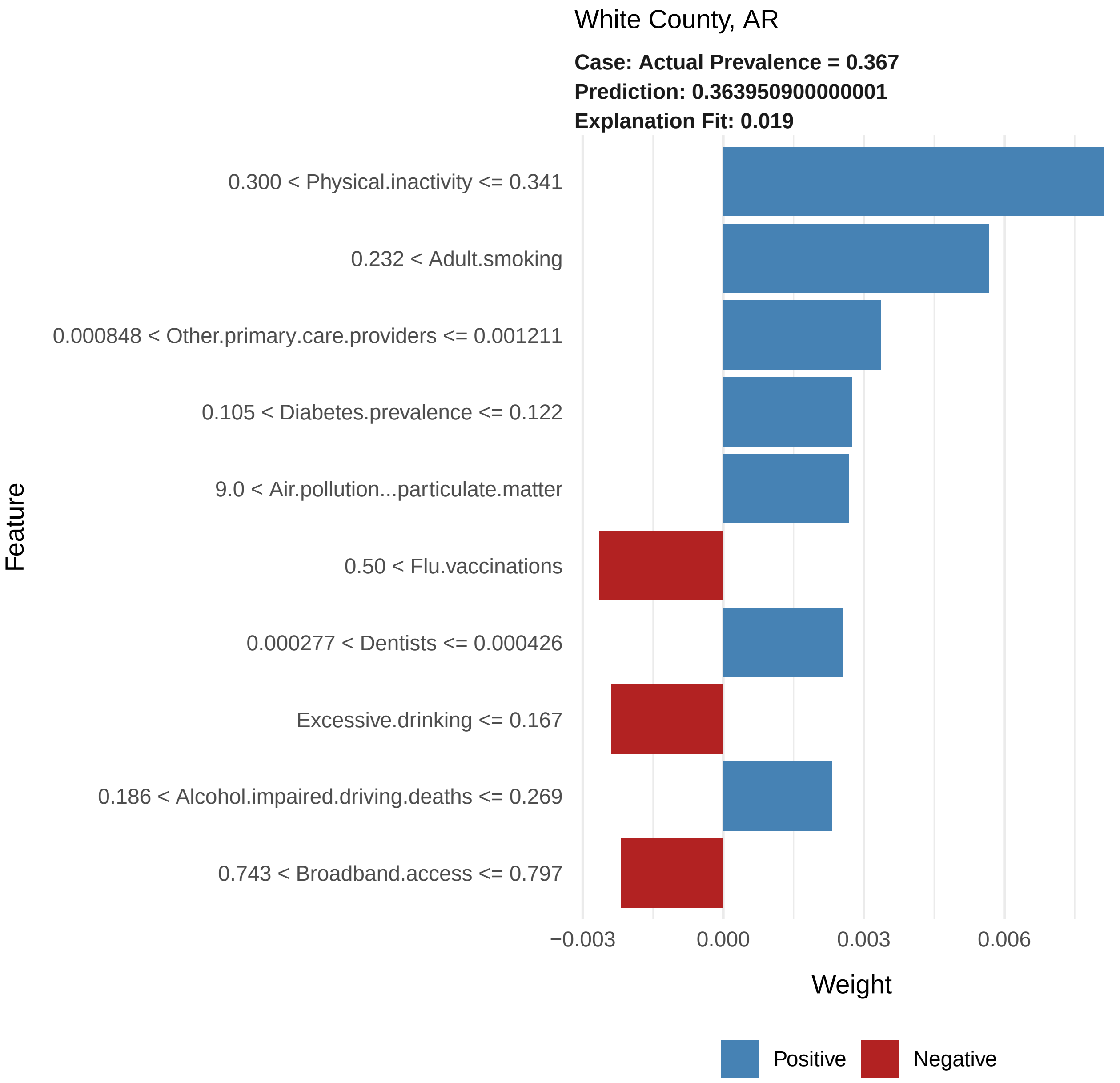
White County, AR
Case: Actual Prevalence = 0.367
Prediction: 0.363950900000001
Explanation Fit: 0.019
0.300 < Physical.inactivity <= 0.341
0.232 < Adult.smoking
0.000848 < Other.primary.care.providers <= 0.001211
0.105 < Diabetes.prevalence <= 0.122
9.0 < Air.pollution...particulate.matter
0.50 < Flu.vaccinations
0.000277 < Dentists <= 0.000426
Excessive.drinking <= 0.167
0.186 < Alcohol.impaired.driving.deaths <= 0.269
0.743 < Broadband.access <= 0.797
Feature
−0.003
0.000
0.003
0.006
Weight
Positive
Negative

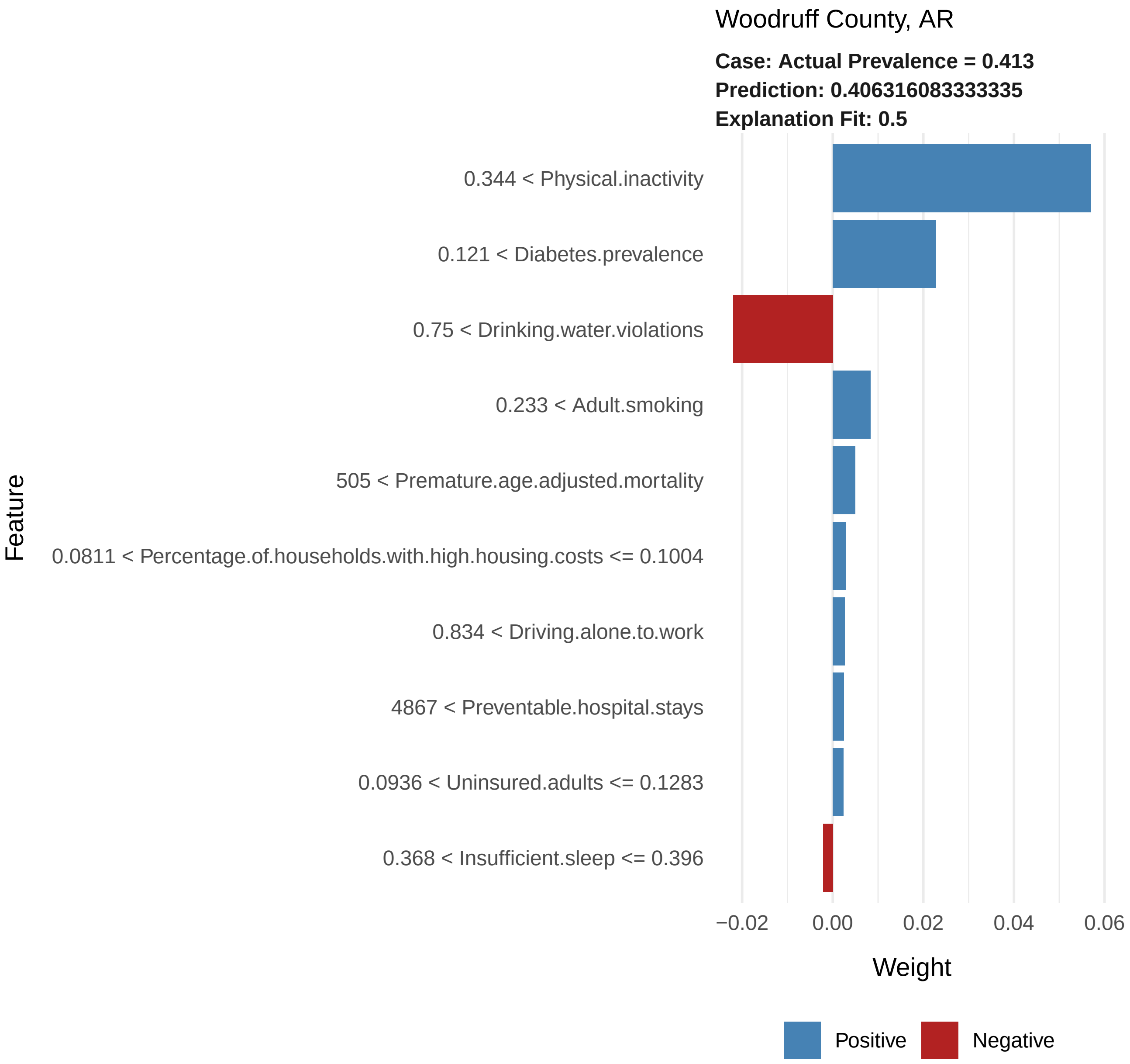

Woodruff County, AR
Case: Actual Prevalence = 0.413
Prediction: 0.406316083333335
Explanation Fit: 0.5
0.344 < Physical.inactivity
0.121 < Diabetes.prevalence
0.75 < Drinking.water.violations
0.233 < Adult.smoking
505 < Premature.age.adjusted.mortality
0.0811 < Percentage.of.households.with.high.housing.costs <= 0.1004
0.834 < Driving.alone.to.work
4867 < Preventable.hospital.stays
0.0936 < Uninsured.adults <= 0.1283
0.368 < Insufficient.sleep <= 0.396
Feature
−0.02
0.00
0.02
0.04
0.06
Weight
Positive
Negative

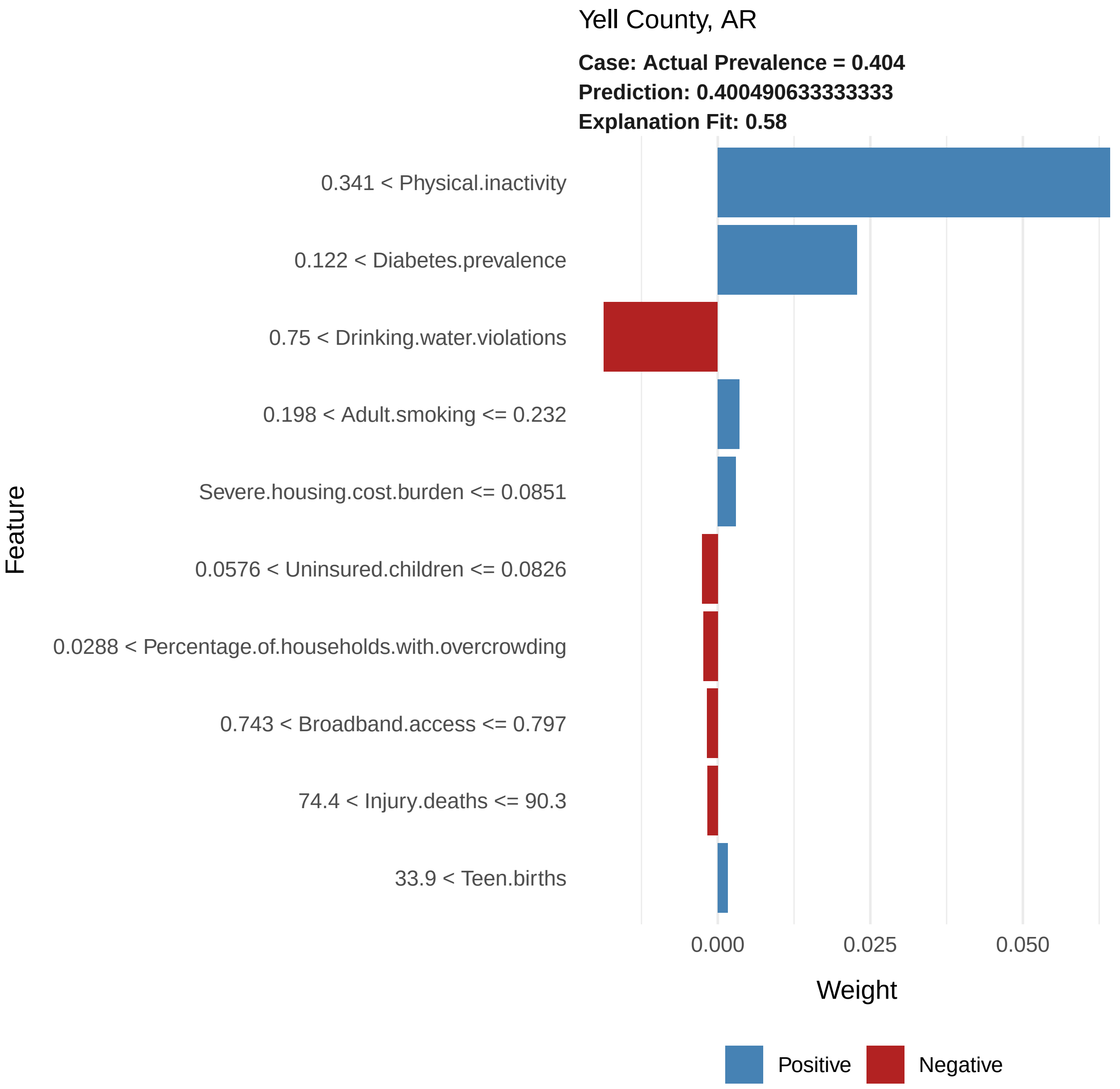

Yell County, AR
Case: Actual Prevalence = 0.404
Prediction: 0.400490633333333
Explanation Fit: 0.58
0.341 < Physical.inactivity
0.122 < Diabetes.prevalence
0.75 < Drinking.water.violations
0.198 < Adult.smoking <= 0.232
Severe.housing.cost.burden <= 0.0851
0.0576 < Uninsured.children <= 0.0826
0.0288 < Percentage.of.households.with.overcrowding
0.743 < Broadband.access <= 0.797
74.4 < Injury.deaths <= 90.3
33.9 < Teen.births
Feature
0.000
0.025
0.050
Weight
Positive
Negative

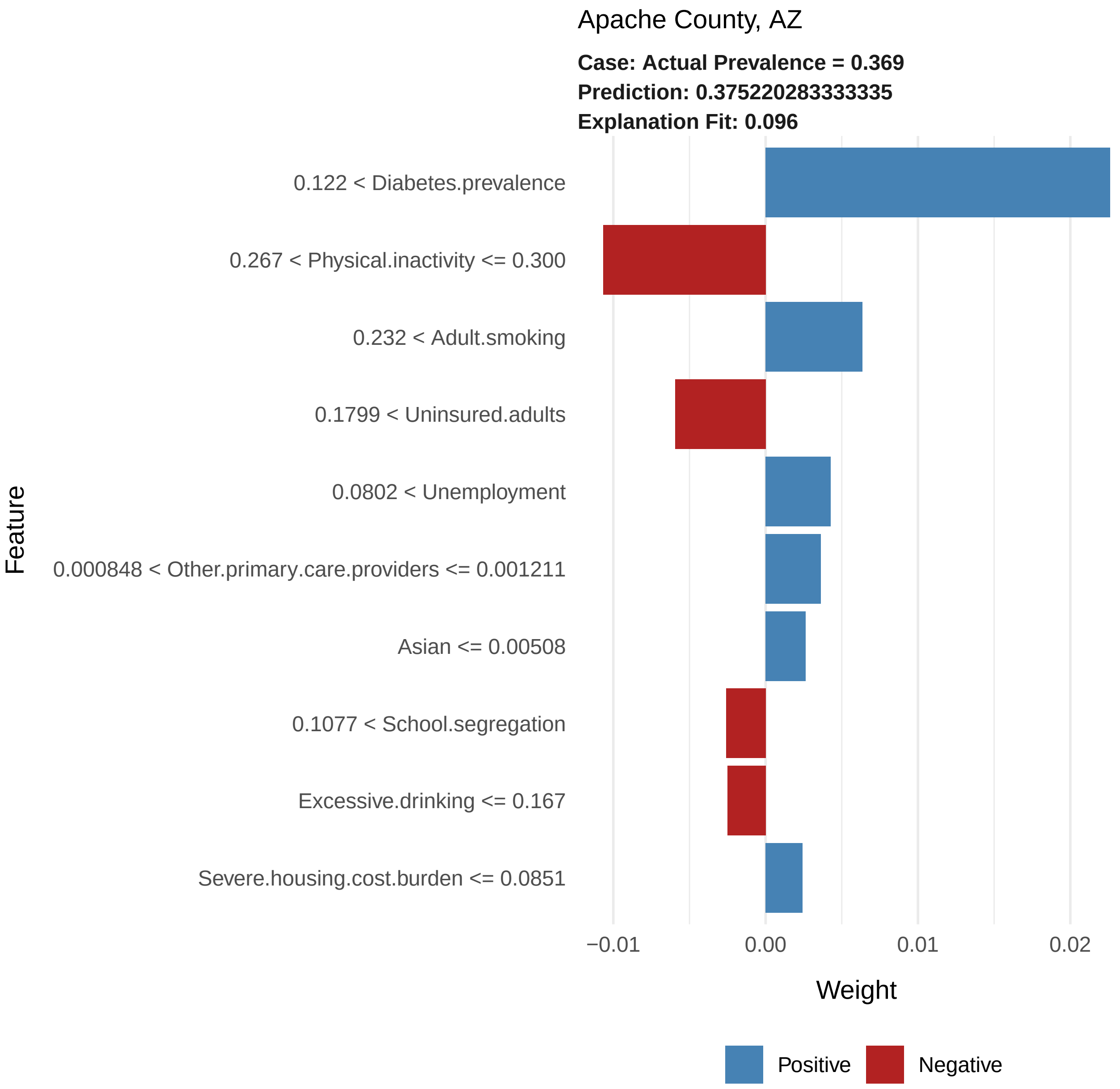

Apache County, AZ
Case: Actual Prevalence = 0.369
Prediction: 0.375220283333335
Explanation Fit: 0.096
0.122 < Diabetes.prevalence
0.267 < Physical.inactivity <= 0.300
0.232 < Adult.smoking
0.1799 < Uninsured.adults
0.0802 < Unemployment
0.000848 < Other.primary.care.providers <= 0.001211
Asian <= 0.00508
0.1077 < School.segregation
Excessive.drinking <= 0.167
Severe.housing.cost.burden <= 0.0851
Feature
−0.01
0.00
0.01
0.02
Weight
Positive
Negative

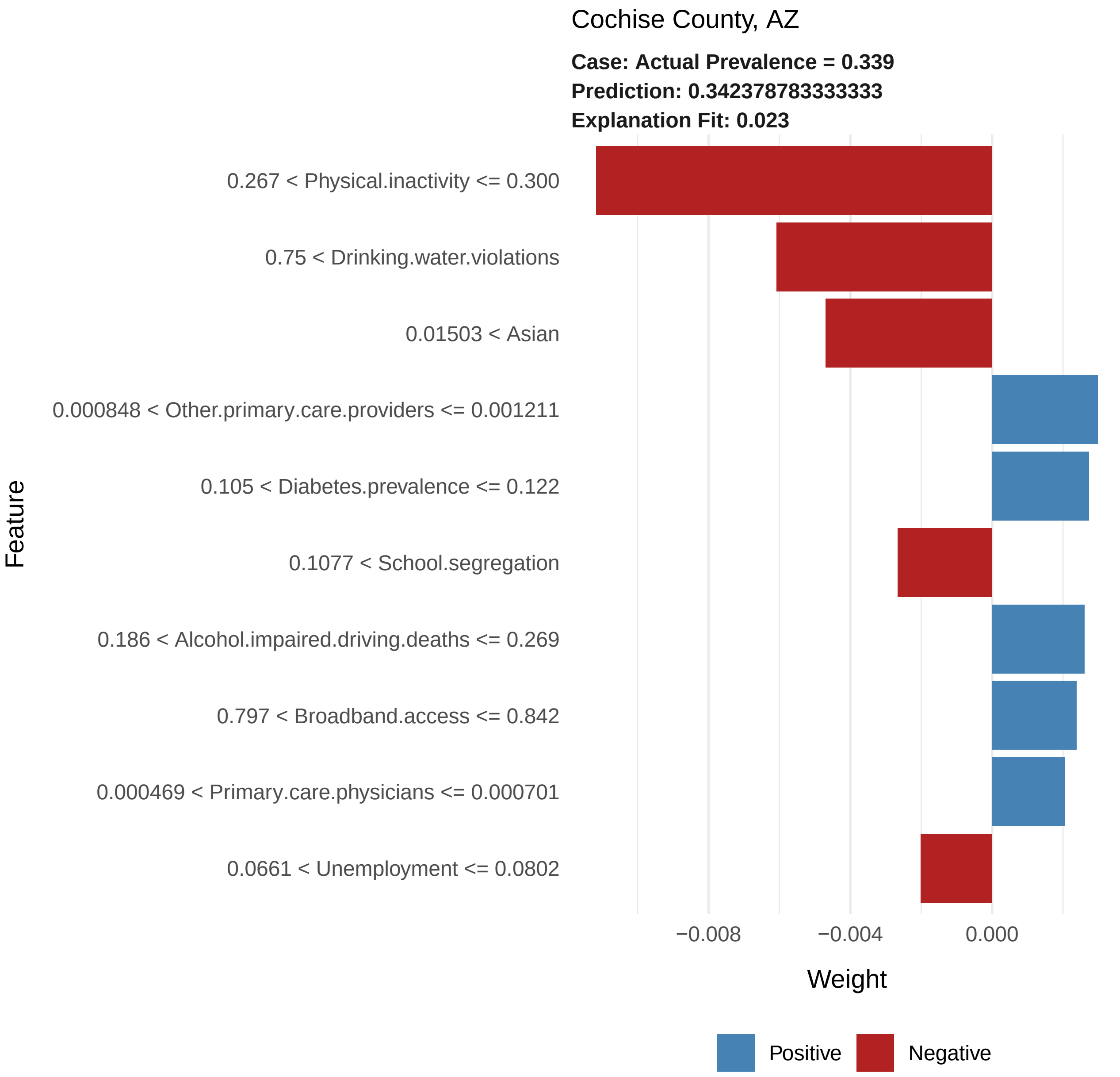

Cochise County, AZ
Case: Actual Prevalence = 0.339
Prediction: 0.342378783333333
Explanation Fit: 0.023
0.267 < Physical.inactivity <= 0.300
0.75 < Drinking.water.violations
0.01503 < Asian
0.000848 < Other.primary.care.providers <= 0.001211
0.105 < Diabetes.prevalence <= 0.122
0.1077 < School.segregation
0.186 < Alcohol.impaired.driving.deaths <= 0.269
0.797 < Broadband.access <= 0.842
0.000469 < Primary.care.physicians <= 0.000701
0.0661 < Unemployment <= 0.0802
Feature
−0.008
−0.004
0.000
Weight
Positive
Negative

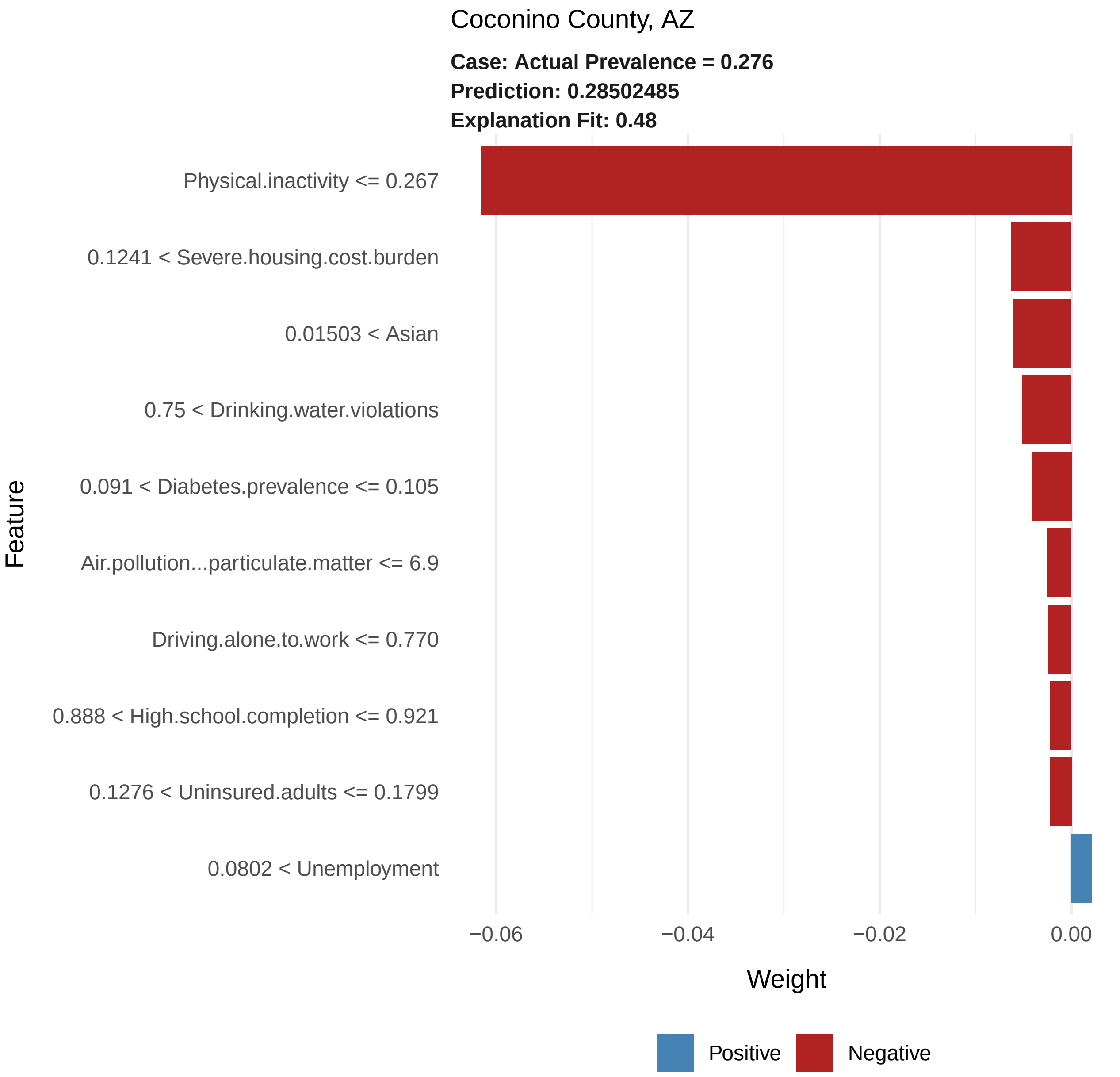
Coconino County, AZ
Case: Actual Prevalence = 0.276
Prediction: 0.28502485
Explanation Fit: 0.48
Physical.inactivity <= 0.267
0.1241 < Severe.housing.cost.burden
0.01503 < Asian
0.75 < Drinking.water.violations
0.091 < Diabetes.prevalence <= 0.105
Air.pollution...particulate.matter <= 6.9
Driving.alone.to.work <= 0.770
0.888 < High.school.completion <= 0.921
0.1276 < Uninsured.adults <= 0.1799
0.0802 < Unemployment
Feature
−0.06
−0.04
−0.02
0.00
Weight
Positive
Negative

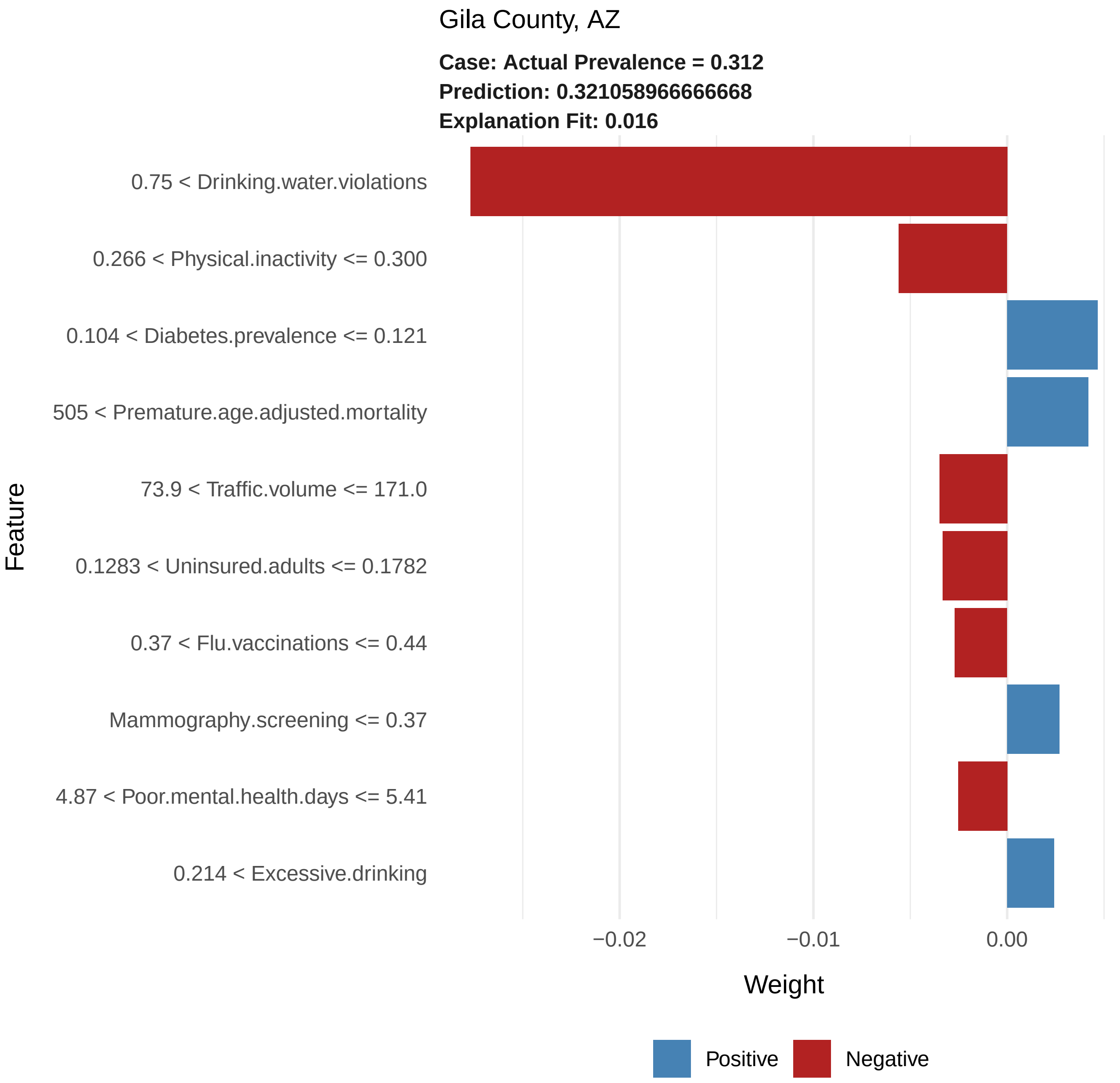

Gila County, AZ
Case: Actual Prevalence = 0.312
Prediction: 0.321058966666668
Explanation Fit: 0.016
0.75 < Drinking.water.violations
0.266 < Physical.inactivity <= 0.300
0.104 < Diabetes.prevalence <= 0.121
505 < Premature.age.adjusted.mortality
73.9 < Traffic.volume <= 171.0
0.1283 < Uninsured.adults <= 0.1782
0.37 < Flu.vaccinations <= 0.44
Mammography.screening <= 0.37
4.87 < Poor.mental.health.days <= 5.41
0.214 < Excessive.drinking
Feature
−0.02
−0.01
0.00
Weight
Positive
Negative

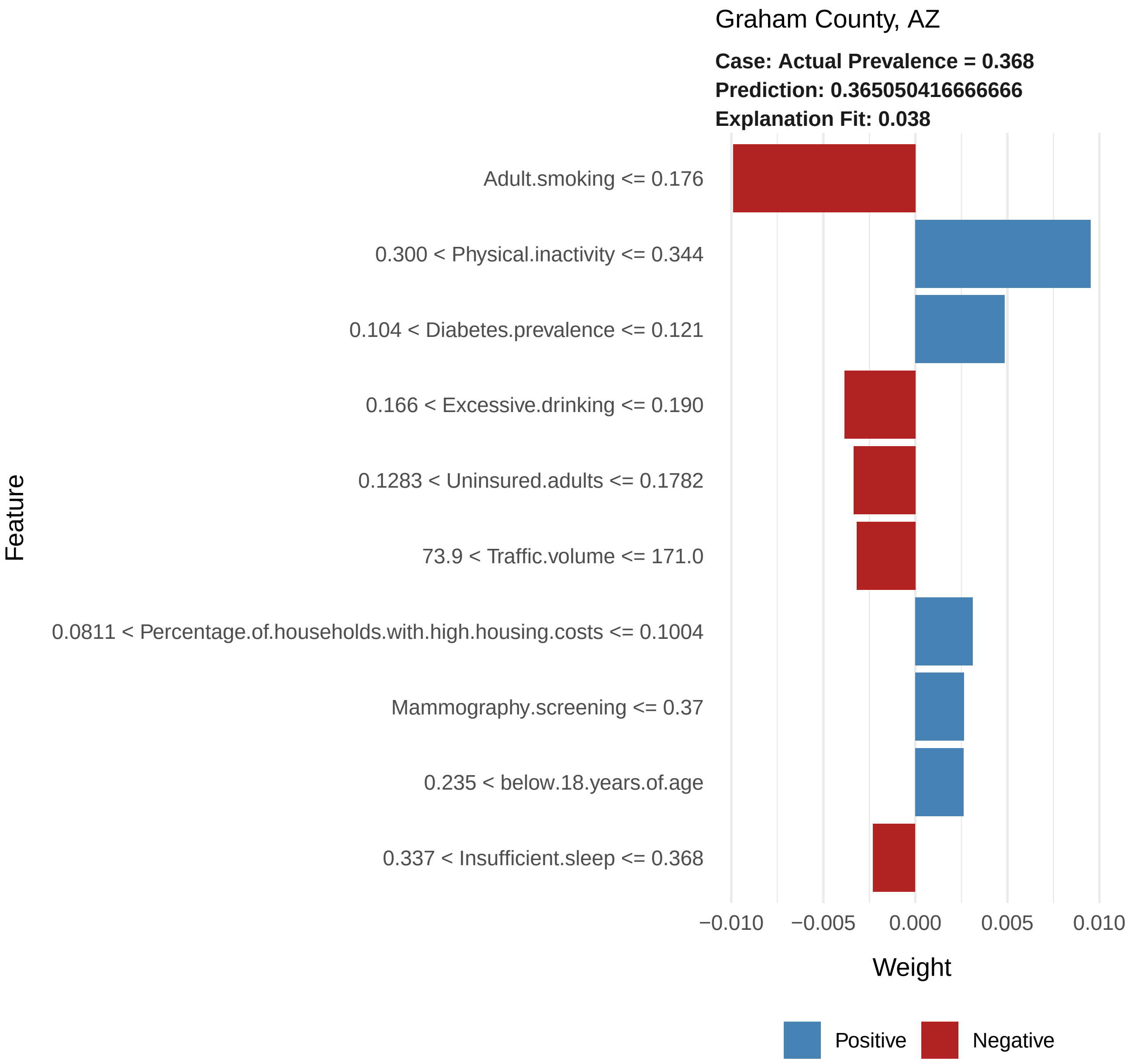

Graham County, AZ
Case: Actual Prevalence = 0.368
Prediction: 0.365050416666666
Explanation Fit: 0.038
Adult.smoking <= 0.176
0.300 < Physical.inactivity <= 0.344
0.104 < Diabetes.prevalence <= 0.121
0.166 < Excessive.drinking <= 0.190
0.1283 < Uninsured.adults <= 0.1782
73.9 < Traffic.volume <= 171.0
0.0811 < Percentage.of.households.with.high.housing.costs <= 0.1004
Mammography.screening <= 0.37
0.235 < below.18.years.of.age
0.337 < Insufficient.sleep <= 0.368
Feature
−0.010
−0.005
0.000
0.005
0.010
Weight
Positive
Negative

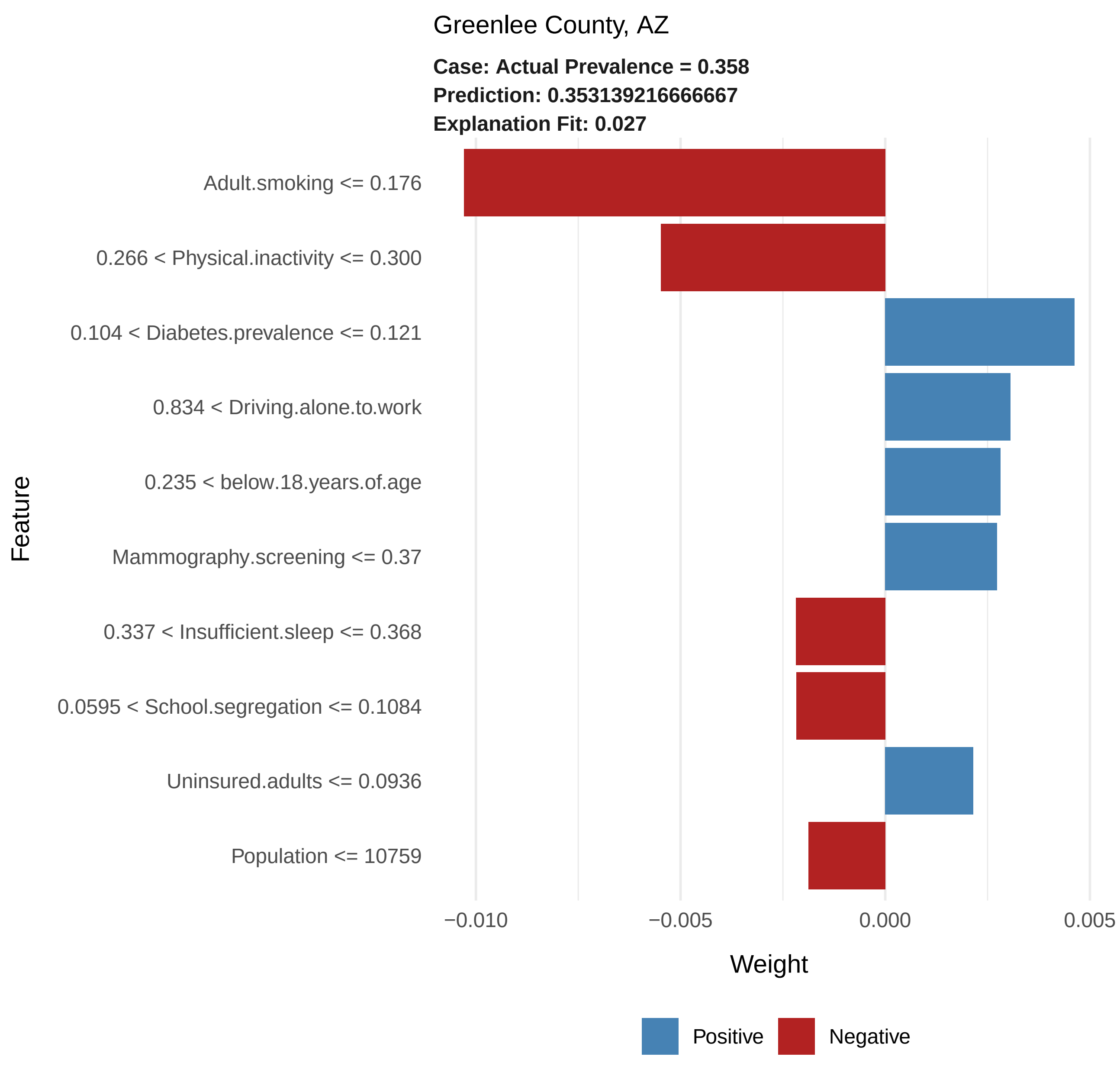
Greenlee County, AZ
Case: Actual Prevalence = 0.358
Prediction: 0.353139216666667
Explanation Fit: 0.027
Adult.smoking <= 0.176
0.266 < Physical.inactivity <= 0.300
0.104 < Diabetes.prevalence <= 0.121
0.834 < Driving.alone.to.work
0.235 < below.18.years.of.age
Mammography.screening <= 0.37
0.337 < Insufficient.sleep <= 0.368
0.0595 < School.segregation <= 0.1084
Uninsured.adults <= 0.0936
Population <= 10759
Feature
−0.010
−0.005
0.000
0.005
Weight
Positive
Negative

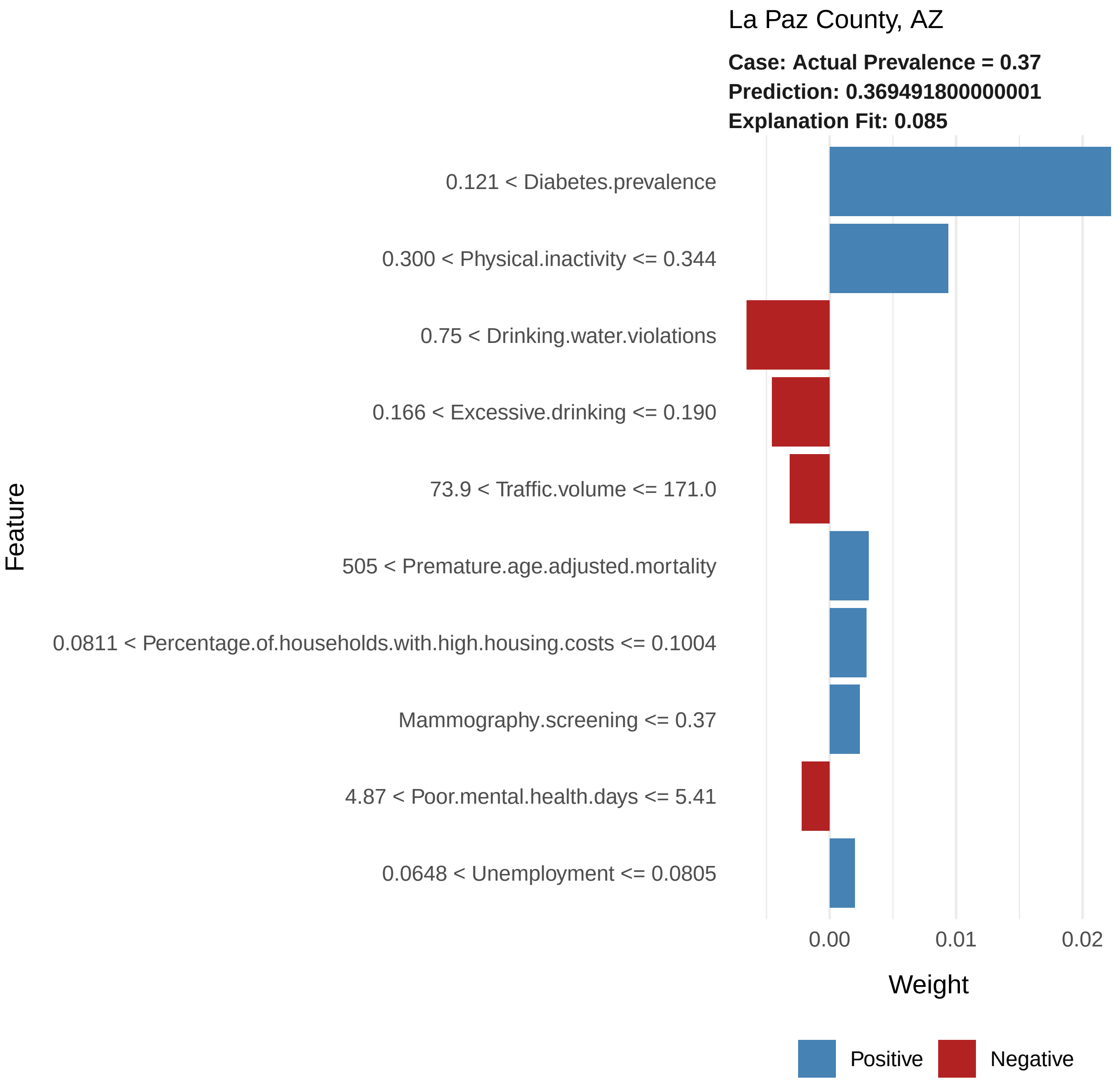
La Paz County, AZ
Case: Actual Prevalence = 0.37
Prediction: 0.369491800000001
Explanation Fit: 0.085
0.121 < Diabetes.prevalence
0.300 < Physical.inactivity <= 0.344
0.75 < Drinking.water.violations
0.166 < Excessive.drinking <= 0.190
73.9 < Traffic.volume <= 171.0
505 < Premature.age.adjusted.mortality
0.0811 < Percentage.of.households.with.high.housing.costs <= 0.1004
Mammography.screening <= 0.37
4.87 < Poor.mental.health.days <= 5.41
0.0648 < Unemployment <= 0.0805
Feature
0.00
0.01
0.02
Weight
Positive
Negative

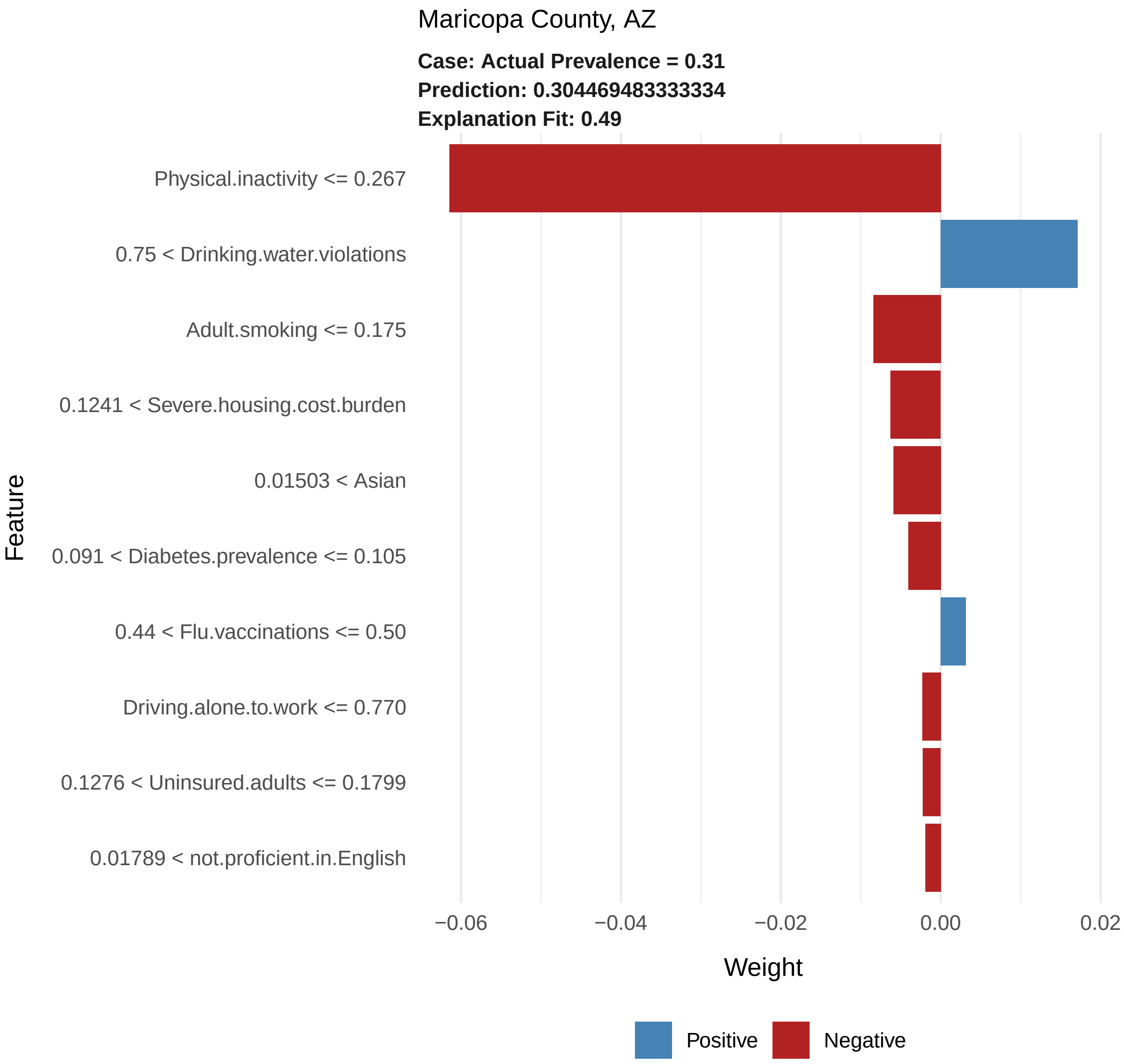

Maricopa County, AZ
Case: Actual Prevalence = 0.31
Prediction: 0.304469483333334
Explanation Fit: 0.49
Physical.inactivity <= 0.267
0.75 < Drinking.water.violations
Adult.smoking <= 0.175
0.1241 < Severe.housing.cost.burden
0.01503 < Asian
0.091 < Diabetes.prevalence <= 0.105
0.44 < Flu.vaccinations <= 0.50
Driving.alone.to.work <= 0.770
0.1276 < Uninsured.adults <= 0.1799
0.01789 < not.proficient.in.English
Feature
−0.06
−0.04
−0.02
0.00
0.02
Weight
Positive
Negative

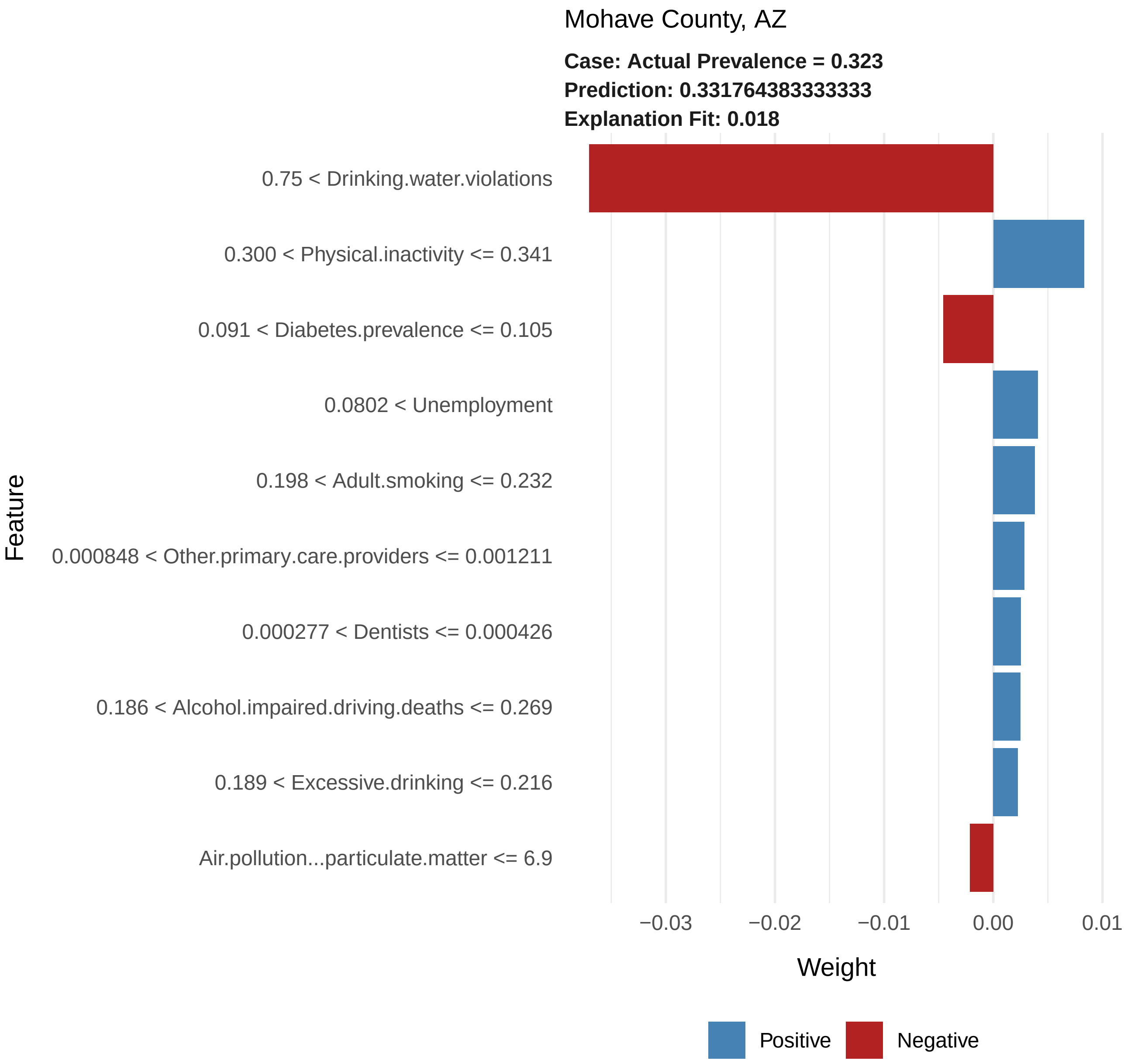

Mohave County, AZ
Case: Actual Prevalence = 0.323
Prediction: 0.331764383333333
Explanation Fit: 0.018
0.75 < Drinking.water.violations
0.300 < Physical.inactivity <= 0.341
0.091 < Diabetes.prevalence <= 0.105
0.0802 < Unemployment
0.198 < Adult.smoking <= 0.232
0.000848 < Other.primary.care.providers <= 0.001211
0.000277 < Dentists <= 0.000426
0.186 < Alcohol.impaired.driving.deaths <= 0.269
0.189 < Excessive.drinking <= 0.216
Air.pollution...particulate.matter <= 6.9
Feature
−0.03
−0.02
−0.01
0.00
0.01
Weight
Positive
Negative

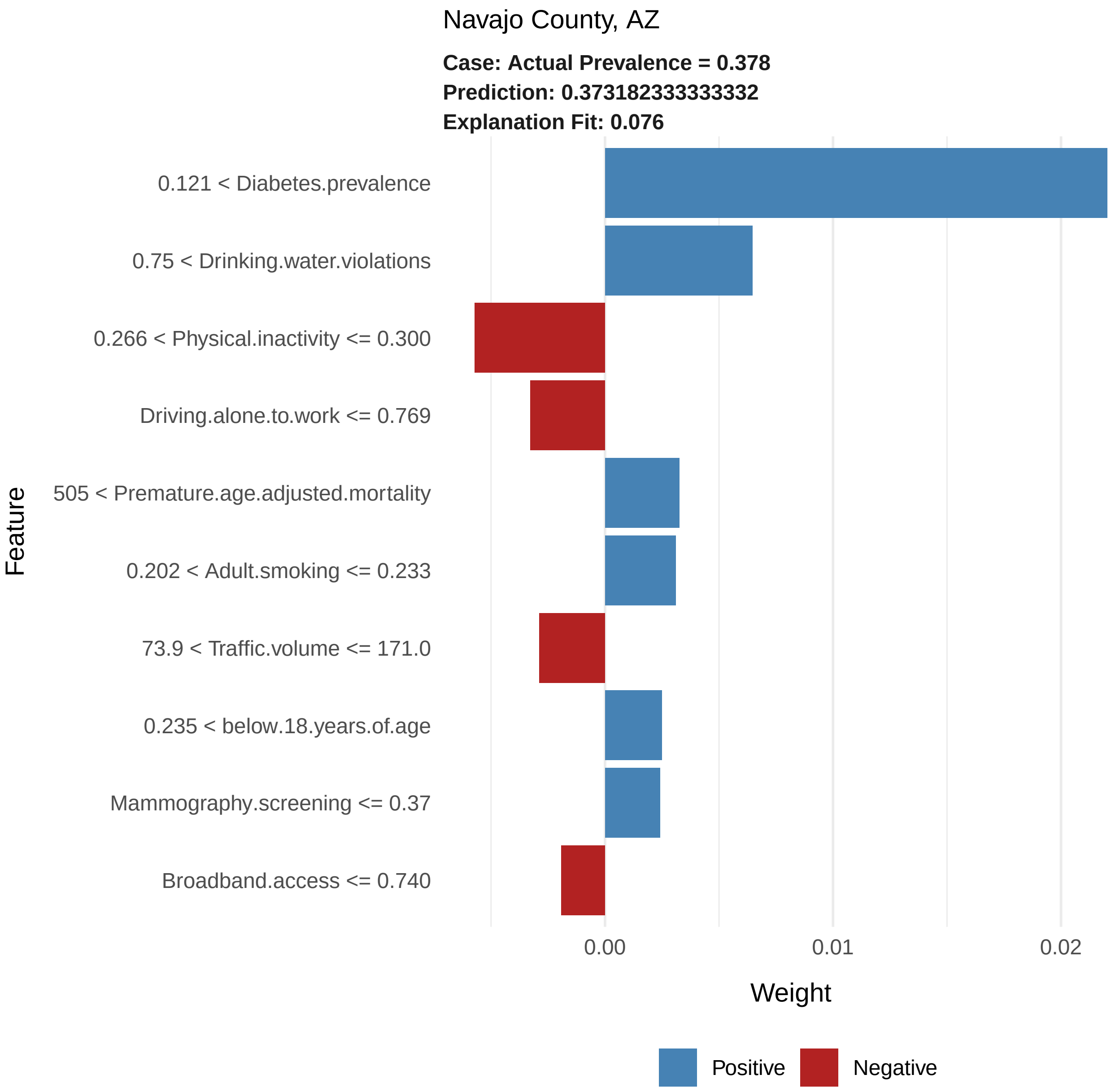
Navajo County, AZ
Case: Actual Prevalence = 0.378
Prediction: 0.373182333333332
Explanation Fit: 0.076
0.121 < Diabetes.prevalence
0.75 < Drinking.water.violations
0.266 < Physical.inactivity <= 0.300
Driving.alone.to.work <= 0.769
505 < Premature.age.adjusted.mortality
0.202 < Adult.smoking <= 0.233
73.9 < Traffic.volume <= 171.0
0.235 < below.18.years.of.age
Mammography.screening <= 0.37
Broadband.access <= 0.740
Feature
0.00
0.01
0.02
Weight
Positive
Negative

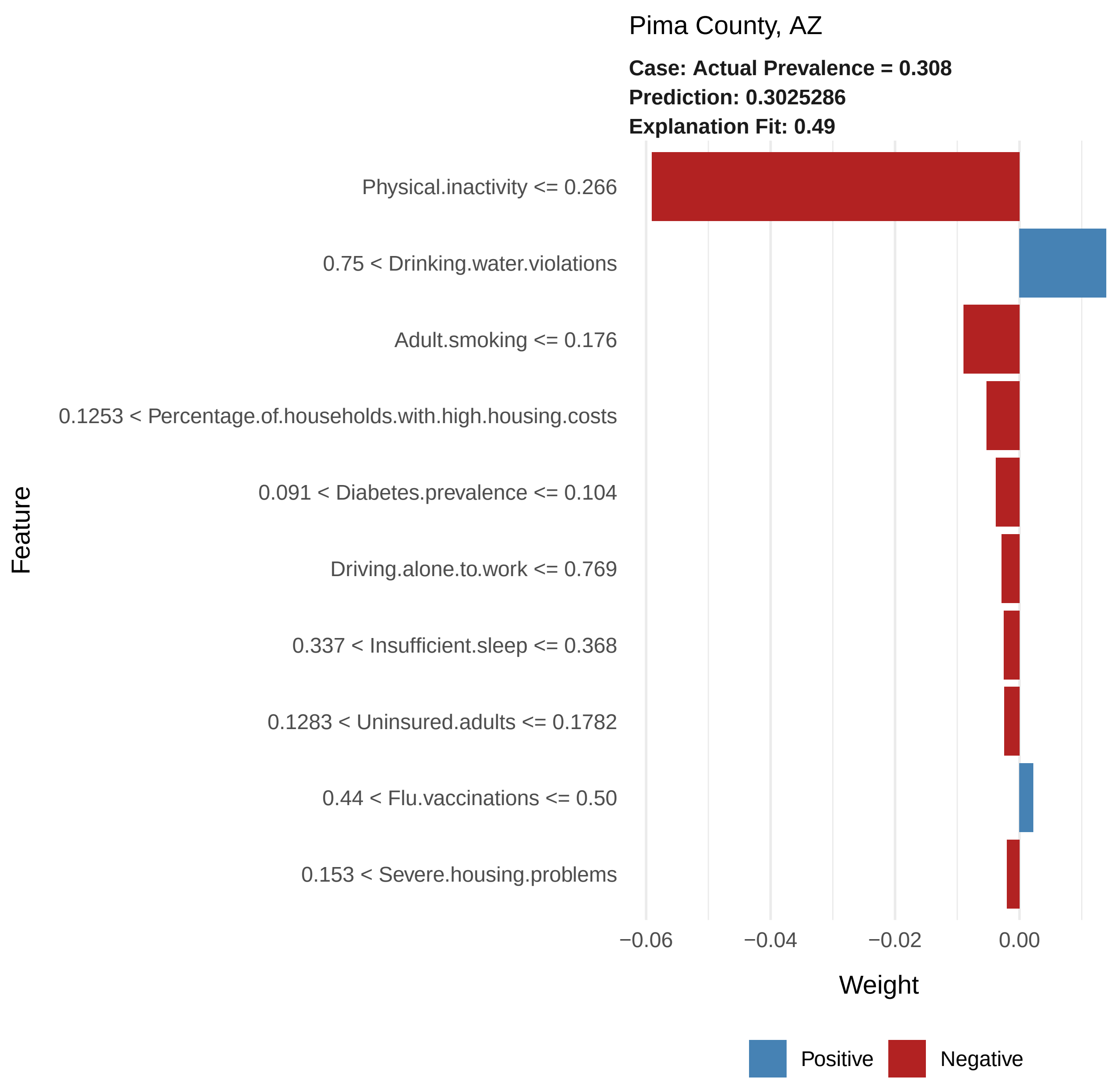

Pima County, AZ
Case: Actual Prevalence = 0.308
Prediction: 0.3025286
Explanation Fit: 0.49
Physical.inactivity <= 0.266
0.75 < Drinking.water.violations
Adult.smoking <= 0.176
0.1253 < Percentage.of.households.with.high.housing.costs
0.091 < Diabetes.prevalence <= 0.104
Driving.alone.to.work <= 0.769
0.337 < Insufficient.sleep <= 0.368
0.1283 < Uninsured.adults <= 0.1782
0.44 < Flu.vaccinations <= 0.50
0.153 < Severe.housing.problems
Feature
−0.06
−0.04
−0.02
0.00
Weight
Positive
Negative

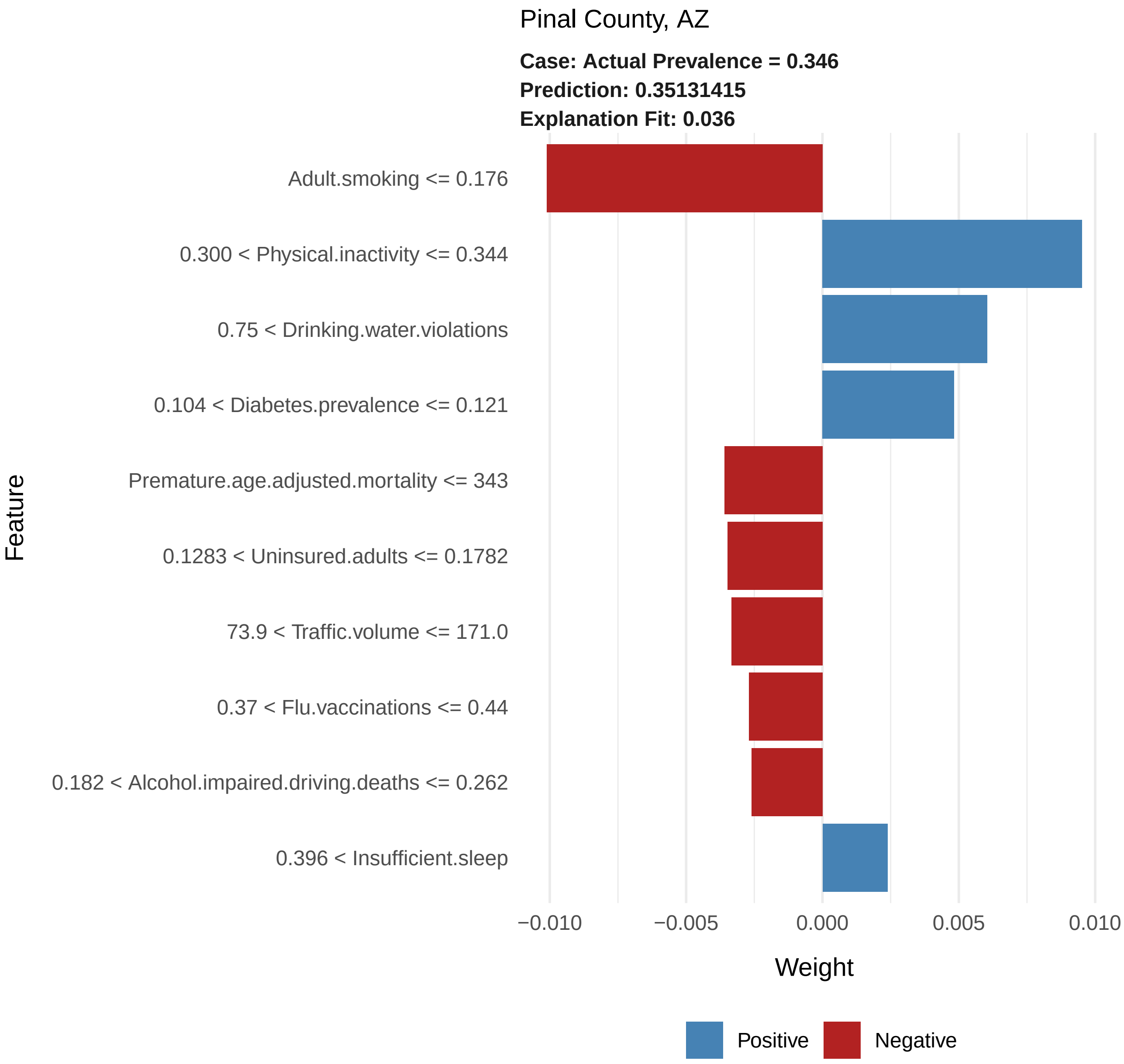
Pinal County, AZ
Case: Actual Prevalence = 0.346
Prediction: 0.35131415
Explanation Fit: 0.036
Adult.smoking <= 0.176
0.300 < Physical.inactivity <= 0.344
0.75 < Drinking.water.violations
0.104 < Diabetes.prevalence <= 0.121
Premature.age.adjusted.mortality <= 343
0.1283 < Uninsured.adults <= 0.1782
73.9 < Traffic.volume <= 171.0
0.37 < Flu.vaccinations <= 0.44
0.182 < Alcohol.impaired.driving.deaths <= 0.262
0.396 < Insufficient.sleep
Feature
−0.010
−0.005
0.000
0.005
0.010
Weight
Positive
Negative

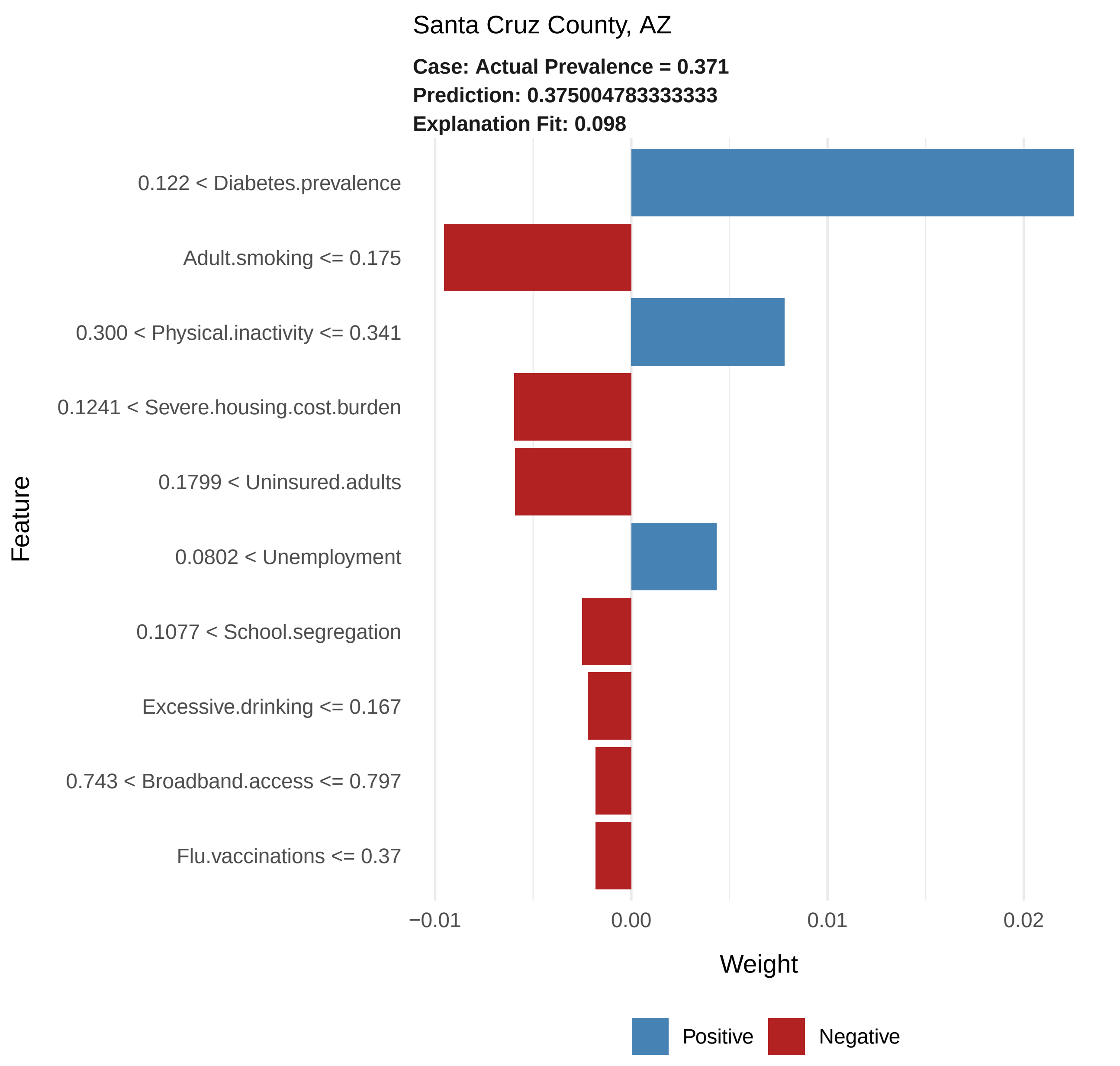
Santa Cruz County, AZ
Case: Actual Prevalence = 0.371
Prediction: 0.375004783333333
Explanation Fit: 0.098
0.122 < Diabetes.prevalence
Adult.smoking <= 0.175
0.300 < Physical.inactivity <= 0.341
0.1241 < Severe.housing.cost.burden
0.1799 < Uninsured.adults
0.0802 < Unemployment
0.1077 < School.segregation
Excessive.drinking <= 0.167
0.743 < Broadband.access <= 0.797
Flu.vaccinations <= 0.37
Feature
−0.01
0.00
0.01
0.02
Weight
Positive
Negative

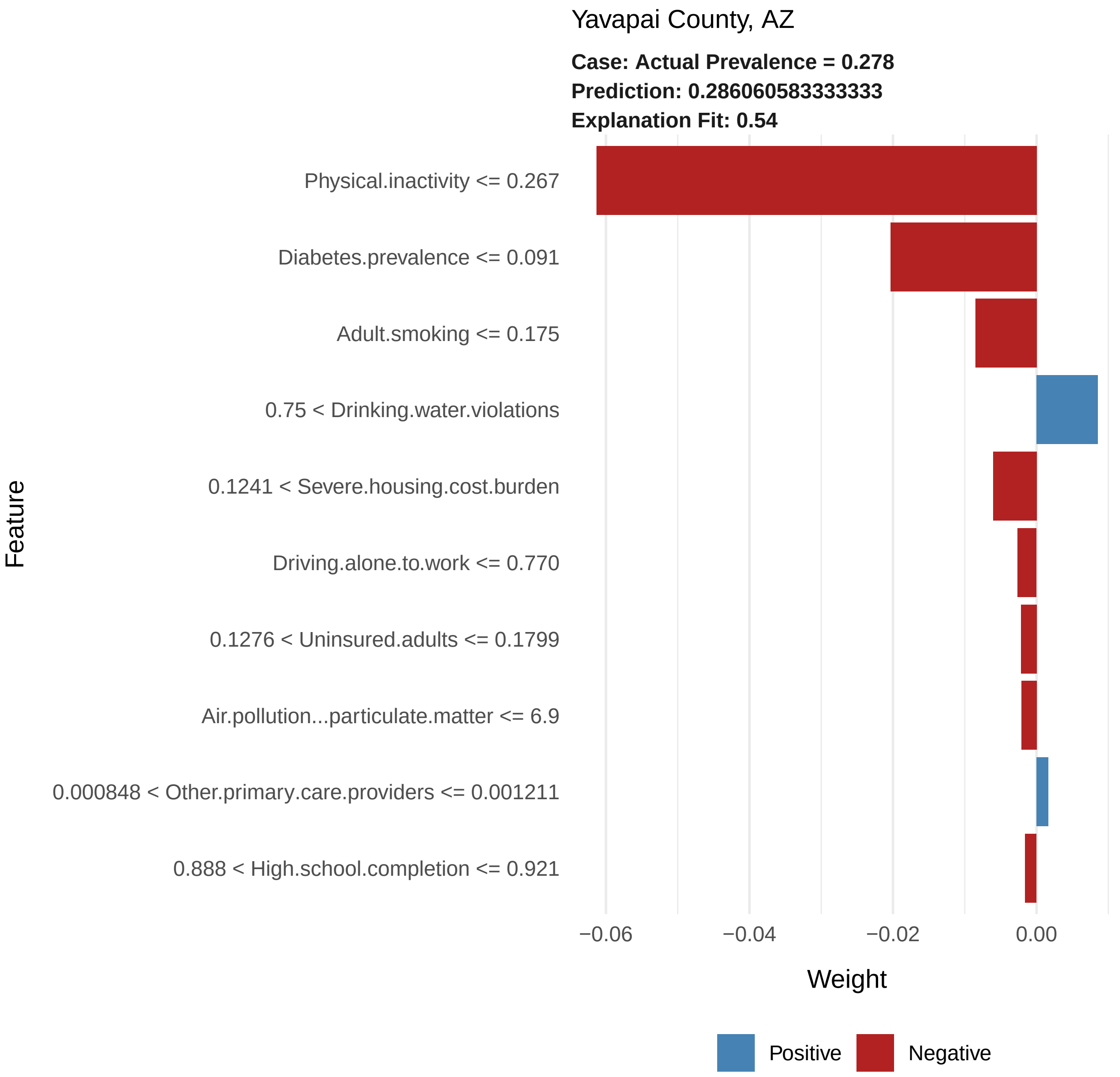

Yavapai County, AZ
Case: Actual Prevalence = 0.278
Prediction: 0.286060583333333
Explanation Fit: 0.54
Physical.inactivity <= 0.267
Diabetes.prevalence <= 0.091
Adult.smoking <= 0.175
0.75 < Drinking.water.violations
0.1241 < Severe.housing.cost.burden
Driving.alone.to.work <= 0.770
0.1276 < Uninsured.adults <= 0.1799
Air.pollution...particulate.matter <= 6.9
0.000848 < Other.primary.care.providers <= 0.001211
0.888 < High.school.completion <= 0.921
Feature
−0.06
−0.04
−0.02
0.00
Weight
Positive
Negative

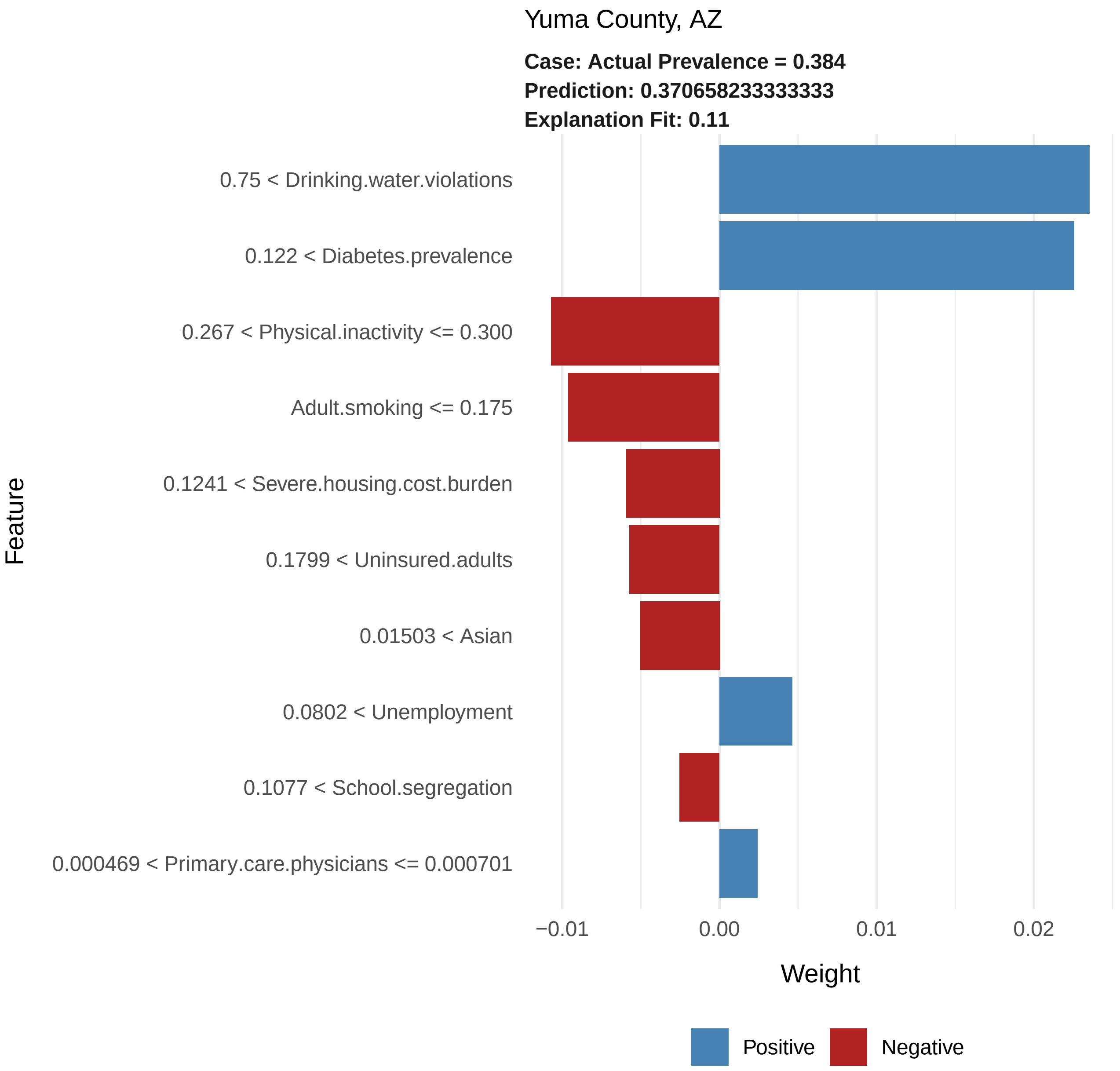
Yuma County, AZ
Case: Actual Prevalence = 0.384
Prediction: 0.370658233333333
Explanation Fit: 0.11
0.75 < Drinking.water.violations
0.122 < Diabetes.prevalence
0.267 < Physical.inactivity <= 0.300
Adult.smoking <= 0.175
0.1241 < Severe.housing.cost.burden
0.1799 < Uninsured.adults
0.01503 < Asian
0.0802 < Unemployment
0.1077 < School.segregation
0.000469 < Primary.care.physicians <= 0.000701
Feature
−0.01
0.00
0.01
0.02
Weight
Positive
Negative

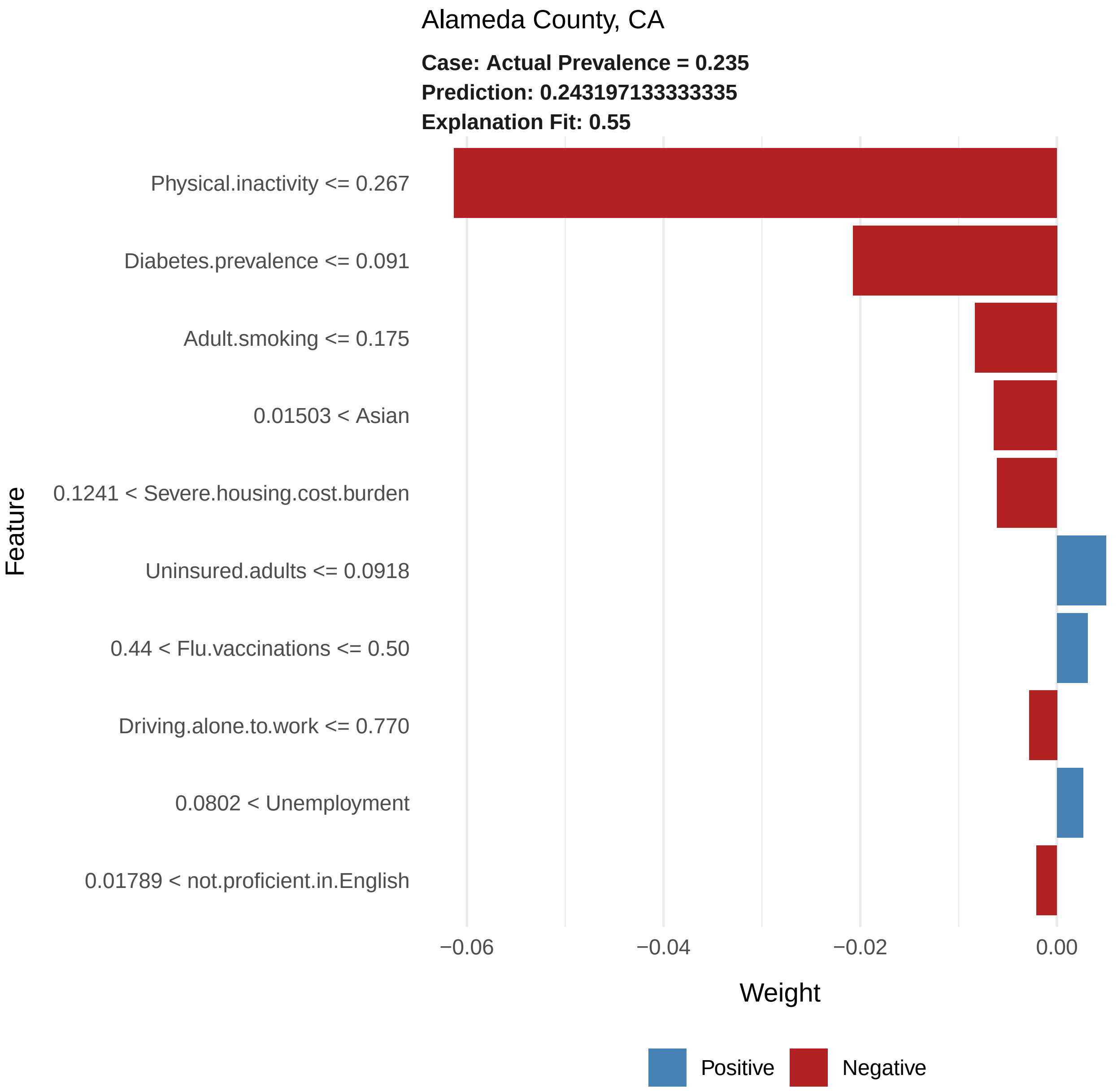
Alameda County, CA
Case: Actual Prevalence = 0.235
Prediction: 0.243197133333335
Explanation Fit: 0.55
Physical.inactivity <= 0.267
Diabetes.prevalence <= 0.091
Adult.smoking <= 0.175
0.01503 < Asian
0.1241 < Severe.housing.cost.burden
Uninsured.adults <= 0.0918
0.44 < Flu.vaccinations <= 0.50
Driving.alone.to.work <= 0.770
0.0802 < Unemployment
0.01789 < not.proficient.in.English
Feature
−0.06
−0.04
−0.02
0.00
Weight
Positive
Negative

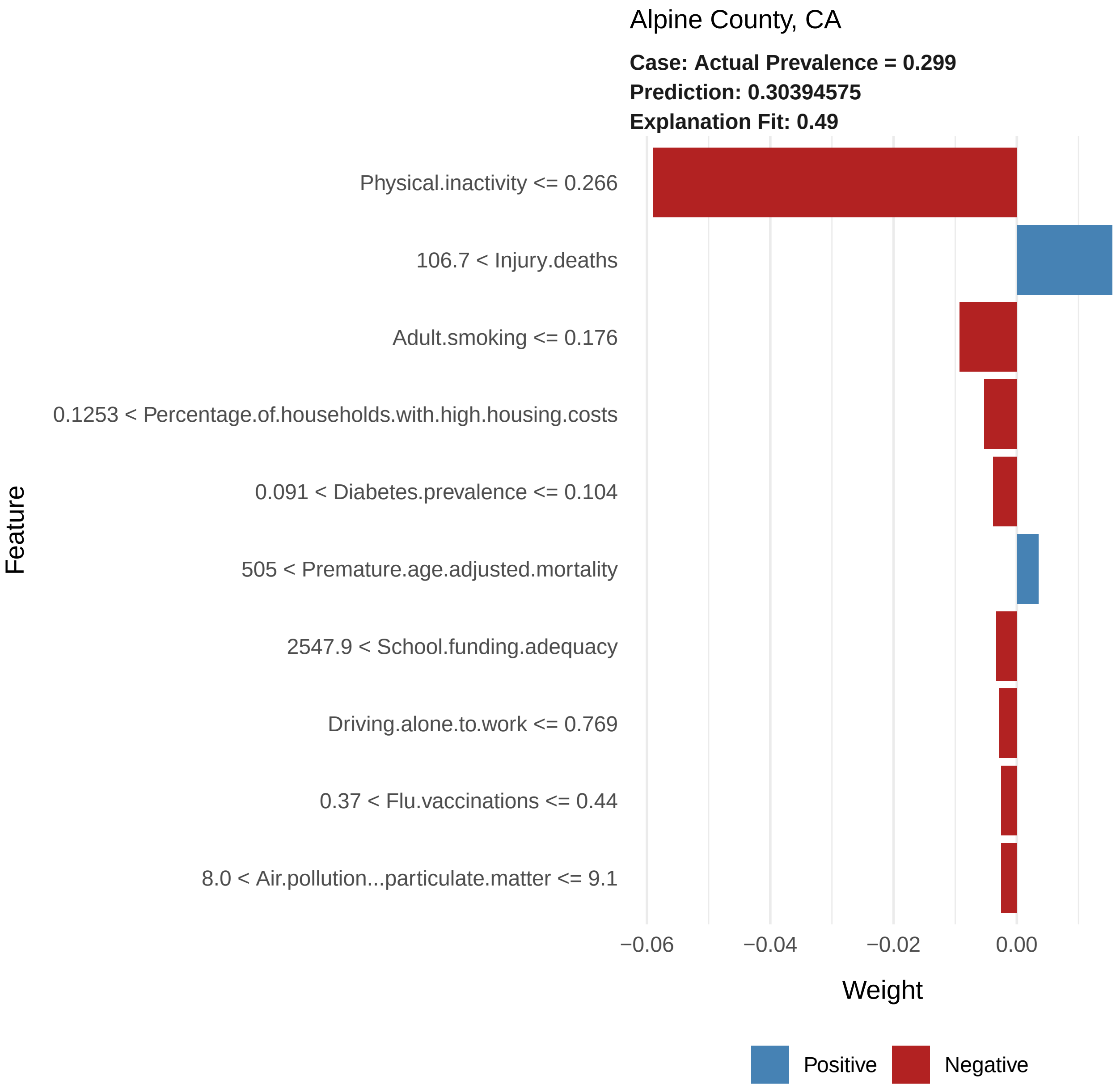

Alpine County, CA
Case: Actual Prevalence = 0.299
Prediction: 0.30394575
Explanation Fit: 0.49
Physical.inactivity <= 0.266
106.7 < Injury.deaths
Adult.smoking <= 0.176
0.1253 < Percentage.of.households.with.high.housing.costs
0.091 < Diabetes.prevalence <= 0.104
505 < Premature.age.adjusted.mortality
2547.9 < School.funding.adequacy
Driving.alone.to.work <= 0.769
0.37 < Flu.vaccinations <= 0.44
8.0 < Air.pollution...particulate.matter <= 9.1
Feature
−0.06
−0.04
−0.02
0.00
Weight
Positive
Negative

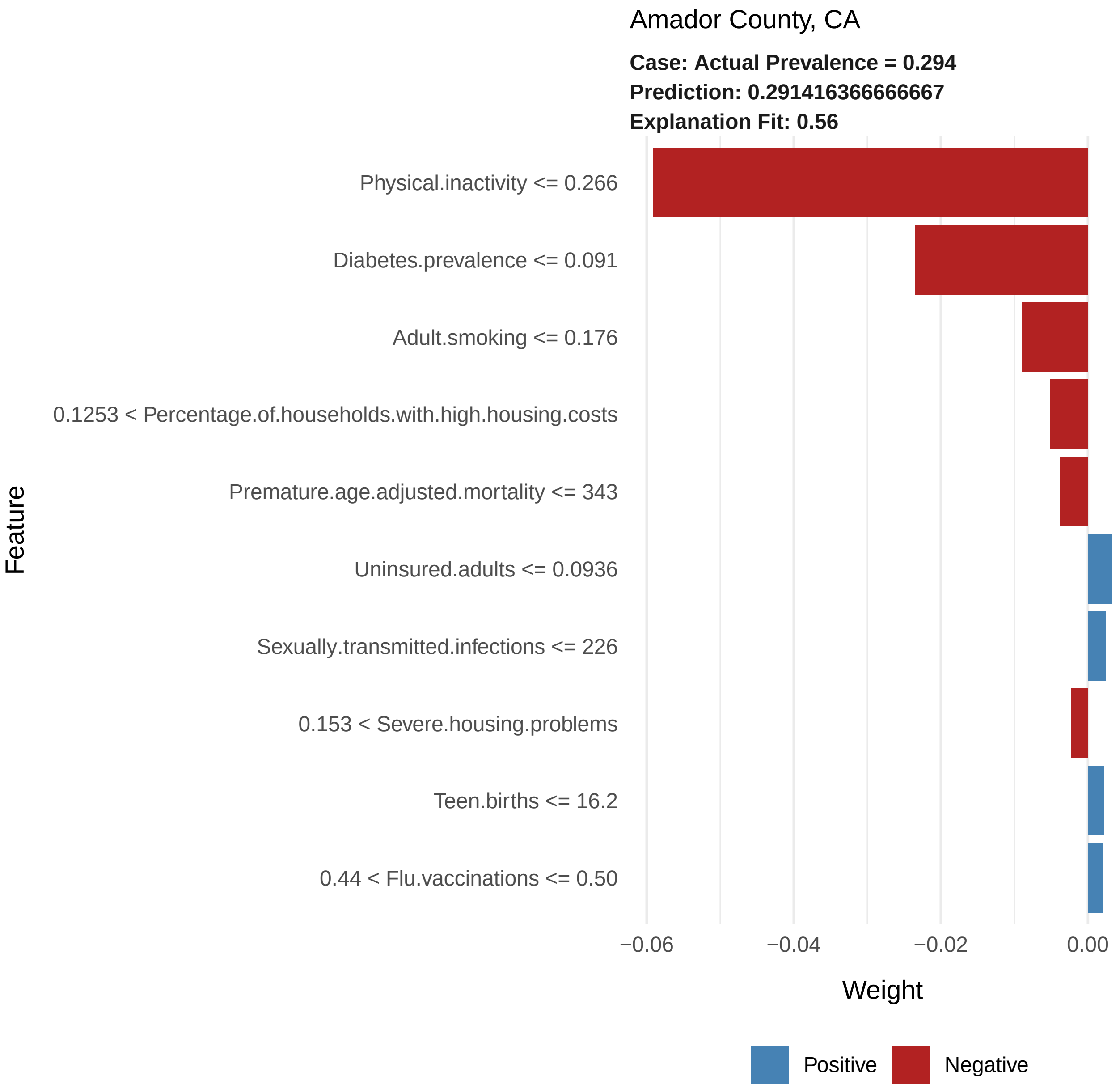

Amador County, CA
Case: Actual Prevalence = 0.294
Prediction: 0.291416366666667
Explanation Fit: 0.56
Physical.inactivity <= 0.266
Diabetes.prevalence <= 0.091
Adult.smoking <= 0.176
0.1253 < Percentage.of.households.with.high.housing.costs
Premature.age.adjusted.mortality <= 343
Uninsured.adults <= 0.0936
Sexually.transmitted.infections <= 226
0.153 < Severe.housing.problems
Teen.births <= 16.2
0.44 < Flu.vaccinations <= 0.50
Feature
−0.06
−0.04
−0.02
0.00
Weight
Positive
Negative

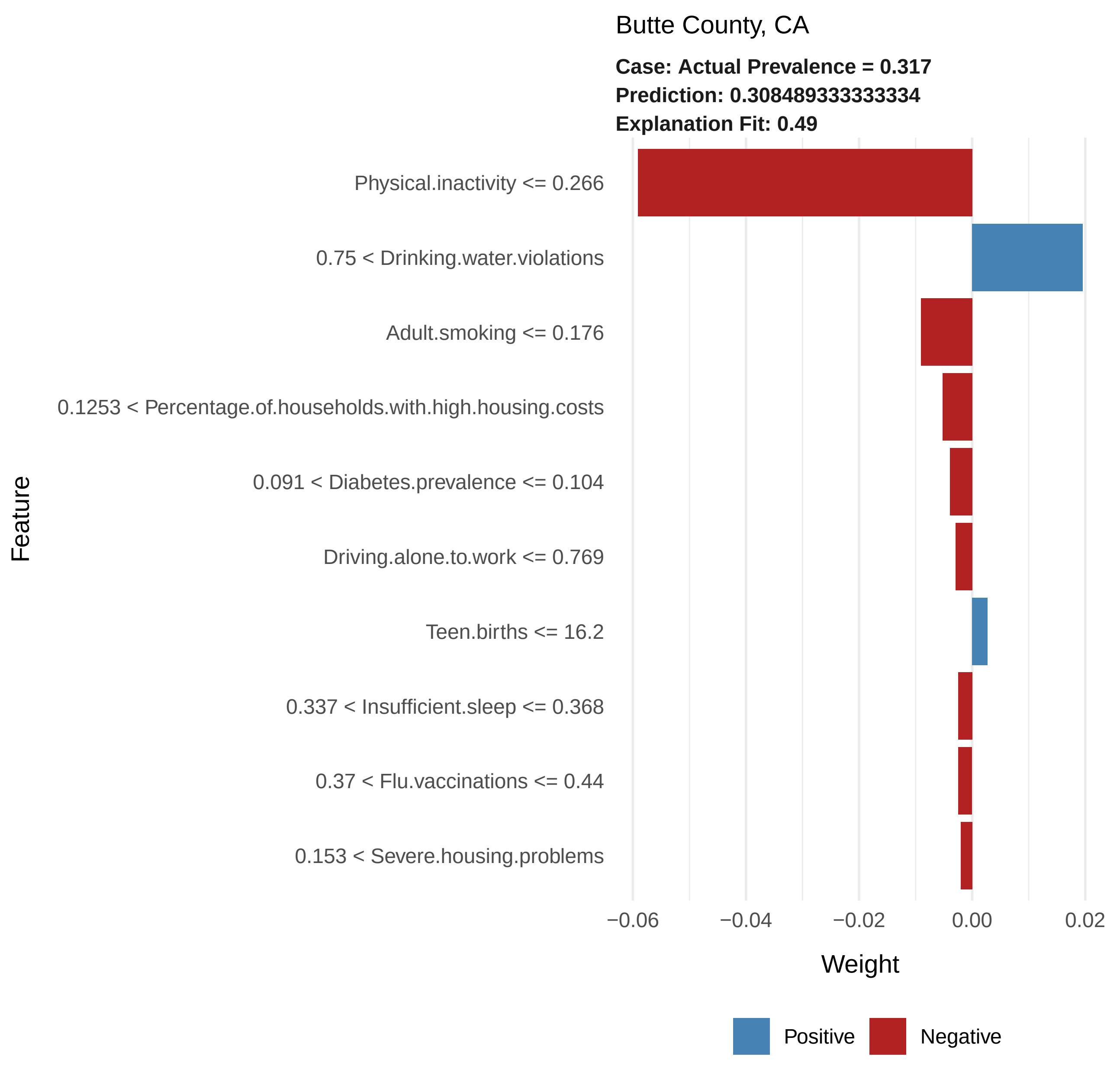
Butte County, CA
Case: Actual Prevalence = 0.317
Prediction: 0.308489333333334
Explanation Fit: 0.49
Physical.inactivity <= 0.266
0.75 < Drinking.water.violations
Adult.smoking <= 0.176
0.1253 < Percentage.of.households.with.high.housing.costs
0.091 < Diabetes.prevalence <= 0.104
Driving.alone.to.work <= 0.769
Teen.births <= 16.2
0.337 < Insufficient.sleep <= 0.368
0.37 < Flu.vaccinations <= 0.44
0.153 < Severe.housing.problems
Feature
−0.06
−0.04
−0.02
0.00
0.02
Weight
Positive
Negative

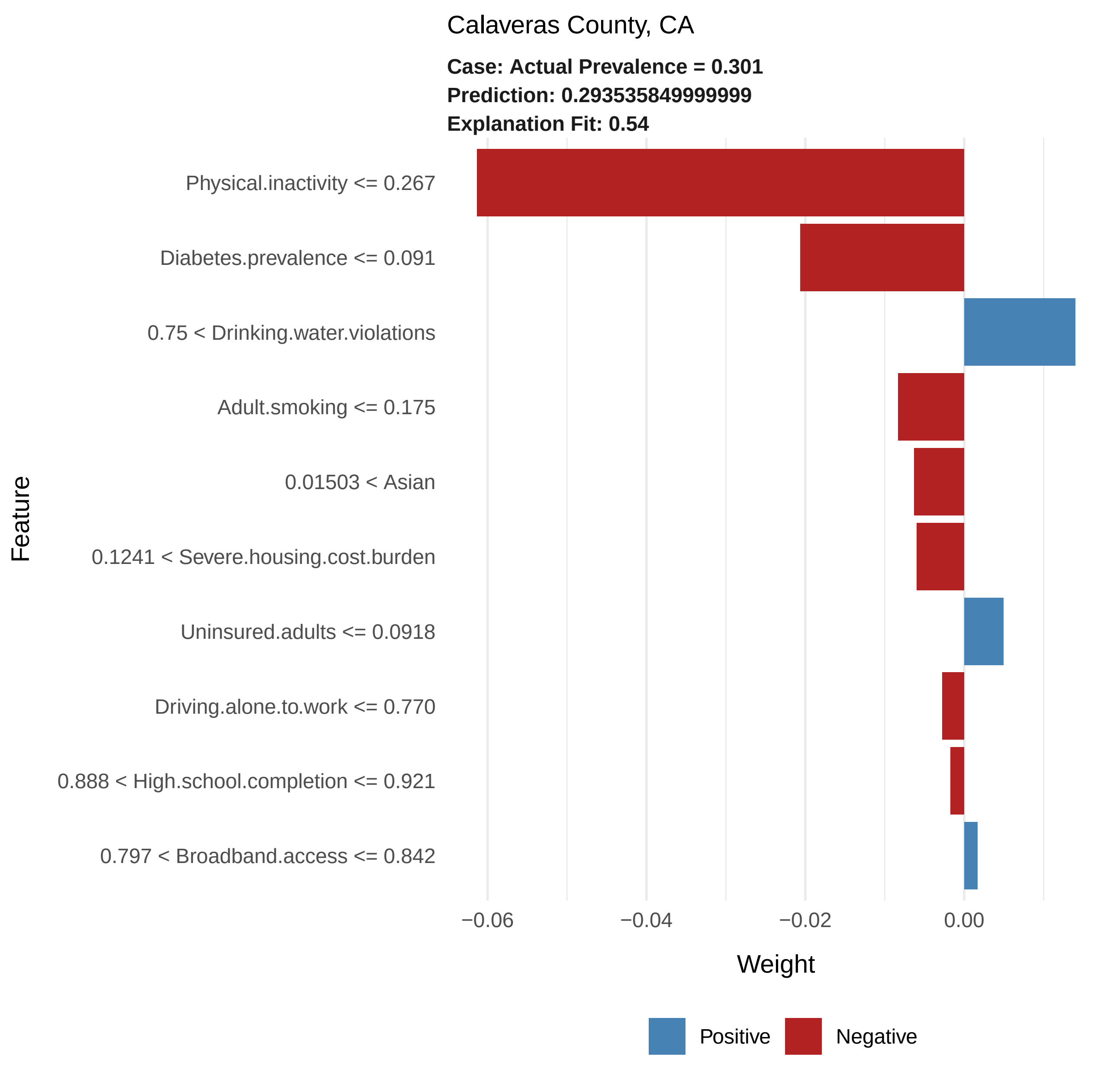

Calaveras County, CA
Case: Actual Prevalence = 0.301
Prediction: 0.293535849999999
Explanation Fit: 0.54
Physical.inactivity <= 0.267
Diabetes.prevalence <= 0.091
0.75 < Drinking.water.violations
Adult.smoking <= 0.175
0.01503 < Asian
0.1241 < Severe.housing.cost.burden
Uninsured.adults <= 0.0918
Driving.alone.to.work <= 0.770
0.888 < High.school.completion <= 0.921
0.797 < Broadband.access <= 0.842
Feature
−0.06
−0.04
−0.02
0.00
Weight
Positive
Negative

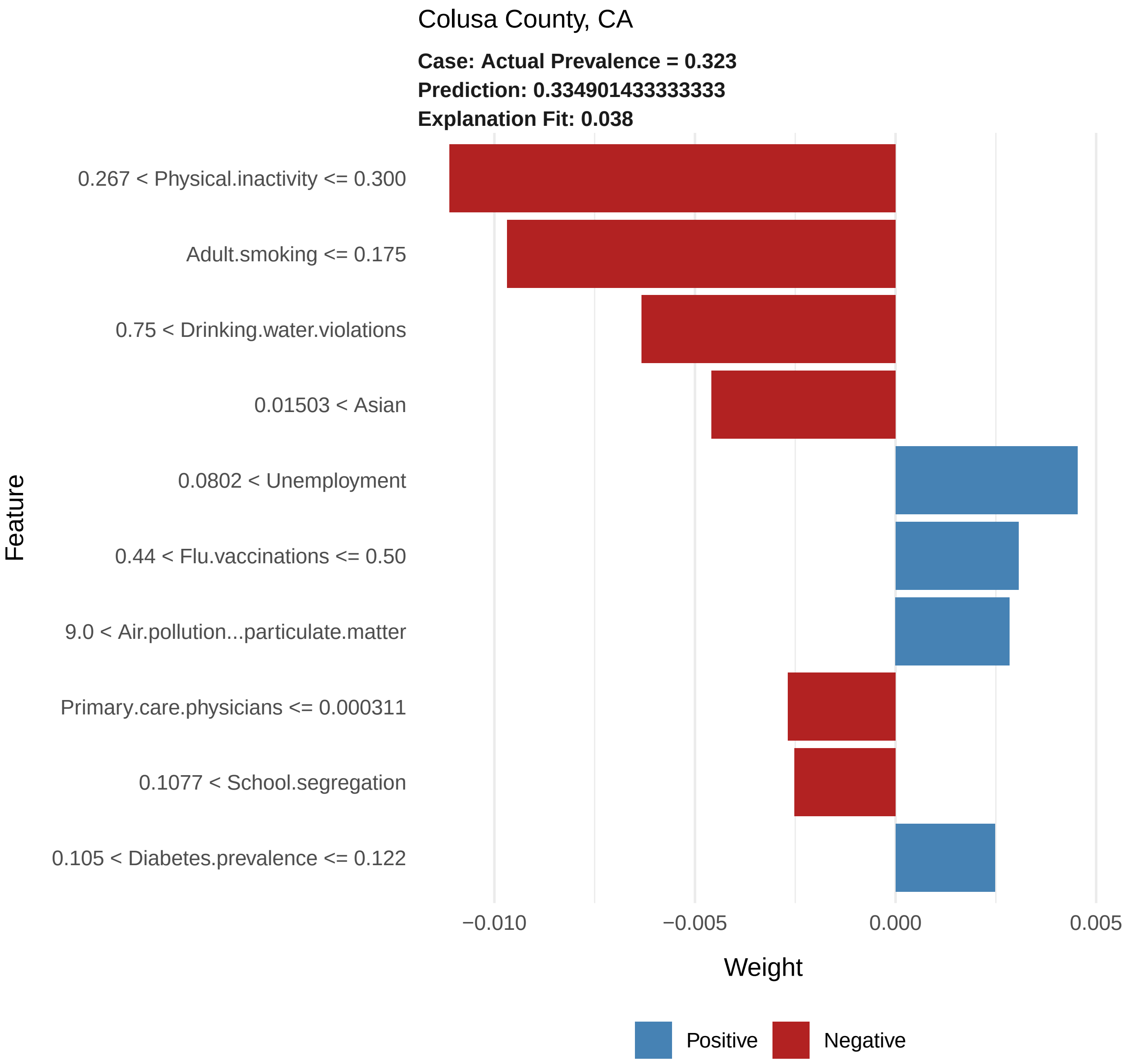
Colusa County, CA
Case: Actual Prevalence = 0.323
Prediction: 0.334901433333333
Explanation Fit: 0.038
0.267 < Physical.inactivity <= 0.300
Adult.smoking <= 0.175
0.75 < Drinking.water.violations
0.01503 < Asian
0.0802 < Unemployment
0.44 < Flu.vaccinations <= 0.50
9.0 < Air.pollution...particulate.matter
Primary.care.physicians <= 0.000311
0.1077 < School.segregation
0.105 < Diabetes.prevalence <= 0.122
Feature
−0.010
−0.005
0.000
0.005
Weight
Positive
Negative

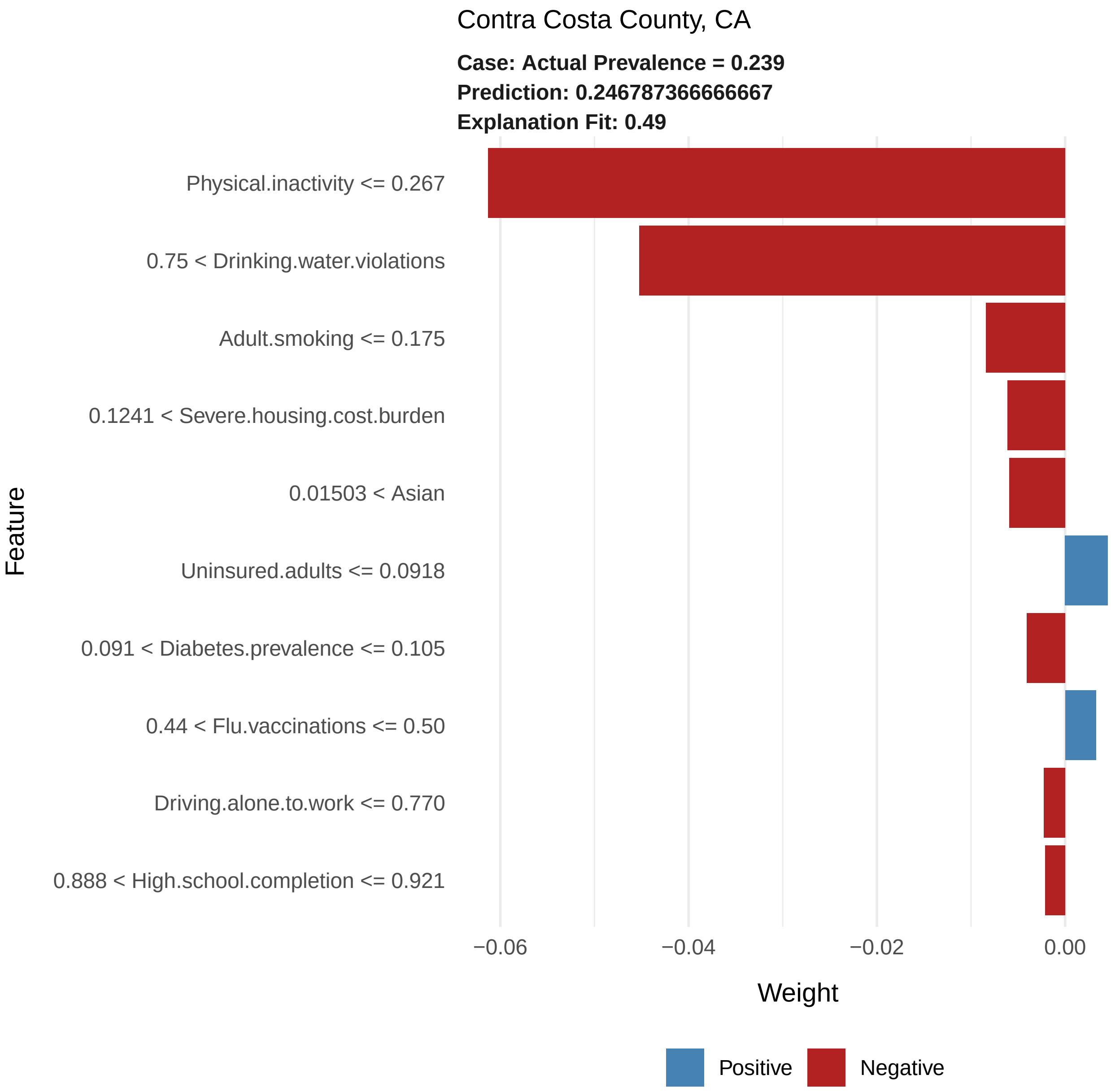
Contra Costa County, CA
Case: Actual Prevalence = 0.239
Prediction: 0.246787366666667
Explanation Fit: 0.49
Physical.inactivity <= 0.267
0.75 < Drinking.water.violations
Adult.smoking <= 0.175
0.1241 < Severe.housing.cost.burden
0.01503 < Asian
Uninsured.adults <= 0.0918
0.091 < Diabetes.prevalence <= 0.105
0.44 < Flu.vaccinations <= 0.50
Driving.alone.to.work <= 0.770
0.888 < High.school.completion <= 0.921
Feature
−0.06
−0.04
−0.02
0.00
Weight
Positive
Negative

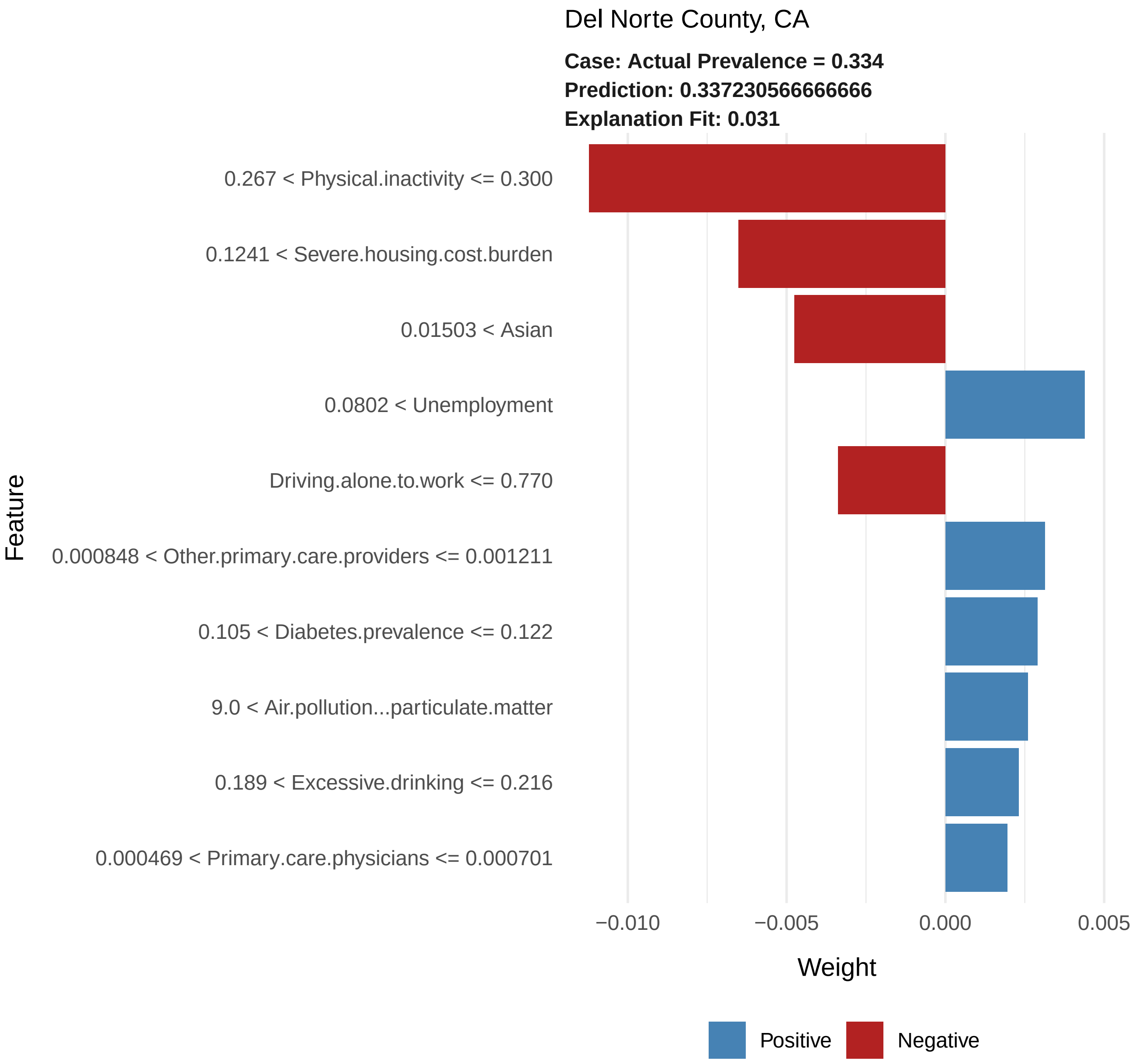

Del Norte County, CA
Case: Actual Prevalence = 0.334
Prediction: 0.337230566666666
Explanation Fit: 0.031
0.267 < Physical.inactivity <= 0.300
0.1241 < Severe.housing.cost.burden
0.01503 < Asian
0.0802 < Unemployment
Driving.alone.to.work <= 0.770
0.000848 < Other.primary.care.providers <= 0.001211
0.105 < Diabetes.prevalence <= 0.122
9.0 < Air.pollution...particulate.matter
0.189 < Excessive.drinking <= 0.216
0.000469 < Primary.care.physicians <= 0.000701
Feature
−0.010
−0.005
0.000
0.005
Weight
Positive
Negative

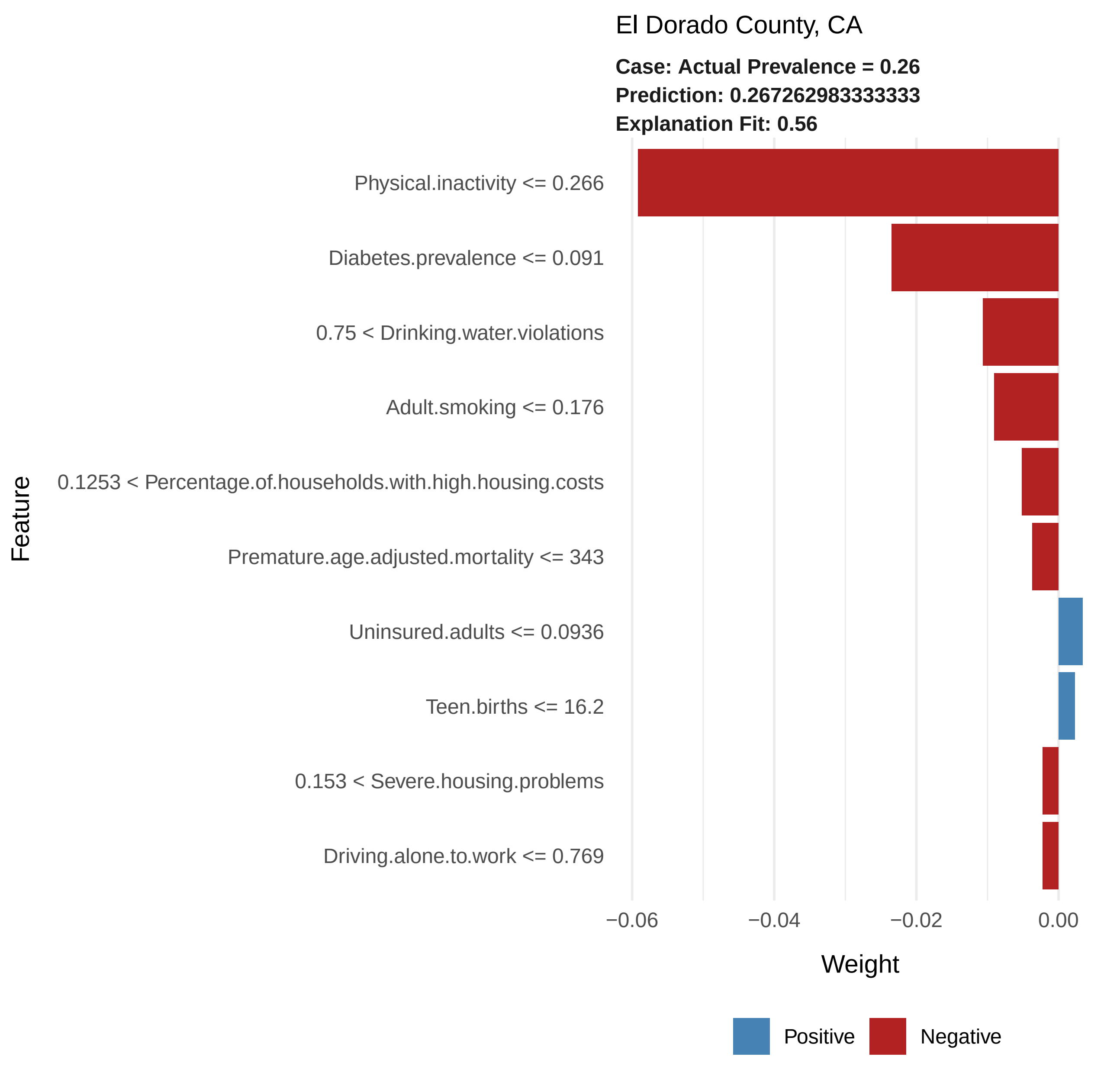
El Dorado County, CA
Case: Actual Prevalence = 0.26
Prediction: 0.267262983333333
Explanation Fit: 0.56
Physical.inactivity <= 0.266
Diabetes.prevalence <= 0.091
0.75 < Drinking.water.violations
Adult.smoking <= 0.176
0.1253 < Percentage.of.households.with.high.housing.costs
Premature.age.adjusted.mortality <= 343
Uninsured.adults <= 0.0936
Teen.births <= 16.2
0.153 < Severe.housing.problems
Driving.alone.to.work <= 0.769
Feature
−0.06
−0.04
−0.02
0.00
Weight
Positive
Negative

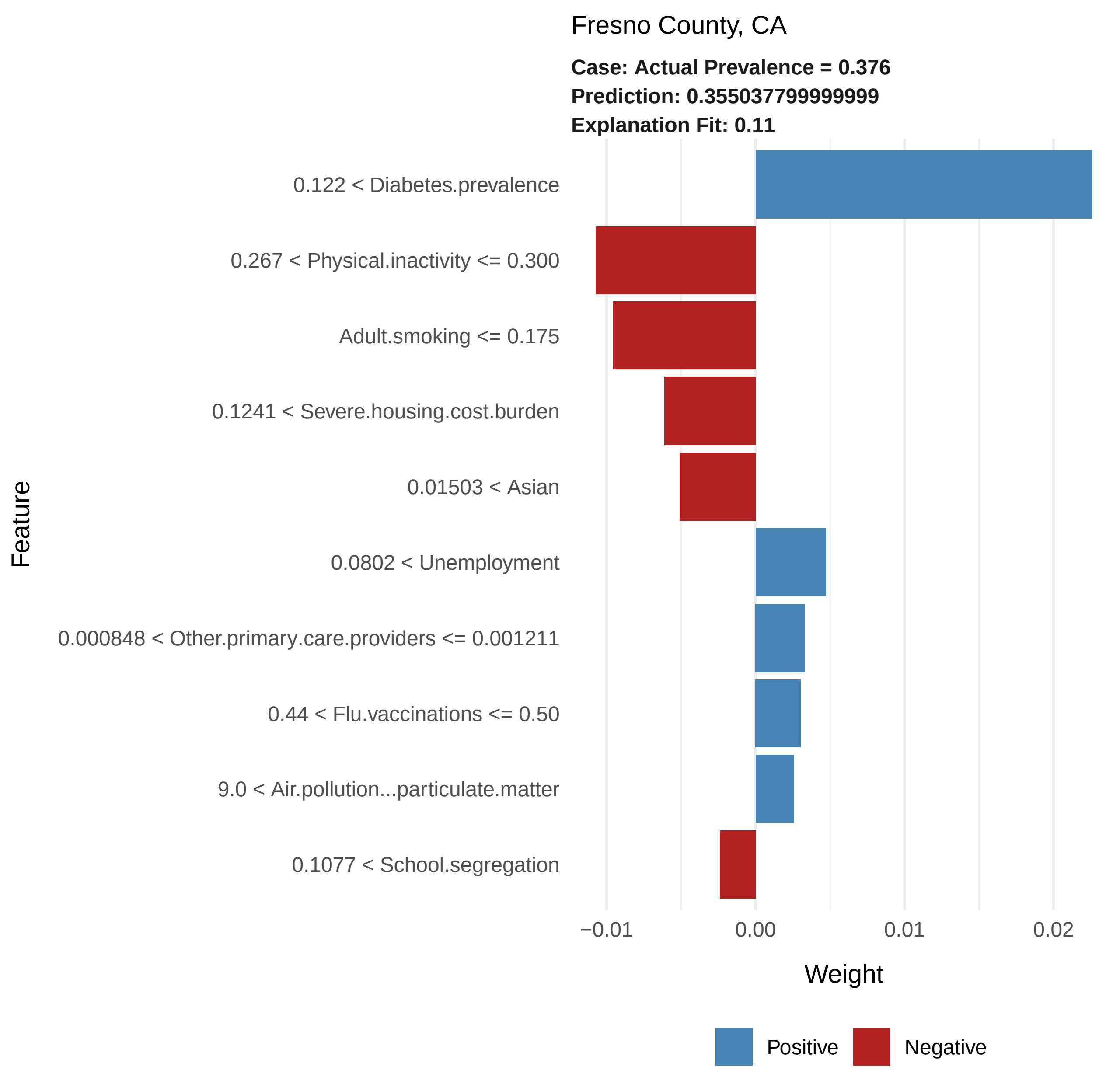

Fresno County, CA
Case: Actual Prevalence = 0.376
Prediction: 0.355037799999999
Explanation Fit: 0.11
0.122 < Diabetes.prevalence
0.267 < Physical.inactivity <= 0.300
Adult.smoking <= 0.175
0.1241 < Severe.housing.cost.burden
0.01503 < Asian
0.0802 < Unemployment
0.000848 < Other.primary.care.providers <= 0.001211
0.44 < Flu.vaccinations <= 0.50
9.0 < Air.pollution...particulate.matter
0.1077 < School.segregation
Feature
−0.01
0.00
0.01
0.02
Weight
Positive
Negative

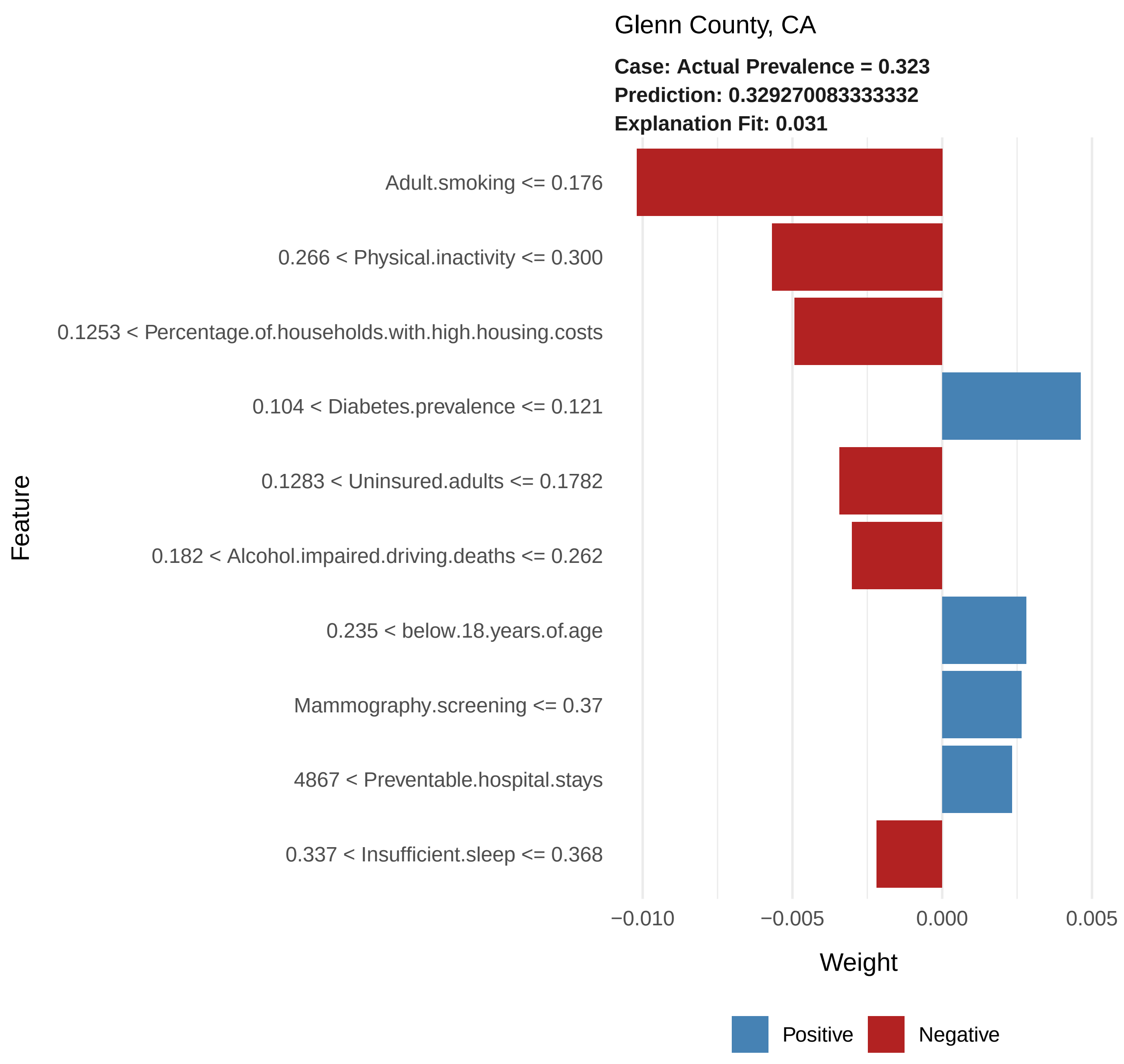

Glenn County, CA
Case: Actual Prevalence = 0.323
Prediction: 0.329270083333332
Explanation Fit: 0.031
Adult.smoking <= 0.176
0.266 < Physical.inactivity <= 0.300
0.1253 < Percentage.of.households.with.high.housing.costs
0.104 < Diabetes.prevalence <= 0.121
0.1283 < Uninsured.adults <= 0.1782
0.182 < Alcohol.impaired.driving.deaths <= 0.262
0.235 < below.18.years.of.age
Mammography.screening <= 0.37
4867 < Preventable.hospital.stays
0.337 < Insufficient.sleep <= 0.368
Feature
−0.010
−0.005
0.000
0.005
Weight
Positive
Negative

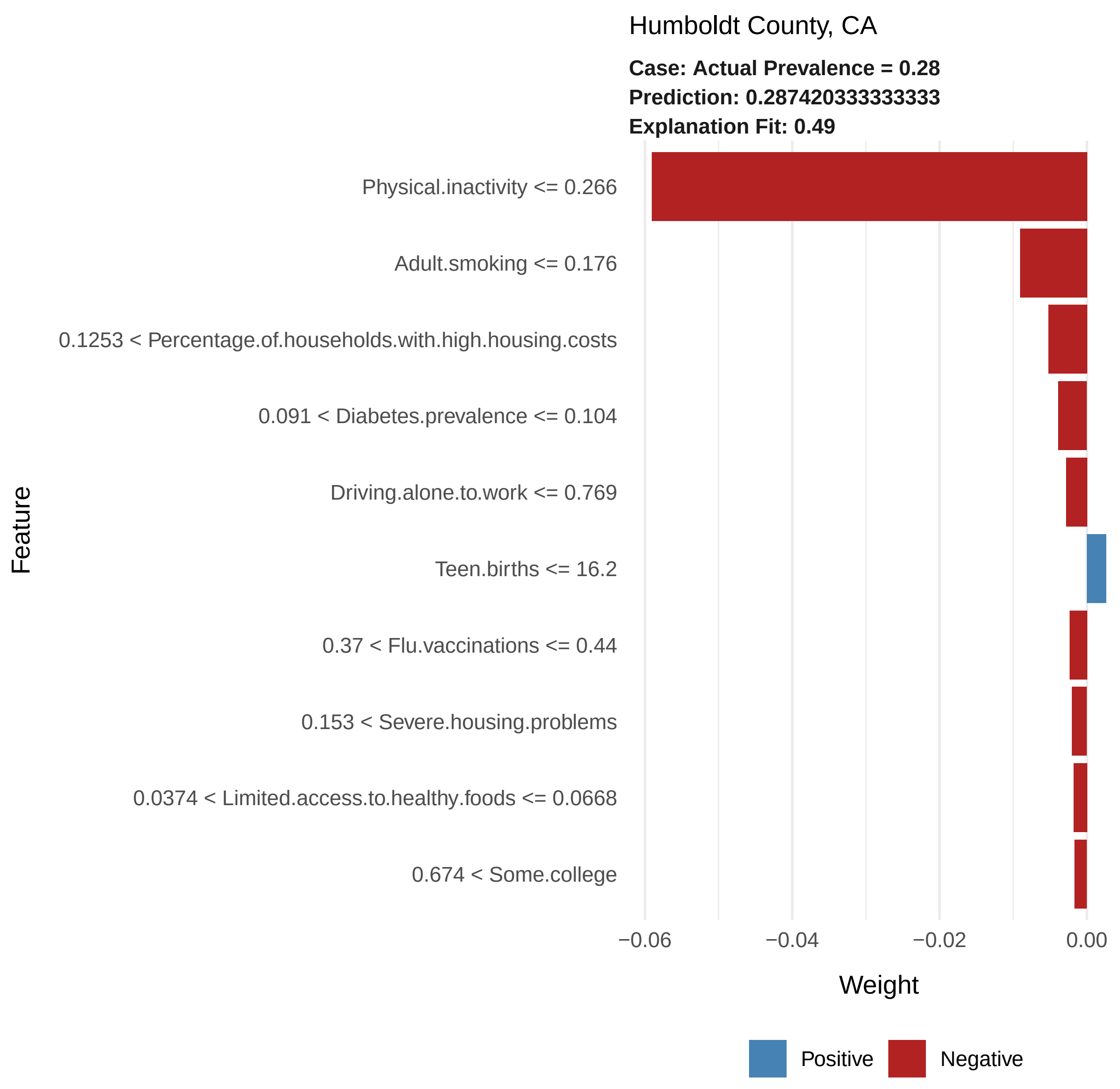

Humboldt County, CA
Case: Actual Prevalence = 0.28
Prediction: 0.287420333333333
Explanation Fit: 0.49
Physical.inactivity <= 0.266
Adult.smoking <= 0.176
0.1253 < Percentage.of.households.with.high.housing.costs
0.091 < Diabetes.prevalence <= 0.104
Driving.alone.to.work <= 0.769
Teen.births <= 16.2
0.37 < Flu.vaccinations <= 0.44
0.153 < Severe.housing.problems
0.0374 < Limited.access.to.healthy.foods <= 0.0668
0.674 < Some.college
Feature
−0.06
−0.04
−0.02
0.00
Weight
Positive
Negative

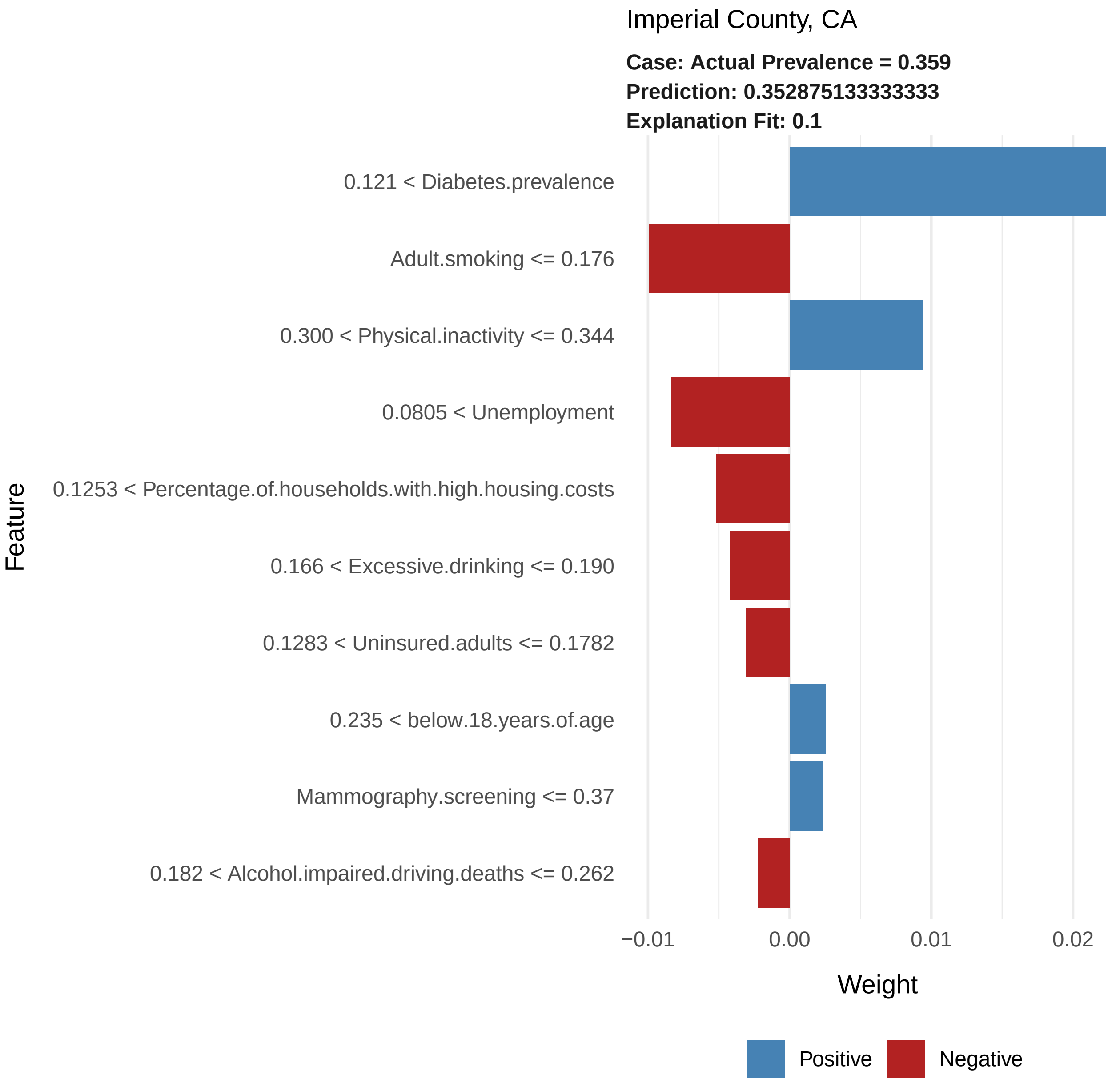
Imperial County, CA
Case: Actual Prevalence = 0.359
Prediction: 0.352875133333333
Explanation Fit: 0.1
0.121 < Diabetes.prevalence
Adult.smoking <= 0.176
0.300 < Physical.inactivity <= 0.344
0.0805 < Unemployment
0.1253 < Percentage.of.households.with.high.housing.costs
0.166 < Excessive.drinking <= 0.190
0.1283 < Uninsured.adults <= 0.1782
0.235 < below.18.years.of.age
Mammography.screening <= 0.37
0.182 < Alcohol.impaired.driving.deaths <= 0.262
Feature
−0.01
0.00
0.01
0.02
Weight
Positive
Negative

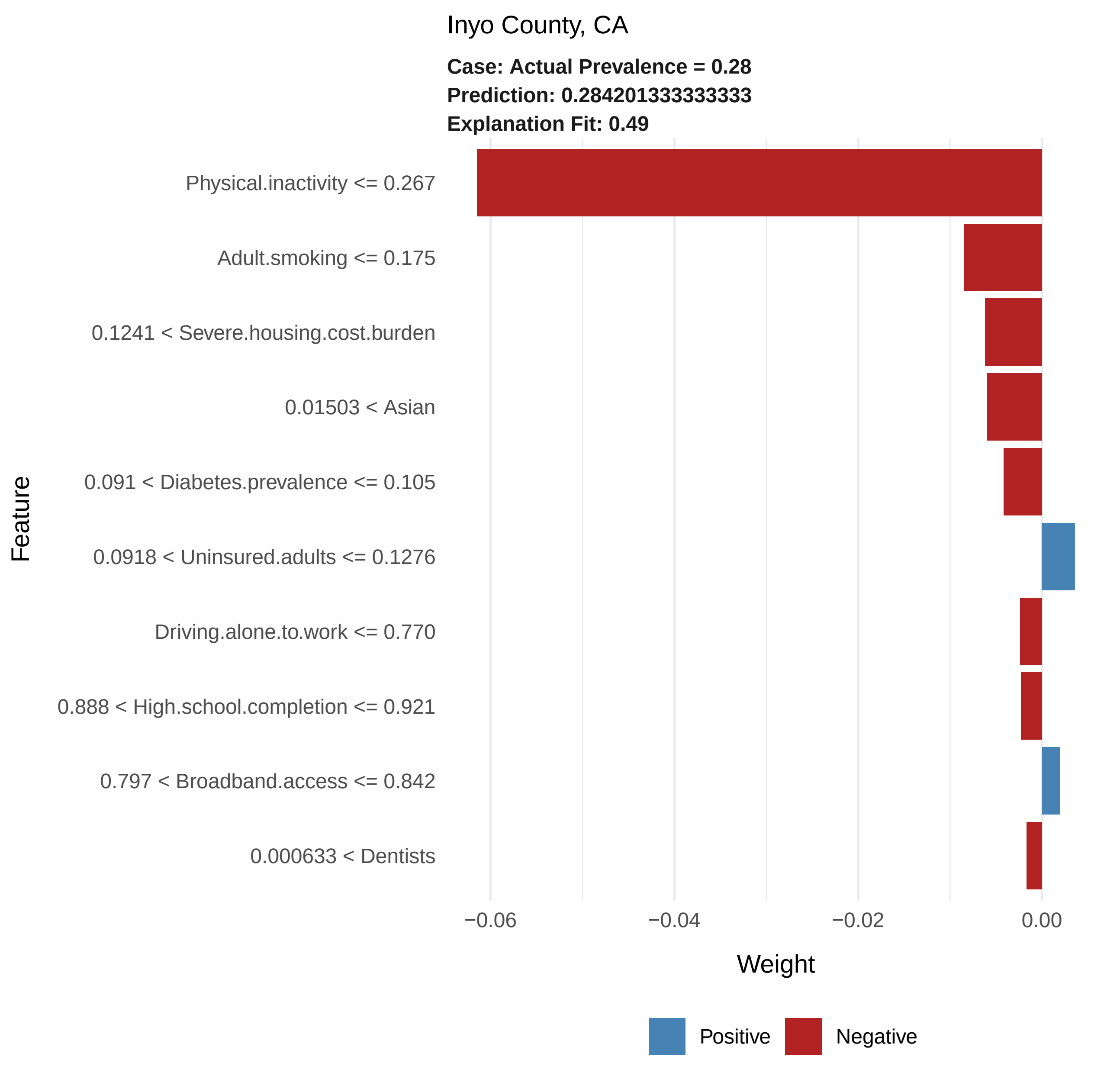

Inyo County, CA
Case: Actual Prevalence = 0.28
Prediction: 0.284201333333333
Explanation Fit: 0.49
Physical.inactivity <= 0.267
Adult.smoking <= 0.175
0.1241 < Severe.housing.cost.burden
0.01503 < Asian
0.091 < Diabetes.prevalence <= 0.105
0.0918 < Uninsured.adults <= 0.1276
Driving.alone.to.work <= 0.770
0.888 < High.school.completion <= 0.921
0.797 < Broadband.access <= 0.842
0.000633 < Dentists
Feature
−0.06
−0.04
−0.02
0.00
Weight
Positive
Negative

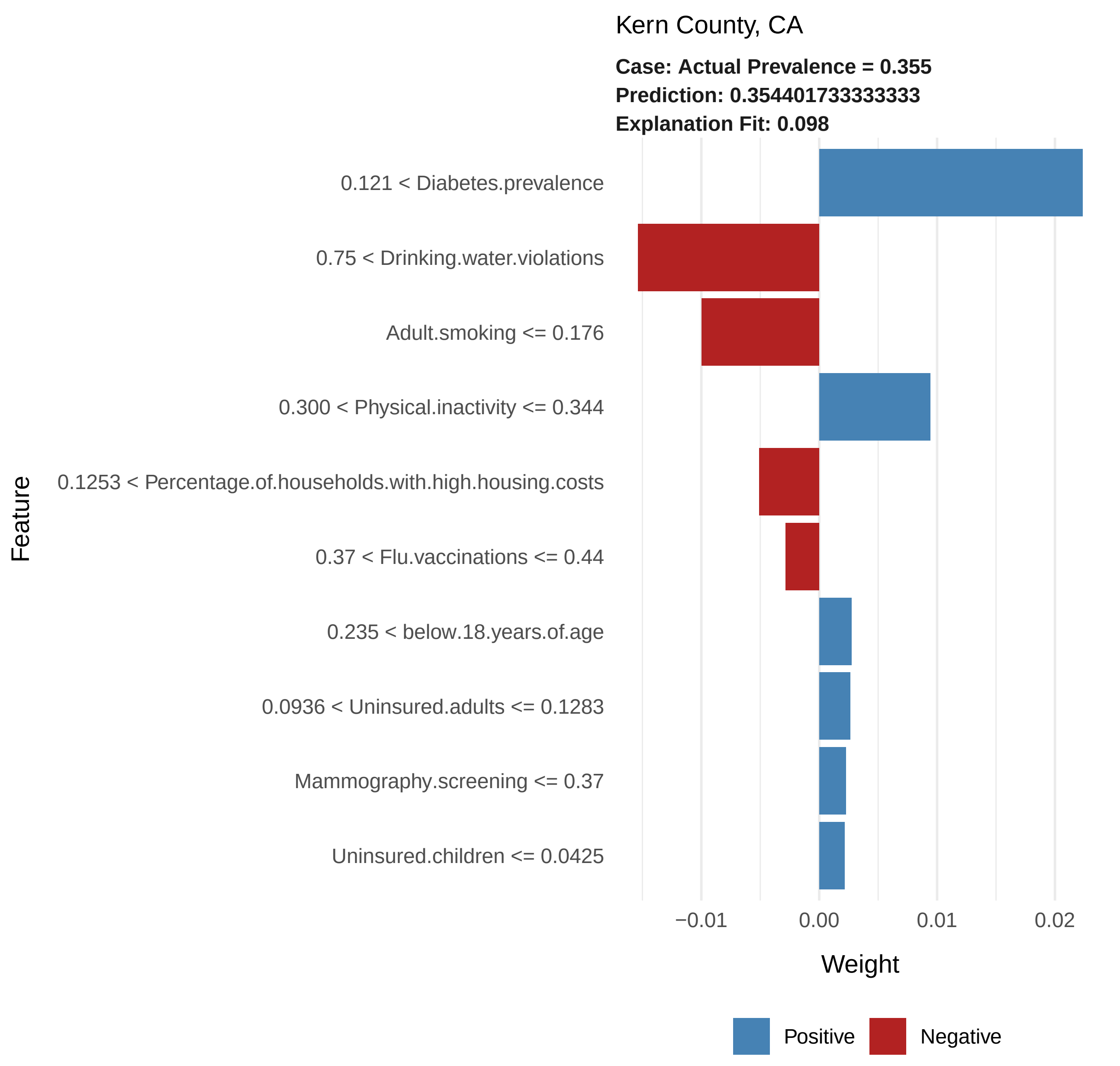

Kern County, CA
Case: Actual Prevalence = 0.355
Prediction: 0.354401733333333
Explanation Fit: 0.098
0.121 < Diabetes.prevalence
0.75 < Drinking.water.violations
Adult.smoking <= 0.176
0.300 < Physical.inactivity <= 0.344
0.1253 < Percentage.of.households.with.high.housing.costs
0.37 < Flu.vaccinations <= 0.44
0.235 < below.18.years.of.age
0.0936 < Uninsured.adults <= 0.1283
Mammography.screening <= 0.37
Uninsured.children <= 0.0425
Feature
−0.01
0.00
0.01
0.02
Weight
Positive
Negative

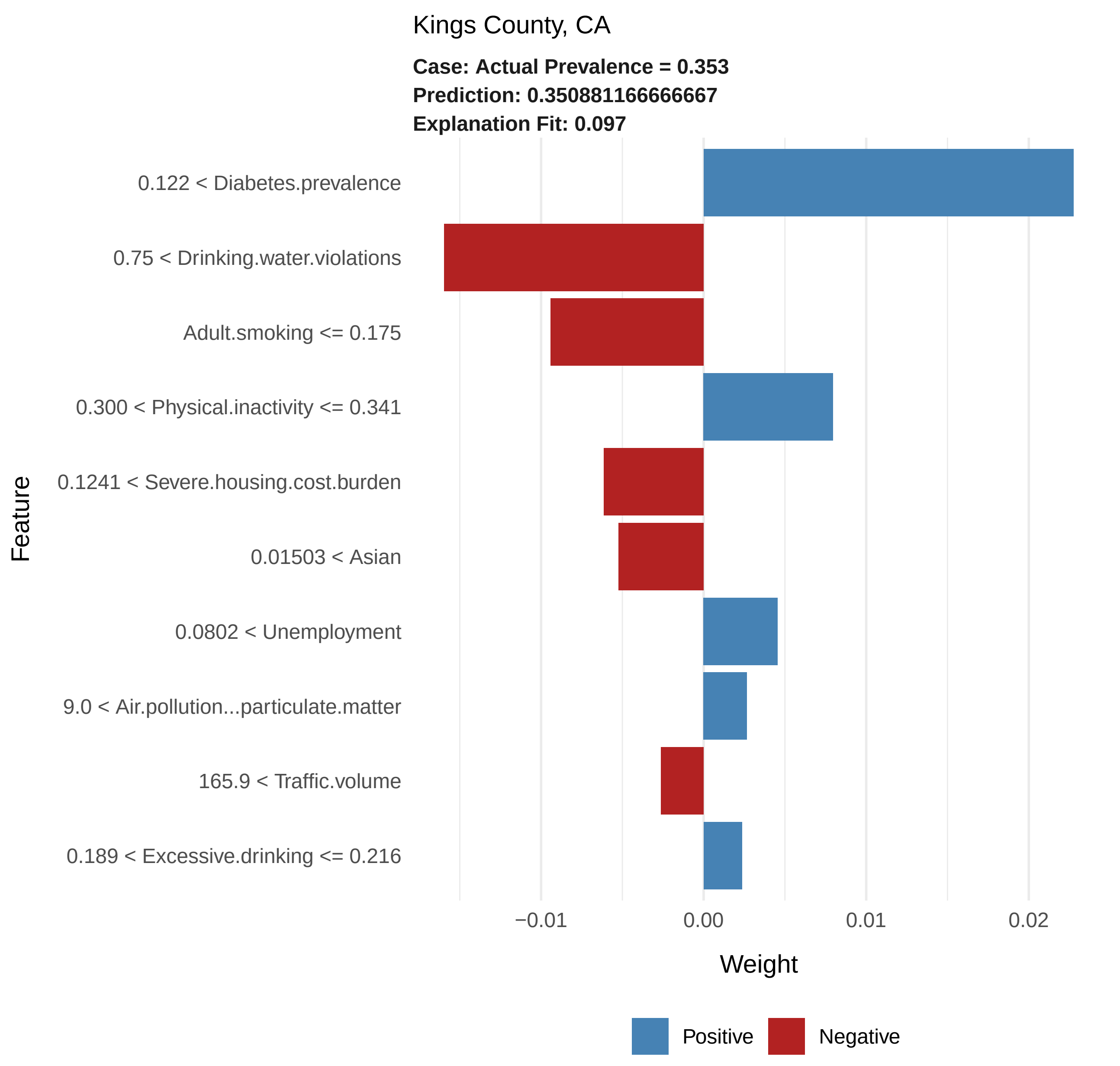

Kings County, CA
Case: Actual Prevalence = 0.353
Prediction: 0.350881166666667
Explanation Fit: 0.097
0.122 < Diabetes.prevalence
0.75 < Drinking.water.violations
Adult.smoking <= 0.175
0.300 < Physical.inactivity <= 0.341
0.1241 < Severe.housing.cost.burden
0.01503 < Asian
0.0802 < Unemployment
9.0 < Air.pollution...particulate.matter
165.9 < Traffic.volume
0.189 < Excessive.drinking <= 0.216
Feature
−0.01
0.00
0.01
0.02
Weight
Positive
Negative

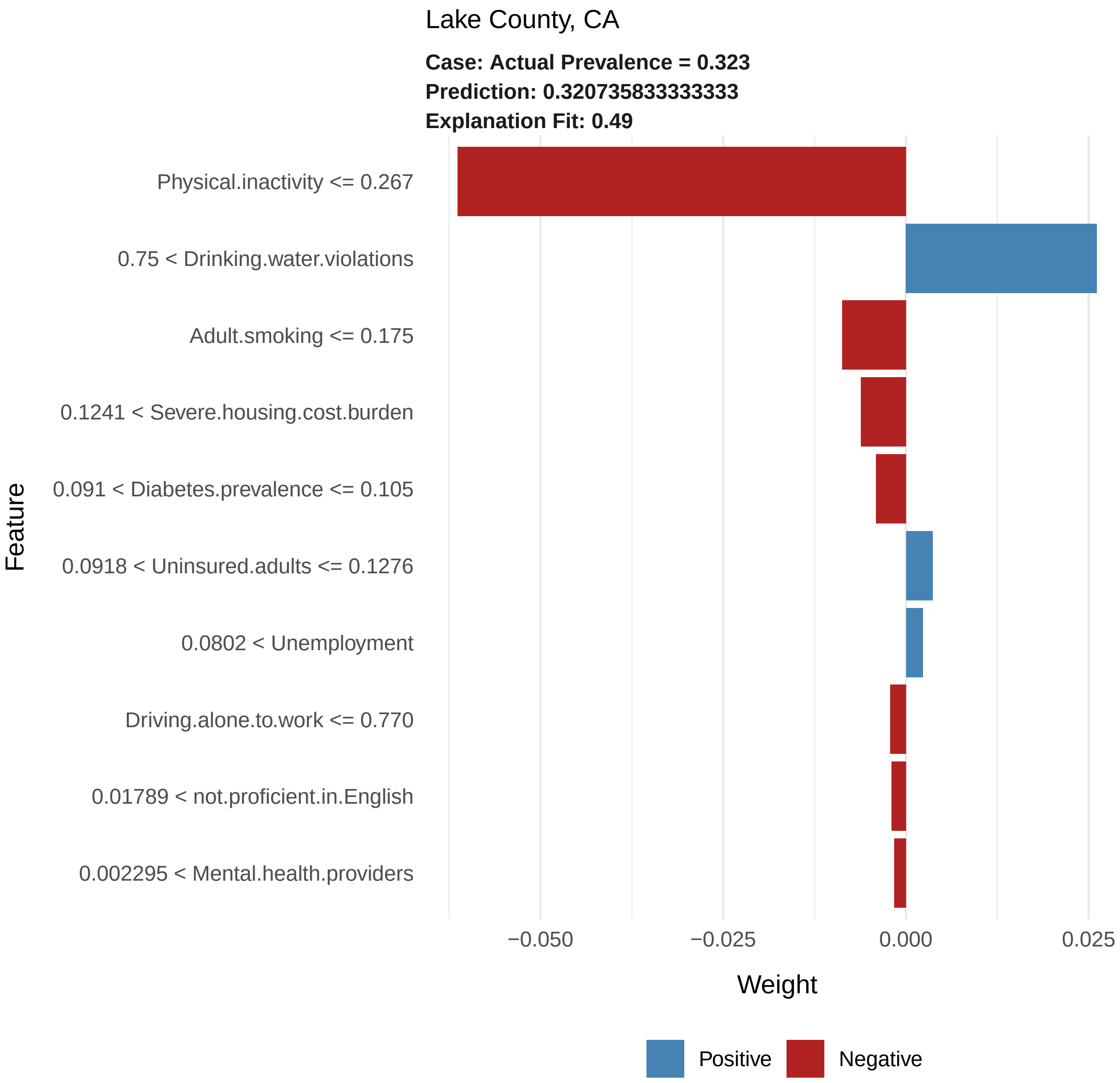

Lake County, CA
Case: Actual Prevalence = 0.323
Prediction: 0.320735833333333
Explanation Fit: 0.49
Physical.inactivity <= 0.267
0.75 < Drinking.water.violations
Adult.smoking <= 0.175
0.1241 < Severe.housing.cost.burden
0.091 < Diabetes.prevalence <= 0.105
0.0918 < Uninsured.adults <= 0.1276
0.0802 < Unemployment
Driving.alone.to.work <= 0.770
0.01789 < not.proficient.in.English
0.002295 < Mental.health.providers
Feature
−0.050
−0.025
0.000
0.025
Weight
Positive
Negative

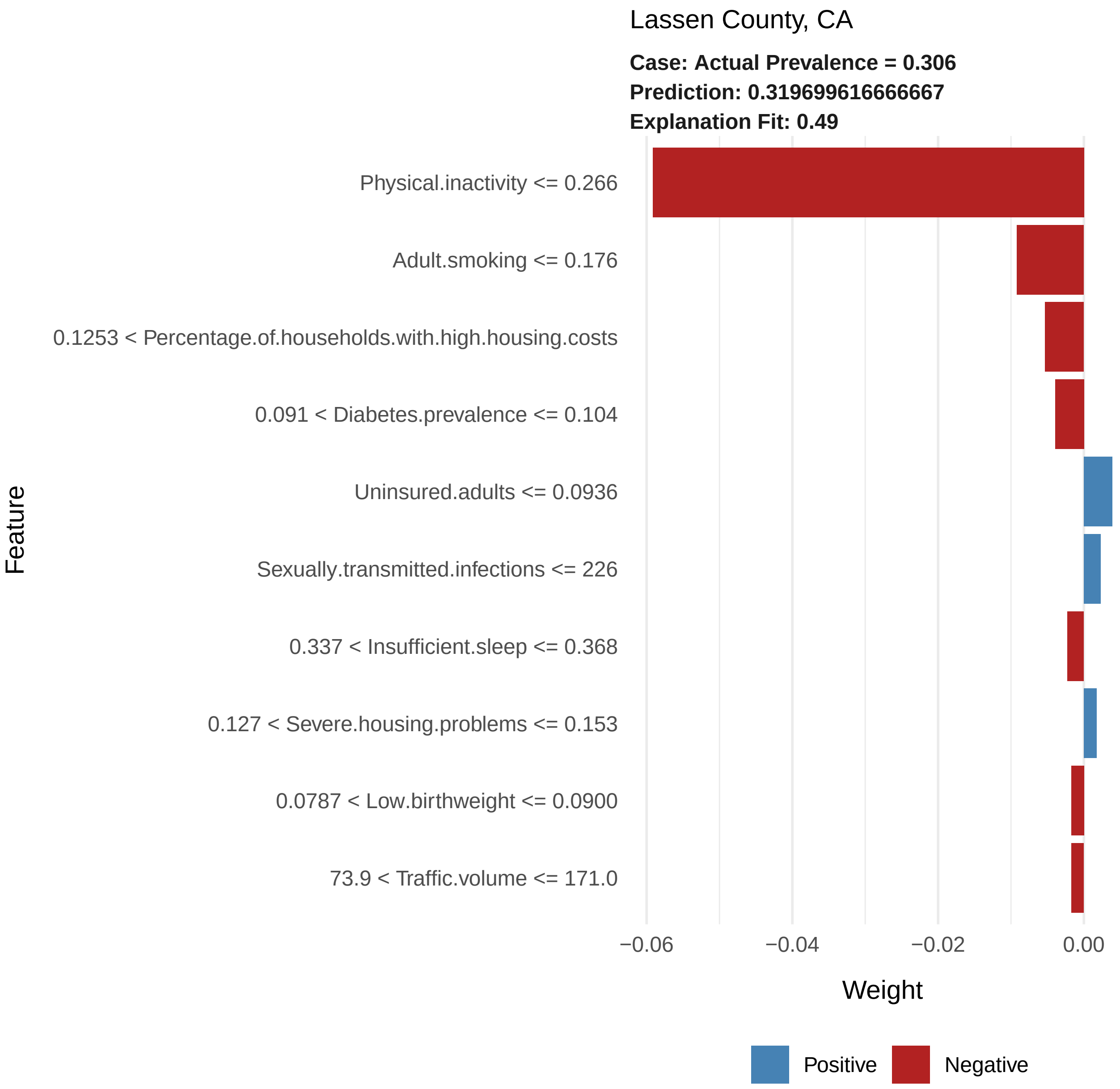

Lassen County, CA
Case: Actual Prevalence = 0.306
Prediction: 0.319699616666667
Explanation Fit: 0.49
Physical.inactivity <= 0.266
Adult.smoking <= 0.176
0.1253 < Percentage.of.households.with.high.housing.costs
0.091 < Diabetes.prevalence <= 0.104
Uninsured.adults <= 0.0936
Sexually.transmitted.infections <= 226
0.337 < Insufficient.sleep <= 0.368
0.127 < Severe.housing.problems <= 0.153
0.0787 < Low.birthweight <= 0.0900
73.9 < Traffic.volume <= 171.0
Feature
−0.06
−0.04
−0.02
0.00
Weight
Positive
Negative

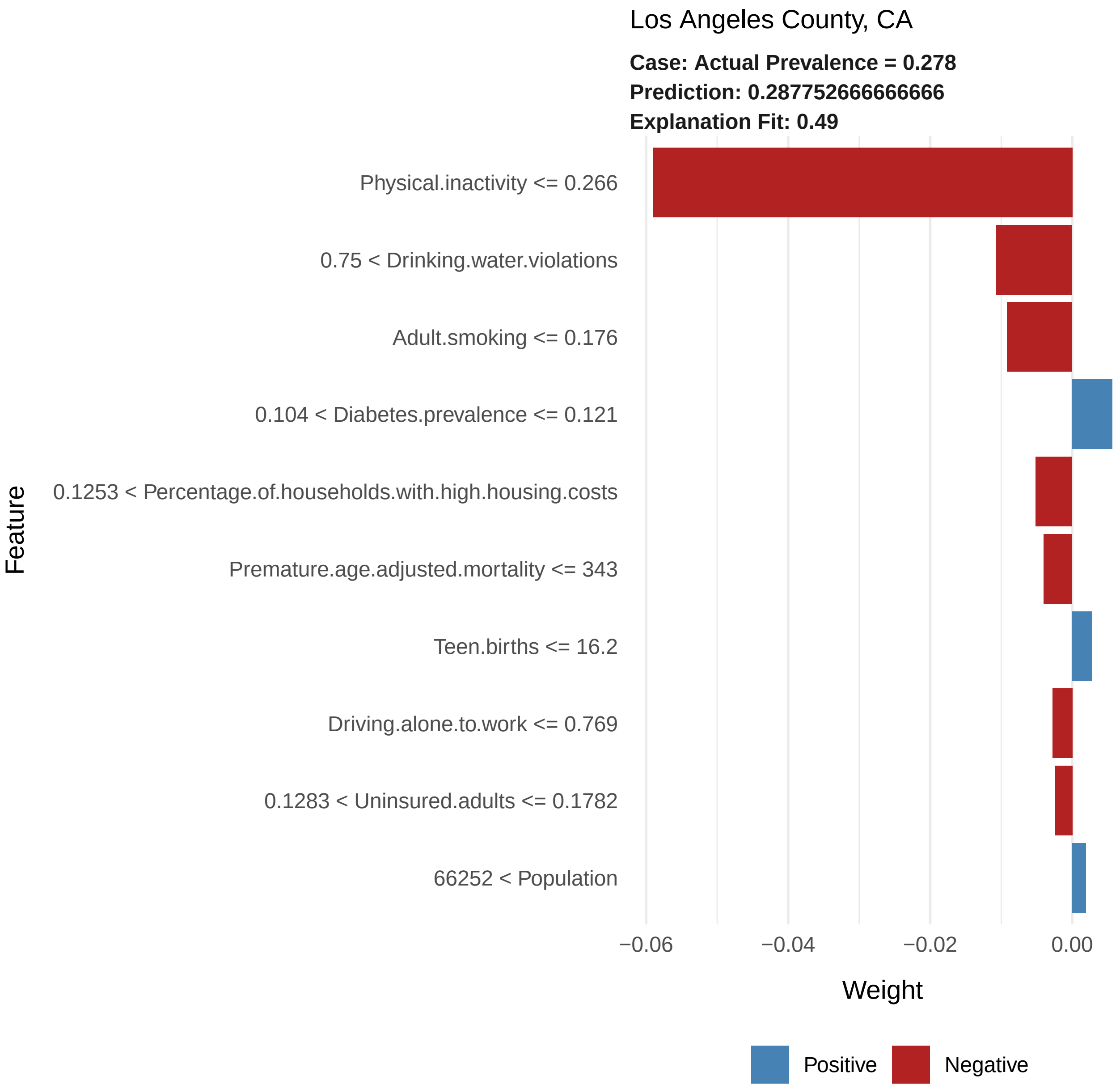
Los Angeles County, CA
Case: Actual Prevalence = 0.278
Prediction: 0.287752666666666
Explanation Fit: 0.49
Physical.inactivity <= 0.266
0.75 < Drinking.water.violations
Adult.smoking <= 0.176
0.104 < Diabetes.prevalence <= 0.121
0.1253 < Percentage.of.households.with.high.housing.costs
Premature.age.adjusted.mortality <= 343
Teen.births <= 16.2
Driving.alone.to.work <= 0.769
0.1283 < Uninsured.adults <= 0.1782
66252 < Population
Feature
−0.06
−0.04
−0.02
0.00
Weight
Positive
Negative

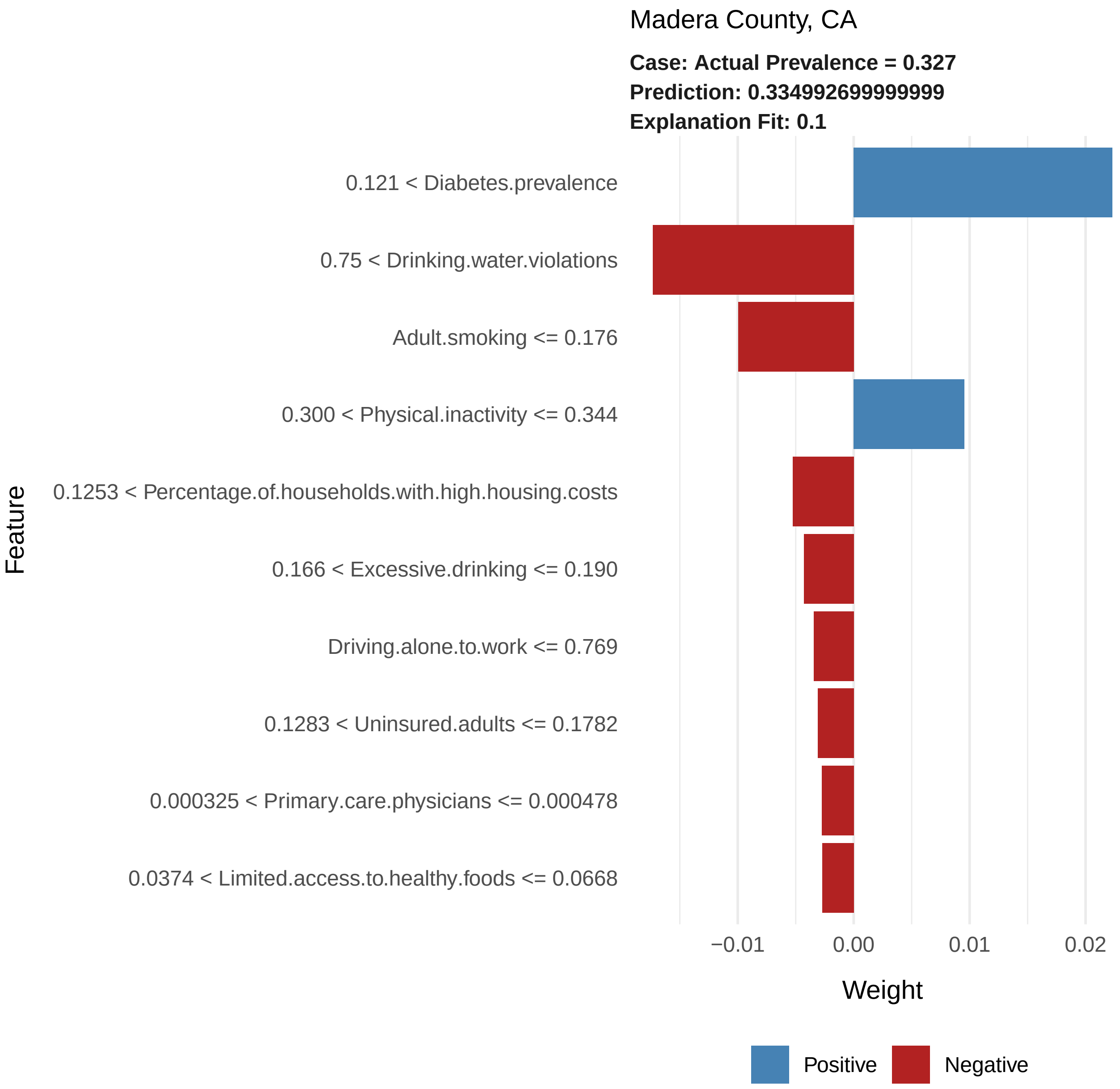

Madera County, CA
Case: Actual Prevalence = 0.327
Prediction: 0.334992699999999
Explanation Fit: 0.1
0.121 < Diabetes.prevalence
0.75 < Drinking.water.violations
Adult.smoking <= 0.176
0.300 < Physical.inactivity <= 0.344
0.1253 < Percentage.of.households.with.high.housing.costs
0.166 < Excessive.drinking <= 0.190
Driving.alone.to.work <= 0.769
0.1283 < Uninsured.adults <= 0.1782
0.000325 < Primary.care.physicians <= 0.000478
0.0374 < Limited.access.to.healthy.foods <= 0.0668
Feature
−0.01
0.00
0.01
0.02
Weight
Positive
Negative

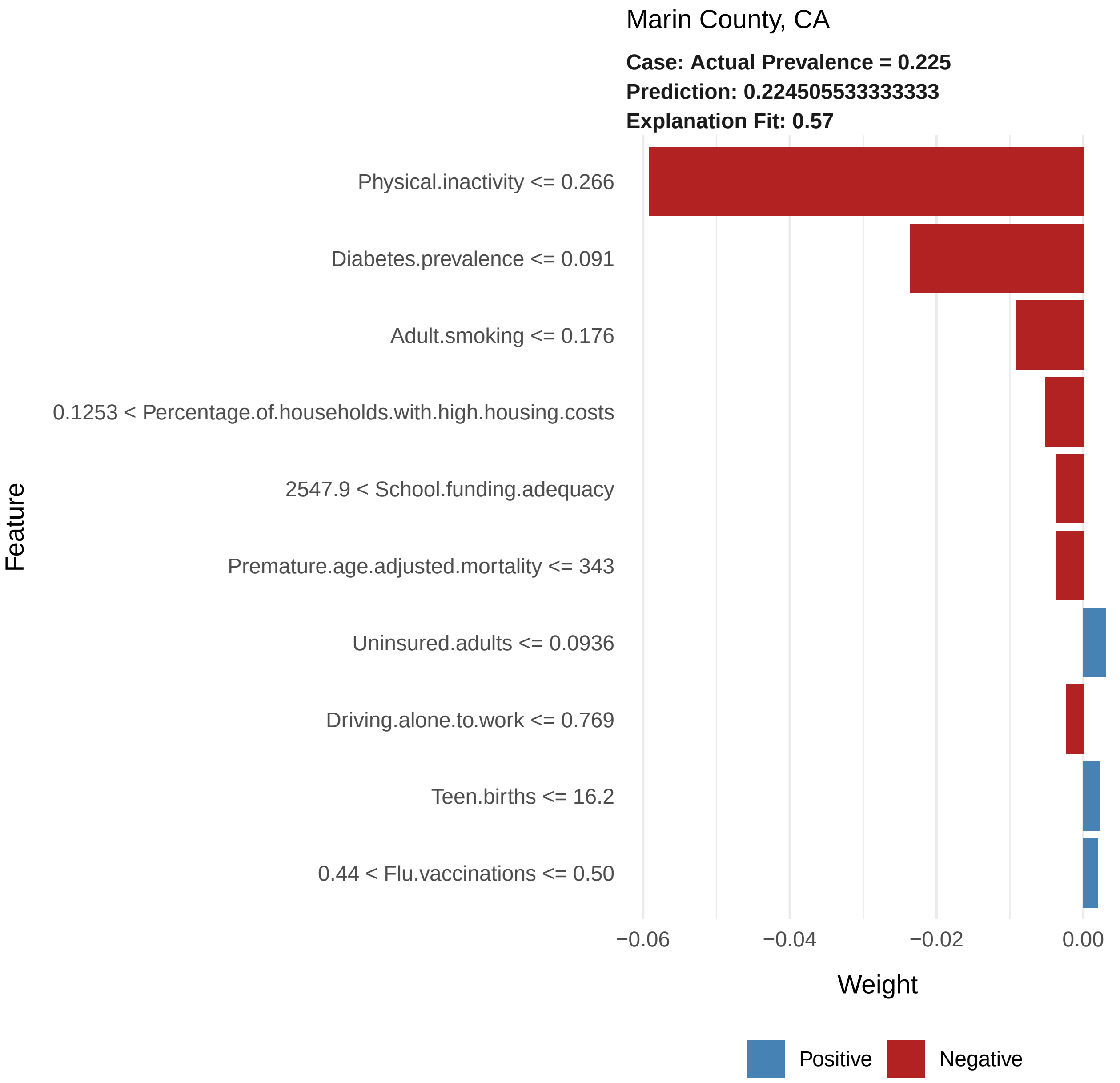
Marin County, CA
Case: Actual Prevalence = 0.225
Prediction: 0.224505533333333
Explanation Fit: 0.57
Physical.inactivity <= 0.266
Diabetes.prevalence <= 0.091
Adult.smoking <= 0.176
0.1253 < Percentage.of.households.with.high.housing.costs
2547.9 < School.funding.adequacy
Premature.age.adjusted.mortality <= 343
Uninsured.adults <= 0.0936
Driving.alone.to.work <= 0.769
Teen.births <= 16.2
0.44 < Flu.vaccinations <= 0.50
Feature
−0.06
−0.04
−0.02
0.00
Weight
Positive
Negative

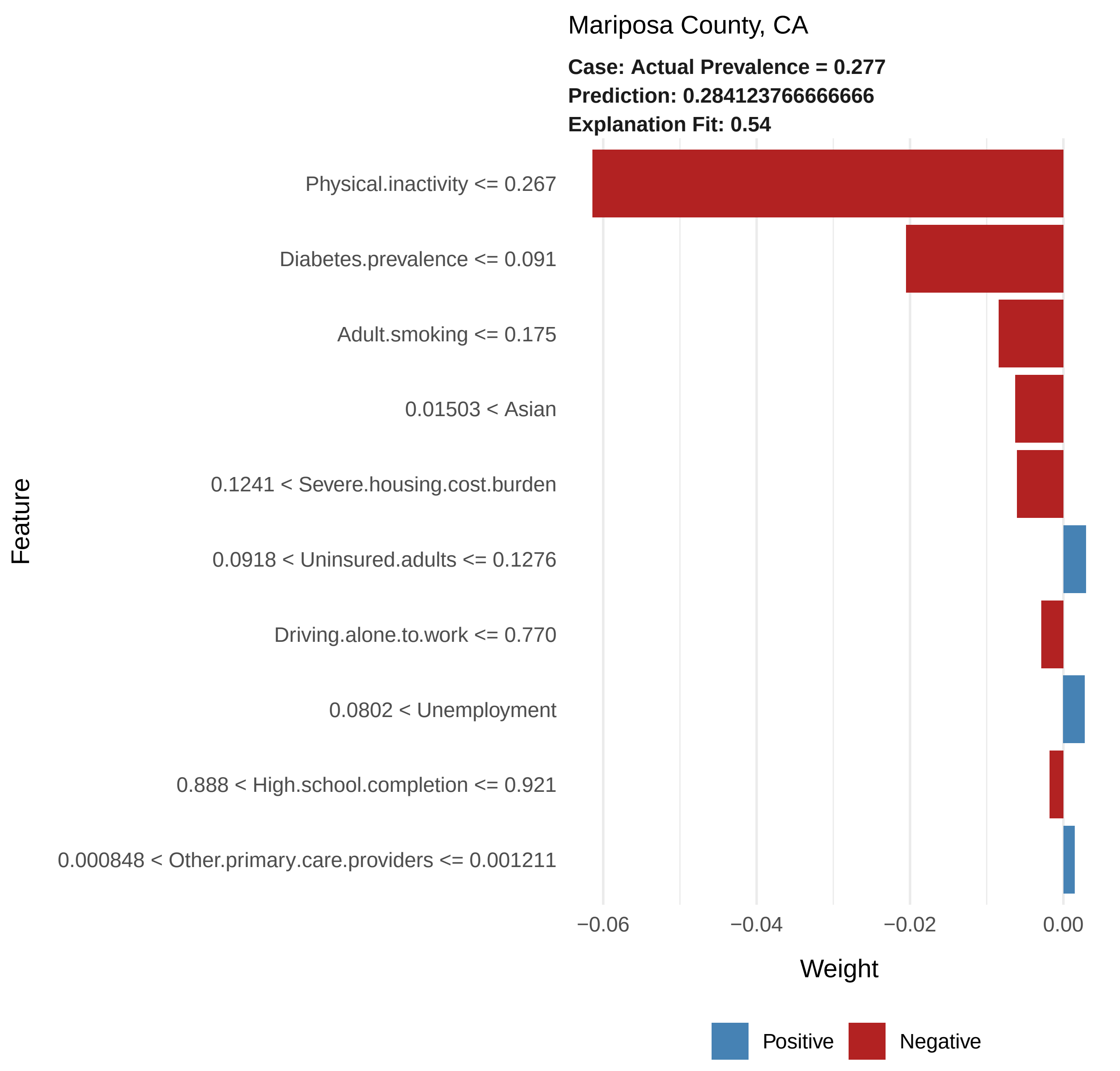

Mariposa County, CA
Case: Actual Prevalence = 0.277
Prediction: 0.284123766666666
Explanation Fit: 0.54
Physical.inactivity <= 0.267
Diabetes.prevalence <= 0.091
Adult.smoking <= 0.175
0.01503 < Asian
0.1241 < Severe.housing.cost.burden
0.0918 < Uninsured.adults <= 0.1276
Driving.alone.to.work <= 0.770
0.0802 < Unemployment
0.888 < High.school.completion <= 0.921
0.000848 < Other.primary.care.providers <= 0.001211
Feature
−0.06
−0.04
−0.02
0.00
Weight
Positive
Negative

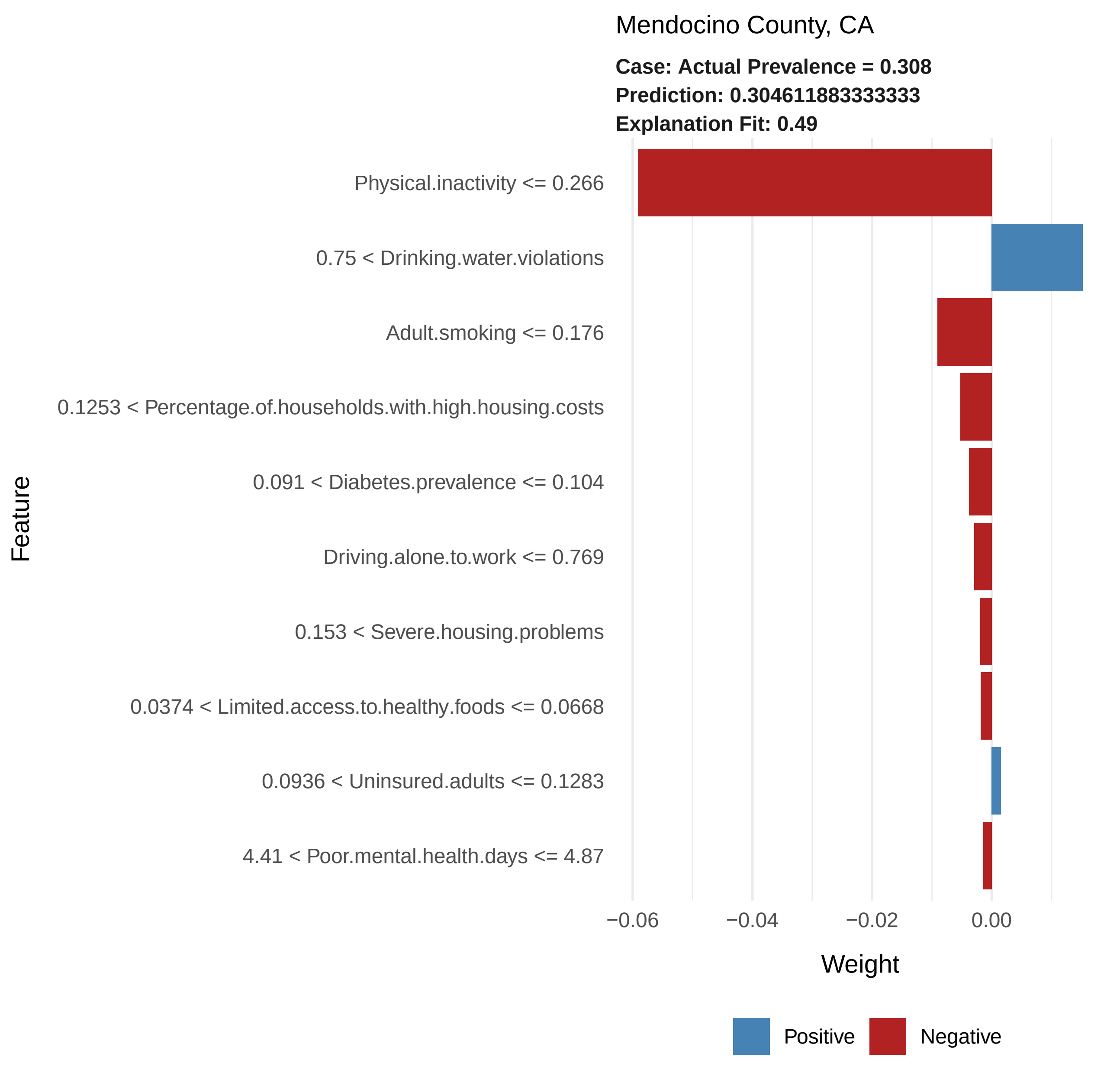

Mendocino County, CA
Case: Actual Prevalence = 0.308
Prediction: 0.304611883333333
Explanation Fit: 0.49
Physical.inactivity <= 0.266
0.75 < Drinking.water.violations
Adult.smoking <= 0.176
0.1253 < Percentage.of.households.with.high.housing.costs
0.091 < Diabetes.prevalence <= 0.104
Driving.alone.to.work <= 0.769
0.153 < Severe.housing.problems
0.0374 < Limited.access.to.healthy.foods <= 0.0668
0.0936 < Uninsured.adults <= 0.1283
4.41 < Poor.mental.health.days <= 4.87
Feature
−0.06
−0.04
−0.02
0.00
Weight
Positive
Negative

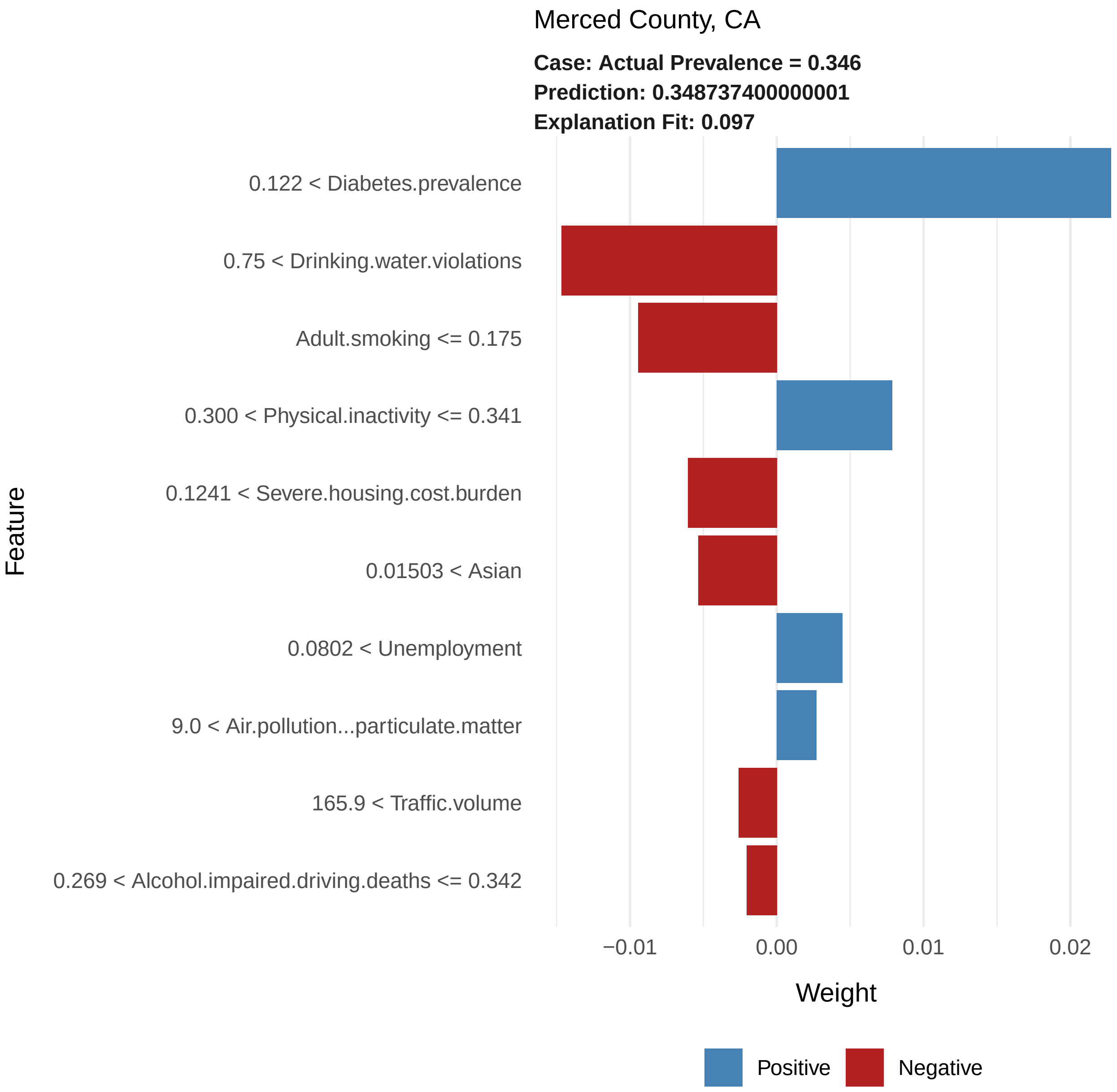

Merced County, CA
Case: Actual Prevalence = 0.346
Prediction: 0.348737400000001
Explanation Fit: 0.097
0.122 < Diabetes.prevalence
0.75 < Drinking.water.violations
Adult.smoking <= 0.175
0.300 < Physical.inactivity <= 0.341
0.1241 < Severe.housing.cost.burden
0.01503 < Asian
0.0802 < Unemployment
9.0 < Air.pollution...particulate.matter
165.9 < Traffic.volume
0.269 < Alcohol.impaired.driving.deaths <= 0.342
Feature
−0.01
0.00
0.01
0.02
Weight
Positive
Negative

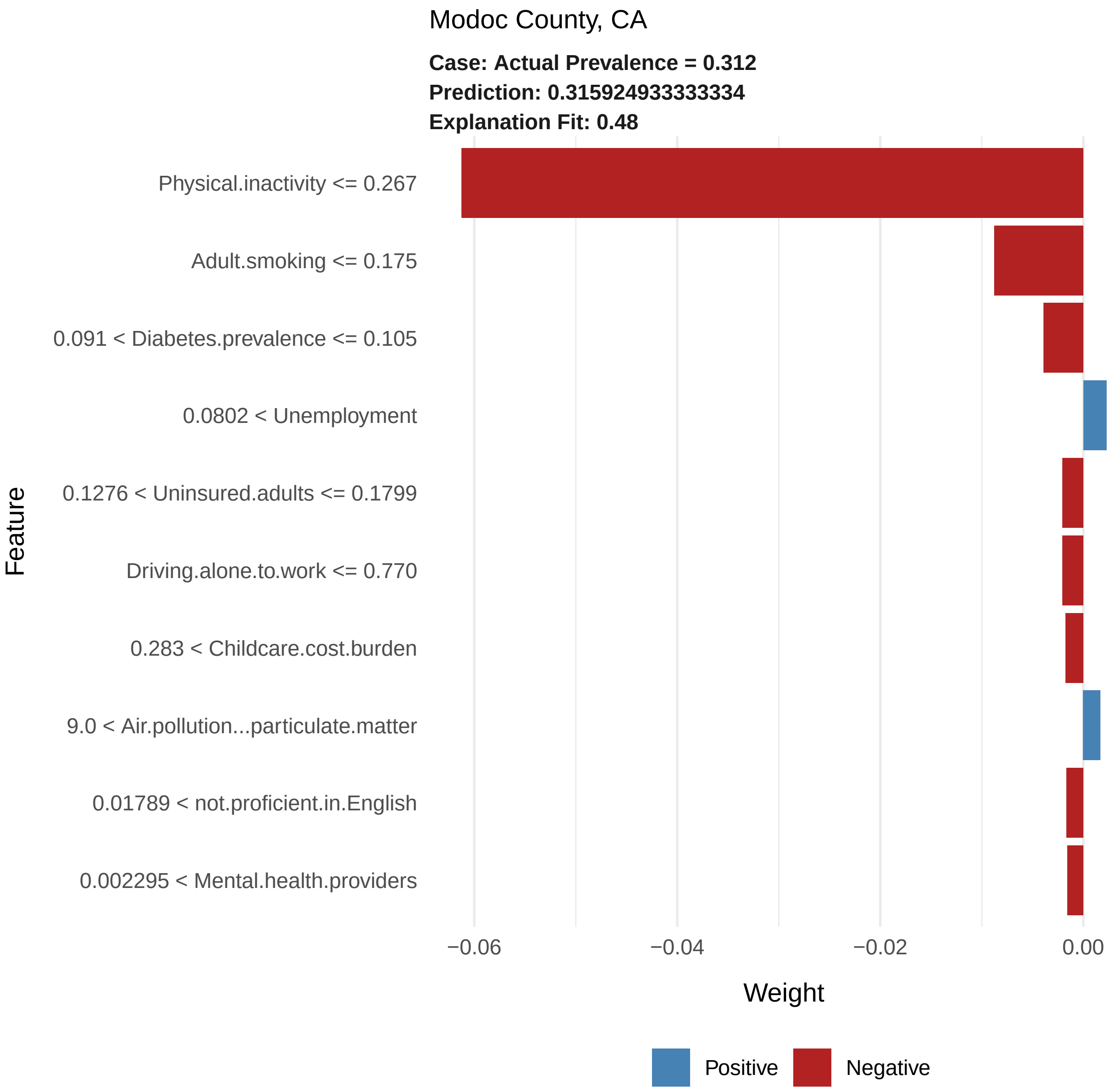

Modoc County, CA
Case: Actual Prevalence = 0.312
Prediction: 0.315924933333334
Explanation Fit: 0.48
Physical.inactivity <= 0.267
Adult.smoking <= 0.175
0.091 < Diabetes.prevalence <= 0.105
0.0802 < Unemployment
0.1276 < Uninsured.adults <= 0.1799
Driving.alone.to.work <= 0.770
0.283 < Childcare.cost.burden
9.0 < Air.pollution...particulate.matter
0.01789 < not.proficient.in.English
0.002295 < Mental.health.providers
Feature
−0.06
−0.04
−0.02
0.00
Weight
Positive
Negative

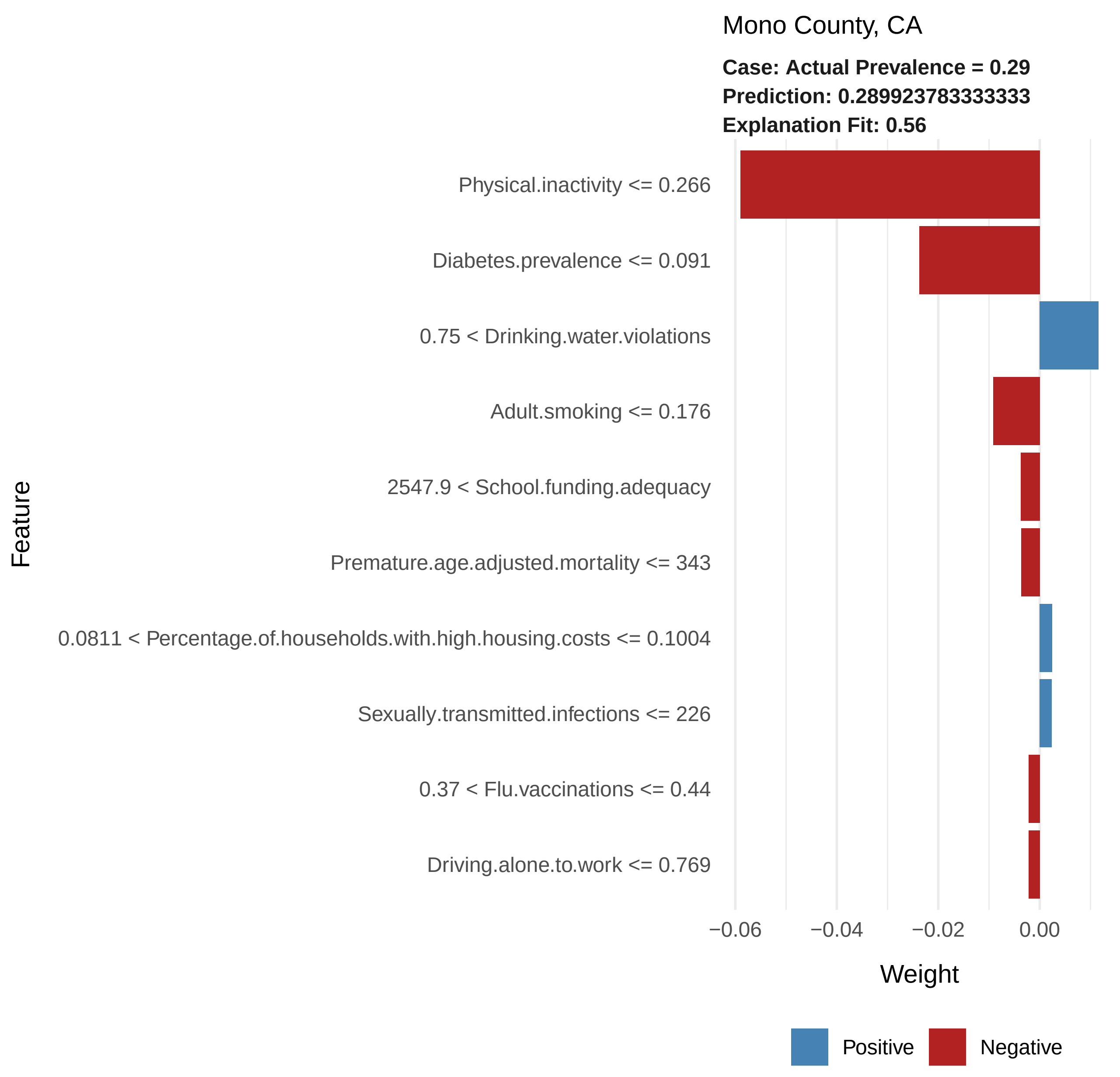

Mono County, CA
Case: Actual Prevalence = 0.29
Prediction: 0.289923783333333
Explanation Fit: 0.56
Physical.inactivity <= 0.266
Diabetes.prevalence <= 0.091
0.75 < Drinking.water.violations
Adult.smoking <= 0.176
2547.9 < School.funding.adequacy
Premature.age.adjusted.mortality <= 343
0.0811 < Percentage.of.households.with.high.housing.costs <= 0.1004
Sexually.transmitted.infections <= 226
0.37 < Flu.vaccinations <= 0.44
Driving.alone.to.work <= 0.769
Feature
−0.06
−0.04
−0.02
0.00
Weight
Positive
Negative

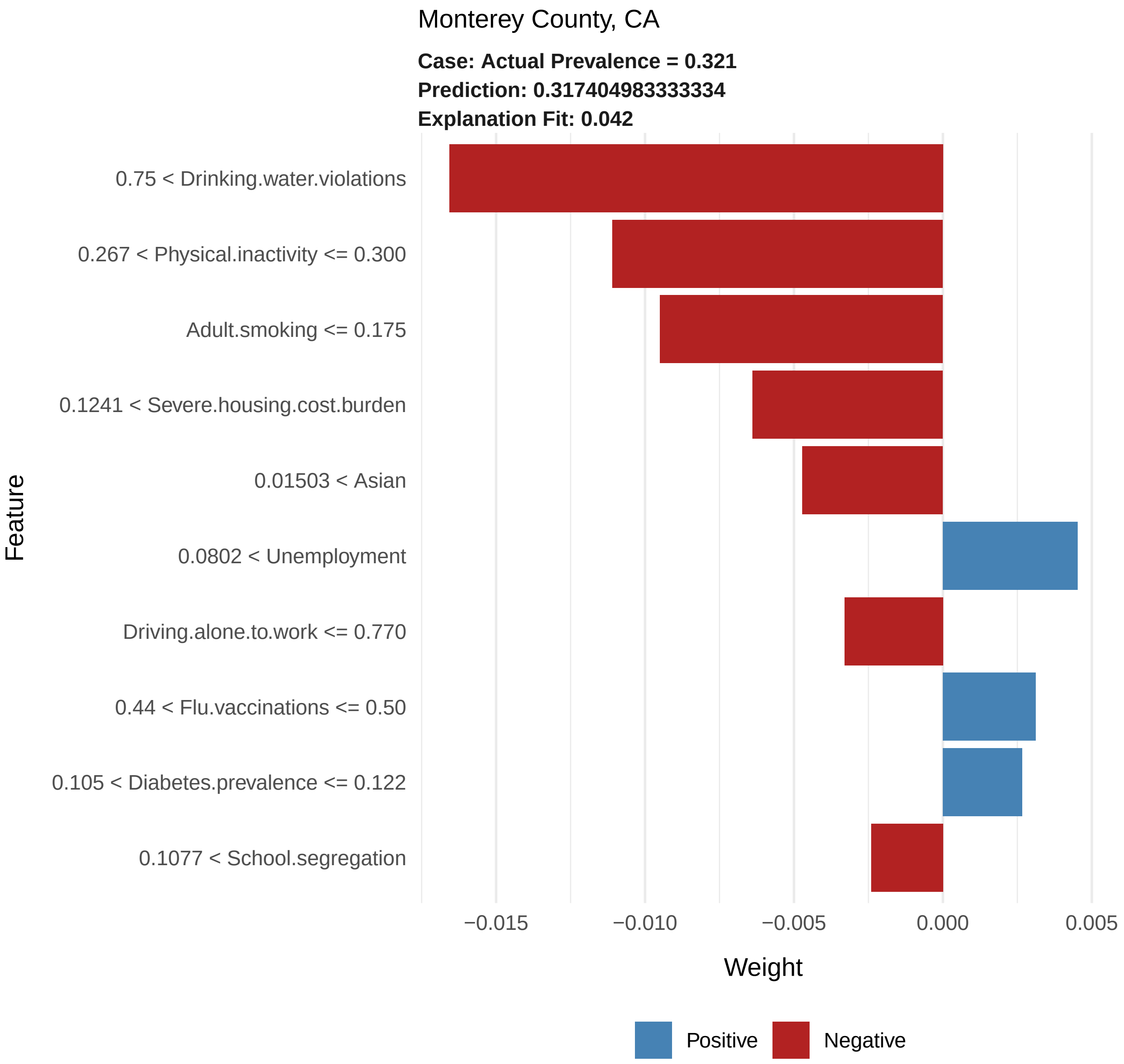

Monterey County, CA
Case: Actual Prevalence = 0.321
Prediction: 0.317404983333334
Explanation Fit: 0.042
0.75 < Drinking.water.violations
0.267 < Physical.inactivity <= 0.300
Adult.smoking <= 0.175
0.1241 < Severe.housing.cost.burden
0.01503 < Asian
0.0802 < Unemployment
Driving.alone.to.work <= 0.770
0.44 < Flu.vaccinations <= 0.50
0.105 < Diabetes.prevalence <= 0.122
0.1077 < School.segregation
Feature
−0.015
−0.010
−0.005
0.000
0.005
Weight
Positive
Negative

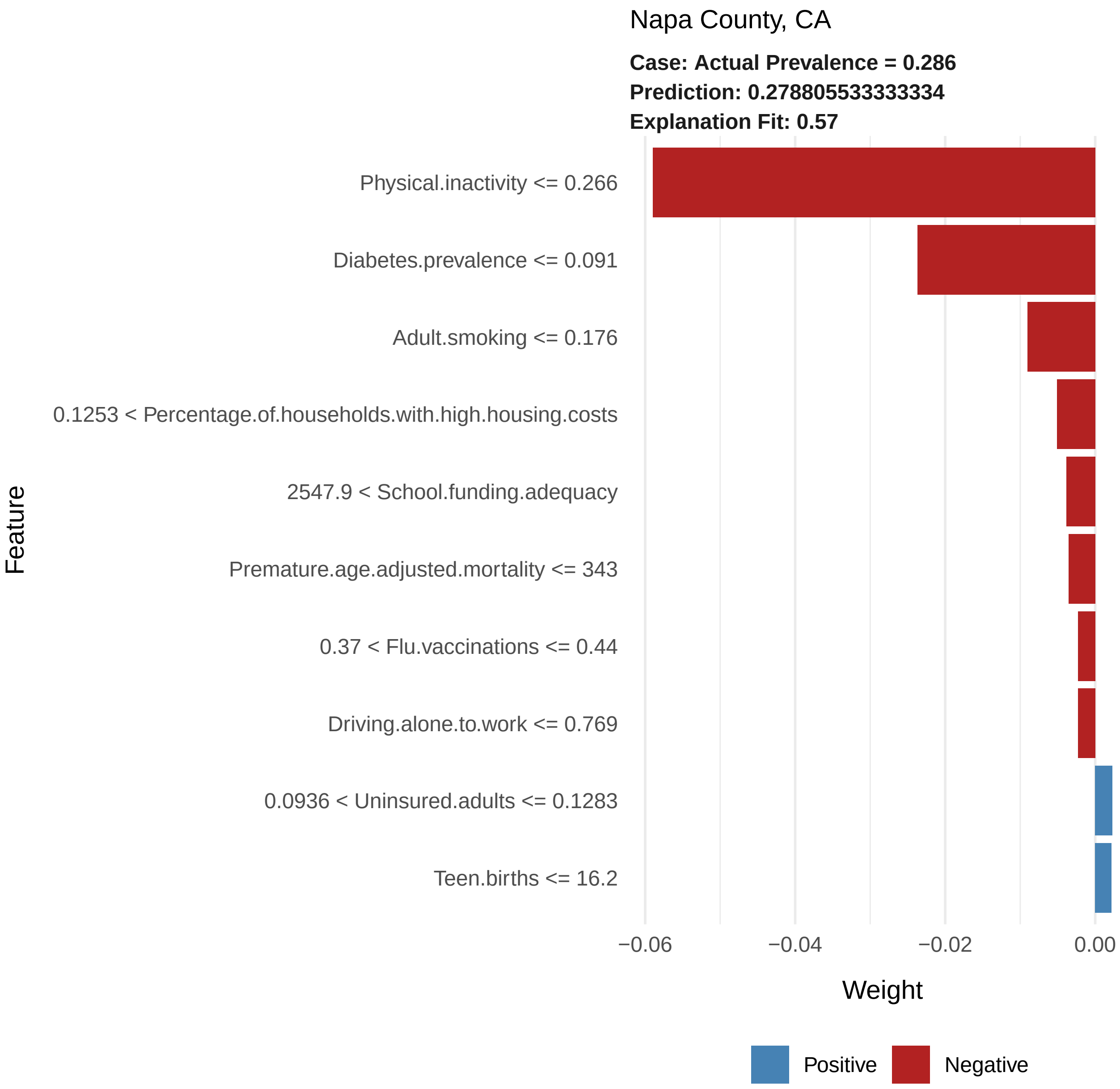
Napa County, CA
Case: Actual Prevalence = 0.286
Prediction: 0.278805533333334
Explanation Fit: 0.57
Physical.inactivity <= 0.266
Diabetes.prevalence <= 0.091
Adult.smoking <= 0.176
0.1253 < Percentage.of.households.with.high.housing.costs
2547.9 < School.funding.adequacy
Premature.age.adjusted.mortality <= 343
0.37 < Flu.vaccinations <= 0.44
Driving.alone.to.work <= 0.769
0.0936 < Uninsured.adults <= 0.1283
Teen.births <= 16.2
Feature
−0.06
−0.04
−0.02
0.00
Weight
Positive
Negative

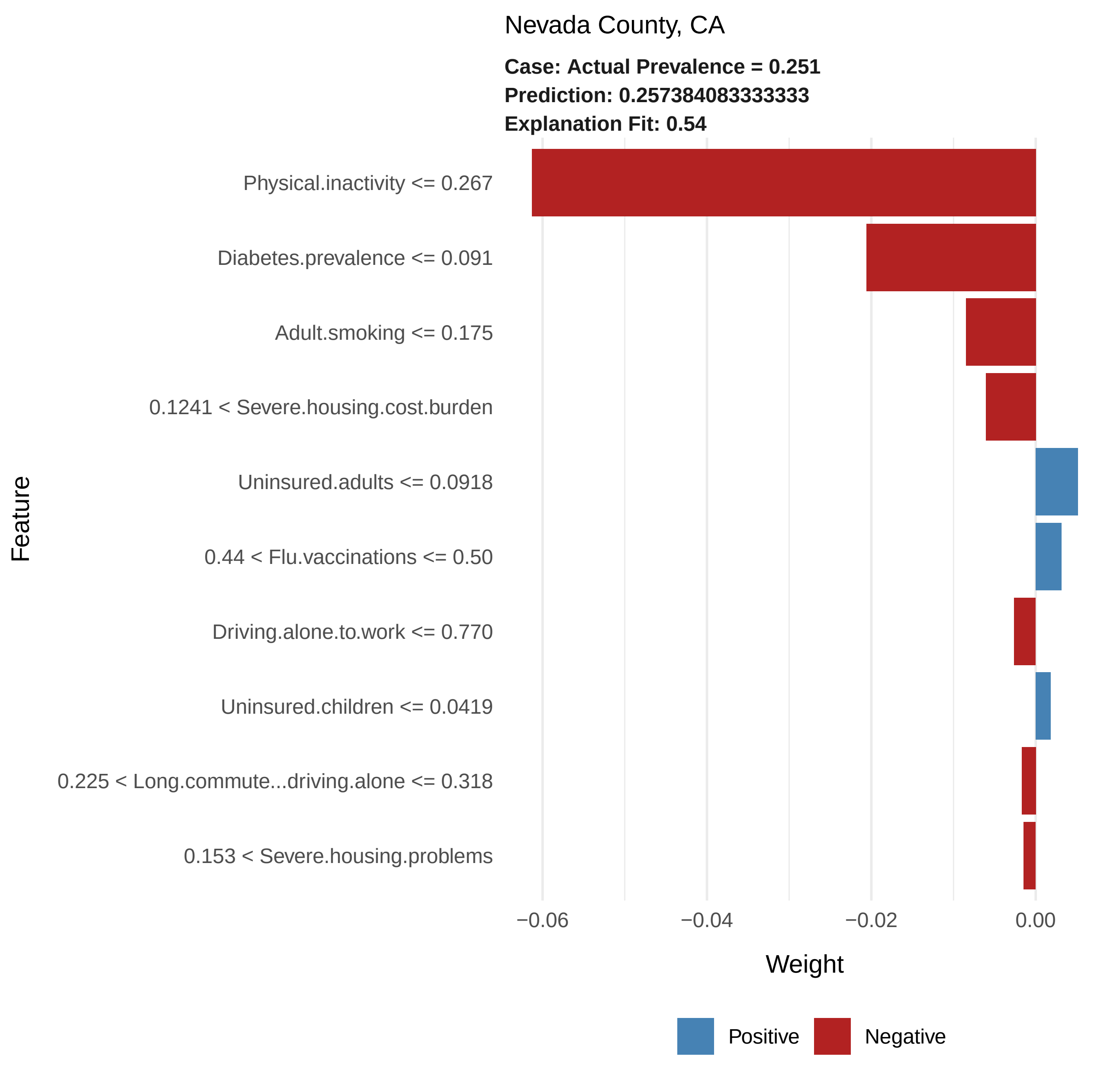
Nevada County, CA
Case: Actual Prevalence = 0.251
Prediction: 0.257384083333333
Explanation Fit: 0.54
Physical.inactivity <= 0.267
Diabetes.prevalence <= 0.091
Adult.smoking <= 0.175
0.1241 < Severe.housing.cost.burden
Uninsured.adults <= 0.0918
0.44 < Flu.vaccinations <= 0.50
Driving.alone.to.work <= 0.770
Uninsured.children <= 0.0419
0.225 < Long.commute...driving.alone <= 0.318
0.153 < Severe.housing.problems
Feature
−0.06
−0.04
−0.02
0.00
Weight
Positive
Negative

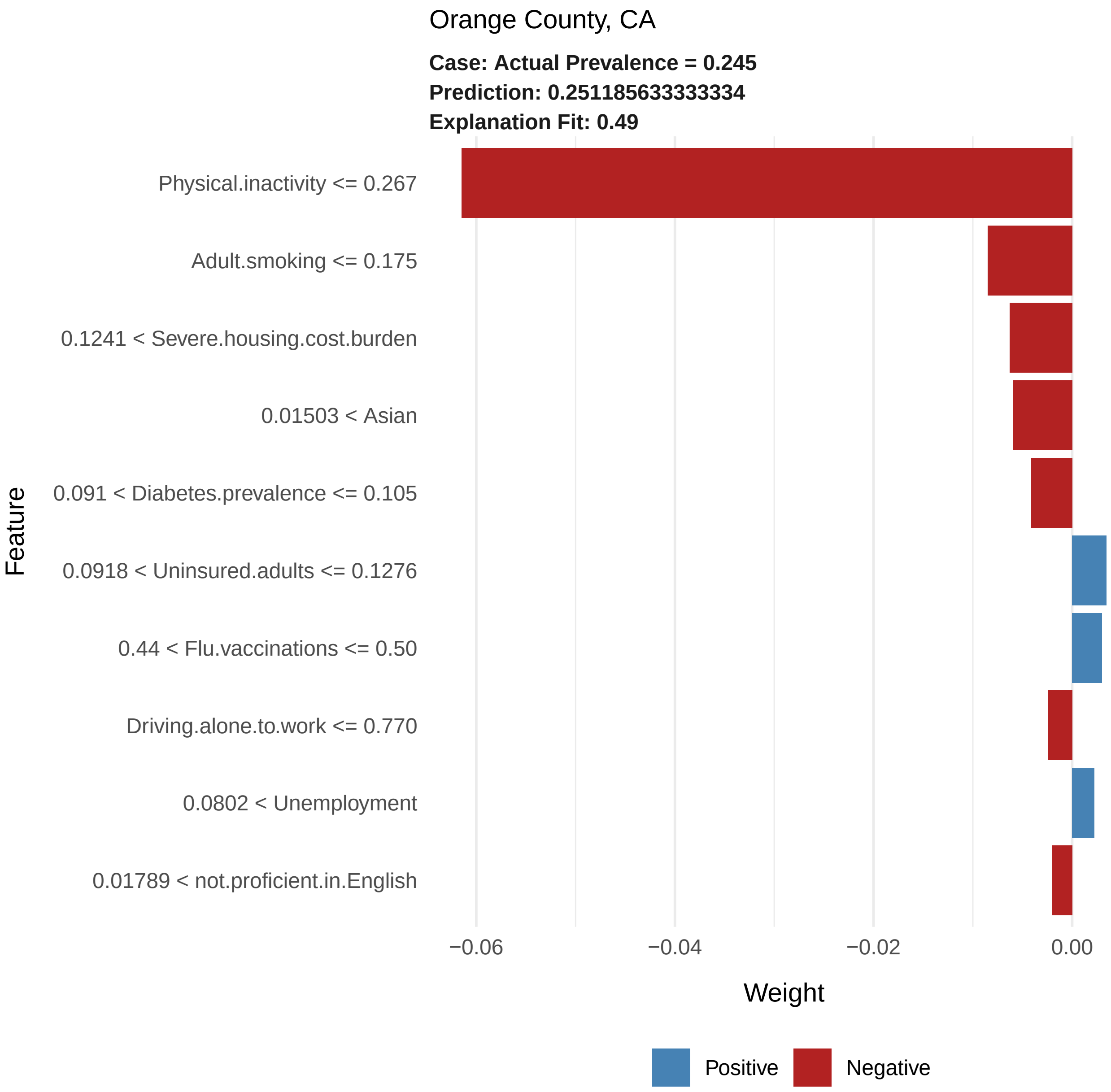
Orange County, CA
Case: Actual Prevalence = 0.245
Prediction: 0.251185633333334
Explanation Fit: 0.49
Physical.inactivity <= 0.267
Adult.smoking <= 0.175
0.1241 < Severe.housing.cost.burden
0.01503 < Asian
0.091 < Diabetes.prevalence <= 0.105
0.0918 < Uninsured.adults <= 0.1276
0.44 < Flu.vaccinations <= 0.50
Driving.alone.to.work <= 0.770
0.0802 < Unemployment
0.01789 < not.proficient.in.English
Feature
−0.06
−0.04
−0.02
0.00
Weight
Positive
Negative

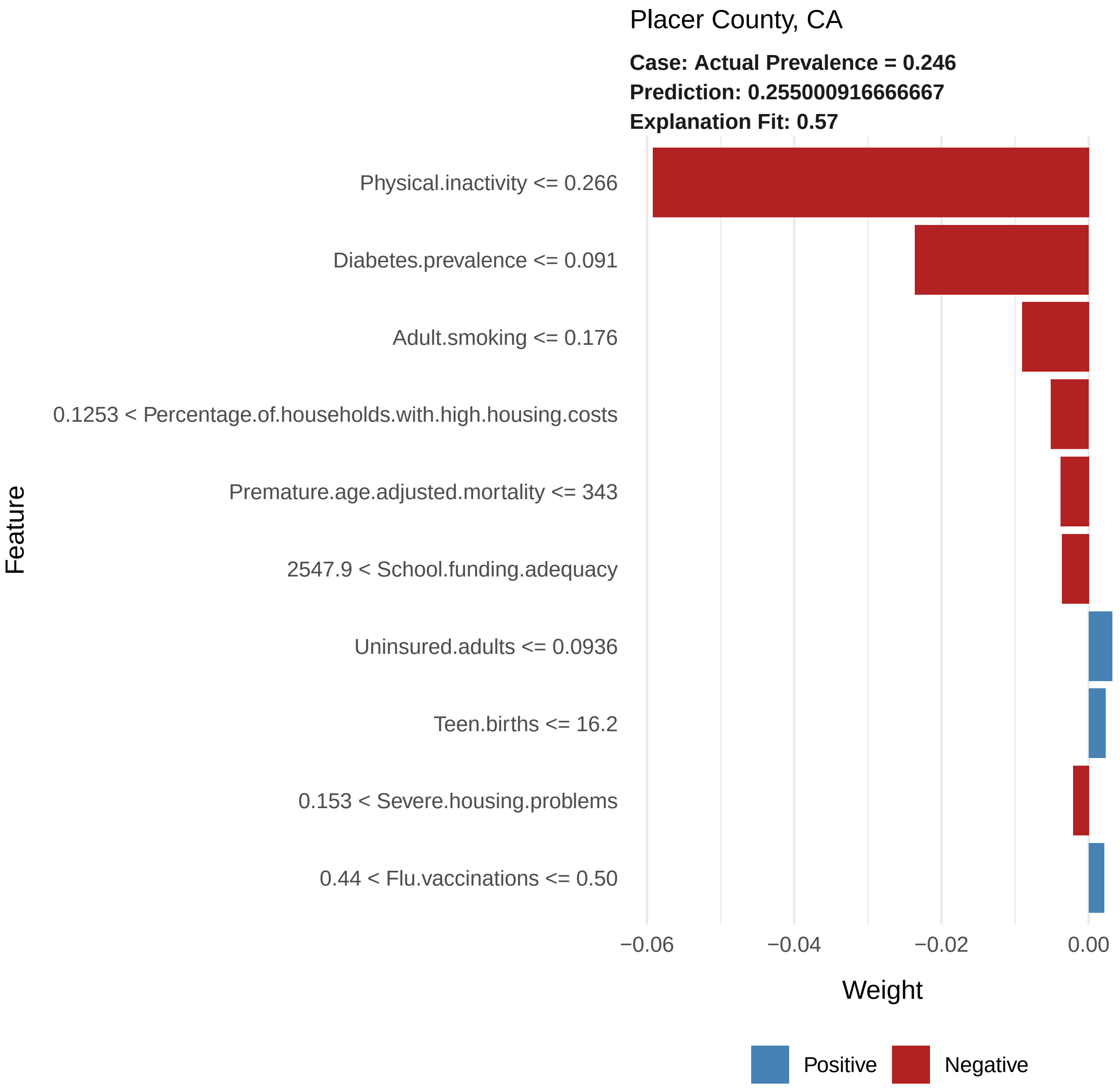
Placer County, CA
Case: Actual Prevalence = 0.246
Prediction: 0.255000916666667
Explanation Fit: 0.57
Physical.inactivity <= 0.266
Diabetes.prevalence <= 0.091
Adult.smoking <= 0.176
0.1253 < Percentage.of.households.with.high.housing.costs
Premature.age.adjusted.mortality <= 343
2547.9 < School.funding.adequacy
Uninsured.adults <= 0.0936
Teen.births <= 16.2
0.153 < Severe.housing.problems
0.44 < Flu.vaccinations <= 0.50
Feature
−0.06
−0.04
−0.02
0.00
Weight
Positive
Negative

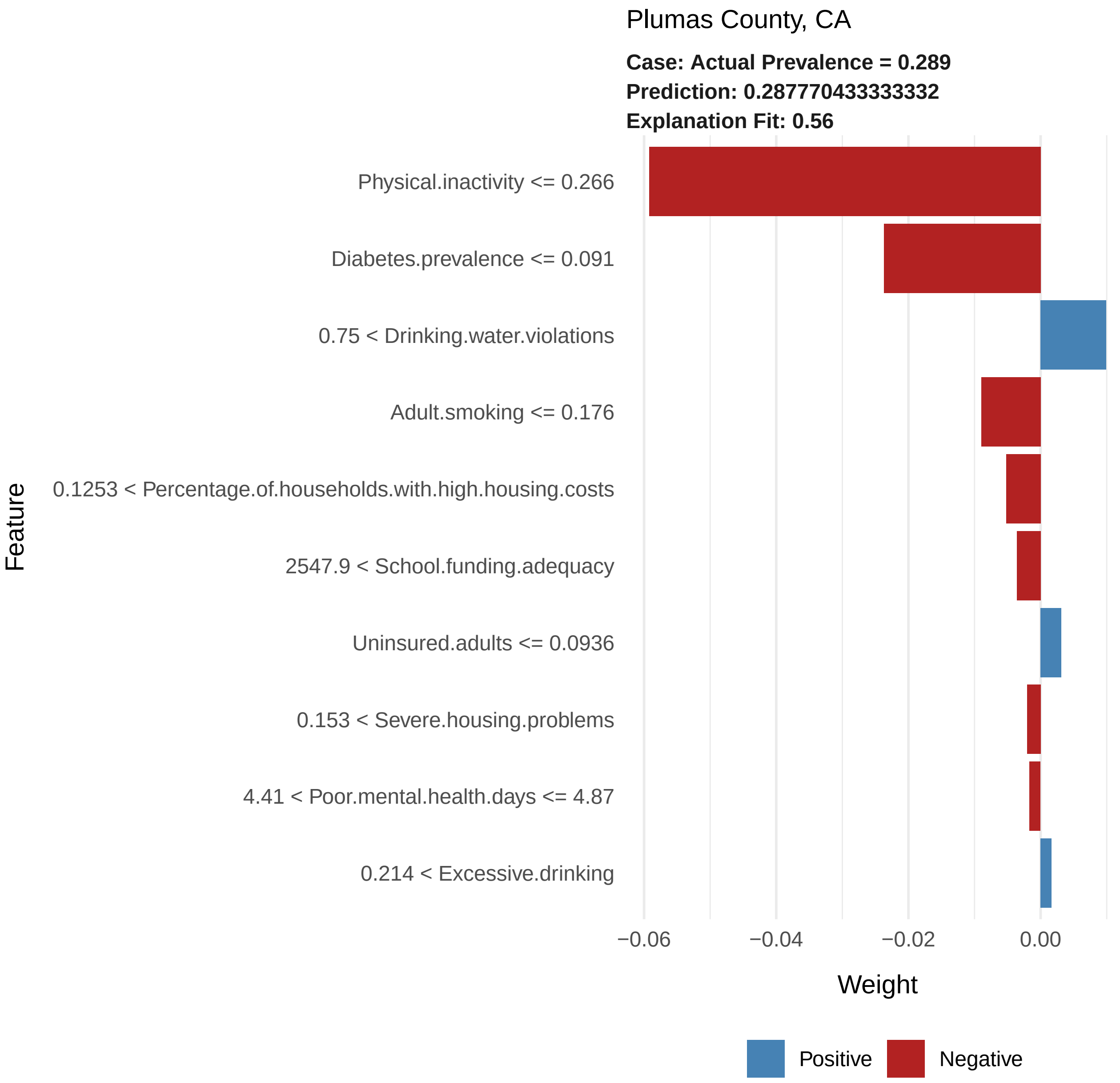

Plumas County, CA
Case: Actual Prevalence = 0.289
Prediction: 0.287770433333332
Explanation Fit: 0.56
Physical.inactivity <= 0.266
Diabetes.prevalence <= 0.091
0.75 < Drinking.water.violations
Adult.smoking <= 0.176
0.1253 < Percentage.of.households.with.high.housing.costs
2547.9 < School.funding.adequacy
Uninsured.adults <= 0.0936
0.153 < Severe.housing.problems
4.41 < Poor.mental.health.days <= 4.87
0.214 < Excessive.drinking
Feature
−0.06
−0.04
−0.02
0.00
Weight
Positive
Negative

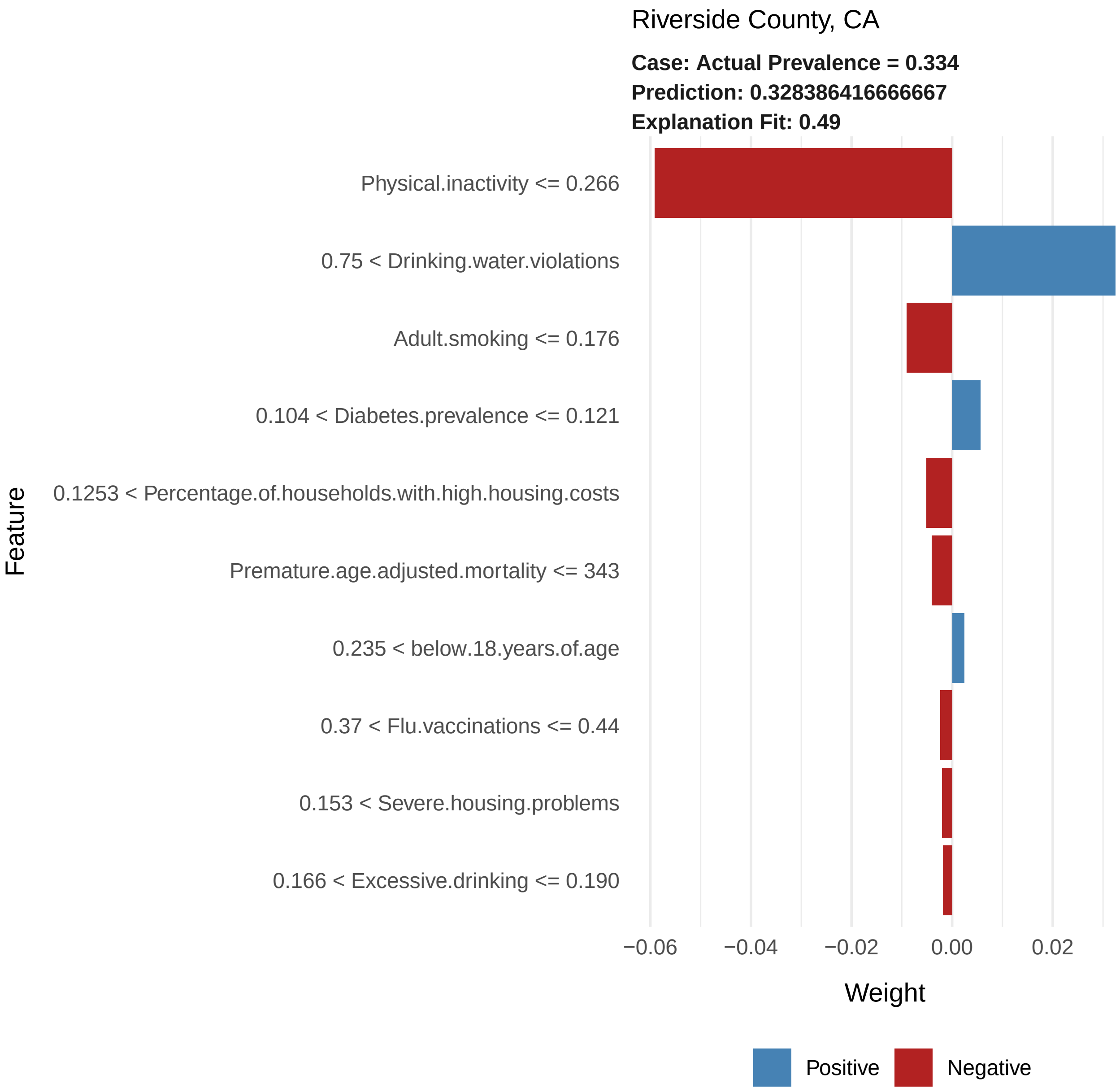

Riverside County, CA
Case: Actual Prevalence = 0.334
Prediction: 0.328386416666667
Explanation Fit: 0.49
Physical.inactivity <= 0.266
0.75 < Drinking.water.violations
Adult.smoking <= 0.176
0.104 < Diabetes.prevalence <= 0.121
0.1253 < Percentage.of.households.with.high.housing.costs
Premature.age.adjusted.mortality <= 343
0.235 < below.18.years.of.age
0.37 < Flu.vaccinations <= 0.44
0.153 < Severe.housing.problems
0.166 < Excessive.drinking <= 0.190
Feature
−0.06
−0.04
−0.02
0.00
0.02
Weight
Positive
Negative

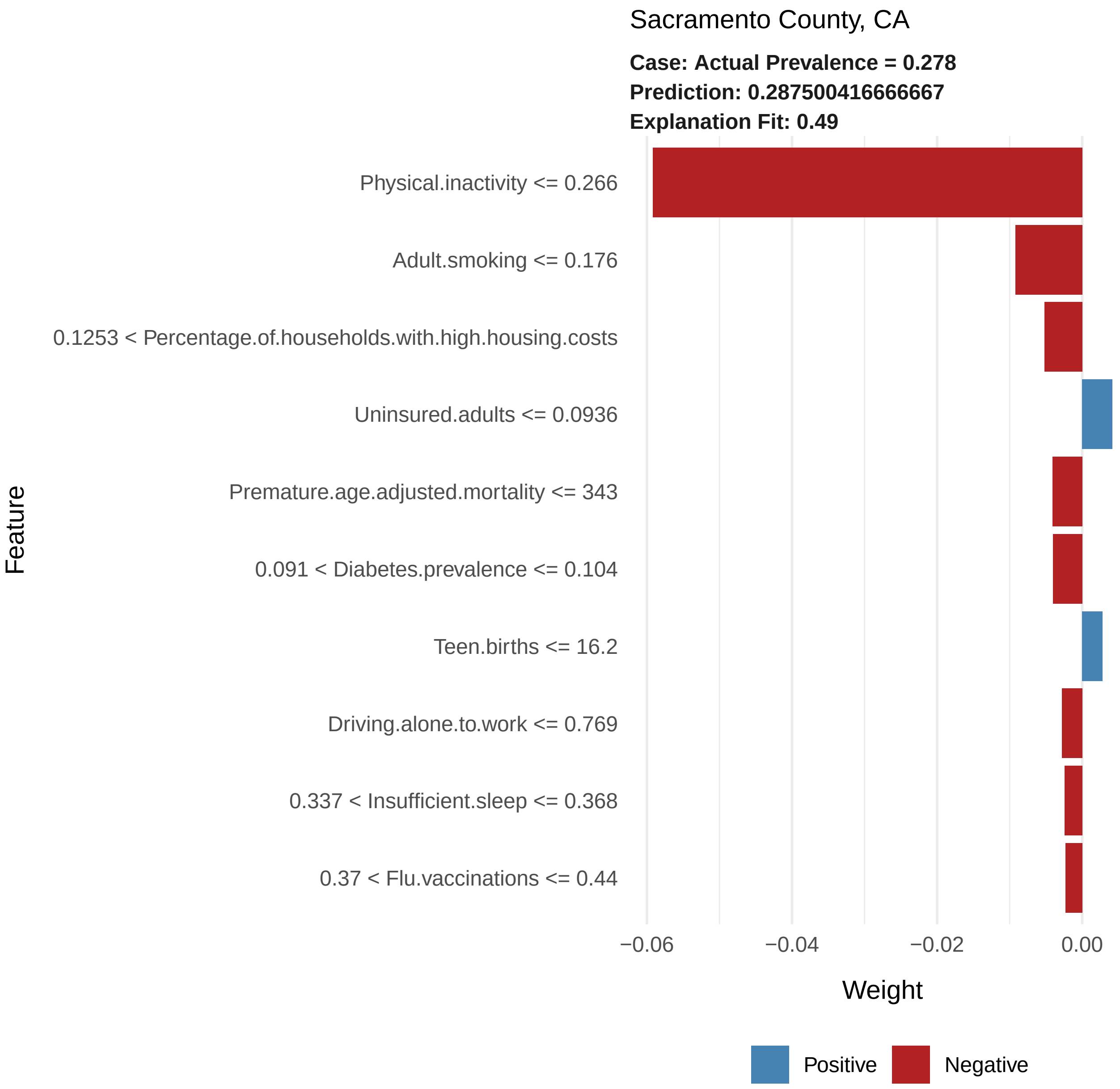

Sacramento County, CA
Case: Actual Prevalence = 0.278
Prediction: 0.287500416666667
Explanation Fit: 0.49
Feature
Physical.inactivity <= 0.266
Adult.smoking <= 0.176
0.1253 < Percentage.of.households.with.high.housing.costs
Uninsured.adults <= 0.0936
Premature.age.adjusted.mortality <= 343
0.091 < Diabetes.prevalence <= 0.104
Teen.births <= 16.2
Driving.alone.to.work <= 0.769
0.337 < Insufficient.sleep <= 0.368
0.37 < Flu.vaccinations <= 0.44
−0.06
−0.04
−0.02
0.00
Weight
Positive
Negative

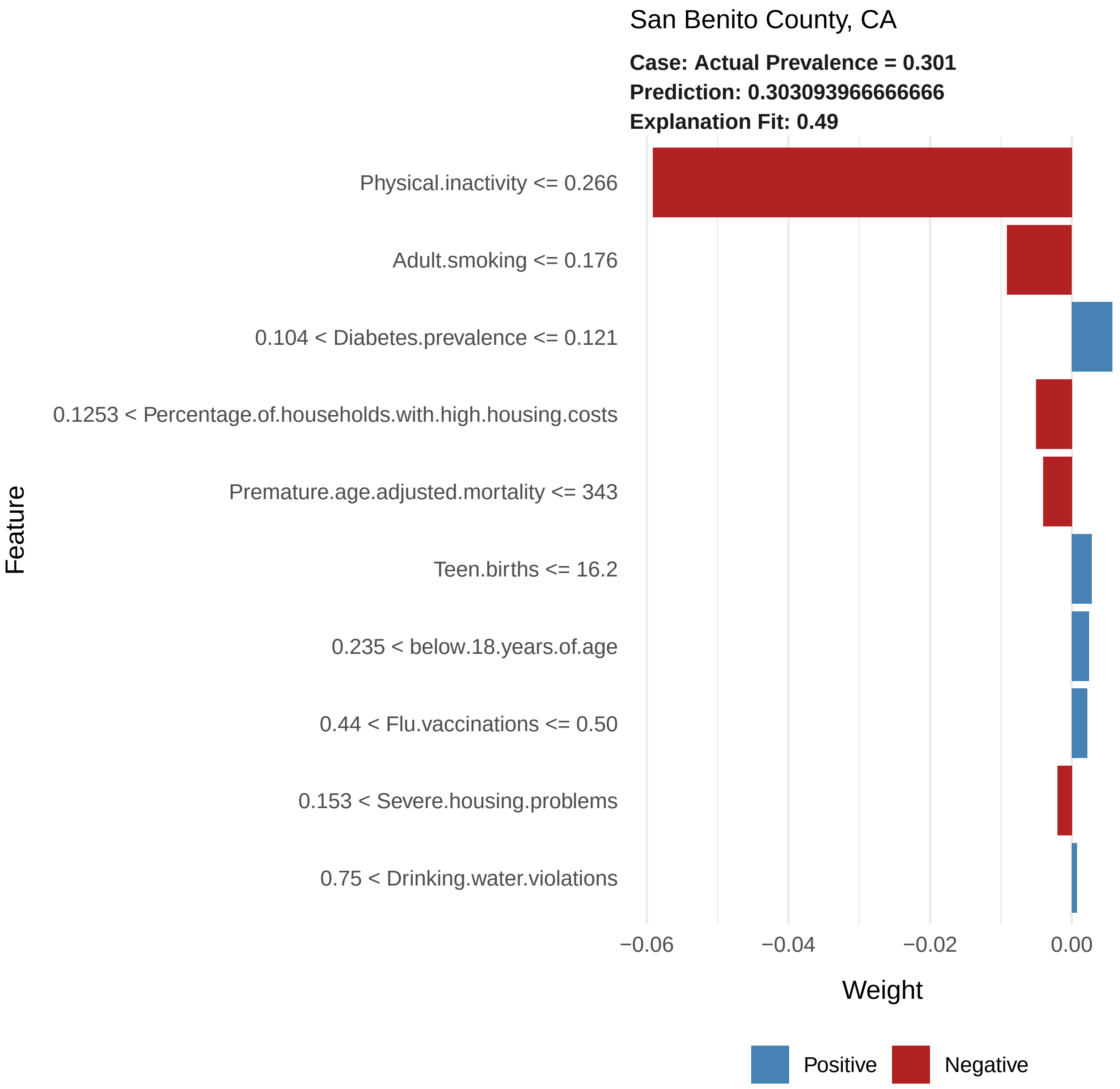
San Benito County, CA
Case: Actual Prevalence = 0.301
Prediction: 0.303093966666666
Explanation Fit: 0.49
Physical.inactivity <= 0.266
Adult.smoking <= 0.176
0.104 < Diabetes.prevalence <= 0.121
0.1253 < Percentage.of.households.with.high.housing.costs
Premature.age.adjusted.mortality <= 343
Teen.births <= 16.2
0.235 < below.18.years.of.age
0.44 < Flu.vaccinations <= 0.50
0.153 < Severe.housing.problems
0.75 < Drinking.water.violations
Feature
−0.06
−0.04
−0.02
0.00
Weight
Positive
Negative

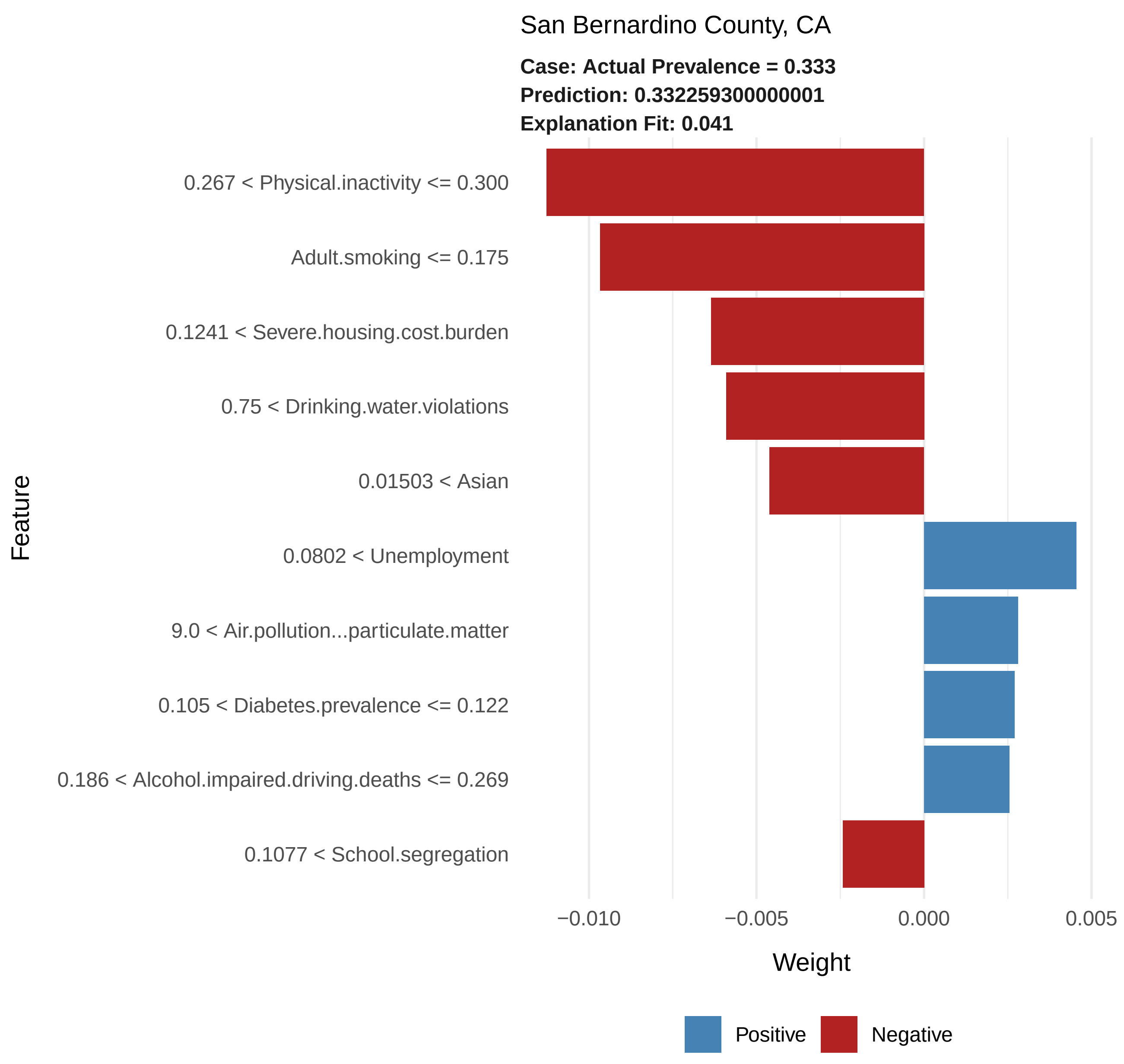

San Bernardino County, CA
Case: Actual Prevalence = 0.333
Prediction: 0.332259300000001
Explanation Fit: 0.041
0.267 < Physical.inactivity <= 0.300
Adult.smoking <= 0.175
0.1241 < Severe.housing.cost.burden
0.75 < Drinking.water.violations
0.01503 < Asian
0.0802 < Unemployment
9.0 < Air.pollution...particulate.matter
0.105 < Diabetes.prevalence <= 0.122
0.186 < Alcohol.impaired.driving.deaths <= 0.269
0.1077 < School.segregation
Feature
−0.010
−0.005
0.000
0.005
Weight
Positive
Negative

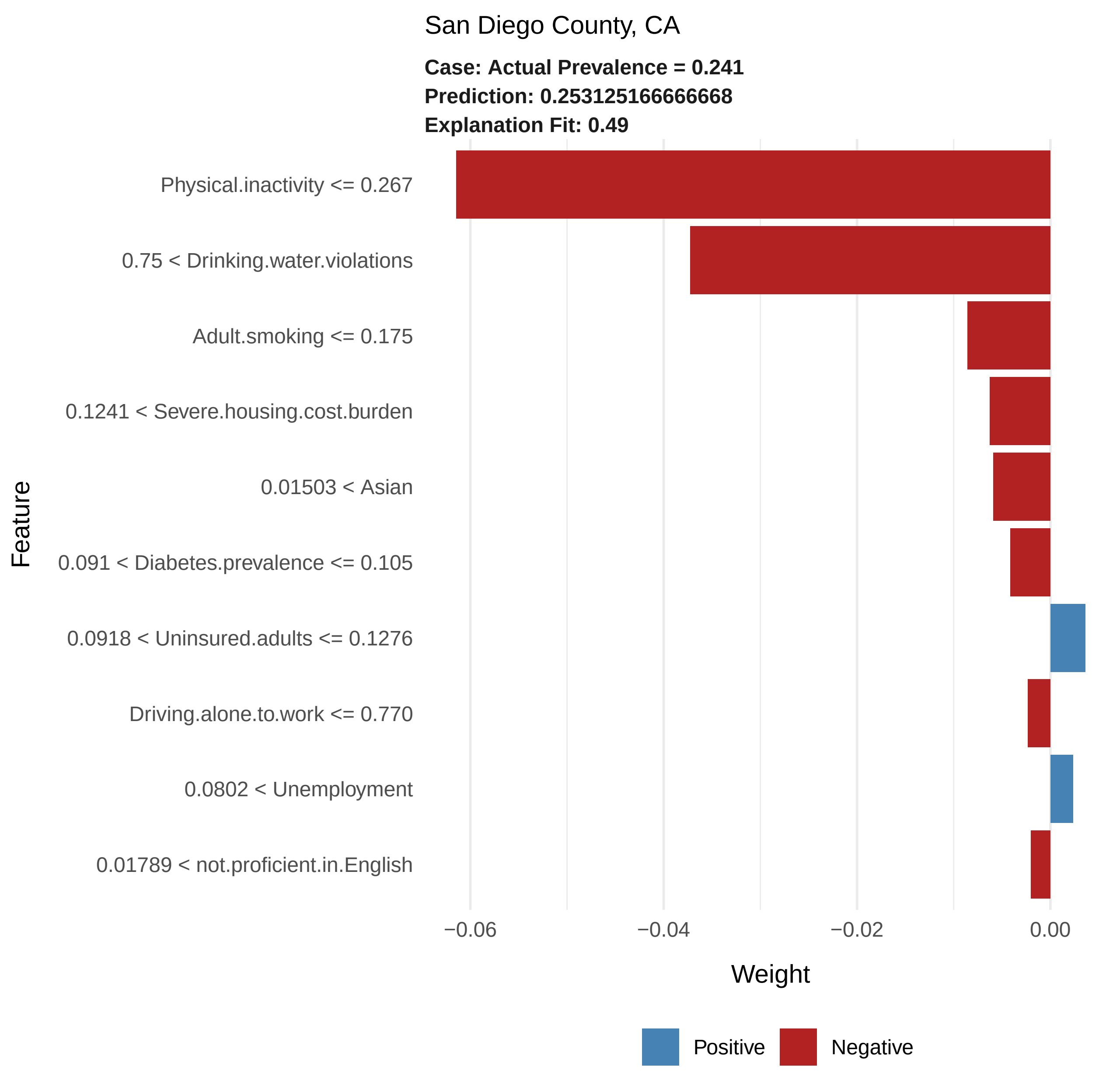
San Diego County, CA
Case: Actual Prevalence = 0.241
Prediction: 0.253125166666668
Explanation Fit: 0.49
Physical.inactivity <= 0.267
0.75 < Drinking.water.violations
Adult.smoking <= 0.175
0.1241 < Severe.housing.cost.burden
0.01503 < Asian
0.091 < Diabetes.prevalence <= 0.105
0.0918 < Uninsured.adults <= 0.1276
Driving.alone.to.work <= 0.770
0.0802 < Unemployment
0.01789 < not.proficient.in.English
Feature
−0.06
−0.04
−0.02
0.00
Weight
Positive
Negative

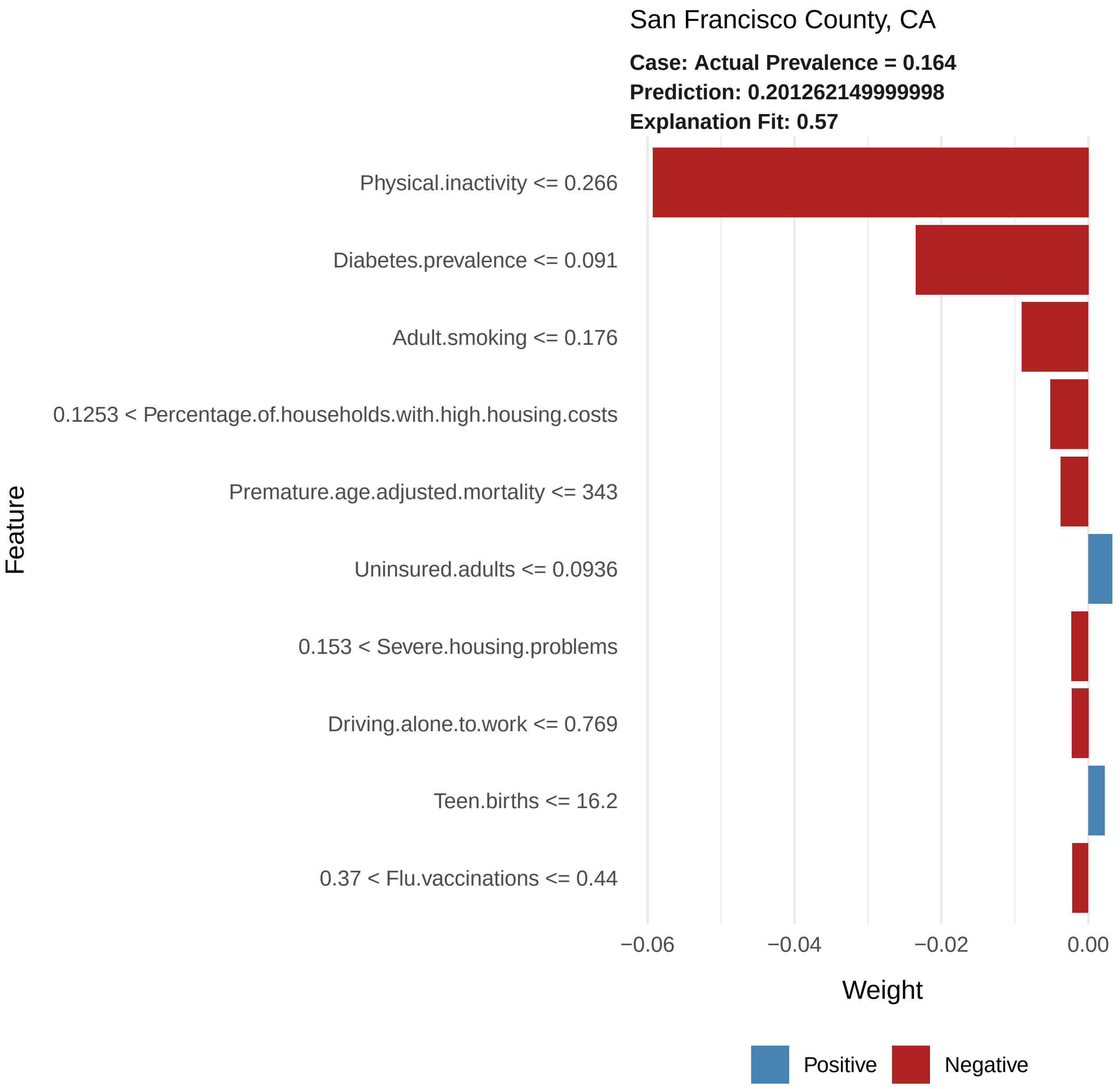
San Francisco County, CA
Case: Actual Prevalence = 0.164
Prediction: 0.201262149999998
Explanation Fit: 0.57
Physical.inactivity <= 0.266
Diabetes.prevalence <= 0.091
Adult.smoking <= 0.176
0.1253 < Percentage.of.households.with.high.housing.costs
Premature.age.adjusted.mortality <= 343
Uninsured.adults <= 0.0936
0.153 < Severe.housing.problems
Driving.alone.to.work <= 0.769
Teen.births <= 16.2
0.37 < Flu.vaccinations <= 0.44
Feature
−0.06
−0.04
−0.02
0.00
Weight
Positive
Negative

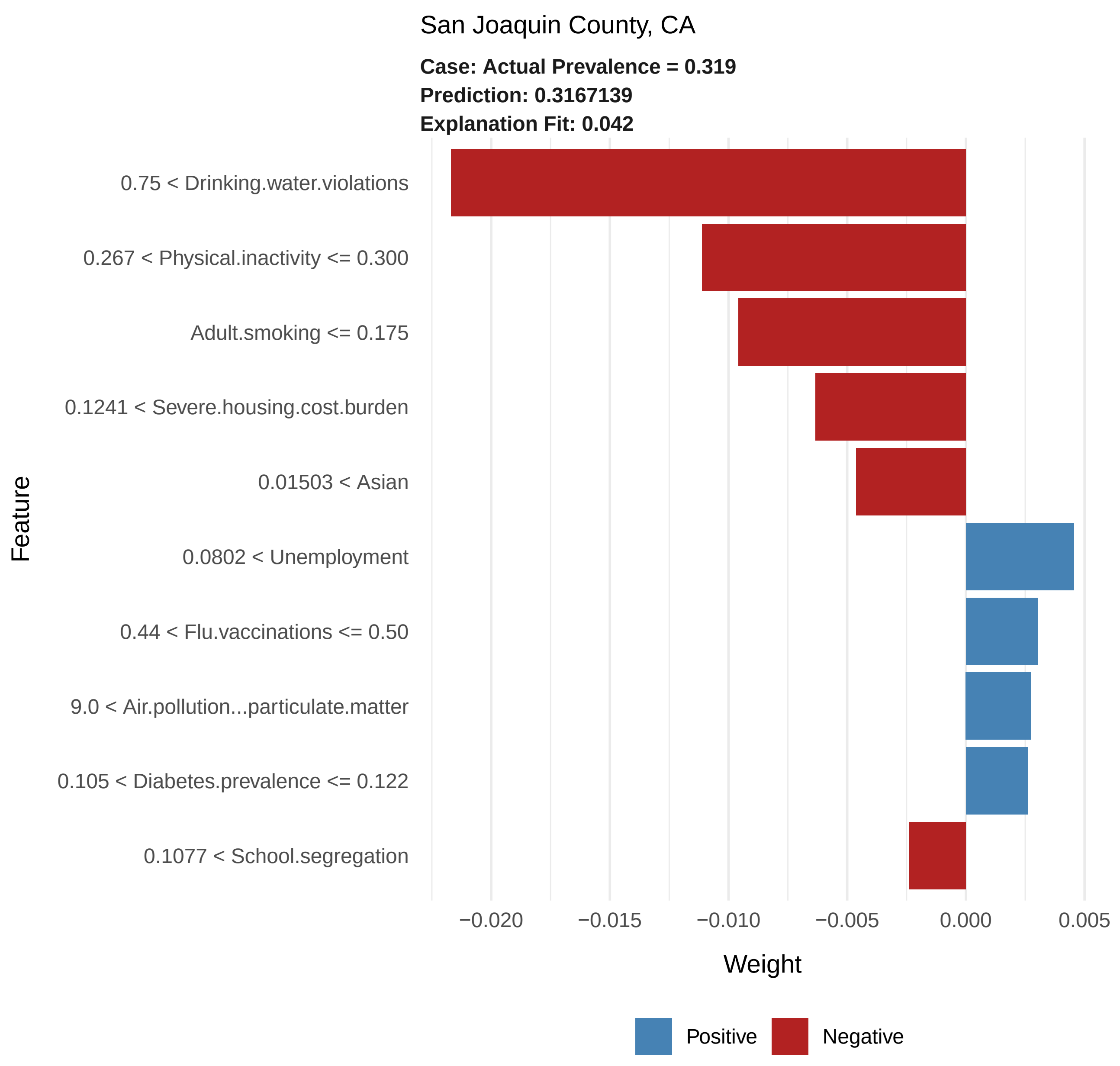

San Joaquin County, CA
Case: Actual Prevalence = 0.319
Prediction: 0.3167139
Explanation Fit: 0.042
0.75 < Drinking.water.violations
0.267 < Physical.inactivity <= 0.300
Adult.smoking <= 0.175
0.1241 < Severe.housing.cost.burden
0.01503 < Asian
0.0802 < Unemployment
0.44 < Flu.vaccinations <= 0.50
9.0 < Air.pollution...particulate.matter
0.105 < Diabetes.prevalence <= 0.122
0.1077 < School.segregation
Feature
−0.020
−0.015
−0.010
−0.005
0.000
0.005
Weight
Positive
Negative

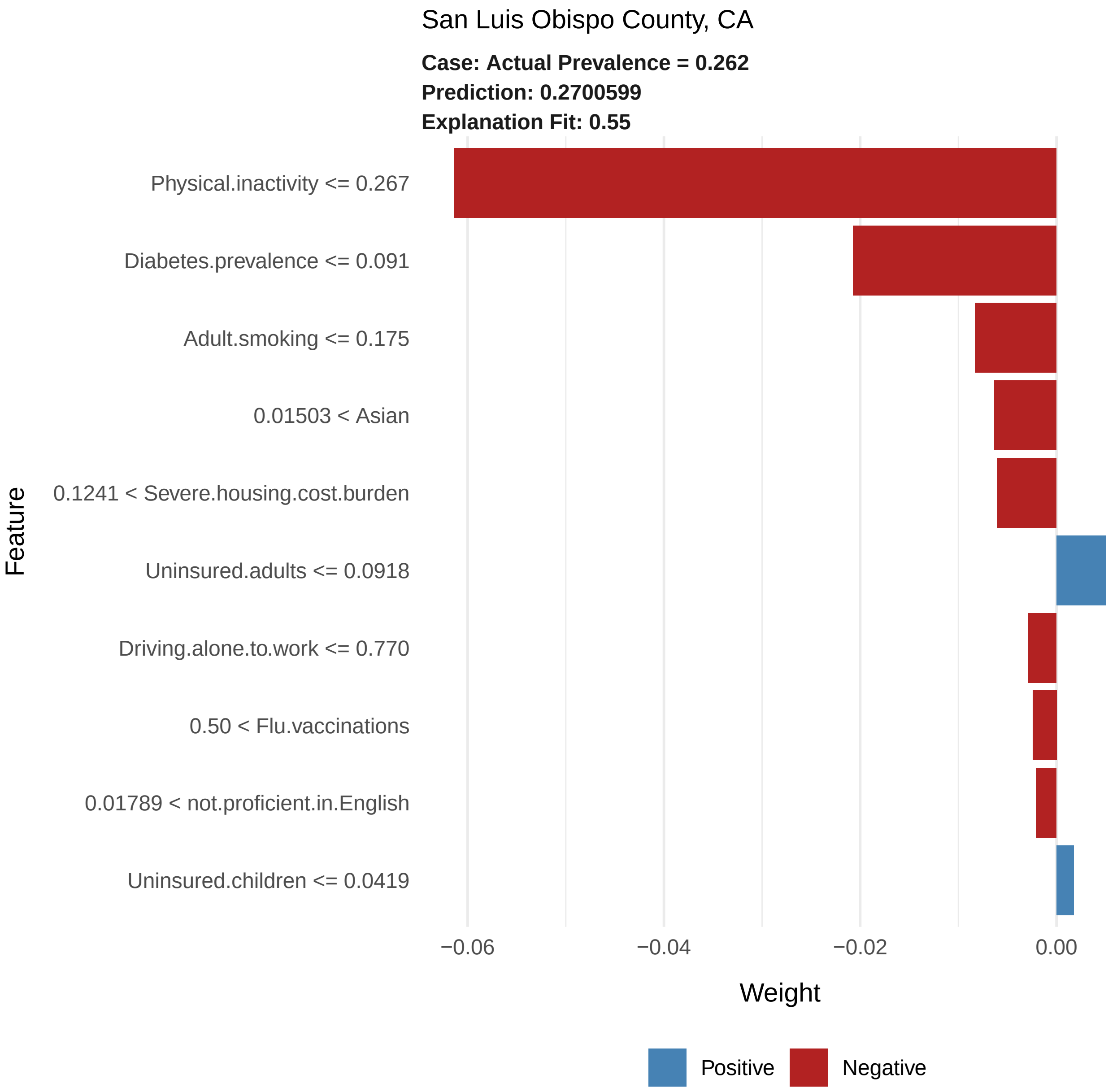
San Luis Obispo County, CA
Case: Actual Prevalence = 0.262
Prediction: 0.2700599
Explanation Fit: 0.55
Feature
Physical.inactivity <= 0.267
Diabetes.prevalence <= 0.091
Adult.smoking <= 0.175
0.01503 < Asian
0.1241 < Severe.housing.cost.burden
Uninsured.adults <= 0.0918
Driving.alone.to.work <= 0.770
0.50 < Flu.vaccinations
0.01789 < not.proficient.in.English
Uninsured.children <= 0.0419
−0.06
−0.04
−0.02
0.00
Weight
Positive
Negative

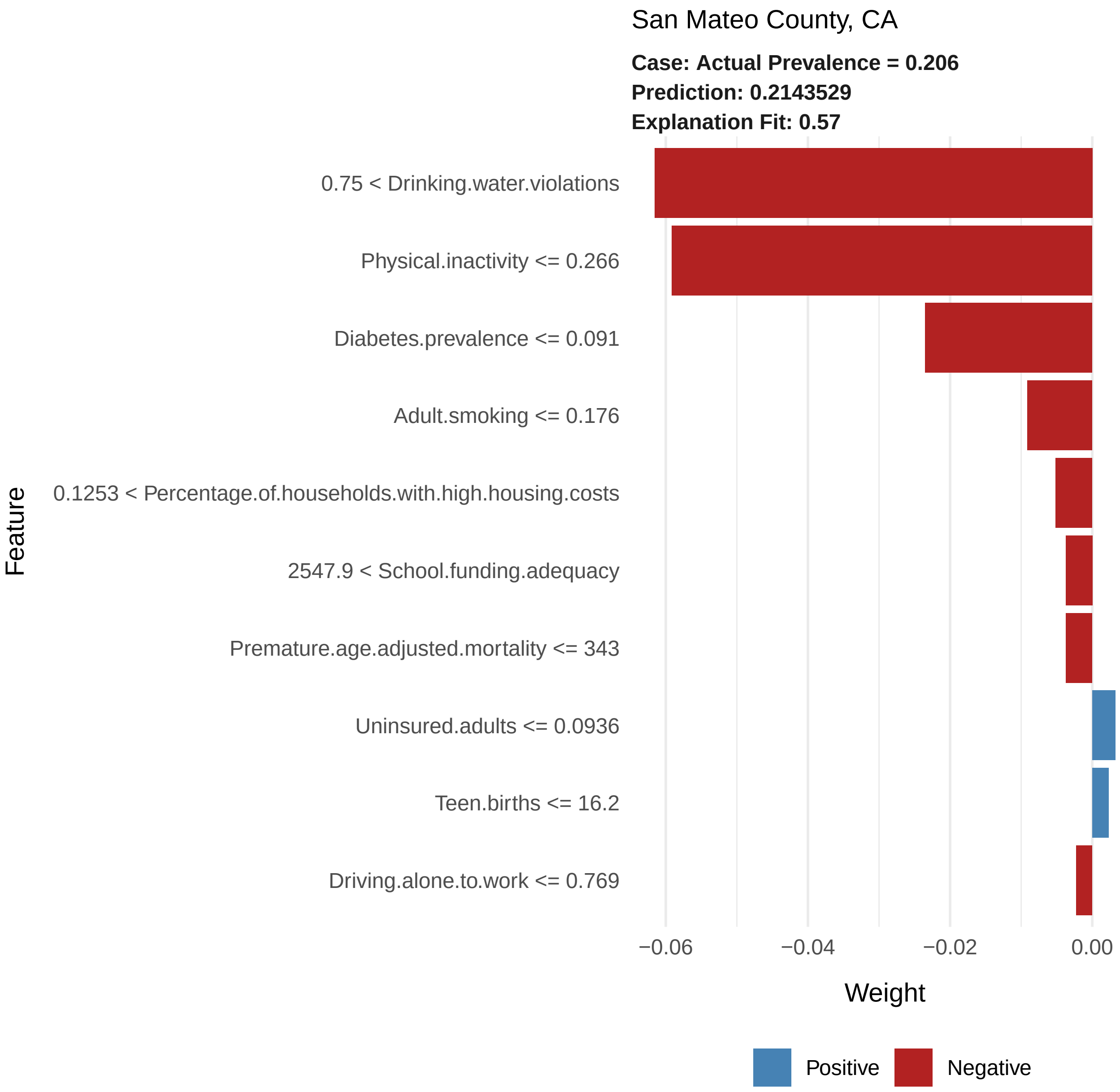

San Mateo County, CA
Case: Actual Prevalence = 0.206
Prediction: 0.2143529
Explanation Fit: 0.57
0.75 < Drinking.water.violations
Physical.inactivity <= 0.266
Diabetes.prevalence <= 0.091
Adult.smoking <= 0.176
0.1253 < Percentage.of.households.with.high.housing.costs
2547.9 < School.funding.adequacy
Premature.age.adjusted.mortality <= 343
Uninsured.adults <= 0.0936
Teen.births <= 16.2
Driving.alone.to.work <= 0.769
Feature
−0.06
−0.04
−0.02
0.00
Weight
Positive
Negative

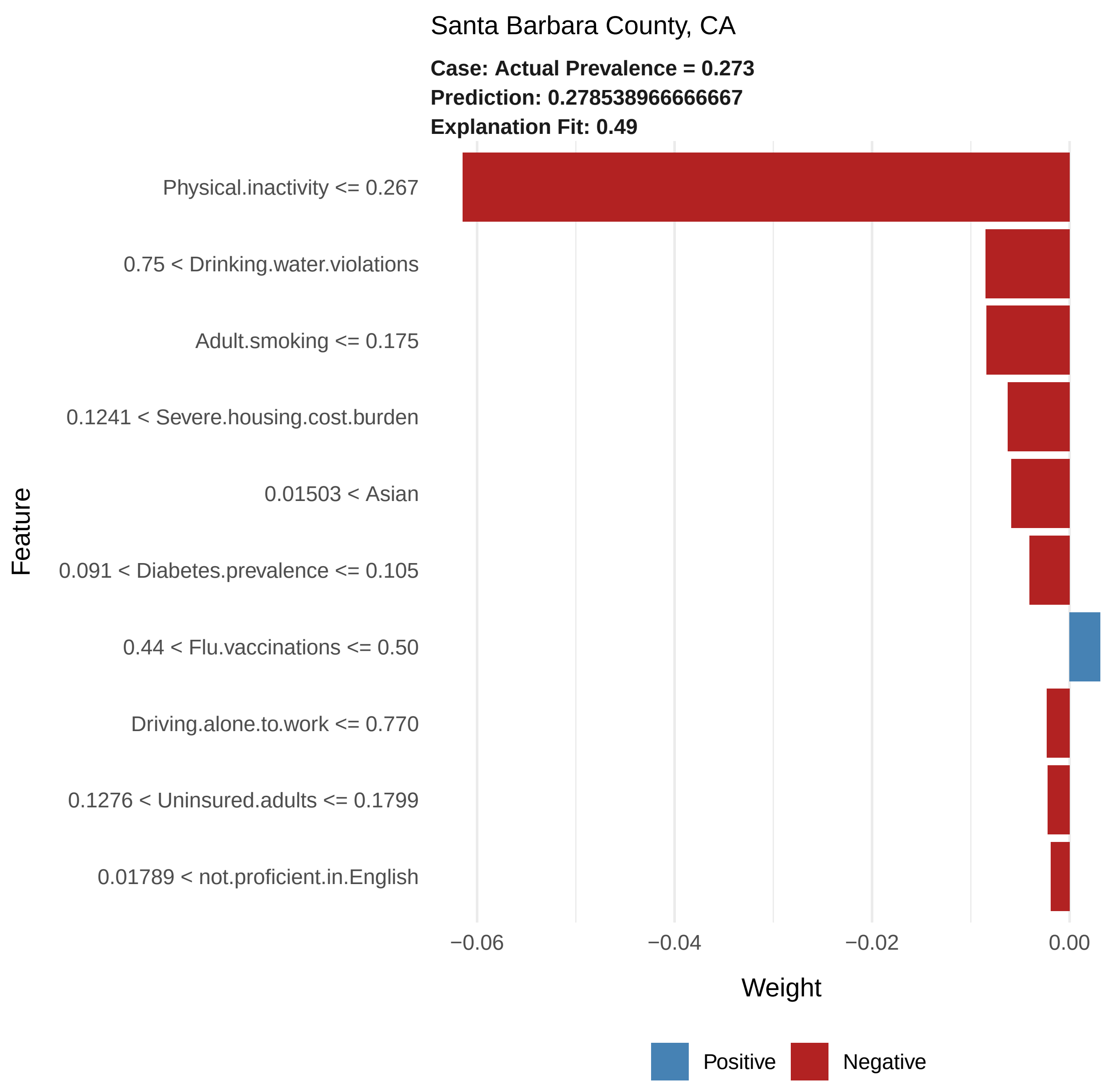

Santa Barbara County, CA
Case: Actual Prevalence = 0.273
Prediction: 0.278538966666667
Explanation Fit: 0.49
Physical.inactivity <= 0.267
0.75 < Drinking.water.violations
Adult.smoking <= 0.175
0.1241 < Severe.housing.cost.burden
0.01503 < Asian
0.091 < Diabetes.prevalence <= 0.105
0.44 < Flu.vaccinations <= 0.50
Driving.alone.to.work <= 0.770
0.1276 < Uninsured.adults <= 0.1799
0.01789 < not.proficient.in.English
Feature
−0.06
−0.04
−0.02
0.00
Weight
Positive
Negative

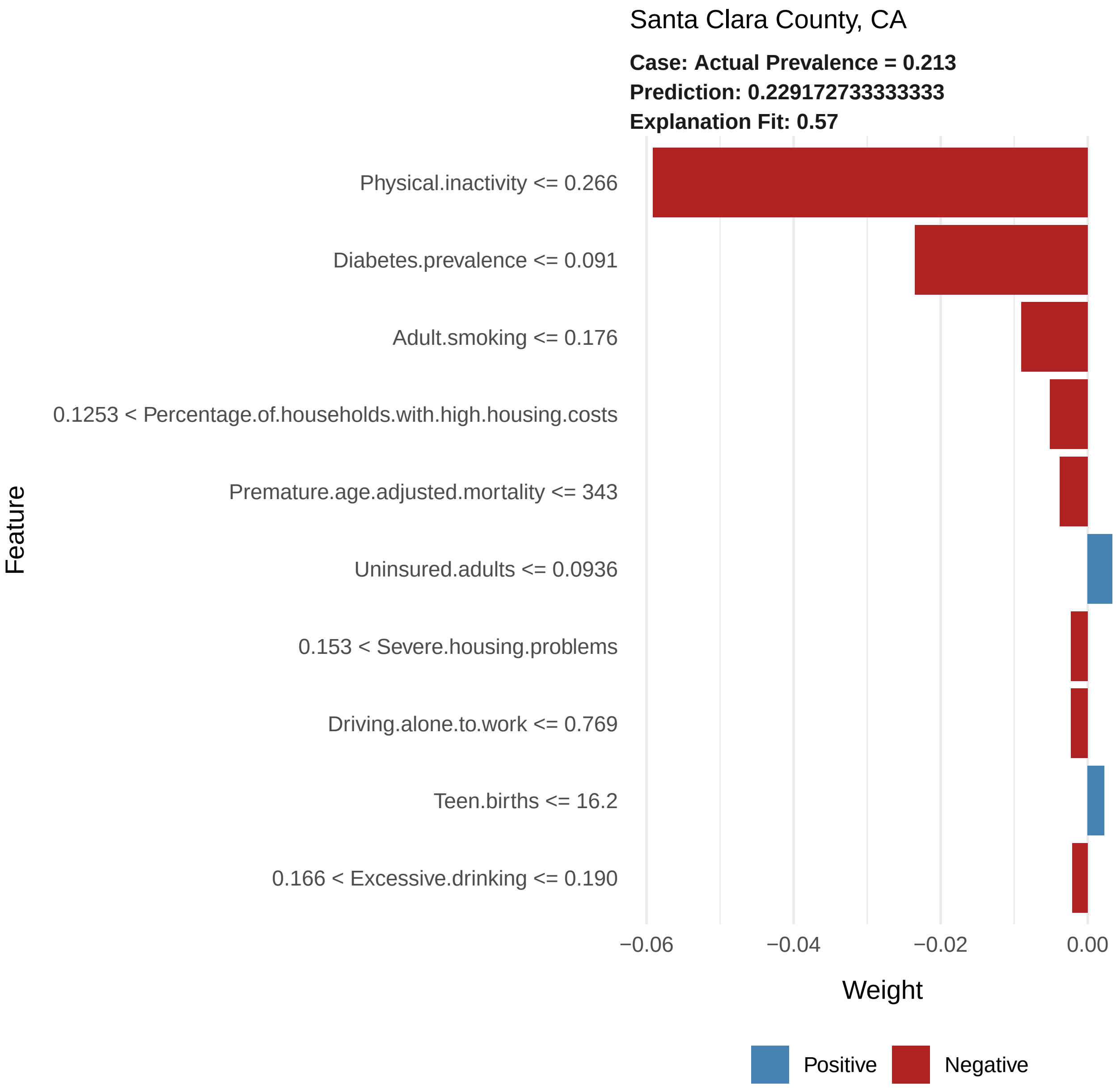

Santa Clara County, CA
Case: Actual Prevalence = 0.213
Prediction: 0.229172733333333
Explanation Fit: 0.57
Physical.inactivity <= 0.266
Diabetes.prevalence <= 0.091
Adult.smoking <= 0.176
0.1253 < Percentage.of.households.with.high.housing.costs
Premature.age.adjusted.mortality <= 343
Uninsured.adults <= 0.0936
0.153 < Severe.housing.problems
Driving.alone.to.work <= 0.769
Teen.births <= 16.2
0.166 < Excessive.drinking <= 0.190
Feature
−0.06
−0.04
−0.02
0.00
Weight
Positive
Negative

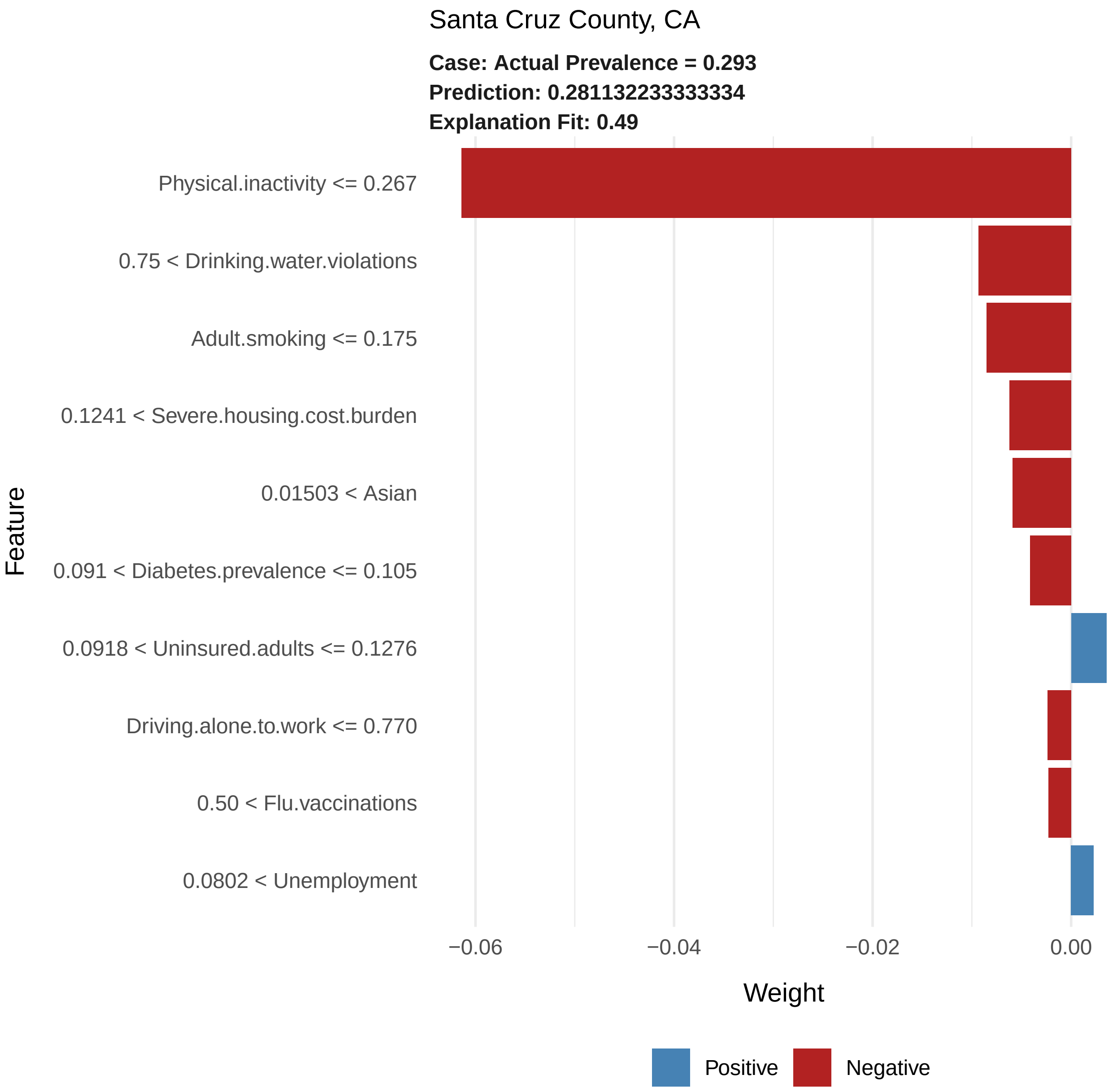

Santa Cruz County, CA
Case: Actual Prevalence = 0.293
Prediction: 0.281132233333334
Explanation Fit: 0.49
Physical.inactivity <= 0.267
0.75 < Drinking.water.violations
Adult.smoking <= 0.175
0.1241 < Severe.housing.cost.burden
0.01503 < Asian
0.091 < Diabetes.prevalence <= 0.105
0.0918 < Uninsured.adults <= 0.1276
Driving.alone.to.work <= 0.770
0.50 < Flu.vaccinations
0.0802 < Unemployment
Feature
−0.06
−0.04
−0.02
0.00
Weight
Positive
Negative

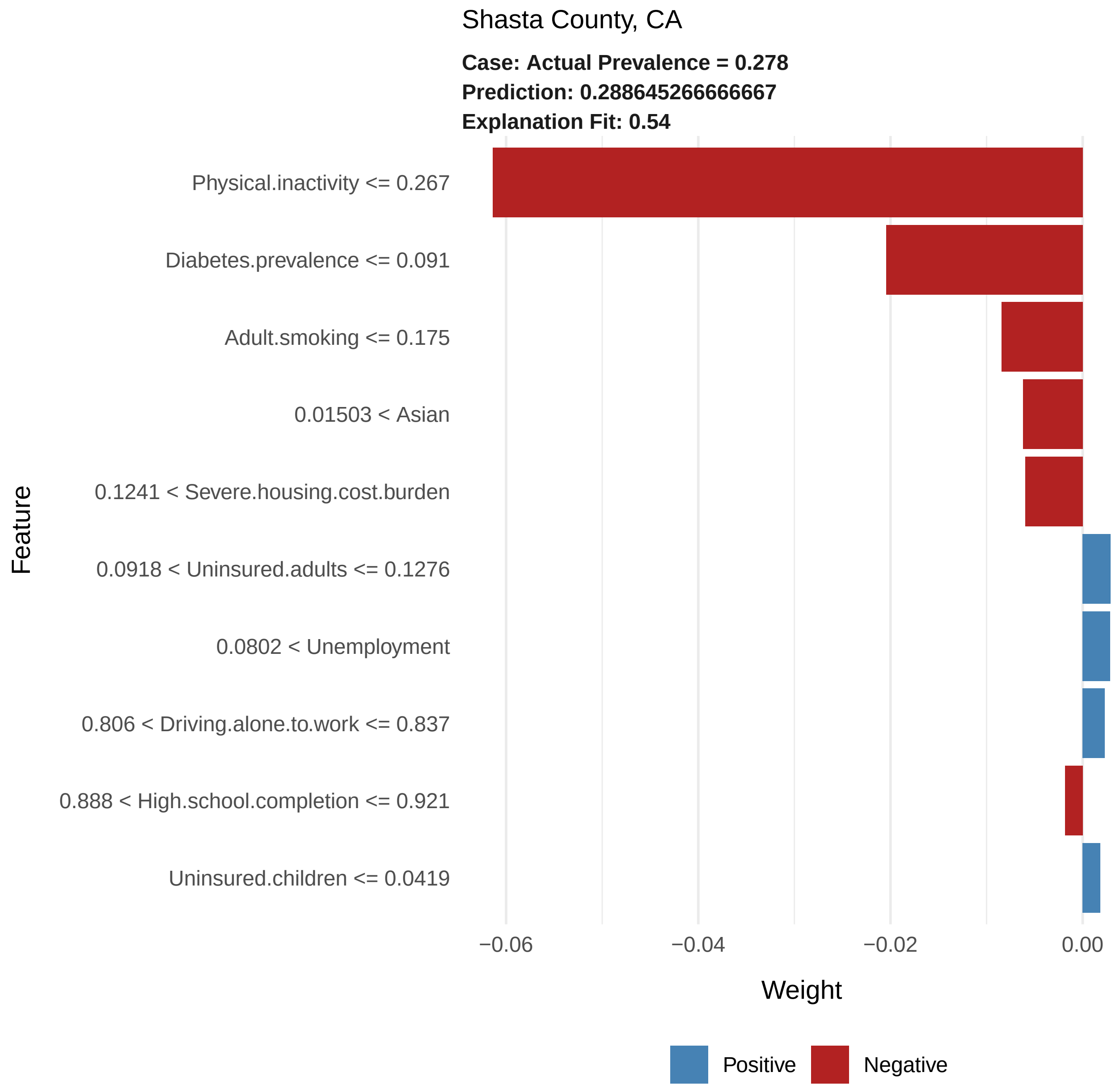
Shasta County, CA
Case: Actual Prevalence = 0.278
Prediction: 0.288645266666667
Explanation Fit: 0.54
Physical.inactivity <= 0.267
Diabetes.prevalence <= 0.091
Adult.smoking <= 0.175
0.01503 < Asian
0.1241 < Severe.housing.cost.burden
0.0918 < Uninsured.adults <= 0.1276
0.0802 < Unemployment
0.806 < Driving.alone.to.work <= 0.837
0.888 < High.school.completion <= 0.921
Uninsured.children <= 0.0419
Feature
−0.06
−0.04
−0.02
0.00
Weight
Positive
Negative

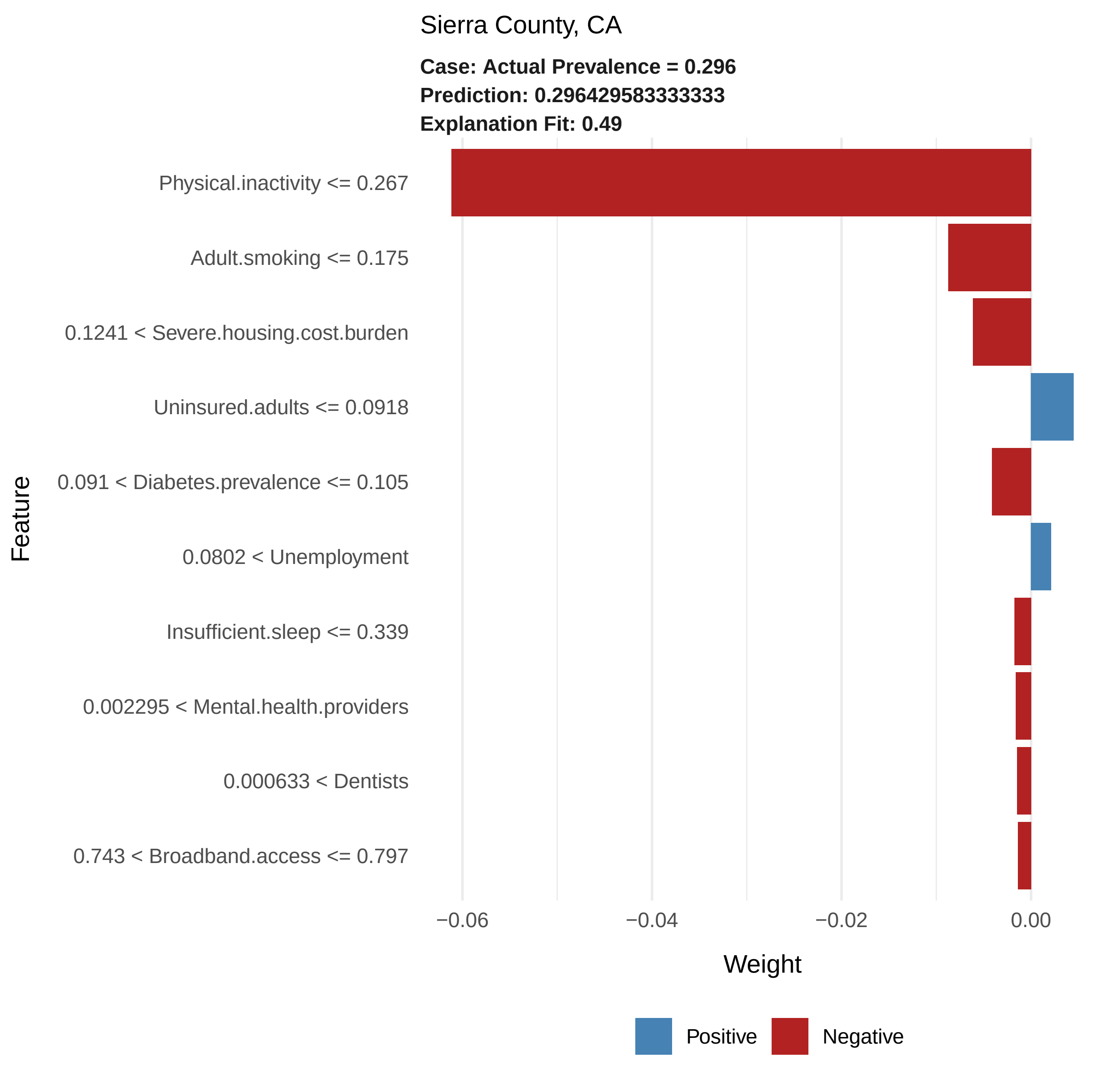
Sierra County, CA
Case: Actual Prevalence = 0.296
Prediction: 0.296429583333333
Explanation Fit: 0.49
Physical.inactivity <= 0.267
Adult.smoking <= 0.175
0.1241 < Severe.housing.cost.burden
Uninsured.adults <= 0.0918
0.091 < Diabetes.prevalence <= 0.105
0.0802 < Unemployment
Insufficient.sleep <= 0.339
0.002295 < Mental.health.providers
0.000633 < Dentists
0.743 < Broadband.access <= 0.797
Feature
−0.06
−0.04
−0.02
0.00
Weight
Positive
Negative

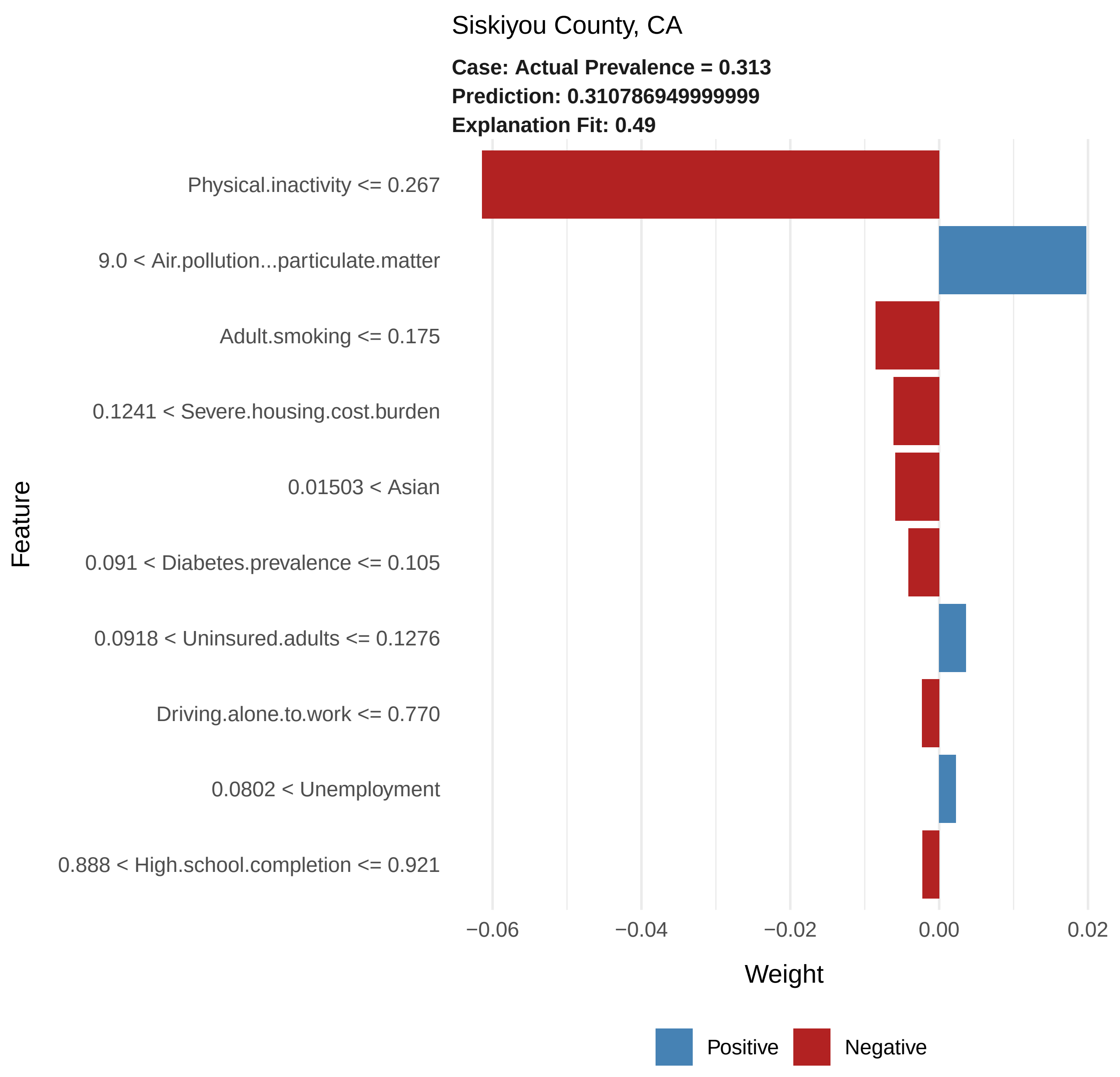

Siskiyou County, CA
Case: Actual Prevalence = 0.313
Prediction: 0.310786949999999
Explanation Fit: 0.49
Physical.inactivity <= 0.267
9.0 < Air.pollution...particulate.matter
Adult.smoking <= 0.175
0.1241 < Severe.housing.cost.burden
0.01503 < Asian
0.091 < Diabetes.prevalence <= 0.105
0.0918 < Uninsured.adults <= 0.1276
Driving.alone.to.work <= 0.770
0.0802 < Unemployment
0.888 < High.school.completion <= 0.921
Feature
−0.06
−0.04
−0.02
0.00
0.02
Weight
Positive
Negative

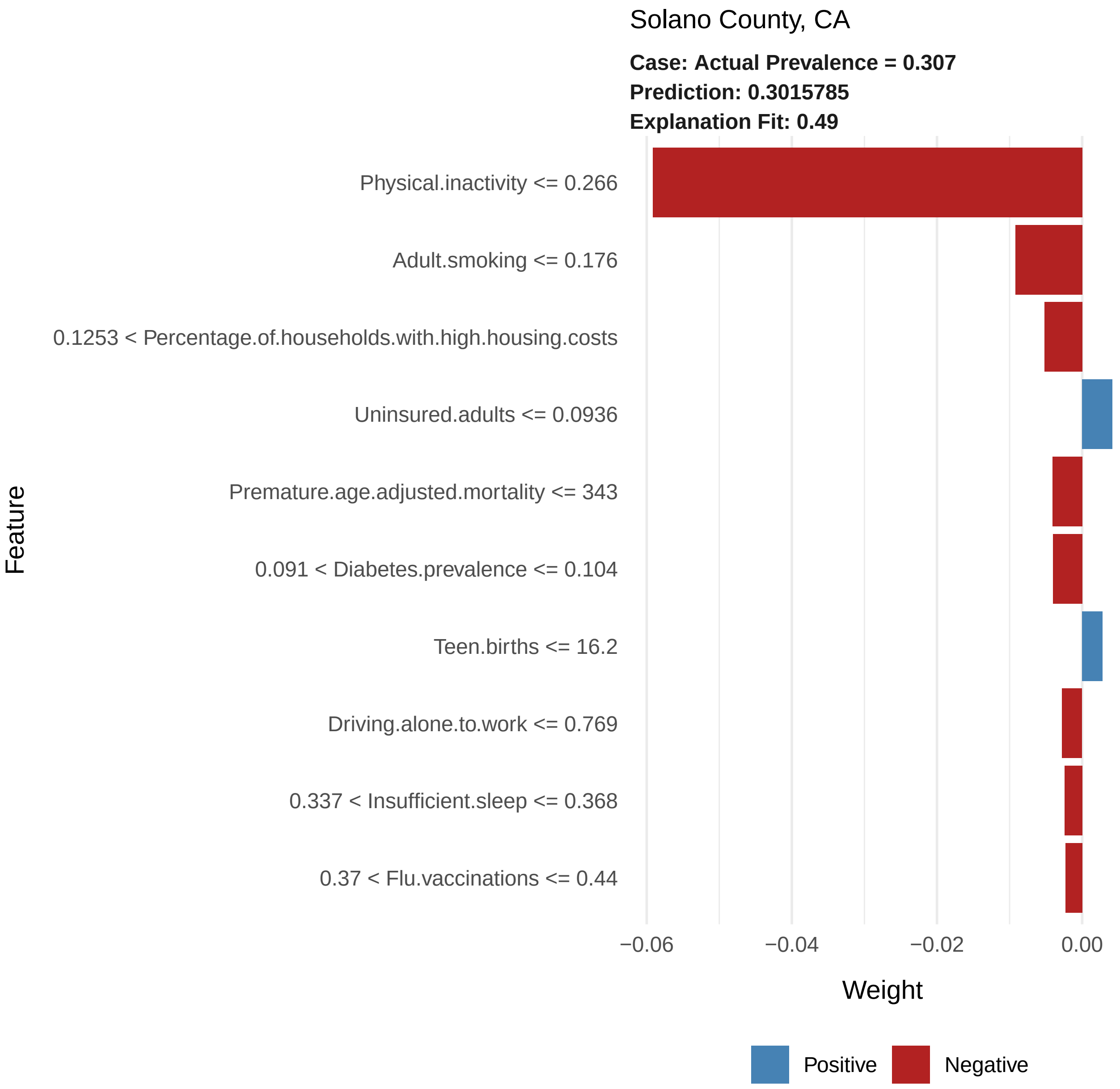

Solano County, CA
Case: Actual Prevalence = 0.307
Prediction: 0.3015785
Explanation Fit: 0.49
Physical.inactivity <= 0.266
Adult.smoking <= 0.176
0.1253 < Percentage.of.households.with.high.housing.costs
Uninsured.adults <= 0.0936
Premature.age.adjusted.mortality <= 343
0.091 < Diabetes.prevalence <= 0.104
Teen.births <= 16.2
Driving.alone.to.work <= 0.769
0.337 < Insufficient.sleep <= 0.368
0.37 < Flu.vaccinations <= 0.44
Feature
−0.06
−0.04
−0.02
0.00
Weight
Positive
Negative

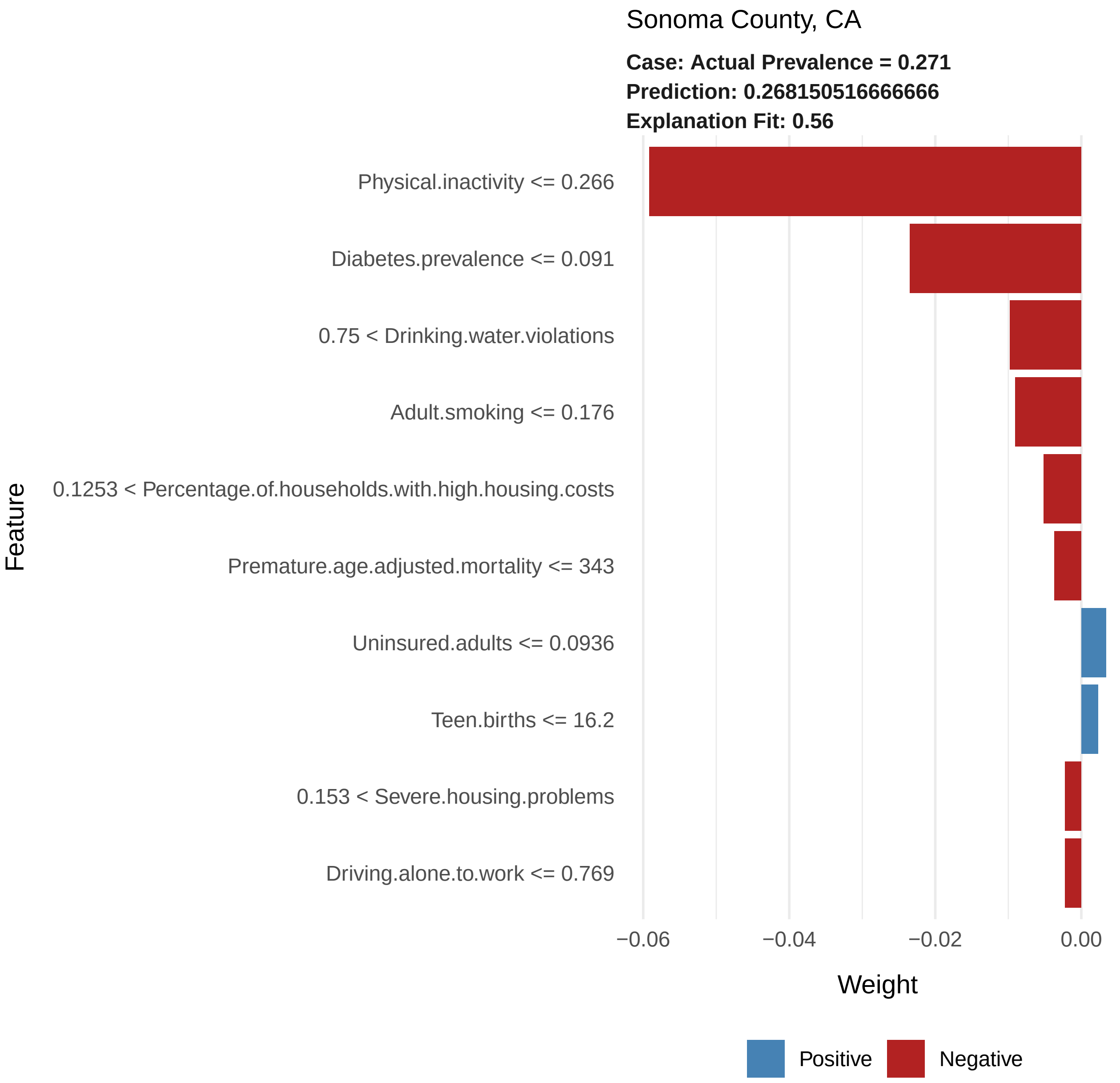

Sonoma County, CA
Case: Actual Prevalence = 0.271
Prediction: 0.268150516666666
Explanation Fit: 0.56
Physical.inactivity <= 0.266
Diabetes.prevalence <= 0.091
0.75 < Drinking.water.violations
Adult.smoking <= 0.176
0.1253 < Percentage.of.households.with.high.housing.costs
Premature.age.adjusted.mortality <= 343
Uninsured.adults <= 0.0936
Teen.births <= 16.2
0.153 < Severe.housing.problems
Driving.alone.to.work <= 0.769
Feature
−0.06
−0.04
−0.02
0.00
Weight
Positive
Negative

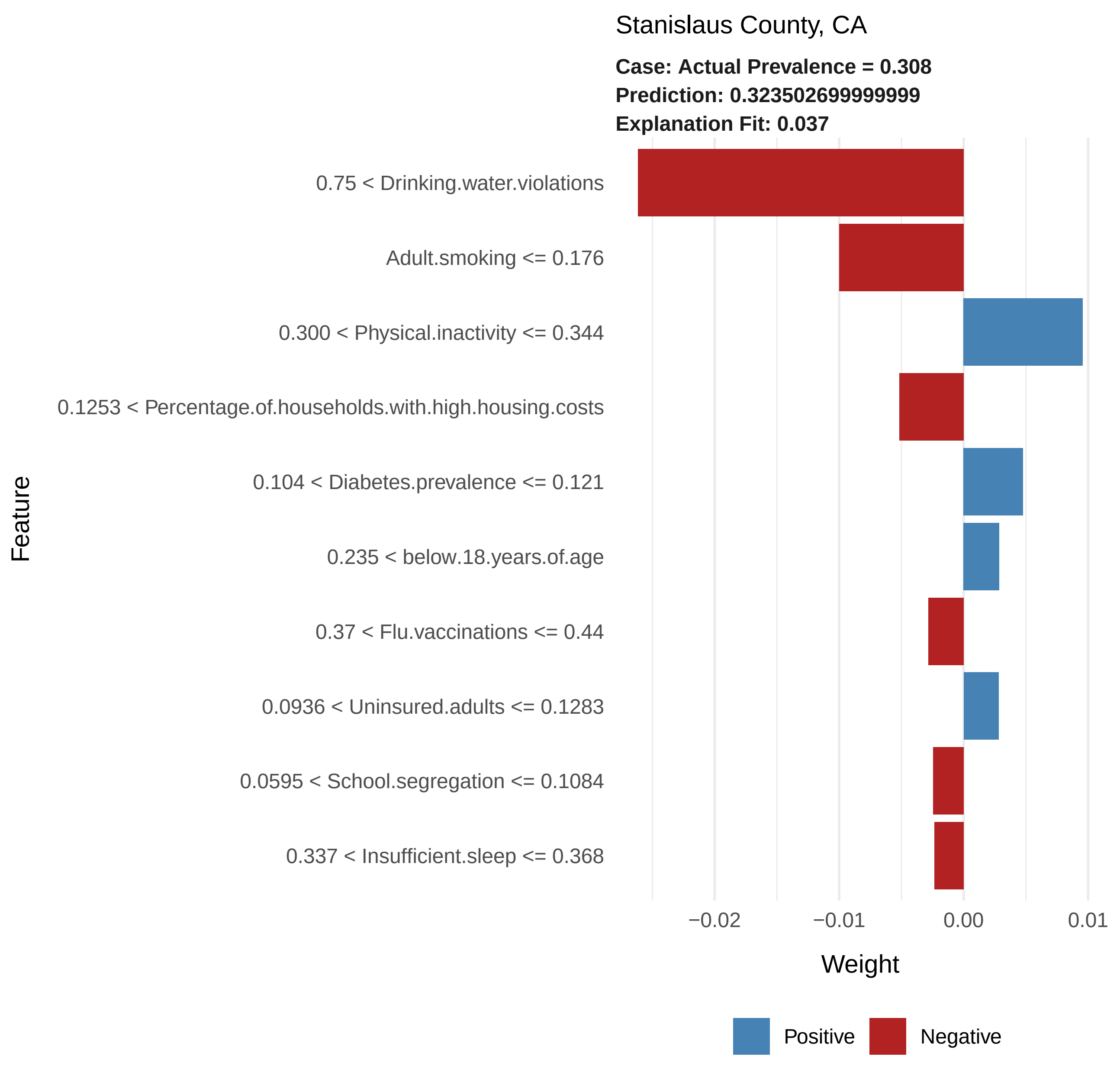

Stanislaus County, CA
Case: Actual Prevalence = 0.308
Prediction: 0.323502699999999
Explanation Fit: 0.037
0.75 < Drinking.water.violations
Adult.smoking <= 0.176
0.300 < Physical.inactivity <= 0.344
0.1253 < Percentage.of.households.with.high.housing.costs
0.104 < Diabetes.prevalence <= 0.121
0.235 < below.18.years.of.age
0.37 < Flu.vaccinations <= 0.44
0.0936 < Uninsured.adults <= 0.1283
0.0595 < School.segregation <= 0.1084
0.337 < Insufficient.sleep <= 0.368
Feature
−0.02
−0.01
0.00
0.01
Weight
Positive
Negative

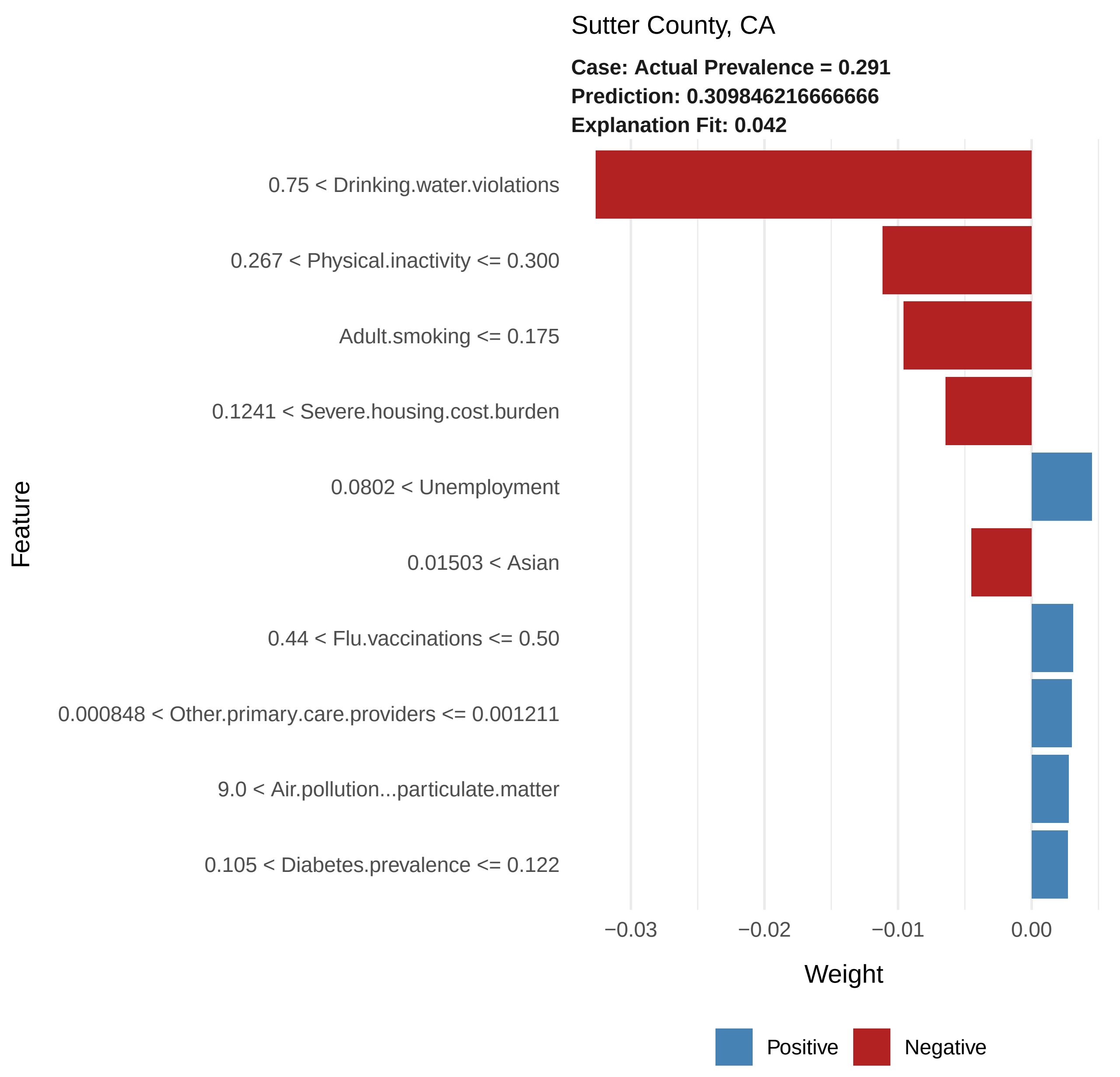

Sutter County, CA
Case: Actual Prevalence = 0.291
Prediction: 0.309846216666666
Explanation Fit: 0.042
0.75 < Drinking.water.violations
0.267 < Physical.inactivity <= 0.300
Adult.smoking <= 0.175
0.1241 < Severe.housing.cost.burden
0.0802 < Unemployment
0.01503 < Asian
0.44 < Flu.vaccinations <= 0.50
0.000848 < Other.primary.care.providers <= 0.001211
9.0 < Air.pollution...particulate.matter
0.105 < Diabetes.prevalence <= 0.122
Feature
−0.03
−0.02
−0.01
0.00
Weight
Positive
Negative

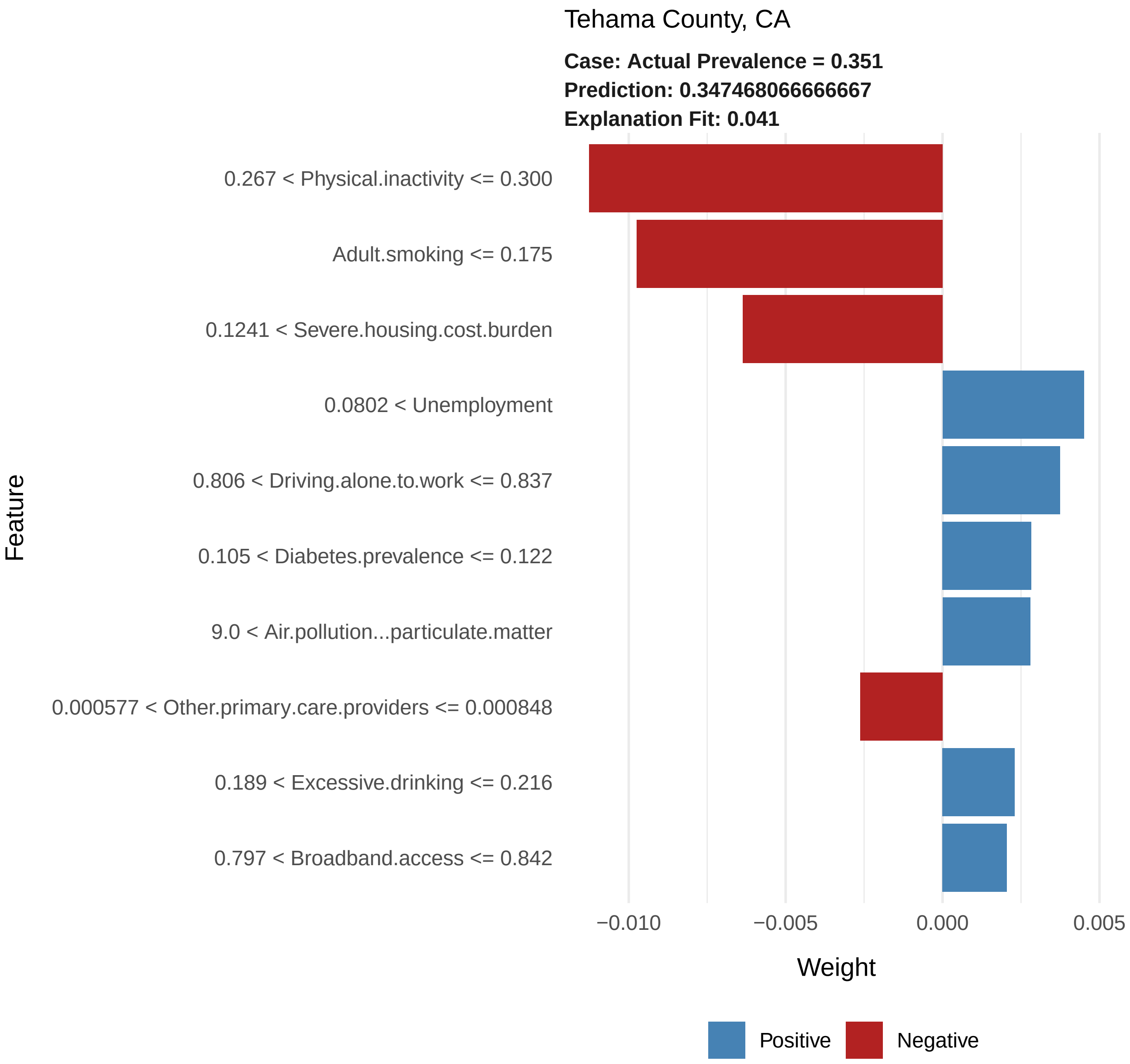
Tehama County, CA
Case: Actual Prevalence = 0.351
Prediction: 0.347468066666667
Explanation Fit: 0.041
0.267 < Physical.inactivity <= 0.300
Adult.smoking <= 0.175
0.1241 < Severe.housing.cost.burden
0.0802 < Unemployment
0.806 < Driving.alone.to.work <= 0.837
0.105 < Diabetes.prevalence <= 0.122
9.0 < Air.pollution...particulate.matter
0.000577 < Other.primary.care.providers <= 0.000848
0.189 < Excessive.drinking <= 0.216
0.797 < Broadband.access <= 0.842
Feature
−0.010
−0.005
0.000
0.005
Weight
Positive
Negative

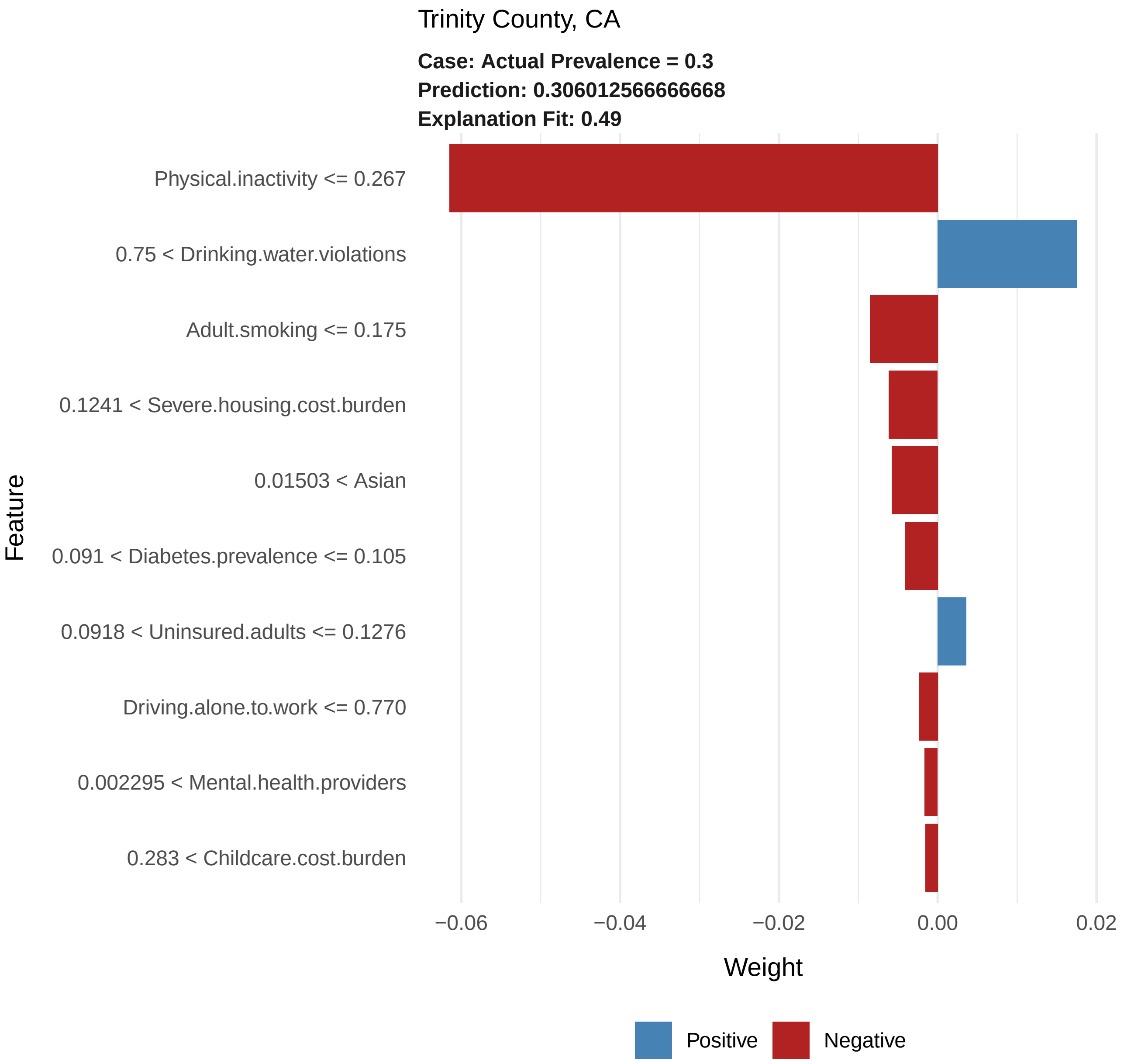

Trinity County, CA
Case: Actual Prevalence = 0.3
Prediction: 0.306012566666668
Explanation Fit: 0.49
Physical.inactivity <= 0.267
0.75 < Drinking.water.violations
Adult.smoking <= 0.175
0.1241 < Severe.housing.cost.burden
0.01503 < Asian
0.091 < Diabetes.prevalence <= 0.105
0.0918 < Uninsured.adults <= 0.1276
Driving.alone.to.work <= 0.770
0.002295 < Mental.health.providers
0.283 < Childcare.cost.burden
Feature
−0.06
−0.04
−0.02
0.00
0.02
Weight
Positive
Negative

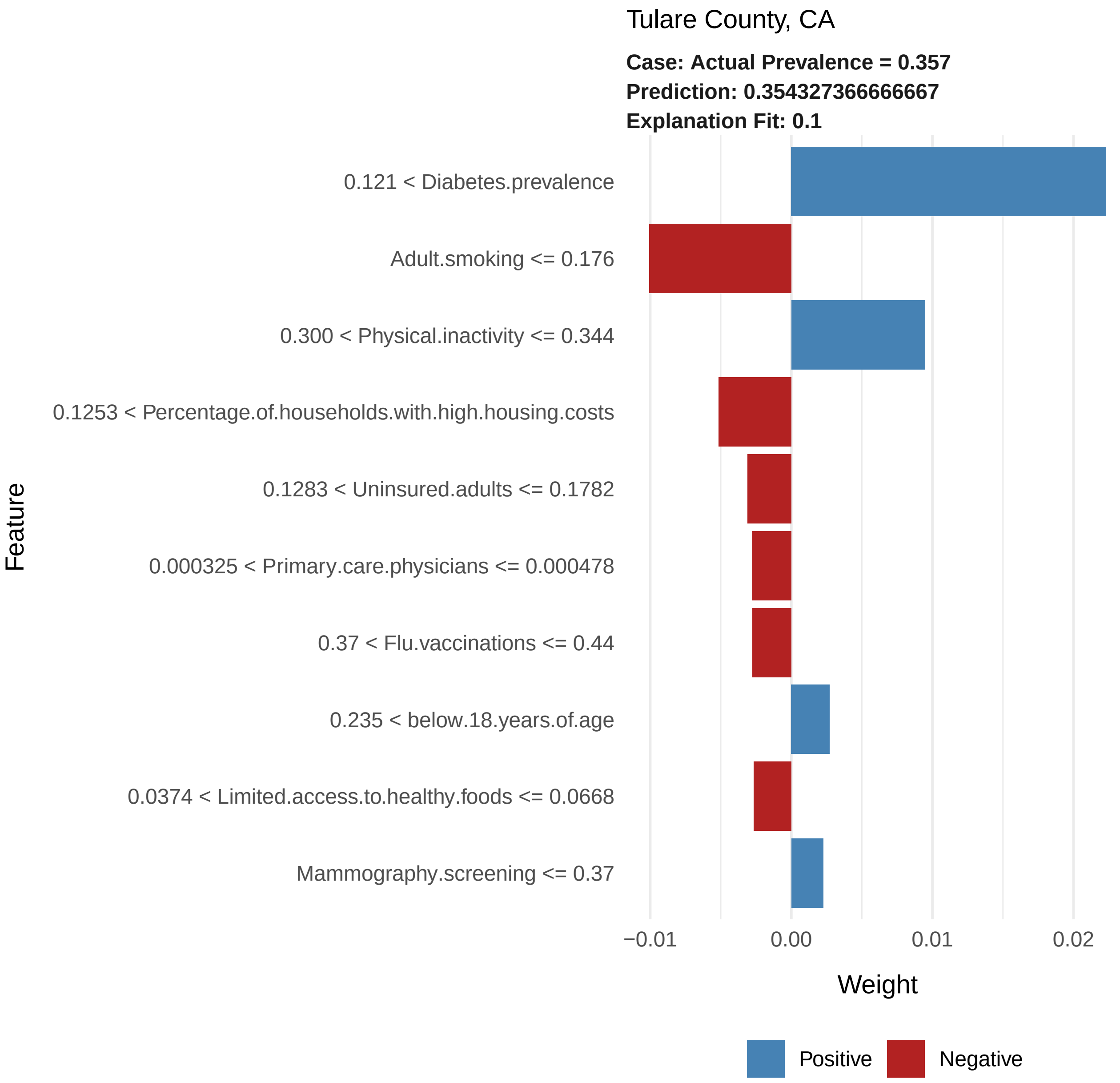

Tulare County, CA
Case: Actual Prevalence = 0.357
Prediction: 0.354327366666667
Explanation Fit: 0.1
0.121 < Diabetes.prevalence
Adult.smoking <= 0.176
0.300 < Physical.inactivity <= 0.344
0.1253 < Percentage.of.households.with.high.housing.costs
0.1283 < Uninsured.adults <= 0.1782
0.000325 < Primary.care.physicians <= 0.000478
0.37 < Flu.vaccinations <= 0.44
0.235 < below.18.years.of.age
0.0374 < Limited.access.to.healthy.foods <= 0.0668
Mammography.screening <= 0.37
Feature
−0.01
0.00
0.01
0.02
Weight
Positive
Negative

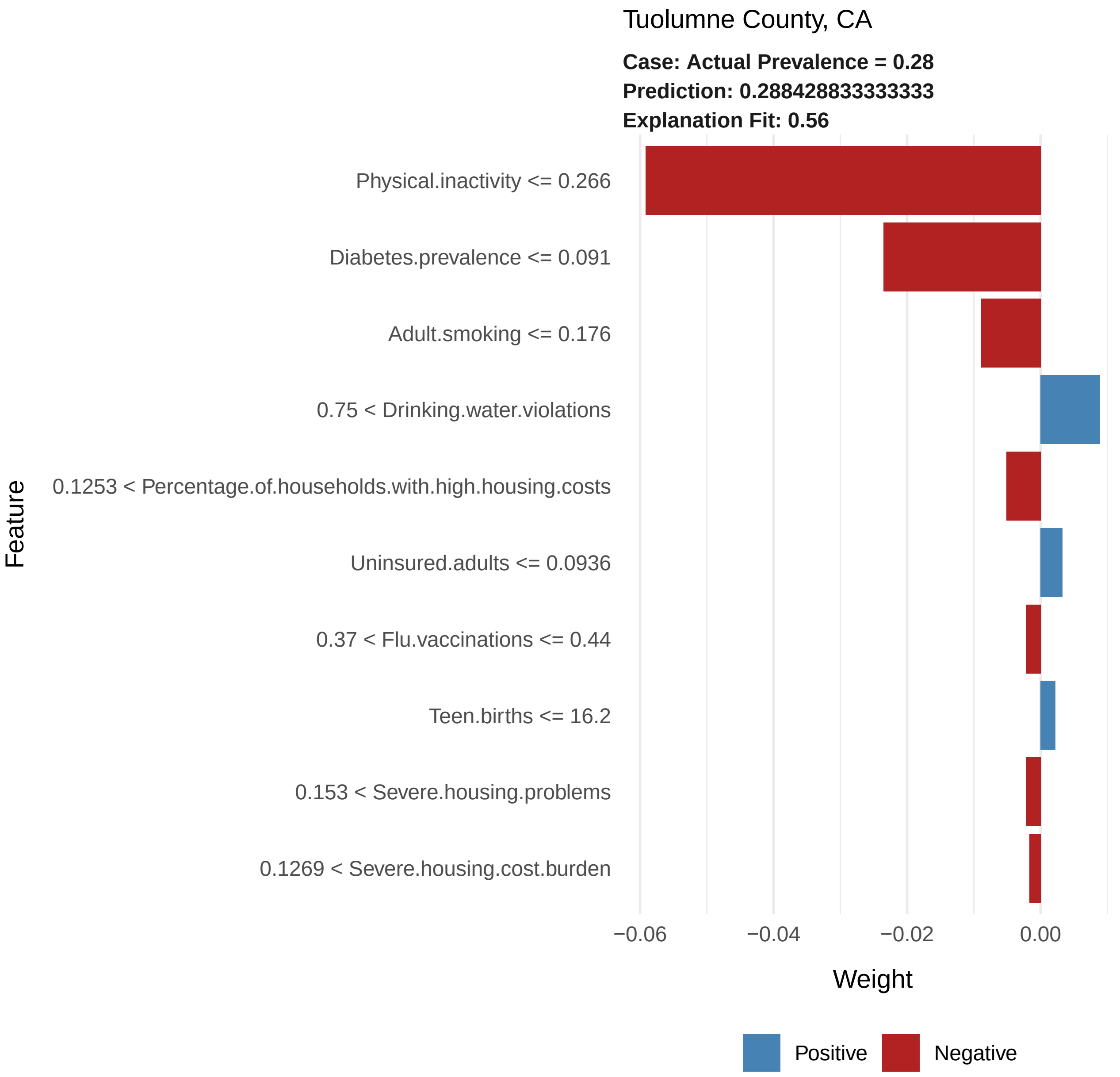

Tuolumne County, CA
Case: Actual Prevalence = 0.28
Prediction: 0.288428833333333
Explanation Fit: 0.56
Physical.inactivity <= 0.266
Diabetes.prevalence <= 0.091
Adult.smoking <= 0.176
0.75 < Drinking.water.violations
0.1253 < Percentage.of.households.with.high.housing.costs
Uninsured.adults <= 0.0936
0.37 < Flu.vaccinations <= 0.44
Teen.births <= 16.2
0.153 < Severe.housing.problems
0.1269 < Severe.housing.cost.burden
Feature
−0.06
−0.04
−0.02
0.00
Weight
Positive
Negative

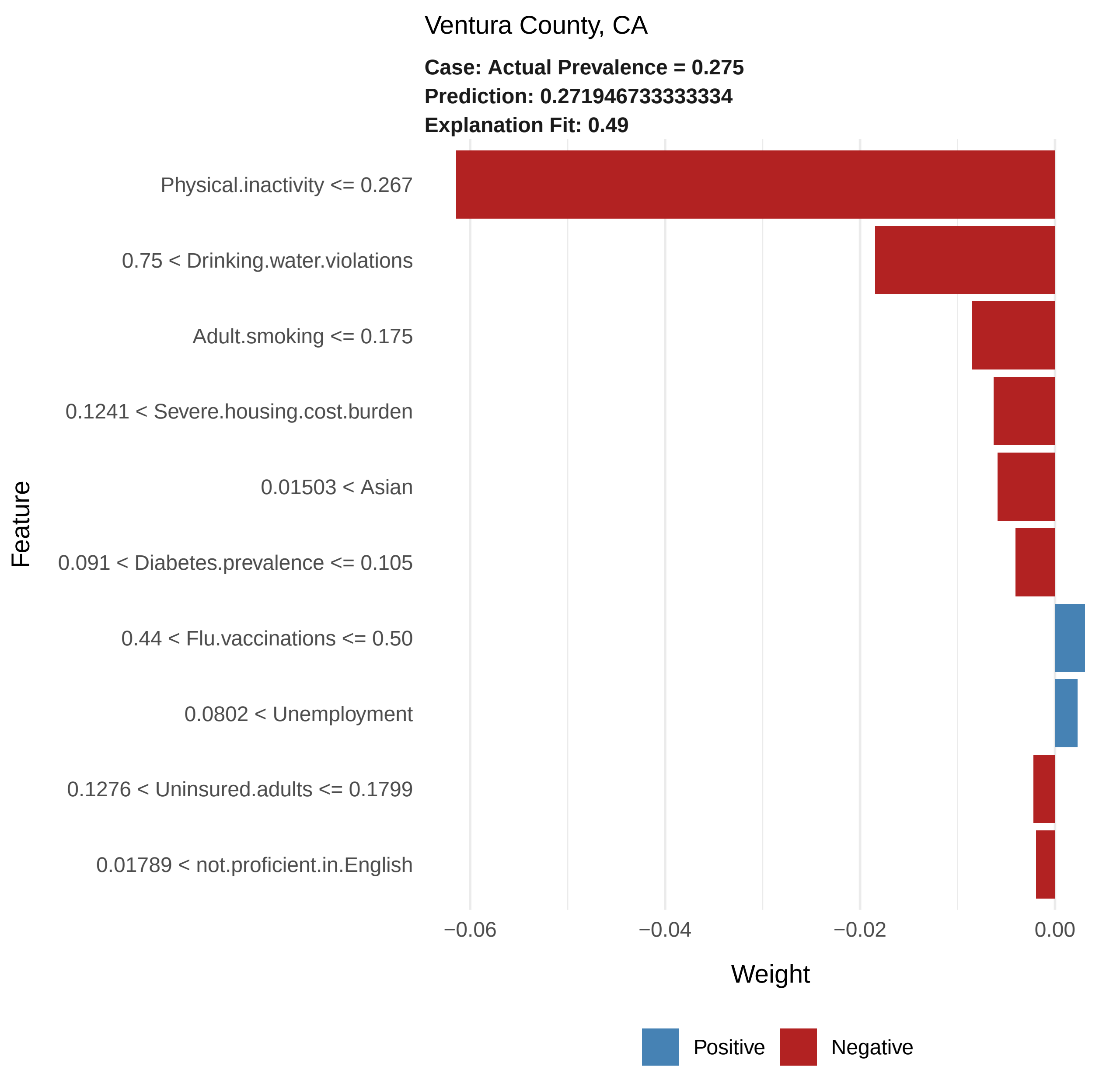
Ventura County, CA
Case: Actual Prevalence = 0.275
Prediction: 0.271946733333334
Explanation Fit: 0.49
Physical.inactivity <= 0.267
0.75 < Drinking.water.violations
Adult.smoking <= 0.175
0.1241 < Severe.housing.cost.burden
0.01503 < Asian
0.091 < Diabetes.prevalence <= 0.105
0.44 < Flu.vaccinations <= 0.50
0.0802 < Unemployment
0.1276 < Uninsured.adults <= 0.1799
0.01789 < not.proficient.in.English
Feature
−0.06
−0.04
−0.02
0.00
Weight
Positive
Negative

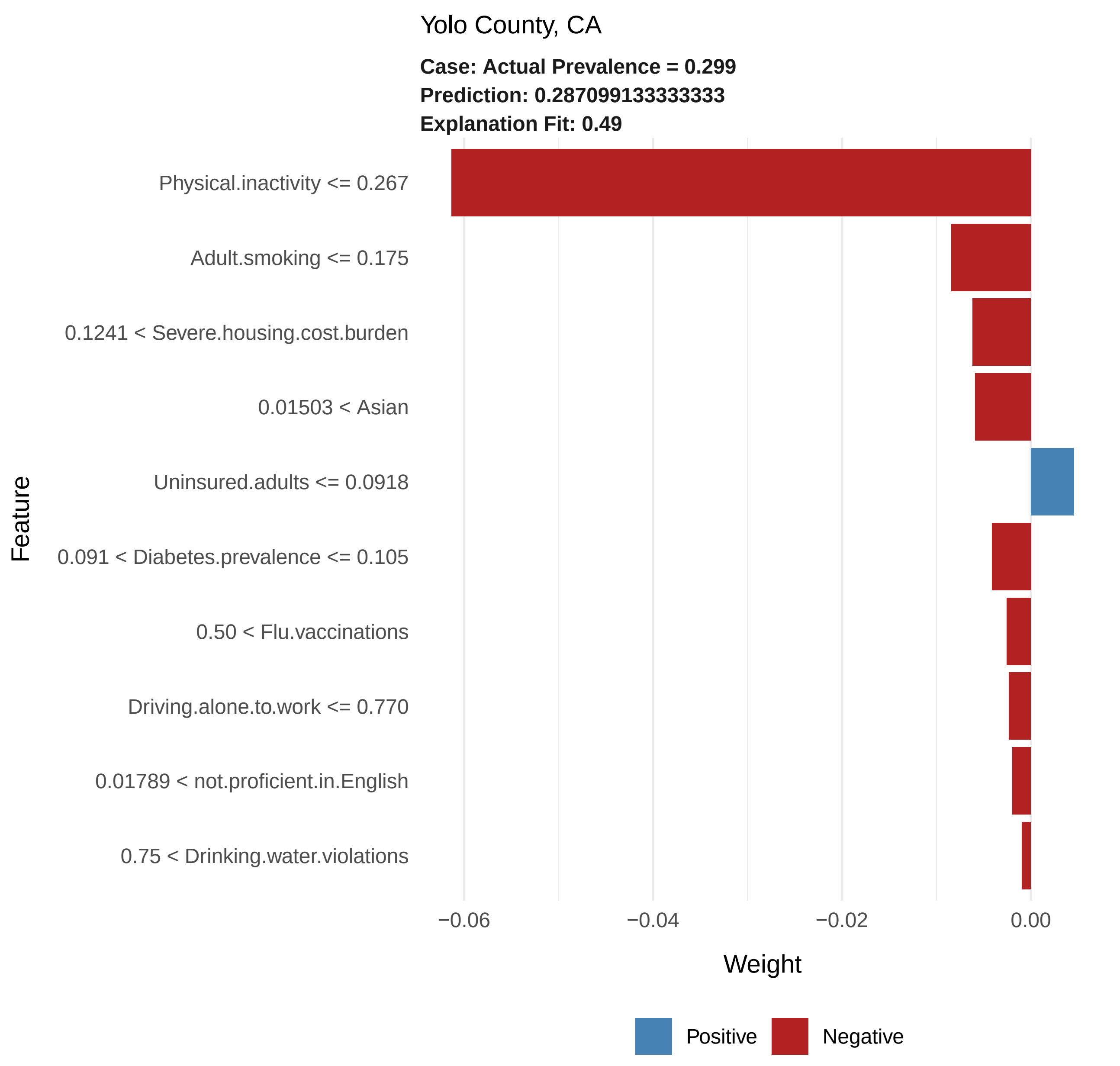
Yolo County, CA
Case: Actual Prevalence = 0.299
Prediction: 0.287099133333333
Explanation Fit: 0.49
Feature
Physical.inactivity <= 0.267
Adult.smoking <= 0.175
0.1241 < Severe.housing.cost.burden
0.01503 < Asian
Uninsured.adults <= 0.0918
0.091 < Diabetes.prevalence <= 0.105
0.50 < Flu.vaccinations
Driving.alone.to.work <= 0.770
0.01789 < not.proficient.in.English
0.75 < Drinking.water.violations
−0.06
−0.04
−0.02
0.00
Weight
Positive
Negative

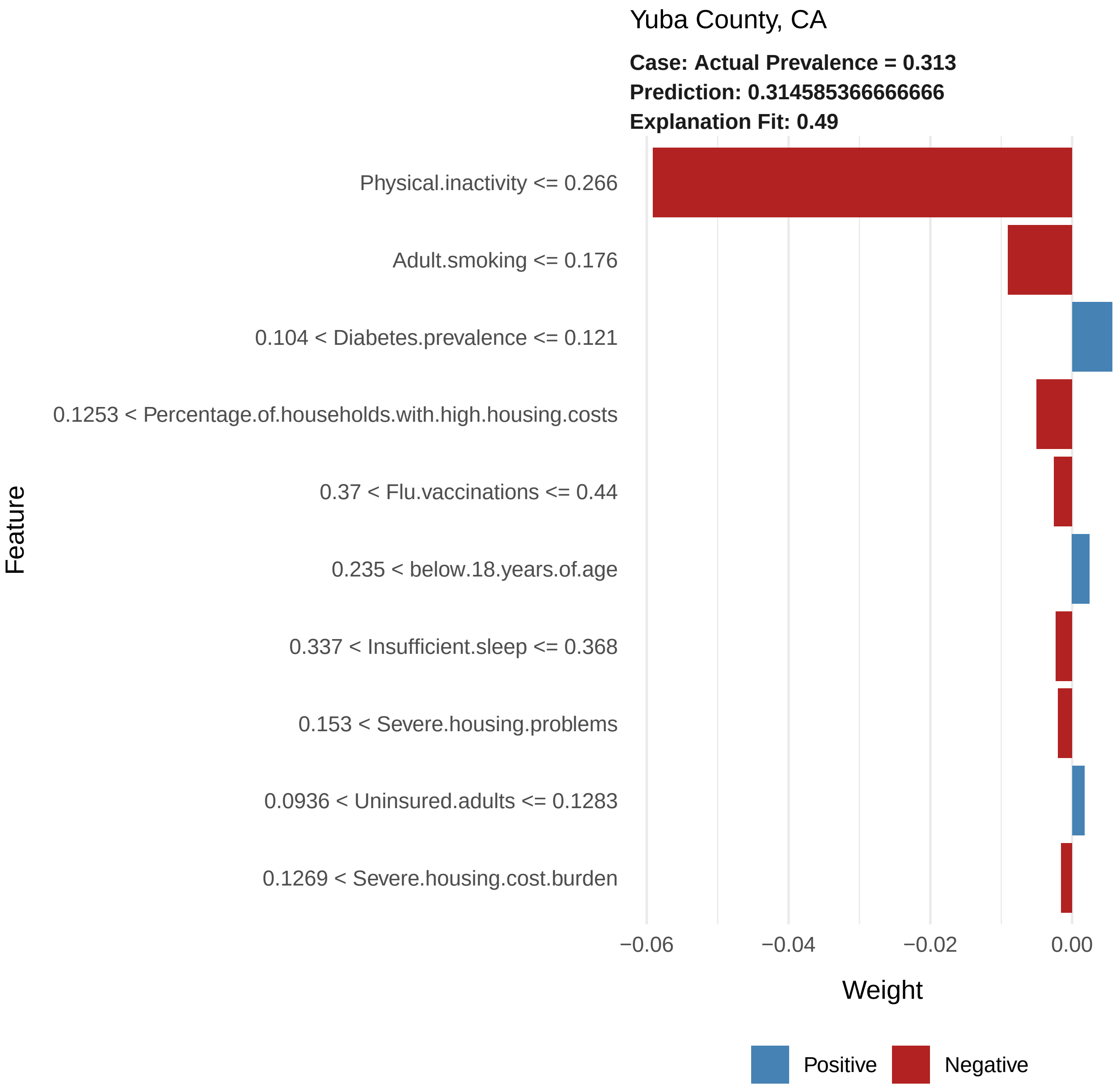
Yuba County, CA
Case: Actual Prevalence = 0.313
Prediction: 0.314585366666666
Explanation Fit: 0.49
Physical.inactivity <= 0.266
Adult.smoking <= 0.176
0.104 < Diabetes.prevalence <= 0.121
0.1253 < Percentage.of.households.with.high.housing.costs
0.37 < Flu.vaccinations <= 0.44
0.235 < below.18.years.of.age
0.337 < Insufficient.sleep <= 0.368
0.153 < Severe.housing.problems
0.0936 < Uninsured.adults <= 0.1283
0.1269 < Severe.housing.cost.burden
Feature
−0.06
−0.04
−0.02
0.00
Weight
Positive
Negative

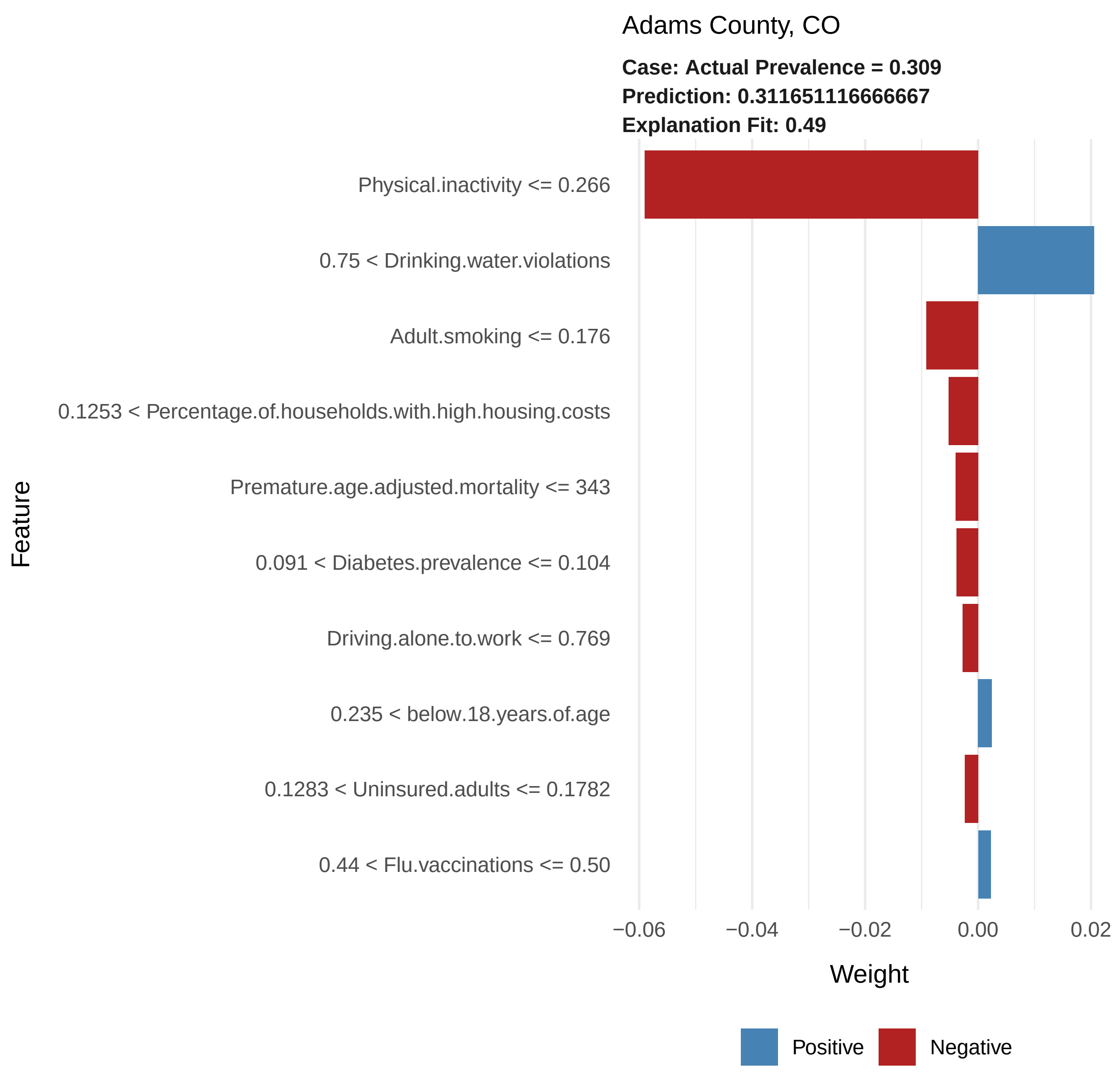
Adams County, CO
Case: Actual Prevalence = 0.309
Prediction: 0.311651116666667
Explanation Fit: 0.49
Physical.inactivity <= 0.266
0.75 < Drinking.water.violations
Adult.smoking <= 0.176
0.1253 < Percentage.of.households.with.high.housing.costs
Premature.age.adjusted.mortality <= 343
0.091 < Diabetes.prevalence <= 0.104
Driving.alone.to.work <= 0.769
0.235 < below.18.years.of.age
0.1283 < Uninsured.adults <= 0.1782
0.44 < Flu.vaccinations <= 0.50
Feature
−0.06
−0.04
−0.02
0.00
0.02
Weight
Positive
Negative

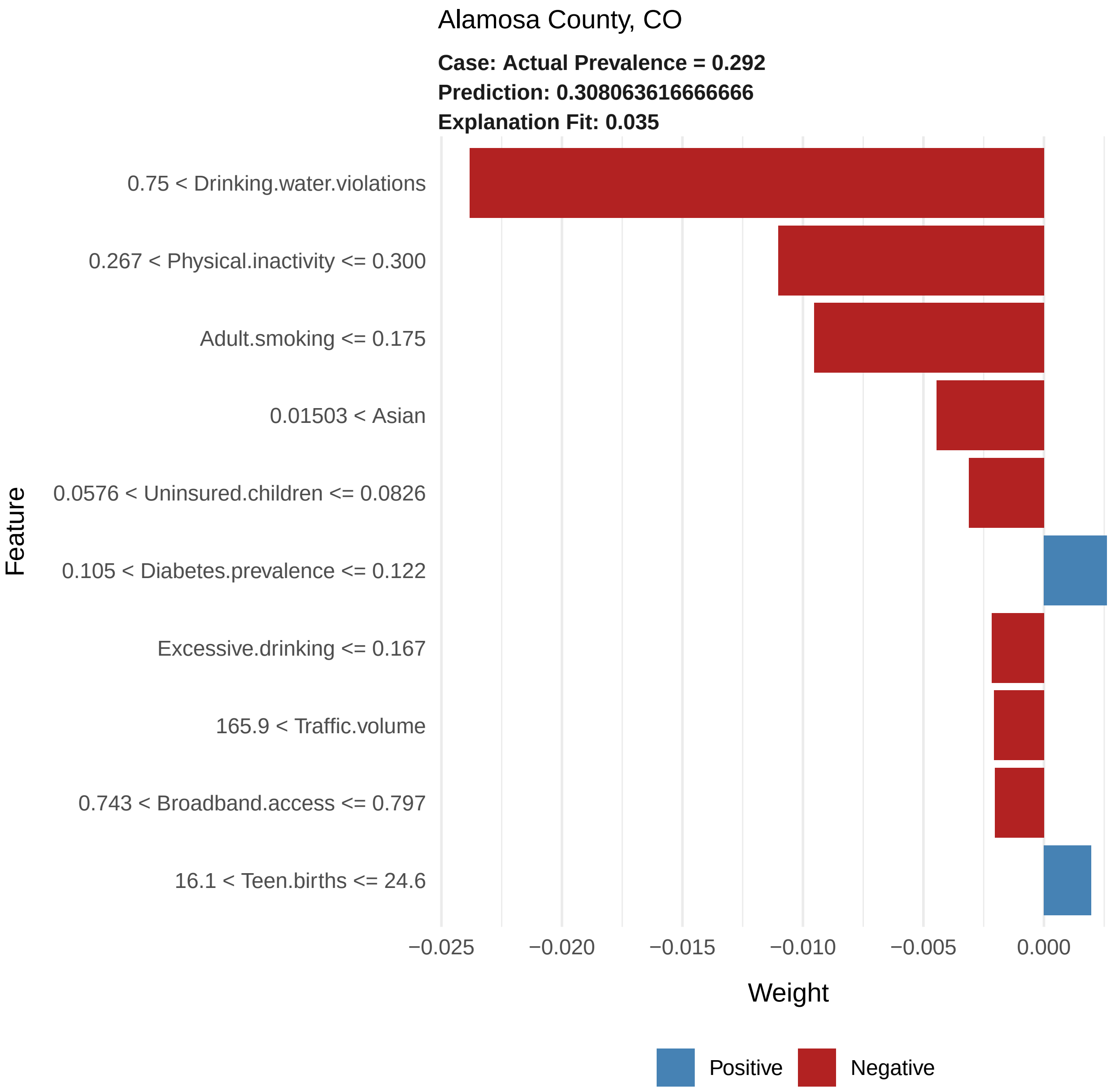

Alamosa County, CO
Case: Actual Prevalence = 0.292
Prediction: 0.308063616666666
Explanation Fit: 0.035
0.75 < Drinking.water.violations
0.267 < Physical.inactivity <= 0.300
Adult.smoking <= 0.175
0.01503 < Asian
0.0576 < Uninsured.children <= 0.0826
0.105 < Diabetes.prevalence <= 0.122
Excessive.drinking <= 0.167
165.9 < Traffic.volume
0.743 < Broadband.access <= 0.797
16.1 < Teen.births <= 24.6
Feature
−0.025
−0.020
−0.015
−0.010
−0.005
0.000
Weight
Positive
Negative

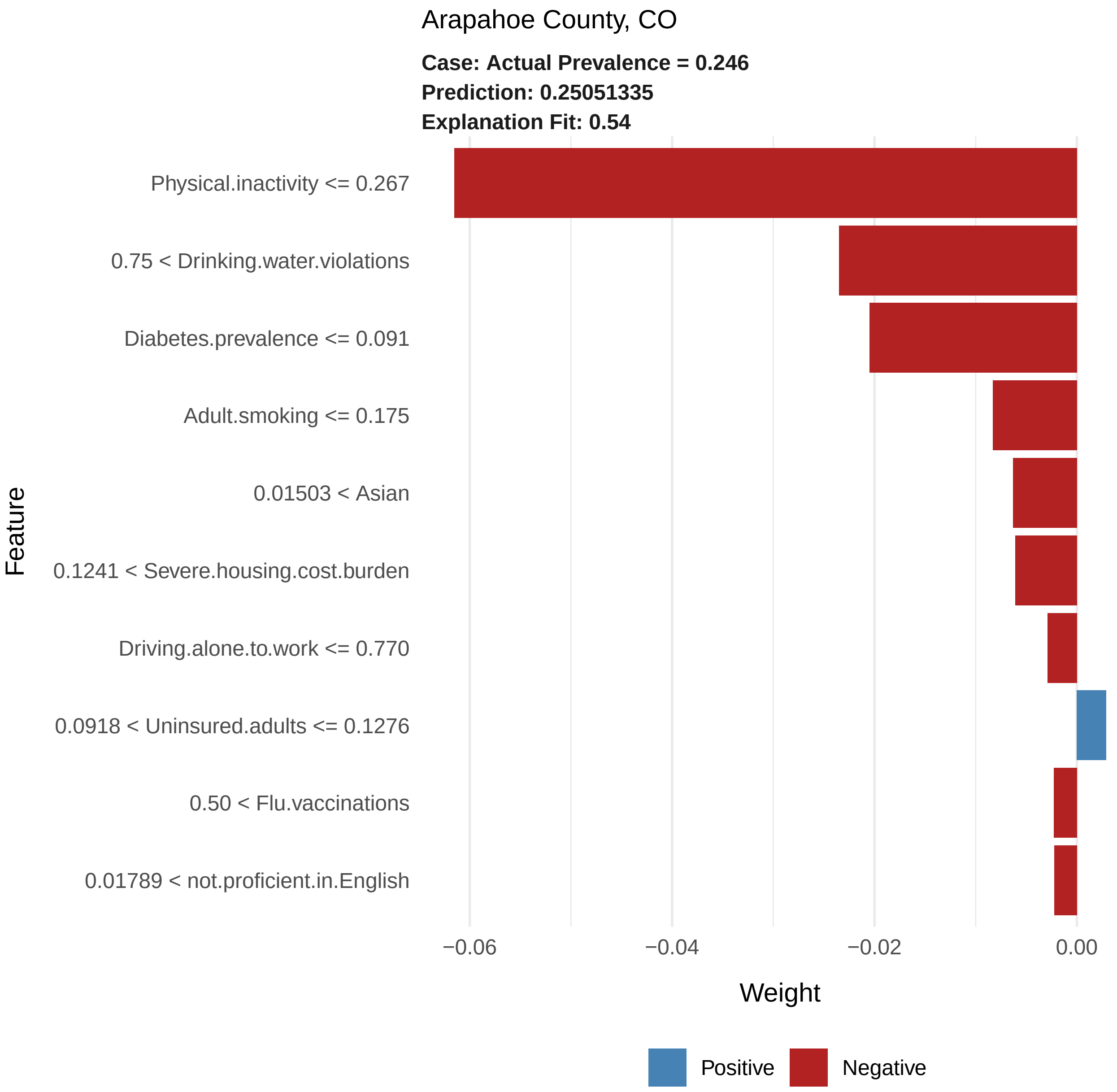

Arapahoe County, CO
Case: Actual Prevalence = 0.246
Prediction: 0.25051335
Explanation Fit: 0.54
Physical.inactivity <= 0.267
0.75 < Drinking.water.violations
Diabetes.prevalence <= 0.091
Adult.smoking <= 0.175
0.01503 < Asian
0.1241 < Severe.housing.cost.burden
Driving.alone.to.work <= 0.770
0.0918 < Uninsured.adults <= 0.1276
0.50 < Flu.vaccinations
0.01789 < not.proficient.in.English
Feature
−0.06
−0.04
−0.02
0.00
Weight
Positive
Negative

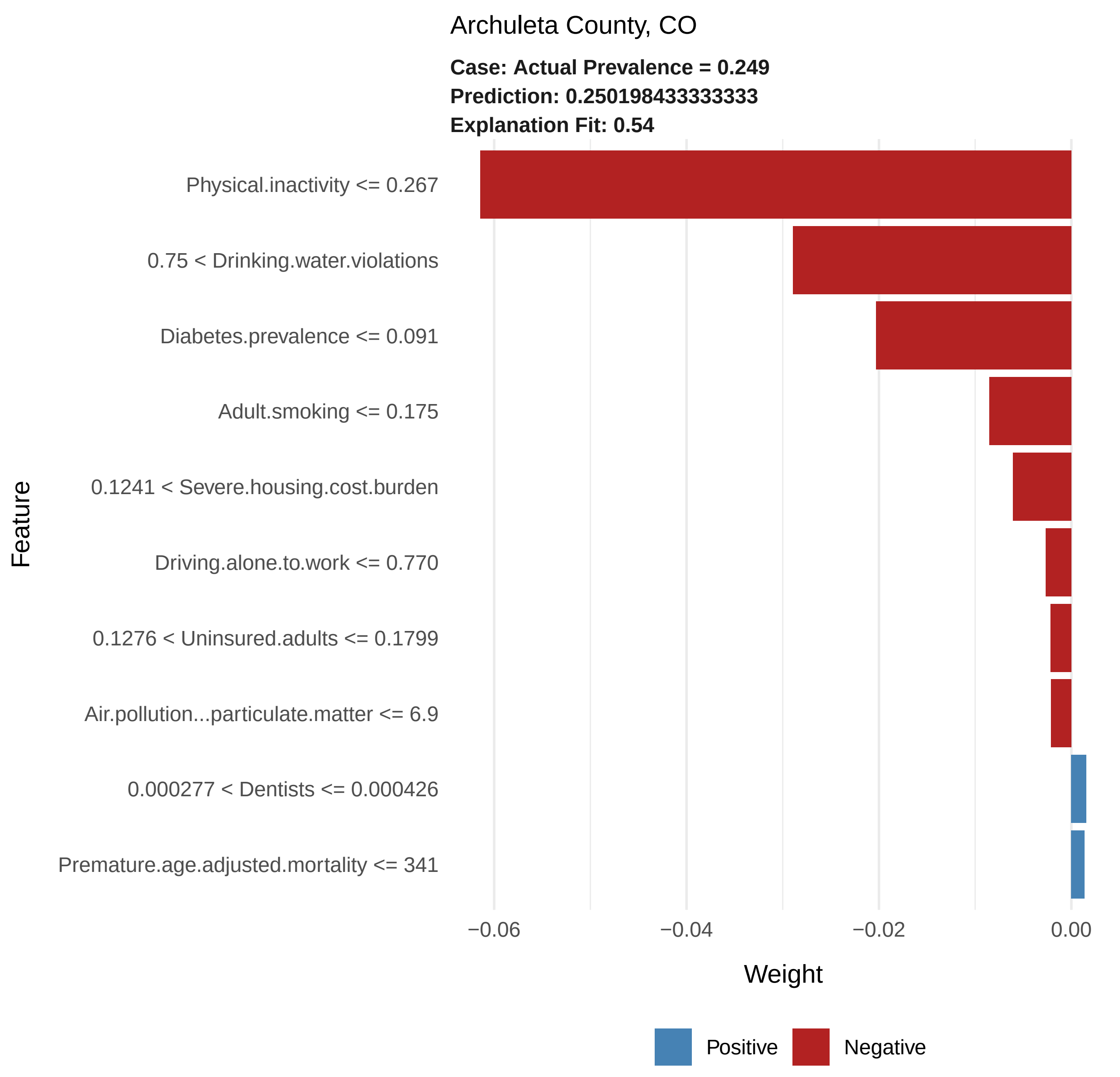

Archuleta County, CO
Case: Actual Prevalence = 0.249
Prediction: 0.250198433333333
Explanation Fit: 0.54
Physical.inactivity <= 0.267
0.75 < Drinking.water.violations
Diabetes.prevalence <= 0.091
Adult.smoking <= 0.175
0.1241 < Severe.housing.cost.burden
Driving.alone.to.work <= 0.770
0.1276 < Uninsured.adults <= 0.1799
Air.pollution...particulate.matter <= 6.9
0.000277 < Dentists <= 0.000426
Premature.age.adjusted.mortality <= 341
Feature
−0.06
−0.04
−0.02
0.00
Weight
Positive
Negative

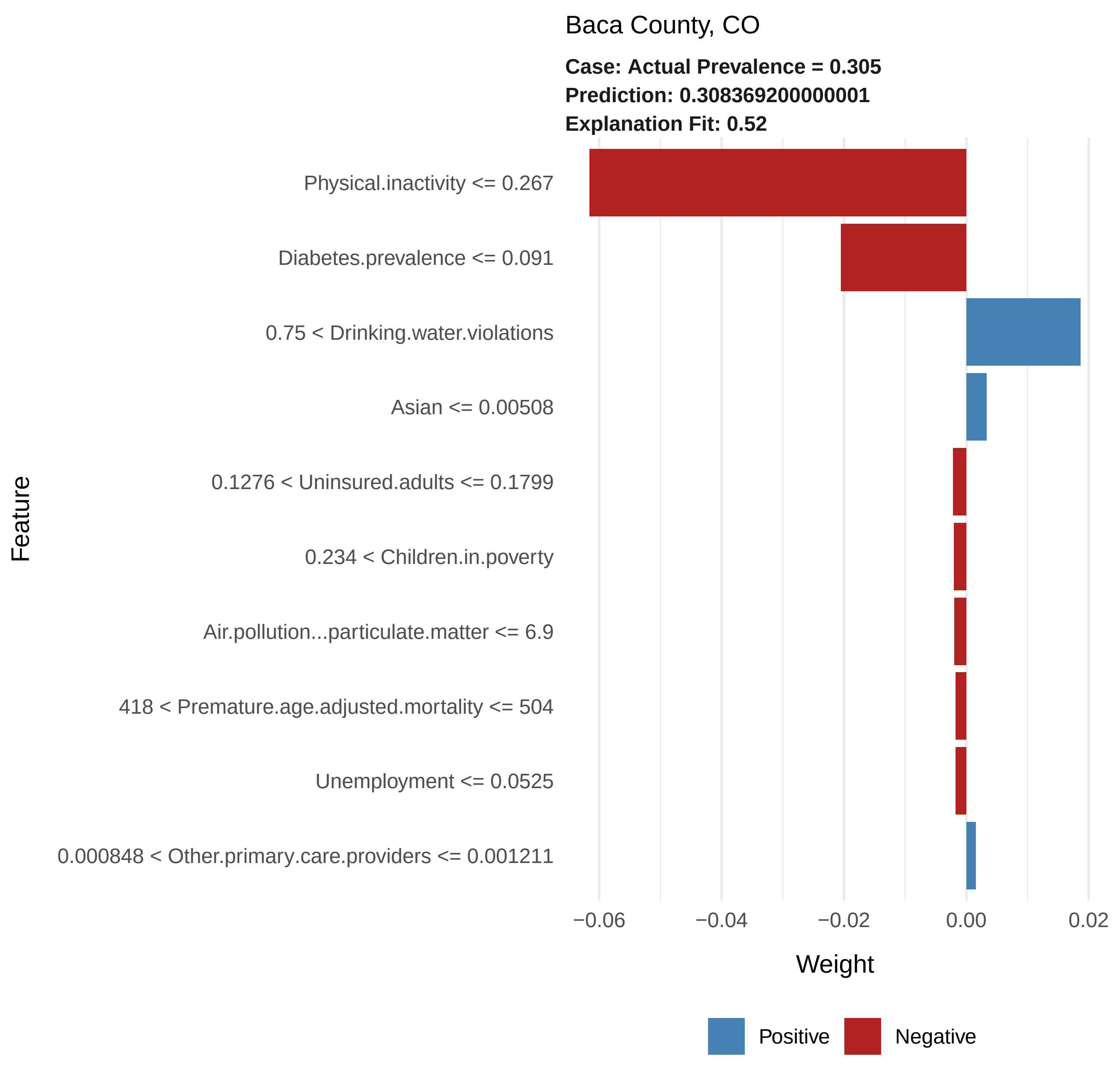

Baca County, CO
Case: Actual Prevalence = 0.305
Prediction: 0.308369200000001
Explanation Fit: 0.52
Physical.inactivity <= 0.267
Diabetes.prevalence <= 0.091
0.75 < Drinking.water.violations
Asian <= 0.00508
0.1276 < Uninsured.adults <= 0.1799
0.234 < Children.in.poverty
Air.pollution...particulate.matter <= 6.9
418 < Premature.age.adjusted.mortality <= 504
Unemployment <= 0.0525
0.000848 < Other.primary.care.providers <= 0.001211
Feature
−0.06
−0.04
−0.02
0.00
0.02
Weight
Positive
Negative

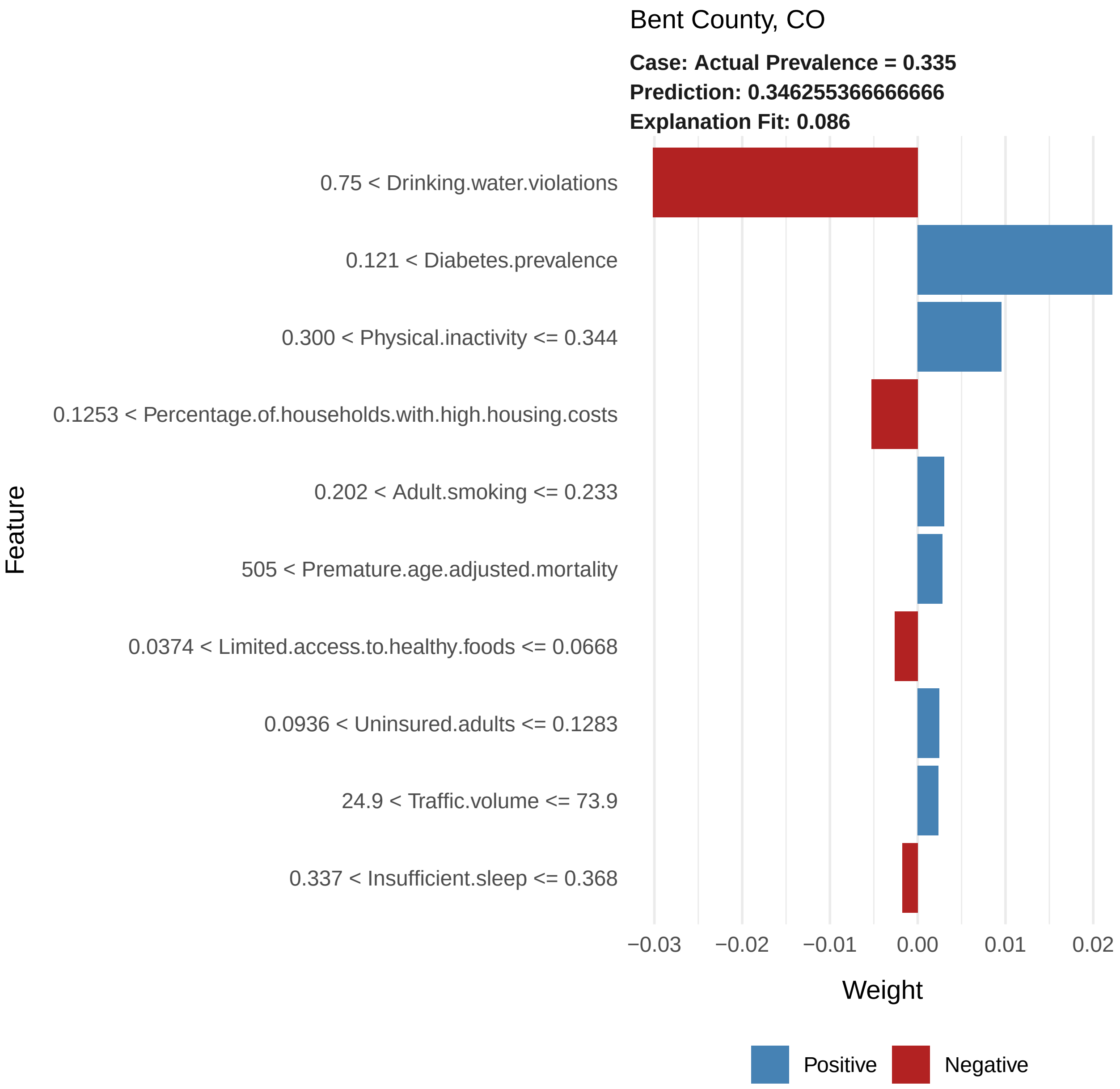

Bent County, CO
Case: Actual Prevalence = 0.335
Prediction: 0.346255366666666
Explanation Fit: 0.086
0.75 < Drinking.water.violations
0.121 < Diabetes.prevalence
0.300 < Physical.inactivity <= 0.344
0.1253 < Percentage.of.households.with.high.housing.costs
0.202 < Adult.smoking <= 0.233
505 < Premature.age.adjusted.mortality
0.0374 < Limited.access.to.healthy.foods <= 0.0668
0.0936 < Uninsured.adults <= 0.1283
24.9 < Traffic.volume <= 73.9
0.337 < Insufficient.sleep <= 0.368
Feature
−0.03
−0.02
−0.01
0.00
0.01
0.02
Weight
Positive
Negative

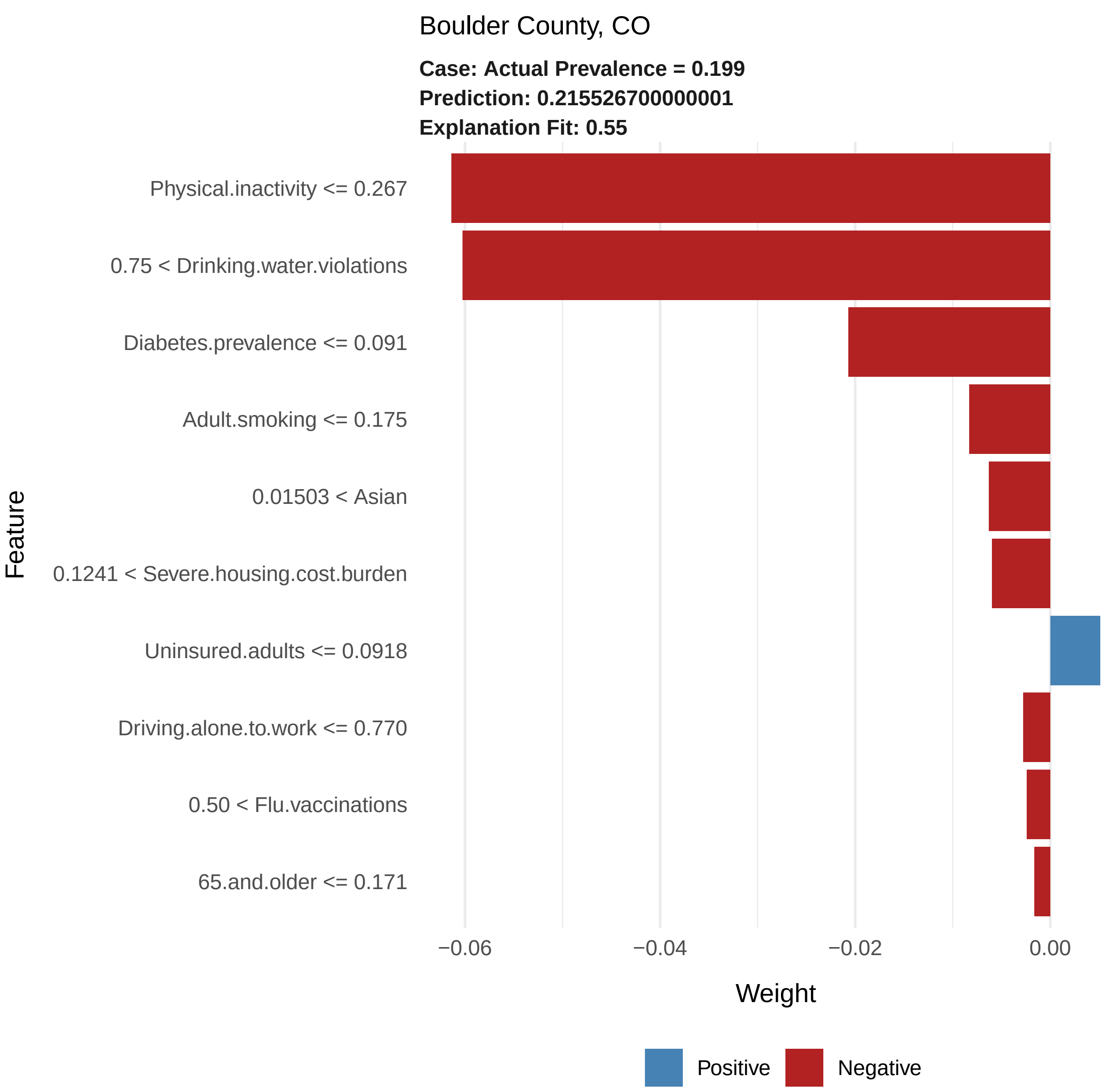

Boulder County, CO
Case: Actual Prevalence = 0.199
Prediction: 0.215526700000001
Explanation Fit: 0.55
Physical.inactivity <= 0.267
0.75 < Drinking.water.violations
Diabetes.prevalence <= 0.091
Adult.smoking <= 0.175
0.01503 < Asian
0.1241 < Severe.housing.cost.burden
Uninsured.adults <= 0.0918
Driving.alone.to.work <= 0.770
0.50 < Flu.vaccinations
65.and.older <= 0.171
Feature
−0.06
−0.04
−0.02
0.00
Weight
Positive
Negative

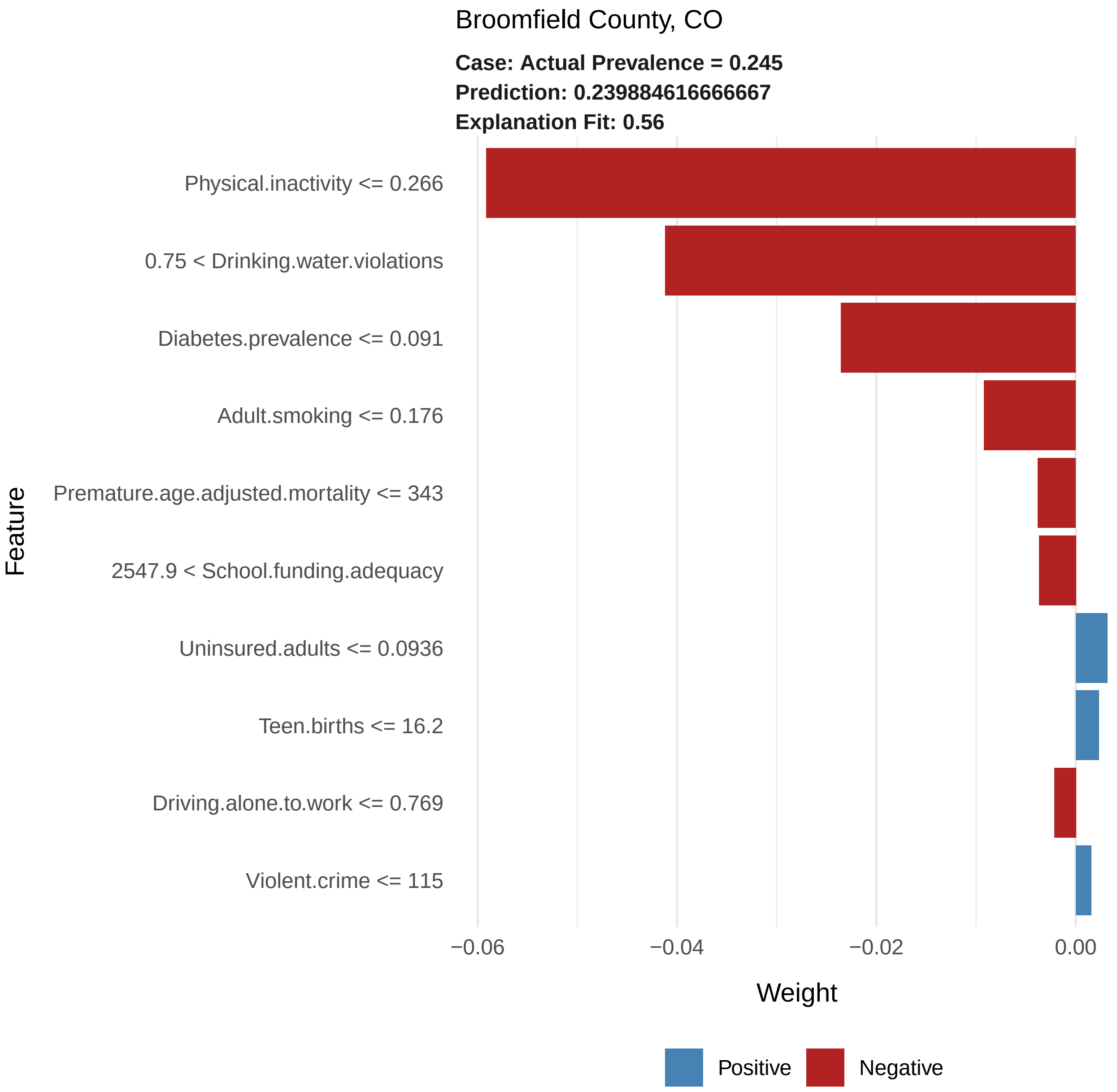

Broomfield County, CO
Case: Actual Prevalence = 0.245
Prediction: 0.239884616666667
Explanation Fit: 0.56
Physical.inactivity <= 0.266
0.75 < Drinking.water.violations
Diabetes.prevalence <= 0.091
Adult.smoking <= 0.176
Premature.age.adjusted.mortality <= 343
2547.9 < School.funding.adequacy
Uninsured.adults <= 0.0936
Teen.births <= 16.2
Driving.alone.to.work <= 0.769
Violent.crime <= 115
Feature
−0.06
−0.04
−0.02
0.00
Weight
Positive
Negative

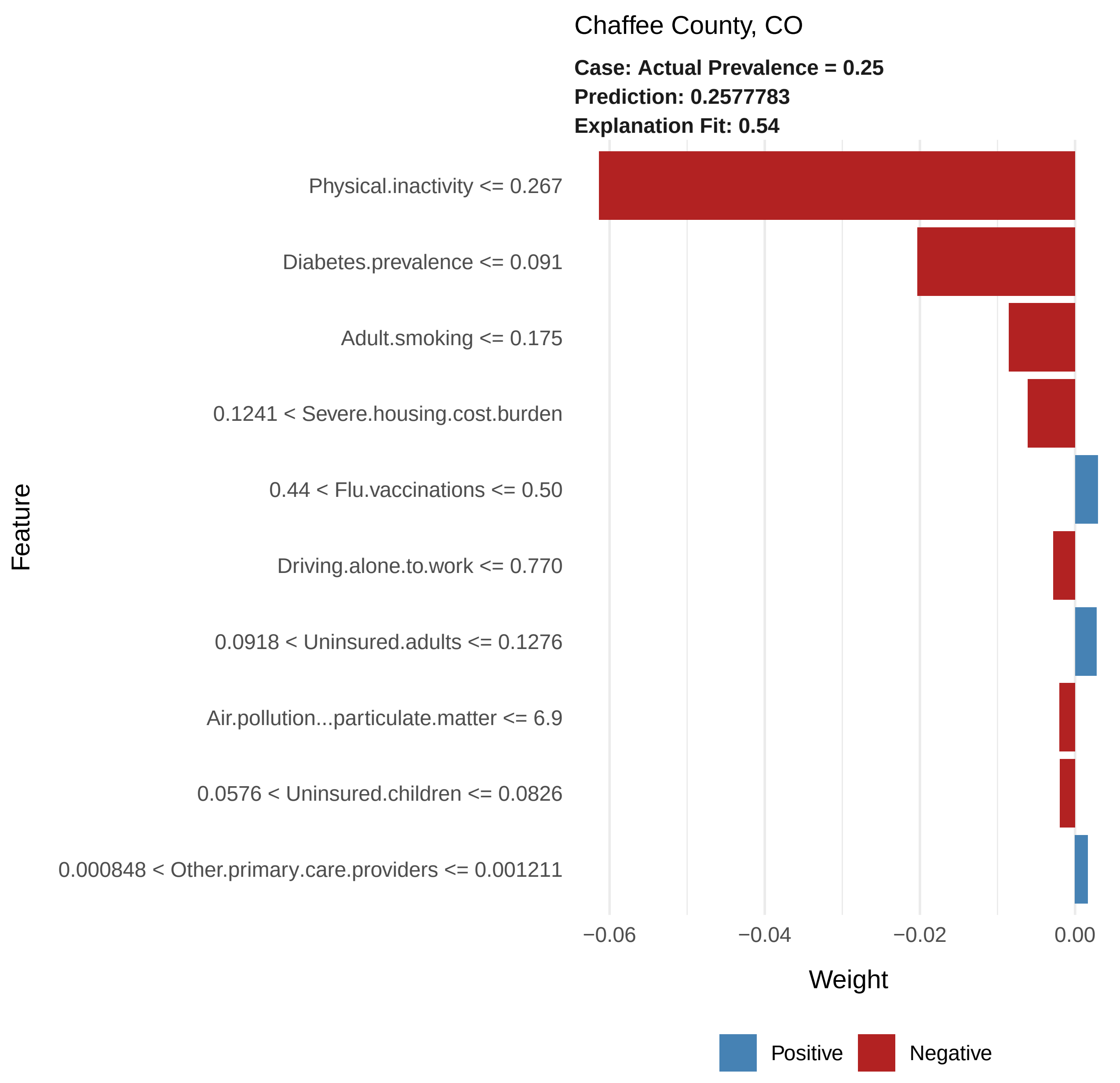

Chaffee County, CO
Case: Actual Prevalence = 0.25
Prediction: 0.2577783
Explanation Fit: 0.54
Physical.inactivity <= 0.267
Diabetes.prevalence <= 0.091
Adult.smoking <= 0.175
0.1241 < Severe.housing.cost.burden
0.44 < Flu.vaccinations <= 0.50
Driving.alone.to.work <= 0.770
0.0918 < Uninsured.adults <= 0.1276
Air.pollution...particulate.matter <= 6.9
0.0576 < Uninsured.children <= 0.0826
0.000848 < Other.primary.care.providers <= 0.001211
Feature
−0.06
−0.04
−0.02
0.00
Weight
Positive
Negative

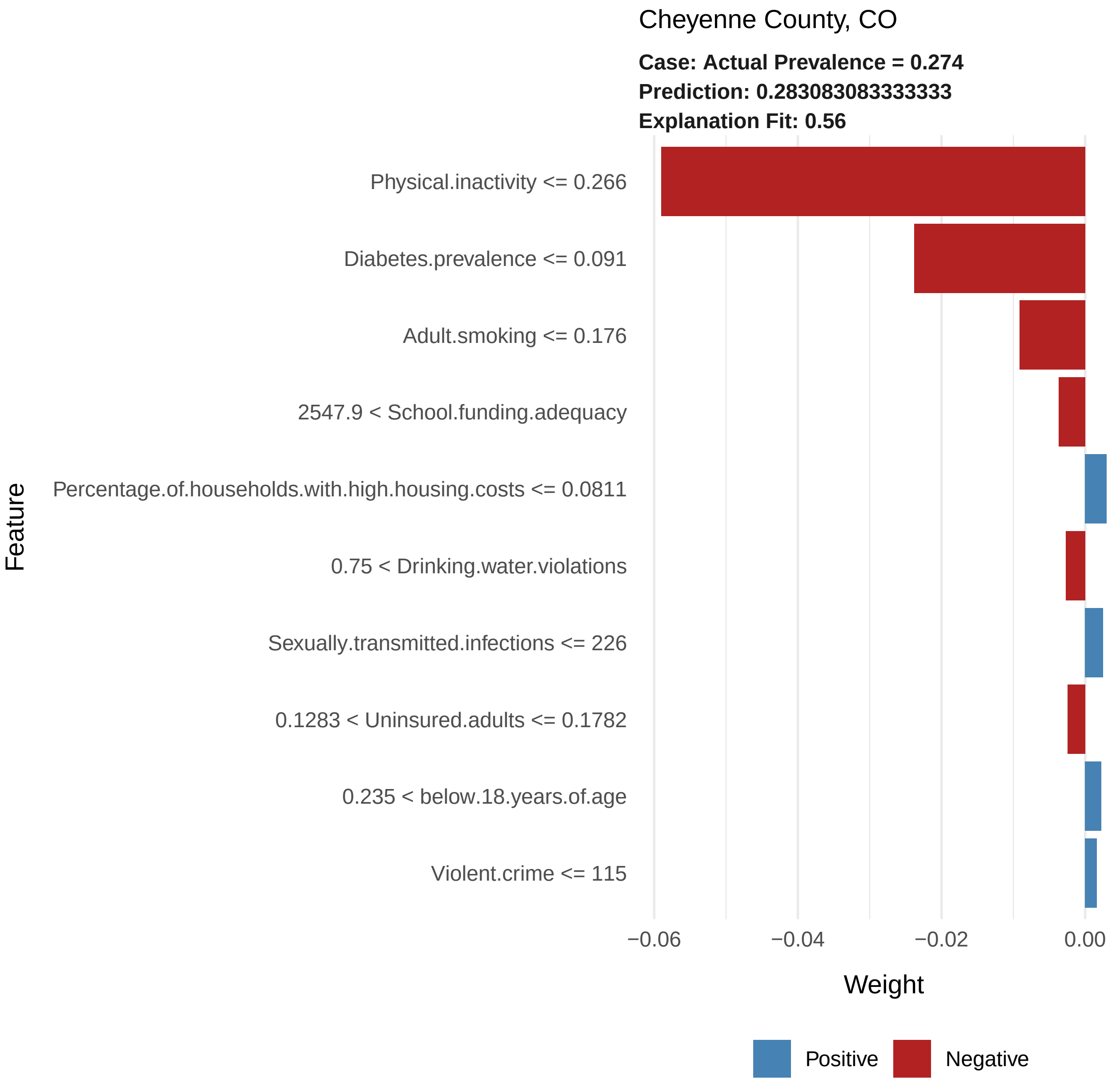
Cheyenne County, CO
Case: Actual Prevalence = 0.274
Prediction: 0.283083083333333
Explanation Fit: 0.56
Physical.inactivity <= 0.266
Diabetes.prevalence <= 0.091
Adult.smoking <= 0.176
2547.9 < School.funding.adequacy
Percentage.of.households.with.high.housing.costs <= 0.0811
0.75 < Drinking.water.violations
Sexually.transmitted.infections <= 226
0.1283 < Uninsured.adults <= 0.1782
0.235 < below.18.years.of.age
Violent.crime <= 115
Feature
−0.06
−0.04
−0.02
0.00
Weight
Positive
Negative

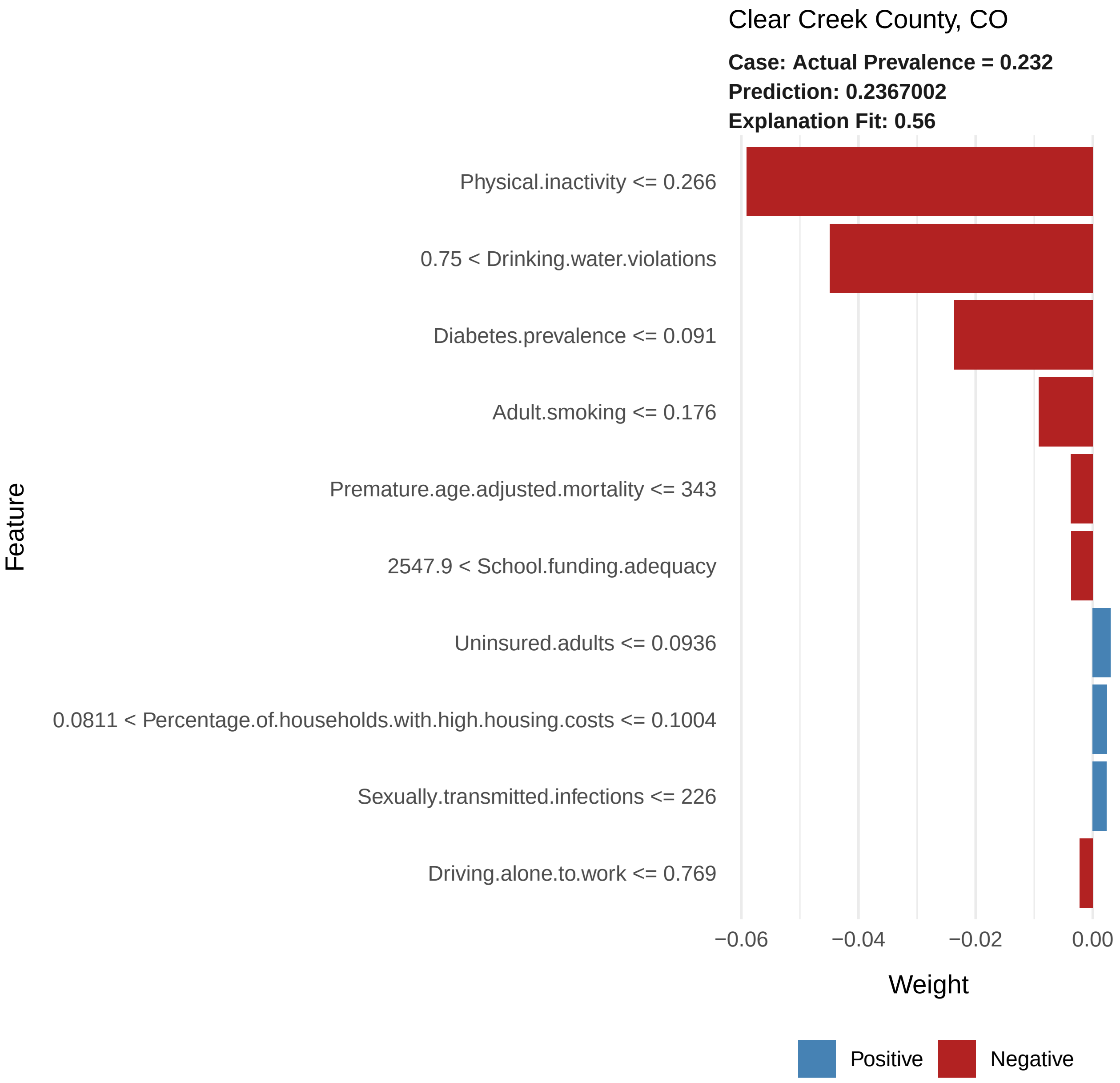

Clear Creek County, CO
Case: Actual Prevalence = 0.232
Prediction: 0.2367002
Explanation Fit: 0.56
Physical.inactivity <= 0.266
0.75 < Drinking.water.violations
Diabetes.prevalence <= 0.091
Adult.smoking <= 0.176
Premature.age.adjusted.mortality <= 343
2547.9 < School.funding.adequacy
Uninsured.adults <= 0.0936
0.0811 < Percentage.of.households.with.high.housing.costs <= 0.1004
Sexually.transmitted.infections <= 226
Driving.alone.to.work <= 0.769
Feature
−0.06
−0.04
−0.02
0.00
Weight
Positive
Negative

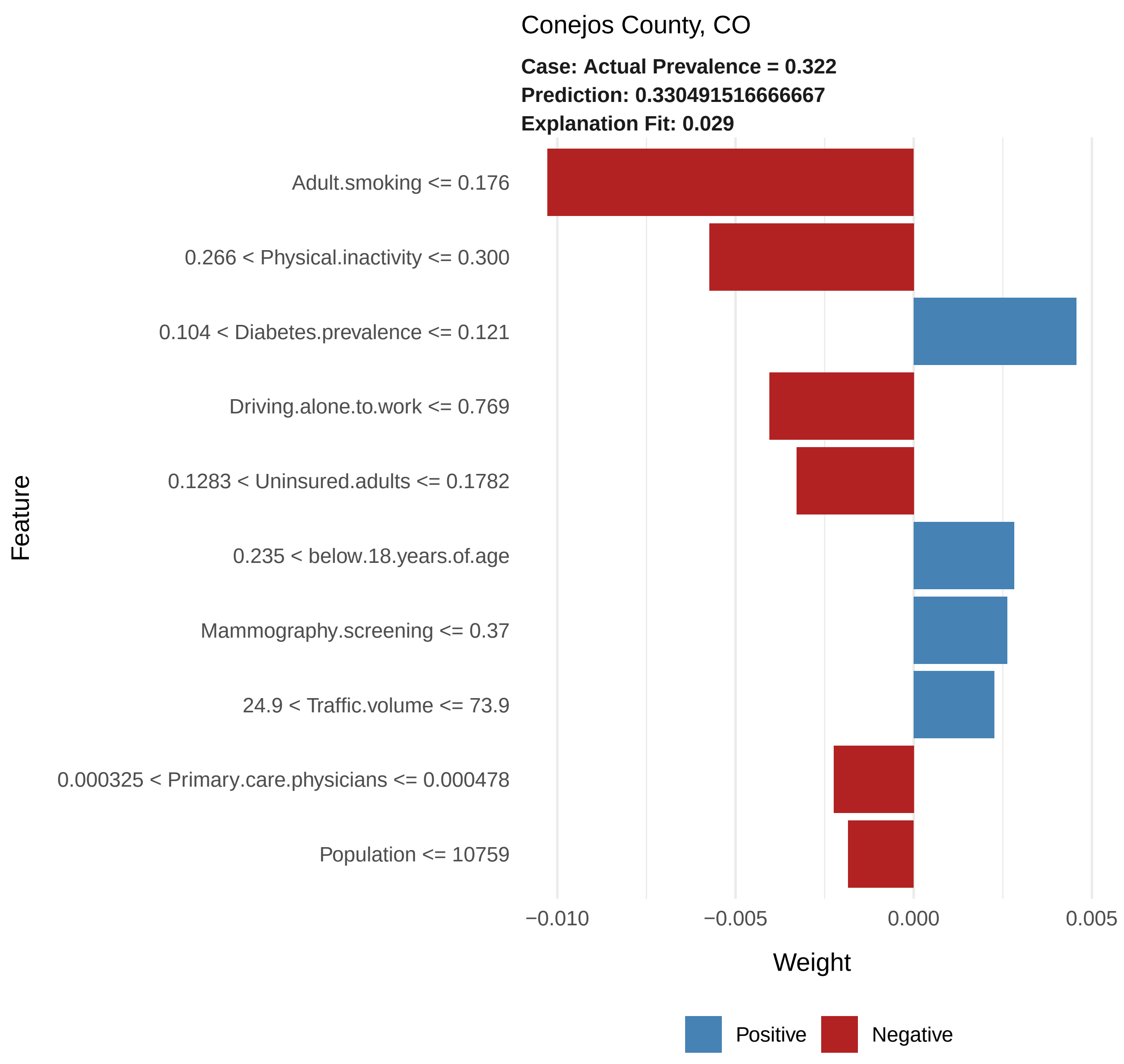
Conejos County, CO
Case: Actual Prevalence = 0.322
Prediction: 0.330491516666667
Explanation Fit: 0.029
Adult.smoking <= 0.176
0.266 < Physical.inactivity <= 0.300
0.104 < Diabetes.prevalence <= 0.121
Driving.alone.to.work <= 0.769
0.1283 < Uninsured.adults <= 0.1782
0.235 < below.18.years.of.age
Mammography.screening <= 0.37
24.9 < Traffic.volume <= 73.9
0.000325 < Primary.care.physicians <= 0.000478
Population <= 10759
Feature
−0.010
−0.005
0.000
0.005
Weight
Positive
Negative

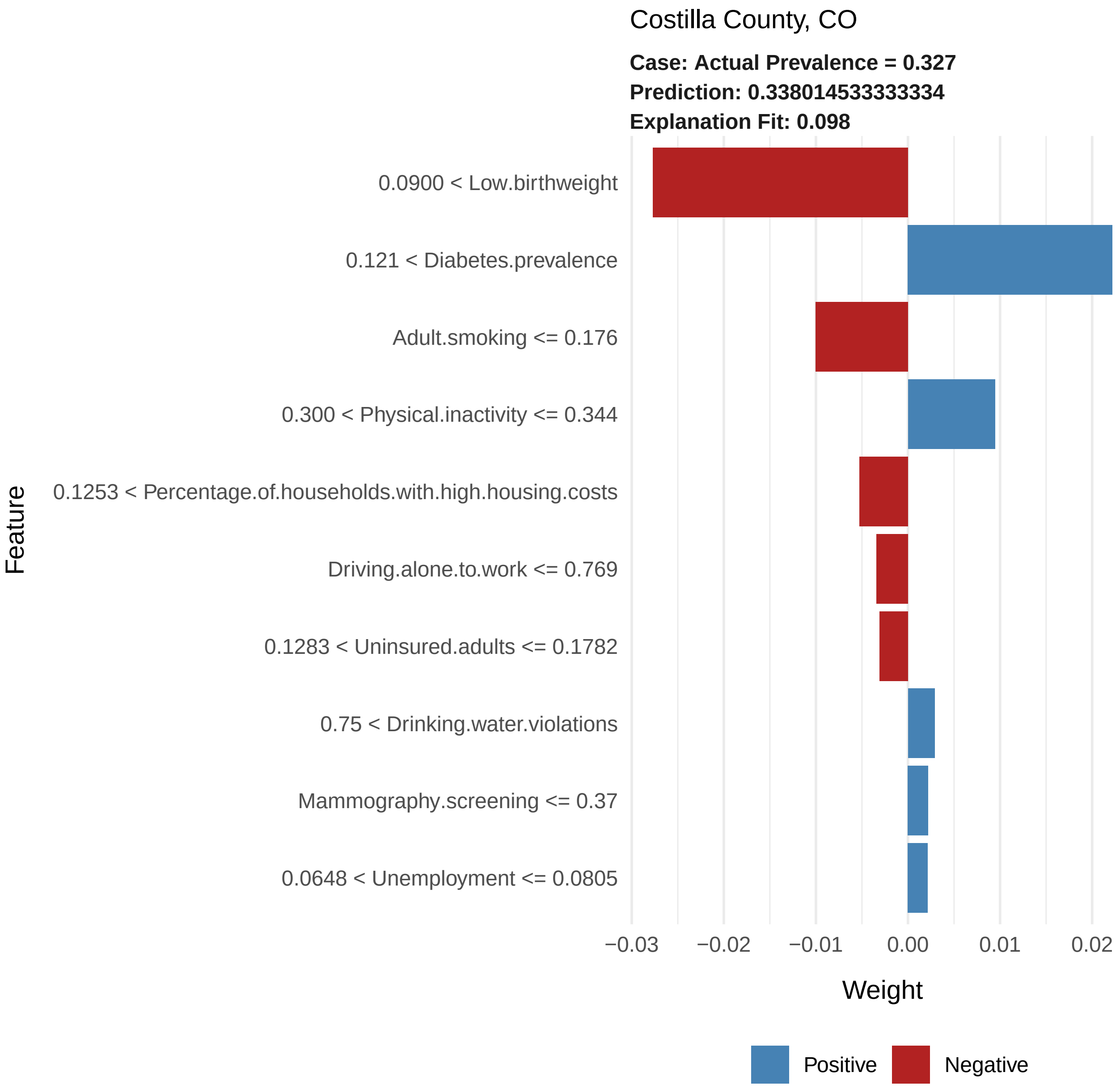

Costilla County, CO
Case: Actual Prevalence = 0.327
Prediction: 0.338014533333334
Explanation Fit: 0.098
0.0900 < Low.birthweight
0.121 < Diabetes.prevalence
Adult.smoking <= 0.176
0.300 < Physical.inactivity <= 0.344
0.1253 < Percentage.of.households.with.high.housing.costs
Driving.alone.to.work <= 0.769
0.1283 < Uninsured.adults <= 0.1782
0.75 < Drinking.water.violations
Mammography.screening <= 0.37
0.0648 < Unemployment <= 0.0805
Feature
−0.03
−0.02
−0.01
0.00
0.01
0.02
Weight
Positive
Negative

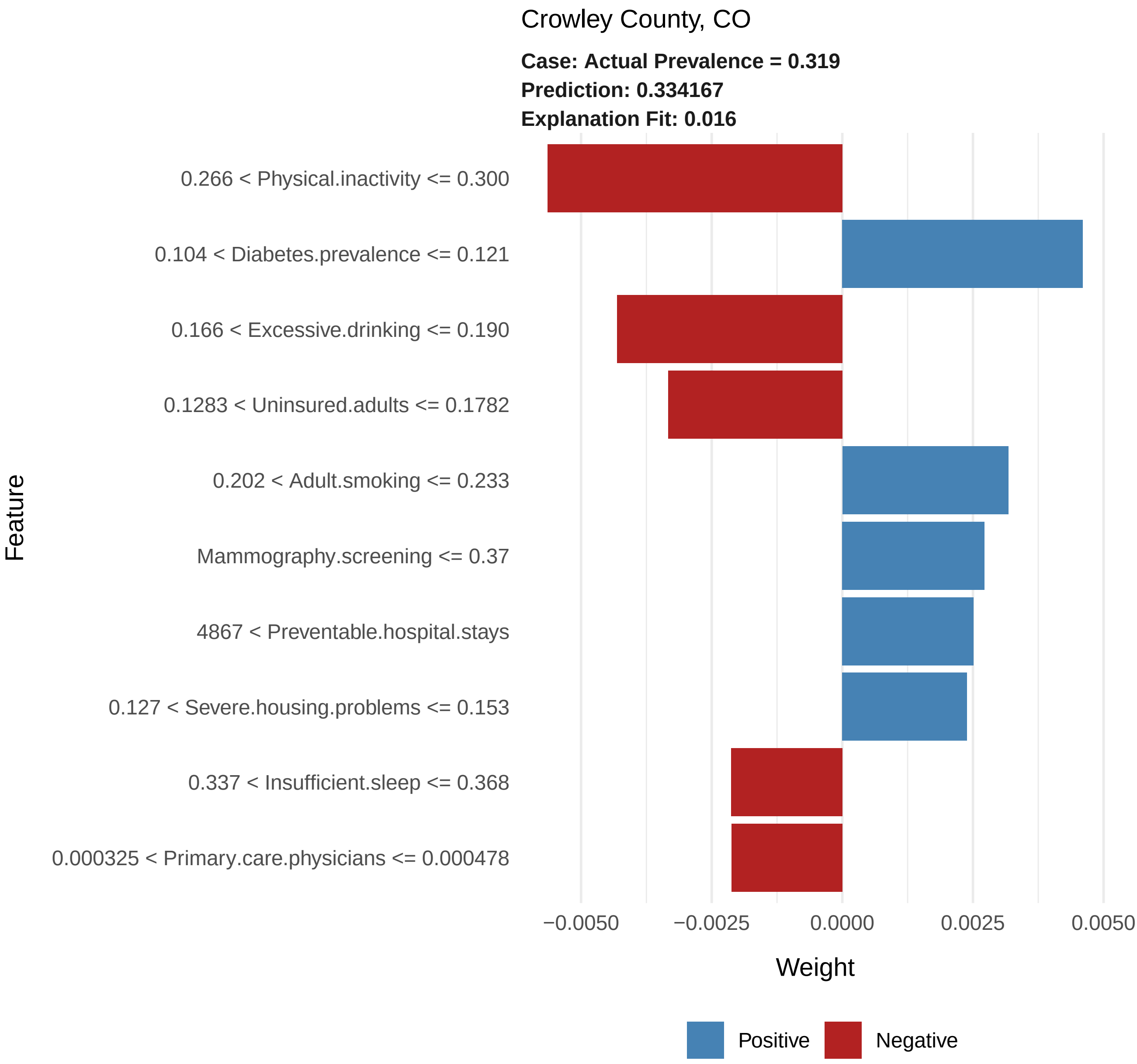

Crowley County, CO
Case: Actual Prevalence = 0.319
Prediction: 0.334167
Explanation Fit: 0.016
0.266 < Physical.inactivity <= 0.300
0.104 < Diabetes.prevalence <= 0.121
0.166 < Excessive.drinking <= 0.190
0.1283 < Uninsured.adults <= 0.1782
0.202 < Adult.smoking <= 0.233
Mammography.screening <= 0.37
4867 < Preventable.hospital.stays
0.127 < Severe.housing.problems <= 0.153
0.337 < Insufficient.sleep <= 0.368
0.000325 < Primary.care.physicians <= 0.000478
Feature
−0.0050
−0.0025
0.0000
0.0025
0.0050
Weight
Positive
Negative

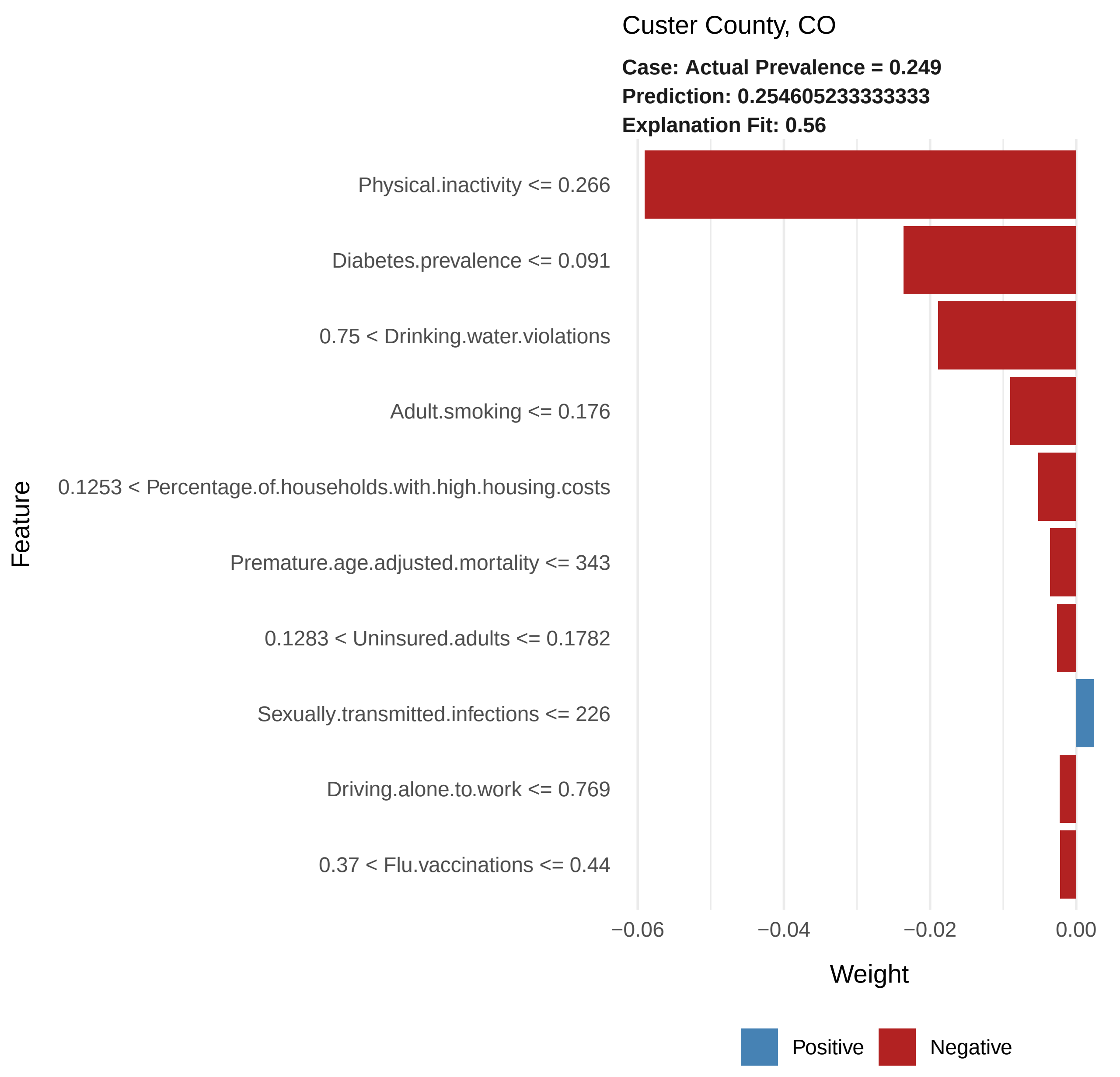

Custer County, CO
Case: Actual Prevalence = 0.249
Prediction: 0.254605233333333
Explanation Fit: 0.56
Physical.inactivity <= 0.266
Diabetes.prevalence <= 0.091
0.75 < Drinking.water.violations
Adult.smoking <= 0.176
0.1253 < Percentage.of.households.with.high.housing.costs
Premature.age.adjusted.mortality <= 343
0.1283 < Uninsured.adults <= 0.1782
Sexually.transmitted.infections <= 226
Driving.alone.to.work <= 0.769
0.37 < Flu.vaccinations <= 0.44
Feature
−0.06
−0.04
−0.02
0.00
Weight
Positive
Negative

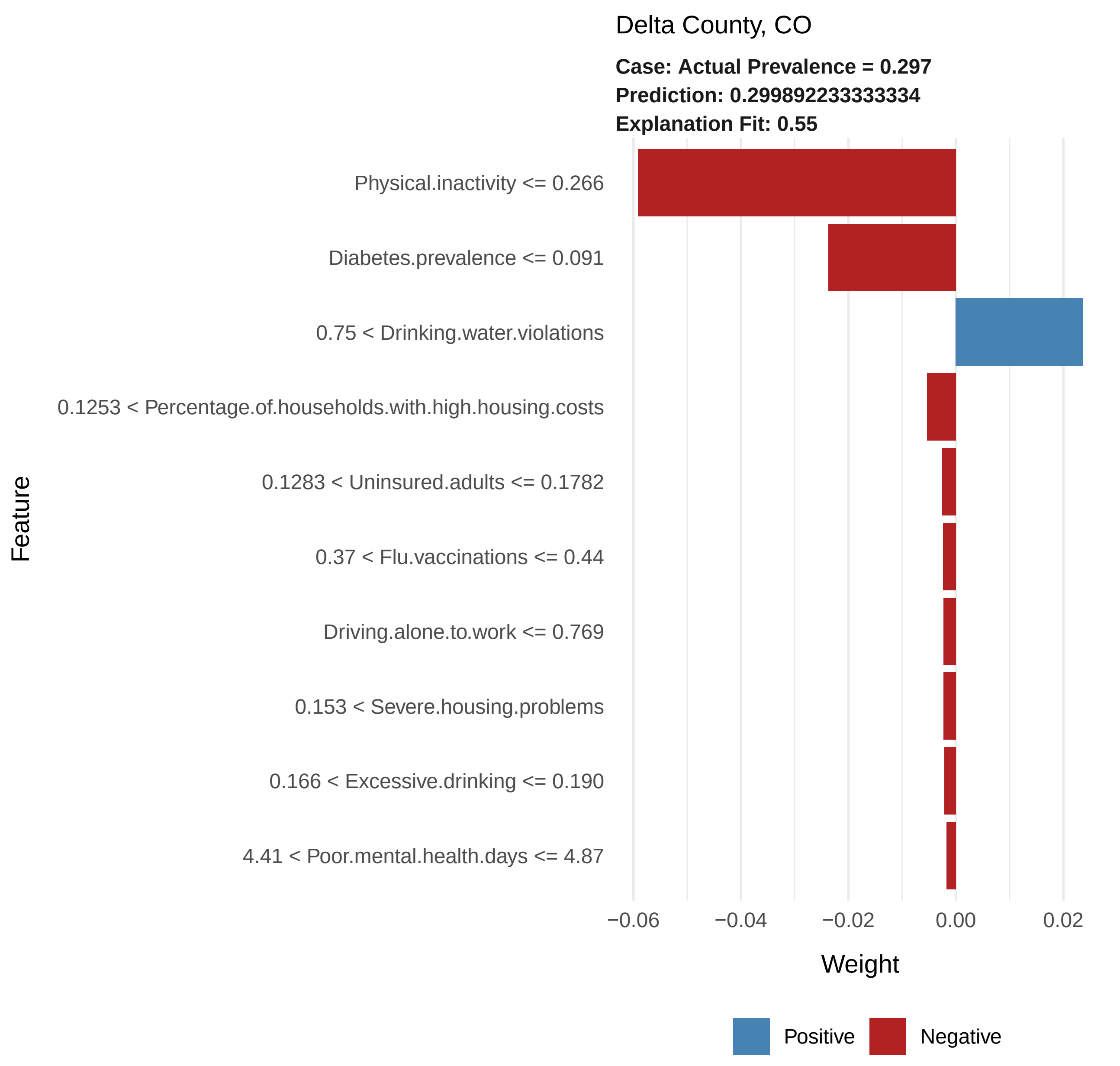
Delta County, CO
Case: Actual Prevalence = 0.297
Prediction: 0.299892233333334
Explanation Fit: 0.55
Physical.inactivity <= 0.266
Diabetes.prevalence <= 0.091
0.75 < Drinking.water.violations
0.1253 < Percentage.of.households.with.high.housing.costs
0.1283 < Uninsured.adults <= 0.1782
0.37 < Flu.vaccinations <= 0.44
Driving.alone.to.work <= 0.769
0.153 < Severe.housing.problems
0.166 < Excessive.drinking <= 0.190
4.41 < Poor.mental.health.days <= 4.87
Feature
−0.06
−0.04
−0.02
0.00
0.02
Weight
Positive
Negative

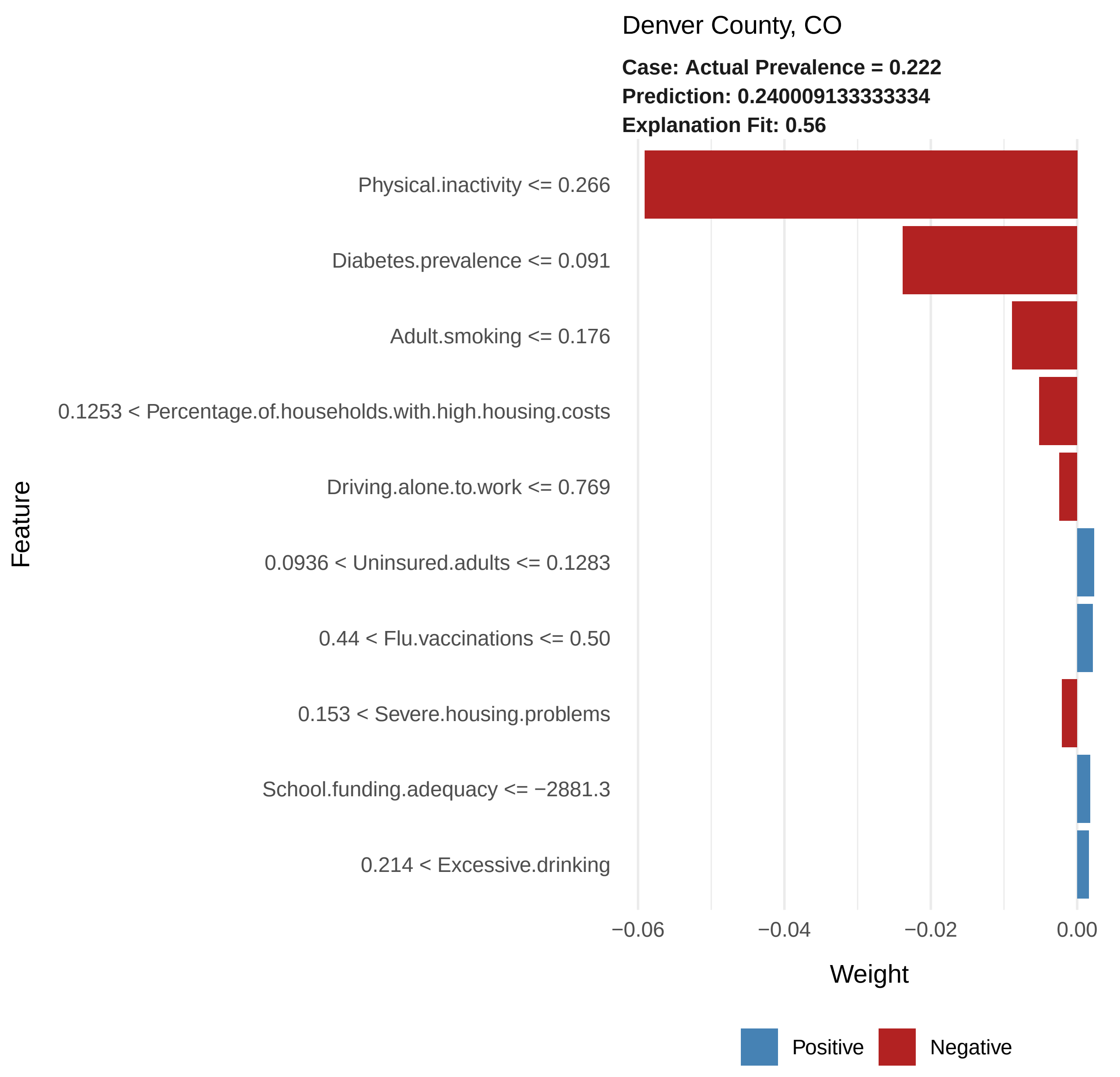

Denver County, CO
Case: Actual Prevalence = 0.222
Prediction: 0.240009133333334
Explanation Fit: 0.56
Physical.inactivity <= 0.266
Diabetes.prevalence <= 0.091
Adult.smoking <= 0.176
0.1253 < Percentage.of.households.with.high.housing.costs
Driving.alone.to.work <= 0.769
0.0936 < Uninsured.adults <= 0.1283
0.44 < Flu.vaccinations <= 0.50
0.153 < Severe.housing.problems
School.funding.adequacy <= −2881.3
0.214 < Excessive.drinking
Feature
−0.06
−0.04
−0.02
0.00
Weight
Positive
Negative

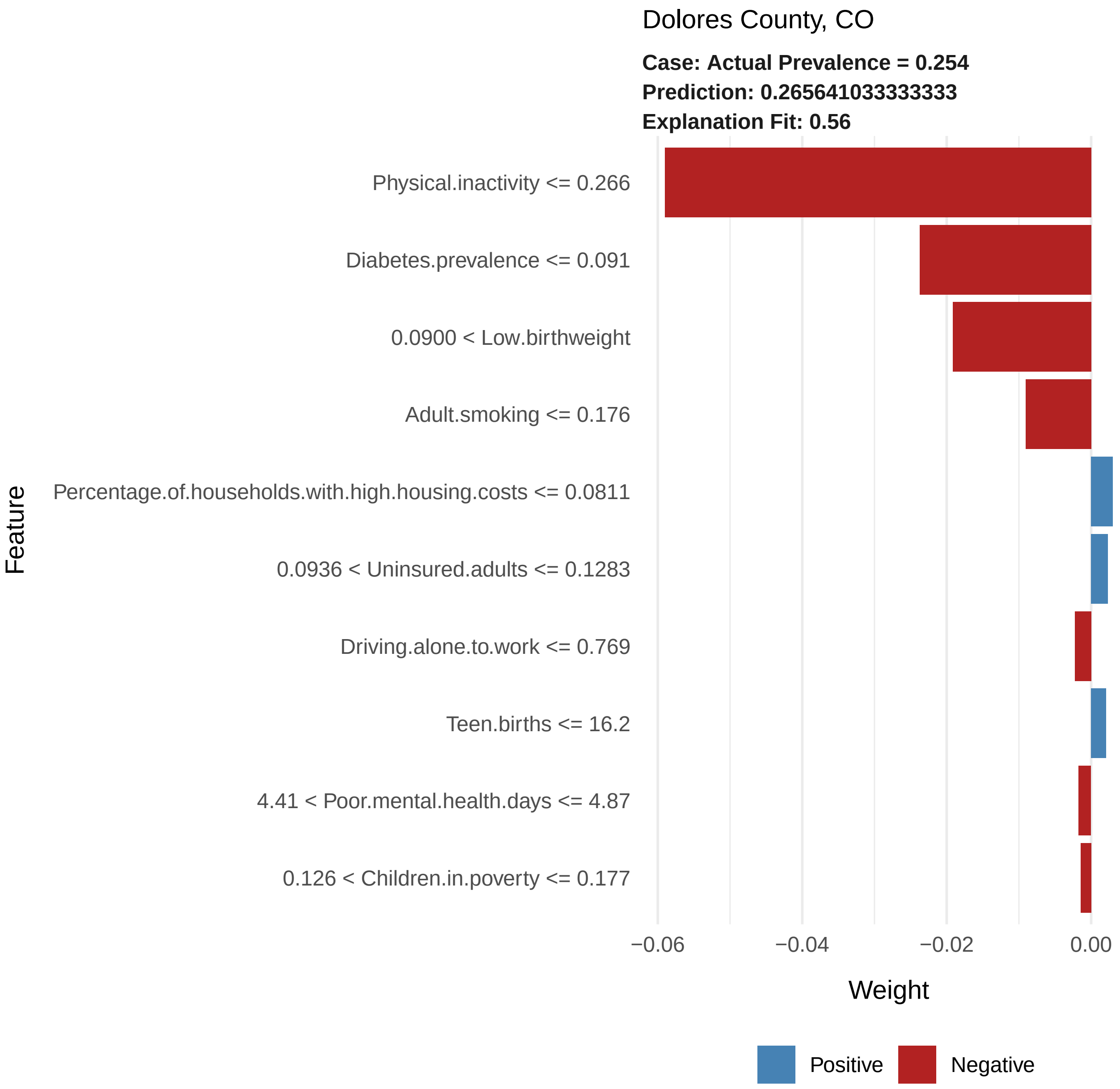
Dolores County, CO
Case: Actual Prevalence = 0.254
Prediction: 0.265641033333333
Explanation Fit: 0.56
Physical.inactivity <= 0.266
Diabetes.prevalence <= 0.091
0.0900 < Low.birthweight
Adult.smoking <= 0.176
Percentage.of.households.with.high.housing.costs <= 0.0811
0.0936 < Uninsured.adults <= 0.1283
Driving.alone.to.work <= 0.769
Teen.births <= 16.2
4.41 < Poor.mental.health.days <= 4.87
0.126 < Children.in.poverty <= 0.177
Feature
−0.06
−0.04
−0.02
0.00
Weight
Positive
Negative

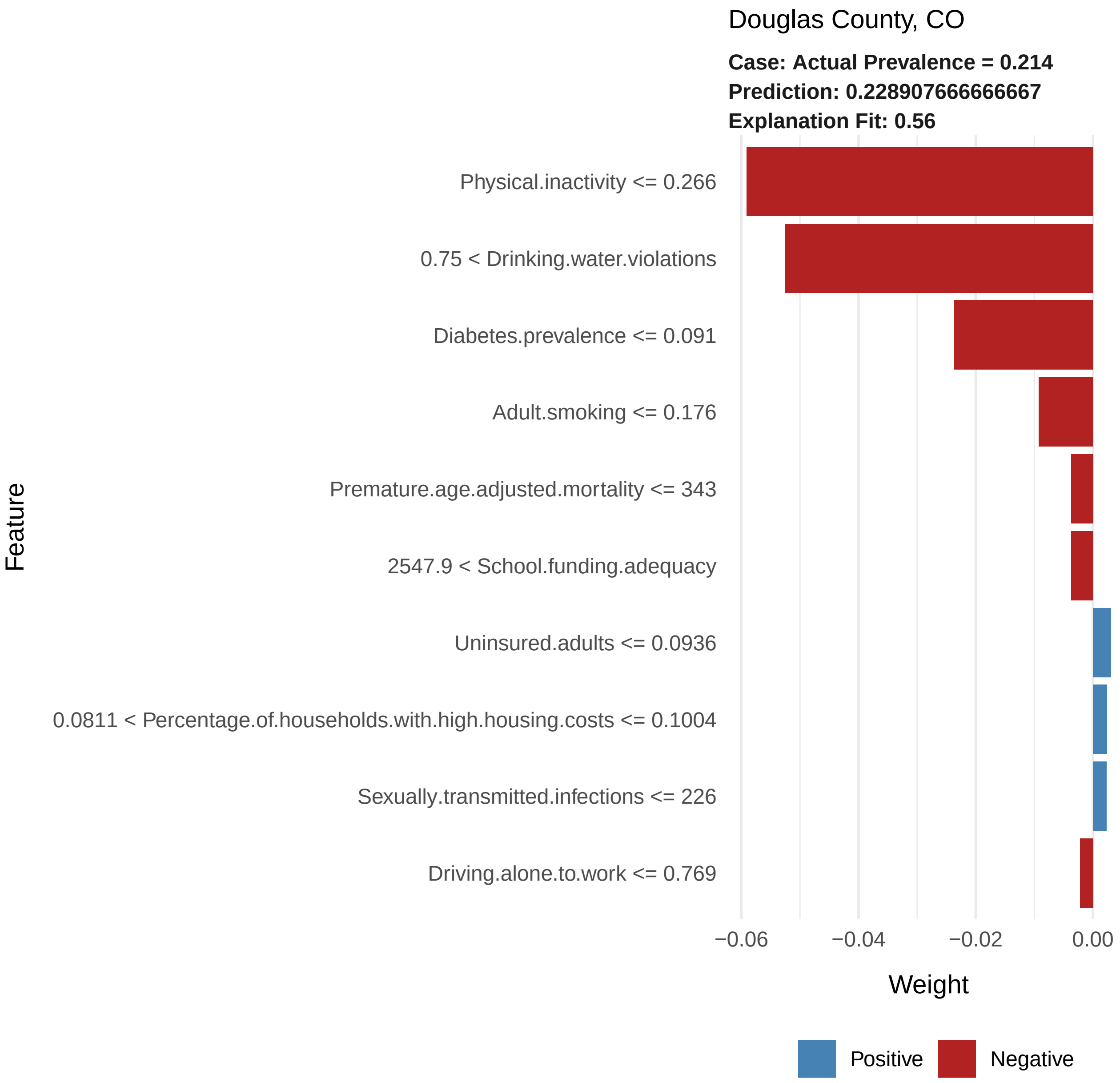
Douglas County, CO
Case: Actual Prevalence = 0.214
Prediction: 0.228907666666667
Explanation Fit: 0.56
Physical.inactivity <= 0.266
0.75 < Drinking.water.violations
Diabetes.prevalence <= 0.091
Adult.smoking <= 0.176
Premature.age.adjusted.mortality <= 343
2547.9 < School.funding.adequacy
Uninsured.adults <= 0.0936
0.0811 < Percentage.of.households.with.high.housing.costs <= 0.1004
Sexually.transmitted.infections <= 226
Driving.alone.to.work <= 0.769
Feature
−0.06
−0.04
−0.02
0.00
Weight
Positive
Negative

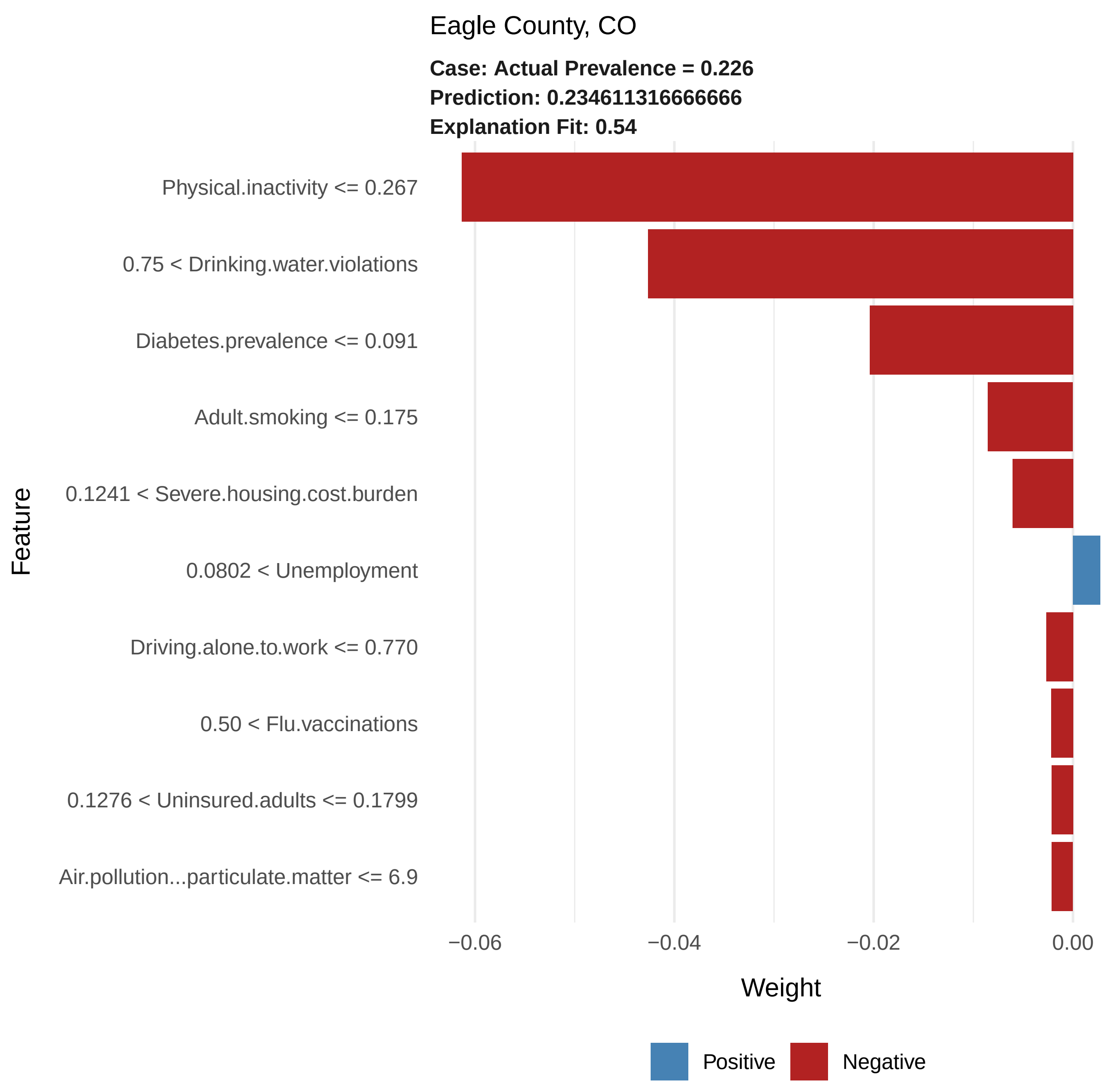

Eagle County, CO
Case: Actual Prevalence = 0.226
Prediction: 0.234611316666666
Explanation Fit: 0.54
Physical.inactivity <= 0.267
0.75 < Drinking.water.violations
Diabetes.prevalence <= 0.091
Adult.smoking <= 0.175
0.1241 < Severe.housing.cost.burden
0.0802 < Unemployment
Driving.alone.to.work <= 0.770
0.50 < Flu.vaccinations
0.1276 < Uninsured.adults <= 0.1799
Air.pollution...particulate.matter <= 6.9
Feature
−0.06
−0.04
−0.02
0.00
Weight
Positive
Negative

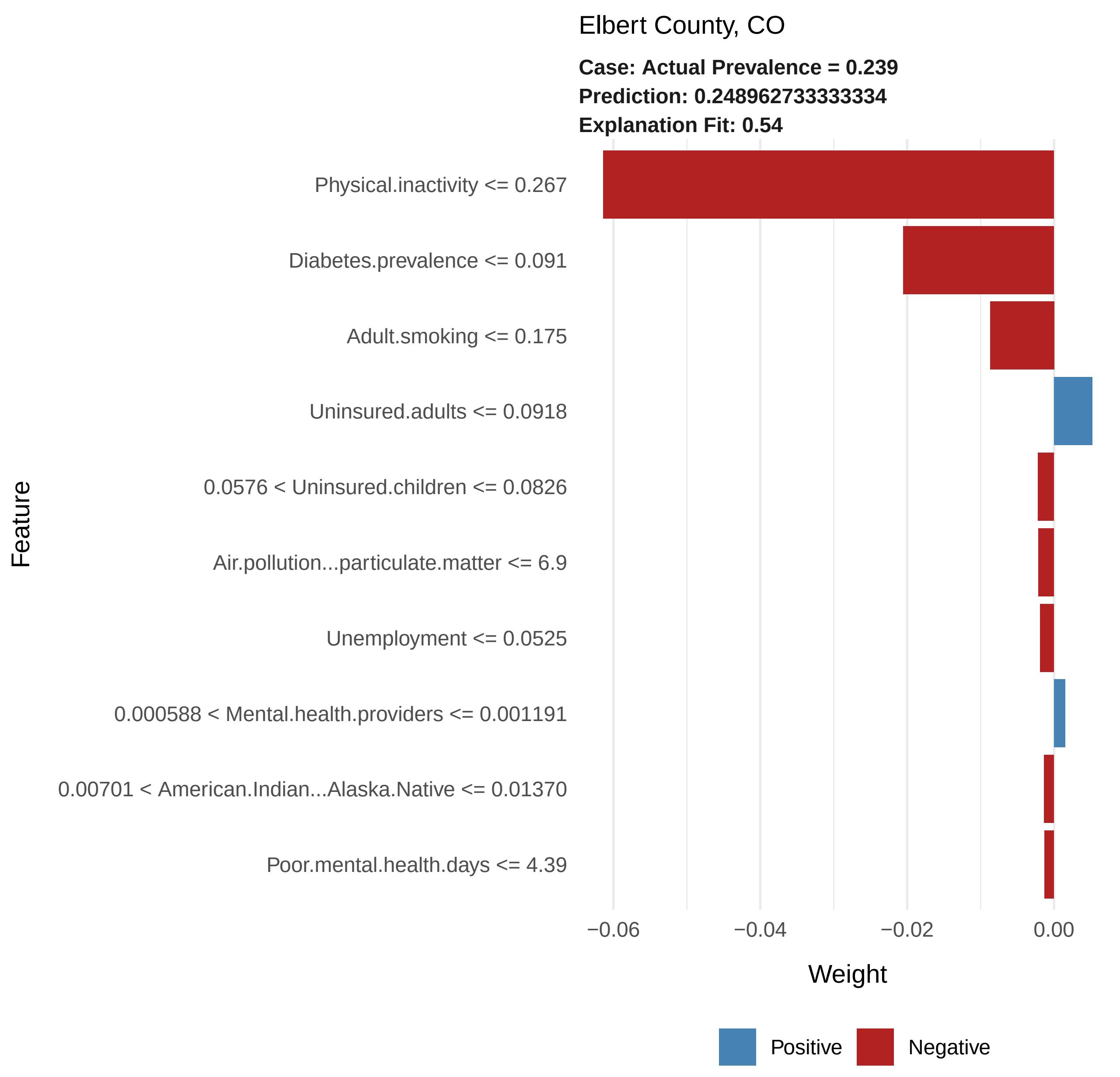

Elbert County, CO
Case: Actual Prevalence = 0.239
Prediction: 0.248962733333334
Explanation Fit: 0.54
Physical.inactivity <= 0.267
Diabetes.prevalence <= 0.091
Adult.smoking <= 0.175
Uninsured.adults <= 0.0918
0.0576 < Uninsured.children <= 0.0826
Air.pollution...particulate.matter <= 6.9
Unemployment <= 0.0525
0.000588 < Mental.health.providers <= 0.001191
0.00701 < American.Indian...Alaska.Native <= 0.01370
Poor.mental.health.days <= 4.39
Feature
−0.06
−0.04
−0.02
0.00
Weight
Positive
Negative

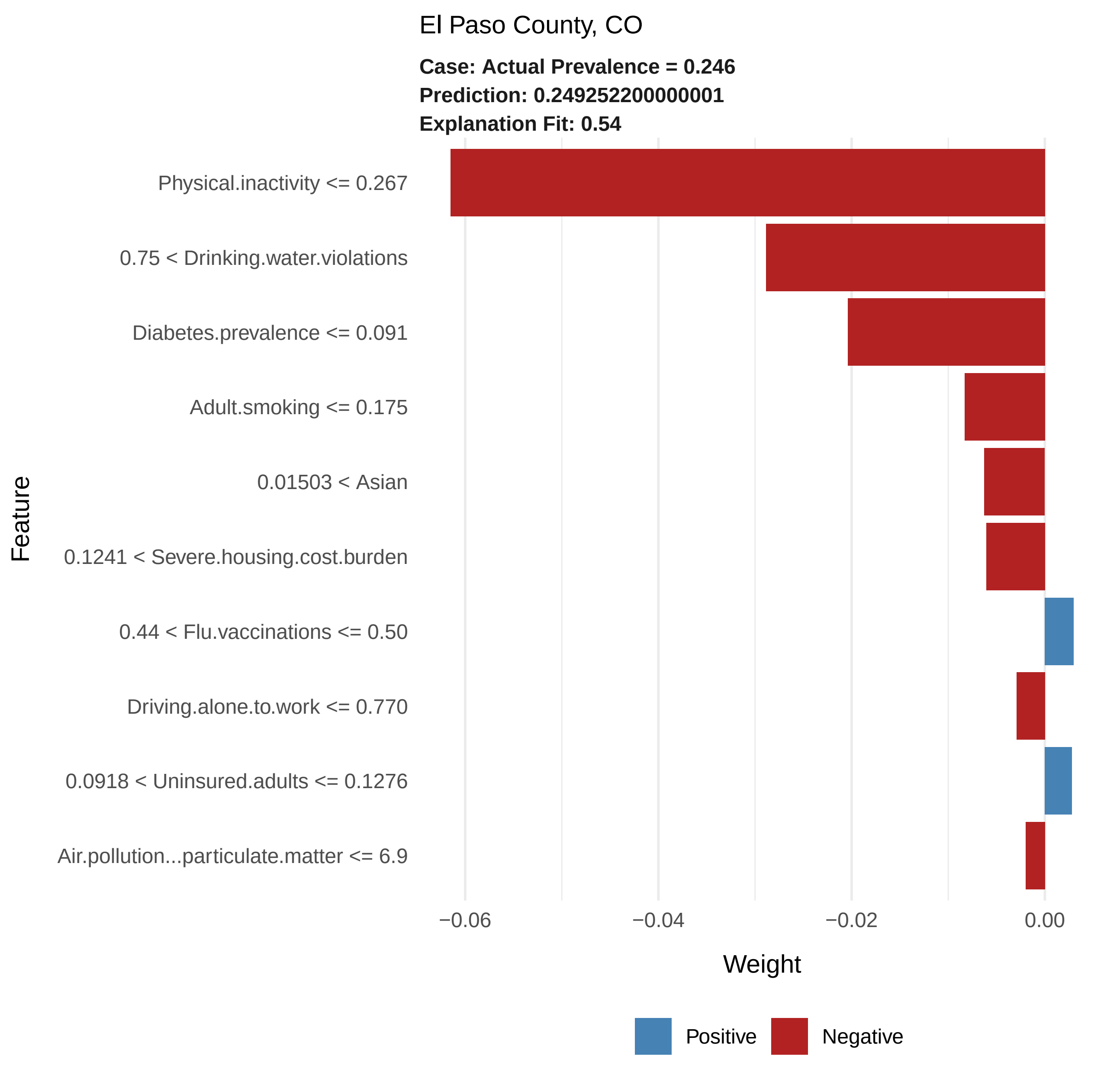

El Paso County, CO
Case: Actual Prevalence = 0.246
Prediction: 0.249252200000001
Explanation Fit: 0.54
Physical.inactivity <= 0.267
0.75 < Drinking.water.violations
Diabetes.prevalence <= 0.091
Adult.smoking <= 0.175
0.01503 < Asian
0.1241 < Severe.housing.cost.burden
0.44 < Flu.vaccinations <= 0.50
Driving.alone.to.work <= 0.770
0.0918 < Uninsured.adults <= 0.1276
Air.pollution...particulate.matter <= 6.9
Feature
−0.06
−0.04
−0.02
0.00
Weight
Positive
Negative

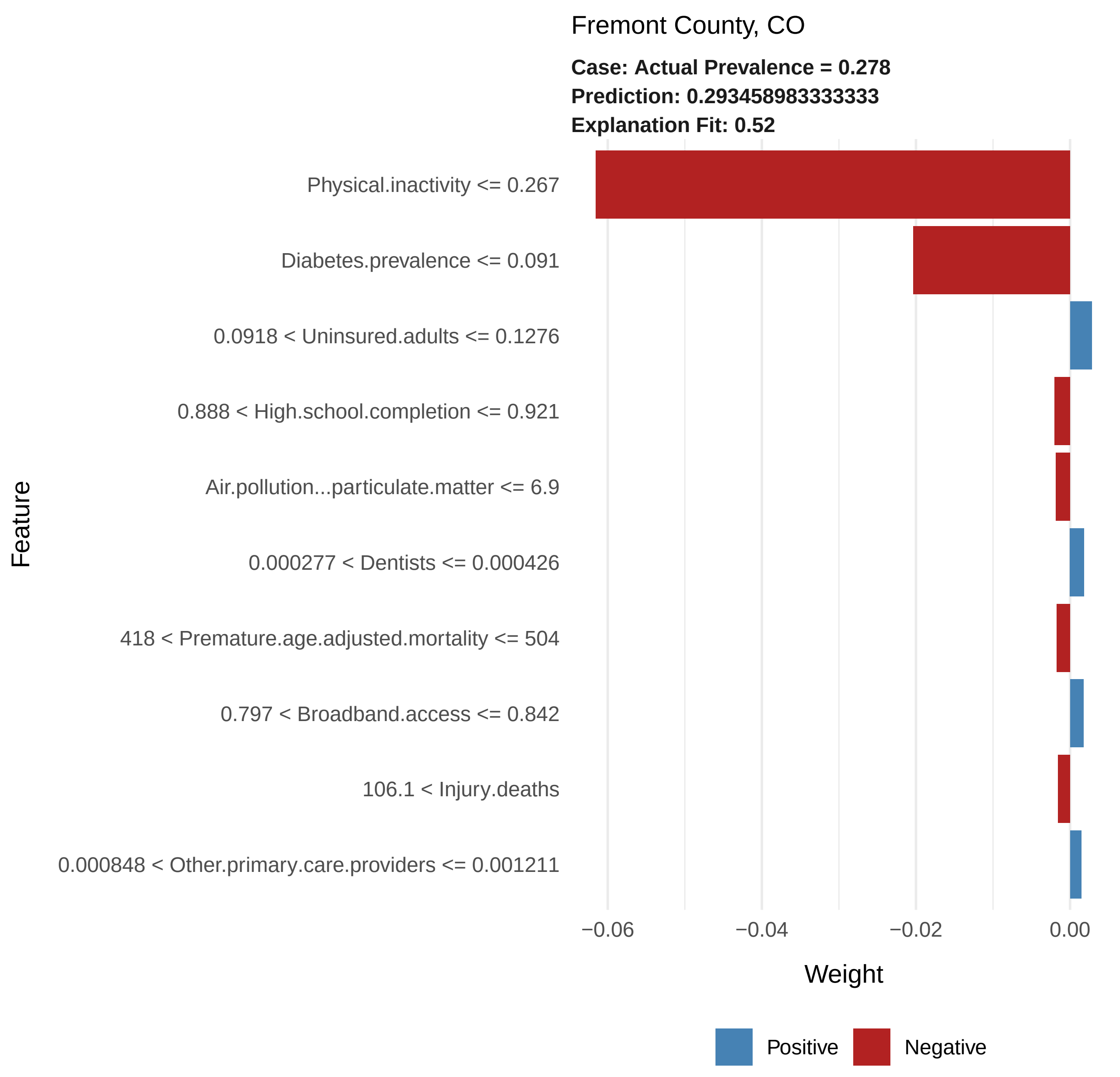

Fremont County, CO
Case: Actual Prevalence = 0.278
Prediction: 0.293458983333333
Explanation Fit: 0.52
Physical.inactivity <= 0.267
Diabetes.prevalence <= 0.091
0.0918 < Uninsured.adults <= 0.1276
0.888 < High.school.completion <= 0.921
Air.pollution...particulate.matter <= 6.9
0.000277 < Dentists <= 0.000426
418 < Premature.age.adjusted.mortality <= 504
0.797 < Broadband.access <= 0.842
106.1 < Injury.deaths
0.000848 < Other.primary.care.providers <= 0.001211
Feature
−0.06
−0.04
−0.02
0.00
Weight
Positive
Negative

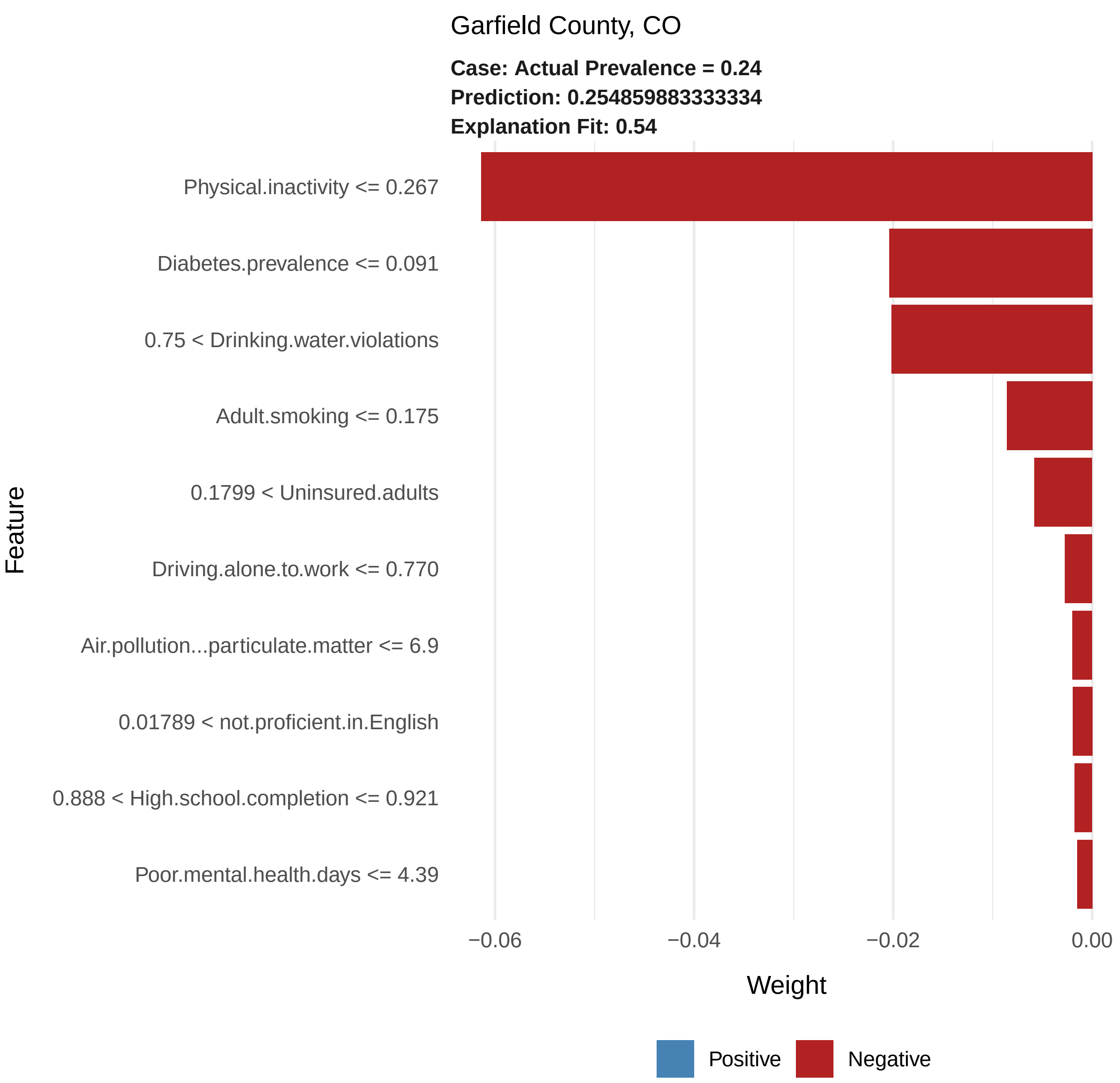

Garfield County, CO
Case: Actual Prevalence = 0.24
Prediction: 0.254859883333334
Explanation Fit: 0.54
Physical.inactivity <= 0.267
Diabetes.prevalence <= 0.091
0.75 < Drinking.water.violations
Adult.smoking <= 0.175
0.1799 < Uninsured.adults
Driving.alone.to.work <= 0.770
Air.pollution...particulate.matter <= 6.9
0.01789 < not.proficient.in.English
0.888 < High.school.completion <= 0.921
Poor.mental.health.days <= 4.39
Feature
−0.06
−0.04
−0.02
0.00
Weight
Positive
Negative

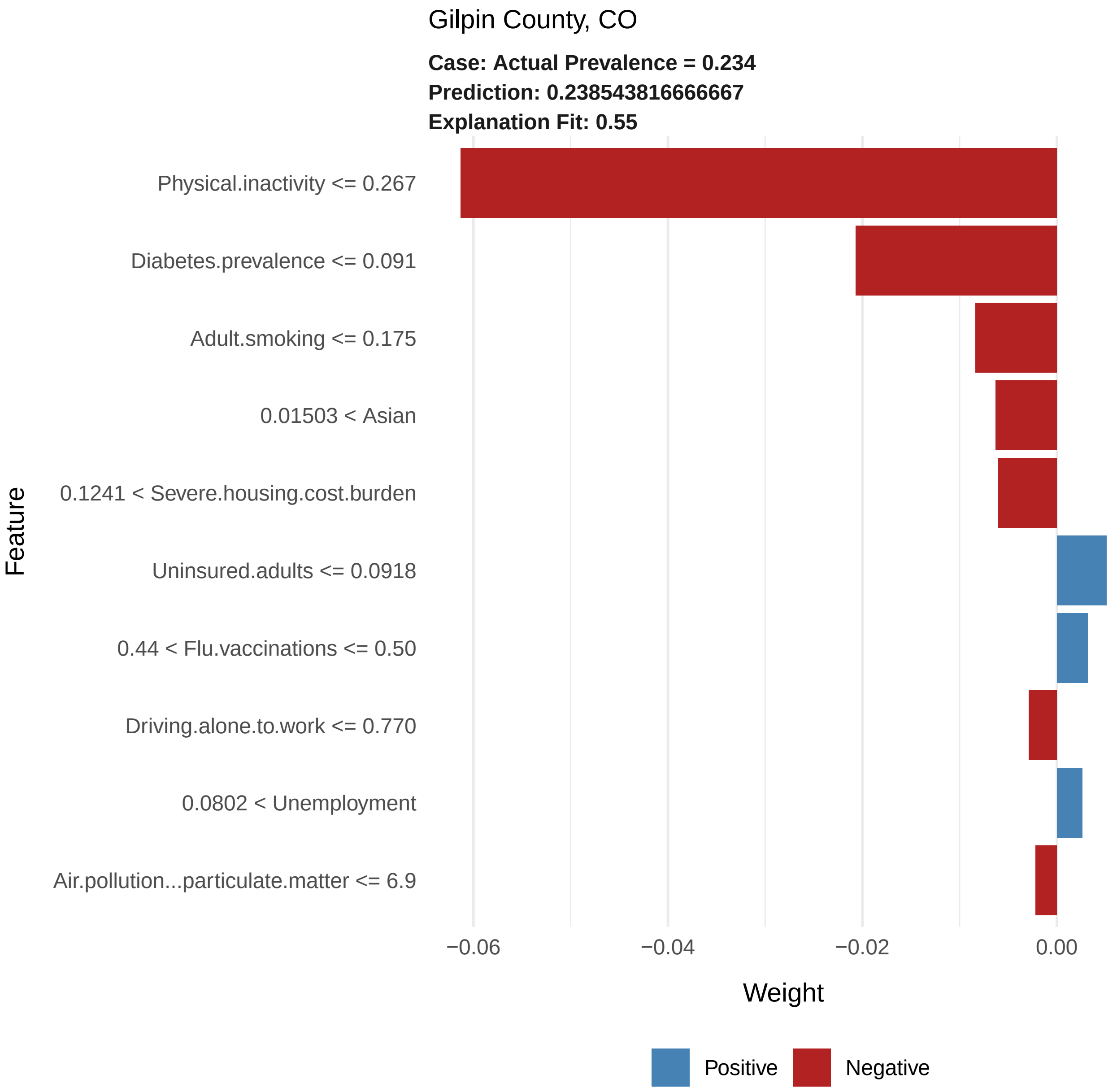
Gilpin County, CO
Case: Actual Prevalence = 0.234
Prediction: 0.238543816666667
Explanation Fit: 0.55
Physical.inactivity <= 0.267
Diabetes.prevalence <= 0.091
Adult.smoking <= 0.175
0.01503 < Asian
0.1241 < Severe.housing.cost.burden
Uninsured.adults <= 0.0918
0.44 < Flu.vaccinations <= 0.50
Driving.alone.to.work <= 0.770
0.0802 < Unemployment
Air.pollution...particulate.matter <= 6.9
Feature
−0.06
−0.04
−0.02
0.00
Weight
Positive
Negative

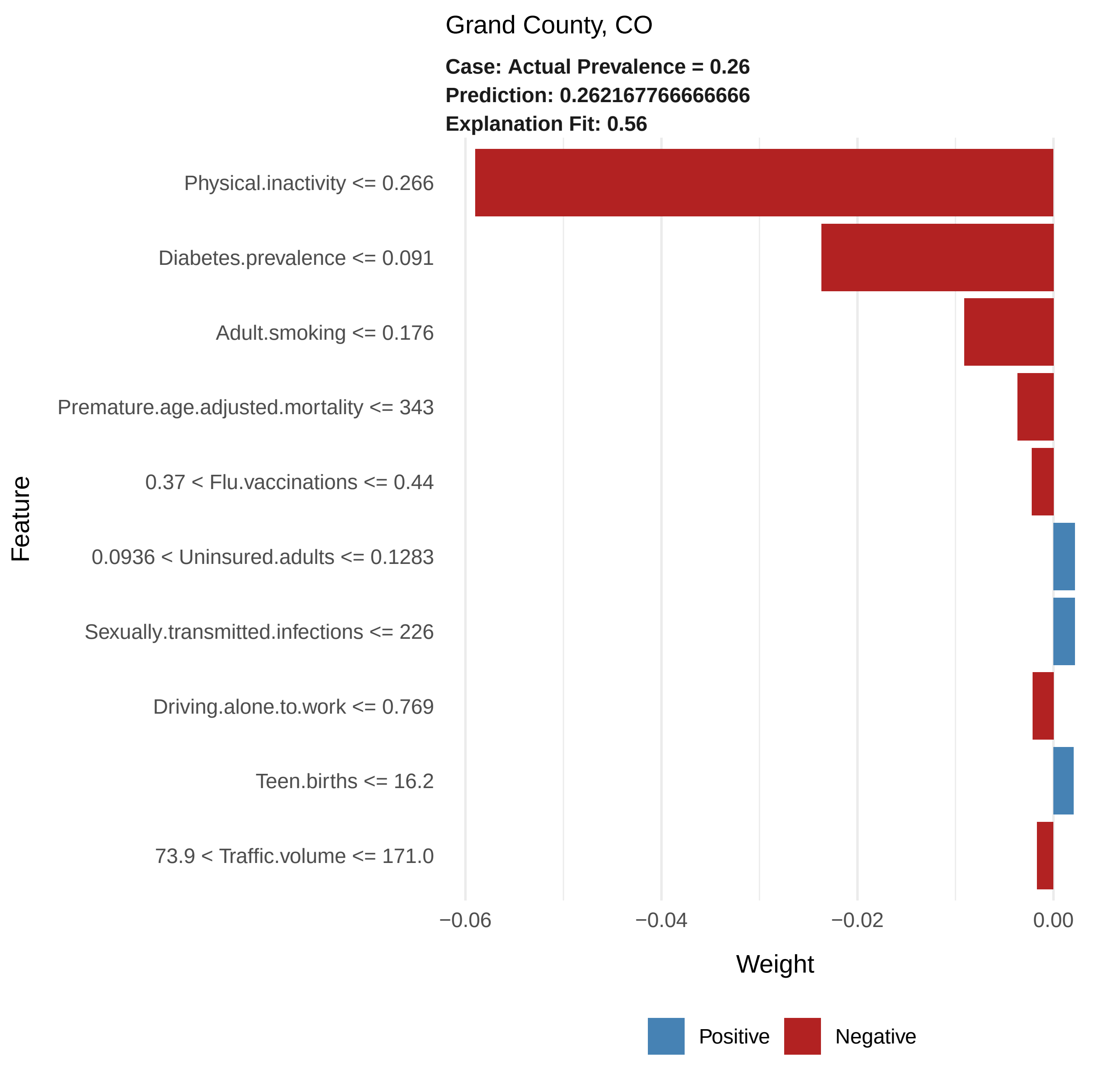

Grand County, CO
Case: Actual Prevalence = 0.26
Prediction: 0.262167766666666
Explanation Fit: 0.56
Physical.inactivity <= 0.266
Diabetes.prevalence <= 0.091
Adult.smoking <= 0.176
Premature.age.adjusted.mortality <= 343
0.37 < Flu.vaccinations <= 0.44
0.0936 < Uninsured.adults <= 0.1283
Sexually.transmitted.infections <= 226
Driving.alone.to.work <= 0.769
Teen.births <= 16.2
73.9 < Traffic.volume <= 171.0
Feature
−0.06
−0.04
−0.02
0.00
Weight
Positive
Negative

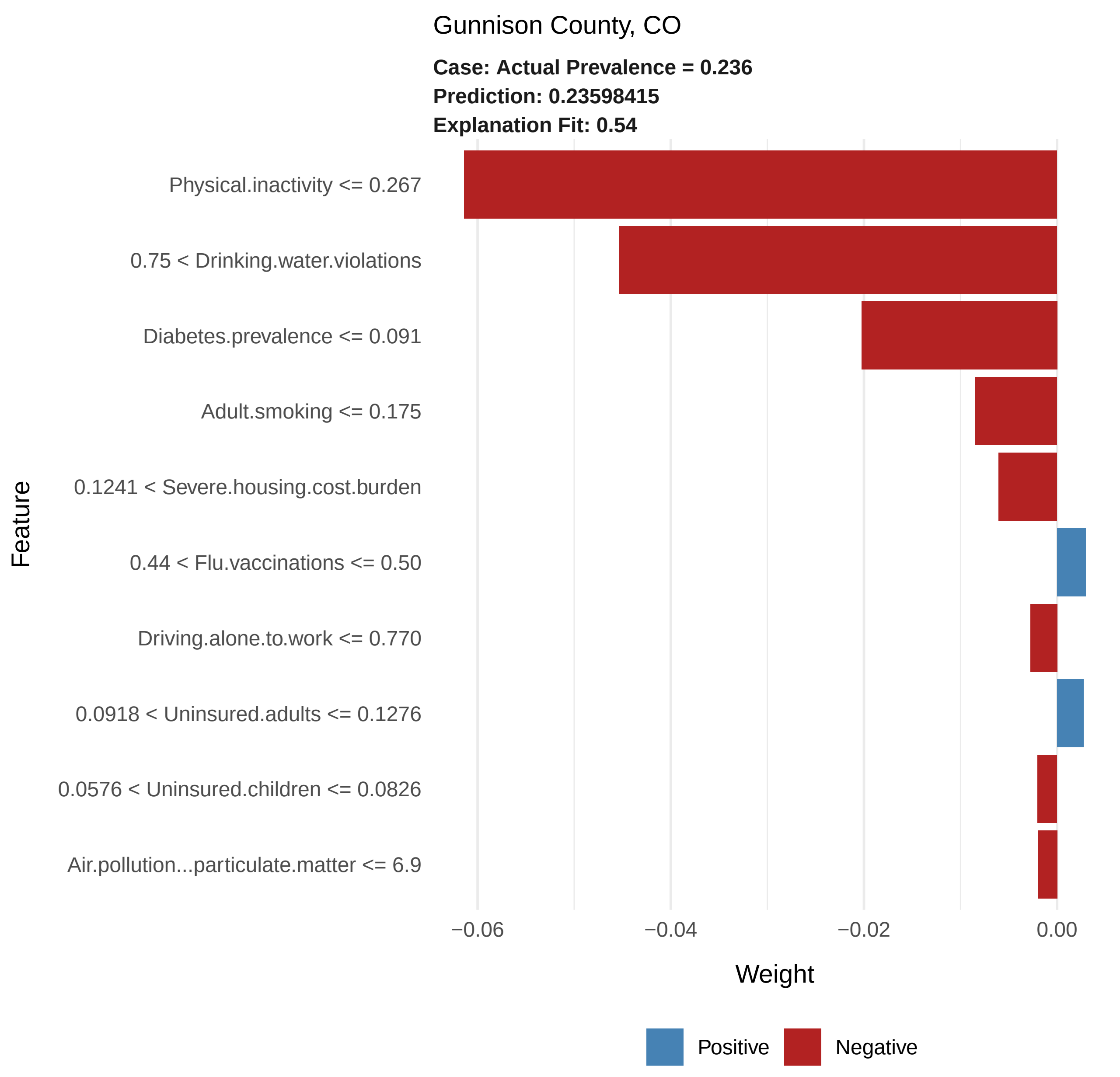

Gunnison County, CO
Case: Actual Prevalence = 0.236
Prediction: 0.23598415
Explanation Fit: 0.54
Feature
Physical.inactivity <= 0.267
0.75 < Drinking.water.violations
Diabetes.prevalence <= 0.091
Adult.smoking <= 0.175
0.1241 < Severe.housing.cost.burden
0.44 < Flu.vaccinations <= 0.50
Driving.alone.to.work <= 0.770
0.0918 < Uninsured.adults <= 0.1276
0.0576 < Uninsured.children <= 0.0826
Air.pollution...particulate.matter <= 6.9
−0.06
−0.04
−0.02
0.00
Weight
Positive
Negative

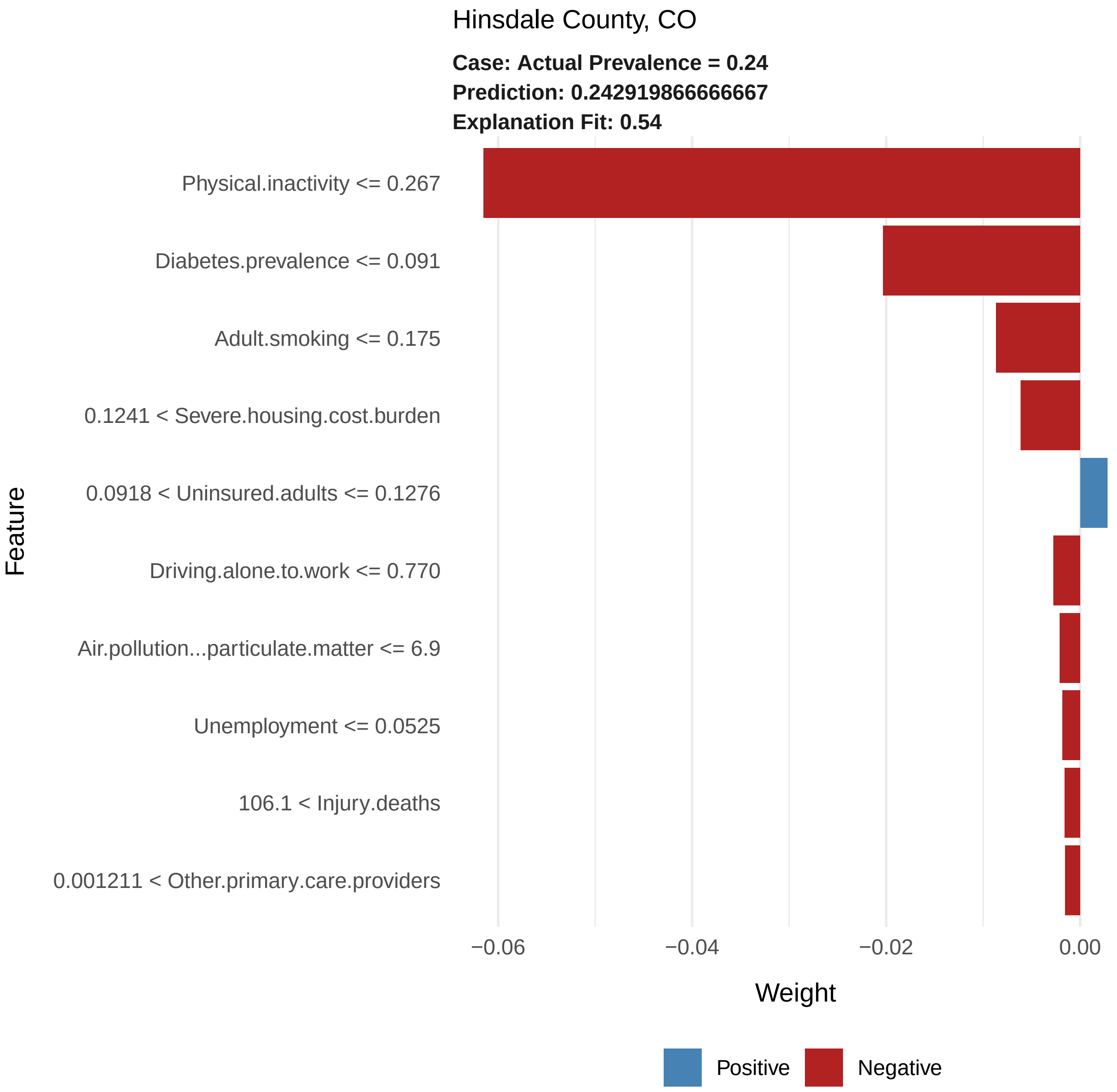

Hinsdale County, CO
Case: Actual Prevalence = 0.24
Prediction: 0.242919866666667
Explanation Fit: 0.54
Physical.inactivity <= 0.267
Diabetes.prevalence <= 0.091
Adult.smoking <= 0.175
0.1241 < Severe.housing.cost.burden
0.0918 < Uninsured.adults <= 0.1276
Driving.alone.to.work <= 0.770
Air.pollution...particulate.matter <= 6.9
Unemployment <= 0.0525
106.1 < Injury.deaths
0.001211 < Other.primary.care.providers
Feature
−0.06
−0.04
−0.02
0.00
Weight
Positive
Negative

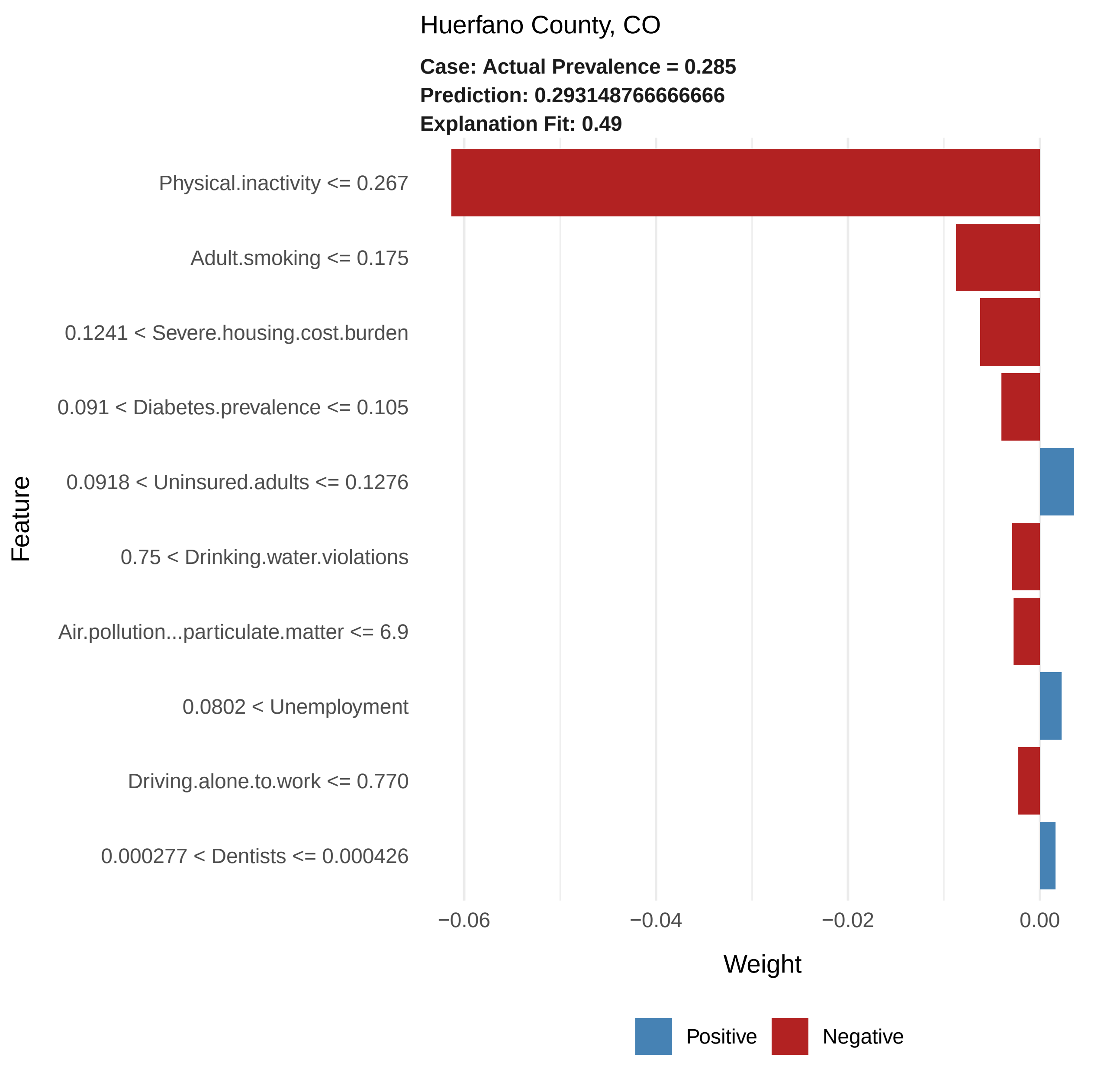
Huerfano County, CO
Case: Actual Prevalence = 0.285
Prediction: 0.293148766666666
Explanation Fit: 0.49
Feature
Physical.inactivity <= 0.267
Adult.smoking <= 0.175
0.1241 < Severe.housing.cost.burden
0.091 < Diabetes.prevalence <= 0.105
0.0918 < Uninsured.adults <= 0.1276
0.75 < Drinking.water.violations
Air.pollution...particulate.matter <= 6.9
0.0802 < Unemployment
Driving.alone.to.work <= 0.770
0.000277 < Dentists <= 0.000426
−0.06
−0.04
−0.02
0.00
Weight
Positive
Negative

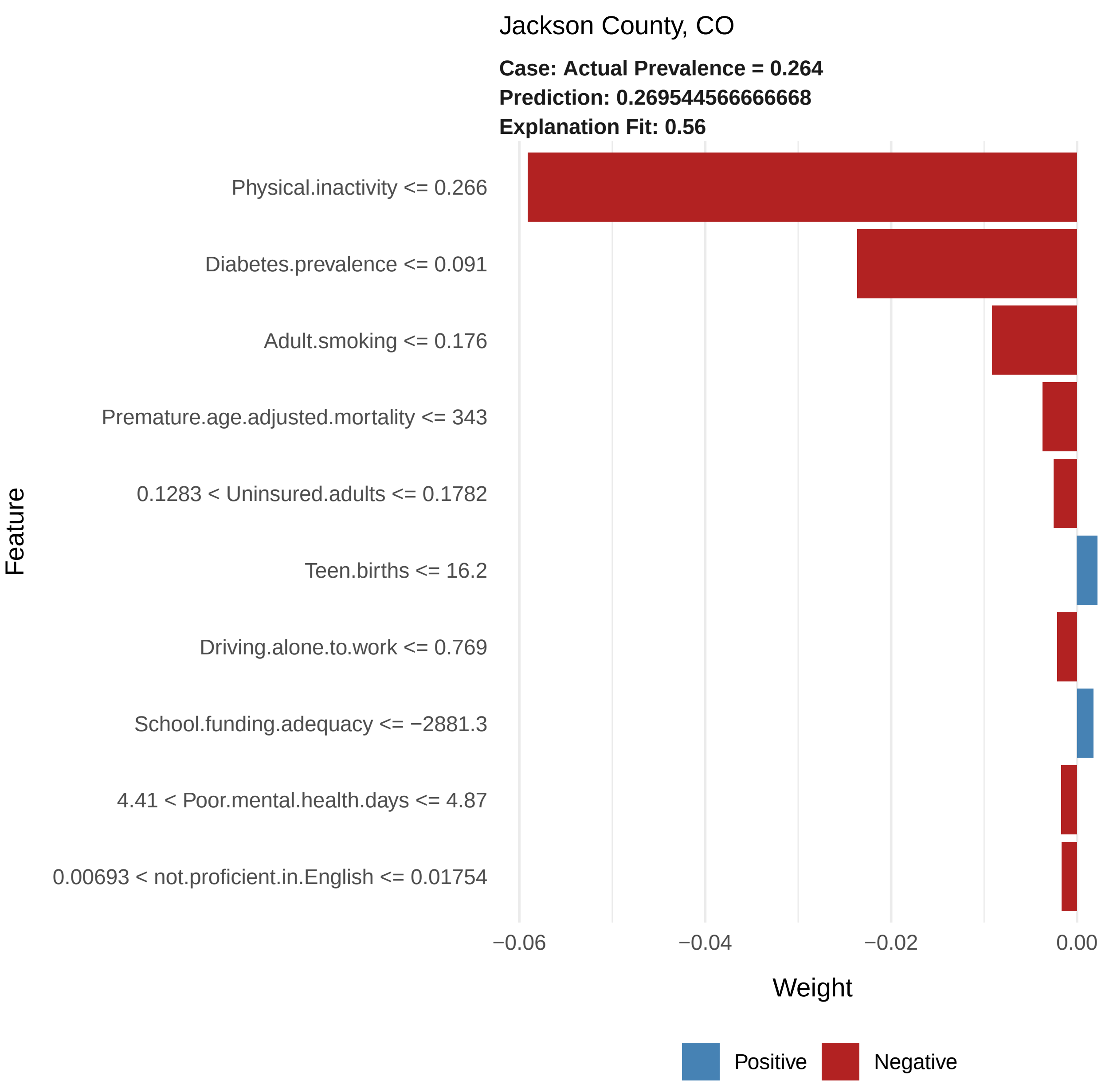

Jackson County, CO
Case: Actual Prevalence = 0.264
Prediction: 0.269544566666668
Explanation Fit: 0.56
Physical.inactivity <= 0.266
Diabetes.prevalence <= 0.091
Adult.smoking <= 0.176
Premature.age.adjusted.mortality <= 343
0.1283 < Uninsured.adults <= 0.1782
Teen.births <= 16.2
Driving.alone.to.work <= 0.769
School.funding.adequacy <= −2881.3
4.41 < Poor.mental.health.days <= 4.87
0.00693 < not.proficient.in.English <= 0.01754
Feature
−0.06
−0.04
−0.02
0.00
Weight
Positive
Negative

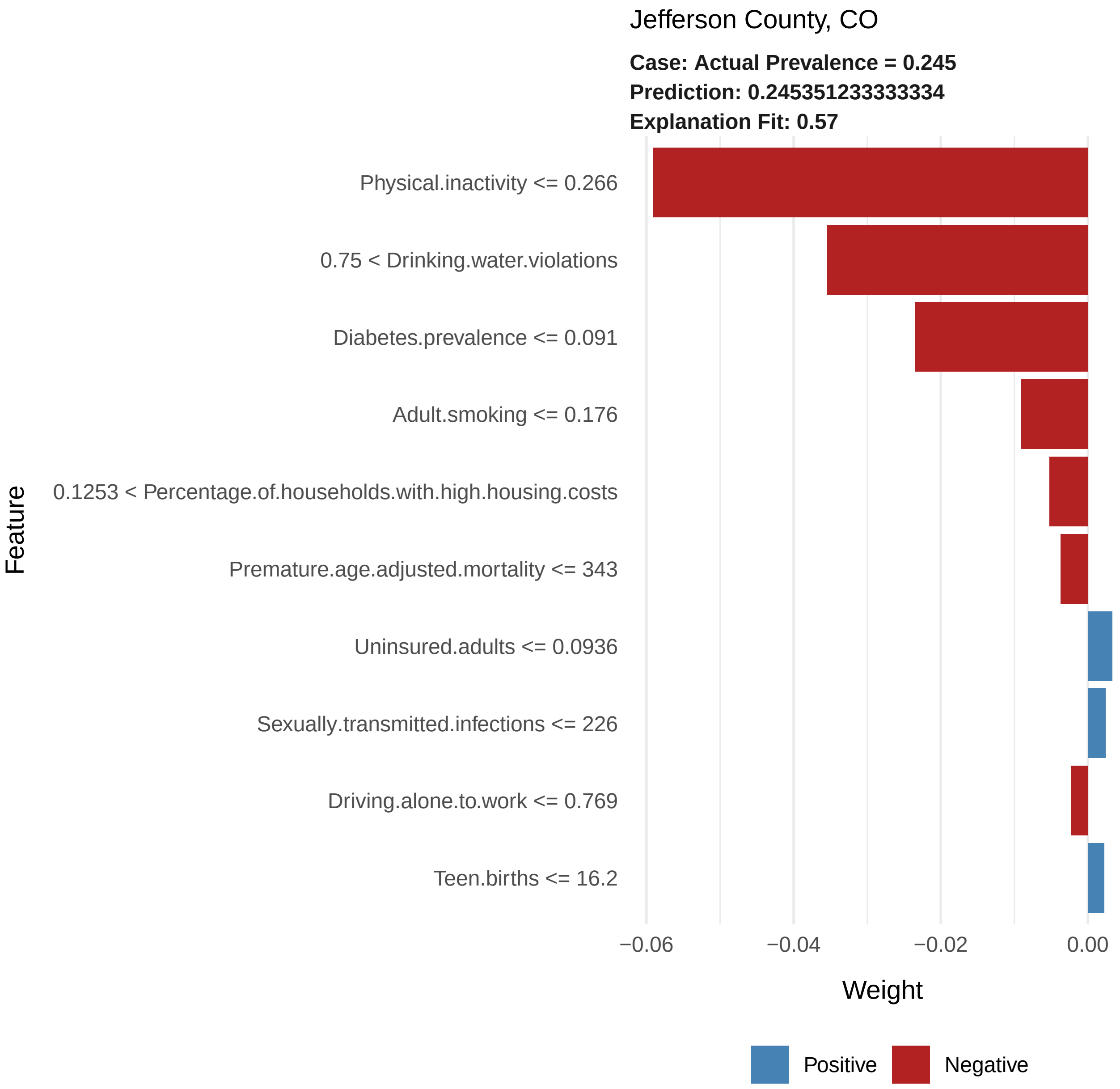
Jefferson County, CO
Case: Actual Prevalence = 0.245
Prediction: 0.245351233333334
Explanation Fit: 0.57
Feature
Physical.inactivity <= 0.266
0.75 < Drinking.water.violations
Diabetes.prevalence <= 0.091
Adult.smoking <= 0.176
0.1253 < Percentage.of.households.with.high.housing.costs
Premature.age.adjusted.mortality <= 343
Uninsured.adults <= 0.0936
Sexually.transmitted.infections <= 226
Driving.alone.to.work <= 0.769
Teen.births <= 16.2
−0.06
−0.04
−0.02
0.00
Weight
Positive
Negative

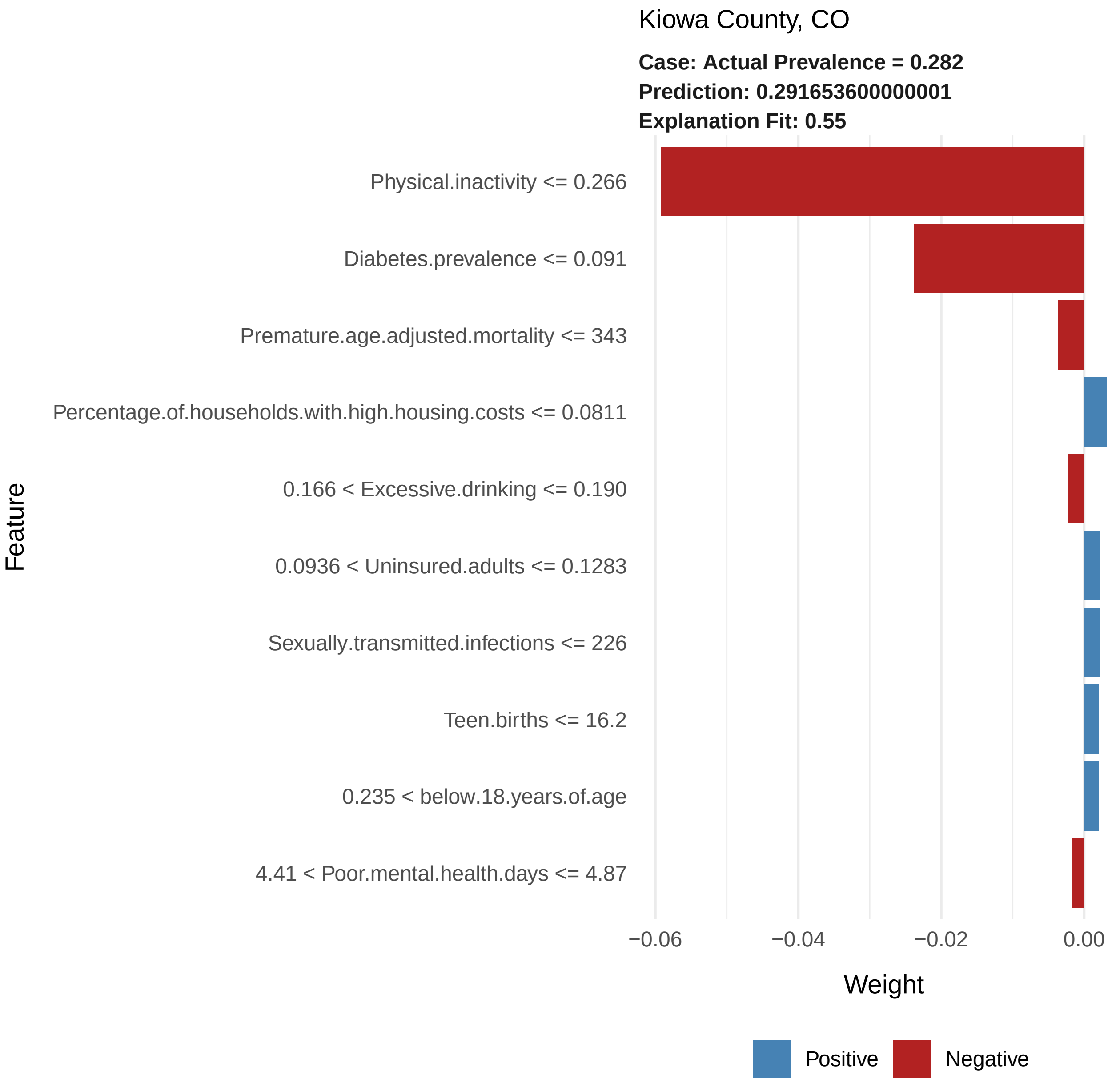

Kiowa County, CO
Case: Actual Prevalence = 0.282
Prediction: 0.291653600000001
Explanation Fit: 0.55
Physical.inactivity <= 0.266
Diabetes.prevalence <= 0.091
Premature.age.adjusted.mortality <= 343
Percentage.of.households.with.high.housing.costs <= 0.0811
0.166 < Excessive.drinking <= 0.190
0.0936 < Uninsured.adults <= 0.1283
Sexually.transmitted.infections <= 226
Teen.births <= 16.2
0.235 < below.18.years.of.age
4.41 < Poor.mental.health.days <= 4.87
Feature
−0.06
−0.04
−0.02
0.00
Weight
Positive
Negative

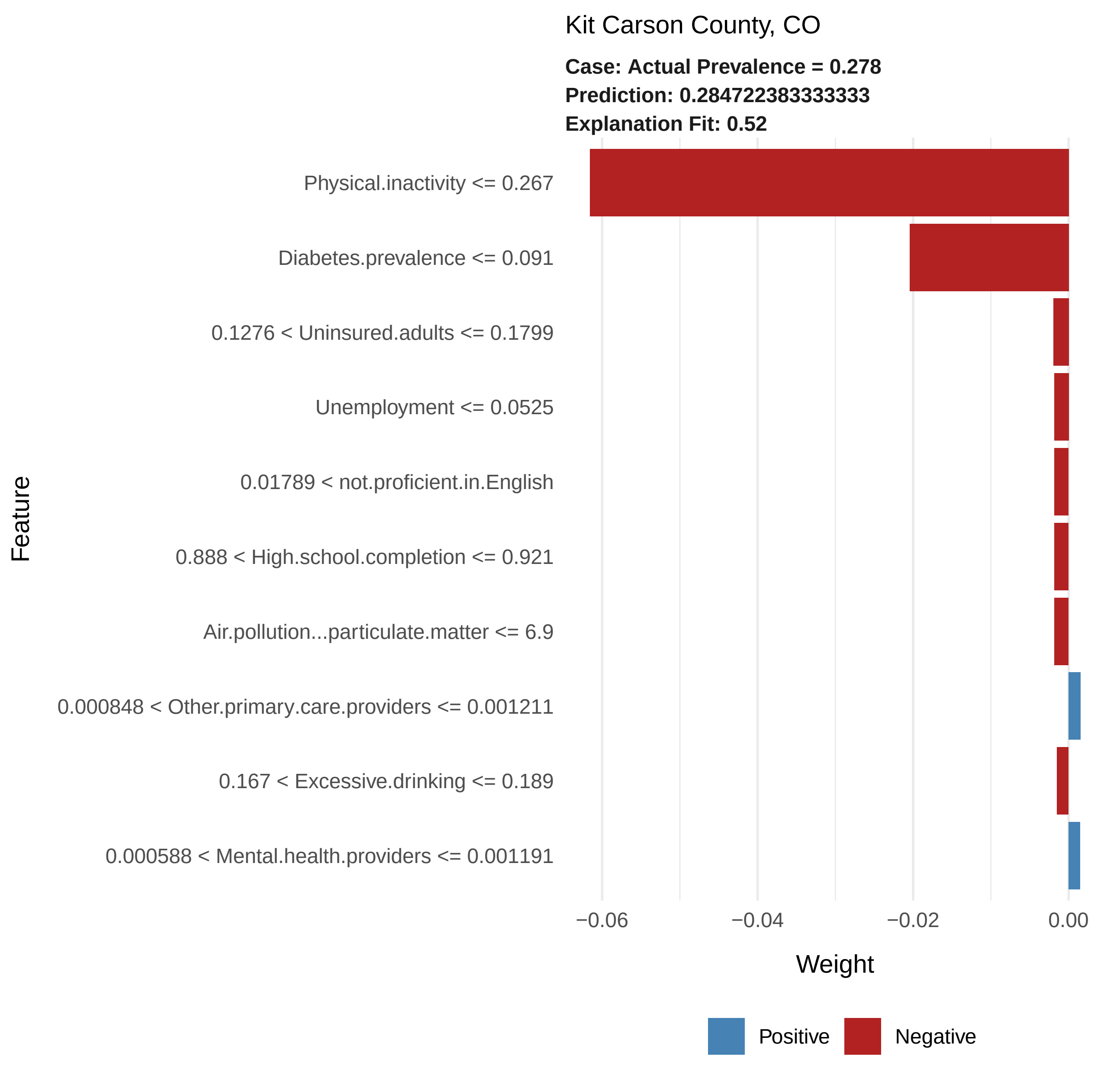

Kit Carson County, CO
Case: Actual Prevalence = 0.278
Prediction: 0.284722383333333
Explanation Fit: 0.52
Physical.inactivity <= 0.267
Diabetes.prevalence <= 0.091
0.1276 < Uninsured.adults <= 0.1799
Unemployment <= 0.0525
0.01789 < not.proficient.in.English
0.888 < High.school.completion <= 0.921
Air.pollution...particulate.matter <= 6.9
0.000848 < Other.primary.care.providers <= 0.001211
0.167 < Excessive.drinking <= 0.189
0.000588 < Mental.health.providers <= 0.001191
Feature
−0.06
−0.04
−0.02
0.00
Weight
Positive
Negative

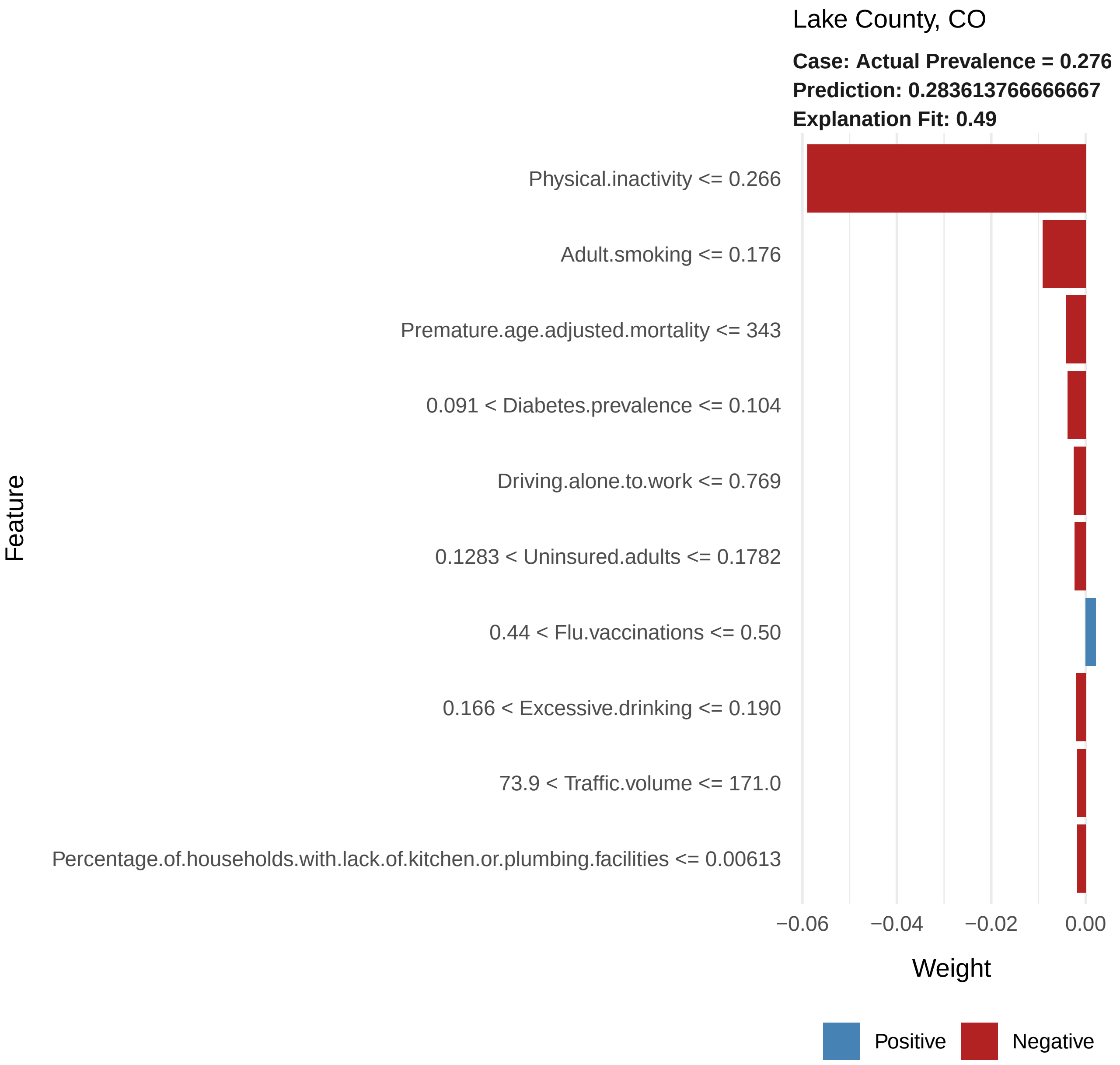

Lake County, CO
Case: Actual Prevalence = 0.276
Prediction: 0.283613766666667
Explanation Fit: 0.49
Physical.inactivity <= 0.266
Adult.smoking <= 0.176
Premature.age.adjusted.mortality <= 343
0.091 < Diabetes.prevalence <= 0.104
Driving.alone.to.work <= 0.769
0.1283 < Uninsured.adults <= 0.1782
0.44 < Flu.vaccinations <= 0.50
0.166 < Excessive.drinking <= 0.190
73.9 < Traffic.volume <= 171.0
Percentage.of.households.with.lack.of.kitchen.or.plumbing.facilities <= 0.00613
Feature
−0.06
−0.04
−0.02
0.00
Weight
Positive
Negative

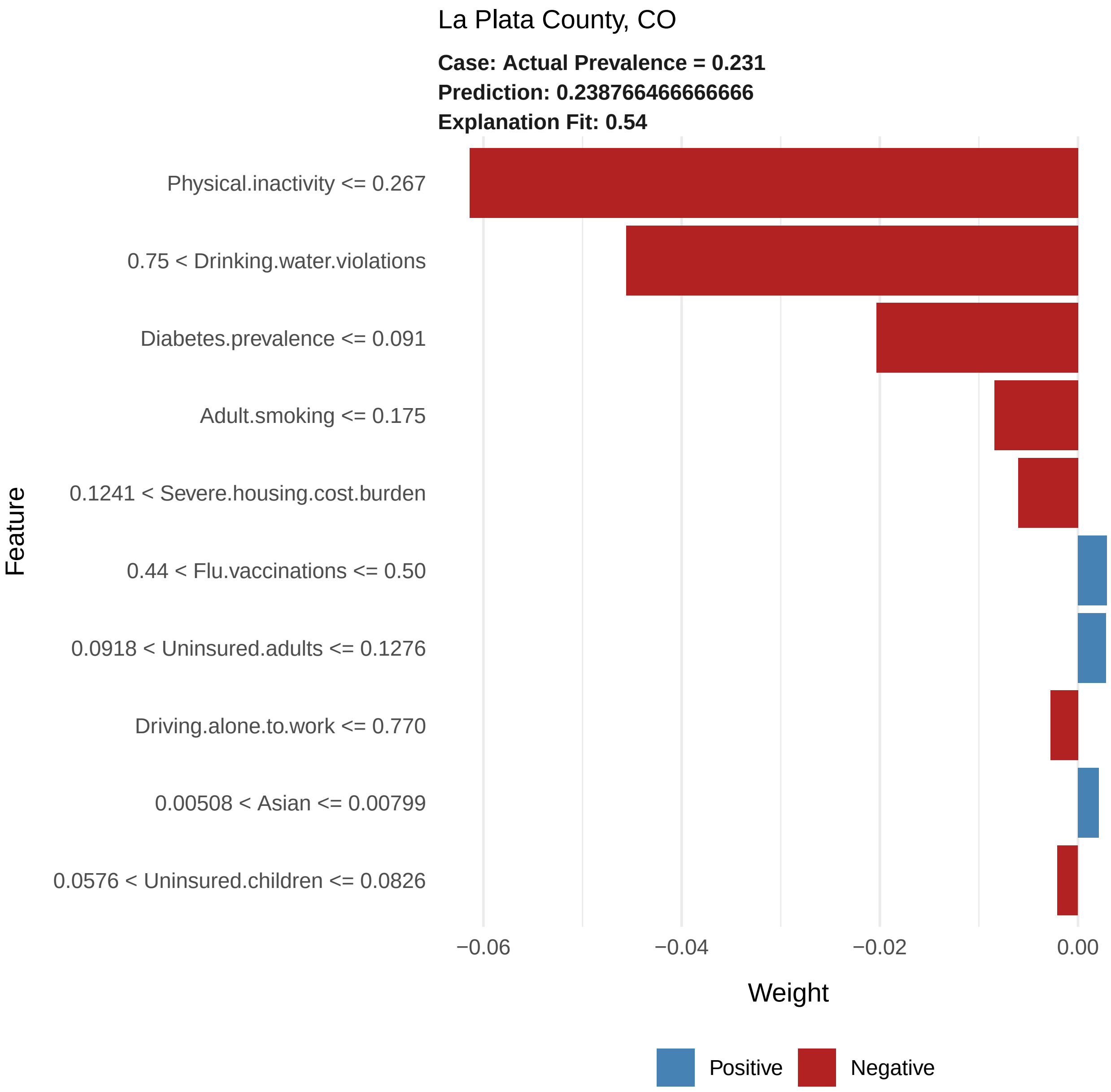
La Plata County, CO
Case: Actual Prevalence = 0.231
Prediction: 0.238766466666666
Explanation Fit: 0.54
Physical.inactivity <= 0.267
0.75 < Drinking.water.violations
Diabetes.prevalence <= 0.091
Adult.smoking <= 0.175
0.1241 < Severe.housing.cost.burden
0.44 < Flu.vaccinations <= 0.50
0.0918 < Uninsured.adults <= 0.1276
Driving.alone.to.work <= 0.770
0.00508 < Asian <= 0.00799
0.0576 < Uninsured.children <= 0.0826
Feature
−0.06
−0.04
−0.02
0.00
Weight
Positive
Negative

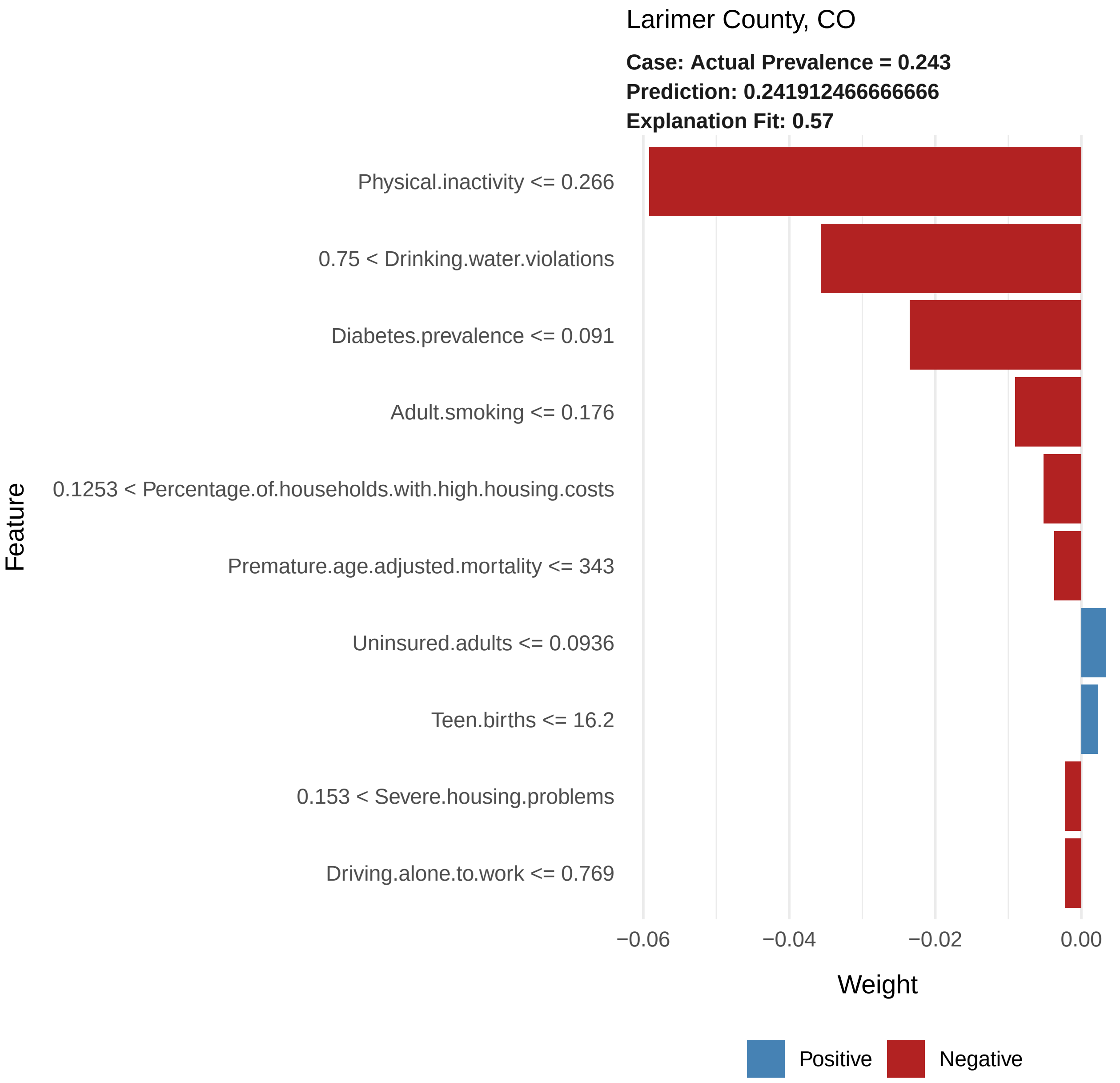

Larimer County, CO
Case: Actual Prevalence = 0.243
Prediction: 0.241912466666666
Explanation Fit: 0.57
Physical.inactivity <= 0.266
0.75 < Drinking.water.violations
Diabetes.prevalence <= 0.091
Adult.smoking <= 0.176
0.1253 < Percentage.of.households.with.high.housing.costs
Premature.age.adjusted.mortality <= 343
Uninsured.adults <= 0.0936
Teen.births <= 16.2
0.153 < Severe.housing.problems
Driving.alone.to.work <= 0.769
Feature
−0.06
−0.04
−0.02
0.00
Weight
Positive
Negative

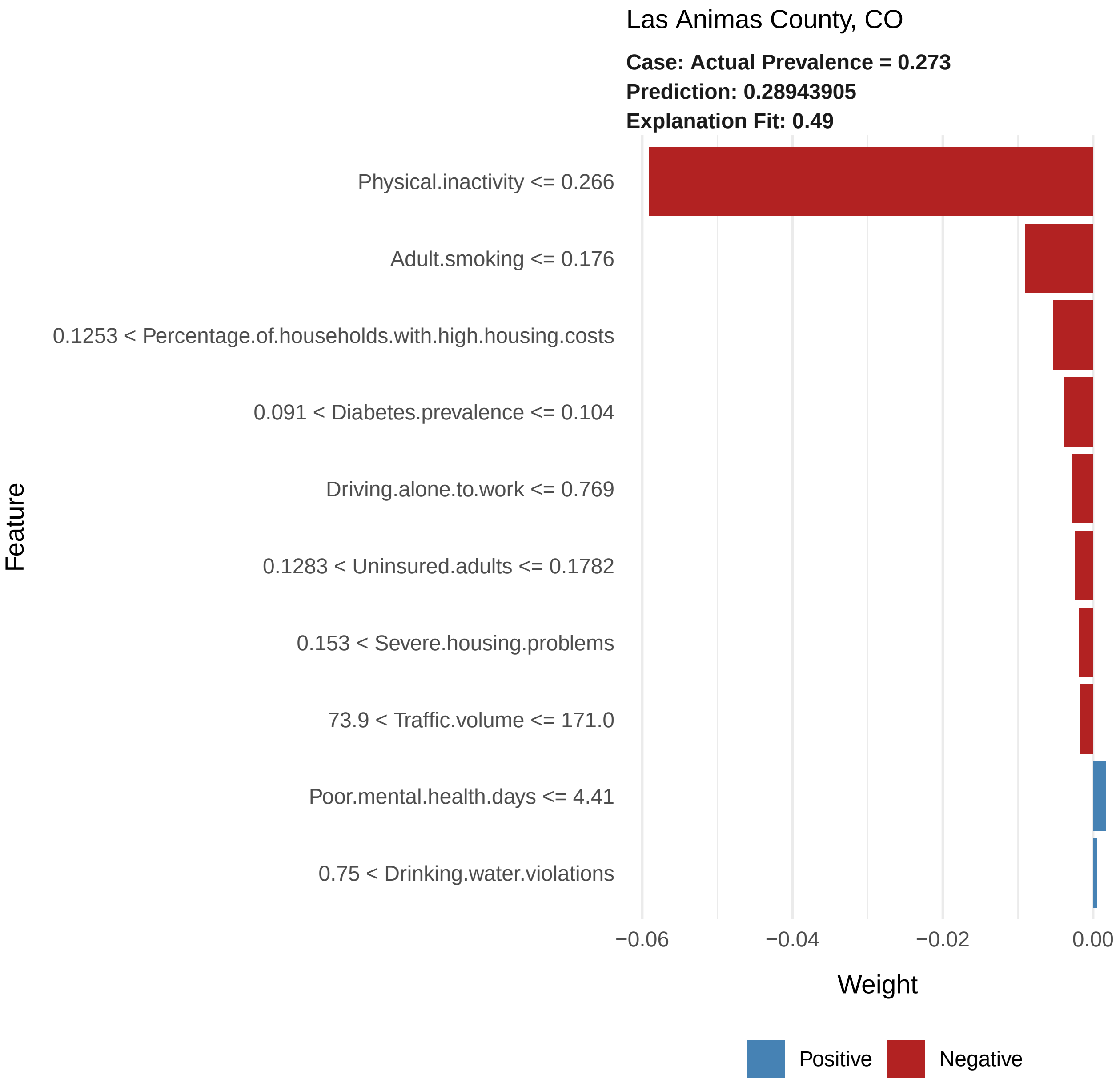

Las Animas County, CO
Case: Actual Prevalence = 0.273
Prediction: 0.28943905
Explanation Fit: 0.49
Physical.inactivity <= 0.266
Adult.smoking <= 0.176
0.1253 < Percentage.of.households.with.high.housing.costs
0.091 < Diabetes.prevalence <= 0.104
Driving.alone.to.work <= 0.769
0.1283 < Uninsured.adults <= 0.1782
0.153 < Severe.housing.problems
73.9 < Traffic.volume <= 171.0
Poor.mental.health.days <= 4.41
0.75 < Drinking.water.violations
Feature
−0.06
−0.04
−0.02
0.00
Weight
Positive
Negative

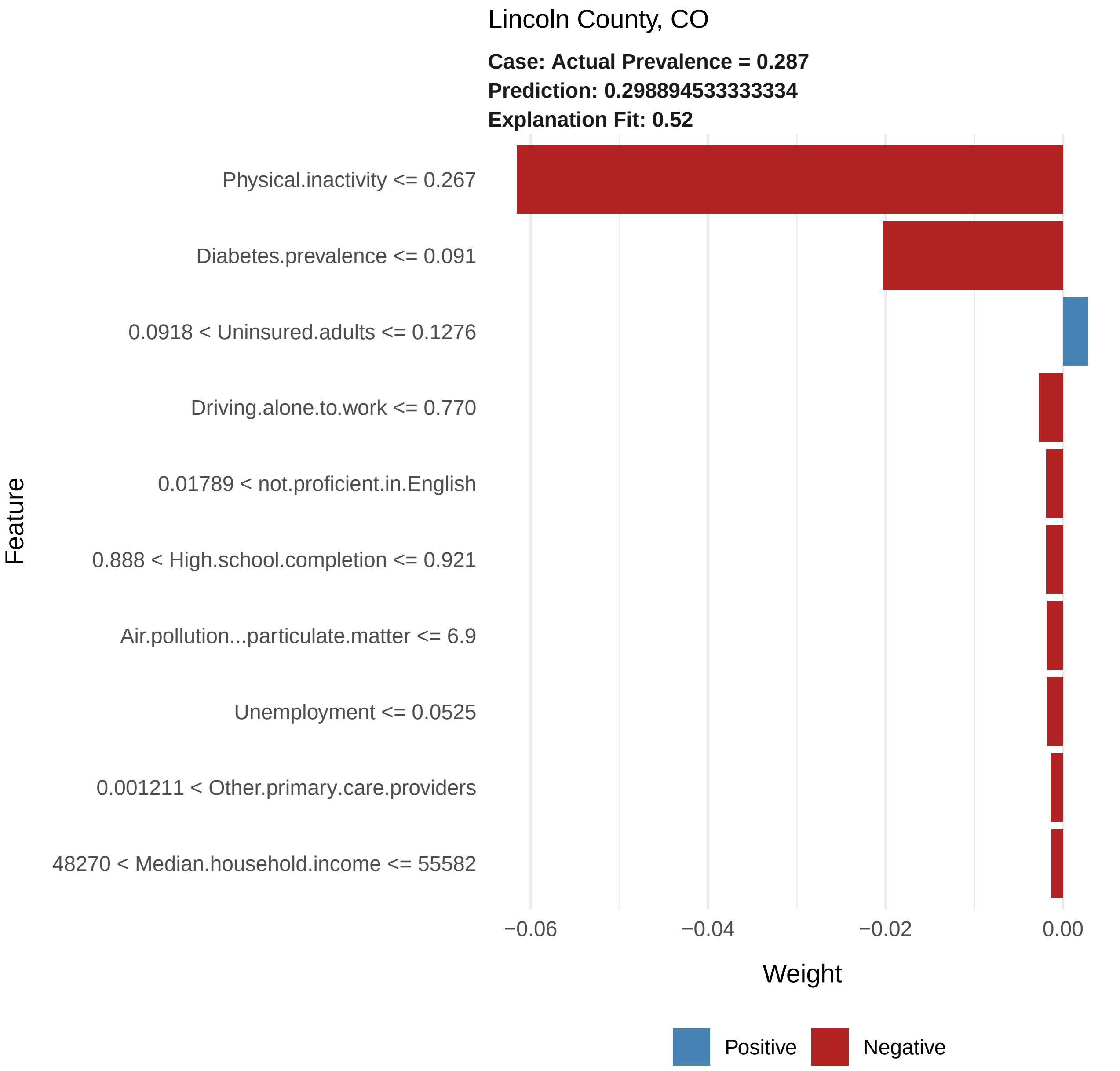

Lincoln County, CO
Case: Actual Prevalence = 0.287
Prediction: 0.298894533333334
Explanation Fit: 0.52
Physical.inactivity <= 0.267
Diabetes.prevalence <= 0.091
0.0918 < Uninsured.adults <= 0.1276
Driving.alone.to.work <= 0.770
0.01789 < not.proficient.in.English
0.888 < High.school.completion <= 0.921
Air.pollution...particulate.matter <= 6.9
Unemployment <= 0.0525
0.001211 < Other.primary.care.providers
48270 < Median.household.income <= 55582
Feature
−0.06
−0.04
−0.02
0.00
Weight
Positive
Negative

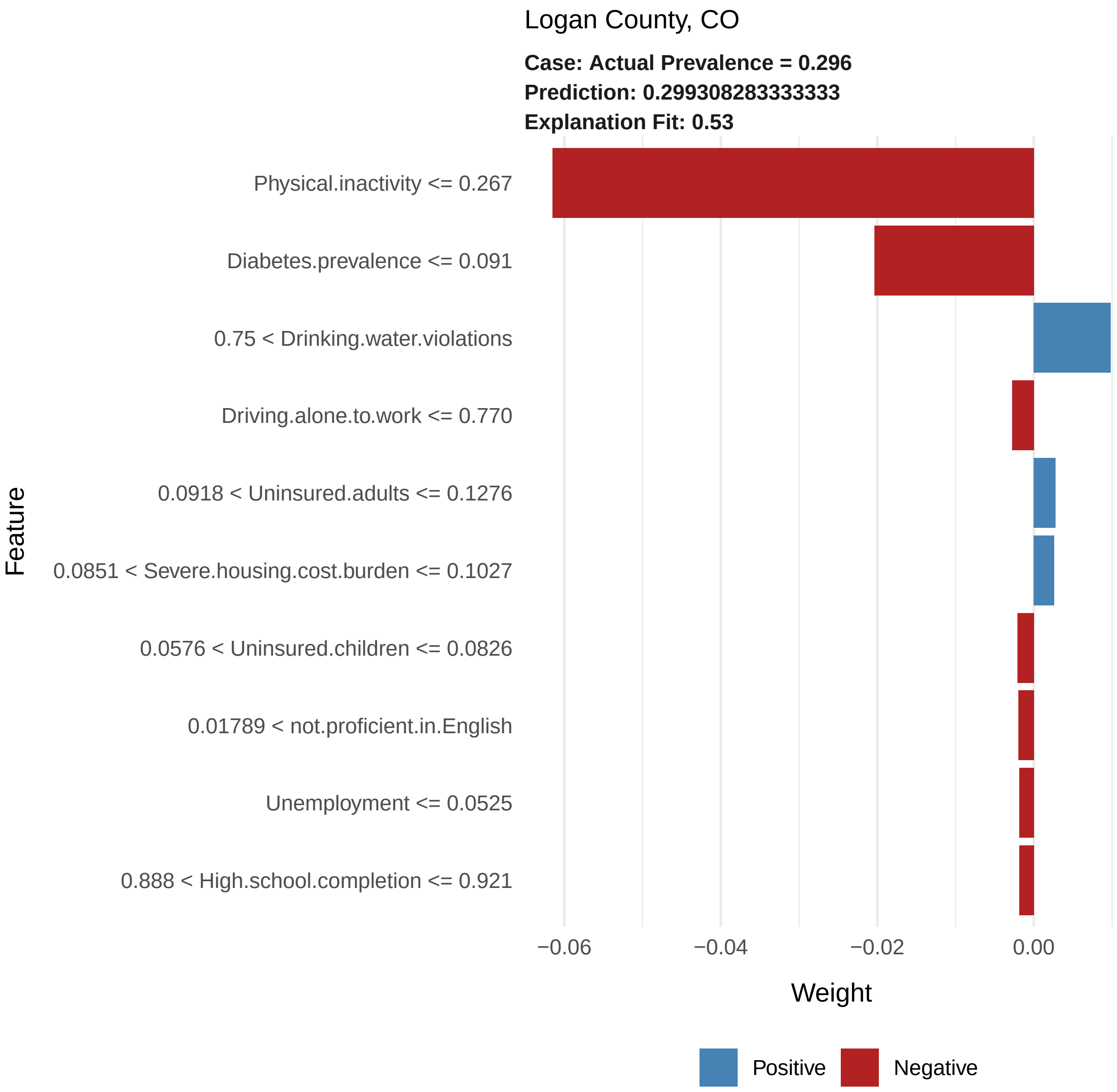
Logan County, CO
Case: Actual Prevalence = 0.296
Prediction: 0.299308283333333
Explanation Fit: 0.53
Physical.inactivity <= 0.267
Diabetes.prevalence <= 0.091
0.75 < Drinking.water.violations
Driving.alone.to.work <= 0.770
0.0918 < Uninsured.adults <= 0.1276
0.0851 < Severe.housing.cost.burden <= 0.1027
0.0576 < Uninsured.children <= 0.0826
0.01789 < not.proficient.in.English
Unemployment <= 0.0525
0.888 < High.school.completion <= 0.921
Feature
−0.06
−0.04
−0.02
0.00
Weight
Positive
Negative

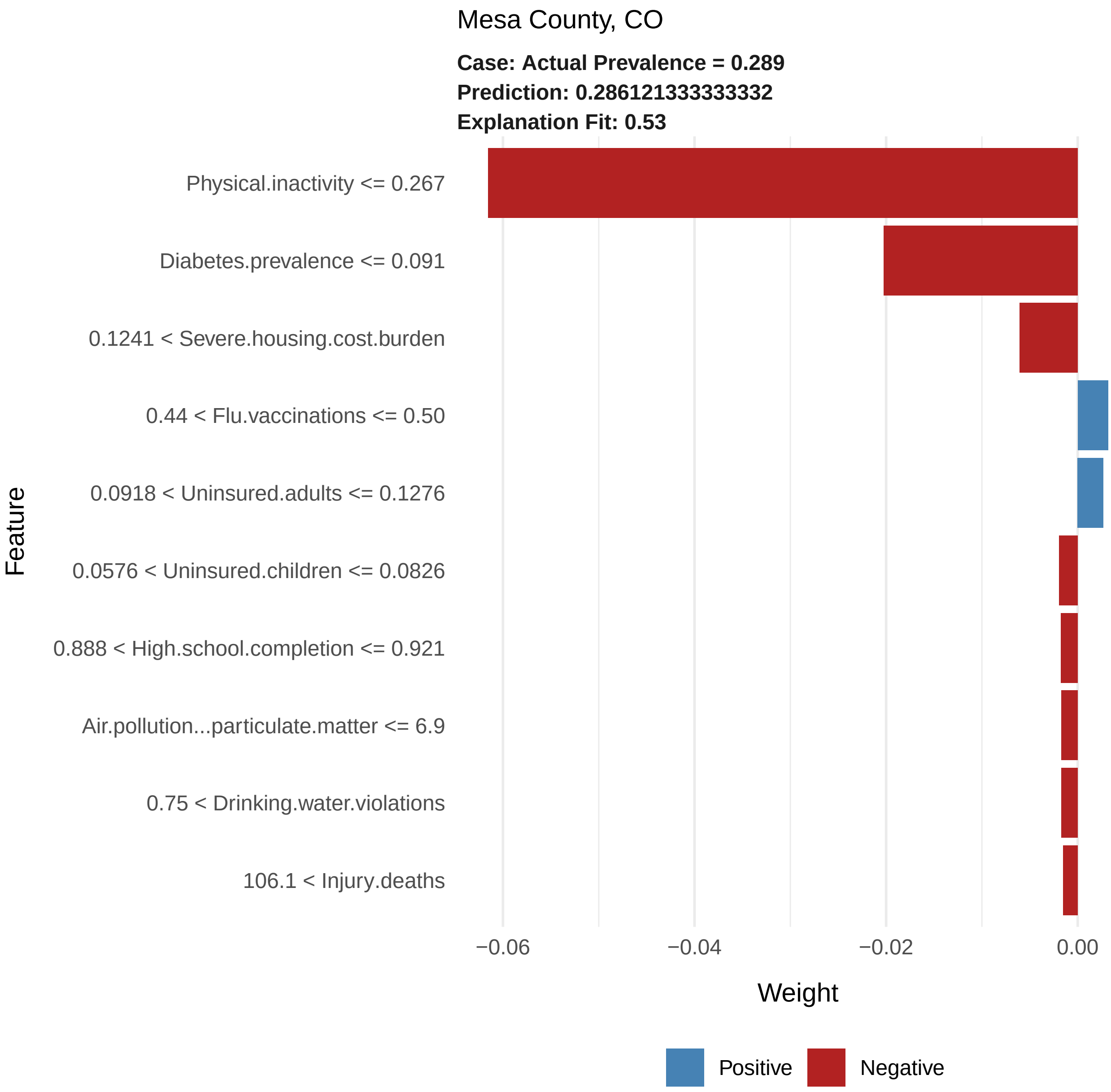

Mesa County, CO
Case: Actual Prevalence = 0.289
Prediction: 0.286121333333332
Explanation Fit: 0.53
Physical.inactivity <= 0.267
Diabetes.prevalence <= 0.091
0.1241 < Severe.housing.cost.burden
0.44 < Flu.vaccinations <= 0.50
0.0918 < Uninsured.adults <= 0.1276
0.0576 < Uninsured.children <= 0.0826
0.888 < High.school.completion <= 0.921
Air.pollution...particulate.matter <= 6.9
0.75 < Drinking.water.violations
106.1 < Injury.deaths
Feature
−0.06
−0.04
−0.02
0.00
Weight
Positive
Negative

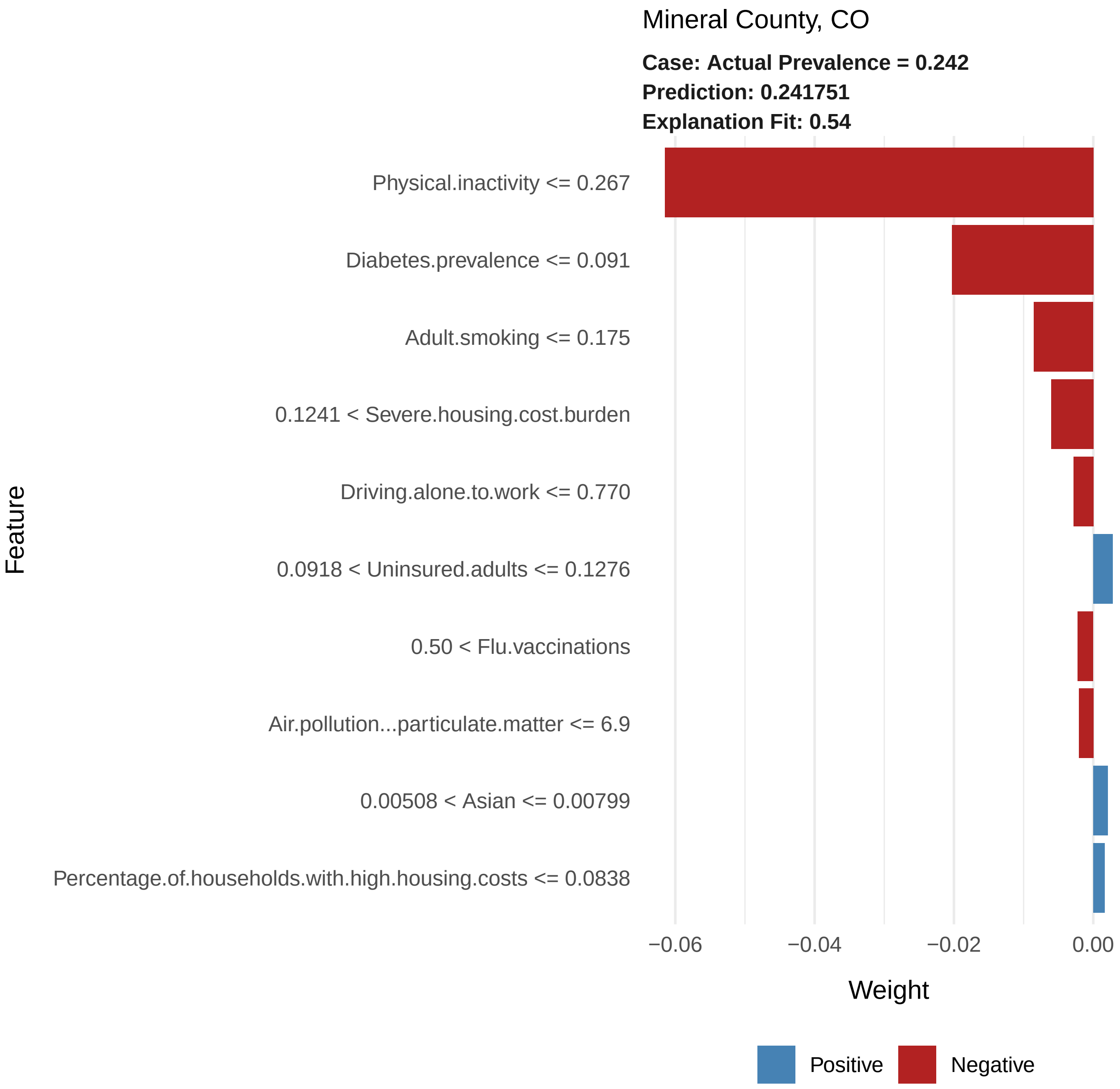
Mineral County, CO
Case: Actual Prevalence = 0.242
Prediction: 0.241751
Explanation Fit: 0.54
Physical.inactivity <= 0.267
Diabetes.prevalence <= 0.091
Adult.smoking <= 0.175
0.1241 < Severe.housing.cost.burden
Driving.alone.to.work <= 0.770
0.0918 < Uninsured.adults <= 0.1276
0.50 < Flu.vaccinations
Air.pollution...particulate.matter <= 6.9
0.00508 < Asian <= 0.00799
Percentage.of.households.with.high.housing.costs <= 0.0838
Feature
−0.06
−0.04
−0.02
0.00
Weight
Positive
Negative

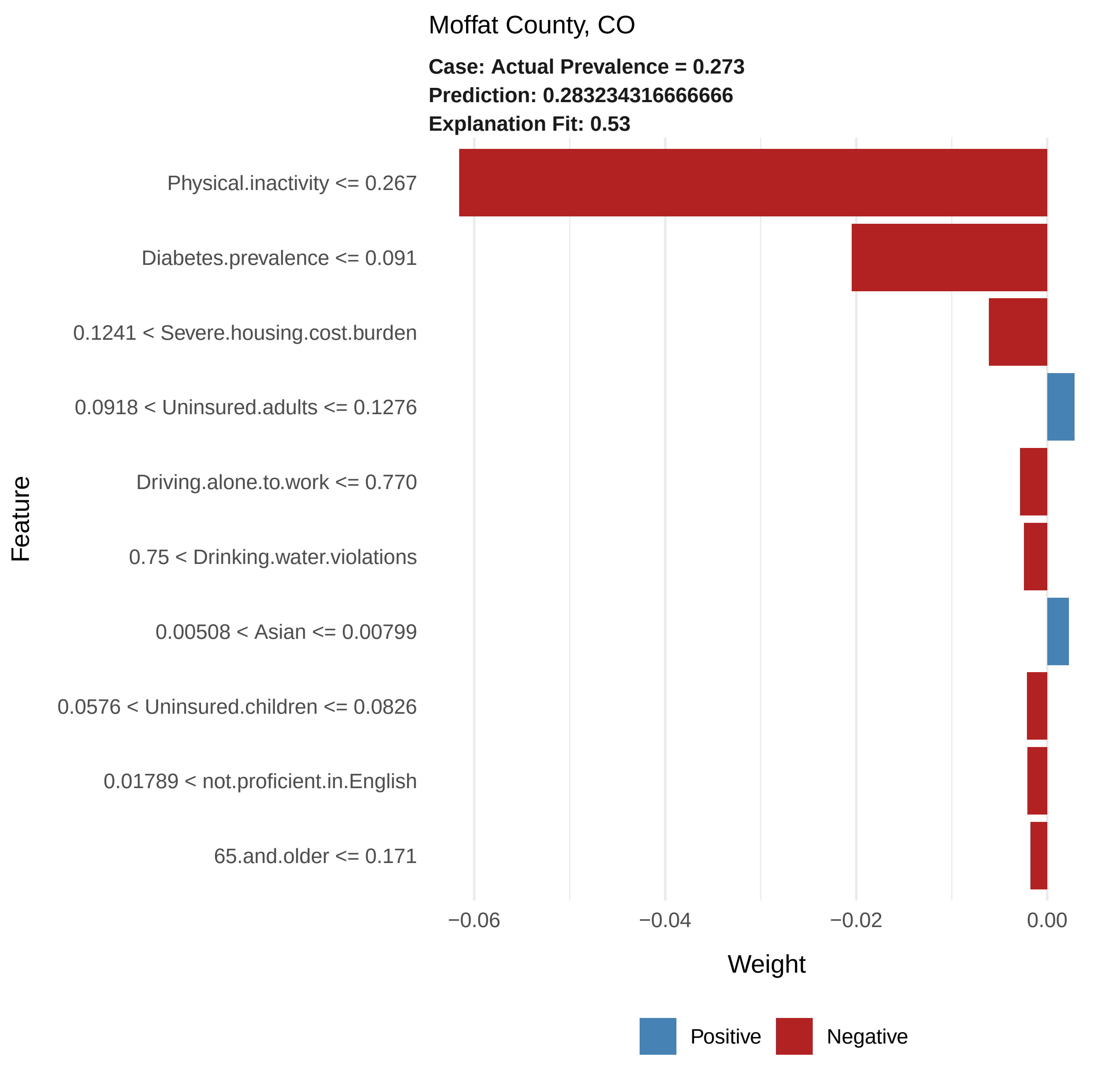

Moffat County, CO
Case: Actual Prevalence = 0.273
Prediction: 0.283234316666666
Explanation Fit: 0.53
Physical.inactivity <= 0.267
Diabetes.prevalence <= 0.091
0.1241 < Severe.housing.cost.burden
0.0918 < Uninsured.adults <= 0.1276
Driving.alone.to.work <= 0.770
0.75 < Drinking.water.violations
0.00508 < Asian <= 0.00799
0.0576 < Uninsured.children <= 0.0826
0.01789 < not.proficient.in.English
65.and.older <= 0.171
Feature
−0.06
−0.04
−0.02
0.00
Weight
Positive
Negative

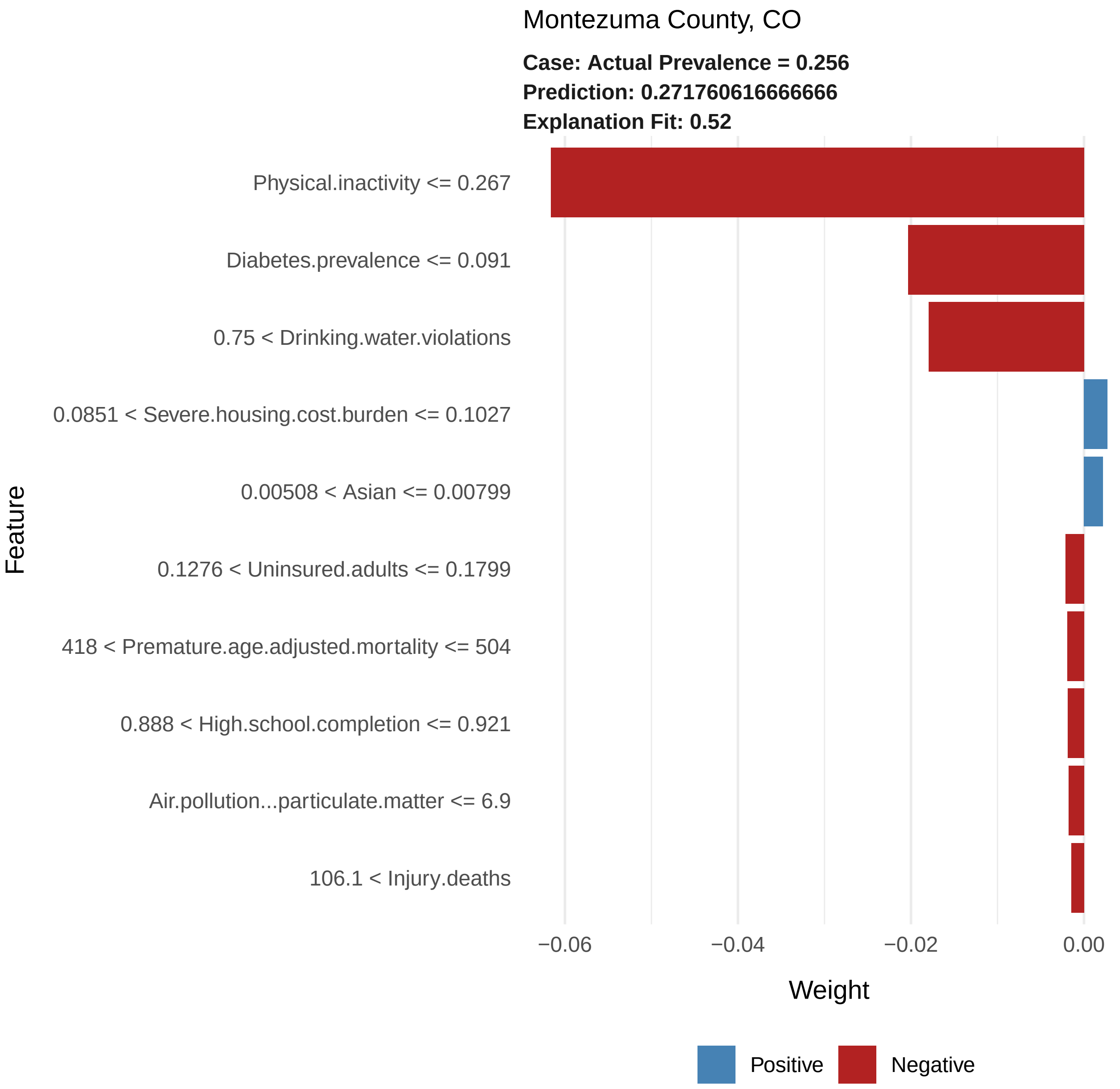

Montezuma County, CO
Case: Actual Prevalence = 0.256
Prediction: 0.271760616666666
Explanation Fit: 0.52
Physical.inactivity <= 0.267
Diabetes.prevalence <= 0.091
0.75 < Drinking.water.violations
0.0851 < Severe.housing.cost.burden <= 0.1027
0.00508 < Asian <= 0.00799
0.1276 < Uninsured.adults <= 0.1799
418 < Premature.age.adjusted.mortality <= 504
0.888 < High.school.completion <= 0.921
Air.pollution...particulate.matter <= 6.9
106.1 < Injury.deaths
Feature
−0.06
−0.04
−0.02
0.00
Weight
Positive
Negative

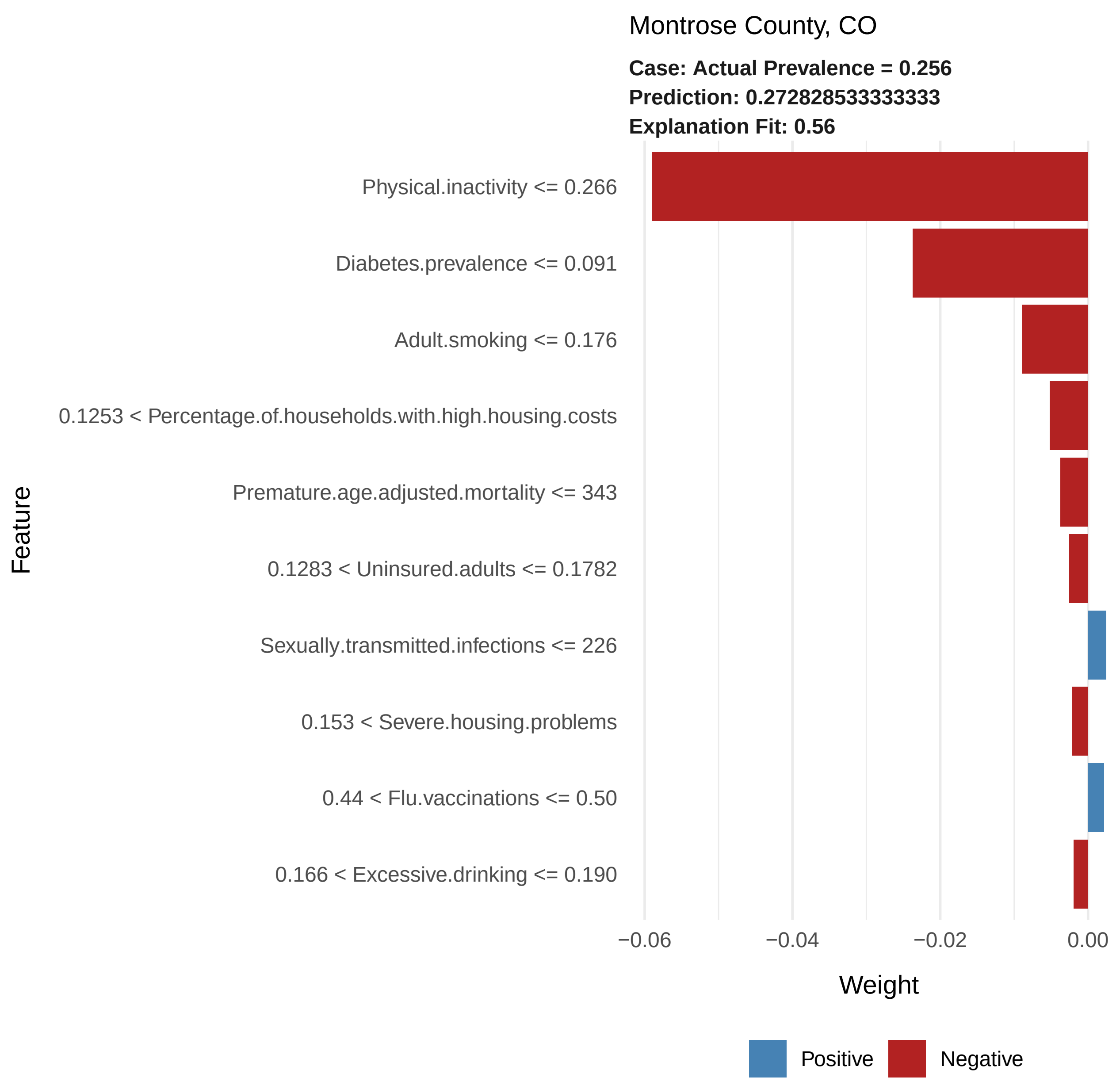

Montrose County, CO
Case: Actual Prevalence = 0.256
Prediction: 0.272828533333333
Explanation Fit: 0.56
Physical.inactivity <= 0.266
Diabetes.prevalence <= 0.091
Adult.smoking <= 0.176
0.1253 < Percentage.of.households.with.high.housing.costs
Premature.age.adjusted.mortality <= 343
0.1283 < Uninsured.adults <= 0.1782
Sexually.transmitted.infections <= 226
0.153 < Severe.housing.problems
0.44 < Flu.vaccinations <= 0.50
0.166 < Excessive.drinking <= 0.190
Feature
−0.06
−0.04
−0.02
0.00
Weight
Positive
Negative

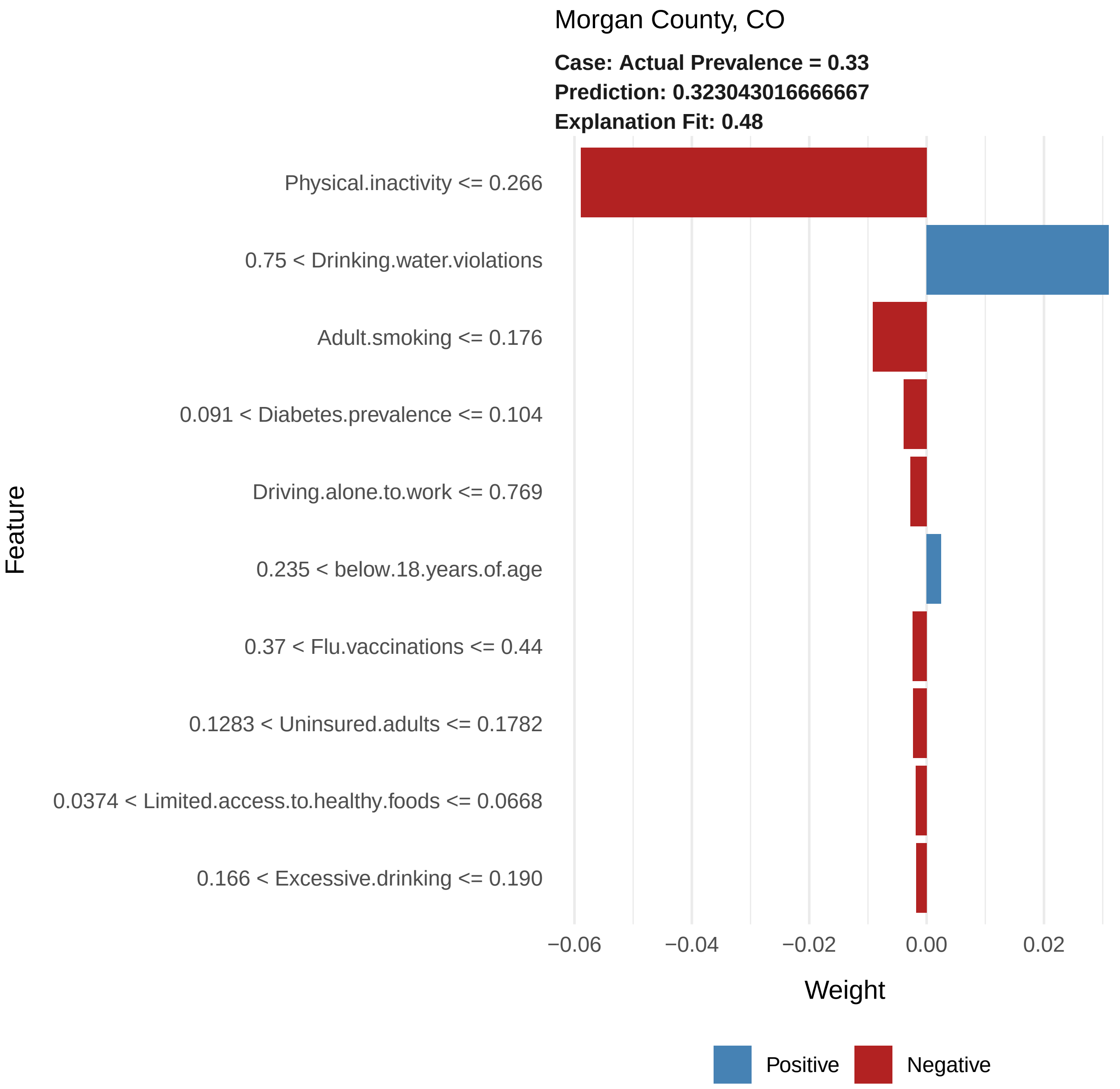

Morgan County, CO
Case: Actual Prevalence = 0.33
Prediction: 0.323043016666667
Explanation Fit: 0.48
Physical.inactivity <= 0.266
0.75 < Drinking.water.violations
Adult.smoking <= 0.176
0.091 < Diabetes.prevalence <= 0.104
Driving.alone.to.work <= 0.769
0.235 < below.18.years.of.age
0.37 < Flu.vaccinations <= 0.44
0.1283 < Uninsured.adults <= 0.1782
0.0374 < Limited.access.to.healthy.foods <= 0.0668
0.166 < Excessive.drinking <= 0.190
Feature
−0.06
−0.04
−0.02
0.00
0.02
Weight
Positive
Negative

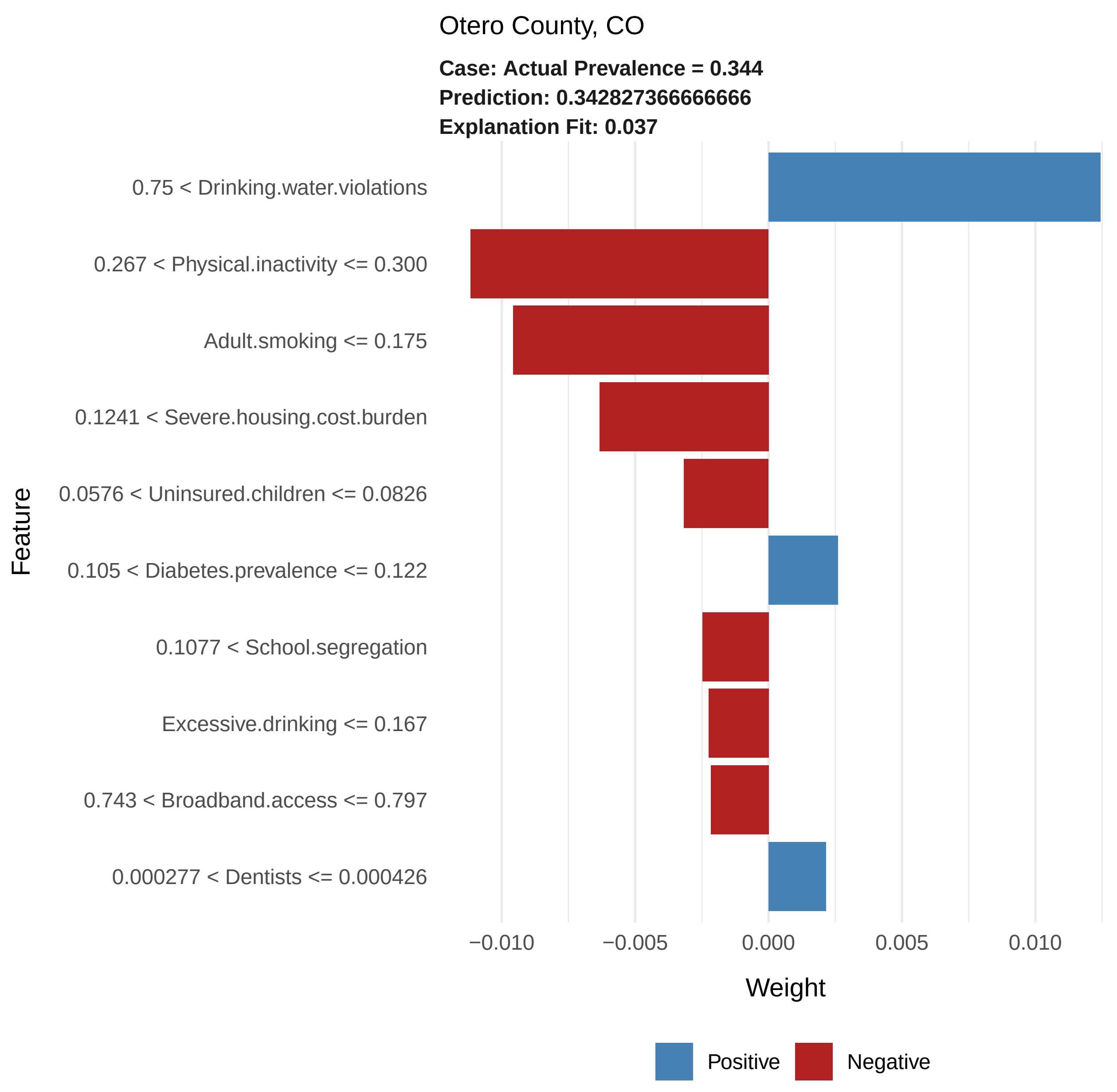
Otero County, CO
Case: Actual Prevalence = 0.344
Prediction: 0.342827366666666
Explanation Fit: 0.037
0.75 < Drinking.water.violations
0.267 < Physical.inactivity <= 0.300
Adult.smoking <= 0.175
0.1241 < Severe.housing.cost.burden
0.0576 < Uninsured.children <= 0.0826
0.105 < Diabetes.prevalence <= 0.122
0.1077 < School.segregation
Excessive.drinking <= 0.167
0.743 < Broadband.access <= 0.797
0.000277 < Dentists <= 0.000426
Feature
−0.010
−0.005
0.000
0.005
0.010
Weight
Positive
Negative

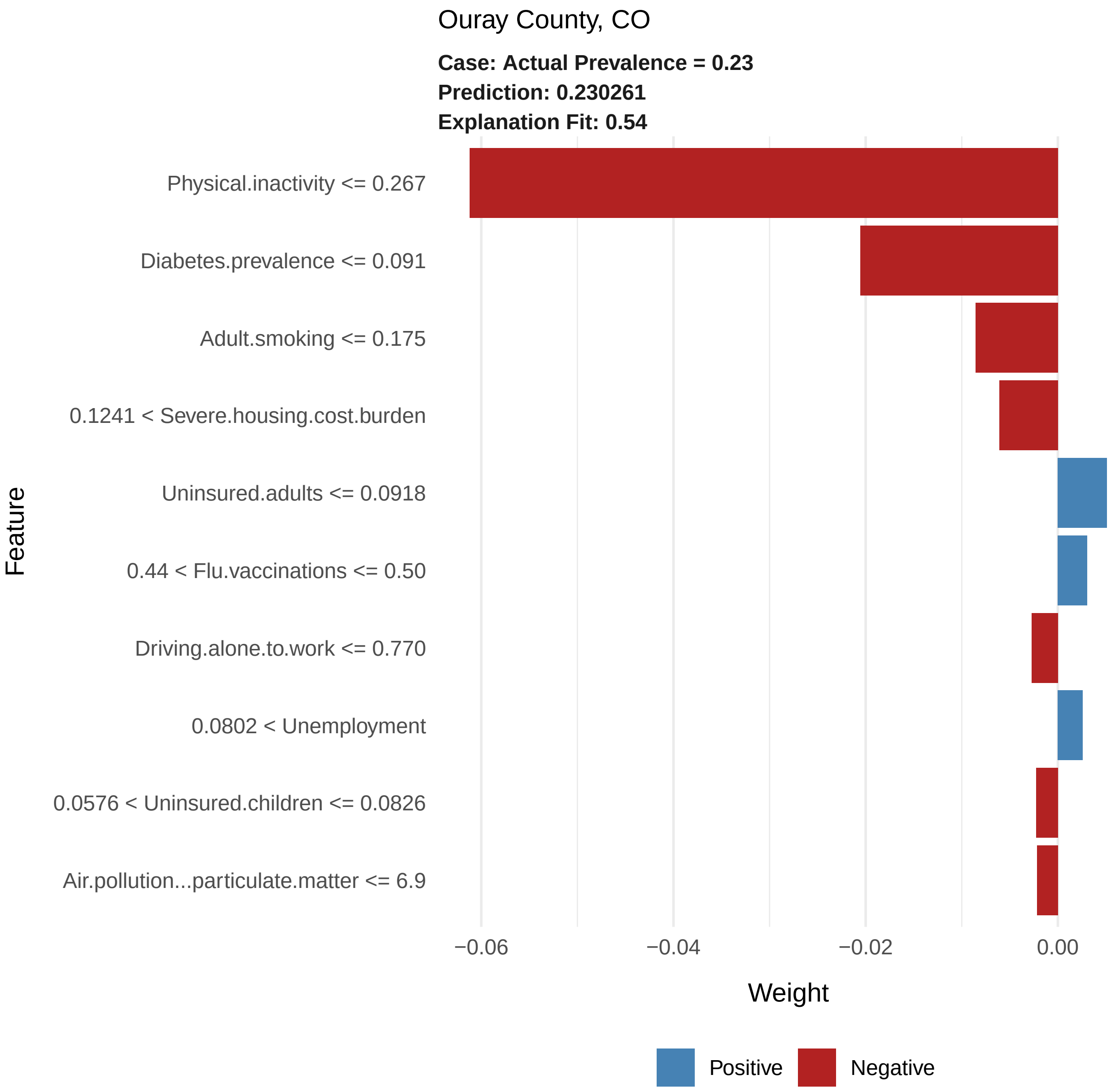
Ouray County, CO
Case: Actual Prevalence = 0.23
Prediction: 0.230261
Explanation Fit: 0.54
Physical.inactivity <= 0.267
Diabetes.prevalence <= 0.091
Adult.smoking <= 0.175
0.1241 < Severe.housing.cost.burden
Uninsured.adults <= 0.0918
0.44 < Flu.vaccinations <= 0.50
Driving.alone.to.work <= 0.770
0.0802 < Unemployment
0.0576 < Uninsured.children <= 0.0826
Air.pollution...particulate.matter <= 6.9
Feature
−0.06
−0.04
−0.02
0.00
Weight
Positive
Negative

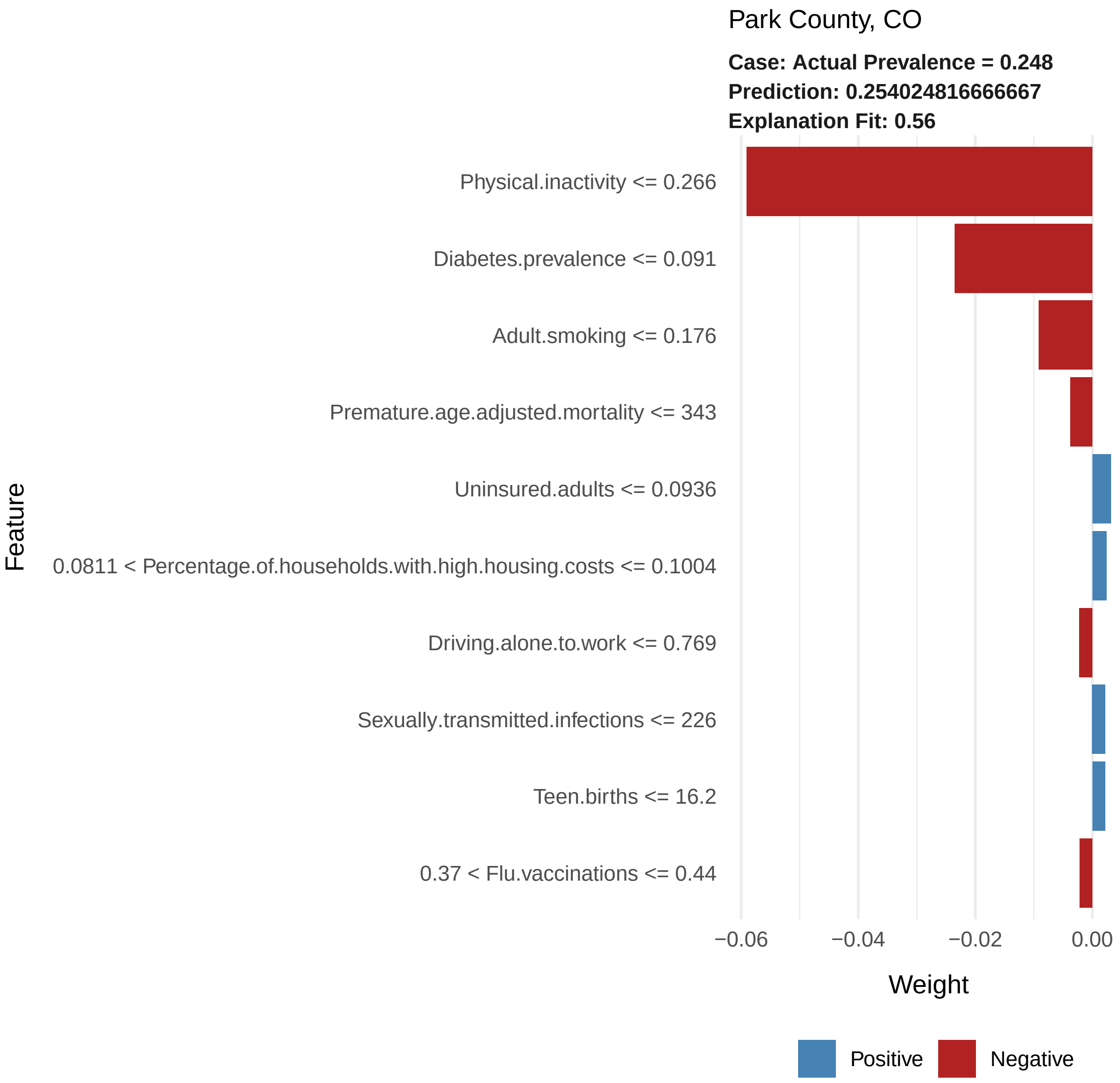

Park County, CO
Case: Actual Prevalence = 0.248
Prediction: 0.254024816666667
Explanation Fit: 0.56
Physical.inactivity <= 0.266
Diabetes.prevalence <= 0.091
Adult.smoking <= 0.176
Premature.age.adjusted.mortality <= 343
Uninsured.adults <= 0.0936
0.0811 < Percentage.of.households.with.high.housing.costs <= 0.1004
Driving.alone.to.work <= 0.769
Sexually.transmitted.infections <= 226
Teen.births <= 16.2
0.37 < Flu.vaccinations <= 0.44
Feature
−0.06
−0.04
−0.02
0.00
Weight
Positive
Negative

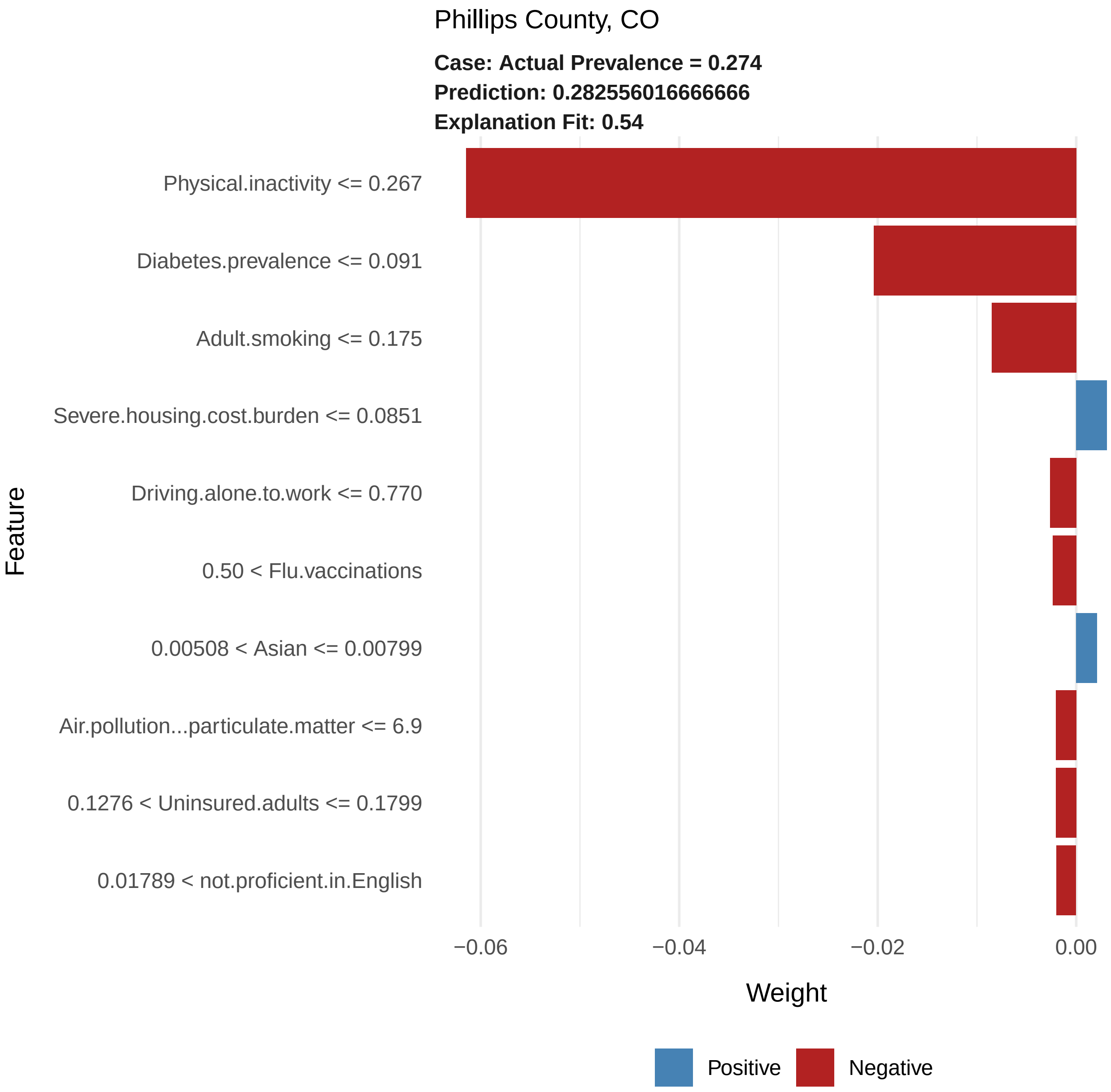

Phillips County, CO
Case: Actual Prevalence = 0.274
Prediction: 0.282556016666666
Explanation Fit: 0.54
Physical.inactivity <= 0.267
Diabetes.prevalence <= 0.091
Adult.smoking <= 0.175
Severe.housing.cost.burden <= 0.0851
Driving.alone.to.work <= 0.770
0.50 < Flu.vaccinations
0.00508 < Asian <= 0.00799
Air.pollution...particulate.matter <= 6.9
0.1276 < Uninsured.adults <= 0.1799
0.01789 < not.proficient.in.English
Feature
−0.06
−0.04
−0.02
0.00
Weight
Positive
Negative

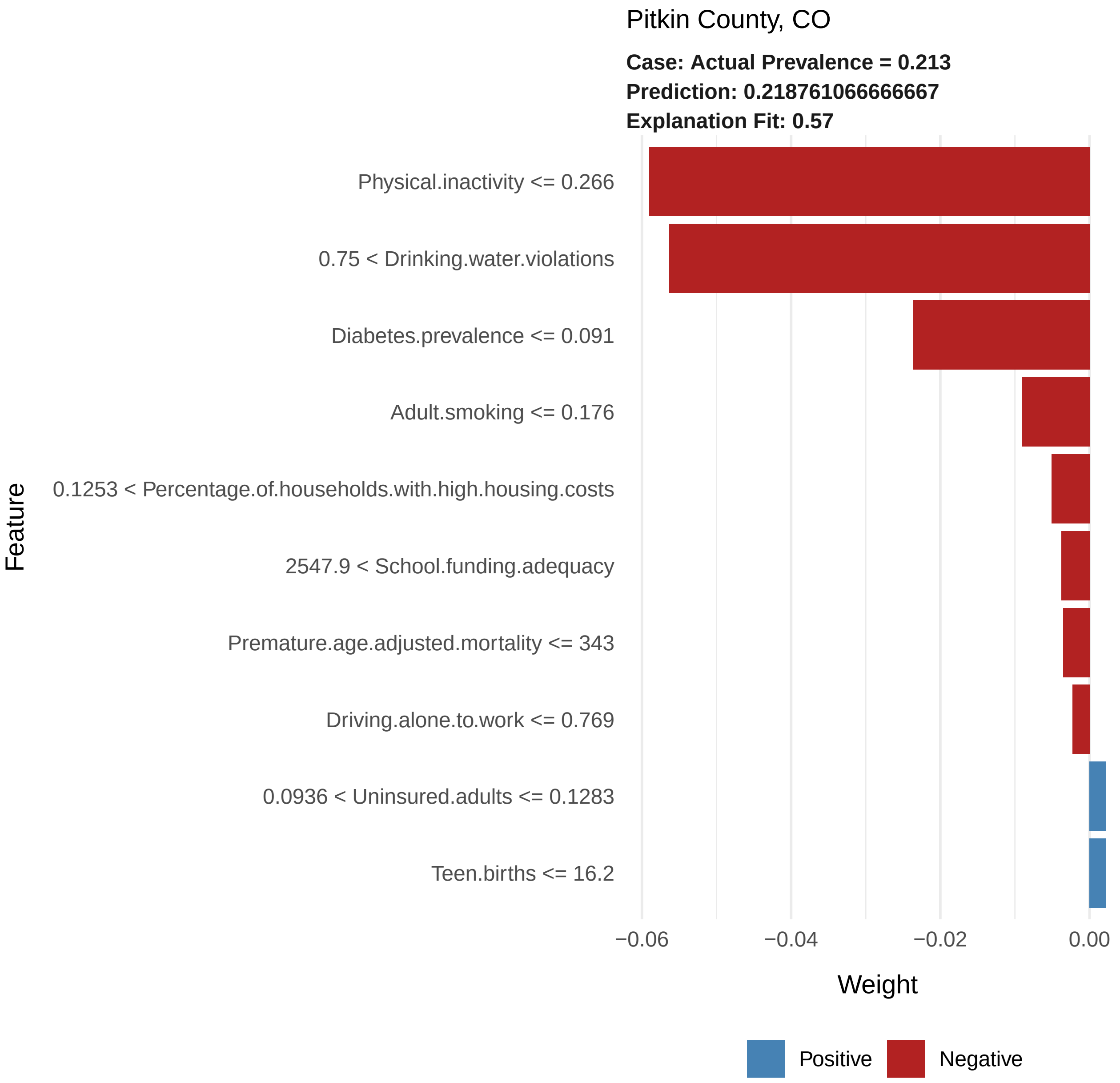

Pitkin County, CO
Case: Actual Prevalence = 0.213
Prediction: 0.218761066666667
Explanation Fit: 0.57
Physical.inactivity <= 0.266
0.75 < Drinking.water.violations
Diabetes.prevalence <= 0.091
Adult.smoking <= 0.176
0.1253 < Percentage.of.households.with.high.housing.costs
2547.9 < School.funding.adequacy
Premature.age.adjusted.mortality <= 343
Driving.alone.to.work <= 0.769
0.0936 < Uninsured.adults <= 0.1283
Teen.births <= 16.2
Feature
−0.06
−0.04
−0.02
0.00
Weight
Positive
Negative

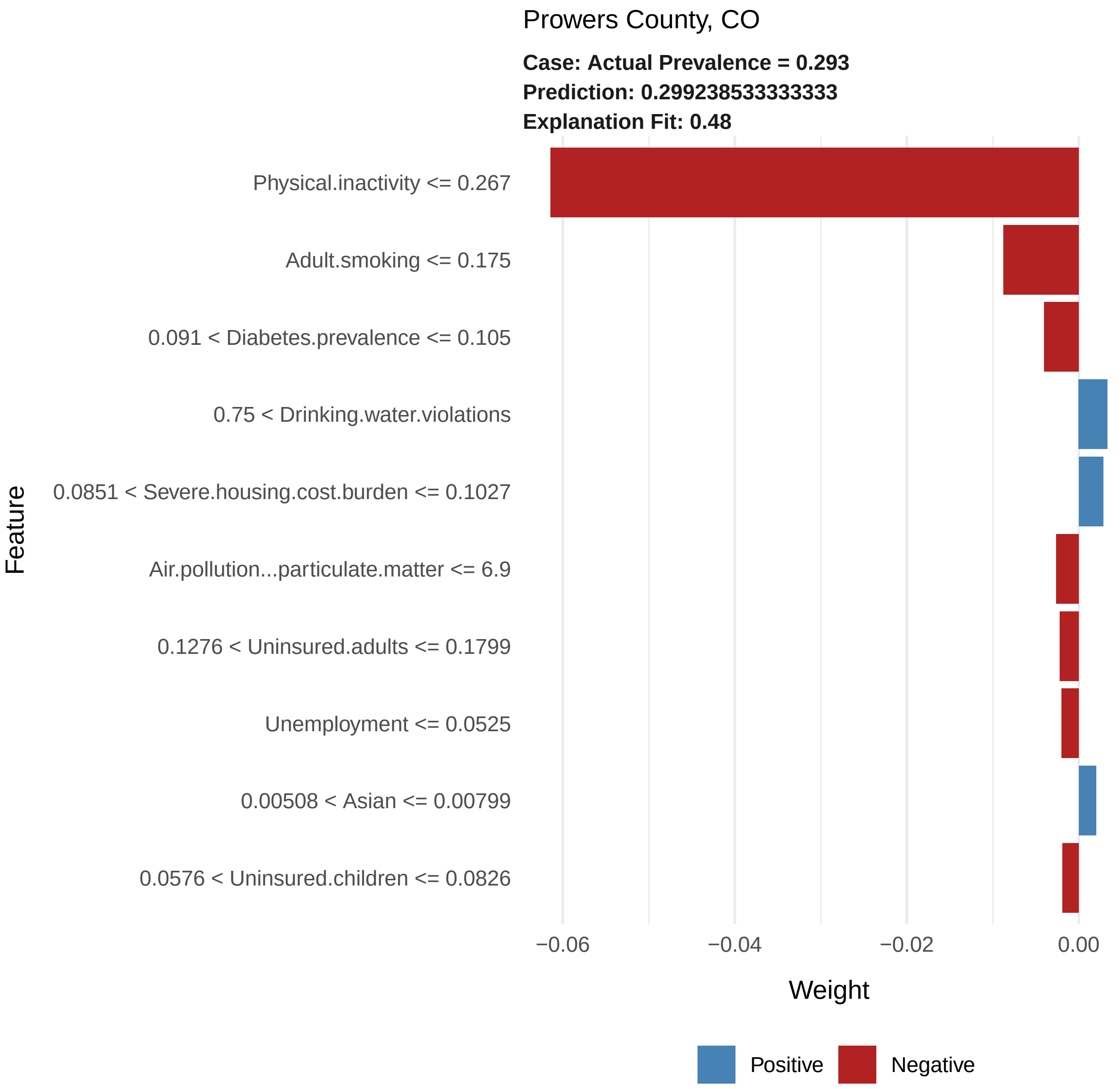
Prowers County, CO
Case: Actual Prevalence = 0.293
Prediction: 0.299238533333333
Explanation Fit: 0.48
Physical.inactivity <= 0.267
Adult.smoking <= 0.175
0.091 < Diabetes.prevalence <= 0.105
0.75 < Drinking.water.violations
0.0851 < Severe.housing.cost.burden <= 0.1027
Air.pollution...particulate.matter <= 6.9
0.1276 < Uninsured.adults <= 0.1799
Unemployment <= 0.0525
0.00508 < Asian <= 0.00799
0.0576 < Uninsured.children <= 0.0826
Feature
−0.06
−0.04
−0.02
0.00
Weight
Positive
Negative

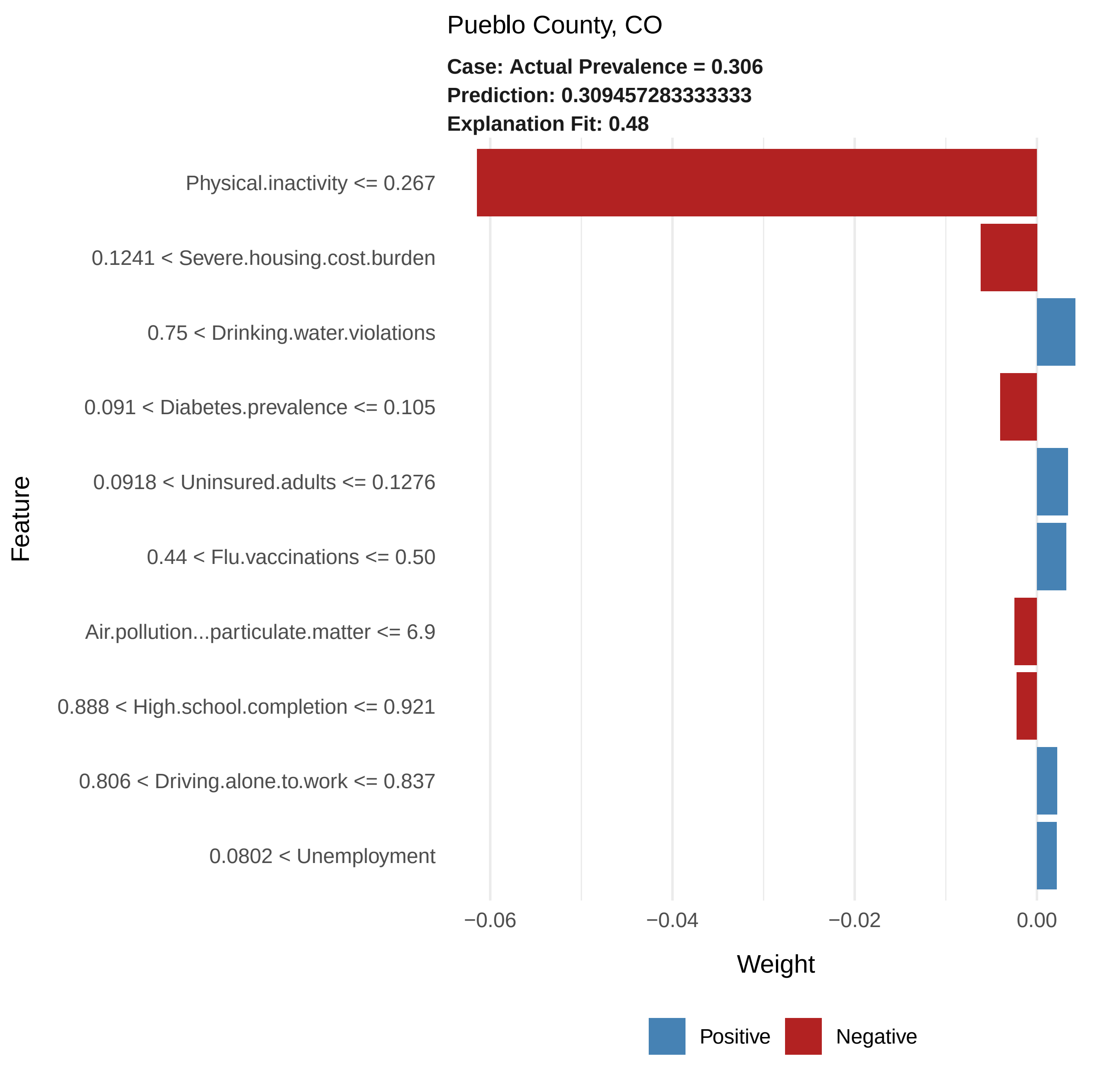
Pueblo County, CO
Case: Actual Prevalence = 0.306
Prediction: 0.309457283333333
Explanation Fit: 0.48
Physical.inactivity <= 0.267
0.1241 < Severe.housing.cost.burden
0.75 < Drinking.water.violations
0.091 < Diabetes.prevalence <= 0.105
0.0918 < Uninsured.adults <= 0.1276
0.44 < Flu.vaccinations <= 0.50
Air.pollution...particulate.matter <= 6.9
0.888 < High.school.completion <= 0.921
0.806 < Driving.alone.to.work <= 0.837
0.0802 < Unemployment
Feature
−0.06
−0.04
−0.02
0.00
Weight
Positive
Negative

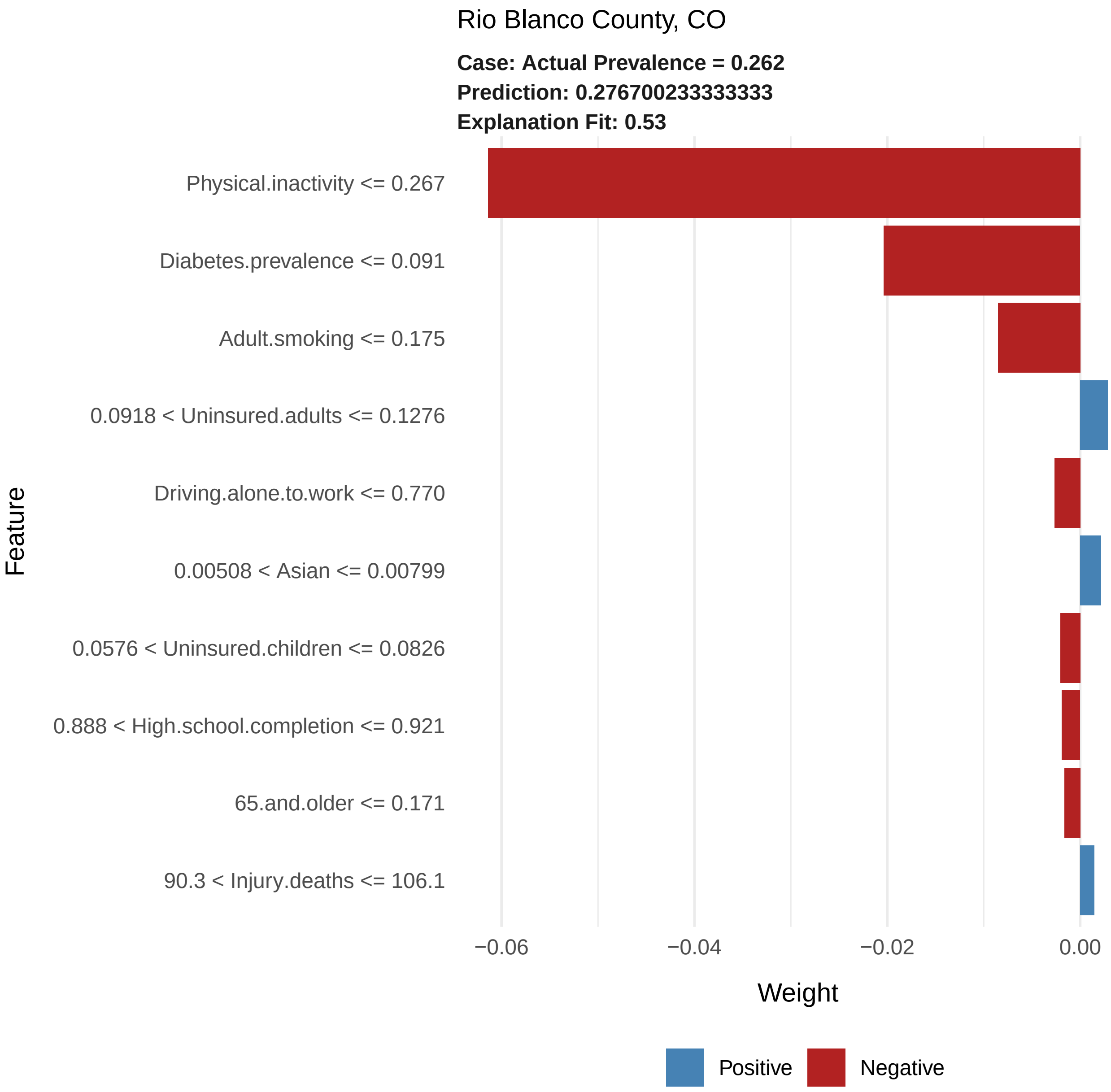
Rio Blanco County, CO
Case: Actual Prevalence = 0.262
Prediction: 0.276700233333333
Explanation Fit: 0.53
Physical.inactivity <= 0.267
Diabetes.prevalence <= 0.091
Adult.smoking <= 0.175
0.0918 < Uninsured.adults <= 0.1276
Driving.alone.to.work <= 0.770
0.00508 < Asian <= 0.00799
0.0576 < Uninsured.children <= 0.0826
0.888 < High.school.completion <= 0.921
65.and.older <= 0.171
90.3 < Injury.deaths <= 106.1
Feature
−0.06
−0.04
−0.02
0.00
Weight
Positive
Negative

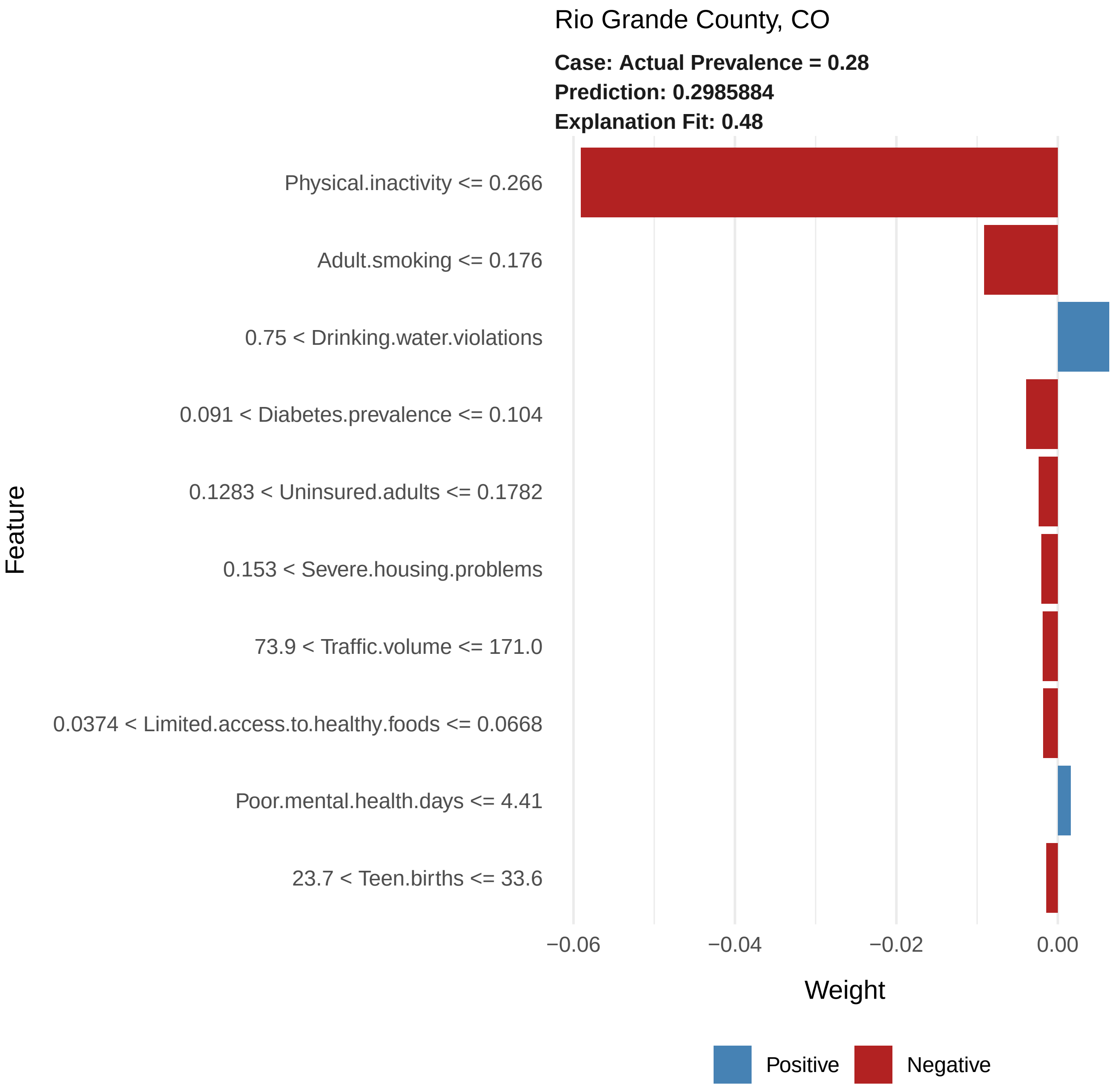
Rio Grande County, CO
Case: Actual Prevalence = 0.28
Prediction: 0.2985884
Explanation Fit: 0.48
Physical.inactivity <= 0.266
Adult.smoking <= 0.176
0.75 < Drinking.water.violations
0.091 < Diabetes.prevalence <= 0.104
0.1283 < Uninsured.adults <= 0.1782
0.153 < Severe.housing.problems
73.9 < Traffic.volume <= 171.0
0.0374 < Limited.access.to.healthy.foods <= 0.0668
Poor.mental.health.days <= 4.41
23.7 < Teen.births <= 33.6
Feature
−0.06
−0.04
−0.02
0.00
Weight
Positive
Negative

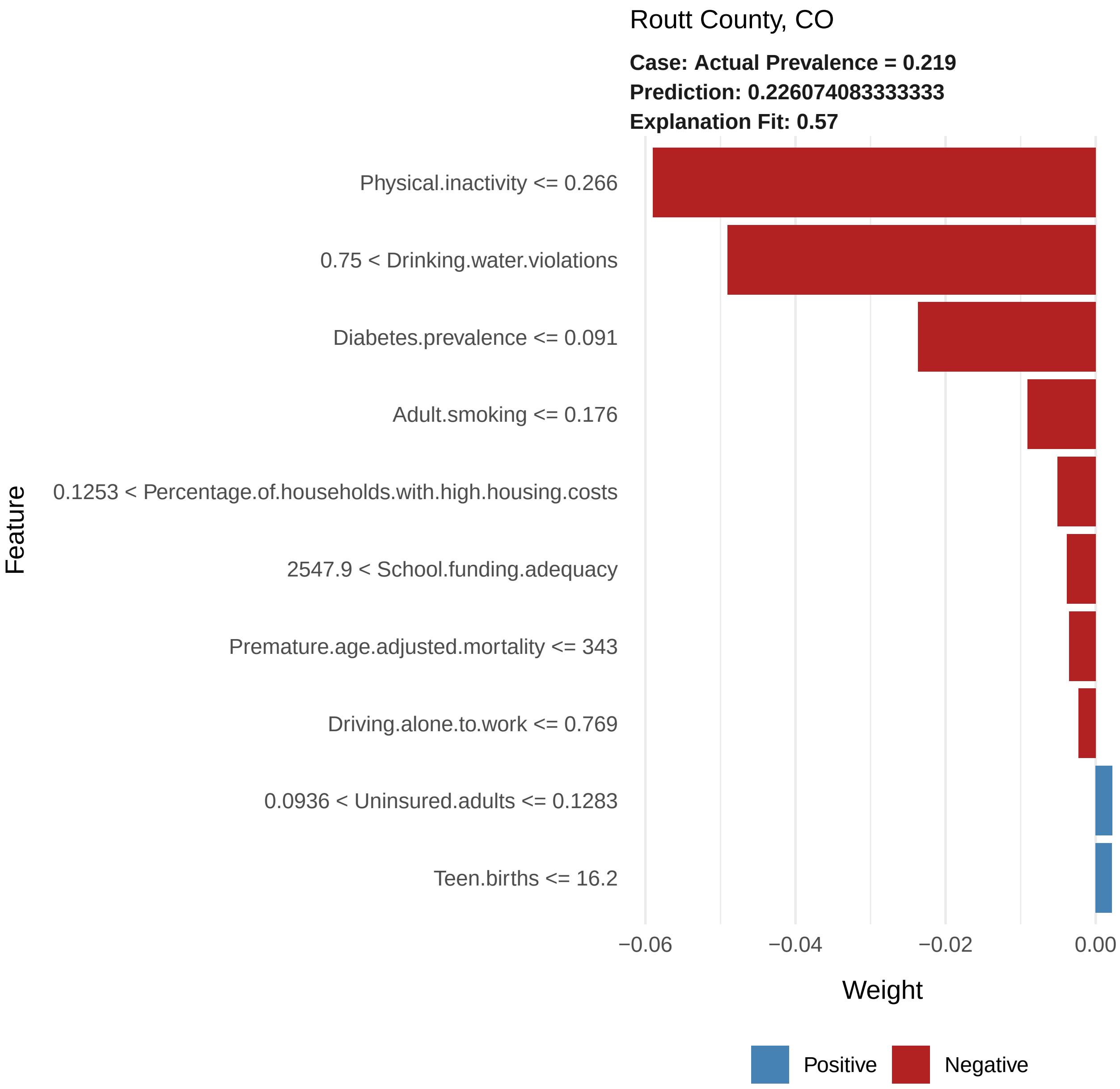
Routt County, CO
Case: Actual Prevalence = 0.219
Prediction: 0.226074083333333
Explanation Fit: 0.57
Physical.inactivity <= 0.266
0.75 < Drinking.water.violations
Diabetes.prevalence <= 0.091
Adult.smoking <= 0.176
0.1253 < Percentage.of.households.with.high.housing.costs
2547.9 < School.funding.adequacy
Premature.age.adjusted.mortality <= 343
Driving.alone.to.work <= 0.769
0.0936 < Uninsured.adults <= 0.1283
Teen.births <= 16.2
Feature
−0.06
−0.04
−0.02
0.00
Weight
Positive
Negative

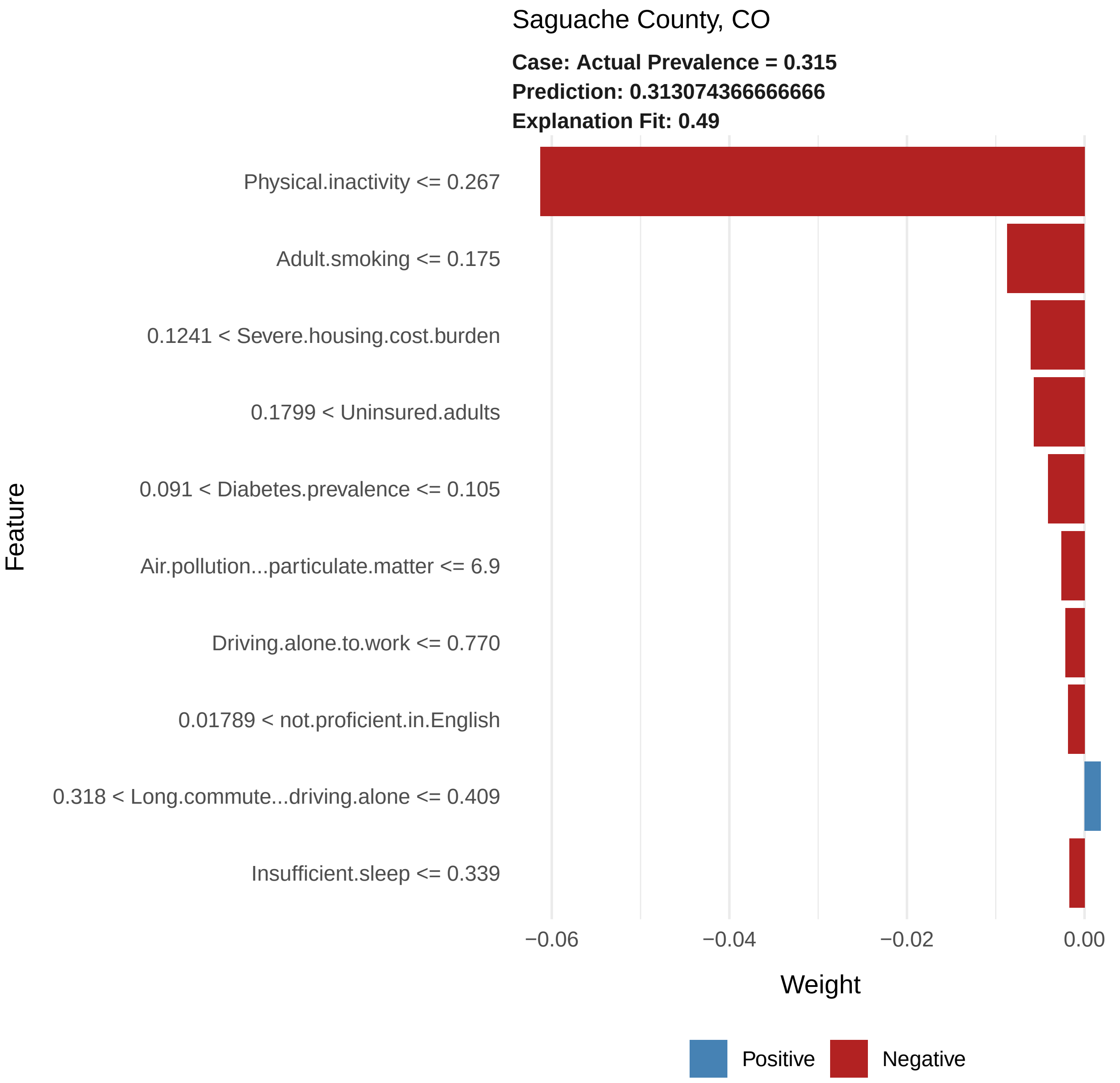
Saguache County, CO
Case: Actual Prevalence = 0.315
Prediction: 0.313074366666666
Explanation Fit: 0.49
Physical.inactivity <= 0.267
Adult.smoking <= 0.175
0.1241 < Severe.housing.cost.burden
0.1799 < Uninsured.adults
0.091 < Diabetes.prevalence <= 0.105
Air.pollution...particulate.matter <= 6.9
Driving.alone.to.work <= 0.770
0.01789 < not.proficient.in.English
0.318 < Long.commute...driving.alone <= 0.409
Insufficient.sleep <= 0.339
Feature
−0.06
−0.04
−0.02
0.00
Weight
Positive
Negative

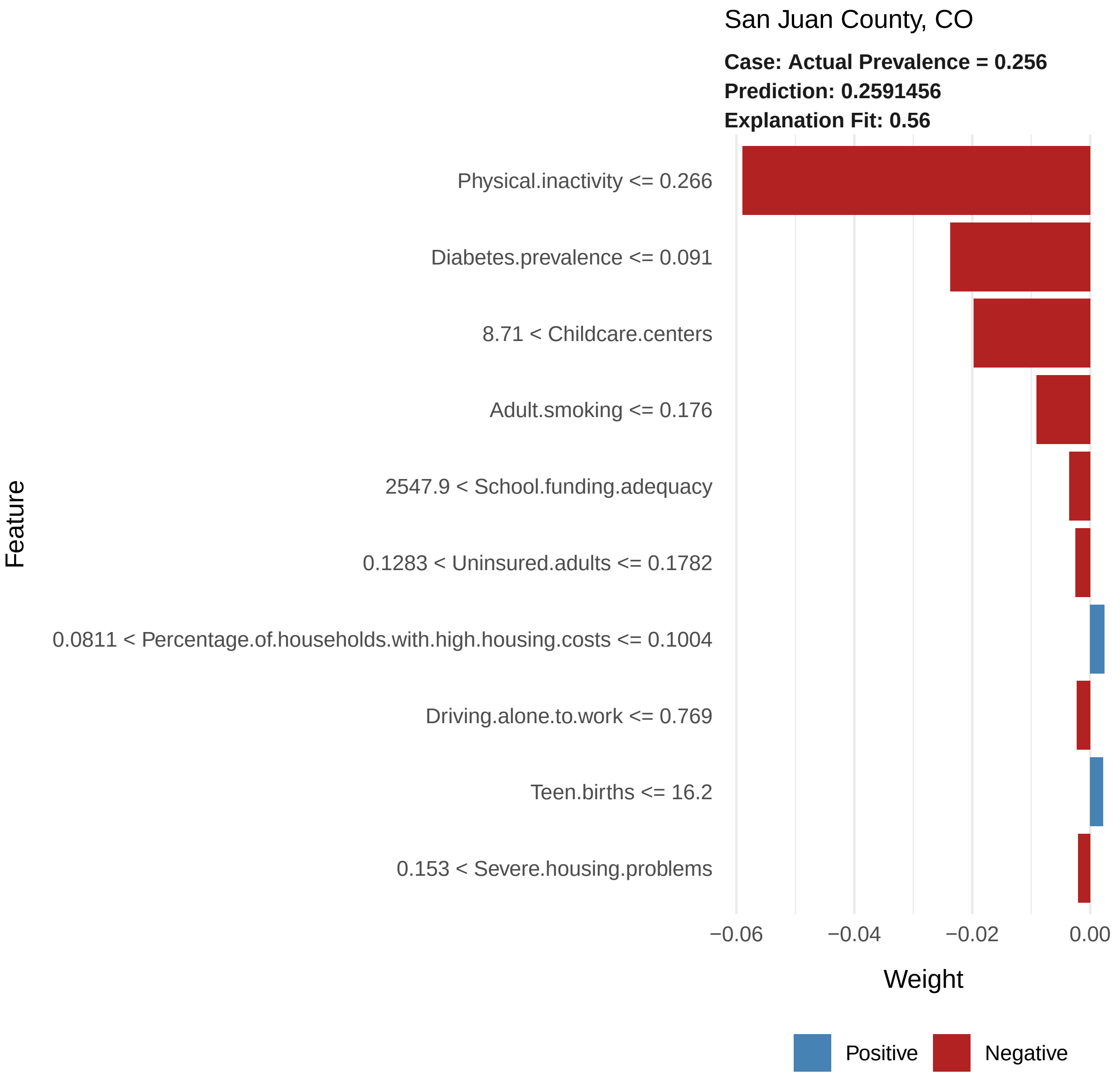
San Juan County, CO
Case: Actual Prevalence = 0.256
Prediction: 0.2591456
Explanation Fit: 0.56
Physical.inactivity <= 0.266
Diabetes.prevalence <= 0.091
8.71 < Childcare.centers
Adult.smoking <= 0.176
2547.9 < School.funding.adequacy
0.1283 < Uninsured.adults <= 0.1782
0.0811 < Percentage.of.households.with.high.housing.costs <= 0.1004
Driving.alone.to.work <= 0.769
Teen.births <= 16.2
0.153 < Severe.housing.problems
Feature
−0.06
−0.04
−0.02
0.00
Weight
Positive
Negative

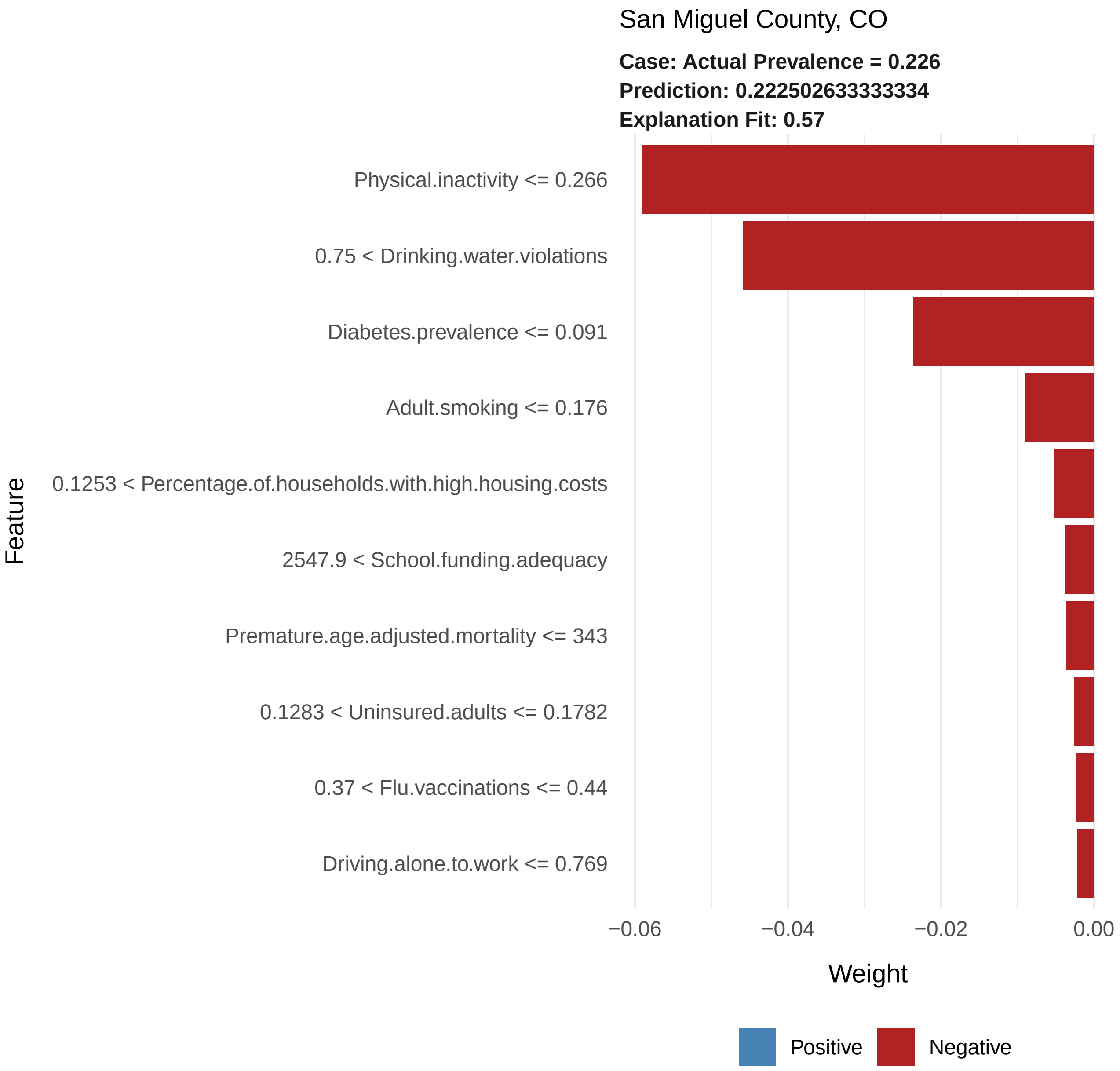

San Miguel County, CO
Case: Actual Prevalence = 0.226
Prediction: 0.222502633333334
Explanation Fit: 0.57
Physical.inactivity <= 0.266
0.75 < Drinking.water.violations
Diabetes.prevalence <= 0.091
Adult.smoking <= 0.176
0.1253 < Percentage.of.households.with.high.housing.costs
2547.9 < School.funding.adequacy
Premature.age.adjusted.mortality <= 343
0.1283 < Uninsured.adults <= 0.1782
0.37 < Flu.vaccinations <= 0.44
Driving.alone.to.work <= 0.769
Feature
−0.06
−0.04
−0.02
0.00
Weight
Positive
Negative

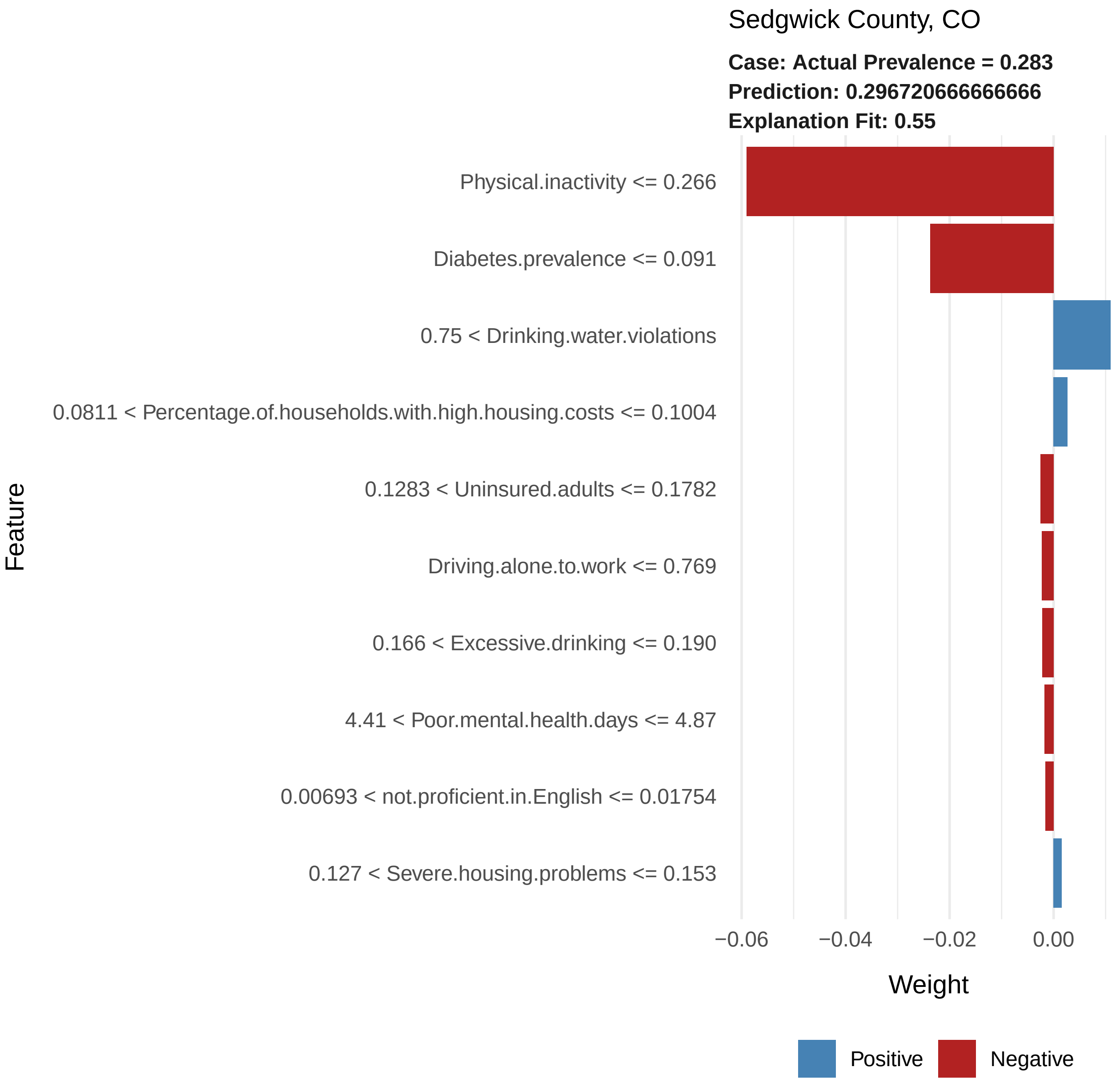

Sedgwick County, CO
Case: Actual Prevalence = 0.283
Prediction: 0.296720666666666
Explanation Fit: 0.55
Physical.inactivity <= 0.266
Diabetes.prevalence <= 0.091
0.75 < Drinking.water.violations
0.0811 < Percentage.of.households.with.high.housing.costs <= 0.1004
0.1283 < Uninsured.adults <= 0.1782
Driving.alone.to.work <= 0.769
0.166 < Excessive.drinking <= 0.190
4.41 < Poor.mental.health.days <= 4.87
0.00693 < not.proficient.in.English <= 0.01754
0.127 < Severe.housing.problems <= 0.153
Feature
−0.06
−0.04
−0.02
0.00
Weight
Positive
Negative

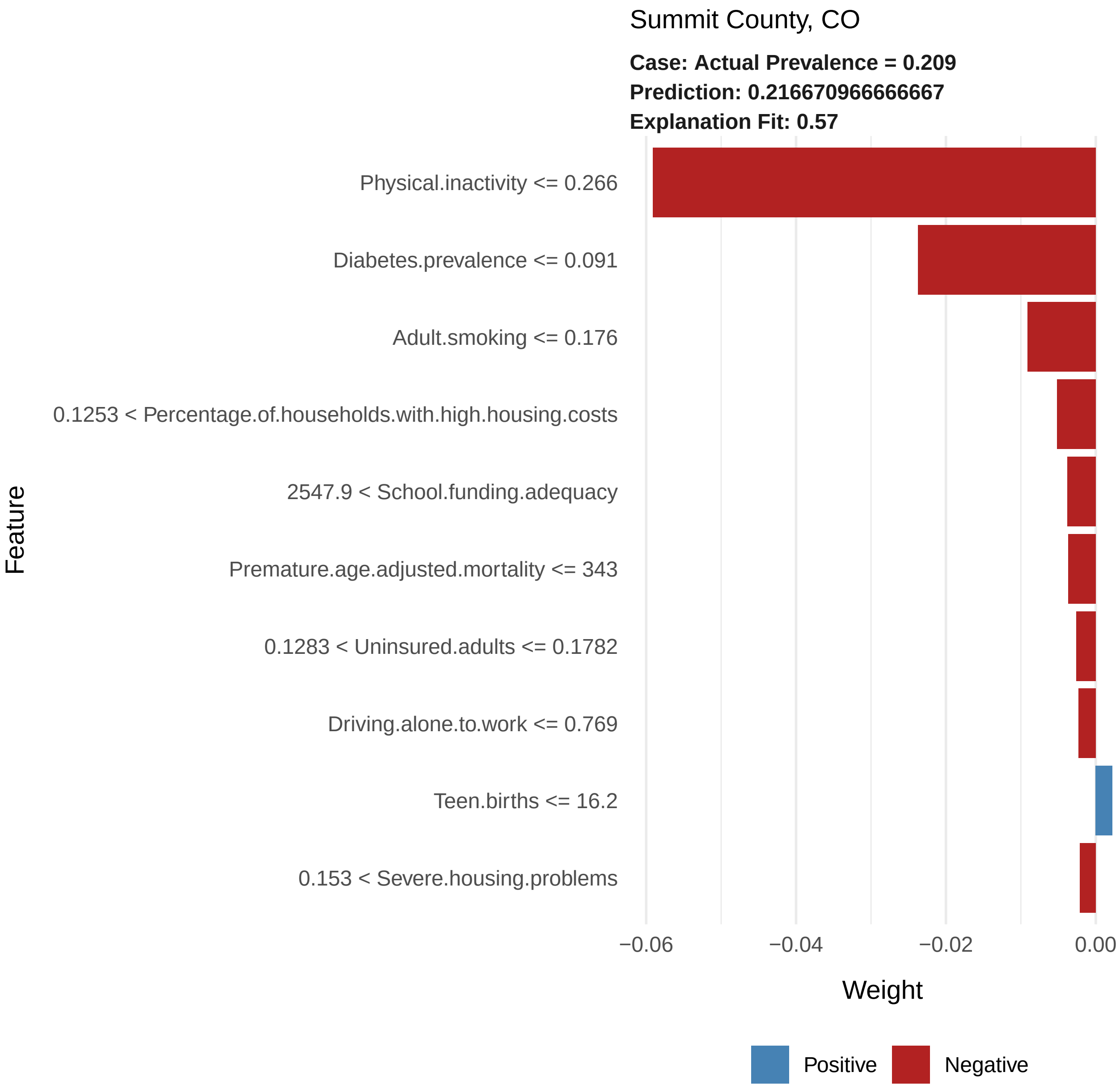

Summit County, CO
Case: Actual Prevalence = 0.209
Prediction: 0.216670966666667
Explanation Fit: 0.57
Physical.inactivity <= 0.266
Diabetes.prevalence <= 0.091
Adult.smoking <= 0.176
0.1253 < Percentage.of.households.with.high.housing.costs
2547.9 < School.funding.adequacy
Premature.age.adjusted.mortality <= 343
0.1283 < Uninsured.adults <= 0.1782
Driving.alone.to.work <= 0.769
Teen.births <= 16.2
0.153 < Severe.housing.problems
Feature
−0.06
−0.04
−0.02
0.00
Weight
Positive
Negative

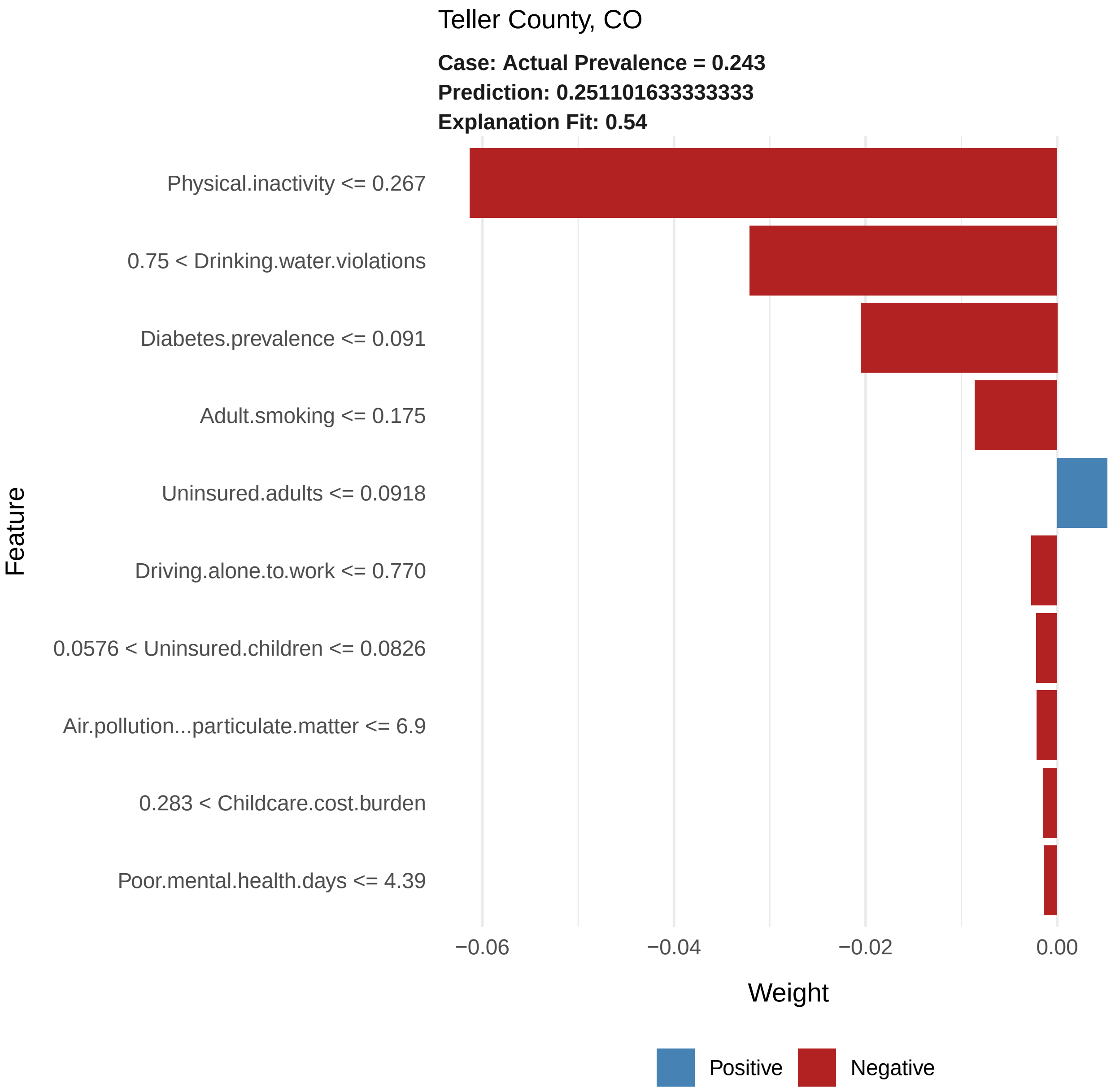

Teller County, CO
Case: Actual Prevalence = 0.243
Prediction: 0.251101633333333
Explanation Fit: 0.54
Physical.inactivity <= 0.267
0.75 < Drinking.water.violations
Diabetes.prevalence <= 0.091
Adult.smoking <= 0.175
Uninsured.adults <= 0.0918
Driving.alone.to.work <= 0.770
0.0576 < Uninsured.children <= 0.0826
Air.pollution...particulate.matter <= 6.9
0.283 < Childcare.cost.burden
Poor.mental.health.days <= 4.39
Feature
−0.06
−0.04
−0.02
0.00
Weight
Positive
Negative

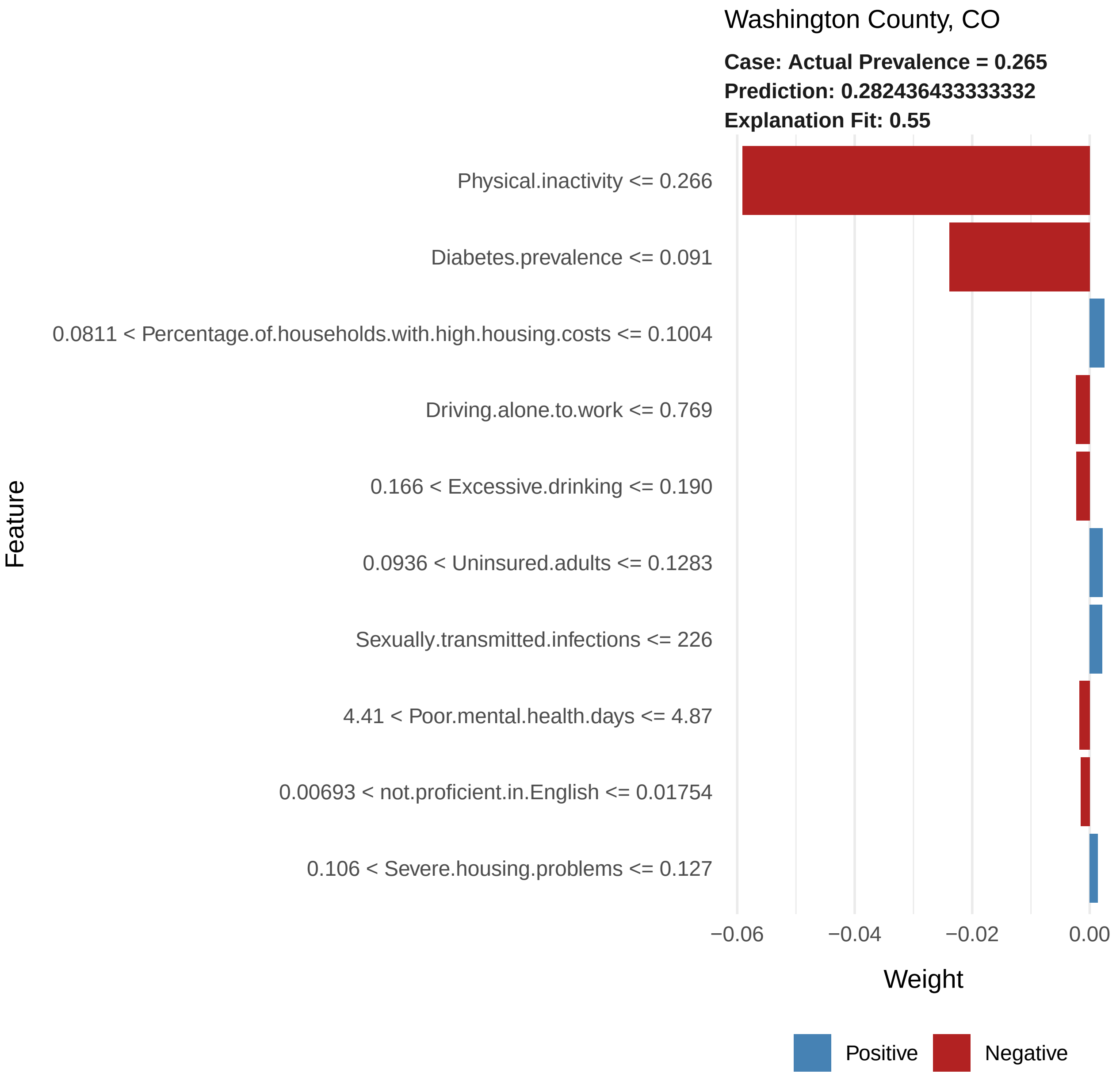

Washington County, CO
Case: Actual Prevalence = 0.265
Prediction: 0.282436433333332
Explanation Fit: 0.55
Physical.inactivity <= 0.266
Diabetes.prevalence <= 0.091
0.0811 < Percentage.of.households.with.high.housing.costs <= 0.1004
Driving.alone.to.work <= 0.769
0.166 < Excessive.drinking <= 0.190
0.0936 < Uninsured.adults <= 0.1283
Sexually.transmitted.infections <= 226
4.41 < Poor.mental.health.days <= 4.87
0.00693 < not.proficient.in.English <= 0.01754
0.106 < Severe.housing.problems <= 0.127
Feature
−0.06
−0.04
−0.02
0.00
Weight
Positive
Negative

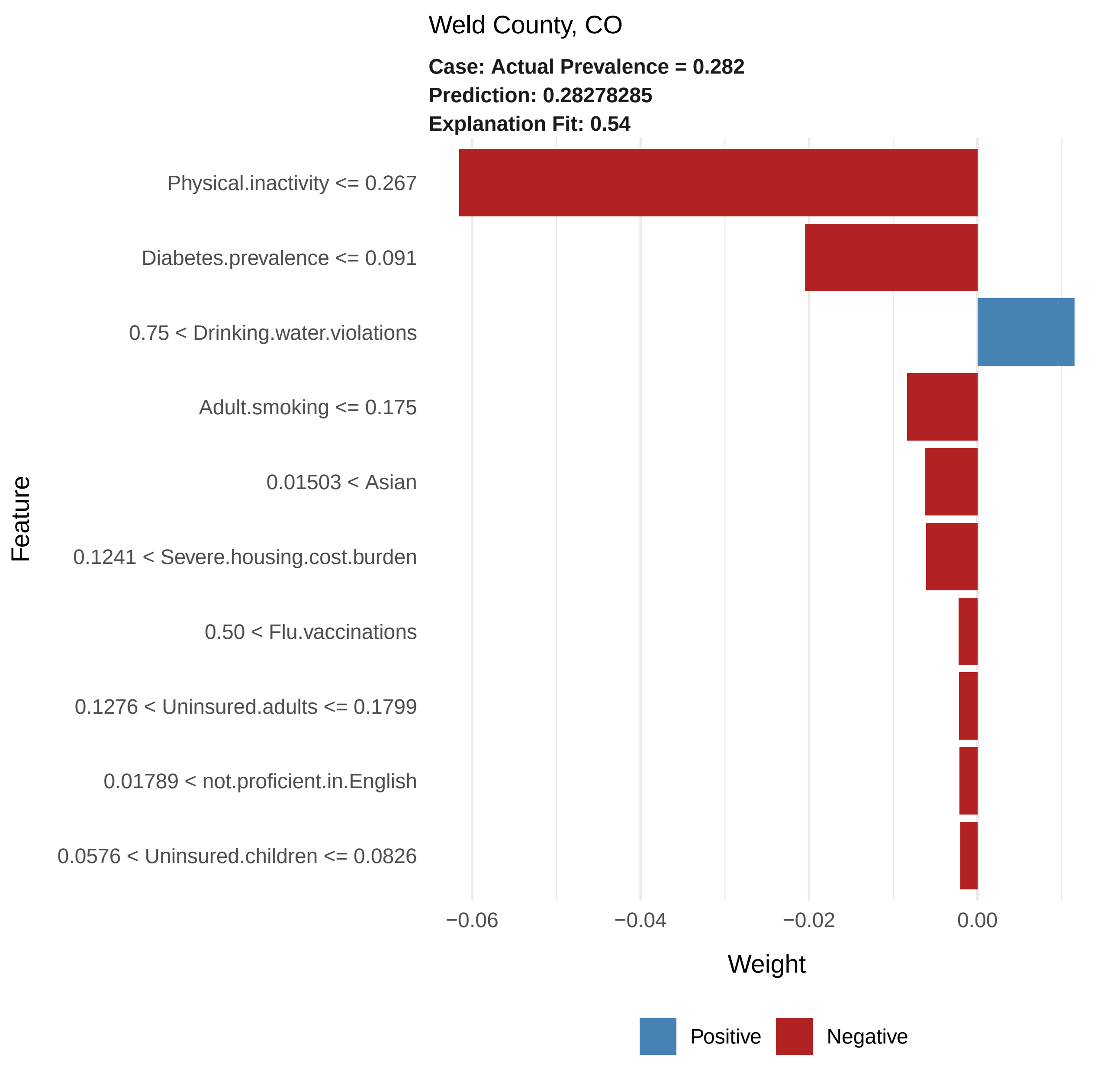

Weld County, CO
Case: Actual Prevalence = 0.282
Prediction: 0.28278285
Explanation Fit: 0.54
Physical.inactivity <= 0.267
Diabetes.prevalence <= 0.091
0.75 < Drinking.water.violations
Adult.smoking <= 0.175
0.01503 < Asian
0.1241 < Severe.housing.cost.burden
0.50 < Flu.vaccinations
0.1276 < Uninsured.adults <= 0.1799
0.01789 < not.proficient.in.English
0.0576 < Uninsured.children <= 0.0826
Feature
−0.06
−0.04
−0.02
0.00
Weight
Positive
Negative

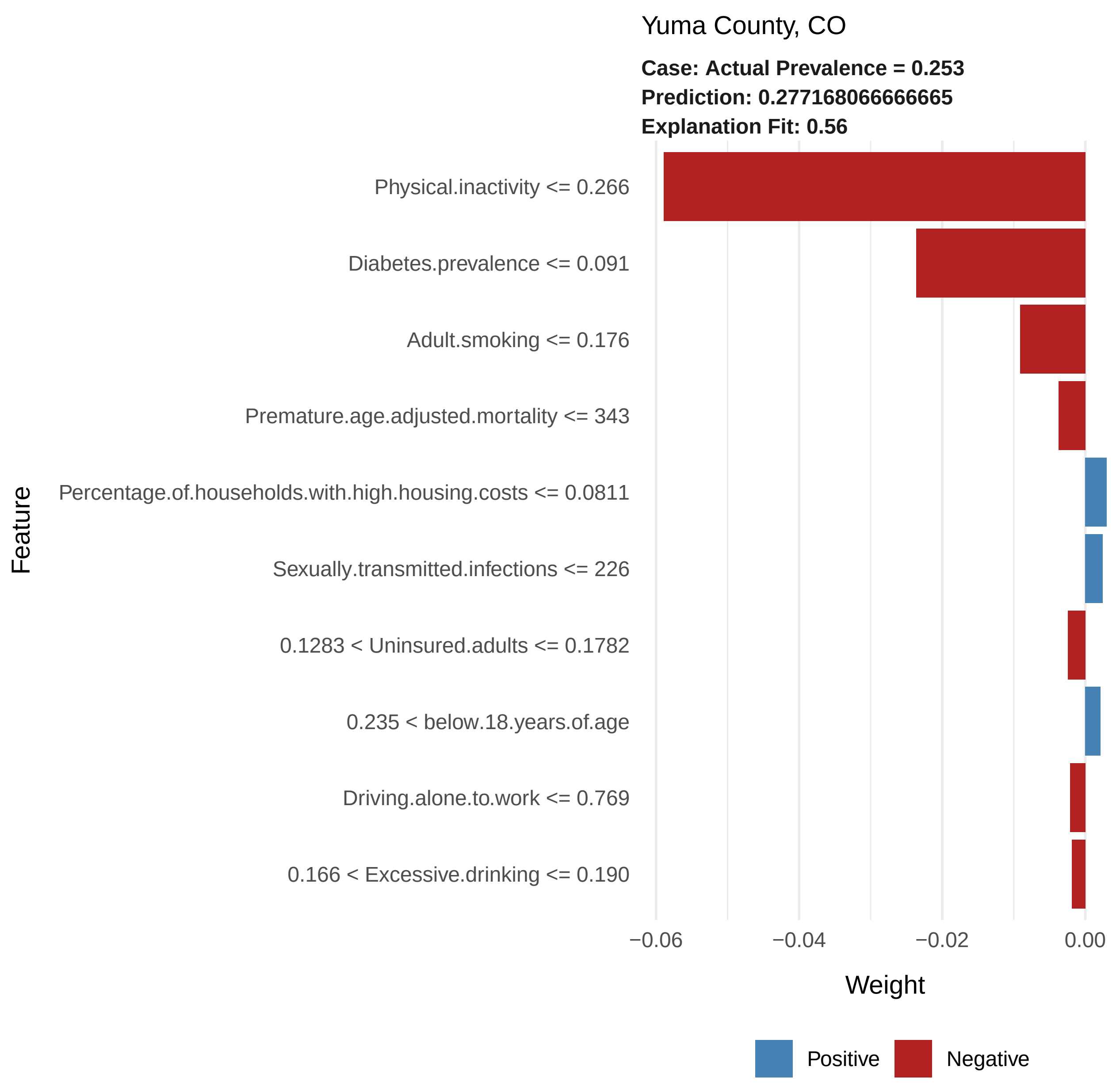

Yuma County, CO
Case: Actual Prevalence = 0.253
Prediction: 0.277168066666665
Explanation Fit: 0.56
Physical.inactivity <= 0.266
Diabetes.prevalence <= 0.091
Adult.smoking <= 0.176
Premature.age.adjusted.mortality <= 343
Percentage.of.households.with.high.housing.costs <= 0.0811
Sexually.transmitted.infections <= 226
0.1283 < Uninsured.adults <= 0.1782
0.235 < below.18.years.of.age
Driving.alone.to.work <= 0.769
0.166 < Excessive.drinking <= 0.190
Feature
−0.06
−0.04
−0.02
0.00
Weight
Positive
Negative

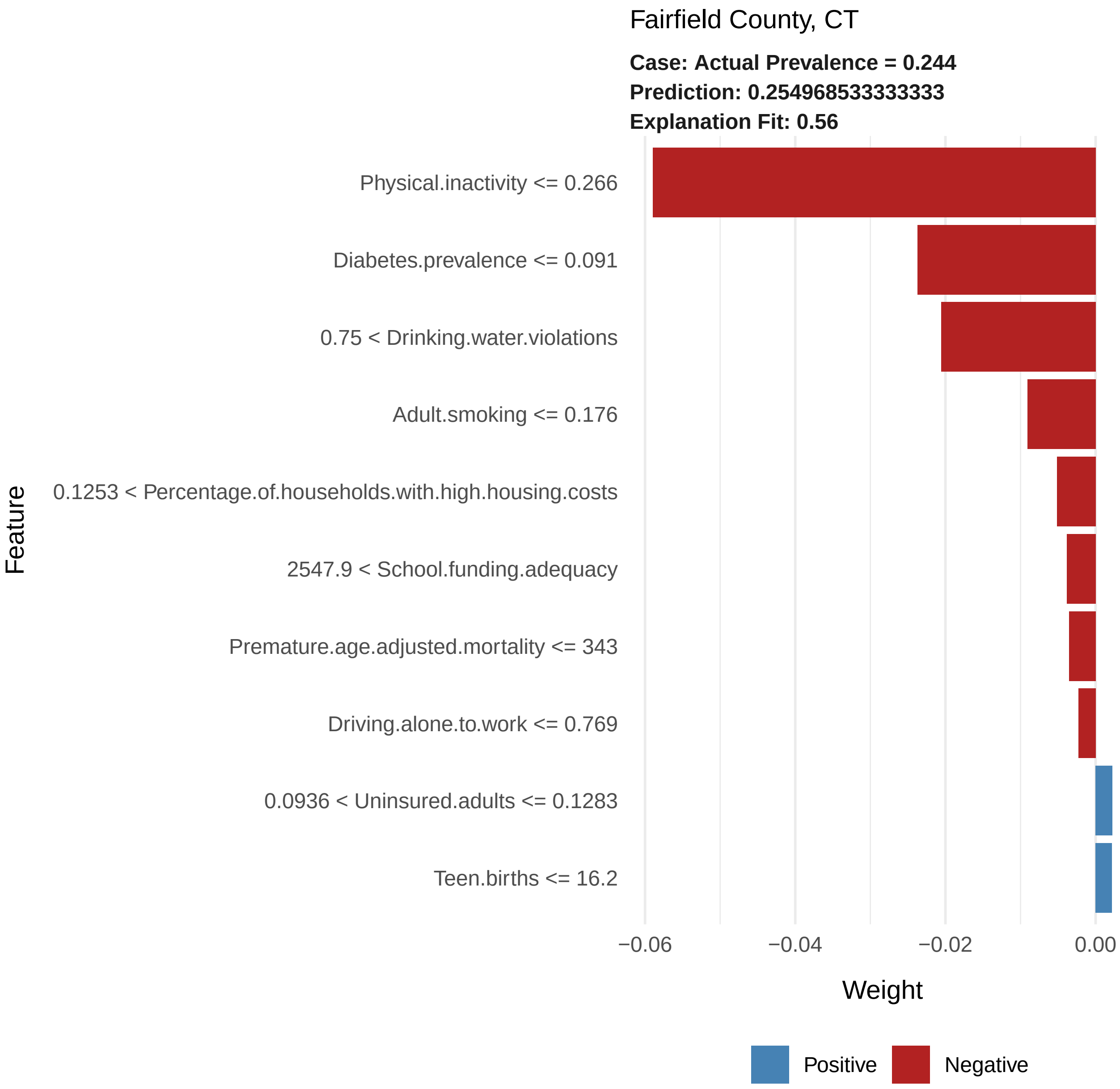
Fairfield County, CT
Case: Actual Prevalence = 0.244
Prediction: 0.254968533333333
Explanation Fit: 0.56
Physical.inactivity <= 0.266
Diabetes.prevalence <= 0.091
0.75 < Drinking.water.violations
Adult.smoking <= 0.176
0.1253 < Percentage.of.households.with.high.housing.costs
2547.9 < School.funding.adequacy
Premature.age.adjusted.mortality <= 343
Driving.alone.to.work <= 0.769
0.0936 < Uninsured.adults <= 0.1283
Teen.births <= 16.2
Feature
−0.06
−0.04
−0.02
0.00
Weight
Positive
Negative

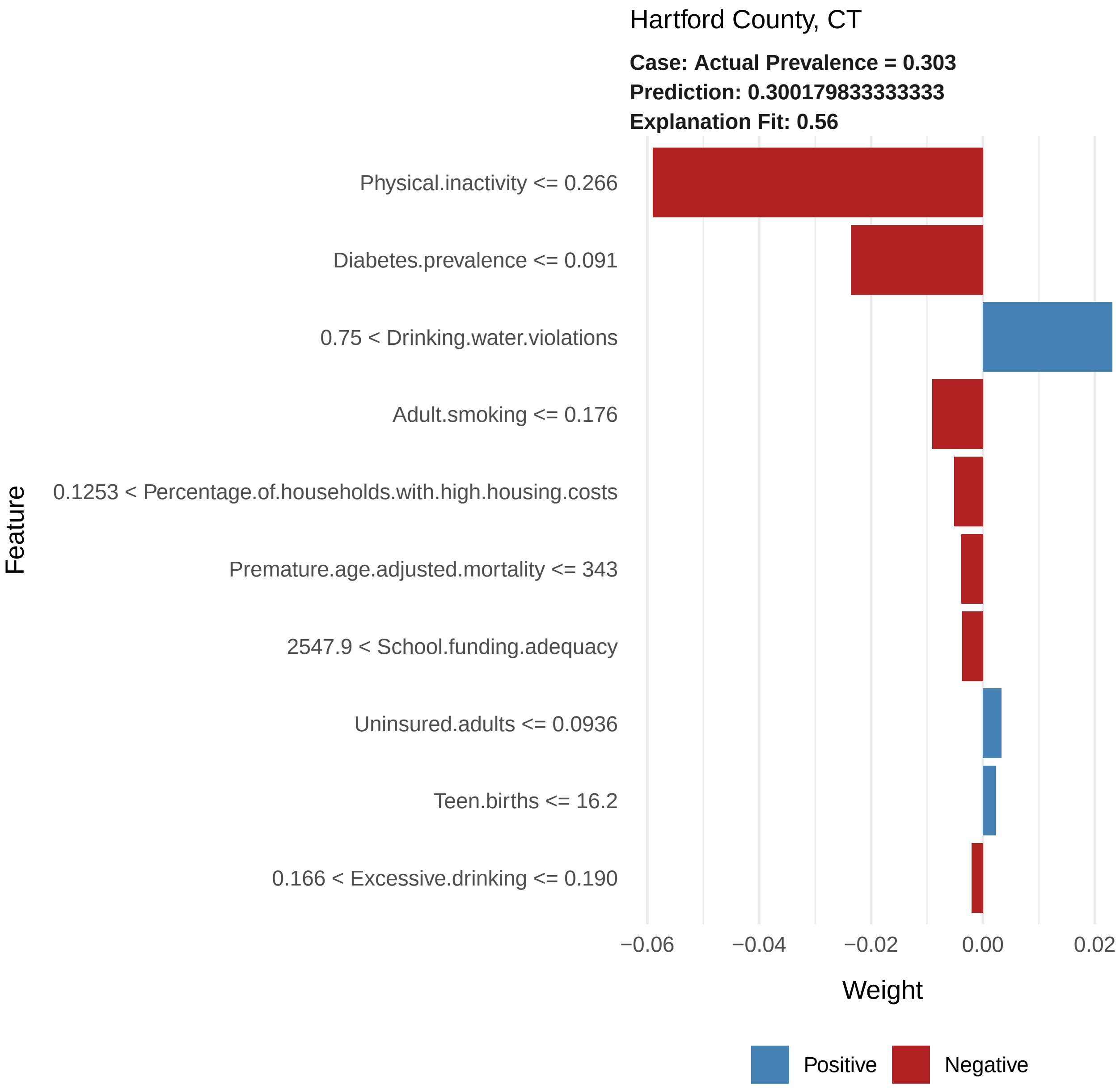

Hartford County, CT
Case: Actual Prevalence = 0.303
Prediction: 0.300179833333333
Explanation Fit: 0.56
Physical.inactivity <= 0.266
Diabetes.prevalence <= 0.091
0.75 < Drinking.water.violations
Adult.smoking <= 0.176
0.1253 < Percentage.of.households.with.high.housing.costs
Premature.age.adjusted.mortality <= 343
2547.9 < School.funding.adequacy
Uninsured.adults <= 0.0936
Teen.births <= 16.2
0.166 < Excessive.drinking <= 0.190
Feature
−0.06
−0.04
−0.02
0.00
0.02
Weight
Positive
Negative

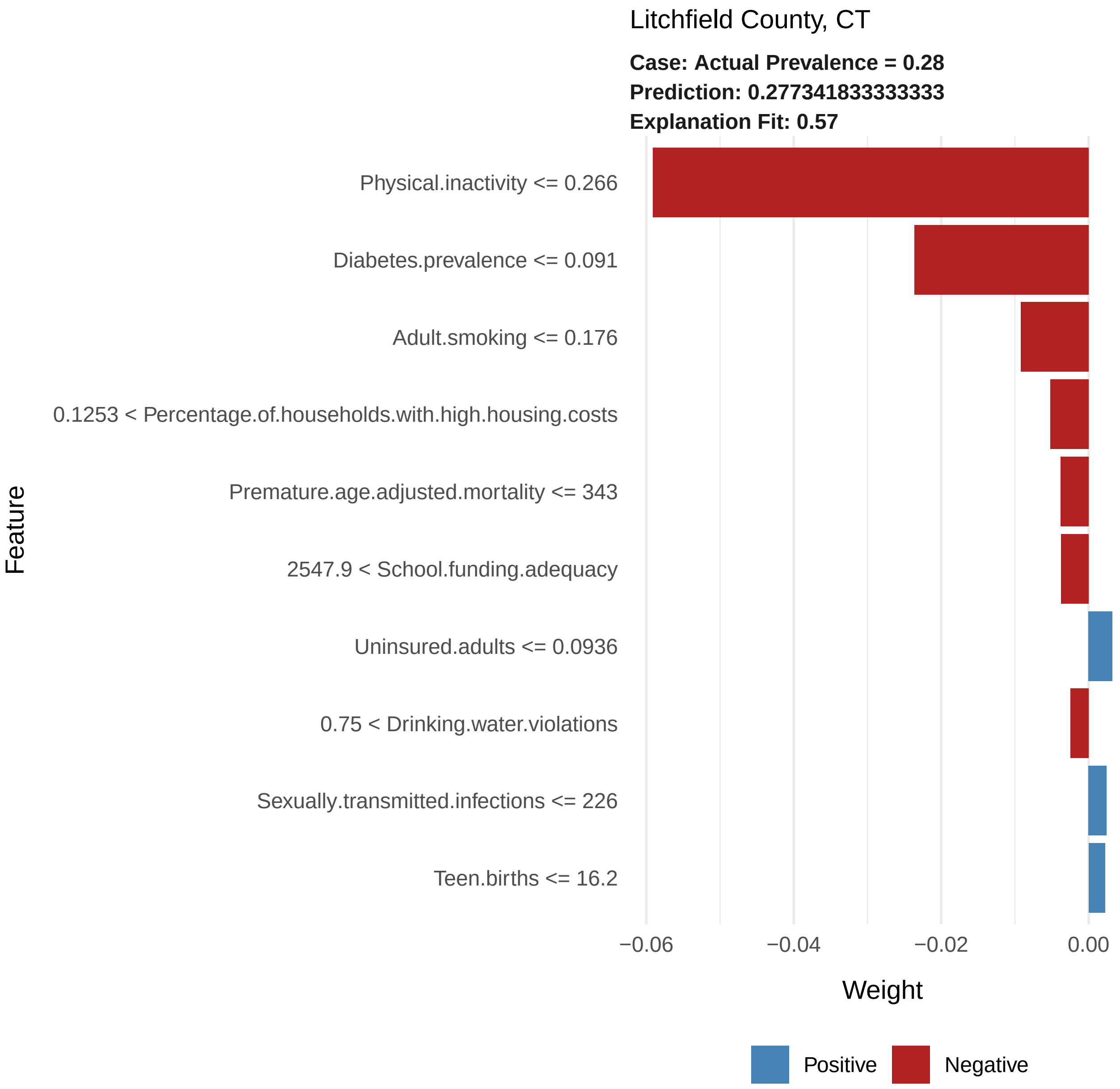

Litchfield County, CT
Case: Actual Prevalence = 0.28
Prediction: 0.277341833333333
Explanation Fit: 0.57
Physical.inactivity <= 0.266
Diabetes.prevalence <= 0.091
Adult.smoking <= 0.176
0.1253 < Percentage.of.households.with.high.housing.costs
Premature.age.adjusted.mortality <= 343
2547.9 < School.funding.adequacy
Uninsured.adults <= 0.0936
0.75 < Drinking.water.violations
Sexually.transmitted.infections <= 226
Teen.births <= 16.2
Feature
−0.06
−0.04
−0.02
0.00
Weight
Positive
Negative

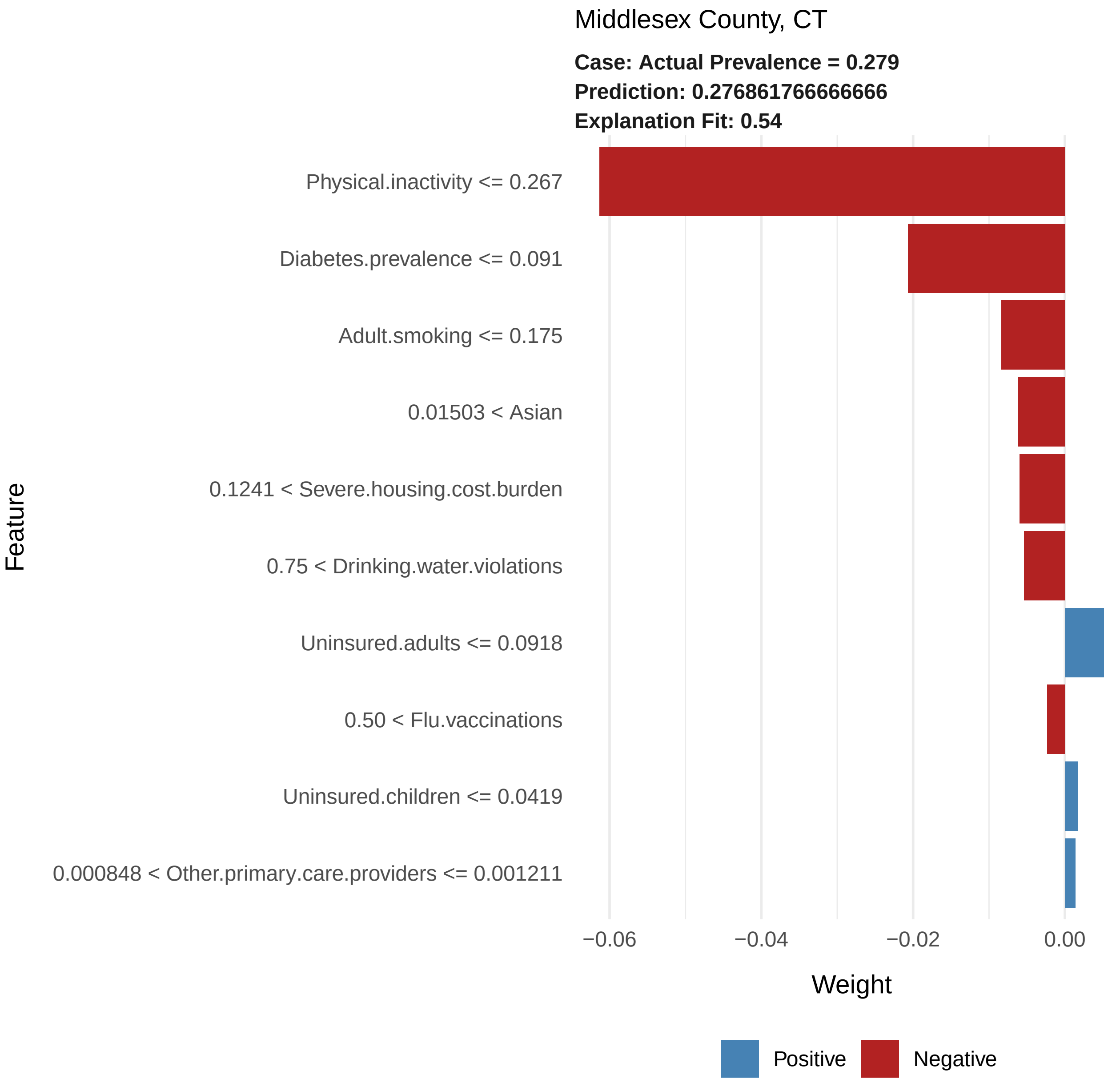

Middlesex County, CT
Case: Actual Prevalence = 0.279
Prediction: 0.276861766666666
Explanation Fit: 0.54
Physical.inactivity <= 0.267
Diabetes.prevalence <= 0.091
Adult.smoking <= 0.175
0.01503 < Asian
0.1241 < Severe.housing.cost.burden
0.75 < Drinking.water.violations
Uninsured.adults <= 0.0918
0.50 < Flu.vaccinations
Uninsured.children <= 0.0419
0.000848 < Other.primary.care.providers <= 0.001211
Feature
−0.06
−0.04
−0.02
0.00
Weight
Positive
Negative

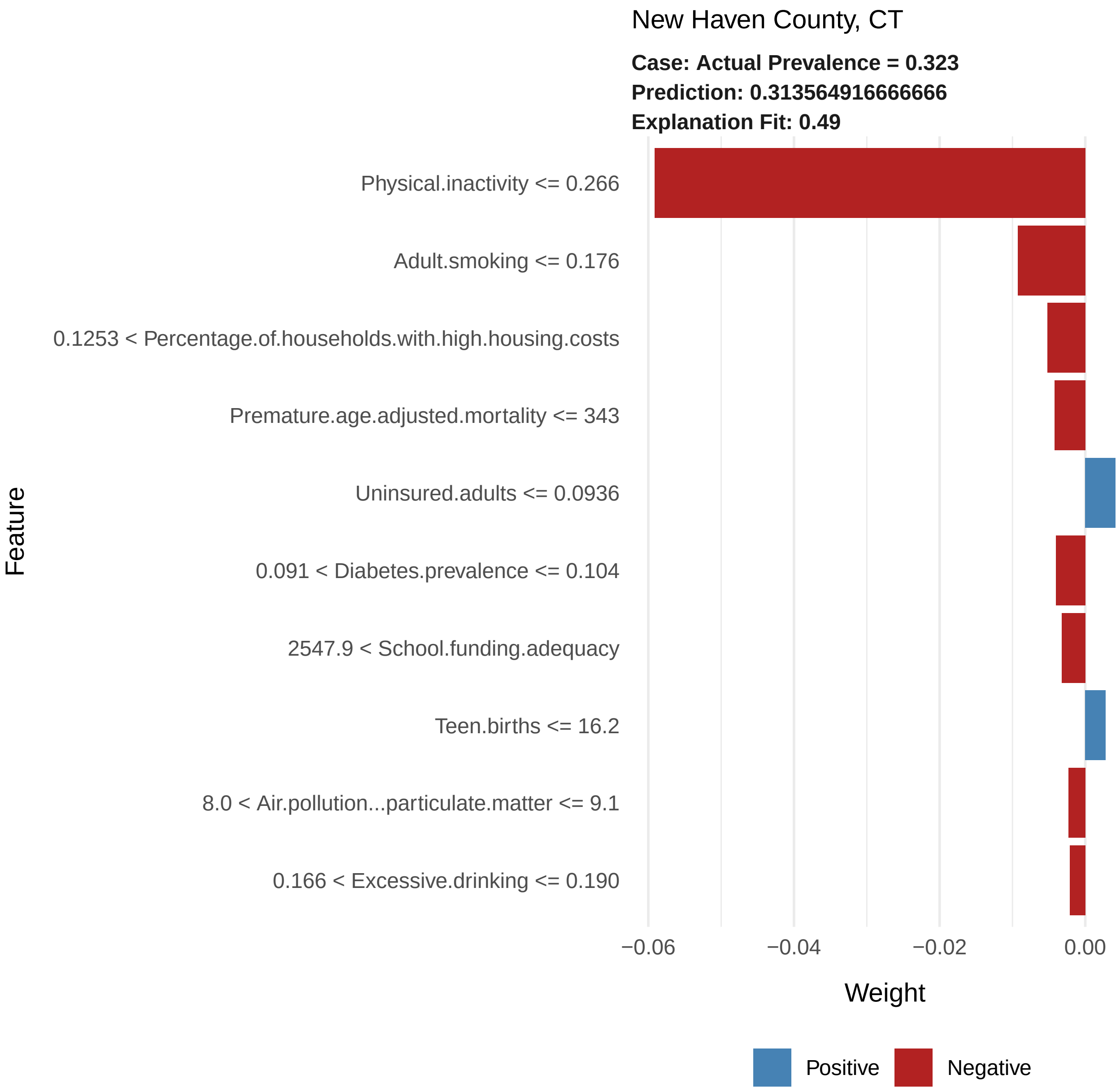

New Haven County, CT
Case: Actual Prevalence = 0.323
Prediction: 0.313564916666666
Explanation Fit: 0.49
Physical.inactivity <= 0.266
Adult.smoking <= 0.176
0.1253 < Percentage.of.households.with.high.housing.costs
Premature.age.adjusted.mortality <= 343
Uninsured.adults <= 0.0936
0.091 < Diabetes.prevalence <= 0.104
2547.9 < School.funding.adequacy
Teen.births <= 16.2
8.0 < Air.pollution...particulate.matter <= 9.1
0.166 < Excessive.drinking <= 0.190
Feature
−0.06
−0.04
−0.02
0.00
Weight
Positive
Negative

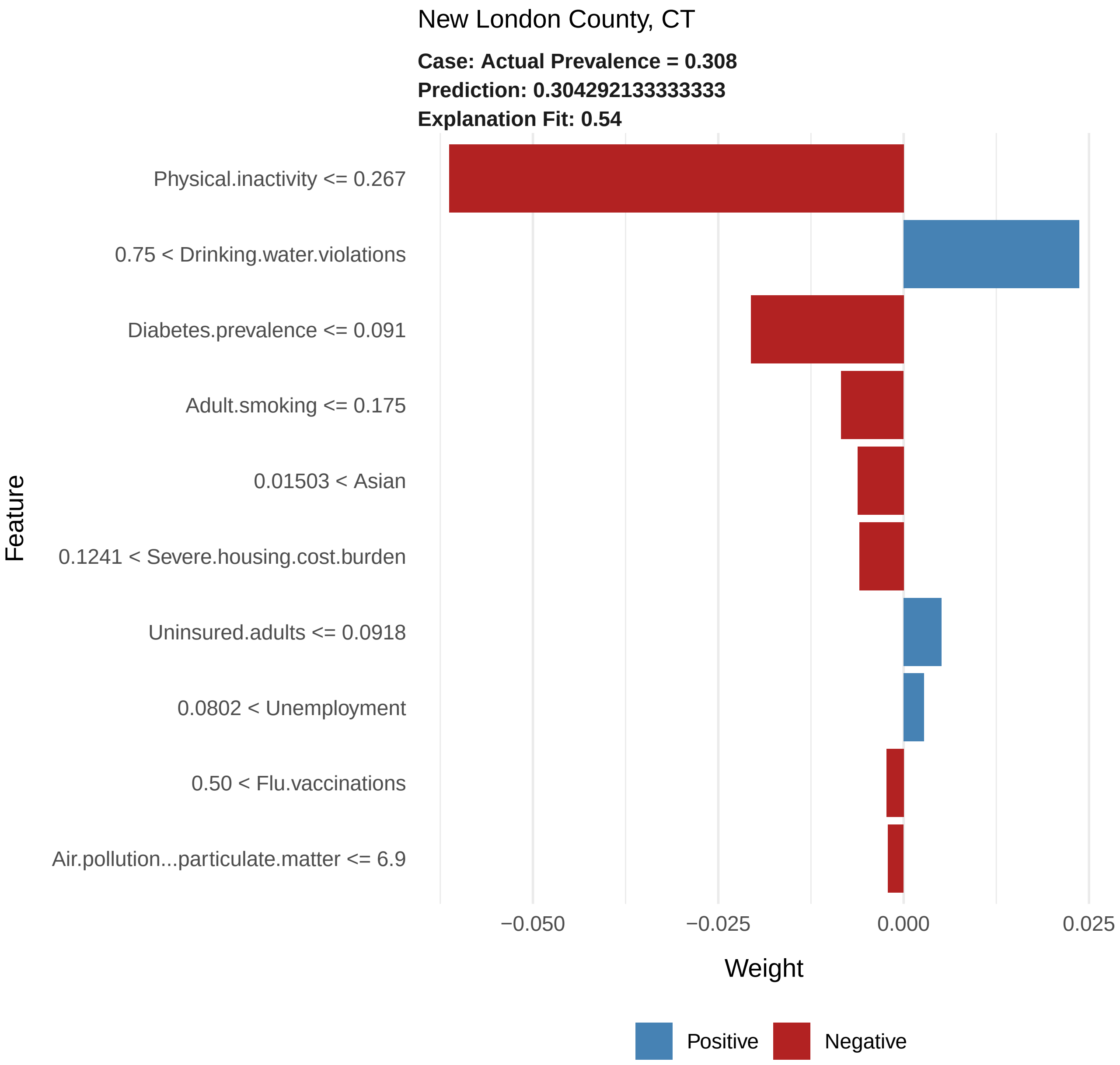

New London County, CT
Case: Actual Prevalence = 0.308
Prediction: 0.304292133333333
Explanation Fit: 0.54
Physical.inactivity <= 0.267
0.75 < Drinking.water.violations
Diabetes.prevalence <= 0.091
Adult.smoking <= 0.175
0.01503 < Asian
0.1241 < Severe.housing.cost.burden
Uninsured.adults <= 0.0918
0.0802 < Unemployment
0.50 < Flu.vaccinations
Air.pollution...particulate.matter <= 6.9
Feature
−0.050
−0.025
0.000
0.025
Weight
Positive
Negative

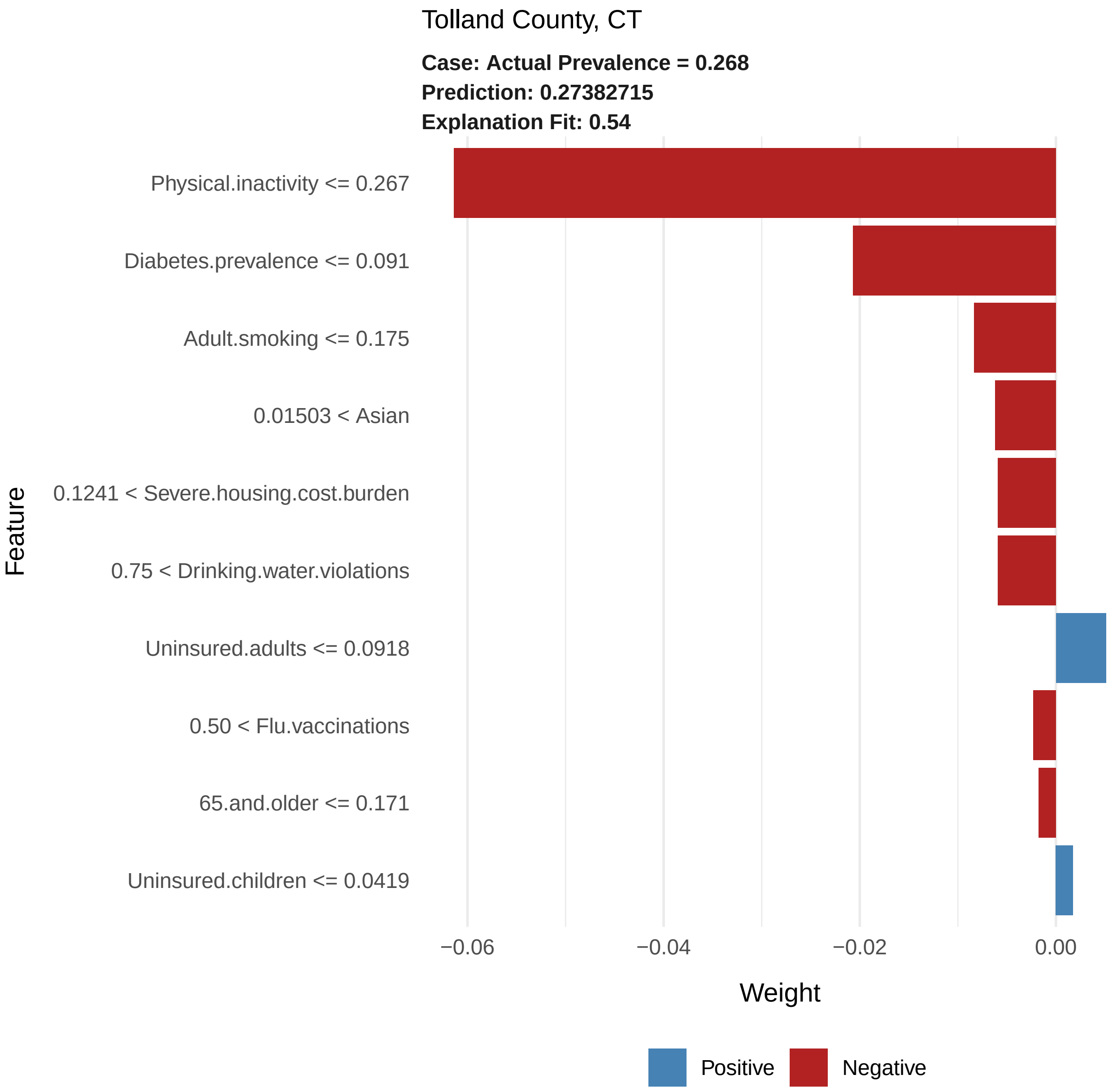
Tolland County, CT
Case: Actual Prevalence = 0.268
Prediction: 0.27382715
Explanation Fit: 0.54
Physical.inactivity <= 0.267
Diabetes.prevalence <= 0.091
Adult.smoking <= 0.175
0.01503 < Asian
0.1241 < Severe.housing.cost.burden
0.75 < Drinking.water.violations
Uninsured.adults <= 0.0918
0.50 < Flu.vaccinations
65.and.older <= 0.171
Uninsured.children <= 0.0419
Feature
−0.06
−0.04
−0.02
0.00
Weight
Positive
Negative

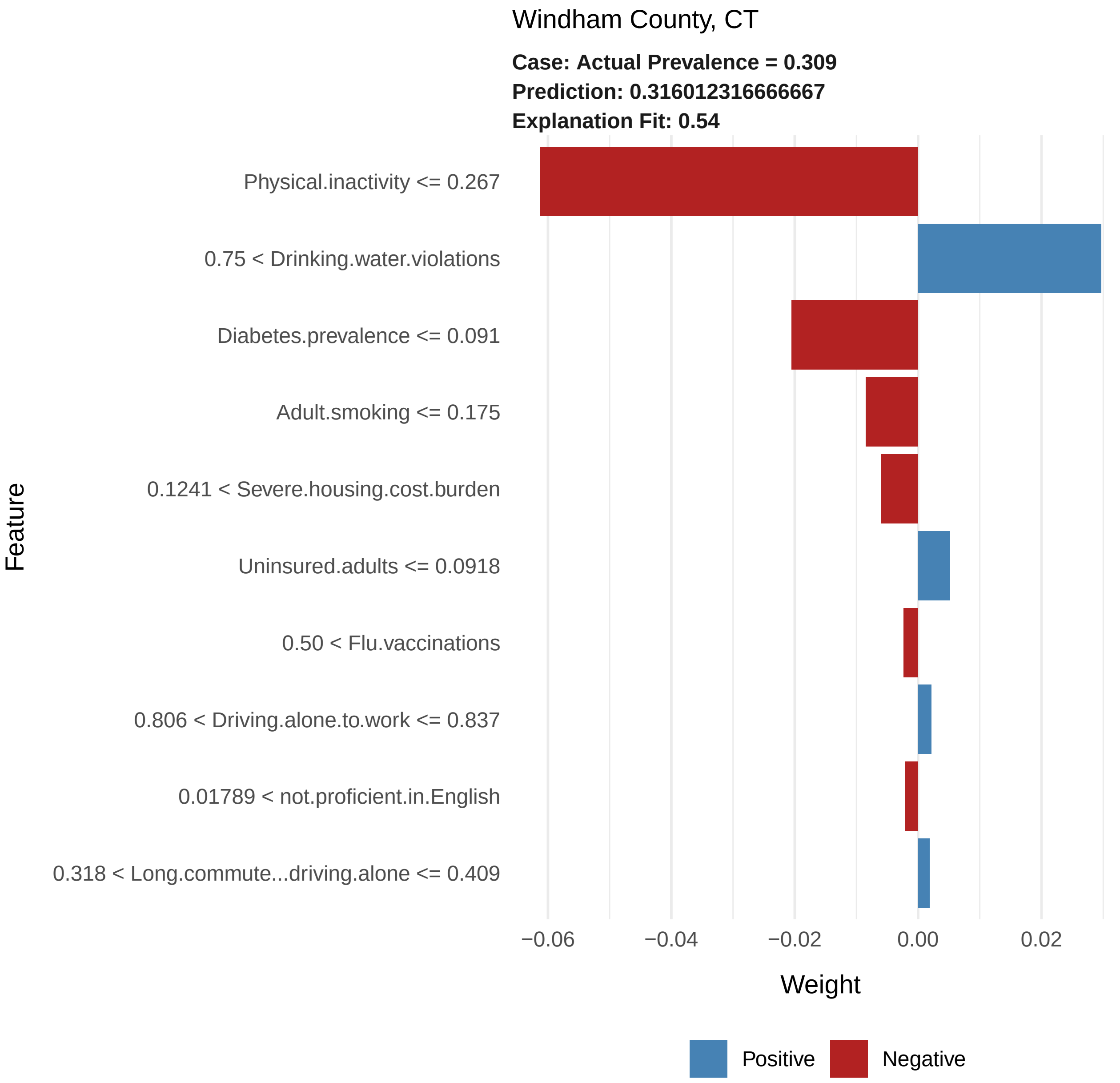
Windham County, CT
Case: Actual Prevalence = 0.309
Prediction: 0.316012316666667
Explanation Fit: 0.54
Physical.inactivity <= 0.267
0.75 < Drinking.water.violations
Diabetes.prevalence <= 0.091
Adult.smoking <= 0.175
0.1241 < Severe.housing.cost.burden
Uninsured.adults <= 0.0918
0.50 < Flu.vaccinations
0.806 < Driving.alone.to.work <= 0.837
0.01789 < not.proficient.in.English
0.318 < Long.commute...driving.alone <= 0.409
Feature
−0.06
−0.04
−0.02
0.00
0.02
Weight
Positive
Negative

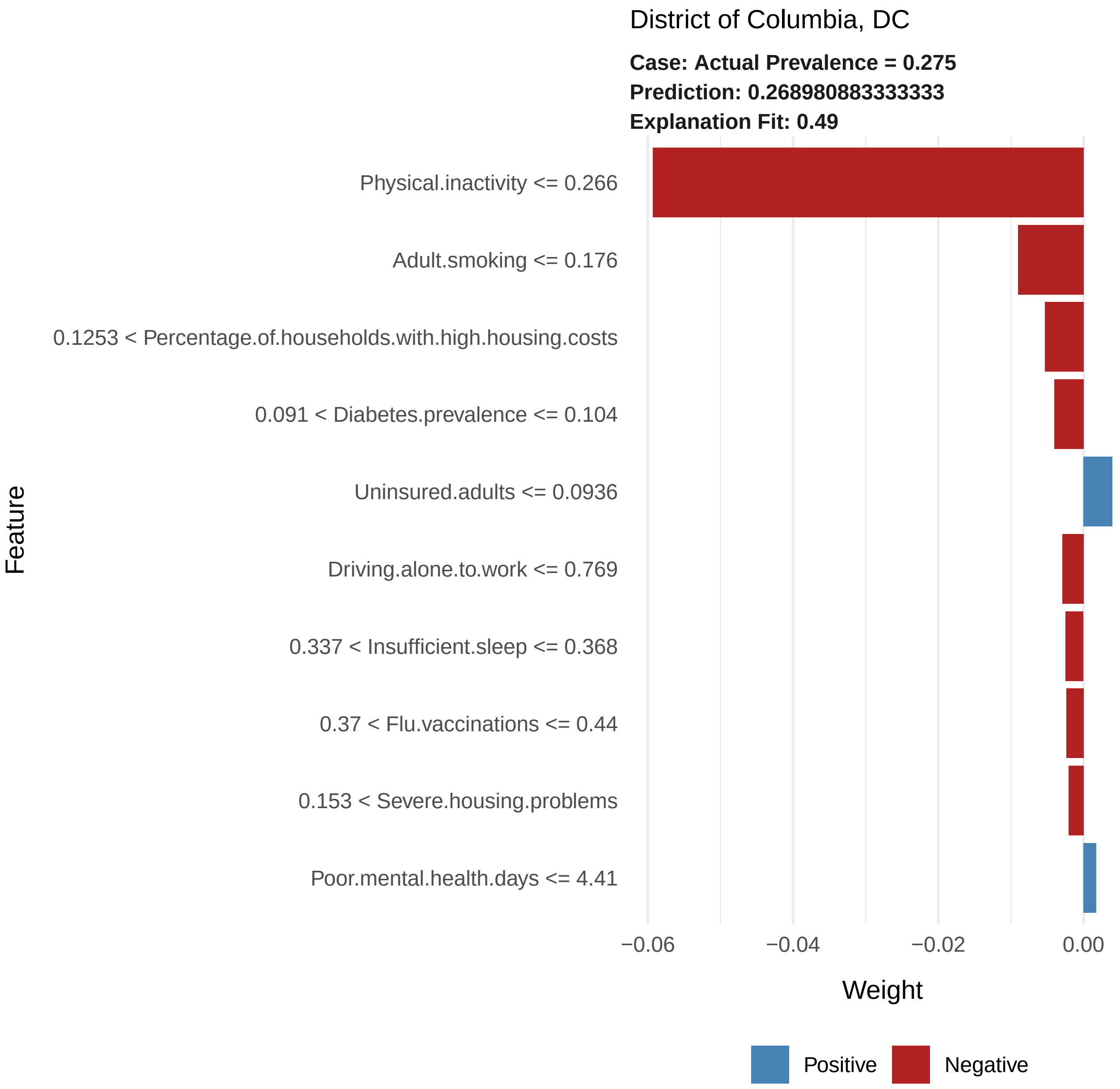
District of Columbia, DC
Case: Actual Prevalence = 0.275
Prediction: 0.268980883333333
Explanation Fit: 0.49
Feature
Physical.inactivity <= 0.266
Adult.smoking <= 0.176
0.1253 < Percentage.of.households.with.high.housing.costs
0.091 < Diabetes.prevalence <= 0.104
Uninsured.adults <= 0.0936
Driving.alone.to.work <= 0.769
0.337 < Insufficient.sleep <= 0.368
0.37 < Flu.vaccinations <= 0.44
0.153 < Severe.housing.problems
Poor.mental.health.days <= 4.41
−0.06
−0.04
−0.02
0.00
Weight
Positive
Negative

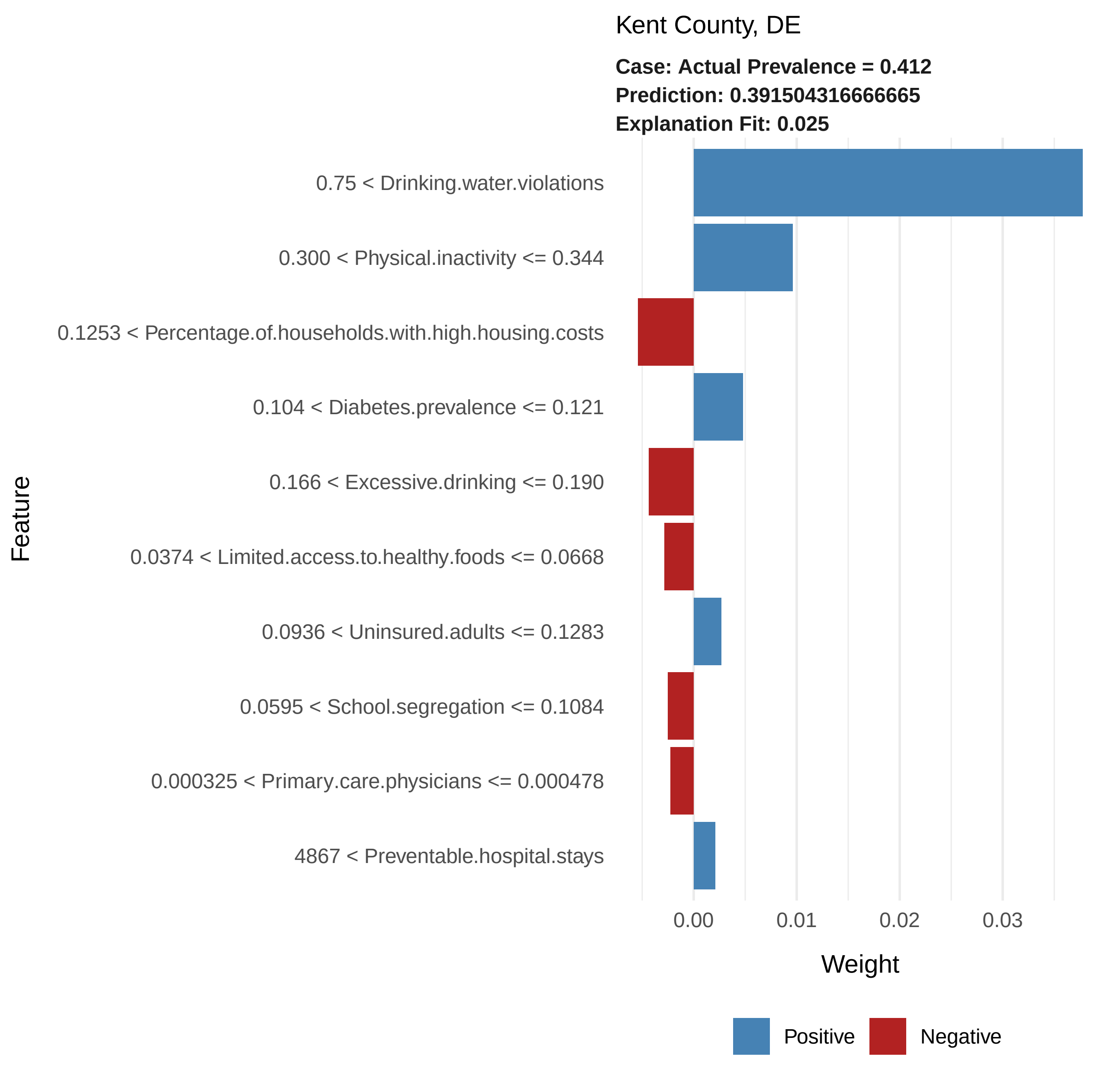
Kent County, DE
Case: Actual Prevalence = 0.412
Prediction: 0.391504316666665
Explanation Fit: 0.025
0.75 < Drinking.water.violations
0.300 < Physical.inactivity <= 0.344
0.1253 < Percentage.of.households.with.high.housing.costs
0.104 < Diabetes.prevalence <= 0.121
0.166 < Excessive.drinking <= 0.190
0.0374 < Limited.access.to.healthy.foods <= 0.0668
0.0936 < Uninsured.adults <= 0.1283
0.0595 < School.segregation <= 0.1084
0.000325 < Primary.care.physicians <= 0.000478
4867 < Preventable.hospital.stays
Feature
0.00
0.01
0.02
0.03
Weight
Positive
Negative

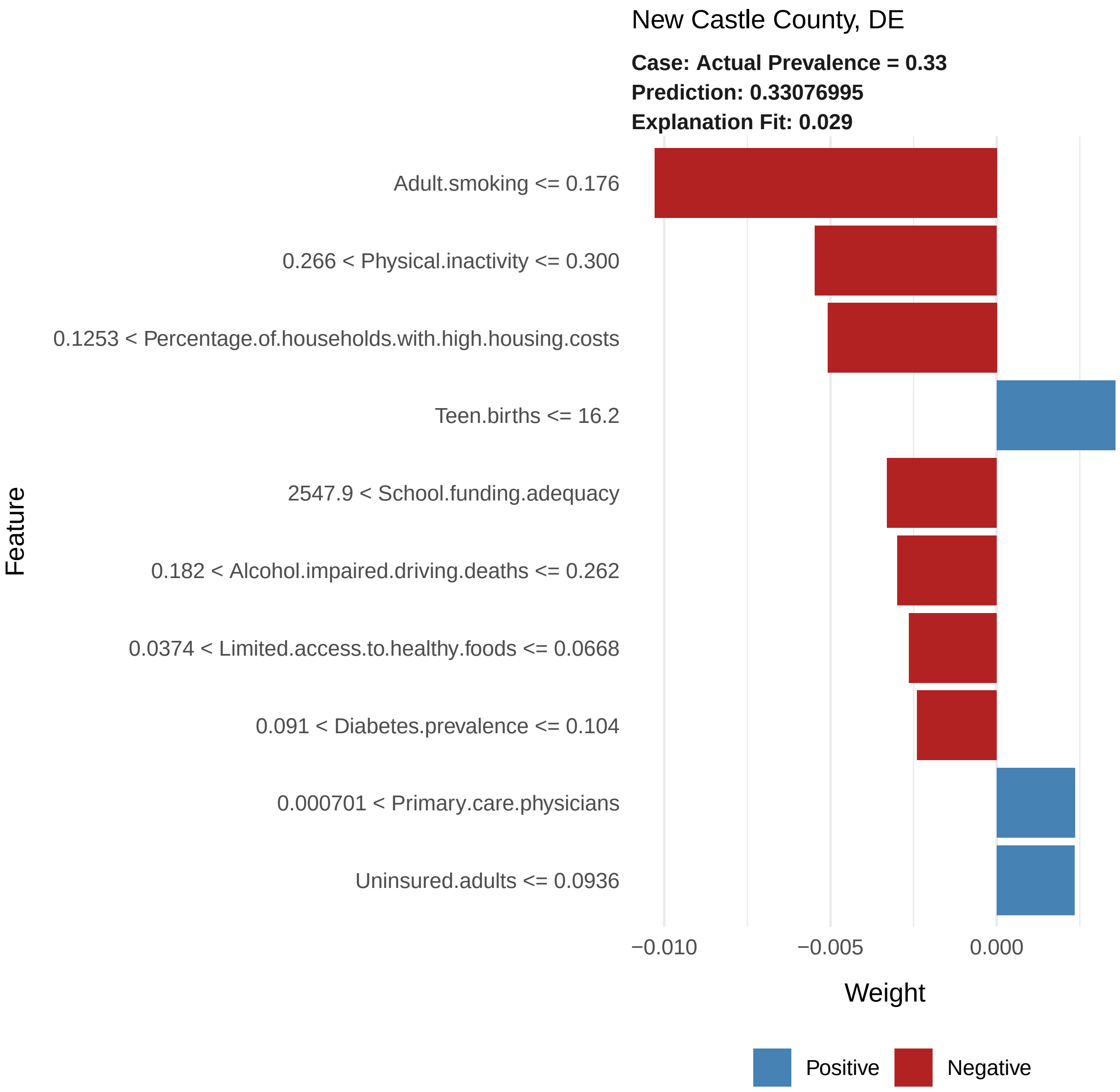
New Castle County, DE
Case: Actual Prevalence = 0.33
Prediction: 0.33076995
Explanation Fit: 0.029
Adult.smoking <= 0.176
0.266 < Physical.inactivity <= 0.300
0.1253 < Percentage.of.households.with.high.housing.costs
Teen.births <= 16.2
2547.9 < School.funding.adequacy
0.182 < Alcohol.impaired.driving.deaths <= 0.262
0.0374 < Limited.access.to.healthy.foods <= 0.0668
0.091 < Diabetes.prevalence <= 0.104
0.000701 < Primary.care.physicians
Uninsured.adults <= 0.0936
Feature
−0.010
−0.005
0.000
Weight
Positive
Negative

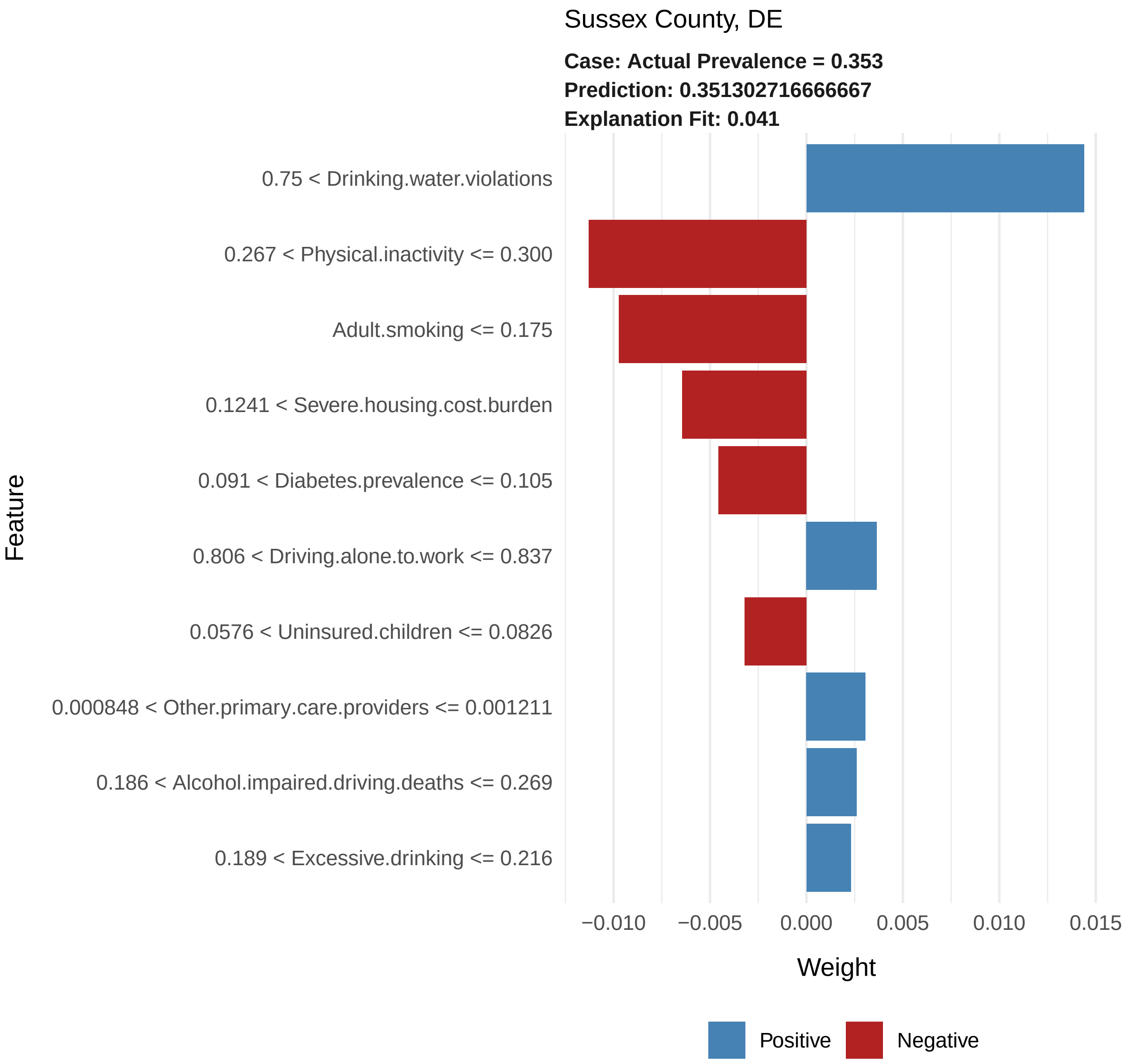

Sussex County, DE
Case: Actual Prevalence = 0.353
Prediction: 0.351302716666667
Explanation Fit: 0.041
0.75 < Drinking.water.violations
0.267 < Physical.inactivity <= 0.300
Adult.smoking <= 0.175
0.1241 < Severe.housing.cost.burden
0.091 < Diabetes.prevalence <= 0.105
0.806 < Driving.alone.to.work <= 0.837
0.0576 < Uninsured.children <= 0.0826
0.000848 < Other.primary.care.providers <= 0.001211
0.186 < Alcohol.impaired.driving.deaths <= 0.269
0.189 < Excessive.drinking <= 0.216
Feature
−0.010
−0.005
0.000
0.005
0.010
0.015
Weight
Positive
Negative

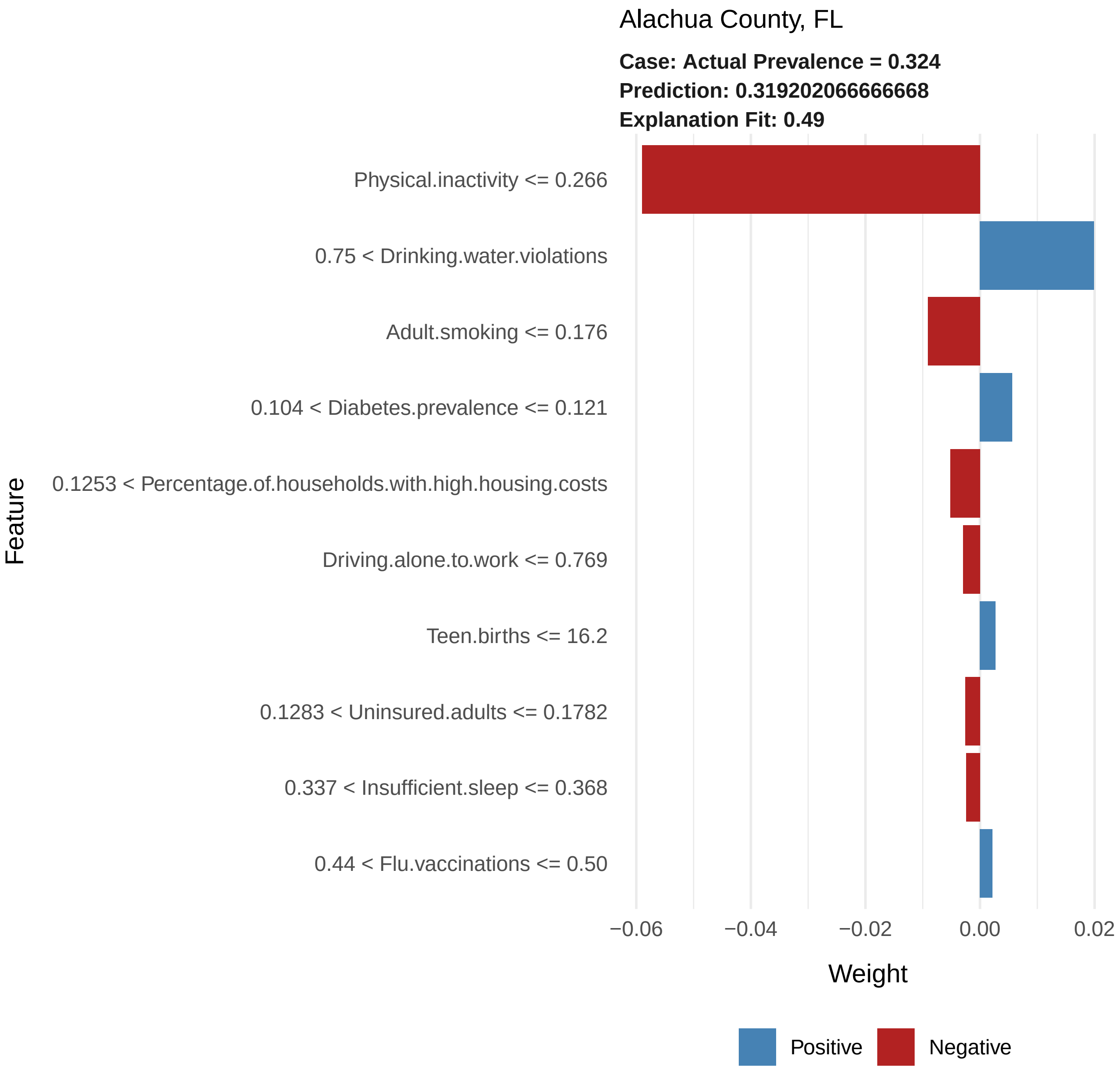
Alachua County, FL
Case: Actual Prevalence = 0.324
Prediction: 0.319202066666668
Explanation Fit: 0.49
Physical.inactivity <= 0.266
0.75 < Drinking.water.violations
Adult.smoking <= 0.176
0.104 < Diabetes.prevalence <= 0.121
0.1253 < Percentage.of.households.with.high.housing.costs
Driving.alone.to.work <= 0.769
Teen.births <= 16.2
0.1283 < Uninsured.adults <= 0.1782
0.337 < Insufficient.sleep <= 0.368
0.44 < Flu.vaccinations <= 0.50
Feature
−0.06
−0.04
−0.02
0.00
0.02
Weight
Positive
Negative

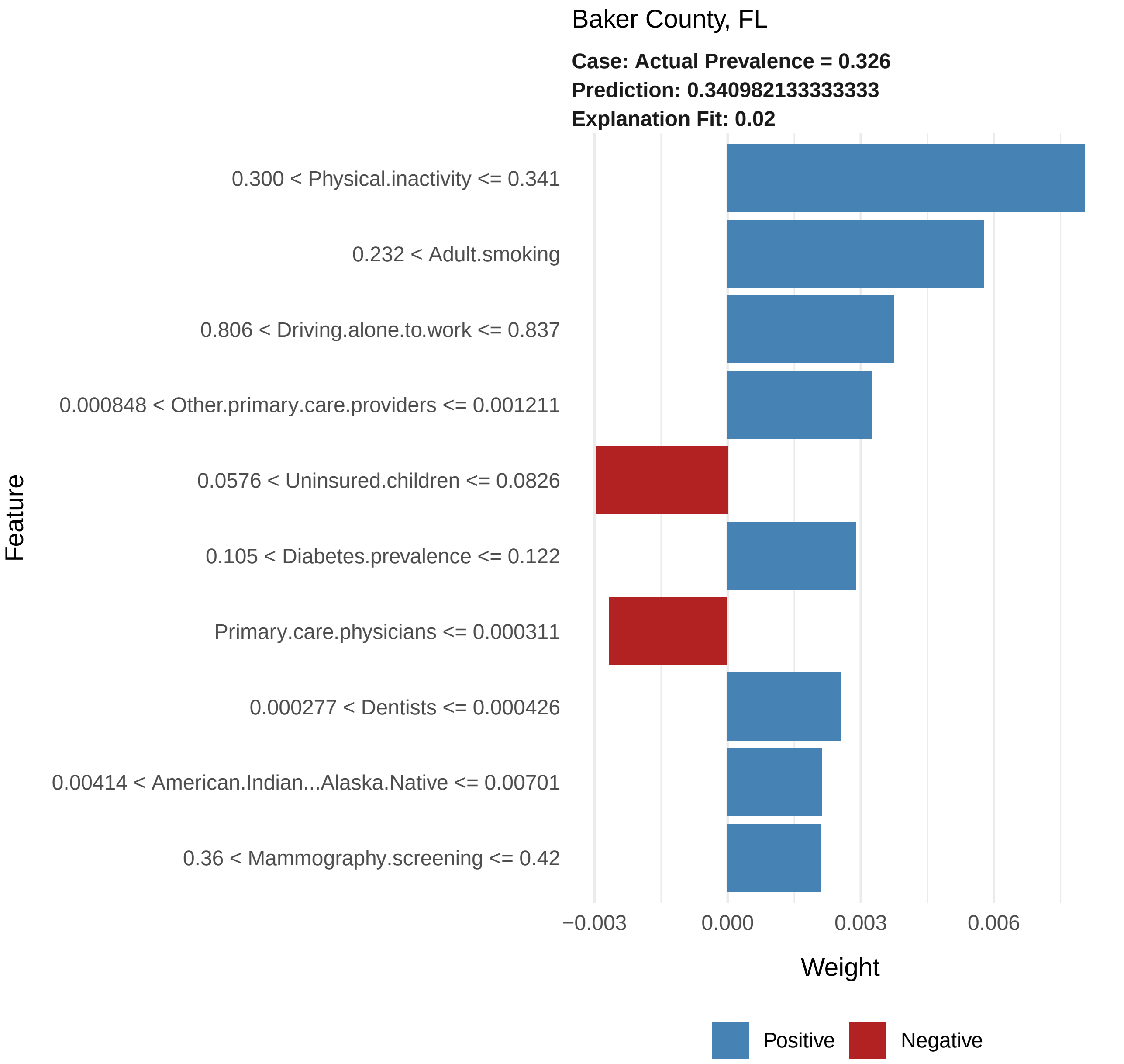

Baker County, FL
Case: Actual Prevalence = 0.326
Prediction: 0.340982133333333
Explanation Fit: 0.02
0.300 < Physical.inactivity <= 0.341
0.232 < Adult.smoking
0.806 < Driving.alone.to.work <= 0.837
0.000848 < Other.primary.care.providers <= 0.001211
0.0576 < Uninsured.children <= 0.0826
0.105 < Diabetes.prevalence <= 0.122
Primary.care.physicians <= 0.000311
0.000277 < Dentists <= 0.000426
0.00414 < American.Indian...Alaska.Native <= 0.00701
0.36 < Mammography.screening <= 0.42
Feature
−0.003
0.000
0.003
0.006
Weight
Positive
Negative

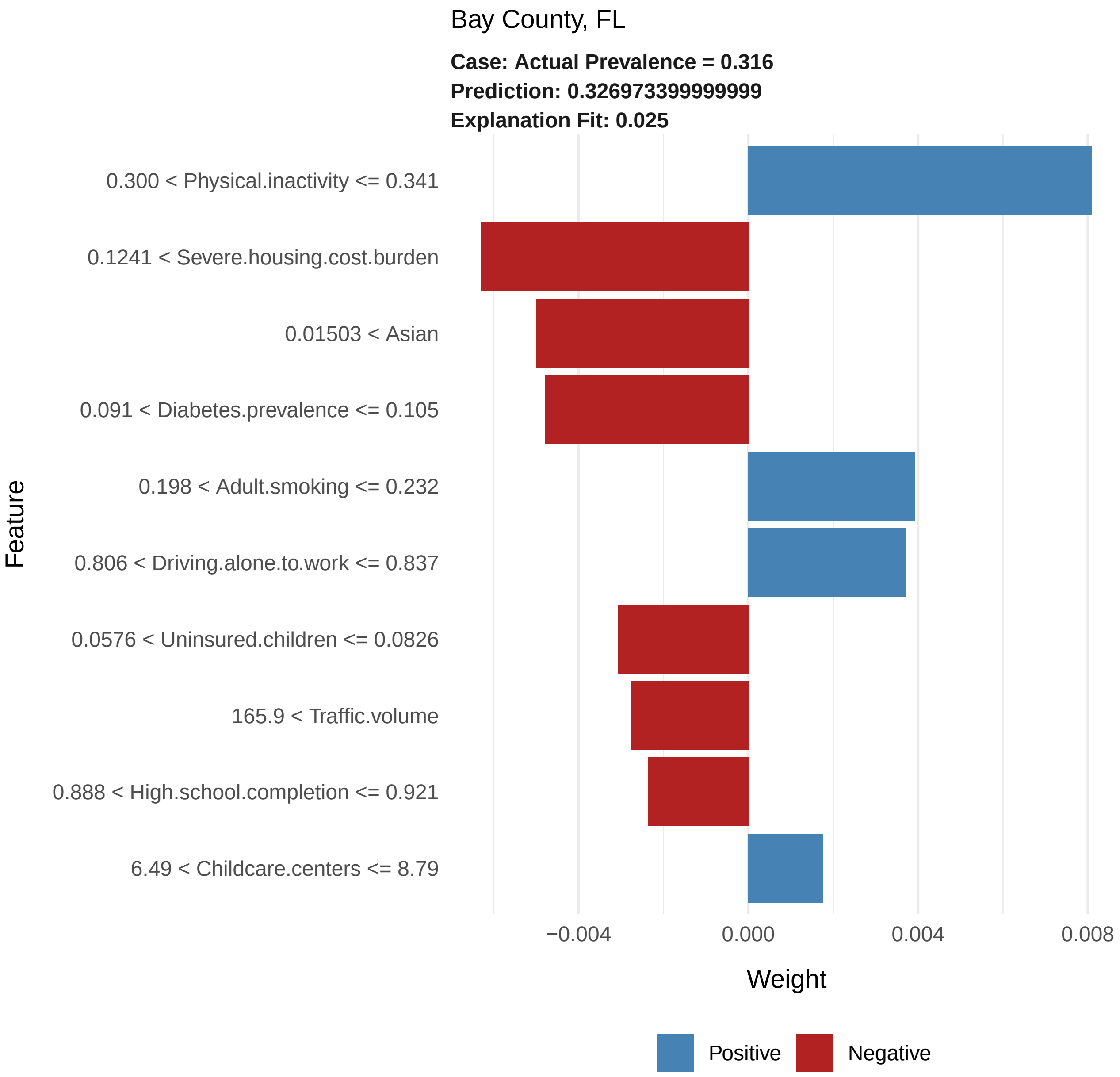

Bay County, FL
Case: Actual Prevalence = 0.316
Prediction: 0.326973399999999
Explanation Fit: 0.025
0.300 < Physical.inactivity <= 0.341
0.1241 < Severe.housing.cost.burden
0.01503 < Asian
0.091 < Diabetes.prevalence <= 0.105
0.198 < Adult.smoking <= 0.232
0.806 < Driving.alone.to.work <= 0.837
0.0576 < Uninsured.children <= 0.0826
165.9 < Traffic.volume
0.888 < High.school.completion <= 0.921
6.49 < Childcare.centers <= 8.79
Feature
−0.004
0.000
0.004
0.008
Weight
Positive
Negative

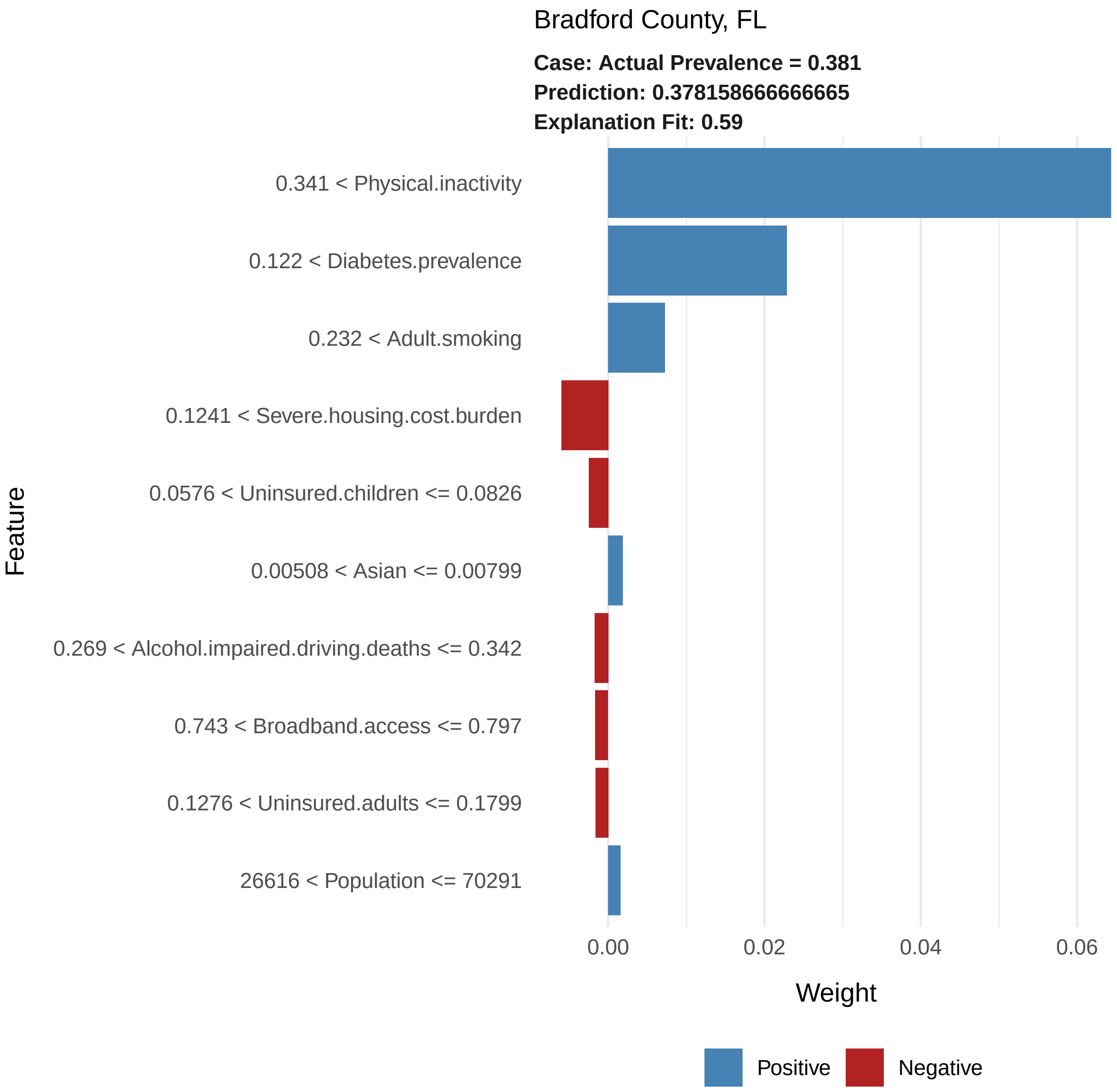

Bradford County, FL
Case: Actual Prevalence = 0.381
Prediction: 0.378158666666665
Explanation Fit: 0.59
0.341 < Physical.inactivity
0.122 < Diabetes.prevalence
0.232 < Adult.smoking
0.1241 < Severe.housing.cost.burden
0.0576 < Uninsured.children <= 0.0826
0.00508 < Asian <= 0.00799
0.269 < Alcohol.impaired.driving.deaths <= 0.342
0.743 < Broadband.access <= 0.797
0.1276 < Uninsured.adults <= 0.1799
26616 < Population <= 70291
Feature
0.00
0.02
0.04
0.06
Weight
Positive
Negative

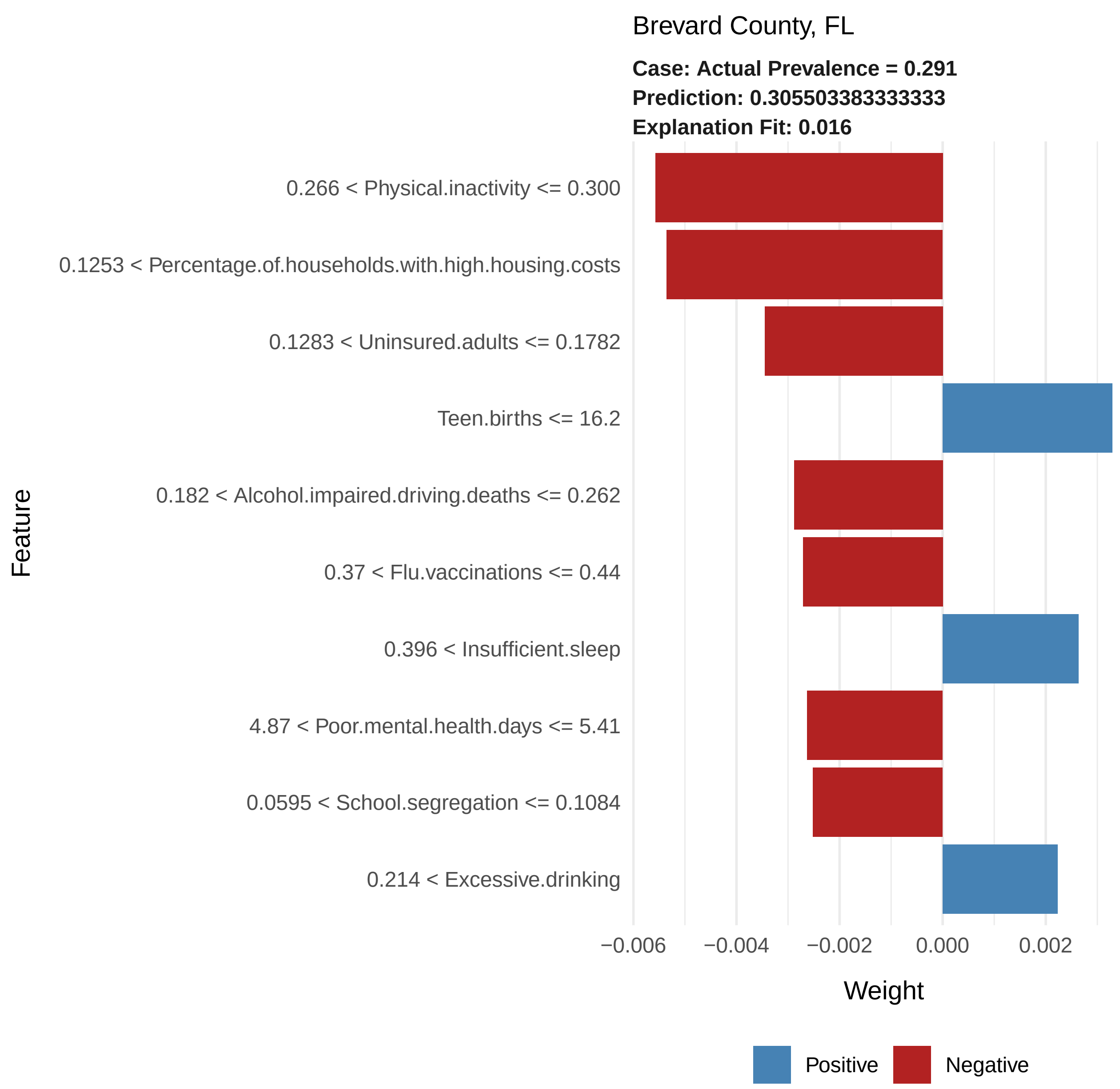
Brevard County, FL
Case: Actual Prevalence = 0.291
Prediction: 0.305503383333333
Explanation Fit: 0.016
0.266 < Physical.inactivity <= 0.300
0.1253 < Percentage.of.households.with.high.housing.costs
0.1283 < Uninsured.adults <= 0.1782
Teen.births <= 16.2
0.182 < Alcohol.impaired.driving.deaths <= 0.262
0.37 < Flu.vaccinations <= 0.44
0.396 < Insufficient.sleep
4.87 < Poor.mental.health.days <= 5.41
0.0595 < School.segregation <= 0.1084
0.214 < Excessive.drinking
Feature
−0.006
−0.004
−0.002
0.000
0.002
Weight
Positive
Negative

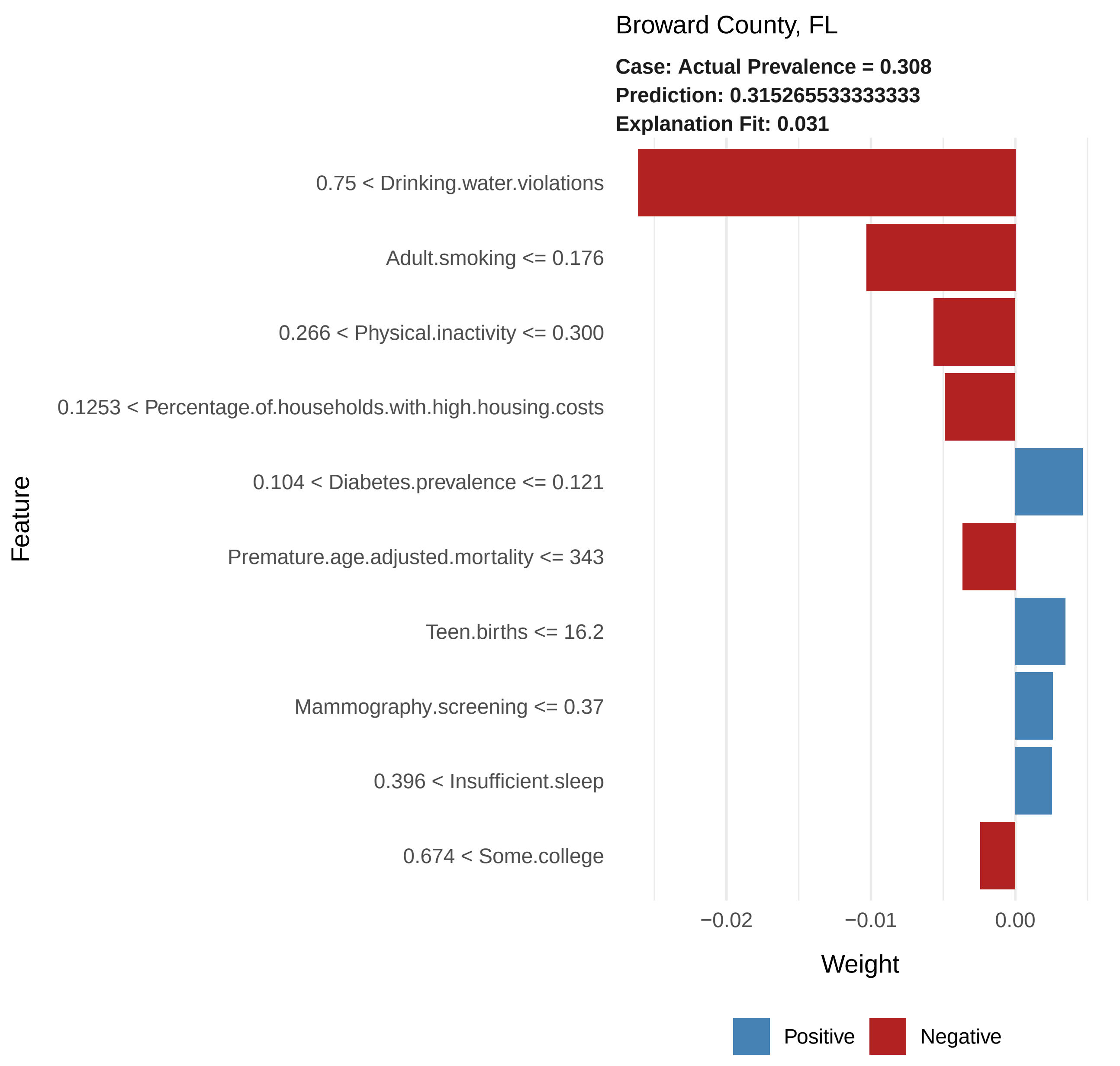

Broward County, FL
Case: Actual Prevalence = 0.308
Prediction: 0.315265533333333
Explanation Fit: 0.031
0.75 < Drinking.water.violations
Adult.smoking <= 0.176
0.266 < Physical.inactivity <= 0.300
0.1253 < Percentage.of.households.with.high.housing.costs
0.104 < Diabetes.prevalence <= 0.121
Premature.age.adjusted.mortality <= 343
Teen.births <= 16.2
Mammography.screening <= 0.37
0.396 < Insufficient.sleep
0.674 < Some.college
Feature
−0.02
−0.01
0.00
Weight
Positive
Negative

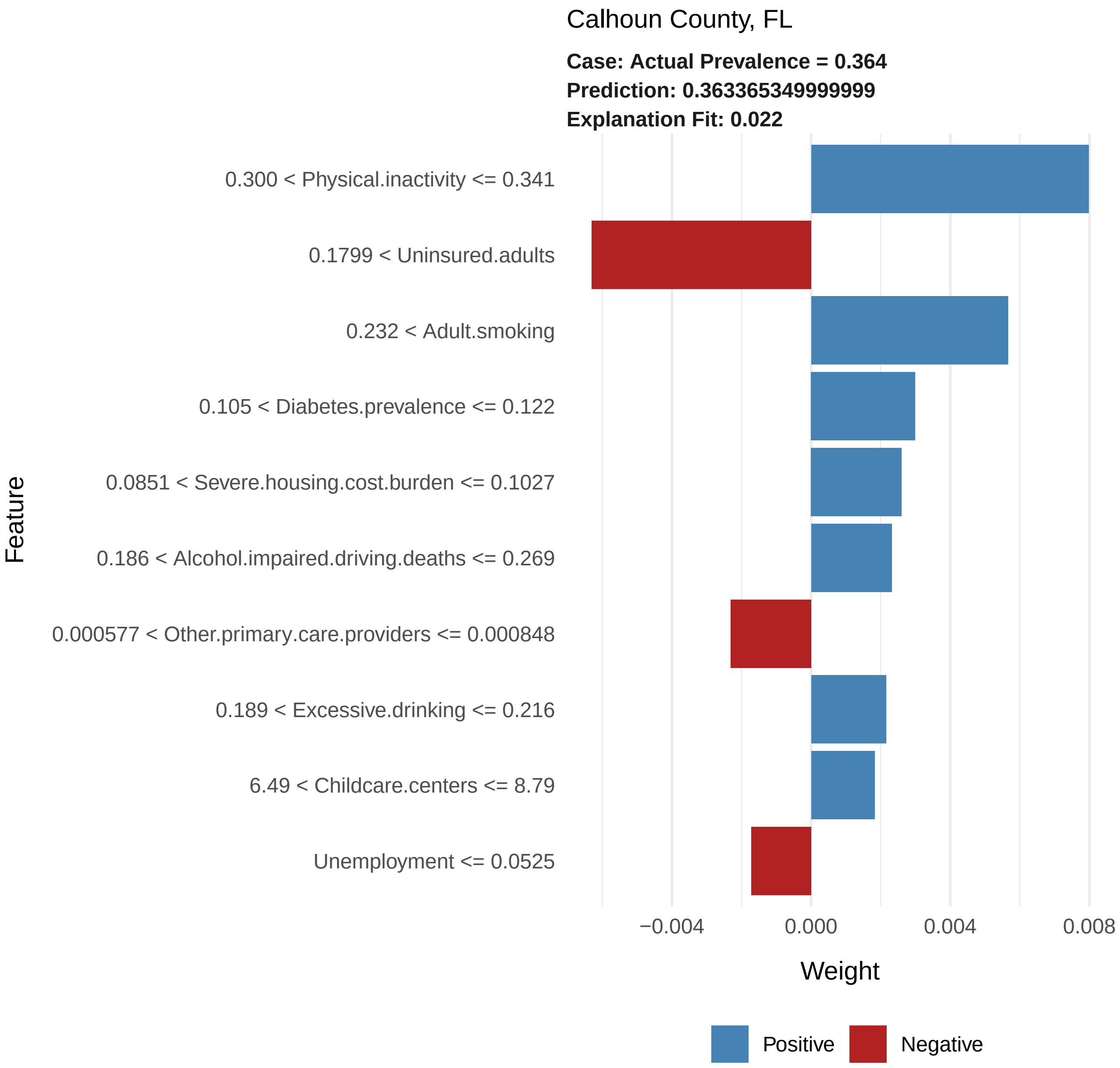

Calhoun County, FL
Case: Actual Prevalence = 0.364
Prediction: 0.363365349999999
Explanation Fit: 0.022
0.300 < Physical.inactivity <= 0.341
0.1799 < Uninsured.adults
0.232 < Adult.smoking
0.105 < Diabetes.prevalence <= 0.122
0.0851 < Severe.housing.cost.burden <= 0.1027
0.186 < Alcohol.impaired.driving.deaths <= 0.269
0.000577 < Other.primary.care.providers <= 0.000848
0.189 < Excessive.drinking <= 0.216
6.49 < Childcare.centers <= 8.79
Unemployment <= 0.0525
Feature
−0.004
0.000
0.004
0.008
Weight
Positive
Negative

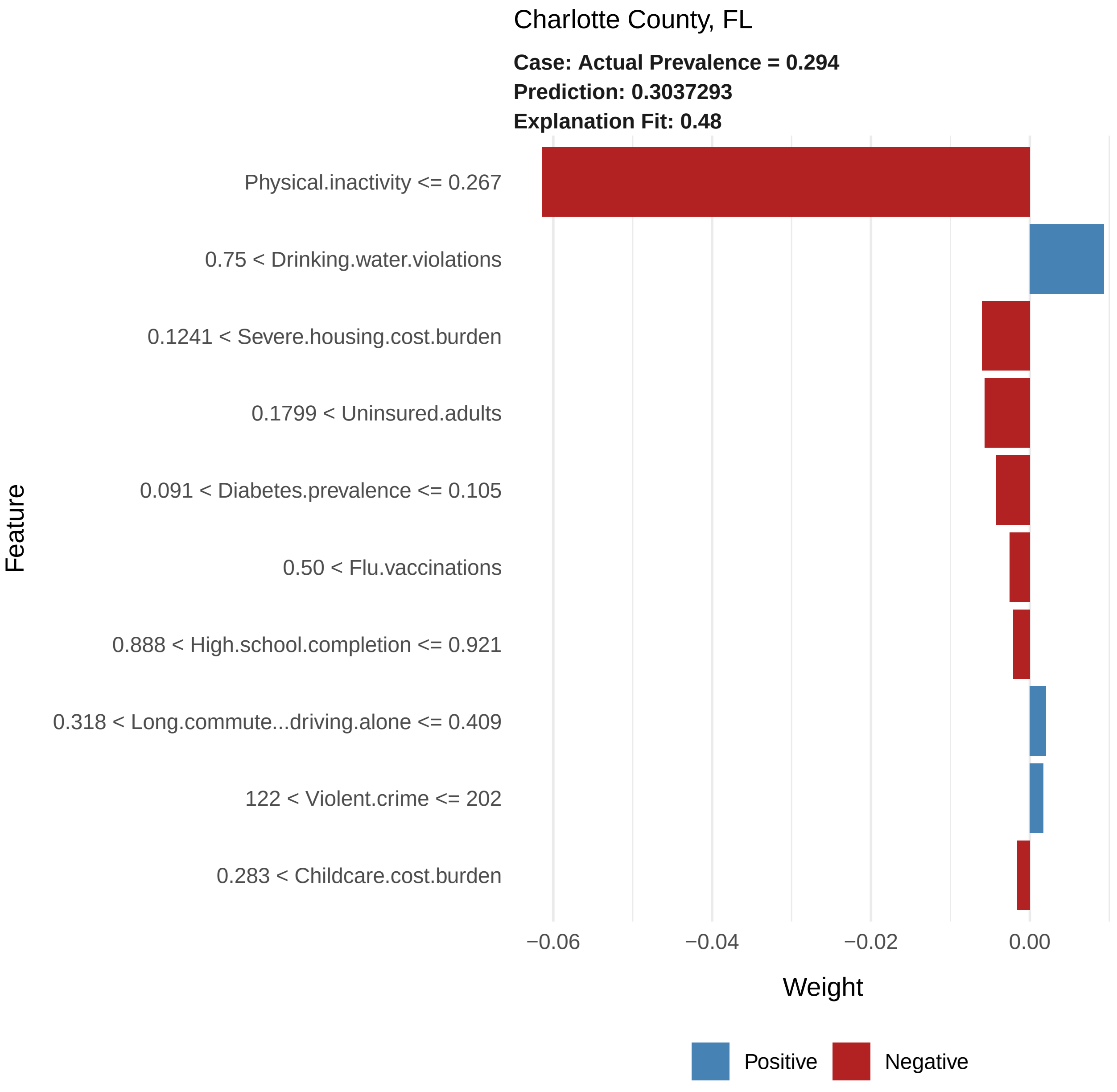
Charlotte County, FL
Case: Actual Prevalence = 0.294
Prediction: 0.3037293
Explanation Fit: 0.48
Physical.inactivity <= 0.267
0.75 < Drinking.water.violations
0.1241 < Severe.housing.cost.burden
0.1799 < Uninsured.adults
0.091 < Diabetes.prevalence <= 0.105
0.50 < Flu.vaccinations
0.888 < High.school.completion <= 0.921
0.318 < Long.commute...driving.alone <= 0.409
122 < Violent.crime <= 202
0.283 < Childcare.cost.burden
Feature
−0.06
−0.04
−0.02
0.00
Weight
Positive
Negative

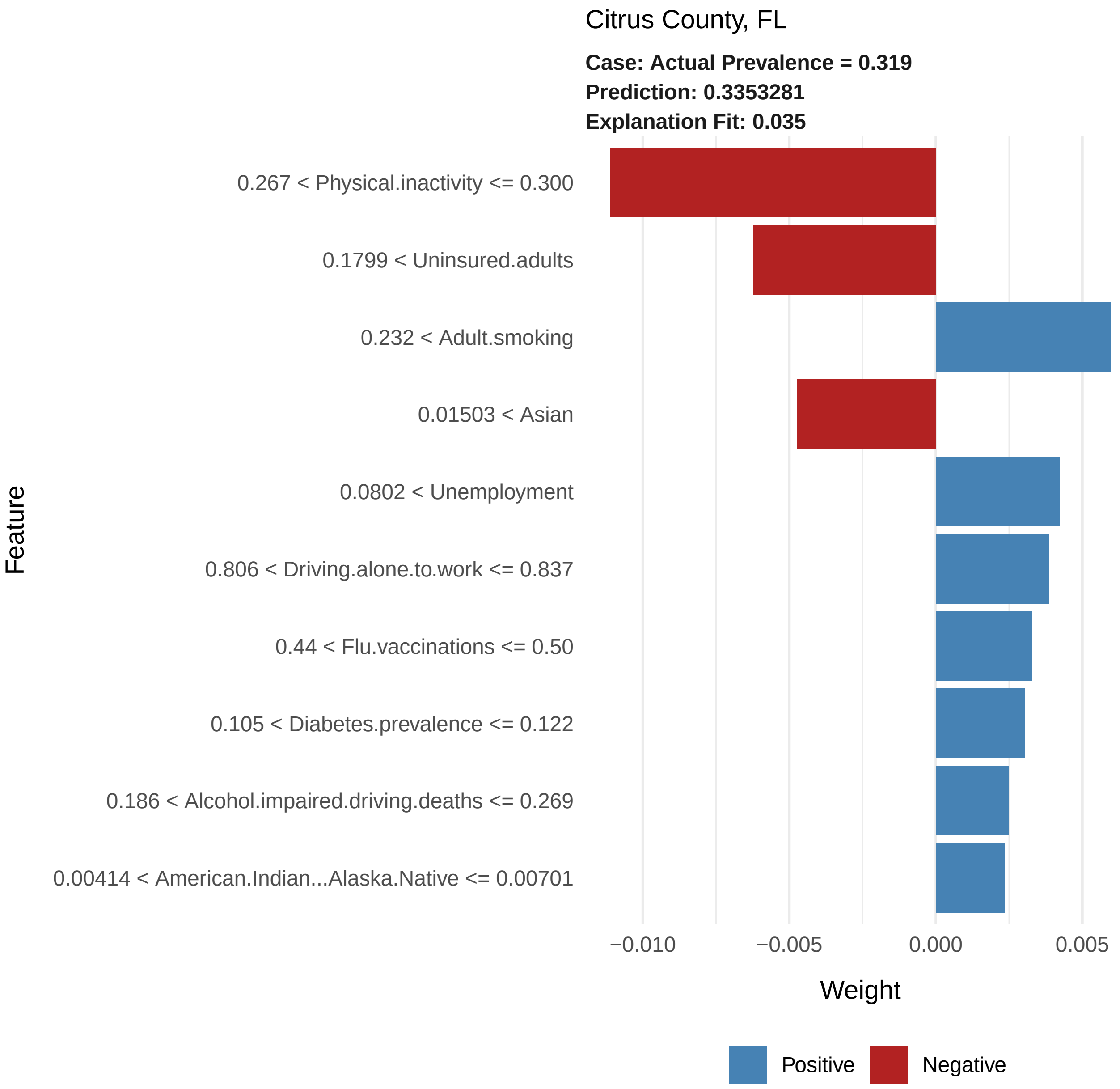

Citrus County, FL
Case: Actual Prevalence = 0.319
Prediction: 0.3353281
Explanation Fit: 0.035
0.267 < Physical.inactivity <= 0.300
0.1799 < Uninsured.adults
0.232 < Adult.smoking
0.01503 < Asian
0.0802 < Unemployment
0.806 < Driving.alone.to.work <= 0.837
0.44 < Flu.vaccinations <= 0.50
0.105 < Diabetes.prevalence <= 0.122
0.186 < Alcohol.impaired.driving.deaths <= 0.269
0.00414 < American.Indian...Alaska.Native <= 0.00701
Feature
−0.010
−0.005
0.000
0.005
Weight
Positive
Negative

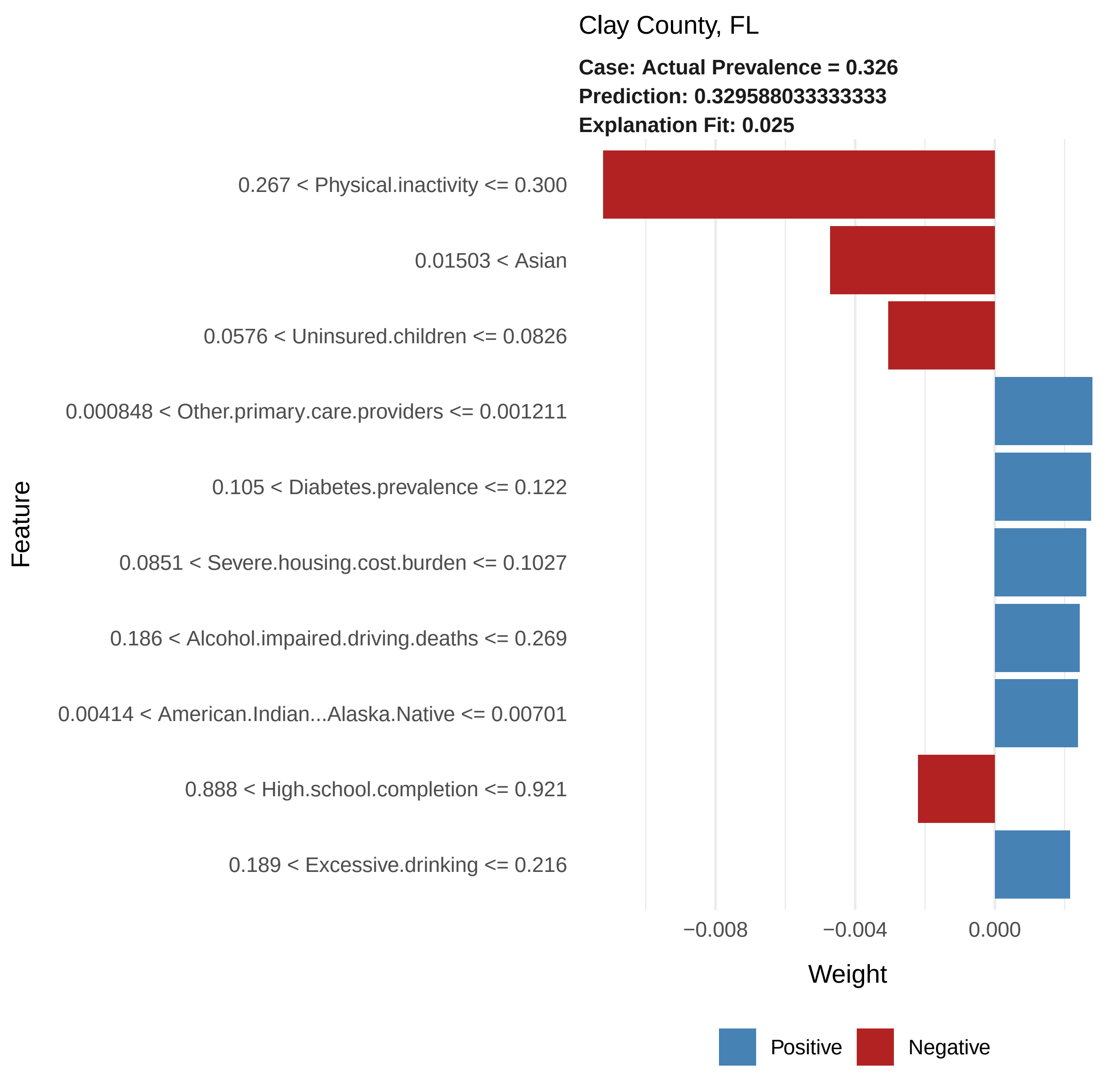
Clay County, FL
Case: Actual Prevalence = 0.326
Prediction: 0.329588033333333
Explanation Fit: 0.025
0.267 < Physical.inactivity <= 0.300
0.01503 < Asian
0.0576 < Uninsured.children <= 0.0826
0.000848 < Other.primary.care.providers <= 0.001211
0.105 < Diabetes.prevalence <= 0.122
0.0851 < Severe.housing.cost.burden <= 0.1027
0.186 < Alcohol.impaired.driving.deaths <= 0.269
0.00414 < American.Indian...Alaska.Native <= 0.00701
0.888 < High.school.completion <= 0.921
0.189 < Excessive.drinking <= 0.216
Feature
−0.008
−0.004
0.000
Weight
Positive
Negative

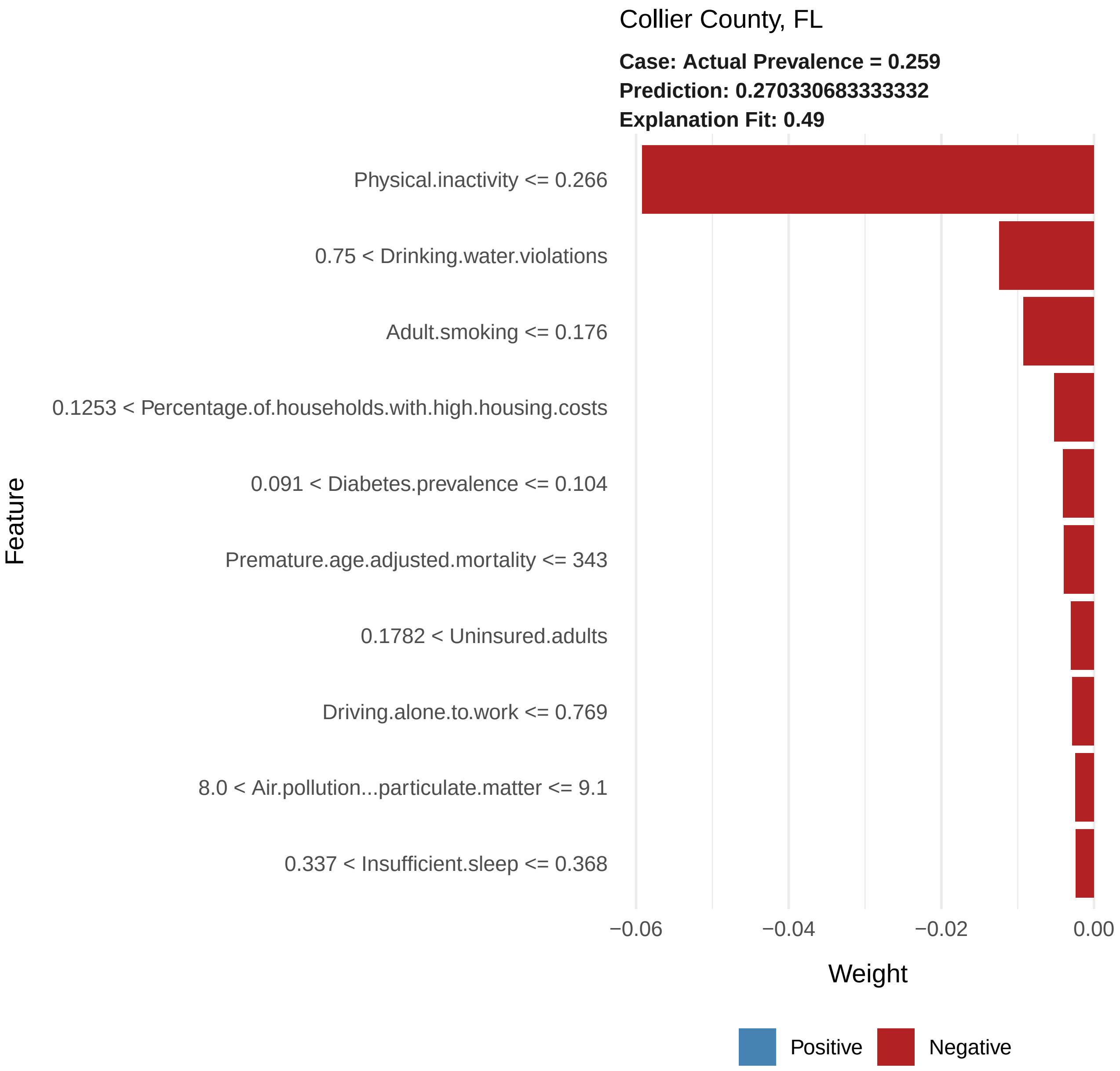

Collier County, FL
Case: Actual Prevalence = 0.259
Prediction: 0.270330683333332
Explanation Fit: 0.49
Physical.inactivity <= 0.266
0.75 < Drinking.water.violations
Adult.smoking <= 0.176
0.1253 < Percentage.of.households.with.high.housing.costs
0.091 < Diabetes.prevalence <= 0.104
Premature.age.adjusted.mortality <= 343
0.1782 < Uninsured.adults
Driving.alone.to.work <= 0.769
8.0 < Air.pollution...particulate.matter <= 9.1
0.337 < Insufficient.sleep <= 0.368
Feature
−0.06
−0.04
−0.02
0.00
Weight
Positive
Negative

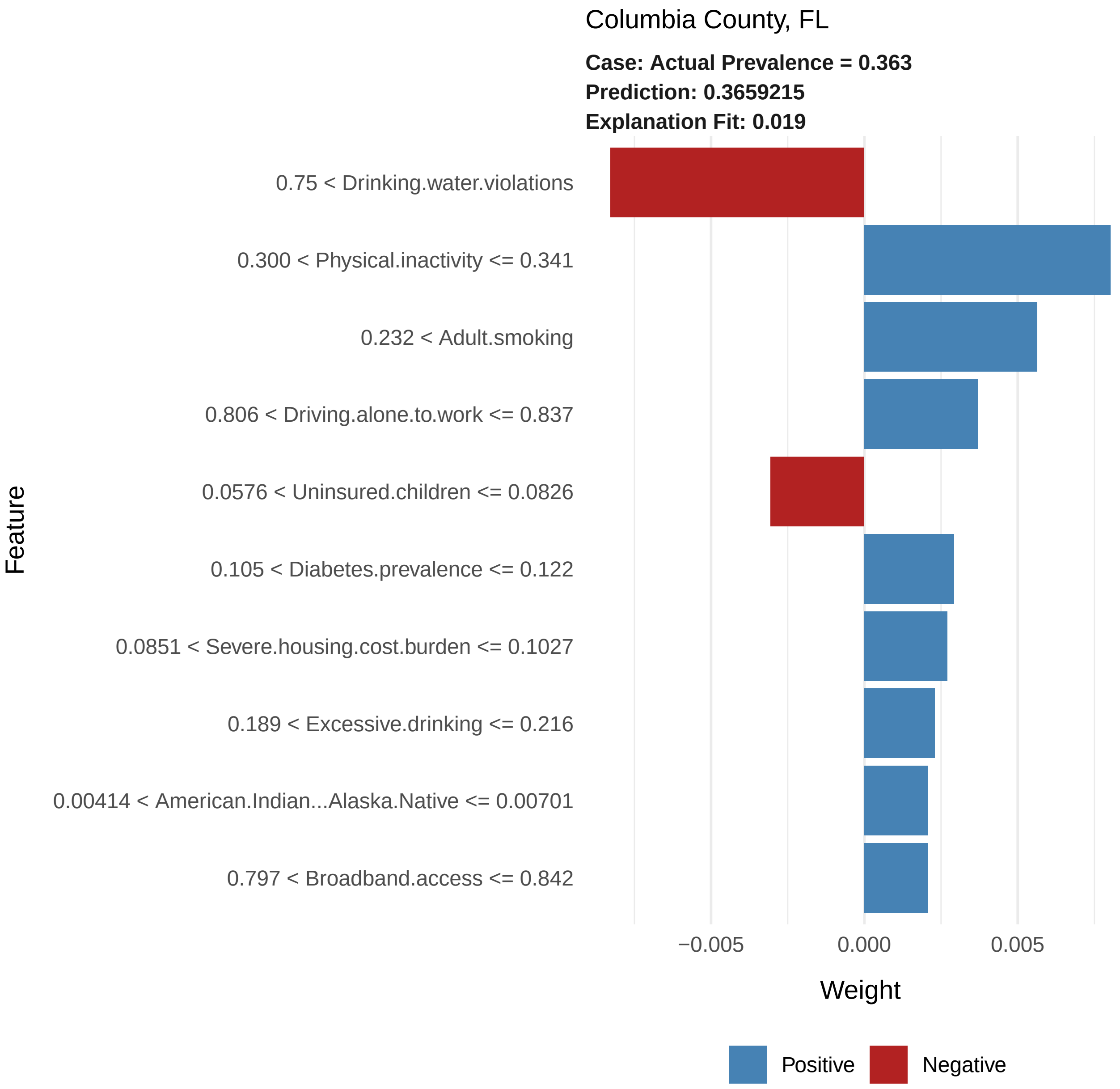

Columbia County, FL
Case: Actual Prevalence = 0.363
Prediction: 0.3659215
Explanation Fit: 0.019
0.75 < Drinking.water.violations
0.300 < Physical.inactivity <= 0.341
0.232 < Adult.smoking
0.806 < Driving.alone.to.work <= 0.837
0.0576 < Uninsured.children <= 0.0826
0.105 < Diabetes.prevalence <= 0.122
0.0851 < Severe.housing.cost.burden <= 0.1027
0.189 < Excessive.drinking <= 0.216
0.00414 < American.Indian...Alaska.Native <= 0.00701
0.797 < Broadband.access <= 0.842
Feature
−0.005
0.000
0.005
Weight
Positive
Negative

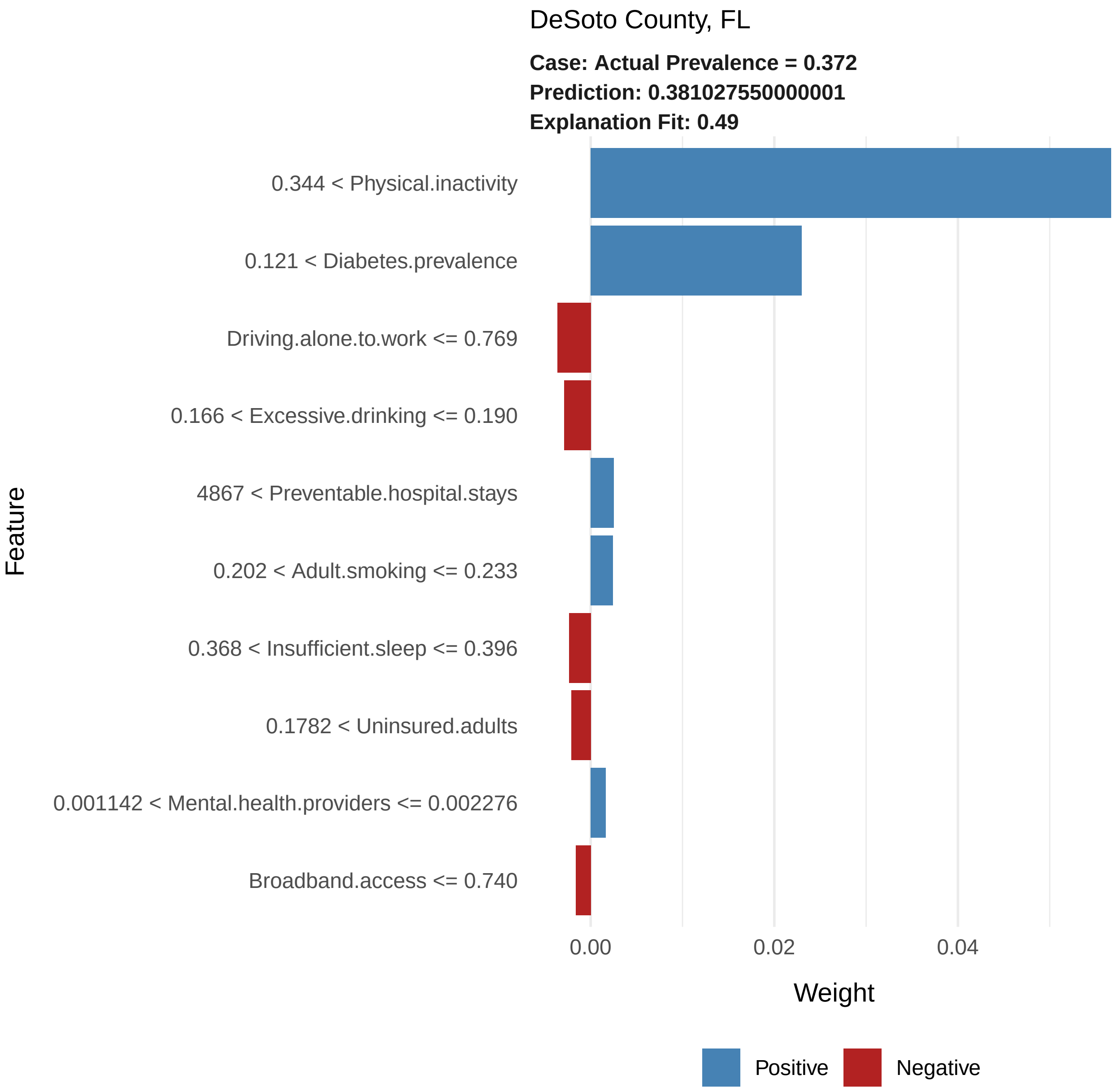

DeSoto County, FL
Case: Actual Prevalence = 0.372
Prediction: 0.381027550000001
Explanation Fit: 0.49
0.344 < Physical.inactivity
0.121 < Diabetes.prevalence
Driving.alone.to.work <= 0.769
0.166 < Excessive.drinking <= 0.190
4867 < Preventable.hospital.stays
0.202 < Adult.smoking <= 0.233
0.368 < Insufficient.sleep <= 0.396
0.1782 < Uninsured.adults
0.001142 < Mental.health.providers <= 0.002276
Broadband.access <= 0.740
Feature
0.00
0.02
0.04
Weight
Positive
Negative

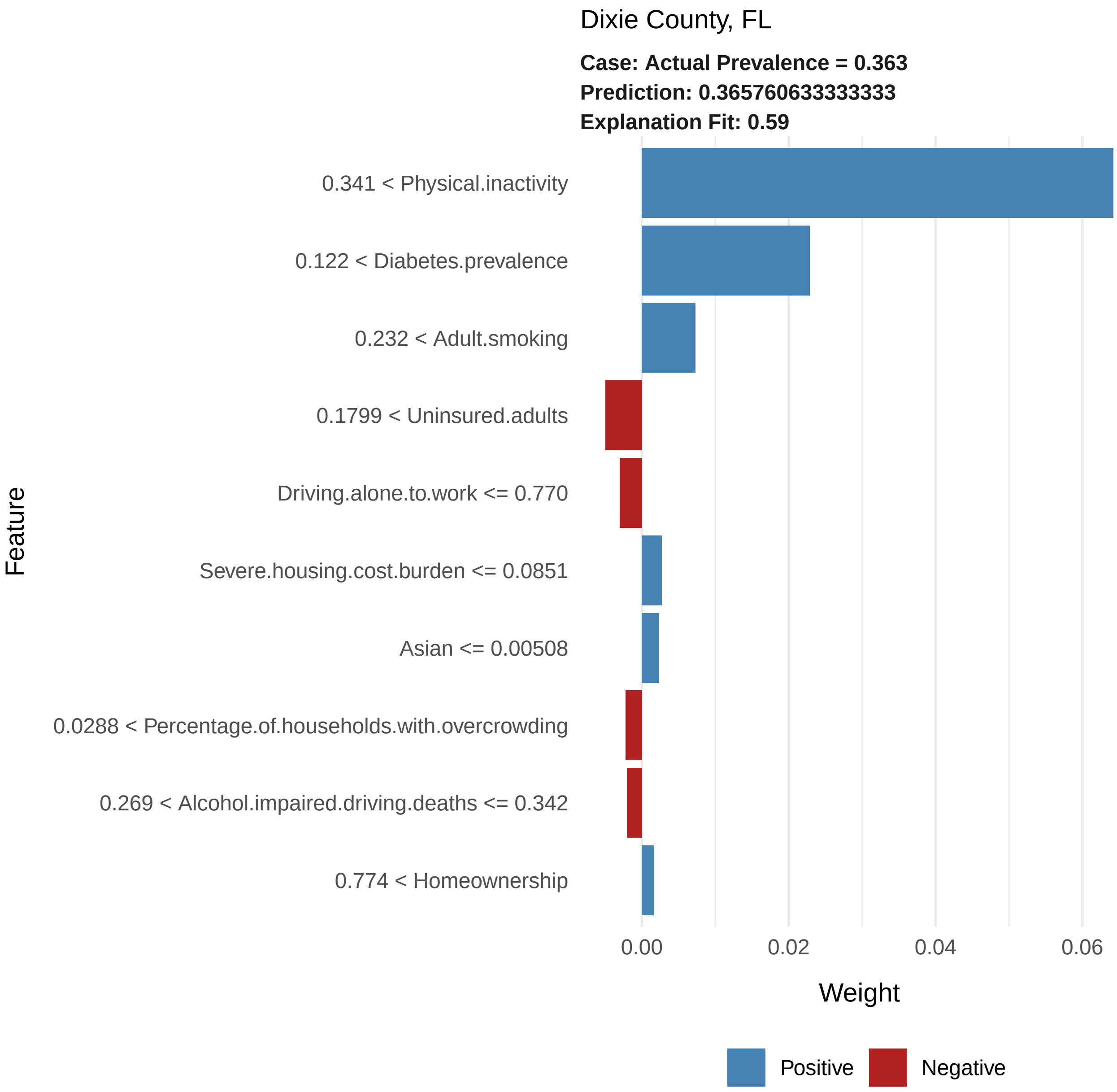

Dixie County, FL
Case: Actual Prevalence = 0.363
Prediction: 0.365760633333333
Explanation Fit: 0.59
0.341 < Physical.inactivity
0.122 < Diabetes.prevalence
0.232 < Adult.smoking
0.1799 < Uninsured.adults
Driving.alone.to.work <= 0.770
Severe.housing.cost.burden <= 0.0851
Asian <= 0.00508
0.0288 < Percentage.of.households.with.overcrowding
0.269 < Alcohol.impaired.driving.deaths <= 0.342
0.774 < Homeownership
Feature
0.00
0.02
0.04
0.06
Weight
Positive
Negative

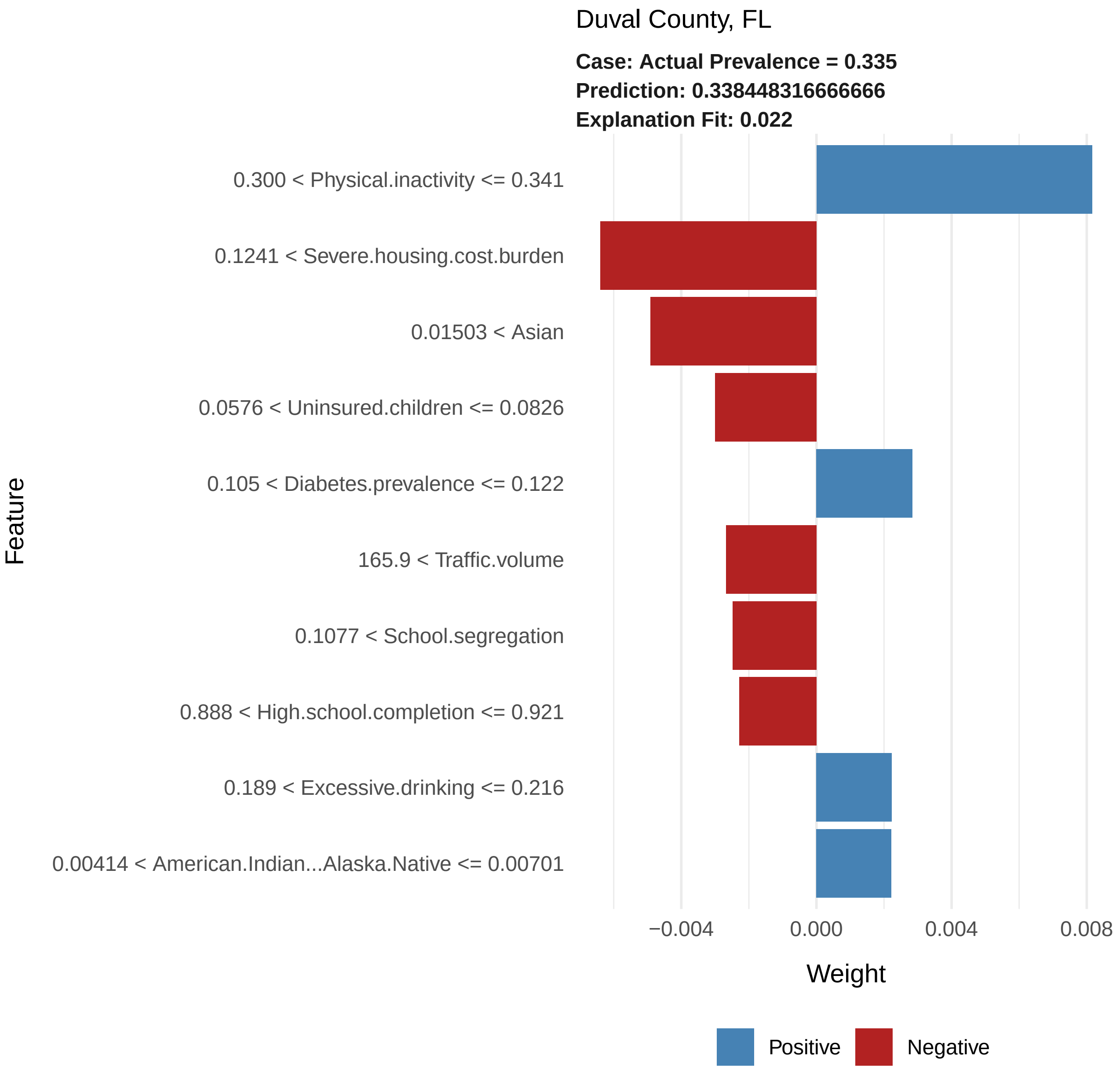
Duval County, FL
Case: Actual Prevalence = 0.335
Prediction: 0.338448316666666
Explanation Fit: 0.022
0.300 < Physical.inactivity <= 0.341
0.1241 < Severe.housing.cost.burden
0.01503 < Asian
0.0576 < Uninsured.children <= 0.0826
0.105 < Diabetes.prevalence <= 0.122
165.9 < Traffic.volume
0.1077 < School.segregation
0.888 < High.school.completion <= 0.921
0.189 < Excessive.drinking <= 0.216
0.00414 < American.Indian...Alaska.Native <= 0.00701
Feature
−0.004
0.000
0.004
0.008
Weight
Positive
Negative

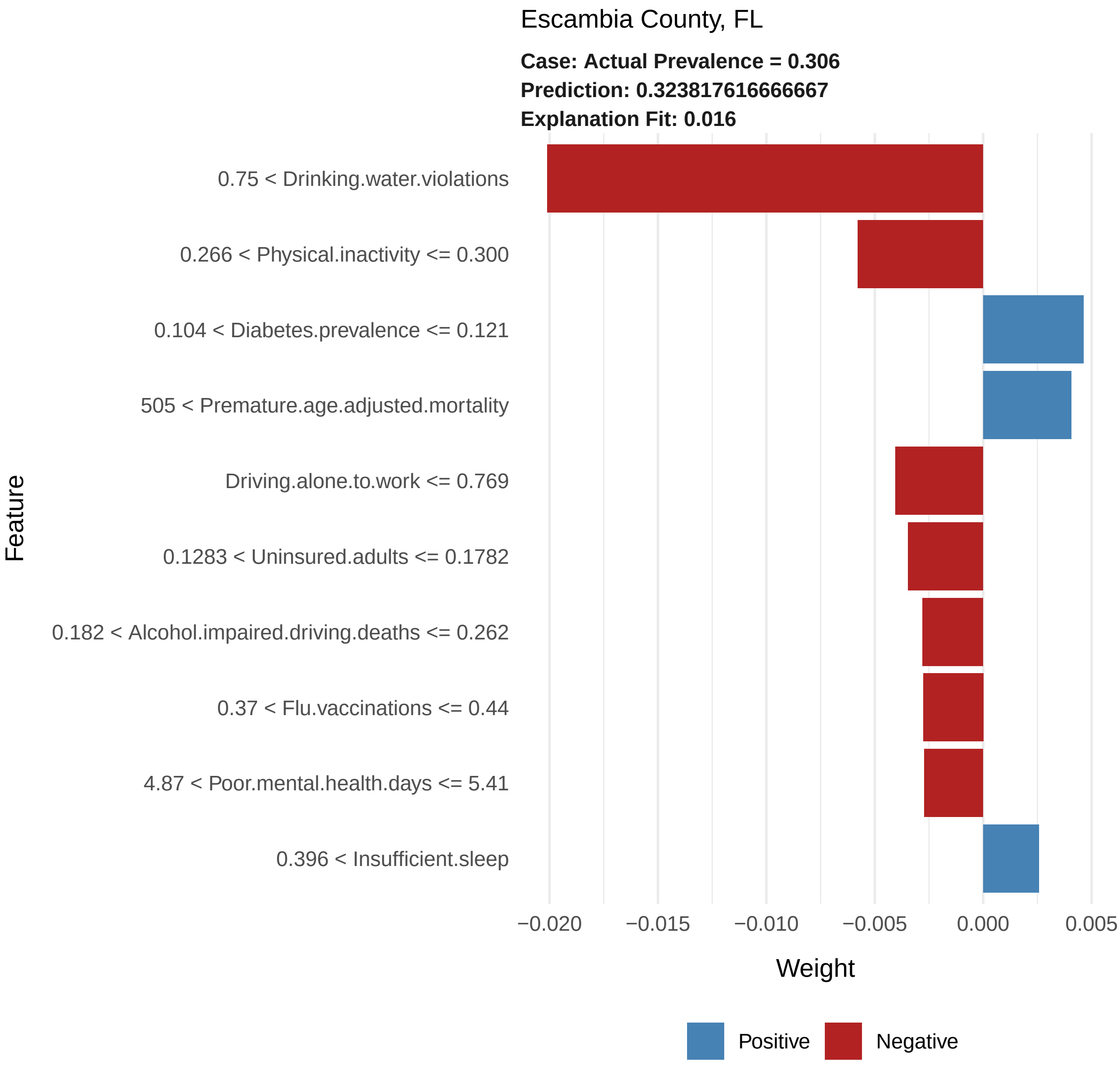
Escambia County, FL
Case: Actual Prevalence = 0.306
Prediction: 0.323817616666667
Explanation Fit: 0.016
0.75 < Drinking.water.violations
0.266 < Physical.inactivity <= 0.300
0.104 < Diabetes.prevalence <= 0.121
505 < Premature.age.adjusted.mortality
Driving.alone.to.work <= 0.769
0.1283 < Uninsured.adults <= 0.1782
0.182 < Alcohol.impaired.driving.deaths <= 0.262
0.37 < Flu.vaccinations <= 0.44
4.87 < Poor.mental.health.days <= 5.41
0.396 < Insufficient.sleep
Feature
−0.020
−0.015
−0.010
−0.005
0.000
0.005
Weight
Positive
Negative

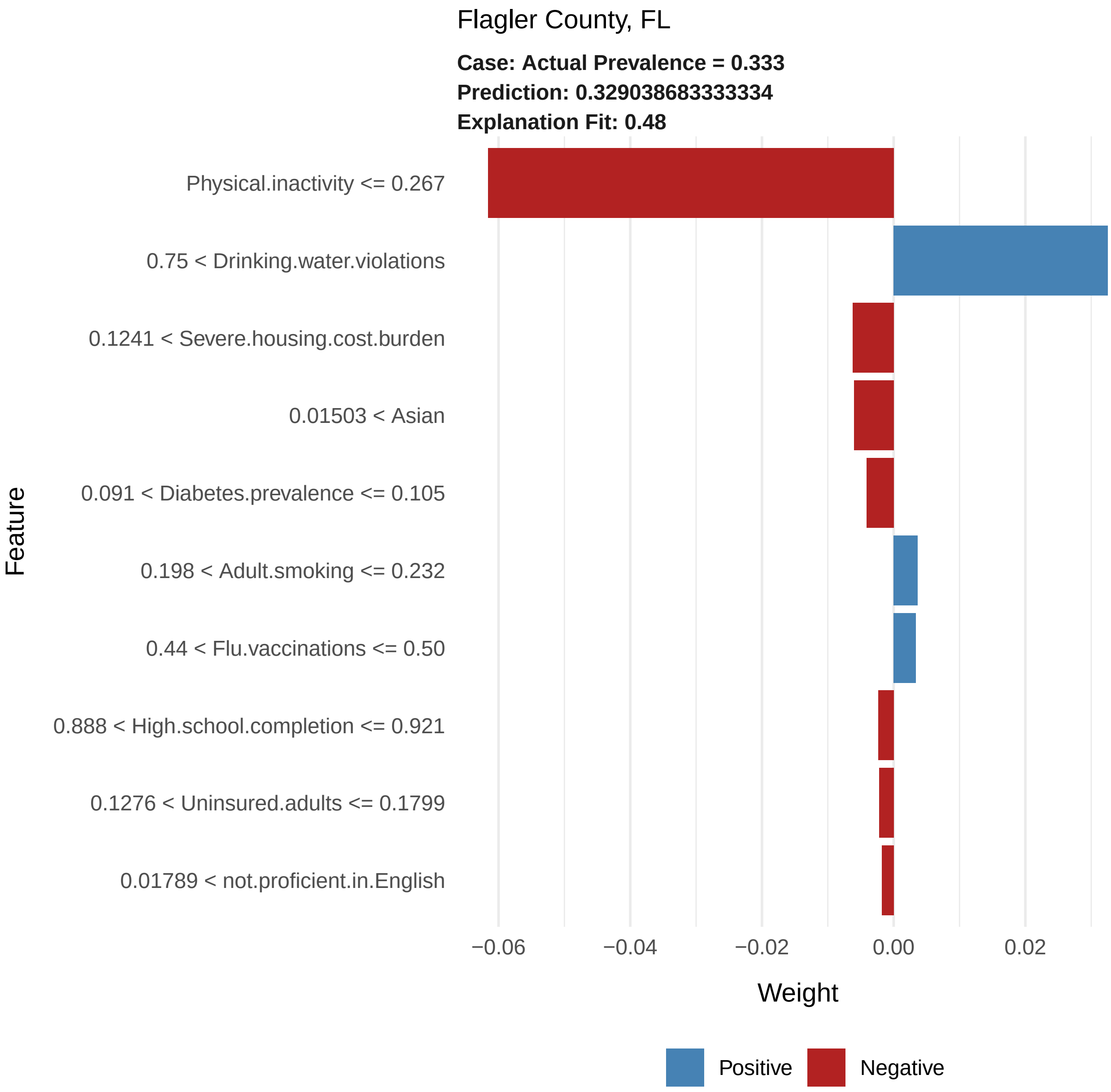
Flagler County, FL
Case: Actual Prevalence = 0.333
Prediction: 0.329038683333334
Explanation Fit: 0.48
Physical.inactivity <= 0.267
0.75 < Drinking.water.violations
0.1241 < Severe.housing.cost.burden
0.01503 < Asian
0.091 < Diabetes.prevalence <= 0.105
0.198 < Adult.smoking <= 0.232
0.44 < Flu.vaccinations <= 0.50
0.888 < High.school.completion <= 0.921
0.1276 < Uninsured.adults <= 0.1799
0.01789 < not.proficient.in.English
Feature
−0.06
−0.04
−0.02
0.00
0.02
Weight
Positive
Negative

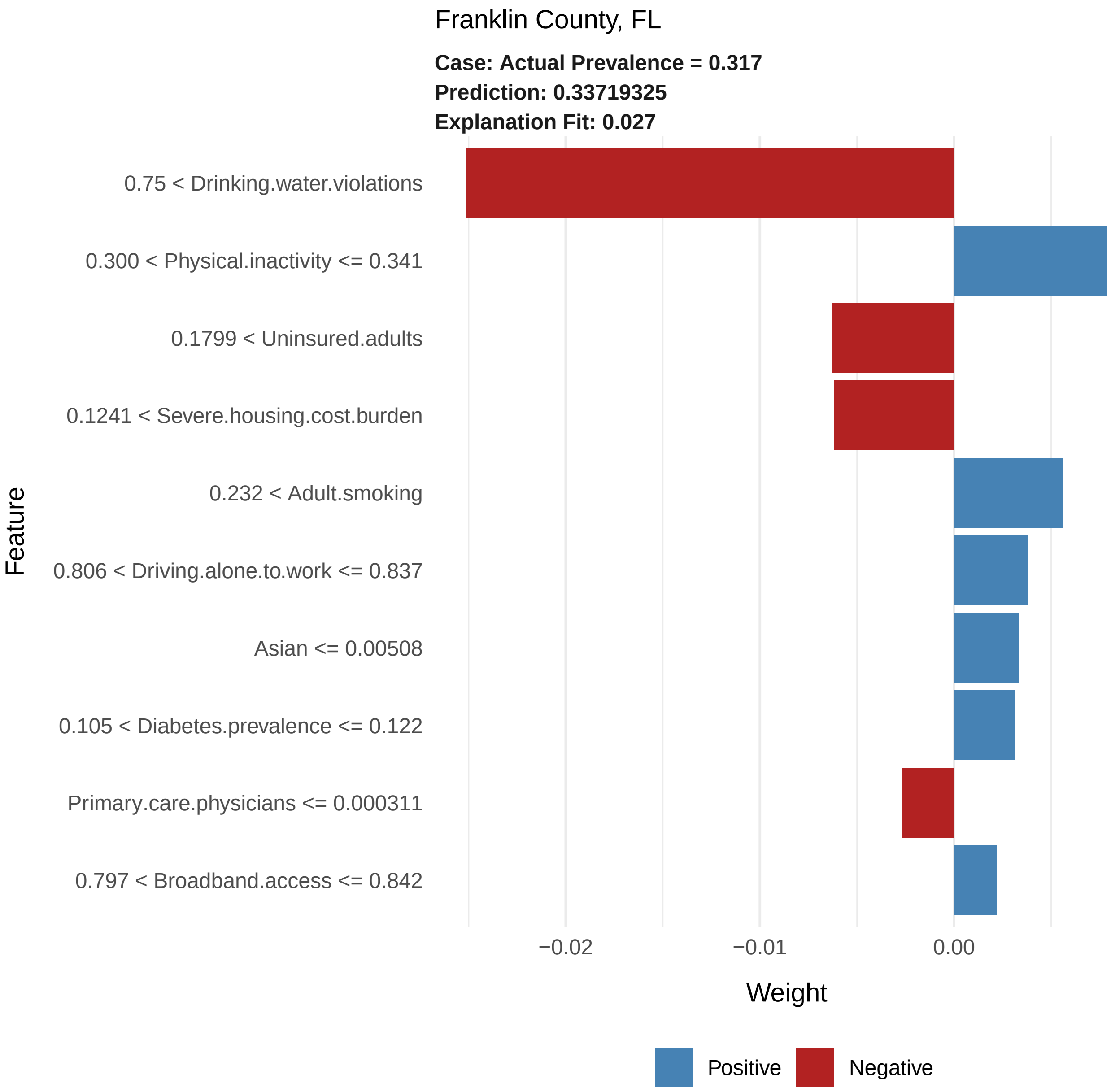
Franklin County, FL
Case: Actual Prevalence = 0.317
Prediction: 0.33719325
Explanation Fit: 0.027
Feature
0.75 < Drinking.water.violations
0.300 < Physical.inactivity <= 0.341
0.1799 < Uninsured.adults
0.1241 < Severe.housing.cost.burden
0.232 < Adult.smoking
0.806 < Driving.alone.to.work <= 0.837
Asian <= 0.00508
0.105 < Diabetes.prevalence <= 0.122
Primary.care.physicians <= 0.000311
0.797 < Broadband.access <= 0.842
−0.02
−0.01
0.00
Weight
Positive
Negative

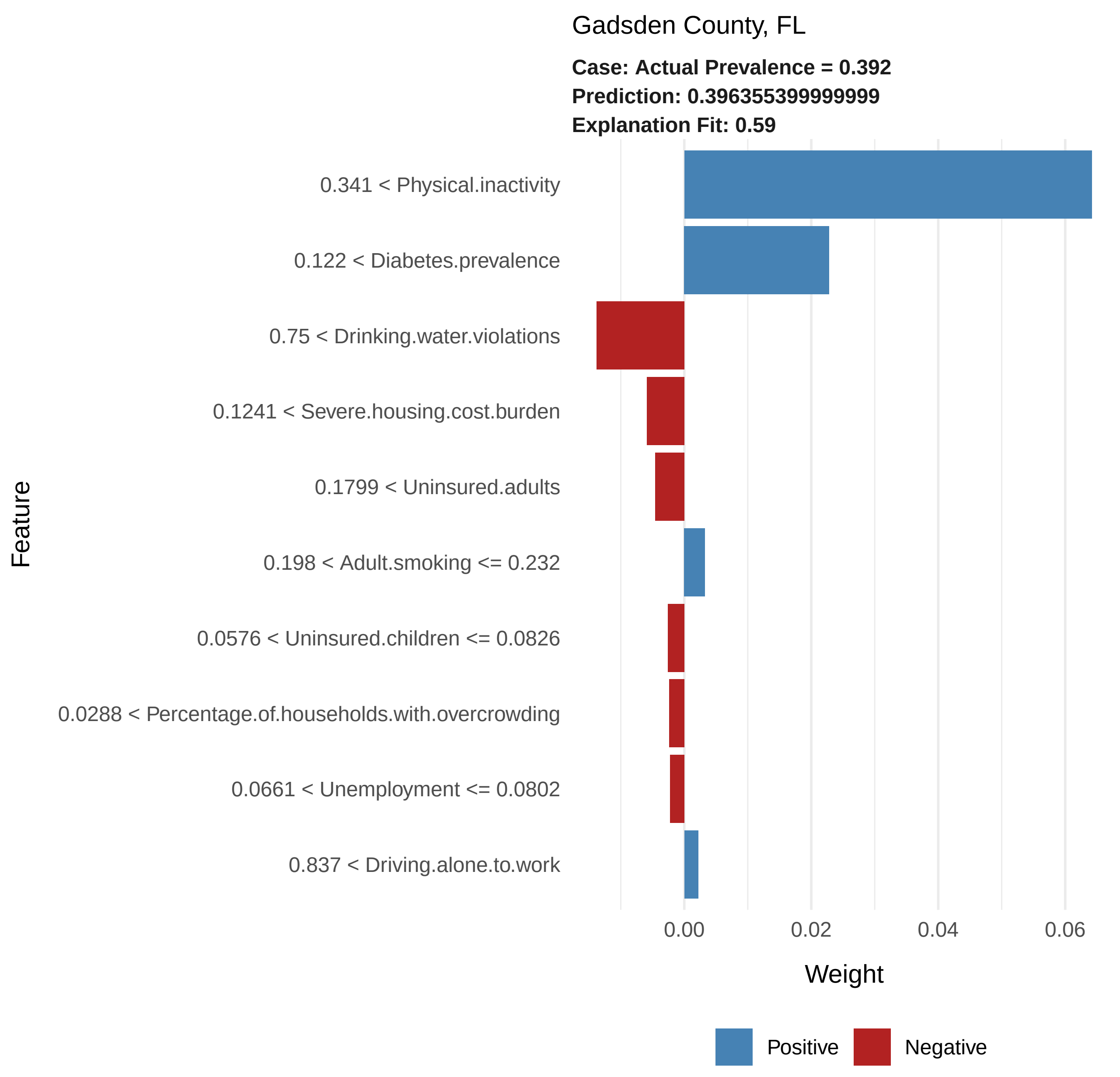

Gadsden County, FL
Case: Actual Prevalence = 0.392
Prediction: 0.396355399999999
Explanation Fit: 0.59
0.341 < Physical.inactivity
0.122 < Diabetes.prevalence
0.75 < Drinking.water.violations
0.1241 < Severe.housing.cost.burden
0.1799 < Uninsured.adults
0.198 < Adult.smoking <= 0.232
0.0576 < Uninsured.children <= 0.0826
0.0288 < Percentage.of.households.with.overcrowding
0.0661 < Unemployment <= 0.0802
0.837 < Driving.alone.to.work
Feature
0.00
0.02
0.04
0.06
Weight
Positive
Negative

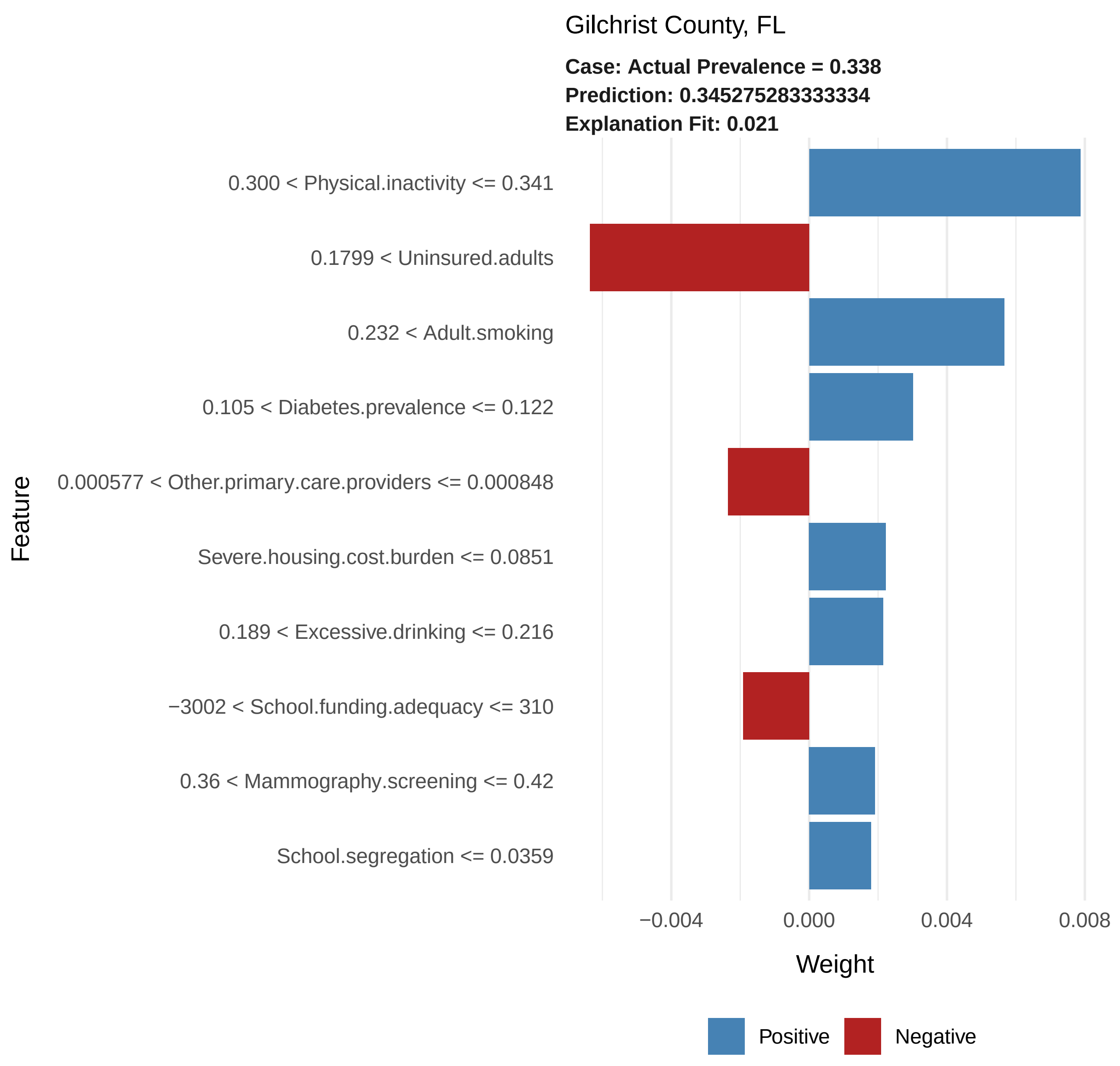

Gilchrist County, FL
Case: Actual Prevalence = 0.338
Prediction: 0.345275283333334
Explanation Fit: 0.021
0.300 < Physical.inactivity <= 0.341
0.1799 < Uninsured.adults
0.232 < Adult.smoking
0.105 < Diabetes.prevalence <= 0.122
0.000577 < Other.primary.care.providers <= 0.000848
Severe.housing.cost.burden <= 0.0851
0.189 < Excessive.drinking <= 0.216
−3002 < School.funding.adequacy <= 310
0.36 < Mammography.screening <= 0.42
School.segregation <= 0.0359
Feature
−0.004
0.000
0.004
0.008
Weight
Positive
Negative

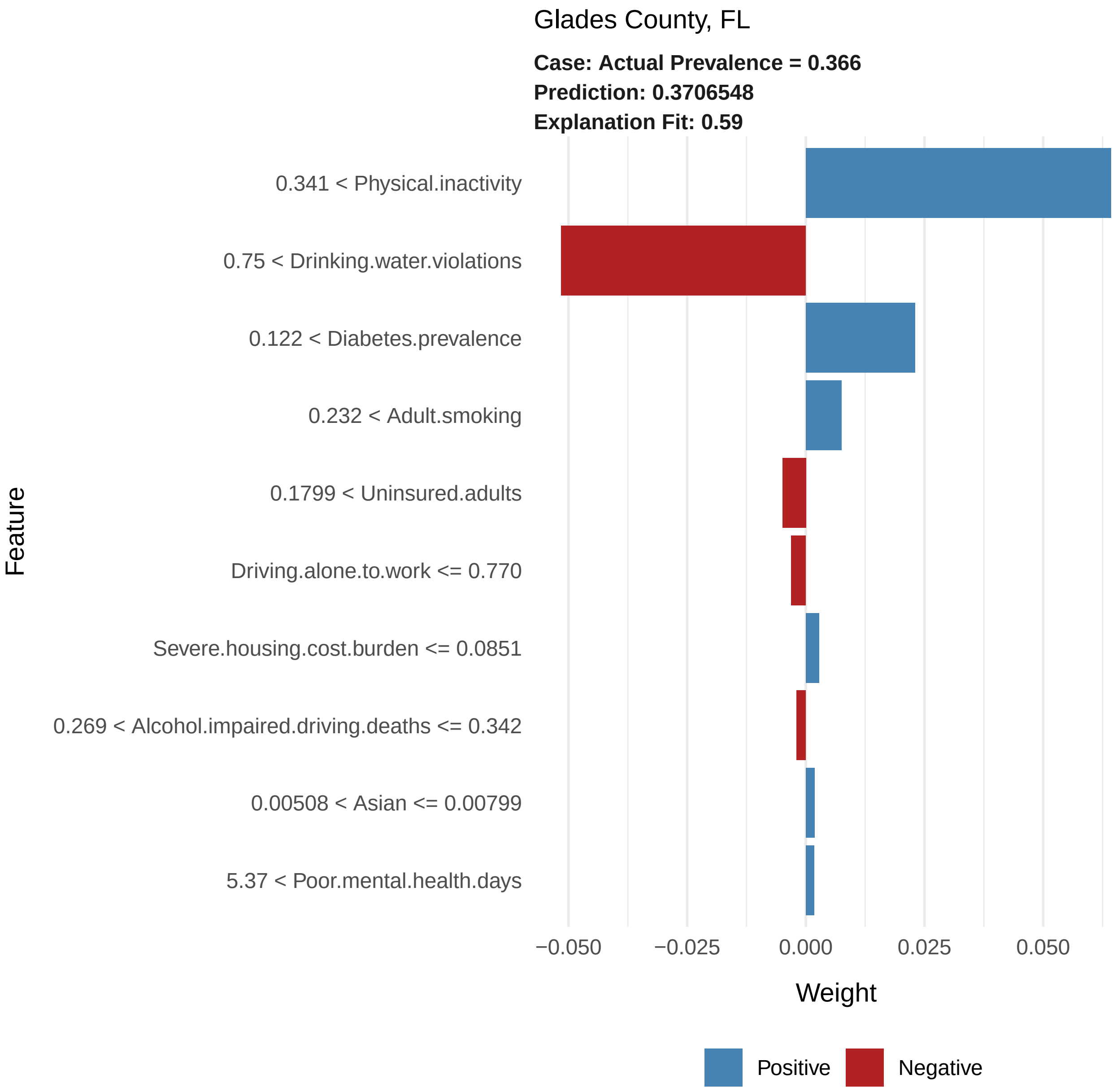
Glades County, FL
Case: Actual Prevalence = 0.366
Prediction: 0.3706548
Explanation Fit: 0.59
0.341 < Physical.inactivity
0.75 < Drinking.water.violations
0.122 < Diabetes.prevalence
0.232 < Adult.smoking
0.1799 < Uninsured.adults
Driving.alone.to.work <= 0.770
Severe.housing.cost.burden <= 0.0851
0.269 < Alcohol.impaired.driving.deaths <= 0.342
0.00508 < Asian <= 0.00799
5.37 < Poor.mental.health.days
Feature
−0.050
−0.025
0.000
0.025
0.050
Weight
Positive
Negative

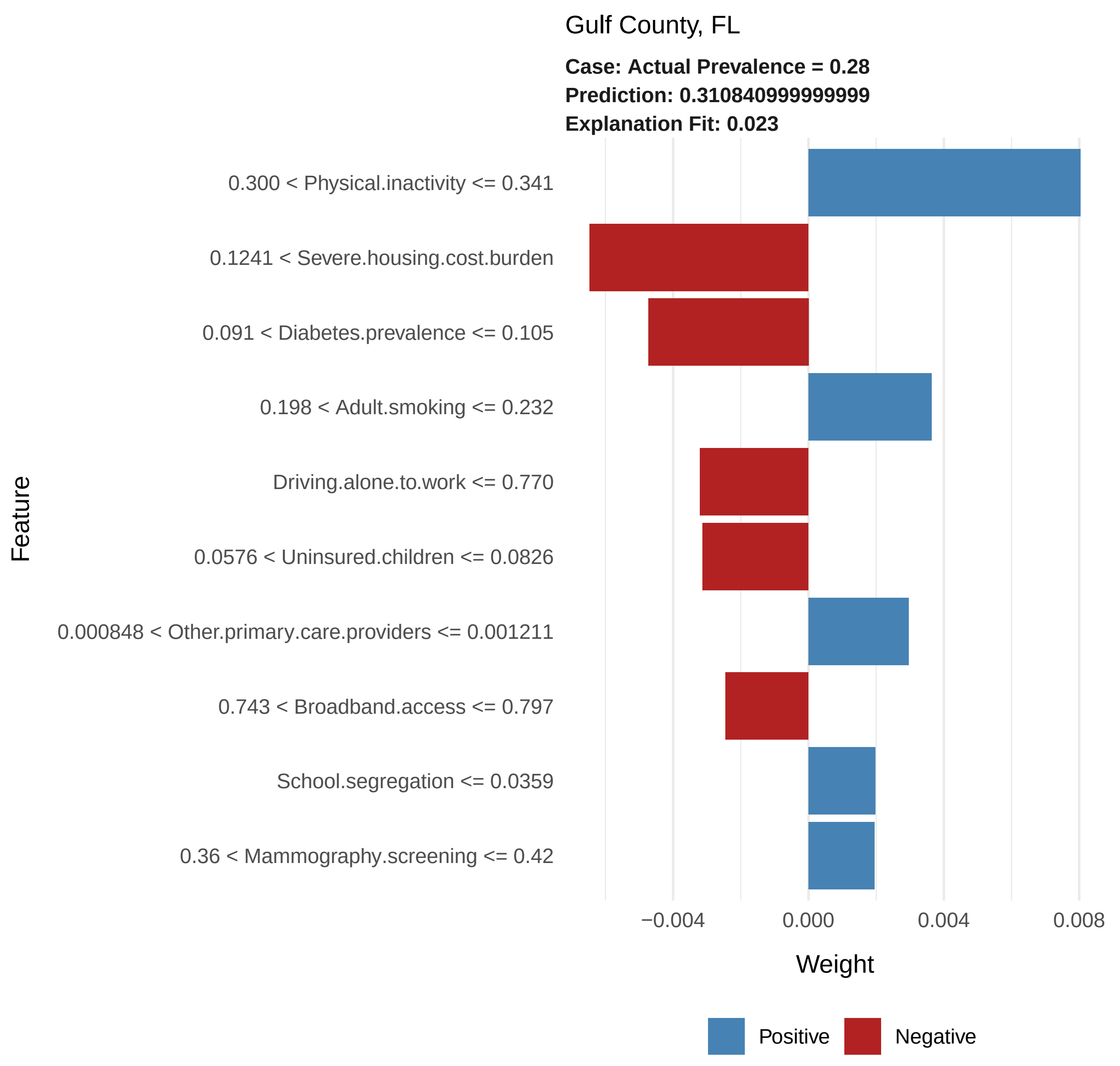
Gulf County, FL
Case: Actual Prevalence = 0.28
Prediction: 0.310840999999999
Explanation Fit: 0.023
0.300 < Physical.inactivity <= 0.341
0.1241 < Severe.housing.cost.burden
0.091 < Diabetes.prevalence <= 0.105
0.198 < Adult.smoking <= 0.232
Driving.alone.to.work <= 0.770
0.0576 < Uninsured.children <= 0.0826
0.000848 < Other.primary.care.providers <= 0.001211
0.743 < Broadband.access <= 0.797
School.segregation <= 0.0359
0.36 < Mammography.screening <= 0.42
Feature
−0.004
0.000
0.004
0.008
Weight
Positive
Negative

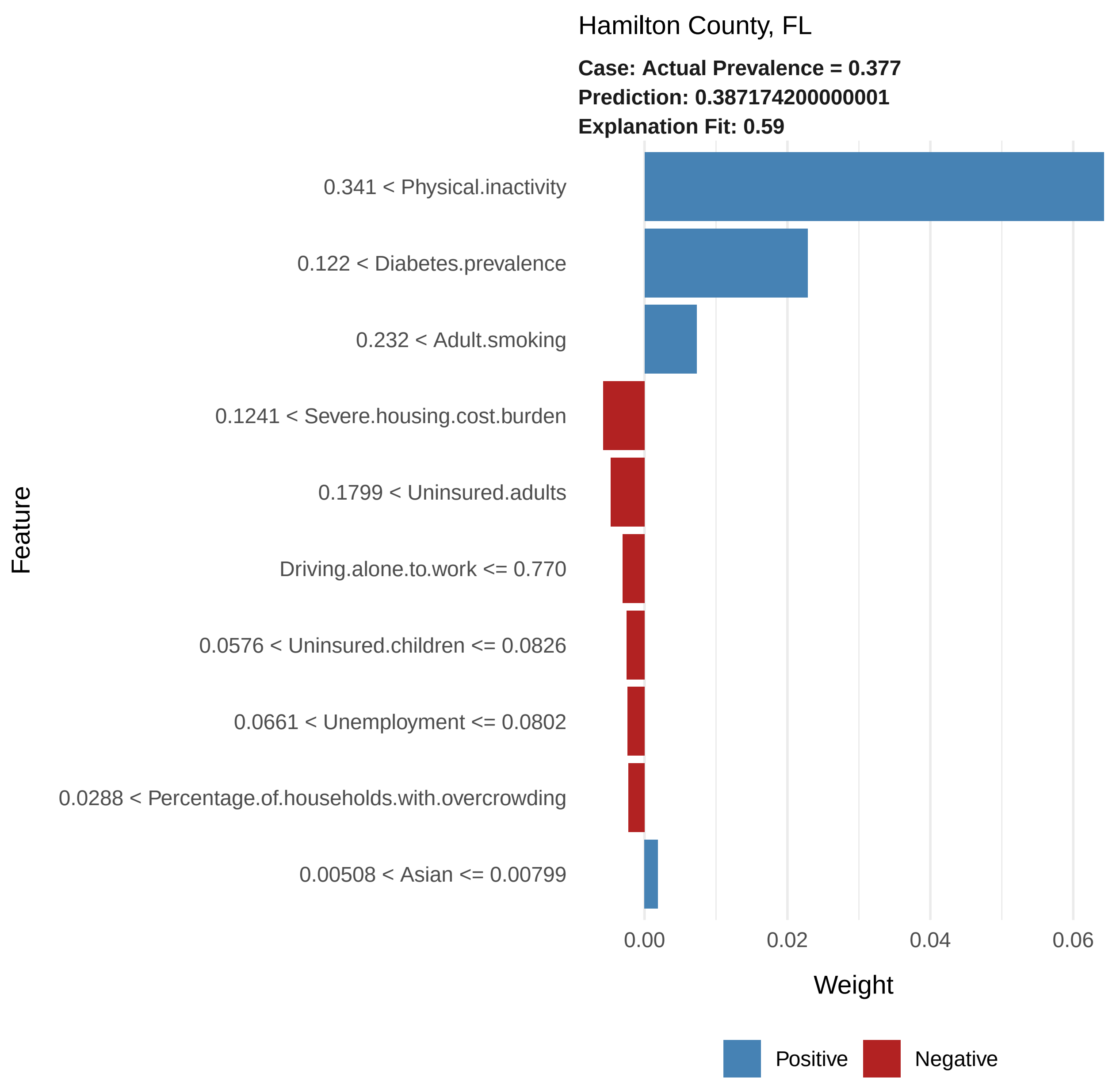
Hamilton County, FL
Case: Actual Prevalence = 0.377
Prediction: 0.387174200000001
Explanation Fit: 0.59
0.341 < Physical.inactivity
0.122 < Diabetes.prevalence
0.232 < Adult.smoking
0.1241 < Severe.housing.cost.burden
0.1799 < Uninsured.adults
Driving.alone.to.work <= 0.770
0.0576 < Uninsured.children <= 0.0826
0.0661 < Unemployment <= 0.0802
0.0288 < Percentage.of.households.with.overcrowding
0.00508 < Asian <= 0.00799
Feature
0.00
0.02
0.04
0.06
Weight
Positive
Negative

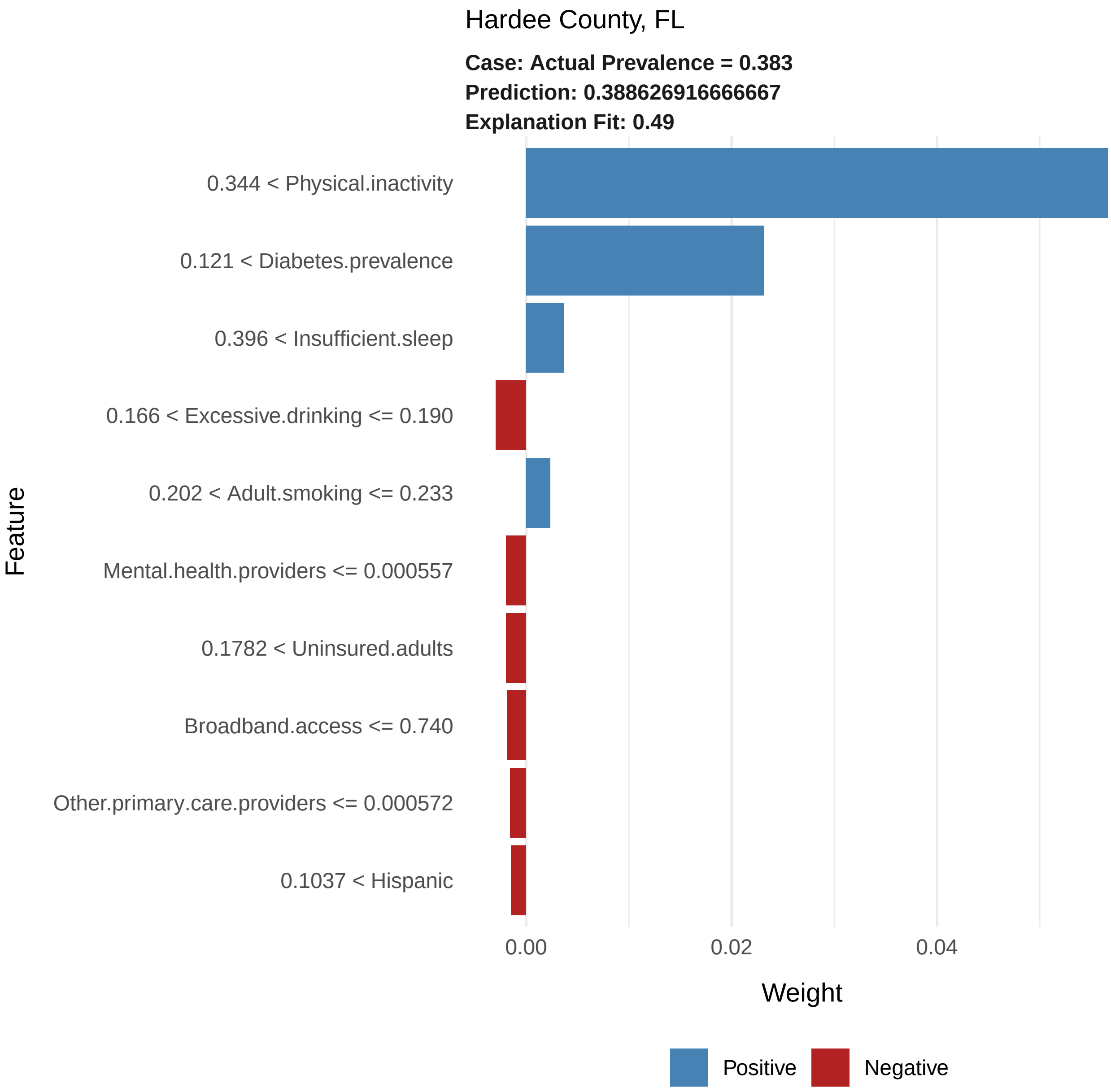

Hardee County, FL
Case: Actual Prevalence = 0.383
Prediction: 0.388626916666667
Explanation Fit: 0.49
0.344 < Physical.inactivity
0.121 < Diabetes.prevalence
0.396 < Insufficient.sleep
0.166 < Excessive.drinking <= 0.190
0.202 < Adult.smoking <= 0.233
Mental.health.providers <= 0.000557
0.1782 < Uninsured.adults
Broadband.access <= 0.740
Other.primary.care.providers <= 0.000572
0.1037 < Hispanic
Feature
0.00
0.02
0.04
Weight
Positive
Negative

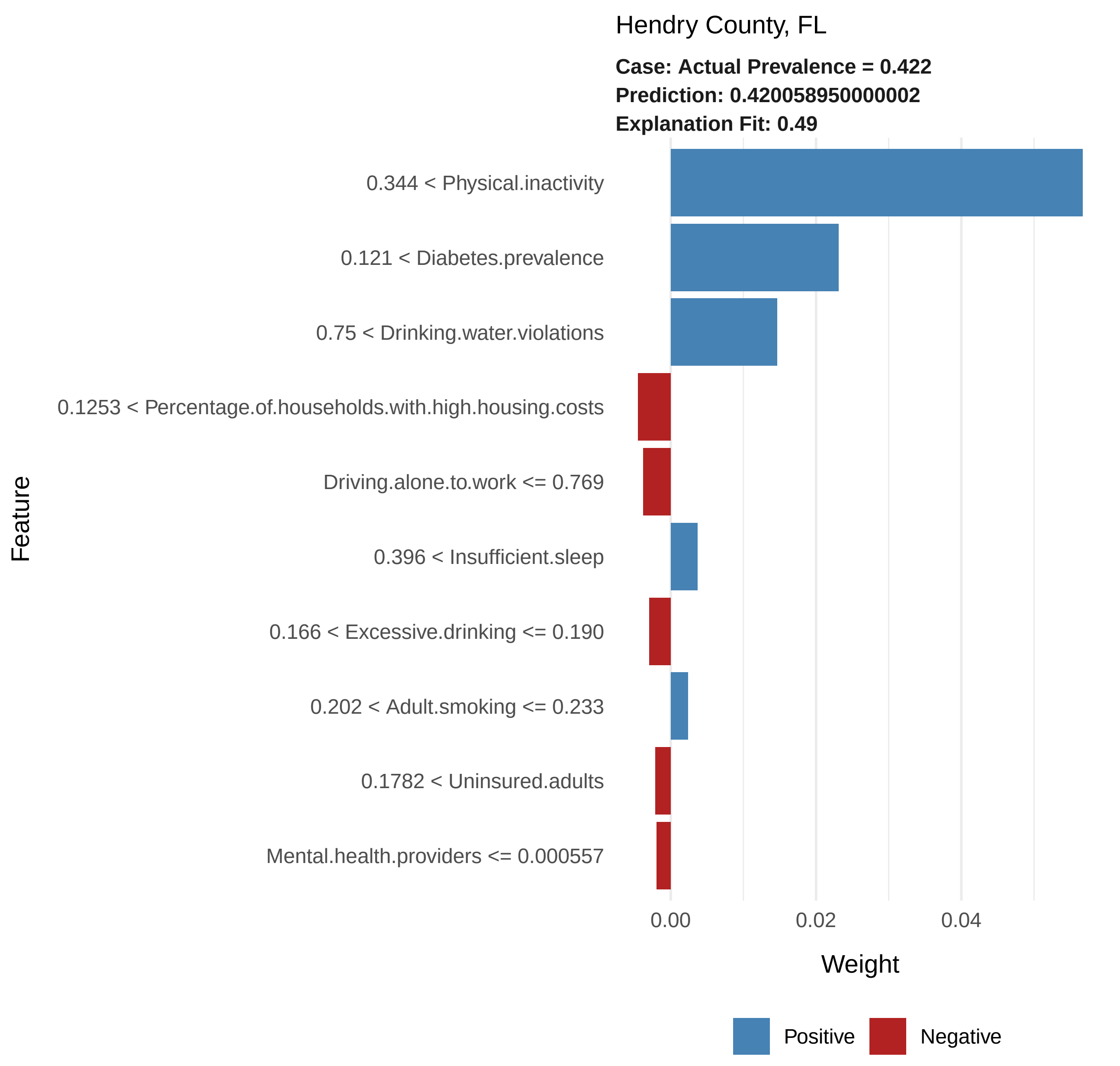
Hendry County, FL
Case: Actual Prevalence = 0.422
Prediction: 0.420058950000002
Explanation Fit: 0.49
0.344 < Physical.inactivity
0.121 < Diabetes.prevalence
0.75 < Drinking.water.violations
0.1253 < Percentage.of.households.with.high.housing.costs
Driving.alone.to.work <= 0.769
0.396 < Insufficient.sleep
0.166 < Excessive.drinking <= 0.190
0.202 < Adult.smoking <= 0.233
0.1782 < Uninsured.adults
Mental.health.providers <= 0.000557
Feature
0.00
0.02
0.04
Weight
Positive
Negative

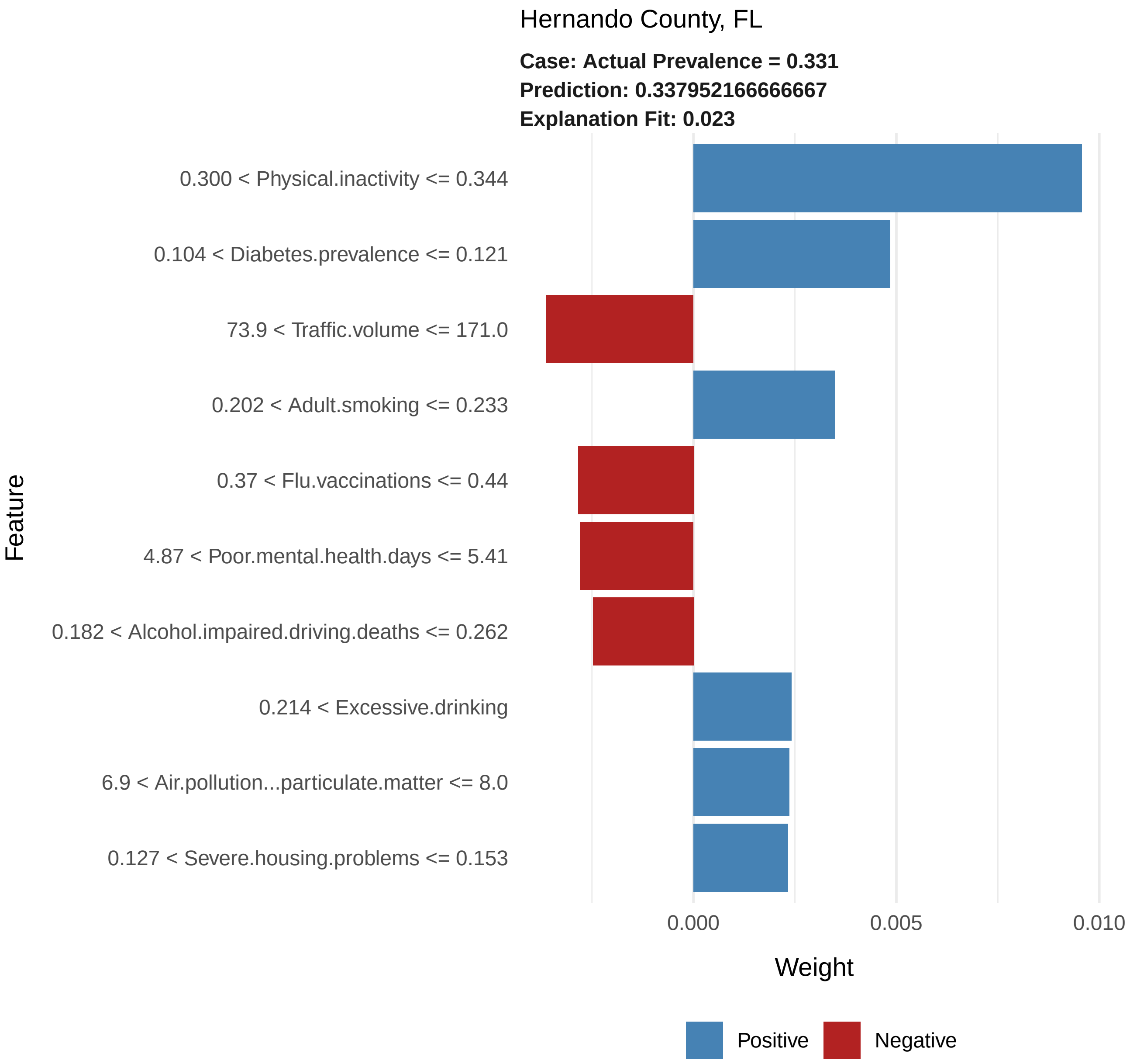

Hernando County, FL
Case: Actual Prevalence = 0.331
Prediction: 0.337952166666667
Explanation Fit: 0.023
0.300 < Physical.inactivity <= 0.344
0.104 < Diabetes.prevalence <= 0.121
73.9 < Traffic.volume <= 171.0
0.202 < Adult.smoking <= 0.233
0.37 < Flu.vaccinations <= 0.44
4.87 < Poor.mental.health.days <= 5.41
0.182 < Alcohol.impaired.driving.deaths <= 0.262
0.214 < Excessive.drinking
6.9 < Air.pollution...particulate.matter <= 8.0
0.127 < Severe.housing.problems <= 0.153
Feature
0.000
0.005
0.010
Weight
Positive
Negative

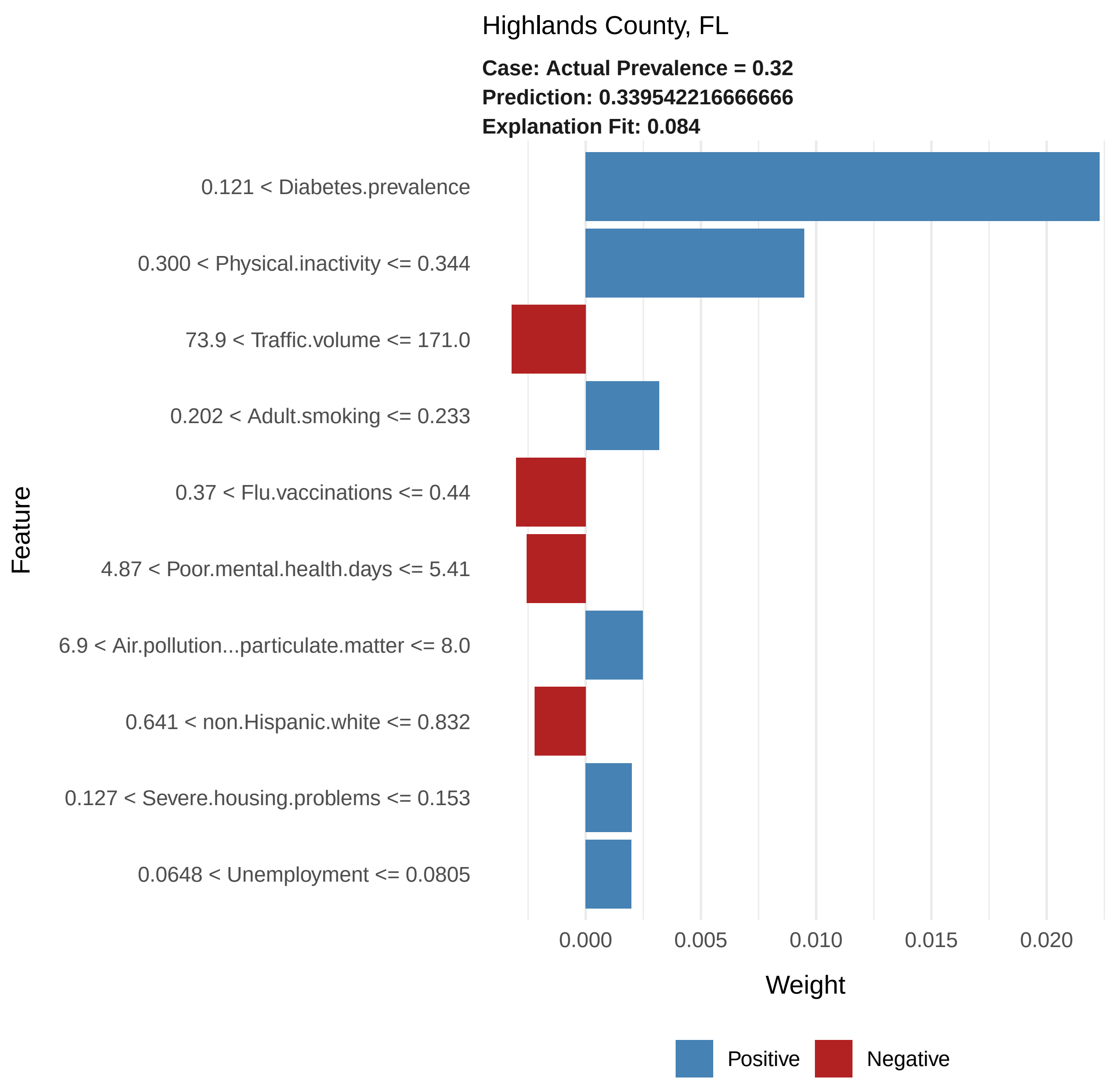

Highlands County, FL
Case: Actual Prevalence = 0.32
Prediction: 0.339542216666666
Explanation Fit: 0.084
0.121 < Diabetes.prevalence
0.300 < Physical.inactivity <= 0.344
73.9 < Traffic.volume <= 171.0
0.202 < Adult.smoking <= 0.233
0.37 < Flu.vaccinations <= 0.44
4.87 < Poor.mental.health.days <= 5.41
6.9 < Air.pollution...particulate.matter <= 8.0
0.641 < non.Hispanic.white <= 0.832
0.127 < Severe.housing.problems <= 0.153
0.0648 < Unemployment <= 0.0805
Feature
0.000
0.005
0.010
0.015
0.020
Weight
Positive
Negative

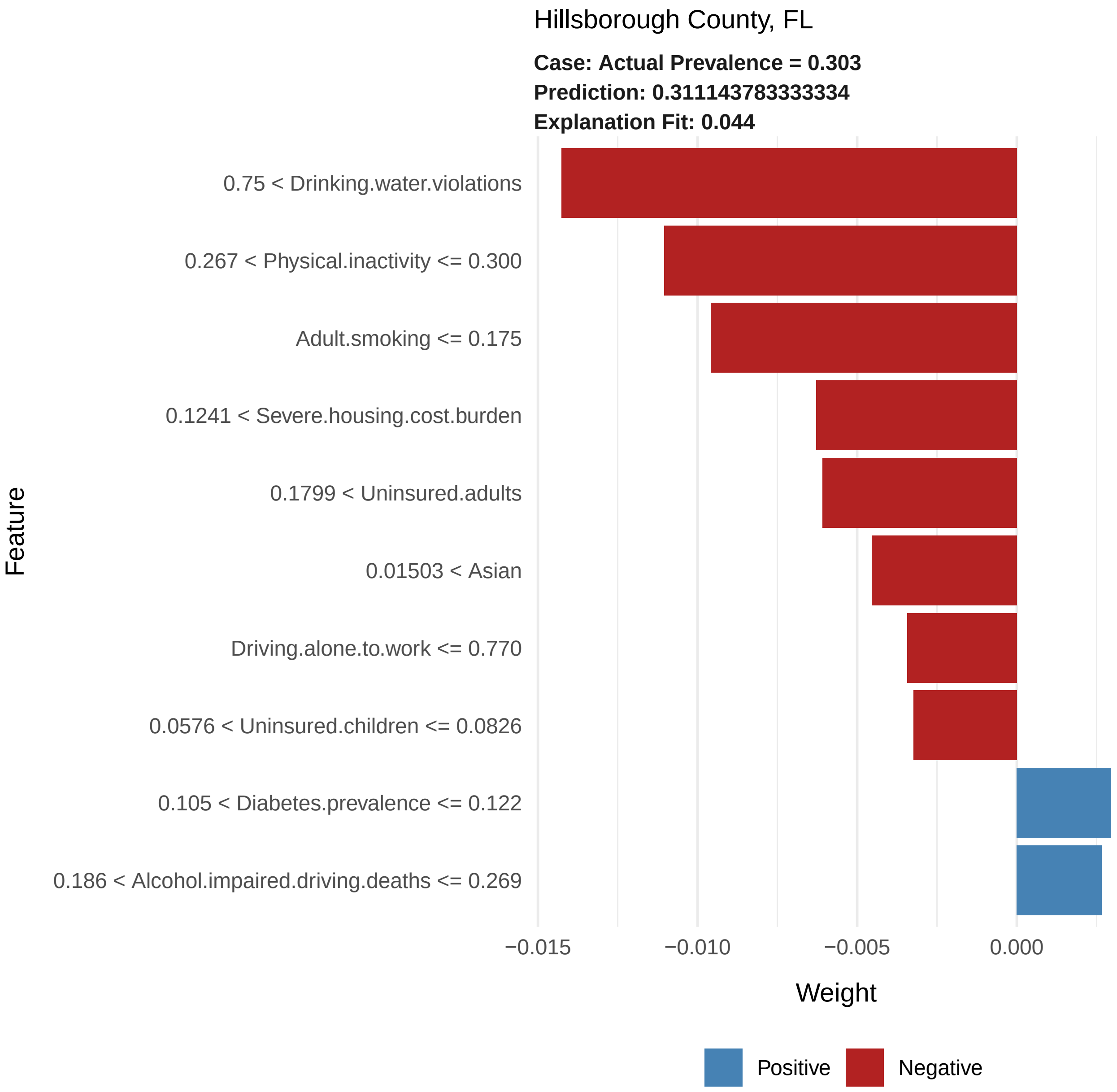
Hillsborough County, FL
Case: Actual Prevalence = 0.303
Prediction: 0.311143783333334
Explanation Fit: 0.044
0.75 < Drinking.water.violations
0.267 < Physical.inactivity <= 0.300
Adult.smoking <= 0.175
0.1241 < Severe.housing.cost.burden
0.1799 < Uninsured.adults
0.01503 < Asian
Driving.alone.to.work <= 0.770
0.0576 < Uninsured.children <= 0.0826
0.105 < Diabetes.prevalence <= 0.122
0.186 < Alcohol.impaired.driving.deaths <= 0.269
Feature
−0.015
−0.010
−0.005
0.000
Weight
Positive
Negative

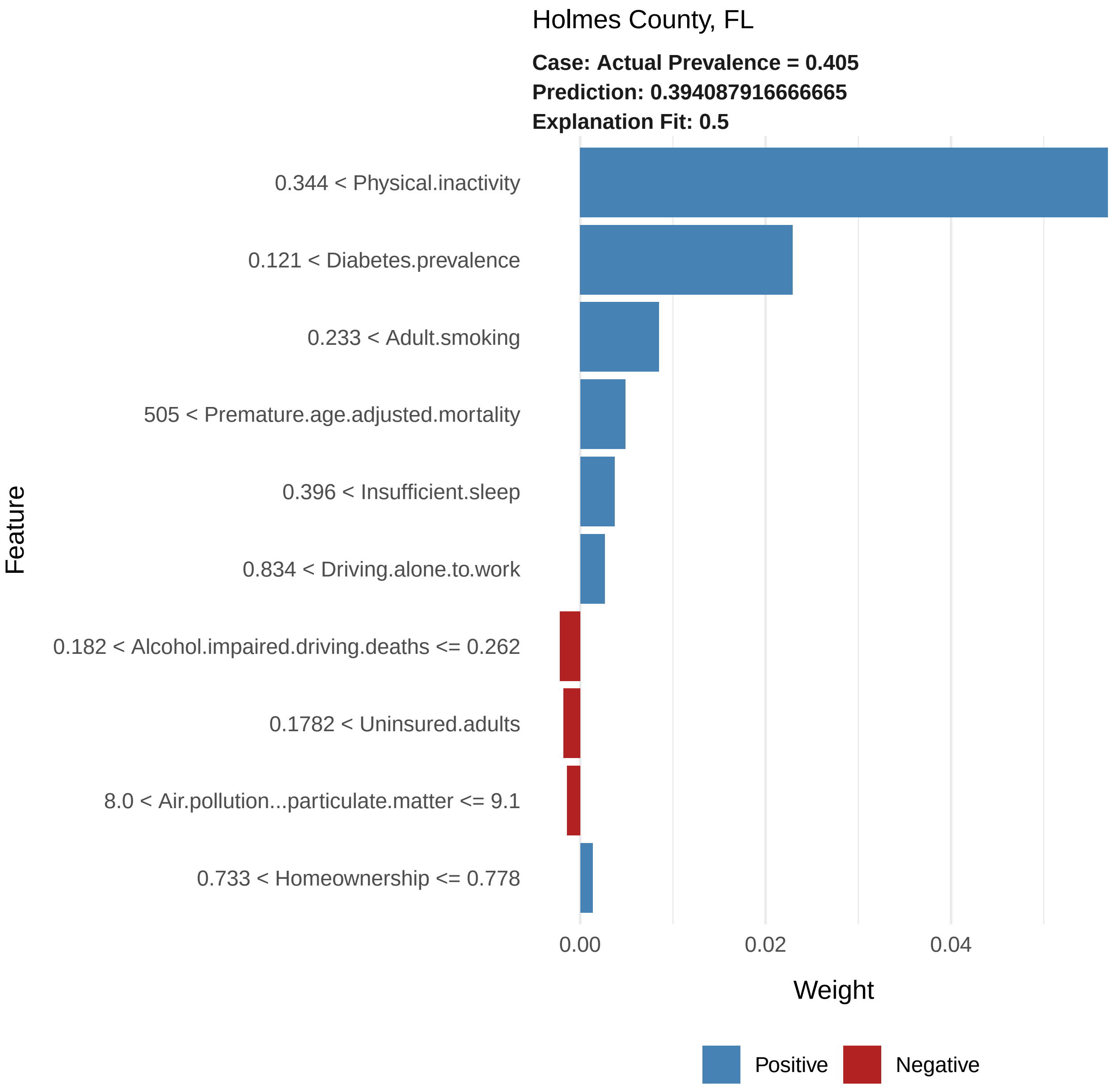

Holmes County, FL
Case: Actual Prevalence = 0.405
Prediction: 0.394087916666665
Explanation Fit: 0.5
0.344 < Physical.inactivity
0.121 < Diabetes.prevalence
0.233 < Adult.smoking
505 < Premature.age.adjusted.mortality
0.396 < Insufficient.sleep
0.834 < Driving.alone.to.work
0.182 < Alcohol.impaired.driving.deaths <= 0.262
0.1782 < Uninsured.adults
8.0 < Air.pollution...particulate.matter <= 9.1
0.733 < Homeownership <= 0.778
Feature
0.00
0.02
0.04
Weight
Positive
Negative

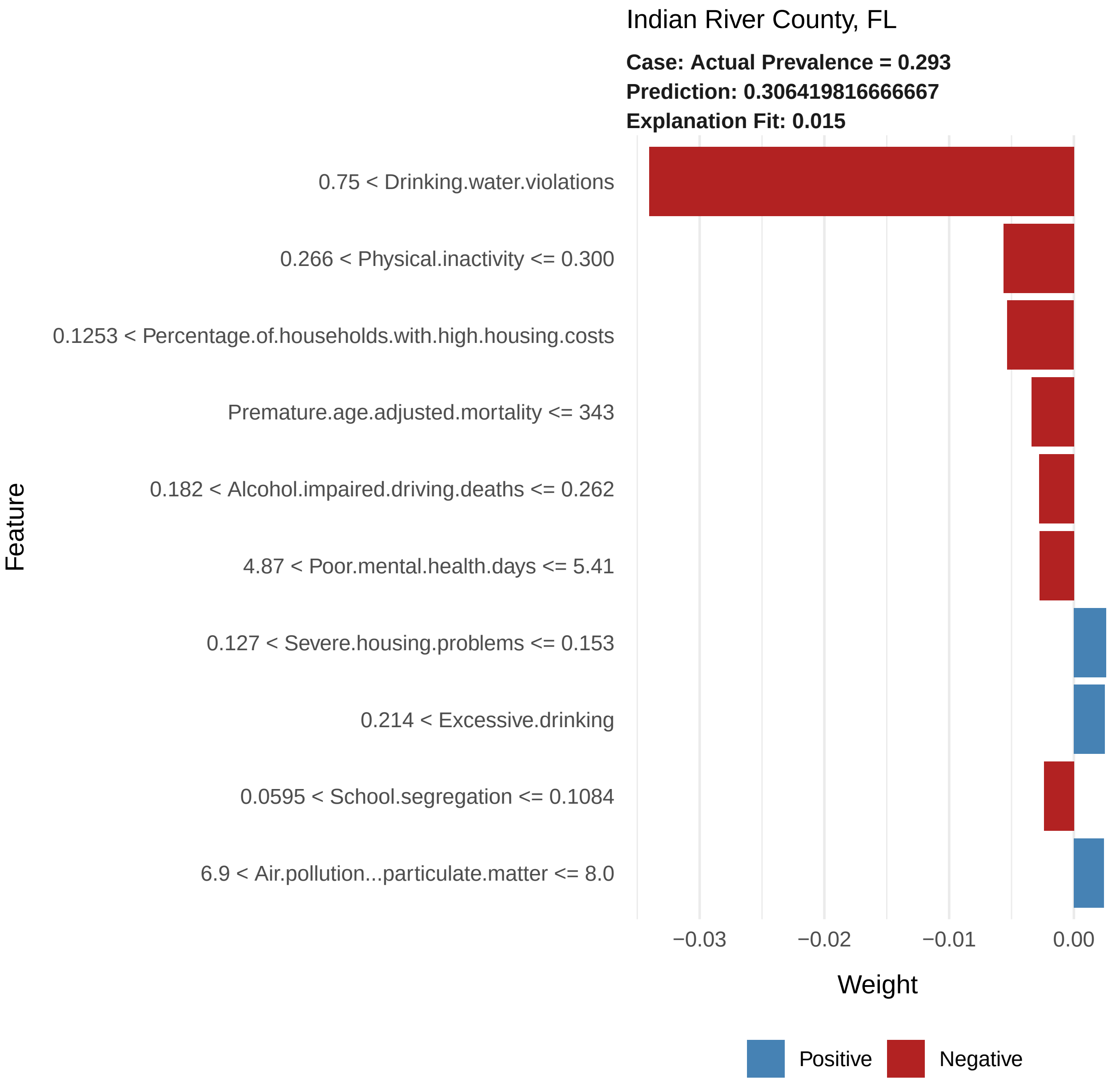
Indian River County, FL
Case: Actual Prevalence = 0.293
Prediction: 0.306419816666667
Explanation Fit: 0.015
0.75 < Drinking.water.violations
0.266 < Physical.inactivity <= 0.300
0.1253 < Percentage.of.households.with.high.housing.costs
Premature.age.adjusted.mortality <= 343
0.182 < Alcohol.impaired.driving.deaths <= 0.262
4.87 < Poor.mental.health.days <= 5.41
0.127 < Severe.housing.problems <= 0.153
0.214 < Excessive.drinking
0.0595 < School.segregation <= 0.1084
6.9 < Air.pollution...particulate.matter <= 8.0
Feature
−0.03
−0.02
−0.01
0.00
Weight
Positive
Negative

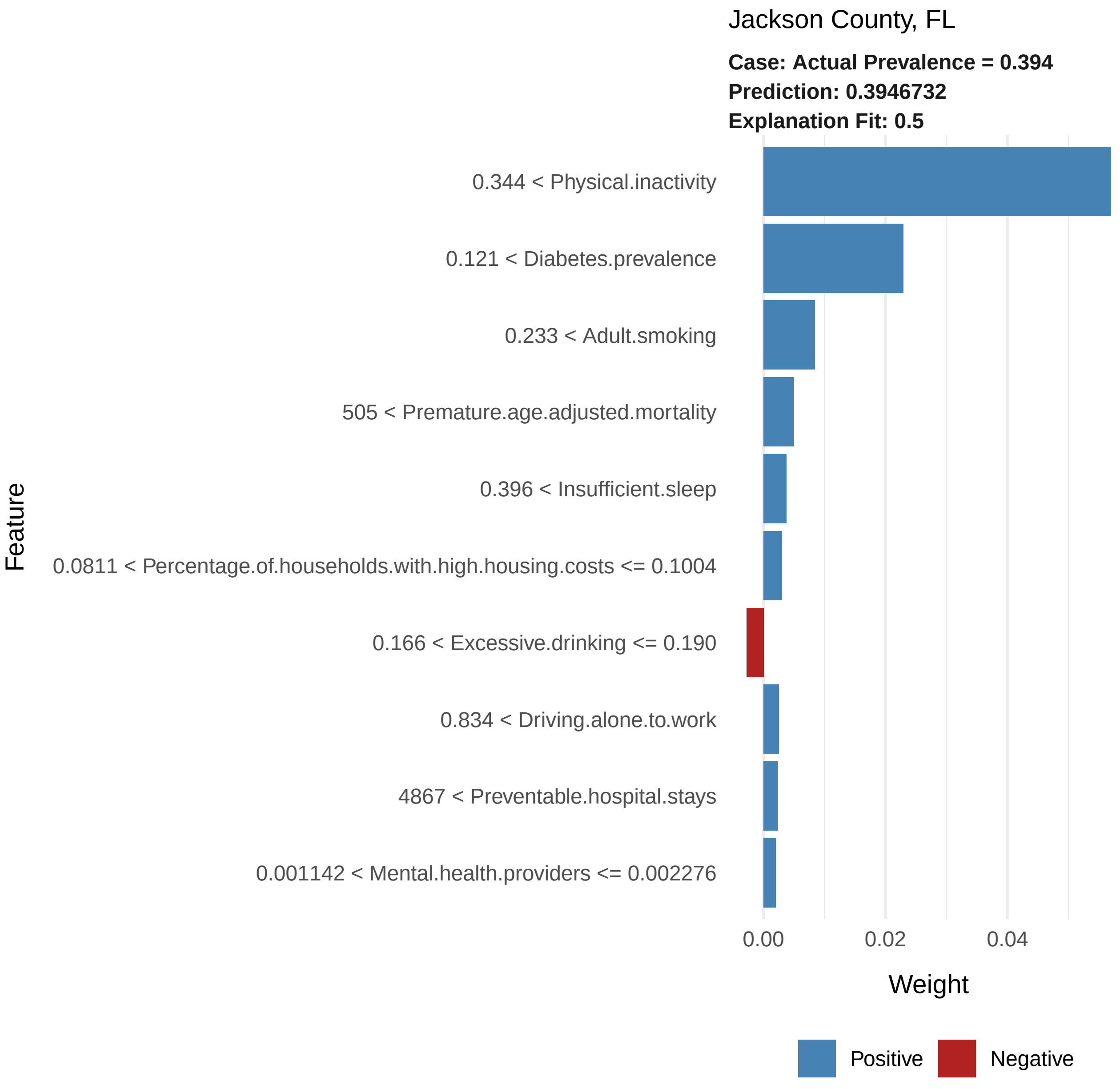

Jackson County, FL
Case: Actual Prevalence = 0.394
Prediction: 0.3946732
Explanation Fit: 0.5
0.344 < Physical.inactivity
0.121 < Diabetes.prevalence
0.233 < Adult.smoking
505 < Premature.age.adjusted.mortality
0.396 < Insufficient.sleep
0.0811 < Percentage.of.households.with.high.housing.costs <= 0.1004
0.166 < Excessive.drinking <= 0.190
0.834 < Driving.alone.to.work
4867 < Preventable.hospital.stays
0.001142 < Mental.health.providers <= 0.002276
Feature
0.00
0.02
0.04
Weight
Positive
Negative

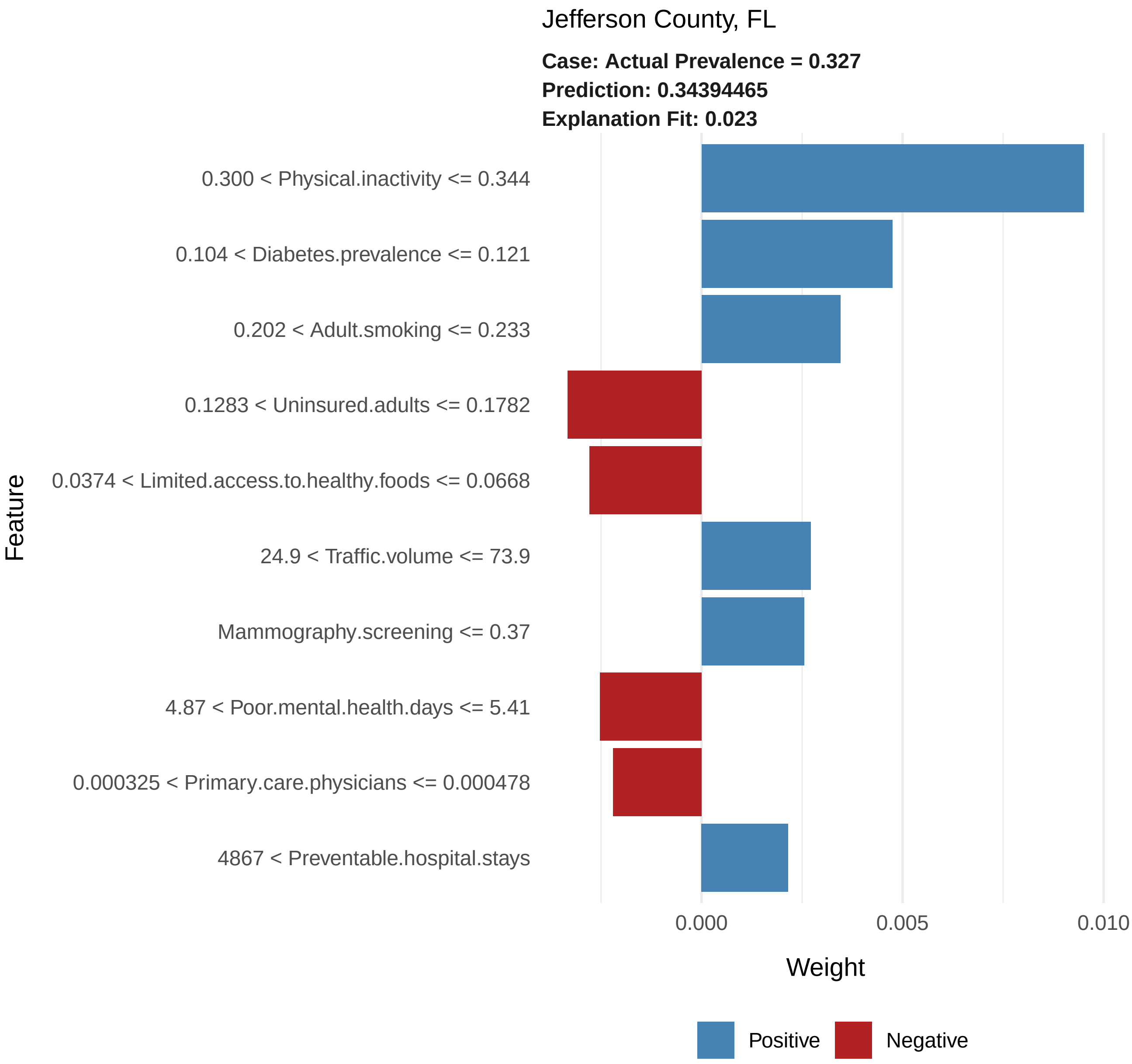

Jefferson County, FL
Case: Actual Prevalence = 0.327
Prediction: 0.34394465
Explanation Fit: 0.023
0.300 < Physical.inactivity <= 0.344
0.104 < Diabetes.prevalence <= 0.121
0.202 < Adult.smoking <= 0.233
0.1283 < Uninsured.adults <= 0.1782
0.0374 < Limited.access.to.healthy.foods <= 0.0668
24.9 < Traffic.volume <= 73.9
Mammography.screening <= 0.37
4.87 < Poor.mental.health.days <= 5.41
0.000325 < Primary.care.physicians <= 0.000478
4867 < Preventable.hospital.stays
Feature
0.000
0.005
0.010
Weight
Positive
Negative

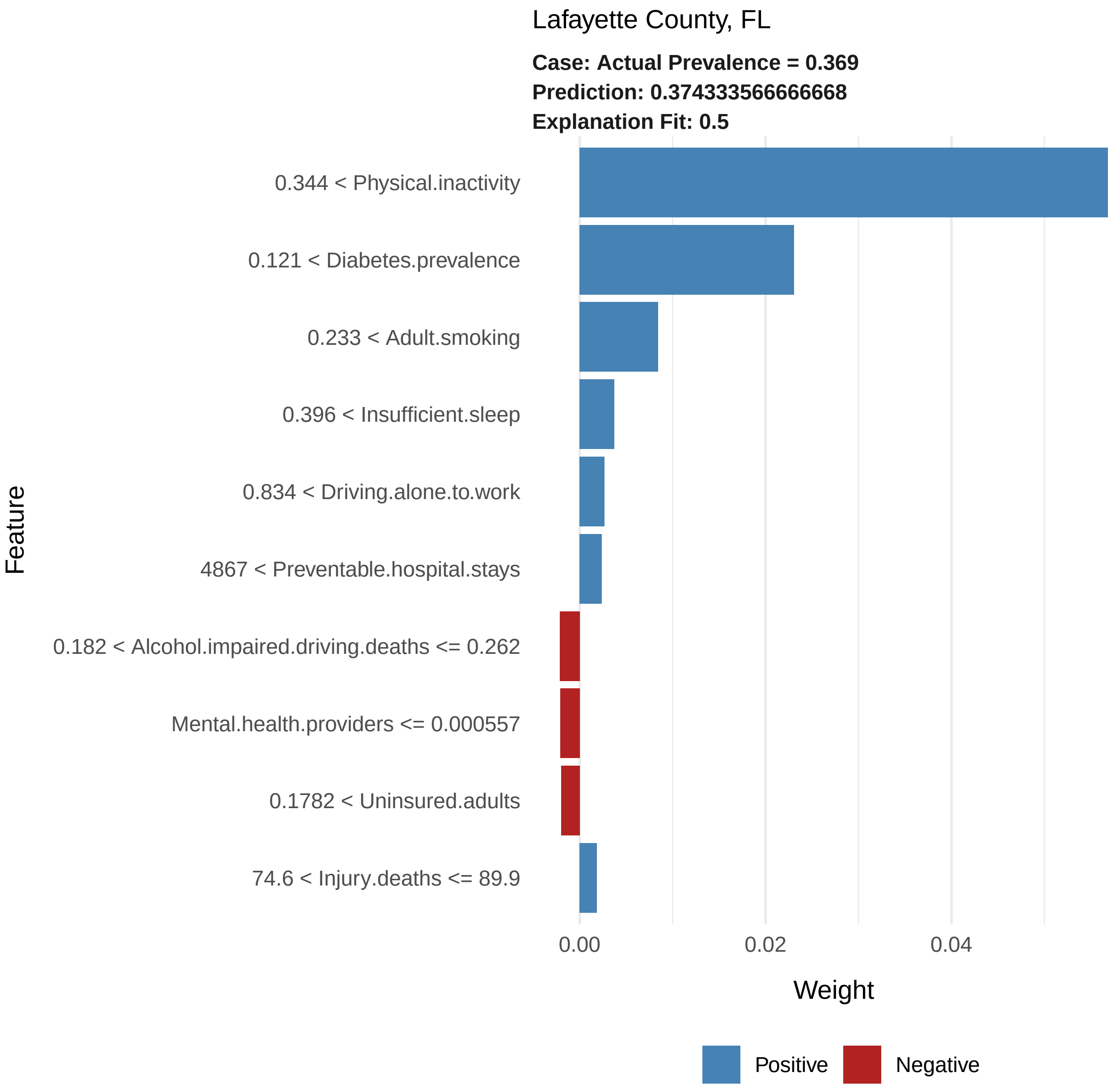

Lafayette County, FL
Case: Actual Prevalence = 0.369
Prediction: 0.374333566666668
Explanation Fit: 0.5
0.344 < Physical.inactivity
0.121 < Diabetes.prevalence
0.233 < Adult.smoking
0.396 < Insufficient.sleep
0.834 < Driving.alone.to.work
4867 < Preventable.hospital.stays
0.182 < Alcohol.impaired.driving.deaths <= 0.262
Mental.health.providers <= 0.000557
0.1782 < Uninsured.adults
74.6 < Injury.deaths <= 89.9
Feature
0.00
0.02
0.04
Weight
Positive
Negative

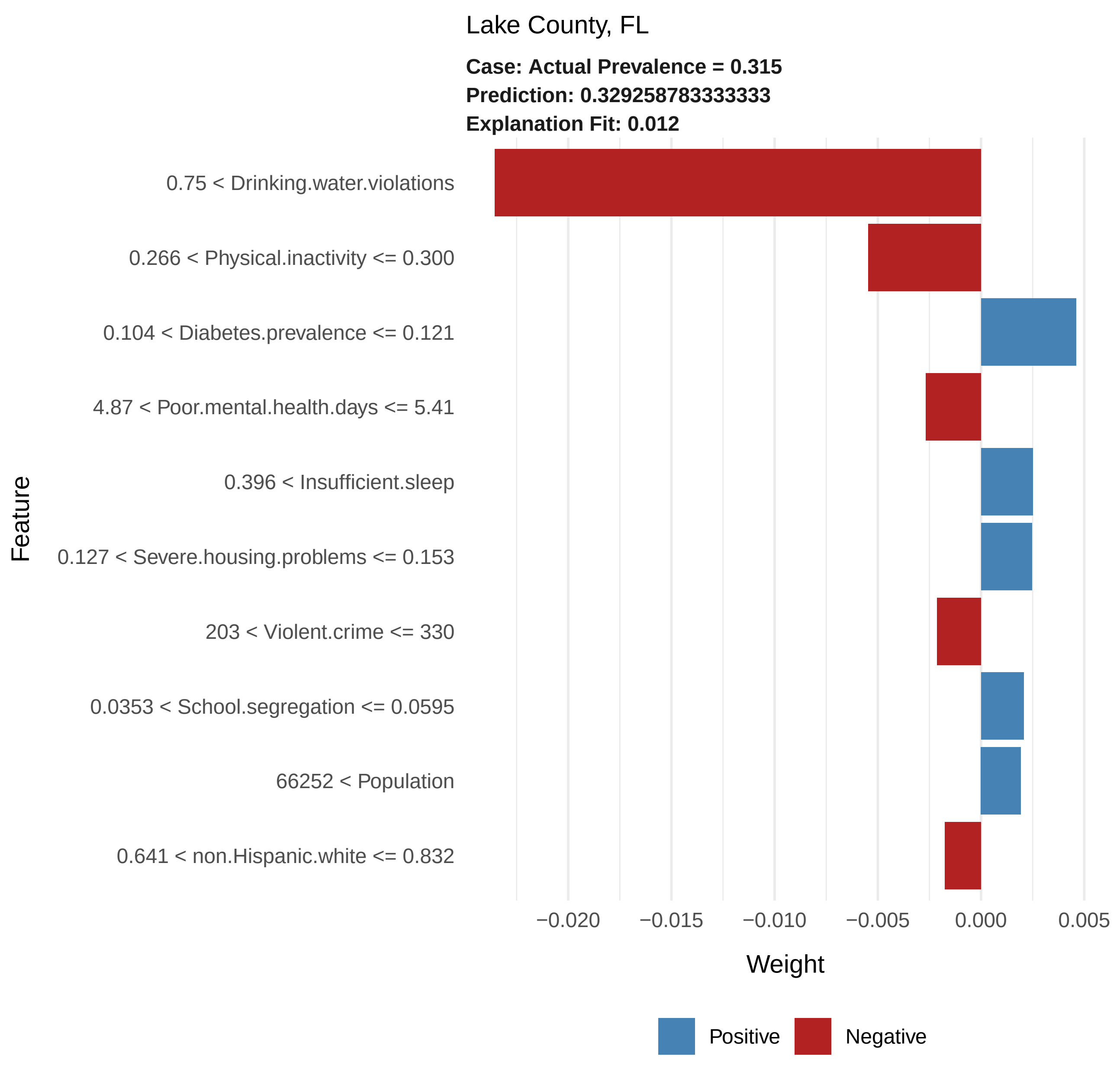
Lake County, FL
Case: Actual Prevalence = 0.315
Prediction: 0.329258783333333
Explanation Fit: 0.012
0.75 < Drinking.water.violations
0.266 < Physical.inactivity <= 0.300
0.104 < Diabetes.prevalence <= 0.121
4.87 < Poor.mental.health.days <= 5.41
0.396 < Insufficient.sleep
0.127 < Severe.housing.problems <= 0.153
203 < Violent.crime <= 330
0.0353 < School.segregation <= 0.0595
66252 < Population
0.641 < non.Hispanic.white <= 0.832
Feature
−0.020
−0.015
−0.010
−0.005
0.000
0.005
Weight
Positive
Negative

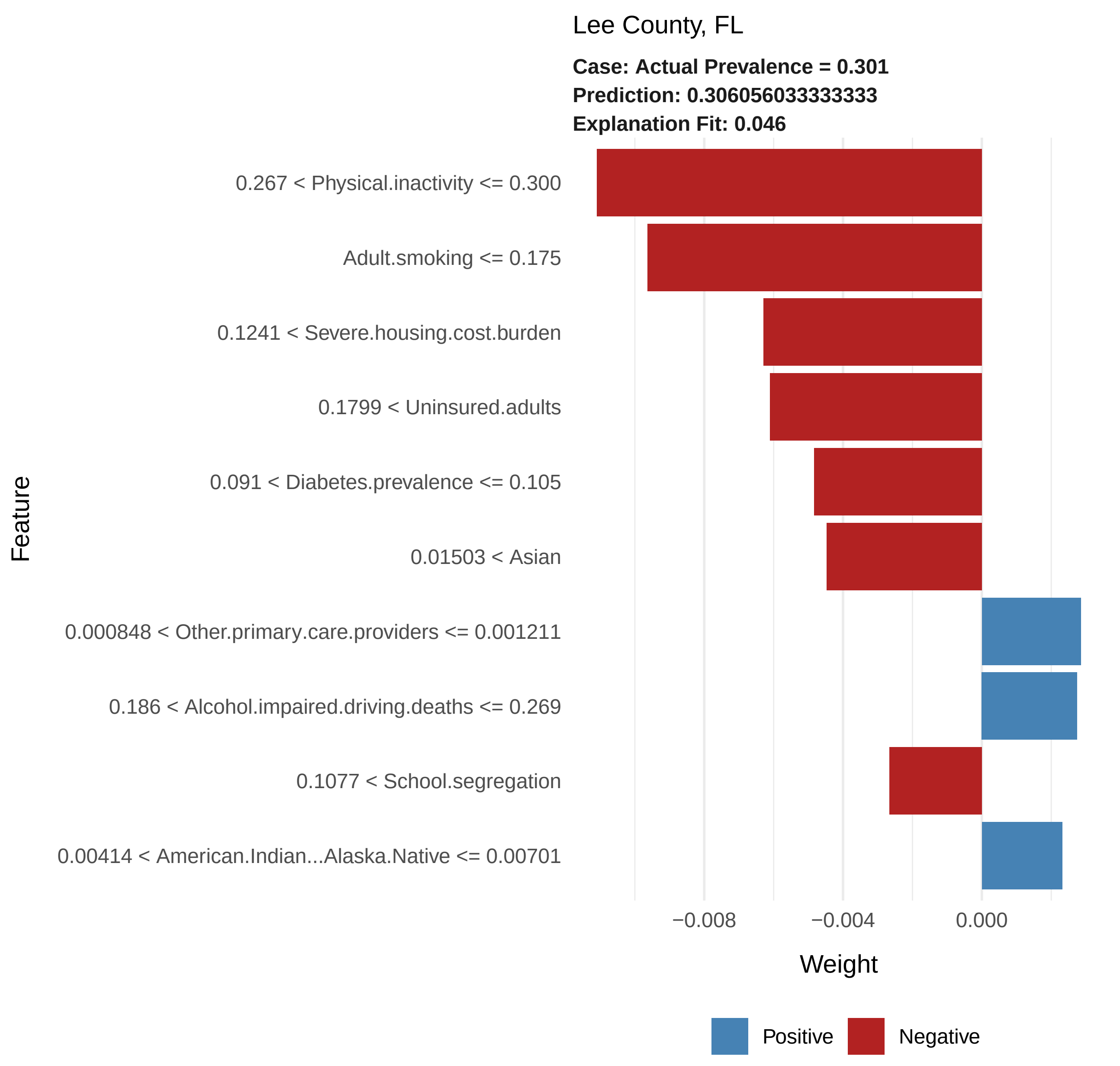

Lee County, FL
Case: Actual Prevalence = 0.301
Prediction: 0.306056033333333
Explanation Fit: 0.046
0.267 < Physical.inactivity <= 0.300
Adult.smoking <= 0.175
0.1241 < Severe.housing.cost.burden
0.1799 < Uninsured.adults
0.091 < Diabetes.prevalence <= 0.105
0.01503 < Asian
0.000848 < Other.primary.care.providers <= 0.001211
0.186 < Alcohol.impaired.driving.deaths <= 0.269
0.1077 < School.segregation
0.00414 < American.Indian...Alaska.Native <= 0.00701
Feature
−0.008
−0.004
0.000
Weight
Positive
Negative

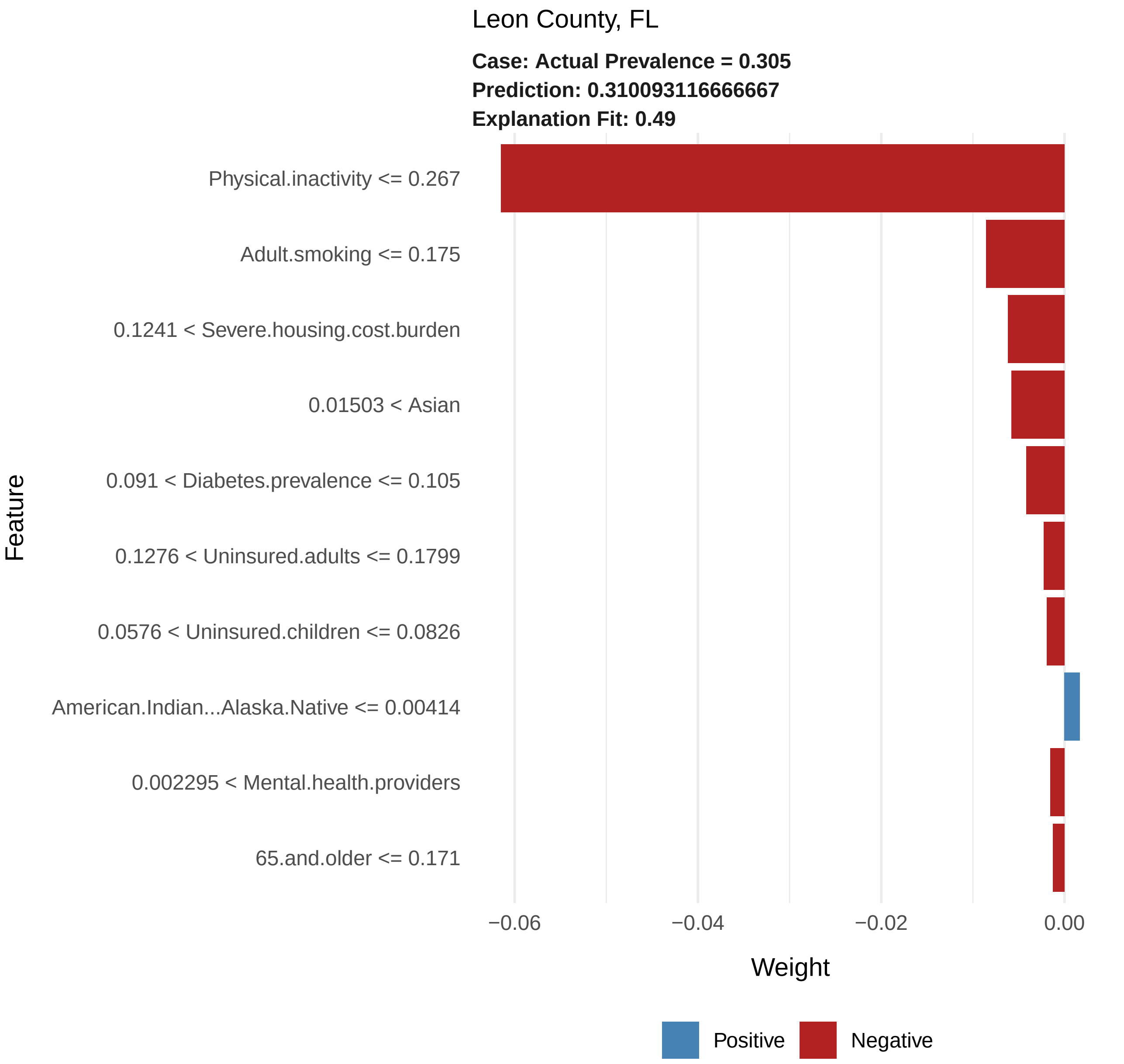
Leon County, FL
Case: Actual Prevalence = 0.305
Prediction: 0.310093116666667
Explanation Fit: 0.49
Physical.inactivity <= 0.267
Adult.smoking <= 0.175
0.1241 < Severe.housing.cost.burden
0.01503 < Asian
0.091 < Diabetes.prevalence <= 0.105
0.1276 < Uninsured.adults <= 0.1799
0.0576 < Uninsured.children <= 0.0826
American.Indian...Alaska.Native <= 0.00414
0.002295 < Mental.health.providers
65.and.older <= 0.171
Feature
−0.06
−0.04
−0.02
0.00
Weight
Positive
Negative

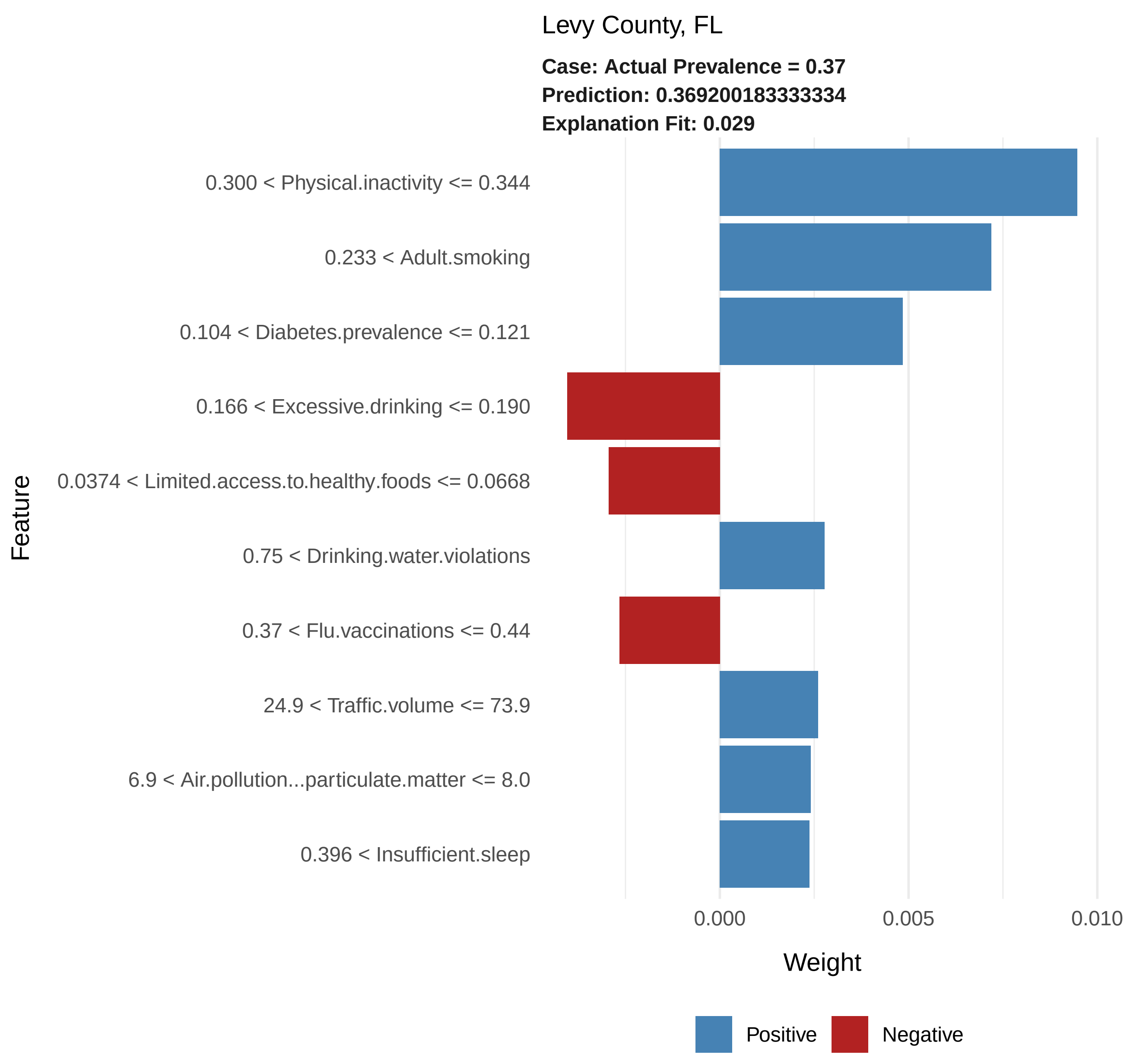
Levy County, FL
Case: Actual Prevalence = 0.37
Prediction: 0.369200183333334
Explanation Fit: 0.029
0.300 < Physical.inactivity <= 0.344
0.233 < Adult.smoking
0.104 < Diabetes.prevalence <= 0.121
0.166 < Excessive.drinking <= 0.190
0.0374 < Limited.access.to.healthy.foods <= 0.0668
0.75 < Drinking.water.violations
0.37 < Flu.vaccinations <= 0.44
24.9 < Traffic.volume <= 73.9
6.9 < Air.pollution...particulate.matter <= 8.0
0.396 < Insufficient.sleep
Feature
0.000
0.005
0.010
Weight
Positive
Negative

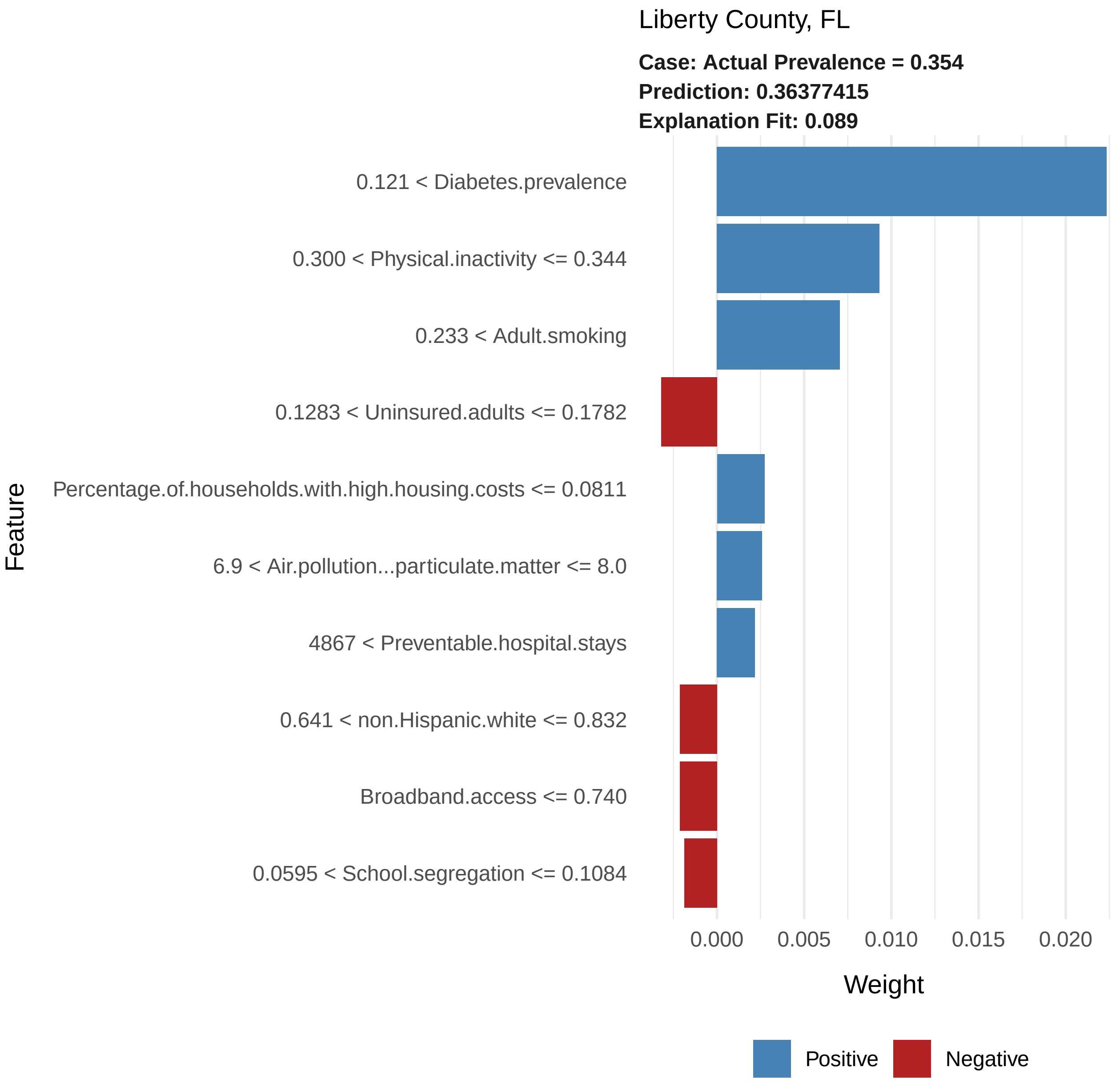

Liberty County, FL
Case: Actual Prevalence = 0.354
Prediction: 0.36377415
Explanation Fit: 0.089
0.121 < Diabetes.prevalence
0.300 < Physical.inactivity <= 0.344
0.233 < Adult.smoking
0.1283 < Uninsured.adults <= 0.1782
Percentage.of.households.with.high.housing.costs <= 0.0811
6.9 < Air.pollution...particulate.matter <= 8.0
4867 < Preventable.hospital.stays
0.641 < non.Hispanic.white <= 0.832
Broadband.access <= 0.740
0.0595 < School.segregation <= 0.1084
Feature
0.000
0.005
0.010
0.015
0.020
Weight
Positive
Negative

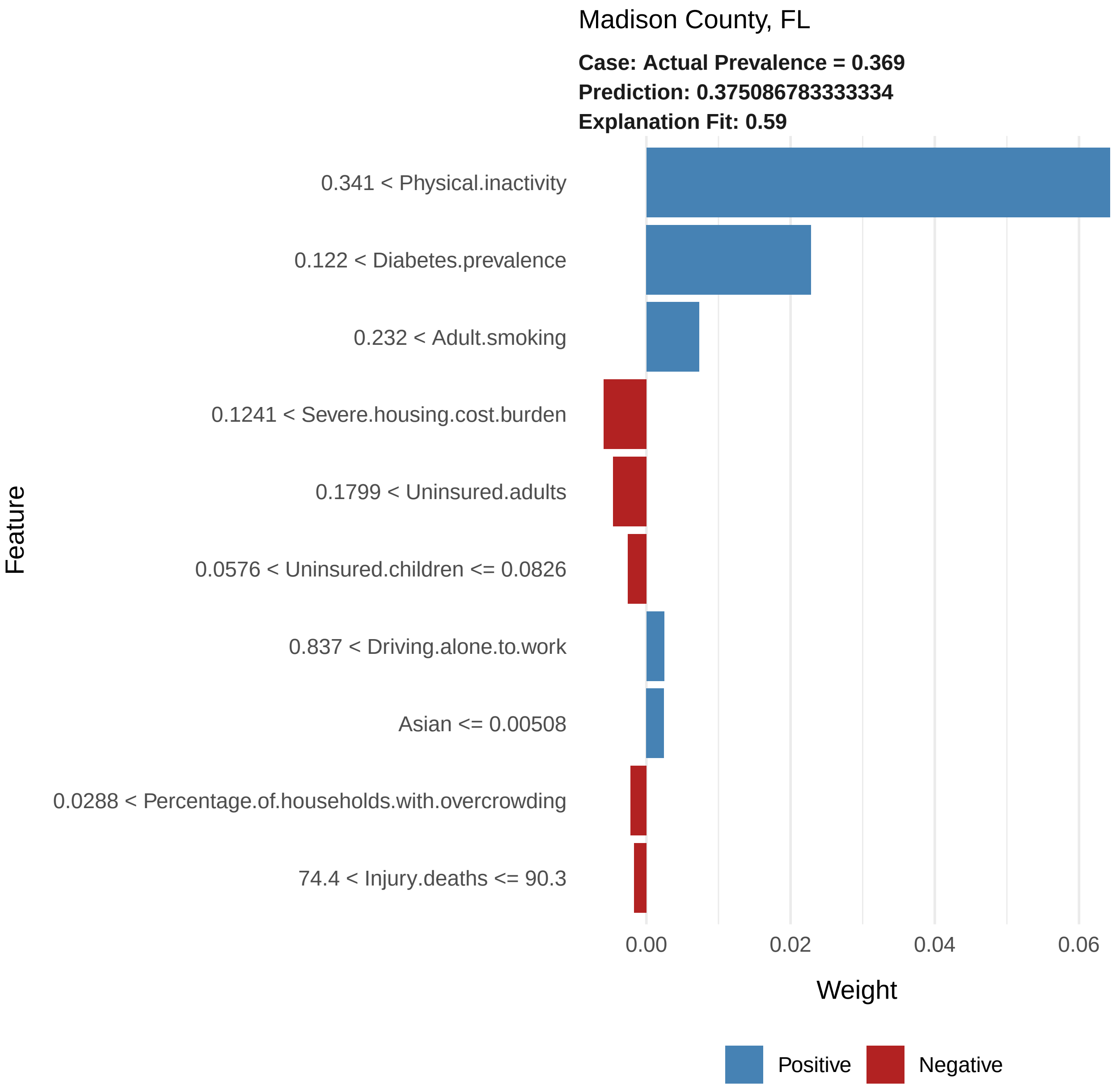
Madison County, FL
Case: Actual Prevalence = 0.369
Prediction: 0.375086783333334
Explanation Fit: 0.59
0.341 < Physical.inactivity
0.122 < Diabetes.prevalence
0.232 < Adult.smoking
0.1241 < Severe.housing.cost.burden
0.1799 < Uninsured.adults
0.0576 < Uninsured.children <= 0.0826
0.837 < Driving.alone.to.work
Asian <= 0.00508
0.0288 < Percentage.of.households.with.overcrowding
74.4 < Injury.deaths <= 90.3
Feature
0.00
0.02
0.04
0.06
Weight
Positive
Negative

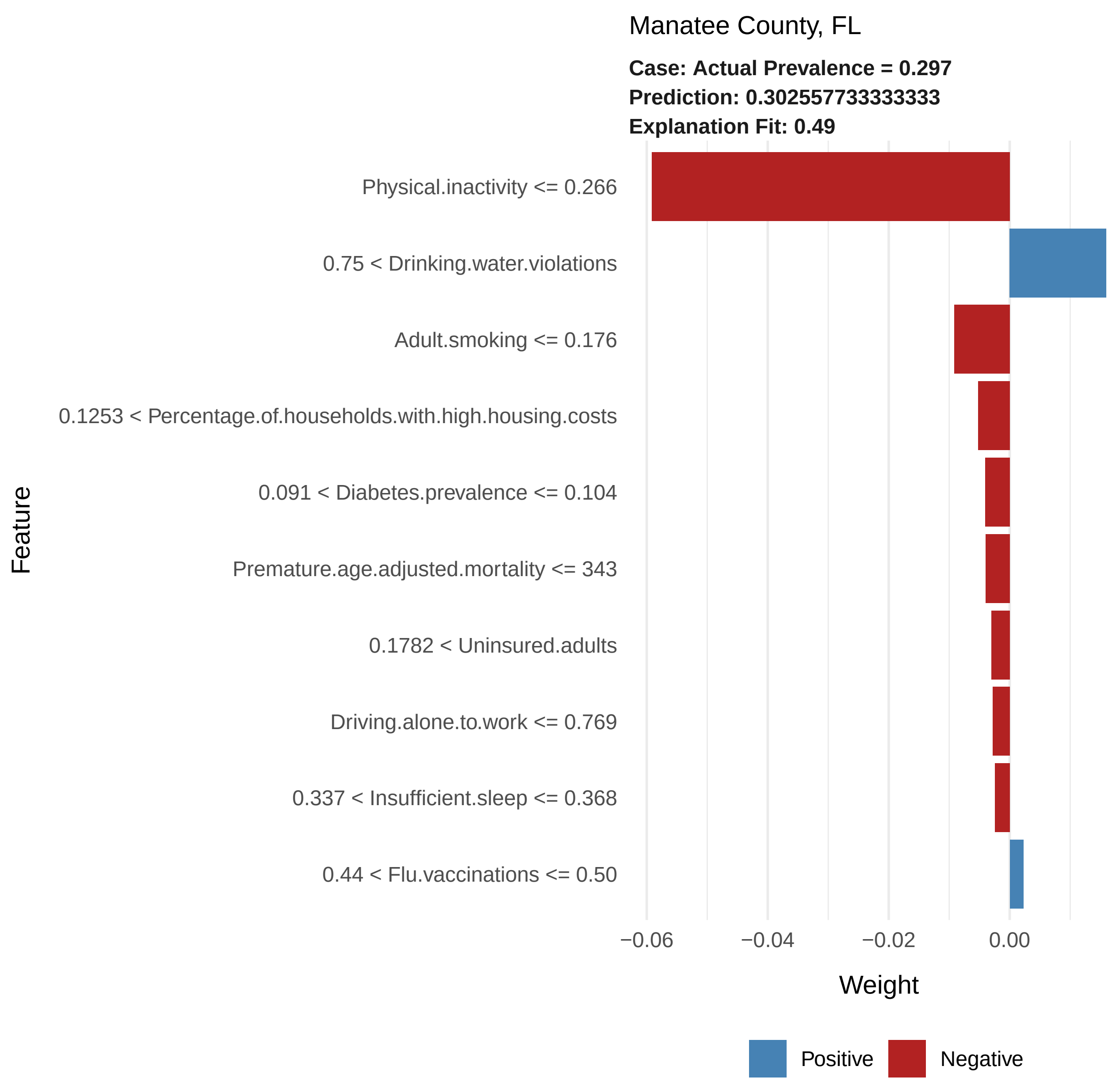

Manatee County, FL
Case: Actual Prevalence = 0.297
Prediction: 0.302557733333333
Explanation Fit: 0.49
Physical.inactivity <= 0.266
0.75 < Drinking.water.violations
Adult.smoking <= 0.176
0.1253 < Percentage.of.households.with.high.housing.costs
0.091 < Diabetes.prevalence <= 0.104
Premature.age.adjusted.mortality <= 343
0.1782 < Uninsured.adults
Driving.alone.to.work <= 0.769
0.337 < Insufficient.sleep <= 0.368
0.44 < Flu.vaccinations <= 0.50
Feature
−0.06
−0.04
−0.02
0.00
Weight
Positive
Negative

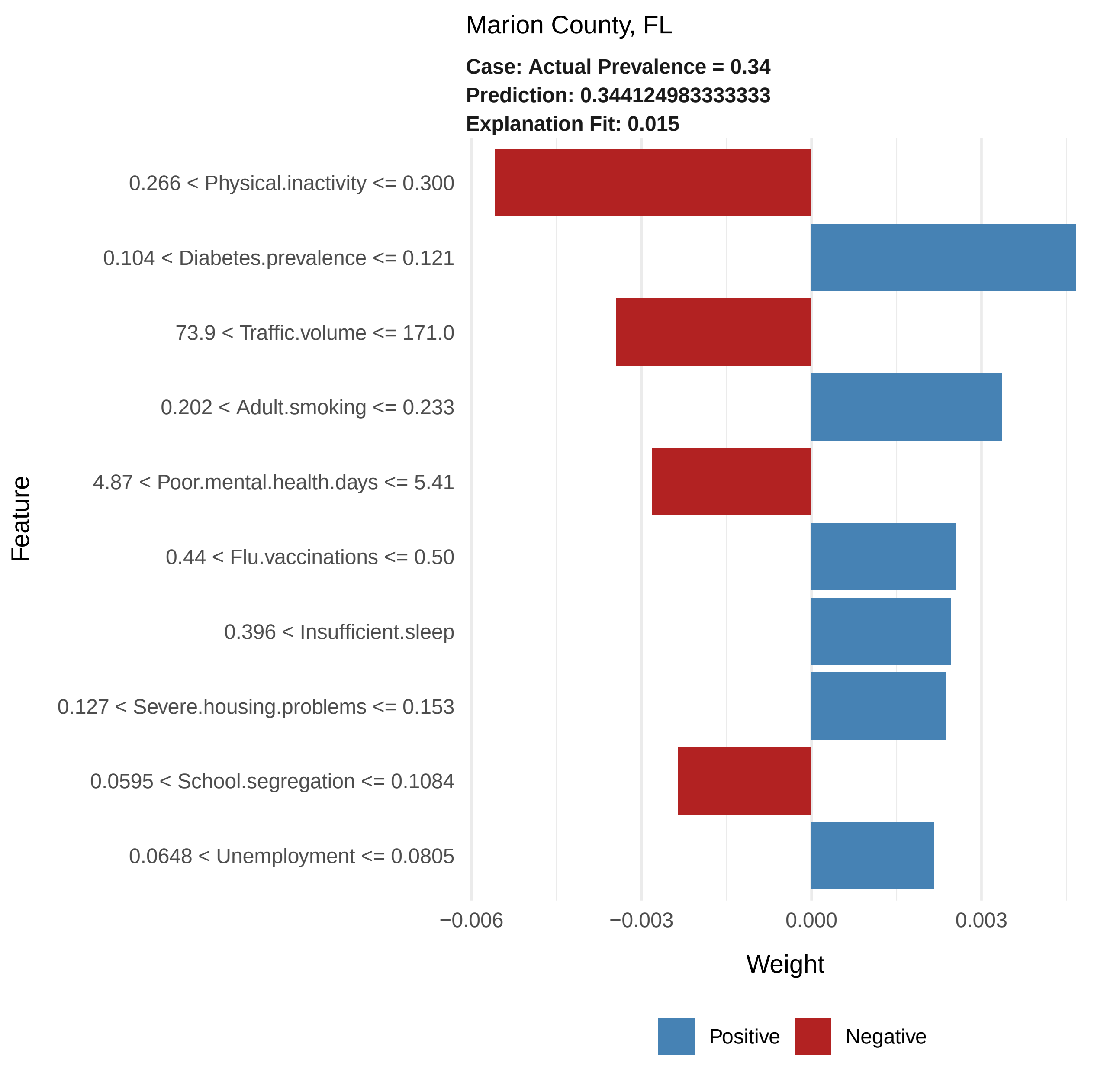
Marion County, FL
Case: Actual Prevalence = 0.34
Prediction: 0.344124983333333
Explanation Fit: 0.015
0.266 < Physical.inactivity <= 0.300
0.104 < Diabetes.prevalence <= 0.121
73.9 < Traffic.volume <= 171.0
0.202 < Adult.smoking <= 0.233
4.87 < Poor.mental.health.days <= 5.41
0.44 < Flu.vaccinations <= 0.50
0.396 < Insufficient.sleep
0.127 < Severe.housing.problems <= 0.153
0.0595 < School.segregation <= 0.1084
0.0648 < Unemployment <= 0.0805
Feature
−0.006
−0.003
0.000
0.003
Weight
Positive
Negative

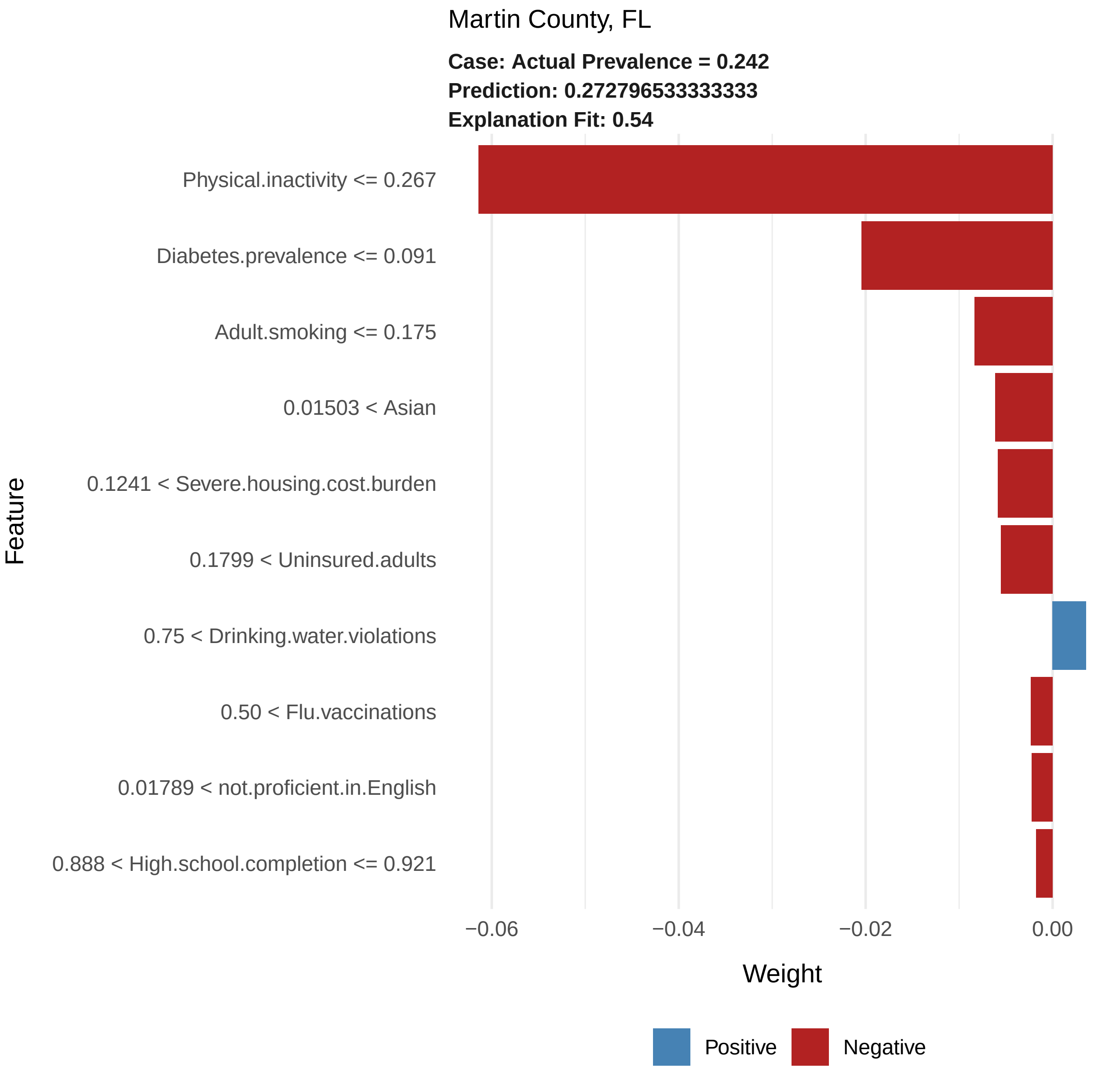

Martin County, FL
Case: Actual Prevalence = 0.242
Prediction: 0.272796533333333
Explanation Fit: 0.54
Physical.inactivity <= 0.267
Diabetes.prevalence <= 0.091
Adult.smoking <= 0.175
0.01503 < Asian
0.1241 < Severe.housing.cost.burden
0.1799 < Uninsured.adults
0.75 < Drinking.water.violations
0.50 < Flu.vaccinations
0.01789 < not.proficient.in.English
0.888 < High.school.completion <= 0.921
Feature
−0.06
−0.04
−0.02
0.00
Weight
Positive
Negative

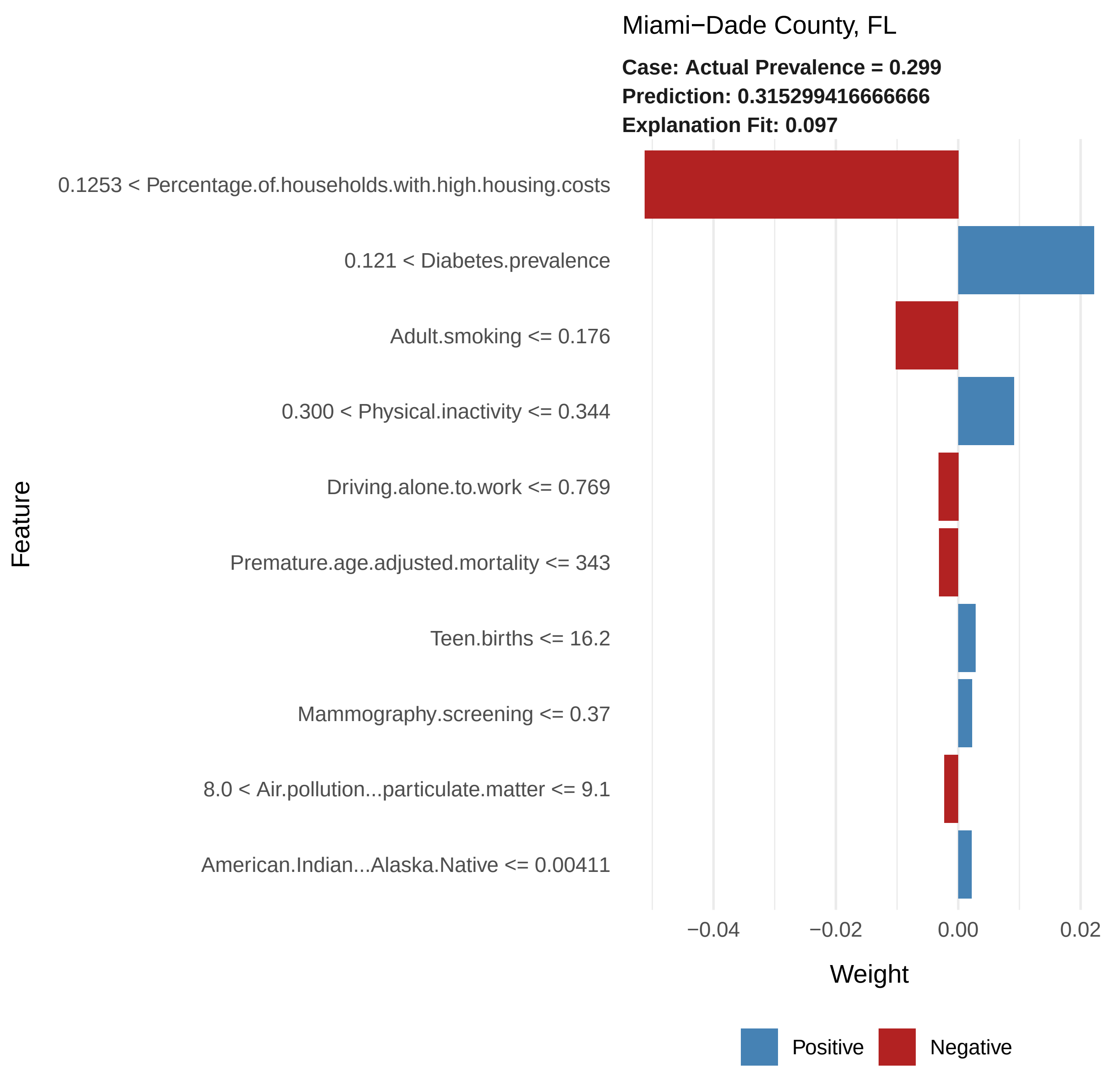
Miami−Dade County, FL
Case: Actual Prevalence = 0.299
Prediction: 0.315299416666666
Explanation Fit: 0.097
0.1253 < Percentage.of.households.with.high.housing.costs
0.121 < Diabetes.prevalence
Adult.smoking <= 0.176
0.300 < Physical.inactivity <= 0.344
Driving.alone.to.work <= 0.769
Premature.age.adjusted.mortality <= 343
Teen.births <= 16.2
Mammography.screening <= 0.37
8.0 < Air.pollution...particulate.matter <= 9.1
American.Indian...Alaska.Native <= 0.00411
Feature
−0.04
−0.02
0.00
0.02
Weight
Positive
Negative

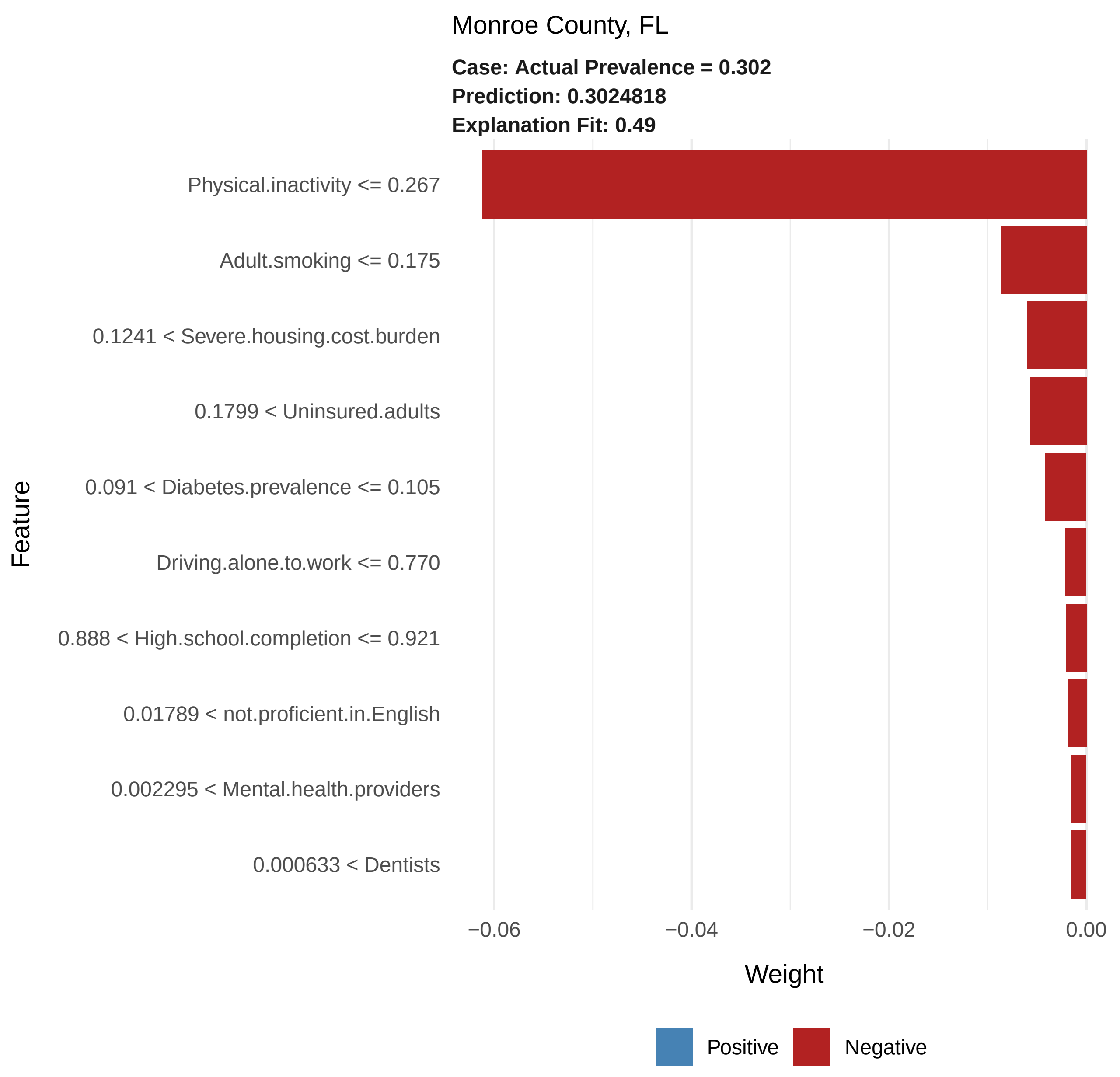

Monroe County, FL
Case: Actual Prevalence = 0.302
Prediction: 0.3024818
Explanation Fit: 0.49
Physical.inactivity <= 0.267
Adult.smoking <= 0.175
0.1241 < Severe.housing.cost.burden
0.1799 < Uninsured.adults
0.091 < Diabetes.prevalence <= 0.105
Driving.alone.to.work <= 0.770
0.888 < High.school.completion <= 0.921
0.01789 < not.proficient.in.English
0.002295 < Mental.health.providers
0.000633 < Dentists
Feature
−0.06
−0.04
−0.02
0.00
Weight
Positive
Negative

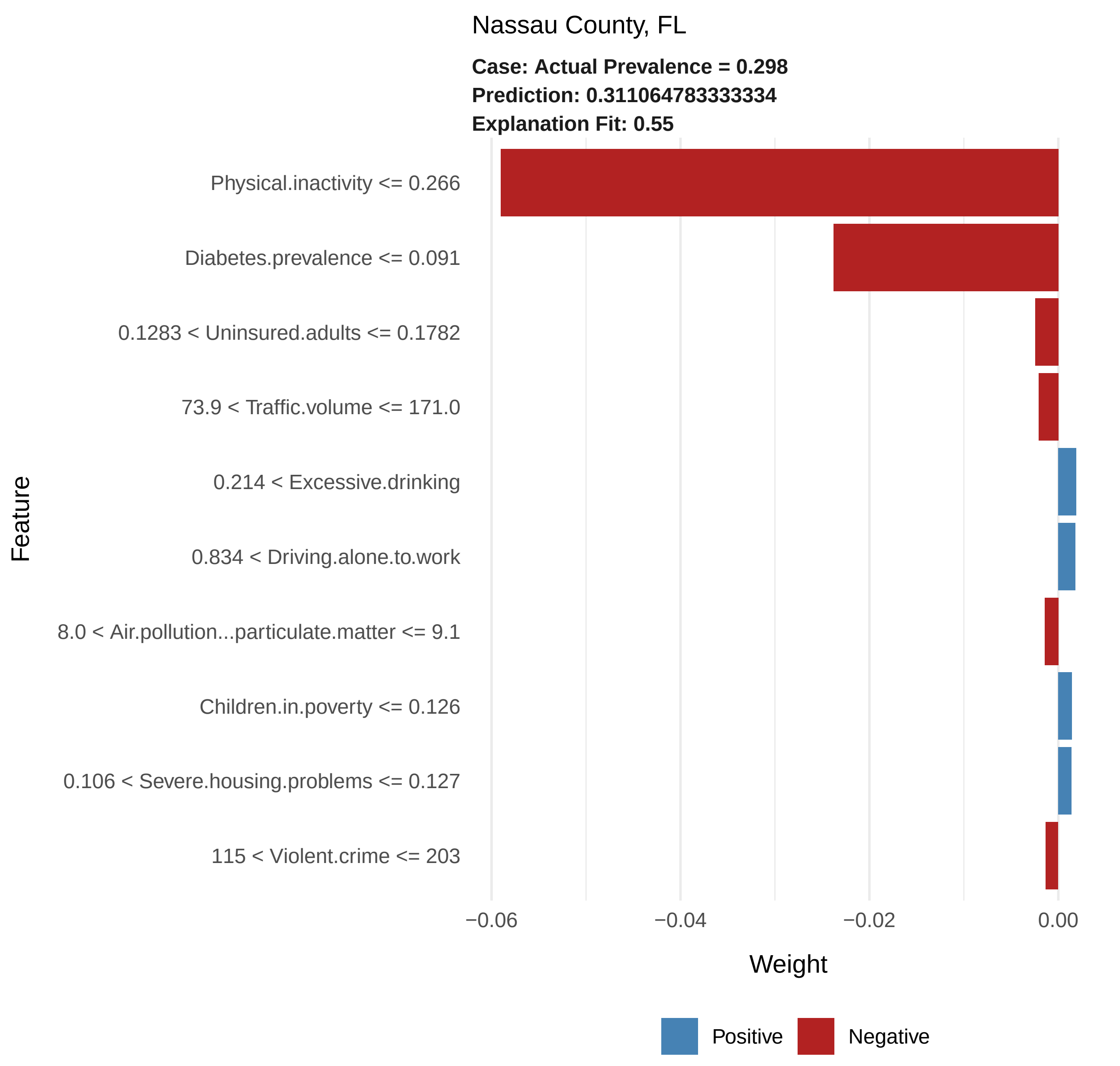
Nassau County, FL
Case: Actual Prevalence = 0.298
Prediction: 0.311064783333334
Explanation Fit: 0.55
Physical.inactivity <= 0.266
Diabetes.prevalence <= 0.091
0.1283 < Uninsured.adults <= 0.1782
73.9 < Traffic.volume <= 171.0
0.214 < Excessive.drinking
0.834 < Driving.alone.to.work
8.0 < Air.pollution...particulate.matter <= 9.1
Children.in.poverty <= 0.126
0.106 < Severe.housing.problems <= 0.127
115 < Violent.crime <= 203
Feature
−0.06
−0.04
−0.02
0.00
Weight
Positive
Negative

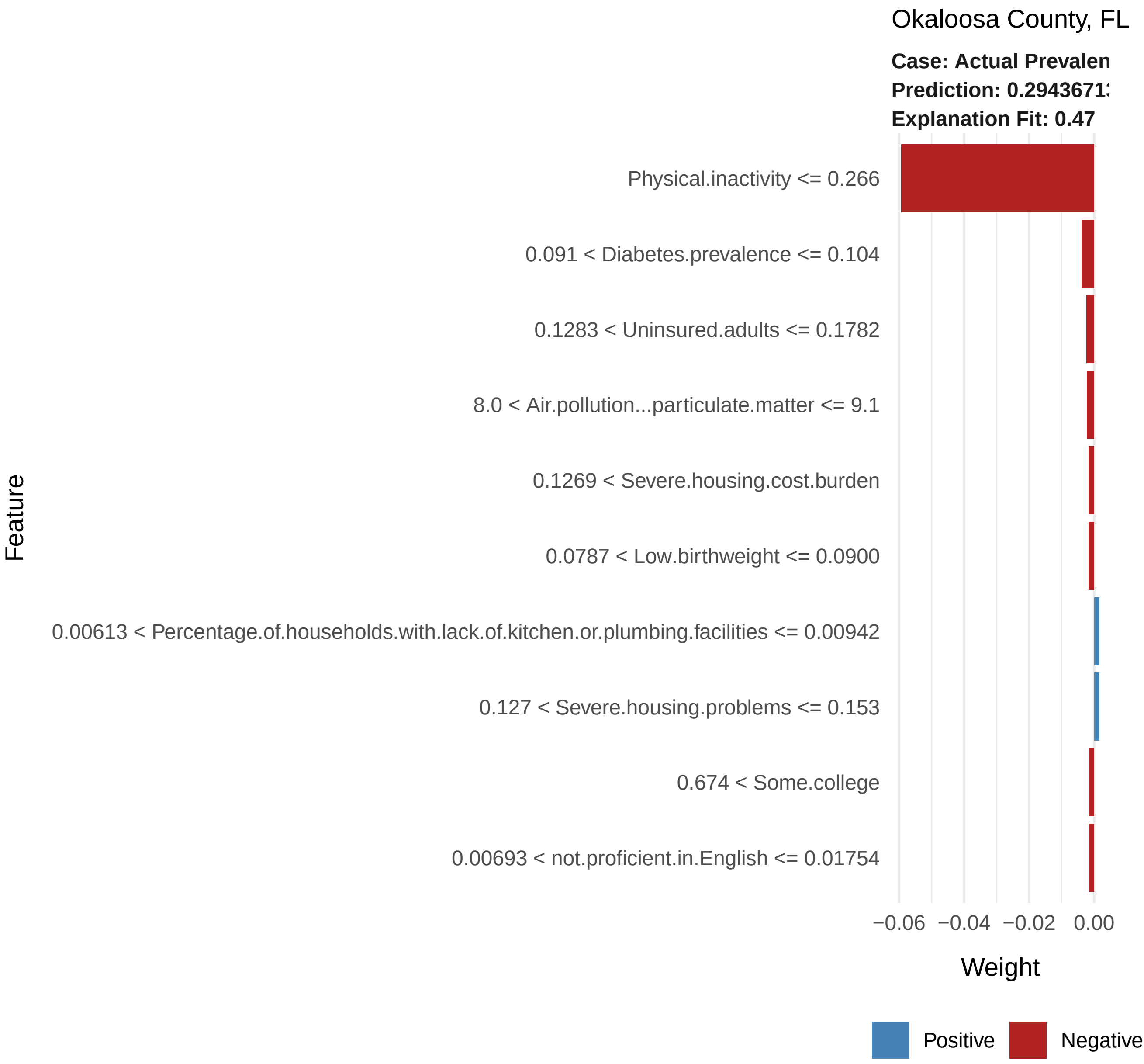

Okaloosa County, FL
Case: Actual Prevalen
Prediction: 0.2943671
Explanation Fit: 0.47
Physical.inactivity <= 0.266
0.091 < Diabetes.prevalence <= 0.104
0.1283 < Uninsured.adults <= 0.1782
8.0 < Air.pollution...particulate.matter <= 9.1
0.1269 < Severe.housing.cost.burden
0.0787 < Low.birthweight <= 0.0900
0.00613 < Percentage.of.households.with.lack.of.kitchen.or.plumbing.facilities <= 0.00942
0.127 < Severe.housing.problems <= 0.153
0.674 < Some.college
0.00693 < not.proficient.in.English <= 0.01754
Feature
−0.06
−0.04
−0.02
0.00
Weight
Positive
Negative

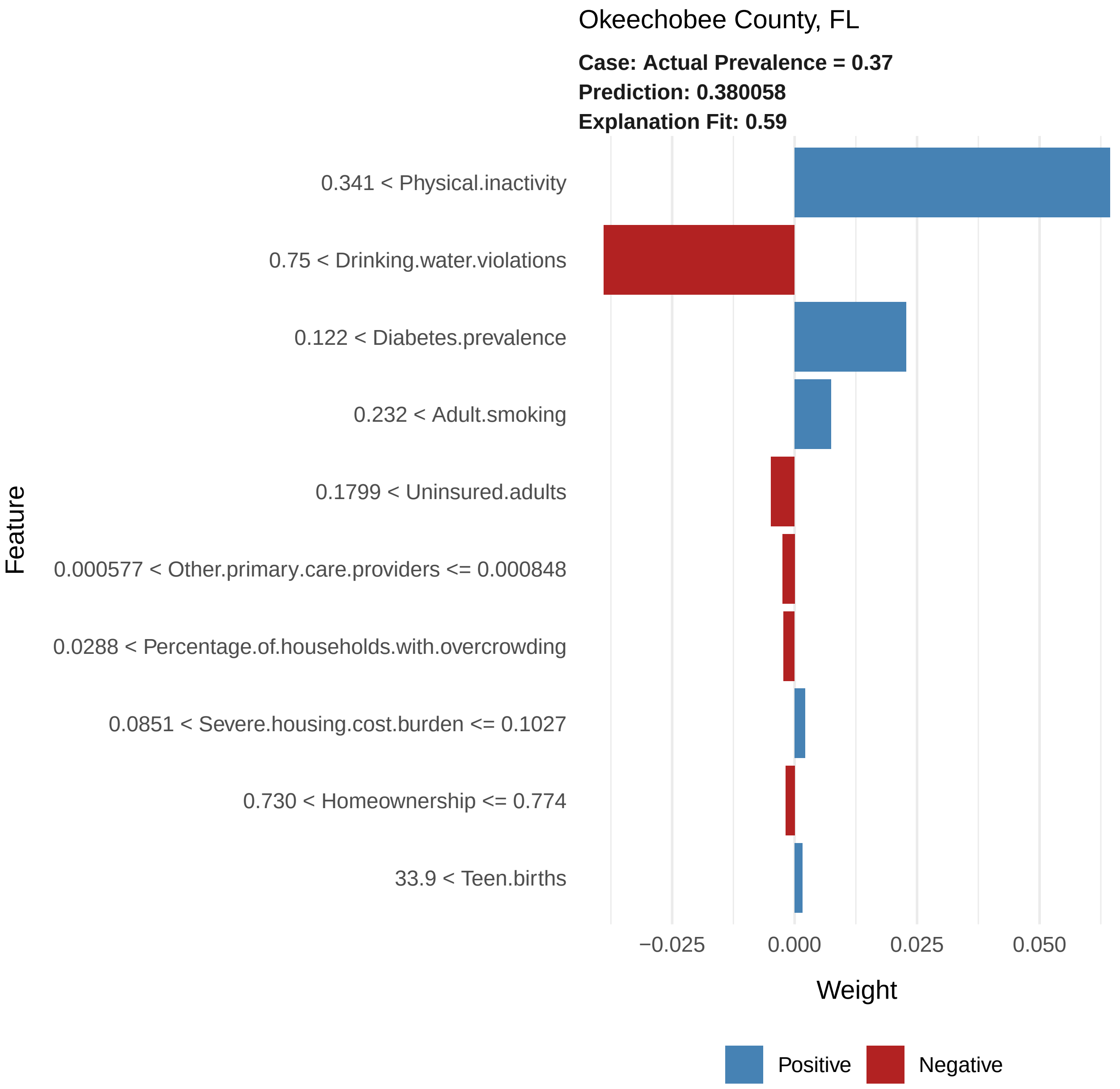

Okeechobee County, FL
Case: Actual Prevalence = 0.37
Prediction: 0.380058
Explanation Fit: 0.59
0.341 < Physical.inactivity
0.75 < Drinking.water.violations
0.122 < Diabetes.prevalence
0.232 < Adult.smoking
0.1799 < Uninsured.adults
0.000577 < Other.primary.care.providers <= 0.000848
0.0288 < Percentage.of.households.with.overcrowding
0.0851 < Severe.housing.cost.burden <= 0.1027
0.730 < Homeownership <= 0.774
33.9 < Teen.births
Feature
−0.025
0.000
0.025
0.050
Weight
Positive
Negative

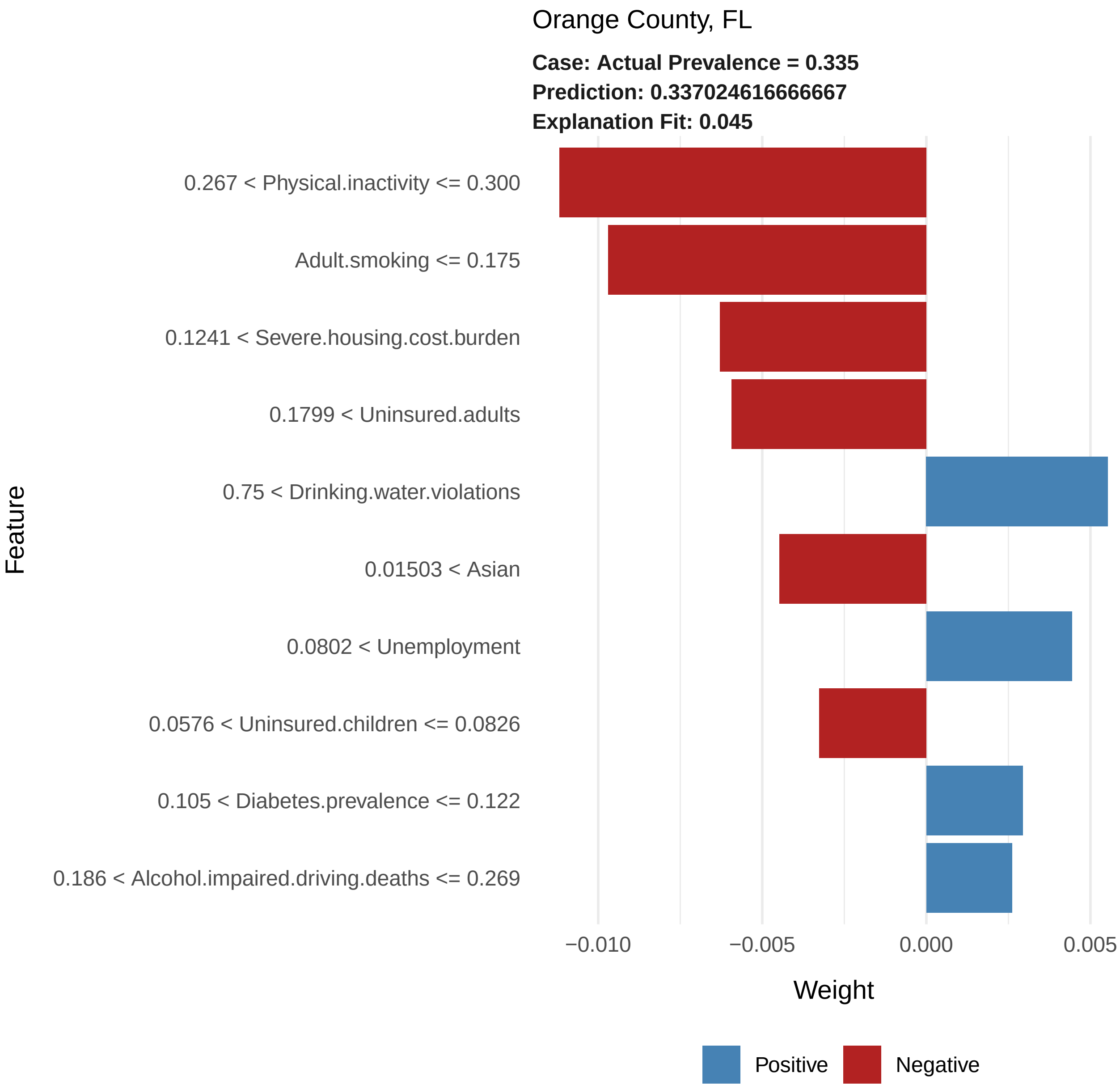
Orange County, FL
Case: Actual Prevalence = 0.335
Prediction: 0.337024616666667
Explanation Fit: 0.045
0.267 < Physical.inactivity <= 0.300
Adult.smoking <= 0.175
0.1241 < Severe.housing.cost.burden
0.1799 < Uninsured.adults
0.75 < Drinking.water.violations
0.01503 < Asian
0.0802 < Unemployment
0.0576 < Uninsured.children <= 0.0826
0.105 < Diabetes.prevalence <= 0.122
0.186 < Alcohol.impaired.driving.deaths <= 0.269
Feature
−0.010
−0.005
0.000
0.005
Weight
Positive
Negative

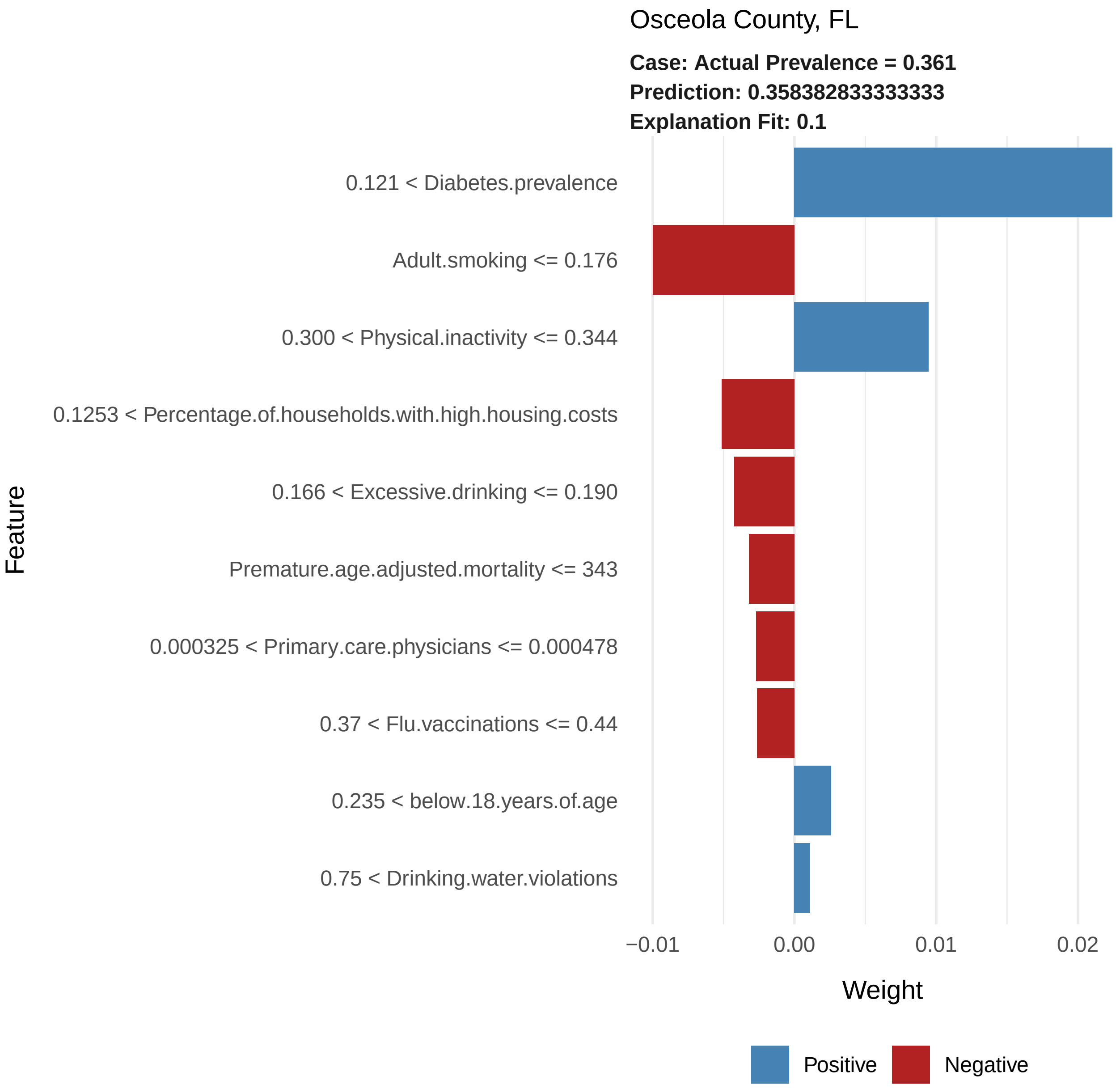
Osceola County, FL
Case: Actual Prevalence = 0.361
Prediction: 0.358382833333333
Explanation Fit: 0.1
0.121 < Diabetes.prevalence
Adult.smoking <= 0.176
0.300 < Physical.inactivity <= 0.344
0.1253 < Percentage.of.households.with.high.housing.costs
0.166 < Excessive.drinking <= 0.190
Premature.age.adjusted.mortality <= 343
0.000325 < Primary.care.physicians <= 0.000478
0.37 < Flu.vaccinations <= 0.44
0.235 < below.18.years.of.age
0.75 < Drinking.water.violations
Feature
−0.01
0.00
0.01
0.02
Weight
Positive
Negative

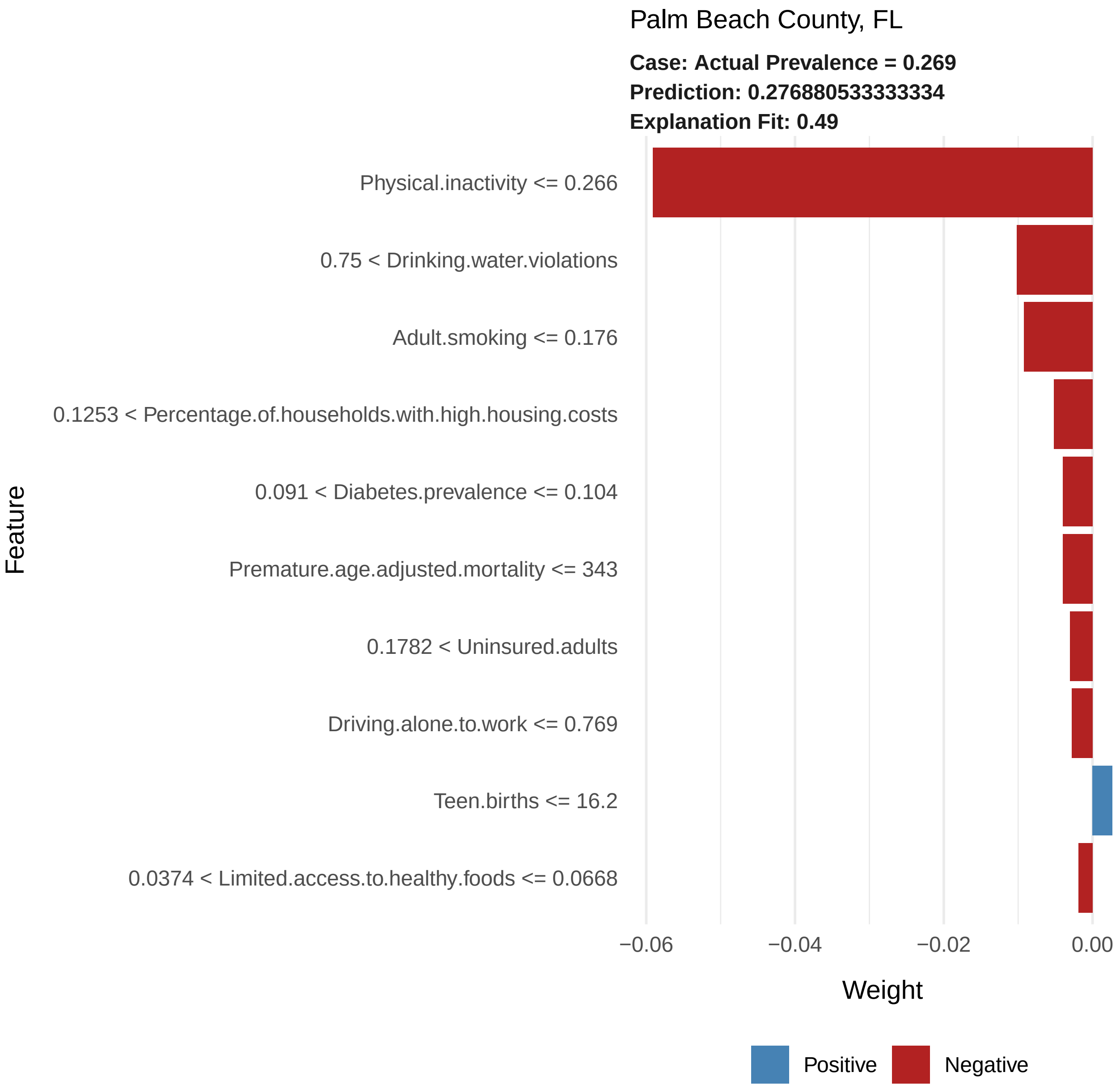
Palm Beach County, FL
Case: Actual Prevalence = 0.269
Prediction: 0.276880533333334
Explanation Fit: 0.49
Physical.inactivity <= 0.266
0.75 < Drinking.water.violations
Adult.smoking <= 0.176
0.1253 < Percentage.of.households.with.high.housing.costs
0.091 < Diabetes.prevalence <= 0.104
Premature.age.adjusted.mortality <= 343
0.1782 < Uninsured.adults
Driving.alone.to.work <= 0.769
Teen.births <= 16.2
0.0374 < Limited.access.to.healthy.foods <= 0.0668
Feature
−0.06
−0.04
−0.02
0.00
Weight
Positive
Negative

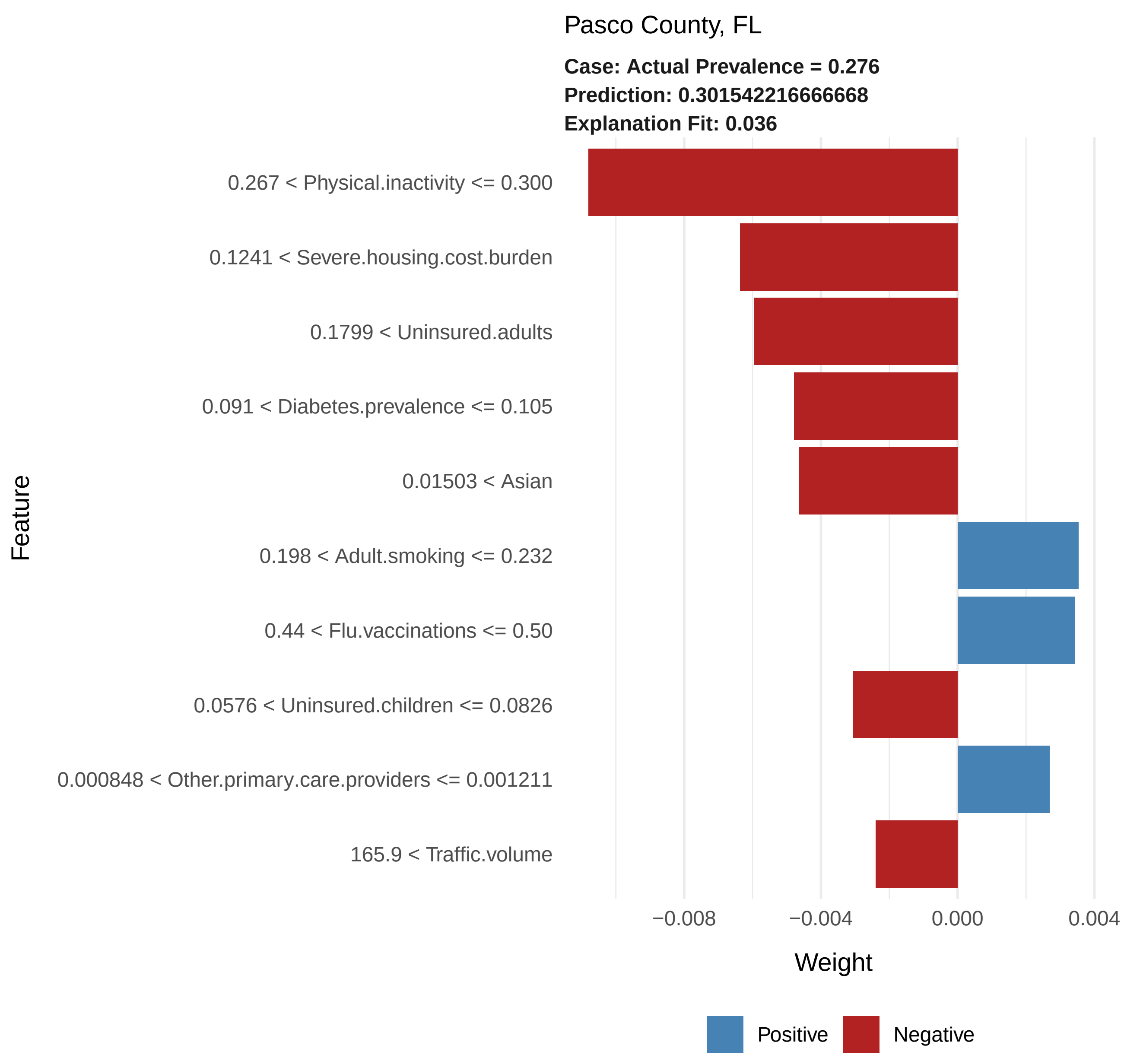

Pasco County, FL
Case: Actual Prevalence = 0.276
Prediction: 0.301542216666668
Explanation Fit: 0.036
0.267 < Physical.inactivity <= 0.300
0.1241 < Severe.housing.cost.burden
0.1799 < Uninsured.adults
0.091 < Diabetes.prevalence <= 0.105
0.01503 < Asian
0.198 < Adult.smoking <= 0.232
0.44 < Flu.vaccinations <= 0.50
0.0576 < Uninsured.children <= 0.0826
0.000848 < Other.primary.care.providers <= 0.001211
165.9 < Traffic.volume
Feature
−0.008
−0.004
0.000
0.004
Weight
Positive
Negative

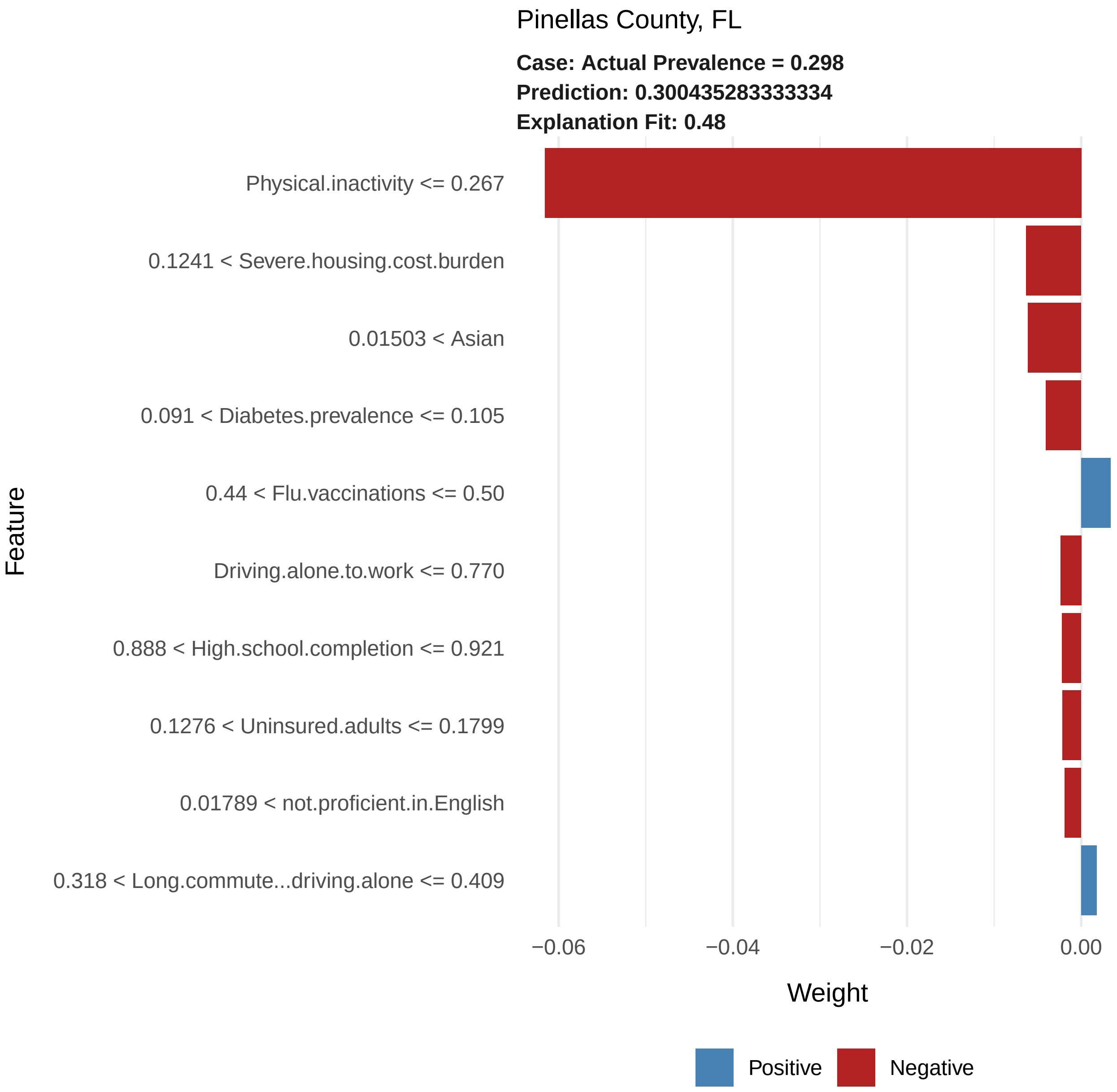
Pinellas County, FL
Case: Actual Prevalence = 0.298
Prediction: 0.300435283333334
Explanation Fit: 0.48
Physical.inactivity <= 0.267
0.1241 < Severe.housing.cost.burden
0.01503 < Asian
0.091 < Diabetes.prevalence <= 0.105
0.44 < Flu.vaccinations <= 0.50
Driving.alone.to.work <= 0.770
0.888 < High.school.completion <= 0.921
0.1276 < Uninsured.adults <= 0.1799
0.01789 < not.proficient.in.English
0.318 < Long.commute...driving.alone <= 0.409
Feature
−0.06
−0.04
−0.02
0.00
Weight
Positive
Negative

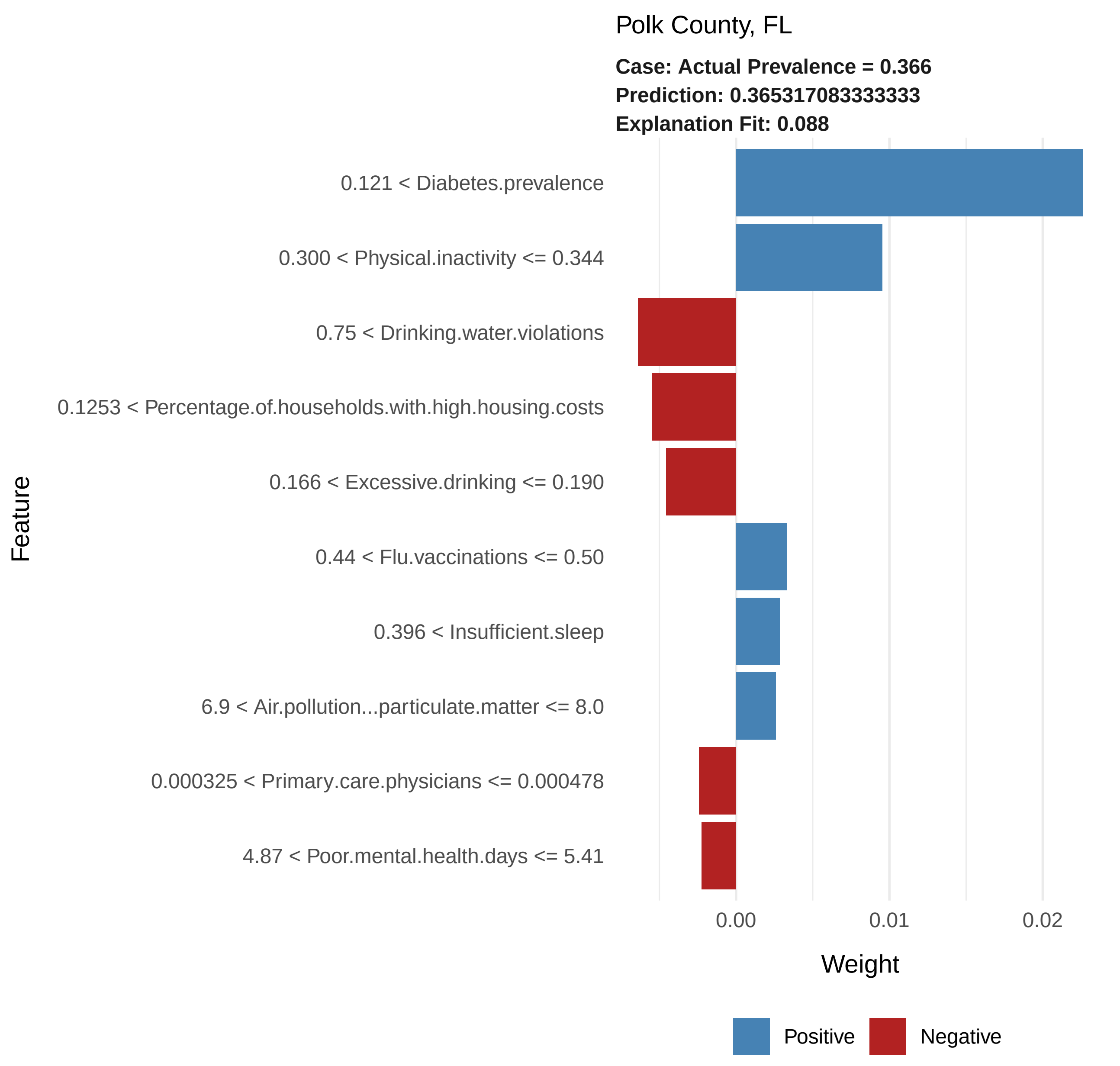

Polk County, FL
Case: Actual Prevalence = 0.366
Prediction: 0.365317083333333
Explanation Fit: 0.088
0.121 < Diabetes.prevalence
0.300 < Physical.inactivity <= 0.344
0.75 < Drinking.water.violations
0.1253 < Percentage.of.households.with.high.housing.costs
0.166 < Excessive.drinking <= 0.190
0.44 < Flu.vaccinations <= 0.50
0.396 < Insufficient.sleep
6.9 < Air.pollution...particulate.matter <= 8.0
0.000325 < Primary.care.physicians <= 0.000478
4.87 < Poor.mental.health.days <= 5.41
Feature
0.00
0.01
0.02
Weight
Positive
Negative

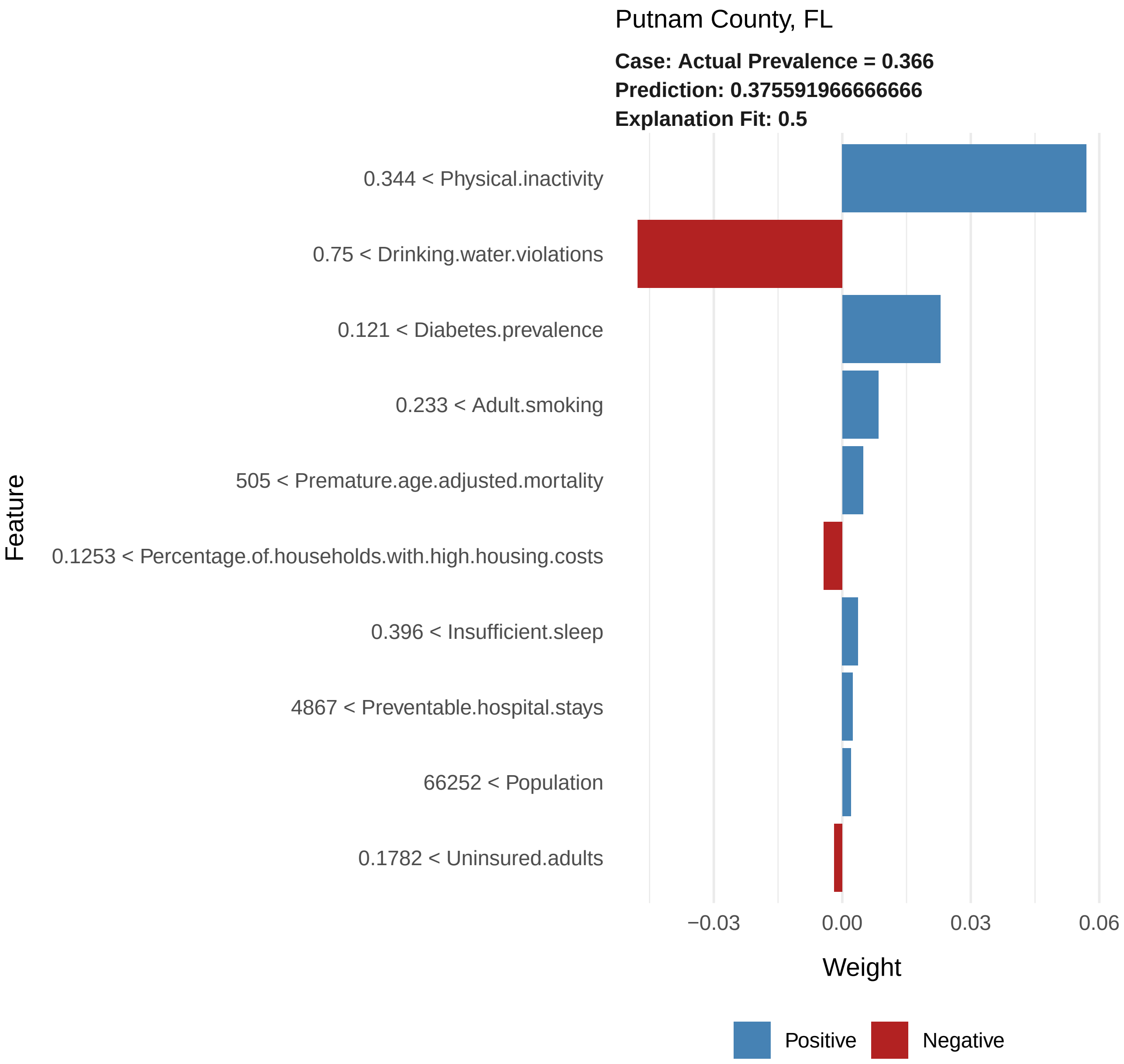

Putnam County, FL
Case: Actual Prevalence = 0.366
Prediction: 0.375591966666666
Explanation Fit: 0.5
0.344 < Physical.inactivity
0.75 < Drinking.water.violations
0.121 < Diabetes.prevalence
0.233 < Adult.smoking
505 < Premature.age.adjusted.mortality
0.1253 < Percentage.of.households.with.high.housing.costs
0.396 < Insufficient.sleep
4867 < Preventable.hospital.stays
66252 < Population
0.1782 < Uninsured.adults
Feature
−0.03
0.00
0.03
0.06
Weight
Positive
Negative

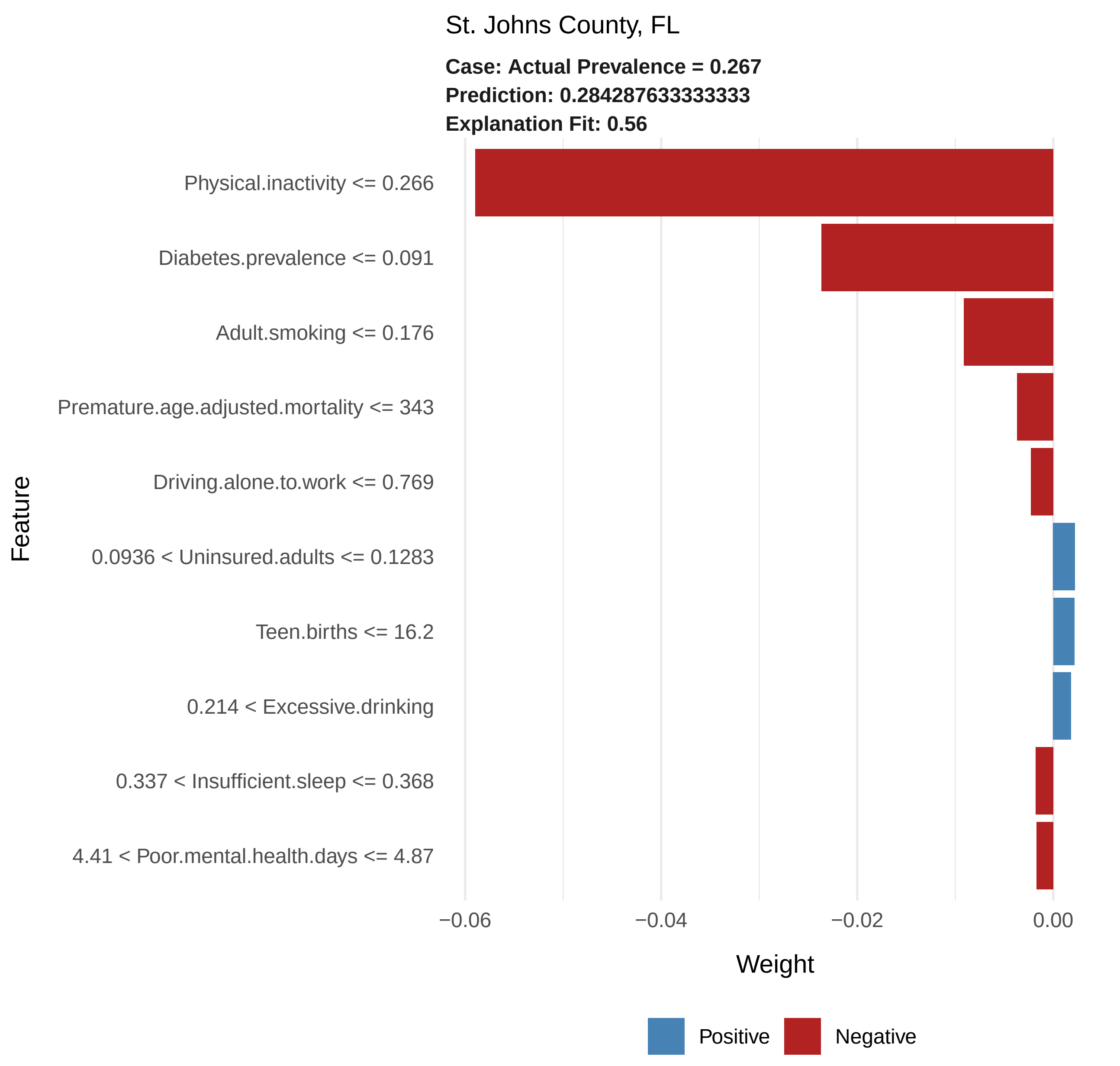

St. Johns County, FL
Case: Actual Prevalence = 0.267
Prediction: 0.284287633333333
Explanation Fit: 0.56
Physical.inactivity <= 0.266
Diabetes.prevalence <= 0.091
Adult.smoking <= 0.176
Premature.age.adjusted.mortality <= 343
Driving.alone.to.work <= 0.769
0.0936 < Uninsured.adults <= 0.1283
Teen.births <= 16.2
0.214 < Excessive.drinking
0.337 < Insufficient.sleep <= 0.368
4.41 < Poor.mental.health.days <= 4.87
Feature
−0.06
−0.04
−0.02
0.00
Weight
Positive
Negative

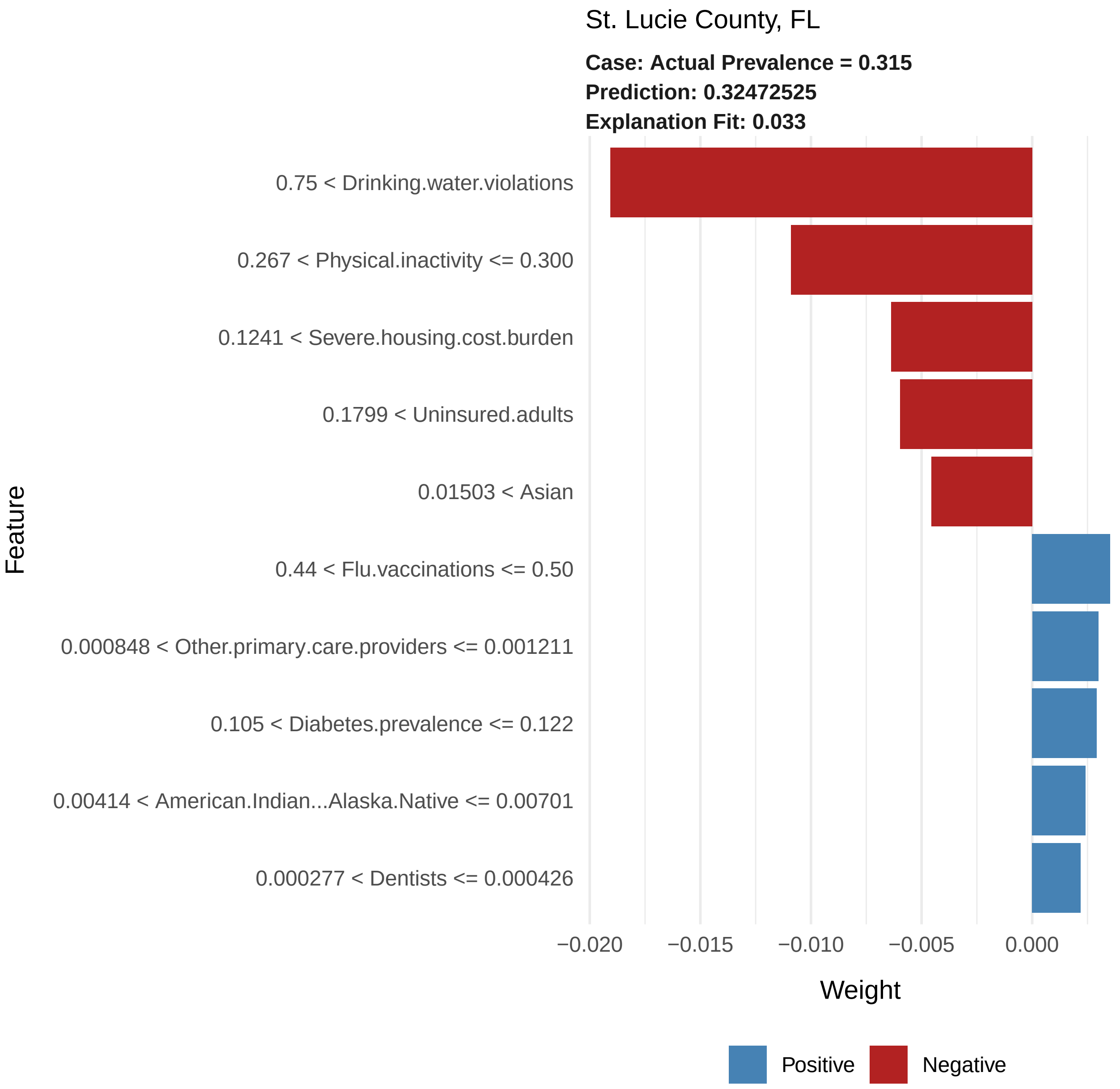
St. Lucie County, FL
Case: Actual Prevalence = 0.315
Prediction: 0.32472525
Explanation Fit: 0.033
Feature
0.75 < Drinking.water.violations
0.267 < Physical.inactivity <= 0.300
0.1241 < Severe.housing.cost.burden
0.1799 < Uninsured.adults
0.01503 < Asian
0.44 < Flu.vaccinations <= 0.50
0.000848 < Other.primary.care.providers <= 0.001211
0.105 < Diabetes.prevalence <= 0.122
0.00414 < American.Indian...Alaska.Native <= 0.00701
0.000277 < Dentists <= 0.000426
−0.020
−0.015
−0.010
−0.005
0.000
Weight
Positive
Negative

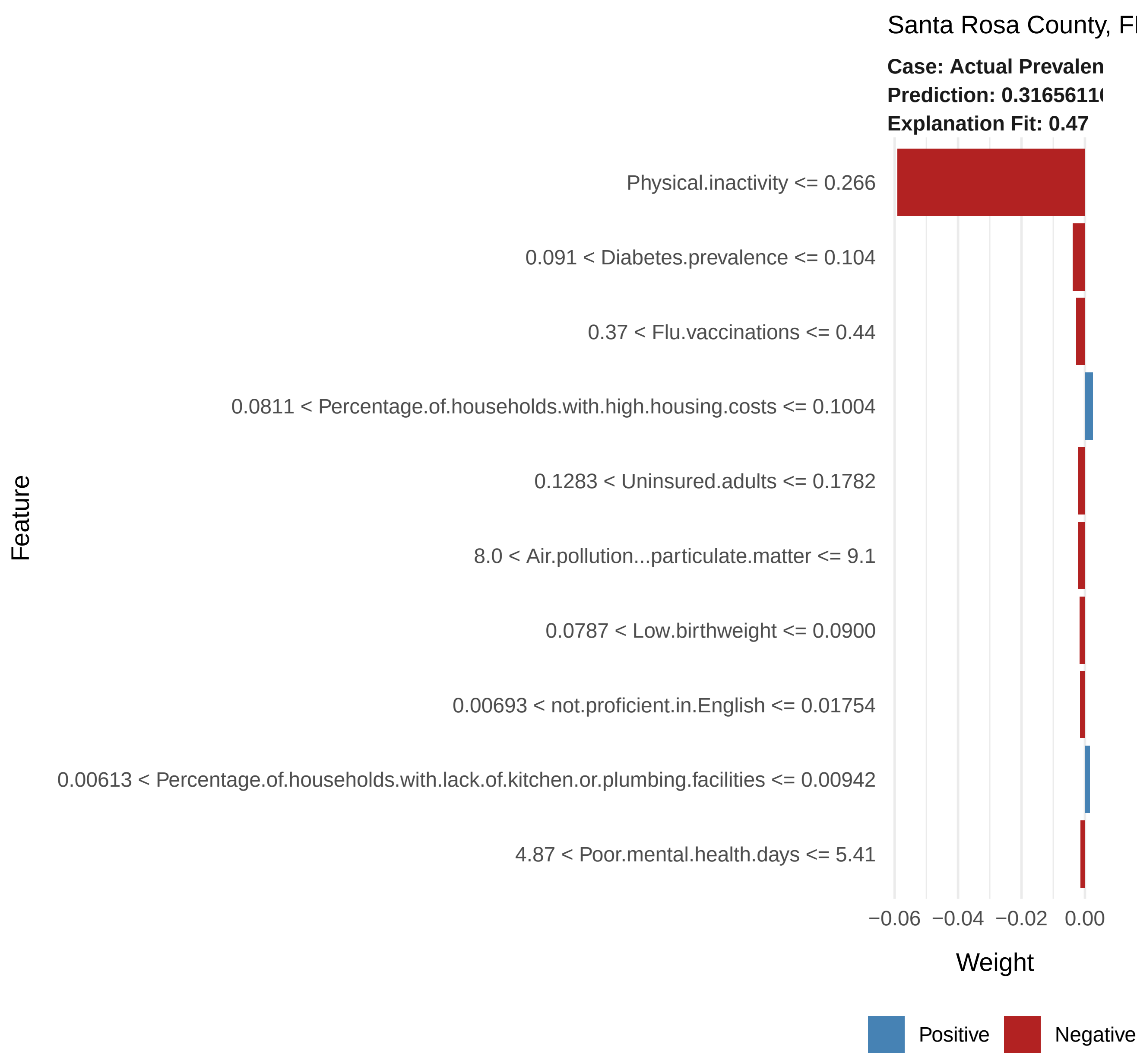

Santa Rosa County, Fl
Case: Actual Prevalen
Prediction: 0.3165611(
Explanation Fit: 0.47
Physical.inactivity <= 0.266
0.091 < Diabetes.prevalence <= 0.104
0.37 < Flu.vaccinations <= 0.44
0.0811 < Percentage.of.households.with.high.housing.costs <= 0.1004
0.1283 < Uninsured.adults <= 0.1782
8.0 < Air.pollution...particulate.matter <= 9.1
0.0787 < Low.birthweight <= 0.0900
0.00693 < not.proficient.in.English <= 0.01754
0.00613 < Percentage.of.households.with.lack.of.kitchen.or.plumbing.facilities <= 0.00942
4.87 < Poor.mental.health.days <= 5.41
Feature
−0.06
−0.04
−0.02
0.00
Weight
Positive
Negative

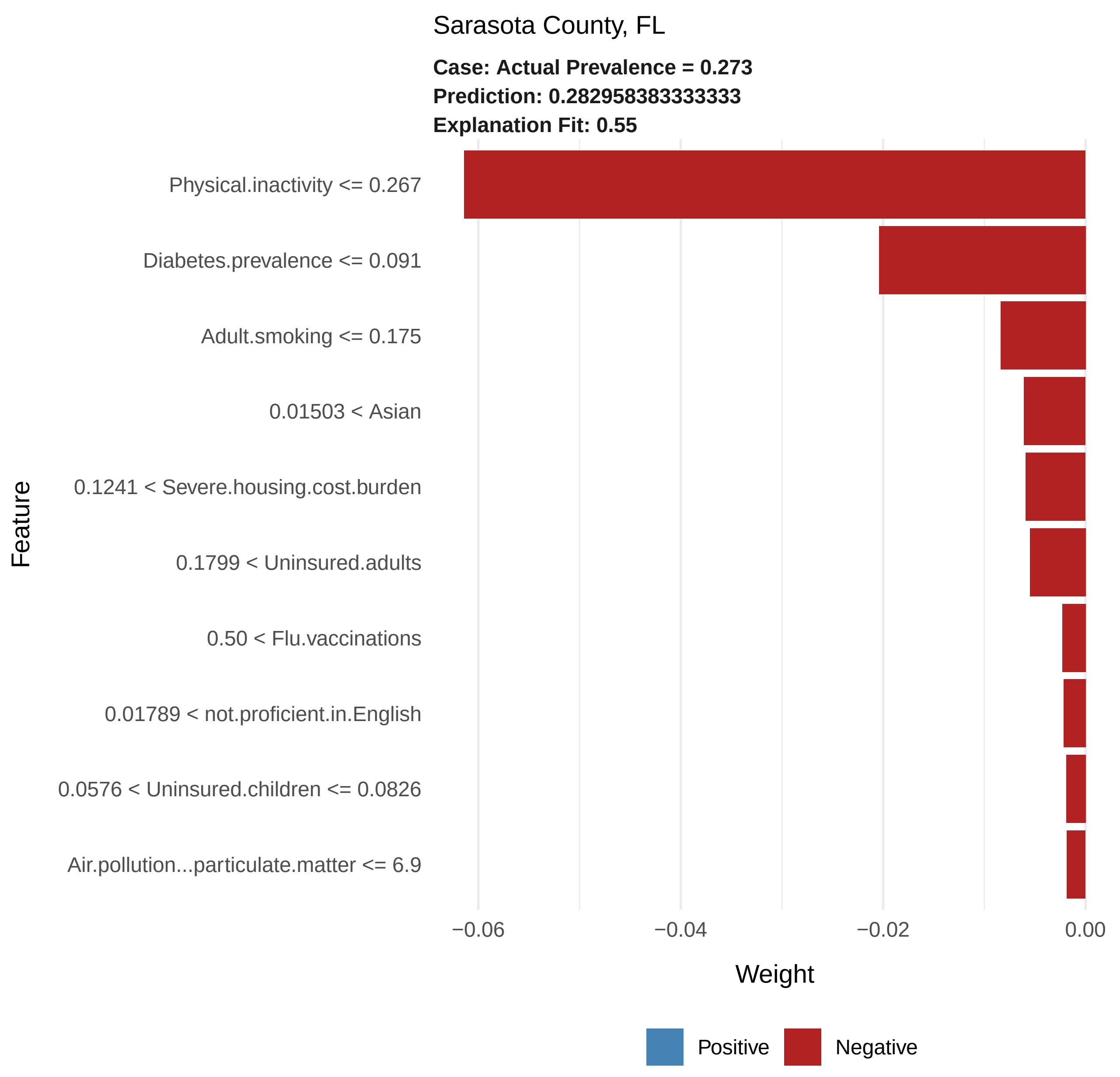

Sarasota County, FL
Case: Actual Prevalence = 0.273
Prediction: 0.282958383333333
Explanation Fit: 0.55
Physical.inactivity <= 0.267
Diabetes.prevalence <= 0.091
Adult.smoking <= 0.175
0.01503 < Asian
0.1241 < Severe.housing.cost.burden
0.1799 < Uninsured.adults
0.50 < Flu.vaccinations
0.01789 < not.proficient.in.English
0.0576 < Uninsured.children <= 0.0826
Air.pollution...particulate.matter <= 6.9
Feature
−0.06
−0.04
−0.02
0.00
Weight
Positive
Negative

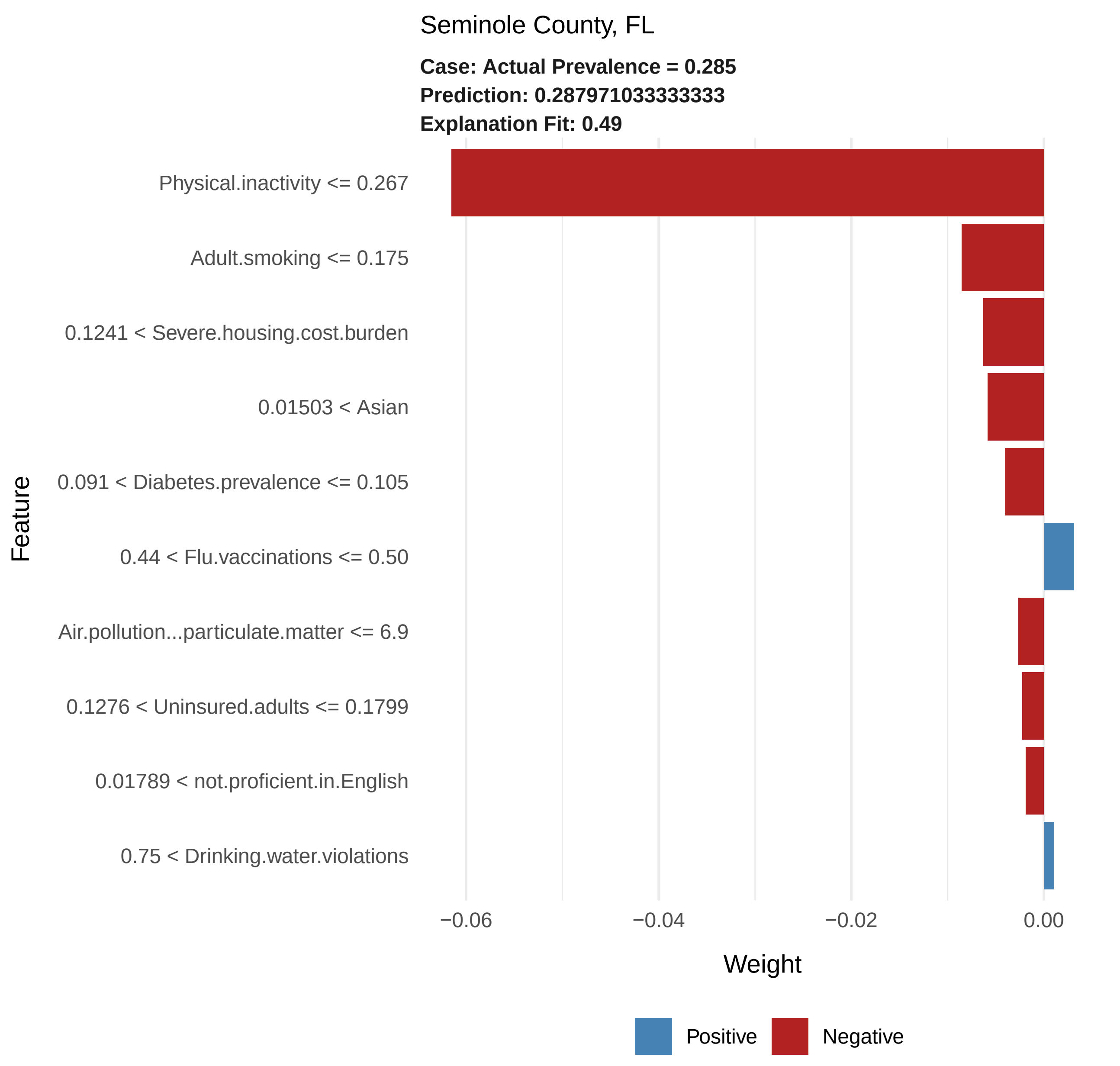
Seminole County, FL
Case: Actual Prevalence = 0.285
Prediction: 0.287971033333333
Explanation Fit: 0.49
Physical.inactivity <= 0.267
Adult.smoking <= 0.175
0.1241 < Severe.housing.cost.burden
0.01503 < Asian
0.091 < Diabetes.prevalence <= 0.105
0.44 < Flu.vaccinations <= 0.50
Air.pollution...particulate.matter <= 6.9
0.1276 < Uninsured.adults <= 0.1799
0.01789 < not.proficient.in.English
0.75 < Drinking.water.violations
Feature
−0.06
−0.04
−0.02
0.00
Weight
Positive
Negative

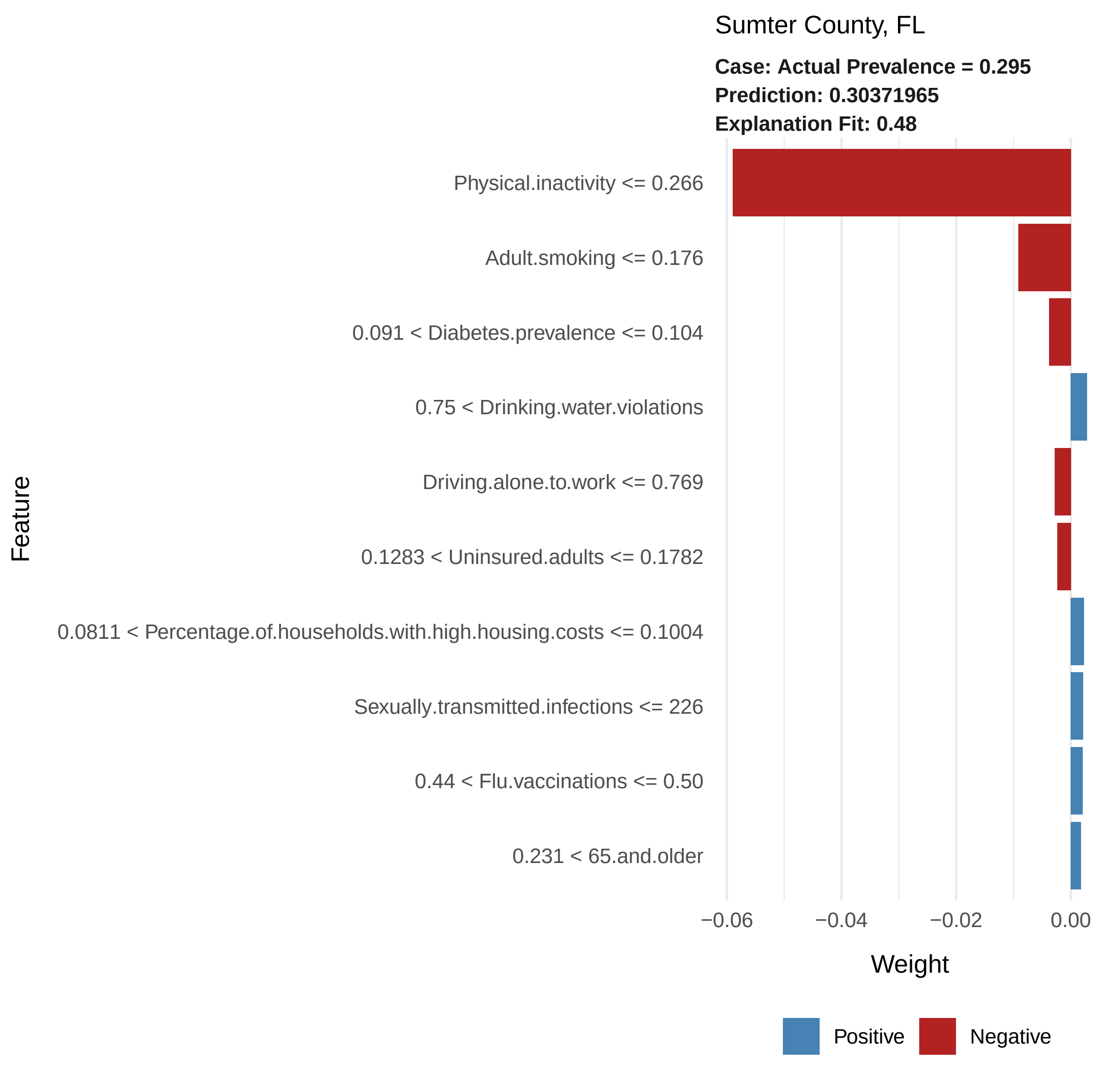

Sumter County, FL
Case: Actual Prevalence = 0.295
Prediction: 0.30371965
Explanation Fit: 0.48
Physical.inactivity <= 0.266
Adult.smoking <= 0.176
0.091 < Diabetes.prevalence <= 0.104
0.75 < Drinking.water.violations
Driving.alone.to.work <= 0.769
0.1283 < Uninsured.adults <= 0.1782
0.0811 < Percentage.of.households.with.high.housing.costs <= 0.1004
Sexually.transmitted.infections <= 226
0.44 < Flu.vaccinations <= 0.50
0.231 < 65.and.older
Feature
−0.06
−0.04
−0.02
0.00
Weight
Positive
Negative

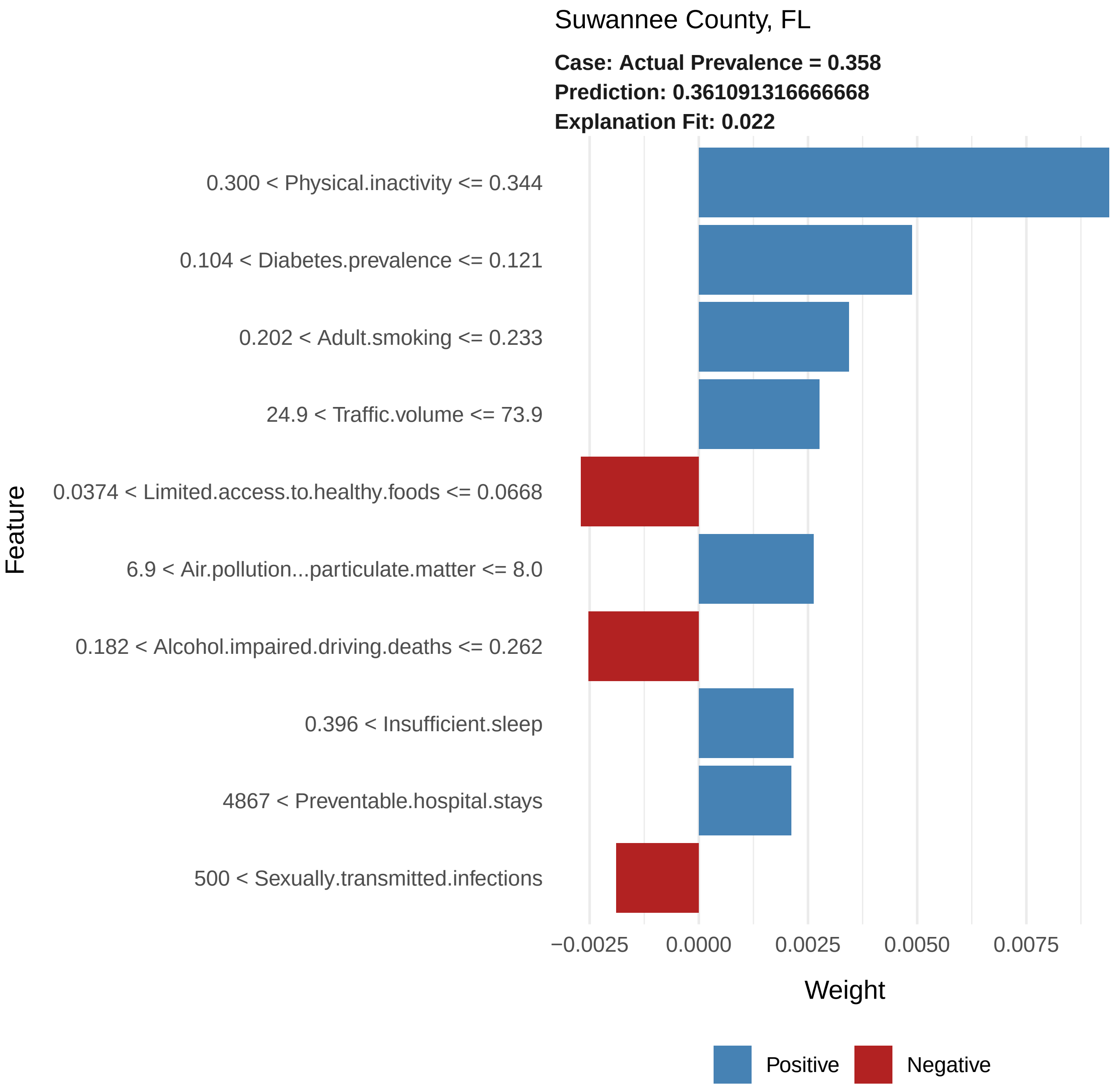

Suwannee County, FL
Case: Actual Prevalence = 0.358
Prediction: 0.361091316666668
Explanation Fit: 0.022
0.300 < Physical.inactivity <= 0.344
0.104 < Diabetes.prevalence <= 0.121
0.202 < Adult.smoking <= 0.233
24.9 < Traffic.volume <= 73.9
0.0374 < Limited.access.to.healthy.foods <= 0.0668
6.9 < Air.pollution...particulate.matter <= 8.0
0.182 < Alcohol.impaired.driving.deaths <= 0.262
0.396 < Insufficient.sleep
4867 < Preventable.hospital.stays
500 < Sexually.transmitted.infections
Feature
−0.0025
0.0000
0.0025
0.0050
0.0075
Weight
Positive
Negative

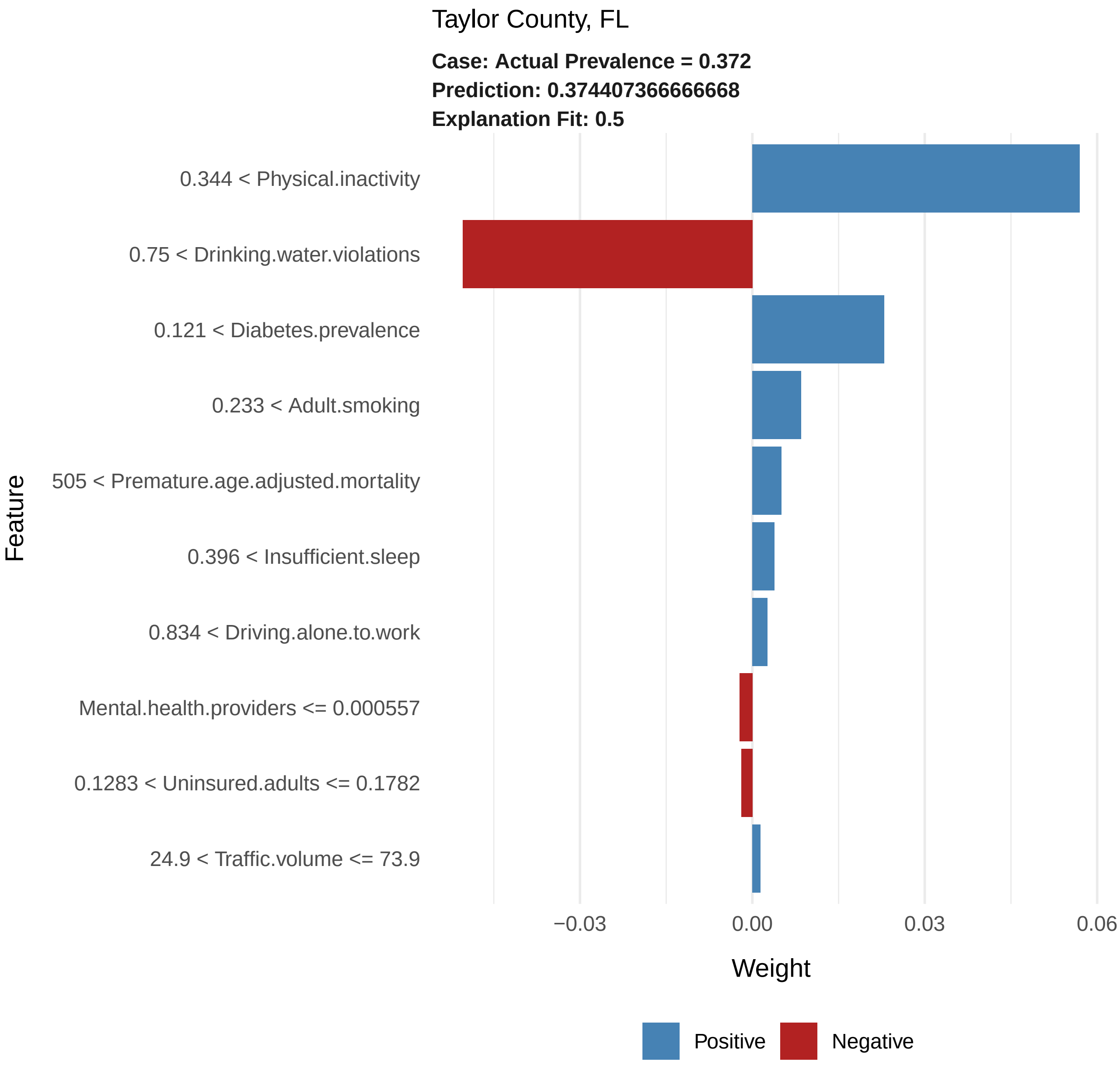
Taylor County, FL
Case: Actual Prevalence = 0.372
Prediction: 0.374407366666668
Explanation Fit: 0.5
Feature
0.344 < Physical.inactivity
0.75 < Drinking.water.violations
0.121 < Diabetes.prevalence
0.233 < Adult.smoking
505 < Premature.age.adjusted.mortality
0.396 < Insufficient.sleep
0.834 < Driving.alone.to.work
Mental.health.providers <= 0.000557
0.1283 < Uninsured.adults <= 0.1782
24.9 < Traffic.volume <= 73.9
−0.03
0.00
0.03
0.06
Weight
Positive
Negative

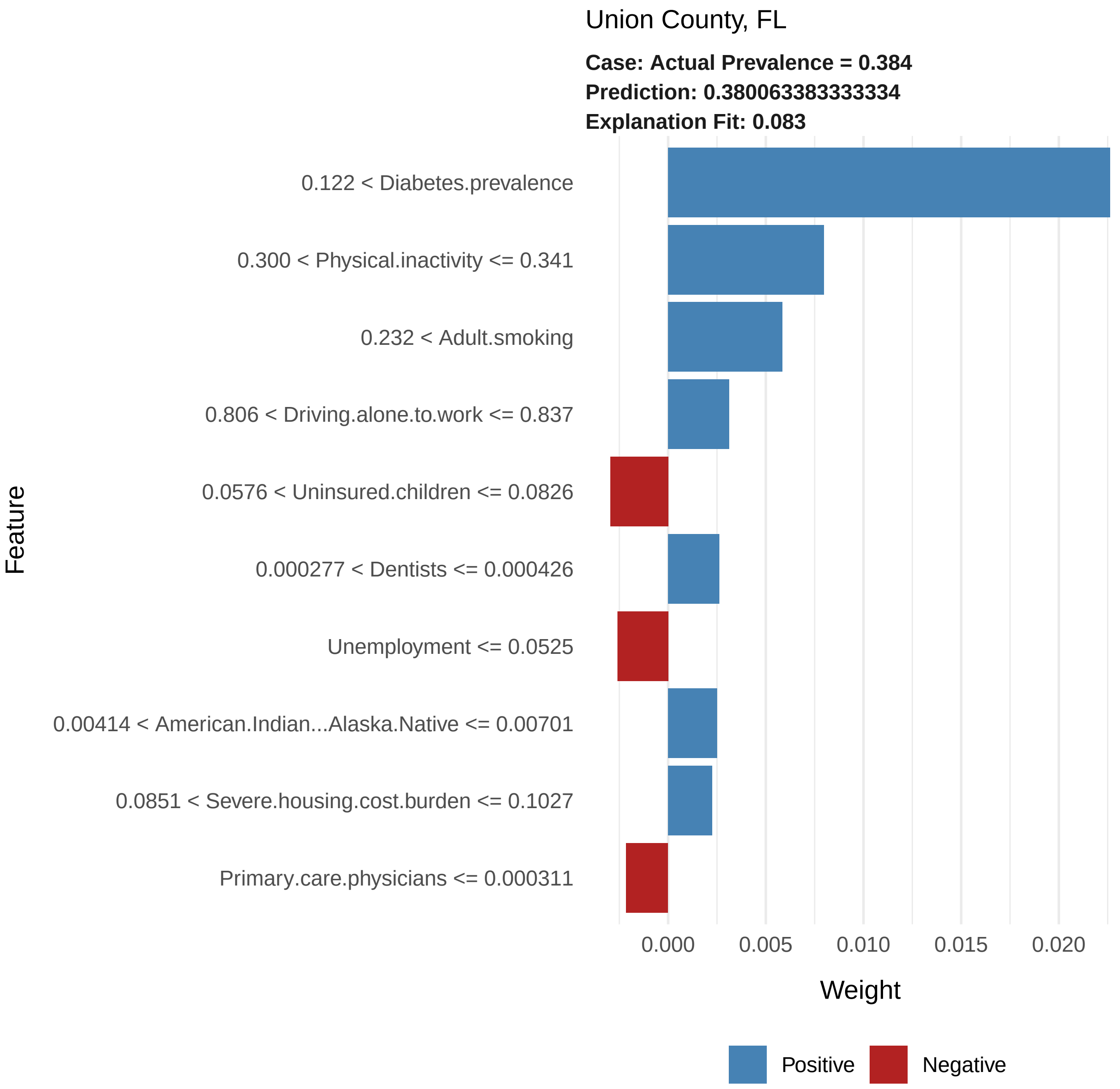

Union County, FL
Case: Actual Prevalence = 0.384
Prediction: 0.380063383333334
Explanation Fit: 0.083
0.122 < Diabetes.prevalence
0.300 < Physical.inactivity <= 0.341
0.232 < Adult.smoking
0.806 < Driving.alone.to.work <= 0.837
0.0576 < Uninsured.children <= 0.0826
0.000277 < Dentists <= 0.000426
Unemployment <= 0.0525
0.00414 < American.Indian...Alaska.Native <= 0.00701
0.0851 < Severe.housing.cost.burden <= 0.1027
Primary.care.physicians <= 0.000311
Feature
0.000
0.005
0.010
0.015
0.020
Weight
Positive
Negative

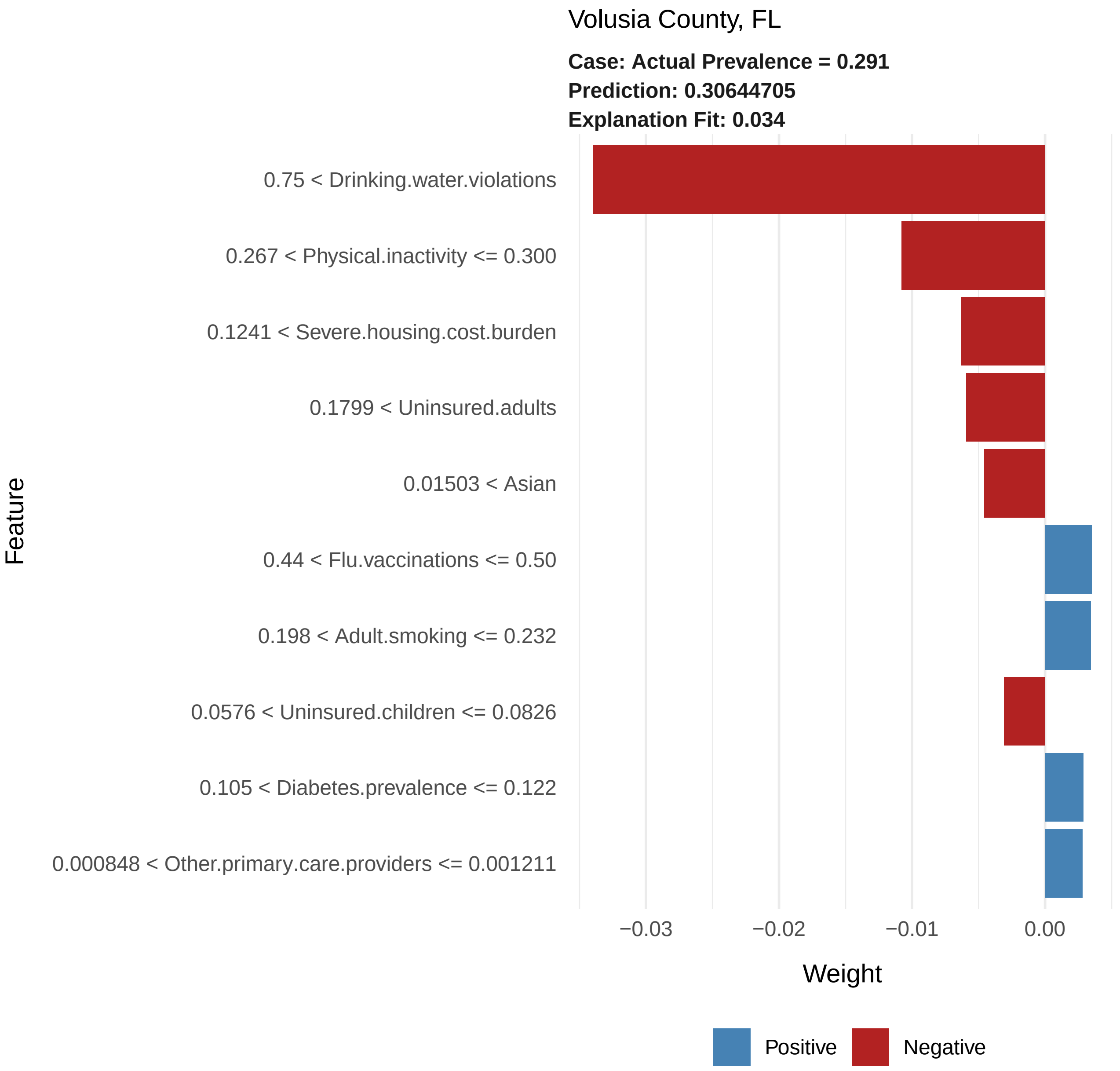
Volusia County, FL
Case: Actual Prevalence = 0.291
Prediction: 0.30644705
Explanation Fit: 0.034
0.75 < Drinking.water.violations
0.267 < Physical.inactivity <= 0.300
0.1241 < Severe.housing.cost.burden
0.1799 < Uninsured.adults
0.01503 < Asian
0.44 < Flu.vaccinations <= 0.50
0.198 < Adult.smoking <= 0.232
0.0576 < Uninsured.children <= 0.0826
0.105 < Diabetes.prevalence <= 0.122
0.000848 < Other.primary.care.providers <= 0.001211
Feature
−0.03
−0.02
−0.01
0.00
Weight
Positive
Negative

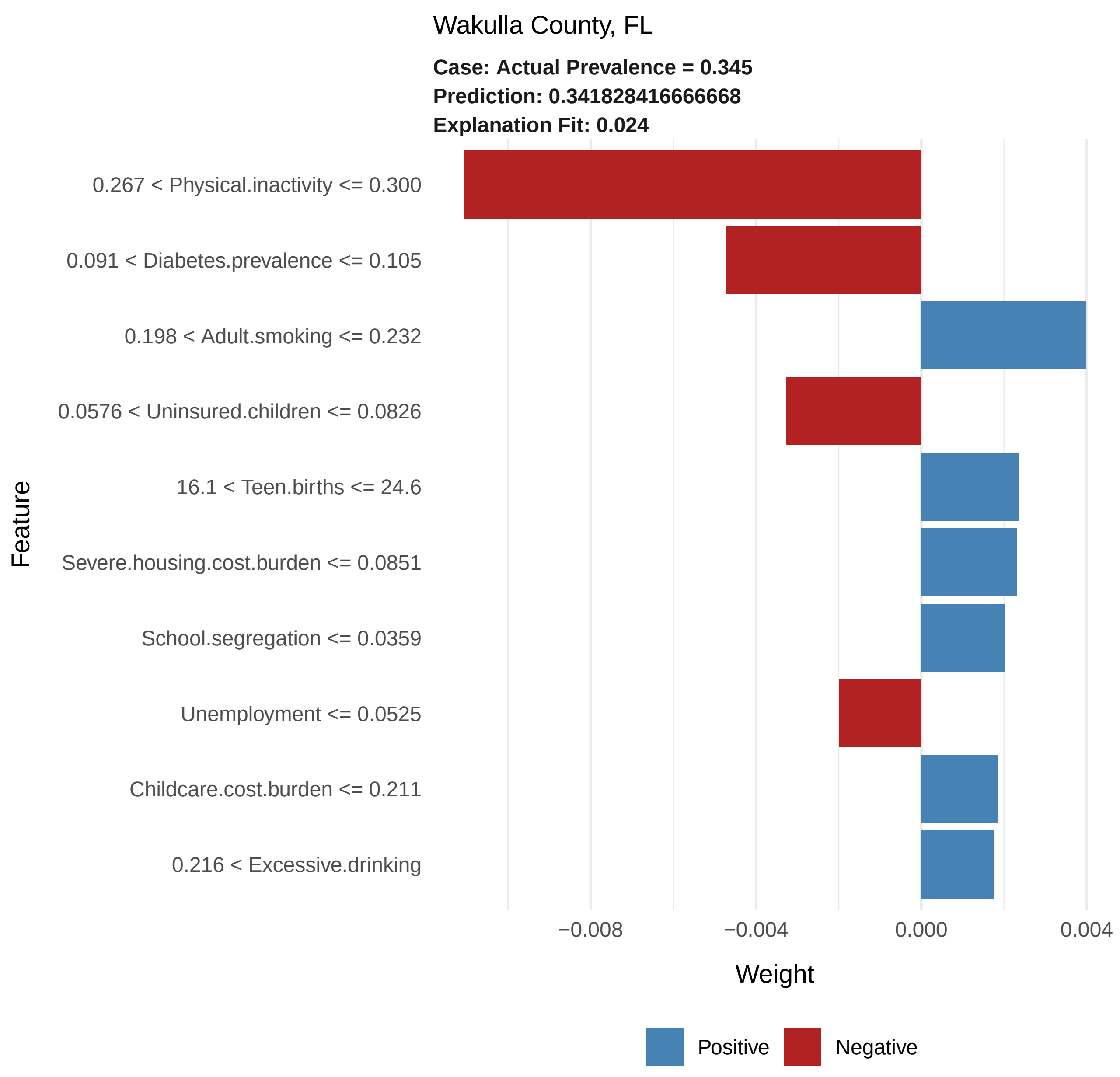
Wakulla County, FL
Case: Actual Prevalence = 0.345
Prediction: 0.341828416666668
Explanation Fit: 0.024
0.267 < Physical.inactivity <= 0.300
0.091 < Diabetes.prevalence <= 0.105
0.198 < Adult.smoking <= 0.232
0.0576 < Uninsured.children <= 0.0826
16.1 < Teen.births <= 24.6
Severe.housing.cost.burden <= 0.0851
School.segregation <= 0.0359
Unemployment <= 0.0525
Childcare.cost.burden <= 0.211
0.216 < Excessive.drinking
Feature
−0.008
−0.004
0.000
0.004
Weight
Positive
Negative

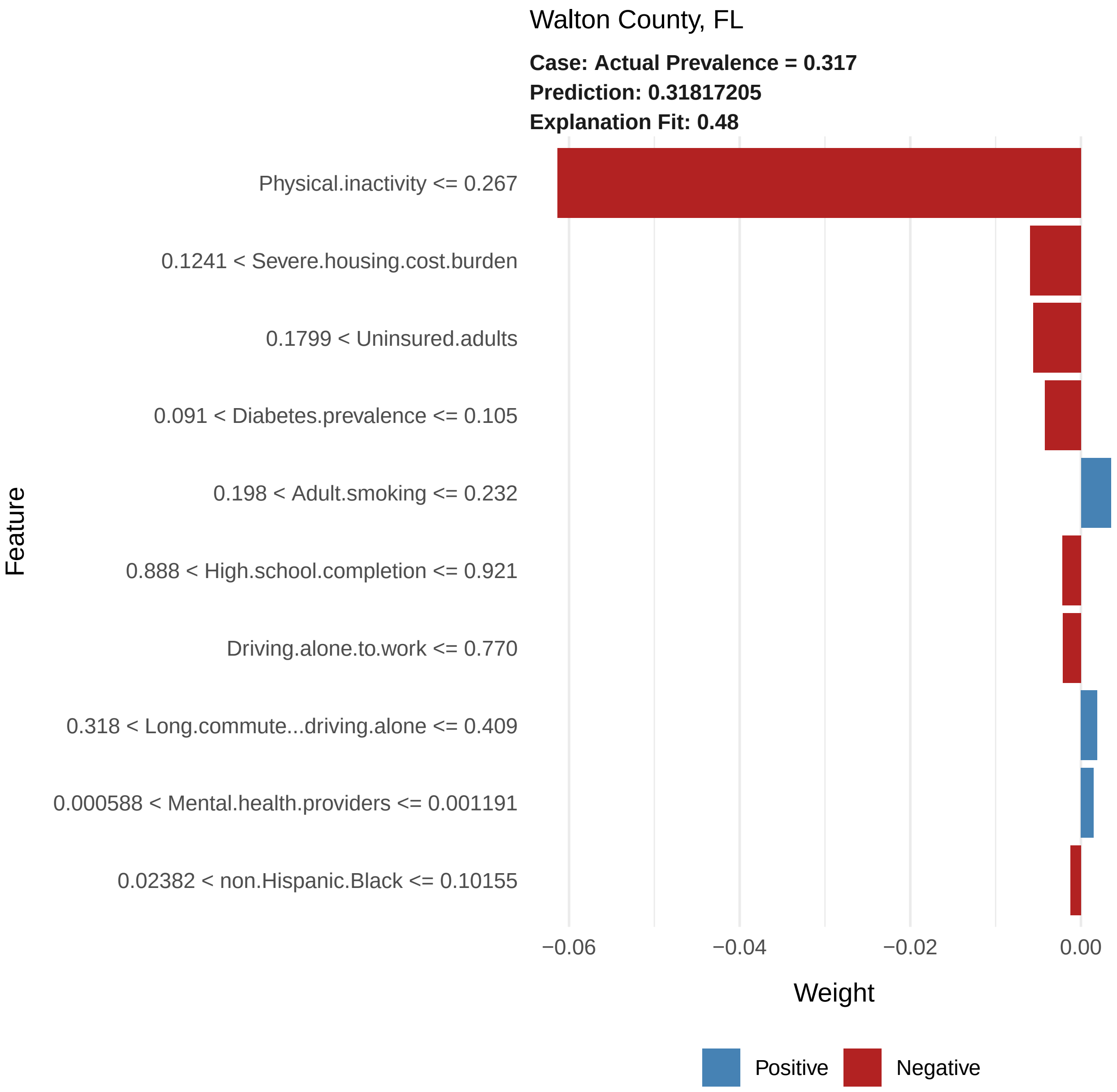
Walton County, FL
Case: Actual Prevalence = 0.317
Prediction: 0.31817205
Explanation Fit: 0.48
Physical.inactivity <= 0.267
0.1241 < Severe.housing.cost.burden
0.1799 < Uninsured.adults
0.091 < Diabetes.prevalence <= 0.105
0.198 < Adult.smoking <= 0.232
0.888 < High.school.completion <= 0.921
Driving.alone.to.work <= 0.770
0.318 < Long.commute...driving.alone <= 0.409
0.000588 < Mental.health.providers <= 0.001191
0.02382 < non.Hispanic.Black <= 0.10155
Feature
−0.06
−0.04
−0.02
0.00
Weight
Positive
Negative

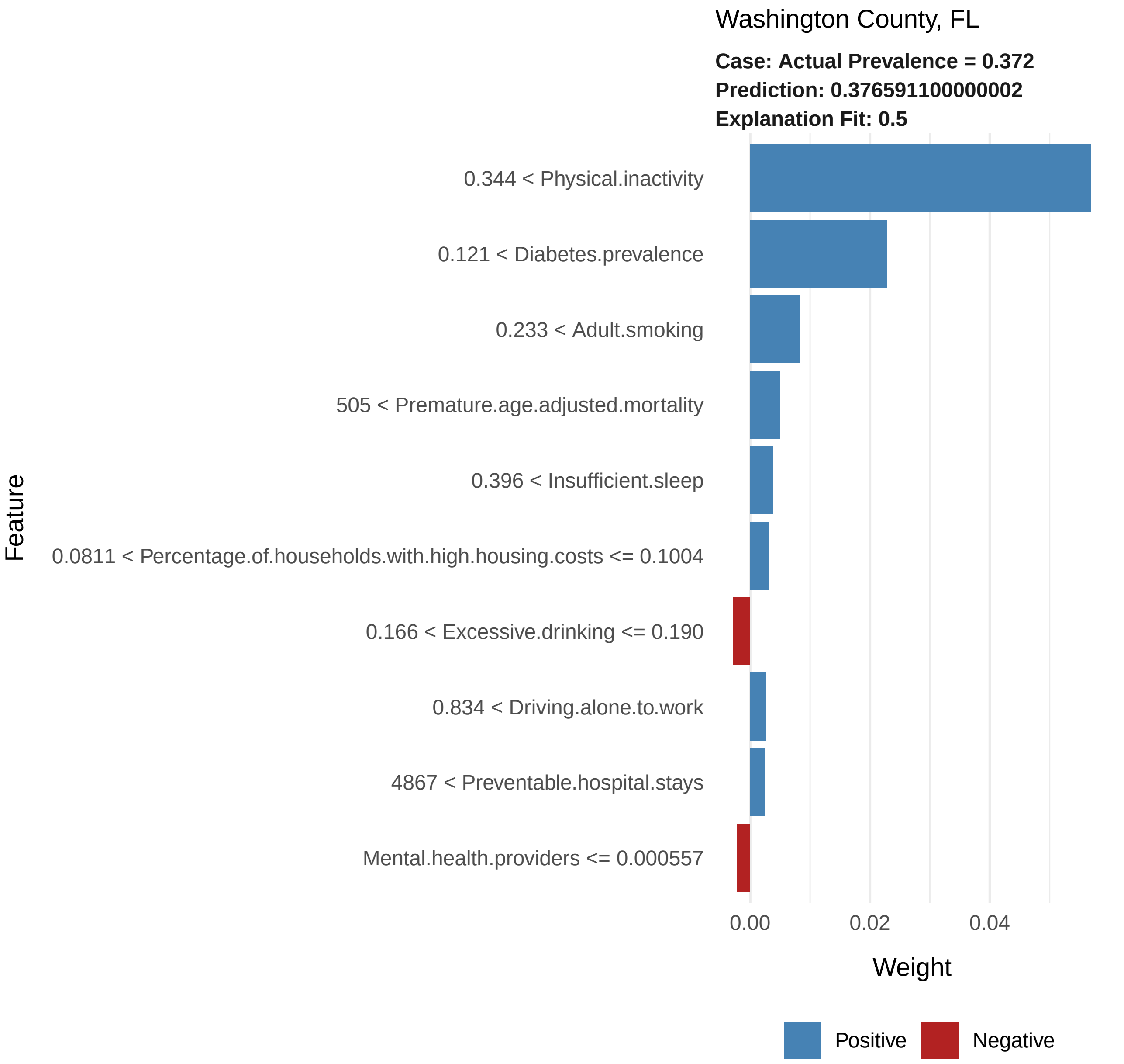

Washington County, FL
Case: Actual Prevalence = 0.372
Prediction: 0.376591100000002
Explanation Fit: 0.5
0.344 < Physical.inactivity
0.121 < Diabetes.prevalence
0.233 < Adult.smoking
505 < Premature.age.adjusted.mortality
0.396 < Insufficient.sleep
0.0811 < Percentage.of.households.with.high.housing.costs <= 0.1004
0.166 < Excessive.drinking <= 0.190
0.834 < Driving.alone.to.work
4867 < Preventable.hospital.stays
Mental.health.providers <= 0.000557
Feature
0.00
0.02
0.04
Weight
Positive
Negative

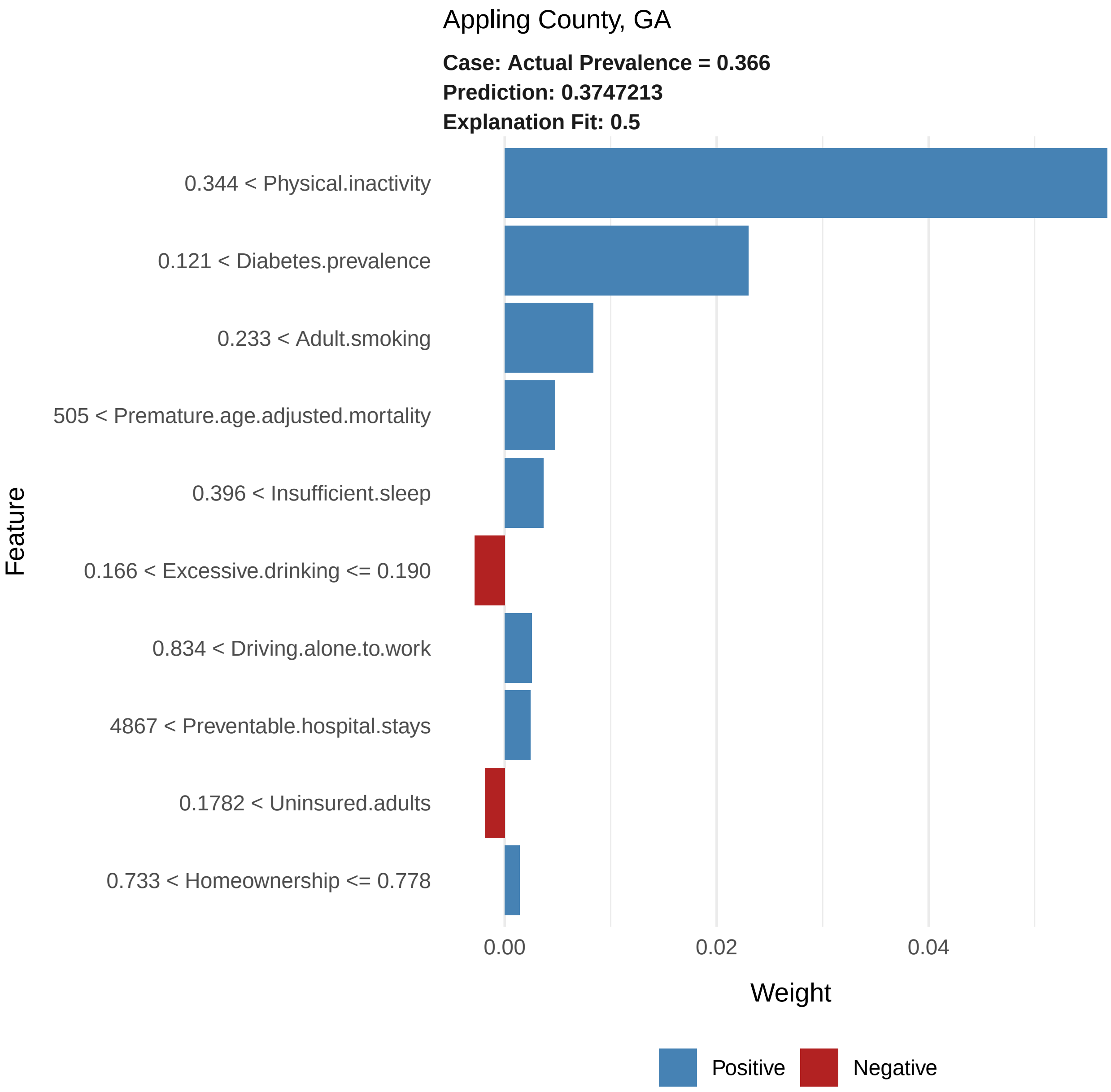

Appling County, GA
Case: Actual Prevalence = 0.366
Prediction: 0.3747213
Explanation Fit: 0.5
0.344 < Physical.inactivity
0.121 < Diabetes.prevalence
0.233 < Adult.smoking
505 < Premature.age.adjusted.mortality
0.396 < Insufficient.sleep
0.166 < Excessive.drinking <= 0.190
0.834 < Driving.alone.to.work
4867 < Preventable.hospital.stays
0.1782 < Uninsured.adults
0.733 < Homeownership <= 0.778
Feature
0.00
0.02
0.04
Weight
Positive
Negative

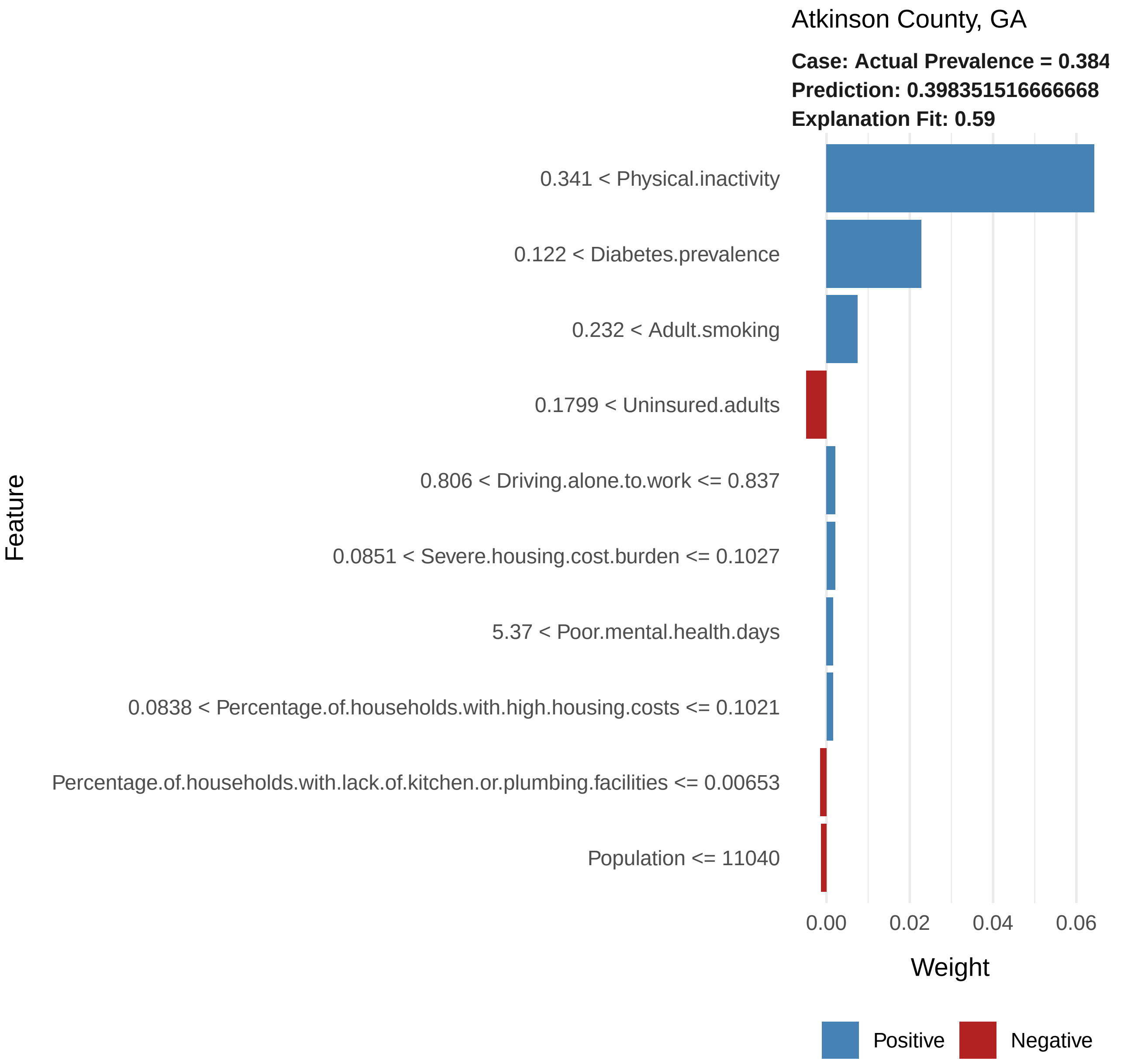

Atkinson County, GA
Case: Actual Prevalence = 0.384
Prediction: 0.398351516666668
Explanation Fit: 0.59
0.341 < Physical.inactivity
0.122 < Diabetes.prevalence
0.232 < Adult.smoking
0.1799 < Uninsured.adults
0.806 < Driving.alone.to.work <= 0.837
0.0851 < Severe.housing.cost.burden <= 0.1027
5.37 < Poor.mental.health.days
0.0838 < Percentage.of.households.with.high.housing.costs <= 0.1021
Percentage.of.households.with.lack.of.kitchen.or.plumbing.facilities <= 0.00653
Population <= 11040
Feature
0.00
0.02
0.04
0.06
Weight
Positive
Negative

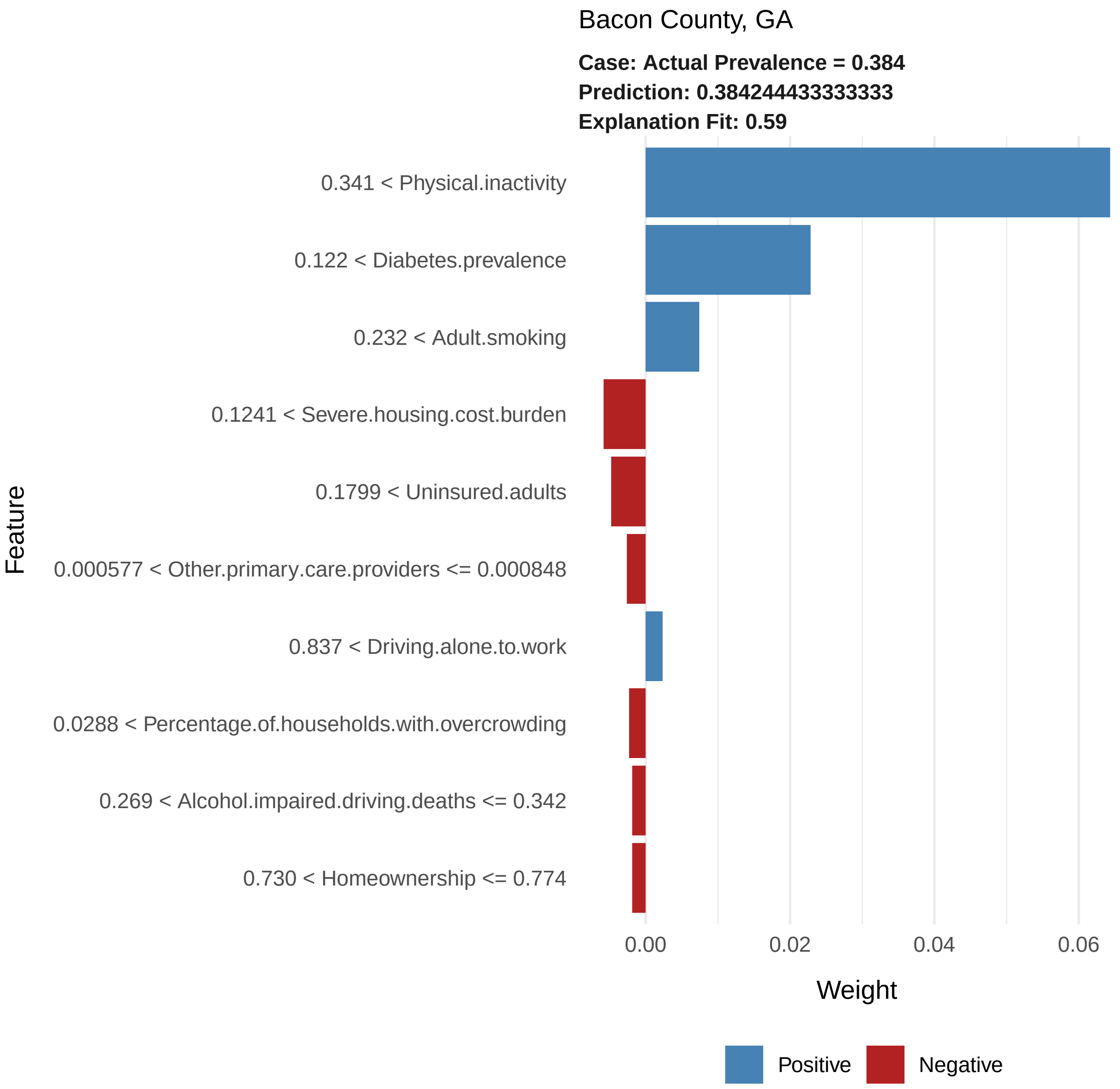
Bacon County, GA
Case: Actual Prevalence = 0.384
Prediction: 0.384244433333333
Explanation Fit: 0.59
0.341 < Physical.inactivity
0.122 < Diabetes.prevalence
0.232 < Adult.smoking
0.1241 < Severe.housing.cost.burden
0.1799 < Uninsured.adults
0.000577 < Other.primary.care.providers <= 0.000848
0.837 < Driving.alone.to.work
0.0288 < Percentage.of.households.with.overcrowding
0.269 < Alcohol.impaired.driving.deaths <= 0.342
0.730 < Homeownership <= 0.774
Feature
0.00
0.02
0.04
0.06
Weight
Positive
Negative

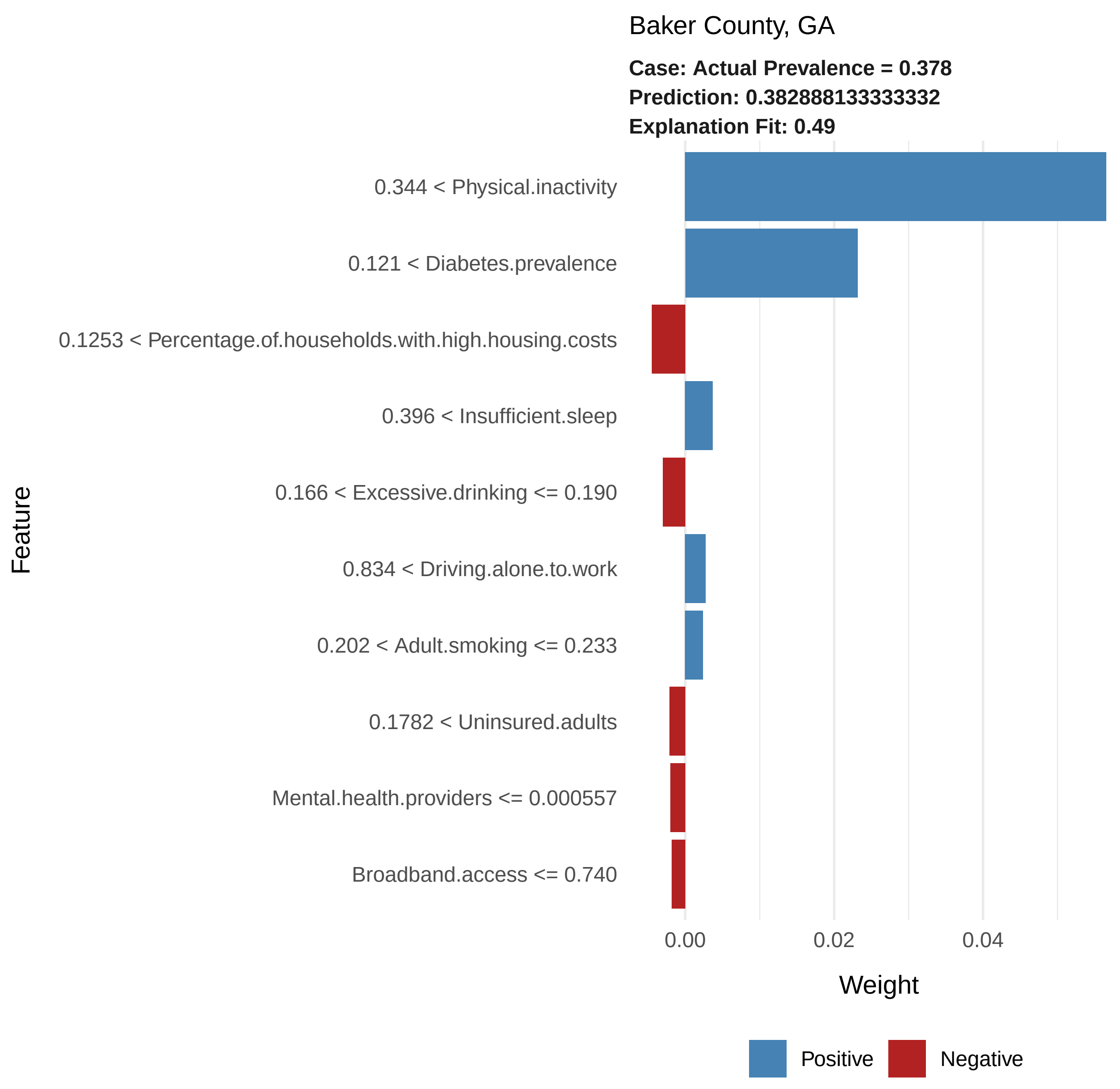

Baker County, GA
Case: Actual Prevalence = 0.378
Prediction: 0.382888133333332
Explanation Fit: 0.49
0.344 < Physical.inactivity
0.121 < Diabetes.prevalence
0.1253 < Percentage.of.households.with.high.housing.costs
0.396 < Insufficient.sleep
0.166 < Excessive.drinking <= 0.190
0.834 < Driving.alone.to.work
0.202 < Adult.smoking <= 0.233
0.1782 < Uninsured.adults
Mental.health.providers <= 0.000557
Broadband.access <= 0.740
Feature
0.00
0.02
0.04
Weight
Positive
Negative

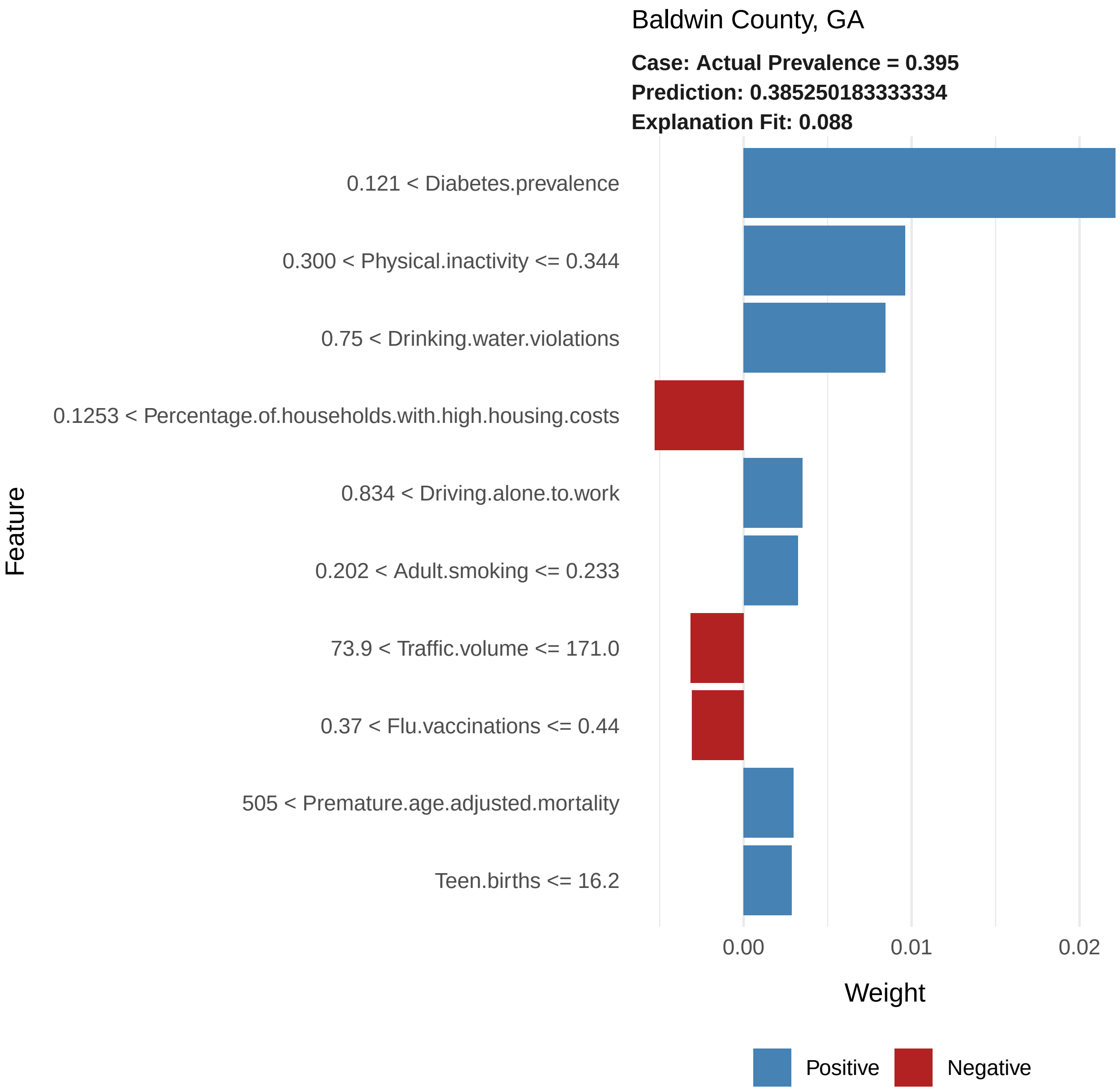
Baldwin County, GA
Case: Actual Prevalence = 0.395
Prediction: 0.385250183333334
Explanation Fit: 0.088
0.121 < Diabetes.prevalence
0.300 < Physical.inactivity <= 0.344
0.75 < Drinking.water.violations
0.1253 < Percentage.of.households.with.high.housing.costs
0.834 < Driving.alone.to.work
0.202 < Adult.smoking <= 0.233
73.9 < Traffic.volume <= 171.0
0.37 < Flu.vaccinations <= 0.44
505 < Premature.age.adjusted.mortality
Teen.births <= 16.2
Feature
0.00
0.01
0.02
Weight
Positive
Negative

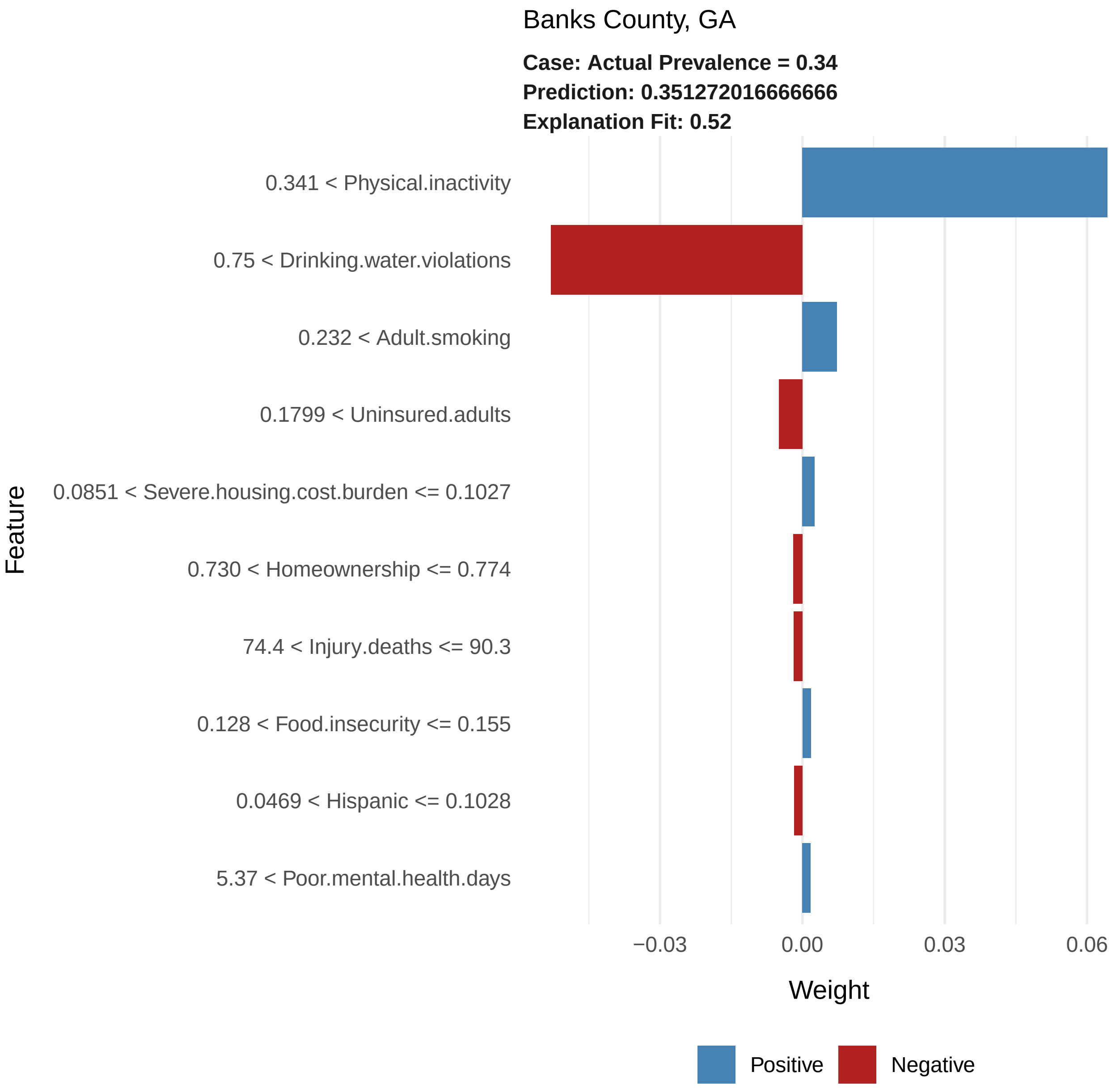

Banks County, GA
Case: Actual Prevalence = 0.34
Prediction: 0.351272016666666
Explanation Fit: 0.52
0.341 < Physical.inactivity
0.75 < Drinking.water.violations
0.232 < Adult.smoking
0.1799 < Uninsured.adults
0.0851 < Severe.housing.cost.burden <= 0.1027
0.730 < Homeownership <= 0.774
74.4 < Injury.deaths <= 90.3
0.128 < Food.insecurity <= 0.155
0.0469 < Hispanic <= 0.1028
5.37 < Poor.mental.health.days
Feature
−0.03
0.00
0.03
0.06
Weight
Positive
Negative

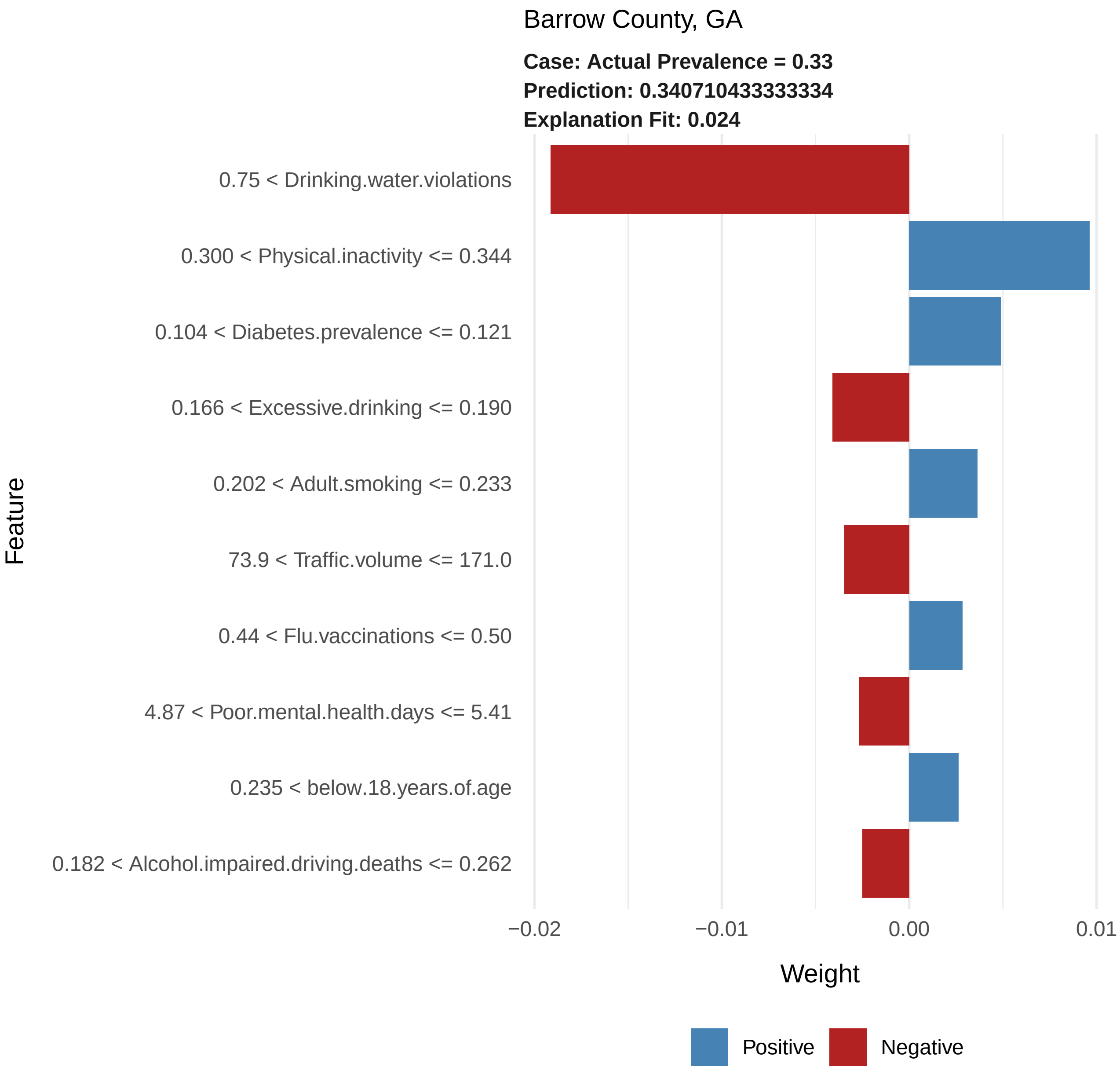

Barrow County, GA
Case: Actual Prevalence = 0.33
Prediction: 0.340710433333334
Explanation Fit: 0.024
0.75 < Drinking.water.violations
0.300 < Physical.inactivity <= 0.344
0.104 < Diabetes.prevalence <= 0.121
0.166 < Excessive.drinking <= 0.190
0.202 < Adult.smoking <= 0.233
73.9 < Traffic.volume <= 171.0
0.44 < Flu.vaccinations <= 0.50
4.87 < Poor.mental.health.days <= 5.41
0.235 < below.18.years.of.age
0.182 < Alcohol.impaired.driving.deaths <= 0.262
Feature
−0.02
−0.01
0.00
0.01
Weight
Positive
Negative

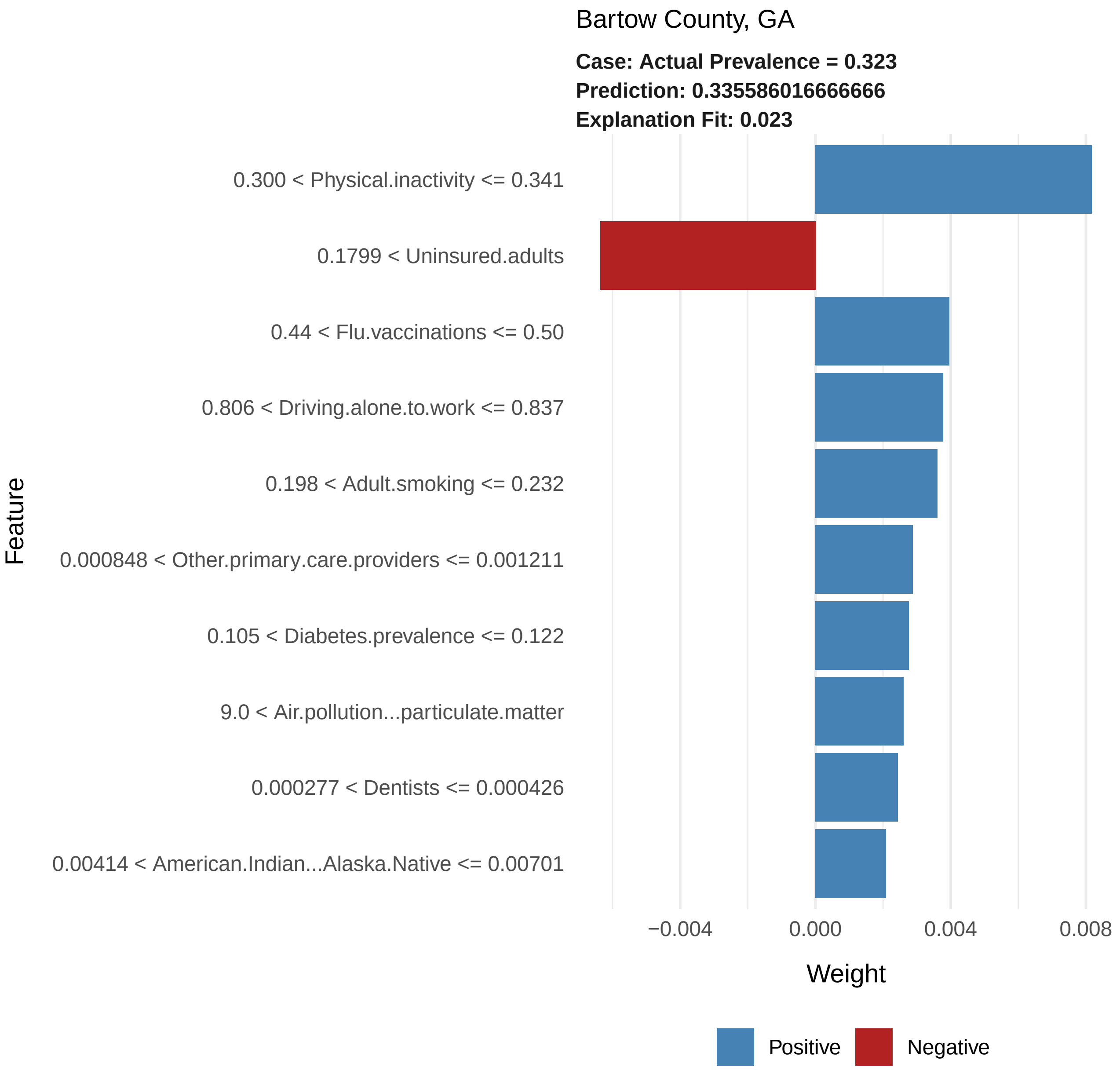

Bartow County, GA
Case: Actual Prevalence = 0.323
Prediction: 0.335586016666666
Explanation Fit: 0.023
0.300 < Physical.inactivity <= 0.341
0.1799 < Uninsured.adults
0.44 < Flu.vaccinations <= 0.50
0.806 < Driving.alone.to.work <= 0.837
0.198 < Adult.smoking <= 0.232
0.000848 < Other.primary.care.providers <= 0.001211
0.105 < Diabetes.prevalence <= 0.122
9.0 < Air.pollution...particulate.matter
0.000277 < Dentists <= 0.000426
0.00414 < American.Indian...Alaska.Native <= 0.00701
Feature
−0.004
0.000
0.004
0.008
Weight
Positive
Negative

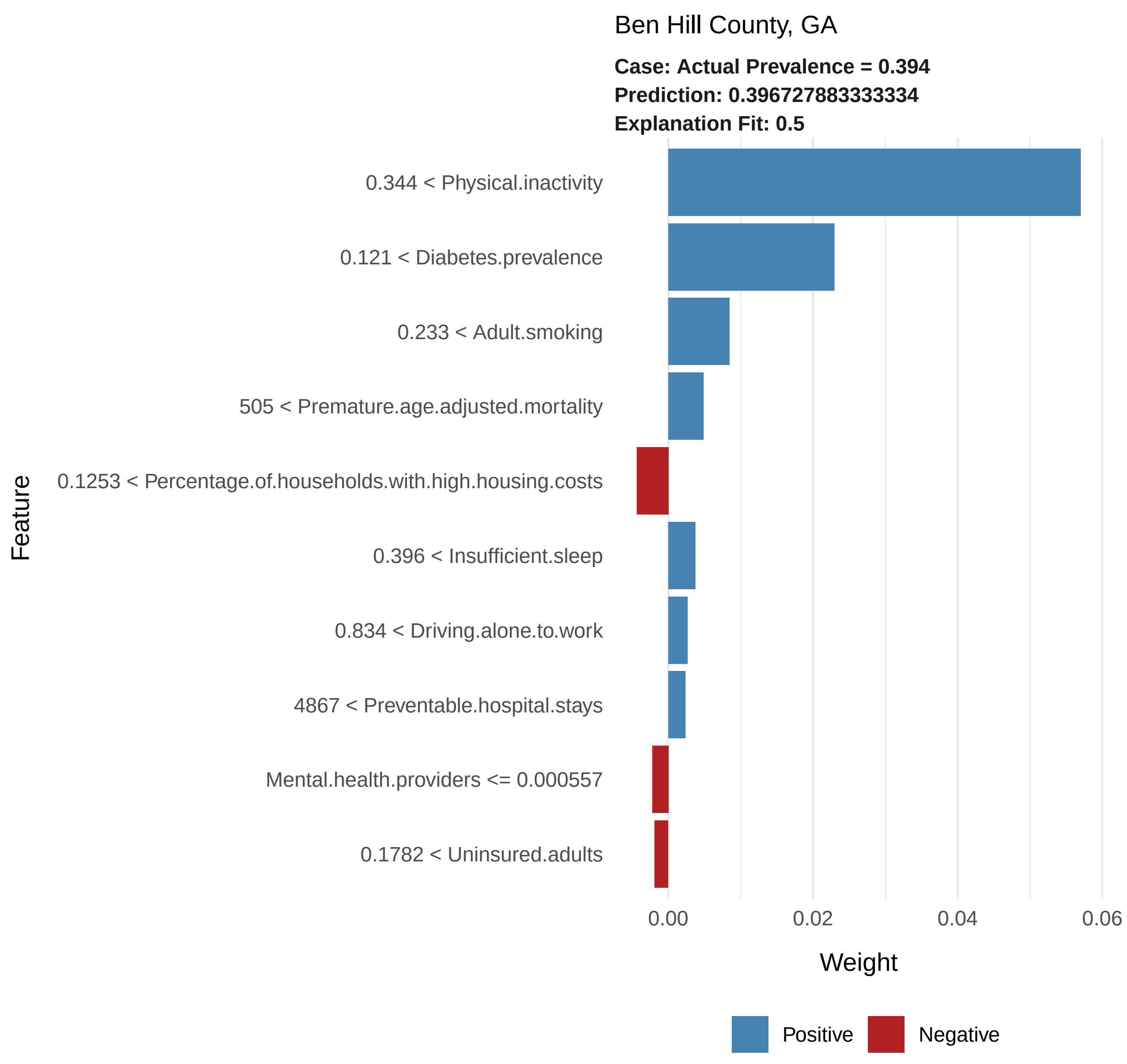
Ben Hill County, GA
Case: Actual Prevalence = 0.394
Prediction: 0.396727883333334
Explanation Fit: 0.5
0.344 < Physical.inactivity
0.121 < Diabetes.prevalence
0.233 < Adult.smoking
505 < Premature.age.adjusted.mortality
0.1253 < Percentage.of.households.with.high.housing.costs
0.396 < Insufficient.sleep
0.834 < Driving.alone.to.work
4867 < Preventable.hospital.stays
Mental.health.providers <= 0.000557
0.1782 < Uninsured.adults
Feature
0.00
0.02
0.04
0.06
Weight
Positive
Negative

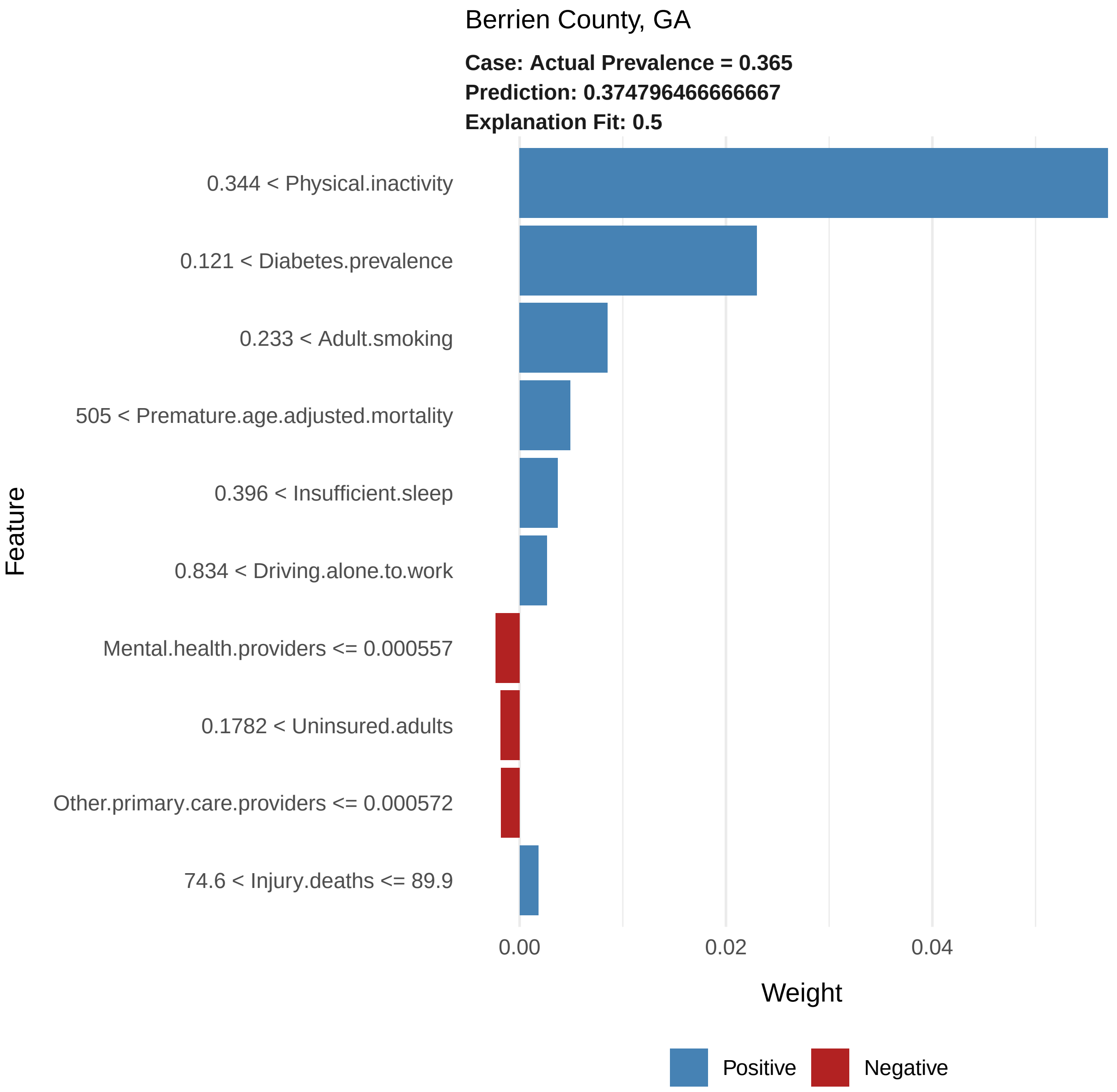

Berrien County, GA
Case: Actual Prevalence = 0.365
Prediction: 0.374796466666667
Explanation Fit: 0.5
0.344 < Physical.inactivity
0.121 < Diabetes.prevalence
0.233 < Adult.smoking
505 < Premature.age.adjusted.mortality
0.396 < Insufficient.sleep
0.834 < Driving.alone.to.work
Mental.health.providers <= 0.000557
0.1782 < Uninsured.adults
Other.primary.care.providers <= 0.000572
74.6 < Injury.deaths <= 89.9
Feature
0.00
0.02
0.04
Weight
Positive
Negative

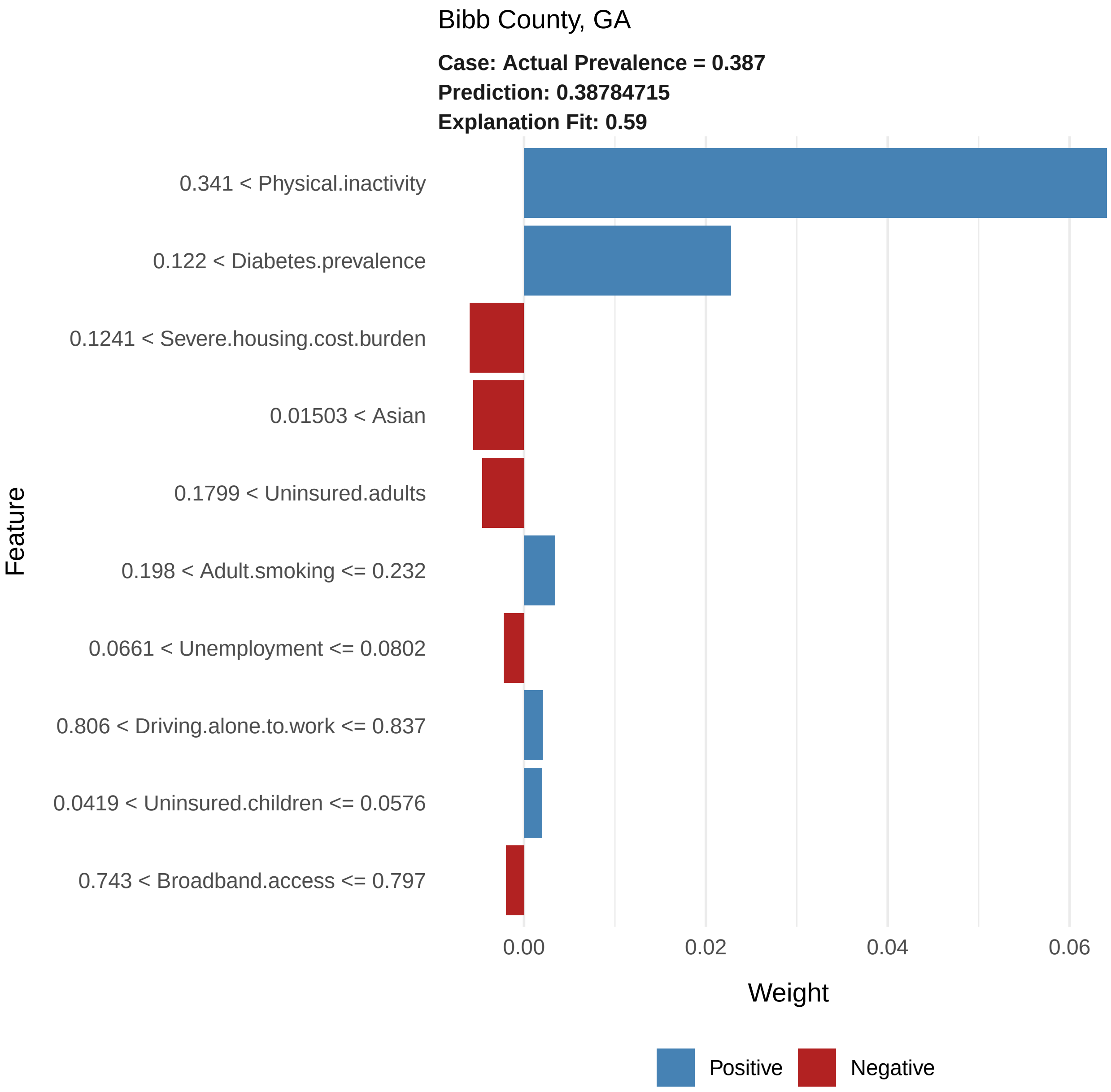
Bibb County, GA
Case: Actual Prevalence = 0.387
Prediction: 0.38784715
Explanation Fit: 0.59
0.341 < Physical.inactivity
0.122 < Diabetes.prevalence
0.1241 < Severe.housing.cost.burden
0.01503 < Asian
0.1799 < Uninsured.adults
0.198 < Adult.smoking <= 0.232
0.0661 < Unemployment <= 0.0802
0.806 < Driving.alone.to.work <= 0.837
0.0419 < Uninsured.children <= 0.0576
0.743 < Broadband.access <= 0.797
Feature
0.00
0.02
0.04
0.06
Weight
Positive
Negative

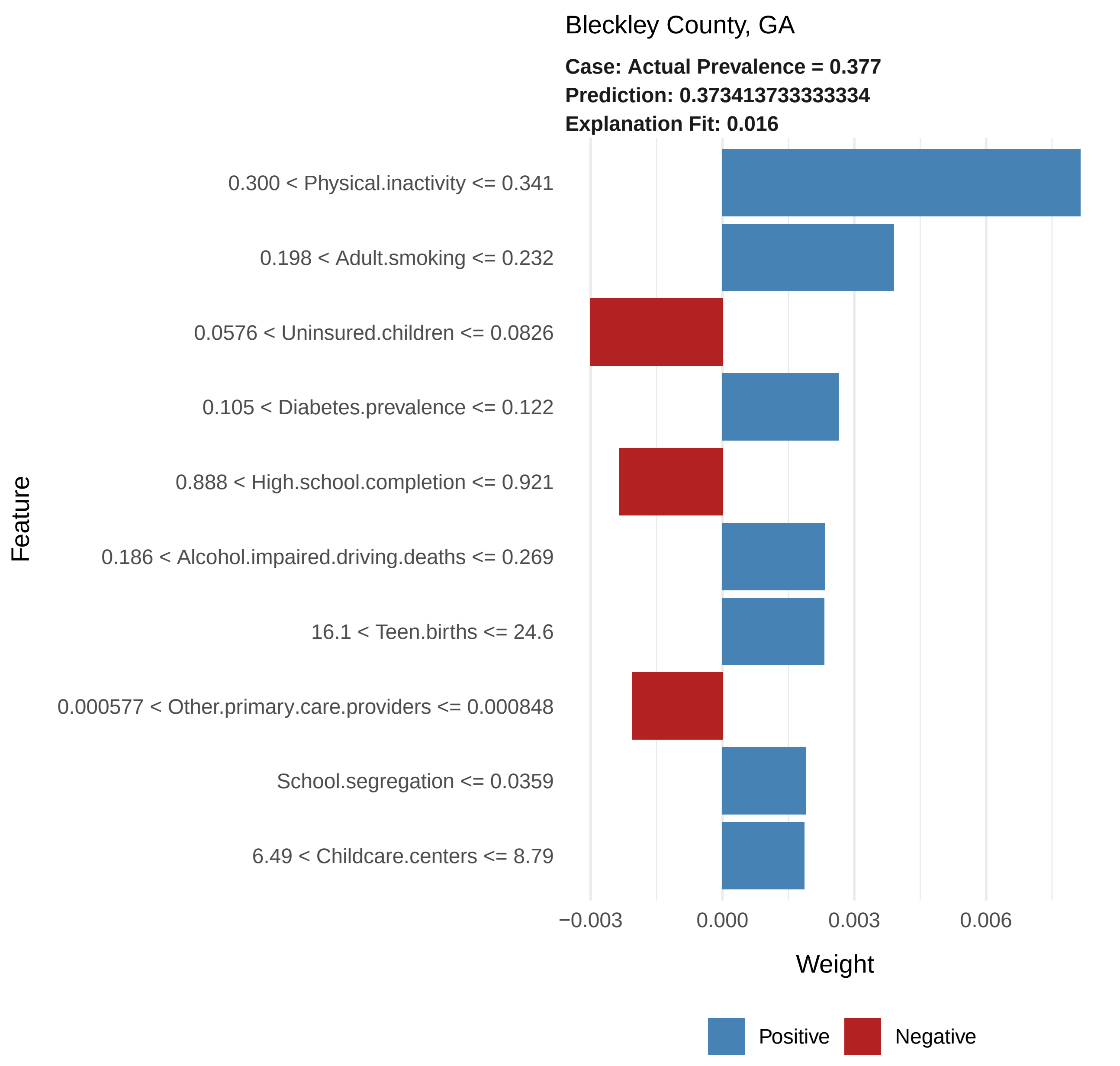

Bleckley County, GA
Case: Actual Prevalence = 0.377
Prediction: 0.373413733333334
Explanation Fit: 0.016
0.300 < Physical.inactivity <= 0.341
0.198 < Adult.smoking <= 0.232
0.0576 < Uninsured.children <= 0.0826
0.105 < Diabetes.prevalence <= 0.122
0.888 < High.school.completion <= 0.921
0.186 < Alcohol.impaired.driving.deaths <= 0.269
16.1 < Teen.births <= 24.6
0.000577 < Other.primary.care.providers <= 0.000848
School.segregation <= 0.0359
6.49 < Childcare.centers <= 8.79
Feature
−0.003
0.000
0.003
0.006
Weight
Positive
Negative

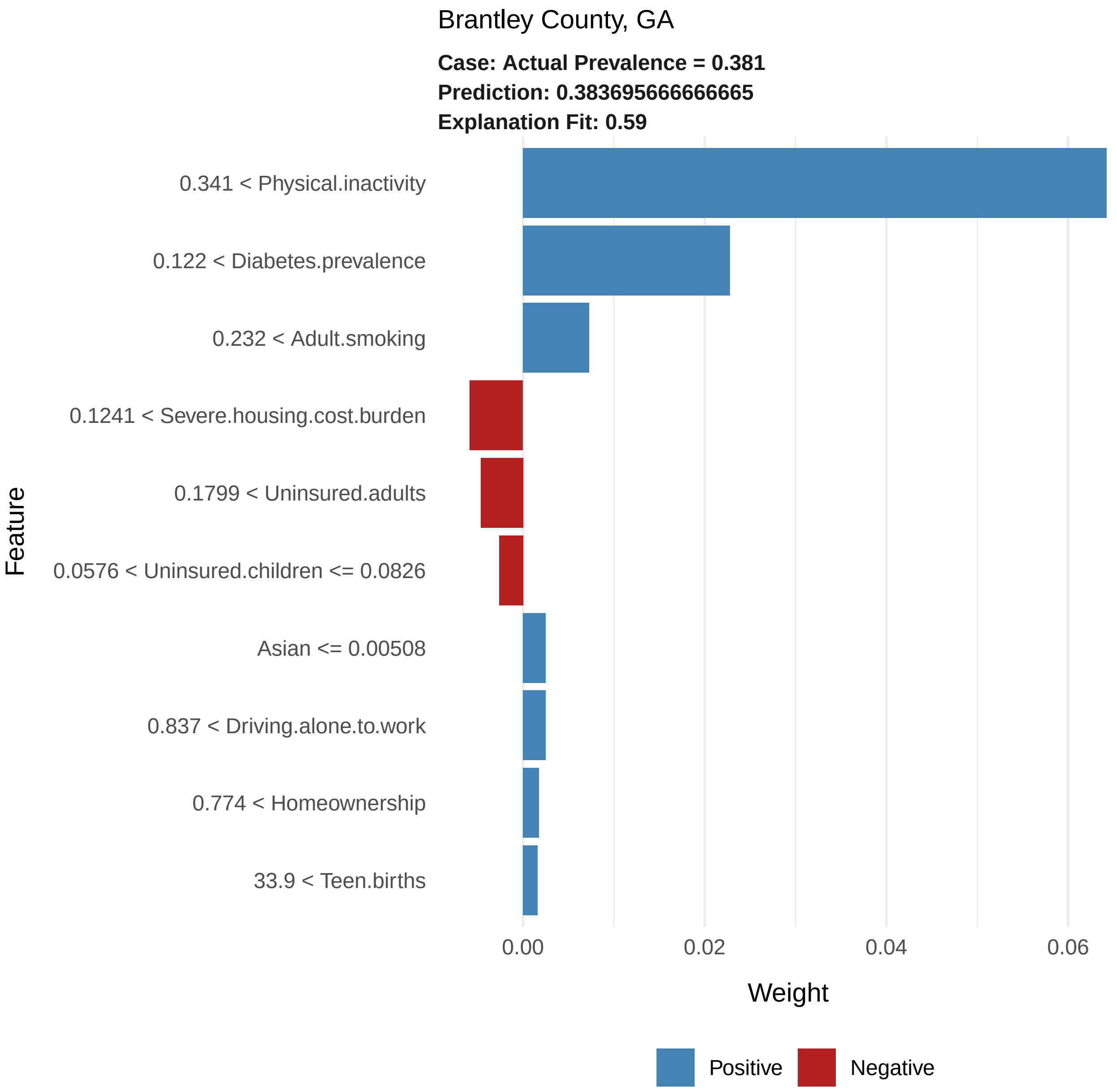
Brantley County, GA
Case: Actual Prevalence = 0.381
Prediction: 0.383695666666665
Explanation Fit: 0.59
0.341 < Physical.inactivity
0.122 < Diabetes.prevalence
0.232 < Adult.smoking
0.1241 < Severe.housing.cost.burden
0.1799 < Uninsured.adults
0.0576 < Uninsured.children <= 0.0826
Asian <= 0.00508
0.837 < Driving.alone.to.work
0.774 < Homeownership
33.9 < Teen.births
Feature
0.00
0.02
0.04
0.06
Weight
Positive
Negative

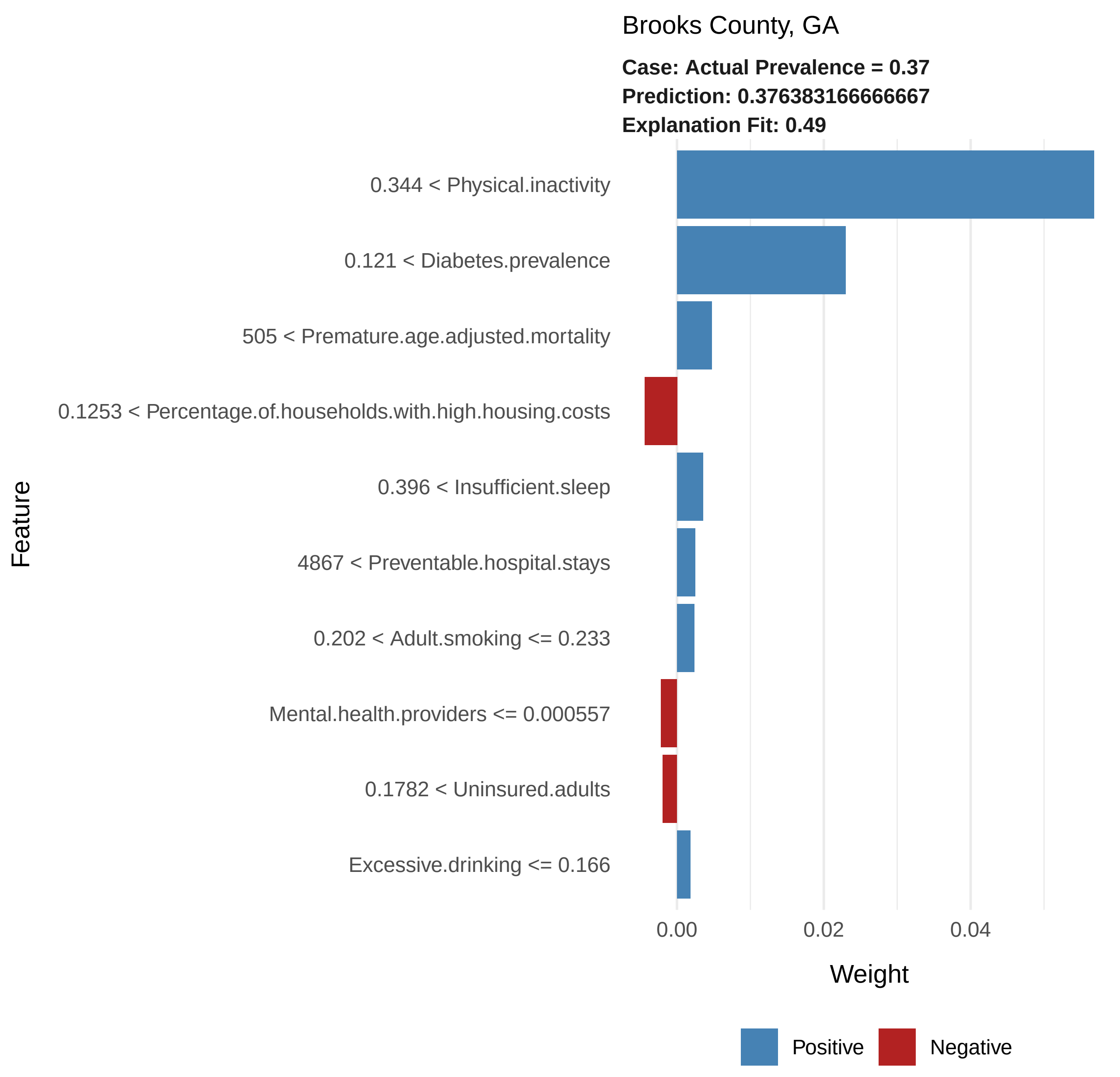
Brooks County, GA
Case: Actual Prevalence = 0.37
Prediction: 0.376383166666667
Explanation Fit: 0.49
0.344 < Physical.inactivity
0.121 < Diabetes.prevalence
505 < Premature.age.adjusted.mortality
0.1253 < Percentage.of.households.with.high.housing.costs
0.396 < Insufficient.sleep
4867 < Preventable.hospital.stays
0.202 < Adult.smoking <= 0.233
Mental.health.providers <= 0.000557
0.1782 < Uninsured.adults
Excessive.drinking <= 0.166
Feature
0.00
0.02
0.04
Weight
Positive
Negative

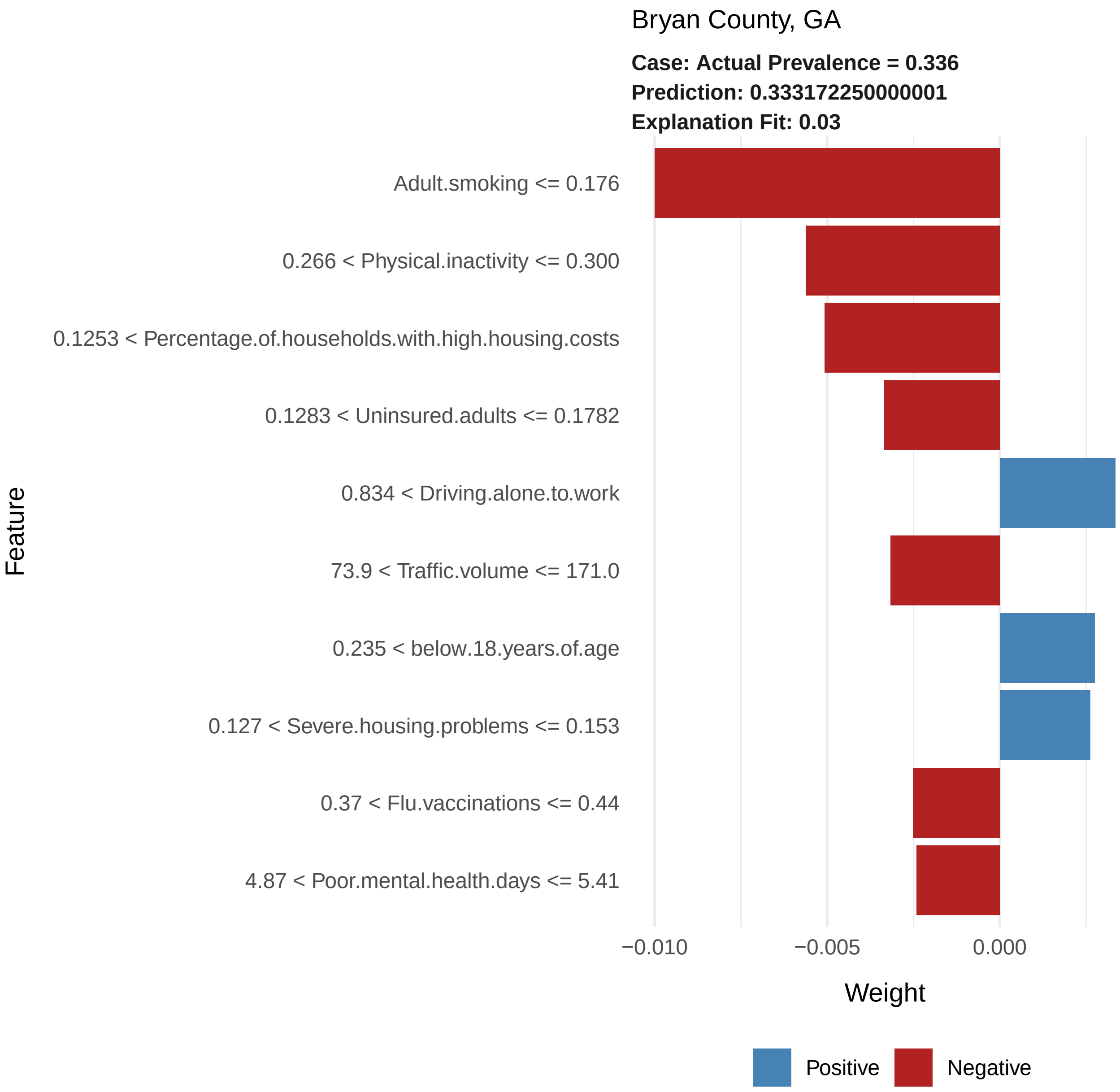

Bryan County, GA
Case: Actual Prevalence = 0.336
Prediction: 0.333172250000001
Explanation Fit: 0.03
Adult.smoking <= 0.176
0.266 < Physical.inactivity <= 0.300
0.1253 < Percentage.of.households.with.high.housing.costs
0.1283 < Uninsured.adults <= 0.1782
0.834 < Driving.alone.to.work
73.9 < Traffic.volume <= 171.0
0.235 < below.18.years.of.age
0.127 < Severe.housing.problems <= 0.153
0.37 < Flu.vaccinations <= 0.44
4.87 < Poor.mental.health.days <= 5.41
Feature
−0.010
−0.005
0.000
Weight
Positive
Negative

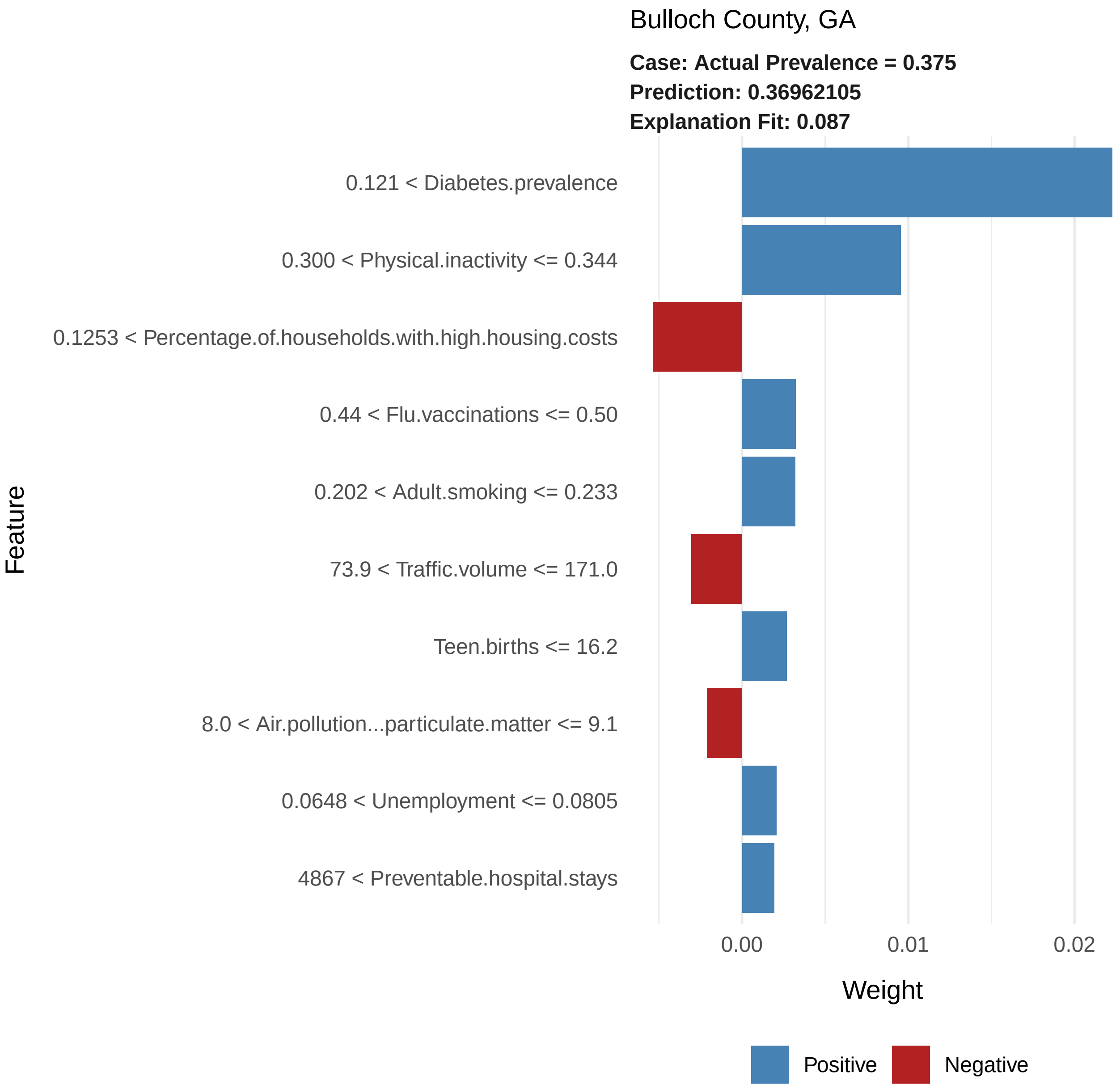

Bulloch County, GA
Case: Actual Prevalence = 0.375
Prediction: 0.36962105
Explanation Fit: 0.087
0.121 < Diabetes.prevalence
0.300 < Physical.inactivity <= 0.344
0.1253 < Percentage.of.households.with.high.housing.costs
0.44 < Flu.vaccinations <= 0.50
0.202 < Adult.smoking <= 0.233
73.9 < Traffic.volume <= 171.0
Teen.births <= 16.2
8.0 < Air.pollution...particulate.matter <= 9.1
0.0648 < Unemployment <= 0.0805
4867 < Preventable.hospital.stays
Feature
0.00
0.01
0.02
Weight
Positive
Negative

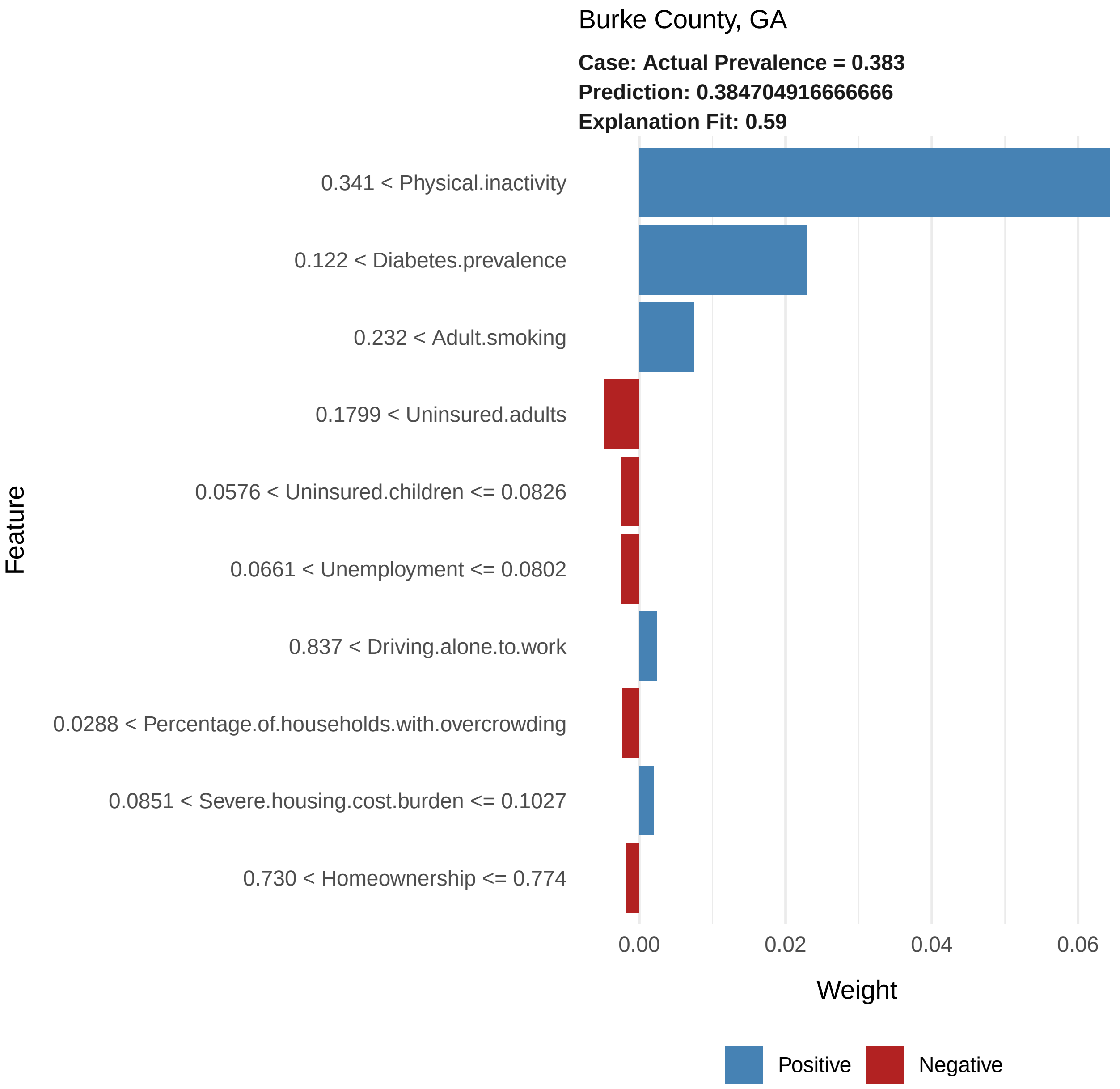

Burke County, GA
Case: Actual Prevalence = 0.383
Prediction: 0.384704916666666
Explanation Fit: 0.59
0.341 < Physical.inactivity
0.122 < Diabetes.prevalence
0.232 < Adult.smoking
0.1799 < Uninsured.adults
0.0576 < Uninsured.children <= 0.0826
0.0661 < Unemployment <= 0.0802
0.837 < Driving.alone.to.work
0.0288 < Percentage.of.households.with.overcrowding
0.0851 < Severe.housing.cost.burden <= 0.1027
0.730 < Homeownership <= 0.774
Feature
0.00
0.02
0.04
0.06
Weight
Positive
Negative

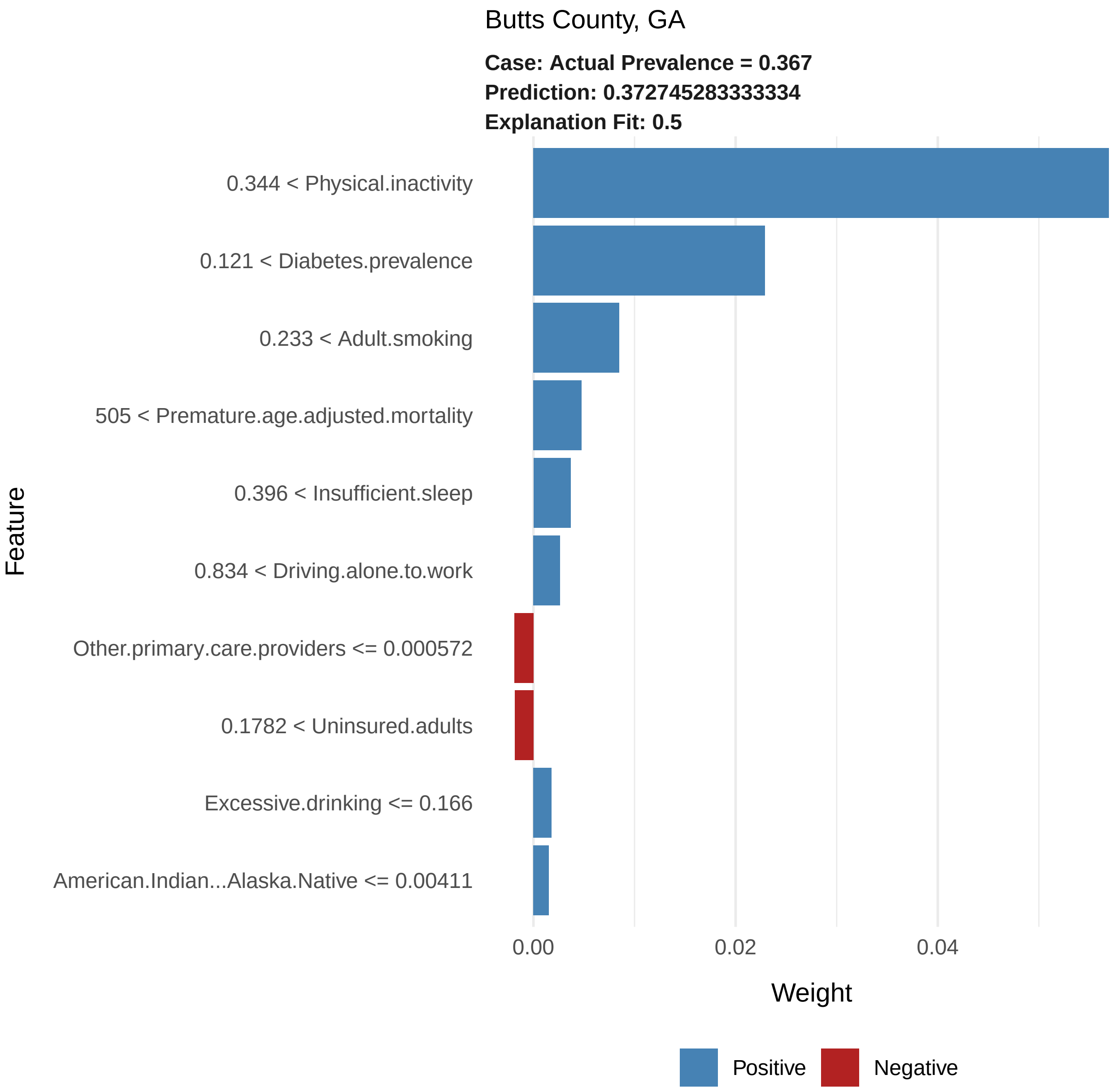
Butts County, GA
Case: Actual Prevalence = 0.367
Prediction: 0.372745283333334
Explanation Fit: 0.5
0.344 < Physical.inactivity
0.121 < Diabetes.prevalence
0.233 < Adult.smoking
505 < Premature.age.adjusted.mortality
0.396 < Insufficient.sleep
0.834 < Driving.alone.to.work
Other.primary.care.providers <= 0.000572
0.1782 < Uninsured.adults
Excessive.drinking <= 0.166
American.Indian...Alaska.Native <= 0.00411
Feature
0.00
0.02
0.04
Weight
Positive
Negative

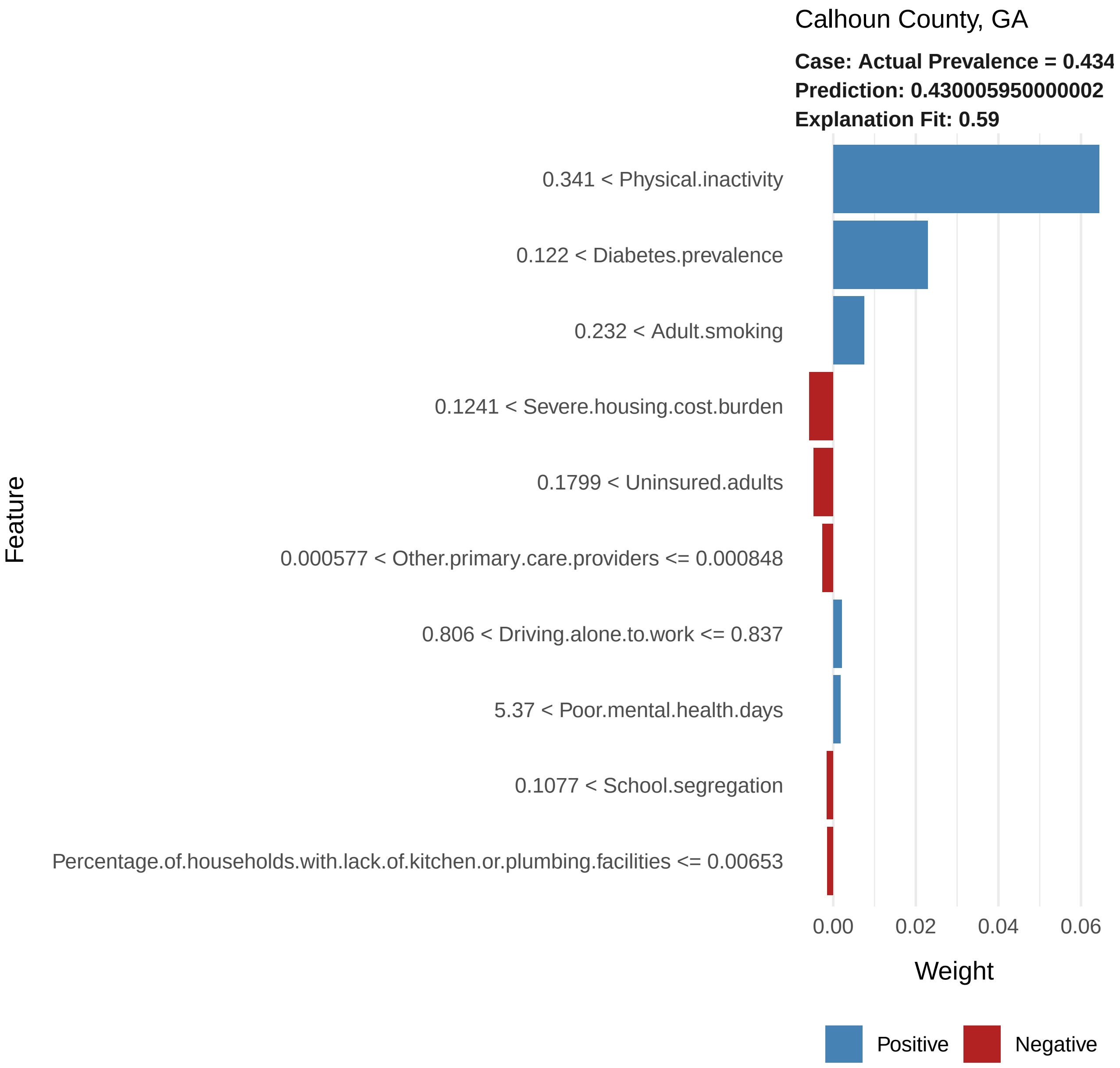

Calhoun County, GA
Case: Actual Prevalence = 0.434
Prediction: 0.430005950000002
Explanation Fit: 0.59
0.341 < Physical.inactivity
0.122 < Diabetes.prevalence
0.232 < Adult.smoking
0.1241 < Severe.housing.cost.burden
0.1799 < Uninsured.adults
0.000577 < Other.primary.care.providers <= 0.000848
0.806 < Driving.alone.to.work <= 0.837
5.37 < Poor.mental.health.days
0.1077 < School.segregation
Percentage.of.households.with.lack.of.kitchen.or.plumbing.facilities <= 0.00653
Feature
0.00
0.02
0.04
0.06
Weight
Positive
Negative

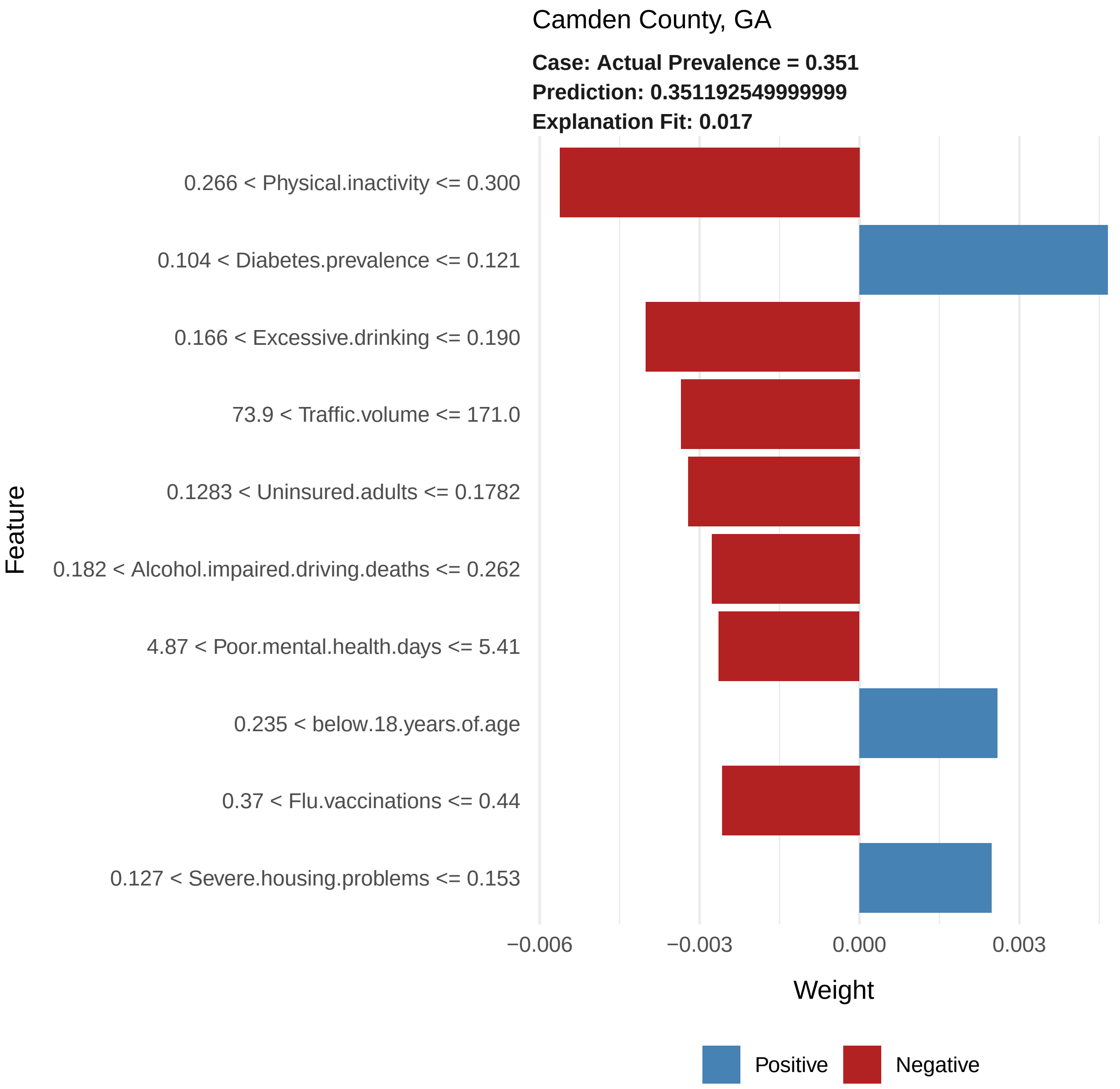

Camden County, GA
Case: Actual Prevalence = 0.351
Prediction: 0.351192549999999
Explanation Fit: 0.017
0.266 < Physical.inactivity <= 0.300
0.104 < Diabetes.prevalence <= 0.121
0.166 < Excessive.drinking <= 0.190
73.9 < Traffic.volume <= 171.0
0.1283 < Uninsured.adults <= 0.1782
0.182 < Alcohol.impaired.driving.deaths <= 0.262
4.87 < Poor.mental.health.days <= 5.41
0.235 < below.18.years.of.age
0.37 < Flu.vaccinations <= 0.44
0.127 < Severe.housing.problems <= 0.153
Feature
−0.006
−0.003
0.000
0.003
Weight
Positive
Negative

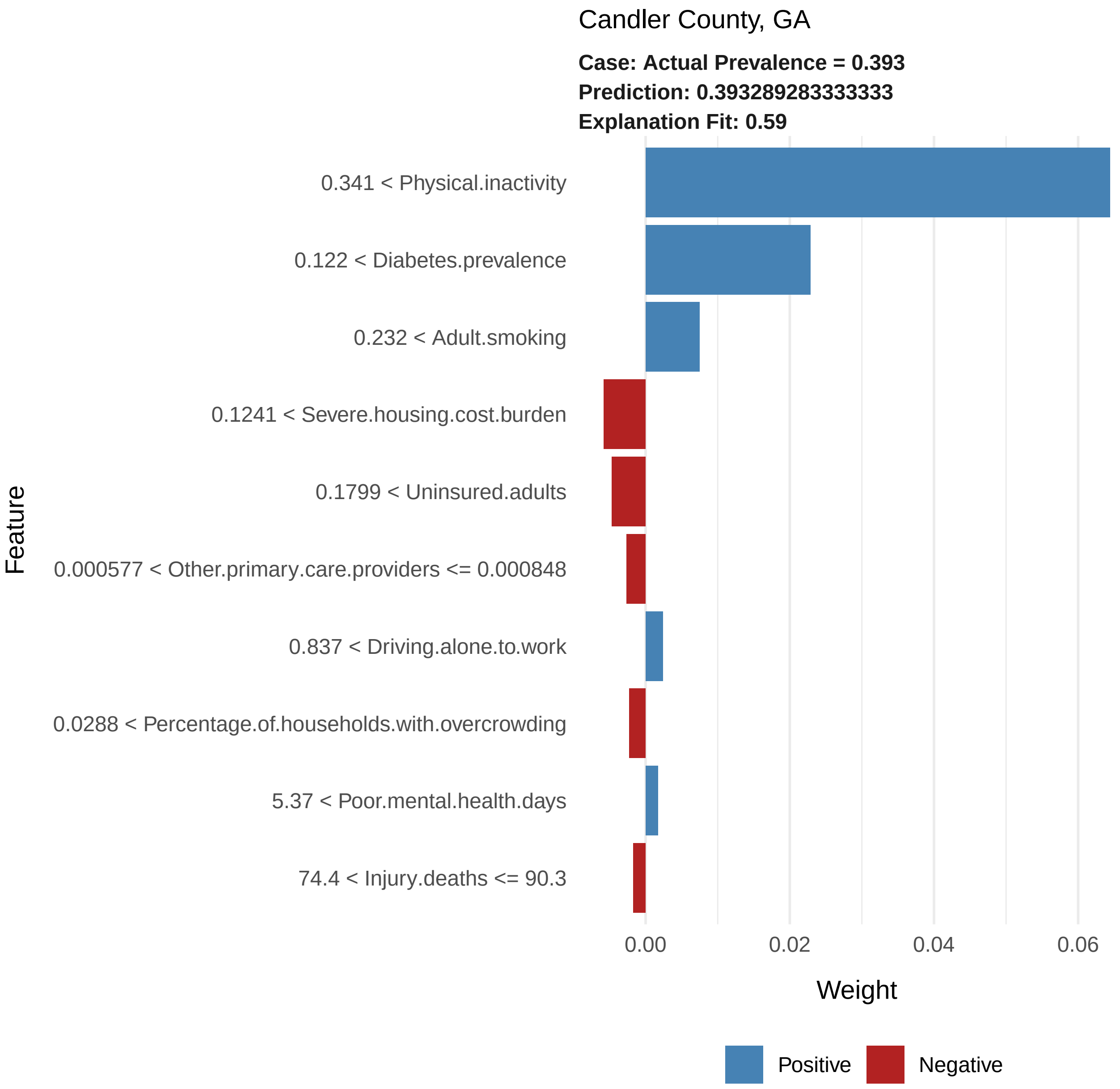
Candler County, GA
Case: Actual Prevalence = 0.393
Prediction: 0.393289283333333
Explanation Fit: 0.59
0.341 < Physical.inactivity
0.122 < Diabetes.prevalence
0.232 < Adult.smoking
0.1241 < Severe.housing.cost.burden
0.1799 < Uninsured.adults
0.000577 < Other.primary.care.providers <= 0.000848
0.837 < Driving.alone.to.work
0.0288 < Percentage.of.households.with.overcrowding
5.37 < Poor.mental.health.days
74.4 < Injury.deaths <= 90.3
Feature
0.00
0.02
0.04
0.06
Weight
Positive
Negative

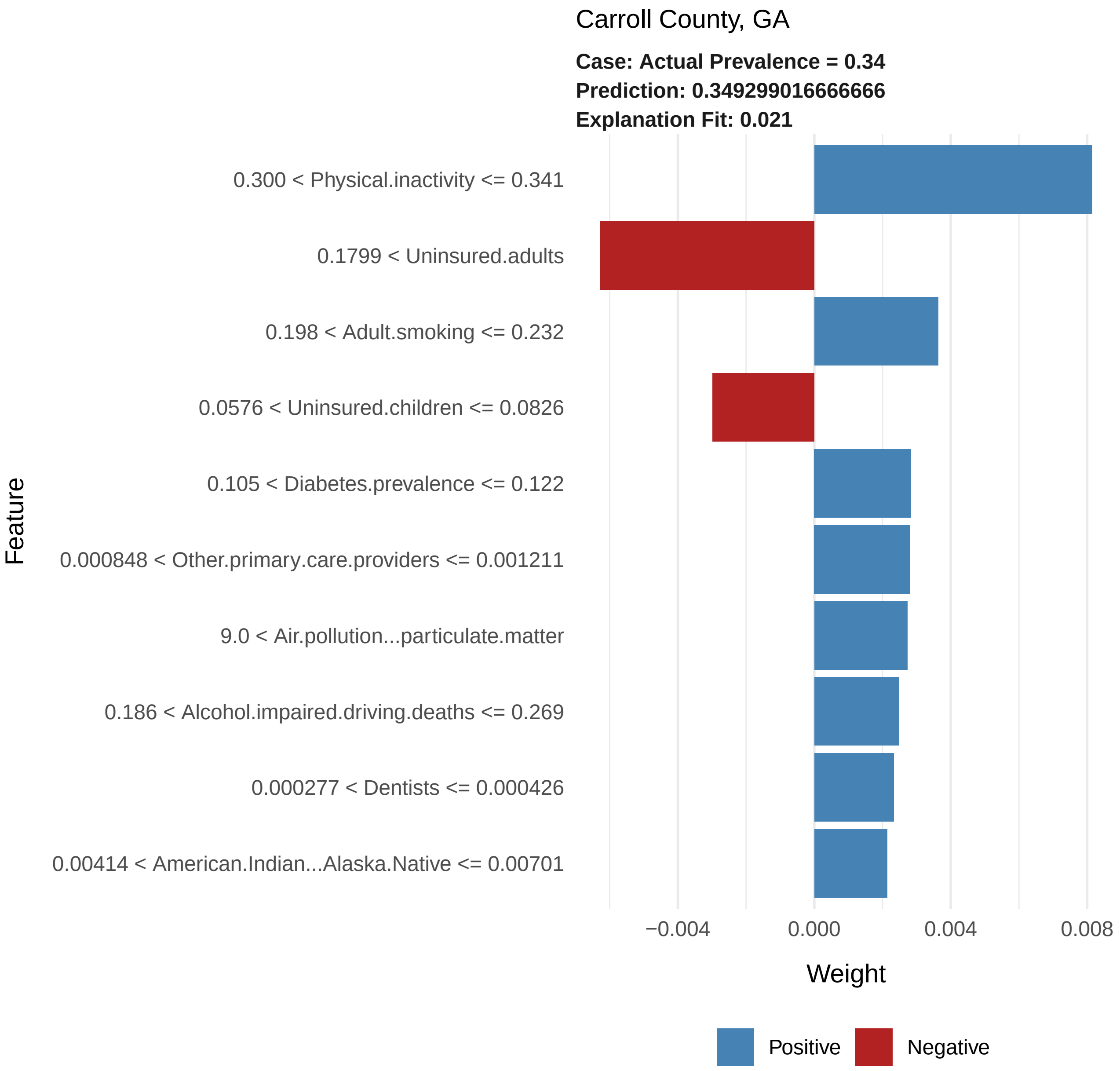
Carroll County, GA
Case: Actual Prevalence = 0.34
Prediction: 0.349299016666666
Explanation Fit: 0.021
0.300 < Physical.inactivity <= 0.341
0.1799 < Uninsured.adults
0.198 < Adult.smoking <= 0.232
0.0576 < Uninsured.children <= 0.0826
0.105 < Diabetes.prevalence <= 0.122
0.000848 < Other.primary.care.providers <= 0.001211
9.0 < Air.pollution...particulate.matter
0.186 < Alcohol.impaired.driving.deaths <= 0.269
0.000277 < Dentists <= 0.000426
0.00414 < American.Indian...Alaska.Native <= 0.00701
Feature
−0.004
0.000
0.004
0.008
Weight
Positive
Negative

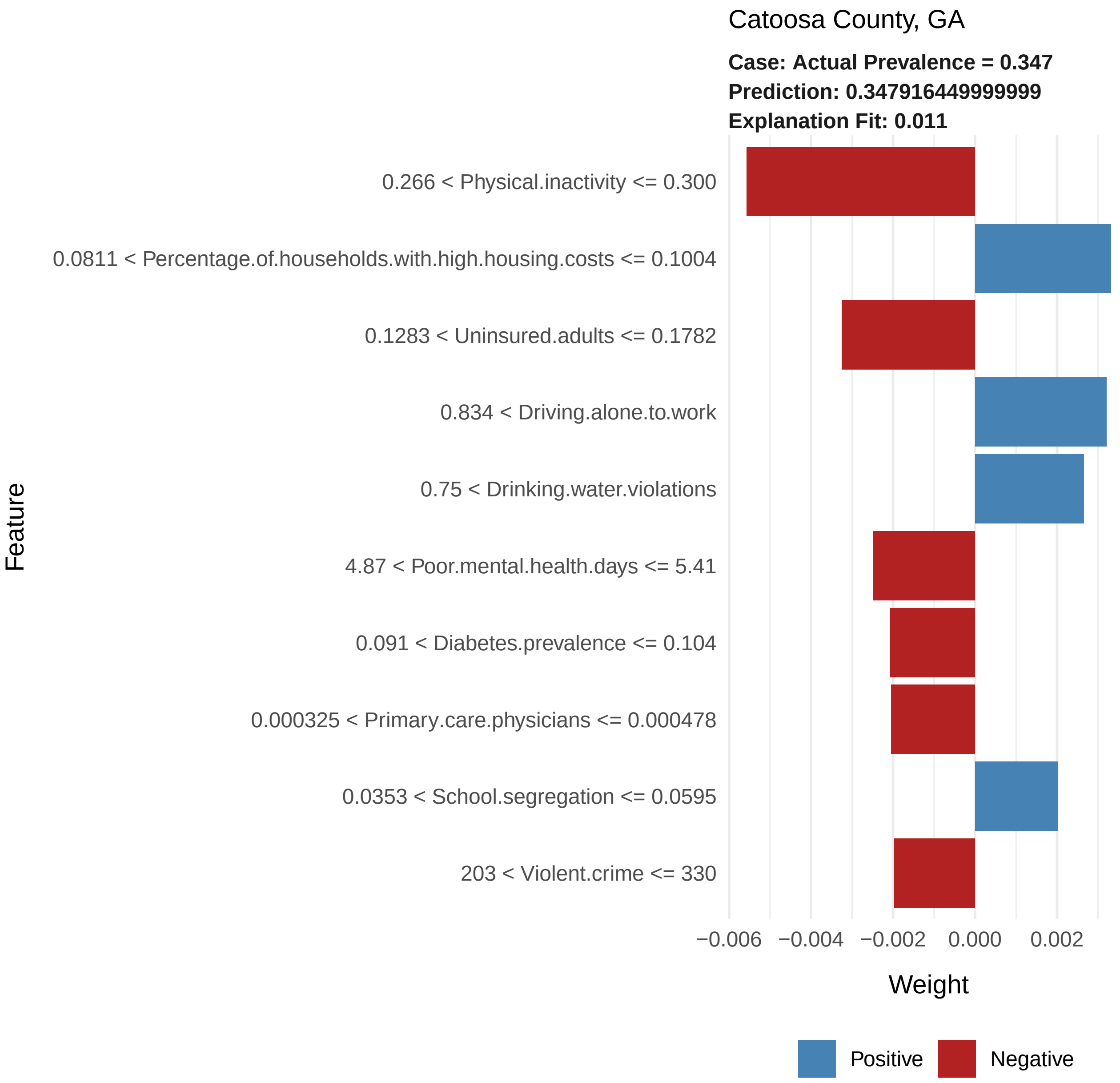
Catoosa County, GA
Case: Actual Prevalence = 0.347
Prediction: 0.347916449999999
Explanation Fit: 0.011
0.266 < Physical.inactivity <= 0.300
0.0811 < Percentage.of.households.with.high.housing.costs <= 0.1004
0.1283 < Uninsured.adults <= 0.1782
0.834 < Driving.alone.to.work
0.75 < Drinking.water.violations
4.87 < Poor.mental.health.days <= 5.41
0.091 < Diabetes.prevalence <= 0.104
0.000325 < Primary.care.physicians <= 0.000478
0.0353 < School.segregation <= 0.0595
203 < Violent.crime <= 330
Feature
−0.006
−0.004
−0.002
0.000
0.002
Weight
Positive
Negative

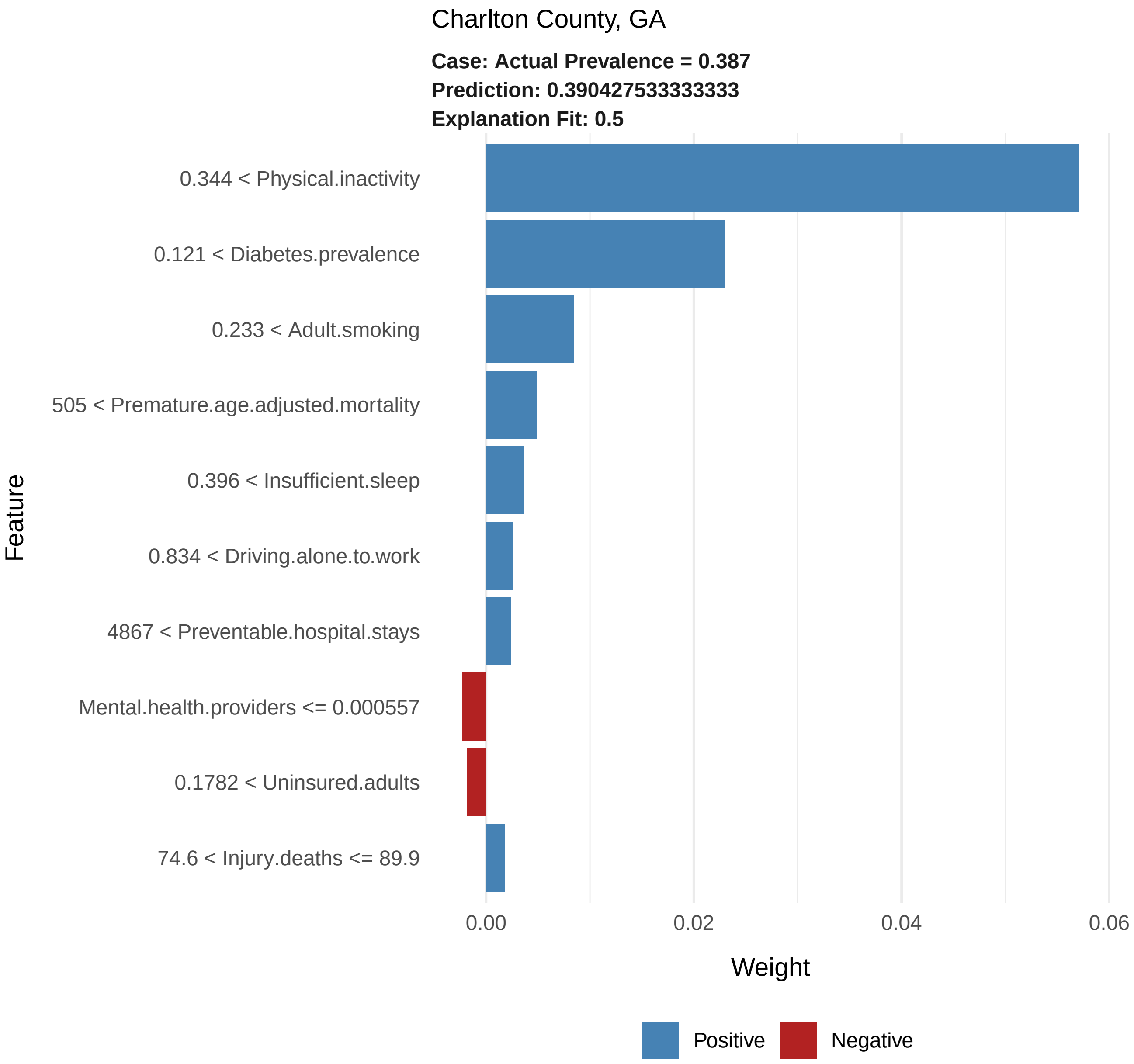
Charlton County, GA
Case: Actual Prevalence = 0.387
Prediction: 0.390427533333333
Explanation Fit: 0.5
0.344 < Physical.inactivity
0.121 < Diabetes.prevalence
0.233 < Adult.smoking
505 < Premature.age.adjusted.mortality
0.396 < Insufficient.sleep
0.834 < Driving.alone.to.work
4867 < Preventable.hospital.stays
Mental.health.providers <= 0.000557
0.1782 < Uninsured.adults
74.6 < Injury.deaths <= 89.9
Feature
0.00
0.02
0.04
0.06
Weight
Positive
Negative

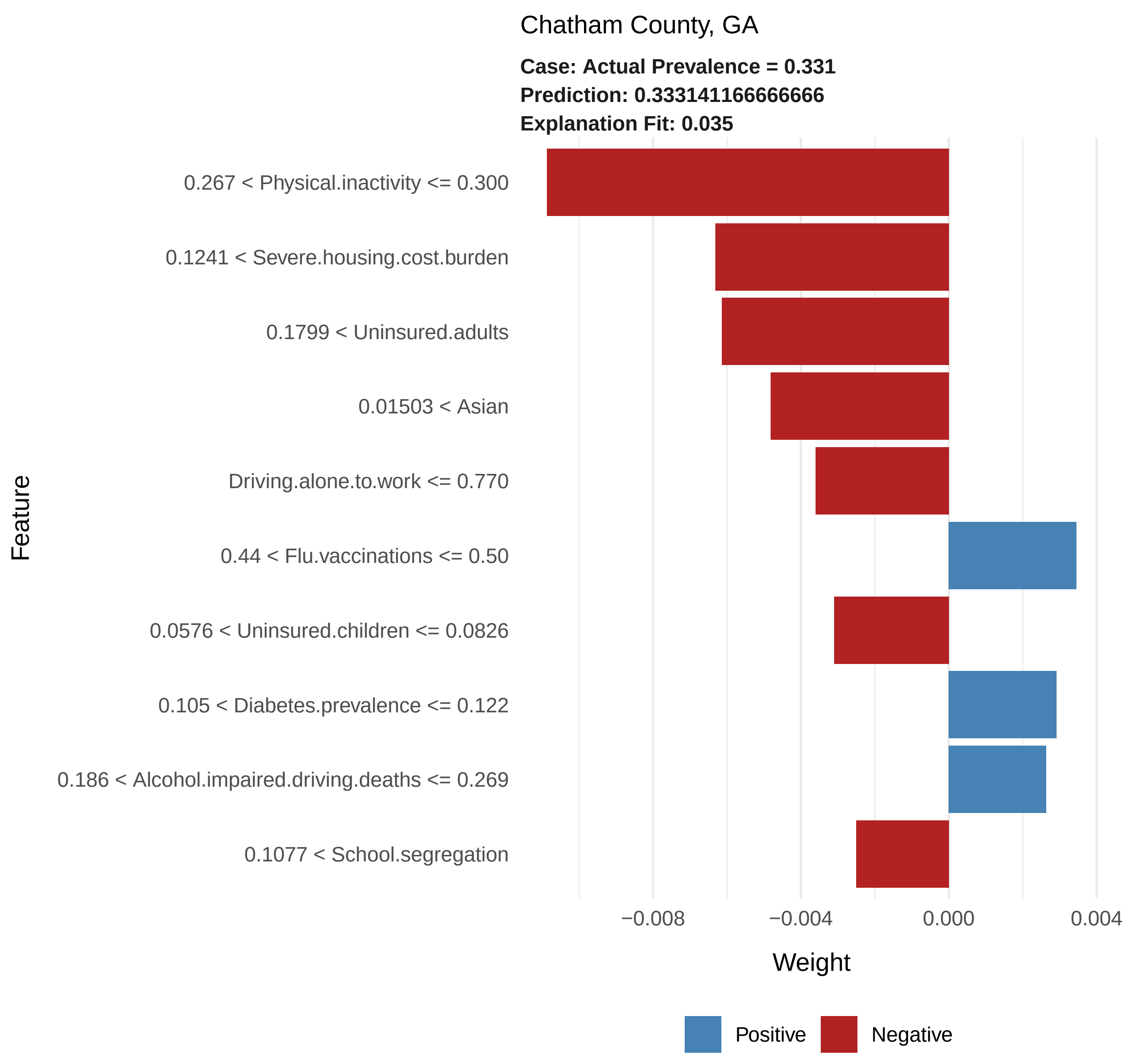
Chatham County, GA
Case: Actual Prevalence = 0.331
Prediction: 0.333141166666666
Explanation Fit: 0.035
0.267 < Physical.inactivity <= 0.300
0.1241 < Severe.housing.cost.burden
0.1799 < Uninsured.adults
0.01503 < Asian
Driving.alone.to.work <= 0.770
0.44 < Flu.vaccinations <= 0.50
0.0576 < Uninsured.children <= 0.0826
0.105 < Diabetes.prevalence <= 0.122
0.186 < Alcohol.impaired.driving.deaths <= 0.269
0.1077 < School.segregation
Feature
−0.008
−0.004
0.000
0.004
Weight
Positive
Negative

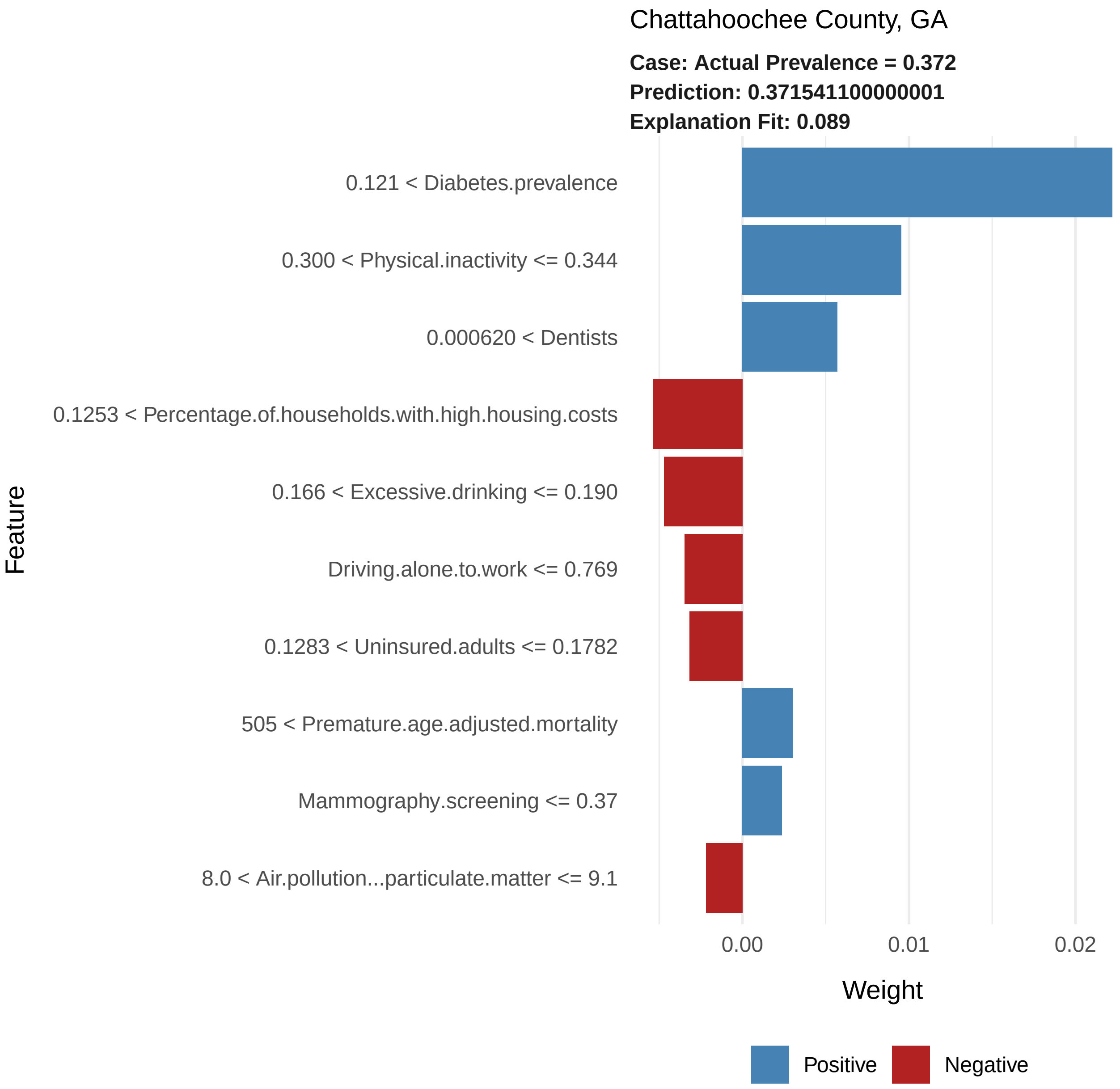
Chattahoochee County, GA
Case: Actual Prevalence = 0.372
Prediction: 0.371541100000001
Explanation Fit: 0.089
0.121 < Diabetes.prevalence
0.300 < Physical.inactivity <= 0.344
0.000620 < Dentists
0.1253 < Percentage.of.households.with.high.housing.costs
0.166 < Excessive.drinking <= 0.190
Driving.alone.to.work <= 0.769
0.1283 < Uninsured.adults <= 0.1782
505 < Premature.age.adjusted.mortality
Mammography.screening <= 0.37
8.0 < Air.pollution...particulate.matter <= 9.1
Feature
0.00
0.01
0.02
Weight
Positive
Negative

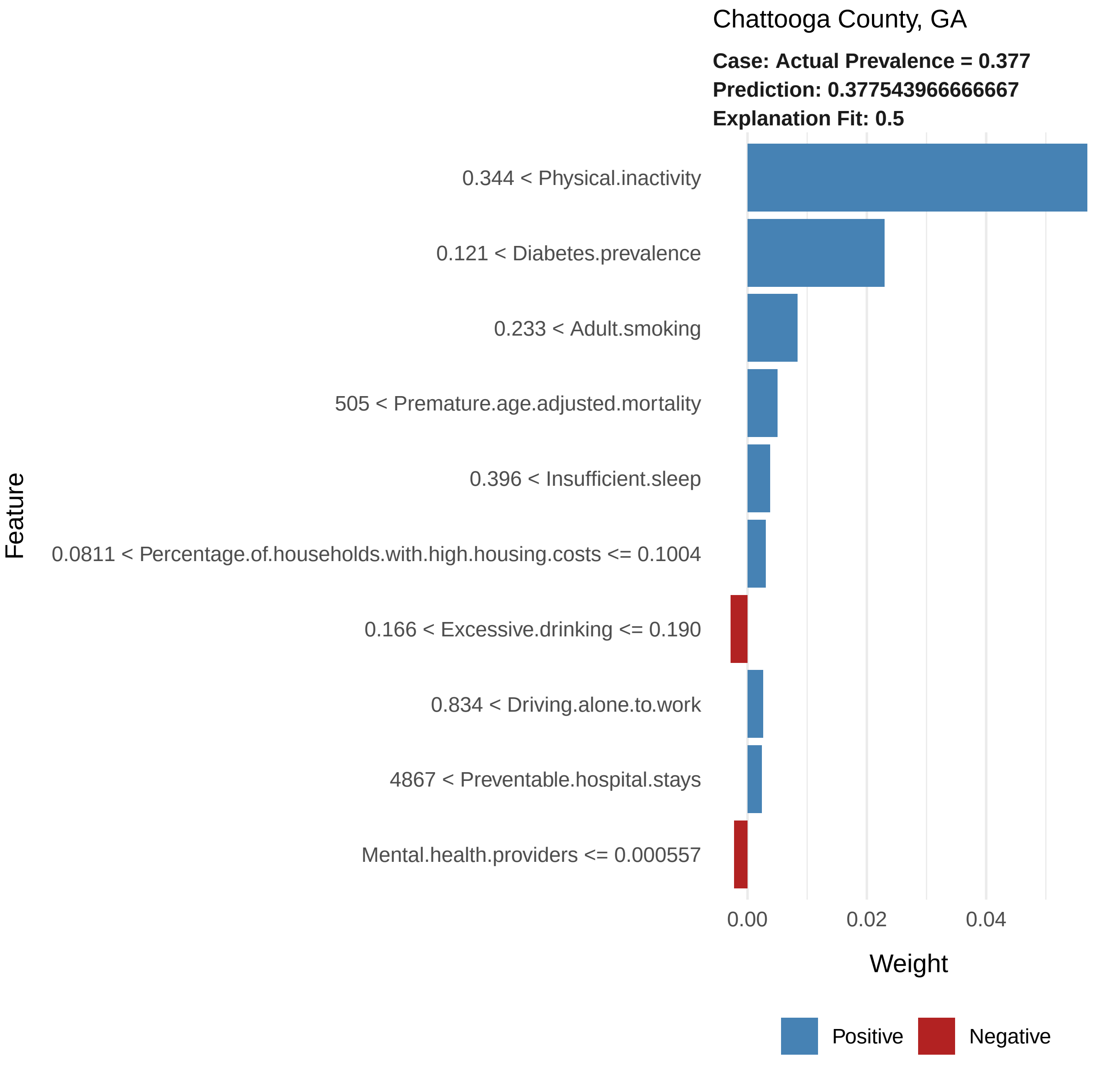
Chattooga County, GA
Case: Actual Prevalence = 0.377
Prediction: 0.377543966666667
Explanation Fit: 0.5
0.344 < Physical.inactivity
0.121 < Diabetes.prevalence
0.233 < Adult.smoking
505 < Premature.age.adjusted.mortality
0.396 < Insufficient.sleep
0.0811 < Percentage.of.households.with.high.housing.costs <= 0.1004
0.166 < Excessive.drinking <= 0.190
0.834 < Driving.alone.to.work
4867 < Preventable.hospital.stays
Mental.health.providers <= 0.000557
Feature
0.00
0.02
0.04
Weight
Positive
Negative

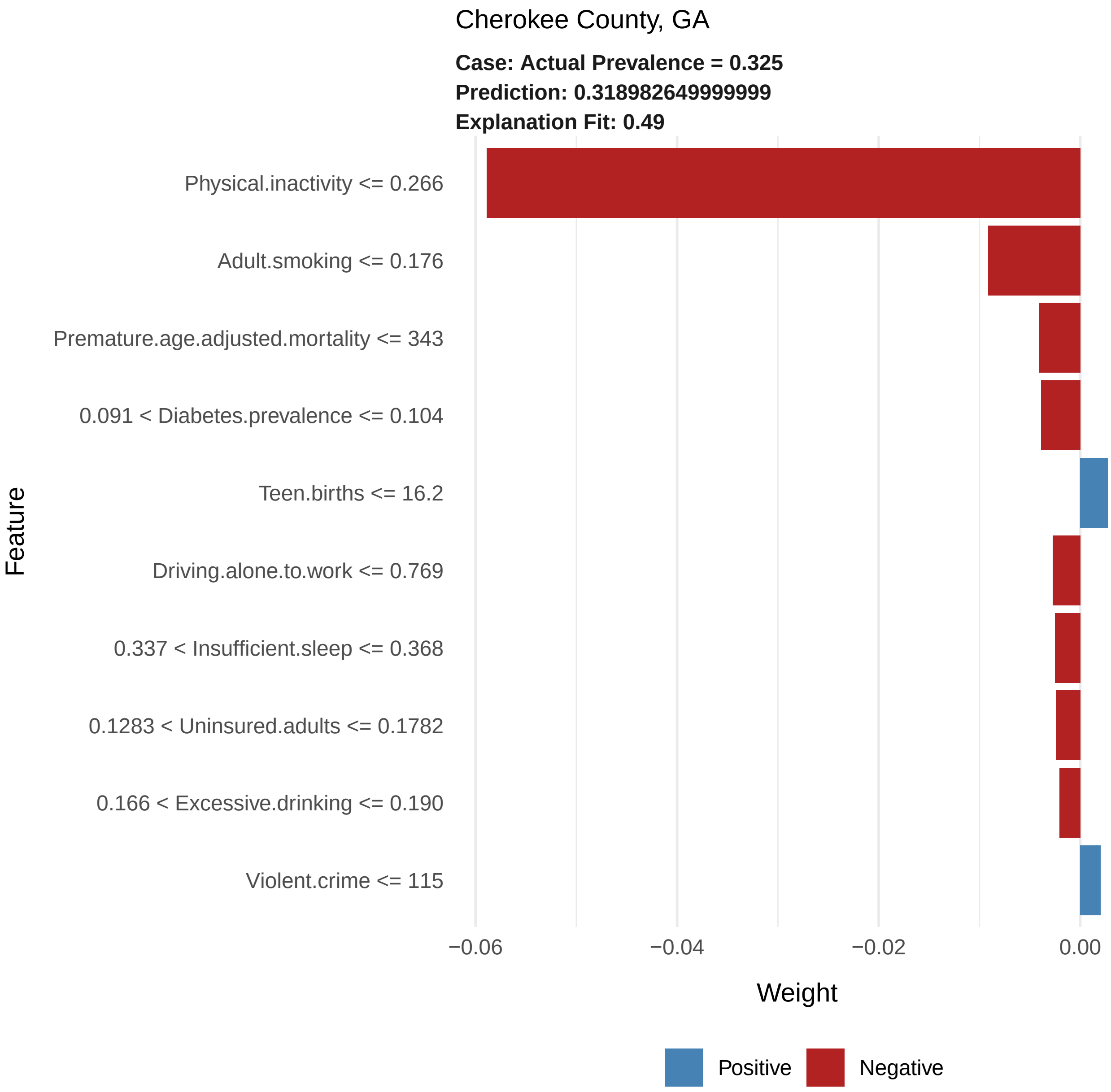

Cherokee County, GA
Case: Actual Prevalence = 0.325
Prediction: 0.318982649999999
Explanation Fit: 0.49
Physical.inactivity <= 0.266
Adult.smoking <= 0.176
Premature.age.adjusted.mortality <= 343
0.091 < Diabetes.prevalence <= 0.104
Teen.births <= 16.2
Driving.alone.to.work <= 0.769
0.337 < Insufficient.sleep <= 0.368
0.1283 < Uninsured.adults <= 0.1782
0.166 < Excessive.drinking <= 0.190
Violent.crime <= 115
Feature
−0.06
−0.04
−0.02
0.00
Weight
Positive
Negative

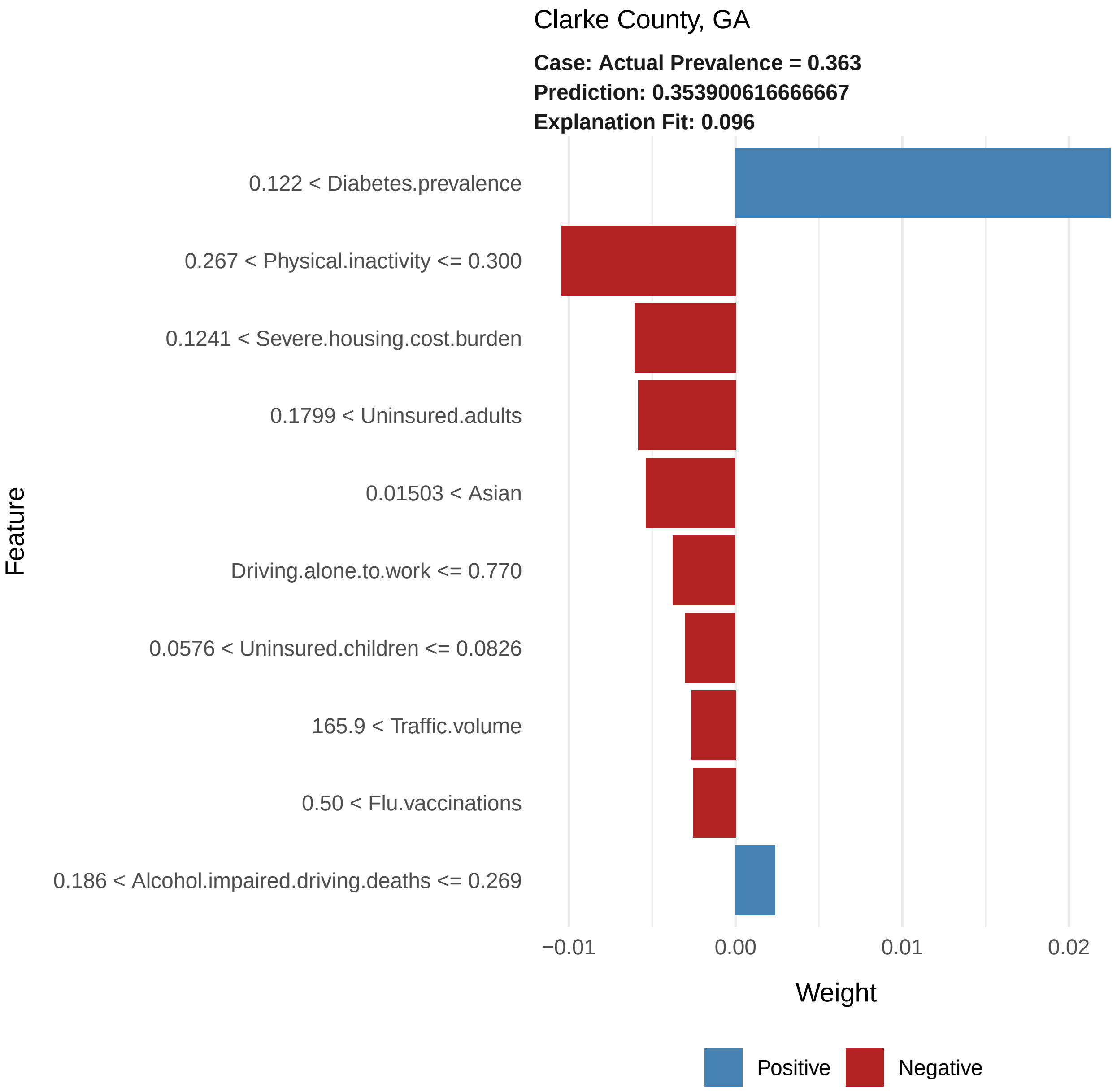

Clarke County, GA
Case: Actual Prevalence = 0.363
Prediction: 0.353900616666667
Explanation Fit: 0.096
0.122 < Diabetes.prevalence
0.267 < Physical.inactivity <= 0.300
0.1241 < Severe.housing.cost.burden
0.1799 < Uninsured.adults
0.01503 < Asian
Driving.alone.to.work <= 0.770
0.0576 < Uninsured.children <= 0.0826
165.9 < Traffic.volume
0.50 < Flu.vaccinations
0.186 < Alcohol.impaired.driving.deaths <= 0.269
Feature
−0.01
0.00
0.01
0.02
Weight
Positive
Negative

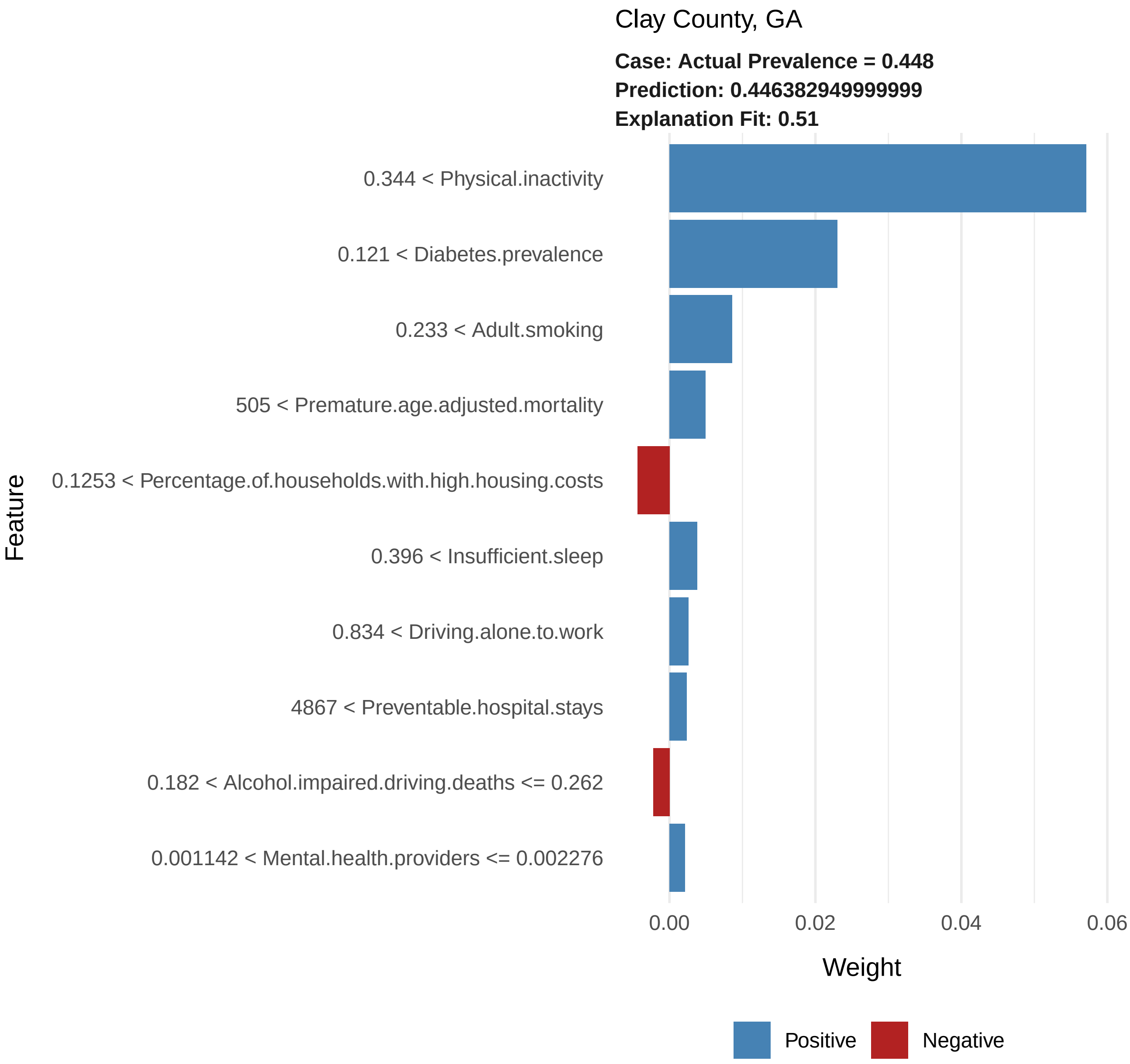
Clay County, GA
Case: Actual Prevalence = 0.448
Prediction: 0.446382949999999
Explanation Fit: 0.51
0.344 < Physical.inactivity
0.121 < Diabetes.prevalence
0.233 < Adult.smoking
505 < Premature.age.adjusted.mortality
0.1253 < Percentage.of.households.with.high.housing.costs
0.396 < Insufficient.sleep
0.834 < Driving.alone.to.work
4867 < Preventable.hospital.stays
0.182 < Alcohol.impaired.driving.deaths <= 0.262
0.001142 < Mental.health.providers <= 0.002276
Feature
0.00
0.02
0.04
0.06
Weight
Positive
Negative

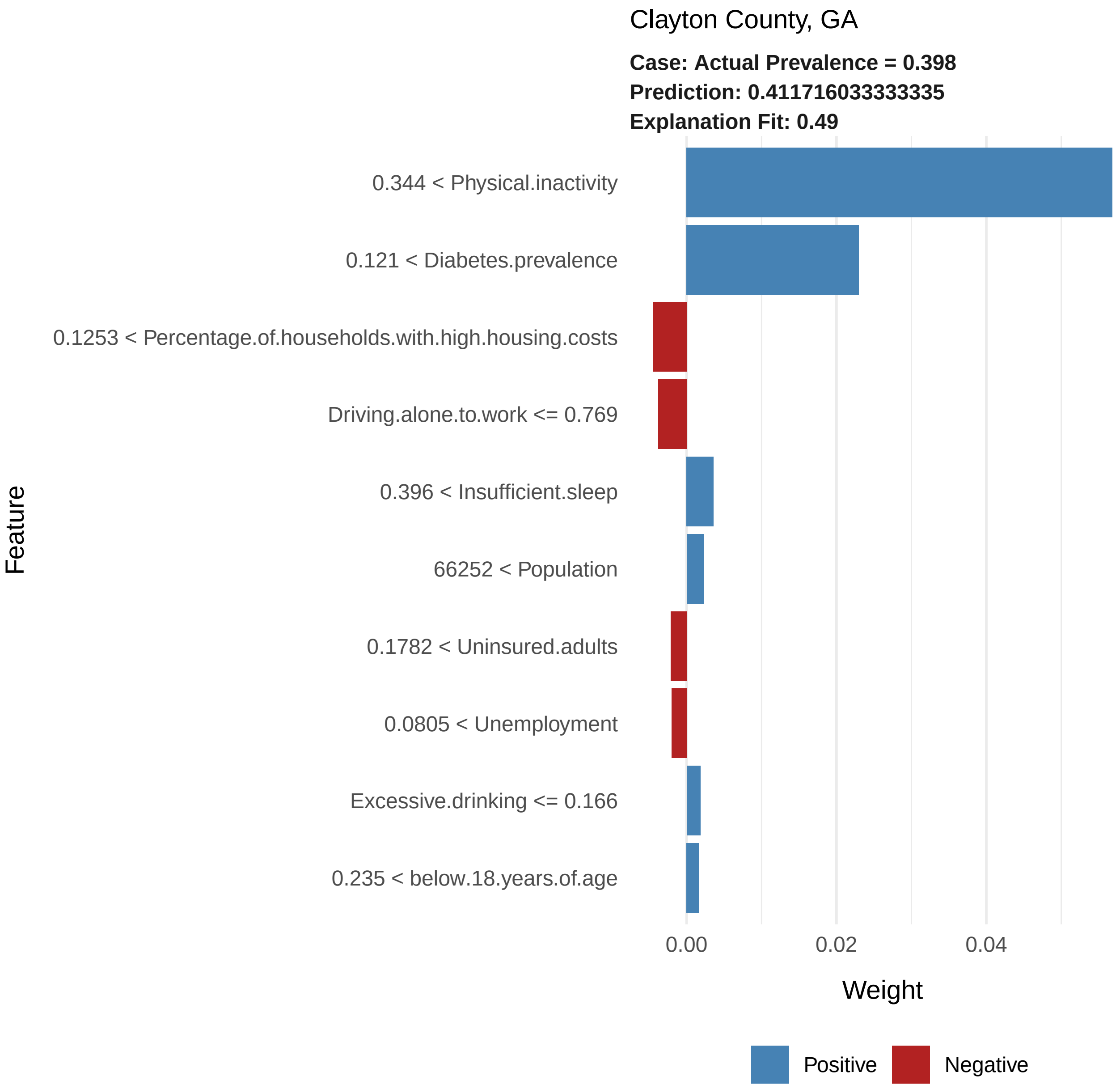

Clayton County, GA
Case: Actual Prevalence = 0.398
Prediction: 0.411716033333335
Explanation Fit: 0.49
0.344 < Physical.inactivity
0.121 < Diabetes.prevalence
0.1253 < Percentage.of.households.with.high.housing.costs
Driving.alone.to.work <= 0.769
0.396 < Insufficient.sleep
66252 < Population
0.1782 < Uninsured.adults
0.0805 < Unemployment
Excessive.drinking <= 0.166
0.235 < below.18.years.of.age
Feature
0.00
0.02
0.04
Weight
Positive
Negative

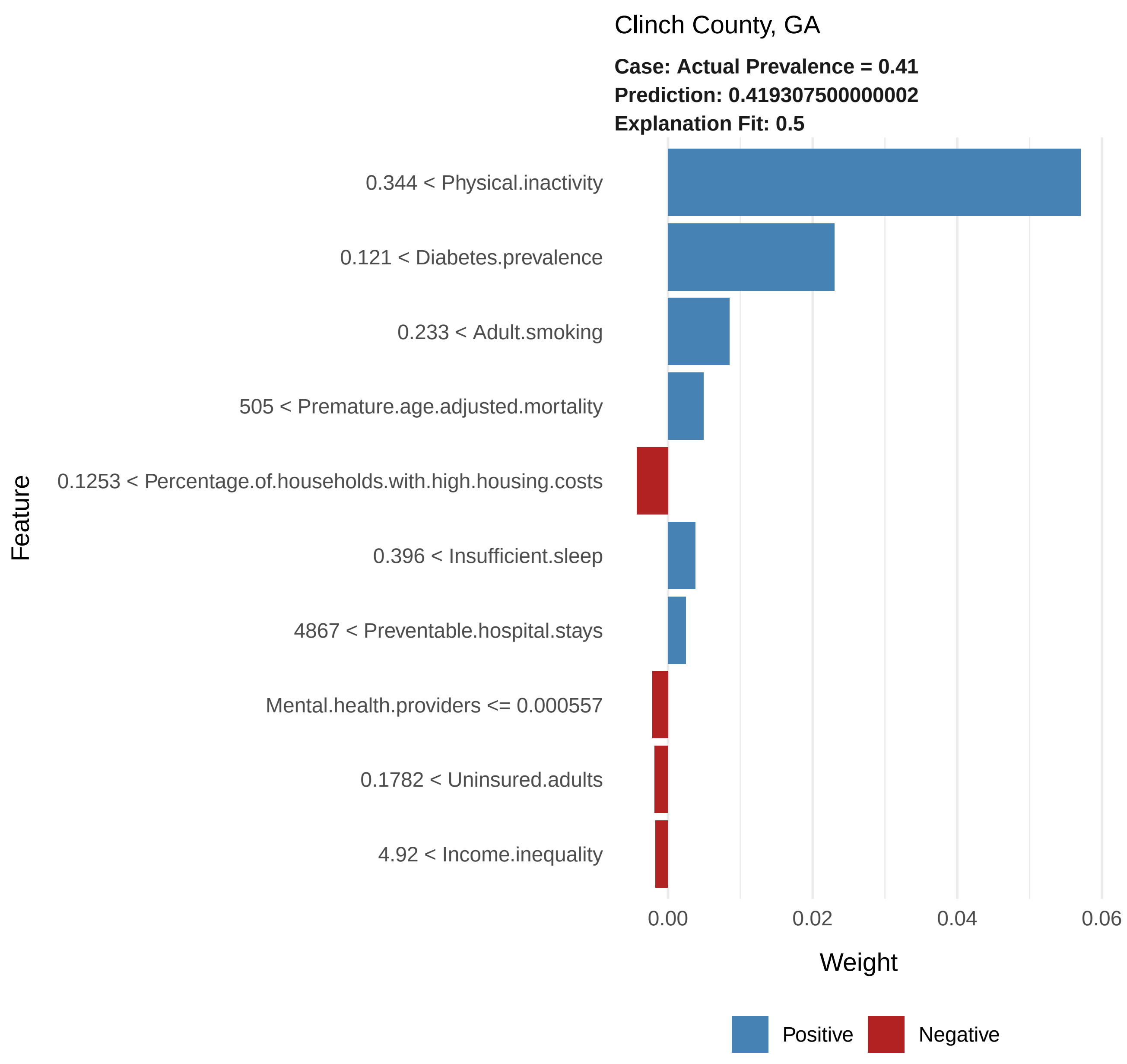
Clinch County, GA
Case: Actual Prevalence = 0.41
Prediction: 0.419307500000002
Explanation Fit: 0.5
0.344 < Physical.inactivity
0.121 < Diabetes.prevalence
0.233 < Adult.smoking
505 < Premature.age.adjusted.mortality
0.1253 < Percentage.of.households.with.high.housing.costs
0.396 < Insufficient.sleep
4867 < Preventable.hospital.stays
Mental.health.providers <= 0.000557
0.1782 < Uninsured.adults
4.92 < Income.inequality
Feature
0.00
0.02
0.04
0.06
Weight
Positive
Negative

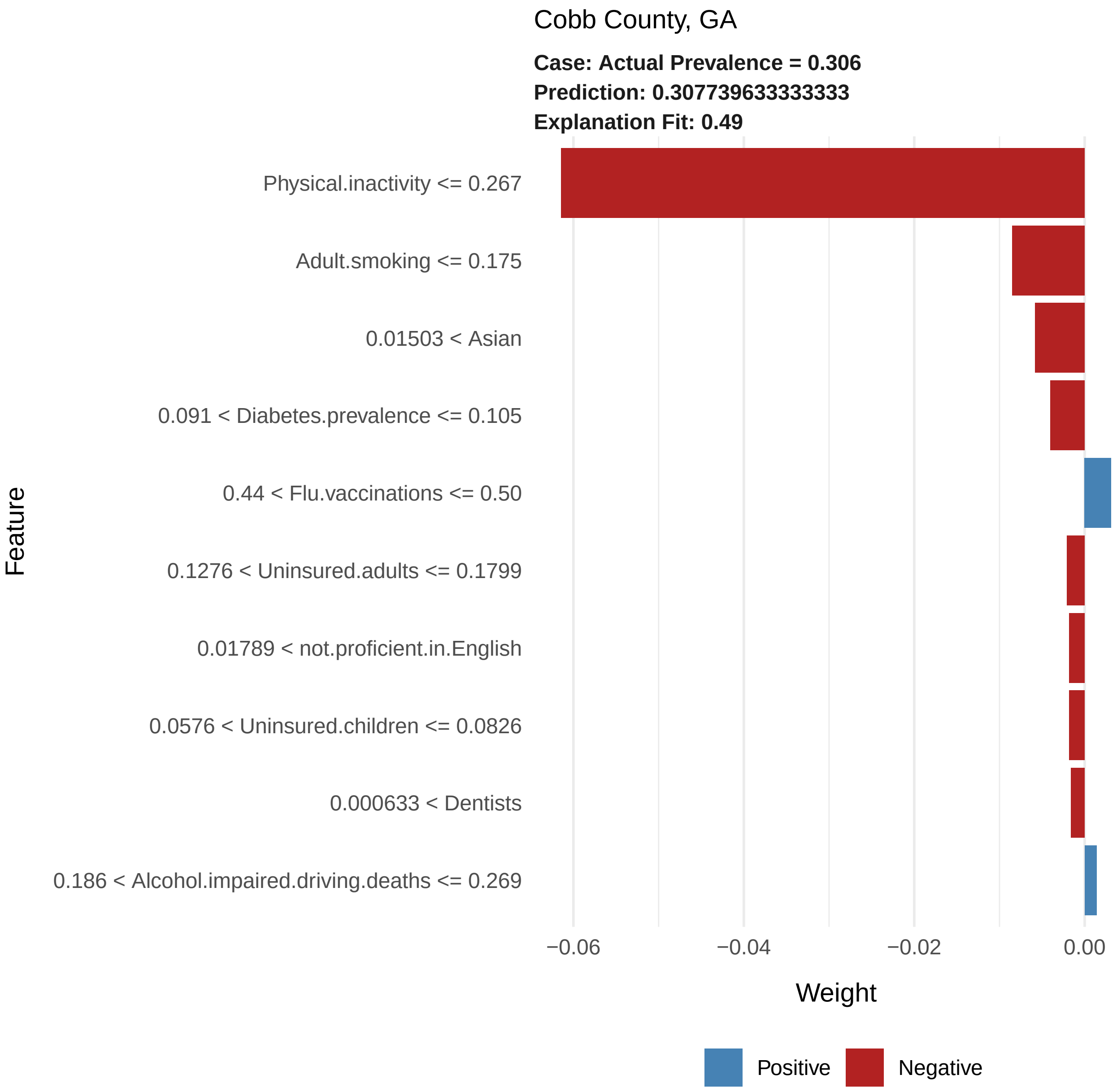

Cobb County, GA
Case: Actual Prevalence = 0.306
Prediction: 0.307739633333333
Explanation Fit: 0.49
Physical.inactivity <= 0.267
Adult.smoking <= 0.175
0.01503 < Asian
0.091 < Diabetes.prevalence <= 0.105
0.44 < Flu.vaccinations <= 0.50
0.1276 < Uninsured.adults <= 0.1799
0.01789 < not.proficient.in.English
0.0576 < Uninsured.children <= 0.0826
0.000633 < Dentists
0.186 < Alcohol.impaired.driving.deaths <= 0.269
Feature
−0.06
−0.04
−0.02
0.00
Weight
Positive
Negative

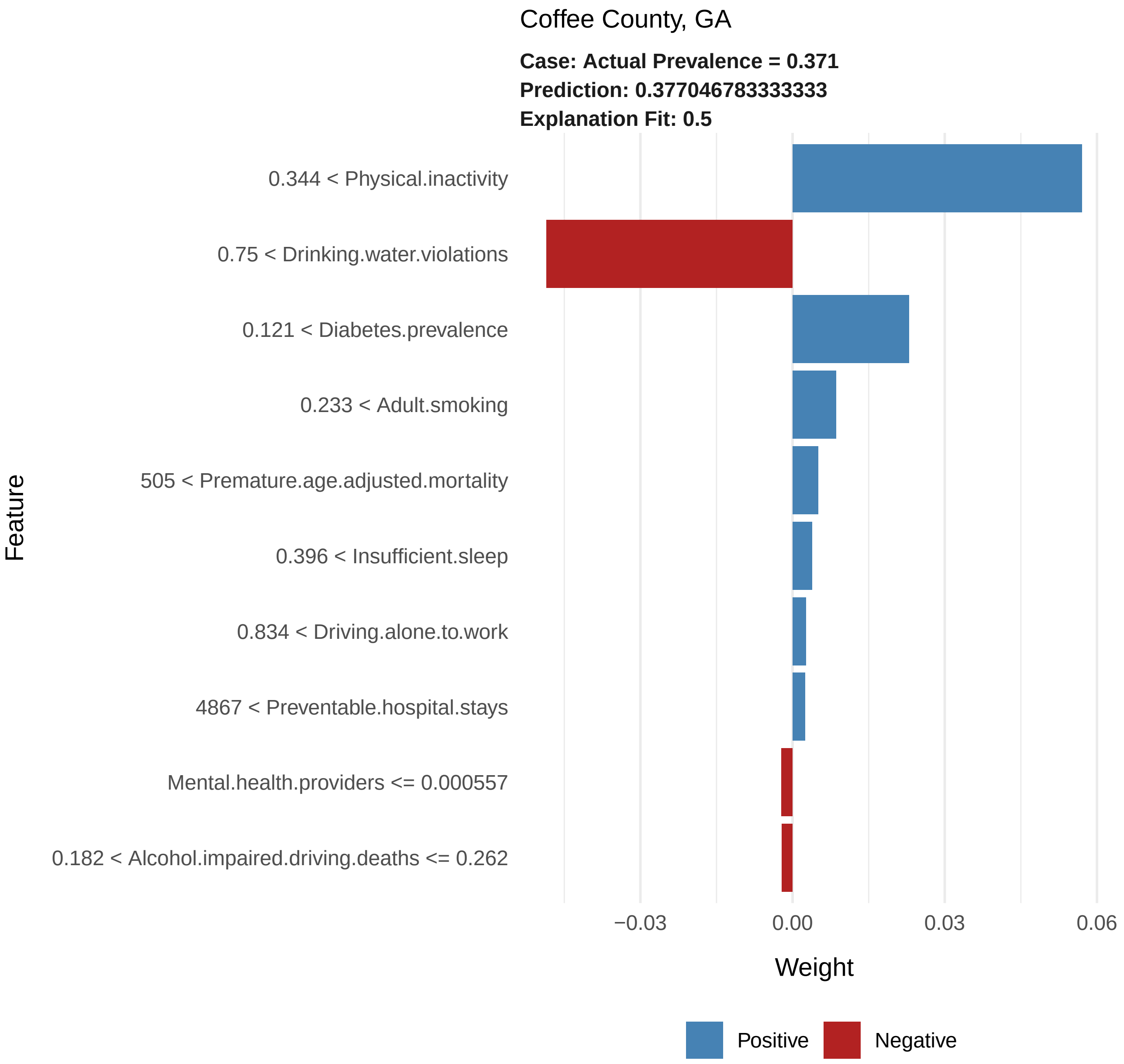

Coffee County, GA
Case: Actual Prevalence = 0.371
Prediction: 0.377046783333333
Explanation Fit: 0.5
0.344 < Physical.inactivity
0.75 < Drinking.water.violations
0.121 < Diabetes.prevalence
0.233 < Adult.smoking
505 < Premature.age.adjusted.mortality
0.396 < Insufficient.sleep
0.834 < Driving.alone.to.work
4867 < Preventable.hospital.stays
Mental.health.providers <= 0.000557
0.182 < Alcohol.impaired.driving.deaths <= 0.262
Feature
−0.03
0.00
0.03
0.06
Weight
Positive
Negative

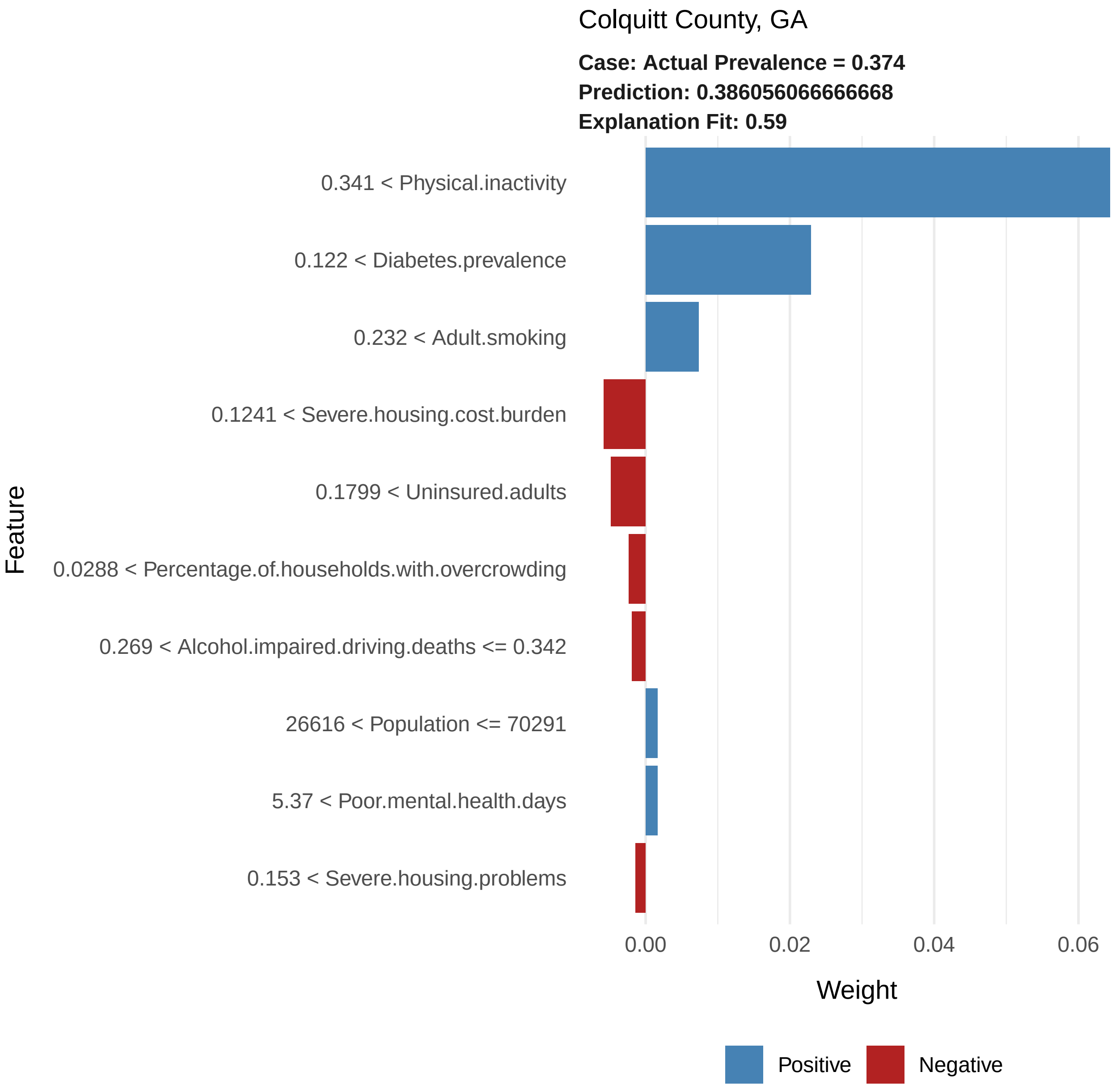

Colquitt County, GA
Case: Actual Prevalence = 0.374
Prediction: 0.386056066666668
Explanation Fit: 0.59
0.341 < Physical.inactivity
0.122 < Diabetes.prevalence
0.232 < Adult.smoking
0.1241 < Severe.housing.cost.burden
0.1799 < Uninsured.adults
0.0288 < Percentage.of.households.with.overcrowding
0.269 < Alcohol.impaired.driving.deaths <= 0.342
26616 < Population <= 70291
5.37 < Poor.mental.health.days
0.153 < Severe.housing.problems
Feature
0.00
0.02
0.04
0.06
Weight
Positive
Negative

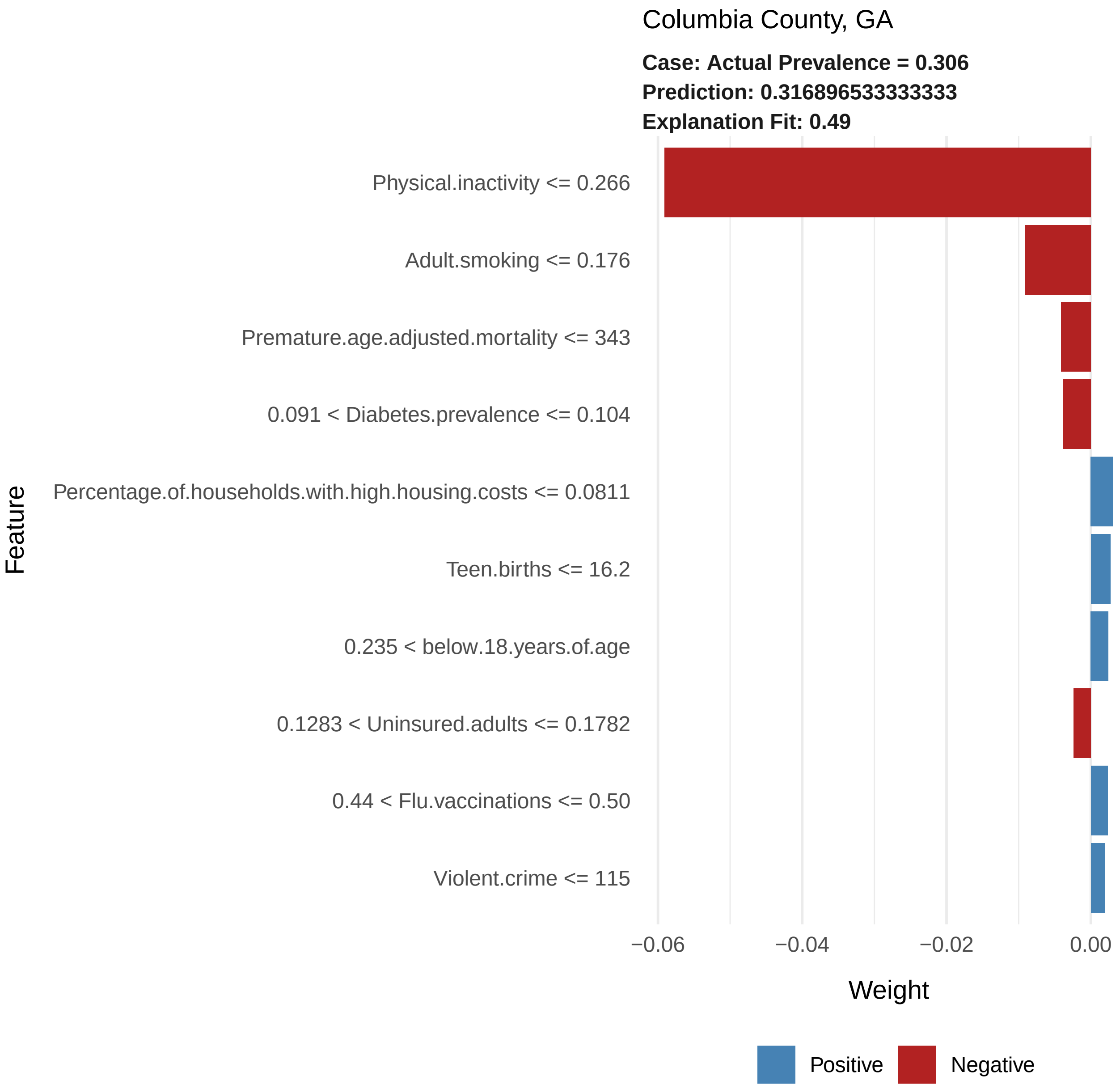

Columbia County, GA
Case: Actual Prevalence = 0.306
Prediction: 0.316896533333333
Explanation Fit: 0.49
Physical.inactivity <= 0.266
Adult.smoking <= 0.176
Premature.age.adjusted.mortality <= 343
0.091 < Diabetes.prevalence <= 0.104
Percentage.of.households.with.high.housing.costs <= 0.0811
Teen.births <= 16.2
0.235 < below.18.years.of.age
0.1283 < Uninsured.adults <= 0.1782
0.44 < Flu.vaccinations <= 0.50
Violent.crime <= 115
Feature
−0.06
−0.04
−0.02
0.00
Weight
Positive
Negative

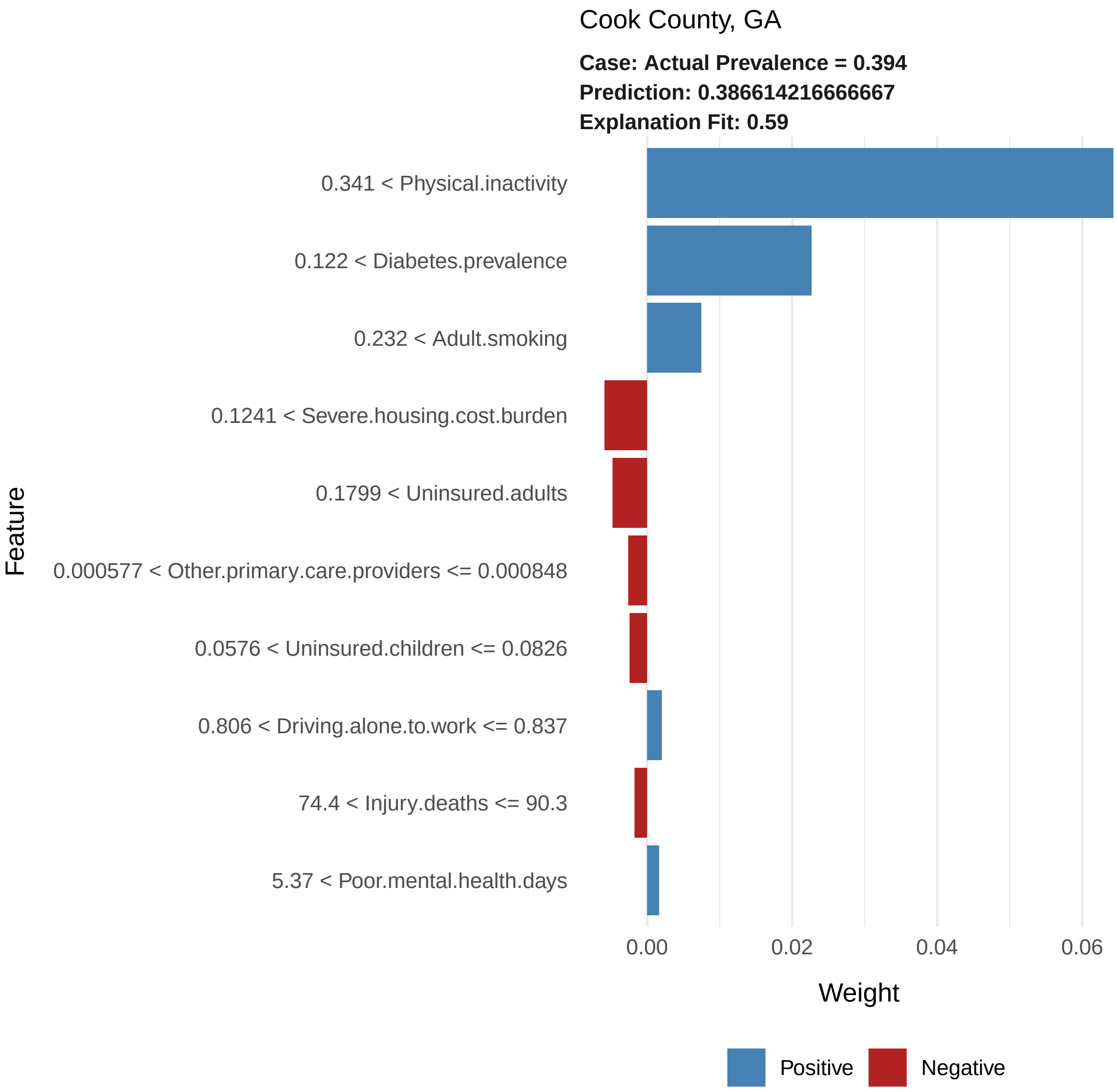

Cook County, GA
Case: Actual Prevalence = 0.394
Prediction: 0.386614216666667
Explanation Fit: 0.59
0.341 < Physical.inactivity
0.122 < Diabetes.prevalence
0.232 < Adult.smoking
0.1241 < Severe.housing.cost.burden
0.1799 < Uninsured.adults
0.000577 < Other.primary.care.providers <= 0.000848
0.0576 < Uninsured.children <= 0.0826
0.806 < Driving.alone.to.work <= 0.837
74.4 < Injury.deaths <= 90.3
5.37 < Poor.mental.health.days
Feature
0.00
0.02
0.04
0.06
Weight
Positive
Negative

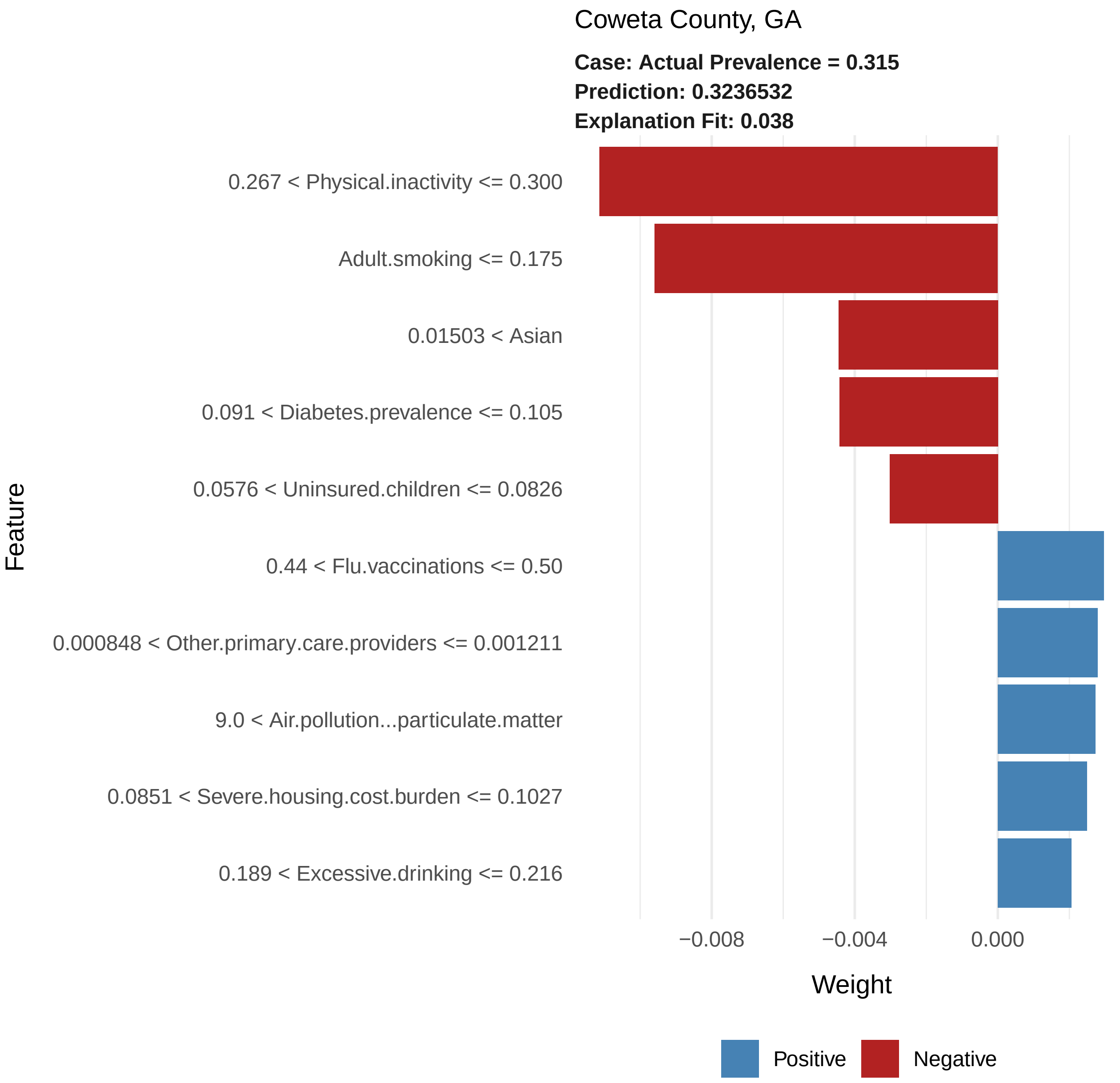
Coweta County, GA
Case: Actual Prevalence = 0.315
Prediction: 0.3236532
Explanation Fit: 0.038
0.267 < Physical.inactivity <= 0.300
Adult.smoking <= 0.175
0.01503 < Asian
0.091 < Diabetes.prevalence <= 0.105
0.0576 < Uninsured.children <= 0.0826
0.44 < Flu.vaccinations <= 0.50
0.000848 < Other.primary.care.providers <= 0.001211
9.0 < Air.pollution...particulate.matter
0.0851 < Severe.housing.cost.burden <= 0.1027
0.189 < Excessive.drinking <= 0.216
Feature
−0.008
−0.004
0.000
Weight
Positive
Negative

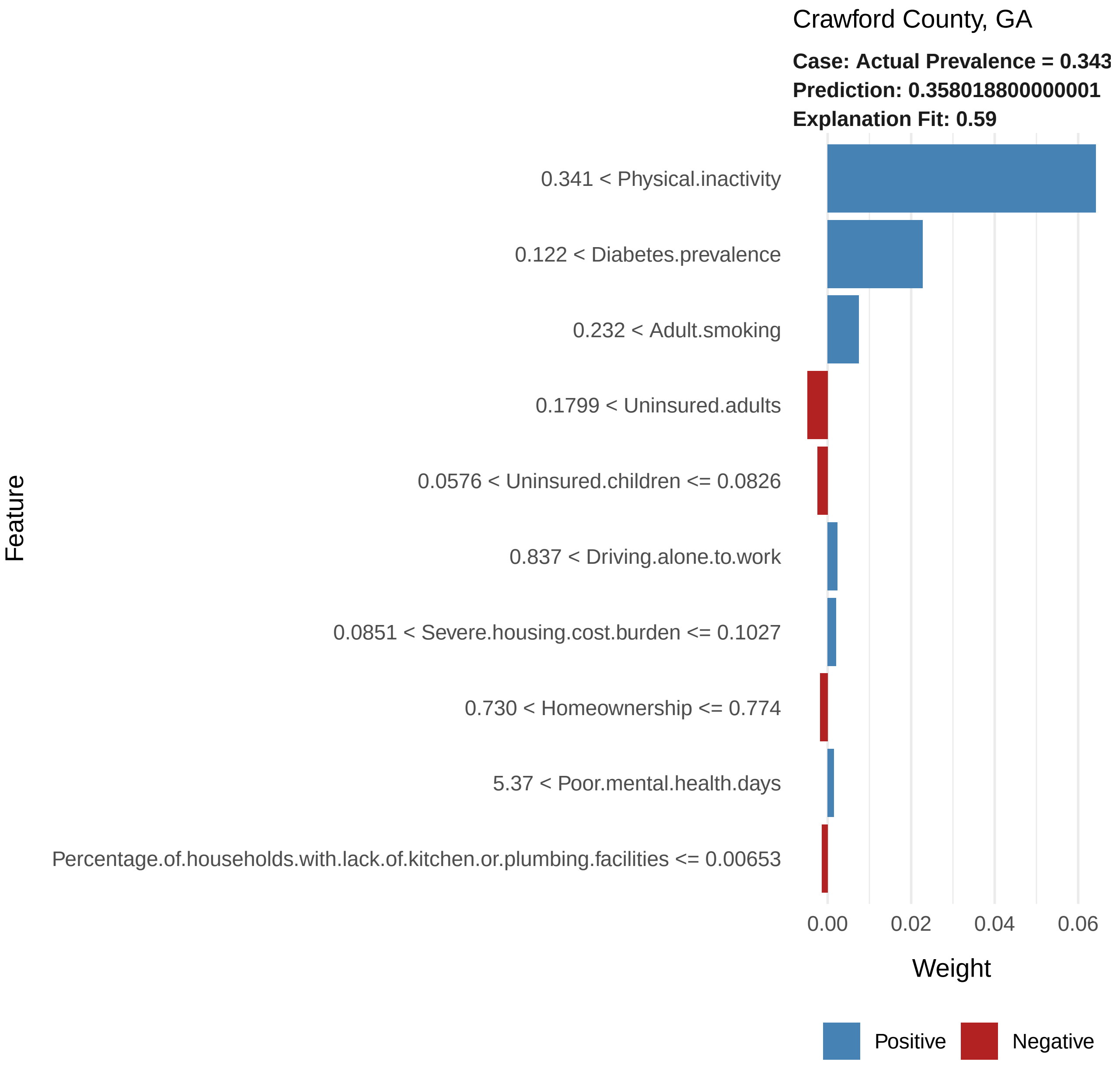
Crawford County, GA
Case: Actual Prevalence = 0.343
Prediction: 0.358018800000001
Explanation Fit: 0.59
0.341 < Physical.inactivity
0.122 < Diabetes.prevalence
0.232 < Adult.smoking
0.1799 < Uninsured.adults
0.0576 < Uninsured.children <= 0.0826
0.837 < Driving.alone.to.work
0.0851 < Severe.housing.cost.burden <= 0.1027
0.730 < Homeownership <= 0.774
5.37 < Poor.mental.health.days
Percentage.of.households.with.lack.of.kitchen.or.plumbing.facilities <= 0.00653
Feature
0.00
0.02
0.04
0.06
Weight
Positive
Negative

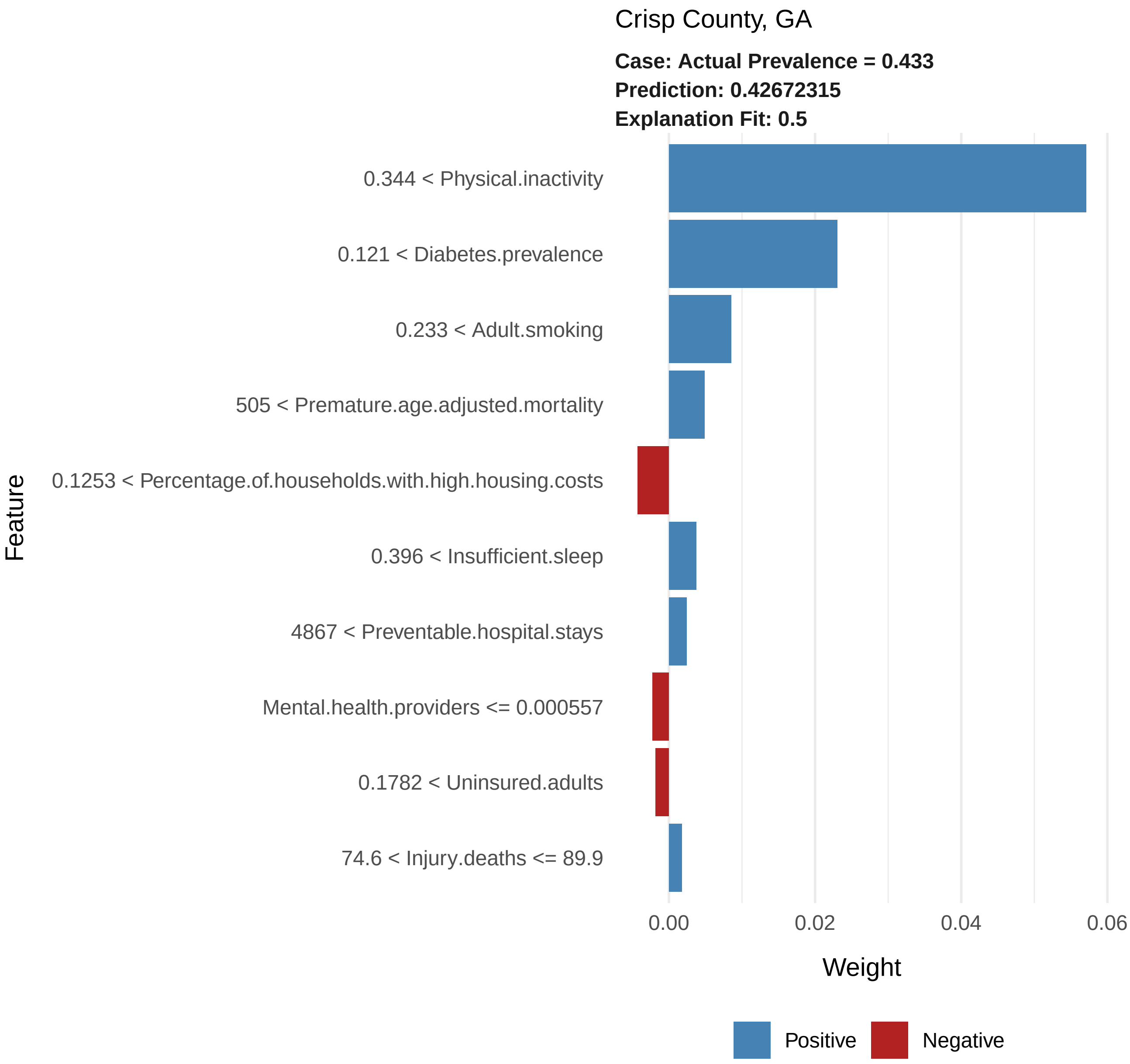

Crisp County, GA
Case: Actual Prevalence = 0.433
Prediction: 0.42672315
Explanation Fit: 0.5
0.344 < Physical.inactivity
0.121 < Diabetes.prevalence
0.233 < Adult.smoking
505 < Premature.age.adjusted.mortality
0.1253 < Percentage.of.households.with.high.housing.costs
0.396 < Insufficient.sleep
4867 < Preventable.hospital.stays
Mental.health.providers <= 0.000557
0.1782 < Uninsured.adults
74.6 < Injury.deaths <= 89.9
Feature
0.00
0.02
0.04
0.06
Weight
Positive
Negative

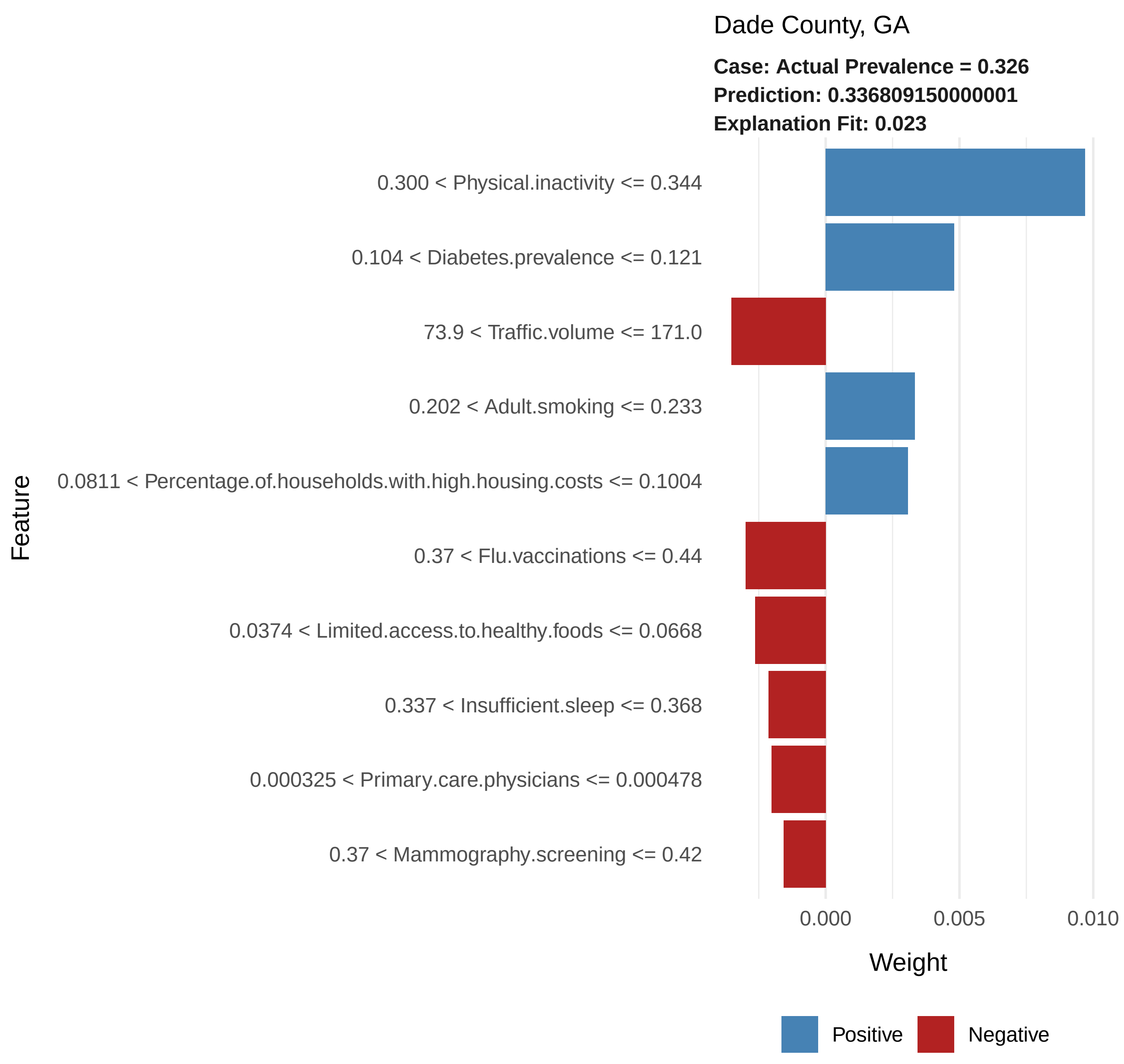
Dade County, GA
Case: Actual Prevalence = 0.326
Prediction: 0.336809150000001
Explanation Fit: 0.023
0.300 < Physical.inactivity <= 0.344
0.104 < Diabetes.prevalence <= 0.121
73.9 < Traffic.volume <= 171.0
0.202 < Adult.smoking <= 0.233
0.0811 < Percentage.of.households.with.high.housing.costs <= 0.1004
0.37 < Flu.vaccinations <= 0.44
0.0374 < Limited.access.to.healthy.foods <= 0.0668
0.337 < Insufficient.sleep <= 0.368
0.000325 < Primary.care.physicians <= 0.000478
0.37 < Mammography.screening <= 0.42
Feature
0.000
0.005
0.010
Weight
Positive
Negative

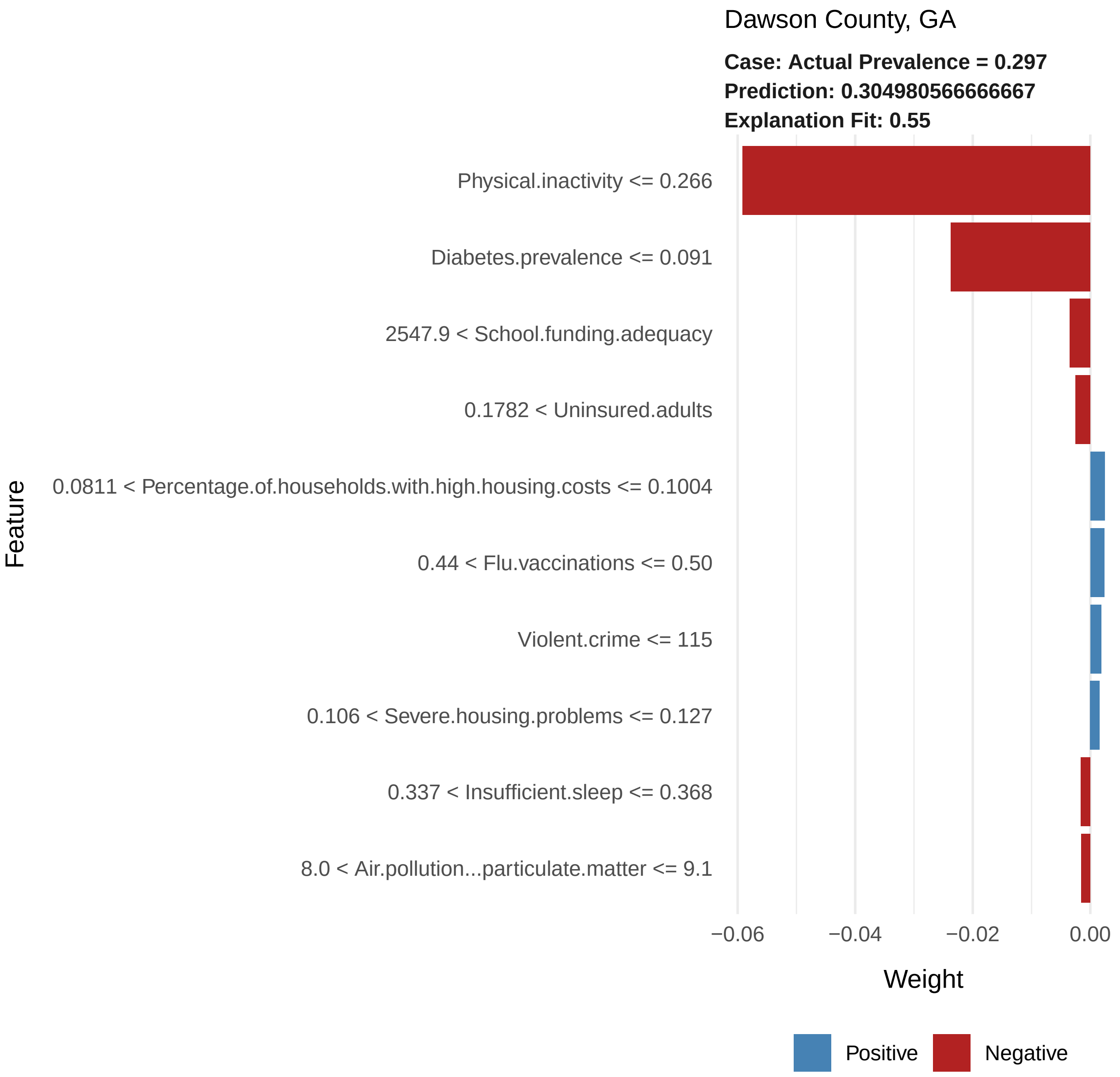
Dawson County, GA
Case: Actual Prevalence = 0.297
Prediction: 0.304980566666667
Explanation Fit: 0.55
Physical.inactivity <= 0.266
Diabetes.prevalence <= 0.091
2547.9 < School.funding.adequacy
0.1782 < Uninsured.adults
0.0811 < Percentage.of.households.with.high.housing.costs <= 0.1004
0.44 < Flu.vaccinations <= 0.50
Violent.crime <= 115
0.106 < Severe.housing.problems <= 0.127
0.337 < Insufficient.sleep <= 0.368
8.0 < Air.pollution...particulate.matter <= 9.1
Feature
−0.06
−0.04
−0.02
0.00
Weight
Positive
Negative

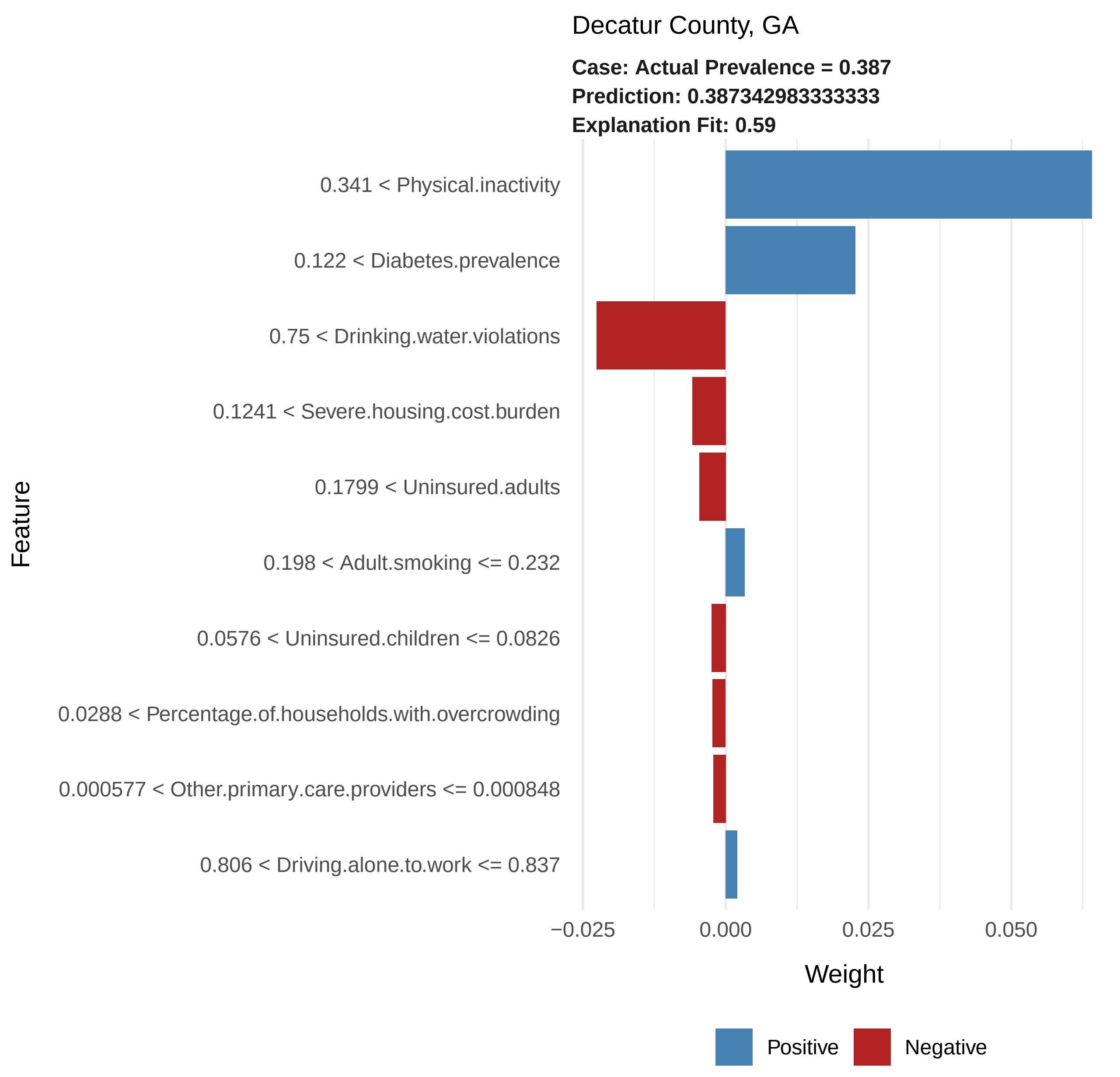
Decatur County, GA
Case: Actual Prevalence = 0.387
Prediction: 0.387342983333333
Explanation Fit: 0.59
0.341 < Physical.inactivity
0.122 < Diabetes.prevalence
0.75 < Drinking.water.violations
0.1241 < Severe.housing.cost.burden
0.1799 < Uninsured.adults
0.198 < Adult.smoking <= 0.232
0.0576 < Uninsured.children <= 0.0826
0.0288 < Percentage.of.households.with.overcrowding
0.000577 < Other.primary.care.providers <= 0.000848
0.806 < Driving.alone.to.work <= 0.837
Feature
−0.025
0.000
0.025
0.050
Weight
Positive
Negative

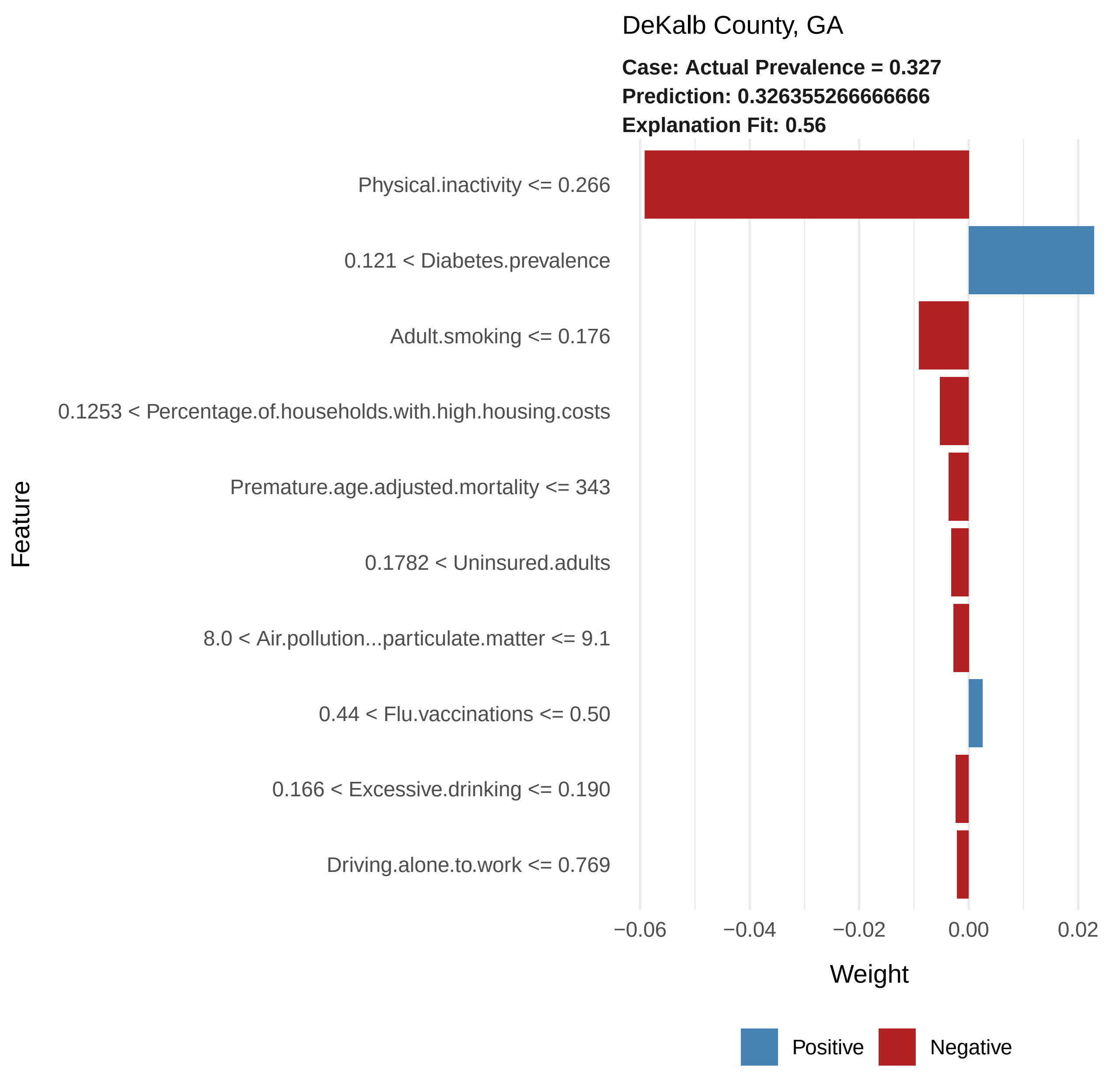
DeKalb County, GA
Case: Actual Prevalence = 0.327
Prediction: 0.326355266666666
Explanation Fit: 0.56
Physical.inactivity <= 0.266
0.121 < Diabetes.prevalence
Adult.smoking <= 0.176
0.1253 < Percentage.of.households.with.high.housing.costs
Premature.age.adjusted.mortality <= 343
0.1782 < Uninsured.adults
8.0 < Air.pollution...particulate.matter <= 9.1
0.44 < Flu.vaccinations <= 0.50
0.166 < Excessive.drinking <= 0.190
Driving.alone.to.work <= 0.769
Feature
−0.06
−0.04
−0.02
0.00
0.02
Weight
Positive
Negative

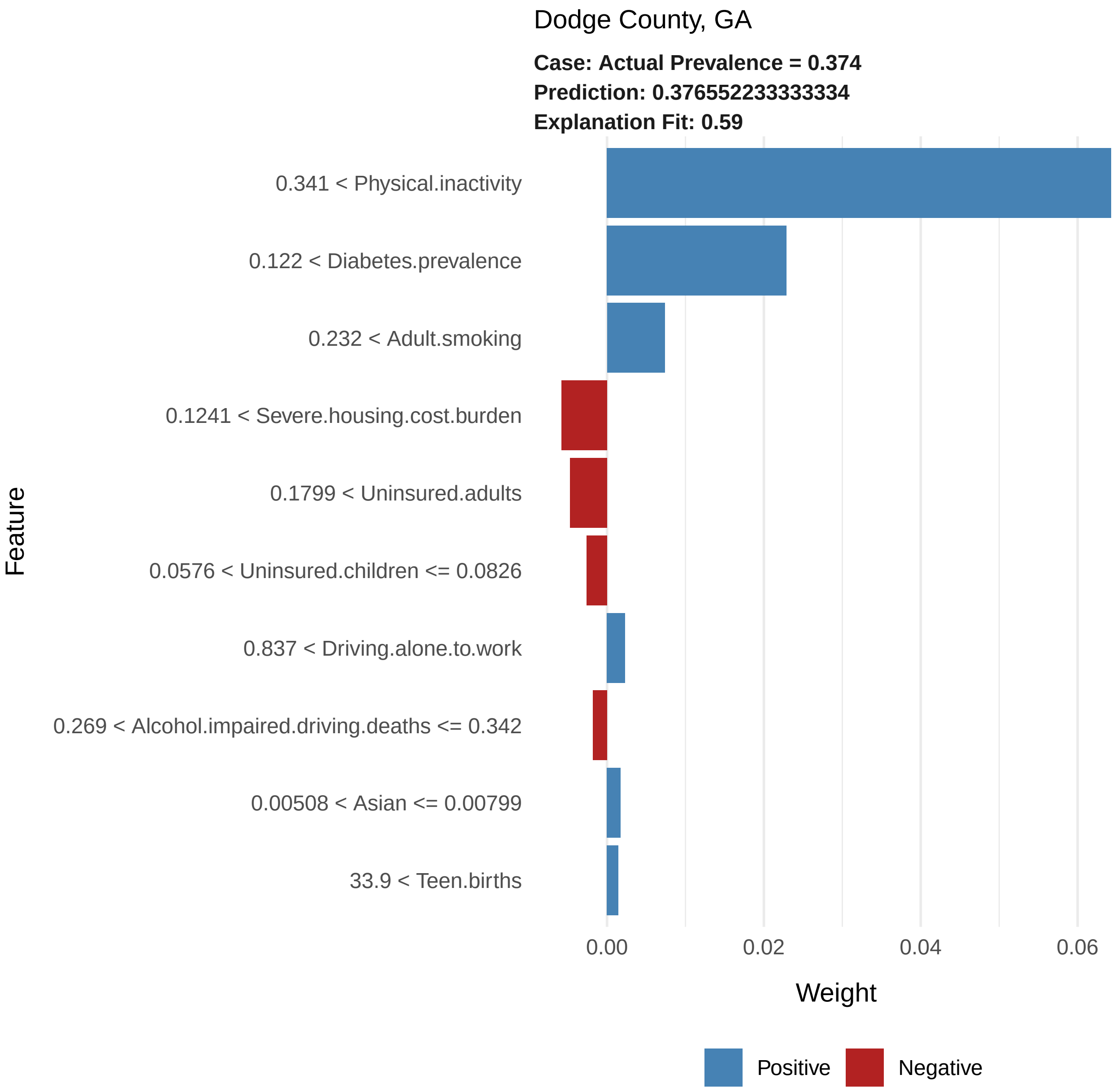
Dodge County, GA
Case: Actual Prevalence = 0.374
Prediction: 0.376552233333334
Explanation Fit: 0.59
0.341 < Physical.inactivity
0.122 < Diabetes.prevalence
0.232 < Adult.smoking
0.1241 < Severe.housing.cost.burden
0.1799 < Uninsured.adults
0.0576 < Uninsured.children <= 0.0826
0.837 < Driving.alone.to.work
0.269 < Alcohol.impaired.driving.deaths <= 0.342
0.00508 < Asian <= 0.00799
33.9 < Teen.births
Feature
0.00
0.02
0.04
0.06
Weight
Positive
Negative

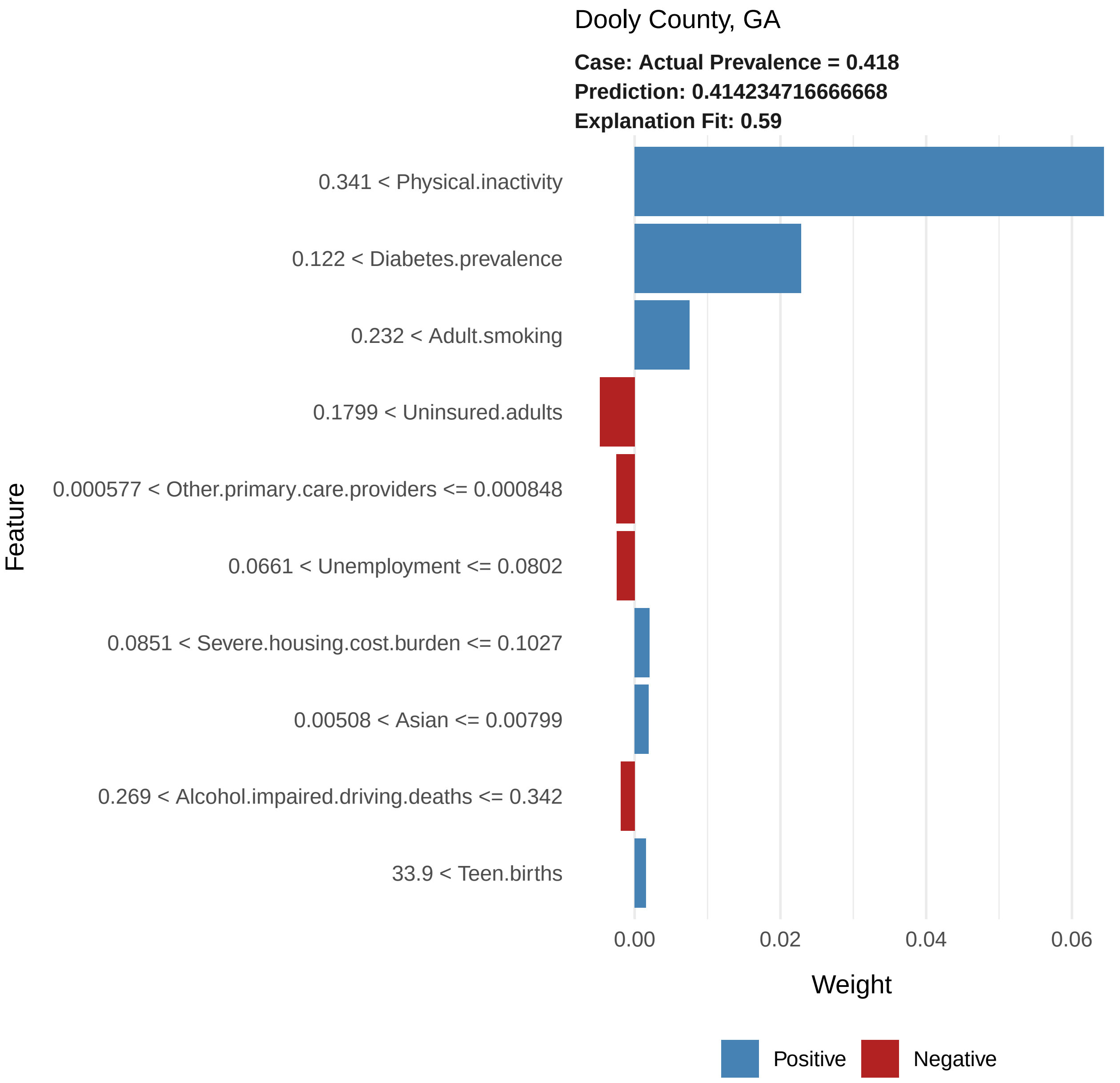
Dooly County, GA
Case: Actual Prevalence = 0.418
Prediction: 0.414234716666668
Explanation Fit: 0.59
0.341 < Physical.inactivity
0.122 < Diabetes.prevalence
0.232 < Adult.smoking
0.1799 < Uninsured.adults
0.000577 < Other.primary.care.providers <= 0.000848
0.0661 < Unemployment <= 0.0802
0.0851 < Severe.housing.cost.burden <= 0.1027
0.00508 < Asian <= 0.00799
0.269 < Alcohol.impaired.driving.deaths <= 0.342
33.9 < Teen.births
Feature
0.00
0.02
0.04
0.06
Weight
Positive
Negative

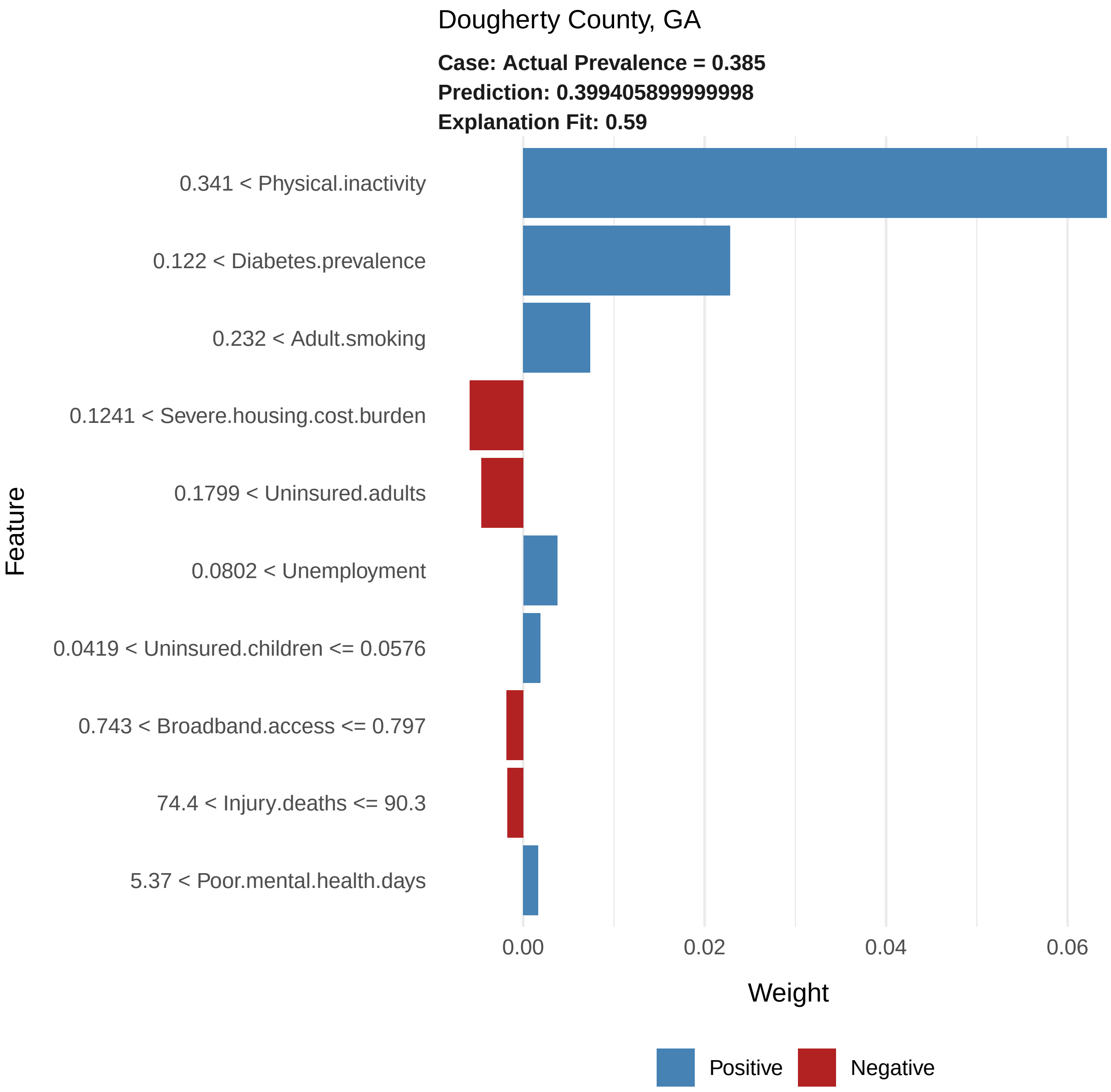

Dougherty County, GA
Case: Actual Prevalence = 0.385
Prediction: 0.399405899999998
Explanation Fit: 0.59
0.341 < Physical.inactivity
0.122 < Diabetes.prevalence
0.232 < Adult.smoking
0.1241 < Severe.housing.cost.burden
0.1799 < Uninsured.adults
0.0802 < Unemployment
0.0419 < Uninsured.children <= 0.0576
0.743 < Broadband.access <= 0.797
74.4 < Injury.deaths <= 90.3
5.37 < Poor.mental.health.days
Feature
0.00
0.02
0.04
0.06
Weight
Positive
Negative

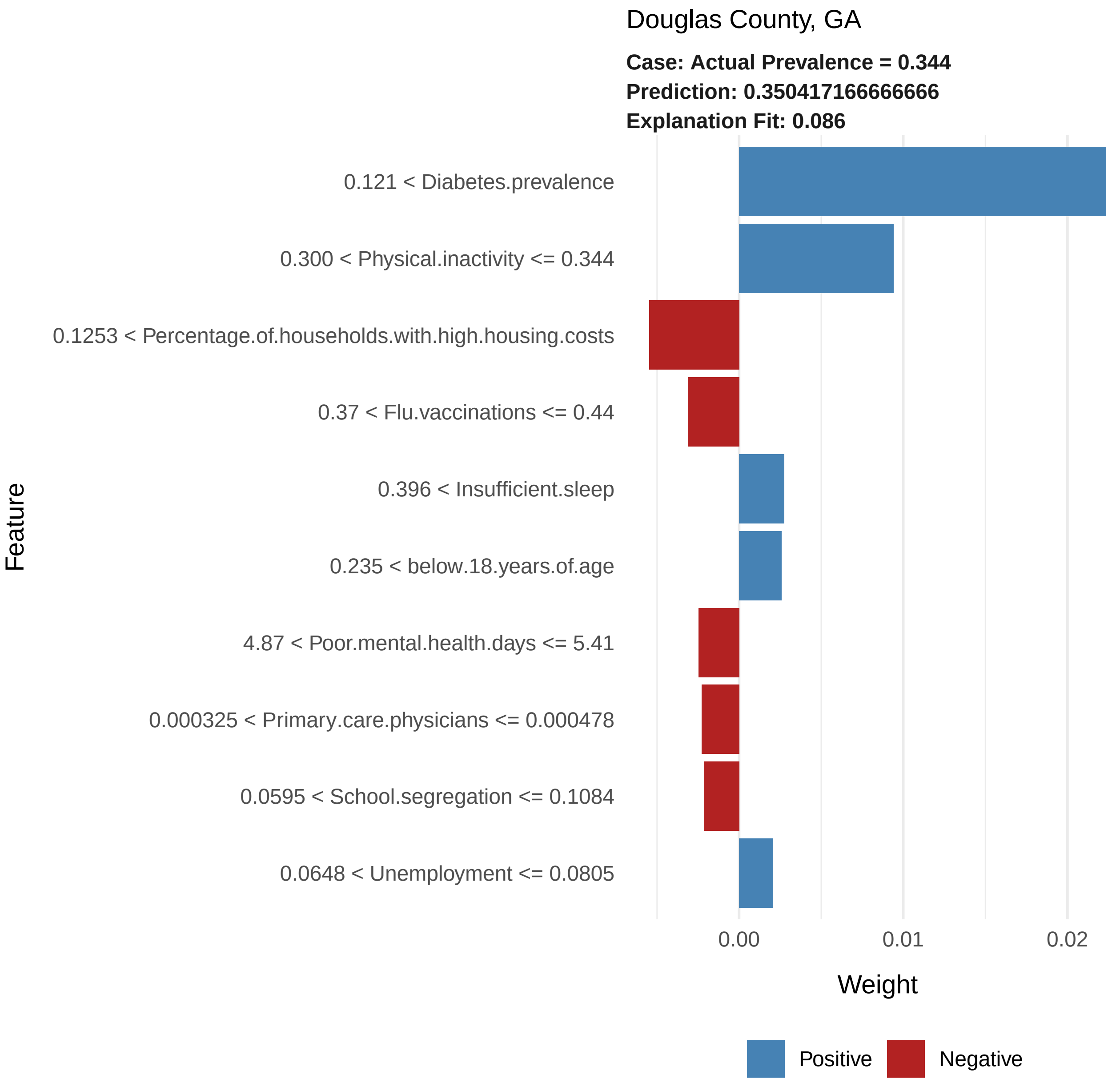
Douglas County, GA
Case: Actual Prevalence = 0.344
Prediction: 0.350417166666666
Explanation Fit: 0.086
0.121 < Diabetes.prevalence
0.300 < Physical.inactivity <= 0.344
0.1253 < Percentage.of.households.with.high.housing.costs
0.37 < Flu.vaccinations <= 0.44
0.396 < Insufficient.sleep
0.235 < below.18.years.of.age
4.87 < Poor.mental.health.days <= 5.41
0.000325 < Primary.care.physicians <= 0.000478
0.0595 < School.segregation <= 0.1084
0.0648 < Unemployment <= 0.0805
Feature
0.00
0.01
0.02
Weight
Positive
Negative

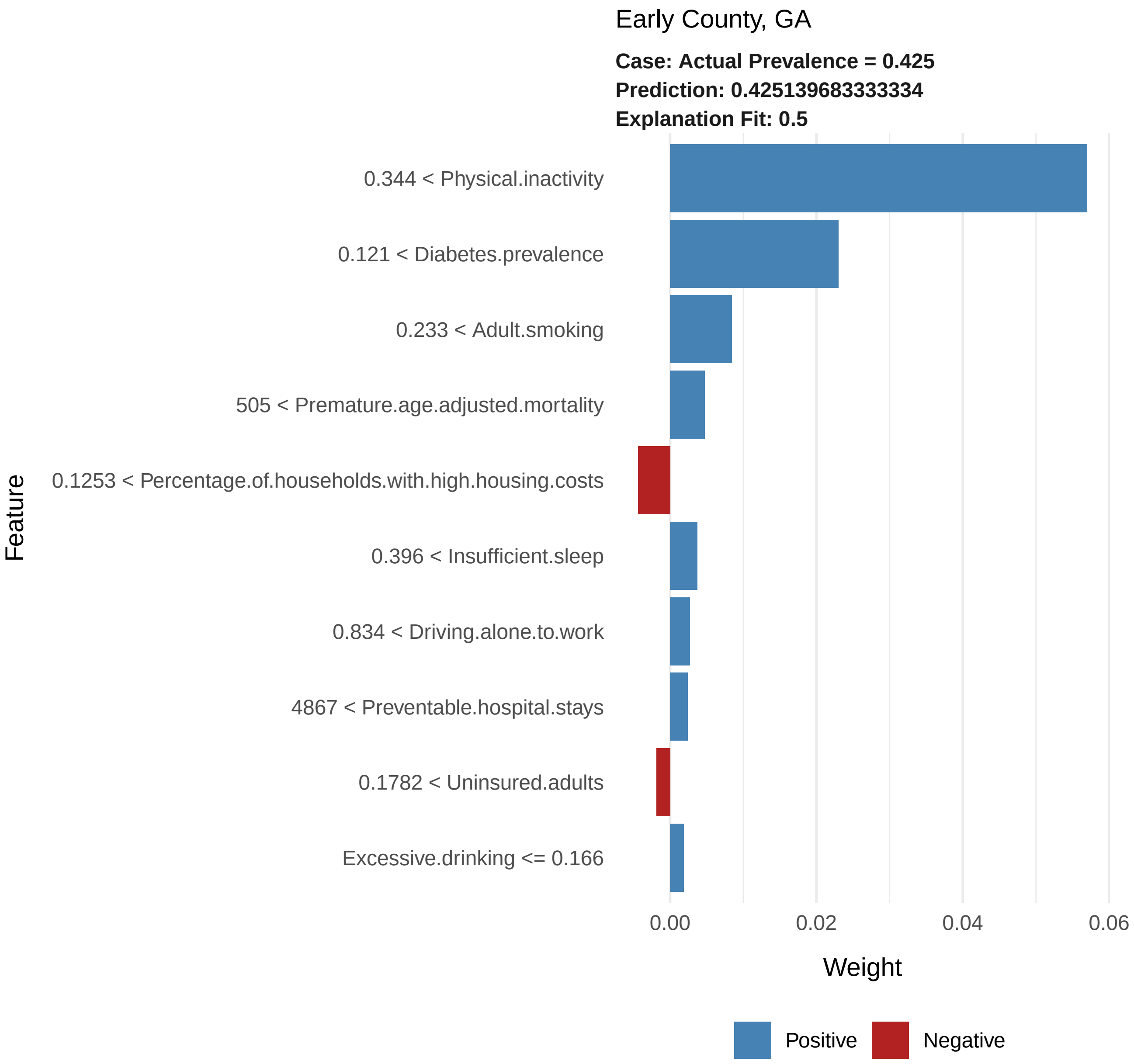

Early County, GA
Case: Actual Prevalence = 0.425
Prediction: 0.425139683333334
Explanation Fit: 0.5
0.344 < Physical.inactivity
0.121 < Diabetes.prevalence
0.233 < Adult.smoking
505 < Premature.age.adjusted.mortality
0.1253 < Percentage.of.households.with.high.housing.costs
0.396 < Insufficient.sleep
0.834 < Driving.alone.to.work
4867 < Preventable.hospital.stays
0.1782 < Uninsured.adults
Excessive.drinking <= 0.166
Feature
0.00
0.02
0.04
0.06
Weight
Positive
Negative

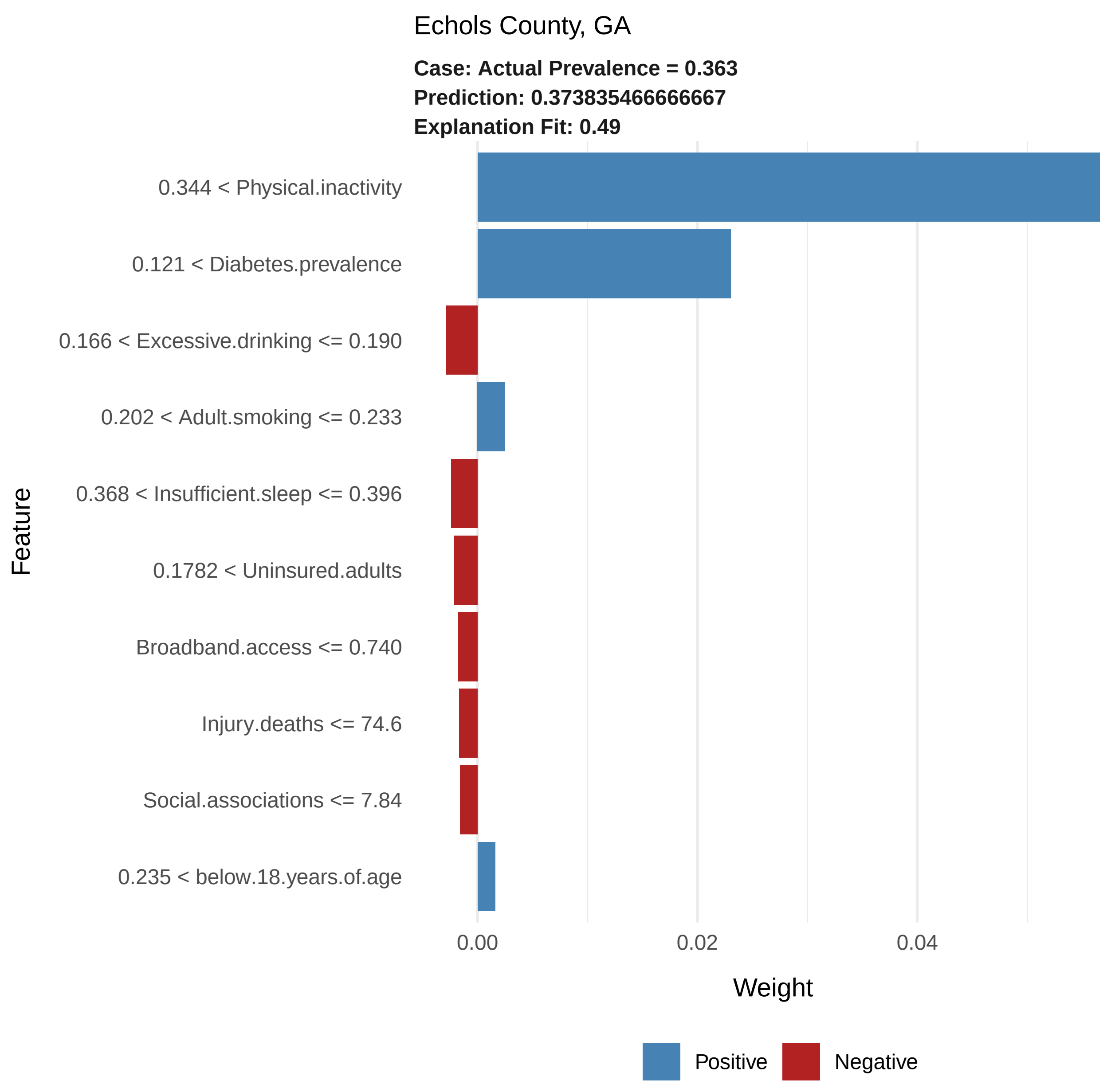

Echols County, GA
Case: Actual Prevalence = 0.363
Prediction: 0.373835466666667
Explanation Fit: 0.49
0.344 < Physical.inactivity
0.121 < Diabetes.prevalence
0.166 < Excessive.drinking <= 0.190
0.202 < Adult.smoking <= 0.233
0.368 < Insufficient.sleep <= 0.396
0.1782 < Uninsured.adults
Broadband.access <= 0.740
Injury.deaths <= 74.6
Social.associations <= 7.84
0.235 < below.18.years.of.age
Feature
0.00
0.02
0.04
Weight
Positive
Negative

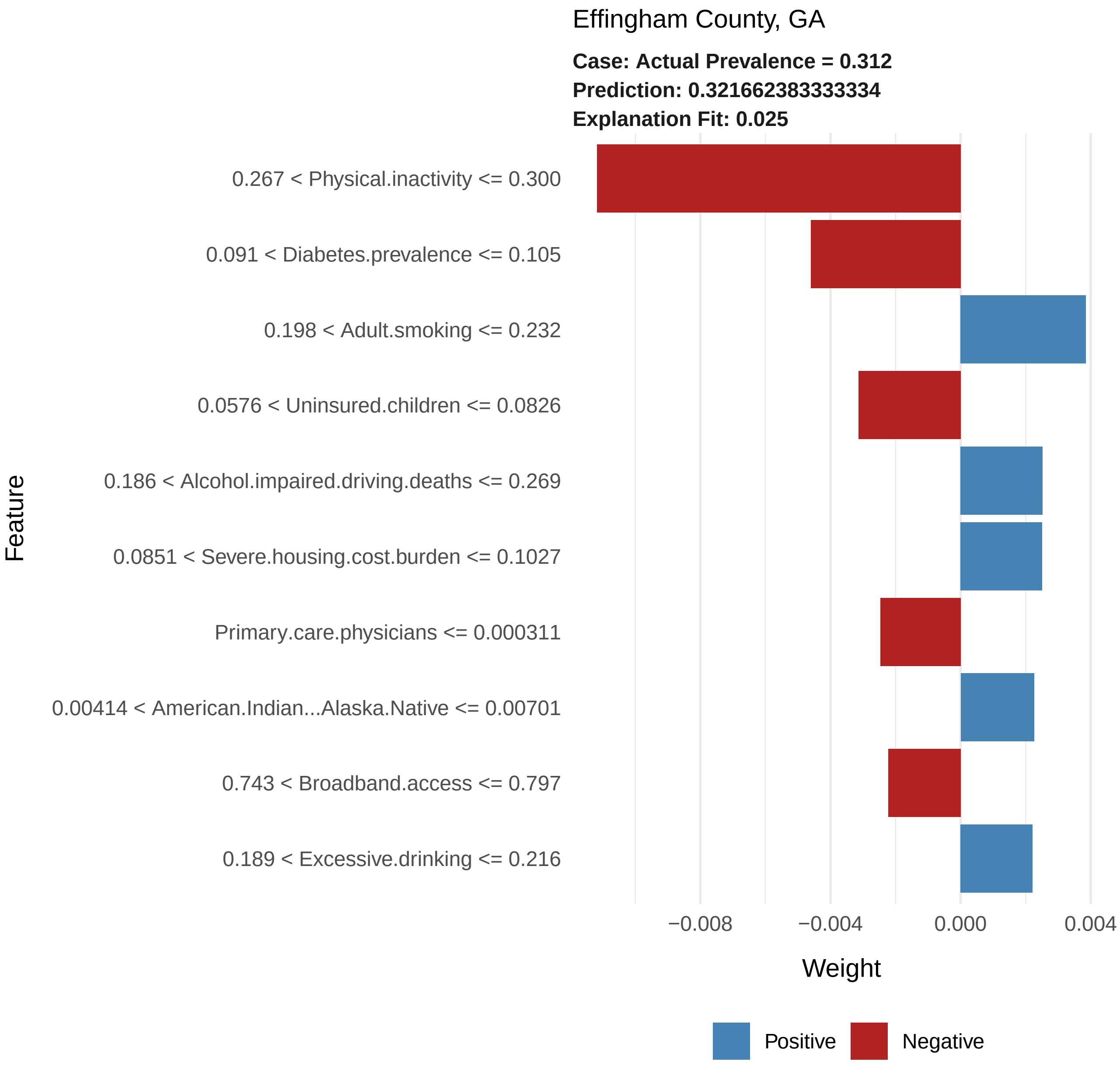

Effingham County, GA
Case: Actual Prevalence = 0.312
Prediction: 0.321662383333334
Explanation Fit: 0.025
0.267 < Physical.inactivity <= 0.300
0.091 < Diabetes.prevalence <= 0.105
0.198 < Adult.smoking <= 0.232
0.0576 < Uninsured.children <= 0.0826
0.186 < Alcohol.impaired.driving.deaths <= 0.269
0.0851 < Severe.housing.cost.burden <= 0.1027
Primary.care.physicians <= 0.000311
0.00414 < American.Indian...Alaska.Native <= 0.00701
0.743 < Broadband.access <= 0.797
0.189 < Excessive.drinking <= 0.216
Feature
−0.008
−0.004
0.000
0.004
Weight
Positive
Negative

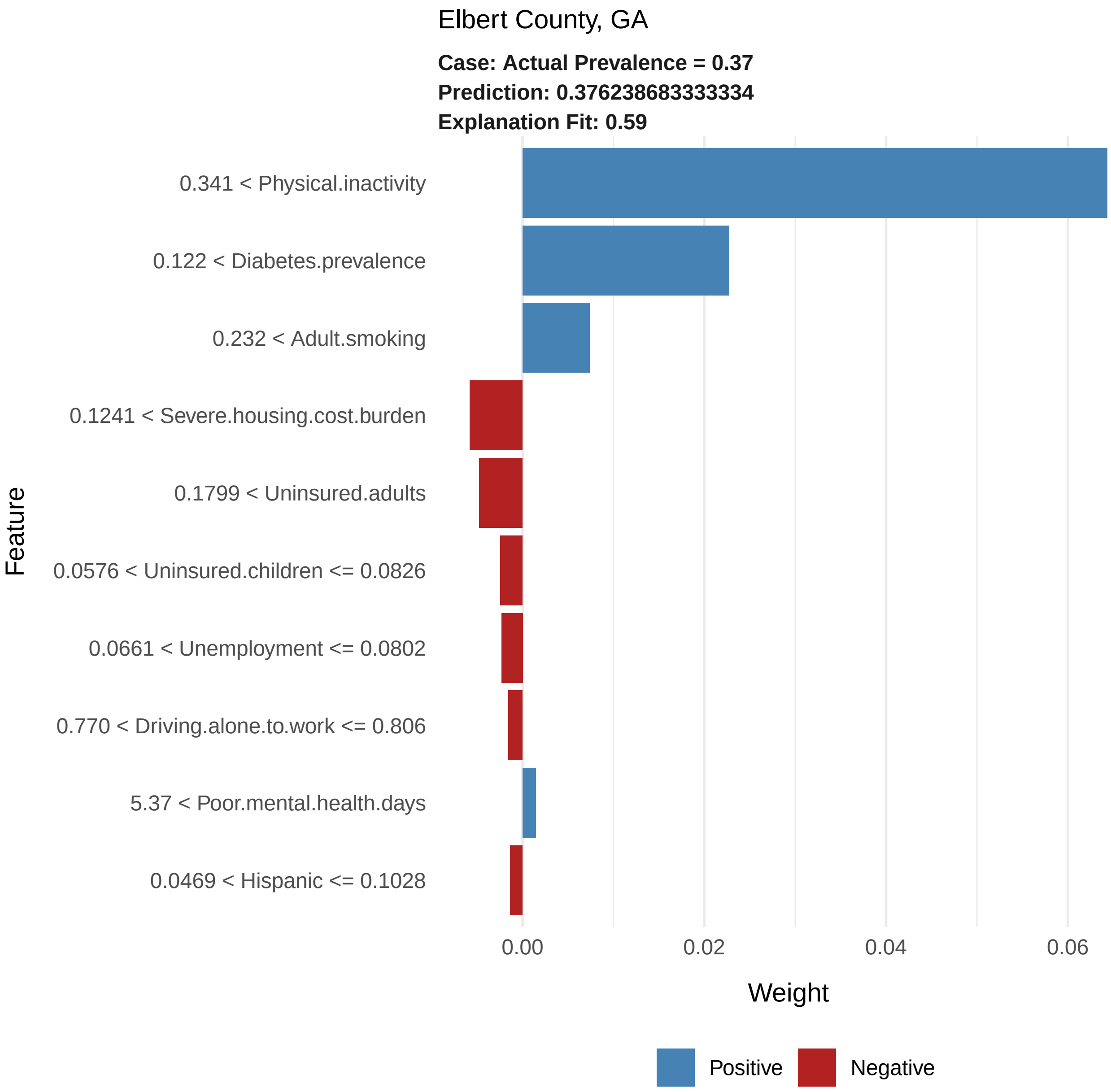

Elbert County, GA
Case: Actual Prevalence = 0.37
Prediction: 0.376238683333334
Explanation Fit: 0.59
0.341 < Physical.inactivity
0.122 < Diabetes.prevalence
0.232 < Adult.smoking
0.1241 < Severe.housing.cost.burden
0.1799 < Uninsured.adults
0.0576 < Uninsured.children <= 0.0826
0.0661 < Unemployment <= 0.0802
0.770 < Driving.alone.to.work <= 0.806
5.37 < Poor.mental.health.days
0.0469 < Hispanic <= 0.1028
Feature
0.00
0.02
0.04
0.06
Weight
Positive
Negative

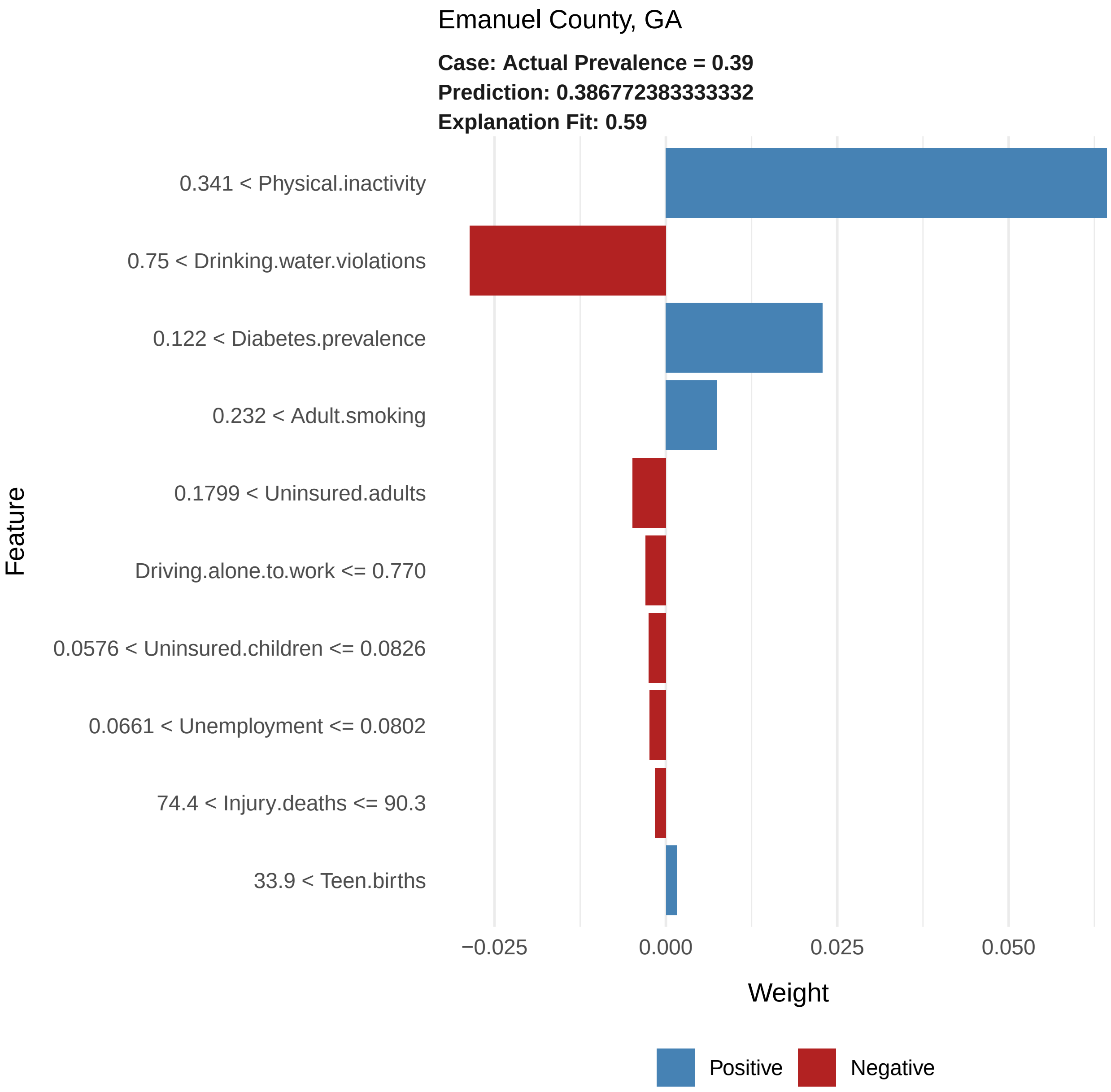
Emanuel County, GA
Case: Actual Prevalence = 0.39
Prediction: 0.386772383333332
Explanation Fit: 0.59
0.341 < Physical.inactivity
0.75 < Drinking.water.violations
0.122 < Diabetes.prevalence
0.232 < Adult.smoking
0.1799 < Uninsured.adults
Driving.alone.to.work <= 0.770
0.0576 < Uninsured.children <= 0.0826
0.0661 < Unemployment <= 0.0802
74.4 < Injury.deaths <= 90.3
33.9 < Teen.births
Feature
−0.025
0.000
0.025
0.050
Weight
Positive
Negative

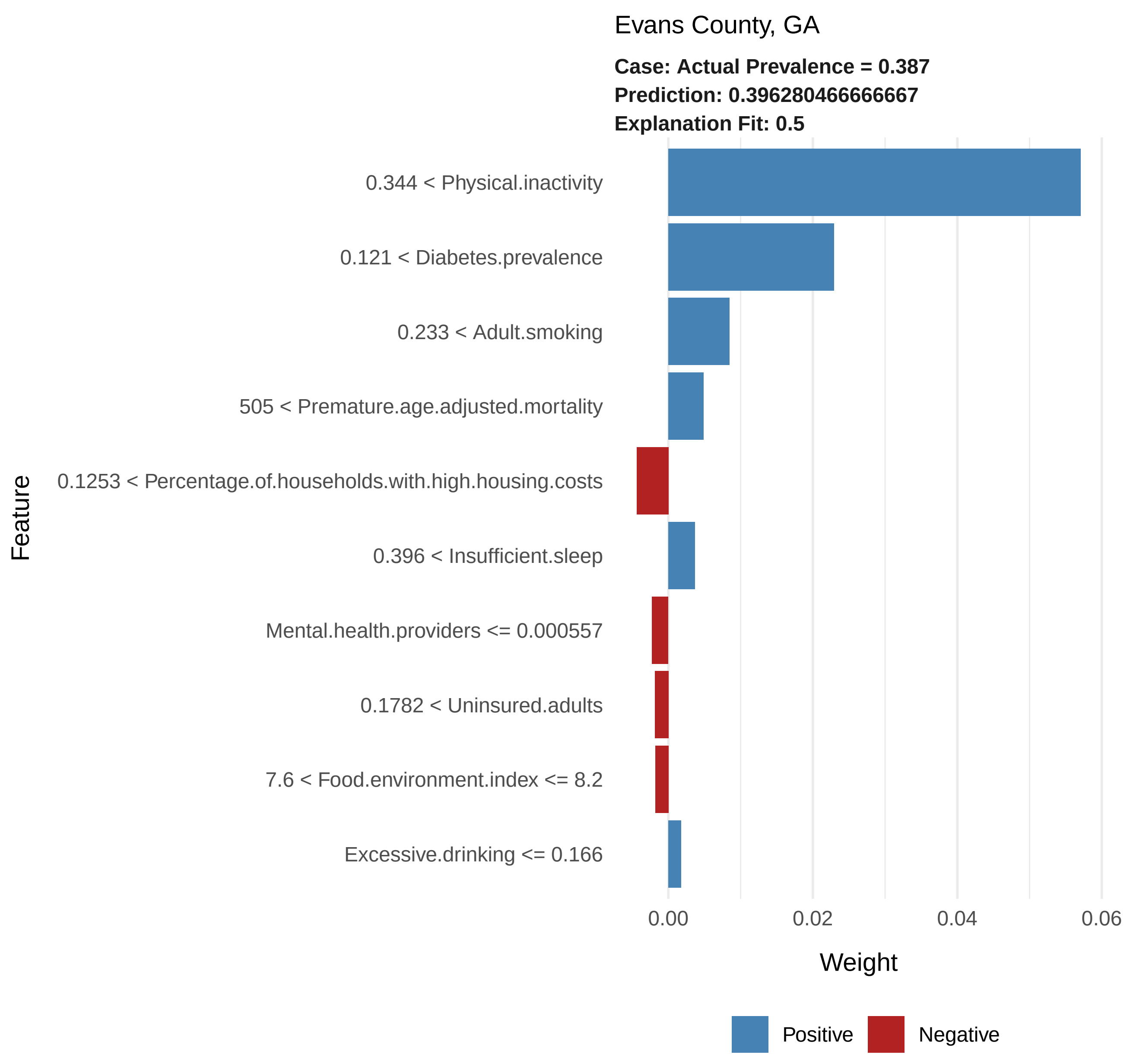

Evans County, GA
Case: Actual Prevalence = 0.387
Prediction: 0.396280466666667
Explanation Fit: 0.5
0.344 < Physical.inactivity
0.121 < Diabetes.prevalence
0.233 < Adult.smoking
505 < Premature.age.adjusted.mortality
0.1253 < Percentage.of.households.with.high.housing.costs
0.396 < Insufficient.sleep
Mental.health.providers <= 0.000557
0.1782 < Uninsured.adults
7.6 < Food.environment.index <= 8.2
Excessive.drinking <= 0.166
Feature
0.00
0.02
0.04
0.06
Weight
Positive
Negative

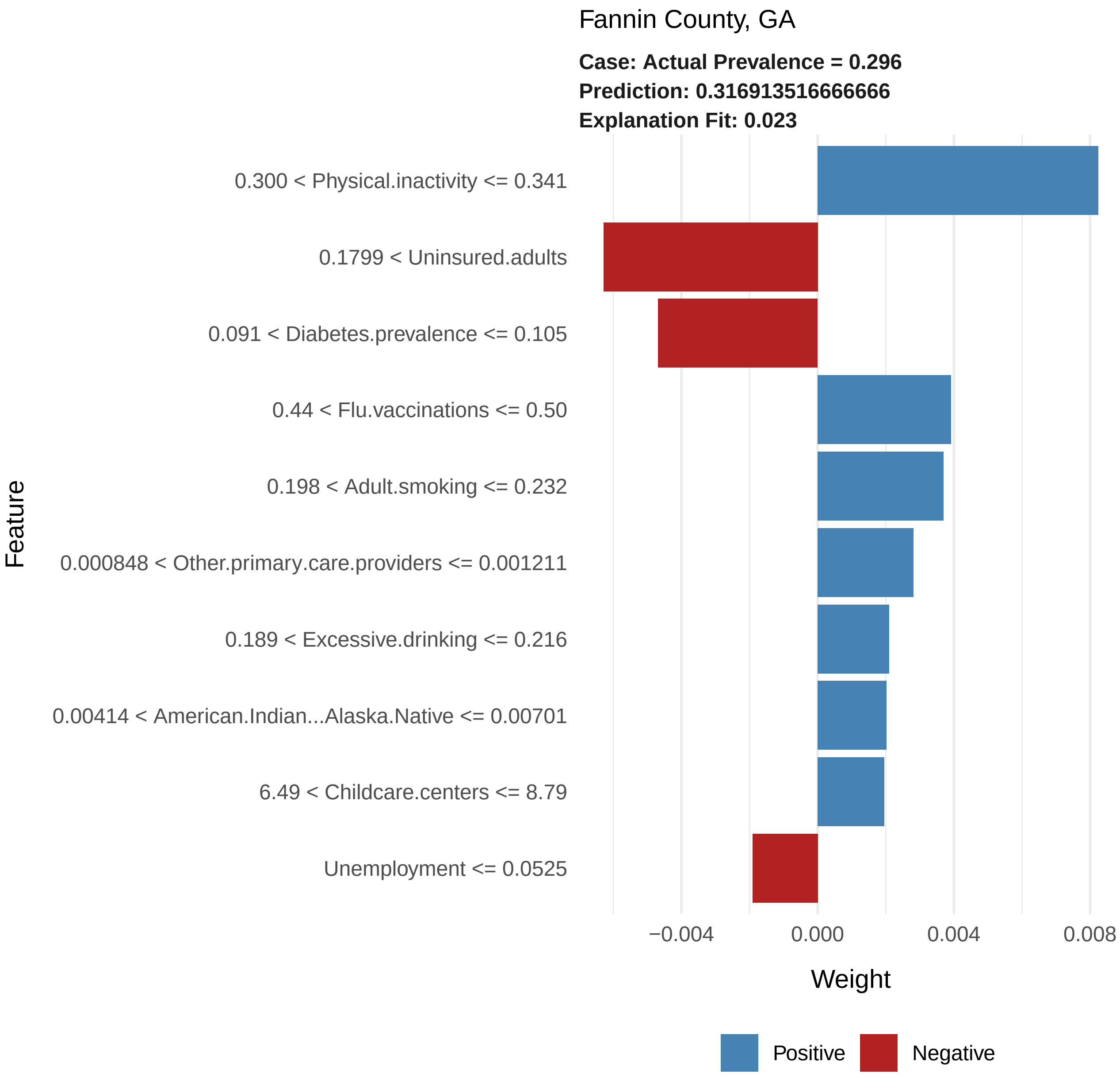

Fannin County, GA
Case: Actual Prevalence = 0.296
Prediction: 0.316913516666666
Explanation Fit: 0.023
0.300 < Physical.inactivity <= 0.341
0.1799 < Uninsured.adults
0.091 < Diabetes.prevalence <= 0.105
0.44 < Flu.vaccinations <= 0.50
0.198 < Adult.smoking <= 0.232
0.000848 < Other.primary.care.providers <= 0.001211
0.189 < Excessive.drinking <= 0.216
0.00414 < American.Indian...Alaska.Native <= 0.00701
6.49 < Childcare.centers <= 8.79
Unemployment <= 0.0525
Feature
−0.004
0.000
0.004
0.008
Weight
Positive
Negative

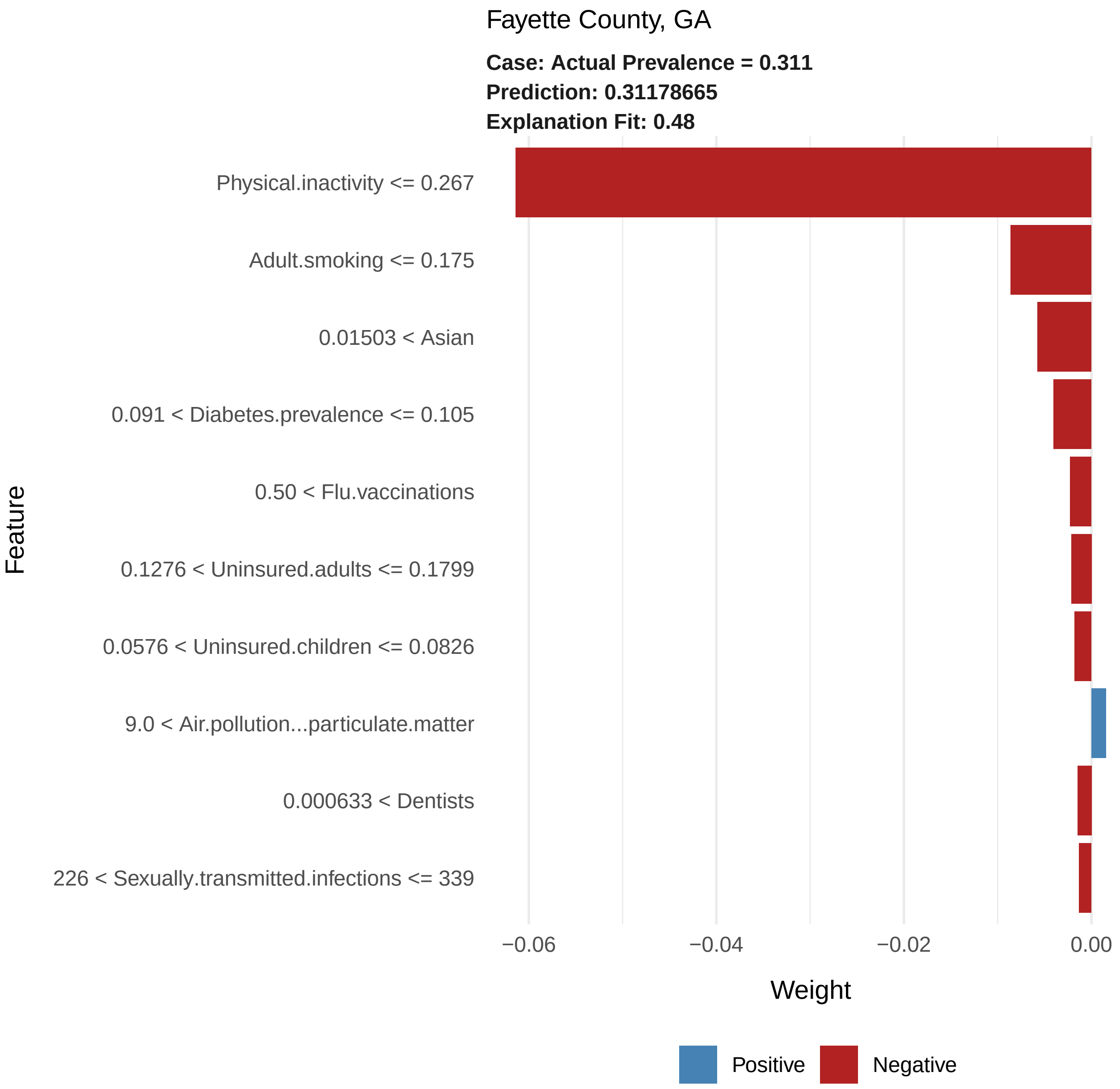
Fayette County, GA
Case: Actual Prevalence = 0.311
Prediction: 0.31178665
Explanation Fit: 0.48
Physical.inactivity <= 0.267
Adult.smoking <= 0.175
0.01503 < Asian
0.091 < Diabetes.prevalence <= 0.105
0.50 < Flu.vaccinations
0.1276 < Uninsured.adults <= 0.1799
0.0576 < Uninsured.children <= 0.0826
9.0 < Air.pollution...particulate.matter
0.000633 < Dentists
226 < Sexually.transmitted.infections <= 339
Feature
−0.06
−0.04
−0.02
0.00
Weight
Positive
Negative

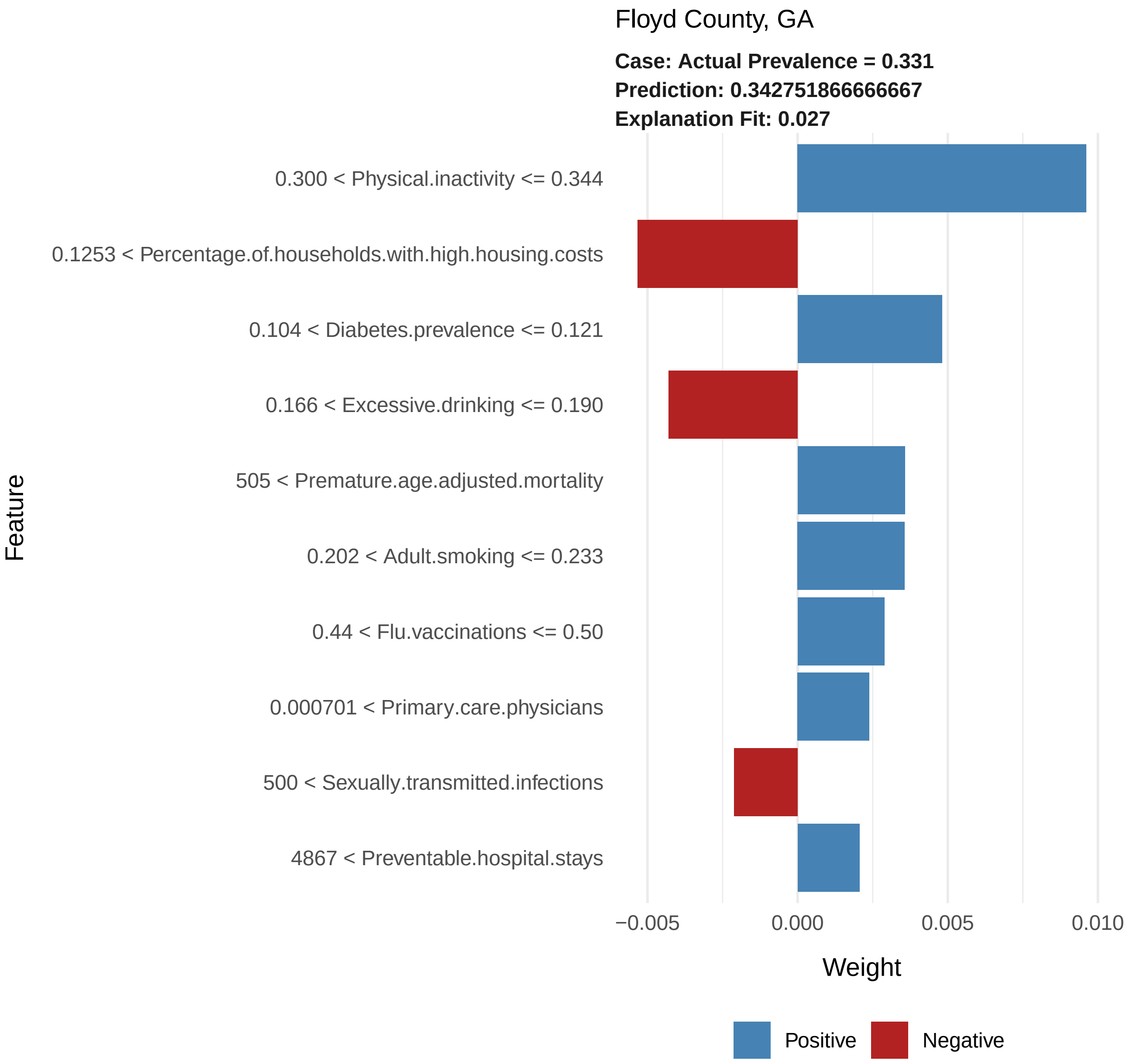

Floyd County, GA
Case: Actual Prevalence = 0.331
Prediction: 0.342751866666667
Explanation Fit: 0.027
0.300 < Physical.inactivity <= 0.344
0.1253 < Percentage.of.households.with.high.housing.costs
0.104 < Diabetes.prevalence <= 0.121
0.166 < Excessive.drinking <= 0.190
505 < Premature.age.adjusted.mortality
0.202 < Adult.smoking <= 0.233
0.44 < Flu.vaccinations <= 0.50
0.000701 < Primary.care.physicians
500 < Sexually.transmitted.infections
4867 < Preventable.hospital.stays
Feature
−0.005
0.000
0.005
0.010
Weight
Positive
Negative

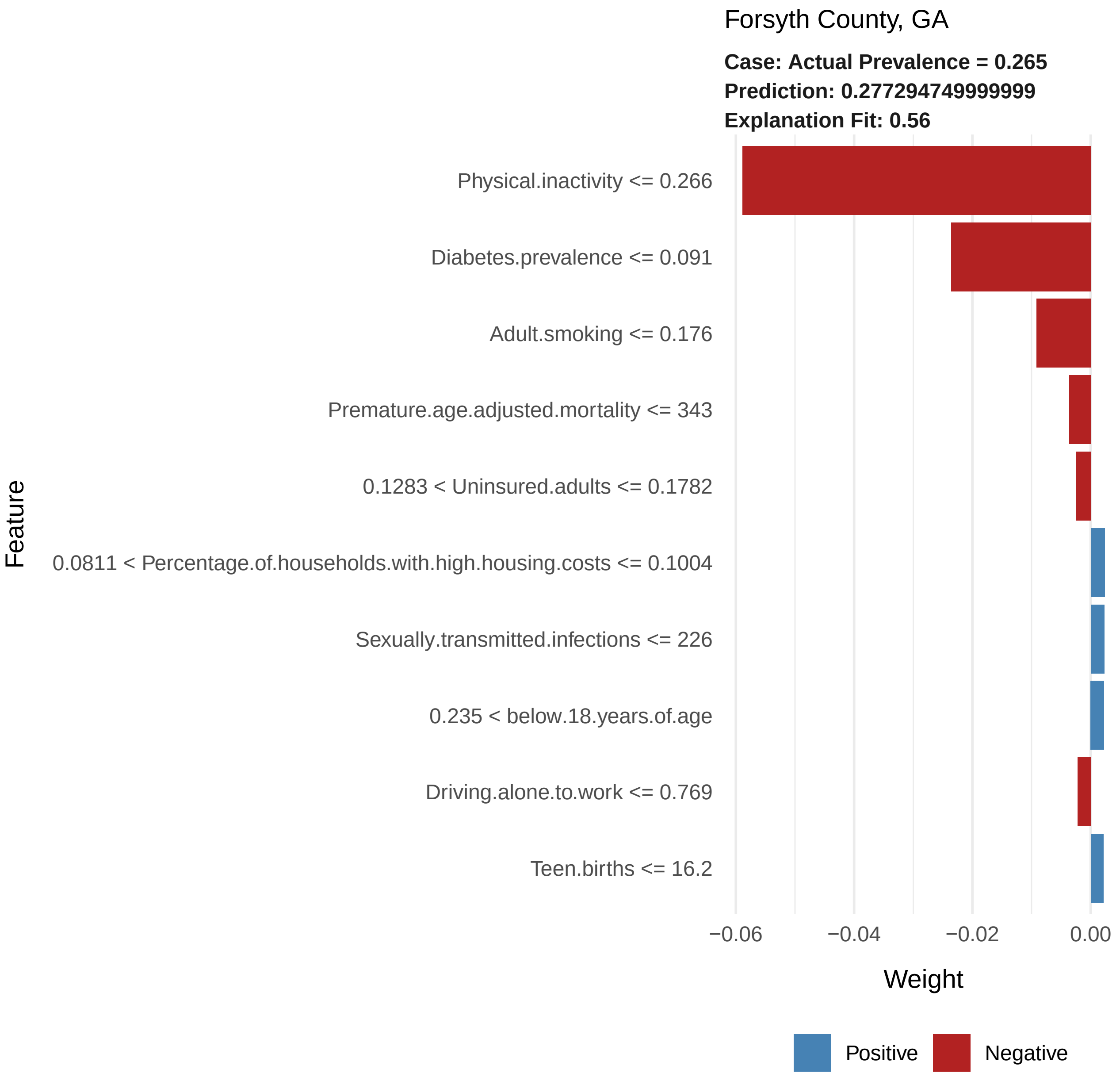

Forsyth County, GA
Case: Actual Prevalence = 0.265
Prediction: 0.277294749999999
Explanation Fit: 0.56
Physical.inactivity <= 0.266
Diabetes.prevalence <= 0.091
Adult.smoking <= 0.176
Premature.age.adjusted.mortality <= 343
0.1283 < Uninsured.adults <= 0.1782
0.0811 < Percentage.of.households.with.high.housing.costs <= 0.1004
Sexually.transmitted.infections <= 226
0.235 < below.18.years.of.age
Driving.alone.to.work <= 0.769
Teen.births <= 16.2
Feature
−0.06
−0.04
−0.02
0.00
Weight
Positive
Negative

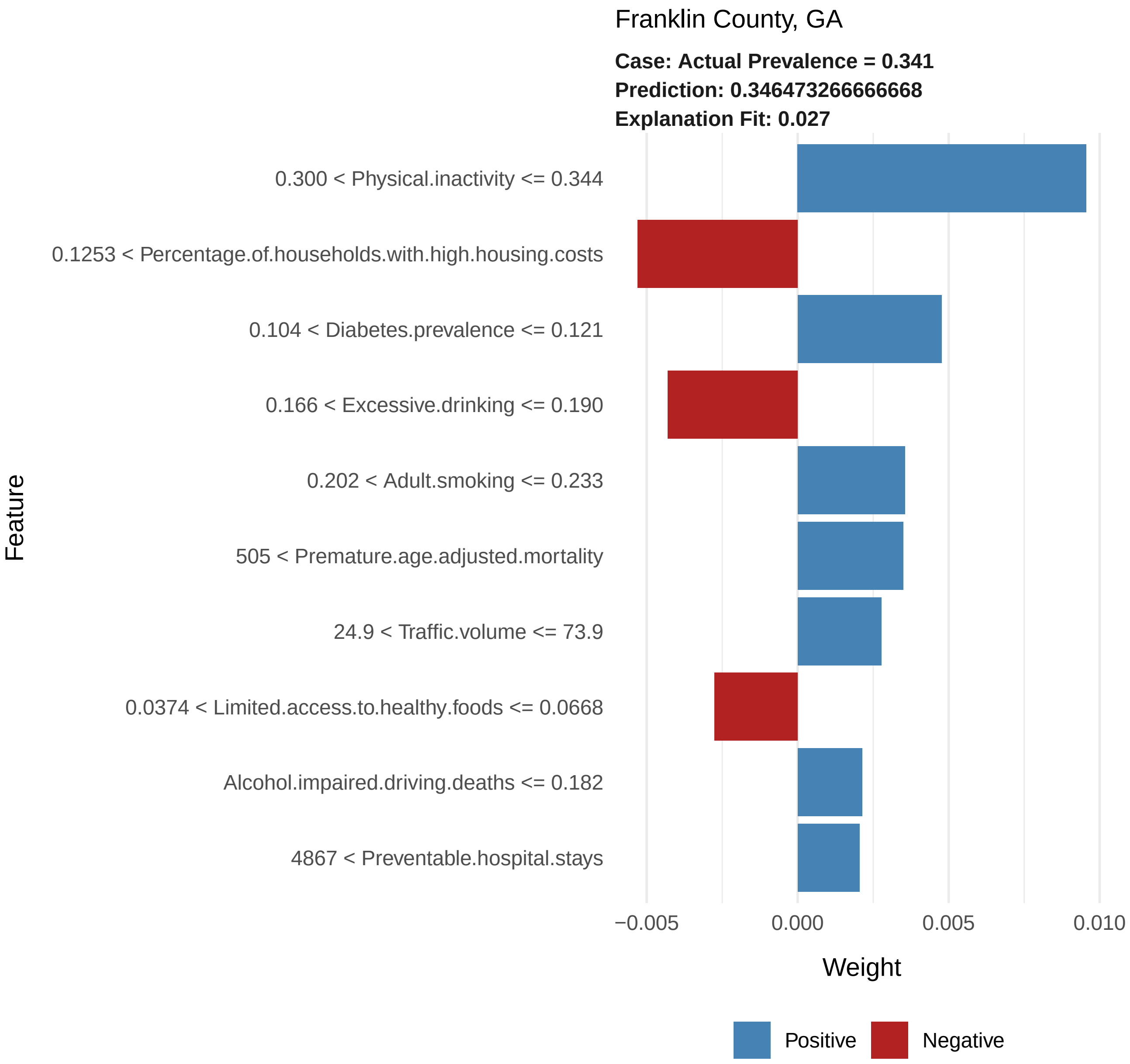

Franklin County, GA
Case: Actual Prevalence = 0.341
Prediction: 0.346473266666668
Explanation Fit: 0.027
0.300 < Physical.inactivity <= 0.344
0.1253 < Percentage.of.households.with.high.housing.costs
0.104 < Diabetes.prevalence <= 0.121
0.166 < Excessive.drinking <= 0.190
0.202 < Adult.smoking <= 0.233
505 < Premature.age.adjusted.mortality
24.9 < Traffic.volume <= 73.9
0.0374 < Limited.access.to.healthy.foods <= 0.0668
Alcohol.impaired.driving.deaths <= 0.182
4867 < Preventable.hospital.stays
Feature
−0.005
0.000
0.005
0.010
Weight
Positive
Negative

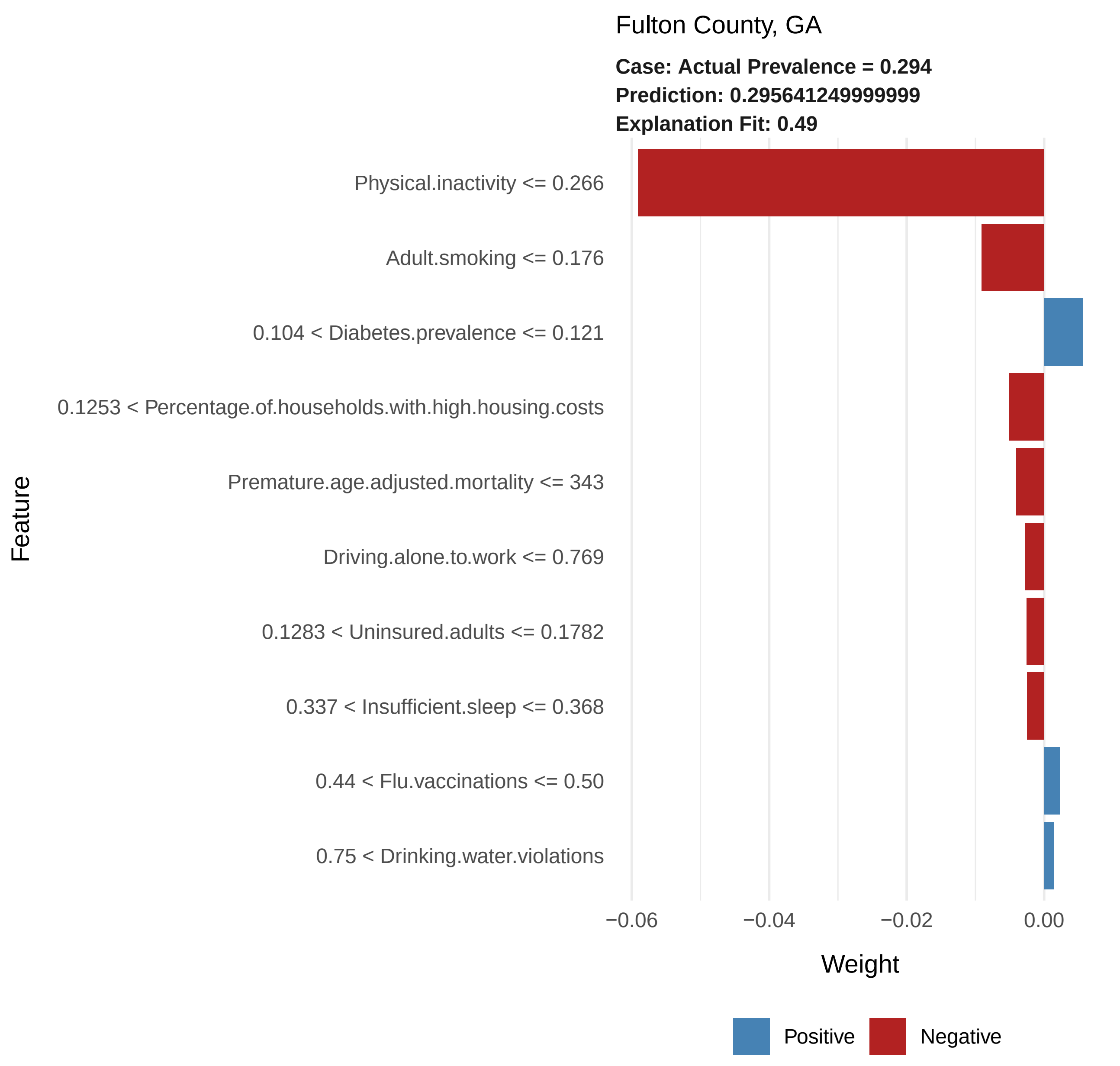
Fulton County, GA
Case: Actual Prevalence = 0.294
Prediction: 0.295641249999999
Explanation Fit: 0.49
Physical.inactivity <= 0.266
Adult.smoking <= 0.176
0.104 < Diabetes.prevalence <= 0.121
0.1253 < Percentage.of.households.with.high.housing.costs
Premature.age.adjusted.mortality <= 343
Driving.alone.to.work <= 0.769
0.1283 < Uninsured.adults <= 0.1782
0.337 < Insufficient.sleep <= 0.368
0.44 < Flu.vaccinations <= 0.50
0.75 < Drinking.water.violations
Feature
−0.06
−0.04
−0.02
0.00
Weight
Positive
Negative

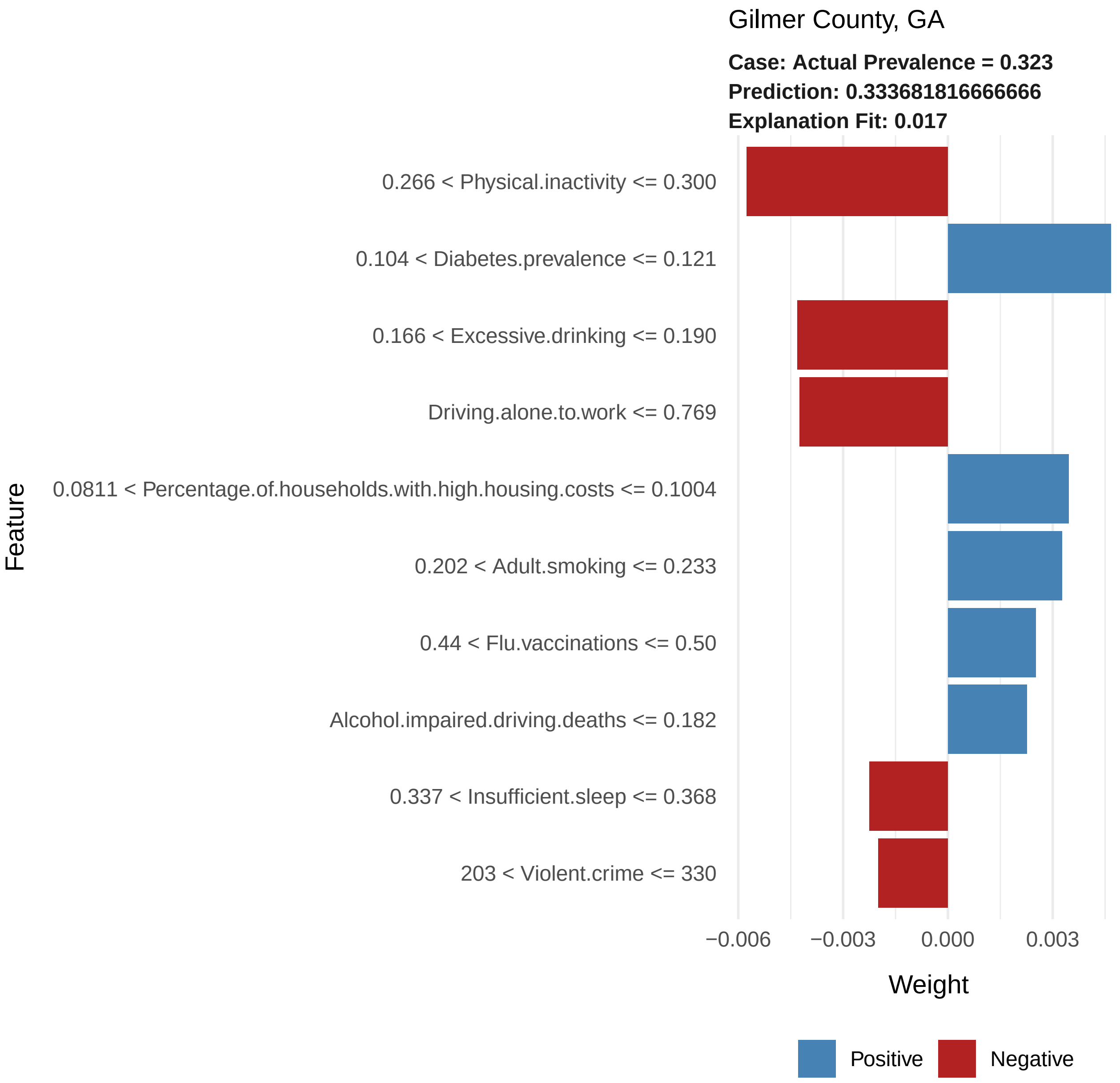

Gilmer County, GA
Case: Actual Prevalence = 0.323
Prediction: 0.333681816666666
Explanation Fit: 0.017
0.266 < Physical.inactivity <= 0.300
0.104 < Diabetes.prevalence <= 0.121
0.166 < Excessive.drinking <= 0.190
Driving.alone.to.work <= 0.769
0.0811 < Percentage.of.households.with.high.housing.costs <= 0.1004
0.202 < Adult.smoking <= 0.233
0.44 < Flu.vaccinations <= 0.50
Alcohol.impaired.driving.deaths <= 0.182
0.337 < Insufficient.sleep <= 0.368
203 < Violent.crime <= 330
Feature
−0.006
−0.003
0.000
0.003
Weight
Positive
Negative

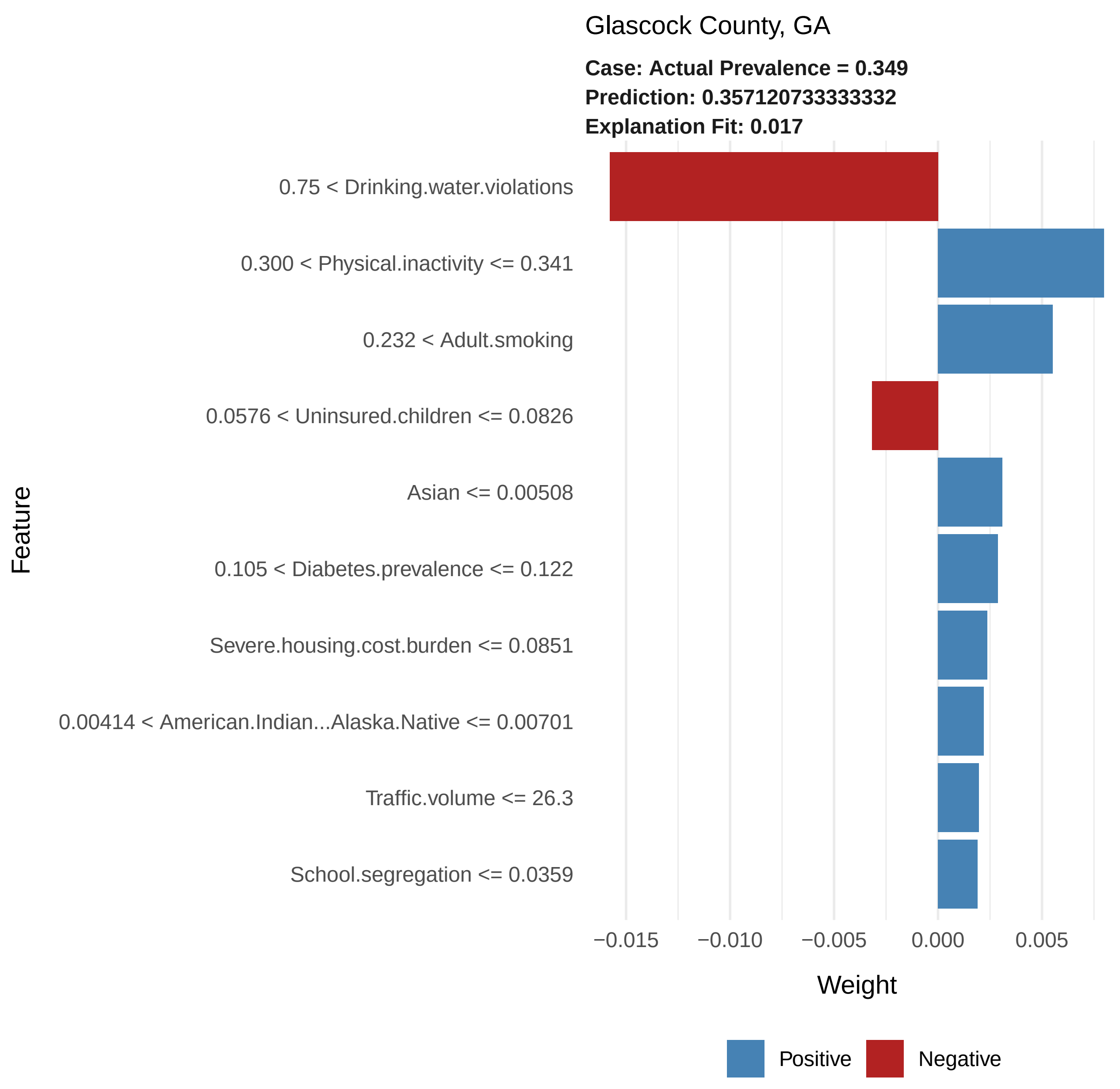
Glascock County, GA
Case: Actual Prevalence = 0.349
Prediction: 0.357120733333332
Explanation Fit: 0.017
0.75 < Drinking.water.violations
0.300 < Physical.inactivity <= 0.341
0.232 < Adult.smoking
0.0576 < Uninsured.children <= 0.0826
Asian <= 0.00508
0.105 < Diabetes.prevalence <= 0.122
Severe.housing.cost.burden <= 0.0851
0.00414 < American.Indian...Alaska.Native <= 0.00701
Traffic.volume <= 26.3
School.segregation <= 0.0359
Feature
−0.015
−0.010
−0.005
0.000
0.005
Weight
Positive
Negative

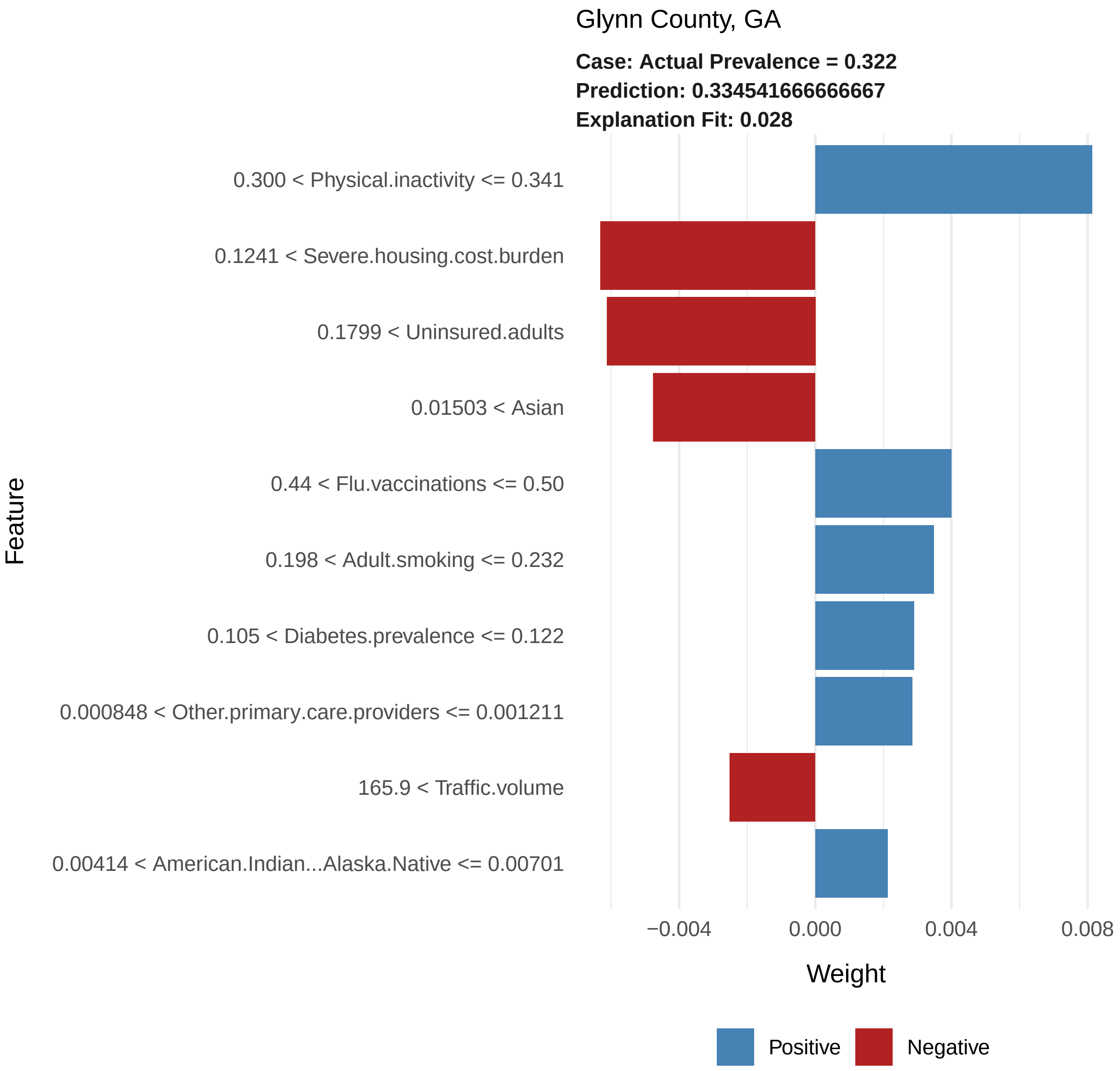
Glynn County, GA
Case: Actual Prevalence = 0.322
Prediction: 0.334541666666667
Explanation Fit: 0.028
0.300 < Physical.inactivity <= 0.341
0.1241 < Severe.housing.cost.burden
0.1799 < Uninsured.adults
0.01503 < Asian
0.44 < Flu.vaccinations <= 0.50
0.198 < Adult.smoking <= 0.232
0.105 < Diabetes.prevalence <= 0.122
0.000848 < Other.primary.care.providers <= 0.001211
165.9 < Traffic.volume
0.00414 < American.Indian...Alaska.Native <= 0.00701
Feature
−0.004
0.000
0.004
0.008
Weight
Positive
Negative

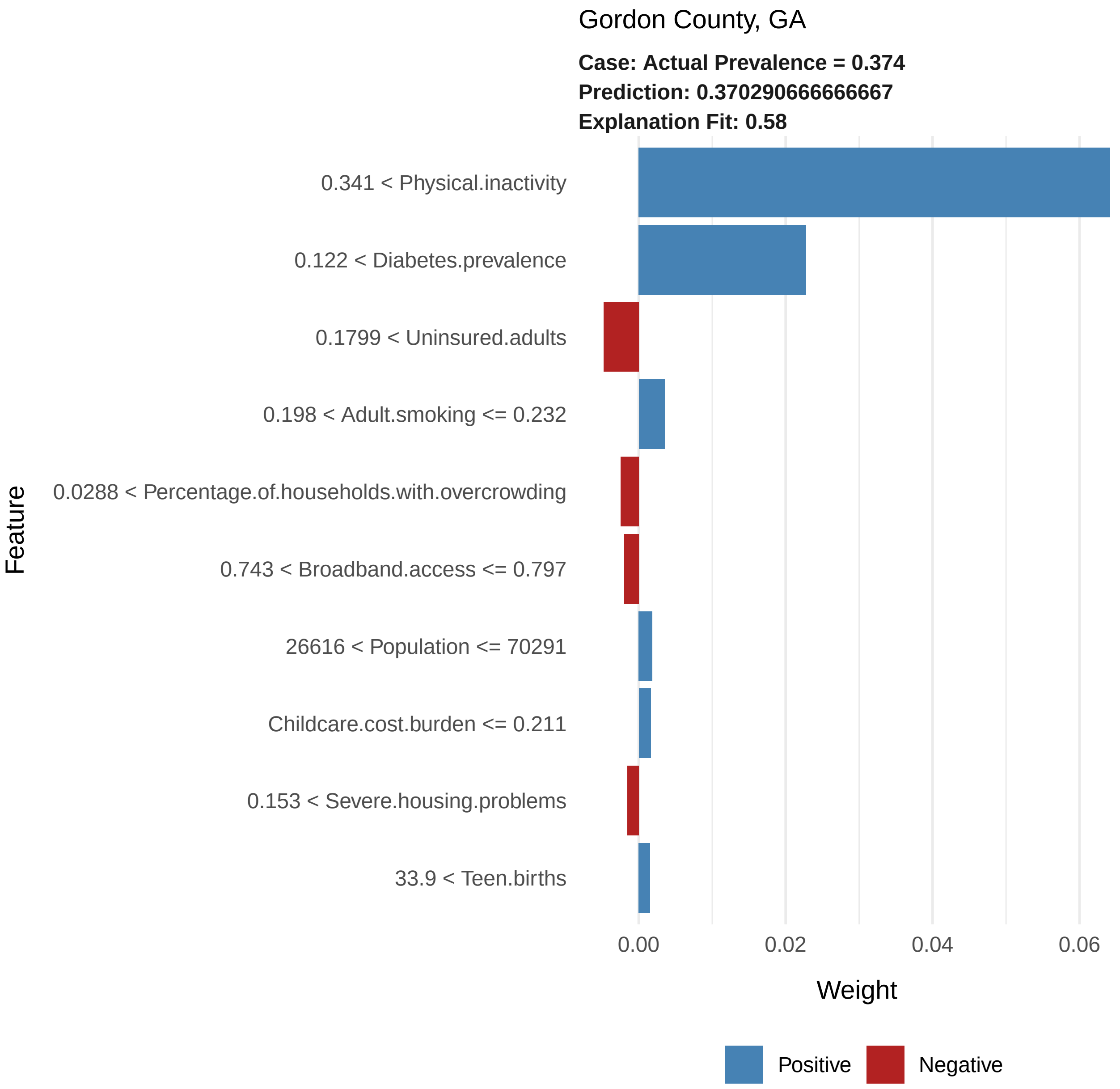

Gordon County, GA
Case: Actual Prevalence = 0.374
Prediction: 0.370290666666667
Explanation Fit: 0.58
0.341 < Physical.inactivity
0.122 < Diabetes.prevalence
0.1799 < Uninsured.adults
0.198 < Adult.smoking <= 0.232
0.0288 < Percentage.of.households.with.overcrowding
0.743 < Broadband.access <= 0.797
26616 < Population <= 70291
Childcare.cost.burden <= 0.211
0.153 < Severe.housing.problems
33.9 < Teen.births
Feature
0.00
0.02
0.04
0.06
Weight
Positive
Negative

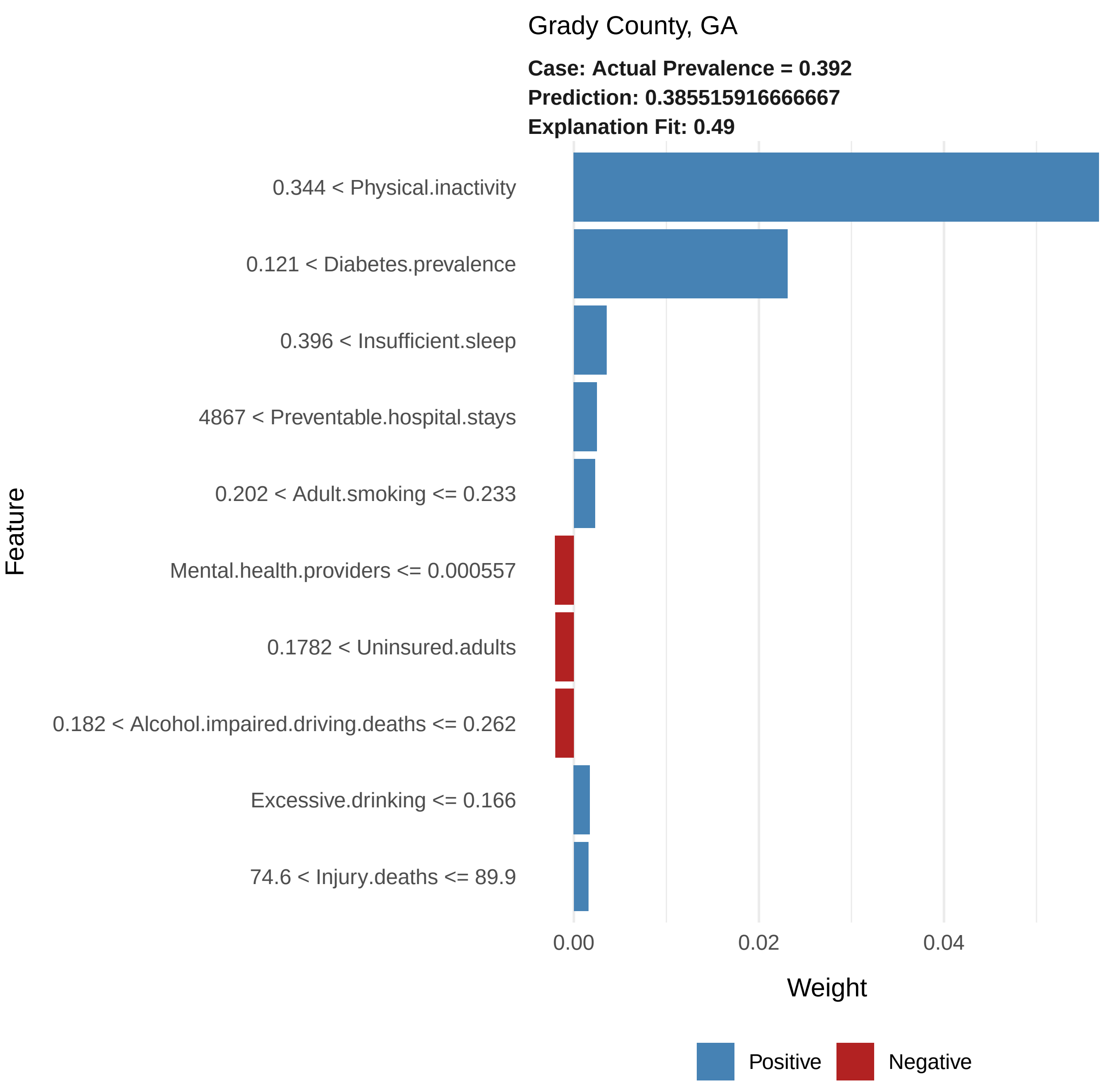
Grady County, GA
Case: Actual Prevalence = 0.392
Prediction: 0.385515916666667
Explanation Fit: 0.49
0.344 < Physical.inactivity
0.121 < Diabetes.prevalence
0.396 < Insufficient.sleep
4867 < Preventable.hospital.stays
0.202 < Adult.smoking <= 0.233
Mental.health.providers <= 0.000557
0.1782 < Uninsured.adults
0.182 < Alcohol.impaired.driving.deaths <= 0.262
Excessive.drinking <= 0.166
74.6 < Injury.deaths <= 89.9
Feature
0.00
0.02
0.04
Weight
Positive
Negative

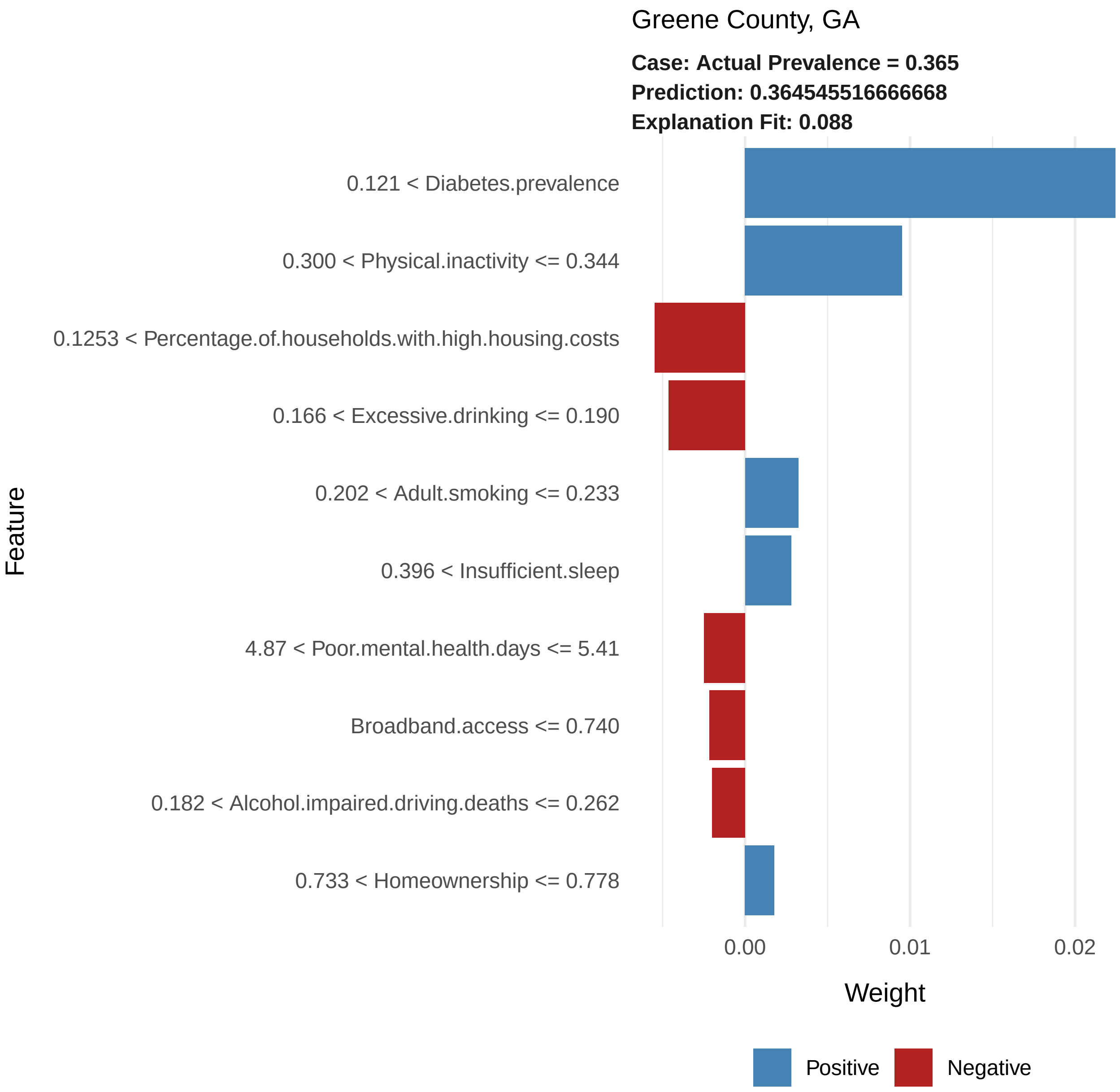

Greene County, GA
Case: Actual Prevalence = 0.365
Prediction: 0.364545516666668
Explanation Fit: 0.088
0.121 < Diabetes.prevalence
0.300 < Physical.inactivity <= 0.344
0.1253 < Percentage.of.households.with.high.housing.costs
0.166 < Excessive.drinking <= 0.190
0.202 < Adult.smoking <= 0.233
0.396 < Insufficient.sleep
4.87 < Poor.mental.health.days <= 5.41
Broadband.access <= 0.740
0.182 < Alcohol.impaired.driving.deaths <= 0.262
0.733 < Homeownership <= 0.778
Feature
0.00
0.01
0.02
Weight
Positive
Negative

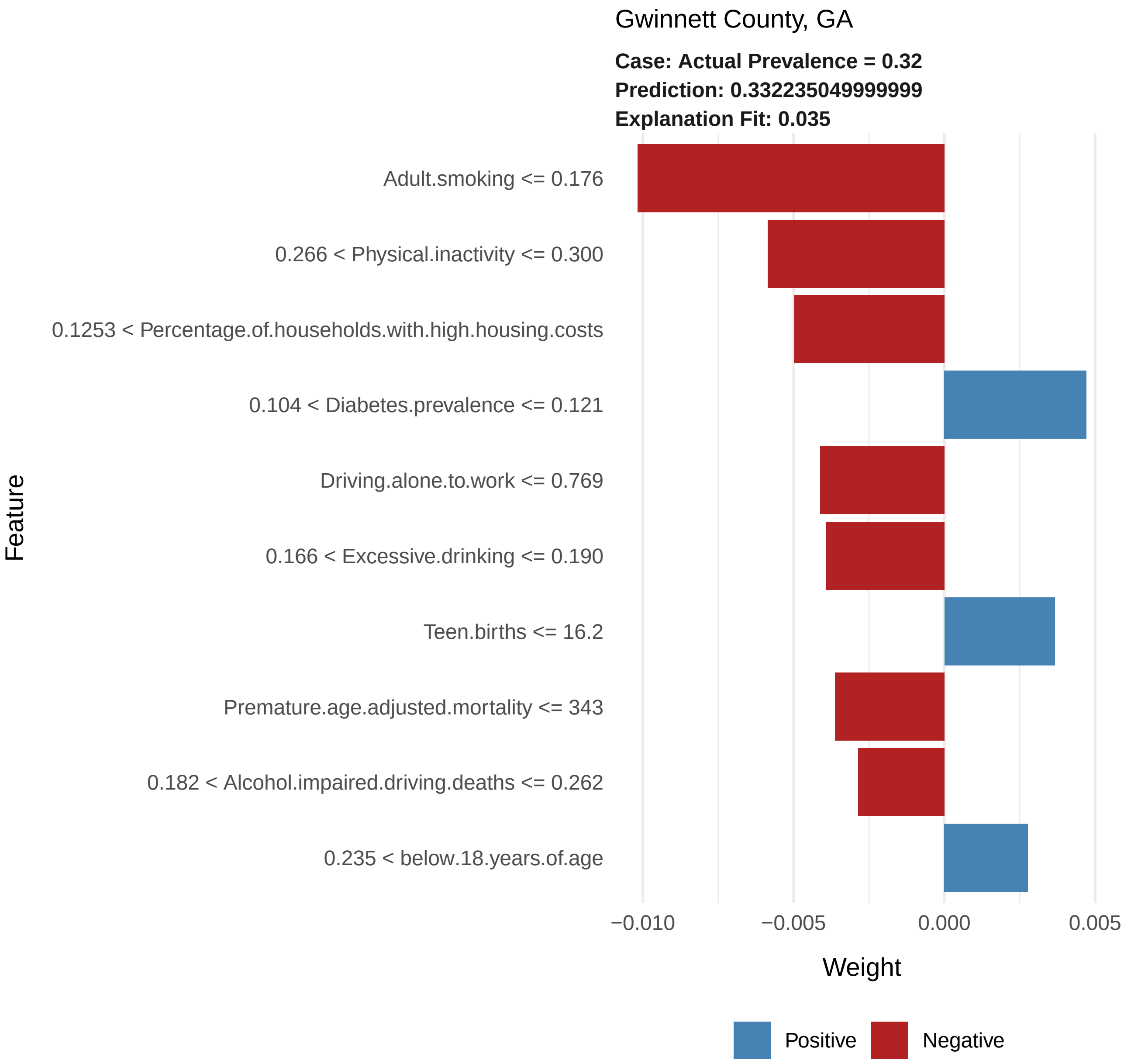

Gwinnett County, GA
Case: Actual Prevalence = 0.32
Prediction: 0.332235049999999
Explanation Fit: 0.035
Adult.smoking <= 0.176
0.266 < Physical.inactivity <= 0.300
0.1253 < Percentage.of.households.with.high.housing.costs
0.104 < Diabetes.prevalence <= 0.121
Driving.alone.to.work <= 0.769
0.166 < Excessive.drinking <= 0.190
Teen.births <= 16.2
Premature.age.adjusted.mortality <= 343
0.182 < Alcohol.impaired.driving.deaths <= 0.262
0.235 < below.18.years.of.age
Feature
−0.010
−0.005
0.000
0.005
Weight
Positive
Negative

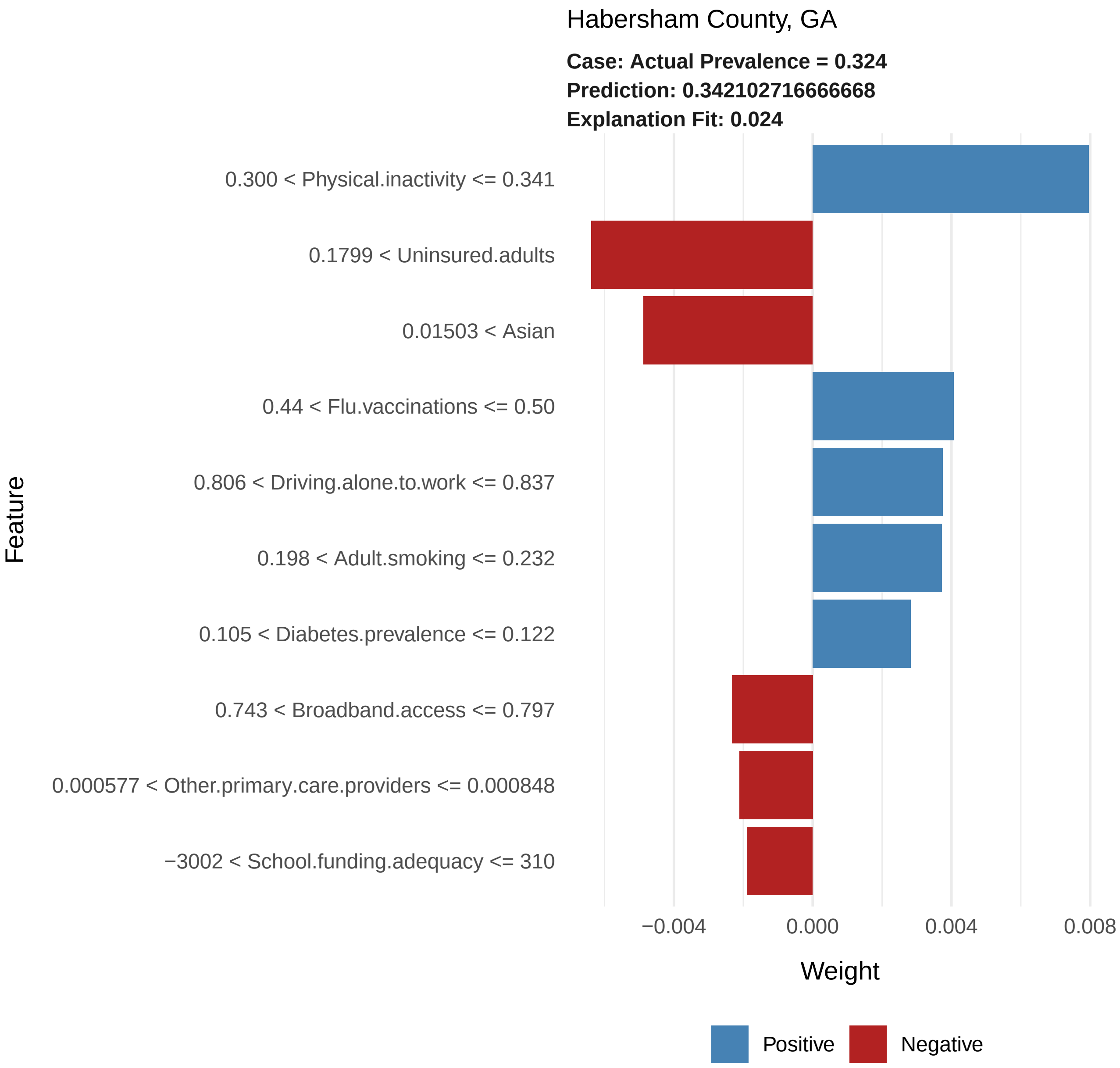

Habersham County, GA
Case: Actual Prevalence = 0.324
Prediction: 0.342102716666668
Explanation Fit: 0.024
0.300 < Physical.inactivity <= 0.341
0.1799 < Uninsured.adults
0.01503 < Asian
0.44 < Flu.vaccinations <= 0.50
0.806 < Driving.alone.to.work <= 0.837
0.198 < Adult.smoking <= 0.232
0.105 < Diabetes.prevalence <= 0.122
0.743 < Broadband.access <= 0.797
0.000577 < Other.primary.care.providers <= 0.000848
−3002 < School.funding.adequacy <= 310
Feature
−0.004
0.000
0.004
0.008
Weight
Positive
Negative

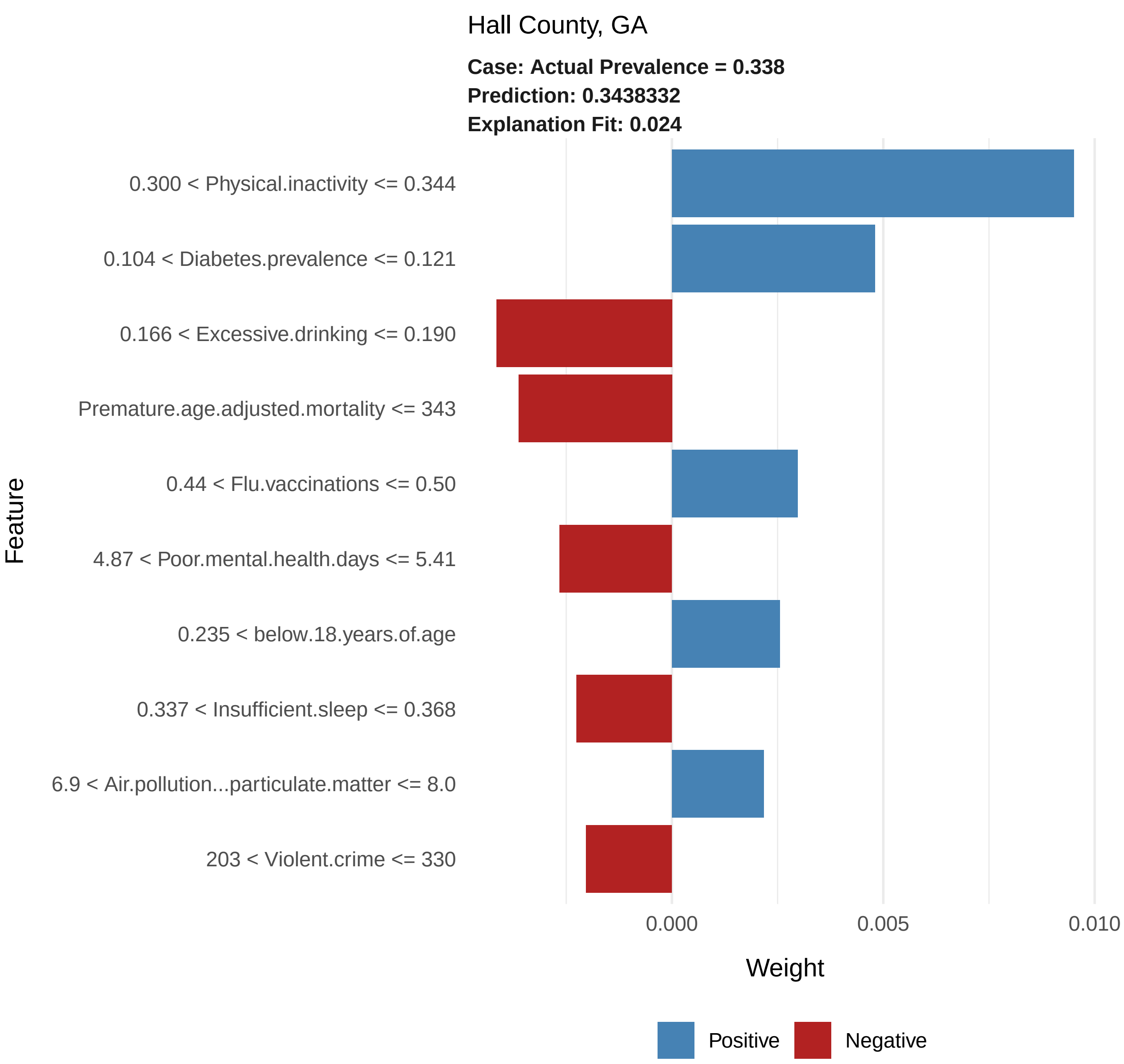

Hall County, GA
Case: Actual Prevalence = 0.338
Prediction: 0.3438332
Explanation Fit: 0.024
0.300 < Physical.inactivity <= 0.344
0.104 < Diabetes.prevalence <= 0.121
0.166 < Excessive.drinking <= 0.190
Premature.age.adjusted.mortality <= 343
0.44 < Flu.vaccinations <= 0.50
4.87 < Poor.mental.health.days <= 5.41
0.235 < below.18.years.of.age
0.337 < Insufficient.sleep <= 0.368
6.9 < Air.pollution...particulate.matter <= 8.0
203 < Violent.crime <= 330
Feature
0.000
0.005
0.010
Weight
Positive
Negative

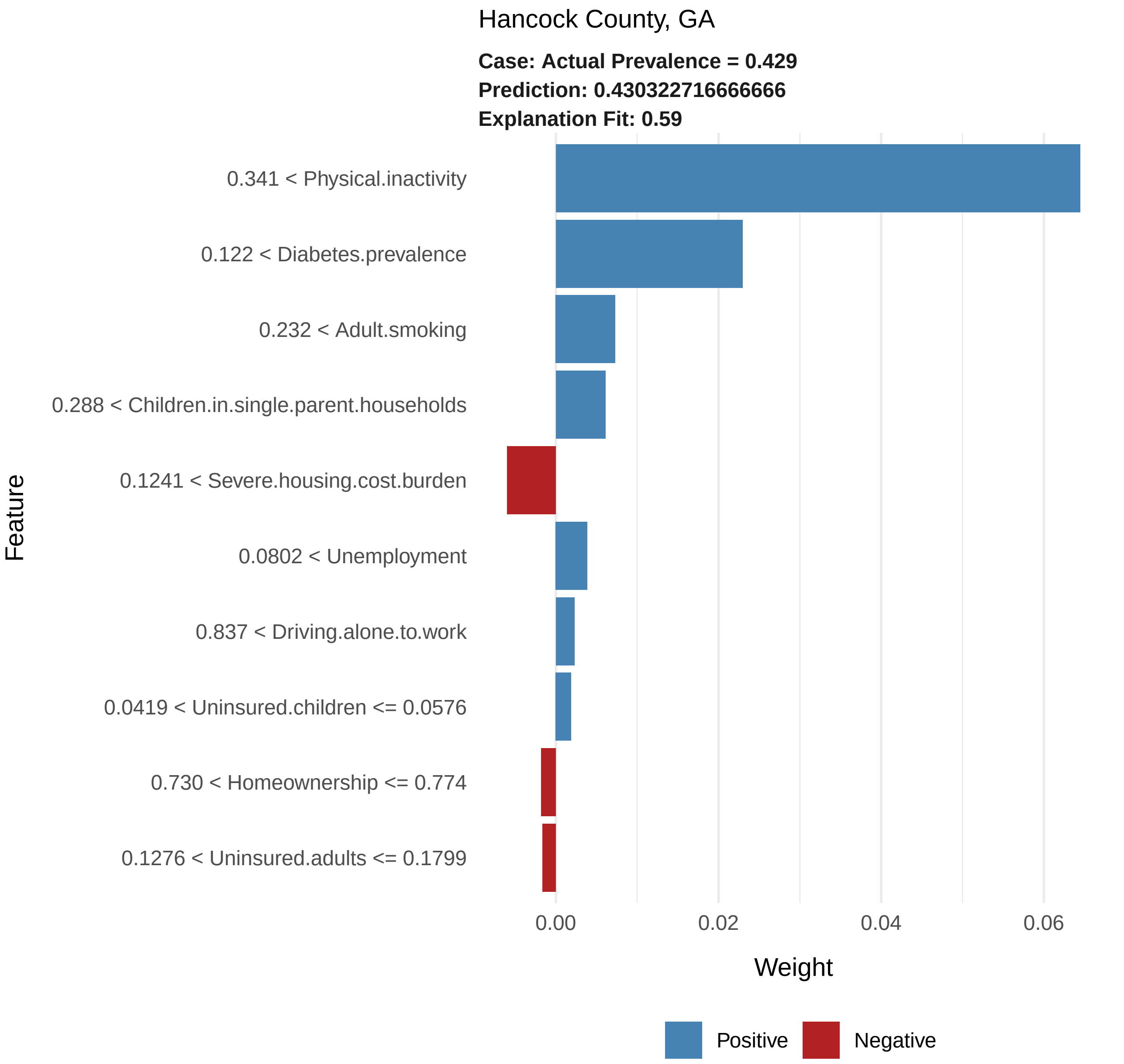

Hancock County, GA
Case: Actual Prevalence = 0.429
Prediction: 0.430322716666666
Explanation Fit: 0.59
0.341 < Physical.inactivity
0.122 < Diabetes.prevalence
0.232 < Adult.smoking
0.288 < Children.in.single.parent.households
0.1241 < Severe.housing.cost.burden
0.0802 < Unemployment
0.837 < Driving.alone.to.work
0.0419 < Uninsured.children <= 0.0576
0.730 < Homeownership <= 0.774
0.1276 < Uninsured.adults <= 0.1799
Feature
0.00
0.02
0.04
0.06
Weight
Positive
Negative

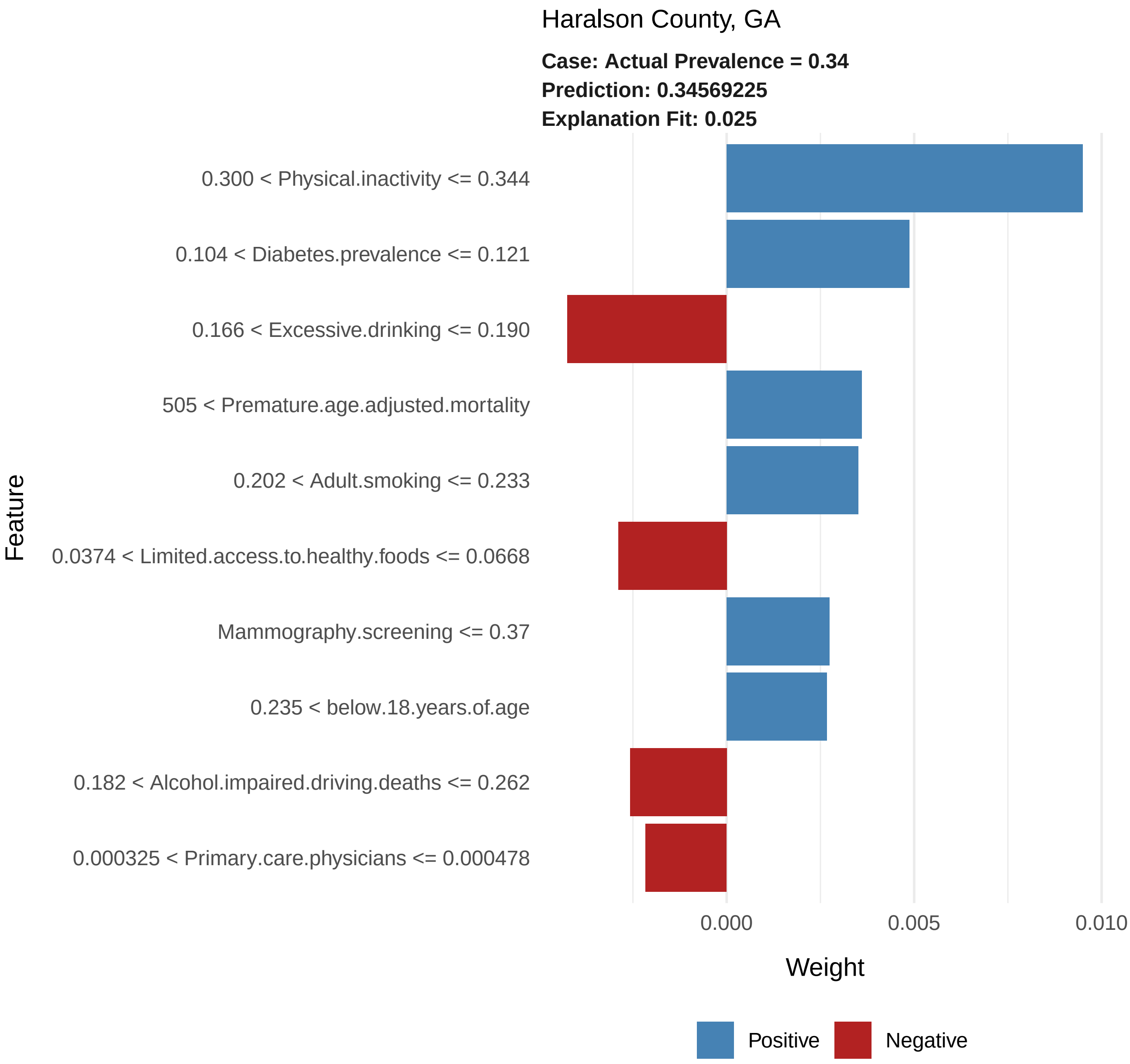
Haralson County, GA
Case: Actual Prevalence = 0.34
Prediction: 0.34569225
Explanation Fit: 0.025
0.300 < Physical.inactivity <= 0.344
0.104 < Diabetes.prevalence <= 0.121
0.166 < Excessive.drinking <= 0.190
505 < Premature.age.adjusted.mortality
0.202 < Adult.smoking <= 0.233
0.0374 < Limited.access.to.healthy.foods <= 0.0668
Mammography.screening <= 0.37
0.235 < below.18.years.of.age
0.182 < Alcohol.impaired.driving.deaths <= 0.262
0.000325 < Primary.care.physicians <= 0.000478
Feature
0.000
0.005
0.010
Weight
Positive
Negative

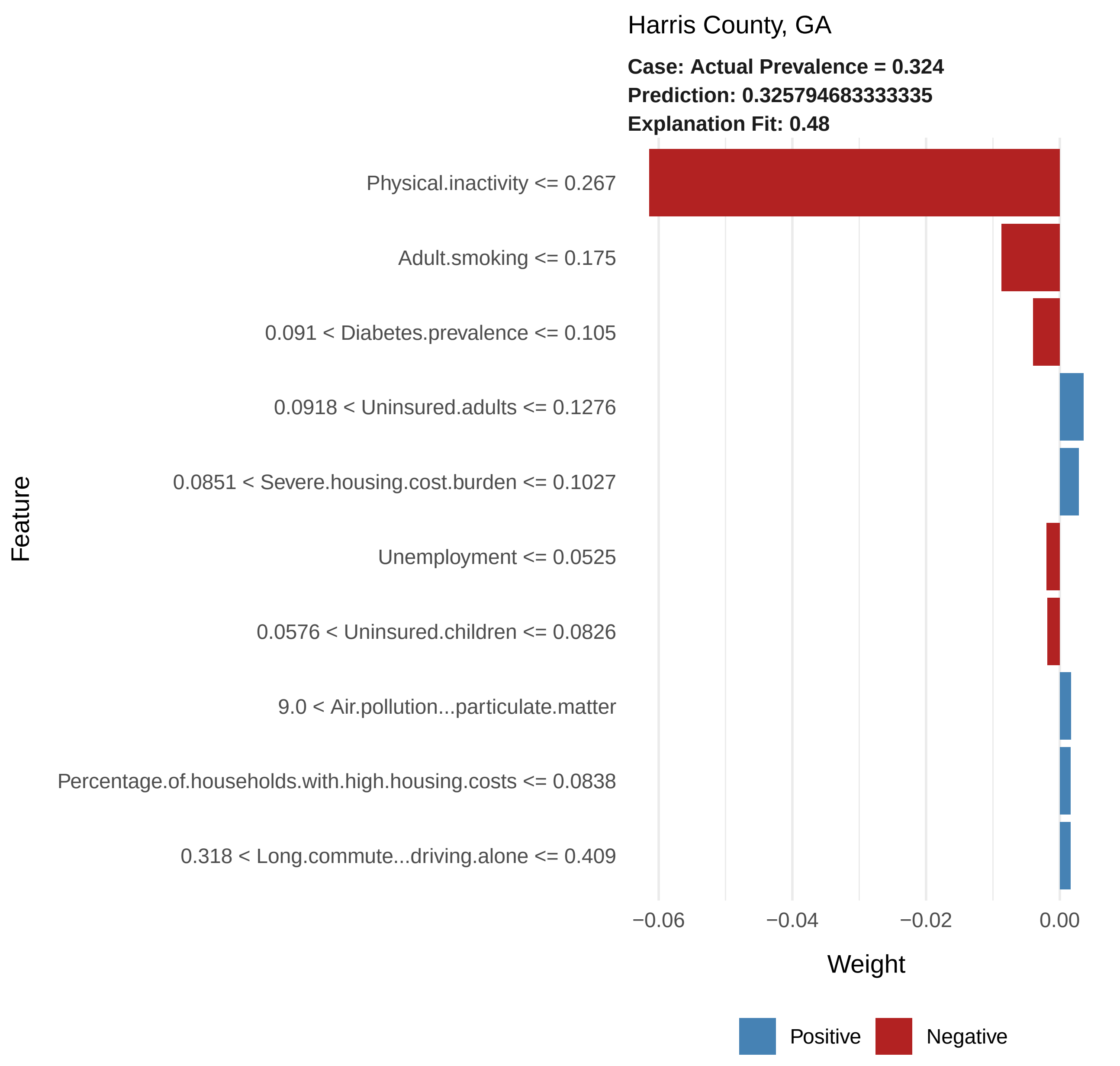

Harris County, GA
Case: Actual Prevalence = 0.324
Prediction: 0.325794683333335
Explanation Fit: 0.48
Physical.inactivity <= 0.267
Adult.smoking <= 0.175
0.091 < Diabetes.prevalence <= 0.105
0.0918 < Uninsured.adults <= 0.1276
0.0851 < Severe.housing.cost.burden <= 0.1027
Unemployment <= 0.0525
0.0576 < Uninsured.children <= 0.0826
9.0 < Air.pollution...particulate.matter
Percentage.of.households.with.high.housing.costs <= 0.0838
0.318 < Long.commute...driving.alone <= 0.409
Feature
−0.06
−0.04
−0.02
0.00
Weight
Positive
Negative

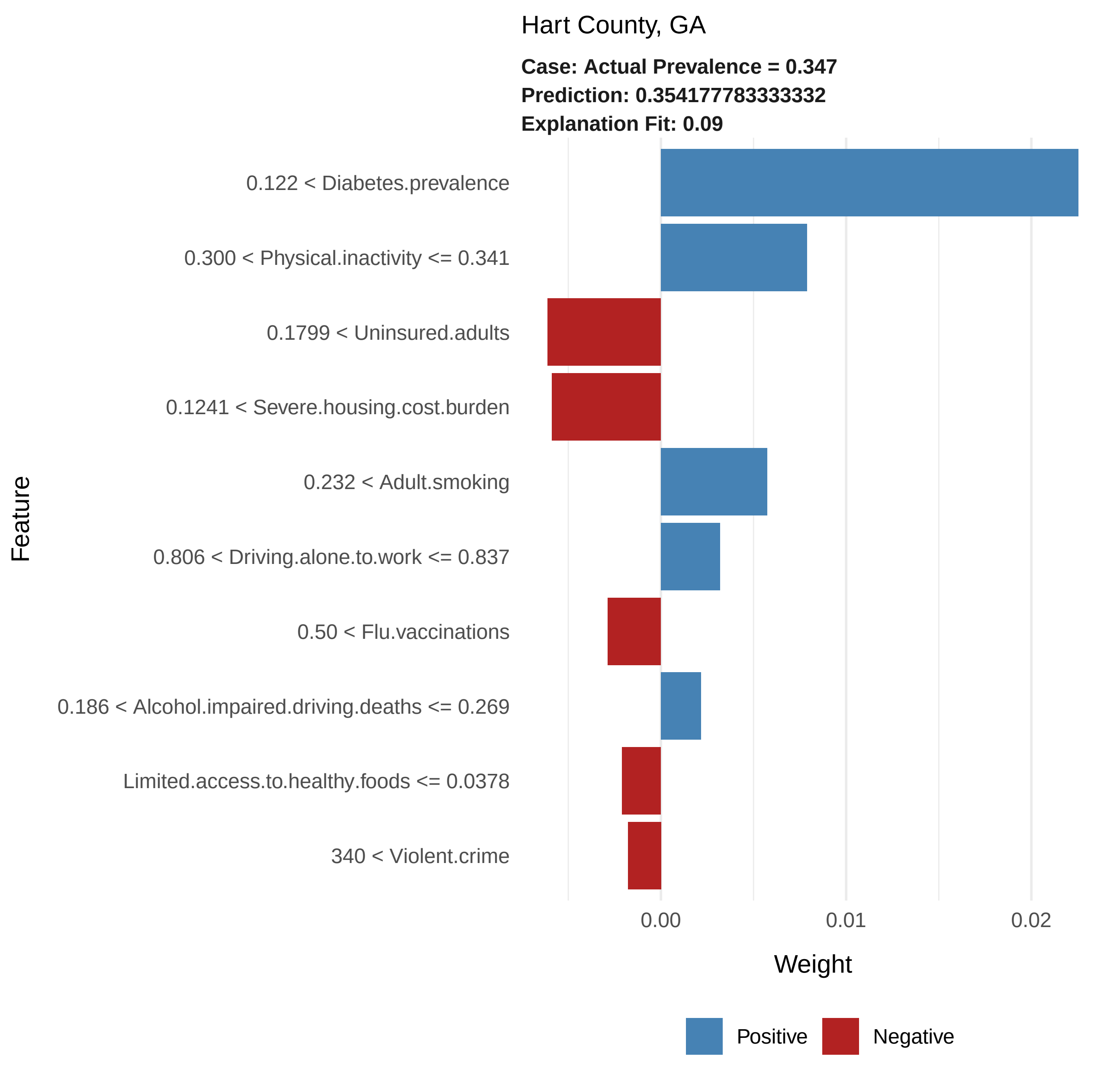

Hart County, GA
Case: Actual Prevalence = 0.347
Prediction: 0.354177783333332
Explanation Fit: 0.09
0.122 < Diabetes.prevalence
0.300 < Physical.inactivity <= 0.341
0.1799 < Uninsured.adults
0.1241 < Severe.housing.cost.burden
0.232 < Adult.smoking
0.806 < Driving.alone.to.work <= 0.837
0.50 < Flu.vaccinations
0.186 < Alcohol.impaired.driving.deaths <= 0.269
Limited.access.to.healthy.foods <= 0.0378
340 < Violent.crime
Feature
0.00
0.01
0.02
Weight
Positive
Negative

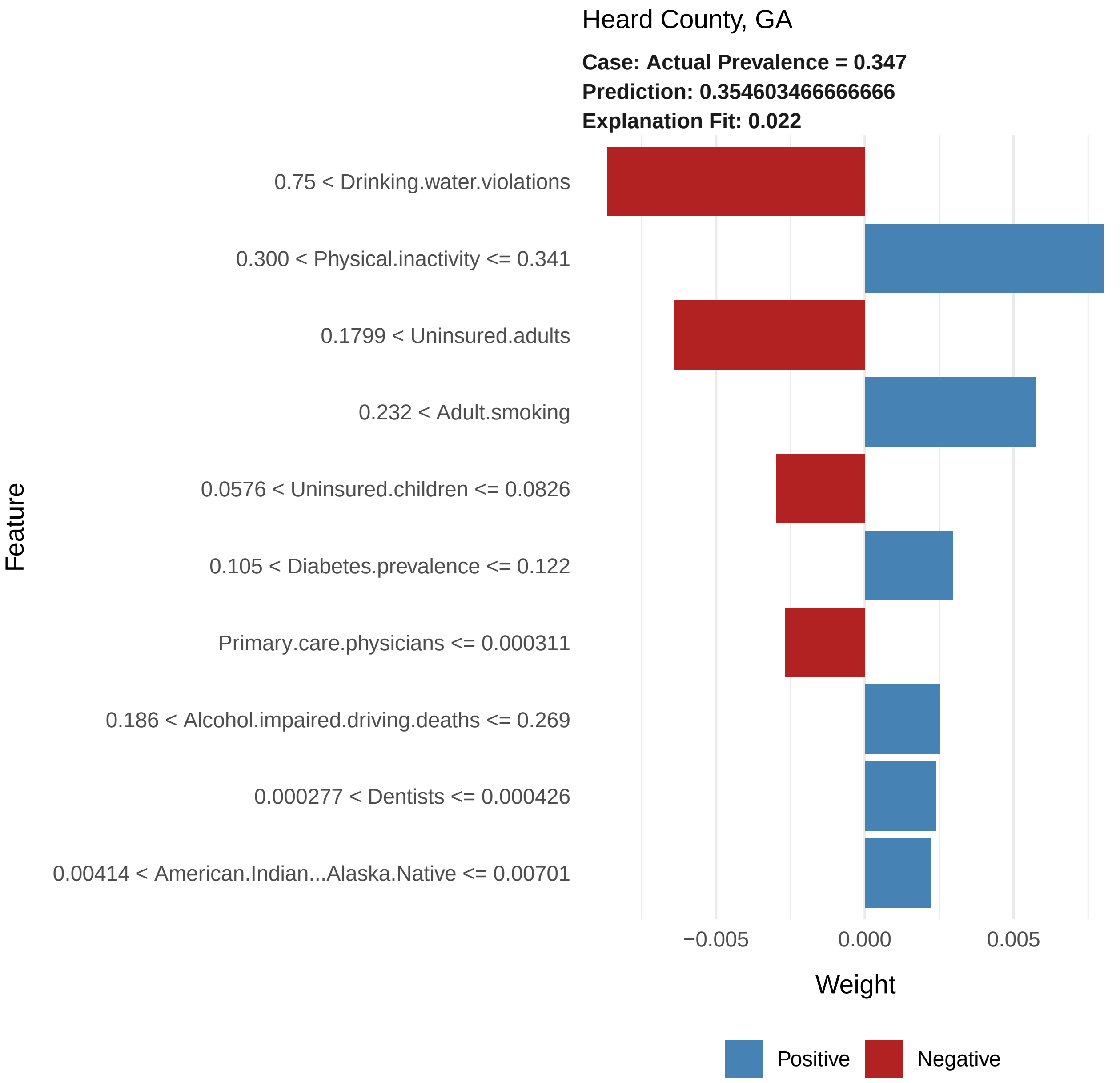

Heard County, GA
Case: Actual Prevalence = 0.347
Prediction: 0.354603466666666
Explanation Fit: 0.022
0.75 < Drinking.water.violations
0.300 < Physical.inactivity <= 0.341
0.1799 < Uninsured.adults
0.232 < Adult.smoking
0.0576 < Uninsured.children <= 0.0826
0.105 < Diabetes.prevalence <= 0.122
Primary.care.physicians <= 0.000311
0.186 < Alcohol.impaired.driving.deaths <= 0.269
0.000277 < Dentists <= 0.000426
0.00414 < American.Indian...Alaska.Native <= 0.00701
Feature
−0.005
0.000
0.005
Weight
Positive
Negative

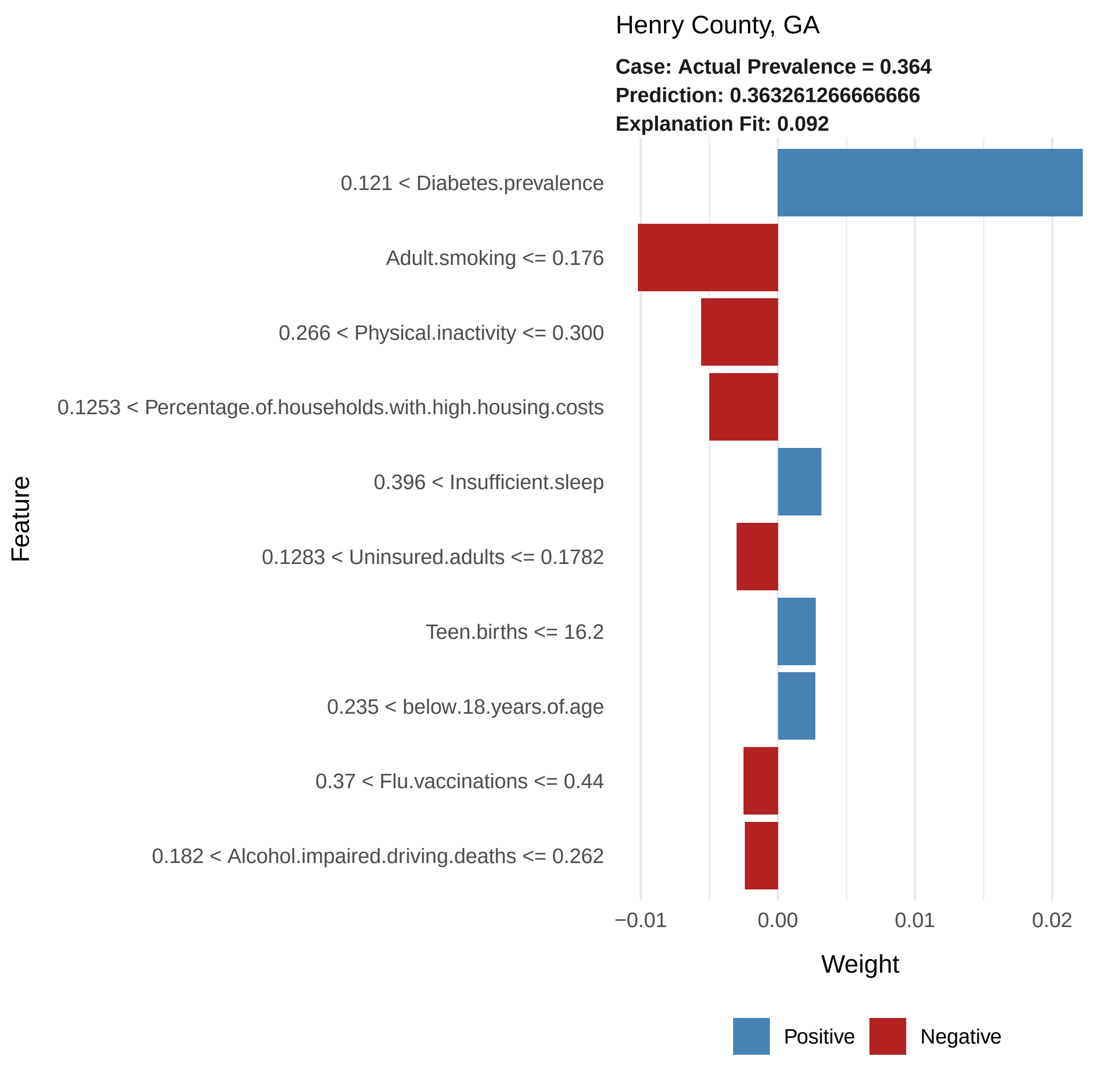

Henry County, GA
Case: Actual Prevalence = 0.364
Prediction: 0.363261266666666
Explanation Fit: 0.092
0.121 < Diabetes.prevalence
Adult.smoking <= 0.176
0.266 < Physical.inactivity <= 0.300
0.1253 < Percentage.of.households.with.high.housing.costs
0.396 < Insufficient.sleep
0.1283 < Uninsured.adults <= 0.1782
Teen.births <= 16.2
0.235 < below.18.years.of.age
0.37 < Flu.vaccinations <= 0.44
0.182 < Alcohol.impaired.driving.deaths <= 0.262
Feature
−0.01
0.00
0.01
0.02
Weight
Positive
Negative

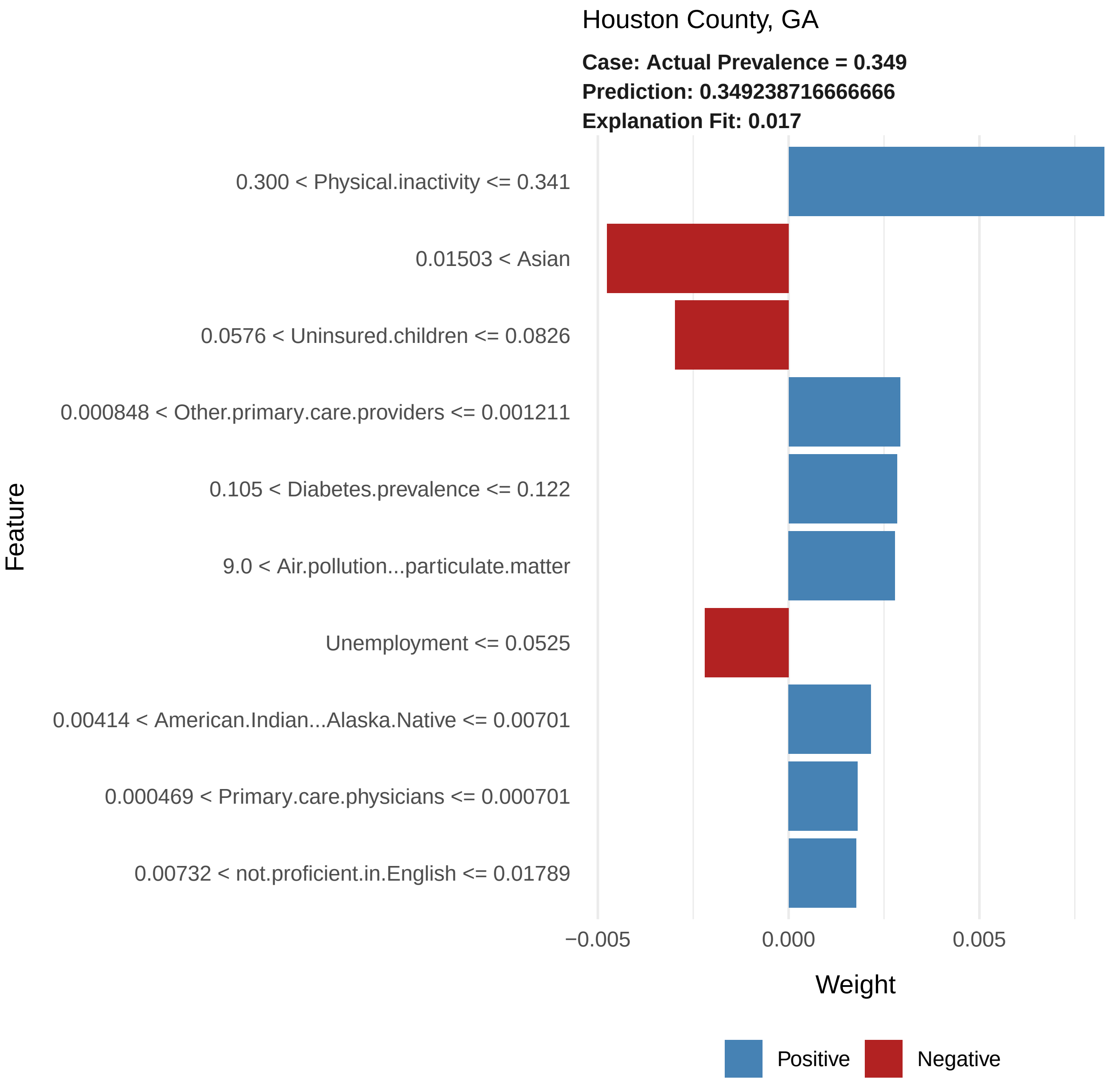
Houston County, GA
Case: Actual Prevalence = 0.349
Prediction: 0.349238716666666
Explanation Fit: 0.017
0.300 < Physical.inactivity <= 0.341
0.01503 < Asian
0.0576 < Uninsured.children <= 0.0826
0.000848 < Other.primary.care.providers <= 0.001211
0.105 < Diabetes.prevalence <= 0.122
9.0 < Air.pollution...particulate.matter
Unemployment <= 0.0525
0.00414 < American.Indian...Alaska.Native <= 0.00701
0.000469 < Primary.care.physicians <= 0.000701
0.00732 < not.proficient.in.English <= 0.01789
Feature
−0.005
0.000
0.005
Weight
Positive
Negative

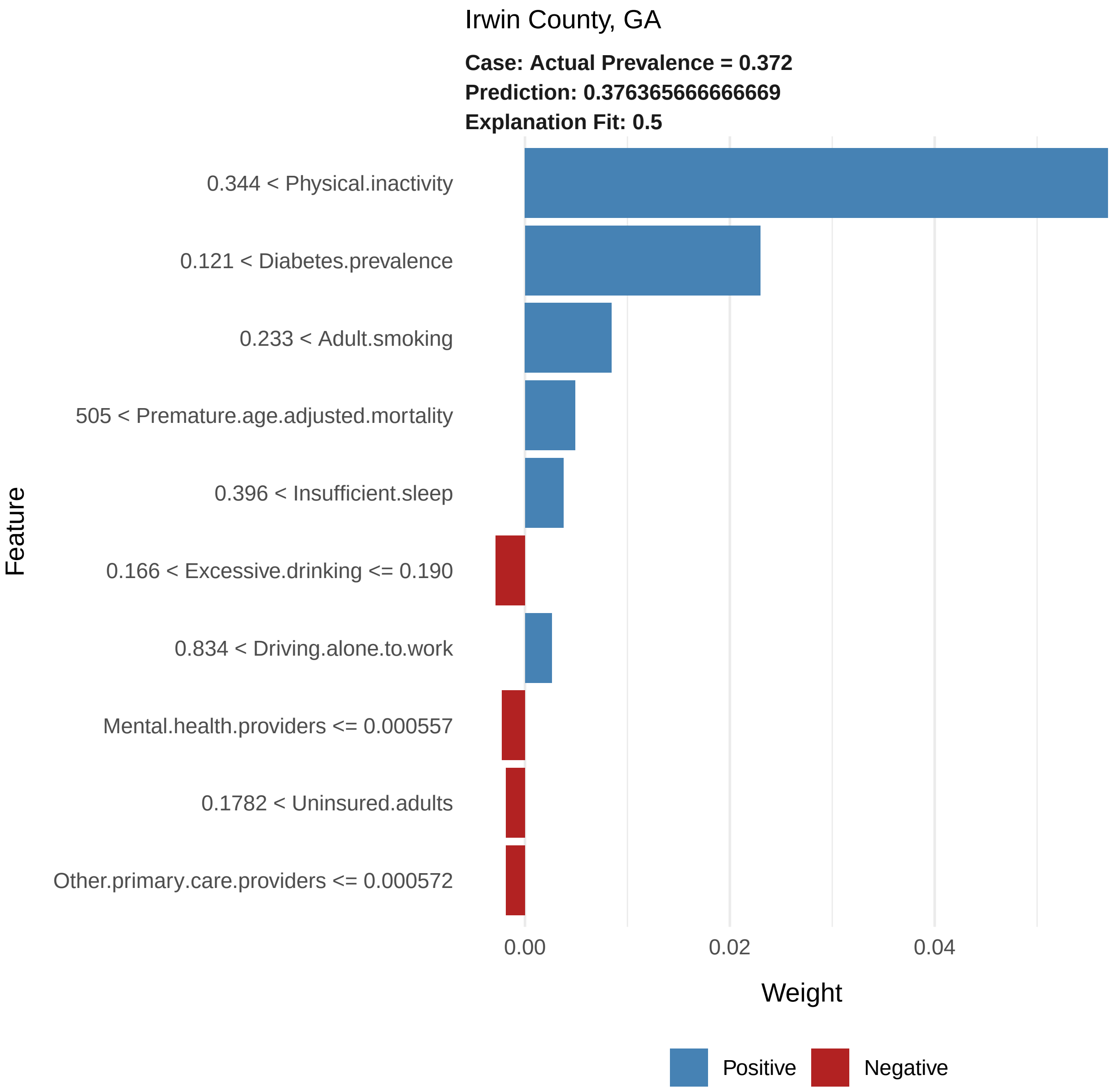

Irwin County, GA
Case: Actual Prevalence = 0.372
Prediction: 0.376365666666669
Explanation Fit: 0.5
0.344 < Physical.inactivity
0.121 < Diabetes.prevalence
0.233 < Adult.smoking
505 < Premature.age.adjusted.mortality
0.396 < Insufficient.sleep
0.166 < Excessive.drinking <= 0.190
0.834 < Driving.alone.to.work
Mental.health.providers <= 0.000557
0.1782 < Uninsured.adults
Other.primary.care.providers <= 0.000572
Feature
0.00
0.02
0.04
Weight
Positive
Negative

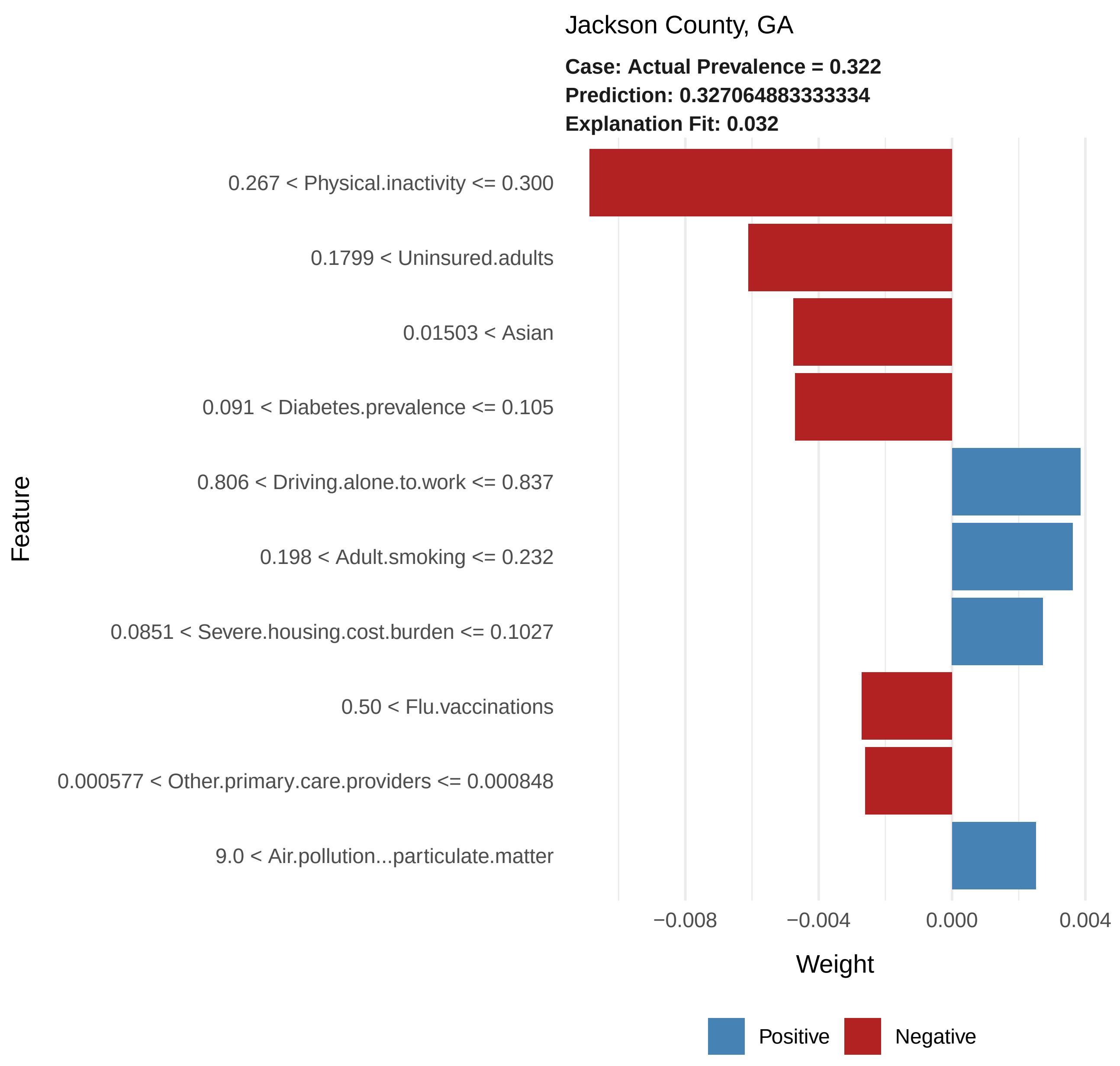
Jackson County, GA
Case: Actual Prevalence = 0.322
Prediction: 0.327064883333334
Explanation Fit: 0.032
0.267 < Physical.inactivity <= 0.300
0.1799 < Uninsured.adults
0.01503 < Asian
0.091 < Diabetes.prevalence <= 0.105
0.806 < Driving.alone.to.work <= 0.837
0.198 < Adult.smoking <= 0.232
0.0851 < Severe.housing.cost.burden <= 0.1027
0.50 < Flu.vaccinations
0.000577 < Other.primary.care.providers <= 0.000848
9.0 < Air.pollution...particulate.matter
Feature
−0.008
−0.004
0.000
0.004
Weight
Positive
Negative

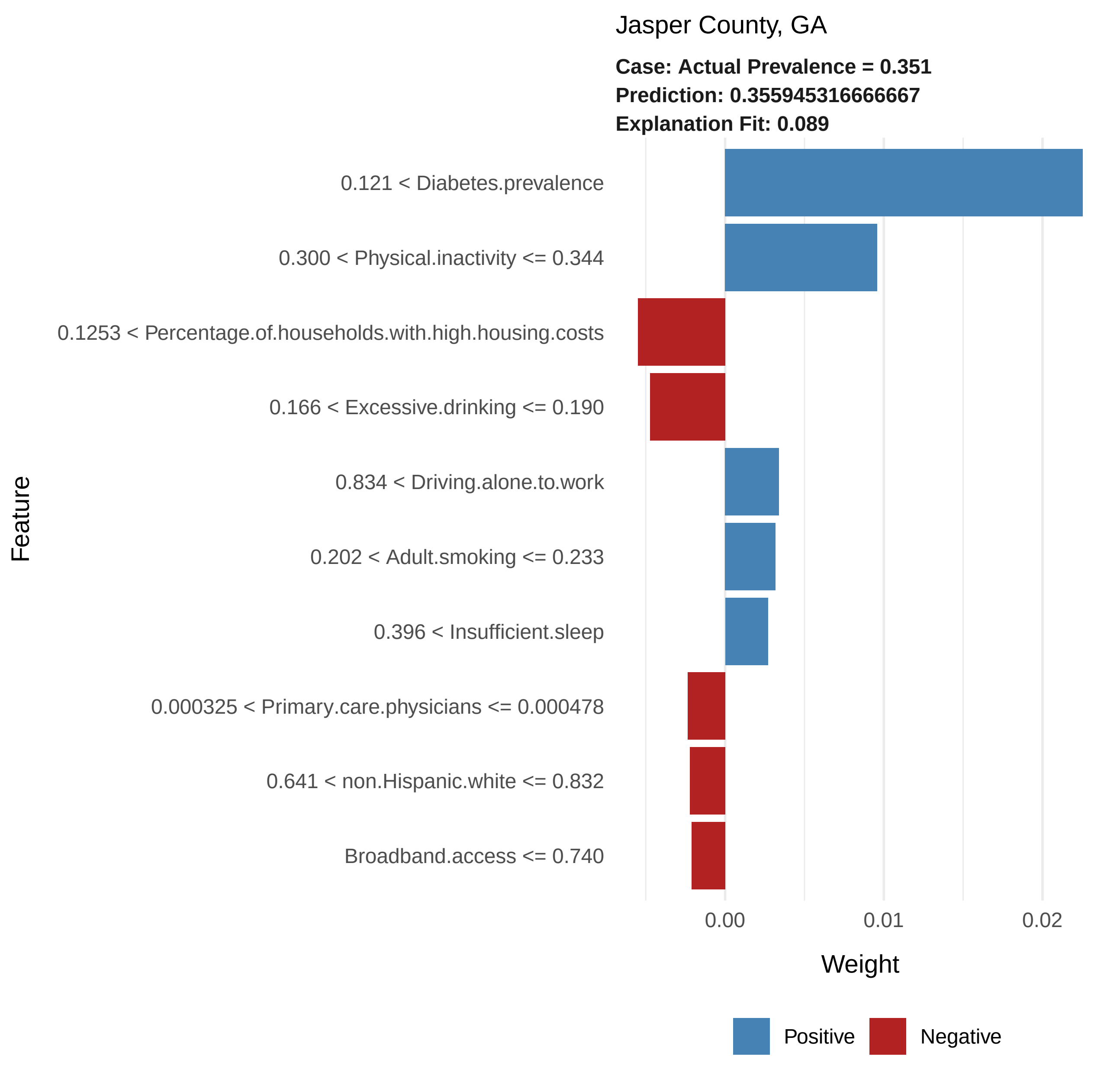

Jasper County, GA
Case: Actual Prevalence = 0.351
Prediction: 0.355945316666667
Explanation Fit: 0.089
0.121 < Diabetes.prevalence
0.300 < Physical.inactivity <= 0.344
0.1253 < Percentage.of.households.with.high.housing.costs
0.166 < Excessive.drinking <= 0.190
0.834 < Driving.alone.to.work
0.202 < Adult.smoking <= 0.233
0.396 < Insufficient.sleep
0.000325 < Primary.care.physicians <= 0.000478
0.641 < non.Hispanic.white <= 0.832
Broadband.access <= 0.740
Feature
0.00
0.01
0.02
Weight
Positive
Negative

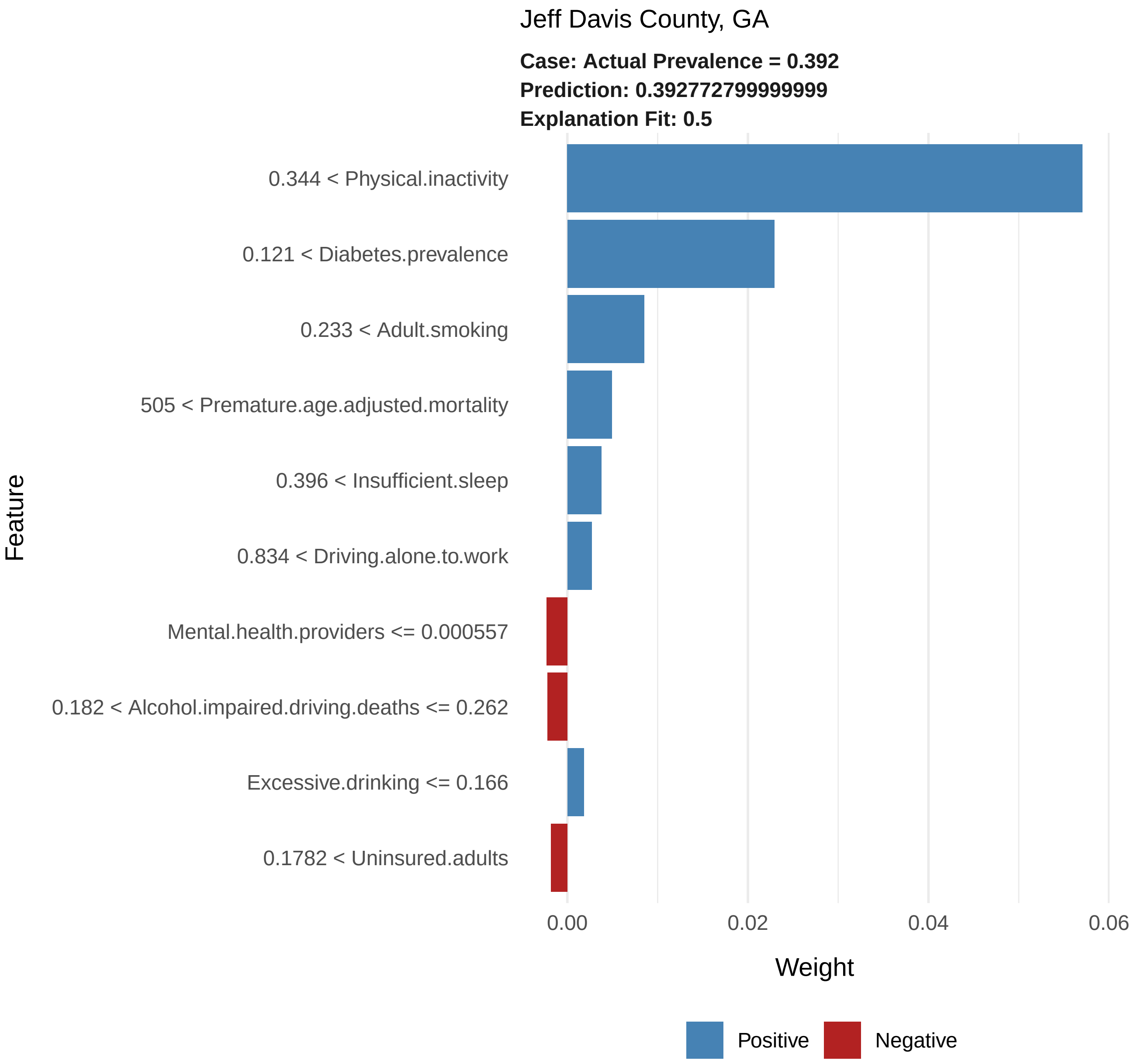

Jeff Davis County, GA
Case: Actual Prevalence = 0.392
Prediction: 0.392772799999999
Explanation Fit: 0.5
Feature
0.344 < Physical.inactivity
0.121 < Diabetes.prevalence
0.233 < Adult.smoking
505 < Premature.age.adjusted.mortality
0.396 < Insufficient.sleep
0.834 < Driving.alone.to.work
Mental.health.providers <= 0.000557
0.182 < Alcohol.impaired.driving.deaths <= 0.262
Excessive.drinking <= 0.166
0.1782 < Uninsured.adults
0.00
0.02
0.04
0.06
Weight
Positive
Negative

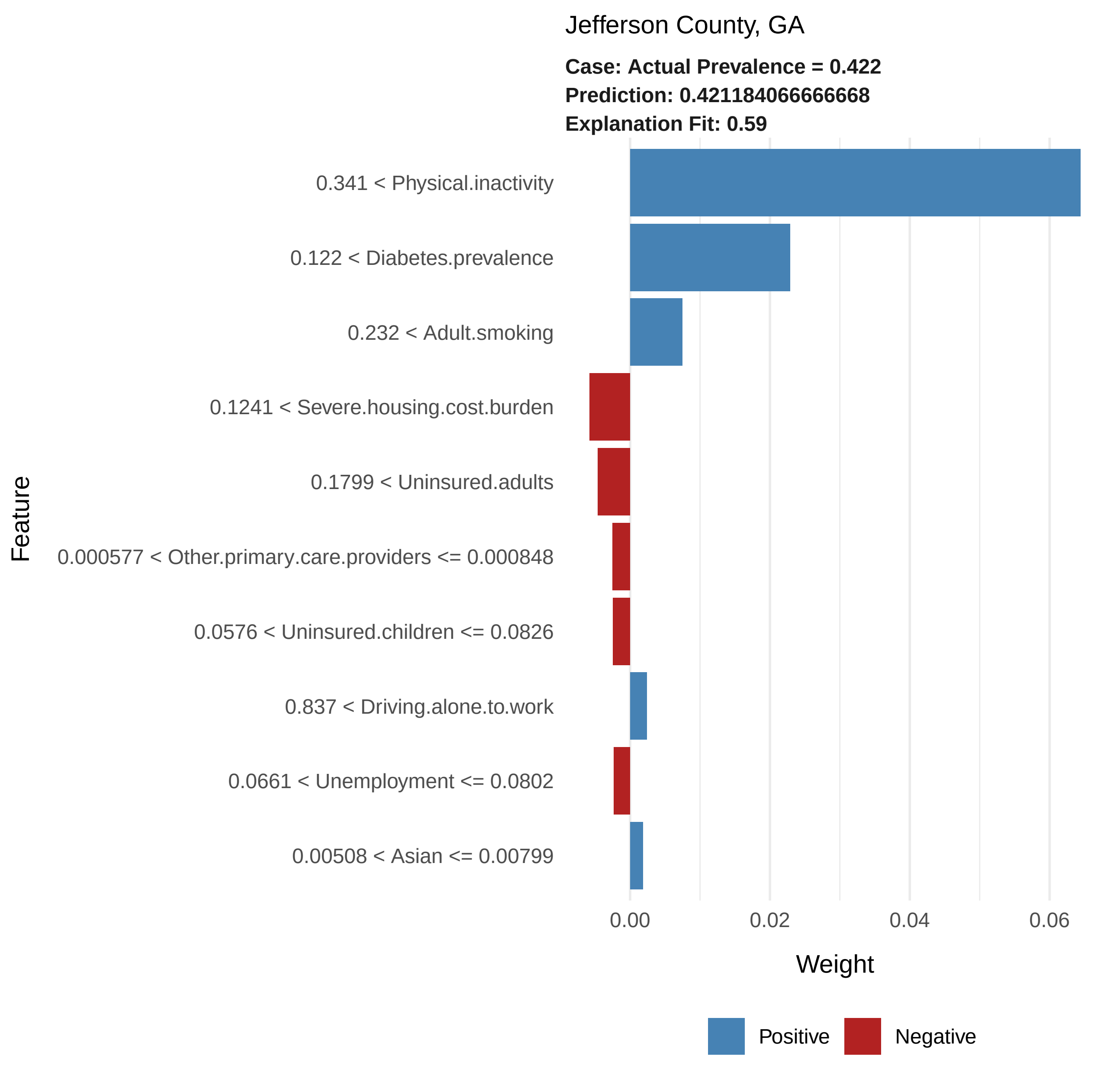
Jefferson County, GA
Case: Actual Prevalence = 0.422
Prediction: 0.421184066666668
Explanation Fit: 0.59
0.341 < Physical.inactivity
0.122 < Diabetes.prevalence
0.232 < Adult.smoking
0.1241 < Severe.housing.cost.burden
0.1799 < Uninsured.adults
0.000577 < Other.primary.care.providers <= 0.000848
0.0576 < Uninsured.children <= 0.0826
0.837 < Driving.alone.to.work
0.0661 < Unemployment <= 0.0802
0.00508 < Asian <= 0.00799
Feature
0.00
0.02
0.04
0.06
Weight
Positive
Negative

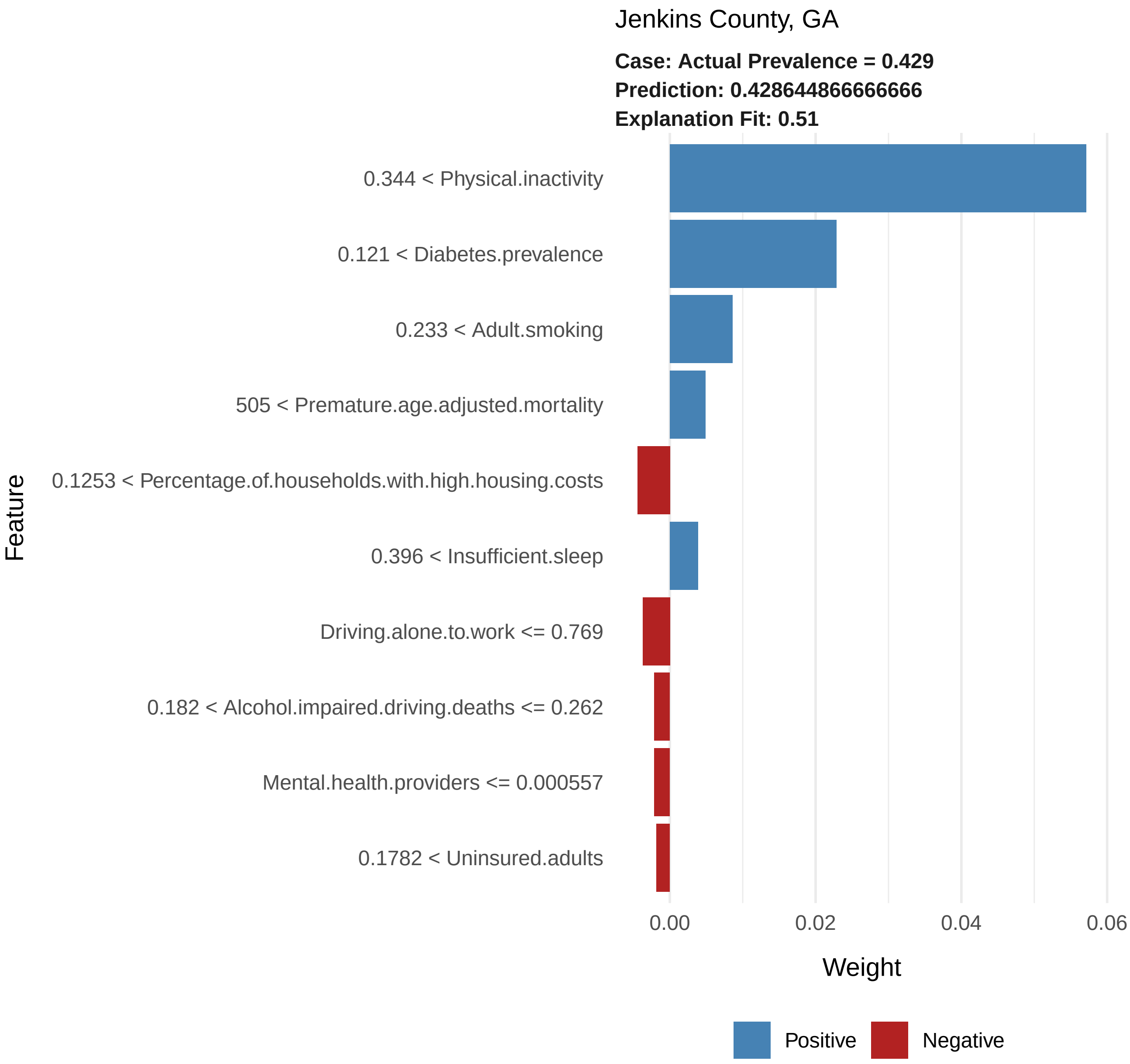
Jenkins County, GA
Case: Actual Prevalence = 0.429
Prediction: 0.428644866666666
Explanation Fit: 0.51
0.344 < Physical.inactivity
0.121 < Diabetes.prevalence
0.233 < Adult.smoking
505 < Premature.age.adjusted.mortality
0.1253 < Percentage.of.households.with.high.housing.costs
0.396 < Insufficient.sleep
Driving.alone.to.work <= 0.769
0.182 < Alcohol.impaired.driving.deaths <= 0.262
Mental.health.providers <= 0.000557
0.1782 < Uninsured.adults
Feature
0.00
0.02
0.04
0.06
Weight
Positive
Negative

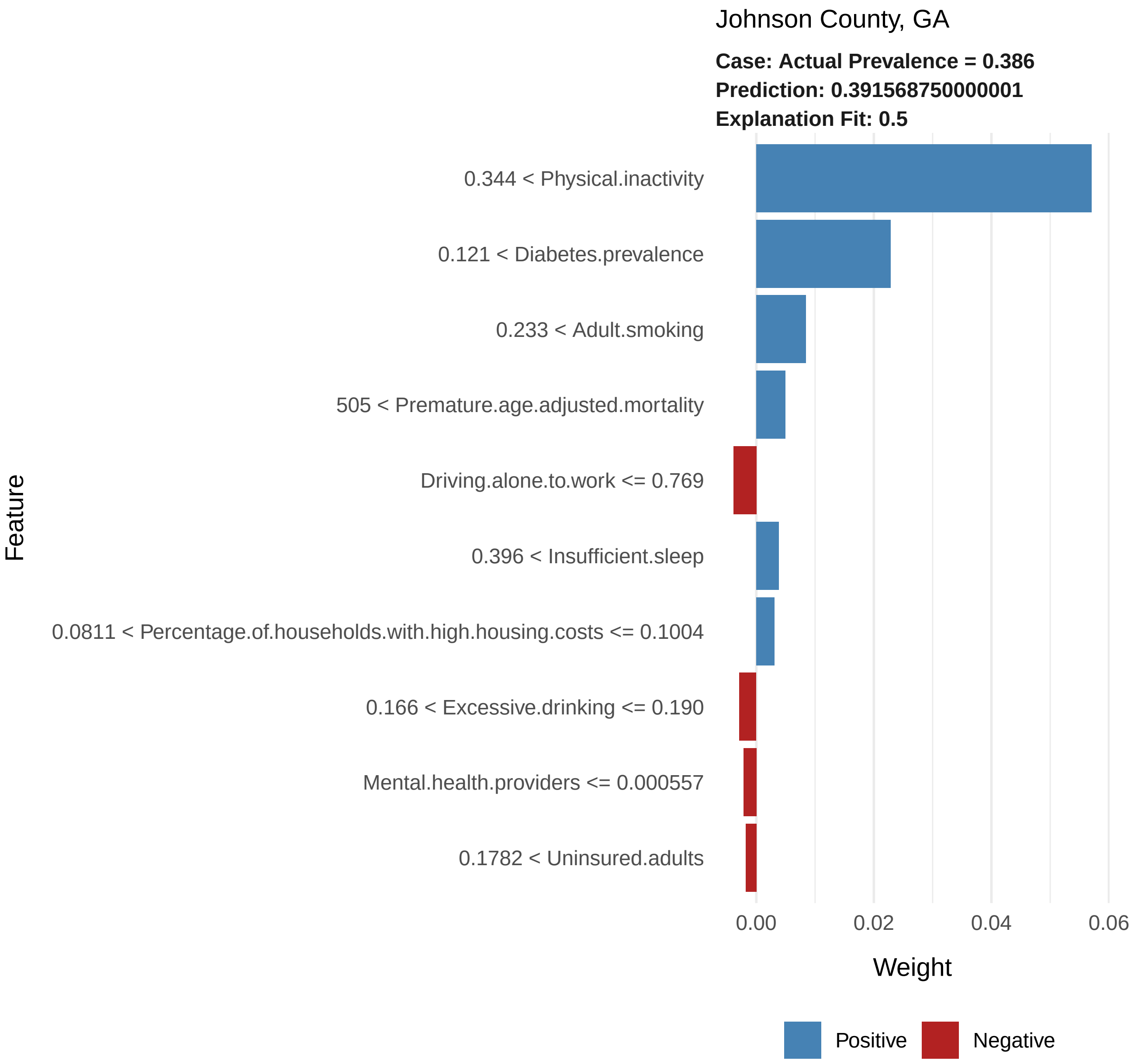

Johnson County, GA
Case: Actual Prevalence = 0.386
Prediction: 0.391568750000001
Explanation Fit: 0.5
Feature
0.344 < Physical.inactivity
0.121 < Diabetes.prevalence
0.233 < Adult.smoking
505 < Premature.age.adjusted.mortality
Driving.alone.to.work <= 0.769
0.396 < Insufficient.sleep
0.0811 < Percentage.of.households.with.high.housing.costs <= 0.1004
0.166 < Excessive.drinking <= 0.190
Mental.health.providers <= 0.000557
0.1782 < Uninsured.adults
0.00
0.02
0.04
0.06
Weight
Positive
Negative

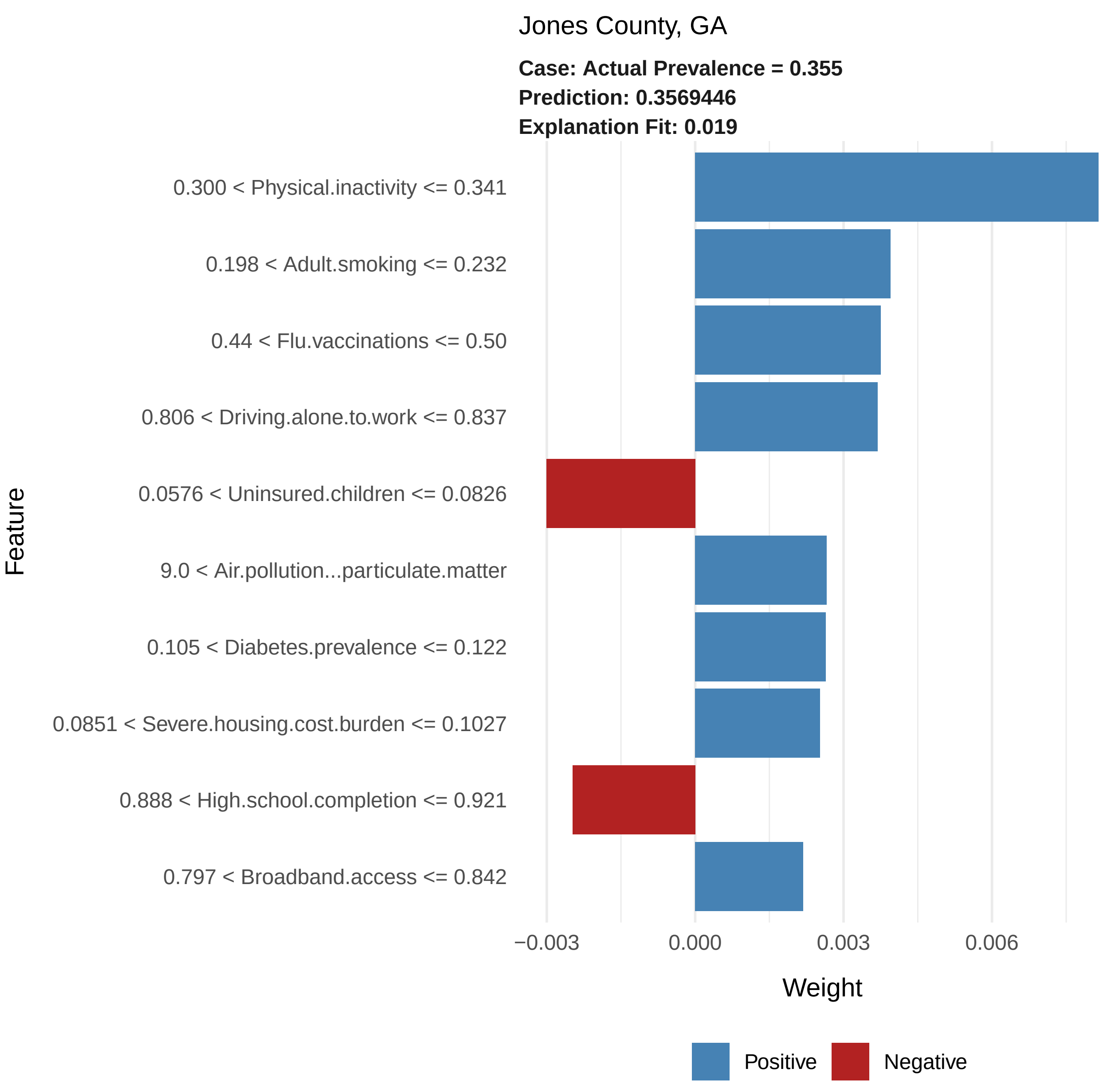

Jones County, GA
Case: Actual Prevalence = 0.355
Prediction: 0.3569446
Explanation Fit: 0.019
0.300 < Physical.inactivity <= 0.341
0.198 < Adult.smoking <= 0.232
0.44 < Flu.vaccinations <= 0.50
0.806 < Driving.alone.to.work <= 0.837
0.0576 < Uninsured.children <= 0.0826
9.0 < Air.pollution...particulate.matter
0.105 < Diabetes.prevalence <= 0.122
0.0851 < Severe.housing.cost.burden <= 0.1027
0.888 < High.school.completion <= 0.921
0.797 < Broadband.access <= 0.842
Feature
−0.003
0.000
0.003
0.006
Weight
Positive
Negative

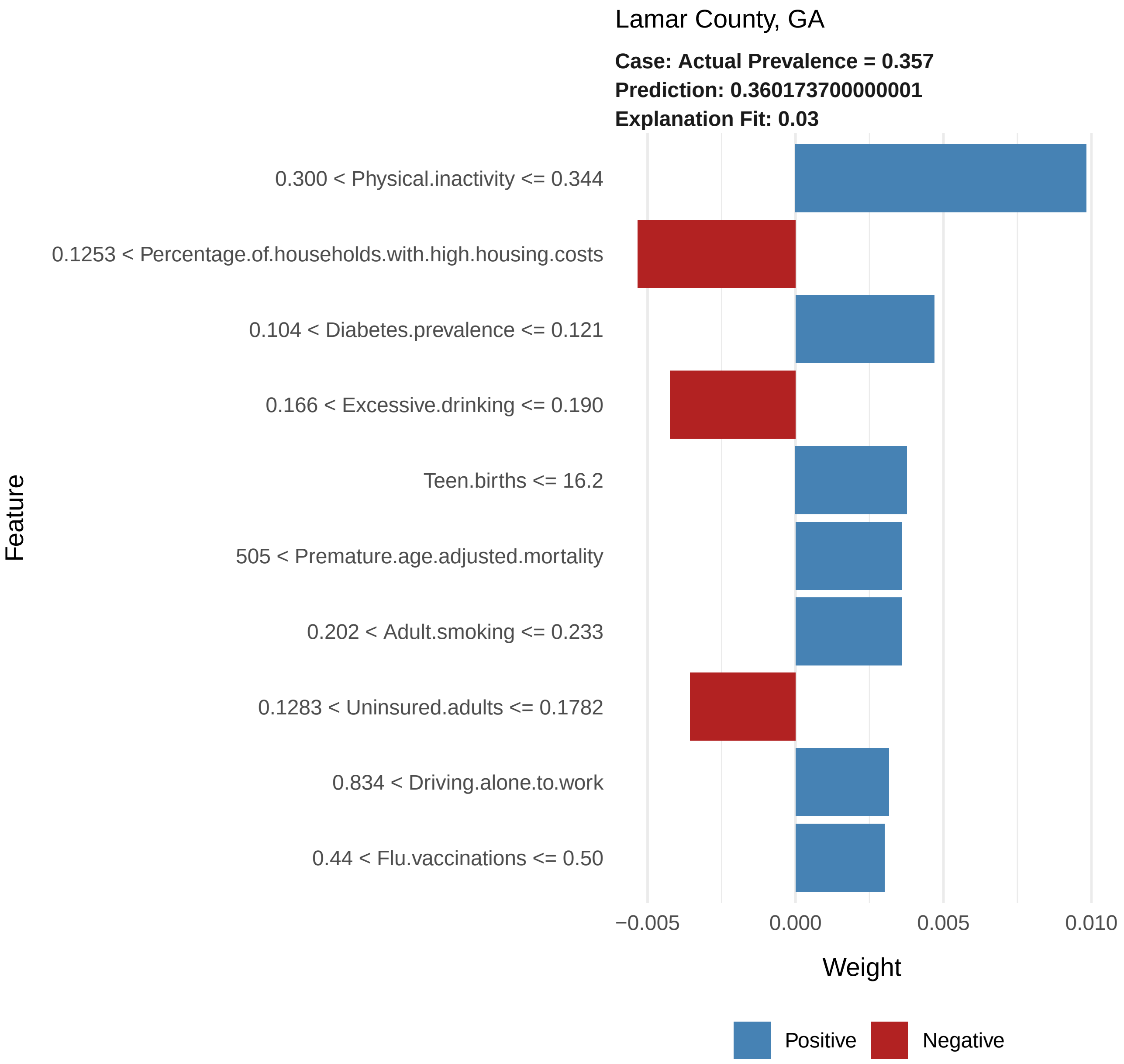
Lamar County, GA
Case: Actual Prevalence = 0.357
Prediction: 0.360173700000001
Explanation Fit: 0.03
0.300 < Physical.inactivity <= 0.344
0.1253 < Percentage.of.households.with.high.housing.costs
0.104 < Diabetes.prevalence <= 0.121
0.166 < Excessive.drinking <= 0.190
Teen.births <= 16.2
505 < Premature.age.adjusted.mortality
0.202 < Adult.smoking <= 0.233
0.1283 < Uninsured.adults <= 0.1782
0.834 < Driving.alone.to.work
0.44 < Flu.vaccinations <= 0.50
Feature
−0.005
0.000
0.005
0.010
Weight
Positive
Negative

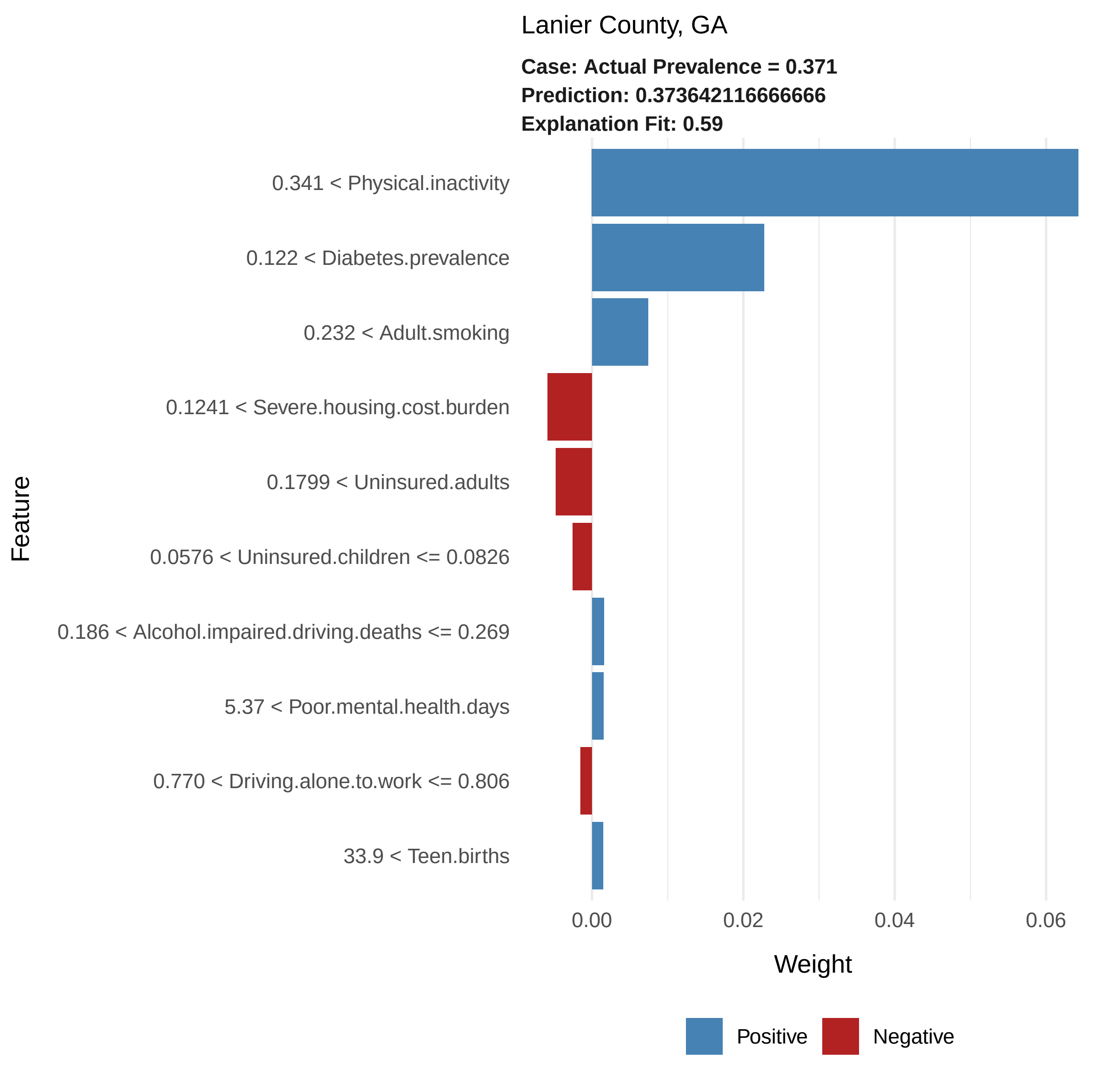

Lanier County, GA
Case: Actual Prevalence = 0.371
Prediction: 0.373642116666666
Explanation Fit: 0.59
0.341 < Physical.inactivity
0.122 < Diabetes.prevalence
0.232 < Adult.smoking
0.1241 < Severe.housing.cost.burden
0.1799 < Uninsured.adults
0.0576 < Uninsured.children <= 0.0826
0.186 < Alcohol.impaired.driving.deaths <= 0.269
5.37 < Poor.mental.health.days
0.770 < Driving.alone.to.work <= 0.806
33.9 < Teen.births
Feature
0.00
0.02
0.04
0.06
Weight
Positive
Negative

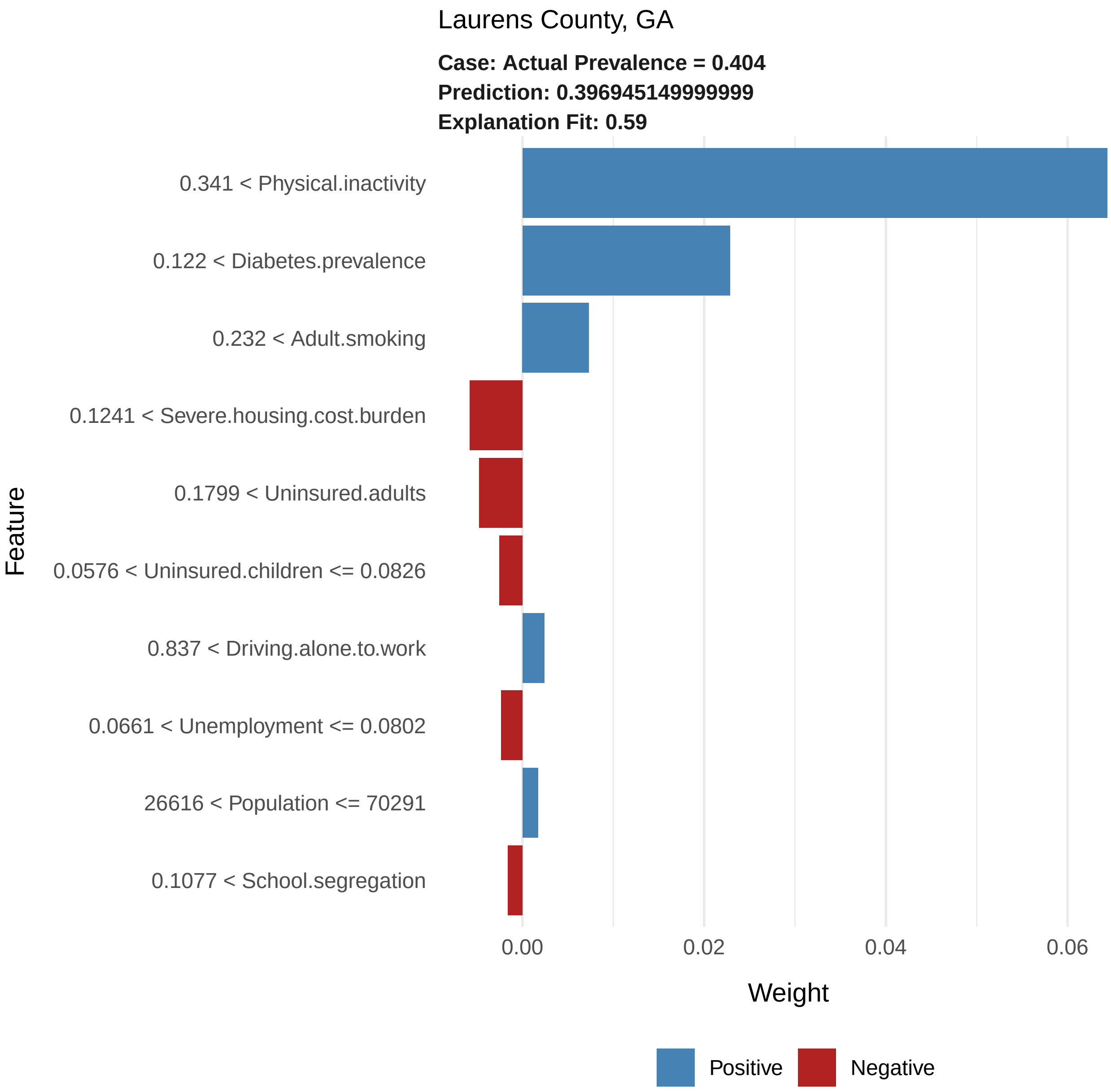
Laurens County, GA
Case: Actual Prevalence = 0.404
Prediction: 0.396945149999999
Explanation Fit: 0.59
0.341 < Physical.inactivity
0.122 < Diabetes.prevalence
0.232 < Adult.smoking
0.1241 < Severe.housing.cost.burden
0.1799 < Uninsured.adults
0.0576 < Uninsured.children <= 0.0826
0.837 < Driving.alone.to.work
0.0661 < Unemployment <= 0.0802
26616 < Population <= 70291
0.1077 < School.segregation
Feature
0.00
0.02
0.04
0.06
Weight
Positive
Negative

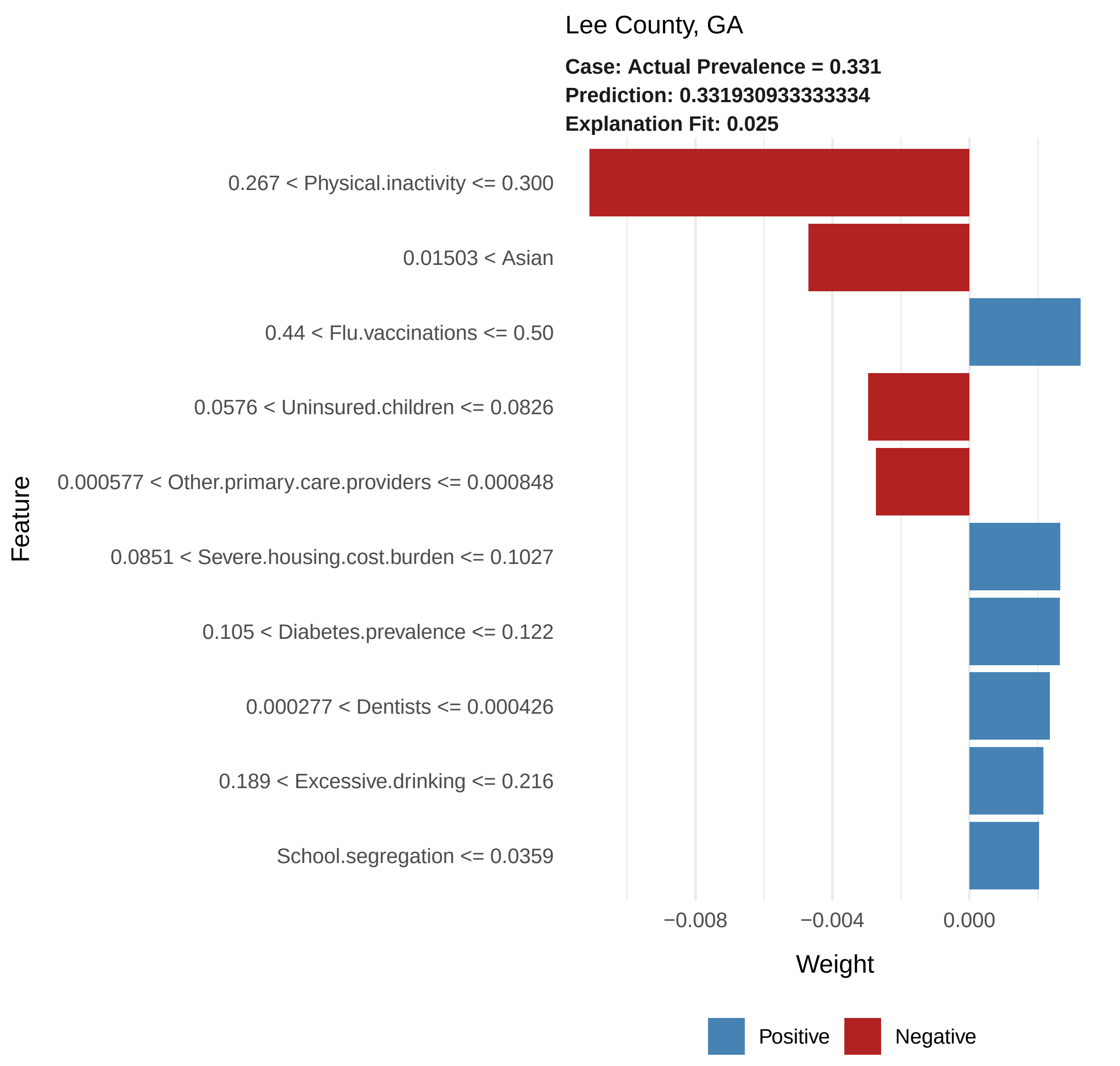
Lee County, GA
Case: Actual Prevalence = 0.331
Prediction: 0.331930933333334
Explanation Fit: 0.025
0.267 < Physical.inactivity <= 0.300
0.01503 < Asian
0.44 < Flu.vaccinations <= 0.50
0.0576 < Uninsured.children <= 0.0826
0.000577 < Other.primary.care.providers <= 0.000848
0.0851 < Severe.housing.cost.burden <= 0.1027
0.105 < Diabetes.prevalence <= 0.122
0.000277 < Dentists <= 0.000426
0.189 < Excessive.drinking <= 0.216
School.segregation <= 0.0359
Feature
−0.008
−0.004
0.000
Weight
Positive
Negative

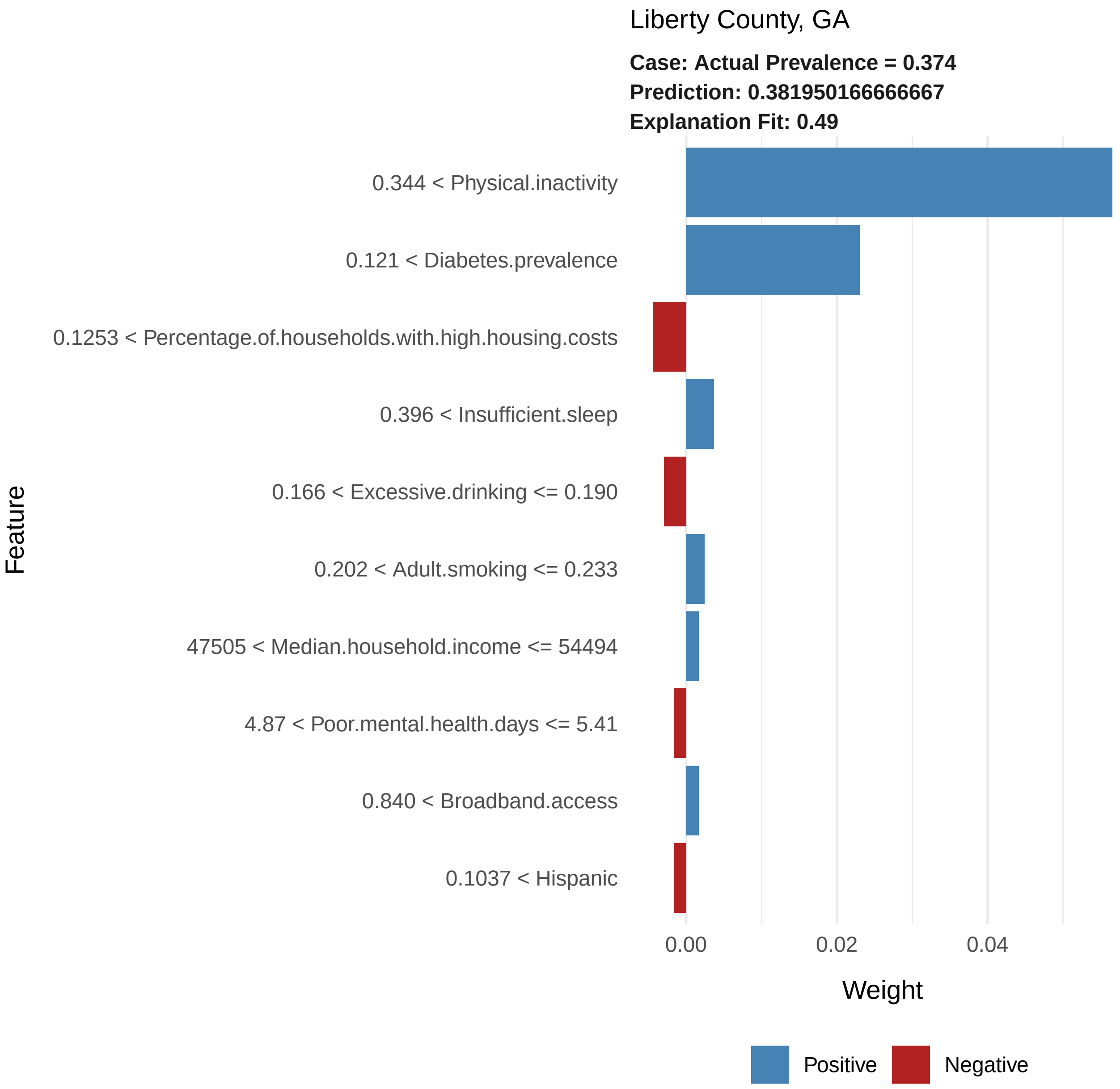
Liberty County, GA
Case: Actual Prevalence = 0.374
Prediction: 0.381950166666667
Explanation Fit: 0.49
0.344 < Physical.inactivity
0.121 < Diabetes.prevalence
0.1253 < Percentage.of.households.with.high.housing.costs
0.396 < Insufficient.sleep
0.166 < Excessive.drinking <= 0.190
0.202 < Adult.smoking <= 0.233
47505 < Median.household.income <= 54494
4.87 < Poor.mental.health.days <= 5.41
0.840 < Broadband.access
0.1037 < Hispanic
Feature
0.00
0.02
0.04
Weight
Positive
Negative

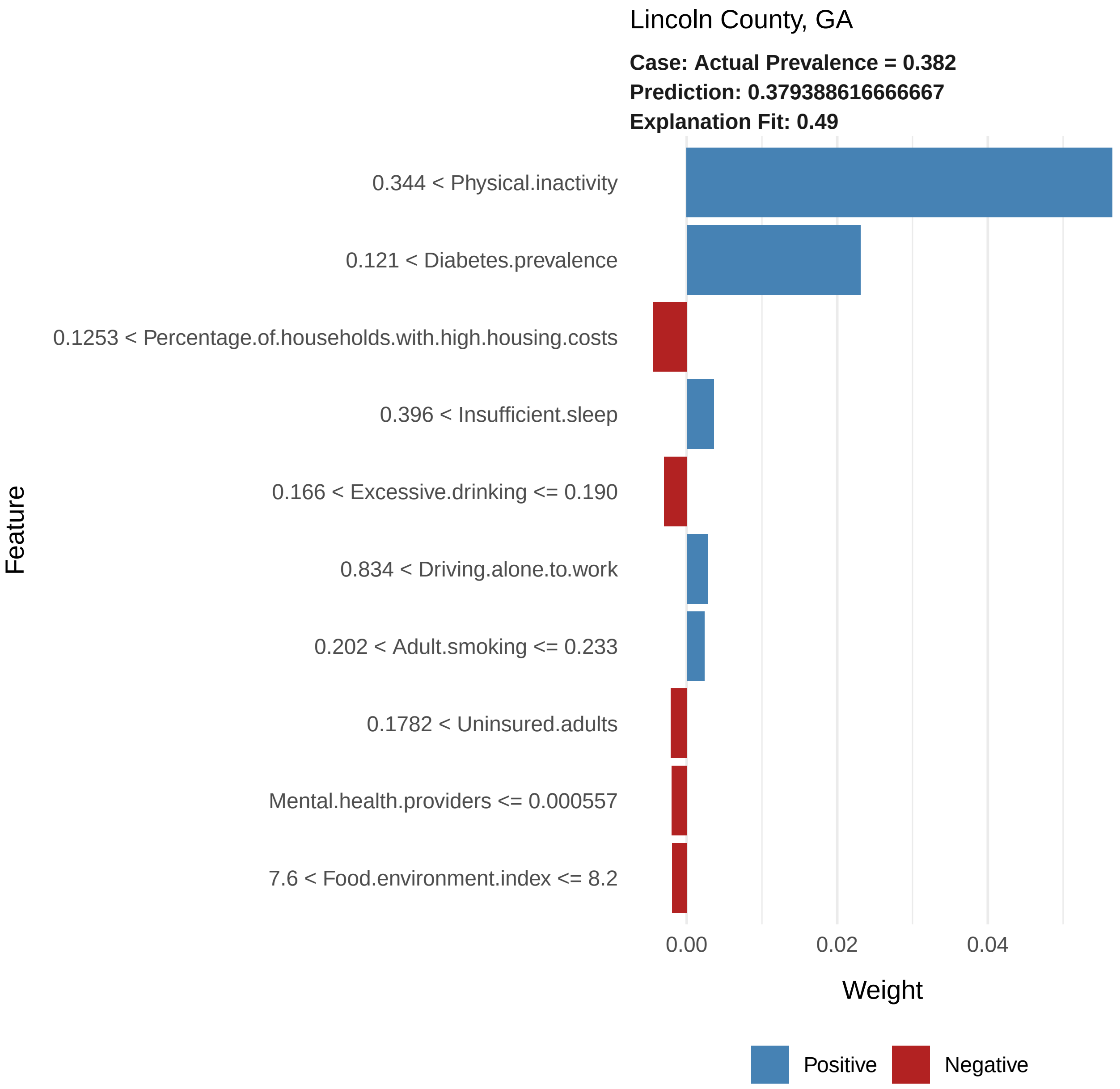
Lincoln County, GA
Case: Actual Prevalence = 0.382
Prediction: 0.379388616666667
Explanation Fit: 0.49
0.344 < Physical.inactivity
0.121 < Diabetes.prevalence
0.1253 < Percentage.of.households.with.high.housing.costs
0.396 < Insufficient.sleep
0.166 < Excessive.drinking <= 0.190
0.834 < Driving.alone.to.work
0.202 < Adult.smoking <= 0.233
0.1782 < Uninsured.adults
Mental.health.providers <= 0.000557
7.6 < Food.environment.index <= 8.2
Feature
0.00
0.02
0.04
Weight
Positive
Negative

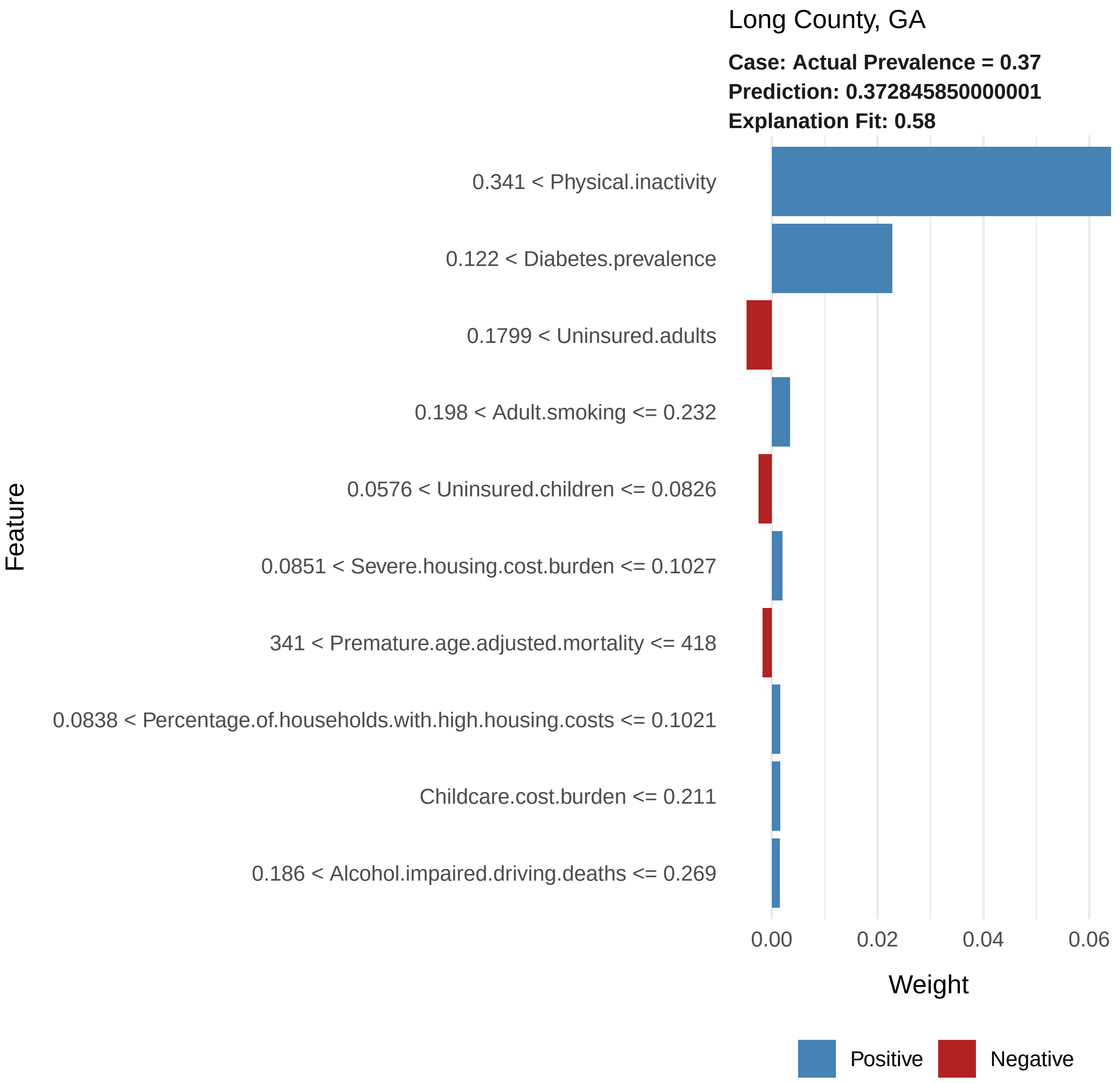

Long County, GA
Case: Actual Prevalence = 0.37
Prediction: 0.372845850000001
Explanation Fit: 0.58
0.341 < Physical.inactivity
0.122 < Diabetes.prevalence
0.1799 < Uninsured.adults
0.198 < Adult.smoking <= 0.232
0.0576 < Uninsured.children <= 0.0826
0.0851 < Severe.housing.cost.burden <= 0.1027
341 < Premature.age.adjusted.mortality <= 418
0.0838 < Percentage.of.households.with.high.housing.costs <= 0.1021
Childcare.cost.burden <= 0.211
0.186 < Alcohol.impaired.driving.deaths <= 0.269
Feature
0.00
0.02
0.04
0.06
Weight
Positive
Negative

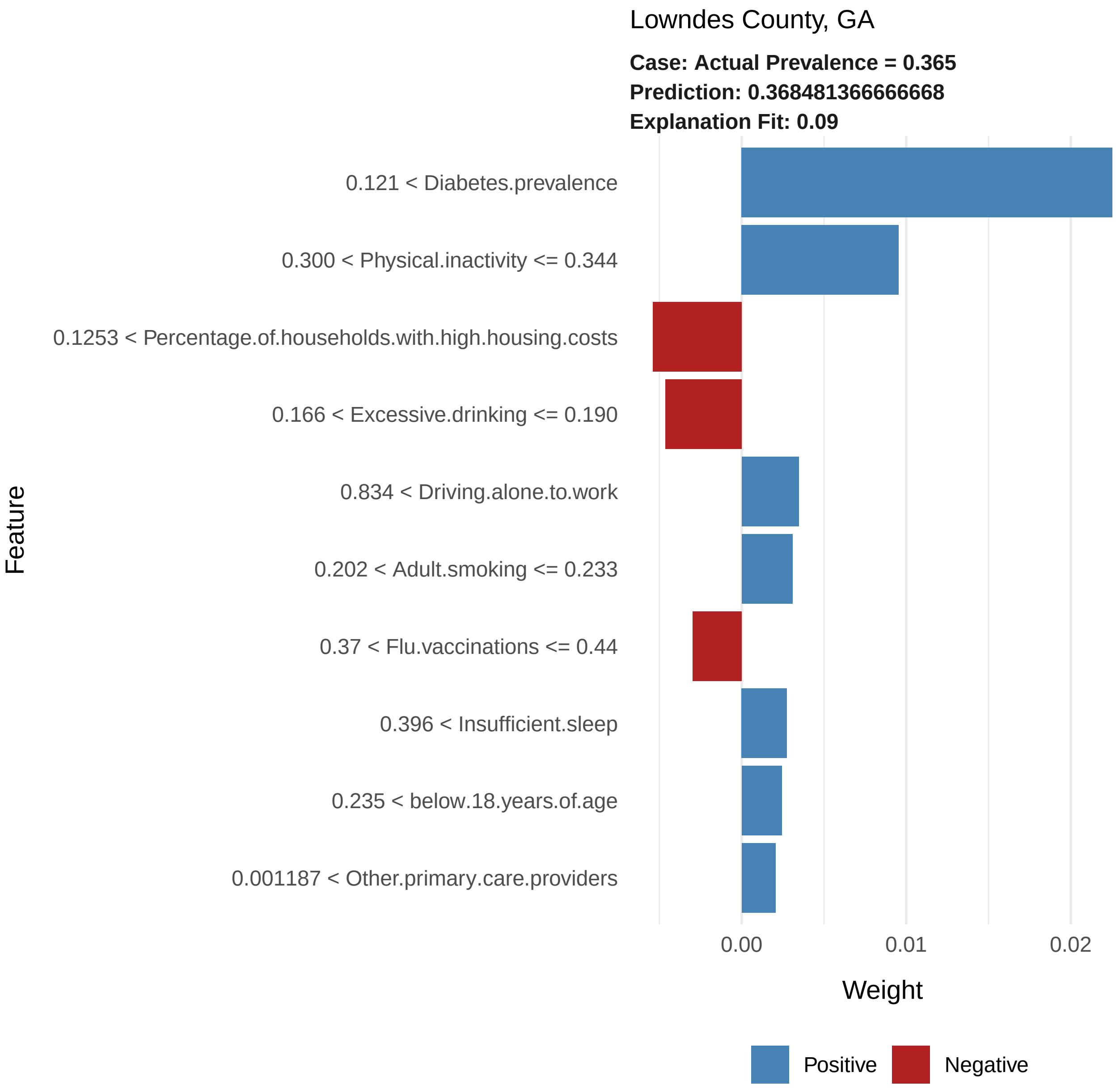

Lowndes County, GA
Case: Actual Prevalence = 0.365
Prediction: 0.368481366666668
Explanation Fit: 0.09
0.121 < Diabetes.prevalence
0.300 < Physical.inactivity <= 0.344
0.1253 < Percentage.of.households.with.high.housing.costs
0.166 < Excessive.drinking <= 0.190
0.834 < Driving.alone.to.work
0.202 < Adult.smoking <= 0.233
0.37 < Flu.vaccinations <= 0.44
0.396 < Insufficient.sleep
0.235 < below.18.years.of.age
0.001187 < Other.primary.care.providers
Feature
0.00
0.01
0.02
Weight
Positive
Negative

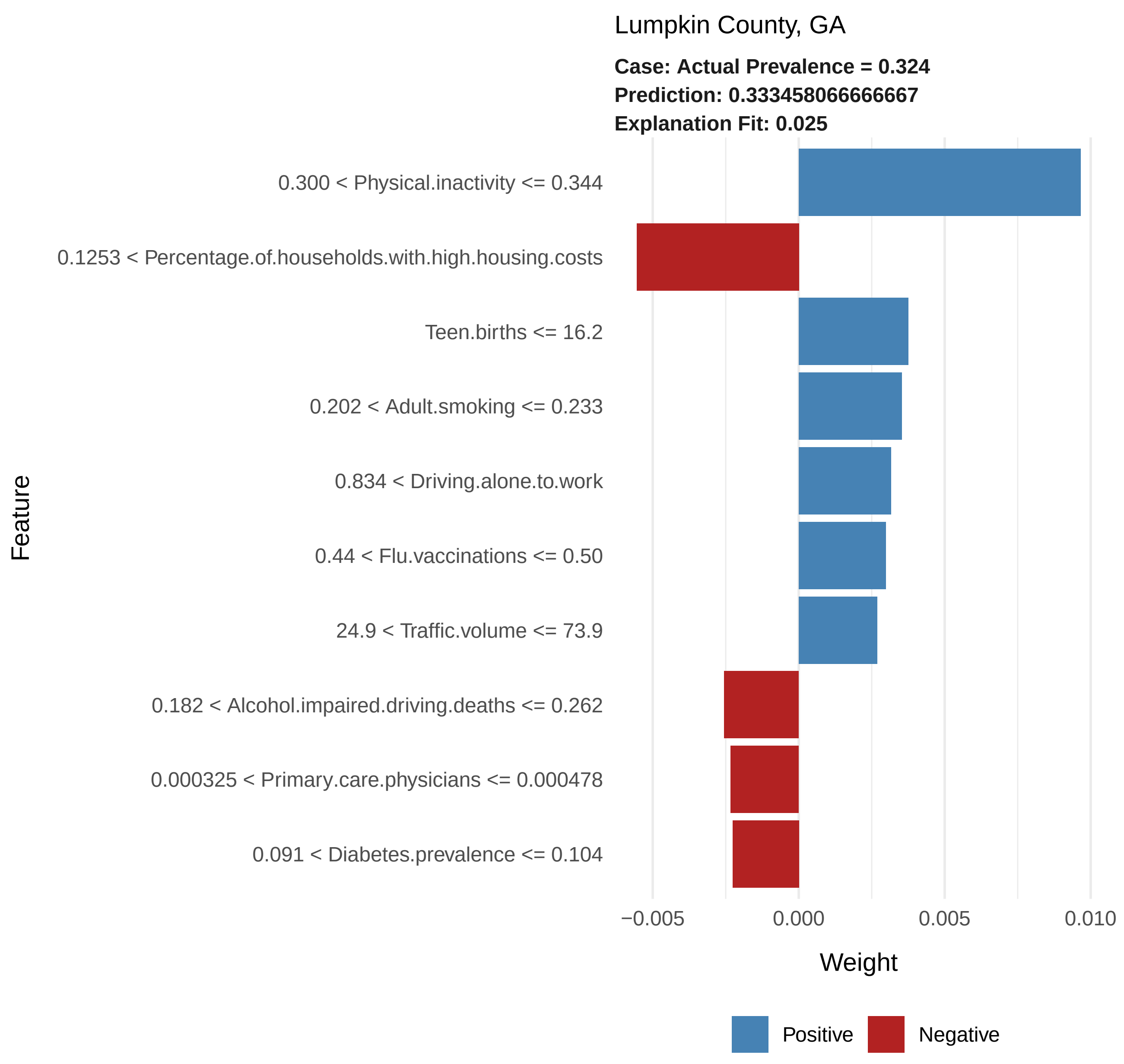
Lumpkin County, GA
Case: Actual Prevalence = 0.324
Prediction: 0.333458066666667
Explanation Fit: 0.025
0.300 < Physical.inactivity <= 0.344
0.1253 < Percentage.of.households.with.high.housing.costs
Teen.births <= 16.2
0.202 < Adult.smoking <= 0.233
0.834 < Driving.alone.to.work
0.44 < Flu.vaccinations <= 0.50
24.9 < Traffic.volume <= 73.9
0.182 < Alcohol.impaired.driving.deaths <= 0.262
0.000325 < Primary.care.physicians <= 0.000478
0.091 < Diabetes.prevalence <= 0.104
Feature
−0.005
0.000
0.005
0.010
Weight
Positive
Negative

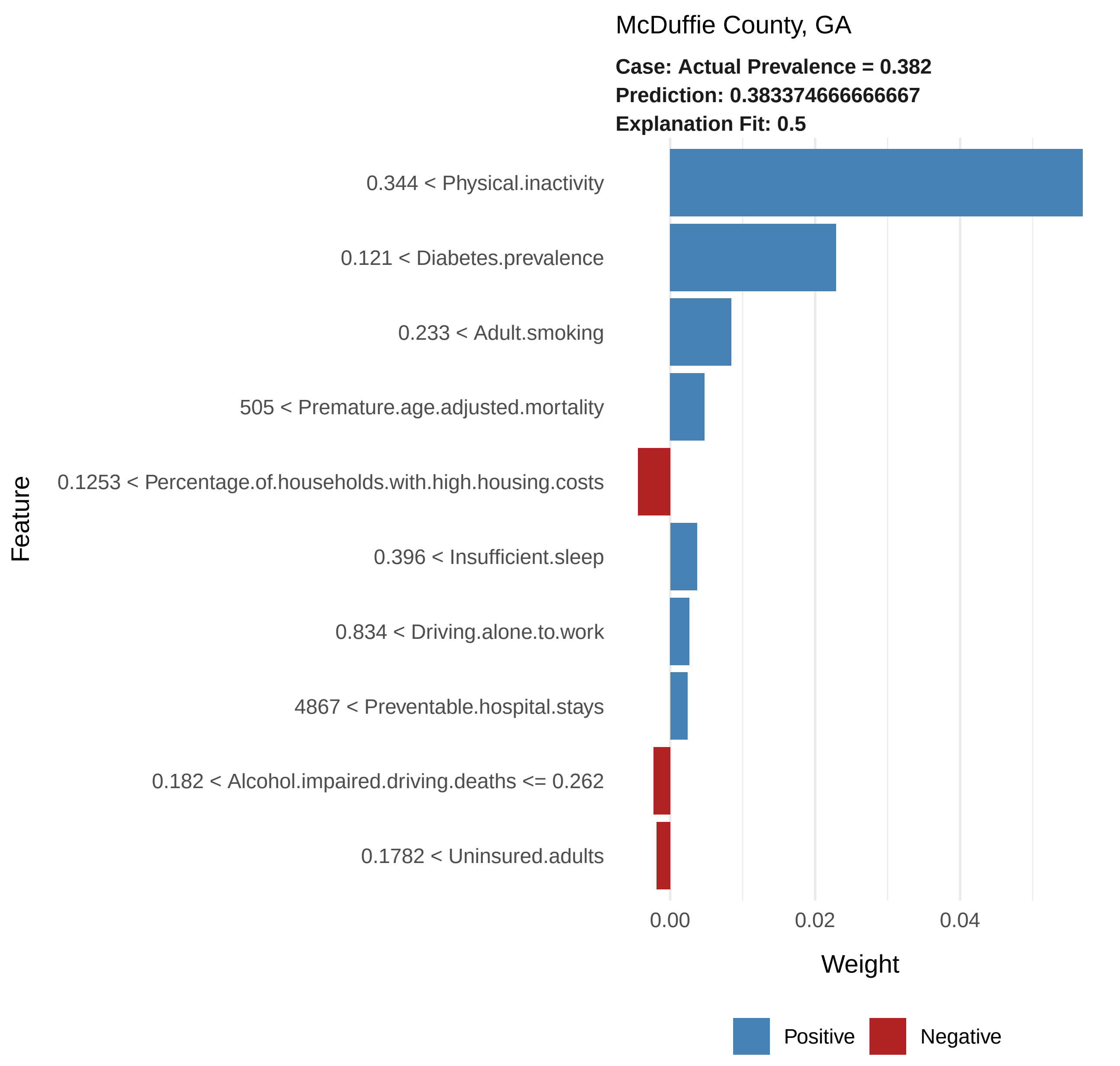
McDuffie County, GA
Case: Actual Prevalence = 0.382
Prediction: 0.383374666666667
Explanation Fit: 0.5
0.344 < Physical.inactivity
0.121 < Diabetes.prevalence
0.233 < Adult.smoking
505 < Premature.age.adjusted.mortality
0.1253 < Percentage.of.households.with.high.housing.costs
0.396 < Insufficient.sleep
0.834 < Driving.alone.to.work
4867 < Preventable.hospital.stays
0.182 < Alcohol.impaired.driving.deaths <= 0.262
0.1782 < Uninsured.adults
Feature
0.00
0.02
0.04
Weight
Positive
Negative

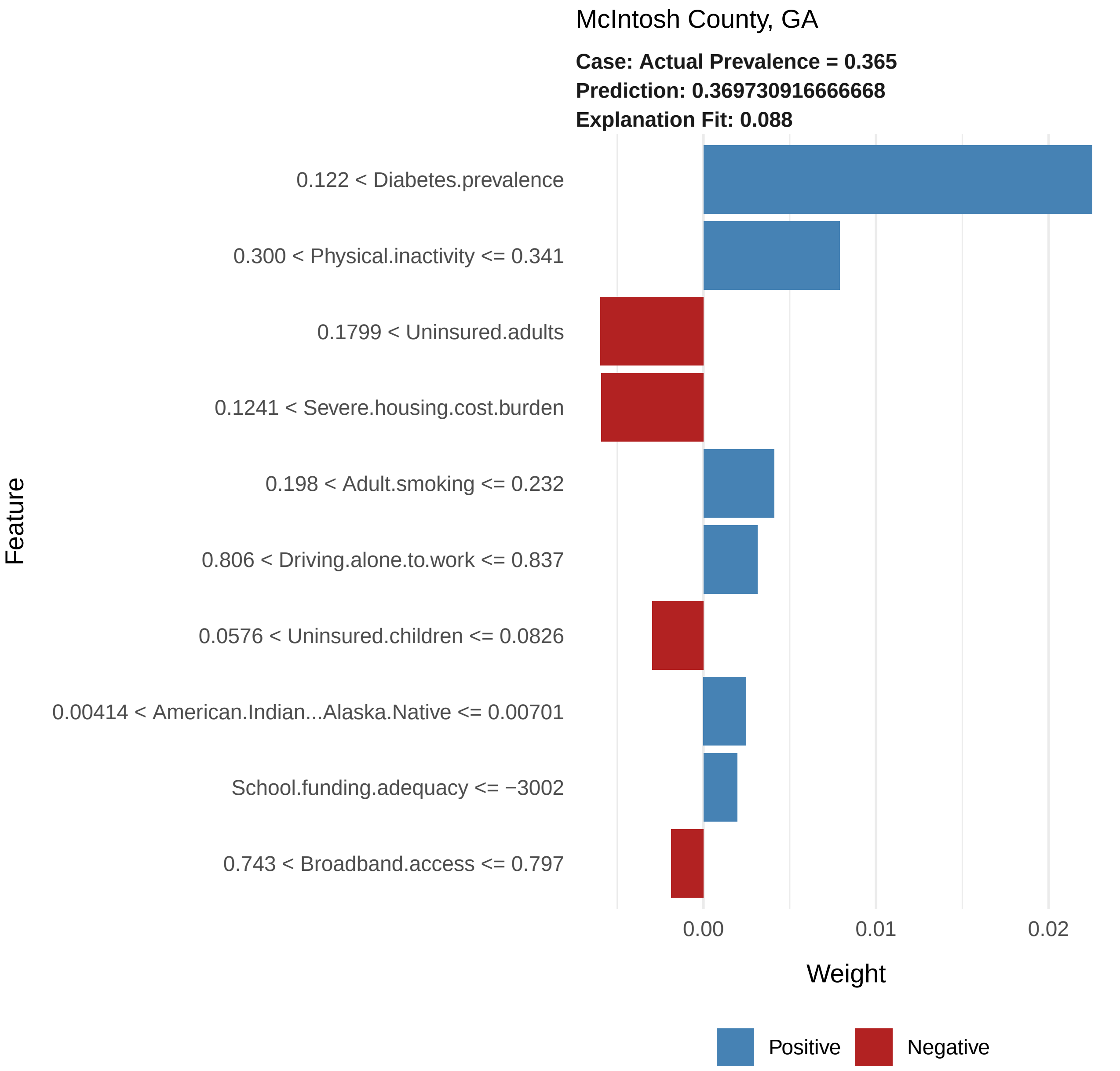
McIntosh County, GA
Case: Actual Prevalence = 0.365
Prediction: 0.369730916666668
Explanation Fit: 0.088
0.122 < Diabetes.prevalence
0.300 < Physical.inactivity <= 0.341
0.1799 < Uninsured.adults
0.1241 < Severe.housing.cost.burden
0.198 < Adult.smoking <= 0.232
0.806 < Driving.alone.to.work <= 0.837
0.0576 < Uninsured.children <= 0.0826
0.00414 < American.Indian...Alaska.Native <= 0.00701
School.funding.adequacy <= −3002
0.743 < Broadband.access <= 0.797
Feature
0.00
0.01
0.02
Weight
Positive
Negative

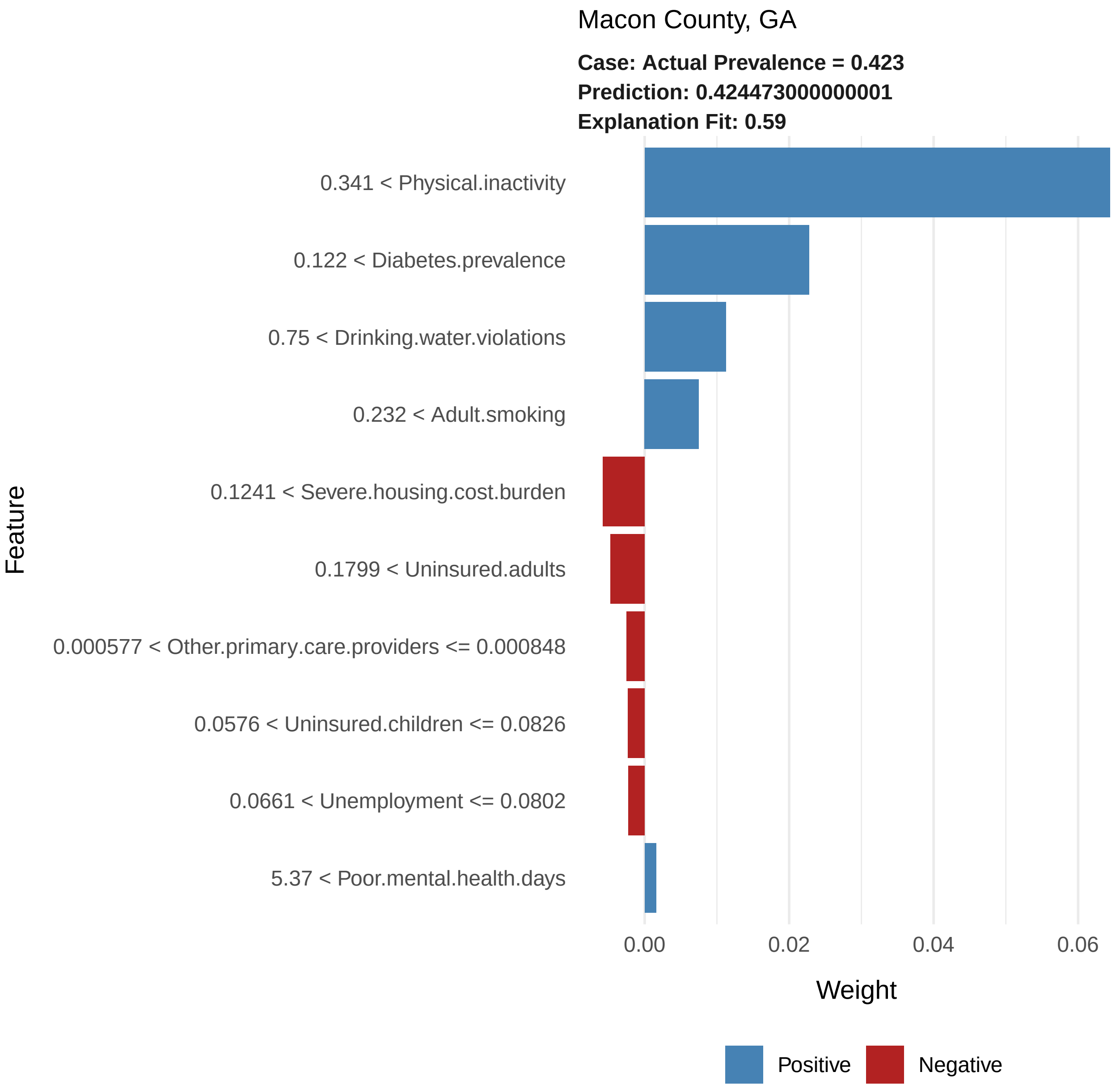

Macon County, GA
Case: Actual Prevalence = 0.423
Prediction: 0.424473000000001
Explanation Fit: 0.59
0.341 < Physical.inactivity
0.122 < Diabetes.prevalence
0.75 < Drinking.water.violations
0.232 < Adult.smoking
0.1241 < Severe.housing.cost.burden
0.1799 < Uninsured.adults
0.000577 < Other.primary.care.providers <= 0.000848
0.0576 < Uninsured.children <= 0.0826
0.0661 < Unemployment <= 0.0802
5.37 < Poor.mental.health.days
Feature
0.00
0.02
0.04
0.06
Weight
Positive
Negative

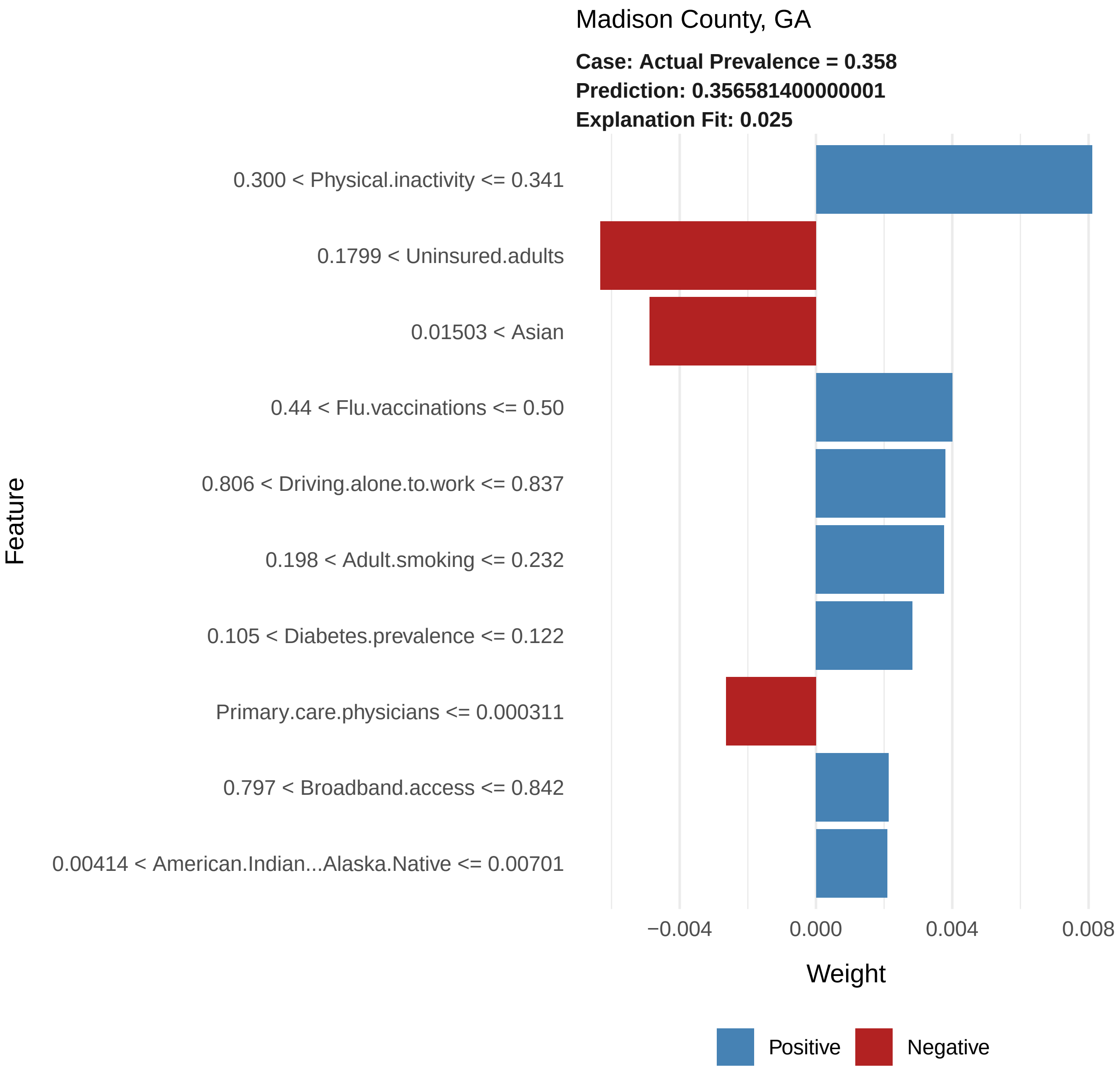

Madison County, GA
Case: Actual Prevalence = 0.358
Prediction: 0.356581400000001
Explanation Fit: 0.025
Feature
0.300 < Physical.inactivity <= 0.341
0.1799 < Uninsured.adults
0.01503 < Asian
0.44 < Flu.vaccinations <= 0.50
0.806 < Driving.alone.to.work <= 0.837
0.198 < Adult.smoking <= 0.232
0.105 < Diabetes.prevalence <= 0.122
Primary.care.physicians <= 0.000311
0.797 < Broadband.access <= 0.842
0.00414 < American.Indian...Alaska.Native <= 0.00701
−0.004
0.000
0.004
0.008
Weight
Positive
Negative

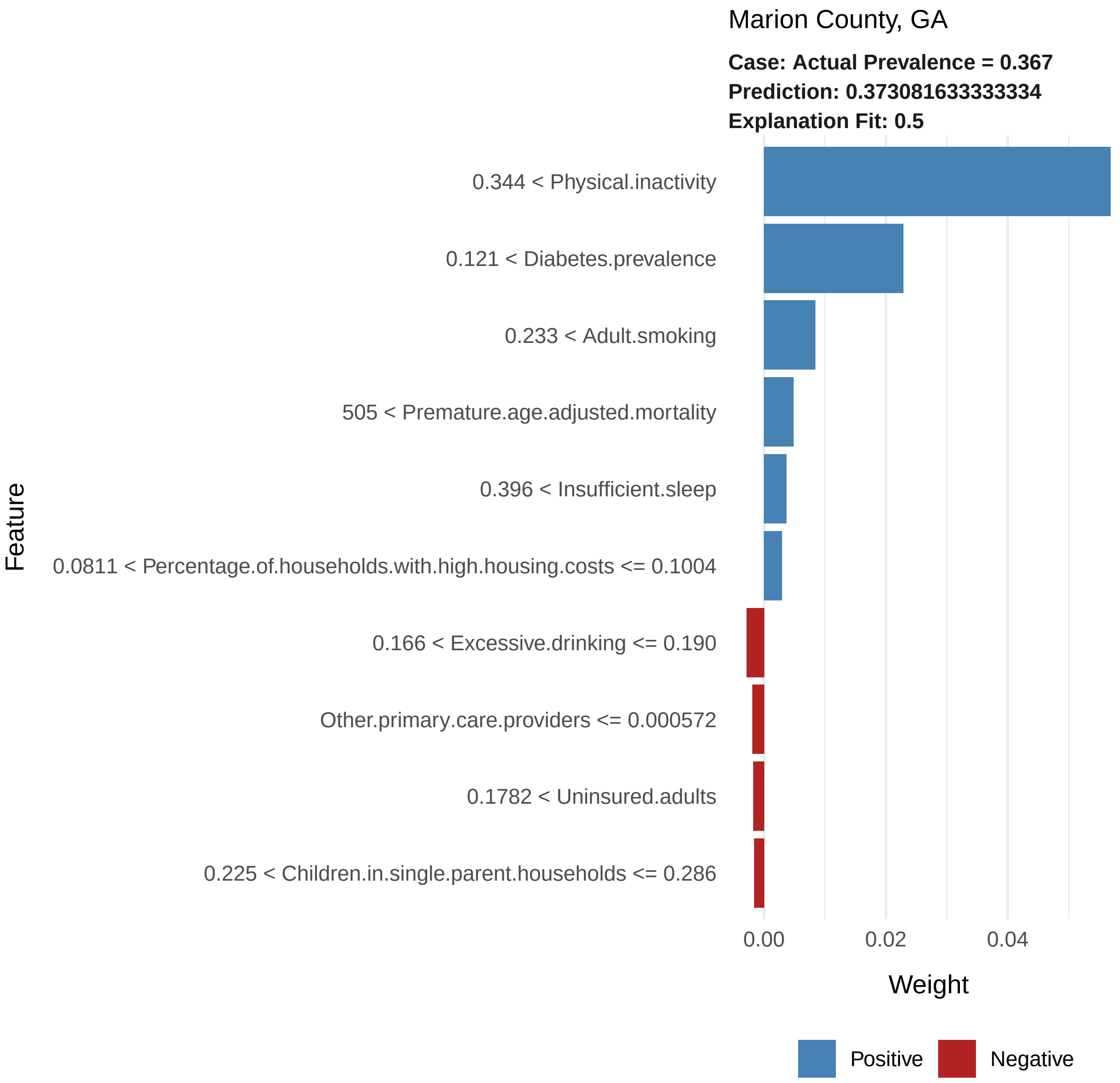

Marion County, GA
Case: Actual Prevalence = 0.367
Prediction: 0.373081633333334
Explanation Fit: 0.5
0.344 < Physical.inactivity
0.121 < Diabetes.prevalence
0.233 < Adult.smoking
505 < Premature.age.adjusted.mortality
0.396 < Insufficient.sleep
0.0811 < Percentage.of.households.with.high.housing.costs <= 0.1004
0.166 < Excessive.drinking <= 0.190
Other.primary.care.providers <= 0.000572
0.1782 < Uninsured.adults
0.225 < Children.in.single.parent.households <= 0.286
Feature
0.00
0.02
0.04
Weight
Positive
Negative

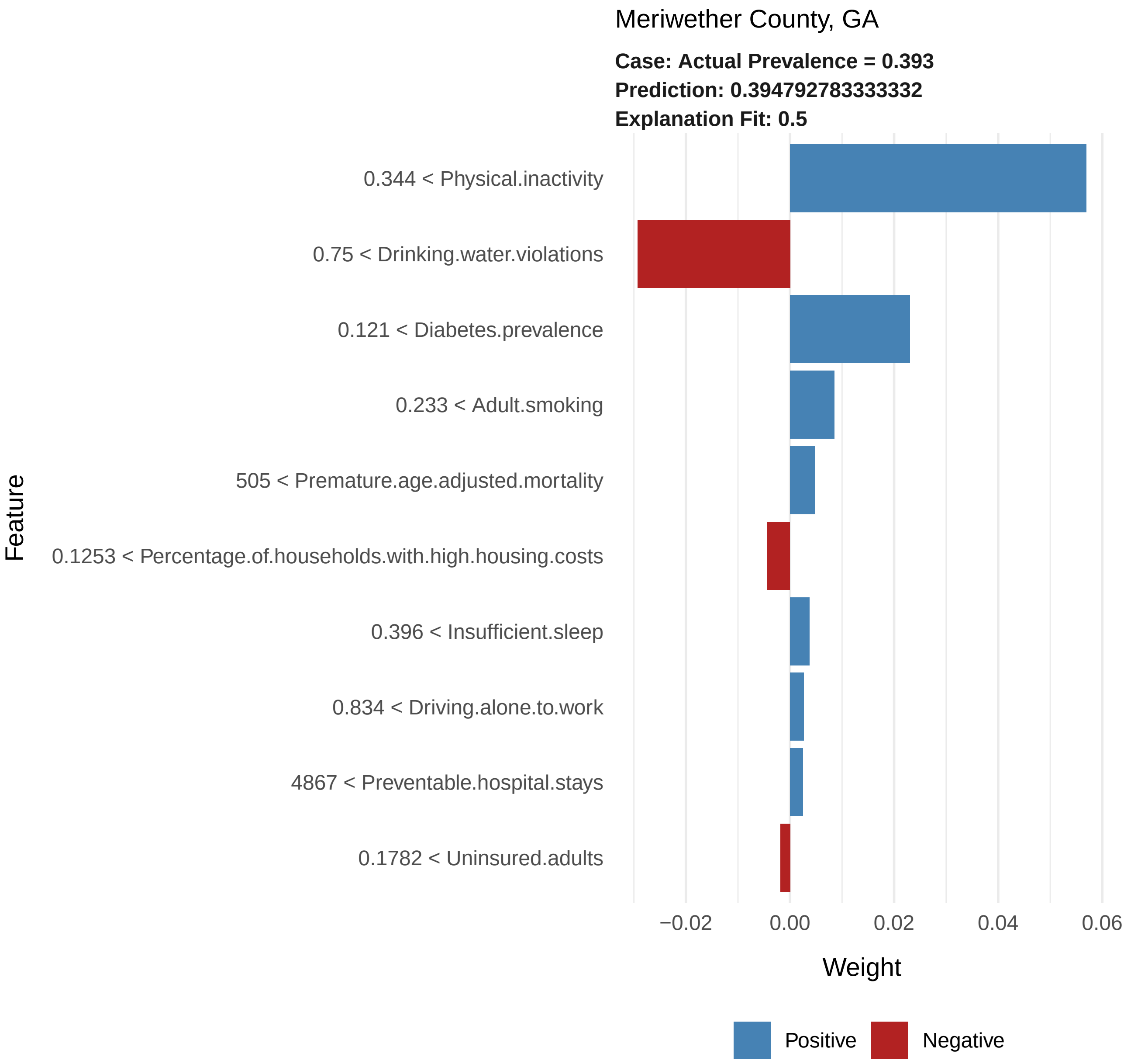

Meriwether County, GA
Case: Actual Prevalence = 0.393
Prediction: 0.394792783333332
Explanation Fit: 0.5
0.344 < Physical.inactivity
0.75 < Drinking.water.violations
0.121 < Diabetes.prevalence
0.233 < Adult.smoking
505 < Premature.age.adjusted.mortality
0.1253 < Percentage.of.households.with.high.housing.costs
0.396 < Insufficient.sleep
0.834 < Driving.alone.to.work
4867 < Preventable.hospital.stays
0.1782 < Uninsured.adults
Feature
−0.02
0.00
0.02
0.04
0.06
Weight
Positive
Negative

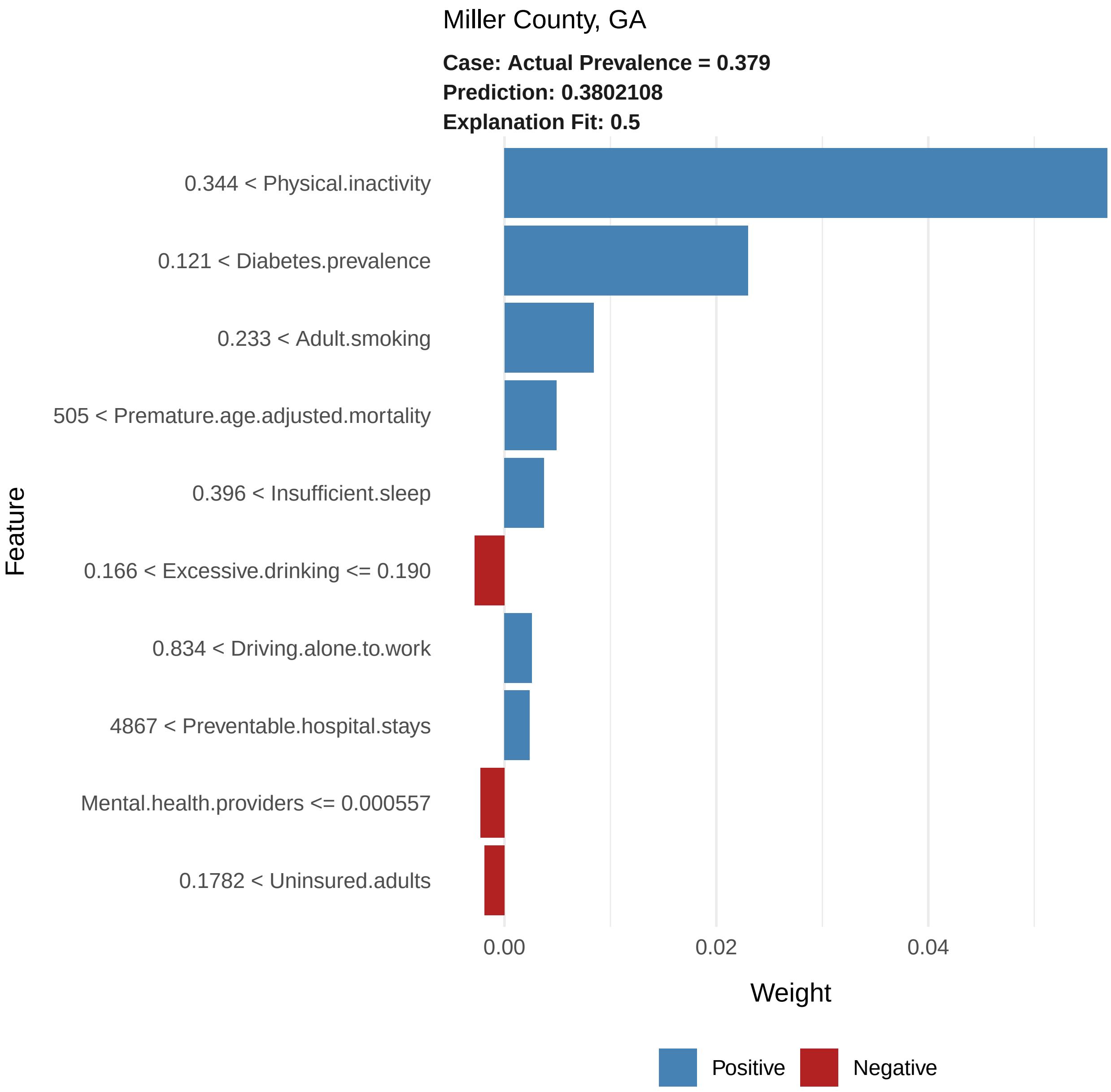
Miller County, GA
Case: Actual Prevalence = 0.379
Prediction: 0.3802108
Explanation Fit: 0.5
0.344 < Physical.inactivity
0.121 < Diabetes.prevalence
0.233 < Adult.smoking
505 < Premature.age.adjusted.mortality
0.396 < Insufficient.sleep
0.166 < Excessive.drinking <= 0.190
0.834 < Driving.alone.to.work
4867 < Preventable.hospital.stays
Mental.health.providers <= 0.000557
0.1782 < Uninsured.adults
Feature
0.00
0.02
0.04
Weight
Positive
Negative

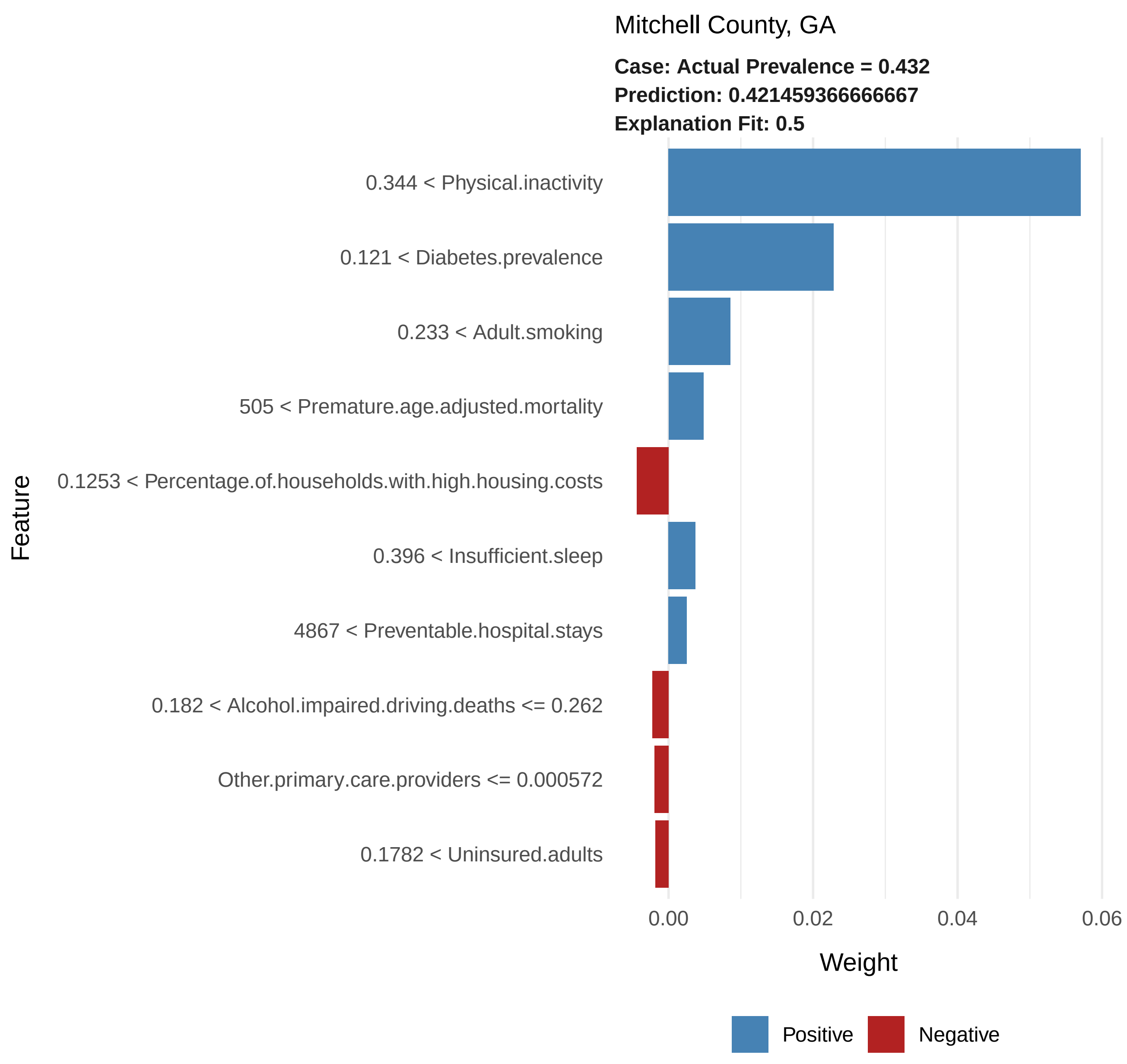

Mitchell County, GA
Case: Actual Prevalence = 0.432
Prediction: 0.421459366666667
Explanation Fit: 0.5
0.344 < Physical.inactivity
0.121 < Diabetes.prevalence
0.233 < Adult.smoking
505 < Premature.age.adjusted.mortality
0.1253 < Percentage.of.households.with.high.housing.costs
0.396 < Insufficient.sleep
4867 < Preventable.hospital.stays
0.182 < Alcohol.impaired.driving.deaths <= 0.262
Other.primary.care.providers <= 0.000572
0.1782 < Uninsured.adults
Feature
0.00
0.02
0.04
0.06
Weight
Positive
Negative

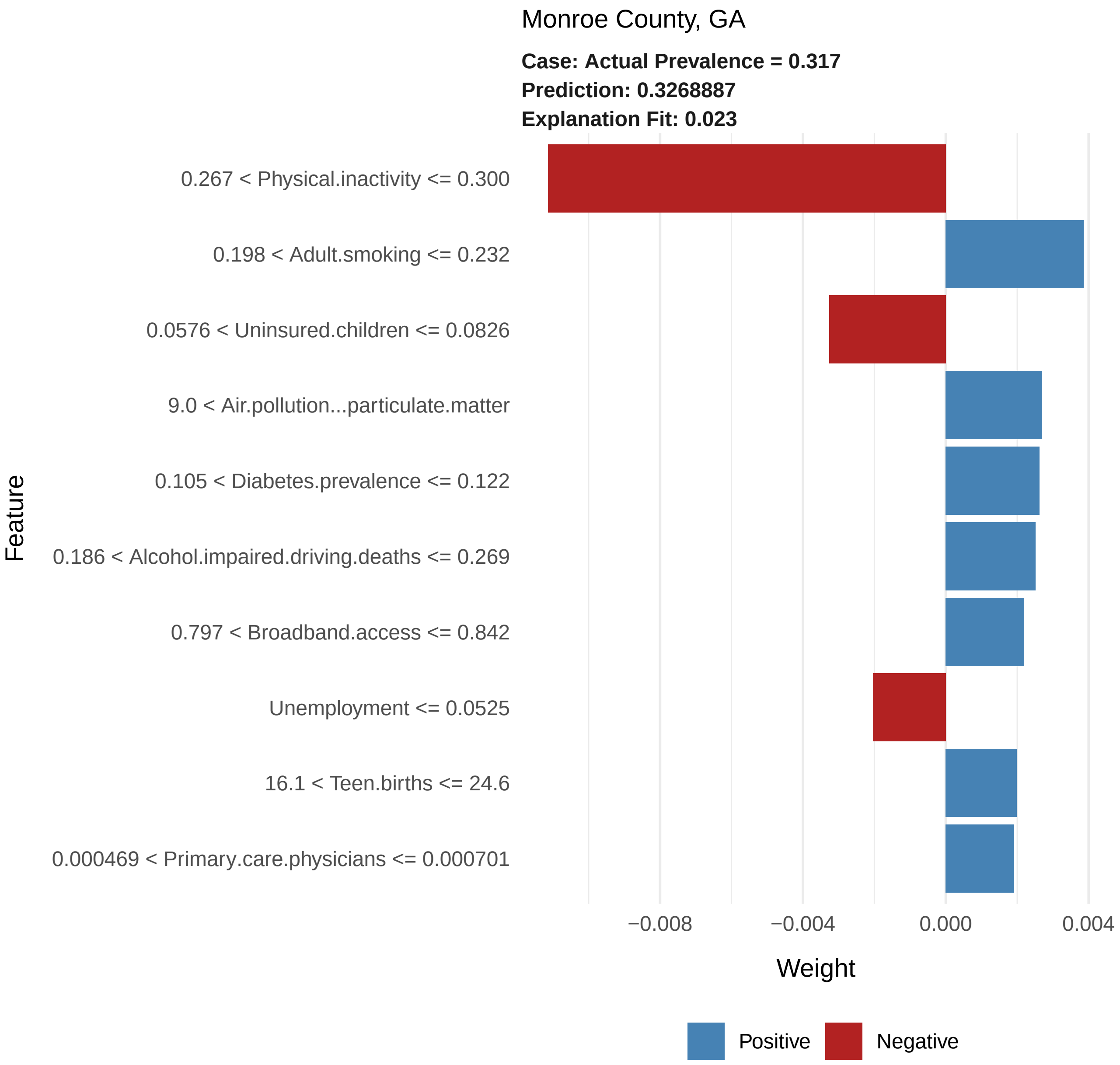

Monroe County, GA
Case: Actual Prevalence = 0.317
Prediction: 0.3268887
Explanation Fit: 0.023
0.267 < Physical.inactivity <= 0.300
0.198 < Adult.smoking <= 0.232
0.0576 < Uninsured.children <= 0.0826
9.0 < Air.pollution...particulate.matter
0.105 < Diabetes.prevalence <= 0.122
0.186 < Alcohol.impaired.driving.deaths <= 0.269
0.797 < Broadband.access <= 0.842
Unemployment <= 0.0525
16.1 < Teen.births <= 24.6
0.000469 < Primary.care.physicians <= 0.000701
Feature
−0.008
−0.004
0.000
0.004
Weight
Positive
Negative

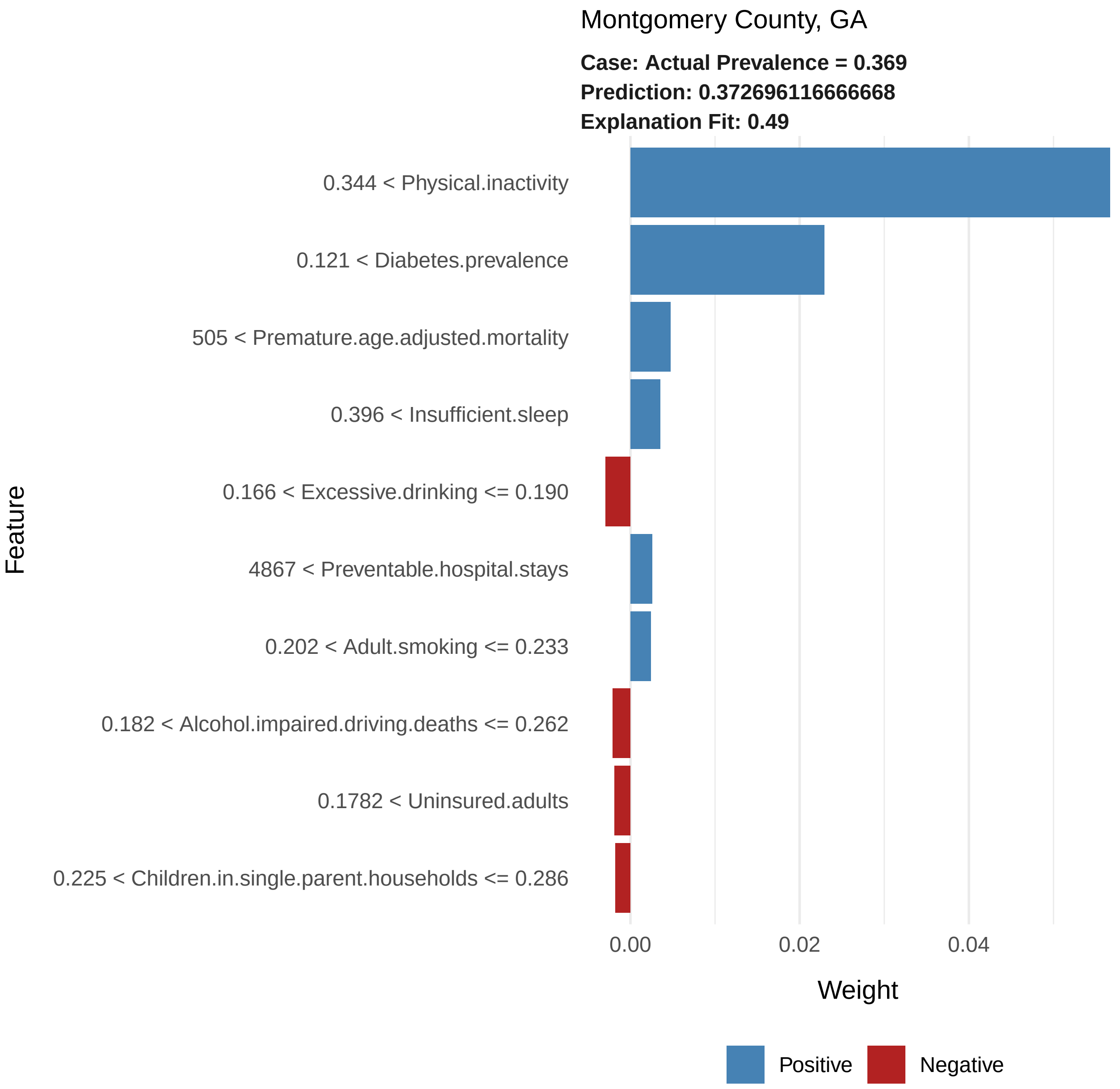

Montgomery County, GA
Case: Actual Prevalence = 0.369
Prediction: 0.372696116666668
Explanation Fit: 0.49
0.344 < Physical.inactivity
0.121 < Diabetes.prevalence
505 < Premature.age.adjusted.mortality
0.396 < Insufficient.sleep
0.166 < Excessive.drinking <= 0.190
4867 < Preventable.hospital.stays
0.202 < Adult.smoking <= 0.233
0.182 < Alcohol.impaired.driving.deaths <= 0.262
0.1782 < Uninsured.adults
0.225 < Children.in.single.parent.households <= 0.286
Feature
0.00
0.02
0.04
Weight
Positive
Negative

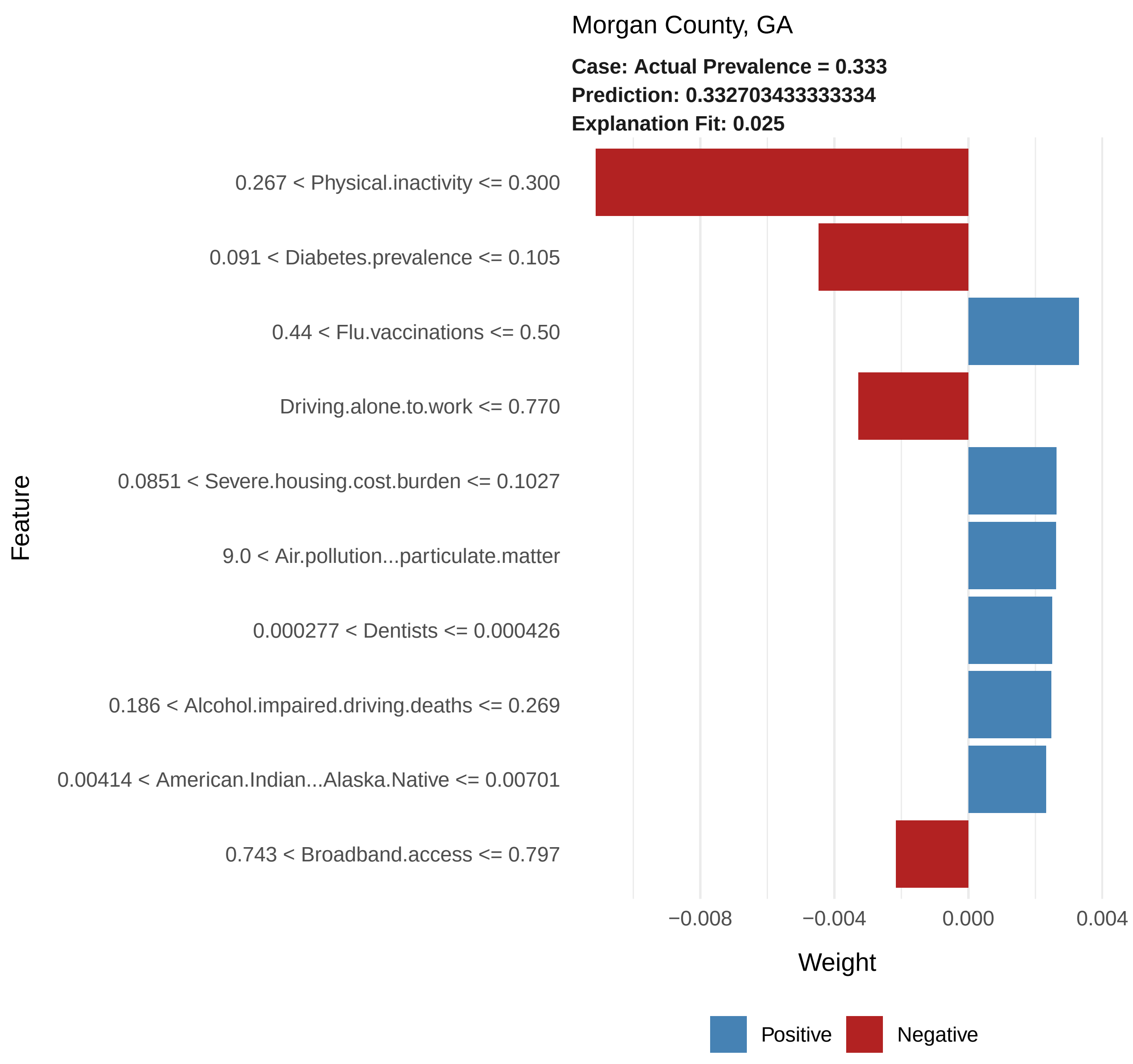

Morgan County, GA
Case: Actual Prevalence = 0.333
Prediction: 0.332703433333334
Explanation Fit: 0.025
0.267 < Physical.inactivity <= 0.300
0.091 < Diabetes.prevalence <= 0.105
0.44 < Flu.vaccinations <= 0.50
Driving.alone.to.work <= 0.770
0.0851 < Severe.housing.cost.burden <= 0.1027
9.0 < Air.pollution...particulate.matter
0.000277 < Dentists <= 0.000426
0.186 < Alcohol.impaired.driving.deaths <= 0.269
0.00414 < American.Indian...Alaska.Native <= 0.00701
0.743 < Broadband.access <= 0.797
Feature
−0.008
−0.004
0.000
0.004
Weight
Positive
Negative

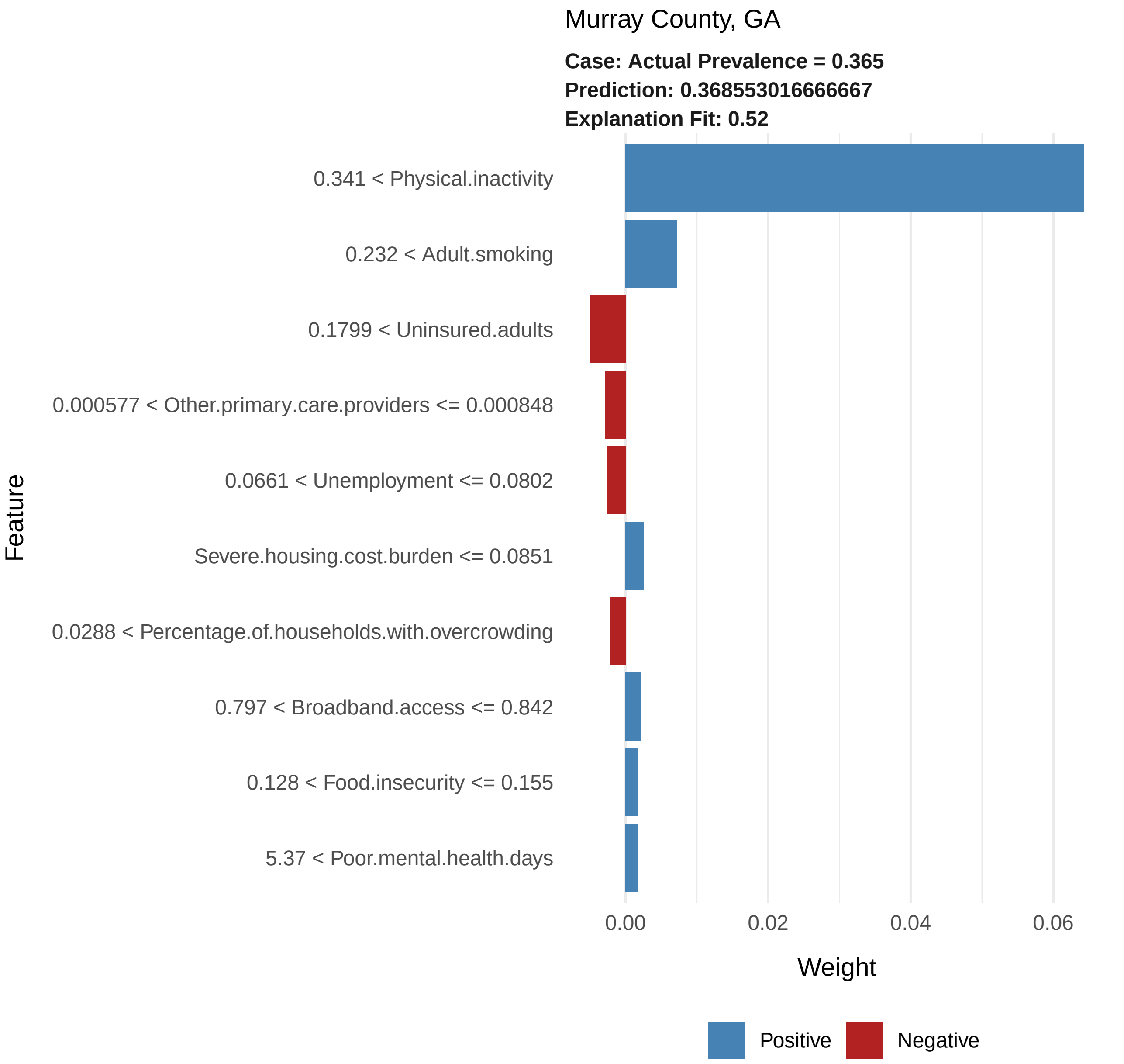

Murray County, GA
Case: Actual Prevalence = 0.365
Prediction: 0.368553016666667
Explanation Fit: 0.52
0.341 < Physical.inactivity
0.232 < Adult.smoking
0.1799 < Uninsured.adults
0.000577 < Other.primary.care.providers <= 0.000848
0.0661 < Unemployment <= 0.0802
Severe.housing.cost.burden <= 0.0851
0.0288 < Percentage.of.households.with.overcrowding
0.797 < Broadband.access <= 0.842
0.128 < Food.insecurity <= 0.155
5.37 < Poor.mental.health.days
Feature
0.00
0.02
0.04
0.06
Weight
Positive
Negative

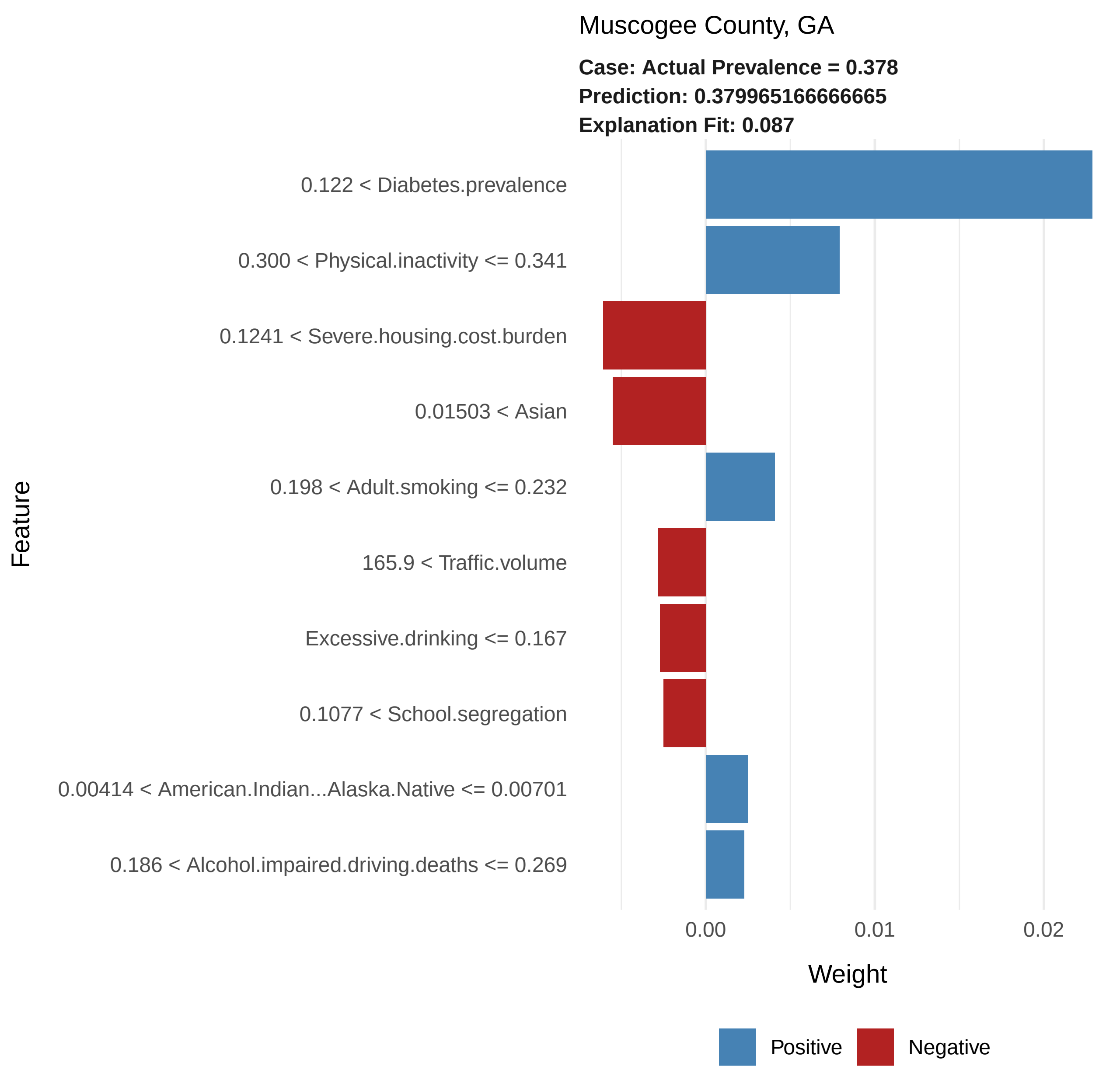

Muscogee County, GA
Case: Actual Prevalence = 0.378
Prediction: 0.379965166666665
Explanation Fit: 0.087
0.122 < Diabetes.prevalence
0.300 < Physical.inactivity <= 0.341
0.1241 < Severe.housing.cost.burden
0.01503 < Asian
0.198 < Adult.smoking <= 0.232
165.9 < Traffic.volume
Excessive.drinking <= 0.167
0.1077 < School.segregation
0.00414 < American.Indian...Alaska.Native <= 0.00701
0.186 < Alcohol.impaired.driving.deaths <= 0.269
Feature
0.00
0.01
0.02
Weight
Positive
Negative

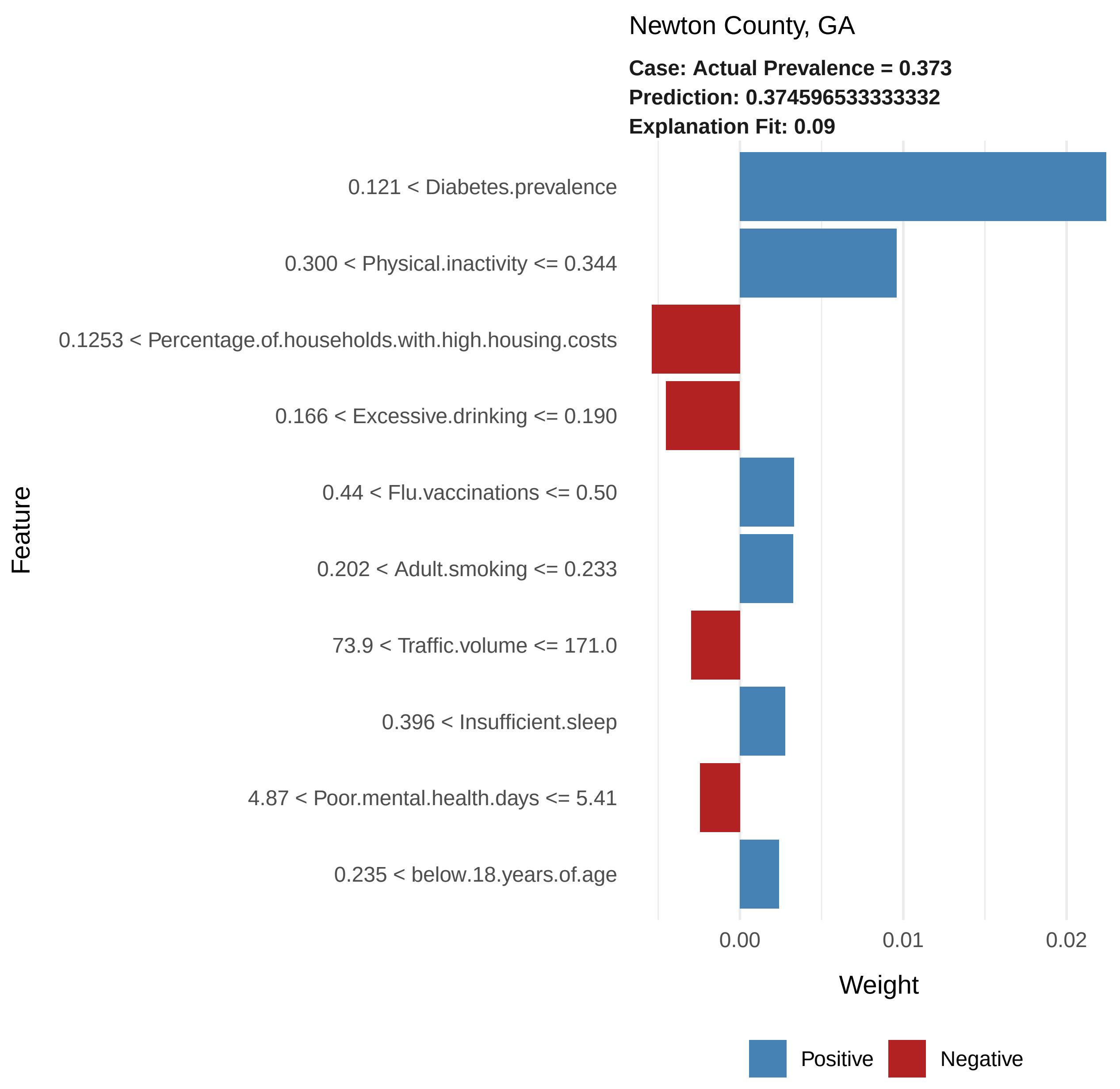

Newton County, GA
Case: Actual Prevalence = 0.373
Prediction: 0.374596533333332
Explanation Fit: 0.09
0.121 < Diabetes.prevalence
0.300 < Physical.inactivity <= 0.344
0.1253 < Percentage.of.households.with.high.housing.costs
0.166 < Excessive.drinking <= 0.190
0.44 < Flu.vaccinations <= 0.50
0.202 < Adult.smoking <= 0.233
73.9 < Traffic.volume <= 171.0
0.396 < Insufficient.sleep
4.87 < Poor.mental.health.days <= 5.41
0.235 < below.18.years.of.age
Feature
0.00
0.01
0.02
Weight
Positive
Negative

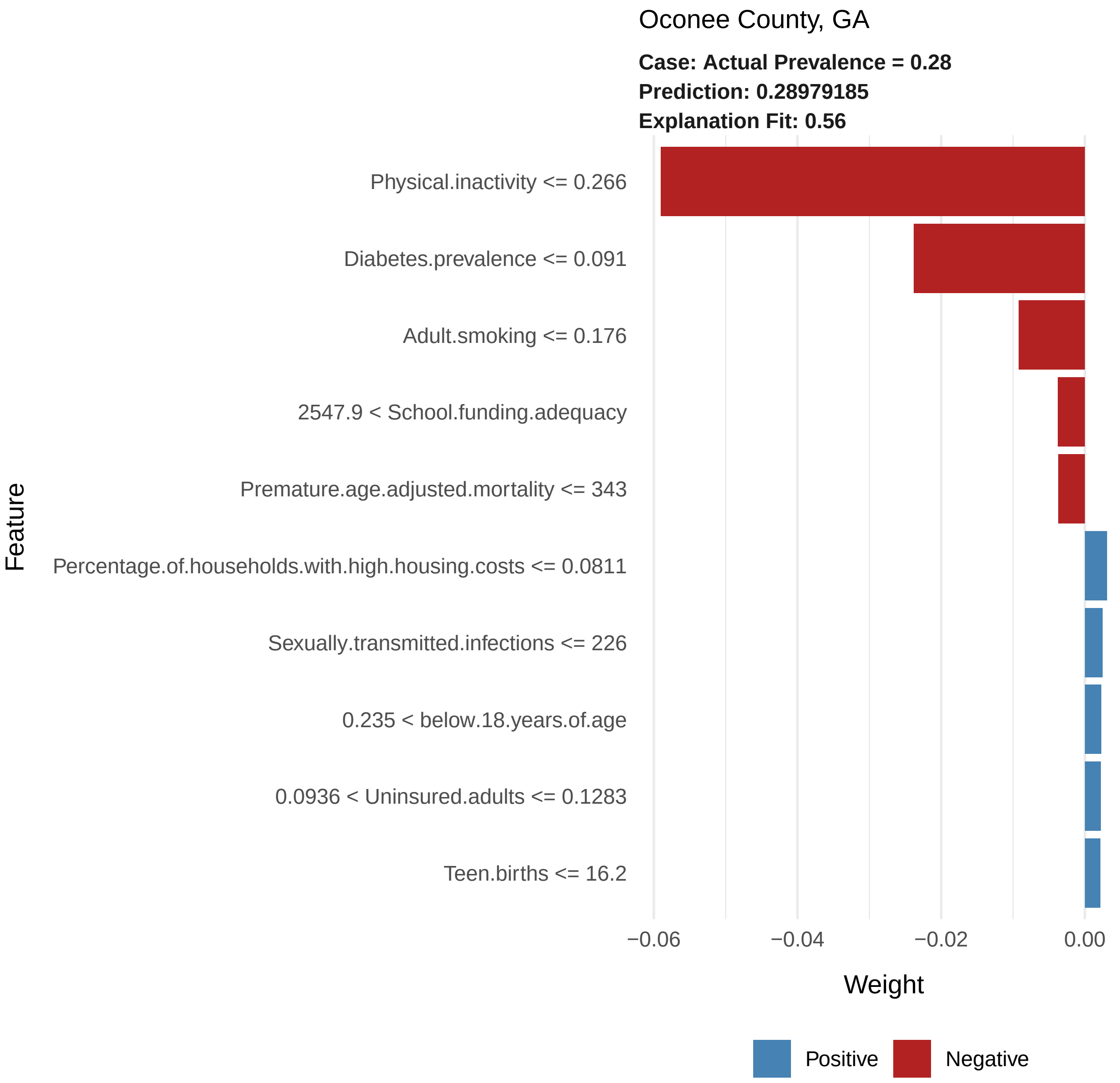

Oconee County, GA
Case: Actual Prevalence = 0.28
Prediction: 0.28979185
Explanation Fit: 0.56
Feature
Physical.inactivity <= 0.266
Diabetes.prevalence <= 0.091
Adult.smoking <= 0.176
2547.9 < School.funding.adequacy
Premature.age.adjusted.mortality <= 343
Percentage.of.households.with.high.housing.costs <= 0.0811
Sexually.transmitted.infections <= 226
0.235 < below.18.years.of.age
0.0936 < Uninsured.adults <= 0.1283
Teen.births <= 16.2
−0.06
−0.04
−0.02
0.00
Weight
Positive
Negative

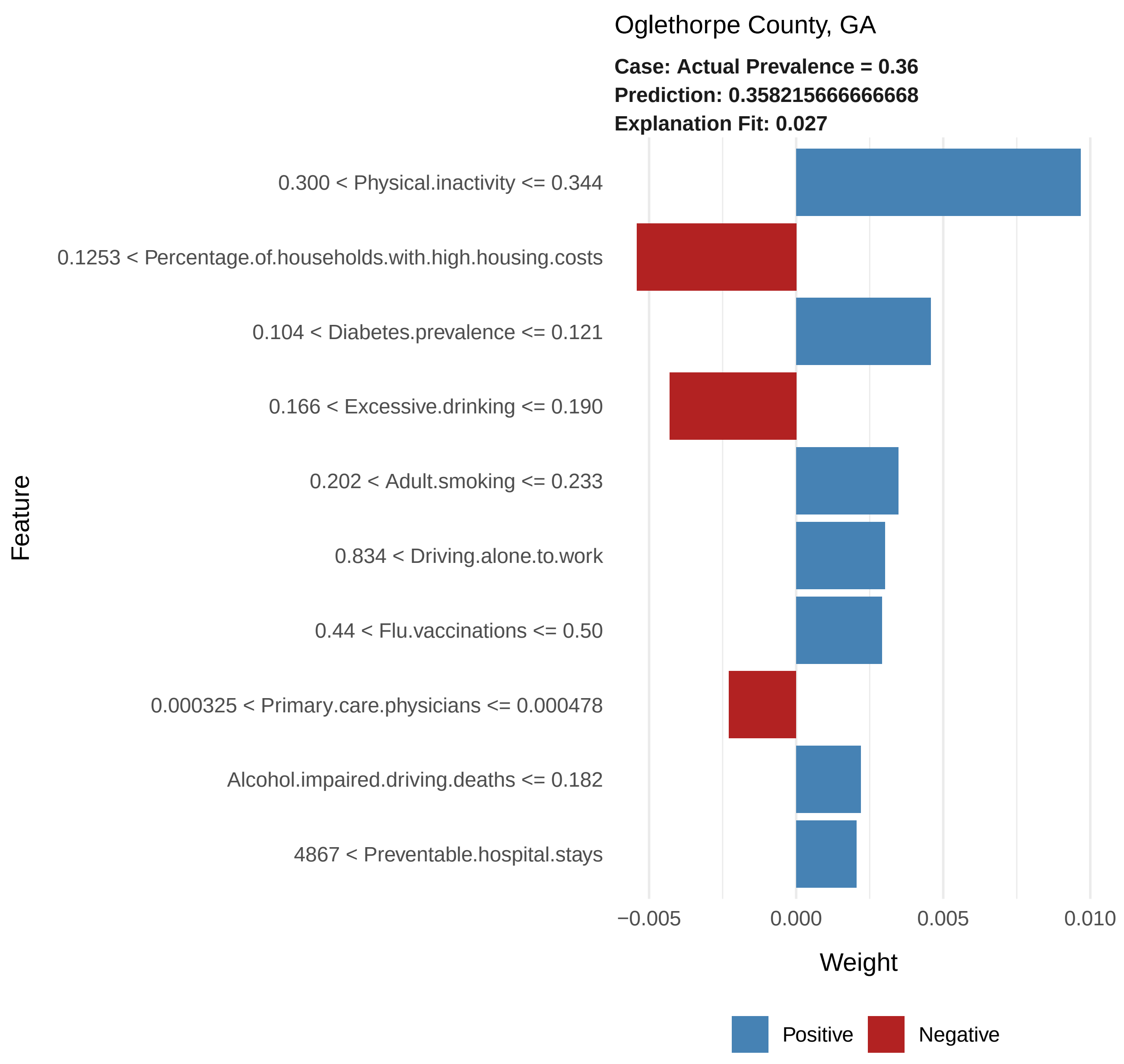

Oglethorpe County, GA
Case: Actual Prevalence = 0.36
Prediction: 0.358215666666668
Explanation Fit: 0.027
0.300 < Physical.inactivity <= 0.344
0.1253 < Percentage.of.households.with.high.housing.costs
0.104 < Diabetes.prevalence <= 0.121
0.166 < Excessive.drinking <= 0.190
0.202 < Adult.smoking <= 0.233
0.834 < Driving.alone.to.work
0.44 < Flu.vaccinations <= 0.50
0.000325 < Primary.care.physicians <= 0.000478
Alcohol.impaired.driving.deaths <= 0.182
4867 < Preventable.hospital.stays
Feature
−0.005
0.000
0.005
0.010
Weight
Positive
Negative

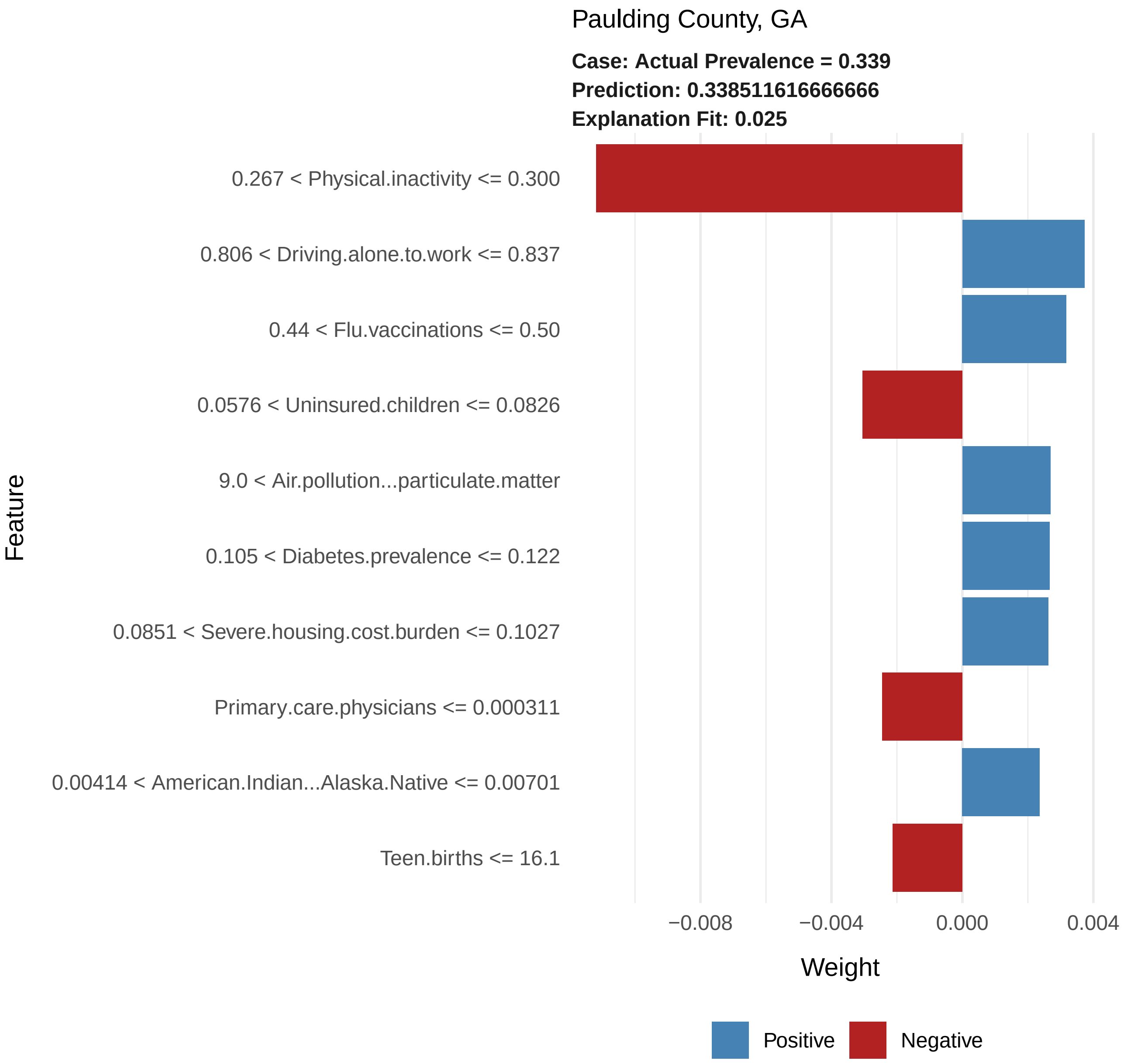

Paulding County, GA
Case: Actual Prevalence = 0.339
Prediction: 0.338511616666666
Explanation Fit: 0.025
0.267 < Physical.inactivity <= 0.300
0.806 < Driving.alone.to.work <= 0.837
0.44 < Flu.vaccinations <= 0.50
0.0576 < Uninsured.children <= 0.0826
9.0 < Air.pollution...particulate.matter
0.105 < Diabetes.prevalence <= 0.122
0.0851 < Severe.housing.cost.burden <= 0.1027
Primary.care.physicians <= 0.000311
0.00414 < American.Indian...Alaska.Native <= 0.00701
Teen.births <= 16.1
Feature
−0.008
−0.004
0.000
0.004
Weight
Positive
Negative

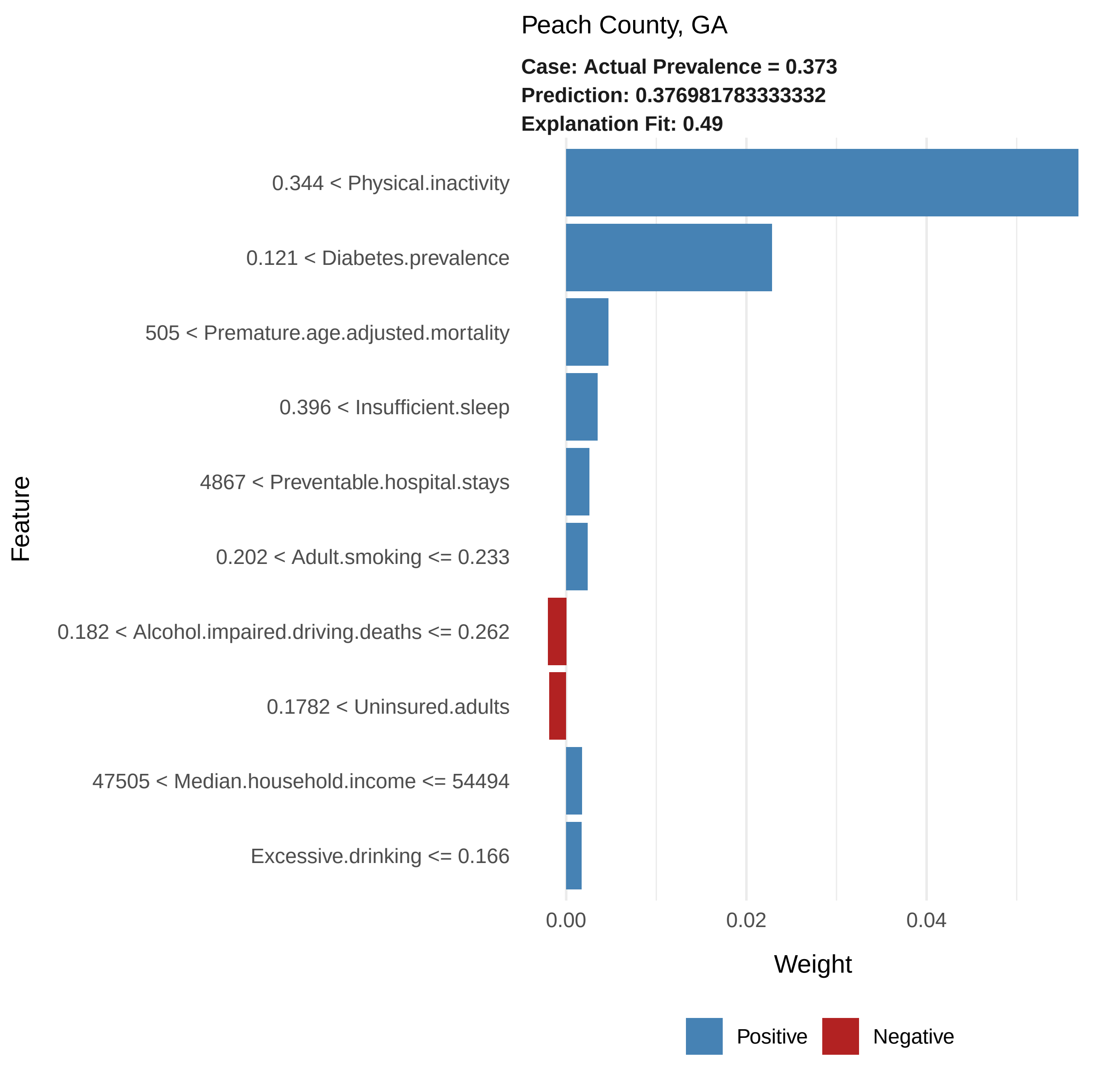
Peach County, GA
Case: Actual Prevalence = 0.373
Prediction: 0.376981783333332
Explanation Fit: 0.49
0.344 < Physical.inactivity
0.121 < Diabetes.prevalence
505 < Premature.age.adjusted.mortality
0.396 < Insufficient.sleep
4867 < Preventable.hospital.stays
0.202 < Adult.smoking <= 0.233
0.182 < Alcohol.impaired.driving.deaths <= 0.262
0.1782 < Uninsured.adults
47505 < Median.household.income <= 54494
Excessive.drinking <= 0.166
Feature
0.00
0.02
0.04
Weight
Positive
Negative

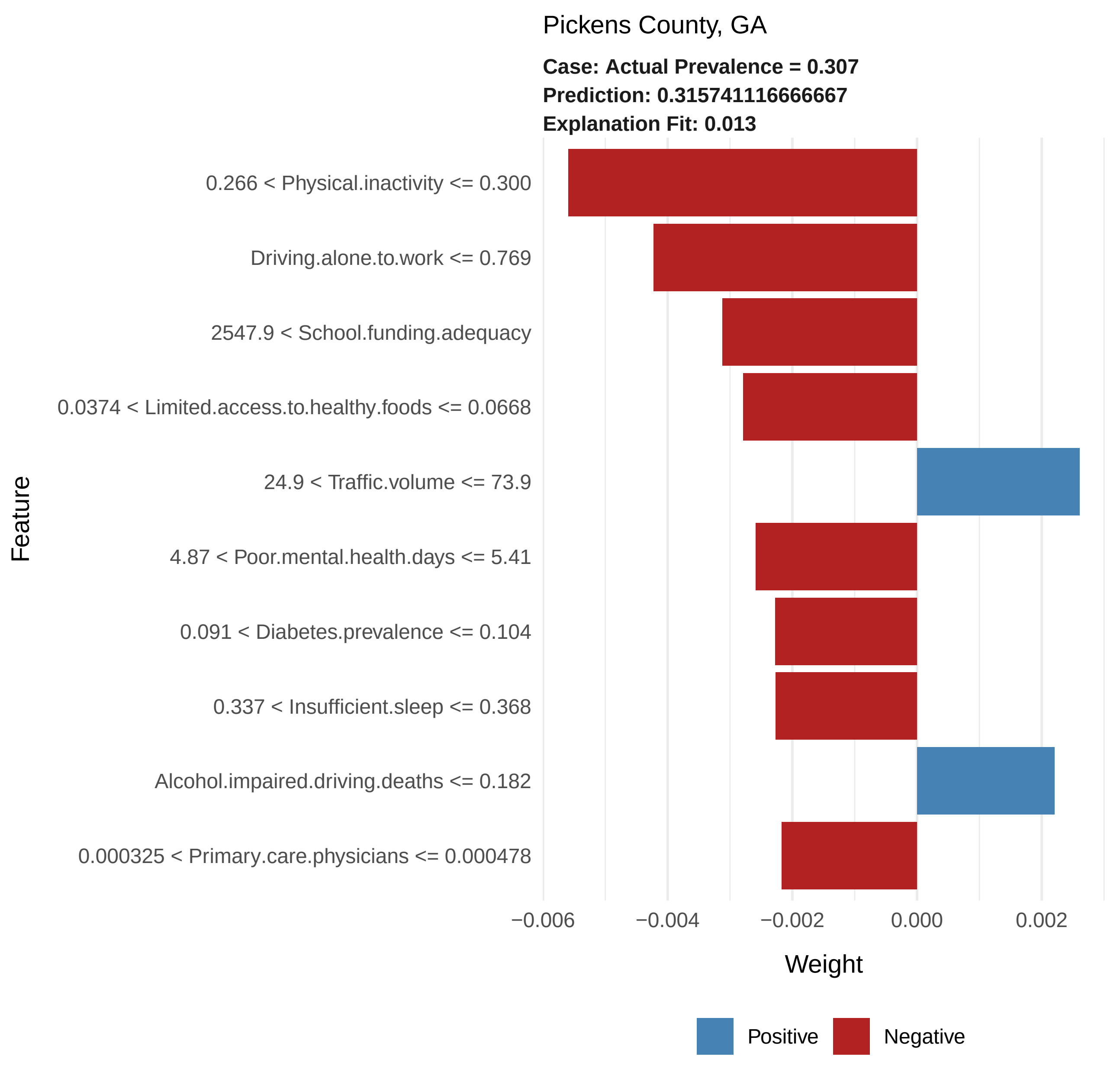
Pickens County, GA
Case: Actual Prevalence = 0.307
Prediction: 0.315741116666667
Explanation Fit: 0.013
0.266 < Physical.inactivity <= 0.300
Driving.alone.to.work <= 0.769
2547.9 < School.funding.adequacy
0.0374 < Limited.access.to.healthy.foods <= 0.0668
24.9 < Traffic.volume <= 73.9
4.87 < Poor.mental.health.days <= 5.41
0.091 < Diabetes.prevalence <= 0.104
0.337 < Insufficient.sleep <= 0.368
Alcohol.impaired.driving.deaths <= 0.182
0.000325 < Primary.care.physicians <= 0.000478
Feature
−0.006
−0.004
−0.002
0.000
0.002
Weight
Positive
Negative

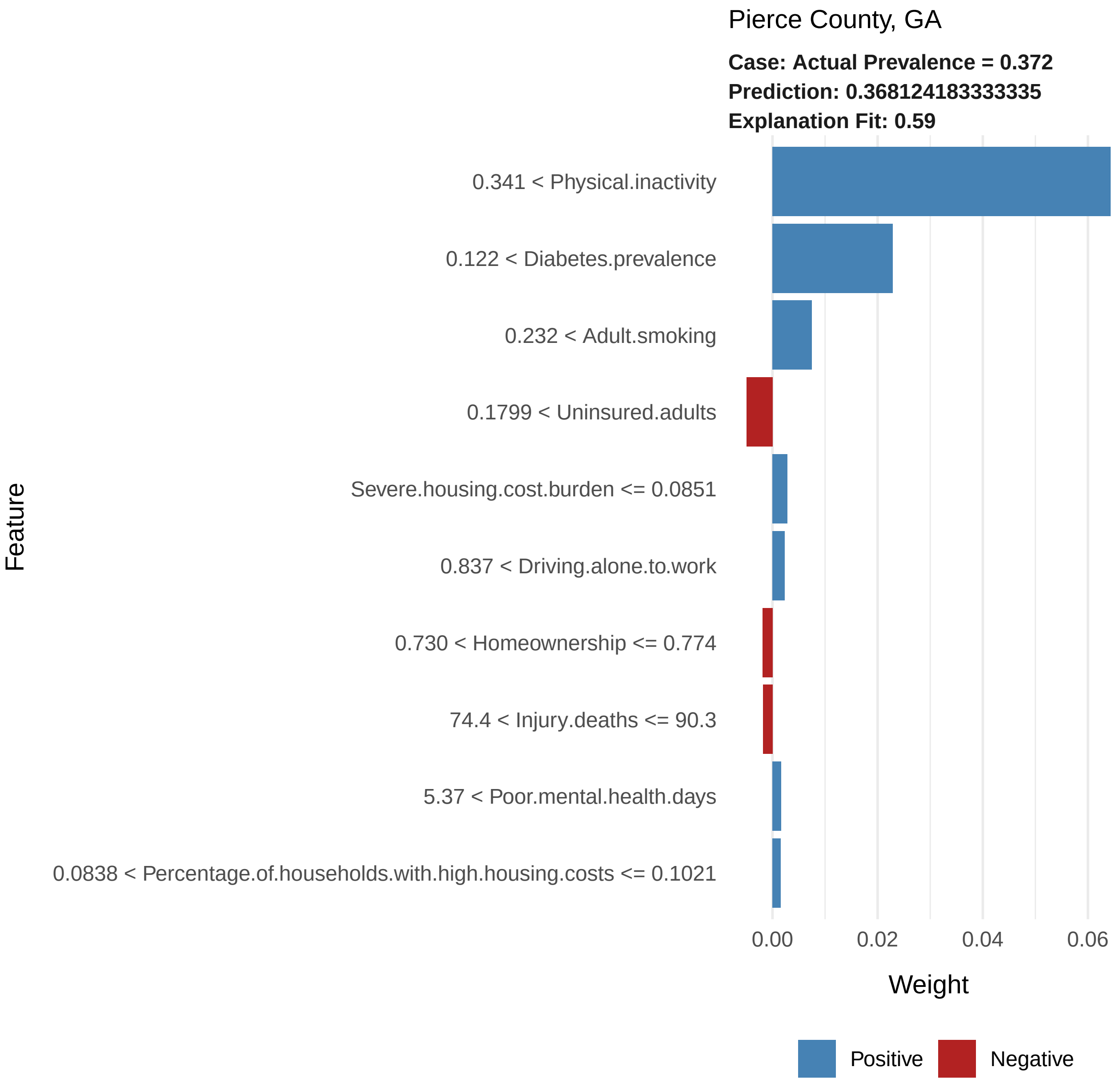
Pierce County, GA
Case: Actual Prevalence = 0.372
Prediction: 0.368124183333335
Explanation Fit: 0.59
0.341 < Physical.inactivity
0.122 < Diabetes.prevalence
0.232 < Adult.smoking
0.1799 < Uninsured.adults
Severe.housing.cost.burden <= 0.0851
0.837 < Driving.alone.to.work
0.730 < Homeownership <= 0.774
74.4 < Injury.deaths <= 90.3
5.37 < Poor.mental.health.days
0.0838 < Percentage.of.households.with.high.housing.costs <= 0.1021
Feature
0.00
0.02
0.04
0.06
Weight
Positive
Negative

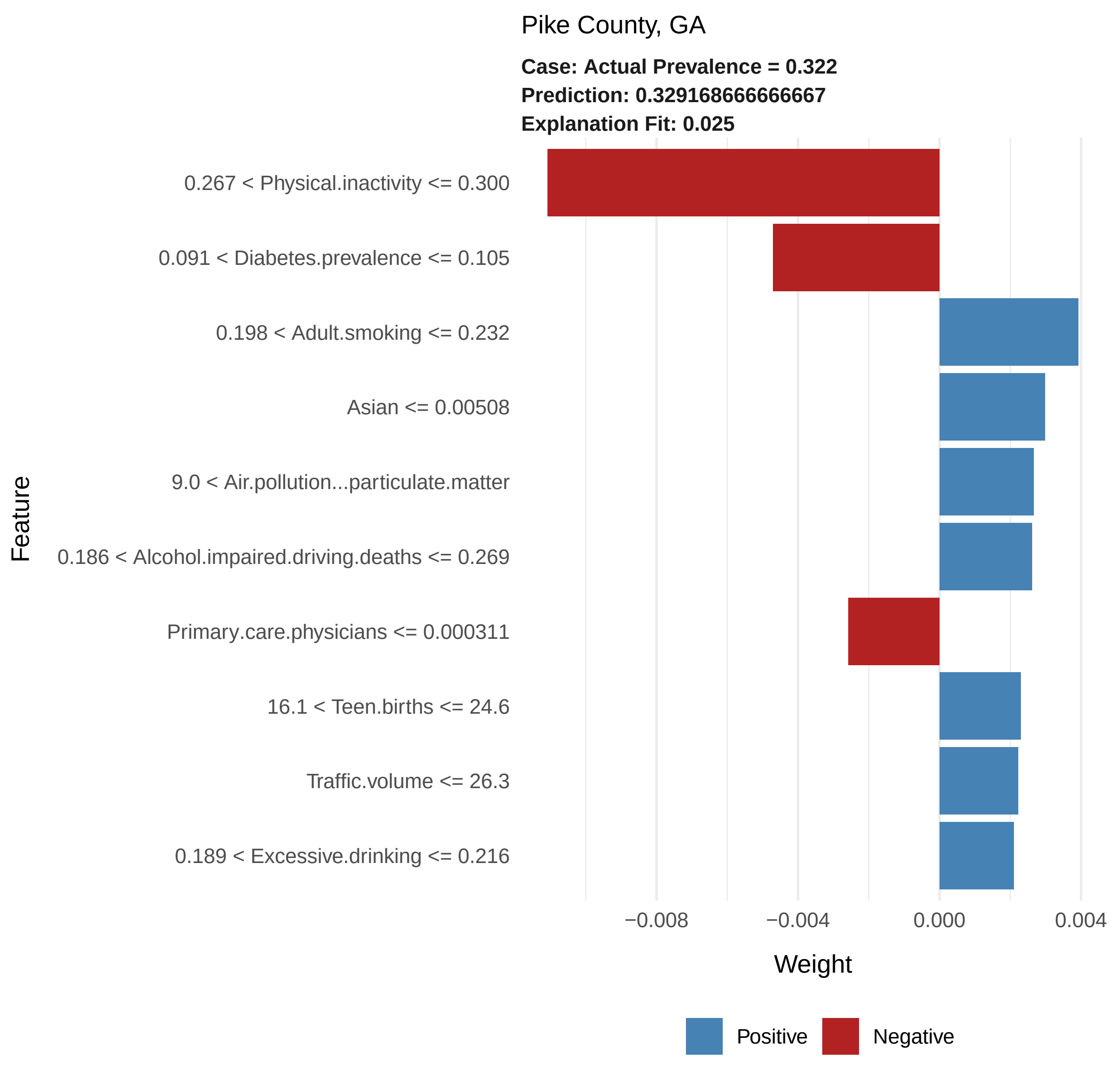
Pike County, GA
Case: Actual Prevalence = 0.322
Prediction: 0.329168666666667
Explanation Fit: 0.025
0.267 < Physical.inactivity <= 0.300
0.091 < Diabetes.prevalence <= 0.105
0.198 < Adult.smoking <= 0.232
Asian <= 0.00508
9.0 < Air.pollution...particulate.matter
0.186 < Alcohol.impaired.driving.deaths <= 0.269
Primary.care.physicians <= 0.000311
16.1 < Teen.births <= 24.6
Traffic.volume <= 26.3
0.189 < Excessive.drinking <= 0.216
Feature
−0.008
−0.004
0.000
0.004
Weight
Positive
Negative

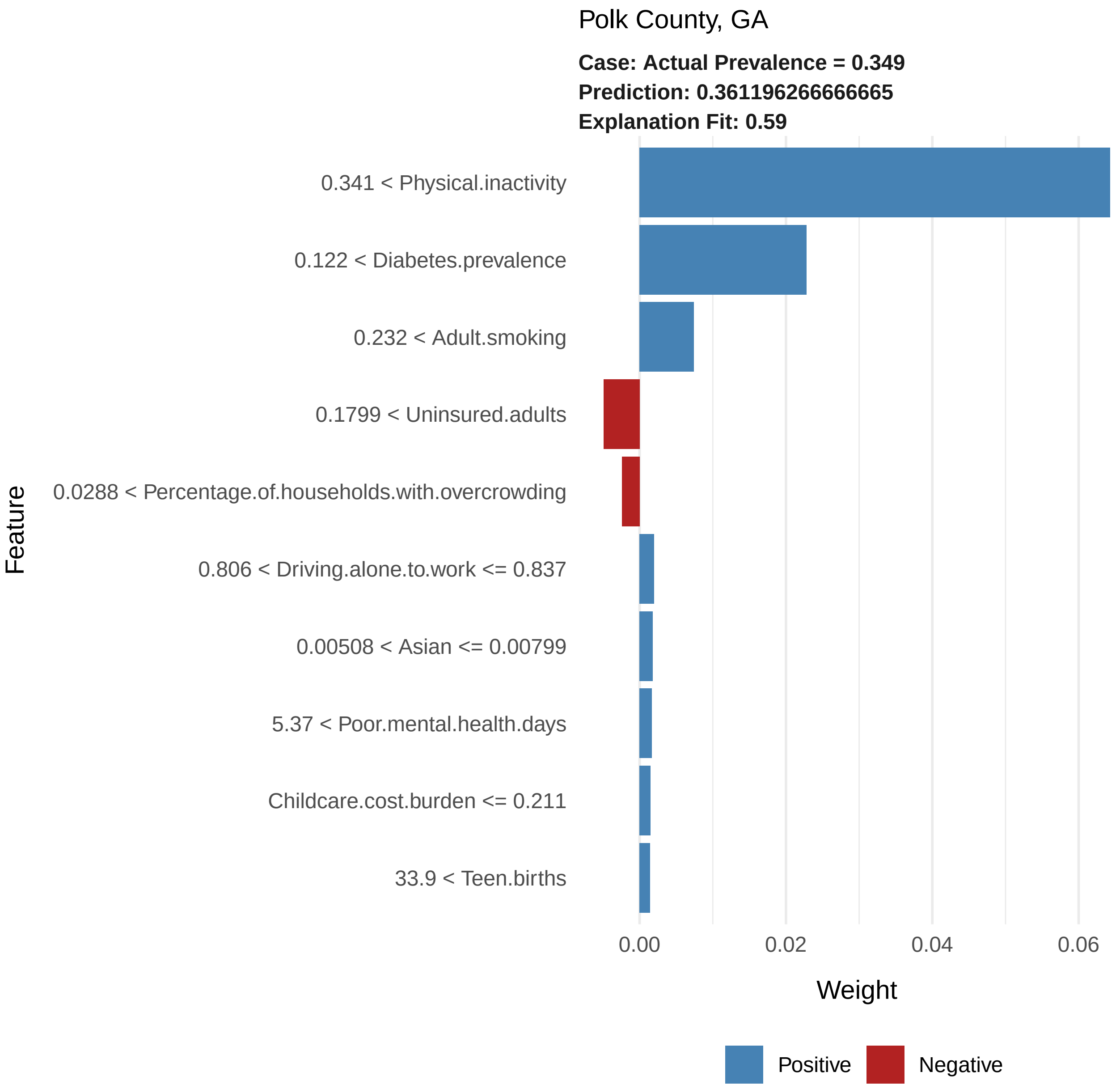

Polk County, GA
Case: Actual Prevalence = 0.349
Prediction: 0.361196266666665
Explanation Fit: 0.59
0.341 < Physical.inactivity
0.122 < Diabetes.prevalence
0.232 < Adult.smoking
0.1799 < Uninsured.adults
0.0288 < Percentage.of.households.with.overcrowding
0.806 < Driving.alone.to.work <= 0.837
0.00508 < Asian <= 0.00799
5.37 < Poor.mental.health.days
Childcare.cost.burden <= 0.211
33.9 < Teen.births
Feature
0.00
0.02
0.04
0.06
Weight
Positive
Negative

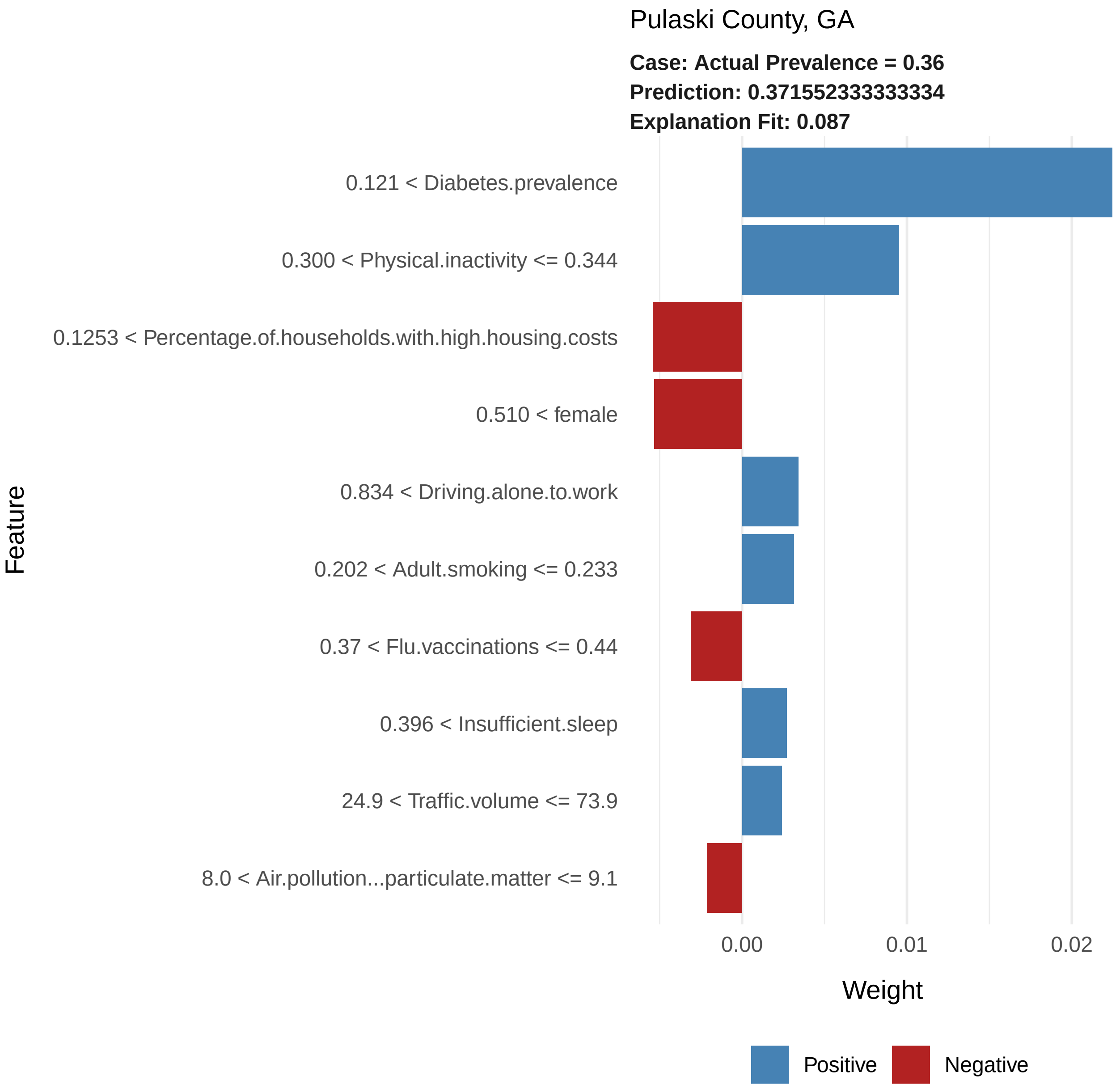

Pulaski County, GA
Case: Actual Prevalence = 0.36
Prediction: 0.371552333333334
Explanation Fit: 0.087
0.121 < Diabetes.prevalence
0.300 < Physical.inactivity <= 0.344
0.1253 < Percentage.of.households.with.high.housing.costs
0.510 < female
0.834 < Driving.alone.to.work
0.202 < Adult.smoking <= 0.233
0.37 < Flu.vaccinations <= 0.44
0.396 < Insufficient.sleep
24.9 < Traffic.volume <= 73.9
8.0 < Air.pollution...particulate.matter <= 9.1
Feature
0.00
0.01
0.02
Weight
Positive
Negative

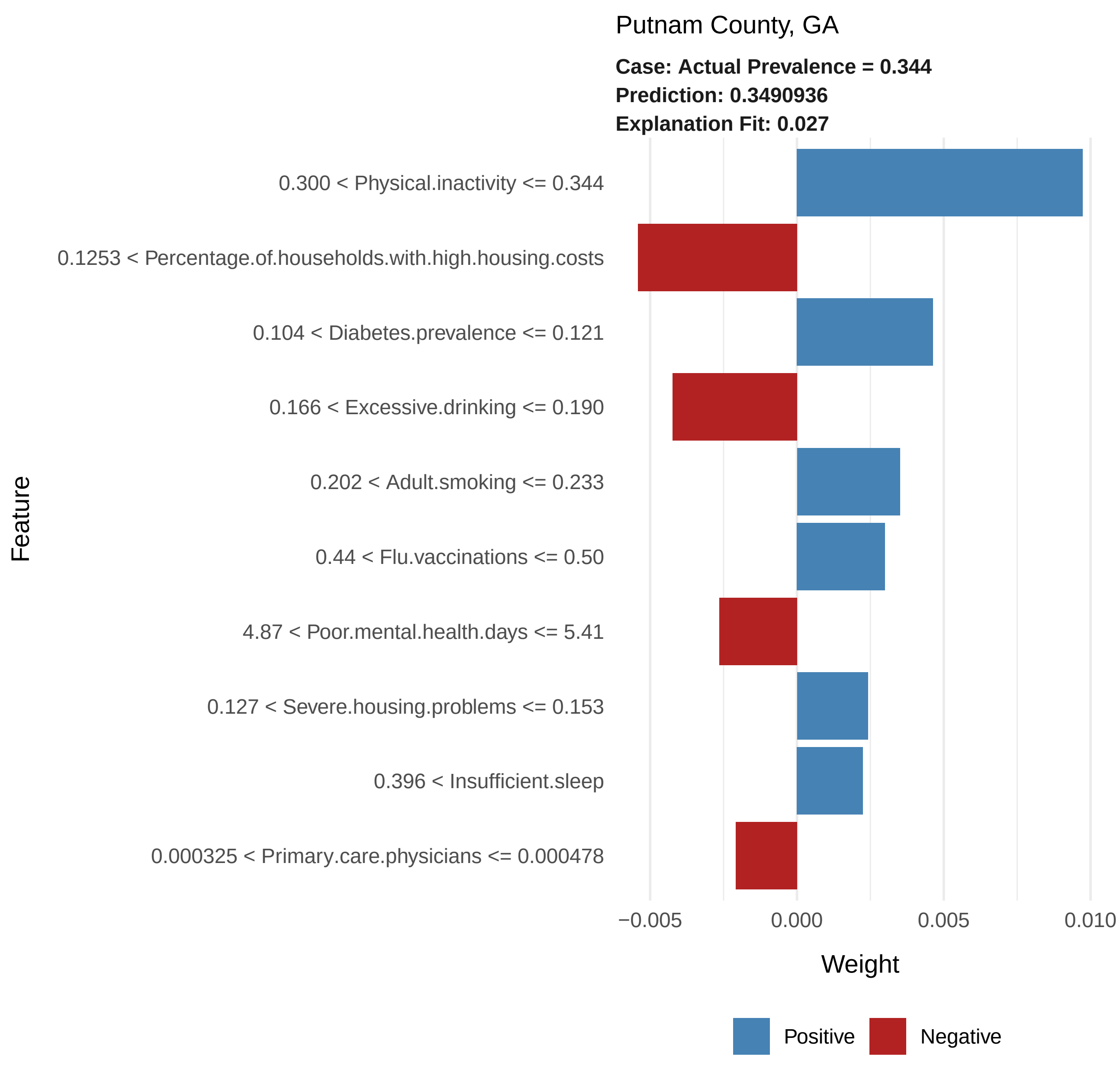
Putnam County, GA
Case: Actual Prevalence = 0.344
Prediction: 0.3490936
Explanation Fit: 0.027
0.300 < Physical.inactivity <= 0.344
0.1253 < Percentage.of.households.with.high.housing.costs
0.104 < Diabetes.prevalence <= 0.121
0.166 < Excessive.drinking <= 0.190
0.202 < Adult.smoking <= 0.233
0.44 < Flu.vaccinations <= 0.50
4.87 < Poor.mental.health.days <= 5.41
0.127 < Severe.housing.problems <= 0.153
0.396 < Insufficient.sleep
0.000325 < Primary.care.physicians <= 0.000478
Feature
−0.005
0.000
0.005
0.010
Weight
Positive
Negative

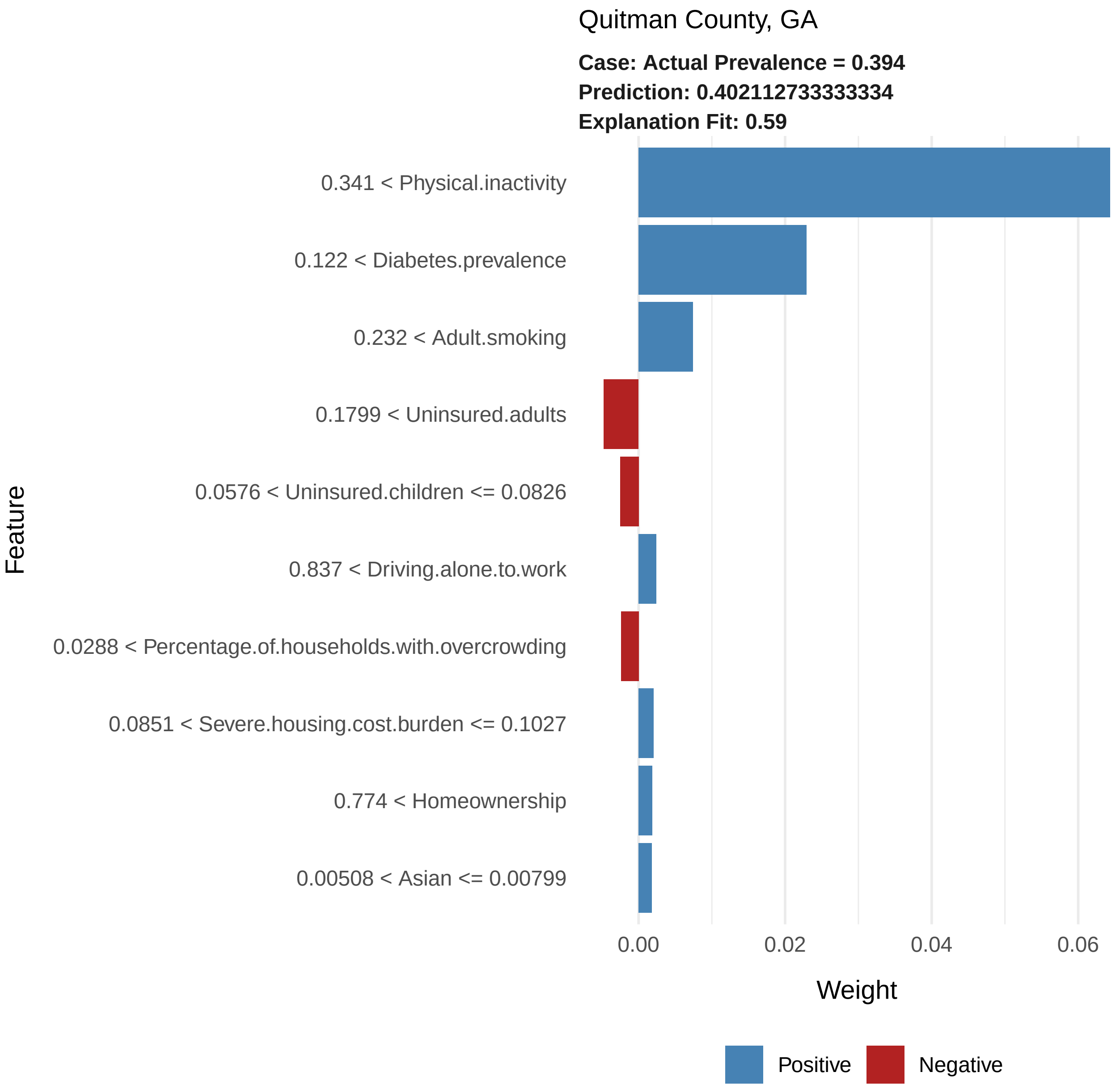

Quitman County, GA
Case: Actual Prevalence = 0.394
Prediction: 0.402112733333334
Explanation Fit: 0.59
0.341 < Physical.inactivity
0.122 < Diabetes.prevalence
0.232 < Adult.smoking
0.1799 < Uninsured.adults
0.0576 < Uninsured.children <= 0.0826
0.837 < Driving.alone.to.work
0.0288 < Percentage.of.households.with.overcrowding
0.0851 < Severe.housing.cost.burden <= 0.1027
0.774 < Homeownership
0.00508 < Asian <= 0.00799
Feature
0.00
0.02
0.04
0.06
Weight
Positive
Negative

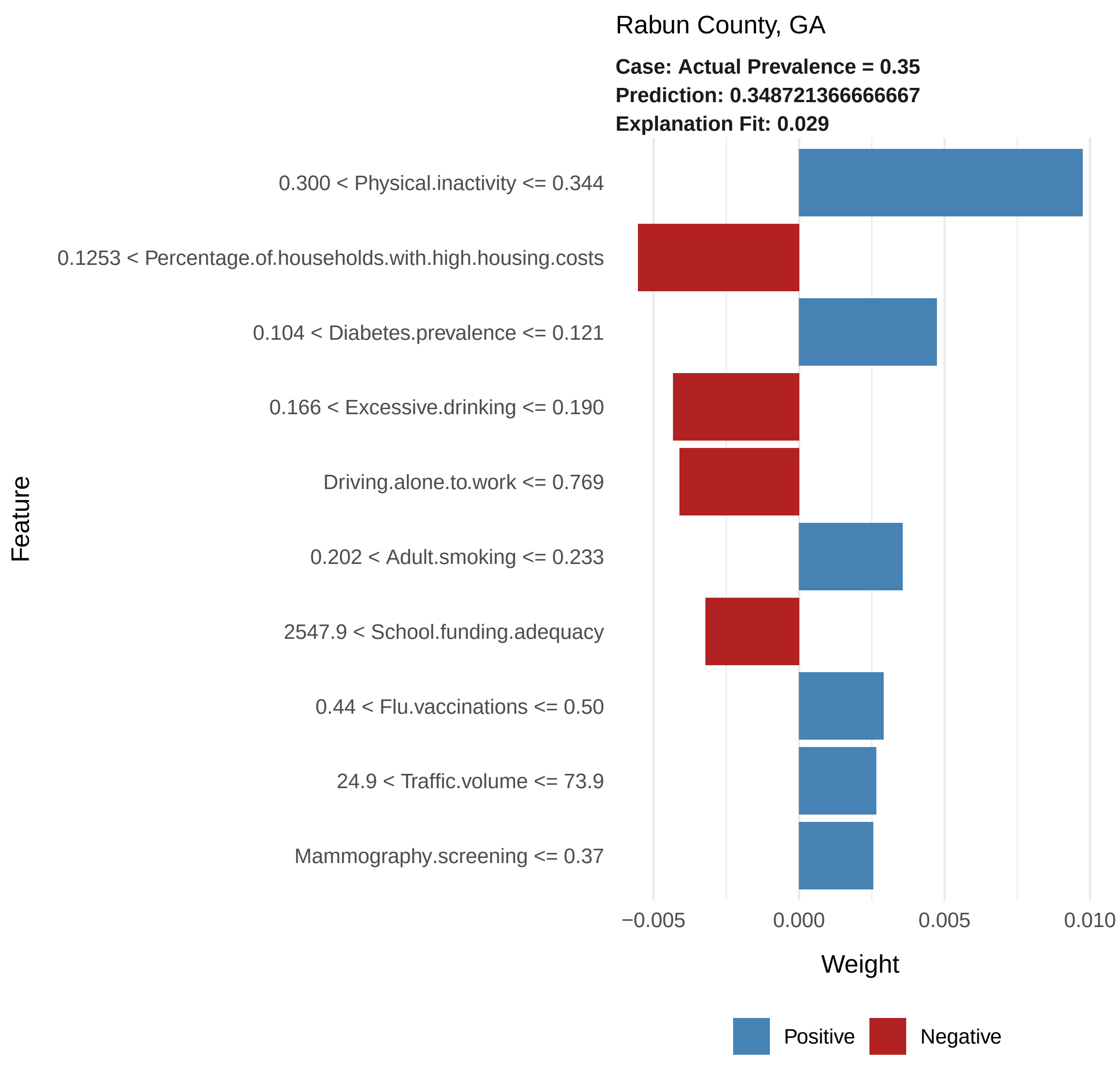
Rabun County, GA
Case: Actual Prevalence = 0.35
Prediction: 0.348721366666667
Explanation Fit: 0.029
0.300 < Physical.inactivity <= 0.344
0.1253 < Percentage.of.households.with.high.housing.costs
0.104 < Diabetes.prevalence <= 0.121
0.166 < Excessive.drinking <= 0.190
Driving.alone.to.work <= 0.769
0.202 < Adult.smoking <= 0.233
2547.9 < School.funding.adequacy
0.44 < Flu.vaccinations <= 0.50
24.9 < Traffic.volume <= 73.9
Mammography.screening <= 0.37
Feature
−0.005
0.000
0.005
0.010
Weight
Positive
Negative

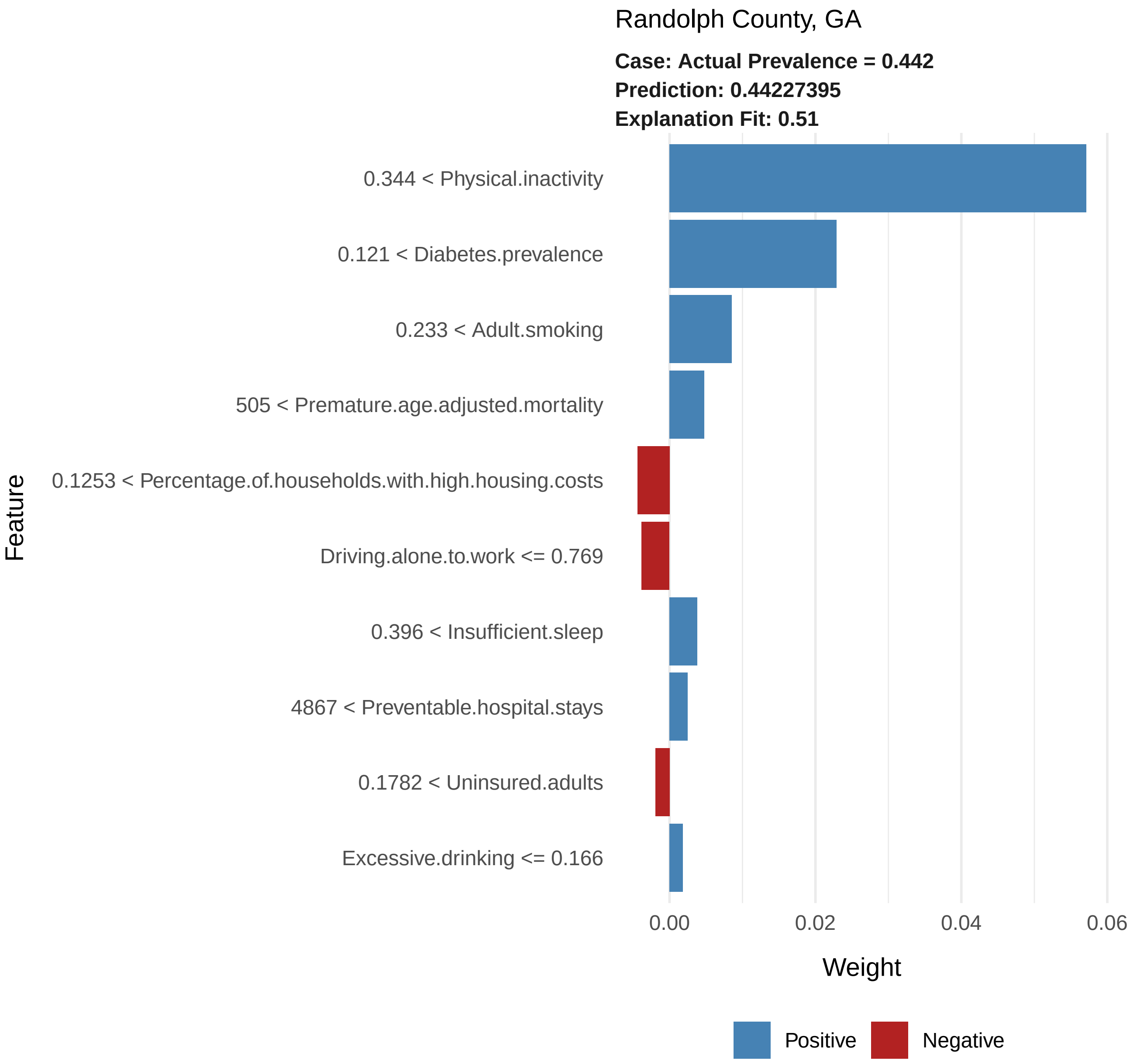

Randolph County, GA
Case: Actual Prevalence = 0.442
Prediction: 0.44227395
Explanation Fit: 0.51
0.344 < Physical.inactivity
0.121 < Diabetes.prevalence
0.233 < Adult.smoking
505 < Premature.age.adjusted.mortality
0.1253 < Percentage.of.households.with.high.housing.costs
Driving.alone.to.work <= 0.769
0.396 < Insufficient.sleep
4867 < Preventable.hospital.stays
0.1782 < Uninsured.adults
Excessive.drinking <= 0.166
Feature
0.00
0.02
0.04
0.06
Weight
Positive
Negative

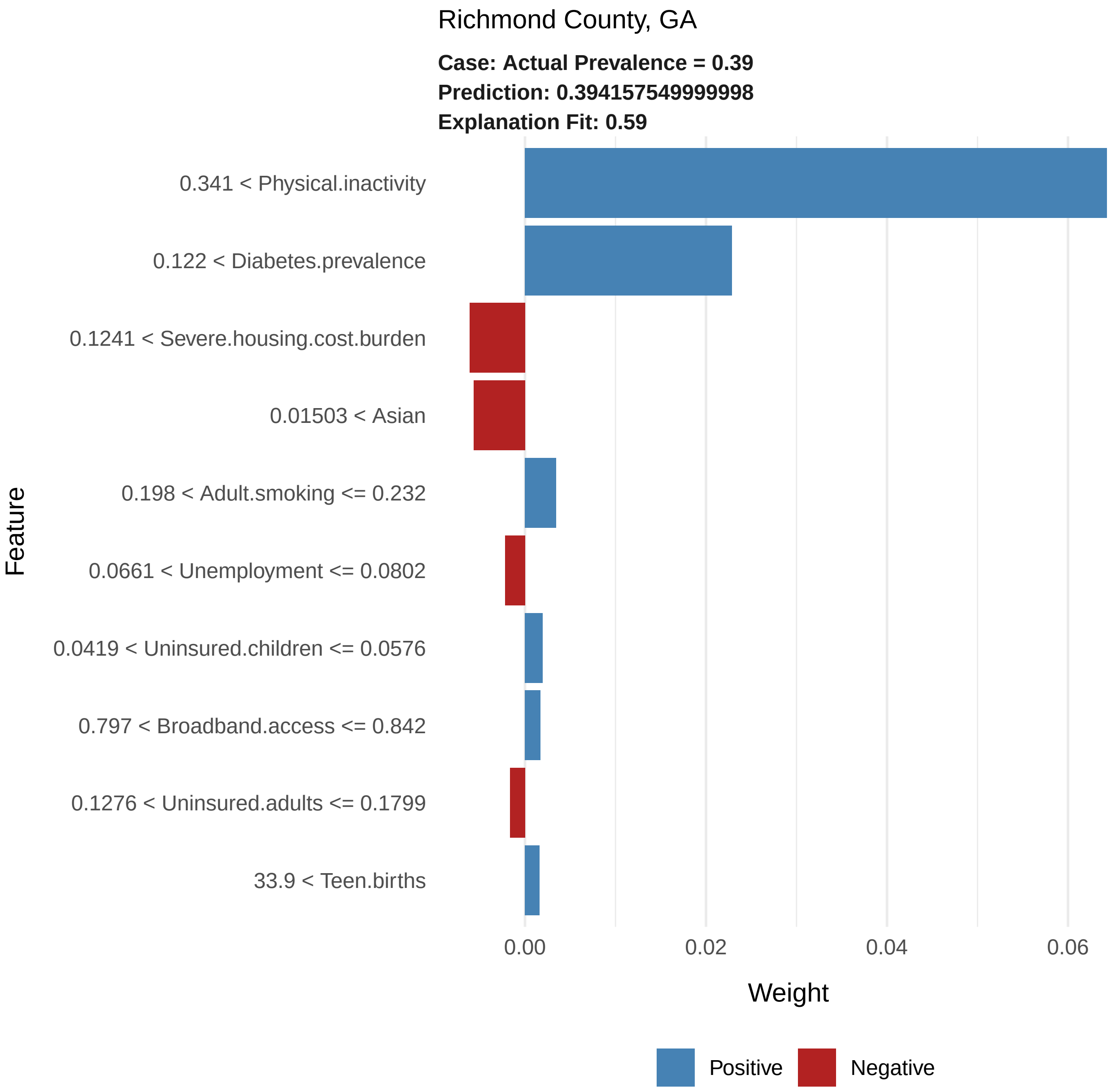

Richmond County, GA
Case: Actual Prevalence = 0.39
Prediction: 0.394157549999998
Explanation Fit: 0.59
0.341 < Physical.inactivity
0.122 < Diabetes.prevalence
0.1241 < Severe.housing.cost.burden
0.01503 < Asian
0.198 < Adult.smoking <= 0.232
0.0661 < Unemployment <= 0.0802
0.0419 < Uninsured.children <= 0.0576
0.797 < Broadband.access <= 0.842
0.1276 < Uninsured.adults <= 0.1799
33.9 < Teen.births
Feature
0.00
0.02
0.04
0.06
Weight
Positive
Negative

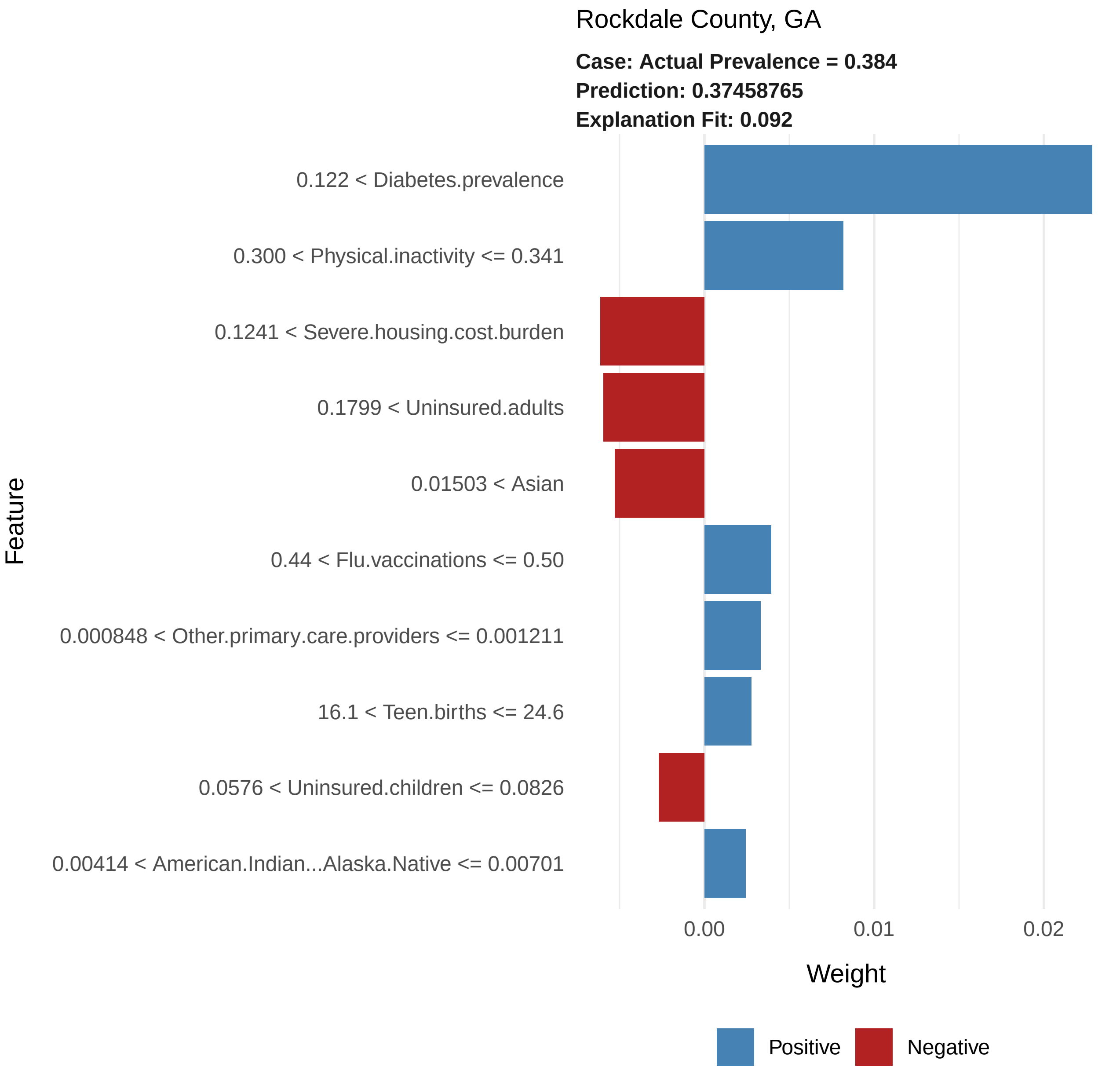

Rockdale County, GA
Case: Actual Prevalence = 0.384
Prediction: 0.37458765
Explanation Fit: 0.092
0.122 < Diabetes.prevalence
0.300 < Physical.inactivity <= 0.341
0.1241 < Severe.housing.cost.burden
0.1799 < Uninsured.adults
0.01503 < Asian
0.44 < Flu.vaccinations <= 0.50
0.000848 < Other.primary.care.providers <= 0.001211
16.1 < Teen.births <= 24.6
0.0576 < Uninsured.children <= 0.0826
0.00414 < American.Indian...Alaska.Native <= 0.00701
Feature
0.00
0.01
0.02
Weight
Positive
Negative

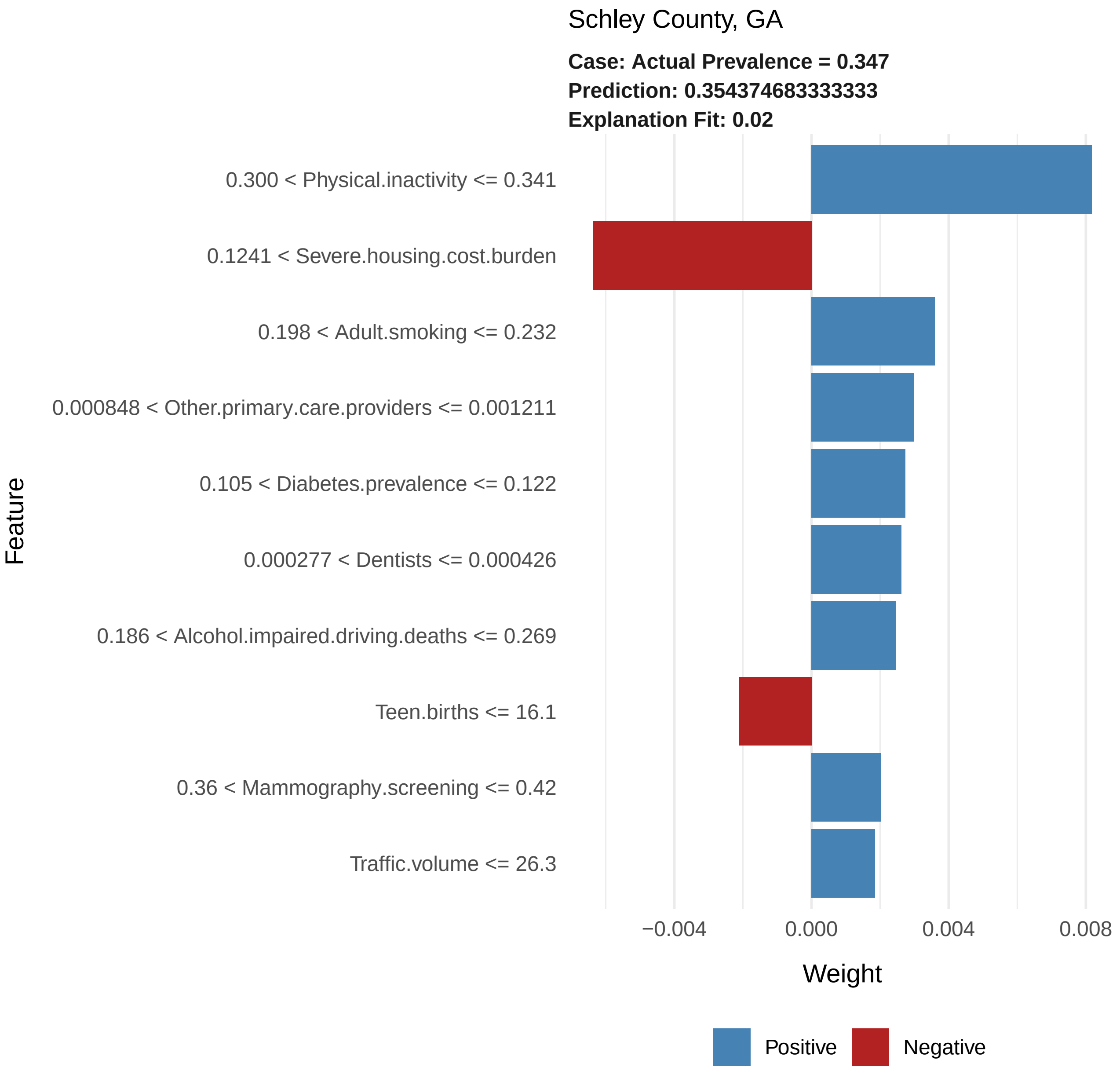
Schley County, GA
Case: Actual Prevalence = 0.347
Prediction: 0.354374683333333
Explanation Fit: 0.02
0.300 < Physical.inactivity <= 0.341
0.1241 < Severe.housing.cost.burden
0.198 < Adult.smoking <= 0.232
0.000848 < Other.primary.care.providers <= 0.001211
0.105 < Diabetes.prevalence <= 0.122
0.000277 < Dentists <= 0.000426
0.186 < Alcohol.impaired.driving.deaths <= 0.269
Teen.births <= 16.1
0.36 < Mammography.screening <= 0.42
Traffic.volume <= 26.3
Feature
−0.004
0.000
0.004
0.008
Weight
Positive
Negative

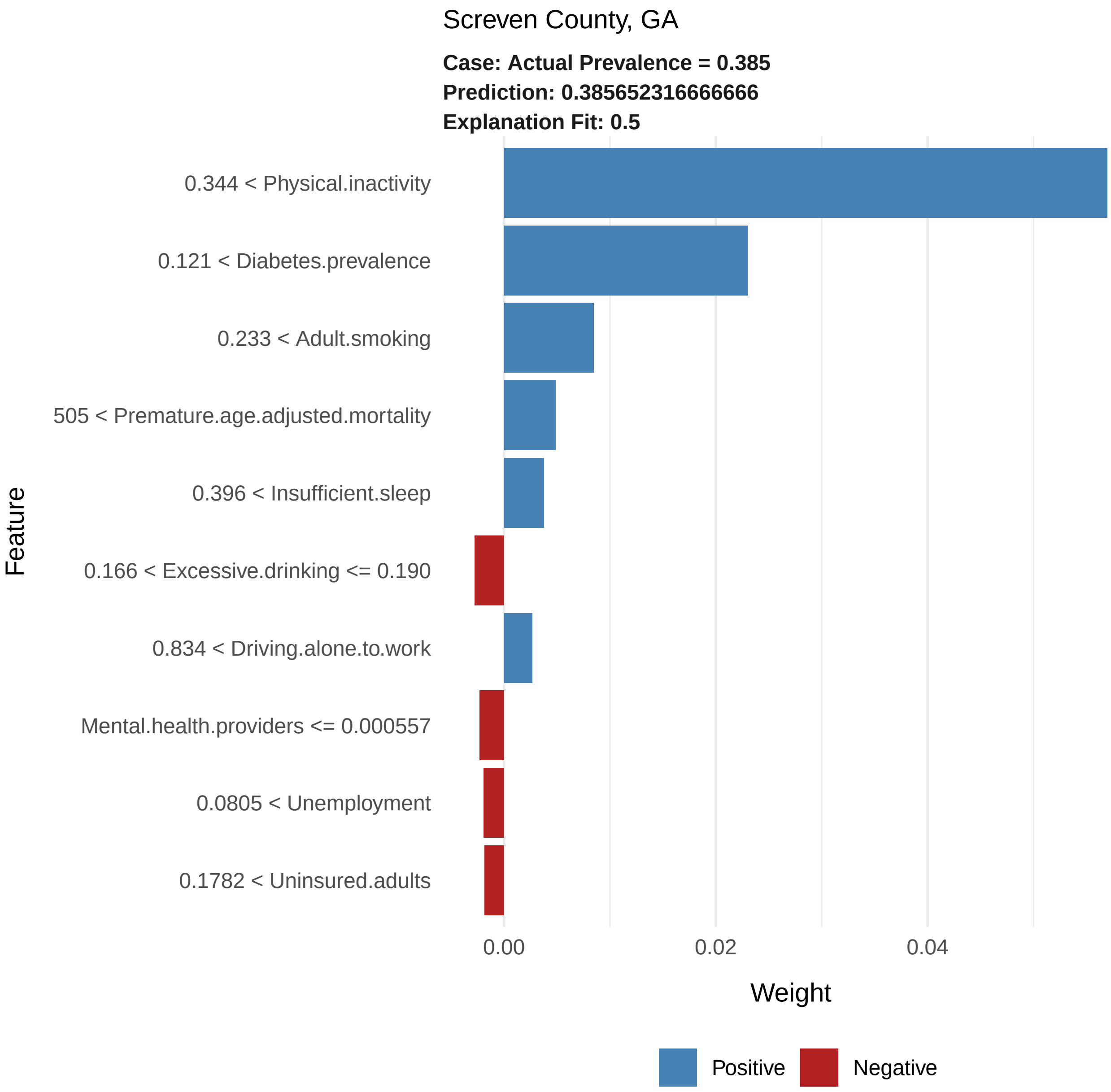
Screven County, GA
Case: Actual Prevalence = 0.385
Prediction: 0.385652316666666
Explanation Fit: 0.5
0.344 < Physical.inactivity
0.121 < Diabetes.prevalence
0.233 < Adult.smoking
505 < Premature.age.adjusted.mortality
0.396 < Insufficient.sleep
0.166 < Excessive.drinking <= 0.190
0.834 < Driving.alone.to.work
Mental.health.providers <= 0.000557
0.0805 < Unemployment
0.1782 < Uninsured.adults
Feature
0.00
0.02
0.04
Weight
Positive
Negative

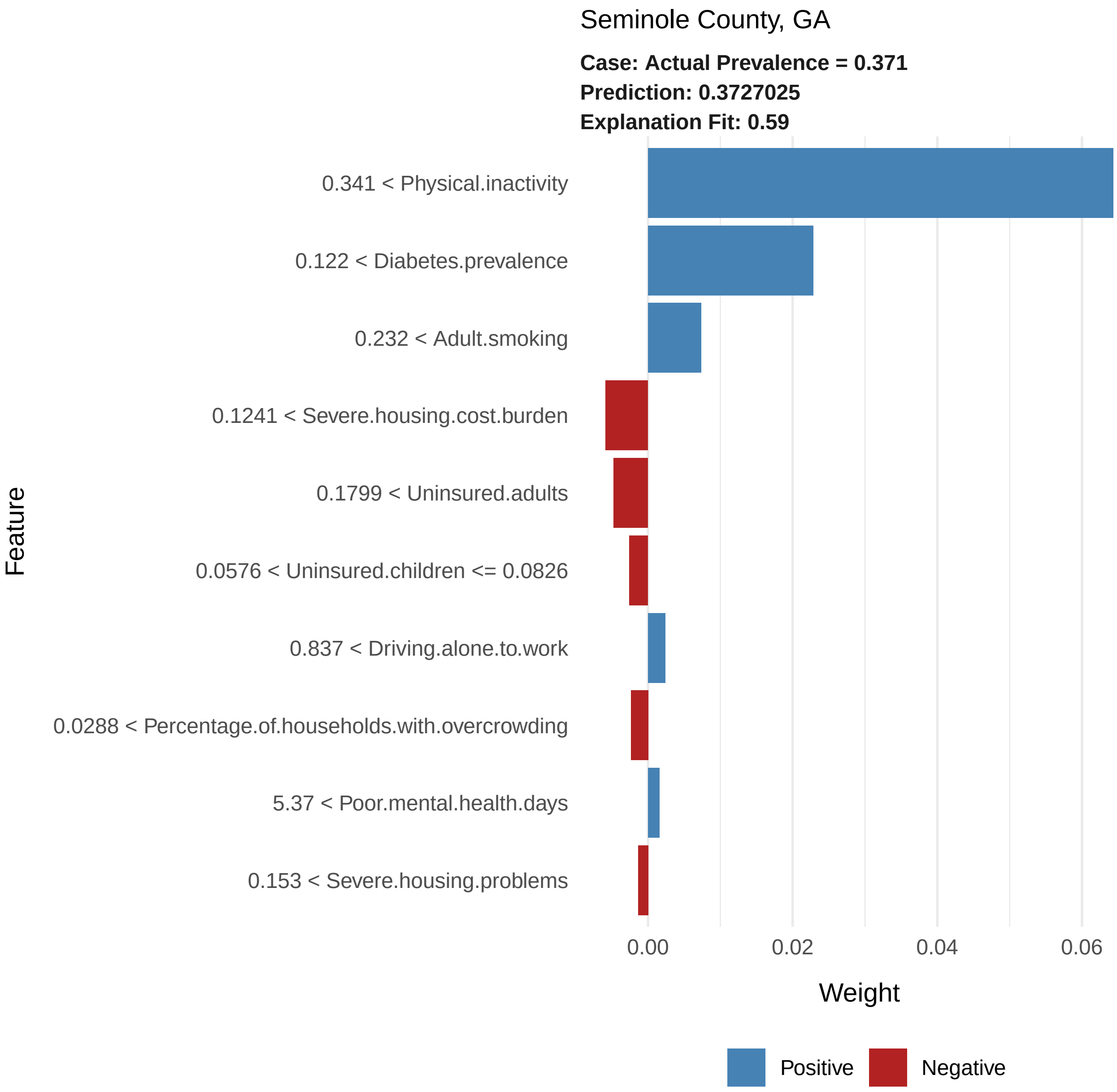

Seminole County, GA
Case: Actual Prevalence = 0.371
Prediction: 0.3727025
Explanation Fit: 0.59
0.341 < Physical.inactivity
0.122 < Diabetes.prevalence
0.232 < Adult.smoking
0.1241 < Severe.housing.cost.burden
0.1799 < Uninsured.adults
0.0576 < Uninsured.children <= 0.0826
0.837 < Driving.alone.to.work
0.0288 < Percentage.of.households.with.overcrowding
5.37 < Poor.mental.health.days
0.153 < Severe.housing.problems
Feature
0.00
0.02
0.04
0.06
Weight
Positive
Negative

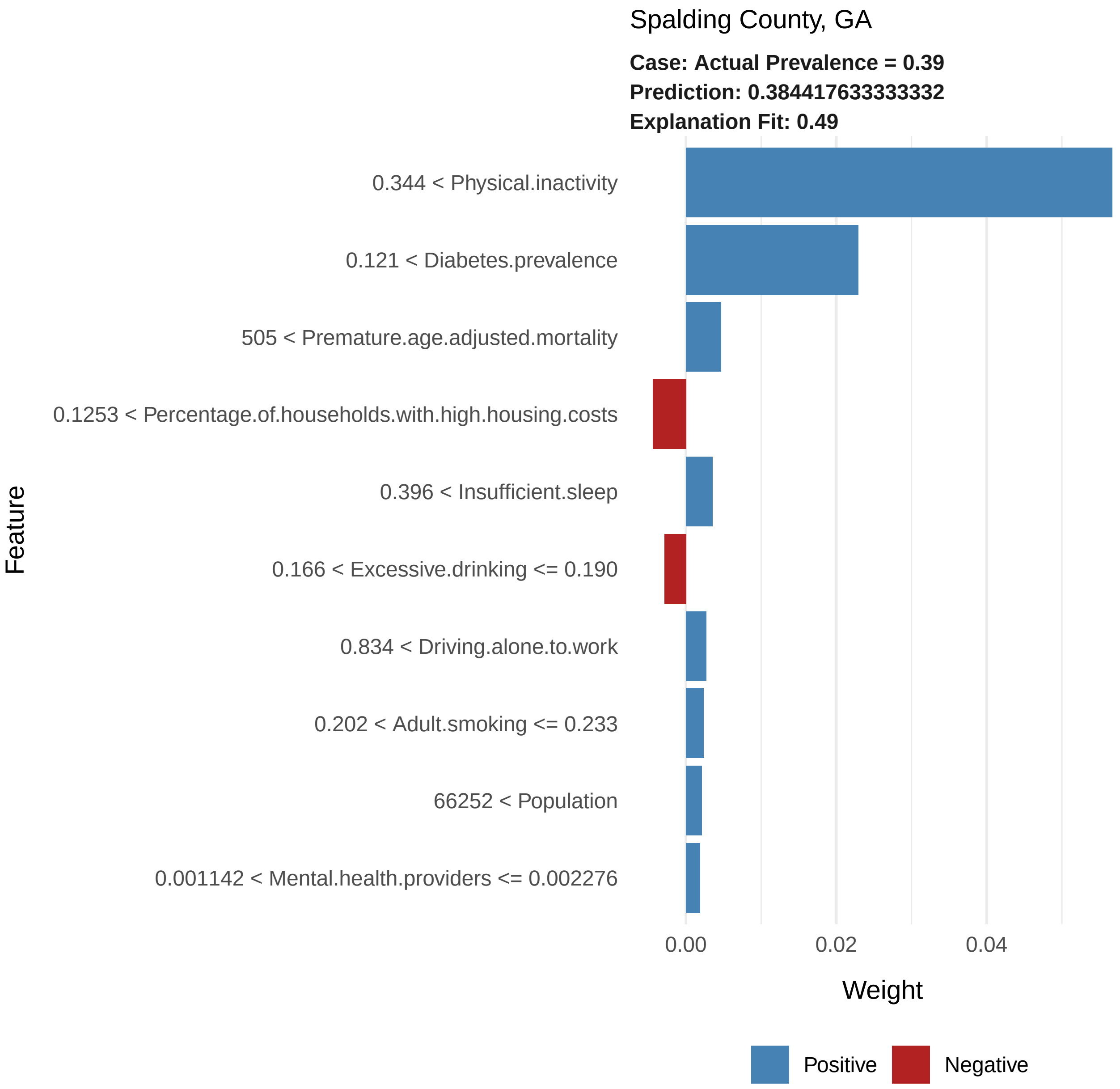

Spalding County, GA
Case: Actual Prevalence = 0.39
Prediction: 0.384417633333332
Explanation Fit: 0.49
0.344 < Physical.inactivity
0.121 < Diabetes.prevalence
505 < Premature.age.adjusted.mortality
0.1253 < Percentage.of.households.with.high.housing.costs
0.396 < Insufficient.sleep
0.166 < Excessive.drinking <= 0.190
0.834 < Driving.alone.to.work
0.202 < Adult.smoking <= 0.233
66252 < Population
0.001142 < Mental.health.providers <= 0.002276
Feature
0.00
0.02
0.04
Weight
Positive
Negative

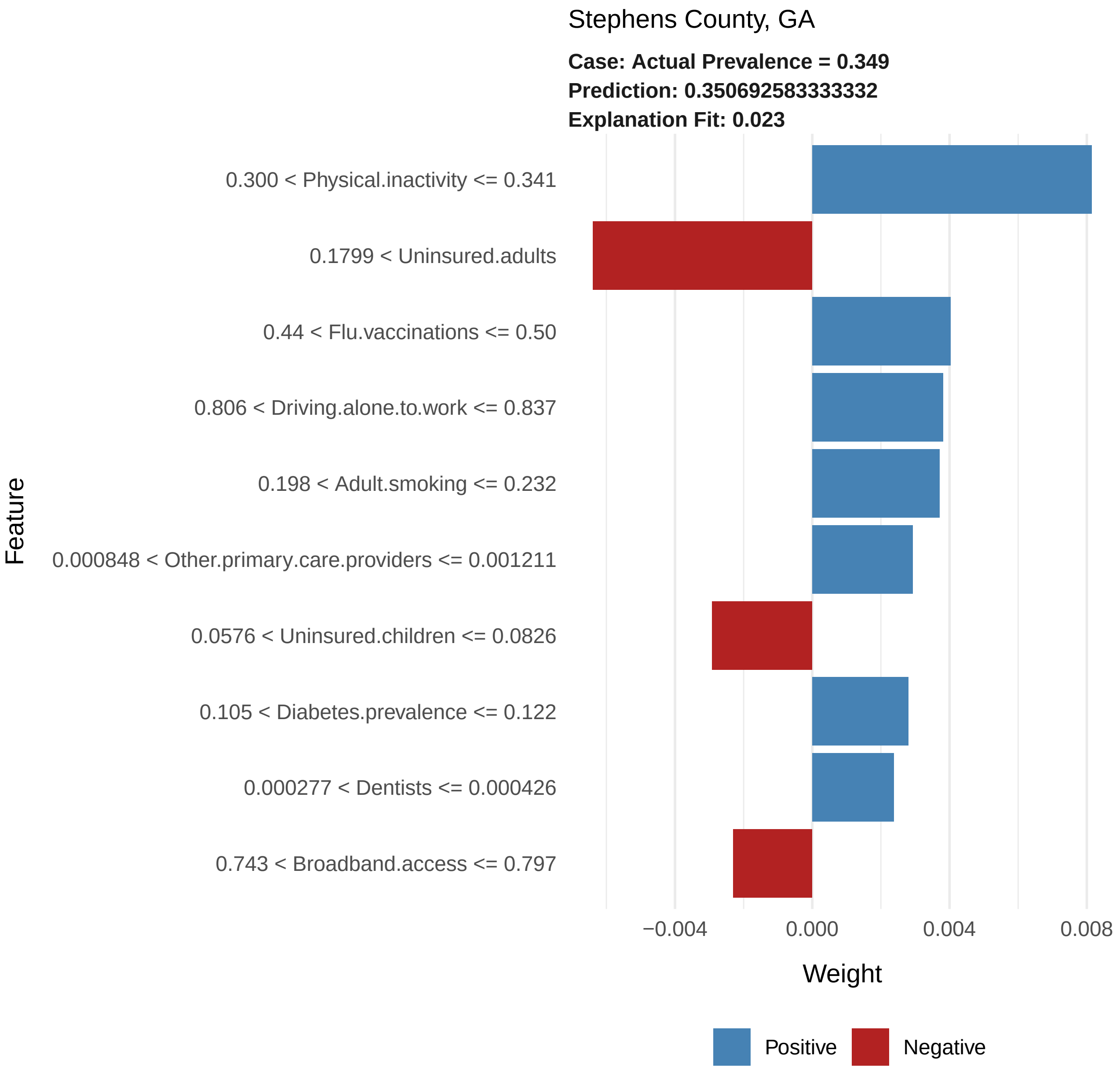

Stephens County, GA
Case: Actual Prevalence = 0.349
Prediction: 0.350692583333332
Explanation Fit: 0.023
0.300 < Physical.inactivity <= 0.341
0.1799 < Uninsured.adults
0.44 < Flu.vaccinations <= 0.50
0.806 < Driving.alone.to.work <= 0.837
0.198 < Adult.smoking <= 0.232
0.000848 < Other.primary.care.providers <= 0.001211
0.0576 < Uninsured.children <= 0.0826
0.105 < Diabetes.prevalence <= 0.122
0.000277 < Dentists <= 0.000426
0.743 < Broadband.access <= 0.797
Feature
−0.004
0.000
0.004
0.008
Weight
Positive
Negative

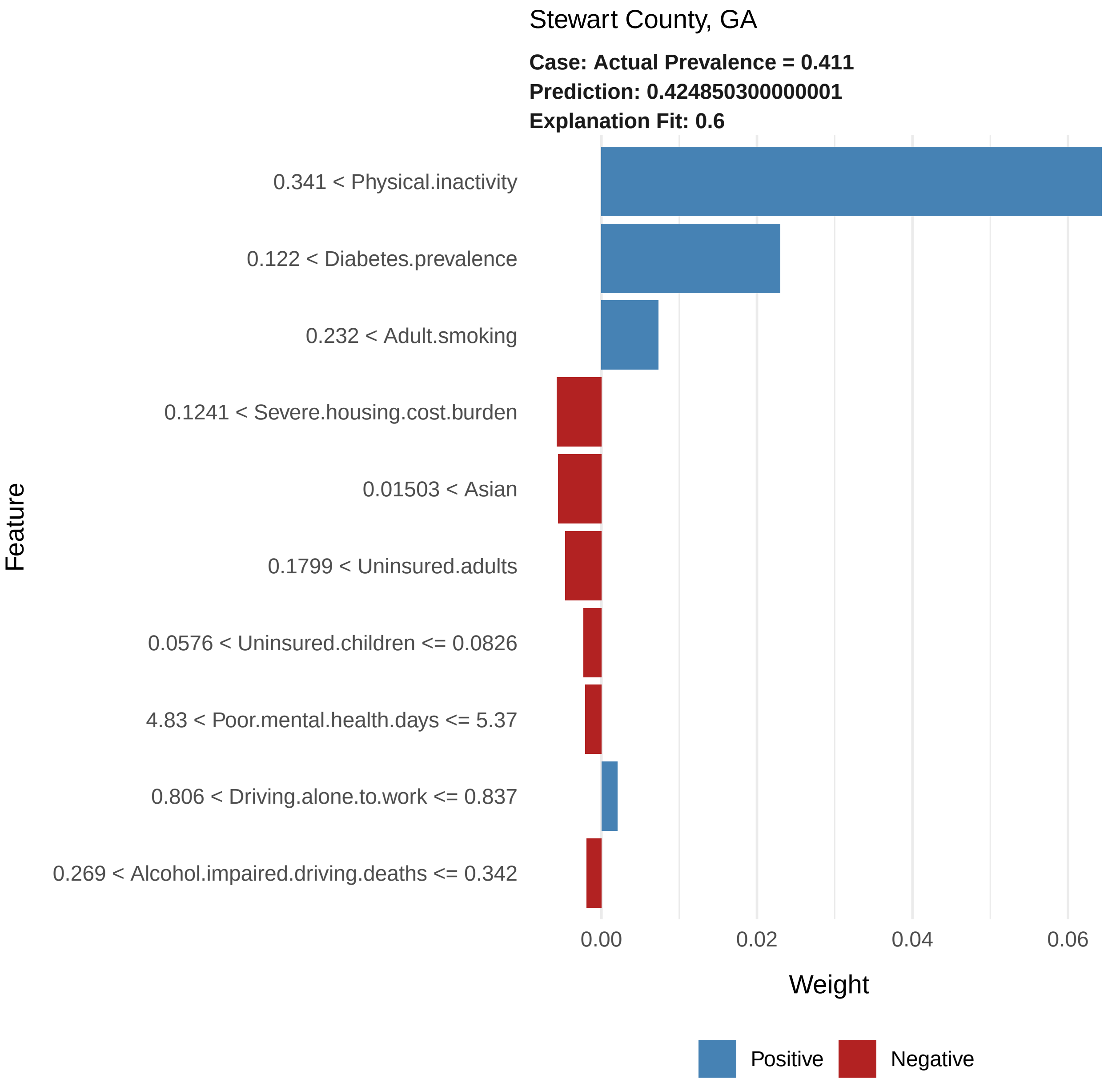

Stewart County, GA
Case: Actual Prevalence = 0.411
Prediction: 0.424850300000001
Explanation Fit: 0.6
0.341 < Physical.inactivity
0.122 < Diabetes.prevalence
0.232 < Adult.smoking
0.1241 < Severe.housing.cost.burden
0.01503 < Asian
0.1799 < Uninsured.adults
0.0576 < Uninsured.children <= 0.0826
4.83 < Poor.mental.health.days <= 5.37
0.806 < Driving.alone.to.work <= 0.837
0.269 < Alcohol.impaired.driving.deaths <= 0.342
Feature
0.00
0.02
0.04
0.06
Weight
Positive
Negative

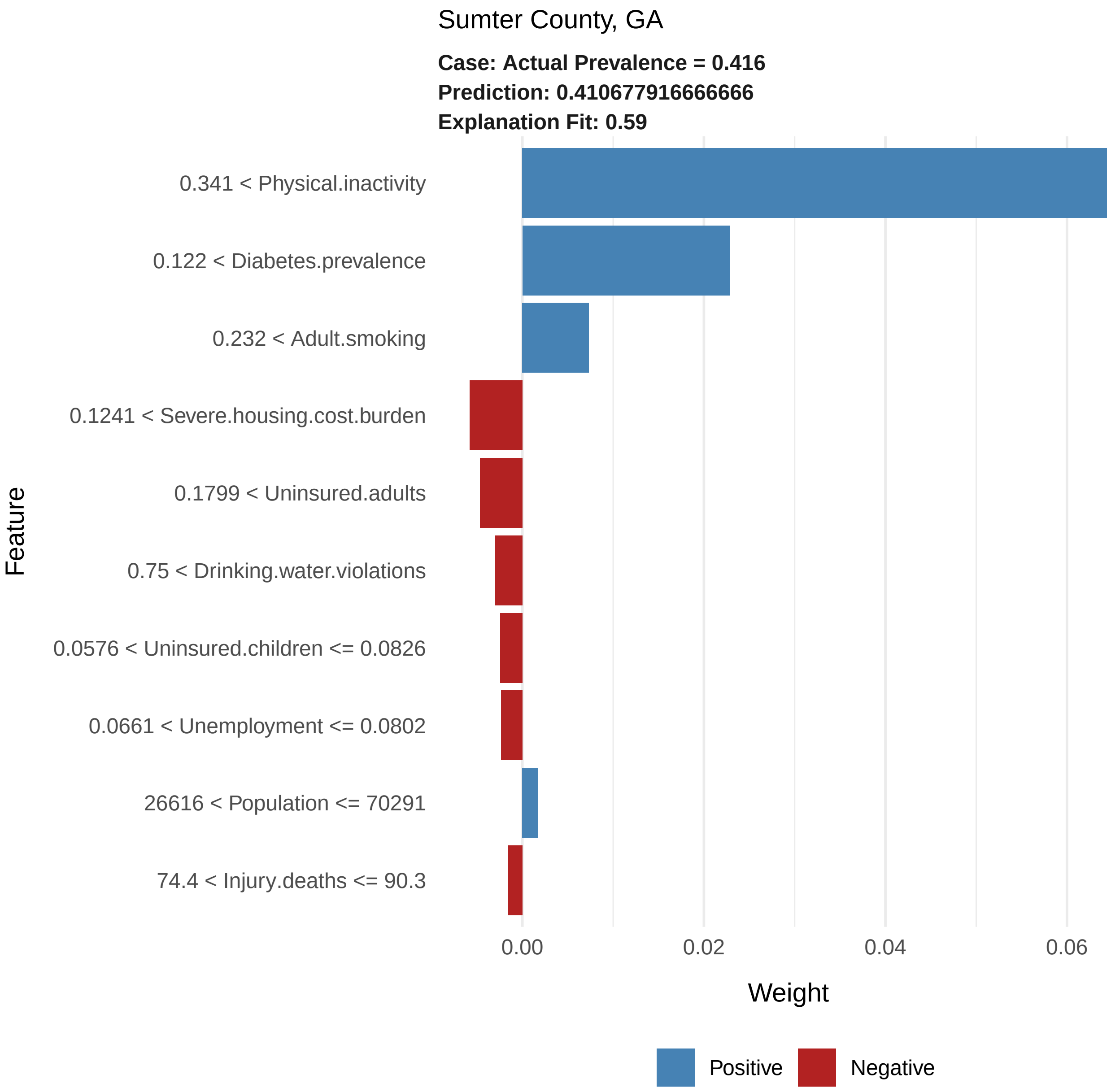

Sumter County, GA
Case: Actual Prevalence = 0.416
Prediction: 0.410677916666666
Explanation Fit: 0.59
0.341 < Physical.inactivity
0.122 < Diabetes.prevalence
0.232 < Adult.smoking
0.1241 < Severe.housing.cost.burden
0.1799 < Uninsured.adults
0.75 < Drinking.water.violations
0.0576 < Uninsured.children <= 0.0826
0.0661 < Unemployment <= 0.0802
26616 < Population <= 70291
74.4 < Injury.deaths <= 90.3
Feature
0.00
0.02
0.04
0.06
Weight
Positive
Negative

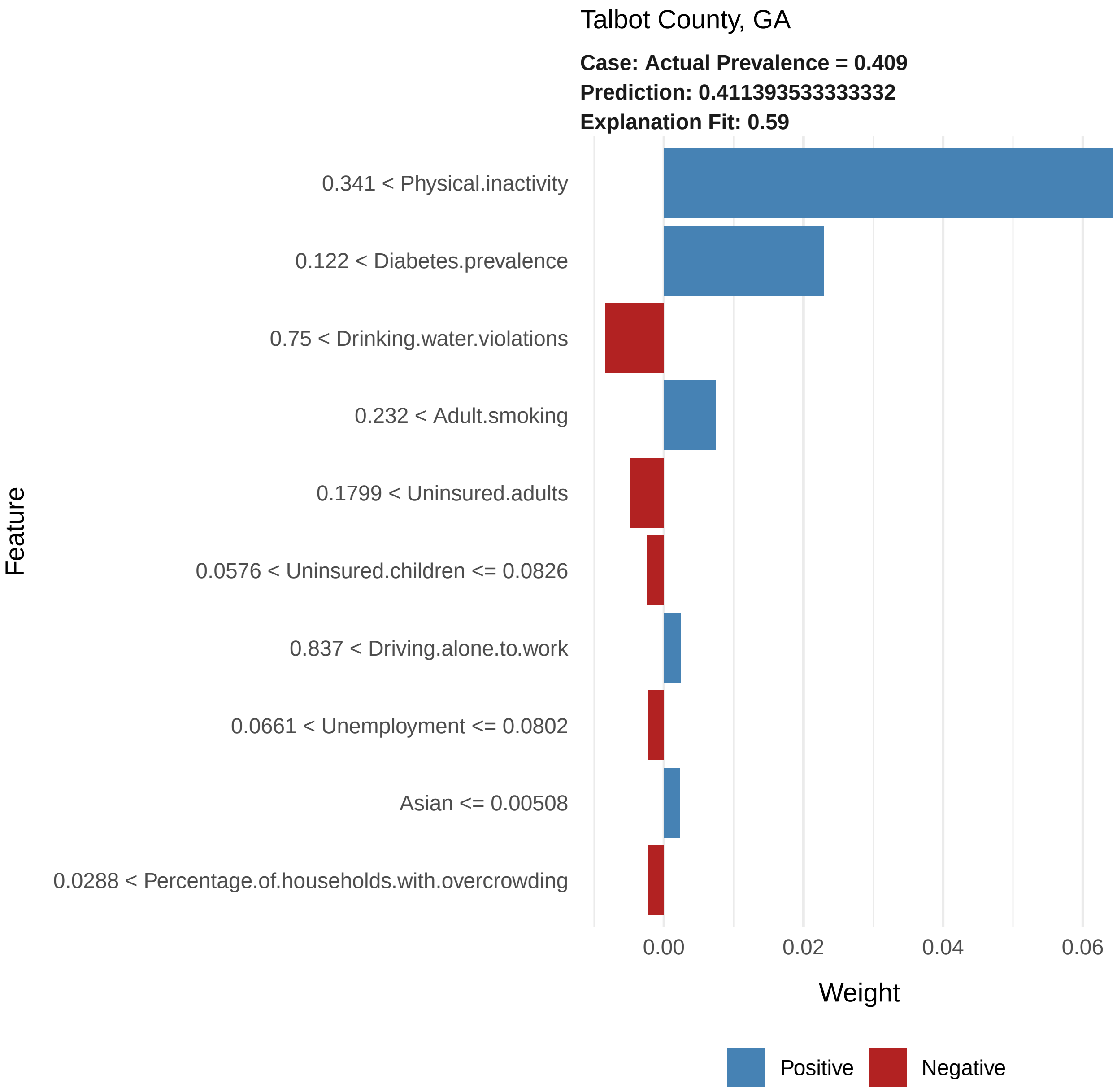

Talbot County, GA
Case: Actual Prevalence = 0.409
Prediction: 0.411393533333332
Explanation Fit: 0.59
0.341 < Physical.inactivity
0.122 < Diabetes.prevalence
0.75 < Drinking.water.violations
0.232 < Adult.smoking
0.1799 < Uninsured.adults
0.0576 < Uninsured.children <= 0.0826
0.837 < Driving.alone.to.work
0.0661 < Unemployment <= 0.0802
Asian <= 0.00508
0.0288 < Percentage.of.households.with.overcrowding
Feature
0.00
0.02
0.04
0.06
Weight
Positive
Negative

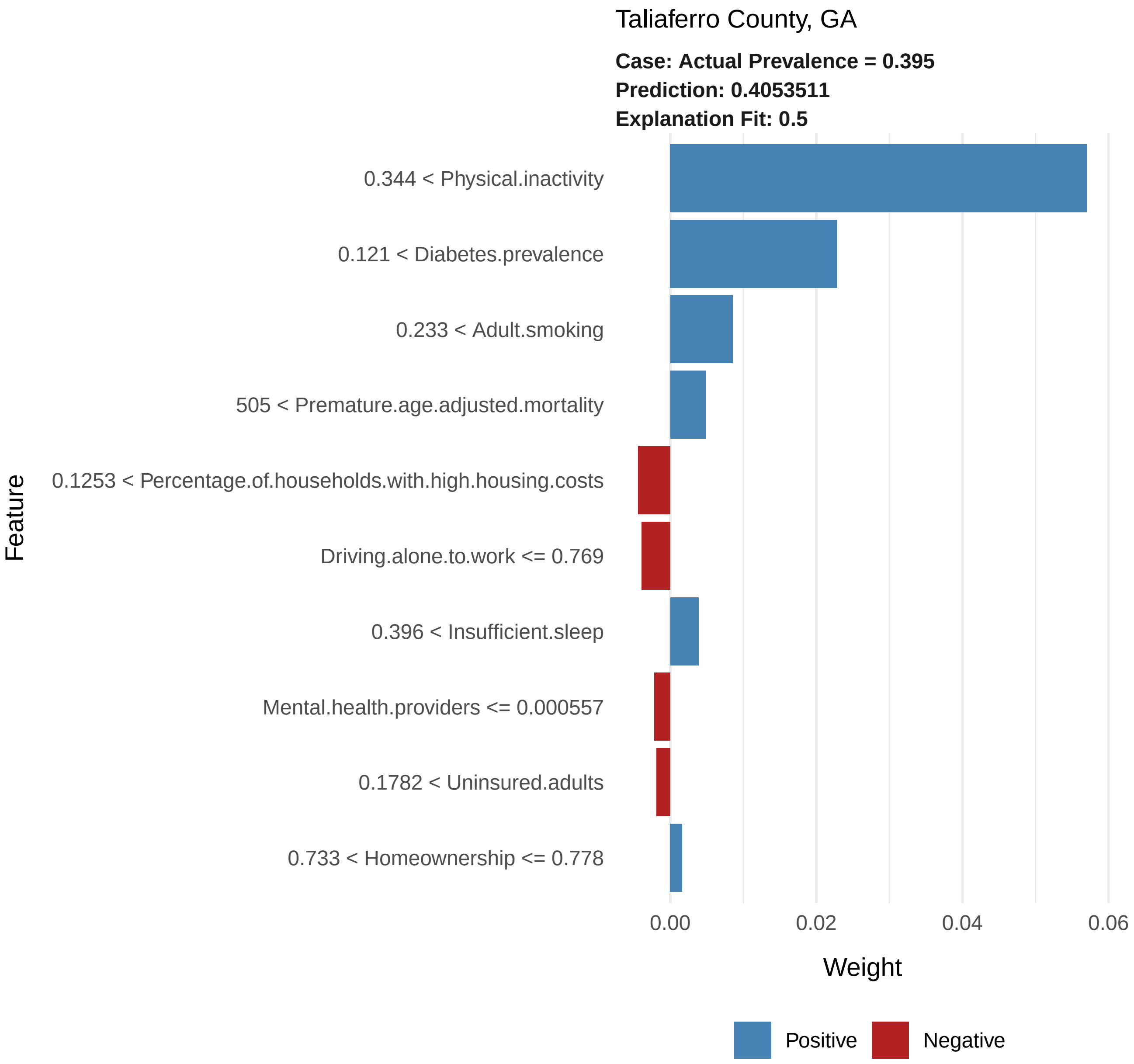
Taliaferro County, GA
Case: Actual Prevalence = 0.395
Prediction: 0.4053511
Explanation Fit: 0.5
0.344 < Physical.inactivity
0.121 < Diabetes.prevalence
0.233 < Adult.smoking
505 < Premature.age.adjusted.mortality
0.1253 < Percentage.of.households.with.high.housing.costs
Driving.alone.to.work <= 0.769
0.396 < Insufficient.sleep
Mental.health.providers <= 0.000557
0.1782 < Uninsured.adults
0.733 < Homeownership <= 0.778
Feature
0.00
0.02
0.04
0.06
Weight
Positive
Negative

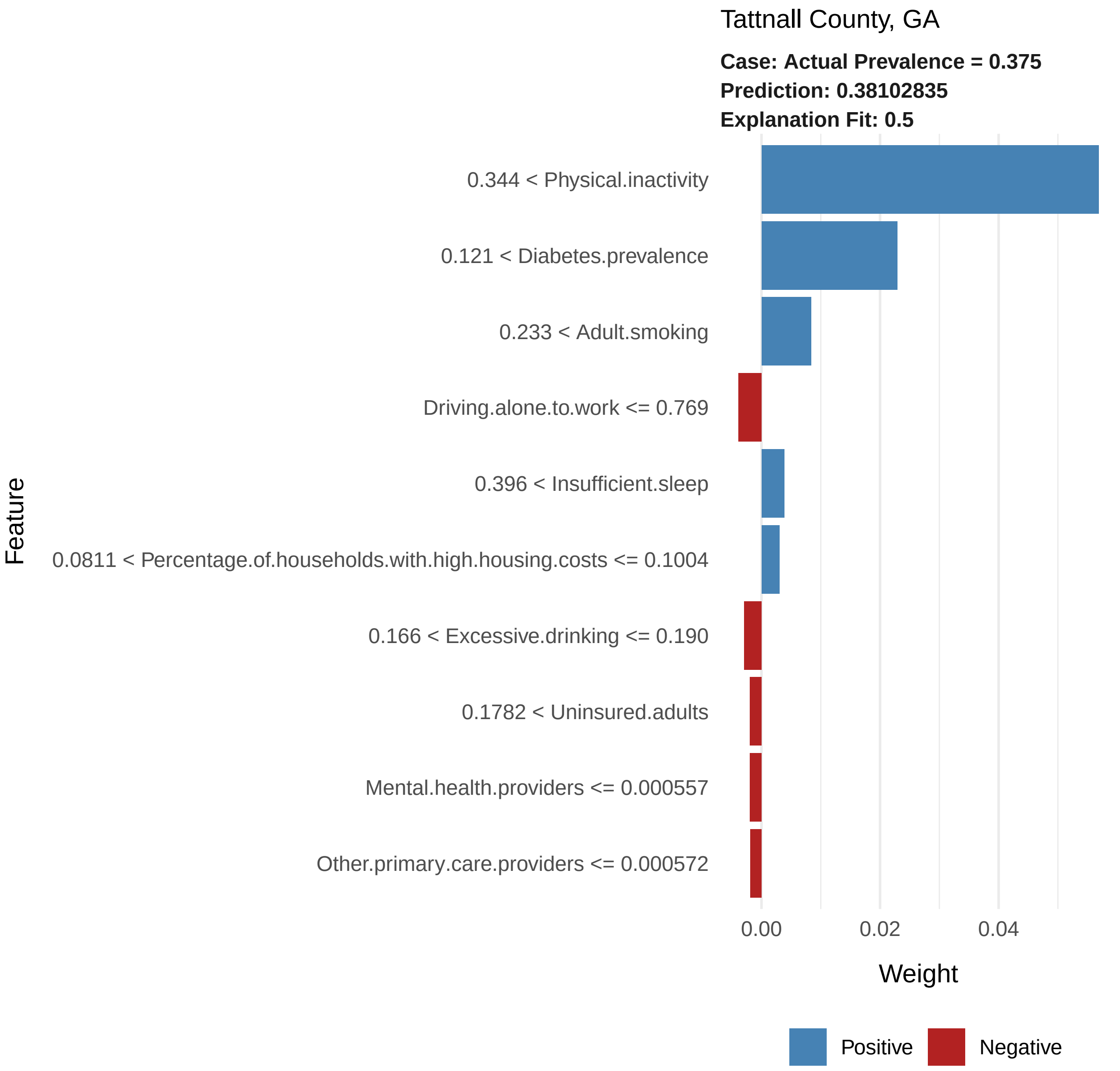
Tattnall County, GA
Case: Actual Prevalence = 0.375
Prediction: 0.38102835
Explanation Fit: 0.5
0.344 < Physical.inactivity
0.121 < Diabetes.prevalence
0.233 < Adult.smoking
Driving.alone.to.work <= 0.769
0.396 < Insufficient.sleep
0.0811 < Percentage.of.households.with.high.housing.costs <= 0.1004
0.166 < Excessive.drinking <= 0.190
0.1782 < Uninsured.adults
Mental.health.providers <= 0.000557
Other.primary.care.providers <= 0.000572
Feature
0.00
0.02
0.04
Weight
Positive
Negative

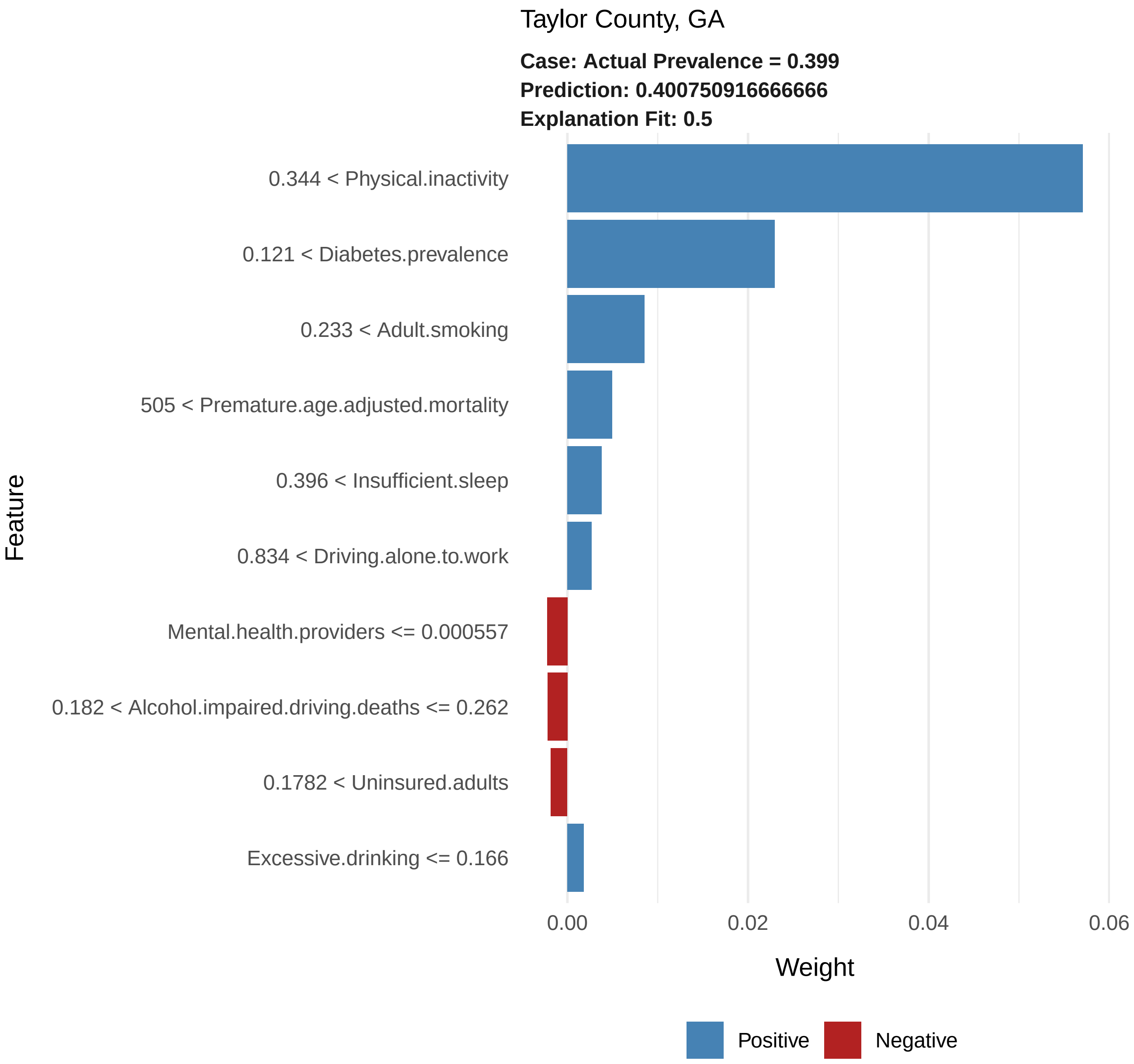

Taylor County, GA
Case: Actual Prevalence = 0.399
Prediction: 0.400750916666666
Explanation Fit: 0.5
0.344 < Physical.inactivity
0.121 < Diabetes.prevalence
0.233 < Adult.smoking
505 < Premature.age.adjusted.mortality
0.396 < Insufficient.sleep
0.834 < Driving.alone.to.work
Mental.health.providers <= 0.000557
0.182 < Alcohol.impaired.driving.deaths <= 0.262
0.1782 < Uninsured.adults
Excessive.drinking <= 0.166
Feature
0.00
0.02
0.04
0.06
Weight
Positive
Negative

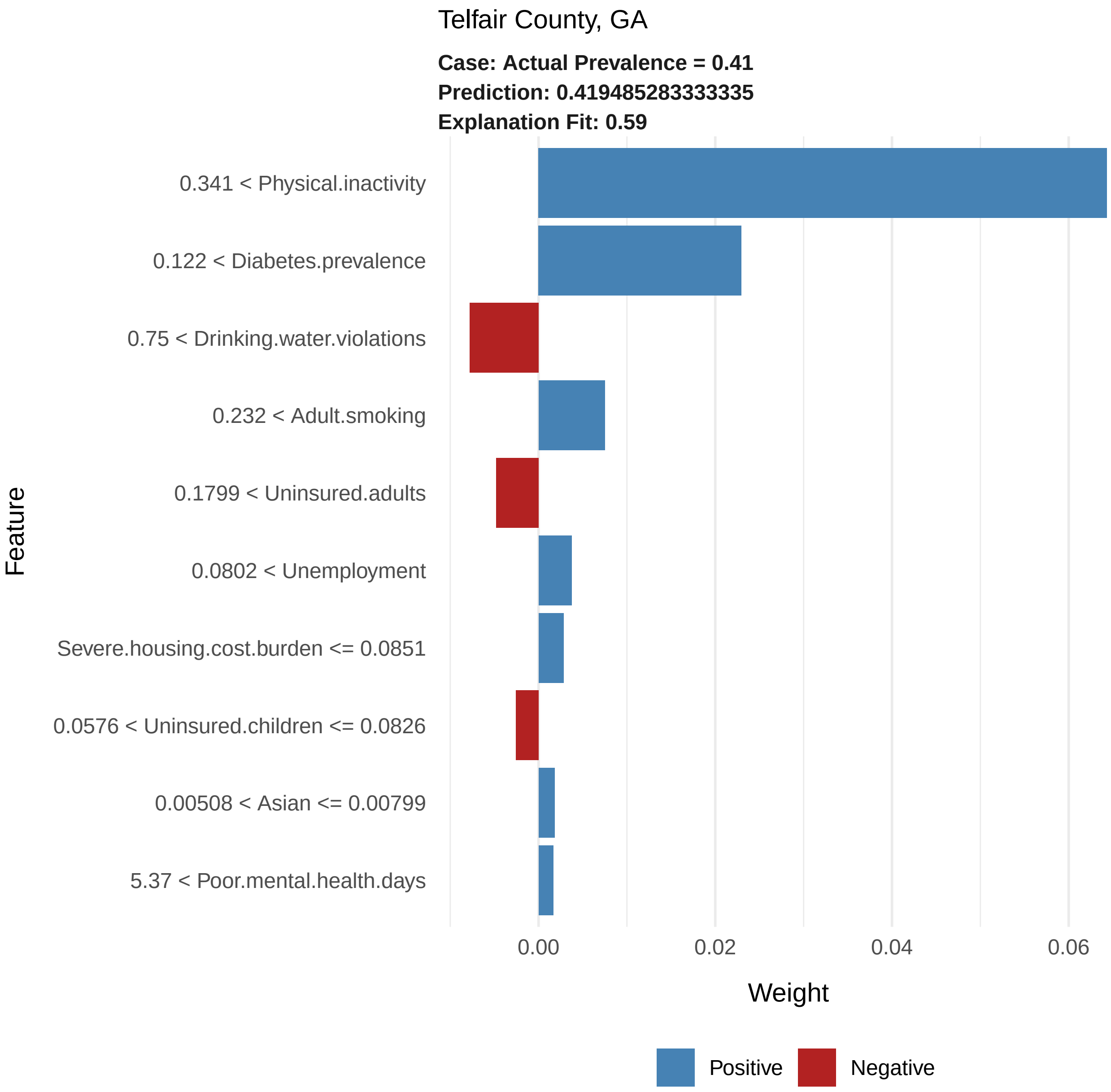

Telfair County, GA
Case: Actual Prevalence = 0.41
Prediction: 0.419485283333335
Explanation Fit: 0.59
0.341 < Physical.inactivity
0.122 < Diabetes.prevalence
0.75 < Drinking.water.violations
0.232 < Adult.smoking
0.1799 < Uninsured.adults
0.0802 < Unemployment
Severe.housing.cost.burden <= 0.0851
0.0576 < Uninsured.children <= 0.0826
0.00508 < Asian <= 0.00799
5.37 < Poor.mental.health.days
Feature
0.00
0.02
0.04
0.06
Weight
Positive
Negative

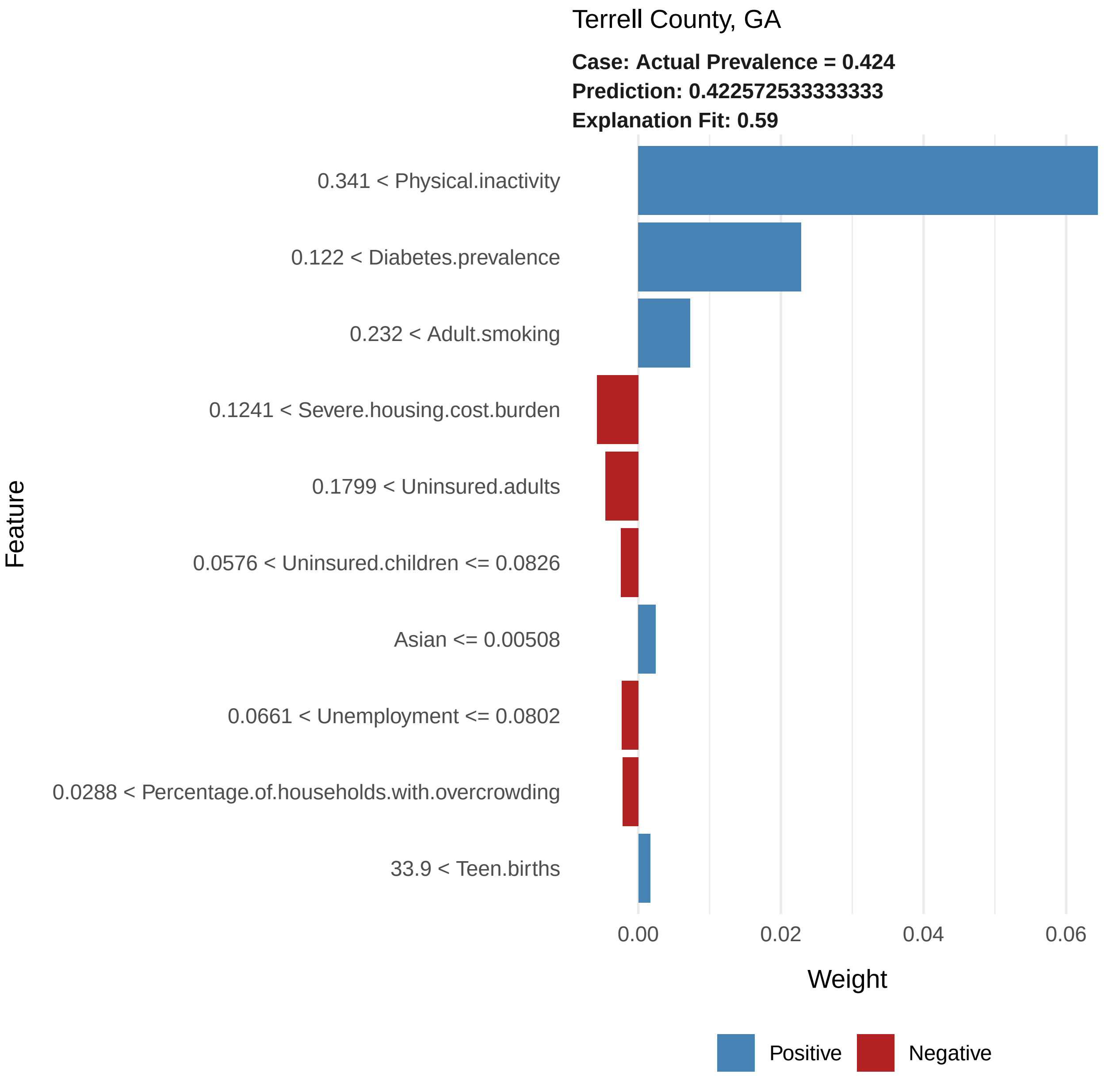
Terrell County, GA
Case: Actual Prevalence = 0.424
Prediction: 0.422572533333333
Explanation Fit: 0.59
0.341 < Physical.inactivity
0.122 < Diabetes.prevalence
0.232 < Adult.smoking
0.1241 < Severe.housing.cost.burden
0.1799 < Uninsured.adults
0.0576 < Uninsured.children <= 0.0826
Asian <= 0.00508
0.0661 < Unemployment <= 0.0802
0.0288 < Percentage.of.households.with.overcrowding
33.9 < Teen.births
Feature
0.00
0.02
0.04
0.06
Weight
Positive
Negative

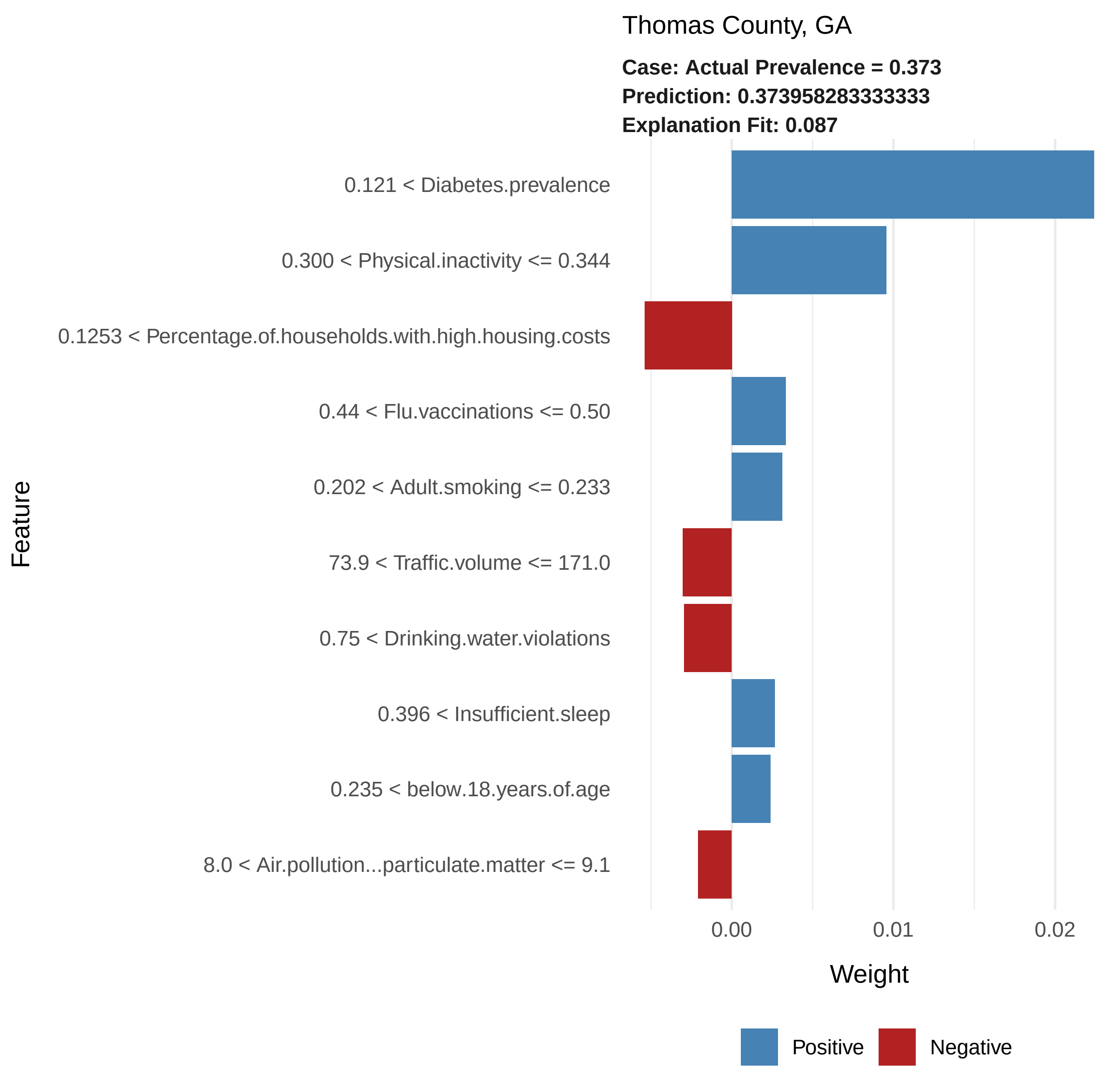

Thomas County, GA
Case: Actual Prevalence = 0.373
Prediction: 0.373958283333333
Explanation Fit: 0.087
0.121 < Diabetes.prevalence
0.300 < Physical.inactivity <= 0.344
0.1253 < Percentage.of.households.with.high.housing.costs
0.44 < Flu.vaccinations <= 0.50
0.202 < Adult.smoking <= 0.233
73.9 < Traffic.volume <= 171.0
0.75 < Drinking.water.violations
0.396 < Insufficient.sleep
0.235 < below.18.years.of.age
8.0 < Air.pollution...particulate.matter <= 9.1
Feature
0.00
0.01
0.02
Weight
Positive
Negative

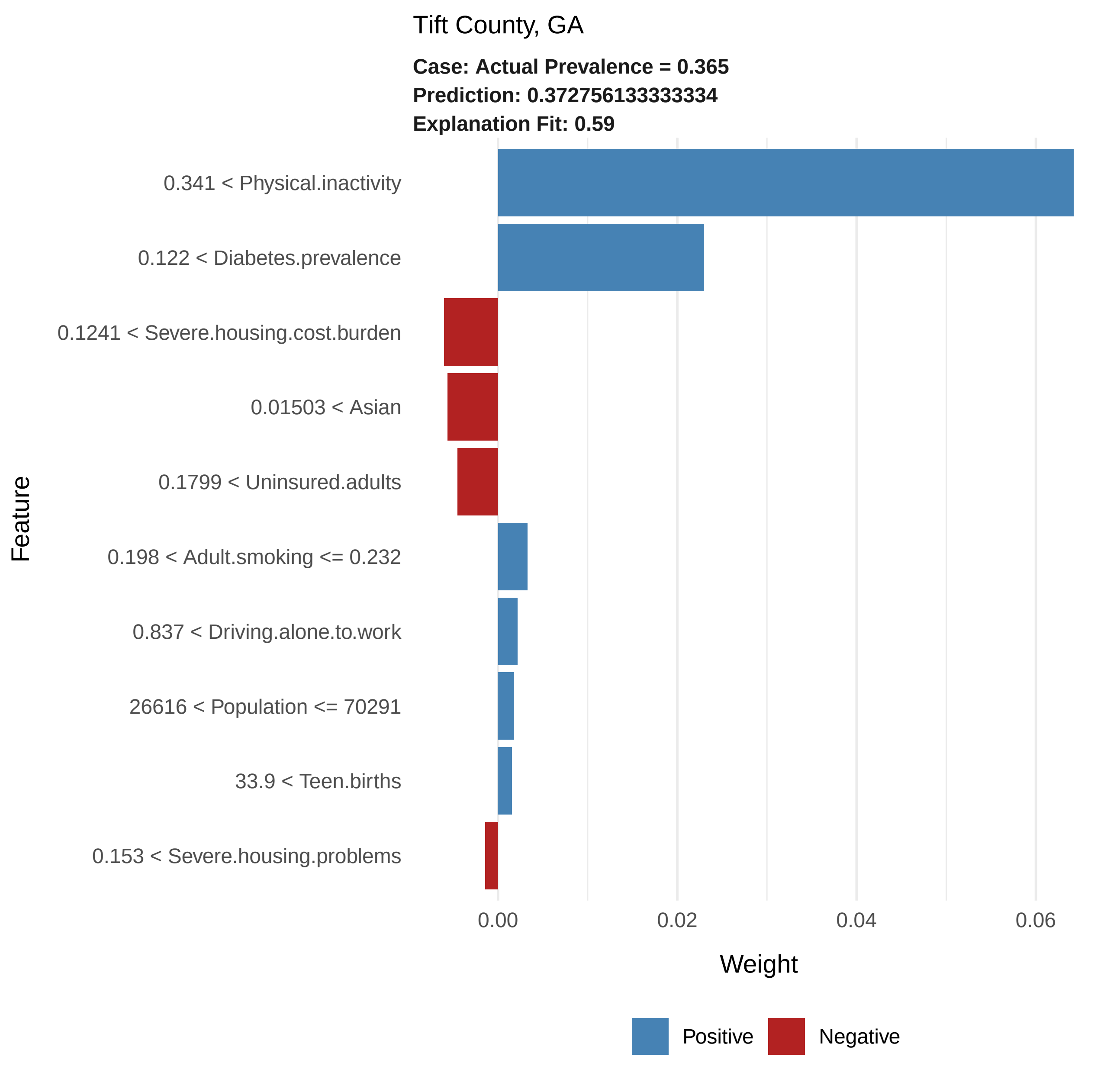

Tift County, GA
Case: Actual Prevalence = 0.365
Prediction: 0.372756133333334
Explanation Fit: 0.59
0.341 < Physical.inactivity
0.122 < Diabetes.prevalence
0.1241 < Severe.housing.cost.burden
0.01503 < Asian
0.1799 < Uninsured.adults
0.198 < Adult.smoking <= 0.232
0.837 < Driving.alone.to.work
26616 < Population <= 70291
33.9 < Teen.births
0.153 < Severe.housing.problems
Feature
0.00
0.02
0.04
0.06
Weight
Positive
Negative

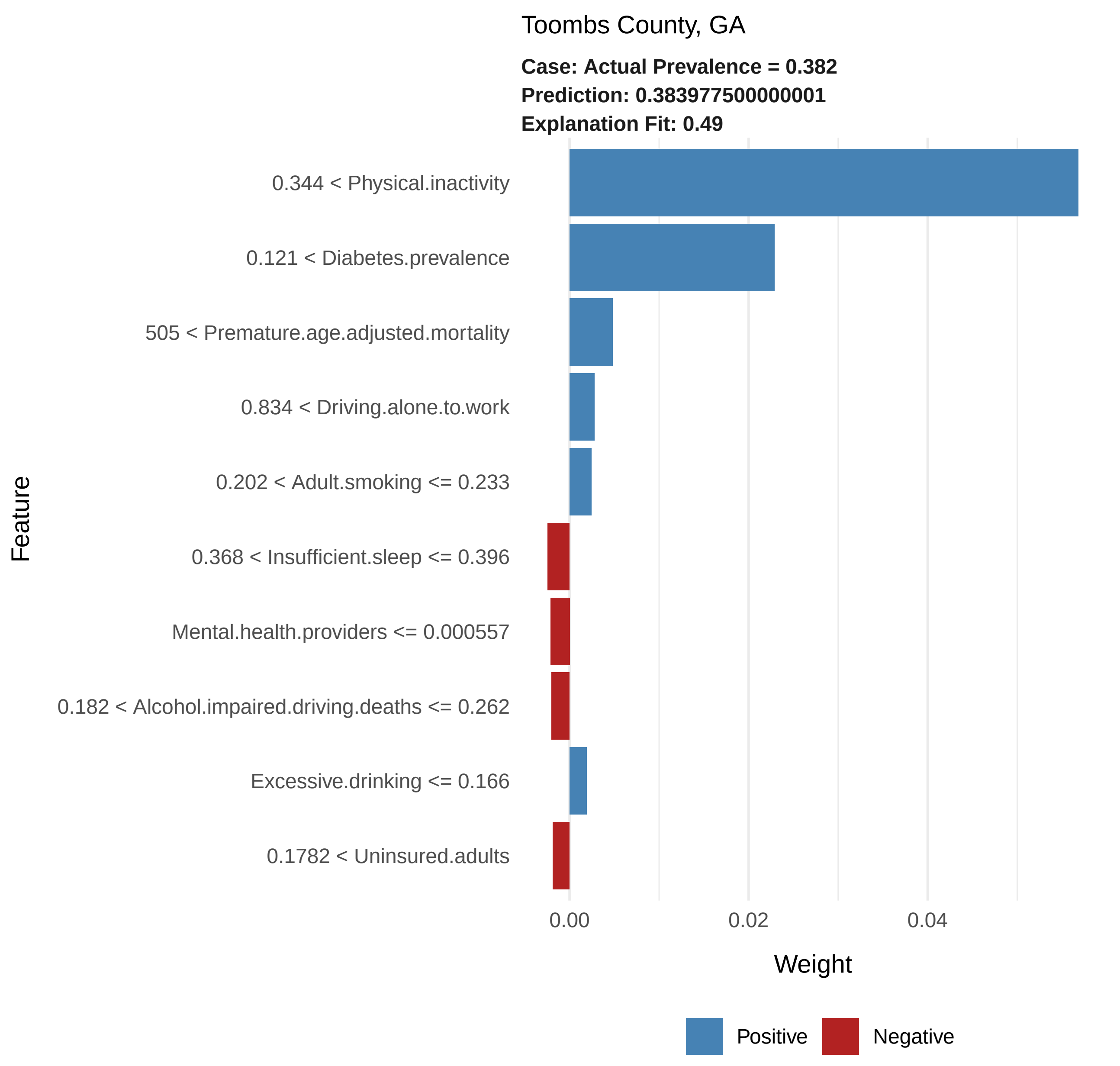
Toombs County, GA
Case: Actual Prevalence = 0.382
Prediction: 0.383977500000001
Explanation Fit: 0.49
0.344 < Physical.inactivity
0.121 < Diabetes.prevalence
505 < Premature.age.adjusted.mortality
0.834 < Driving.alone.to.work
0.202 < Adult.smoking <= 0.233
0.368 < Insufficient.sleep <= 0.396
Mental.health.providers <= 0.000557
0.182 < Alcohol.impaired.driving.deaths <= 0.262
Excessive.drinking <= 0.166
0.1782 < Uninsured.adults
Feature
0.00
0.02
0.04
Weight
Positive
Negative

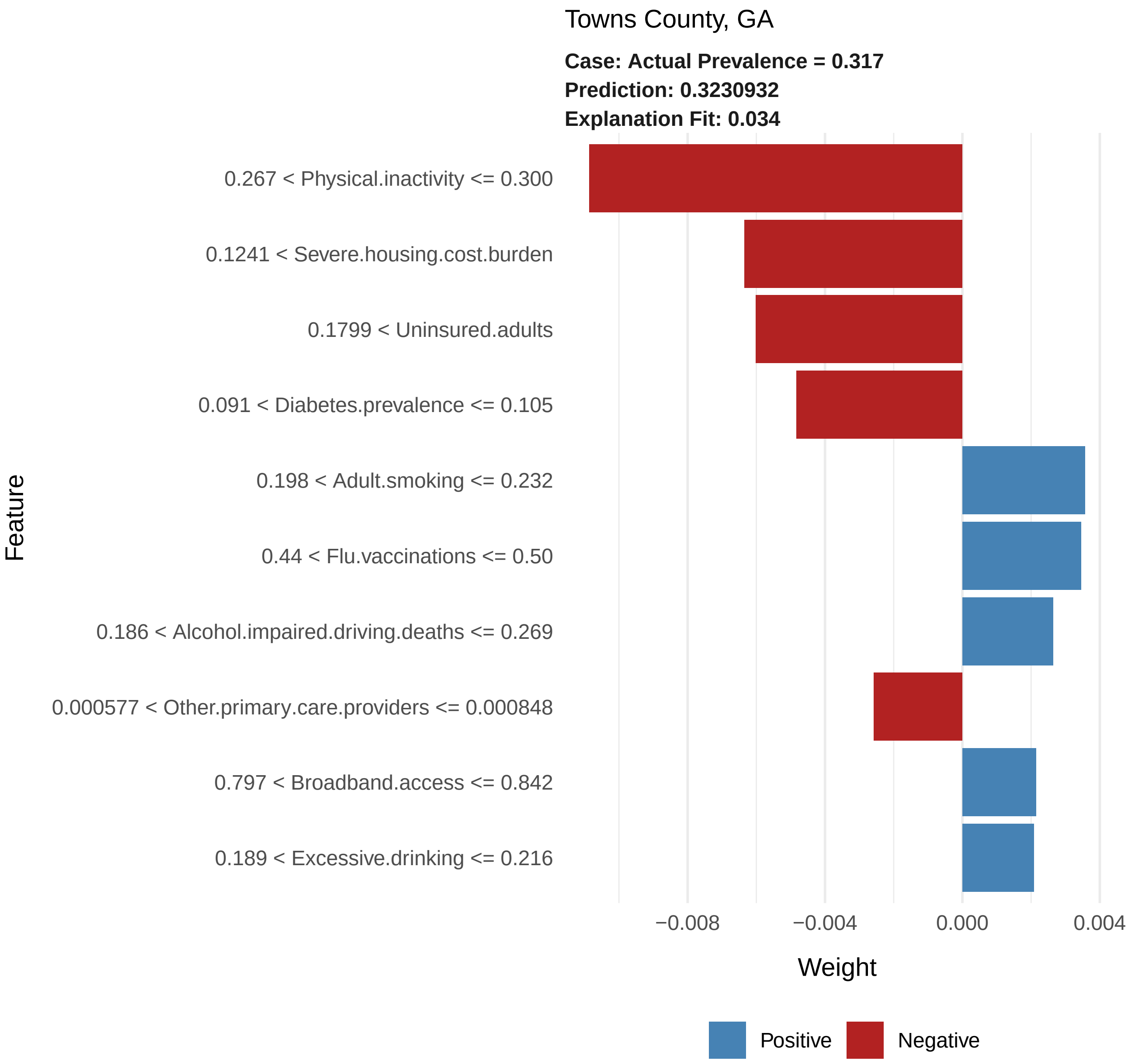

Towns County, GA
Case: Actual Prevalence = 0.317
Prediction: 0.3230932
Explanation Fit: 0.034
0.267 < Physical.inactivity <= 0.300
0.1241 < Severe.housing.cost.burden
0.1799 < Uninsured.adults
0.091 < Diabetes.prevalence <= 0.105
0.198 < Adult.smoking <= 0.232
0.44 < Flu.vaccinations <= 0.50
0.186 < Alcohol.impaired.driving.deaths <= 0.269
0.000577 < Other.primary.care.providers <= 0.000848
0.797 < Broadband.access <= 0.842
0.189 < Excessive.drinking <= 0.216
Feature
−0.008
−0.004
0.000
0.004
Weight
Positive
Negative

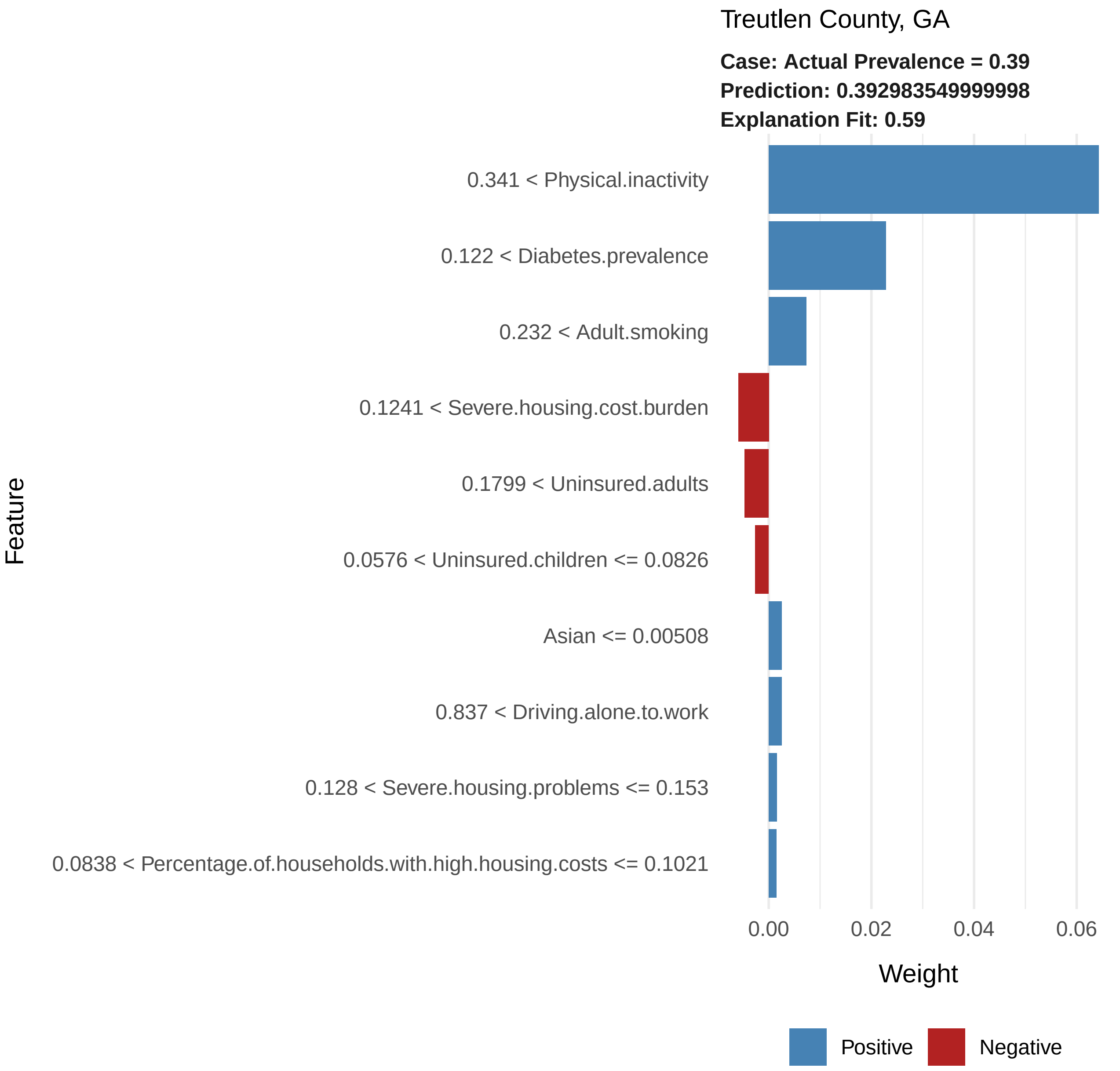
Treutlen County, GA
Case: Actual Prevalence = 0.39
Prediction: 0.392983549999998
Explanation Fit: 0.59
0.341 < Physical.inactivity
0.122 < Diabetes.prevalence
0.232 < Adult.smoking
0.1241 < Severe.housing.cost.burden
0.1799 < Uninsured.adults
0.0576 < Uninsured.children <= 0.0826
Asian <= 0.00508
0.837 < Driving.alone.to.work
0.128 < Severe.housing.problems <= 0.153
0.0838 < Percentage.of.households.with.high.housing.costs <= 0.1021
Feature
0.00
0.02
0.04
0.06
Weight
Positive
Negative

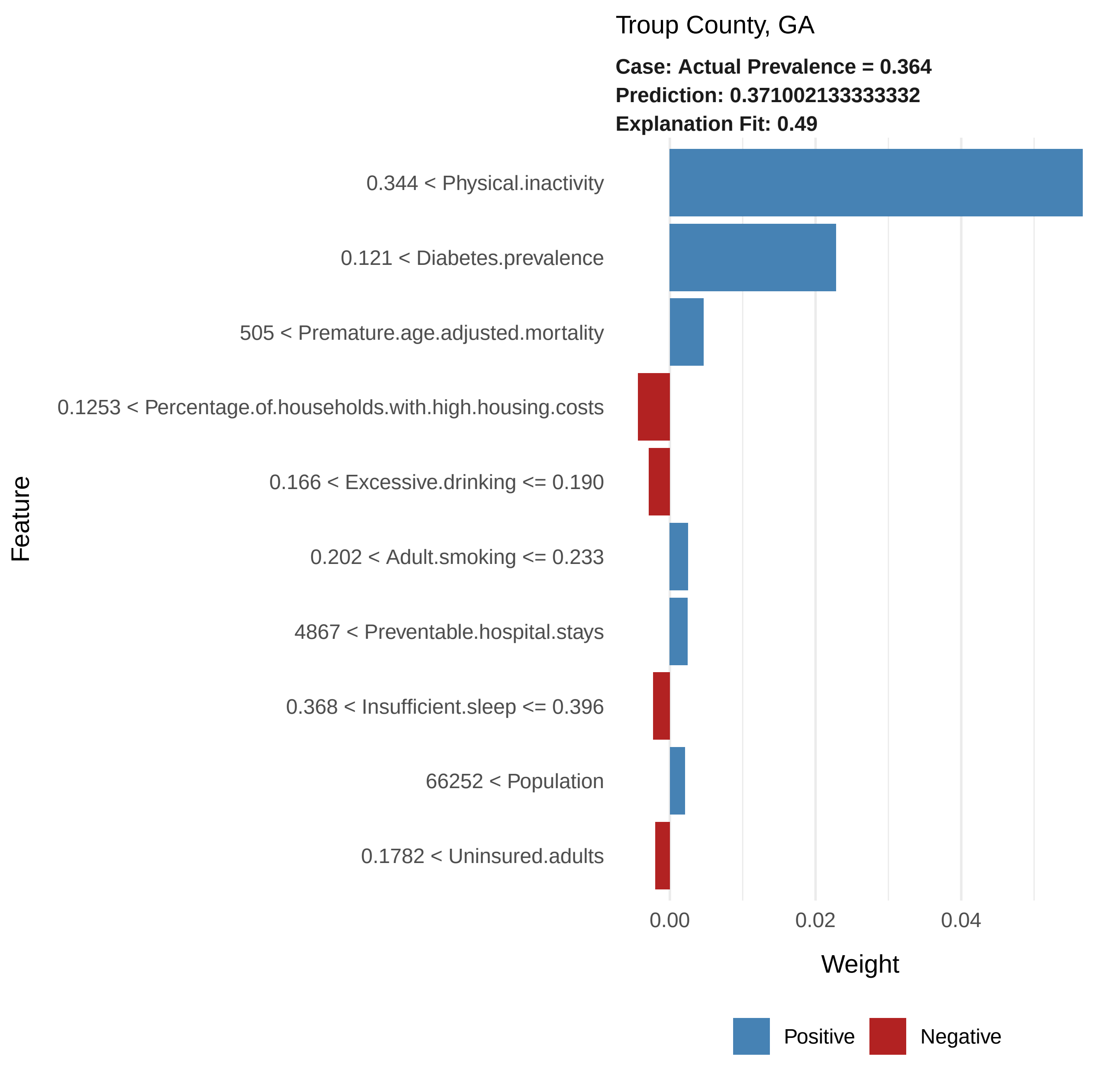
Troup County, GA
Case: Actual Prevalence = 0.364
Prediction: 0.371002133333332
Explanation Fit: 0.49
0.344 < Physical.inactivity
0.121 < Diabetes.prevalence
505 < Premature.age.adjusted.mortality
0.1253 < Percentage.of.households.with.high.housing.costs
0.166 < Excessive.drinking <= 0.190
0.202 < Adult.smoking <= 0.233
4867 < Preventable.hospital.stays
0.368 < Insufficient.sleep <= 0.396
66252 < Population
0.1782 < Uninsured.adults
Feature
0.00
0.02
0.04
Weight
Positive
Negative

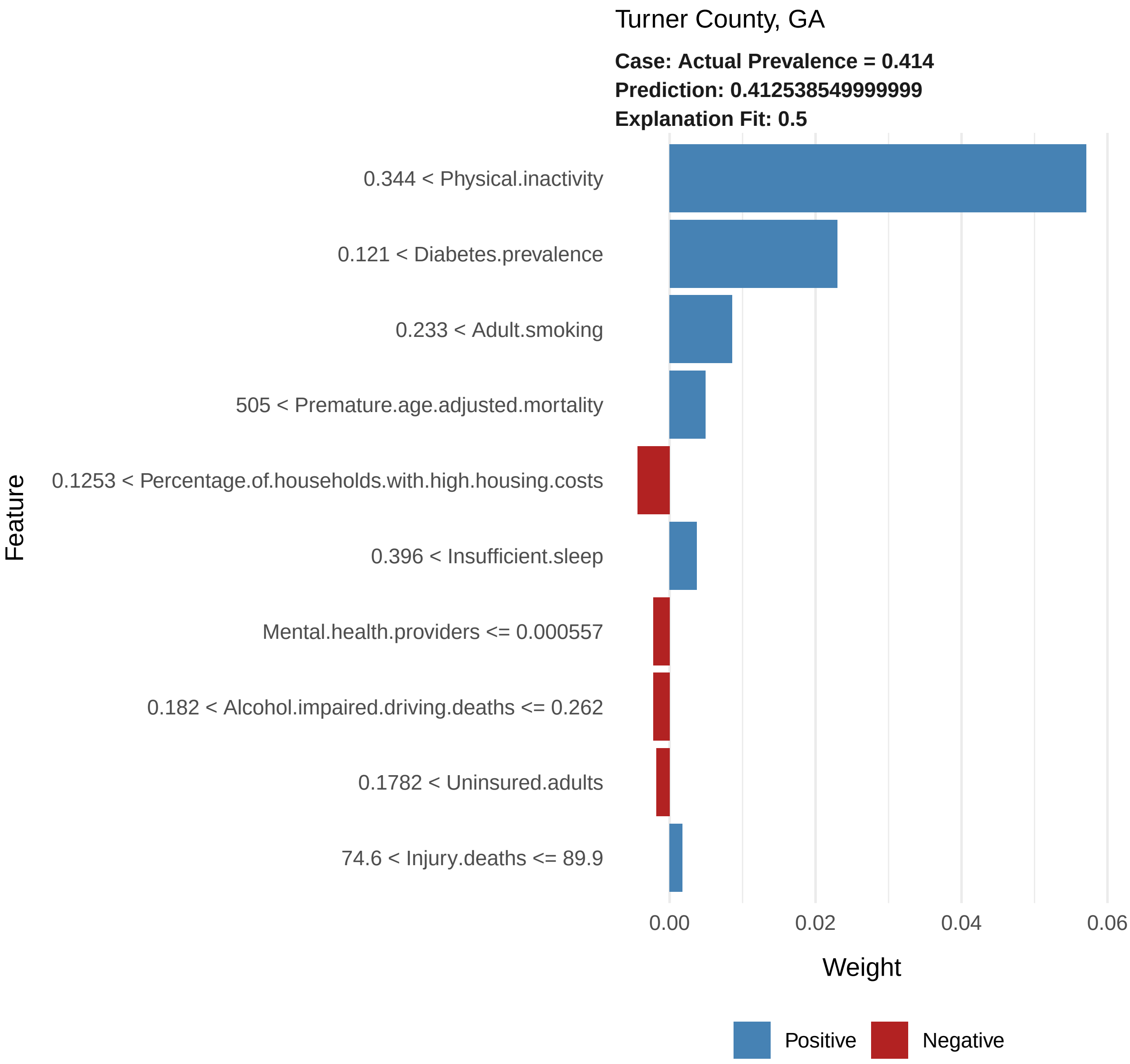

Turner County, GA
Case: Actual Prevalence = 0.414
Prediction: 0.412538549999999
Explanation Fit: 0.5
0.344 < Physical.inactivity
0.121 < Diabetes.prevalence
0.233 < Adult.smoking
505 < Premature.age.adjusted.mortality
0.1253 < Percentage.of.households.with.high.housing.costs
0.396 < Insufficient.sleep
Mental.health.providers <= 0.000557
0.182 < Alcohol.impaired.driving.deaths <= 0.262
0.1782 < Uninsured.adults
74.6 < Injury.deaths <= 89.9
Feature
0.00
0.02
0.04
0.06
Weight
Positive
Negative

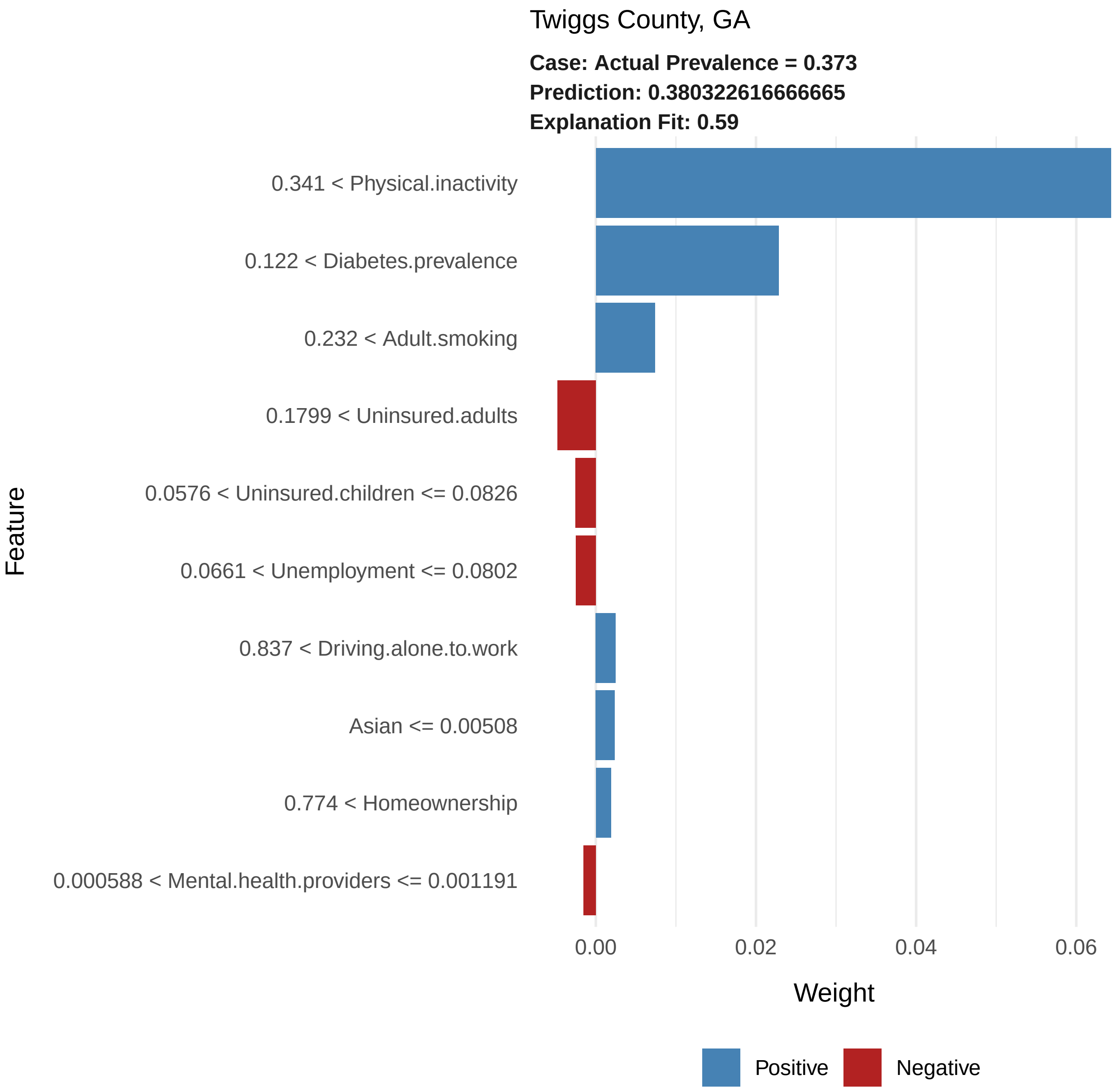
Twiggs County, GA
Case: Actual Prevalence = 0.373
Prediction: 0.380322616666665
Explanation Fit: 0.59
0.341 < Physical.inactivity
0.122 < Diabetes.prevalence
0.232 < Adult.smoking
0.1799 < Uninsured.adults
0.0576 < Uninsured.children <= 0.0826
0.0661 < Unemployment <= 0.0802
0.837 < Driving.alone.to.work
Asian <= 0.00508
0.774 < Homeownership
0.000588 < Mental.health.providers <= 0.001191
Feature
0.00
0.02
0.04
0.06
Weight
Positive
Negative

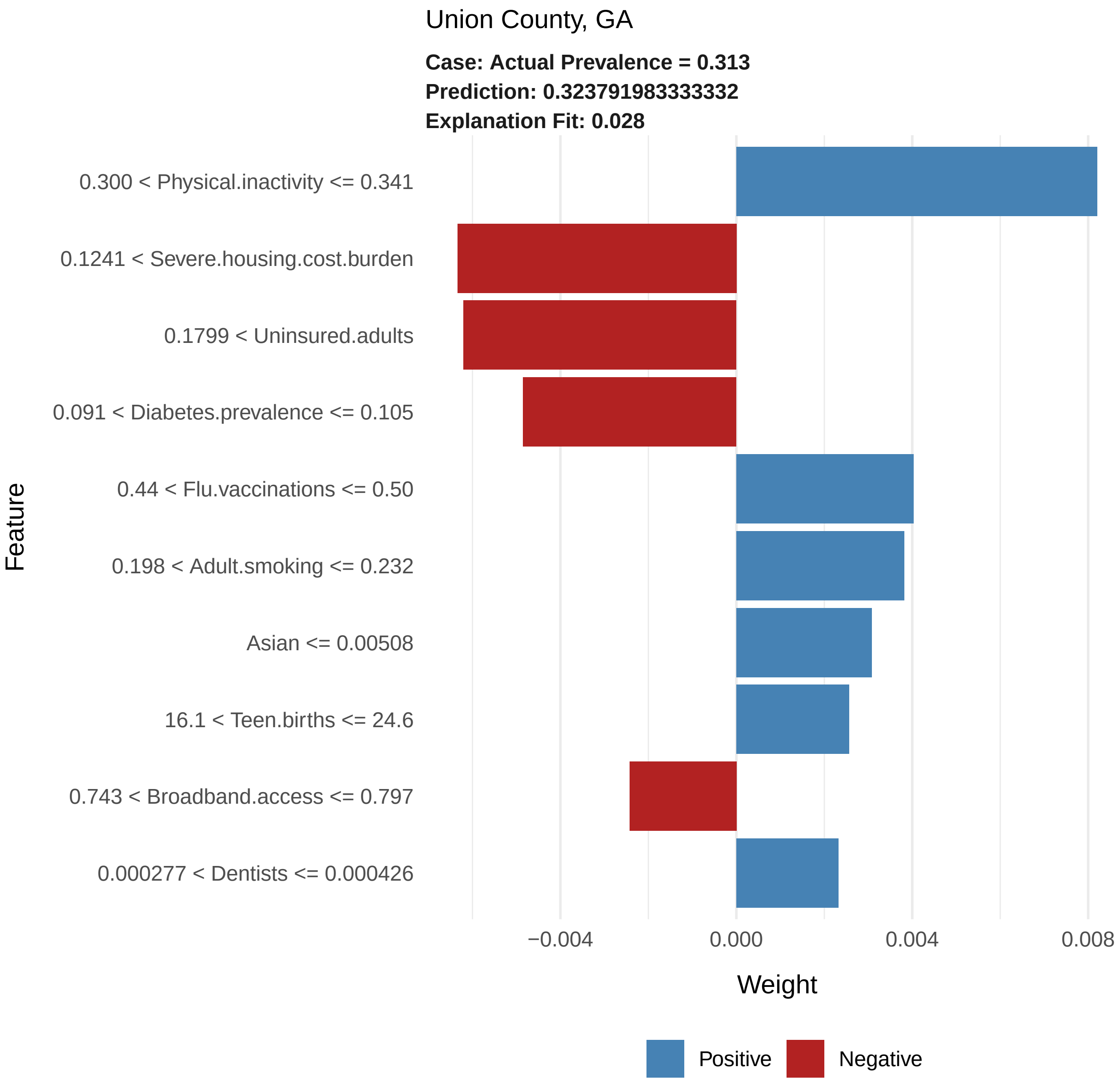
Union County, GA
Case: Actual Prevalence = 0.313
Prediction: 0.323791983333332
Explanation Fit: 0.028
0.300 < Physical.inactivity <= 0.341
0.1241 < Severe.housing.cost.burden
0.1799 < Uninsured.adults
0.091 < Diabetes.prevalence <= 0.105
0.44 < Flu.vaccinations <= 0.50
0.198 < Adult.smoking <= 0.232
Asian <= 0.00508
16.1 < Teen.births <= 24.6
0.743 < Broadband.access <= 0.797
0.000277 < Dentists <= 0.000426
Feature
−0.004
0.000
0.004
0.008
Weight
Positive
Negative

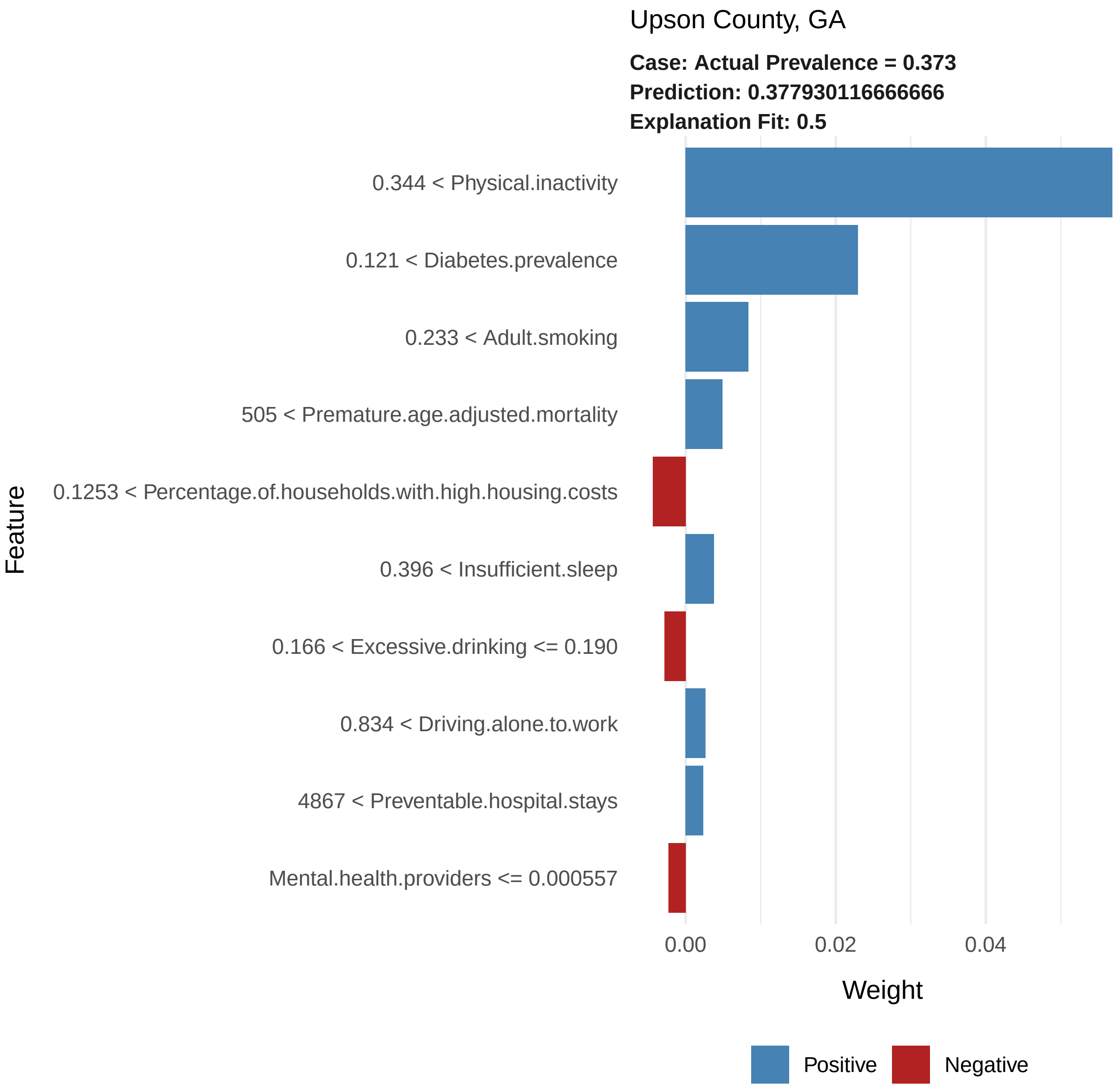

Upson County, GA
Case: Actual Prevalence = 0.373
Prediction: 0.377930116666666
Explanation Fit: 0.5
0.344 < Physical.inactivity
0.121 < Diabetes.prevalence
0.233 < Adult.smoking
505 < Premature.age.adjusted.mortality
0.1253 < Percentage.of.households.with.high.housing.costs
0.396 < Insufficient.sleep
0.166 < Excessive.drinking <= 0.190
0.834 < Driving.alone.to.work
4867 < Preventable.hospital.stays
Mental.health.providers <= 0.000557
Feature
0.00
0.02
0.04
Weight
Positive
Negative

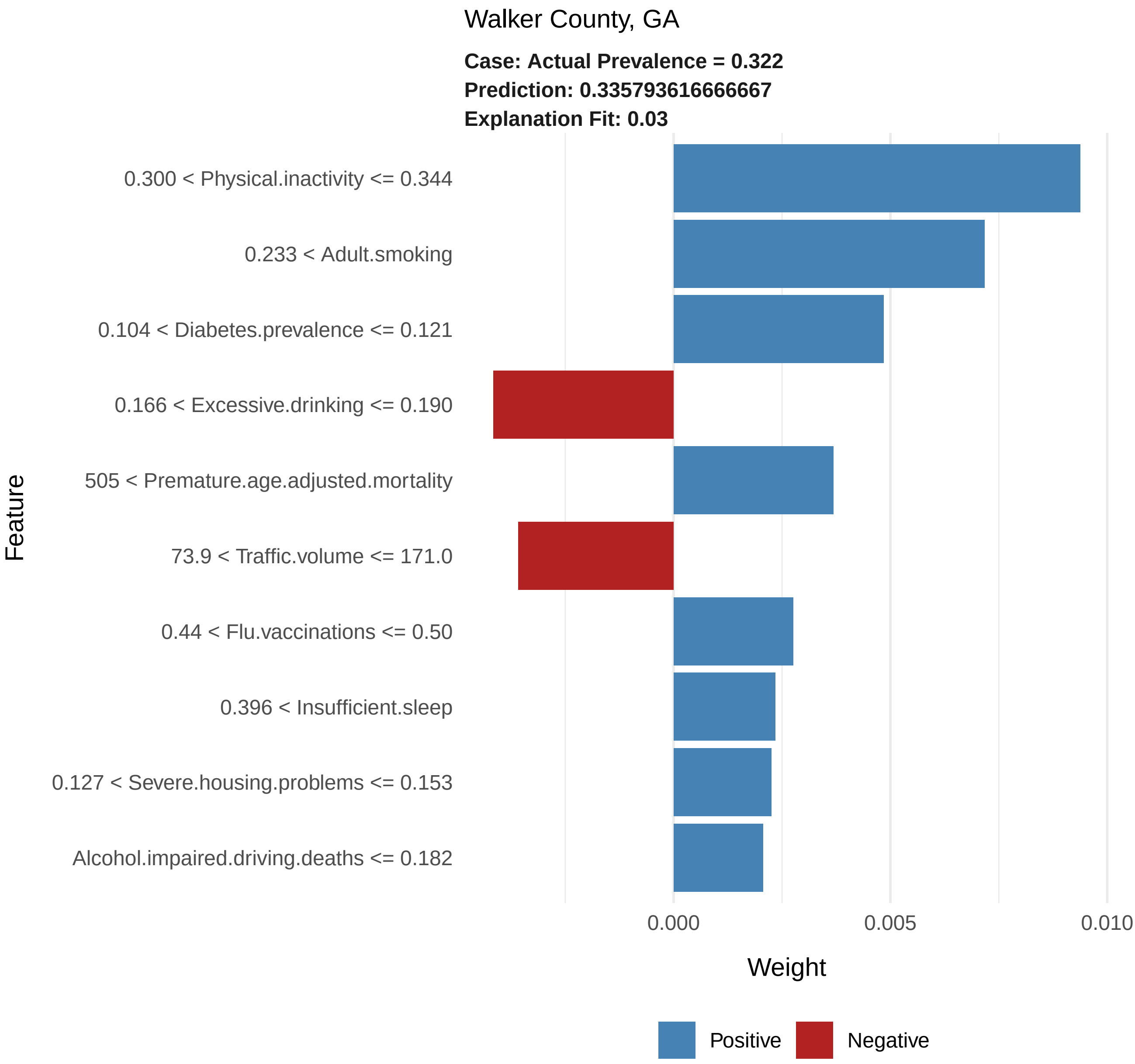

Walker County, GA
Case: Actual Prevalence = 0.322
Prediction: 0.335793616666667
Explanation Fit: 0.03
0.300 < Physical.inactivity <= 0.344
0.233 < Adult.smoking
0.104 < Diabetes.prevalence <= 0.121
0.166 < Excessive.drinking <= 0.190
505 < Premature.age.adjusted.mortality
73.9 < Traffic.volume <= 171.0
0.44 < Flu.vaccinations <= 0.50
0.396 < Insufficient.sleep
0.127 < Severe.housing.problems <= 0.153
Alcohol.impaired.driving.deaths <= 0.182
Feature
0.000
0.005
0.010
Weight
Positive
Negative

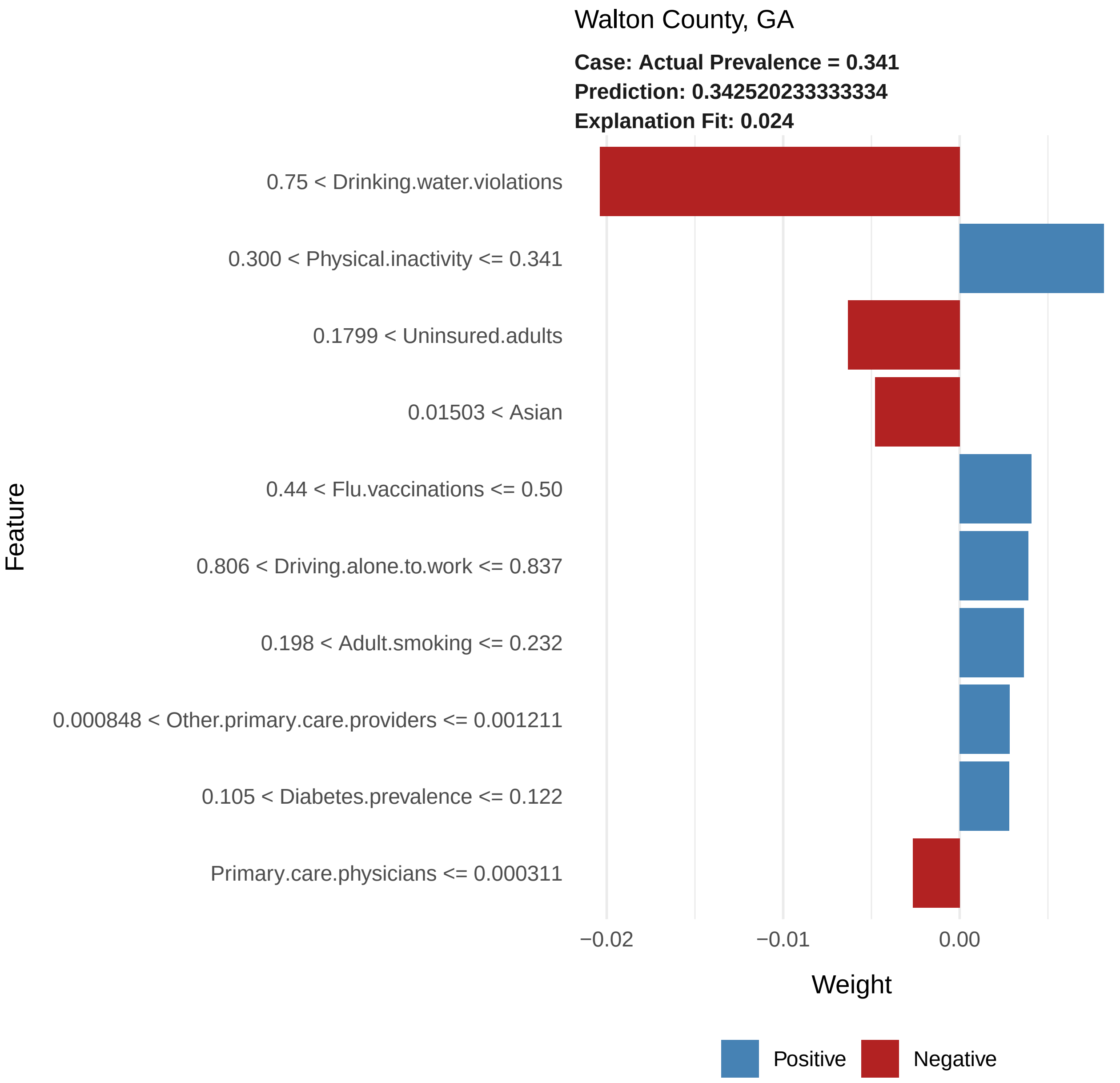

Walton County, GA
Case: Actual Prevalence = 0.341
Prediction: 0.342520233333334
Explanation Fit: 0.024
0.75 < Drinking.water.violations
0.300 < Physical.inactivity <= 0.341
0.1799 < Uninsured.adults
0.01503 < Asian
0.44 < Flu.vaccinations <= 0.50
0.806 < Driving.alone.to.work <= 0.837
0.198 < Adult.smoking <= 0.232
0.000848 < Other.primary.care.providers <= 0.001211
0.105 < Diabetes.prevalence <= 0.122
Primary.care.physicians <= 0.000311
Feature
−0.02
−0.01
0.00
Weight
Positive
Negative

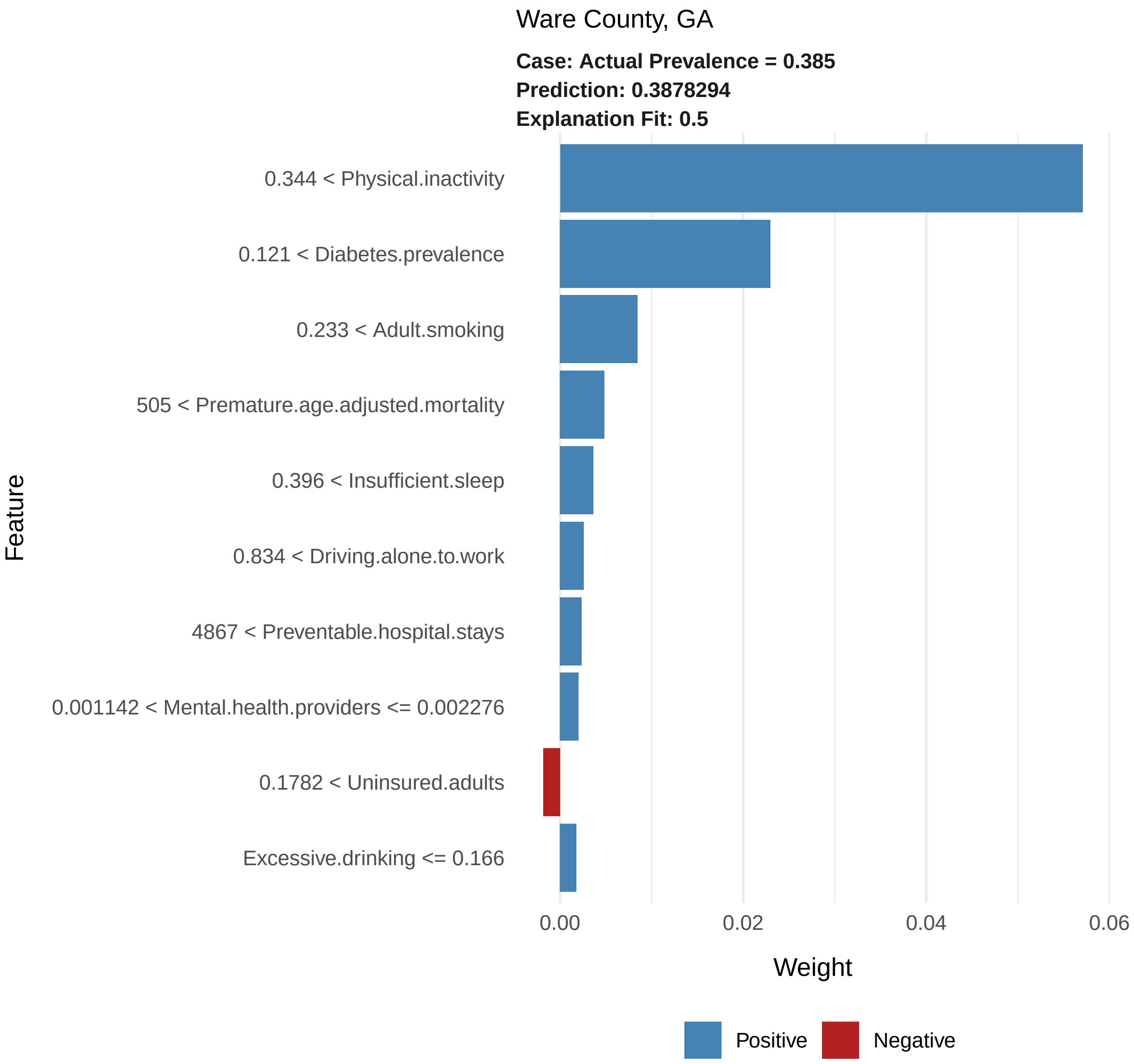

Ware County, GA
Case: Actual Prevalence = 0.385
Prediction: 0.3878294
Explanation Fit: 0.5
0.344 < Physical.inactivity
0.121 < Diabetes.prevalence
0.233 < Adult.smoking
505 < Premature.age.adjusted.mortality
0.396 < Insufficient.sleep
0.834 < Driving.alone.to.work
4867 < Preventable.hospital.stays
0.001142 < Mental.health.providers <= 0.002276
0.1782 < Uninsured.adults
Excessive.drinking <= 0.166
Feature
0.00
0.02
0.04
0.06
Weight
Positive
Negative

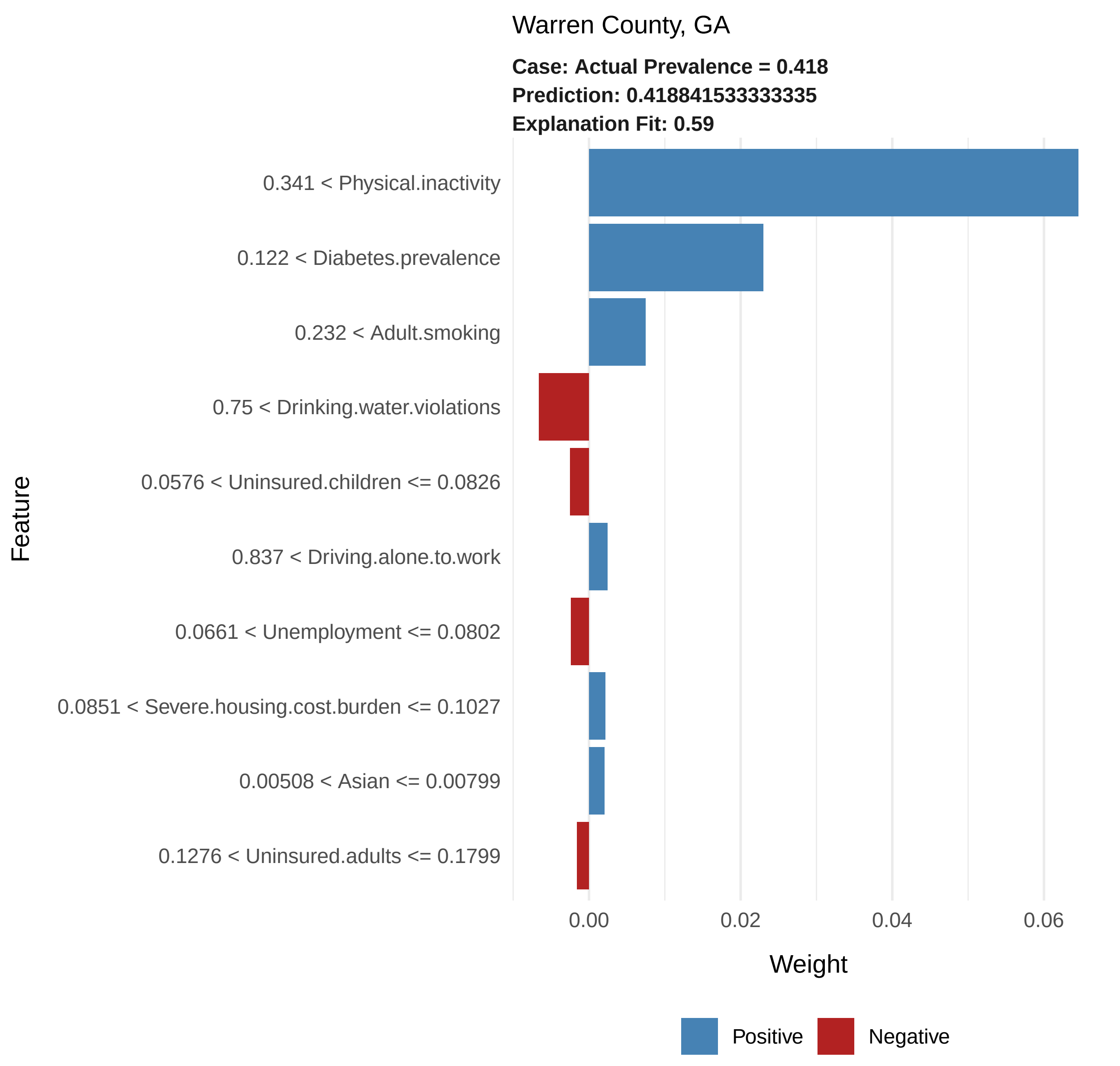

Warren County, GA
Case: Actual Prevalence = 0.418
Prediction: 0.418841533333335
Explanation Fit: 0.59
0.341 < Physical.inactivity
0.122 < Diabetes.prevalence
0.232 < Adult.smoking
0.75 < Drinking.water.violations
0.0576 < Uninsured.children <= 0.0826
0.837 < Driving.alone.to.work
0.0661 < Unemployment <= 0.0802
0.0851 < Severe.housing.cost.burden <= 0.1027
0.00508 < Asian <= 0.00799
0.1276 < Uninsured.adults <= 0.1799
Feature
0.00
0.02
0.04
0.06
Weight
Positive
Negative

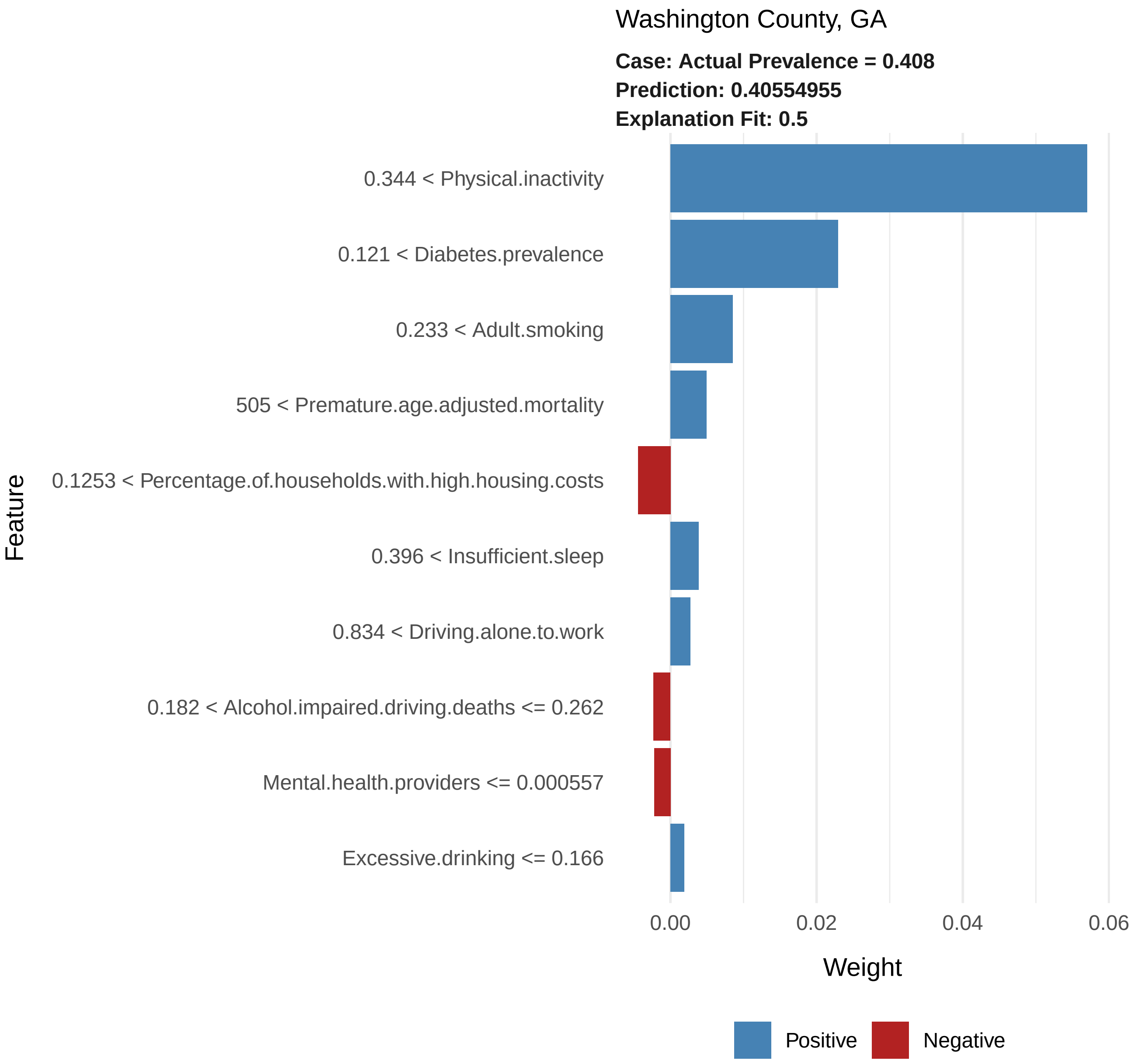
Washington County, GA
Case: Actual Prevalence = 0.408
Prediction: 0.40554955
Explanation Fit: 0.5
0.344 < Physical.inactivity
0.121 < Diabetes.prevalence
0.233 < Adult.smoking
505 < Premature.age.adjusted.mortality
0.1253 < Percentage.of.households.with.high.housing.costs
0.396 < Insufficient.sleep
0.834 < Driving.alone.to.work
0.182 < Alcohol.impaired.driving.deaths <= 0.262
Mental.health.providers <= 0.000557
Excessive.drinking <= 0.166
Feature
0.00
0.02
0.04
0.06
Weight
Positive
Negative

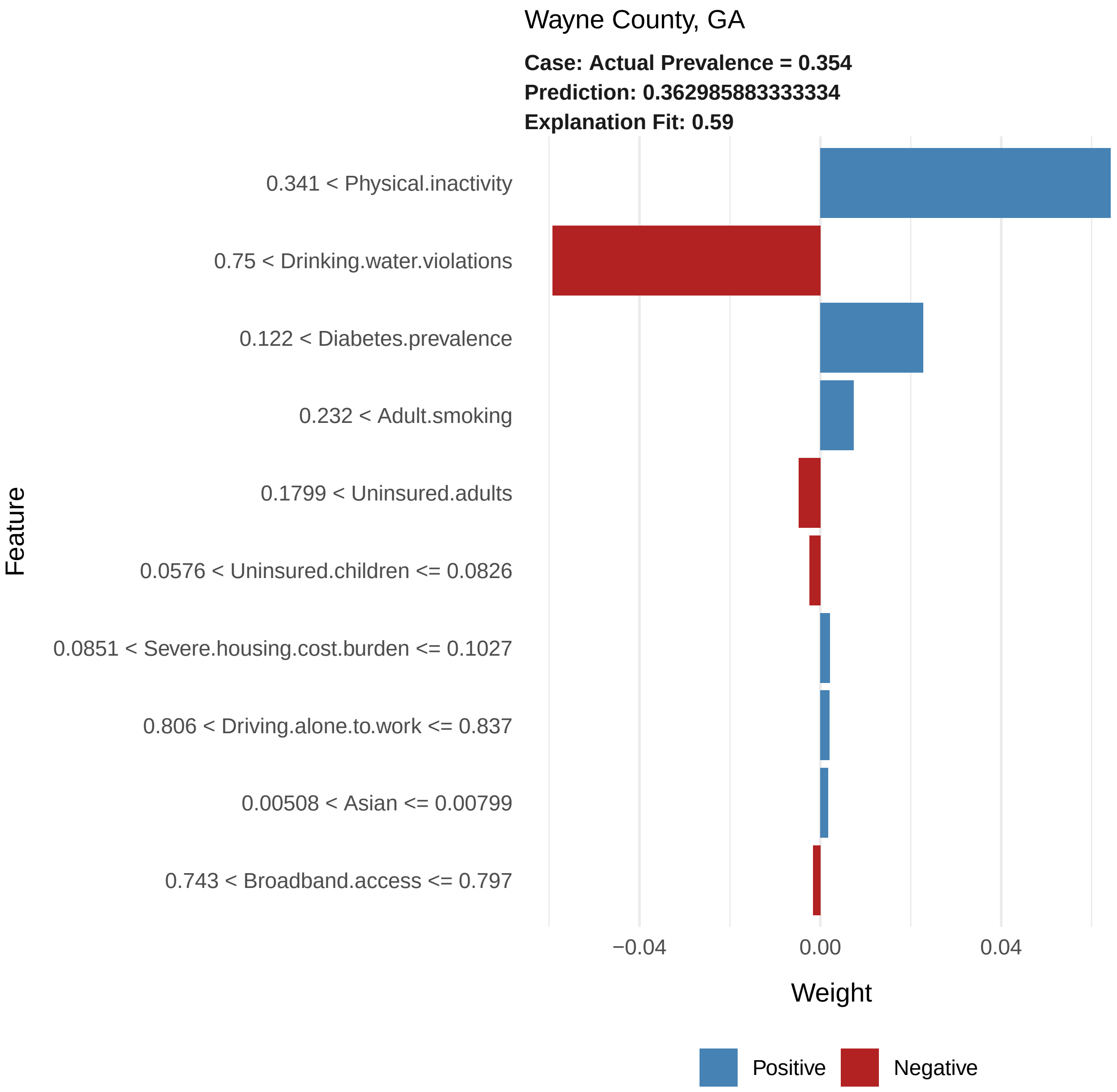

Wayne County, GA
Case: Actual Prevalence = 0.354
Prediction: 0.362985883333334
Explanation Fit: 0.59
0.341 < Physical.inactivity
0.75 < Drinking.water.violations
0.122 < Diabetes.prevalence
0.232 < Adult.smoking
0.1799 < Uninsured.adults
0.0576 < Uninsured.children <= 0.0826
0.0851 < Severe.housing.cost.burden <= 0.1027
0.806 < Driving.alone.to.work <= 0.837
0.00508 < Asian <= 0.00799
0.743 < Broadband.access <= 0.797
Feature
−0.04
0.00
0.04
Weight
Positive
Negative

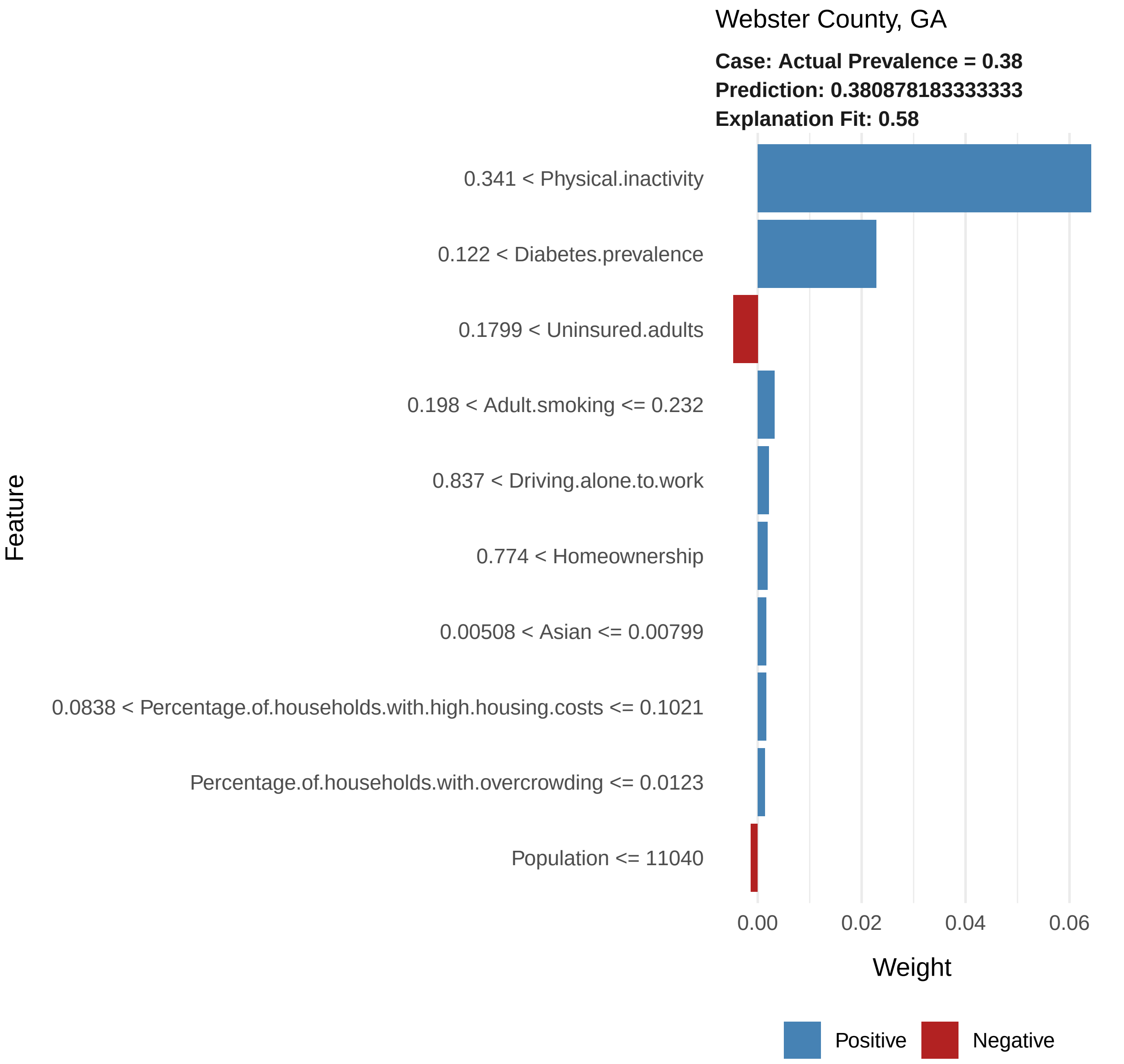

Webster County, GA
Case: Actual Prevalence = 0.38
Prediction: 0.380878183333333
Explanation Fit: 0.58
0.341 < Physical.inactivity
0.122 < Diabetes.prevalence
0.1799 < Uninsured.adults
0.198 < Adult.smoking <= 0.232
0.837 < Driving.alone.to.work
0.774 < Homeownership
0.00508 < Asian <= 0.00799
0.0838 < Percentage.of.households.with.high.housing.costs <= 0.1021
Percentage.of.households.with.overcrowding <= 0.0123
Population <= 11040
Feature
0.00
0.02
0.04
0.06
Weight
Positive
Negative

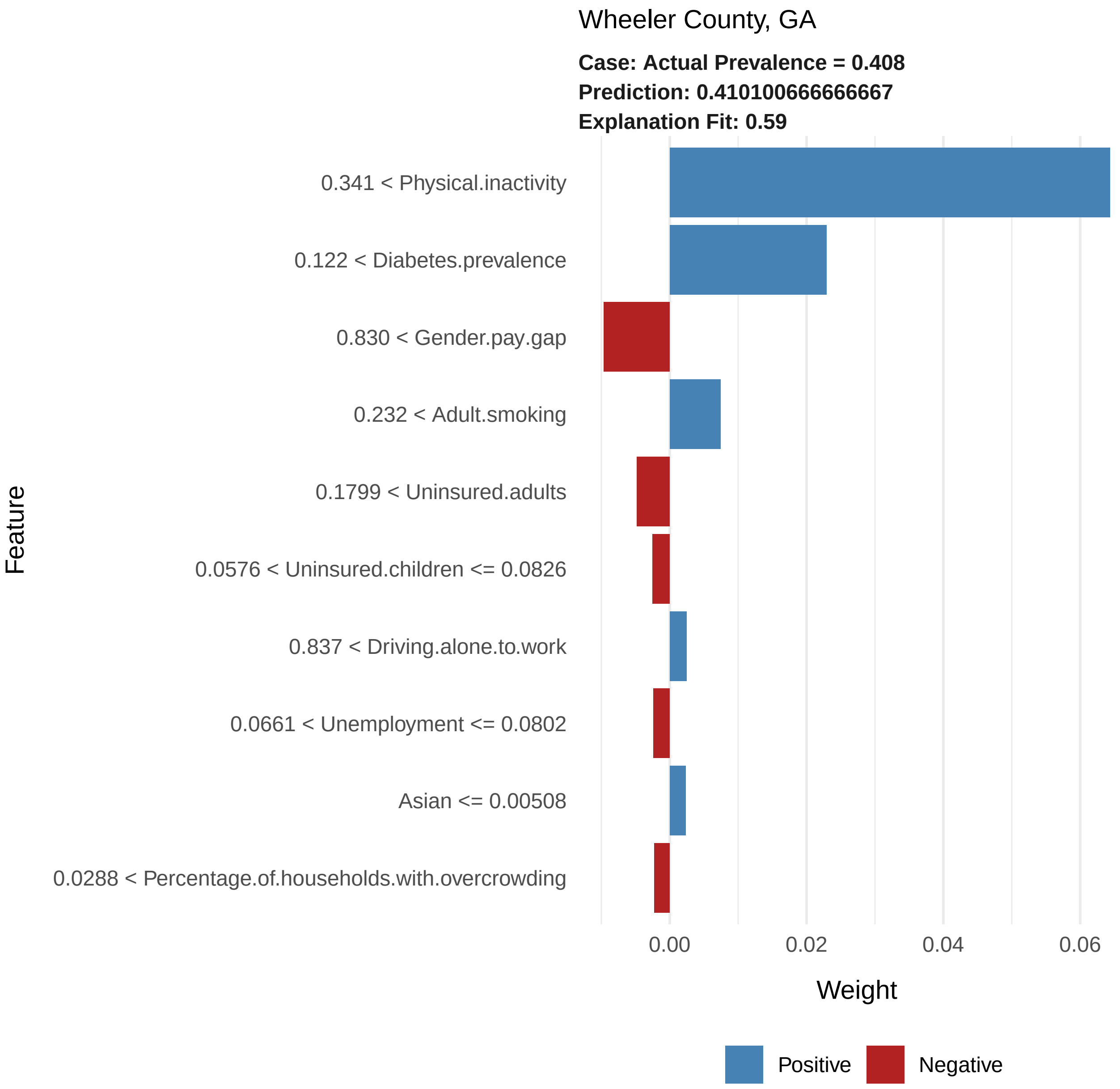

Wheeler County, GA
Case: Actual Prevalence = 0.408
Prediction: 0.410100666666667
Explanation Fit: 0.59
0.341 < Physical.inactivity
0.122 < Diabetes.prevalence
0.830 < Gender.pay.gap
0.232 < Adult.smoking
0.1799 < Uninsured.adults
0.0576 < Uninsured.children <= 0.0826
0.837 < Driving.alone.to.work
0.0661 < Unemployment <= 0.0802
Asian <= 0.00508
0.0288 < Percentage.of.households.with.overcrowding
Feature
0.00
0.02
0.04
0.06
Weight
Positive
Negative

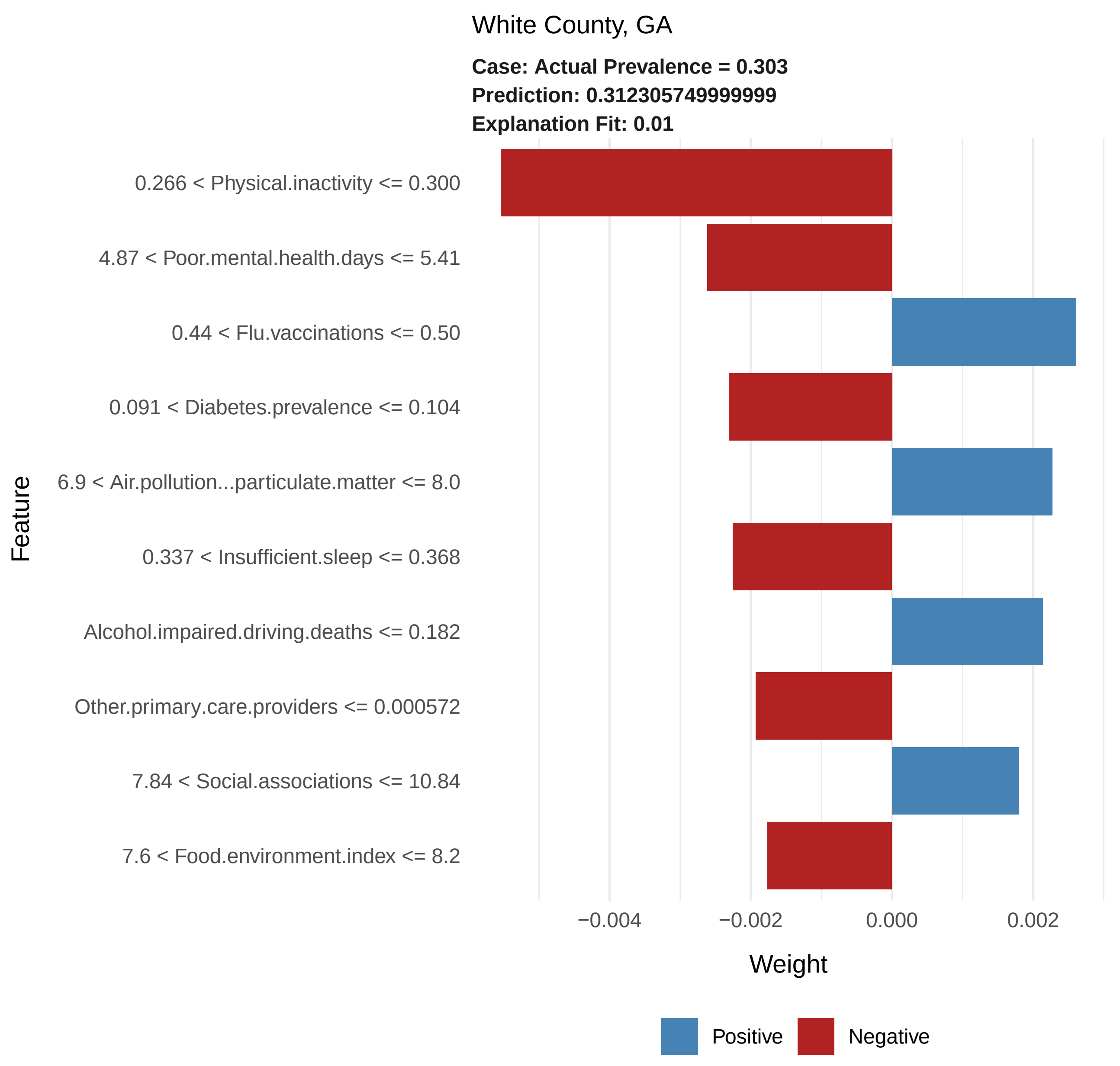

White County, GA
Case: Actual Prevalence = 0.303
Prediction: 0.312305749999999
Explanation Fit: 0.01
0.266 < Physical.inactivity <= 0.300
4.87 < Poor.mental.health.days <= 5.41
0.44 < Flu.vaccinations <= 0.50
0.091 < Diabetes.prevalence <= 0.104
6.9 < Air.pollution...particulate.matter <= 8.0
0.337 < Insufficient.sleep <= 0.368
Alcohol.impaired.driving.deaths <= 0.182
Other.primary.care.providers <= 0.000572
7.84 < Social.associations <= 10.84
7.6 < Food.environment.index <= 8.2
Feature
−0.004
−0.002
0.000
0.002
Weight
Positive
Negative

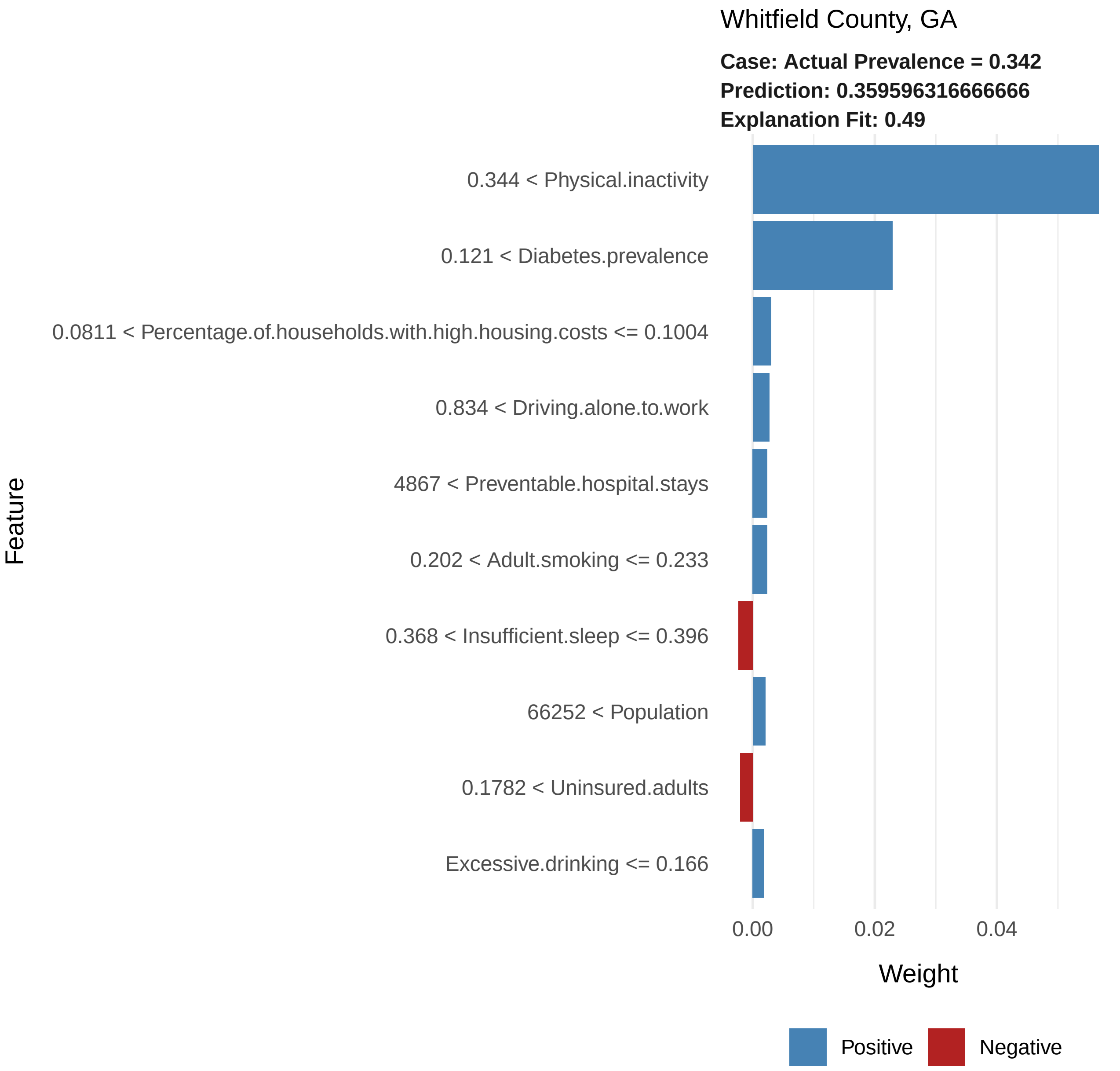

Whitfield County, GA
Case: Actual Prevalence = 0.342
Prediction: 0.359596316666666
Explanation Fit: 0.49
0.344 < Physical.inactivity
0.121 < Diabetes.prevalence
0.0811 < Percentage.of.households.with.high.housing.costs <= 0.1004
0.834 < Driving.alone.to.work
4867 < Preventable.hospital.stays
0.202 < Adult.smoking <= 0.233
0.368 < Insufficient.sleep <= 0.396
66252 < Population
0.1782 < Uninsured.adults
Excessive.drinking <= 0.166
Feature
0.00
0.02
0.04
Weight
Positive
Negative

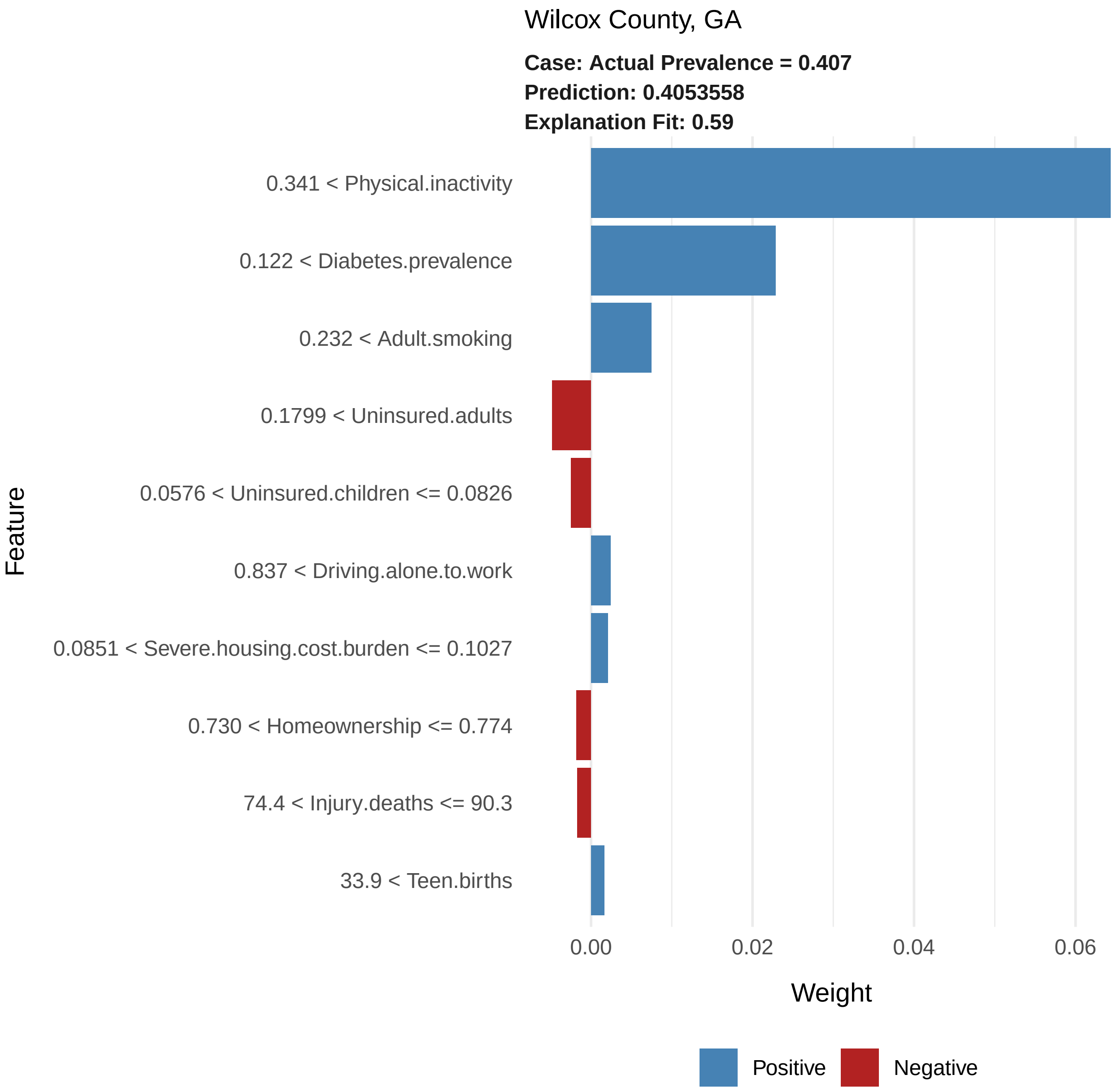
Wilcox County, GA
Case: Actual Prevalence = 0.407
Prediction: 0.4053558
Explanation Fit: 0.59
0.341 < Physical.inactivity
0.122 < Diabetes.prevalence
0.232 < Adult.smoking
0.1799 < Uninsured.adults
0.0576 < Uninsured.children <= 0.0826
0.837 < Driving.alone.to.work
0.0851 < Severe.housing.cost.burden <= 0.1027
0.730 < Homeownership <= 0.774
74.4 < Injury.deaths <= 90.3
33.9 < Teen.births
Feature
0.00
0.02
0.04
0.06
Weight
Positive
Negative

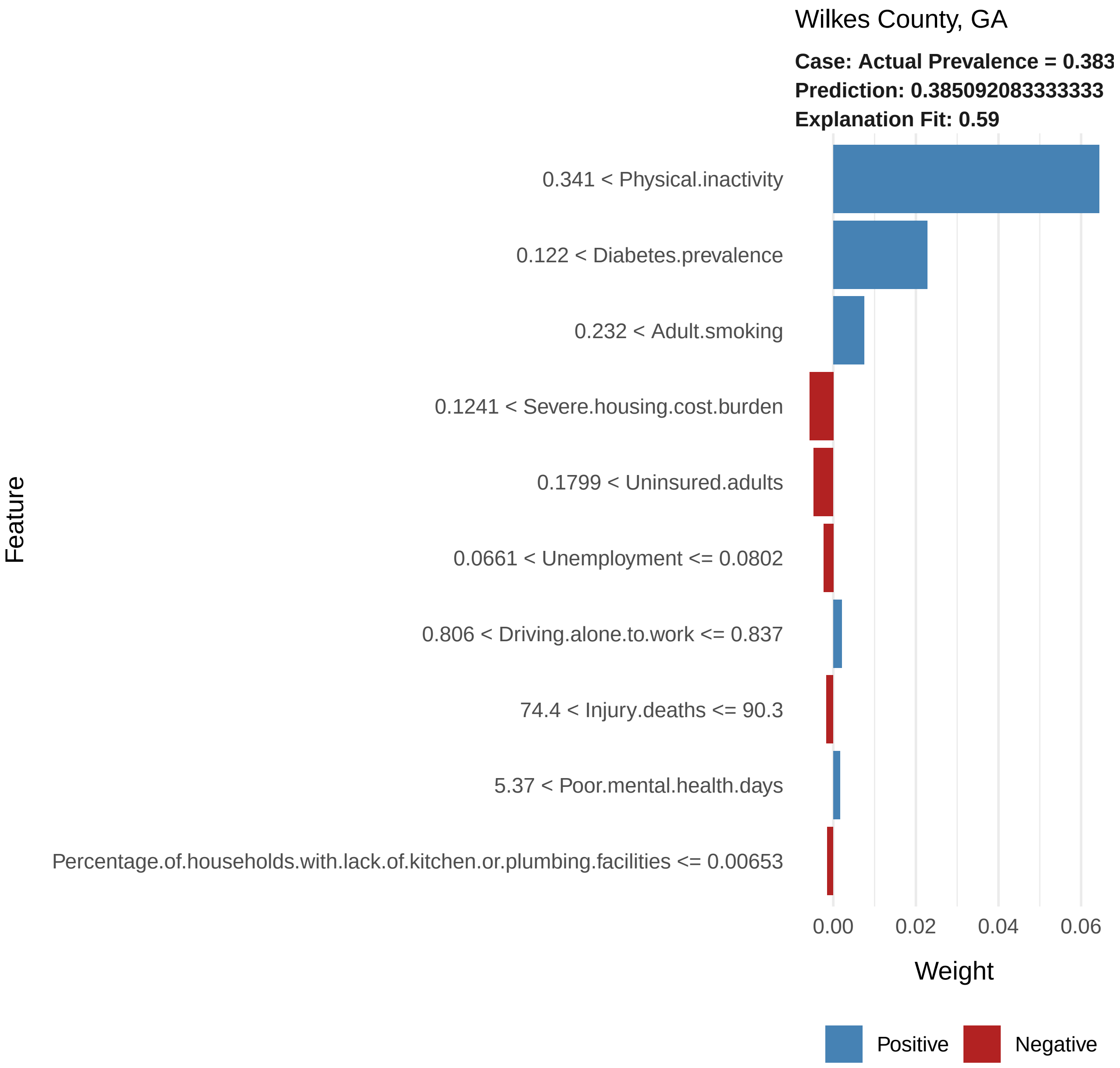

Wilkes County, GA
Case: Actual Prevalence = 0.383
Prediction: 0.385092083333333
Explanation Fit: 0.59
0.341 < Physical.inactivity
0.122 < Diabetes.prevalence
0.232 < Adult.smoking
0.1241 < Severe.housing.cost.burden
0.1799 < Uninsured.adults
0.0661 < Unemployment <= 0.0802
0.806 < Driving.alone.to.work <= 0.837
74.4 < Injury.deaths <= 90.3
5.37 < Poor.mental.health.days
Percentage.of.households.with.lack.of.kitchen.or.plumbing.facilities <= 0.00653
Feature
0.00
0.02
0.04
0.06
Weight
Positive
Negative

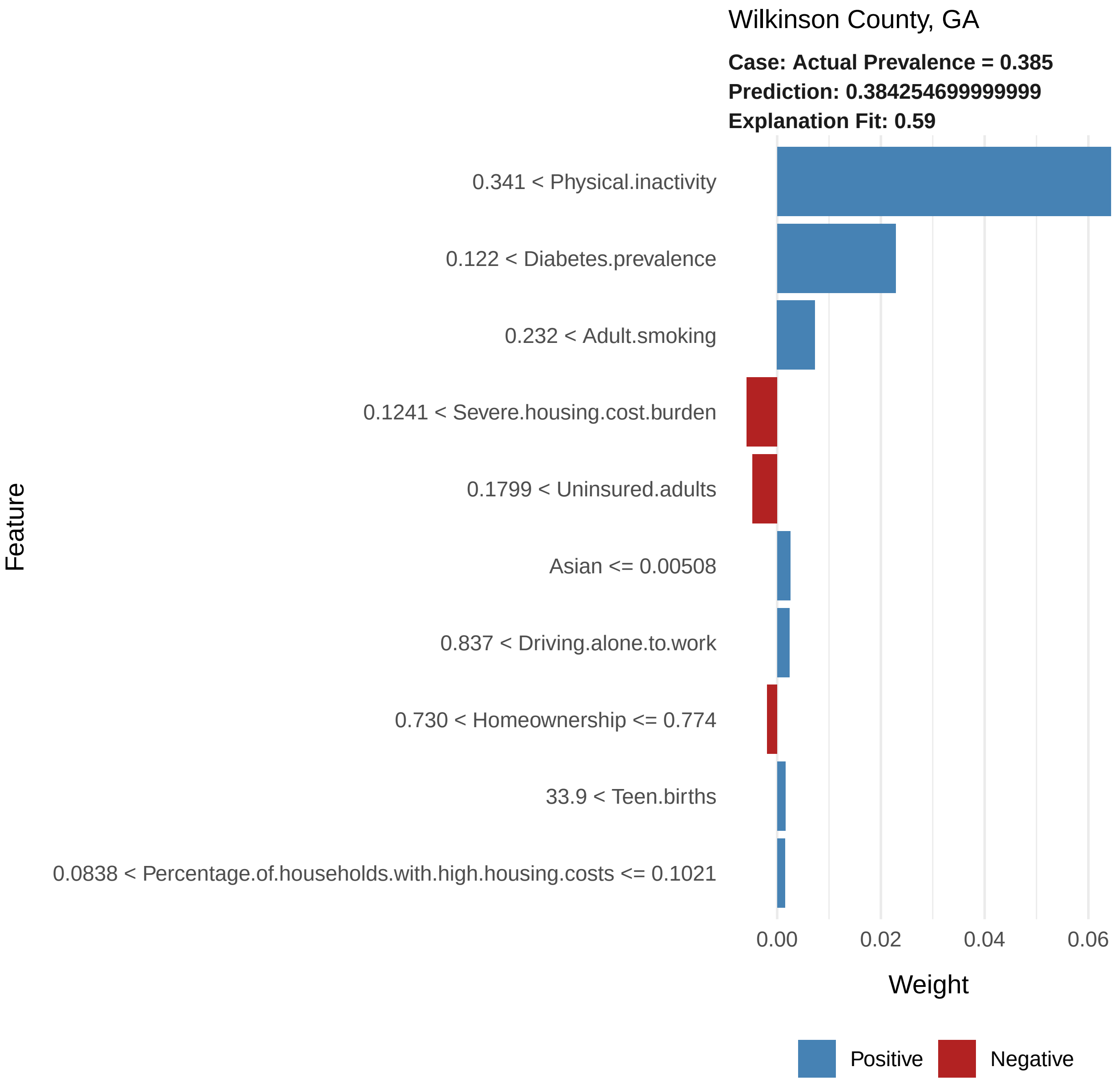

Wilkinson County, GA
Case: Actual Prevalence = 0.385
Prediction: 0.384254699999999
Explanation Fit: 0.59
0.341 < Physical.inactivity
0.122 < Diabetes.prevalence
0.232 < Adult.smoking
0.1241 < Severe.housing.cost.burden
0.1799 < Uninsured.adults
Asian <= 0.00508
0.837 < Driving.alone.to.work
0.730 < Homeownership <= 0.774
33.9 < Teen.births
0.0838 < Percentage.of.households.with.high.housing.costs <= 0.1021
Feature
0.00
0.02
0.04
0.06
Weight
Positive
Negative

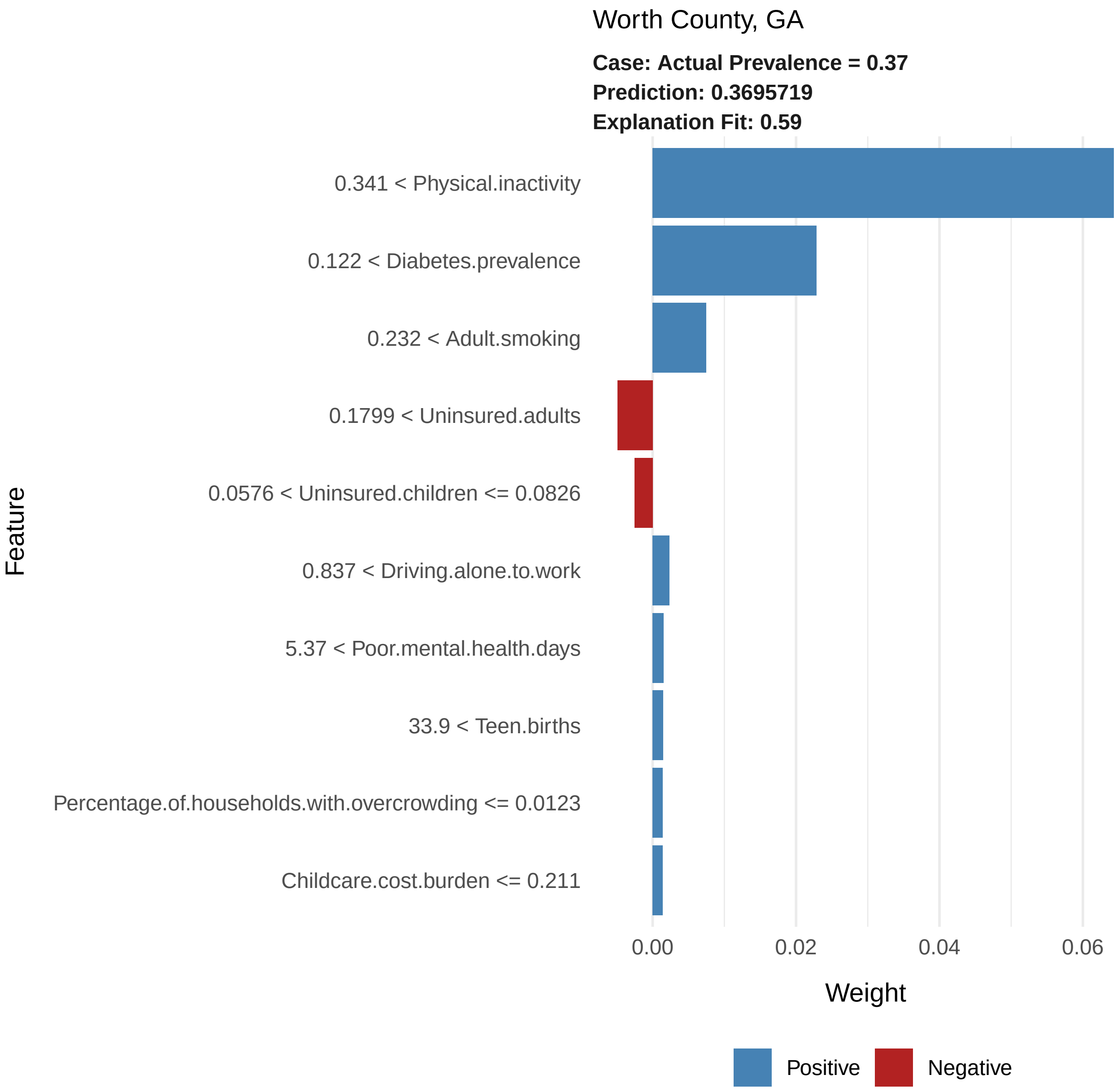

Worth County, GA
Case: Actual Prevalence = 0.37
Prediction: 0.3695719
Explanation Fit: 0.59
0.341 < Physical.inactivity
0.122 < Diabetes.prevalence
0.232 < Adult.smoking
0.1799 < Uninsured.adults
0.0576 < Uninsured.children <= 0.0826
0.837 < Driving.alone.to.work
5.37 < Poor.mental.health.days
33.9 < Teen.births
Percentage.of.households.with.overcrowding <= 0.0123
Childcare.cost.burden <= 0.211
Feature
0.00
0.02
0.04
0.06
Weight
Positive
Negative

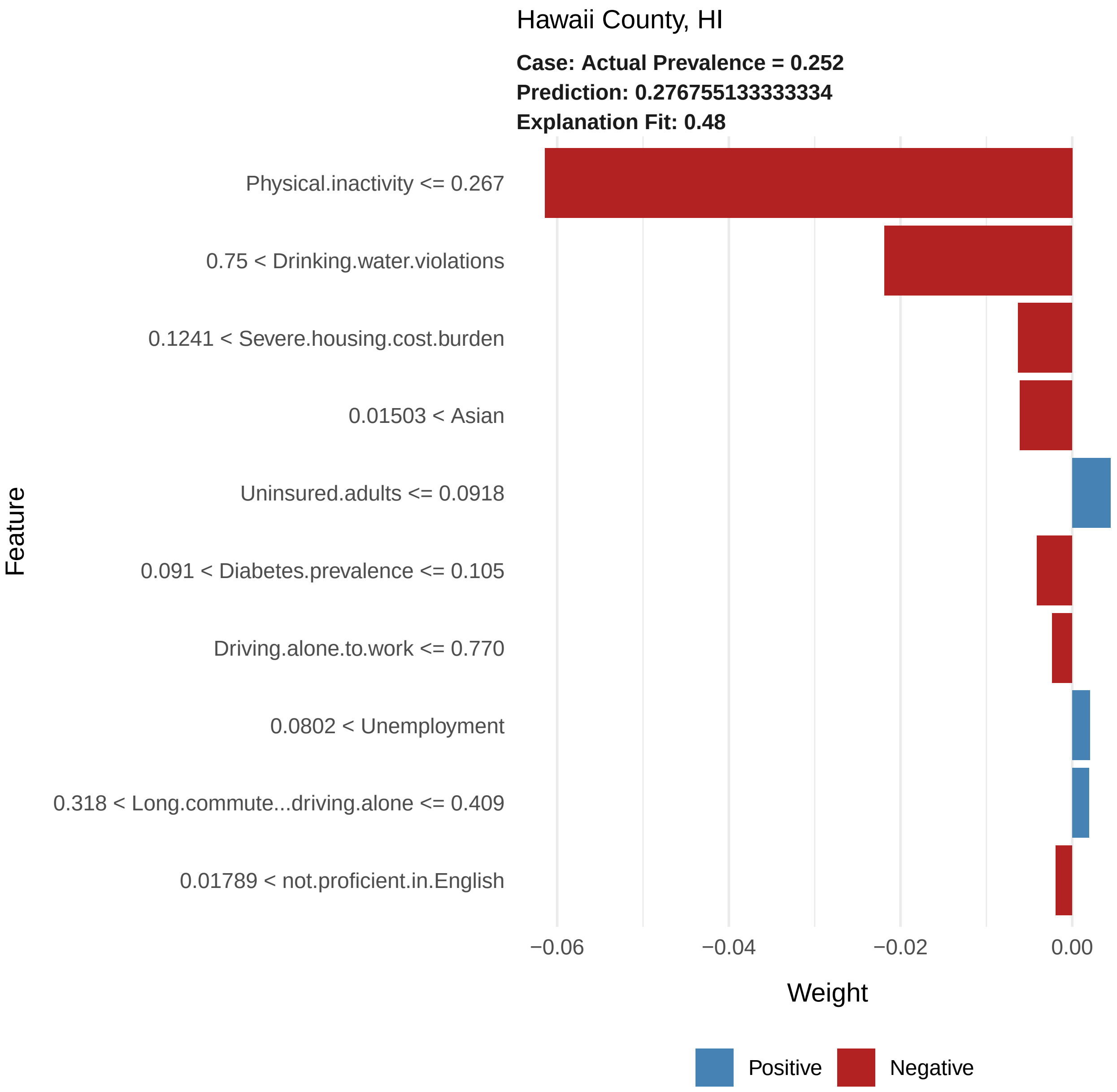
Hawaii County, HI
Case: Actual Prevalence = 0.252
Prediction: 0.276755133333334
Explanation Fit: 0.48
Physical.inactivity <= 0.267
0.75 < Drinking.water.violations
0.1241 < Severe.housing.cost.burden
0.01503 < Asian
Uninsured.adults <= 0.0918
0.091 < Diabetes.prevalence <= 0.105
Driving.alone.to.work <= 0.770
0.0802 < Unemployment
0.318 < Long.commute...driving.alone <= 0.409
0.01789 < not.proficient.in.English
Feature
−0.06
−0.04
−0.02
0.00
Weight
Positive
Negative

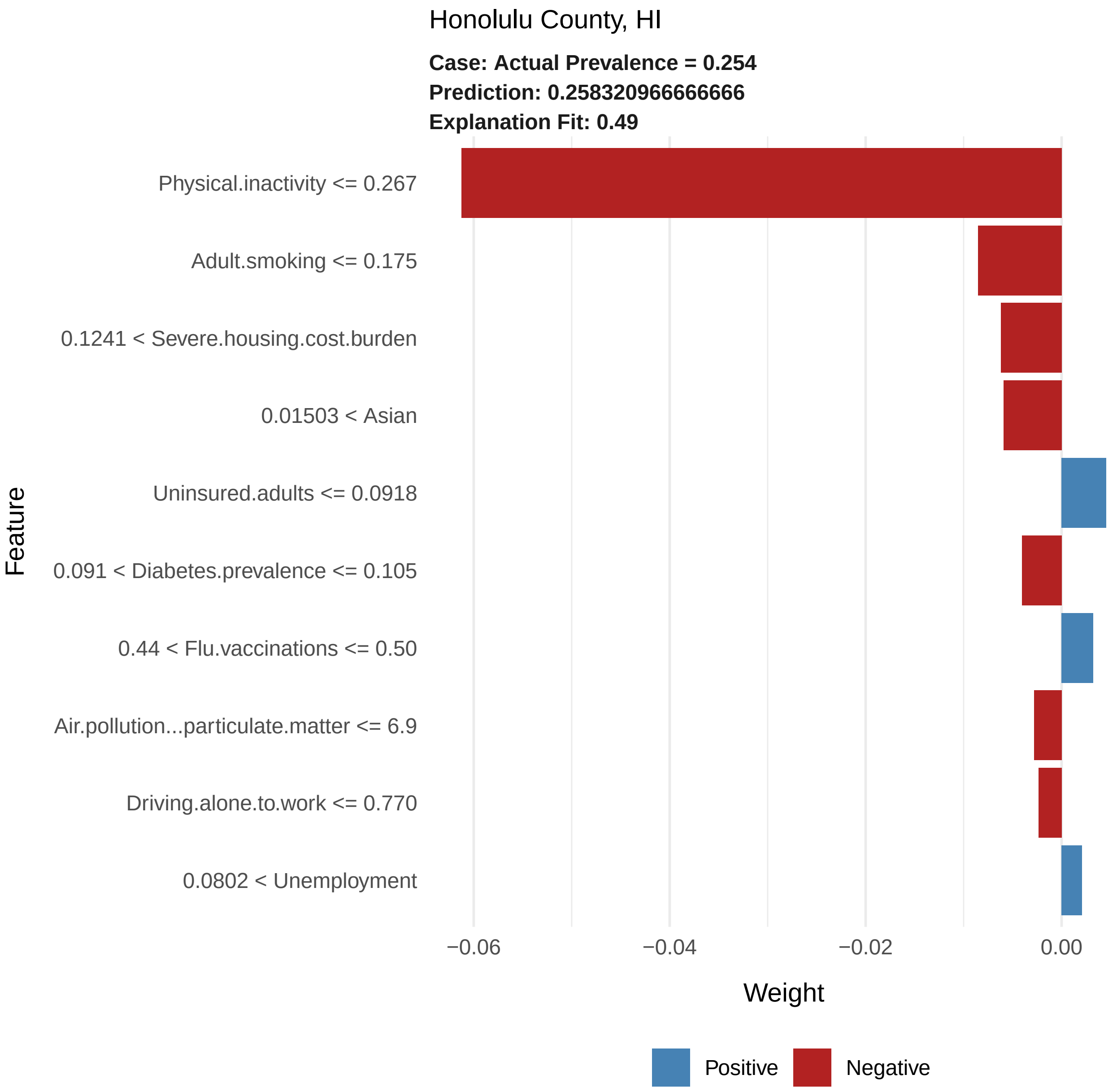

Honolulu County, HI
Case: Actual Prevalence = 0.254
Prediction: 0.258320966666666
Explanation Fit: 0.49
Physical.inactivity <= 0.267
Adult.smoking <= 0.175
0.1241 < Severe.housing.cost.burden
0.01503 < Asian
Uninsured.adults <= 0.0918
0.091 < Diabetes.prevalence <= 0.105
0.44 < Flu.vaccinations <= 0.50
Air.pollution...particulate.matter <= 6.9
Driving.alone.to.work <= 0.770
0.0802 < Unemployment
Feature
−0.06
−0.04
−0.02
0.00
Weight
Positive
Negative

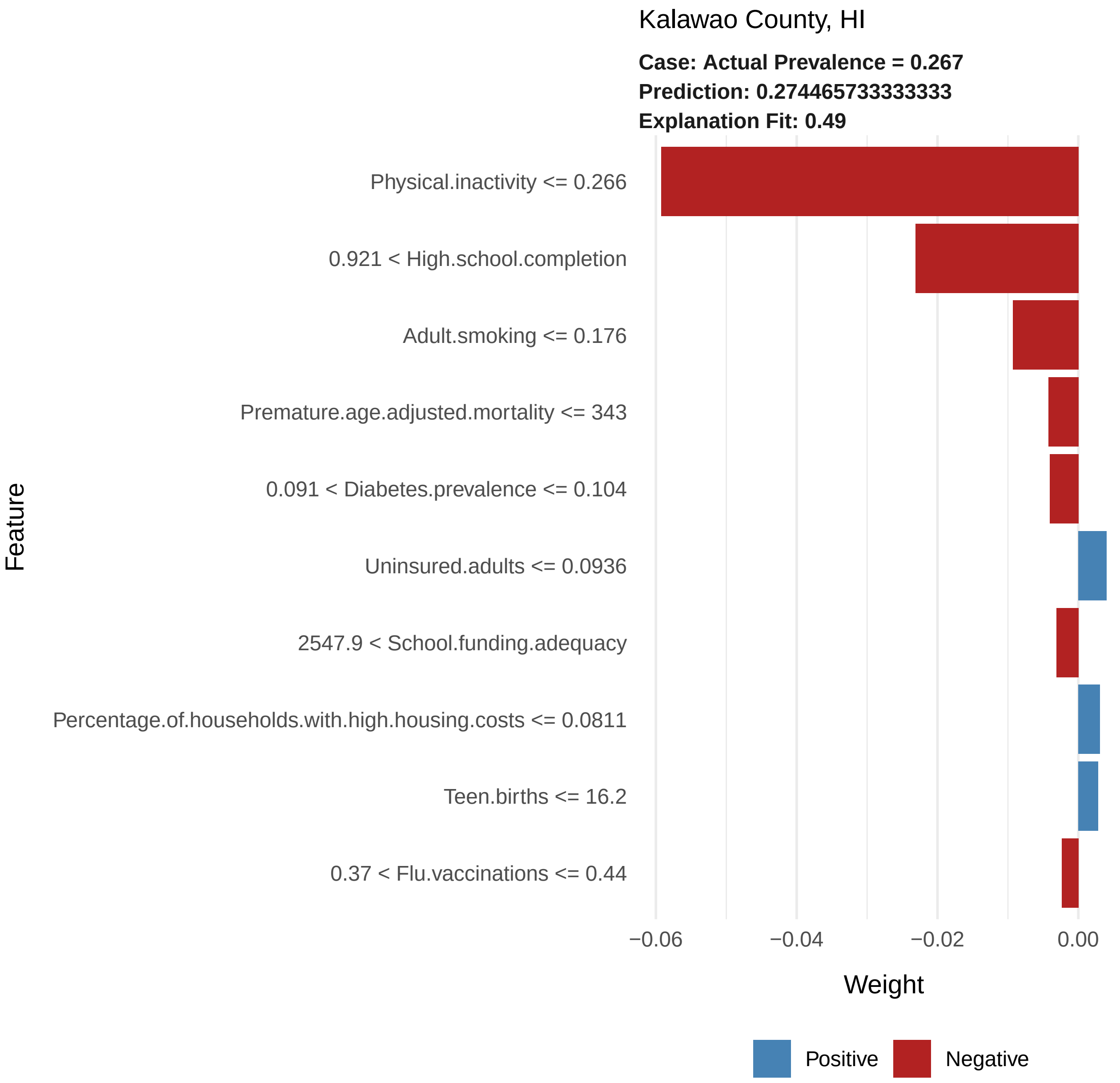

Kalawao County, HI
Case: Actual Prevalence = 0.267
Prediction: 0.274465733333333
Explanation Fit: 0.49
Physical.inactivity <= 0.266
0.921 < High.school.completion
Adult.smoking <= 0.176
Premature.age.adjusted.mortality <= 343
0.091 < Diabetes.prevalence <= 0.104
Uninsured.adults <= 0.0936
2547.9 < School.funding.adequacy
Percentage.of.households.with.high.housing.costs <= 0.0811
Teen.births <= 16.2
0.37 < Flu.vaccinations <= 0.44
Feature
−0.06
−0.04
−0.02
0.00
Weight
Positive
Negative

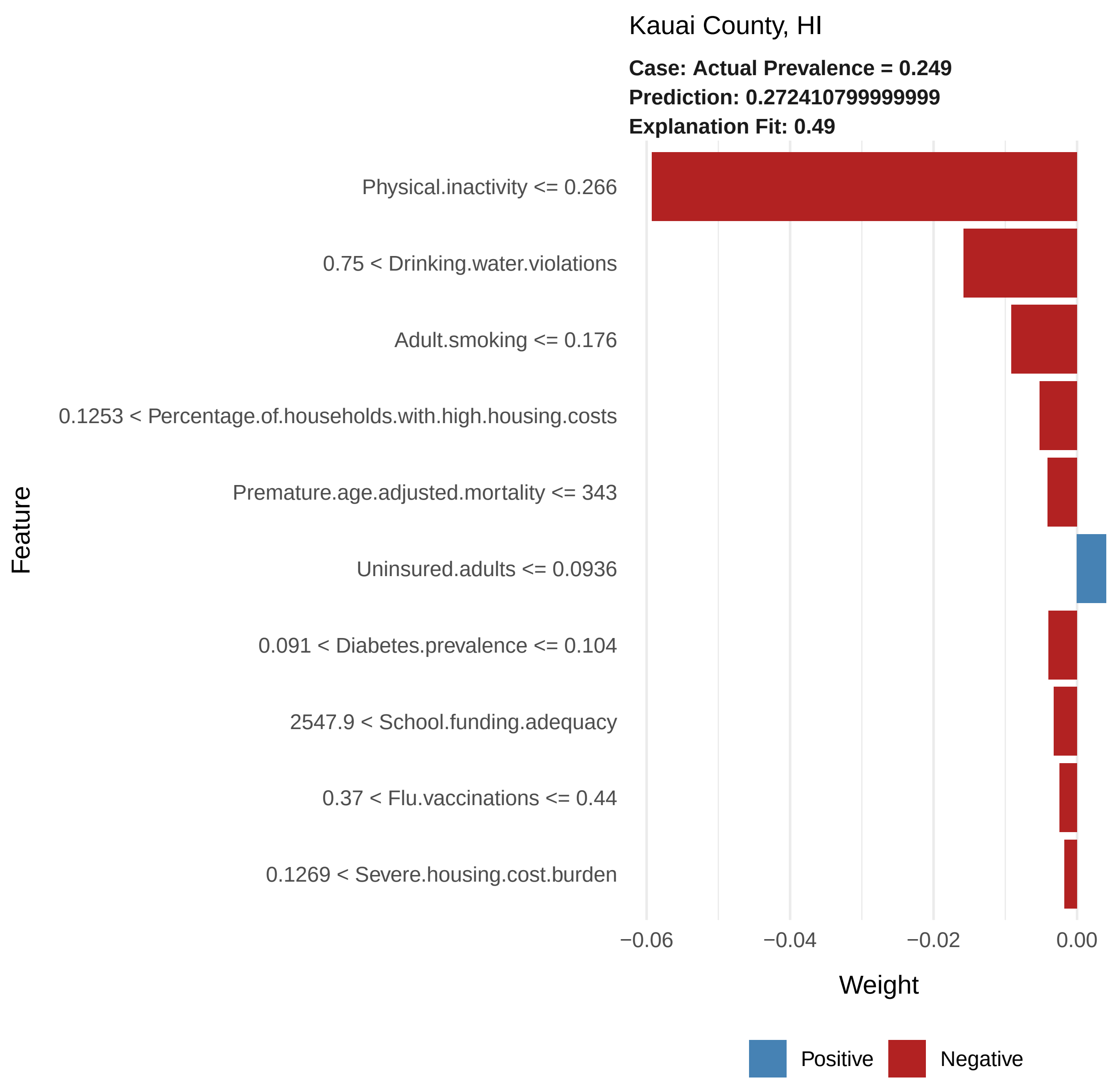
Kauai County, HI
Case: Actual Prevalence = 0.249
Prediction: 0.272410799999999
Explanation Fit: 0.49
Physical.inactivity <= 0.266
0.75 < Drinking.water.violations
Adult.smoking <= 0.176
0.1253 < Percentage.of.households.with.high.housing.costs
Premature.age.adjusted.mortality <= 343
Uninsured.adults <= 0.0936
0.091 < Diabetes.prevalence <= 0.104
2547.9 < School.funding.adequacy
0.37 < Flu.vaccinations <= 0.44
0.1269 < Severe.housing.cost.burden
Feature
−0.06
−0.04
−0.02
0.00
Weight
Positive
Negative

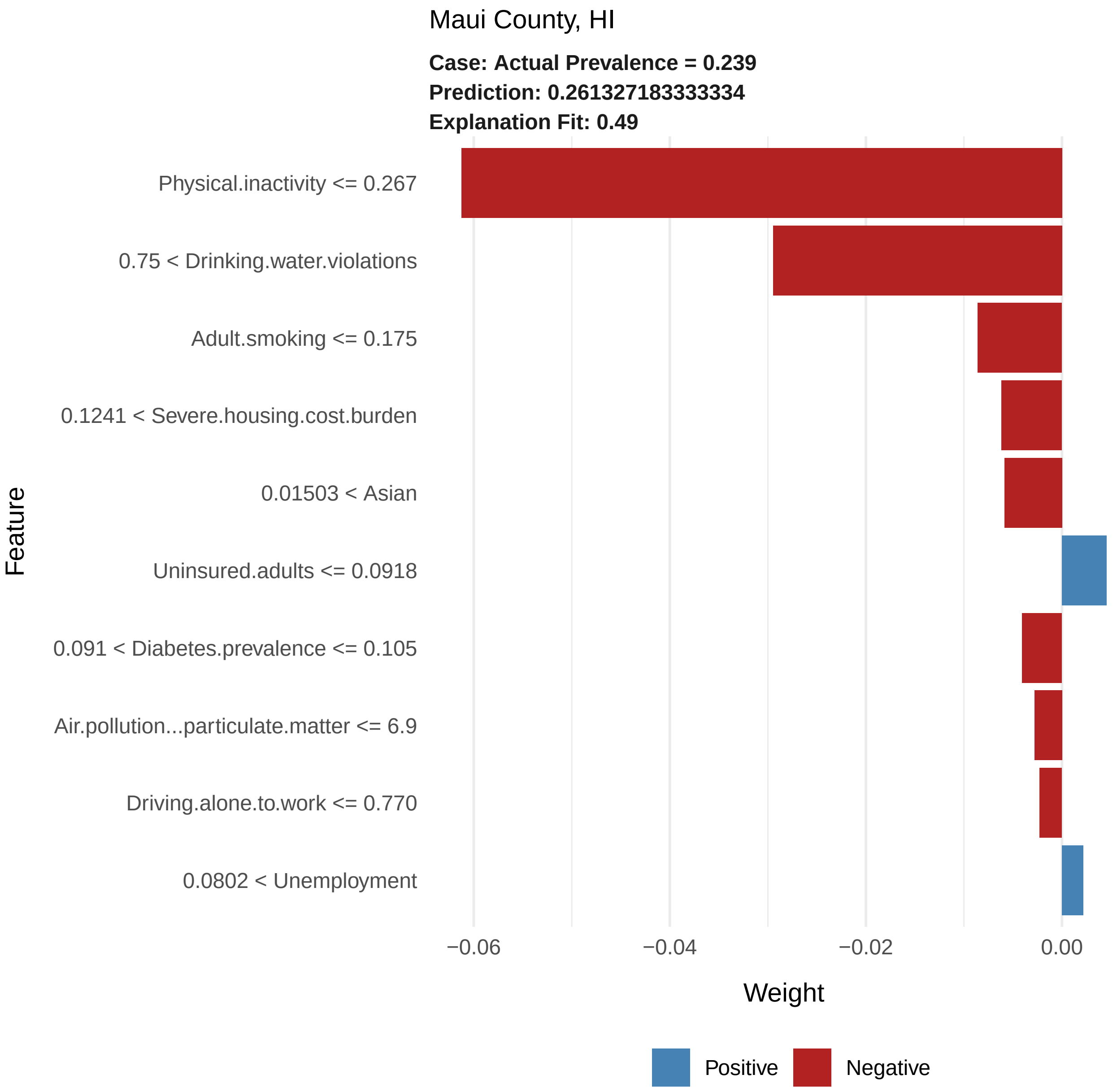

Maui County, HI
Case: Actual Prevalence = 0.239
Prediction: 0.261327183333334
Explanation Fit: 0.49
Physical.inactivity <= 0.267
0.75 < Drinking.water.violations
Adult.smoking <= 0.175
0.1241 < Severe.housing.cost.burden
0.01503 < Asian
Uninsured.adults <= 0.0918
0.091 < Diabetes.prevalence <= 0.105
Air.pollution...particulate.matter <= 6.9
Driving.alone.to.work <= 0.770
0.0802 < Unemployment
Feature
−0.06
−0.04
−0.02
0.00
Weight
Positive
Negative

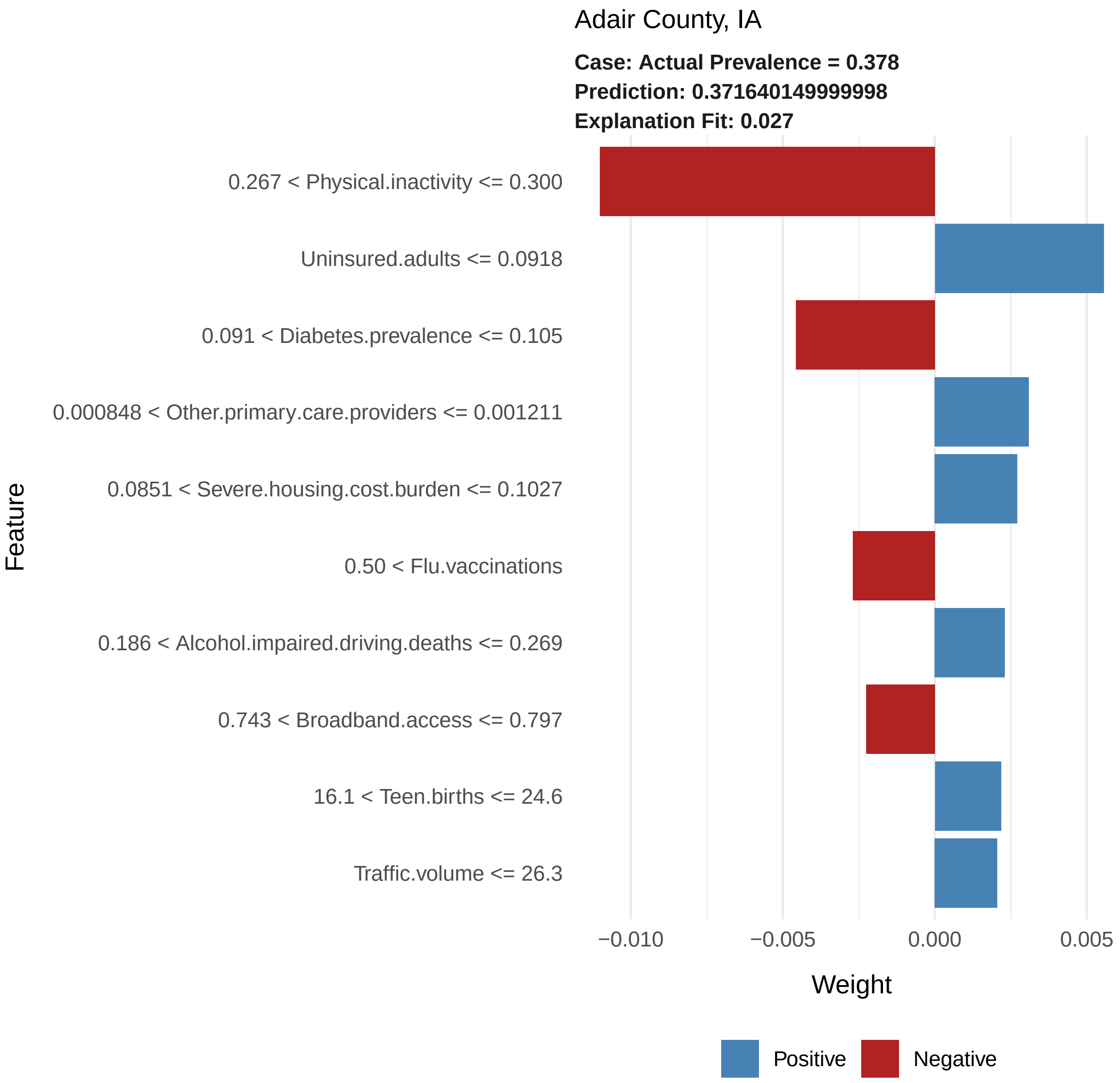

Adair County, IA
Case: Actual Prevalence = 0.378
Prediction: 0.371640149999998
Explanation Fit: 0.027
0.267 < Physical.inactivity <= 0.300
Uninsured.adults <= 0.0918
0.091 < Diabetes.prevalence <= 0.105
0.000848 < Other.primary.care.providers <= 0.001211
0.0851 < Severe.housing.cost.burden <= 0.1027
0.50 < Flu.vaccinations
0.186 < Alcohol.impaired.driving.deaths <= 0.269
0.743 < Broadband.access <= 0.797
16.1 < Teen.births <= 24.6
Traffic.volume <= 26.3
Feature
−0.010
−0.005
0.000
0.005
Weight
Positive
Negative

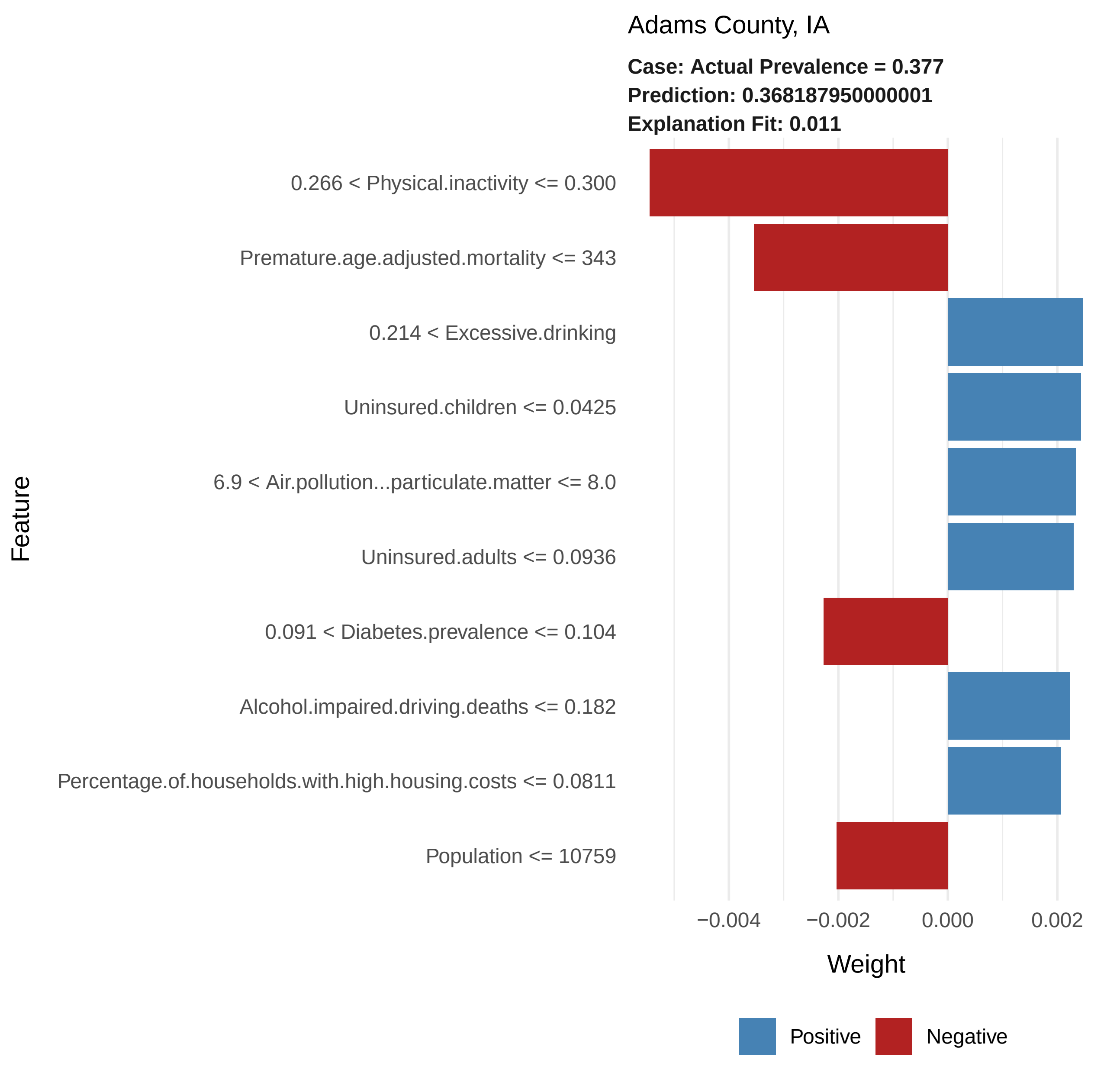
Adams County, IA
Case: Actual Prevalence = 0.377
Prediction: 0.368187950000001
Explanation Fit: 0.011
0.266 < Physical.inactivity <= 0.300
Premature.age.adjusted.mortality <= 343
0.214 < Excessive.drinking
Uninsured.children <= 0.0425
6.9 < Air.pollution...particulate.matter <= 8.0
Uninsured.adults <= 0.0936
0.091 < Diabetes.prevalence <= 0.104
Alcohol.impaired.driving.deaths <= 0.182
Percentage.of.households.with.high.housing.costs <= 0.0811
Population <= 10759
Feature
−0.004
−0.002
0.000
0.002
Weight
Positive
Negative

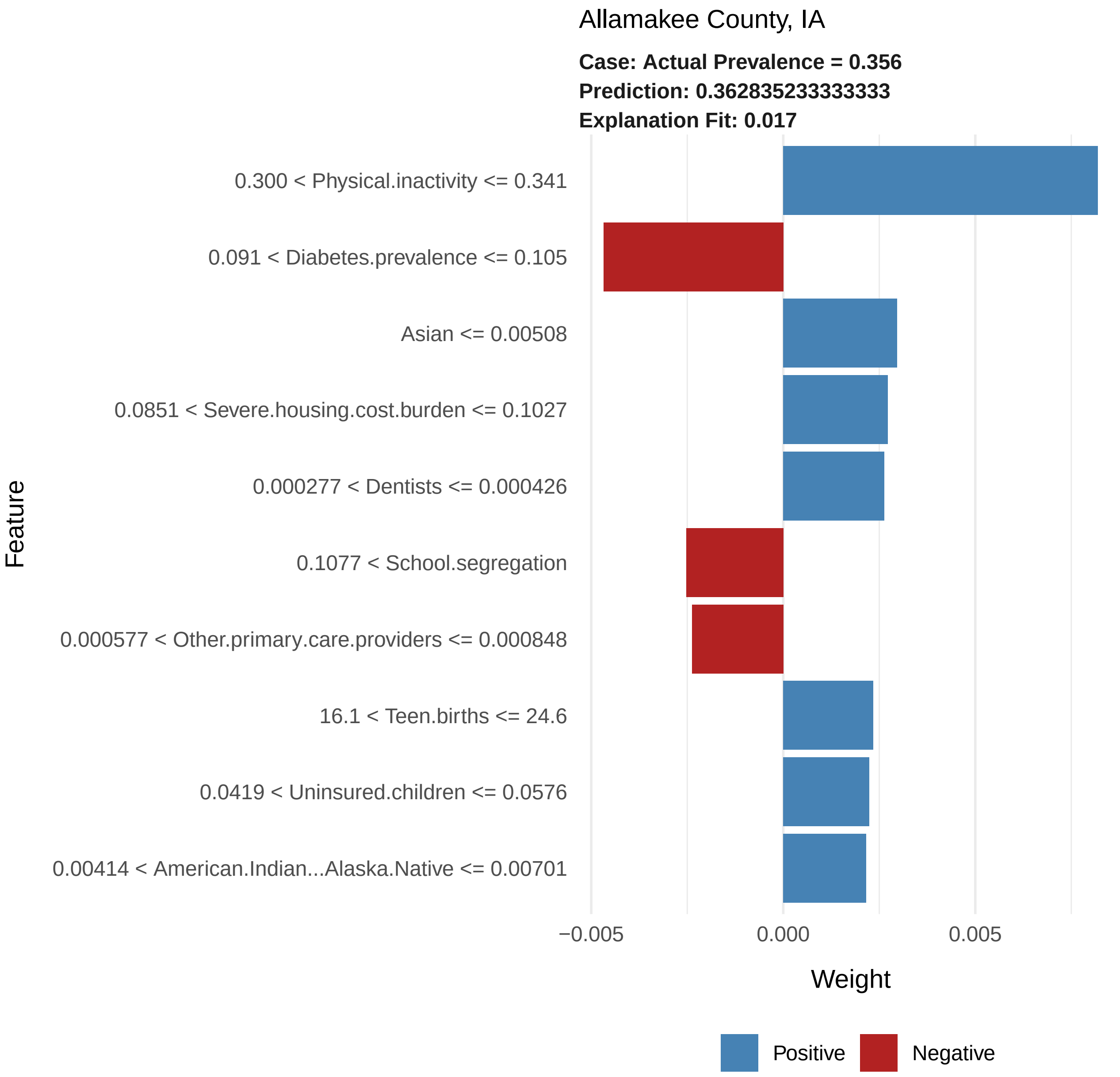
Allamakee County, IA
Case: Actual Prevalence = 0.356
Prediction: 0.362835233333333
Explanation Fit: 0.017
0.300 < Physical.inactivity <= 0.341
0.091 < Diabetes.prevalence <= 0.105
Asian <= 0.00508
0.0851 < Severe.housing.cost.burden <= 0.1027
0.000277 < Dentists <= 0.000426
0.1077 < School.segregation
0.000577 < Other.primary.care.providers <= 0.000848
16.1 < Teen.births <= 24.6
0.0419 < Uninsured.children <= 0.0576
0.00414 < American.Indian...Alaska.Native <= 0.00701
Feature
−0.005
0.000
0.005
Weight
Positive
Negative

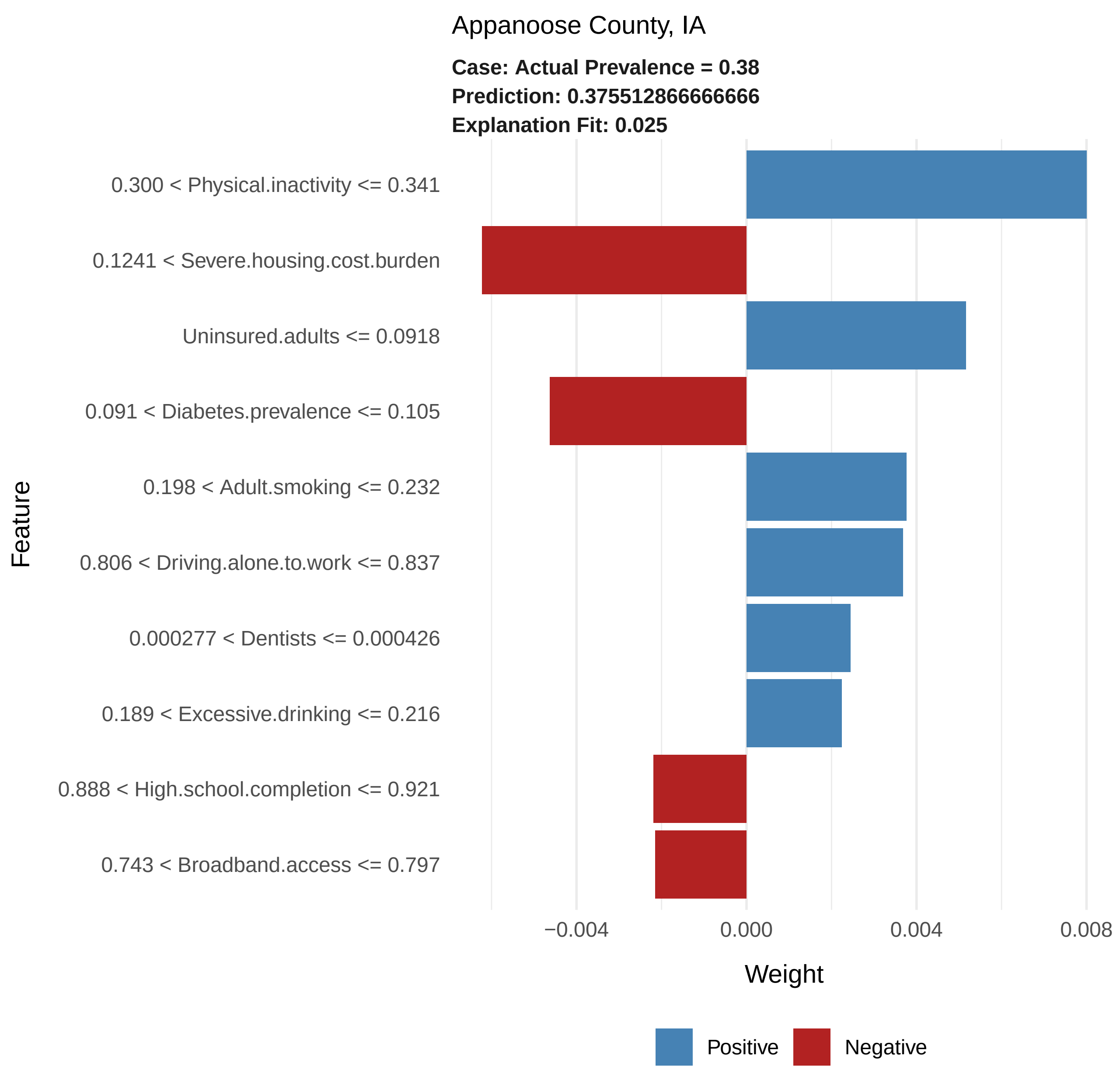
Appanoose County, IA
Case: Actual Prevalence = 0.38
Prediction: 0.375512866666666
Explanation Fit: 0.025
0.300 < Physical.inactivity <= 0.341
0.1241 < Severe.housing.cost.burden
Uninsured.adults <= 0.0918
0.091 < Diabetes.prevalence <= 0.105
0.198 < Adult.smoking <= 0.232
0.806 < Driving.alone.to.work <= 0.837
0.000277 < Dentists <= 0.000426
0.189 < Excessive.drinking <= 0.216
0.888 < High.school.completion <= 0.921
0.743 < Broadband.access <= 0.797
Feature
−0.004
0.000
0.004
0.008
Weight
Positive
Negative

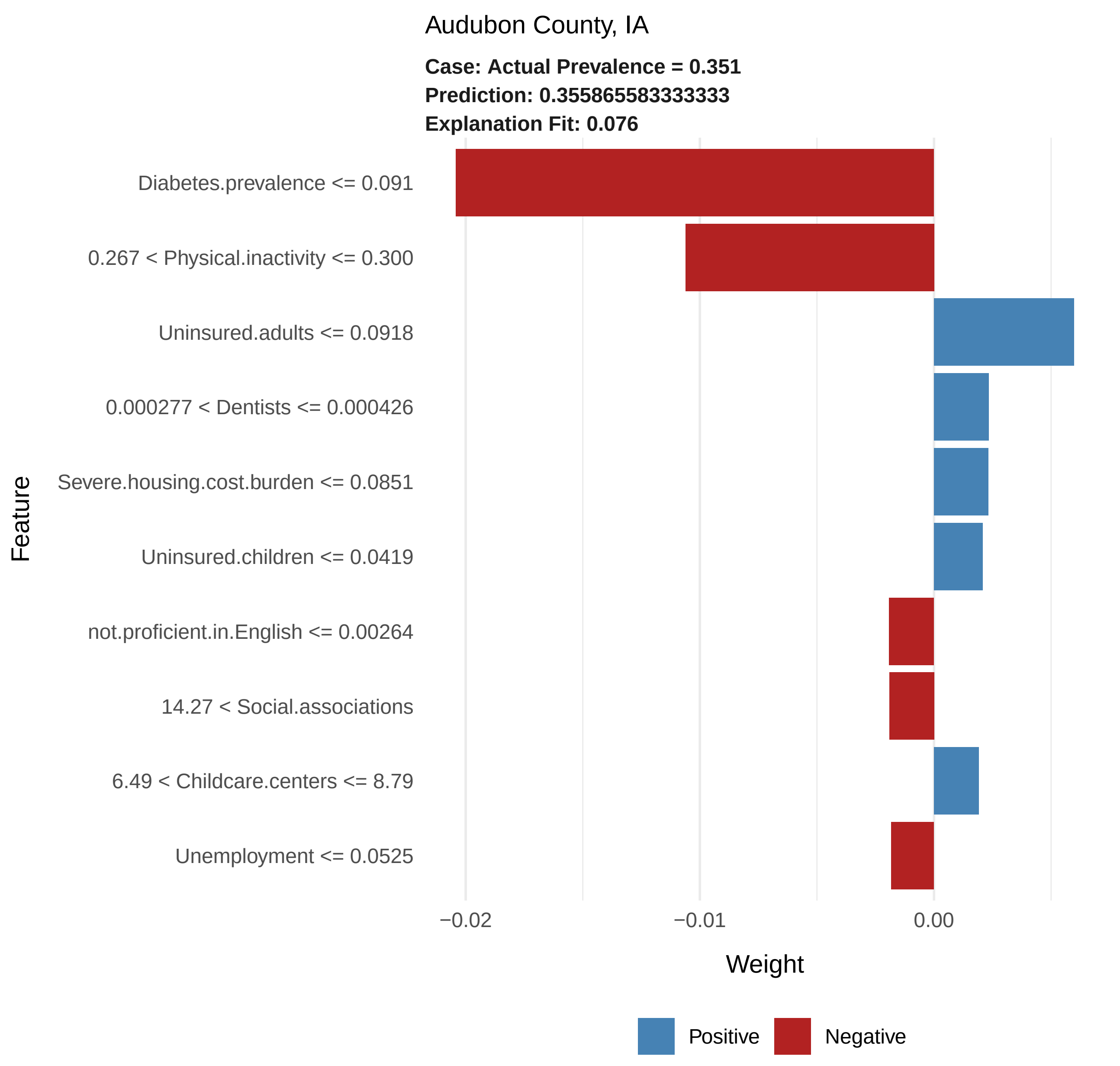
Audubon County, IA
Case: Actual Prevalence = 0.351
Prediction: 0.355865583333333
Explanation Fit: 0.076
Diabetes.prevalence <= 0.091
0.267 < Physical.inactivity <= 0.300
Uninsured.adults <= 0.0918
0.000277 < Dentists <= 0.000426
Severe.housing.cost.burden <= 0.0851
Uninsured.children <= 0.0419
not.proficient.in.English <= 0.00264
14.27 < Social.associations
6.49 < Childcare.centers <= 8.79
Unemployment <= 0.0525
Feature
−0.02
−0.01
0.00
Weight
Positive
Negative

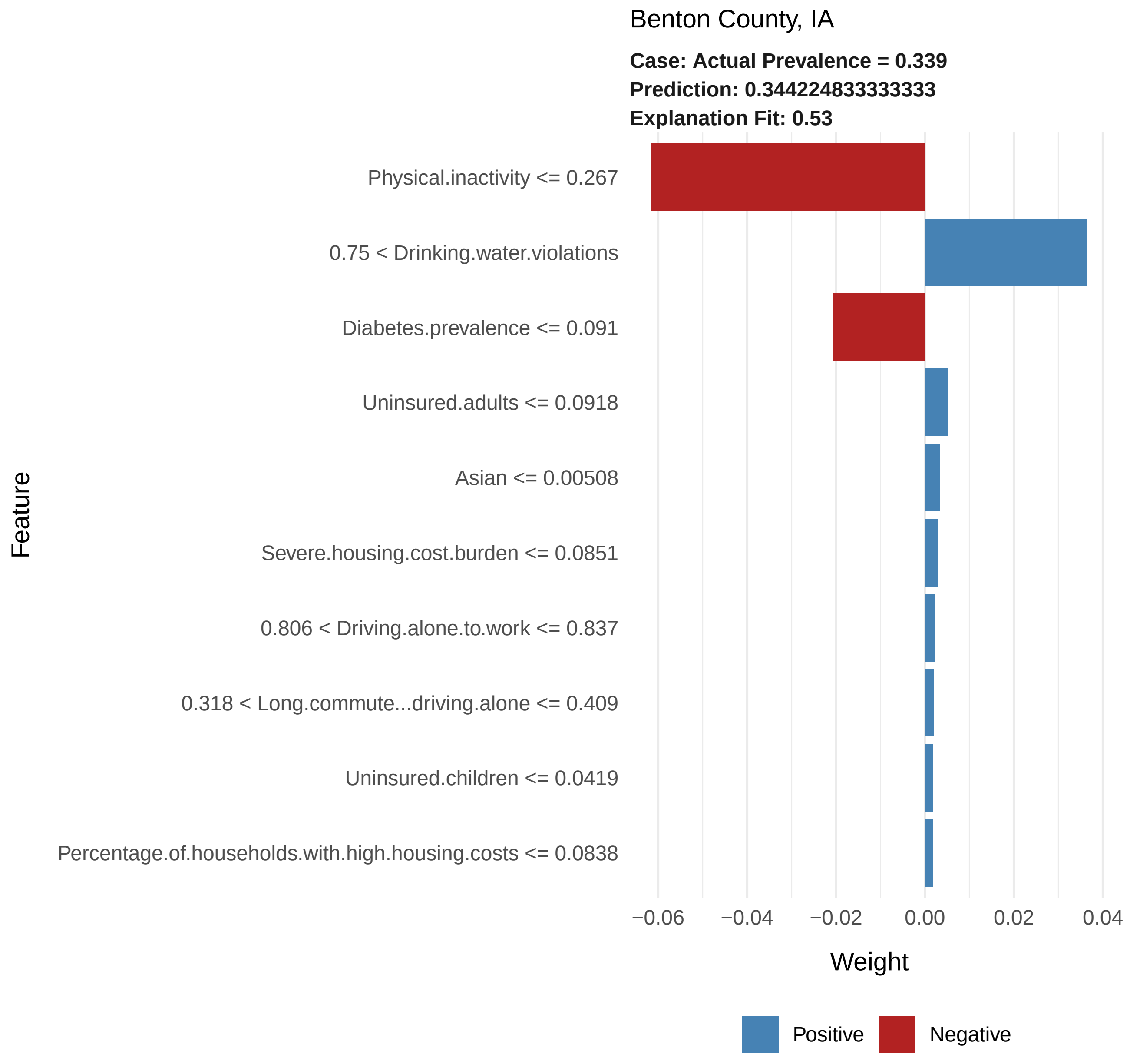

Benton County, IA
Case: Actual Prevalence = 0.339
Prediction: 0.344224833333333
Explanation Fit: 0.53
Physical.inactivity <= 0.267
0.75 < Drinking.water.violations
Diabetes.prevalence <= 0.091
Uninsured.adults <= 0.0918
Asian <= 0.00508
Severe.housing.cost.burden <= 0.0851
0.806 < Driving.alone.to.work <= 0.837
0.318 < Long.commute...driving.alone <= 0.409
Uninsured.children <= 0.0419
Percentage.of.households.with.high.housing.costs <= 0.0838
Feature
−0.06
−0.04
−0.02
0.00
0.02
0.04
Weight
Positive
Negative

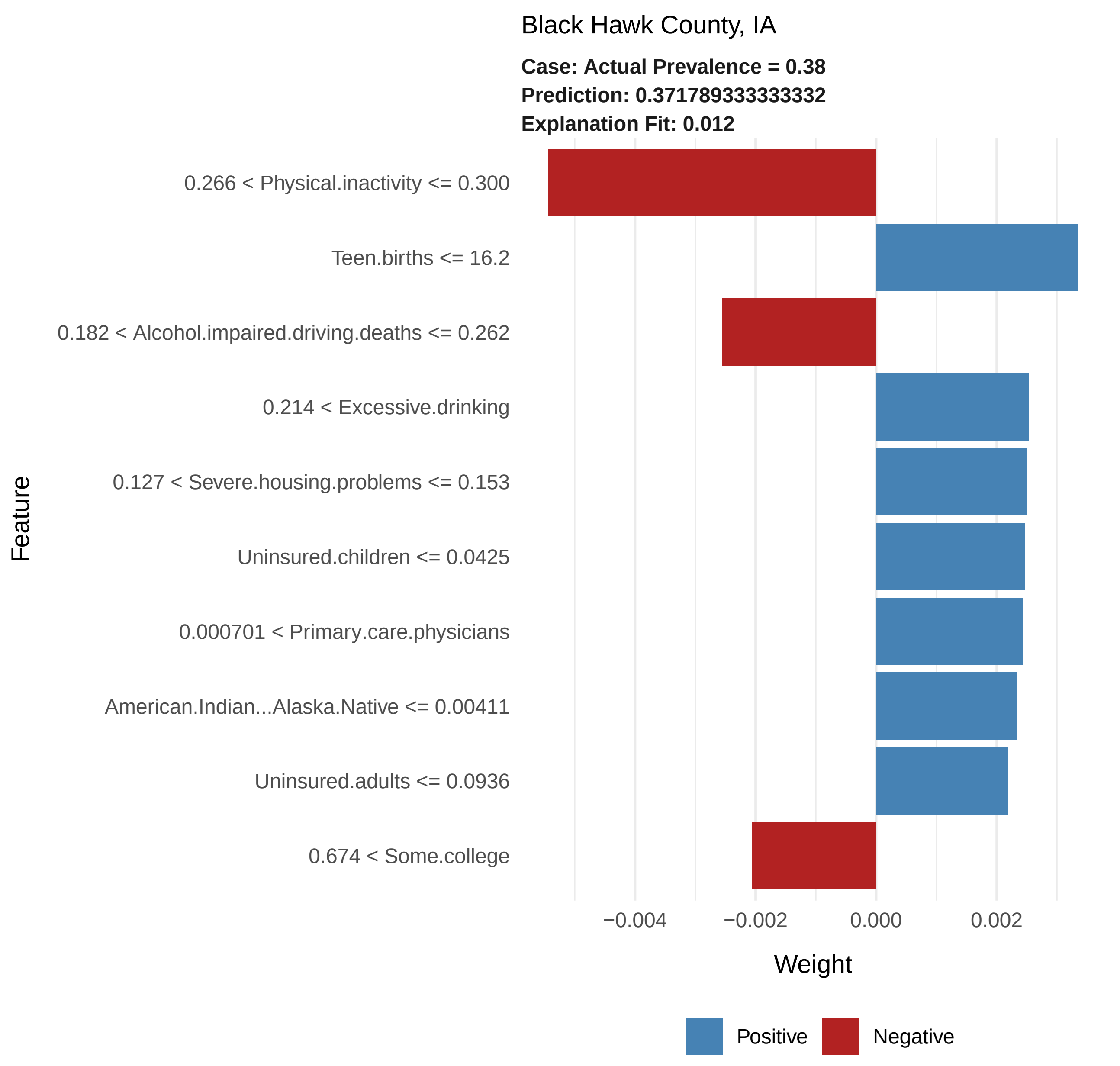
Black Hawk County, IA
Case: Actual Prevalence = 0.38
Prediction: 0.371789333333332
Explanation Fit: 0.012
0.266 < Physical.inactivity <= 0.300
Teen.births <= 16.2
0.182 < Alcohol.impaired.driving.deaths <= 0.262
0.214 < Excessive.drinking
0.127 < Severe.housing.problems <= 0.153
Uninsured.children <= 0.0425
0.000701 < Primary.care.physicians
American.Indian...Alaska.Native <= 0.00411
Uninsured.adults <= 0.0936
0.674 < Some.college
Feature
−0.004
−0.002
0.000
0.002
Weight
Positive
Negative

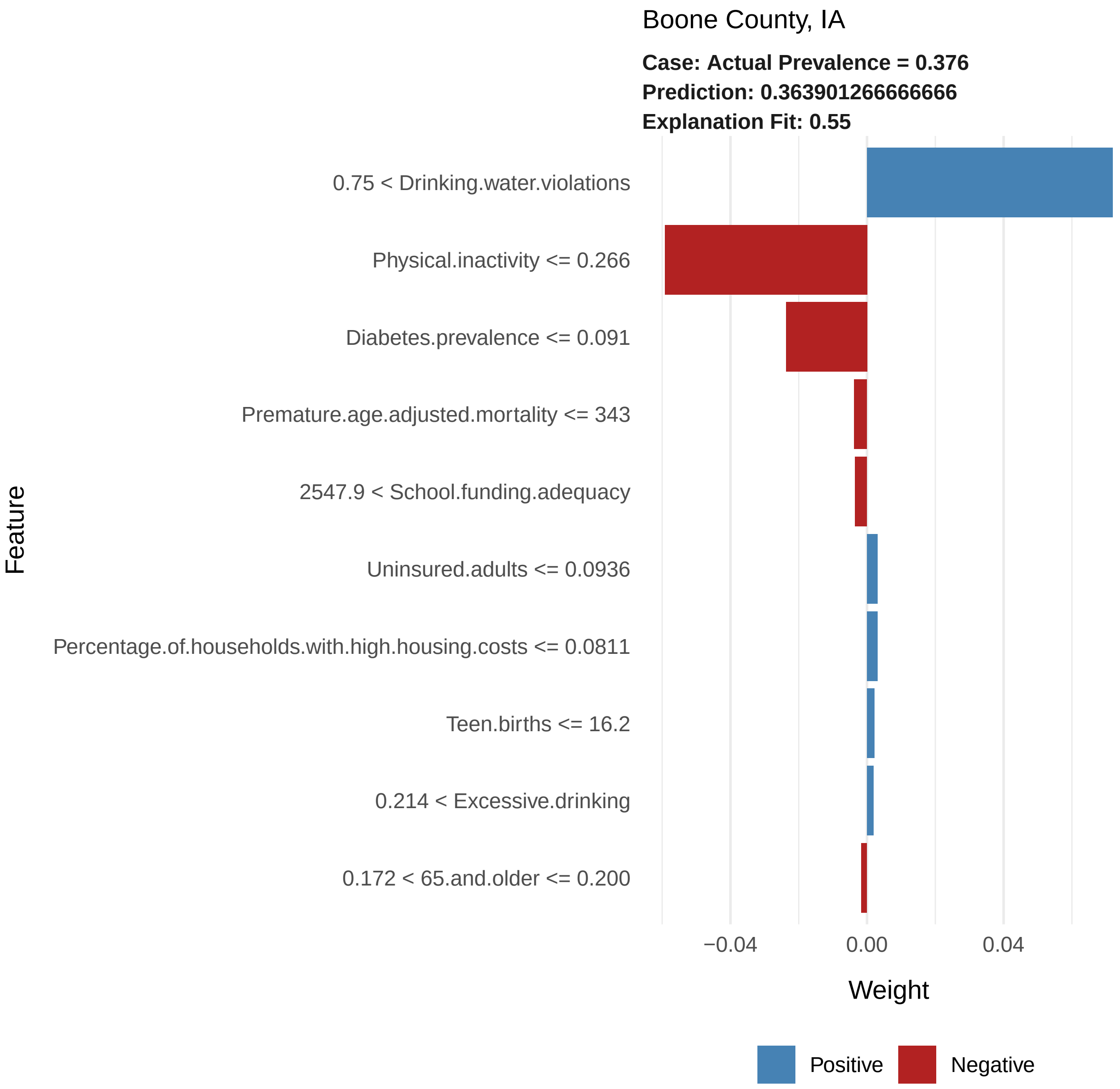

Boone County, IA
Case: Actual Prevalence = 0.376
Prediction: 0.363901266666666
Explanation Fit: 0.55
0.75 < Drinking.water.violations
Physical.inactivity <= 0.266
Diabetes.prevalence <= 0.091
Premature.age.adjusted.mortality <= 343
2547.9 < School.funding.adequacy
Uninsured.adults <= 0.0936
Percentage.of.households.with.high.housing.costs <= 0.0811
Teen.births <= 16.2
0.214 < Excessive.drinking
0.172 < 65.and.older <= 0.200
Feature
−0.04
0.00
0.04
Weight
Positive
Negative

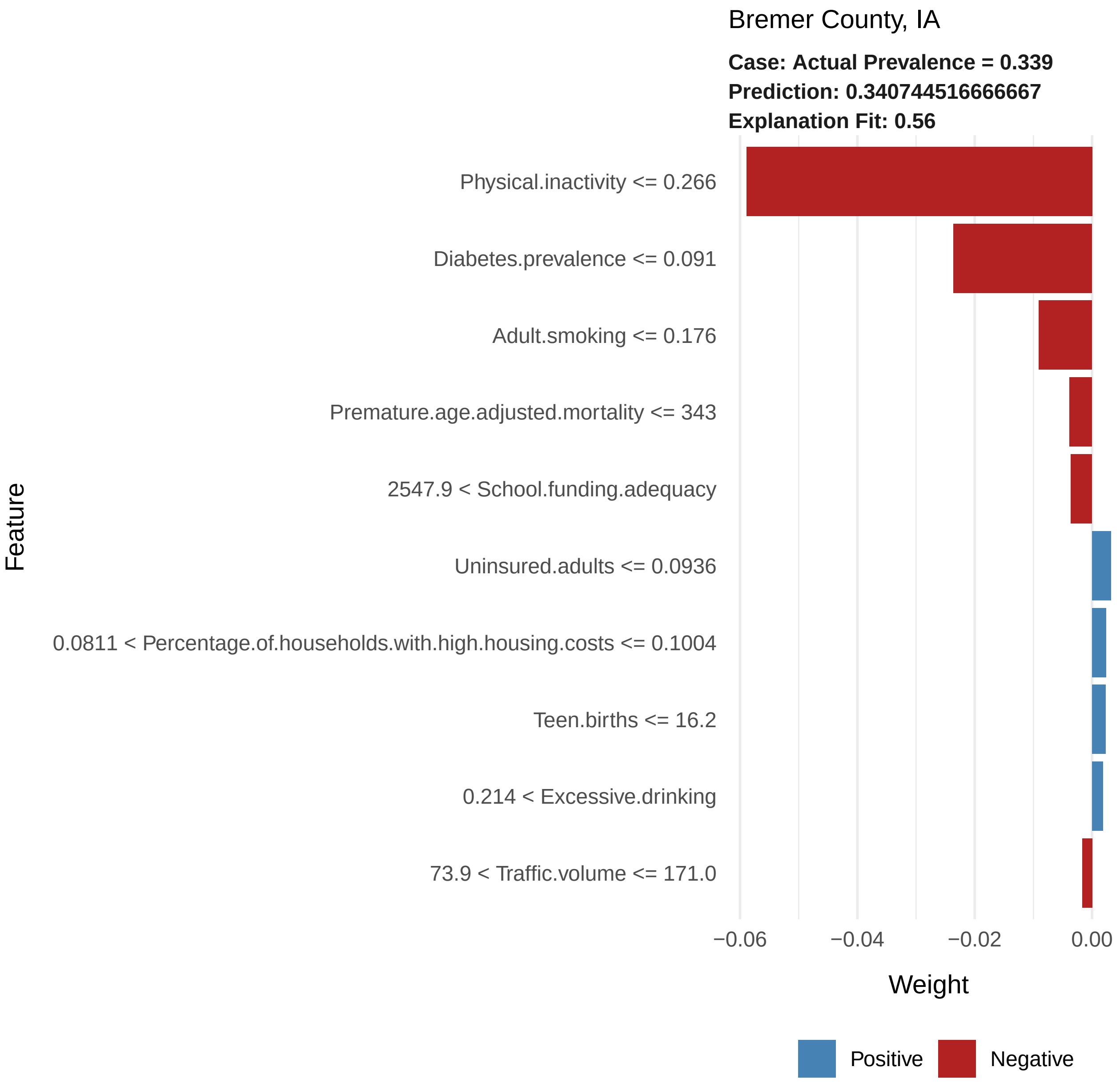
Bremer County, IA
Case: Actual Prevalence = 0.339
Prediction: 0.340744516666667
Explanation Fit: 0.56
Physical.inactivity <= 0.266
Diabetes.prevalence <= 0.091
Adult.smoking <= 0.176
Premature.age.adjusted.mortality <= 343
2547.9 < School.funding.adequacy
Uninsured.adults <= 0.0936
0.0811 < Percentage.of.households.with.high.housing.costs <= 0.1004
Teen.births <= 16.2
0.214 < Excessive.drinking
73.9 < Traffic.volume <= 171.0
Feature
−0.06
−0.04
−0.02
0.00
Weight
Positive
Negative

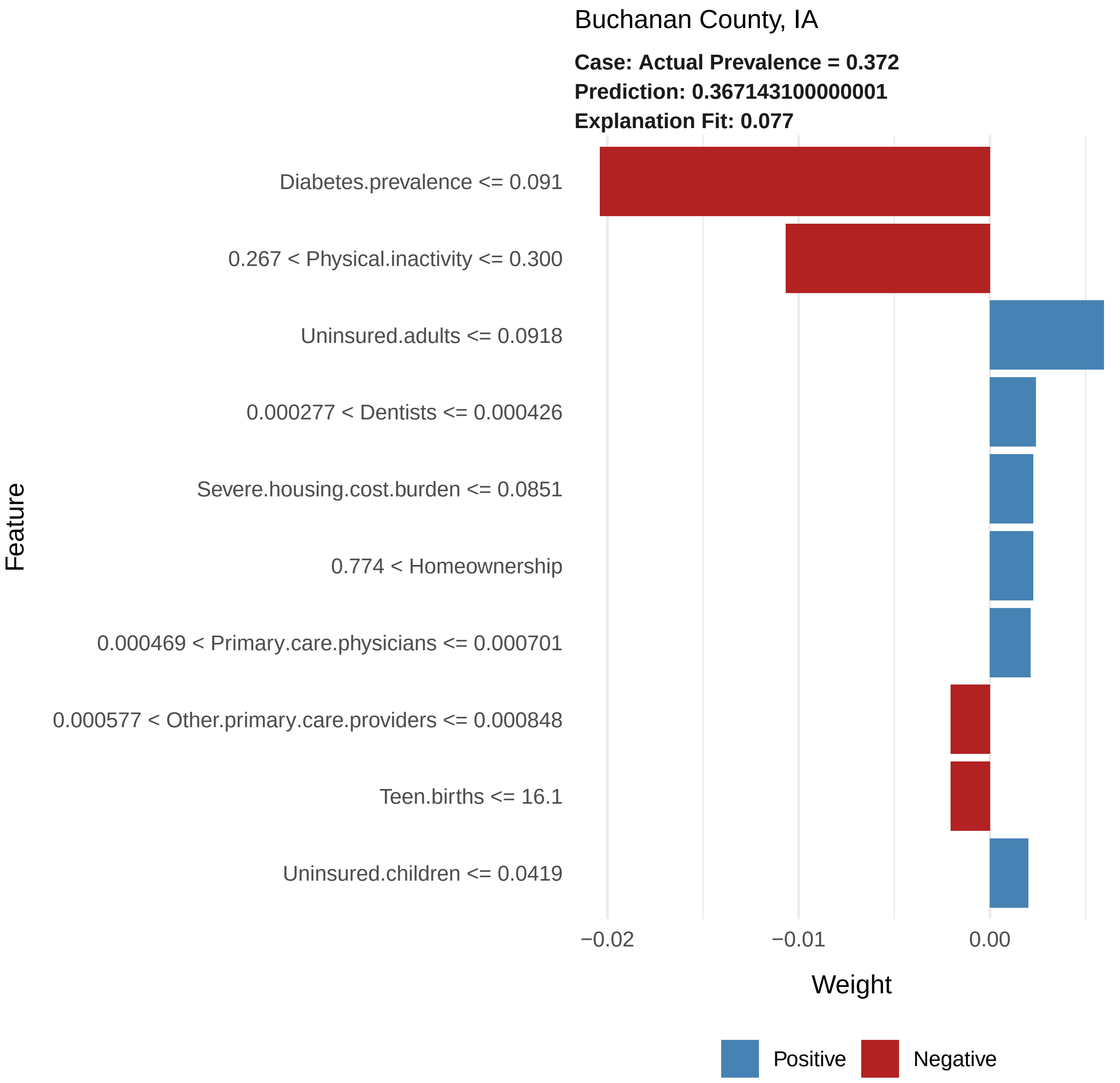

Buchanan County, IA
Case: Actual Prevalence = 0.372
Prediction: 0.367143100000001
Explanation Fit: 0.077
Diabetes.prevalence <= 0.091
0.267 < Physical.inactivity <= 0.300
Uninsured.adults <= 0.0918
0.000277 < Dentists <= 0.000426
Severe.housing.cost.burden <= 0.0851
0.774 < Homeownership
0.000469 < Primary.care.physicians <= 0.000701
0.000577 < Other.primary.care.providers <= 0.000848
Teen.births <= 16.1
Uninsured.children <= 0.0419
Feature
−0.02
−0.01
0.00
Weight
Positive
Negative

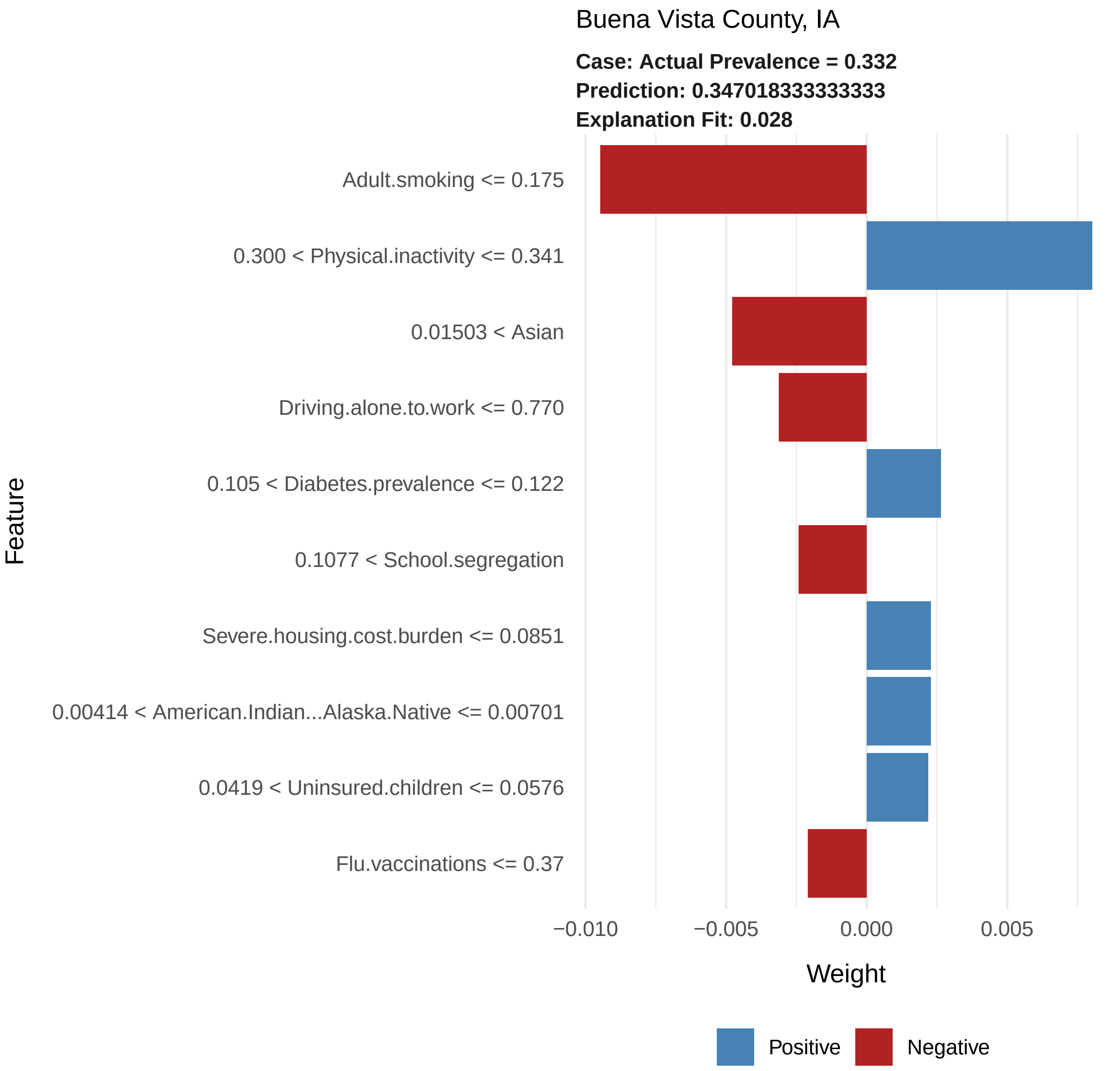
Buena Vista County, IA
Case: Actual Prevalence = 0.332
Prediction: 0.347018333333333
Explanation Fit: 0.028
Adult.smoking <= 0.175
0.300 < Physical.inactivity <= 0.341
0.01503 < Asian
Driving.alone.to.work <= 0.770
0.105 < Diabetes.prevalence <= 0.122
0.1077 < School.segregation
Severe.housing.cost.burden <= 0.0851
0.00414 < American.Indian...Alaska.Native <= 0.00701
0.0419 < Uninsured.children <= 0.0576
Flu.vaccinations <= 0.37
Feature
−0.010
−0.005
0.000
0.005
Weight
Positive
Negative

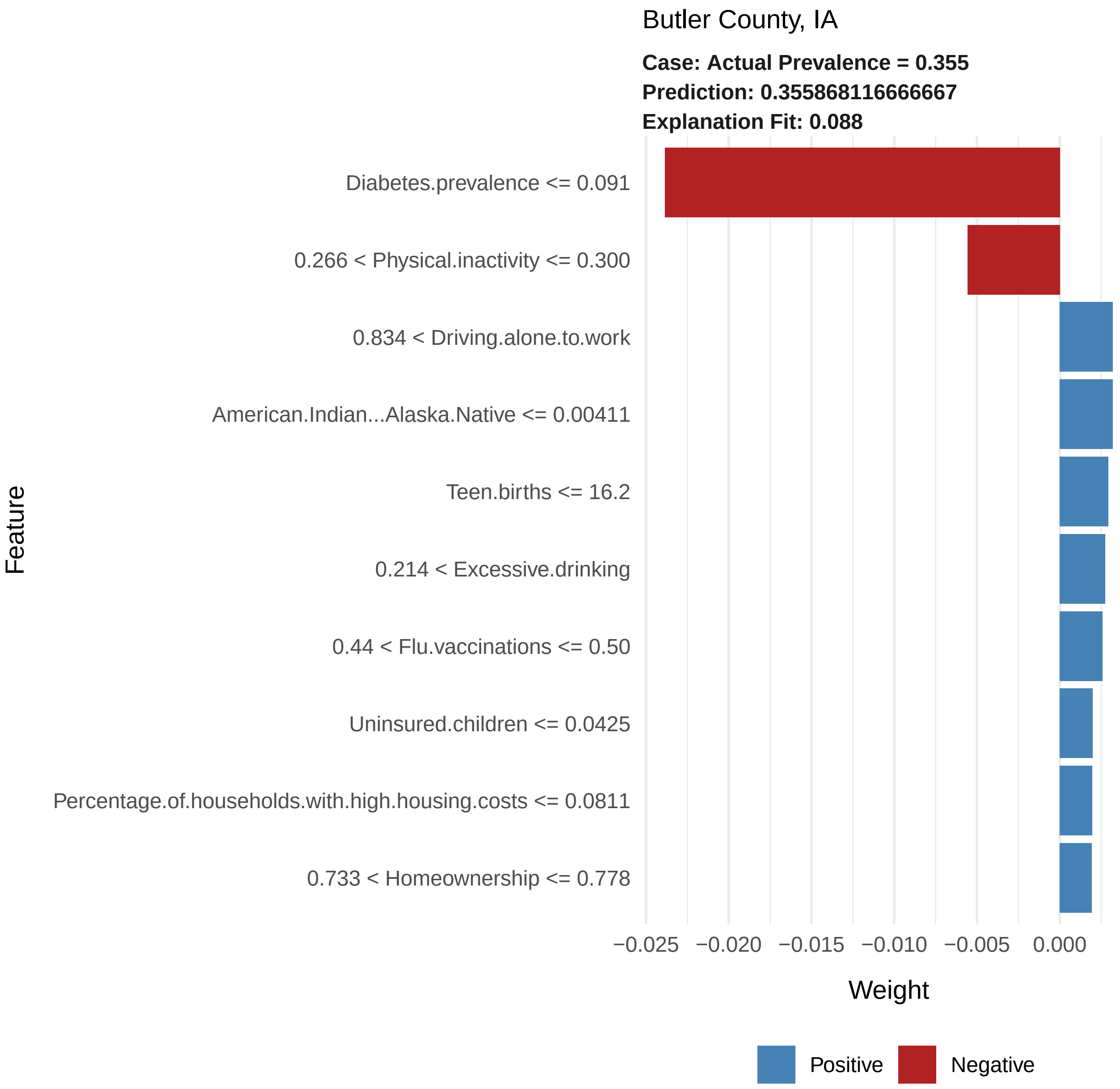
Butler County, IA
Case: Actual Prevalence = 0.355
Prediction: 0.355868116666667
Explanation Fit: 0.088
Diabetes.prevalence <= 0.091
0.266 < Physical.inactivity <= 0.300
0.834 < Driving.alone.to.work
American.Indian...Alaska.Native <= 0.00411
Teen.births <= 16.2
0.214 < Excessive.drinking
0.44 < Flu.vaccinations <= 0.50
Uninsured.children <= 0.0425
Percentage.of.households.with.high.housing.costs <= 0.0811
0.733 < Homeownership <= 0.778
Feature
−0.025
−0.020
−0.015
−0.010
−0.005
0.000
Weight
Positive
Negative

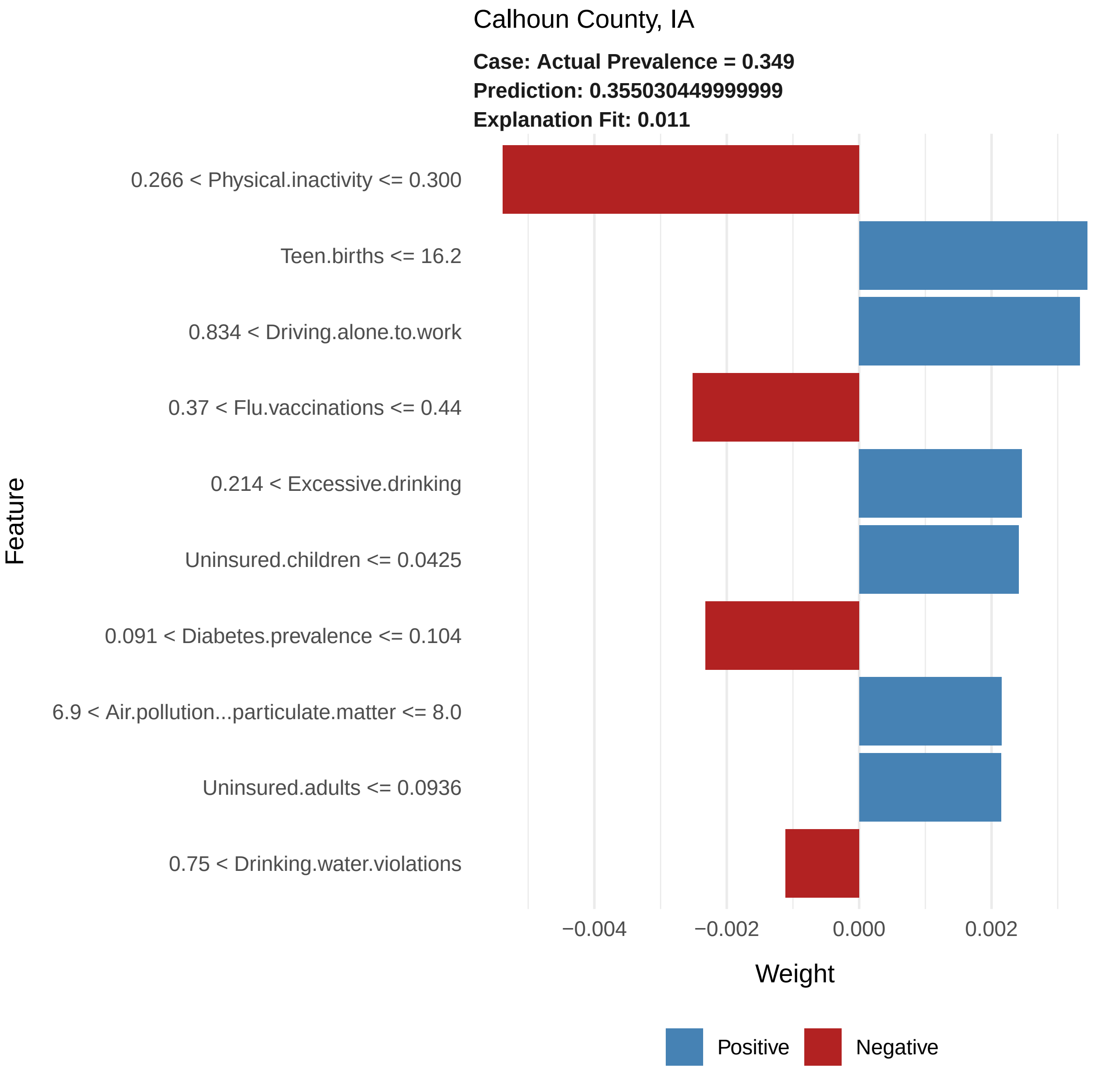

Calhoun County, IA
Case: Actual Prevalence = 0.349
Prediction: 0.355030449999999
Explanation Fit: 0.011
0.266 < Physical.inactivity <= 0.300
Teen.births <= 16.2
0.834 < Driving.alone.to.work
0.37 < Flu.vaccinations <= 0.44
0.214 < Excessive.drinking
Uninsured.children <= 0.0425
0.091 < Diabetes.prevalence <= 0.104
6.9 < Air.pollution...particulate.matter <= 8.0
Uninsured.adults <= 0.0936
0.75 < Drinking.water.violations
Feature
−0.004
−0.002
0.000
0.002
Weight
Positive
Negative

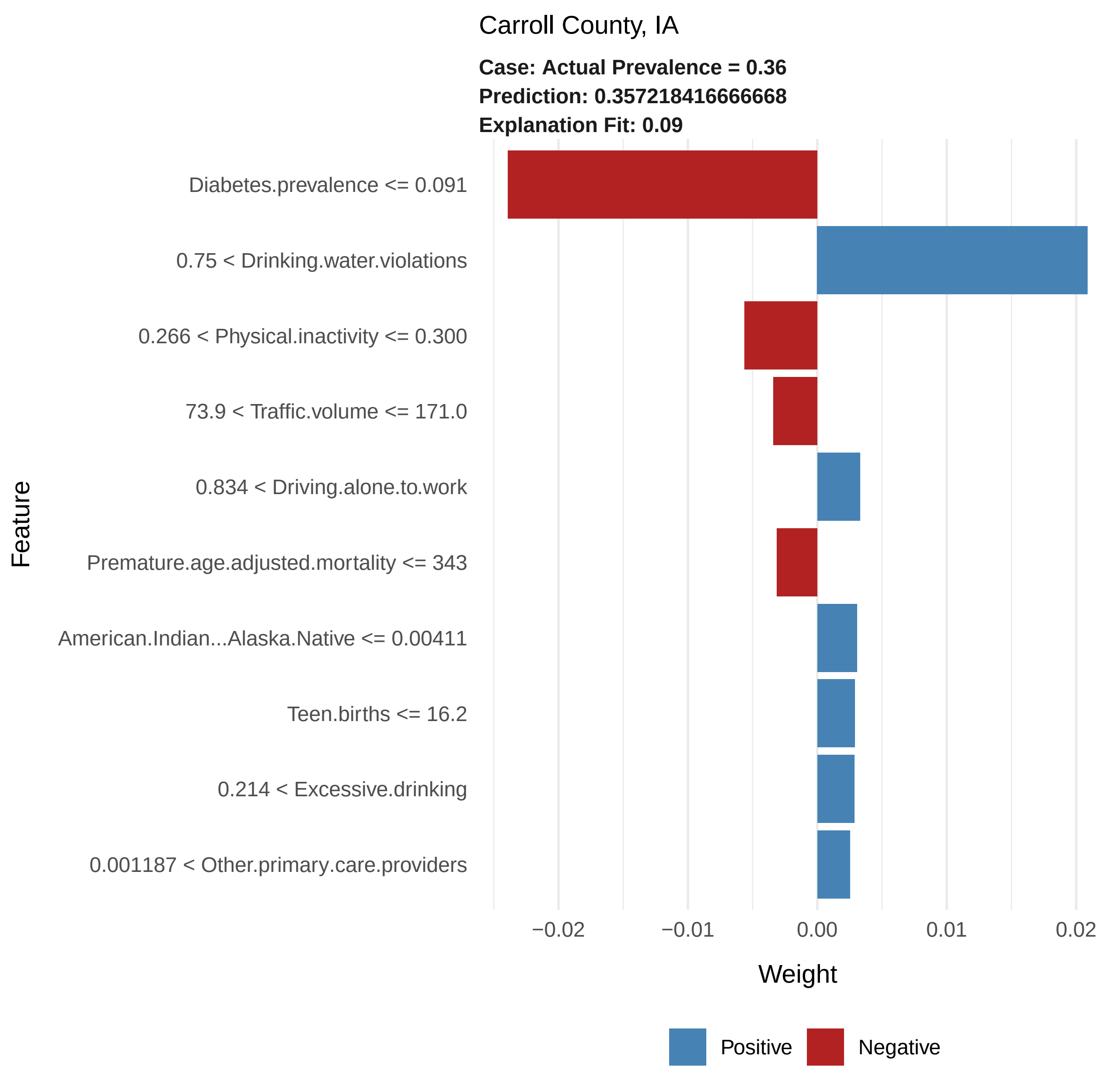

Carroll County, IA
Case: Actual Prevalence = 0.36
Prediction: 0.357218416666668
Explanation Fit: 0.09
Diabetes.prevalence <= 0.091
0.75 < Drinking.water.violations
0.266 < Physical.inactivity <= 0.300
73.9 < Traffic.volume <= 171.0
0.834 < Driving.alone.to.work
Premature.age.adjusted.mortality <= 343
American.Indian...Alaska.Native <= 0.00411
Teen.births <= 16.2
0.214 < Excessive.drinking
0.001187 < Other.primary.care.providers
Feature
−0.02
−0.01
0.00
0.01
0.02
Weight
Positive
Negative

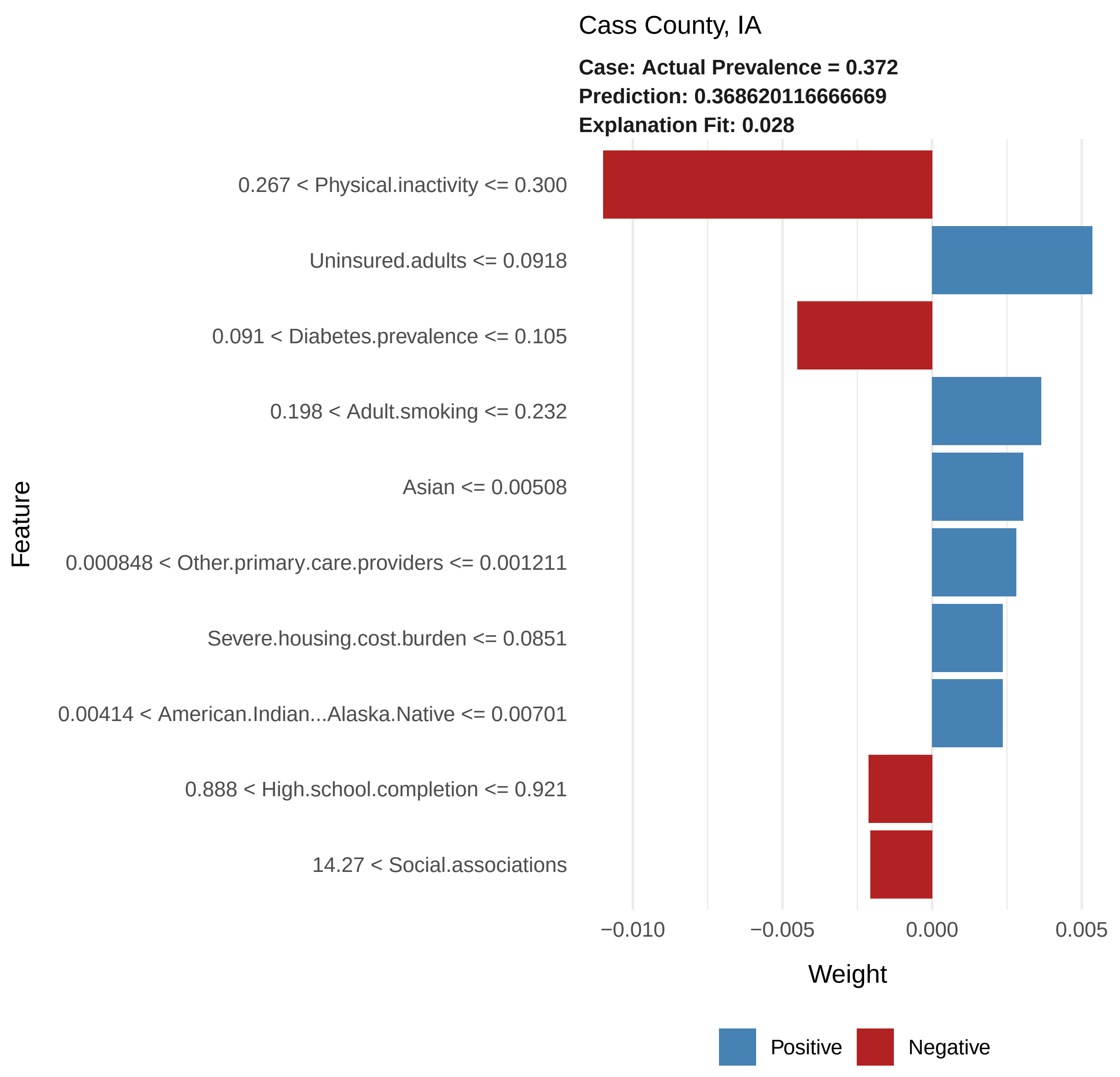

Cass County, IA
Case: Actual Prevalence = 0.372
Prediction: 0.368620116666669
Explanation Fit: 0.028
0.267 < Physical.inactivity <= 0.300
Uninsured.adults <= 0.0918
0.091 < Diabetes.prevalence <= 0.105
0.198 < Adult.smoking <= 0.232
Asian <= 0.00508
0.000848 < Other.primary.care.providers <= 0.001211
Severe.housing.cost.burden <= 0.0851
0.00414 < American.Indian...Alaska.Native <= 0.00701
0.888 < High.school.completion <= 0.921
14.27 < Social.associations
Feature
−0.010
−0.005
0.000
0.005
Weight
Positive
Negative

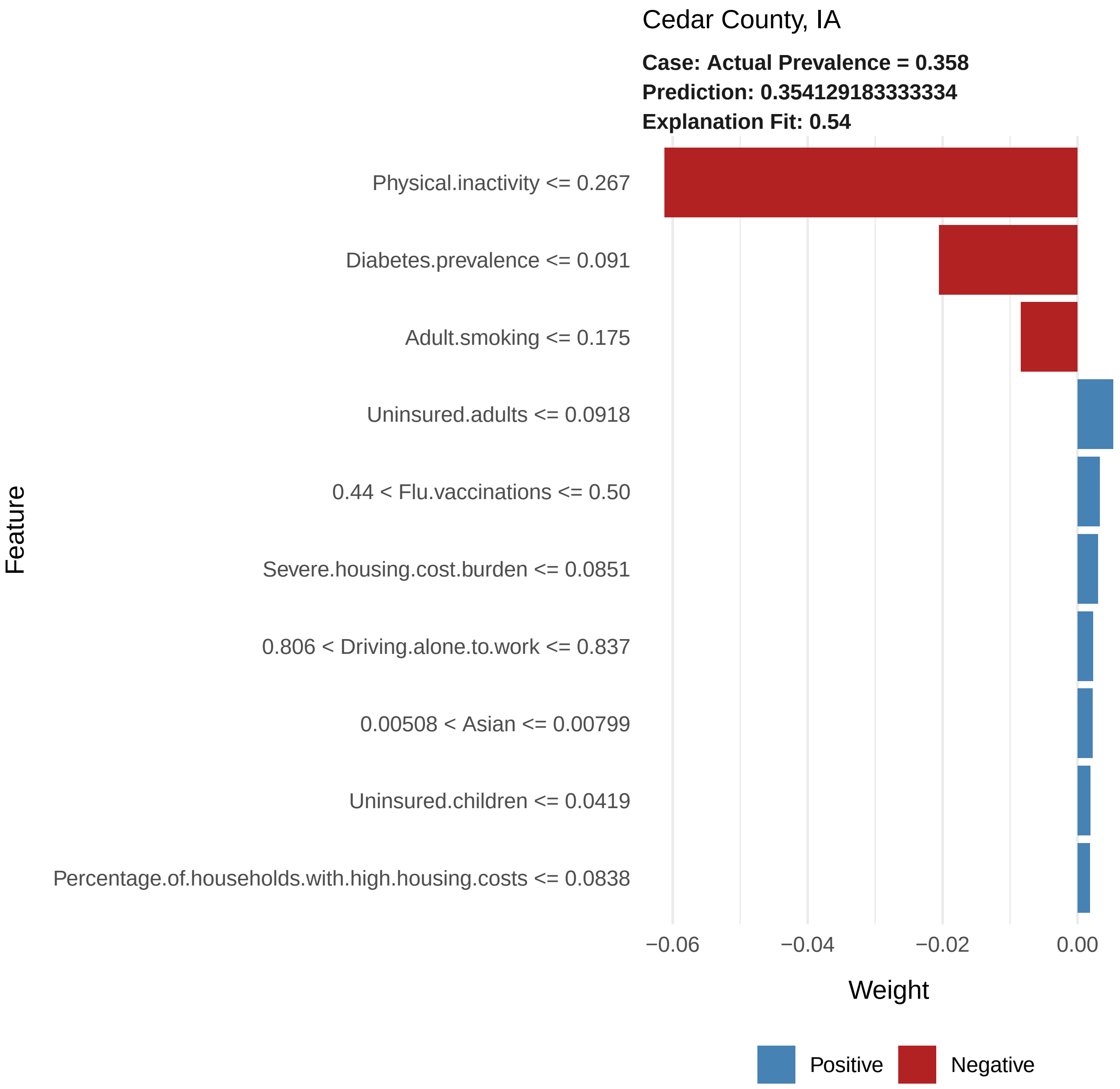

Cedar County, IA
Case: Actual Prevalence = 0.358
Prediction: 0.354129183333334
Explanation Fit: 0.54
Physical.inactivity <= 0.267
Diabetes.prevalence <= 0.091
Adult.smoking <= 0.175
Uninsured.adults <= 0.0918
0.44 < Flu.vaccinations <= 0.50
Severe.housing.cost.burden <= 0.0851
0.806 < Driving.alone.to.work <= 0.837
0.00508 < Asian <= 0.00799
Uninsured.children <= 0.0419
Percentage.of.households.with.high.housing.costs <= 0.0838
Feature
−0.06
−0.04
−0.02
0.00
Weight
Positive
Negative

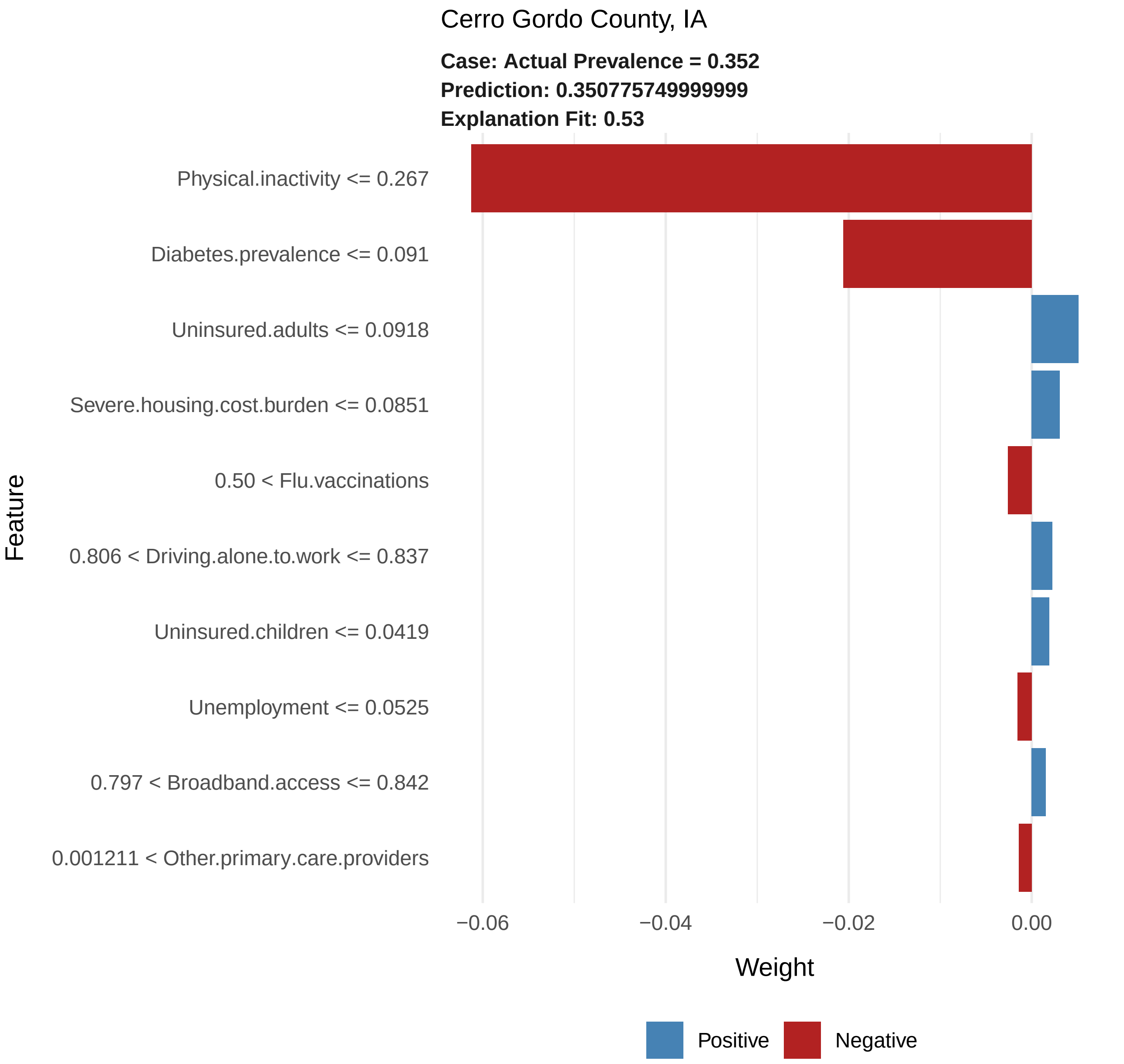

Cerro Gordo County, IA
Case: Actual Prevalence = 0.352
Prediction: 0.350775749999999
Explanation Fit: 0.53
Feature
Physical.inactivity <= 0.267
Diabetes.prevalence <= 0.091
Uninsured.adults <= 0.0918
Severe.housing.cost.burden <= 0.0851
0.50 < Flu.vaccinations
0.806 < Driving.alone.to.work <= 0.837
Uninsured.children <= 0.0419
Unemployment <= 0.0525
0.797 < Broadband.access <= 0.842
0.001211 < Other.primary.care.providers
−0.06
−0.04
−0.02
0.00
Weight
Positive
Negative

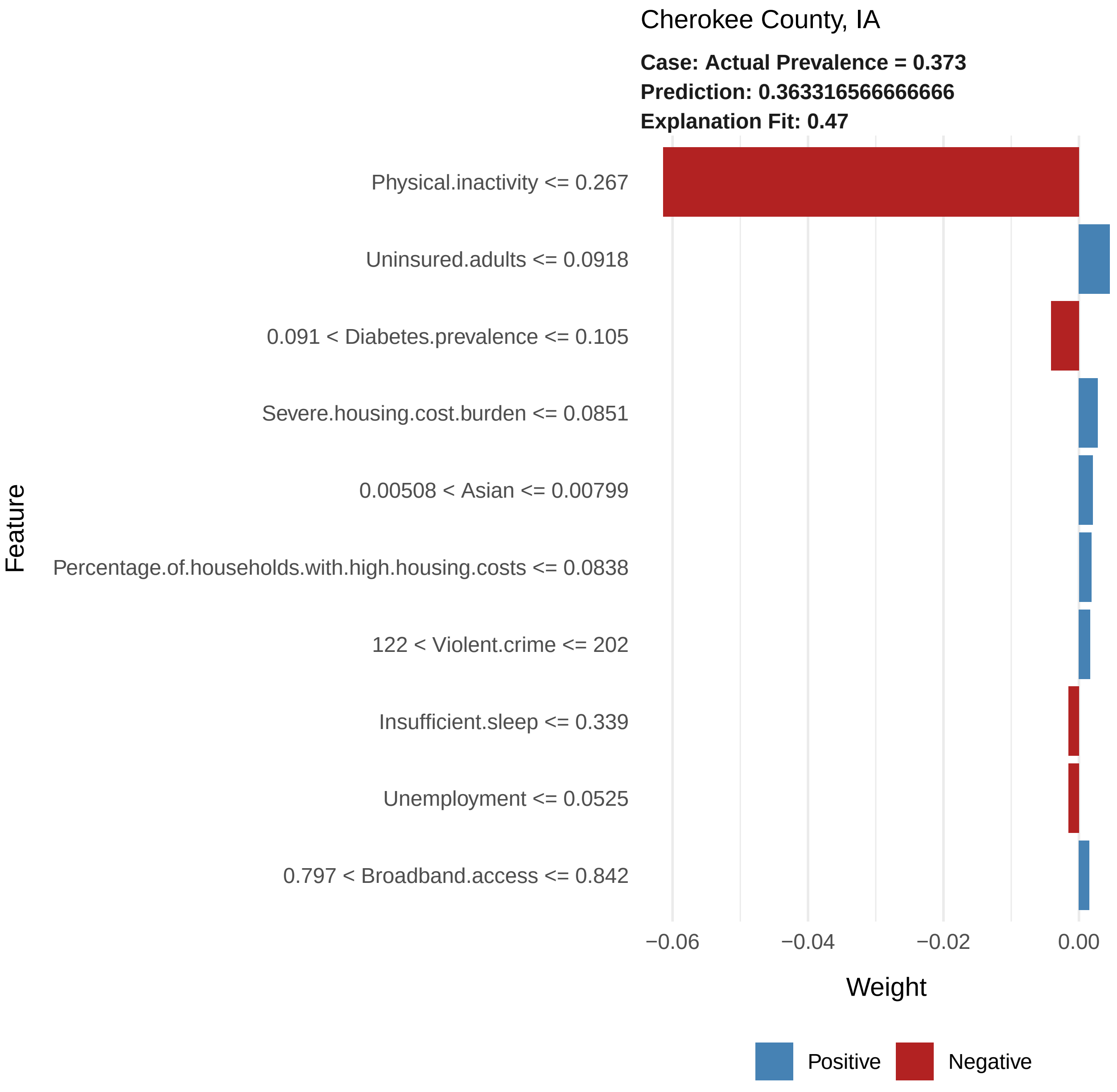

Cherokee County, IA
Case: Actual Prevalence = 0.373
Prediction: 0.363316566666666
Explanation Fit: 0.47
Physical.inactivity <= 0.267
Uninsured.adults <= 0.0918
0.091 < Diabetes.prevalence <= 0.105
Severe.housing.cost.burden <= 0.0851
0.00508 < Asian <= 0.00799
Percentage.of.households.with.high.housing.costs <= 0.0838
122 < Violent.crime <= 202
Insufficient.sleep <= 0.339
Unemployment <= 0.0525
0.797 < Broadband.access <= 0.842
Feature
−0.06
−0.04
−0.02
0.00
Weight
Positive
Negative

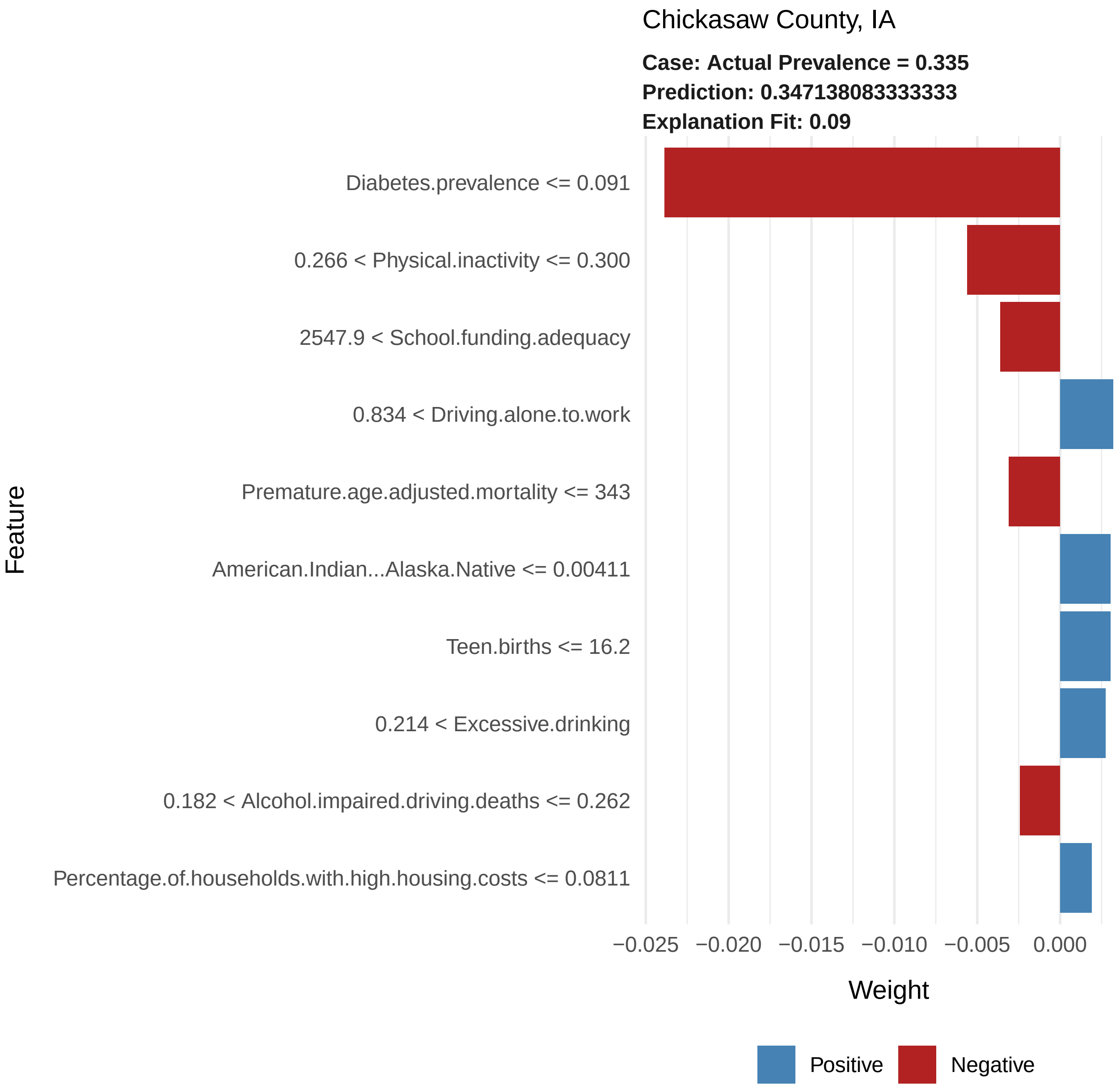
Chickasaw County, IA
Case: Actual Prevalence = 0.335
Prediction: 0.347138083333333
Explanation Fit: 0.09
Diabetes.prevalence <= 0.091
0.266 < Physical.inactivity <= 0.300
2547.9 < School.funding.adequacy
0.834 < Driving.alone.to.work
Premature.age.adjusted.mortality <= 343
American.Indian...Alaska.Native <= 0.00411
Teen.births <= 16.2
0.214 < Excessive.drinking
0.182 < Alcohol.impaired.driving.deaths <= 0.262
Percentage.of.households.with.high.housing.costs <= 0.0811
Feature
−0.025
−0.020
−0.015
−0.010
−0.005
0.000
Weight
Positive
Negative

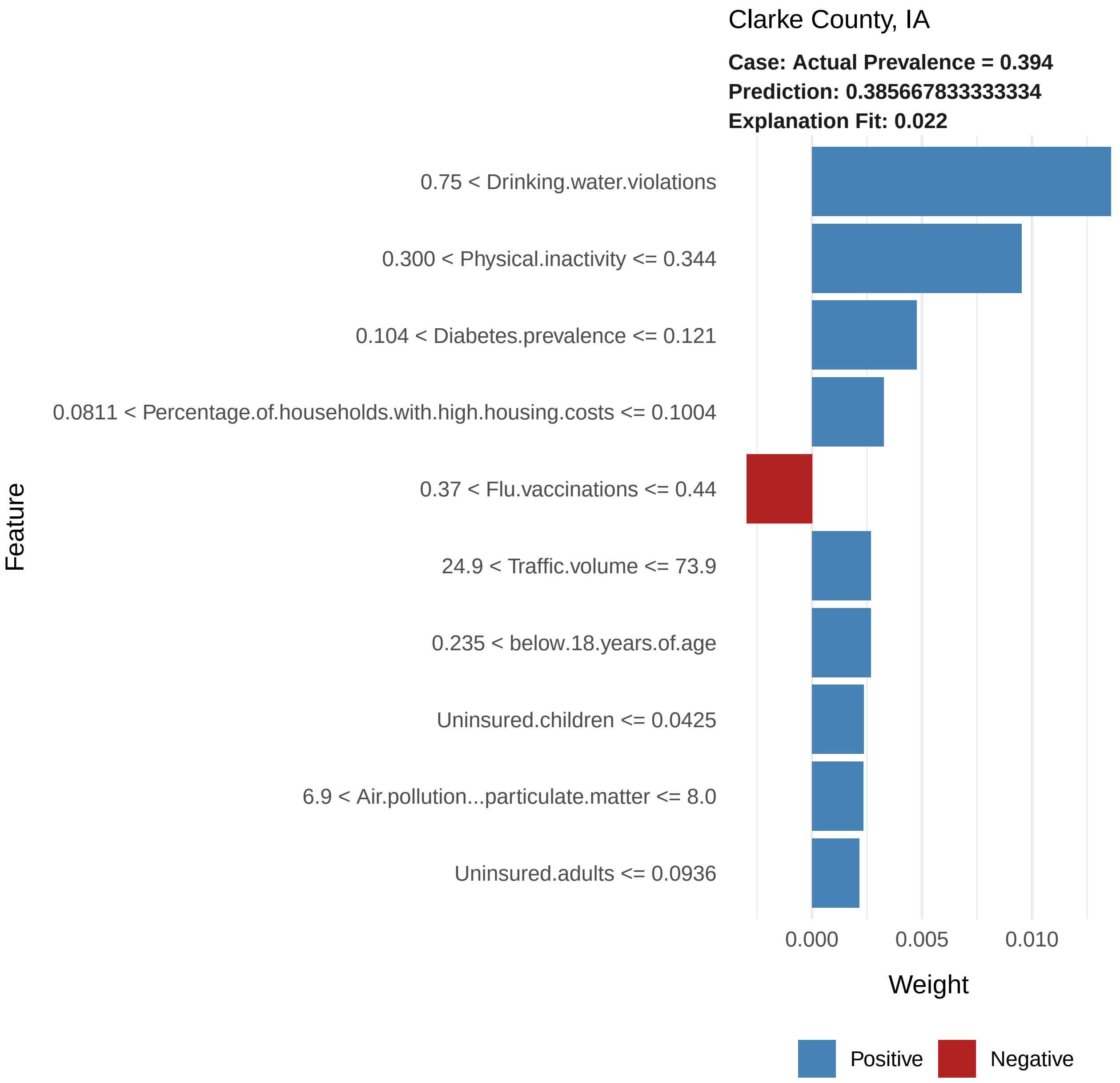

Clarke County, IA
Case: Actual Prevalence = 0.394
Prediction: 0.385667833333334
Explanation Fit: 0.022
0.75 < Drinking.water.violations
0.300 < Physical.inactivity <= 0.344
0.104 < Diabetes.prevalence <= 0.121
0.0811 < Percentage.of.households.with.high.housing.costs <= 0.1004
0.37 < Flu.vaccinations <= 0.44
24.9 < Traffic.volume <= 73.9
0.235 < below.18.years.of.age
Uninsured.children <= 0.0425
6.9 < Air.pollution...particulate.matter <= 8.0
Uninsured.adults <= 0.0936
Feature
0.000
0.005
0.010
Weight
Positive
Negative

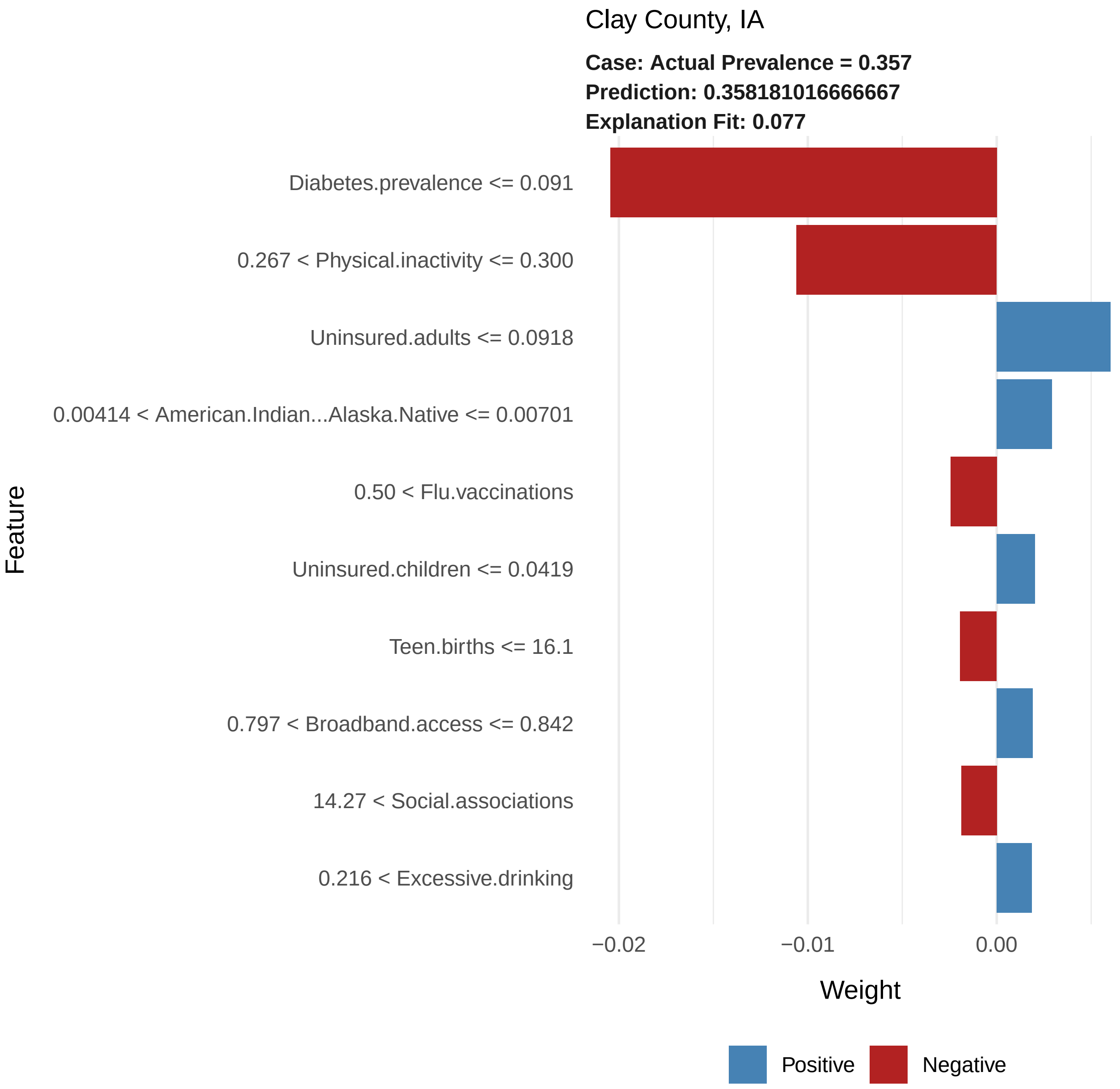

Clay County, IA
Case: Actual Prevalence = 0.357
Prediction: 0.358181016666667
Explanation Fit: 0.077
Diabetes.prevalence <= 0.091
0.267 < Physical.inactivity <= 0.300
Uninsured.adults <= 0.0918
0.00414 < American.Indian...Alaska.Native <= 0.00701
0.50 < Flu.vaccinations
Uninsured.children <= 0.0419
Teen.births <= 16.1
0.797 < Broadband.access <= 0.842
14.27 < Social.associations
0.216 < Excessive.drinking
Feature
−0.02
−0.01
0.00
Weight
Positive
Negative

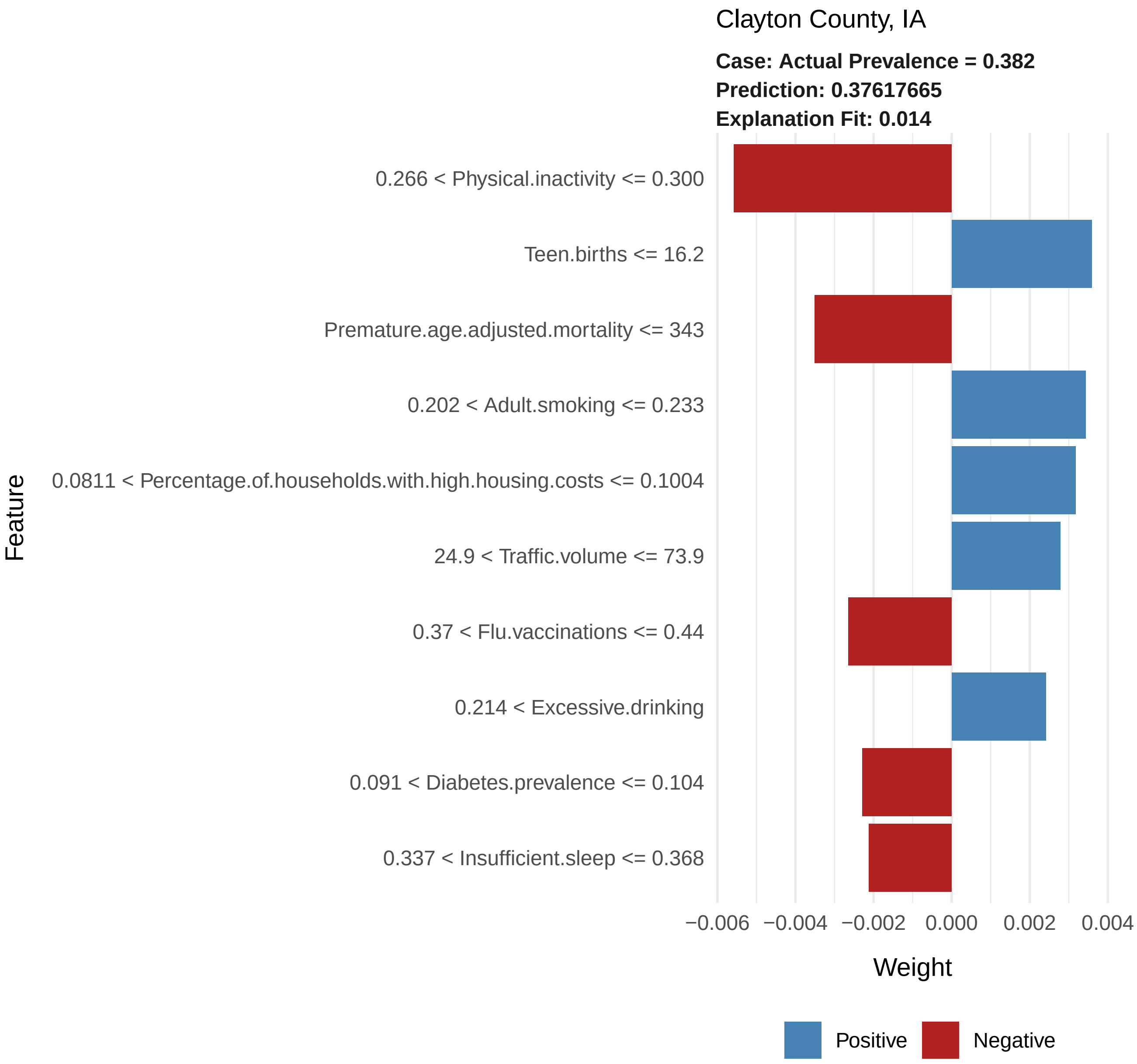

Clayton County, IA
Case: Actual Prevalence = 0.382
Prediction: 0.37617665
Explanation Fit: 0.014
0.266 < Physical.inactivity <= 0.300
Teen.births <= 16.2
Premature.age.adjusted.mortality <= 343
0.202 < Adult.smoking <= 0.233
0.0811 < Percentage.of.households.with.high.housing.costs <= 0.1004
24.9 < Traffic.volume <= 73.9
0.37 < Flu.vaccinations <= 0.44
0.214 < Excessive.drinking
0.091 < Diabetes.prevalence <= 0.104
0.337 < Insufficient.sleep <= 0.368
Feature
−0.006
−0.004
−0.002
0.000
0.002
0.004
Weight
Positive
Negative

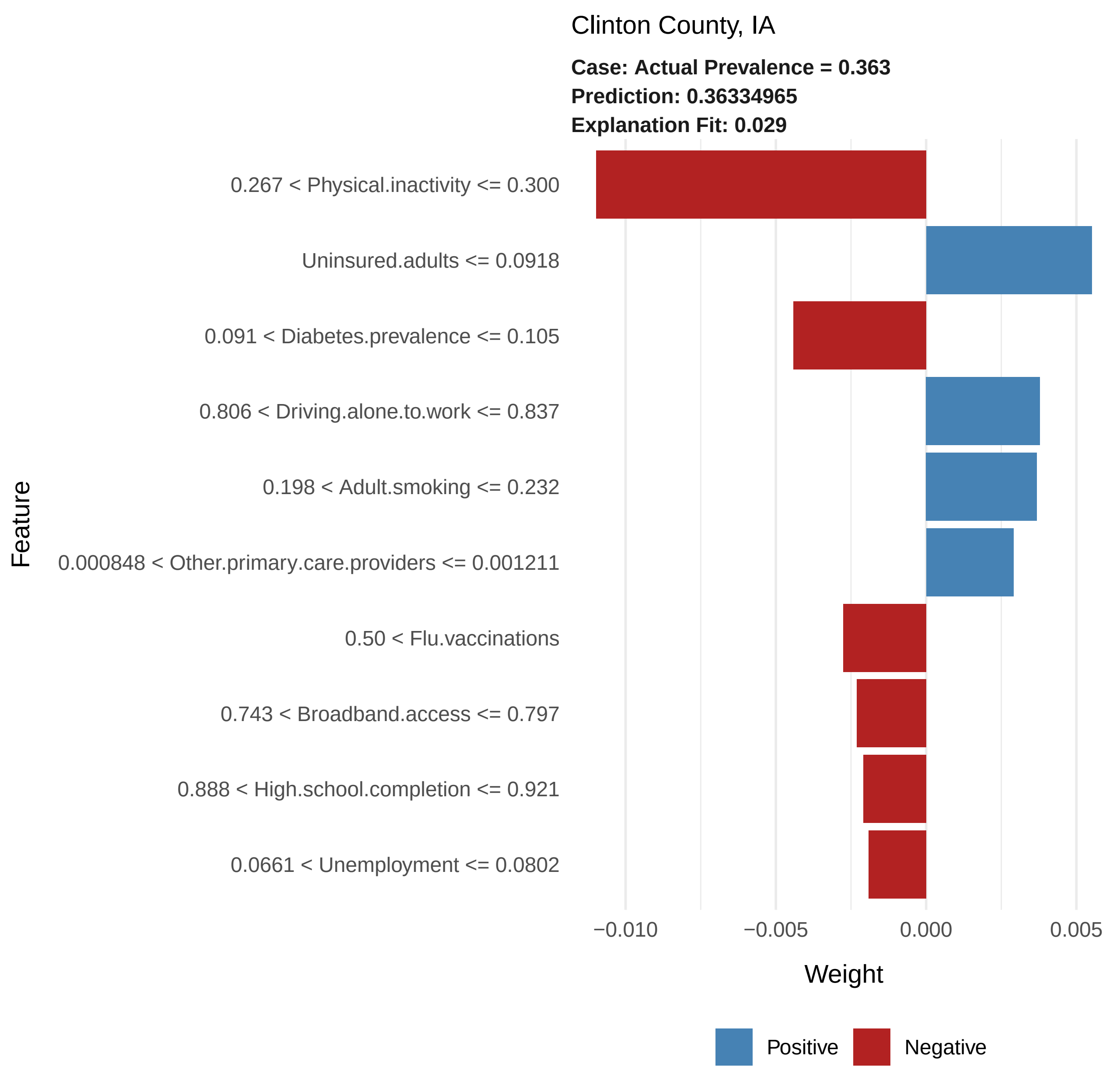
Clinton County, IA
Case: Actual Prevalence = 0.363
Prediction: 0.36334965
Explanation Fit: 0.029
0.267 < Physical.inactivity <= 0.300
Uninsured.adults <= 0.0918
0.091 < Diabetes.prevalence <= 0.105
0.806 < Driving.alone.to.work <= 0.837
0.198 < Adult.smoking <= 0.232
0.000848 < Other.primary.care.providers <= 0.001211
0.50 < Flu.vaccinations
0.743 < Broadband.access <= 0.797
0.888 < High.school.completion <= 0.921
0.0661 < Unemployment <= 0.0802
Feature
−0.010
−0.005
0.000
0.005
Weight
Positive
Negative

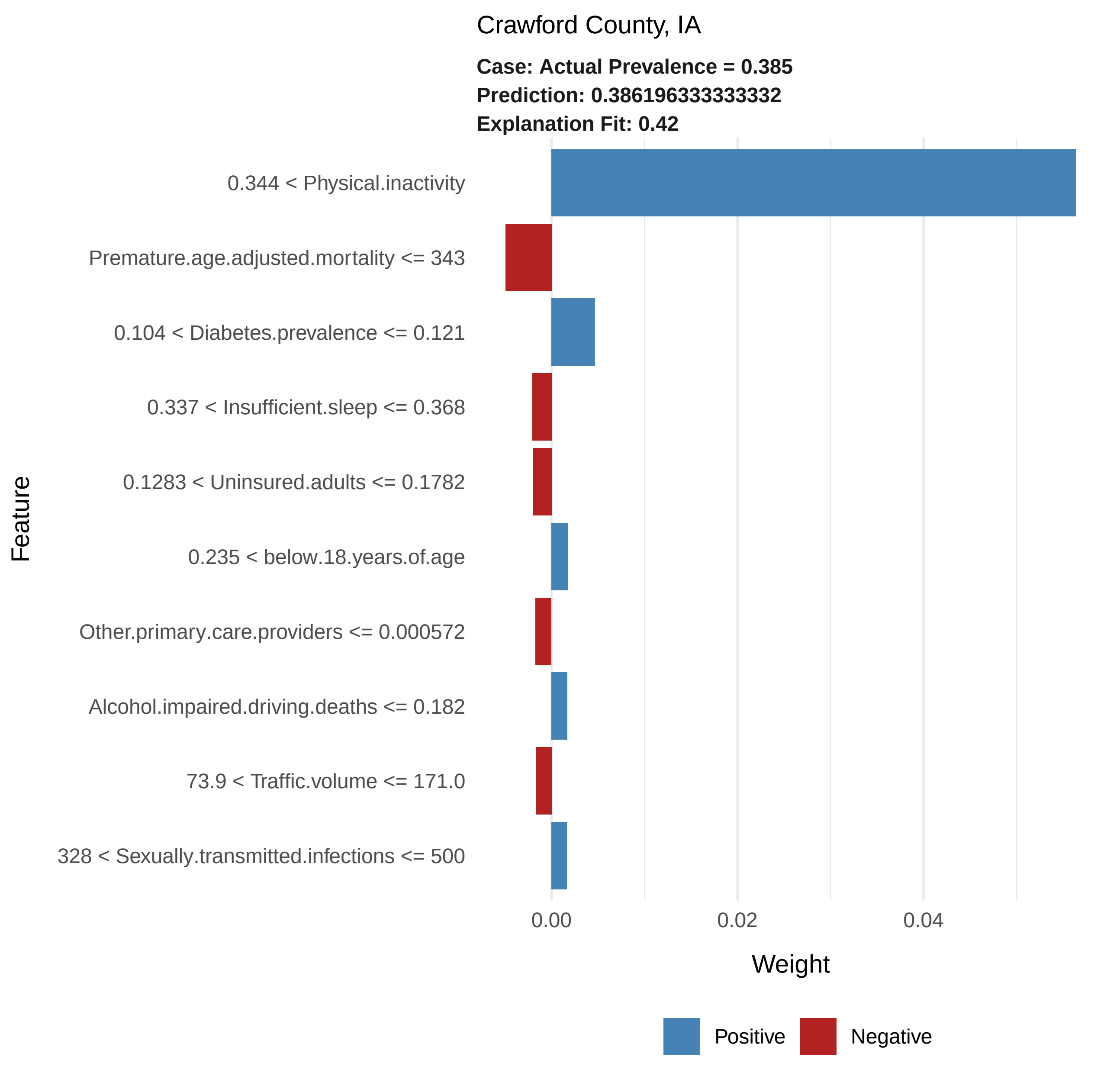
Crawford County, IA
Case: Actual Prevalence = 0.385
Prediction: 0.386196333333332
Explanation Fit: 0.42
0.344 < Physical.inactivity
Premature.age.adjusted.mortality <= 343
0.104 < Diabetes.prevalence <= 0.121
0.337 < Insufficient.sleep <= 0.368
0.1283 < Uninsured.adults <= 0.1782
0.235 < below.18.years.of.age
Other.primary.care.providers <= 0.000572
Alcohol.impaired.driving.deaths <= 0.182
73.9 < Traffic.volume <= 171.0
328 < Sexually.transmitted.infections <= 500
Feature
0.00
0.02
0.04
Weight
Positive
Negative

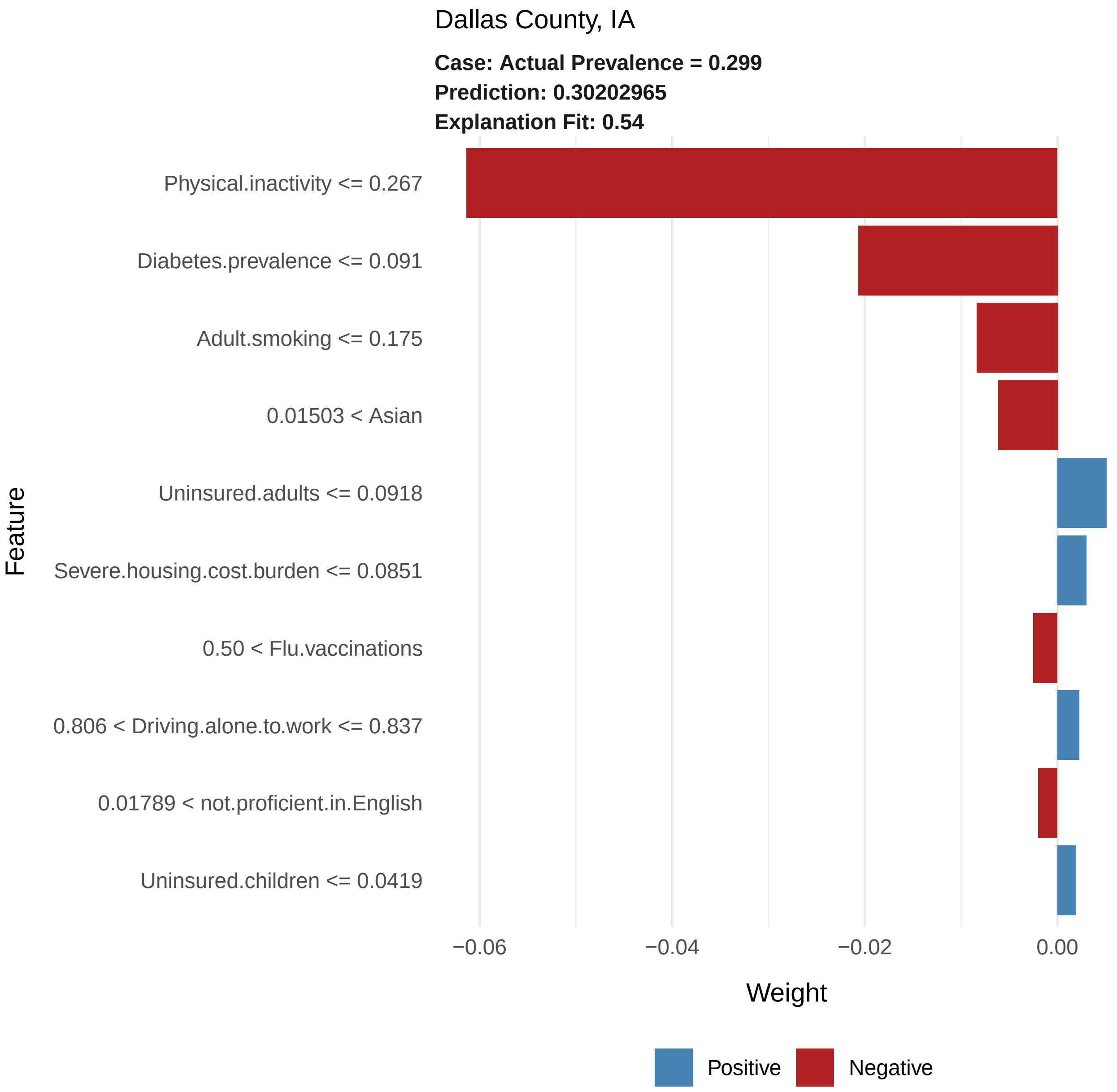

Dallas County, IA
Case: Actual Prevalence = 0.299
Prediction: 0.30202965
Explanation Fit: 0.54
Physical.inactivity <= 0.267
Diabetes.prevalence <= 0.091
Adult.smoking <= 0.175
0.01503 < Asian
Uninsured.adults <= 0.0918
Severe.housing.cost.burden <= 0.0851
0.50 < Flu.vaccinations
0.806 < Driving.alone.to.work <= 0.837
0.01789 < not.proficient.in.English
Uninsured.children <= 0.0419
Feature
−0.06
−0.04
−0.02
0.00
Weight
Positive
Negative

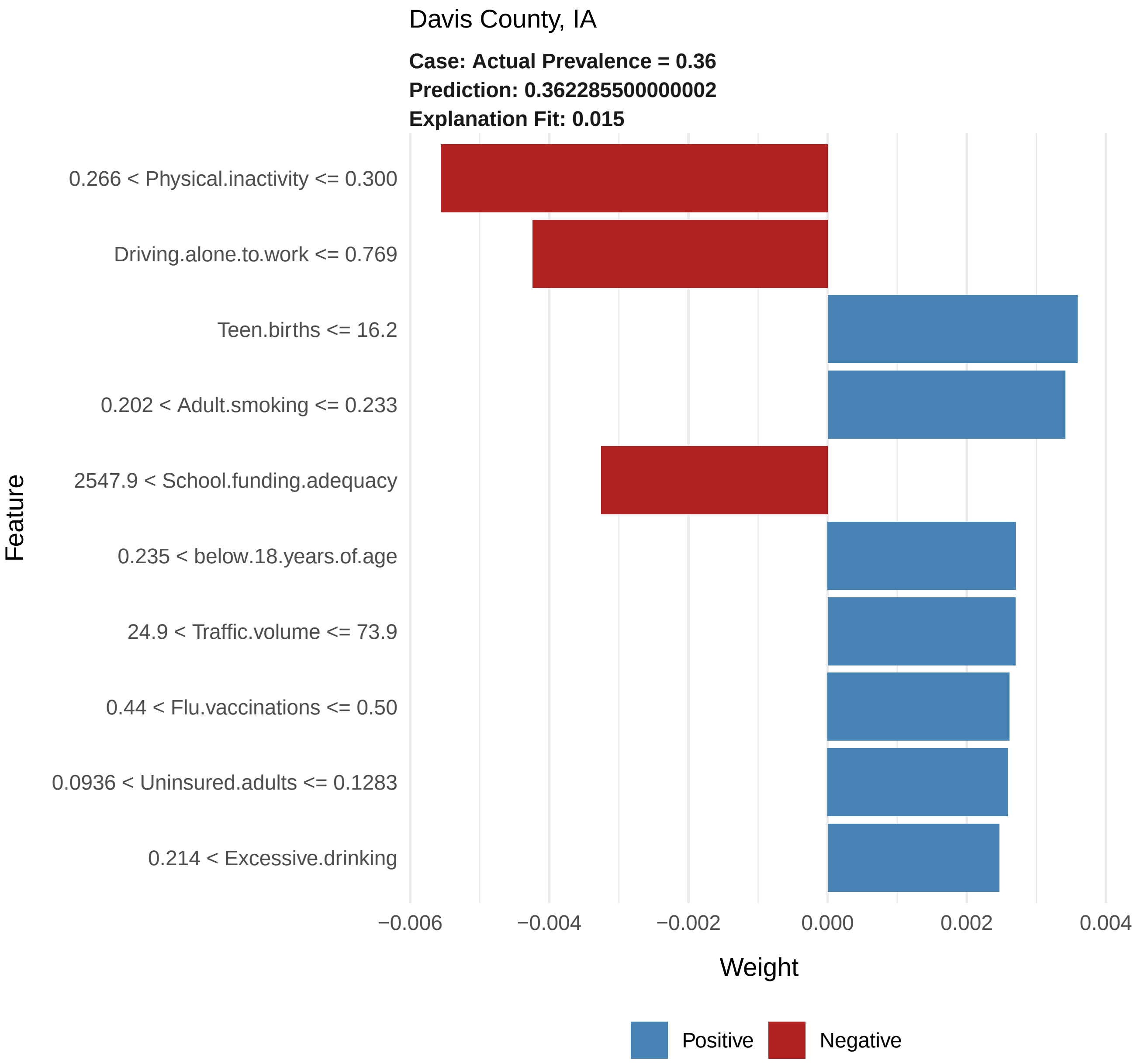

Davis County, IA
Case: Actual Prevalence = 0.36
Prediction: 0.362285500000002
Explanation Fit: 0.015
0.266 < Physical.inactivity <= 0.300
Driving.alone.to.work <= 0.769
Teen.births <= 16.2
0.202 < Adult.smoking <= 0.233
2547.9 < School.funding.adequacy
0.235 < below.18.years.of.age
24.9 < Traffic.volume <= 73.9
0.44 < Flu.vaccinations <= 0.50
0.0936 < Uninsured.adults <= 0.1283
0.214 < Excessive.drinking
Feature
−0.006
−0.004
−0.002
0.000
0.002
0.004
Weight
Positive
Negative

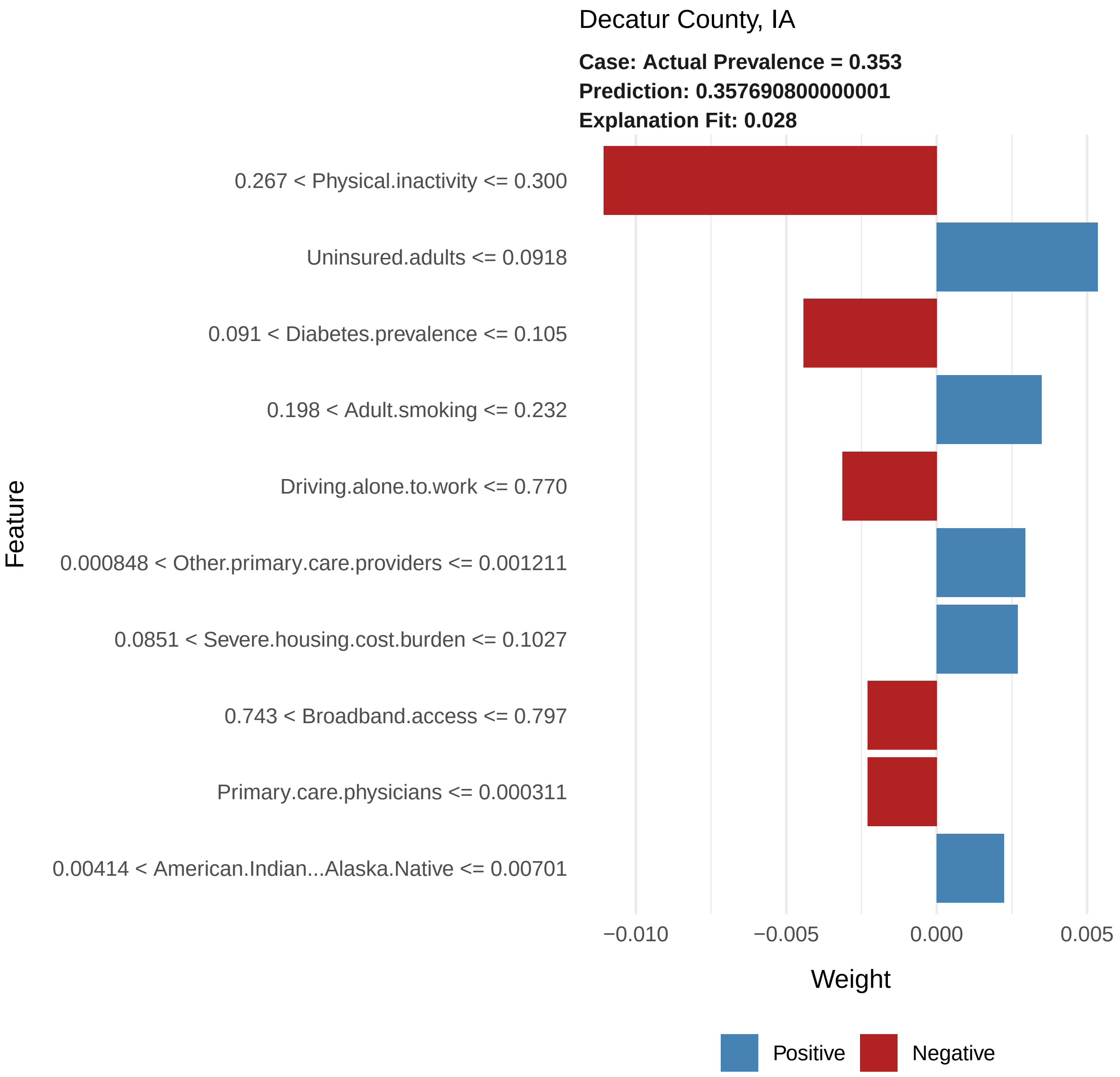
Decatur County, IA
Case: Actual Prevalence = 0.353
Prediction: 0.357690800000001
Explanation Fit: 0.028
0.267 < Physical.inactivity <= 0.300
Uninsured.adults <= 0.0918
0.091 < Diabetes.prevalence <= 0.105
0.198 < Adult.smoking <= 0.232
Driving.alone.to.work <= 0.770
0.000848 < Other.primary.care.providers <= 0.001211
0.0851 < Severe.housing.cost.burden <= 0.1027
0.743 < Broadband.access <= 0.797
Primary.care.physicians <= 0.000311
0.00414 < American.Indian...Alaska.Native <= 0.00701
Feature
−0.010
−0.005
0.000
0.005
Weight
Positive
Negative

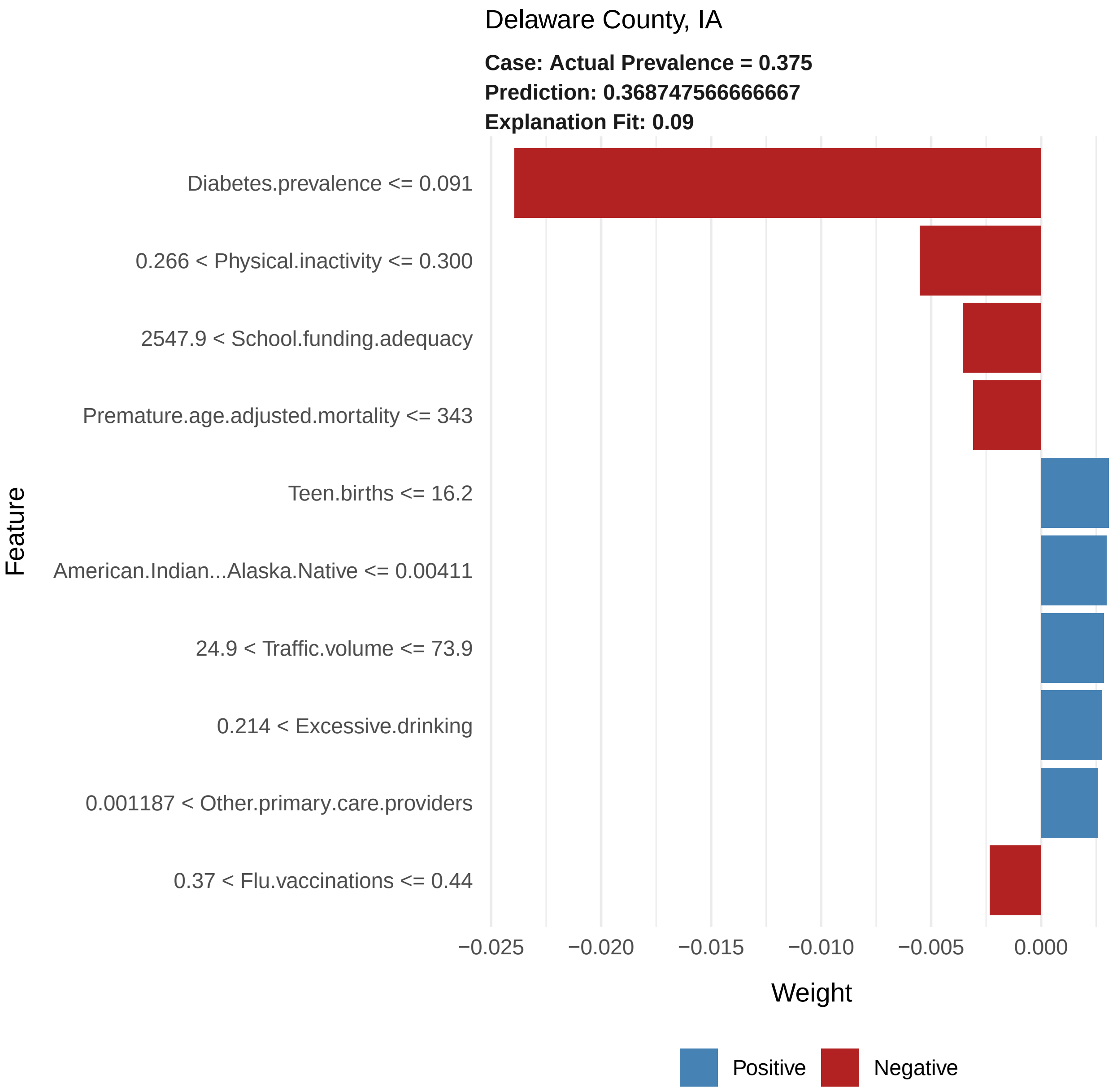

Delaware County, IA
Case: Actual Prevalence = 0.375
Prediction: 0.368747566666667
Explanation Fit: 0.09
Diabetes.prevalence <= 0.091
0.266 < Physical.inactivity <= 0.300
2547.9 < School.funding.adequacy
Premature.age.adjusted.mortality <= 343
Teen.births <= 16.2
American.Indian...Alaska.Native <= 0.00411
24.9 < Traffic.volume <= 73.9
0.214 < Excessive.drinking
0.001187 < Other.primary.care.providers
0.37 < Flu.vaccinations <= 0.44
Feature
−0.025
−0.020
−0.015
−0.010
−0.005
0.000
Weight
Positive
Negative

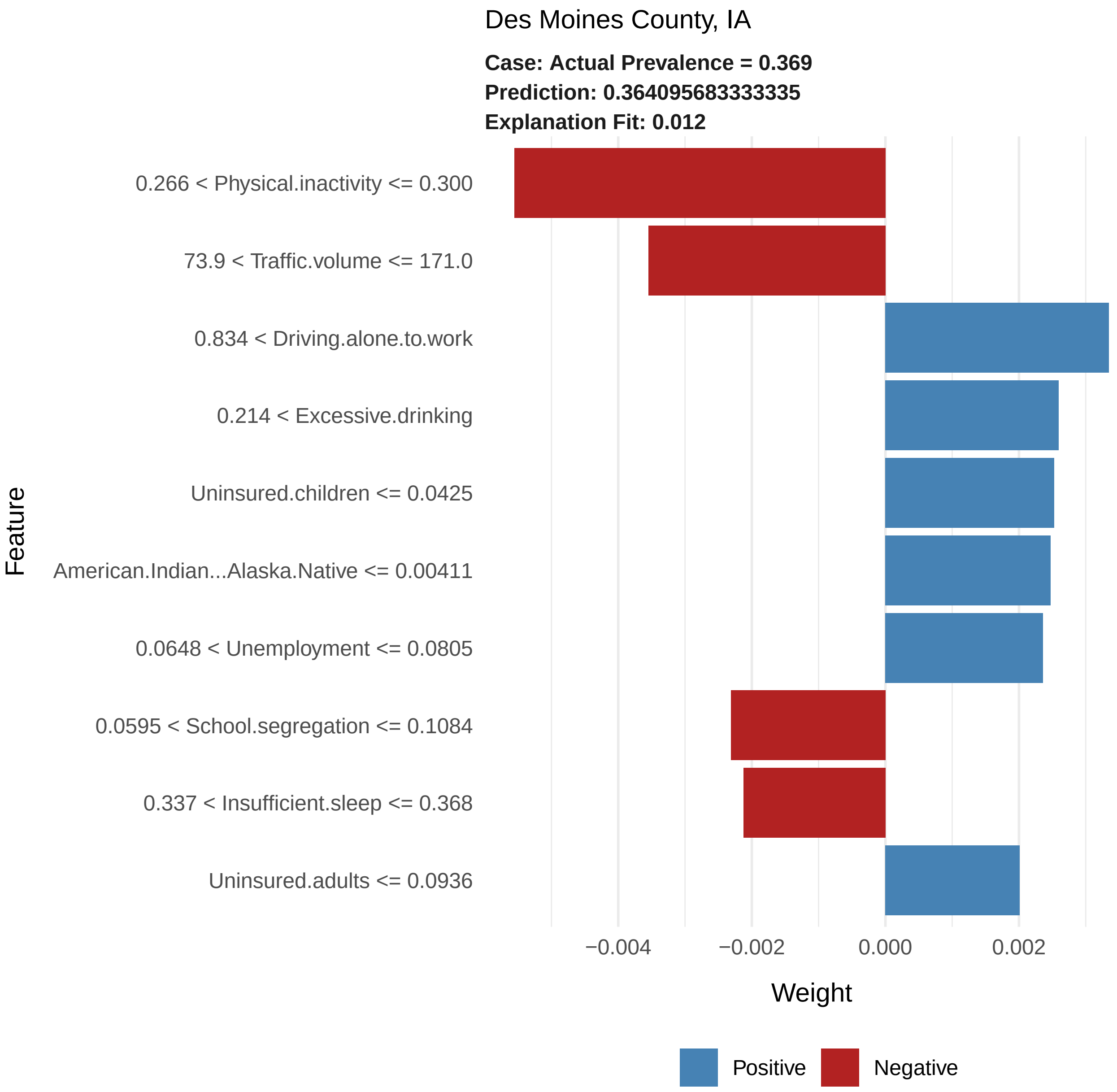

Des Moines County, IA
Case: Actual Prevalence = 0.369
Prediction: 0.364095683333335
Explanation Fit: 0.012
0.266 < Physical.inactivity <= 0.300
73.9 < Traffic.volume <= 171.0
0.834 < Driving.alone.to.work
0.214 < Excessive.drinking
Uninsured.children <= 0.0425
American.Indian...Alaska.Native <= 0.00411
0.0648 < Unemployment <= 0.0805
0.0595 < School.segregation <= 0.1084
0.337 < Insufficient.sleep <= 0.368
Uninsured.adults <= 0.0936
Feature
−0.004
−0.002
0.000
0.002
Weight
Positive
Negative

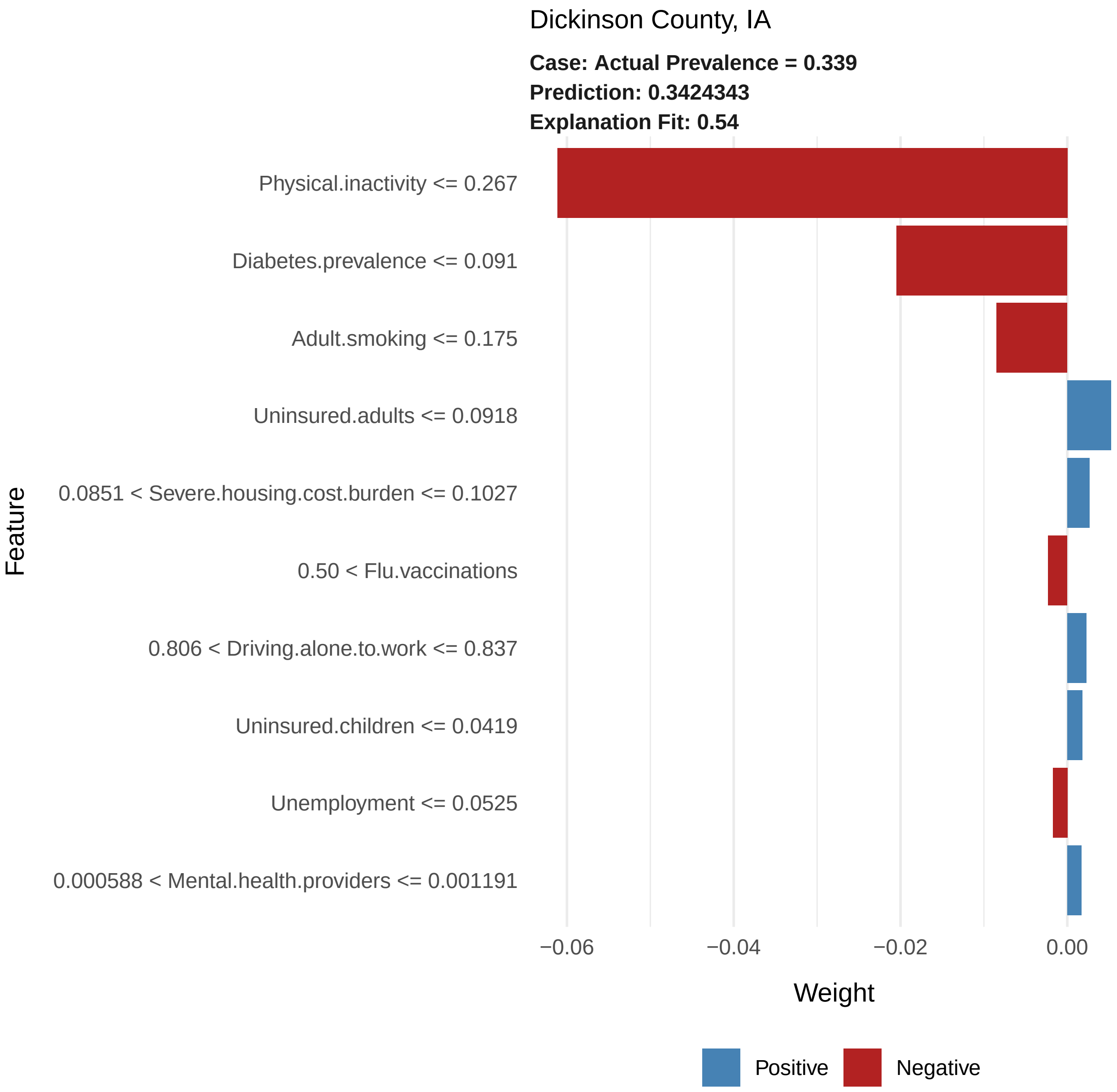

Dickinson County, IA
Case: Actual Prevalence = 0.339
Prediction: 0.3424343
Explanation Fit: 0.54
Physical.inactivity <= 0.267
Diabetes.prevalence <= 0.091
Adult.smoking <= 0.175
Uninsured.adults <= 0.0918
0.0851 < Severe.housing.cost.burden <= 0.1027
0.50 < Flu.vaccinations
0.806 < Driving.alone.to.work <= 0.837
Uninsured.children <= 0.0419
Unemployment <= 0.0525
0.000588 < Mental.health.providers <= 0.001191
Feature
−0.06
−0.04
−0.02
0.00
Weight
Positive
Negative

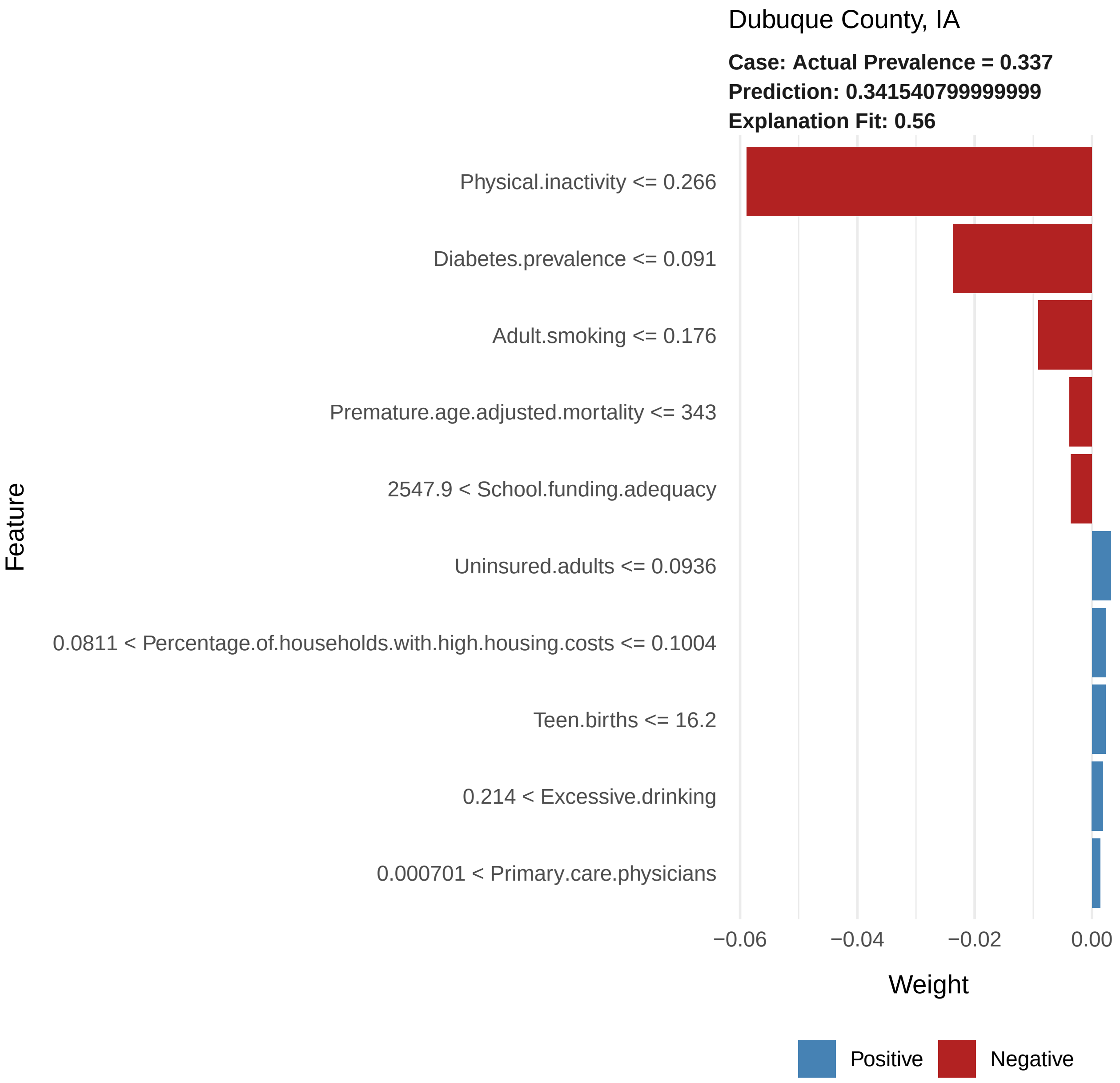

Dubuque County, IA
Case: Actual Prevalence = 0.337
Prediction: 0.341540799999999
Explanation Fit: 0.56
Feature
Physical.inactivity <= 0.266
Diabetes.prevalence <= 0.091
Adult.smoking <= 0.176
Premature.age.adjusted.mortality <= 343
2547.9 < School.funding.adequacy
Uninsured.adults <= 0.0936
0.0811 < Percentage.of.households.with.high.housing.costs <= 0.1004
Teen.births <= 16.2
0.214 < Excessive.drinking
0.000701 < Primary.care.physicians
−0.06
−0.04
−0.02
0.00
Weight
Positive
Negative

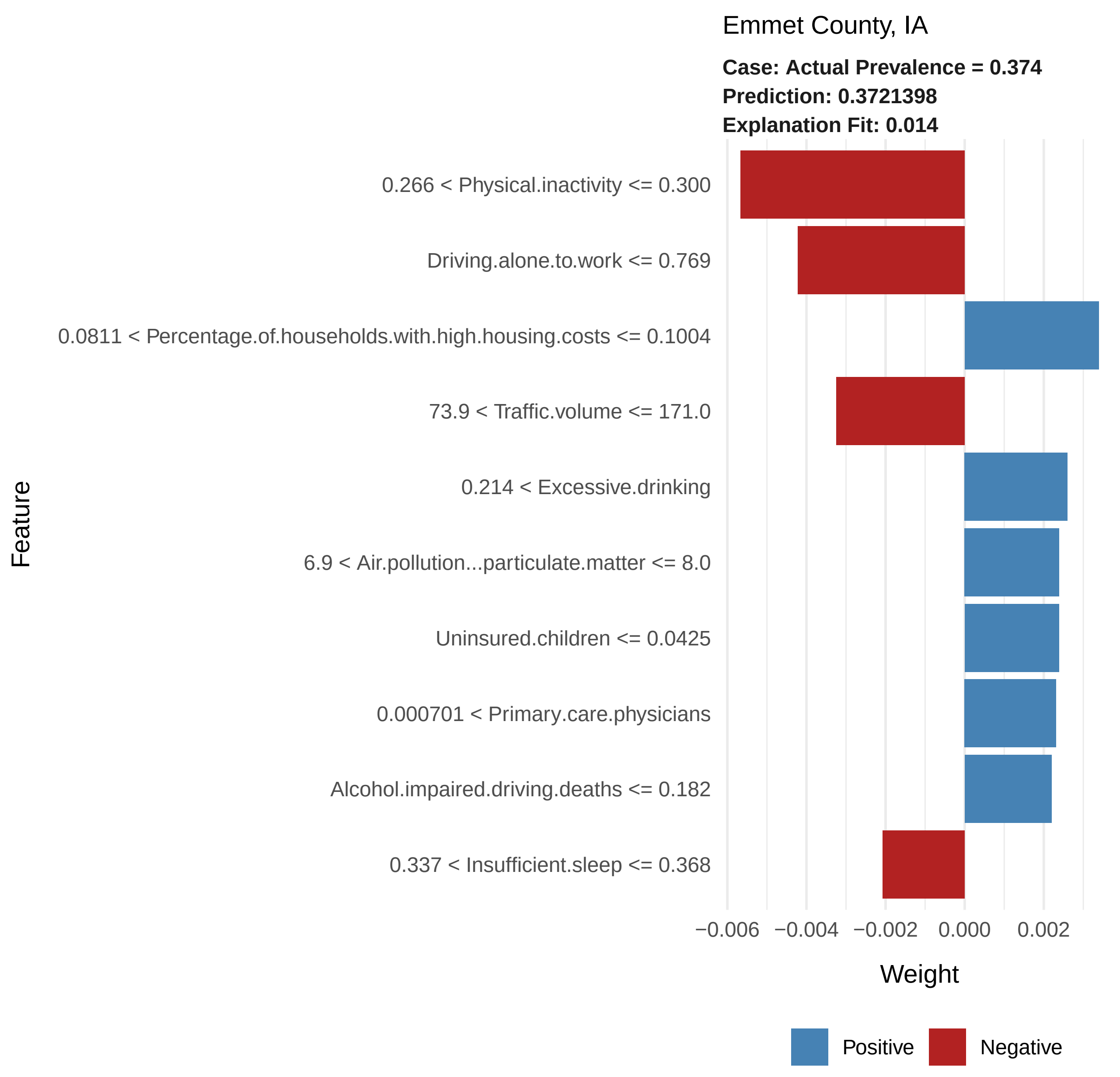

Emmet County, IA
Case: Actual Prevalence = 0.374
Prediction: 0.3721398
Explanation Fit: 0.014
0.266 < Physical.inactivity <= 0.300
Driving.alone.to.work <= 0.769
0.0811 < Percentage.of.households.with.high.housing.costs <= 0.1004
73.9 < Traffic.volume <= 171.0
0.214 < Excessive.drinking
6.9 < Air.pollution...particulate.matter <= 8.0
Uninsured.children <= 0.0425
0.000701 < Primary.care.physicians
Alcohol.impaired.driving.deaths <= 0.182
0.337 < Insufficient.sleep <= 0.368
Feature
−0.006
−0.004
−0.002
0.000
0.002
Weight
Positive
Negative

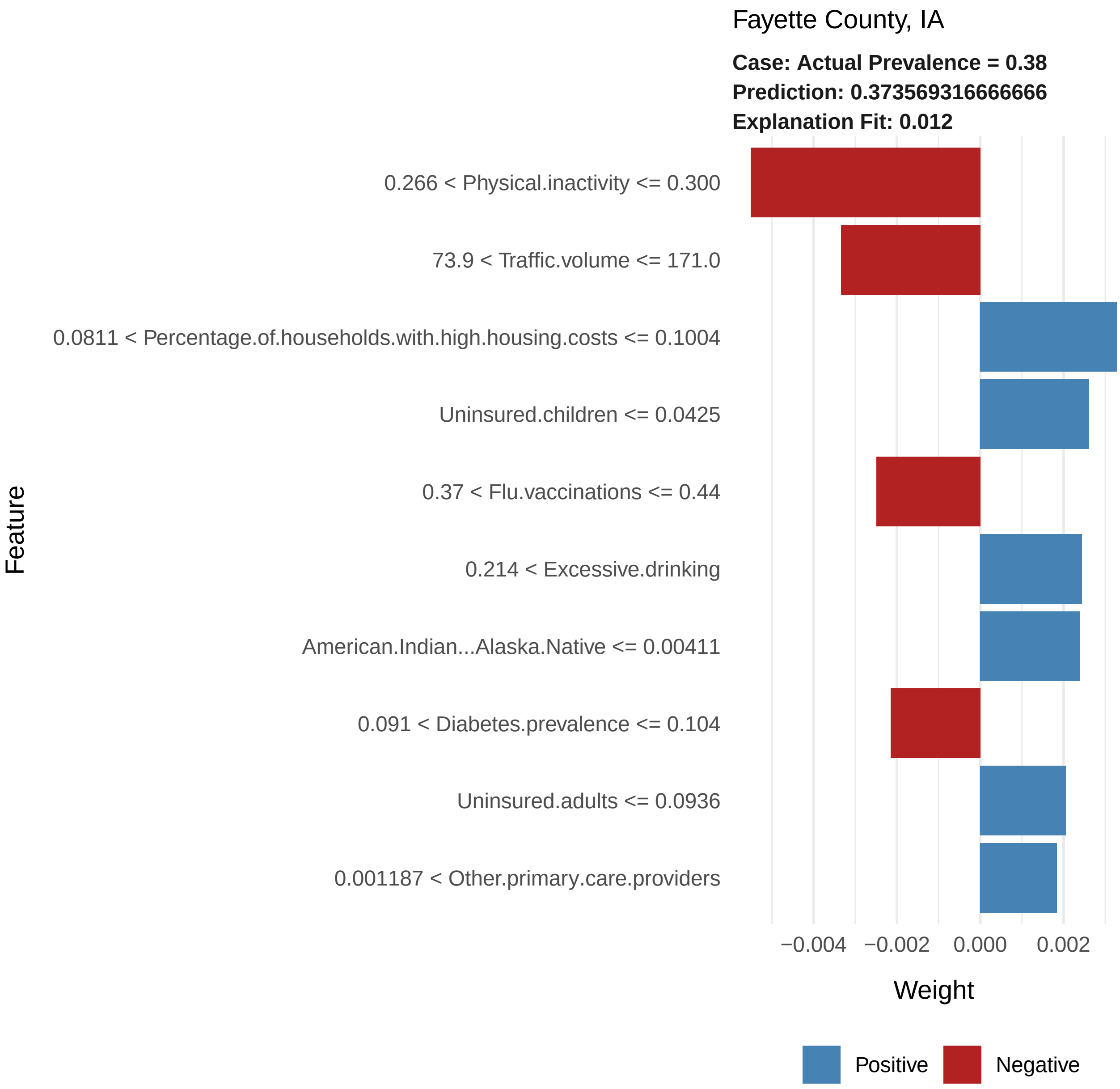

Fayette County, IA
Case: Actual Prevalence = 0.38
Prediction: 0.373569316666666
Explanation Fit: 0.012
0.266 < Physical.inactivity <= 0.300
73.9 < Traffic.volume <= 171.0
0.0811 < Percentage.of.households.with.high.housing.costs <= 0.1004
Uninsured.children <= 0.0425
0.37 < Flu.vaccinations <= 0.44
0.214 < Excessive.drinking
American.Indian...Alaska.Native <= 0.00411
0.091 < Diabetes.prevalence <= 0.104
Uninsured.adults <= 0.0936
0.001187 < Other.primary.care.providers
Feature
−0.004
−0.002
0.000
0.002
Weight
Positive
Negative

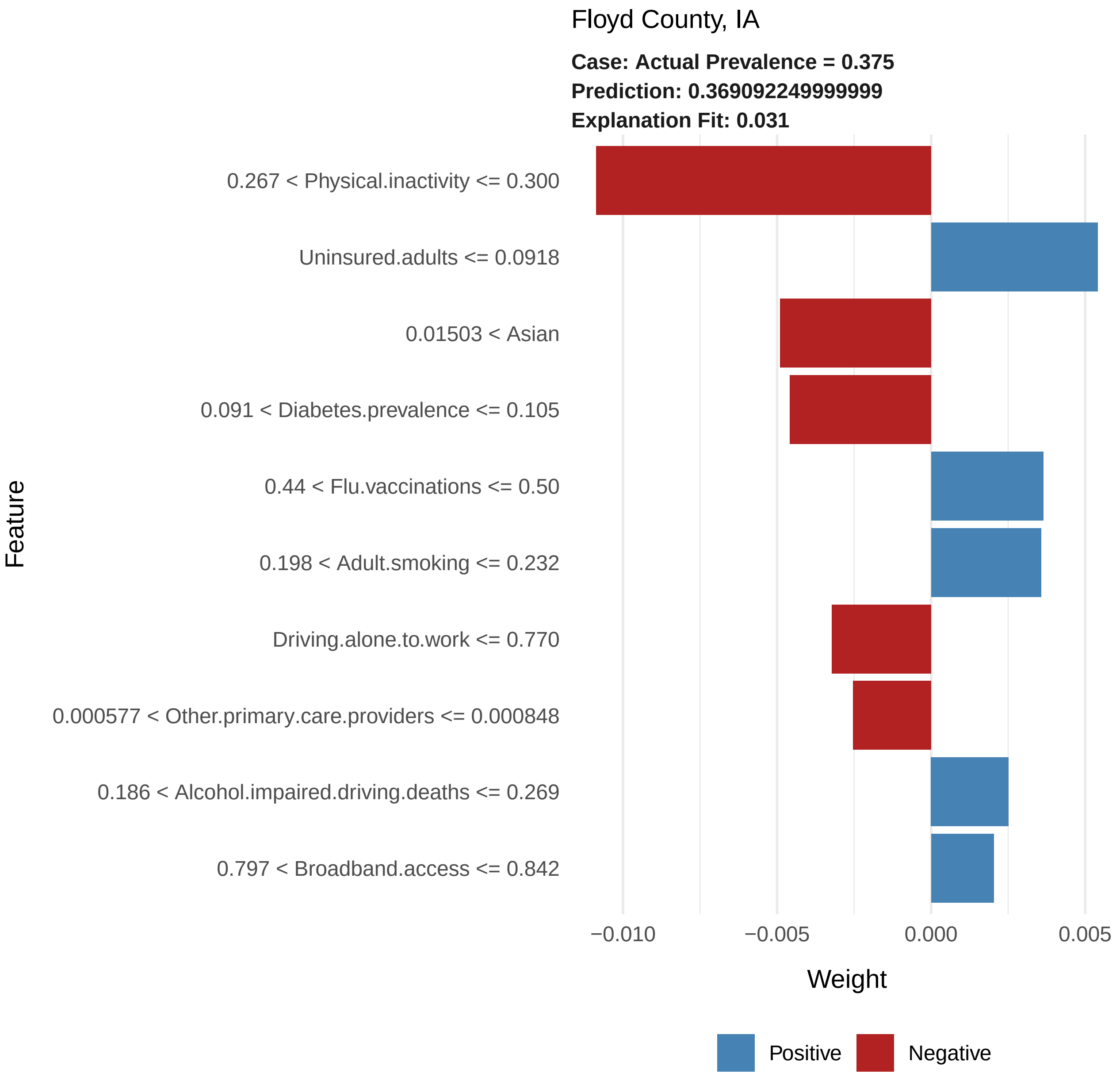

Floyd County, IA
Case: Actual Prevalence = 0.375
Prediction: 0.369092249999999
Explanation Fit: 0.031
0.267 < Physical.inactivity <= 0.300
Uninsured.adults <= 0.0918
0.01503 < Asian
0.091 < Diabetes.prevalence <= 0.105
0.44 < Flu.vaccinations <= 0.50
0.198 < Adult.smoking <= 0.232
Driving.alone.to.work <= 0.770
0.000577 < Other.primary.care.providers <= 0.000848
0.186 < Alcohol.impaired.driving.deaths <= 0.269
0.797 < Broadband.access <= 0.842
Feature
−0.010
−0.005
0.000
0.005
Weight
Positive
Negative

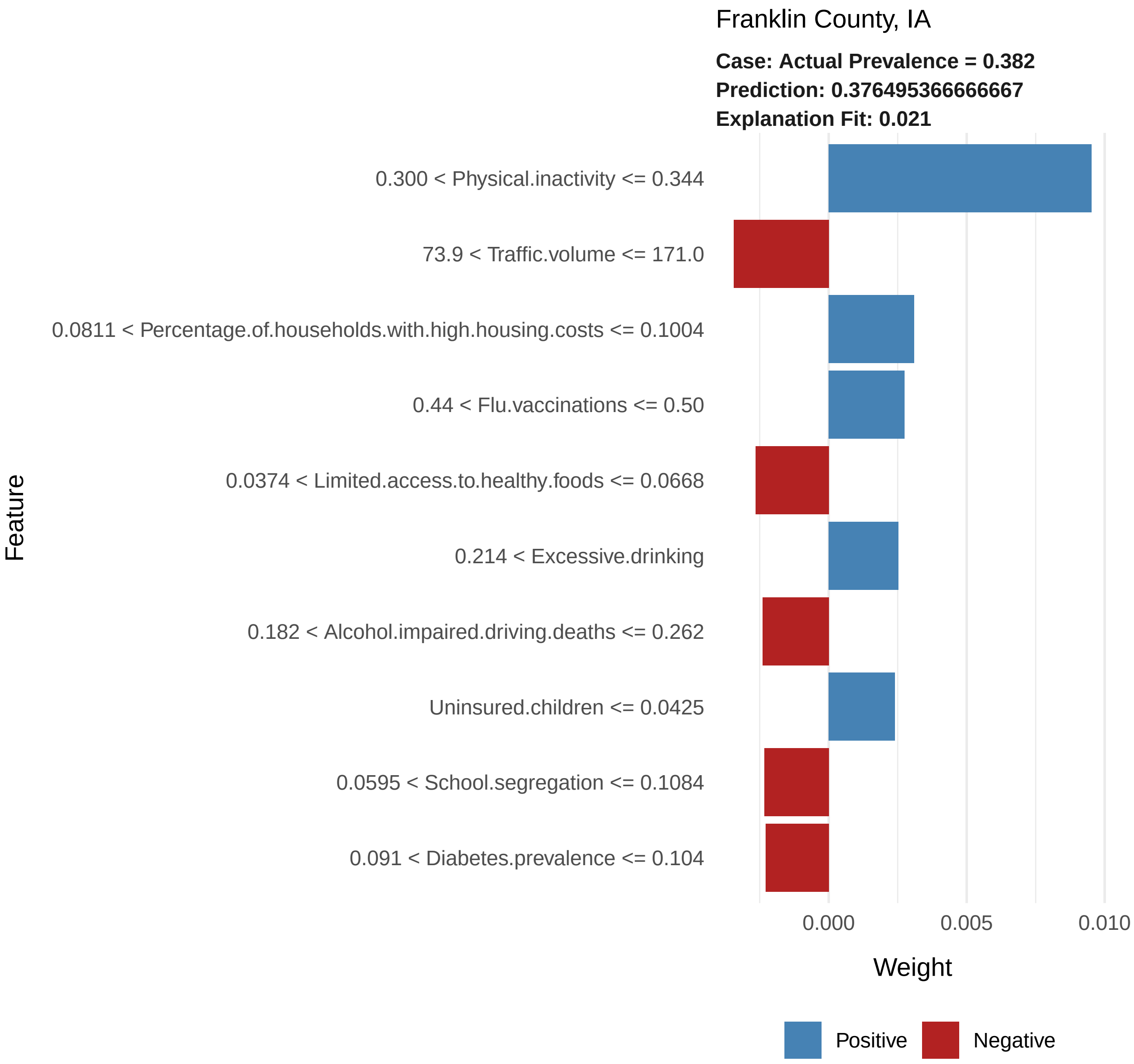
Franklin County, IA
Case: Actual Prevalence = 0.382
Prediction: 0.376495366666667
Explanation Fit: 0.021
0.300 < Physical.inactivity <= 0.344
73.9 < Traffic.volume <= 171.0
0.0811 < Percentage.of.households.with.high.housing.costs <= 0.1004
0.44 < Flu.vaccinations <= 0.50
0.0374 < Limited.access.to.healthy.foods <= 0.0668
0.214 < Excessive.drinking
0.182 < Alcohol.impaired.driving.deaths <= 0.262
Uninsured.children <= 0.0425
0.0595 < School.segregation <= 0.1084
0.091 < Diabetes.prevalence <= 0.104
Feature
0.000
0.005
0.010
Weight
Positive
Negative

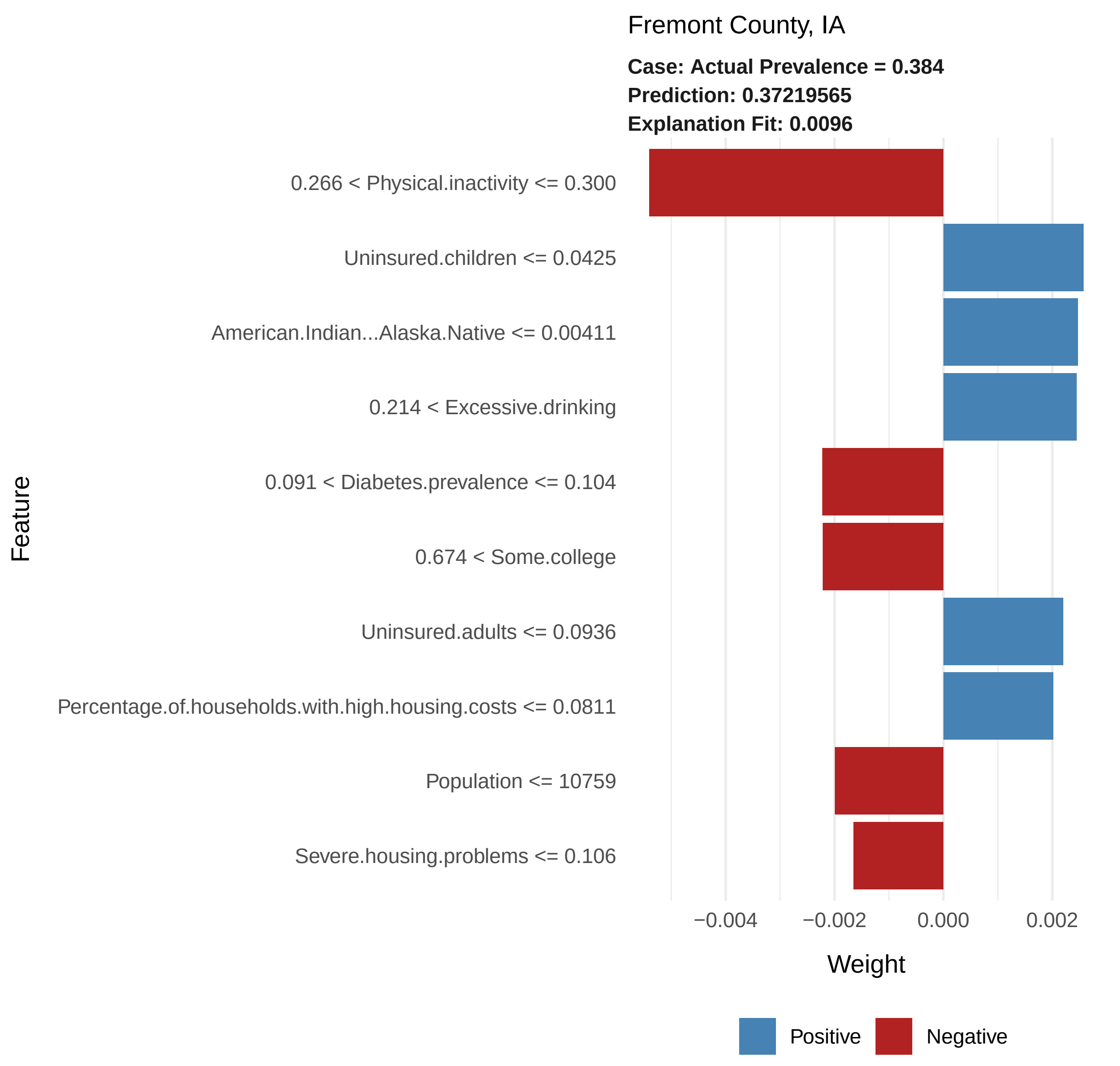

Fremont County, IA
Case: Actual Prevalence = 0.384
Prediction: 0.37219565
Explanation Fit: 0.0096
Feature
0.266 < Physical.inactivity <= 0.300
Uninsured.children <= 0.0425
American.Indian...Alaska.Native <= 0.00411
0.214 < Excessive.drinking
0.091 < Diabetes.prevalence <= 0.104
0.674 < Some.college
Uninsured.adults <= 0.0936
Percentage.of.households.with.high.housing.costs <= 0.0811
Population <= 10759
Severe.housing.problems <= 0.106
−0.004
−0.002
0.000
0.002
Weight
Positive
Negative

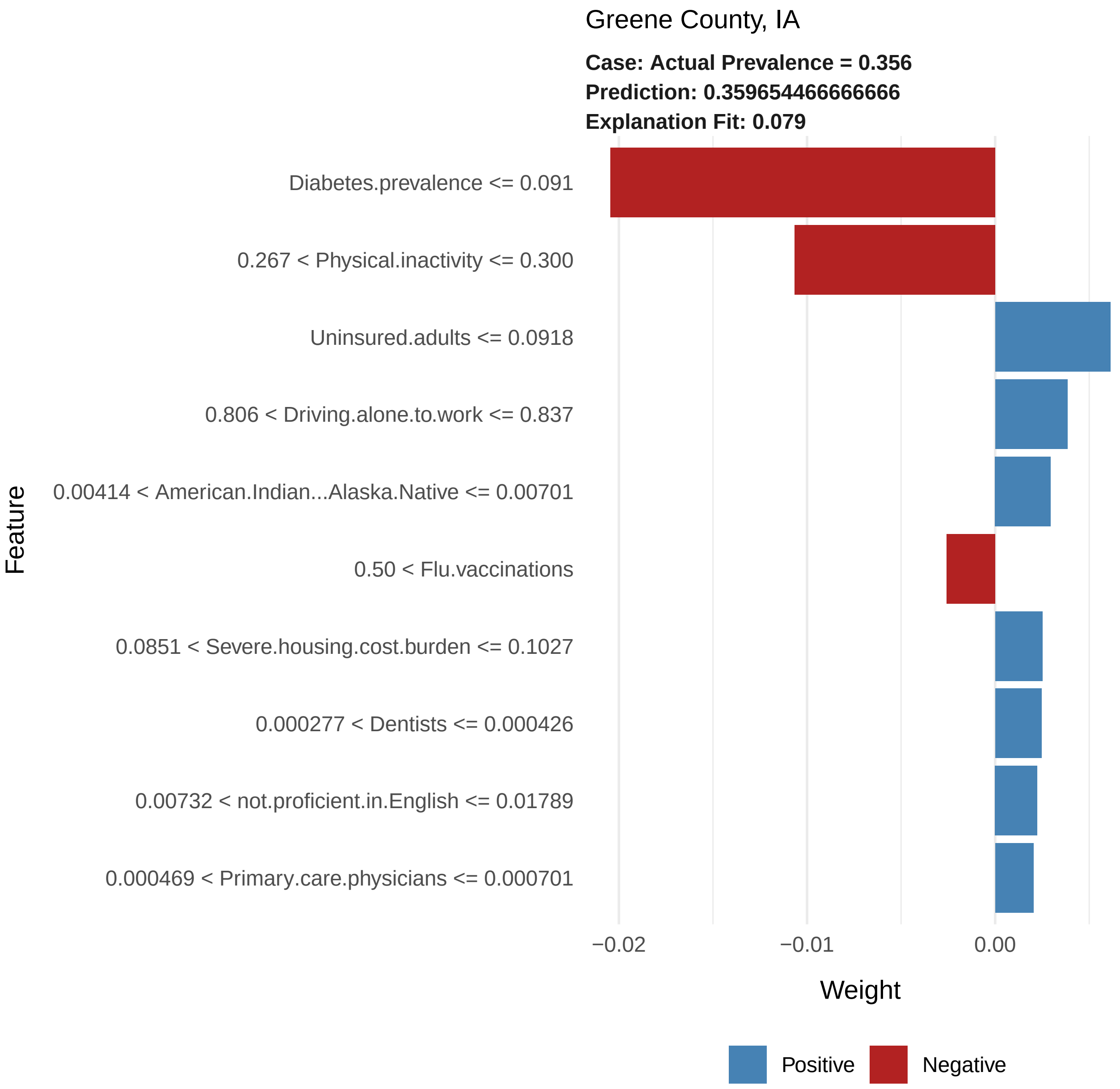

Greene County, IA
Case: Actual Prevalence = 0.356
Prediction: 0.359654466666666
Explanation Fit: 0.079
Diabetes.prevalence <= 0.091
0.267 < Physical.inactivity <= 0.300
Uninsured.adults <= 0.0918
0.806 < Driving.alone.to.work <= 0.837
0.00414 < American.Indian...Alaska.Native <= 0.00701
0.50 < Flu.vaccinations
0.0851 < Severe.housing.cost.burden <= 0.1027
0.000277 < Dentists <= 0.000426
0.00732 < not.proficient.in.English <= 0.01789
0.000469 < Primary.care.physicians <= 0.000701
Feature
−0.02
−0.01
0.00
Weight
Positive
Negative

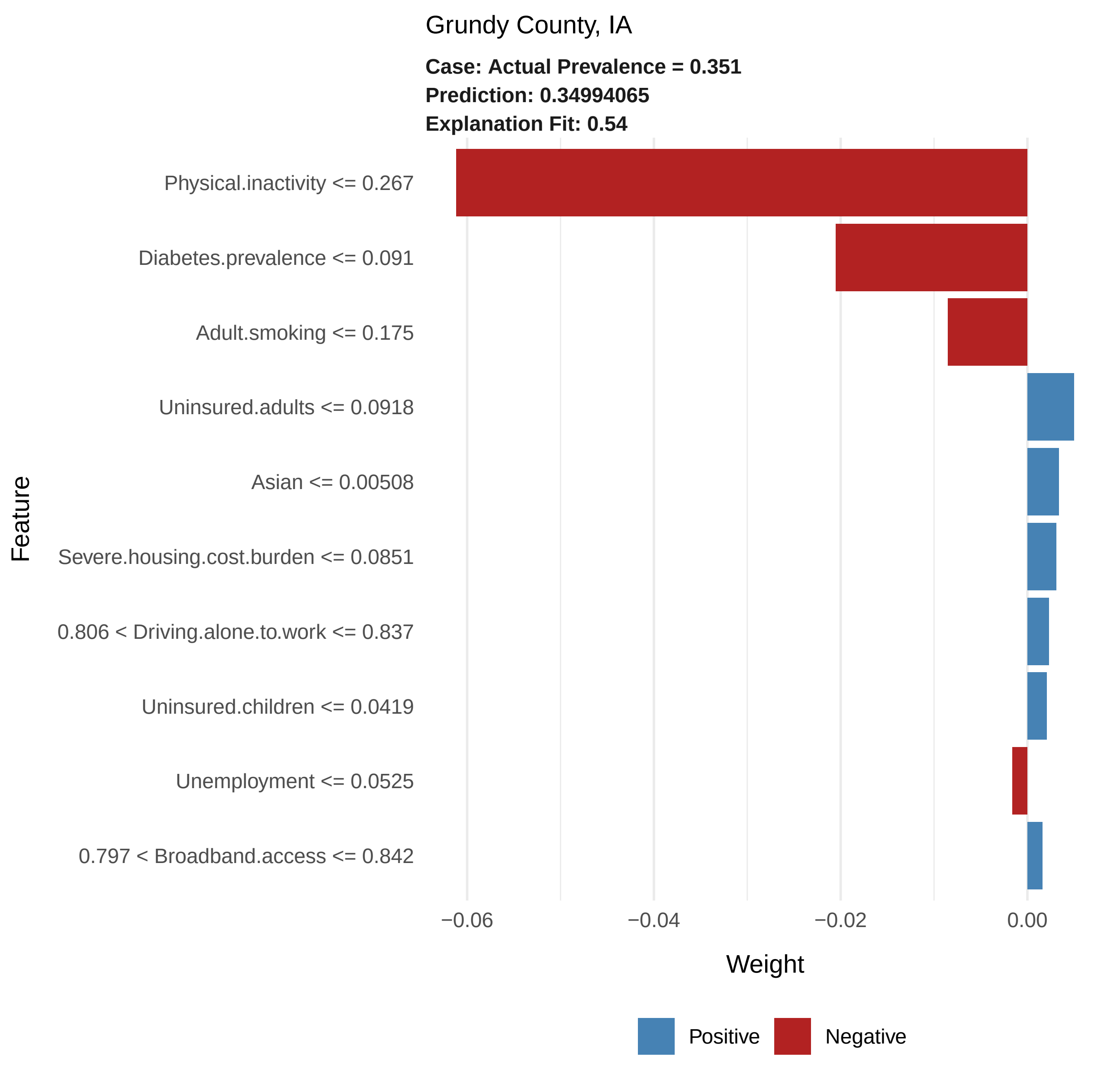

Grundy County, IA
Case: Actual Prevalence = 0.351
Prediction: 0.34994065
Explanation Fit: 0.54
Physical.inactivity <= 0.267
Diabetes.prevalence <= 0.091
Adult.smoking <= 0.175
Uninsured.adults <= 0.0918
Asian <= 0.00508
Severe.housing.cost.burden <= 0.0851
0.806 < Driving.alone.to.work <= 0.837
Uninsured.children <= 0.0419
Unemployment <= 0.0525
0.797 < Broadband.access <= 0.842
Feature
−0.06
−0.04
−0.02
0.00
Weight
Positive
Negative

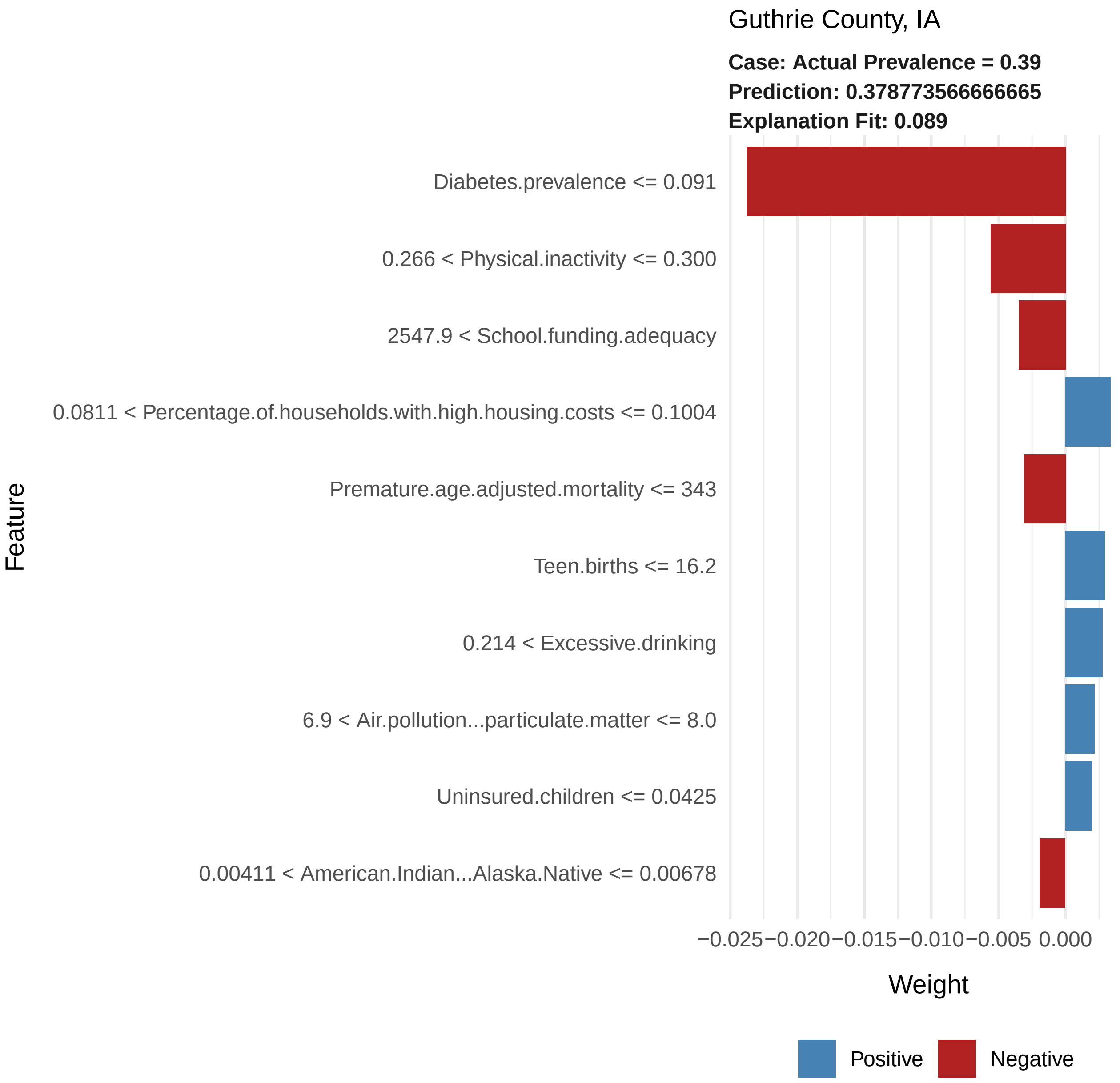
Guthrie County, IA
Case: Actual Prevalence = 0.39
Prediction: 0.378773566666665
Explanation Fit: 0.089
Diabetes.prevalence <= 0.091
0.266 < Physical.inactivity <= 0.300
2547.9 < School.funding.adequacy
0.0811 < Percentage.of.households.with.high.housing.costs <= 0.1004
Premature.age.adjusted.mortality <= 343
Teen.births <= 16.2
0.214 < Excessive.drinking
6.9 < Air.pollution...particulate.matter <= 8.0
Uninsured.children <= 0.0425
0.00411 < American.Indian...Alaska.Native <= 0.00678
Feature
−0.025
−0.020
−0.015
−0.010
−0.005
0.000
Weight
Positive
Negative

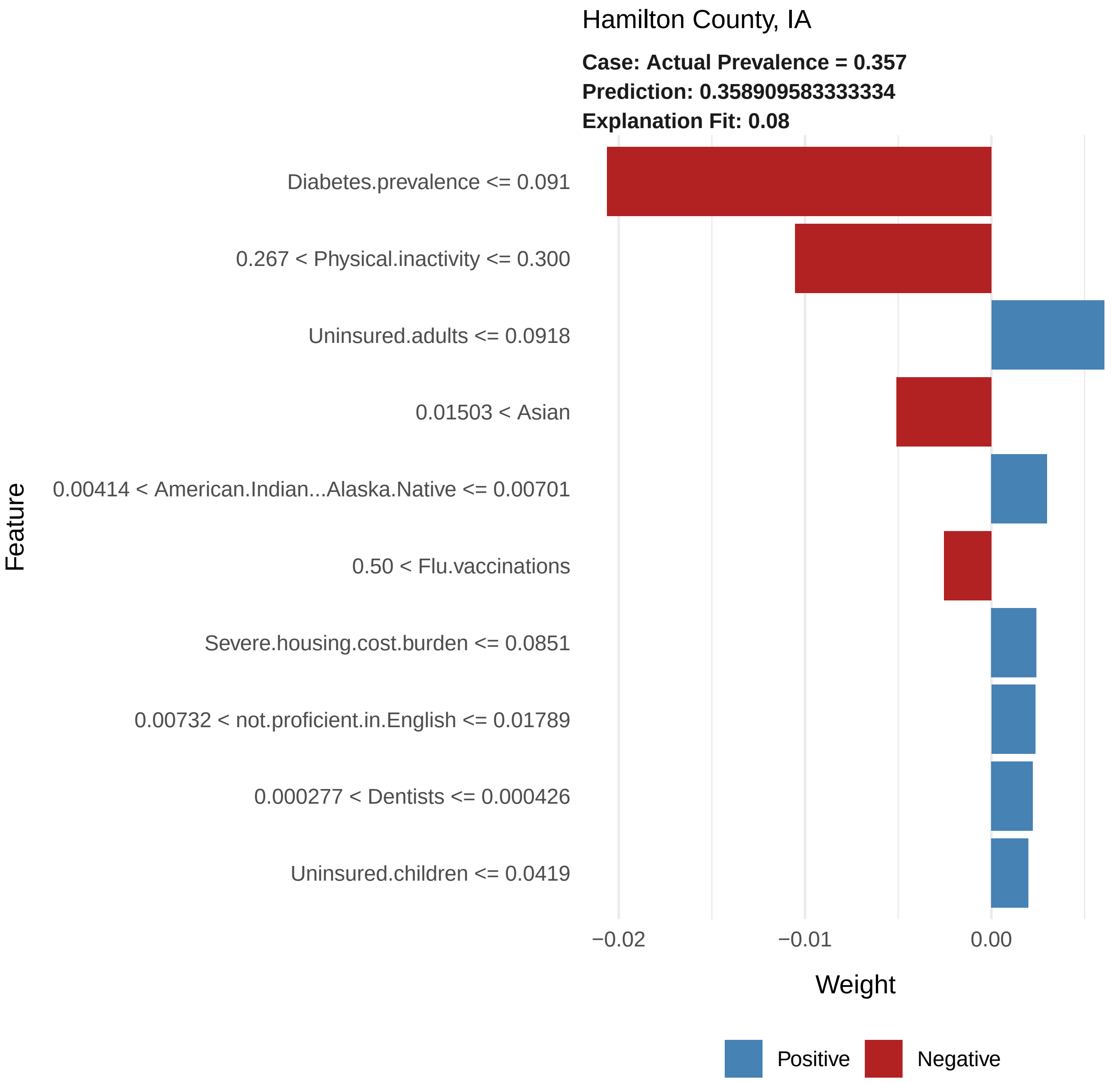

Hamilton County, IA
Case: Actual Prevalence = 0.357
Prediction: 0.358909583333334
Explanation Fit: 0.08
Diabetes.prevalence <= 0.091
0.267 < Physical.inactivity <= 0.300
Uninsured.adults <= 0.0918
0.01503 < Asian
0.00414 < American.Indian...Alaska.Native <= 0.00701
0.50 < Flu.vaccinations
Severe.housing.cost.burden <= 0.0851
0.00732 < not.proficient.in.English <= 0.01789
0.000277 < Dentists <= 0.000426
Uninsured.children <= 0.0419
Feature
−0.02
−0.01
0.00
Weight
Positive
Negative

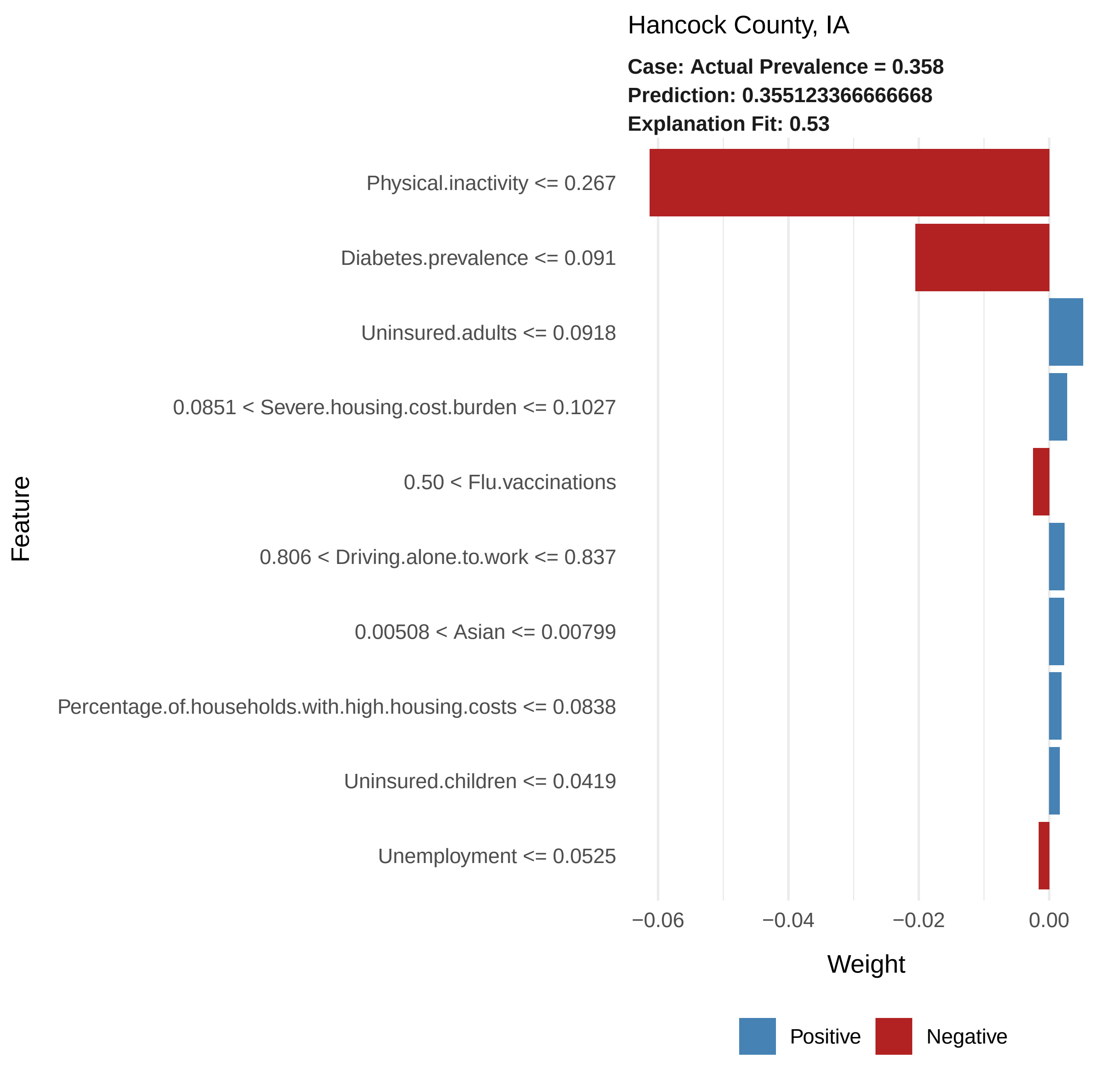

Hancock County, IA
Case: Actual Prevalence = 0.358
Prediction: 0.355123366666668
Explanation Fit: 0.53
Physical.inactivity <= 0.267
Diabetes.prevalence <= 0.091
Uninsured.adults <= 0.0918
0.0851 < Severe.housing.cost.burden <= 0.1027
0.50 < Flu.vaccinations
0.806 < Driving.alone.to.work <= 0.837
0.00508 < Asian <= 0.00799
Percentage.of.households.with.high.housing.costs <= 0.0838
Uninsured.children <= 0.0419
Unemployment <= 0.0525
Feature
−0.06
−0.04
−0.02
0.00
Weight
Positive
Negative

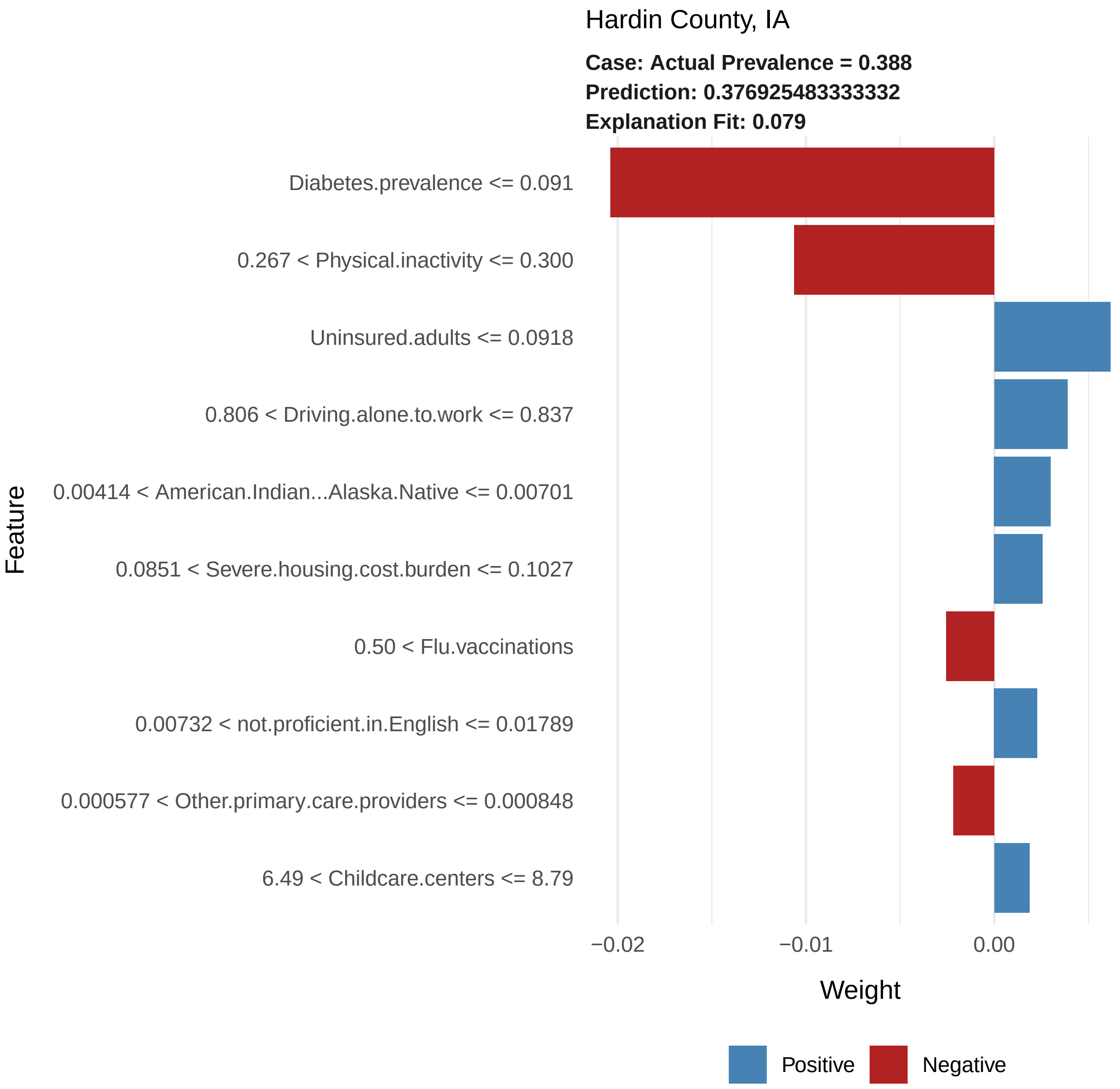

Hardin County, IA
Case: Actual Prevalence = 0.388
Prediction: 0.376925483333332
Explanation Fit: 0.079
Diabetes.prevalence <= 0.091
0.267 < Physical.inactivity <= 0.300
Uninsured.adults <= 0.0918
0.806 < Driving.alone.to.work <= 0.837
0.00414 < American.Indian...Alaska.Native <= 0.00701
0.0851 < Severe.housing.cost.burden <= 0.1027
0.50 < Flu.vaccinations
0.00732 < not.proficient.in.English <= 0.01789
0.000577 < Other.primary.care.providers <= 0.000848
6.49 < Childcare.centers <= 8.79
Feature
−0.02
−0.01
0.00
Weight
Positive
Negative

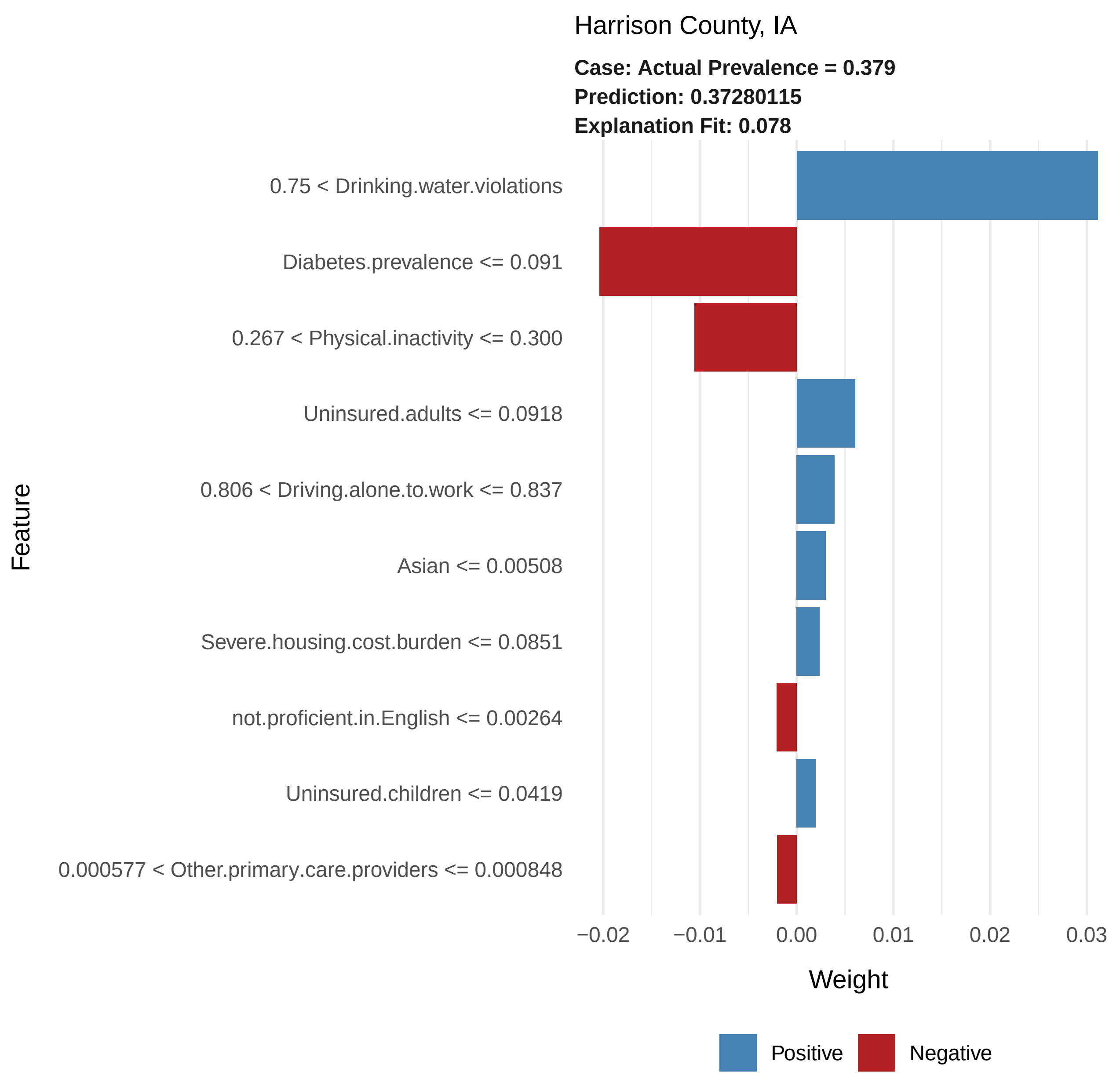

Harrison County, IA
Case: Actual Prevalence = 0.379
Prediction: 0.37280115
Explanation Fit: 0.078
0.75 < Drinking.water.violations
Diabetes.prevalence <= 0.091
0.267 < Physical.inactivity <= 0.300
Uninsured.adults <= 0.0918
0.806 < Driving.alone.to.work <= 0.837
Asian <= 0.00508
Severe.housing.cost.burden <= 0.0851
not.proficient.in.English <= 0.00264
Uninsured.children <= 0.0419
0.000577 < Other.primary.care.providers <= 0.000848
Feature
−0.02
−0.01
0.00
0.01
0.02
0.03
Weight
Positive
Negative

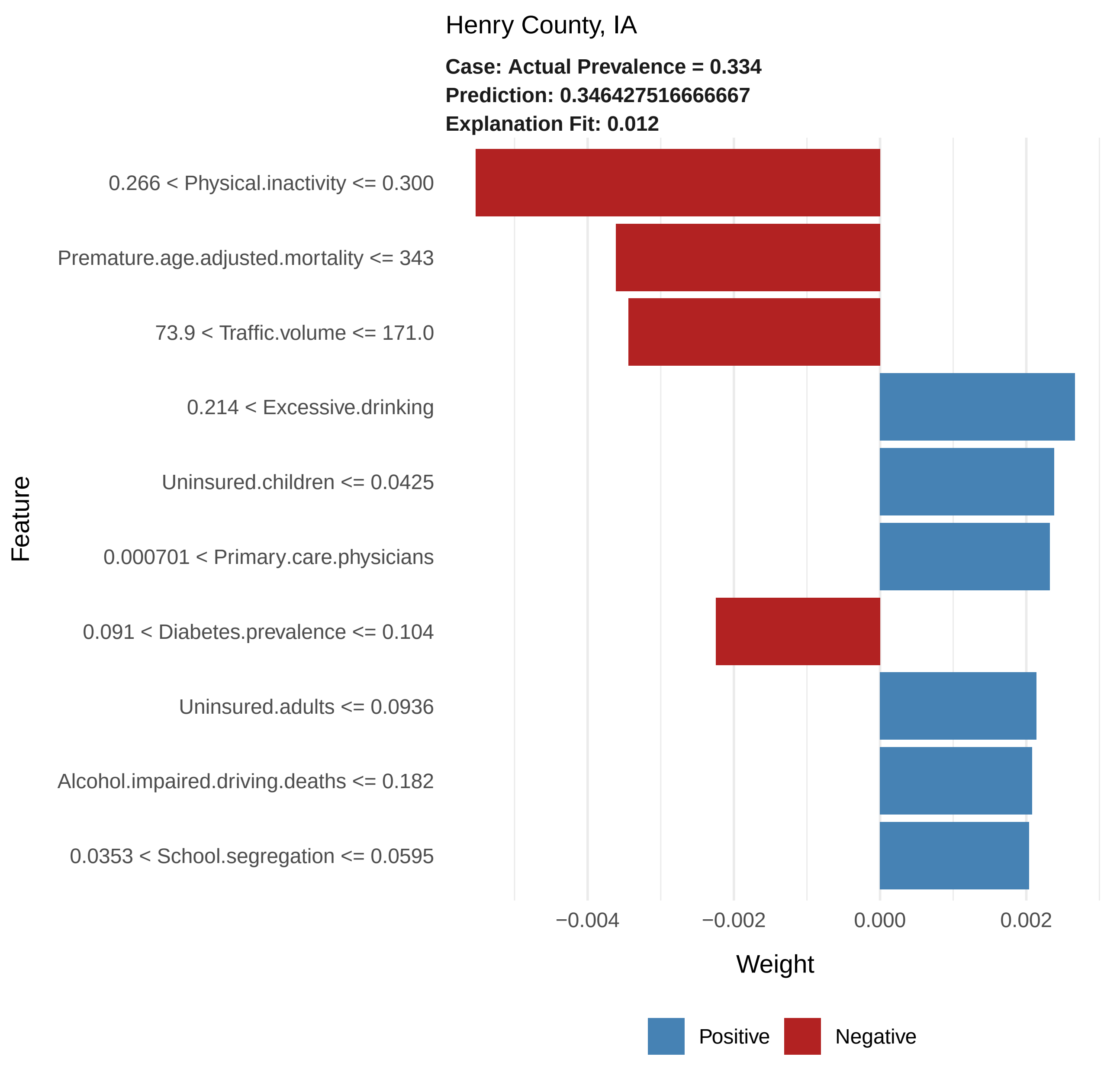

Henry County, IA
Case: Actual Prevalence = 0.334
Prediction: 0.346427516666667
Explanation Fit: 0.012
0.266 < Physical.inactivity <= 0.300
Premature.age.adjusted.mortality <= 343
73.9 < Traffic.volume <= 171.0
0.214 < Excessive.drinking
Uninsured.children <= 0.0425
0.000701 < Primary.care.physicians
0.091 < Diabetes.prevalence <= 0.104
Uninsured.adults <= 0.0936
Alcohol.impaired.driving.deaths <= 0.182
0.0353 < School.segregation <= 0.0595
Feature
−0.004
−0.002
0.000
0.002
Weight
Positive
Negative

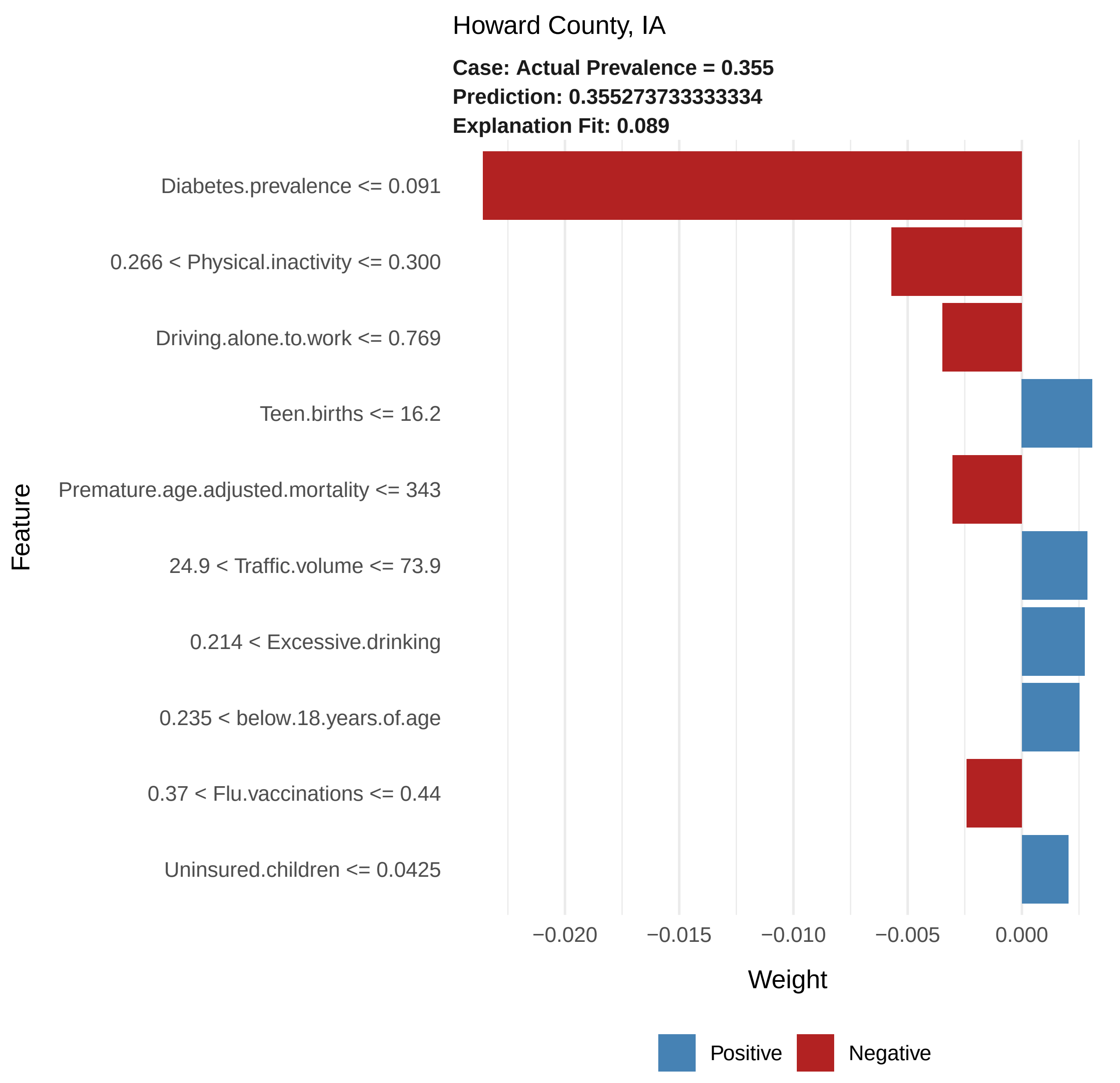
Howard County, IA
Case: Actual Prevalence = 0.355
Prediction: 0.355273733333334
Explanation Fit: 0.089
Diabetes.prevalence <= 0.091
0.266 < Physical.inactivity <= 0.300
Driving.alone.to.work <= 0.769
Teen.births <= 16.2
Premature.age.adjusted.mortality <= 343
24.9 < Traffic.volume <= 73.9
0.214 < Excessive.drinking
0.235 < below.18.years.of.age
0.37 < Flu.vaccinations <= 0.44
Uninsured.children <= 0.0425
Feature
−0.020
−0.015
−0.010
−0.005
0.000
Weight
Positive
Negative

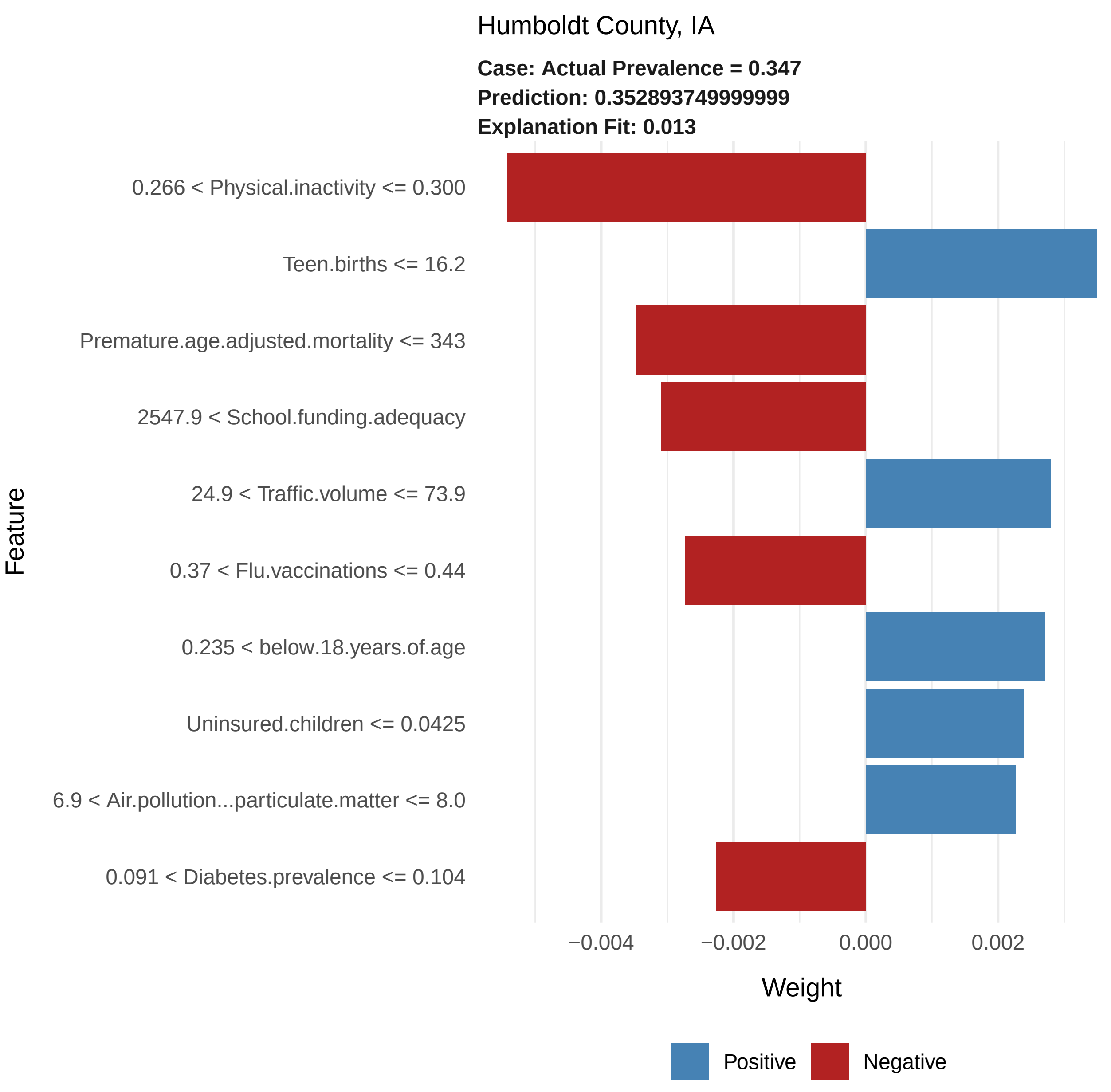
Humboldt County, IA
Case: Actual Prevalence = 0.347
Prediction: 0.352893749999999
Explanation Fit: 0.013
0.266 < Physical.inactivity <= 0.300
Teen.births <= 16.2
Premature.age.adjusted.mortality <= 343
2547.9 < School.funding.adequacy
24.9 < Traffic.volume <= 73.9
0.37 < Flu.vaccinations <= 0.44
0.235 < below.18.years.of.age
Uninsured.children <= 0.0425
6.9 < Air.pollution...particulate.matter <= 8.0
0.091 < Diabetes.prevalence <= 0.104
Feature
−0.004
−0.002
0.000
0.002
Weight
Positive
Negative

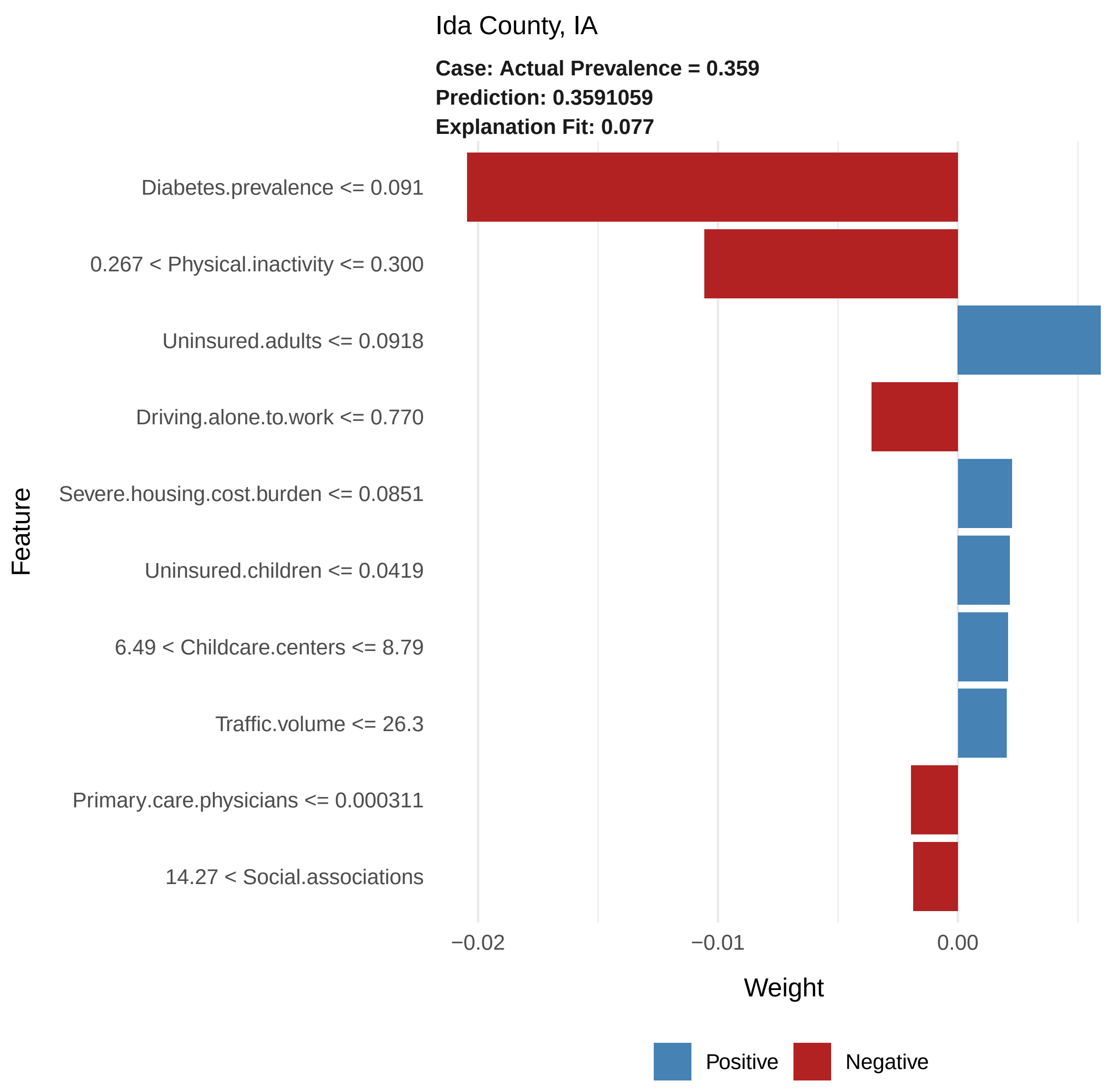

Ida County, IA
Case: Actual Prevalence = 0.359
Prediction: 0.3591059
Explanation Fit: 0.077
Diabetes.prevalence <= 0.091
0.267 < Physical.inactivity <= 0.300
Uninsured.adults <= 0.0918
Driving.alone.to.work <= 0.770
Severe.housing.cost.burden <= 0.0851
Uninsured.children <= 0.0419
6.49 < Childcare.centers <= 8.79
Traffic.volume <= 26.3
Primary.care.physicians <= 0.000311
14.27 < Social.associations
Feature
−0.02
−0.01
0.00
Weight
Positive
Negative

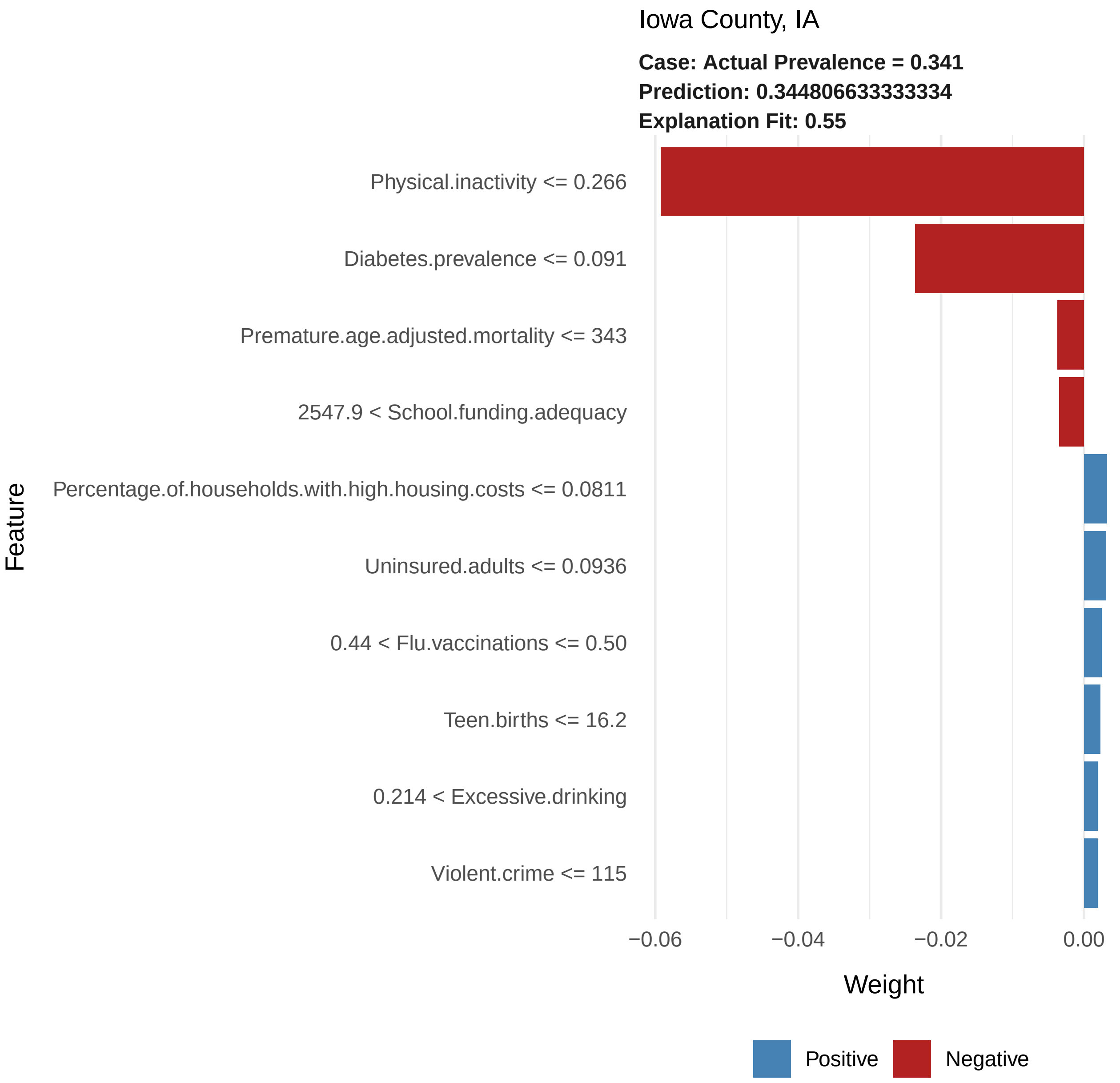
Iowa County, IA
Case: Actual Prevalence = 0.341
Prediction: 0.344806633333334
Explanation Fit: 0.55
Physical.inactivity <= 0.266
Diabetes.prevalence <= 0.091
Premature.age.adjusted.mortality <= 343
2547.9 < School.funding.adequacy
Percentage.of.households.with.high.housing.costs <= 0.0811
Uninsured.adults <= 0.0936
0.44 < Flu.vaccinations <= 0.50
Teen.births <= 16.2
0.214 < Excessive.drinking
Violent.crime <= 115
Feature
−0.06
−0.04
−0.02
0.00
Weight
Positive
Negative

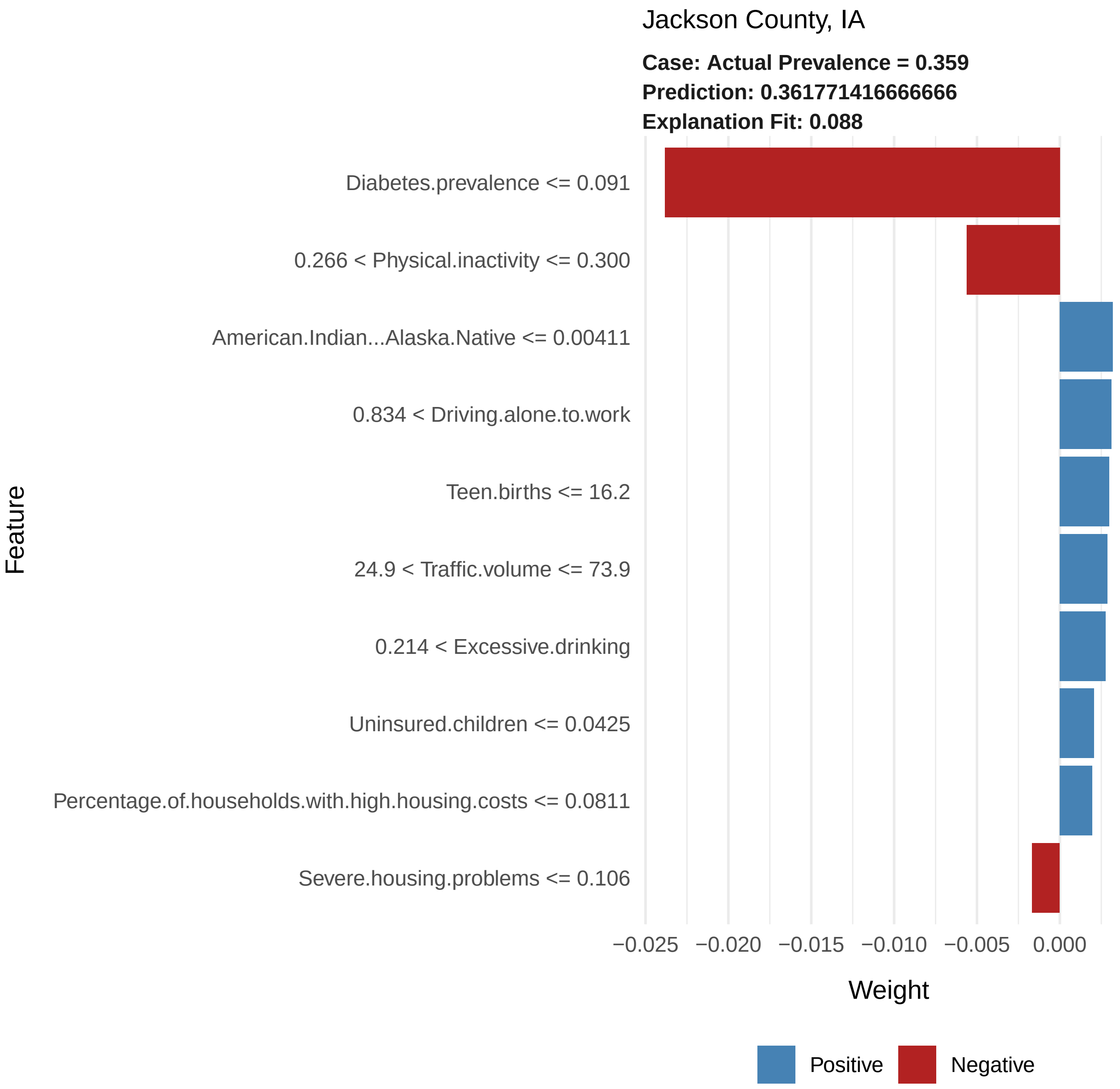

Jackson County, IA
Case: Actual Prevalence = 0.359
Prediction: 0.361771416666666
Explanation Fit: 0.088
Diabetes.prevalence <= 0.091
0.266 < Physical.inactivity <= 0.300
American.Indian...Alaska.Native <= 0.00411
0.834 < Driving.alone.to.work
Teen.births <= 16.2
24.9 < Traffic.volume <= 73.9
0.214 < Excessive.drinking
Uninsured.children <= 0.0425
Percentage.of.households.with.high.housing.costs <= 0.0811
Severe.housing.problems <= 0.106
Feature
−0.025
−0.020
−0.015
−0.010
−0.005
0.000
Weight
Positive
Negative

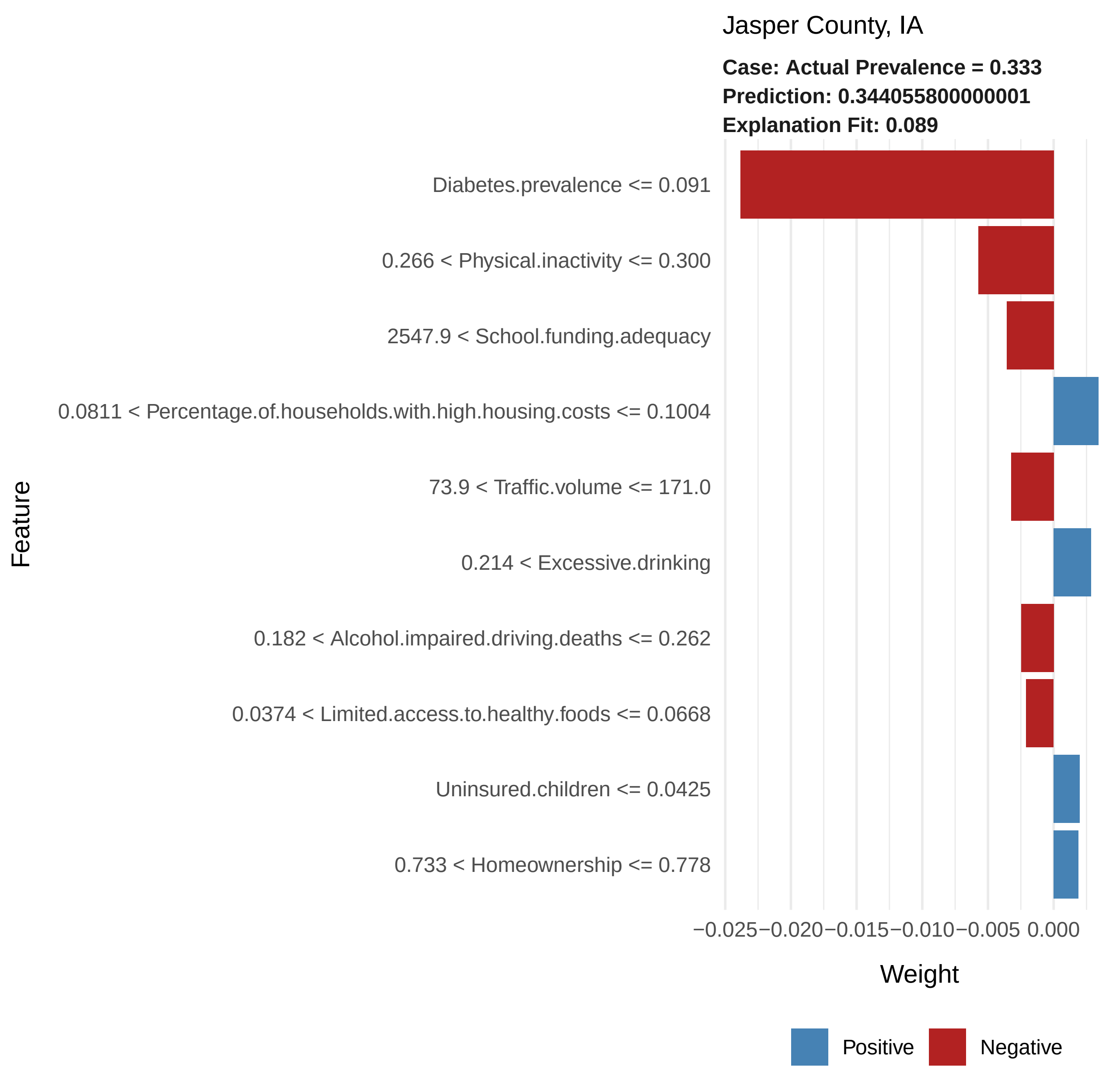
Jasper County, IA
Case: Actual Prevalence = 0.333
Prediction: 0.344055800000001
Explanation Fit: 0.089
Diabetes.prevalence <= 0.091
0.266 < Physical.inactivity <= 0.300
2547.9 < School.funding.adequacy
0.0811 < Percentage.of.households.with.high.housing.costs <= 0.1004
73.9 < Traffic.volume <= 171.0
0.214 < Excessive.drinking
0.182 < Alcohol.impaired.driving.deaths <= 0.262
0.0374 < Limited.access.to.healthy.foods <= 0.0668
Uninsured.children <= 0.0425
0.733 < Homeownership <= 0.778
Feature
−0.025
−0.020
−0.015
−0.010
−0.005
0.000
Weight
Positive
Negative

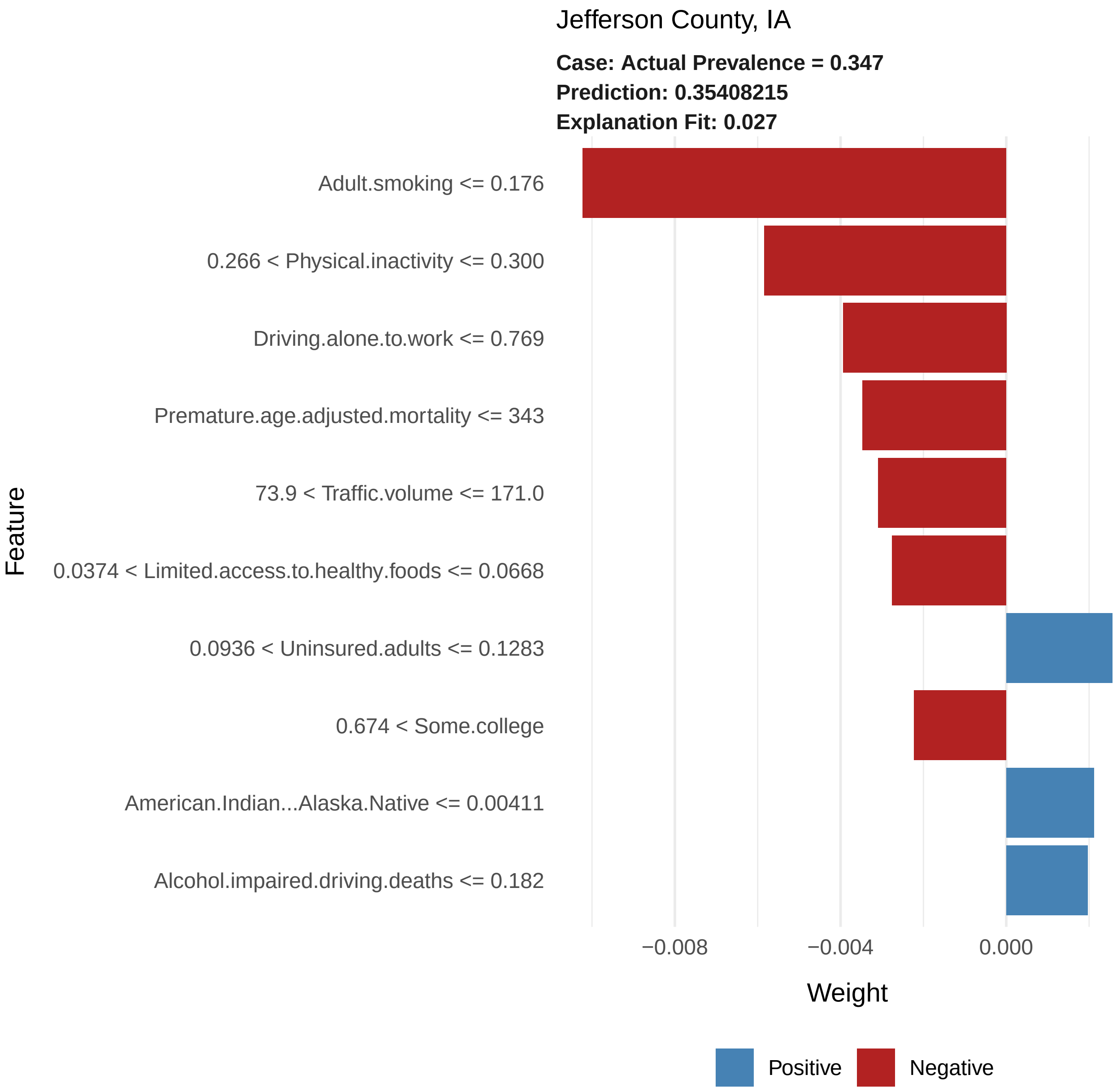

Jefferson County, IA
Case: Actual Prevalence = 0.347
Prediction: 0.35408215
Explanation Fit: 0.027
Adult.smoking <= 0.176
0.266 < Physical.inactivity <= 0.300
Driving.alone.to.work <= 0.769
Premature.age.adjusted.mortality <= 343
73.9 < Traffic.volume <= 171.0
0.0374 < Limited.access.to.healthy.foods <= 0.0668
0.0936 < Uninsured.adults <= 0.1283
0.674 < Some.college
American.Indian...Alaska.Native <= 0.00411
Alcohol.impaired.driving.deaths <= 0.182
Feature
−0.008
−0.004
0.000
Weight
Positive
Negative

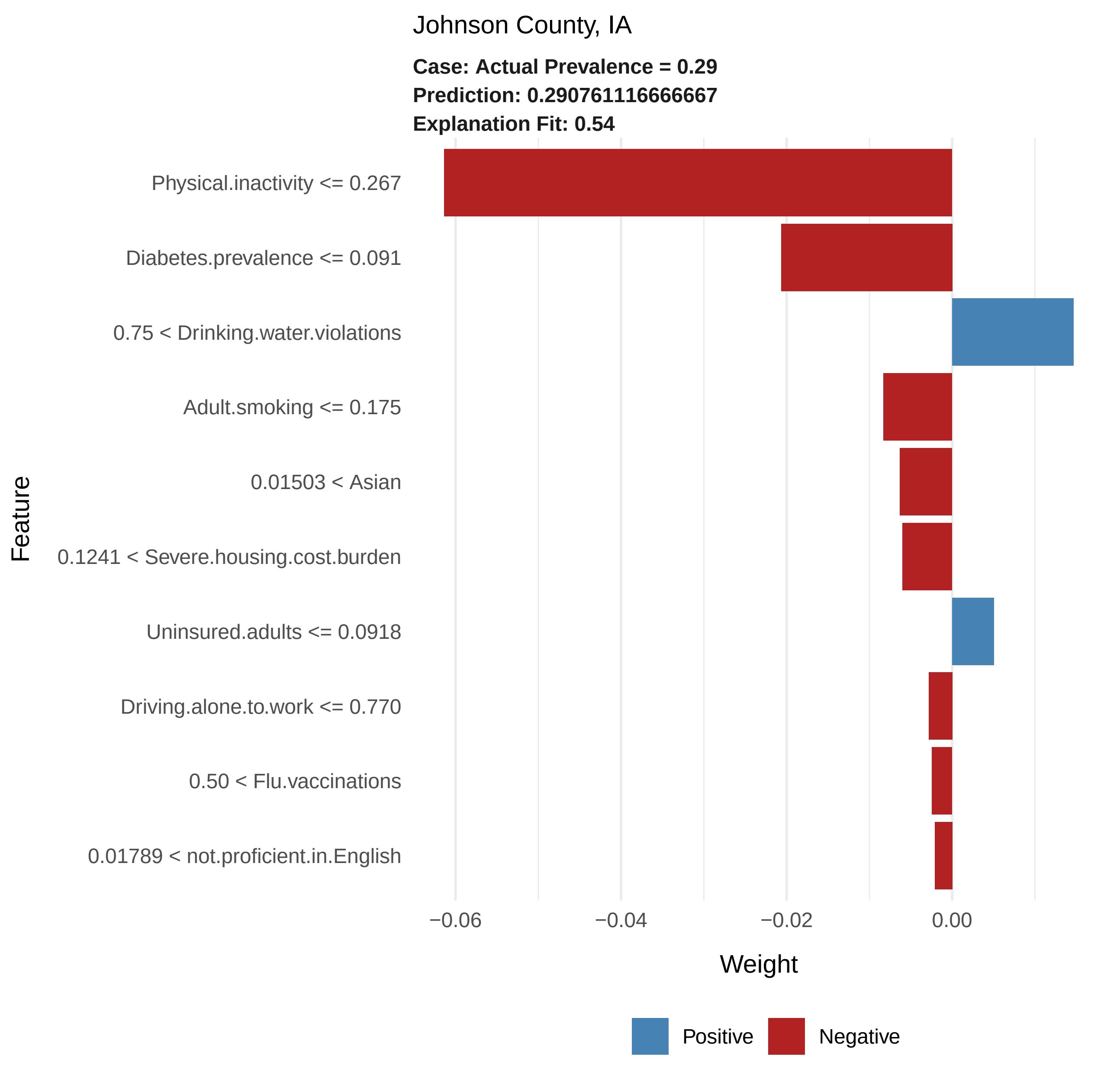

Johnson County, IA
Case: Actual Prevalence = 0.29
Prediction: 0.290761116666667
Explanation Fit: 0.54
Physical.inactivity <= 0.267
Diabetes.prevalence <= 0.091
0.75 < Drinking.water.violations
Adult.smoking <= 0.175
0.01503 < Asian
0.1241 < Severe.housing.cost.burden
Uninsured.adults <= 0.0918
Driving.alone.to.work <= 0.770
0.50 < Flu.vaccinations
0.01789 < not.proficient.in.English
Feature
−0.06
−0.04
−0.02
0.00
Weight
Positive
Negative

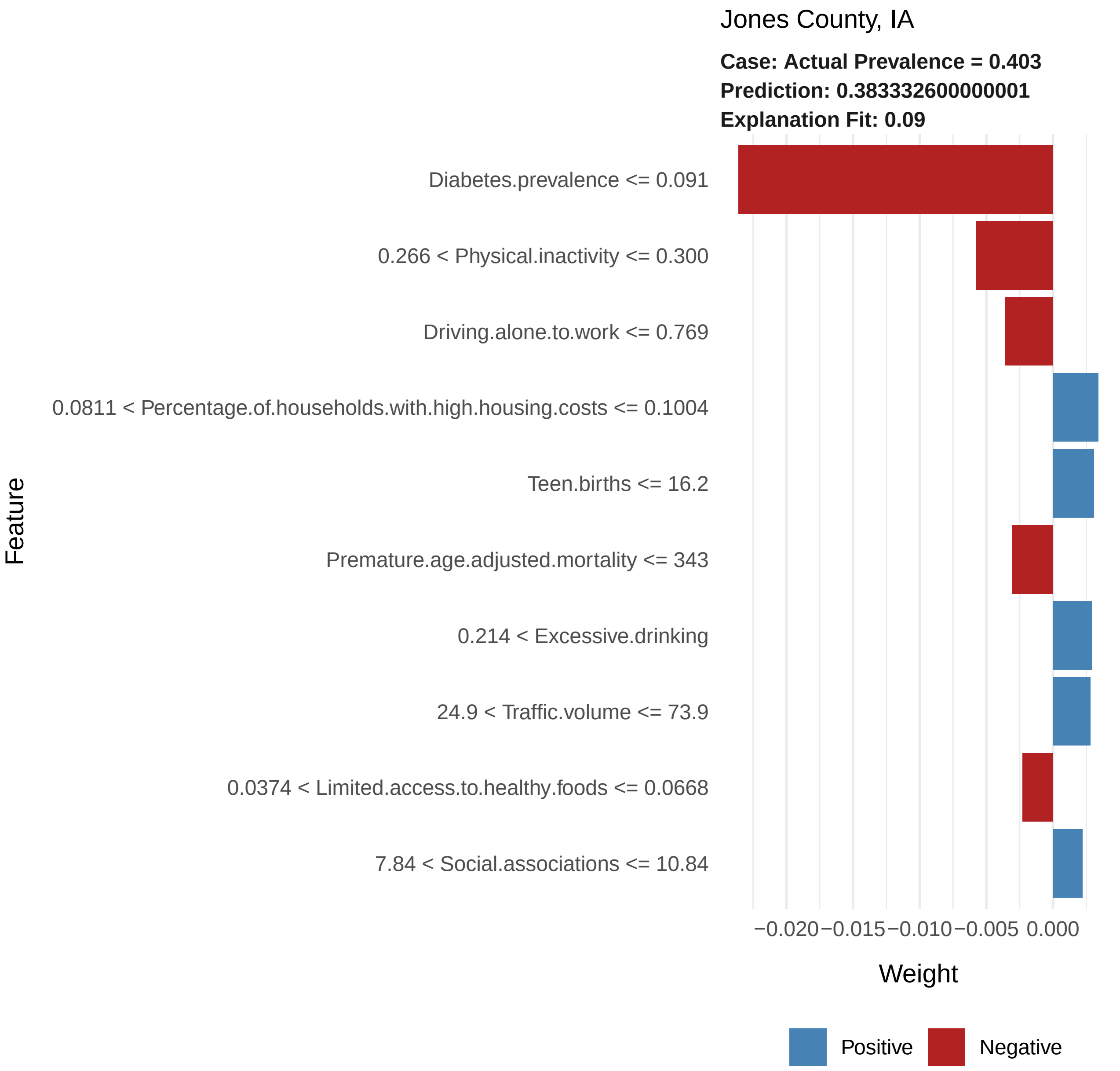

Jones County, IA
Case: Actual Prevalence = 0.403
Prediction: 0.383332600000001
Explanation Fit: 0.09
Diabetes.prevalence <= 0.091
0.266 < Physical.inactivity <= 0.300
Driving.alone.to.work <= 0.769
0.0811 < Percentage.of.households.with.high.housing.costs <= 0.1004
Teen.births <= 16.2
Premature.age.adjusted.mortality <= 343
0.214 < Excessive.drinking
24.9 < Traffic.volume <= 73.9
0.0374 < Limited.access.to.healthy.foods <= 0.0668
7.84 < Social.associations <= 10.84
Feature
−0.020
−0.015
−0.010
−0.005
0.000
Weight
Positive
Negative

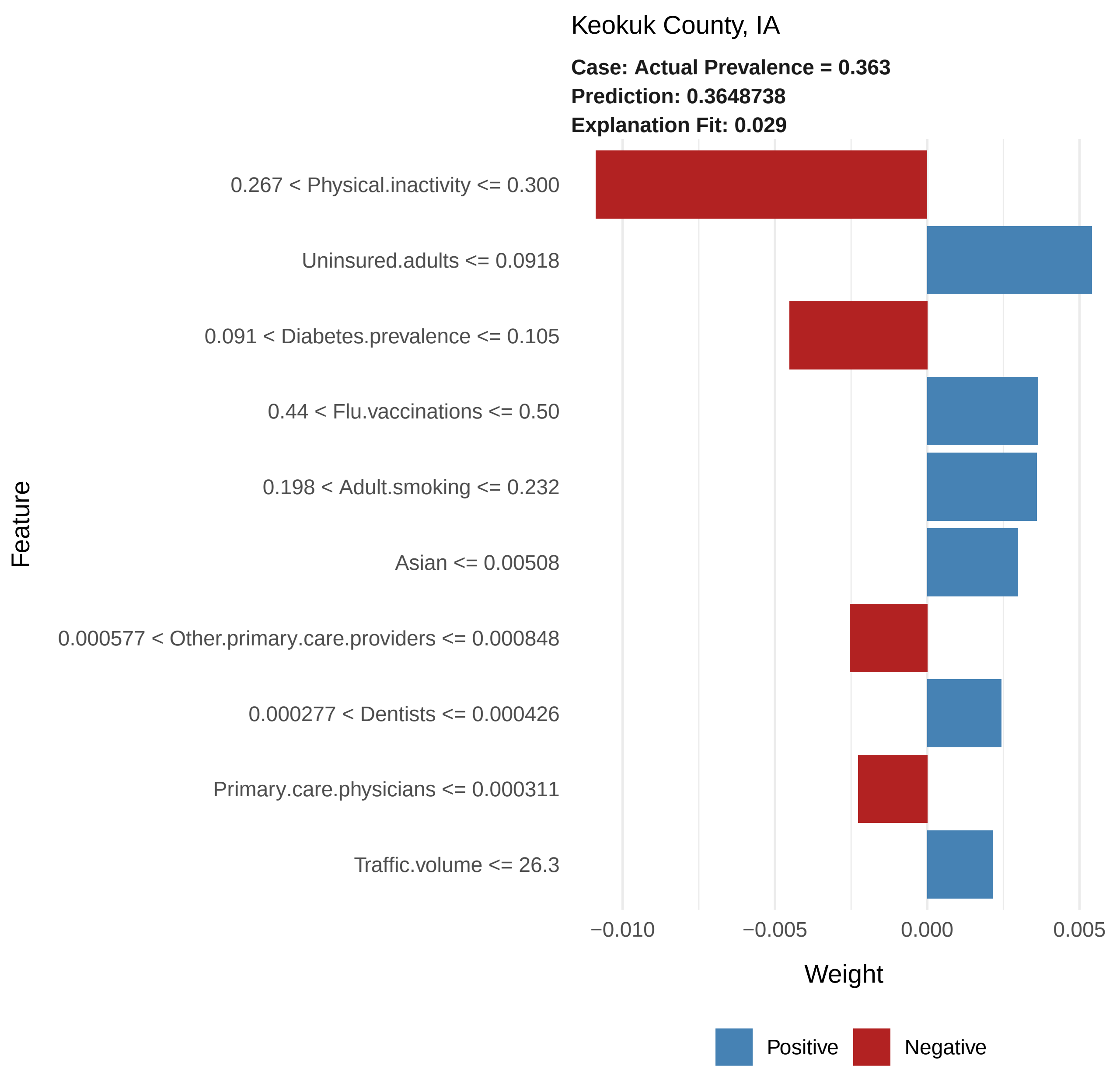
Keokuk County, IA
Case: Actual Prevalence = 0.363
Prediction: 0.3648738
Explanation Fit: 0.029
Feature
0.267 < Physical.inactivity <= 0.300
Uninsured.adults <= 0.0918
0.091 < Diabetes.prevalence <= 0.105
0.44 < Flu.vaccinations <= 0.50
0.198 < Adult.smoking <= 0.232
Asian <= 0.00508
0.000577 < Other.primary.care.providers <= 0.000848
0.000277 < Dentists <= 0.000426
Primary.care.physicians <= 0.000311
Traffic.volume <= 26.3
−0.010
−0.005
0.000
0.005
Weight
Positive
Negative

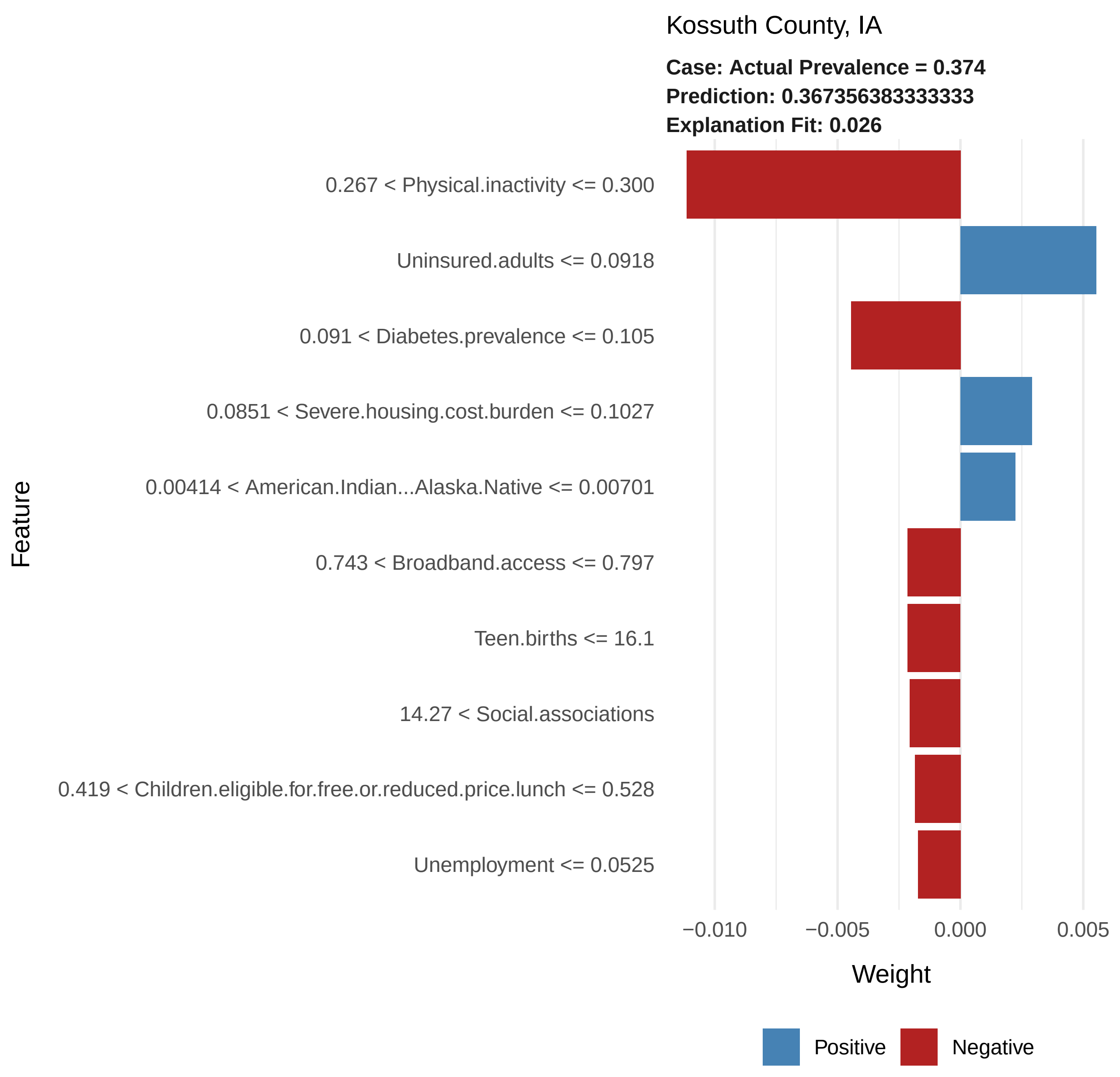

Kossuth County, IA
Case: Actual Prevalence = 0.374
Prediction: 0.367356383333333
Explanation Fit: 0.026
0.267 < Physical.inactivity <= 0.300
Uninsured.adults <= 0.0918
0.091 < Diabetes.prevalence <= 0.105
0.0851 < Severe.housing.cost.burden <= 0.1027
0.00414 < American.Indian...Alaska.Native <= 0.00701
0.743 < Broadband.access <= 0.797
Teen.births <= 16.1
14.27 < Social.associations
0.419 < Children.eligible.for.free.or.reduced.price.lunch <= 0.528
Unemployment <= 0.0525
Feature
−0.010
−0.005
0.000
0.005
Weight
Positive
Negative

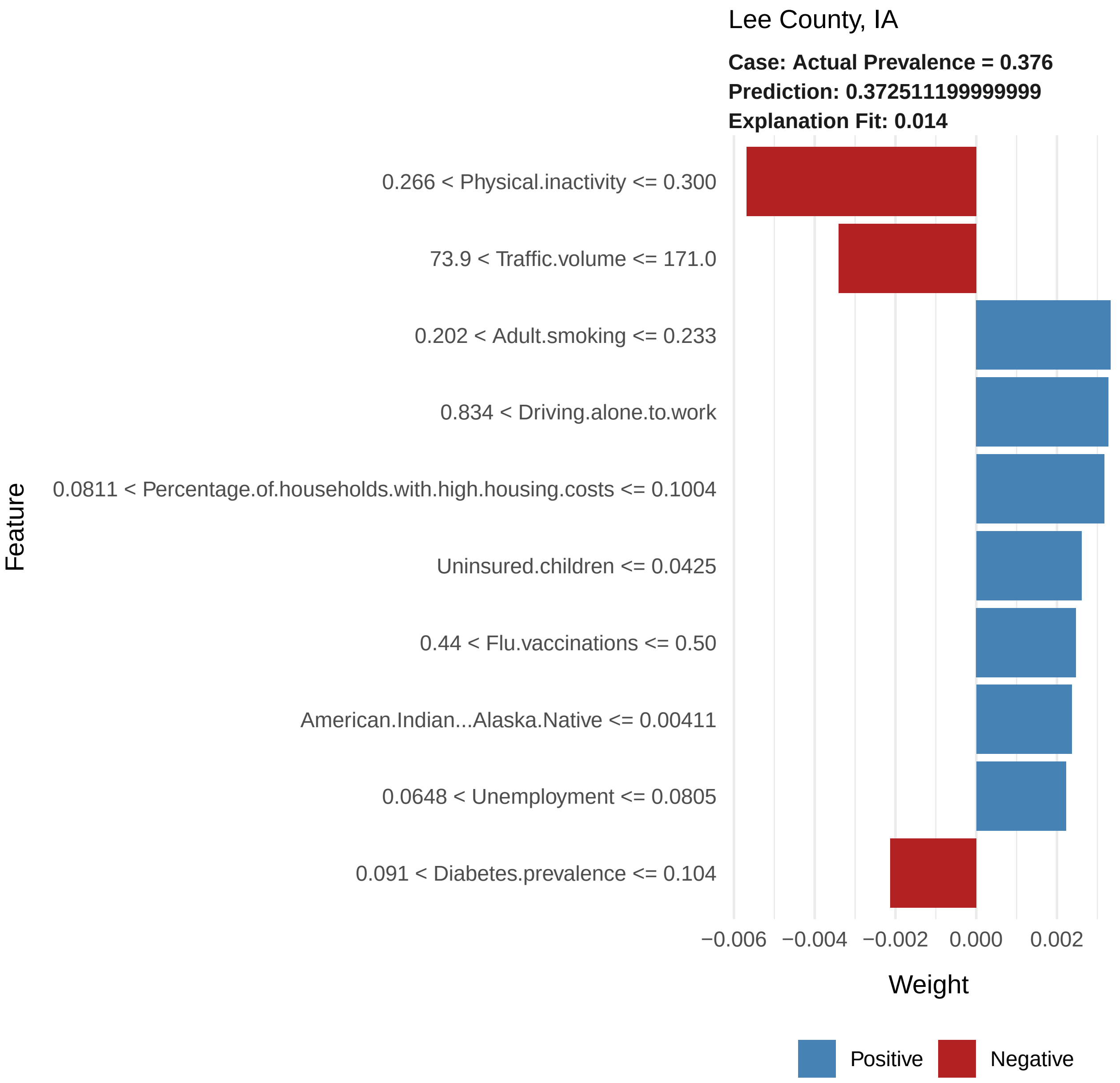

Lee County, IA
Case: Actual Prevalence = 0.376
Prediction: 0.372511199999999
Explanation Fit: 0.014
0.266 < Physical.inactivity <= 0.300
73.9 < Traffic.volume <= 171.0
0.202 < Adult.smoking <= 0.233
0.834 < Driving.alone.to.work
0.0811 < Percentage.of.households.with.high.housing.costs <= 0.1004
Uninsured.children <= 0.0425
0.44 < Flu.vaccinations <= 0.50
American.Indian...Alaska.Native <= 0.00411
0.0648 < Unemployment <= 0.0805
0.091 < Diabetes.prevalence <= 0.104
Feature
−0.006
−0.004
−0.002
0.000
0.002
Weight
Positive
Negative

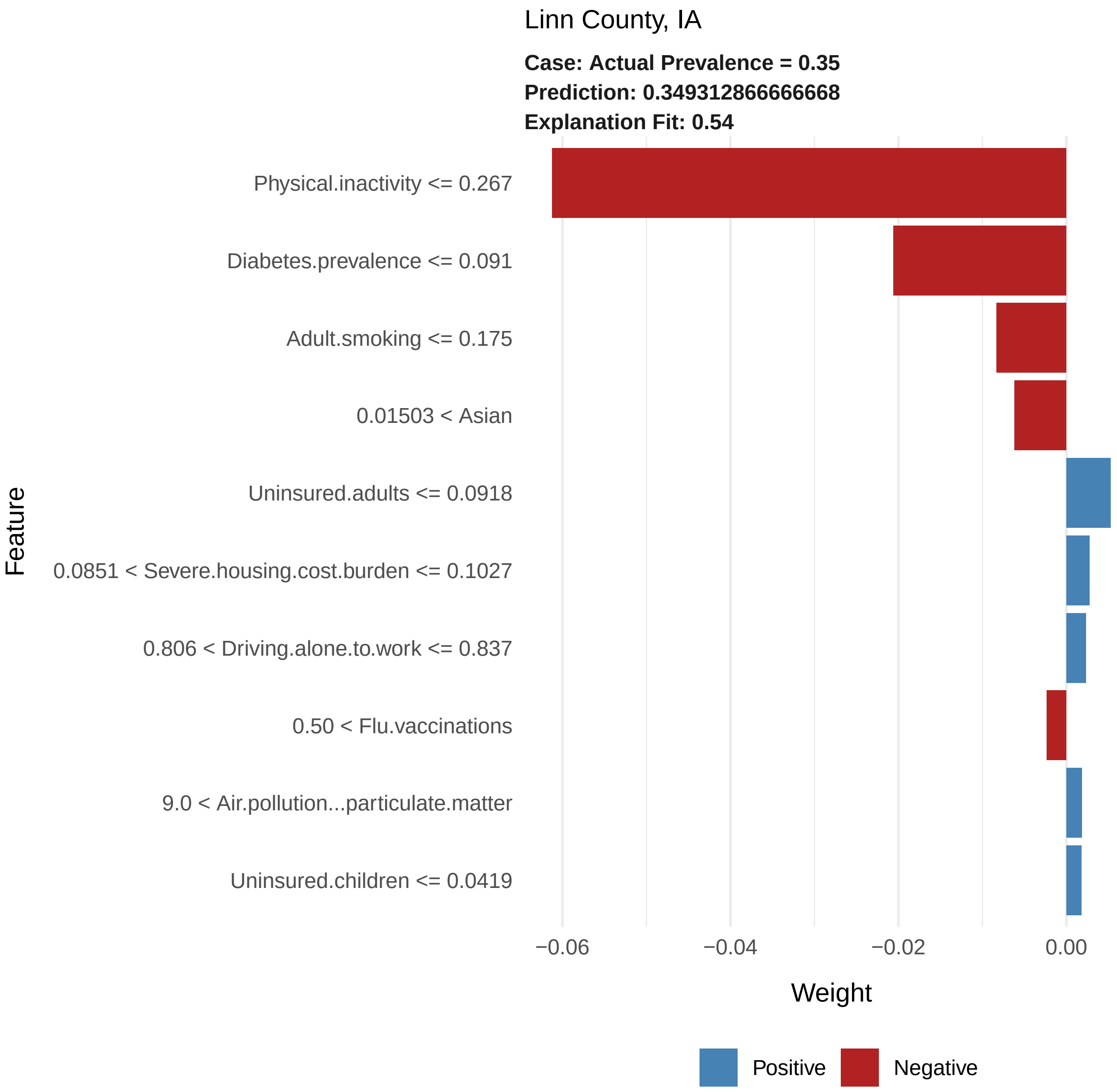

Linn County, IA
Case: Actual Prevalence = 0.35
Prediction: 0.349312866666668
Explanation Fit: 0.54
Physical.inactivity <= 0.267
Diabetes.prevalence <= 0.091
Adult.smoking <= 0.175
0.01503 < Asian
Uninsured.adults <= 0.0918
0.0851 < Severe.housing.cost.burden <= 0.1027
0.806 < Driving.alone.to.work <= 0.837
0.50 < Flu.vaccinations
9.0 < Air.pollution...particulate.matter
Uninsured.children <= 0.0419
Feature
−0.06
−0.04
−0.02
0.00
Weight
Positive
Negative

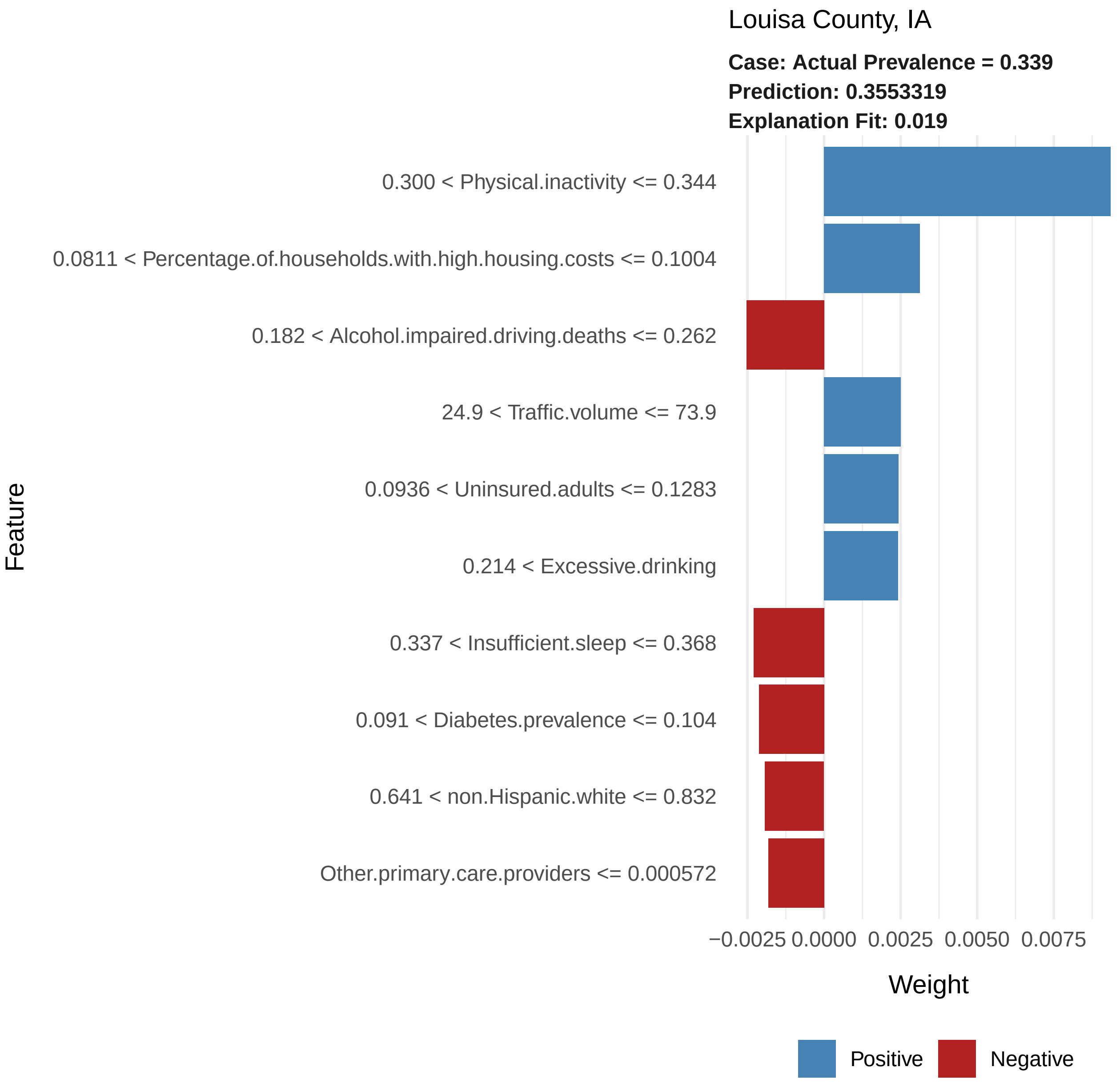
Louisa County, IA
Case: Actual Prevalence = 0.339
Prediction: 0.3553319
Explanation Fit: 0.019
0.300 < Physical.inactivity <= 0.344
0.0811 < Percentage.of.households.with.high.housing.costs <= 0.1004
0.182 < Alcohol.impaired.driving.deaths <= 0.262
24.9 < Traffic.volume <= 73.9
0.0936 < Uninsured.adults <= 0.1283
0.214 < Excessive.drinking
0.337 < Insufficient.sleep <= 0.368
0.091 < Diabetes.prevalence <= 0.104
0.641 < non.Hispanic.white <= 0.832
Other.primary.care.providers <= 0.000572
Feature
−0.0025
0.0000
0.0025
0.0050
0.0075
Weight
Positive
Negative

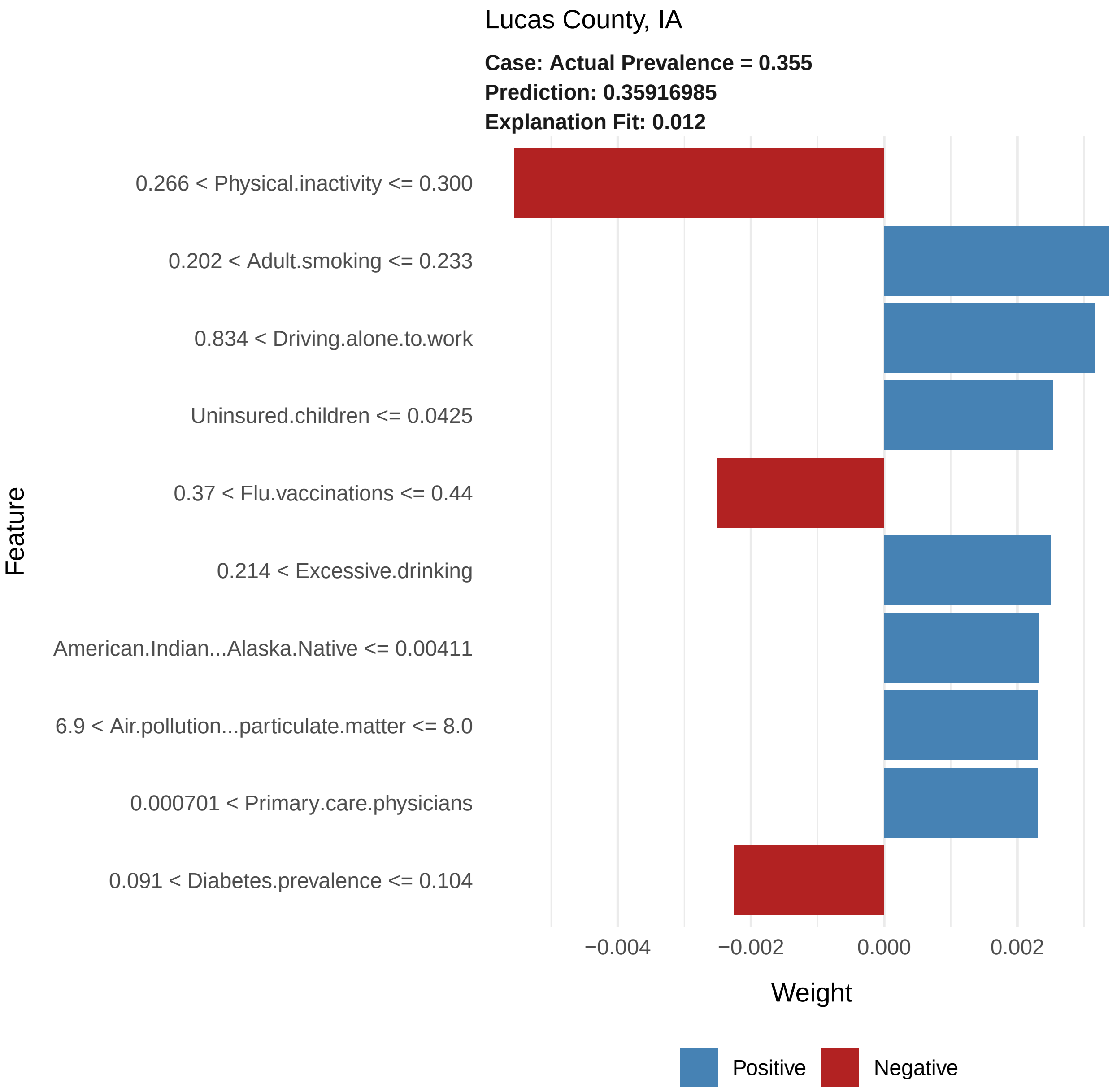

Lucas County, IA
Case: Actual Prevalence = 0.355
Prediction: 0.35916985
Explanation Fit: 0.012
0.266 < Physical.inactivity <= 0.300
0.202 < Adult.smoking <= 0.233
0.834 < Driving.alone.to.work
Uninsured.children <= 0.0425
0.37 < Flu.vaccinations <= 0.44
0.214 < Excessive.drinking
American.Indian...Alaska.Native <= 0.00411
6.9 < Air.pollution...particulate.matter <= 8.0
0.000701 < Primary.care.physicians
0.091 < Diabetes.prevalence <= 0.104
Feature
−0.004
−0.002
0.000
0.002
Weight
Positive
Negative

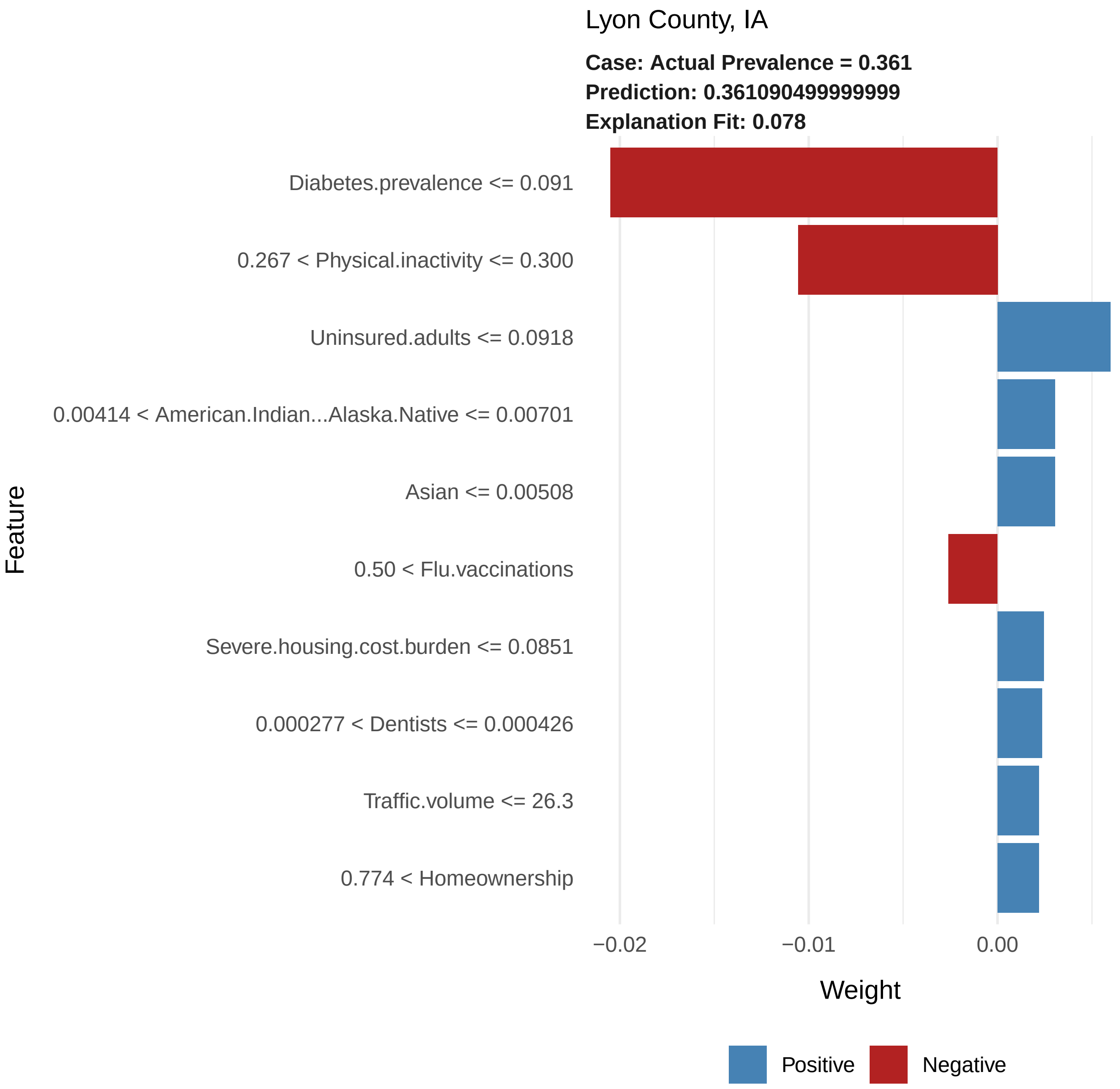

Lyon County, IA
Case: Actual Prevalence = 0.361
Prediction: 0.361090499999999
Explanation Fit: 0.078
Diabetes.prevalence <= 0.091
0.267 < Physical.inactivity <= 0.300
Uninsured.adults <= 0.0918
0.00414 < American.Indian...Alaska.Native <= 0.00701
Asian <= 0.00508
0.50 < Flu.vaccinations
Severe.housing.cost.burden <= 0.0851
0.000277 < Dentists <= 0.000426
Traffic.volume <= 26.3
0.774 < Homeownership
Feature
−0.02
−0.01
0.00
Weight
Positive
Negative

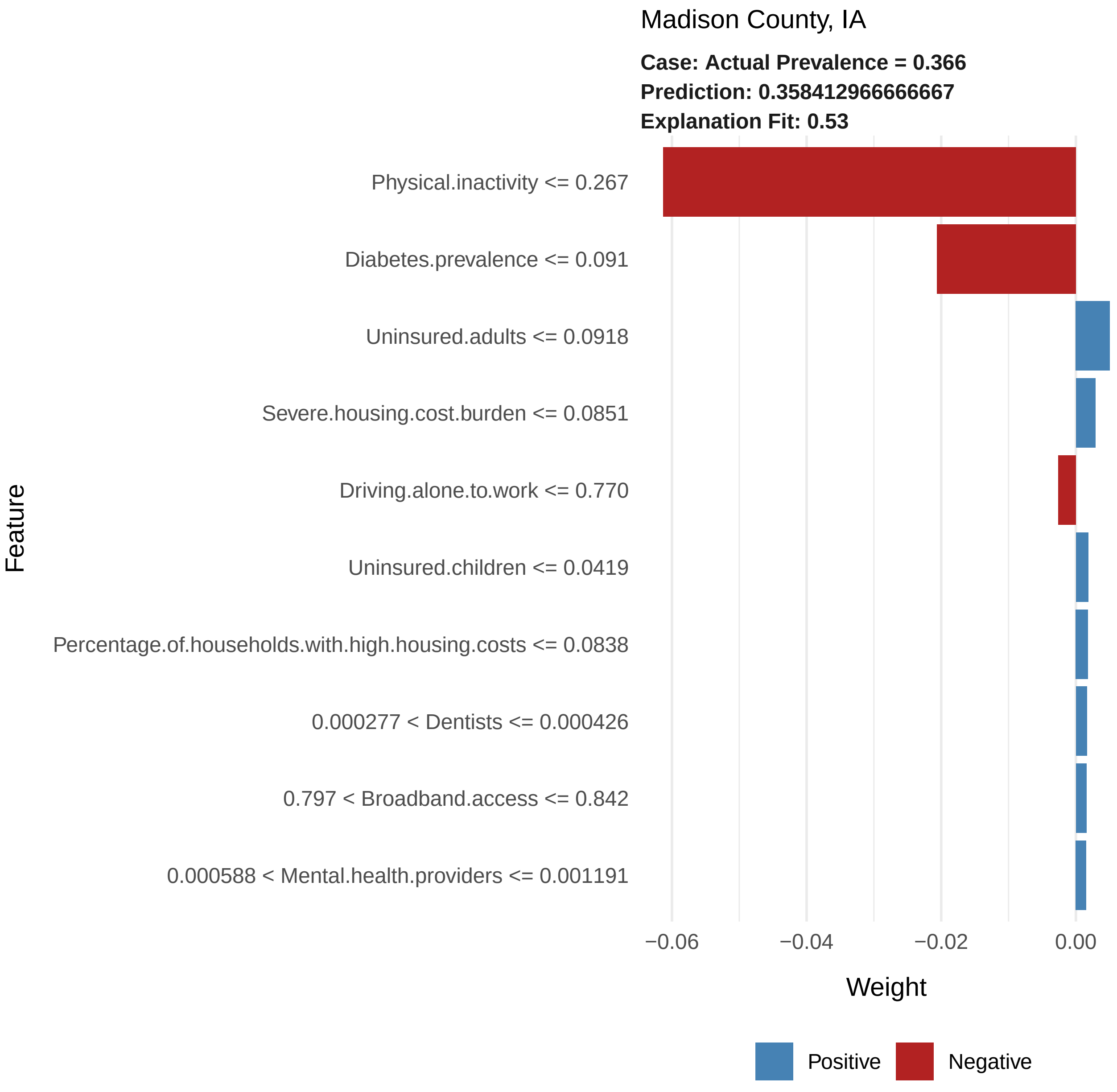

Madison County, IA
Case: Actual Prevalence = 0.366
Prediction: 0.358412966666667
Explanation Fit: 0.53
Physical.inactivity <= 0.267
Diabetes.prevalence <= 0.091
Uninsured.adults <= 0.0918
Severe.housing.cost.burden <= 0.0851
Driving.alone.to.work <= 0.770
Uninsured.children <= 0.0419
Percentage.of.households.with.high.housing.costs <= 0.0838
0.000277 < Dentists <= 0.000426
0.797 < Broadband.access <= 0.842
0.000588 < Mental.health.providers <= 0.001191
Feature
−0.06
−0.04
−0.02
0.00
Weight
Positive
Negative

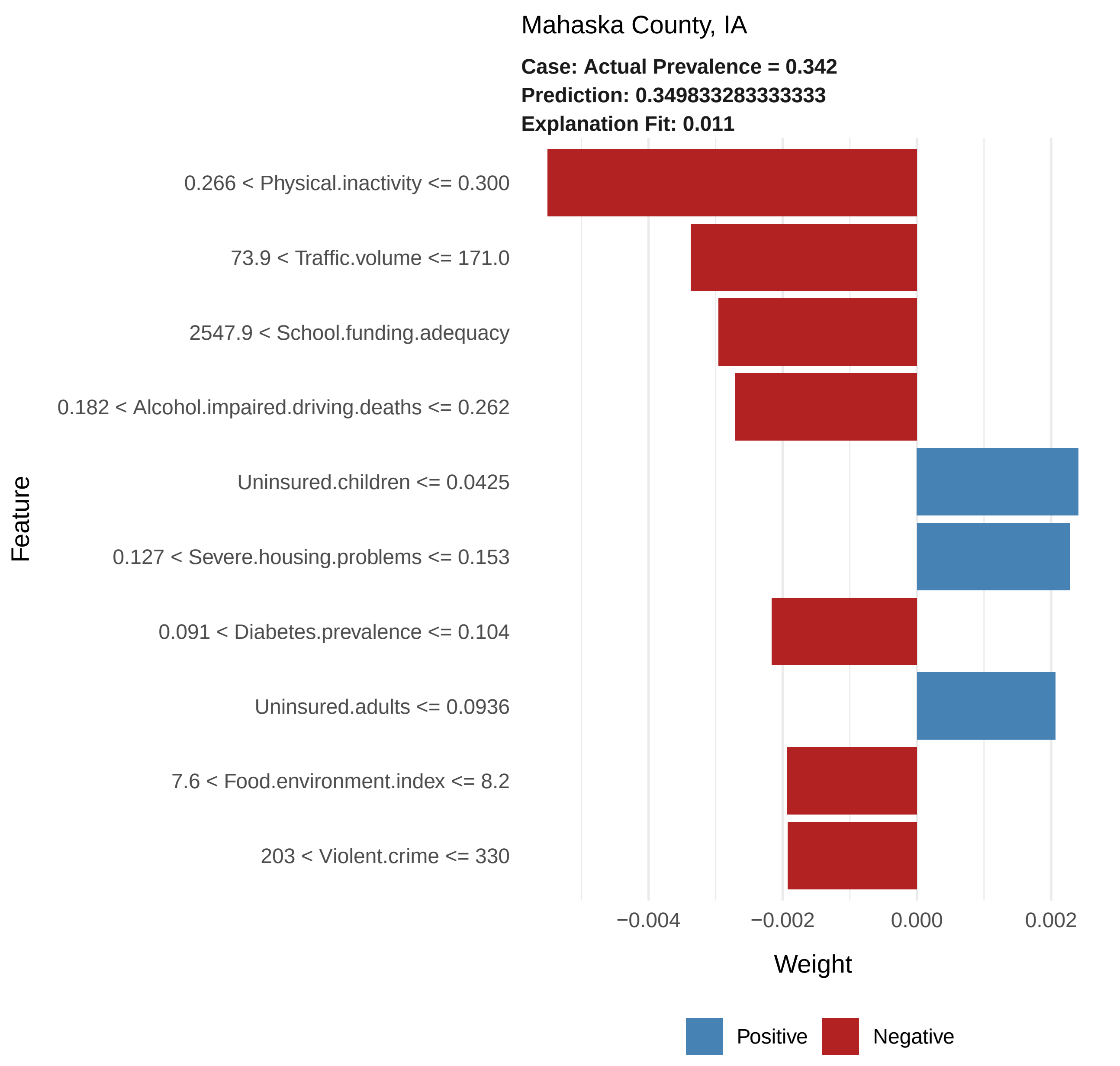
Mahaska County, IA
Case: Actual Prevalence = 0.342
Prediction: 0.349833283333333
Explanation Fit: 0.011
0.266 < Physical.inactivity <= 0.300
73.9 < Traffic.volume <= 171.0
2547.9 < School.funding.adequacy
0.182 < Alcohol.impaired.driving.deaths <= 0.262
Uninsured.children <= 0.0425
0.127 < Severe.housing.problems <= 0.153
0.091 < Diabetes.prevalence <= 0.104
Uninsured.adults <= 0.0936
7.6 < Food.environment.index <= 8.2
203 < Violent.crime <= 330
Feature
−0.004
−0.002
0.000
0.002
Weight
Positive
Negative

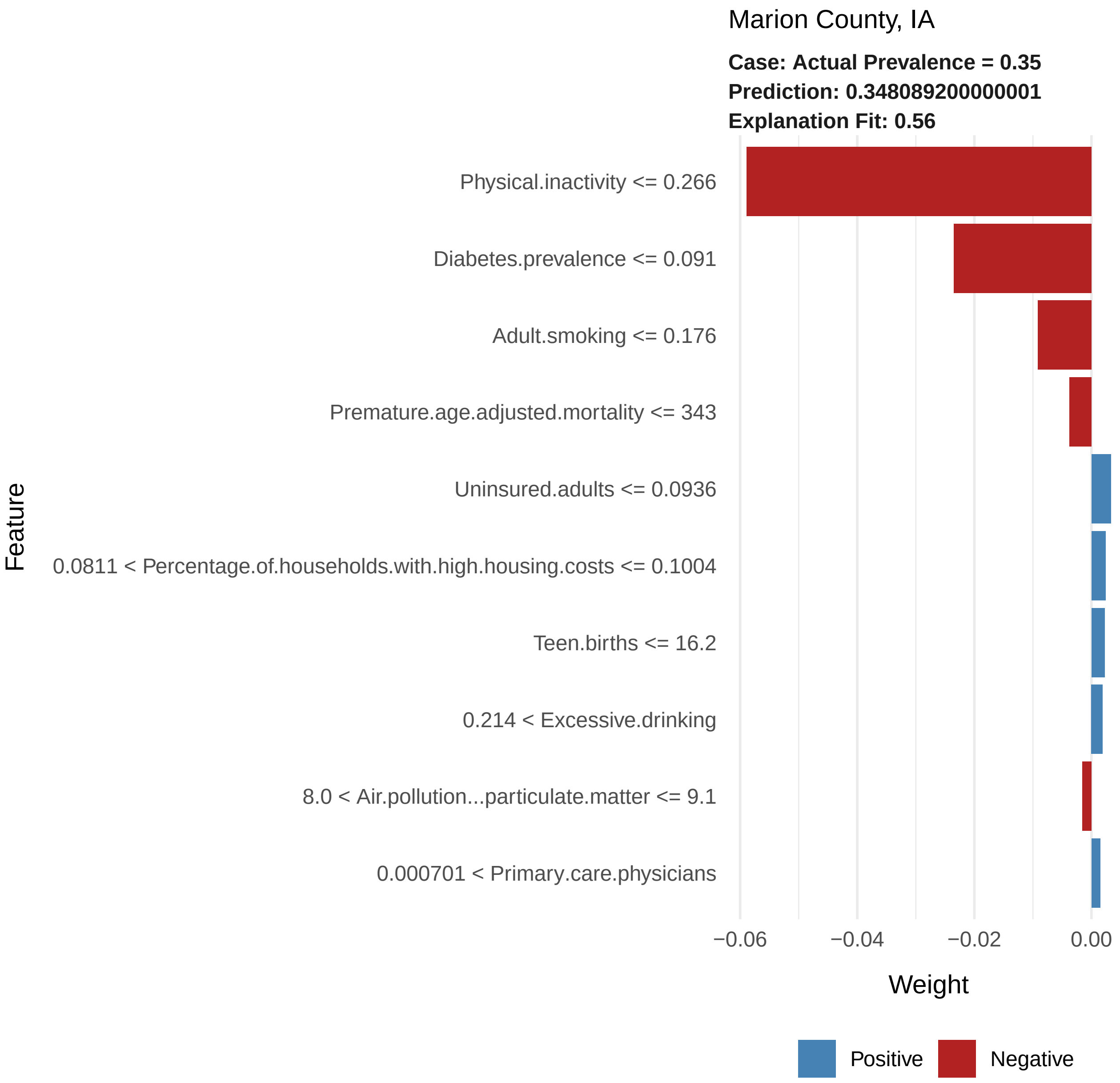
Marion County, IA
Case: Actual Prevalence = 0.35
Prediction: 0.348089200000001
Explanation Fit: 0.56
Physical.inactivity <= 0.266
Diabetes.prevalence <= 0.091
Adult.smoking <= 0.176
Premature.age.adjusted.mortality <= 343
Uninsured.adults <= 0.0936
0.0811 < Percentage.of.households.with.high.housing.costs <= 0.1004
Teen.births <= 16.2
0.214 < Excessive.drinking
8.0 < Air.pollution...particulate.matter <= 9.1
0.000701 < Primary.care.physicians
Feature
−0.06
−0.04
−0.02
0.00
Weight
Positive
Negative

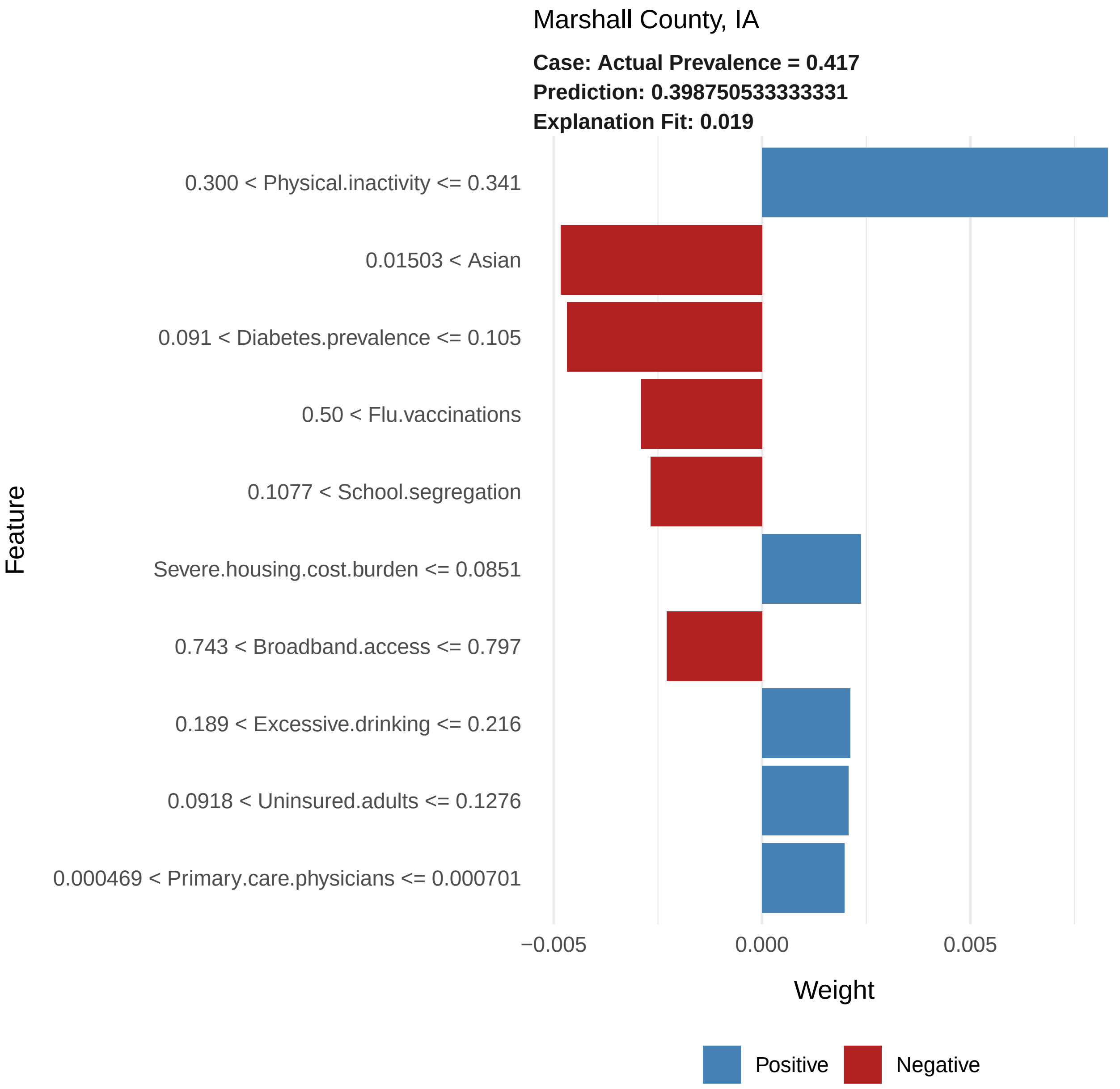

Marshall County, IA
Case: Actual Prevalence = 0.417
Prediction: 0.398750533333331
Explanation Fit: 0.019
Feature
0.300 < Physical.inactivity <= 0.341
0.01503 < Asian
0.091 < Diabetes.prevalence <= 0.105
0.50 < Flu.vaccinations
0.1077 < School.segregation
Severe.housing.cost.burden <= 0.0851
0.743 < Broadband.access <= 0.797
0.189 < Excessive.drinking <= 0.216
0.0918 < Uninsured.adults <= 0.1276
0.000469 < Primary.care.physicians <= 0.000701
−0.005
0.000
0.005
Weight
Positive
Negative

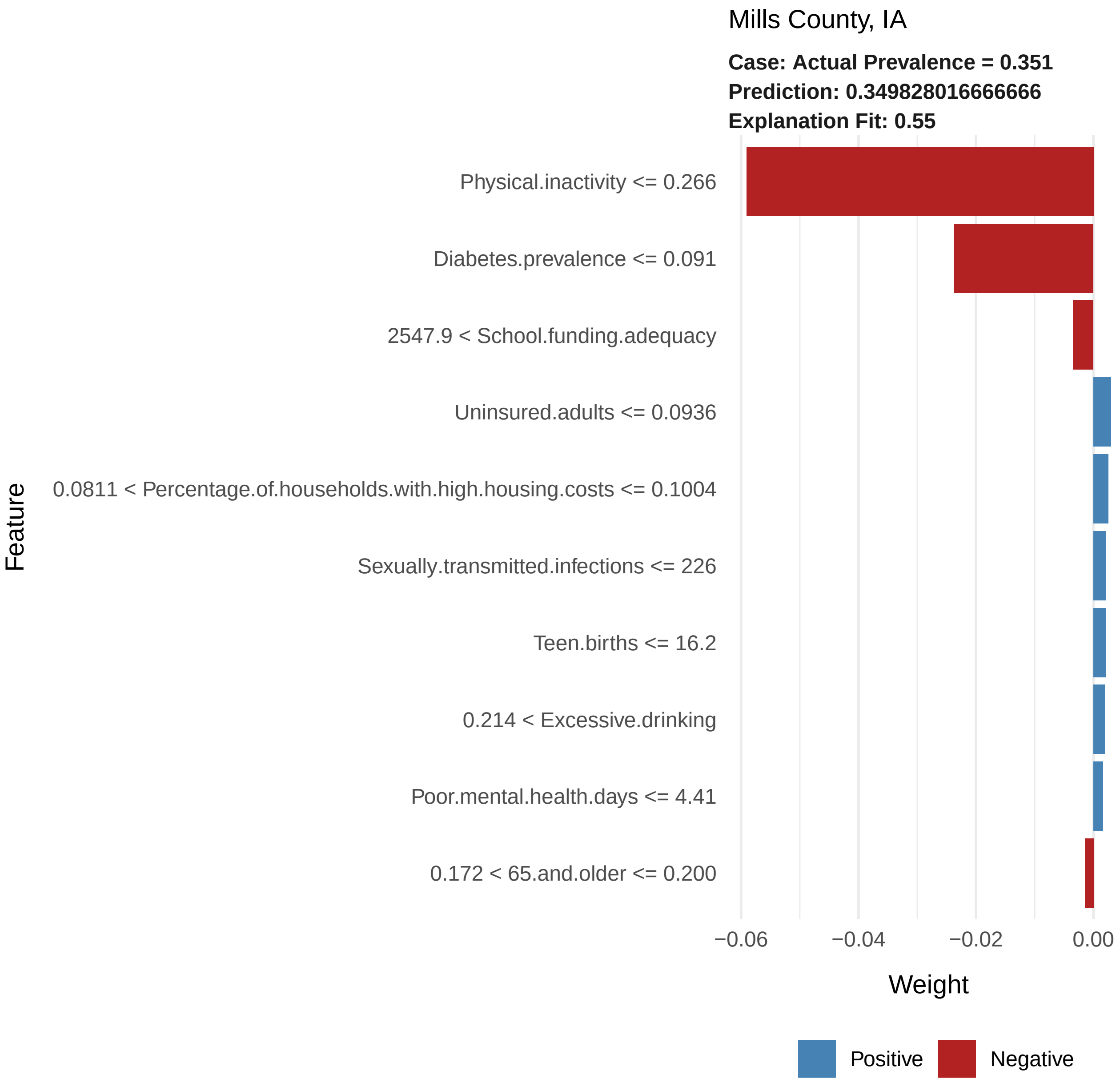

Mills County, IA
Case: Actual Prevalence = 0.351
Prediction: 0.349828016666666
Explanation Fit: 0.55
Physical.inactivity <= 0.266
Diabetes.prevalence <= 0.091
2547.9 < School.funding.adequacy
Uninsured.adults <= 0.0936
0.0811 < Percentage.of.households.with.high.housing.costs <= 0.1004
Sexually.transmitted.infections <= 226
Teen.births <= 16.2
0.214 < Excessive.drinking
Poor.mental.health.days <= 4.41
0.172 < 65.and.older <= 0.200
Feature
−0.06
−0.04
−0.02
0.00
Weight
Positive
Negative

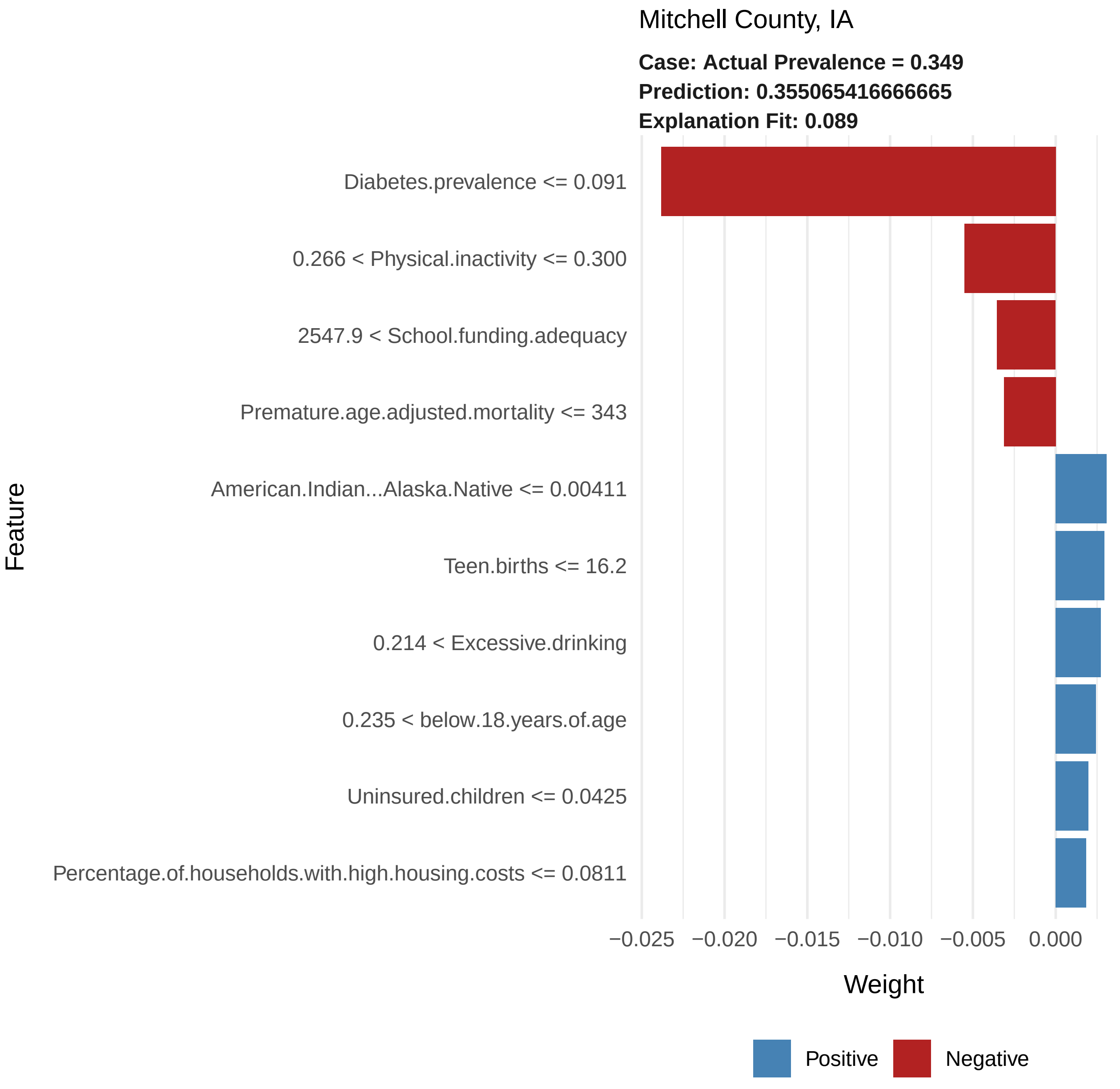

Mitchell County, IA
Case: Actual Prevalence = 0.349
Prediction: 0.355065416666665
Explanation Fit: 0.089
Diabetes.prevalence <= 0.091
0.266 < Physical.inactivity <= 0.300
2547.9 < School.funding.adequacy
Premature.age.adjusted.mortality <= 343
American.Indian...Alaska.Native <= 0.00411
Teen.births <= 16.2
0.214 < Excessive.drinking
0.235 < below.18.years.of.age
Uninsured.children <= 0.0425
Percentage.of.households.with.high.housing.costs <= 0.0811
Feature
−0.025
−0.020
−0.015
−0.010
−0.005
0.000
Weight
Positive
Negative

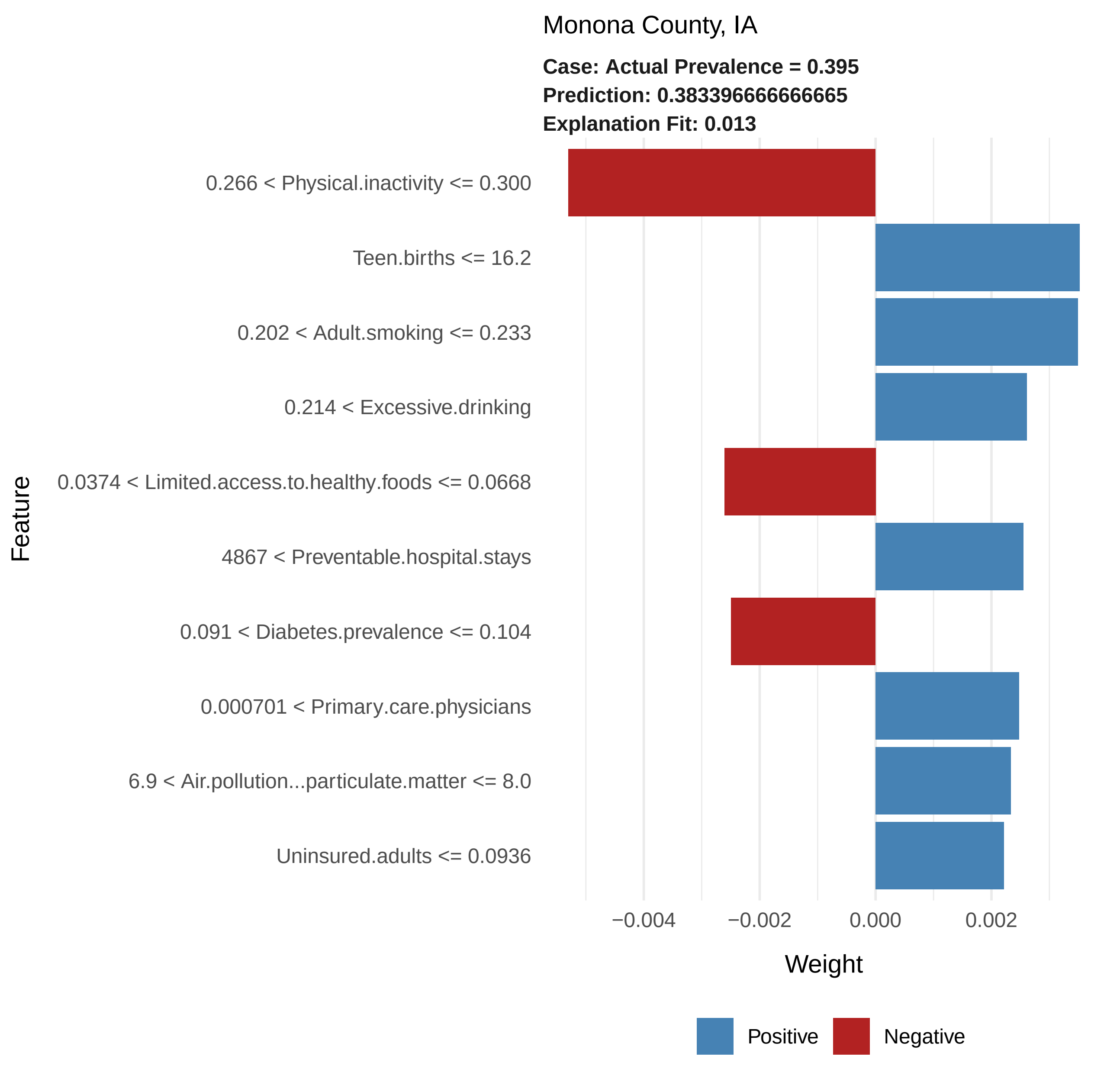

Monona County, IA
Case: Actual Prevalence = 0.395
Prediction: 0.383396666666665
Explanation Fit: 0.013
0.266 < Physical.inactivity <= 0.300
Teen.births <= 16.2
0.202 < Adult.smoking <= 0.233
0.214 < Excessive.drinking
0.0374 < Limited.access.to.healthy.foods <= 0.0668
4867 < Preventable.hospital.stays
0.091 < Diabetes.prevalence <= 0.104
0.000701 < Primary.care.physicians
6.9 < Air.pollution...particulate.matter <= 8.0
Uninsured.adults <= 0.0936
Feature
−0.004
−0.002
0.000
0.002
Weight
Positive
Negative

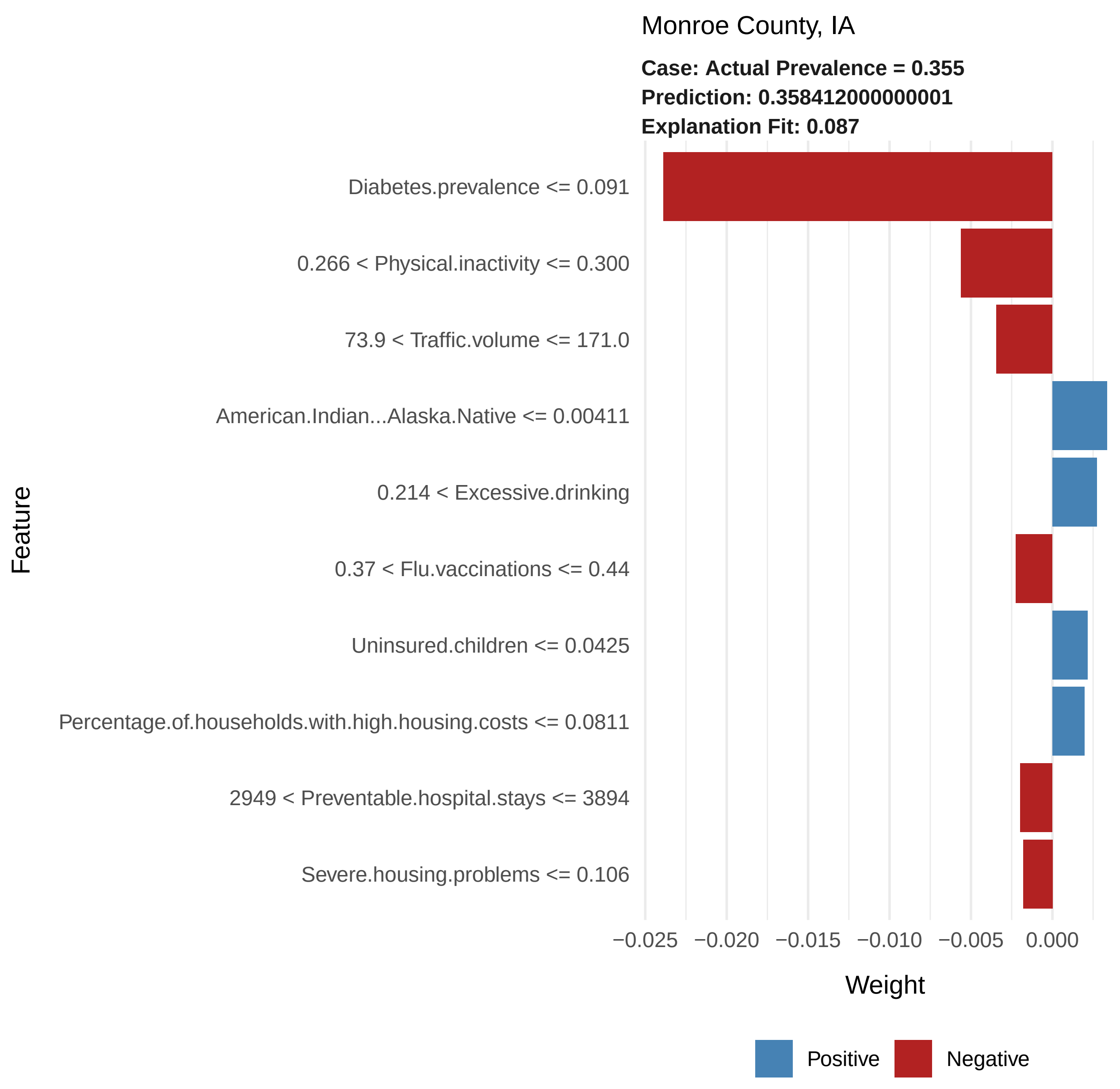

Monroe County, IA
Case: Actual Prevalence = 0.355
Prediction: 0.358412000000001
Explanation Fit: 0.087
Diabetes.prevalence <= 0.091
0.266 < Physical.inactivity <= 0.300
73.9 < Traffic.volume <= 171.0
American.Indian...Alaska.Native <= 0.00411
0.214 < Excessive.drinking
0.37 < Flu.vaccinations <= 0.44
Uninsured.children <= 0.0425
Percentage.of.households.with.high.housing.costs <= 0.0811
2949 < Preventable.hospital.stays <= 3894
Severe.housing.problems <= 0.106
Feature
−0.025
−0.020
−0.015
−0.010
−0.005
0.000
Weight
Positive
Negative

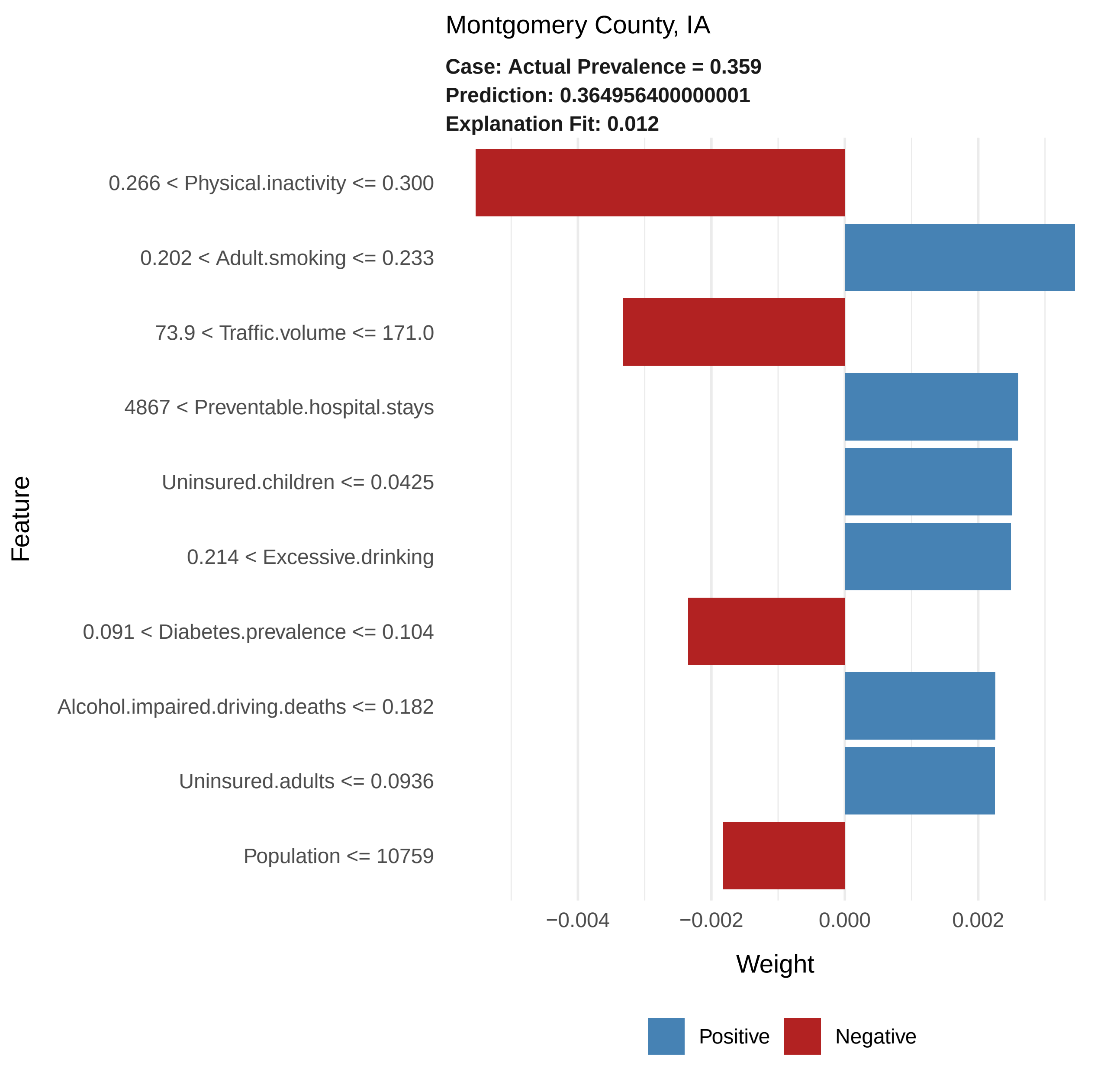

Montgomery County, IA
Case: Actual Prevalence = 0.359
Prediction: 0.364956400000001
Explanation Fit: 0.012
0.266 < Physical.inactivity <= 0.300
0.202 < Adult.smoking <= 0.233
73.9 < Traffic.volume <= 171.0
4867 < Preventable.hospital.stays
Uninsured.children <= 0.0425
0.214 < Excessive.drinking
0.091 < Diabetes.prevalence <= 0.104
Alcohol.impaired.driving.deaths <= 0.182
Uninsured.adults <= 0.0936
Population <= 10759
Feature
−0.004
−0.002
0.000
0.002
Weight
Positive
Negative

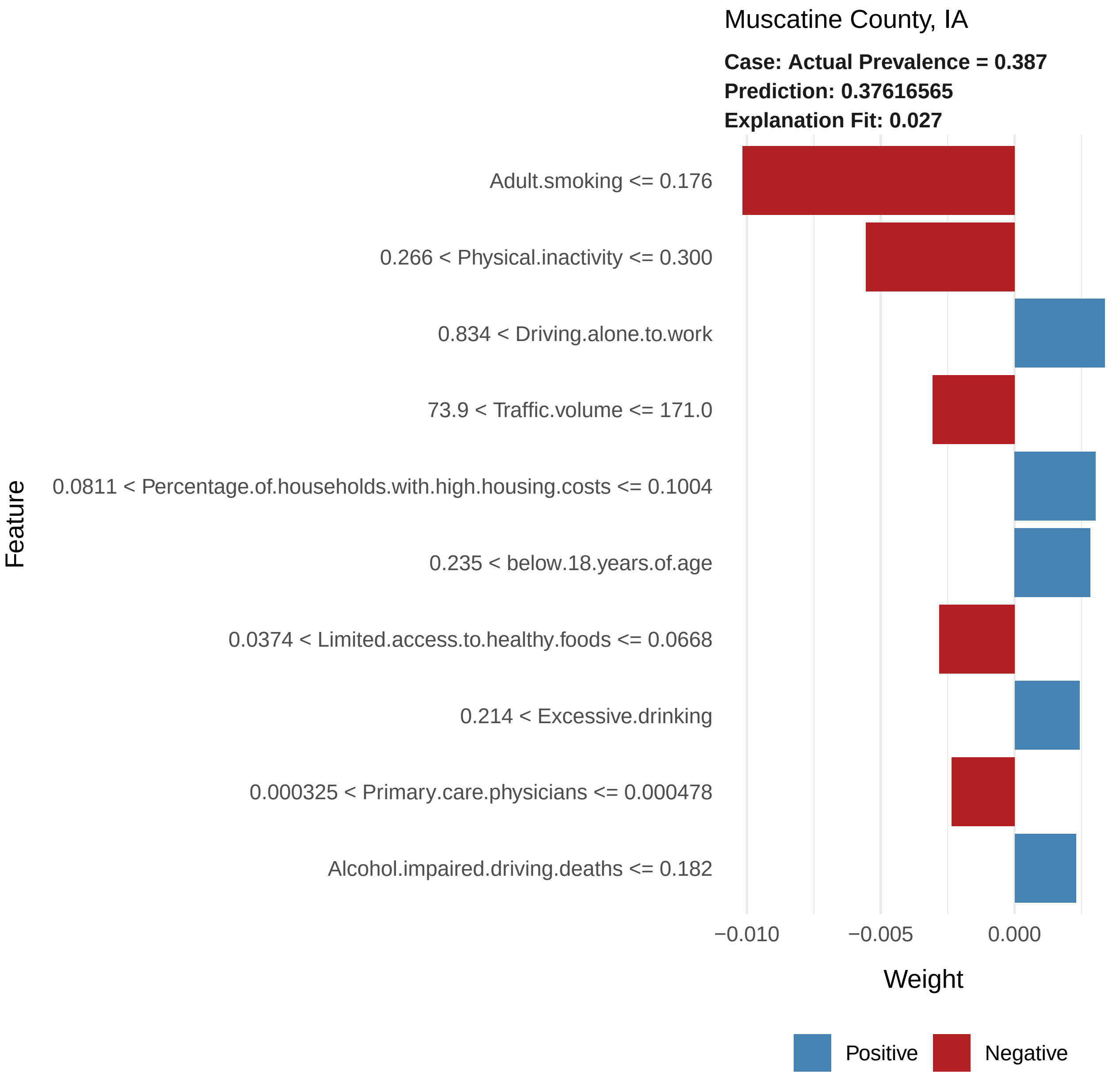

Muscatine County, IA
Case: Actual Prevalence = 0.387
Prediction: 0.37616565
Explanation Fit: 0.027
Adult.smoking <= 0.176
0.266 < Physical.inactivity <= 0.300
0.834 < Driving.alone.to.work
73.9 < Traffic.volume <= 171.0
0.0811 < Percentage.of.households.with.high.housing.costs <= 0.1004
0.235 < below.18.years.of.age
0.0374 < Limited.access.to.healthy.foods <= 0.0668
0.214 < Excessive.drinking
0.000325 < Primary.care.physicians <= 0.000478
Alcohol.impaired.driving.deaths <= 0.182
Feature
−0.010
−0.005
0.000
Weight
Positive
Negative

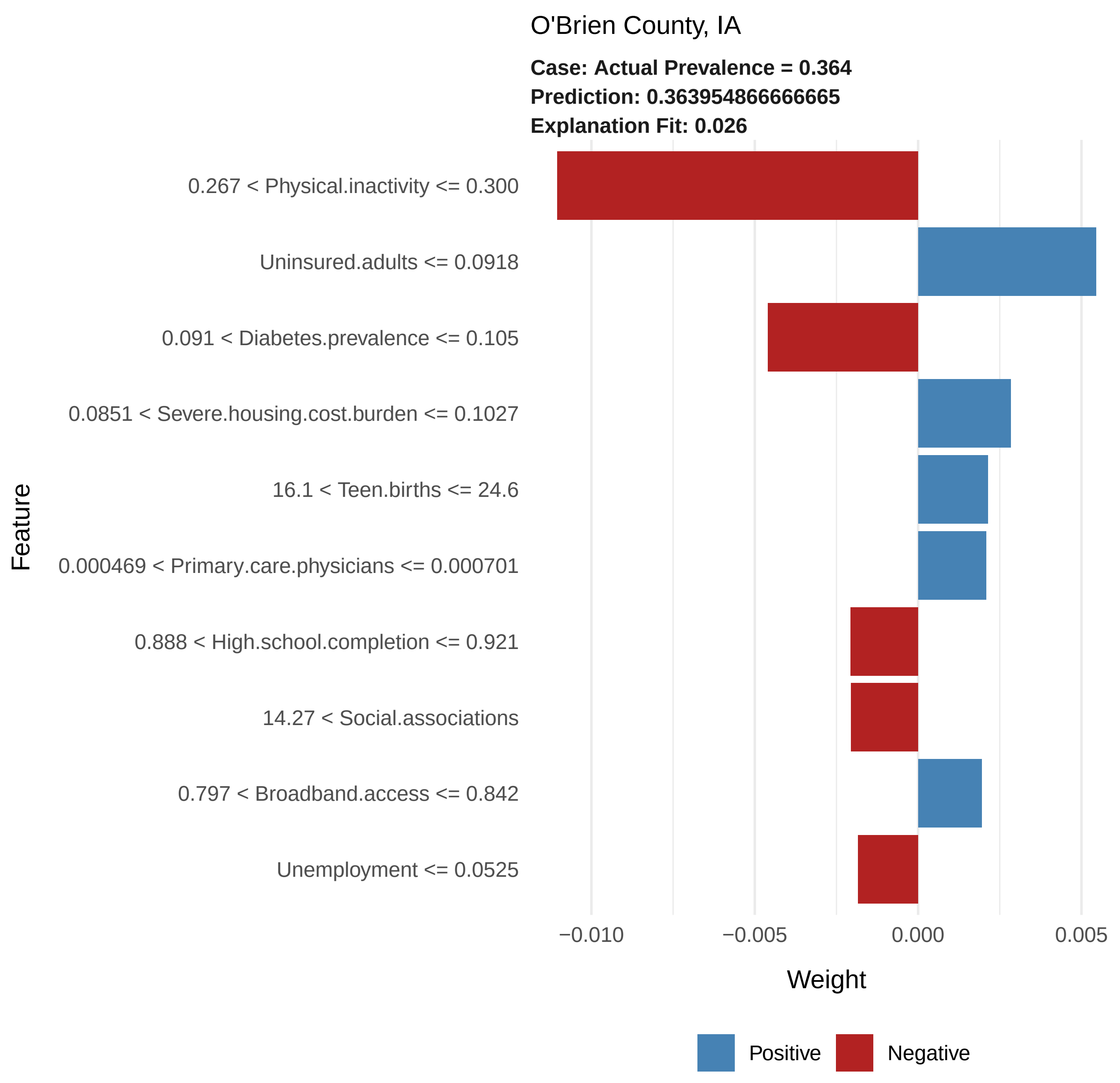
O'Brien County, IA
Case: Actual Prevalence = 0.364
Prediction: 0.363954866666665
Explanation Fit: 0.026
0.267 < Physical.inactivity <= 0.300
Uninsured.adults <= 0.0918
0.091 < Diabetes.prevalence <= 0.105
0.0851 < Severe.housing.cost.burden <= 0.1027
16.1 < Teen.births <= 24.6
0.000469 < Primary.care.physicians <= 0.000701
0.888 < High.school.completion <= 0.921
14.27 < Social.associations
0.797 < Broadband.access <= 0.842
Unemployment <= 0.0525
Feature
−0.010
−0.005
0.000
0.005
Weight
Positive
Negative

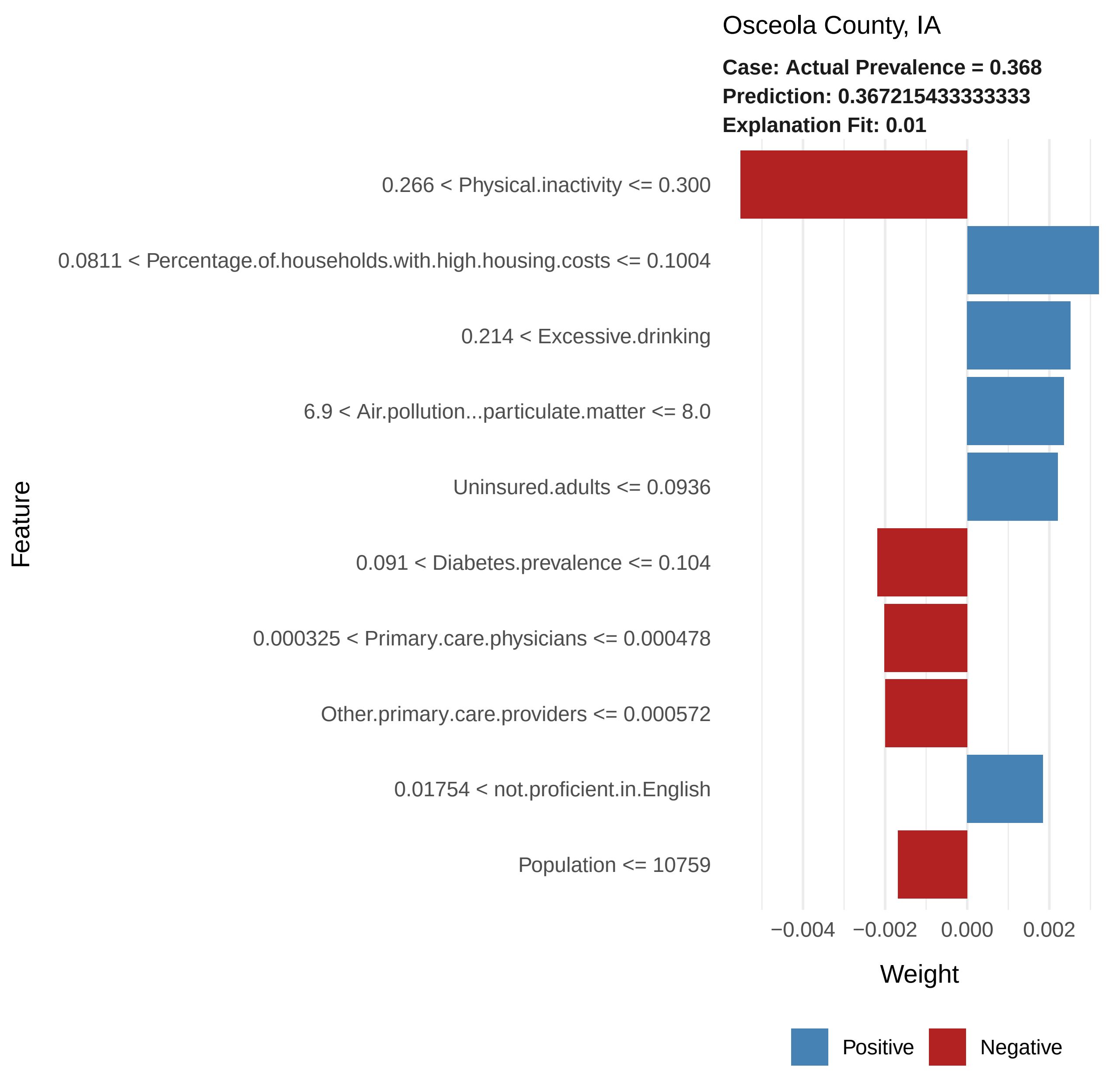

Osceola County, IA
Case: Actual Prevalence = 0.368
Prediction: 0.367215433333333
Explanation Fit: 0.01
0.266 < Physical.inactivity <= 0.300
0.0811 < Percentage.of.households.with.high.housing.costs <= 0.1004
0.214 < Excessive.drinking
6.9 < Air.pollution...particulate.matter <= 8.0
Uninsured.adults <= 0.0936
0.091 < Diabetes.prevalence <= 0.104
0.000325 < Primary.care.physicians <= 0.000478
Other.primary.care.providers <= 0.000572
0.01754 < not.proficient.in.English
Population <= 10759
Feature
−0.004
−0.002
0.000
0.002
Weight
Positive
Negative

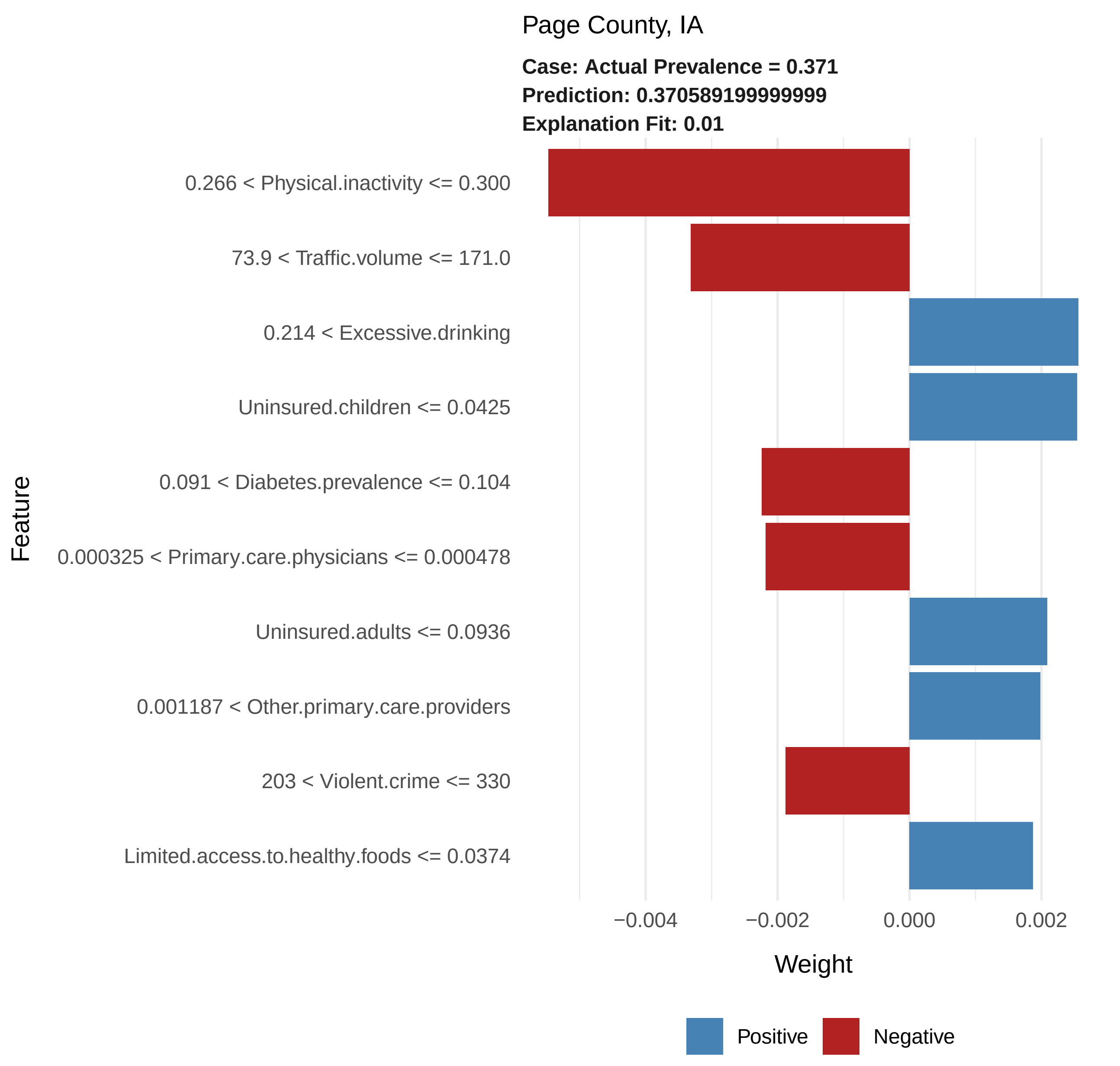

Page County, IA
Case: Actual Prevalence = 0.371
Prediction: 0.370589199999999
Explanation Fit: 0.01
0.266 < Physical.inactivity <= 0.300
73.9 < Traffic.volume <= 171.0
0.214 < Excessive.drinking
Uninsured.children <= 0.0425
0.091 < Diabetes.prevalence <= 0.104
0.000325 < Primary.care.physicians <= 0.000478
Uninsured.adults <= 0.0936
0.001187 < Other.primary.care.providers
203 < Violent.crime <= 330
Limited.access.to.healthy.foods <= 0.0374
Feature
−0.004
−0.002
0.000
0.002
Weight
Positive
Negative

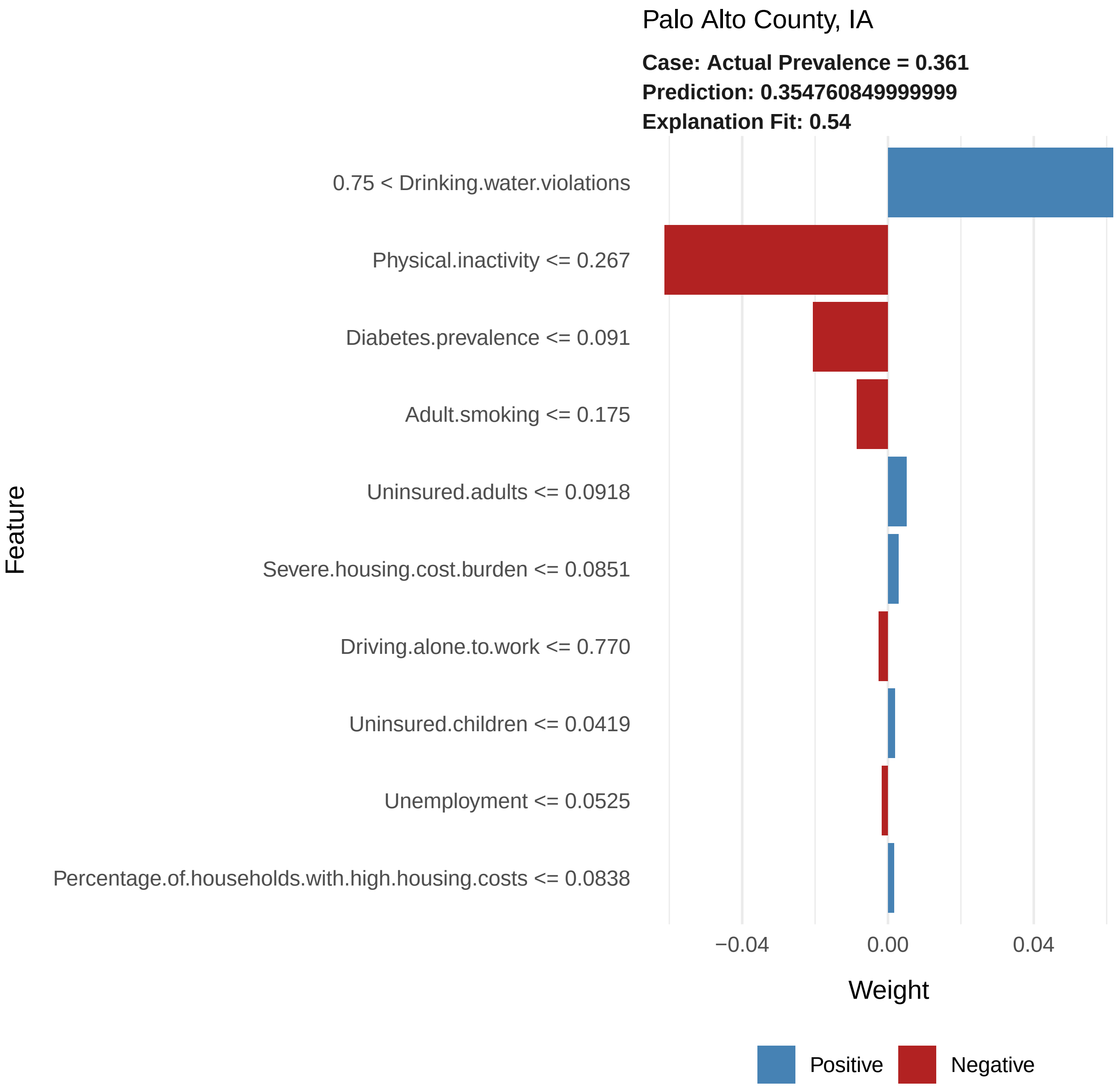

Palo Alto County, IA
Case: Actual Prevalence = 0.361
Prediction: 0.354760849999999
Explanation Fit: 0.54
0.75 < Drinking.water.violations
Physical.inactivity <= 0.267
Diabetes.prevalence <= 0.091
Adult.smoking <= 0.175
Uninsured.adults <= 0.0918
Severe.housing.cost.burden <= 0.0851
Driving.alone.to.work <= 0.770
Uninsured.children <= 0.0419
Unemployment <= 0.0525
Percentage.of.households.with.high.housing.costs <= 0.0838
Feature
−0.04
0.00
0.04
Weight
Positive
Negative

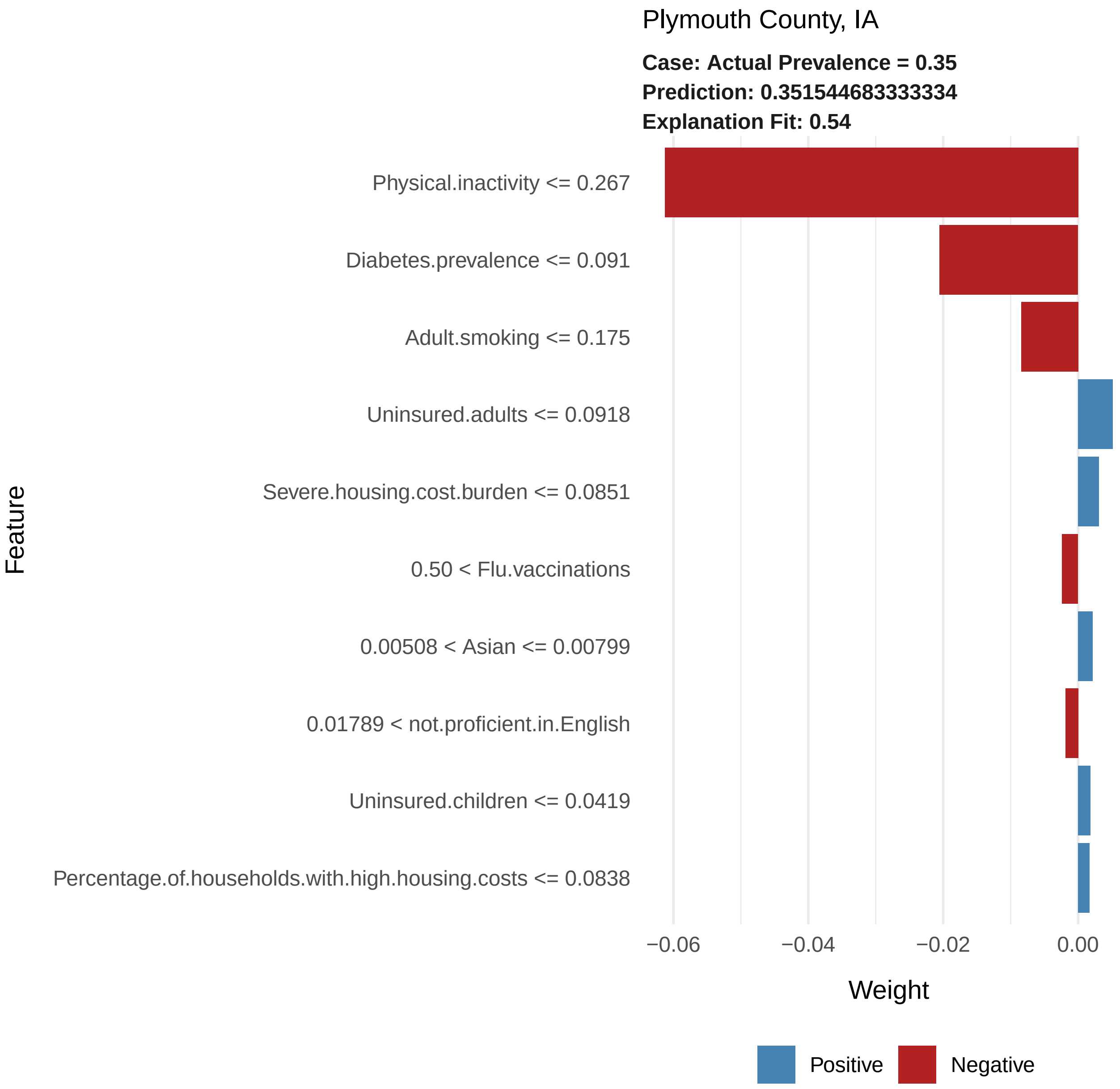

Plymouth County, IA
Case: Actual Prevalence = 0.35
Prediction: 0.351544683333334
Explanation Fit: 0.54
Physical.inactivity <= 0.267
Diabetes.prevalence <= 0.091
Adult.smoking <= 0.175
Uninsured.adults <= 0.0918
Severe.housing.cost.burden <= 0.0851
0.50 < Flu.vaccinations
0.00508 < Asian <= 0.00799
0.01789 < not.proficient.in.English
Uninsured.children <= 0.0419
Percentage.of.households.with.high.housing.costs <= 0.0838
Feature
−0.06
−0.04
−0.02
0.00
Weight
Positive
Negative

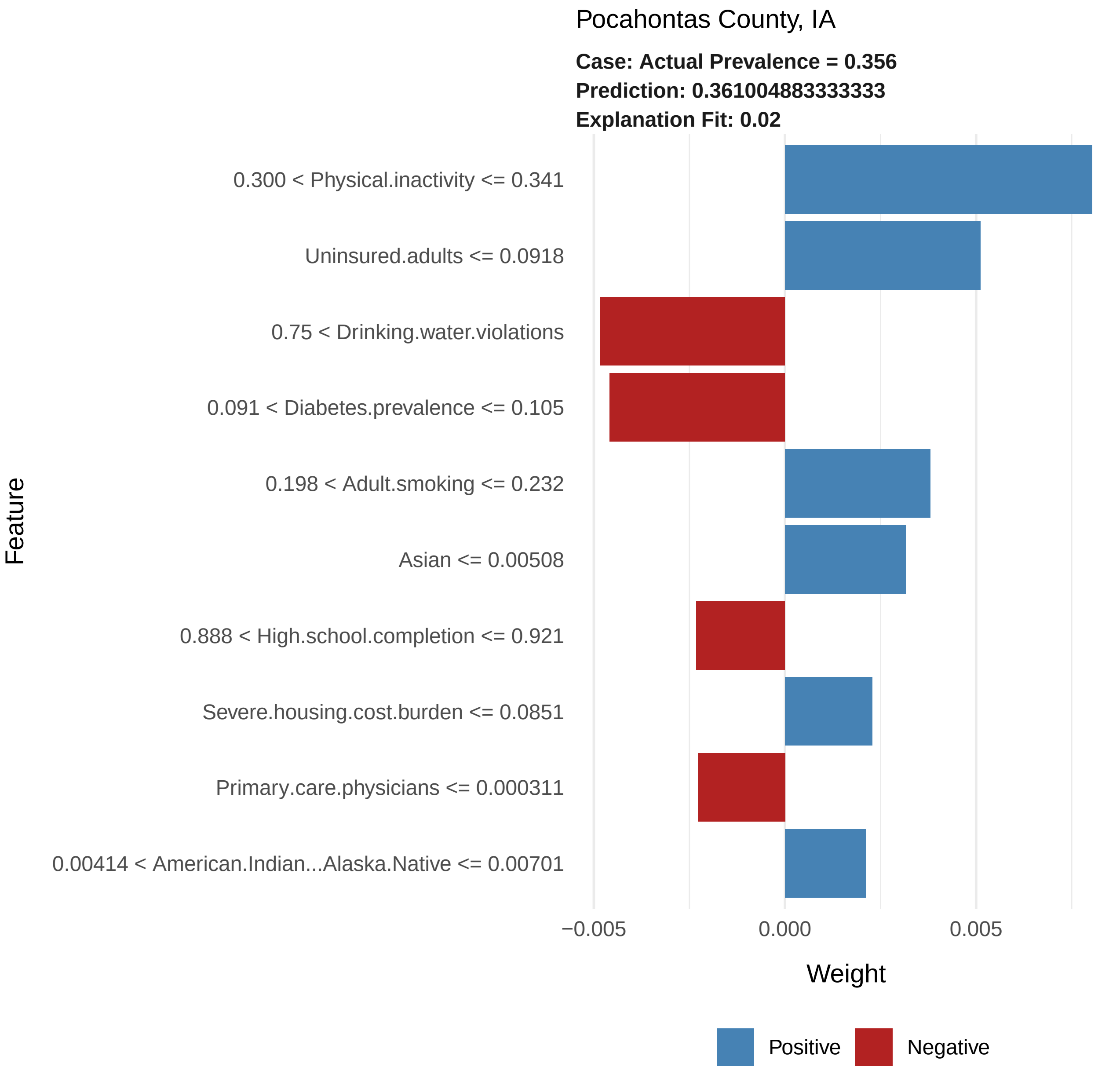

Pocahontas County, IA
Case: Actual Prevalence = 0.356
Prediction: 0.361004883333333
Explanation Fit: 0.02
0.300 < Physical.inactivity <= 0.341
Uninsured.adults <= 0.0918
0.75 < Drinking.water.violations
0.091 < Diabetes.prevalence <= 0.105
0.198 < Adult.smoking <= 0.232
Asian <= 0.00508
0.888 < High.school.completion <= 0.921
Severe.housing.cost.burden <= 0.0851
Primary.care.physicians <= 0.000311
0.00414 < American.Indian...Alaska.Native <= 0.00701
Feature
−0.005
0.000
0.005
Weight
Positive
Negative

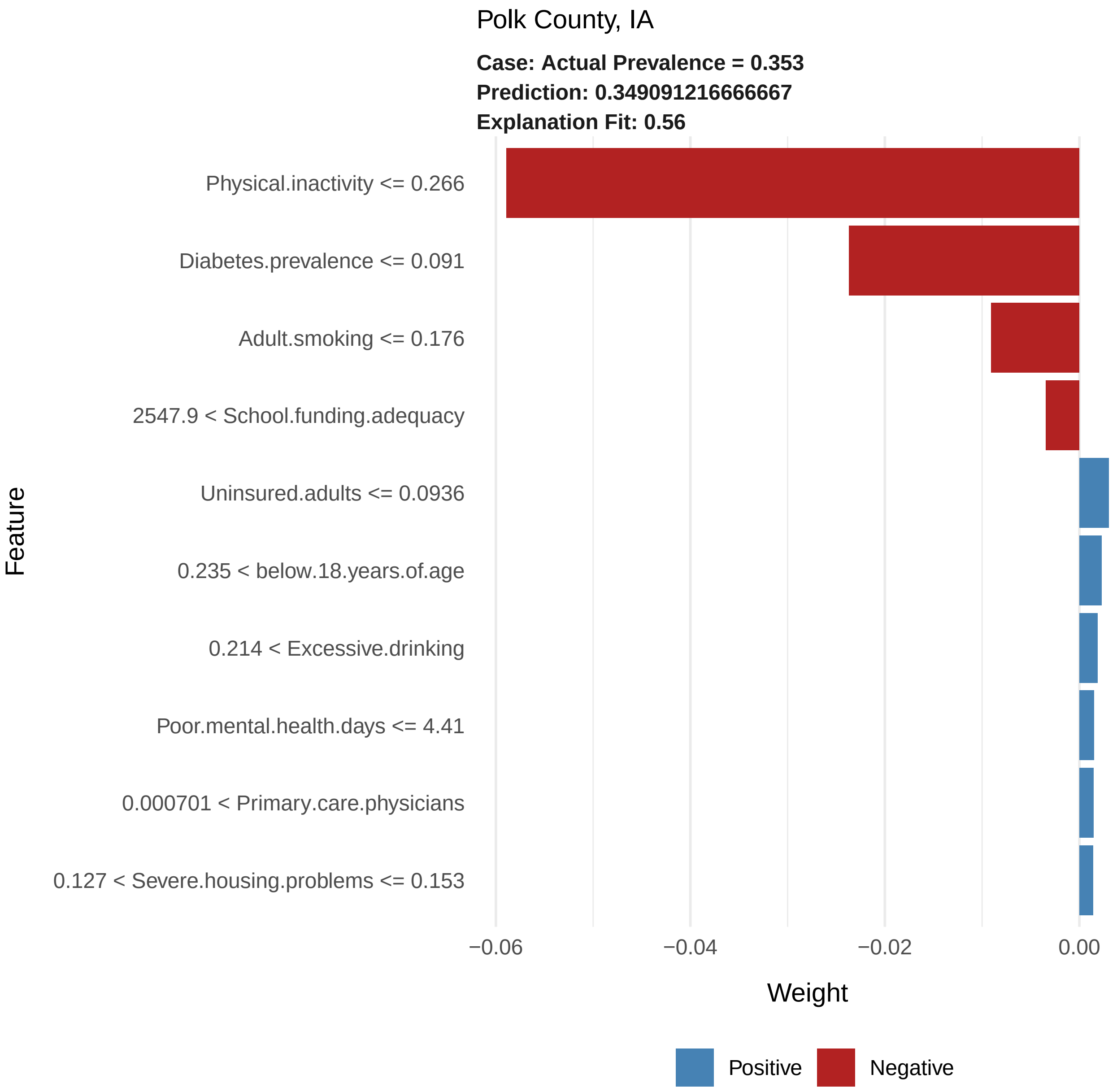

Polk County, IA
Case: Actual Prevalence = 0.353
Prediction: 0.349091216666667
Explanation Fit: 0.56
Physical.inactivity <= 0.266
Diabetes.prevalence <= 0.091
Adult.smoking <= 0.176
2547.9 < School.funding.adequacy
Uninsured.adults <= 0.0936
0.235 < below.18.years.of.age
0.214 < Excessive.drinking
Poor.mental.health.days <= 4.41
0.000701 < Primary.care.physicians
0.127 < Severe.housing.problems <= 0.153
Feature
−0.06
−0.04
−0.02
0.00
Weight
Positive
Negative

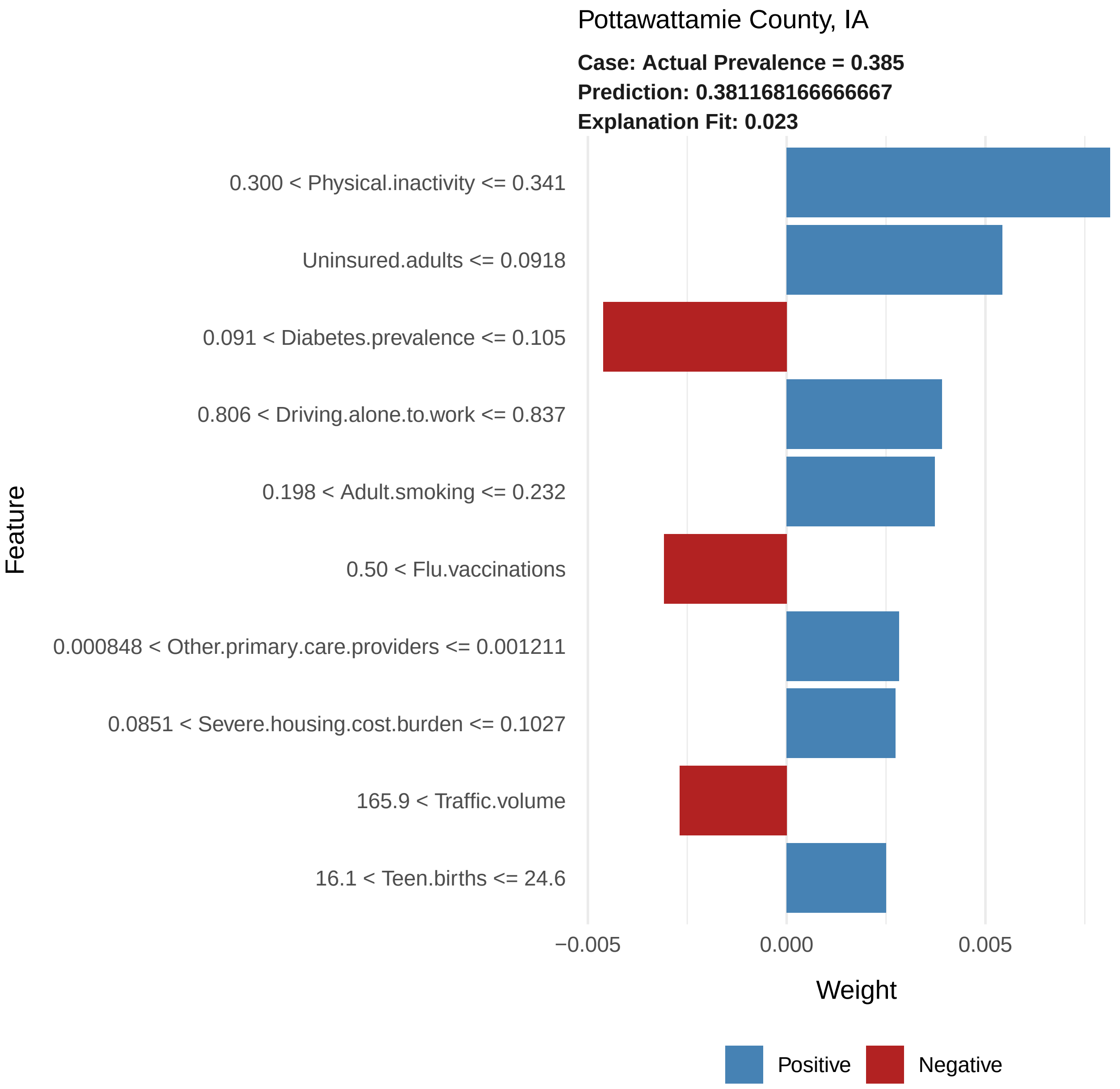

Pottawattamie County, IA
Case: Actual Prevalence = 0.385
Prediction: 0.381168166666667
Explanation Fit: 0.023
0.300 < Physical.inactivity <= 0.341
Uninsured.adults <= 0.0918
0.091 < Diabetes.prevalence <= 0.105
0.806 < Driving.alone.to.work <= 0.837
0.198 < Adult.smoking <= 0.232
0.50 < Flu.vaccinations
0.000848 < Other.primary.care.providers <= 0.001211
0.0851 < Severe.housing.cost.burden <= 0.1027
165.9 < Traffic.volume
16.1 < Teen.births <= 24.6
Feature
−0.005
0.000
0.005
Weight
Positive
Negative

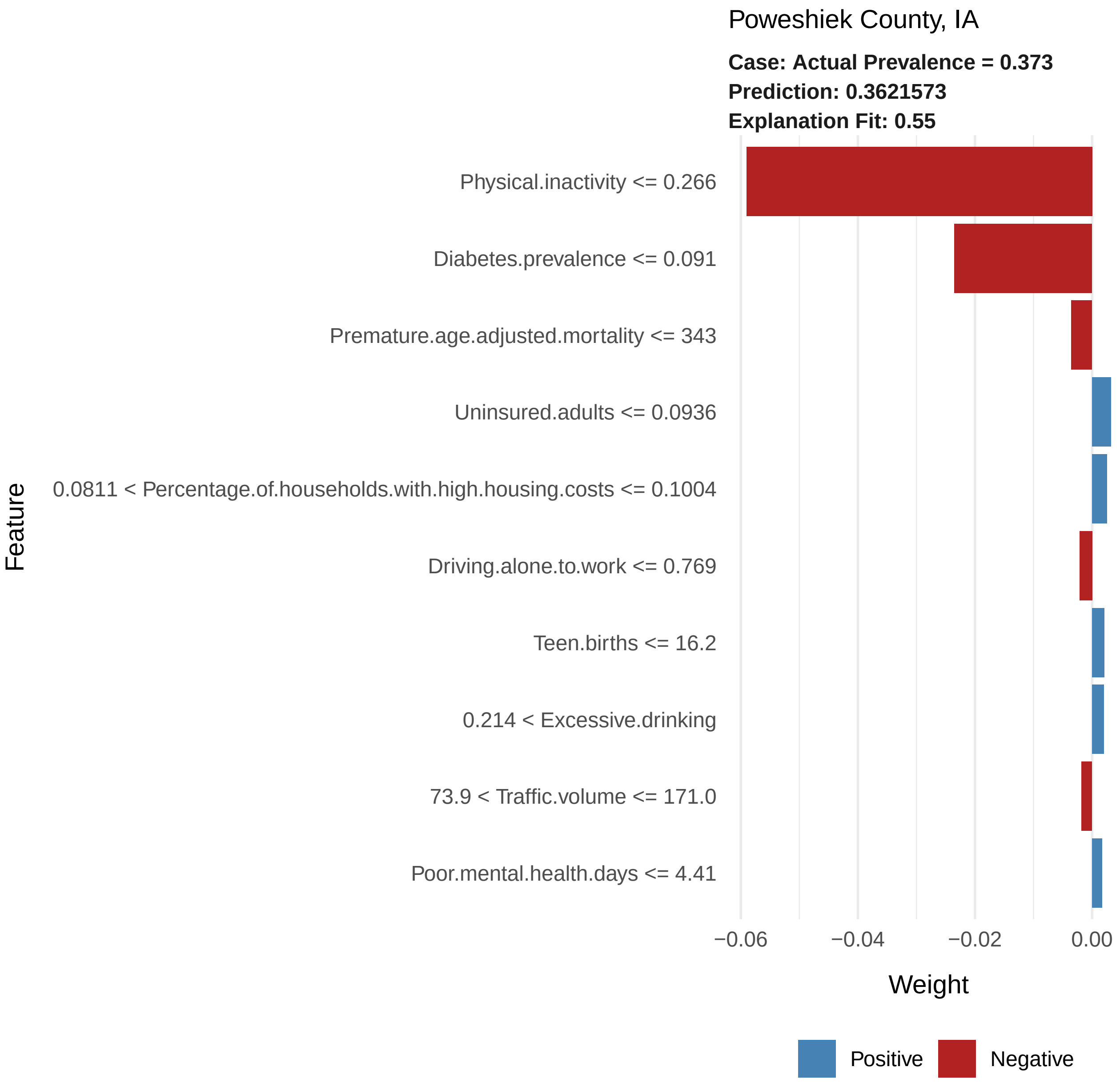

Poweshiek County, IA
Case: Actual Prevalence = 0.373
Prediction: 0.3621573
Explanation Fit: 0.55
Physical.inactivity <= 0.266
Diabetes.prevalence <= 0.091
Premature.age.adjusted.mortality <= 343
Uninsured.adults <= 0.0936
0.0811 < Percentage.of.households.with.high.housing.costs <= 0.1004
Driving.alone.to.work <= 0.769
Teen.births <= 16.2
0.214 < Excessive.drinking
73.9 < Traffic.volume <= 171.0
Poor.mental.health.days <= 4.41
Feature
−0.06
−0.04
−0.02
0.00
Weight
Positive
Negative

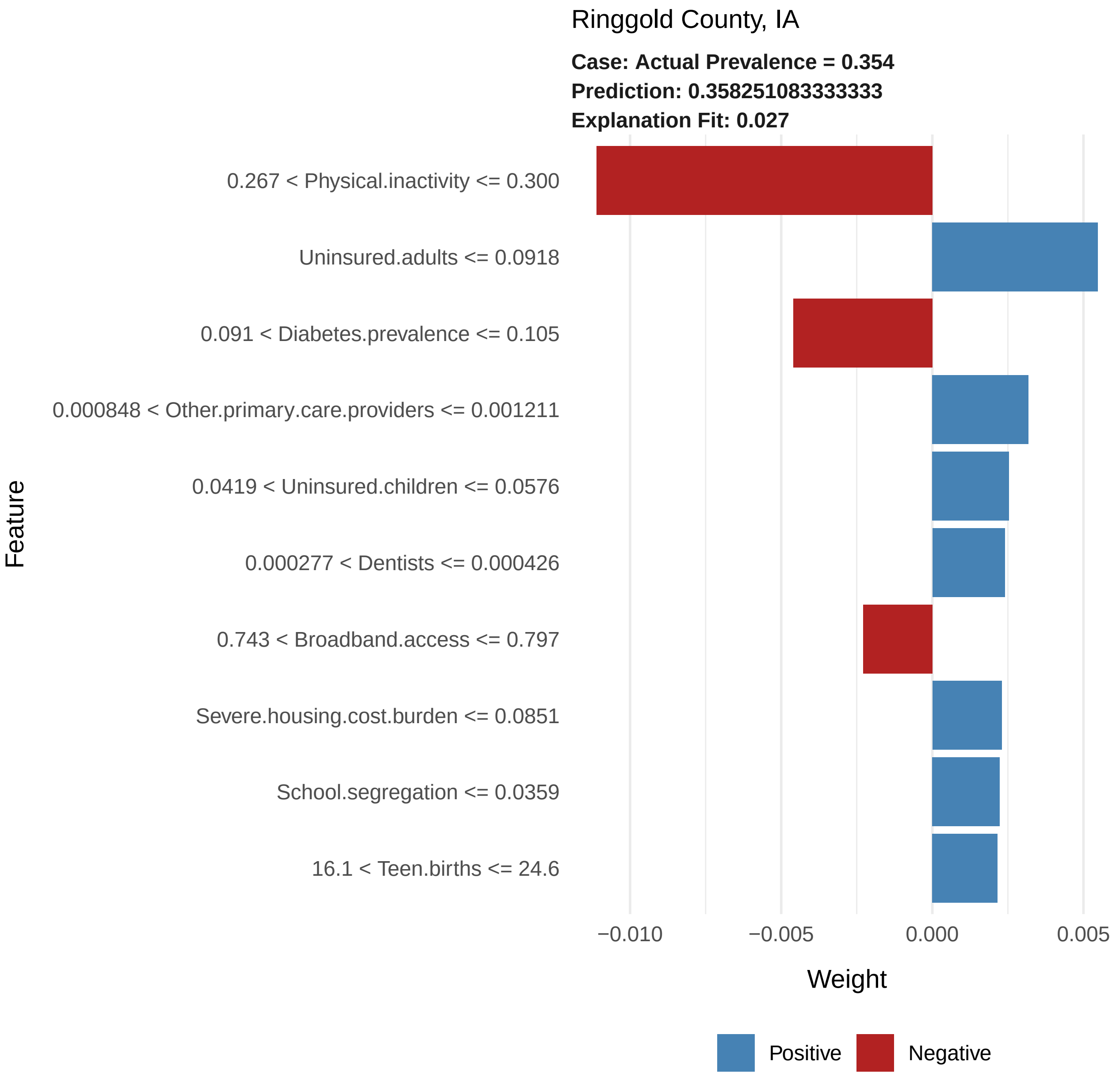

Ringgold County, IA
Case: Actual Prevalence = 0.354
Prediction: 0.358251083333333
Explanation Fit: 0.027
0.267 < Physical.inactivity <= 0.300
Uninsured.adults <= 0.0918
0.091 < Diabetes.prevalence <= 0.105
0.000848 < Other.primary.care.providers <= 0.001211
0.0419 < Uninsured.children <= 0.0576
0.000277 < Dentists <= 0.000426
0.743 < Broadband.access <= 0.797
Severe.housing.cost.burden <= 0.0851
School.segregation <= 0.0359
16.1 < Teen.births <= 24.6
Feature
−0.010
−0.005
0.000
0.005
Weight
Positive
Negative

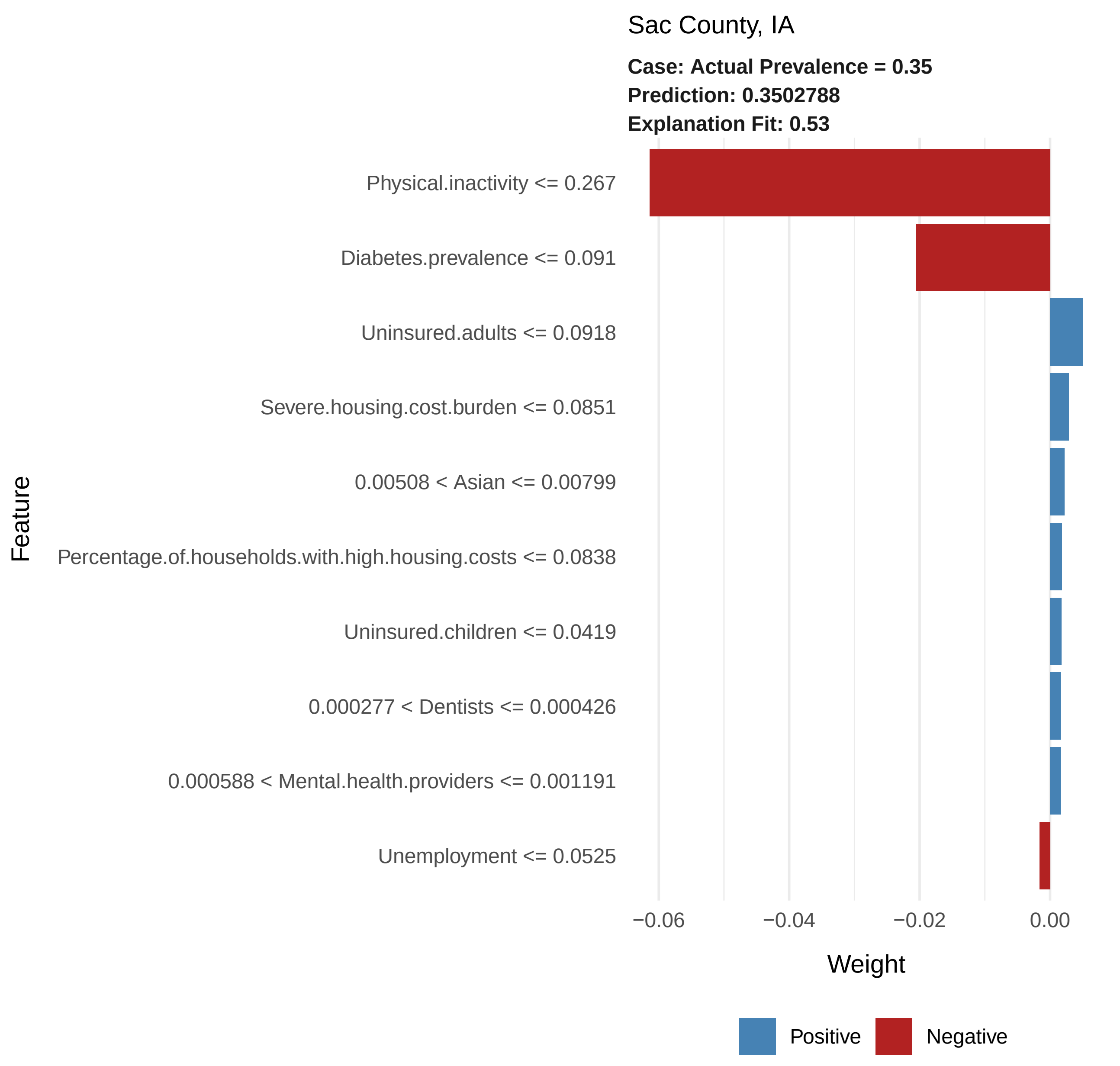

Sac County, IA
Case: Actual Prevalence = 0.35
Prediction: 0.3502788
Explanation Fit: 0.53
Physical.inactivity <= 0.267
Diabetes.prevalence <= 0.091
Uninsured.adults <= 0.0918
Severe.housing.cost.burden <= 0.0851
0.00508 < Asian <= 0.00799
Percentage.of.households.with.high.housing.costs <= 0.0838
Uninsured.children <= 0.0419
0.000277 < Dentists <= 0.000426
0.000588 < Mental.health.providers <= 0.001191
Unemployment <= 0.0525
Feature
−0.06
−0.04
−0.02
0.00
Weight
Positive
Negative

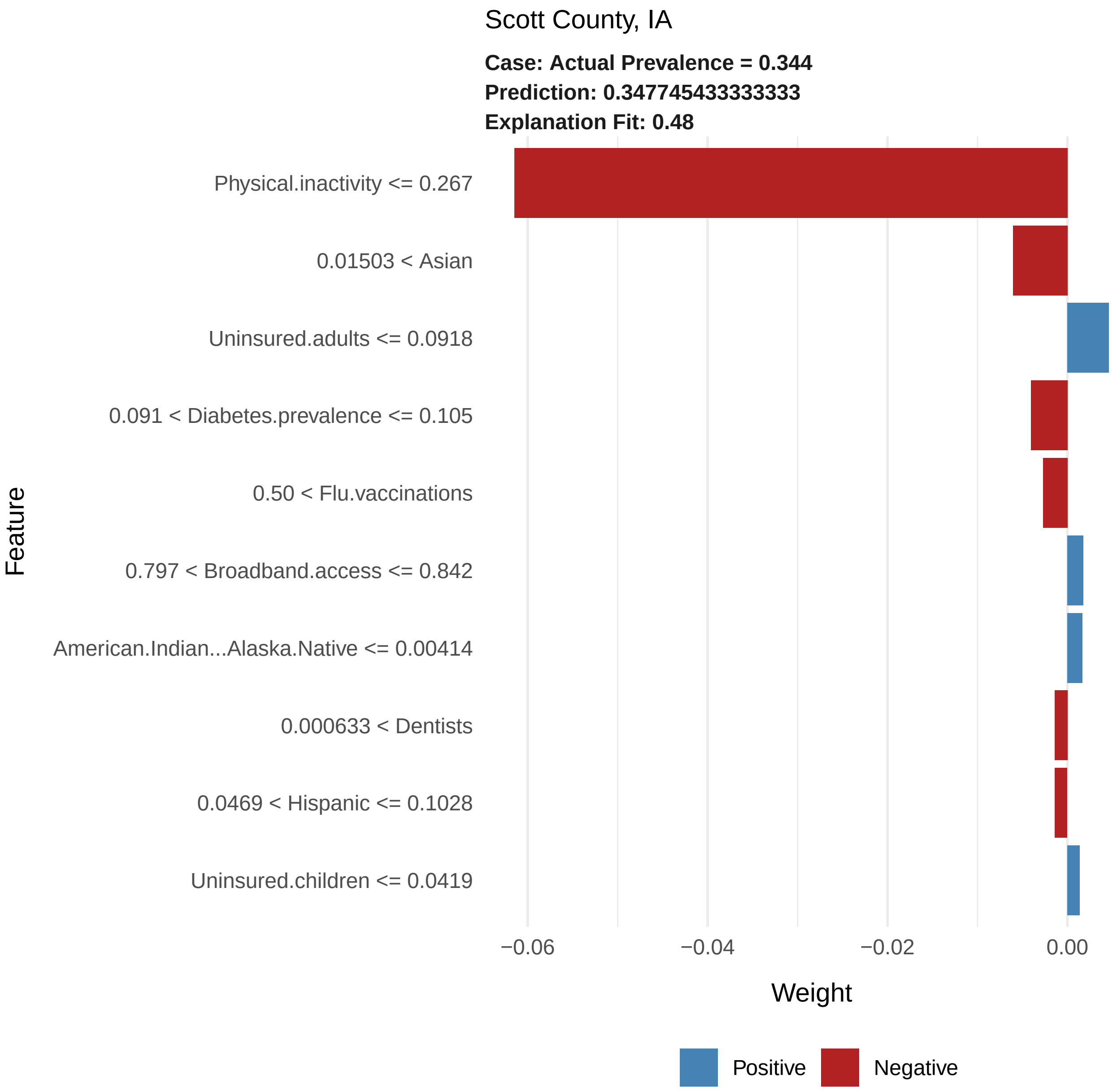

Scott County, IA
Case: Actual Prevalence = 0.344
Prediction: 0.347745433333333
Explanation Fit: 0.48
Physical.inactivity <= 0.267
0.01503 < Asian
Uninsured.adults <= 0.0918
0.091 < Diabetes.prevalence <= 0.105
0.50 < Flu.vaccinations
0.797 < Broadband.access <= 0.842
American.Indian...Alaska.Native <= 0.00414
0.000633 < Dentists
0.0469 < Hispanic <= 0.1028
Uninsured.children <= 0.0419
Feature
−0.06
−0.04
−0.02
0.00
Weight
Positive
Negative

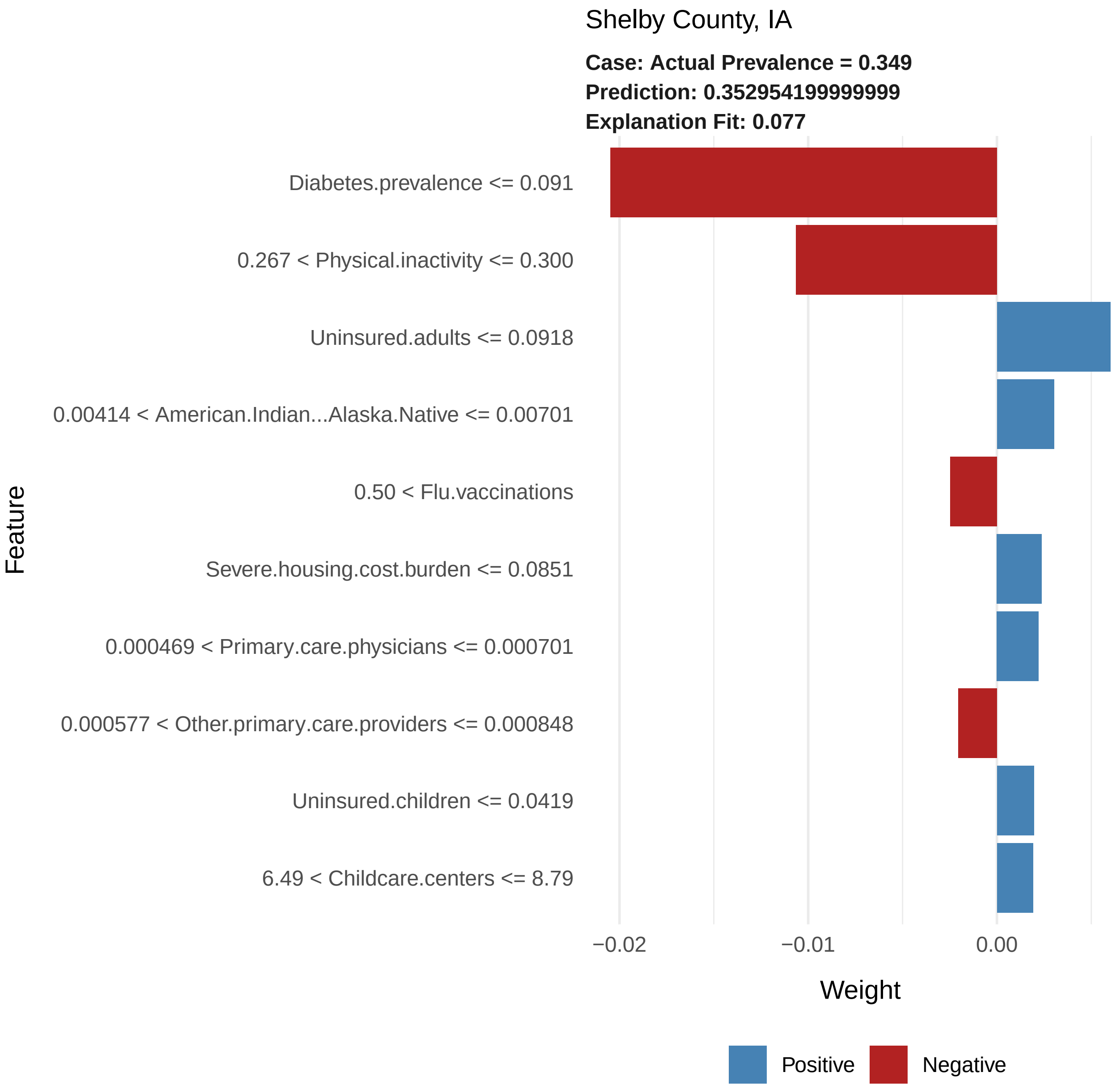
Shelby County, IA
Case: Actual Prevalence = 0.349
Prediction: 0.352954199999999
Explanation Fit: 0.077
Diabetes.prevalence <= 0.091
0.267 < Physical.inactivity <= 0.300
Uninsured.adults <= 0.0918
0.00414 < American.Indian...Alaska.Native <= 0.00701
0.50 < Flu.vaccinations
Severe.housing.cost.burden <= 0.0851
0.000469 < Primary.care.physicians <= 0.000701
0.000577 < Other.primary.care.providers <= 0.000848
Uninsured.children <= 0.0419
6.49 < Childcare.centers <= 8.79
Feature
−0.02
−0.01
0.00
Weight
Positive
Negative

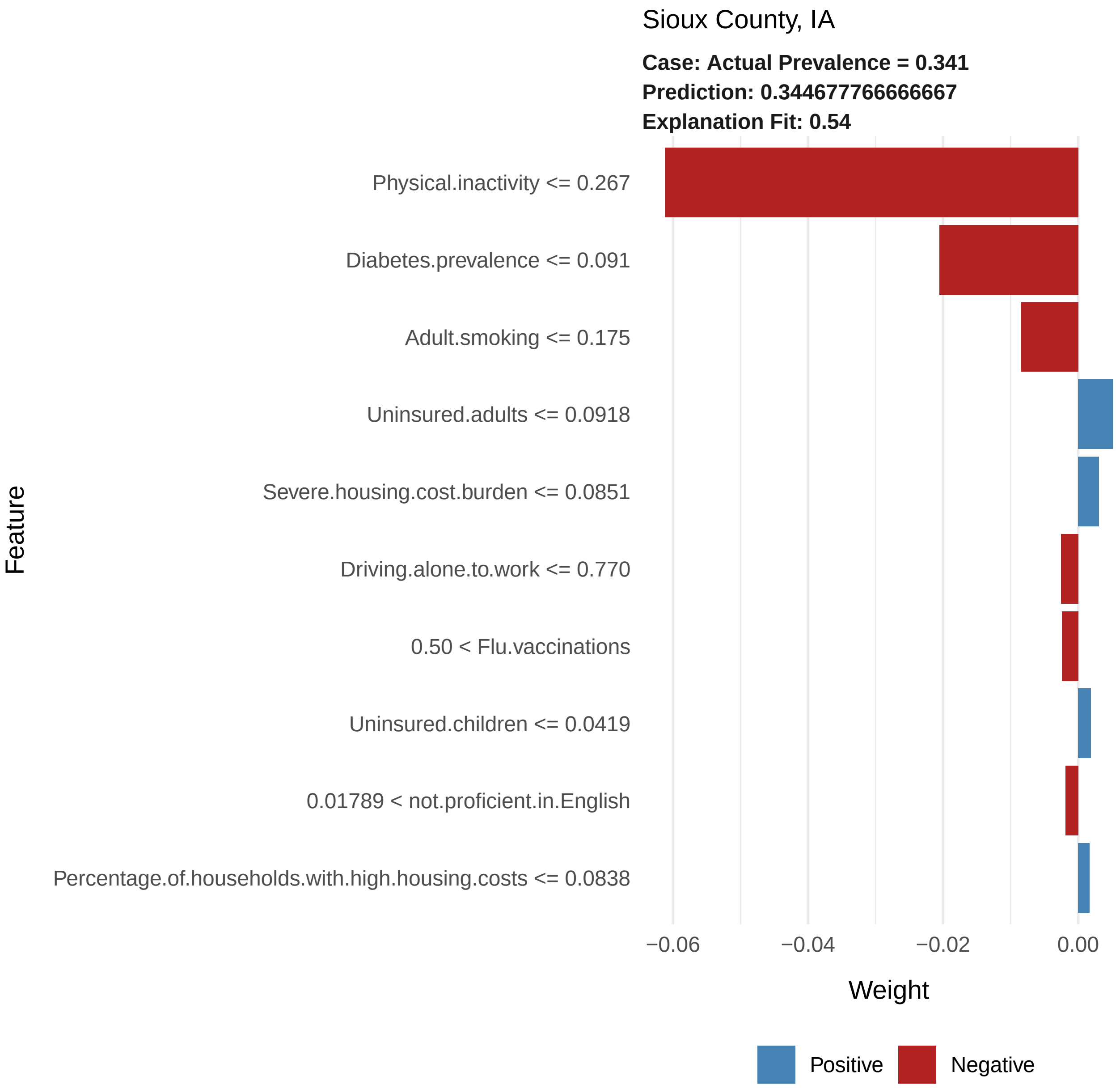
Sioux County, IA
Case: Actual Prevalence = 0.341
Prediction: 0.344677766666667
Explanation Fit: 0.54
Feature
Physical.inactivity <= 0.267
Diabetes.prevalence <= 0.091
Adult.smoking <= 0.175
Uninsured.adults <= 0.0918
Severe.housing.cost.burden <= 0.0851
Driving.alone.to.work <= 0.770
0.50 < Flu.vaccinations
Uninsured.children <= 0.0419
0.01789 < not.proficient.in.English
Percentage.of.households.with.high.housing.costs <= 0.0838
−0.06
−0.04
−0.02
0.00
Weight
Positive
Negative

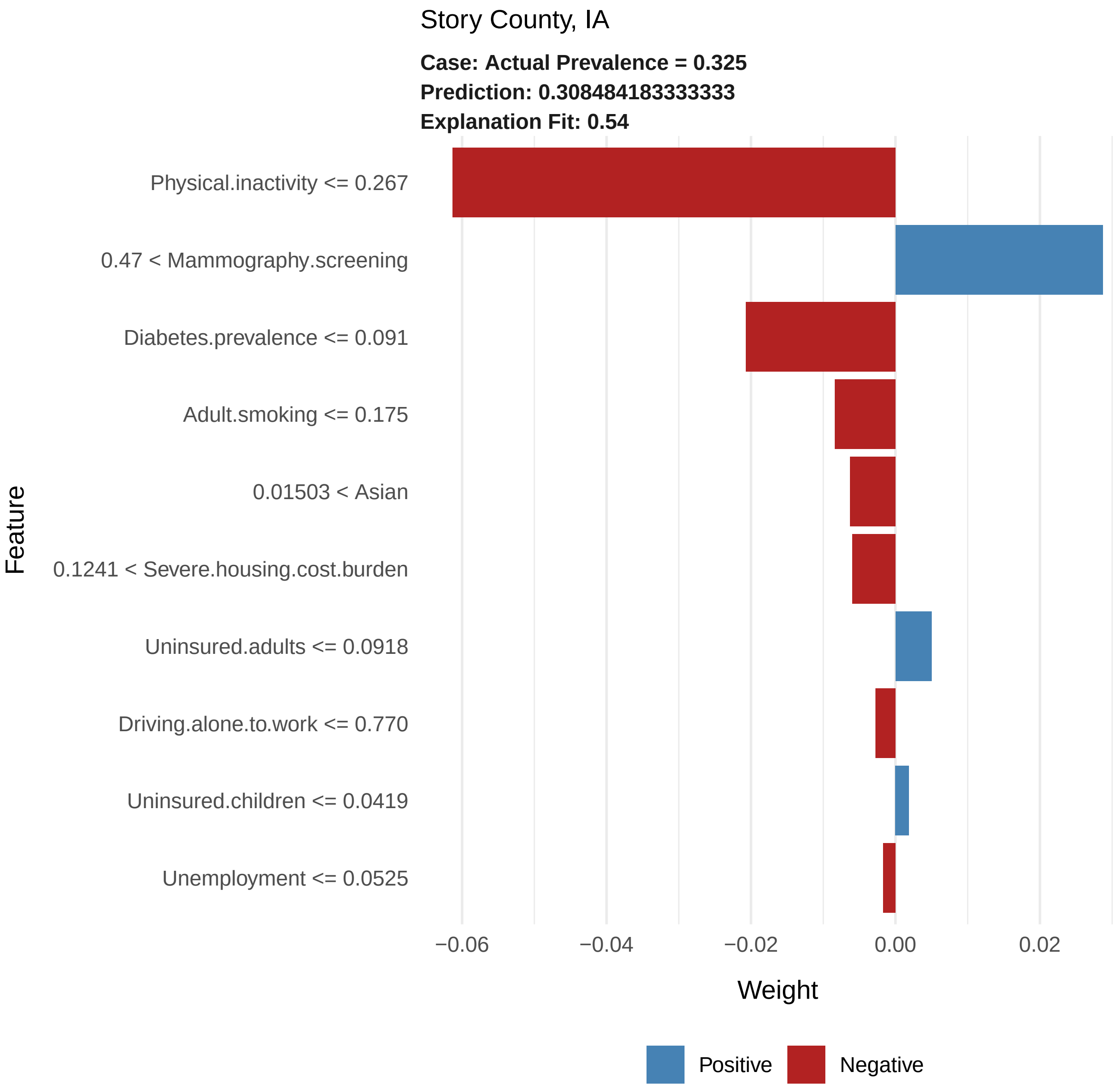

Story County, IA
Case: Actual Prevalence = 0.325
Prediction: 0.308484183333333
Explanation Fit: 0.54
Physical.inactivity <= 0.267
0.47 < Mammography.screening
Diabetes.prevalence <= 0.091
Adult.smoking <= 0.175
0.01503 < Asian
0.1241 < Severe.housing.cost.burden
Uninsured.adults <= 0.0918
Driving.alone.to.work <= 0.770
Uninsured.children <= 0.0419
Unemployment <= 0.0525
Feature
−0.06
−0.04
−0.02
0.00
0.02
Weight
Positive
Negative

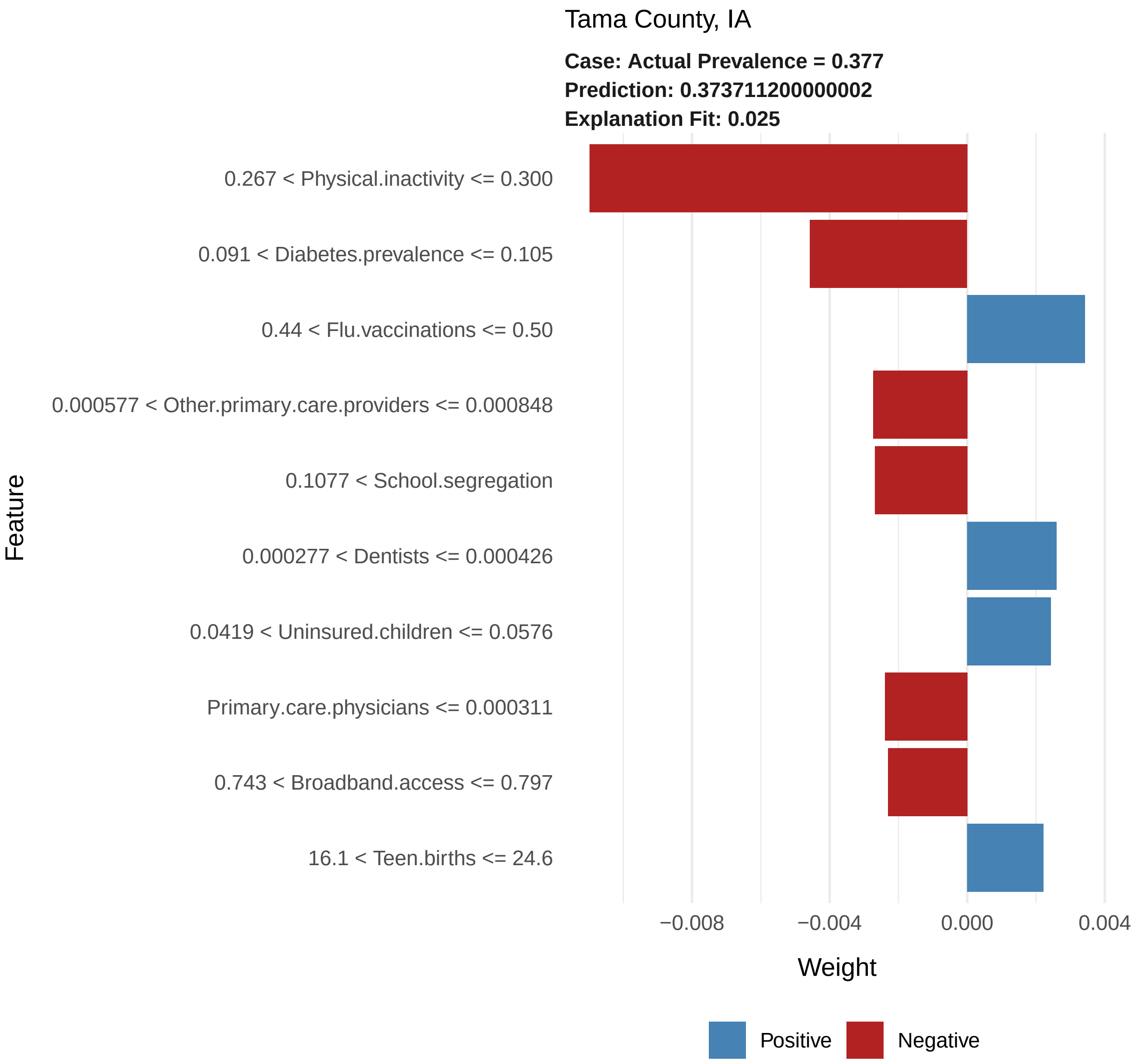

Tama County, IA
Case: Actual Prevalence = 0.377
Prediction: 0.373711200000002
Explanation Fit: 0.025
0.267 < Physical.inactivity <= 0.300
0.091 < Diabetes.prevalence <= 0.105
0.44 < Flu.vaccinations <= 0.50
0.000577 < Other.primary.care.providers <= 0.000848
0.1077 < School.segregation
0.000277 < Dentists <= 0.000426
0.0419 < Uninsured.children <= 0.0576
Primary.care.physicians <= 0.000311
0.743 < Broadband.access <= 0.797
16.1 < Teen.births <= 24.6
Feature
−0.008
−0.004
0.000
0.004
Weight
Positive
Negative

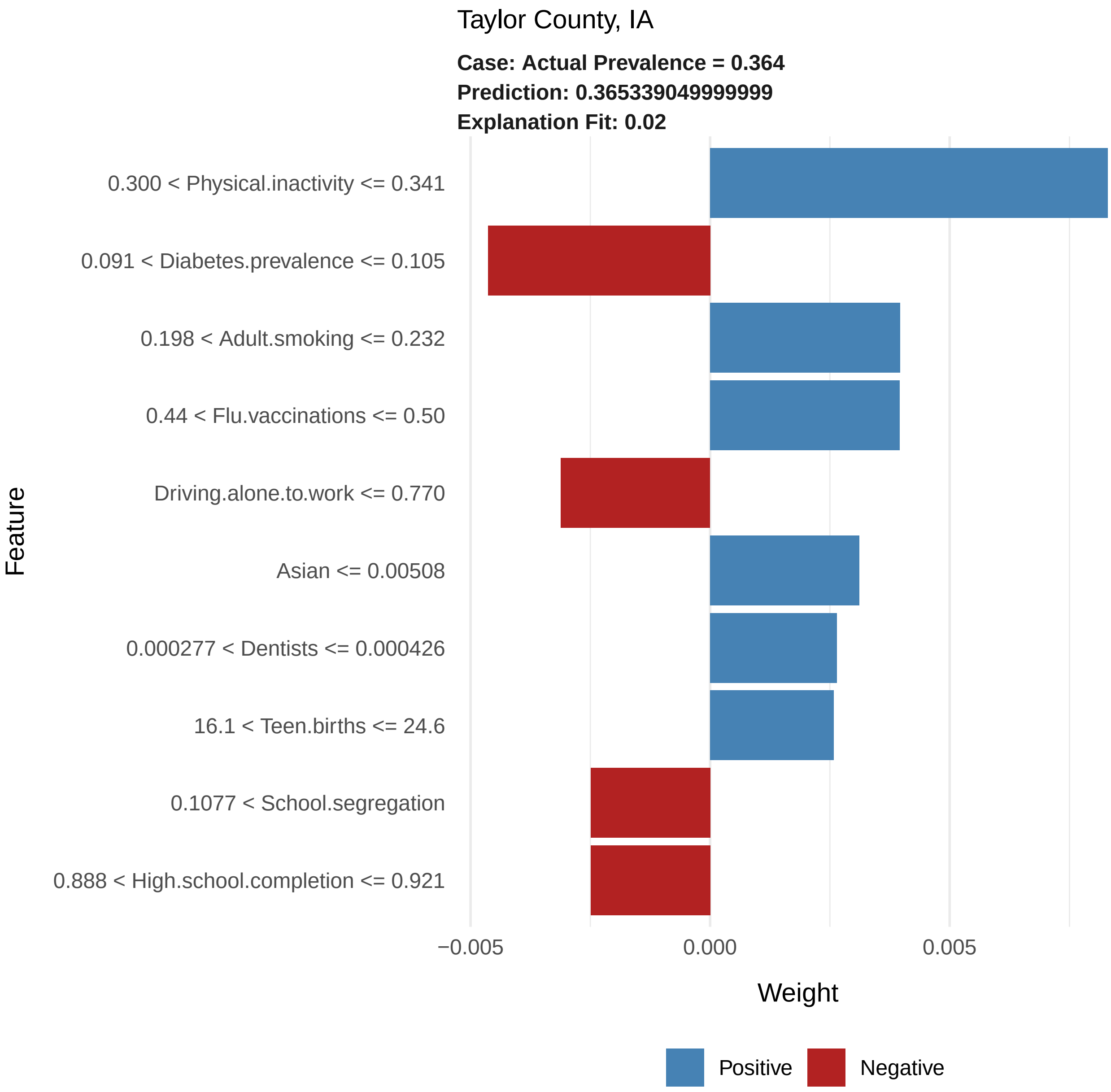
Taylor County, IA
Case: Actual Prevalence = 0.364
Prediction: 0.365339049999999
Explanation Fit: 0.02
0.300 < Physical.inactivity <= 0.341
0.091 < Diabetes.prevalence <= 0.105
0.198 < Adult.smoking <= 0.232
0.44 < Flu.vaccinations <= 0.50
Driving.alone.to.work <= 0.770
Asian <= 0.00508
0.000277 < Dentists <= 0.000426
16.1 < Teen.births <= 24.6
0.1077 < School.segregation
0.888 < High.school.completion <= 0.921
Feature
−0.005
0.000
0.005
Weight
Positive
Negative

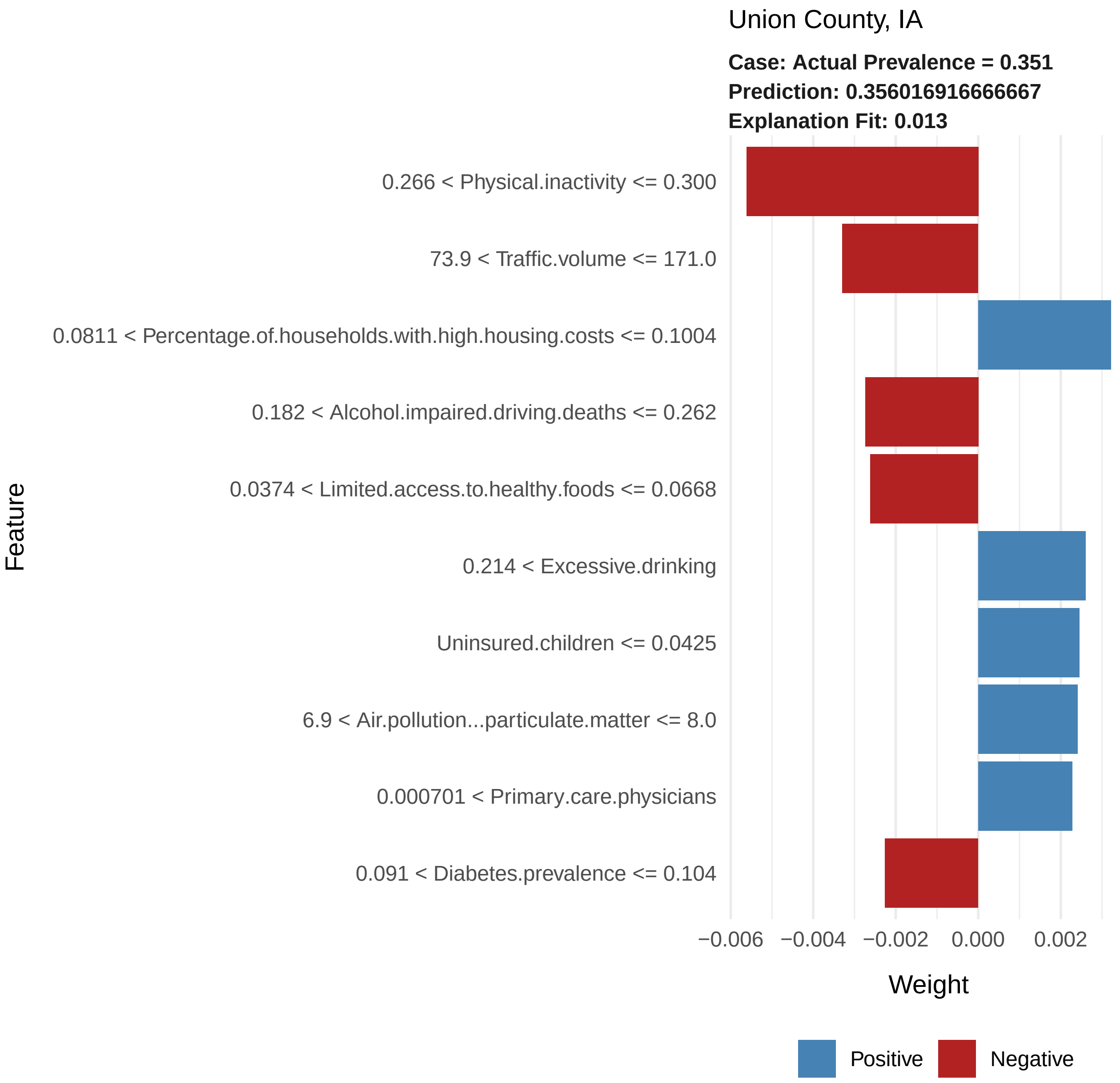

Union County, IA
Case: Actual Prevalence = 0.351
Prediction: 0.356016916666667
Explanation Fit: 0.013
0.266 < Physical.inactivity <= 0.300
73.9 < Traffic.volume <= 171.0
0.0811 < Percentage.of.households.with.high.housing.costs <= 0.1004
0.182 < Alcohol.impaired.driving.deaths <= 0.262
0.0374 < Limited.access.to.healthy.foods <= 0.0668
0.214 < Excessive.drinking
Uninsured.children <= 0.0425
6.9 < Air.pollution...particulate.matter <= 8.0
0.000701 < Primary.care.physicians
0.091 < Diabetes.prevalence <= 0.104
Feature
−0.006
−0.004
−0.002
0.000
0.002
Weight
Positive
Negative

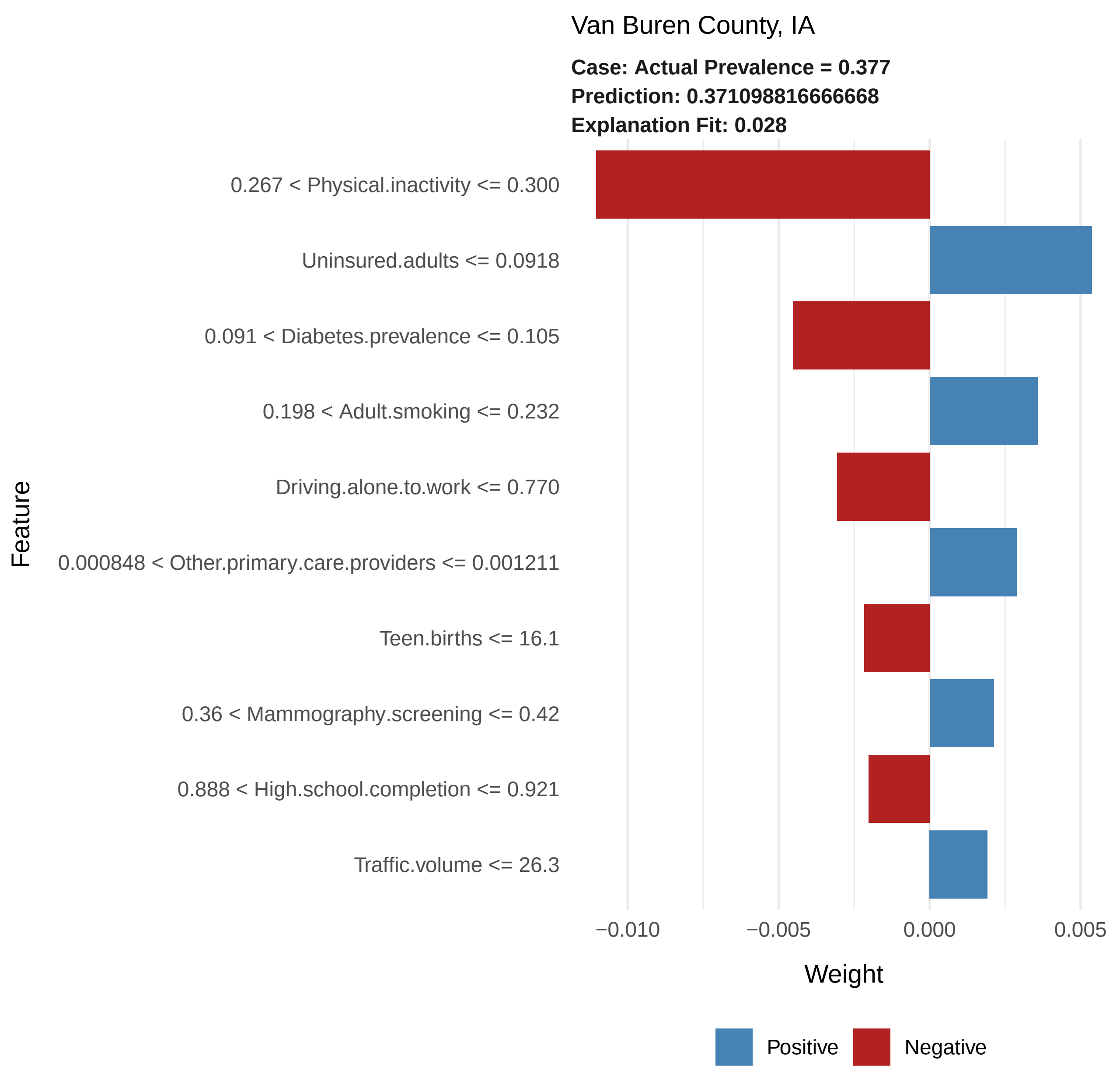

Van Buren County, IA
Case: Actual Prevalence = 0.377
Prediction: 0.371098816666668
Explanation Fit: 0.028
Feature
0.267 < Physical.inactivity <= 0.300
Uninsured.adults <= 0.0918
0.091 < Diabetes.prevalence <= 0.105
0.198 < Adult.smoking <= 0.232
Driving.alone.to.work <= 0.770
0.000848 < Other.primary.care.providers <= 0.001211
Teen.births <= 16.1
0.36 < Mammography.screening <= 0.42
0.888 < High.school.completion <= 0.921
Traffic.volume <= 26.3
−0.010
−0.005
0.000
0.005
Weight
Positive
Negative

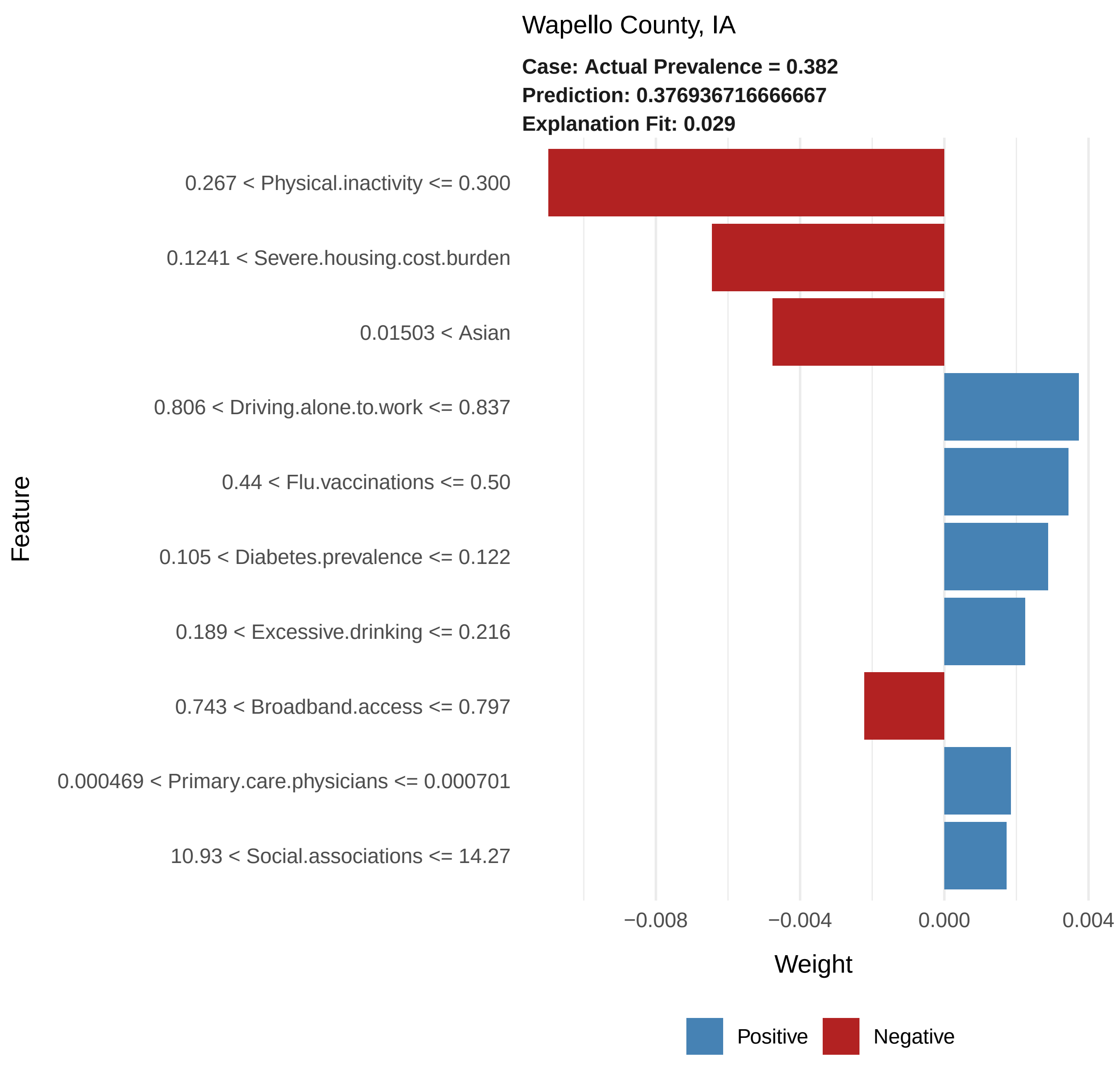

Wapello County, IA
Case: Actual Prevalence = 0.382
Prediction: 0.376936716666667
Explanation Fit: 0.029
0.267 < Physical.inactivity <= 0.300
0.1241 < Severe.housing.cost.burden
0.01503 < Asian
0.806 < Driving.alone.to.work <= 0.837
0.44 < Flu.vaccinations <= 0.50
0.105 < Diabetes.prevalence <= 0.122
0.189 < Excessive.drinking <= 0.216
0.743 < Broadband.access <= 0.797
0.000469 < Primary.care.physicians <= 0.000701
10.93 < Social.associations <= 14.27
Feature
−0.008
−0.004
0.000
0.004
Weight
Positive
Negative

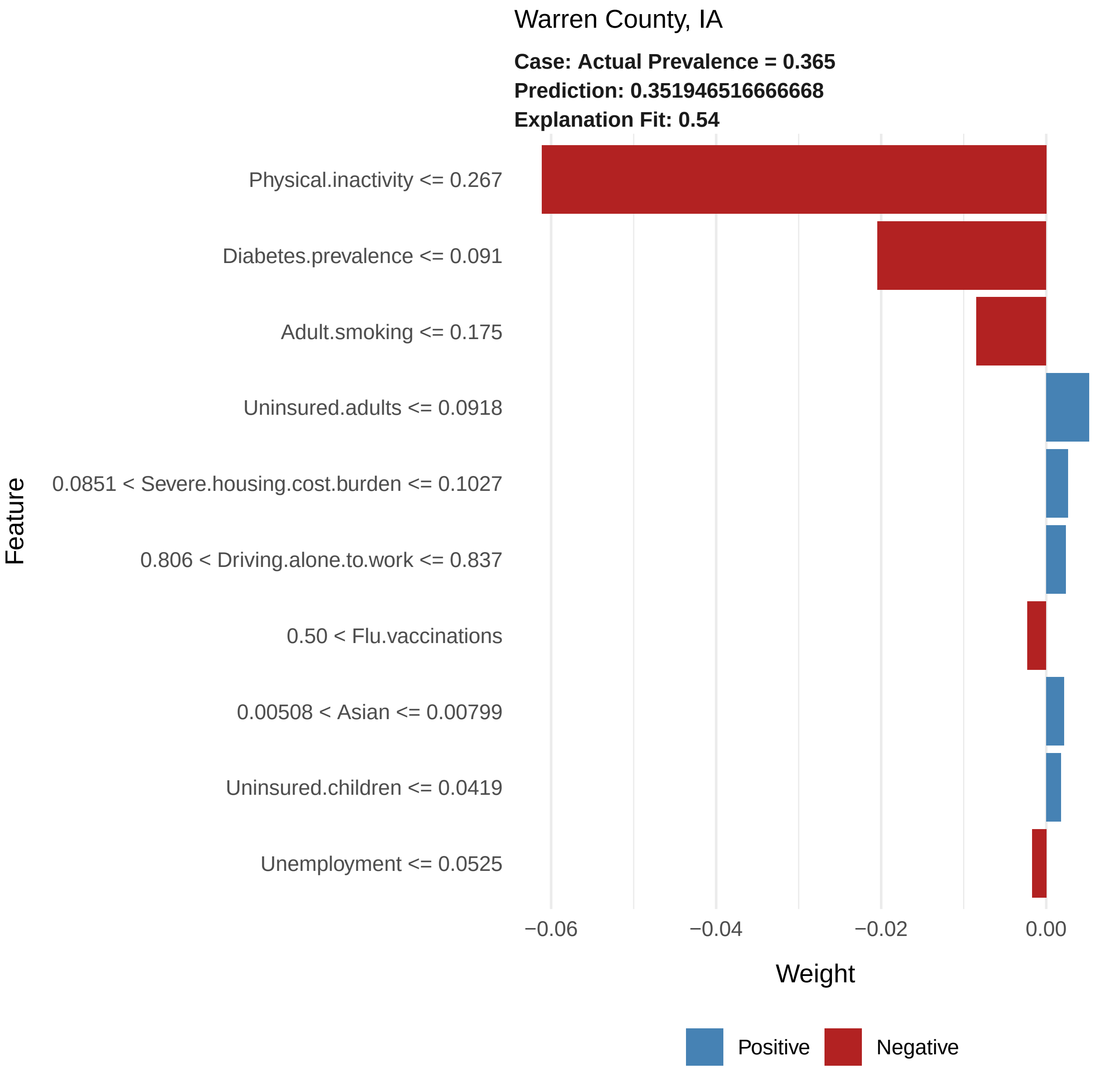

Warren County, IA
Case: Actual Prevalence = 0.365
Prediction: 0.351946516666668
Explanation Fit: 0.54
Physical.inactivity <= 0.267
Diabetes.prevalence <= 0.091
Adult.smoking <= 0.175
Uninsured.adults <= 0.0918
0.0851 < Severe.housing.cost.burden <= 0.1027
0.806 < Driving.alone.to.work <= 0.837
0.50 < Flu.vaccinations
0.00508 < Asian <= 0.00799
Uninsured.children <= 0.0419
Unemployment <= 0.0525
Feature
−0.06
−0.04
−0.02
0.00
Weight
Positive
Negative

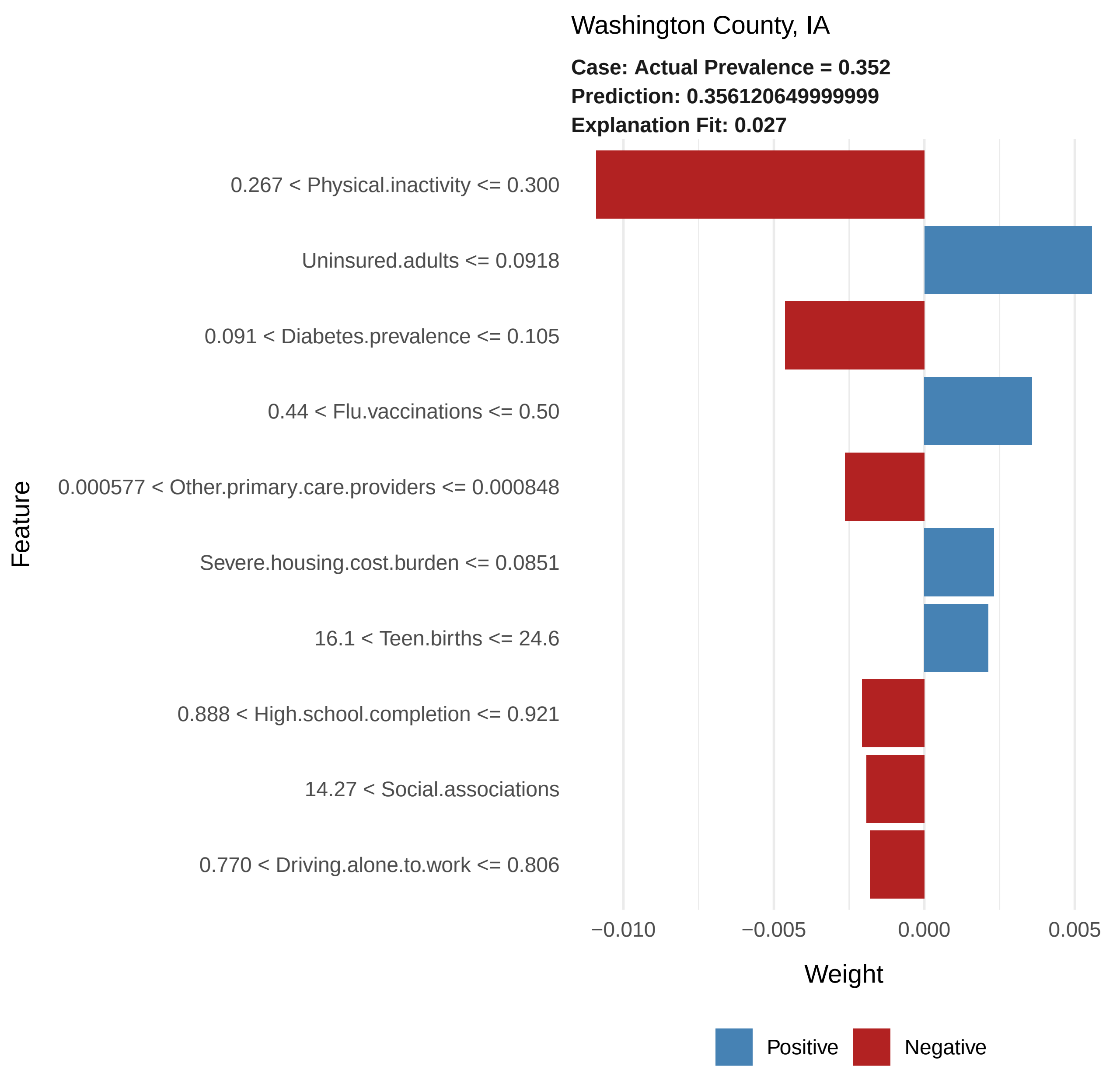

Washington County, IA
Case: Actual Prevalence = 0.352
Prediction: 0.356120649999999
Explanation Fit: 0.027
0.267 < Physical.inactivity <= 0.300
Uninsured.adults <= 0.0918
0.091 < Diabetes.prevalence <= 0.105
0.44 < Flu.vaccinations <= 0.50
0.000577 < Other.primary.care.providers <= 0.000848
Severe.housing.cost.burden <= 0.0851
16.1 < Teen.births <= 24.6
0.888 < High.school.completion <= 0.921
14.27 < Social.associations
0.770 < Driving.alone.to.work <= 0.806
Feature
−0.010
−0.005
0.000
0.005
Weight
Positive
Negative

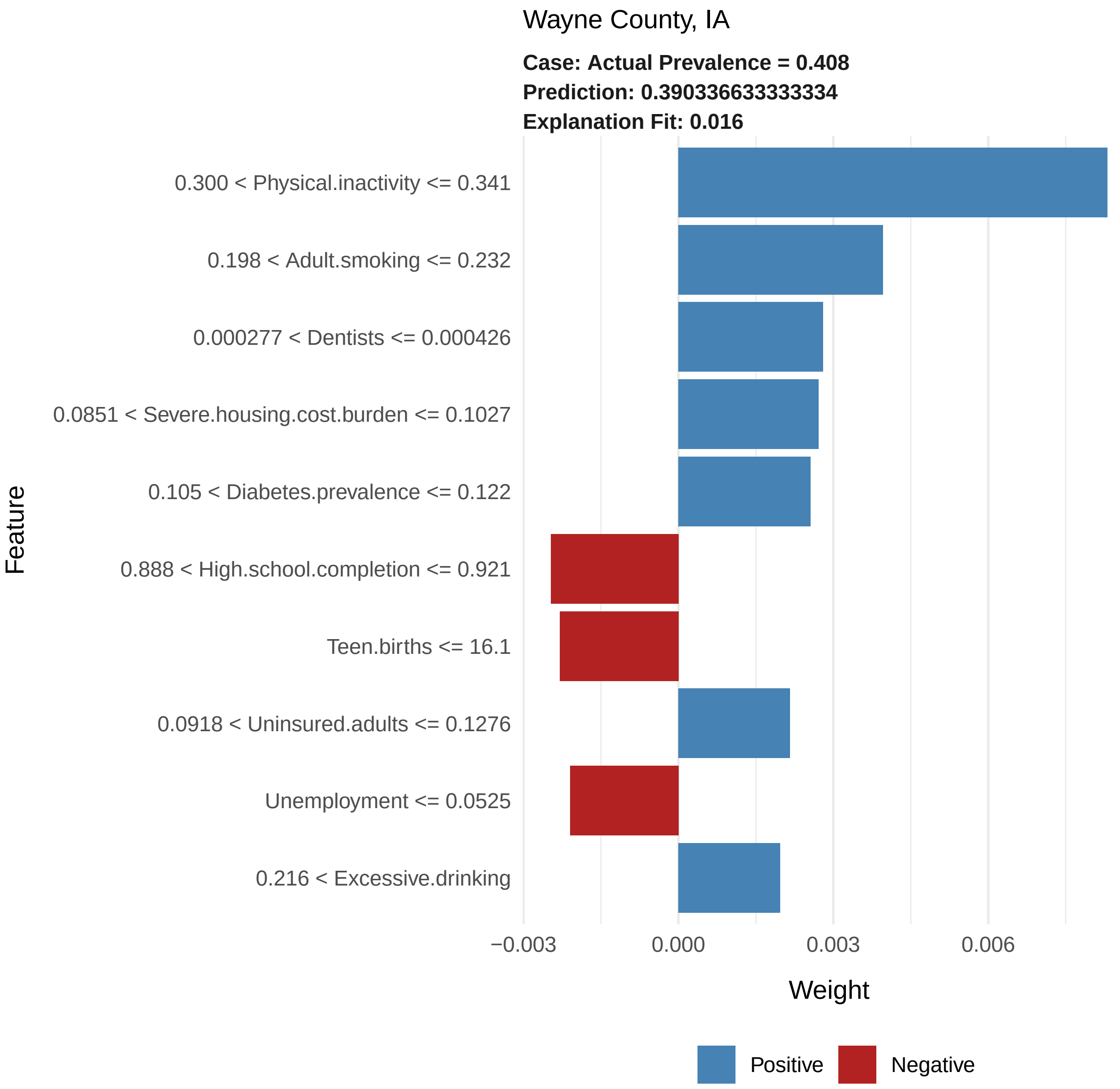

Wayne County, IA
Case: Actual Prevalence = 0.408
Prediction: 0.390336633333334
Explanation Fit: 0.016
0.300 < Physical.inactivity <= 0.341
0.198 < Adult.smoking <= 0.232
0.000277 < Dentists <= 0.000426
0.0851 < Severe.housing.cost.burden <= 0.1027
0.105 < Diabetes.prevalence <= 0.122
0.888 < High.school.completion <= 0.921
Teen.births <= 16.1
0.0918 < Uninsured.adults <= 0.1276
Unemployment <= 0.0525
0.216 < Excessive.drinking
Feature
−0.003
0.000
0.003
0.006
Weight
Positive
Negative

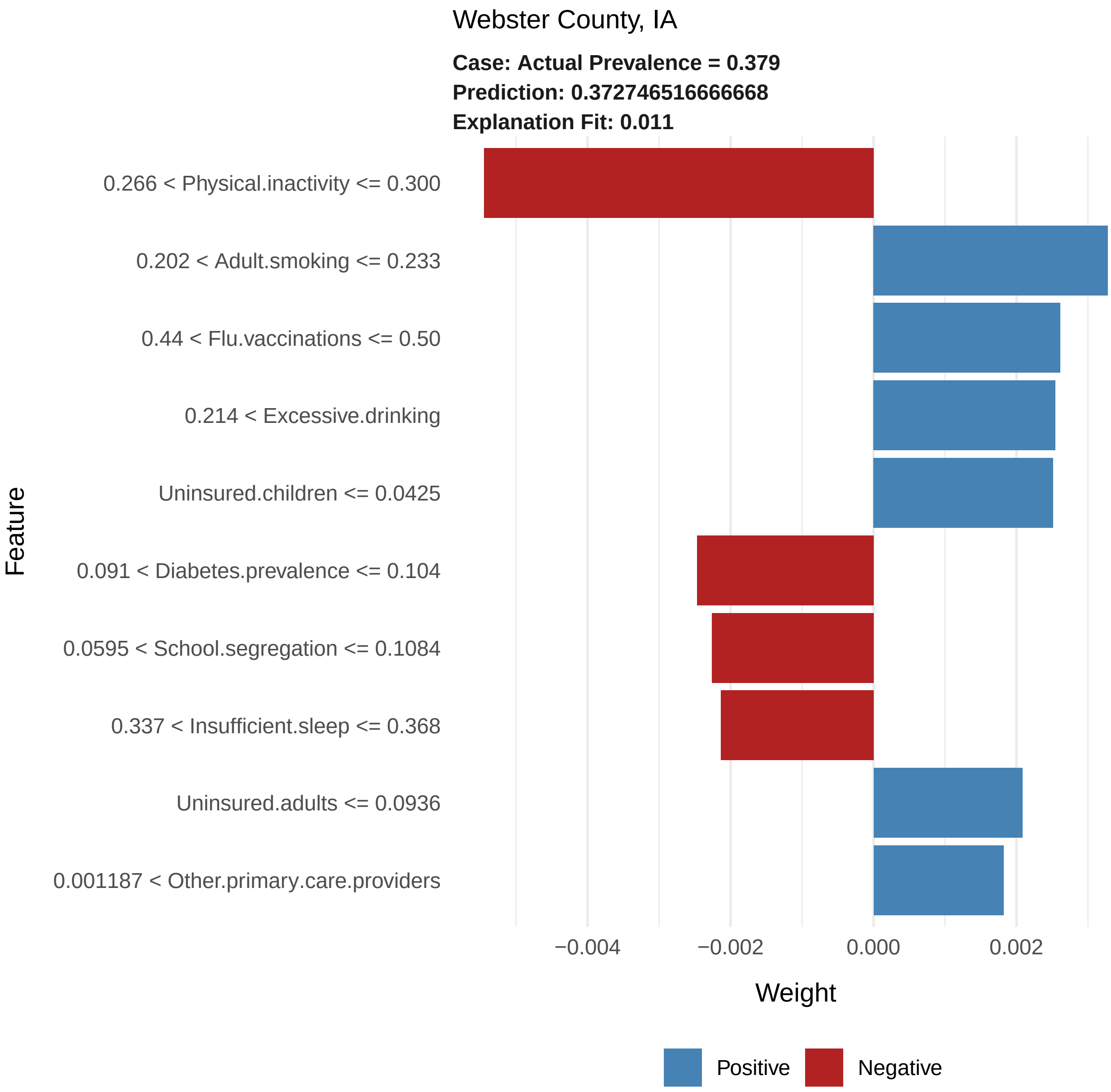
Webster County, IA
Case: Actual Prevalence = 0.379
Prediction: 0.372746516666668
Explanation Fit: 0.011
0.266 < Physical.inactivity <= 0.300
0.202 < Adult.smoking <= 0.233
0.44 < Flu.vaccinations <= 0.50
0.214 < Excessive.drinking
Uninsured.children <= 0.0425
0.091 < Diabetes.prevalence <= 0.104
0.0595 < School.segregation <= 0.1084
0.337 < Insufficient.sleep <= 0.368
Uninsured.adults <= 0.0936
0.001187 < Other.primary.care.providers
Feature
−0.004
−0.002
0.000
0.002
Weight
Positive
Negative

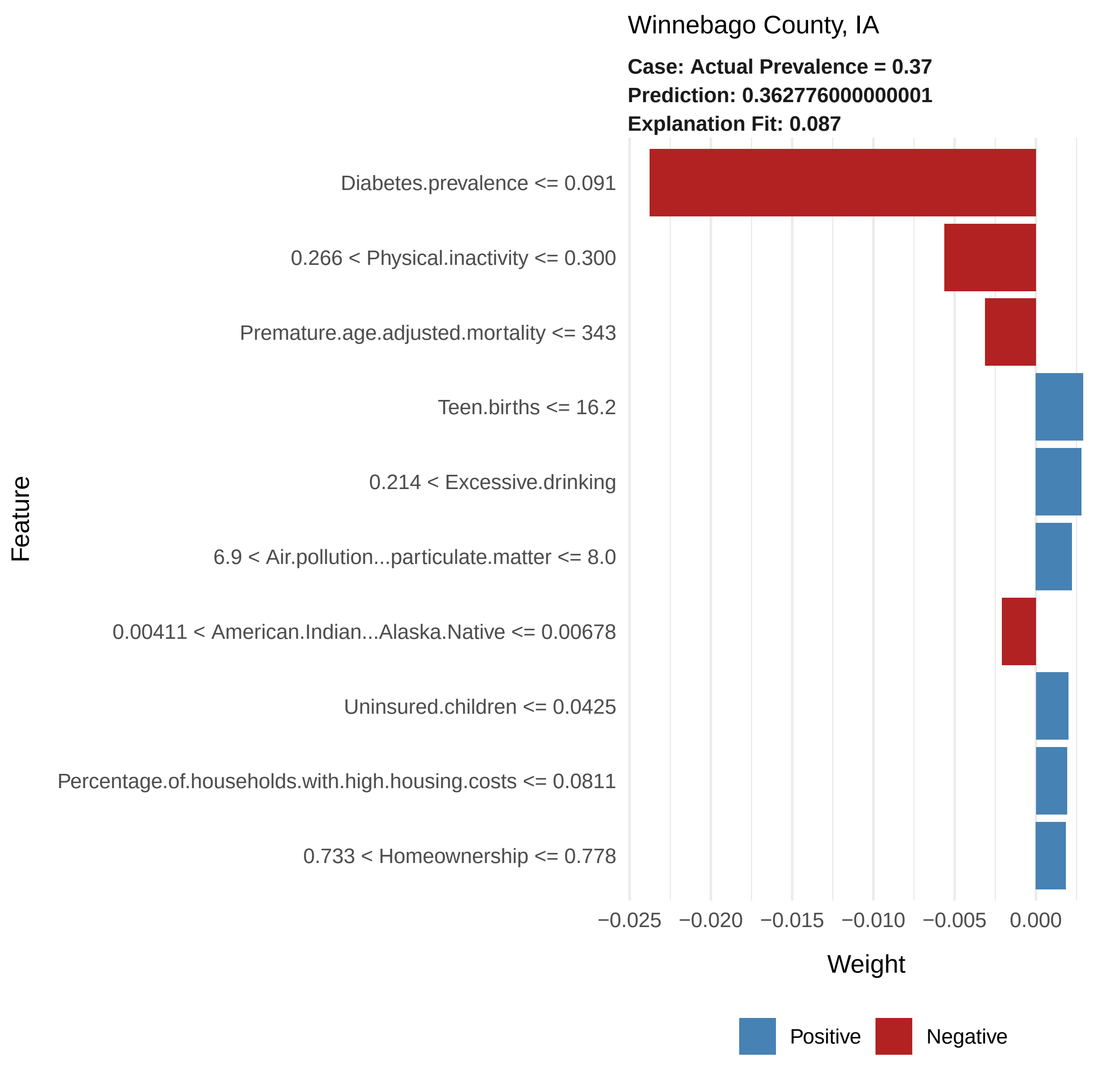

Winnebago County, IA
Case: Actual Prevalence = 0.37
Prediction: 0.362776000000001
Explanation Fit: 0.087
Diabetes.prevalence <= 0.091
0.266 < Physical.inactivity <= 0.300
Premature.age.adjusted.mortality <= 343
Teen.births <= 16.2
0.214 < Excessive.drinking
6.9 < Air.pollution...particulate.matter <= 8.0
0.00411 < American.Indian...Alaska.Native <= 0.00678
Uninsured.children <= 0.0425
Percentage.of.households.with.high.housing.costs <= 0.0811
0.733 < Homeownership <= 0.778
Feature
−0.025
−0.020
−0.015
−0.010
−0.005
0.000
Weight
Positive
Negative

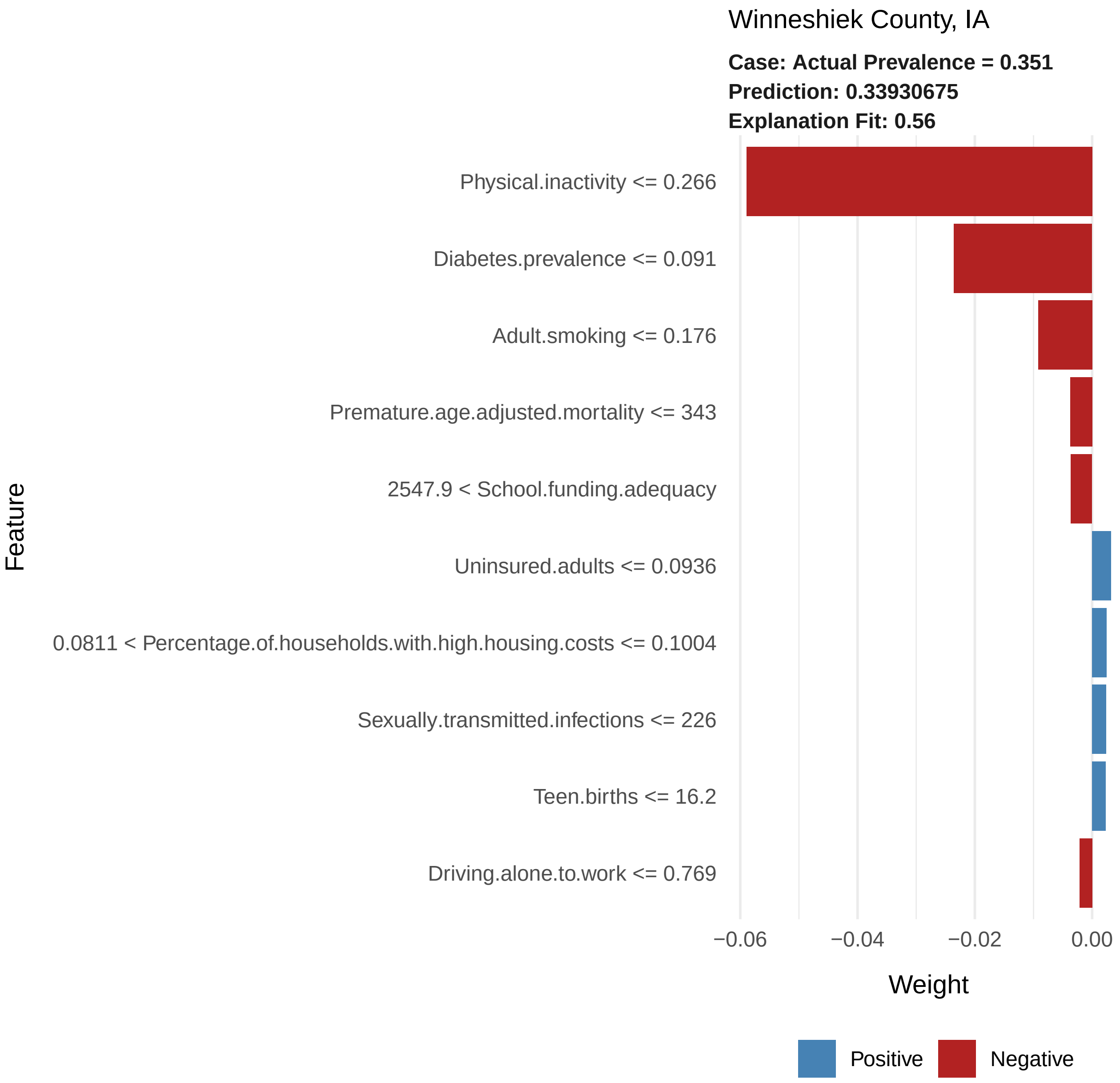

Winneshiek County, IA
Case: Actual Prevalence = 0.351
Prediction: 0.33930675
Explanation Fit: 0.56
Physical.inactivity <= 0.266
Diabetes.prevalence <= 0.091
Adult.smoking <= 0.176
Premature.age.adjusted.mortality <= 343
2547.9 < School.funding.adequacy
Uninsured.adults <= 0.0936
0.0811 < Percentage.of.households.with.high.housing.costs <= 0.1004
Sexually.transmitted.infections <= 226
Teen.births <= 16.2
Driving.alone.to.work <= 0.769
Feature
−0.06
−0.04
−0.02
0.00
Weight
Positive
Negative

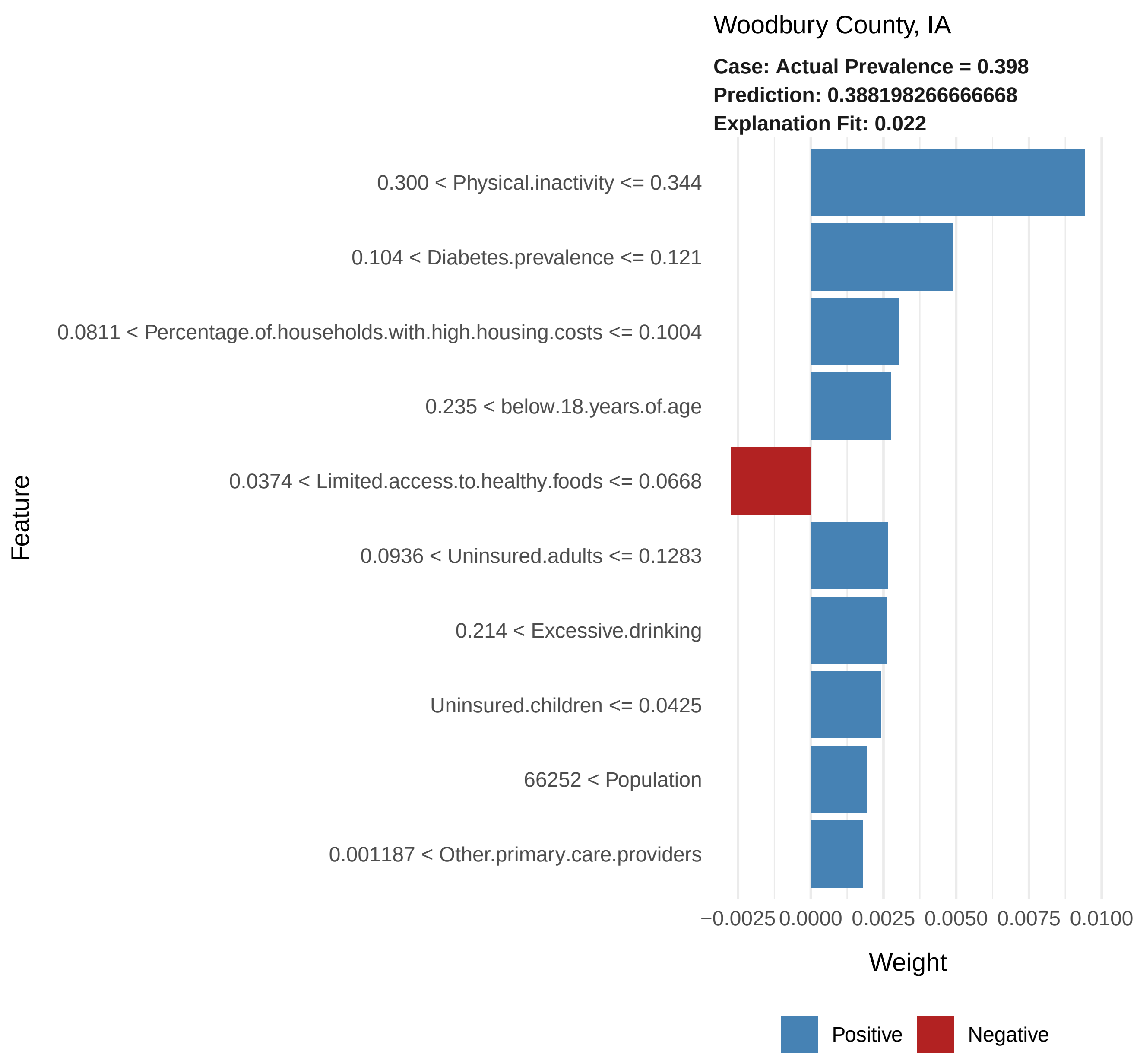
Woodbury County, IA
Case: Actual Prevalence = 0.398
Prediction: 0.388198266666668
Explanation Fit: 0.022
0.300 < Physical.inactivity <= 0.344
0.104 < Diabetes.prevalence <= 0.121
0.0811 < Percentage.of.households.with.high.housing.costs <= 0.1004
0.235 < below.18.years.of.age
0.0374 < Limited.access.to.healthy.foods <= 0.0668
0.0936 < Uninsured.adults <= 0.1283
0.214 < Excessive.drinking
Uninsured.children <= 0.0425
66252 < Population
0.001187 < Other.primary.care.providers
Feature
−0.0025
0.0000
0.0025
0.0050
0.0075
0.0100
Weight
Positive
Negative

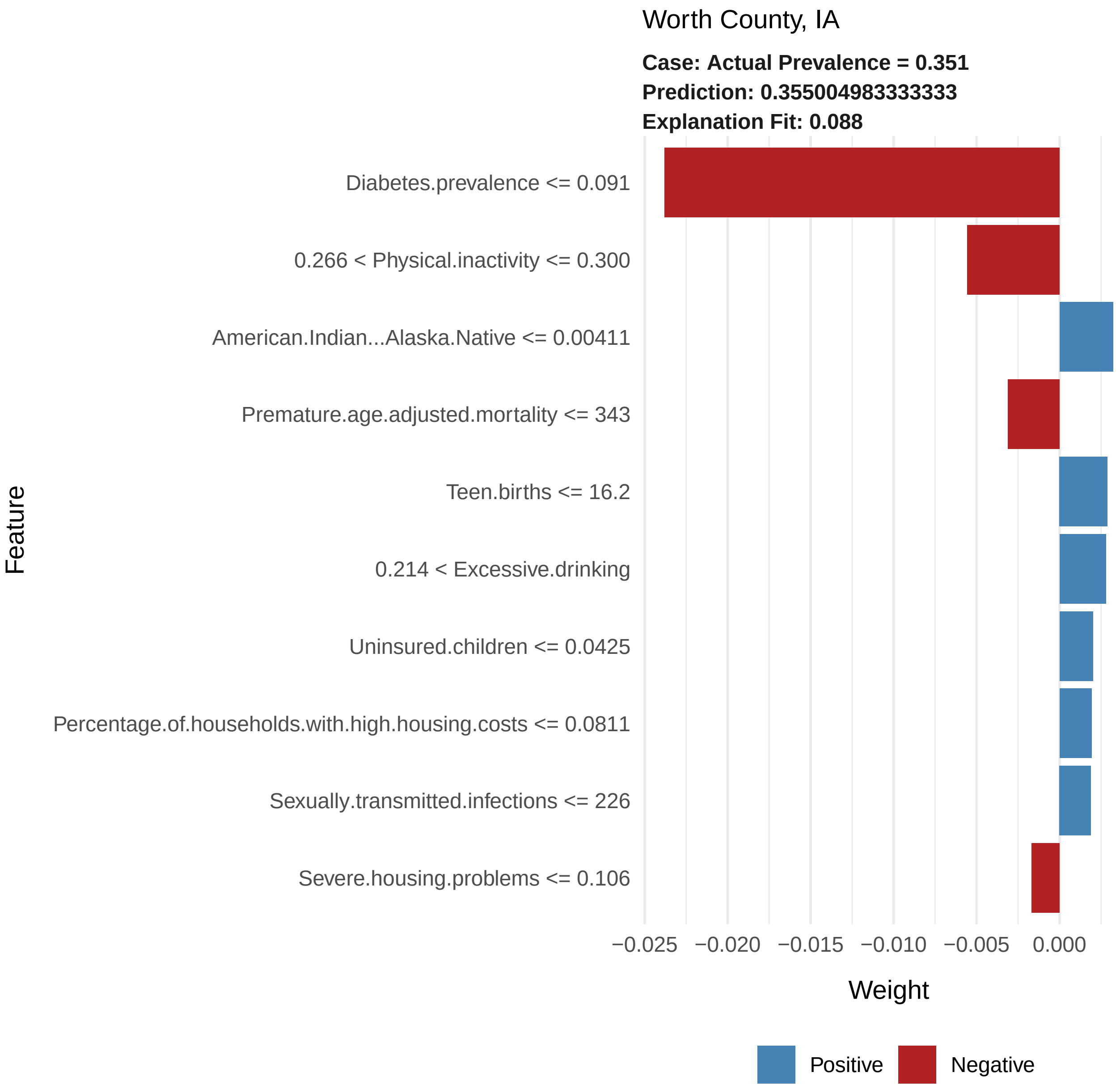
Worth County, IA
Case: Actual Prevalence = 0.351
Prediction: 0.355004983333333
Explanation Fit: 0.088
Diabetes.prevalence <= 0.091
0.266 < Physical.inactivity <= 0.300
American.Indian...Alaska.Native <= 0.00411
Premature.age.adjusted.mortality <= 343
Teen.births <= 16.2
0.214 < Excessive.drinking
Uninsured.children <= 0.0425
Percentage.of.households.with.high.housing.costs <= 0.0811
Sexually.transmitted.infections <= 226
Severe.housing.problems <= 0.106
Feature
−0.025
−0.020
−0.015
−0.010
−0.005
0.000
Weight
Positive
Negative

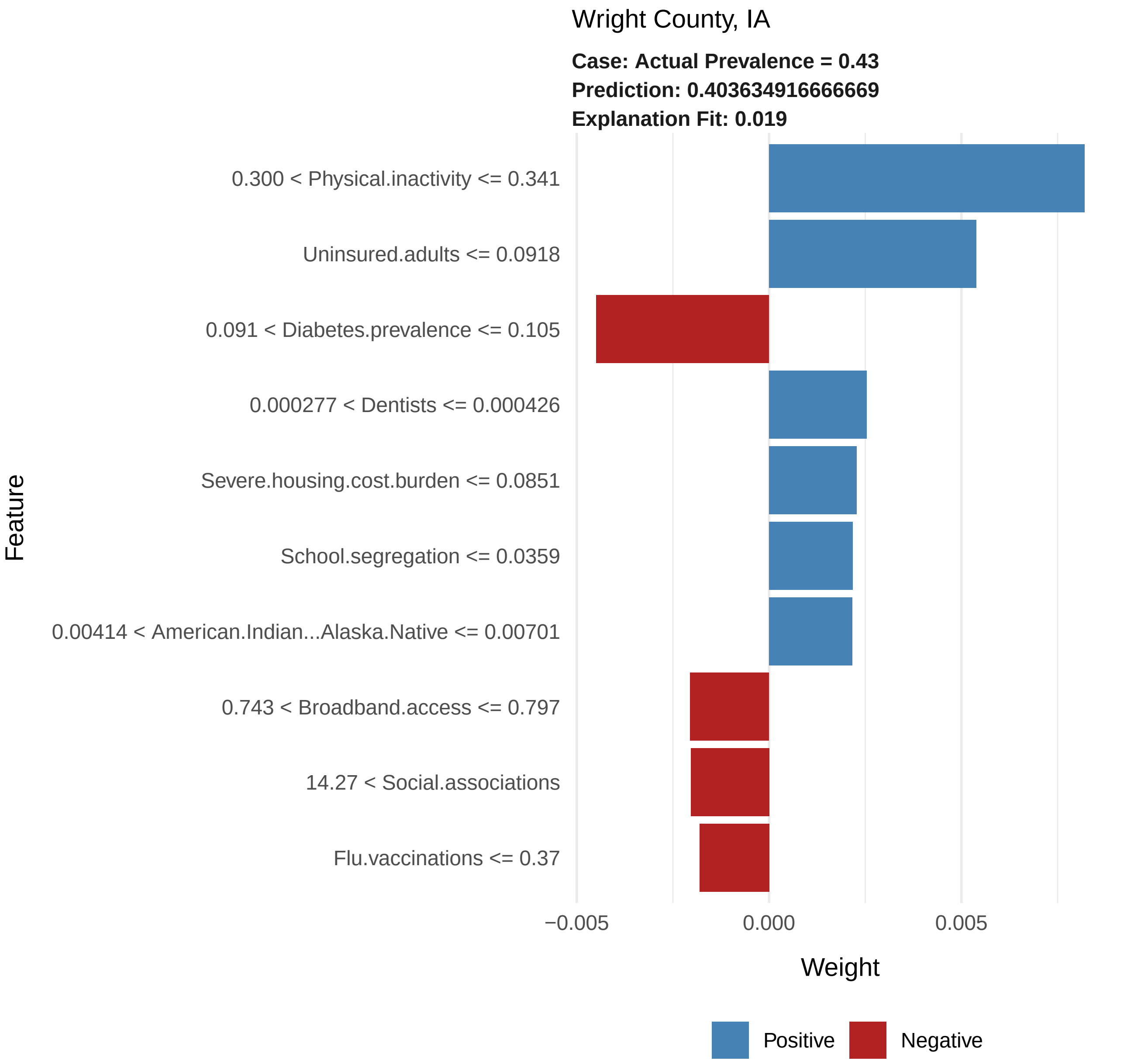

Wright County, IA
Case: Actual Prevalence = 0.43
Prediction: 0.403634916666669
Explanation Fit: 0.019
0.300 < Physical.inactivity <= 0.341
Uninsured.adults <= 0.0918
0.091 < Diabetes.prevalence <= 0.105
0.000277 < Dentists <= 0.000426
Severe.housing.cost.burden <= 0.0851
School.segregation <= 0.0359
0.00414 < American.Indian...Alaska.Native <= 0.00701
0.743 < Broadband.access <= 0.797
14.27 < Social.associations
Flu.vaccinations <= 0.37
Feature
−0.005
0.000
0.005
Weight
Positive
Negative

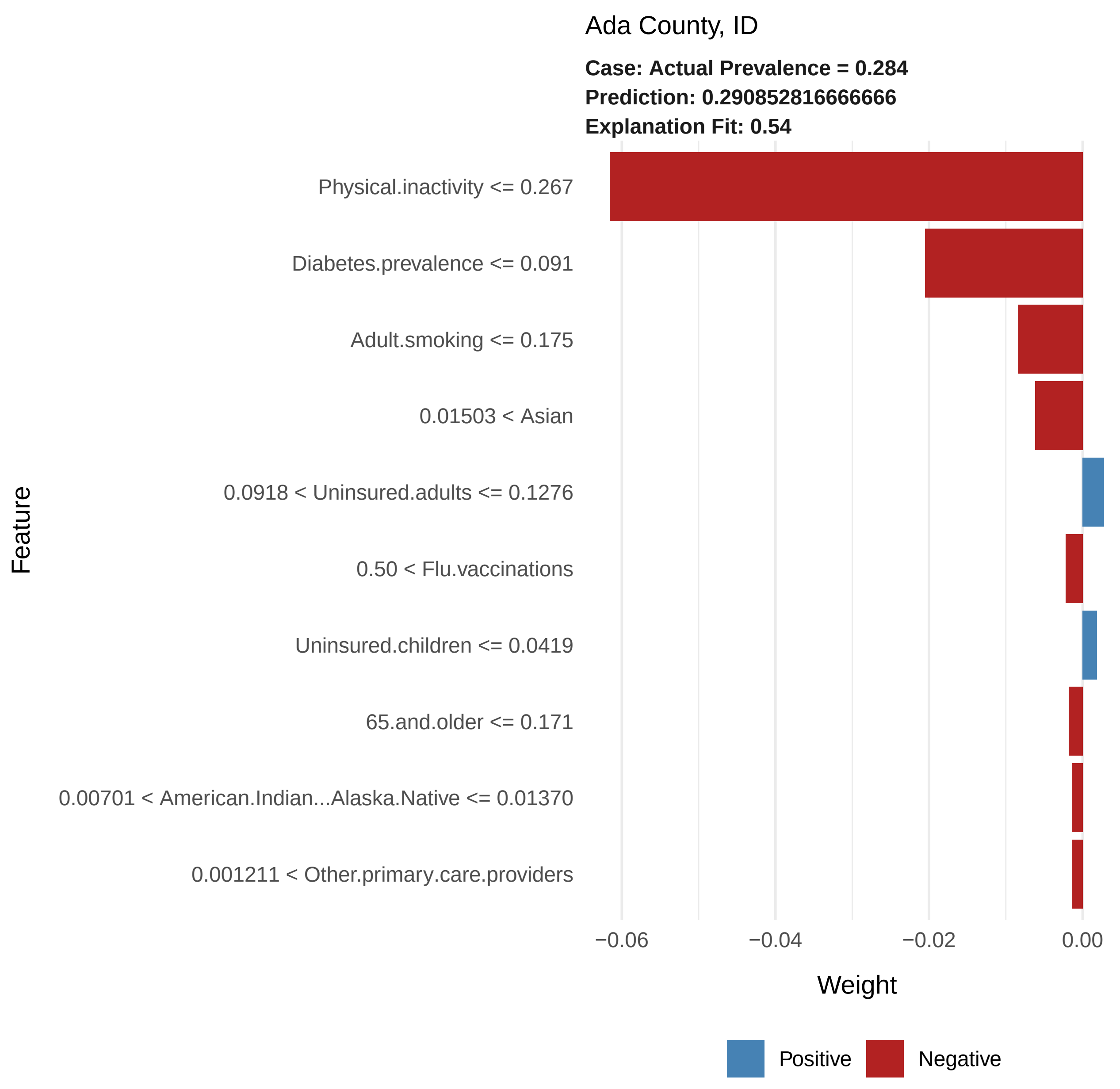

Ada County, ID
Case: Actual Prevalence = 0.284
Prediction: 0.290852816666666
Explanation Fit: 0.54
Physical.inactivity <= 0.267
Diabetes.prevalence <= 0.091
Adult.smoking <= 0.175
0.01503 < Asian
0.0918 < Uninsured.adults <= 0.1276
0.50 < Flu.vaccinations
Uninsured.children <= 0.0419
65.and.older <= 0.171
0.00701 < American.Indian...Alaska.Native <= 0.01370
0.001211 < Other.primary.care.providers
Feature
−0.06
−0.04
−0.02
0.00
Weight
Positive
Negative

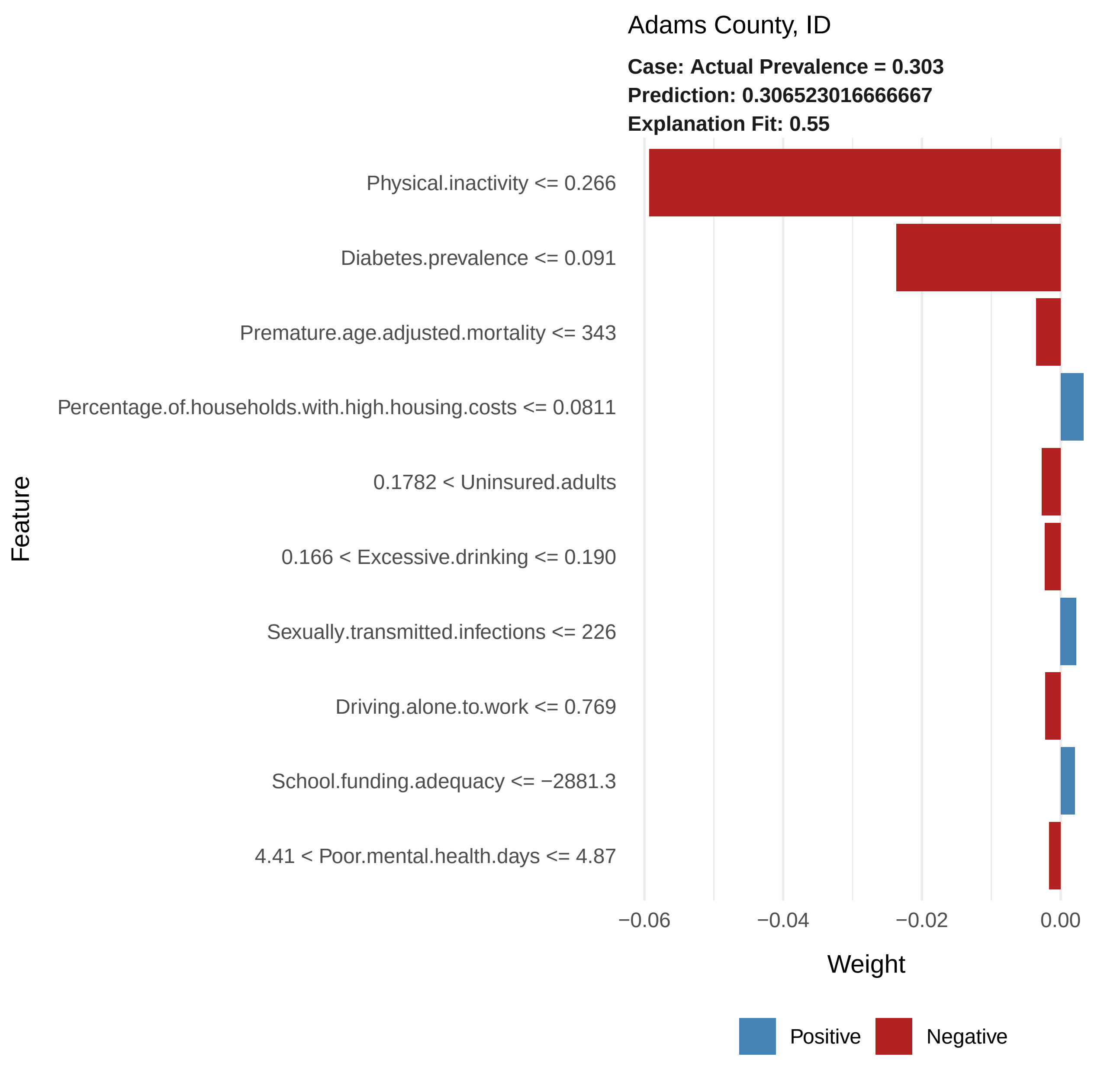

Adams County, ID
Case: Actual Prevalence = 0.303
Prediction: 0.306523016666667
Explanation Fit: 0.55
Physical.inactivity <= 0.266
Diabetes.prevalence <= 0.091
Premature.age.adjusted.mortality <= 343
Percentage.of.households.with.high.housing.costs <= 0.0811
0.1782 < Uninsured.adults
0.166 < Excessive.drinking <= 0.190
Sexually.transmitted.infections <= 226
Driving.alone.to.work <= 0.769
School.funding.adequacy <= −2881.3
4.41 < Poor.mental.health.days <= 4.87
Feature
−0.06
−0.04
−0.02
0.00
Weight
Positive
Negative

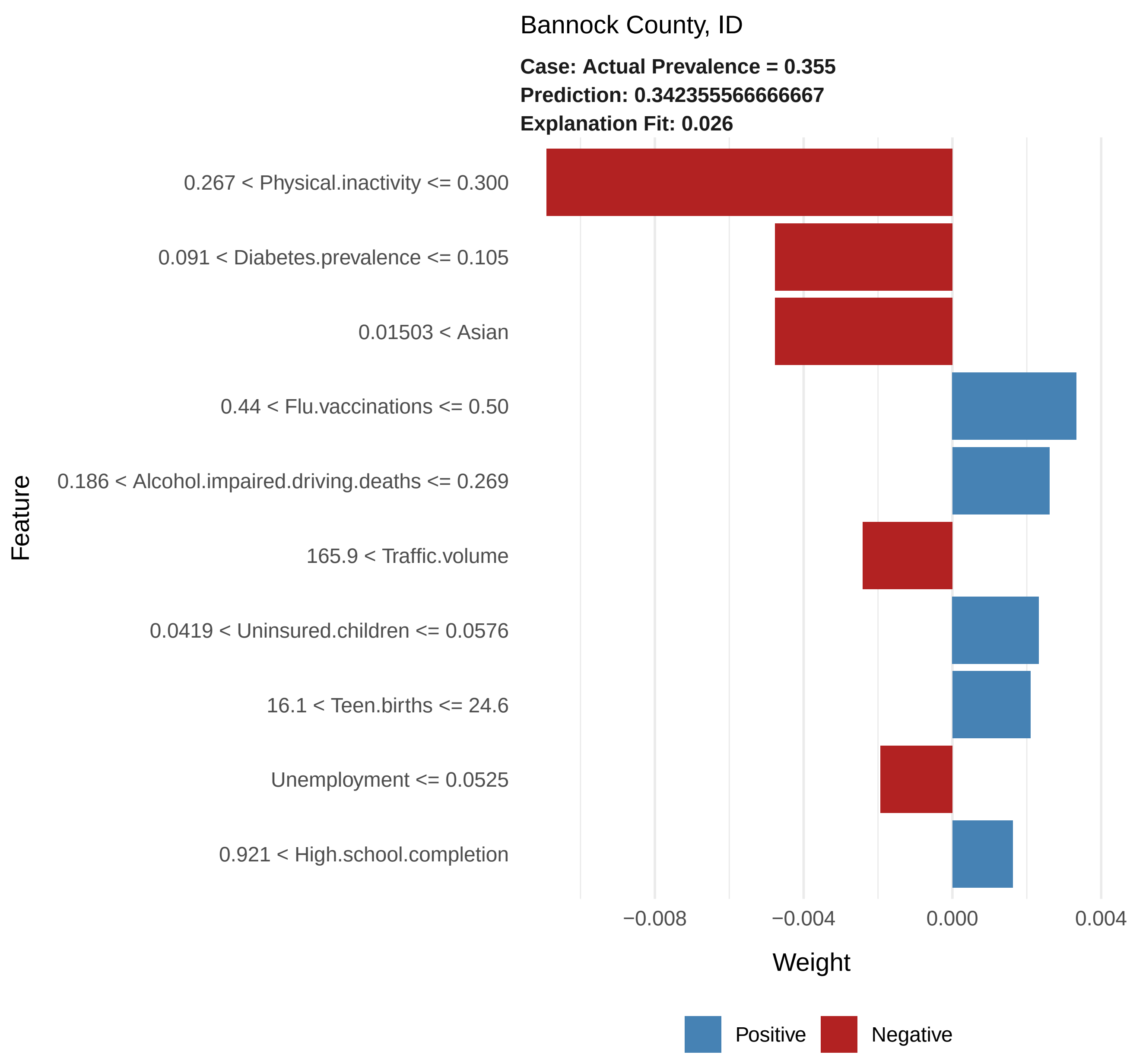

Bannock County, ID
Case: Actual Prevalence = 0.355
Prediction: 0.342355566666667
Explanation Fit: 0.026
0.267 < Physical.inactivity <= 0.300
0.091 < Diabetes.prevalence <= 0.105
0.01503 < Asian
0.44 < Flu.vaccinations <= 0.50
0.186 < Alcohol.impaired.driving.deaths <= 0.269
165.9 < Traffic.volume
0.0419 < Uninsured.children <= 0.0576
16.1 < Teen.births <= 24.6
Unemployment <= 0.0525
0.921 < High.school.completion
Feature
−0.008
−0.004
0.000
0.004
Weight
Positive
Negative

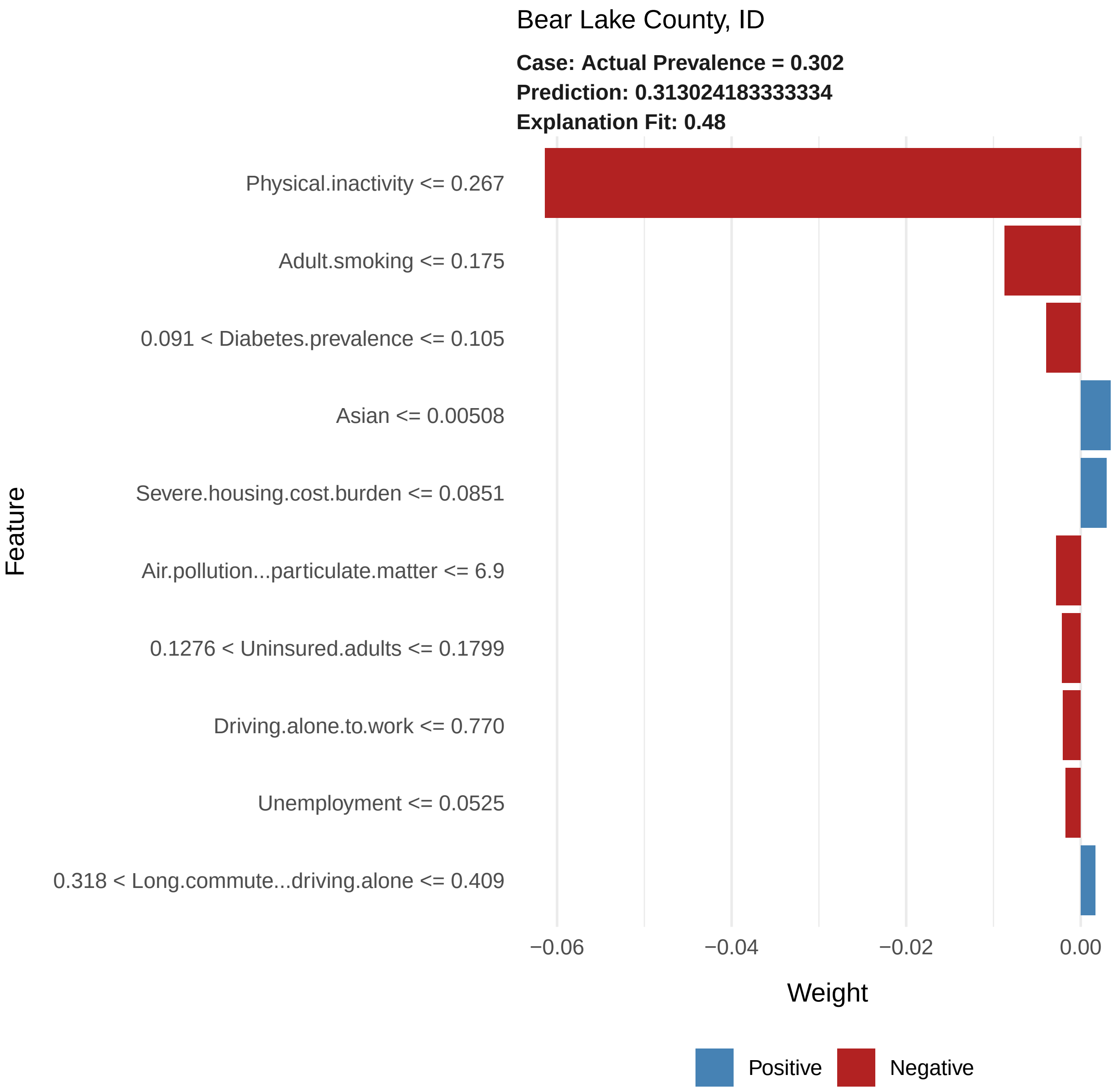

Bear Lake County, ID
Case: Actual Prevalence = 0.302
Prediction: 0.313024183333334
Explanation Fit: 0.48
Physical.inactivity <= 0.267
Adult.smoking <= 0.175
0.091 < Diabetes.prevalence <= 0.105
Asian <= 0.00508
Severe.housing.cost.burden <= 0.0851
Air.pollution...particulate.matter <= 6.9
0.1276 < Uninsured.adults <= 0.1799
Driving.alone.to.work <= 0.770
Unemployment <= 0.0525
0.318 < Long.commute...driving.alone <= 0.409
Feature
−0.06
−0.04
−0.02
0.00
Weight
Positive
Negative

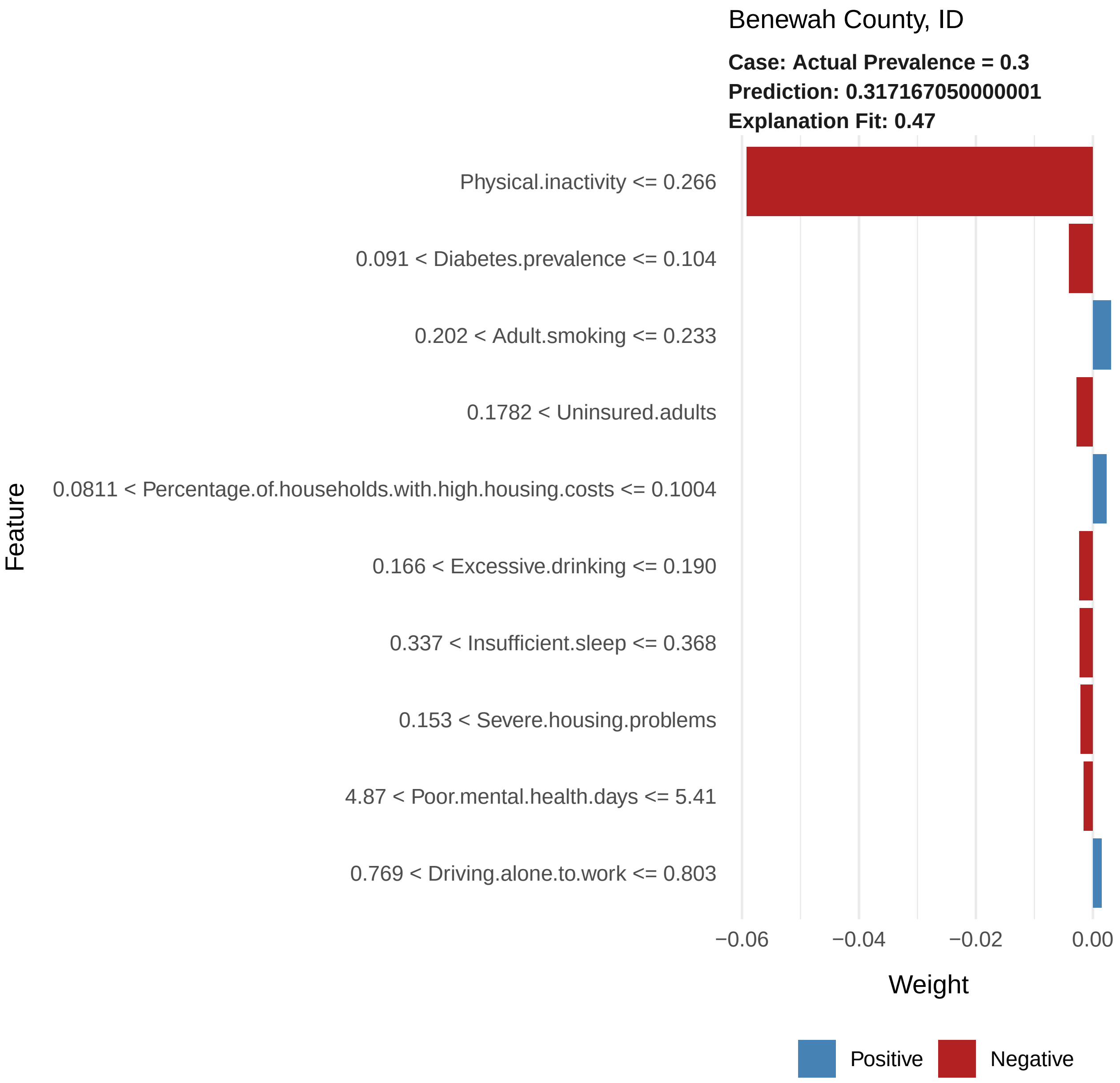

Benewah County, ID
Case: Actual Prevalence = 0.3
Prediction: 0.317167050000001
Explanation Fit: 0.47
Physical.inactivity <= 0.266
0.091 < Diabetes.prevalence <= 0.104
0.202 < Adult.smoking <= 0.233
0.1782 < Uninsured.adults
0.0811 < Percentage.of.households.with.high.housing.costs <= 0.1004
0.166 < Excessive.drinking <= 0.190
0.337 < Insufficient.sleep <= 0.368
0.153 < Severe.housing.problems
4.87 < Poor.mental.health.days <= 5.41
0.769 < Driving.alone.to.work <= 0.803
Feature
−0.06
−0.04
−0.02
0.00
Weight
Positive
Negative

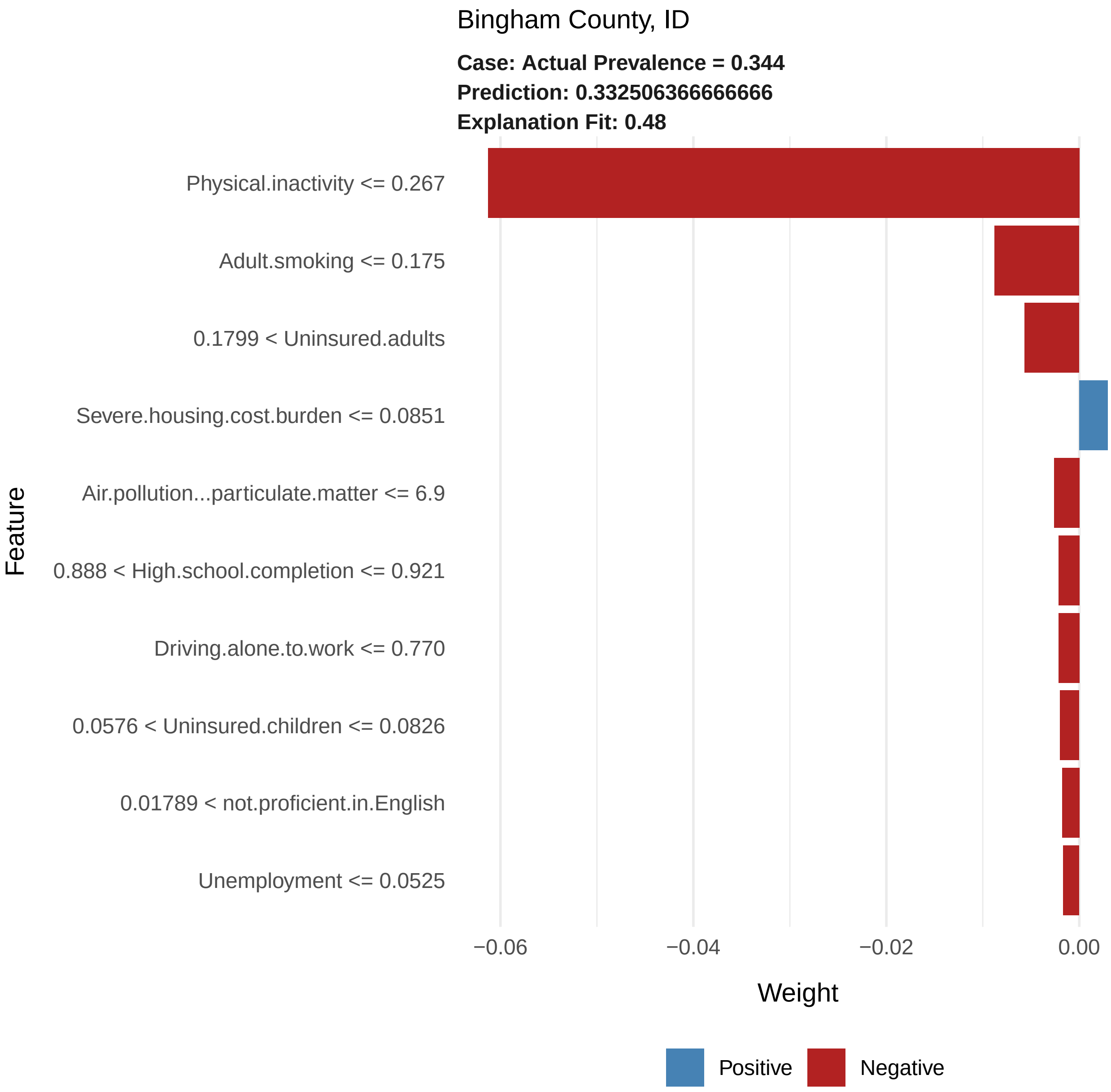
Bingham County, ID
Case: Actual Prevalence = 0.344
Prediction: 0.332506366666666
Explanation Fit: 0.48
Physical.inactivity <= 0.267
Adult.smoking <= 0.175
0.1799 < Uninsured.adults
Severe.housing.cost.burden <= 0.0851
Air.pollution...particulate.matter <= 6.9
0.888 < High.school.completion <= 0.921
Driving.alone.to.work <= 0.770
0.0576 < Uninsured.children <= 0.0826
0.01789 < not.proficient.in.English
Unemployment <= 0.0525
Feature
−0.06
−0.04
−0.02
0.00
Weight
Positive
Negative

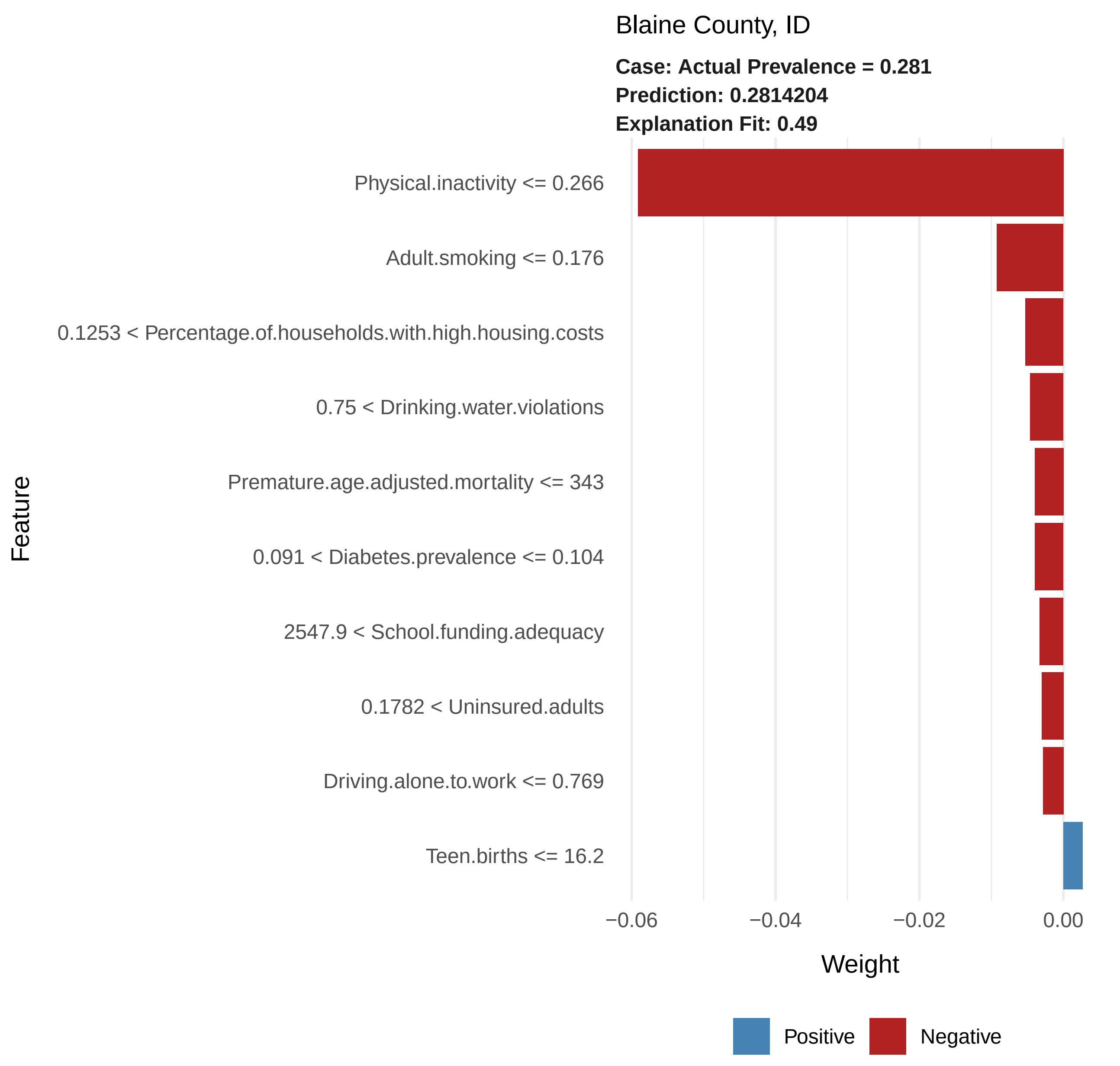

Blaine County, ID
Case: Actual Prevalence = 0.281
Prediction: 0.2814204
Explanation Fit: 0.49
Physical.inactivity <= 0.266
Adult.smoking <= 0.176
0.1253 < Percentage.of.households.with.high.housing.costs
0.75 < Drinking.water.violations
Premature.age.adjusted.mortality <= 343
0.091 < Diabetes.prevalence <= 0.104
2547.9 < School.funding.adequacy
0.1782 < Uninsured.adults
Driving.alone.to.work <= 0.769
Teen.births <= 16.2
Feature
−0.06
−0.04
−0.02
0.00
Weight
Positive
Negative

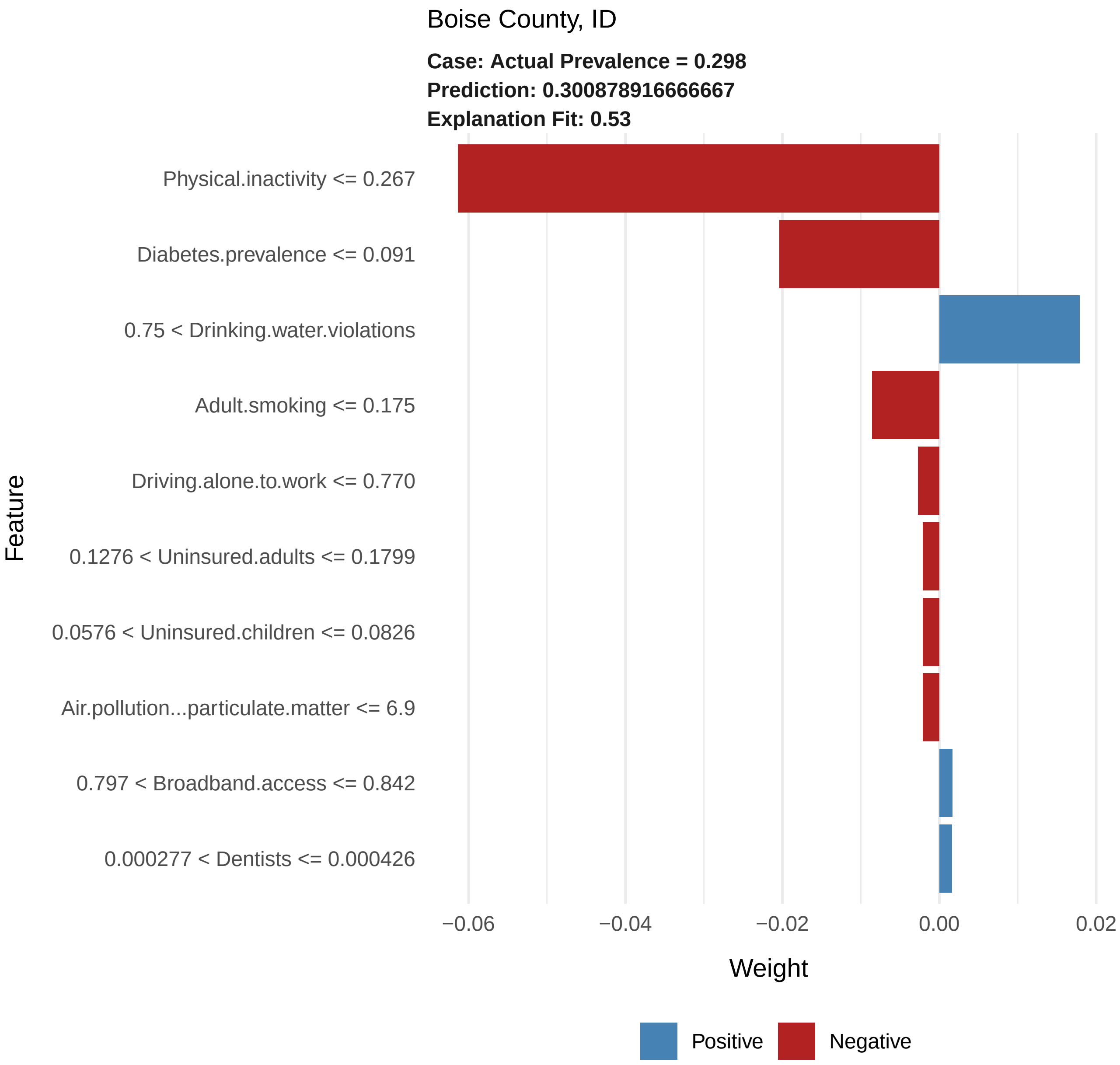

Boise County, ID
Case: Actual Prevalence = 0.298
Prediction: 0.300878916666667
Explanation Fit: 0.53
Physical.inactivity <= 0.267
Diabetes.prevalence <= 0.091
0.75 < Drinking.water.violations
Adult.smoking <= 0.175
Driving.alone.to.work <= 0.770
0.1276 < Uninsured.adults <= 0.1799
0.0576 < Uninsured.children <= 0.0826
Air.pollution...particulate.matter <= 6.9
0.797 < Broadband.access <= 0.842
0.000277 < Dentists <= 0.000426
Feature
−0.06
−0.04
−0.02
0.00
0.02
Weight
Positive
Negative

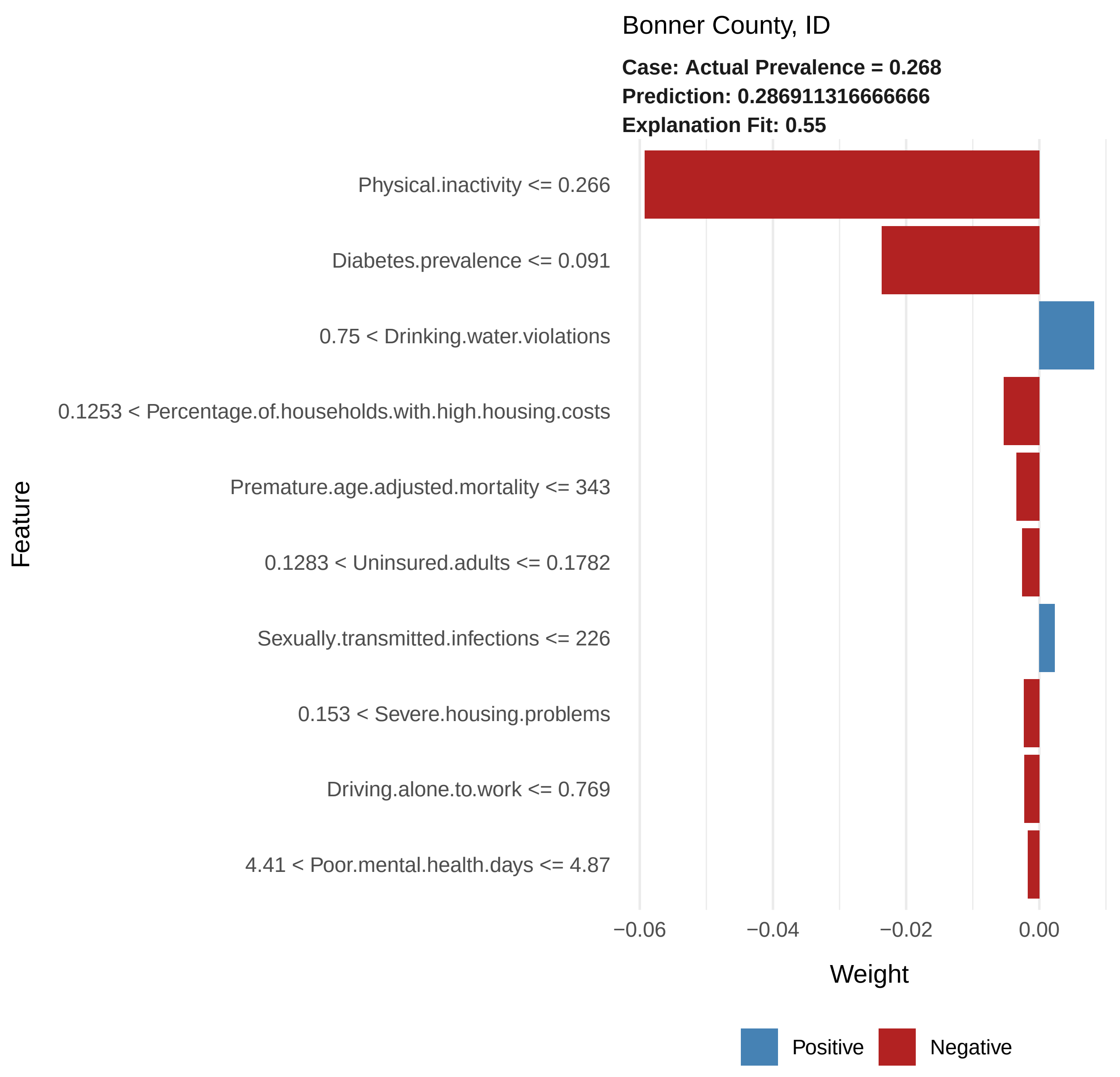
Bonner County, ID
Case: Actual Prevalence = 0.268
Prediction: 0.286911316666666
Explanation Fit: 0.55
Physical.inactivity <= 0.266
Diabetes.prevalence <= 0.091
0.75 < Drinking.water.violations
0.1253 < Percentage.of.households.with.high.housing.costs
Premature.age.adjusted.mortality <= 343
0.1283 < Uninsured.adults <= 0.1782
Sexually.transmitted.infections <= 226
0.153 < Severe.housing.problems
Driving.alone.to.work <= 0.769
4.41 < Poor.mental.health.days <= 4.87
Feature
−0.06
−0.04
−0.02
0.00
Weight
Positive
Negative

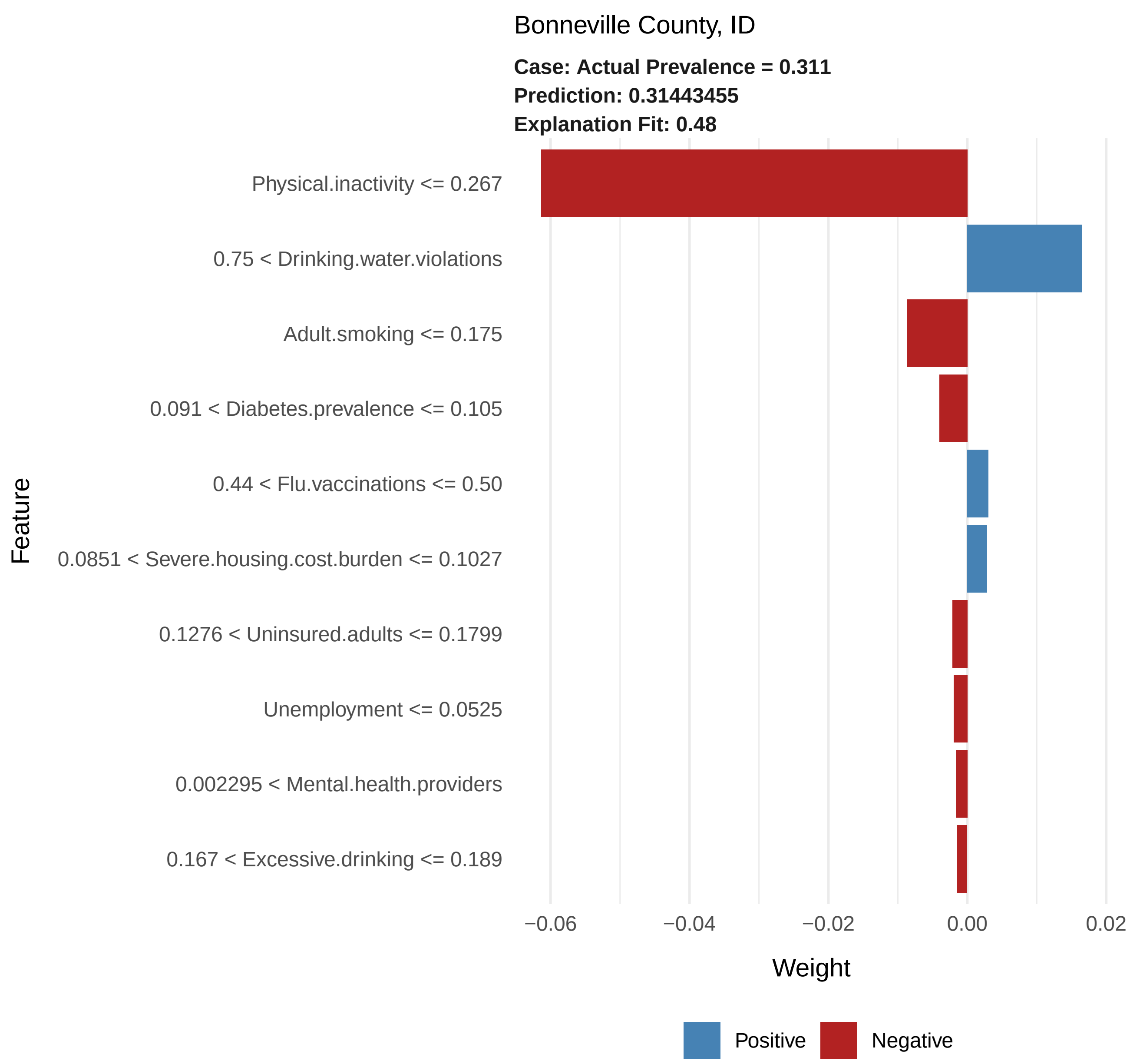

Bonneville County, ID
Case: Actual Prevalence = 0.311
Prediction: 0.31443455
Explanation Fit: 0.48
Physical.inactivity <= 0.267
0.75 < Drinking.water.violations
Adult.smoking <= 0.175
0.091 < Diabetes.prevalence <= 0.105
0.44 < Flu.vaccinations <= 0.50
0.0851 < Severe.housing.cost.burden <= 0.1027
0.1276 < Uninsured.adults <= 0.1799
Unemployment <= 0.0525
0.002295 < Mental.health.providers
0.167 < Excessive.drinking <= 0.189
Feature
−0.06
−0.04
−0.02
0.00
0.02
Weight
Positive
Negative

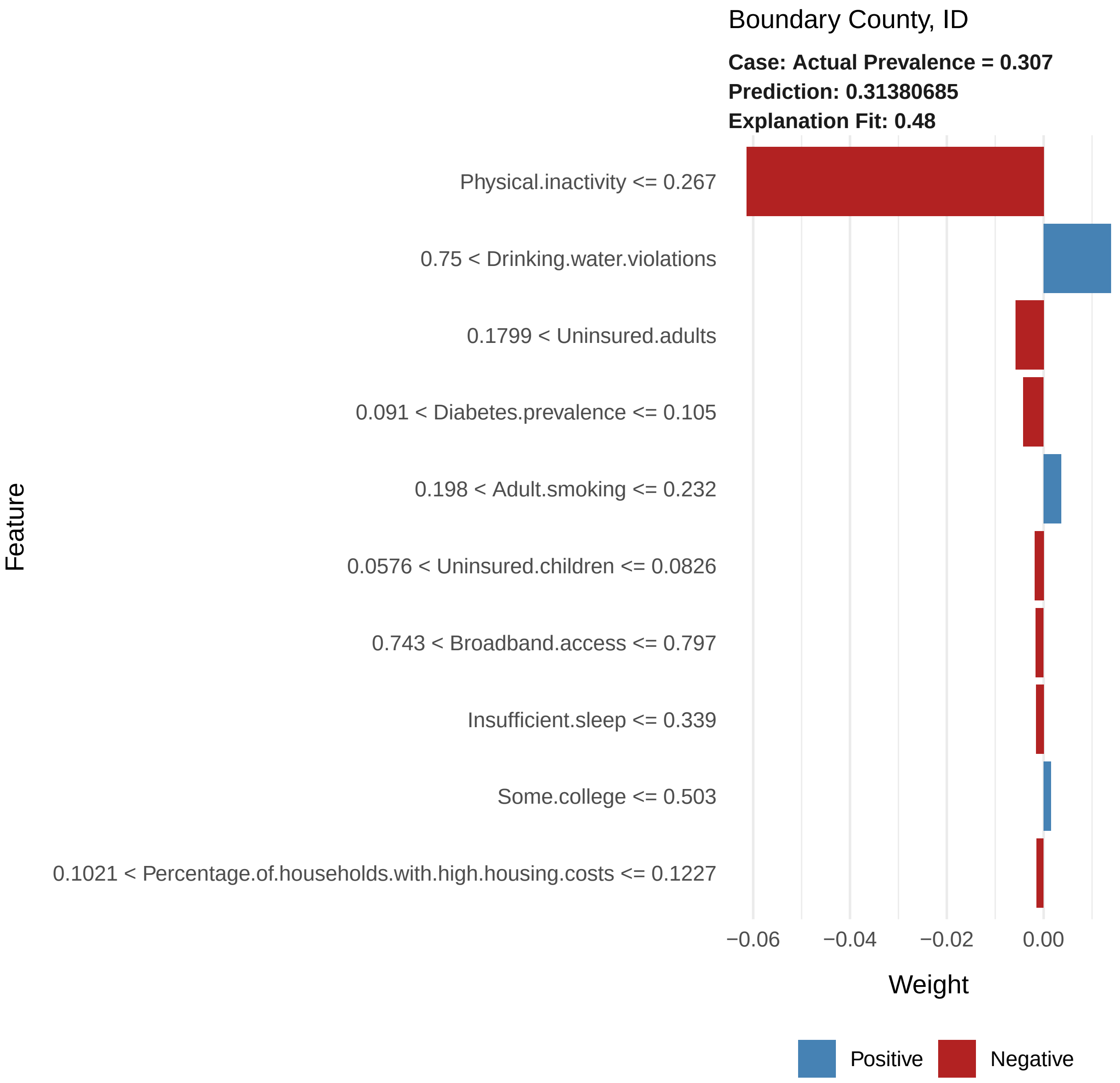

Boundary County, ID
Case: Actual Prevalence = 0.307
Prediction: 0.31380685
Explanation Fit: 0.48
Physical.inactivity <= 0.267
0.75 < Drinking.water.violations
0.1799 < Uninsured.adults
0.091 < Diabetes.prevalence <= 0.105
0.198 < Adult.smoking <= 0.232
0.0576 < Uninsured.children <= 0.0826
0.743 < Broadband.access <= 0.797
Insufficient.sleep <= 0.339
Some.college <= 0.503
0.1021 < Percentage.of.households.with.high.housing.costs <= 0.1227
Feature
−0.06
−0.04
−0.02
0.00
Weight
Positive
Negative

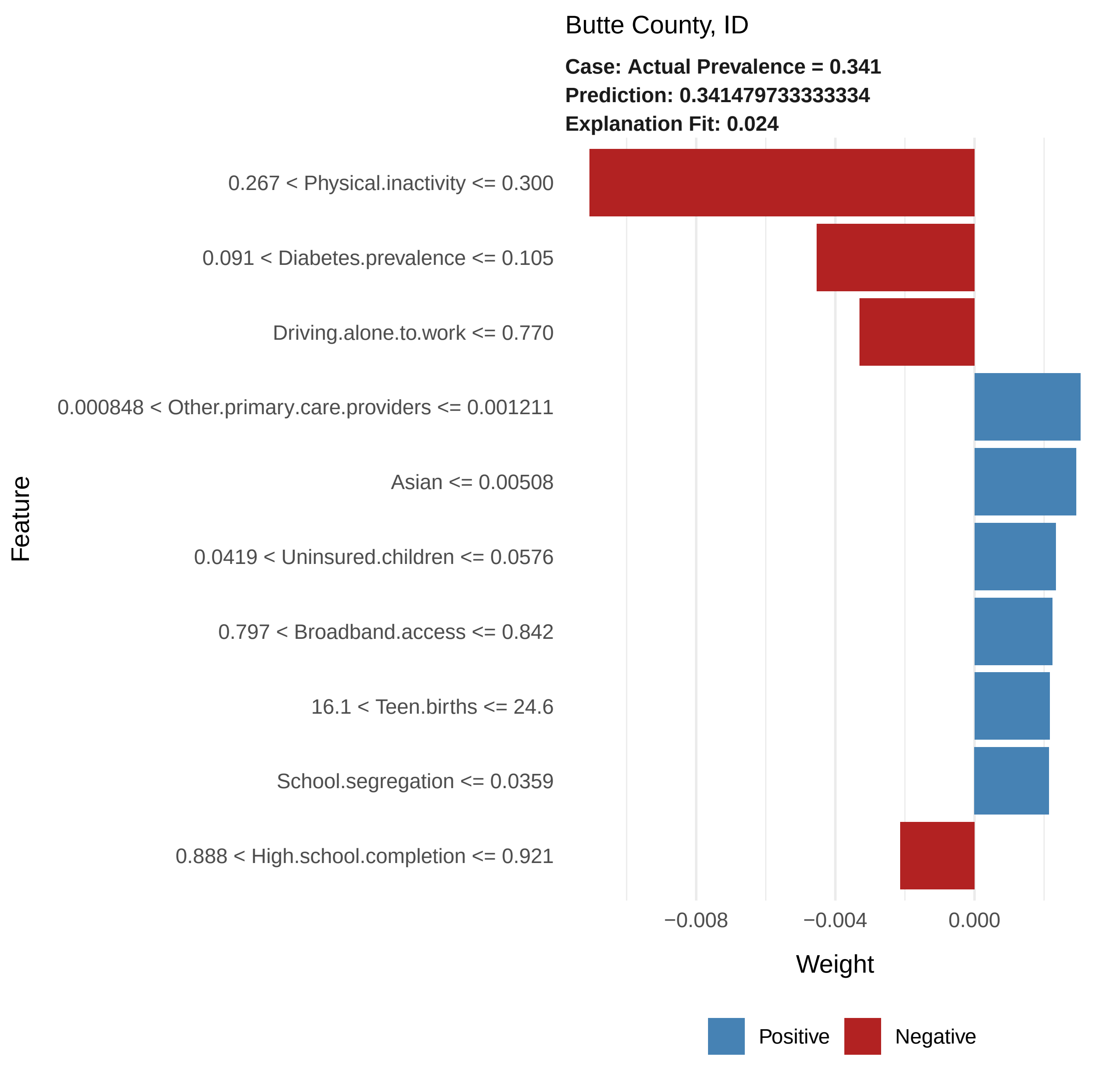

Butte County, ID
Case: Actual Prevalence = 0.341
Prediction: 0.341479733333334
Explanation Fit: 0.024
0.267 < Physical.inactivity <= 0.300
0.091 < Diabetes.prevalence <= 0.105
Driving.alone.to.work <= 0.770
0.000848 < Other.primary.care.providers <= 0.001211
Asian <= 0.00508
0.0419 < Uninsured.children <= 0.0576
0.797 < Broadband.access <= 0.842
16.1 < Teen.births <= 24.6
School.segregation <= 0.0359
0.888 < High.school.completion <= 0.921
Feature
−0.008
−0.004
0.000
Weight
Positive
Negative

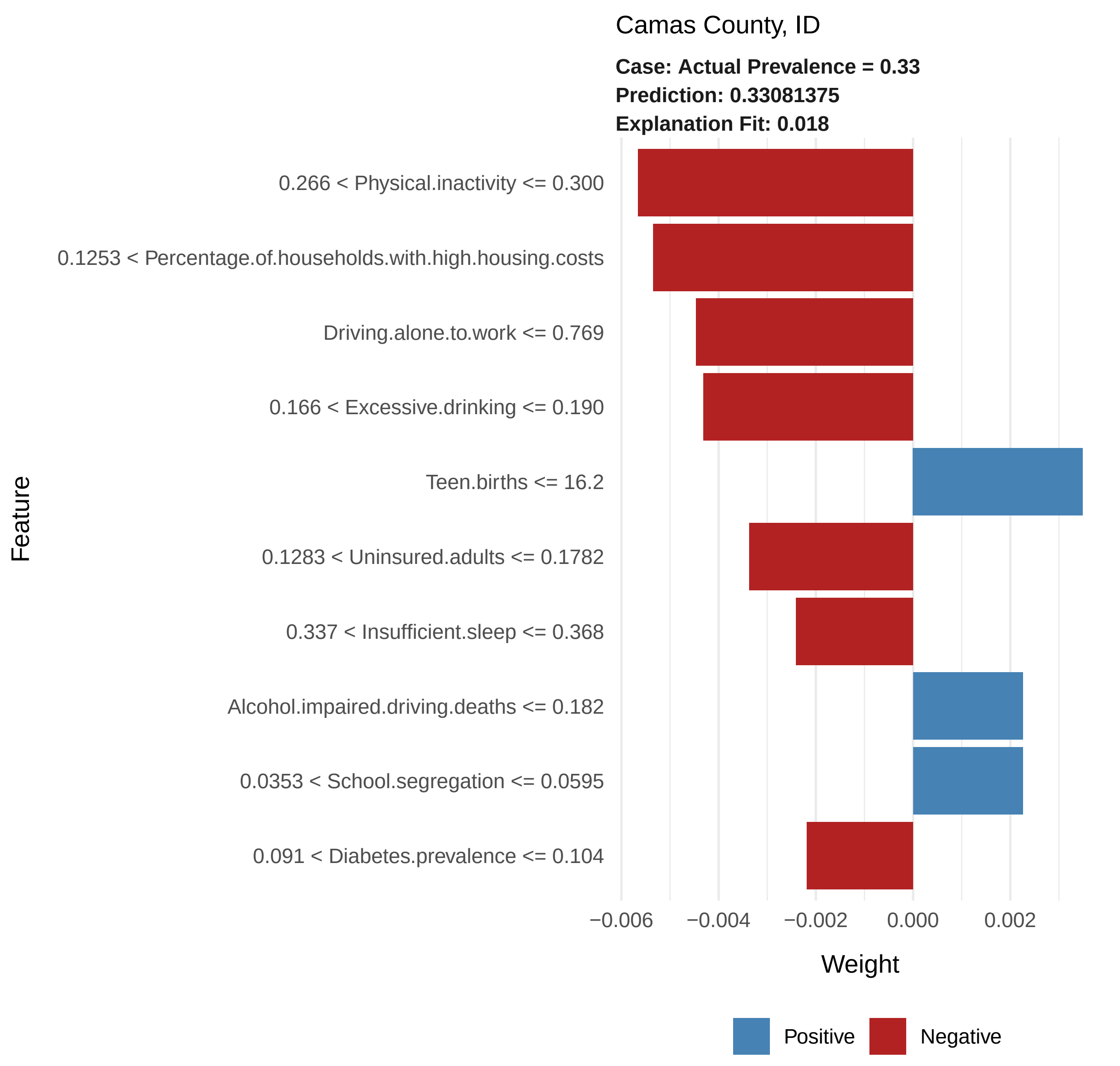

Camas County, ID
Case: Actual Prevalence = 0.33
Prediction: 0.33081375
Explanation Fit: 0.018
0.266 < Physical.inactivity <= 0.300
0.1253 < Percentage.of.households.with.high.housing.costs
Driving.alone.to.work <= 0.769
0.166 < Excessive.drinking <= 0.190
Teen.births <= 16.2
0.1283 < Uninsured.adults <= 0.1782
0.337 < Insufficient.sleep <= 0.368
Alcohol.impaired.driving.deaths <= 0.182
0.0353 < School.segregation <= 0.0595
0.091 < Diabetes.prevalence <= 0.104
Feature
−0.006
−0.004
−0.002
0.000
0.002
Weight
Positive
Negative

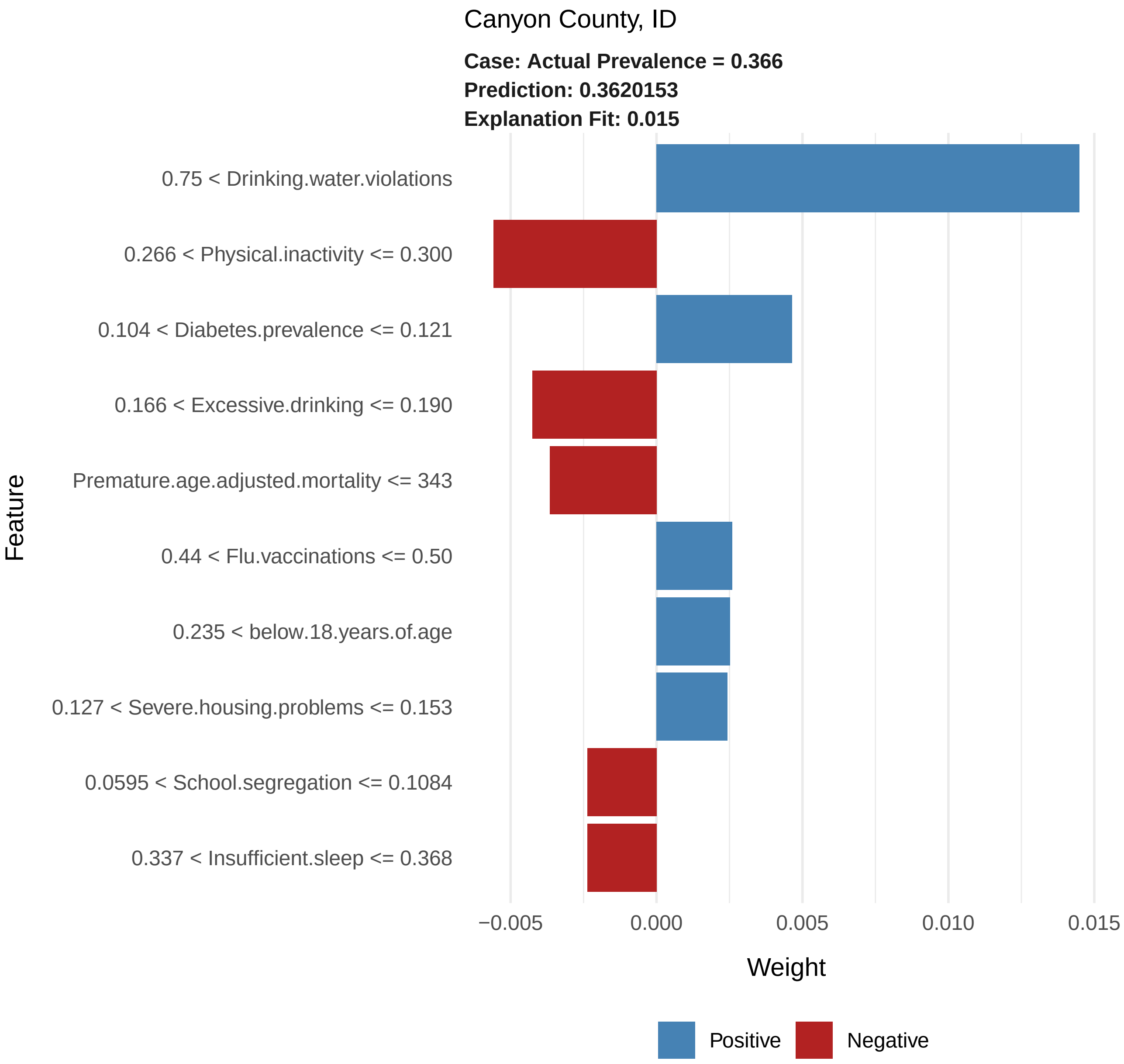

Canyon County, ID
Case: Actual Prevalence = 0.366
Prediction: 0.3620153
Explanation Fit: 0.015
0.75 < Drinking.water.violations
0.266 < Physical.inactivity <= 0.300
0.104 < Diabetes.prevalence <= 0.121
0.166 < Excessive.drinking <= 0.190
Premature.age.adjusted.mortality <= 343
0.44 < Flu.vaccinations <= 0.50
0.235 < below.18.years.of.age
0.127 < Severe.housing.problems <= 0.153
0.0595 < School.segregation <= 0.1084
0.337 < Insufficient.sleep <= 0.368
Feature
−0.005
0.000
0.005
0.010
0.015
Weight
Positive
Negative

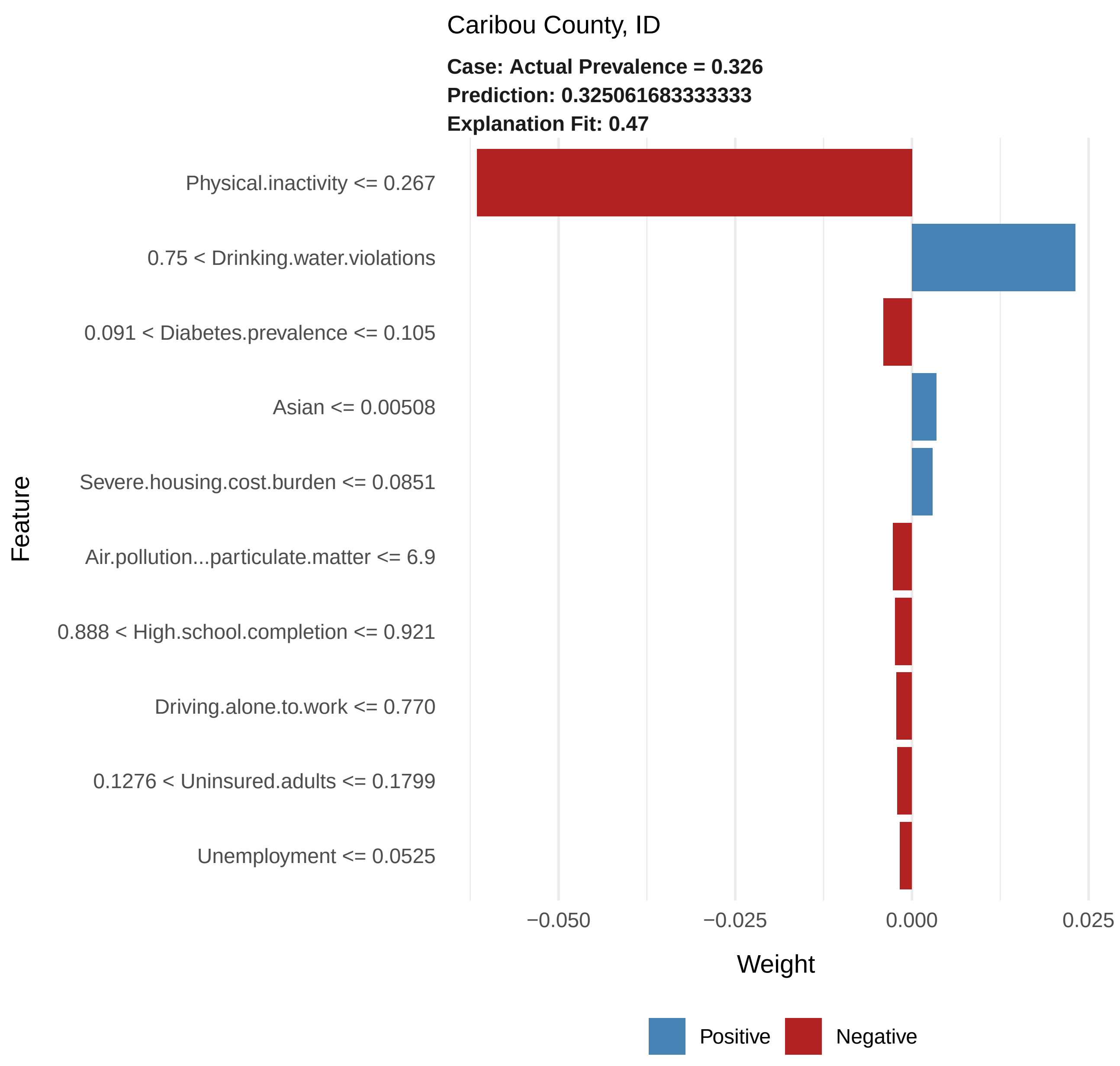

Caribou County, ID
Case: Actual Prevalence = 0.326
Prediction: 0.325061683333333
Explanation Fit: 0.47
Physical.inactivity <= 0.267
0.75 < Drinking.water.violations
0.091 < Diabetes.prevalence <= 0.105
Asian <= 0.00508
Severe.housing.cost.burden <= 0.0851
Air.pollution...particulate.matter <= 6.9
0.888 < High.school.completion <= 0.921
Driving.alone.to.work <= 0.770
0.1276 < Uninsured.adults <= 0.1799
Unemployment <= 0.0525
Feature
−0.050
−0.025
0.000
0.025
Weight
Positive
Negative

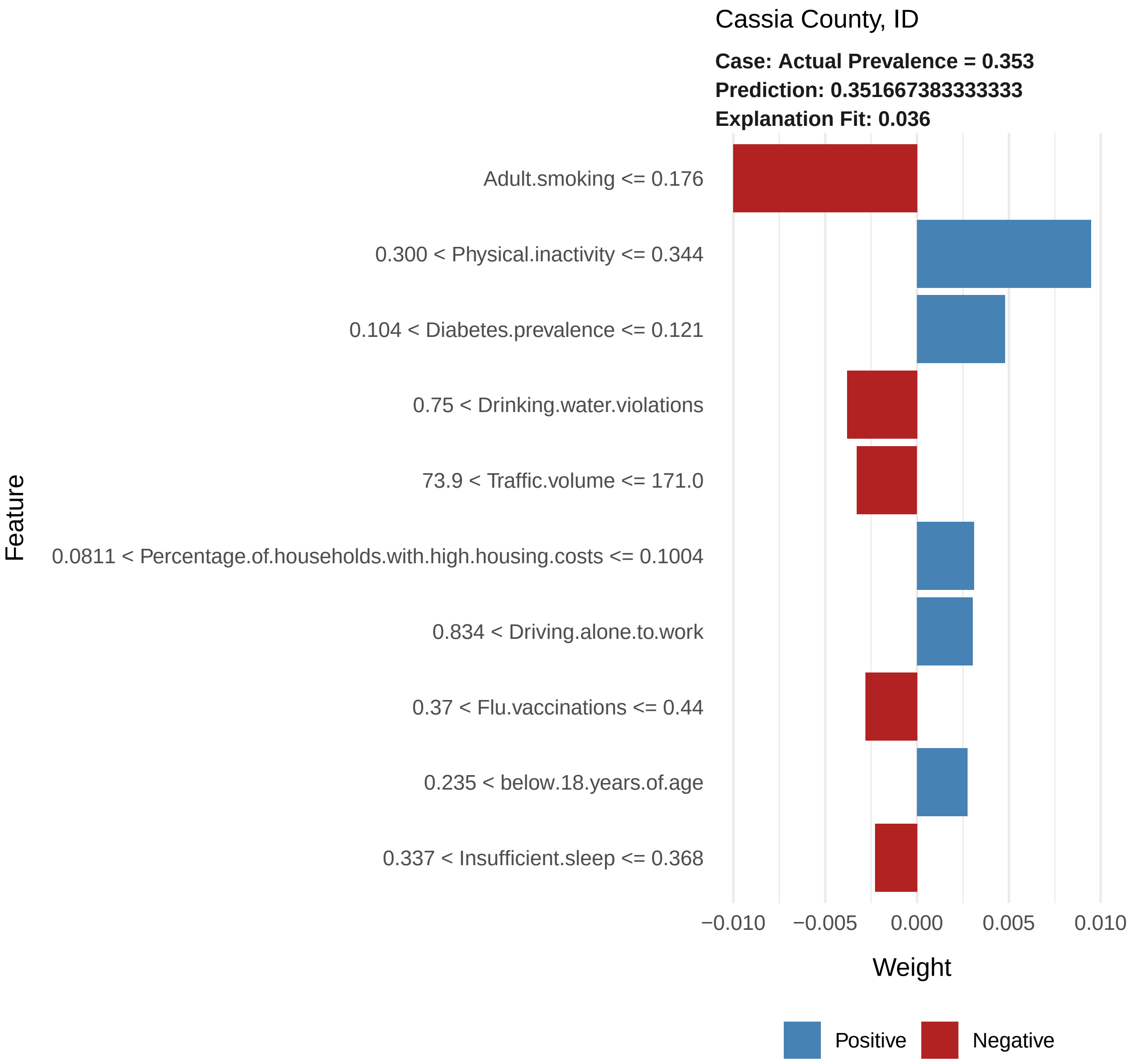

Cassia County, ID
Case: Actual Prevalence = 0.353
Prediction: 0.351667383333333
Explanation Fit: 0.036
Adult.smoking <= 0.176
0.300 < Physical.inactivity <= 0.344
0.104 < Diabetes.prevalence <= 0.121
0.75 < Drinking.water.violations
73.9 < Traffic.volume <= 171.0
0.0811 < Percentage.of.households.with.high.housing.costs <= 0.1004
0.834 < Driving.alone.to.work
0.37 < Flu.vaccinations <= 0.44
0.235 < below.18.years.of.age
0.337 < Insufficient.sleep <= 0.368
Feature
−0.010
−0.005
0.000
0.005
0.010
Weight
Positive
Negative

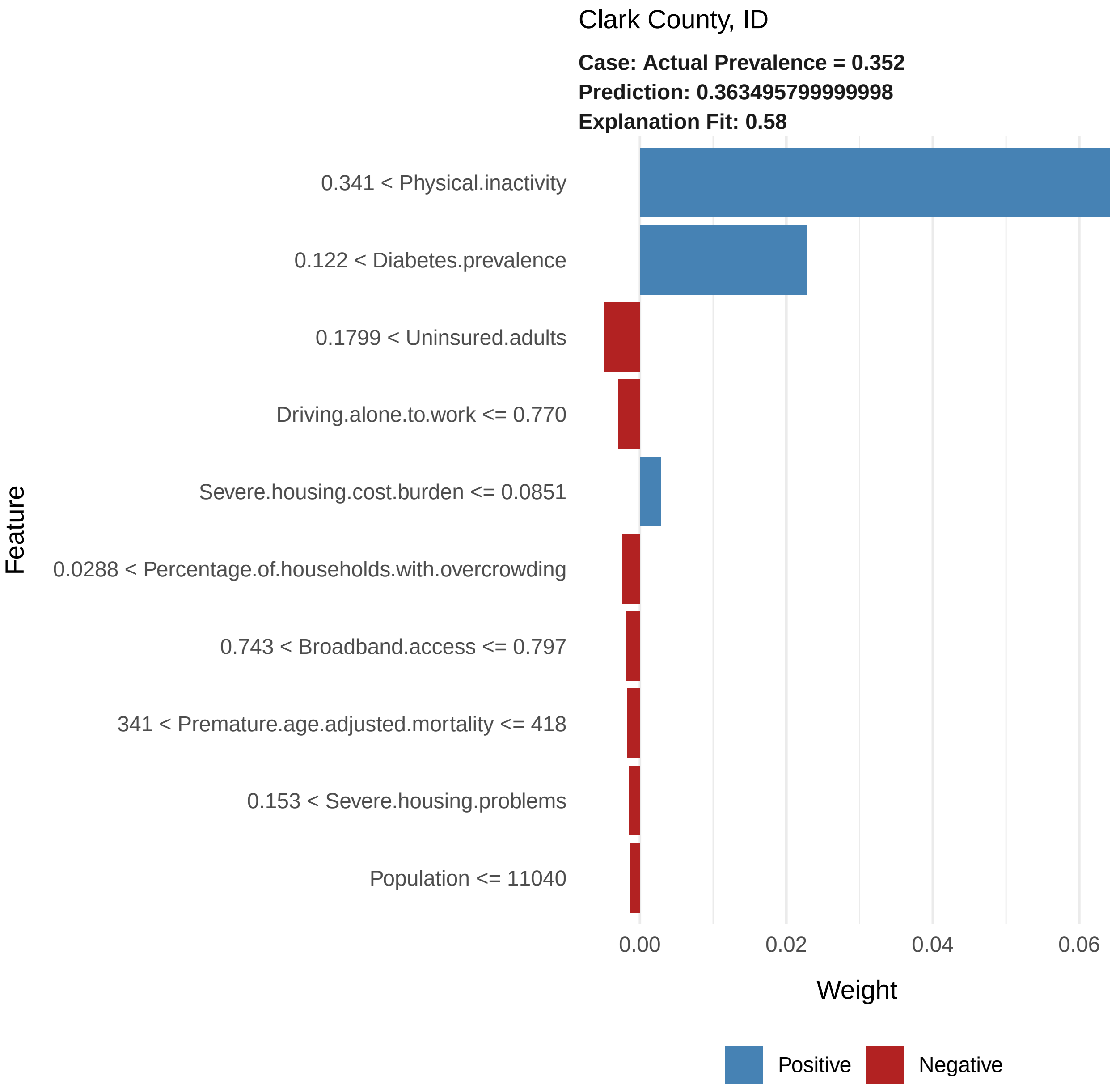
Clark County, ID
Case: Actual Prevalence = 0.352
Prediction: 0.363495799999998
Explanation Fit: 0.58
0.341 < Physical.inactivity
0.122 < Diabetes.prevalence
0.1799 < Uninsured.adults
Driving.alone.to.work <= 0.770
Severe.housing.cost.burden <= 0.0851
0.0288 < Percentage.of.households.with.overcrowding
0.743 < Broadband.access <= 0.797
341 < Premature.age.adjusted.mortality <= 418
0.153 < Severe.housing.problems
Population <= 11040
Feature
0.00
0.02
0.04
0.06
Weight
Positive
Negative

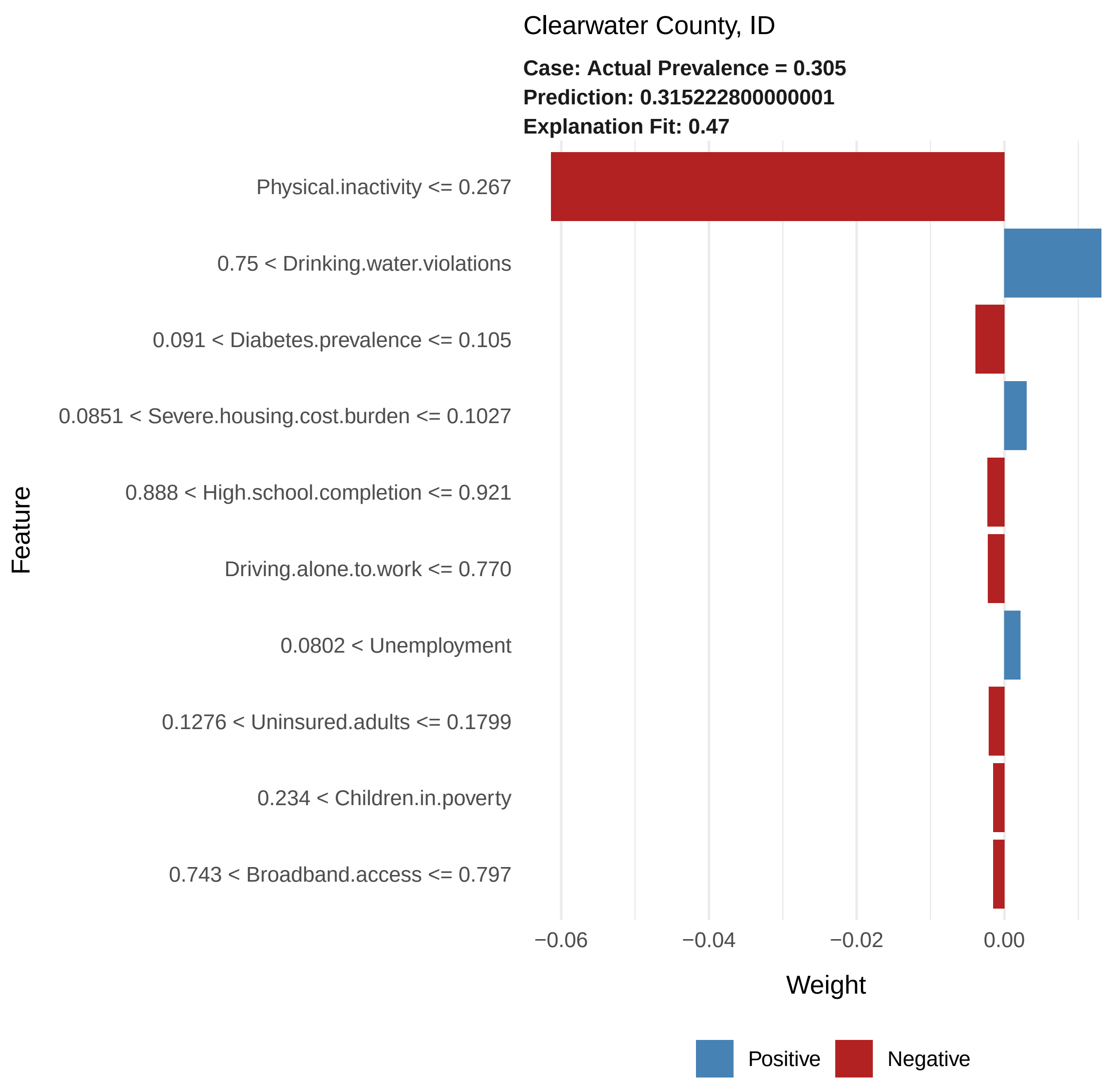

Clearwater County, ID
Case: Actual Prevalence = 0.305
Prediction: 0.315222800000001
Explanation Fit: 0.47
Physical.inactivity <= 0.267
0.75 < Drinking.water.violations
0.091 < Diabetes.prevalence <= 0.105
0.0851 < Severe.housing.cost.burden <= 0.1027
0.888 < High.school.completion <= 0.921
Driving.alone.to.work <= 0.770
0.0802 < Unemployment
0.1276 < Uninsured.adults <= 0.1799
0.234 < Children.in.poverty
0.743 < Broadband.access <= 0.797
Feature
−0.06
−0.04
−0.02
0.00
Weight
Positive
Negative

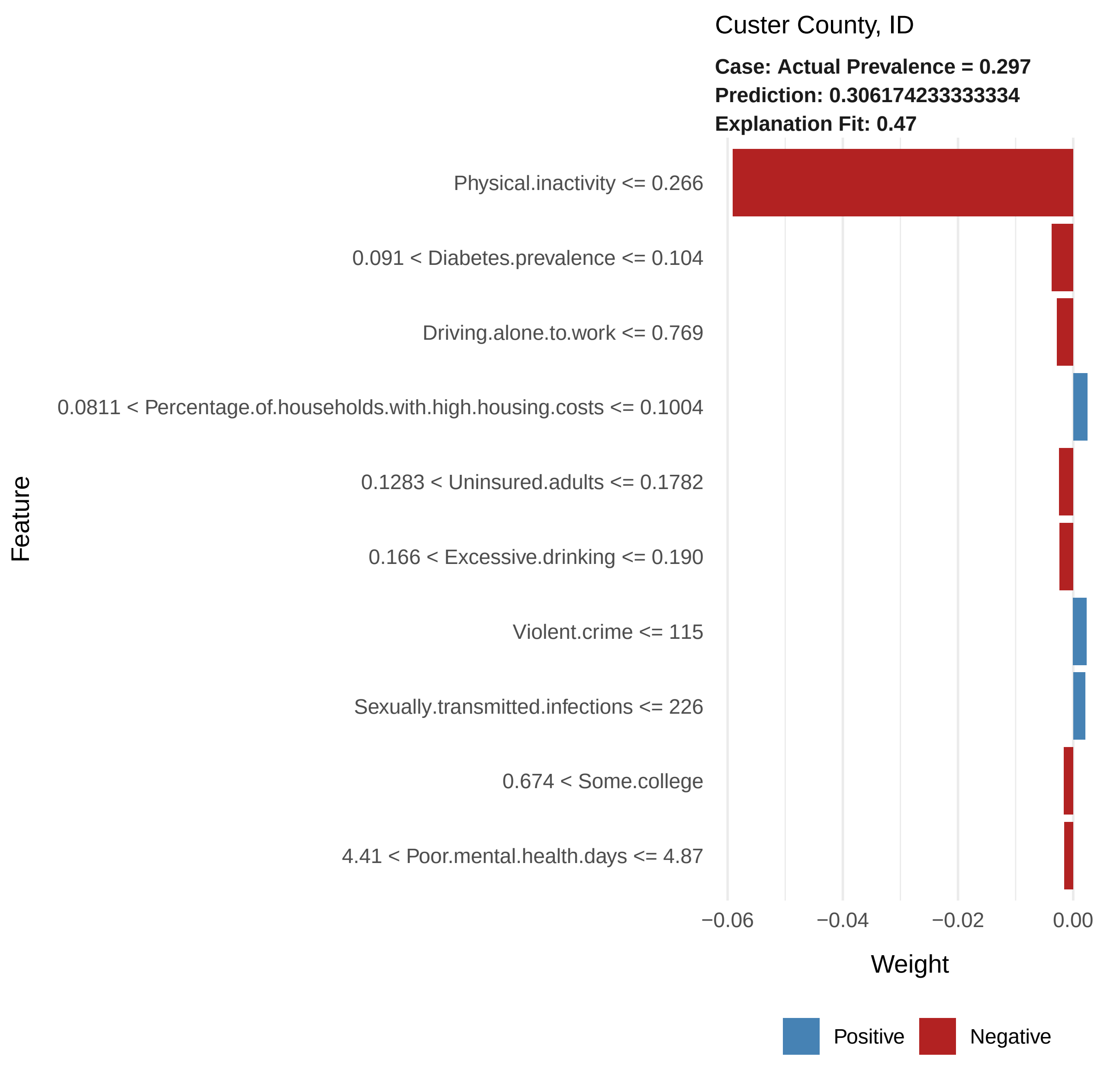

Custer County, ID
Case: Actual Prevalence = 0.297
Prediction: 0.306174233333334
Explanation Fit: 0.47
Physical.inactivity <= 0.266
0.091 < Diabetes.prevalence <= 0.104
Driving.alone.to.work <= 0.769
0.0811 < Percentage.of.households.with.high.housing.costs <= 0.1004
0.1283 < Uninsured.adults <= 0.1782
0.166 < Excessive.drinking <= 0.190
Violent.crime <= 115
Sexually.transmitted.infections <= 226
0.674 < Some.college
4.41 < Poor.mental.health.days <= 4.87
Feature
−0.06
−0.04
−0.02
0.00
Weight
Positive
Negative

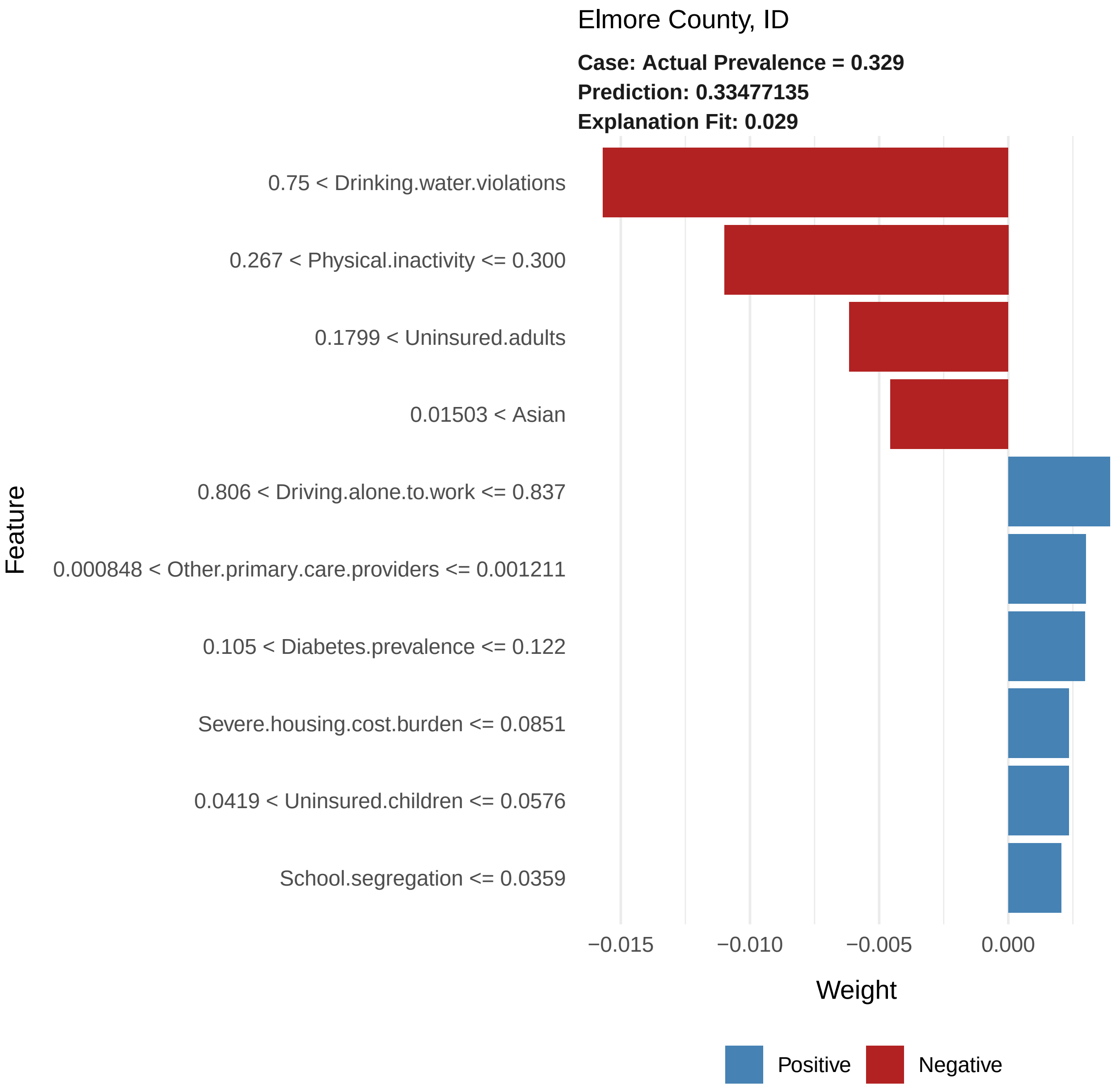

Elmore County, ID
Case: Actual Prevalence = 0.329
Prediction: 0.33477135
Explanation Fit: 0.029
0.75 < Drinking.water.violations
0.267 < Physical.inactivity <= 0.300
0.1799 < Uninsured.adults
0.01503 < Asian
0.806 < Driving.alone.to.work <= 0.837
0.000848 < Other.primary.care.providers <= 0.001211
0.105 < Diabetes.prevalence <= 0.122
Severe.housing.cost.burden <= 0.0851
0.0419 < Uninsured.children <= 0.0576
School.segregation <= 0.0359
Feature
−0.015
−0.010
−0.005
0.000
Weight
Positive
Negative

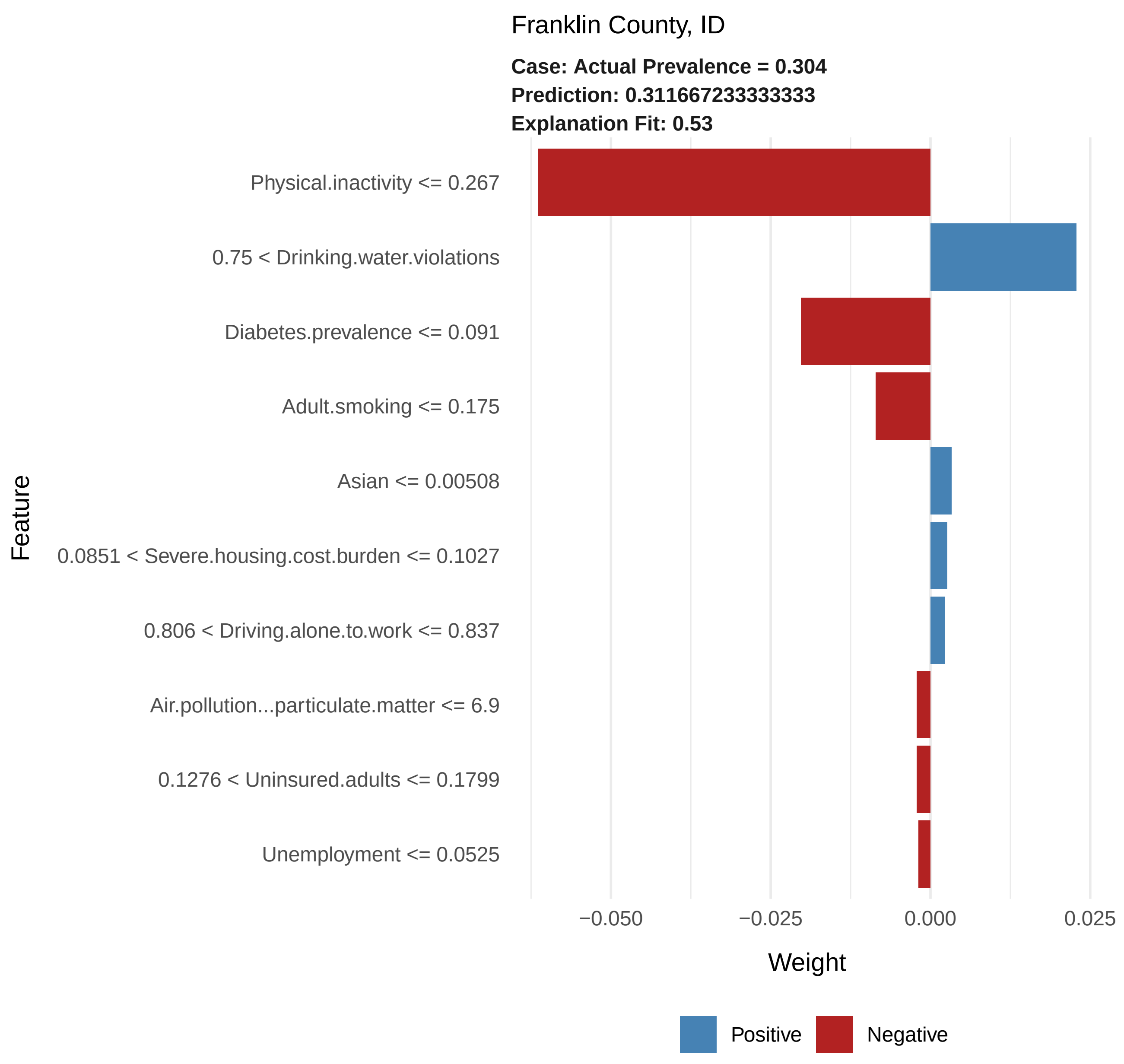

Franklin County, ID
Case: Actual Prevalence = 0.304
Prediction: 0.311667233333333
Explanation Fit: 0.53
Physical.inactivity <= 0.267
0.75 < Drinking.water.violations
Diabetes.prevalence <= 0.091
Adult.smoking <= 0.175
Asian <= 0.00508
0.0851 < Severe.housing.cost.burden <= 0.1027
0.806 < Driving.alone.to.work <= 0.837
Air.pollution...particulate.matter <= 6.9
0.1276 < Uninsured.adults <= 0.1799
Unemployment <= 0.0525
Feature
−0.050
−0.025
0.000
0.025
Weight
Positive
Negative

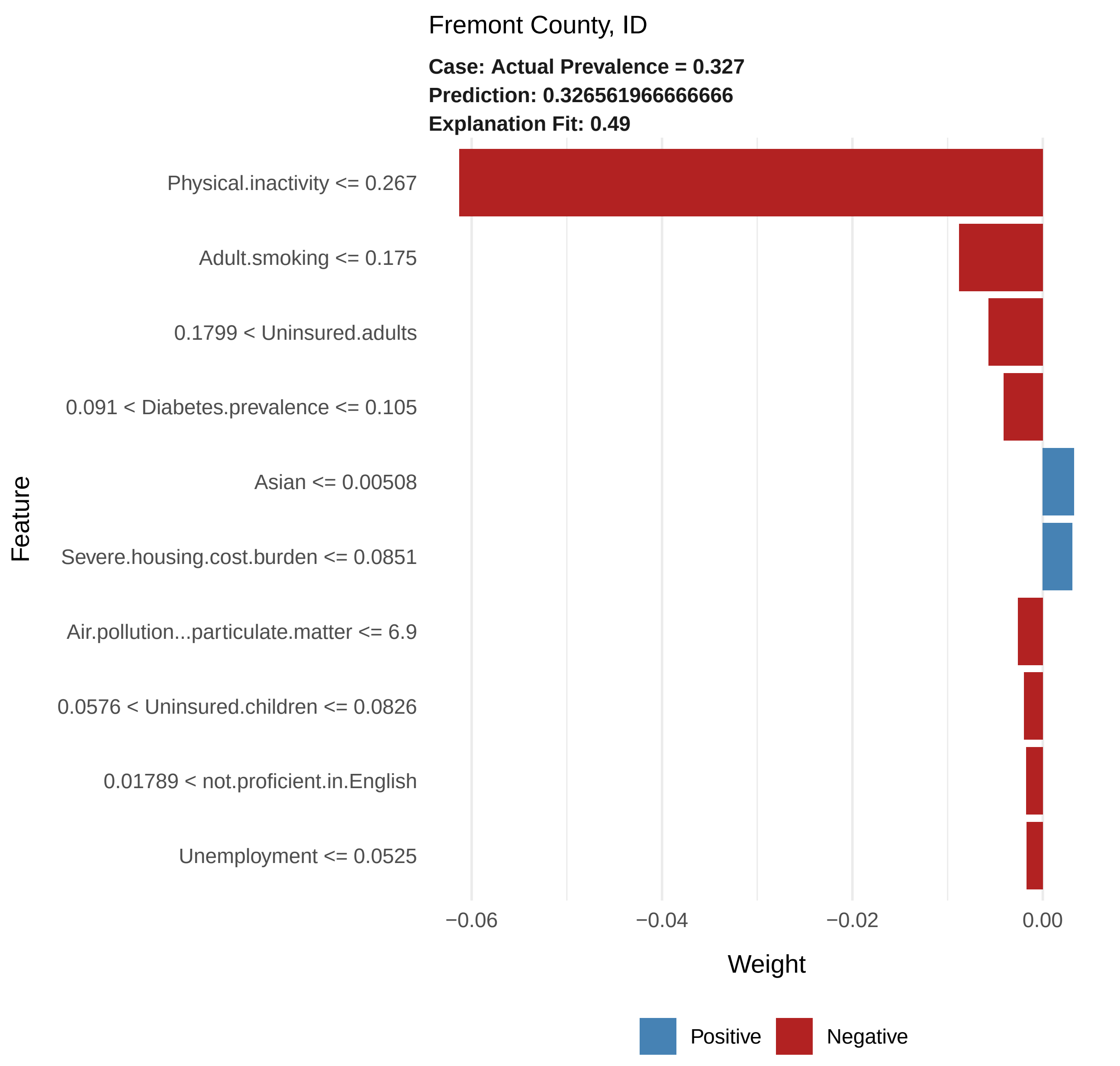

Fremont County, ID
Case: Actual Prevalence = 0.327
Prediction: 0.326561966666666
Explanation Fit: 0.49
Physical.inactivity <= 0.267
Adult.smoking <= 0.175
0.1799 < Uninsured.adults
0.091 < Diabetes.prevalence <= 0.105
Asian <= 0.00508
Severe.housing.cost.burden <= 0.0851
Air.pollution...particulate.matter <= 6.9
0.0576 < Uninsured.children <= 0.0826
0.01789 < not.proficient.in.English
Unemployment <= 0.0525
Feature
−0.06
−0.04
−0.02
0.00
Weight
Positive
Negative

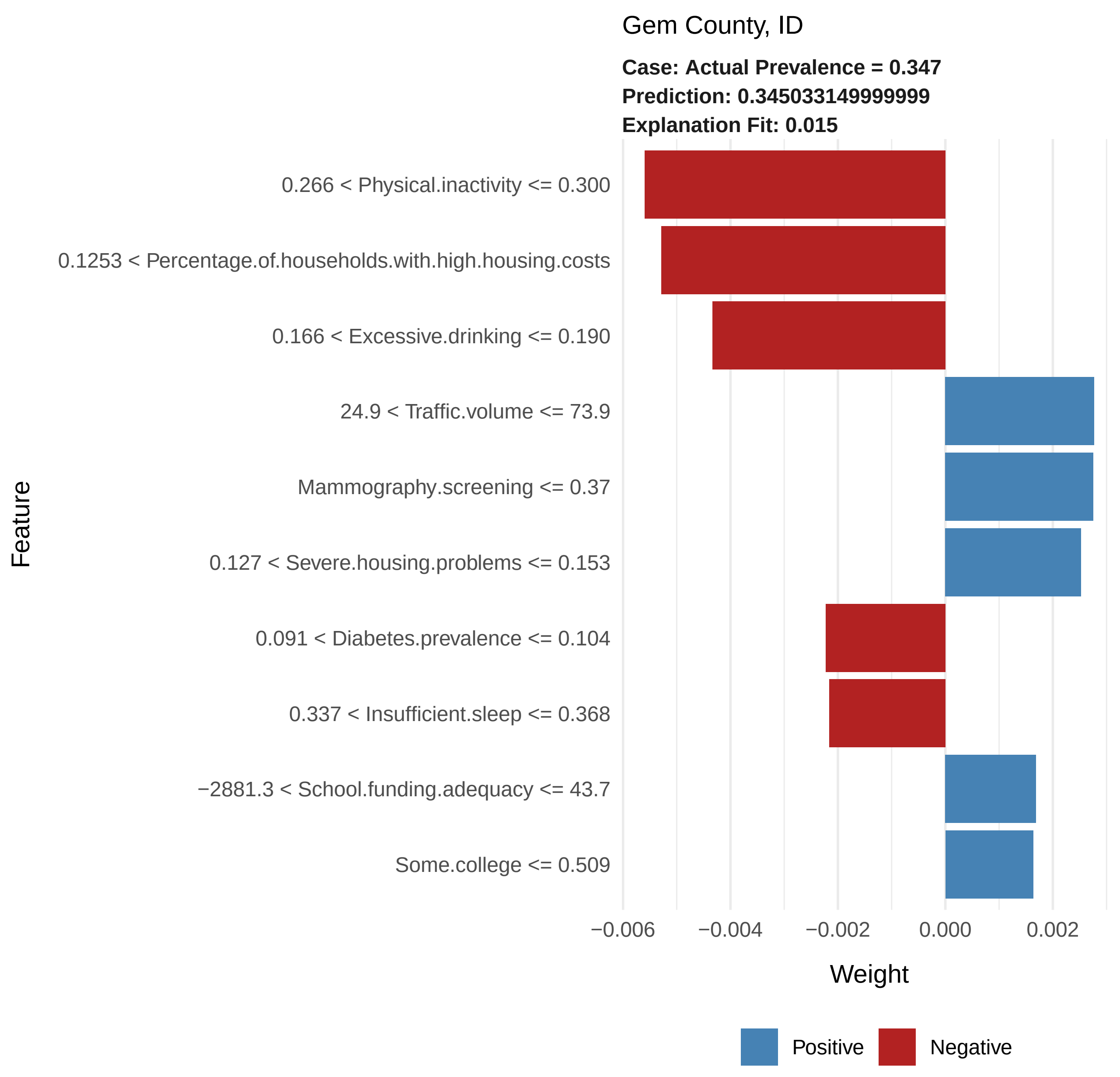

Gem County, ID
Case: Actual Prevalence = 0.347
Prediction: 0.345033149999999
Explanation Fit: 0.015
0.266 < Physical.inactivity <= 0.300
0.1253 < Percentage.of.households.with.high.housing.costs
0.166 < Excessive.drinking <= 0.190
24.9 < Traffic.volume <= 73.9
Mammography.screening <= 0.37
0.127 < Severe.housing.problems <= 0.153
0.091 < Diabetes.prevalence <= 0.104
0.337 < Insufficient.sleep <= 0.368
−2881.3 < School.funding.adequacy <= 43.7
Some.college <= 0.509
Feature
−0.006
−0.004
−0.002
0.000
0.002
Weight
Positive
Negative

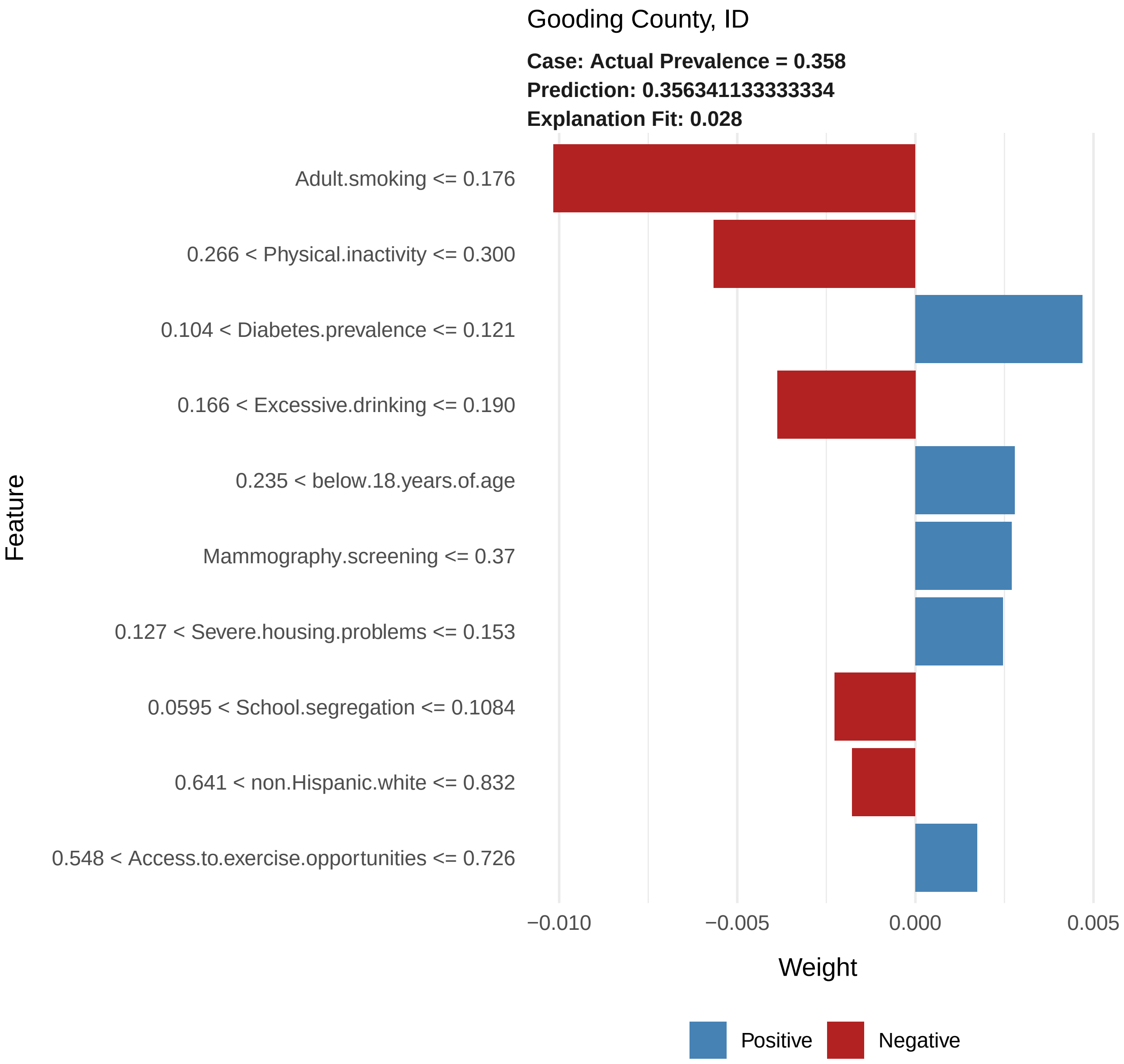

Gooding County, ID
Case: Actual Prevalence = 0.358
Prediction: 0.356341133333334
Explanation Fit: 0.028
Adult.smoking <= 0.176
0.266 < Physical.inactivity <= 0.300
0.104 < Diabetes.prevalence <= 0.121
0.166 < Excessive.drinking <= 0.190
0.235 < below.18.years.of.age
Mammography.screening <= 0.37
0.127 < Severe.housing.problems <= 0.153
0.0595 < School.segregation <= 0.1084
0.641 < non.Hispanic.white <= 0.832
0.548 < Access.to.exercise.opportunities <= 0.726
Feature
−0.010
−0.005
0.000
0.005
Weight
Positive
Negative

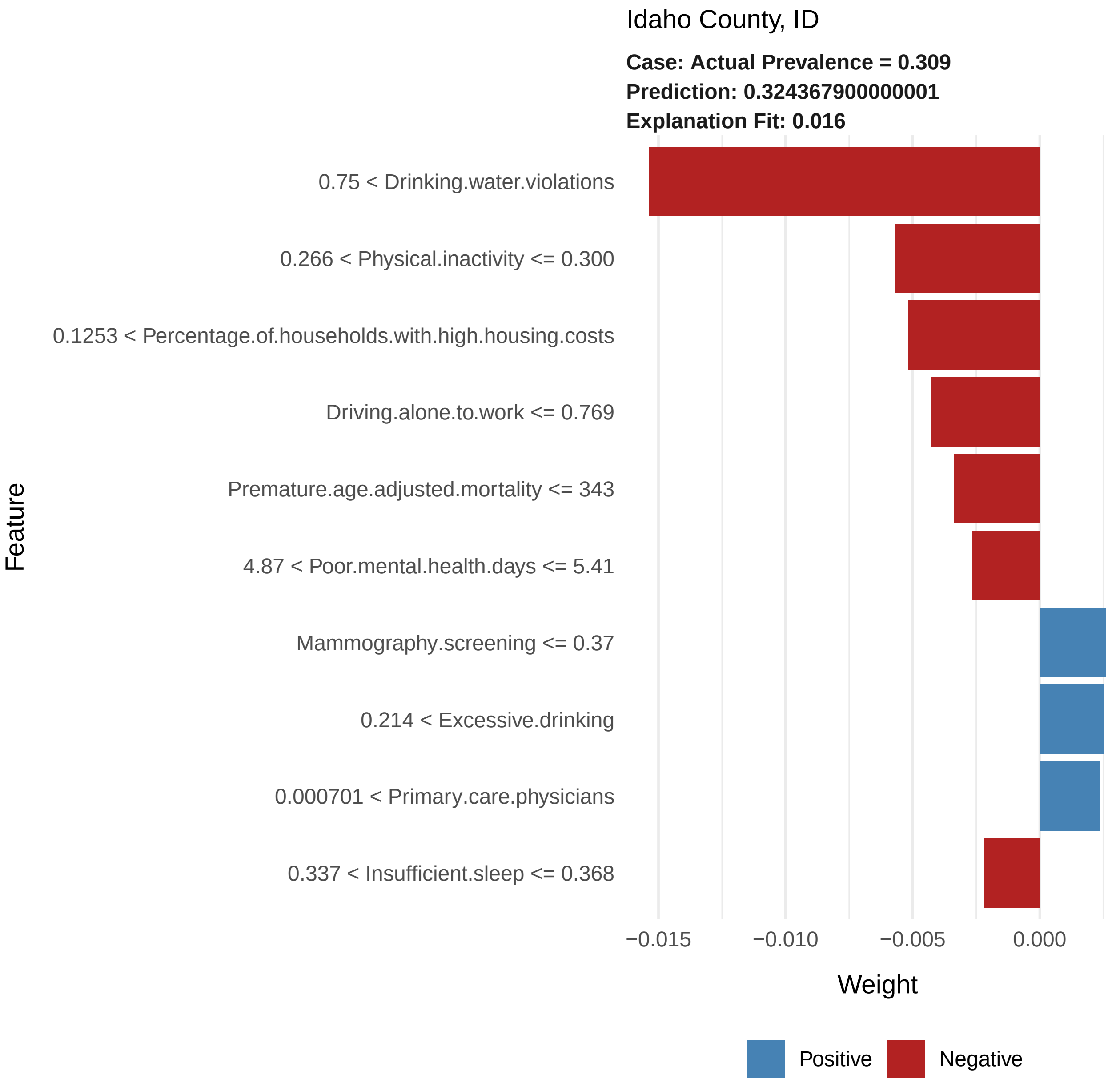

Idaho County, ID
Case: Actual Prevalence = 0.309
Prediction: 0.324367900000001
Explanation Fit: 0.016
Feature
0.75 < Drinking.water.violations
0.266 < Physical.inactivity <= 0.300
0.1253 < Percentage.of.households.with.high.housing.costs
Driving.alone.to.work <= 0.769
Premature.age.adjusted.mortality <= 343
4.87 < Poor.mental.health.days <= 5.41
Mammography.screening <= 0.37
0.214 < Excessive.drinking
0.000701 < Primary.care.physicians
0.337 < Insufficient.sleep <= 0.368
−0.015
−0.010
−0.005
0.000
Weight
Positive
Negative

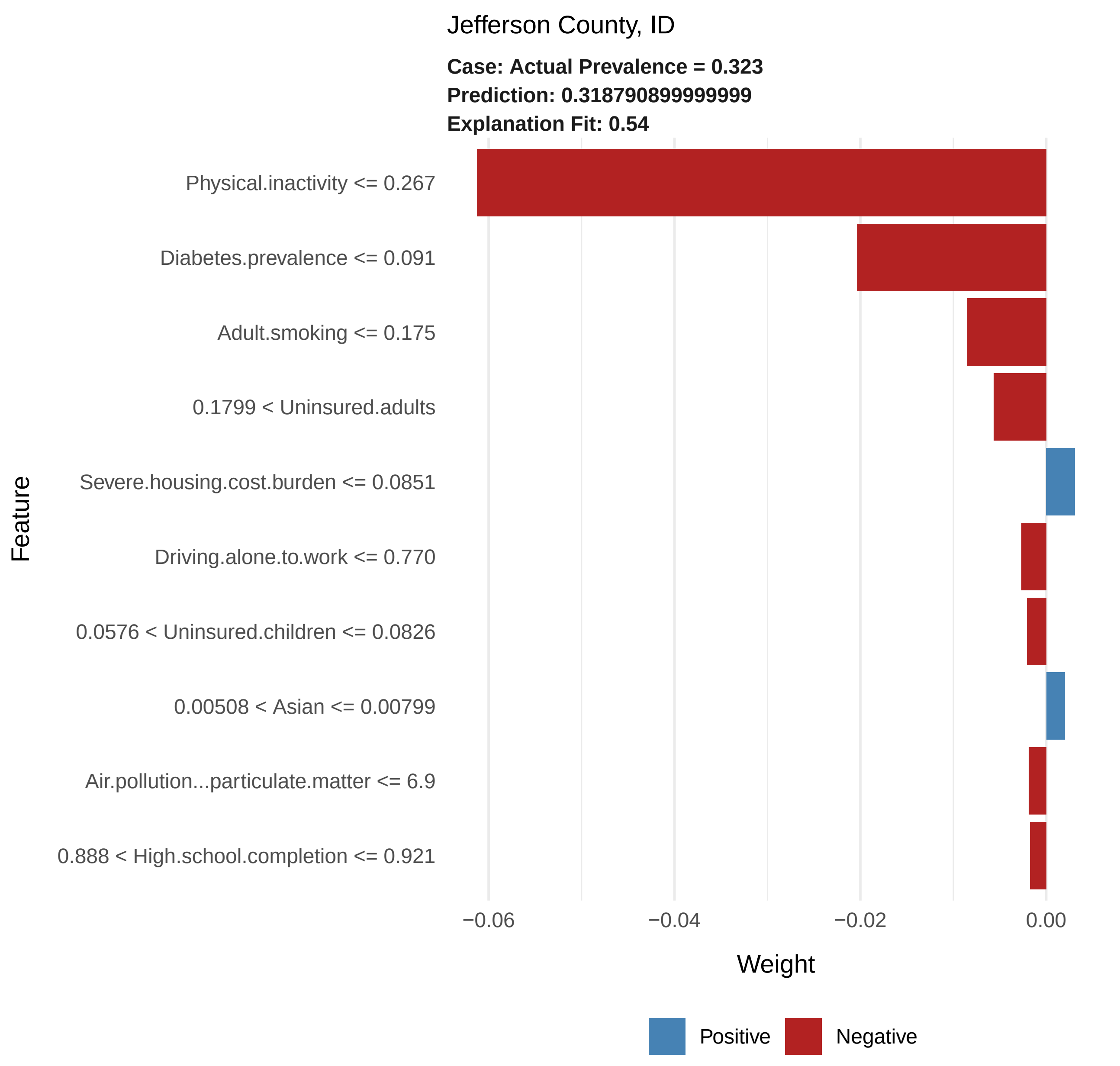
Jefferson County, ID
Case: Actual Prevalence = 0.323
Prediction: 0.318790899999999
Explanation Fit: 0.54
Physical.inactivity <= 0.267
Diabetes.prevalence <= 0.091
Adult.smoking <= 0.175
0.1799 < Uninsured.adults
Severe.housing.cost.burden <= 0.0851
Driving.alone.to.work <= 0.770
0.0576 < Uninsured.children <= 0.0826
0.00508 < Asian <= 0.00799
Air.pollution...particulate.matter <= 6.9
0.888 < High.school.completion <= 0.921
Feature
−0.06
−0.04
−0.02
0.00
Weight
Positive
Negative

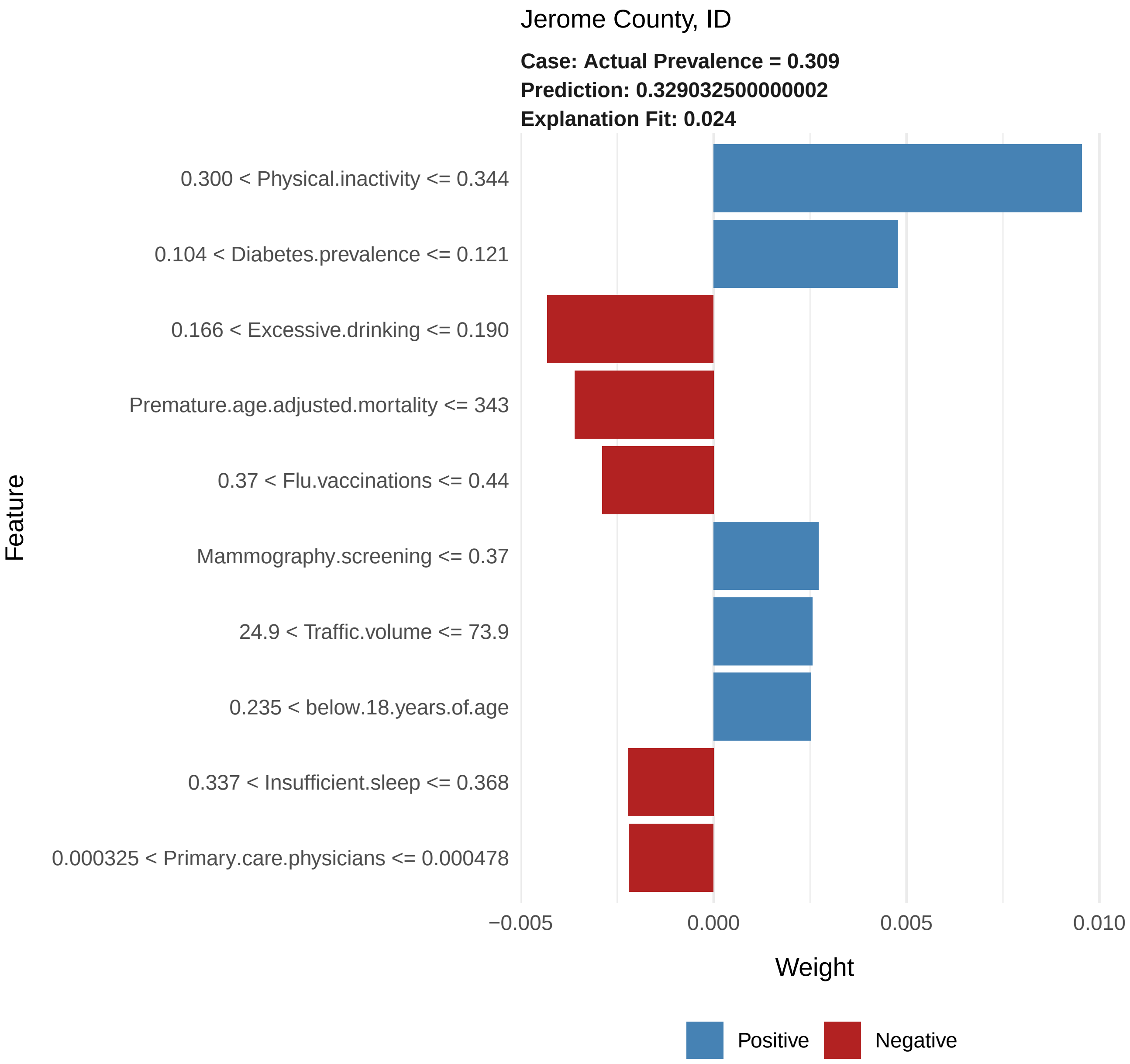

Jerome County, ID
Case: Actual Prevalence = 0.309
Prediction: 0.329032500000002
Explanation Fit: 0.024
Feature
0.300 < Physical.inactivity <= 0.344
0.104 < Diabetes.prevalence <= 0.121
0.166 < Excessive.drinking <= 0.190
Premature.age.adjusted.mortality <= 343
0.37 < Flu.vaccinations <= 0.44
Mammography.screening <= 0.37
24.9 < Traffic.volume <= 73.9
0.235 < below.18.years.of.age
0.337 < Insufficient.sleep <= 0.368
0.000325 < Primary.care.physicians <= 0.000478
−0.005
0.000
0.005
0.010
Weight
Positive
Negative

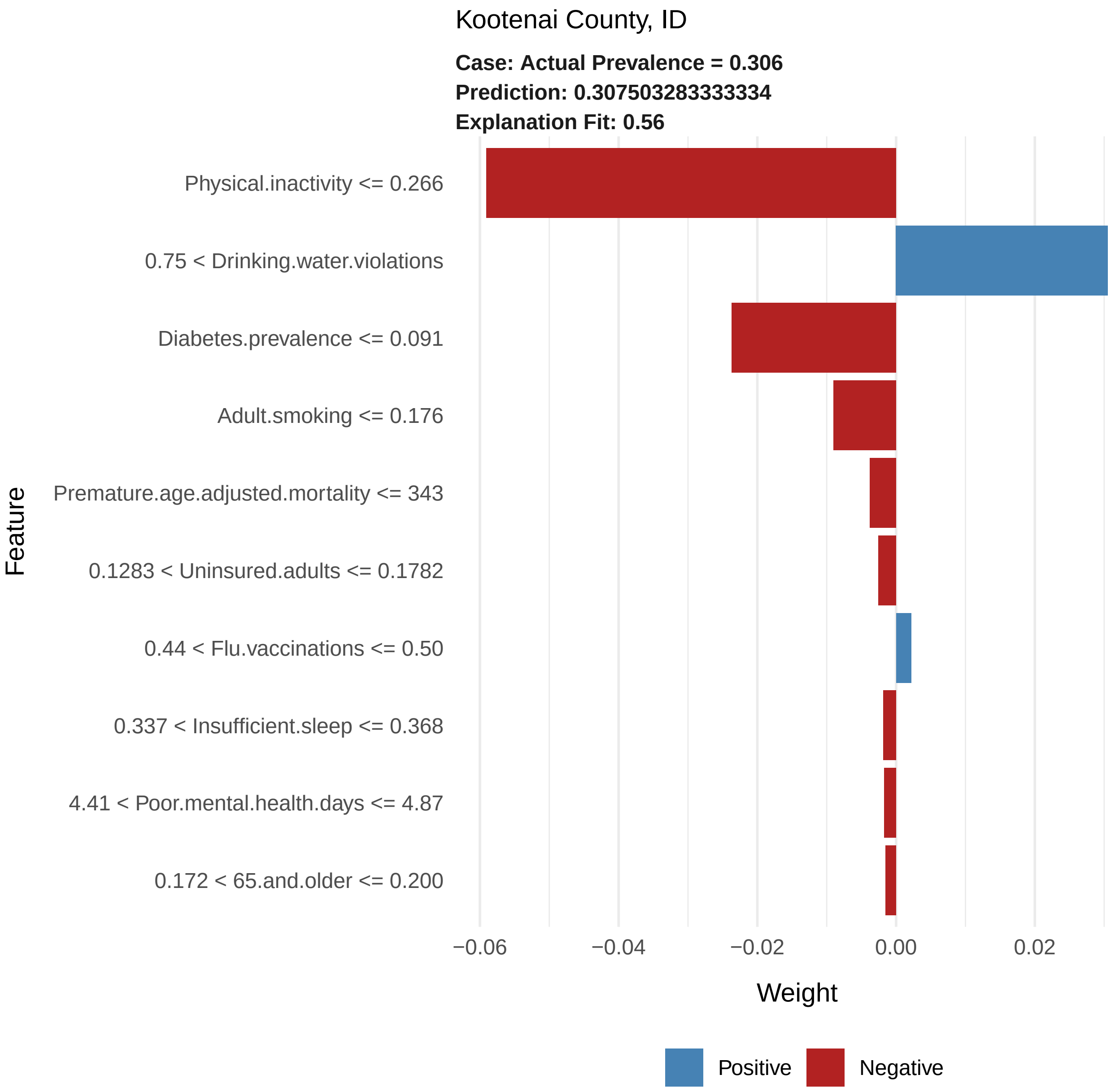
Kootenai County, ID
Case: Actual Prevalence = 0.306
Prediction: 0.307503283333334
Explanation Fit: 0.56
Physical.inactivity <= 0.266
0.75 < Drinking.water.violations
Diabetes.prevalence <= 0.091
Adult.smoking <= 0.176
Premature.age.adjusted.mortality <= 343
0.1283 < Uninsured.adults <= 0.1782
0.44 < Flu.vaccinations <= 0.50
0.337 < Insufficient.sleep <= 0.368
4.41 < Poor.mental.health.days <= 4.87
0.172 < 65.and.older <= 0.200
Feature
−0.06
−0.04
−0.02
0.00
0.02
Weight
Positive
Negative

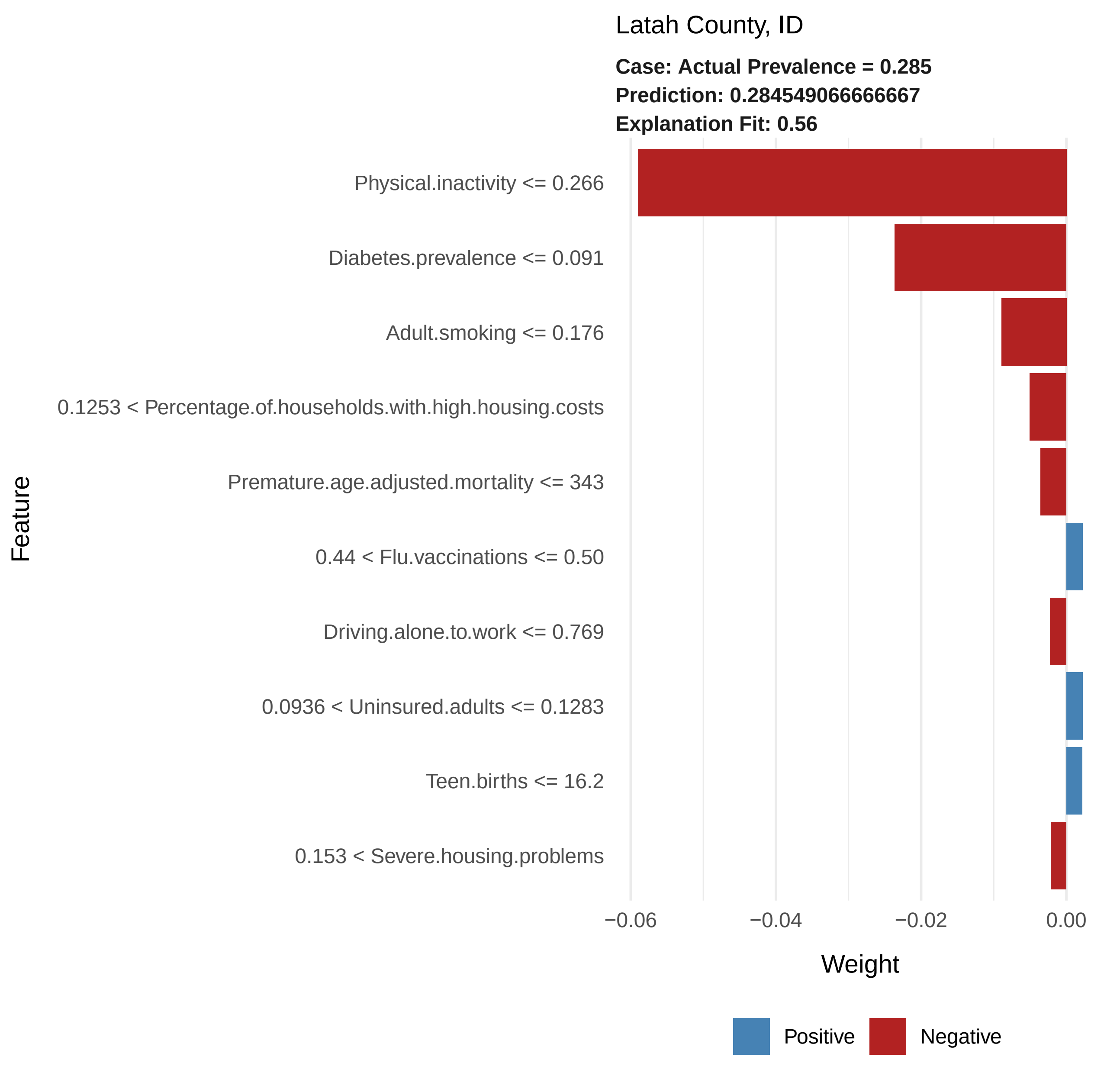
Latah County, ID
Case: Actual Prevalence = 0.285
Prediction: 0.284549066666667
Explanation Fit: 0.56
Physical.inactivity <= 0.266
Diabetes.prevalence <= 0.091
Adult.smoking <= 0.176
0.1253 < Percentage.of.households.with.high.housing.costs
Premature.age.adjusted.mortality <= 343
0.44 < Flu.vaccinations <= 0.50
Driving.alone.to.work <= 0.769
0.0936 < Uninsured.adults <= 0.1283
Teen.births <= 16.2
0.153 < Severe.housing.problems
Feature
−0.06
−0.04
−0.02
0.00
Weight
Positive
Negative

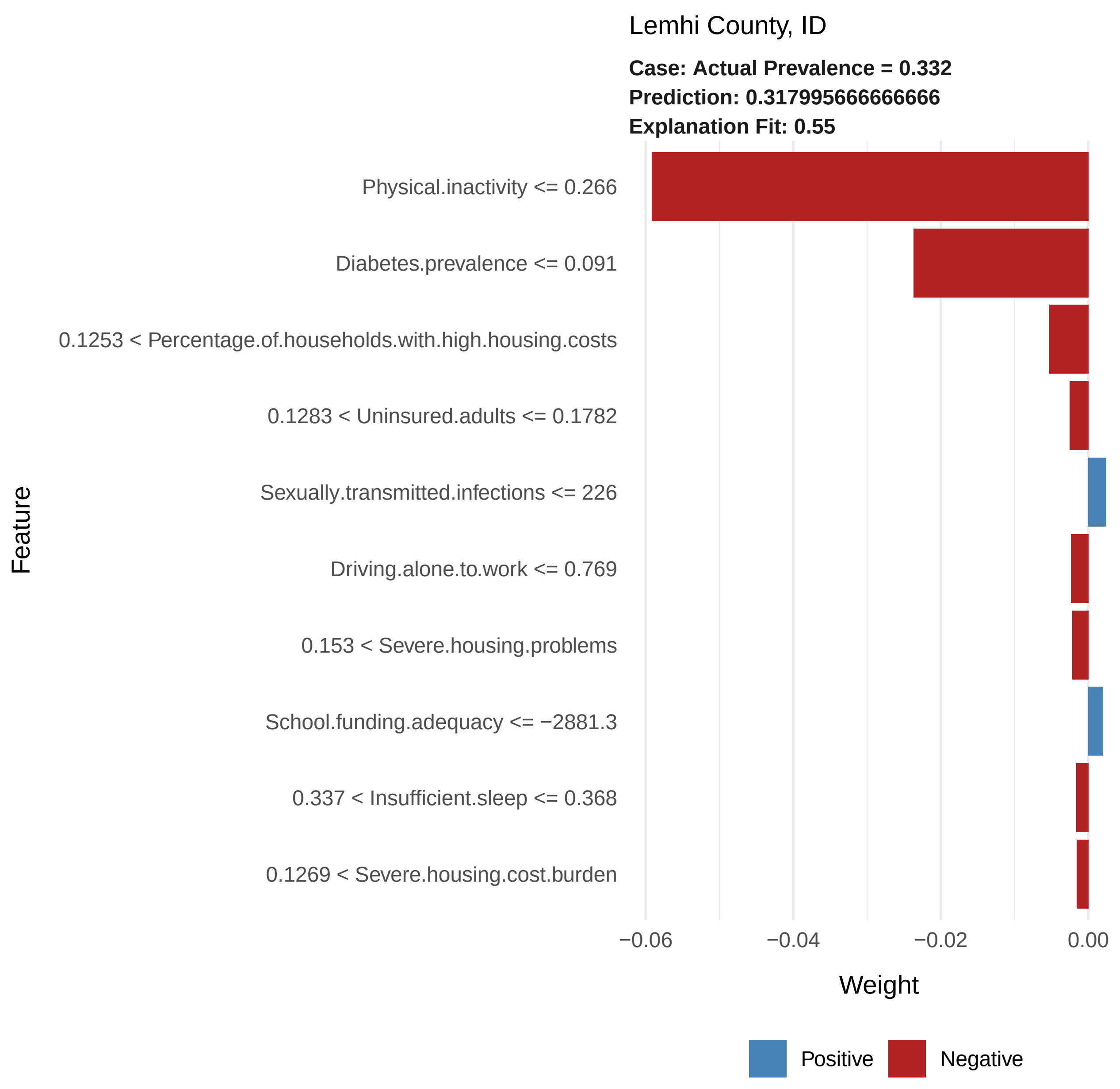

Lemhi County, ID
Case: Actual Prevalence = 0.332
Prediction: 0.317995666666666
Explanation Fit: 0.55
Physical.inactivity <= 0.266
Diabetes.prevalence <= 0.091
0.1253 < Percentage.of.households.with.high.housing.costs
0.1283 < Uninsured.adults <= 0.1782
Sexually.transmitted.infections <= 226
Driving.alone.to.work <= 0.769
0.153 < Severe.housing.problems
School.funding.adequacy <= −2881.3
0.337 < Insufficient.sleep <= 0.368
0.1269 < Severe.housing.cost.burden
Feature
−0.06
−0.04
−0.02
0.00
Weight
Positive
Negative

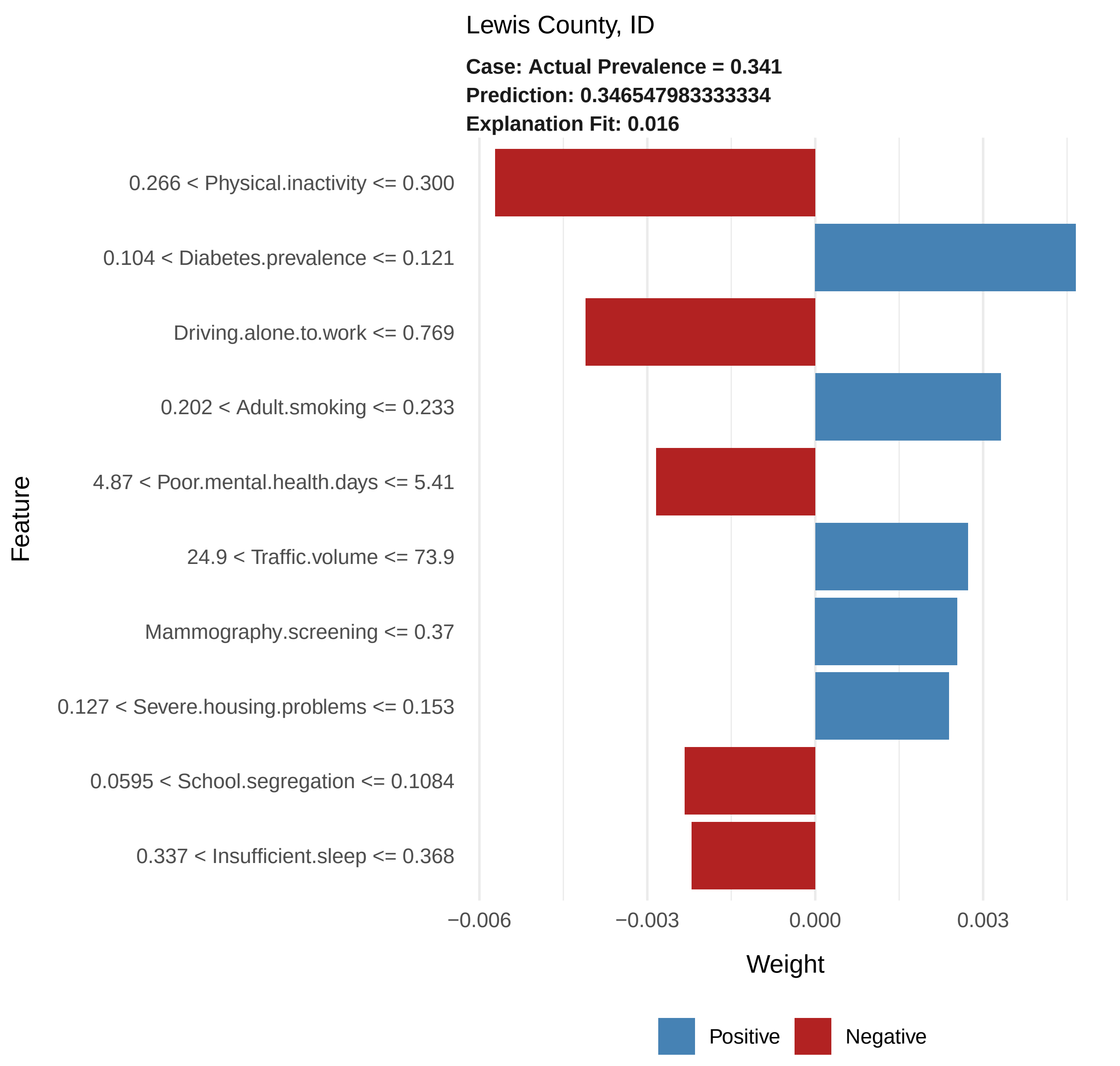
Lewis County, ID
Case: Actual Prevalence = 0.341
Prediction: 0.346547983333334
Explanation Fit: 0.016
0.266 < Physical.inactivity <= 0.300
0.104 < Diabetes.prevalence <= 0.121
Driving.alone.to.work <= 0.769
0.202 < Adult.smoking <= 0.233
4.87 < Poor.mental.health.days <= 5.41
24.9 < Traffic.volume <= 73.9
Mammography.screening <= 0.37
0.127 < Severe.housing.problems <= 0.153
0.0595 < School.segregation <= 0.1084
0.337 < Insufficient.sleep <= 0.368
Feature
−0.006
−0.003
0.000
0.003
Weight
Positive
Negative

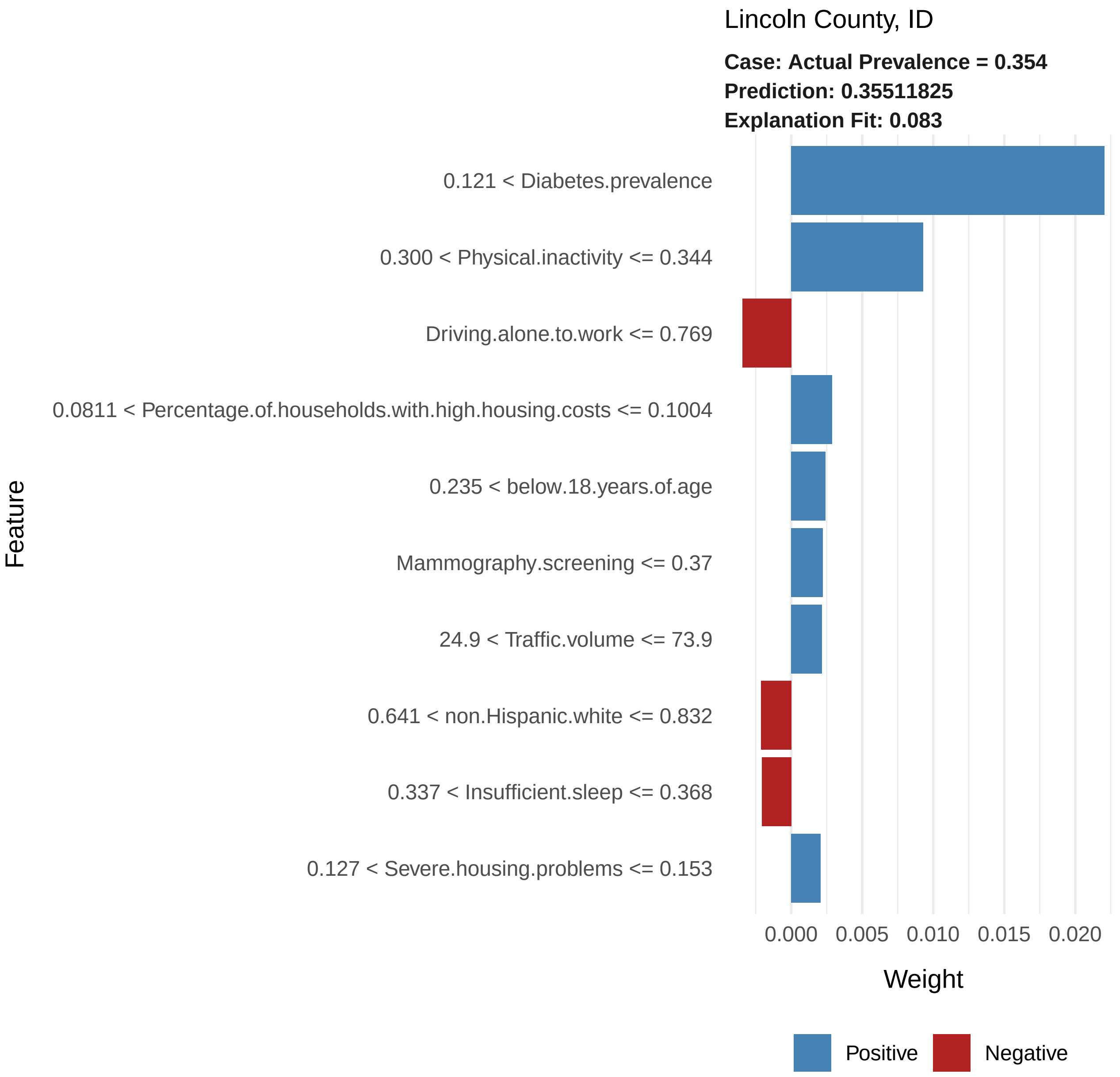
Lincoln County, ID
Case: Actual Prevalence = 0.354
Prediction: 0.35511825
Explanation Fit: 0.083
0.121 < Diabetes.prevalence
0.300 < Physical.inactivity <= 0.344
Driving.alone.to.work <= 0.769
0.0811 < Percentage.of.households.with.high.housing.costs <= 0.1004
0.235 < below.18.years.of.age
Mammography.screening <= 0.37
24.9 < Traffic.volume <= 73.9
0.641 < non.Hispanic.white <= 0.832
0.337 < Insufficient.sleep <= 0.368
0.127 < Severe.housing.problems <= 0.153
Feature
0.000
0.005
0.010
0.015
0.020
Weight
Positive
Negative

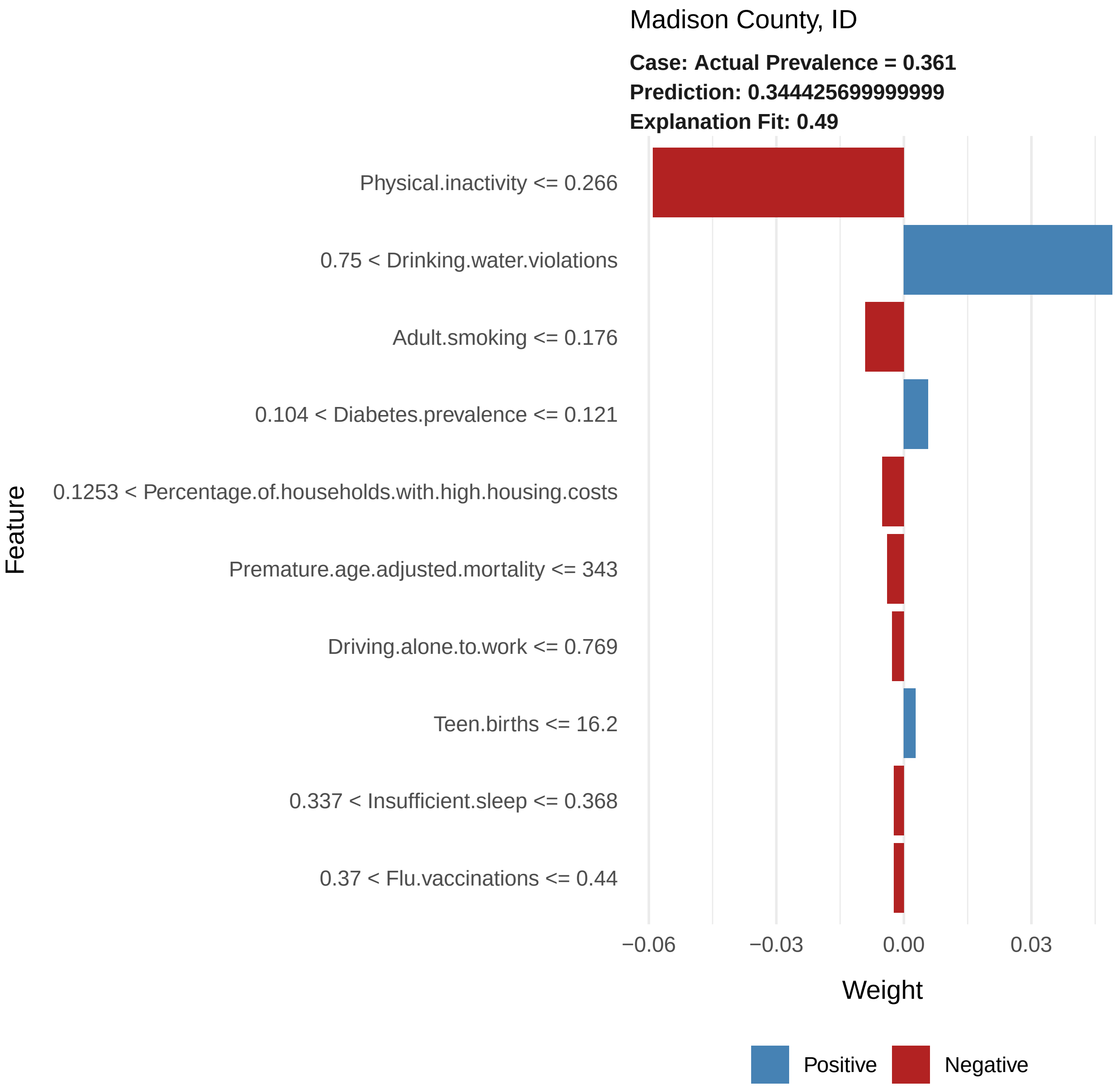

Madison County, ID
Case: Actual Prevalence = 0.361
Prediction: 0.344425699999999
Explanation Fit: 0.49
Physical.inactivity <= 0.266
0.75 < Drinking.water.violations
Adult.smoking <= 0.176
0.104 < Diabetes.prevalence <= 0.121
0.1253 < Percentage.of.households.with.high.housing.costs
Premature.age.adjusted.mortality <= 343
Driving.alone.to.work <= 0.769
Teen.births <= 16.2
0.337 < Insufficient.sleep <= 0.368
0.37 < Flu.vaccinations <= 0.44
Feature
−0.06
−0.03
0.00
0.03
Weight
Positive
Negative

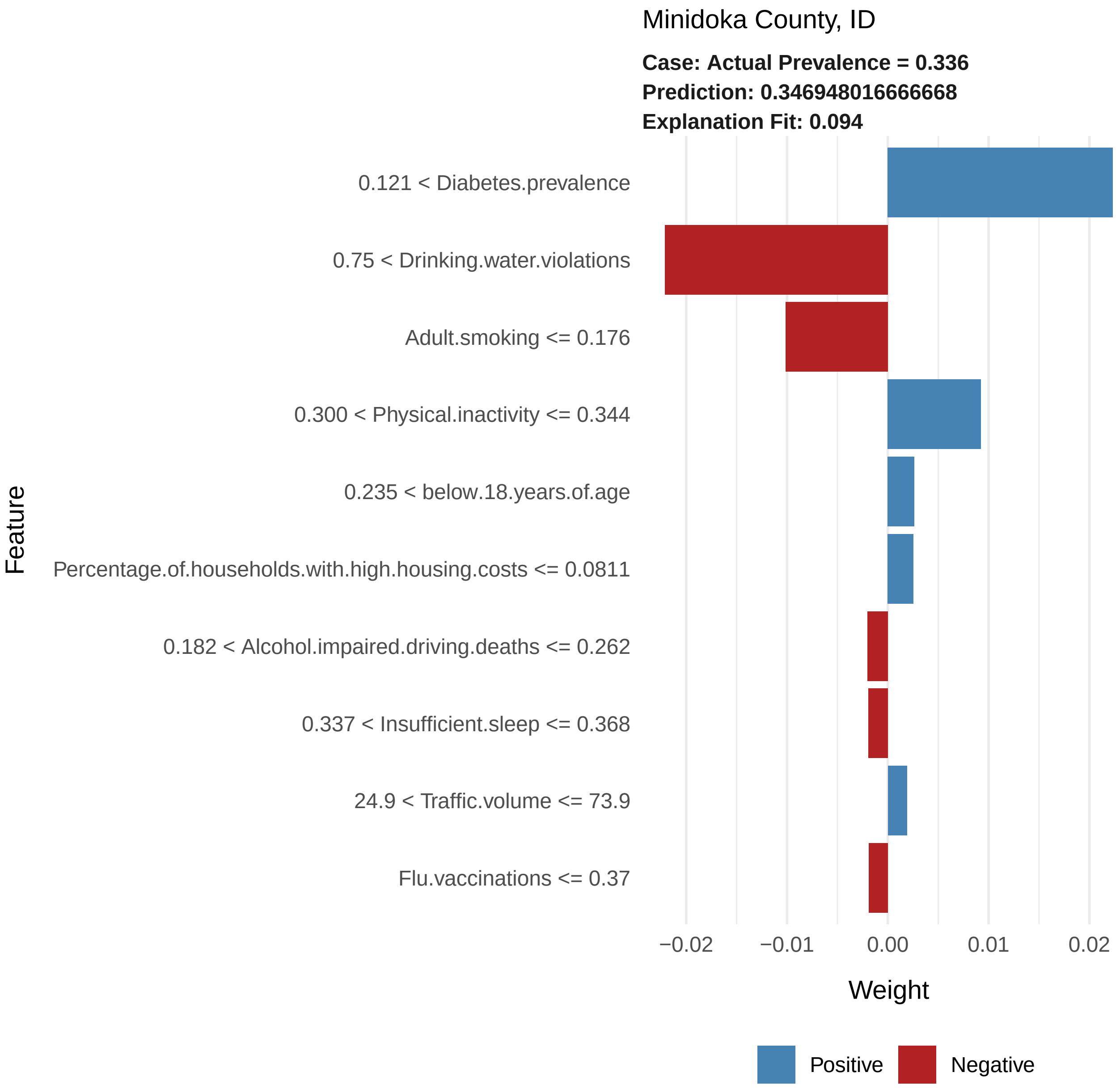

Minidoka County, ID
Case: Actual Prevalence = 0.336
Prediction: 0.346948016666668
Explanation Fit: 0.094
0.121 < Diabetes.prevalence
0.75 < Drinking.water.violations
Adult.smoking <= 0.176
0.300 < Physical.inactivity <= 0.344
0.235 < below.18.years.of.age
Percentage.of.households.with.high.housing.costs <= 0.0811
0.182 < Alcohol.impaired.driving.deaths <= 0.262
0.337 < Insufficient.sleep <= 0.368
24.9 < Traffic.volume <= 73.9
Flu.vaccinations <= 0.37
Feature
−0.02
−0.01
0.00
0.01
0.02
Weight
Positive
Negative

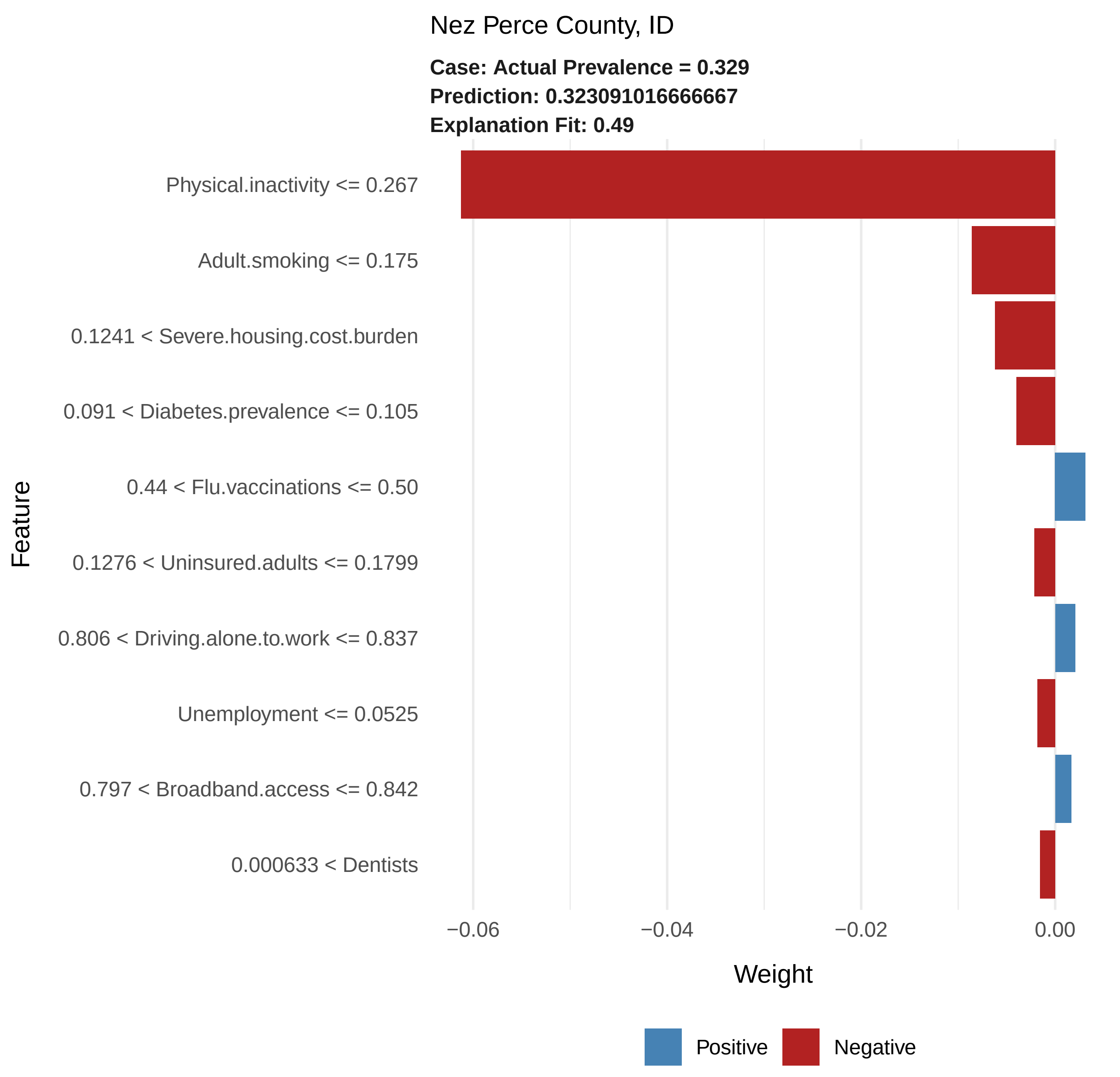

Nez Perce County, ID
Case: Actual Prevalence = 0.329
Prediction: 0.323091016666667
Explanation Fit: 0.49
Physical.inactivity <= 0.267
Adult.smoking <= 0.175
0.1241 < Severe.housing.cost.burden
0.091 < Diabetes.prevalence <= 0.105
0.44 < Flu.vaccinations <= 0.50
0.1276 < Uninsured.adults <= 0.1799
0.806 < Driving.alone.to.work <= 0.837
Unemployment <= 0.0525
0.797 < Broadband.access <= 0.842
0.000633 < Dentists
Feature
−0.06
−0.04
−0.02
0.00
Weight
Positive
Negative

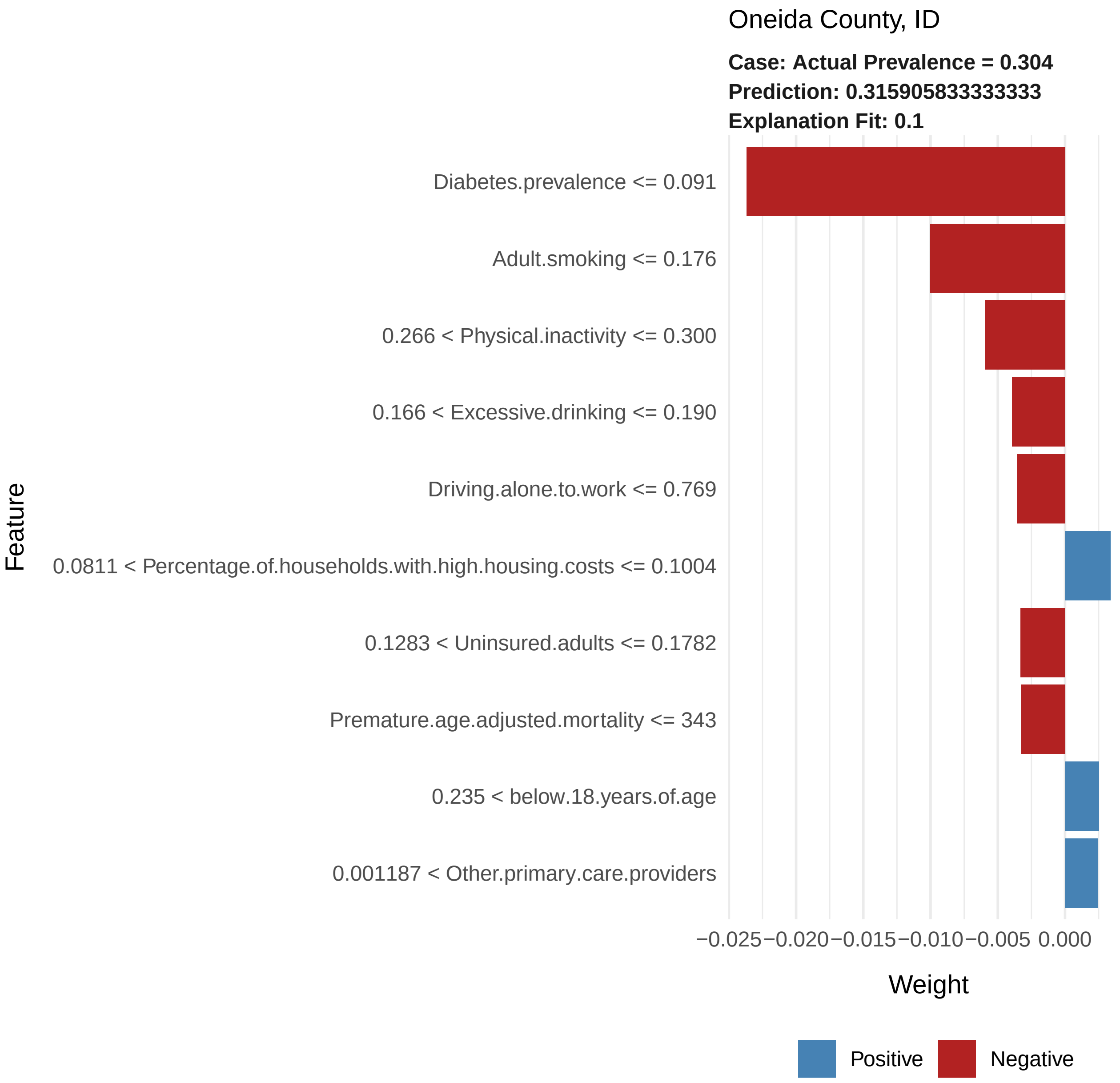
Oneida County, ID
Case: Actual Prevalence = 0.304
Prediction: 0.315905833333333
Explanation Fit: 0.1
Feature
Diabetes.prevalence <= 0.091
Adult.smoking <= 0.176
0.266 < Physical.inactivity <= 0.300
0.166 < Excessive.drinking <= 0.190
Driving.alone.to.work <= 0.769
0.0811 < Percentage.of.households.with.high.housing.costs <= 0.1004
0.1283 < Uninsured.adults <= 0.1782
Premature.age.adjusted.mortality <= 343
0.235 < below.18.years.of.age
0.001187 < Other.primary.care.providers
−0.025
−0.020
−0.015
−0.010
−0.005
0.000
Weight
Positive
Negative

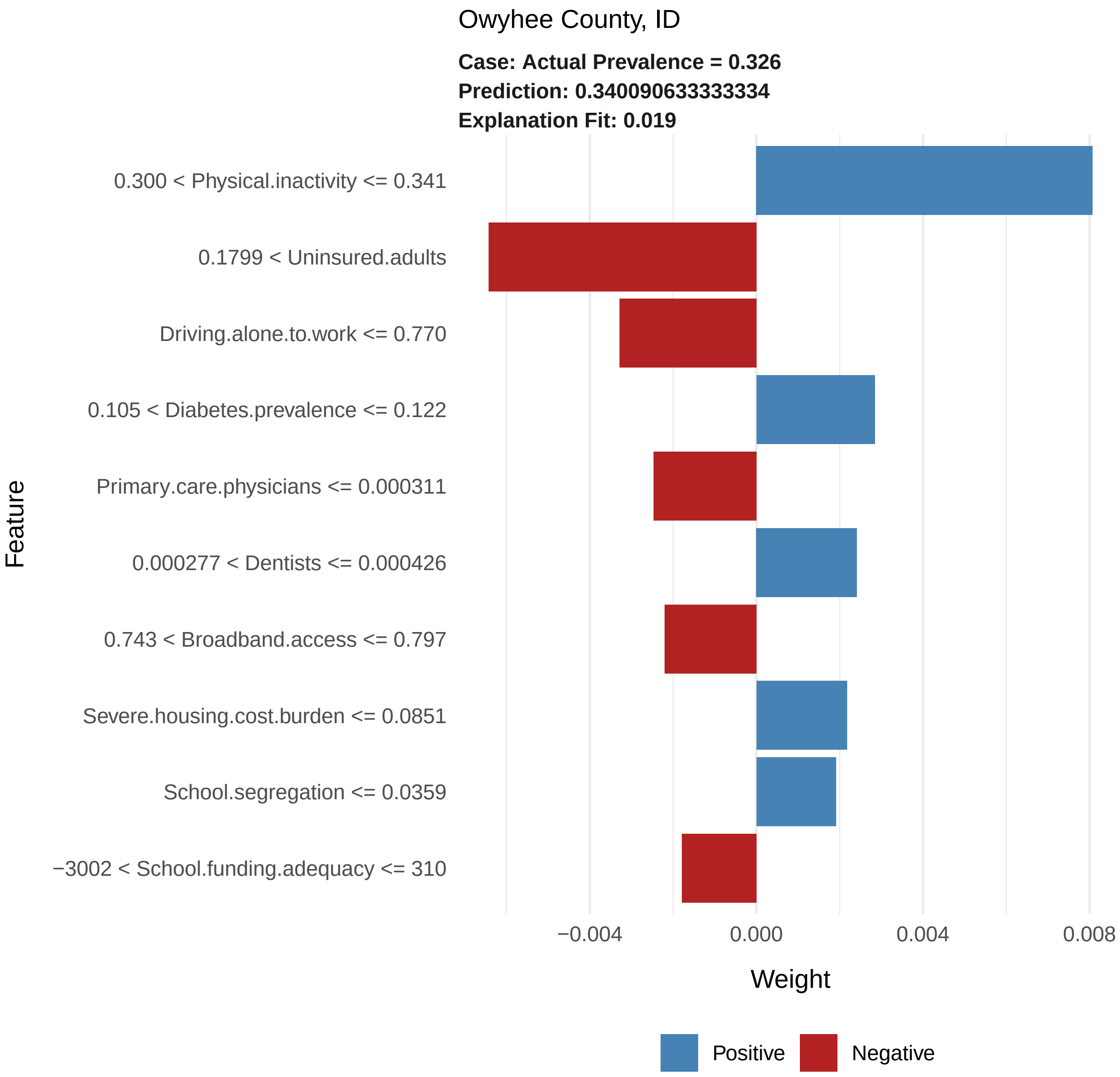

Owyhee County, ID
Case: Actual Prevalence = 0.326
Prediction: 0.340090633333334
Explanation Fit: 0.019
0.300 < Physical.inactivity <= 0.341
0.1799 < Uninsured.adults
Driving.alone.to.work <= 0.770
0.105 < Diabetes.prevalence <= 0.122
Primary.care.physicians <= 0.000311
0.000277 < Dentists <= 0.000426
0.743 < Broadband.access <= 0.797
Severe.housing.cost.burden <= 0.0851
School.segregation <= 0.0359
−3002 < School.funding.adequacy <= 310
Feature
−0.004
0.000
0.004
0.008
Weight
Positive
Negative

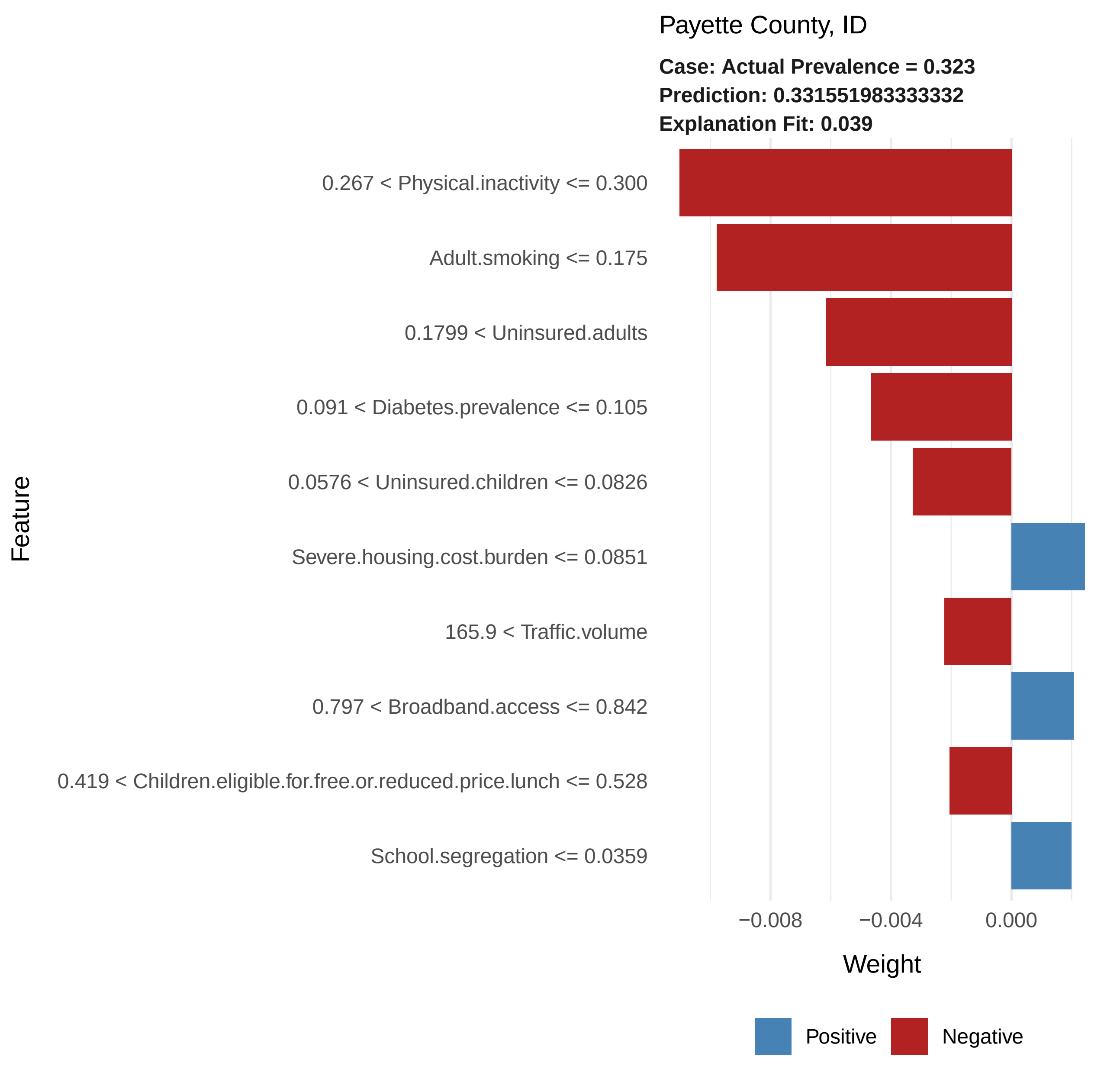

Payette County, ID
Case: Actual Prevalence = 0.323
Prediction: 0.331551983333332
Explanation Fit: 0.039
0.267 < Physical.inactivity <= 0.300
Adult.smoking <= 0.175
0.1799 < Uninsured.adults
0.091 < Diabetes.prevalence <= 0.105
0.0576 < Uninsured.children <= 0.0826
Severe.housing.cost.burden <= 0.0851
165.9 < Traffic.volume
0.797 < Broadband.access <= 0.842
0.419 < Children.eligible.for.free.or.reduced.price.lunch <= 0.528
School.segregation <= 0.0359
Feature
−0.008
−0.004
0.000
Weight
Positive
Negative

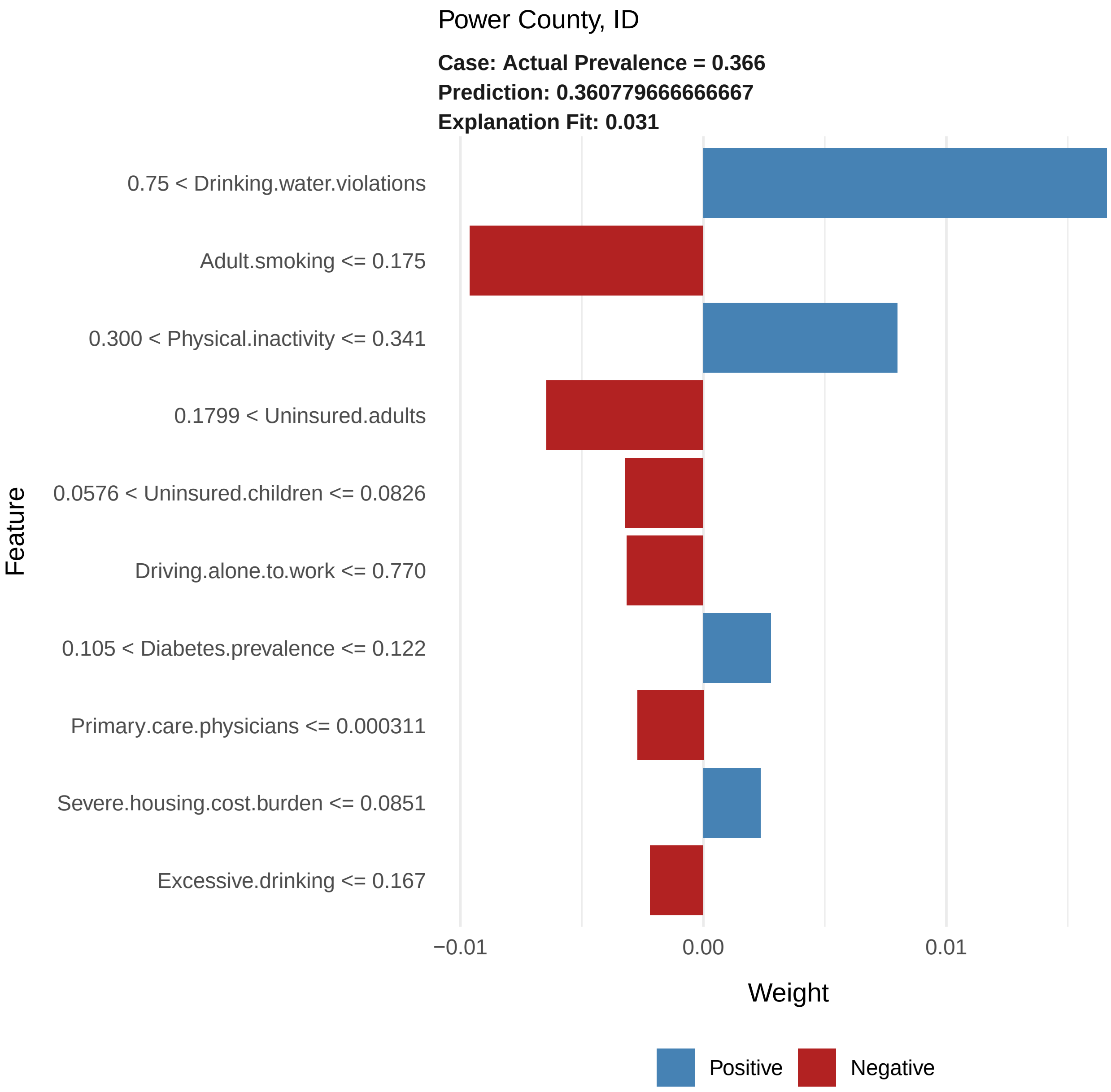

Power County, ID
Case: Actual Prevalence = 0.366
Prediction: 0.360779666666667
Explanation Fit: 0.031
0.75 < Drinking.water.violations
Adult.smoking <= 0.175
0.300 < Physical.inactivity <= 0.341
0.1799 < Uninsured.adults
0.0576 < Uninsured.children <= 0.0826
Driving.alone.to.work <= 0.770
0.105 < Diabetes.prevalence <= 0.122
Primary.care.physicians <= 0.000311
Severe.housing.cost.burden <= 0.0851
Excessive.drinking <= 0.167
Feature
−0.01
0.00
0.01
Weight
Positive
Negative

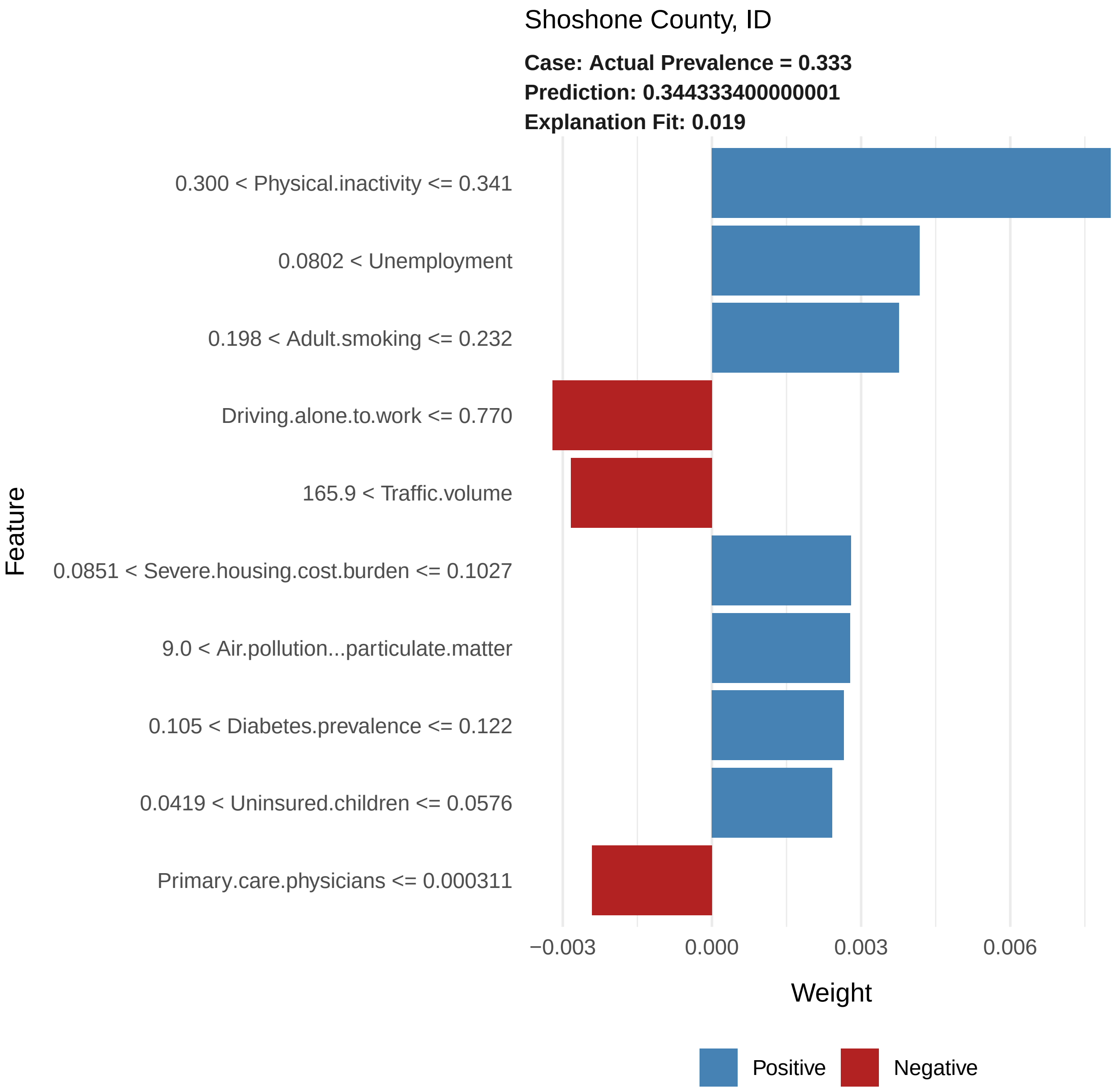

Shoshone County, ID
Case: Actual Prevalence = 0.333
Prediction: 0.344333400000001
Explanation Fit: 0.019
0.300 < Physical.inactivity <= 0.341
0.0802 < Unemployment
0.198 < Adult.smoking <= 0.232
Driving.alone.to.work <= 0.770
165.9 < Traffic.volume
0.0851 < Severe.housing.cost.burden <= 0.1027
9.0 < Air.pollution...particulate.matter
0.105 < Diabetes.prevalence <= 0.122
0.0419 < Uninsured.children <= 0.0576
Primary.care.physicians <= 0.000311
Feature
−0.003
0.000
0.003
0.006
Weight
Positive
Negative

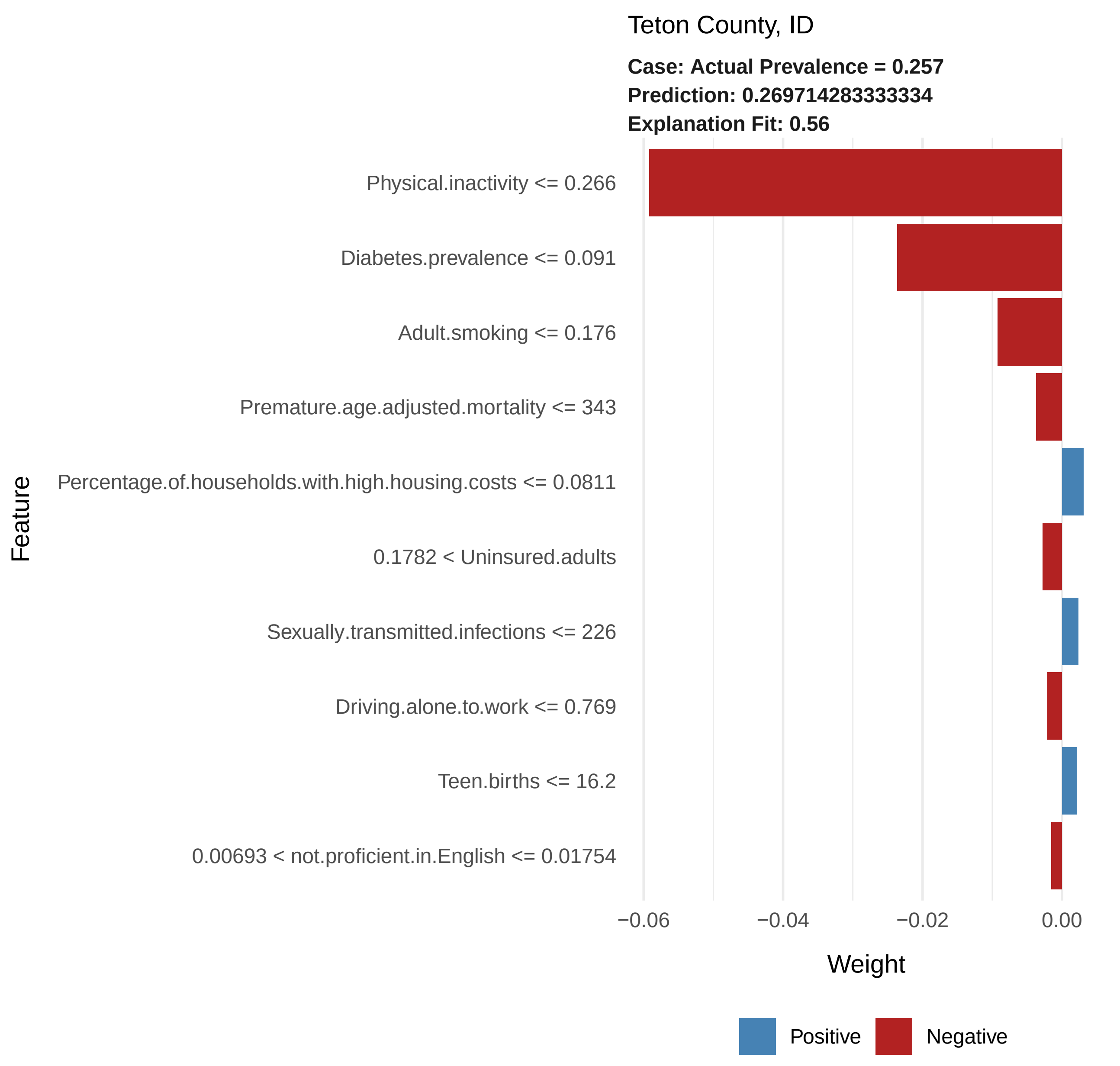

Teton County, ID
Case: Actual Prevalence = 0.257
Prediction: 0.269714283333334
Explanation Fit: 0.56
Physical.inactivity <= 0.266
Diabetes.prevalence <= 0.091
Adult.smoking <= 0.176
Premature.age.adjusted.mortality <= 343
Percentage.of.households.with.high.housing.costs <= 0.0811
0.1782 < Uninsured.adults
Sexually.transmitted.infections <= 226
Driving.alone.to.work <= 0.769
Teen.births <= 16.2
0.00693 < not.proficient.in.English <= 0.01754
Feature
−0.06
−0.04
−0.02
0.00
Weight
Positive
Negative

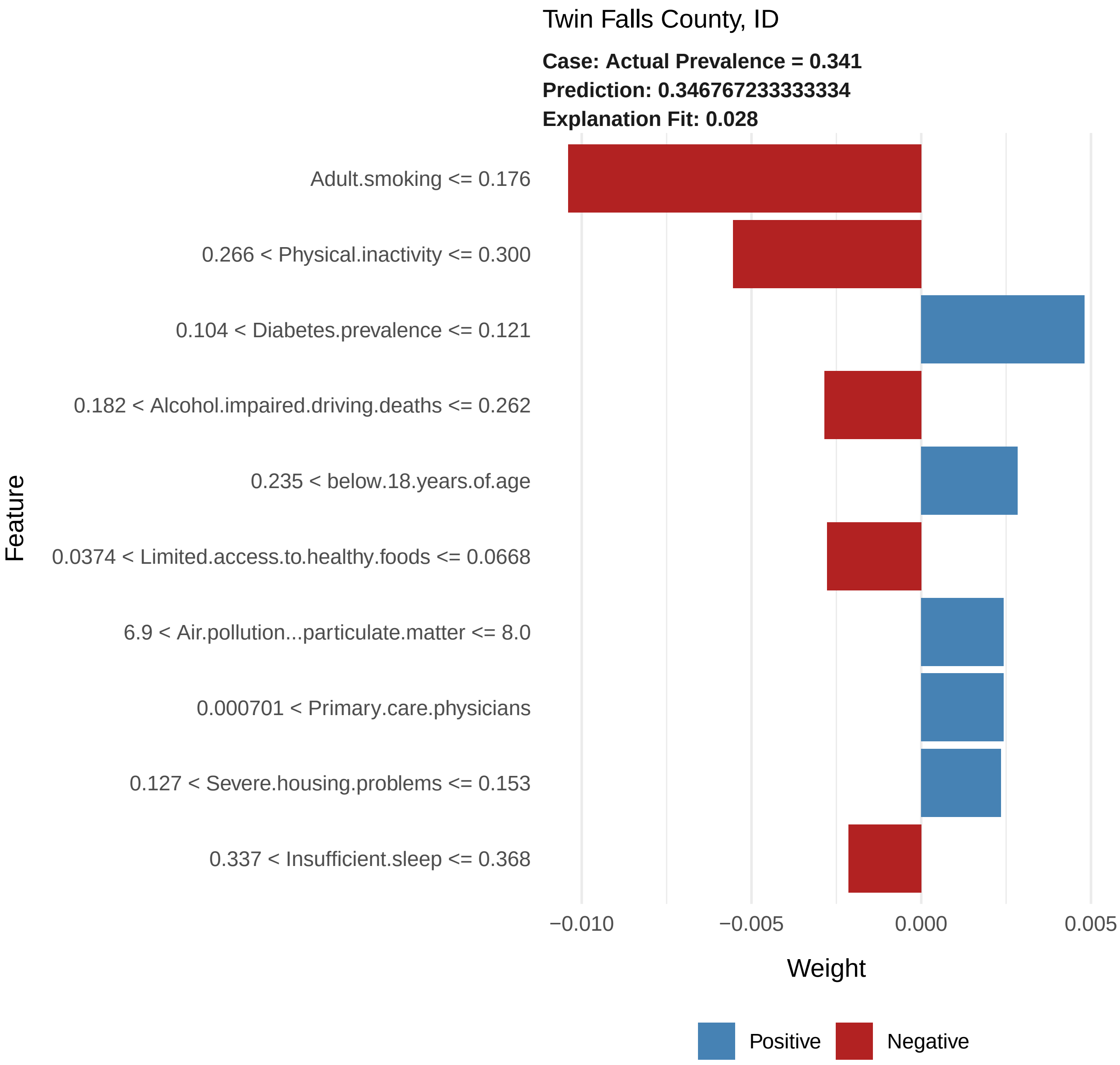
Twin Falls County, ID
Case: Actual Prevalence = 0.341
Prediction: 0.346767233333334
Explanation Fit: 0.028
Adult.smoking <= 0.176
0.266 < Physical.inactivity <= 0.300
0.104 < Diabetes.prevalence <= 0.121
0.182 < Alcohol.impaired.driving.deaths <= 0.262
0.235 < below.18.years.of.age
0.0374 < Limited.access.to.healthy.foods <= 0.0668
6.9 < Air.pollution...particulate.matter <= 8.0
0.000701 < Primary.care.physicians
0.127 < Severe.housing.problems <= 0.153
0.337 < Insufficient.sleep <= 0.368
Feature
−0.010
−0.005
0.000
0.005
Weight
Positive
Negative

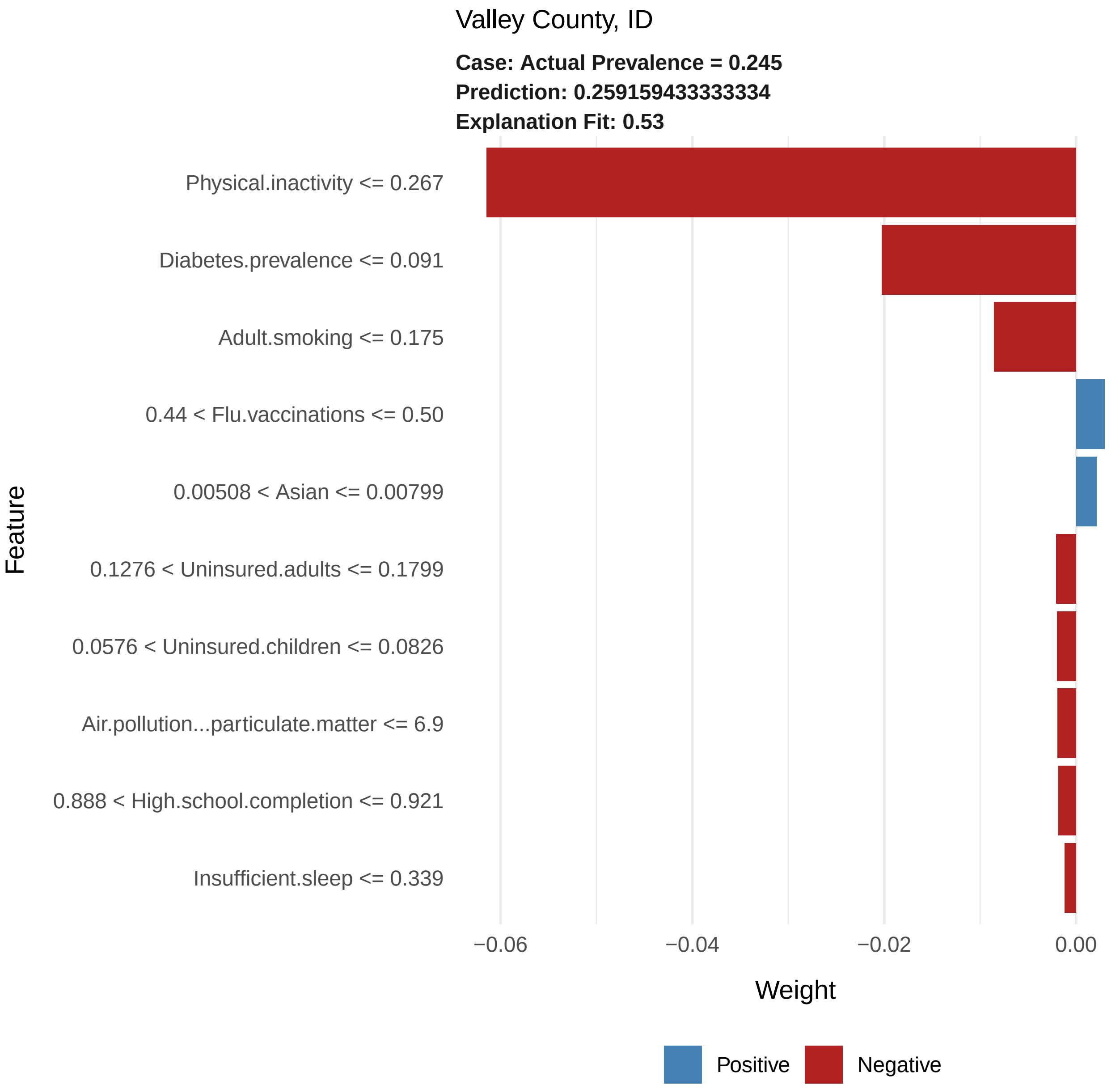

Valley County, ID
Case: Actual Prevalence = 0.245
Prediction: 0.259159433333334
Explanation Fit: 0.53
Physical.inactivity <= 0.267
Diabetes.prevalence <= 0.091
Adult.smoking <= 0.175
0.44 < Flu.vaccinations <= 0.50
0.00508 < Asian <= 0.00799
0.1276 < Uninsured.adults <= 0.1799
0.0576 < Uninsured.children <= 0.0826
Air.pollution...particulate.matter <= 6.9
0.888 < High.school.completion <= 0.921
Insufficient.sleep <= 0.339
Feature
−0.06
−0.04
−0.02
0.00
Weight
Positive
Negative

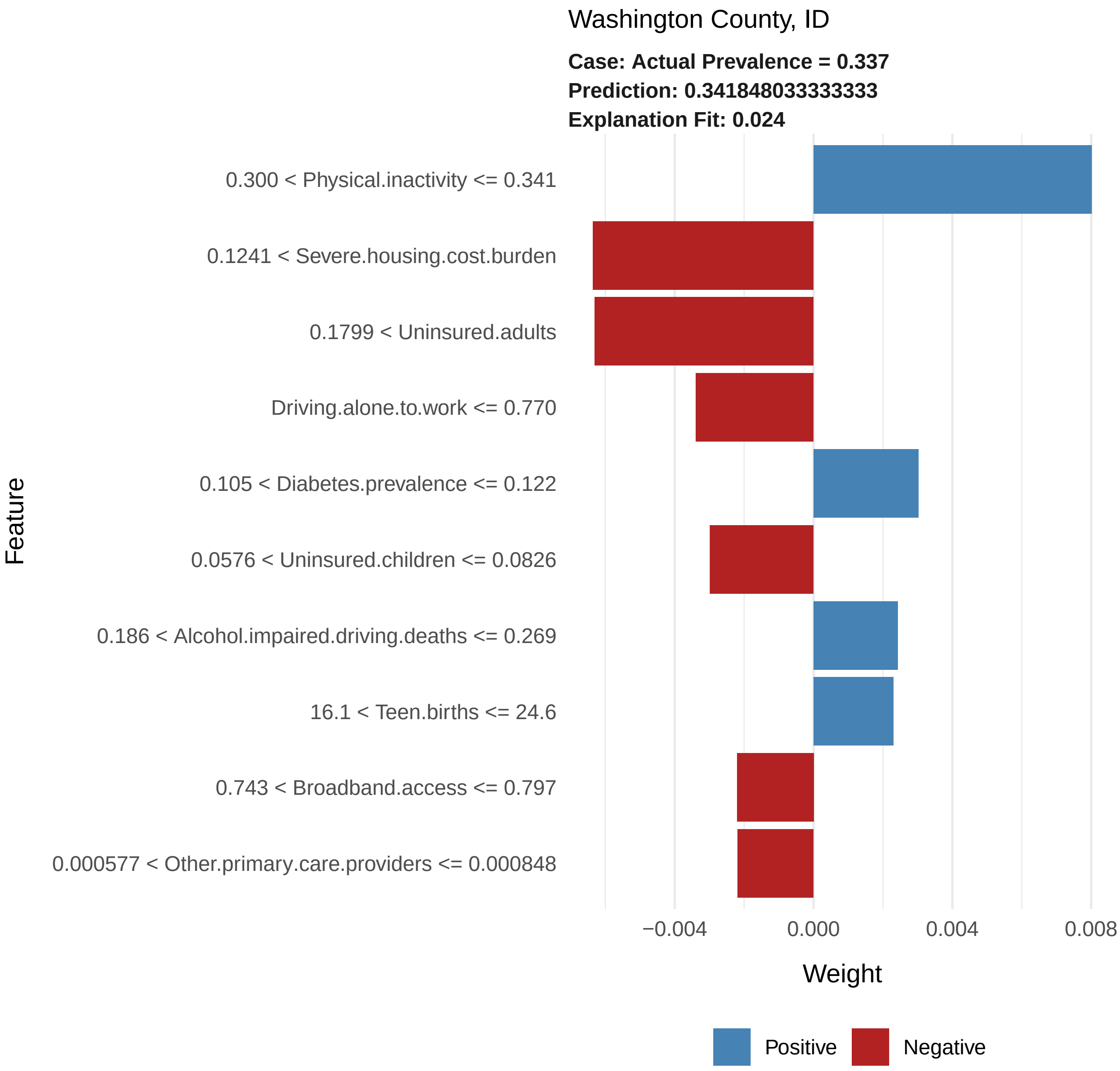

Washington County, ID
Case: Actual Prevalence = 0.337
Prediction: 0.341848033333333
Explanation Fit: 0.024
0.300 < Physical.inactivity <= 0.341
0.1241 < Severe.housing.cost.burden
0.1799 < Uninsured.adults
Driving.alone.to.work <= 0.770
0.105 < Diabetes.prevalence <= 0.122
0.0576 < Uninsured.children <= 0.0826
0.186 < Alcohol.impaired.driving.deaths <= 0.269
16.1 < Teen.births <= 24.6
0.743 < Broadband.access <= 0.797
0.000577 < Other.primary.care.providers <= 0.000848
Feature
−0.004
0.000
0.004
0.008
Weight
Positive
Negative

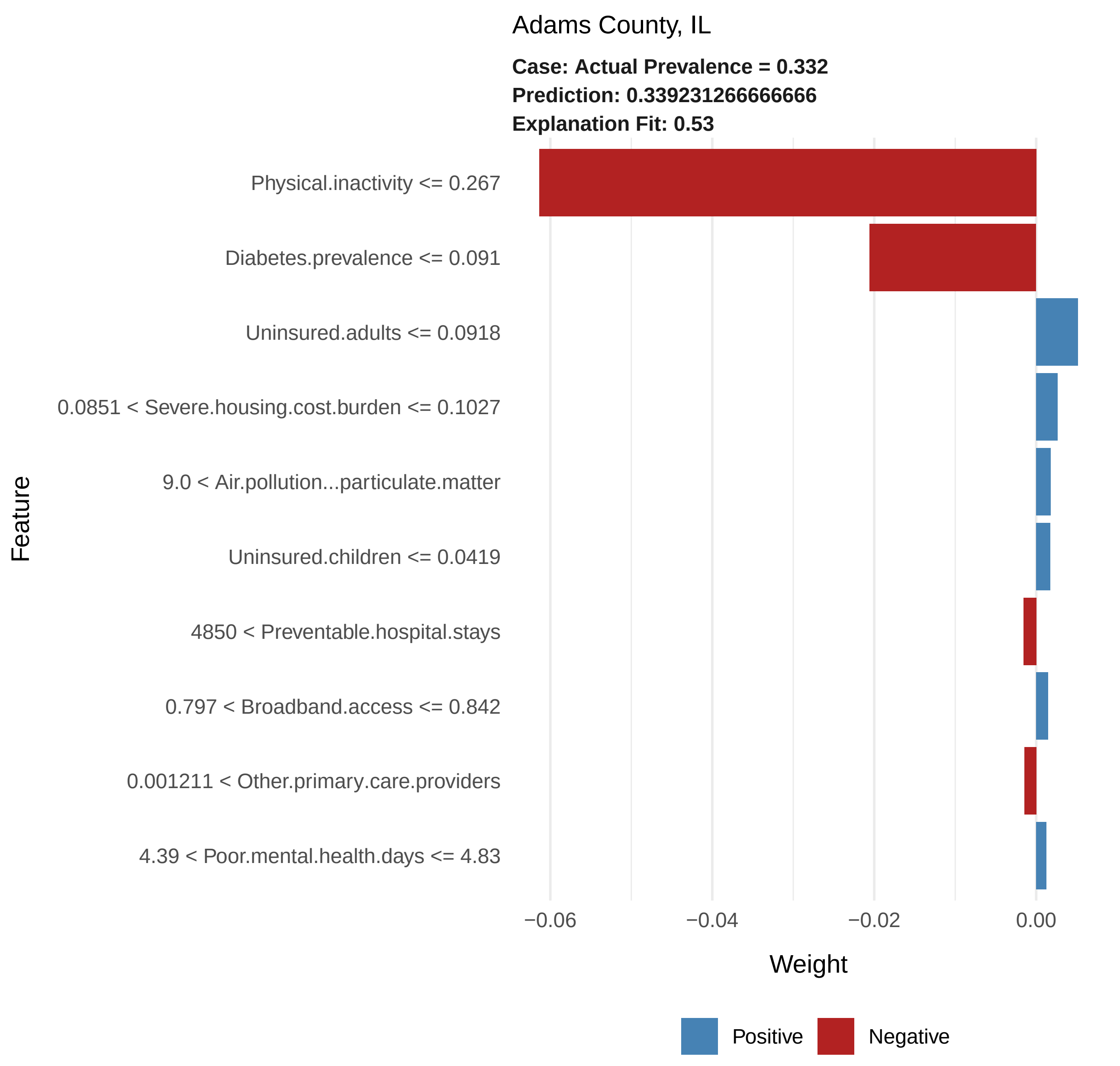

Adams County, IL
Case: Actual Prevalence = 0.332
Prediction: 0.339231266666666
Explanation Fit: 0.53
Physical.inactivity <= 0.267
Diabetes.prevalence <= 0.091
Uninsured.adults <= 0.0918
0.0851 < Severe.housing.cost.burden <= 0.1027
9.0 < Air.pollution...particulate.matter
Uninsured.children <= 0.0419
4850 < Preventable.hospital.stays
0.797 < Broadband.access <= 0.842
0.001211 < Other.primary.care.providers
4.39 < Poor.mental.health.days <= 4.83
Feature
−0.06
−0.04
−0.02
0.00
Weight
Positive
Negative

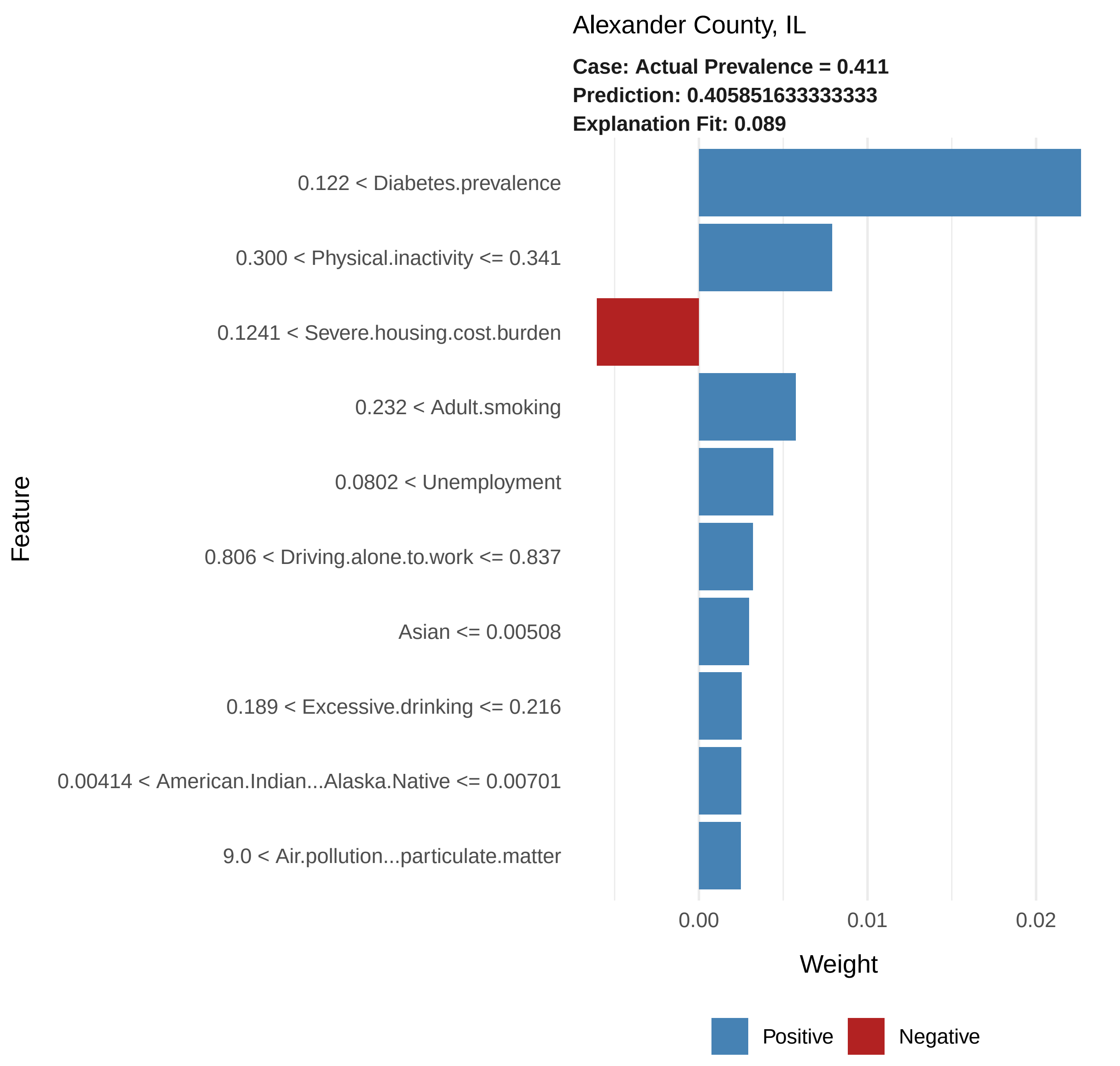

Alexander County, IL
Case: Actual Prevalence = 0.411
Prediction: 0.405851633333333
Explanation Fit: 0.089
0.122 < Diabetes.prevalence
0.300 < Physical.inactivity <= 0.341
0.1241 < Severe.housing.cost.burden
0.232 < Adult.smoking
0.0802 < Unemployment
0.806 < Driving.alone.to.work <= 0.837
Asian <= 0.00508
0.189 < Excessive.drinking <= 0.216
0.00414 < American.Indian...Alaska.Native <= 0.00701
9.0 < Air.pollution...particulate.matter
Feature
0.00
0.01
0.02
Weight
Positive
Negative

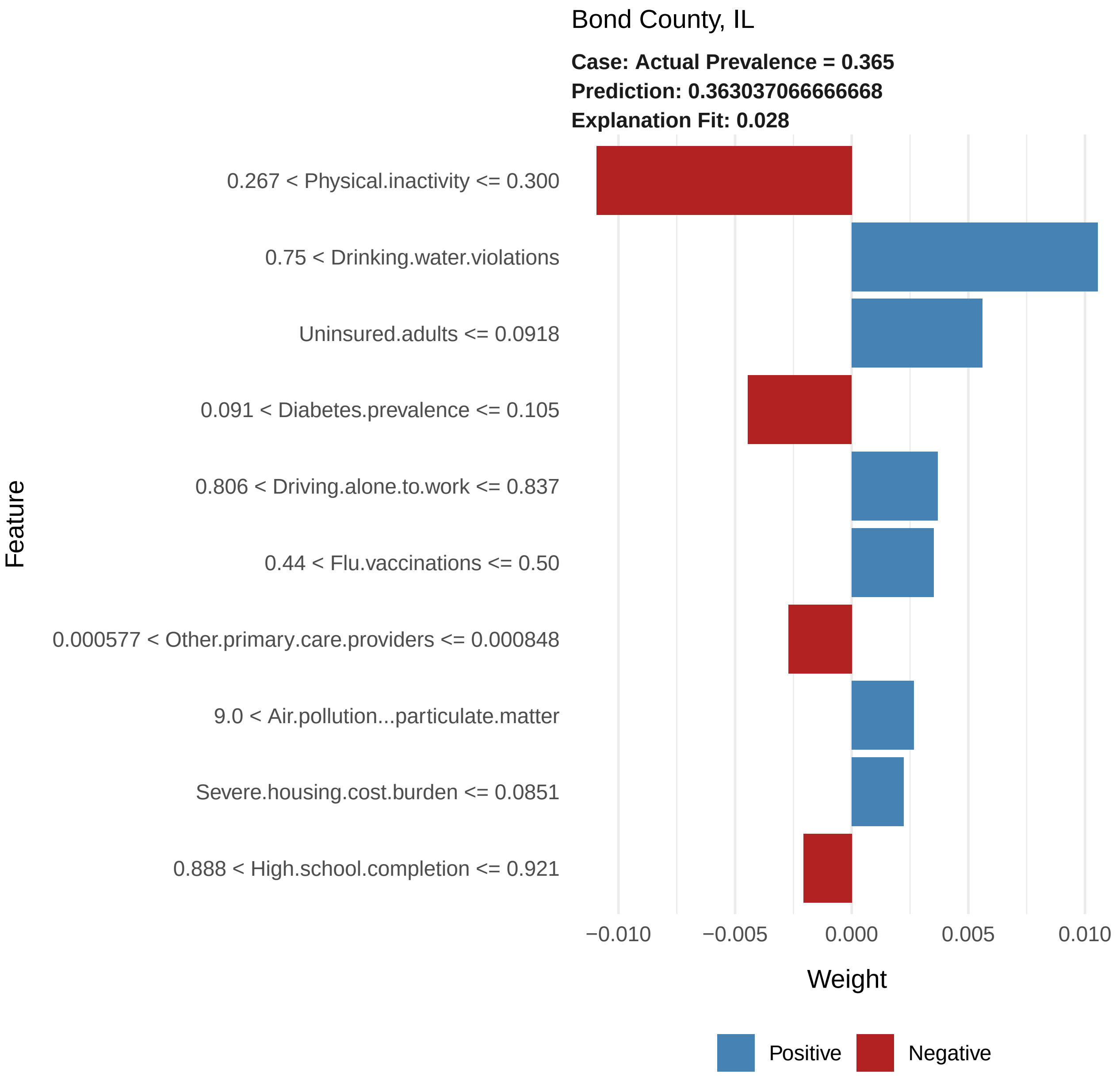

Bond County, IL
Case: Actual Prevalence = 0.365
Prediction: 0.363037066666668
Explanation Fit: 0.028
0.267 < Physical.inactivity <= 0.300
0.75 < Drinking.water.violations
Uninsured.adults <= 0.0918
0.091 < Diabetes.prevalence <= 0.105
0.806 < Driving.alone.to.work <= 0.837
0.44 < Flu.vaccinations <= 0.50
0.000577 < Other.primary.care.providers <= 0.000848
9.0 < Air.pollution...particulate.matter
Severe.housing.cost.burden <= 0.0851
0.888 < High.school.completion <= 0.921
Feature
−0.010
−0.005
0.000
0.005
0.010
Weight
Positive
Negative

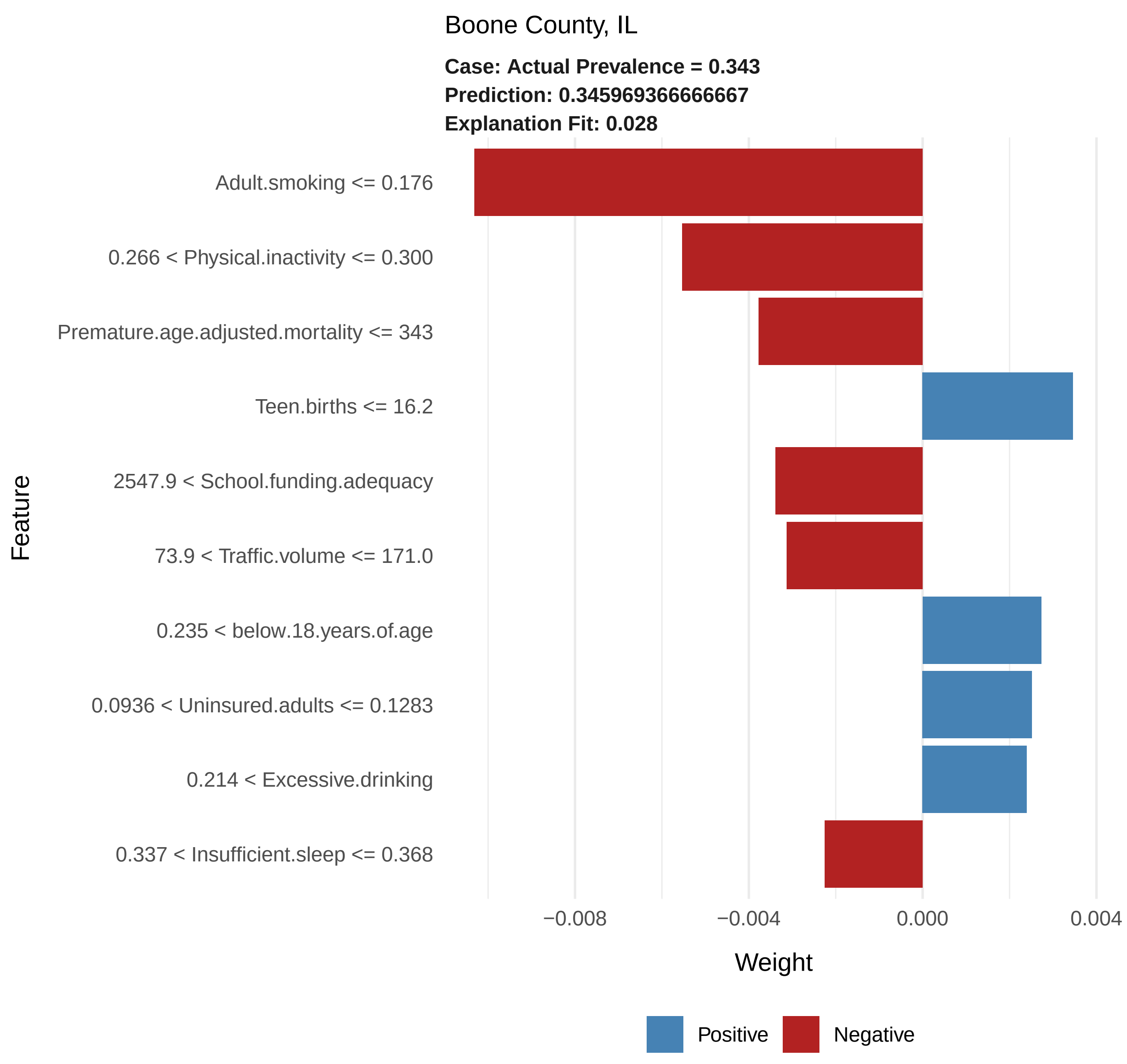

Boone County, IL
Case: Actual Prevalence = 0.343
Prediction: 0.345969366666667
Explanation Fit: 0.028
Feature
Adult.smoking <= 0.176
0.266 < Physical.inactivity <= 0.300
Premature.age.adjusted.mortality <= 343
Teen.births <= 16.2
2547.9 < School.funding.adequacy
73.9 < Traffic.volume <= 171.0
0.235 < below.18.years.of.age
0.0936 < Uninsured.adults <= 0.1283
0.214 < Excessive.drinking
0.337 < Insufficient.sleep <= 0.368
−0.008
−0.004
0.000
0.004
Weight
Positive
Negative

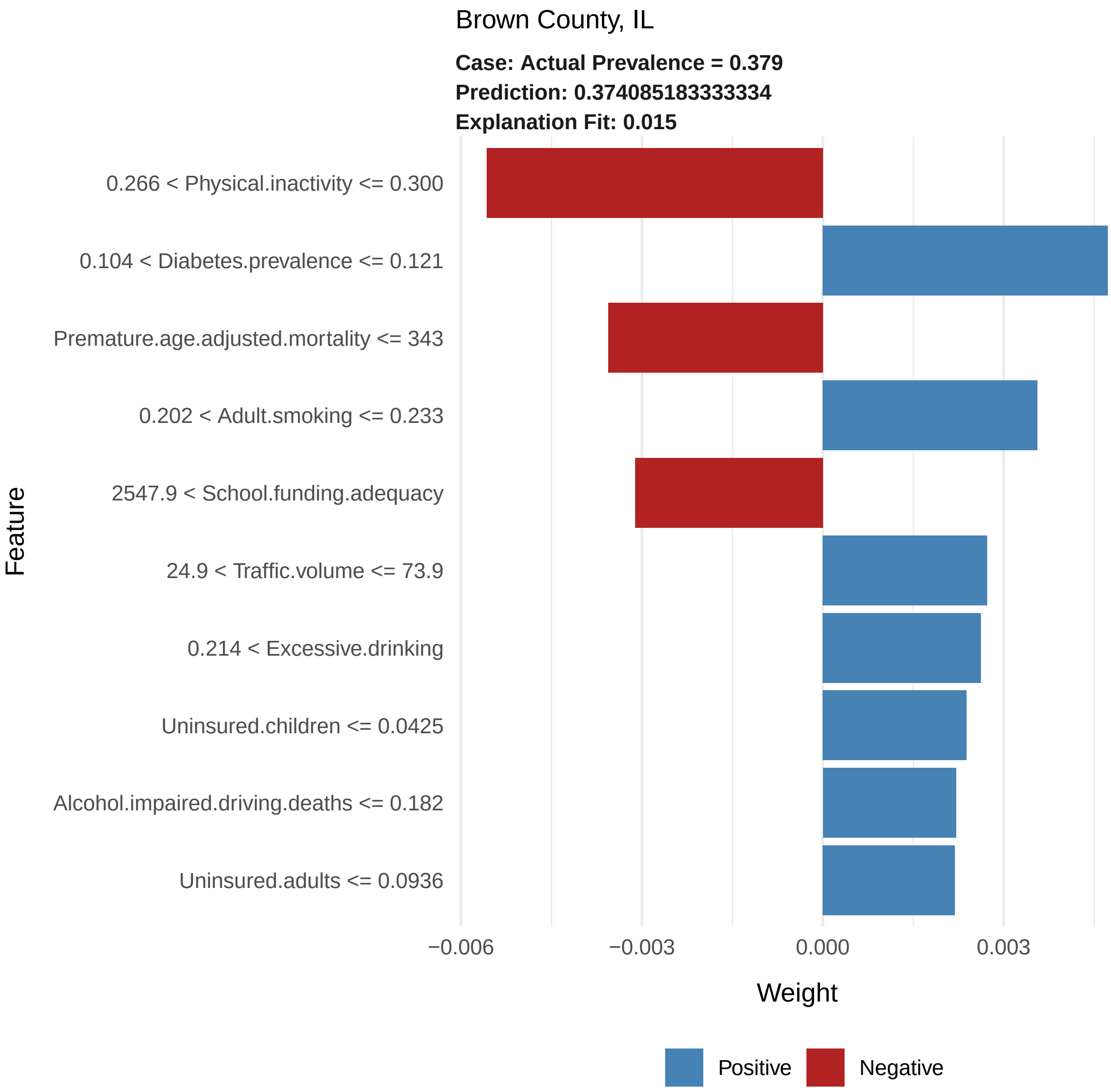

Brown County, IL
Case: Actual Prevalence = 0.379
Prediction: 0.374085183333334
Explanation Fit: 0.015
0.266 < Physical.inactivity <= 0.300
0.104 < Diabetes.prevalence <= 0.121
Premature.age.adjusted.mortality <= 343
0.202 < Adult.smoking <= 0.233
2547.9 < School.funding.adequacy
24.9 < Traffic.volume <= 73.9
0.214 < Excessive.drinking
Uninsured.children <= 0.0425
Alcohol.impaired.driving.deaths <= 0.182
Uninsured.adults <= 0.0936
Feature
−0.006
−0.003
0.000
0.003
Weight
Positive
Negative

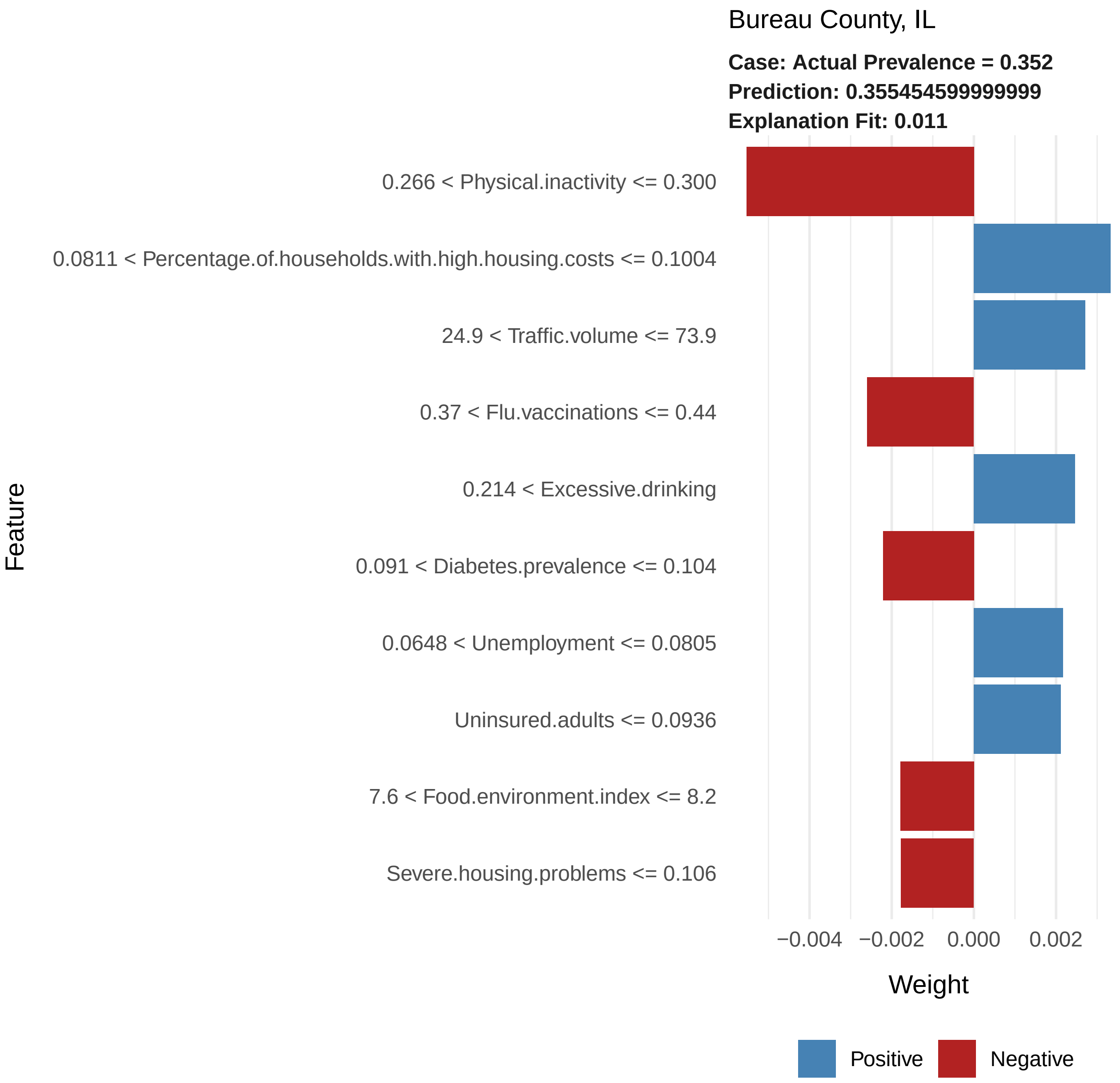
Bureau County, IL
Case: Actual Prevalence = 0.352
Prediction: 0.355454599999999
Explanation Fit: 0.011
0.266 < Physical.inactivity <= 0.300
0.0811 < Percentage.of.households.with.high.housing.costs <= 0.1004
24.9 < Traffic.volume <= 73.9
0.37 < Flu.vaccinations <= 0.44
0.214 < Excessive.drinking
0.091 < Diabetes.prevalence <= 0.104
0.0648 < Unemployment <= 0.0805
Uninsured.adults <= 0.0936
7.6 < Food.environment.index <= 8.2
Severe.housing.problems <= 0.106
Feature
−0.004
−0.002
0.000
0.002
Weight
Positive
Negative

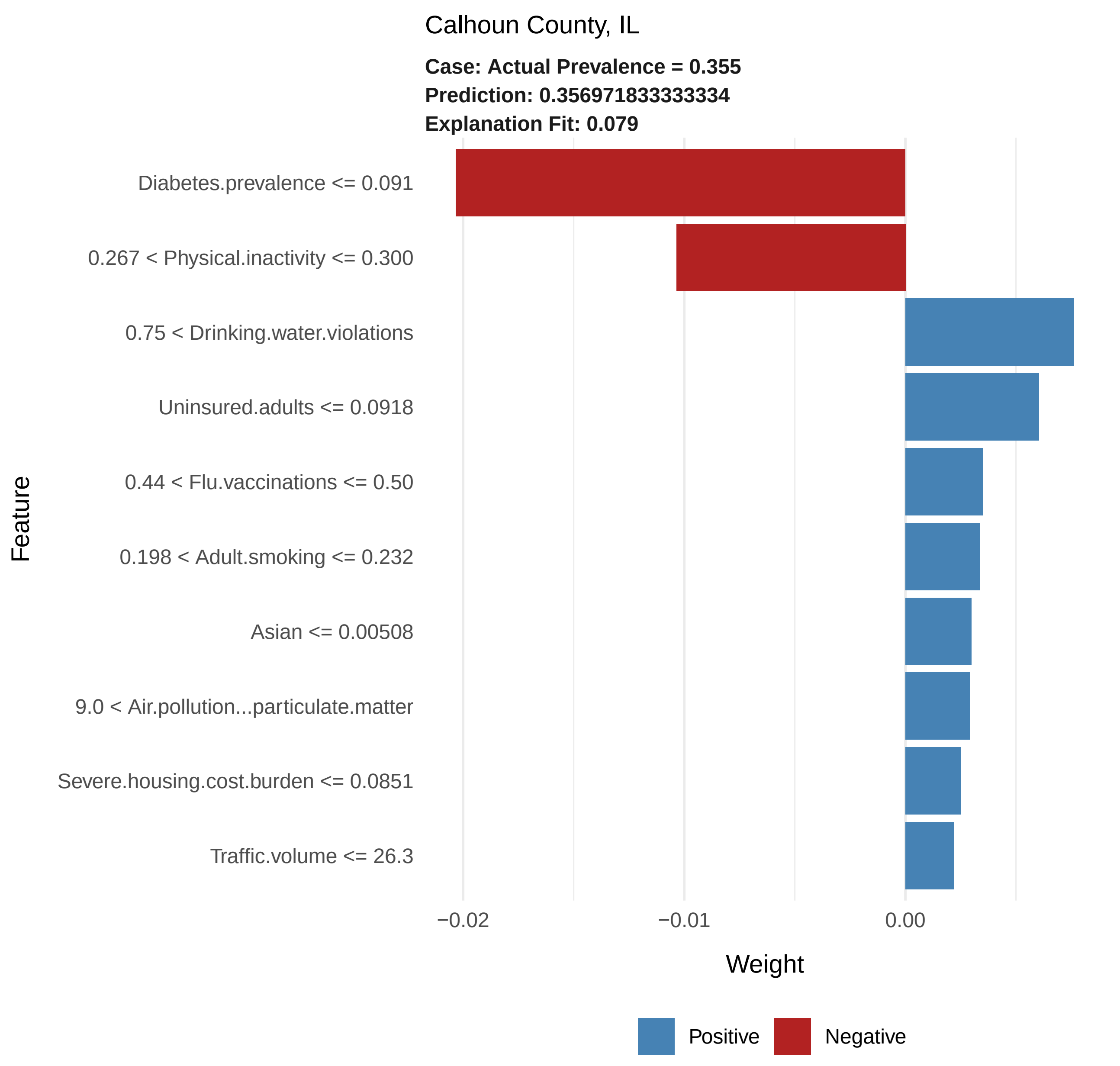

Calhoun County, IL
Case: Actual Prevalence = 0.355
Prediction: 0.356971833333334
Explanation Fit: 0.079
Diabetes.prevalence <= 0.091
0.267 < Physical.inactivity <= 0.300
0.75 < Drinking.water.violations
Uninsured.adults <= 0.0918
0.44 < Flu.vaccinations <= 0.50
0.198 < Adult.smoking <= 0.232
Asian <= 0.00508
9.0 < Air.pollution...particulate.matter
Severe.housing.cost.burden <= 0.0851
Traffic.volume <= 26.3
Feature
−0.02
−0.01
0.00
Weight
Positive
Negative

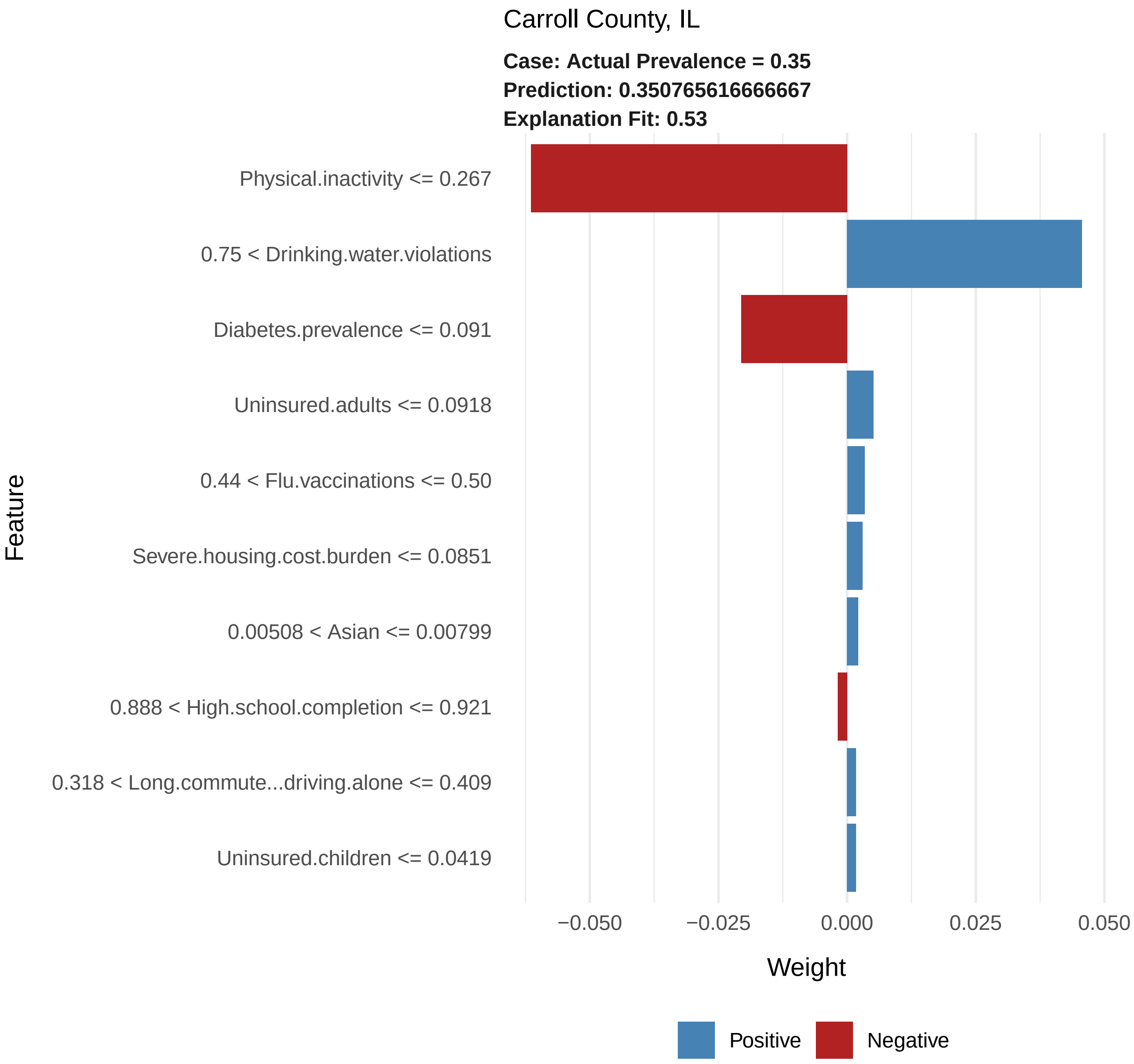
Carroll County, IL
Case: Actual Prevalence = 0.35
Prediction: 0.350765616666667
Explanation Fit: 0.53
Physical.inactivity <= 0.267
0.75 < Drinking.water.violations
Diabetes.prevalence <= 0.091
Uninsured.adults <= 0.0918
0.44 < Flu.vaccinations <= 0.50
Severe.housing.cost.burden <= 0.0851
0.00508 < Asian <= 0.00799
0.888 < High.school.completion <= 0.921
0.318 < Long.commute...driving.alone <= 0.409
Uninsured.children <= 0.0419
Feature
−0.050
−0.025
0.000
0.025
0.050
Weight
Positive
Negative

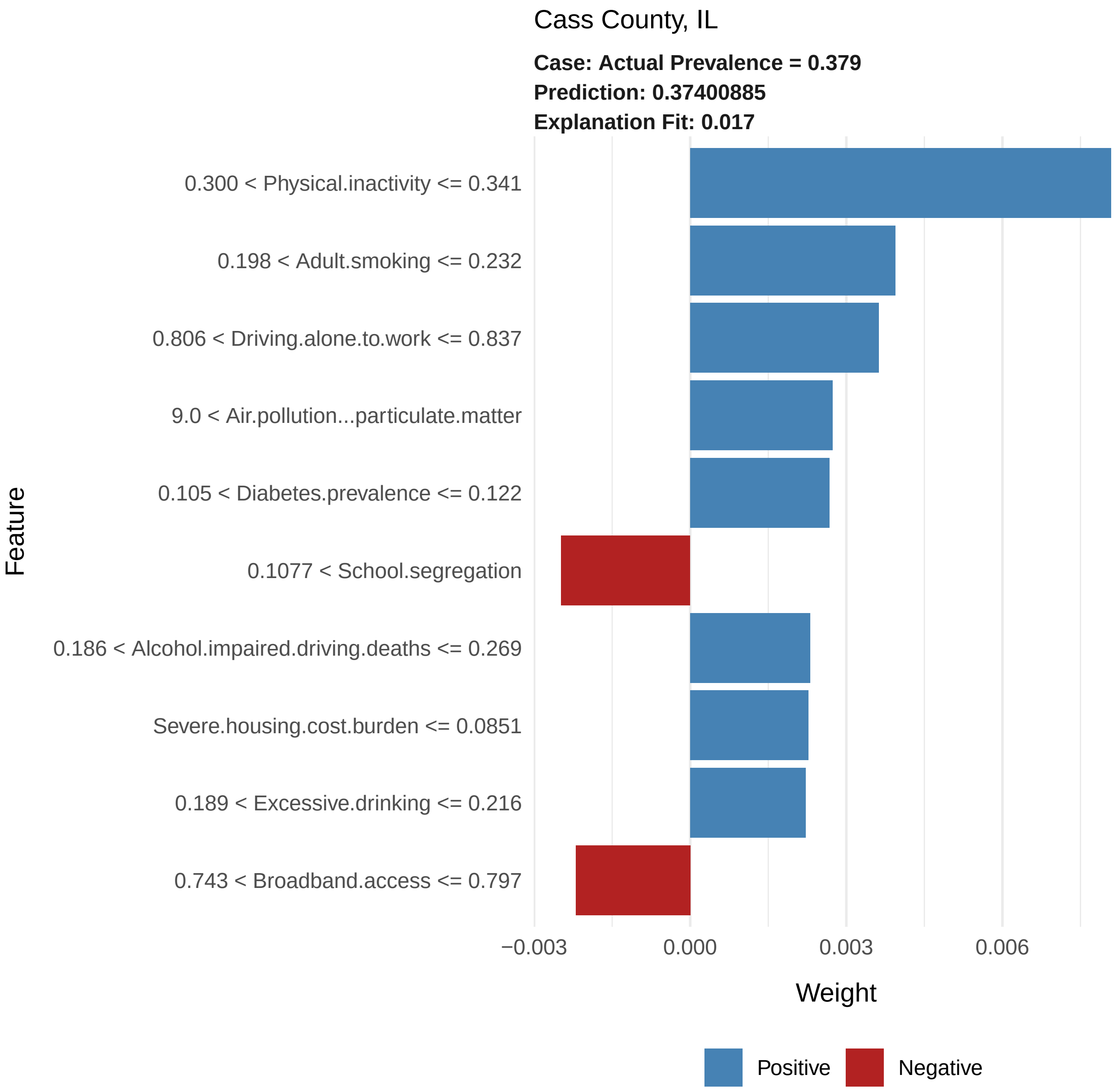

Cass County, IL
Case: Actual Prevalence = 0.379
Prediction: 0.37400885
Explanation Fit: 0.017
0.300 < Physical.inactivity <= 0.341
0.198 < Adult.smoking <= 0.232
0.806 < Driving.alone.to.work <= 0.837
9.0 < Air.pollution...particulate.matter
0.105 < Diabetes.prevalence <= 0.122
0.1077 < School.segregation
0.186 < Alcohol.impaired.driving.deaths <= 0.269
Severe.housing.cost.burden <= 0.0851
0.189 < Excessive.drinking <= 0.216
0.743 < Broadband.access <= 0.797
Feature
−0.003
0.000
0.003
0.006
Weight
Positive
Negative

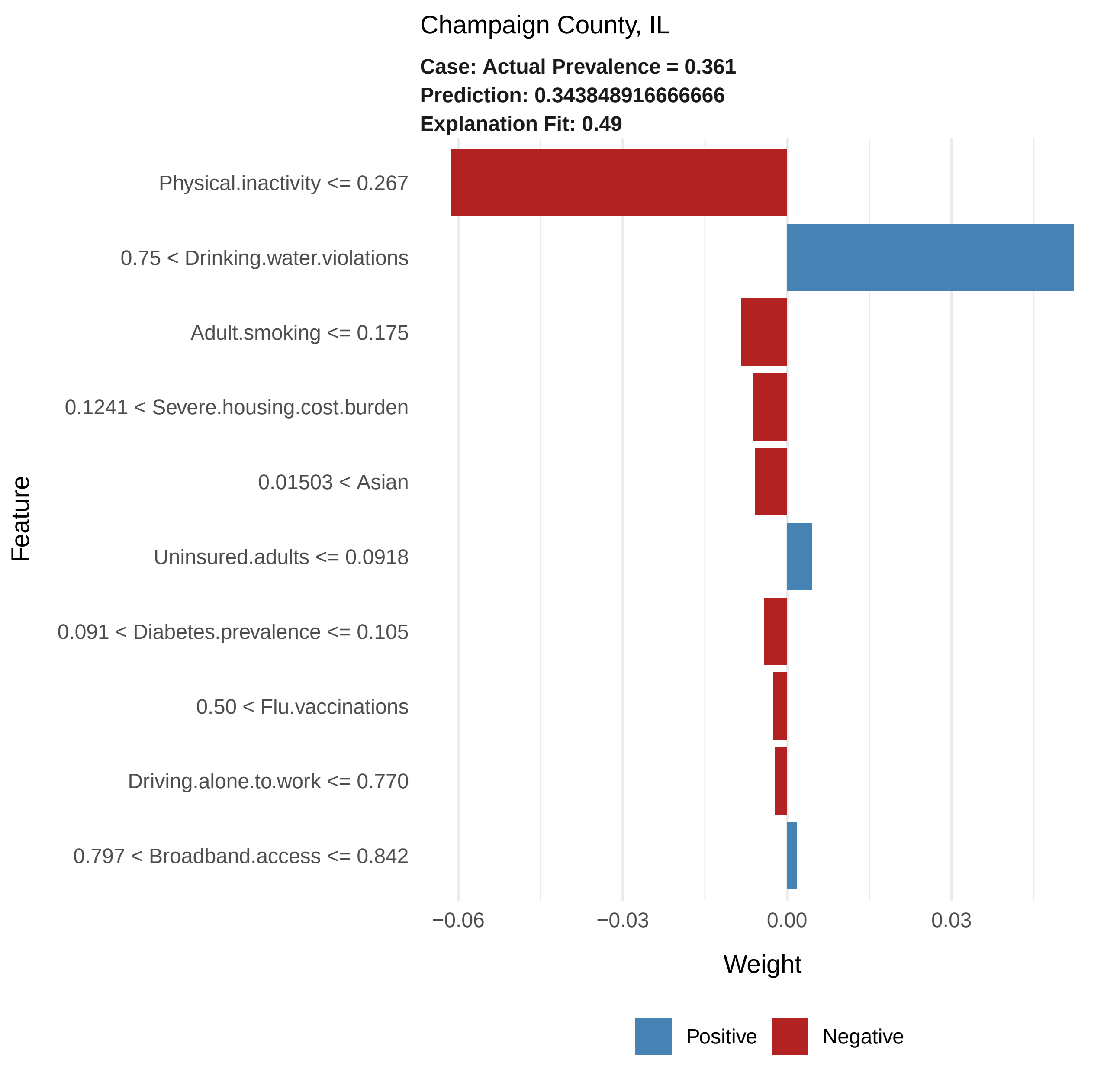

Champaign County, IL
Case: Actual Prevalence = 0.361
Prediction: 0.343848916666666
Explanation Fit: 0.49
Physical.inactivity <= 0.267
0.75 < Drinking.water.violations
Adult.smoking <= 0.175
0.1241 < Severe.housing.cost.burden
0.01503 < Asian
Uninsured.adults <= 0.0918
0.091 < Diabetes.prevalence <= 0.105
0.50 < Flu.vaccinations
Driving.alone.to.work <= 0.770
0.797 < Broadband.access <= 0.842
Feature
−0.06
−0.03
0.00
0.03
Weight
Positive
Negative

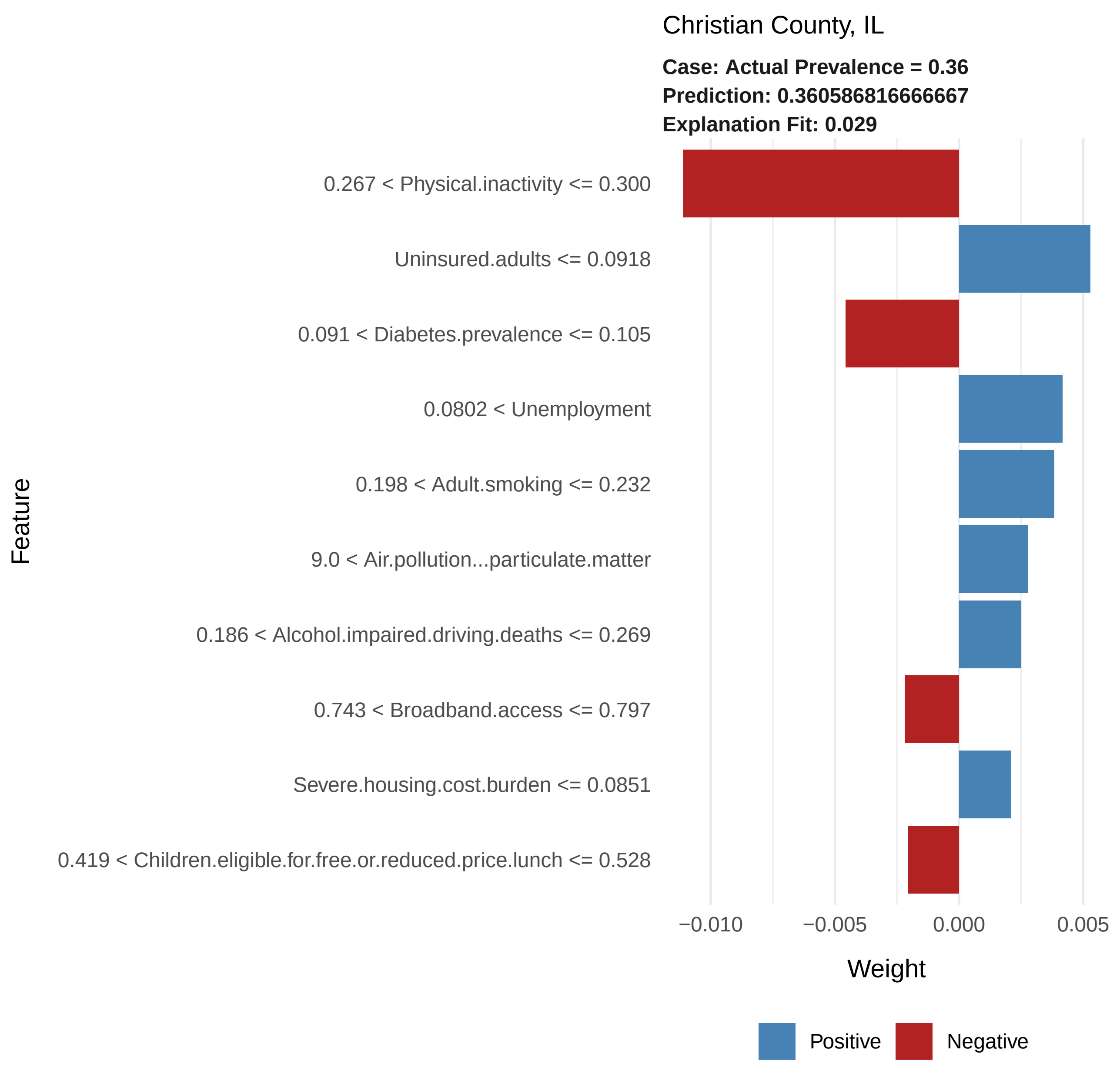
Christian County, IL
Case: Actual Prevalence = 0.36
Prediction: 0.360586816666667
Explanation Fit: 0.029
Feature
0.267 < Physical.inactivity <= 0.300
Uninsured.adults <= 0.0918
0.091 < Diabetes.prevalence <= 0.105
0.0802 < Unemployment
0.198 < Adult.smoking <= 0.232
9.0 < Air.pollution...particulate.matter
0.186 < Alcohol.impaired.driving.deaths <= 0.269
0.743 < Broadband.access <= 0.797
Severe.housing.cost.burden <= 0.0851
0.419 < Children.eligible.for.free.or.reduced.price.lunch <= 0.528
−0.010
−0.005
0.000
0.005
Weight
Positive
Negative

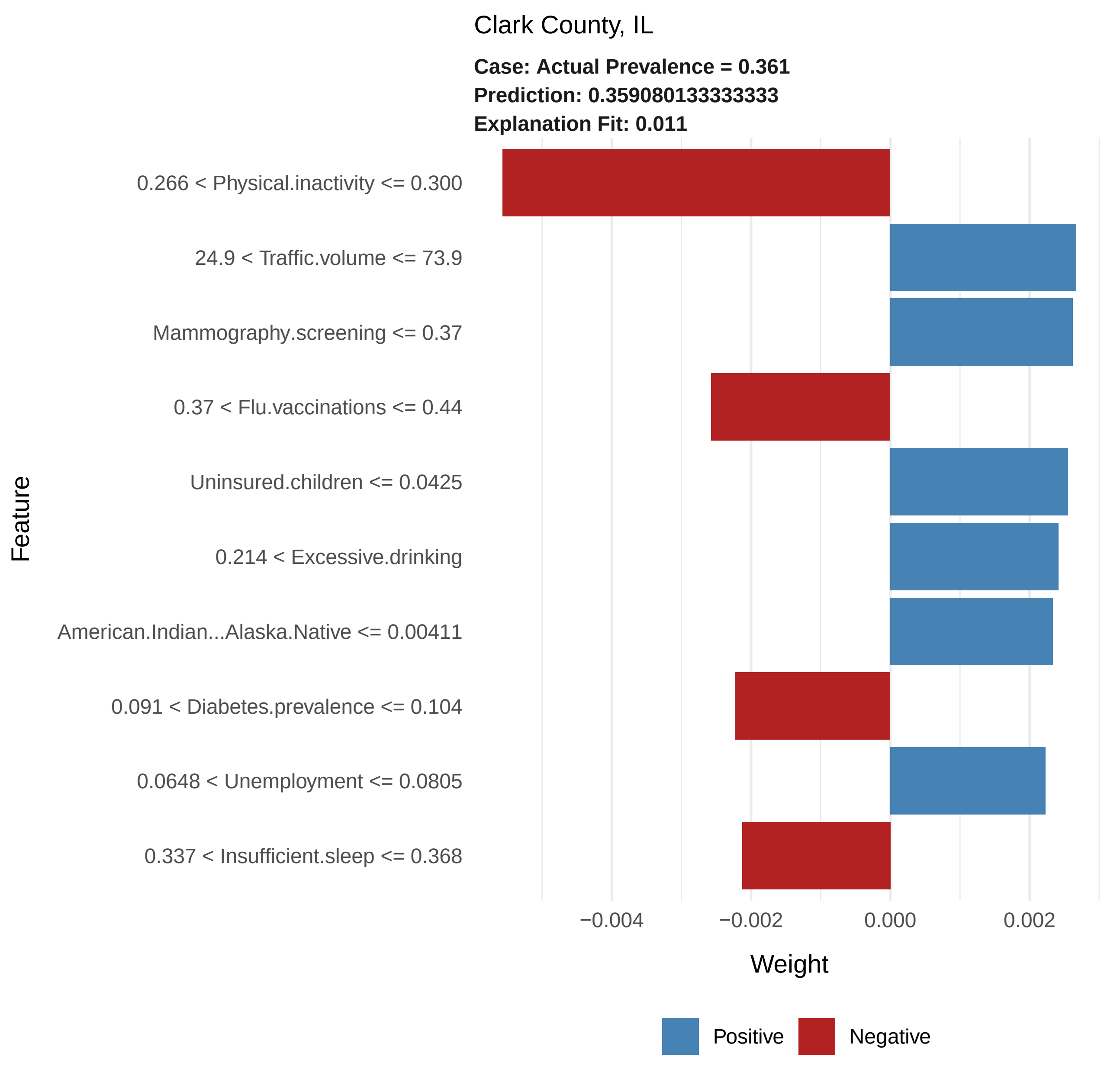
Clark County, IL
Case: Actual Prevalence = 0.361
Prediction: 0.359080133333333
Explanation Fit: 0.011
0.266 < Physical.inactivity <= 0.300
24.9 < Traffic.volume <= 73.9
Mammography.screening <= 0.37
0.37 < Flu.vaccinations <= 0.44
Uninsured.children <= 0.0425
0.214 < Excessive.drinking
American.Indian...Alaska.Native <= 0.00411
0.091 < Diabetes.prevalence <= 0.104
0.0648 < Unemployment <= 0.0805
0.337 < Insufficient.sleep <= 0.368
Feature
−0.004
−0.002
0.000
0.002
Weight
Positive
Negative

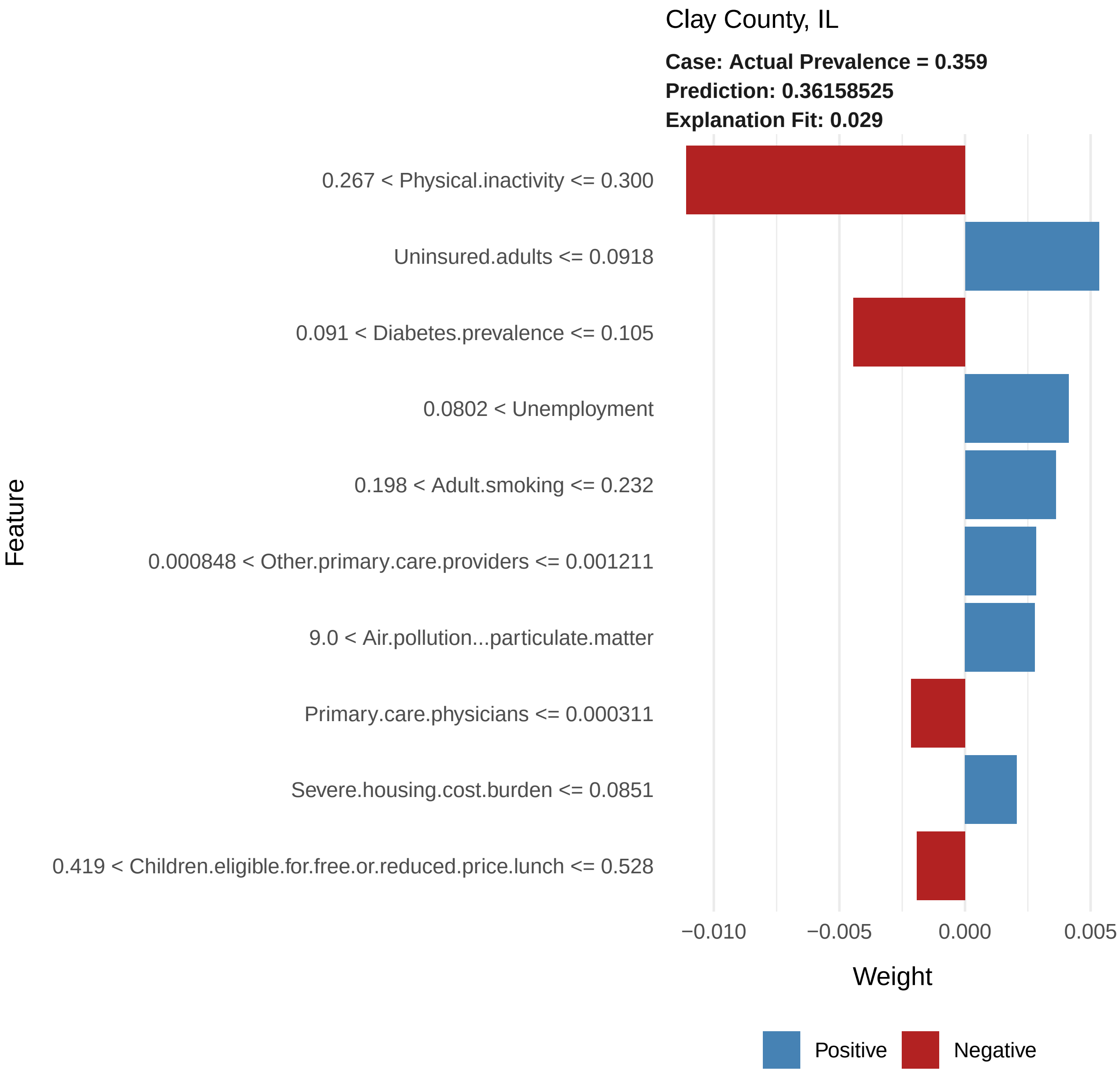

Clay County, IL
Case: Actual Prevalence = 0.359
Prediction: 0.36158525
Explanation Fit: 0.029
Feature
0.267 < Physical.inactivity <= 0.300
Uninsured.adults <= 0.0918
0.091 < Diabetes.prevalence <= 0.105
0.0802 < Unemployment
0.198 < Adult.smoking <= 0.232
0.000848 < Other.primary.care.providers <= 0.001211
9.0 < Air.pollution...particulate.matter
Primary.care.physicians <= 0.000311
Severe.housing.cost.burden <= 0.0851
0.419 < Children.eligible.for.free.or.reduced.price.lunch <= 0.528
−0.010
−0.005
0.000
0.005
Weight
Positive
Negative

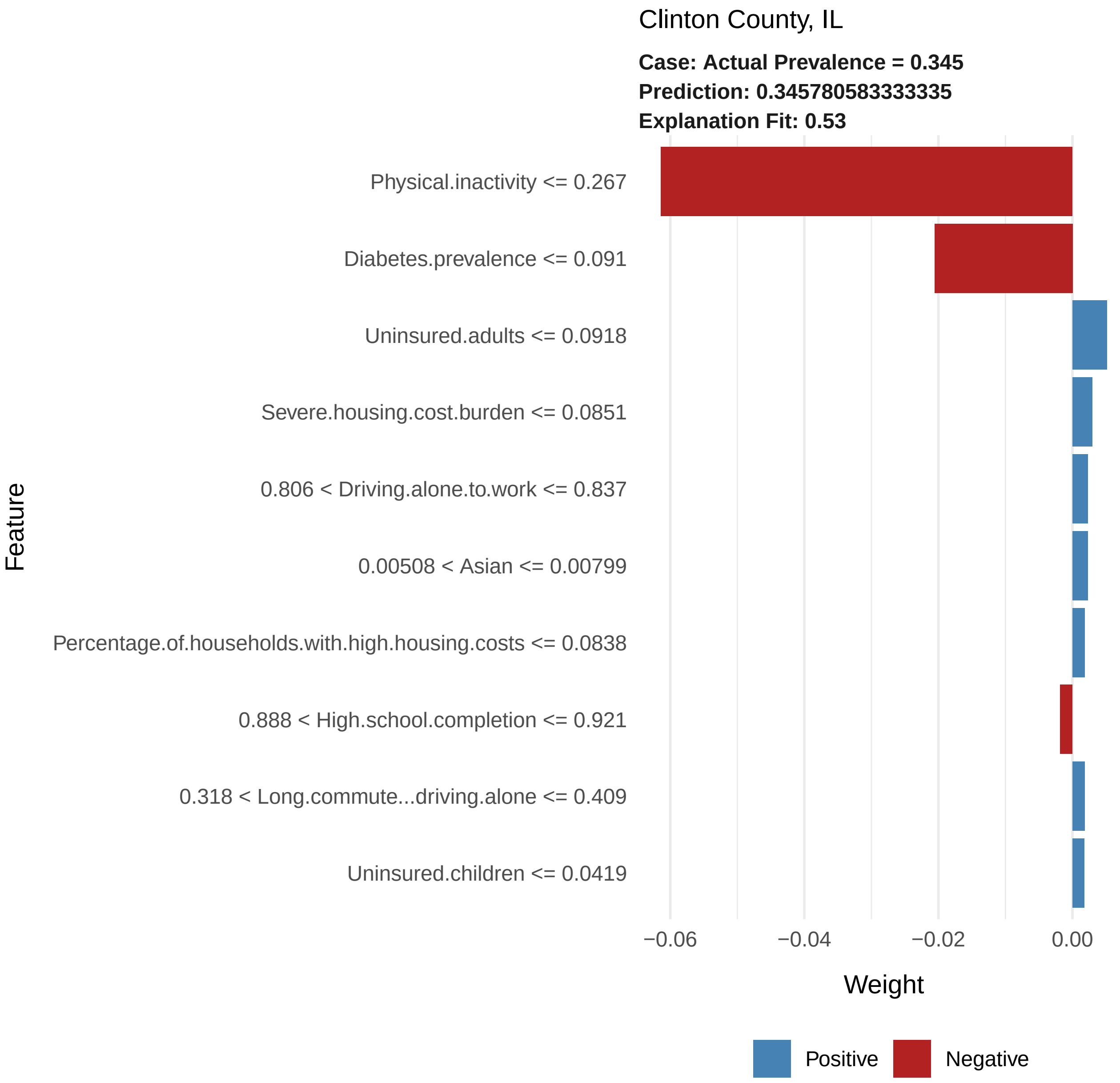
Clinton County, IL
Case: Actual Prevalence = 0.345
Prediction: 0.345780583333335
Explanation Fit: 0.53
Physical.inactivity <= 0.267
Diabetes.prevalence <= 0.091
Uninsured.adults <= 0.0918
Severe.housing.cost.burden <= 0.0851
0.806 < Driving.alone.to.work <= 0.837
0.00508 < Asian <= 0.00799
Percentage.of.households.with.high.housing.costs <= 0.0838
0.888 < High.school.completion <= 0.921
0.318 < Long.commute...driving.alone <= 0.409
Uninsured.children <= 0.0419
Feature
−0.06
−0.04
−0.02
0.00
Weight
Positive
Negative

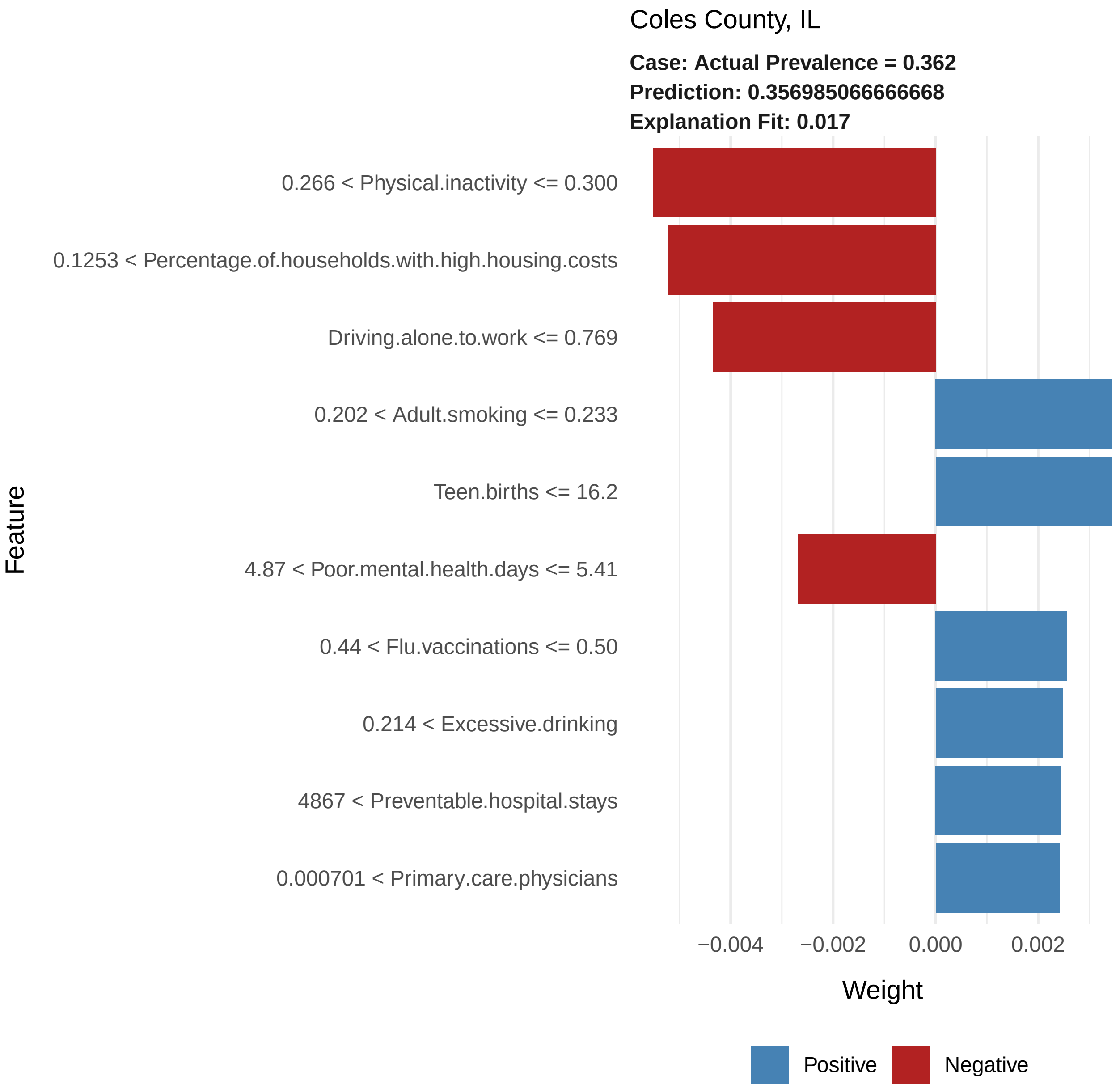
Coles County, IL
Case: Actual Prevalence = 0.362
Prediction: 0.356985066666668
Explanation Fit: 0.017
0.266 < Physical.inactivity <= 0.300
0.1253 < Percentage.of.households.with.high.housing.costs
Driving.alone.to.work <= 0.769
0.202 < Adult.smoking <= 0.233
Teen.births <= 16.2
4.87 < Poor.mental.health.days <= 5.41
0.44 < Flu.vaccinations <= 0.50
0.214 < Excessive.drinking
4867 < Preventable.hospital.stays
0.000701 < Primary.care.physicians
Feature
−0.004
−0.002
0.000
0.002
Weight
Positive
Negative

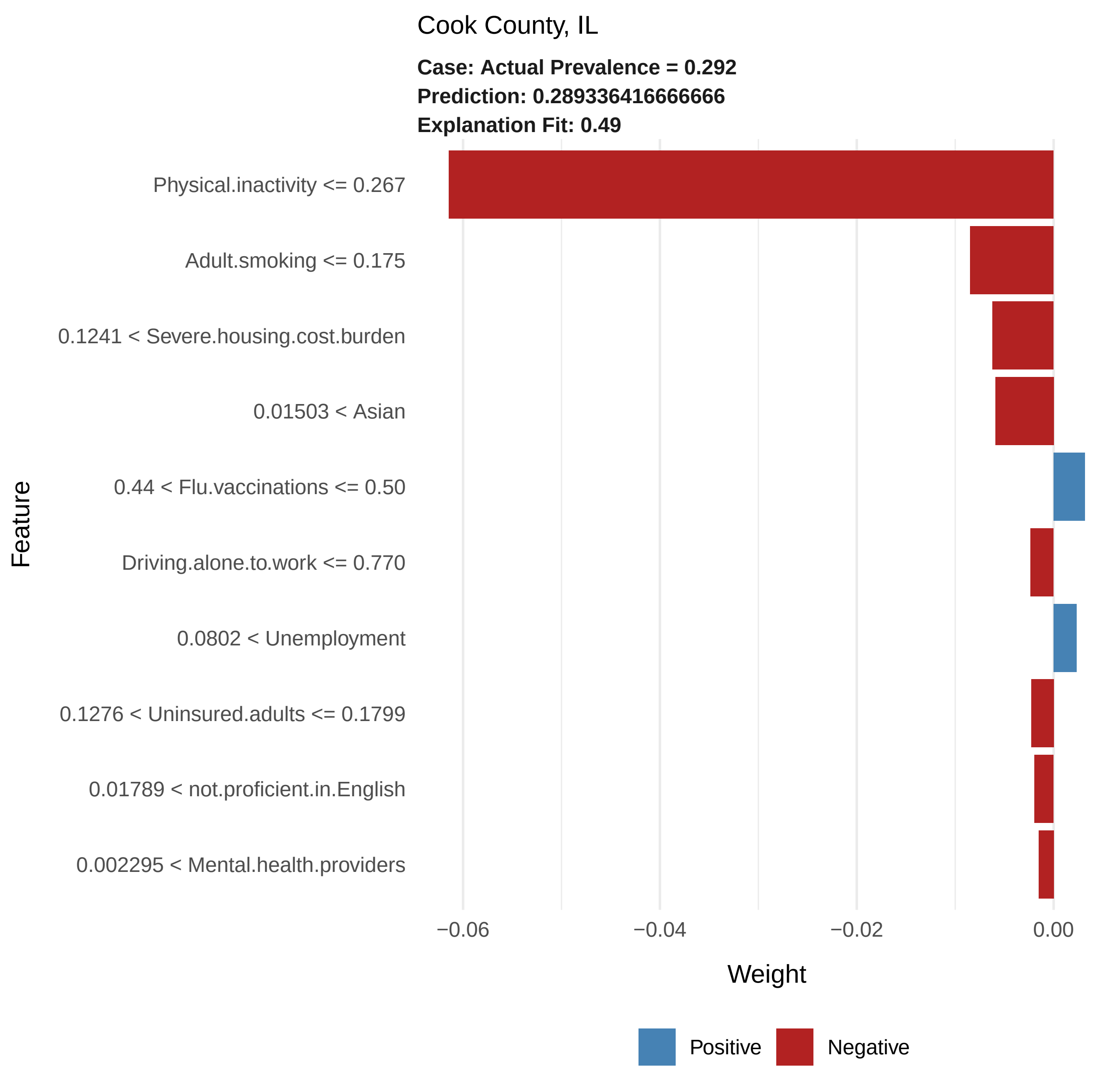

Cook County, IL
Case: Actual Prevalence = 0.292
Prediction: 0.289336416666666
Explanation Fit: 0.49
Physical.inactivity <= 0.267
Adult.smoking <= 0.175
0.1241 < Severe.housing.cost.burden
0.01503 < Asian
0.44 < Flu.vaccinations <= 0.50
Driving.alone.to.work <= 0.770
0.0802 < Unemployment
0.1276 < Uninsured.adults <= 0.1799
0.01789 < not.proficient.in.English
0.002295 < Mental.health.providers
Feature
−0.06
−0.04
−0.02
0.00
Weight
Positive
Negative

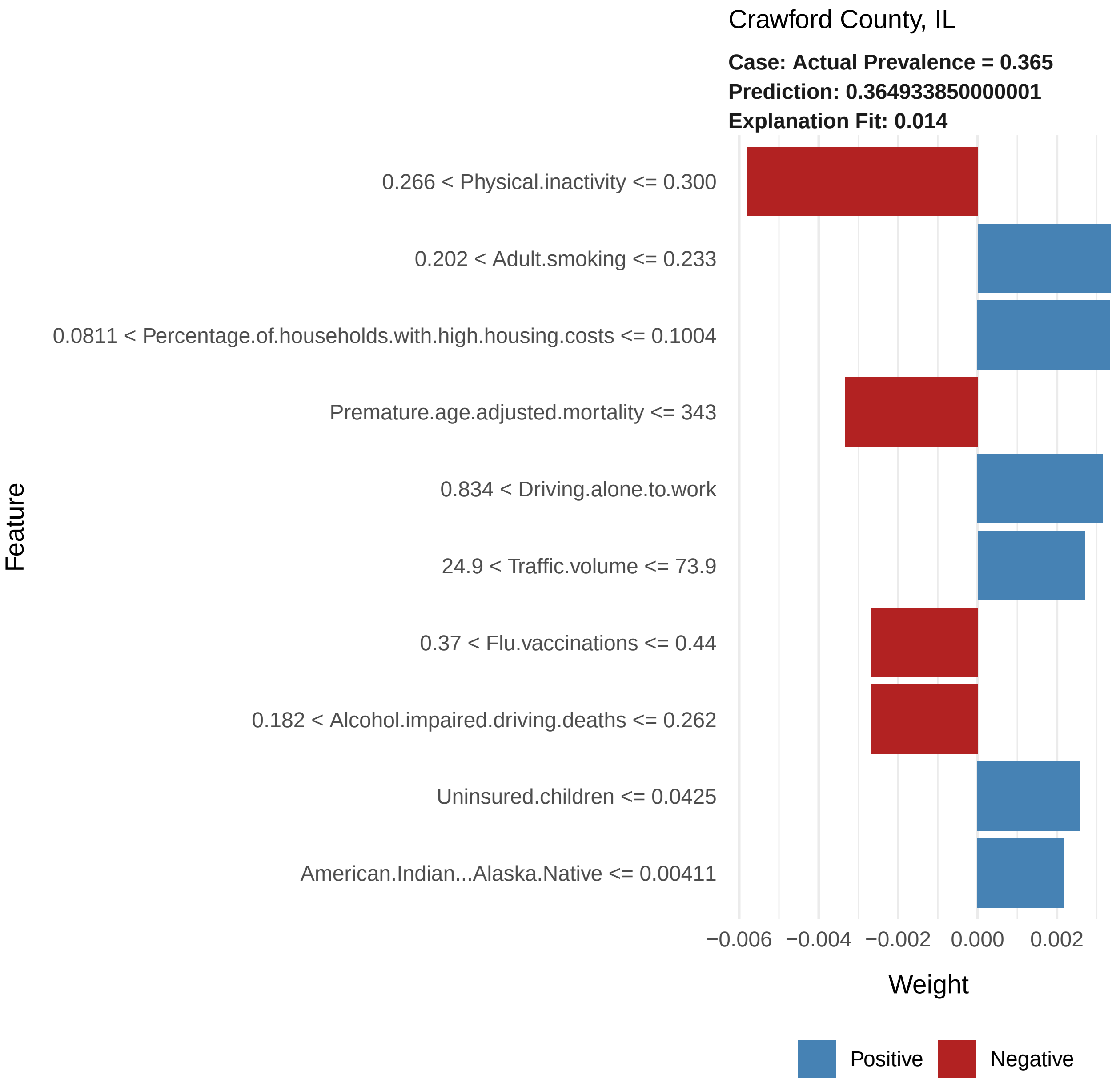

Crawford County, IL
Case: Actual Prevalence = 0.365
Prediction: 0.364933850000001
Explanation Fit: 0.014
0.266 < Physical.inactivity <= 0.300
0.202 < Adult.smoking <= 0.233
0.0811 < Percentage.of.households.with.high.housing.costs <= 0.1004
Premature.age.adjusted.mortality <= 343
0.834 < Driving.alone.to.work
24.9 < Traffic.volume <= 73.9
0.37 < Flu.vaccinations <= 0.44
0.182 < Alcohol.impaired.driving.deaths <= 0.262
Uninsured.children <= 0.0425
American.Indian...Alaska.Native <= 0.00411
Feature
−0.006
−0.004
−0.002
0.000
0.002
Weight
Positive
Negative

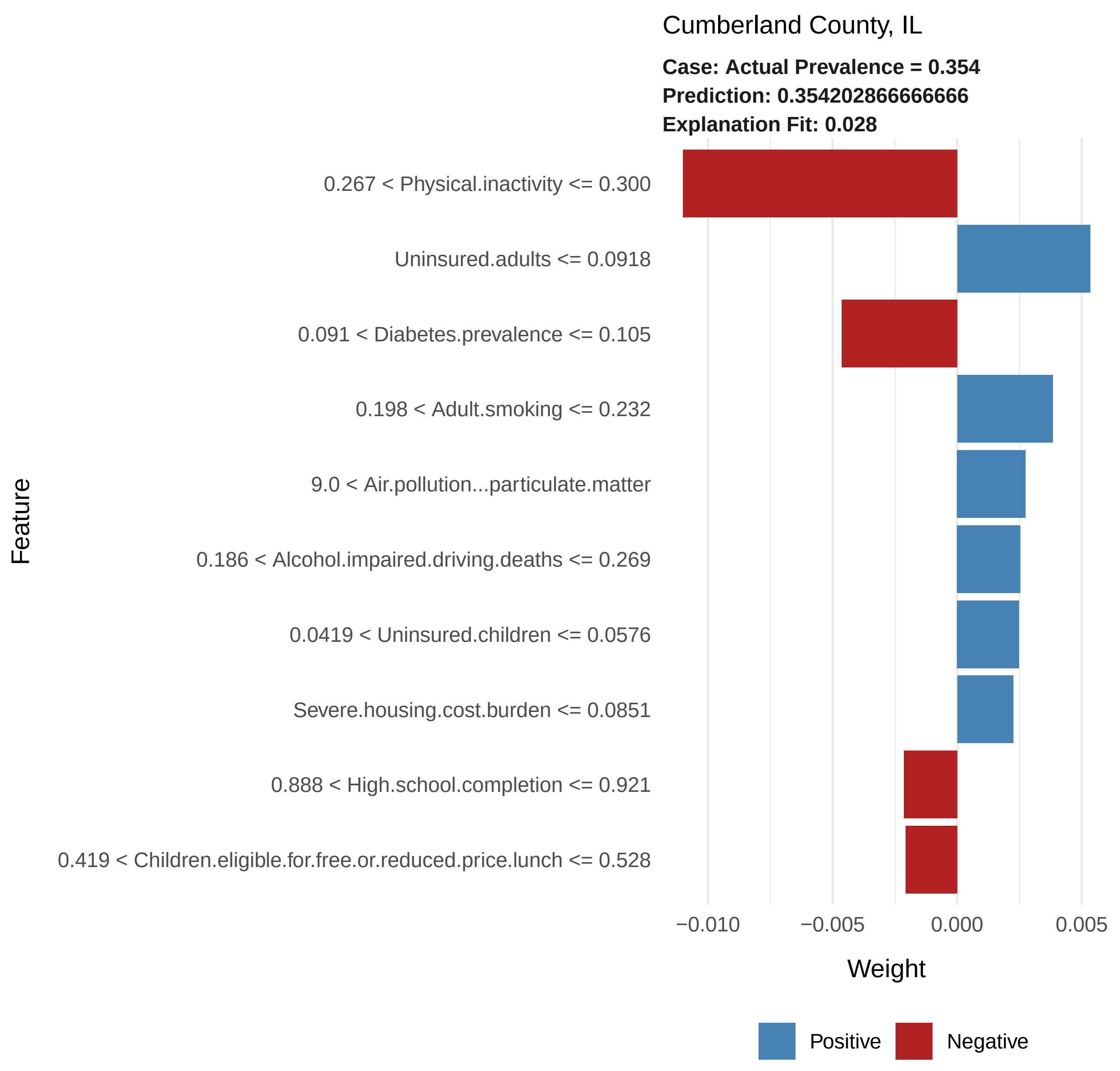
Cumberland County, IL
Case: Actual Prevalence = 0.354
Prediction: 0.354202866666666
Explanation Fit: 0.028
Feature
0.267 < Physical.inactivity <= 0.300
Uninsured.adults <= 0.0918
0.091 < Diabetes.prevalence <= 0.105
0.198 < Adult.smoking <= 0.232
9.0 < Air.pollution...particulate.matter
0.186 < Alcohol.impaired.driving.deaths <= 0.269
0.0419 < Uninsured.children <= 0.0576
Severe.housing.cost.burden <= 0.0851
0.888 < High.school.completion <= 0.921
0.419 < Children.eligible.for.free.or.reduced.price.lunch <= 0.528
−0.010
−0.005
0.000
0.005
Weight
Positive
Negative

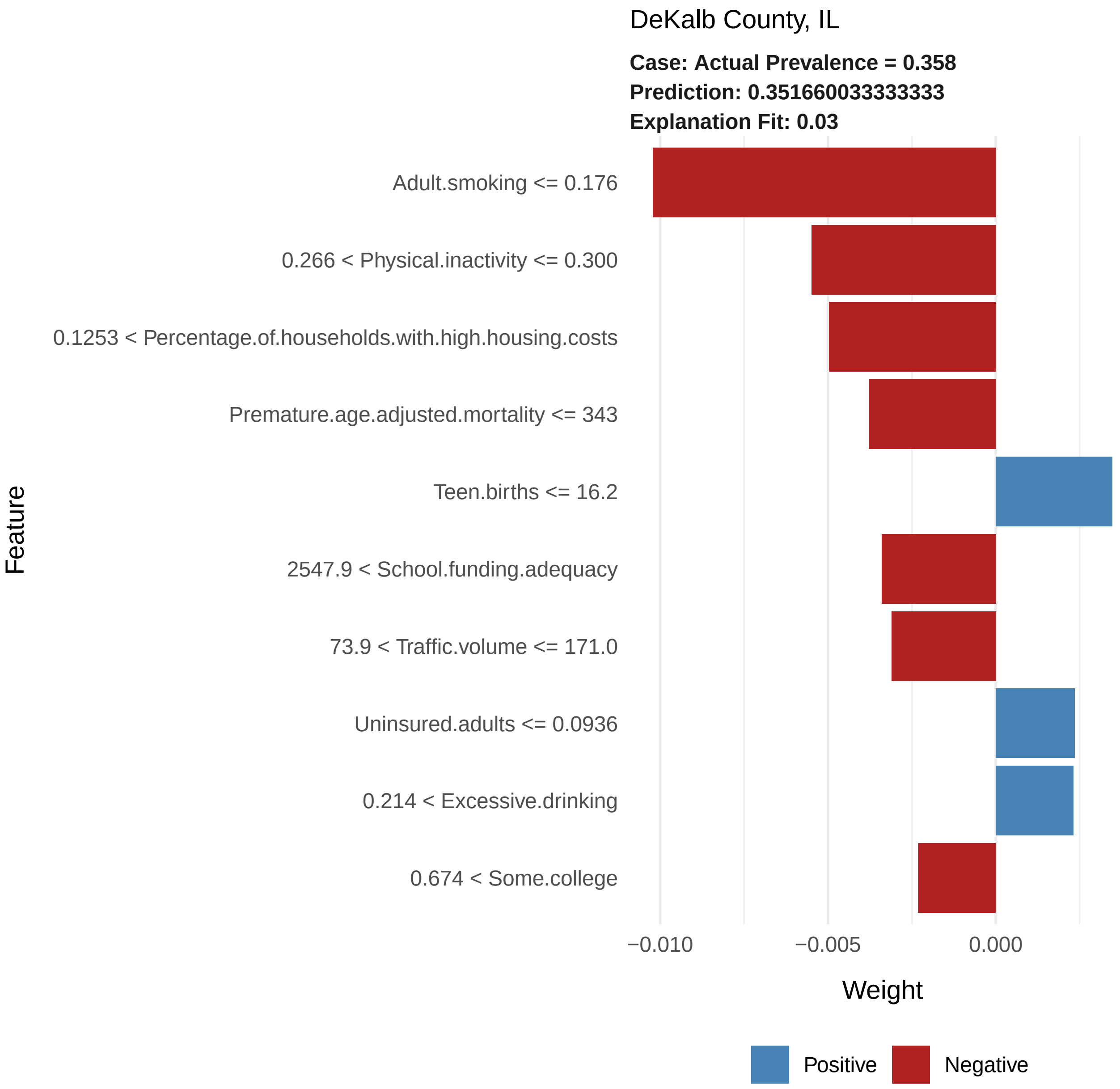

DeKalb County, IL
Case: Actual Prevalence = 0.358
Prediction: 0.351660033333333
Explanation Fit: 0.03
Adult.smoking <= 0.176
0.266 < Physical.inactivity <= 0.300
0.1253 < Percentage.of.households.with.high.housing.costs
Premature.age.adjusted.mortality <= 343
Teen.births <= 16.2
2547.9 < School.funding.adequacy
73.9 < Traffic.volume <= 171.0
Uninsured.adults <= 0.0936
0.214 < Excessive.drinking
0.674 < Some.college
Feature
−0.010
−0.005
0.000
Weight
Positive
Negative

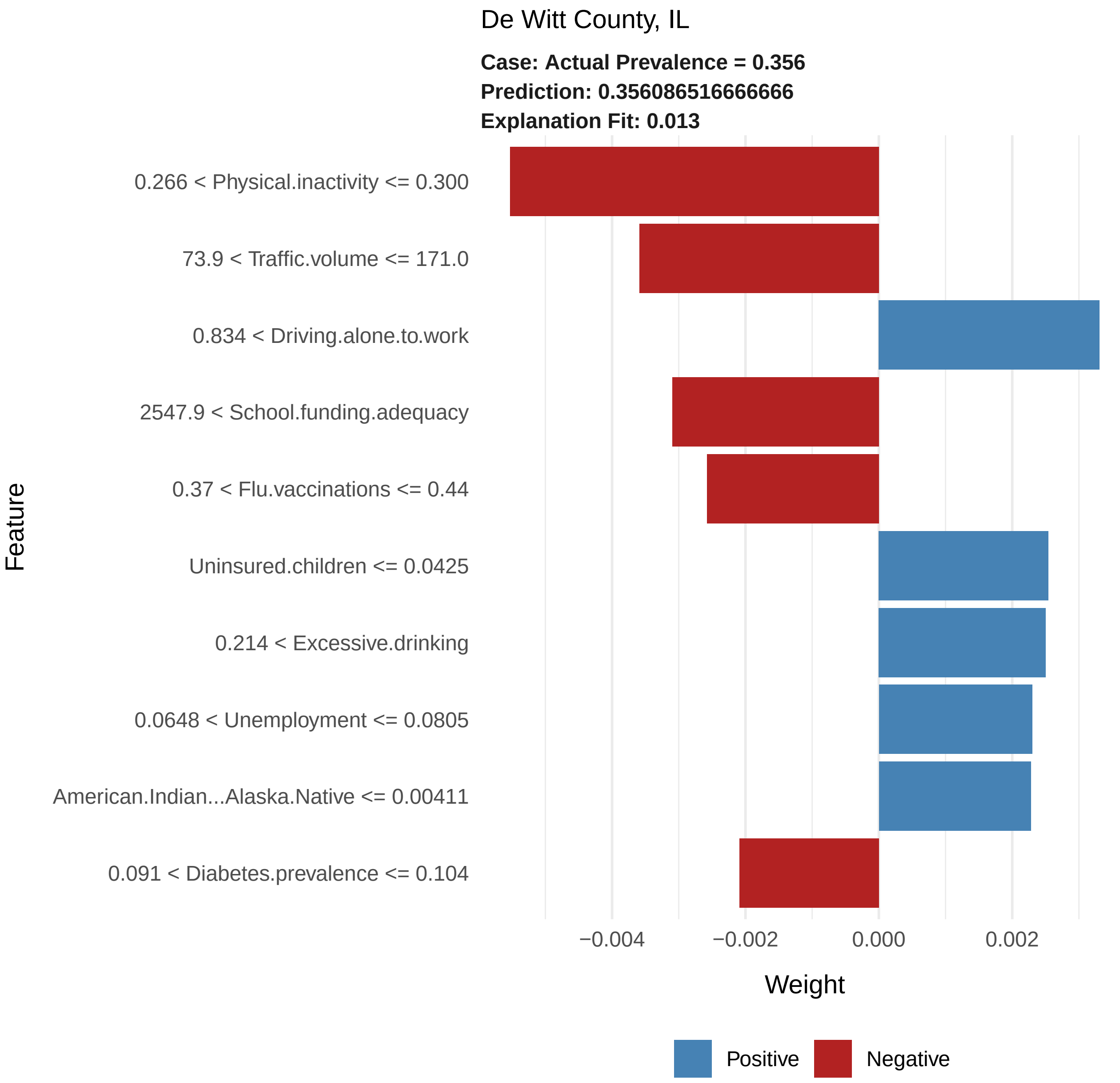

De Witt County, IL
Case: Actual Prevalence = 0.356
Prediction: 0.356086516666666
Explanation Fit: 0.013
0.266 < Physical.inactivity <= 0.300
73.9 < Traffic.volume <= 171.0
0.834 < Driving.alone.to.work
2547.9 < School.funding.adequacy
0.37 < Flu.vaccinations <= 0.44
Uninsured.children <= 0.0425
0.214 < Excessive.drinking
0.0648 < Unemployment <= 0.0805
American.Indian...Alaska.Native <= 0.00411
0.091 < Diabetes.prevalence <= 0.104
Feature
−0.004
−0.002
0.000
0.002
Weight
Positive
Negative

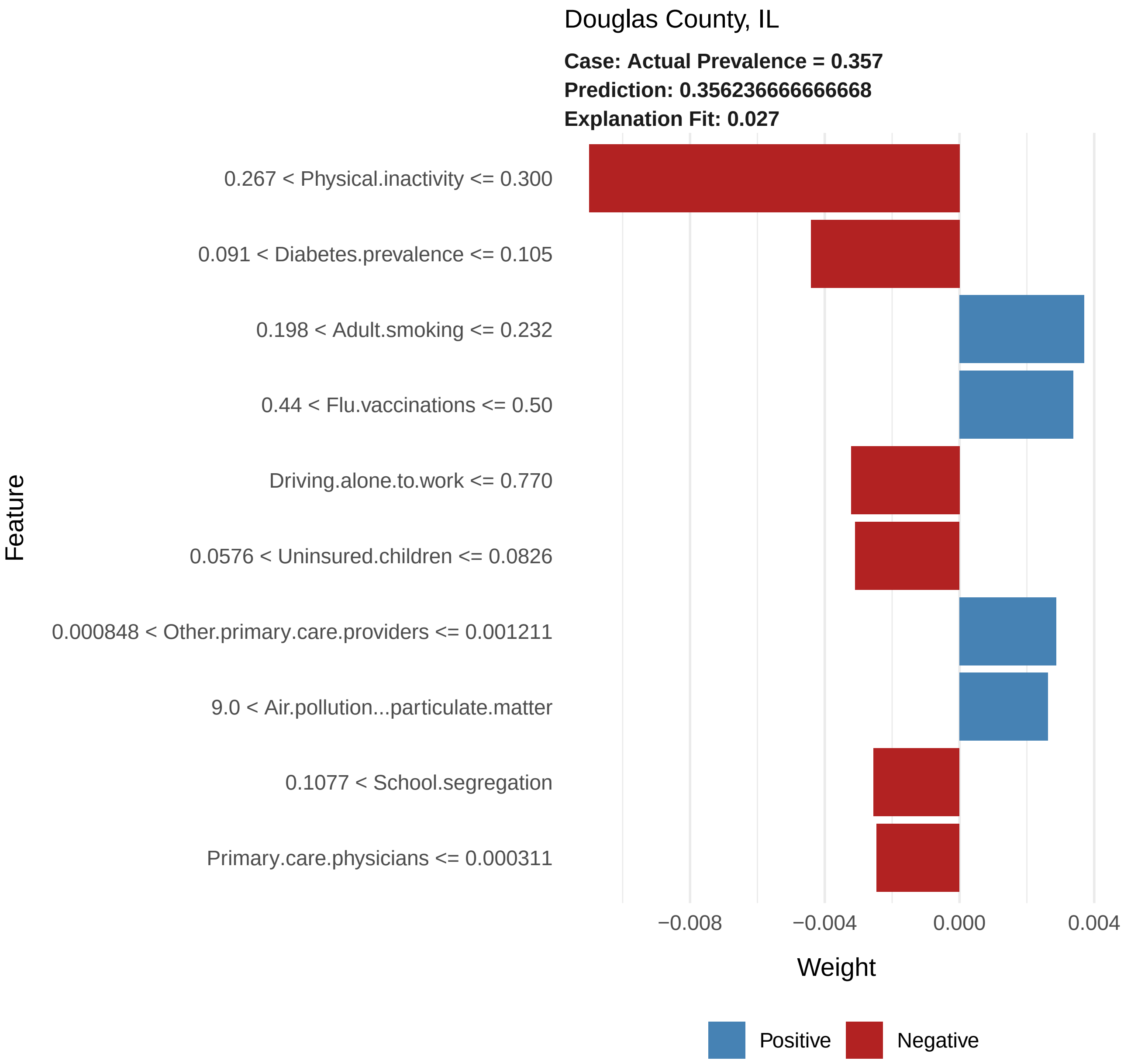

Douglas County, IL
Case: Actual Prevalence = 0.357
Prediction: 0.356236666666668
Explanation Fit: 0.027
0.267 < Physical.inactivity <= 0.300
0.091 < Diabetes.prevalence <= 0.105
0.198 < Adult.smoking <= 0.232
0.44 < Flu.vaccinations <= 0.50
Driving.alone.to.work <= 0.770
0.0576 < Uninsured.children <= 0.0826
0.000848 < Other.primary.care.providers <= 0.001211
9.0 < Air.pollution...particulate.matter
0.1077 < School.segregation
Primary.care.physicians <= 0.000311
Feature
−0.008
−0.004
0.000
0.004
Weight
Positive
Negative

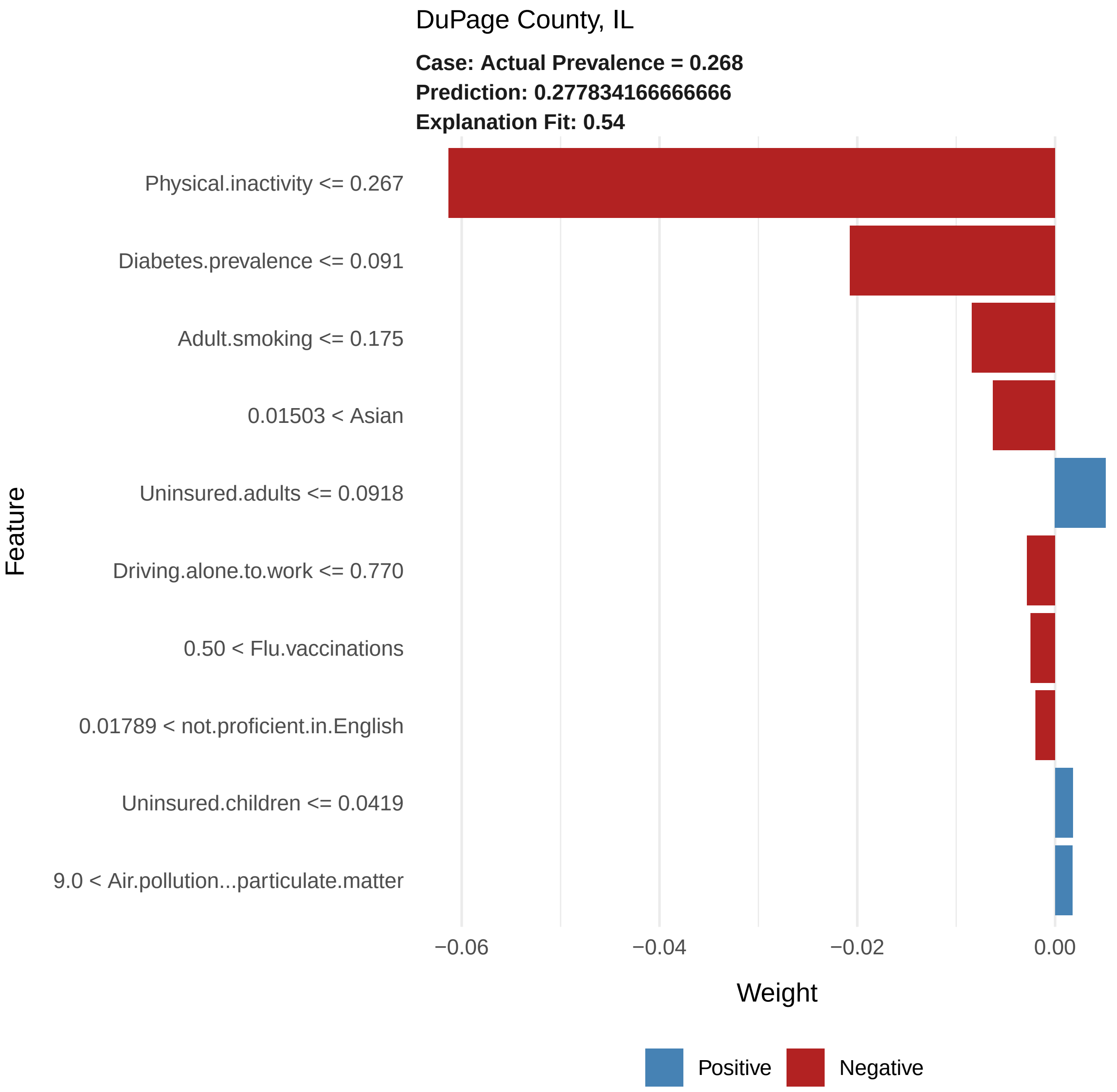

DuPage County, IL
Case: Actual Prevalence = 0.268
Prediction: 0.277834166666666
Explanation Fit: 0.54
Physical.inactivity <= 0.267
Diabetes.prevalence <= 0.091
Adult.smoking <= 0.175
0.01503 < Asian
Uninsured.adults <= 0.0918
Driving.alone.to.work <= 0.770
0.50 < Flu.vaccinations
0.01789 < not.proficient.in.English
Uninsured.children <= 0.0419
9.0 < Air.pollution...particulate.matter
Feature
−0.06
−0.04
−0.02
0.00
Weight
Positive
Negative

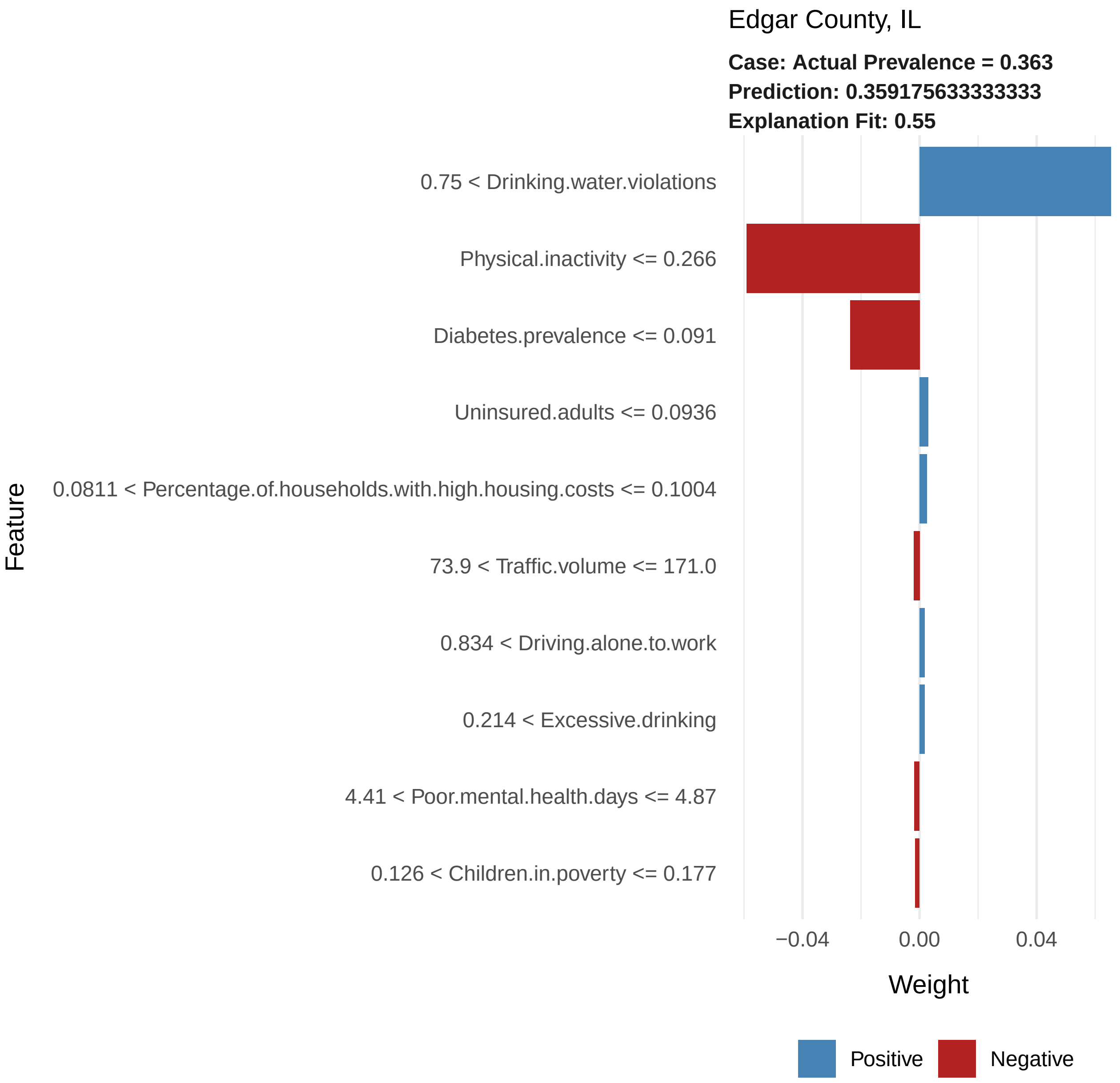

Edgar County, IL
Case: Actual Prevalence = 0.363
Prediction: 0.359175633333333
Explanation Fit: 0.55
0.75 < Drinking.water.violations
Physical.inactivity <= 0.266
Diabetes.prevalence <= 0.091
Uninsured.adults <= 0.0936
0.0811 < Percentage.of.households.with.high.housing.costs <= 0.1004
73.9 < Traffic.volume <= 171.0
0.834 < Driving.alone.to.work
0.214 < Excessive.drinking
4.41 < Poor.mental.health.days <= 4.87
0.126 < Children.in.poverty <= 0.177
Feature
−0.04
0.00
0.04
Weight
Positive
Negative

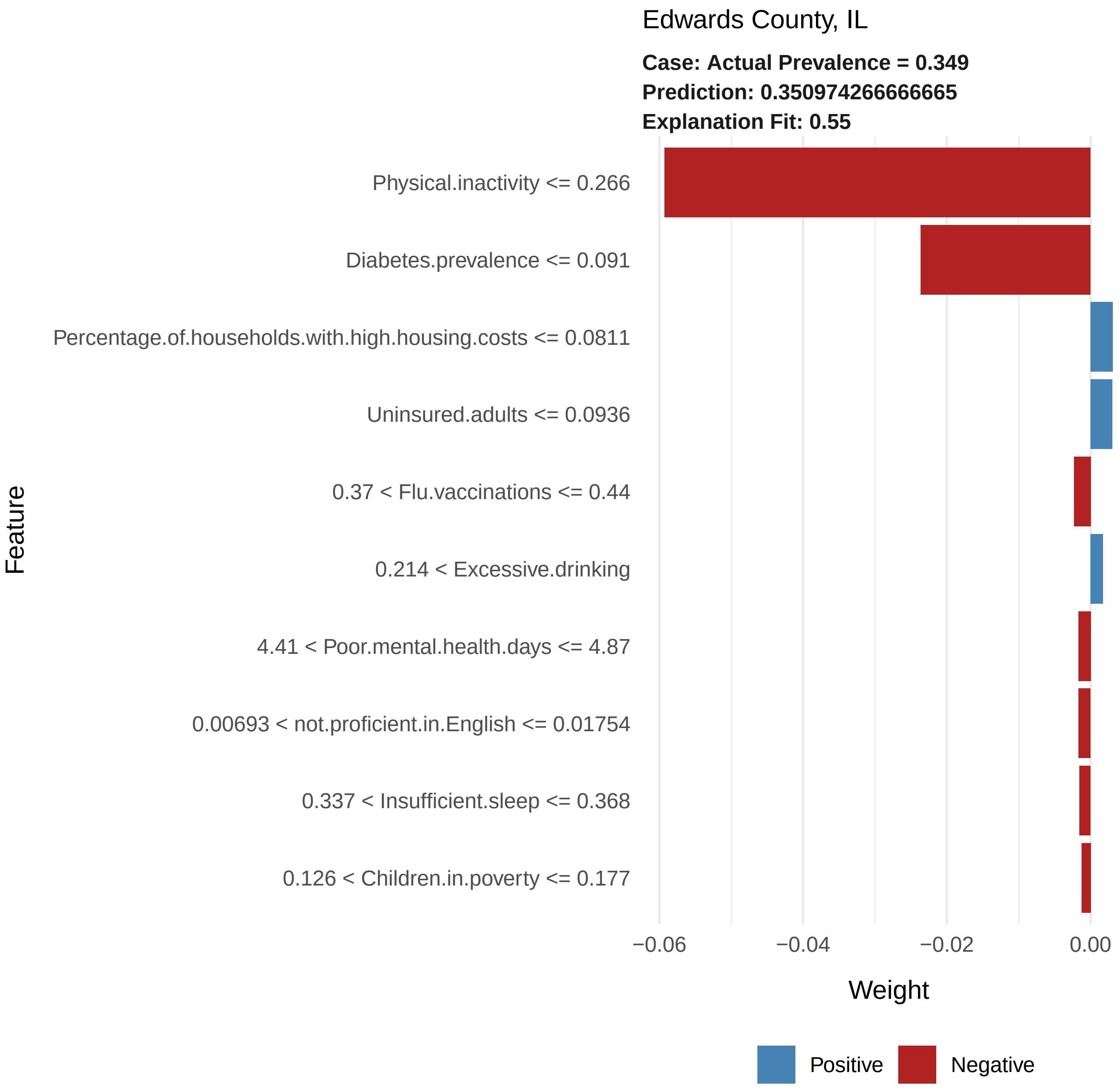

Edwards County, IL
Case: Actual Prevalence = 0.349
Prediction: 0.350974266666665
Explanation Fit: 0.55
Physical.inactivity <= 0.266
Diabetes.prevalence <= 0.091
Percentage.of.households.with.high.housing.costs <= 0.0811
Uninsured.adults <= 0.0936
0.37 < Flu.vaccinations <= 0.44
0.214 < Excessive.drinking
4.41 < Poor.mental.health.days <= 4.87
0.00693 < not.proficient.in.English <= 0.01754
0.337 < Insufficient.sleep <= 0.368
0.126 < Children.in.poverty <= 0.177
Feature
−0.06
−0.04
−0.02
0.00
Weight
Positive
Negative

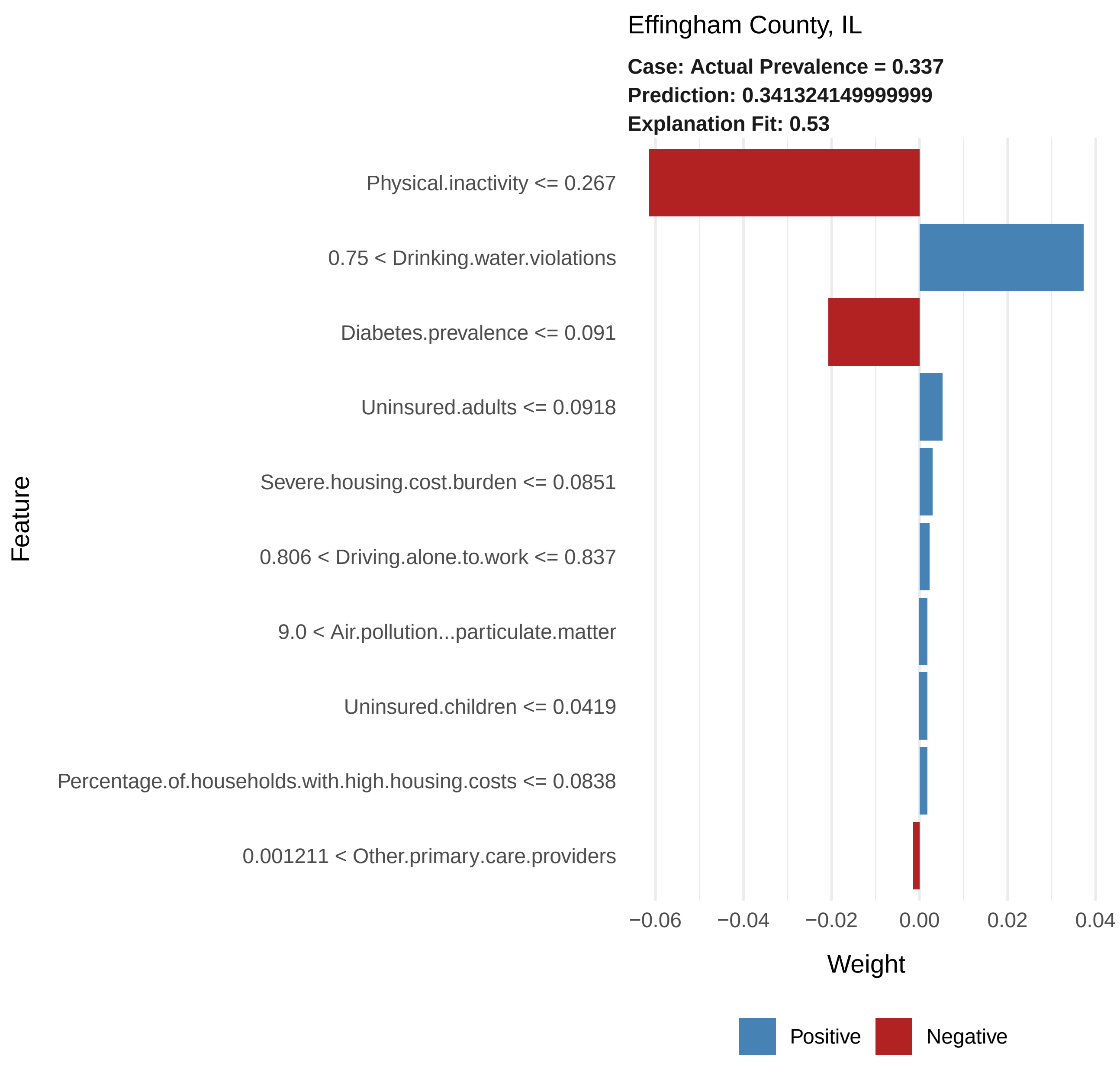
Effingham County, IL
Case: Actual Prevalence = 0.337
Prediction: 0.341324149999999
Explanation Fit: 0.53
Physical.inactivity <= 0.267
0.75 < Drinking.water.violations
Diabetes.prevalence <= 0.091
Uninsured.adults <= 0.0918
Severe.housing.cost.burden <= 0.0851
0.806 < Driving.alone.to.work <= 0.837
9.0 < Air.pollution...particulate.matter
Uninsured.children <= 0.0419
Percentage.of.households.with.high.housing.costs <= 0.0838
0.001211 < Other.primary.care.providers
Feature
−0.06
−0.04
−0.02
0.00
0.02
0.04
Weight
Positive
Negative

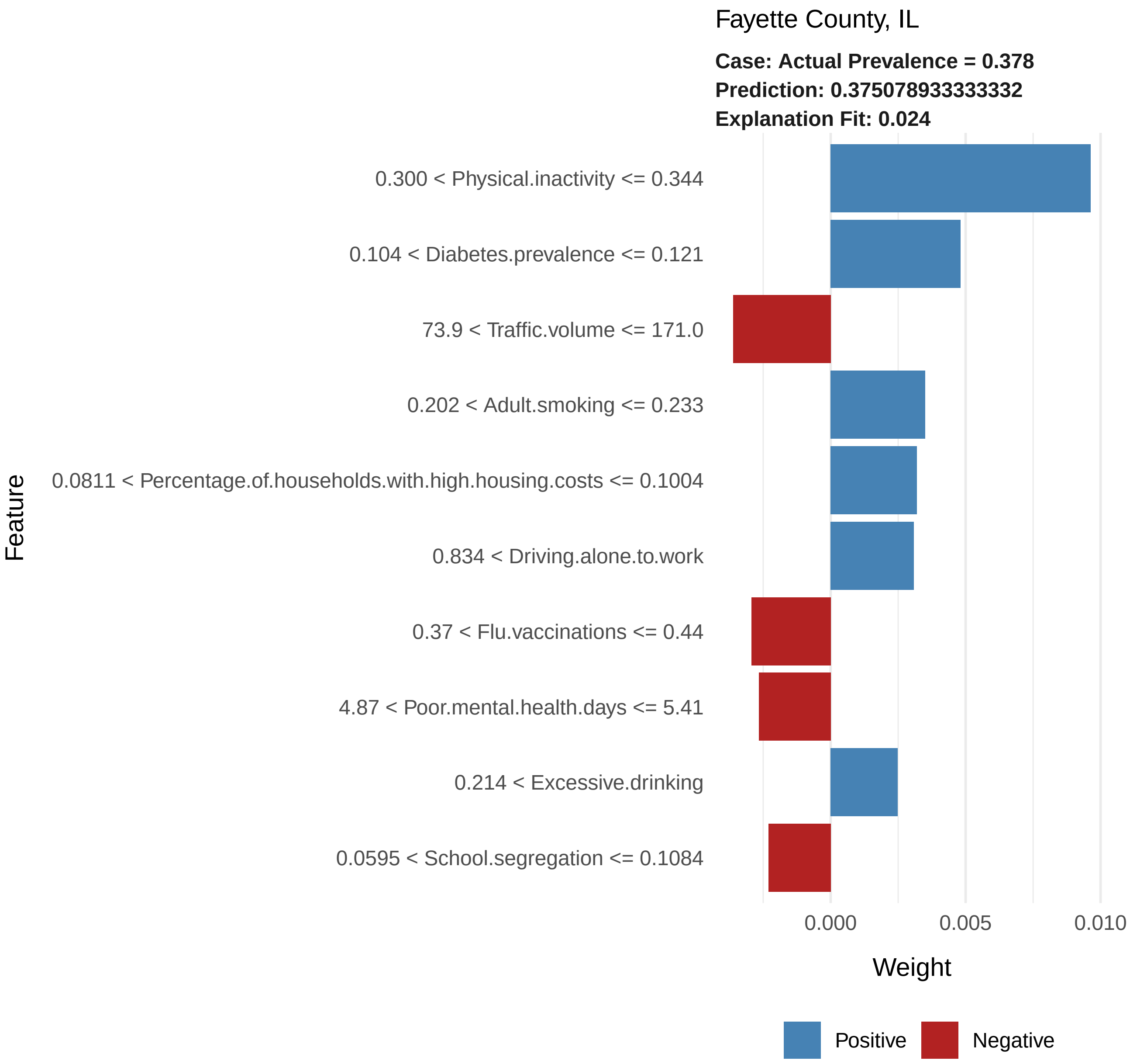

Fayette County, IL
Case: Actual Prevalence = 0.378
Prediction: 0.375078933333332
Explanation Fit: 0.024
0.300 < Physical.inactivity <= 0.344
0.104 < Diabetes.prevalence <= 0.121
73.9 < Traffic.volume <= 171.0
0.202 < Adult.smoking <= 0.233
0.0811 < Percentage.of.households.with.high.housing.costs <= 0.1004
0.834 < Driving.alone.to.work
0.37 < Flu.vaccinations <= 0.44
4.87 < Poor.mental.health.days <= 5.41
0.214 < Excessive.drinking
0.0595 < School.segregation <= 0.1084
Feature
0.000
0.005
0.010
Weight
Positive
Negative

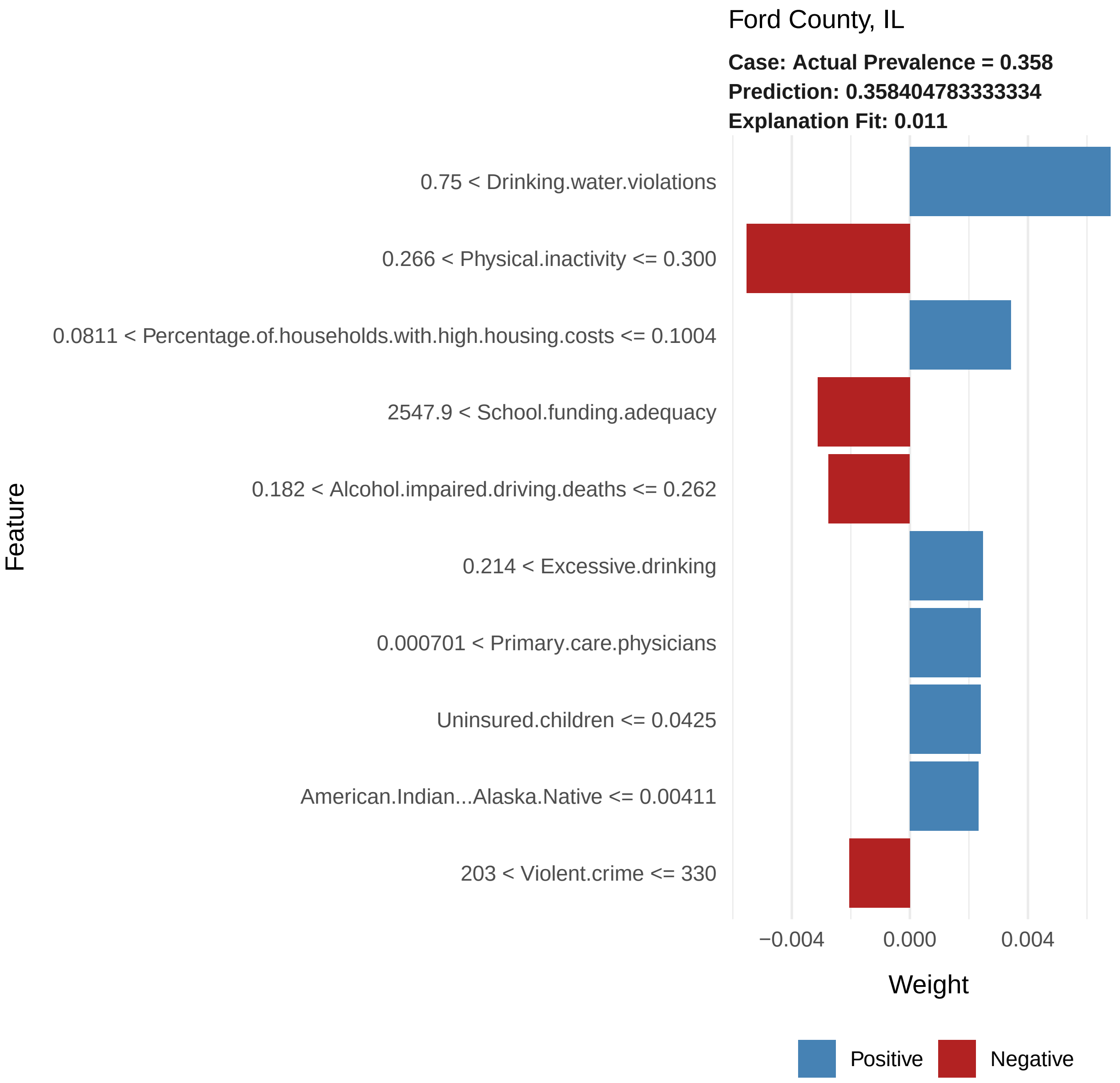

Ford County, IL
Case: Actual Prevalence = 0.358
Prediction: 0.358404783333334
Explanation Fit: 0.011
0.75 < Drinking.water.violations
0.266 < Physical.inactivity <= 0.300
0.0811 < Percentage.of.households.with.high.housing.costs <= 0.1004
2547.9 < School.funding.adequacy
0.182 < Alcohol.impaired.driving.deaths <= 0.262
0.214 < Excessive.drinking
0.000701 < Primary.care.physicians
Uninsured.children <= 0.0425
American.Indian...Alaska.Native <= 0.00411
203 < Violent.crime <= 330
Feature
−0.004
0.000
0.004
Weight
Positive
Negative

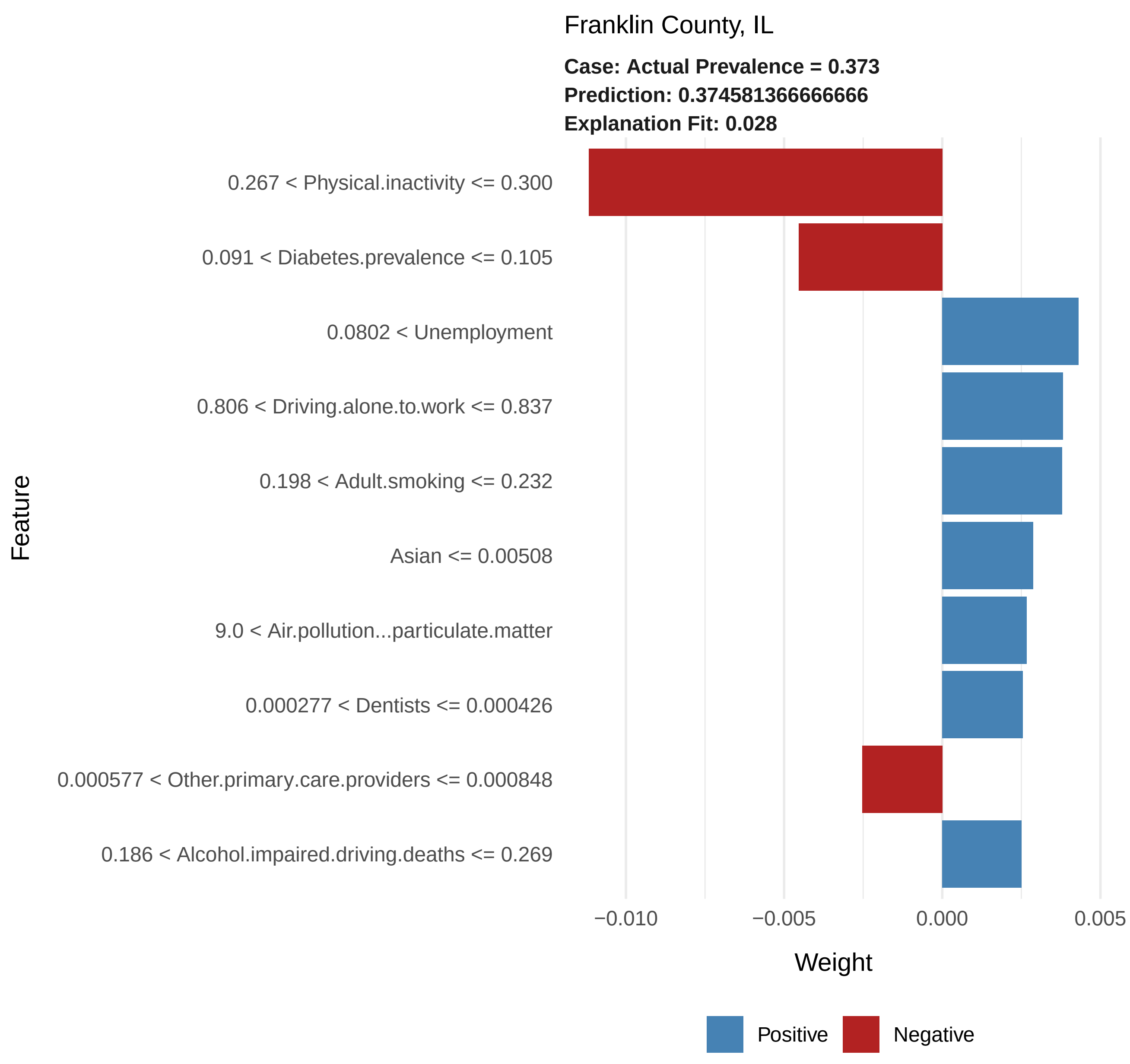
Franklin County, IL
Case: Actual Prevalence = 0.373
Prediction: 0.374581366666666
Explanation Fit: 0.028
0.267 < Physical.inactivity <= 0.300
0.091 < Diabetes.prevalence <= 0.105
0.0802 < Unemployment
0.806 < Driving.alone.to.work <= 0.837
0.198 < Adult.smoking <= 0.232
Asian <= 0.00508
9.0 < Air.pollution...particulate.matter
0.000277 < Dentists <= 0.000426
0.000577 < Other.primary.care.providers <= 0.000848
0.186 < Alcohol.impaired.driving.deaths <= 0.269
Feature
−0.010
−0.005
0.000
0.005
Weight
Positive
Negative

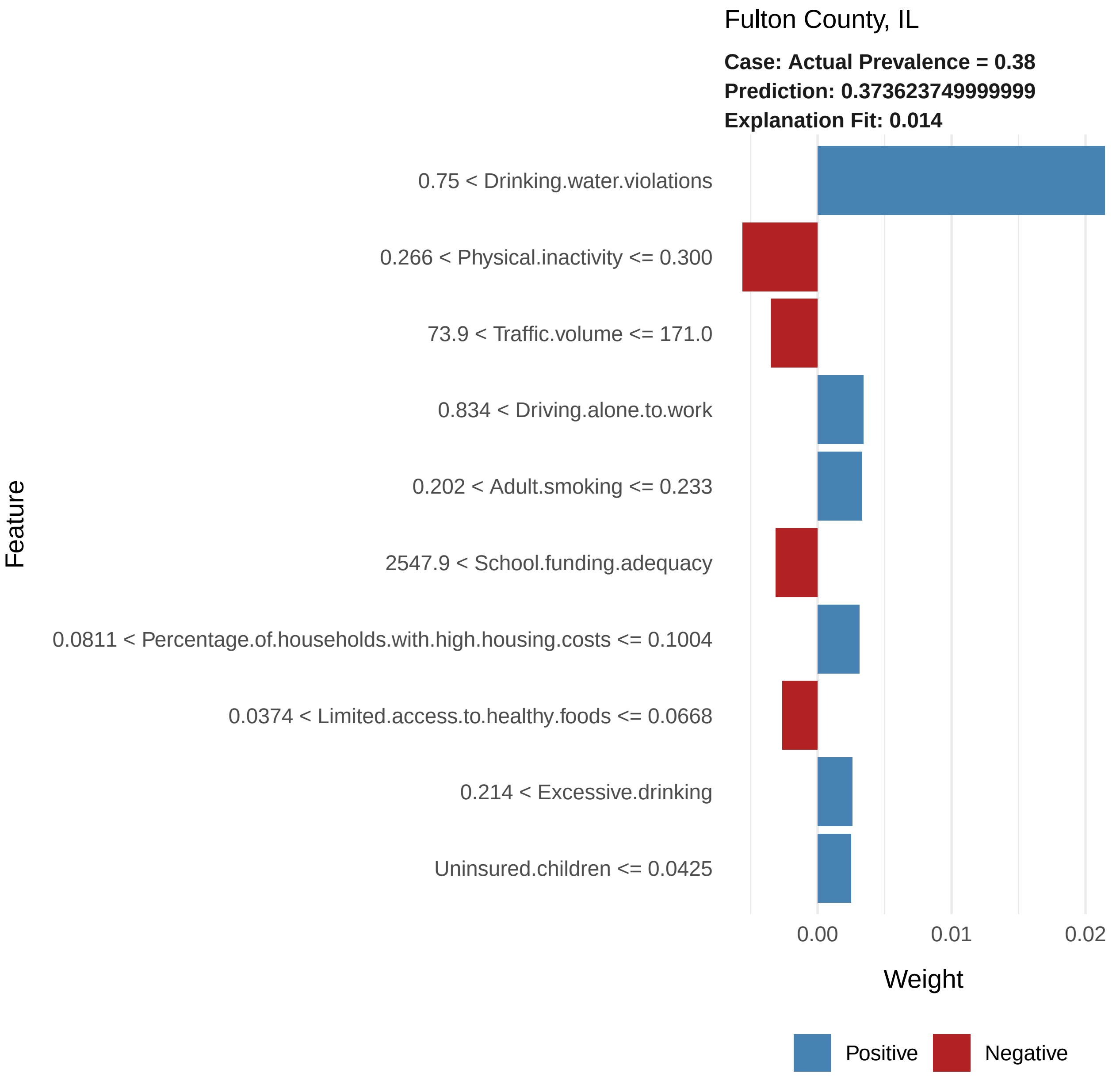
Fulton County, IL
Case: Actual Prevalence = 0.38
Prediction: 0.373623749999999
Explanation Fit: 0.014
0.75 < Drinking.water.violations
0.266 < Physical.inactivity <= 0.300
73.9 < Traffic.volume <= 171.0
0.834 < Driving.alone.to.work
0.202 < Adult.smoking <= 0.233
2547.9 < School.funding.adequacy
0.0811 < Percentage.of.households.with.high.housing.costs <= 0.1004
0.0374 < Limited.access.to.healthy.foods <= 0.0668
0.214 < Excessive.drinking
Uninsured.children <= 0.0425
Feature
0.00
0.01
0.02
Weight
Positive
Negative

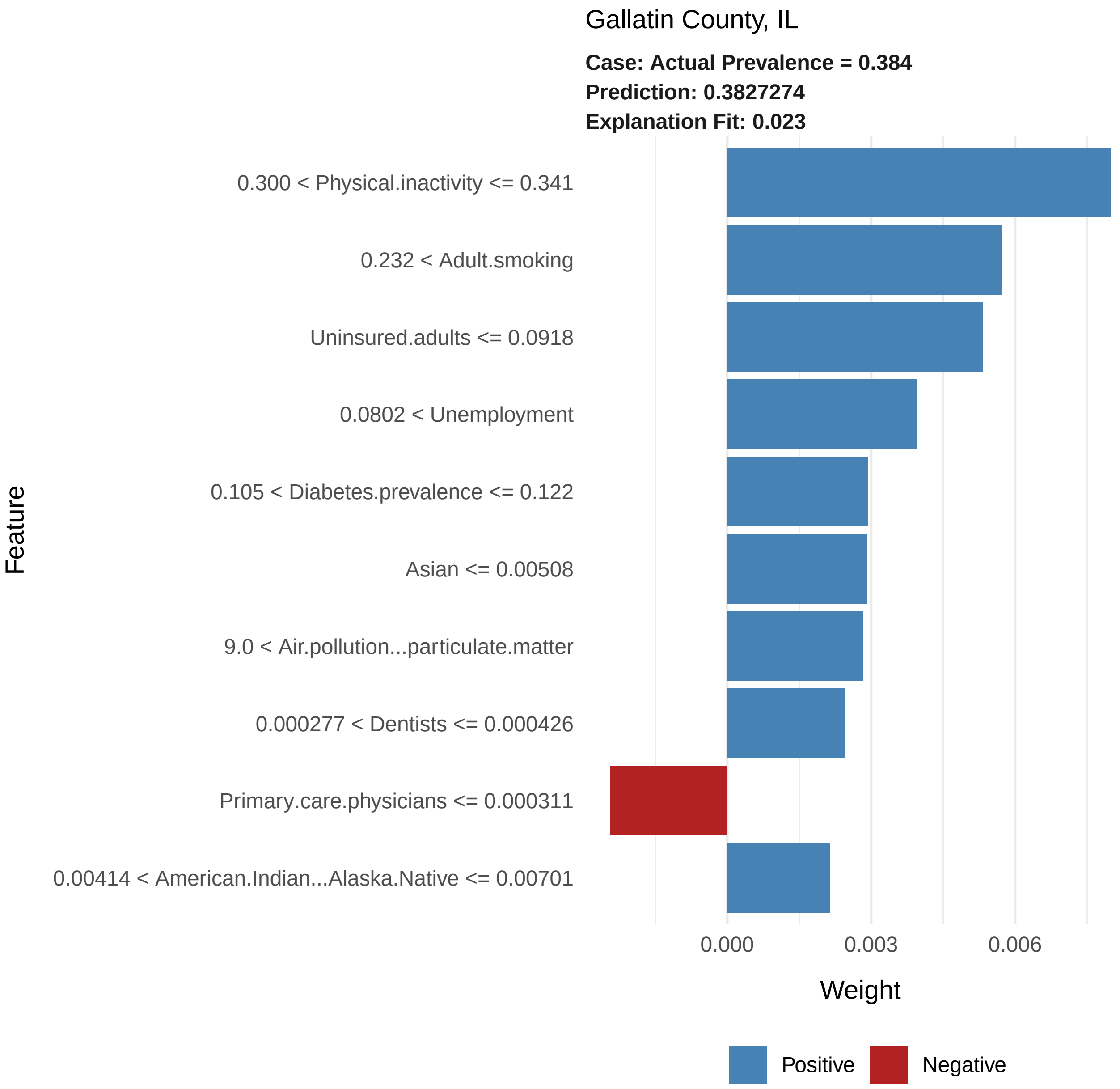
Gallatin County, IL
Case: Actual Prevalence = 0.384
Prediction: 0.3827274
Explanation Fit: 0.023
0.300 < Physical.inactivity <= 0.341
0.232 < Adult.smoking
Uninsured.adults <= 0.0918
0.0802 < Unemployment
0.105 < Diabetes.prevalence <= 0.122
Asian <= 0.00508
9.0 < Air.pollution...particulate.matter
0.000277 < Dentists <= 0.000426
Primary.care.physicians <= 0.000311
0.00414 < American.Indian...Alaska.Native <= 0.00701
Feature
0.000
0.003
0.006
Weight
Positive
Negative

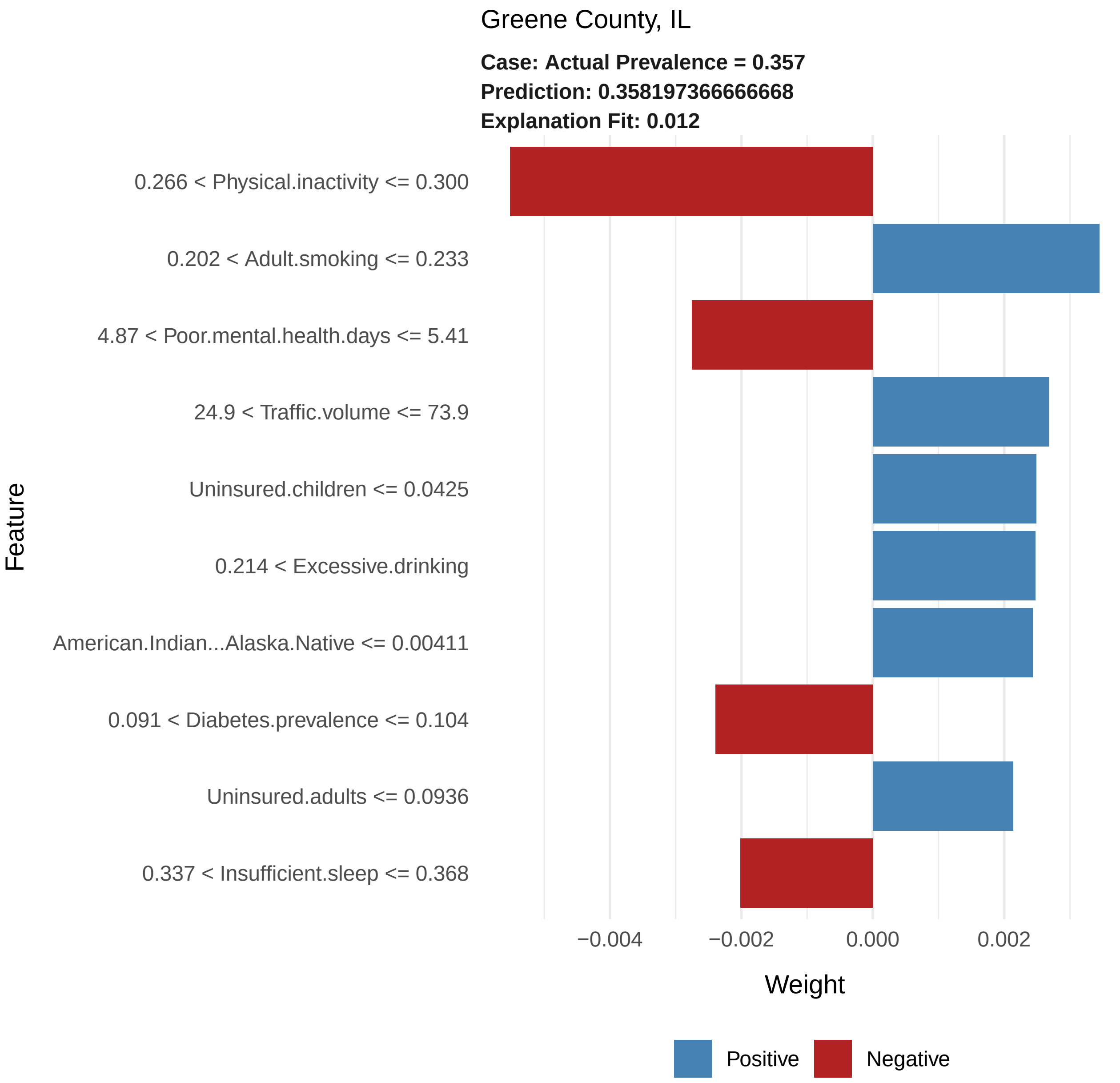

Greene County, IL
Case: Actual Prevalence = 0.357
Prediction: 0.358197366666668
Explanation Fit: 0.012
0.266 < Physical.inactivity <= 0.300
0.202 < Adult.smoking <= 0.233
4.87 < Poor.mental.health.days <= 5.41
24.9 < Traffic.volume <= 73.9
Uninsured.children <= 0.0425
0.214 < Excessive.drinking
American.Indian...Alaska.Native <= 0.00411
0.091 < Diabetes.prevalence <= 0.104
Uninsured.adults <= 0.0936
0.337 < Insufficient.sleep <= 0.368
Feature
−0.004
−0.002
0.000
0.002
Weight
Positive
Negative

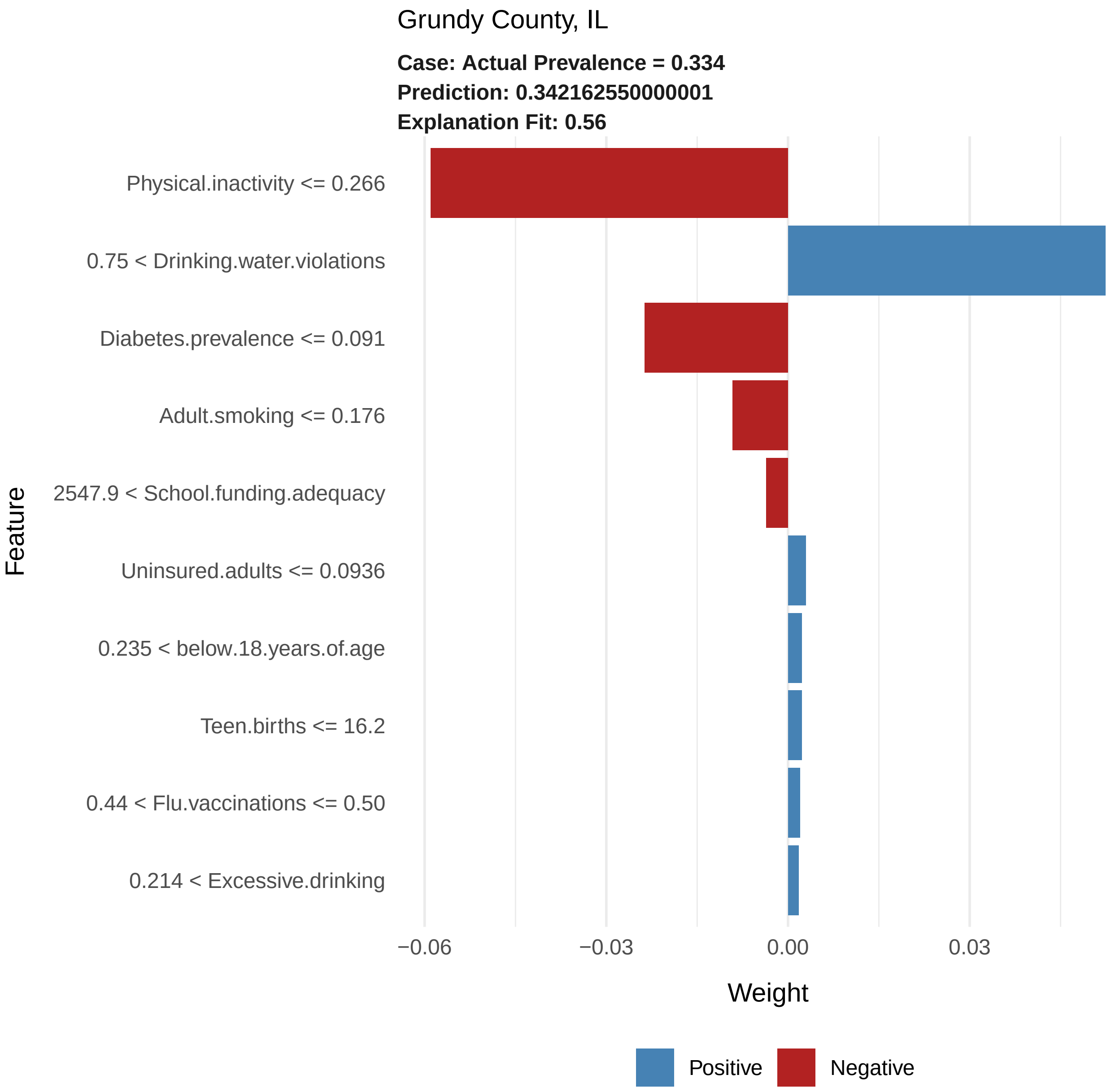

Grundy County, IL
Case: Actual Prevalence = 0.334
Prediction: 0.342162550000001
Explanation Fit: 0.56
Physical.inactivity <= 0.266
0.75 < Drinking.water.violations
Diabetes.prevalence <= 0.091
Adult.smoking <= 0.176
2547.9 < School.funding.adequacy
Uninsured.adults <= 0.0936
0.235 < below.18.years.of.age
Teen.births <= 16.2
0.44 < Flu.vaccinations <= 0.50
0.214 < Excessive.drinking
Feature
−0.06
−0.03
0.00
0.03
Weight
Positive
Negative

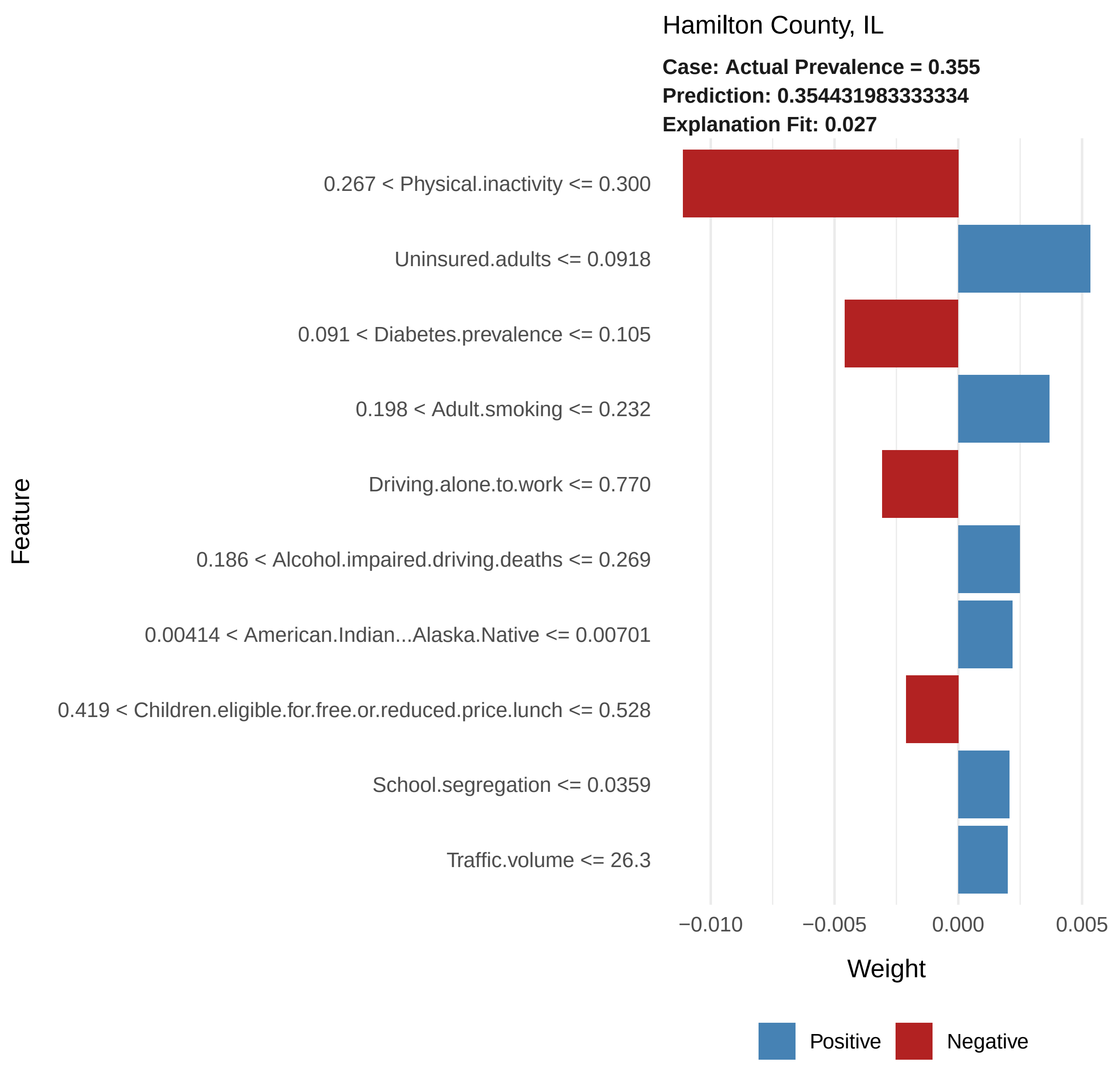

Hamilton County, IL
Case: Actual Prevalence = 0.355
Prediction: 0.354431983333334
Explanation Fit: 0.027
0.267 < Physical.inactivity <= 0.300
Uninsured.adults <= 0.0918
0.091 < Diabetes.prevalence <= 0.105
0.198 < Adult.smoking <= 0.232
Driving.alone.to.work <= 0.770
0.186 < Alcohol.impaired.driving.deaths <= 0.269
0.00414 < American.Indian...Alaska.Native <= 0.00701
0.419 < Children.eligible.for.free.or.reduced.price.lunch <= 0.528
School.segregation <= 0.0359
Traffic.volume <= 26.3
Feature
−0.010
−0.005
0.000
0.005
Weight
Positive
Negative

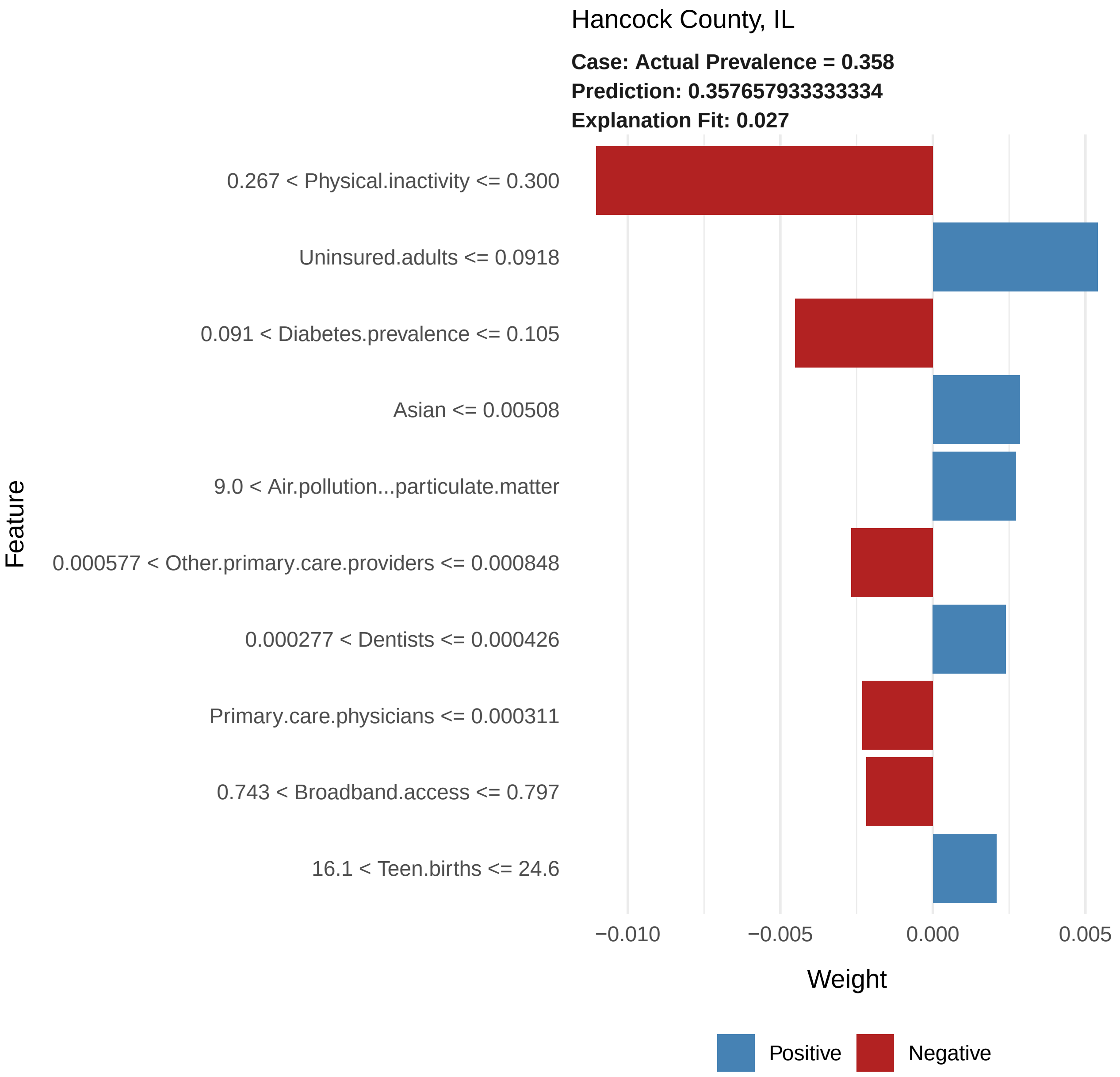

Hancock County, IL
Case: Actual Prevalence = 0.358
Prediction: 0.357657933333334
Explanation Fit: 0.027
0.267 < Physical.inactivity <= 0.300
Uninsured.adults <= 0.0918
0.091 < Diabetes.prevalence <= 0.105
Asian <= 0.00508
9.0 < Air.pollution...particulate.matter
0.000577 < Other.primary.care.providers <= 0.000848
0.000277 < Dentists <= 0.000426
Primary.care.physicians <= 0.000311
0.743 < Broadband.access <= 0.797
16.1 < Teen.births <= 24.6
Feature
−0.010
−0.005
0.000
0.005
Weight
Positive
Negative

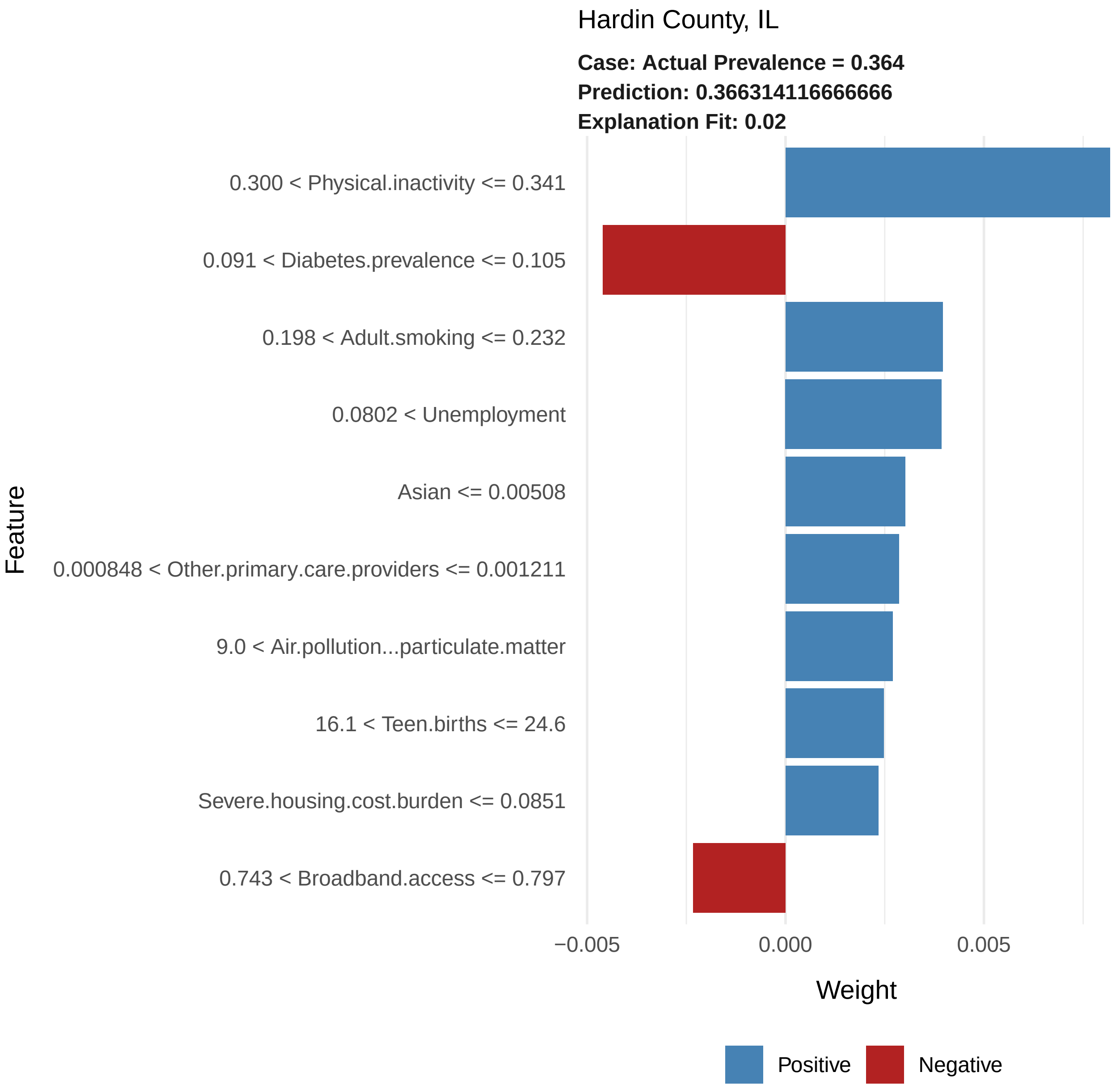

Hardin County, IL
Case: Actual Prevalence = 0.364
Prediction: 0.366314116666666
Explanation Fit: 0.02
0.300 < Physical.inactivity <= 0.341
0.091 < Diabetes.prevalence <= 0.105
0.198 < Adult.smoking <= 0.232
0.0802 < Unemployment
Asian <= 0.00508
0.000848 < Other.primary.care.providers <= 0.001211
9.0 < Air.pollution...particulate.matter
16.1 < Teen.births <= 24.6
Severe.housing.cost.burden <= 0.0851
0.743 < Broadband.access <= 0.797
Feature
−0.005
0.000
0.005
Weight
Positive
Negative

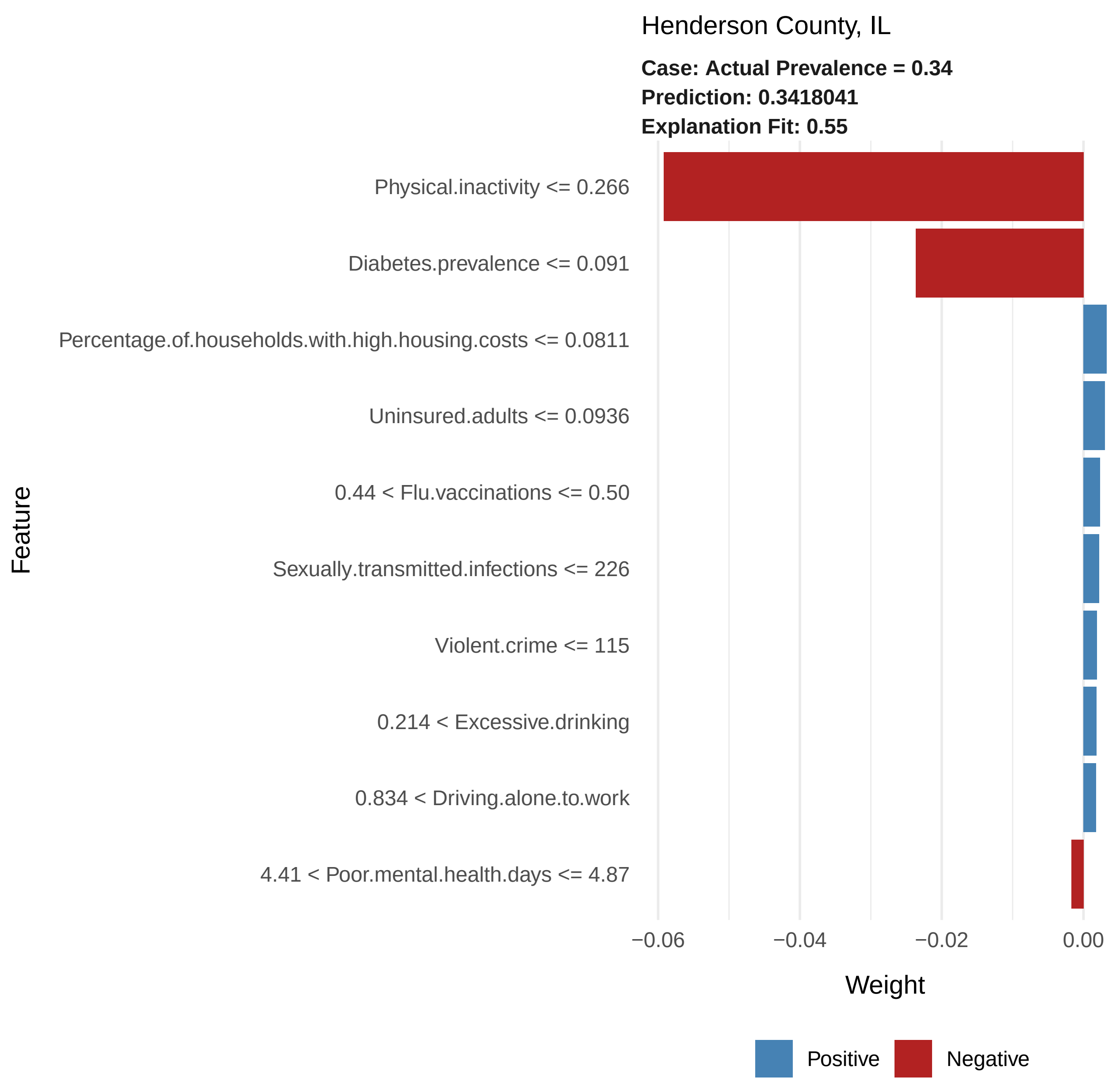
Henderson County, IL
Case: Actual Prevalence = 0.34
Prediction: 0.3418041
Explanation Fit: 0.55
Physical.inactivity <= 0.266
Diabetes.prevalence <= 0.091
Percentage.of.households.with.high.housing.costs <= 0.0811
Uninsured.adults <= 0.0936
0.44 < Flu.vaccinations <= 0.50
Sexually.transmitted.infections <= 226
Violent.crime <= 115
0.214 < Excessive.drinking
0.834 < Driving.alone.to.work
4.41 < Poor.mental.health.days <= 4.87
Feature
−0.06
−0.04
−0.02
0.00
Weight
Positive
Negative

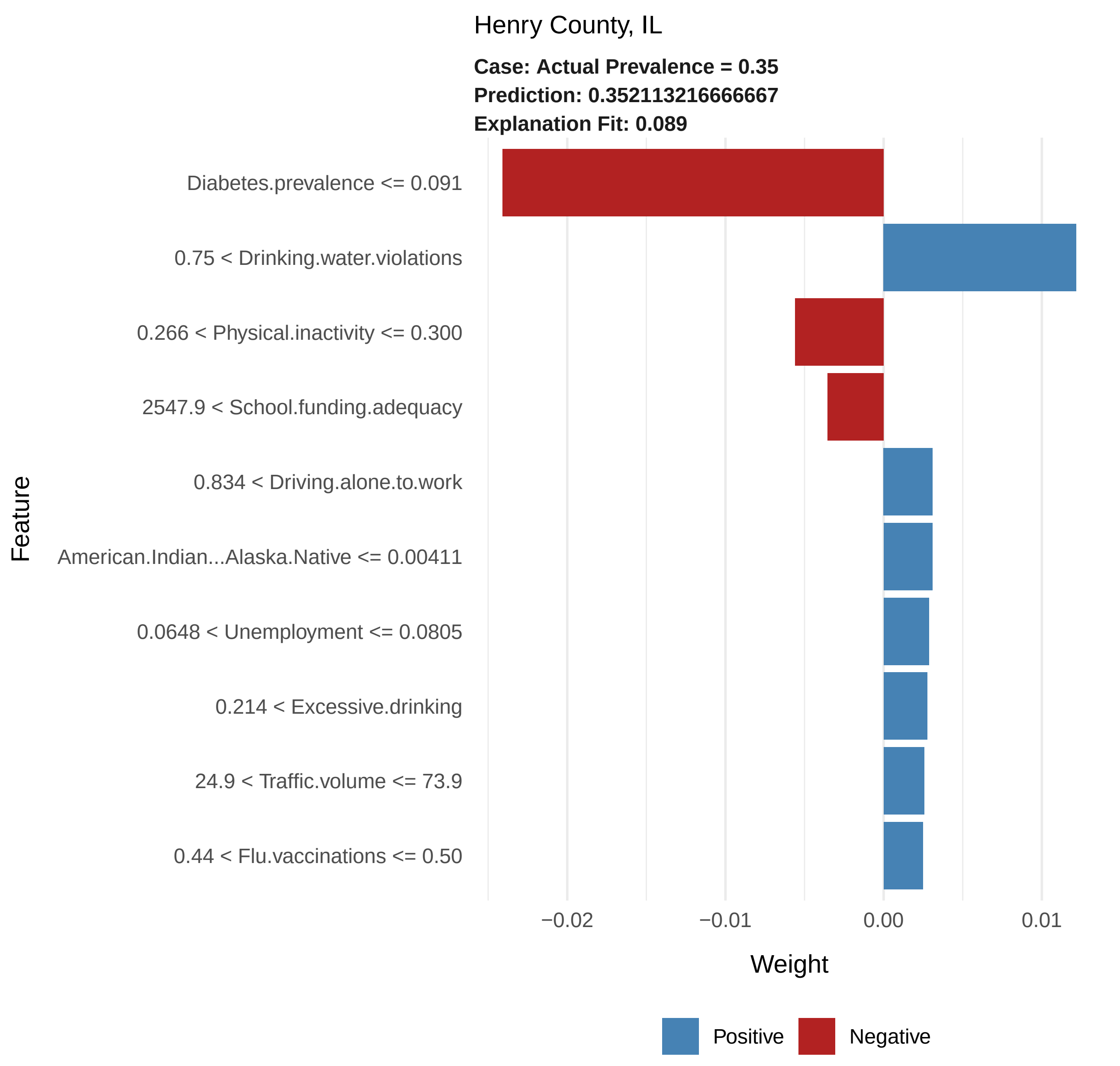

Henry County, IL
Case: Actual Prevalence = 0.35
Prediction: 0.352113216666667
Explanation Fit: 0.089
Diabetes.prevalence <= 0.091
0.75 < Drinking.water.violations
0.266 < Physical.inactivity <= 0.300
2547.9 < School.funding.adequacy
0.834 < Driving.alone.to.work
American.Indian...Alaska.Native <= 0.00411
0.0648 < Unemployment <= 0.0805
0.214 < Excessive.drinking
24.9 < Traffic.volume <= 73.9
0.44 < Flu.vaccinations <= 0.50
Feature
−0.02
−0.01
0.00
0.01
Weight
Positive
Negative

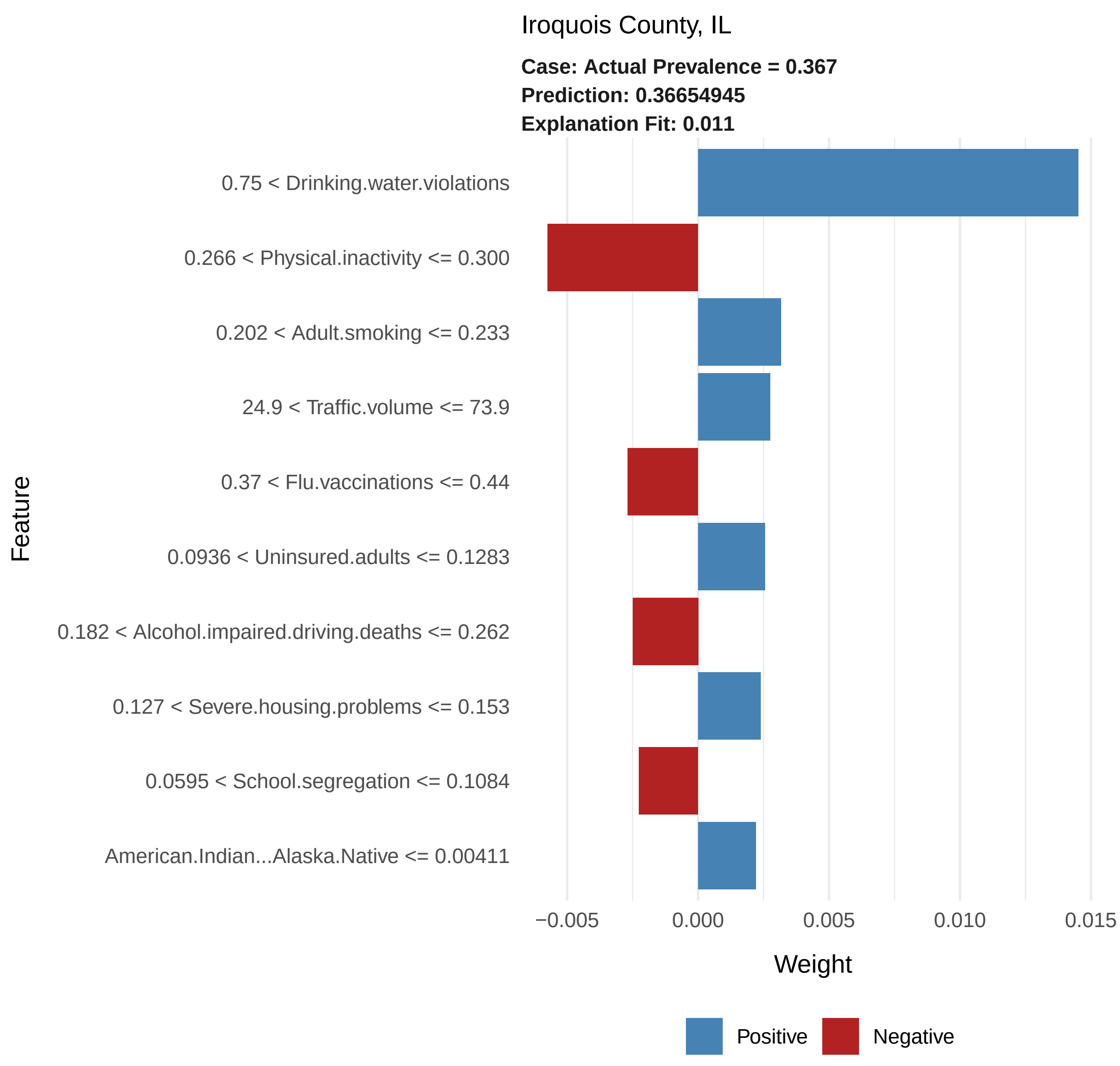
Iroquois County, IL
Case: Actual Prevalence = 0.367
Prediction: 0.36654945
Explanation Fit: 0.011
0.75 < Drinking.water.violations
0.266 < Physical.inactivity <= 0.300
0.202 < Adult.smoking <= 0.233
24.9 < Traffic.volume <= 73.9
0.37 < Flu.vaccinations <= 0.44
0.0936 < Uninsured.adults <= 0.1283
0.182 < Alcohol.impaired.driving.deaths <= 0.262
0.127 < Severe.housing.problems <= 0.153
0.0595 < School.segregation <= 0.1084
American.Indian...Alaska.Native <= 0.00411
Feature
−0.005
0.000
0.005
0.010
0.015
Weight
Positive
Negative

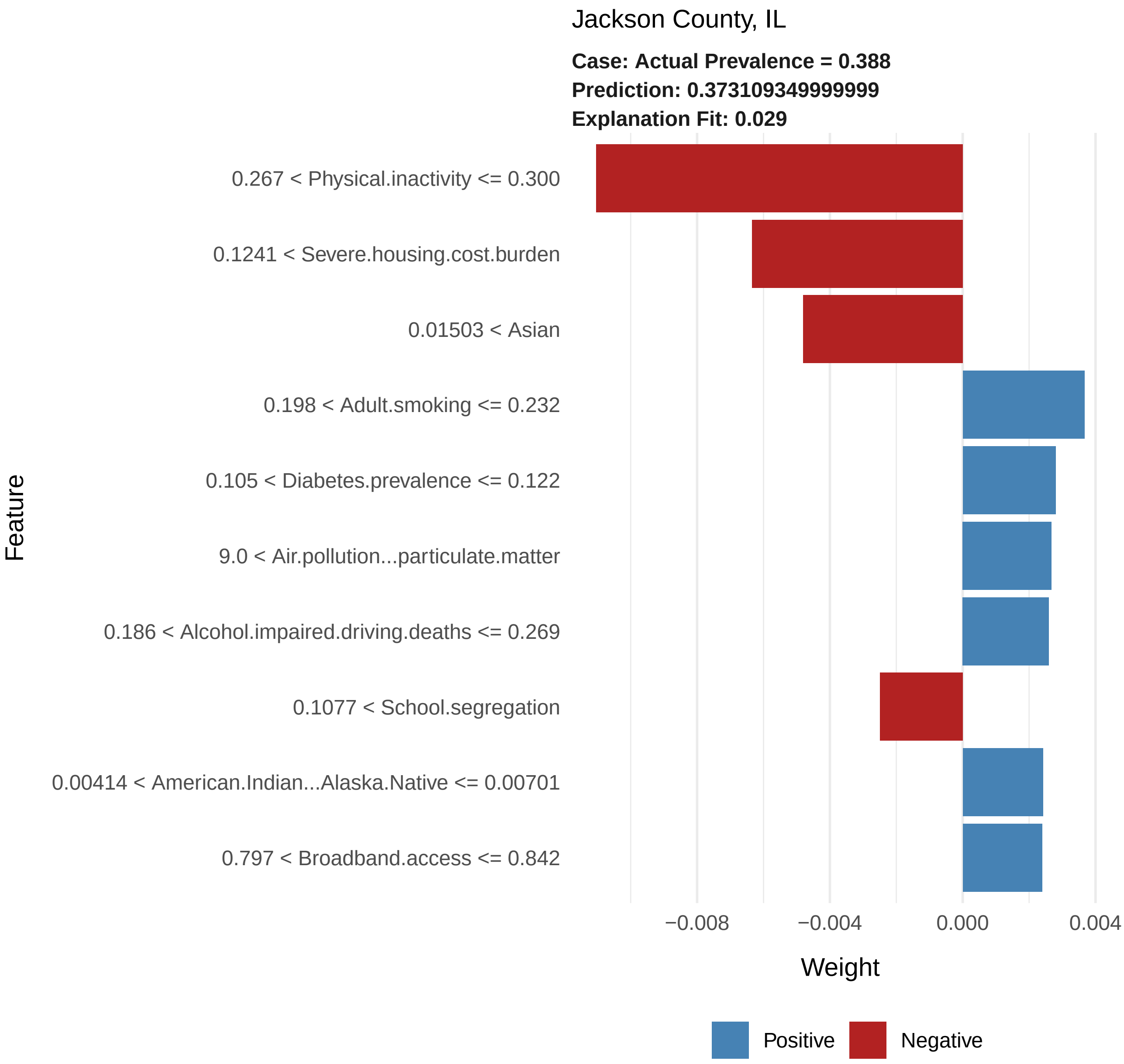
Jackson County, IL
Case: Actual Prevalence = 0.388
Prediction: 0.373109349999999
Explanation Fit: 0.029
0.267 < Physical.inactivity <= 0.300
0.1241 < Severe.housing.cost.burden
0.01503 < Asian
0.198 < Adult.smoking <= 0.232
0.105 < Diabetes.prevalence <= 0.122
9.0 < Air.pollution...particulate.matter
0.186 < Alcohol.impaired.driving.deaths <= 0.269
0.1077 < School.segregation
0.00414 < American.Indian...Alaska.Native <= 0.00701
0.797 < Broadband.access <= 0.842
Feature
−0.008
−0.004
0.000
0.004
Weight
Positive
Negative

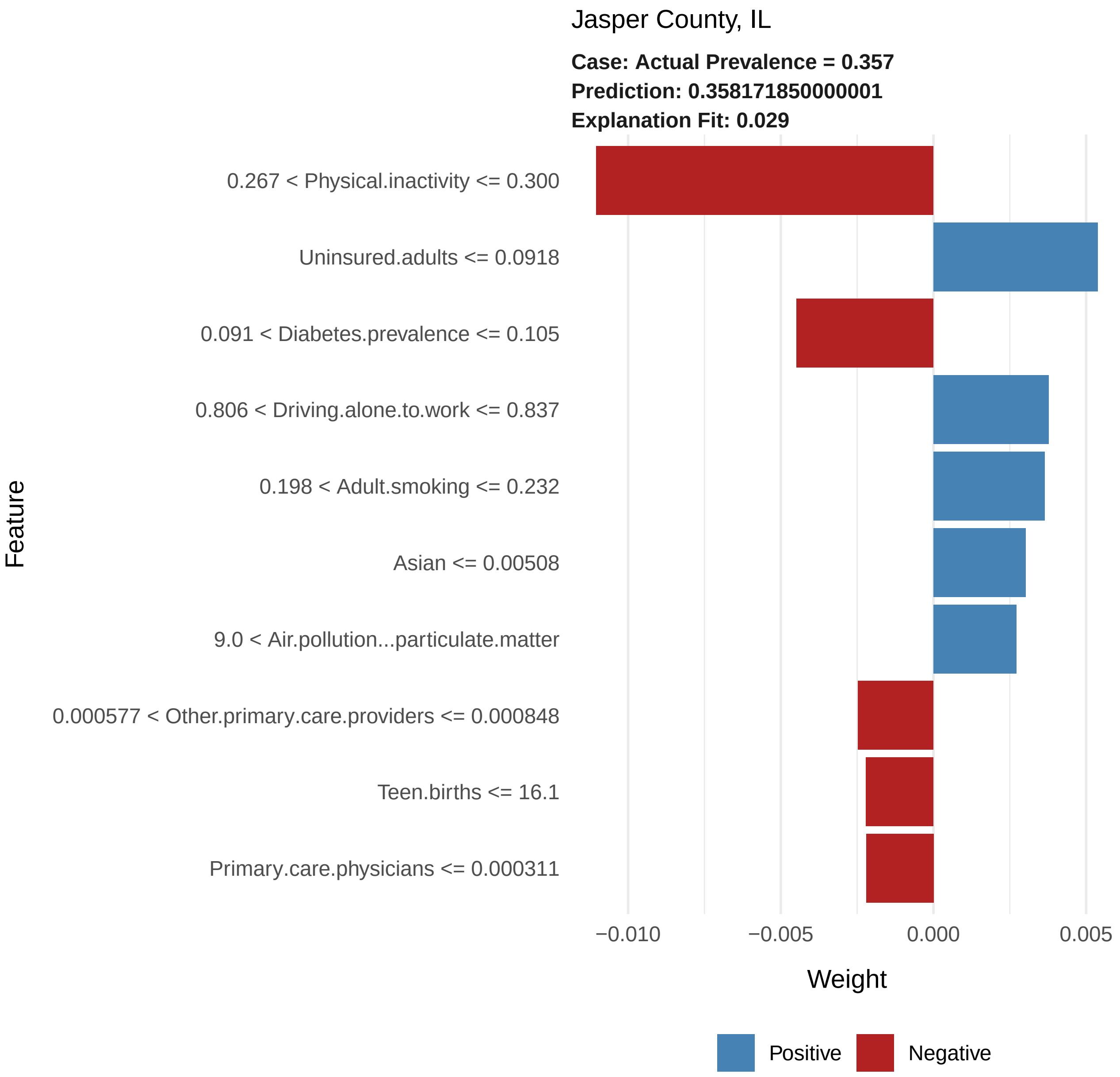

Jasper County, IL
Case: Actual Prevalence = 0.357
Prediction: 0.358171850000001
Explanation Fit: 0.029
0.267 < Physical.inactivity <= 0.300
Uninsured.adults <= 0.0918
0.091 < Diabetes.prevalence <= 0.105
0.806 < Driving.alone.to.work <= 0.837
0.198 < Adult.smoking <= 0.232
Asian <= 0.00508
9.0 < Air.pollution...particulate.matter
0.000577 < Other.primary.care.providers <= 0.000848
Teen.births <= 16.1
Primary.care.physicians <= 0.000311
Feature
−0.010
−0.005
0.000
0.005
Weight
Positive
Negative

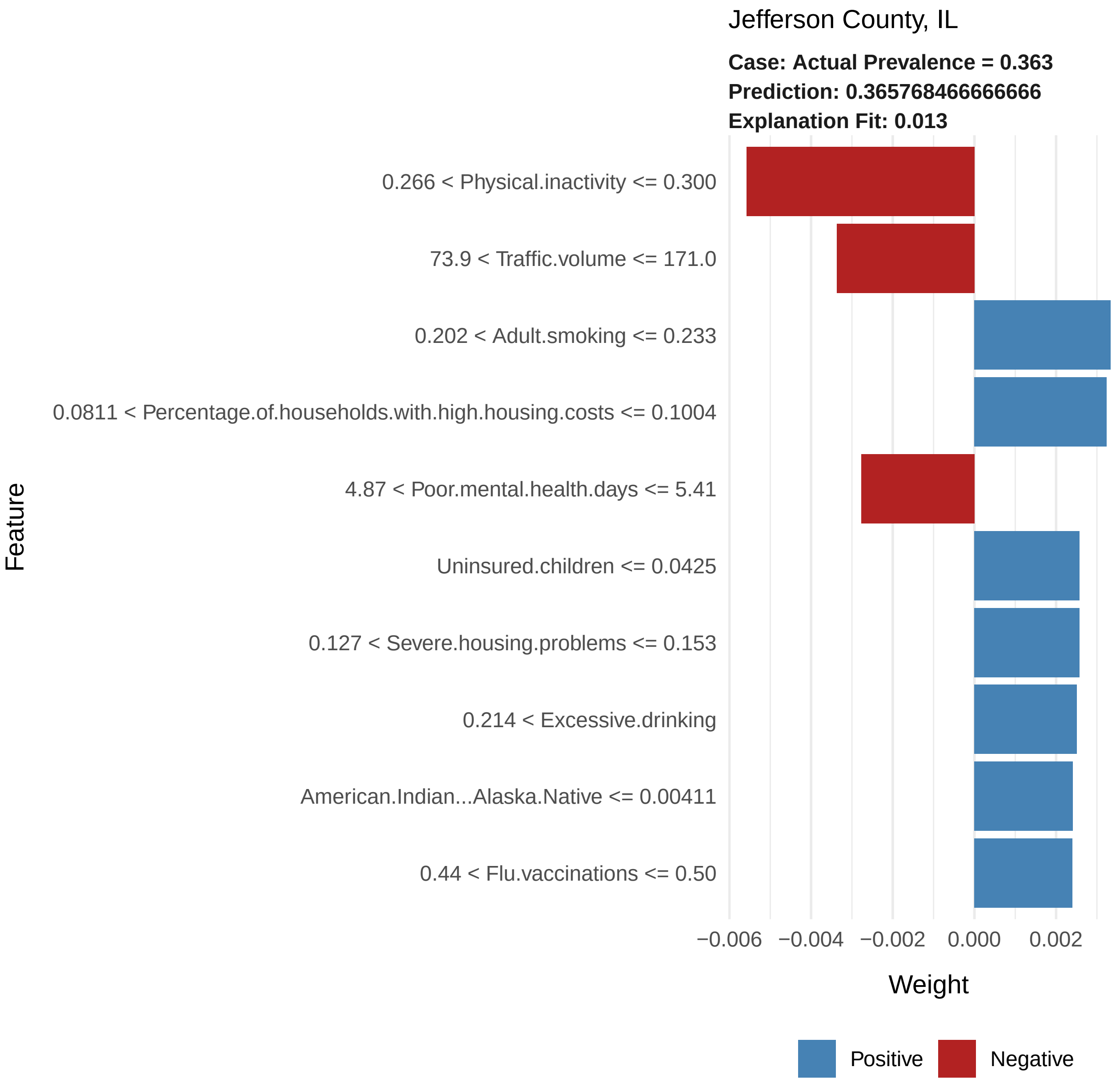

Jefferson County, IL
Case: Actual Prevalence = 0.363
Prediction: 0.365768466666666
Explanation Fit: 0.013
0.266 < Physical.inactivity <= 0.300
73.9 < Traffic.volume <= 171.0
0.202 < Adult.smoking <= 0.233
0.0811 < Percentage.of.households.with.high.housing.costs <= 0.1004
4.87 < Poor.mental.health.days <= 5.41
Uninsured.children <= 0.0425
0.127 < Severe.housing.problems <= 0.153
0.214 < Excessive.drinking
American.Indian...Alaska.Native <= 0.00411
0.44 < Flu.vaccinations <= 0.50
Feature
−0.006
−0.004
−0.002
0.000
0.002
Weight
Positive
Negative

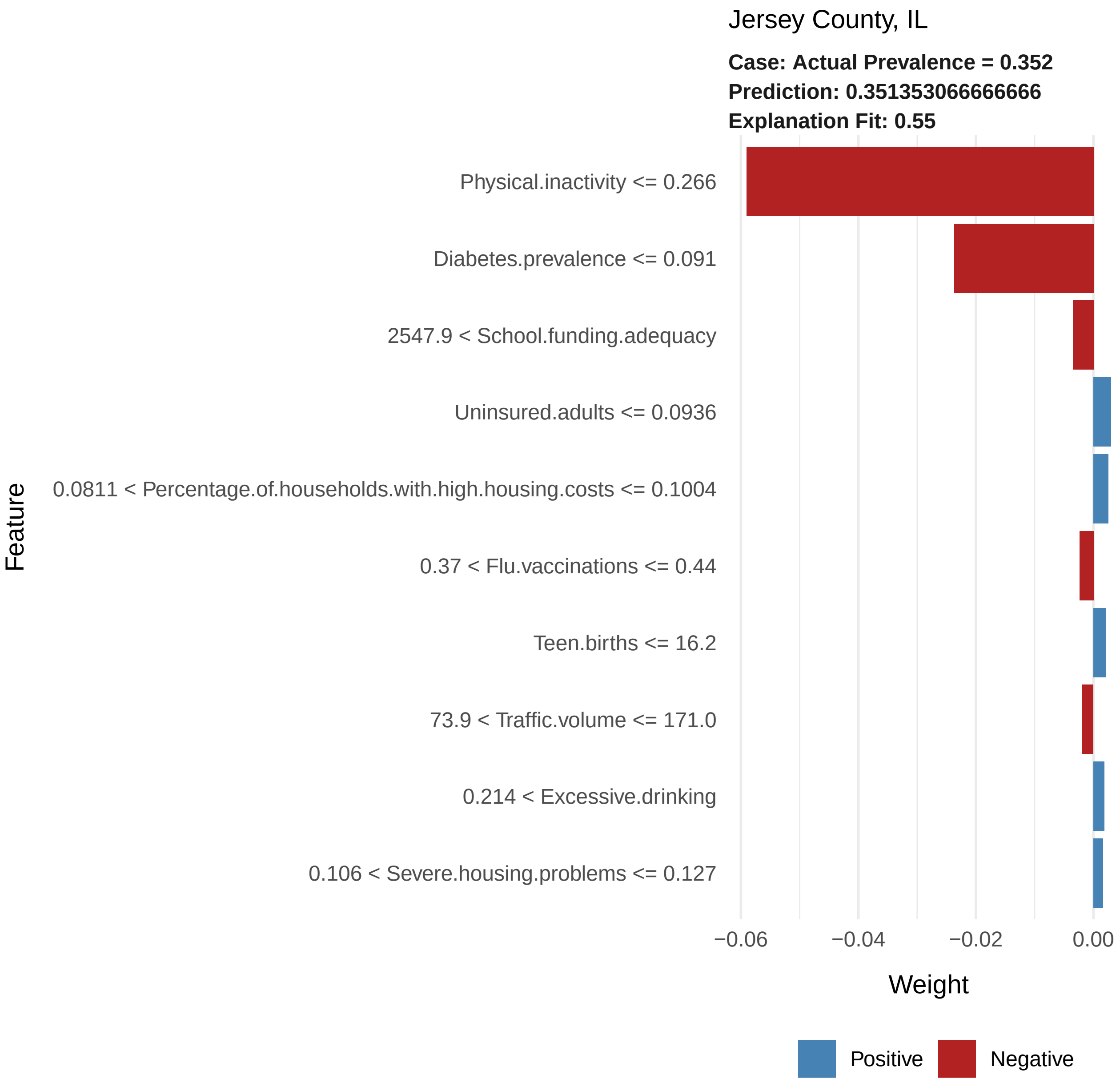

Jersey County, IL
Case: Actual Prevalence = 0.352
Prediction: 0.351353066666666
Explanation Fit: 0.55
Physical.inactivity <= 0.266
Diabetes.prevalence <= 0.091
2547.9 < School.funding.adequacy
Uninsured.adults <= 0.0936
0.0811 < Percentage.of.households.with.high.housing.costs <= 0.1004
0.37 < Flu.vaccinations <= 0.44
Teen.births <= 16.2
73.9 < Traffic.volume <= 171.0
0.214 < Excessive.drinking
0.106 < Severe.housing.problems <= 0.127
Feature
−0.06
−0.04
−0.02
0.00
Weight
Positive
Negative

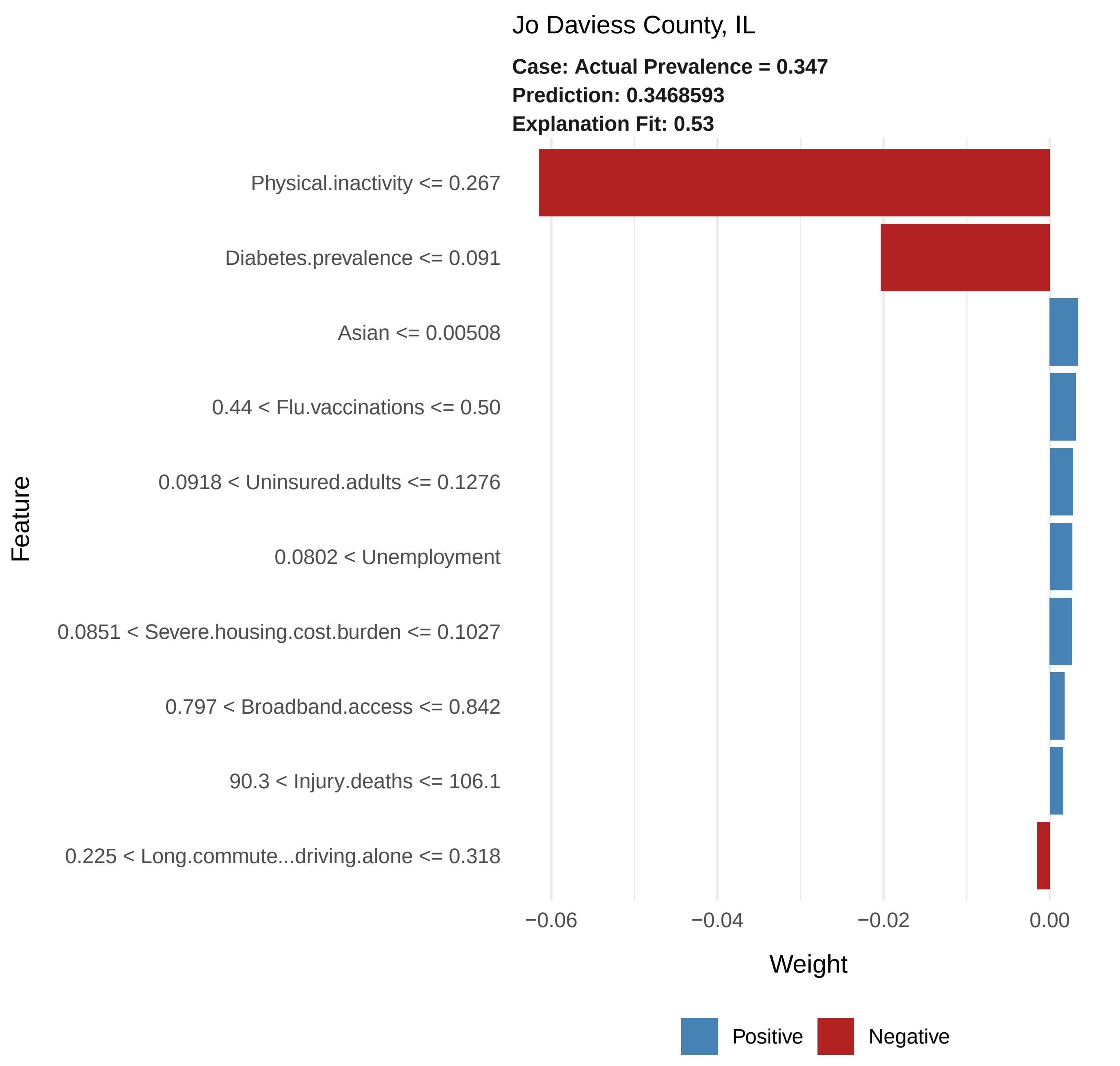

Jo Daviess County, IL
Case: Actual Prevalence = 0.347
Prediction: 0.3468593
Explanation Fit: 0.53
Physical.inactivity <= 0.267
Diabetes.prevalence <= 0.091
Asian <= 0.00508
0.44 < Flu.vaccinations <= 0.50
0.0918 < Uninsured.adults <= 0.1276
0.0802 < Unemployment
0.0851 < Severe.housing.cost.burden <= 0.1027
0.797 < Broadband.access <= 0.842
90.3 < Injury.deaths <= 106.1
0.225 < Long.commute...driving.alone <= 0.318
Feature
−0.06
−0.04
−0.02
0.00
Weight
Positive
Negative

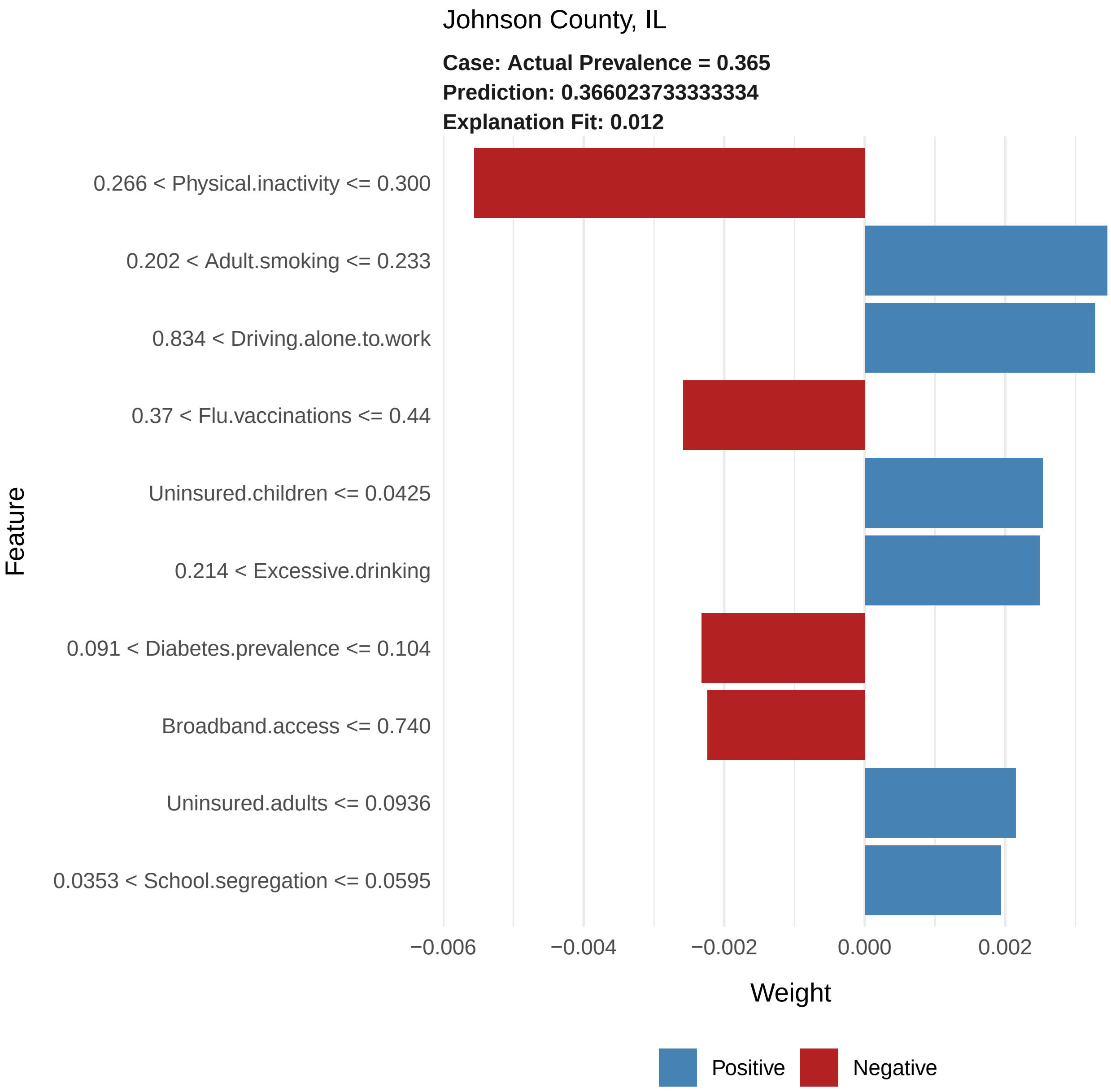

Johnson County, IL
Case: Actual Prevalence = 0.365
Prediction: 0.366023733333334
Explanation Fit: 0.012
0.266 < Physical.inactivity <= 0.300
0.202 < Adult.smoking <= 0.233
0.834 < Driving.alone.to.work
0.37 < Flu.vaccinations <= 0.44
Uninsured.children <= 0.0425
0.214 < Excessive.drinking
0.091 < Diabetes.prevalence <= 0.104
Broadband.access <= 0.740
Uninsured.adults <= 0.0936
0.0353 < School.segregation <= 0.0595
Feature
−0.006
−0.004
−0.002
0.000
0.002
Weight
Positive
Negative

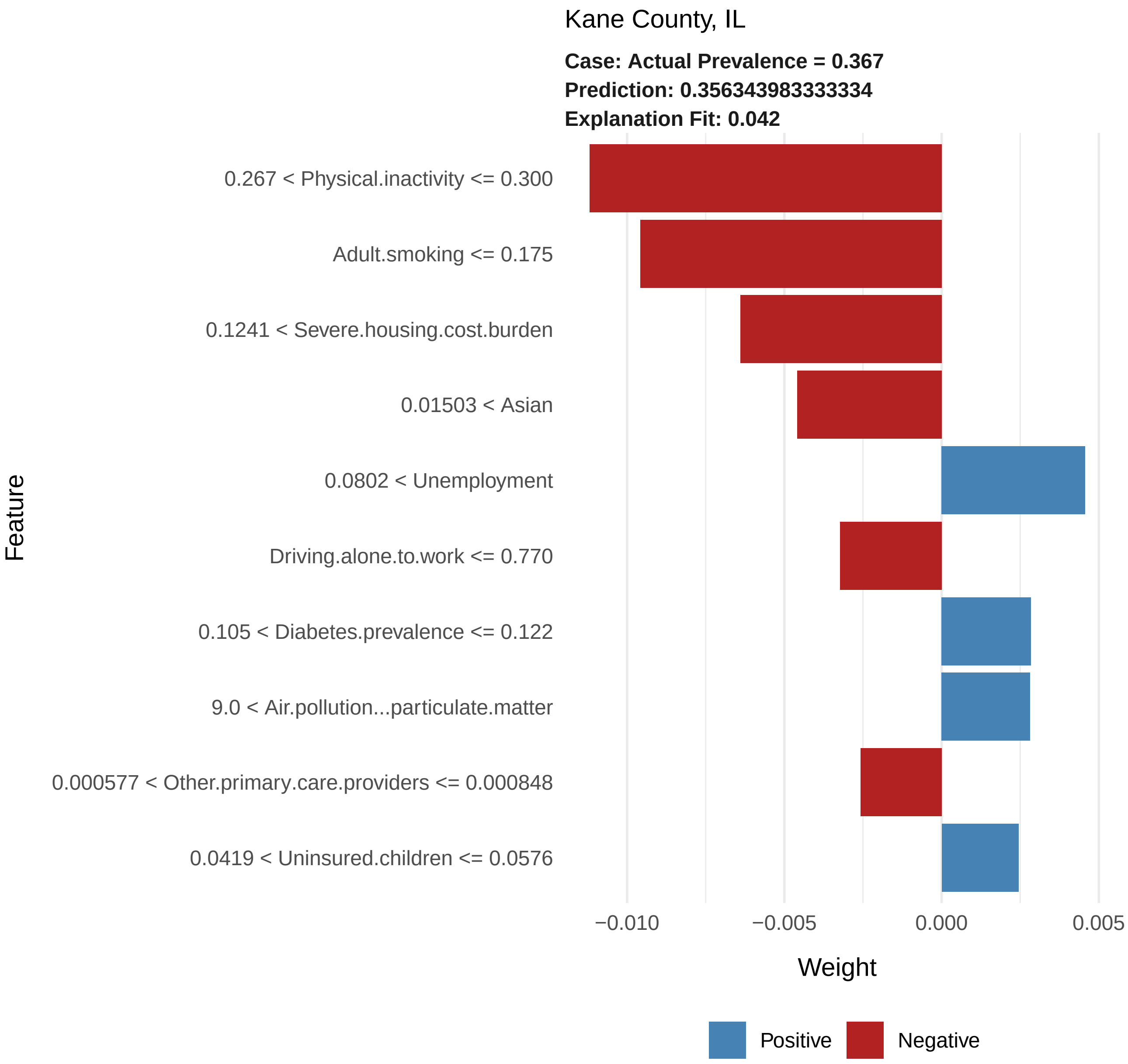

Kane County, IL
Case: Actual Prevalence = 0.367
Prediction: 0.356343983333334
Explanation Fit: 0.042
0.267 < Physical.inactivity <= 0.300
Adult.smoking <= 0.175
0.1241 < Severe.housing.cost.burden
0.01503 < Asian
0.0802 < Unemployment
Driving.alone.to.work <= 0.770
0.105 < Diabetes.prevalence <= 0.122
9.0 < Air.pollution...particulate.matter
0.000577 < Other.primary.care.providers <= 0.000848
0.0419 < Uninsured.children <= 0.0576
Feature
−0.010
−0.005
0.000
0.005
Weight
Positive
Negative

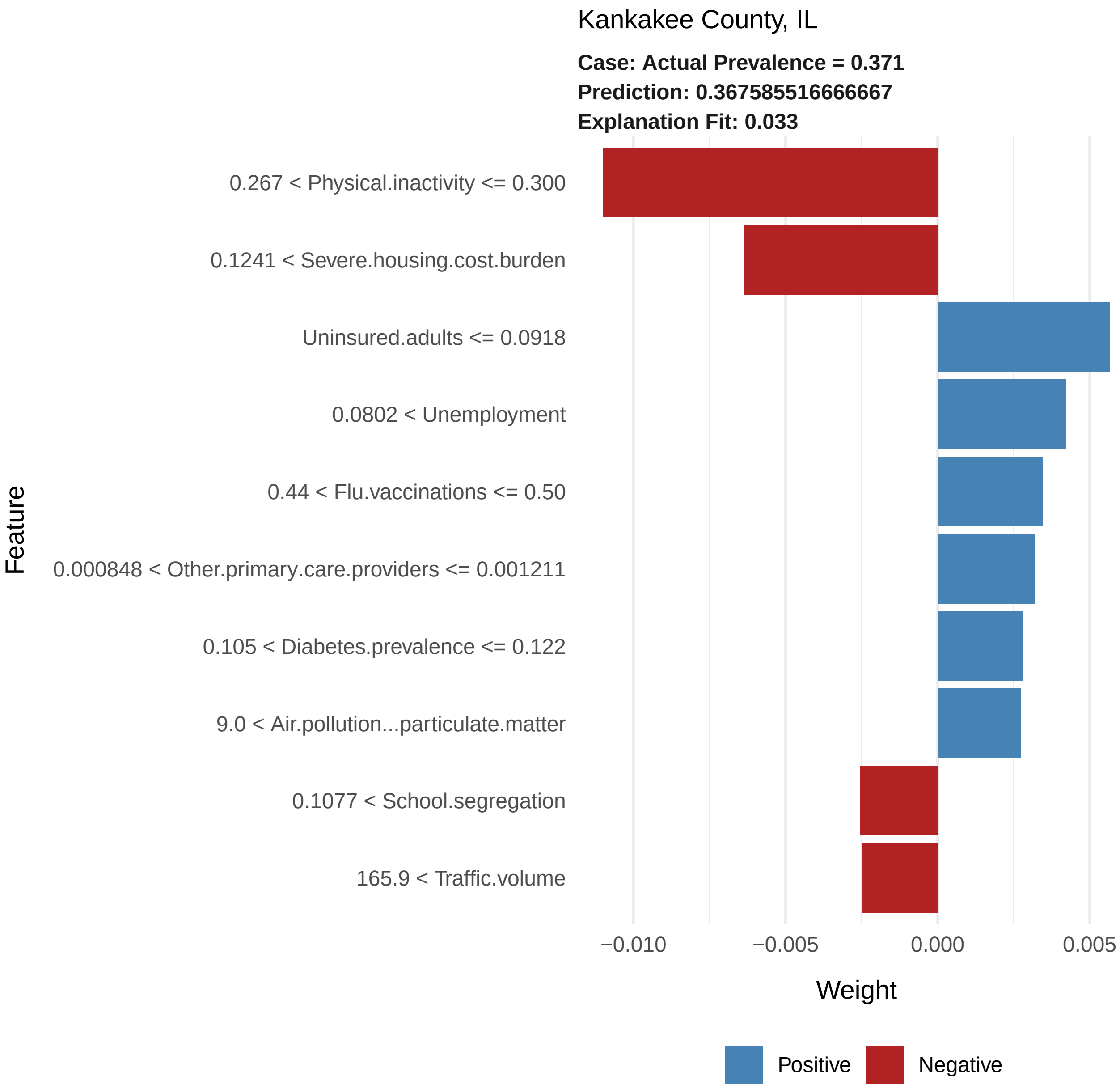

Kankakee County, IL
Case: Actual Prevalence = 0.371
Prediction: 0.367585516666667
Explanation Fit: 0.033
0.267 < Physical.inactivity <= 0.300
0.1241 < Severe.housing.cost.burden
Uninsured.adults <= 0.0918
0.0802 < Unemployment
0.44 < Flu.vaccinations <= 0.50
0.000848 < Other.primary.care.providers <= 0.001211
0.105 < Diabetes.prevalence <= 0.122
9.0 < Air.pollution...particulate.matter
0.1077 < School.segregation
165.9 < Traffic.volume
Feature
−0.010
−0.005
0.000
0.005
Weight
Positive
Negative

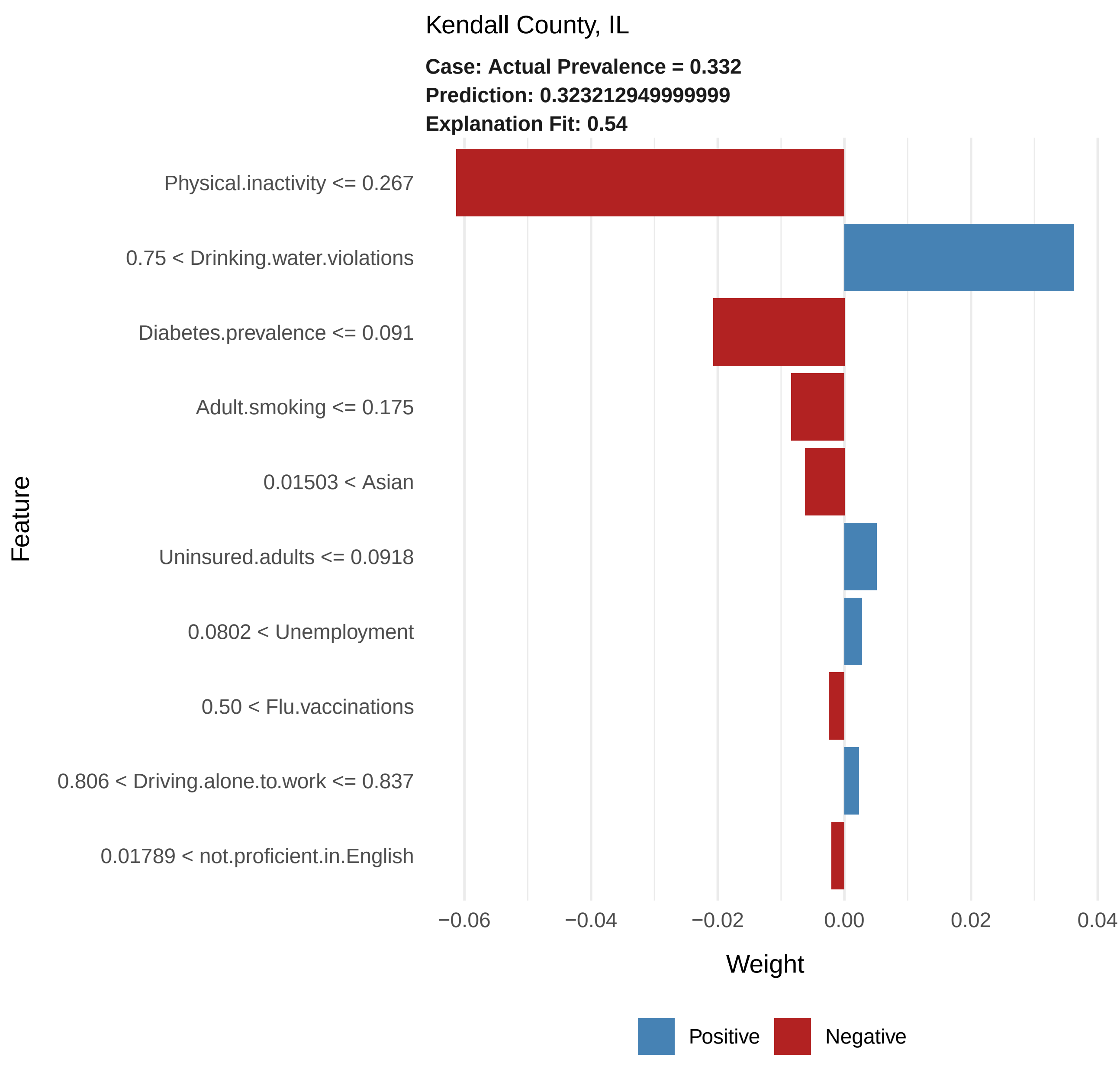

Kendall County, IL
Case: Actual Prevalence = 0.332
Prediction: 0.323212949999999
Explanation Fit: 0.54
Physical.inactivity <= 0.267
0.75 < Drinking.water.violations
Diabetes.prevalence <= 0.091
Adult.smoking <= 0.175
0.01503 < Asian
Uninsured.adults <= 0.0918
0.0802 < Unemployment
0.50 < Flu.vaccinations
0.806 < Driving.alone.to.work <= 0.837
0.01789 < not.proficient.in.English
Feature
−0.06
−0.04
−0.02
0.00
0.02
0.04
Weight
Positive
Negative

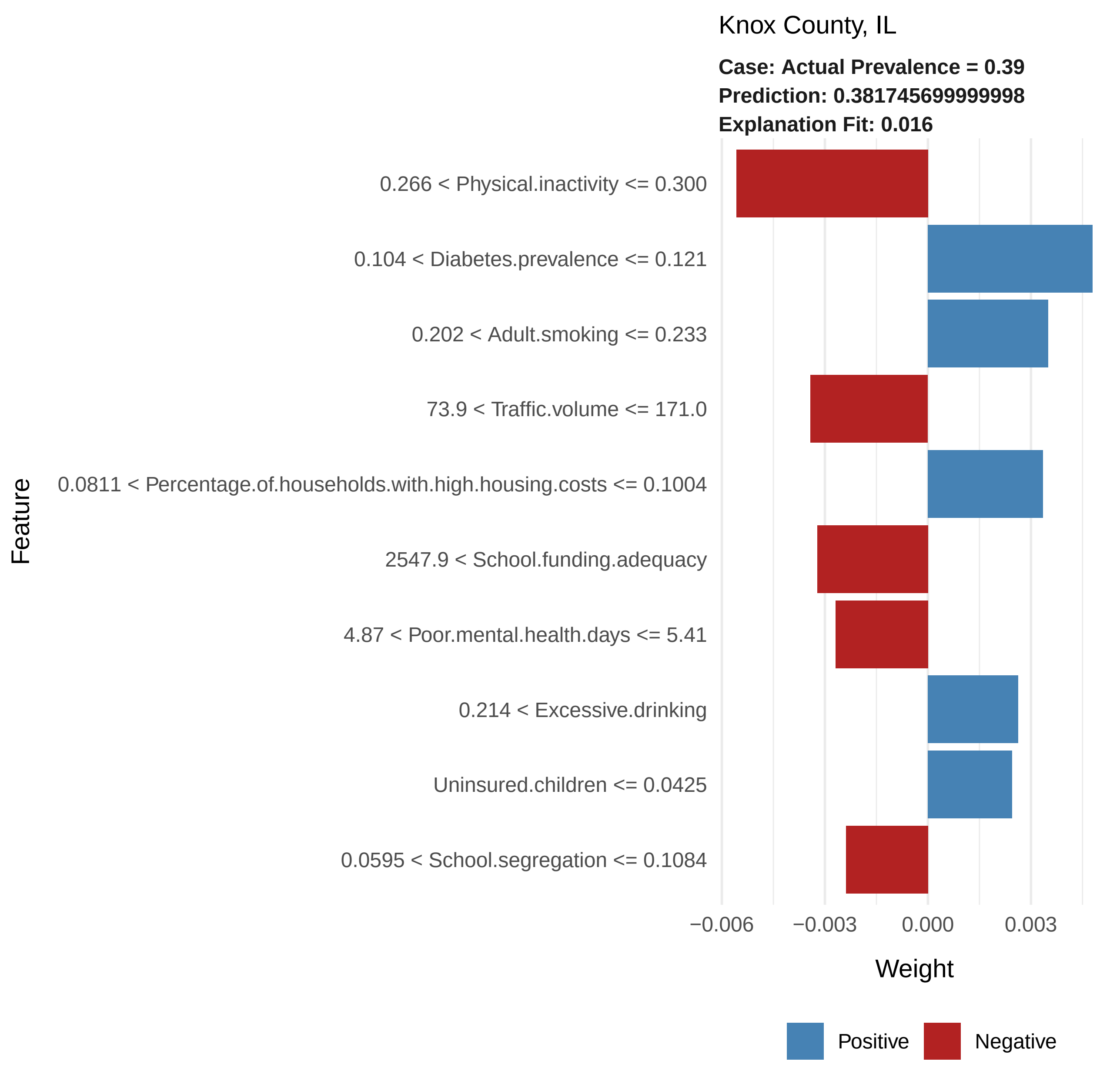

Knox County, IL
Case: Actual Prevalence = 0.39
Prediction: 0.381745699999998
Explanation Fit: 0.016
0.266 < Physical.inactivity <= 0.300
0.104 < Diabetes.prevalence <= 0.121
0.202 < Adult.smoking <= 0.233
73.9 < Traffic.volume <= 171.0
0.0811 < Percentage.of.households.with.high.housing.costs <= 0.1004
2547.9 < School.funding.adequacy
4.87 < Poor.mental.health.days <= 5.41
0.214 < Excessive.drinking
Uninsured.children <= 0.0425
0.0595 < School.segregation <= 0.1084
Feature
−0.006
−0.003
0.000
0.003
Weight
Positive
Negative

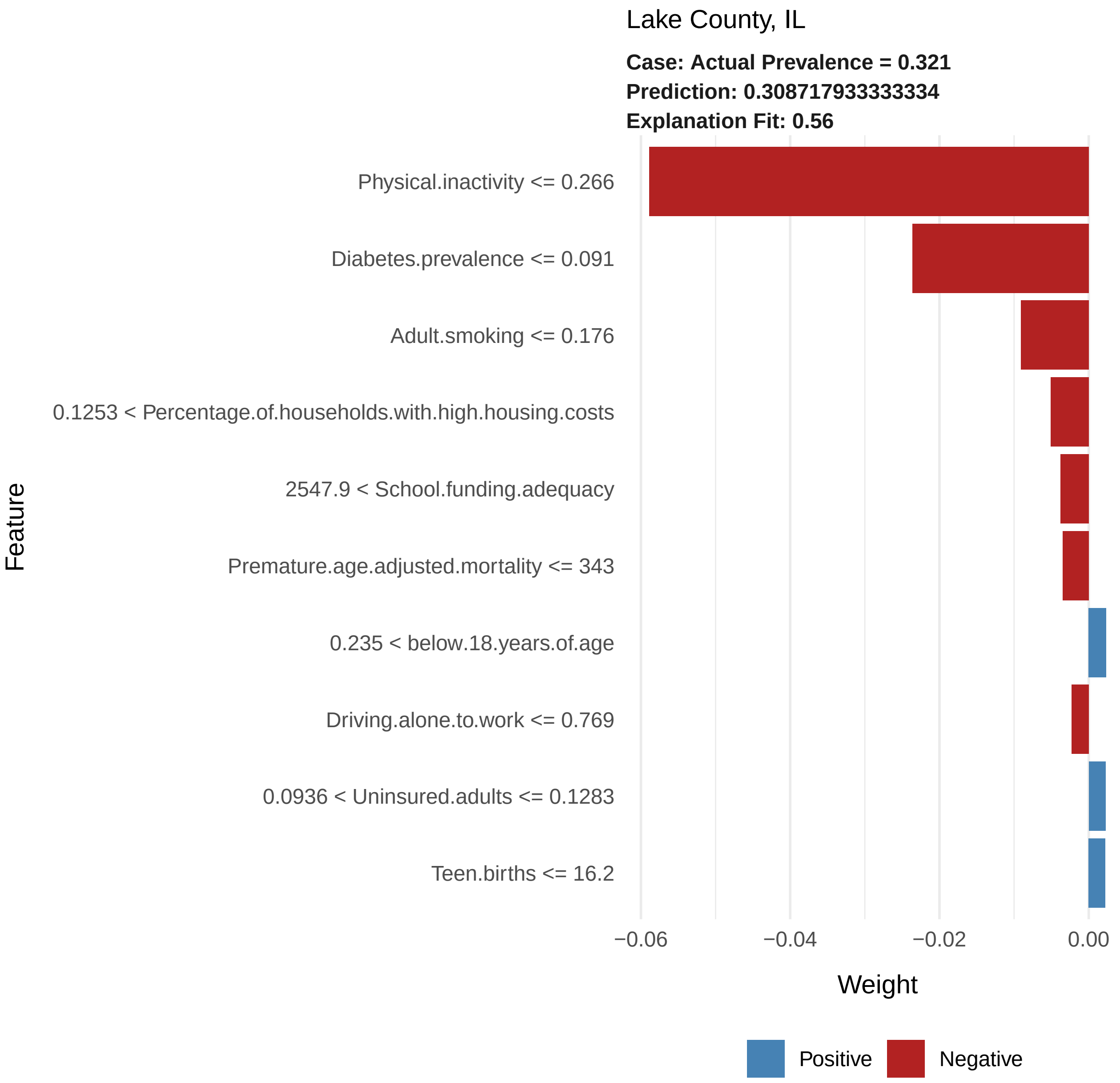
Lake County, IL
Case: Actual Prevalence = 0.321
Prediction: 0.308717933333334
Explanation Fit: 0.56
Physical.inactivity <= 0.266
Diabetes.prevalence <= 0.091
Adult.smoking <= 0.176
0.1253 < Percentage.of.households.with.high.housing.costs
2547.9 < School.funding.adequacy
Premature.age.adjusted.mortality <= 343
0.235 < below.18.years.of.age
Driving.alone.to.work <= 0.769
0.0936 < Uninsured.adults <= 0.1283
Teen.births <= 16.2
Feature
−0.06
−0.04
−0.02
0.00
Weight
Positive
Negative

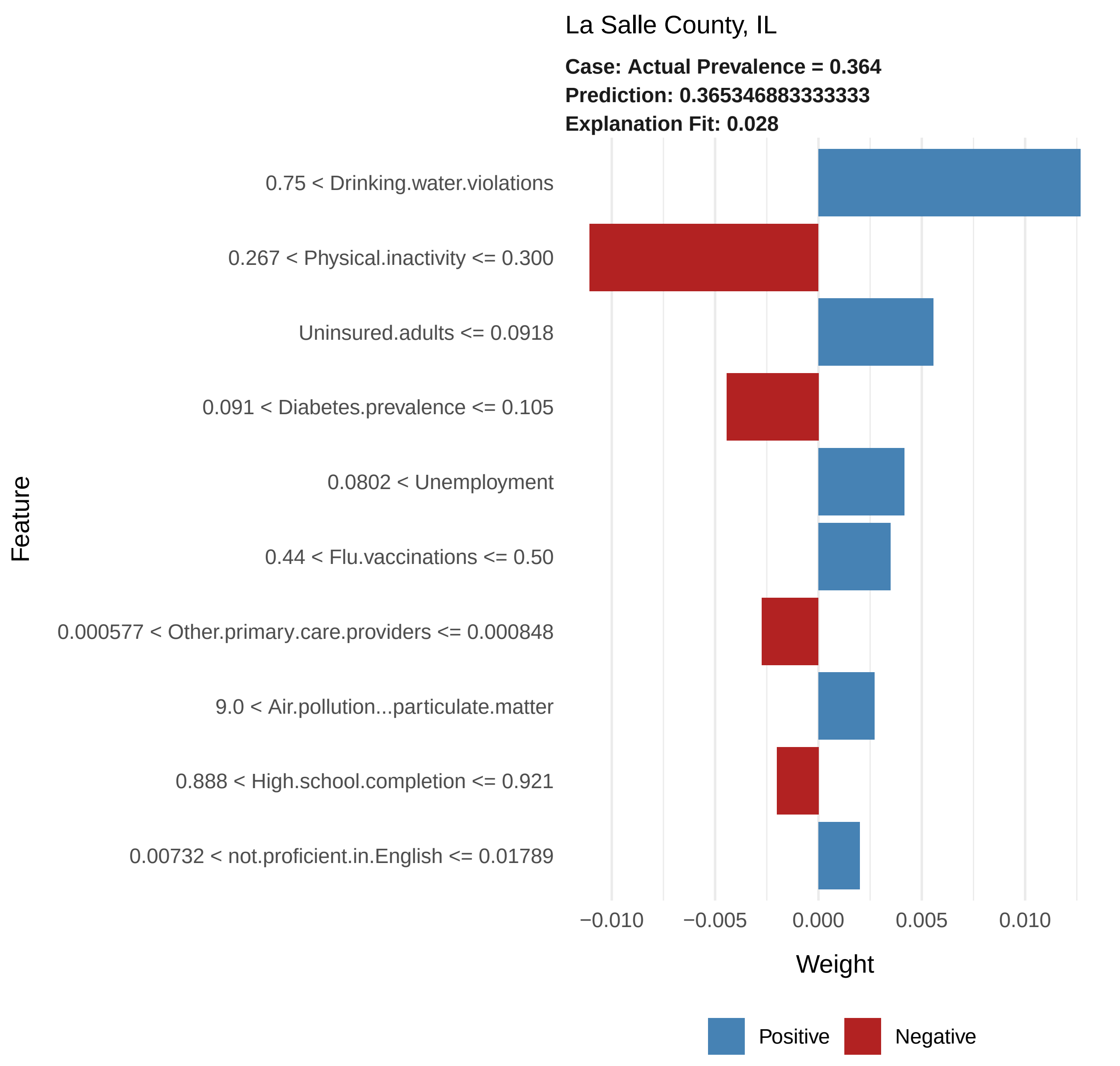

La Salle County, IL
Case: Actual Prevalence = 0.364
Prediction: 0.365346883333333
Explanation Fit: 0.028
0.75 < Drinking.water.violations
0.267 < Physical.inactivity <= 0.300
Uninsured.adults <= 0.0918
0.091 < Diabetes.prevalence <= 0.105
0.0802 < Unemployment
0.44 < Flu.vaccinations <= 0.50
0.000577 < Other.primary.care.providers <= 0.000848
9.0 < Air.pollution...particulate.matter
0.888 < High.school.completion <= 0.921
0.00732 < not.proficient.in.English <= 0.01789
Feature
−0.010
−0.005
0.000
0.005
0.010
Weight
Positive
Negative

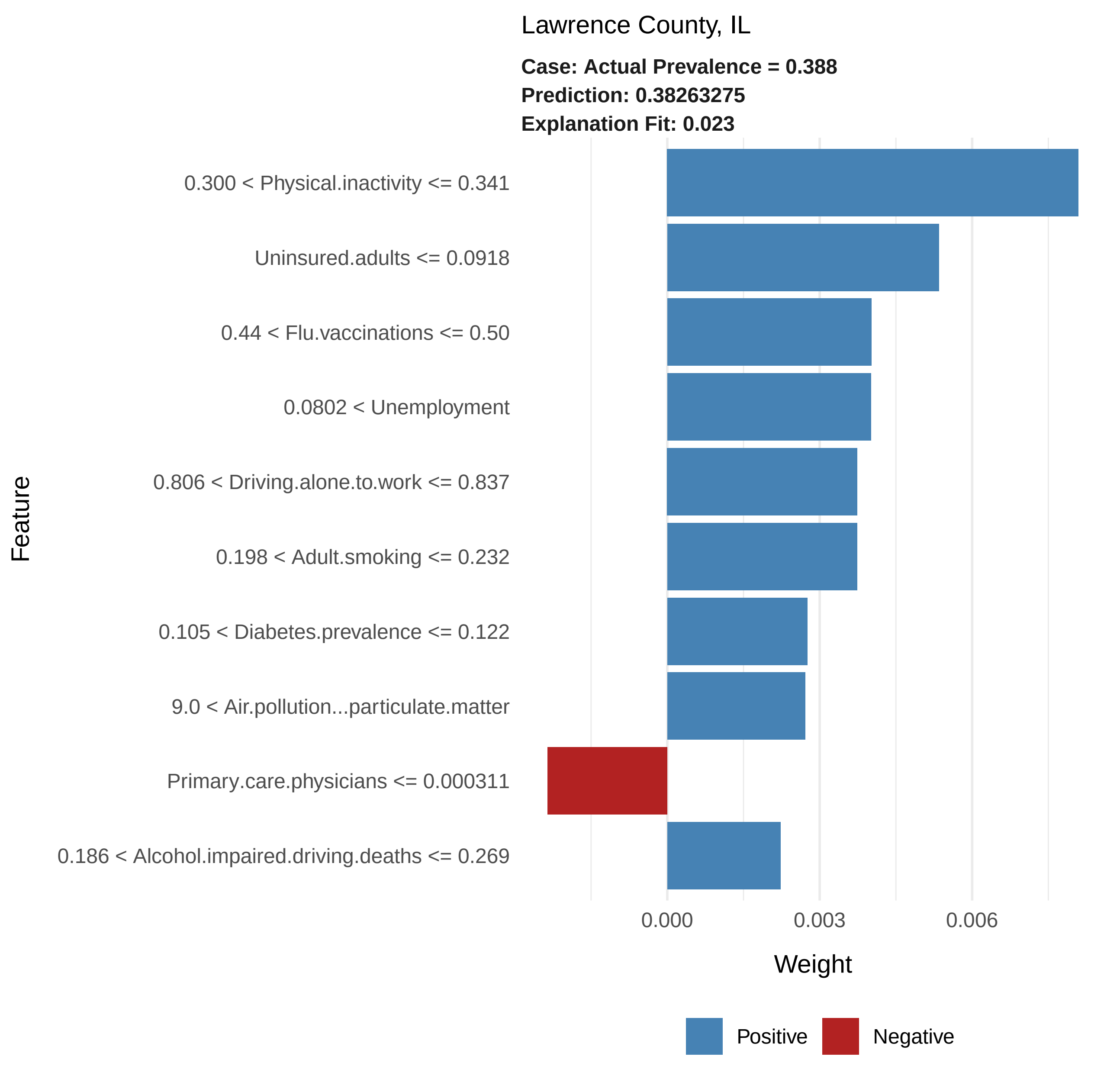
Lawrence County, IL
Case: Actual Prevalence = 0.388
Prediction: 0.38263275
Explanation Fit: 0.023
0.300 < Physical.inactivity <= 0.341
Uninsured.adults <= 0.0918
0.44 < Flu.vaccinations <= 0.50
0.0802 < Unemployment
0.806 < Driving.alone.to.work <= 0.837
0.198 < Adult.smoking <= 0.232
0.105 < Diabetes.prevalence <= 0.122
9.0 < Air.pollution...particulate.matter
Primary.care.physicians <= 0.000311
0.186 < Alcohol.impaired.driving.deaths <= 0.269
Feature
0.000
0.003
0.006
Weight
Positive
Negative

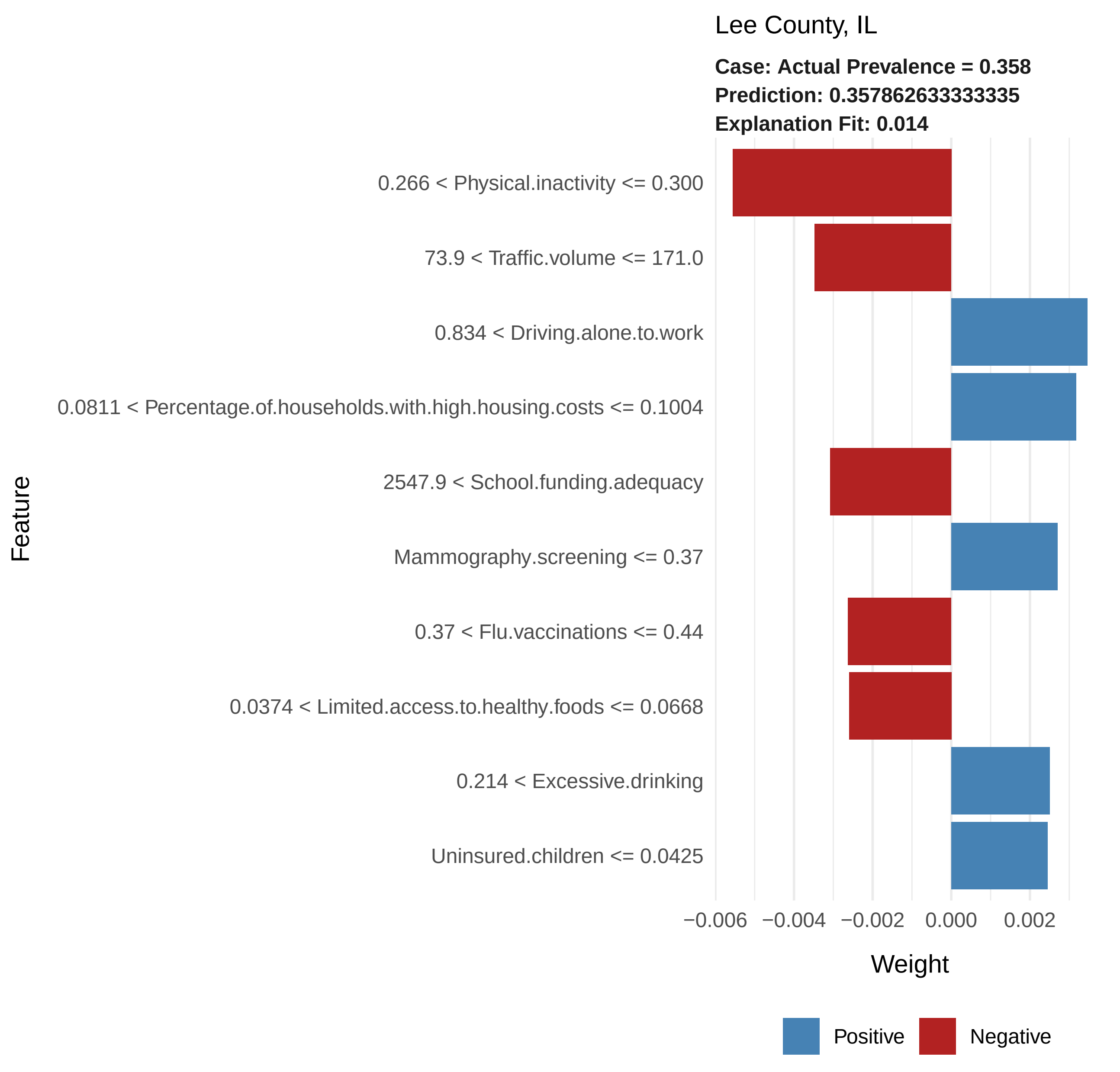

Lee County, IL
Case: Actual Prevalence = 0.358
Prediction: 0.357862633333335
Explanation Fit: 0.014
0.266 < Physical.inactivity <= 0.300
73.9 < Traffic.volume <= 171.0
0.834 < Driving.alone.to.work
0.0811 < Percentage.of.households.with.high.housing.costs <= 0.1004
2547.9 < School.funding.adequacy
Mammography.screening <= 0.37
0.37 < Flu.vaccinations <= 0.44
0.0374 < Limited.access.to.healthy.foods <= 0.0668
0.214 < Excessive.drinking
Uninsured.children <= 0.0425
Feature
−0.006
−0.004
−0.002
0.000
0.002
Weight
Positive
Negative

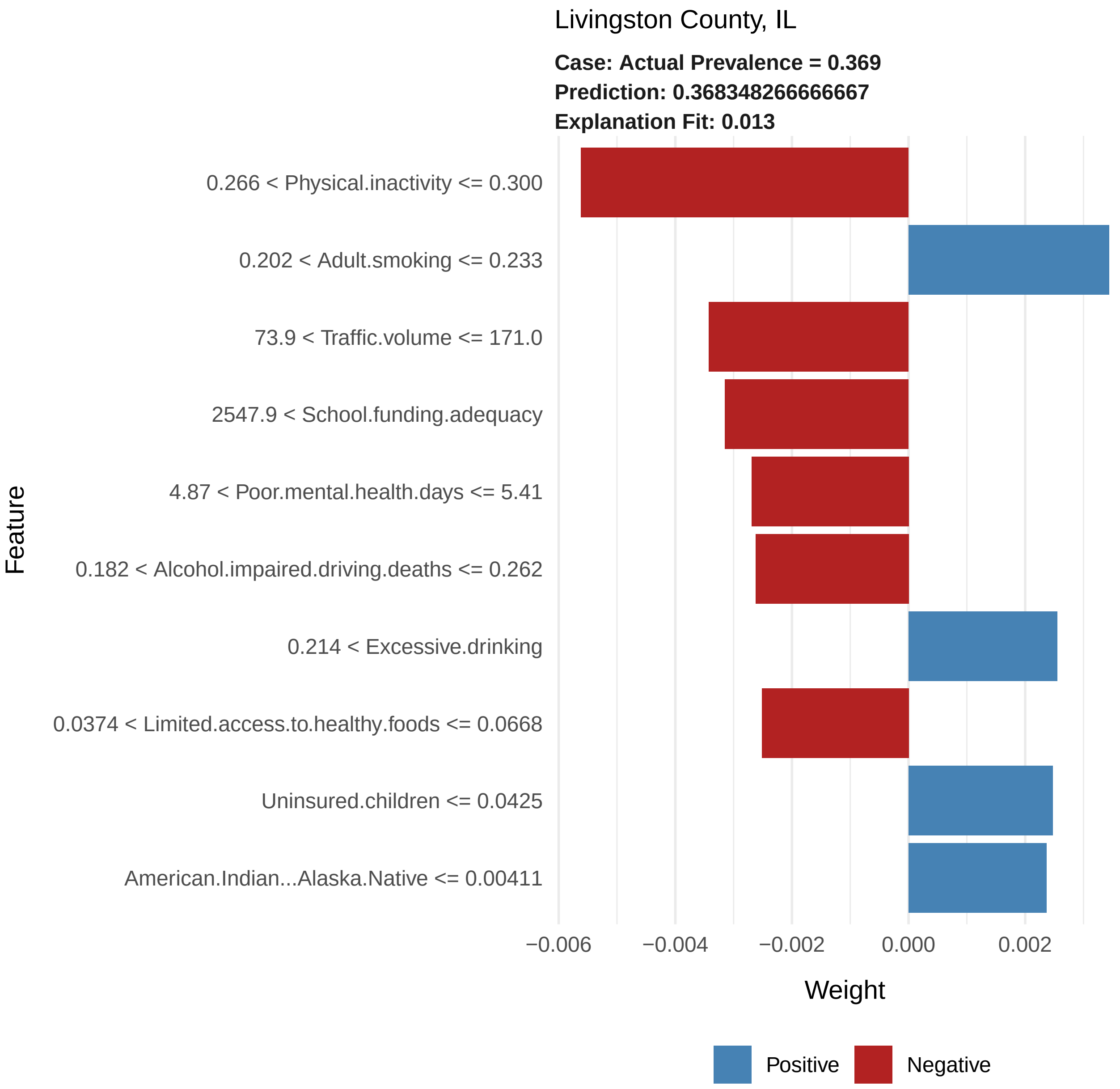
Livingston County, IL
Case: Actual Prevalence = 0.369
Prediction: 0.368348266666667
Explanation Fit: 0.013
0.266 < Physical.inactivity <= 0.300
0.202 < Adult.smoking <= 0.233
73.9 < Traffic.volume <= 171.0
2547.9 < School.funding.adequacy
4.87 < Poor.mental.health.days <= 5.41
0.182 < Alcohol.impaired.driving.deaths <= 0.262
0.214 < Excessive.drinking
0.0374 < Limited.access.to.healthy.foods <= 0.0668
Uninsured.children <= 0.0425
American.Indian...Alaska.Native <= 0.00411
Feature
−0.006
−0.004
−0.002
0.000
0.002
Weight
Positive
Negative

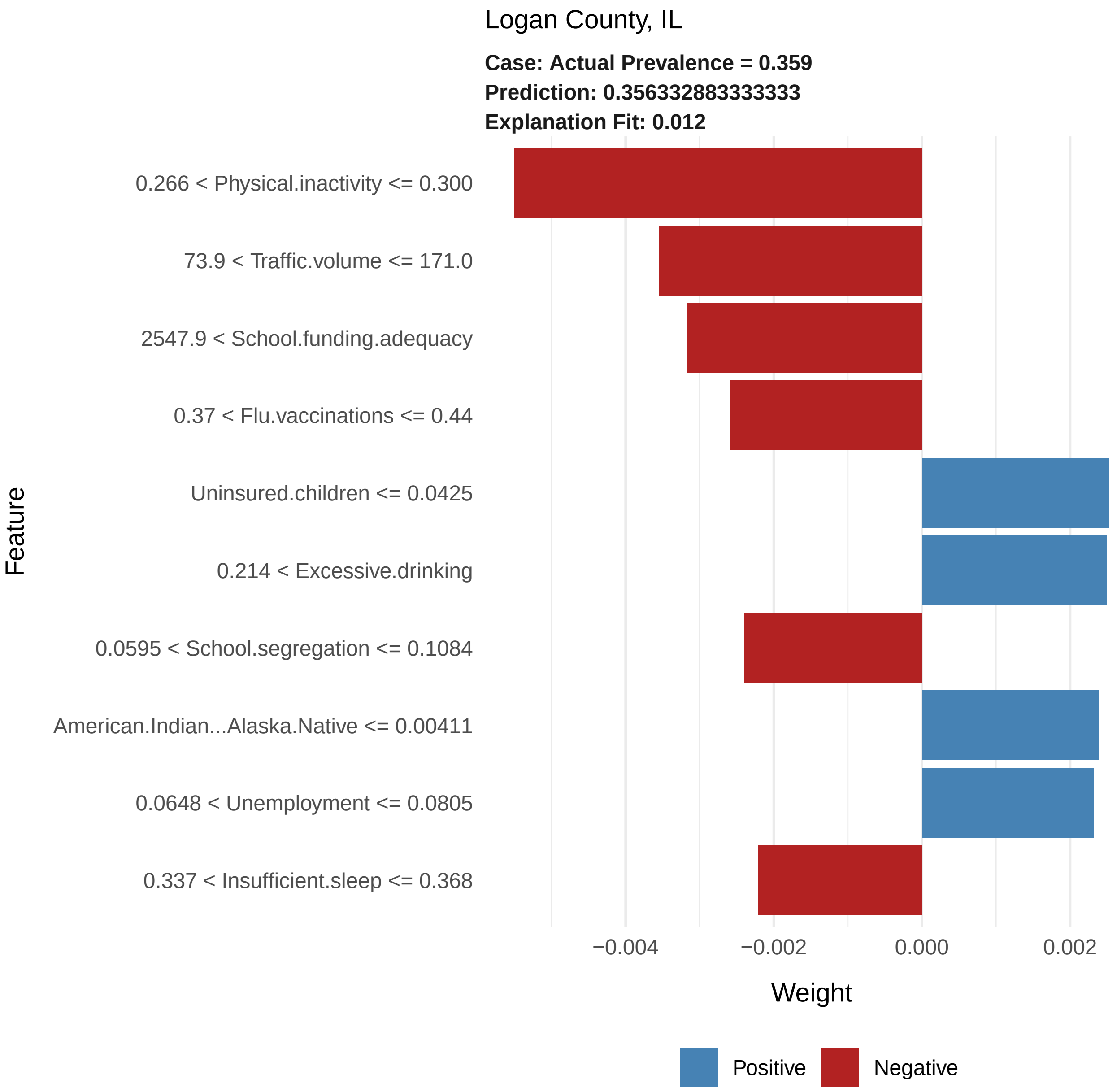

Logan County, IL
Case: Actual Prevalence = 0.359
Prediction: 0.356332883333333
Explanation Fit: 0.012
0.266 < Physical.inactivity <= 0.300
73.9 < Traffic.volume <= 171.0
2547.9 < School.funding.adequacy
0.37 < Flu.vaccinations <= 0.44
Uninsured.children <= 0.0425
0.214 < Excessive.drinking
0.0595 < School.segregation <= 0.1084
American.Indian...Alaska.Native <= 0.00411
0.0648 < Unemployment <= 0.0805
0.337 < Insufficient.sleep <= 0.368
Feature
−0.004
−0.002
0.000
0.002
Weight
Positive
Negative

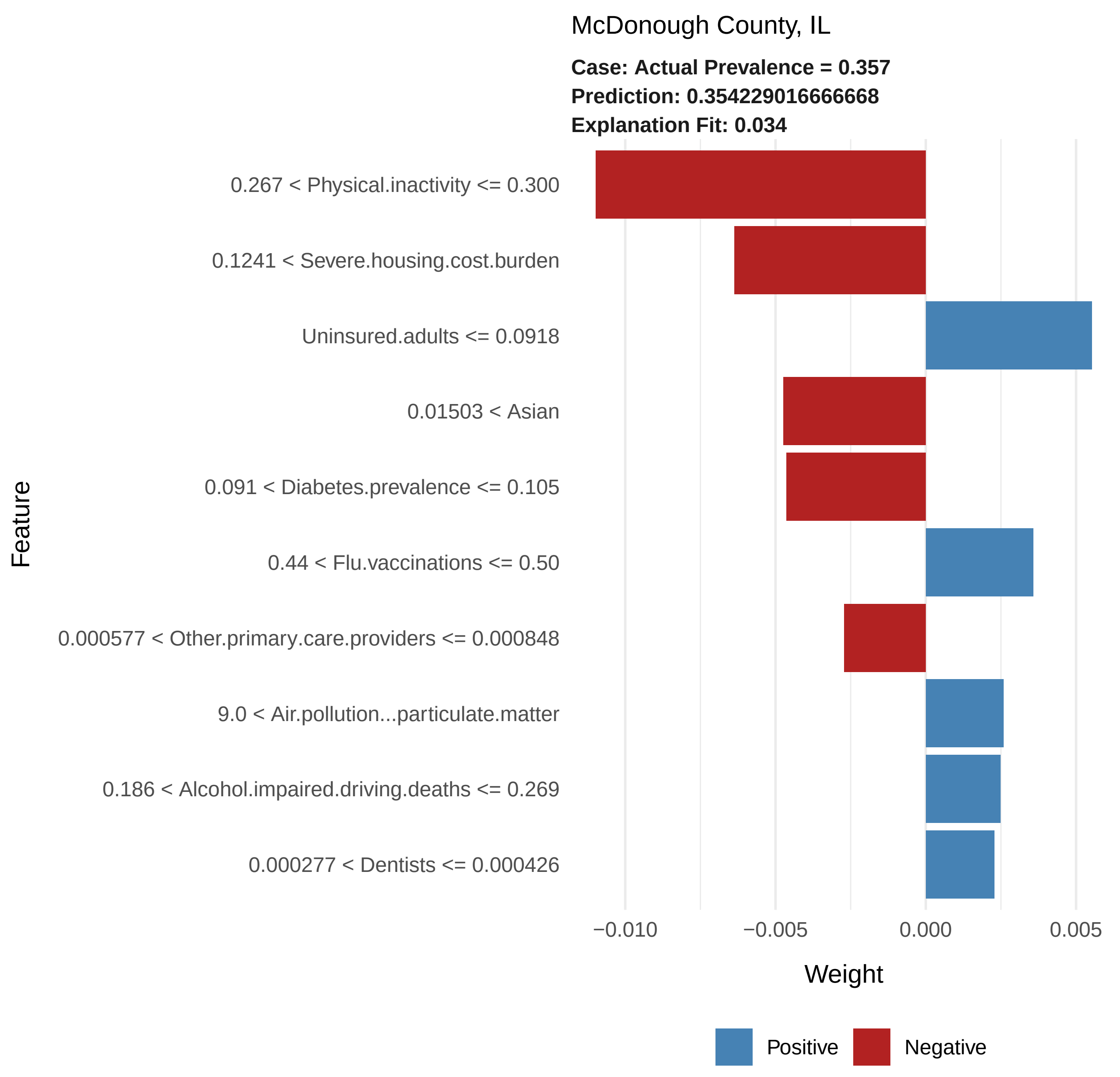

McDonough County, IL
Case: Actual Prevalence = 0.357
Prediction: 0.354229016666668
Explanation Fit: 0.034
0.267 < Physical.inactivity <= 0.300
0.1241 < Severe.housing.cost.burden
Uninsured.adults <= 0.0918
0.01503 < Asian
0.091 < Diabetes.prevalence <= 0.105
0.44 < Flu.vaccinations <= 0.50
0.000577 < Other.primary.care.providers <= 0.000848
9.0 < Air.pollution...particulate.matter
0.186 < Alcohol.impaired.driving.deaths <= 0.269
0.000277 < Dentists <= 0.000426
Feature
−0.010
−0.005
0.000
0.005
Weight
Positive
Negative

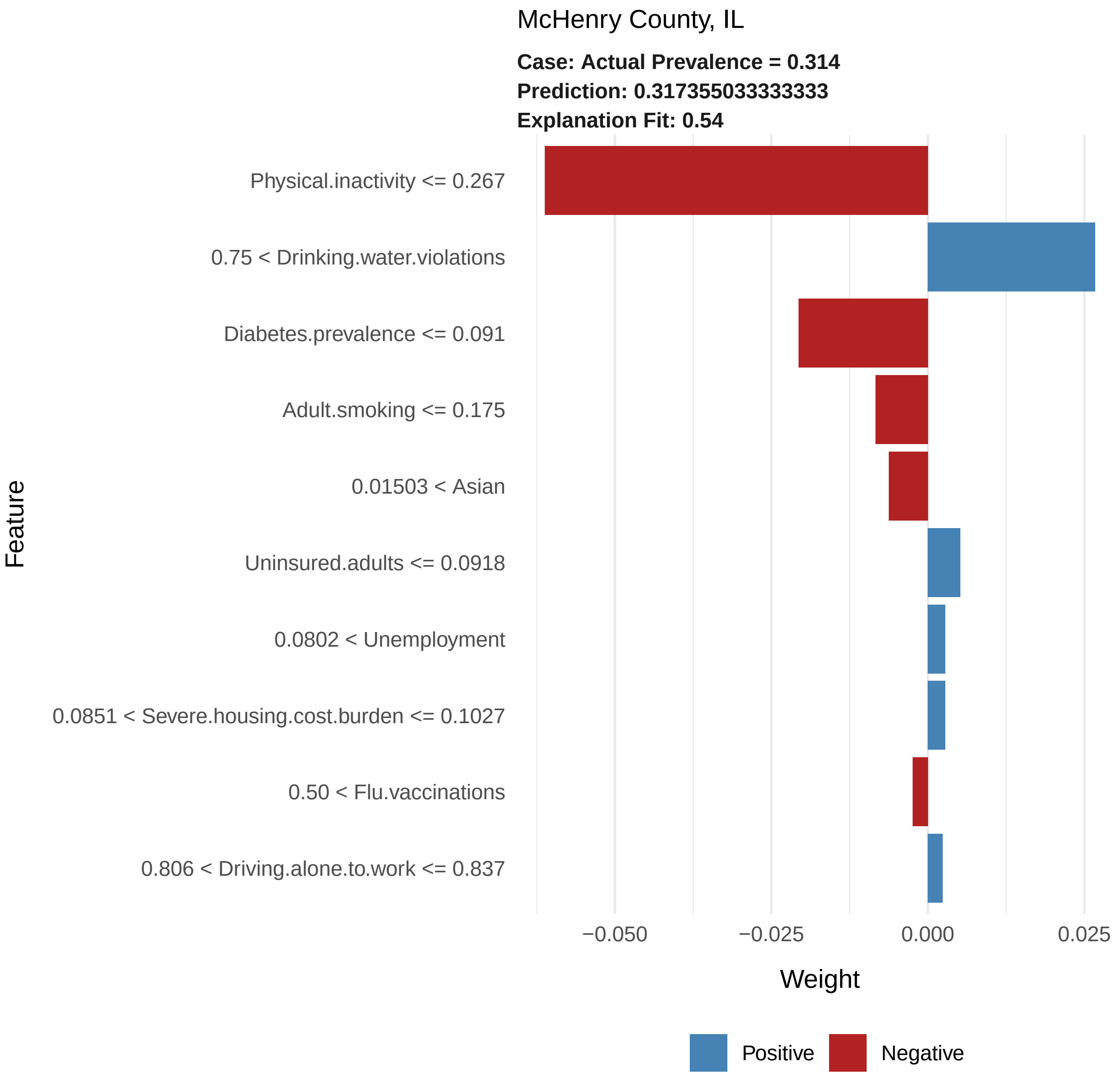

McHenry County, IL
Case: Actual Prevalence = 0.314
Prediction: 0.317355033333333
Explanation Fit: 0.54
Physical.inactivity <= 0.267
0.75 < Drinking.water.violations
Diabetes.prevalence <= 0.091
Adult.smoking <= 0.175
0.01503 < Asian
Uninsured.adults <= 0.0918
0.0802 < Unemployment
0.0851 < Severe.housing.cost.burden <= 0.1027
0.50 < Flu.vaccinations
0.806 < Driving.alone.to.work <= 0.837
Feature
−0.050
−0.025
0.000
0.025
Weight
Positive
Negative

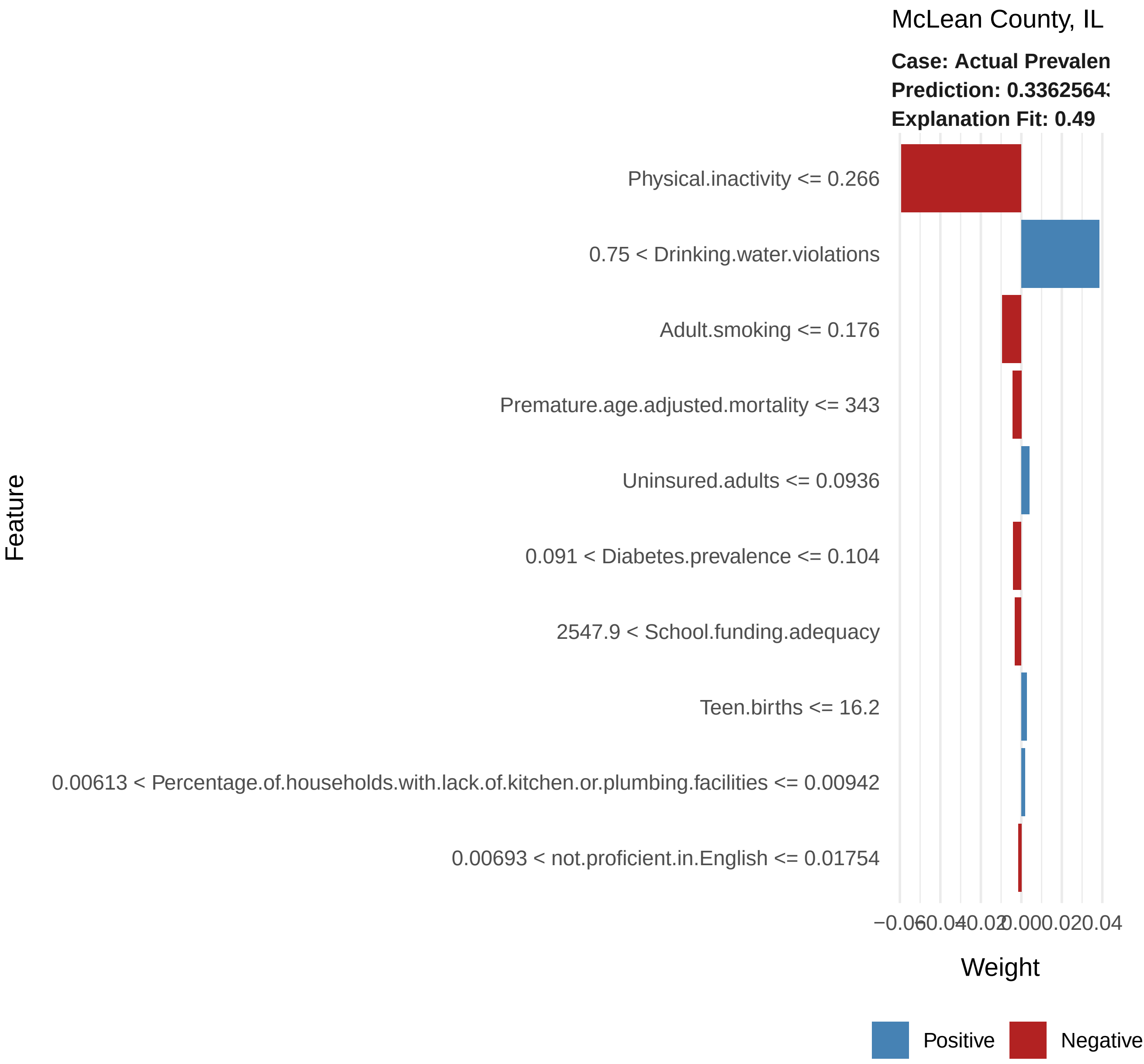

McLean County, IL
Case: Actual Prevalen
Prediction: 0.3362564
Explanation Fit: 0.49
Physical.inactivity <= 0.266
0.75 < Drinking.water.violations
Adult.smoking <= 0.176
Premature.age.adjusted.mortality <= 343
Uninsured.adults <= 0.0936
0.091 < Diabetes.prevalence <= 0.104
2547.9 < School.funding.adequacy
Teen.births <= 16.2
0.00613 < Percentage.of.households.with.lack.of.kitchen.or.plumbing.facilities <= 0.00942
0.00693 < not.proficient.in.English <= 0.01754
Feature
Weight
Positive
Negative

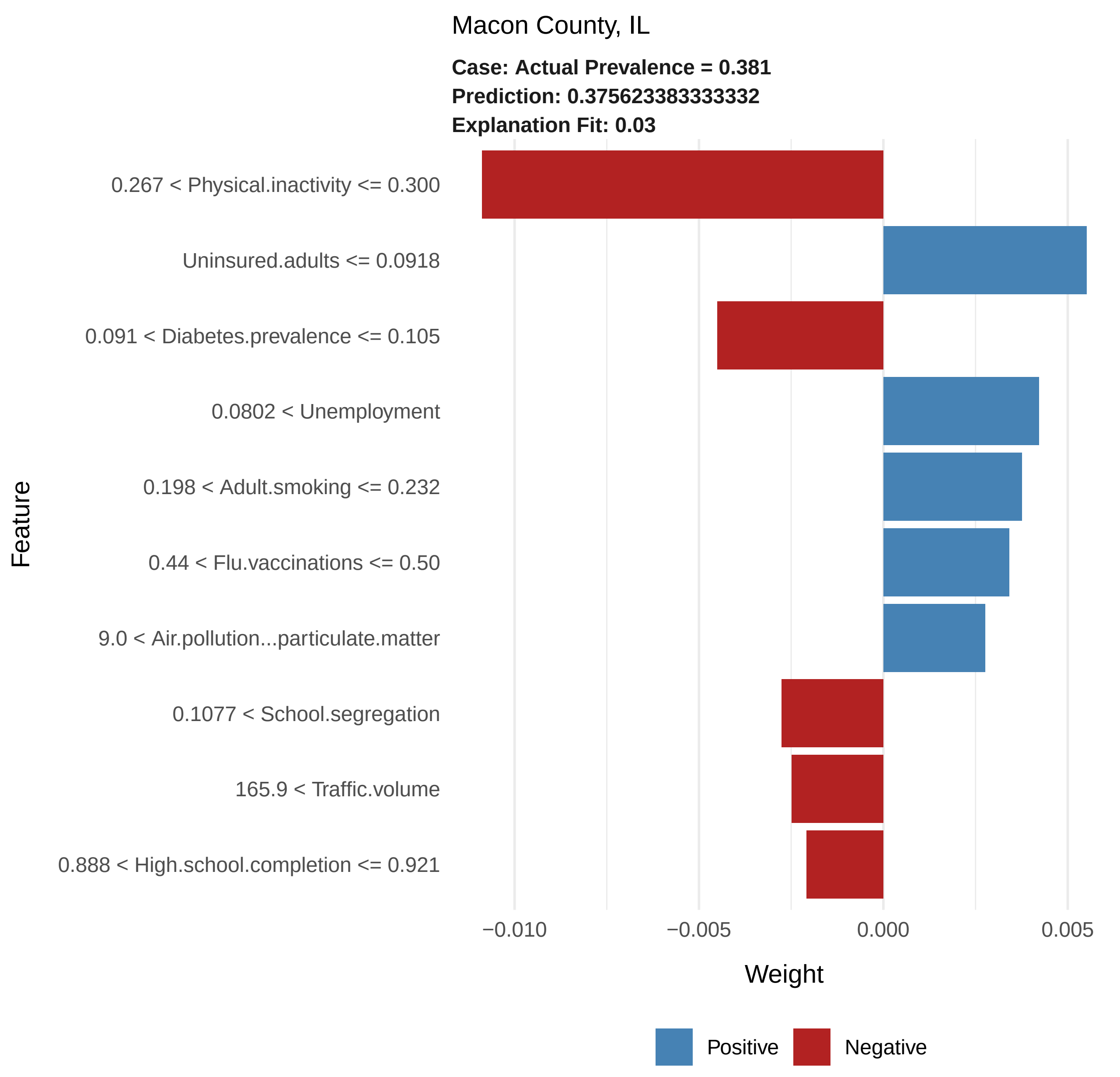

Macon County, IL
Case: Actual Prevalence = 0.381
Prediction: 0.375623383333332
Explanation Fit: 0.03
0.267 < Physical.inactivity <= 0.300
Uninsured.adults <= 0.0918
0.091 < Diabetes.prevalence <= 0.105
0.0802 < Unemployment
0.198 < Adult.smoking <= 0.232
0.44 < Flu.vaccinations <= 0.50
9.0 < Air.pollution...particulate.matter
0.1077 < School.segregation
165.9 < Traffic.volume
0.888 < High.school.completion <= 0.921
Feature
−0.010
−0.005
0.000
0.005
Weight
Positive
Negative

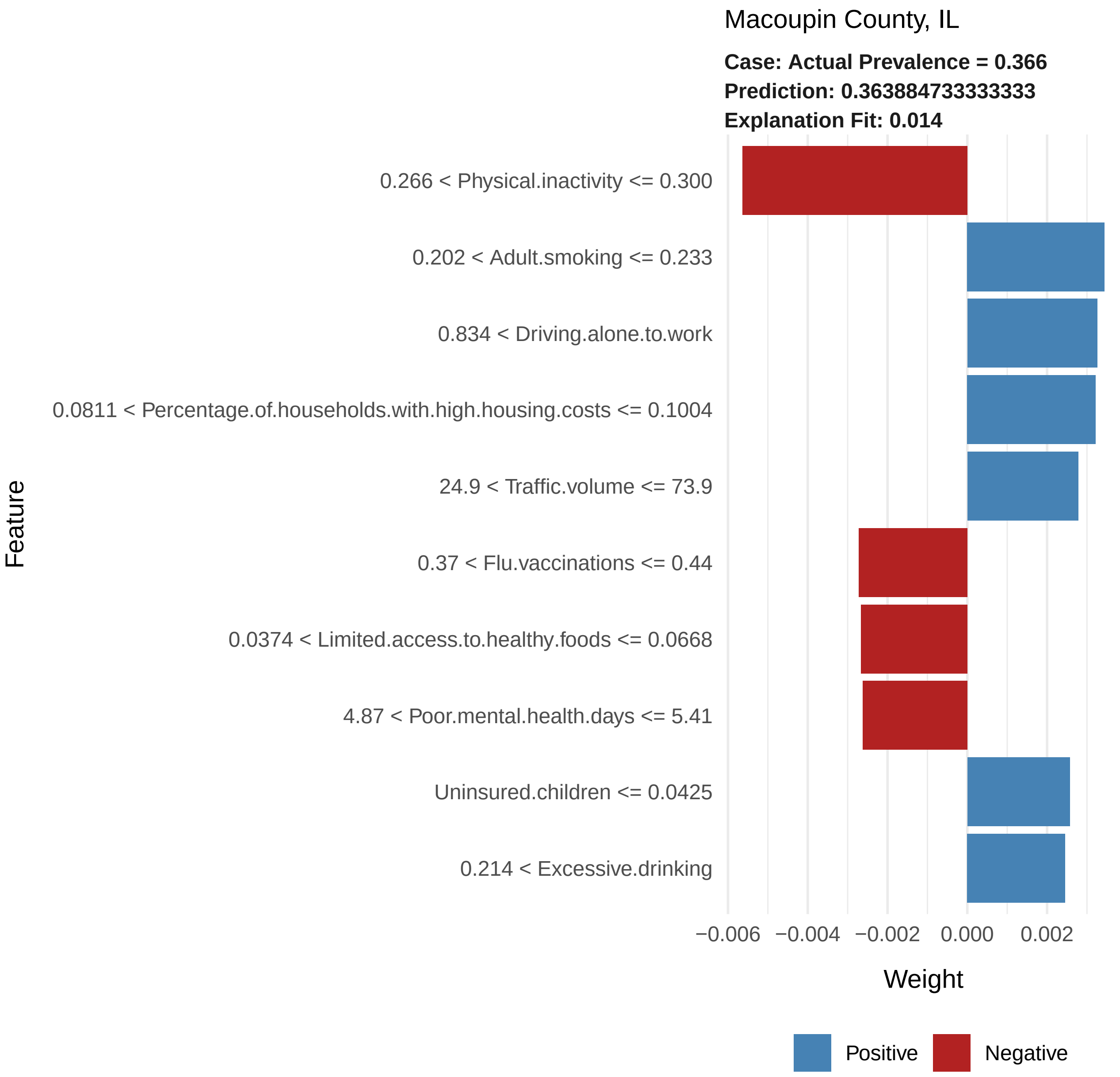

Macoupin County, IL
Case: Actual Prevalence = 0.366
Prediction: 0.363884733333333
Explanation Fit: 0.014
0.266 < Physical.inactivity <= 0.300
0.202 < Adult.smoking <= 0.233
0.834 < Driving.alone.to.work
0.0811 < Percentage.of.households.with.high.housing.costs <= 0.1004
24.9 < Traffic.volume <= 73.9
0.37 < Flu.vaccinations <= 0.44
0.0374 < Limited.access.to.healthy.foods <= 0.0668
4.87 < Poor.mental.health.days <= 5.41
Uninsured.children <= 0.0425
0.214 < Excessive.drinking
Feature
−0.006
−0.004
−0.002
0.000
0.002
Weight
Positive
Negative

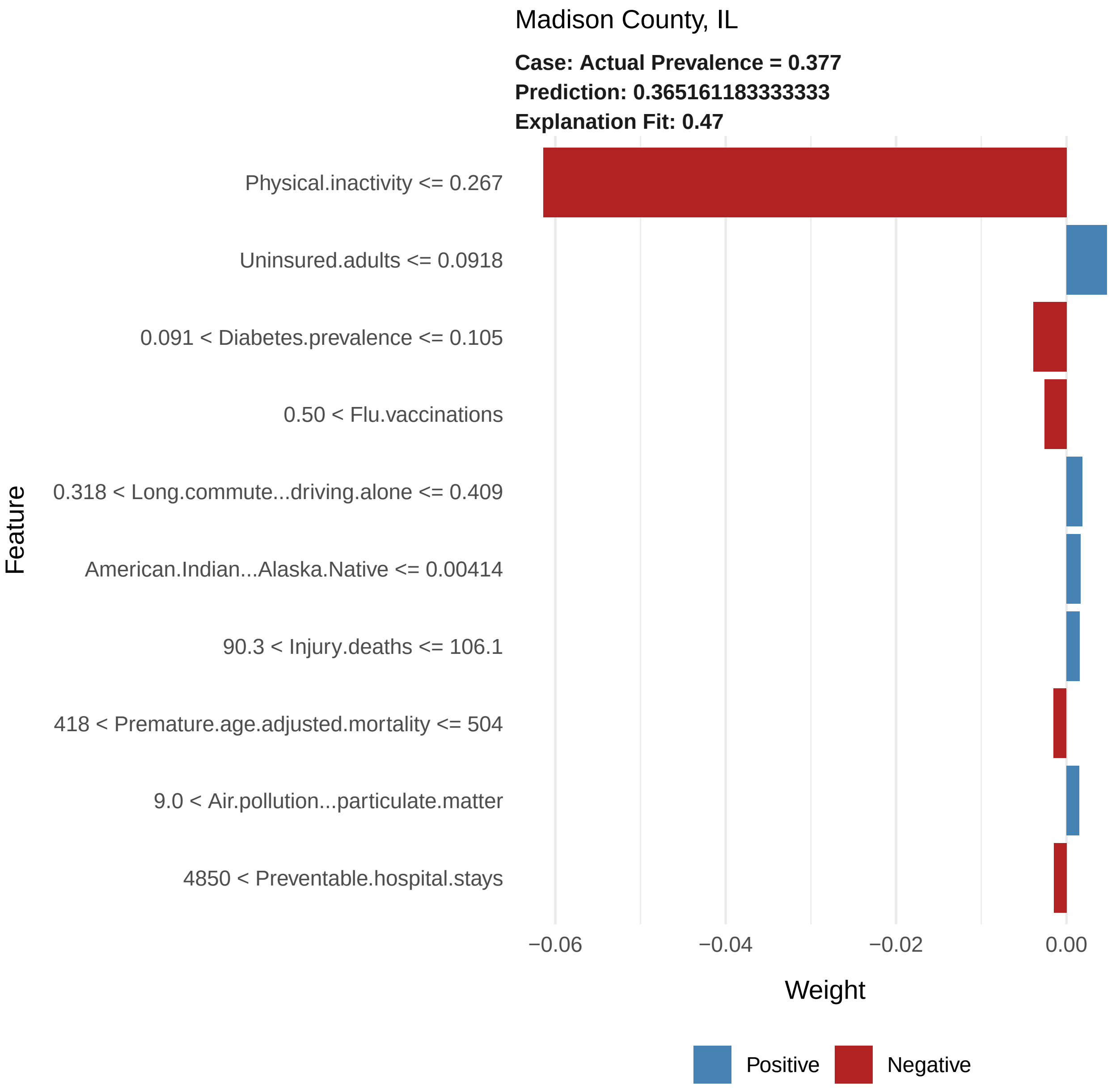
Madison County, IL
Case: Actual Prevalence = 0.377
Prediction: 0.365161183333333
Explanation Fit: 0.47
Physical.inactivity <= 0.267
Uninsured.adults <= 0.0918
0.091 < Diabetes.prevalence <= 0.105
0.50 < Flu.vaccinations
0.318 < Long.commute...driving.alone <= 0.409
American.Indian...Alaska.Native <= 0.00414
90.3 < Injury.deaths <= 106.1
418 < Premature.age.adjusted.mortality <= 504
9.0 < Air.pollution...particulate.matter
4850 < Preventable.hospital.stays
Feature
−0.06
−0.04
−0.02
0.00
Weight
Positive
Negative

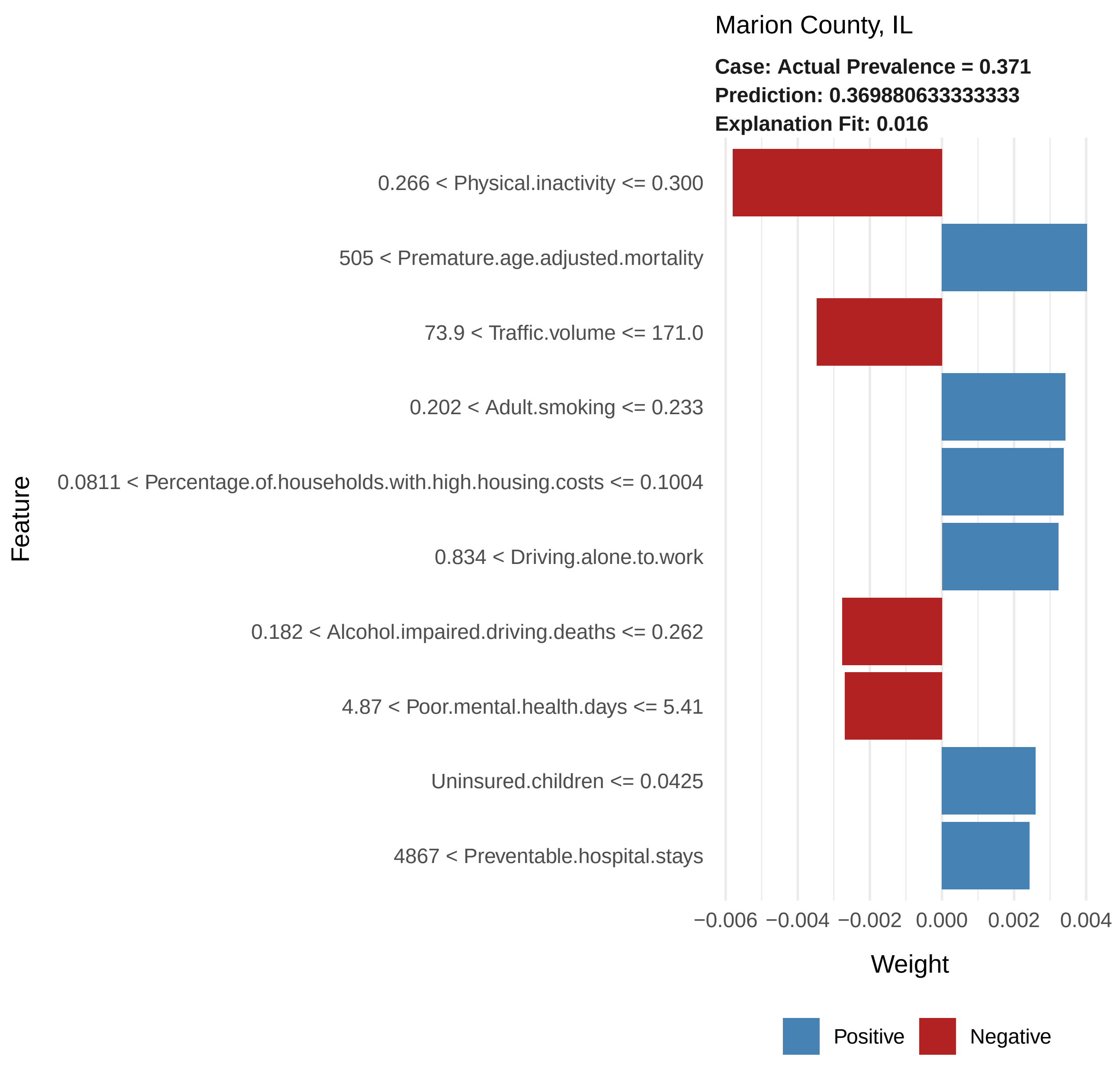
Marion County, IL
Case: Actual Prevalence = 0.371
Prediction: 0.369880633333333
Explanation Fit: 0.016
0.266 < Physical.inactivity <= 0.300
505 < Premature.age.adjusted.mortality
73.9 < Traffic.volume <= 171.0
0.202 < Adult.smoking <= 0.233
0.0811 < Percentage.of.households.with.high.housing.costs <= 0.1004
0.834 < Driving.alone.to.work
0.182 < Alcohol.impaired.driving.deaths <= 0.262
4.87 < Poor.mental.health.days <= 5.41
Uninsured.children <= 0.0425
4867 < Preventable.hospital.stays
Feature
−0.006
−0.004
−0.002
0.000
0.002
0.004
Weight
Positive
Negative

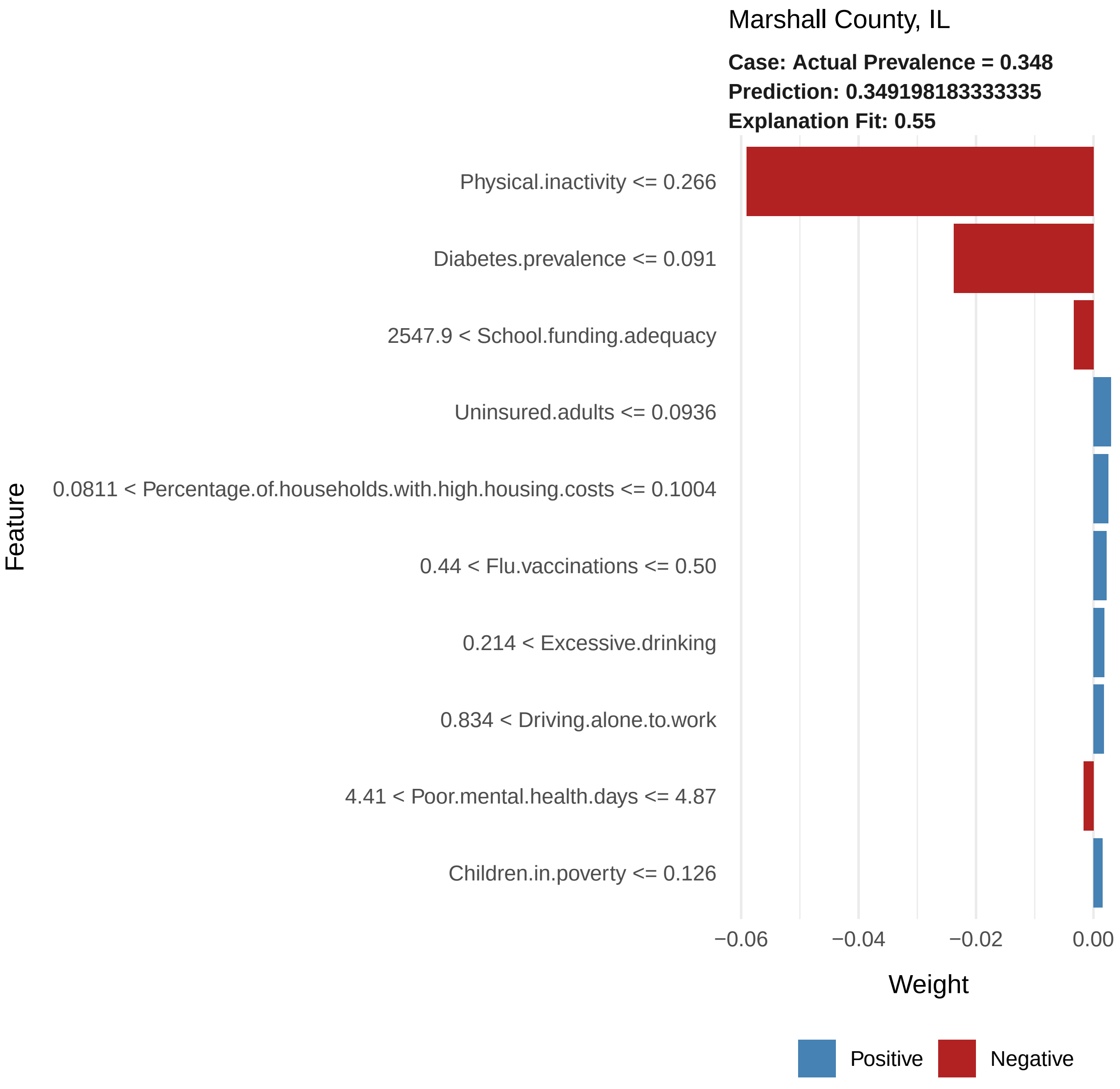

Marshall County, IL
Case: Actual Prevalence = 0.348
Prediction: 0.349198183333335
Explanation Fit: 0.55
Physical.inactivity <= 0.266
Diabetes.prevalence <= 0.091
2547.9 < School.funding.adequacy
Uninsured.adults <= 0.0936
0.0811 < Percentage.of.households.with.high.housing.costs <= 0.1004
0.44 < Flu.vaccinations <= 0.50
0.214 < Excessive.drinking
0.834 < Driving.alone.to.work
4.41 < Poor.mental.health.days <= 4.87
Children.in.poverty <= 0.126
Feature
−0.06
−0.04
−0.02
0.00
Weight
Positive
Negative

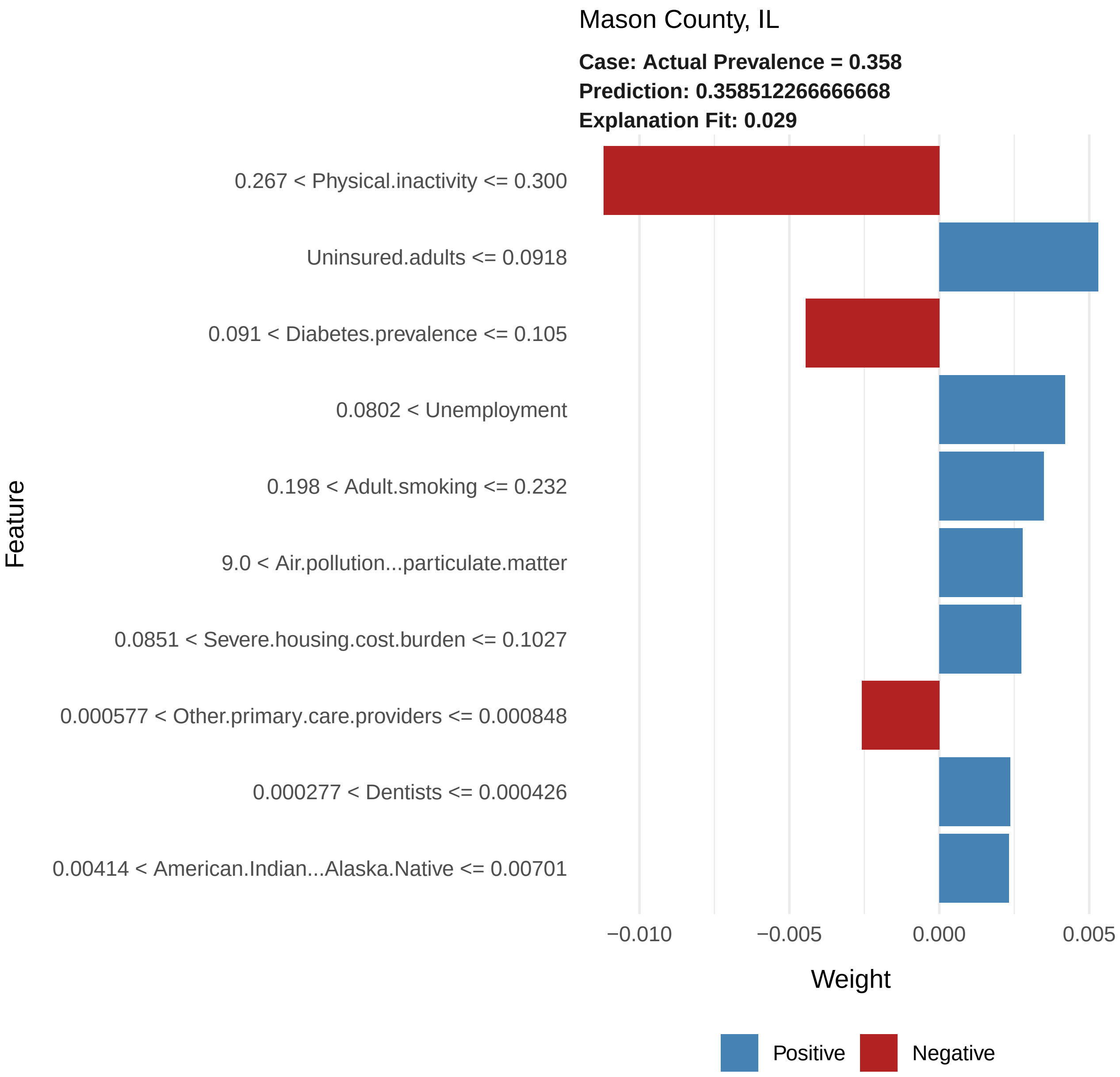

Mason County, IL
Case: Actual Prevalence = 0.358
Prediction: 0.358512266666668
Explanation Fit: 0.029
0.267 < Physical.inactivity <= 0.300
Uninsured.adults <= 0.0918
0.091 < Diabetes.prevalence <= 0.105
0.0802 < Unemployment
0.198 < Adult.smoking <= 0.232
9.0 < Air.pollution...particulate.matter
0.0851 < Severe.housing.cost.burden <= 0.1027
0.000577 < Other.primary.care.providers <= 0.000848
0.000277 < Dentists <= 0.000426
0.00414 < American.Indian...Alaska.Native <= 0.00701
Feature
−0.010
−0.005
0.000
0.005
Weight
Positive
Negative

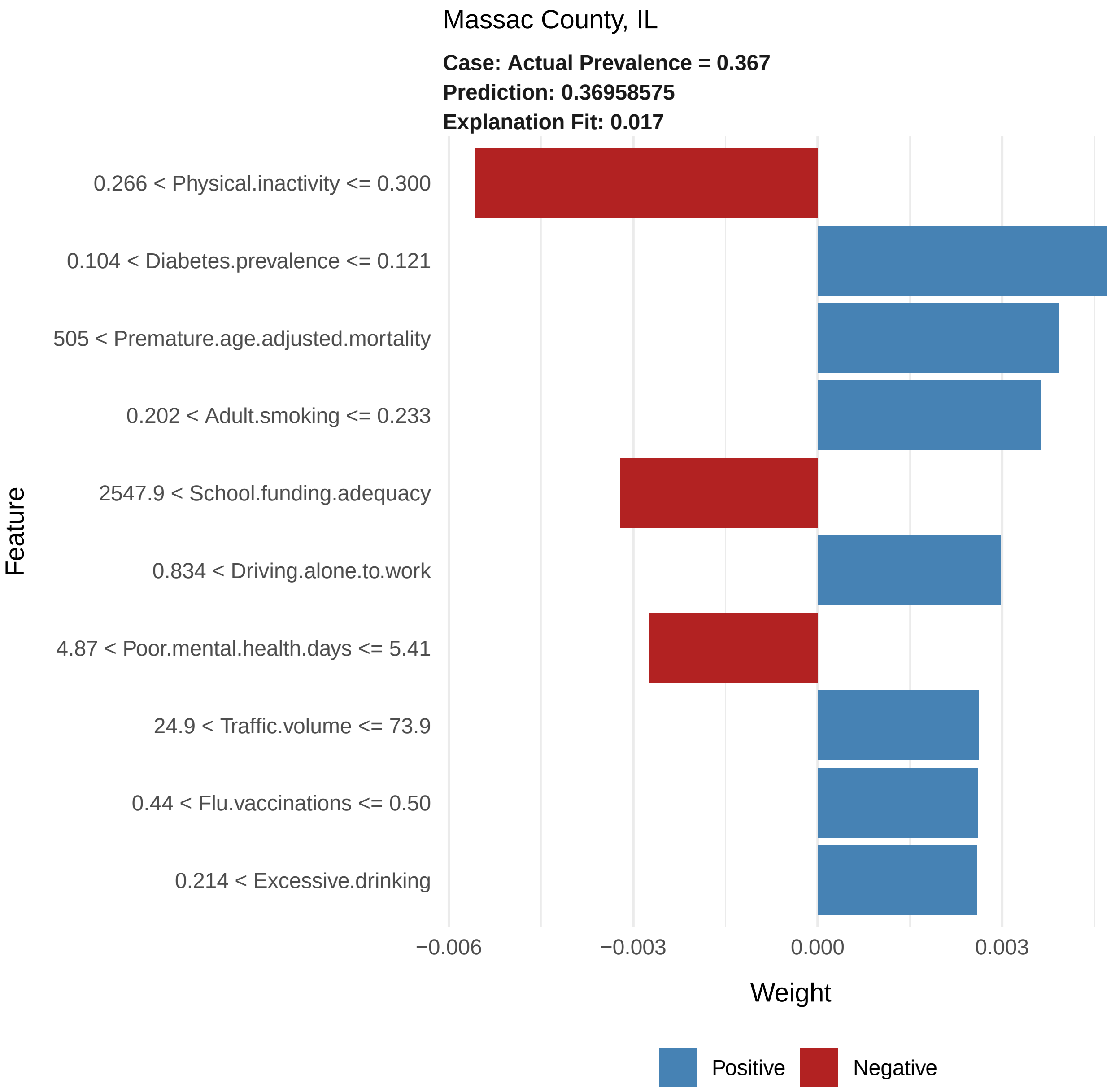

Massac County, IL
Case: Actual Prevalence = 0.367
Prediction: 0.36958575
Explanation Fit: 0.017
0.266 < Physical.inactivity <= 0.300
0.104 < Diabetes.prevalence <= 0.121
505 < Premature.age.adjusted.mortality
0.202 < Adult.smoking <= 0.233
2547.9 < School.funding.adequacy
0.834 < Driving.alone.to.work
4.87 < Poor.mental.health.days <= 5.41
24.9 < Traffic.volume <= 73.9
0.44 < Flu.vaccinations <= 0.50
0.214 < Excessive.drinking
Feature
−0.006
−0.003
0.000
0.003
Weight
Positive
Negative

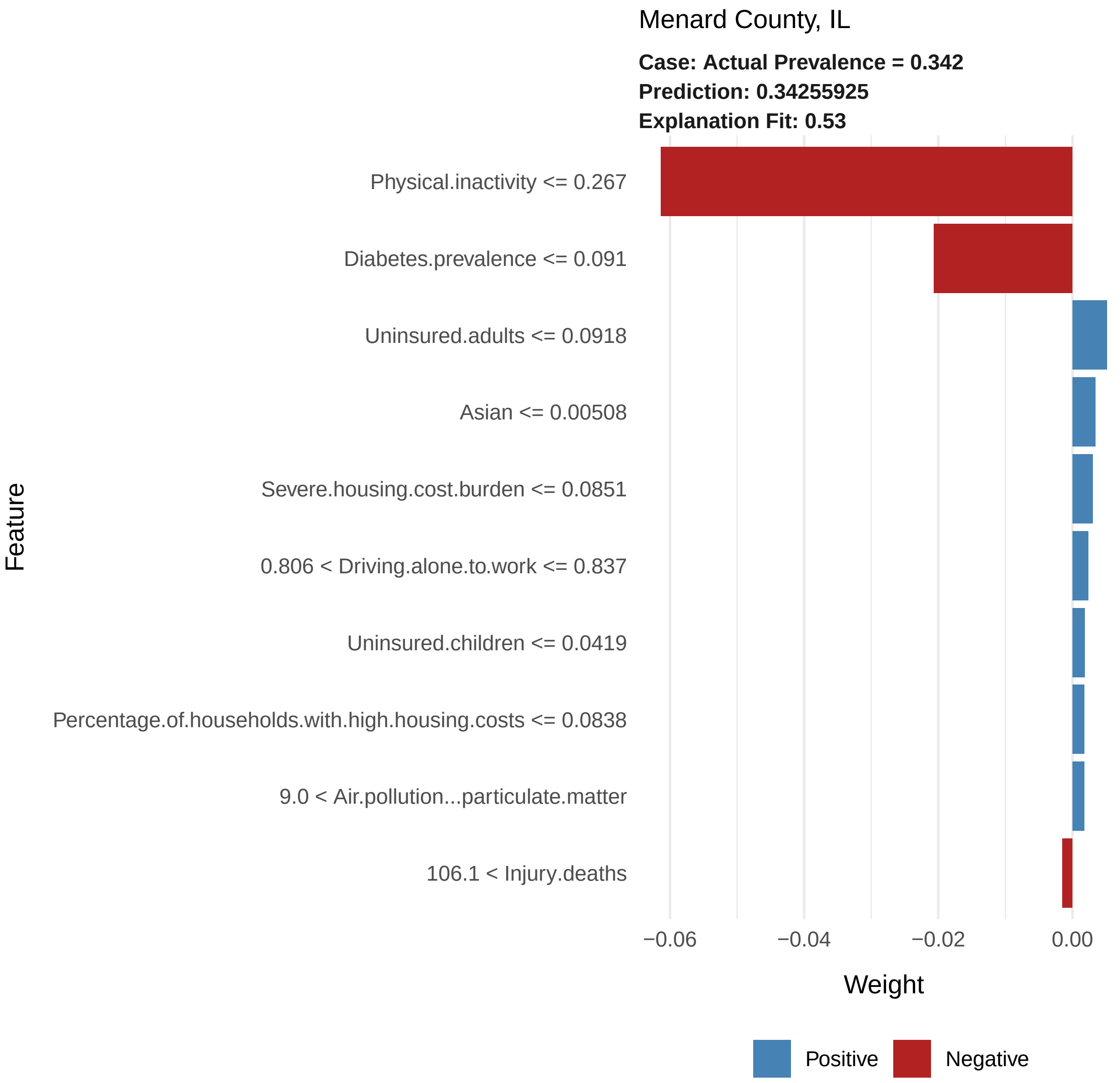

Menard County, IL
Case: Actual Prevalence = 0.342
Prediction: 0.34255925
Explanation Fit: 0.53
Physical.inactivity <= 0.267
Diabetes.prevalence <= 0.091
Uninsured.adults <= 0.0918
Asian <= 0.00508
Severe.housing.cost.burden <= 0.0851
0.806 < Driving.alone.to.work <= 0.837
Uninsured.children <= 0.0419
Percentage.of.households.with.high.housing.costs <= 0.0838
9.0 < Air.pollution...particulate.matter
106.1 < Injury.deaths
Feature
−0.06
−0.04
−0.02
0.00
Weight
Positive
Negative

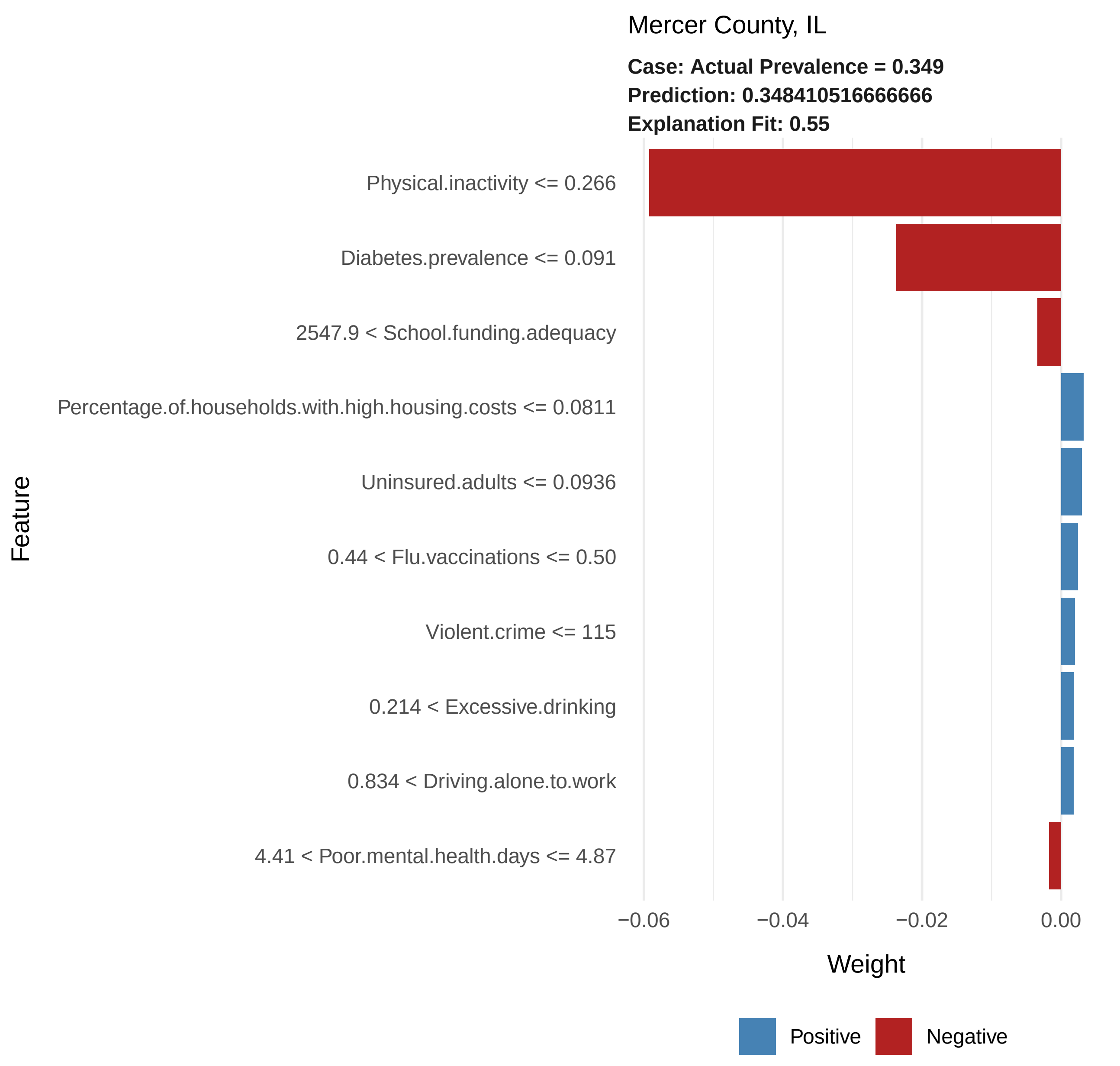

Mercer County, IL
Case: Actual Prevalence = 0.349
Prediction: 0.348410516666666
Explanation Fit: 0.55
Physical.inactivity <= 0.266
Diabetes.prevalence <= 0.091
2547.9 < School.funding.adequacy
Percentage.of.households.with.high.housing.costs <= 0.0811
Uninsured.adults <= 0.0936
0.44 < Flu.vaccinations <= 0.50
Violent.crime <= 115
0.214 < Excessive.drinking
0.834 < Driving.alone.to.work
4.41 < Poor.mental.health.days <= 4.87
Feature
−0.06
−0.04
−0.02
0.00
Weight
Positive
Negative

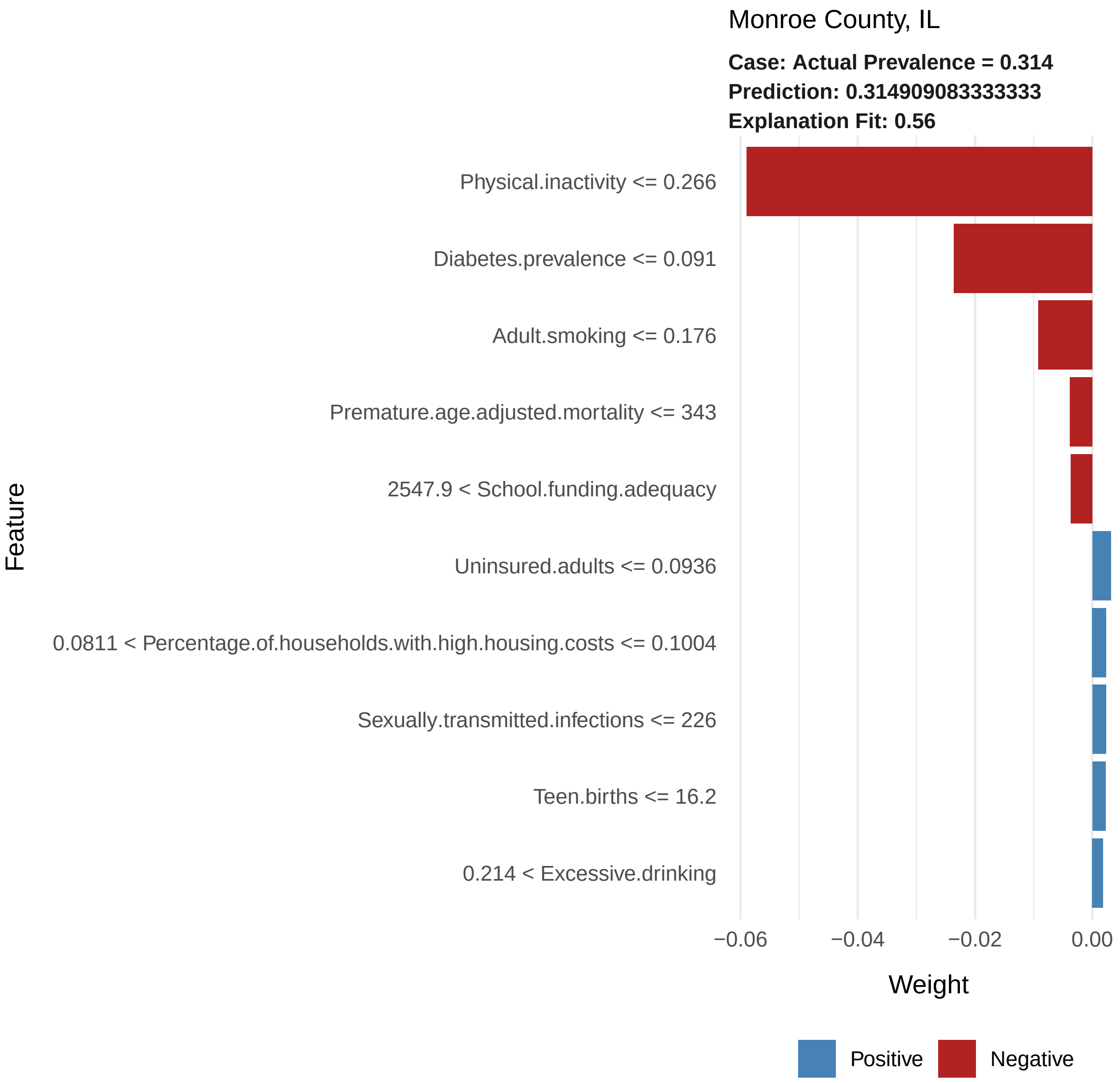

Monroe County, IL
Case: Actual Prevalence = 0.314
Prediction: 0.314909083333333
Explanation Fit: 0.56
Physical.inactivity <= 0.266
Diabetes.prevalence <= 0.091
Adult.smoking <= 0.176
Premature.age.adjusted.mortality <= 343
2547.9 < School.funding.adequacy
Uninsured.adults <= 0.0936
0.0811 < Percentage.of.households.with.high.housing.costs <= 0.1004
Sexually.transmitted.infections <= 226
Teen.births <= 16.2
0.214 < Excessive.drinking
Feature
−0.06
−0.04
−0.02
0.00
Weight
Positive
Negative

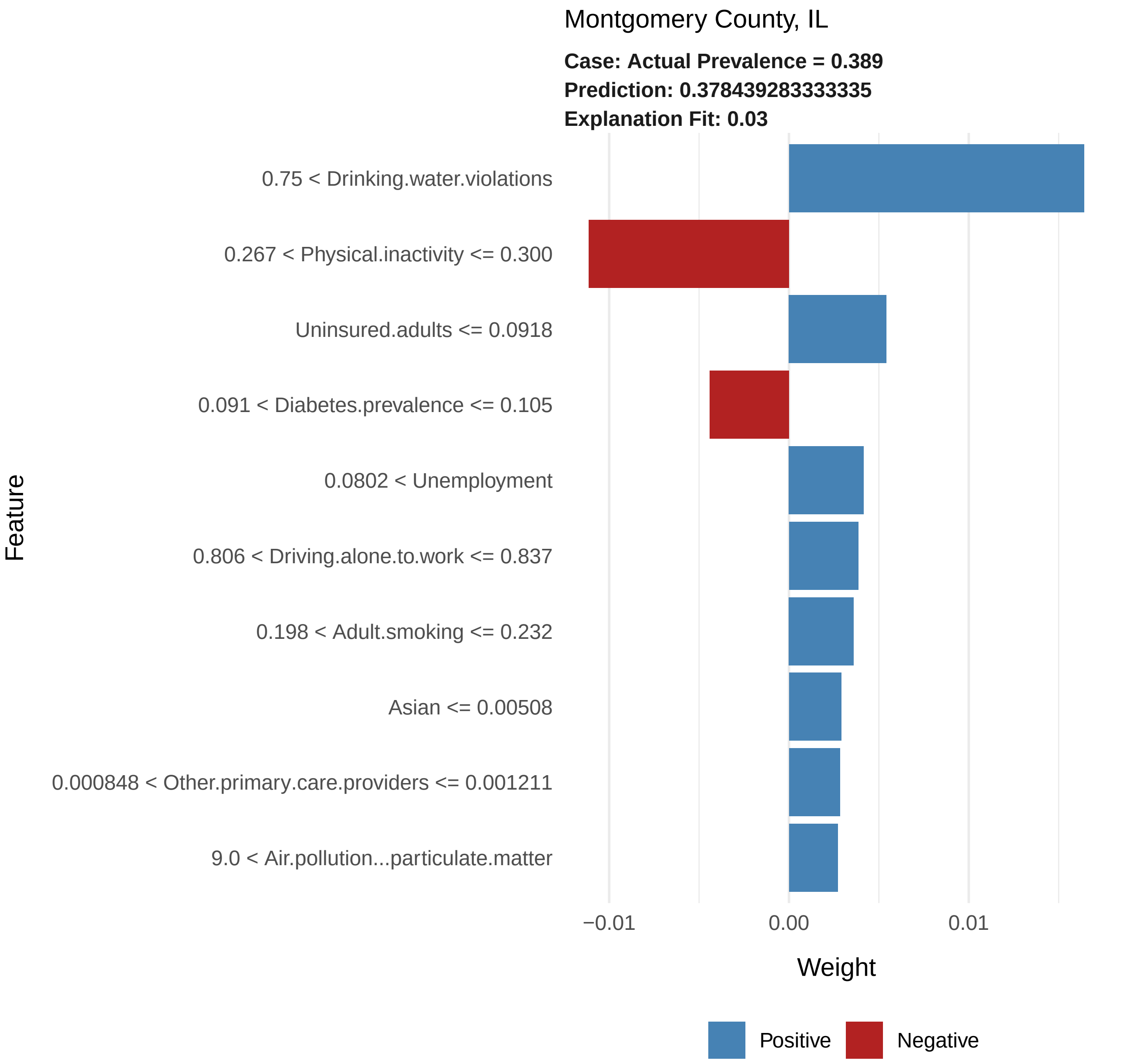
Montgomery County, IL
Case: Actual Prevalence = 0.389
Prediction: 0.378439283333335
Explanation Fit: 0.03
0.75 < Drinking.water.violations
0.267 < Physical.inactivity <= 0.300
Uninsured.adults <= 0.0918
0.091 < Diabetes.prevalence <= 0.105
0.0802 < Unemployment
0.806 < Driving.alone.to.work <= 0.837
0.198 < Adult.smoking <= 0.232
Asian <= 0.00508
0.000848 < Other.primary.care.providers <= 0.001211
9.0 < Air.pollution...particulate.matter
Feature
−0.01
0.00
0.01
Weight
Positive
Negative

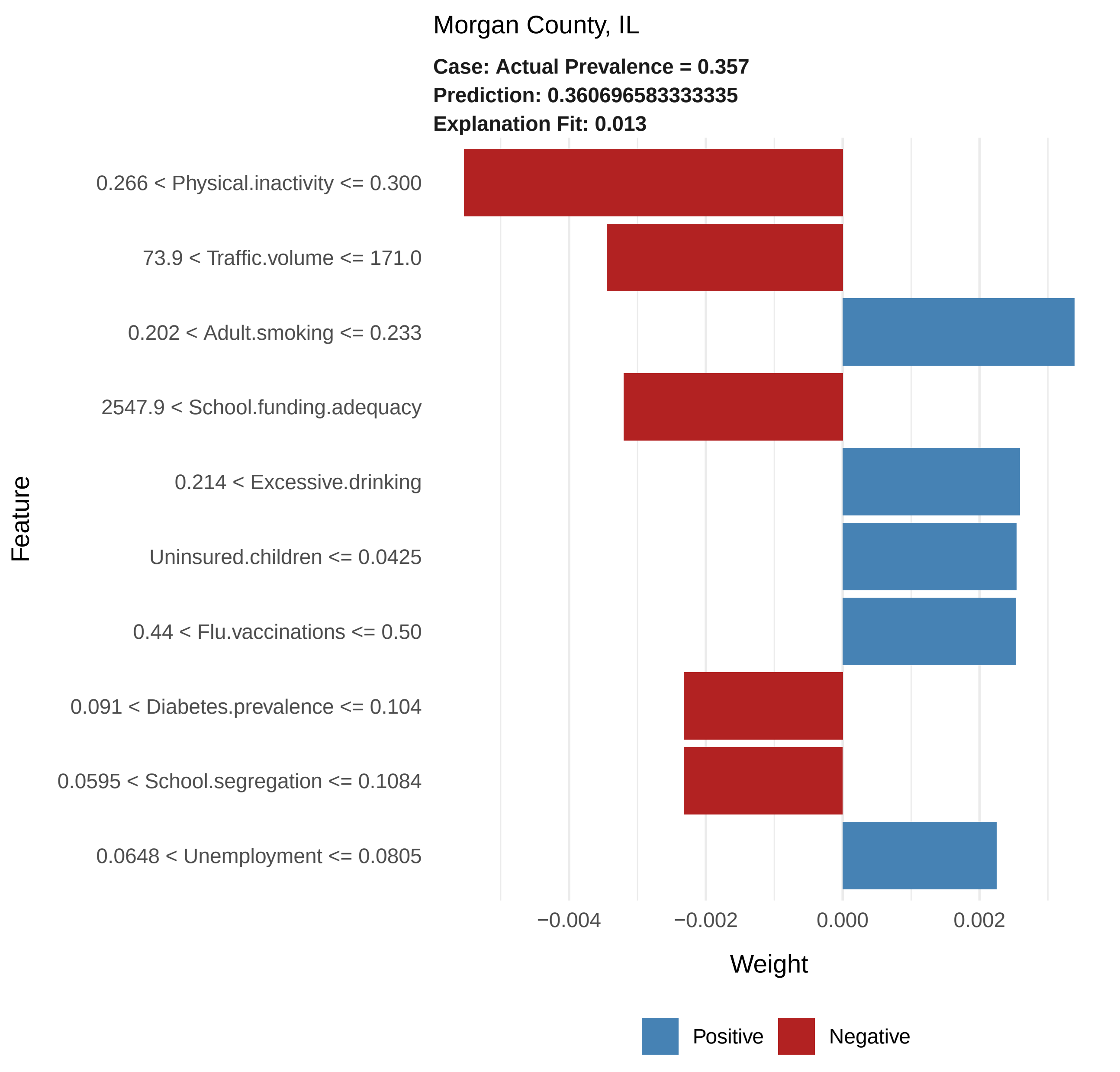

Morgan County, IL
Case: Actual Prevalence = 0.357
Prediction: 0.360696583333335
Explanation Fit: 0.013
0.266 < Physical.inactivity <= 0.300
73.9 < Traffic.volume <= 171.0
0.202 < Adult.smoking <= 0.233
2547.9 < School.funding.adequacy
0.214 < Excessive.drinking
Uninsured.children <= 0.0425
0.44 < Flu.vaccinations <= 0.50
0.091 < Diabetes.prevalence <= 0.104
0.0595 < School.segregation <= 0.1084
0.0648 < Unemployment <= 0.0805
Feature
−0.004
−0.002
0.000
0.002
Weight
Positive
Negative

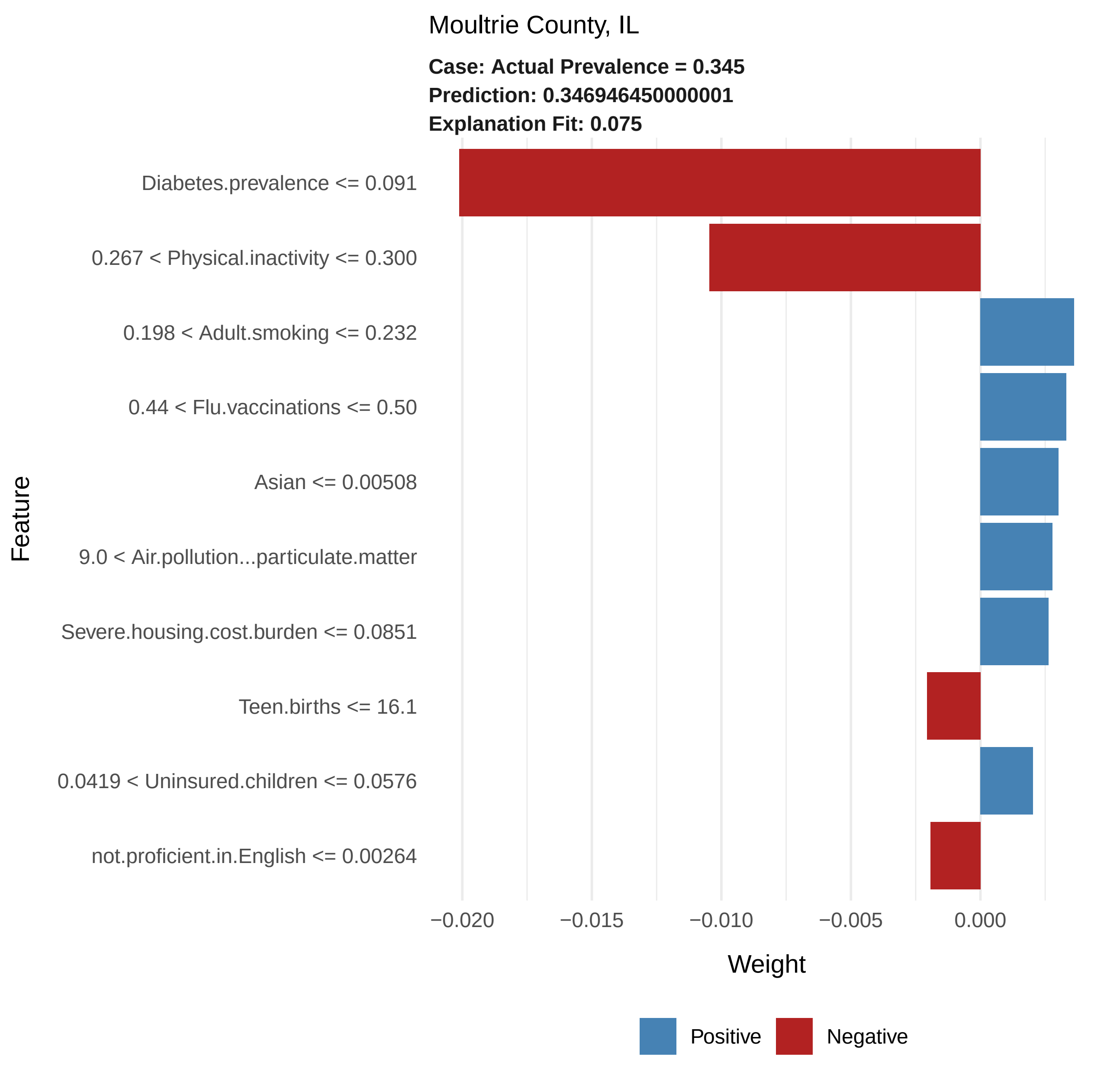

Moultrie County, IL
Case: Actual Prevalence = 0.345
Prediction: 0.346946450000001
Explanation Fit: 0.075
Diabetes.prevalence <= 0.091
0.267 < Physical.inactivity <= 0.300
0.198 < Adult.smoking <= 0.232
0.44 < Flu.vaccinations <= 0.50
Asian <= 0.00508
9.0 < Air.pollution...particulate.matter
Severe.housing.cost.burden <= 0.0851
Teen.births <= 16.1
0.0419 < Uninsured.children <= 0.0576
not.proficient.in.English <= 0.00264
Feature
−0.020
−0.015
−0.010
−0.005
0.000
Weight
Positive
Negative

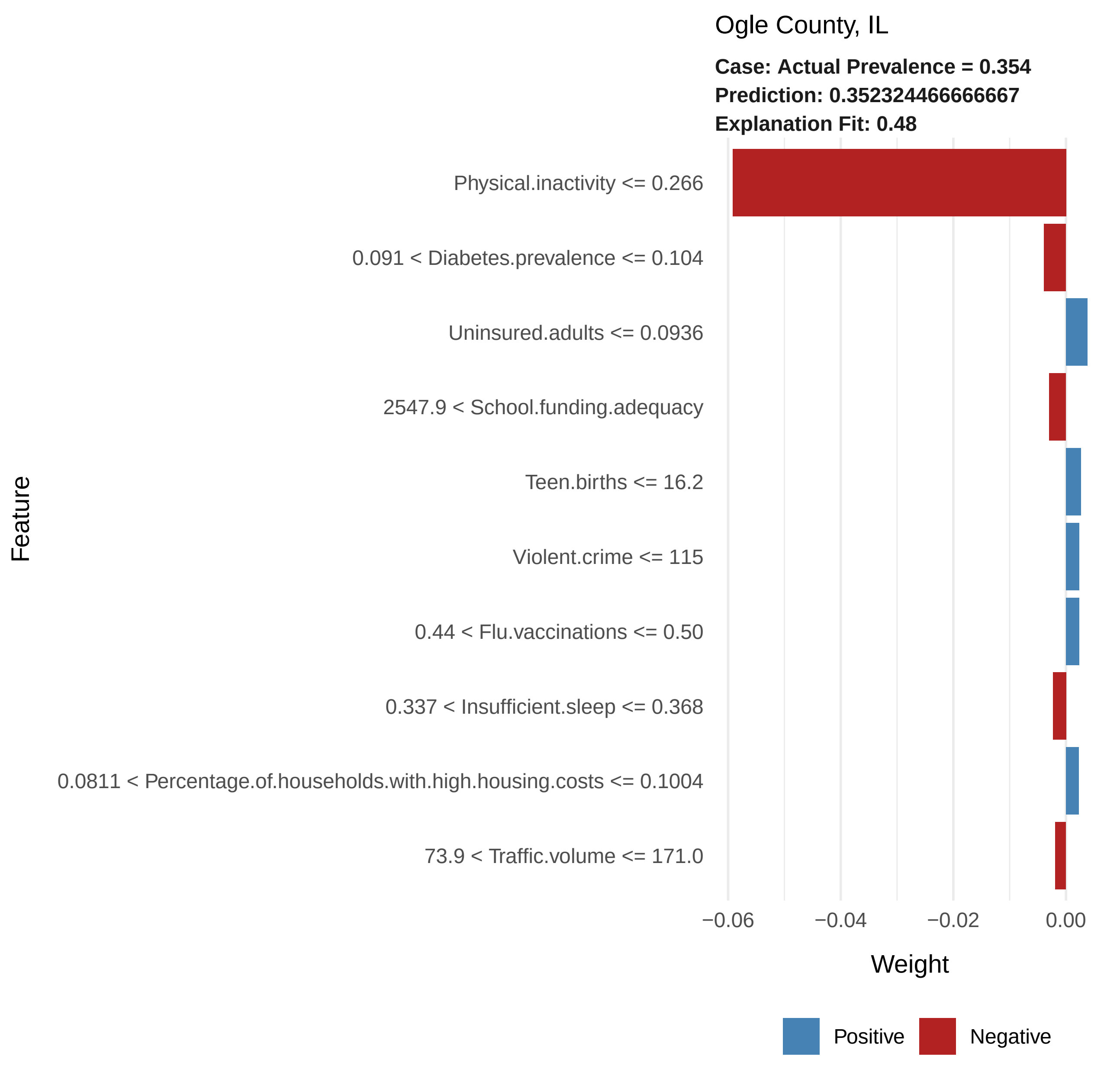

Ogle County, IL
Case: Actual Prevalence = 0.354
Prediction: 0.352324466666667
Explanation Fit: 0.48
Physical.inactivity <= 0.266
0.091 < Diabetes.prevalence <= 0.104
Uninsured.adults <= 0.0936
2547.9 < School.funding.adequacy
Teen.births <= 16.2
Violent.crime <= 115
0.44 < Flu.vaccinations <= 0.50
0.337 < Insufficient.sleep <= 0.368
0.0811 < Percentage.of.households.with.high.housing.costs <= 0.1004
73.9 < Traffic.volume <= 171.0
Feature
−0.06
−0.04
−0.02
0.00
Weight
Positive
Negative

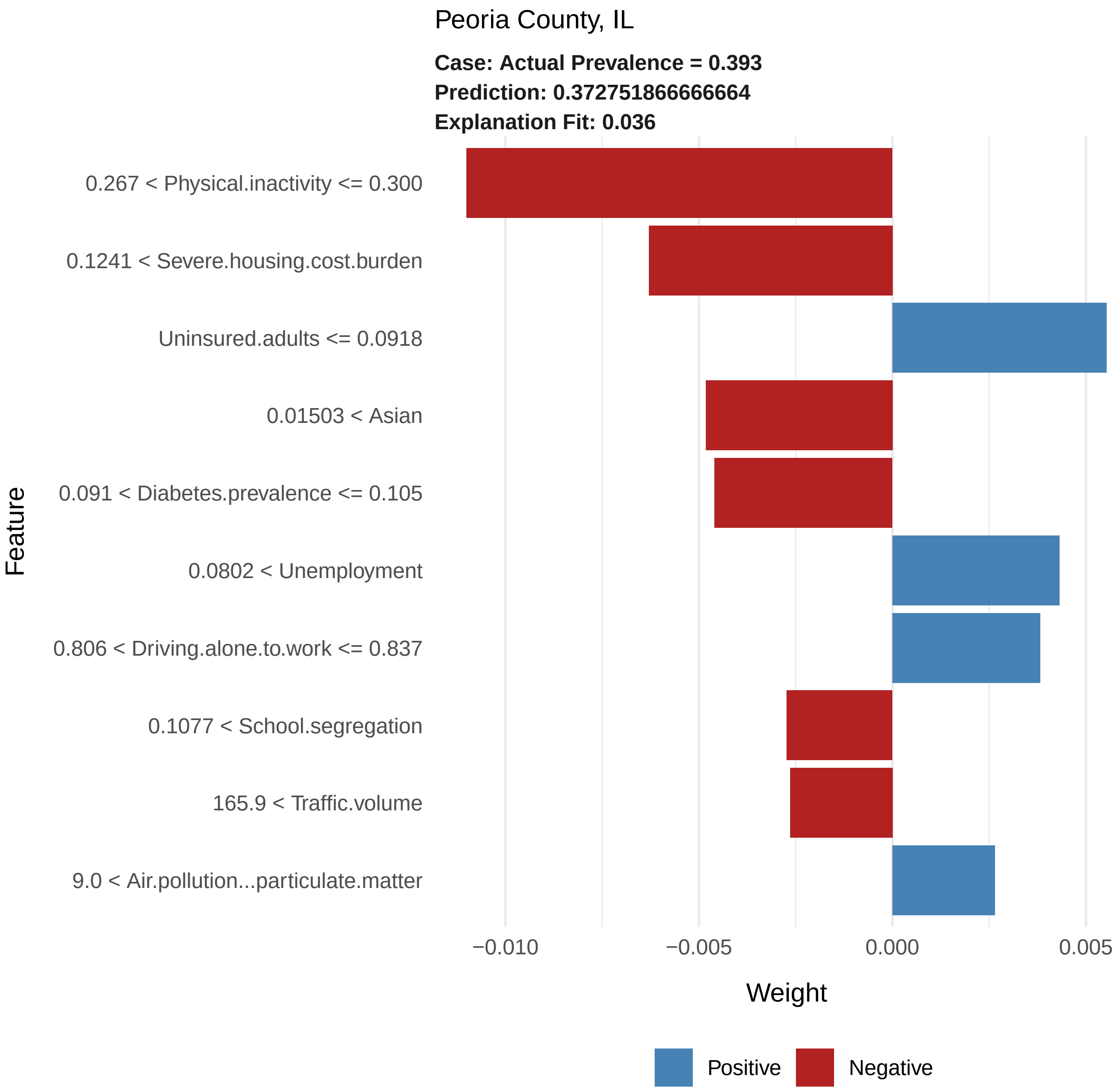

Peoria County, IL
Case: Actual Prevalence = 0.393
Prediction: 0.372751866666664
Explanation Fit: 0.036
0.267 < Physical.inactivity <= 0.300
0.1241 < Severe.housing.cost.burden
Uninsured.adults <= 0.0918
0.01503 < Asian
0.091 < Diabetes.prevalence <= 0.105
0.0802 < Unemployment
0.806 < Driving.alone.to.work <= 0.837
0.1077 < School.segregation
165.9 < Traffic.volume
9.0 < Air.pollution...particulate.matter
Feature
−0.010
−0.005
0.000
0.005
Weight
Positive
Negative

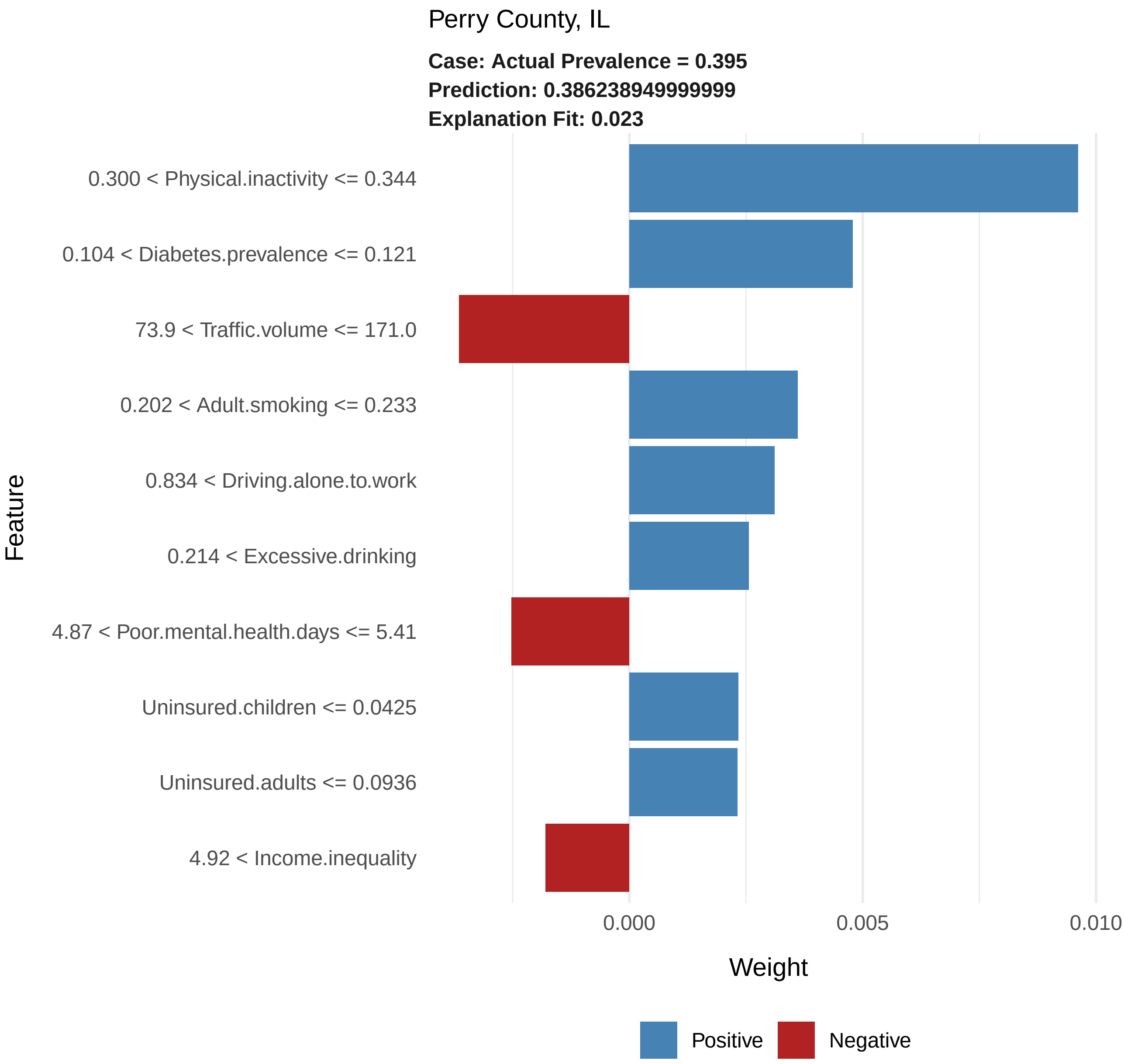

Perry County, IL
Case: Actual Prevalence = 0.395
Prediction: 0.386238949999999
Explanation Fit: 0.023
0.300 < Physical.inactivity <= 0.344
0.104 < Diabetes.prevalence <= 0.121
73.9 < Traffic.volume <= 171.0
0.202 < Adult.smoking <= 0.233
0.834 < Driving.alone.to.work
0.214 < Excessive.drinking
4.87 < Poor.mental.health.days <= 5.41
Uninsured.children <= 0.0425
Uninsured.adults <= 0.0936
4.92 < Income.inequality
Feature
0.000
0.005
0.010
Weight
Positive
Negative

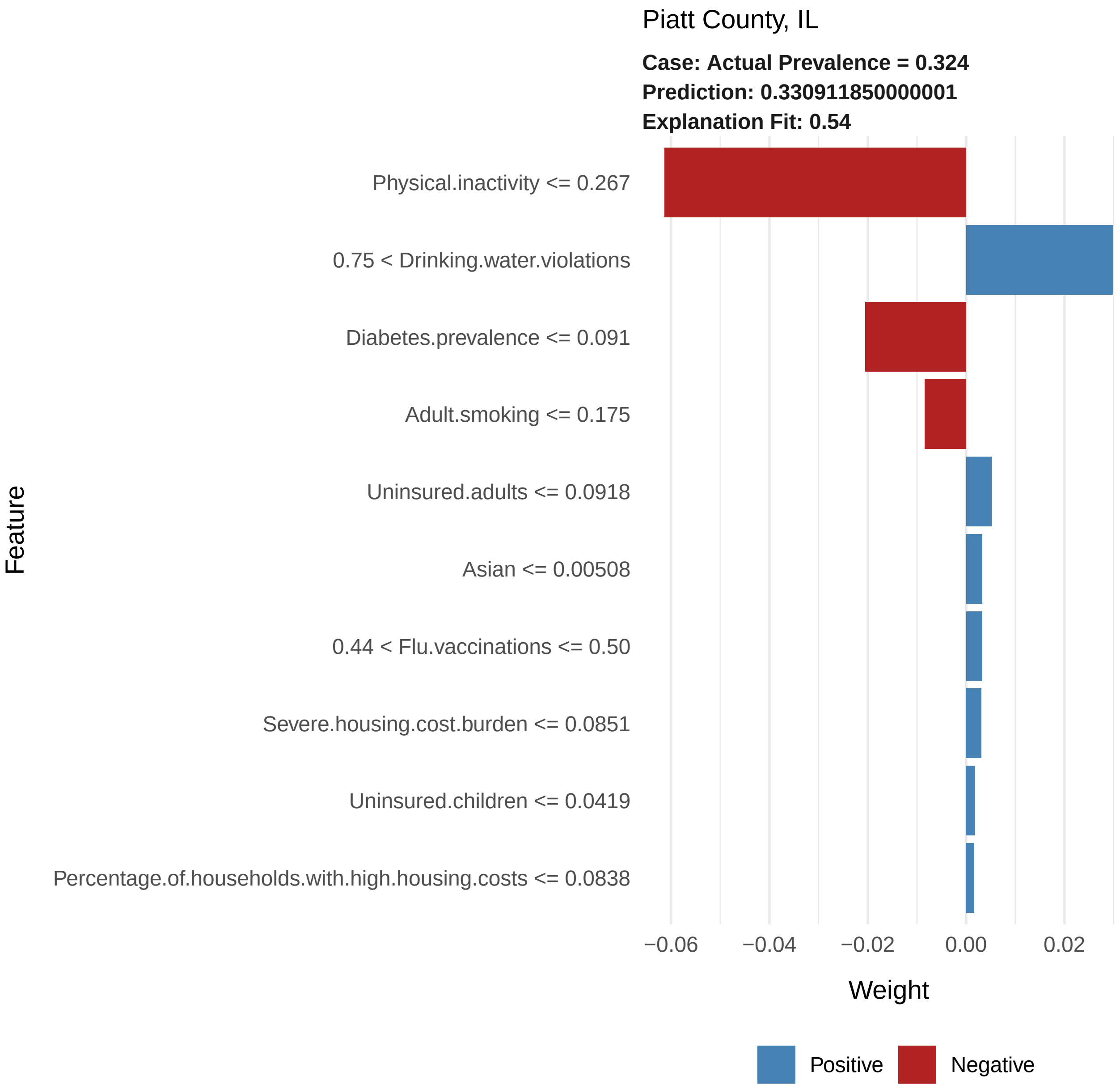
Piatt County, IL
Case: Actual Prevalence = 0.324
Prediction: 0.330911850000001
Explanation Fit: 0.54
Physical.inactivity <= 0.267
0.75 < Drinking.water.violations
Diabetes.prevalence <= 0.091
Adult.smoking <= 0.175
Uninsured.adults <= 0.0918
Asian <= 0.00508
0.44 < Flu.vaccinations <= 0.50
Severe.housing.cost.burden <= 0.0851
Uninsured.children <= 0.0419
Percentage.of.households.with.high.housing.costs <= 0.0838
Feature
−0.06
−0.04
−0.02
0.00
0.02
Weight
Positive
Negative

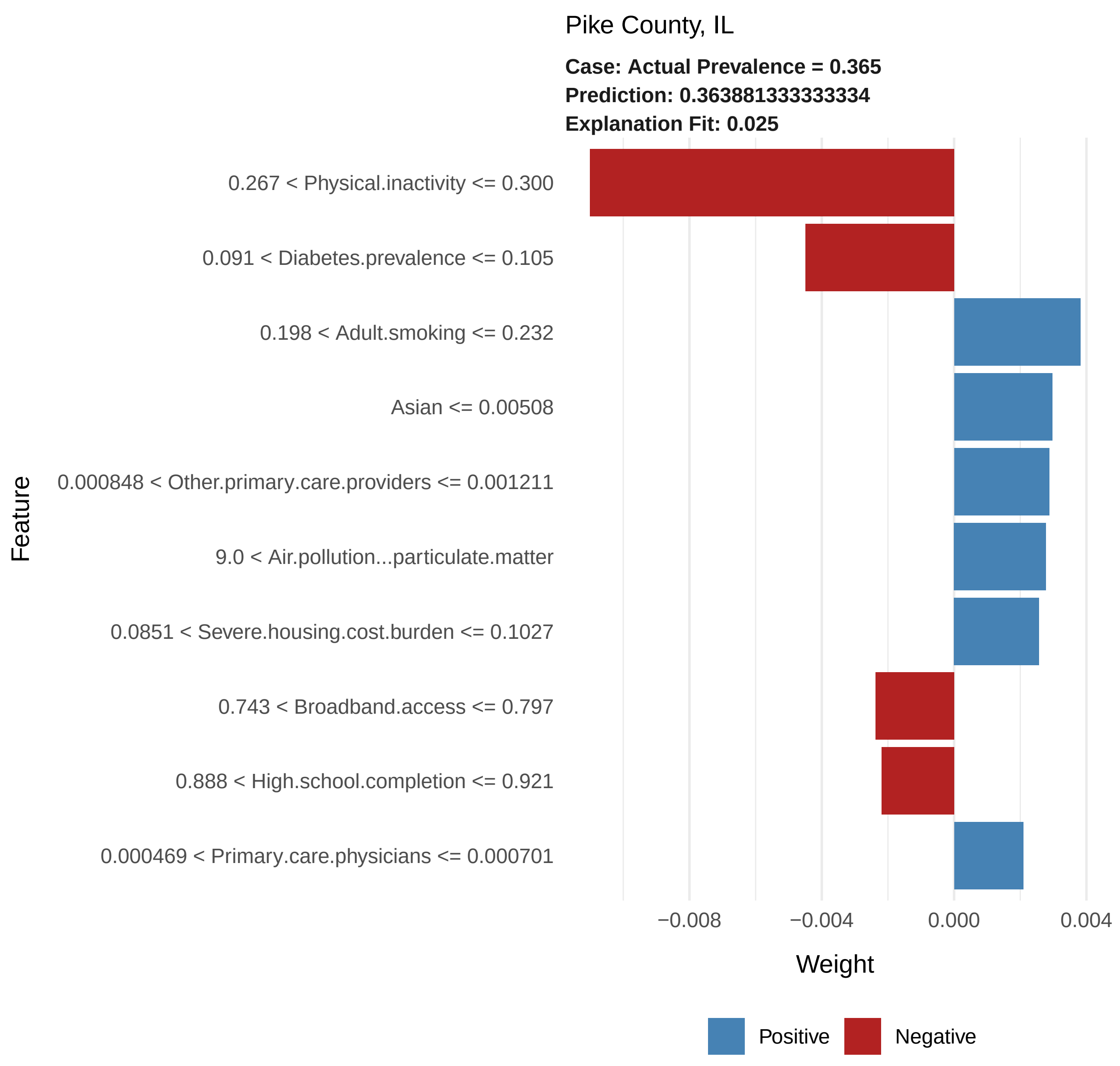

Pike County, IL
Case: Actual Prevalence = 0.365
Prediction: 0.363881333333334
Explanation Fit: 0.025
0.267 < Physical.inactivity <= 0.300
0.091 < Diabetes.prevalence <= 0.105
0.198 < Adult.smoking <= 0.232
Asian <= 0.00508
0.000848 < Other.primary.care.providers <= 0.001211
9.0 < Air.pollution...particulate.matter
0.0851 < Severe.housing.cost.burden <= 0.1027
0.743 < Broadband.access <= 0.797
0.888 < High.school.completion <= 0.921
0.000469 < Primary.care.physicians <= 0.000701
Feature
−0.008
−0.004
0.000
0.004
Weight
Positive
Negative

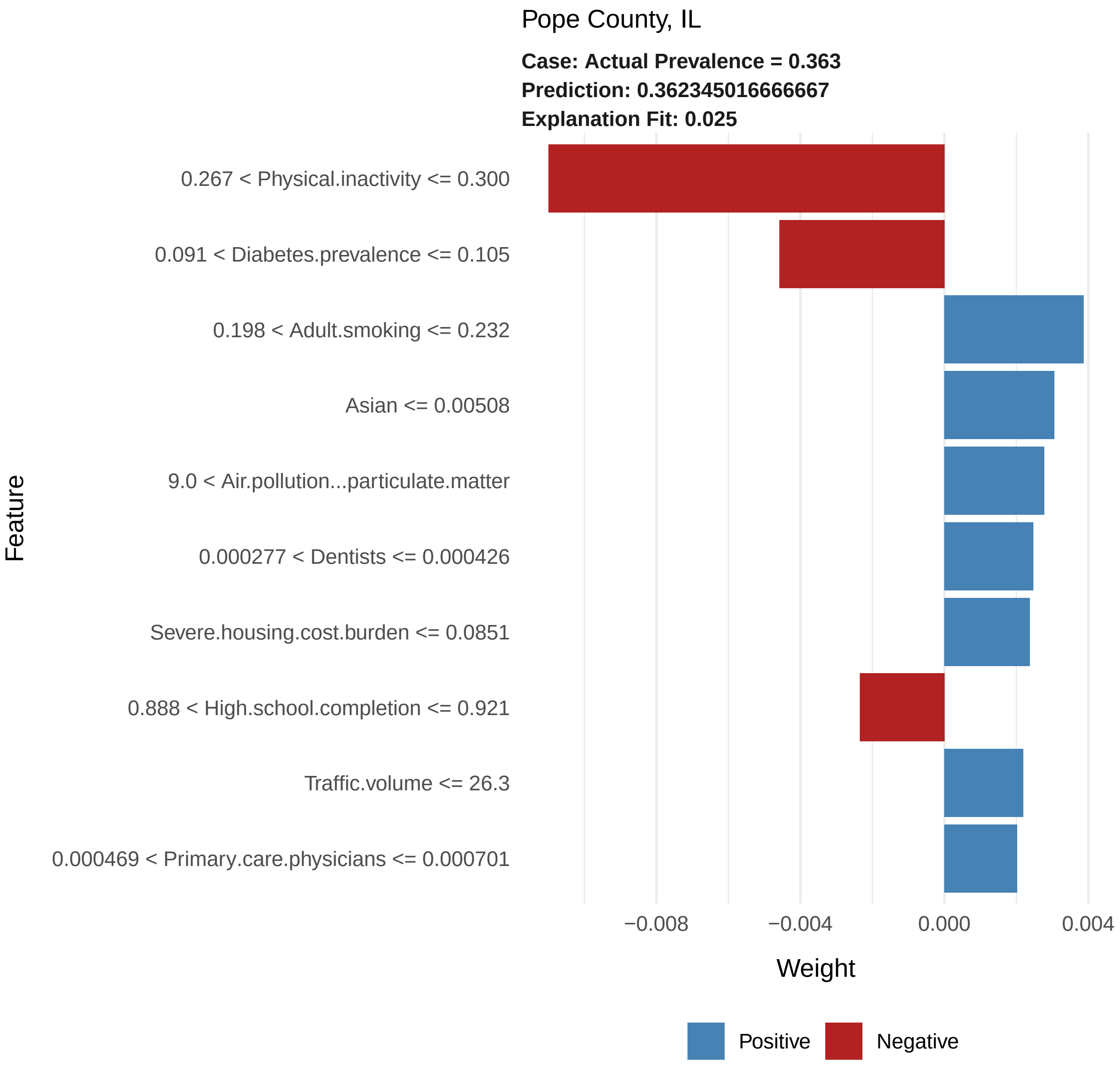
Pope County, IL
Case: Actual Prevalence = 0.363
Prediction: 0.362345016666667
Explanation Fit: 0.025
0.267 < Physical.inactivity <= 0.300
0.091 < Diabetes.prevalence <= 0.105
0.198 < Adult.smoking <= 0.232
Asian <= 0.00508
9.0 < Air.pollution...particulate.matter
0.000277 < Dentists <= 0.000426
Severe.housing.cost.burden <= 0.0851
0.888 < High.school.completion <= 0.921
Traffic.volume <= 26.3
0.000469 < Primary.care.physicians <= 0.000701
Feature
−0.008
−0.004
0.000
0.004
Weight
Positive
Negative

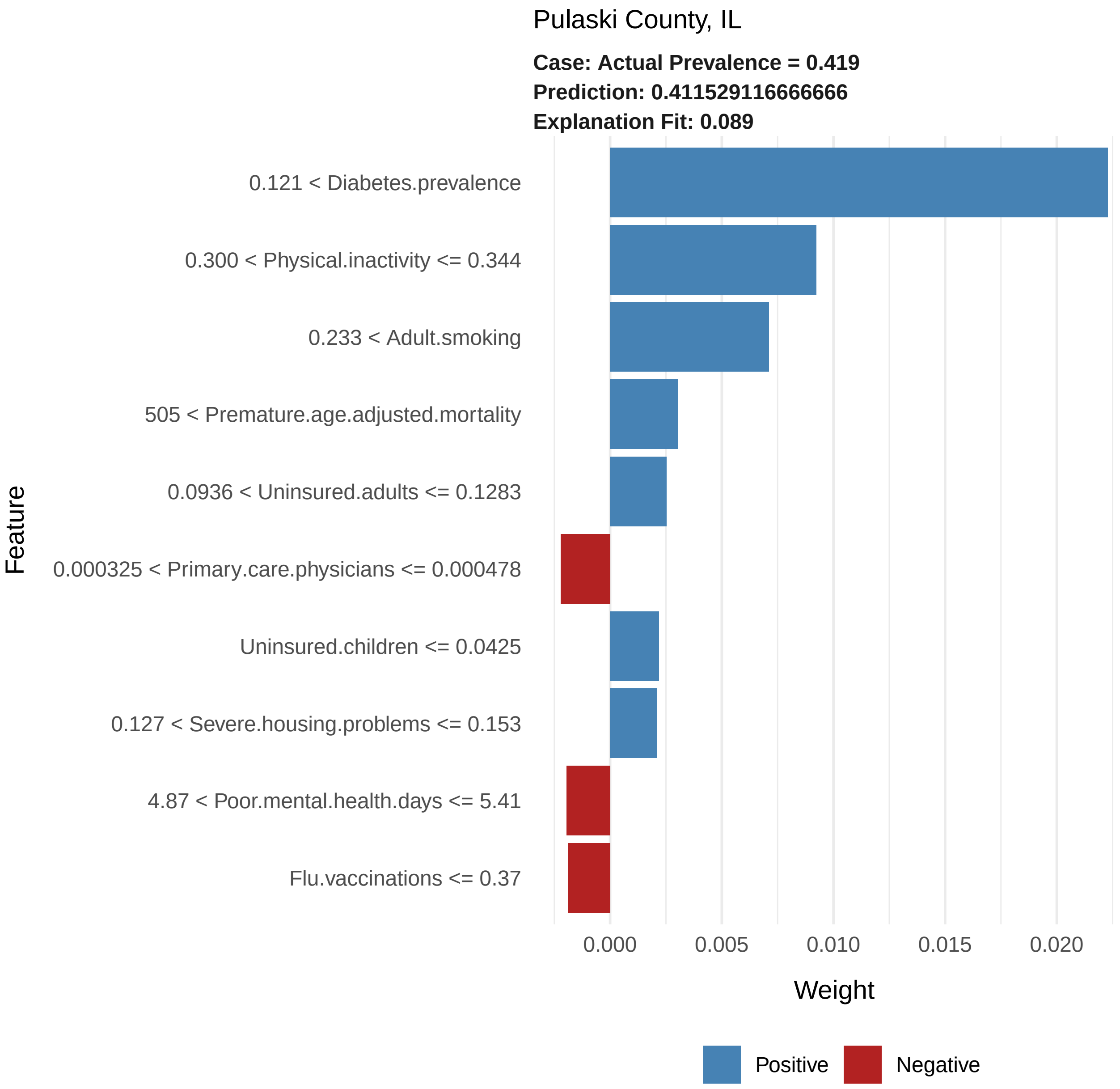
Pulaski County, IL
Case: Actual Prevalence = 0.419
Prediction: 0.411529116666666
Explanation Fit: 0.089
0.121 < Diabetes.prevalence
0.300 < Physical.inactivity <= 0.344
0.233 < Adult.smoking
505 < Premature.age.adjusted.mortality
0.0936 < Uninsured.adults <= 0.1283
0.000325 < Primary.care.physicians <= 0.000478
Uninsured.children <= 0.0425
0.127 < Severe.housing.problems <= 0.153
4.87 < Poor.mental.health.days <= 5.41
Flu.vaccinations <= 0.37
Feature
0.000
0.005
0.010
0.015
0.020
Weight
Positive
Negative

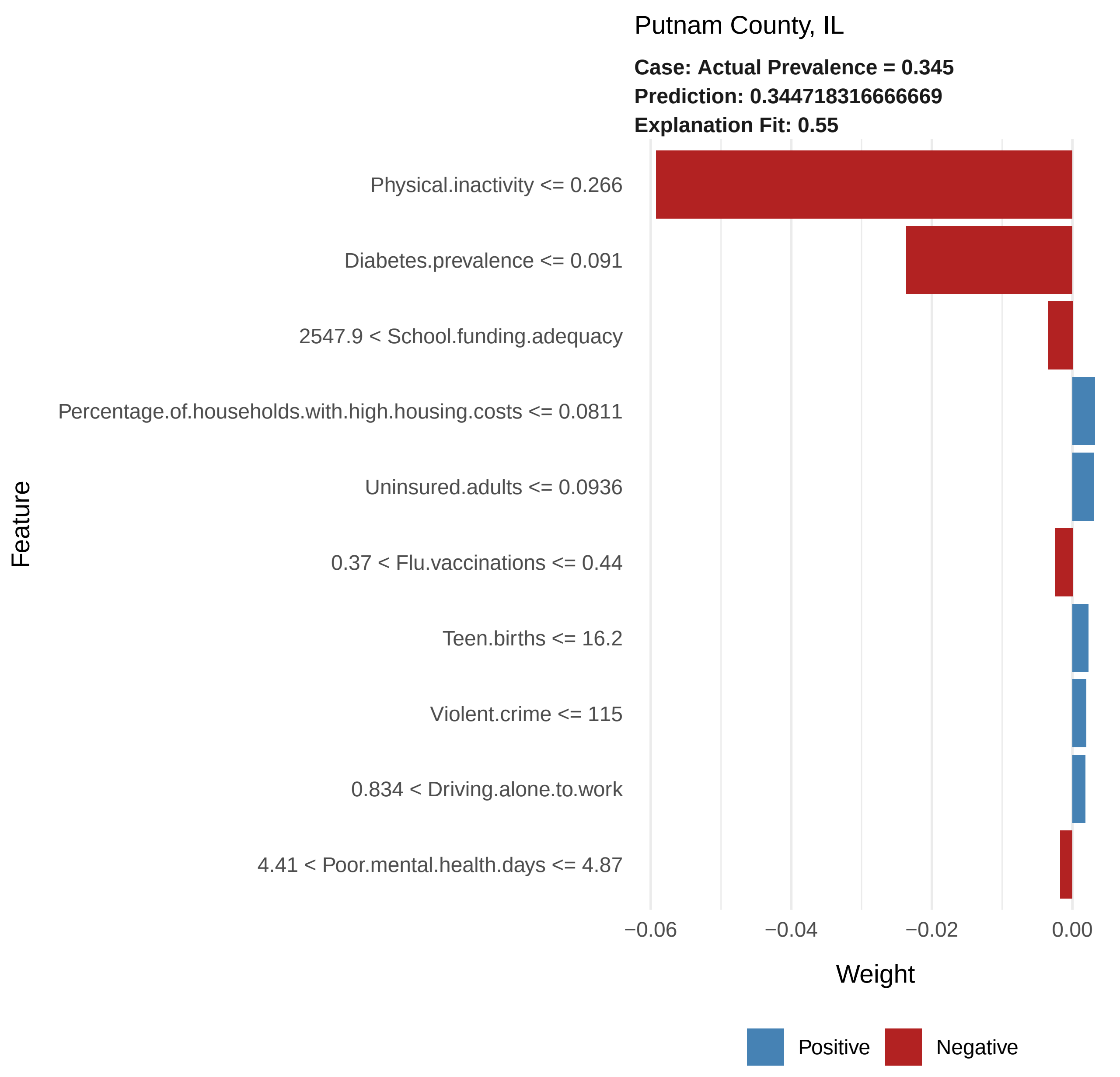

Putnam County, IL
Case: Actual Prevalence = 0.345
Prediction: 0.344718316666669
Explanation Fit: 0.55
Physical.inactivity <= 0.266
Diabetes.prevalence <= 0.091
2547.9 < School.funding.adequacy
Percentage.of.households.with.high.housing.costs <= 0.0811
Uninsured.adults <= 0.0936
0.37 < Flu.vaccinations <= 0.44
Teen.births <= 16.2
Violent.crime <= 115
0.834 < Driving.alone.to.work
4.41 < Poor.mental.health.days <= 4.87
Feature
−0.06
−0.04
−0.02
0.00
Weight
Positive
Negative

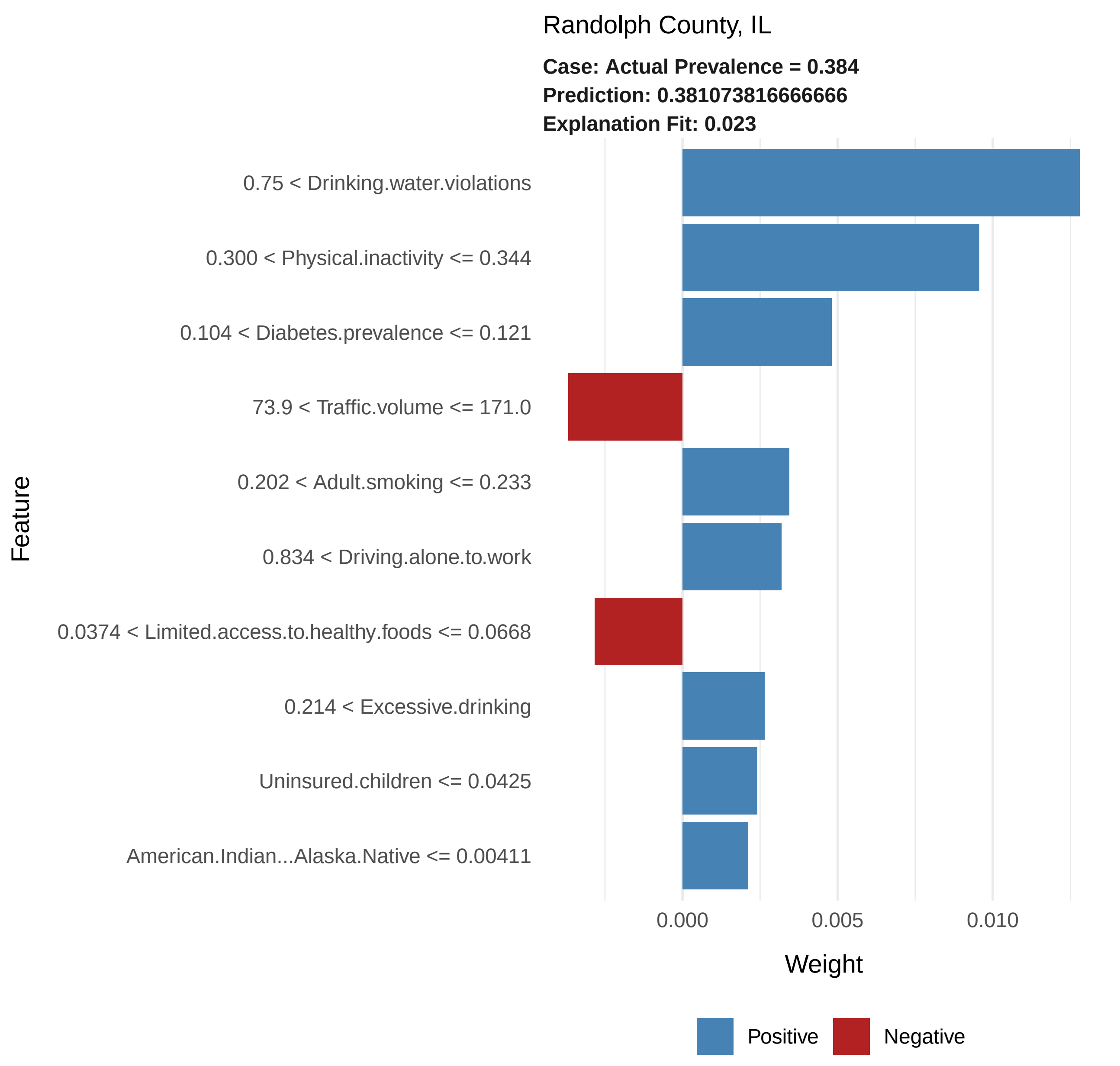
Randolph County, IL
Case: Actual Prevalence = 0.384
Prediction: 0.381073816666666
Explanation Fit: 0.023
0.75 < Drinking.water.violations
0.300 < Physical.inactivity <= 0.344
0.104 < Diabetes.prevalence <= 0.121
73.9 < Traffic.volume <= 171.0
0.202 < Adult.smoking <= 0.233
0.834 < Driving.alone.to.work
0.0374 < Limited.access.to.healthy.foods <= 0.0668
0.214 < Excessive.drinking
Uninsured.children <= 0.0425
American.Indian...Alaska.Native <= 0.00411
Feature
0.000
0.005
0.010
Weight
Positive
Negative

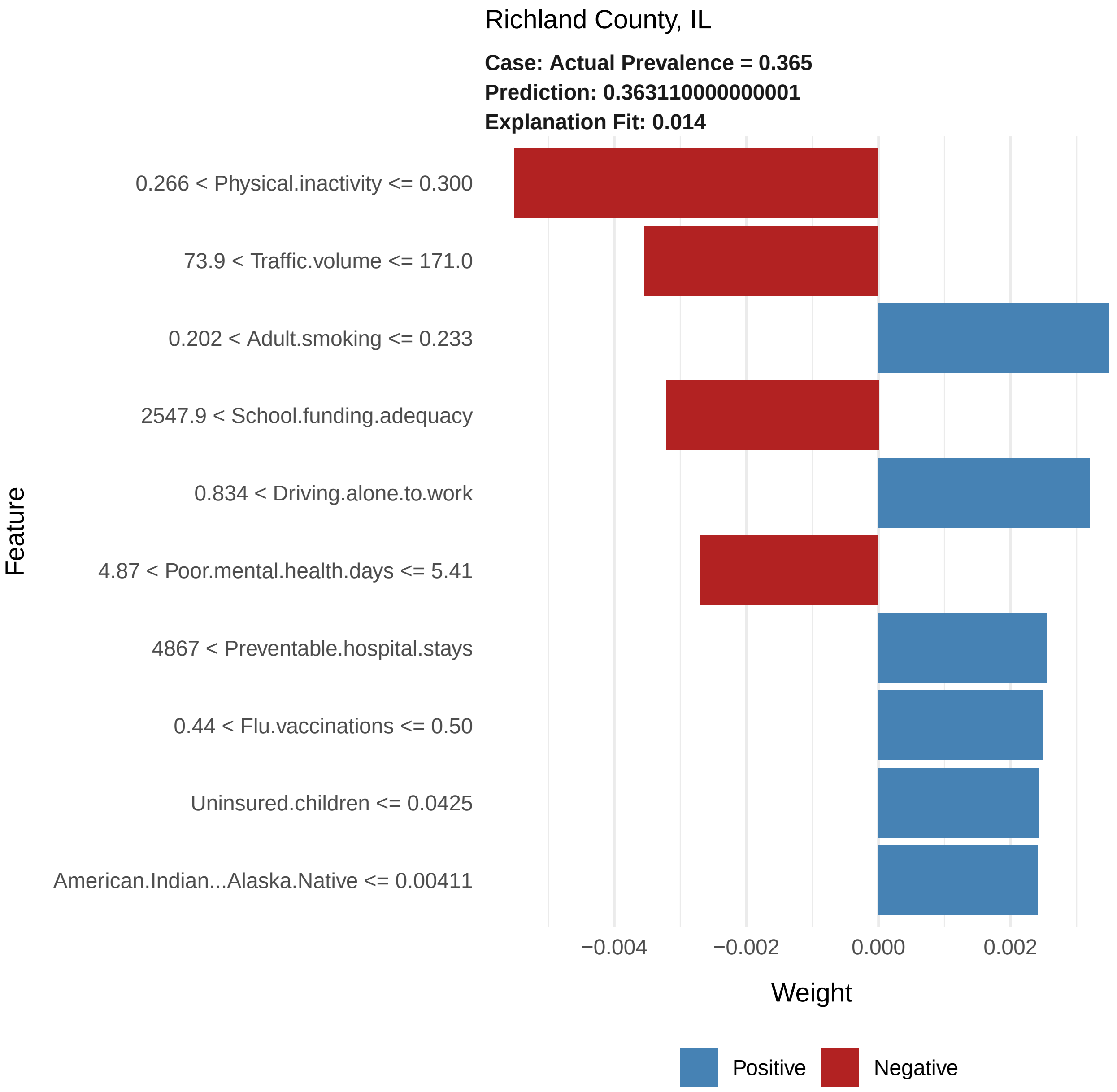

Richland County, IL
Case: Actual Prevalence = 0.365
Prediction: 0.363110000000001
Explanation Fit: 0.014
0.266 < Physical.inactivity <= 0.300
73.9 < Traffic.volume <= 171.0
0.202 < Adult.smoking <= 0.233
2547.9 < School.funding.adequacy
0.834 < Driving.alone.to.work
4.87 < Poor.mental.health.days <= 5.41
4867 < Preventable.hospital.stays
0.44 < Flu.vaccinations <= 0.50
Uninsured.children <= 0.0425
American.Indian...Alaska.Native <= 0.00411
Feature
−0.004
−0.002
0.000
0.002
Weight
Positive
Negative

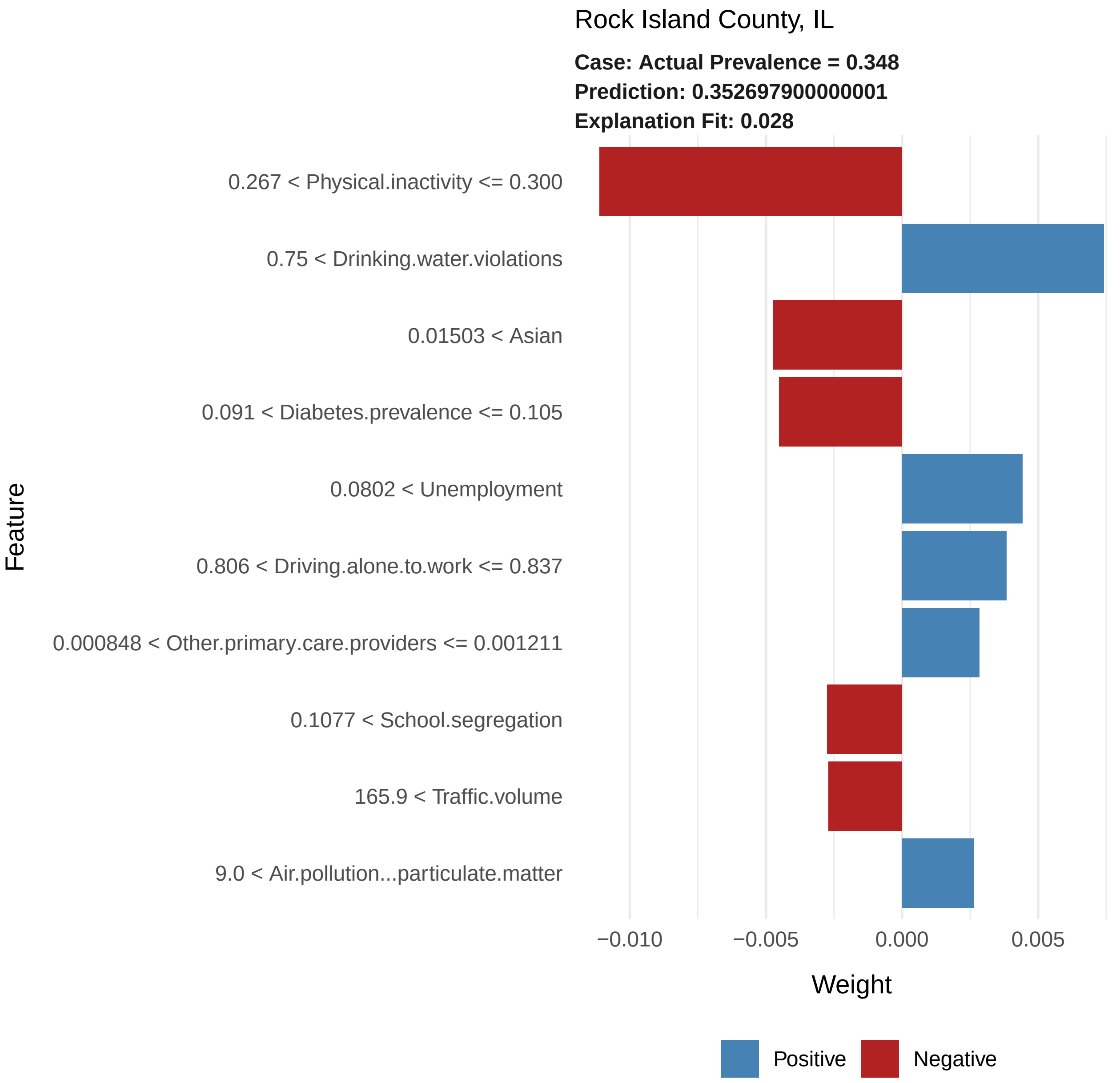

Rock Island County, IL
Case: Actual Prevalence = 0.348
Prediction: 0.352697900000001
Explanation Fit: 0.028
0.267 < Physical.inactivity <= 0.300
0.75 < Drinking.water.violations
0.01503 < Asian
0.091 < Diabetes.prevalence <= 0.105
0.0802 < Unemployment
0.806 < Driving.alone.to.work <= 0.837
0.000848 < Other.primary.care.providers <= 0.001211
0.1077 < School.segregation
165.9 < Traffic.volume
9.0 < Air.pollution...particulate.matter
Feature
−0.010
−0.005
0.000
0.005
Weight
Positive
Negative

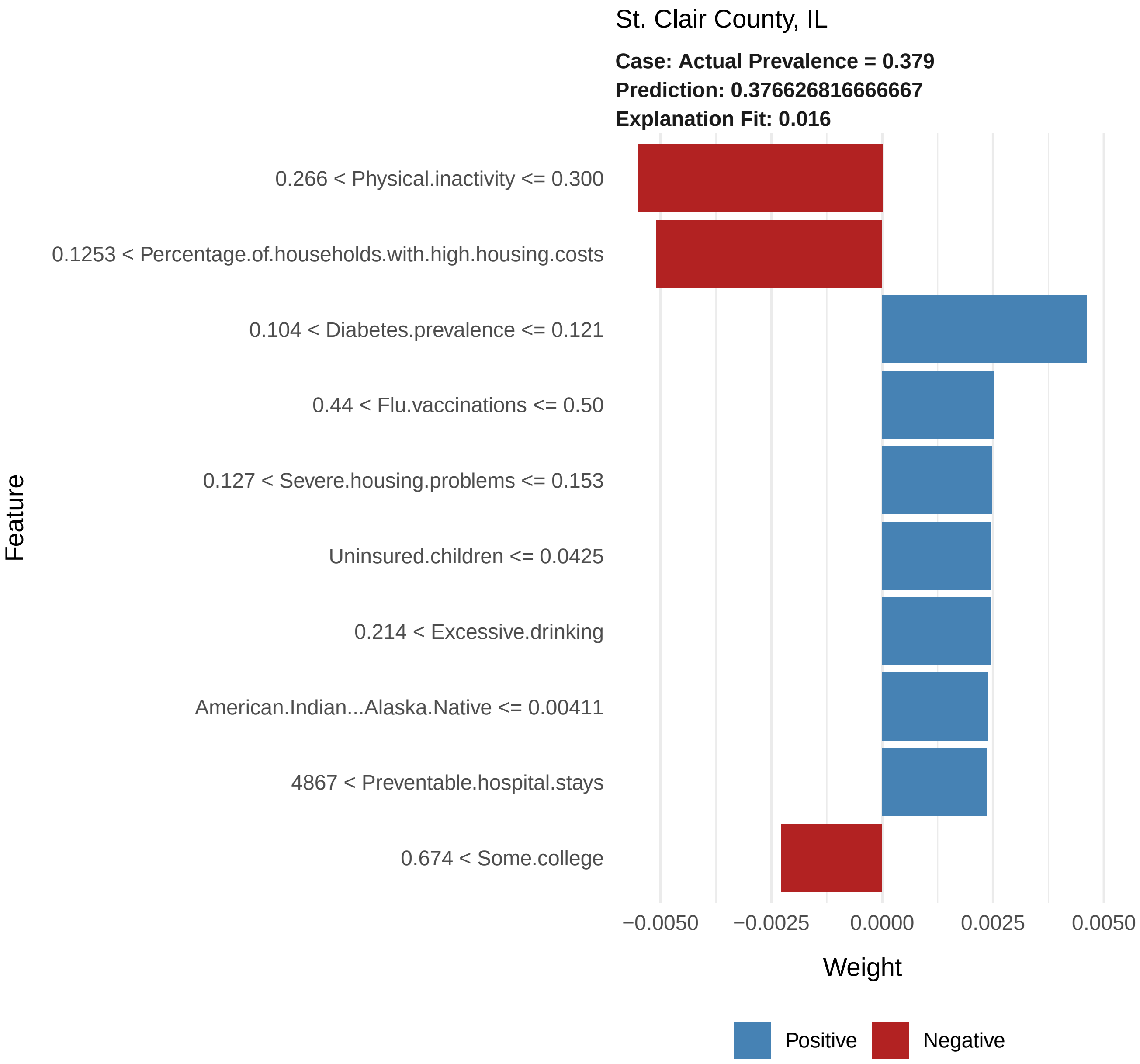

St. Clair County, IL
Case: Actual Prevalence = 0.379
Prediction: 0.376626816666667
Explanation Fit: 0.016
0.266 < Physical.inactivity <= 0.300
0.1253 < Percentage.of.households.with.high.housing.costs
0.104 < Diabetes.prevalence <= 0.121
0.44 < Flu.vaccinations <= 0.50
0.127 < Severe.housing.problems <= 0.153
Uninsured.children <= 0.0425
0.214 < Excessive.drinking
American.Indian...Alaska.Native <= 0.00411
4867 < Preventable.hospital.stays
0.674 < Some.college
Feature
−0.0050
−0.0025
0.0000
0.0025
0.0050
Weight
Positive
Negative

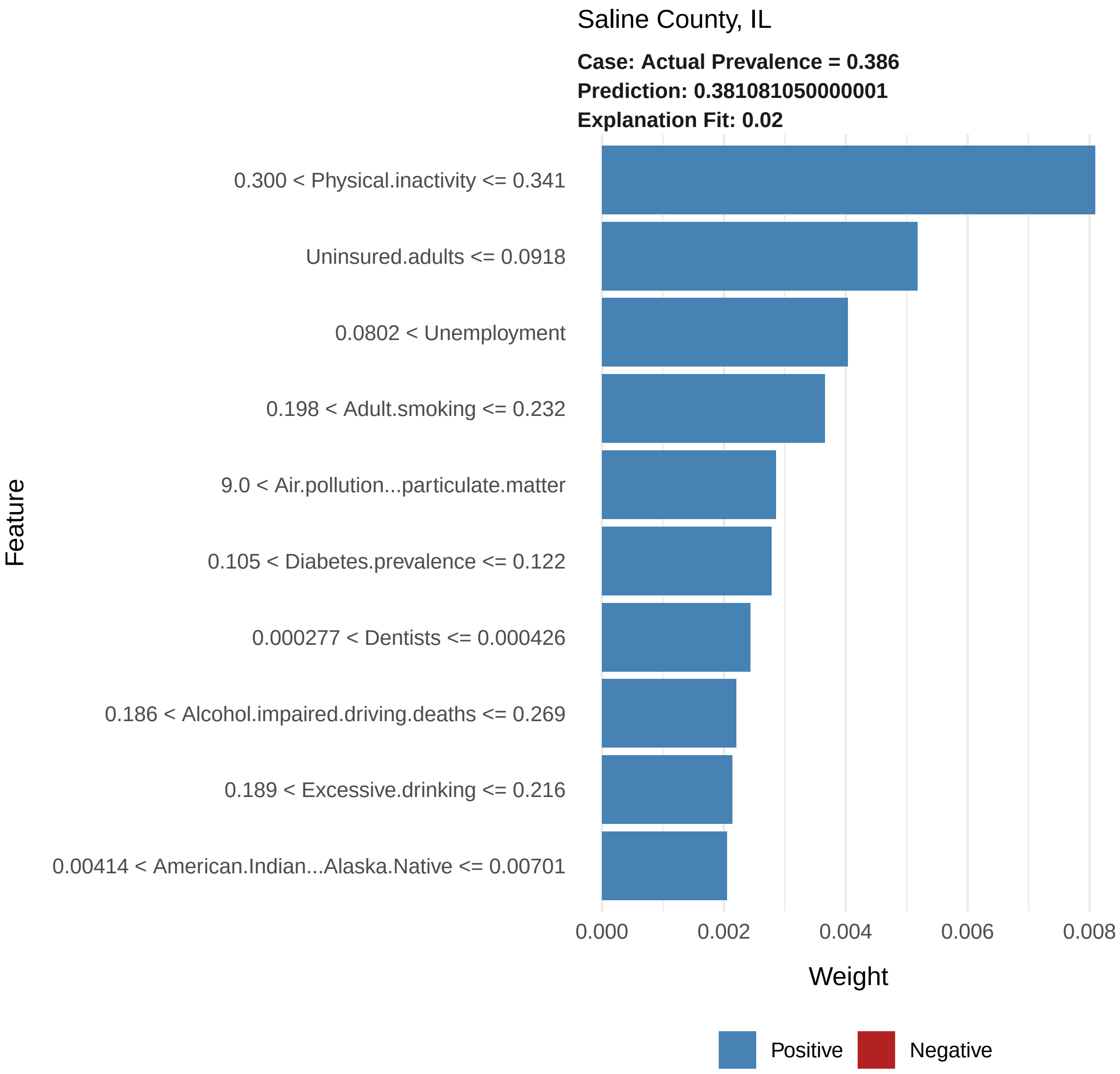

Saline County, IL
Case: Actual Prevalence = 0.386
Prediction: 0.381081050000001
Explanation Fit: 0.02
0.300 < Physical.inactivity <= 0.341
Uninsured.adults <= 0.0918
0.0802 < Unemployment
0.198 < Adult.smoking <= 0.232
9.0 < Air.pollution...particulate.matter
0.105 < Diabetes.prevalence <= 0.122
0.000277 < Dentists <= 0.000426
0.186 < Alcohol.impaired.driving.deaths <= 0.269
0.189 < Excessive.drinking <= 0.216
0.00414 < American.Indian...Alaska.Native <= 0.00701
Feature
0.000
0.002
0.004
0.006
0.008
Weight
Positive
Negative

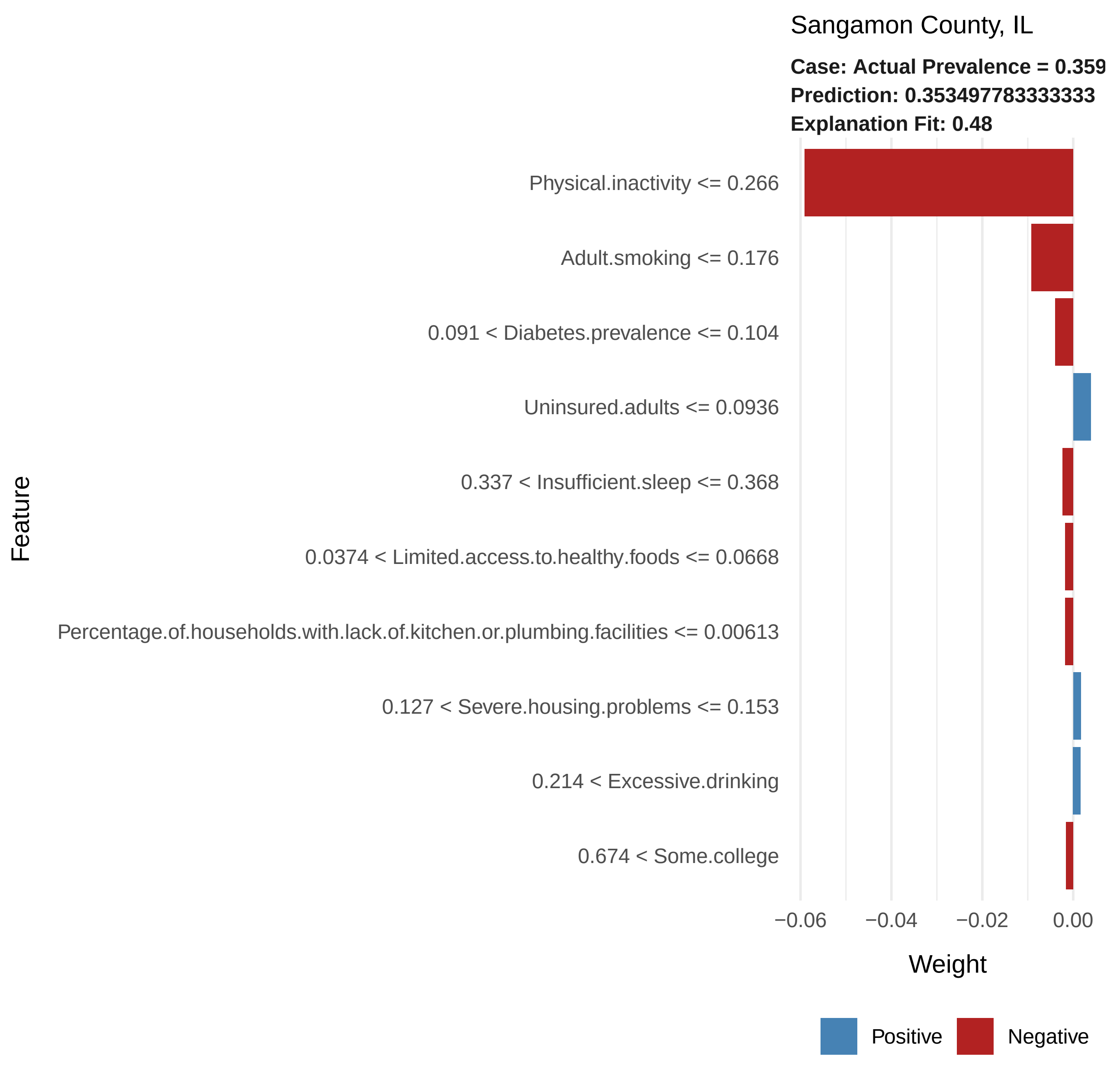

Sangamon County, IL
Case: Actual Prevalence = 0.359
Prediction: 0.353497783333333
Explanation Fit: 0.48
Physical.inactivity <= 0.266
Adult.smoking <= 0.176
0.091 < Diabetes.prevalence <= 0.104
Uninsured.adults <= 0.0936
0.337 < Insufficient.sleep <= 0.368
0.0374 < Limited.access.to.healthy.foods <= 0.0668
Percentage.of.households.with.lack.of.kitchen.or.plumbing.facilities <= 0.00613
0.127 < Severe.housing.problems <= 0.153
0.214 < Excessive.drinking
0.674 < Some.college
Feature
−0.06
−0.04
−0.02
0.00
Weight
Positive
Negative

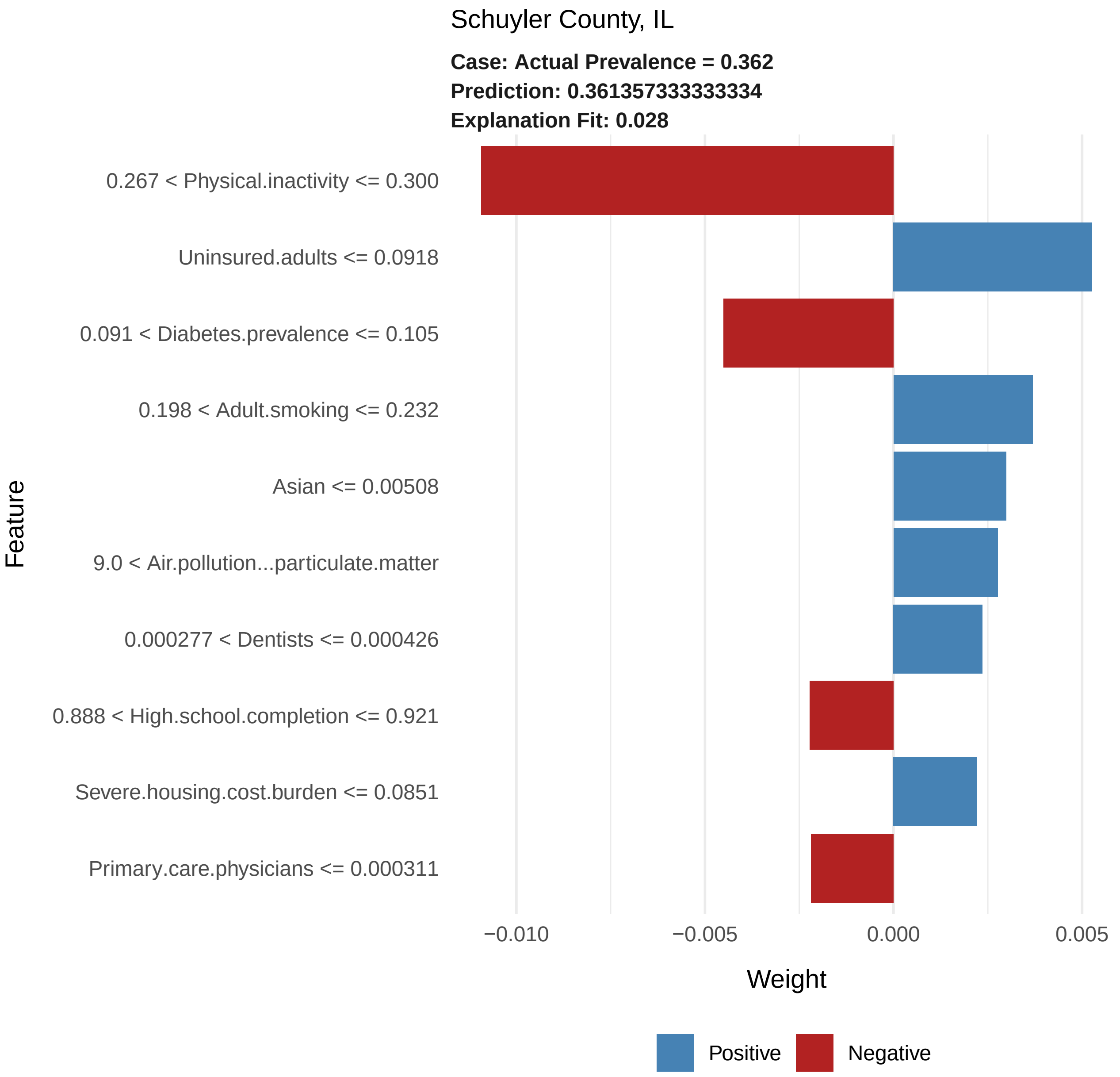
Schuyler County, IL
Case: Actual Prevalence = 0.362
Prediction: 0.361357333333334
Explanation Fit: 0.028
0.267 < Physical.inactivity <= 0.300
Uninsured.adults <= 0.0918
0.091 < Diabetes.prevalence <= 0.105
0.198 < Adult.smoking <= 0.232
Asian <= 0.00508
9.0 < Air.pollution...particulate.matter
0.000277 < Dentists <= 0.000426
0.888 < High.school.completion <= 0.921
Severe.housing.cost.burden <= 0.0851
Primary.care.physicians <= 0.000311
Feature
−0.010
−0.005
0.000
0.005
Weight
Positive
Negative

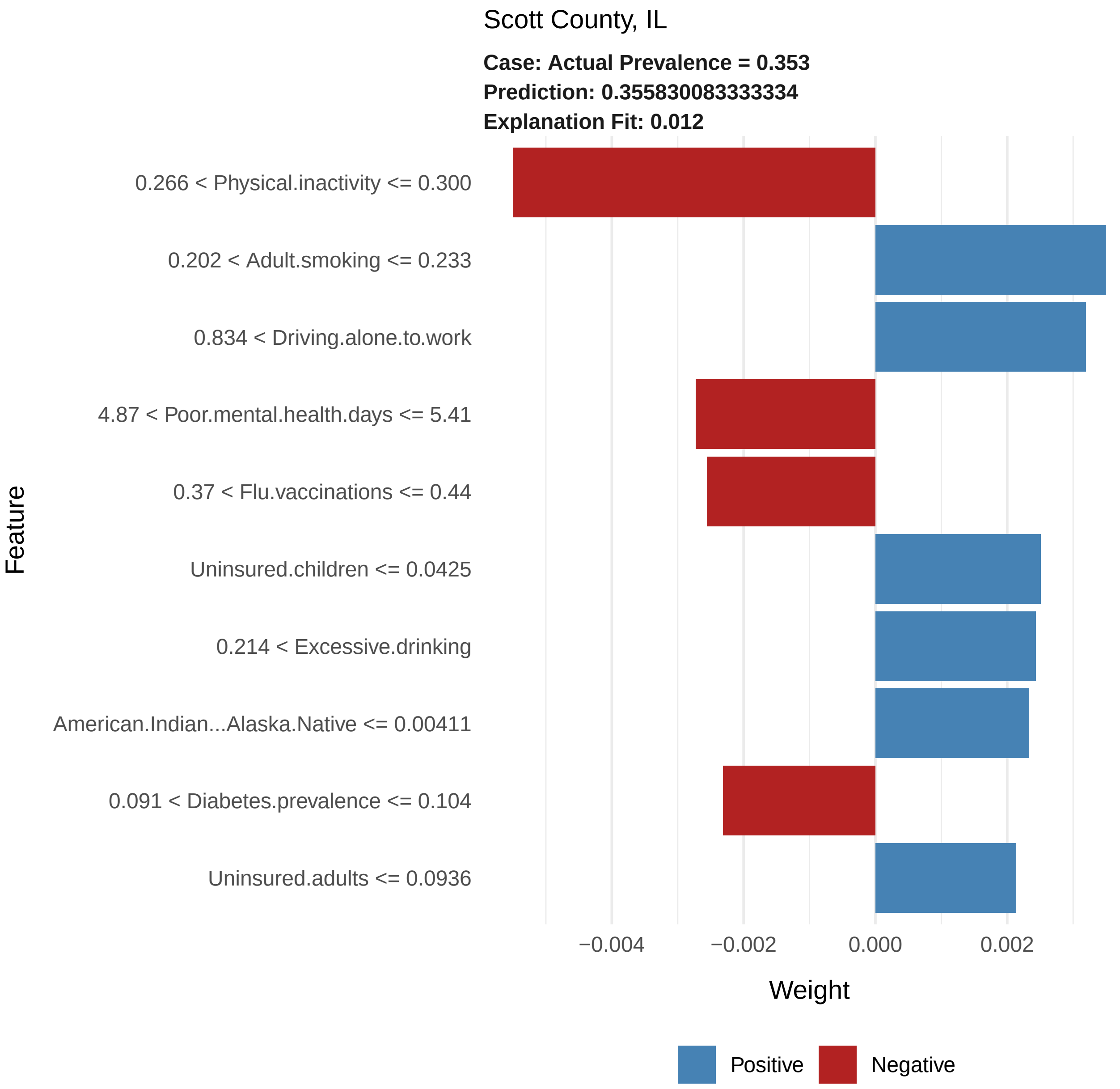
Scott County, IL
Case: Actual Prevalence = 0.353
Prediction: 0.355830083333334
Explanation Fit: 0.012
0.266 < Physical.inactivity <= 0.300
0.202 < Adult.smoking <= 0.233
0.834 < Driving.alone.to.work
4.87 < Poor.mental.health.days <= 5.41
0.37 < Flu.vaccinations <= 0.44
Uninsured.children <= 0.0425
0.214 < Excessive.drinking
American.Indian...Alaska.Native <= 0.00411
0.091 < Diabetes.prevalence <= 0.104
Uninsured.adults <= 0.0936
Feature
−0.004
−0.002
0.000
0.002
Weight
Positive
Negative

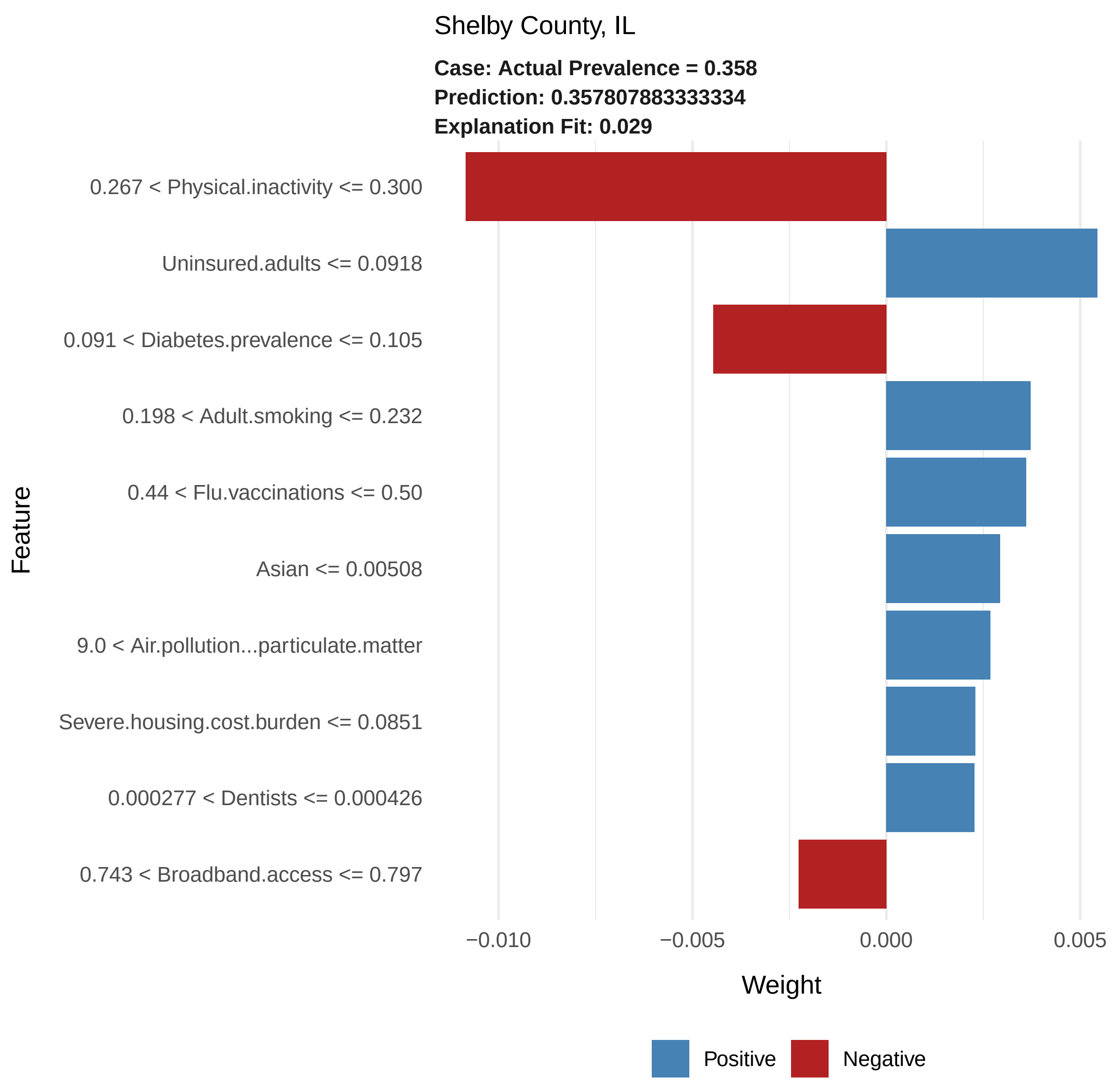

Shelby County, IL
Case: Actual Prevalence = 0.358
Prediction: 0.357807883333334
Explanation Fit: 0.029
0.267 < Physical.inactivity <= 0.300
Uninsured.adults <= 0.0918
0.091 < Diabetes.prevalence <= 0.105
0.198 < Adult.smoking <= 0.232
0.44 < Flu.vaccinations <= 0.50
Asian <= 0.00508
9.0 < Air.pollution...particulate.matter
Severe.housing.cost.burden <= 0.0851
0.000277 < Dentists <= 0.000426
0.743 < Broadband.access <= 0.797
Feature
−0.010
−0.005
0.000
0.005
Weight
Positive
Negative

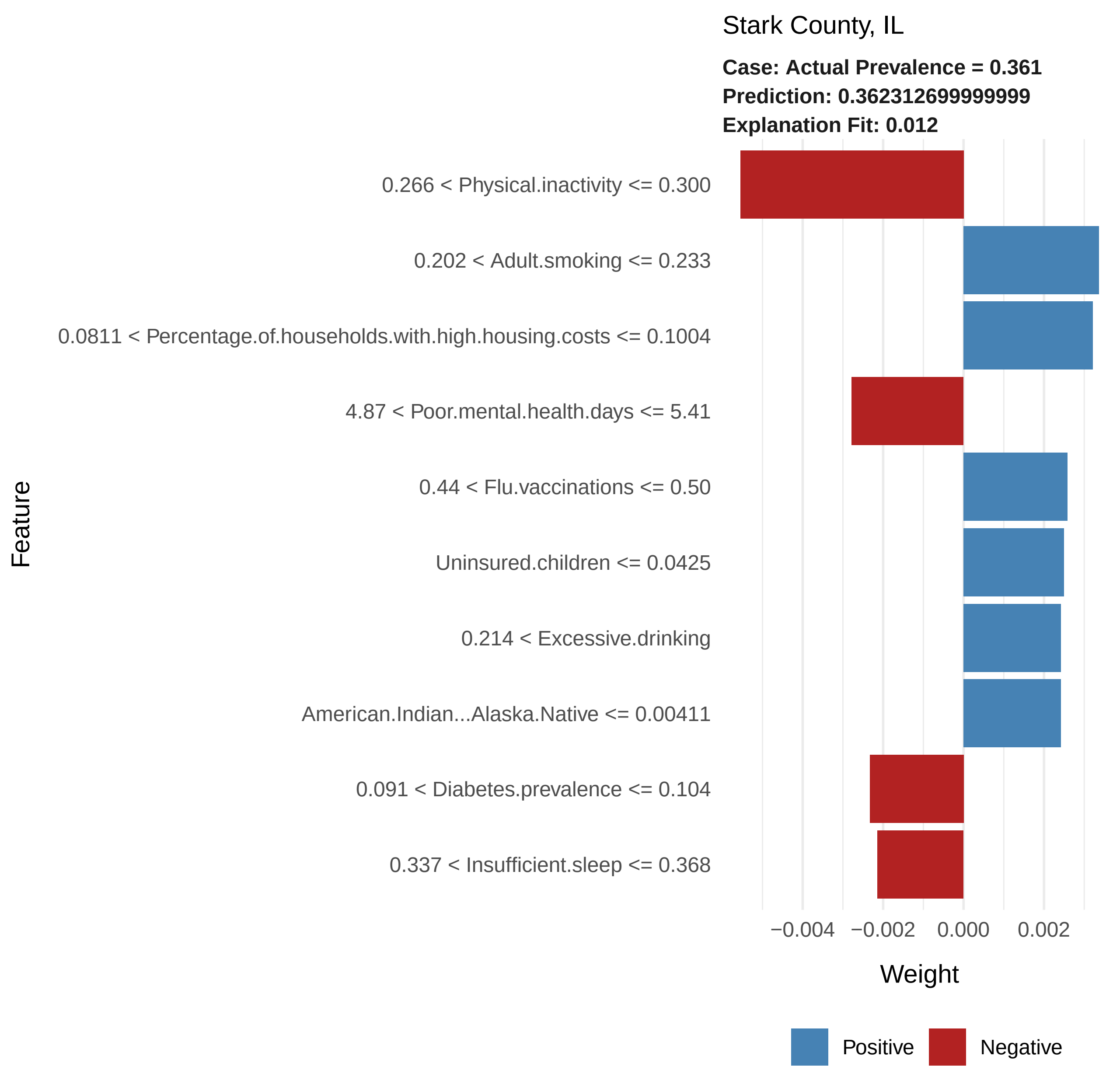
Stark County, IL
Case: Actual Prevalence = 0.361
Prediction: 0.362312699999999
Explanation Fit: 0.012
0.266 < Physical.inactivity <= 0.300
0.202 < Adult.smoking <= 0.233
0.0811 < Percentage.of.households.with.high.housing.costs <= 0.1004
4.87 < Poor.mental.health.days <= 5.41
0.44 < Flu.vaccinations <= 0.50
Uninsured.children <= 0.0425
0.214 < Excessive.drinking
American.Indian...Alaska.Native <= 0.00411
0.091 < Diabetes.prevalence <= 0.104
0.337 < Insufficient.sleep <= 0.368
Feature
−0.004
−0.002
0.000
0.002
Weight
Positive
Negative

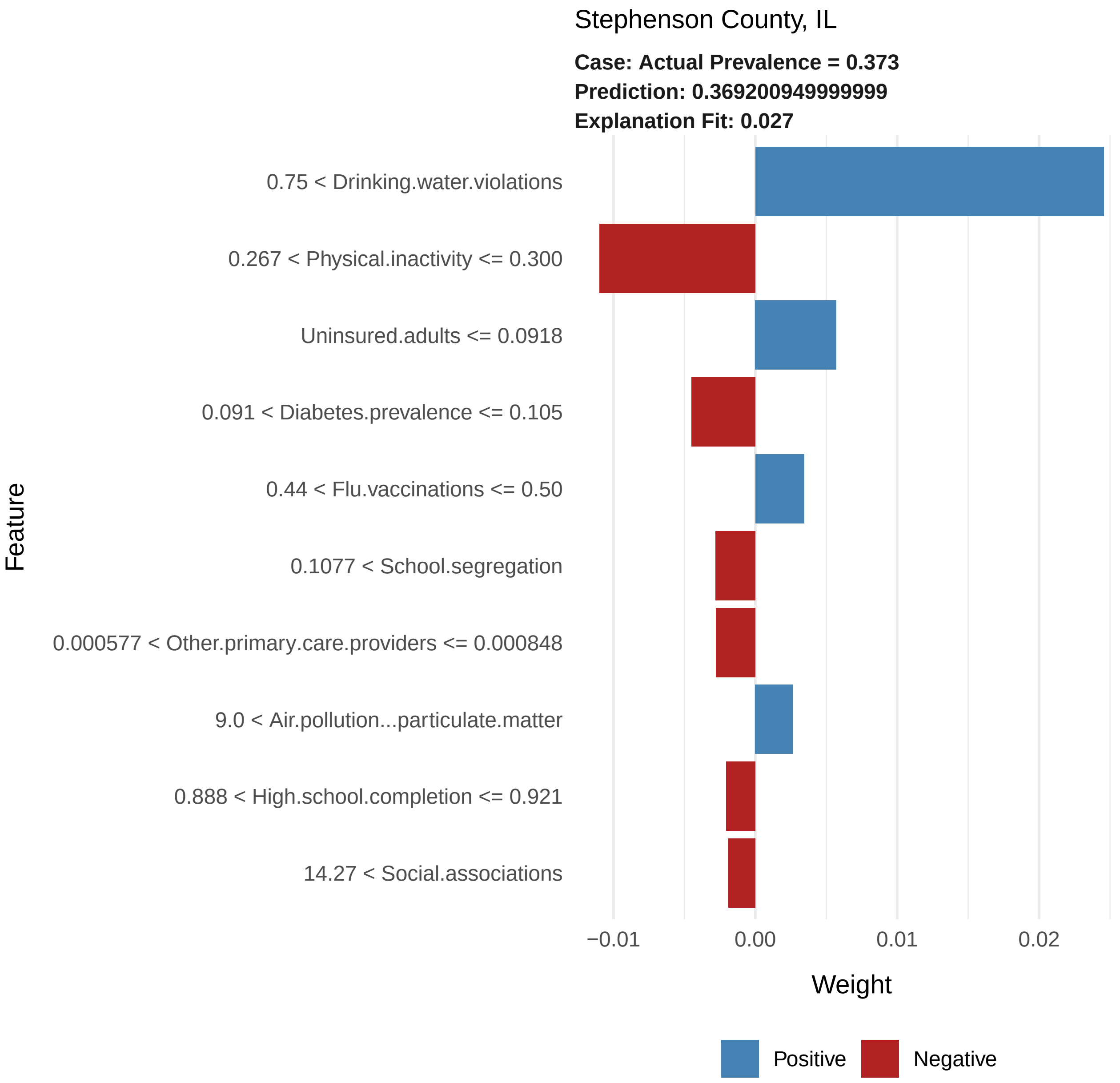

Stephenson County, IL
Case: Actual Prevalence = 0.373
Prediction: 0.369200949999999
Explanation Fit: 0.027
0.75 < Drinking.water.violations
0.267 < Physical.inactivity <= 0.300
Uninsured.adults <= 0.0918
0.091 < Diabetes.prevalence <= 0.105
0.44 < Flu.vaccinations <= 0.50
0.1077 < School.segregation
0.000577 < Other.primary.care.providers <= 0.000848
9.0 < Air.pollution...particulate.matter
0.888 < High.school.completion <= 0.921
14.27 < Social.associations
Feature
−0.01
0.00
0.01
0.02
Weight
Positive
Negative

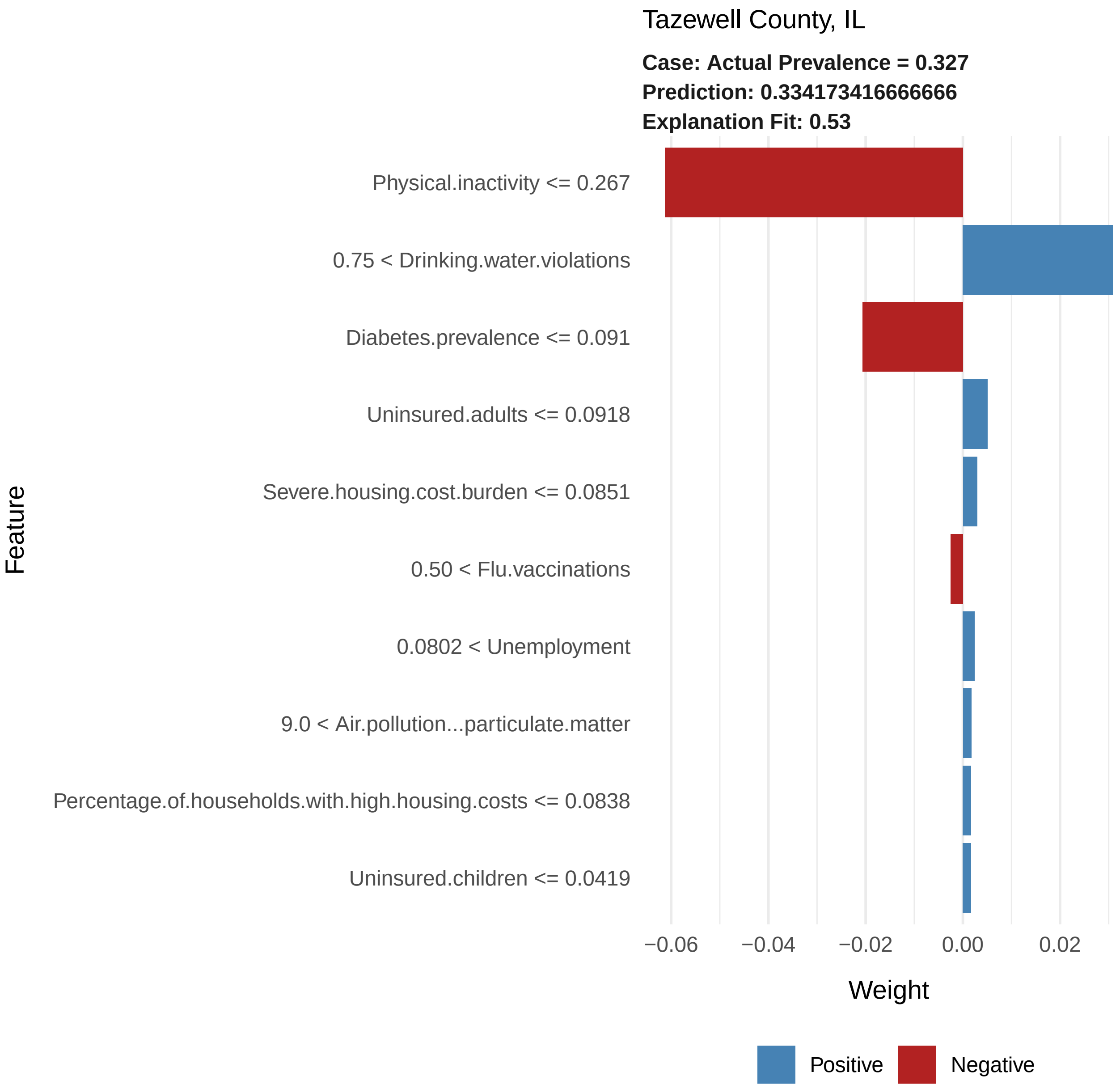
Tazewell County, IL
Case: Actual Prevalence = 0.327
Prediction: 0.334173416666666
Explanation Fit: 0.53
Physical.inactivity <= 0.267
0.75 < Drinking.water.violations
Diabetes.prevalence <= 0.091
Uninsured.adults <= 0.0918
Severe.housing.cost.burden <= 0.0851
0.50 < Flu.vaccinations
0.0802 < Unemployment
9.0 < Air.pollution...particulate.matter
Percentage.of.households.with.high.housing.costs <= 0.0838
Uninsured.children <= 0.0419
Feature
−0.06
−0.04
−0.02
0.00
0.02
Weight
Positive
Negative

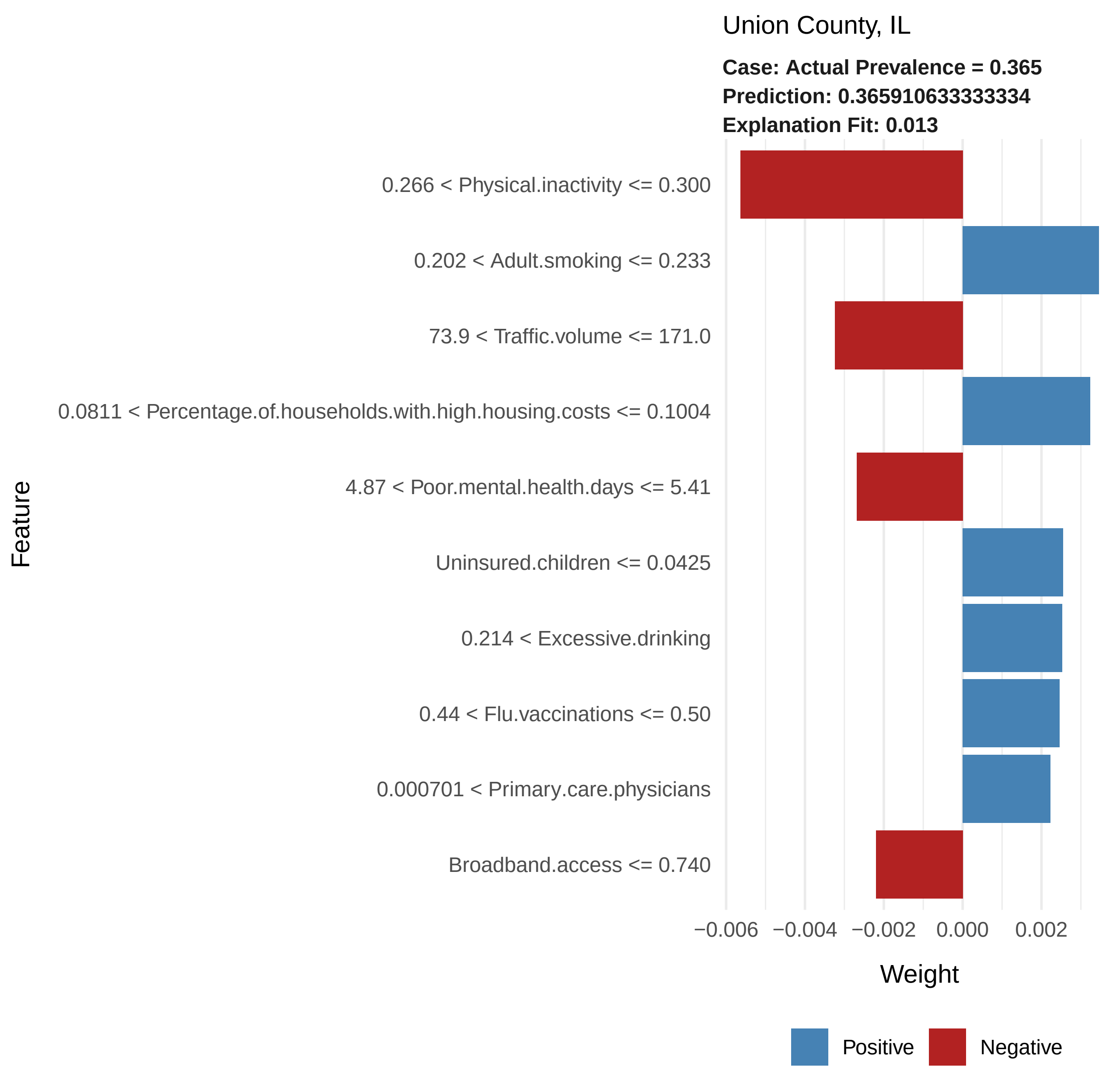

Union County, IL
Case: Actual Prevalence = 0.365
Prediction: 0.365910633333334
Explanation Fit: 0.013
0.266 < Physical.inactivity <= 0.300
0.202 < Adult.smoking <= 0.233
73.9 < Traffic.volume <= 171.0
0.0811 < Percentage.of.households.with.high.housing.costs <= 0.1004
4.87 < Poor.mental.health.days <= 5.41
Uninsured.children <= 0.0425
0.214 < Excessive.drinking
0.44 < Flu.vaccinations <= 0.50
0.000701 < Primary.care.physicians
Broadband.access <= 0.740
Feature
−0.006
−0.004
−0.002
0.000
0.002
Weight
Positive
Negative

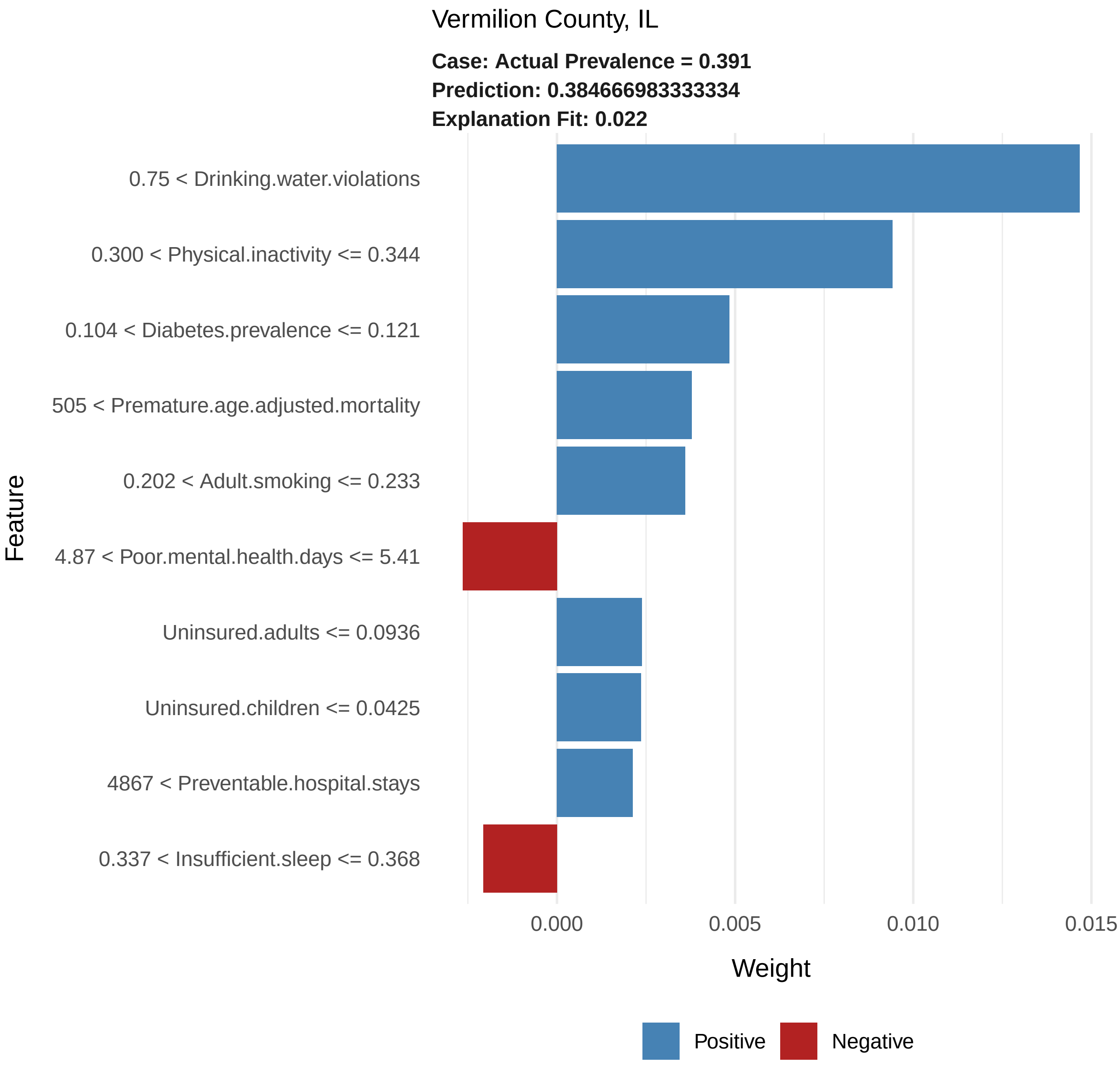
Vermilion County, IL
Case: Actual Prevalence = 0.391
Prediction: 0.384666983333334
Explanation Fit: 0.022
0.75 < Drinking.water.violations
0.300 < Physical.inactivity <= 0.344
0.104 < Diabetes.prevalence <= 0.121
505 < Premature.age.adjusted.mortality
0.202 < Adult.smoking <= 0.233
4.87 < Poor.mental.health.days <= 5.41
Uninsured.adults <= 0.0936
Uninsured.children <= 0.0425
4867 < Preventable.hospital.stays
0.337 < Insufficient.sleep <= 0.368
Feature
0.000
0.005
0.010
0.015
Weight
Positive
Negative

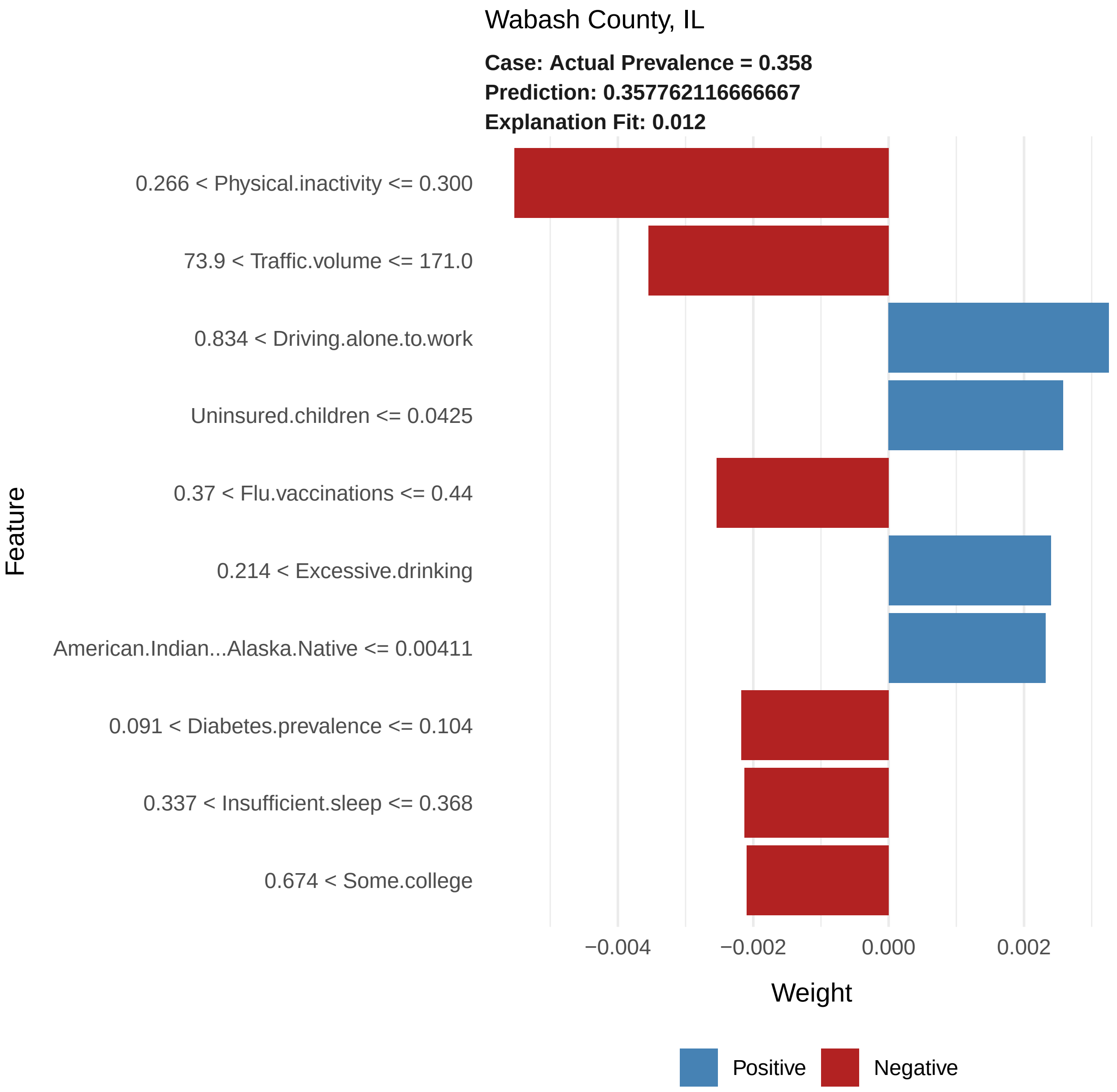
Wabash County, IL
Case: Actual Prevalence = 0.358
Prediction: 0.357762116666667
Explanation Fit: 0.012
0.266 < Physical.inactivity <= 0.300
73.9 < Traffic.volume <= 171.0
0.834 < Driving.alone.to.work
Uninsured.children <= 0.0425
0.37 < Flu.vaccinations <= 0.44
0.214 < Excessive.drinking
American.Indian...Alaska.Native <= 0.00411
0.091 < Diabetes.prevalence <= 0.104
0.337 < Insufficient.sleep <= 0.368
0.674 < Some.college
Feature
−0.004
−0.002
0.000
0.002
Weight
Positive
Negative

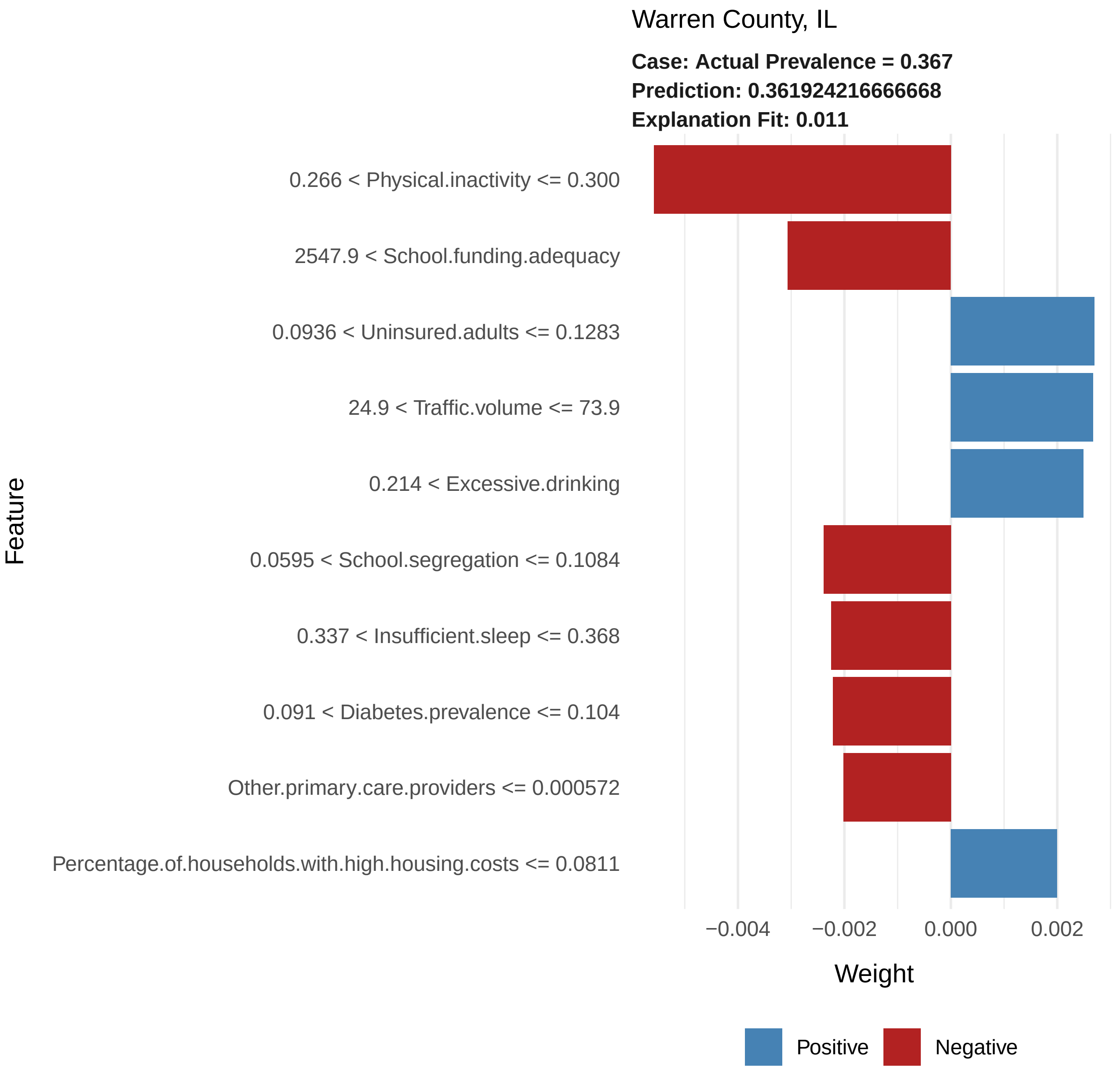
Warren County, IL
Case: Actual Prevalence = 0.367
Prediction: 0.361924216666668
Explanation Fit: 0.011
0.266 < Physical.inactivity <= 0.300
2547.9 < School.funding.adequacy
0.0936 < Uninsured.adults <= 0.1283
24.9 < Traffic.volume <= 73.9
0.214 < Excessive.drinking
0.0595 < School.segregation <= 0.1084
0.337 < Insufficient.sleep <= 0.368
0.091 < Diabetes.prevalence <= 0.104
Other.primary.care.providers <= 0.000572
Percentage.of.households.with.high.housing.costs <= 0.0811
Feature
−0.004
−0.002
0.000
0.002
Weight
Positive
Negative

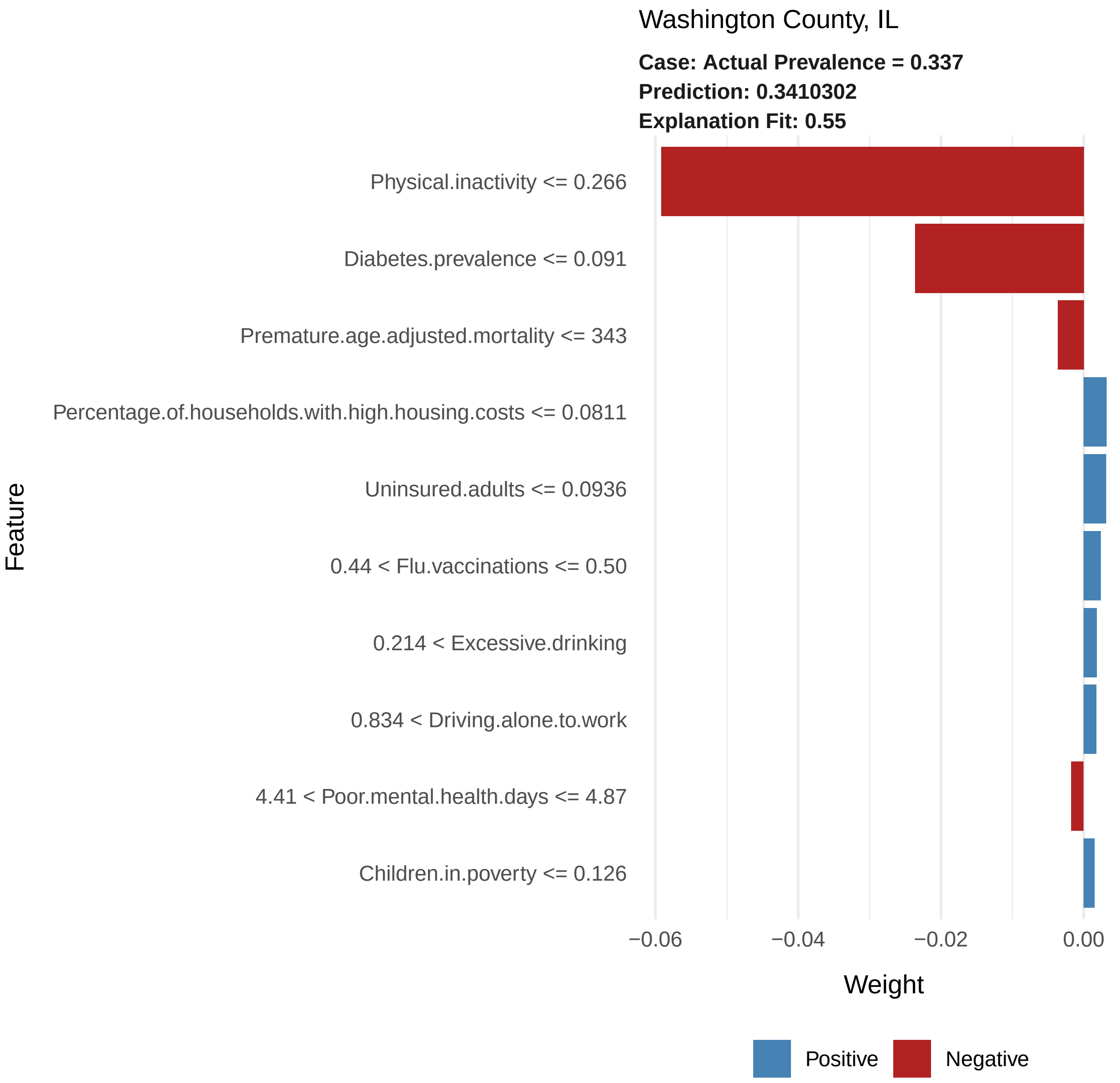

Washington County, IL
Case: Actual Prevalence = 0.337
Prediction: 0.3410302
Explanation Fit: 0.55
Physical.inactivity <= 0.266
Diabetes.prevalence <= 0.091
Premature.age.adjusted.mortality <= 343
Percentage.of.households.with.high.housing.costs <= 0.0811
Uninsured.adults <= 0.0936
0.44 < Flu.vaccinations <= 0.50
0.214 < Excessive.drinking
0.834 < Driving.alone.to.work
4.41 < Poor.mental.health.days <= 4.87
Children.in.poverty <= 0.126
Feature
−0.06
−0.04
−0.02
0.00
Weight
Positive
Negative

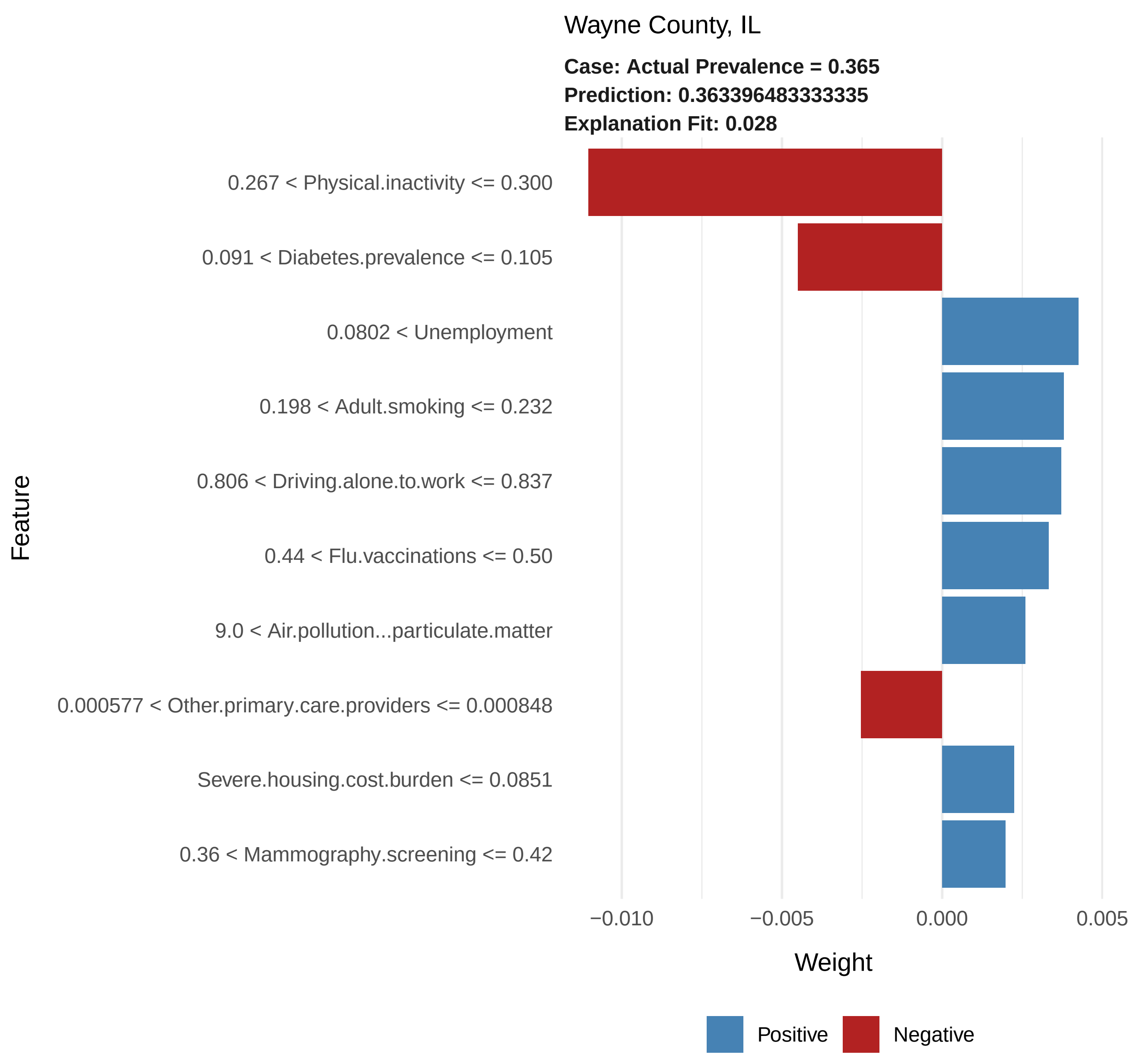
Wayne County, IL
Case: Actual Prevalence = 0.365
Prediction: 0.363396483333335
Explanation Fit: 0.028
0.267 < Physical.inactivity <= 0.300
0.091 < Diabetes.prevalence <= 0.105
0.0802 < Unemployment
0.198 < Adult.smoking <= 0.232
0.806 < Driving.alone.to.work <= 0.837
0.44 < Flu.vaccinations <= 0.50
9.0 < Air.pollution...particulate.matter
0.000577 < Other.primary.care.providers <= 0.000848
Severe.housing.cost.burden <= 0.0851
0.36 < Mammography.screening <= 0.42
Feature
−0.010
−0.005
0.000
0.005
Weight
Positive
Negative

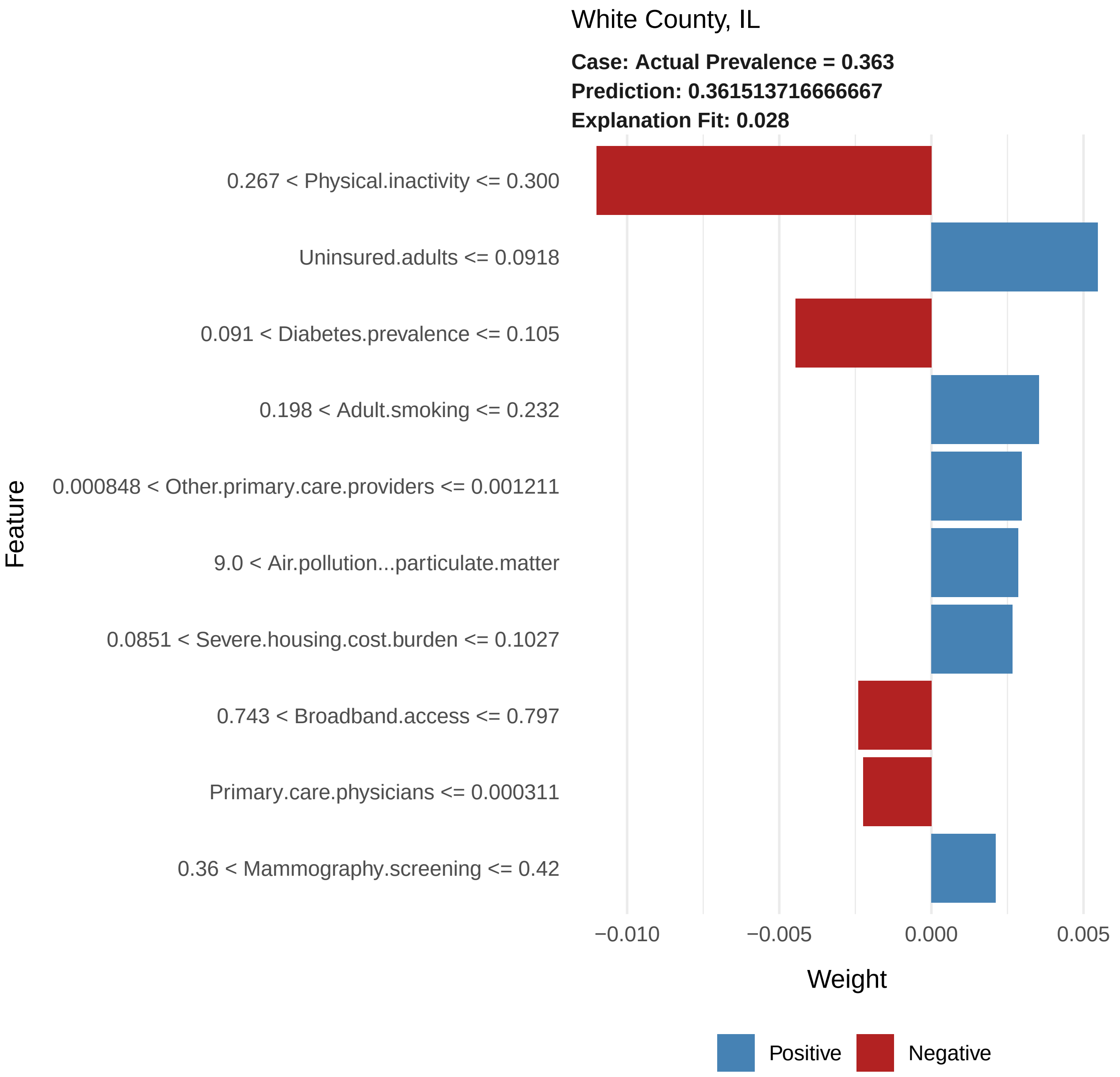

White County, IL
Case: Actual Prevalence = 0.363
Prediction: 0.361513716666667
Explanation Fit: 0.028
0.267 < Physical.inactivity <= 0.300
Uninsured.adults <= 0.0918
0.091 < Diabetes.prevalence <= 0.105
0.198 < Adult.smoking <= 0.232
0.000848 < Other.primary.care.providers <= 0.001211
9.0 < Air.pollution...particulate.matter
0.0851 < Severe.housing.cost.burden <= 0.1027
0.743 < Broadband.access <= 0.797
Primary.care.physicians <= 0.000311
0.36 < Mammography.screening <= 0.42
Feature
−0.010
−0.005
0.000
0.005
Weight
Positive
Negative

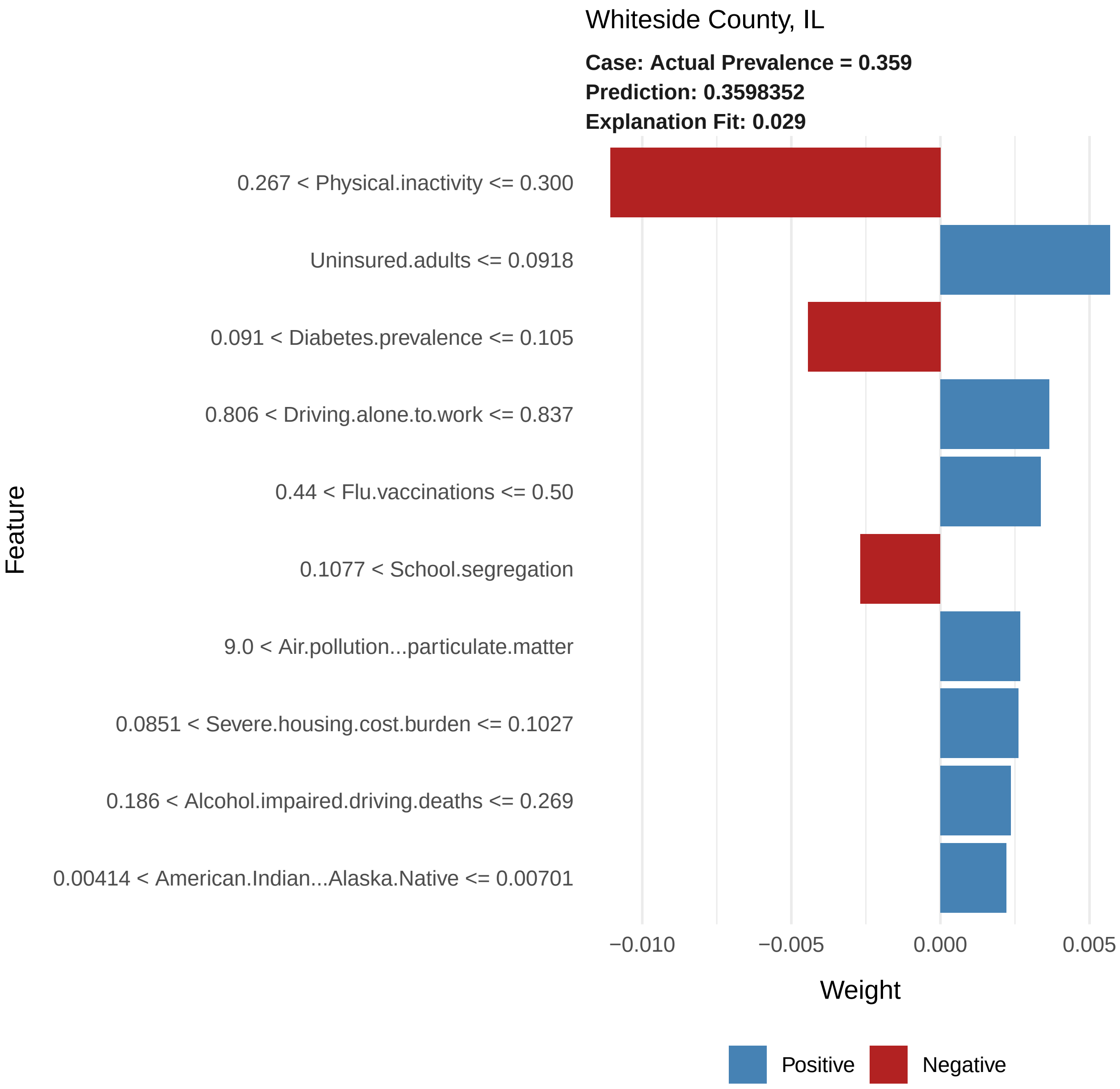

Whiteside County, IL
Case: Actual Prevalence = 0.359
Prediction: 0.3598352
Explanation Fit: 0.029
0.267 < Physical.inactivity <= 0.300
Uninsured.adults <= 0.0918
0.091 < Diabetes.prevalence <= 0.105
0.806 < Driving.alone.to.work <= 0.837
0.44 < Flu.vaccinations <= 0.50
0.1077 < School.segregation
9.0 < Air.pollution...particulate.matter
0.0851 < Severe.housing.cost.burden <= 0.1027
0.186 < Alcohol.impaired.driving.deaths <= 0.269
0.00414 < American.Indian...Alaska.Native <= 0.00701
Feature
−0.010
−0.005
0.000
0.005
Weight
Positive
Negative

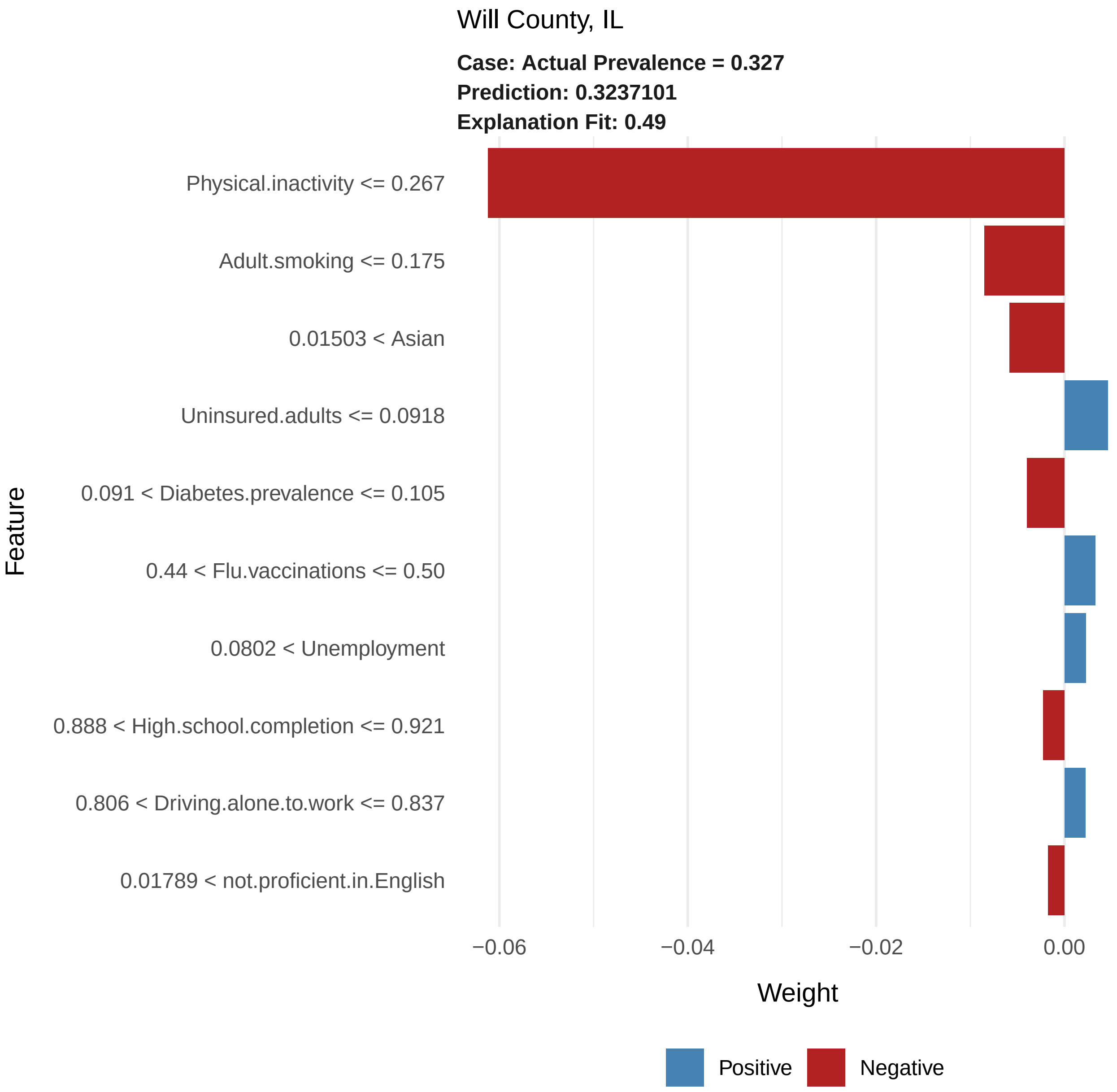
Will County, IL
Case: Actual Prevalence = 0.327
Prediction: 0.3237101
Explanation Fit: 0.49
Physical.inactivity <= 0.267
Adult.smoking <= 0.175
0.01503 < Asian
Uninsured.adults <= 0.0918
0.091 < Diabetes.prevalence <= 0.105
0.44 < Flu.vaccinations <= 0.50
0.0802 < Unemployment
0.888 < High.school.completion <= 0.921
0.806 < Driving.alone.to.work <= 0.837
0.01789 < not.proficient.in.English
Feature
−0.06
−0.04
−0.02
0.00
Weight
Positive
Negative

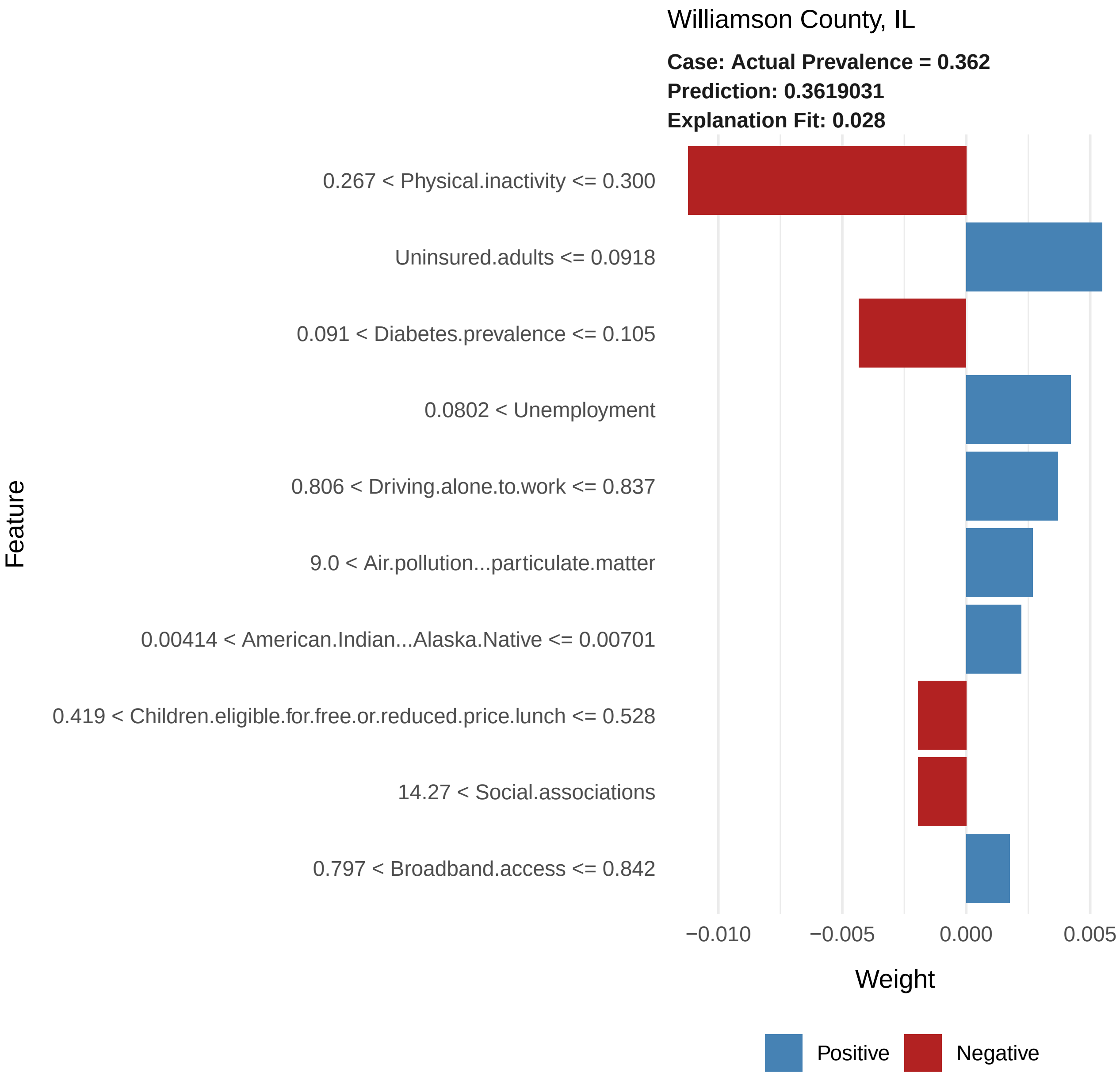

Williamson County, IL
Case: Actual Prevalence = 0.362
Prediction: 0.3619031
Explanation Fit: 0.028
0.267 < Physical.inactivity <= 0.300
Uninsured.adults <= 0.0918
0.091 < Diabetes.prevalence <= 0.105
0.0802 < Unemployment
0.806 < Driving.alone.to.work <= 0.837
9.0 < Air.pollution...particulate.matter
0.00414 < American.Indian...Alaska.Native <= 0.00701
0.419 < Children.eligible.for.free.or.reduced.price.lunch <= 0.528
14.27 < Social.associations
0.797 < Broadband.access <= 0.842
Feature
−0.010
−0.005
0.000
0.005
Weight
Positive
Negative

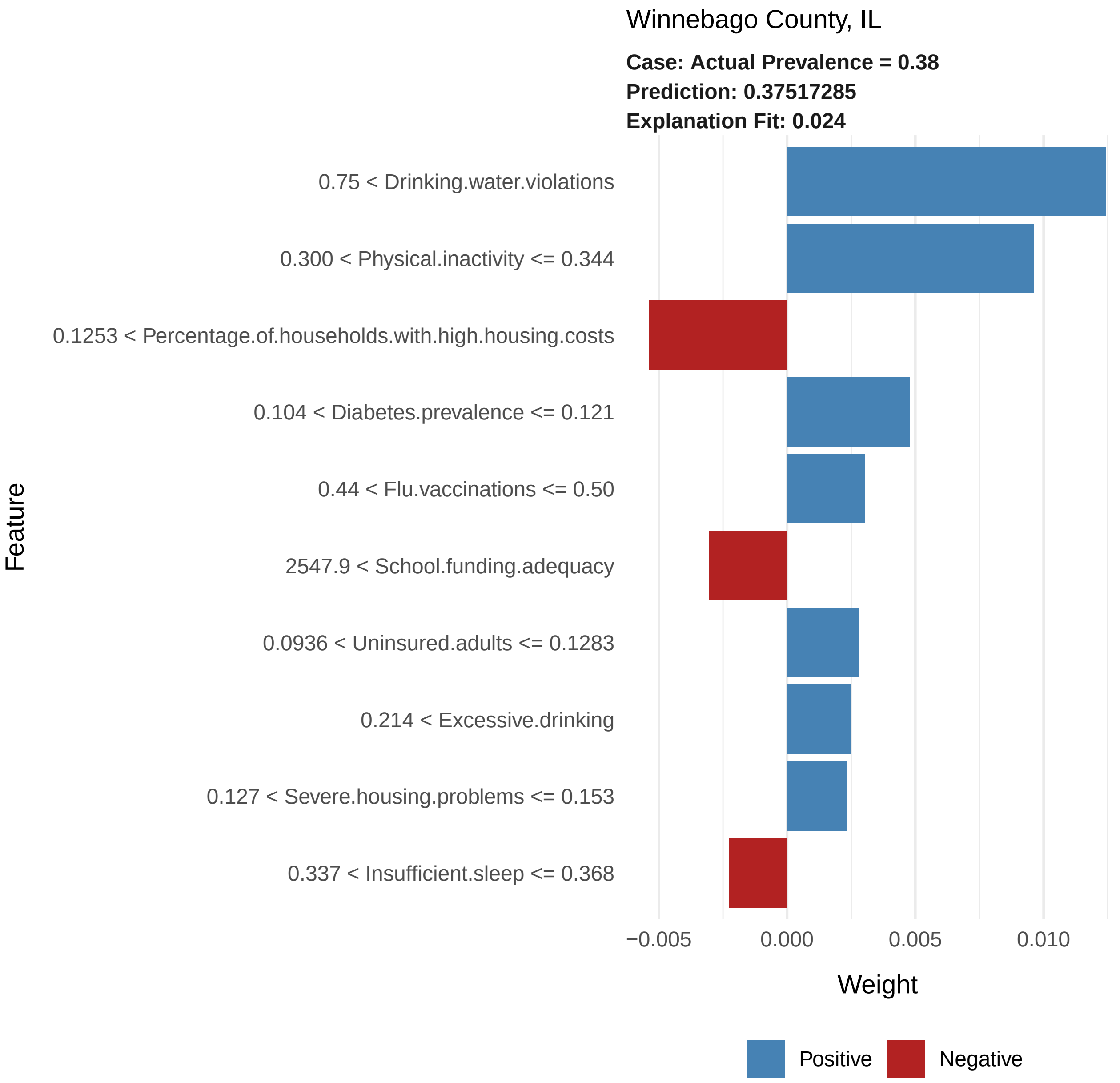

Winnebago County, IL
Case: Actual Prevalence = 0.38
Prediction: 0.37517285
Explanation Fit: 0.024
0.75 < Drinking.water.violations
0.300 < Physical.inactivity <= 0.344
0.1253 < Percentage.of.households.with.high.housing.costs
0.104 < Diabetes.prevalence <= 0.121
0.44 < Flu.vaccinations <= 0.50
2547.9 < School.funding.adequacy
0.0936 < Uninsured.adults <= 0.1283
0.214 < Excessive.drinking
0.127 < Severe.housing.problems <= 0.153
0.337 < Insufficient.sleep <= 0.368
Feature
−0.005
0.000
0.005
0.010
Weight
Positive
Negative

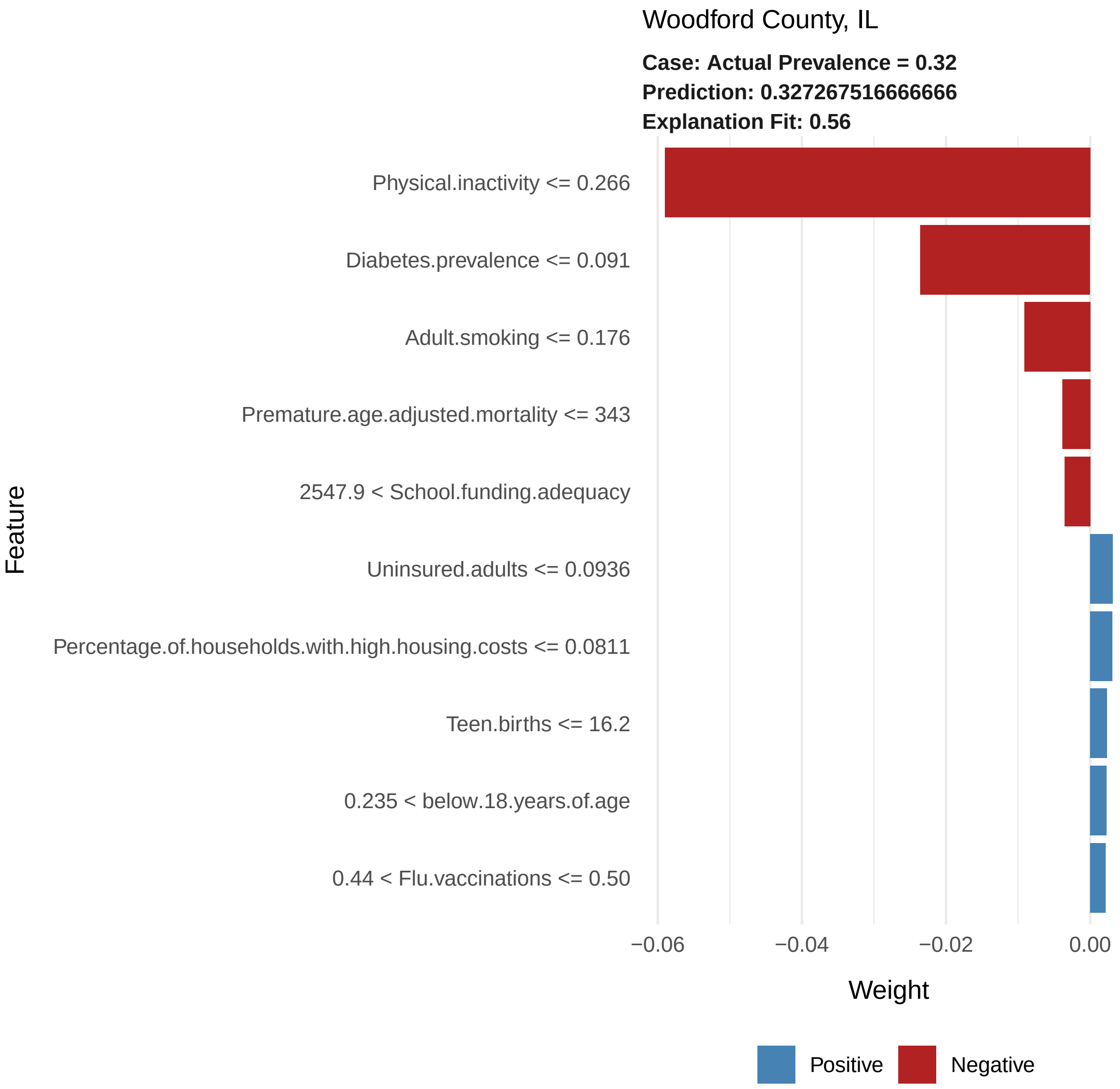

Woodford County, IL
Case: Actual Prevalence = 0.32
Prediction: 0.327267516666666
Explanation Fit: 0.56
Physical.inactivity <= 0.266
Diabetes.prevalence <= 0.091
Adult.smoking <= 0.176
Premature.age.adjusted.mortality <= 343
2547.9 < School.funding.adequacy
Uninsured.adults <= 0.0936
Percentage.of.households.with.high.housing.costs <= 0.0811
Teen.births <= 16.2
0.235 < below.18.years.of.age
0.44 < Flu.vaccinations <= 0.50
Feature
−0.06
−0.04
−0.02
0.00
Weight
Positive
Negative

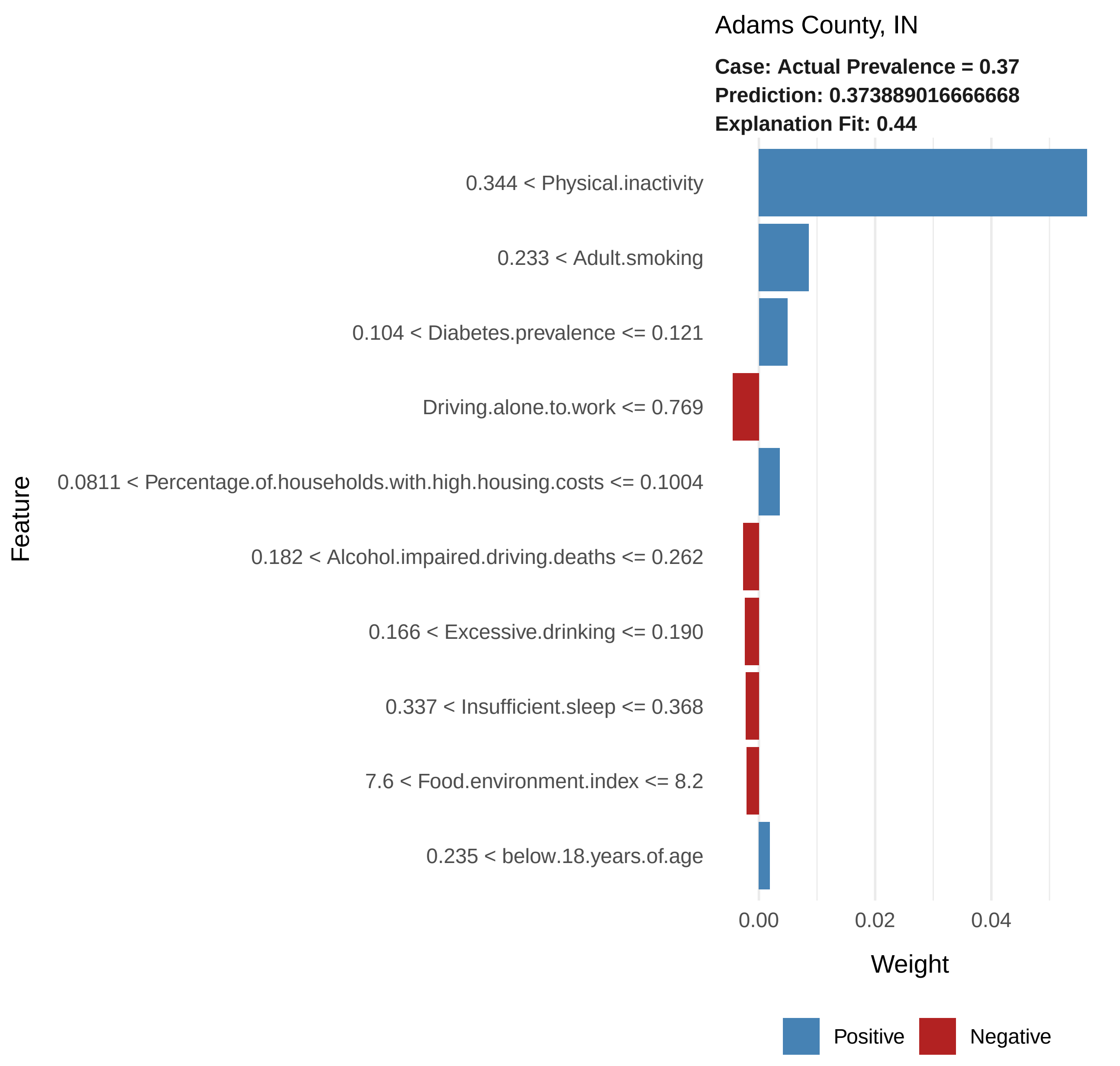
Adams County, IN
Case: Actual Prevalence = 0.37
Prediction: 0.373889016666668
Explanation Fit: 0.44
0.344 < Physical.inactivity
0.233 < Adult.smoking
0.104 < Diabetes.prevalence <= 0.121
Driving.alone.to.work <= 0.769
0.0811 < Percentage.of.households.with.high.housing.costs <= 0.1004
0.182 < Alcohol.impaired.driving.deaths <= 0.262
0.166 < Excessive.drinking <= 0.190
0.337 < Insufficient.sleep <= 0.368
7.6 < Food.environment.index <= 8.2
0.235 < below.18.years.of.age
Feature
0.00
0.02
0.04
Weight
Positive
Negative

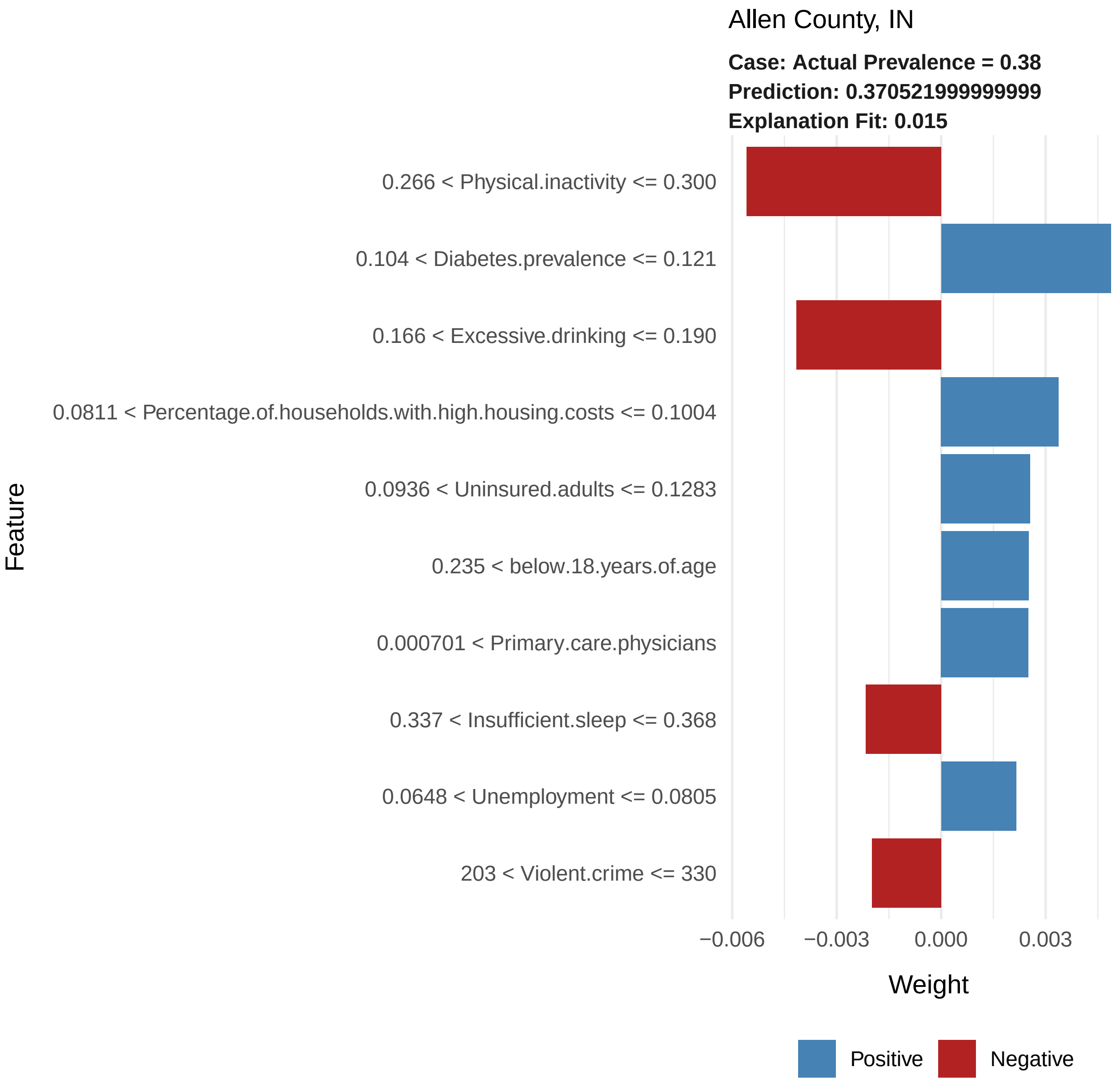

Allen County, IN
Case: Actual Prevalence = 0.38
Prediction: 0.370521999999999
Explanation Fit: 0.015
Feature
0.266 < Physical.inactivity <= 0.300
0.104 < Diabetes.prevalence <= 0.121
0.166 < Excessive.drinking <= 0.190
0.0811 < Percentage.of.households.with.high.housing.costs <= 0.1004
0.0936 < Uninsured.adults <= 0.1283
0.235 < below.18.years.of.age
0.000701 < Primary.care.physicians
0.337 < Insufficient.sleep <= 0.368
0.0648 < Unemployment <= 0.0805
203 < Violent.crime <= 330
−0.006
−0.003
0.000
0.003
Weight
Positive
Negative

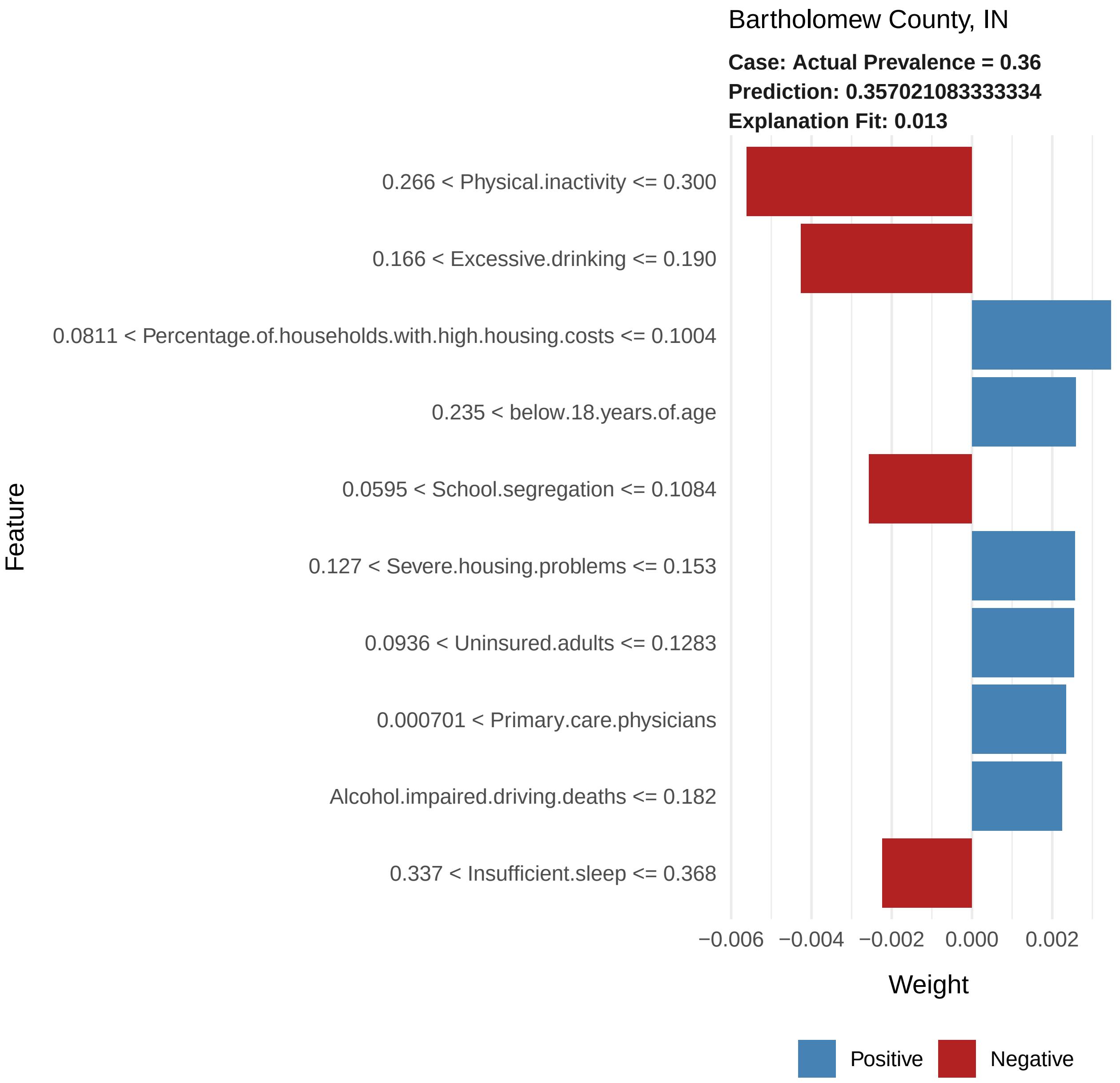

Bartholomew County, IN
Case: Actual Prevalence = 0.36
Prediction: 0.357021083333334
Explanation Fit: 0.013
0.266 < Physical.inactivity <= 0.300
0.166 < Excessive.drinking <= 0.190
0.0811 < Percentage.of.households.with.high.housing.costs <= 0.1004
0.235 < below.18.years.of.age
0.0595 < School.segregation <= 0.1084
0.127 < Severe.housing.problems <= 0.153
0.0936 < Uninsured.adults <= 0.1283
0.000701 < Primary.care.physicians
Alcohol.impaired.driving.deaths <= 0.182
0.337 < Insufficient.sleep <= 0.368
Feature
−0.006
−0.004
−0.002
0.000
0.002
Weight
Positive
Negative

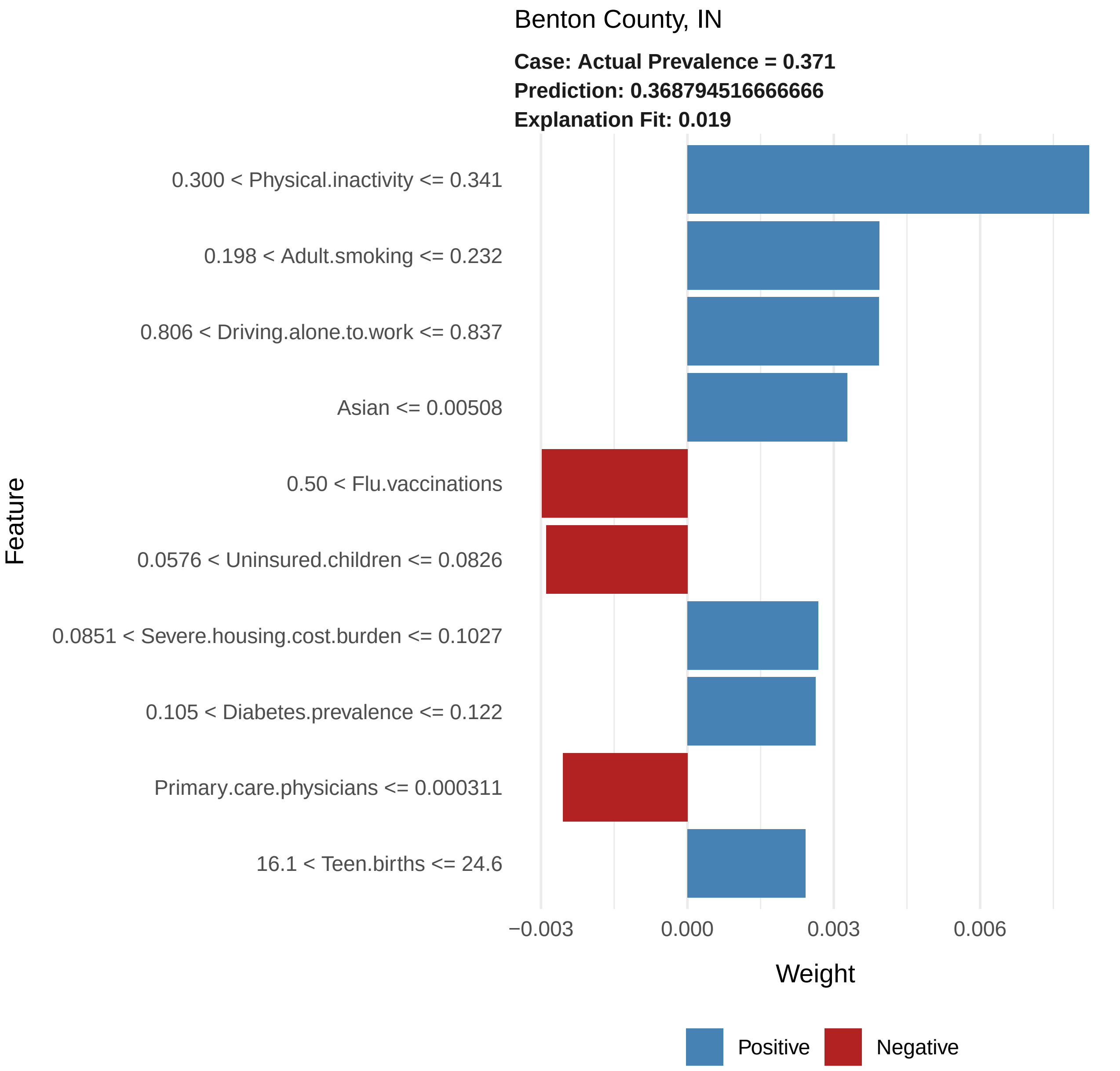
Benton County, IN
Case: Actual Prevalence = 0.371
Prediction: 0.368794516666666
Explanation Fit: 0.019
Feature
0.300 < Physical.inactivity <= 0.341
0.198 < Adult.smoking <= 0.232
0.806 < Driving.alone.to.work <= 0.837
Asian <= 0.00508
0.50 < Flu.vaccinations
0.0576 < Uninsured.children <= 0.0826
0.0851 < Severe.housing.cost.burden <= 0.1027
0.105 < Diabetes.prevalence <= 0.122
Primary.care.physicians <= 0.000311
16.1 < Teen.births <= 24.6
−0.003
0.000
0.003
0.006
Weight
Positive
Negative

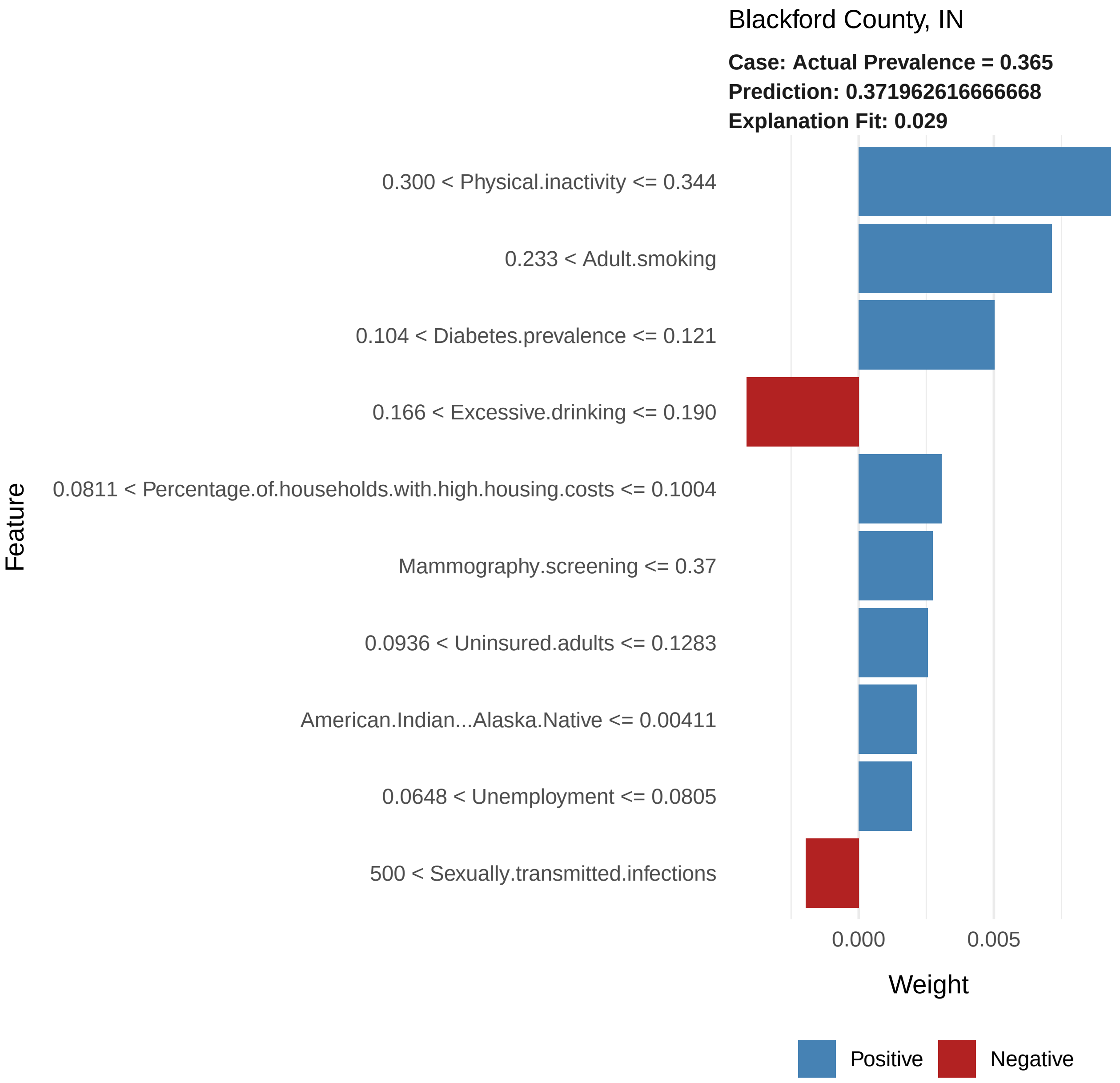
Blackford County, IN
Case: Actual Prevalence = 0.365
Prediction: 0.371962616666668
Explanation Fit: 0.029
0.300 < Physical.inactivity <= 0.344
0.233 < Adult.smoking
0.104 < Diabetes.prevalence <= 0.121
0.166 < Excessive.drinking <= 0.190
0.0811 < Percentage.of.households.with.high.housing.costs <= 0.1004
Mammography.screening <= 0.37
0.0936 < Uninsured.adults <= 0.1283
American.Indian...Alaska.Native <= 0.00411
0.0648 < Unemployment <= 0.0805
500 < Sexually.transmitted.infections
Feature
0.000
0.005
Weight
Positive
Negative

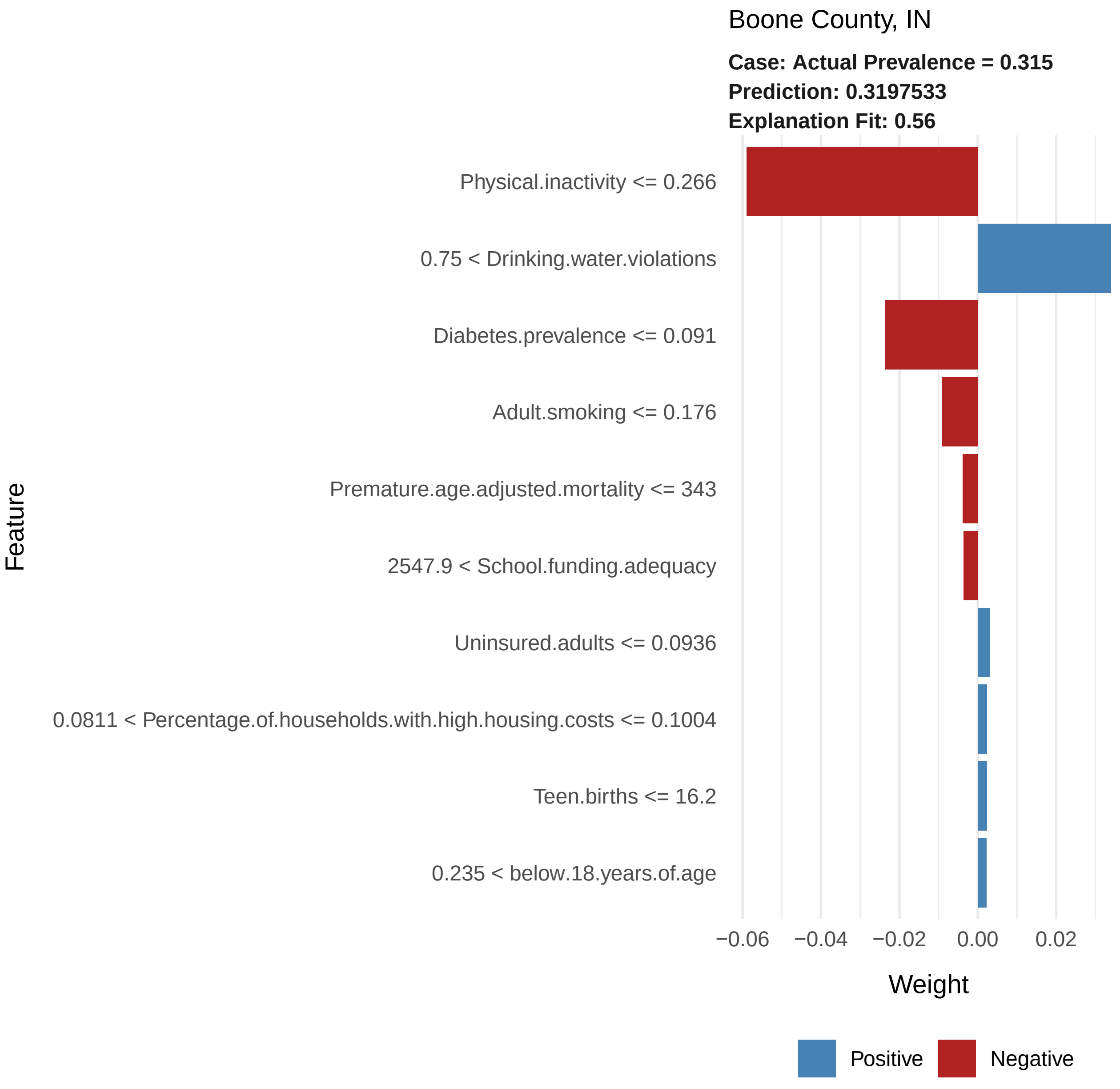

Boone County, IN
Case: Actual Prevalence = 0.315
Prediction: 0.3197533
Explanation Fit: 0.56
Physical.inactivity <= 0.266
0.75 < Drinking.water.violations
Diabetes.prevalence <= 0.091
Adult.smoking <= 0.176
Premature.age.adjusted.mortality <= 343
2547.9 < School.funding.adequacy
Uninsured.adults <= 0.0936
0.0811 < Percentage.of.households.with.high.housing.costs <= 0.1004
Teen.births <= 16.2
0.235 < below.18.years.of.age
Feature
−0.06
−0.04
−0.02
0.00
0.02
Weight
Positive
Negative

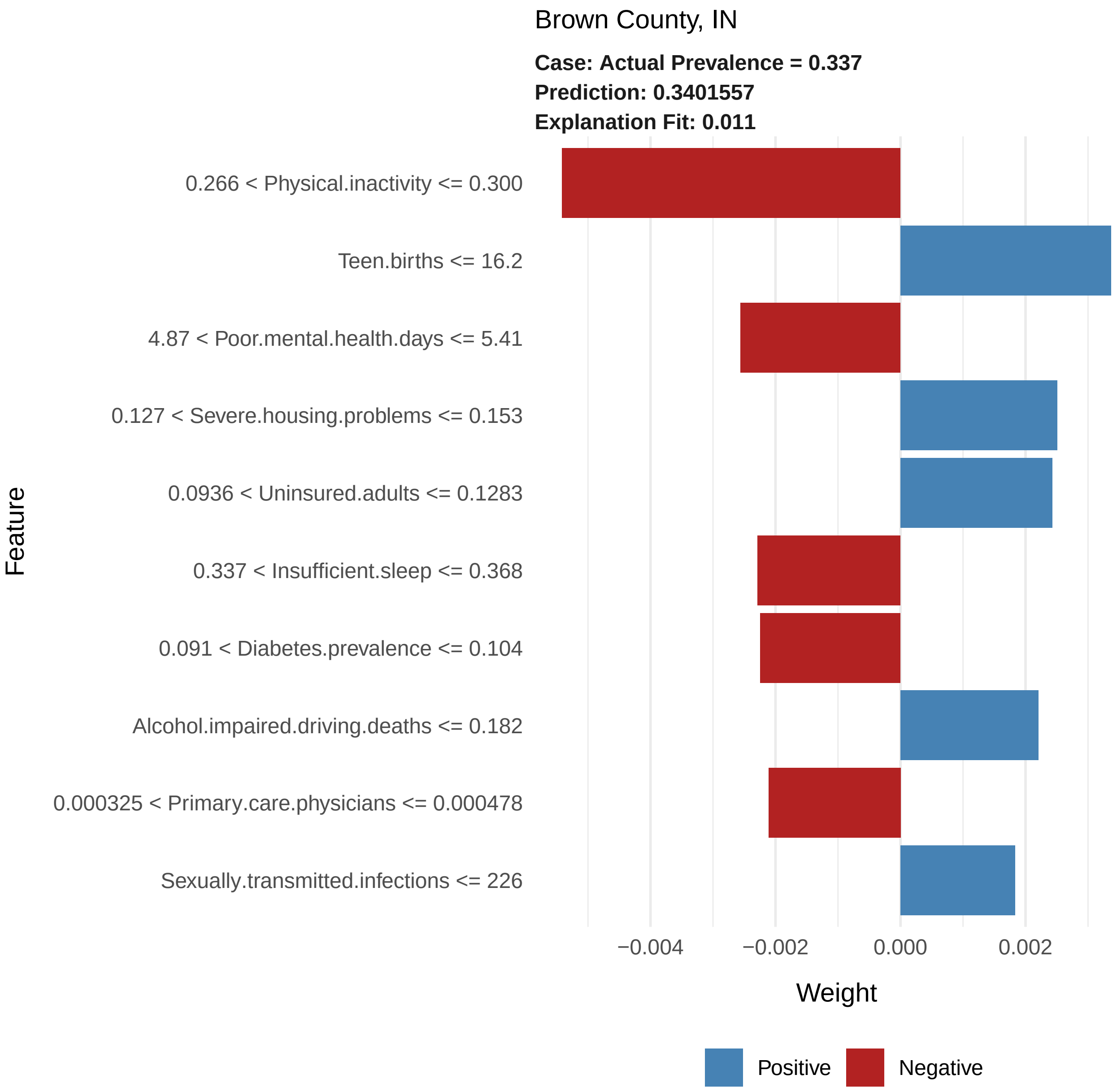

Brown County, IN
Case: Actual Prevalence = 0.337
Prediction: 0.3401557
Explanation Fit: 0.011
0.266 < Physical.inactivity <= 0.300
Teen.births <= 16.2
4.87 < Poor.mental.health.days <= 5.41
0.127 < Severe.housing.problems <= 0.153
0.0936 < Uninsured.adults <= 0.1283
0.337 < Insufficient.sleep <= 0.368
0.091 < Diabetes.prevalence <= 0.104
Alcohol.impaired.driving.deaths <= 0.182
0.000325 < Primary.care.physicians <= 0.000478
Sexually.transmitted.infections <= 226
Feature
−0.004
−0.002
0.000
0.002
Weight
Positive
Negative

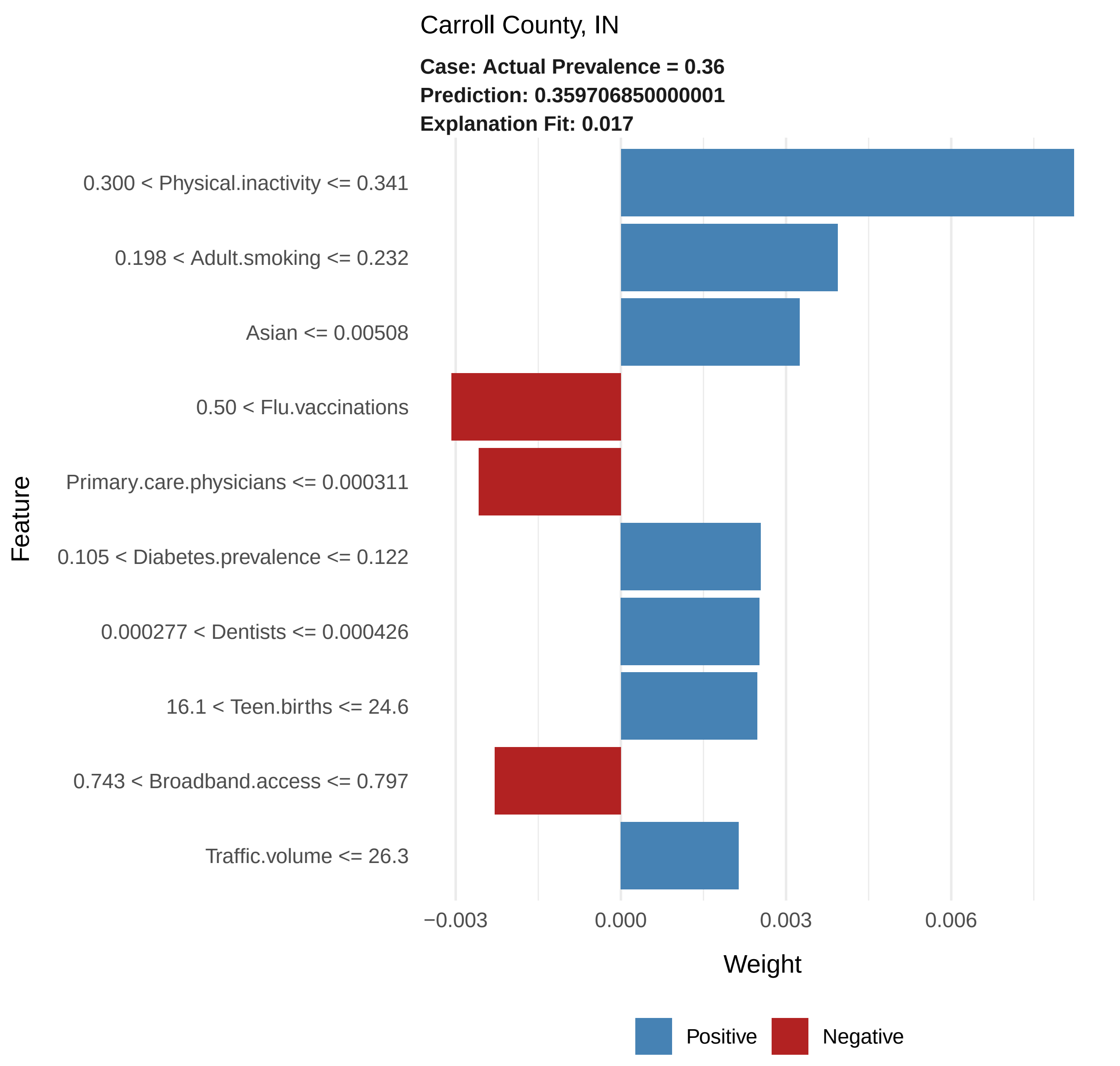

Carroll County, IN
Case: Actual Prevalence = 0.36
Prediction: 0.359706850000001
Explanation Fit: 0.017
0.300 < Physical.inactivity <= 0.341
0.198 < Adult.smoking <= 0.232
Asian <= 0.00508
0.50 < Flu.vaccinations
Primary.care.physicians <= 0.000311
0.105 < Diabetes.prevalence <= 0.122
0.000277 < Dentists <= 0.000426
16.1 < Teen.births <= 24.6
0.743 < Broadband.access <= 0.797
Traffic.volume <= 26.3
Feature
−0.003
0.000
0.003
0.006
Weight
Positive
Negative

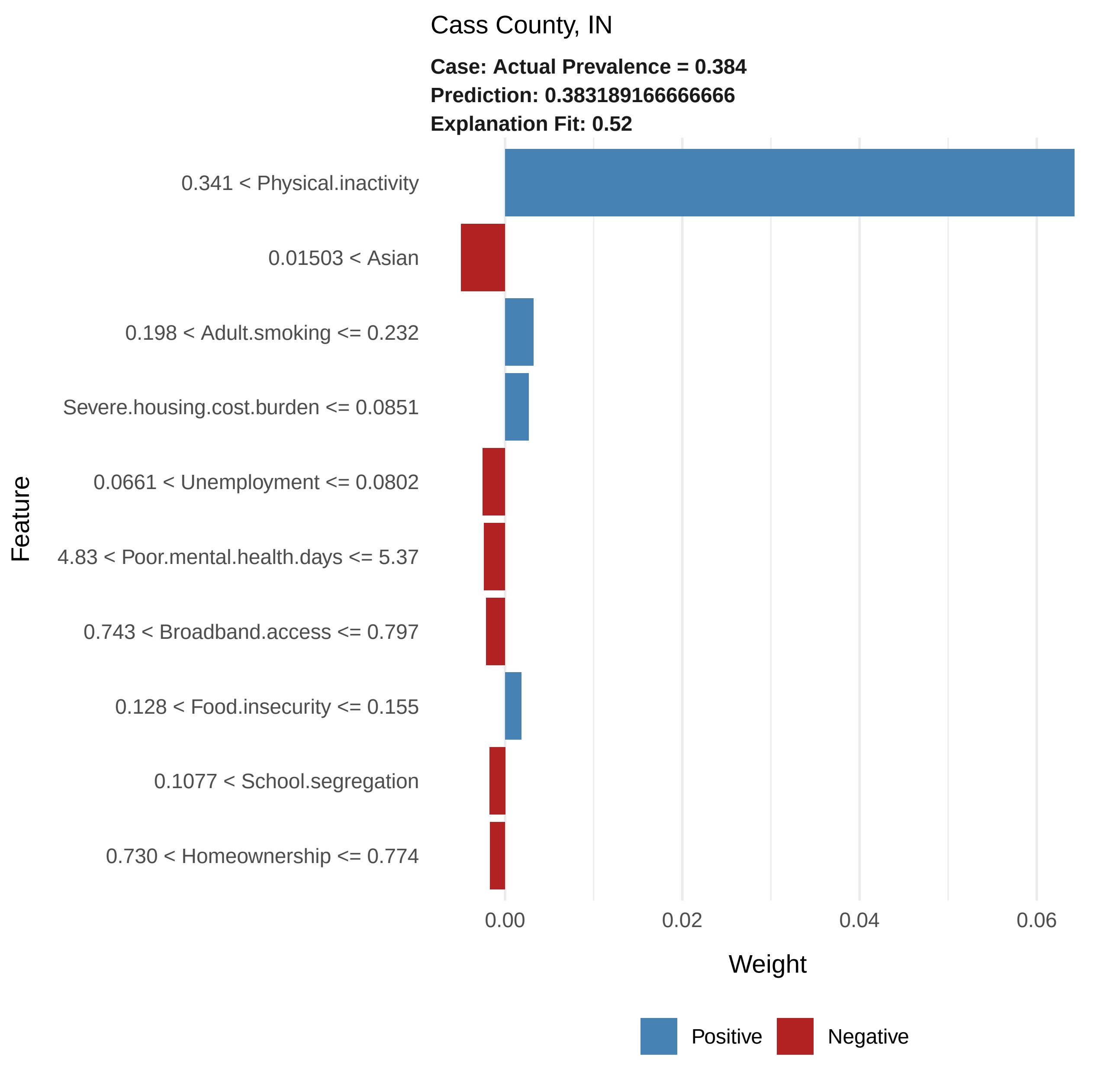
Cass County, IN
Case: Actual Prevalence = 0.384
Prediction: 0.383189166666666
Explanation Fit: 0.52
0.341 < Physical.inactivity
0.01503 < Asian
0.198 < Adult.smoking <= 0.232
Severe.housing.cost.burden <= 0.0851
0.0661 < Unemployment <= 0.0802
4.83 < Poor.mental.health.days <= 5.37
0.743 < Broadband.access <= 0.797
0.128 < Food.insecurity <= 0.155
0.1077 < School.segregation
0.730 < Homeownership <= 0.774
Feature
0.00
0.02
0.04
0.06
Weight
Positive
Negative

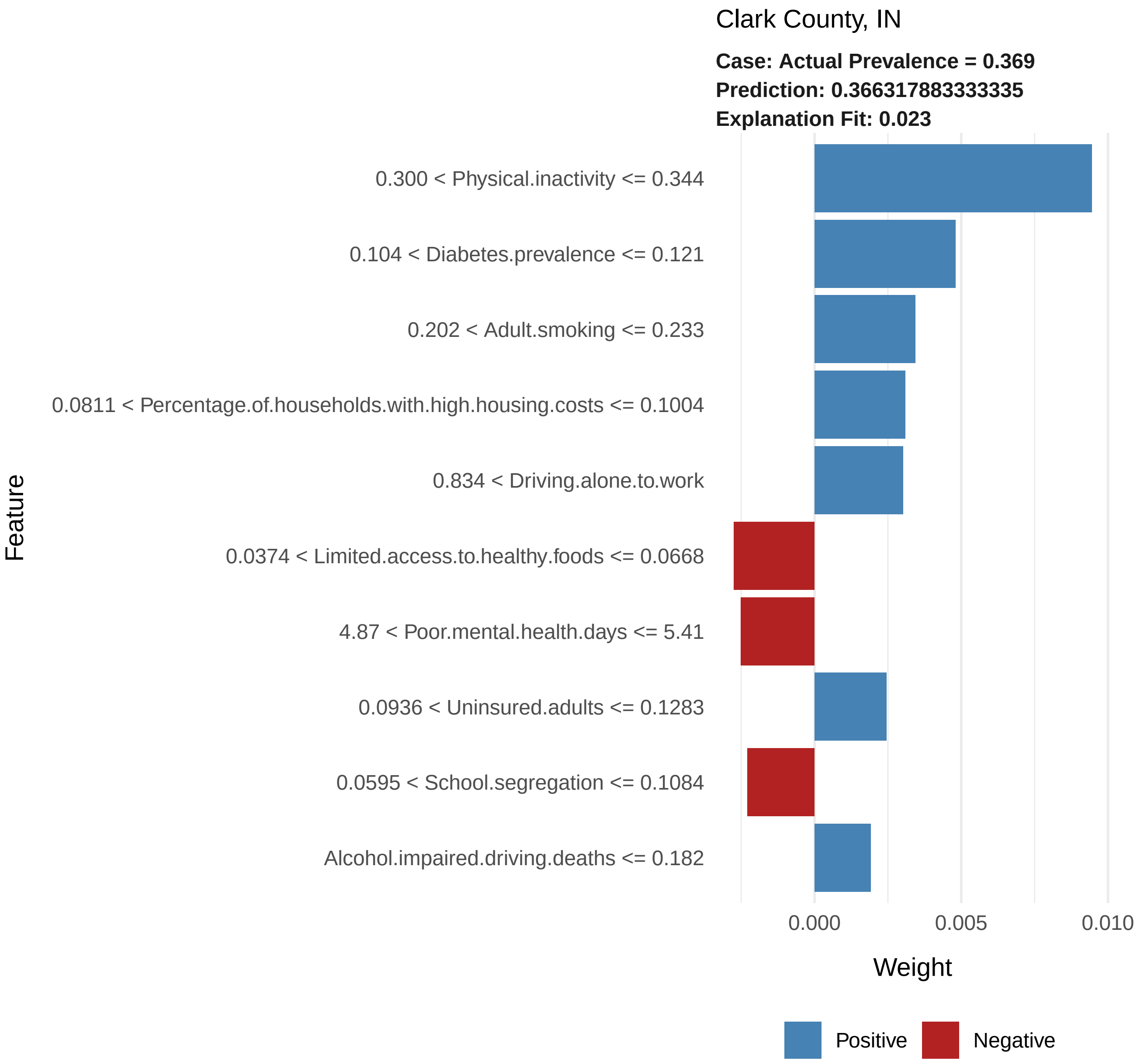

Clark County, IN
Case: Actual Prevalence = 0.369
Prediction: 0.366317883333335
Explanation Fit: 0.023
0.300 < Physical.inactivity <= 0.344
0.104 < Diabetes.prevalence <= 0.121
0.202 < Adult.smoking <= 0.233
0.0811 < Percentage.of.households.with.high.housing.costs <= 0.1004
0.834 < Driving.alone.to.work
0.0374 < Limited.access.to.healthy.foods <= 0.0668
4.87 < Poor.mental.health.days <= 5.41
0.0936 < Uninsured.adults <= 0.1283
0.0595 < School.segregation <= 0.1084
Alcohol.impaired.driving.deaths <= 0.182
Feature
0.000
0.005
0.010
Weight
Positive
Negative

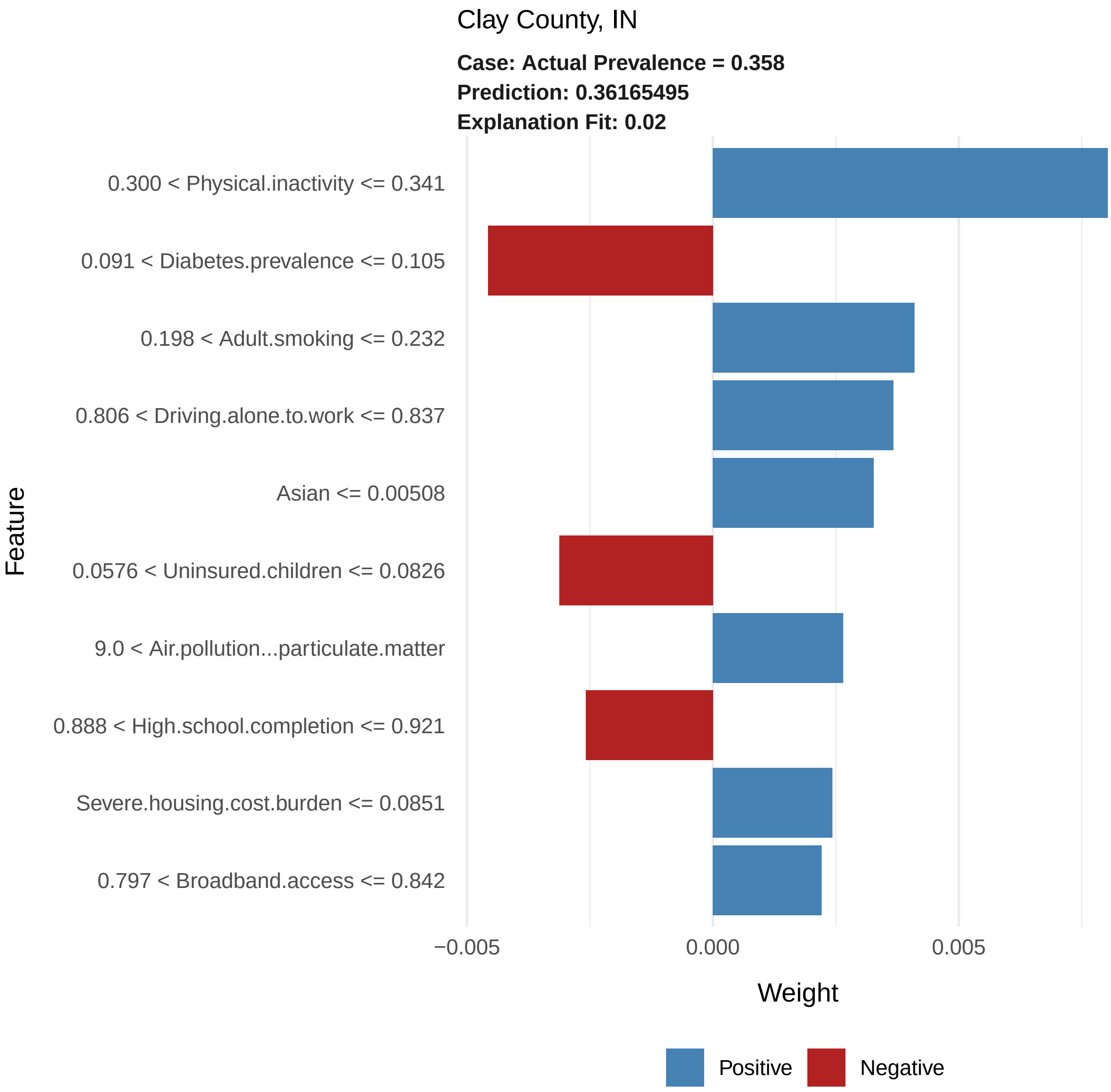

Clay County, IN
Case: Actual Prevalence = 0.358
Prediction: 0.36165495
Explanation Fit: 0.02
0.300 < Physical.inactivity <= 0.341
0.091 < Diabetes.prevalence <= 0.105
0.198 < Adult.smoking <= 0.232
0.806 < Driving.alone.to.work <= 0.837
Asian <= 0.00508
0.0576 < Uninsured.children <= 0.0826
9.0 < Air.pollution...particulate.matter
0.888 < High.school.completion <= 0.921
Severe.housing.cost.burden <= 0.0851
0.797 < Broadband.access <= 0.842
Feature
−0.005
0.000
0.005
Weight
Positive
Negative

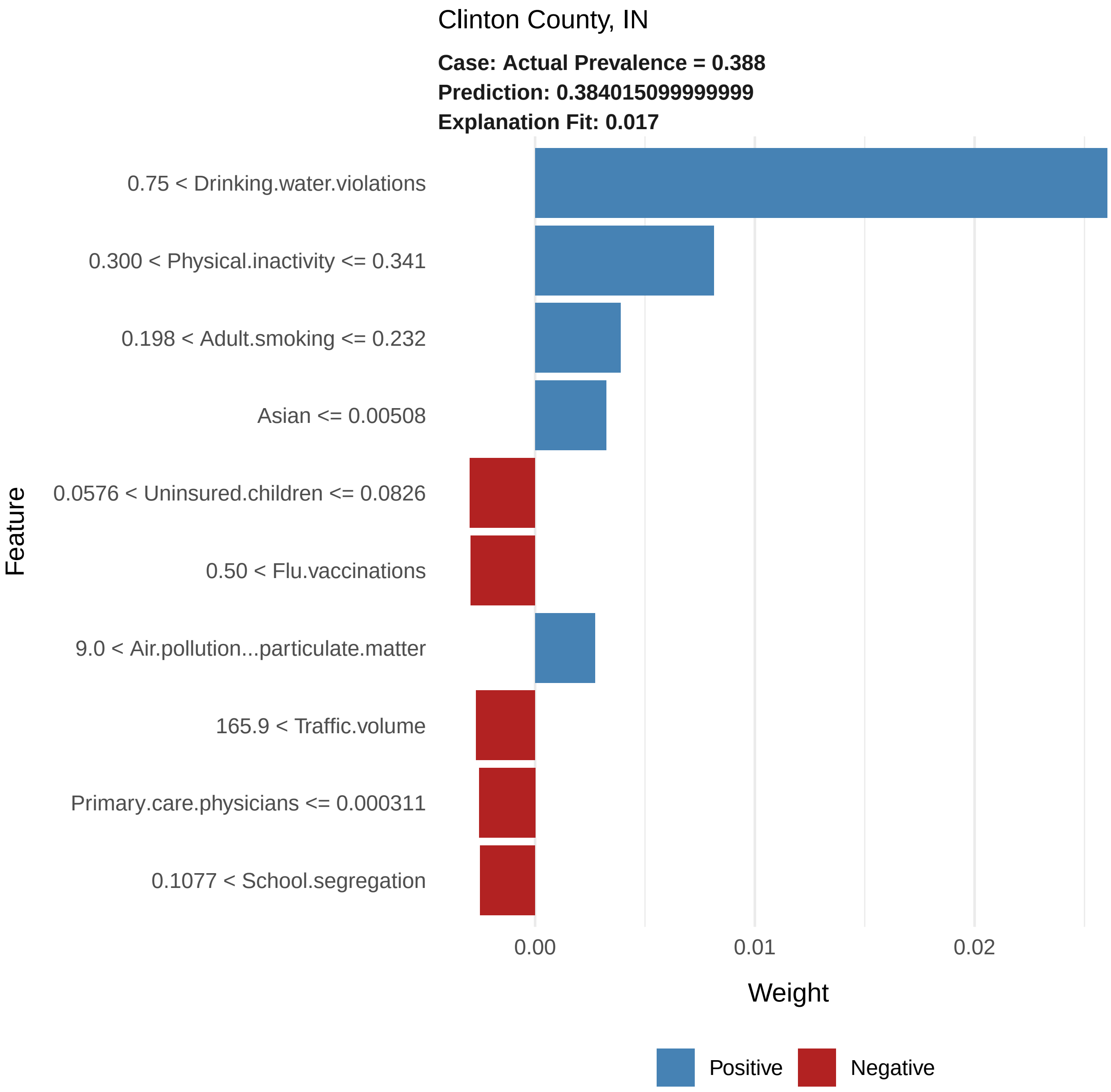

Clinton County, IN
Case: Actual Prevalence = 0.388
Prediction: 0.384015099999999
Explanation Fit: 0.017
0.75 < Drinking.water.violations
0.300 < Physical.inactivity <= 0.341
0.198 < Adult.smoking <= 0.232
Asian <= 0.00508
0.0576 < Uninsured.children <= 0.0826
0.50 < Flu.vaccinations
9.0 < Air.pollution...particulate.matter
165.9 < Traffic.volume
Primary.care.physicians <= 0.000311
0.1077 < School.segregation
Feature
0.00
0.01
0.02
Weight
Positive
Negative

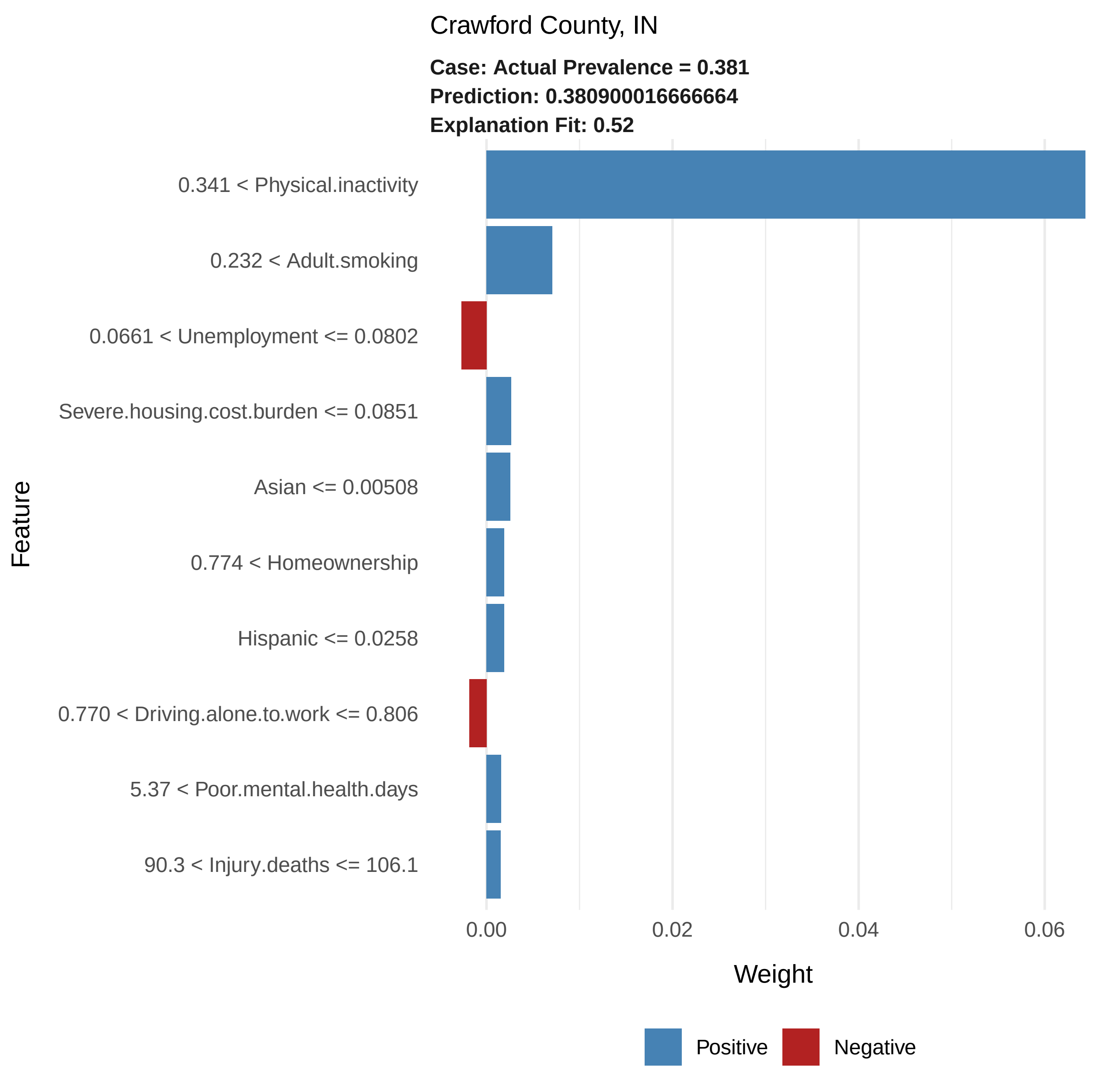

Crawford County, IN
Case: Actual Prevalence = 0.381
Prediction: 0.380900016666664
Explanation Fit: 0.52
0.341 < Physical.inactivity
0.232 < Adult.smoking
0.0661 < Unemployment <= 0.0802
Severe.housing.cost.burden <= 0.0851
Asian <= 0.00508
0.774 < Homeownership
Hispanic <= 0.0258
0.770 < Driving.alone.to.work <= 0.806
5.37 < Poor.mental.health.days
90.3 < Injury.deaths <= 106.1
Feature
0.00
0.02
0.04
0.06
Weight
Positive
Negative

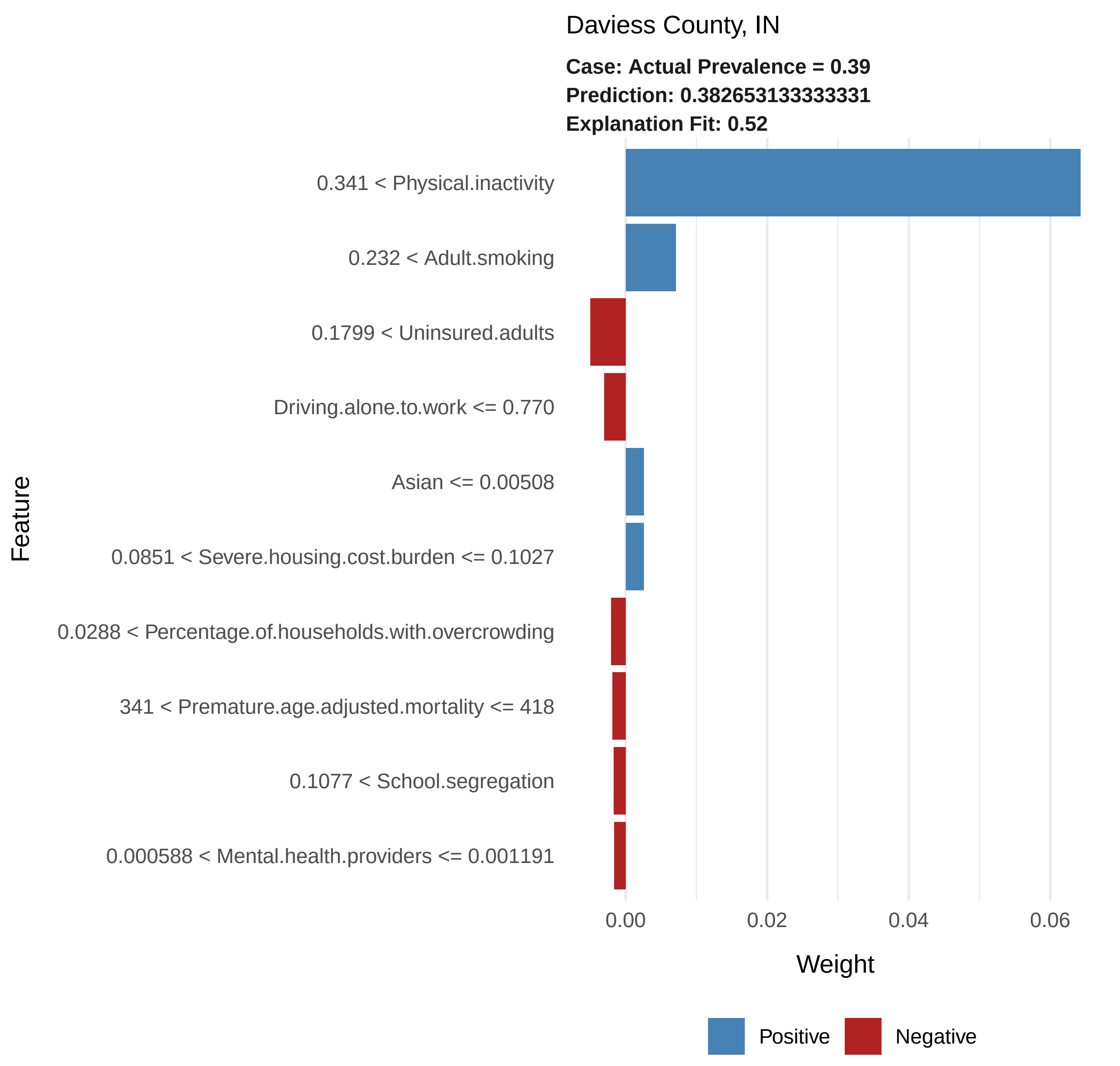

Daviess County, IN
Case: Actual Prevalence = 0.39
Prediction: 0.382653133333331
Explanation Fit: 0.52
0.341 < Physical.inactivity
0.232 < Adult.smoking
0.1799 < Uninsured.adults
Driving.alone.to.work <= 0.770
Asian <= 0.00508
0.0851 < Severe.housing.cost.burden <= 0.1027
0.0288 < Percentage.of.households.with.overcrowding
341 < Premature.age.adjusted.mortality <= 418
0.1077 < School.segregation
0.000588 < Mental.health.providers <= 0.001191
Feature
0.00
0.02
0.04
0.06
Weight
Positive
Negative

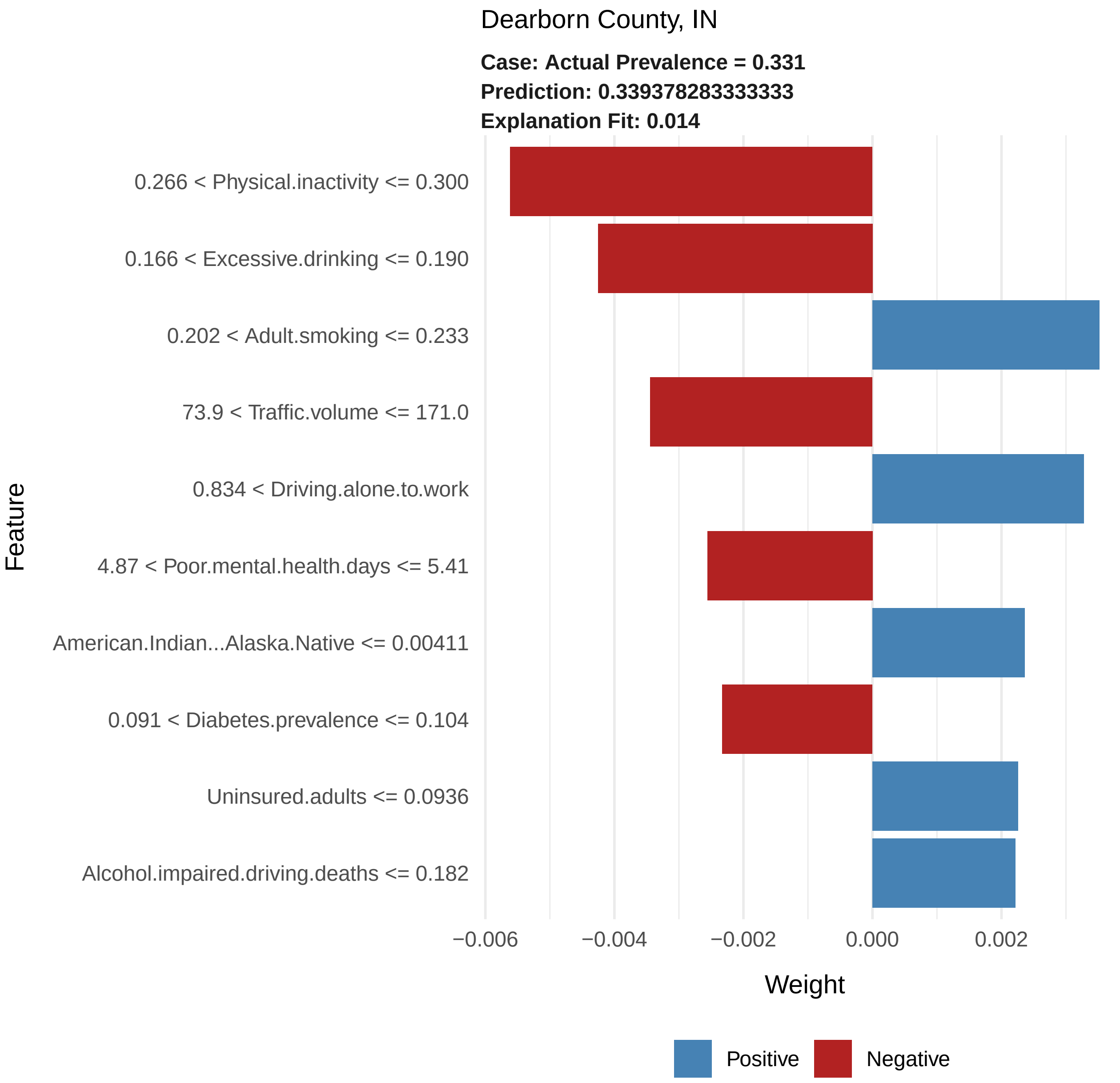

Dearborn County, IN
Case: Actual Prevalence = 0.331
Prediction: 0.339378283333333
Explanation Fit: 0.014
0.266 < Physical.inactivity <= 0.300
0.166 < Excessive.drinking <= 0.190
0.202 < Adult.smoking <= 0.233
73.9 < Traffic.volume <= 171.0
0.834 < Driving.alone.to.work
4.87 < Poor.mental.health.days <= 5.41
American.Indian...Alaska.Native <= 0.00411
0.091 < Diabetes.prevalence <= 0.104
Uninsured.adults <= 0.0936
Alcohol.impaired.driving.deaths <= 0.182
Feature
−0.006
−0.004
−0.002
0.000
0.002
Weight
Positive
Negative

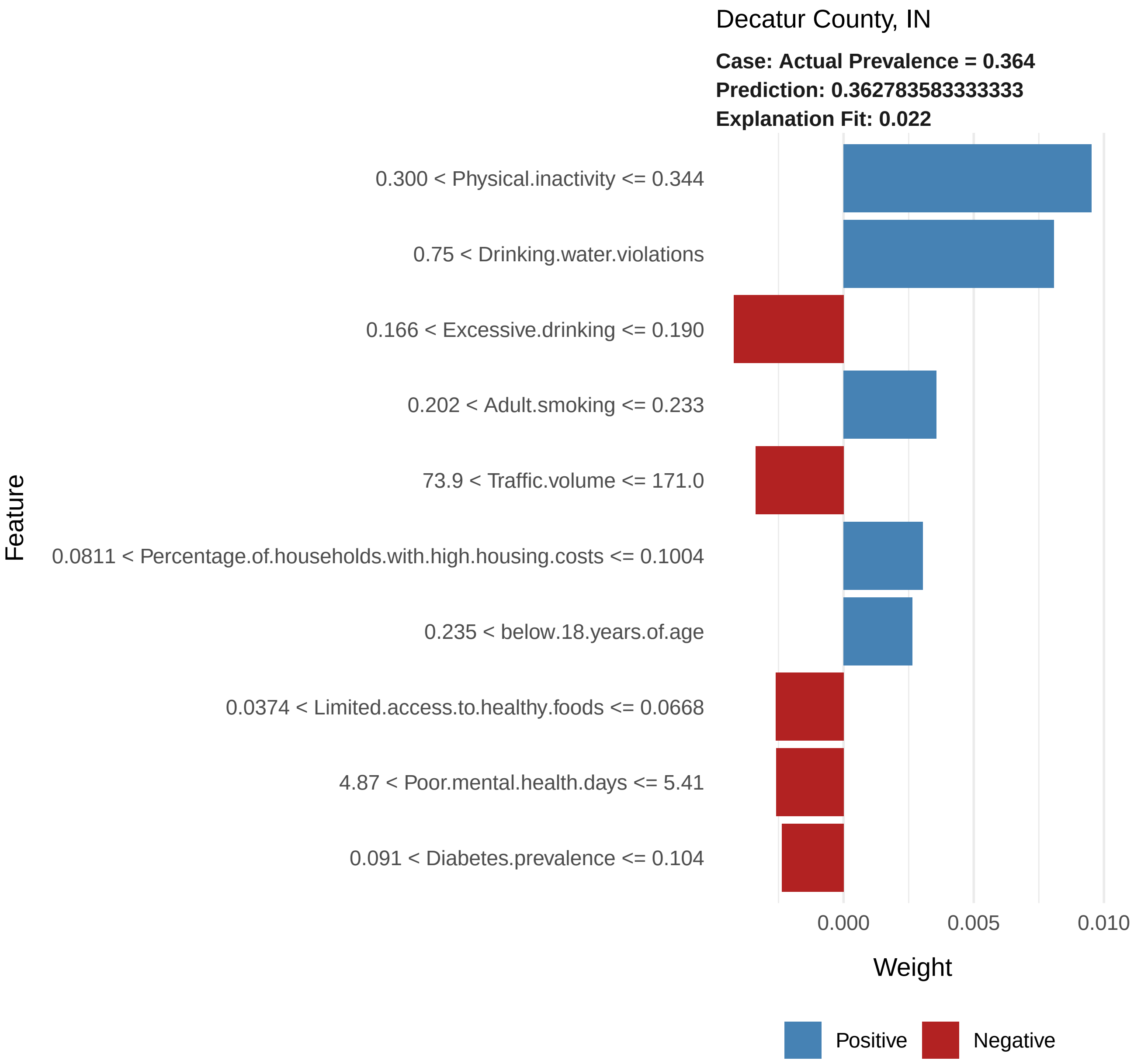
Decatur County, IN
Case: Actual Prevalence = 0.364
Prediction: 0.362783583333333
Explanation Fit: 0.022
0.300 < Physical.inactivity <= 0.344
0.75 < Drinking.water.violations
0.166 < Excessive.drinking <= 0.190
0.202 < Adult.smoking <= 0.233
73.9 < Traffic.volume <= 171.0
0.0811 < Percentage.of.households.with.high.housing.costs <= 0.1004
0.235 < below.18.years.of.age
0.0374 < Limited.access.to.healthy.foods <= 0.0668
4.87 < Poor.mental.health.days <= 5.41
0.091 < Diabetes.prevalence <= 0.104
Feature
0.000
0.005
0.010
Weight
Positive
Negative

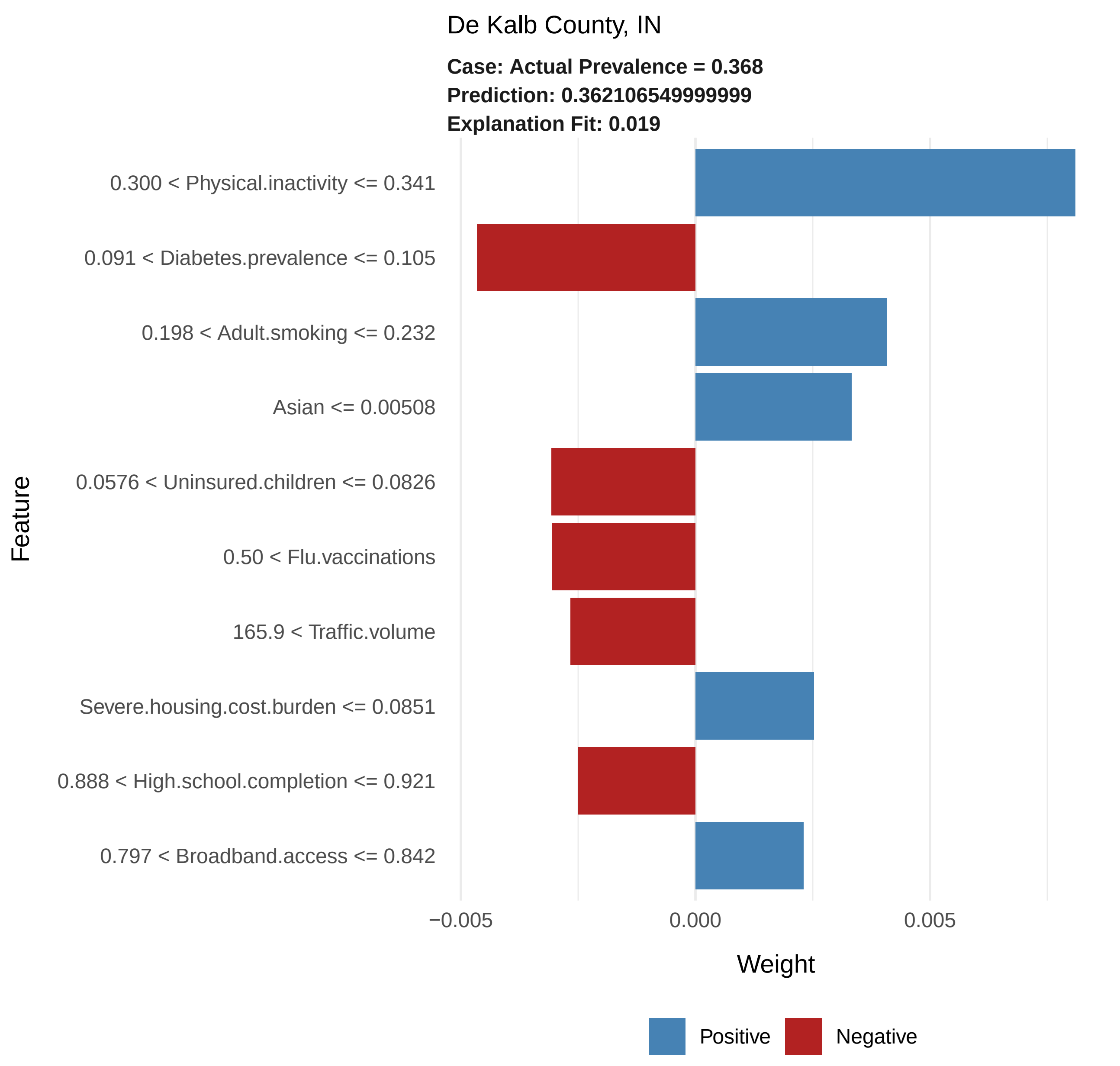

De Kalb County, IN
Case: Actual Prevalence = 0.368
Prediction: 0.362106549999999
Explanation Fit: 0.019
0.300 < Physical.inactivity <= 0.341
0.091 < Diabetes.prevalence <= 0.105
0.198 < Adult.smoking <= 0.232
Asian <= 0.00508
0.0576 < Uninsured.children <= 0.0826
0.50 < Flu.vaccinations
165.9 < Traffic.volume
Severe.housing.cost.burden <= 0.0851
0.888 < High.school.completion <= 0.921
0.797 < Broadband.access <= 0.842
Feature
−0.005
0.000
0.005
Weight
Positive
Negative

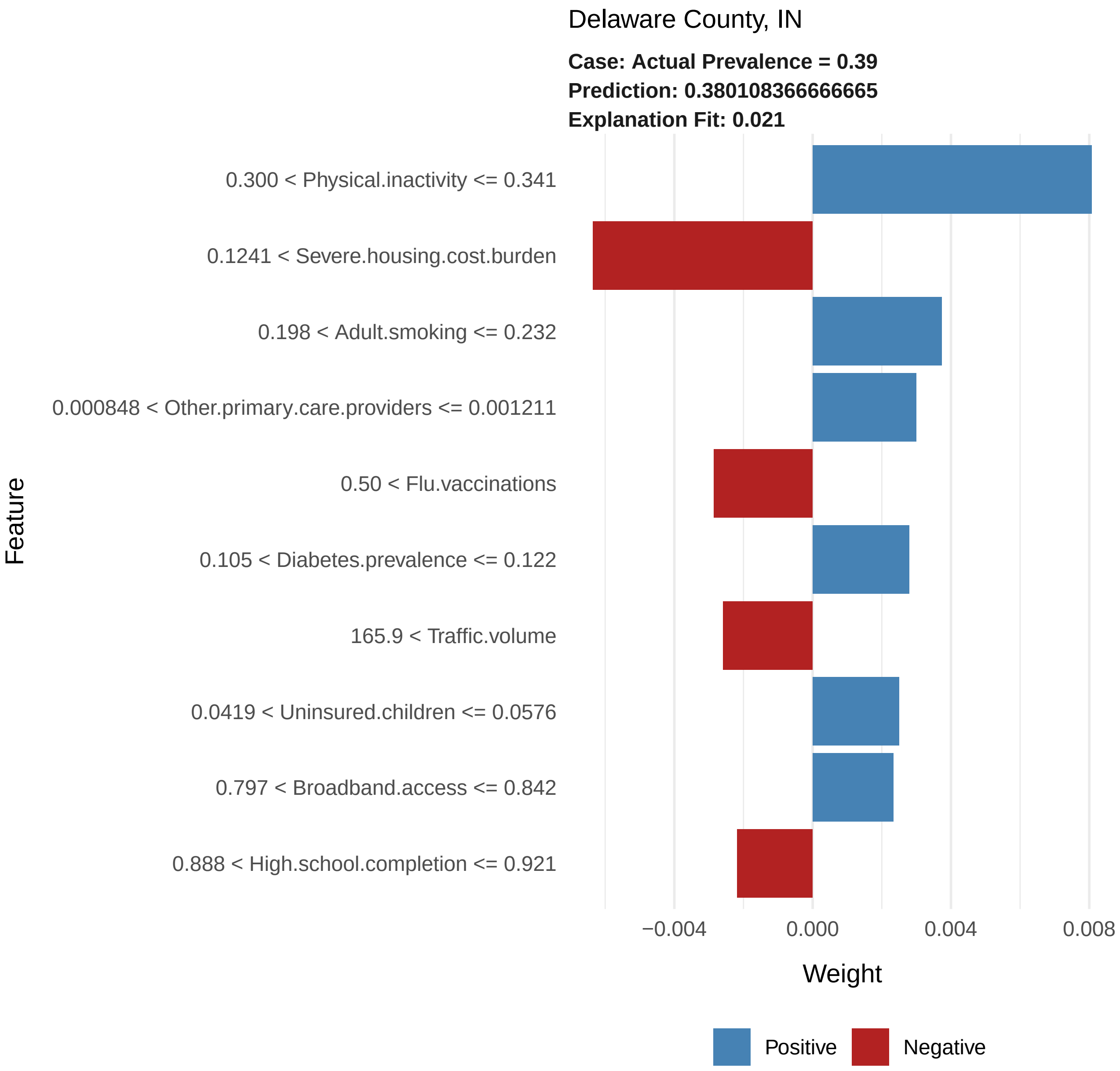

Delaware County, IN
Case: Actual Prevalence = 0.39
Prediction: 0.380108366666665
Explanation Fit: 0.021
0.300 < Physical.inactivity <= 0.341
0.1241 < Severe.housing.cost.burden
0.198 < Adult.smoking <= 0.232
0.000848 < Other.primary.care.providers <= 0.001211
0.50 < Flu.vaccinations
0.105 < Diabetes.prevalence <= 0.122
165.9 < Traffic.volume
0.0419 < Uninsured.children <= 0.0576
0.797 < Broadband.access <= 0.842
0.888 < High.school.completion <= 0.921
Feature
−0.004
0.000
0.004
0.008
Weight
Positive
Negative

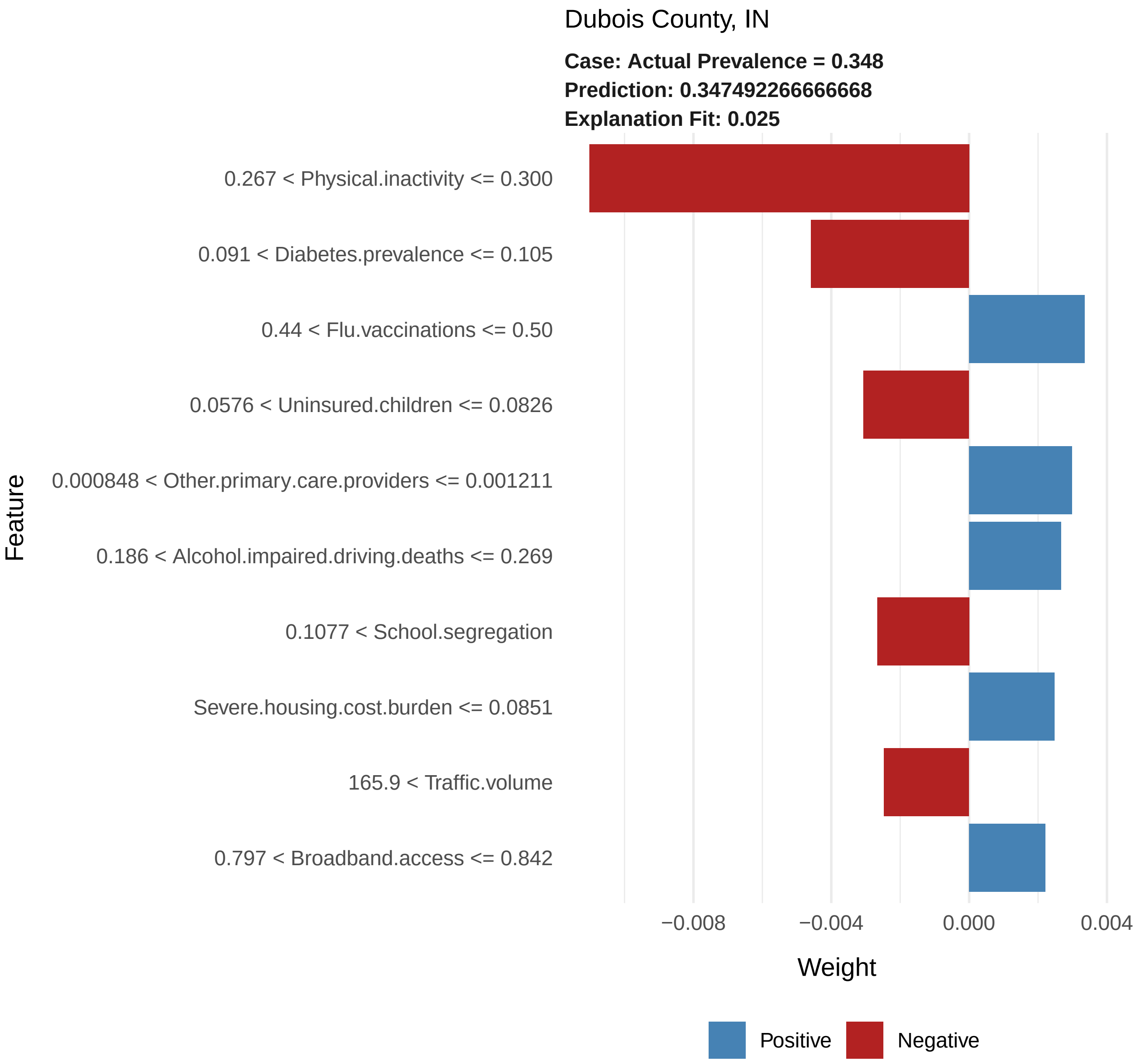

Dubois County, IN
Case: Actual Prevalence = 0.348
Prediction: 0.347492266666668
Explanation Fit: 0.025
0.267 < Physical.inactivity <= 0.300
0.091 < Diabetes.prevalence <= 0.105
0.44 < Flu.vaccinations <= 0.50
0.0576 < Uninsured.children <= 0.0826
0.000848 < Other.primary.care.providers <= 0.001211
0.186 < Alcohol.impaired.driving.deaths <= 0.269
0.1077 < School.segregation
Severe.housing.cost.burden <= 0.0851
165.9 < Traffic.volume
0.797 < Broadband.access <= 0.842
Feature
−0.008
−0.004
0.000
0.004
Weight
Positive
Negative

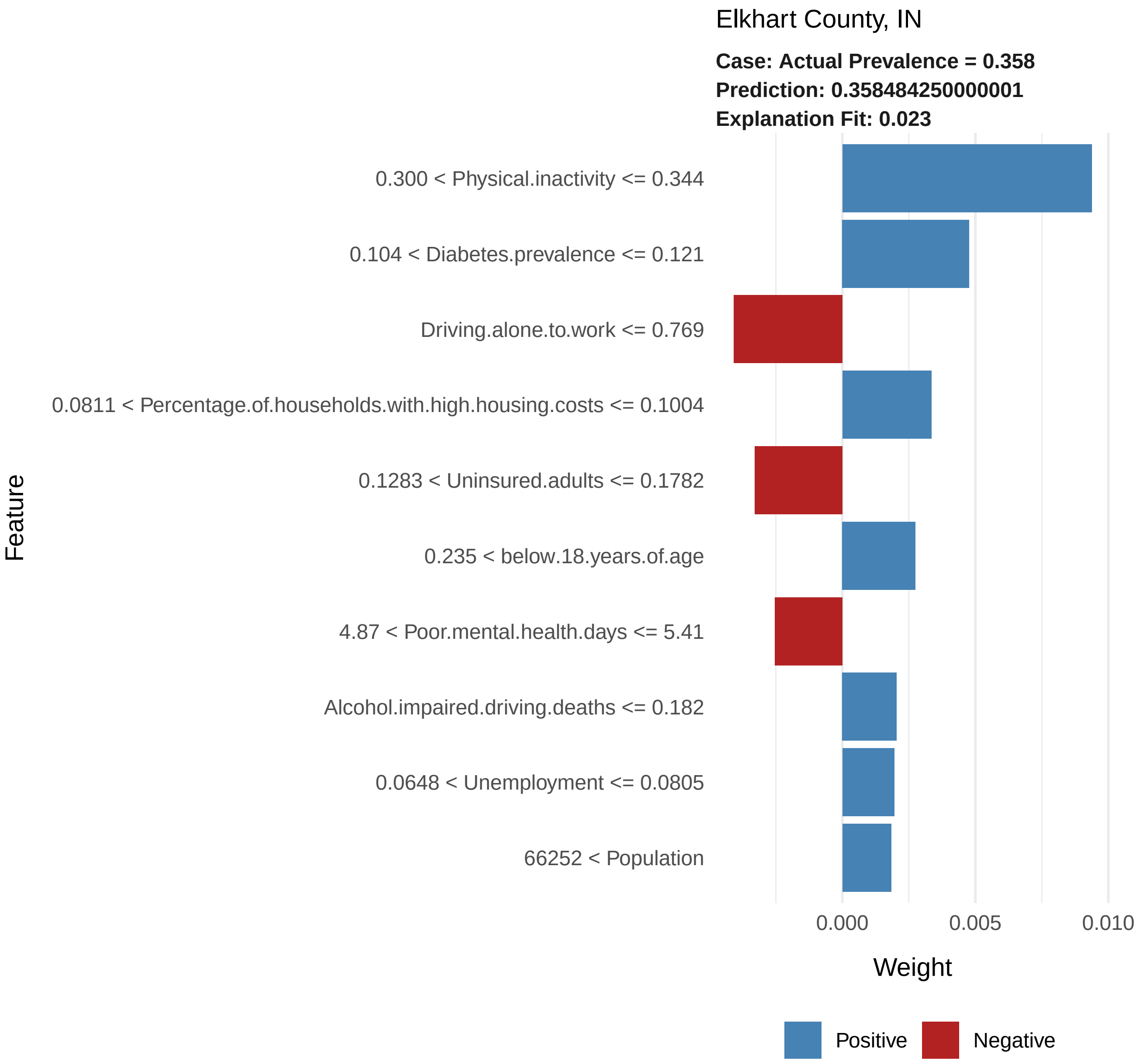

Elkhart County, IN
Case: Actual Prevalence = 0.358
Prediction: 0.358484250000001
Explanation Fit: 0.023
0.300 < Physical.inactivity <= 0.344
0.104 < Diabetes.prevalence <= 0.121
Driving.alone.to.work <= 0.769
0.0811 < Percentage.of.households.with.high.housing.costs <= 0.1004
0.1283 < Uninsured.adults <= 0.1782
0.235 < below.18.years.of.age
4.87 < Poor.mental.health.days <= 5.41
Alcohol.impaired.driving.deaths <= 0.182
0.0648 < Unemployment <= 0.0805
66252 < Population
Feature
0.000
0.005
0.010
Weight
Positive
Negative

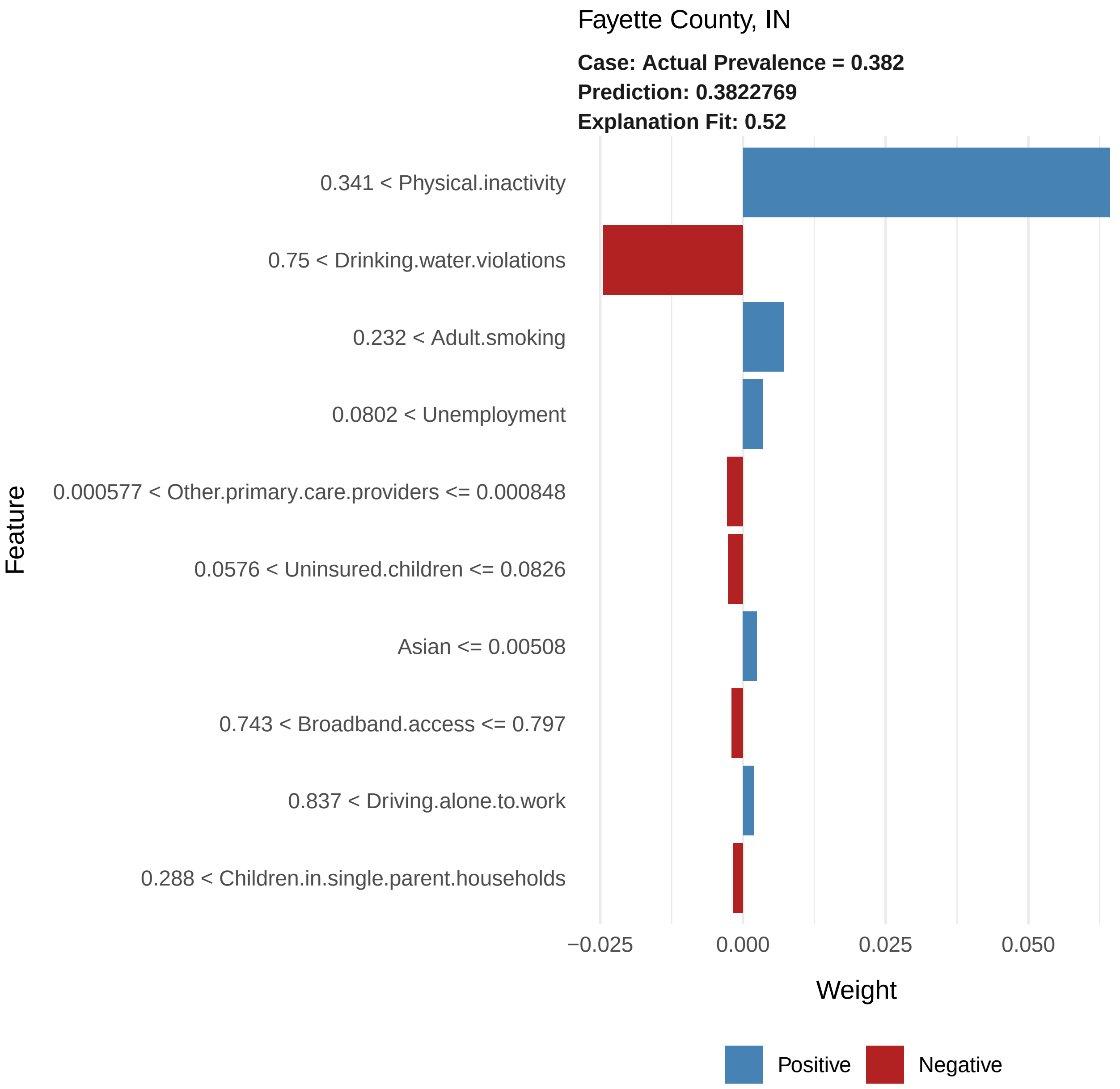

Fayette County, IN
Case: Actual Prevalence = 0.382
Prediction: 0.3822769
Explanation Fit: 0.52
0.341 < Physical.inactivity
0.75 < Drinking.water.violations
0.232 < Adult.smoking
0.0802 < Unemployment
0.000577 < Other.primary.care.providers <= 0.000848
0.0576 < Uninsured.children <= 0.0826
Asian <= 0.00508
0.743 < Broadband.access <= 0.797
0.837 < Driving.alone.to.work
0.288 < Children.in.single.parent.households
Feature
−0.025
0.000
0.025
0.050
Weight
Positive
Negative

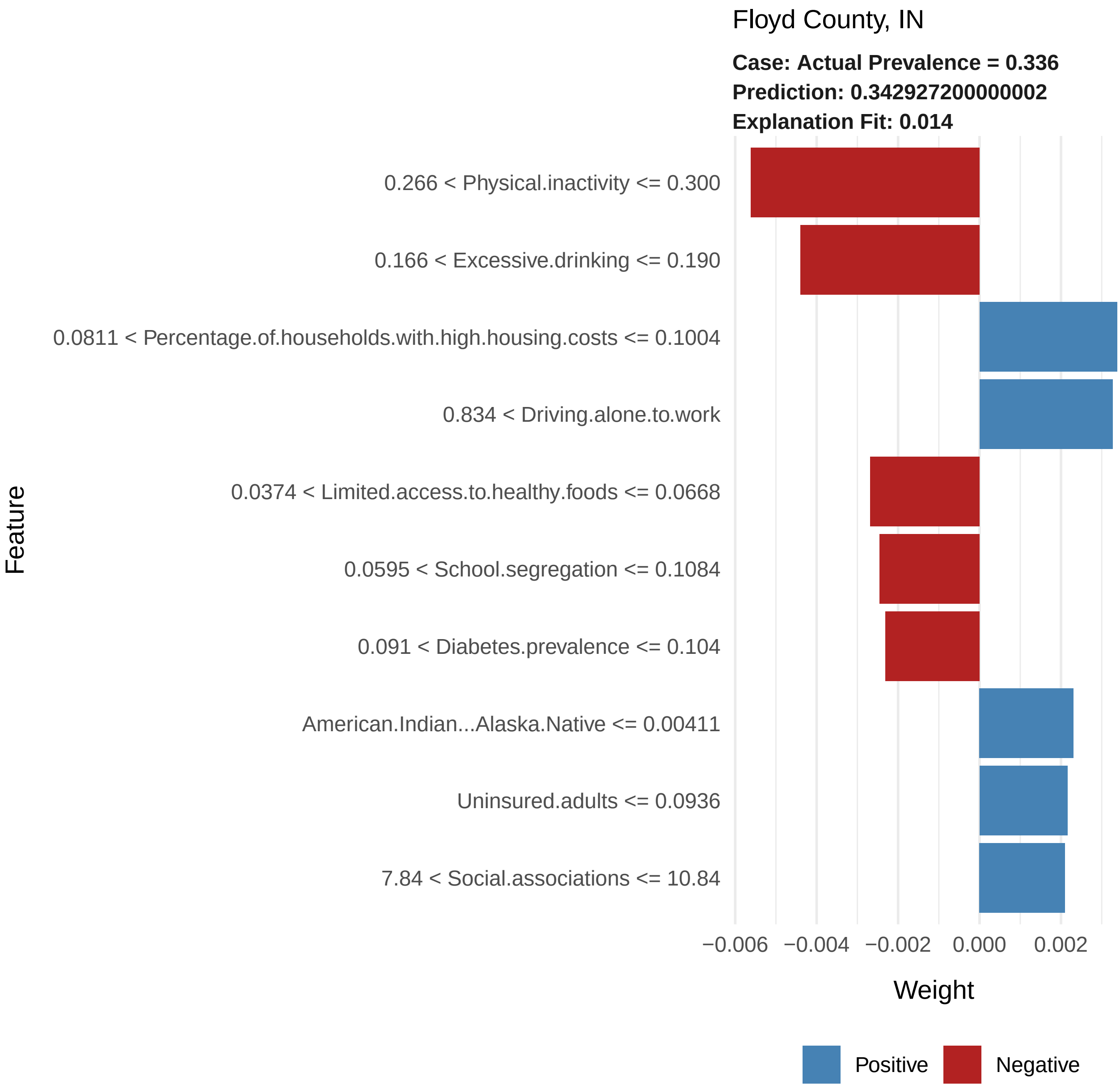

Floyd County, IN
Case: Actual Prevalence = 0.336
Prediction: 0.342927200000002
Explanation Fit: 0.014
0.266 < Physical.inactivity <= 0.300
0.166 < Excessive.drinking <= 0.190
0.0811 < Percentage.of.households.with.high.housing.costs <= 0.1004
0.834 < Driving.alone.to.work
0.0374 < Limited.access.to.healthy.foods <= 0.0668
0.0595 < School.segregation <= 0.1084
0.091 < Diabetes.prevalence <= 0.104
American.Indian...Alaska.Native <= 0.00411
Uninsured.adults <= 0.0936
7.84 < Social.associations <= 10.84
Feature
−0.006
−0.004
−0.002
0.000
0.002
Weight
Positive
Negative

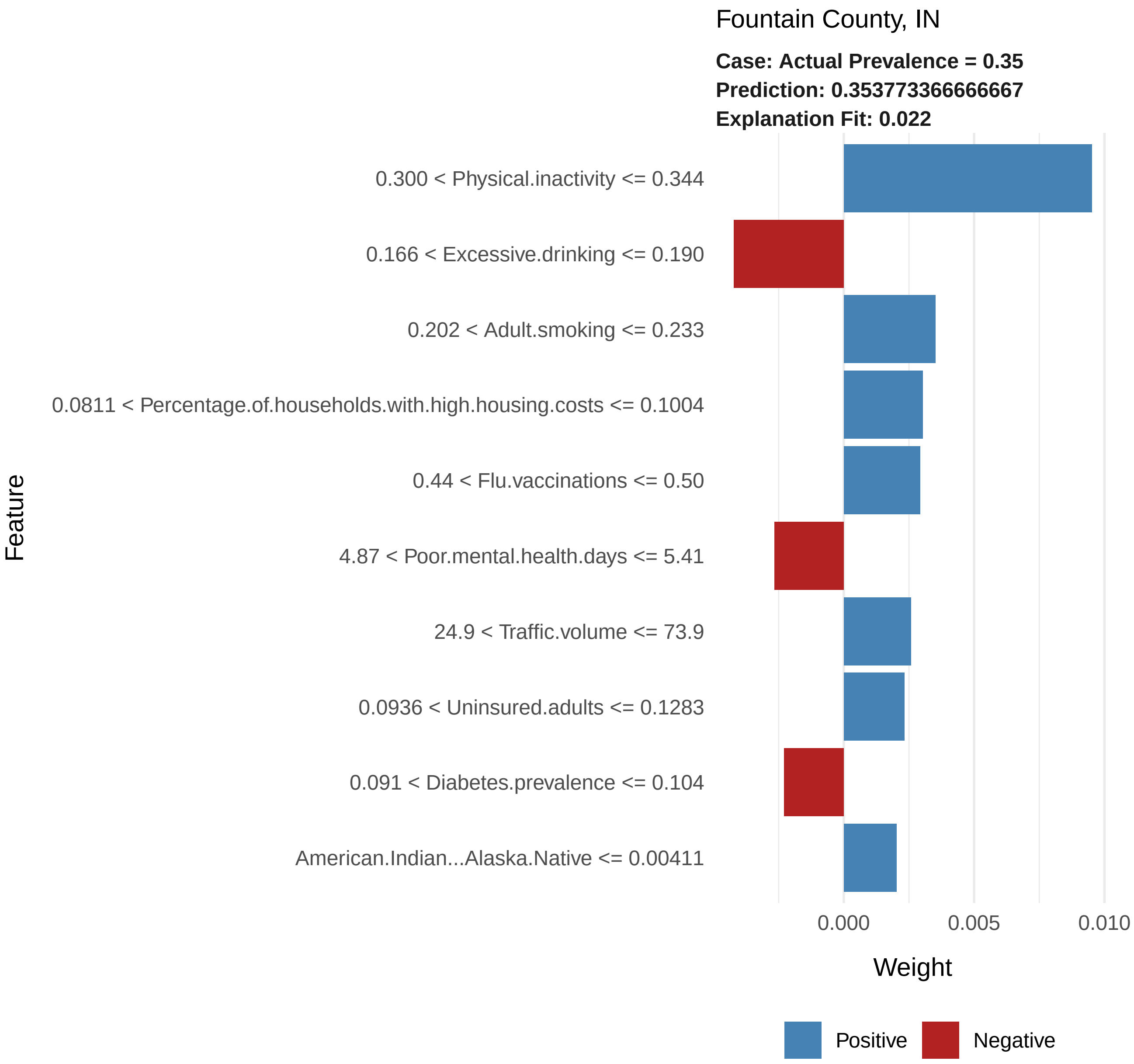
Fountain County, IN
Case: Actual Prevalence = 0.35
Prediction: 0.353773366666667
Explanation Fit: 0.022
0.300 < Physical.inactivity <= 0.344
0.166 < Excessive.drinking <= 0.190
0.202 < Adult.smoking <= 0.233
0.0811 < Percentage.of.households.with.high.housing.costs <= 0.1004
0.44 < Flu.vaccinations <= 0.50
4.87 < Poor.mental.health.days <= 5.41
24.9 < Traffic.volume <= 73.9
0.0936 < Uninsured.adults <= 0.1283
0.091 < Diabetes.prevalence <= 0.104
American.Indian...Alaska.Native <= 0.00411
Feature
0.000
0.005
0.010
Weight
Positive
Negative

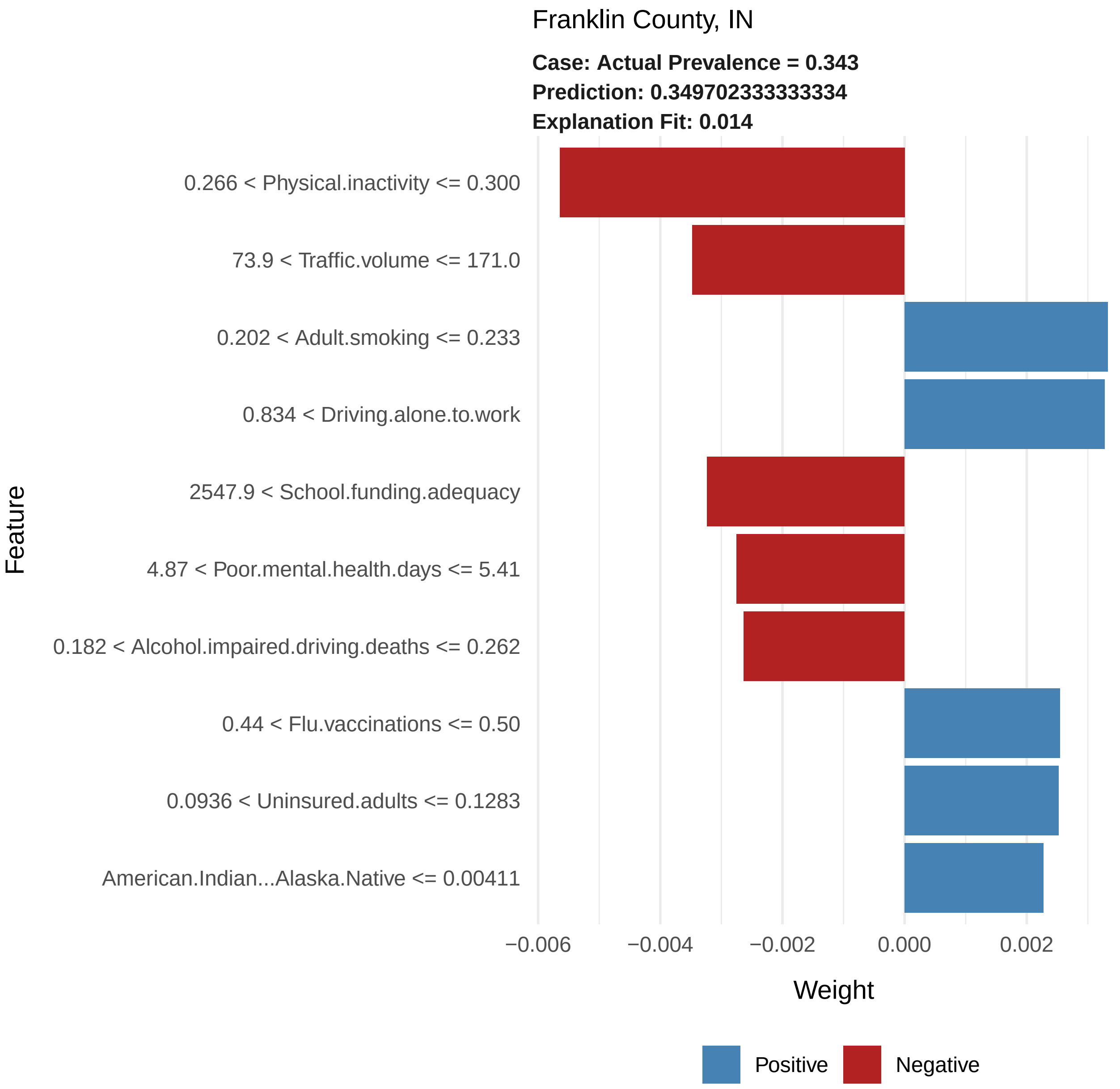

Franklin County, IN
Case: Actual Prevalence = 0.343
Prediction: 0.349702333333334
Explanation Fit: 0.014
0.266 < Physical.inactivity <= 0.300
73.9 < Traffic.volume <= 171.0
0.202 < Adult.smoking <= 0.233
0.834 < Driving.alone.to.work
2547.9 < School.funding.adequacy
4.87 < Poor.mental.health.days <= 5.41
0.182 < Alcohol.impaired.driving.deaths <= 0.262
0.44 < Flu.vaccinations <= 0.50
0.0936 < Uninsured.adults <= 0.1283
American.Indian...Alaska.Native <= 0.00411
Feature
−0.006
−0.004
−0.002
0.000
0.002
Weight
Positive
Negative

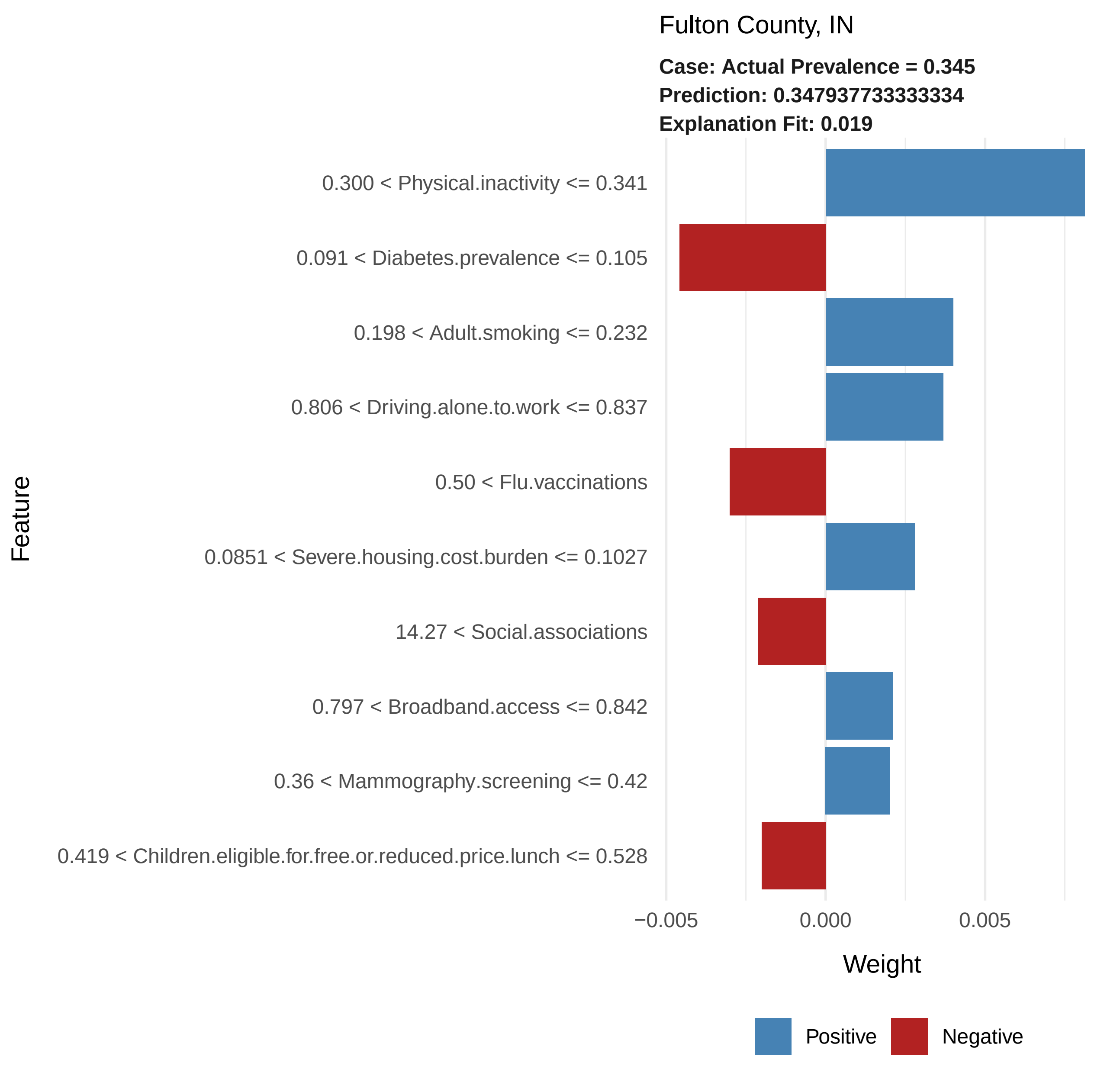

Fulton County, IN
Case: Actual Prevalence = 0.345
Prediction: 0.347937733333334
Explanation Fit: 0.019
0.300 < Physical.inactivity <= 0.341
0.091 < Diabetes.prevalence <= 0.105
0.198 < Adult.smoking <= 0.232
0.806 < Driving.alone.to.work <= 0.837
0.50 < Flu.vaccinations
0.0851 < Severe.housing.cost.burden <= 0.1027
14.27 < Social.associations
0.797 < Broadband.access <= 0.842
0.36 < Mammography.screening <= 0.42
0.419 < Children.eligible.for.free.or.reduced.price.lunch <= 0.528
Feature
−0.005
0.000
0.005
Weight
Positive
Negative

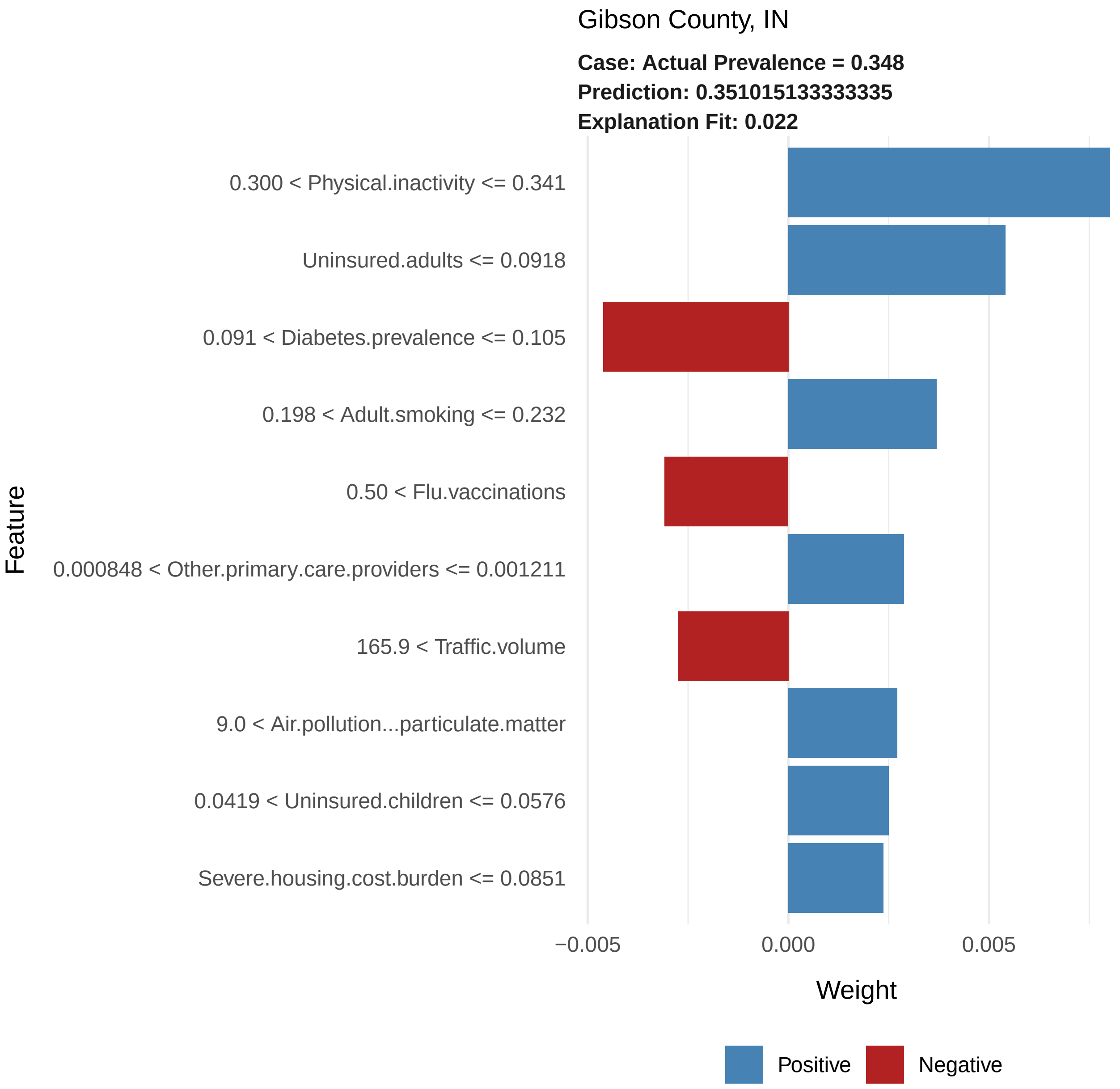
Gibson County, IN
Case: Actual Prevalence = 0.348
Prediction: 0.351015133333335
Explanation Fit: 0.022
0.300 < Physical.inactivity <= 0.341
Uninsured.adults <= 0.0918
0.091 < Diabetes.prevalence <= 0.105
0.198 < Adult.smoking <= 0.232
0.50 < Flu.vaccinations
0.000848 < Other.primary.care.providers <= 0.001211
165.9 < Traffic.volume
9.0 < Air.pollution...particulate.matter
0.0419 < Uninsured.children <= 0.0576
Severe.housing.cost.burden <= 0.0851
Feature
−0.005
0.000
0.005
Weight
Positive
Negative

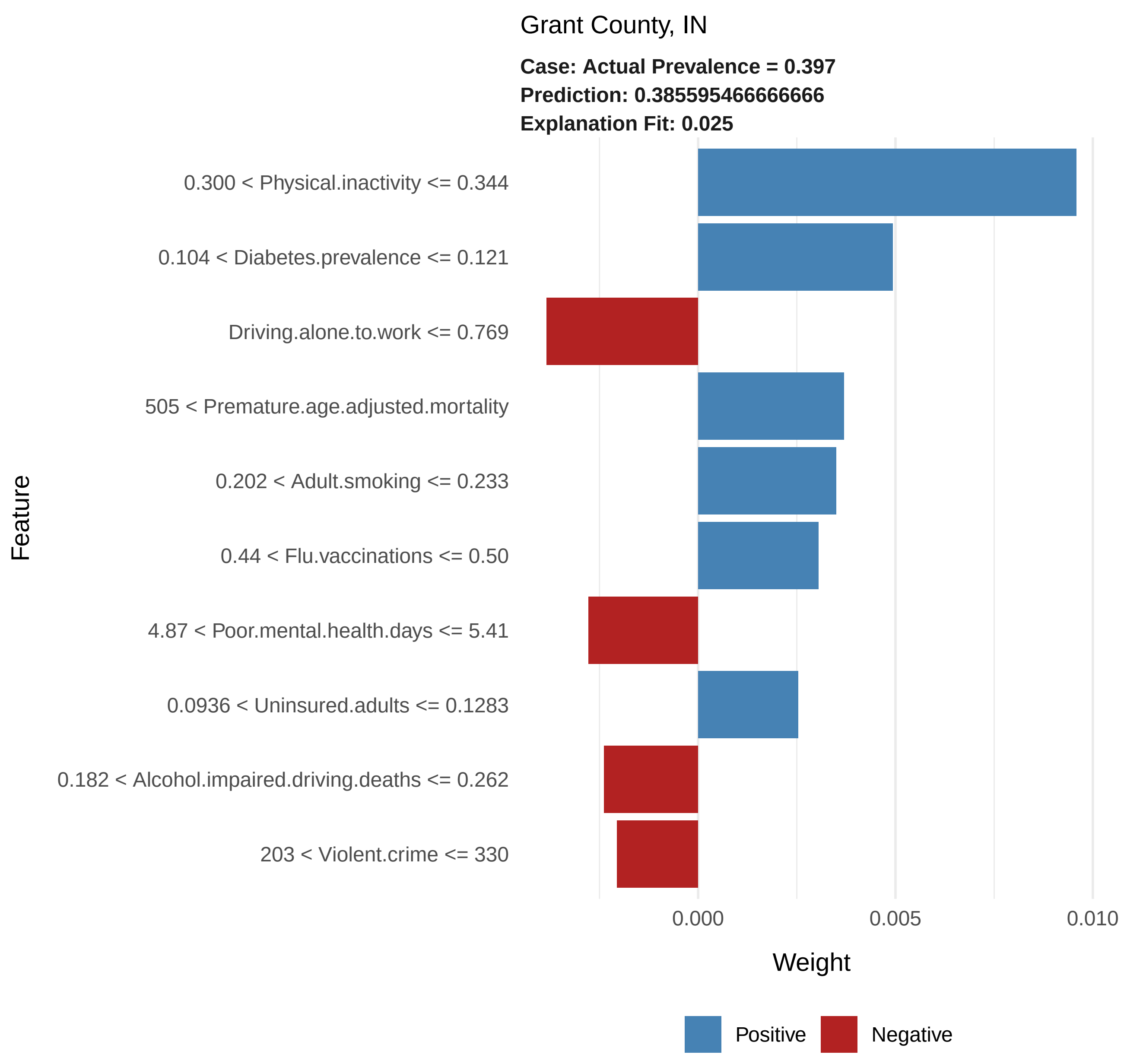

Grant County, IN
Case: Actual Prevalence = 0.397
Prediction: 0.385595466666666
Explanation Fit: 0.025
0.300 < Physical.inactivity <= 0.344
0.104 < Diabetes.prevalence <= 0.121
Driving.alone.to.work <= 0.769
505 < Premature.age.adjusted.mortality
0.202 < Adult.smoking <= 0.233
0.44 < Flu.vaccinations <= 0.50
4.87 < Poor.mental.health.days <= 5.41
0.0936 < Uninsured.adults <= 0.1283
0.182 < Alcohol.impaired.driving.deaths <= 0.262
203 < Violent.crime <= 330
Feature
0.000
0.005
0.010
Weight
Positive
Negative

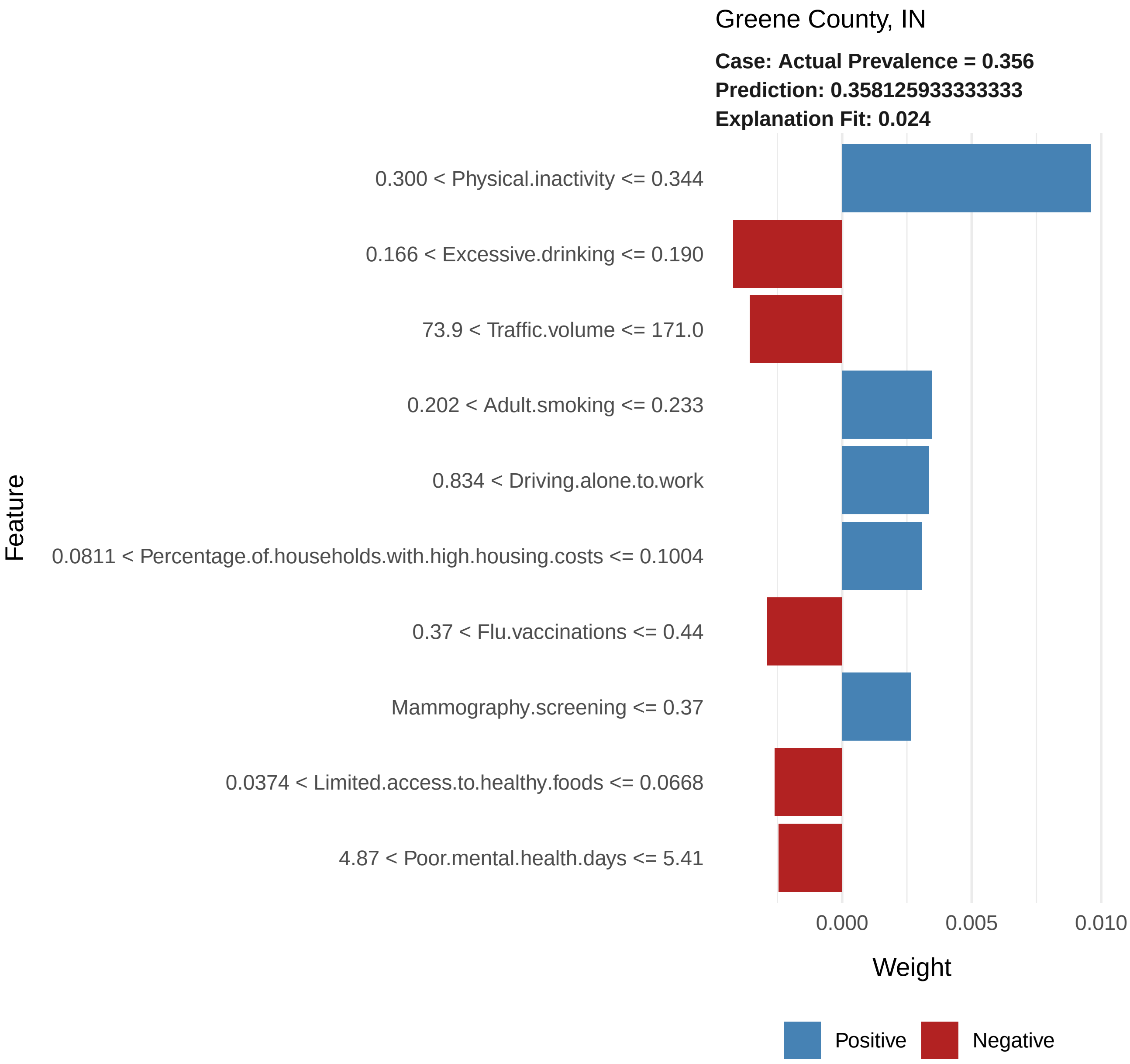
Greene County, IN
Case: Actual Prevalence = 0.356
Prediction: 0.358125933333333
Explanation Fit: 0.024
0.300 < Physical.inactivity <= 0.344
0.166 < Excessive.drinking <= 0.190
73.9 < Traffic.volume <= 171.0
0.202 < Adult.smoking <= 0.233
0.834 < Driving.alone.to.work
0.0811 < Percentage.of.households.with.high.housing.costs <= 0.1004
0.37 < Flu.vaccinations <= 0.44
Mammography.screening <= 0.37
0.0374 < Limited.access.to.healthy.foods <= 0.0668
4.87 < Poor.mental.health.days <= 5.41
Feature
0.000
0.005
0.010
Weight
Positive
Negative

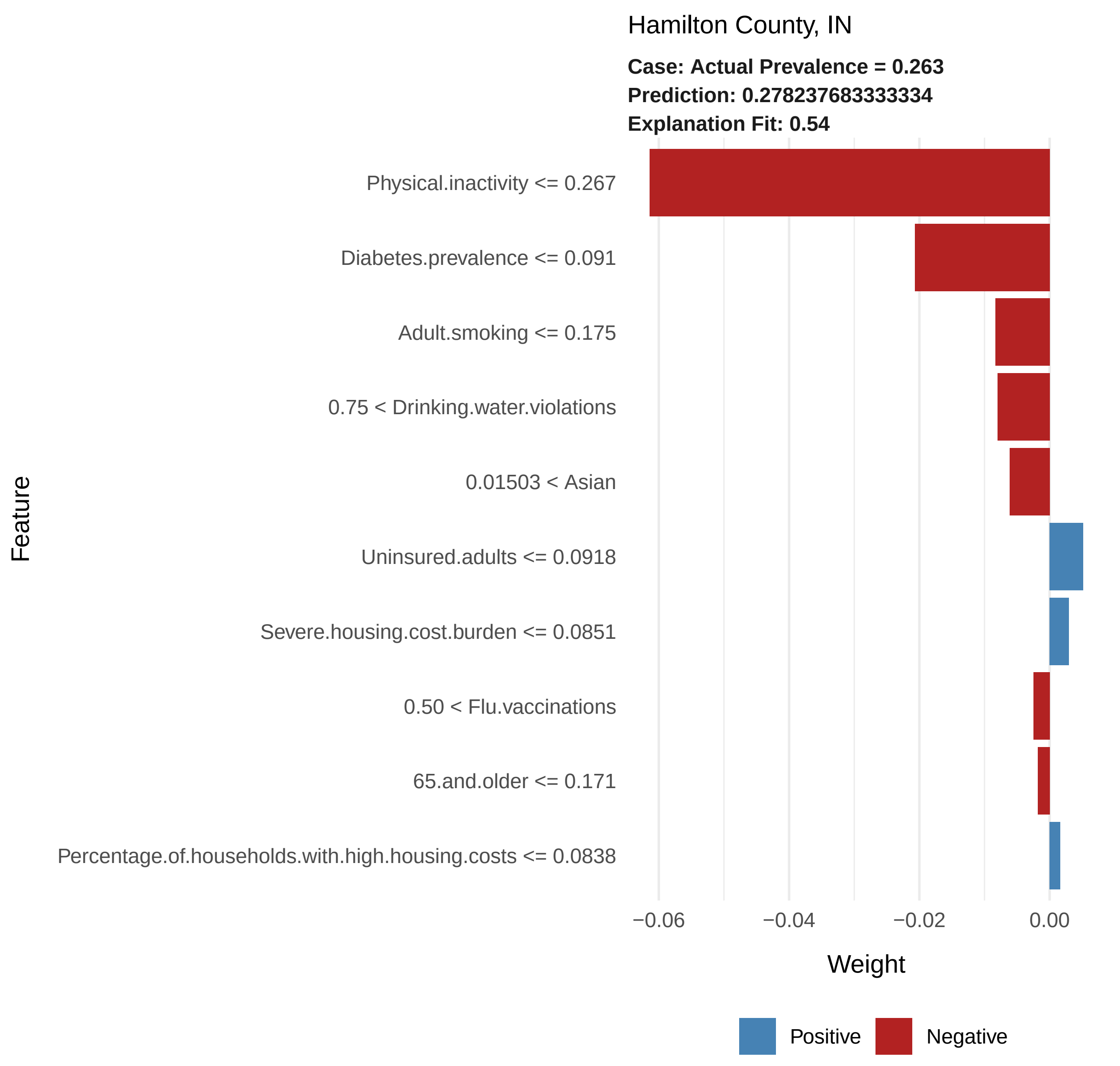

Hamilton County, IN
Case: Actual Prevalence = 0.263
Prediction: 0.278237683333334
Explanation Fit: 0.54
Physical.inactivity <= 0.267
Diabetes.prevalence <= 0.091
Adult.smoking <= 0.175
0.75 < Drinking.water.violations
0.01503 < Asian
Uninsured.adults <= 0.0918
Severe.housing.cost.burden <= 0.0851
0.50 < Flu.vaccinations
65.and.older <= 0.171
Percentage.of.households.with.high.housing.costs <= 0.0838
Feature
−0.06
−0.04
−0.02
0.00
Weight
Positive
Negative

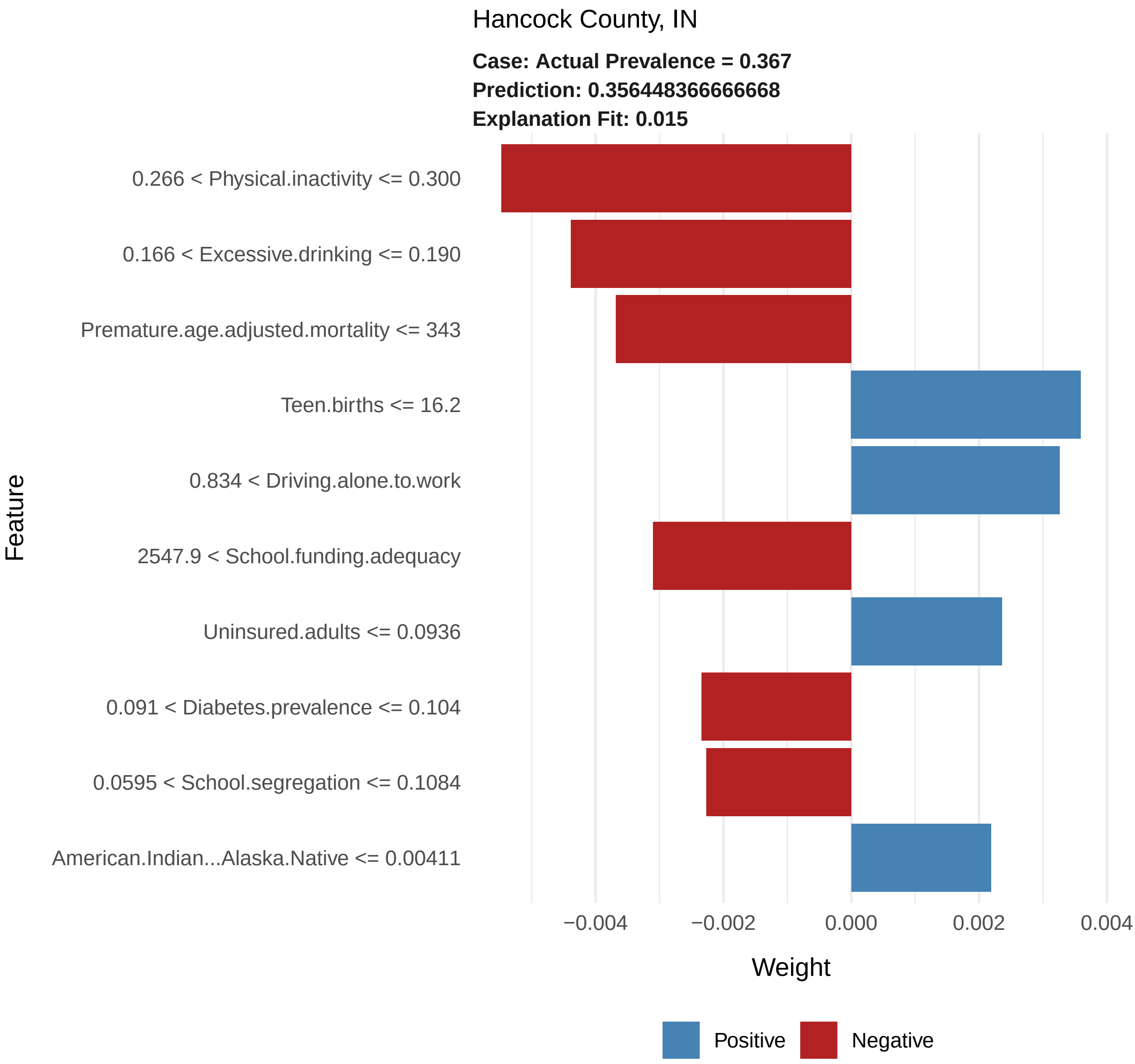

Hancock County, IN
Case: Actual Prevalence = 0.367
Prediction: 0.356448366666668
Explanation Fit: 0.015
0.266 < Physical.inactivity <= 0.300
0.166 < Excessive.drinking <= 0.190
Premature.age.adjusted.mortality <= 343
Teen.births <= 16.2
0.834 < Driving.alone.to.work
2547.9 < School.funding.adequacy
Uninsured.adults <= 0.0936
0.091 < Diabetes.prevalence <= 0.104
0.0595 < School.segregation <= 0.1084
American.Indian...Alaska.Native <= 0.00411
Feature
−0.004
−0.002
0.000
0.002
0.004
Weight
Positive
Negative

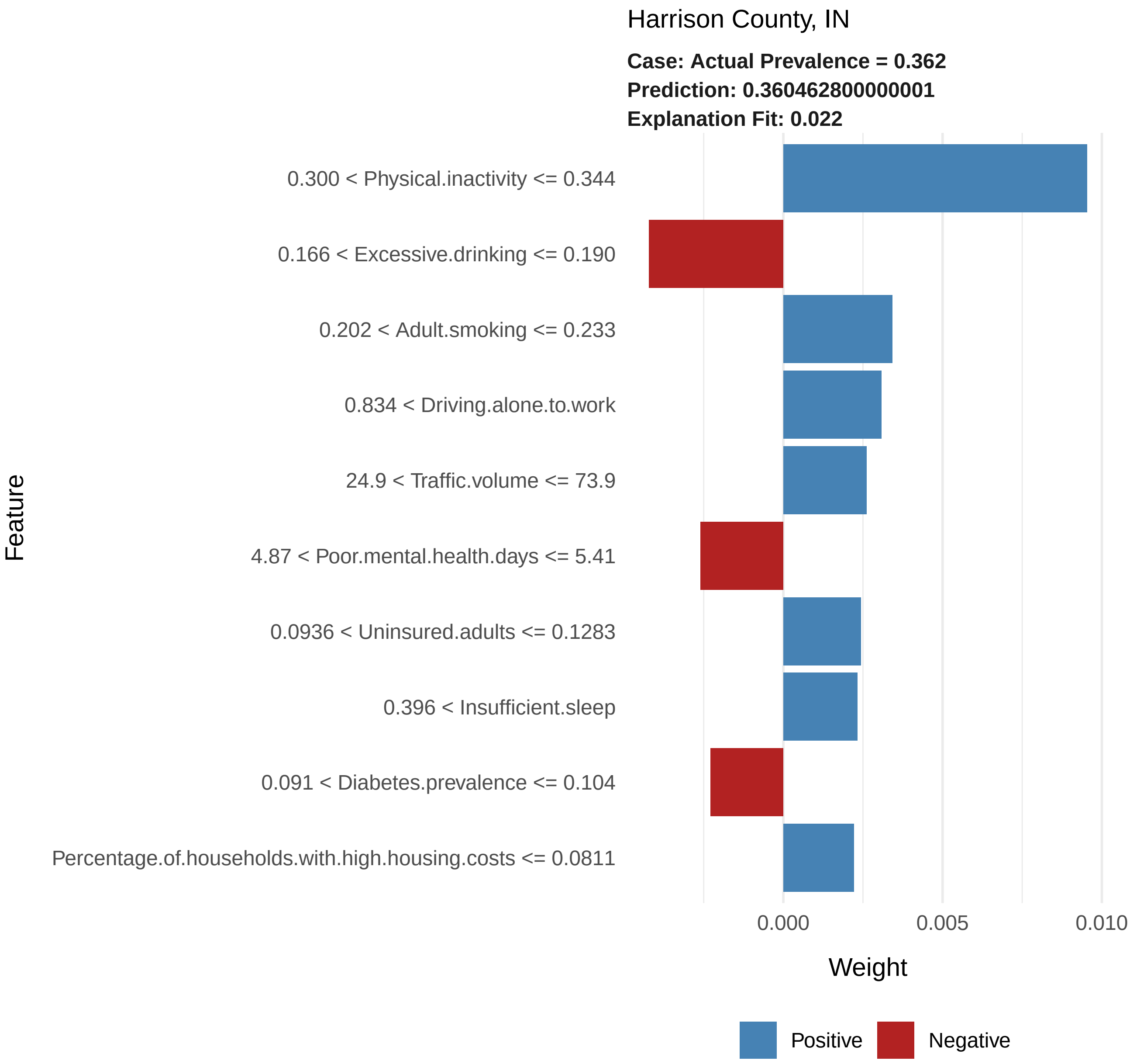

Harrison County, IN
Case: Actual Prevalence = 0.362
Prediction: 0.360462800000001
Explanation Fit: 0.022
0.300 < Physical.inactivity <= 0.344
0.166 < Excessive.drinking <= 0.190
0.202 < Adult.smoking <= 0.233
0.834 < Driving.alone.to.work
24.9 < Traffic.volume <= 73.9
4.87 < Poor.mental.health.days <= 5.41
0.0936 < Uninsured.adults <= 0.1283
0.396 < Insufficient.sleep
0.091 < Diabetes.prevalence <= 0.104
Percentage.of.households.with.high.housing.costs <= 0.0811
Feature
0.000
0.005
0.010
Weight
Positive
Negative

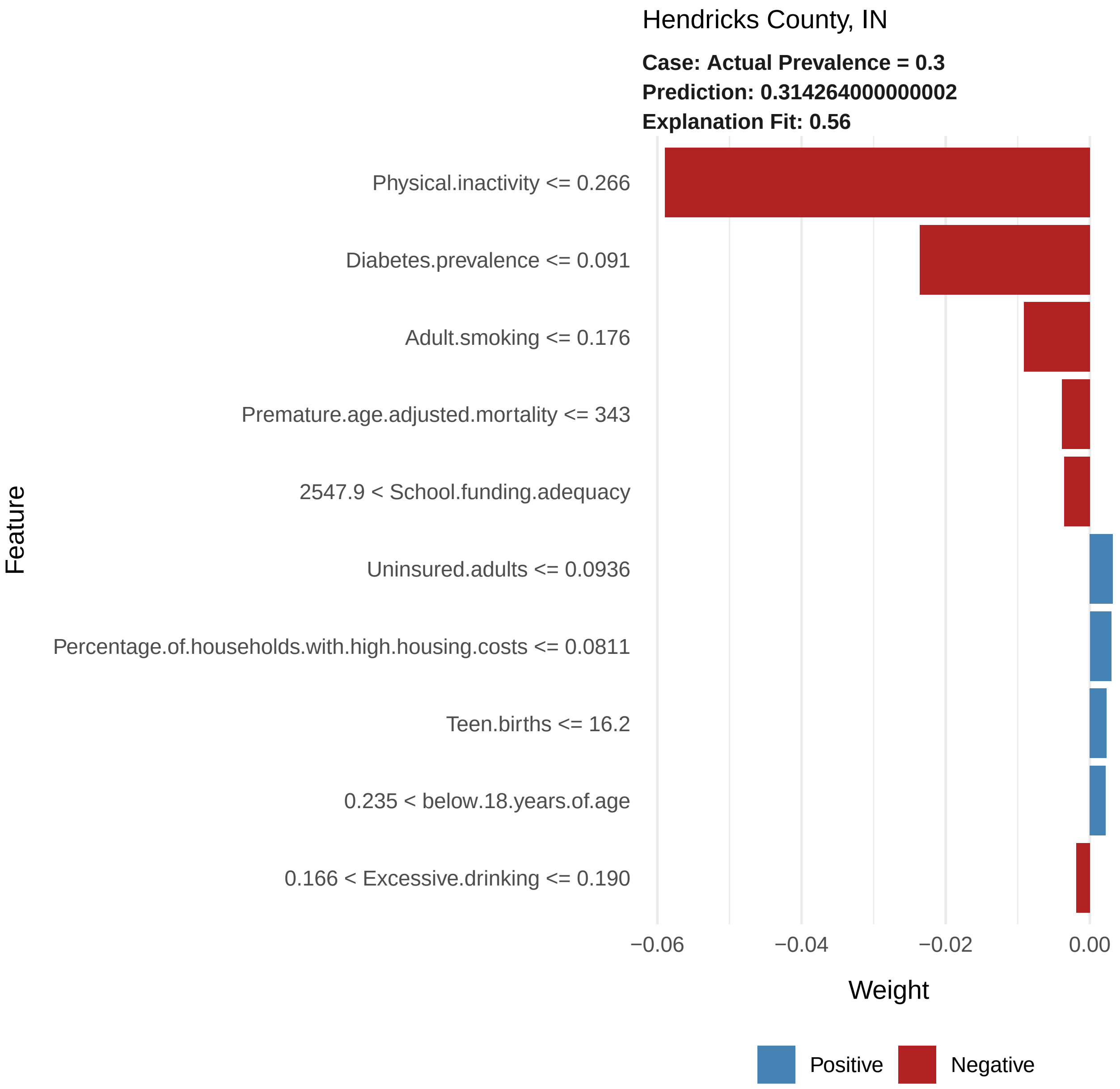
Hendricks County, IN
Case: Actual Prevalence = 0.3
Prediction: 0.314264000000002
Explanation Fit: 0.56
Physical.inactivity <= 0.266
Diabetes.prevalence <= 0.091
Adult.smoking <= 0.176
Premature.age.adjusted.mortality <= 343
2547.9 < School.funding.adequacy
Uninsured.adults <= 0.0936
Percentage.of.households.with.high.housing.costs <= 0.0811
Teen.births <= 16.2
0.235 < below.18.years.of.age
0.166 < Excessive.drinking <= 0.190
Feature
−0.06
−0.04
−0.02
0.00
Weight
Positive
Negative

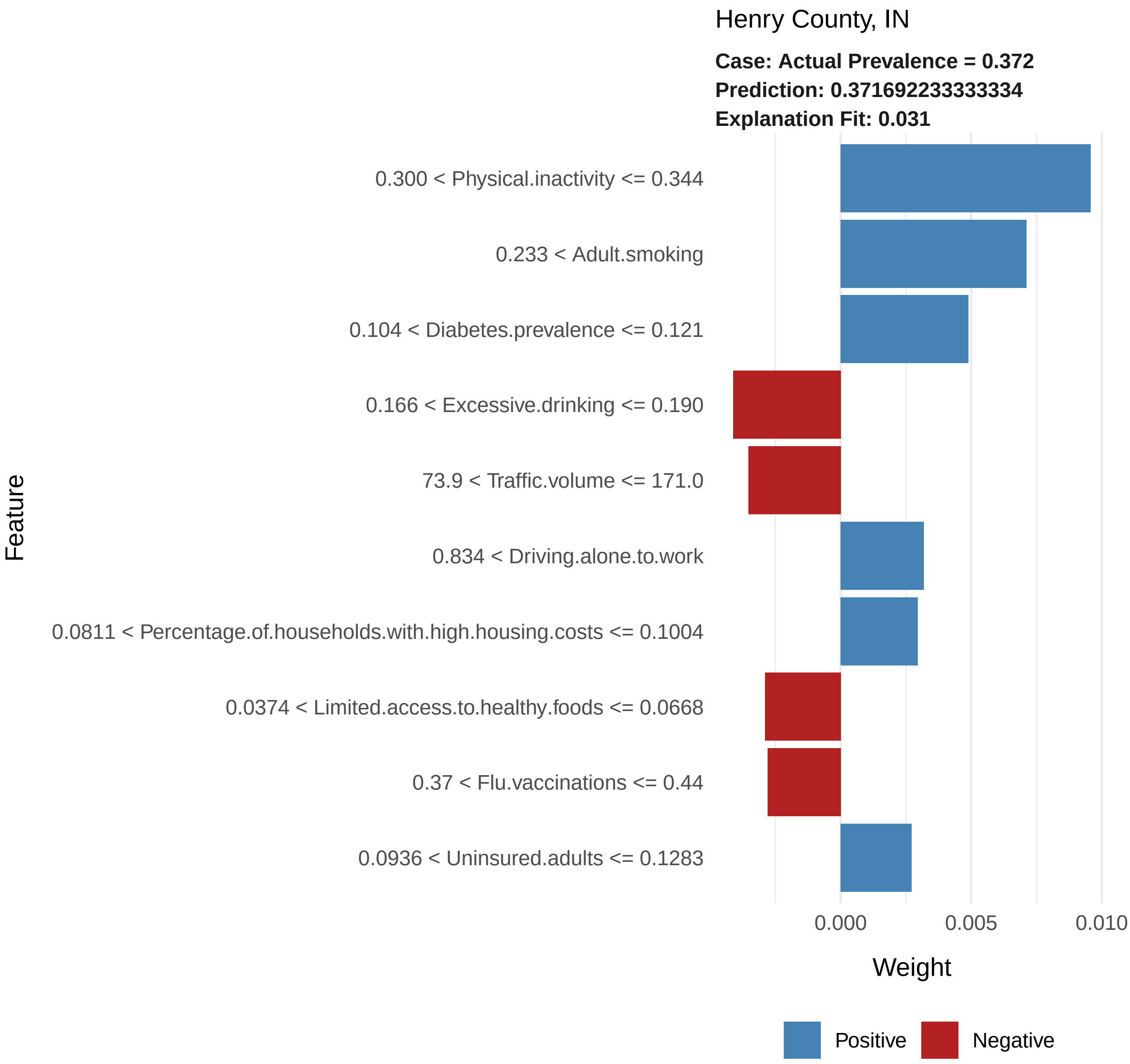

Henry County, IN
Case: Actual Prevalence = 0.372
Prediction: 0.371692233333334
Explanation Fit: 0.031
0.300 < Physical.inactivity <= 0.344
0.233 < Adult.smoking
0.104 < Diabetes.prevalence <= 0.121
0.166 < Excessive.drinking <= 0.190
73.9 < Traffic.volume <= 171.0
0.834 < Driving.alone.to.work
0.0811 < Percentage.of.households.with.high.housing.costs <= 0.1004
0.0374 < Limited.access.to.healthy.foods <= 0.0668
0.37 < Flu.vaccinations <= 0.44
0.0936 < Uninsured.adults <= 0.1283
Feature
0.000
0.005
0.010
Weight
Positive
Negative

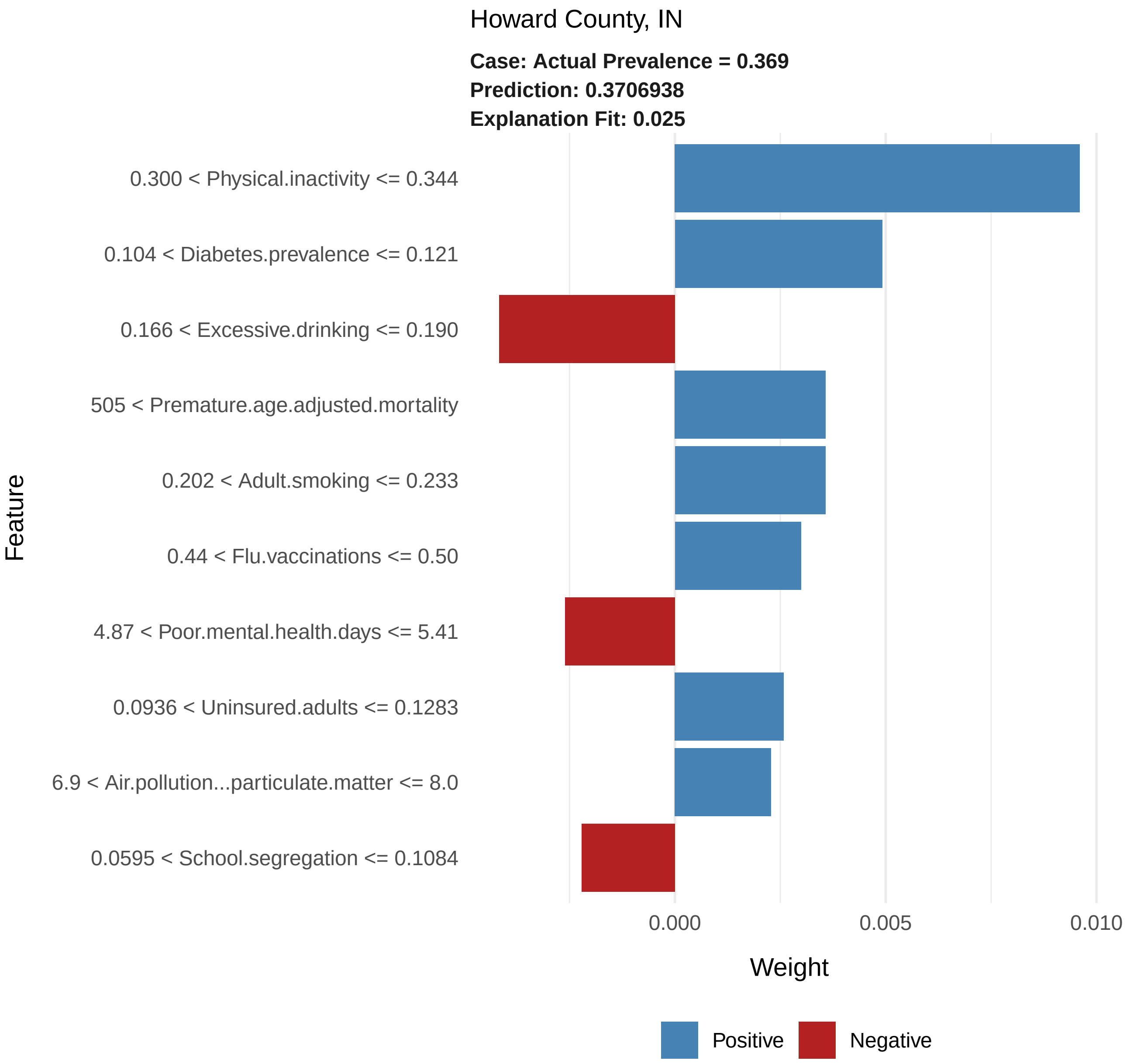
Howard County, IN
Case: Actual Prevalence = 0.369
Prediction: 0.3706938
Explanation Fit: 0.025
0.300 < Physical.inactivity <= 0.344
0.104 < Diabetes.prevalence <= 0.121
0.166 < Excessive.drinking <= 0.190
505 < Premature.age.adjusted.mortality
0.202 < Adult.smoking <= 0.233
0.44 < Flu.vaccinations <= 0.50
4.87 < Poor.mental.health.days <= 5.41
0.0936 < Uninsured.adults <= 0.1283
6.9 < Air.pollution...particulate.matter <= 8.0
0.0595 < School.segregation <= 0.1084
Feature
0.000
0.005
0.010
Weight
Positive
Negative

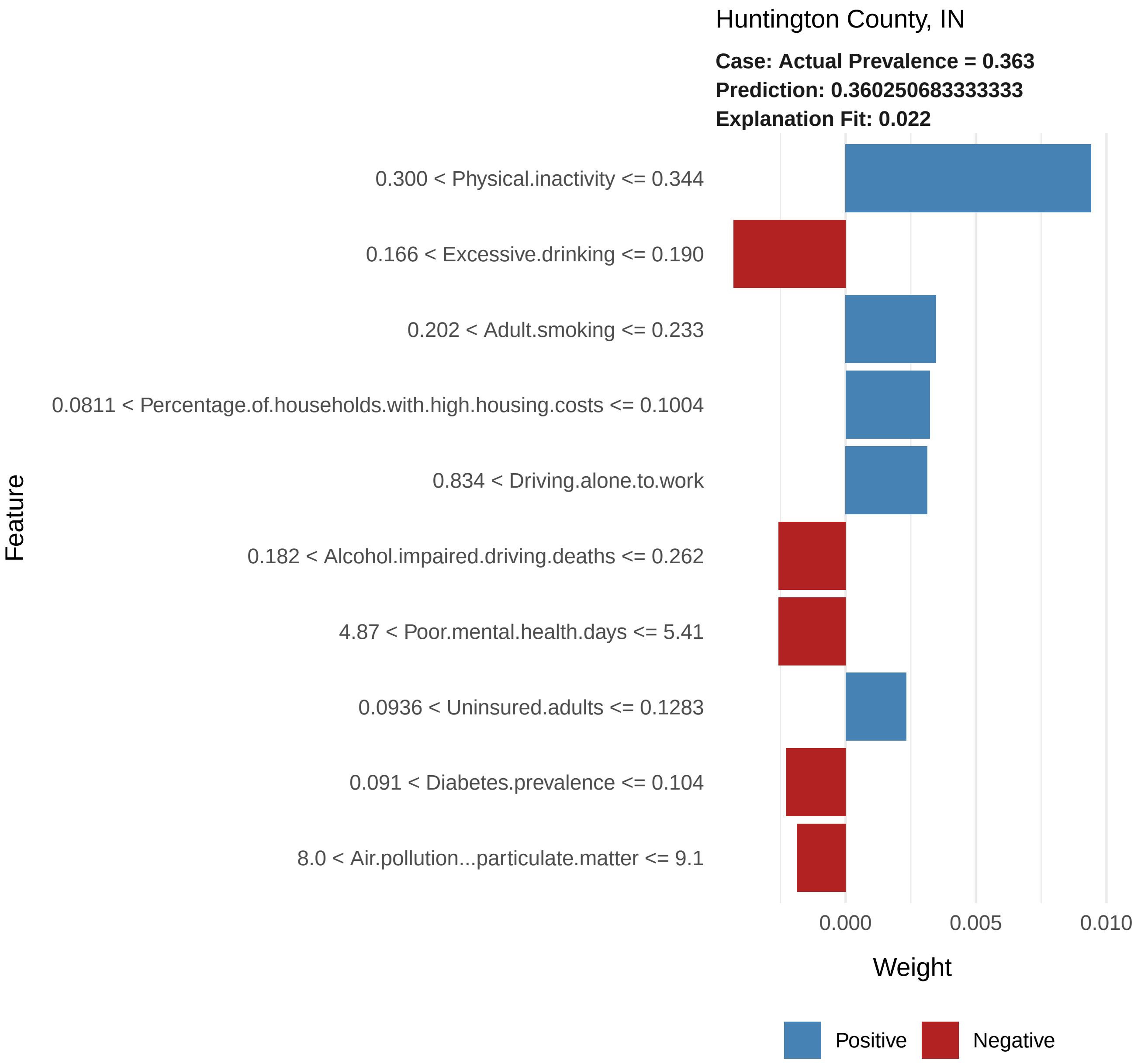
Huntington County, IN
Case: Actual Prevalence = 0.363
Prediction: 0.360250683333333
Explanation Fit: 0.022
0.300 < Physical.inactivity <= 0.344
0.166 < Excessive.drinking <= 0.190
0.202 < Adult.smoking <= 0.233
0.0811 < Percentage.of.households.with.high.housing.costs <= 0.1004
0.834 < Driving.alone.to.work
0.182 < Alcohol.impaired.driving.deaths <= 0.262
4.87 < Poor.mental.health.days <= 5.41
0.0936 < Uninsured.adults <= 0.1283
0.091 < Diabetes.prevalence <= 0.104
8.0 < Air.pollution...particulate.matter <= 9.1
Feature
0.000
0.005
0.010
Weight
Positive
Negative

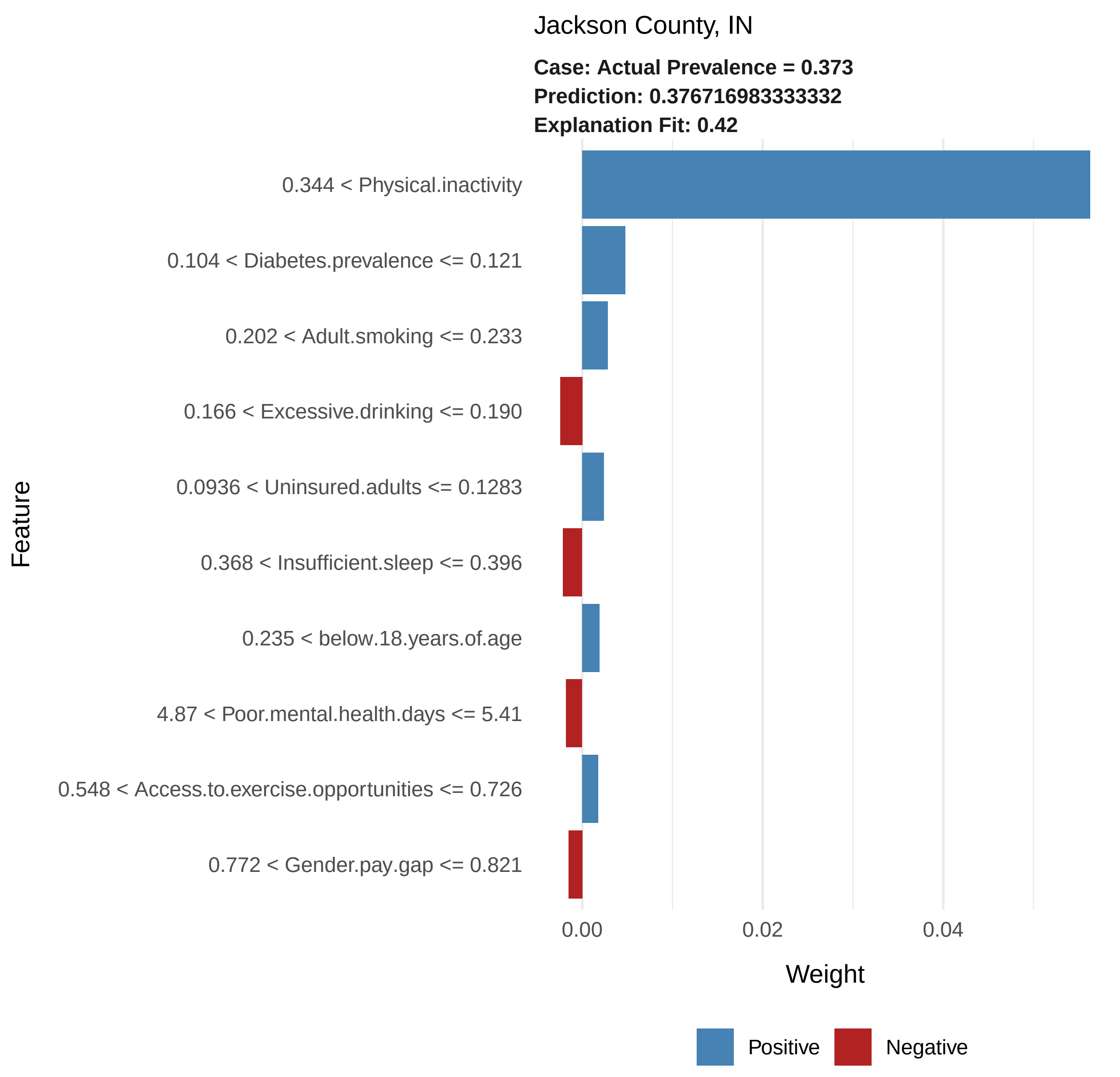
Jackson County, IN
Case: Actual Prevalence = 0.373
Prediction: 0.376716983333332
Explanation Fit: 0.42
0.344 < Physical.inactivity
0.104 < Diabetes.prevalence <= 0.121
0.202 < Adult.smoking <= 0.233
0.166 < Excessive.drinking <= 0.190
0.0936 < Uninsured.adults <= 0.1283
0.368 < Insufficient.sleep <= 0.396
0.235 < below.18.years.of.age
4.87 < Poor.mental.health.days <= 5.41
0.548 < Access.to.exercise.opportunities <= 0.726
0.772 < Gender.pay.gap <= 0.821
Feature
0.00
0.02
0.04
Weight
Positive
Negative

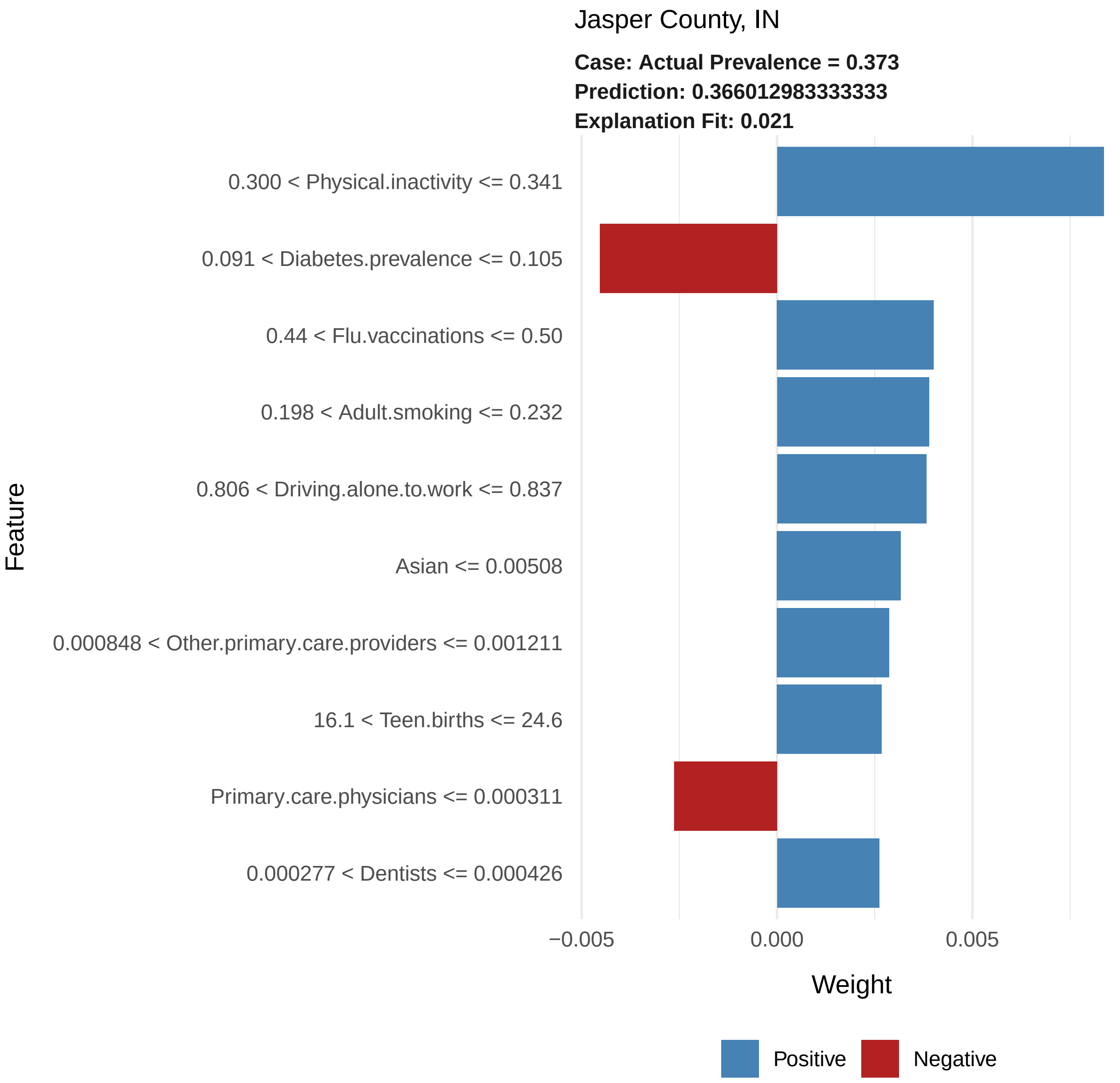
Jasper County, IN
Case: Actual Prevalence = 0.373
Prediction: 0.366012983333333
Explanation Fit: 0.021
0.300 < Physical.inactivity <= 0.341
0.091 < Diabetes.prevalence <= 0.105
0.44 < Flu.vaccinations <= 0.50
0.198 < Adult.smoking <= 0.232
0.806 < Driving.alone.to.work <= 0.837
Asian <= 0.00508
0.000848 < Other.primary.care.providers <= 0.001211
16.1 < Teen.births <= 24.6
Primary.care.physicians <= 0.000311
0.000277 < Dentists <= 0.000426
Feature
−0.005
0.000
0.005
Weight
Positive
Negative

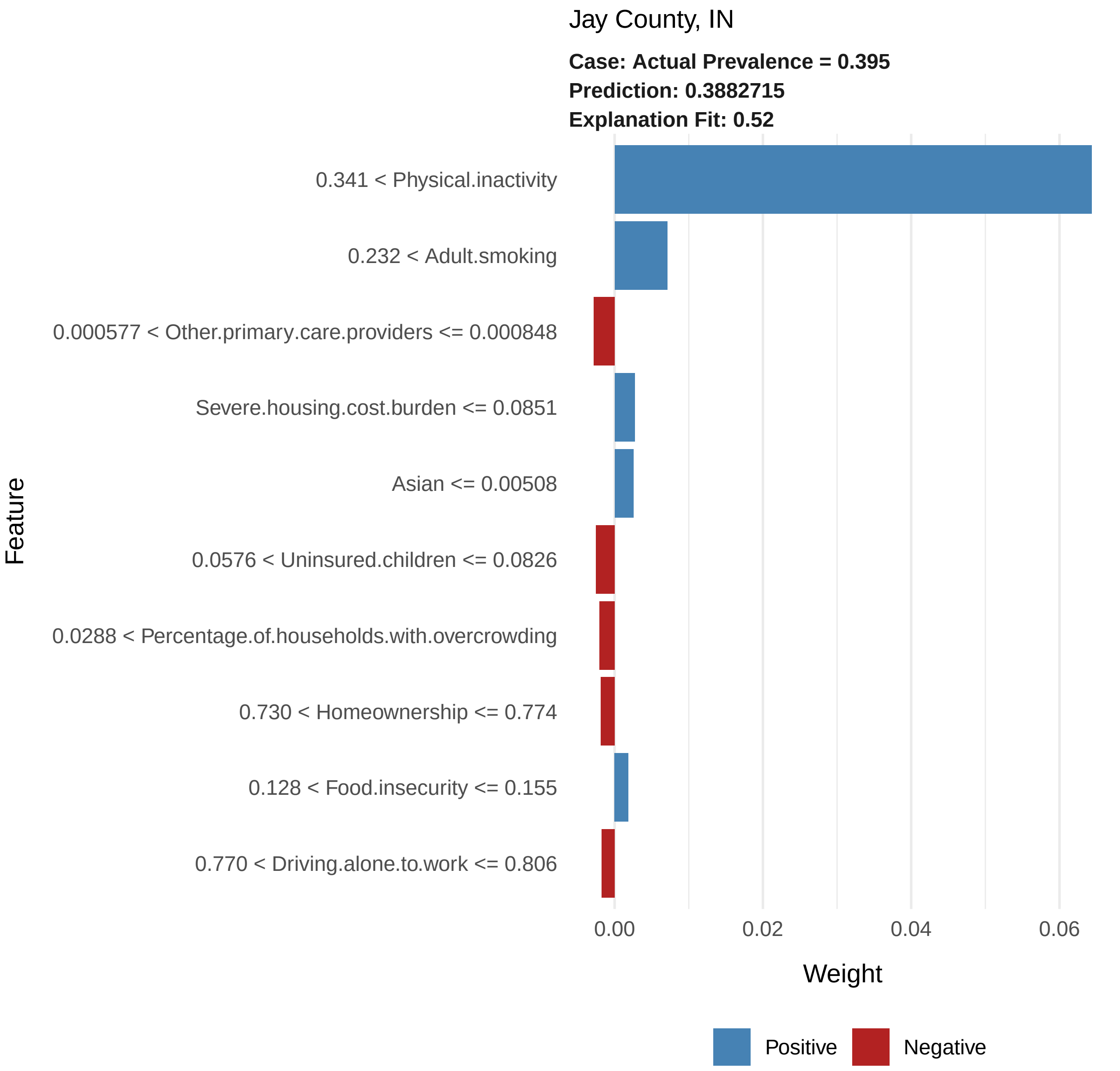

Jay County, IN
Case: Actual Prevalence = 0.395
Prediction: 0.3882715
Explanation Fit: 0.52
0.341 < Physical.inactivity
0.232 < Adult.smoking
0.000577 < Other.primary.care.providers <= 0.000848
Severe.housing.cost.burden <= 0.0851
Asian <= 0.00508
0.0576 < Uninsured.children <= 0.0826
0.0288 < Percentage.of.households.with.overcrowding
0.730 < Homeownership <= 0.774
0.128 < Food.insecurity <= 0.155
0.770 < Driving.alone.to.work <= 0.806
Feature
0.00
0.02
0.04
0.06
Weight
Positive
Negative

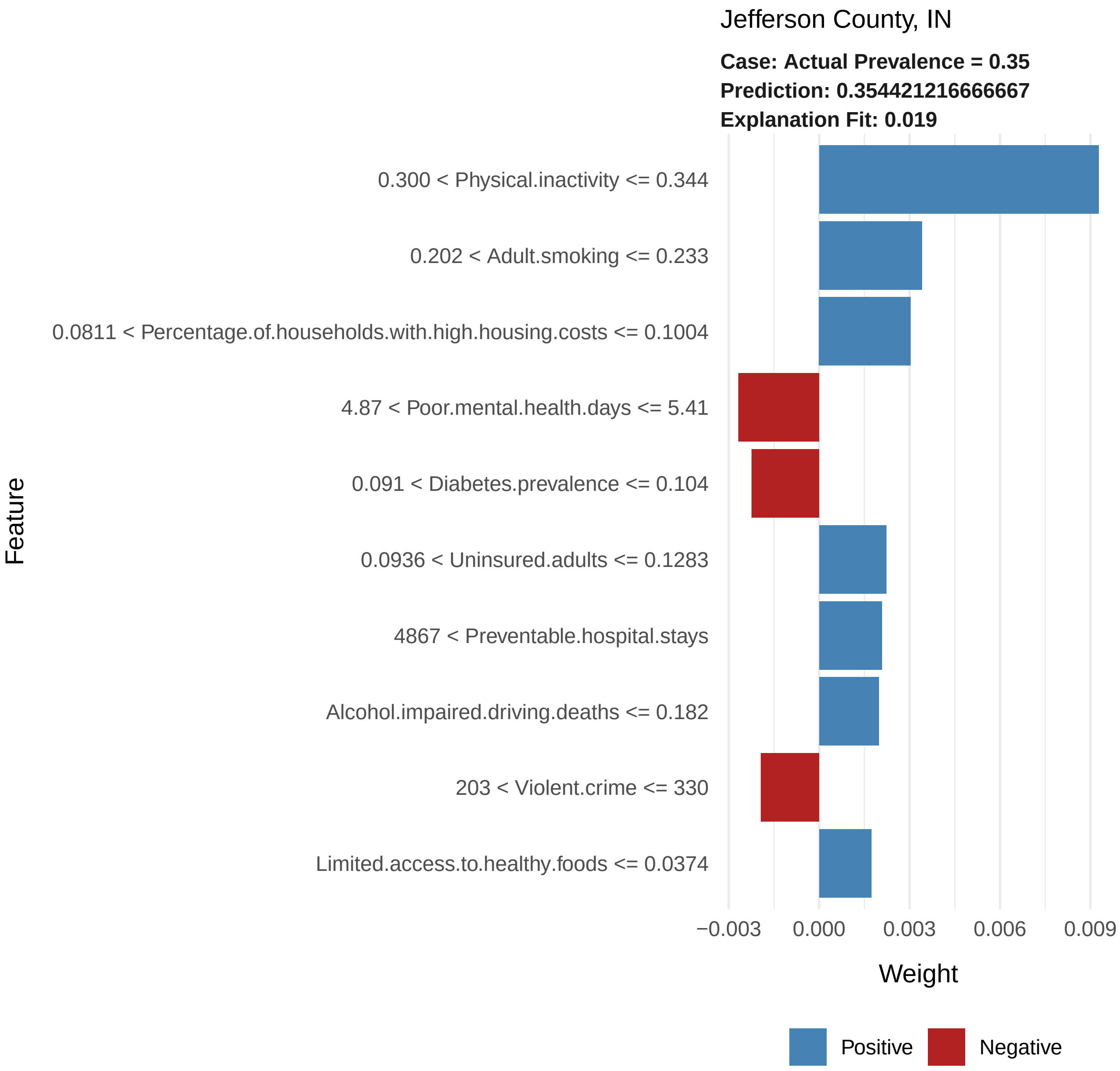

Jefferson County, IN
Case: Actual Prevalence = 0.35
Prediction: 0.354421216666667
Explanation Fit: 0.019
0.300 < Physical.inactivity <= 0.344
0.202 < Adult.smoking <= 0.233
0.0811 < Percentage.of.households.with.high.housing.costs <= 0.1004
4.87 < Poor.mental.health.days <= 5.41
0.091 < Diabetes.prevalence <= 0.104
0.0936 < Uninsured.adults <= 0.1283
4867 < Preventable.hospital.stays
Alcohol.impaired.driving.deaths <= 0.182
203 < Violent.crime <= 330
Limited.access.to.healthy.foods <= 0.0374
Feature
−0.003
0.000
0.003
0.006
0.009
Weight
Positive
Negative

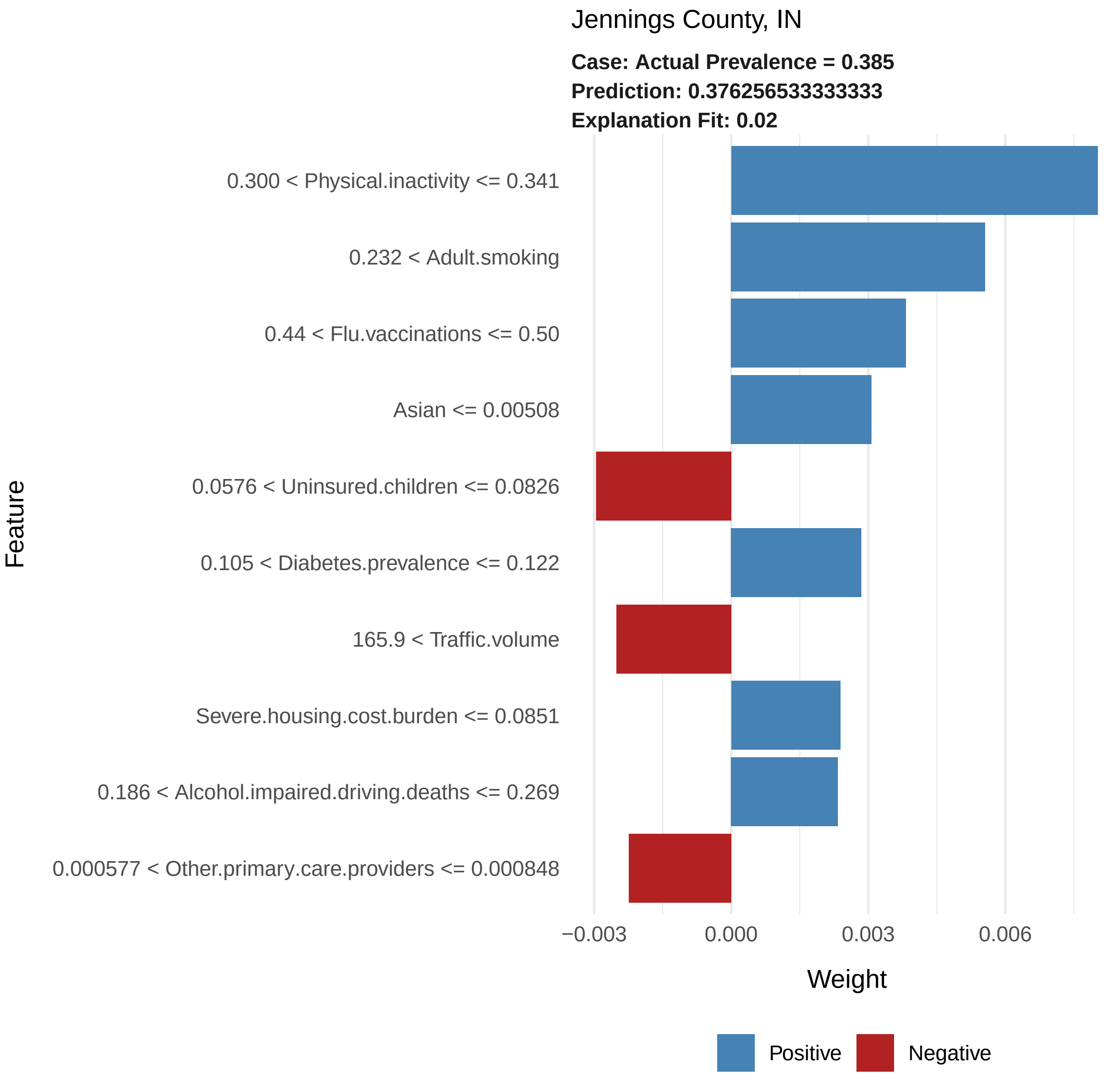

Jennings County, IN
Case: Actual Prevalence = 0.385
Prediction: 0.376256533333333
Explanation Fit: 0.02
0.300 < Physical.inactivity <= 0.341
0.232 < Adult.smoking
0.44 < Flu.vaccinations <= 0.50
Asian <= 0.00508
0.0576 < Uninsured.children <= 0.0826
0.105 < Diabetes.prevalence <= 0.122
165.9 < Traffic.volume
Severe.housing.cost.burden <= 0.0851
0.186 < Alcohol.impaired.driving.deaths <= 0.269
0.000577 < Other.primary.care.providers <= 0.000848
Feature
−0.003
0.000
0.003
0.006
Weight
Positive
Negative

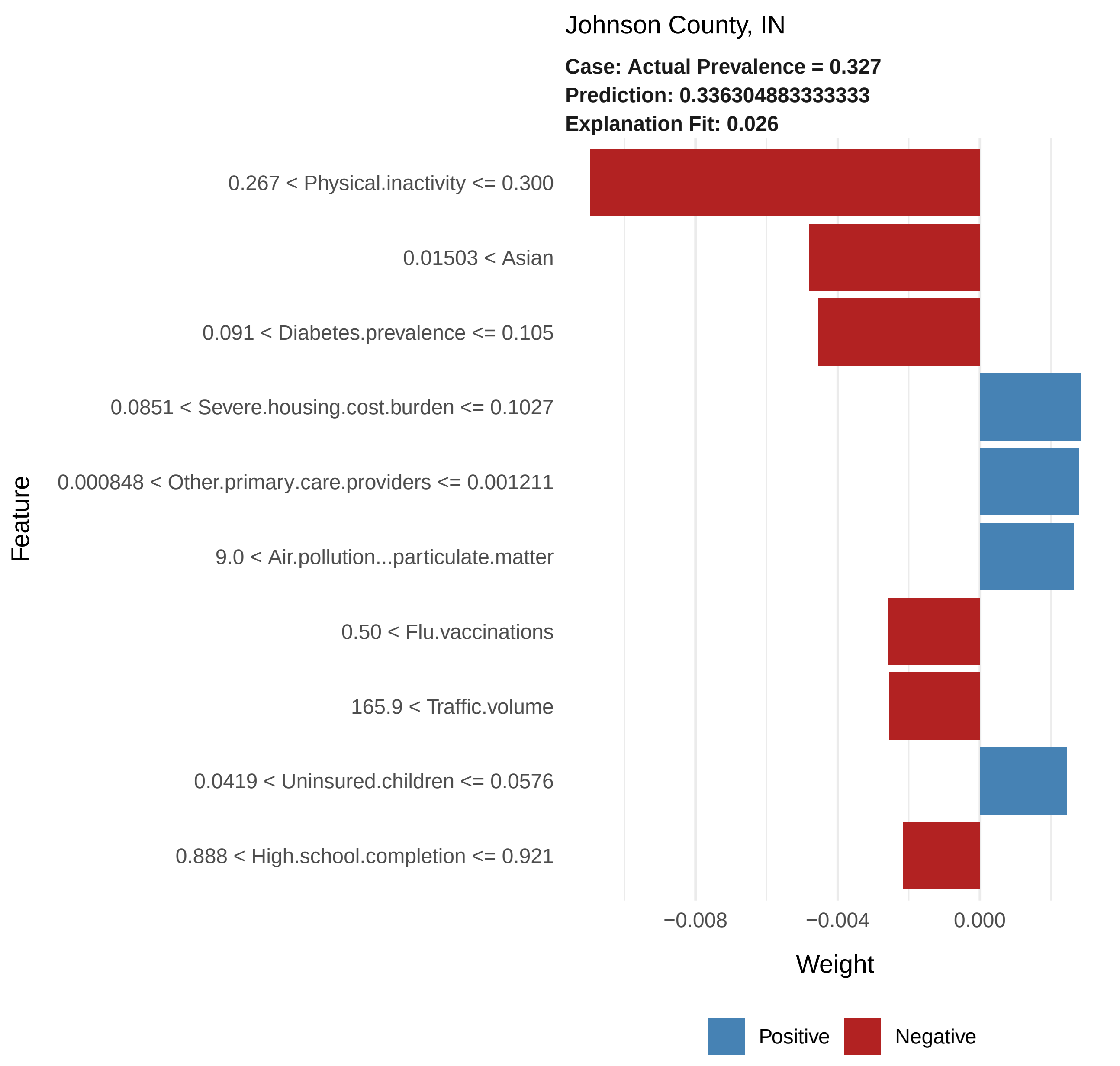
Johnson County, IN
Case: Actual Prevalence = 0.327
Prediction: 0.336304883333333
Explanation Fit: 0.026
0.267 < Physical.inactivity <= 0.300
0.01503 < Asian
0.091 < Diabetes.prevalence <= 0.105
0.0851 < Severe.housing.cost.burden <= 0.1027
0.000848 < Other.primary.care.providers <= 0.001211
9.0 < Air.pollution...particulate.matter
0.50 < Flu.vaccinations
165.9 < Traffic.volume
0.0419 < Uninsured.children <= 0.0576
0.888 < High.school.completion <= 0.921
Feature
−0.008
−0.004
0.000
Weight
Positive
Negative

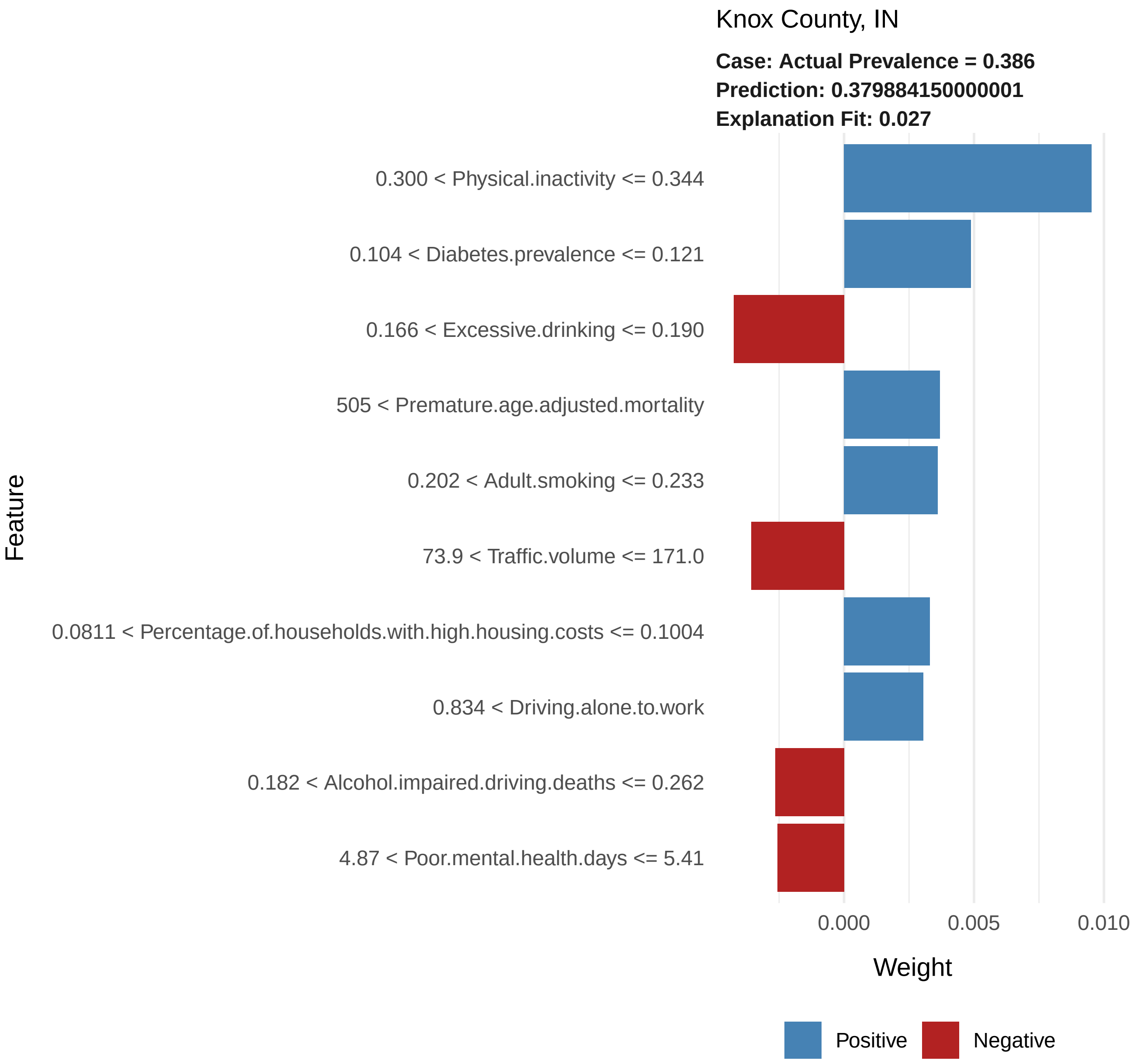
Knox County, IN
Case: Actual Prevalence = 0.386
Prediction: 0.379884150000001
Explanation Fit: 0.027
0.300 < Physical.inactivity <= 0.344
0.104 < Diabetes.prevalence <= 0.121
0.166 < Excessive.drinking <= 0.190
505 < Premature.age.adjusted.mortality
0.202 < Adult.smoking <= 0.233
73.9 < Traffic.volume <= 171.0
0.0811 < Percentage.of.households.with.high.housing.costs <= 0.1004
0.834 < Driving.alone.to.work
0.182 < Alcohol.impaired.driving.deaths <= 0.262
4.87 < Poor.mental.health.days <= 5.41
Feature
0.000
0.005
0.010
Weight
Positive
Negative

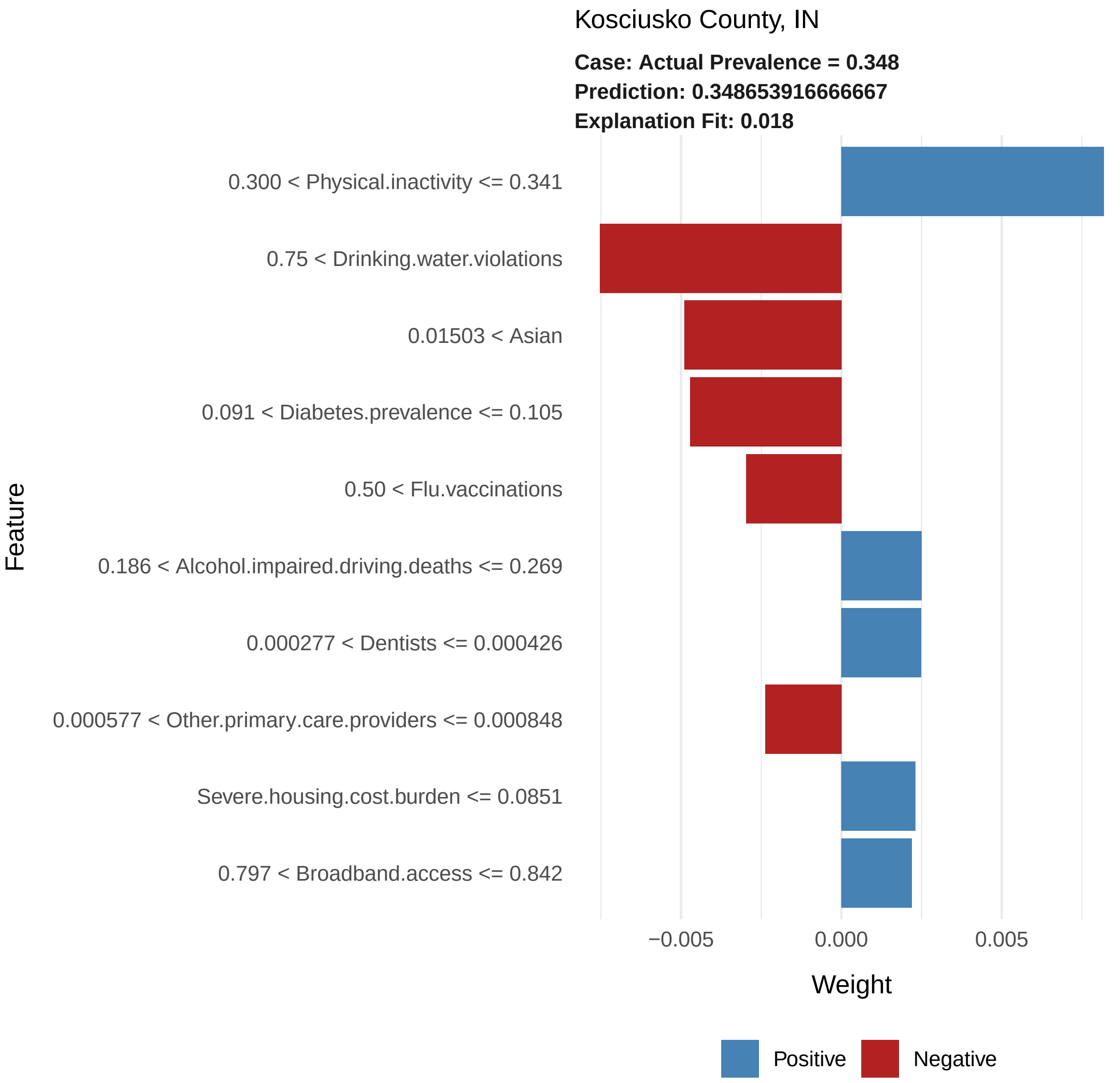
Kosciusko County, IN
Case: Actual Prevalence = 0.348
Prediction: 0.348653916666667
Explanation Fit: 0.018
0.300 < Physical.inactivity <= 0.341
0.75 < Drinking.water.violations
0.01503 < Asian
0.091 < Diabetes.prevalence <= 0.105
0.50 < Flu.vaccinations
0.186 < Alcohol.impaired.driving.deaths <= 0.269
0.000277 < Dentists <= 0.000426
0.000577 < Other.primary.care.providers <= 0.000848
Severe.housing.cost.burden <= 0.0851
0.797 < Broadband.access <= 0.842
Feature
−0.005
0.000
0.005
Weight
Positive
Negative

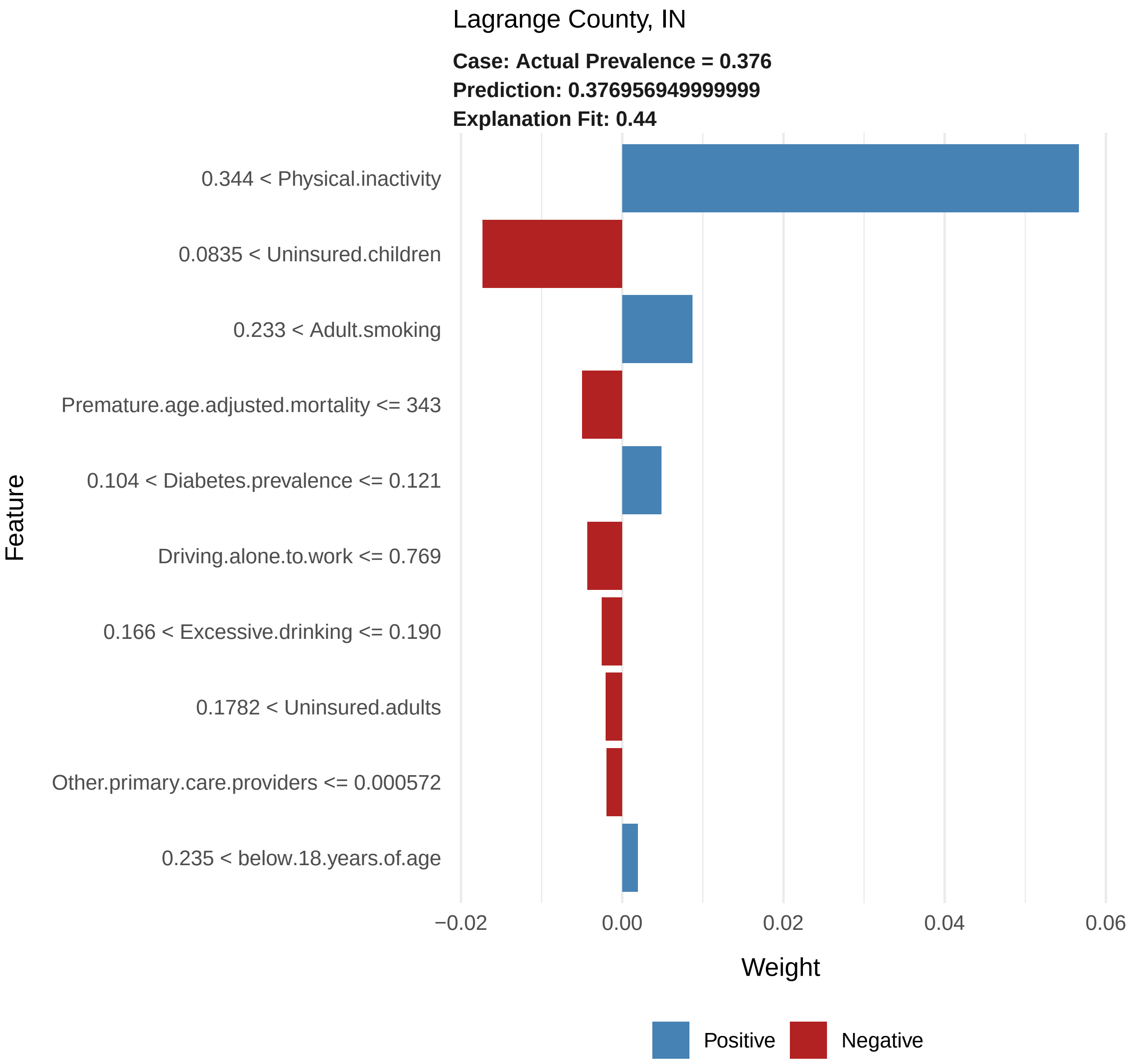

Lagrange County, IN
Case: Actual Prevalence = 0.376
Prediction: 0.376956949999999
Explanation Fit: 0.44
0.344 < Physical.inactivity
0.0835 < Uninsured.children
0.233 < Adult.smoking
Premature.age.adjusted.mortality <= 343
0.104 < Diabetes.prevalence <= 0.121
Driving.alone.to.work <= 0.769
0.166 < Excessive.drinking <= 0.190
0.1782 < Uninsured.adults
Other.primary.care.providers <= 0.000572
0.235 < below.18.years.of.age
Feature
−0.02
0.00
0.02
0.04
0.06
Weight
Positive
Negative

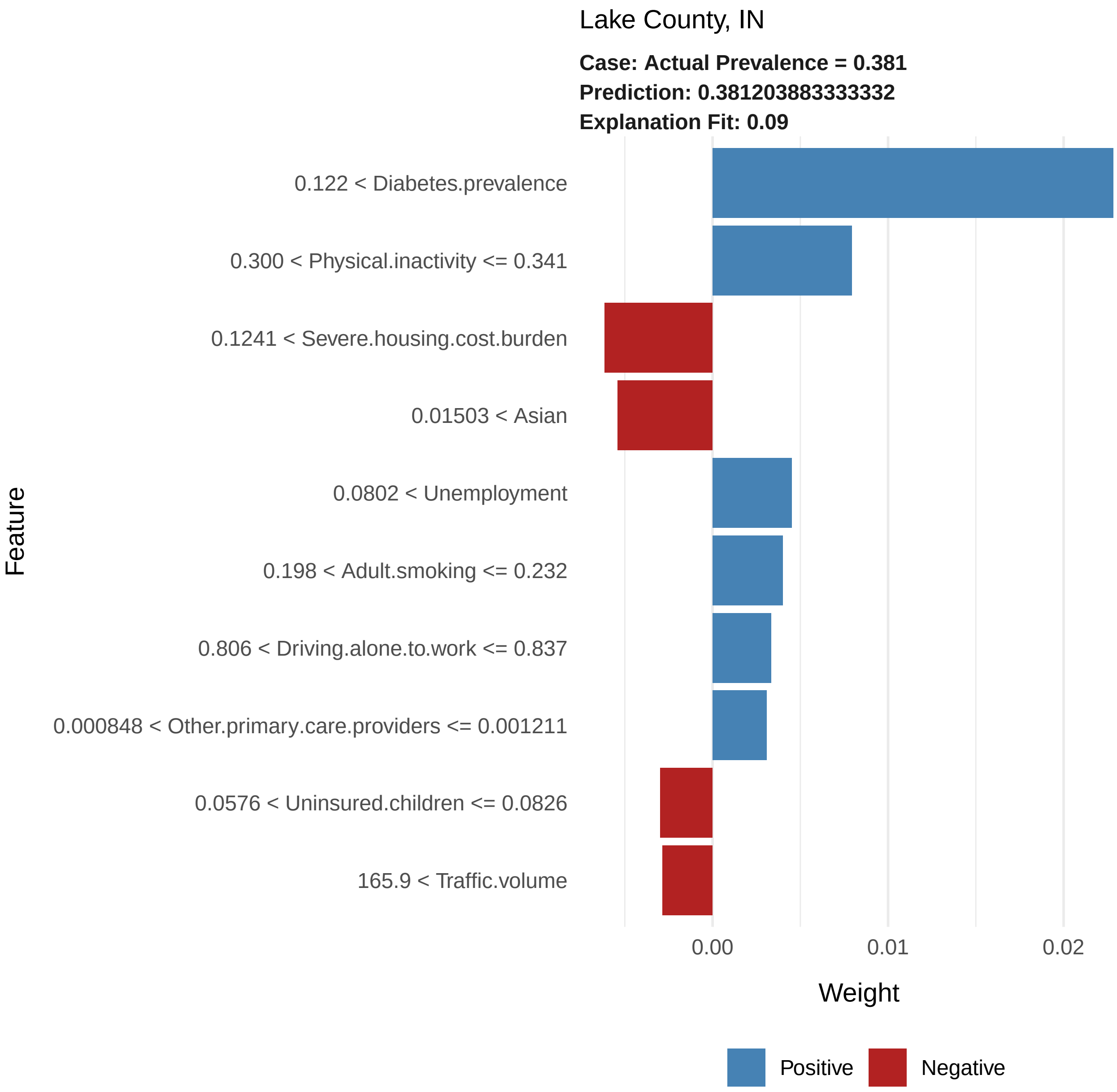
Lake County, IN
Case: Actual Prevalence = 0.381
Prediction: 0.381203883333332
Explanation Fit: 0.09
Feature
0.122 < Diabetes.prevalence
0.300 < Physical.inactivity <= 0.341
0.1241 < Severe.housing.cost.burden
0.01503 < Asian
0.0802 < Unemployment
0.198 < Adult.smoking <= 0.232
0.806 < Driving.alone.to.work <= 0.837
0.000848 < Other.primary.care.providers <= 0.001211
0.0576 < Uninsured.children <= 0.0826
165.9 < Traffic.volume
0.00
0.01
0.02
Weight
Positive
Negative

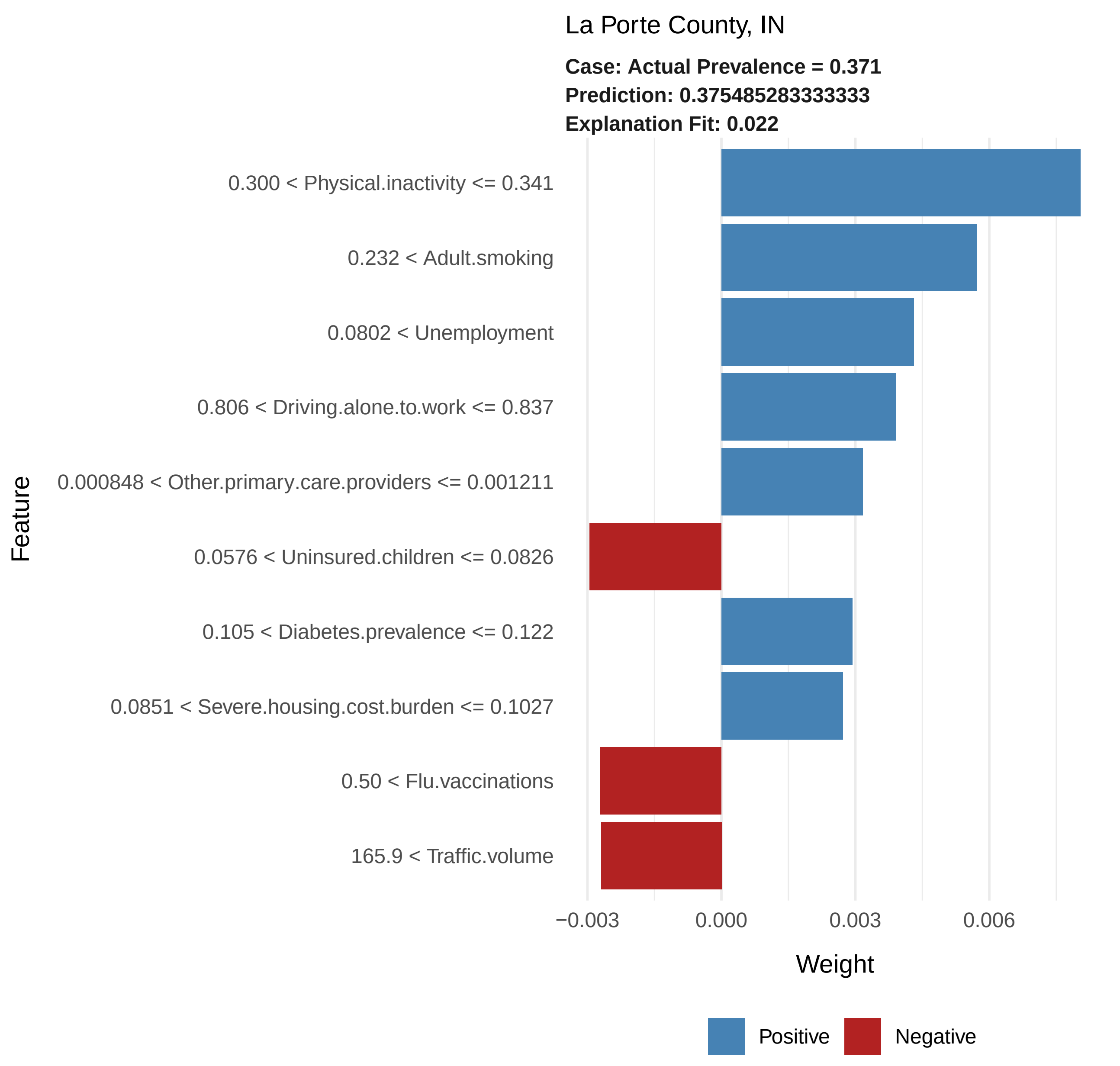

La Porte County, IN
Case: Actual Prevalence = 0.371
Prediction: 0.375485283333333
Explanation Fit: 0.022
0.300 < Physical.inactivity <= 0.341
0.232 < Adult.smoking
0.0802 < Unemployment
0.806 < Driving.alone.to.work <= 0.837
0.000848 < Other.primary.care.providers <= 0.001211
0.0576 < Uninsured.children <= 0.0826
0.105 < Diabetes.prevalence <= 0.122
0.0851 < Severe.housing.cost.burden <= 0.1027
0.50 < Flu.vaccinations
165.9 < Traffic.volume
Feature
−0.003
0.000
0.003
0.006
Weight
Positive
Negative

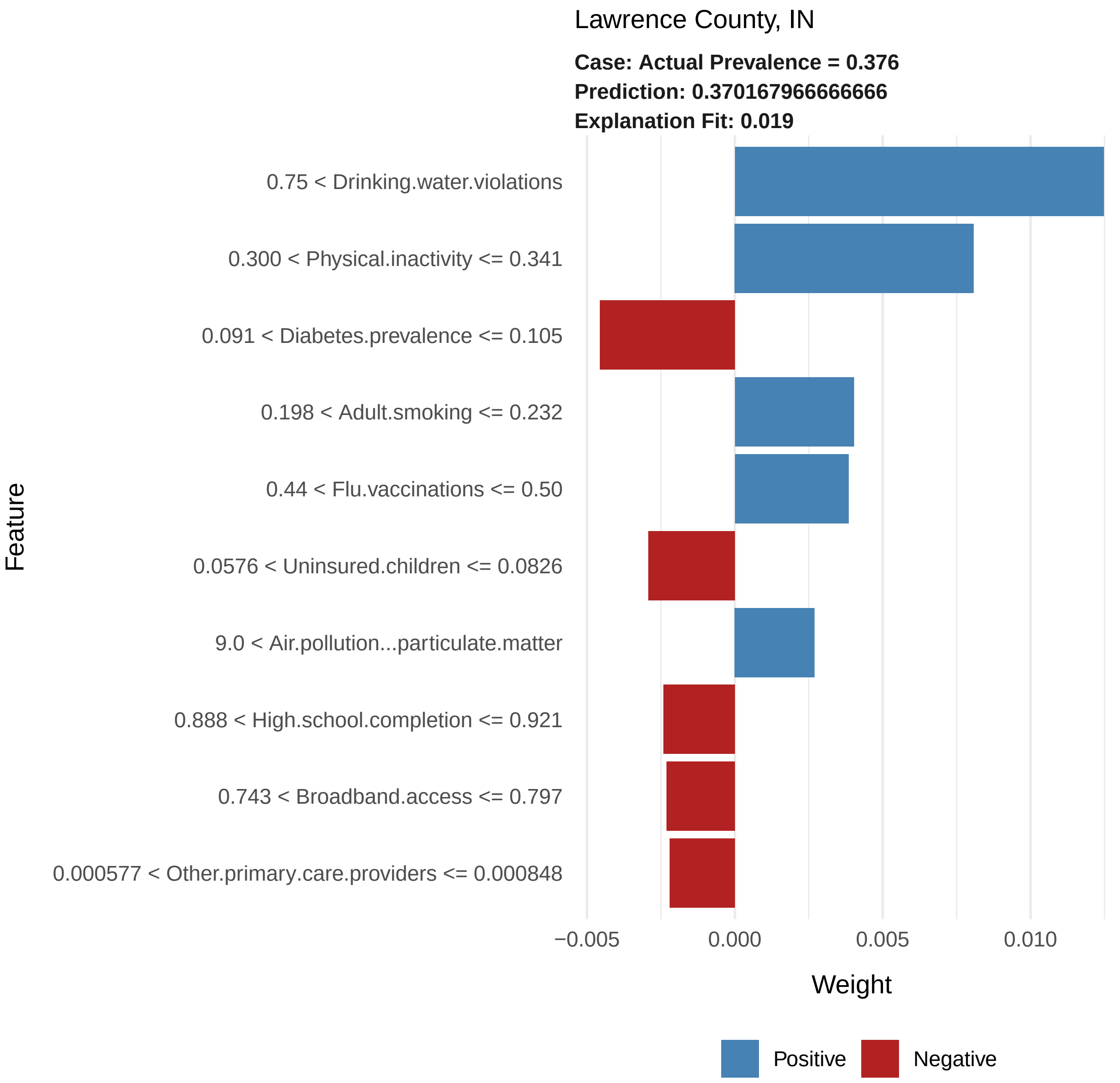

Lawrence County, IN
Case: Actual Prevalence = 0.376
Prediction: 0.370167966666666
Explanation Fit: 0.019
0.75 < Drinking.water.violations
0.300 < Physical.inactivity <= 0.341
0.091 < Diabetes.prevalence <= 0.105
0.198 < Adult.smoking <= 0.232
0.44 < Flu.vaccinations <= 0.50
0.0576 < Uninsured.children <= 0.0826
9.0 < Air.pollution...particulate.matter
0.888 < High.school.completion <= 0.921
0.743 < Broadband.access <= 0.797
0.000577 < Other.primary.care.providers <= 0.000848
Feature
−0.005
0.000
0.005
0.010
Weight
Positive
Negative

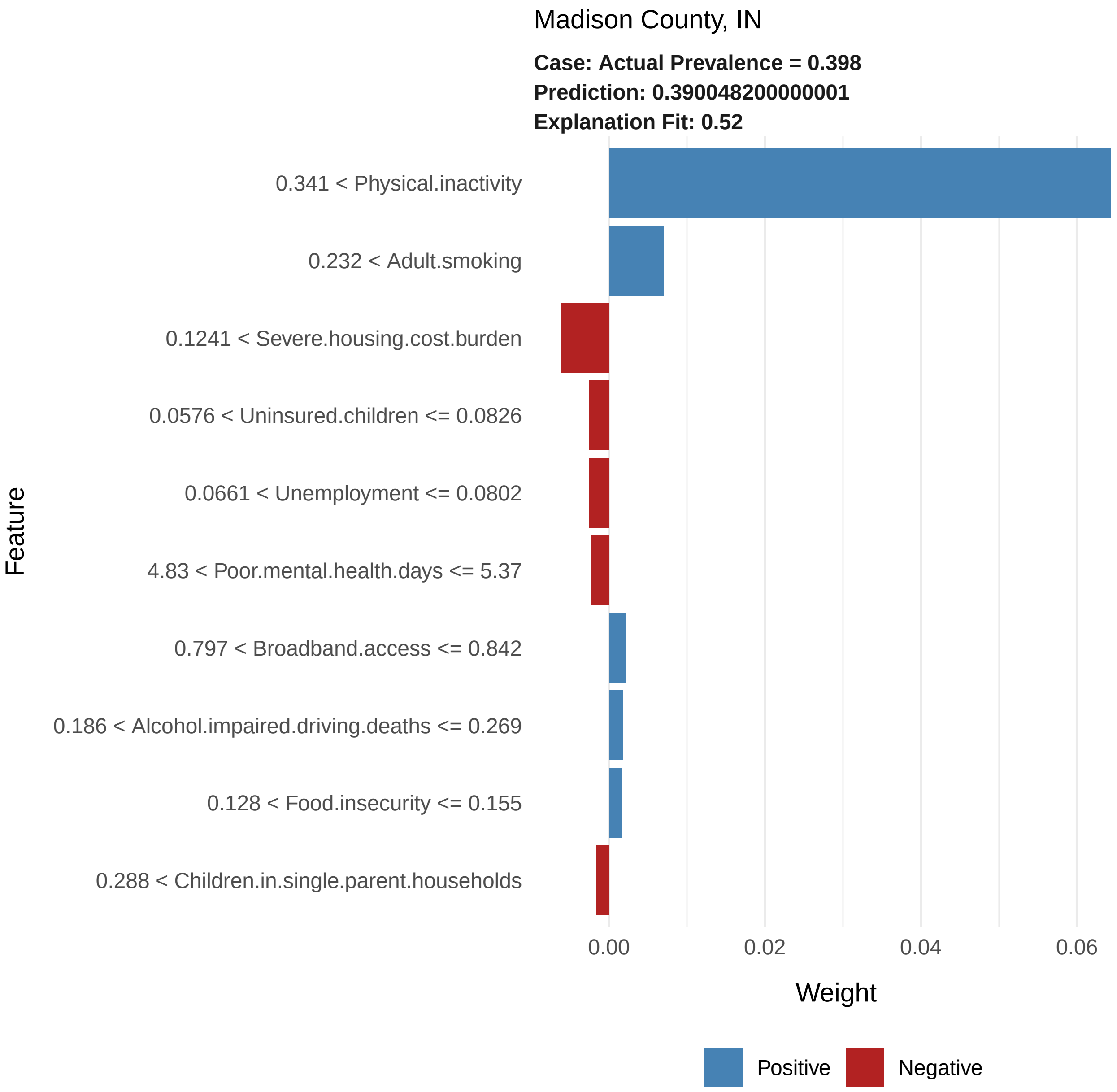
Madison County, IN
Case: Actual Prevalence = 0.398
Prediction: 0.390048200000001
Explanation Fit: 0.52
0.341 < Physical.inactivity
0.232 < Adult.smoking
0.1241 < Severe.housing.cost.burden
0.0576 < Uninsured.children <= 0.0826
0.0661 < Unemployment <= 0.0802
4.83 < Poor.mental.health.days <= 5.37
0.797 < Broadband.access <= 0.842
0.186 < Alcohol.impaired.driving.deaths <= 0.269
0.128 < Food.insecurity <= 0.155
0.288 < Children.in.single.parent.households
Feature
0.00
0.02
0.04
0.06
Weight
Positive
Negative

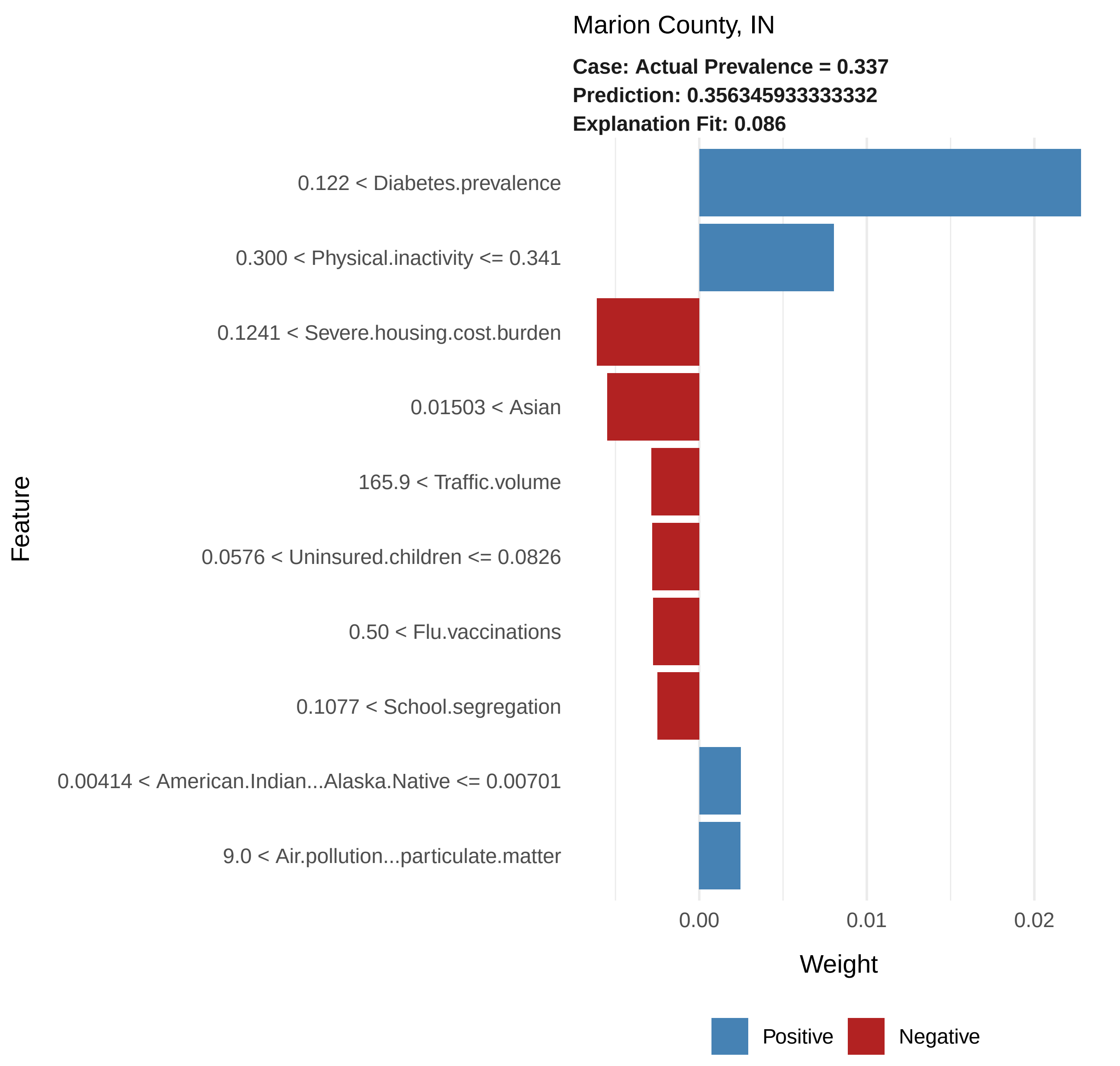
Marion County, IN
Case: Actual Prevalence = 0.337
Prediction: 0.356345933333332
Explanation Fit: 0.086
0.122 < Diabetes.prevalence
0.300 < Physical.inactivity <= 0.341
0.1241 < Severe.housing.cost.burden
0.01503 < Asian
165.9 < Traffic.volume
0.0576 < Uninsured.children <= 0.0826
0.50 < Flu.vaccinations
0.1077 < School.segregation
0.00414 < American.Indian...Alaska.Native <= 0.00701
9.0 < Air.pollution...particulate.matter
Feature
0.00
0.01
0.02
Weight
Positive
Negative

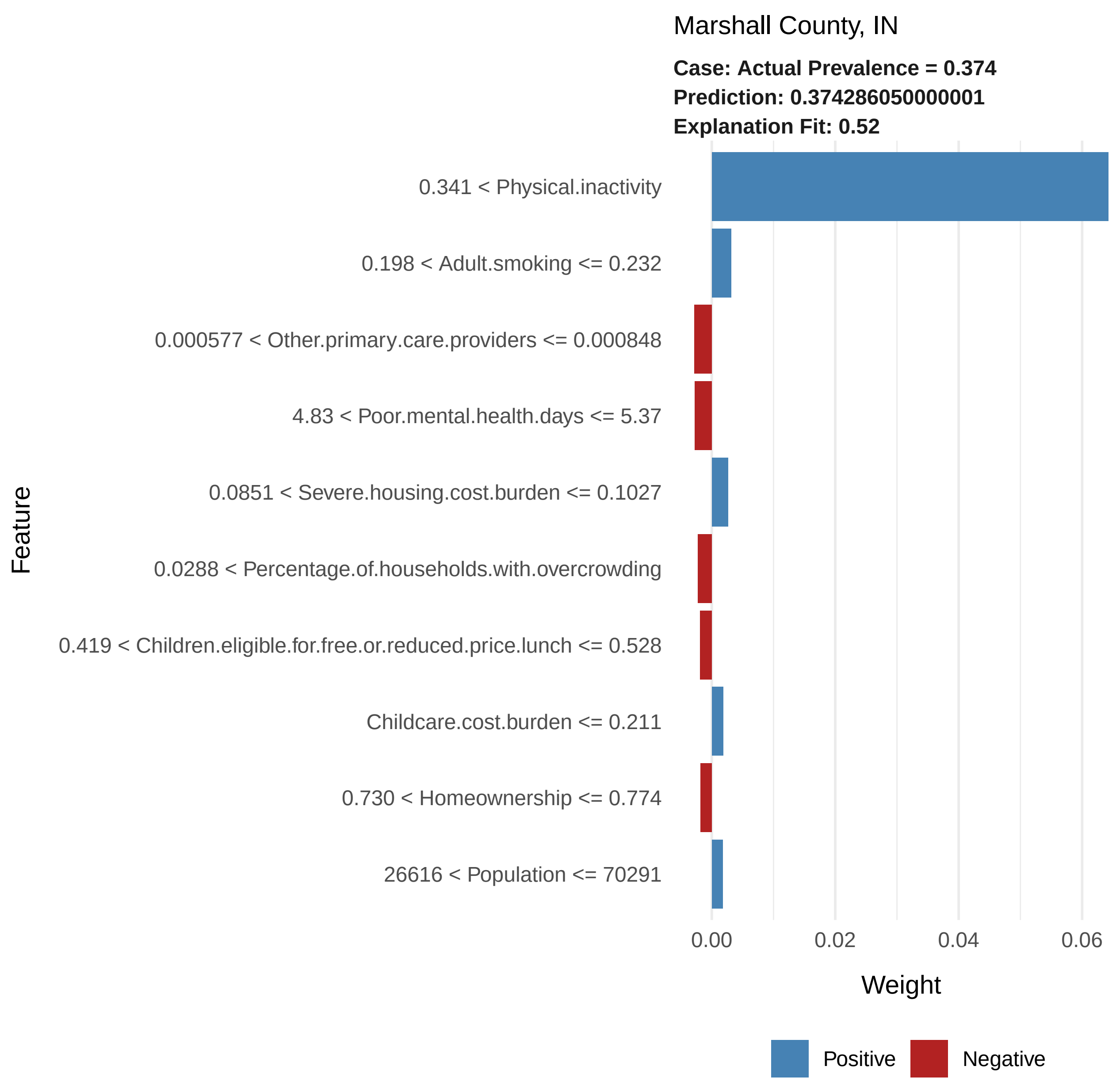
Marshall County, IN
Case: Actual Prevalence = 0.374
Prediction: 0.374286050000001
Explanation Fit: 0.52
0.341 < Physical.inactivity
0.198 < Adult.smoking <= 0.232
0.000577 < Other.primary.care.providers <= 0.000848
4.83 < Poor.mental.health.days <= 5.37
0.0851 < Severe.housing.cost.burden <= 0.1027
0.0288 < Percentage.of.households.with.overcrowding
0.419 < Children.eligible.for.free.or.reduced.price.lunch <= 0.528
Childcare.cost.burden <= 0.211
0.730 < Homeownership <= 0.774
26616 < Population <= 70291
Feature
0.00
0.02
0.04
0.06
Weight
Positive
Negative

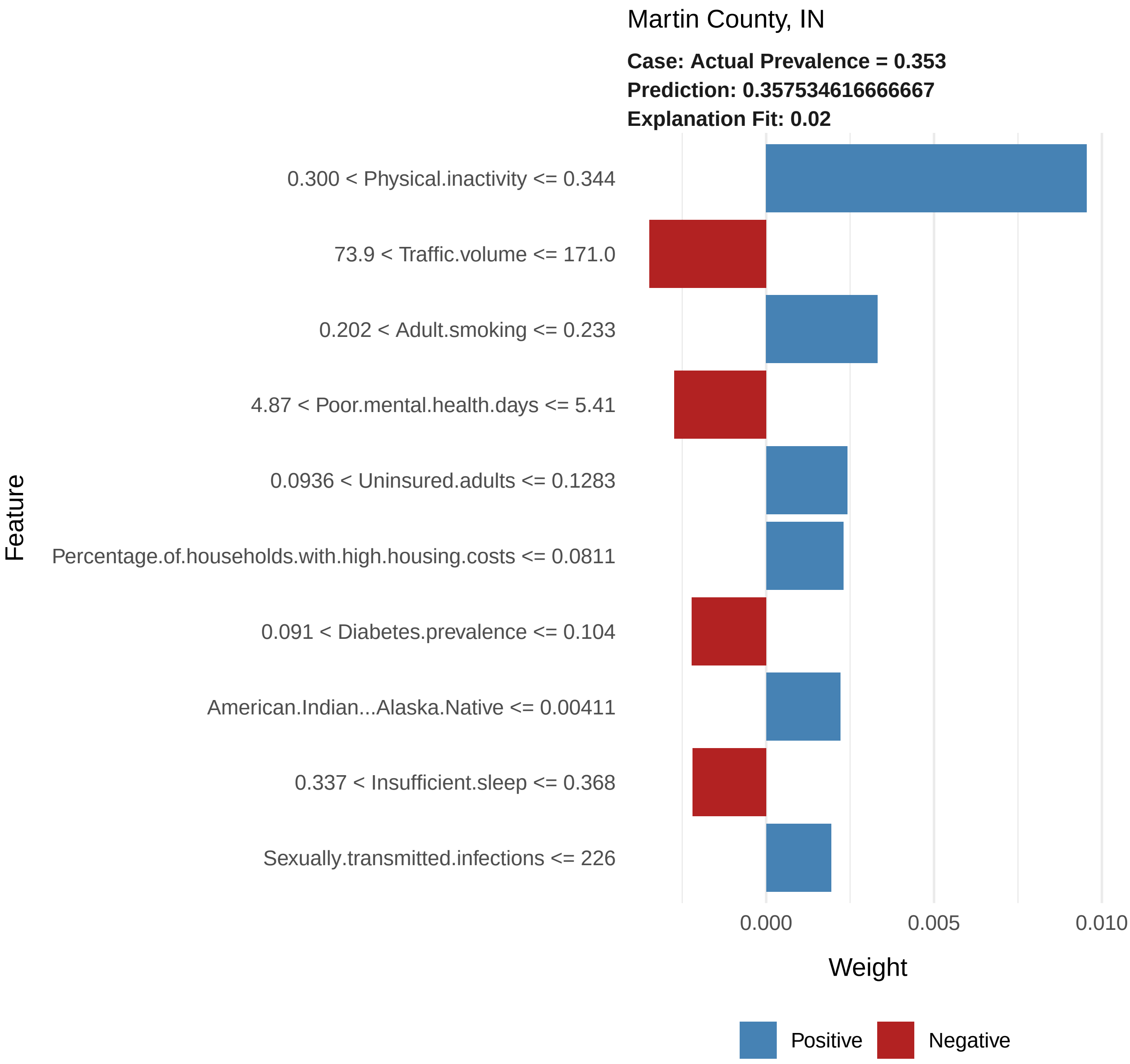
Martin County, IN
Case: Actual Prevalence = 0.353
Prediction: 0.357534616666667
Explanation Fit: 0.02
0.300 < Physical.inactivity <= 0.344
73.9 < Traffic.volume <= 171.0
0.202 < Adult.smoking <= 0.233
4.87 < Poor.mental.health.days <= 5.41
0.0936 < Uninsured.adults <= 0.1283
Percentage.of.households.with.high.housing.costs <= 0.0811
0.091 < Diabetes.prevalence <= 0.104
American.Indian...Alaska.Native <= 0.00411
0.337 < Insufficient.sleep <= 0.368
Sexually.transmitted.infections <= 226
Feature
0.000
0.005
0.010
Weight
Positive
Negative

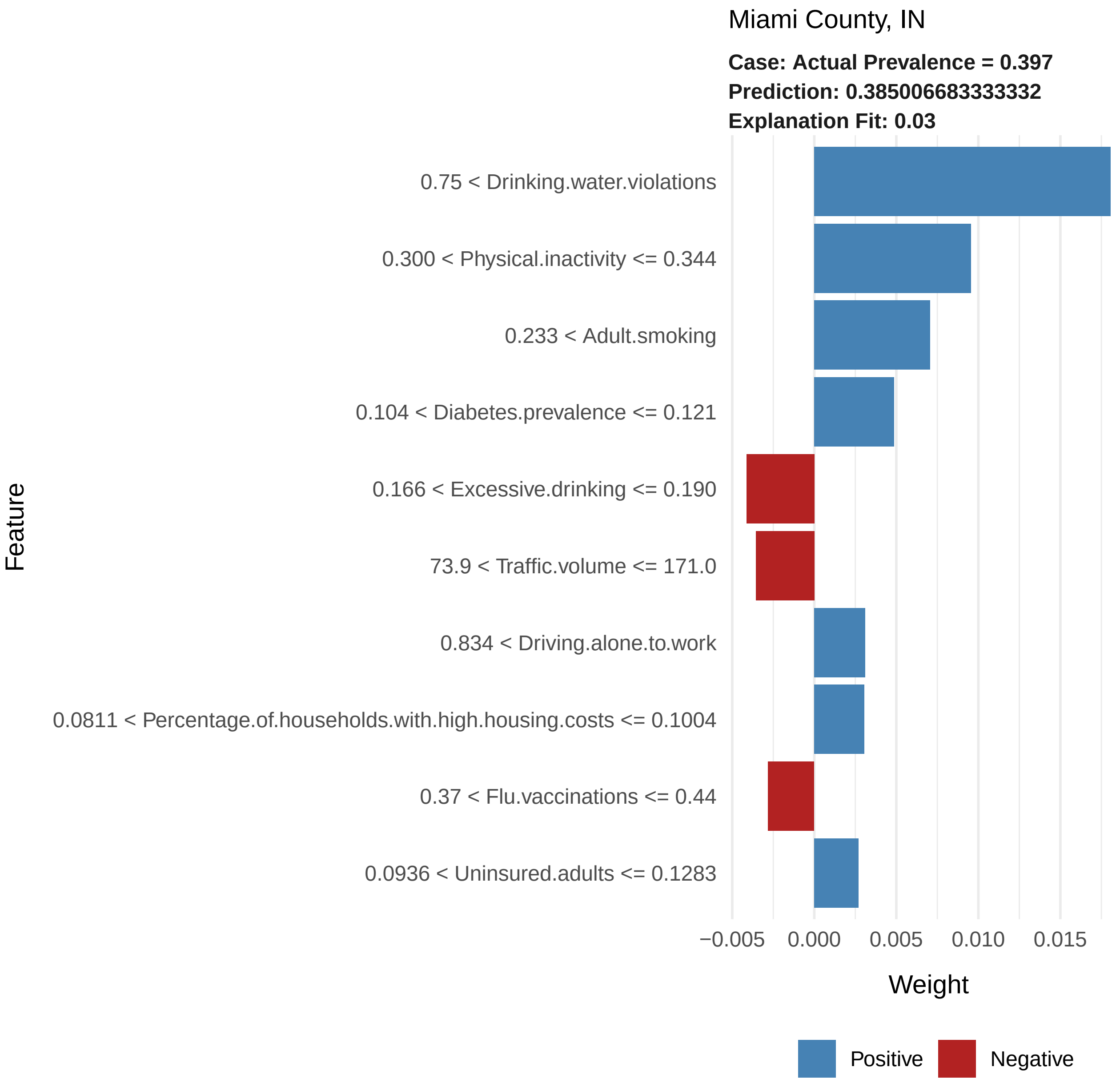

Miami County, IN
Case: Actual Prevalence = 0.397
Prediction: 0.385006683333332
Explanation Fit: 0.03
0.75 < Drinking.water.violations
0.300 < Physical.inactivity <= 0.344
0.233 < Adult.smoking
0.104 < Diabetes.prevalence <= 0.121
0.166 < Excessive.drinking <= 0.190
73.9 < Traffic.volume <= 171.0
0.834 < Driving.alone.to.work
0.0811 < Percentage.of.households.with.high.housing.costs <= 0.1004
0.37 < Flu.vaccinations <= 0.44
0.0936 < Uninsured.adults <= 0.1283
Feature
−0.005
0.000
0.005
0.010
0.015
Weight
Positive
Negative

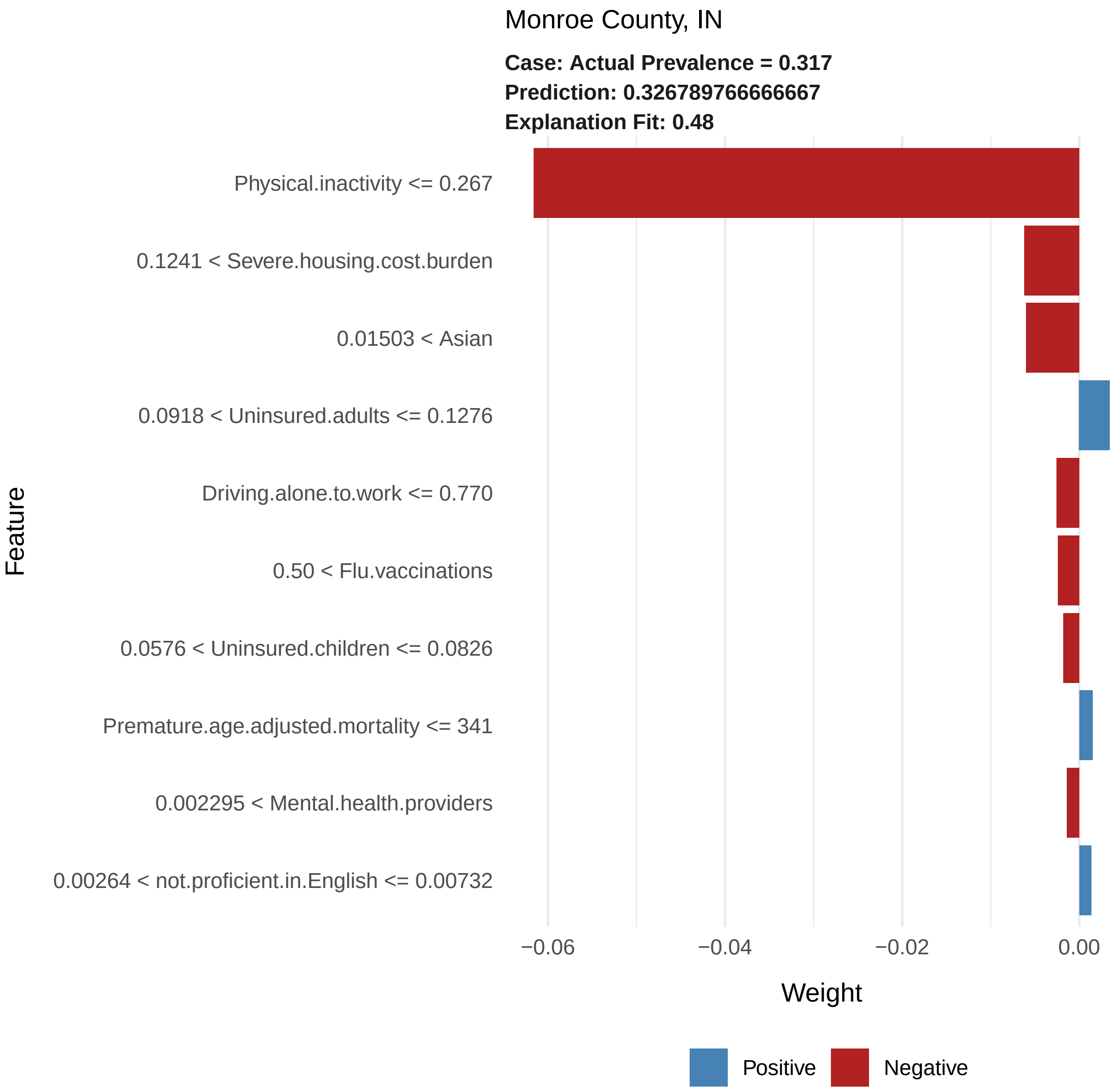

Monroe County, IN
Case: Actual Prevalence = 0.317
Prediction: 0.326789766666667
Explanation Fit: 0.48
Physical.inactivity <= 0.267
0.1241 < Severe.housing.cost.burden
0.01503 < Asian
0.0918 < Uninsured.adults <= 0.1276
Driving.alone.to.work <= 0.770
0.50 < Flu.vaccinations
0.0576 < Uninsured.children <= 0.0826
Premature.age.adjusted.mortality <= 341
0.002295 < Mental.health.providers
0.00264 < not.proficient.in.English <= 0.00732
Feature
−0.06
−0.04
−0.02
0.00
Weight
Positive
Negative

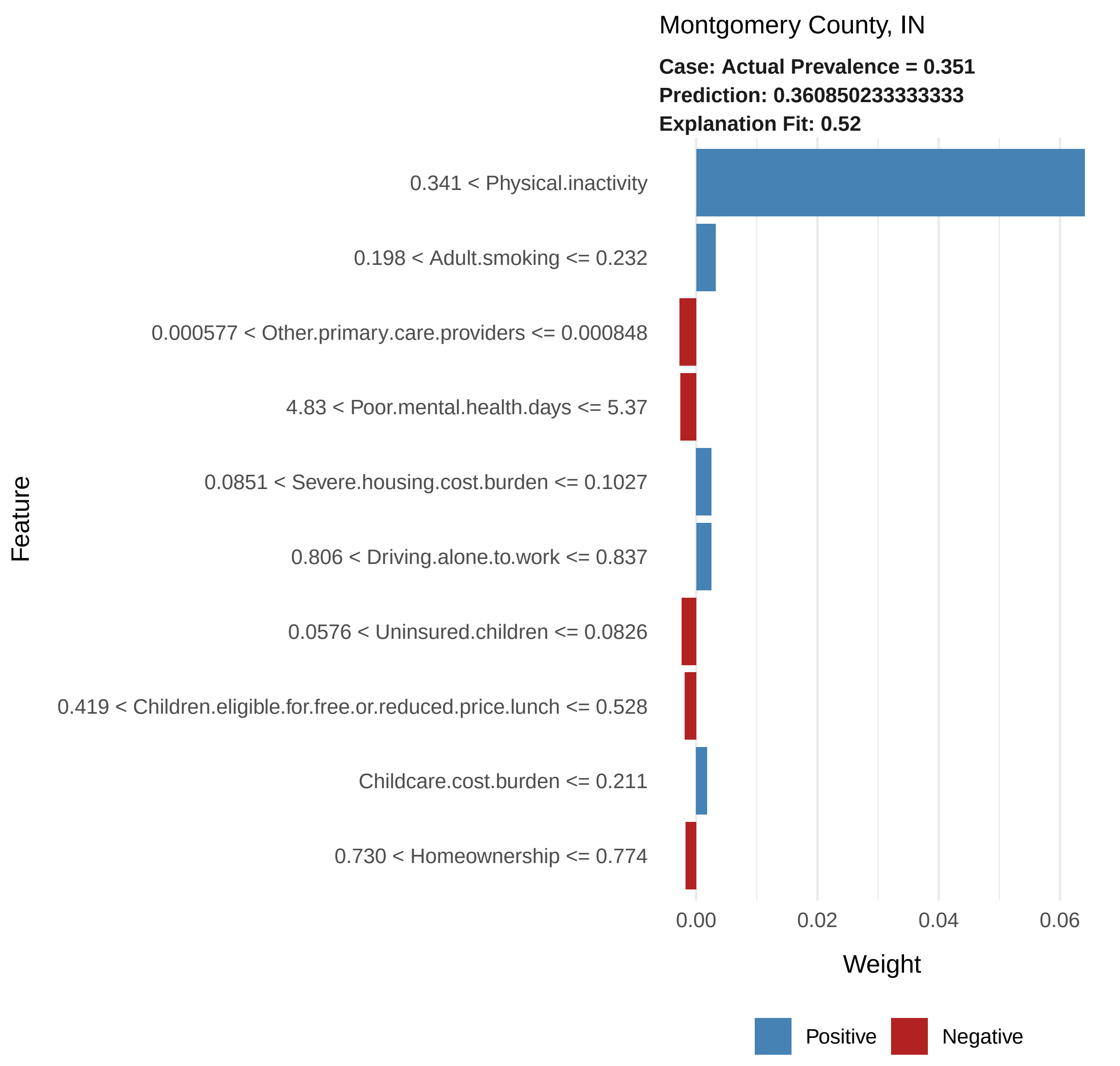

Montgomery County, IN
Case: Actual Prevalence = 0.351
Prediction: 0.360850233333333
Explanation Fit: 0.52
0.341 < Physical.inactivity
0.198 < Adult.smoking <= 0.232
0.000577 < Other.primary.care.providers <= 0.000848
4.83 < Poor.mental.health.days <= 5.37
0.0851 < Severe.housing.cost.burden <= 0.1027
0.806 < Driving.alone.to.work <= 0.837
0.0576 < Uninsured.children <= 0.0826
0.419 < Children.eligible.for.free.or.reduced.price.lunch <= 0.528
Childcare.cost.burden <= 0.211
0.730 < Homeownership <= 0.774
Feature
0.00
0.02
0.04
0.06
Weight
Positive
Negative

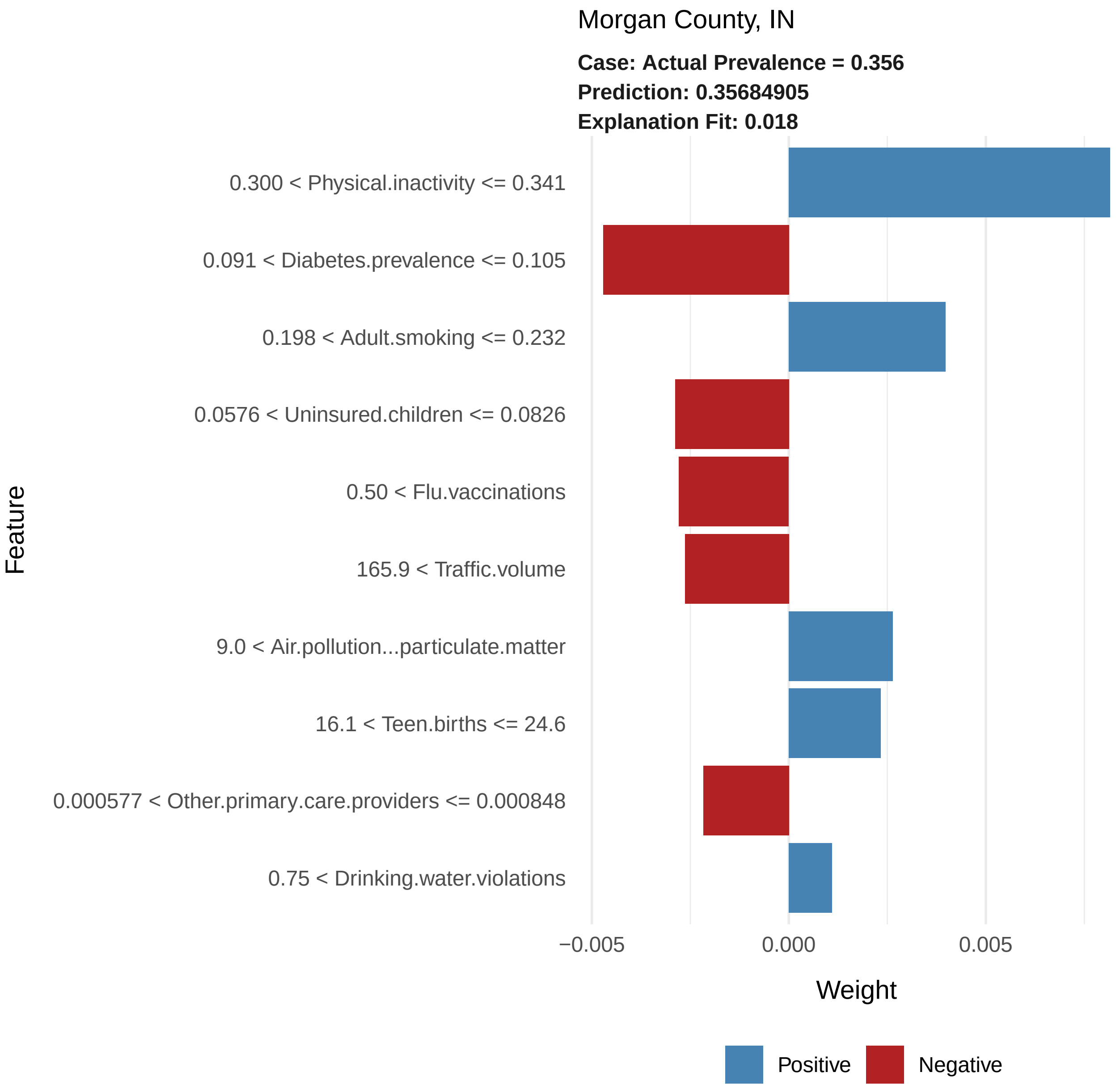
Morgan County, IN
Case: Actual Prevalence = 0.356
Prediction: 0.35684905
Explanation Fit: 0.018
0.300 < Physical.inactivity <= 0.341
0.091 < Diabetes.prevalence <= 0.105
0.198 < Adult.smoking <= 0.232
0.0576 < Uninsured.children <= 0.0826
0.50 < Flu.vaccinations
165.9 < Traffic.volume
9.0 < Air.pollution...particulate.matter
16.1 < Teen.births <= 24.6
0.000577 < Other.primary.care.providers <= 0.000848
0.75 < Drinking.water.violations
Feature
−0.005
0.000
0.005
Weight
Positive
Negative

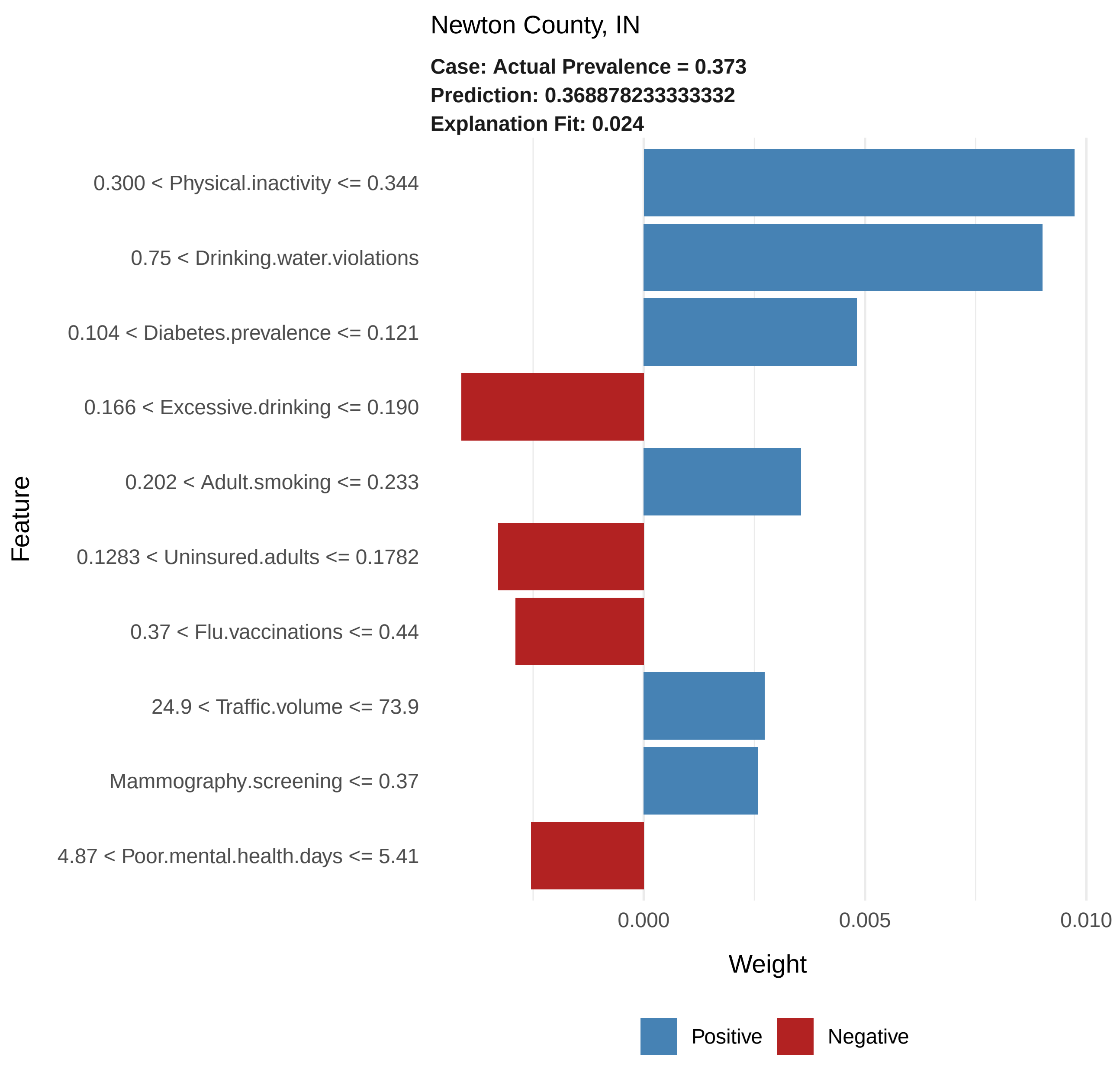

Newton County, IN
Case: Actual Prevalence = 0.373
Prediction: 0.368878233333332
Explanation Fit: 0.024
0.300 < Physical.inactivity <= 0.344
0.75 < Drinking.water.violations
0.104 < Diabetes.prevalence <= 0.121
0.166 < Excessive.drinking <= 0.190
0.202 < Adult.smoking <= 0.233
0.1283 < Uninsured.adults <= 0.1782
0.37 < Flu.vaccinations <= 0.44
24.9 < Traffic.volume <= 73.9
Mammography.screening <= 0.37
4.87 < Poor.mental.health.days <= 5.41
Feature
0.000
0.005
0.010
Weight
Positive
Negative

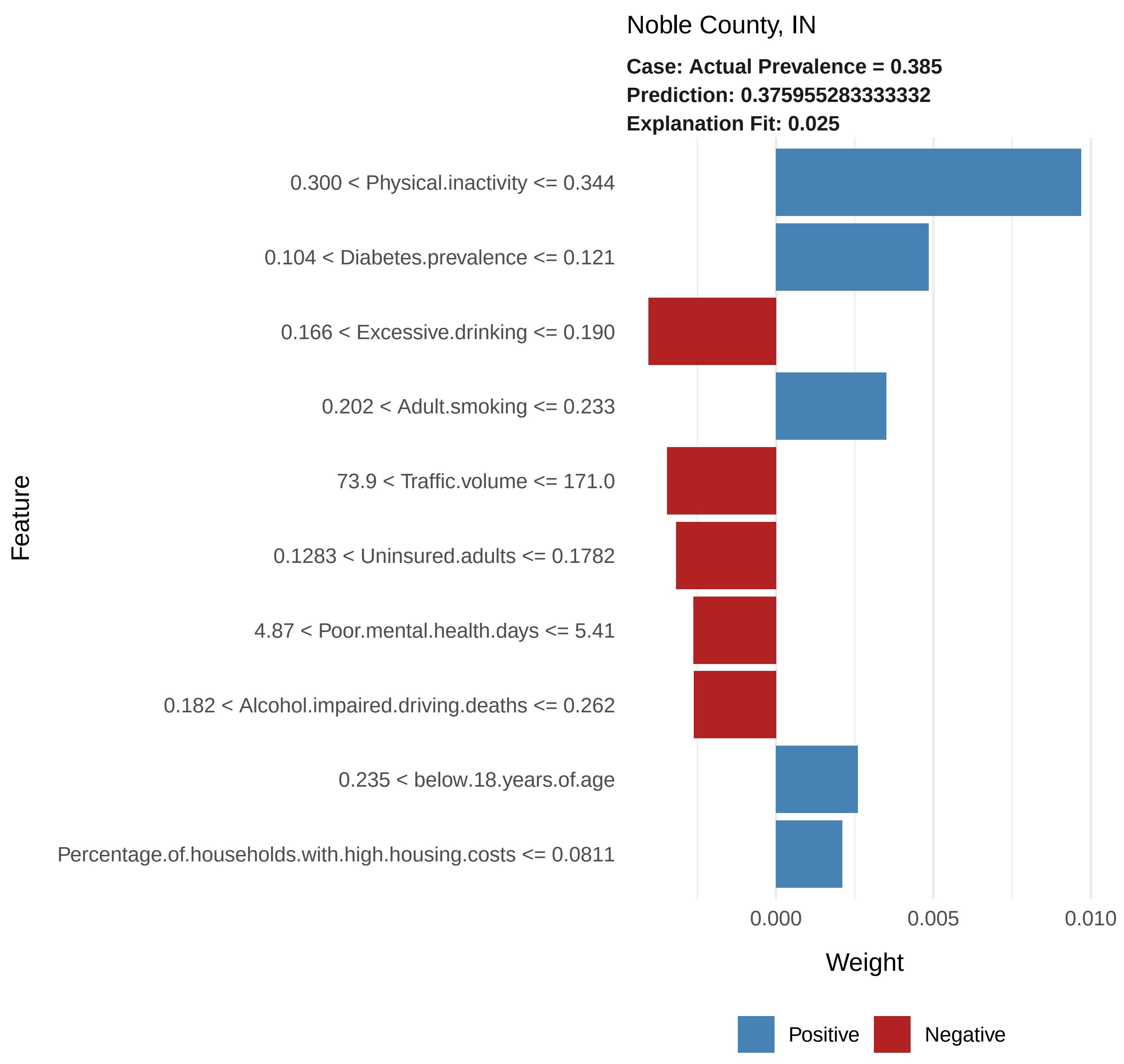

Noble County, IN
Case: Actual Prevalence = 0.385
Prediction: 0.375955283333332
Explanation Fit: 0.025
0.300 < Physical.inactivity <= 0.344
0.104 < Diabetes.prevalence <= 0.121
0.166 < Excessive.drinking <= 0.190
0.202 < Adult.smoking <= 0.233
73.9 < Traffic.volume <= 171.0
0.1283 < Uninsured.adults <= 0.1782
4.87 < Poor.mental.health.days <= 5.41
0.182 < Alcohol.impaired.driving.deaths <= 0.262
0.235 < below.18.years.of.age
Percentage.of.households.with.high.housing.costs <= 0.0811
Feature
0.000
0.005
0.010
Weight
Positive
Negative

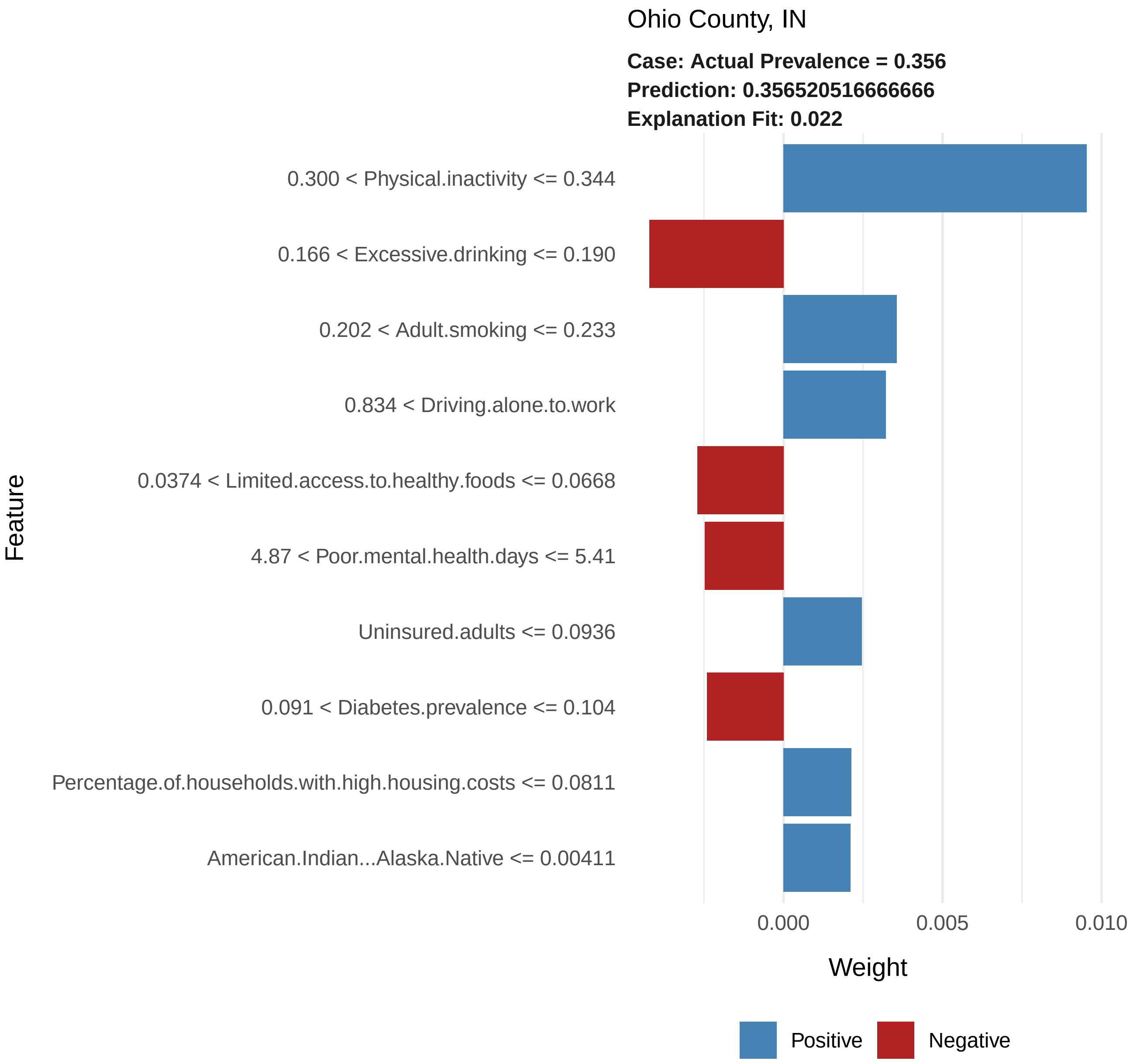

Ohio County, IN
Case: Actual Prevalence = 0.356
Prediction: 0.356520516666666
Explanation Fit: 0.022
0.300 < Physical.inactivity <= 0.344
0.166 < Excessive.drinking <= 0.190
0.202 < Adult.smoking <= 0.233
0.834 < Driving.alone.to.work
0.0374 < Limited.access.to.healthy.foods <= 0.0668
4.87 < Poor.mental.health.days <= 5.41
Uninsured.adults <= 0.0936
0.091 < Diabetes.prevalence <= 0.104
Percentage.of.households.with.high.housing.costs <= 0.0811
American.Indian...Alaska.Native <= 0.00411
Feature
0.000
0.005
0.010
Weight
Positive
Negative

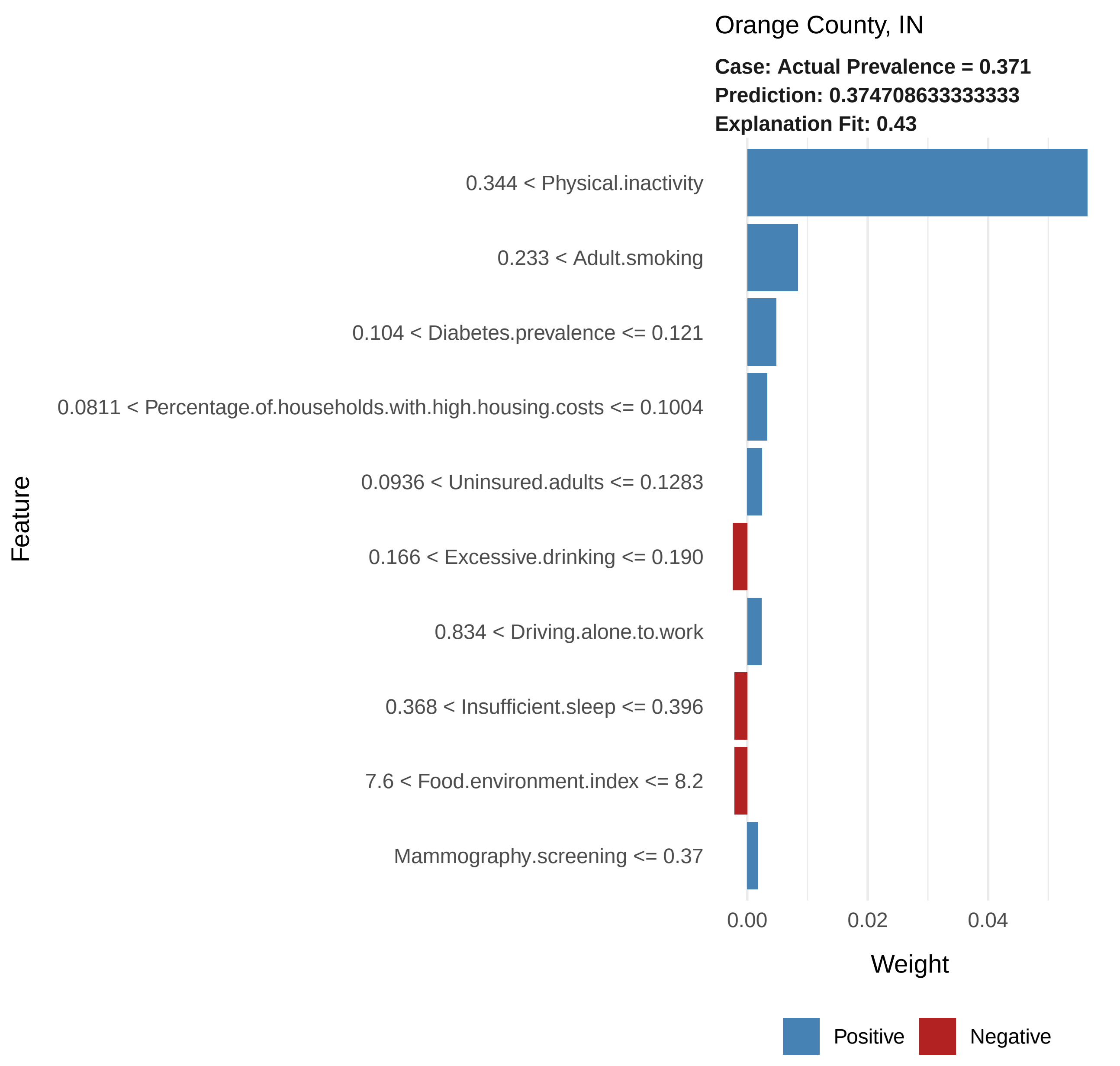

Orange County, IN
Case: Actual Prevalence = 0.371
Prediction: 0.374708633333333
Explanation Fit: 0.43
0.344 < Physical.inactivity
0.233 < Adult.smoking
0.104 < Diabetes.prevalence <= 0.121
0.0811 < Percentage.of.households.with.high.housing.costs <= 0.1004
0.0936 < Uninsured.adults <= 0.1283
0.166 < Excessive.drinking <= 0.190
0.834 < Driving.alone.to.work
0.368 < Insufficient.sleep <= 0.396
7.6 < Food.environment.index <= 8.2
Mammography.screening <= 0.37
Feature
0.00
0.02
0.04
Weight
Positive
Negative

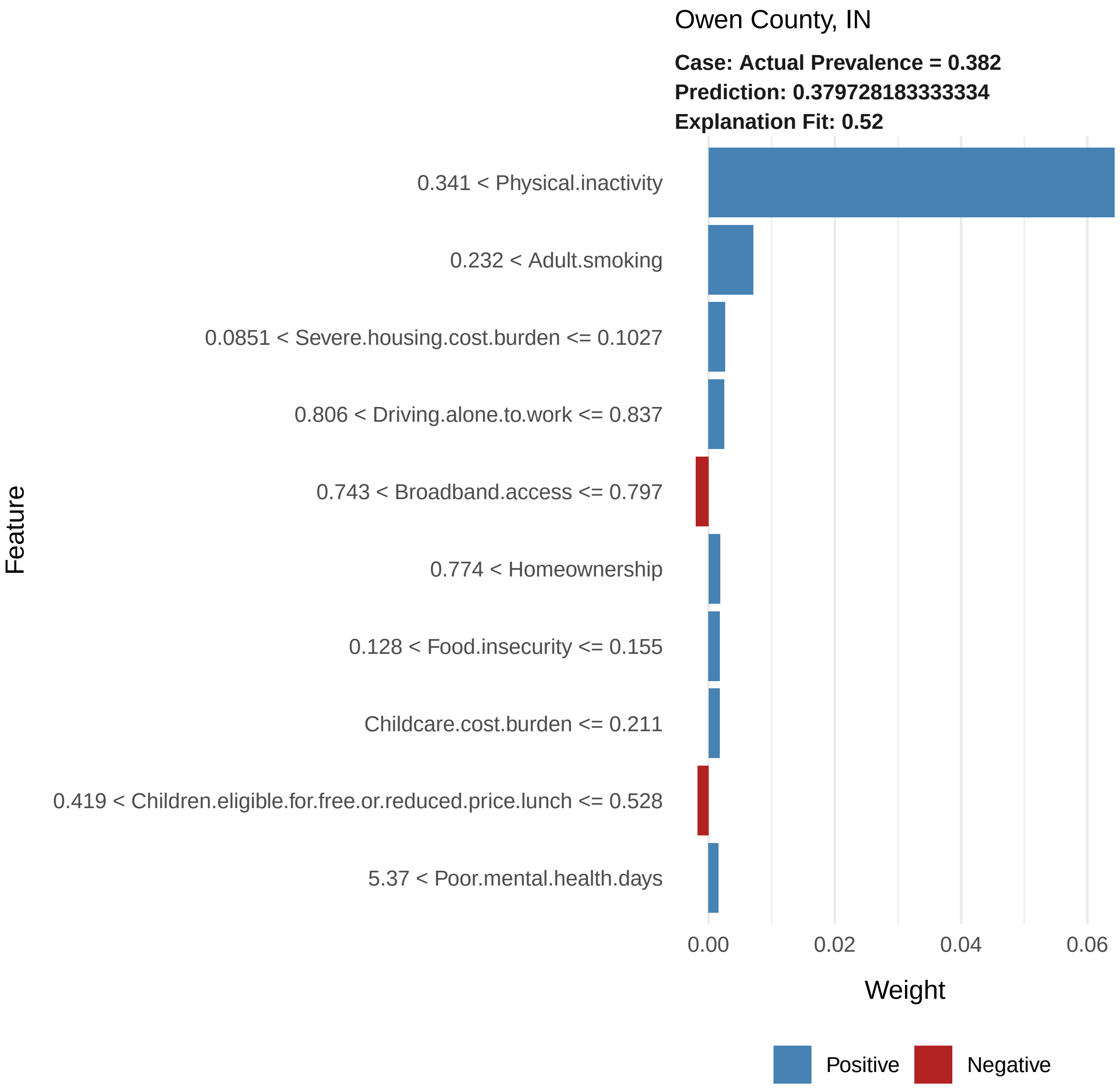

Owen County, IN
Case: Actual Prevalence = 0.382
Prediction: 0.379728183333334
Explanation Fit: 0.52
0.341 < Physical.inactivity
0.232 < Adult.smoking
0.0851 < Severe.housing.cost.burden <= 0.1027
0.806 < Driving.alone.to.work <= 0.837
0.743 < Broadband.access <= 0.797
0.774 < Homeownership
0.128 < Food.insecurity <= 0.155
Childcare.cost.burden <= 0.211
0.419 < Children.eligible.for.free.or.reduced.price.lunch <= 0.528
5.37 < Poor.mental.health.days
Feature
0.00
0.02
0.04
0.06
Weight
Positive
Negative

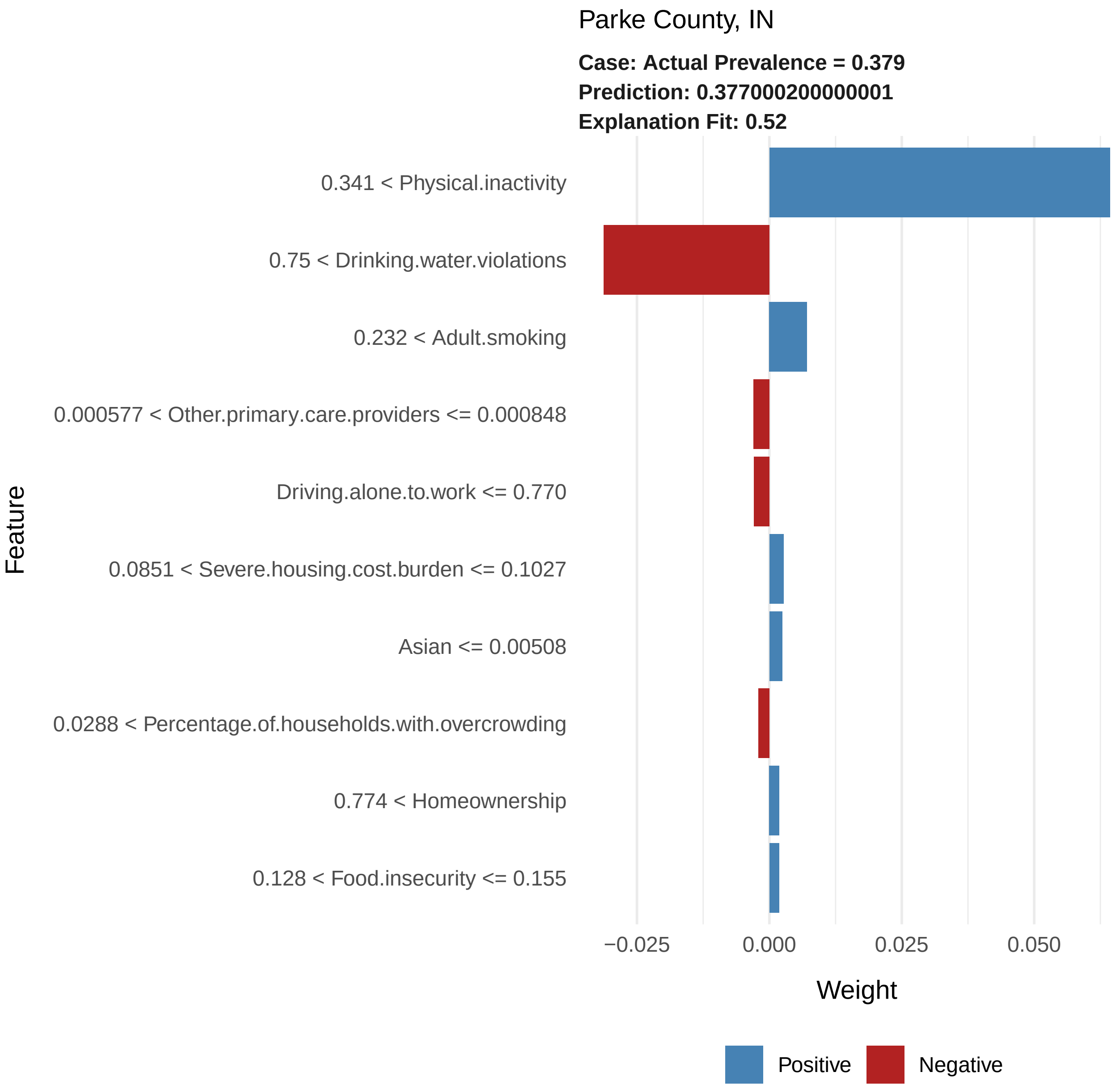

Parke County, IN
Case: Actual Prevalence = 0.379
Prediction: 0.377000200000001
Explanation Fit: 0.52
0.341 < Physical.inactivity
0.75 < Drinking.water.violations
0.232 < Adult.smoking
0.000577 < Other.primary.care.providers <= 0.000848
Driving.alone.to.work <= 0.770
0.0851 < Severe.housing.cost.burden <= 0.1027
Asian <= 0.00508
0.0288 < Percentage.of.households.with.overcrowding
0.774 < Homeownership
0.128 < Food.insecurity <= 0.155
Feature
−0.025
0.000
0.025
0.050
Weight
Positive
Negative

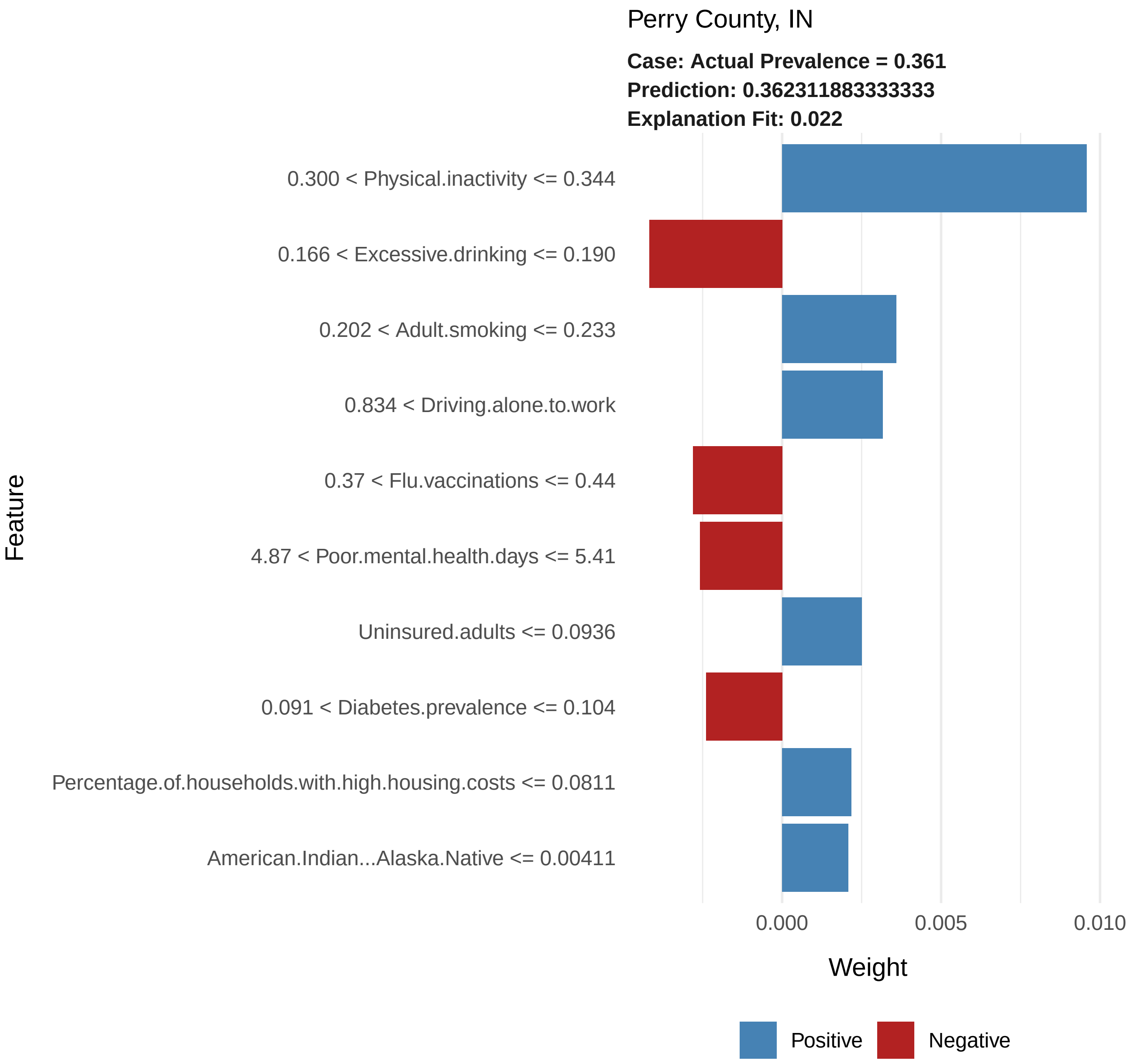
Perry County, IN
Case: Actual Prevalence = 0.361
Prediction: 0.362311883333333
Explanation Fit: 0.022
0.300 < Physical.inactivity <= 0.344
0.166 < Excessive.drinking <= 0.190
0.202 < Adult.smoking <= 0.233
0.834 < Driving.alone.to.work
0.37 < Flu.vaccinations <= 0.44
4.87 < Poor.mental.health.days <= 5.41
Uninsured.adults <= 0.0936
0.091 < Diabetes.prevalence <= 0.104
Percentage.of.households.with.high.housing.costs <= 0.0811
American.Indian...Alaska.Native <= 0.00411
Feature
0.000
0.005
0.010
Weight
Positive
Negative

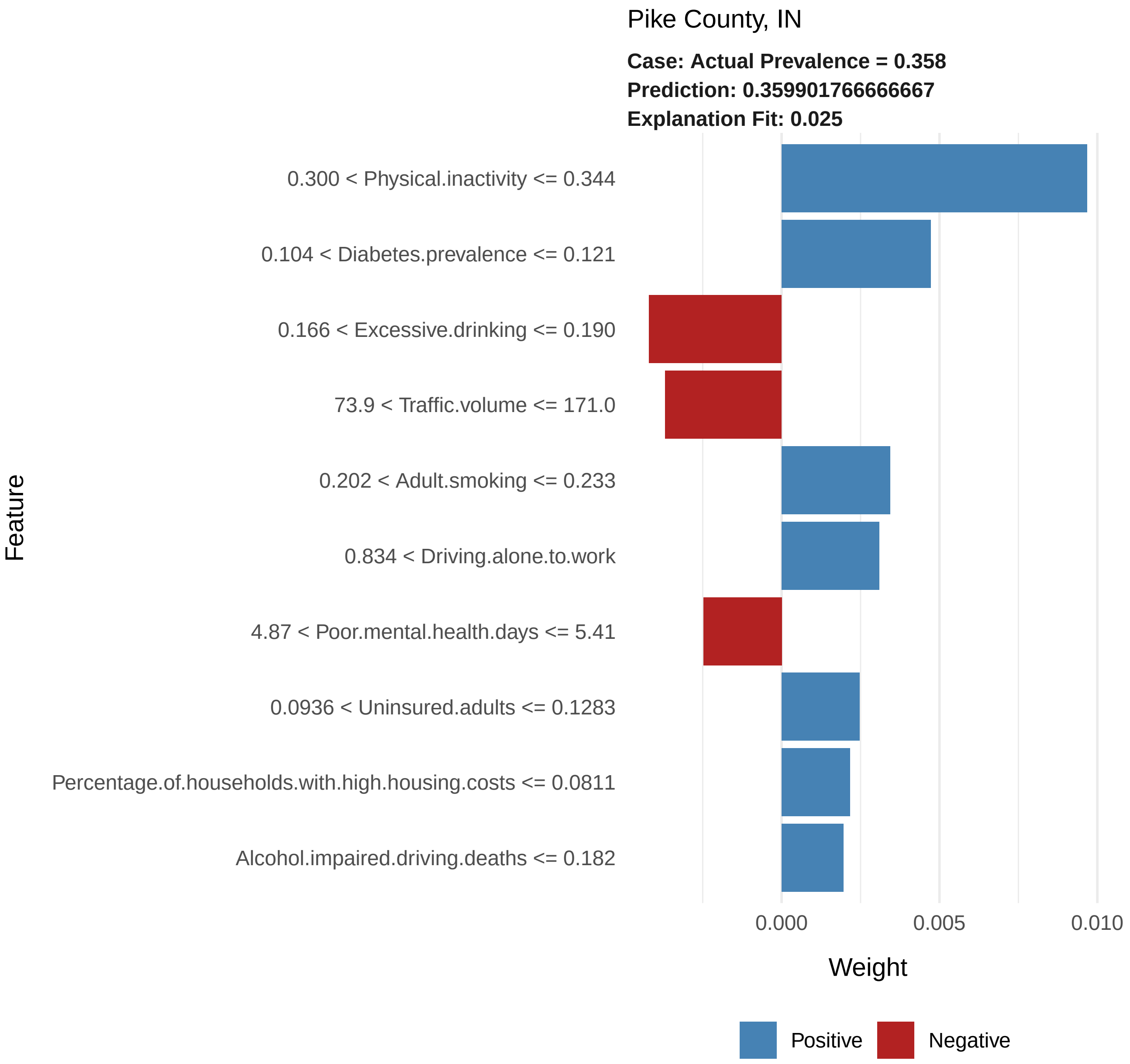

Pike County, IN
Case: Actual Prevalence = 0.358
Prediction: 0.359901766666667
Explanation Fit: 0.025
0.300 < Physical.inactivity <= 0.344
0.104 < Diabetes.prevalence <= 0.121
0.166 < Excessive.drinking <= 0.190
73.9 < Traffic.volume <= 171.0
0.202 < Adult.smoking <= 0.233
0.834 < Driving.alone.to.work
4.87 < Poor.mental.health.days <= 5.41
0.0936 < Uninsured.adults <= 0.1283
Percentage.of.households.with.high.housing.costs <= 0.0811
Alcohol.impaired.driving.deaths <= 0.182
Feature
0.000
0.005
0.010
Weight
Positive
Negative

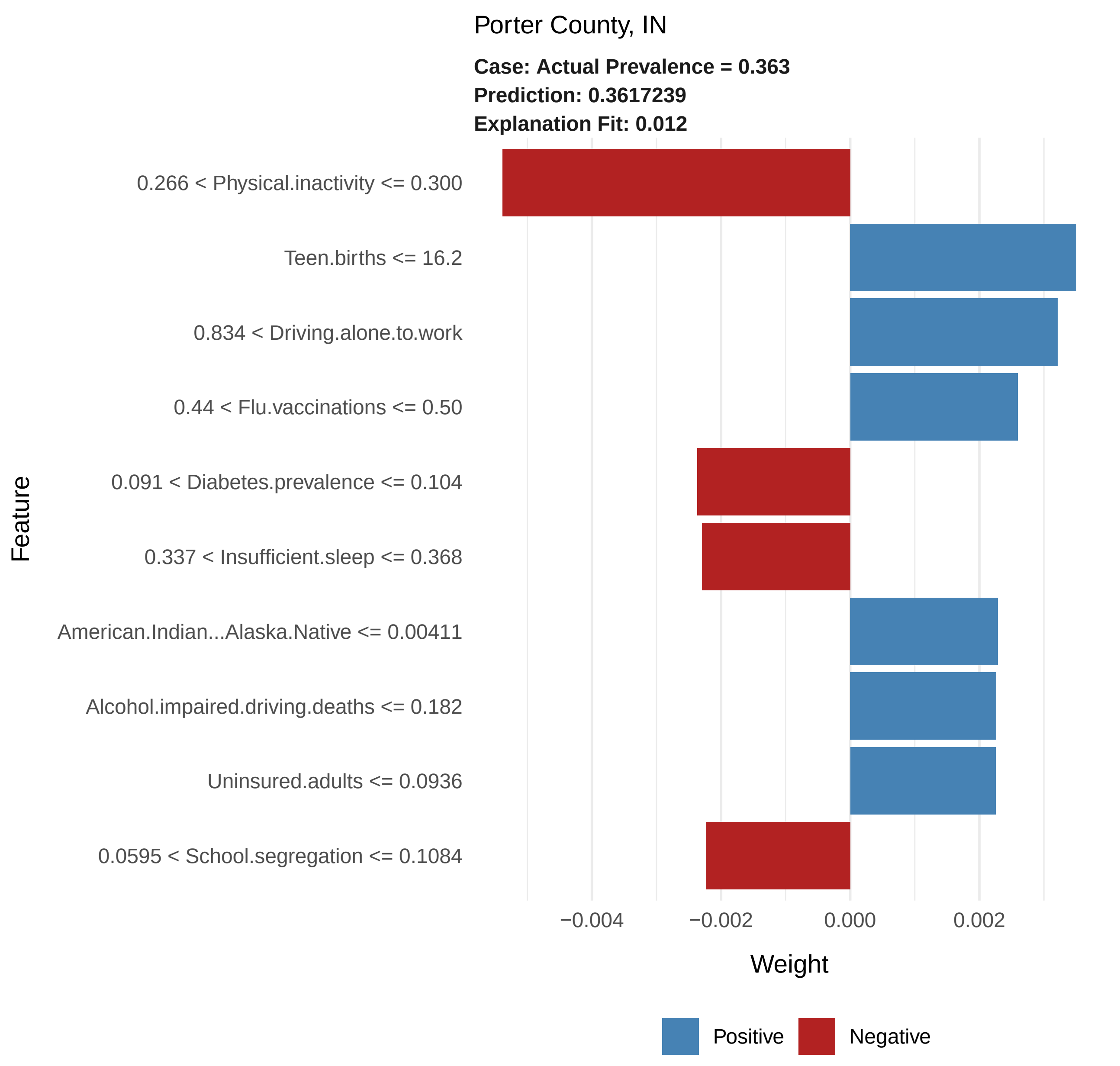

Porter County, IN
Case: Actual Prevalence = 0.363
Prediction: 0.3617239
Explanation Fit: 0.012
0.266 < Physical.inactivity <= 0.300
Teen.births <= 16.2
0.834 < Driving.alone.to.work
0.44 < Flu.vaccinations <= 0.50
0.091 < Diabetes.prevalence <= 0.104
0.337 < Insufficient.sleep <= 0.368
American.Indian...Alaska.Native <= 0.00411
Alcohol.impaired.driving.deaths <= 0.182
Uninsured.adults <= 0.0936
0.0595 < School.segregation <= 0.1084
Feature
−0.004
−0.002
0.000
0.002
Weight
Positive
Negative

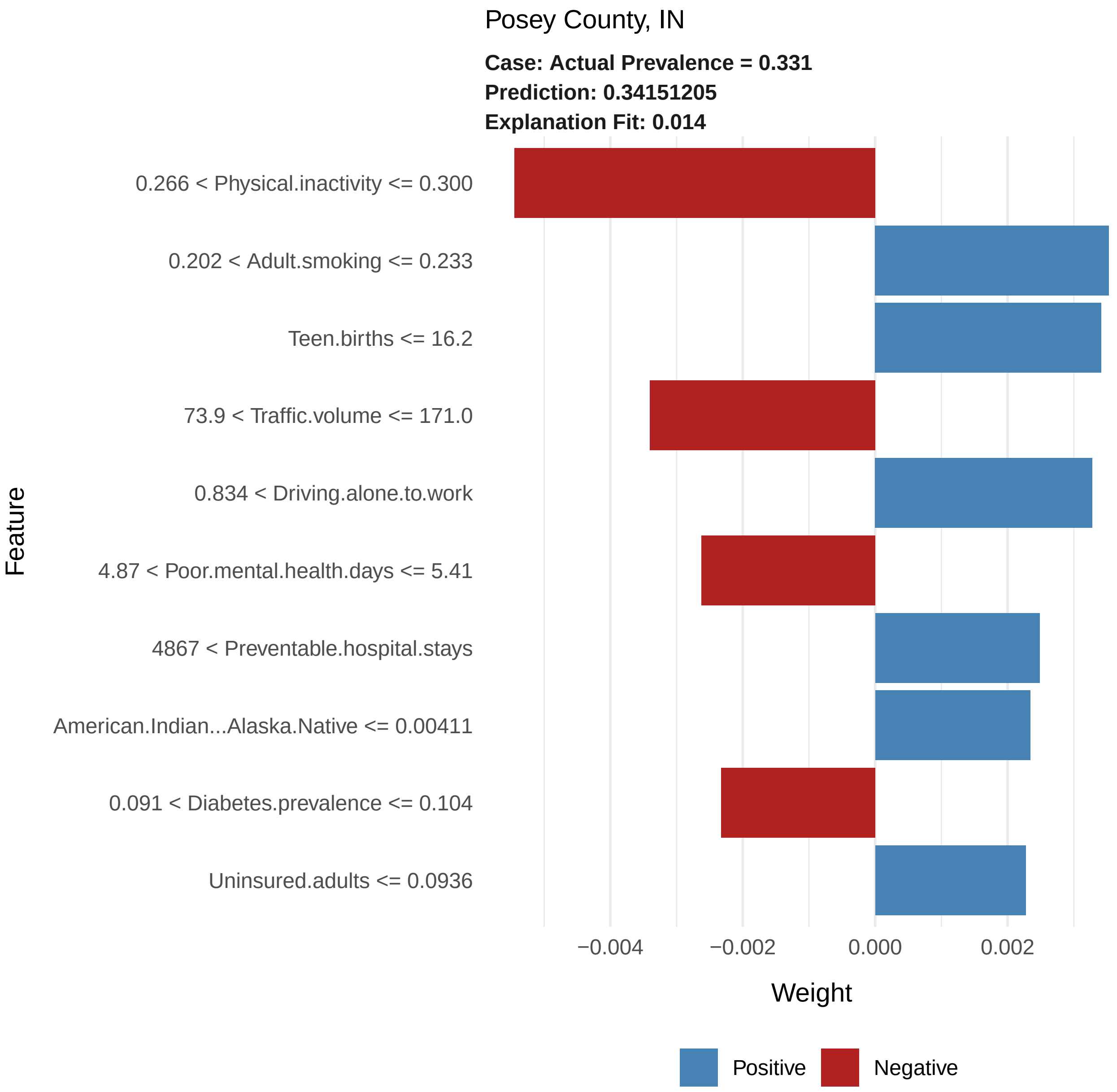

Posey County, IN
Case: Actual Prevalence = 0.331
Prediction: 0.34151205
Explanation Fit: 0.014
0.266 < Physical.inactivity <= 0.300
0.202 < Adult.smoking <= 0.233
Teen.births <= 16.2
73.9 < Traffic.volume <= 171.0
0.834 < Driving.alone.to.work
4.87 < Poor.mental.health.days <= 5.41
4867 < Preventable.hospital.stays
American.Indian...Alaska.Native <= 0.00411
0.091 < Diabetes.prevalence <= 0.104
Uninsured.adults <= 0.0936
Feature
−0.004
−0.002
0.000
0.002
Weight
Positive
Negative

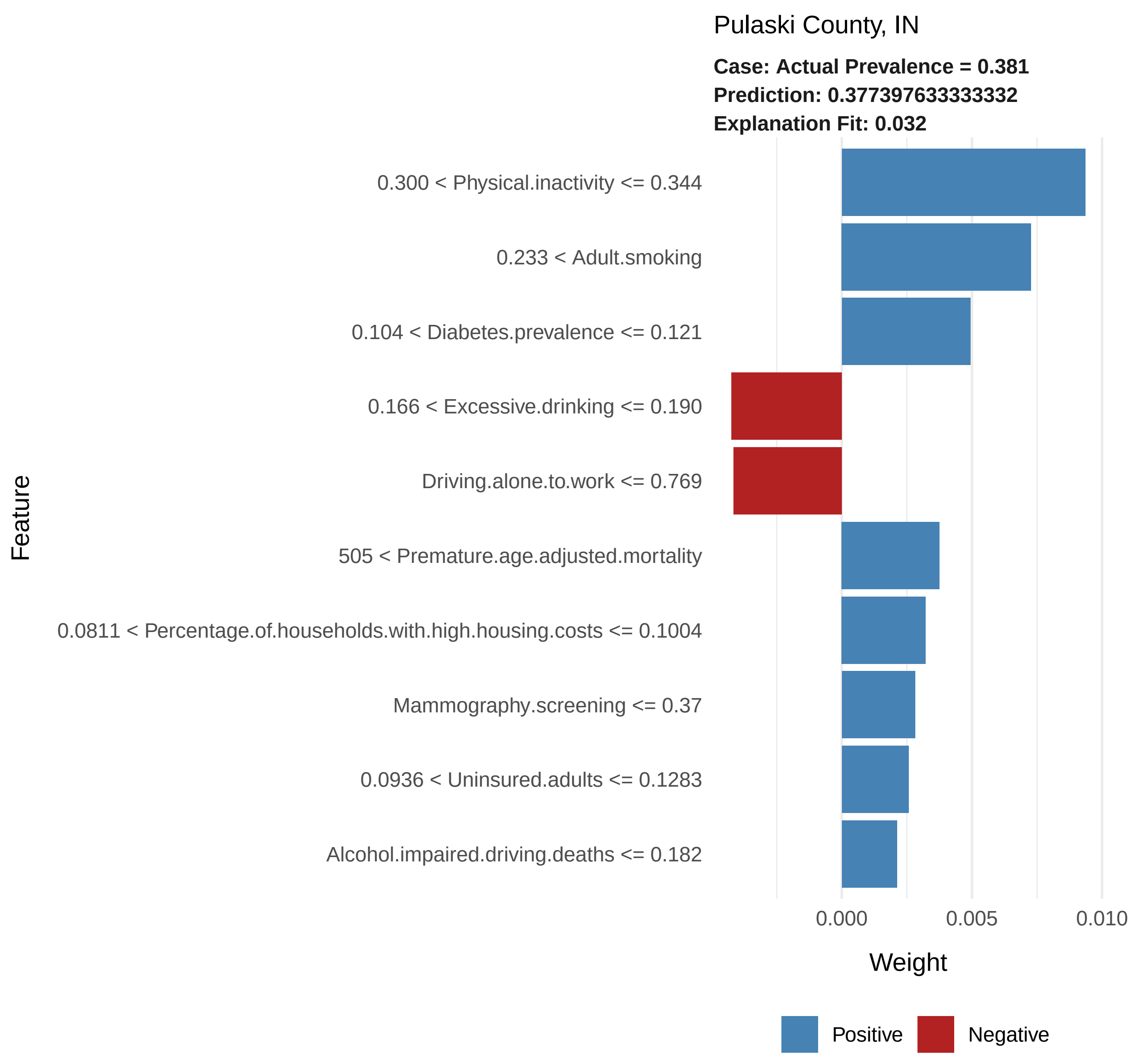
Pulaski County, IN
Case: Actual Prevalence = 0.381
Prediction: 0.377397633333332
Explanation Fit: 0.032
0.300 < Physical.inactivity <= 0.344
0.233 < Adult.smoking
0.104 < Diabetes.prevalence <= 0.121
0.166 < Excessive.drinking <= 0.190
Driving.alone.to.work <= 0.769
505 < Premature.age.adjusted.mortality
0.0811 < Percentage.of.households.with.high.housing.costs <= 0.1004
Mammography.screening <= 0.37
0.0936 < Uninsured.adults <= 0.1283
Alcohol.impaired.driving.deaths <= 0.182
Feature
0.000
0.005
0.010
Weight
Positive
Negative

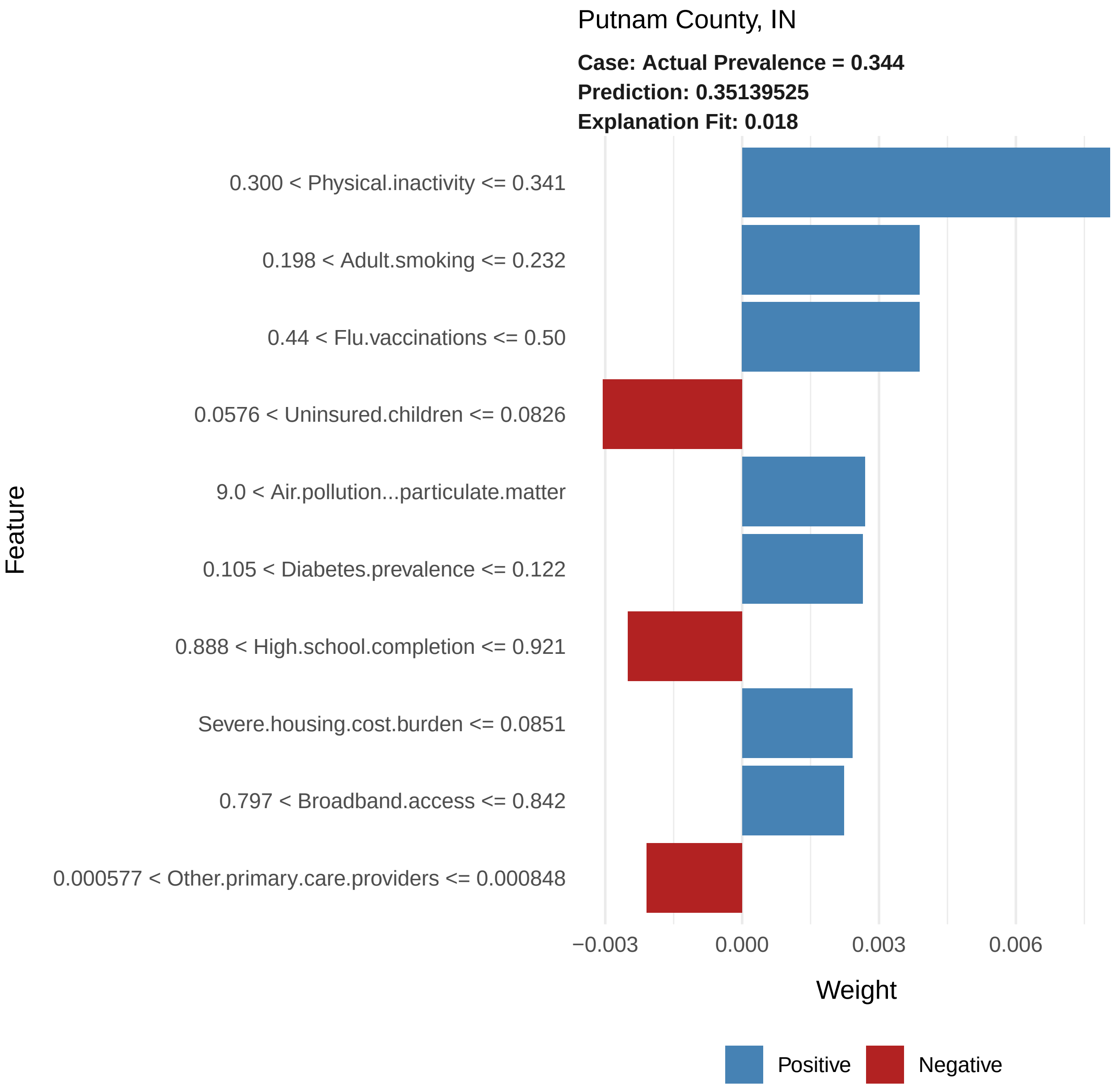
Putnam County, IN
Case: Actual Prevalence = 0.344
Prediction: 0.35139525
Explanation Fit: 0.018
0.300 < Physical.inactivity <= 0.341
0.198 < Adult.smoking <= 0.232
0.44 < Flu.vaccinations <= 0.50
0.0576 < Uninsured.children <= 0.0826
9.0 < Air.pollution...particulate.matter
0.105 < Diabetes.prevalence <= 0.122
0.888 < High.school.completion <= 0.921
Severe.housing.cost.burden <= 0.0851
0.797 < Broadband.access <= 0.842
0.000577 < Other.primary.care.providers <= 0.000848
Feature
−0.003
0.000
0.003
0.006
Weight
Positive
Negative

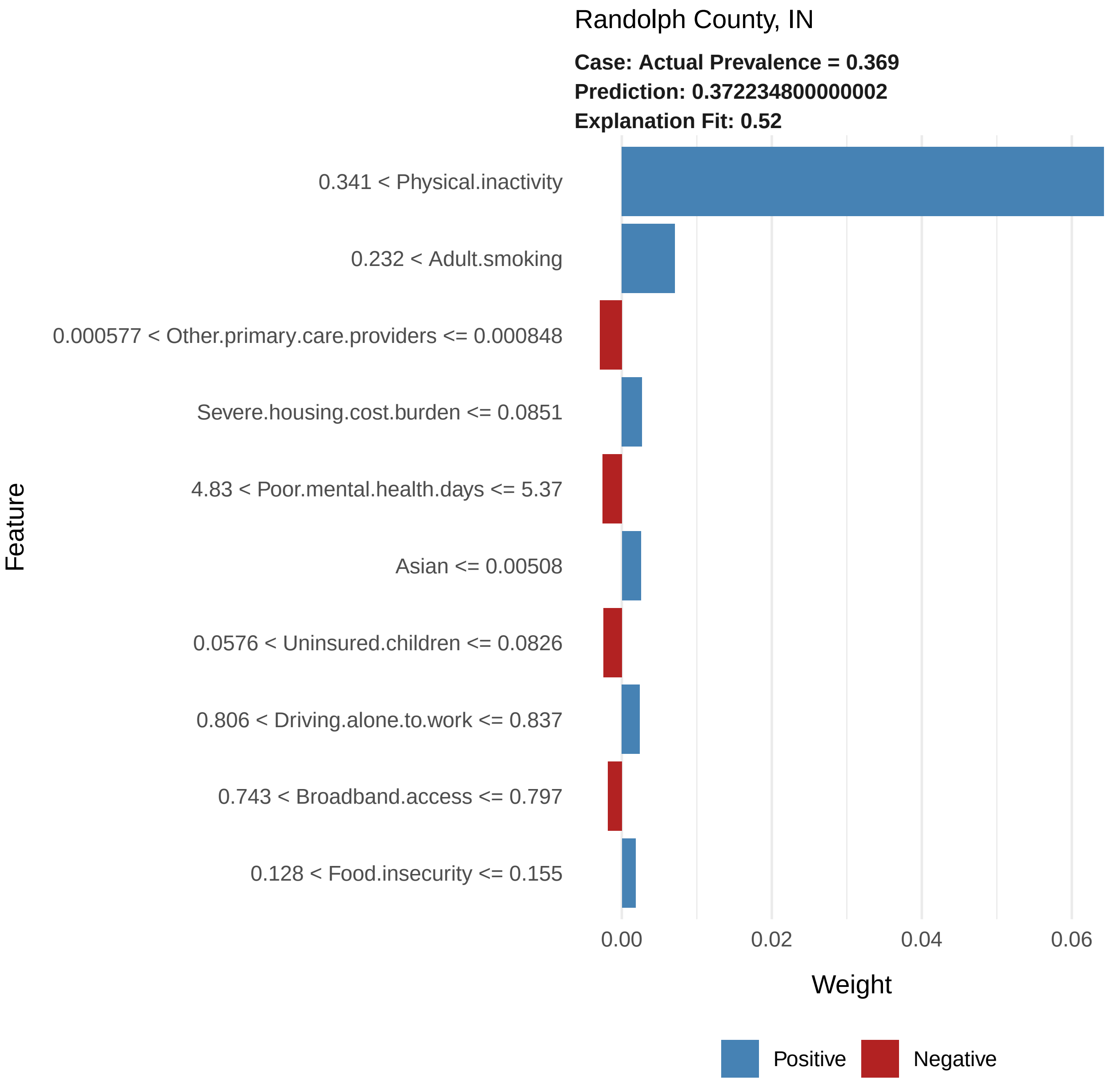
Randolph County, IN
Case: Actual Prevalence = 0.369
Prediction: 0.372234800000002
Explanation Fit: 0.52
0.341 < Physical.inactivity
0.232 < Adult.smoking
0.000577 < Other.primary.care.providers <= 0.000848
Severe.housing.cost.burden <= 0.0851
4.83 < Poor.mental.health.days <= 5.37
Asian <= 0.00508
0.0576 < Uninsured.children <= 0.0826
0.806 < Driving.alone.to.work <= 0.837
0.743 < Broadband.access <= 0.797
0.128 < Food.insecurity <= 0.155
Feature
0.00
0.02
0.04
0.06
Weight
Positive
Negative

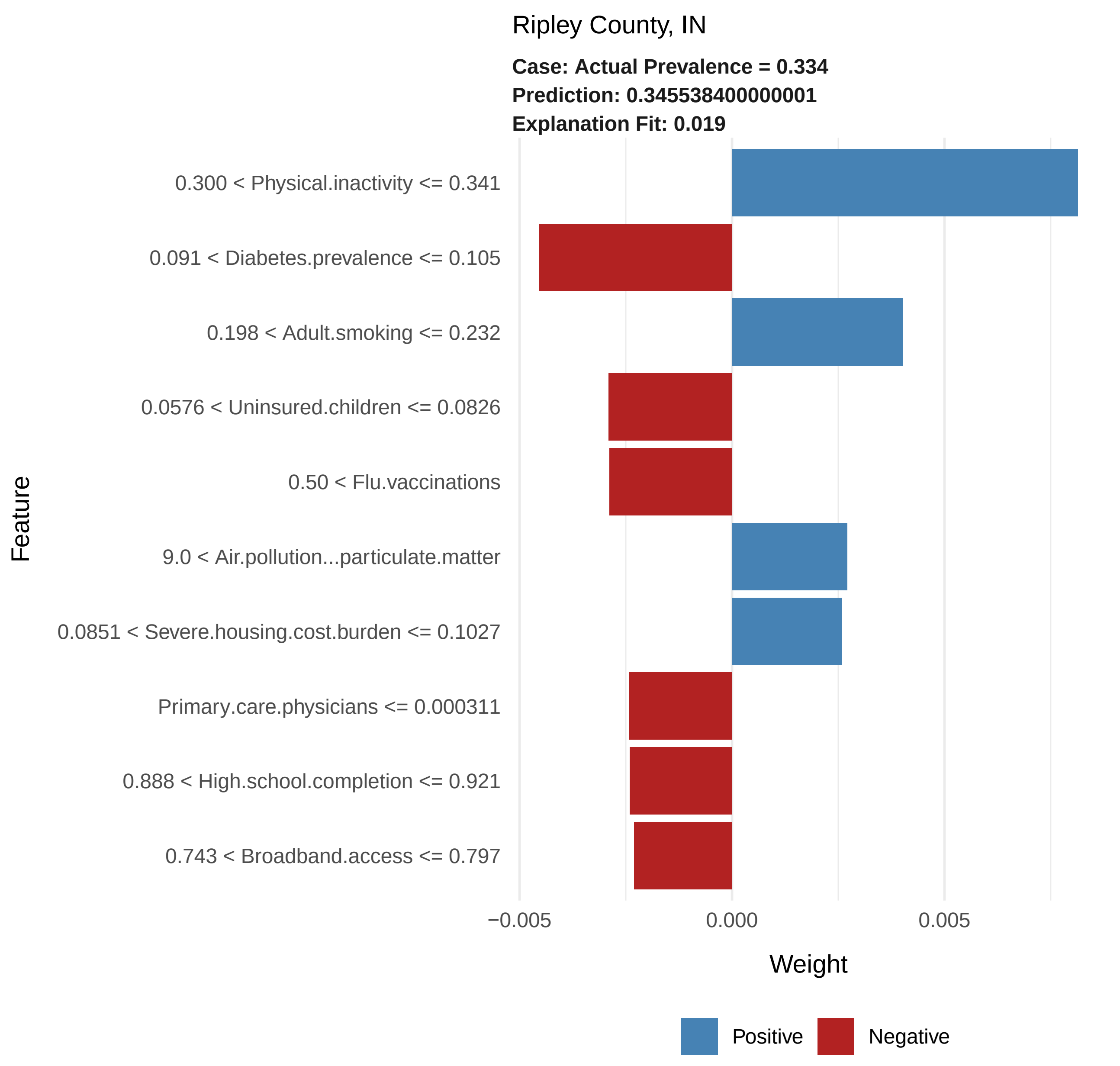
Ripley County, IN
Case: Actual Prevalence = 0.334
Prediction: 0.345538400000001
Explanation Fit: 0.019
0.300 < Physical.inactivity <= 0.341
0.091 < Diabetes.prevalence <= 0.105
0.198 < Adult.smoking <= 0.232
0.0576 < Uninsured.children <= 0.0826
0.50 < Flu.vaccinations
9.0 < Air.pollution...particulate.matter
0.0851 < Severe.housing.cost.burden <= 0.1027
Primary.care.physicians <= 0.000311
0.888 < High.school.completion <= 0.921
0.743 < Broadband.access <= 0.797
Feature
−0.005
0.000
0.005
Weight
Positive
Negative

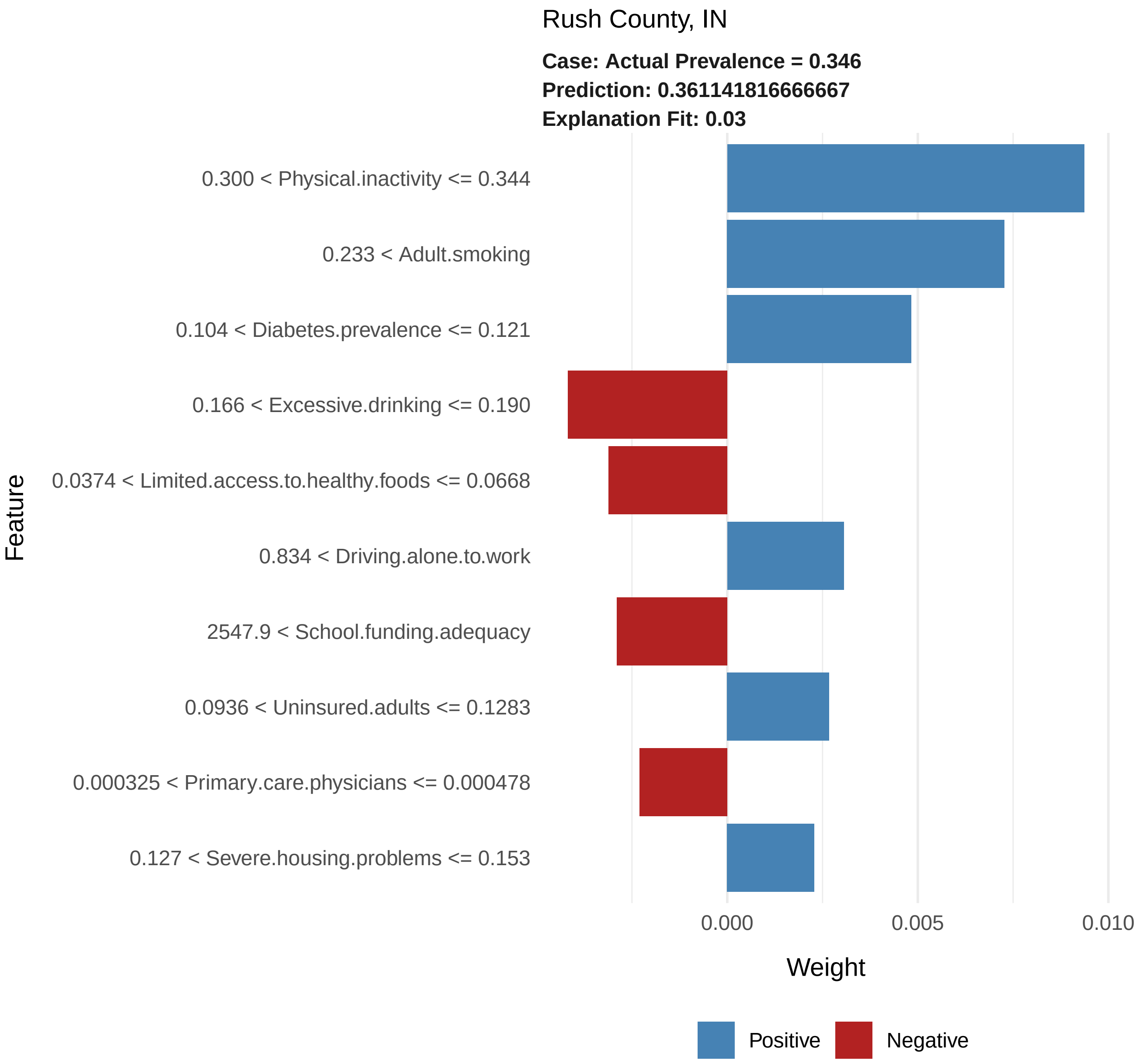

Rush County, IN
Case: Actual Prevalence = 0.346
Prediction: 0.361141816666667
Explanation Fit: 0.03
0.300 < Physical.inactivity <= 0.344
0.233 < Adult.smoking
0.104 < Diabetes.prevalence <= 0.121
0.166 < Excessive.drinking <= 0.190
0.0374 < Limited.access.to.healthy.foods <= 0.0668
0.834 < Driving.alone.to.work
2547.9 < School.funding.adequacy
0.0936 < Uninsured.adults <= 0.1283
0.000325 < Primary.care.physicians <= 0.000478
0.127 < Severe.housing.problems <= 0.153
Feature
0.000
0.005
0.010
Weight
Positive
Negative

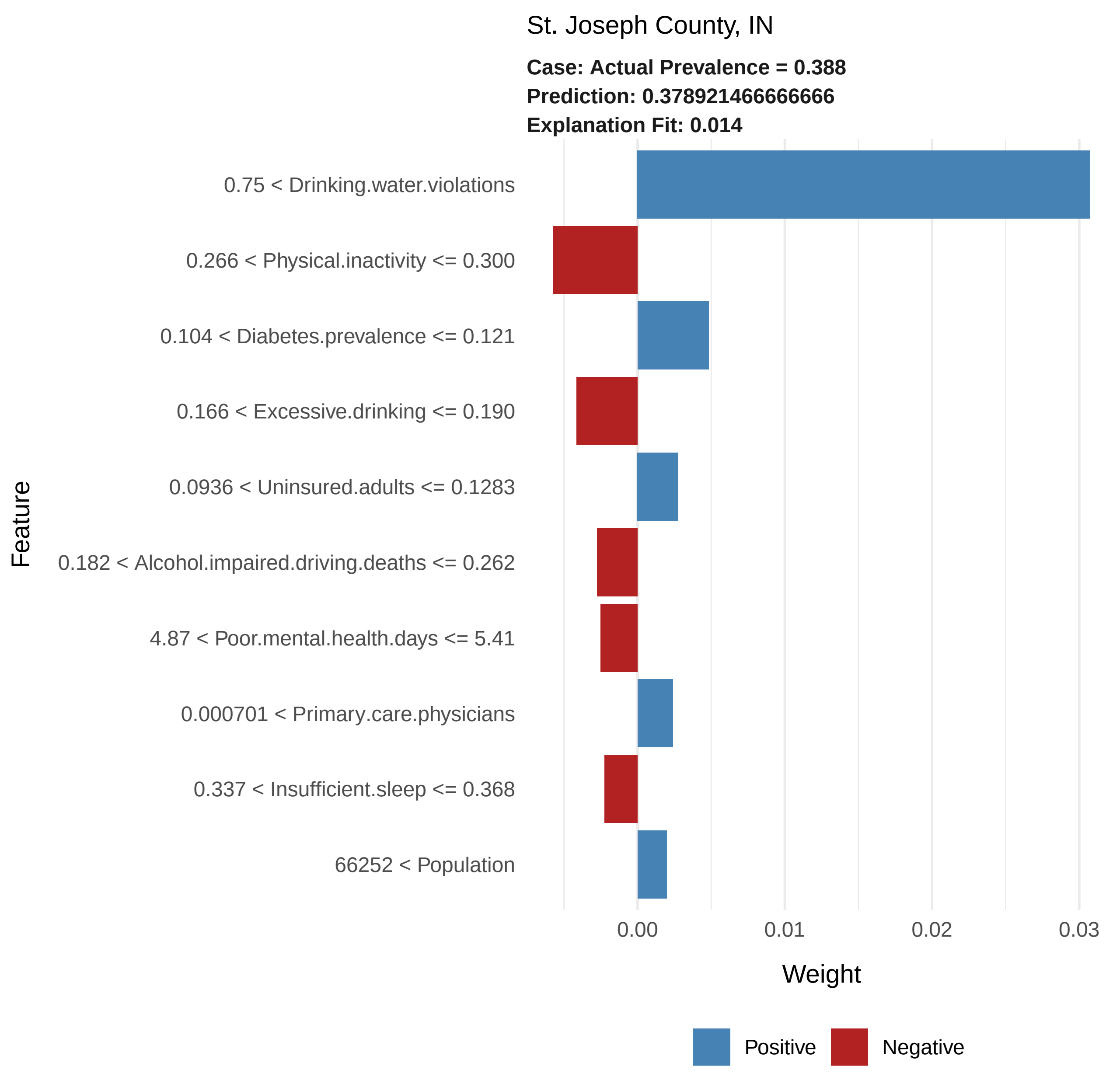
St. Joseph County, IN
Case: Actual Prevalence = 0.388
Prediction: 0.378921466666666
Explanation Fit: 0.014
0.75 < Drinking.water.violations
0.266 < Physical.inactivity <= 0.300
0.104 < Diabetes.prevalence <= 0.121
0.166 < Excessive.drinking <= 0.190
0.0936 < Uninsured.adults <= 0.1283
0.182 < Alcohol.impaired.driving.deaths <= 0.262
4.87 < Poor.mental.health.days <= 5.41
0.000701 < Primary.care.physicians
0.337 < Insufficient.sleep <= 0.368
66252 < Population
Feature
0.00
0.01
0.02
0.03
Weight
Positive
Negative

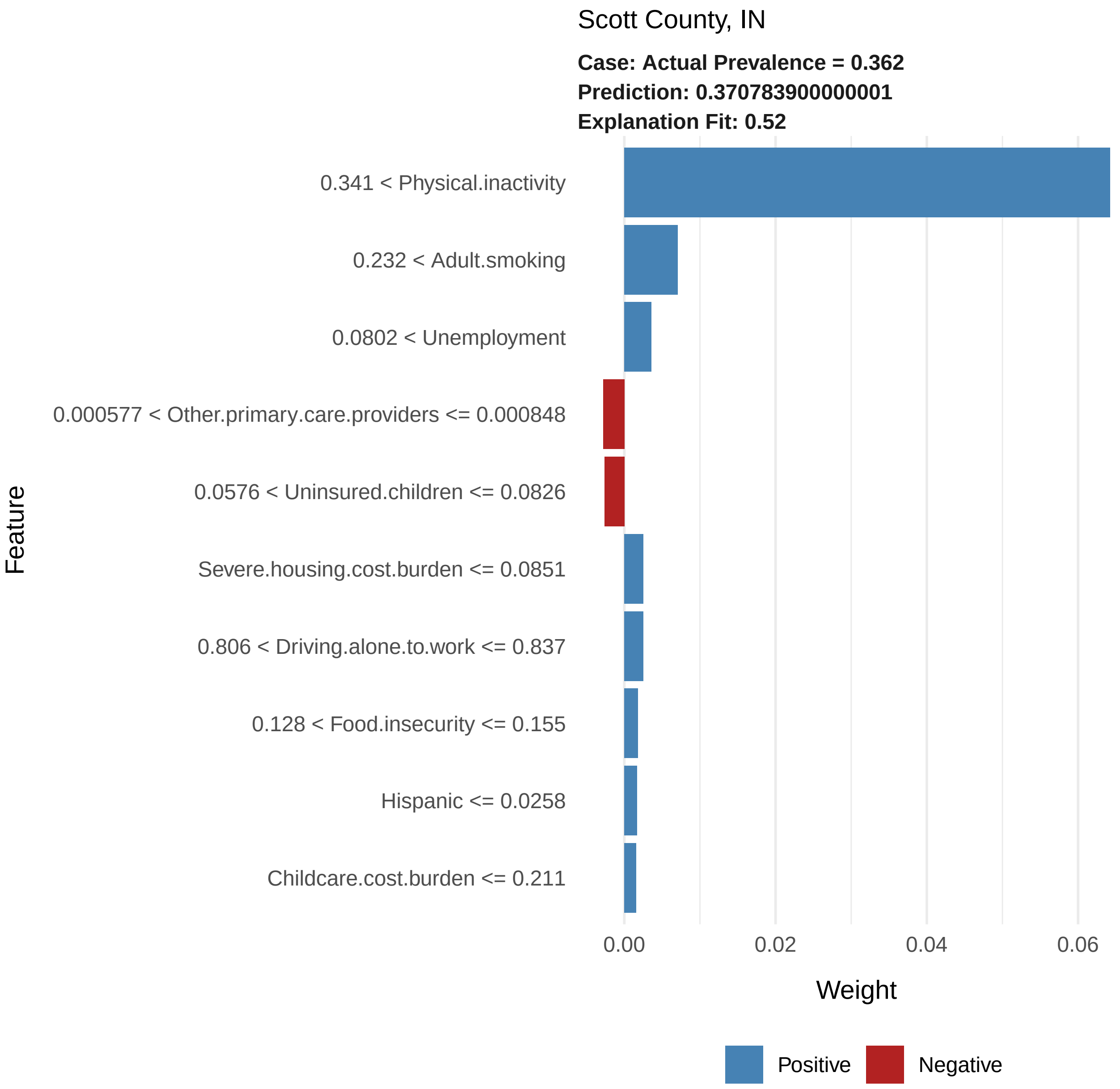

Scott County, IN
Case: Actual Prevalence = 0.362
Prediction: 0.370783900000001
Explanation Fit: 0.52
0.341 < Physical.inactivity
0.232 < Adult.smoking
0.0802 < Unemployment
0.000577 < Other.primary.care.providers <= 0.000848
0.0576 < Uninsured.children <= 0.0826
Severe.housing.cost.burden <= 0.0851
0.806 < Driving.alone.to.work <= 0.837
0.128 < Food.insecurity <= 0.155
Hispanic <= 0.0258
Childcare.cost.burden <= 0.211
Feature
0.00
0.02
0.04
0.06
Weight
Positive
Negative

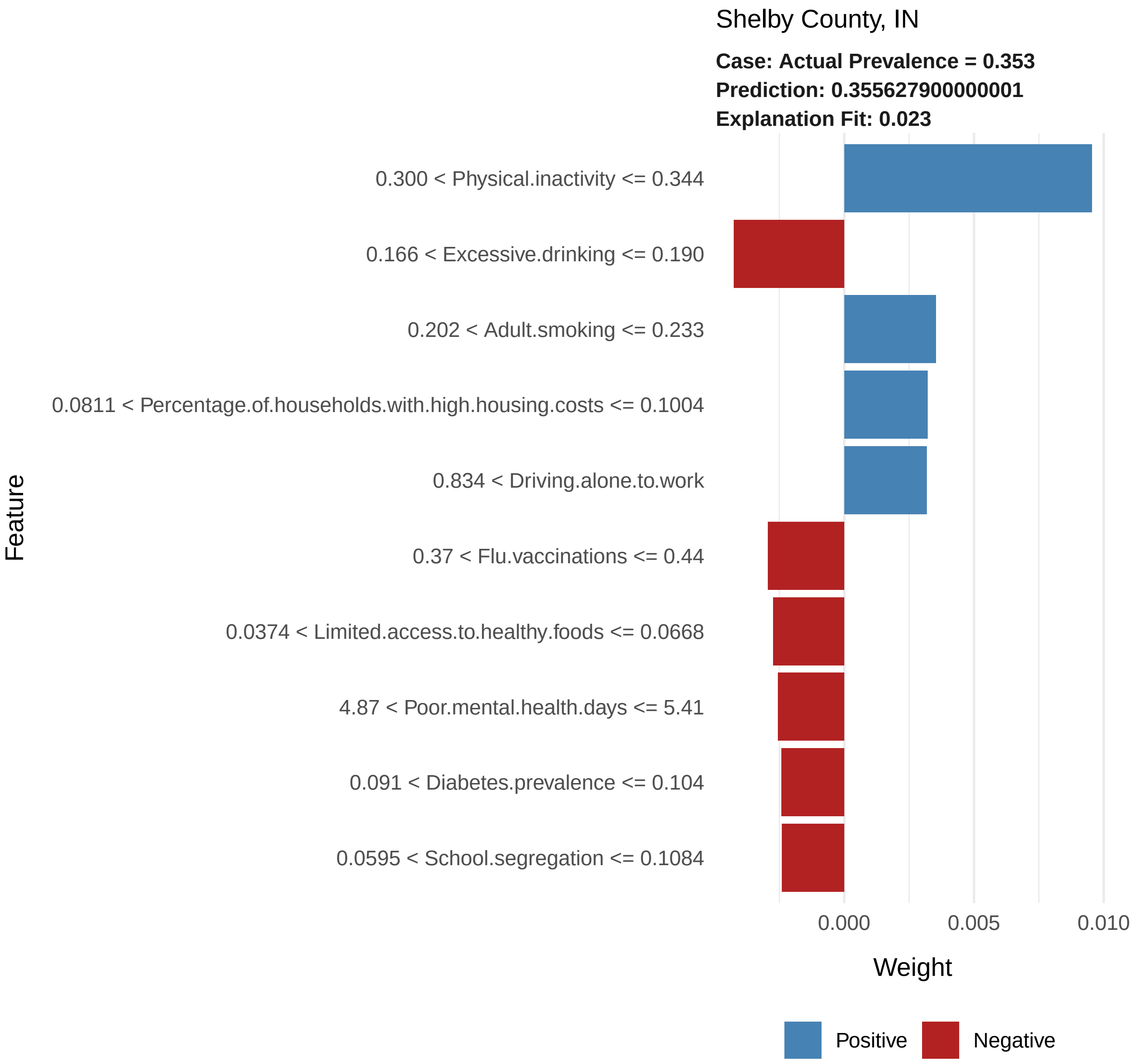
Shelby County, IN
Case: Actual Prevalence = 0.353
Prediction: 0.355627900000001
Explanation Fit: 0.023
0.300 < Physical.inactivity <= 0.344
0.166 < Excessive.drinking <= 0.190
0.202 < Adult.smoking <= 0.233
0.0811 < Percentage.of.households.with.high.housing.costs <= 0.1004
0.834 < Driving.alone.to.work
0.37 < Flu.vaccinations <= 0.44
0.0374 < Limited.access.to.healthy.foods <= 0.0668
4.87 < Poor.mental.health.days <= 5.41
0.091 < Diabetes.prevalence <= 0.104
0.0595 < School.segregation <= 0.1084
Feature
0.000
0.005
0.010
Weight
Positive
Negative

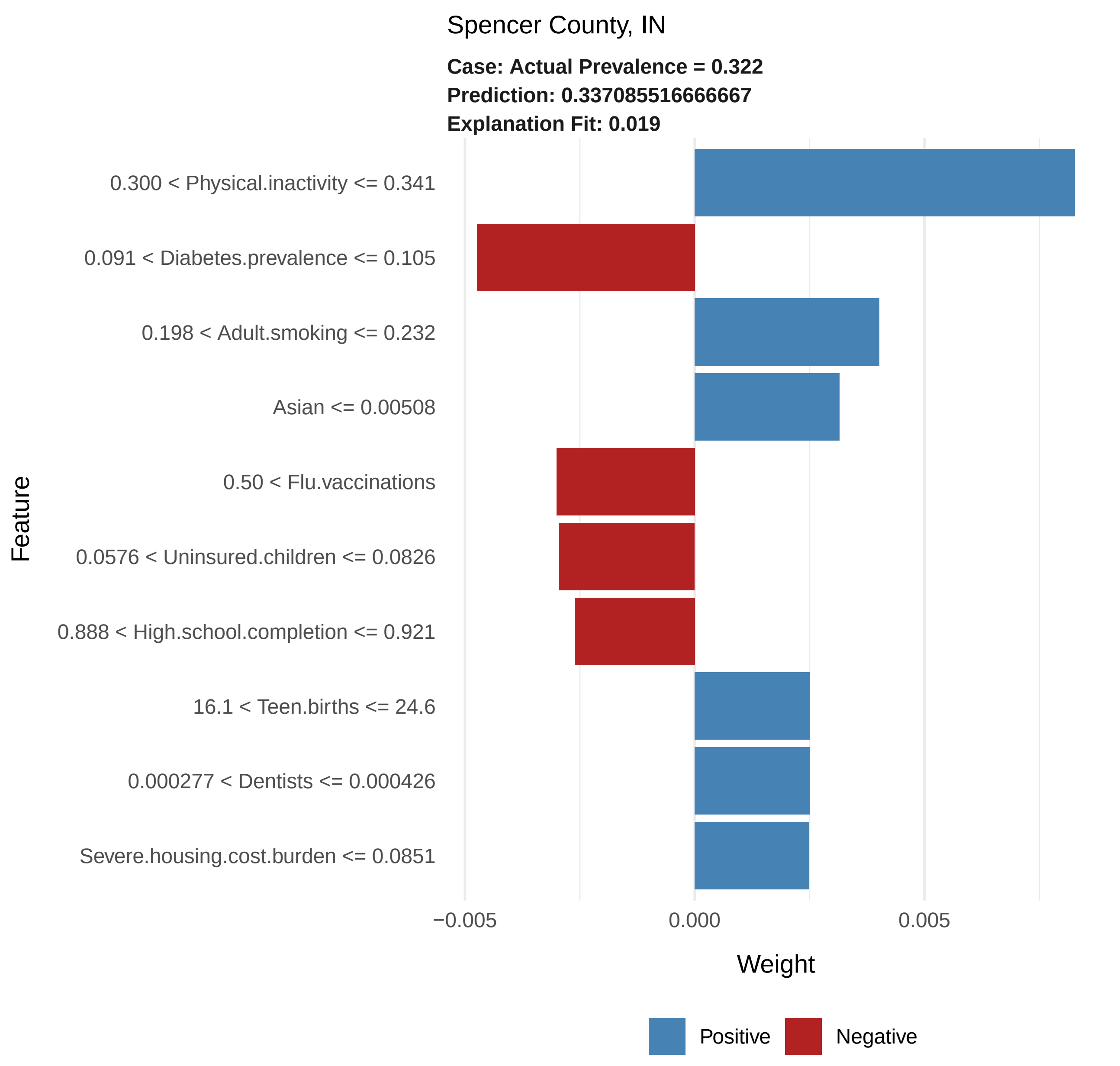
Spencer County, IN
Case: Actual Prevalence = 0.322
Prediction: 0.337085516666667
Explanation Fit: 0.019
0.300 < Physical.inactivity <= 0.341
0.091 < Diabetes.prevalence <= 0.105
0.198 < Adult.smoking <= 0.232
Asian <= 0.00508
0.50 < Flu.vaccinations
0.0576 < Uninsured.children <= 0.0826
0.888 < High.school.completion <= 0.921
16.1 < Teen.births <= 24.6
0.000277 < Dentists <= 0.000426
Severe.housing.cost.burden <= 0.0851
Feature
−0.005
0.000
0.005
Weight
Positive
Negative

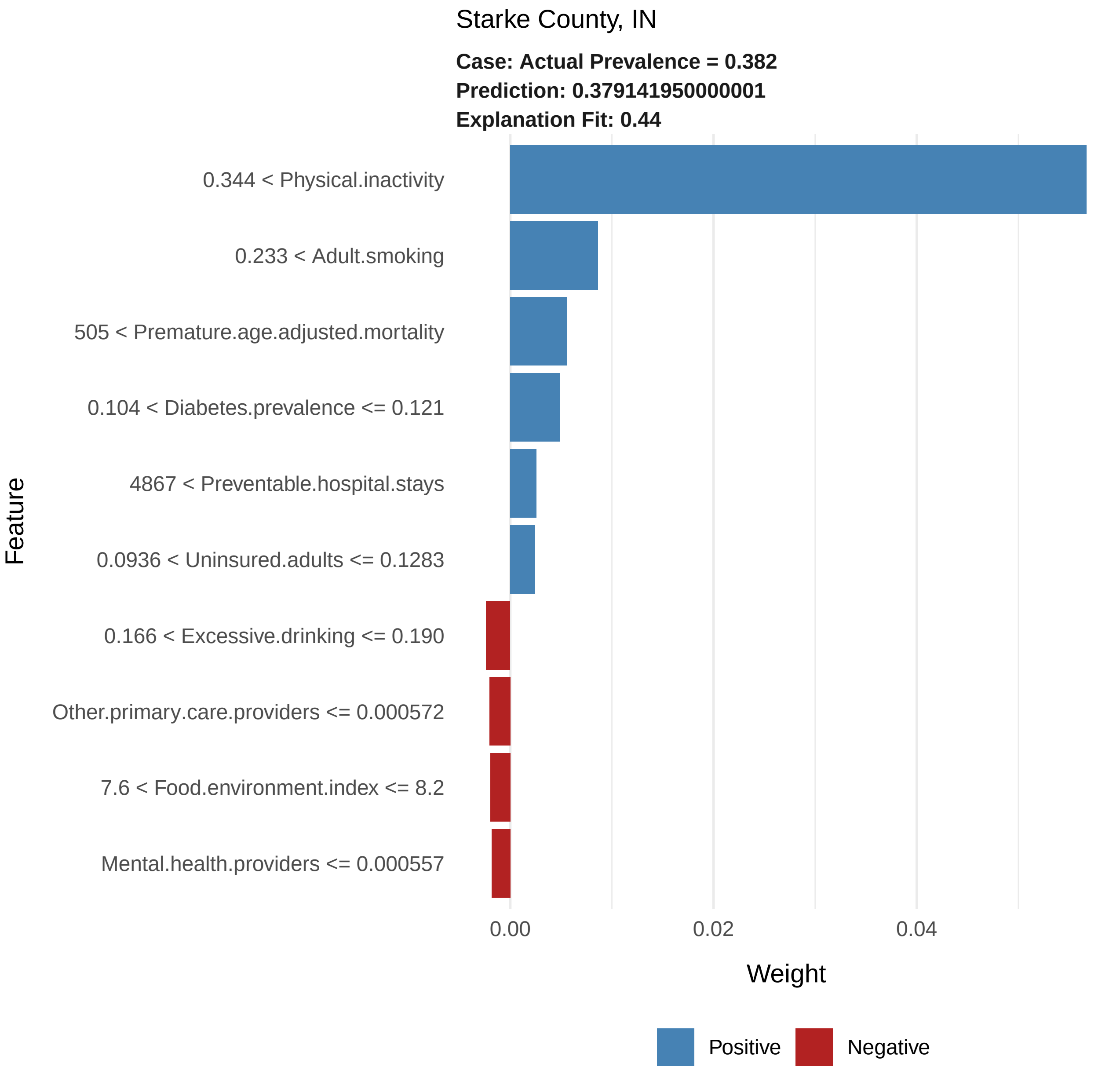

Starke County, IN
Case: Actual Prevalence = 0.382
Prediction: 0.379141950000001
Explanation Fit: 0.44
0.344 < Physical.inactivity
0.233 < Adult.smoking
505 < Premature.age.adjusted.mortality
0.104 < Diabetes.prevalence <= 0.121
4867 < Preventable.hospital.stays
0.0936 < Uninsured.adults <= 0.1283
0.166 < Excessive.drinking <= 0.190
Other.primary.care.providers <= 0.000572
7.6 < Food.environment.index <= 8.2
Mental.health.providers <= 0.000557
Feature
0.00
0.02
0.04
Weight
Positive
Negative

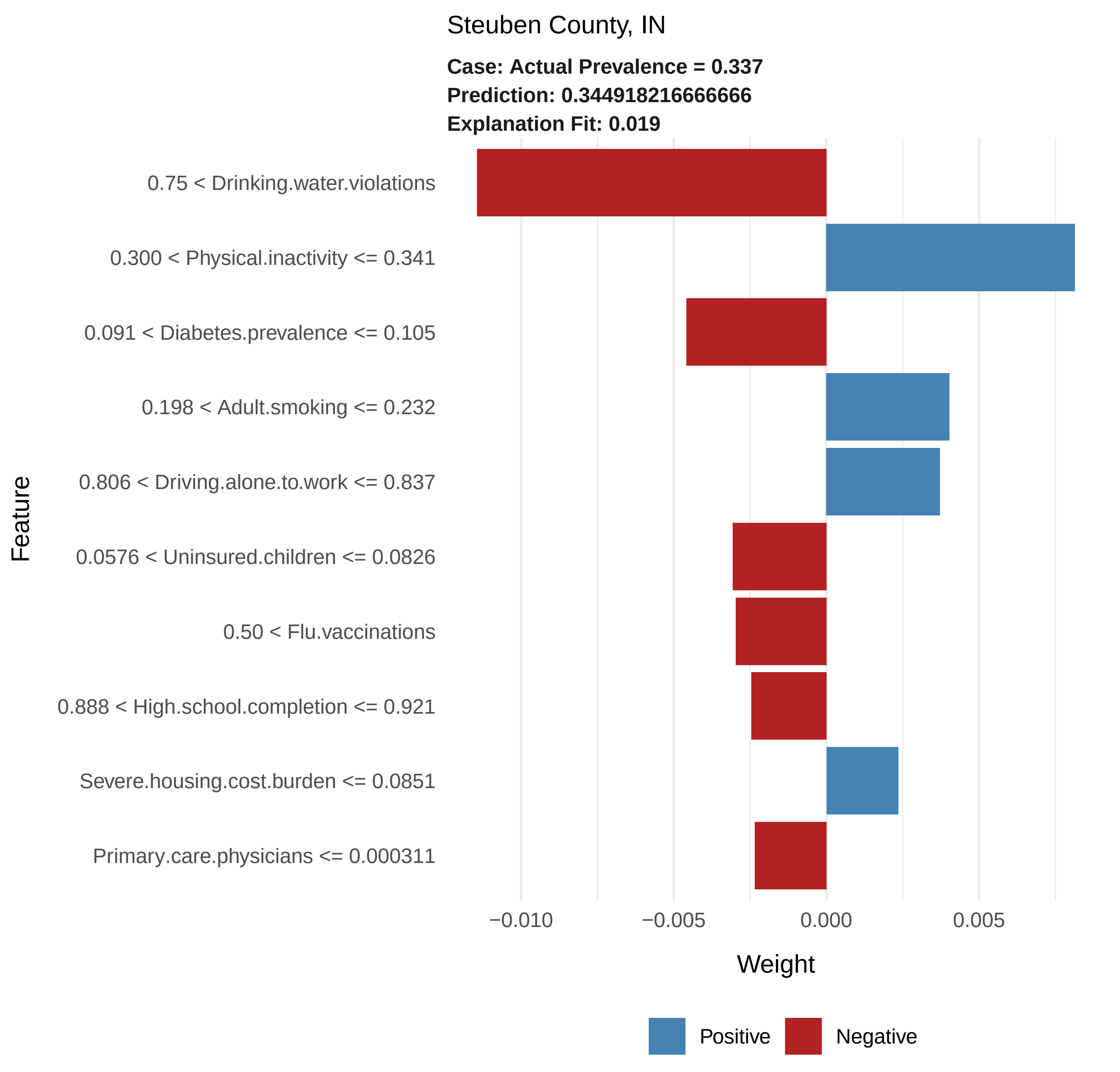
Steuben County, IN
Case: Actual Prevalence = 0.337
Prediction: 0.344918216666666
Explanation Fit: 0.019
0.75 < Drinking.water.violations
0.300 < Physical.inactivity <= 0.341
0.091 < Diabetes.prevalence <= 0.105
0.198 < Adult.smoking <= 0.232
0.806 < Driving.alone.to.work <= 0.837
0.0576 < Uninsured.children <= 0.0826
0.50 < Flu.vaccinations
0.888 < High.school.completion <= 0.921
Severe.housing.cost.burden <= 0.0851
Primary.care.physicians <= 0.000311
Feature
−0.010
−0.005
0.000
0.005
Weight
Positive
Negative

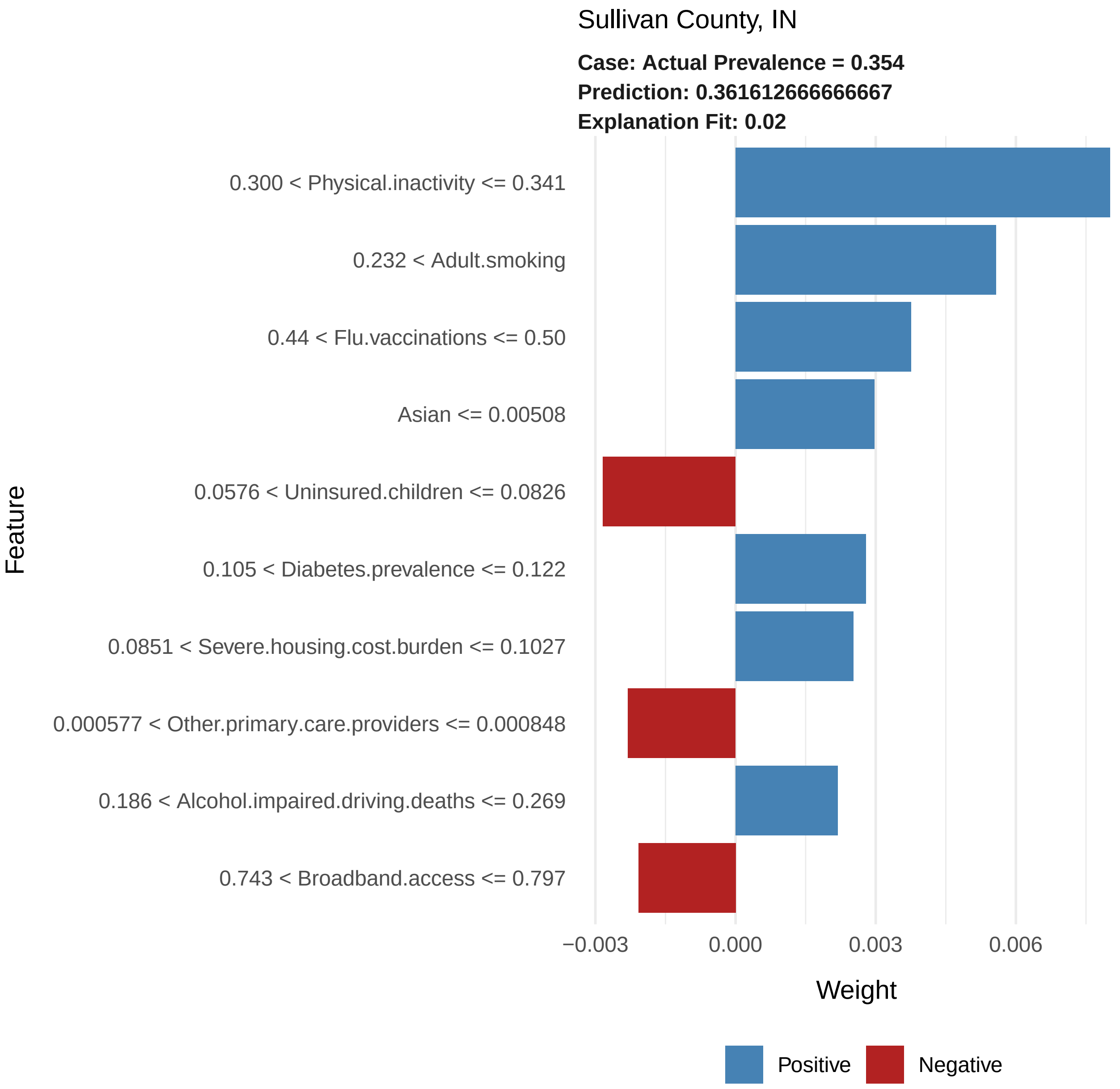
Sullivan County, IN
Case: Actual Prevalence = 0.354
Prediction: 0.361612666666667
Explanation Fit: 0.02
0.300 < Physical.inactivity <= 0.341
0.232 < Adult.smoking
0.44 < Flu.vaccinations <= 0.50
Asian <= 0.00508
0.0576 < Uninsured.children <= 0.0826
0.105 < Diabetes.prevalence <= 0.122
0.0851 < Severe.housing.cost.burden <= 0.1027
0.000577 < Other.primary.care.providers <= 0.000848
0.186 < Alcohol.impaired.driving.deaths <= 0.269
0.743 < Broadband.access <= 0.797
Feature
−0.003
0.000
0.003
0.006
Weight
Positive
Negative

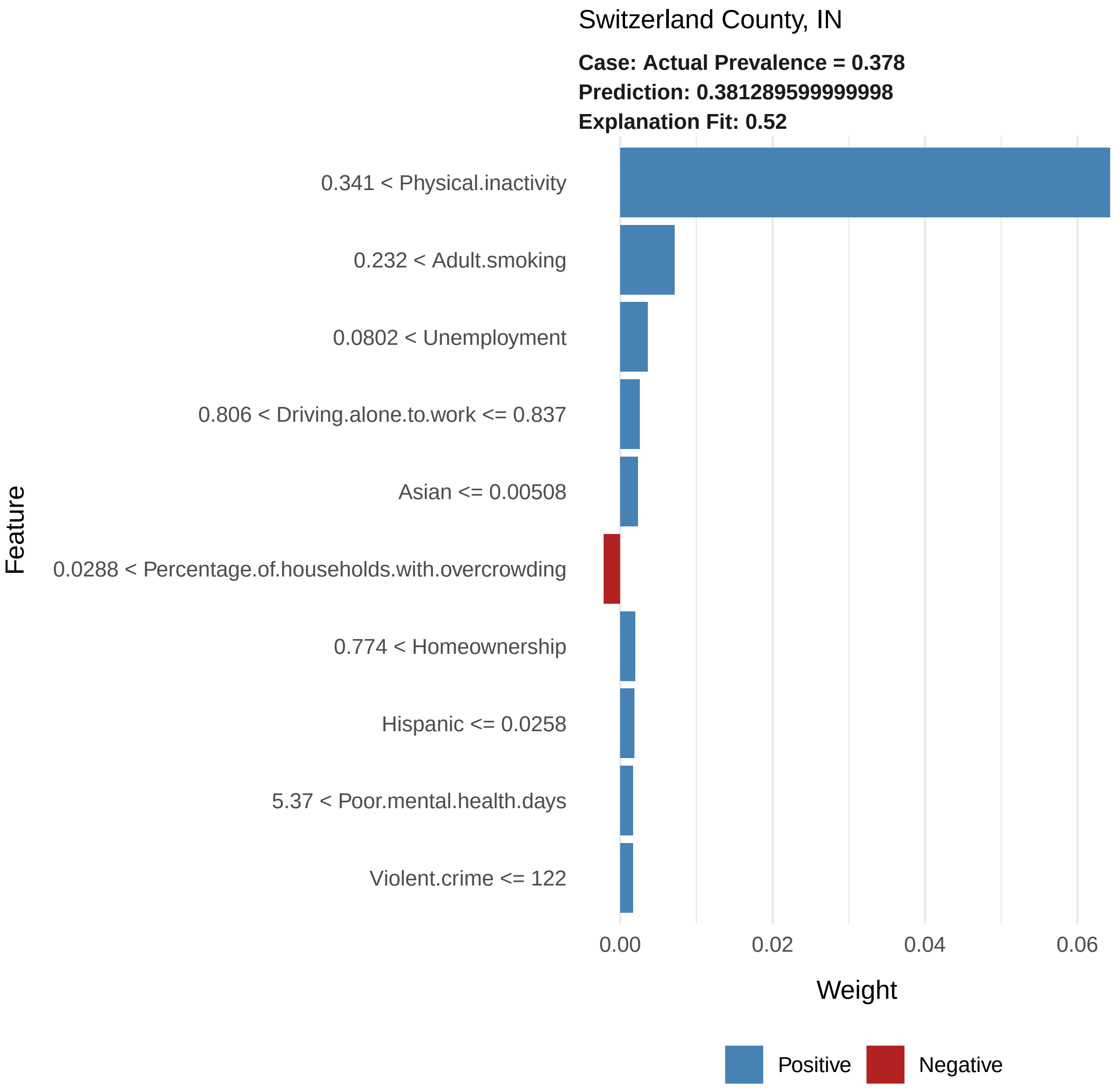

Switzerland County, IN
Case: Actual Prevalence = 0.378
Prediction: 0.381289599999998
Explanation Fit: 0.52
0.341 < Physical.inactivity
0.232 < Adult.smoking
0.0802 < Unemployment
0.806 < Driving.alone.to.work <= 0.837
Asian <= 0.00508
0.0288 < Percentage.of.households.with.overcrowding
0.774 < Homeownership
Hispanic <= 0.0258
5.37 < Poor.mental.health.days
Violent.crime <= 122
Feature
0.00
0.02
0.04
0.06
Weight
Positive
Negative

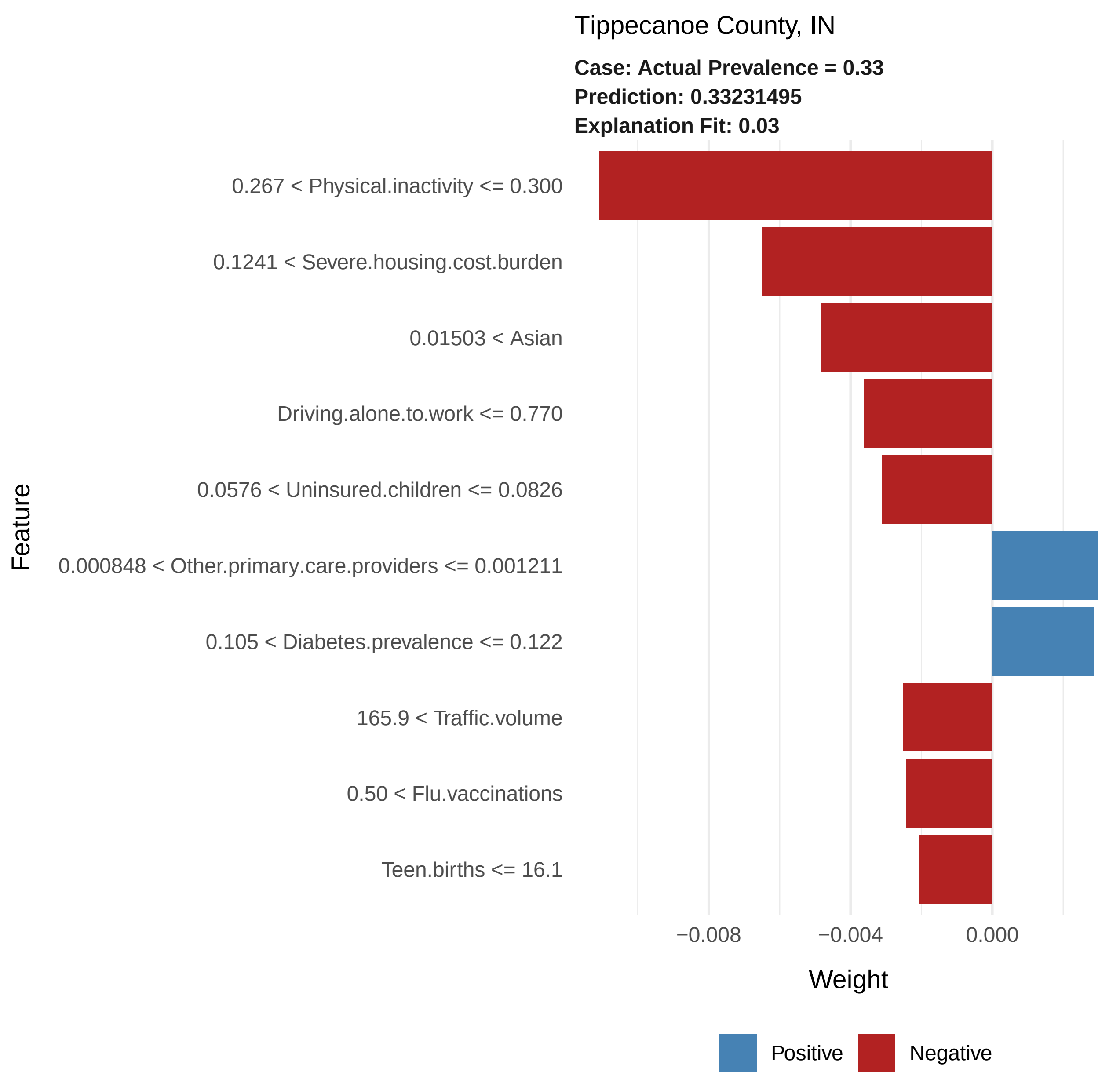

Tippecanoe County, IN
Case: Actual Prevalence = 0.33
Prediction: 0.33231495
Explanation Fit: 0.03
0.267 < Physical.inactivity <= 0.300
0.1241 < Severe.housing.cost.burden
0.01503 < Asian
Driving.alone.to.work <= 0.770
0.0576 < Uninsured.children <= 0.0826
0.000848 < Other.primary.care.providers <= 0.001211
0.105 < Diabetes.prevalence <= 0.122
165.9 < Traffic.volume
0.50 < Flu.vaccinations
Teen.births <= 16.1
Feature
−0.008
−0.004
0.000
Weight
Positive
Negative

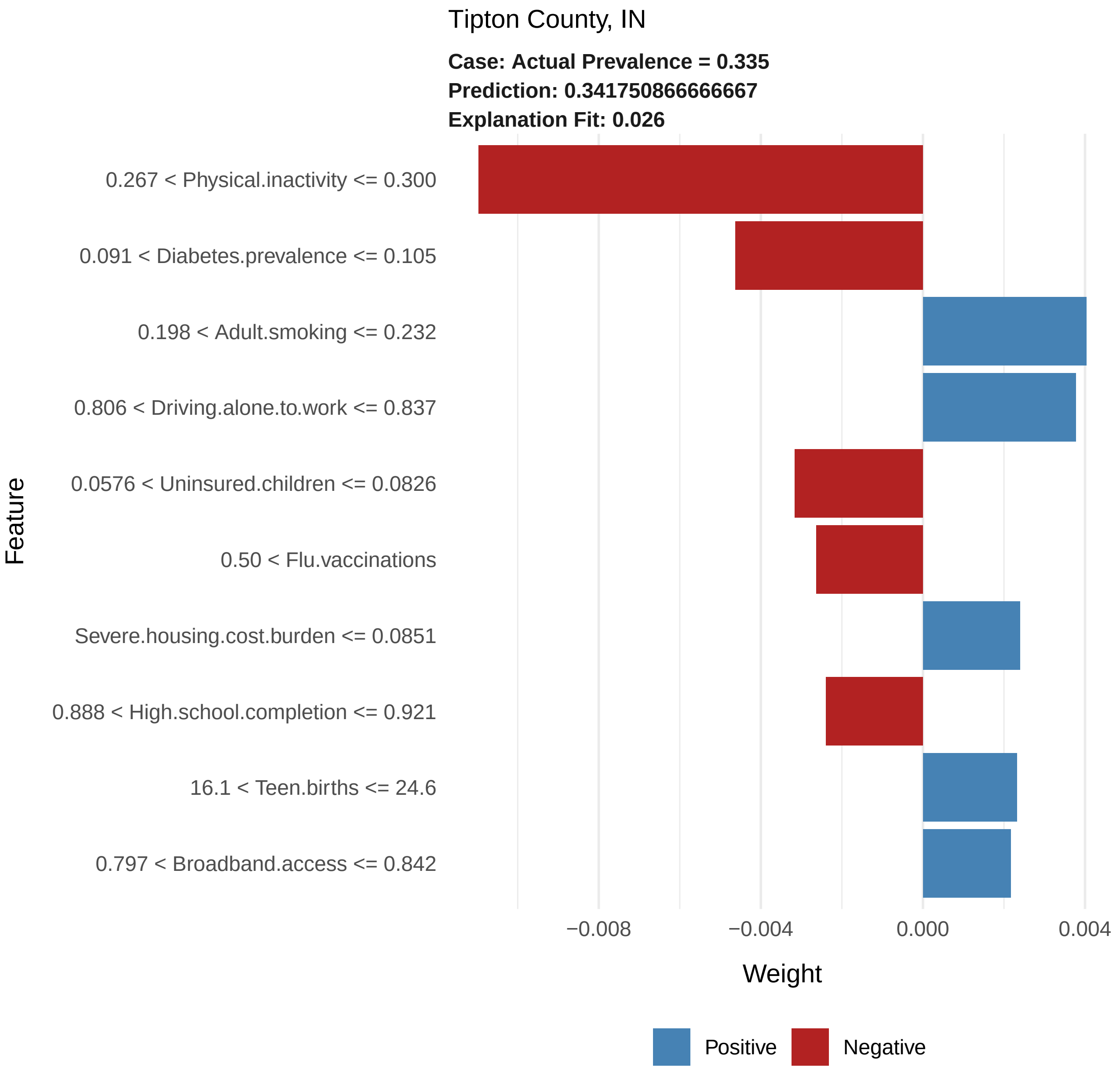

Tipton County, IN
Case: Actual Prevalence = 0.335
Prediction: 0.341750866666667
Explanation Fit: 0.026
0.267 < Physical.inactivity <= 0.300
0.091 < Diabetes.prevalence <= 0.105
0.198 < Adult.smoking <= 0.232
0.806 < Driving.alone.to.work <= 0.837
0.0576 < Uninsured.children <= 0.0826
0.50 < Flu.vaccinations
Severe.housing.cost.burden <= 0.0851
0.888 < High.school.completion <= 0.921
16.1 < Teen.births <= 24.6
0.797 < Broadband.access <= 0.842
Feature
−0.008
−0.004
0.000
0.004
Weight
Positive
Negative

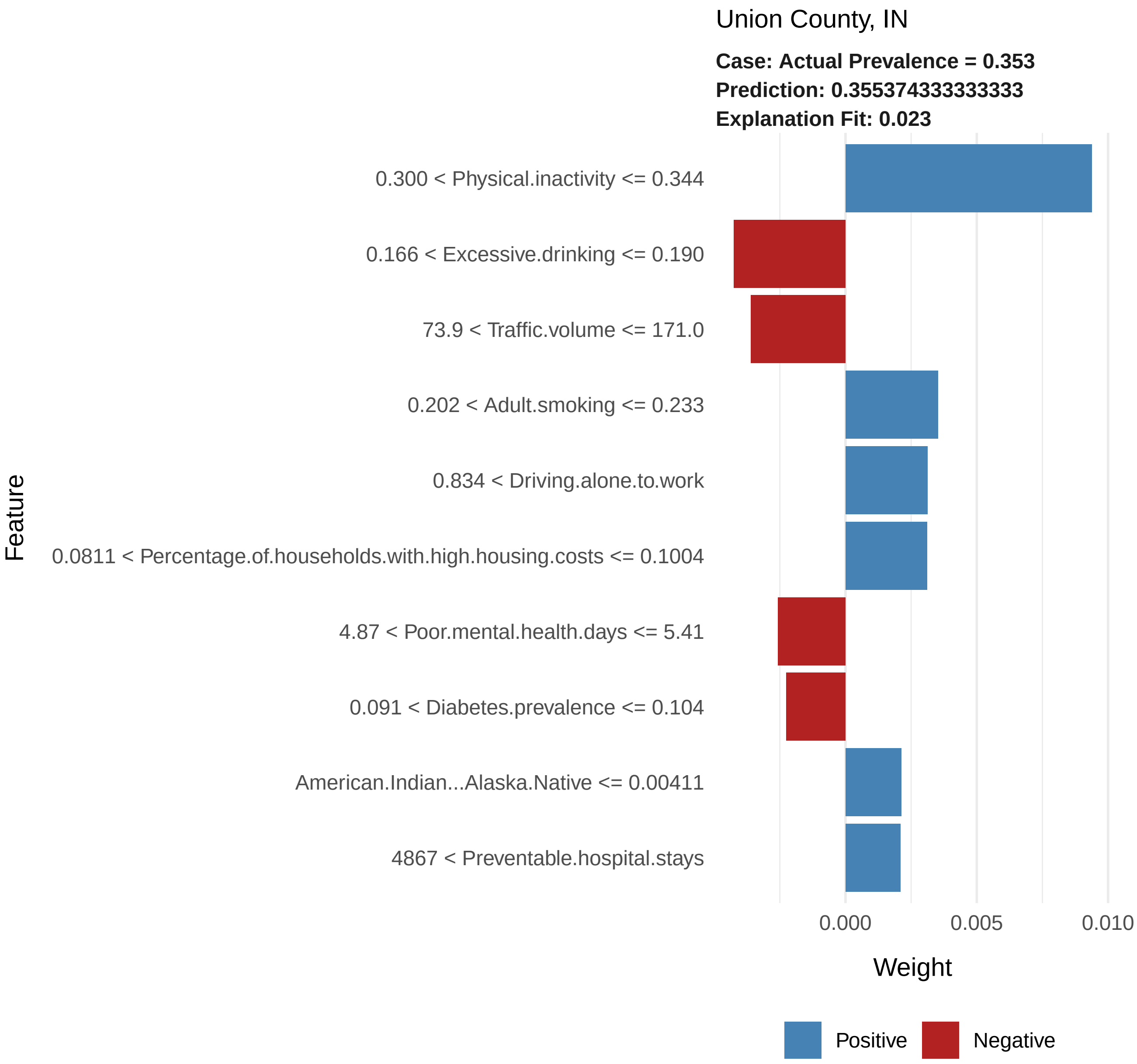
Union County, IN
Case: Actual Prevalence = 0.353
Prediction: 0.355374333333333
Explanation Fit: 0.023
0.300 < Physical.inactivity <= 0.344
0.166 < Excessive.drinking <= 0.190
73.9 < Traffic.volume <= 171.0
0.202 < Adult.smoking <= 0.233
0.834 < Driving.alone.to.work
0.0811 < Percentage.of.households.with.high.housing.costs <= 0.1004
4.87 < Poor.mental.health.days <= 5.41
0.091 < Diabetes.prevalence <= 0.104
American.Indian...Alaska.Native <= 0.00411
4867 < Preventable.hospital.stays
Feature
0.000
0.005
0.010
Weight
Positive
Negative

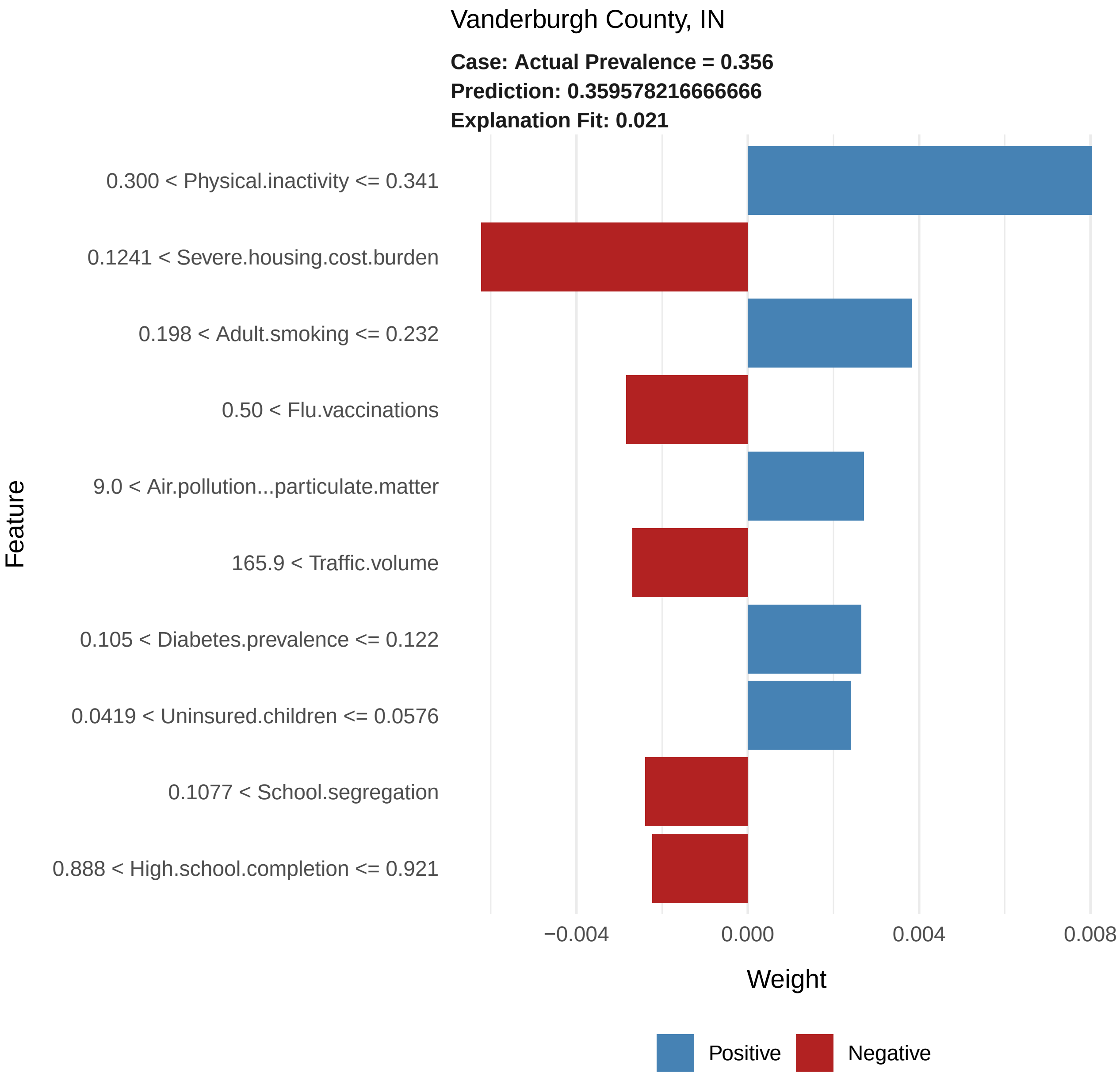
Vanderburgh County, IN
Case: Actual Prevalence = 0.356
Prediction: 0.359578216666666
Explanation Fit: 0.021
0.300 < Physical.inactivity <= 0.341
0.1241 < Severe.housing.cost.burden
0.198 < Adult.smoking <= 0.232
0.50 < Flu.vaccinations
9.0 < Air.pollution...particulate.matter
165.9 < Traffic.volume
0.105 < Diabetes.prevalence <= 0.122
0.0419 < Uninsured.children <= 0.0576
0.1077 < School.segregation
0.888 < High.school.completion <= 0.921
Feature
−0.004
0.000
0.004
0.008
Weight
Positive
Negative

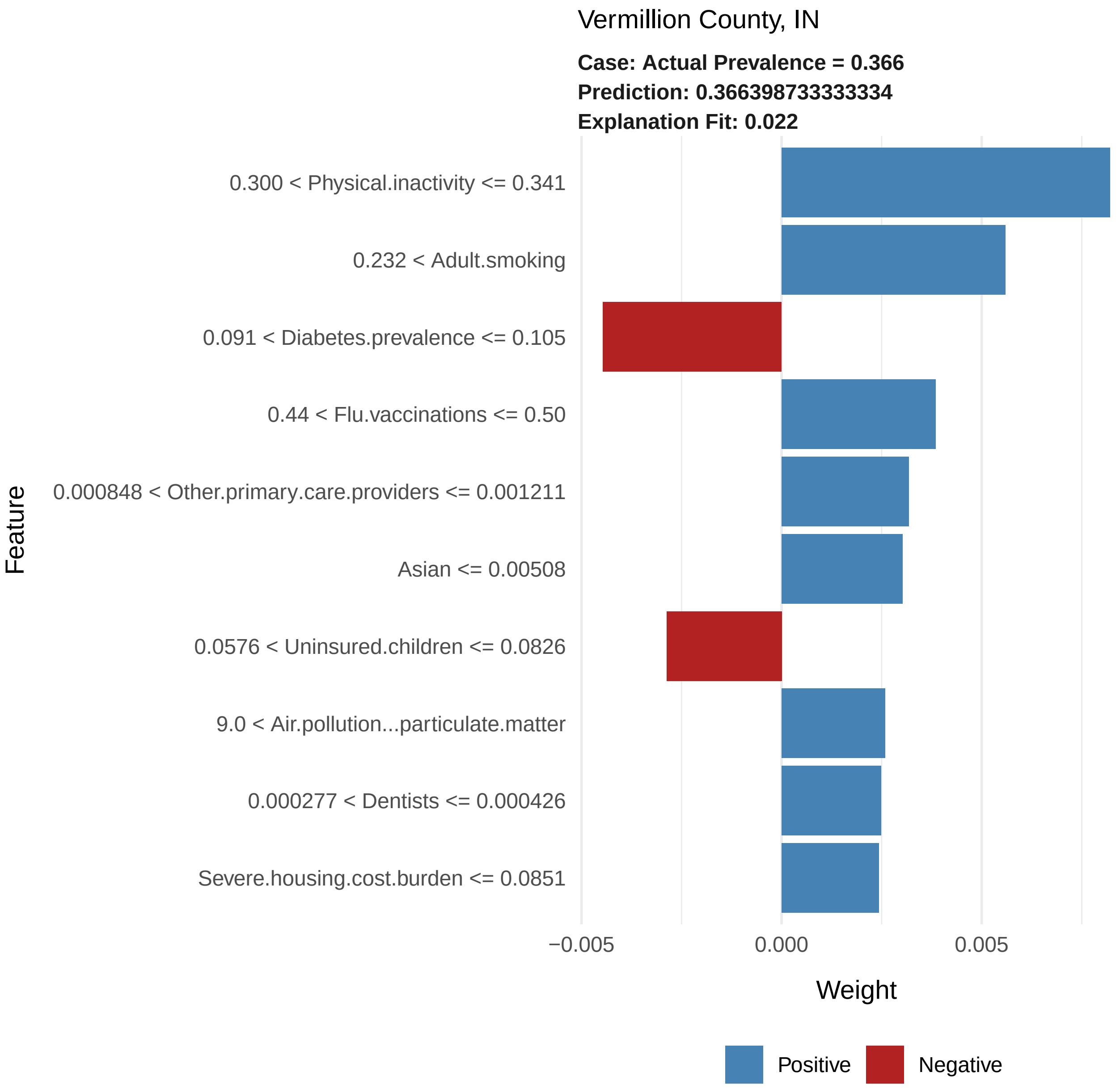

Vermillion County, IN
Case: Actual Prevalence = 0.366
Prediction: 0.366398733333334
Explanation Fit: 0.022
0.300 < Physical.inactivity <= 0.341
0.232 < Adult.smoking
0.091 < Diabetes.prevalence <= 0.105
0.44 < Flu.vaccinations <= 0.50
0.000848 < Other.primary.care.providers <= 0.001211
Asian <= 0.00508
0.0576 < Uninsured.children <= 0.0826
9.0 < Air.pollution...particulate.matter
0.000277 < Dentists <= 0.000426
Severe.housing.cost.burden <= 0.0851
Feature
−0.005
0.000
0.005
Weight
Positive
Negative

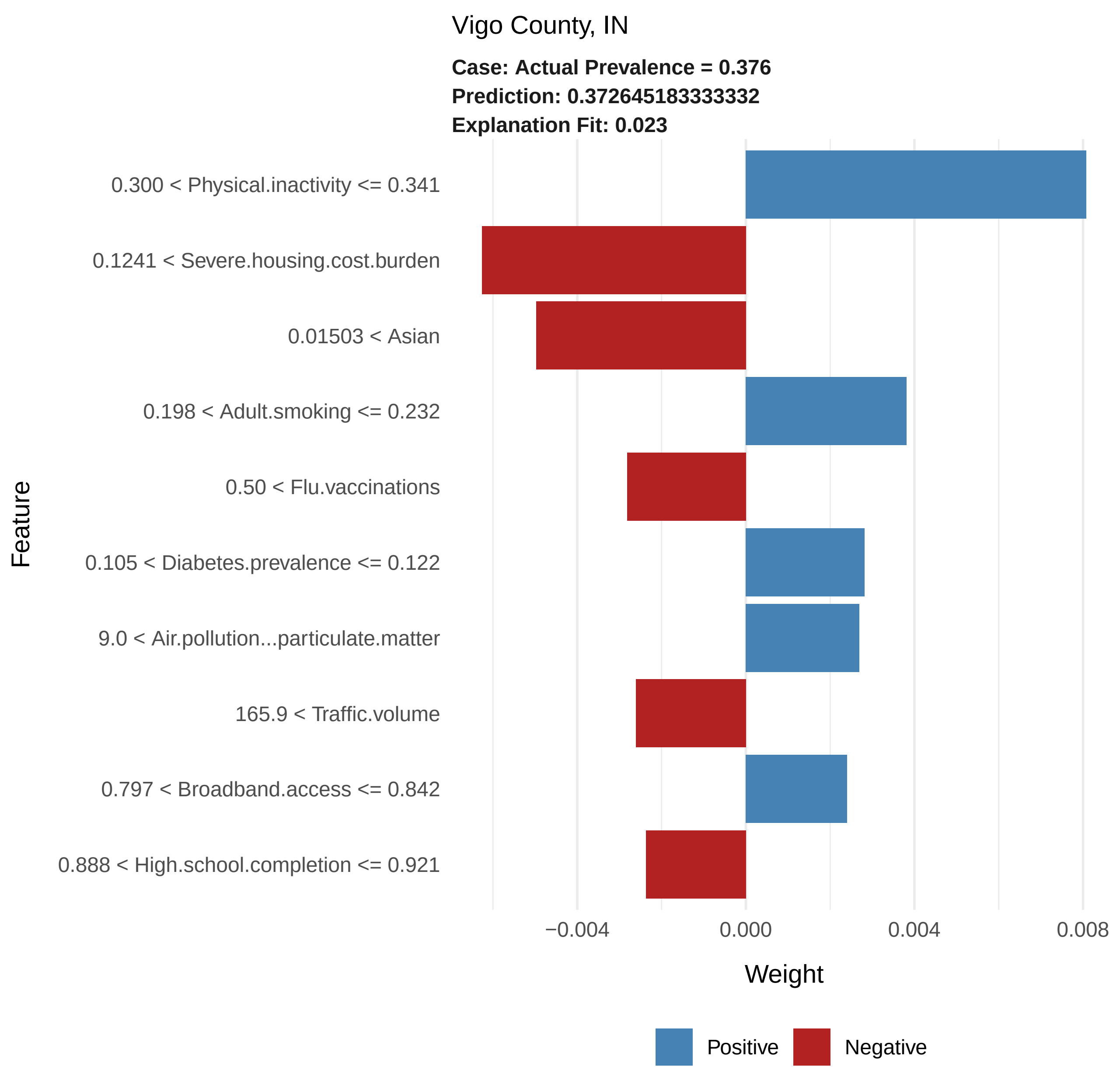

Vigo County, IN
Case: Actual Prevalence = 0.376
Prediction: 0.372645183333332
Explanation Fit: 0.023
0.300 < Physical.inactivity <= 0.341
0.1241 < Severe.housing.cost.burden
0.01503 < Asian
0.198 < Adult.smoking <= 0.232
0.50 < Flu.vaccinations
0.105 < Diabetes.prevalence <= 0.122
9.0 < Air.pollution...particulate.matter
165.9 < Traffic.volume
0.797 < Broadband.access <= 0.842
0.888 < High.school.completion <= 0.921
Feature
−0.004
0.000
0.004
0.008
Weight
Positive
Negative

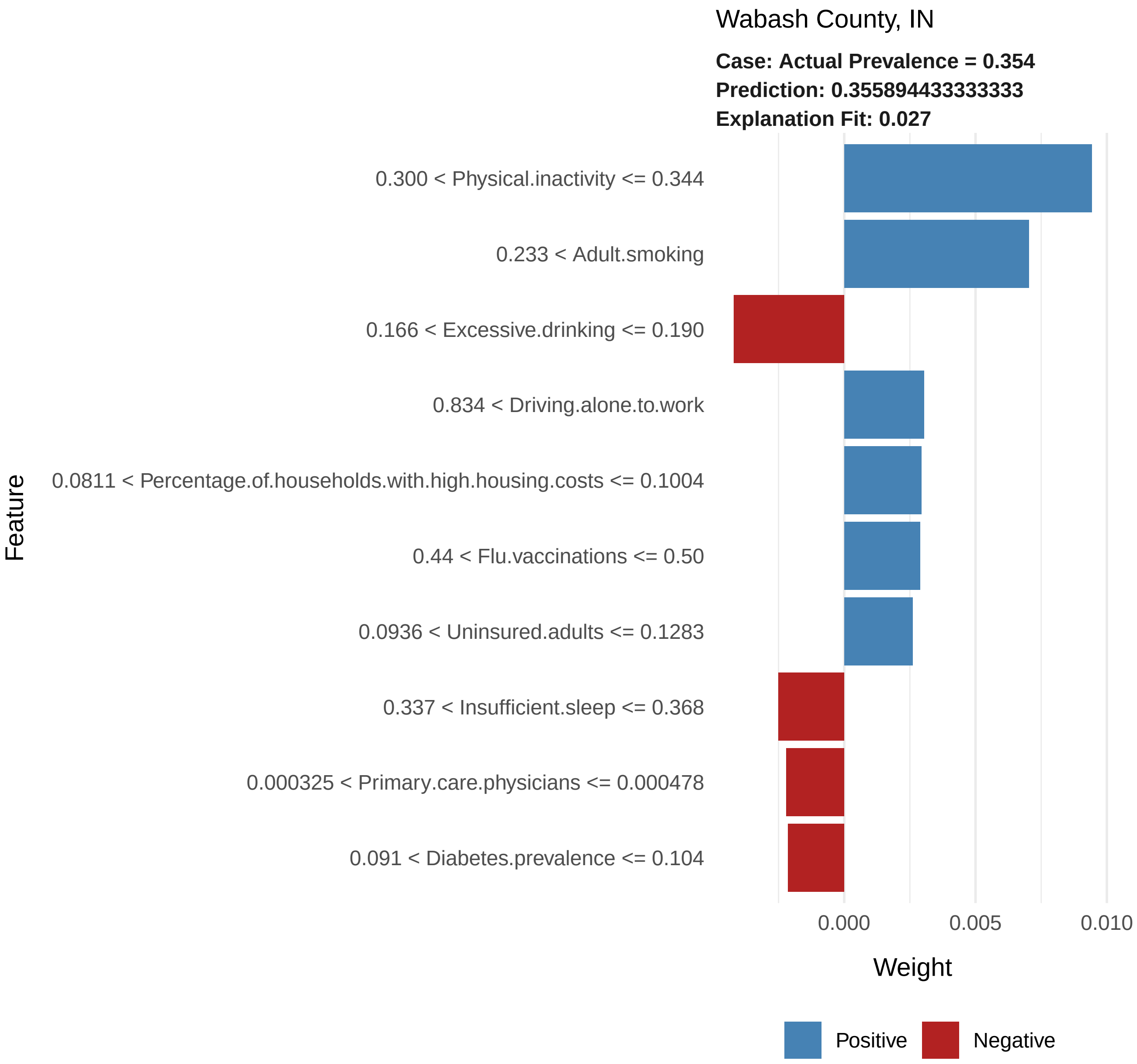

Wabash County, IN
Case: Actual Prevalence = 0.354
Prediction: 0.355894433333333
Explanation Fit: 0.027
0.300 < Physical.inactivity <= 0.344
0.233 < Adult.smoking
0.166 < Excessive.drinking <= 0.190
0.834 < Driving.alone.to.work
0.0811 < Percentage.of.households.with.high.housing.costs <= 0.1004
0.44 < Flu.vaccinations <= 0.50
0.0936 < Uninsured.adults <= 0.1283
0.337 < Insufficient.sleep <= 0.368
0.000325 < Primary.care.physicians <= 0.000478
0.091 < Diabetes.prevalence <= 0.104
Feature
0.000
0.005
0.010
Weight
Positive
Negative

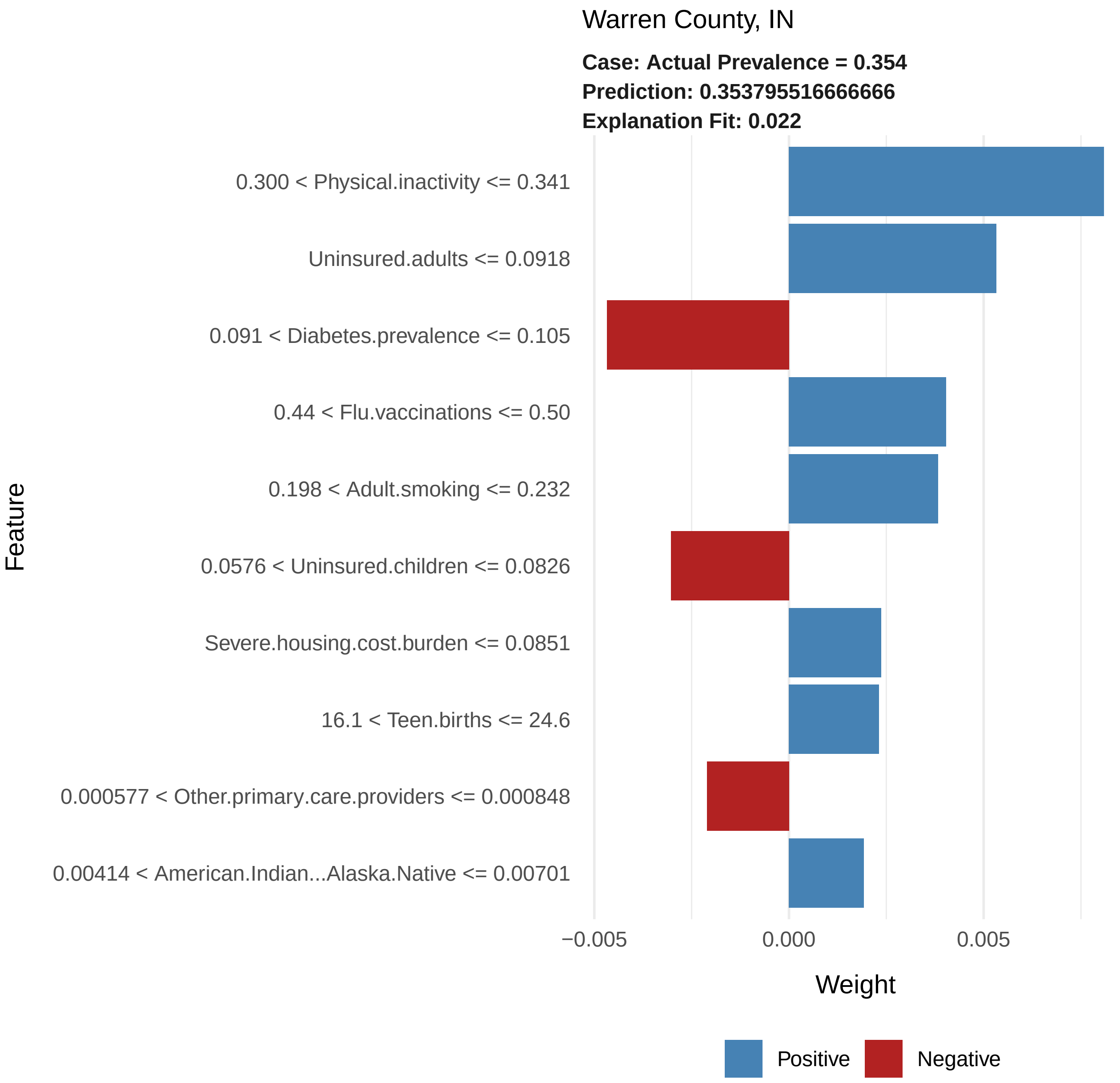

Warren County, IN
Case: Actual Prevalence = 0.354
Prediction: 0.353795516666666
Explanation Fit: 0.022
0.300 < Physical.inactivity <= 0.341
Uninsured.adults <= 0.0918
0.091 < Diabetes.prevalence <= 0.105
0.44 < Flu.vaccinations <= 0.50
0.198 < Adult.smoking <= 0.232
0.0576 < Uninsured.children <= 0.0826
Severe.housing.cost.burden <= 0.0851
16.1 < Teen.births <= 24.6
0.000577 < Other.primary.care.providers <= 0.000848
0.00414 < American.Indian...Alaska.Native <= 0.00701
Feature
−0.005
0.000
0.005
Weight
Positive
Negative

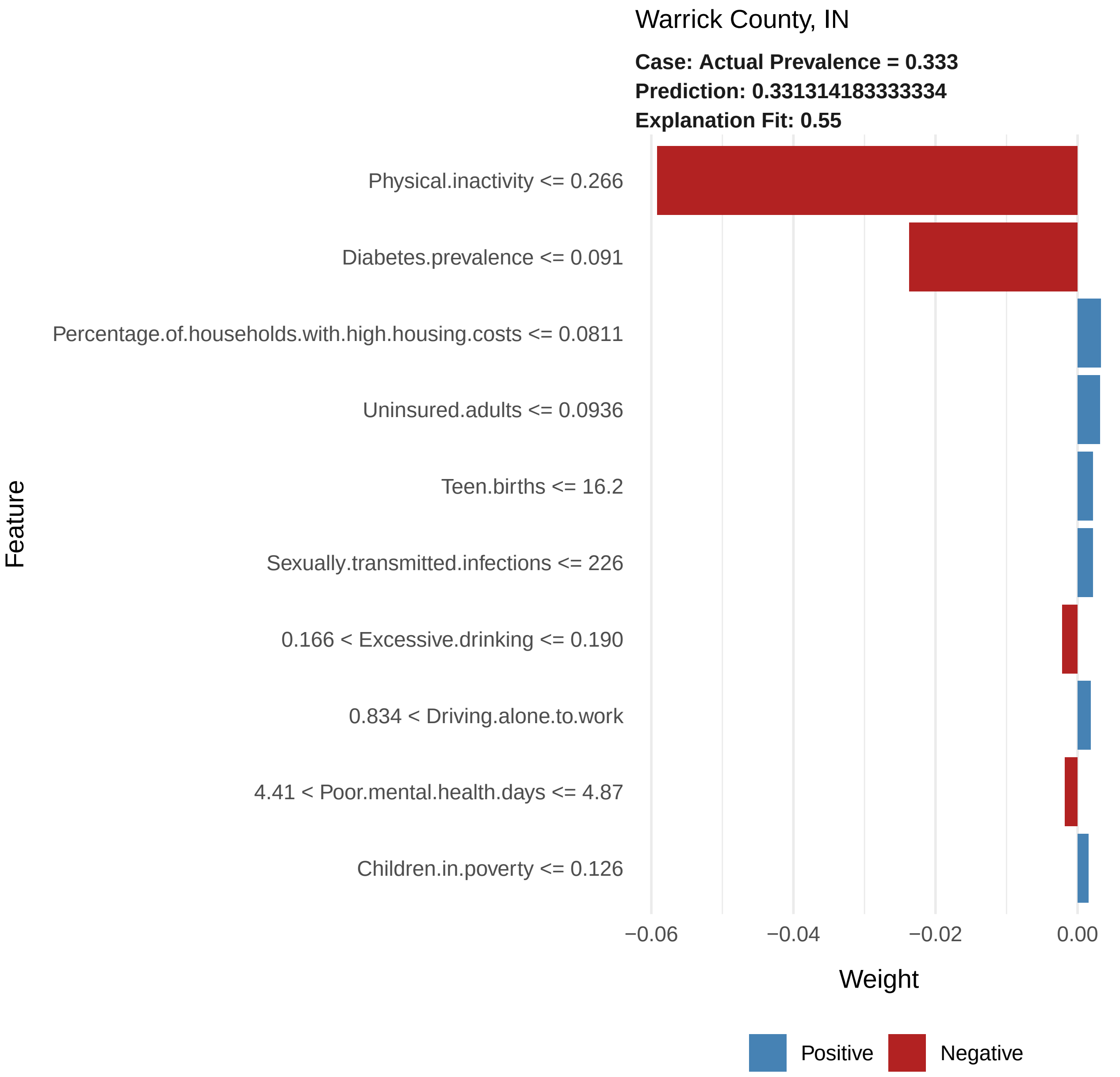

Warrick County, IN
Case: Actual Prevalence = 0.333
Prediction: 0.331314183333334
Explanation Fit: 0.55
Physical.inactivity <= 0.266
Diabetes.prevalence <= 0.091
Percentage.of.households.with.high.housing.costs <= 0.0811
Uninsured.adults <= 0.0936
Teen.births <= 16.2
Sexually.transmitted.infections <= 226
0.166 < Excessive.drinking <= 0.190
0.834 < Driving.alone.to.work
4.41 < Poor.mental.health.days <= 4.87
Children.in.poverty <= 0.126
Feature
−0.06
−0.04
−0.02
0.00
Weight
Positive
Negative

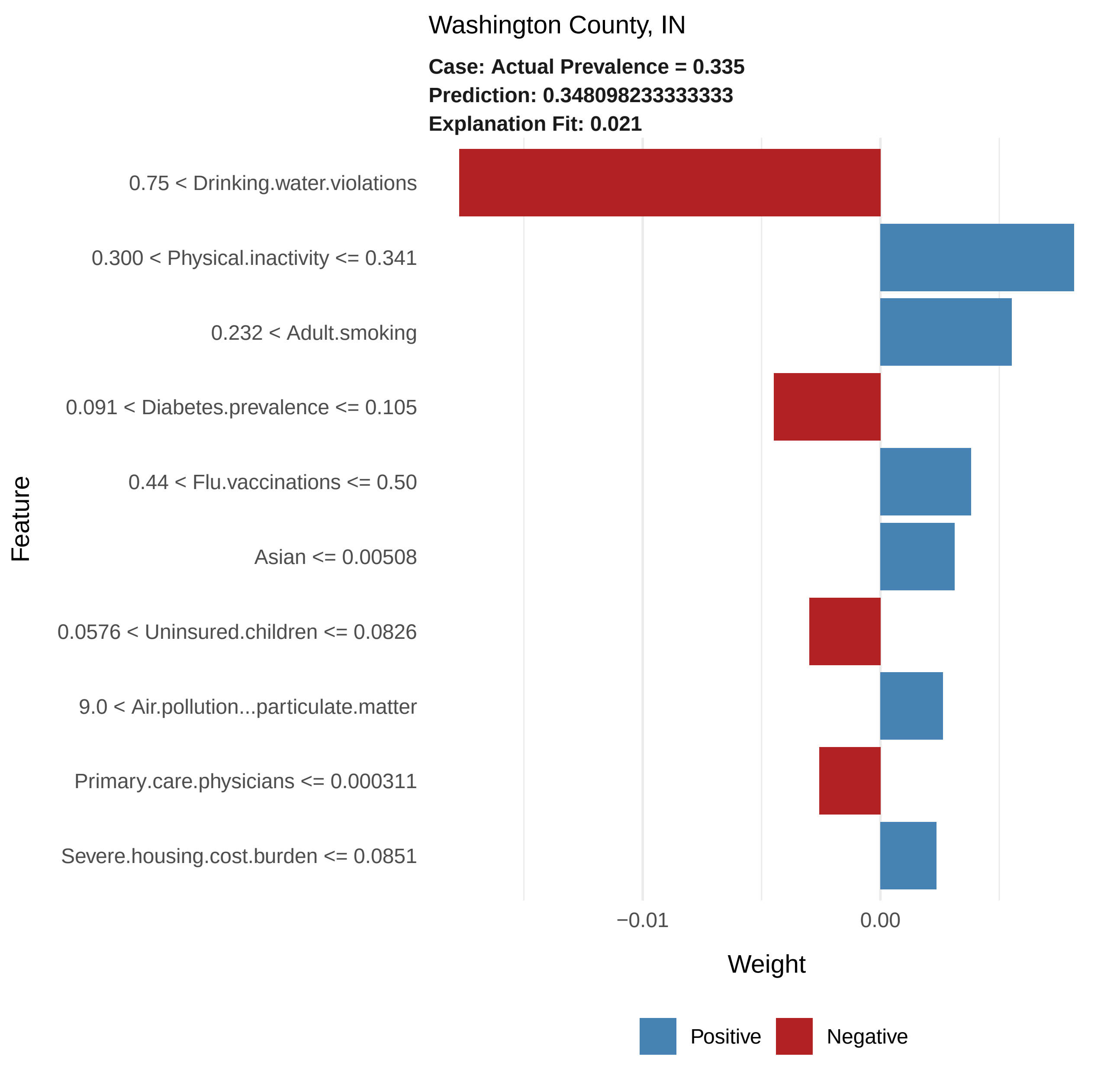
Washington County, IN
Case: Actual Prevalence = 0.335
Prediction: 0.348098233333333
Explanation Fit: 0.021
0.75 < Drinking.water.violations
0.300 < Physical.inactivity <= 0.341
0.232 < Adult.smoking
0.091 < Diabetes.prevalence <= 0.105
0.44 < Flu.vaccinations <= 0.50
Asian <= 0.00508
0.0576 < Uninsured.children <= 0.0826
9.0 < Air.pollution...particulate.matter
Primary.care.physicians <= 0.000311
Severe.housing.cost.burden <= 0.0851
Feature
−0.01
0.00
Weight
Positive
Negative

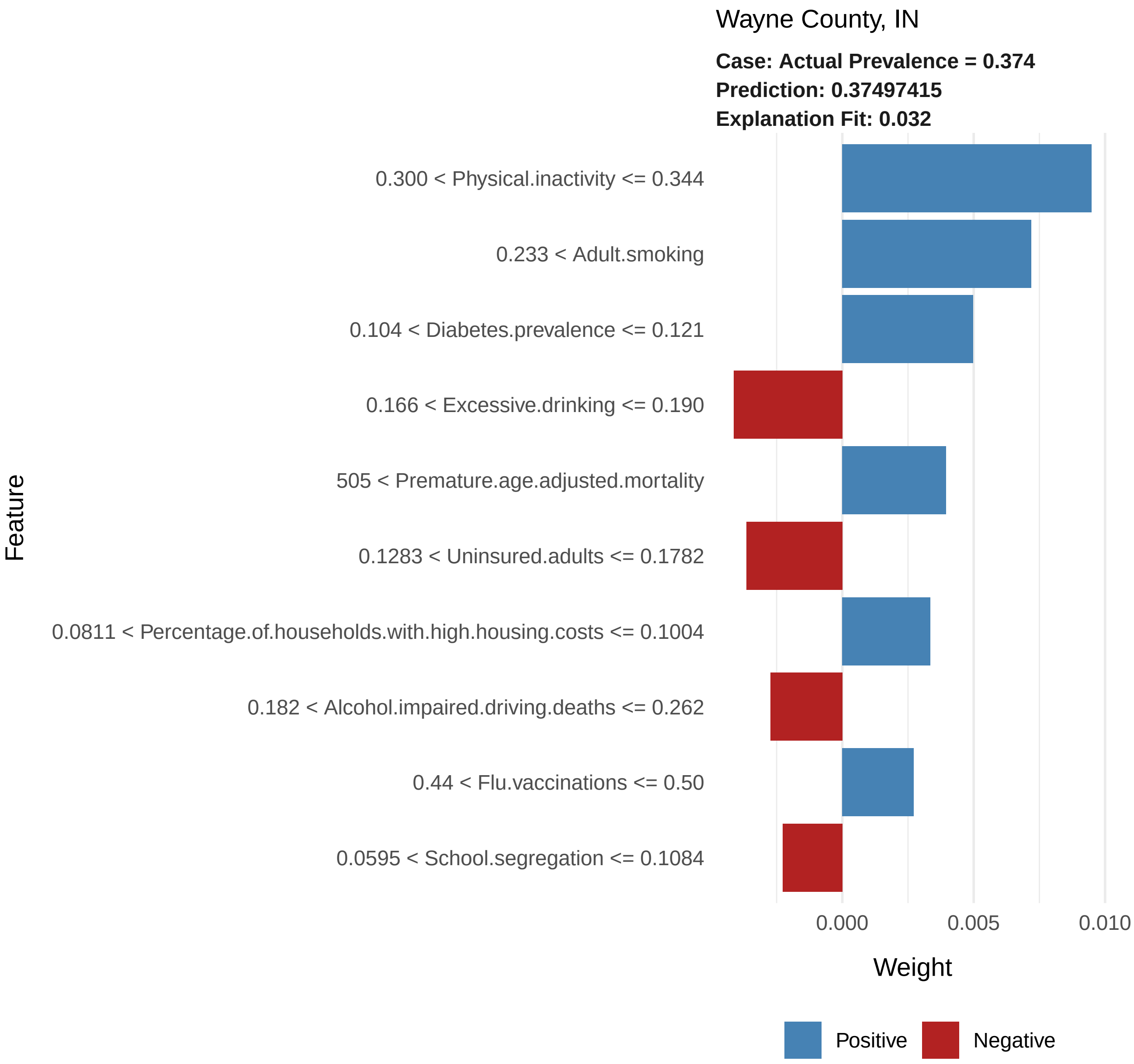
Wayne County, IN
Case: Actual Prevalence = 0.374
Prediction: 0.37497415
Explanation Fit: 0.032
0.300 < Physical.inactivity <= 0.344
0.233 < Adult.smoking
0.104 < Diabetes.prevalence <= 0.121
0.166 < Excessive.drinking <= 0.190
505 < Premature.age.adjusted.mortality
0.1283 < Uninsured.adults <= 0.1782
0.0811 < Percentage.of.households.with.high.housing.costs <= 0.1004
0.182 < Alcohol.impaired.driving.deaths <= 0.262
0.44 < Flu.vaccinations <= 0.50
0.0595 < School.segregation <= 0.1084
Feature
0.000
0.005
0.010
Weight
Positive
Negative

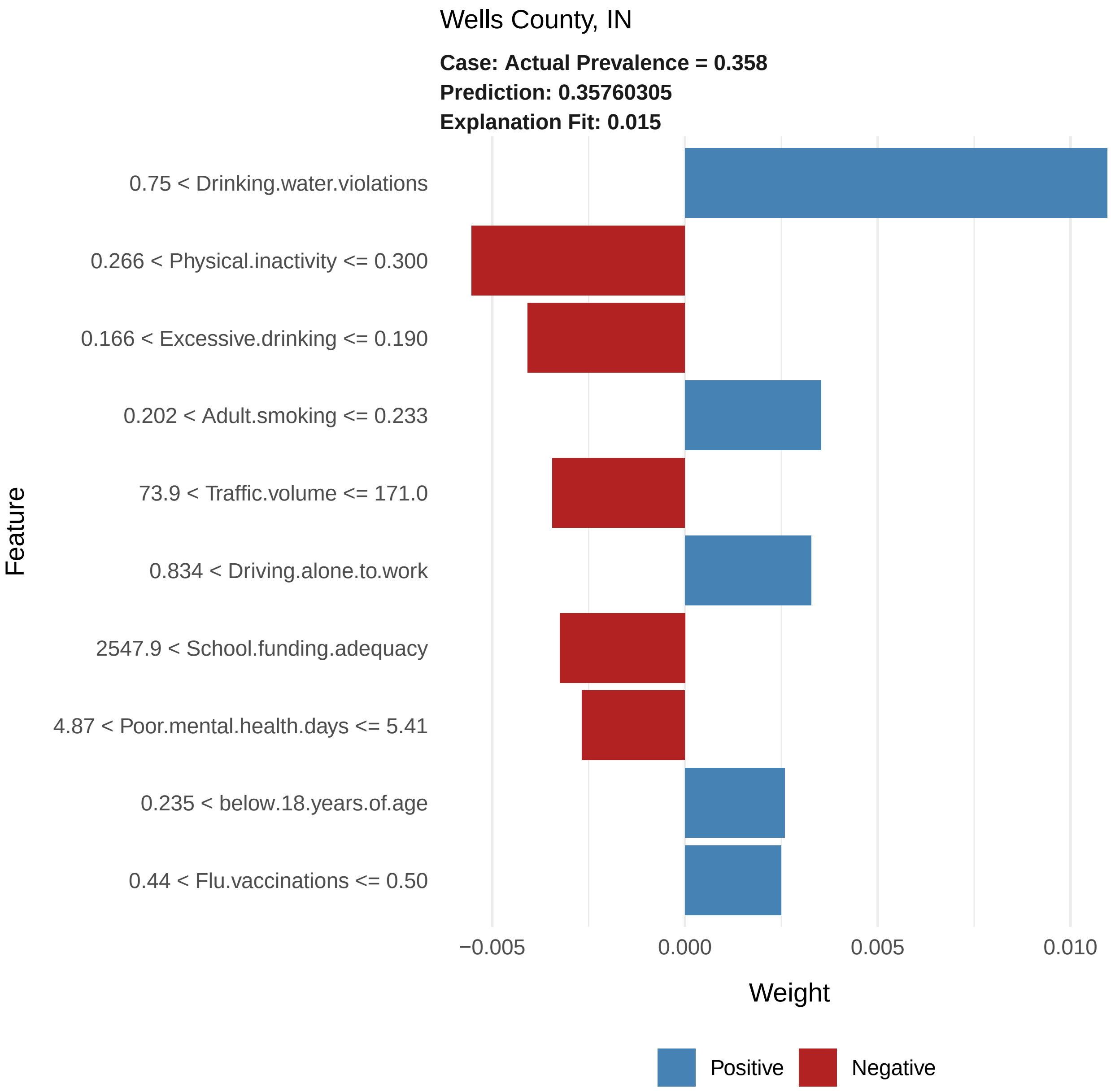
Wells County, IN
Case: Actual Prevalence = 0.358
Prediction: 0.35760305
Explanation Fit: 0.015
0.75 < Drinking.water.violations
0.266 < Physical.inactivity <= 0.300
0.166 < Excessive.drinking <= 0.190
0.202 < Adult.smoking <= 0.233
73.9 < Traffic.volume <= 171.0
0.834 < Driving.alone.to.work
2547.9 < School.funding.adequacy
4.87 < Poor.mental.health.days <= 5.41
0.235 < below.18.years.of.age
0.44 < Flu.vaccinations <= 0.50
Feature
−0.005
0.000
0.005
0.010
Weight
Positive
Negative

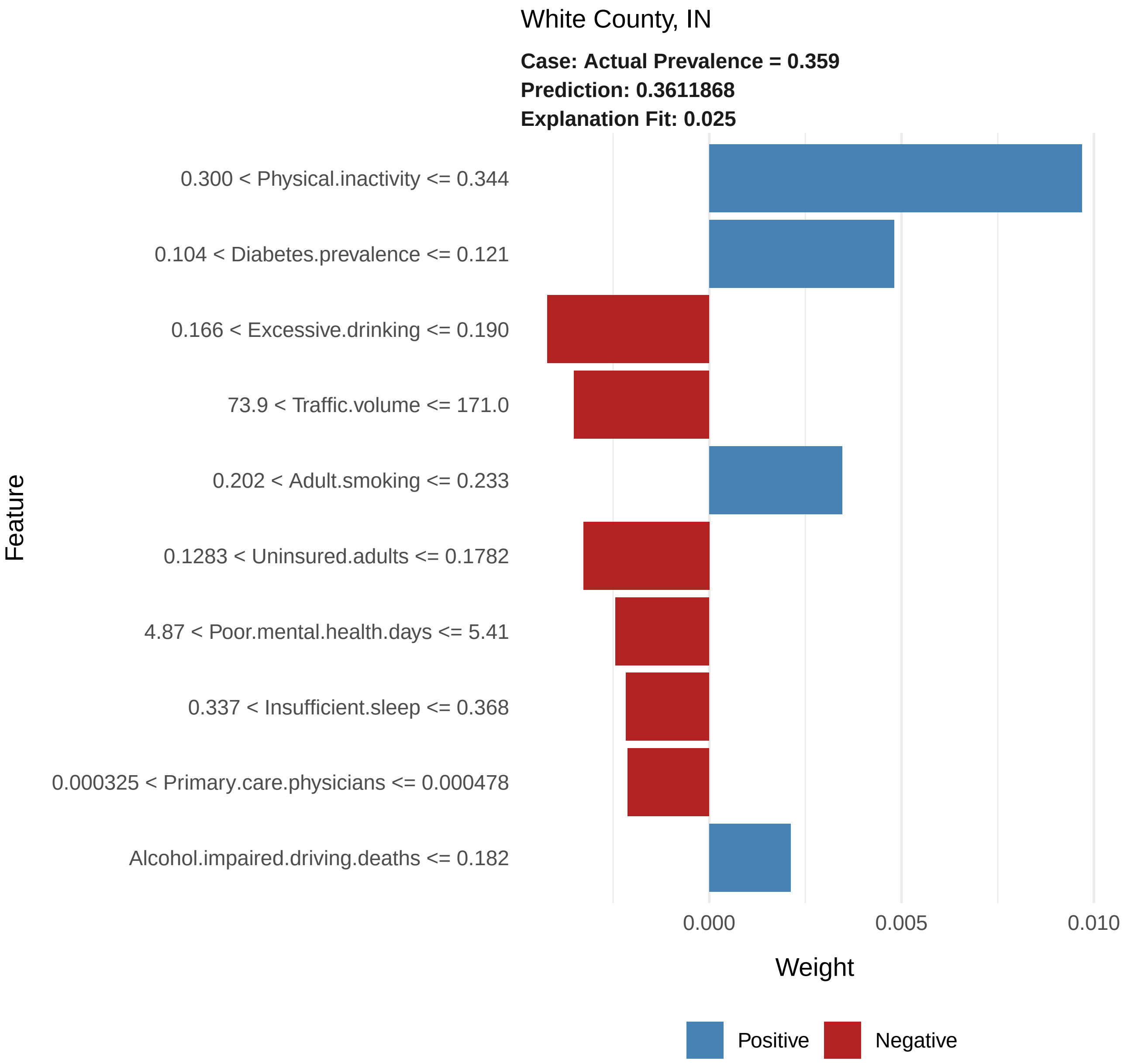
White County, IN
Case: Actual Prevalence = 0.359
Prediction: 0.3611868
Explanation Fit: 0.025
0.300 < Physical.inactivity <= 0.344
0.104 < Diabetes.prevalence <= 0.121
0.166 < Excessive.drinking <= 0.190
73.9 < Traffic.volume <= 171.0
0.202 < Adult.smoking <= 0.233
0.1283 < Uninsured.adults <= 0.1782
4.87 < Poor.mental.health.days <= 5.41
0.337 < Insufficient.sleep <= 0.368
0.000325 < Primary.care.physicians <= 0.000478
Alcohol.impaired.driving.deaths <= 0.182
Feature
0.000
0.005
0.010
Weight
Positive
Negative

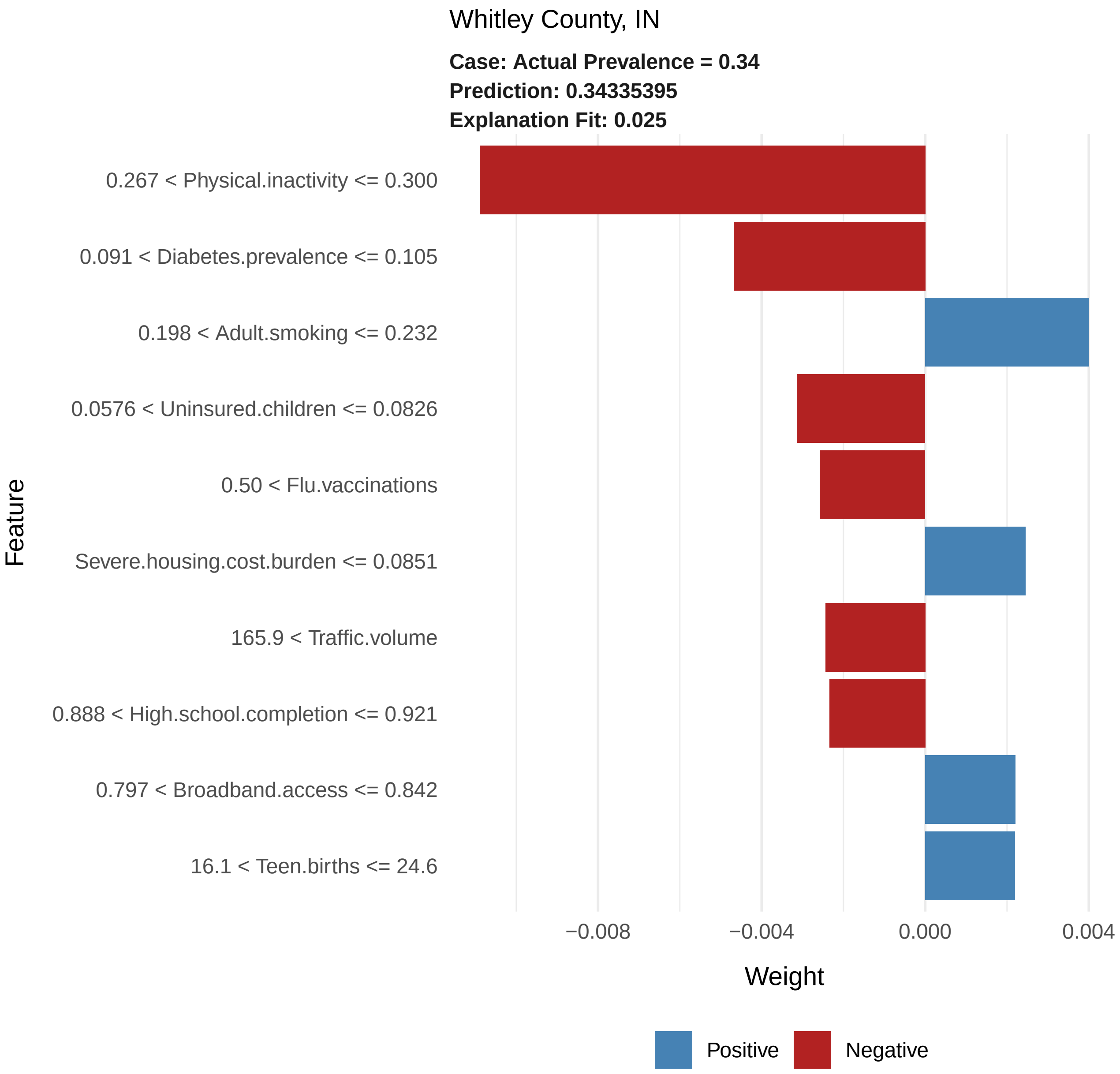
Whitley County, IN
Case: Actual Prevalence = 0.34
Prediction: 0.34335395
Explanation Fit: 0.025
0.267 < Physical.inactivity <= 0.300
0.091 < Diabetes.prevalence <= 0.105
0.198 < Adult.smoking <= 0.232
0.0576 < Uninsured.children <= 0.0826
0.50 < Flu.vaccinations
Severe.housing.cost.burden <= 0.0851
165.9 < Traffic.volume
0.888 < High.school.completion <= 0.921
0.797 < Broadband.access <= 0.842
16.1 < Teen.births <= 24.6
Feature
−0.008
−0.004
0.000
0.004
Weight
Positive
Negative

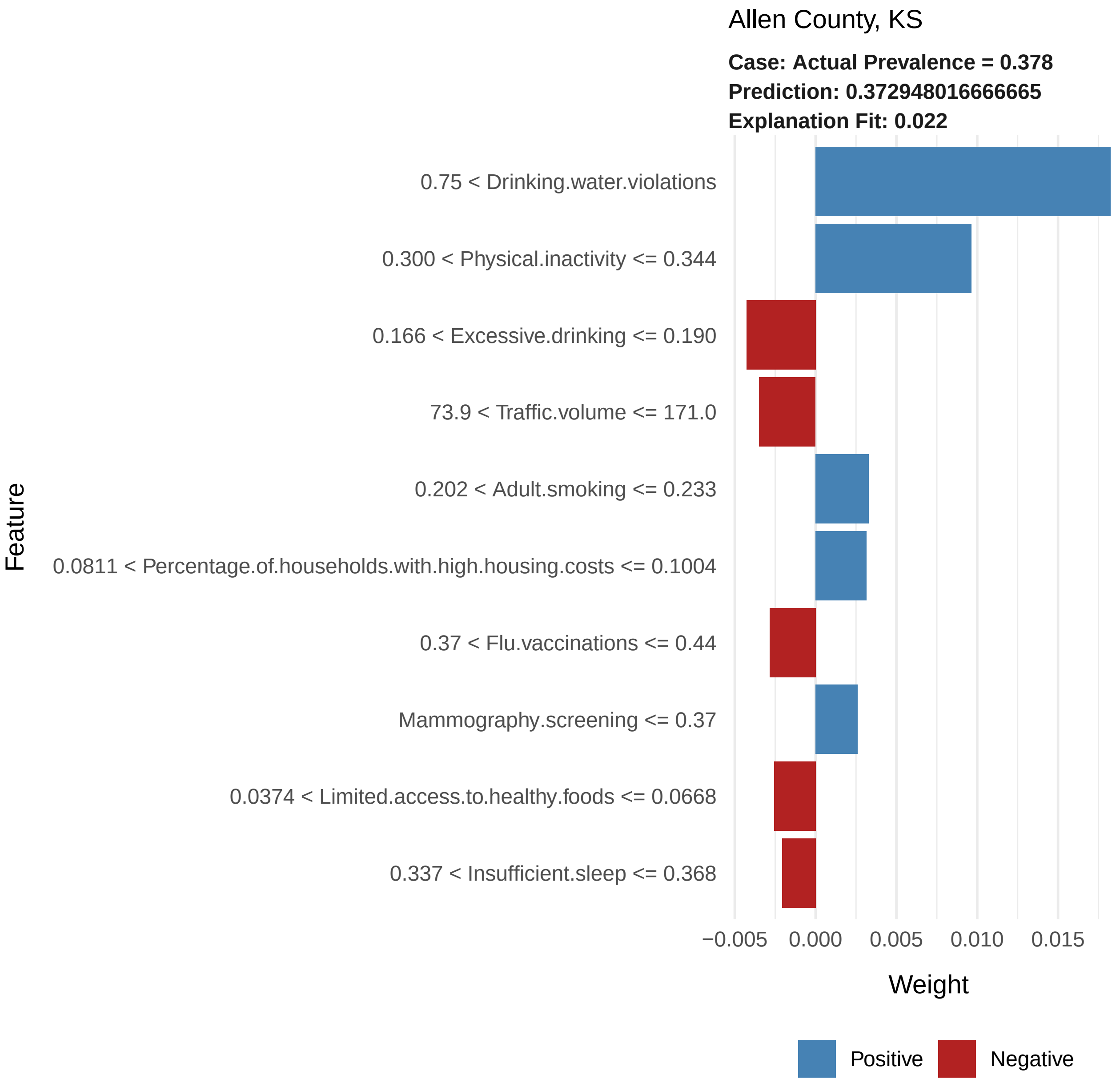

Allen County, KS
Case: Actual Prevalence = 0.378
Prediction: 0.372948016666665
Explanation Fit: 0.022
0.75 < Drinking.water.violations
0.300 < Physical.inactivity <= 0.344
0.166 < Excessive.drinking <= 0.190
73.9 < Traffic.volume <= 171.0
0.202 < Adult.smoking <= 0.233
0.0811 < Percentage.of.households.with.high.housing.costs <= 0.1004
0.37 < Flu.vaccinations <= 0.44
Mammography.screening <= 0.37
0.0374 < Limited.access.to.healthy.foods <= 0.0668
0.337 < Insufficient.sleep <= 0.368
Feature
−0.005
0.000
0.005
0.010
0.015
Weight
Positive
Negative

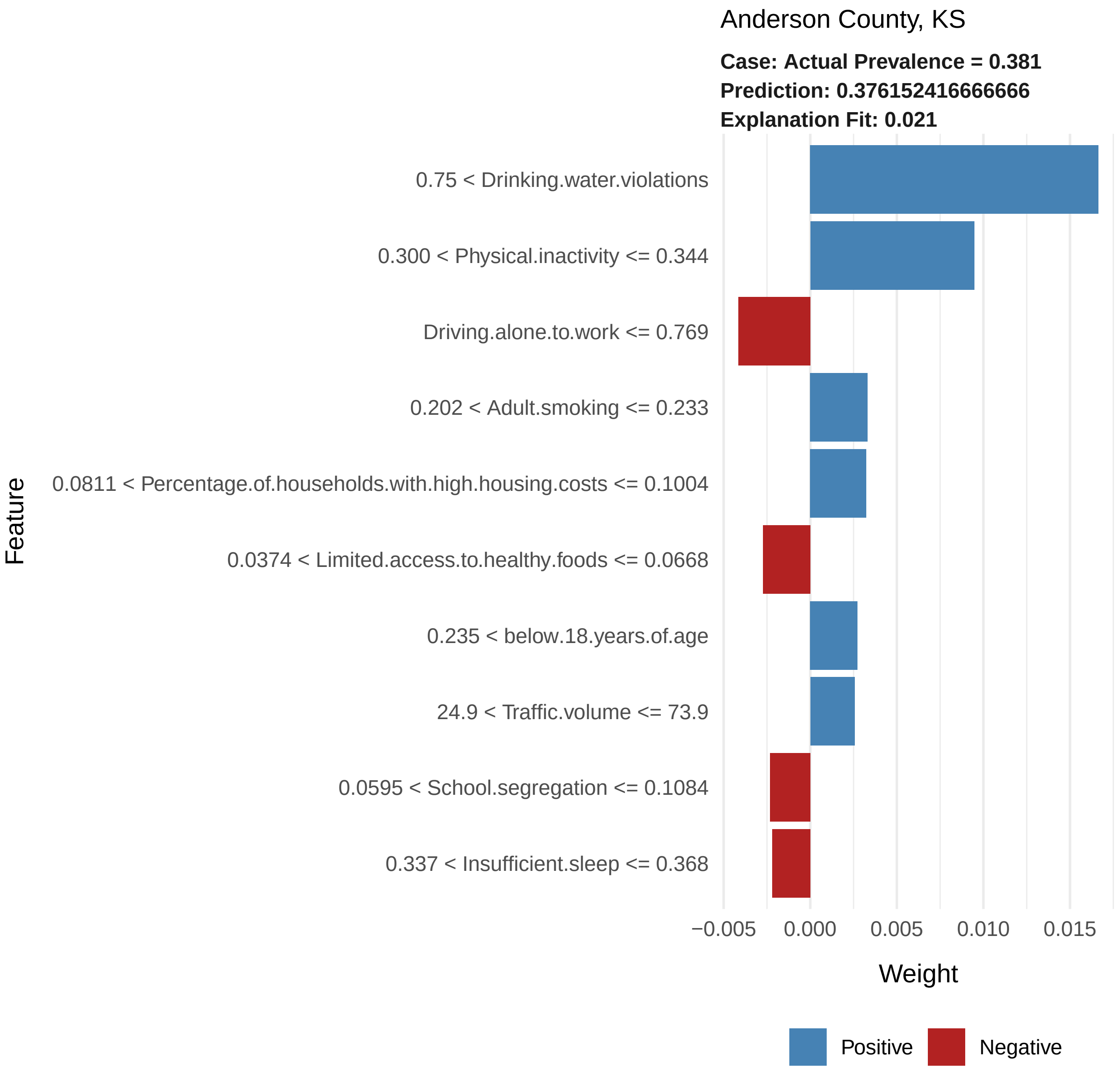

Anderson County, KS
Case: Actual Prevalence = 0.381
Prediction: 0.376152416666666
Explanation Fit: 0.021
Feature
0.75 < Drinking.water.violations
0.300 < Physical.inactivity <= 0.344
Driving.alone.to.work <= 0.769
0.202 < Adult.smoking <= 0.233
0.0811 < Percentage.of.households.with.high.housing.costs <= 0.1004
0.0374 < Limited.access.to.healthy.foods <= 0.0668
0.235 < below.18.years.of.age
24.9 < Traffic.volume <= 73.9
0.0595 < School.segregation <= 0.1084
0.337 < Insufficient.sleep <= 0.368
−0.005
0.000
0.005
0.010
0.015
Weight
Positive
Negative

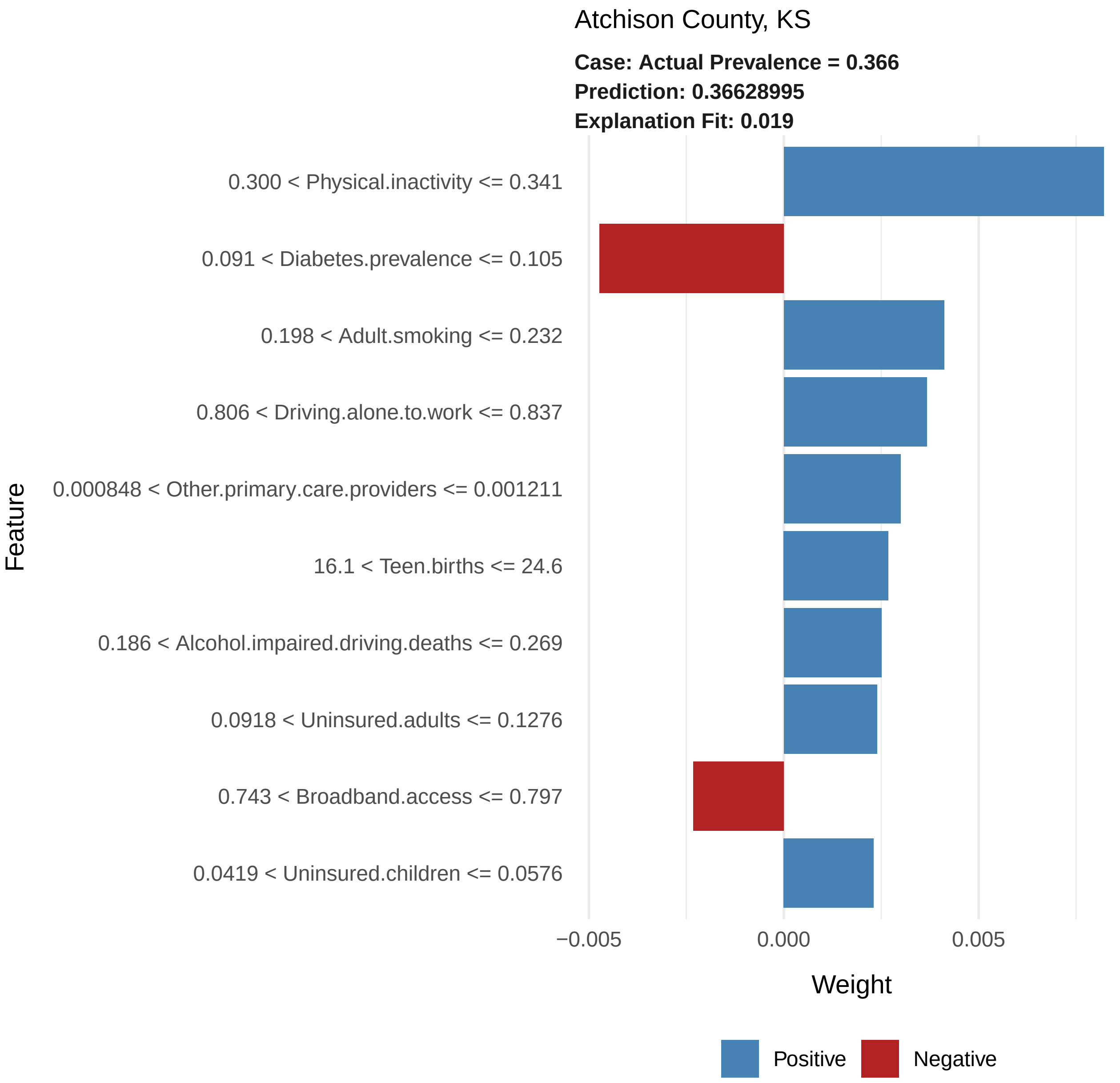

Atchison County, KS
Case: Actual Prevalence = 0.366
Prediction: 0.36628995
Explanation Fit: 0.019
0.300 < Physical.inactivity <= 0.341
0.091 < Diabetes.prevalence <= 0.105
0.198 < Adult.smoking <= 0.232
0.806 < Driving.alone.to.work <= 0.837
0.000848 < Other.primary.care.providers <= 0.001211
16.1 < Teen.births <= 24.6
0.186 < Alcohol.impaired.driving.deaths <= 0.269
0.0918 < Uninsured.adults <= 0.1276
0.743 < Broadband.access <= 0.797
0.0419 < Uninsured.children <= 0.0576
Feature
−0.005
0.000
0.005
Weight
Positive
Negative

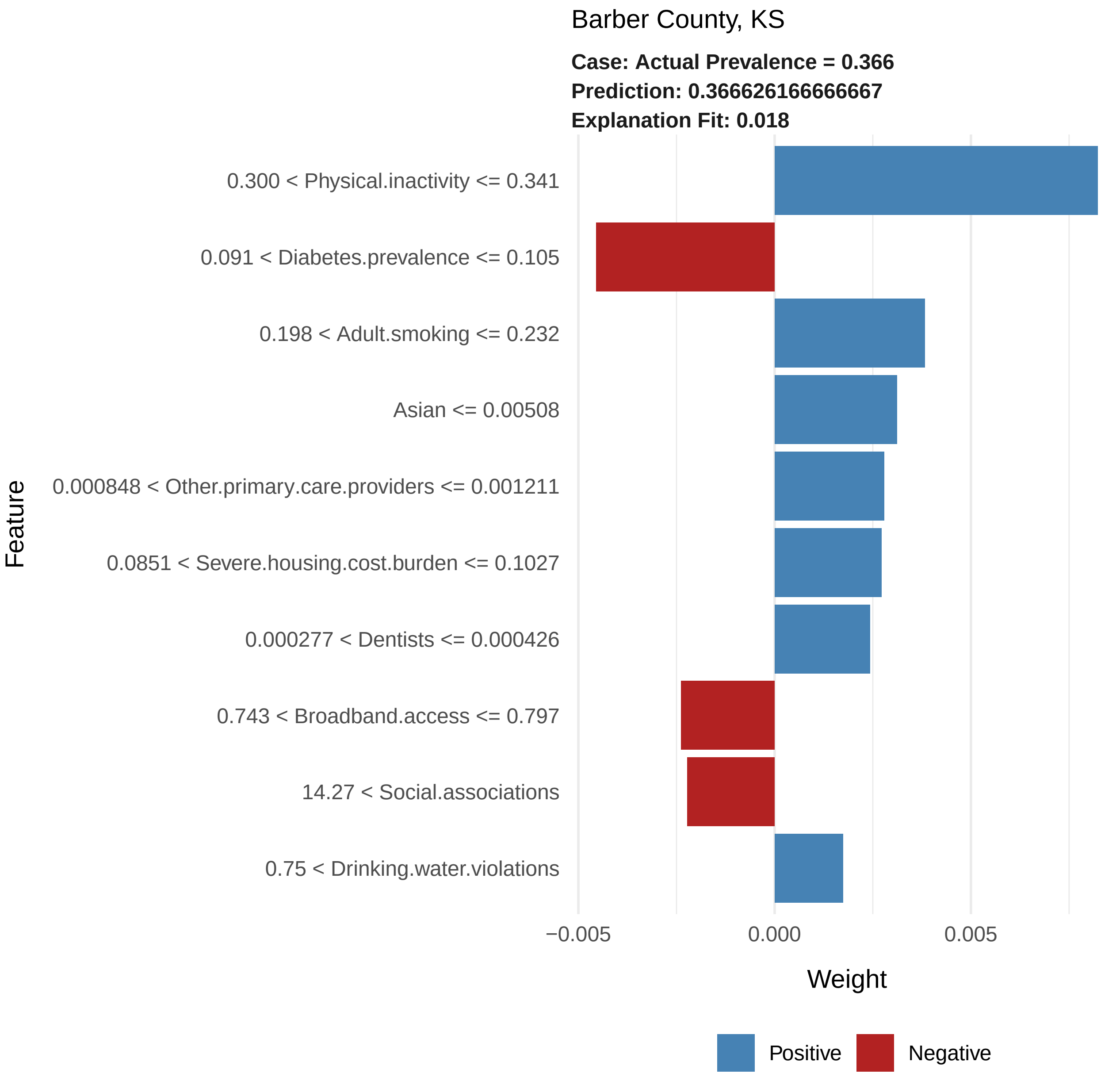
Barber County, KS
Case: Actual Prevalence = 0.366
Prediction: 0.366626166666667
Explanation Fit: 0.018
0.300 < Physical.inactivity <= 0.341
0.091 < Diabetes.prevalence <= 0.105
0.198 < Adult.smoking <= 0.232
Asian <= 0.00508
0.000848 < Other.primary.care.providers <= 0.001211
0.0851 < Severe.housing.cost.burden <= 0.1027
0.000277 < Dentists <= 0.000426
0.743 < Broadband.access <= 0.797
14.27 < Social.associations
0.75 < Drinking.water.violations
Feature
−0.005
0.000
0.005
Weight
Positive
Negative

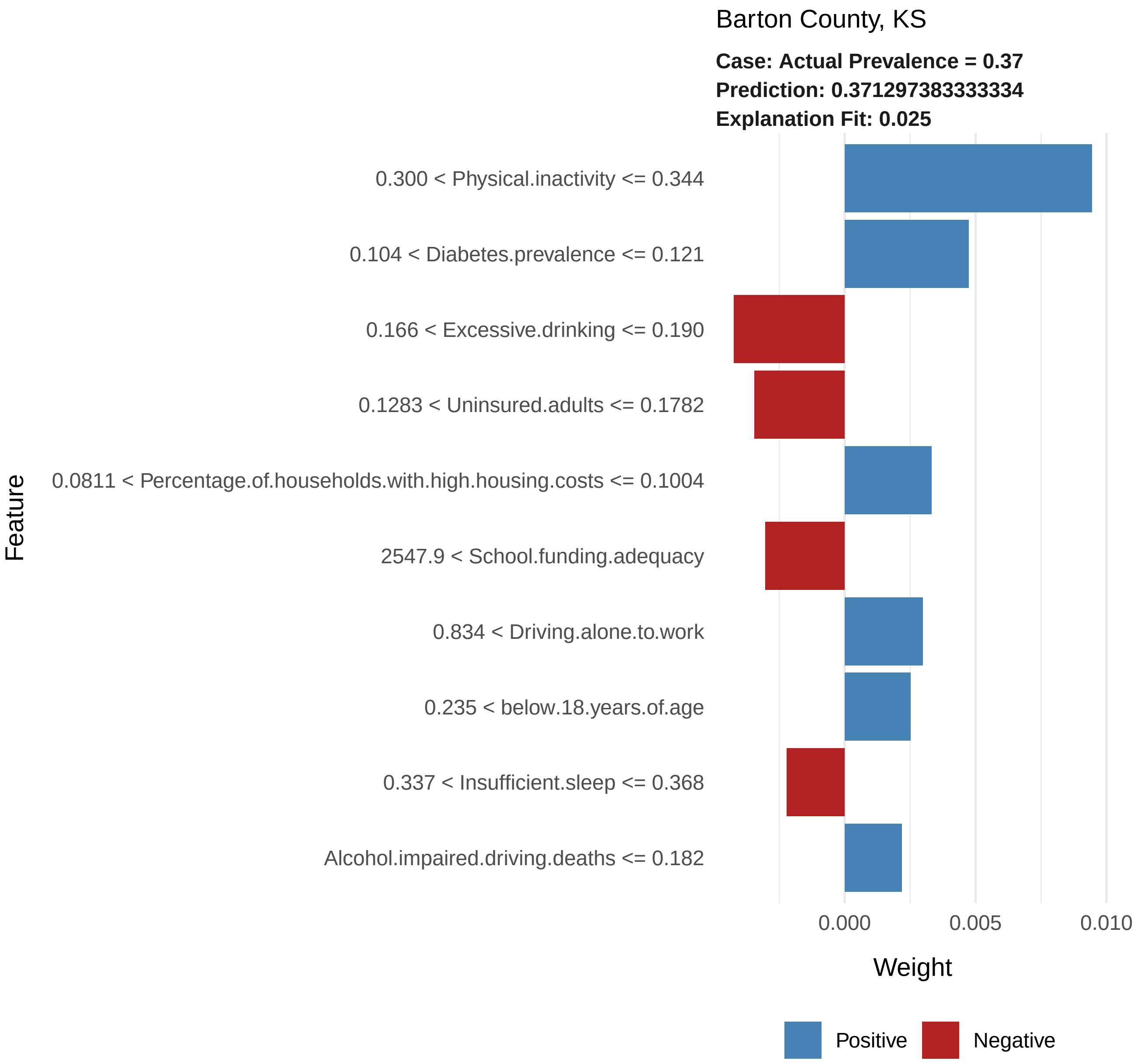
Barton County, KS
Case: Actual Prevalence = 0.37
Prediction: 0.371297383333334
Explanation Fit: 0.025
0.300 < Physical.inactivity <= 0.344
0.104 < Diabetes.prevalence <= 0.121
0.166 < Excessive.drinking <= 0.190
0.1283 < Uninsured.adults <= 0.1782
0.0811 < Percentage.of.households.with.high.housing.costs <= 0.1004
2547.9 < School.funding.adequacy
0.834 < Driving.alone.to.work
0.235 < below.18.years.of.age
0.337 < Insufficient.sleep <= 0.368
Alcohol.impaired.driving.deaths <= 0.182
Feature
0.000
0.005
0.010
Weight
Positive
Negative

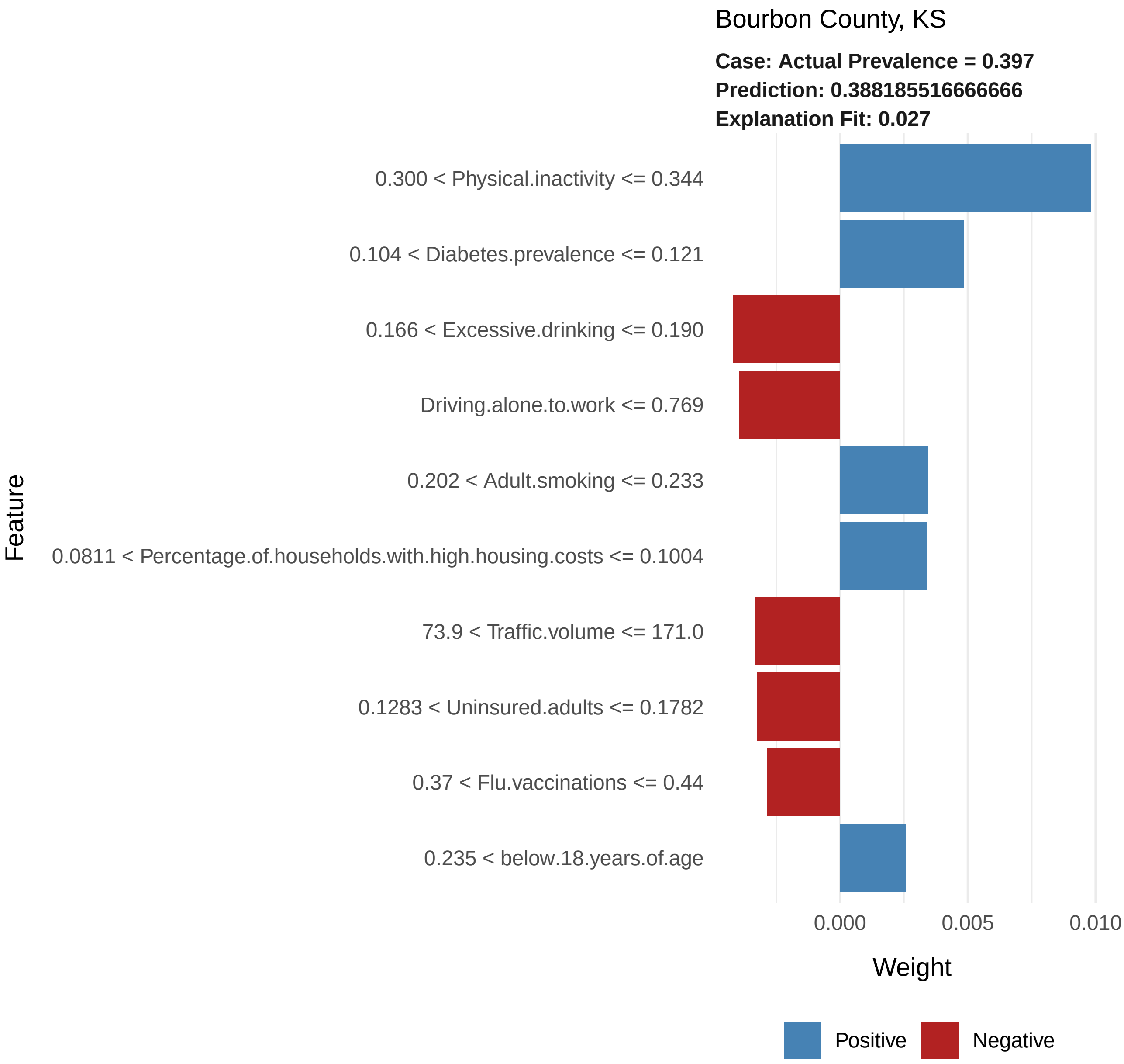

Bourbon County, KS
Case: Actual Prevalence = 0.397
Prediction: 0.388185516666666
Explanation Fit: 0.027
0.300 < Physical.inactivity <= 0.344
0.104 < Diabetes.prevalence <= 0.121
0.166 < Excessive.drinking <= 0.190
Driving.alone.to.work <= 0.769
0.202 < Adult.smoking <= 0.233
0.0811 < Percentage.of.households.with.high.housing.costs <= 0.1004
73.9 < Traffic.volume <= 171.0
0.1283 < Uninsured.adults <= 0.1782
0.37 < Flu.vaccinations <= 0.44
0.235 < below.18.years.of.age
Feature
0.000
0.005
0.010
Weight
Positive
Negative

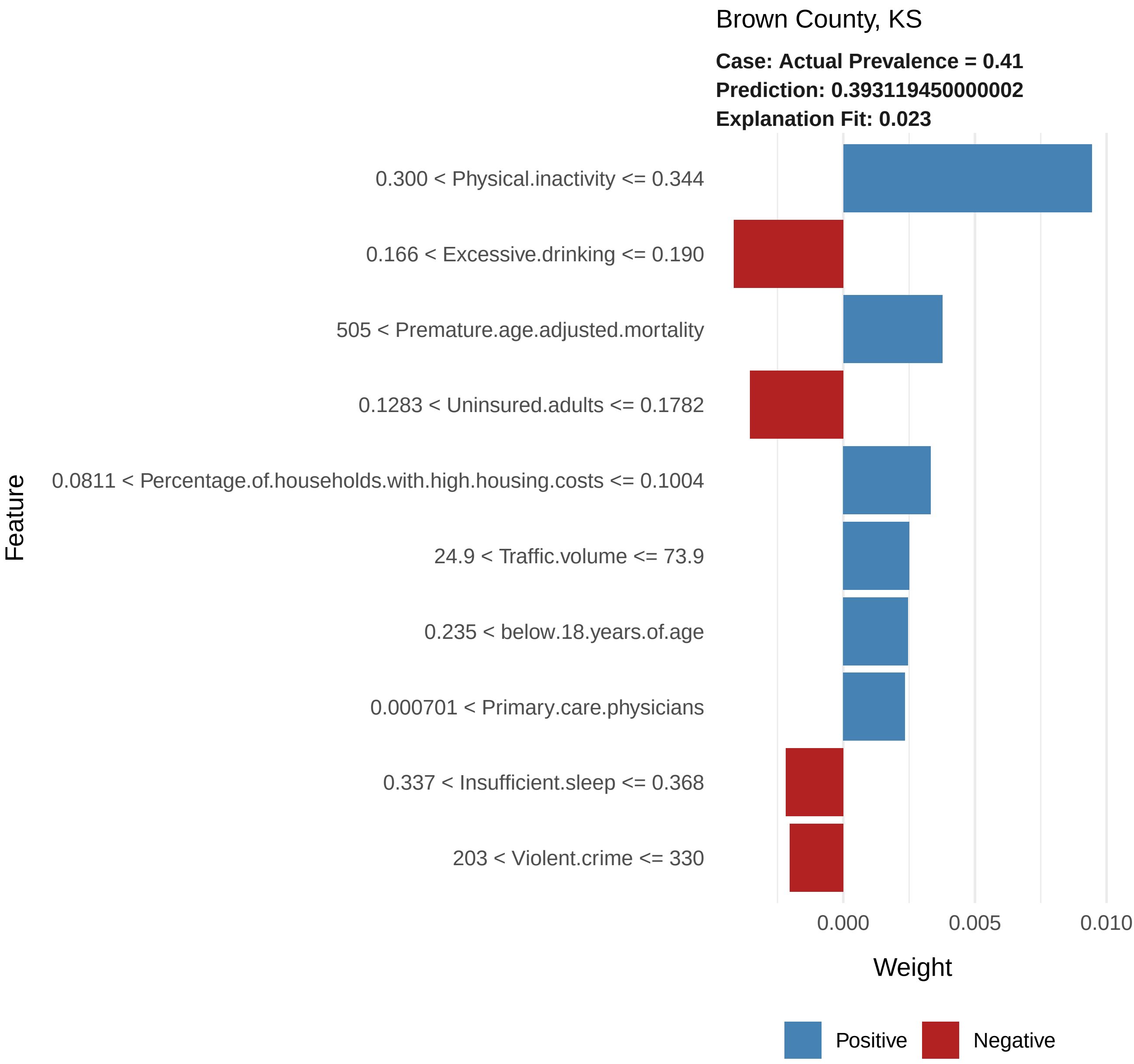
Brown County, KS
Case: Actual Prevalence = 0.41
Prediction: 0.393119450000002
Explanation Fit: 0.023
0.300 < Physical.inactivity <= 0.344
0.166 < Excessive.drinking <= 0.190
505 < Premature.age.adjusted.mortality
0.1283 < Uninsured.adults <= 0.1782
0.0811 < Percentage.of.households.with.high.housing.costs <= 0.1004
24.9 < Traffic.volume <= 73.9
0.235 < below.18.years.of.age
0.000701 < Primary.care.physicians
0.337 < Insufficient.sleep <= 0.368
203 < Violent.crime <= 330
Feature
0.000
0.005
0.010
Weight
Positive
Negative

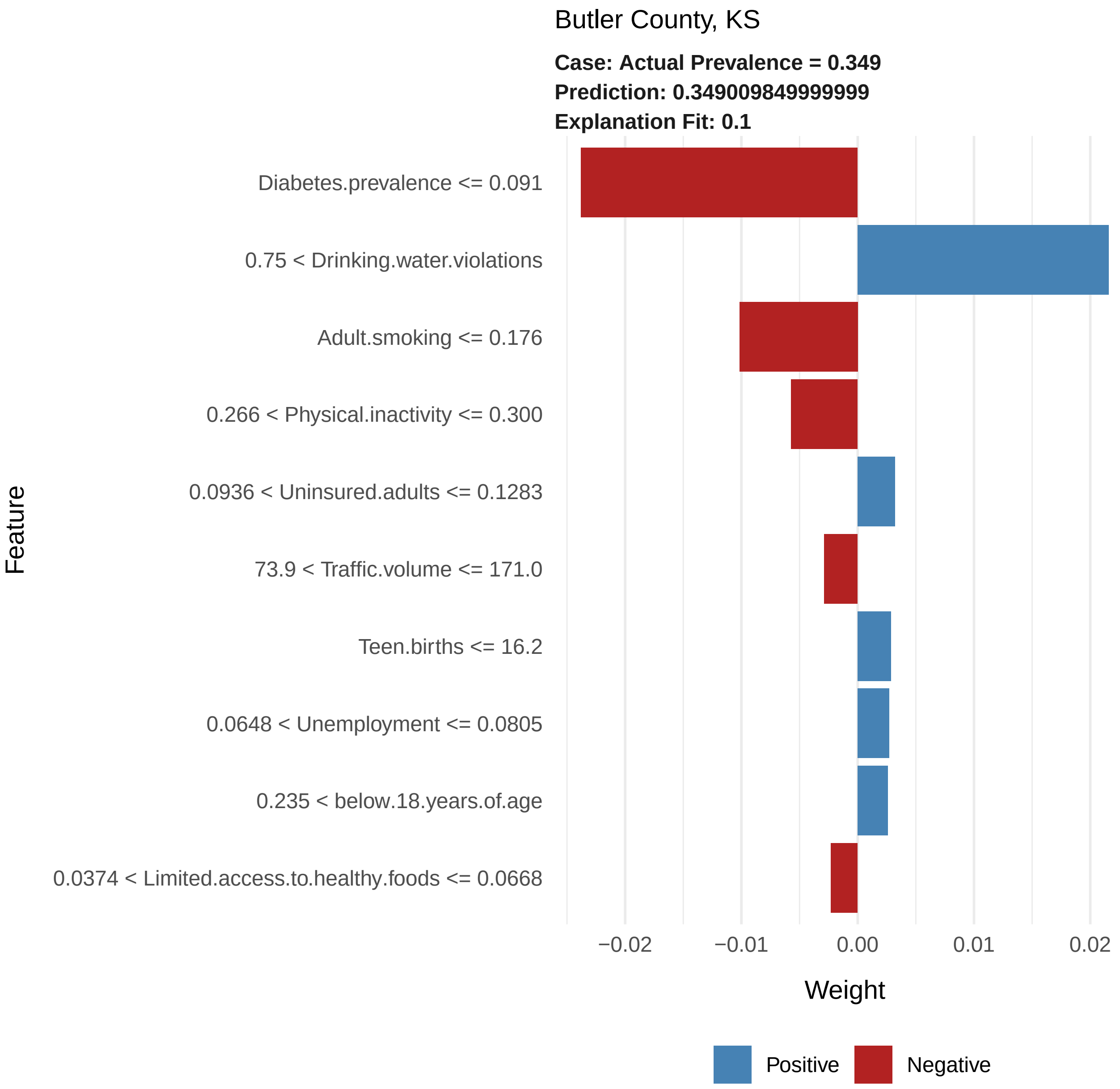
Butler County, KS
Case: Actual Prevalence = 0.349
Prediction: 0.349009849999999
Explanation Fit: 0.1
Diabetes.prevalence <= 0.091
0.75 < Drinking.water.violations
Adult.smoking <= 0.176
0.266 < Physical.inactivity <= 0.300
0.0936 < Uninsured.adults <= 0.1283
73.9 < Traffic.volume <= 171.0
Teen.births <= 16.2
0.0648 < Unemployment <= 0.0805
0.235 < below.18.years.of.age
0.0374 < Limited.access.to.healthy.foods <= 0.0668
Feature
−0.02
−0.01
0.00
0.01
0.02
Weight
Positive
Negative

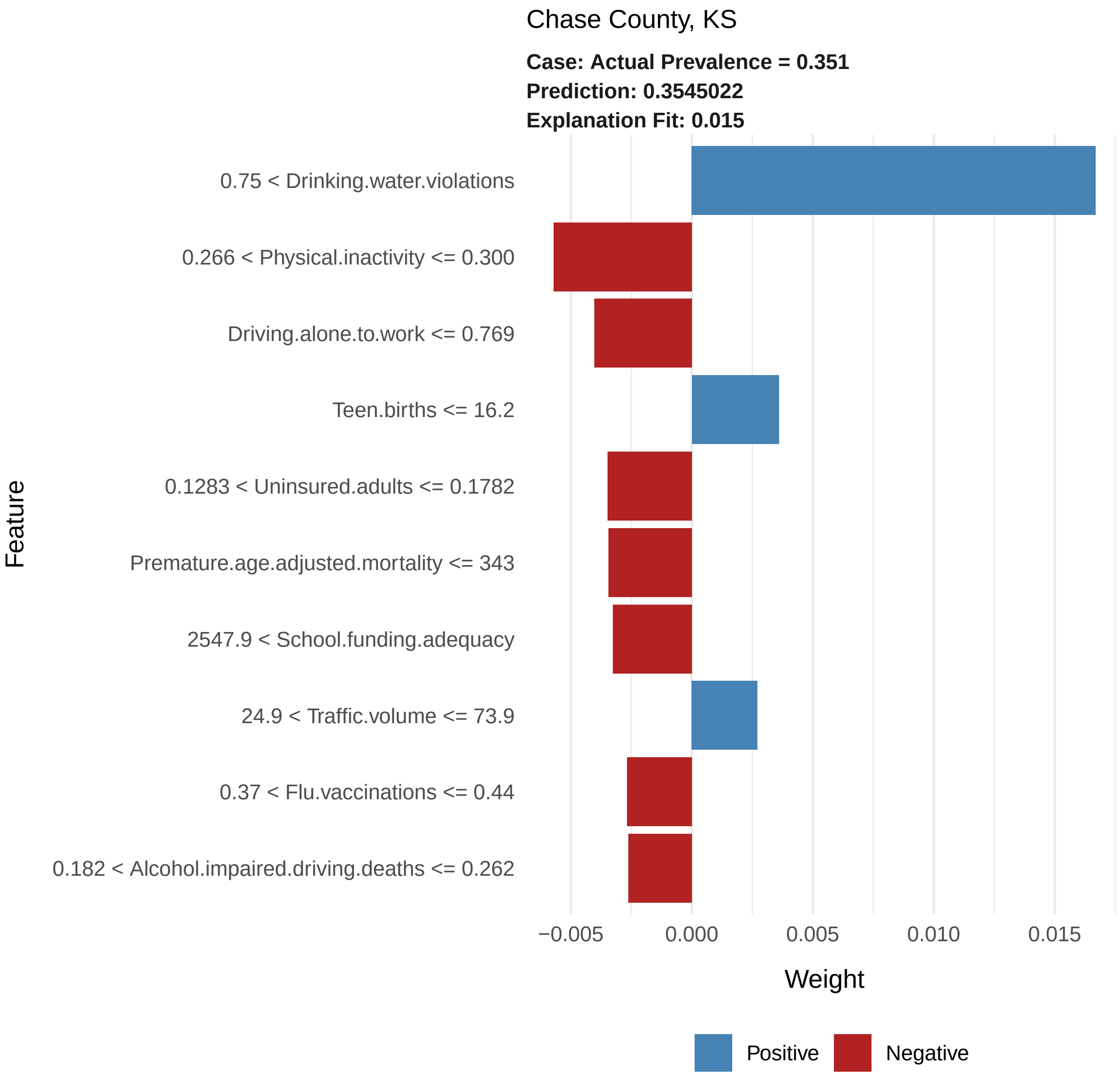
Chase County, KS
Case: Actual Prevalence = 0.351
Prediction: 0.3545022
Explanation Fit: 0.015
0.75 < Drinking.water.violations
0.266 < Physical.inactivity <= 0.300
Driving.alone.to.work <= 0.769
Teen.births <= 16.2
0.1283 < Uninsured.adults <= 0.1782
Premature.age.adjusted.mortality <= 343
2547.9 < School.funding.adequacy
24.9 < Traffic.volume <= 73.9
0.37 < Flu.vaccinations <= 0.44
0.182 < Alcohol.impaired.driving.deaths <= 0.262
Feature
−0.005
0.000
0.005
0.010
0.015
Weight
Positive
Negative

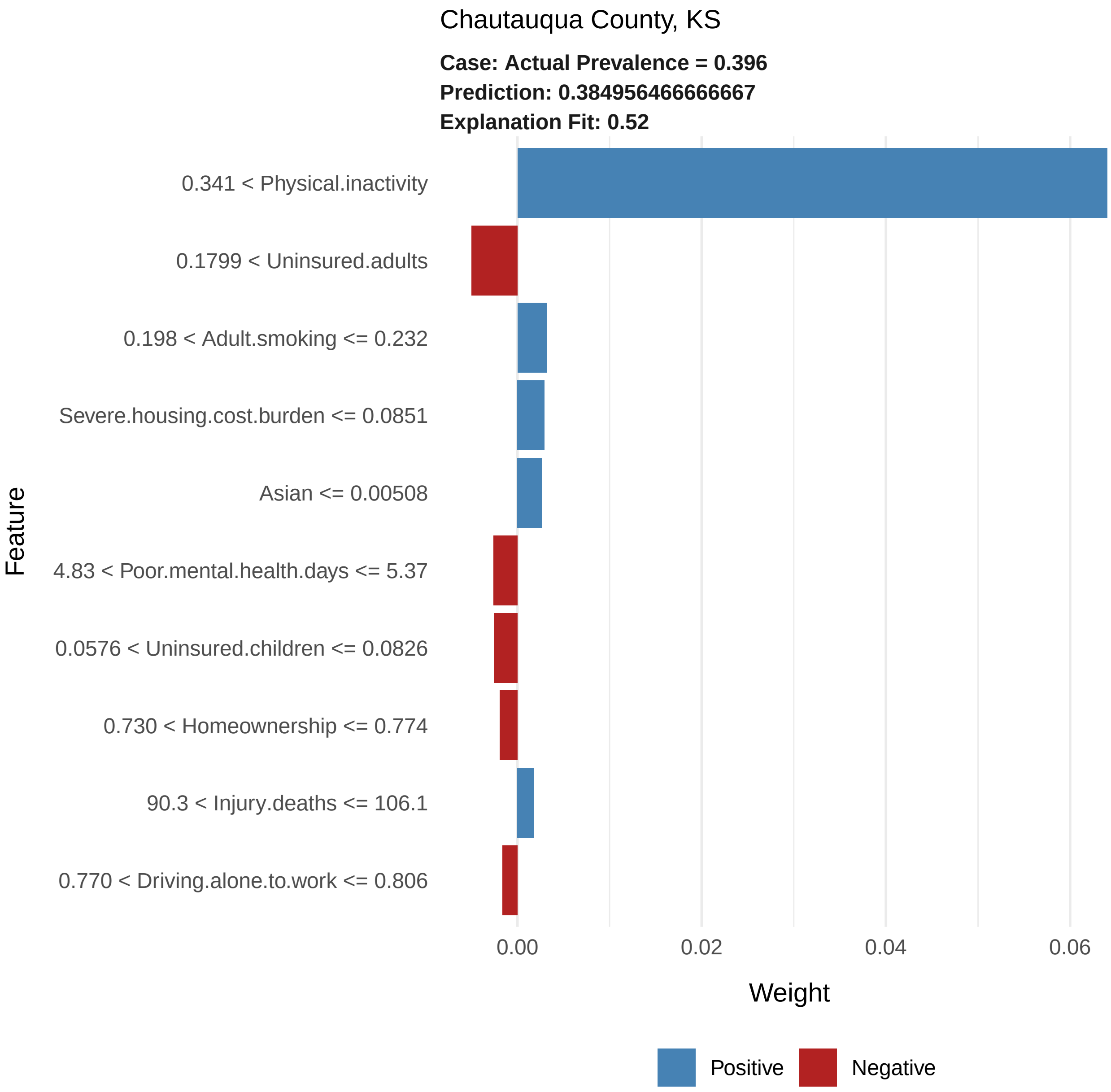

Chautauqua County, KS
Case: Actual Prevalence = 0.396
Prediction: 0.384956466666667
Explanation Fit: 0.52
0.341 < Physical.inactivity
0.1799 < Uninsured.adults
0.198 < Adult.smoking <= 0.232
Severe.housing.cost.burden <= 0.0851
Asian <= 0.00508
4.83 < Poor.mental.health.days <= 5.37
0.0576 < Uninsured.children <= 0.0826
0.730 < Homeownership <= 0.774
90.3 < Injury.deaths <= 106.1
0.770 < Driving.alone.to.work <= 0.806
Feature
0.00
0.02
0.04
0.06
Weight
Positive
Negative

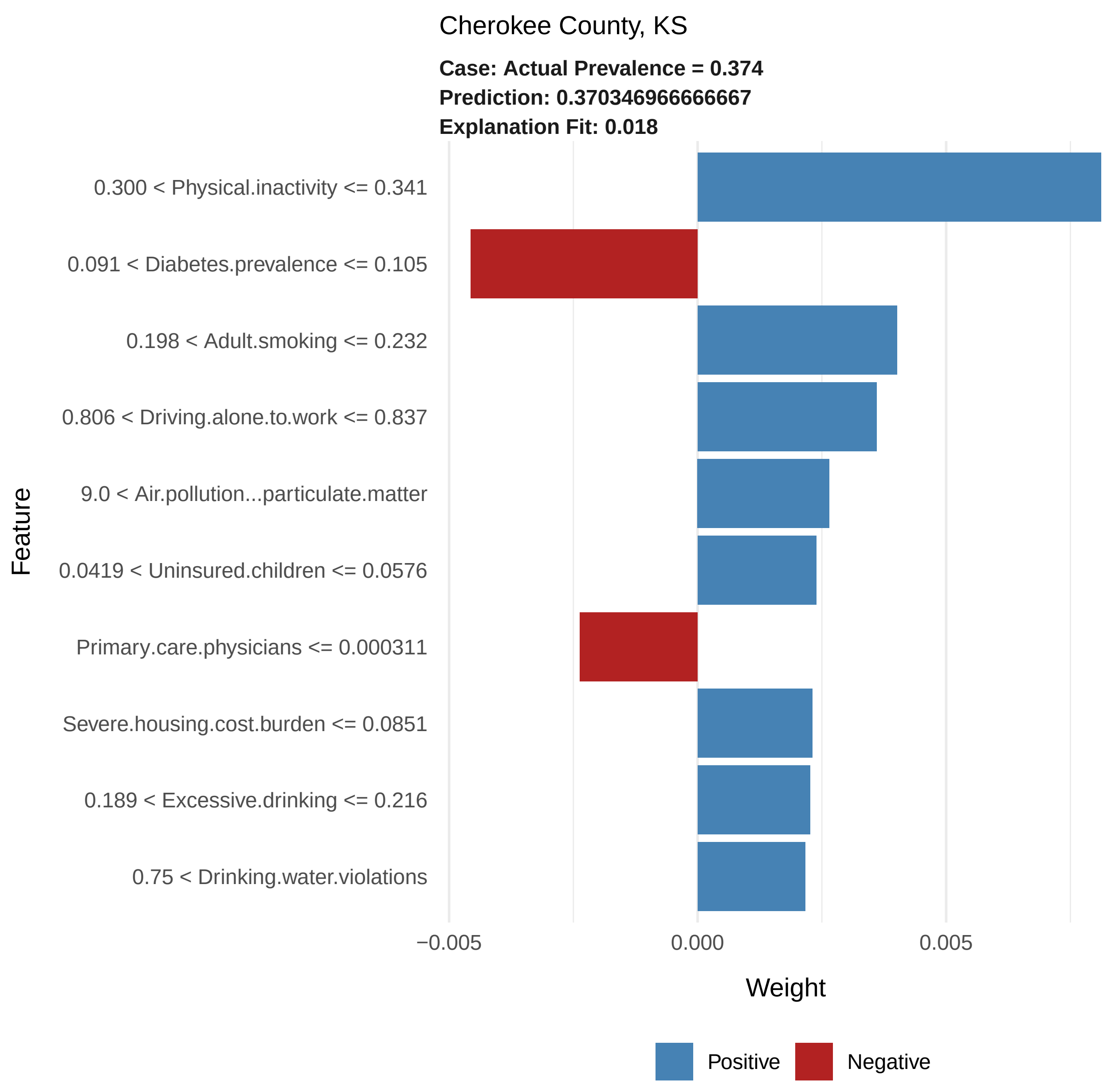

Cherokee County, KS
Case: Actual Prevalence = 0.374
Prediction: 0.370346966666667
Explanation Fit: 0.018
0.300 < Physical.inactivity <= 0.341
0.091 < Diabetes.prevalence <= 0.105
0.198 < Adult.smoking <= 0.232
0.806 < Driving.alone.to.work <= 0.837
9.0 < Air.pollution...particulate.matter
0.0419 < Uninsured.children <= 0.0576
Primary.care.physicians <= 0.000311
Severe.housing.cost.burden <= 0.0851
0.189 < Excessive.drinking <= 0.216
0.75 < Drinking.water.violations
Feature
−0.005
0.000
0.005
Weight
Positive
Negative

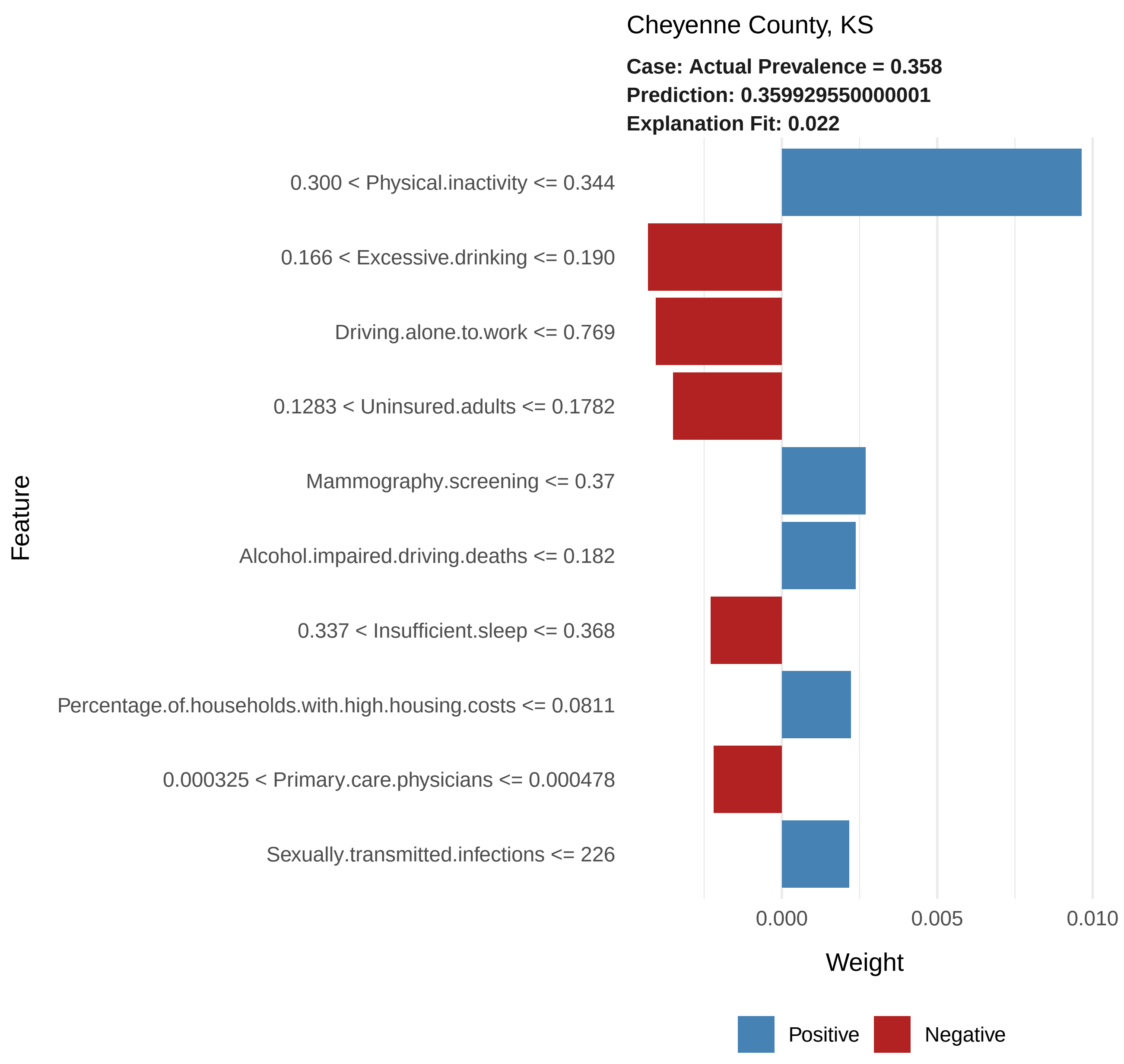
Cheyenne County, KS
Case: Actual Prevalence = 0.358
Prediction: 0.359929550000001
Explanation Fit: 0.022
0.300 < Physical.inactivity <= 0.344
0.166 < Excessive.drinking <= 0.190
Driving.alone.to.work <= 0.769
0.1283 < Uninsured.adults <= 0.1782
Mammography.screening <= 0.37
Alcohol.impaired.driving.deaths <= 0.182
0.337 < Insufficient.sleep <= 0.368
Percentage.of.households.with.high.housing.costs <= 0.0811
0.000325 < Primary.care.physicians <= 0.000478
Sexually.transmitted.infections <= 226
Feature
0.000
0.005
0.010
Weight
Positive
Negative

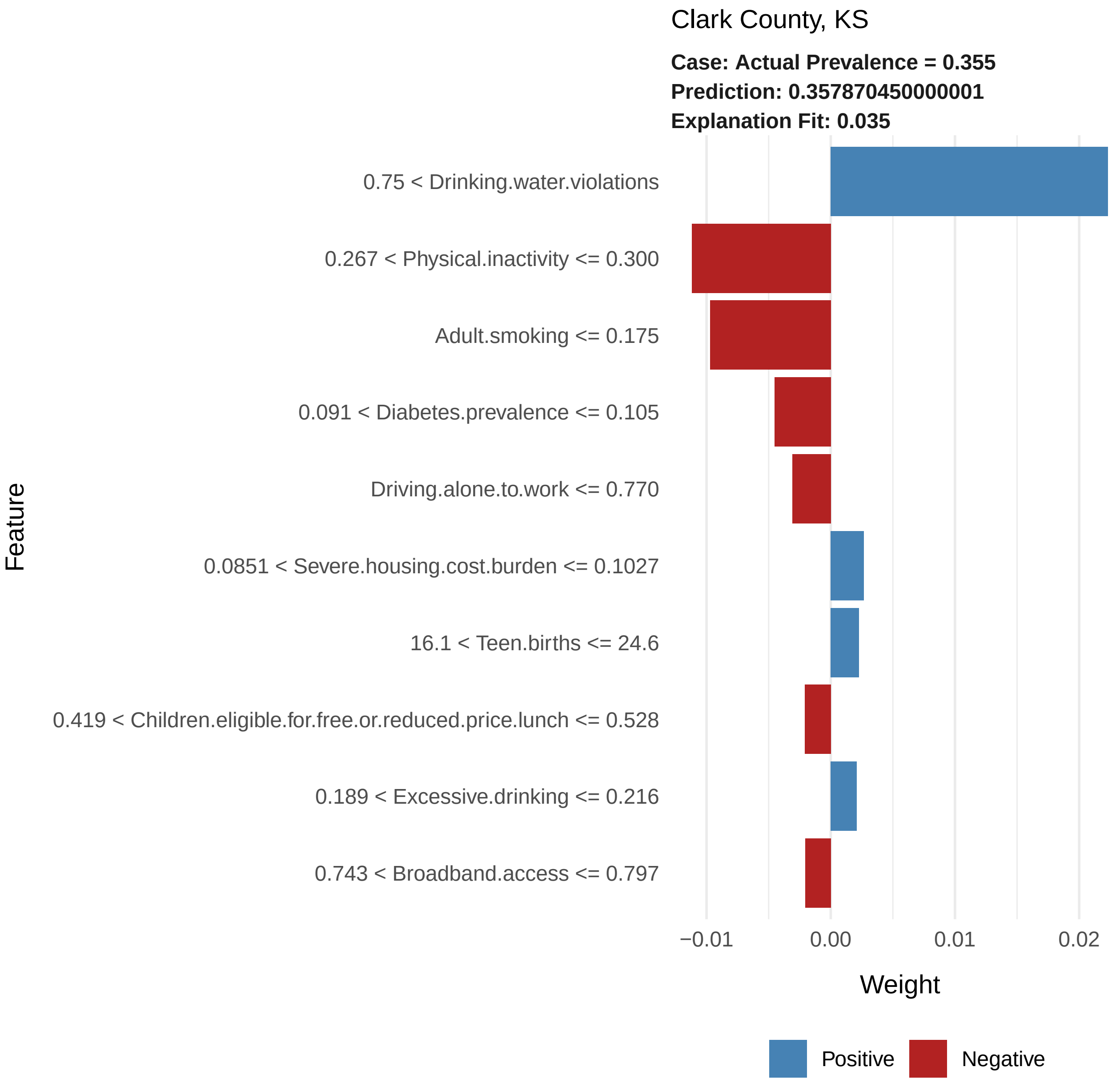

Clark County, KS
Case: Actual Prevalence = 0.355
Prediction: 0.357870450000001
Explanation Fit: 0.035
0.75 < Drinking.water.violations
0.267 < Physical.inactivity <= 0.300
Adult.smoking <= 0.175
0.091 < Diabetes.prevalence <= 0.105
Driving.alone.to.work <= 0.770
0.0851 < Severe.housing.cost.burden <= 0.1027
16.1 < Teen.births <= 24.6
0.419 < Children.eligible.for.free.or.reduced.price.lunch <= 0.528
0.189 < Excessive.drinking <= 0.216
0.743 < Broadband.access <= 0.797
Feature
−0.01
0.00
0.01
0.02
Weight
Positive
Negative

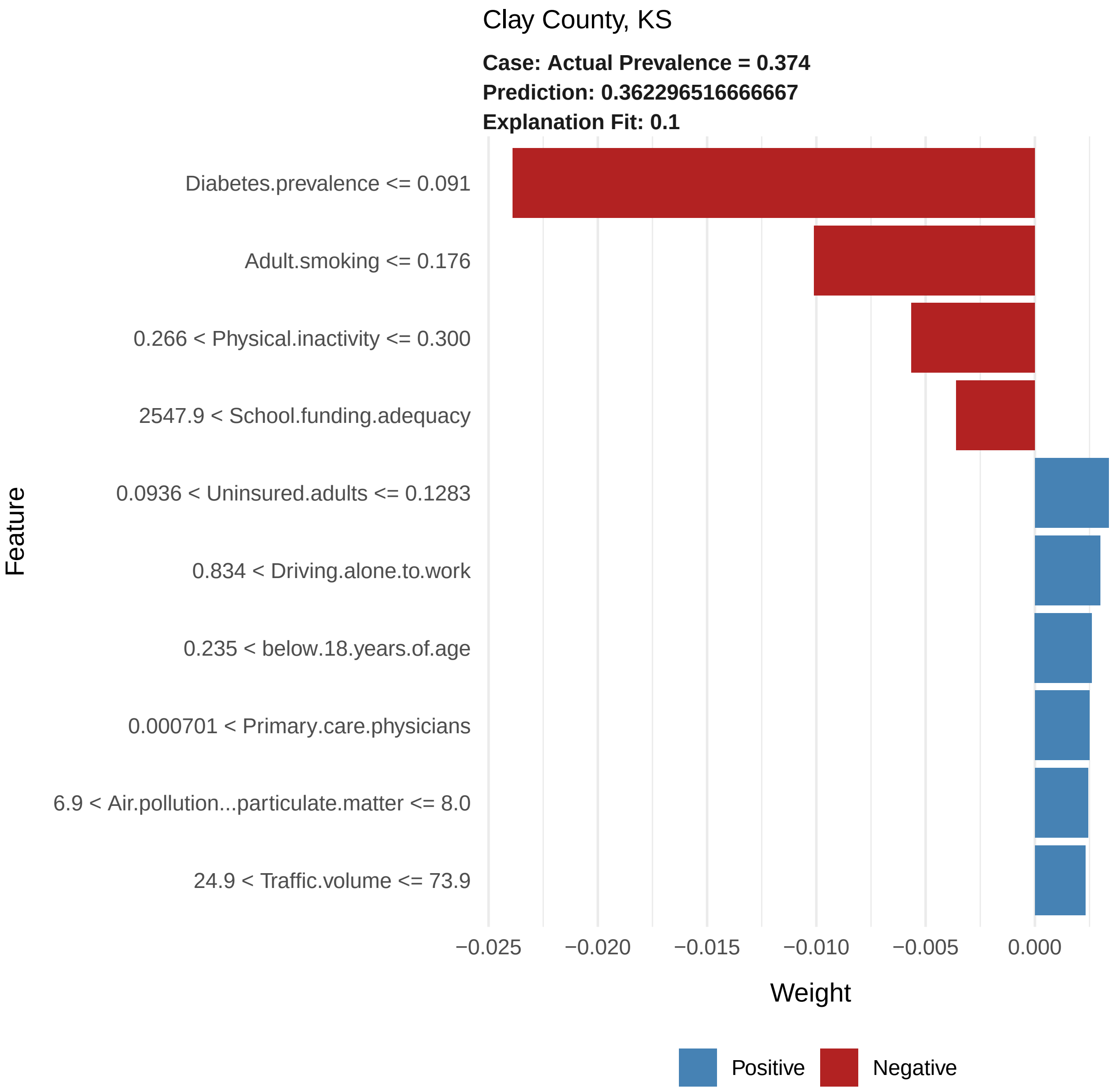

Clay County, KS
Case: Actual Prevalence = 0.374
Prediction: 0.362296516666667
Explanation Fit: 0.1
Diabetes.prevalence <= 0.091
Adult.smoking <= 0.176
0.266 < Physical.inactivity <= 0.300
2547.9 < School.funding.adequacy
0.0936 < Uninsured.adults <= 0.1283
0.834 < Driving.alone.to.work
0.235 < below.18.years.of.age
0.000701 < Primary.care.physicians
6.9 < Air.pollution...particulate.matter <= 8.0
24.9 < Traffic.volume <= 73.9
Feature
−0.025
−0.020
−0.015
−0.010
−0.005
0.000
Weight
Positive
Negative

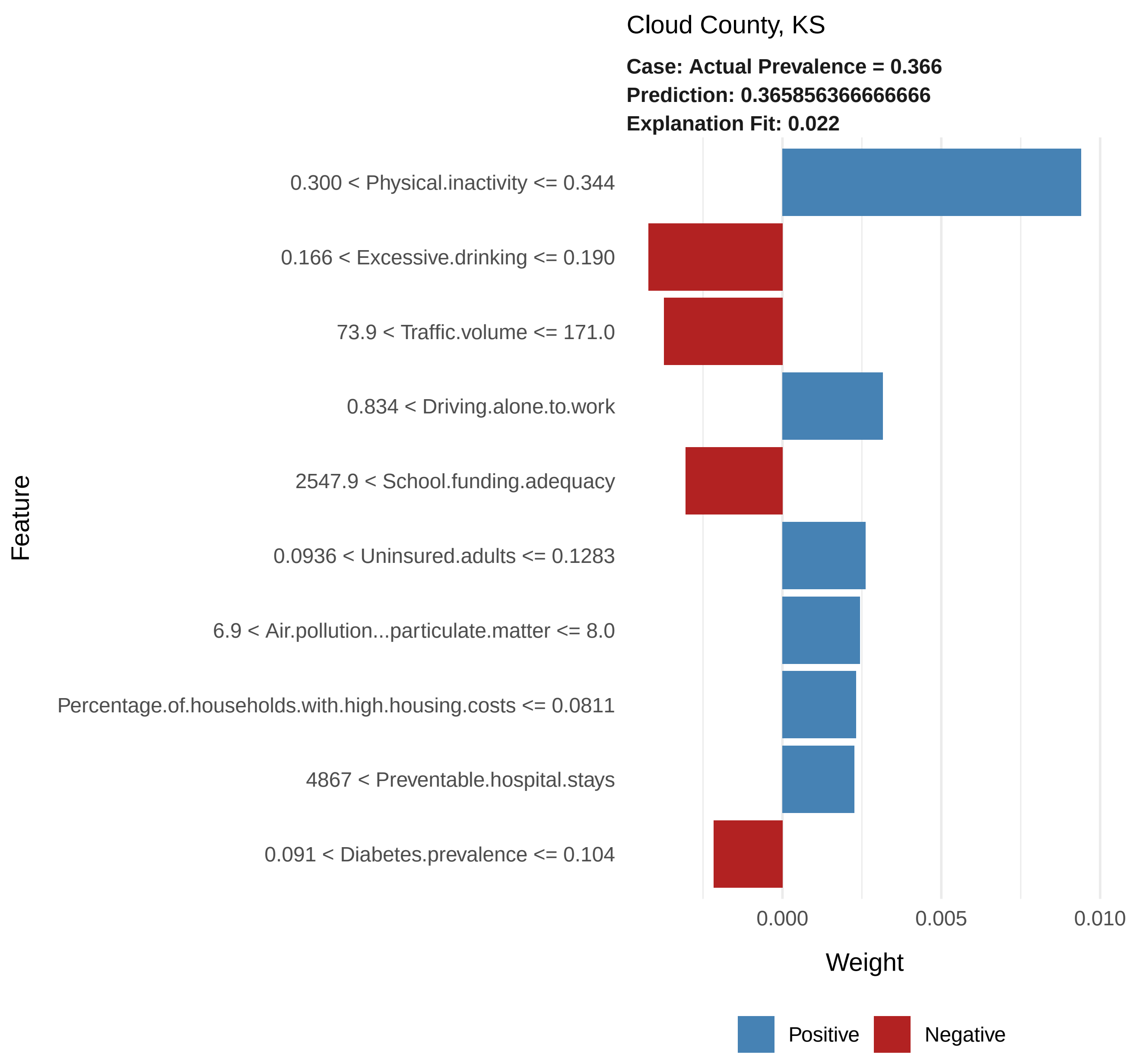

Cloud County, KS
Case: Actual Prevalence = 0.366
Prediction: 0.365856366666666
Explanation Fit: 0.022
0.300 < Physical.inactivity <= 0.344
0.166 < Excessive.drinking <= 0.190
73.9 < Traffic.volume <= 171.0
0.834 < Driving.alone.to.work
2547.9 < School.funding.adequacy
0.0936 < Uninsured.adults <= 0.1283
6.9 < Air.pollution...particulate.matter <= 8.0
Percentage.of.households.with.high.housing.costs <= 0.0811
4867 < Preventable.hospital.stays
0.091 < Diabetes.prevalence <= 0.104
Feature
0.000
0.005
0.010
Weight
Positive
Negative

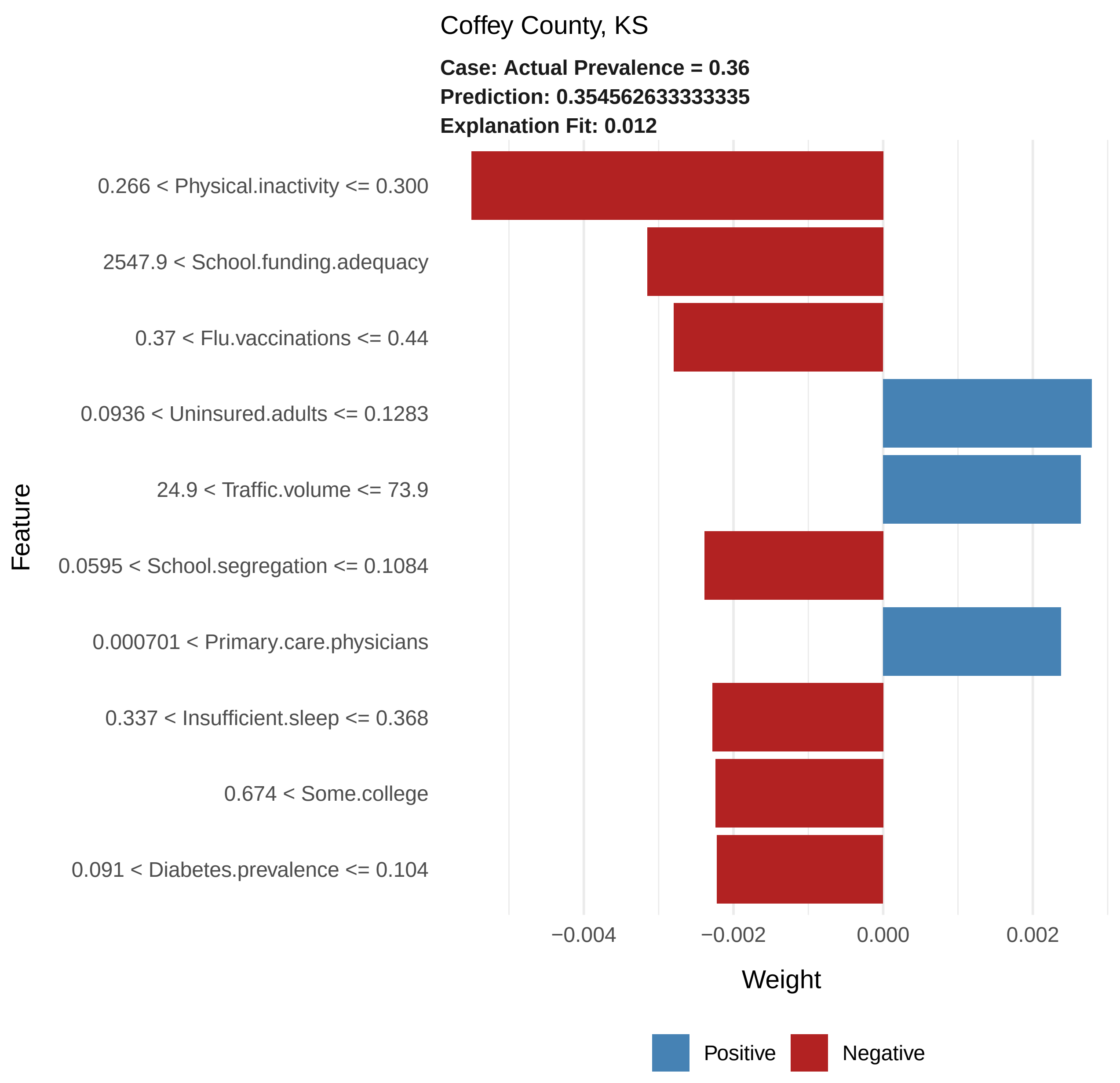

Coffey County, KS
Case: Actual Prevalence = 0.36
Prediction: 0.354562633333335
Explanation Fit: 0.012
0.266 < Physical.inactivity <= 0.300
2547.9 < School.funding.adequacy
0.37 < Flu.vaccinations <= 0.44
0.0936 < Uninsured.adults <= 0.1283
24.9 < Traffic.volume <= 73.9
0.0595 < School.segregation <= 0.1084
0.000701 < Primary.care.physicians
0.337 < Insufficient.sleep <= 0.368
0.674 < Some.college
0.091 < Diabetes.prevalence <= 0.104
Feature
−0.004
−0.002
0.000
0.002
Weight
Positive
Negative

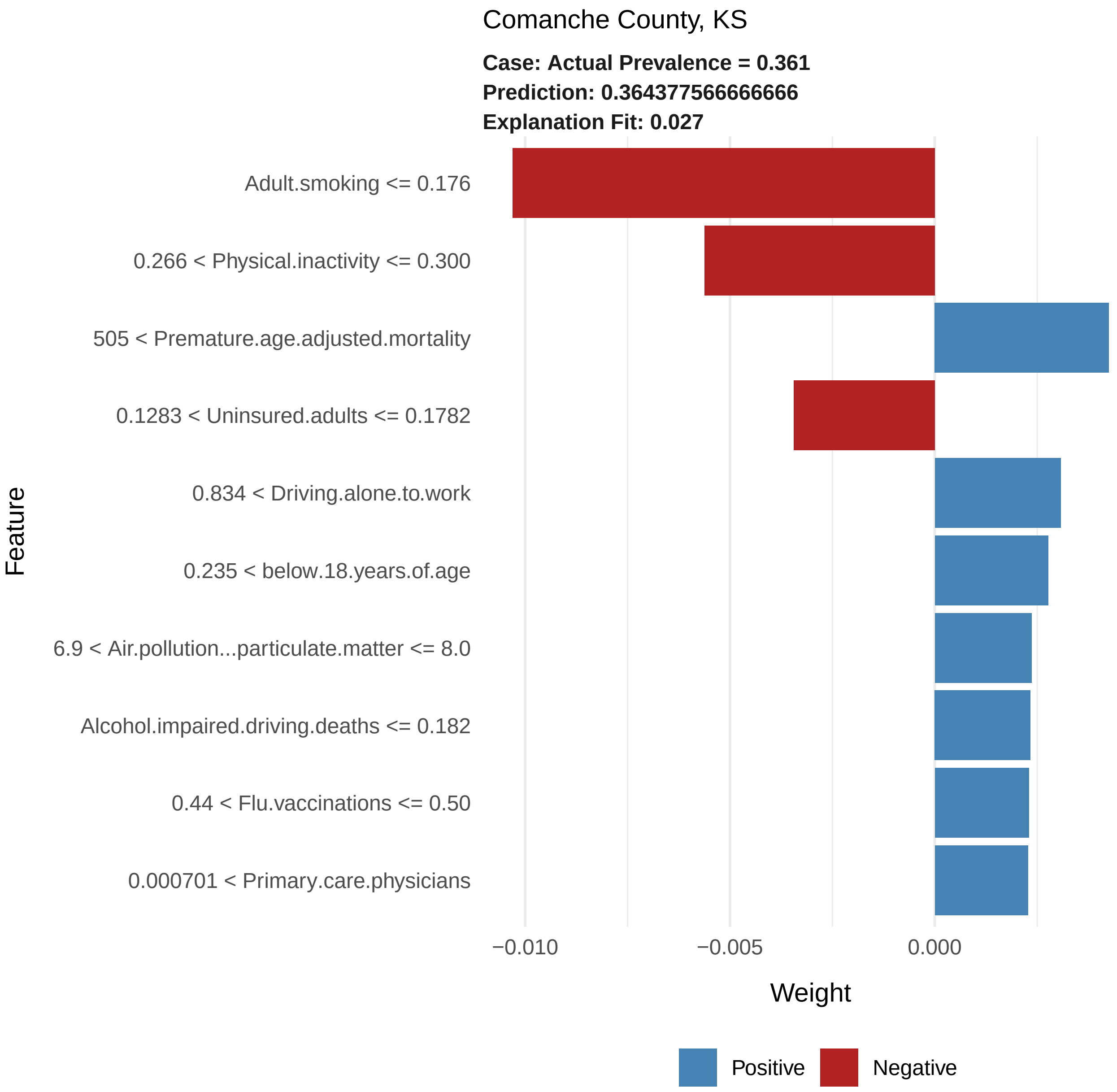

Comanche County, KS
Case: Actual Prevalence = 0.361
Prediction: 0.364377566666666
Explanation Fit: 0.027
Adult.smoking <= 0.176
0.266 < Physical.inactivity <= 0.300
505 < Premature.age.adjusted.mortality
0.1283 < Uninsured.adults <= 0.1782
0.834 < Driving.alone.to.work
0.235 < below.18.years.of.age
6.9 < Air.pollution...particulate.matter <= 8.0
Alcohol.impaired.driving.deaths <= 0.182
0.44 < Flu.vaccinations <= 0.50
0.000701 < Primary.care.physicians
Feature
−0.010
−0.005
0.000
Weight
Positive
Negative

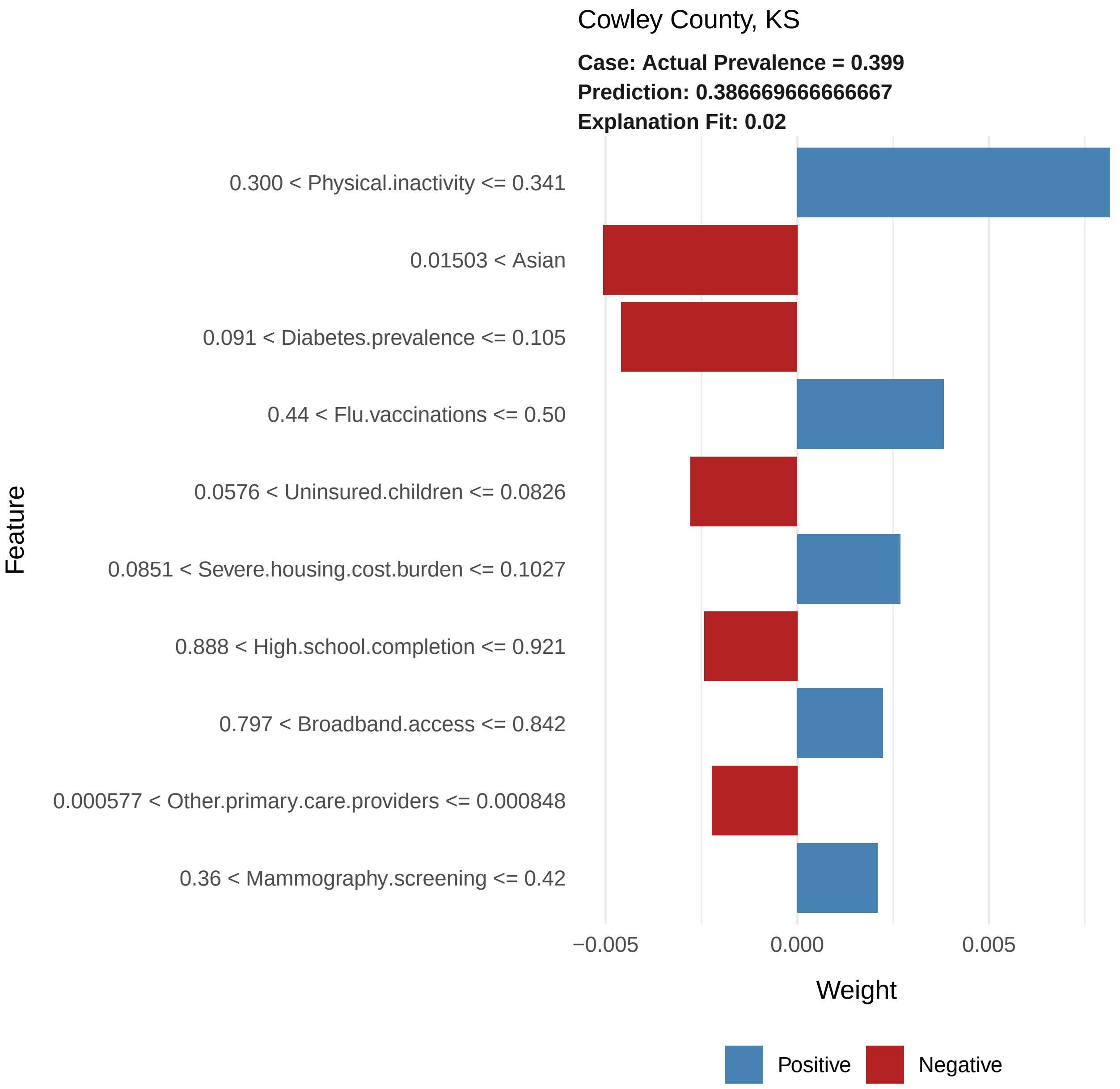

Cowley County, KS
Case: Actual Prevalence = 0.399
Prediction: 0.386669666666667
Explanation Fit: 0.02
0.300 < Physical.inactivity <= 0.341
0.01503 < Asian
0.091 < Diabetes.prevalence <= 0.105
0.44 < Flu.vaccinations <= 0.50
0.0576 < Uninsured.children <= 0.0826
0.0851 < Severe.housing.cost.burden <= 0.1027
0.888 < High.school.completion <= 0.921
0.797 < Broadband.access <= 0.842
0.000577 < Other.primary.care.providers <= 0.000848
0.36 < Mammography.screening <= 0.42
Feature
−0.005
0.000
0.005
Weight
Positive
Negative

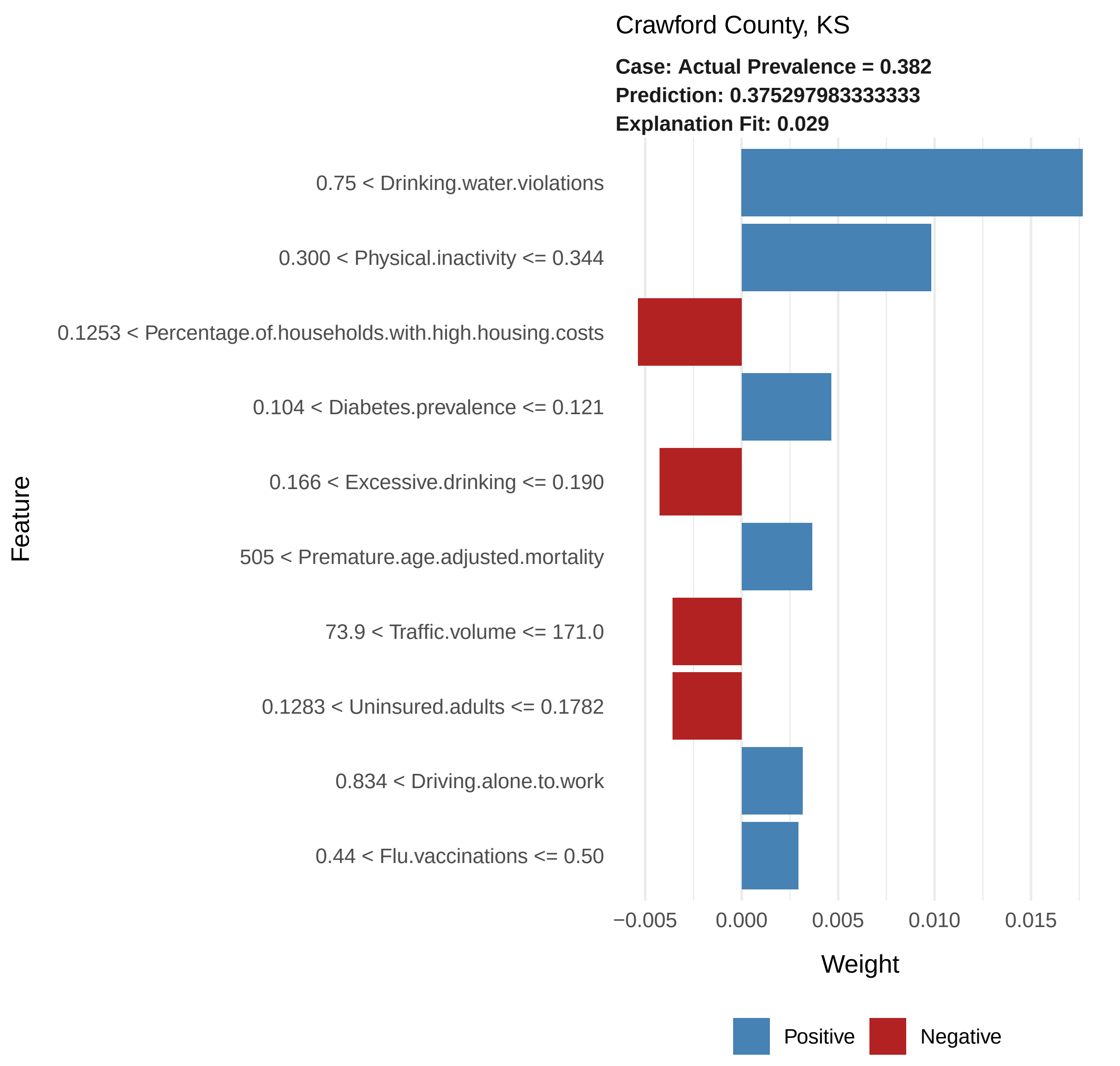

Crawford County, KS
Case: Actual Prevalence = 0.382
Prediction: 0.375297983333333
Explanation Fit: 0.029
0.75 < Drinking.water.violations
0.300 < Physical.inactivity <= 0.344
0.1253 < Percentage.of.households.with.high.housing.costs
0.104 < Diabetes.prevalence <= 0.121
0.166 < Excessive.drinking <= 0.190
505 < Premature.age.adjusted.mortality
73.9 < Traffic.volume <= 171.0
0.1283 < Uninsured.adults <= 0.1782
0.834 < Driving.alone.to.work
0.44 < Flu.vaccinations <= 0.50
Feature
−0.005
0.000
0.005
0.010
0.015
Weight
Positive
Negative

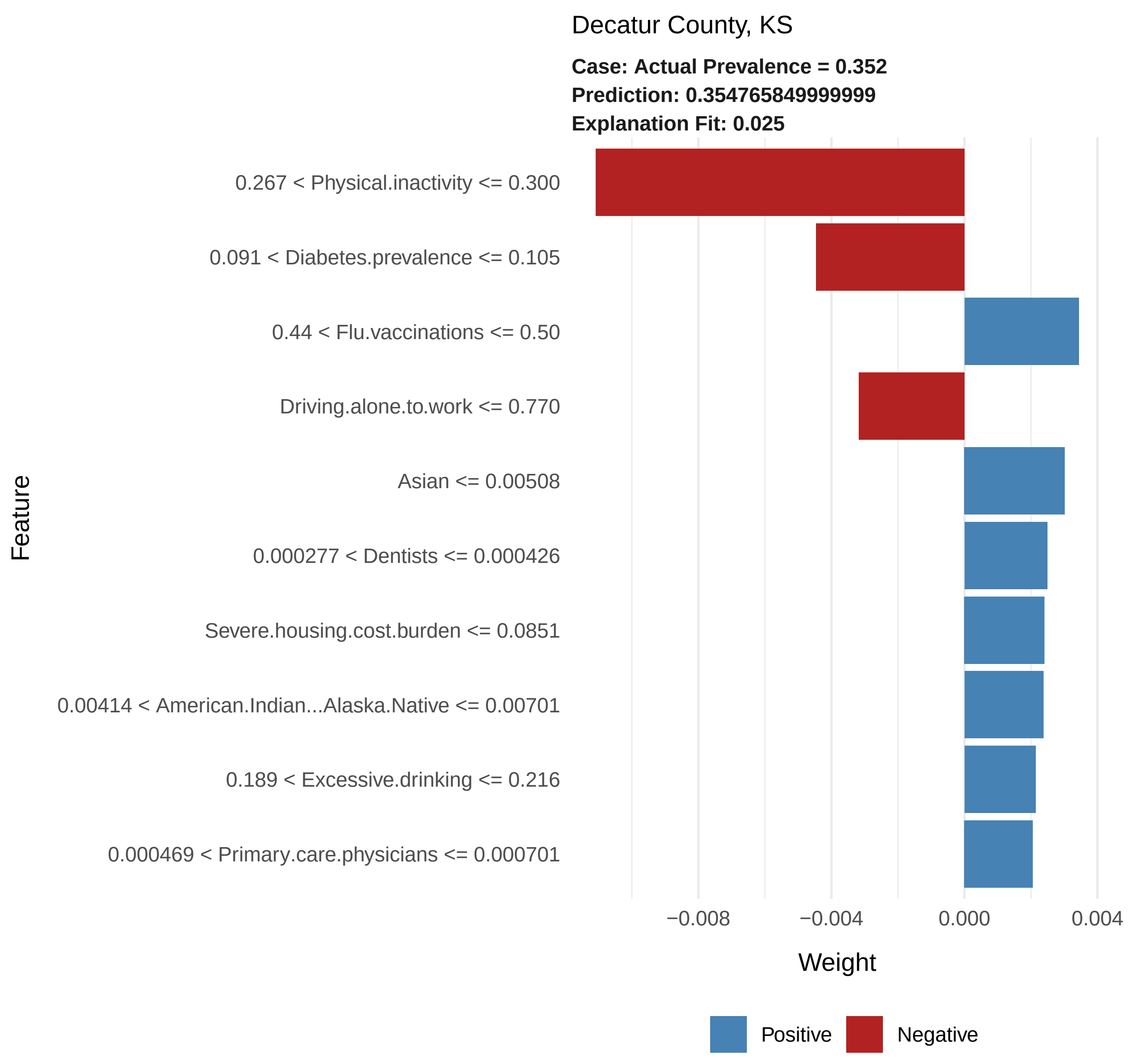

Decatur County, KS
Case: Actual Prevalence = 0.352
Prediction: 0.354765849999999
Explanation Fit: 0.025
0.267 < Physical.inactivity <= 0.300
0.091 < Diabetes.prevalence <= 0.105
0.44 < Flu.vaccinations <= 0.50
Driving.alone.to.work <= 0.770
Asian <= 0.00508
0.000277 < Dentists <= 0.000426
Severe.housing.cost.burden <= 0.0851
0.00414 < American.Indian...Alaska.Native <= 0.00701
0.189 < Excessive.drinking <= 0.216
0.000469 < Primary.care.physicians <= 0.000701
Feature
−0.008
−0.004
0.000
0.004
Weight
Positive
Negative

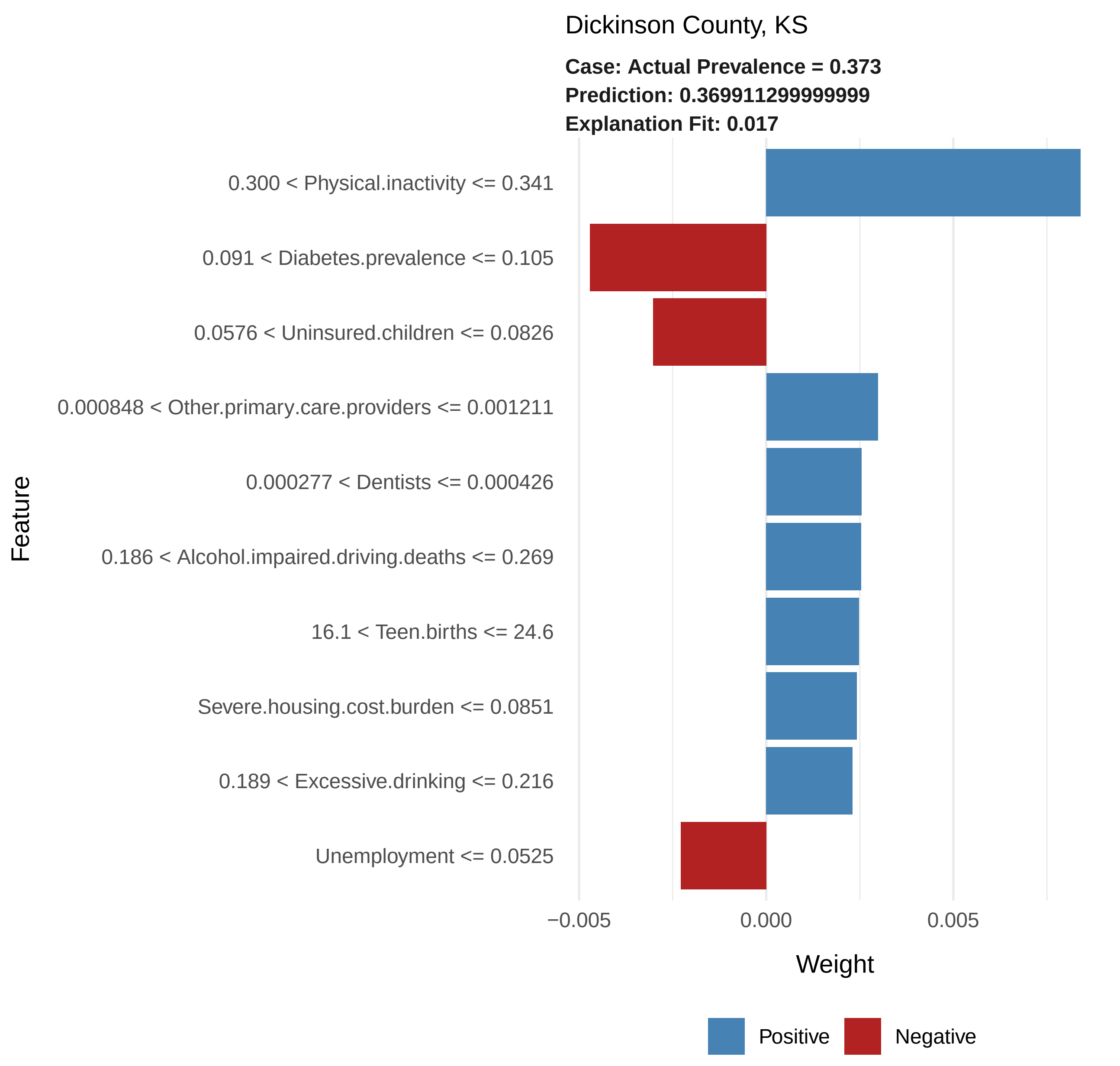
Dickinson County, KS
Case: Actual Prevalence = 0.373
Prediction: 0.369911299999999
Explanation Fit: 0.017
0.300 < Physical.inactivity <= 0.341
0.091 < Diabetes.prevalence <= 0.105
0.0576 < Uninsured.children <= 0.0826
0.000848 < Other.primary.care.providers <= 0.001211
0.000277 < Dentists <= 0.000426
0.186 < Alcohol.impaired.driving.deaths <= 0.269
16.1 < Teen.births <= 24.6
Severe.housing.cost.burden <= 0.0851
0.189 < Excessive.drinking <= 0.216
Unemployment <= 0.0525
Feature
−0.005
0.000
0.005
Weight
Positive
Negative

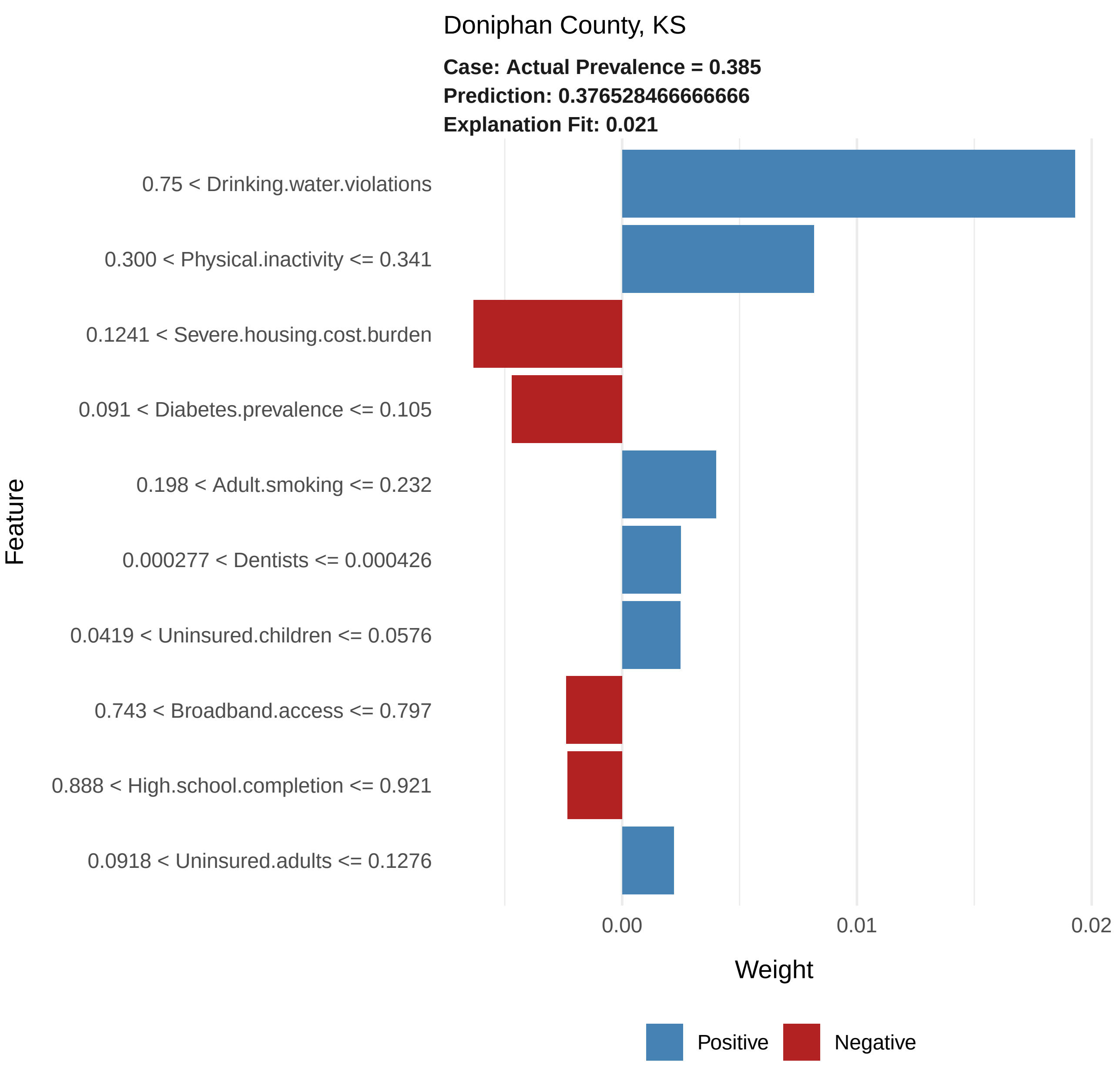

Doniphan County, KS
Case: Actual Prevalence = 0.385
Prediction: 0.376528466666666
Explanation Fit: 0.021
0.75 < Drinking.water.violations
0.300 < Physical.inactivity <= 0.341
0.1241 < Severe.housing.cost.burden
0.091 < Diabetes.prevalence <= 0.105
0.198 < Adult.smoking <= 0.232
0.000277 < Dentists <= 0.000426
0.0419 < Uninsured.children <= 0.0576
0.743 < Broadband.access <= 0.797
0.888 < High.school.completion <= 0.921
0.0918 < Uninsured.adults <= 0.1276
Feature
0.00
0.01
0.02
Weight
Positive
Negative

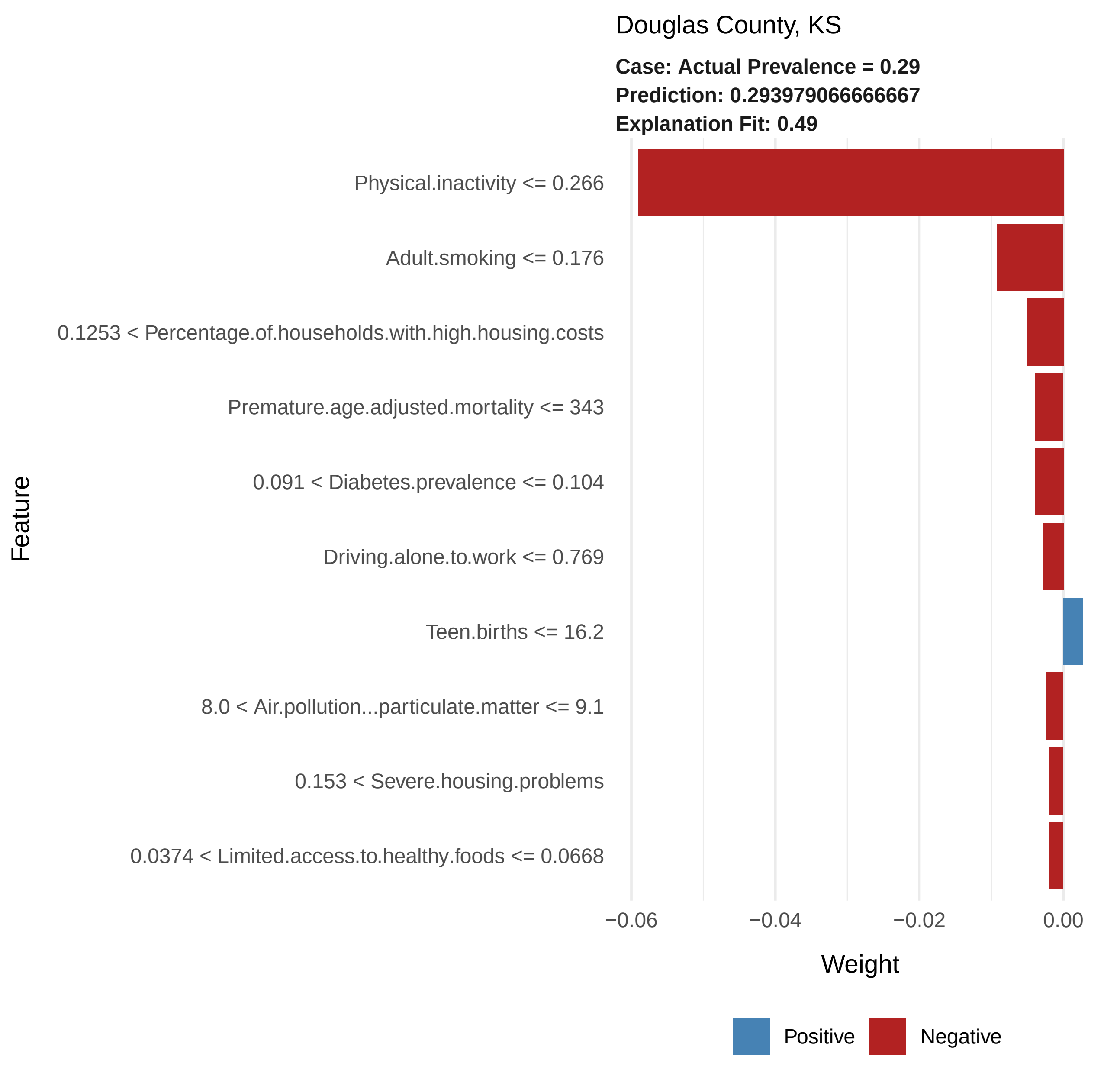

Douglas County, KS
Case: Actual Prevalence = 0.29
Prediction: 0.293979066666667
Explanation Fit: 0.49
Feature
Physical.inactivity <= 0.266
Adult.smoking <= 0.176
0.1253 < Percentage.of.households.with.high.housing.costs
Premature.age.adjusted.mortality <= 343
0.091 < Diabetes.prevalence <= 0.104
Driving.alone.to.work <= 0.769
Teen.births <= 16.2
8.0 < Air.pollution...particulate.matter <= 9.1
0.153 < Severe.housing.problems
0.0374 < Limited.access.to.healthy.foods <= 0.0668
−0.06
−0.04
−0.02
0.00
Weight
Positive
Negative

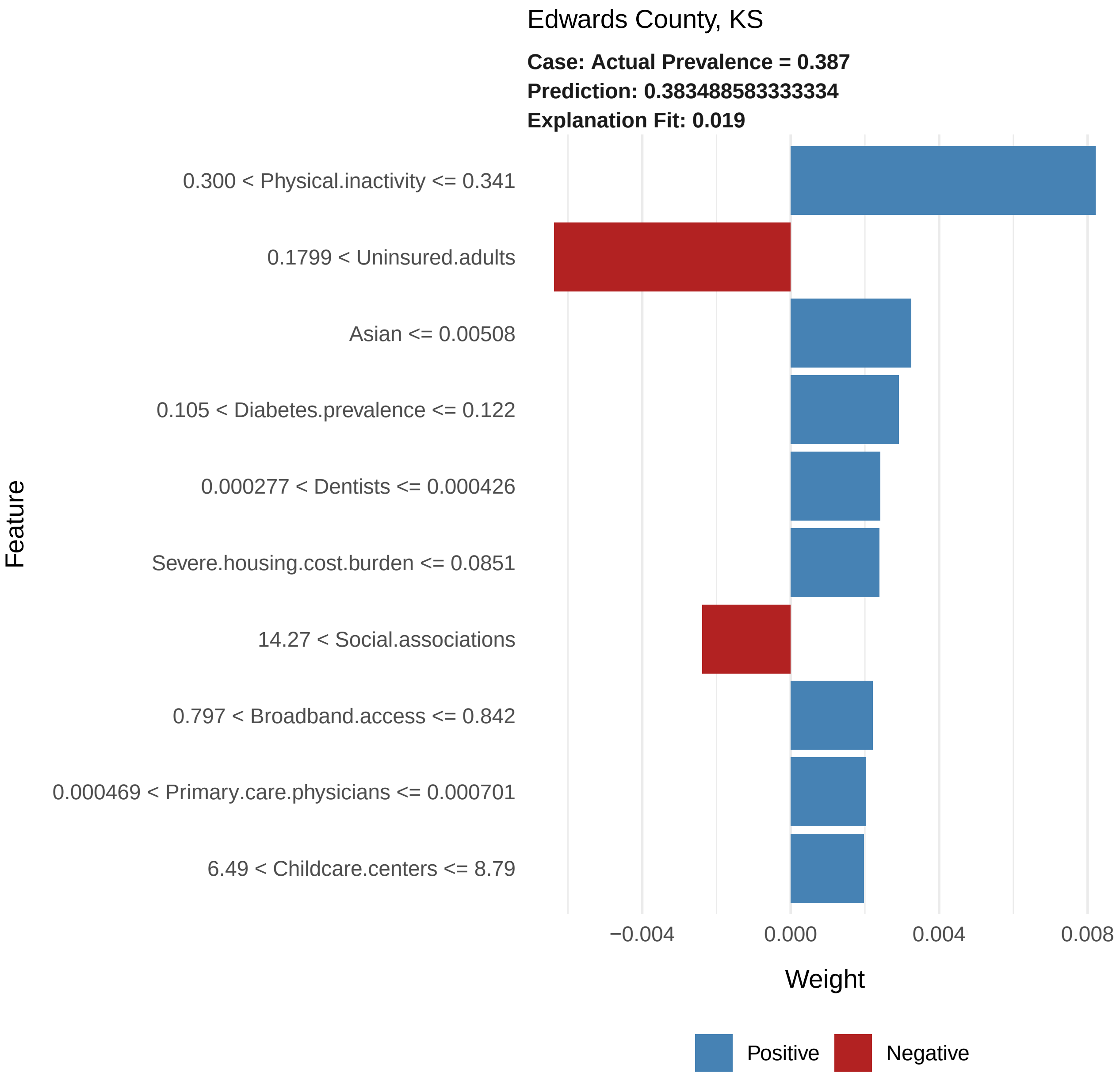
Edwards County, KS
Case: Actual Prevalence = 0.387
Prediction: 0.383488583333334
Explanation Fit: 0.019
0.300 < Physical.inactivity <= 0.341
0.1799 < Uninsured.adults
Asian <= 0.00508
0.105 < Diabetes.prevalence <= 0.122
0.000277 < Dentists <= 0.000426
Severe.housing.cost.burden <= 0.0851
14.27 < Social.associations
0.797 < Broadband.access <= 0.842
0.000469 < Primary.care.physicians <= 0.000701
6.49 < Childcare.centers <= 8.79
Feature
−0.004
0.000
0.004
0.008
Weight
Positive
Negative

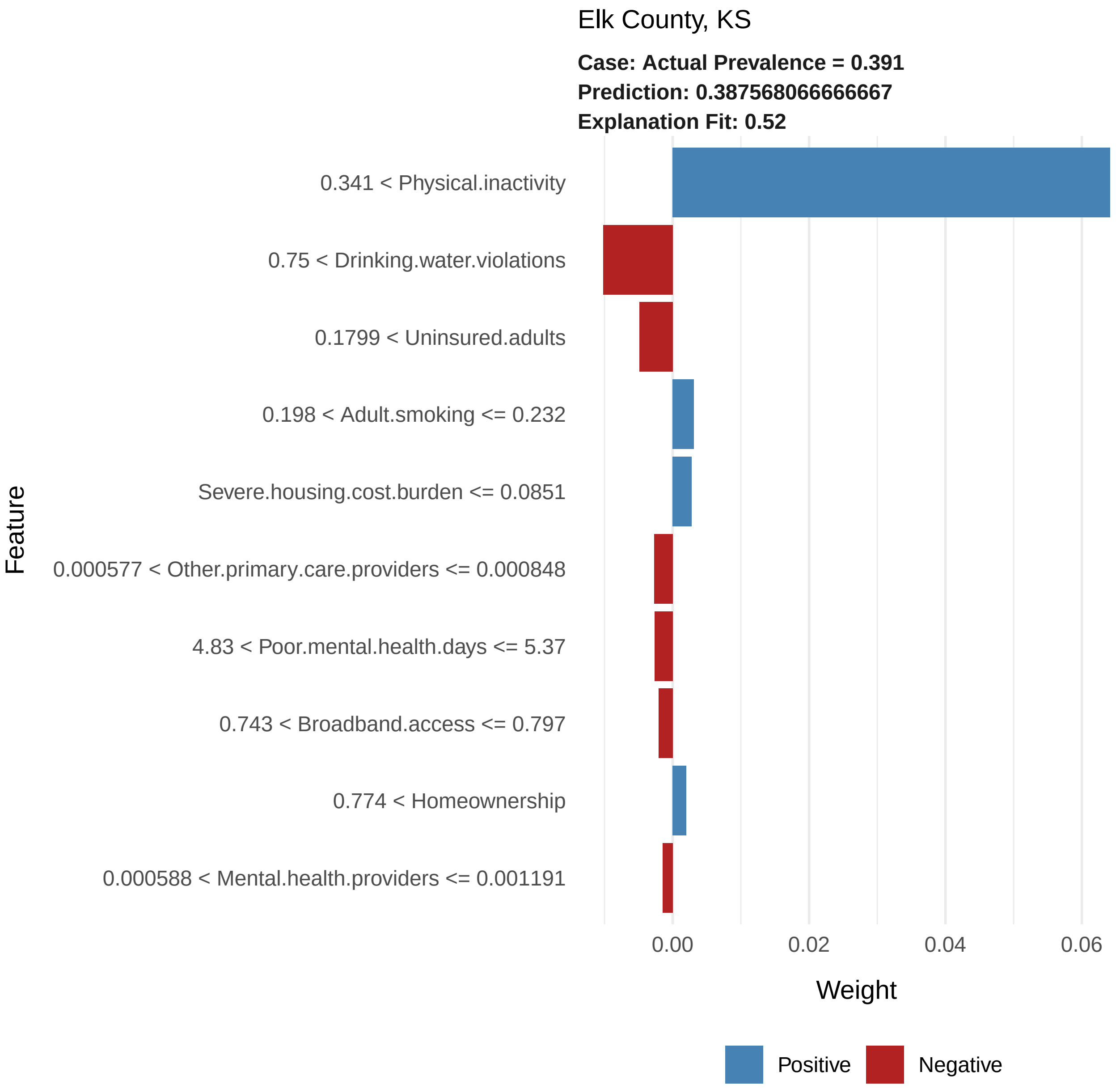
Elk County, KS
Case: Actual Prevalence = 0.391
Prediction: 0.387568066666667
Explanation Fit: 0.52
Feature
0.341 < Physical.inactivity
0.75 < Drinking.water.violations
0.1799 < Uninsured.adults
0.198 < Adult.smoking <= 0.232
Severe.housing.cost.burden <= 0.0851
0.000577 < Other.primary.care.providers <= 0.000848
4.83 < Poor.mental.health.days <= 5.37
0.743 < Broadband.access <= 0.797
0.774 < Homeownership
0.000588 < Mental.health.providers <= 0.001191
0.00
0.02
0.04
0.06
Weight
Positive
Negative

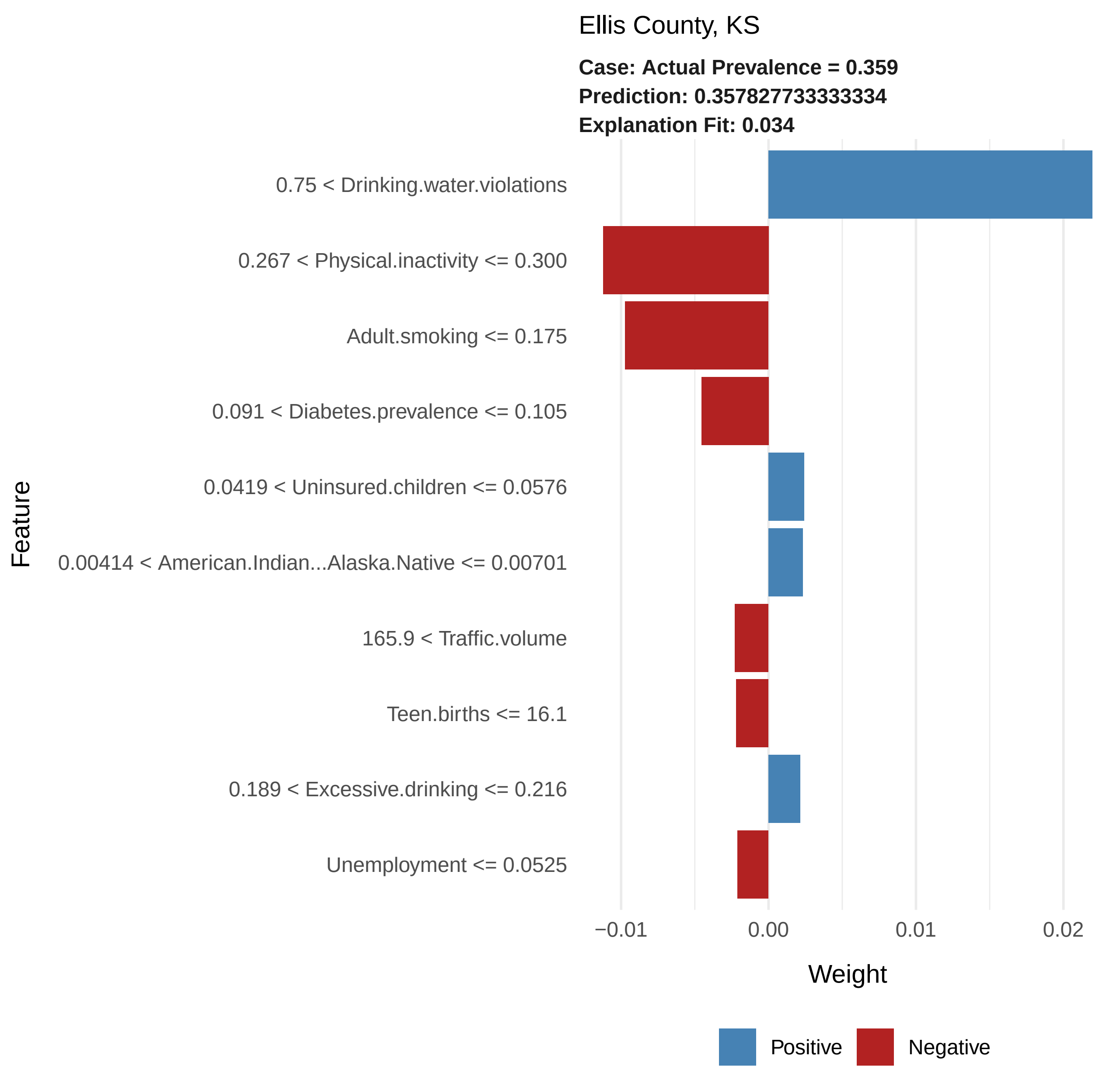

Ellis County, KS
Case: Actual Prevalence = 0.359
Prediction: 0.357827733333334
Explanation Fit: 0.034
0.75 < Drinking.water.violations
0.267 < Physical.inactivity <= 0.300
Adult.smoking <= 0.175
0.091 < Diabetes.prevalence <= 0.105
0.0419 < Uninsured.children <= 0.0576
0.00414 < American.Indian...Alaska.Native <= 0.00701
165.9 < Traffic.volume
Teen.births <= 16.1
0.189 < Excessive.drinking <= 0.216
Unemployment <= 0.0525
Feature
−0.01
0.00
0.01
0.02
Weight
Positive
Negative

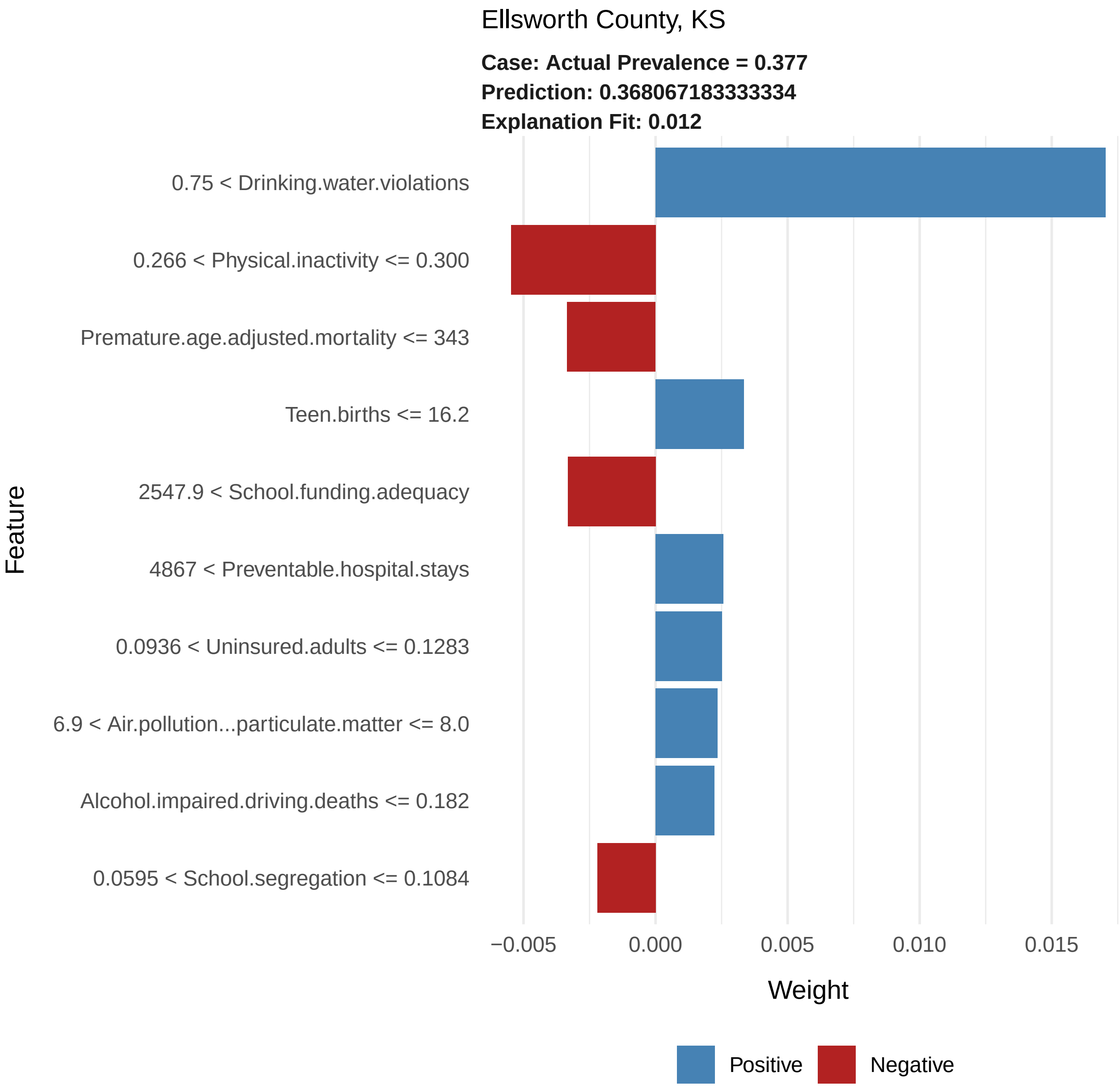

Ellsworth County, KS
Case: Actual Prevalence = 0.377
Prediction: 0.368067183333334
Explanation Fit: 0.012
0.75 < Drinking.water.violations
0.266 < Physical.inactivity <= 0.300
Premature.age.adjusted.mortality <= 343
Teen.births <= 16.2
2547.9 < School.funding.adequacy
4867 < Preventable.hospital.stays
0.0936 < Uninsured.adults <= 0.1283
6.9 < Air.pollution...particulate.matter <= 8.0
Alcohol.impaired.driving.deaths <= 0.182
0.0595 < School.segregation <= 0.1084
Feature
−0.005
0.000
0.005
0.010
0.015
Weight
Positive
Negative

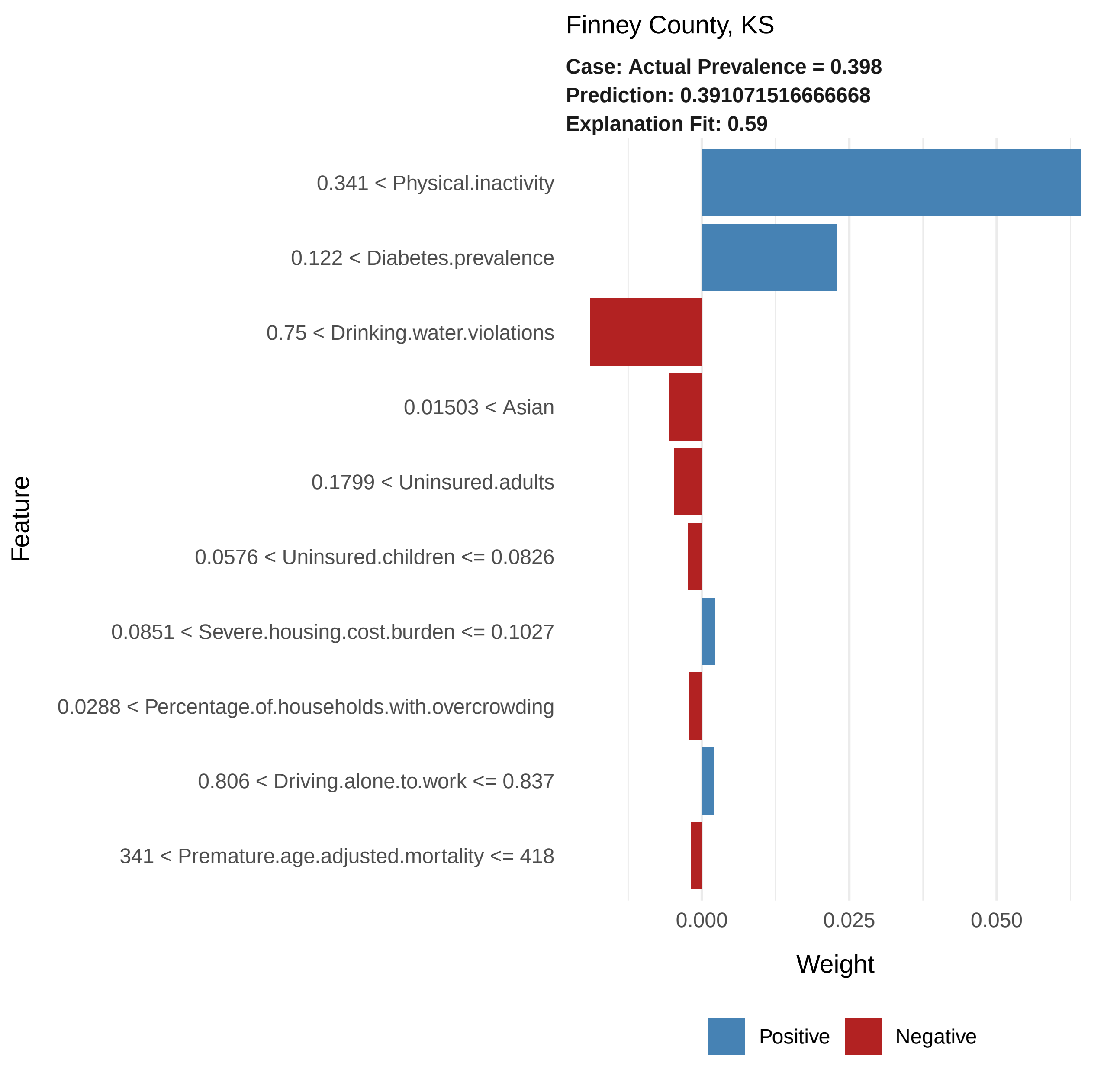

Finney County, KS
Case: Actual Prevalence = 0.398
Prediction: 0.391071516666668
Explanation Fit: 0.59
0.341 < Physical.inactivity
0.122 < Diabetes.prevalence
0.75 < Drinking.water.violations
0.01503 < Asian
0.1799 < Uninsured.adults
0.0576 < Uninsured.children <= 0.0826
0.0851 < Severe.housing.cost.burden <= 0.1027
0.0288 < Percentage.of.households.with.overcrowding
0.806 < Driving.alone.to.work <= 0.837
341 < Premature.age.adjusted.mortality <= 418
Feature
0.000
0.025
0.050
Weight
Positive
Negative

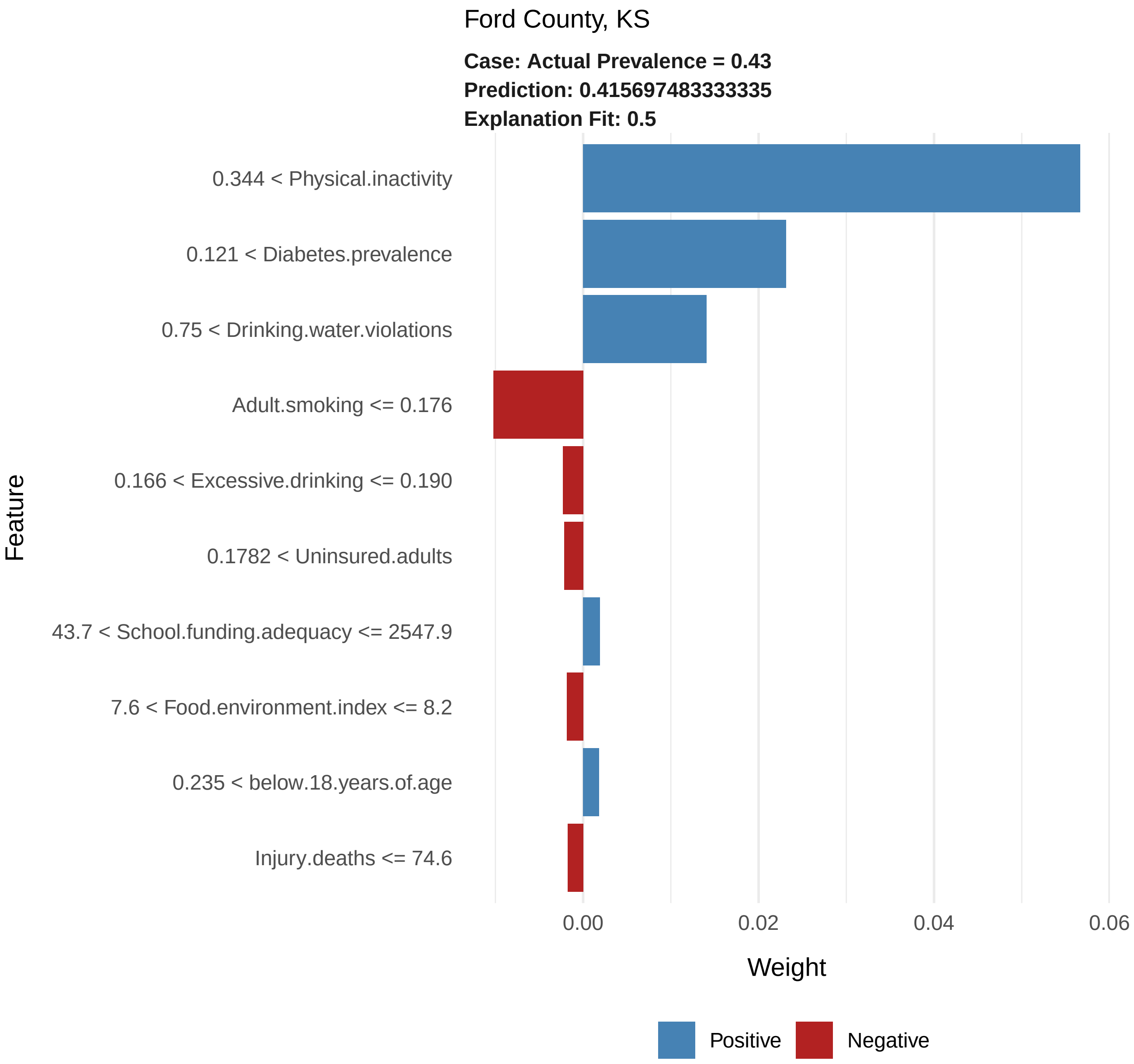
Ford County, KS
Case: Actual Prevalence = 0.43
Prediction: 0.415697483333335
Explanation Fit: 0.5
0.344 < Physical.inactivity
0.121 < Diabetes.prevalence
0.75 < Drinking.water.violations
Adult.smoking <= 0.176
0.166 < Excessive.drinking <= 0.190
0.1782 < Uninsured.adults
43.7 < School.funding.adequacy <= 2547.9
7.6 < Food.environment.index <= 8.2
0.235 < below.18.years.of.age
Injury.deaths <= 74.6
Feature
0.00
0.02
0.04
0.06
Weight
Positive
Negative

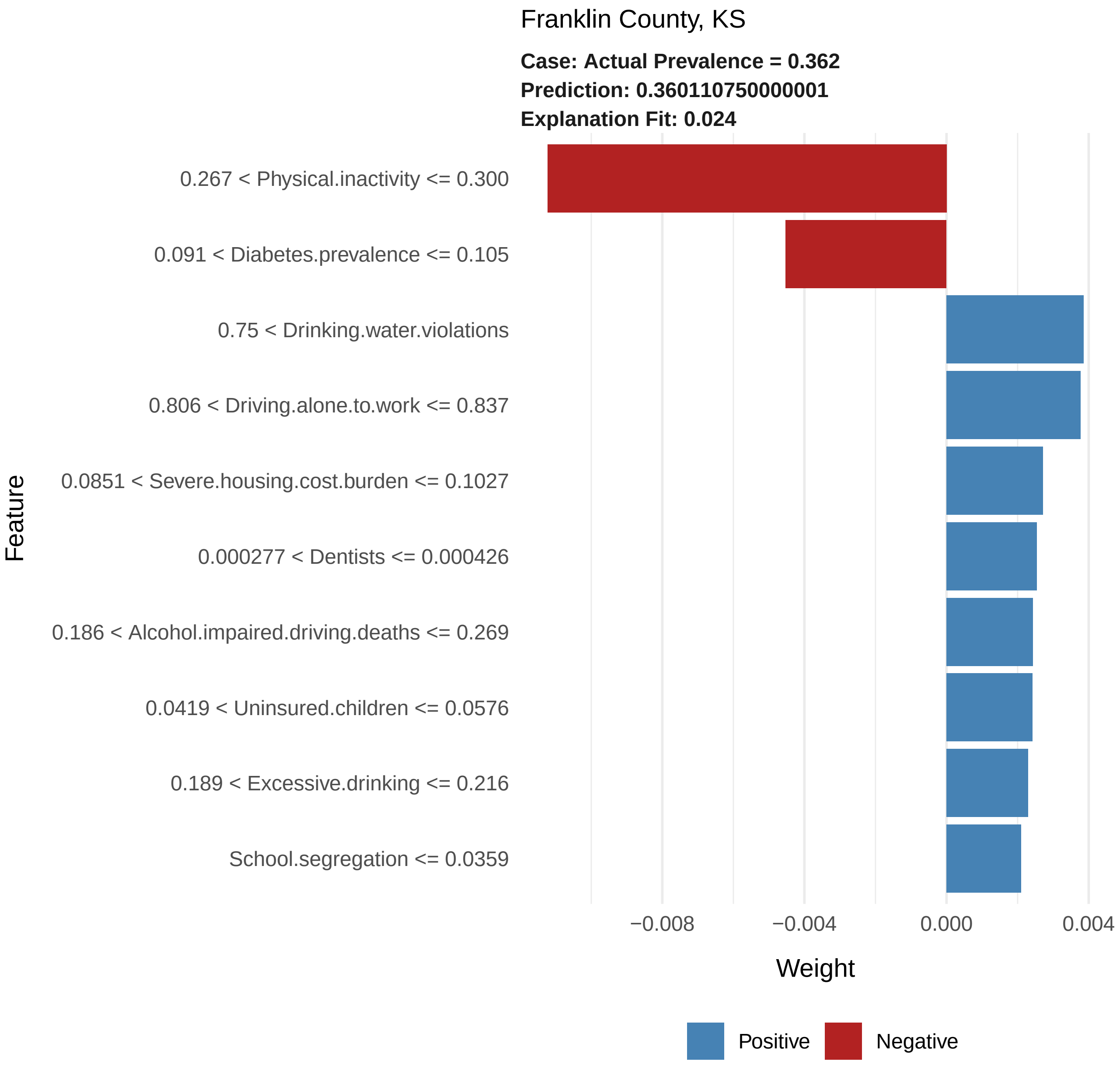

Franklin County, KS
Case: Actual Prevalence = 0.362
Prediction: 0.360110750000001
Explanation Fit: 0.024
0.267 < Physical.inactivity <= 0.300
0.091 < Diabetes.prevalence <= 0.105
0.75 < Drinking.water.violations
0.806 < Driving.alone.to.work <= 0.837
0.0851 < Severe.housing.cost.burden <= 0.1027
0.000277 < Dentists <= 0.000426
0.186 < Alcohol.impaired.driving.deaths <= 0.269
0.0419 < Uninsured.children <= 0.0576
0.189 < Excessive.drinking <= 0.216
School.segregation <= 0.0359
Feature
−0.008
−0.004
0.000
0.004
Weight
Positive
Negative

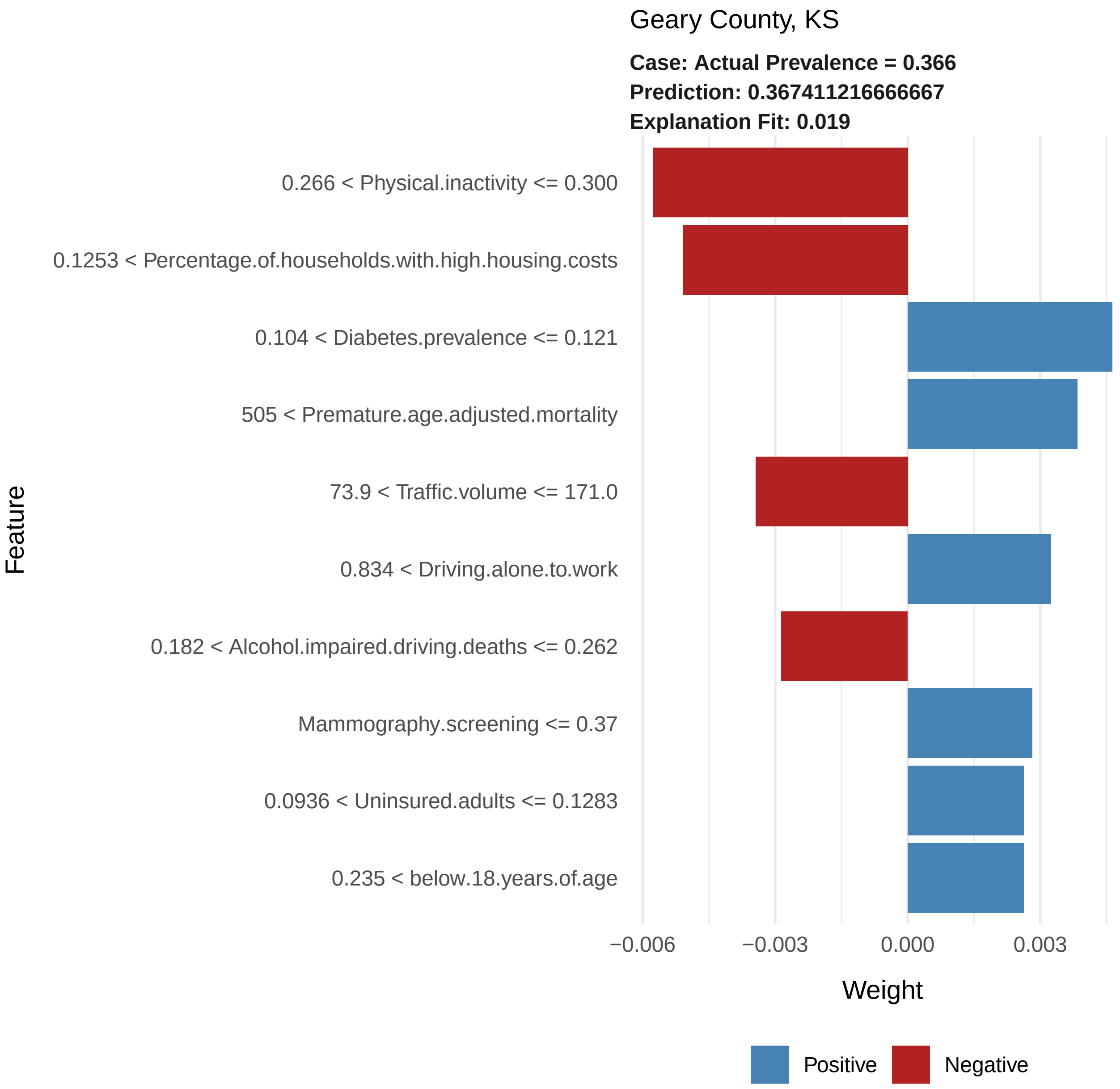

Geary County, KS
Case: Actual Prevalence = 0.366
Prediction: 0.367411216666667
Explanation Fit: 0.019
0.266 < Physical.inactivity <= 0.300
0.1253 < Percentage.of.households.with.high.housing.costs
0.104 < Diabetes.prevalence <= 0.121
505 < Premature.age.adjusted.mortality
73.9 < Traffic.volume <= 171.0
0.834 < Driving.alone.to.work
0.182 < Alcohol.impaired.driving.deaths <= 0.262
Mammography.screening <= 0.37
0.0936 < Uninsured.adults <= 0.1283
0.235 < below.18.years.of.age
Feature
−0.006
−0.003
0.000
0.003
Weight
Positive
Negative

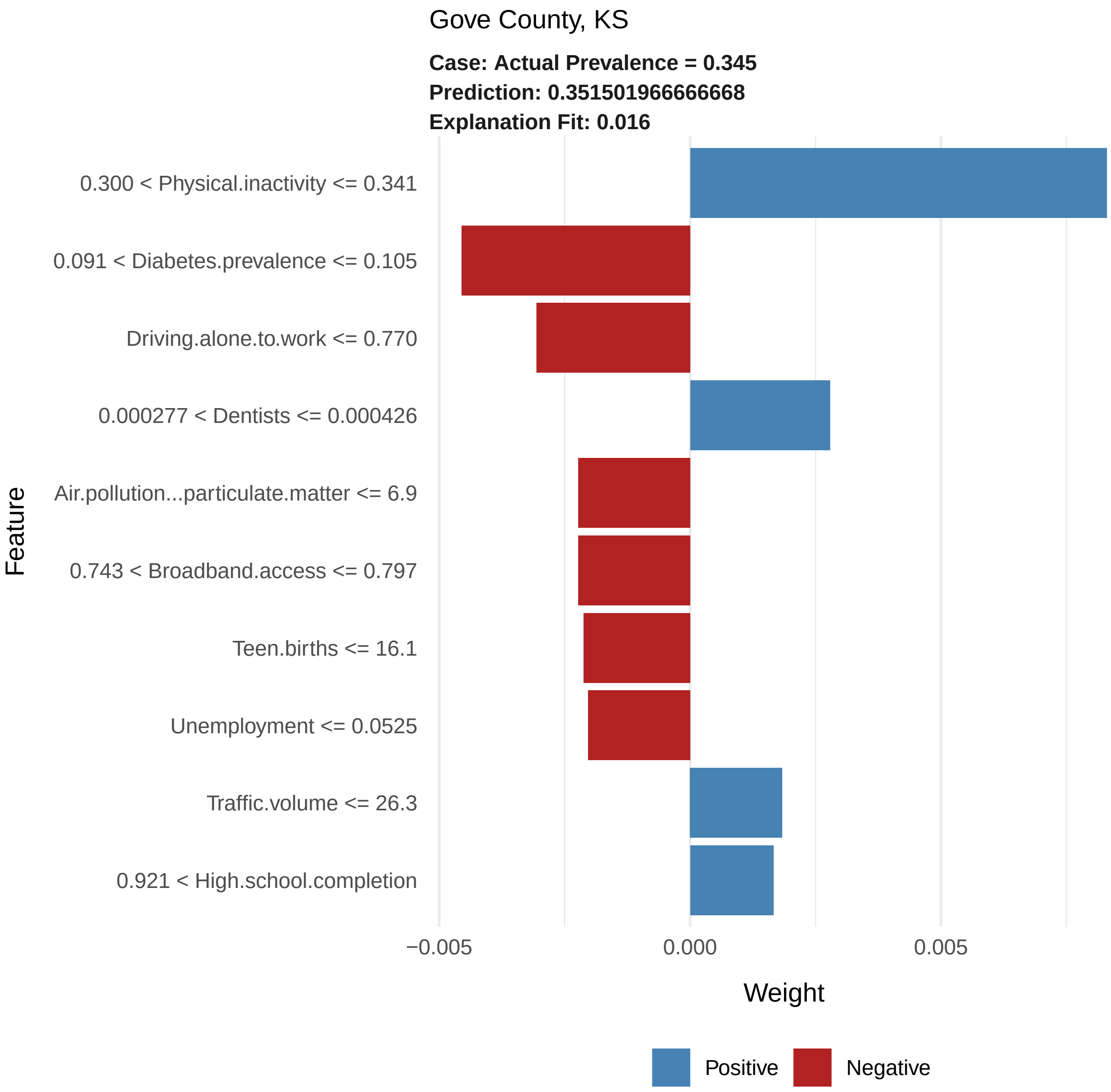

Gove County, KS
Case: Actual Prevalence = 0.345
Prediction: 0.351501966666668
Explanation Fit: 0.016
0.300 < Physical.inactivity <= 0.341
0.091 < Diabetes.prevalence <= 0.105
Driving.alone.to.work <= 0.770
0.000277 < Dentists <= 0.000426
Air.pollution...particulate.matter <= 6.9
0.743 < Broadband.access <= 0.797
Teen.births <= 16.1
Unemployment <= 0.0525
Traffic.volume <= 26.3
0.921 < High.school.completion
Feature
−0.005
0.000
0.005
Weight
Positive
Negative

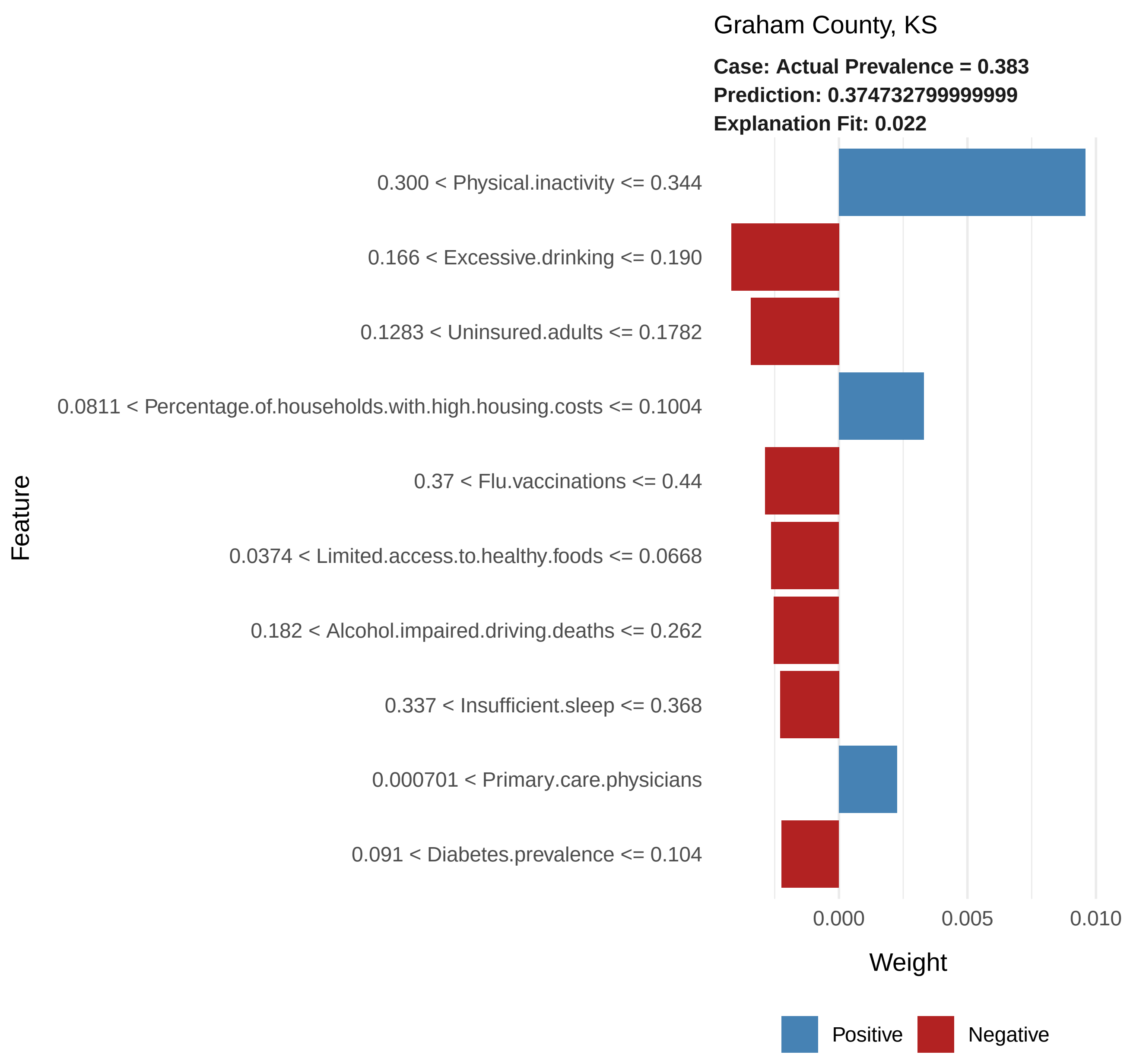

Graham County, KS
Case: Actual Prevalence = 0.383
Prediction: 0.374732799999999
Explanation Fit: 0.022
0.300 < Physical.inactivity <= 0.344
0.166 < Excessive.drinking <= 0.190
0.1283 < Uninsured.adults <= 0.1782
0.0811 < Percentage.of.households.with.high.housing.costs <= 0.1004
0.37 < Flu.vaccinations <= 0.44
0.0374 < Limited.access.to.healthy.foods <= 0.0668
0.182 < Alcohol.impaired.driving.deaths <= 0.262
0.337 < Insufficient.sleep <= 0.368
0.000701 < Primary.care.physicians
0.091 < Diabetes.prevalence <= 0.104
Feature
0.000
0.005
0.010
Weight
Positive
Negative

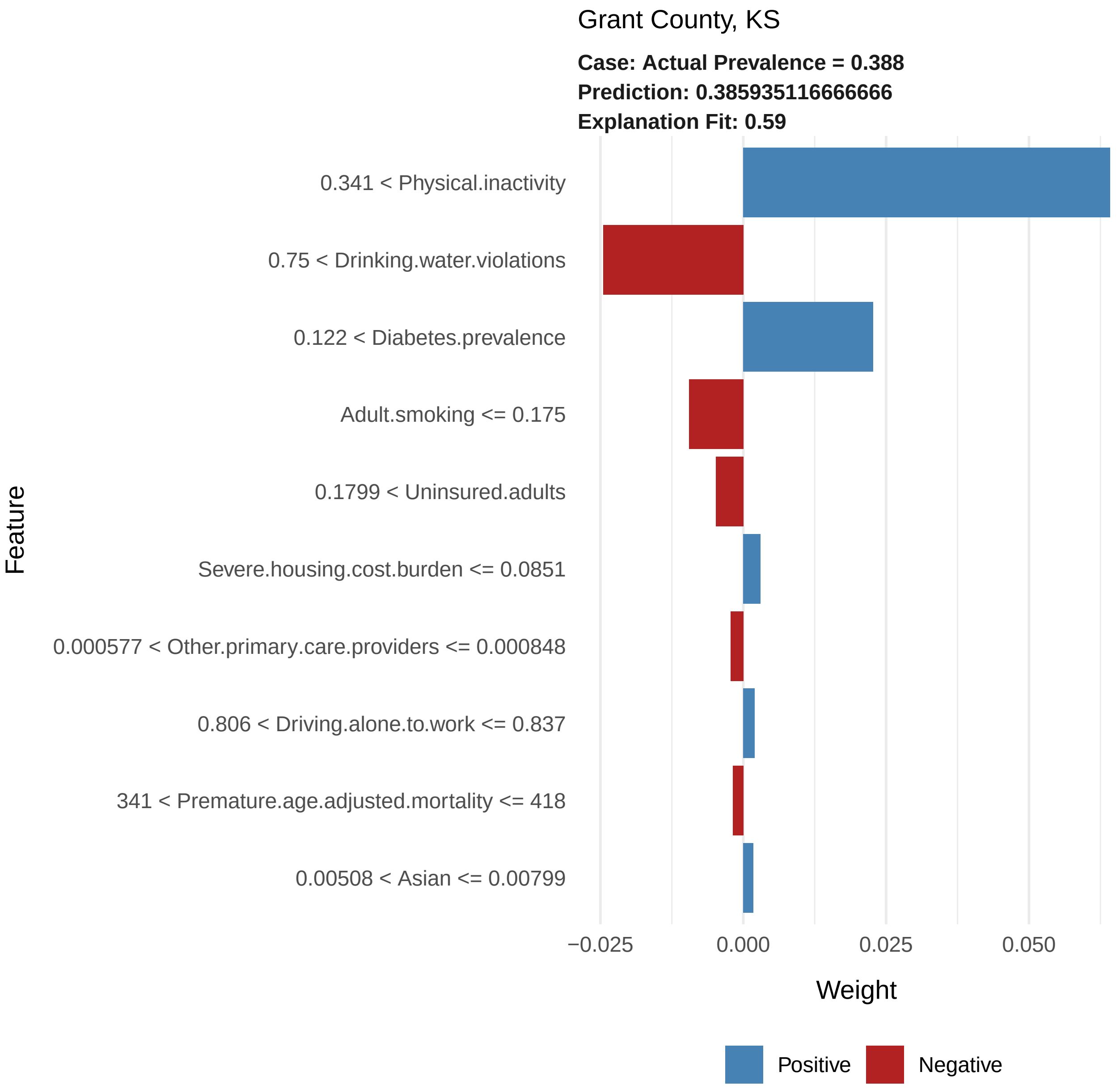
Grant County, KS
Case: Actual Prevalence = 0.388
Prediction: 0.385935116666666
Explanation Fit: 0.59
0.341 < Physical.inactivity
0.75 < Drinking.water.violations
0.122 < Diabetes.prevalence
Adult.smoking <= 0.175
0.1799 < Uninsured.adults
Severe.housing.cost.burden <= 0.0851
0.000577 < Other.primary.care.providers <= 0.000848
0.806 < Driving.alone.to.work <= 0.837
341 < Premature.age.adjusted.mortality <= 418
0.00508 < Asian <= 0.00799
Feature
−0.025
0.000
0.025
0.050
Weight
Positive
Negative

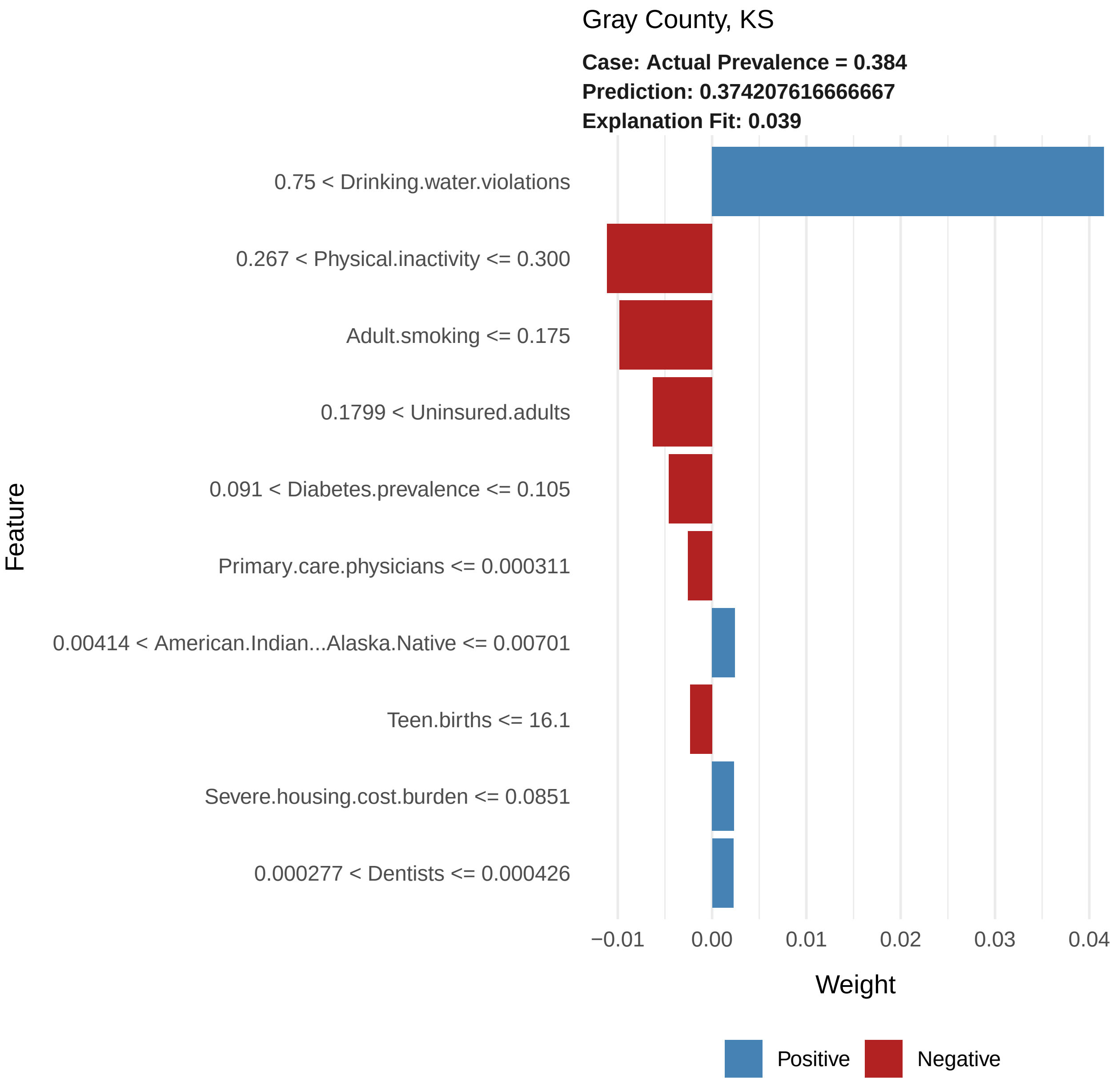

Gray County, KS
Case: Actual Prevalence = 0.384
Prediction: 0.374207616666667
Explanation Fit: 0.039
0.75 < Drinking.water.violations
0.267 < Physical.inactivity <= 0.300
Adult.smoking <= 0.175
0.1799 < Uninsured.adults
0.091 < Diabetes.prevalence <= 0.105
Primary.care.physicians <= 0.000311
0.00414 < American.Indian...Alaska.Native <= 0.00701
Teen.births <= 16.1
Severe.housing.cost.burden <= 0.0851
0.000277 < Dentists <= 0.000426
Feature
−0.01
0.00
0.01
0.02
0.03
0.04
Weight
Positive
Negative

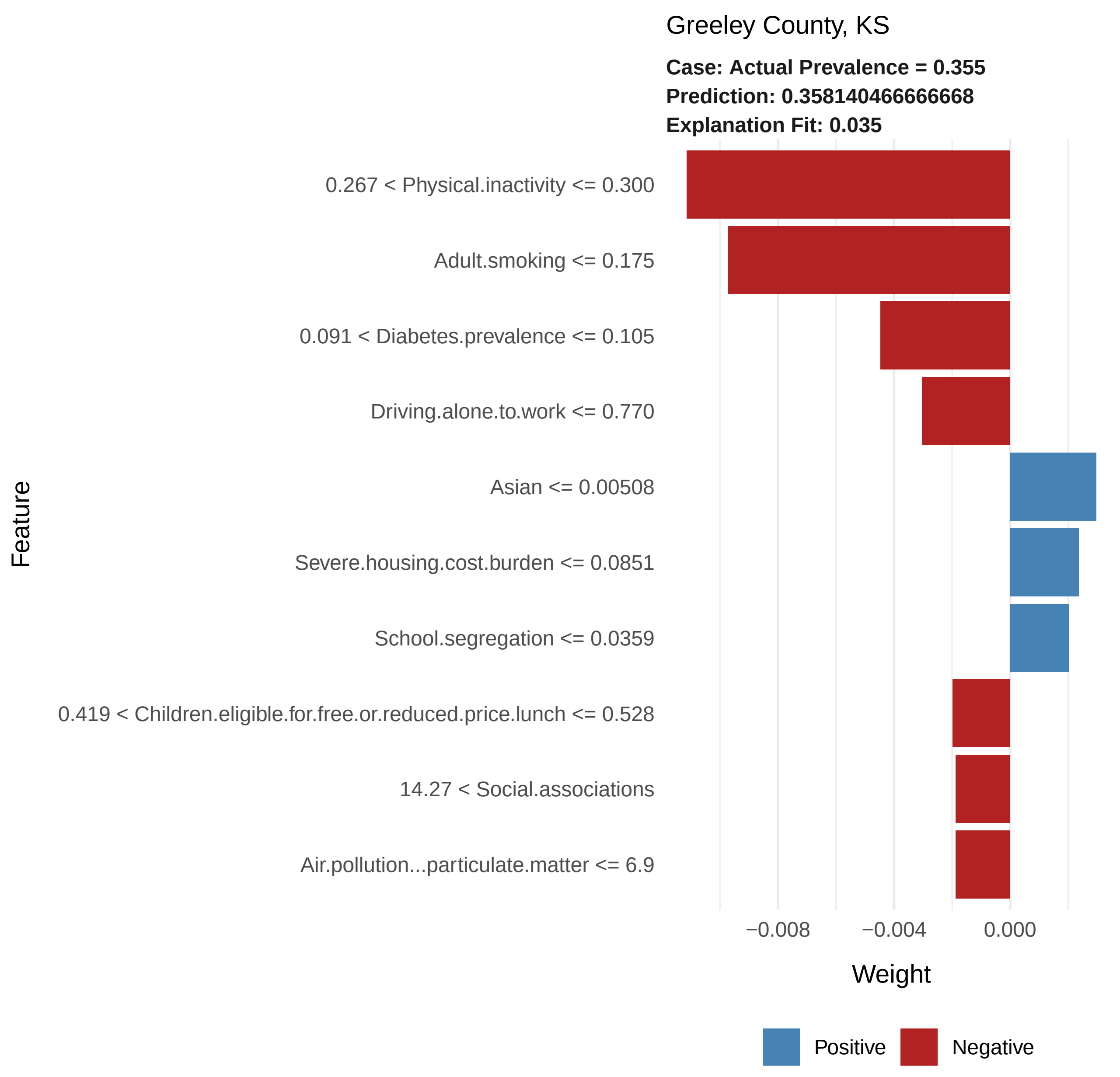

Greeley County, KS
Case: Actual Prevalence = 0.355
Prediction: 0.358140466666668
Explanation Fit: 0.035
0.267 < Physical.inactivity <= 0.300
Adult.smoking <= 0.175
0.091 < Diabetes.prevalence <= 0.105
Driving.alone.to.work <= 0.770
Asian <= 0.00508
Severe.housing.cost.burden <= 0.0851
School.segregation <= 0.0359
0.419 < Children.eligible.for.free.or.reduced.price.lunch <= 0.528
14.27 < Social.associations
Air.pollution...particulate.matter <= 6.9
Feature
−0.008
−0.004
0.000
Weight
Positive
Negative

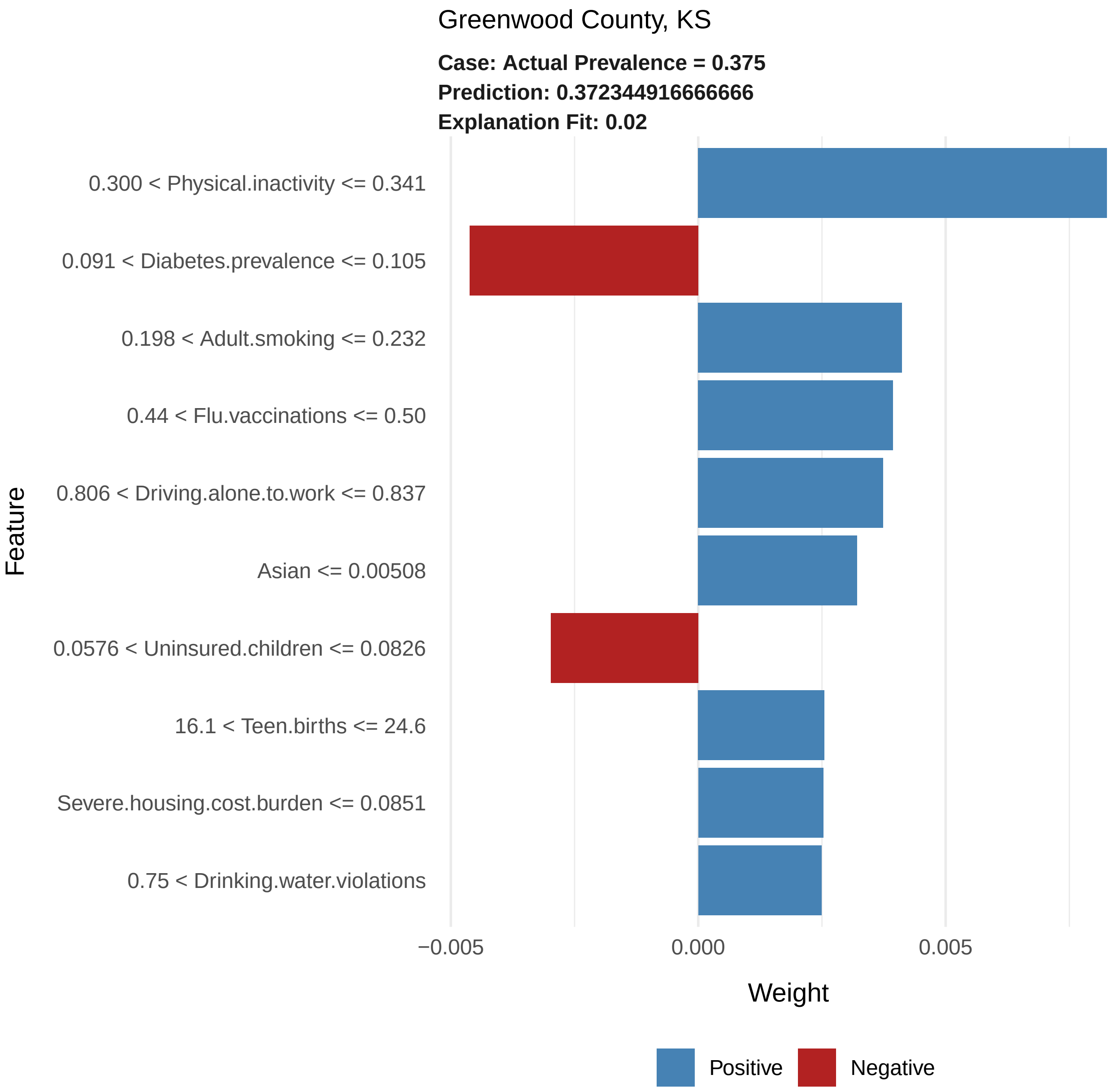

Greenwood County, KS
Case: Actual Prevalence = 0.375
Prediction: 0.372344916666666
Explanation Fit: 0.02
0.300 < Physical.inactivity <= 0.341
0.091 < Diabetes.prevalence <= 0.105
0.198 < Adult.smoking <= 0.232
0.44 < Flu.vaccinations <= 0.50
0.806 < Driving.alone.to.work <= 0.837
Asian <= 0.00508
0.0576 < Uninsured.children <= 0.0826
16.1 < Teen.births <= 24.6
Severe.housing.cost.burden <= 0.0851
0.75 < Drinking.water.violations
Feature
−0.005
0.000
0.005
Weight
Positive
Negative

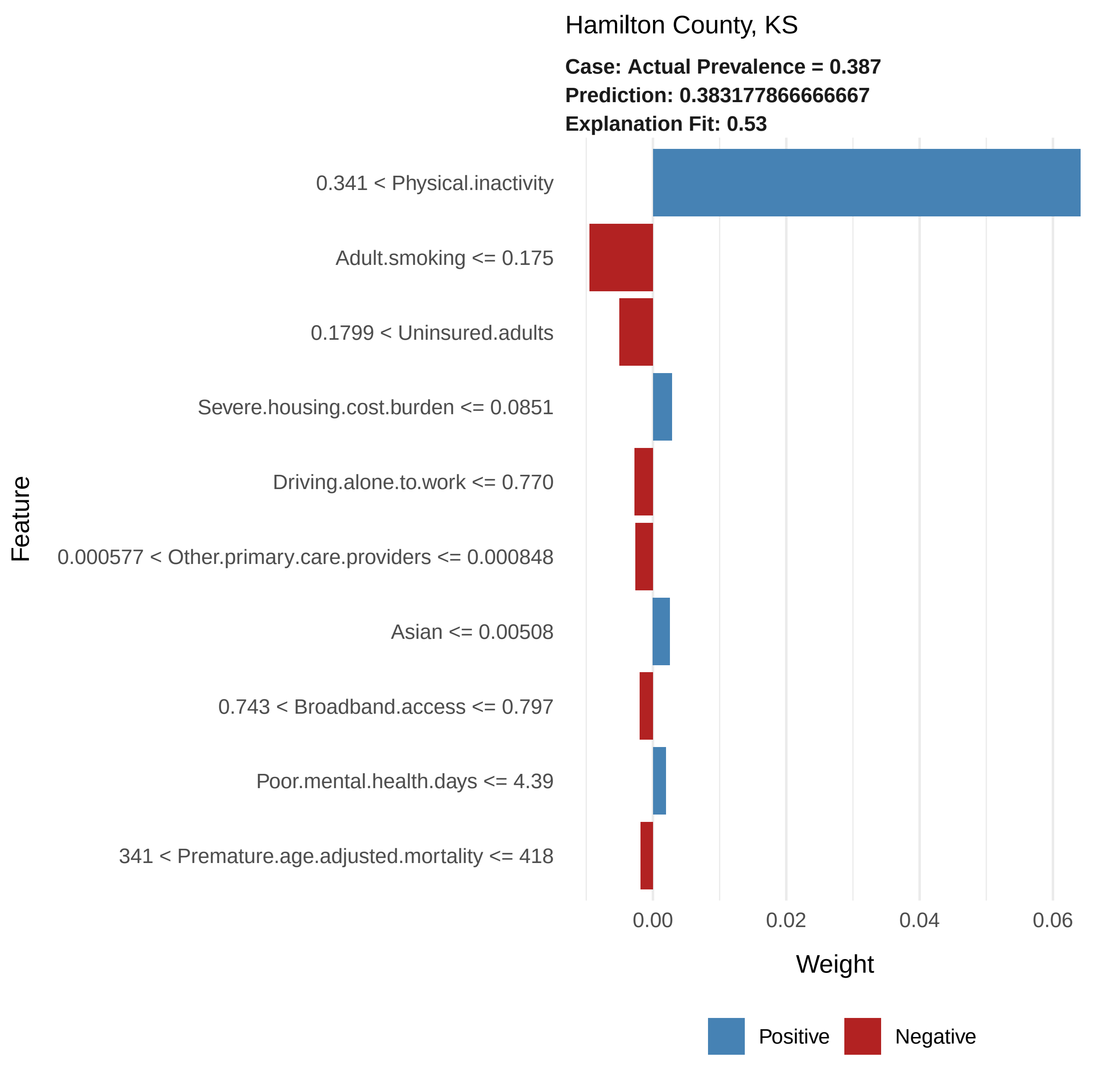
Hamilton County, KS
Case: Actual Prevalence = 0.387
Prediction: 0.383177866666667
Explanation Fit: 0.53
0.341 < Physical.inactivity
Adult.smoking <= 0.175
0.1799 < Uninsured.adults
Severe.housing.cost.burden <= 0.0851
Driving.alone.to.work <= 0.770
0.000577 < Other.primary.care.providers <= 0.000848
Asian <= 0.00508
0.743 < Broadband.access <= 0.797
Poor.mental.health.days <= 4.39
341 < Premature.age.adjusted.mortality <= 418
Feature
0.00
0.02
0.04
0.06
Weight
Positive
Negative

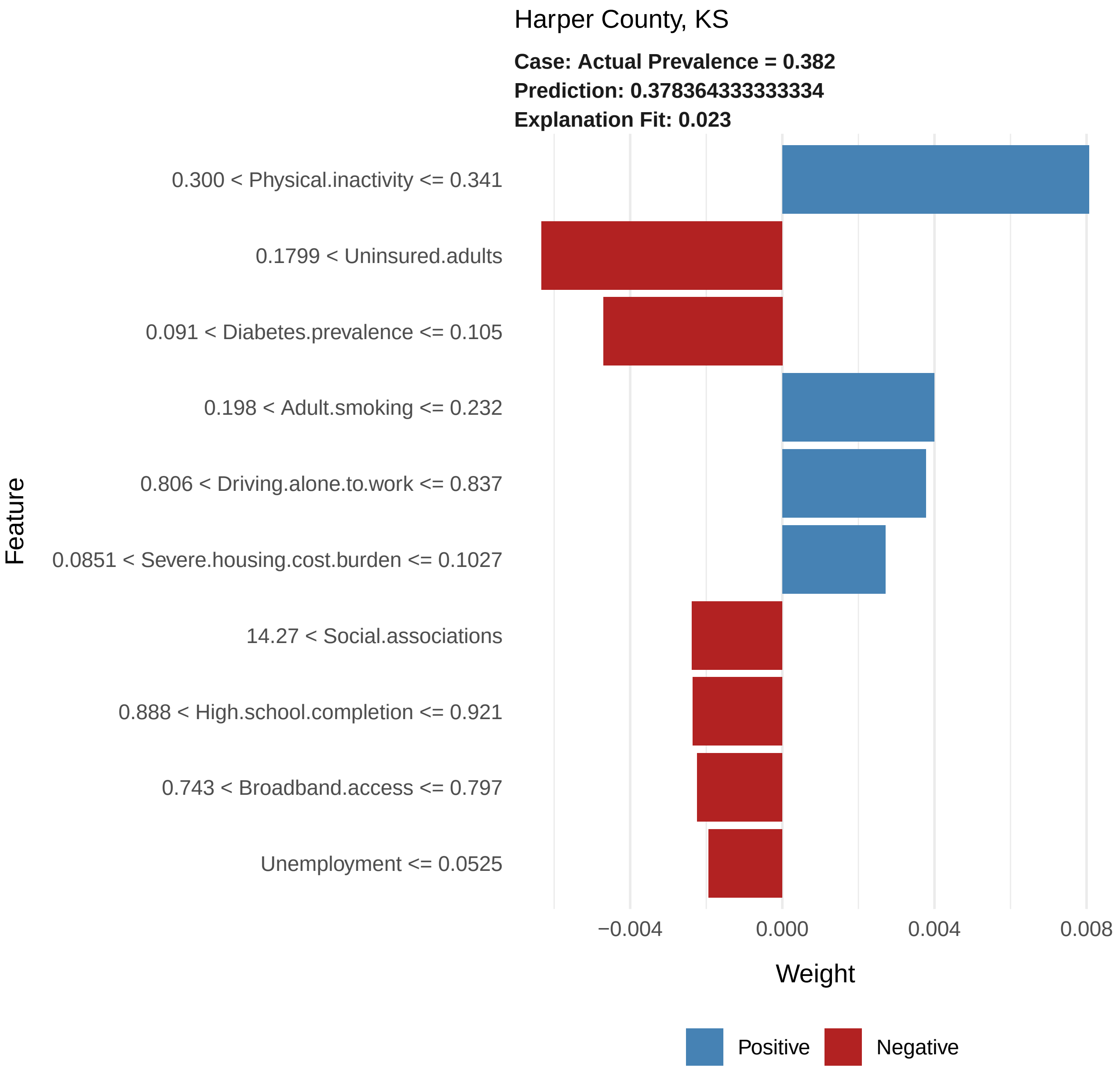

Harper County, KS
Case: Actual Prevalence = 0.382
Prediction: 0.378364333333334
Explanation Fit: 0.023
0.300 < Physical.inactivity <= 0.341
0.1799 < Uninsured.adults
0.091 < Diabetes.prevalence <= 0.105
0.198 < Adult.smoking <= 0.232
0.806 < Driving.alone.to.work <= 0.837
0.0851 < Severe.housing.cost.burden <= 0.1027
14.27 < Social.associations
0.888 < High.school.completion <= 0.921
0.743 < Broadband.access <= 0.797
Unemployment <= 0.0525
Feature
−0.004
0.000
0.004
0.008
Weight
Positive
Negative

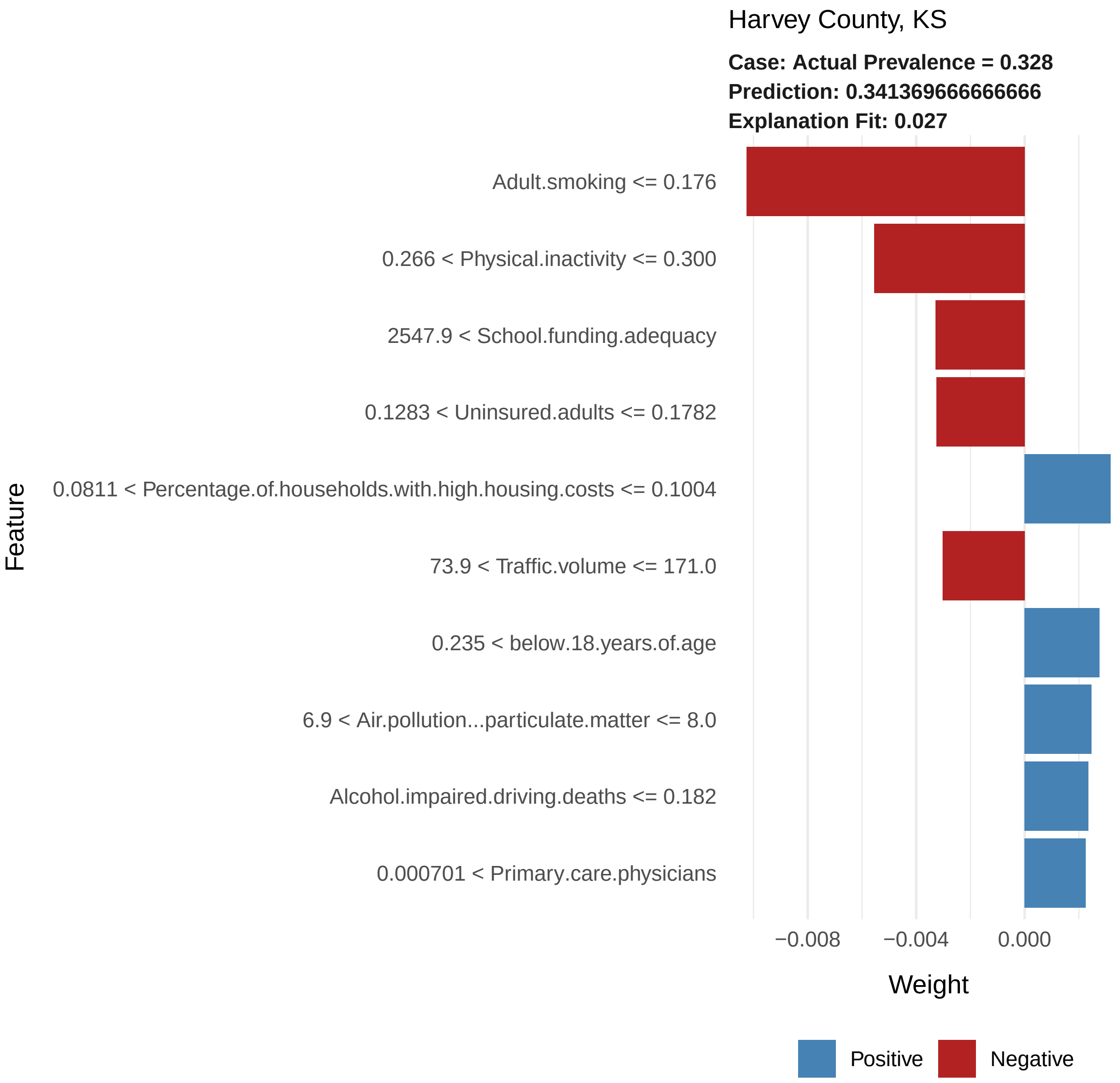
Harvey County, KS
Case: Actual Prevalence = 0.328
Prediction: 0.341369666666666
Explanation Fit: 0.027
Adult.smoking <= 0.176
0.266 < Physical.inactivity <= 0.300
2547.9 < School.funding.adequacy
0.1283 < Uninsured.adults <= 0.1782
0.0811 < Percentage.of.households.with.high.housing.costs <= 0.1004
73.9 < Traffic.volume <= 171.0
0.235 < below.18.years.of.age
6.9 < Air.pollution...particulate.matter <= 8.0
Alcohol.impaired.driving.deaths <= 0.182
0.000701 < Primary.care.physicians
Feature
−0.008
−0.004
0.000
Weight
Positive
Negative

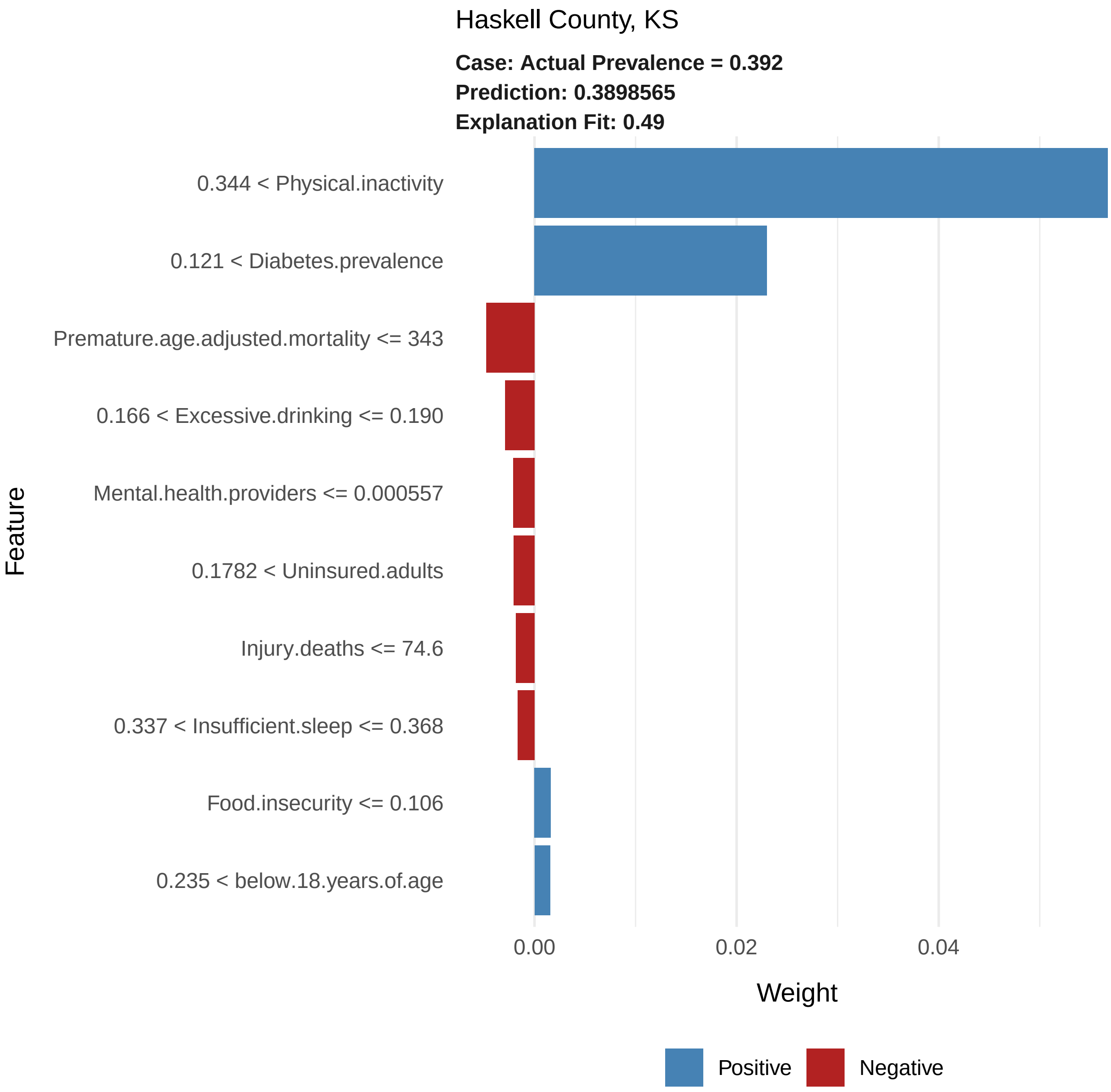

Haskell County, KS
Case: Actual Prevalence = 0.392
Prediction: 0.3898565
Explanation Fit: 0.49
0.344 < Physical.inactivity
0.121 < Diabetes.prevalence
Premature.age.adjusted.mortality <= 343
0.166 < Excessive.drinking <= 0.190
Mental.health.providers <= 0.000557
0.1782 < Uninsured.adults
Injury.deaths <= 74.6
0.337 < Insufficient.sleep <= 0.368
Food.insecurity <= 0.106
0.235 < below.18.years.of.age
Feature
0.00
0.02
0.04
Weight
Positive
Negative

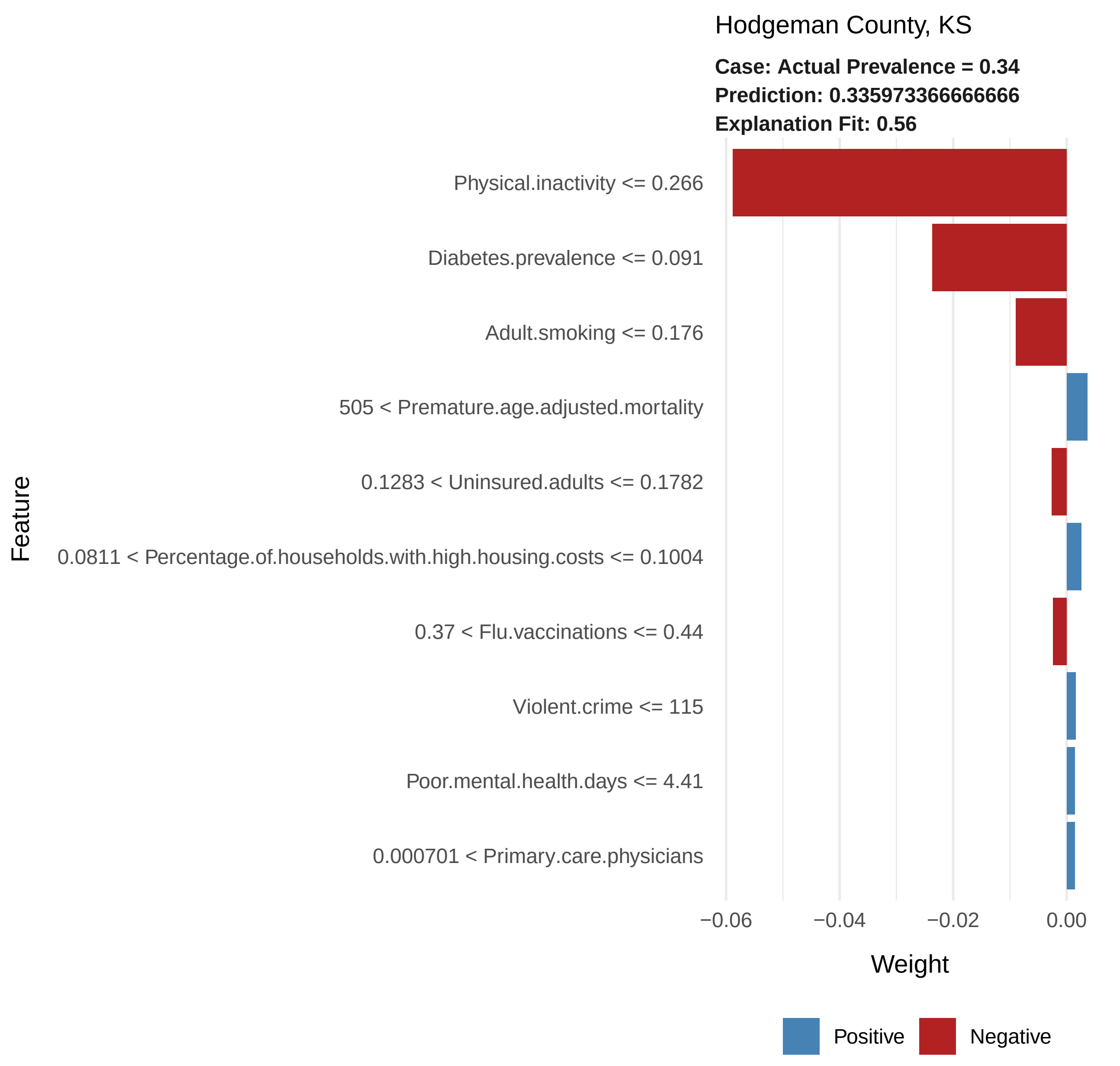

Hodgeman County, KS
Case: Actual Prevalence = 0.34
Prediction: 0.335973366666666
Explanation Fit: 0.56
Physical.inactivity <= 0.266
Diabetes.prevalence <= 0.091
Adult.smoking <= 0.176
505 < Premature.age.adjusted.mortality
0.1283 < Uninsured.adults <= 0.1782
0.0811 < Percentage.of.households.with.high.housing.costs <= 0.1004
0.37 < Flu.vaccinations <= 0.44
Violent.crime <= 115
Poor.mental.health.days <= 4.41
0.000701 < Primary.care.physicians
Feature
−0.06
−0.04
−0.02
0.00
Weight
Positive
Negative

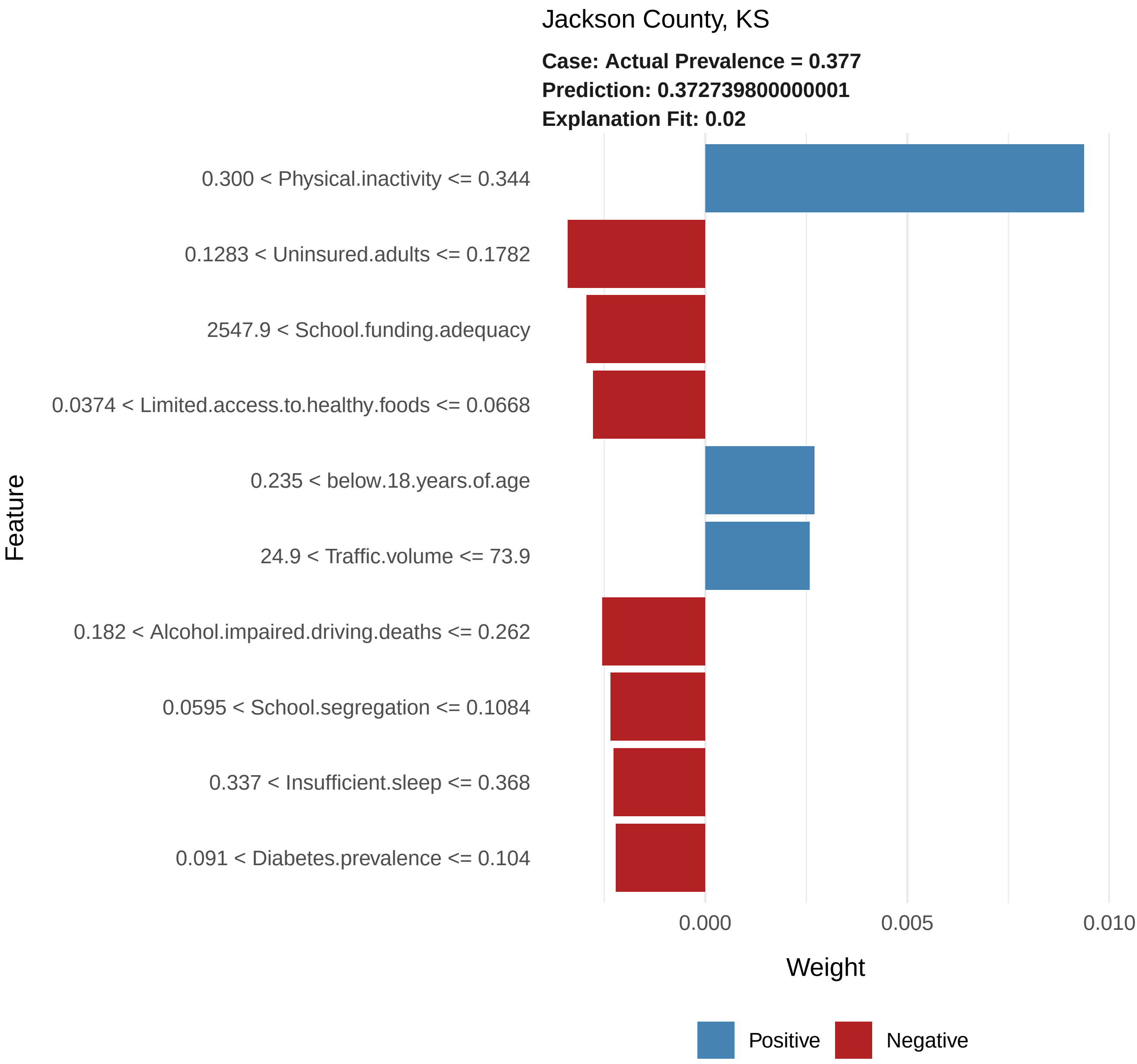

Jackson County, KS
Case: Actual Prevalence = 0.377
Prediction: 0.372739800000001
Explanation Fit: 0.02
0.300 < Physical.inactivity <= 0.344
0.1283 < Uninsured.adults <= 0.1782
2547.9 < School.funding.adequacy
0.0374 < Limited.access.to.healthy.foods <= 0.0668
0.235 < below.18.years.of.age
24.9 < Traffic.volume <= 73.9
0.182 < Alcohol.impaired.driving.deaths <= 0.262
0.0595 < School.segregation <= 0.1084
0.337 < Insufficient.sleep <= 0.368
0.091 < Diabetes.prevalence <= 0.104
Feature
0.000
0.005
0.010
Weight
Positive
Negative

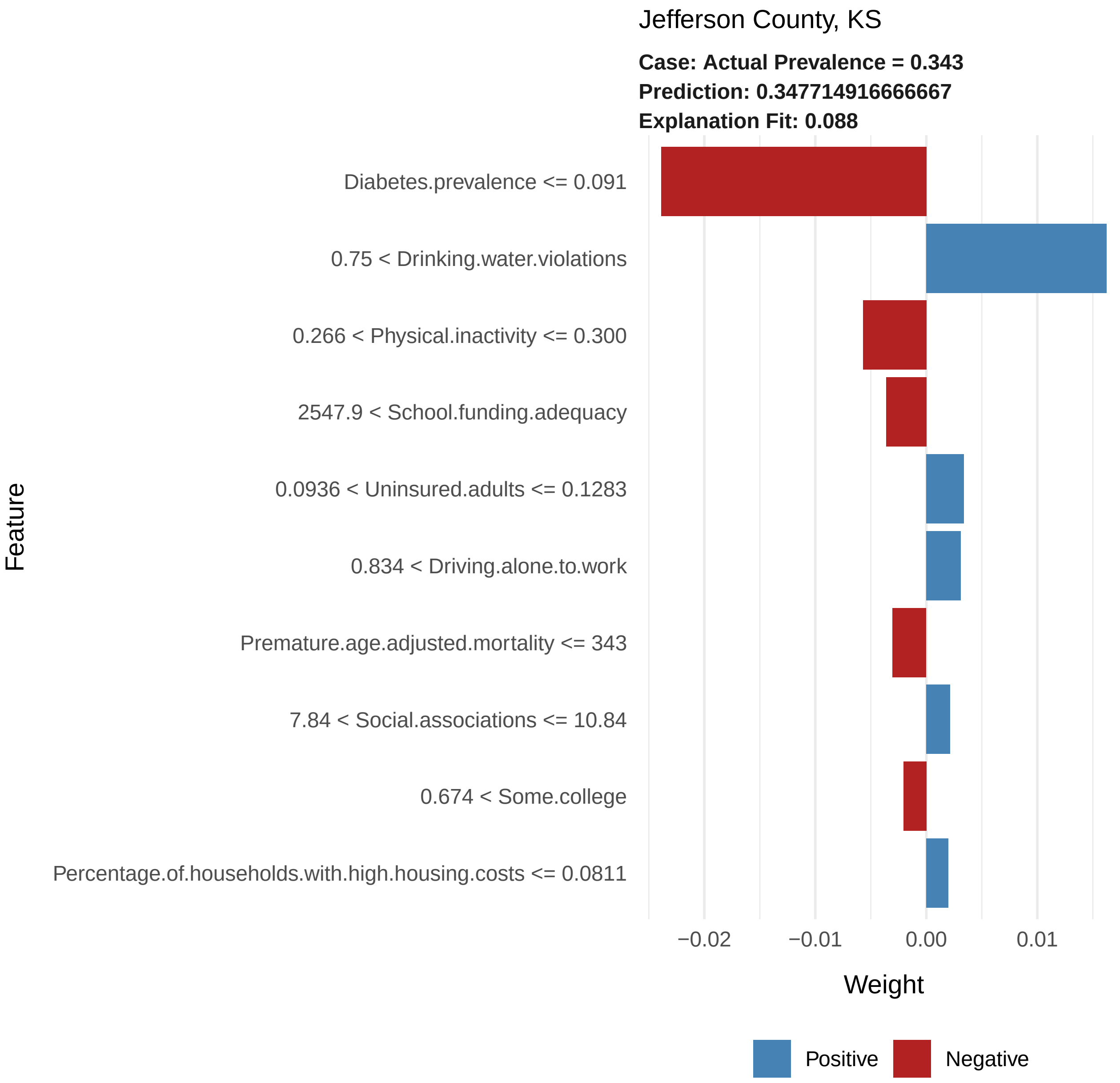
Jefferson County, KS
Case: Actual Prevalence = 0.343
Prediction: 0.347714916666667
Explanation Fit: 0.088
Feature
Diabetes.prevalence <= 0.091
0.75 < Drinking.water.violations
0.266 < Physical.inactivity <= 0.300
2547.9 < School.funding.adequacy
0.0936 < Uninsured.adults <= 0.1283
0.834 < Driving.alone.to.work
Premature.age.adjusted.mortality <= 343
7.84 < Social.associations <= 10.84
0.674 < Some.college
Percentage.of.households.with.high.housing.costs <= 0.0811
−0.02
−0.01
0.00
0.01
Weight
Positive
Negative

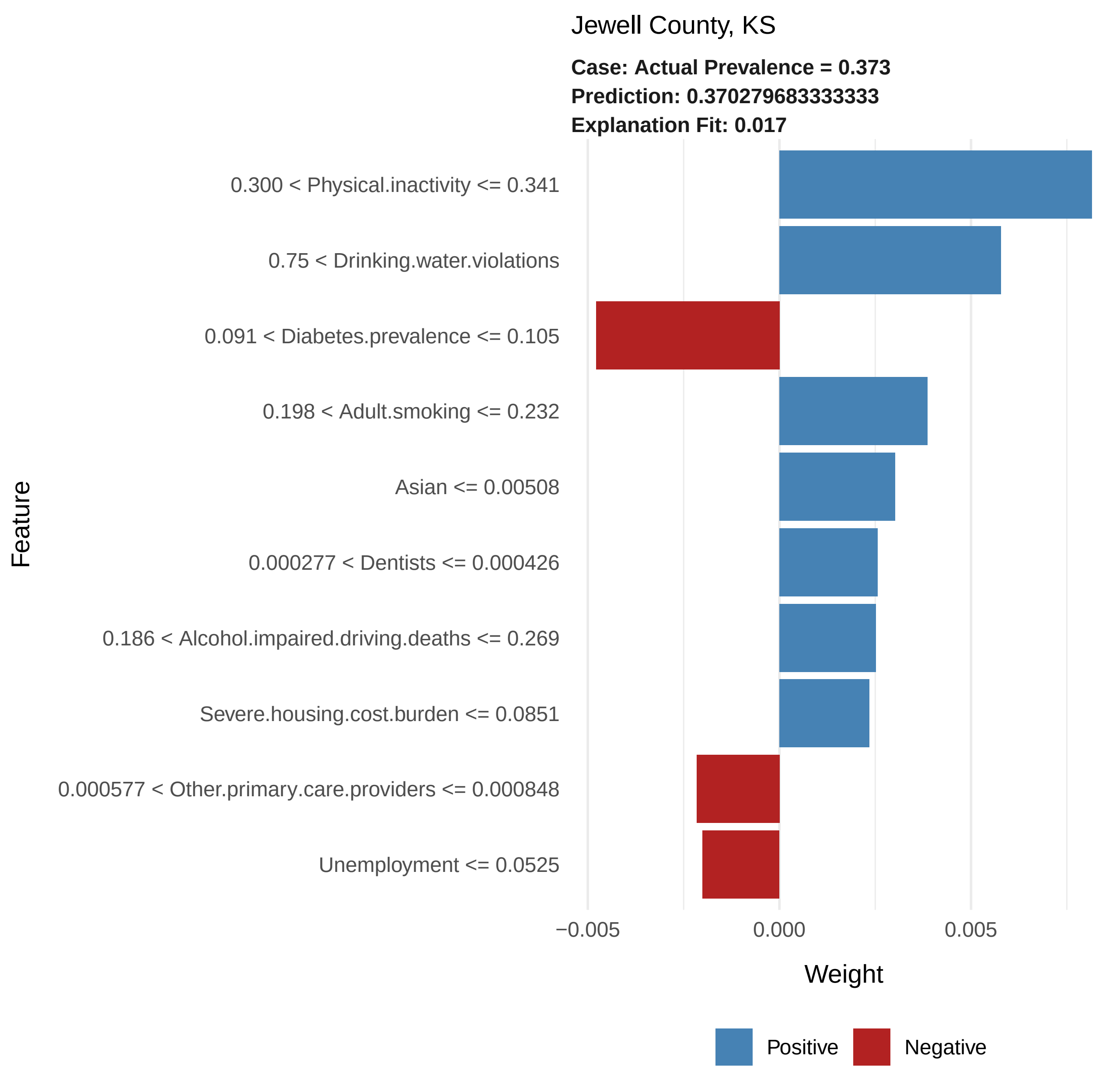
Jewell County, KS
Case: Actual Prevalence = 0.373
Prediction: 0.370279683333333
Explanation Fit: 0.017
0.300 < Physical.inactivity <= 0.341
0.75 < Drinking.water.violations
0.091 < Diabetes.prevalence <= 0.105
0.198 < Adult.smoking <= 0.232
Asian <= 0.00508
0.000277 < Dentists <= 0.000426
0.186 < Alcohol.impaired.driving.deaths <= 0.269
Severe.housing.cost.burden <= 0.0851
0.000577 < Other.primary.care.providers <= 0.000848
Unemployment <= 0.0525
Feature
−0.005
0.000
0.005
Weight
Positive
Negative

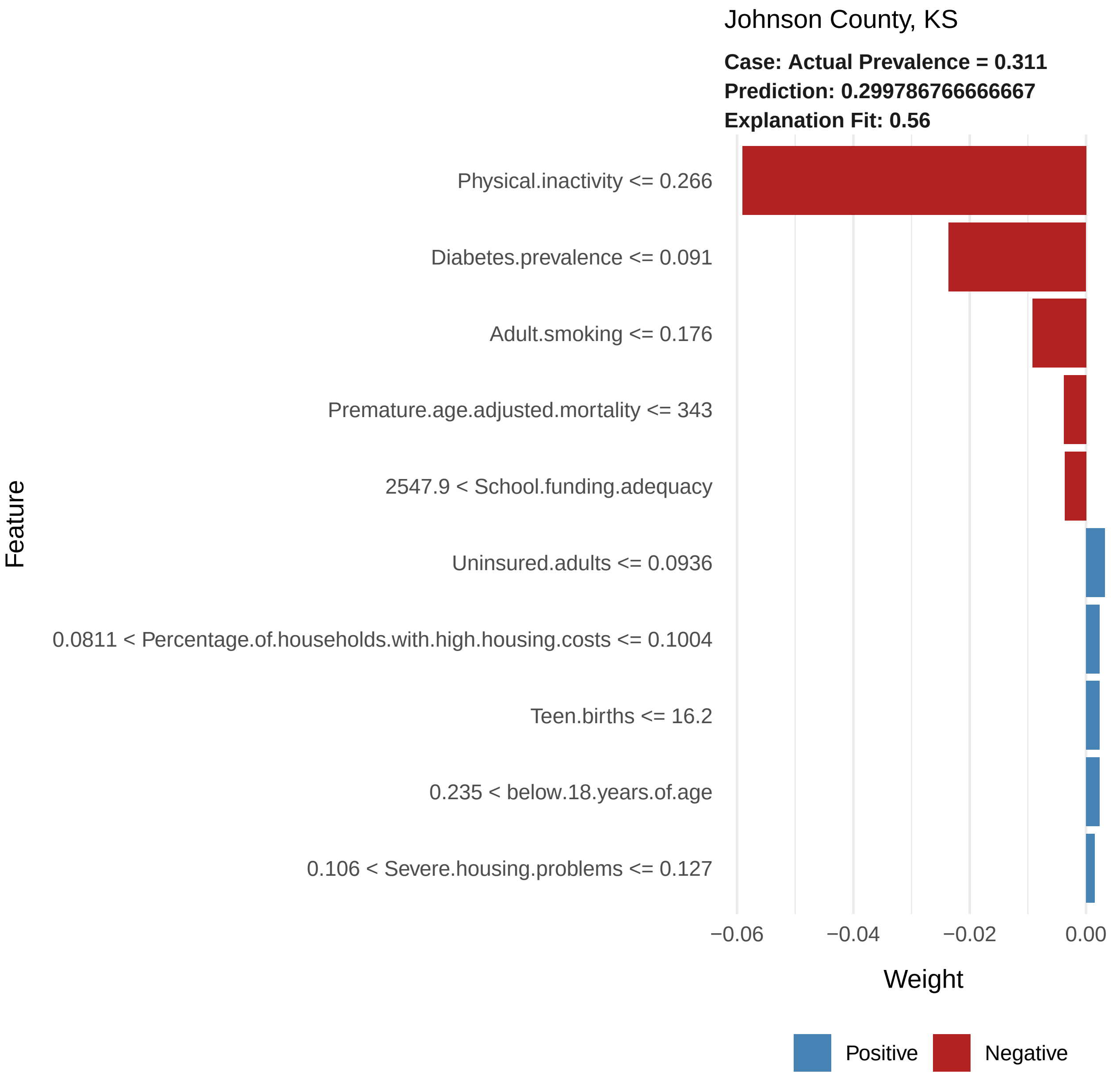

Johnson County, KS
Case: Actual Prevalence = 0.311
Prediction: 0.299786766666667
Explanation Fit: 0.56
Physical.inactivity <= 0.266
Diabetes.prevalence <= 0.091
Adult.smoking <= 0.176
Premature.age.adjusted.mortality <= 343
2547.9 < School.funding.adequacy
Uninsured.adults <= 0.0936
0.0811 < Percentage.of.households.with.high.housing.costs <= 0.1004
Teen.births <= 16.2
0.235 < below.18.years.of.age
0.106 < Severe.housing.problems <= 0.127
Feature
−0.06
−0.04
−0.02
0.00
Weight
Positive
Negative

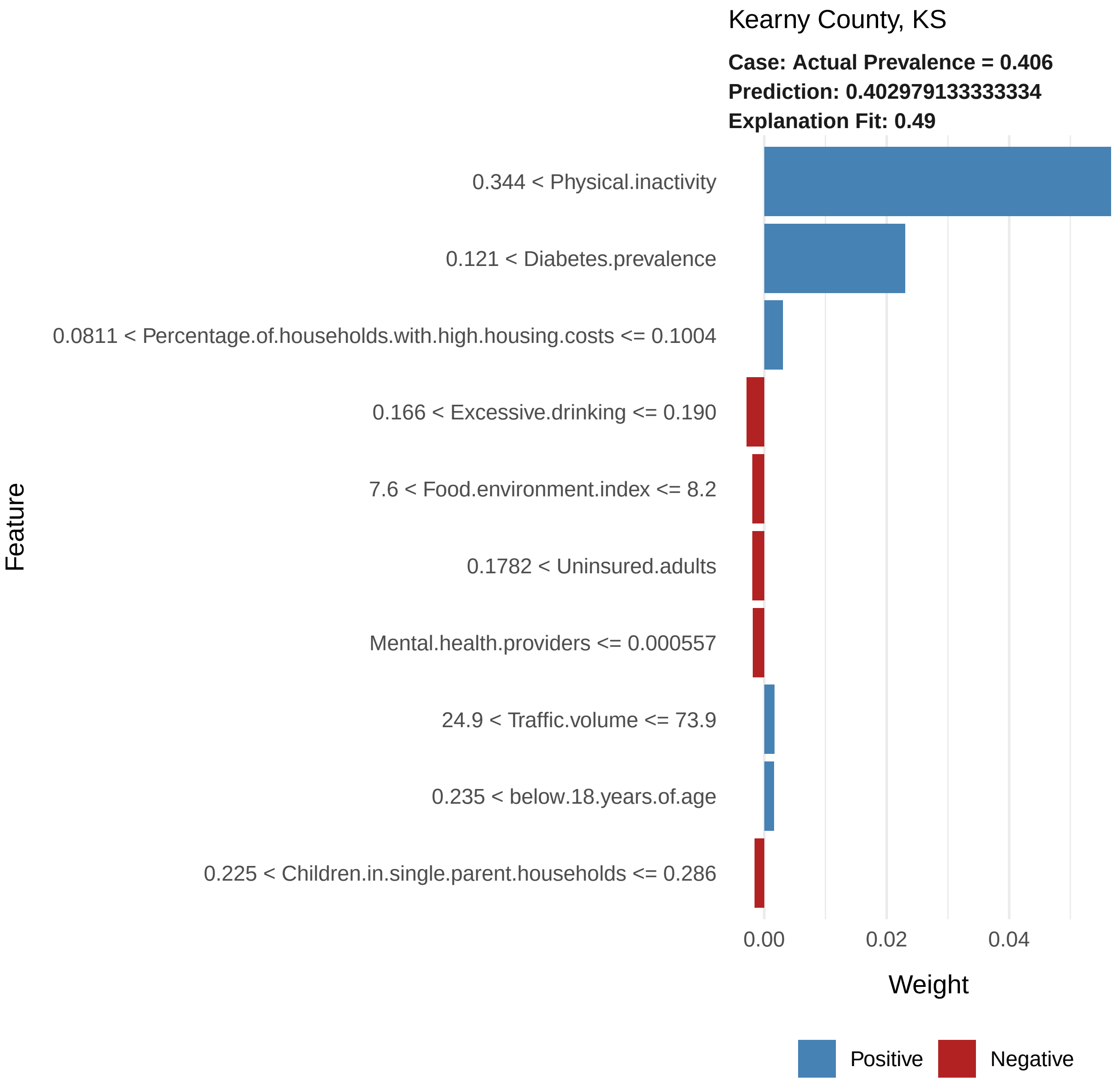

Kearny County, KS
Case: Actual Prevalence = 0.406
Prediction: 0.402979133333334
Explanation Fit: 0.49
0.344 < Physical.inactivity
0.121 < Diabetes.prevalence
0.0811 < Percentage.of.households.with.high.housing.costs <= 0.1004
0.166 < Excessive.drinking <= 0.190
7.6 < Food.environment.index <= 8.2
0.1782 < Uninsured.adults
Mental.health.providers <= 0.000557
24.9 < Traffic.volume <= 73.9
0.235 < below.18.years.of.age
0.225 < Children.in.single.parent.households <= 0.286
Feature
0.00
0.02
0.04
Weight
Positive
Negative

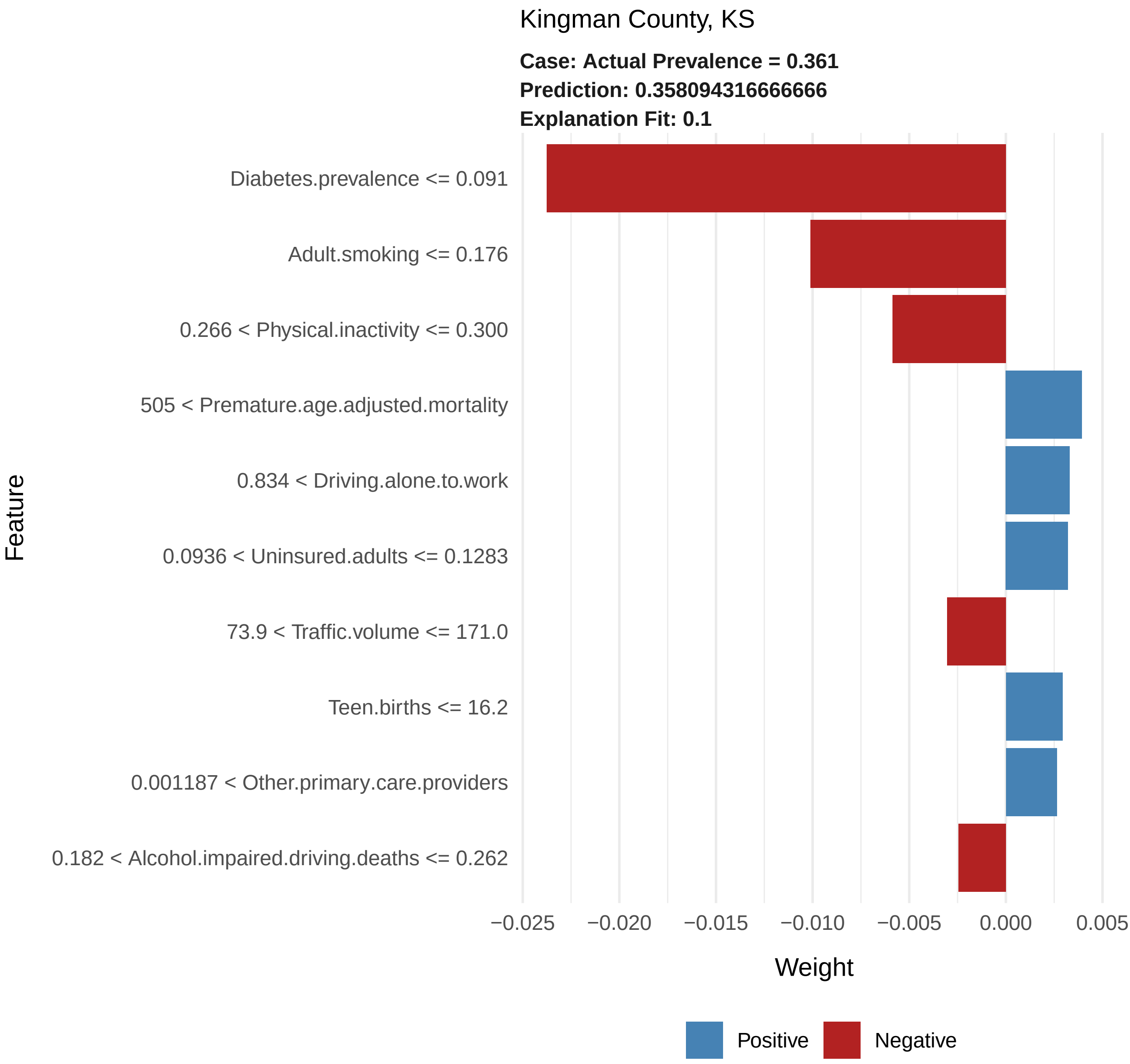

Kingman County, KS
Case: Actual Prevalence = 0.361
Prediction: 0.358094316666666
Explanation Fit: 0.1
Diabetes.prevalence <= 0.091
Adult.smoking <= 0.176
0.266 < Physical.inactivity <= 0.300
505 < Premature.age.adjusted.mortality
0.834 < Driving.alone.to.work
0.0936 < Uninsured.adults <= 0.1283
73.9 < Traffic.volume <= 171.0
Teen.births <= 16.2
0.001187 < Other.primary.care.providers
0.182 < Alcohol.impaired.driving.deaths <= 0.262
Feature
−0.025
−0.020
−0.015
−0.010
−0.005
0.000
0.005
Weight
Positive
Negative

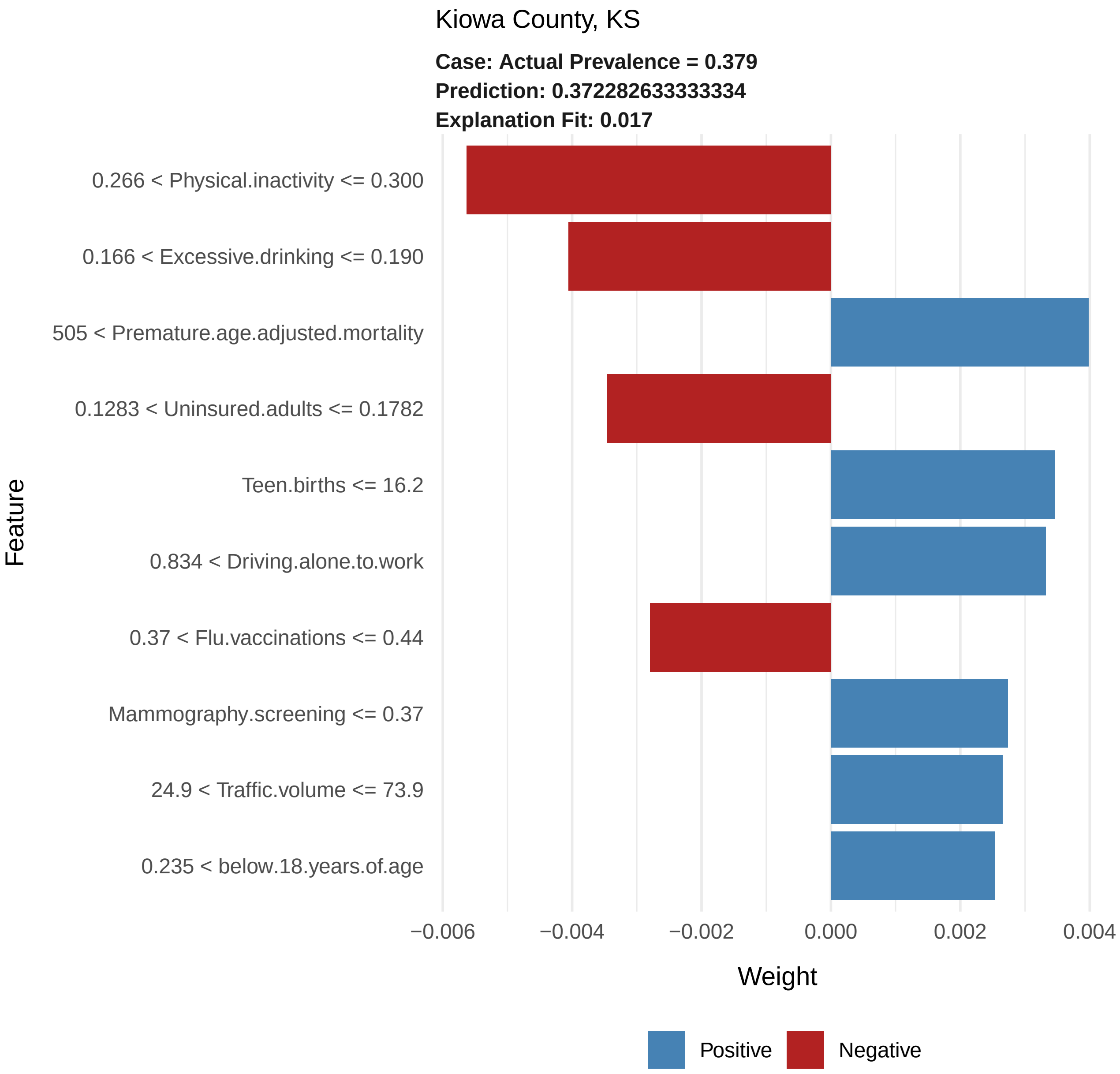
Kiowa County, KS
Case: Actual Prevalence = 0.379
Prediction: 0.372282633333334
Explanation Fit: 0.017
0.266 < Physical.inactivity <= 0.300
0.166 < Excessive.drinking <= 0.190
505 < Premature.age.adjusted.mortality
0.1283 < Uninsured.adults <= 0.1782
Teen.births <= 16.2
0.834 < Driving.alone.to.work
0.37 < Flu.vaccinations <= 0.44
Mammography.screening <= 0.37
24.9 < Traffic.volume <= 73.9
0.235 < below.18.years.of.age
Feature
−0.006
−0.004
−0.002
0.000
0.002
0.004
Weight
Positive
Negative

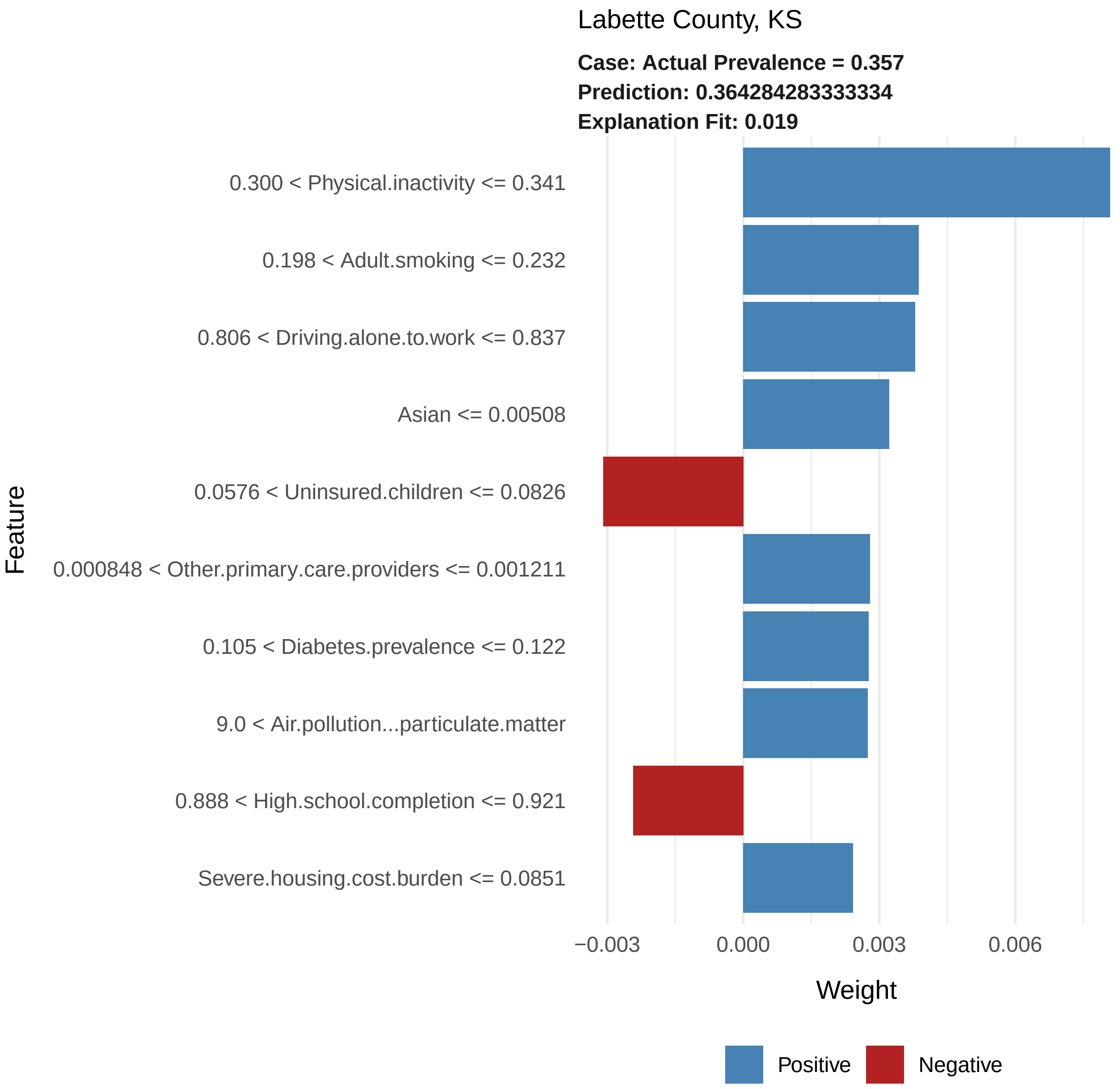
Labette County, KS
Case: Actual Prevalence = 0.357
Prediction: 0.364284283333334
Explanation Fit: 0.019
0.300 < Physical.inactivity <= 0.341
0.198 < Adult.smoking <= 0.232
0.806 < Driving.alone.to.work <= 0.837
Asian <= 0.00508
0.0576 < Uninsured.children <= 0.0826
0.000848 < Other.primary.care.providers <= 0.001211
0.105 < Diabetes.prevalence <= 0.122
9.0 < Air.pollution...particulate.matter
0.888 < High.school.completion <= 0.921
Severe.housing.cost.burden <= 0.0851
Feature
−0.003
0.000
0.003
0.006
Weight
Positive
Negative

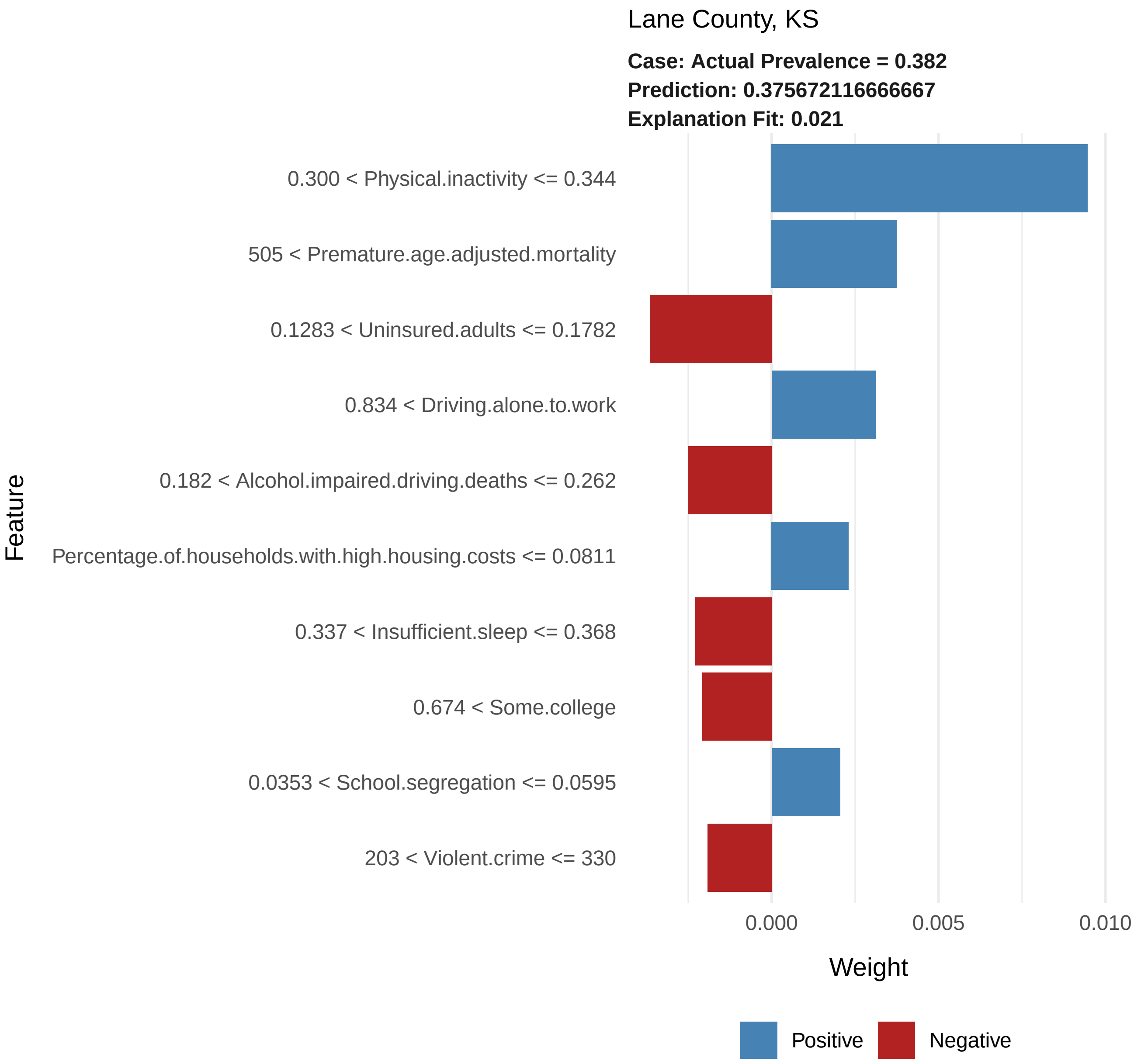

Lane County, KS
Case: Actual Prevalence = 0.382
Prediction: 0.375672116666667
Explanation Fit: 0.021
0.300 < Physical.inactivity <= 0.344
505 < Premature.age.adjusted.mortality
0.1283 < Uninsured.adults <= 0.1782
0.834 < Driving.alone.to.work
0.182 < Alcohol.impaired.driving.deaths <= 0.262
Percentage.of.households.with.high.housing.costs <= 0.0811
0.337 < Insufficient.sleep <= 0.368
0.674 < Some.college
0.0353 < School.segregation <= 0.0595
203 < Violent.crime <= 330
Feature
0.000
0.005
0.010
Weight
Positive
Negative

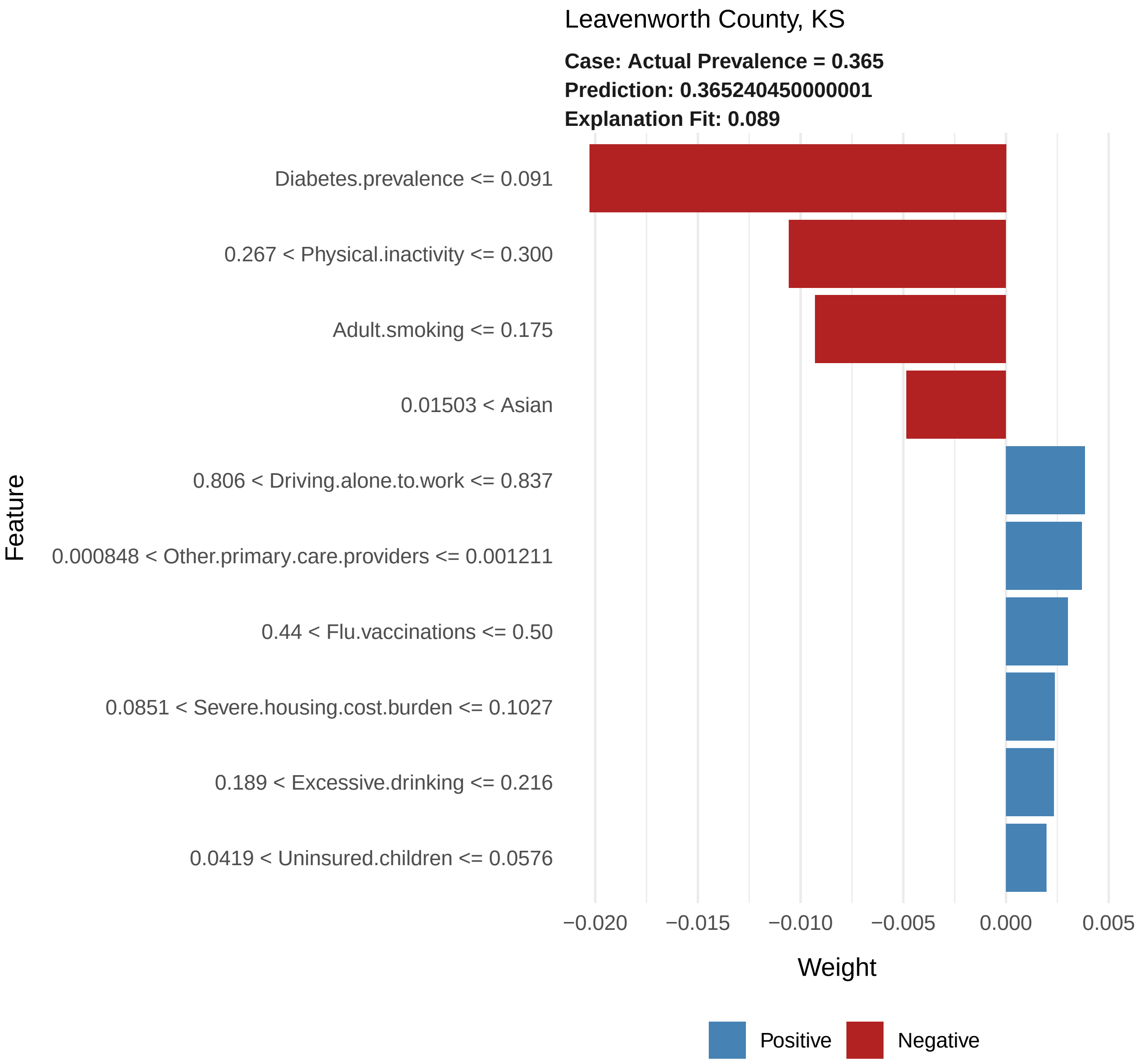

Leavenworth County, KS
Case: Actual Prevalence = 0.365
Prediction: 0.365240450000001
Explanation Fit: 0.089
Diabetes.prevalence <= 0.091
0.267 < Physical.inactivity <= 0.300
Adult.smoking <= 0.175
0.01503 < Asian
0.806 < Driving.alone.to.work <= 0.837
0.000848 < Other.primary.care.providers <= 0.001211
0.44 < Flu.vaccinations <= 0.50
0.0851 < Severe.housing.cost.burden <= 0.1027
0.189 < Excessive.drinking <= 0.216
0.0419 < Uninsured.children <= 0.0576
Feature
−0.020
−0.015
−0.010
−0.005
0.000
0.005
Weight
Positive
Negative

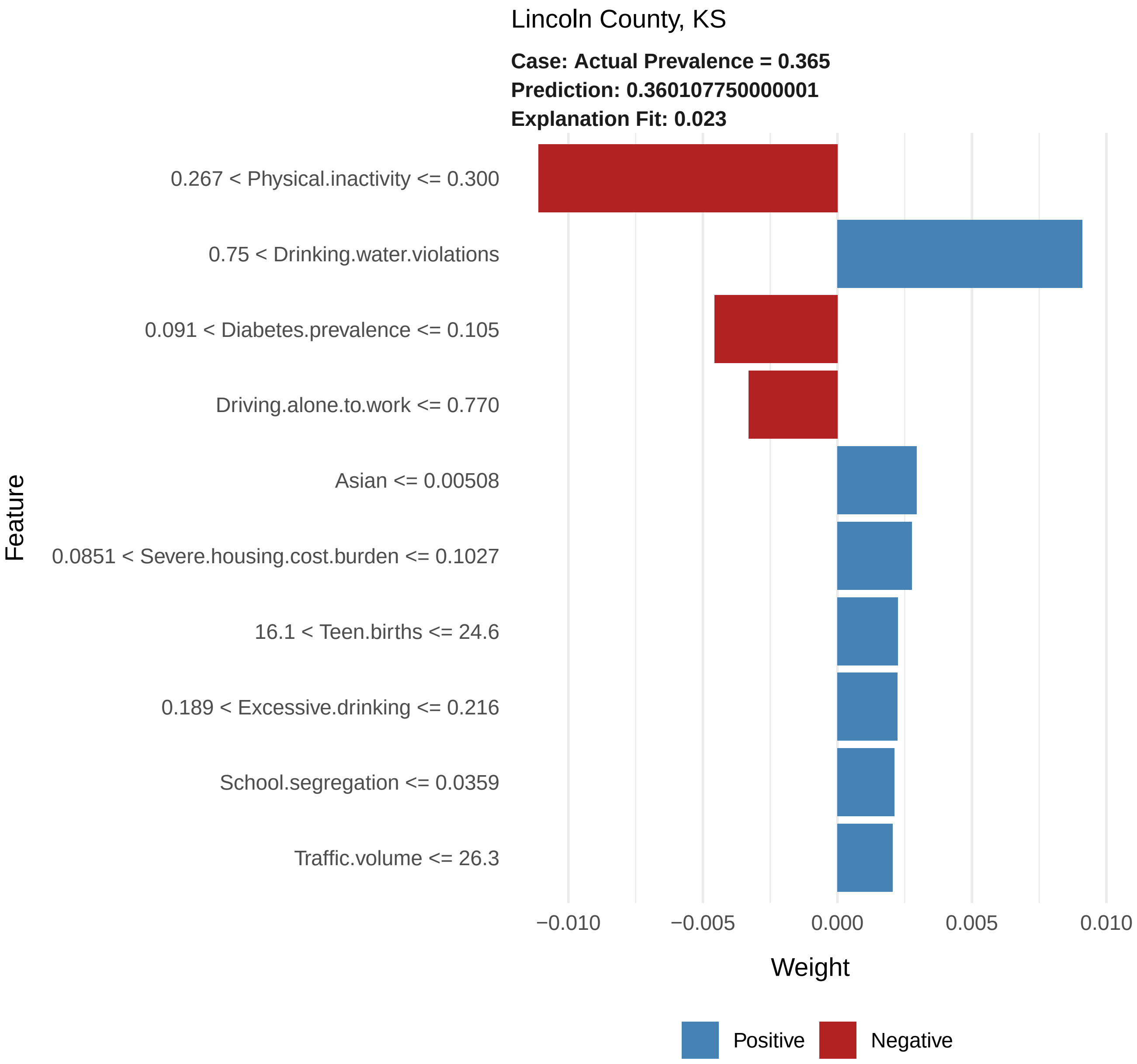
Lincoln County, KS
Case: Actual Prevalence = 0.365
Prediction: 0.360107750000001
Explanation Fit: 0.023
0.267 < Physical.inactivity <= 0.300
0.75 < Drinking.water.violations
0.091 < Diabetes.prevalence <= 0.105
Driving.alone.to.work <= 0.770
Asian <= 0.00508
0.0851 < Severe.housing.cost.burden <= 0.1027
16.1 < Teen.births <= 24.6
0.189 < Excessive.drinking <= 0.216
School.segregation <= 0.0359
Traffic.volume <= 26.3
Feature
−0.010
−0.005
0.000
0.005
0.010
Weight
Positive
Negative

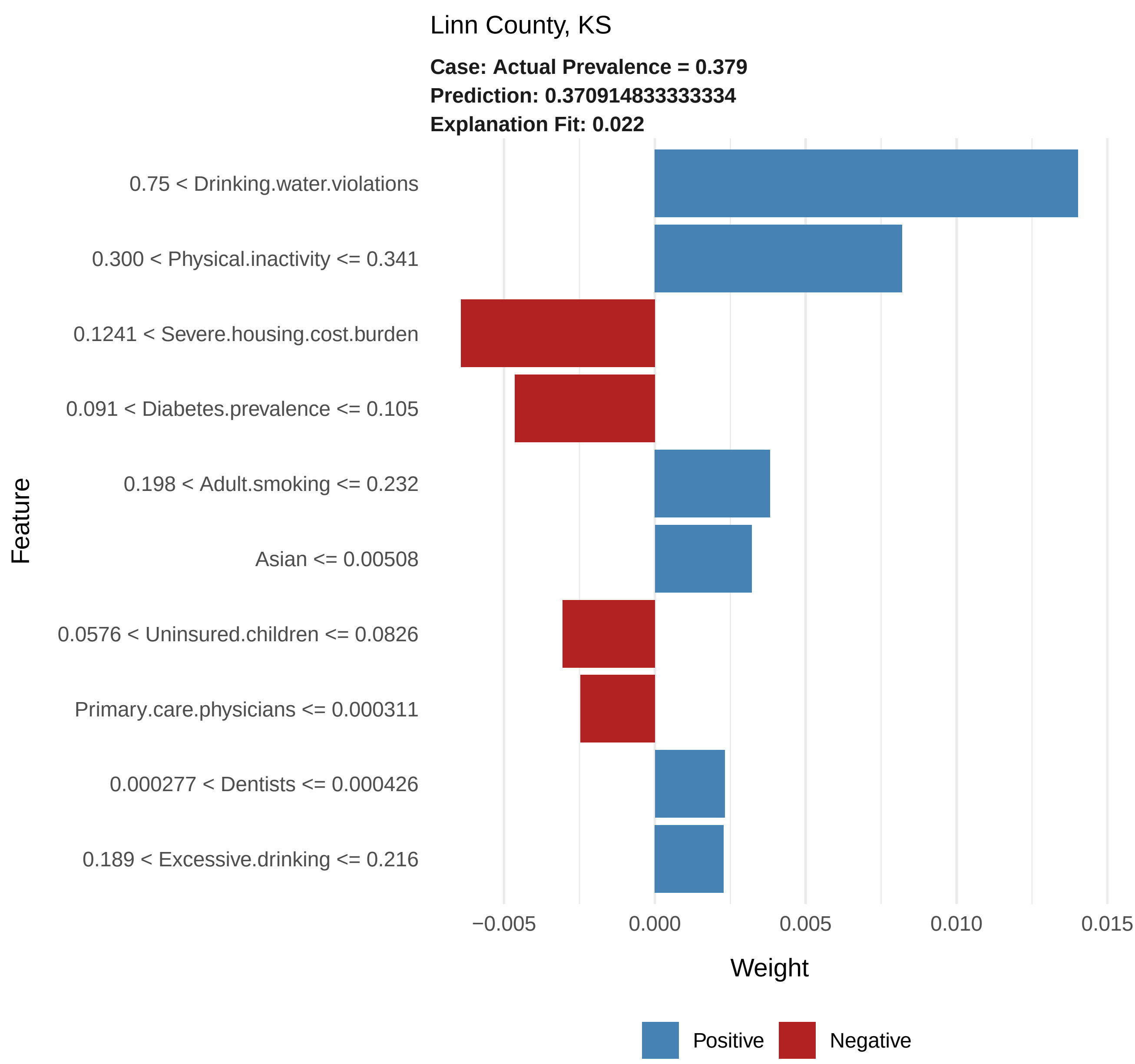

Linn County, KS
Case: Actual Prevalence = 0.379
Prediction: 0.370914833333334
Explanation Fit: 0.022
Feature
0.75 < Drinking.water.violations
0.300 < Physical.inactivity <= 0.341
0.1241 < Severe.housing.cost.burden
0.091 < Diabetes.prevalence <= 0.105
0.198 < Adult.smoking <= 0.232
Asian <= 0.00508
0.0576 < Uninsured.children <= 0.0826
Primary.care.physicians <= 0.000311
0.000277 < Dentists <= 0.000426
0.189 < Excessive.drinking <= 0.216
−0.005
0.000
0.005
0.010
0.015
Weight
Positive
Negative

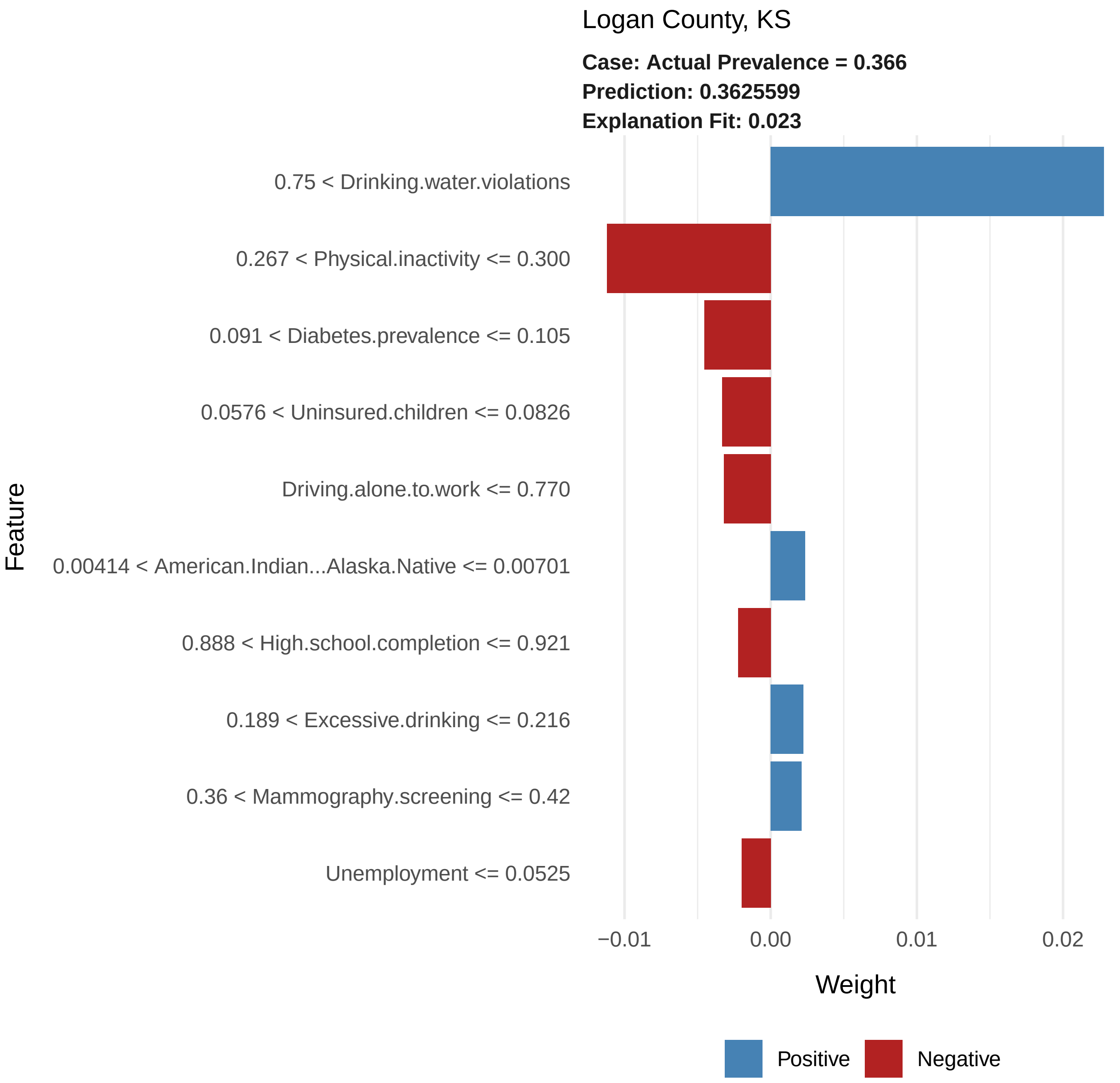

Logan County, KS
Case: Actual Prevalence = 0.366
Prediction: 0.3625599
Explanation Fit: 0.023
Feature
0.75 < Drinking.water.violations
0.267 < Physical.inactivity <= 0.300
0.091 < Diabetes.prevalence <= 0.105
0.0576 < Uninsured.children <= 0.0826
Driving.alone.to.work <= 0.770
0.00414 < American.Indian...Alaska.Native <= 0.00701
0.888 < High.school.completion <= 0.921
0.189 < Excessive.drinking <= 0.216
0.36 < Mammography.screening <= 0.42
Unemployment <= 0.0525
−0.01
0.00
0.01
0.02
Weight
Positive
Negative

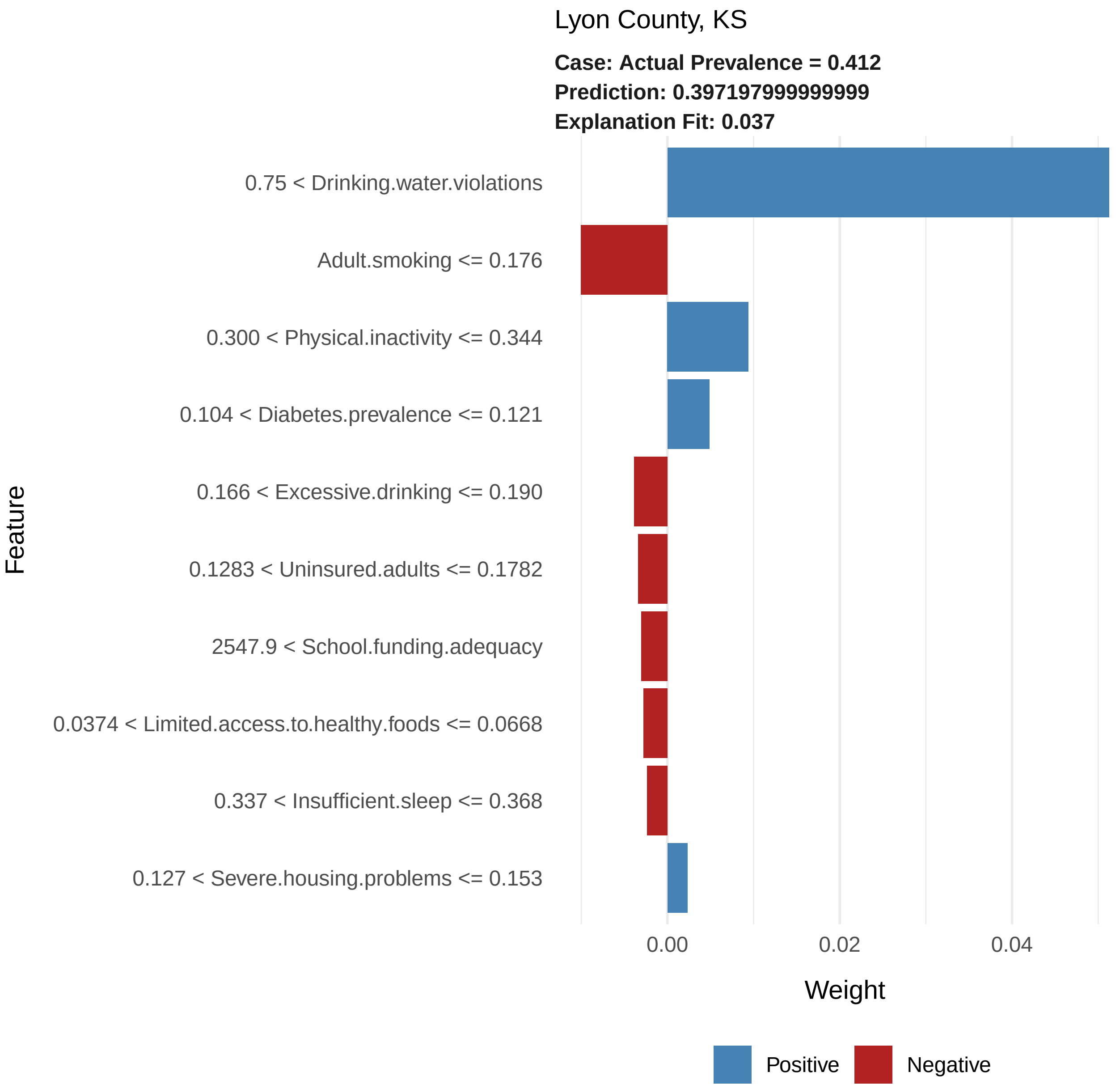

Lyon County, KS
Case: Actual Prevalence = 0.412
Prediction: 0.397197999999999
Explanation Fit: 0.037
0.75 < Drinking.water.violations
Adult.smoking <= 0.176
0.300 < Physical.inactivity <= 0.344
0.104 < Diabetes.prevalence <= 0.121
0.166 < Excessive.drinking <= 0.190
0.1283 < Uninsured.adults <= 0.1782
2547.9 < School.funding.adequacy
0.0374 < Limited.access.to.healthy.foods <= 0.0668
0.337 < Insufficient.sleep <= 0.368
0.127 < Severe.housing.problems <= 0.153
Feature
0.00
0.02
0.04
Weight
Positive
Negative

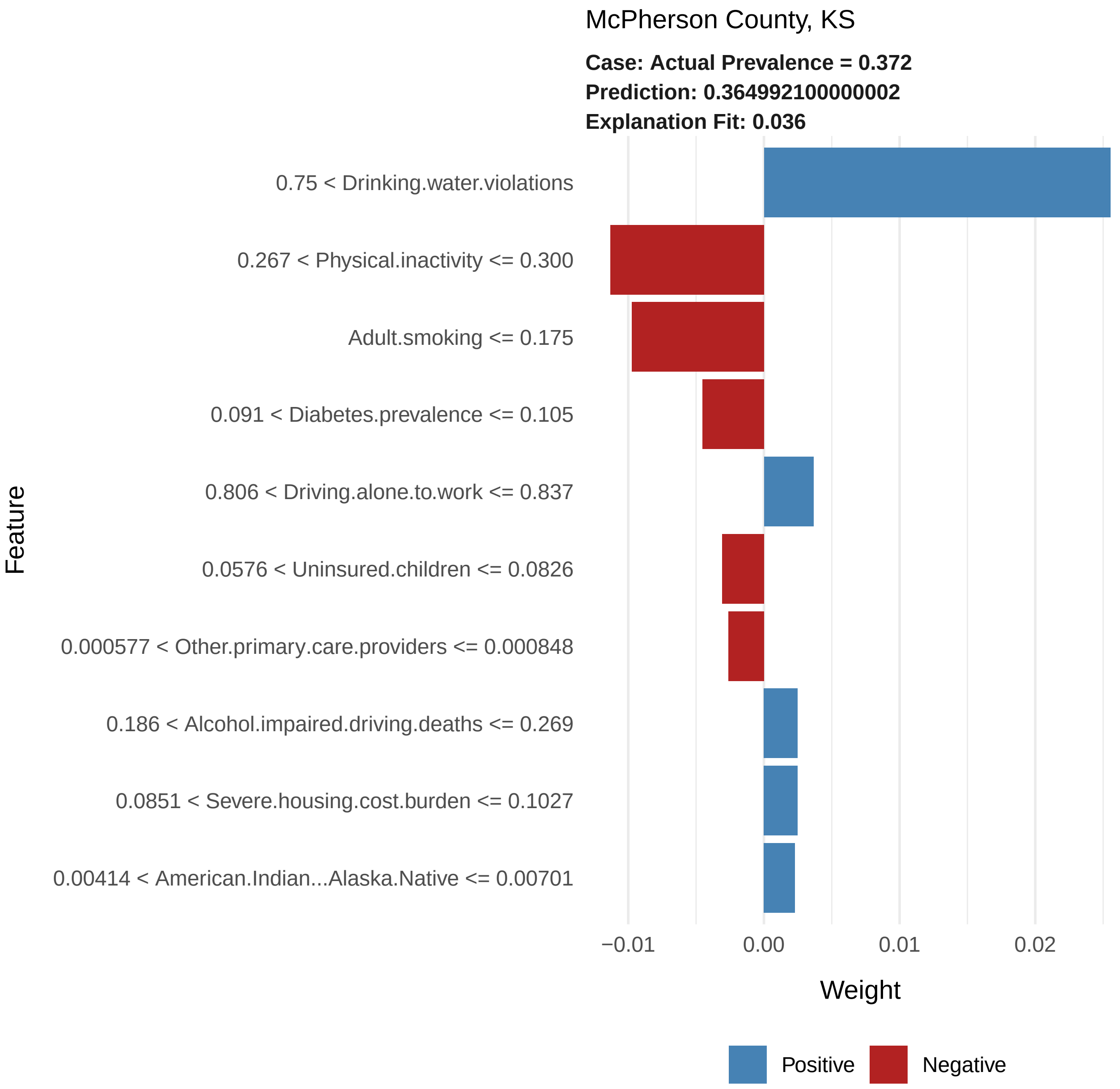

McPherson County, KS
Case: Actual Prevalence = 0.372
Prediction: 0.364992100000002
Explanation Fit: 0.036
0.75 < Drinking.water.violations
0.267 < Physical.inactivity <= 0.300
Adult.smoking <= 0.175
0.091 < Diabetes.prevalence <= 0.105
0.806 < Driving.alone.to.work <= 0.837
0.0576 < Uninsured.children <= 0.0826
0.000577 < Other.primary.care.providers <= 0.000848
0.186 < Alcohol.impaired.driving.deaths <= 0.269
0.0851 < Severe.housing.cost.burden <= 0.1027
0.00414 < American.Indian...Alaska.Native <= 0.00701
Feature
−0.01
0.00
0.01
0.02
Weight
Positive
Negative

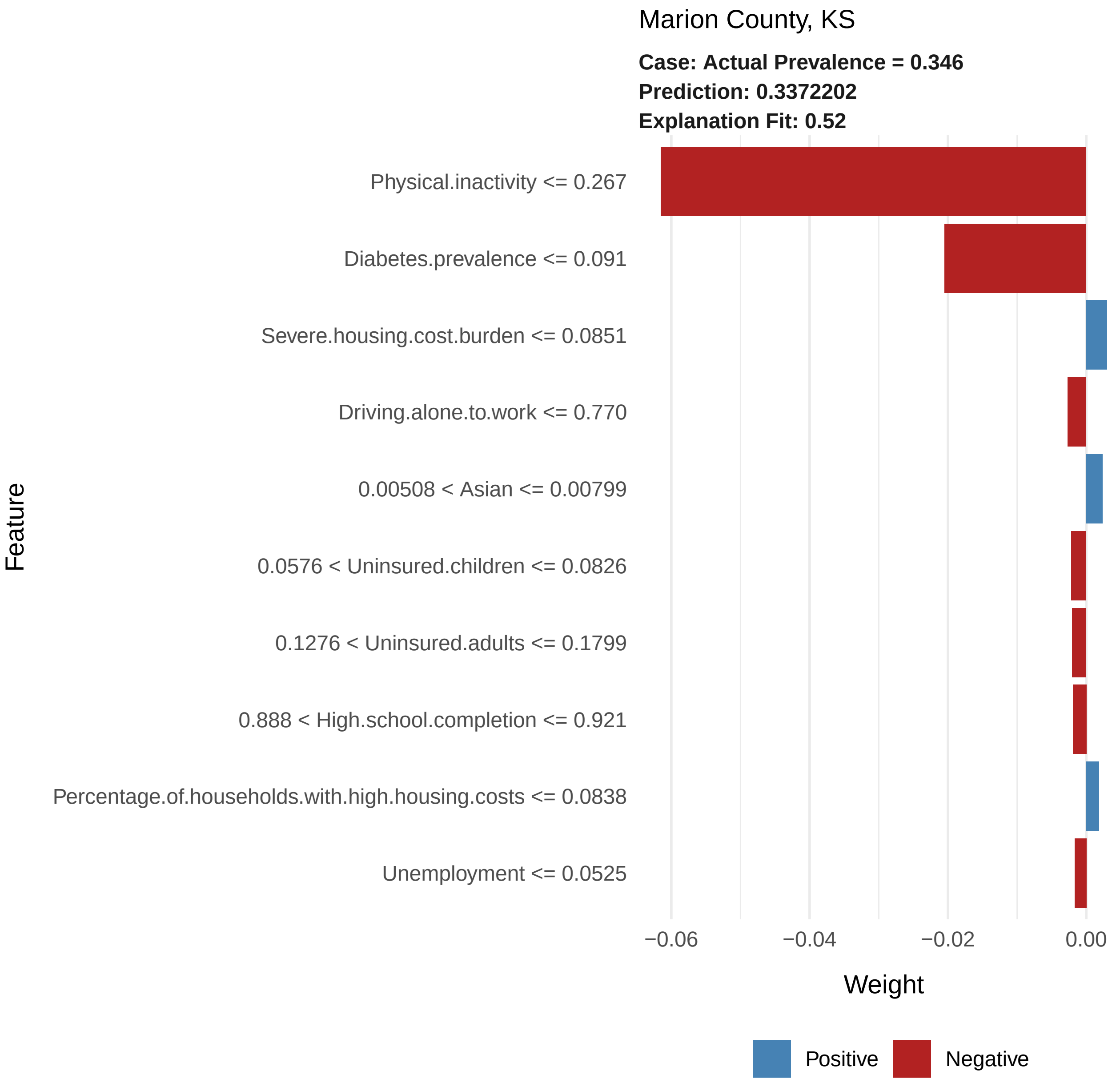

Marion County, KS
Case: Actual Prevalence = 0.346
Prediction: 0.3372202
Explanation Fit: 0.52
Physical.inactivity <= 0.267
Diabetes.prevalence <= 0.091
Severe.housing.cost.burden <= 0.0851
Driving.alone.to.work <= 0.770
0.00508 < Asian <= 0.00799
0.0576 < Uninsured.children <= 0.0826
0.1276 < Uninsured.adults <= 0.1799
0.888 < High.school.completion <= 0.921
Percentage.of.households.with.high.housing.costs <= 0.0838
Unemployment <= 0.0525
Feature
−0.06
−0.04
−0.02
0.00
Weight
Positive
Negative

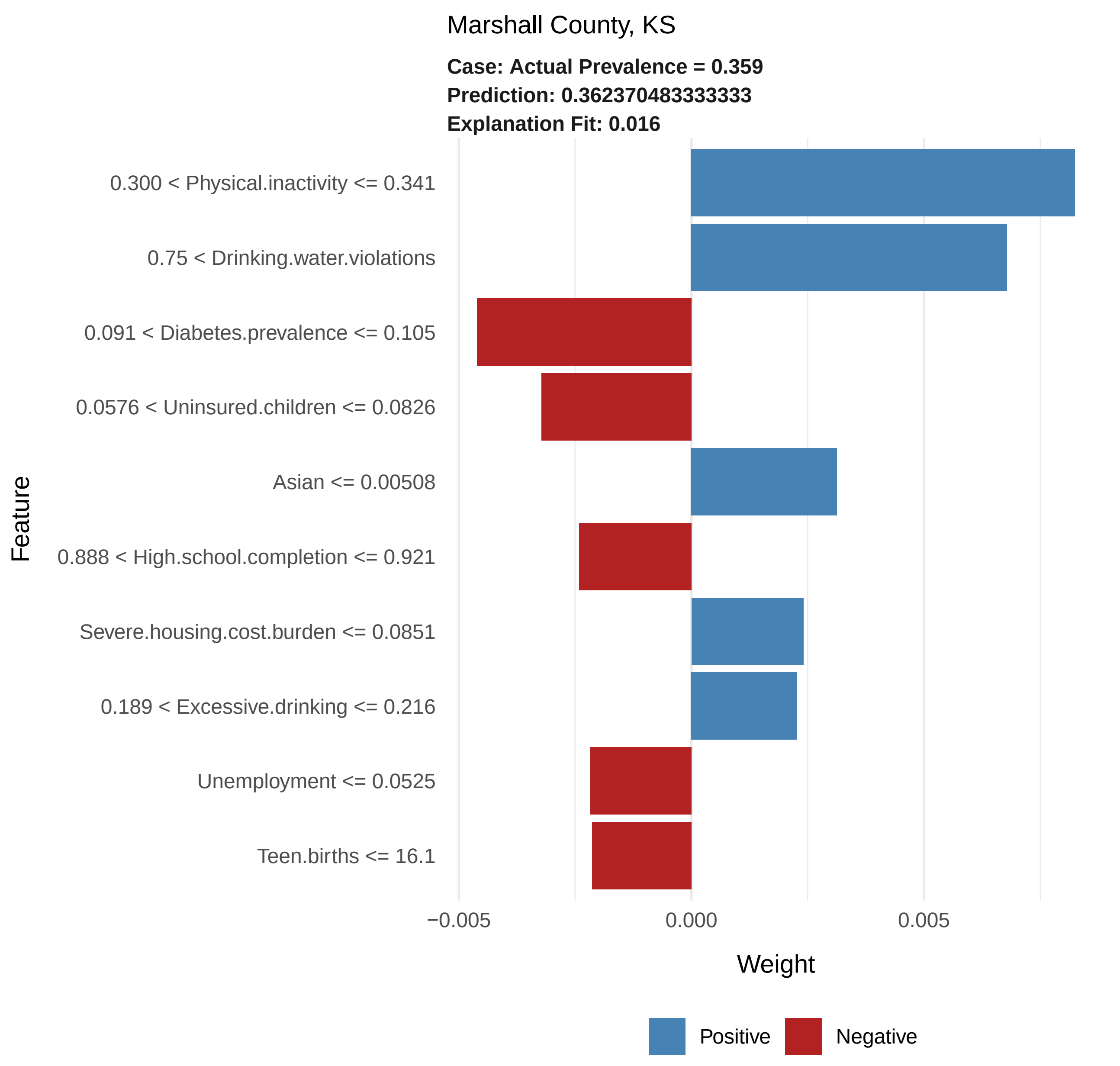
Marshall County, KS
Case: Actual Prevalence = 0.359
Prediction: 0.362370483333333
Explanation Fit: 0.016
0.300 < Physical.inactivity <= 0.341
0.75 < Drinking.water.violations
0.091 < Diabetes.prevalence <= 0.105
0.0576 < Uninsured.children <= 0.0826
Asian <= 0.00508
0.888 < High.school.completion <= 0.921
Severe.housing.cost.burden <= 0.0851
0.189 < Excessive.drinking <= 0.216
Unemployment <= 0.0525
Teen.births <= 16.1
Feature
−0.005
0.000
0.005
Weight
Positive
Negative

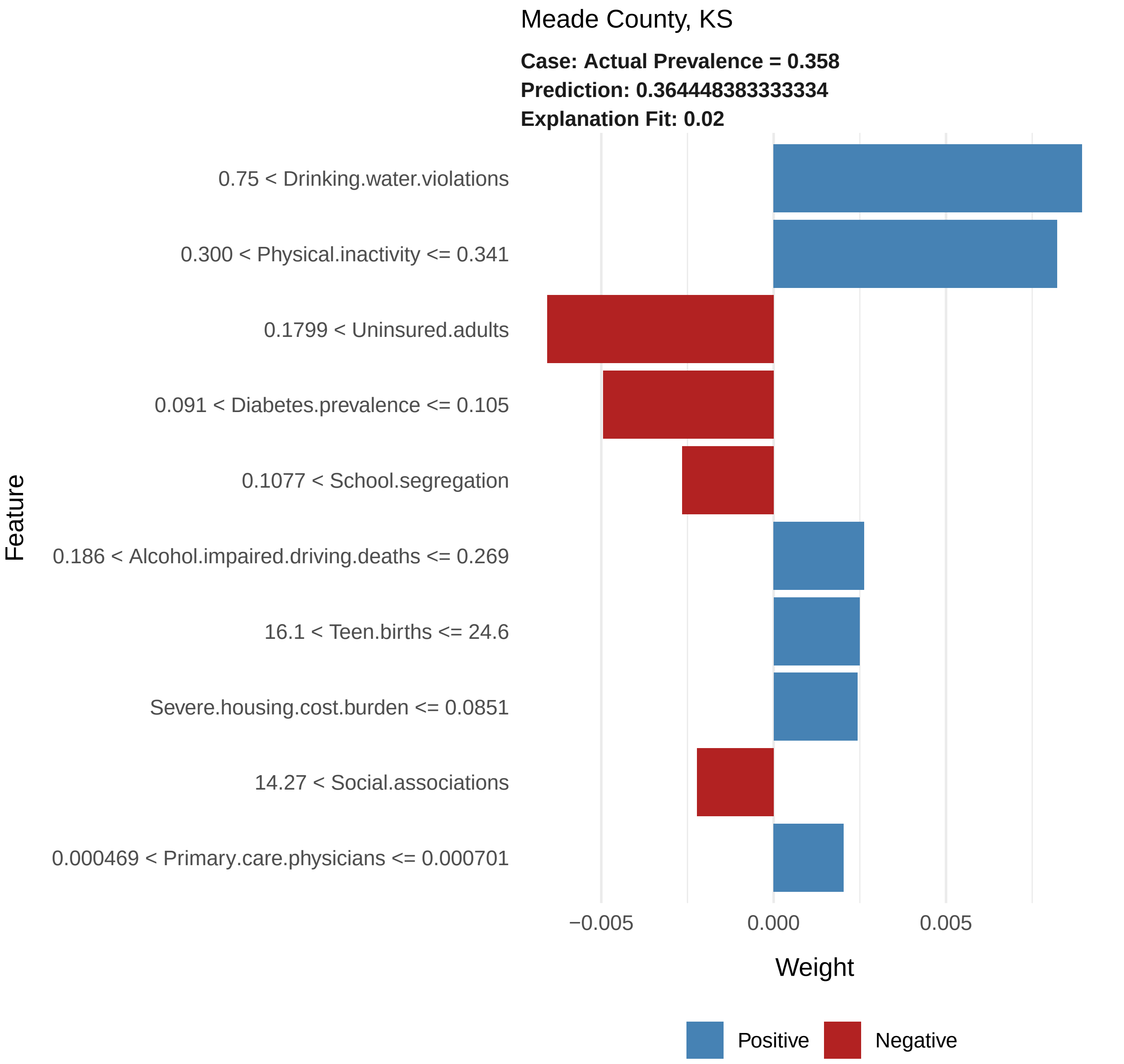

Meade County, KS
Case: Actual Prevalence = 0.358
Prediction: 0.364448383333334
Explanation Fit: 0.02
0.75 < Drinking.water.violations
0.300 < Physical.inactivity <= 0.341
0.1799 < Uninsured.adults
0.091 < Diabetes.prevalence <= 0.105
0.1077 < School.segregation
0.186 < Alcohol.impaired.driving.deaths <= 0.269
16.1 < Teen.births <= 24.6
Severe.housing.cost.burden <= 0.0851
14.27 < Social.associations
0.000469 < Primary.care.physicians <= 0.000701
Feature
−0.005
0.000
0.005
Weight
Positive
Negative

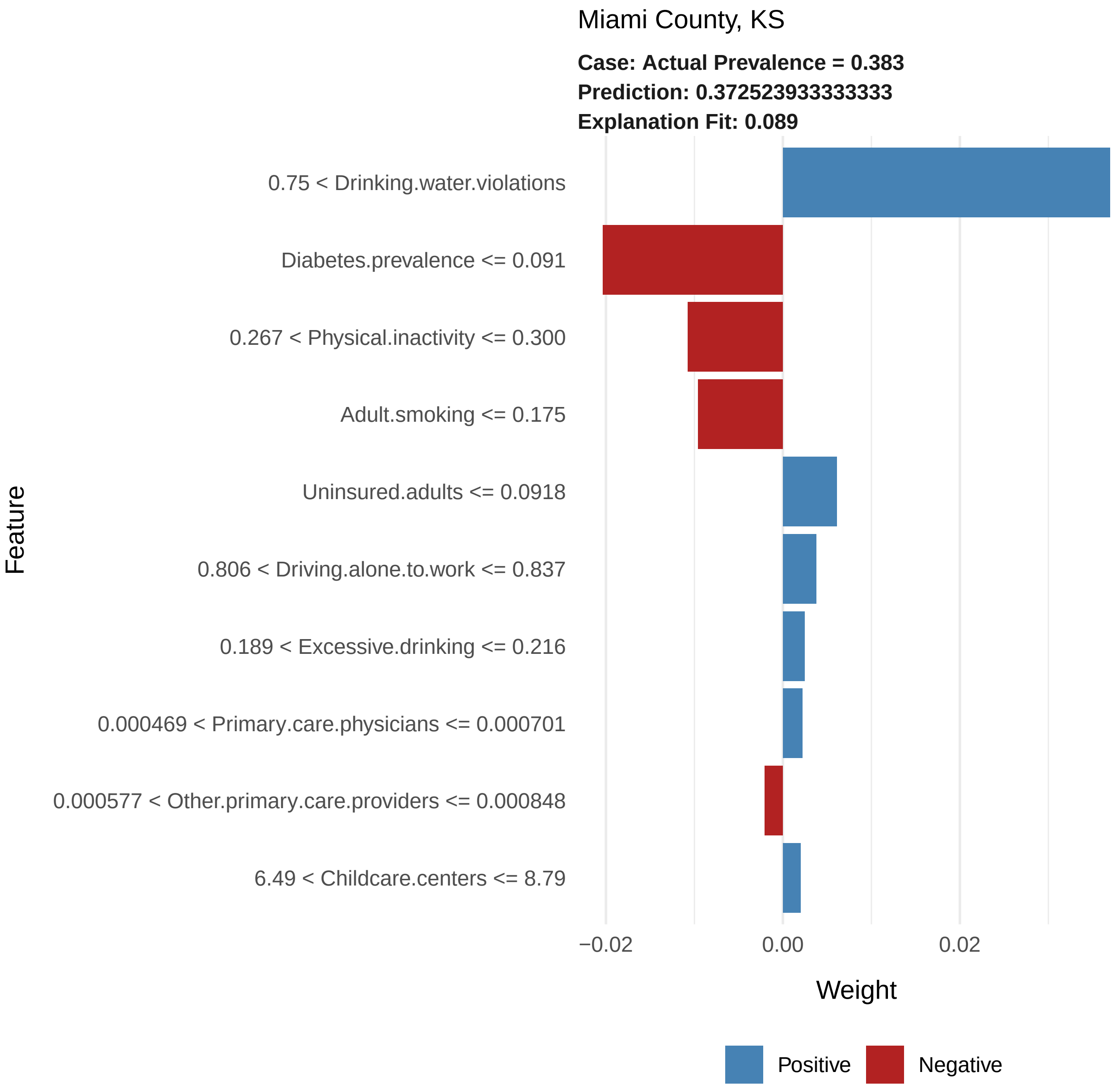

Miami County, KS
Case: Actual Prevalence = 0.383
Prediction: 0.372523933333333
Explanation Fit: 0.089
0.75 < Drinking.water.violations
Diabetes.prevalence <= 0.091
0.267 < Physical.inactivity <= 0.300
Adult.smoking <= 0.175
Uninsured.adults <= 0.0918
0.806 < Driving.alone.to.work <= 0.837
0.189 < Excessive.drinking <= 0.216
0.000469 < Primary.care.physicians <= 0.000701
0.000577 < Other.primary.care.providers <= 0.000848
6.49 < Childcare.centers <= 8.79
Feature
−0.02
0.00
0.02
Weight
Positive
Negative

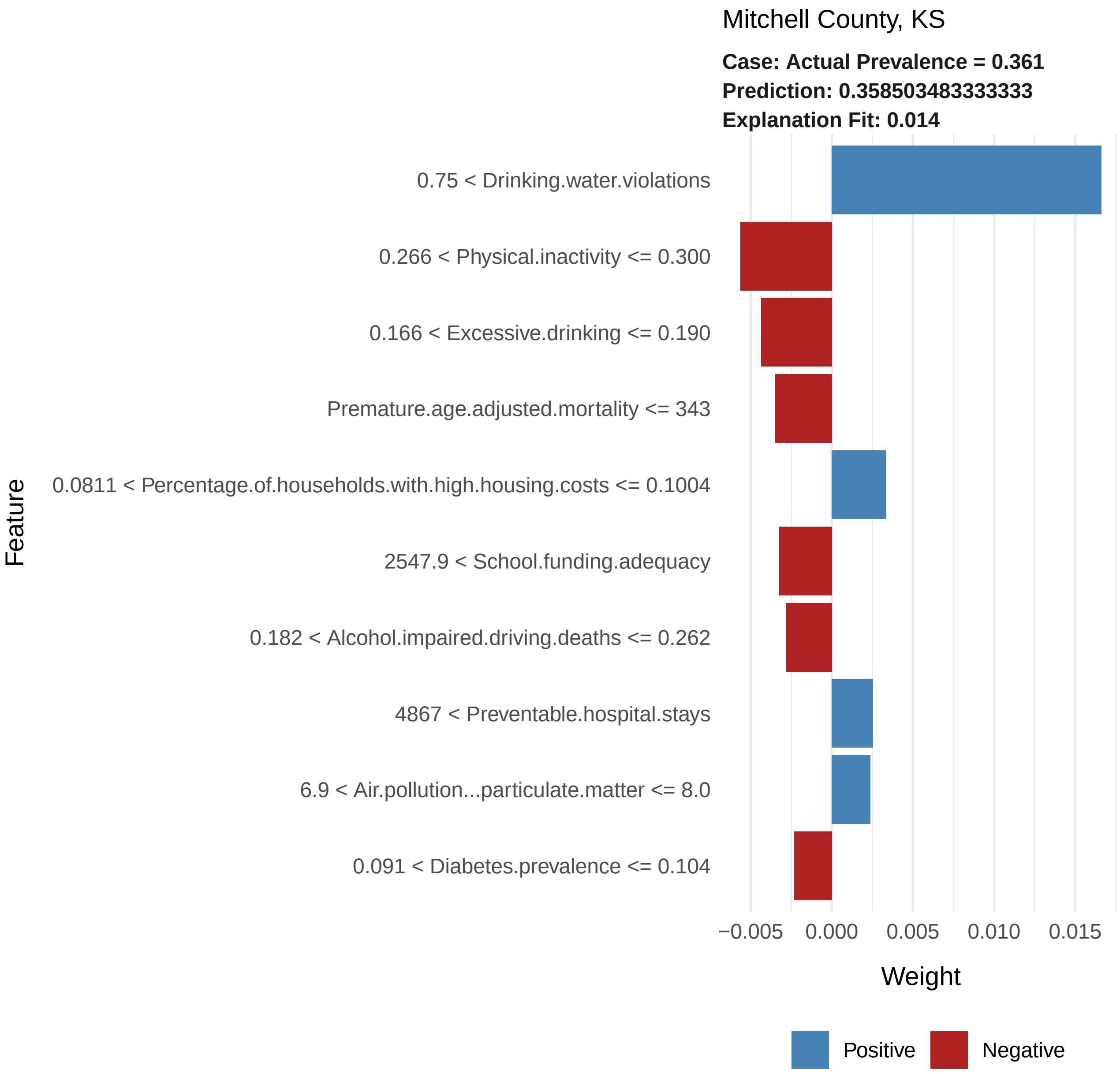
Mitchell County, KS
Case: Actual Prevalence = 0.361
Prediction: 0.358503483333333
Explanation Fit: 0.014
0.75 < Drinking.water.violations
0.266 < Physical.inactivity <= 0.300
0.166 < Excessive.drinking <= 0.190
Premature.age.adjusted.mortality <= 343
0.0811 < Percentage.of.households.with.high.housing.costs <= 0.1004
2547.9 < School.funding.adequacy
0.182 < Alcohol.impaired.driving.deaths <= 0.262
4867 < Preventable.hospital.stays
6.9 < Air.pollution...particulate.matter <= 8.0
0.091 < Diabetes.prevalence <= 0.104
Feature
−0.005
0.000
0.005
0.010
0.015
Weight
Positive
Negative

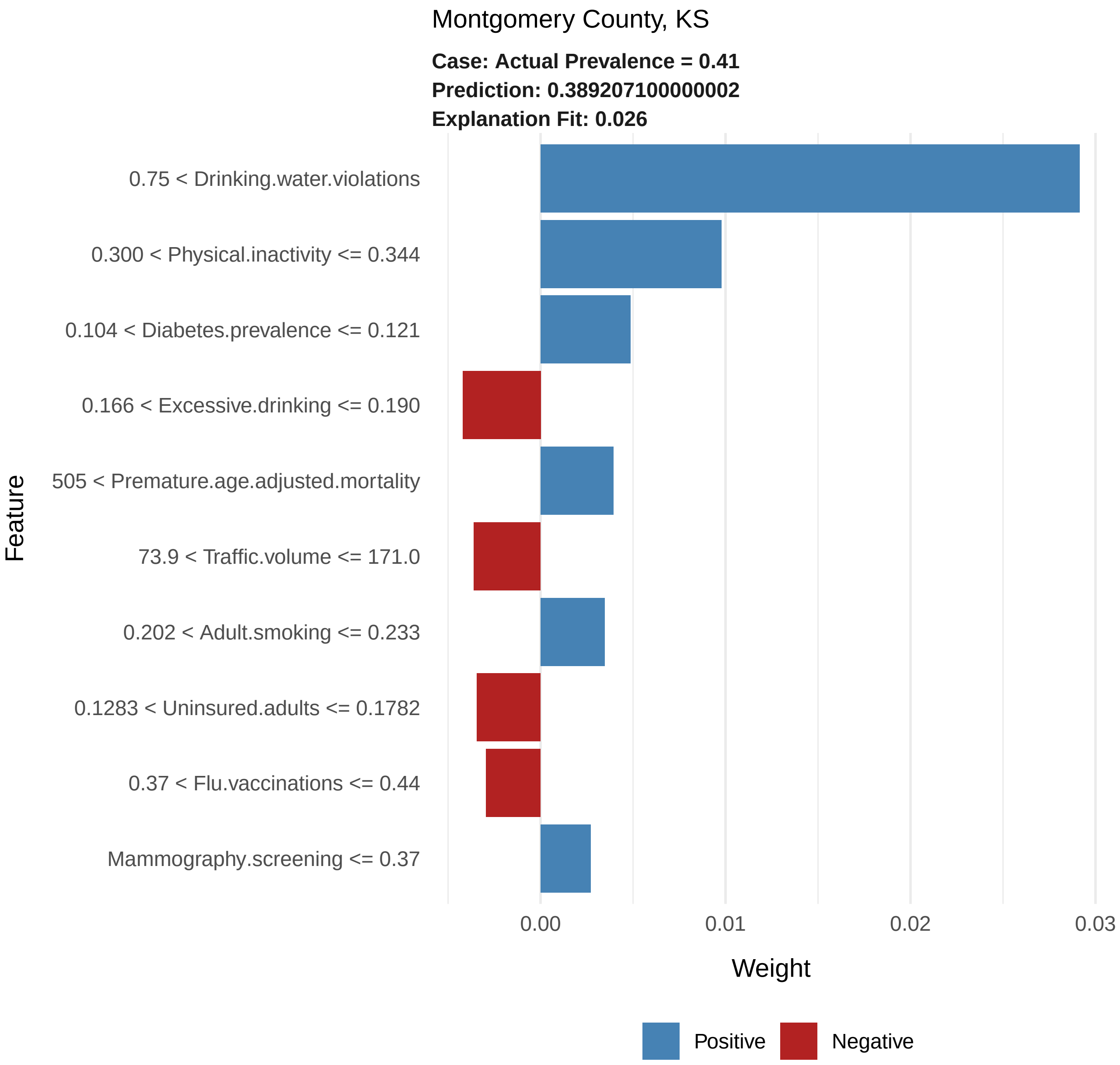
Montgomery County, KS
Case: Actual Prevalence = 0.41
Prediction: 0.389207100000002
Explanation Fit: 0.026
0.75 < Drinking.water.violations
0.300 < Physical.inactivity <= 0.344
0.104 < Diabetes.prevalence <= 0.121
0.166 < Excessive.drinking <= 0.190
505 < Premature.age.adjusted.mortality
73.9 < Traffic.volume <= 171.0
0.202 < Adult.smoking <= 0.233
0.1283 < Uninsured.adults <= 0.1782
0.37 < Flu.vaccinations <= 0.44
Mammography.screening <= 0.37
Feature
0.00
0.01
0.02
0.03
Weight
Positive
Negative

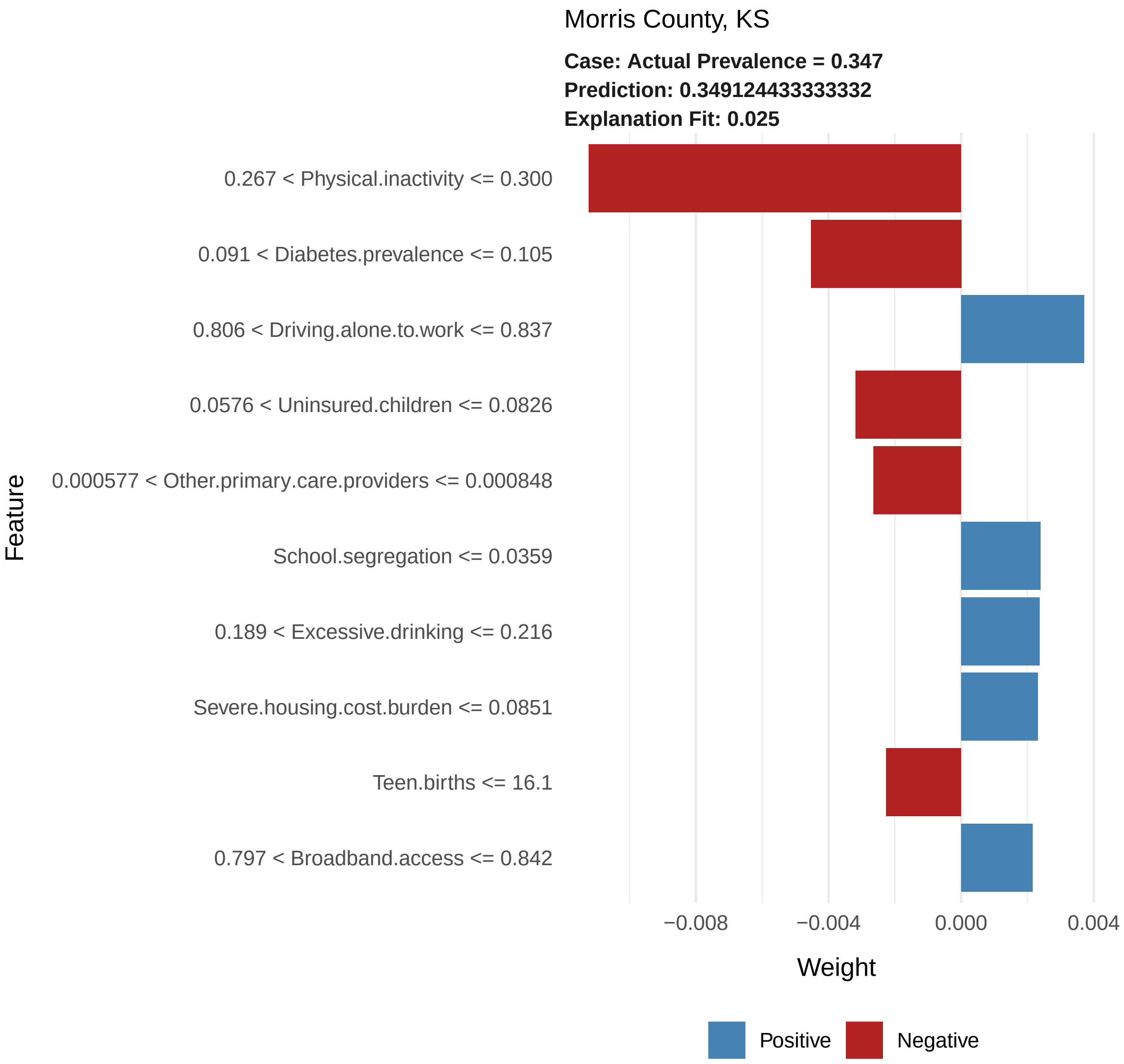

Morris County, KS
Case: Actual Prevalence = 0.347
Prediction: 0.349124433333332
Explanation Fit: 0.025
0.267 < Physical.inactivity <= 0.300
0.091 < Diabetes.prevalence <= 0.105
0.806 < Driving.alone.to.work <= 0.837
0.0576 < Uninsured.children <= 0.0826
0.000577 < Other.primary.care.providers <= 0.000848
School.segregation <= 0.0359
0.189 < Excessive.drinking <= 0.216
Severe.housing.cost.burden <= 0.0851
Teen.births <= 16.1
0.797 < Broadband.access <= 0.842
Feature
−0.008
−0.004
0.000
0.004
Weight
Positive
Negative

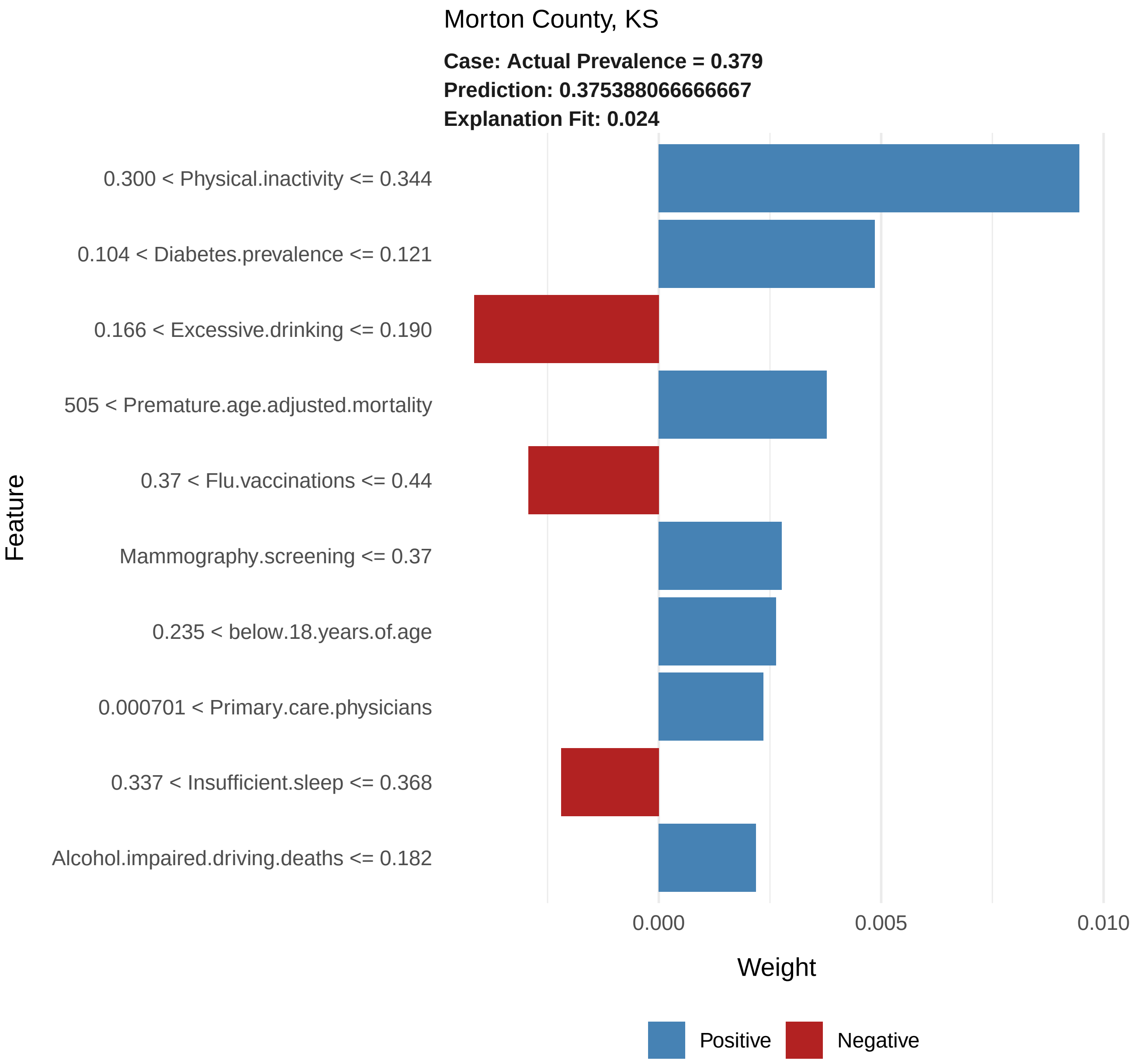
Morton County, KS
Case: Actual Prevalence = 0.379
Prediction: 0.375388066666667
Explanation Fit: 0.024
0.300 < Physical.inactivity <= 0.344
0.104 < Diabetes.prevalence <= 0.121
0.166 < Excessive.drinking <= 0.190
505 < Premature.age.adjusted.mortality
0.37 < Flu.vaccinations <= 0.44
Mammography.screening <= 0.37
0.235 < below.18.years.of.age
0.000701 < Primary.care.physicians
0.337 < Insufficient.sleep <= 0.368
Alcohol.impaired.driving.deaths <= 0.182
Feature
0.000
0.005
0.010
Weight
Positive
Negative

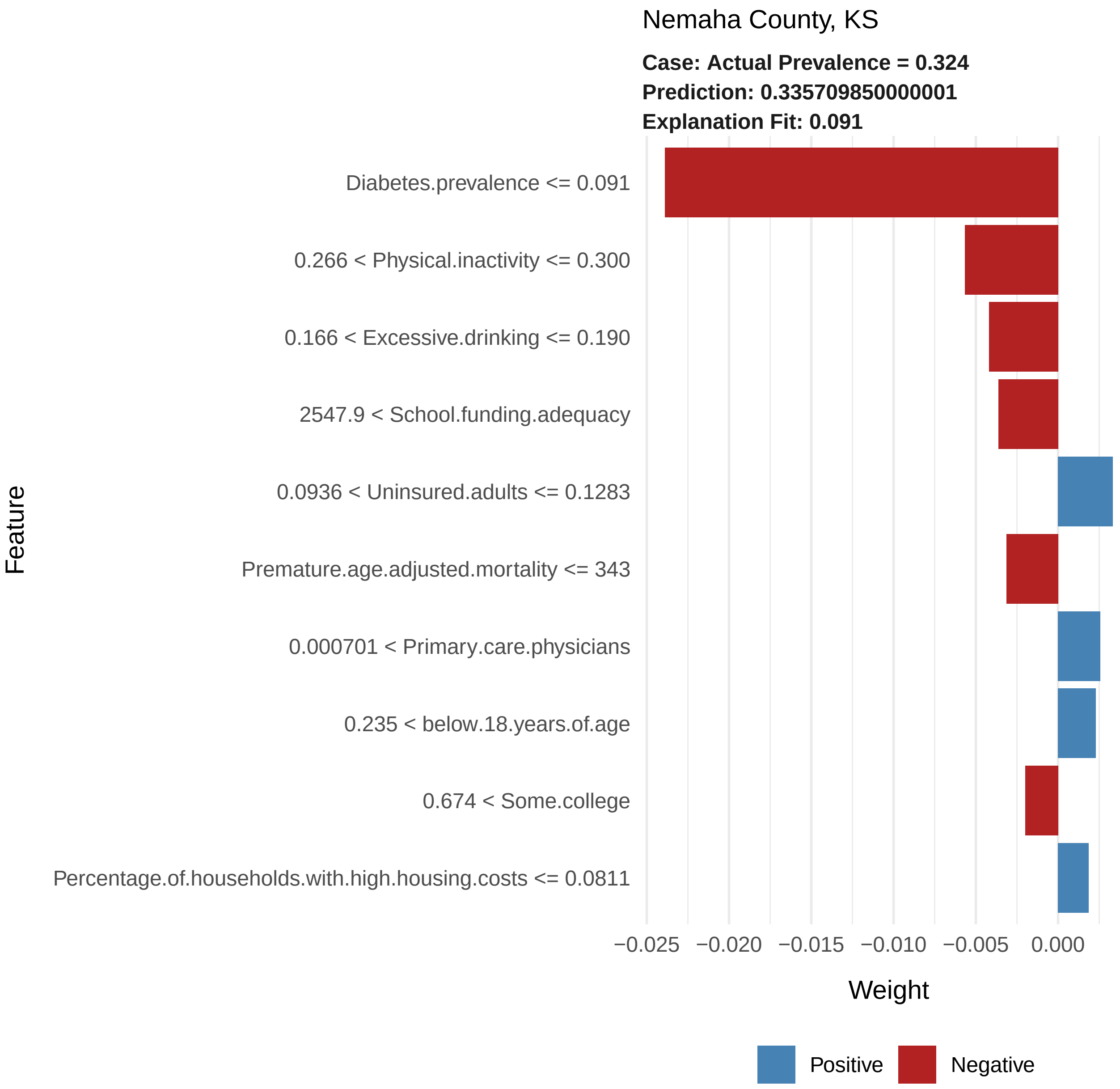

Nemaha County, KS
Case: Actual Prevalence = 0.324
Prediction: 0.335709850000001
Explanation Fit: 0.091
Feature
Diabetes.prevalence <= 0.091
0.266 < Physical.inactivity <= 0.300
0.166 < Excessive.drinking <= 0.190
2547.9 < School.funding.adequacy
0.0936 < Uninsured.adults <= 0.1283
Premature.age.adjusted.mortality <= 343
0.000701 < Primary.care.physicians
0.235 < below.18.years.of.age
0.674 < Some.college
Percentage.of.households.with.high.housing.costs <= 0.0811
−0.025
−0.020
−0.015
−0.010
−0.005
0.000
Weight
Positive
Negative

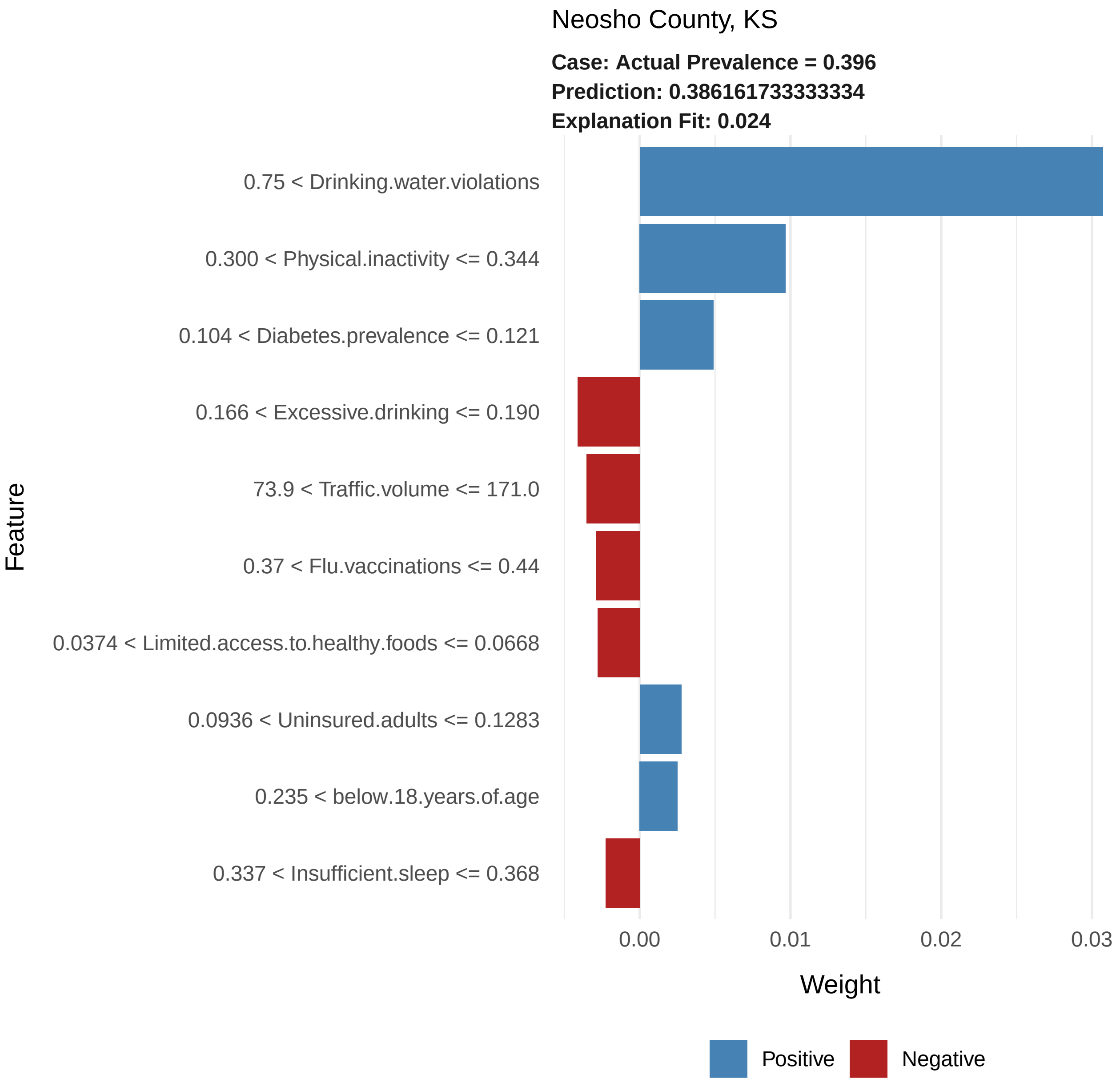
Neosho County, KS
Case: Actual Prevalence = 0.396
Prediction: 0.386161733333334
Explanation Fit: 0.024
0.75 < Drinking.water.violations
0.300 < Physical.inactivity <= 0.344
0.104 < Diabetes.prevalence <= 0.121
0.166 < Excessive.drinking <= 0.190
73.9 < Traffic.volume <= 171.0
0.37 < Flu.vaccinations <= 0.44
0.0374 < Limited.access.to.healthy.foods <= 0.0668
0.0936 < Uninsured.adults <= 0.1283
0.235 < below.18.years.of.age
0.337 < Insufficient.sleep <= 0.368
Feature
0.00
0.01
0.02
0.03
Weight
Positive
Negative

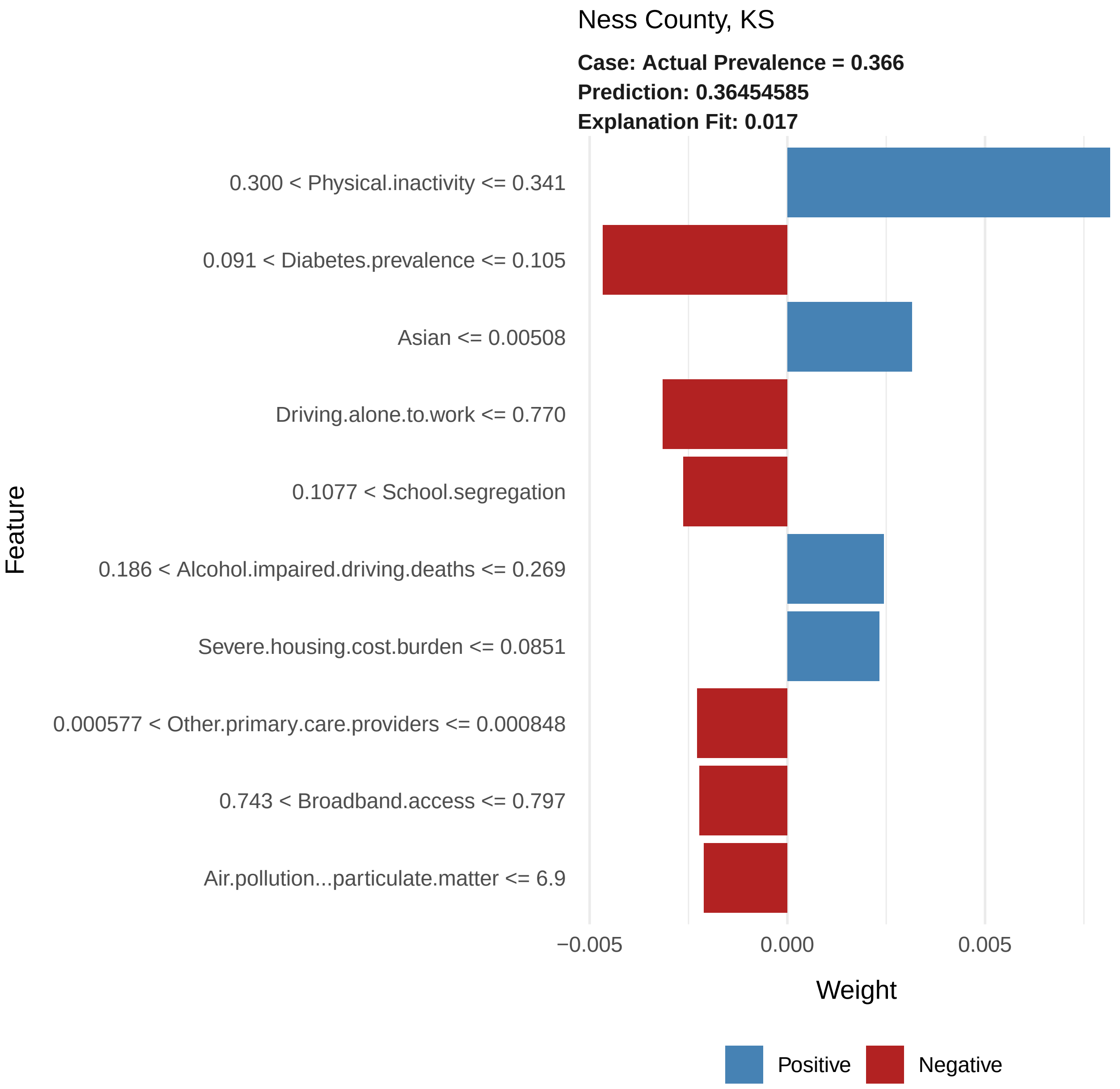

Ness County, KS
Case: Actual Prevalence = 0.366
Prediction: 0.36454585
Explanation Fit: 0.017
0.300 < Physical.inactivity <= 0.341
0.091 < Diabetes.prevalence <= 0.105
Asian <= 0.00508
Driving.alone.to.work <= 0.770
0.1077 < School.segregation
0.186 < Alcohol.impaired.driving.deaths <= 0.269
Severe.housing.cost.burden <= 0.0851
0.000577 < Other.primary.care.providers <= 0.000848
0.743 < Broadband.access <= 0.797
Air.pollution...particulate.matter <= 6.9
Feature
−0.005
0.000
0.005
Weight
Positive
Negative

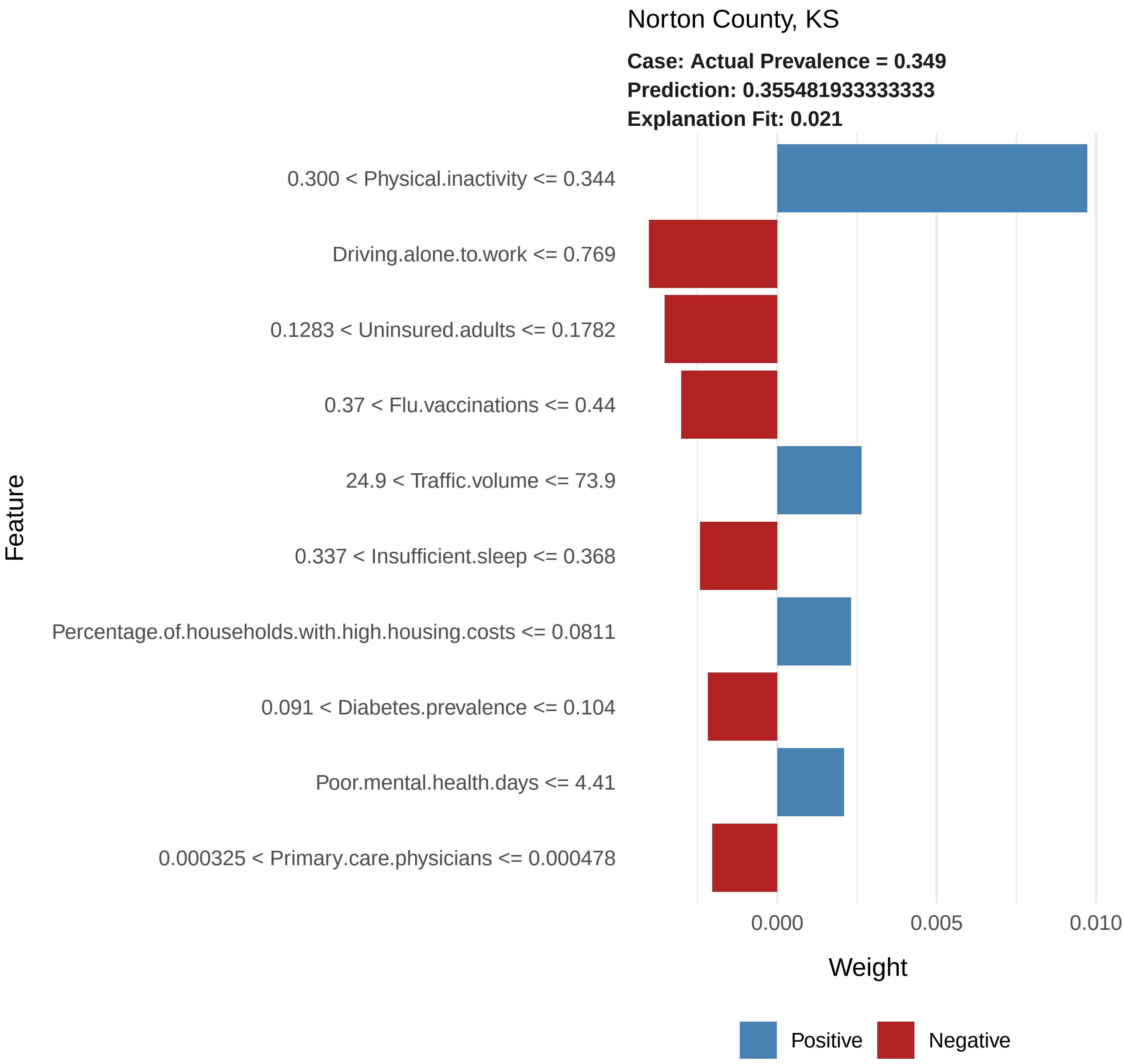

Norton County, KS
Case: Actual Prevalence = 0.349
Prediction: 0.355481933333333
Explanation Fit: 0.021
0.300 < Physical.inactivity <= 0.344
Driving.alone.to.work <= 0.769
0.1283 < Uninsured.adults <= 0.1782
0.37 < Flu.vaccinations <= 0.44
24.9 < Traffic.volume <= 73.9
0.337 < Insufficient.sleep <= 0.368
Percentage.of.households.with.high.housing.costs <= 0.0811
0.091 < Diabetes.prevalence <= 0.104
Poor.mental.health.days <= 4.41
0.000325 < Primary.care.physicians <= 0.000478
Feature
0.000
0.005
0.010
Weight
Positive
Negative

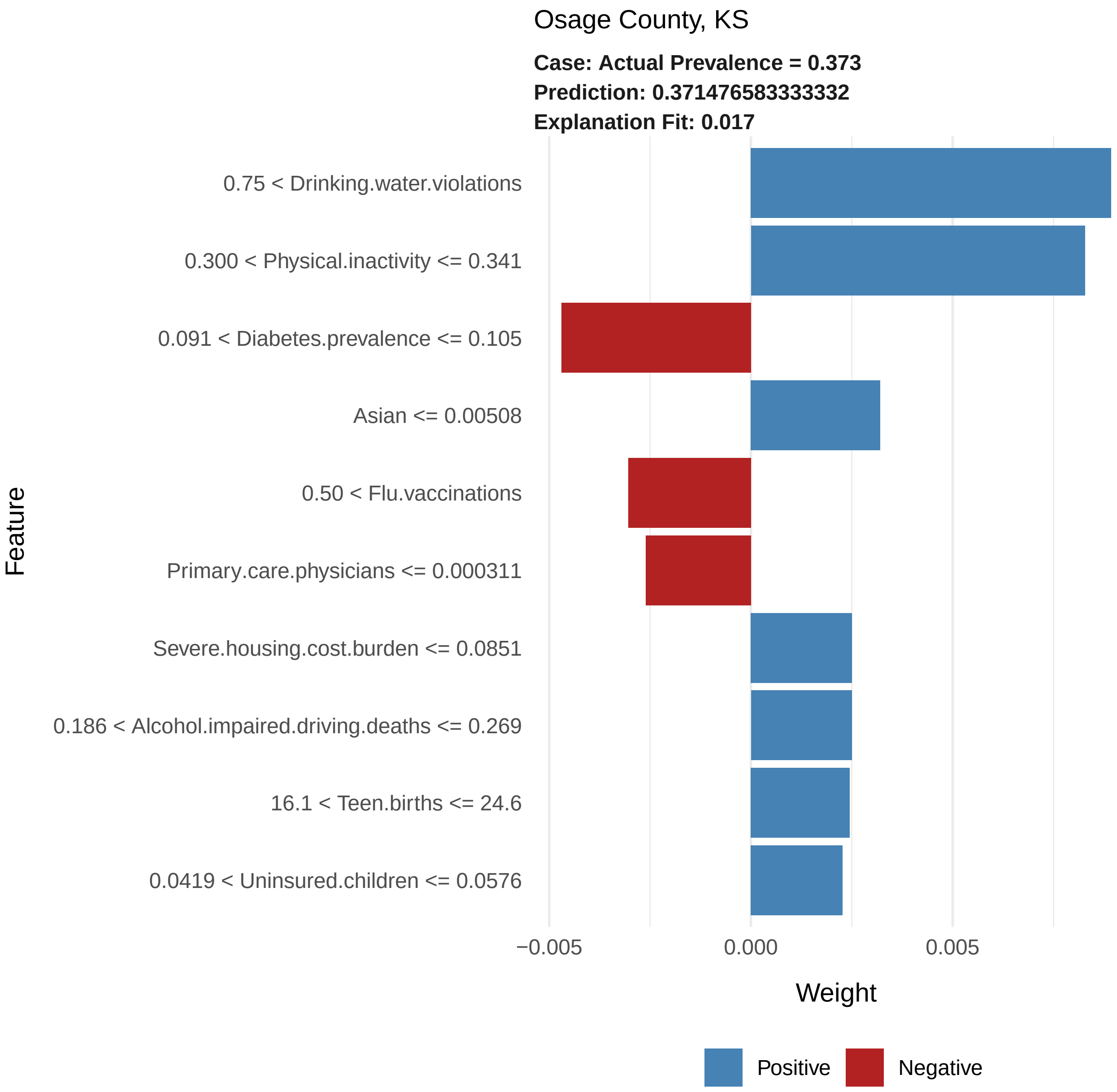

Osage County, KS
Case: Actual Prevalence = 0.373
Prediction: 0.371476583333332
Explanation Fit: 0.017
0.75 < Drinking.water.violations
0.300 < Physical.inactivity <= 0.341
0.091 < Diabetes.prevalence <= 0.105
Asian <= 0.00508
0.50 < Flu.vaccinations
Primary.care.physicians <= 0.000311
Severe.housing.cost.burden <= 0.0851
0.186 < Alcohol.impaired.driving.deaths <= 0.269
16.1 < Teen.births <= 24.6
0.0419 < Uninsured.children <= 0.0576
Feature
−0.005
0.000
0.005
Weight
Positive
Negative

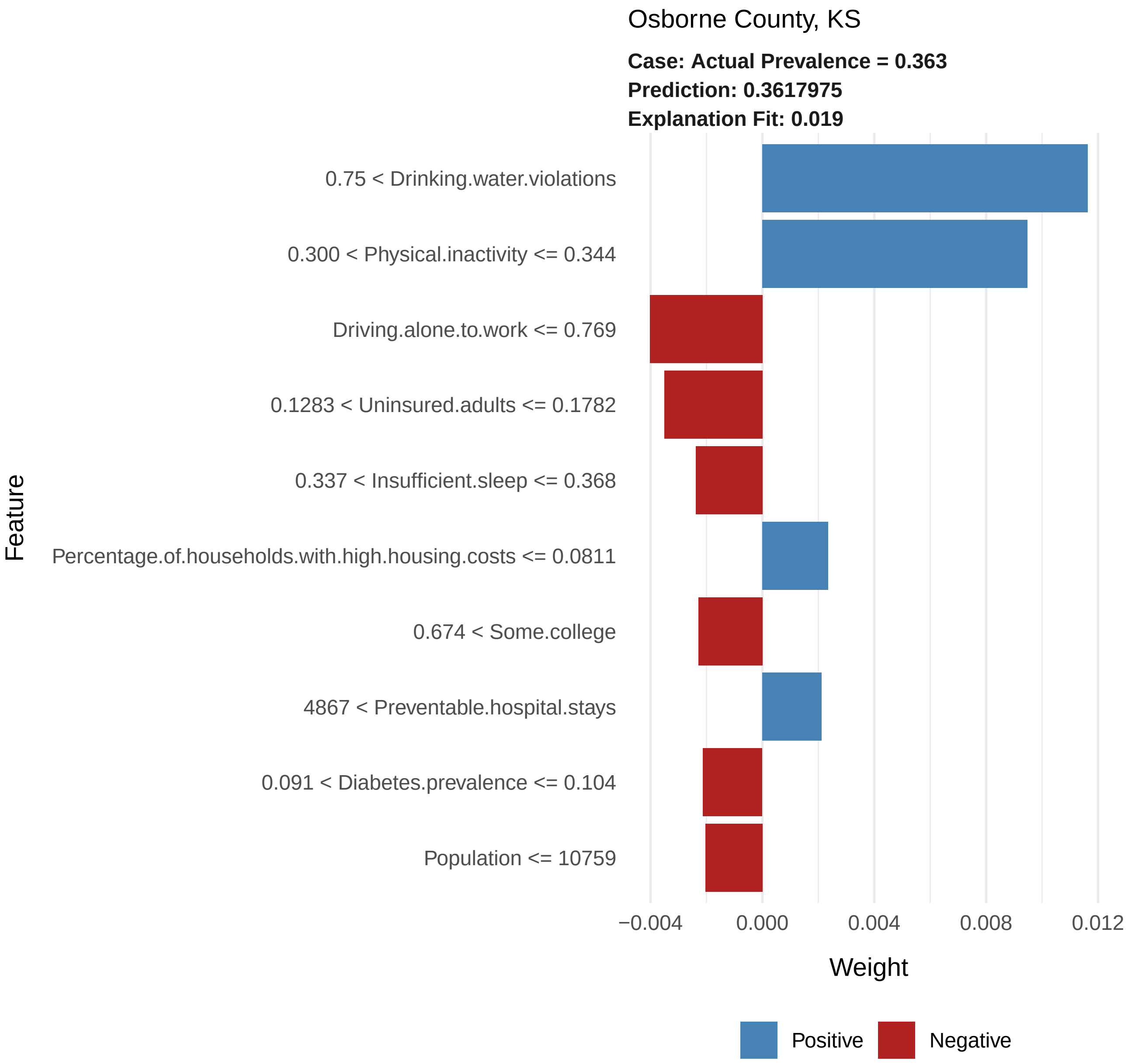

Osborne County, KS
Case: Actual Prevalence = 0.363
Prediction: 0.3617975
Explanation Fit: 0.019
0.75 < Drinking.water.violations
0.300 < Physical.inactivity <= 0.344
Driving.alone.to.work <= 0.769
0.1283 < Uninsured.adults <= 0.1782
0.337 < Insufficient.sleep <= 0.368
Percentage.of.households.with.high.housing.costs <= 0.0811
0.674 < Some.college
4867 < Preventable.hospital.stays
0.091 < Diabetes.prevalence <= 0.104
Population <= 10759
Feature
−0.004
0.000
0.004
0.008
0.012
Weight
Positive
Negative

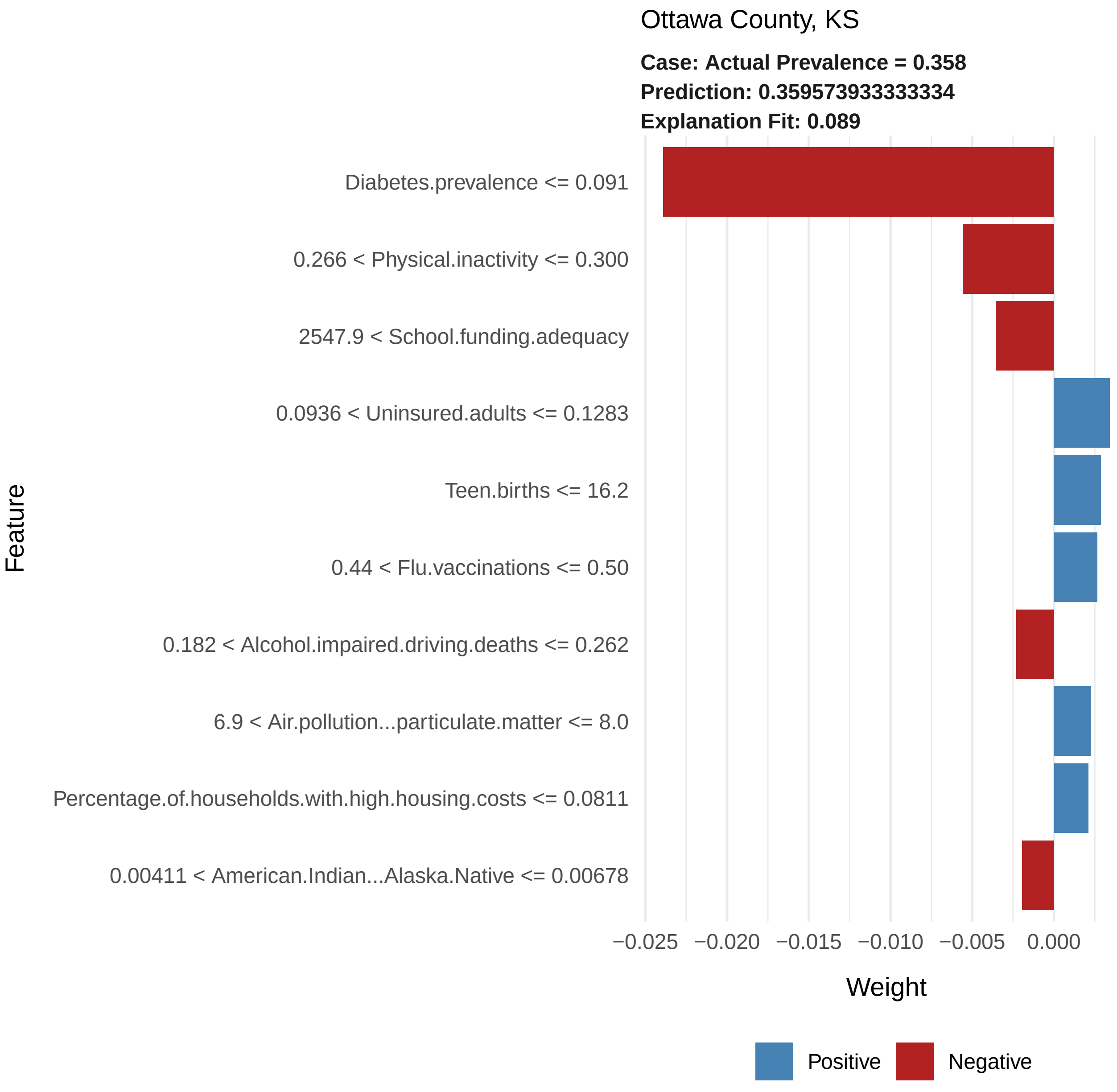

Ottawa County, KS
Case: Actual Prevalence = 0.358
Prediction: 0.359573933333334
Explanation Fit: 0.089
Feature
Diabetes.prevalence <= 0.091
0.266 < Physical.inactivity <= 0.300
2547.9 < School.funding.adequacy
0.0936 < Uninsured.adults <= 0.1283
Teen.births <= 16.2
0.44 < Flu.vaccinations <= 0.50
0.182 < Alcohol.impaired.driving.deaths <= 0.262
6.9 < Air.pollution...particulate.matter <= 8.0
Percentage.of.households.with.high.housing.costs <= 0.0811
0.00411 < American.Indian...Alaska.Native <= 0.00678
−0.025
−0.020
−0.015
−0.010
−0.005
0.000
Weight
Positive
Negative

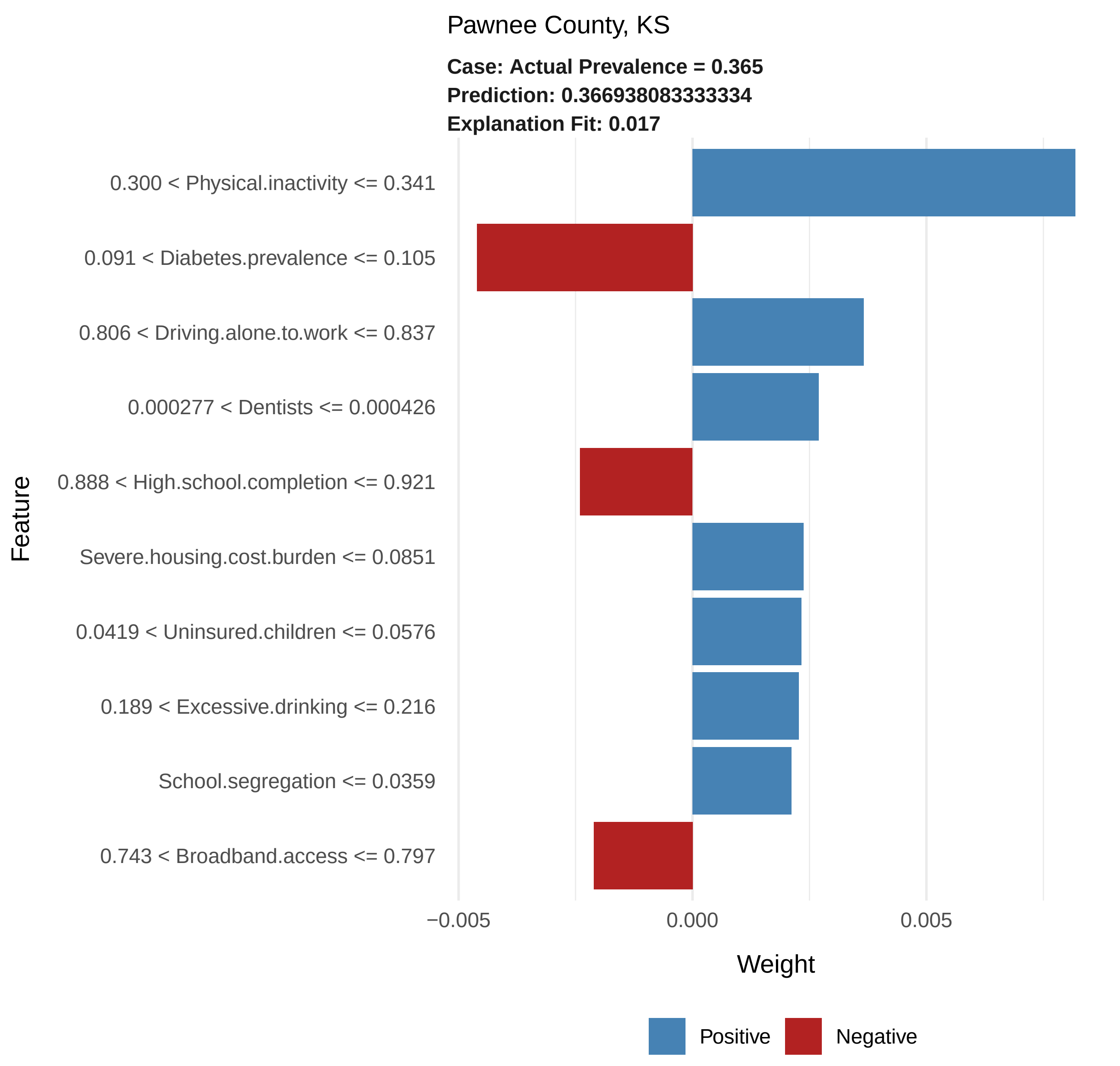

Pawnee County, KS
Case: Actual Prevalence = 0.365
Prediction: 0.366938083333334
Explanation Fit: 0.017
0.300 < Physical.inactivity <= 0.341
0.091 < Diabetes.prevalence <= 0.105
0.806 < Driving.alone.to.work <= 0.837
0.000277 < Dentists <= 0.000426
0.888 < High.school.completion <= 0.921
Severe.housing.cost.burden <= 0.0851
0.0419 < Uninsured.children <= 0.0576
0.189 < Excessive.drinking <= 0.216
School.segregation <= 0.0359
0.743 < Broadband.access <= 0.797
Feature
−0.005
0.000
0.005
Weight
Positive
Negative

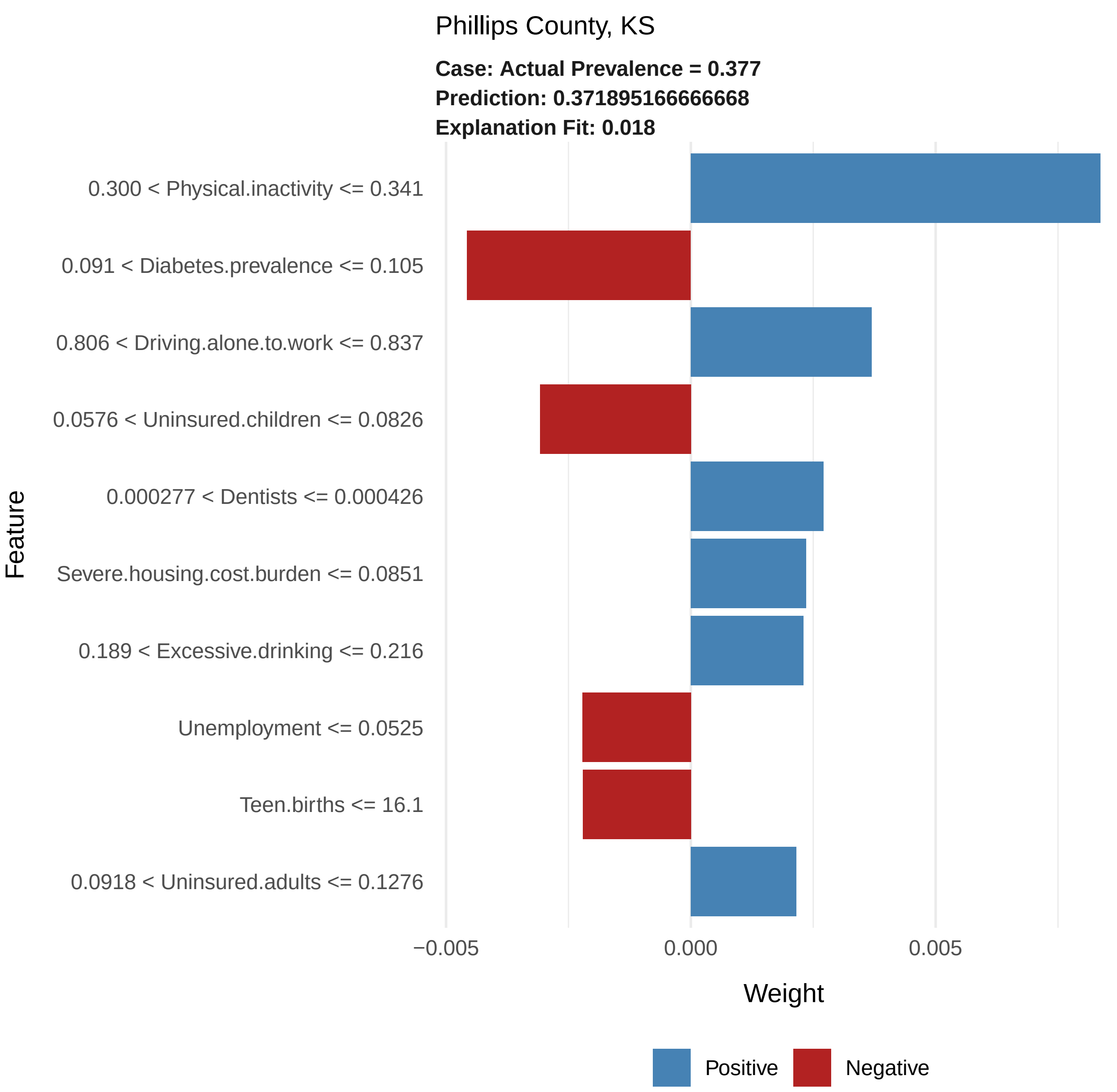
Phillips County, KS
Case: Actual Prevalence = 0.377
Prediction: 0.371895166666668
Explanation Fit: 0.018
0.300 < Physical.inactivity <= 0.341
0.091 < Diabetes.prevalence <= 0.105
0.806 < Driving.alone.to.work <= 0.837
0.0576 < Uninsured.children <= 0.0826
0.000277 < Dentists <= 0.000426
Severe.housing.cost.burden <= 0.0851
0.189 < Excessive.drinking <= 0.216
Unemployment <= 0.0525
Teen.births <= 16.1
0.0918 < Uninsured.adults <= 0.1276
Feature
−0.005
0.000
0.005
Weight
Positive
Negative

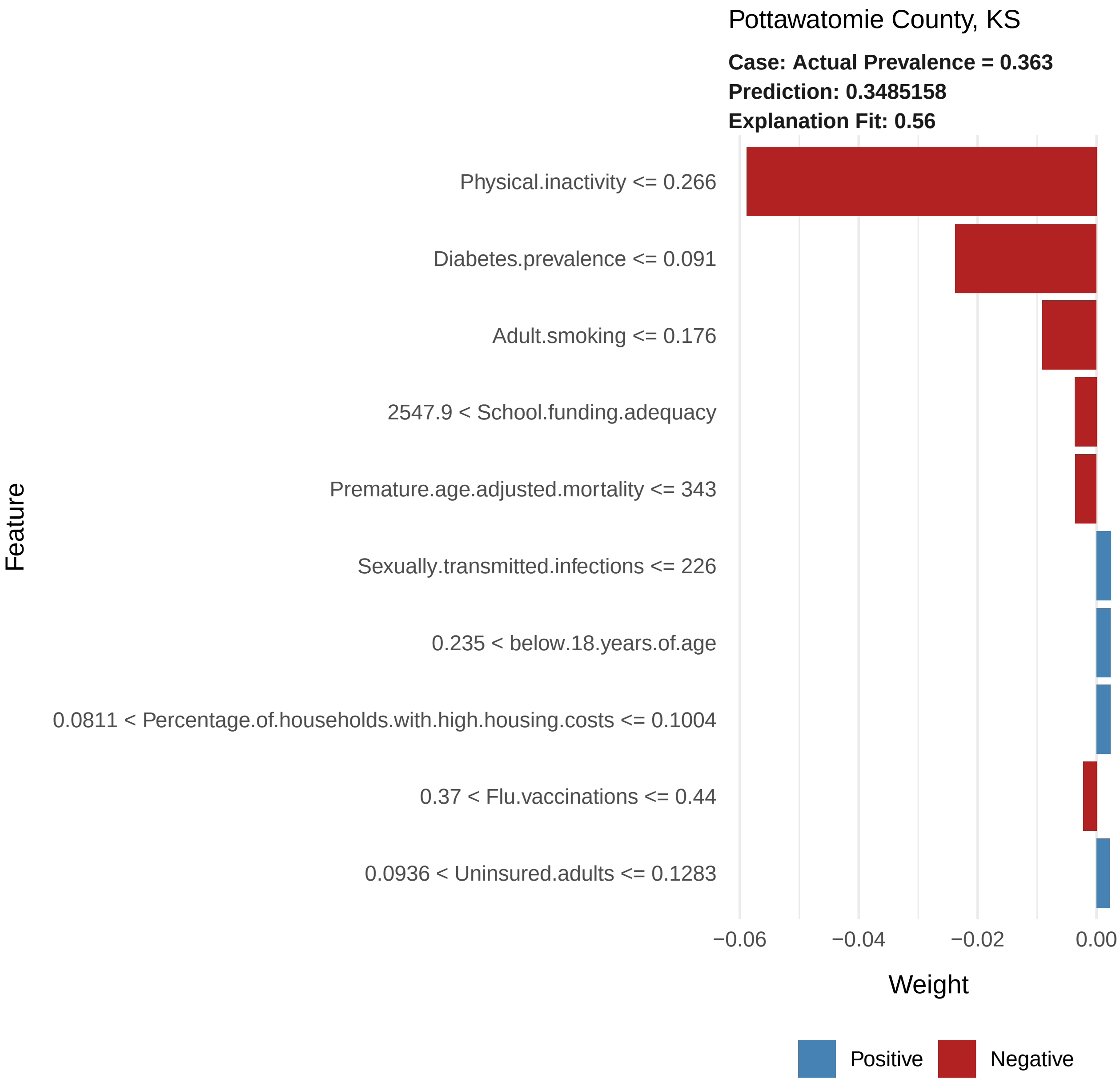
Pottawatomie County, KS
Case: Actual Prevalence = 0.363
Prediction: 0.3485158
Explanation Fit: 0.56
Physical.inactivity <= 0.266
Diabetes.prevalence <= 0.091
Adult.smoking <= 0.176
2547.9 < School.funding.adequacy
Premature.age.adjusted.mortality <= 343
Sexually.transmitted.infections <= 226
0.235 < below.18.years.of.age
0.0811 < Percentage.of.households.with.high.housing.costs <= 0.1004
0.37 < Flu.vaccinations <= 0.44
0.0936 < Uninsured.adults <= 0.1283
Feature
−0.06
−0.04
−0.02
0.00
Weight
Positive
Negative

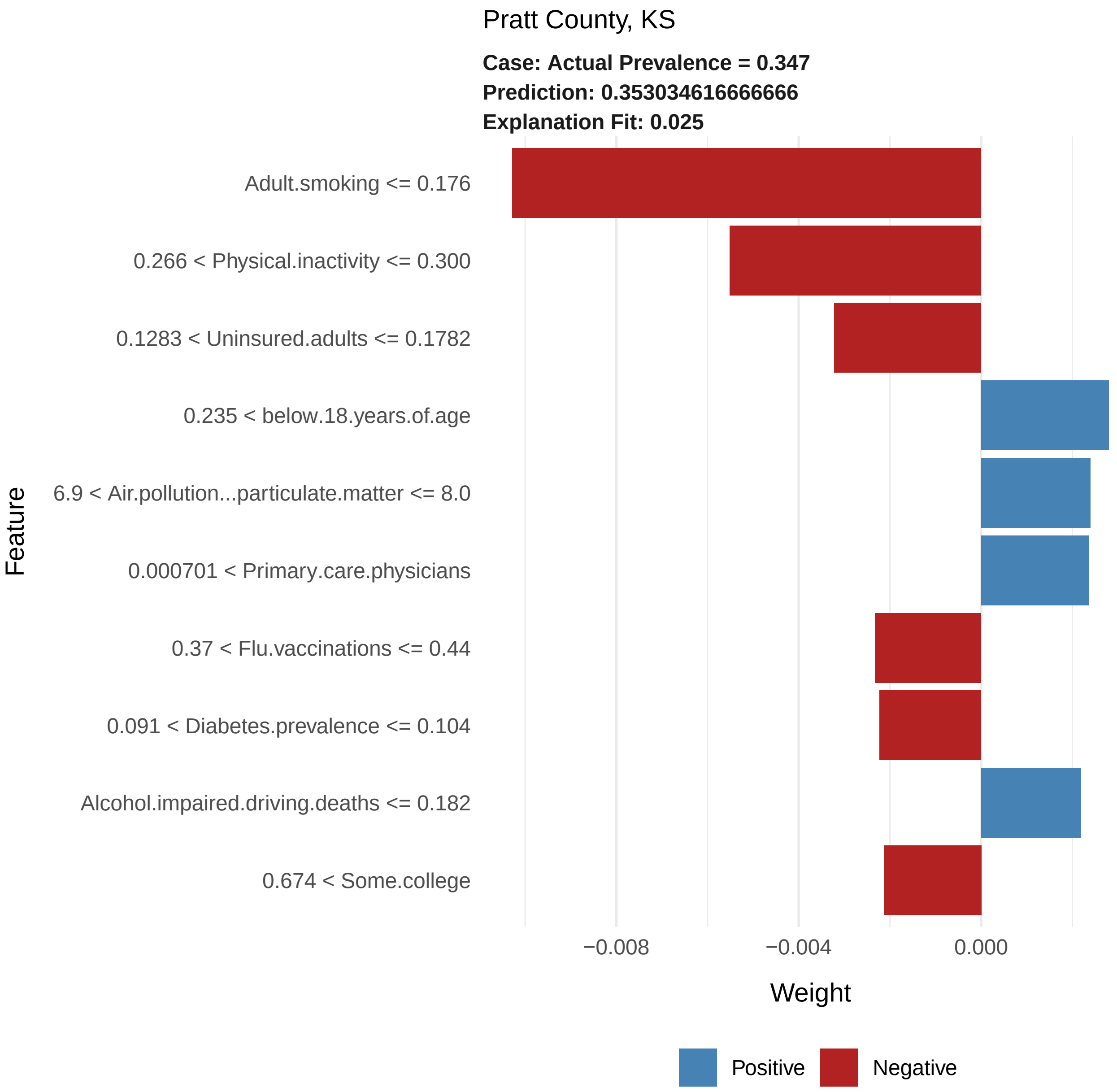
Pratt County, KS
Case: Actual Prevalence = 0.347
Prediction: 0.353034616666666
Explanation Fit: 0.025
Adult.smoking <= 0.176
0.266 < Physical.inactivity <= 0.300
0.1283 < Uninsured.adults <= 0.1782
0.235 < below.18.years.of.age
6.9 < Air.pollution...particulate.matter <= 8.0
0.000701 < Primary.care.physicians
0.37 < Flu.vaccinations <= 0.44
0.091 < Diabetes.prevalence <= 0.104
Alcohol.impaired.driving.deaths <= 0.182
0.674 < Some.college
Feature
−0.008
−0.004
0.000
Weight
Positive
Negative

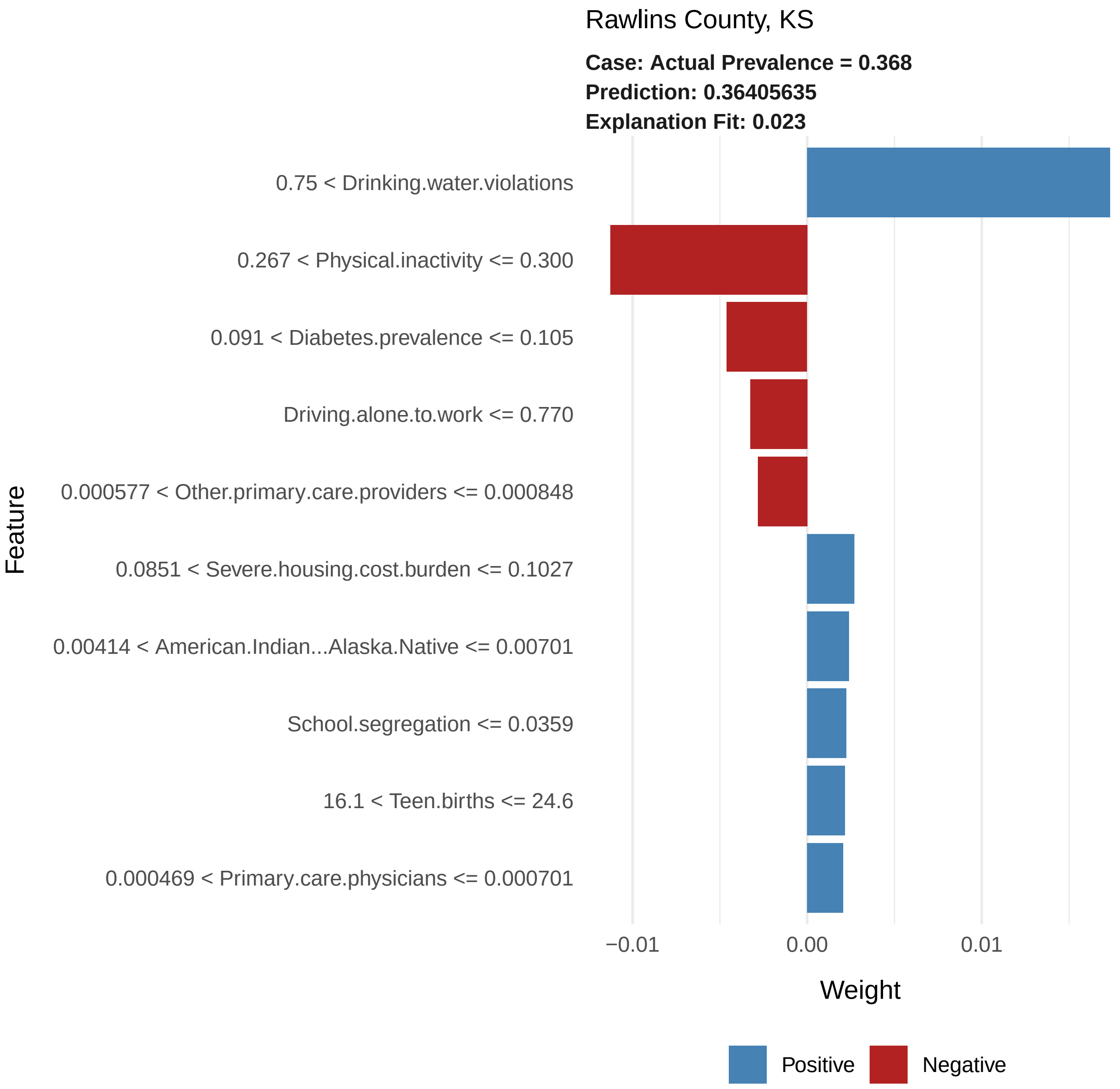

Rawlins County, KS
Case: Actual Prevalence = 0.368
Prediction: 0.36405635
Explanation Fit: 0.023
0.75 < Drinking.water.violations
0.267 < Physical.inactivity <= 0.300
0.091 < Diabetes.prevalence <= 0.105
Driving.alone.to.work <= 0.770
0.000577 < Other.primary.care.providers <= 0.000848
0.0851 < Severe.housing.cost.burden <= 0.1027
0.00414 < American.Indian...Alaska.Native <= 0.00701
School.segregation <= 0.0359
16.1 < Teen.births <= 24.6
0.000469 < Primary.care.physicians <= 0.000701
Feature
−0.01
0.00
0.01
Weight
Positive
Negative

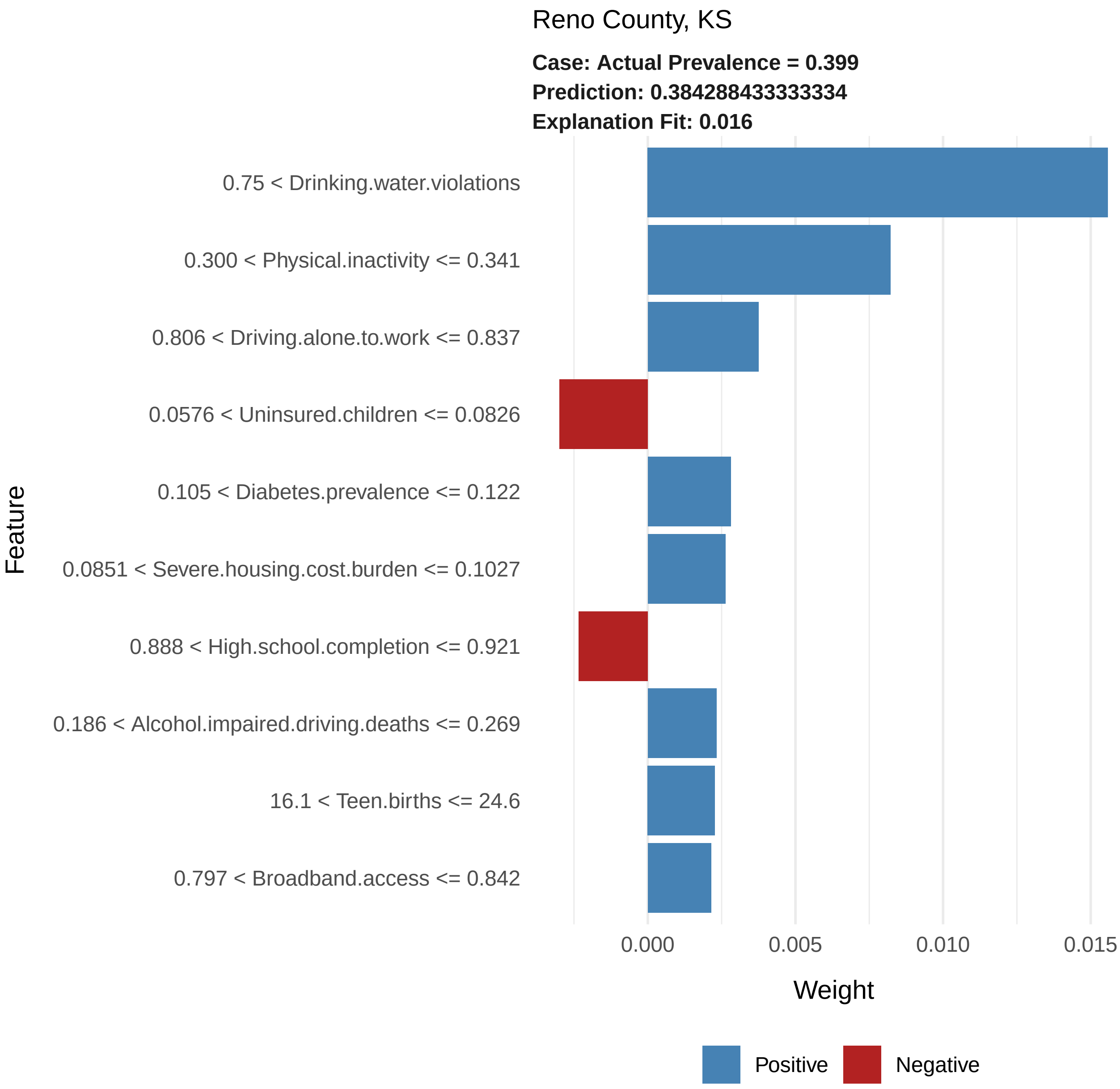

Reno County, KS
Case: Actual Prevalence = 0.399
Prediction: 0.384288433333334
Explanation Fit: 0.016
0.75 < Drinking.water.violations
0.300 < Physical.inactivity <= 0.341
0.806 < Driving.alone.to.work <= 0.837
0.0576 < Uninsured.children <= 0.0826
0.105 < Diabetes.prevalence <= 0.122
0.0851 < Severe.housing.cost.burden <= 0.1027
0.888 < High.school.completion <= 0.921
0.186 < Alcohol.impaired.driving.deaths <= 0.269
16.1 < Teen.births <= 24.6
0.797 < Broadband.access <= 0.842
Feature
0.000
0.005
0.010
0.015
Weight
Positive
Negative

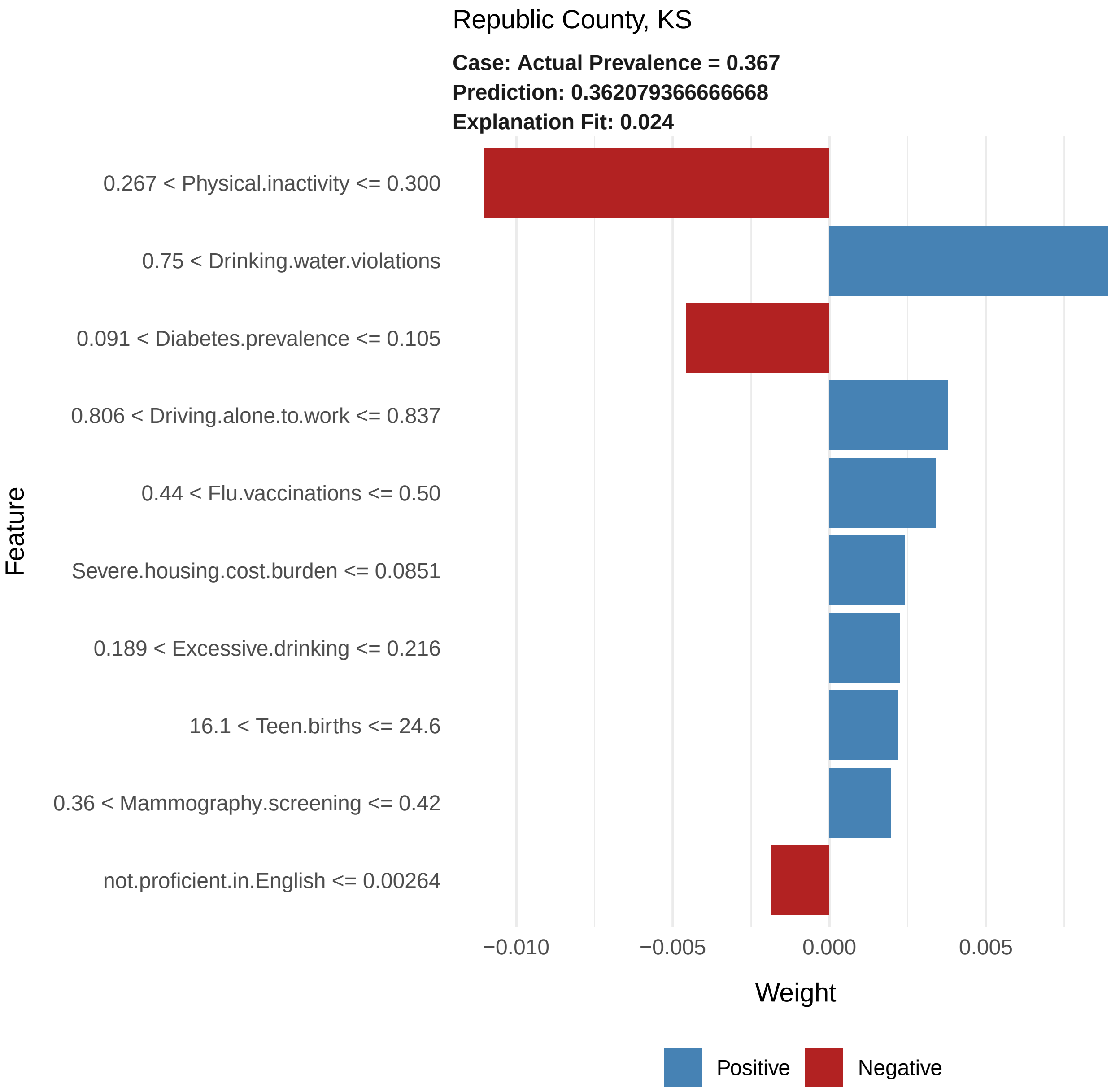

Republic County, KS
Case: Actual Prevalence = 0.367
Prediction: 0.362079366666668
Explanation Fit: 0.024
0.267 < Physical.inactivity <= 0.300
0.75 < Drinking.water.violations
0.091 < Diabetes.prevalence <= 0.105
0.806 < Driving.alone.to.work <= 0.837
0.44 < Flu.vaccinations <= 0.50
Severe.housing.cost.burden <= 0.0851
0.189 < Excessive.drinking <= 0.216
16.1 < Teen.births <= 24.6
0.36 < Mammography.screening <= 0.42
not.proficient.in.English <= 0.00264
Feature
−0.010
−0.005
0.000
0.005
Weight
Positive
Negative

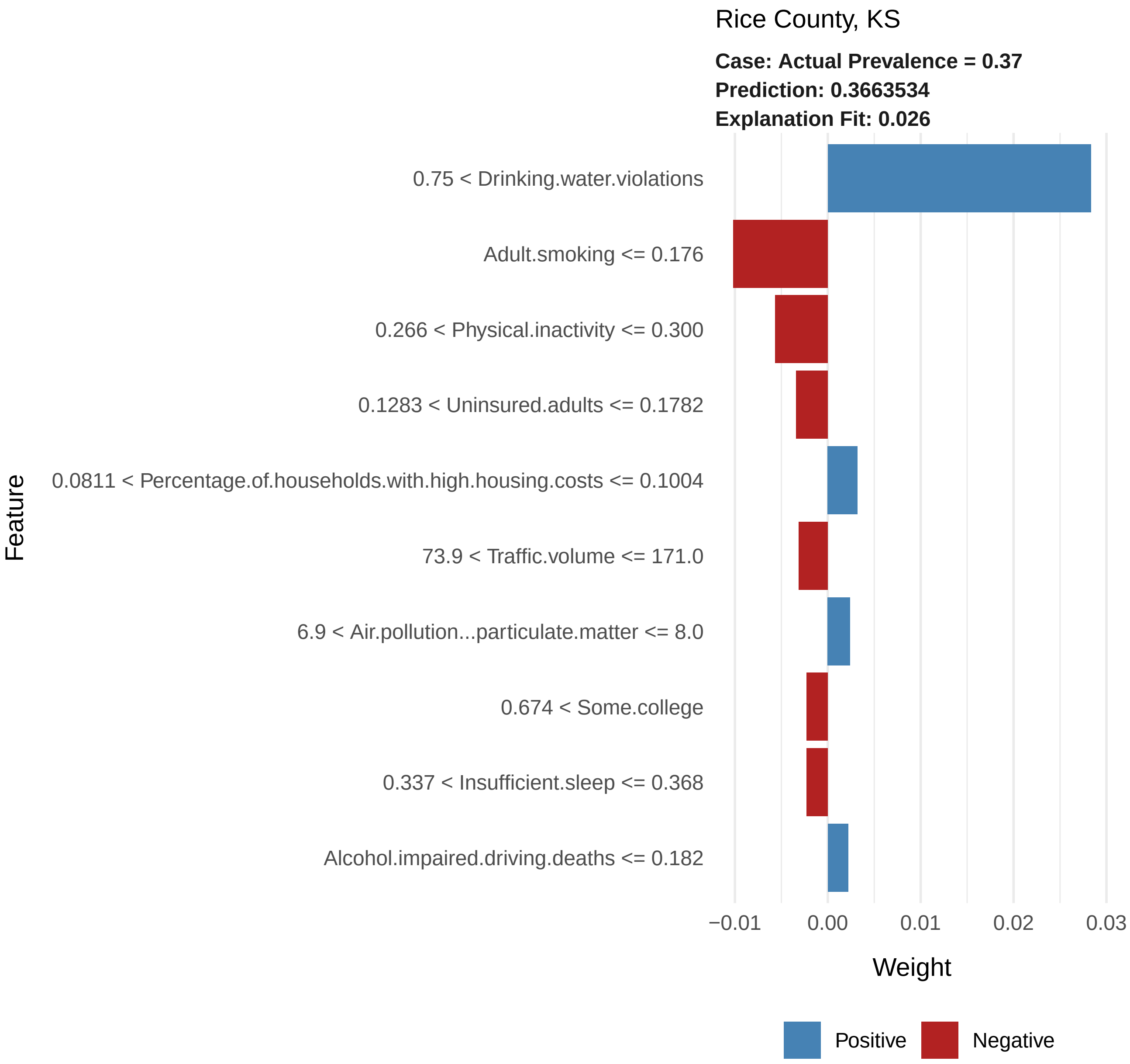

Rice County, KS
Case: Actual Prevalence = 0.37
Prediction: 0.3663534
Explanation Fit: 0.026
0.75 < Drinking.water.violations
Adult.smoking <= 0.176
0.266 < Physical.inactivity <= 0.300
0.1283 < Uninsured.adults <= 0.1782
0.0811 < Percentage.of.households.with.high.housing.costs <= 0.1004
73.9 < Traffic.volume <= 171.0
6.9 < Air.pollution...particulate.matter <= 8.0
0.674 < Some.college
0.337 < Insufficient.sleep <= 0.368
Alcohol.impaired.driving.deaths <= 0.182
Feature
−0.01
0.00
0.01
0.02
0.03
Weight
Positive
Negative

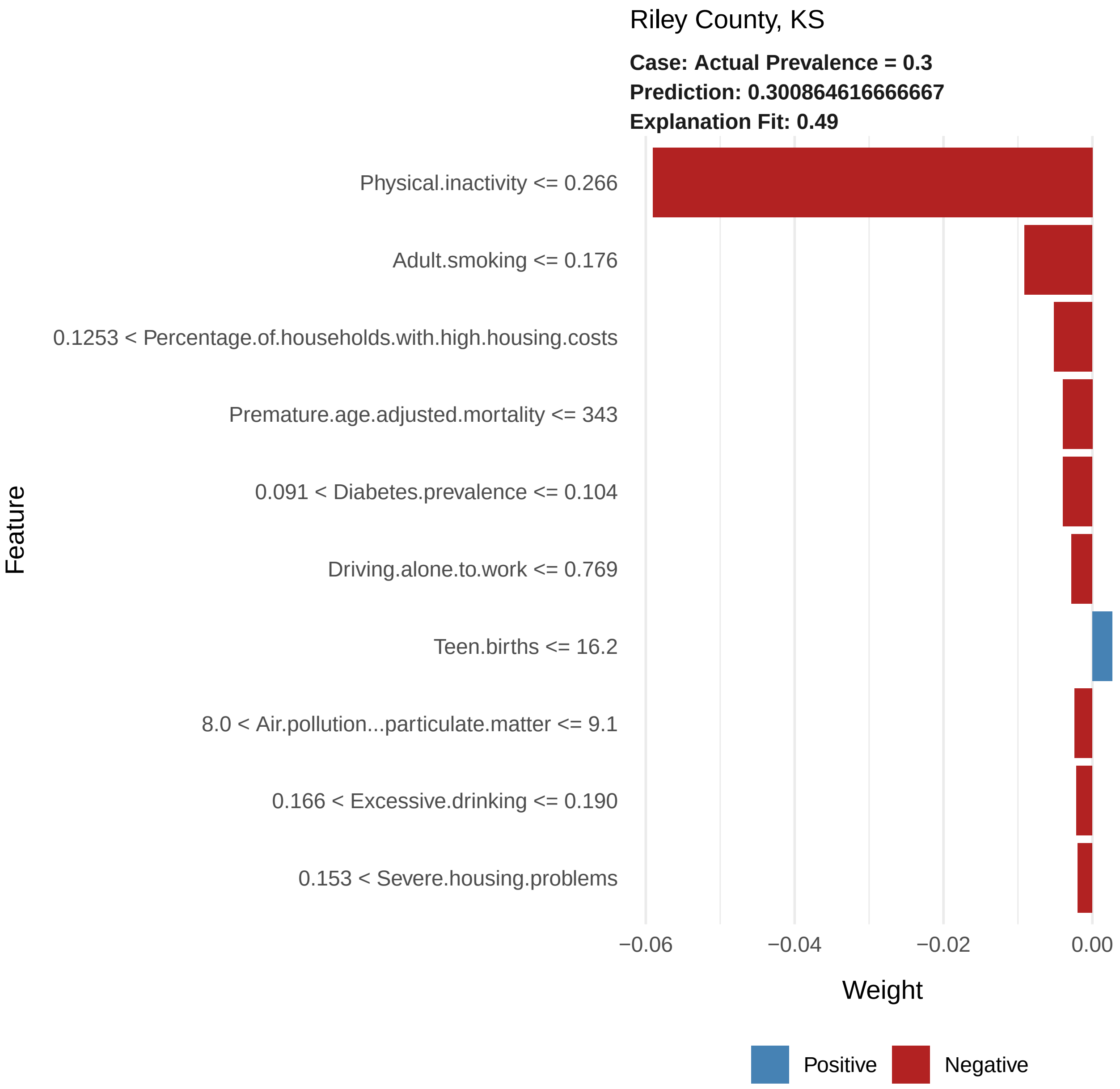
Riley County, KS
Case: Actual Prevalence = 0.3
Prediction: 0.300864616666667
Explanation Fit: 0.49
Physical.inactivity <= 0.266
Adult.smoking <= 0.176
0.1253 < Percentage.of.households.with.high.housing.costs
Premature.age.adjusted.mortality <= 343
0.091 < Diabetes.prevalence <= 0.104
Driving.alone.to.work <= 0.769
Teen.births <= 16.2
8.0 < Air.pollution...particulate.matter <= 9.1
0.166 < Excessive.drinking <= 0.190
0.153 < Severe.housing.problems
Feature
−0.06
−0.04
−0.02
0.00
Weight
Positive
Negative

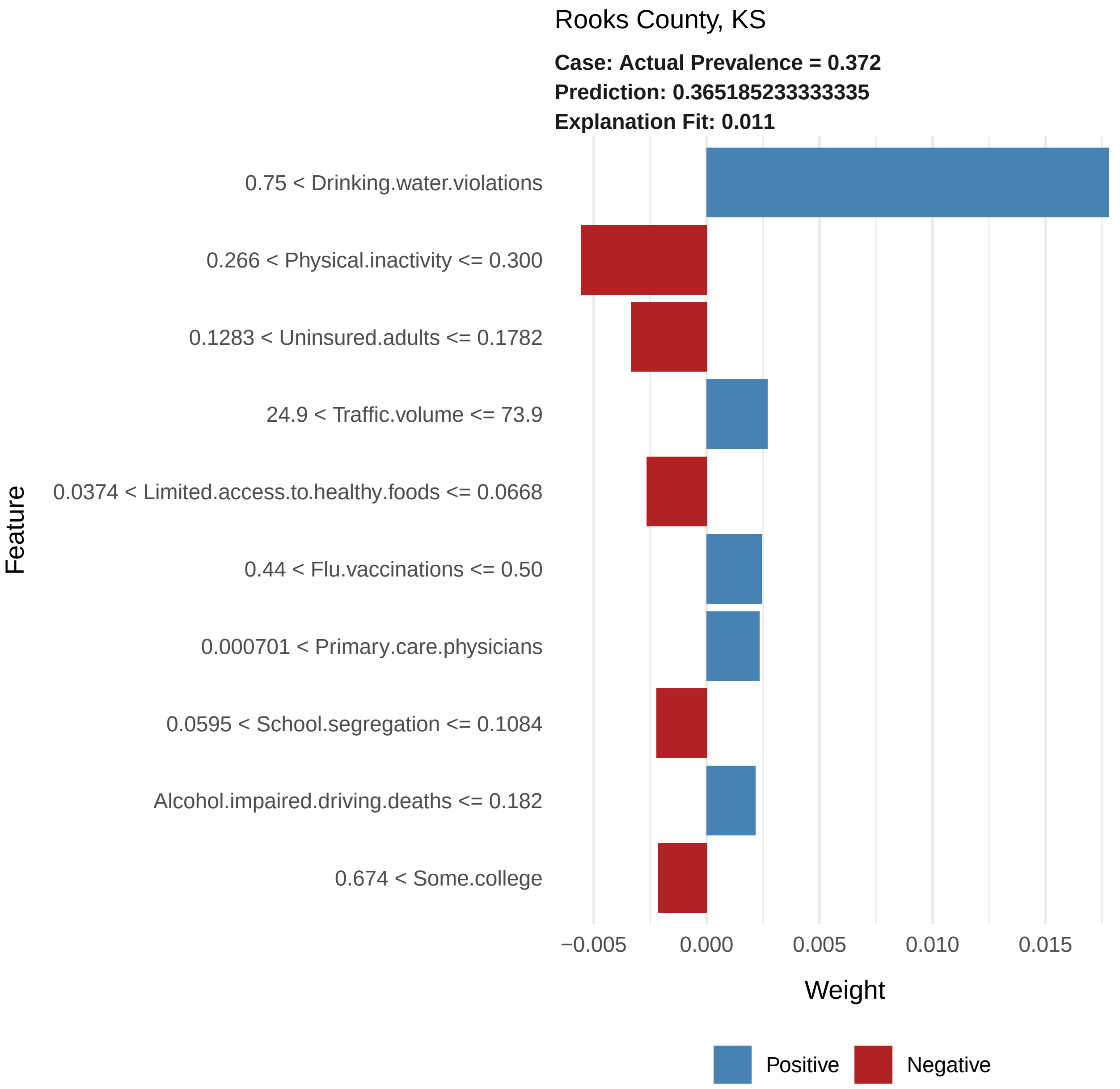
Rooks County, KS
Case: Actual Prevalence = 0.372
Prediction: 0.365185233333335
Explanation Fit: 0.011
0.75 < Drinking.water.violations
0.266 < Physical.inactivity <= 0.300
0.1283 < Uninsured.adults <= 0.1782
24.9 < Traffic.volume <= 73.9
0.0374 < Limited.access.to.healthy.foods <= 0.0668
0.44 < Flu.vaccinations <= 0.50
0.000701 < Primary.care.physicians
0.0595 < School.segregation <= 0.1084
Alcohol.impaired.driving.deaths <= 0.182
0.674 < Some.college
Feature
−0.005
0.000
0.005
0.010
0.015
Weight
Positive
Negative

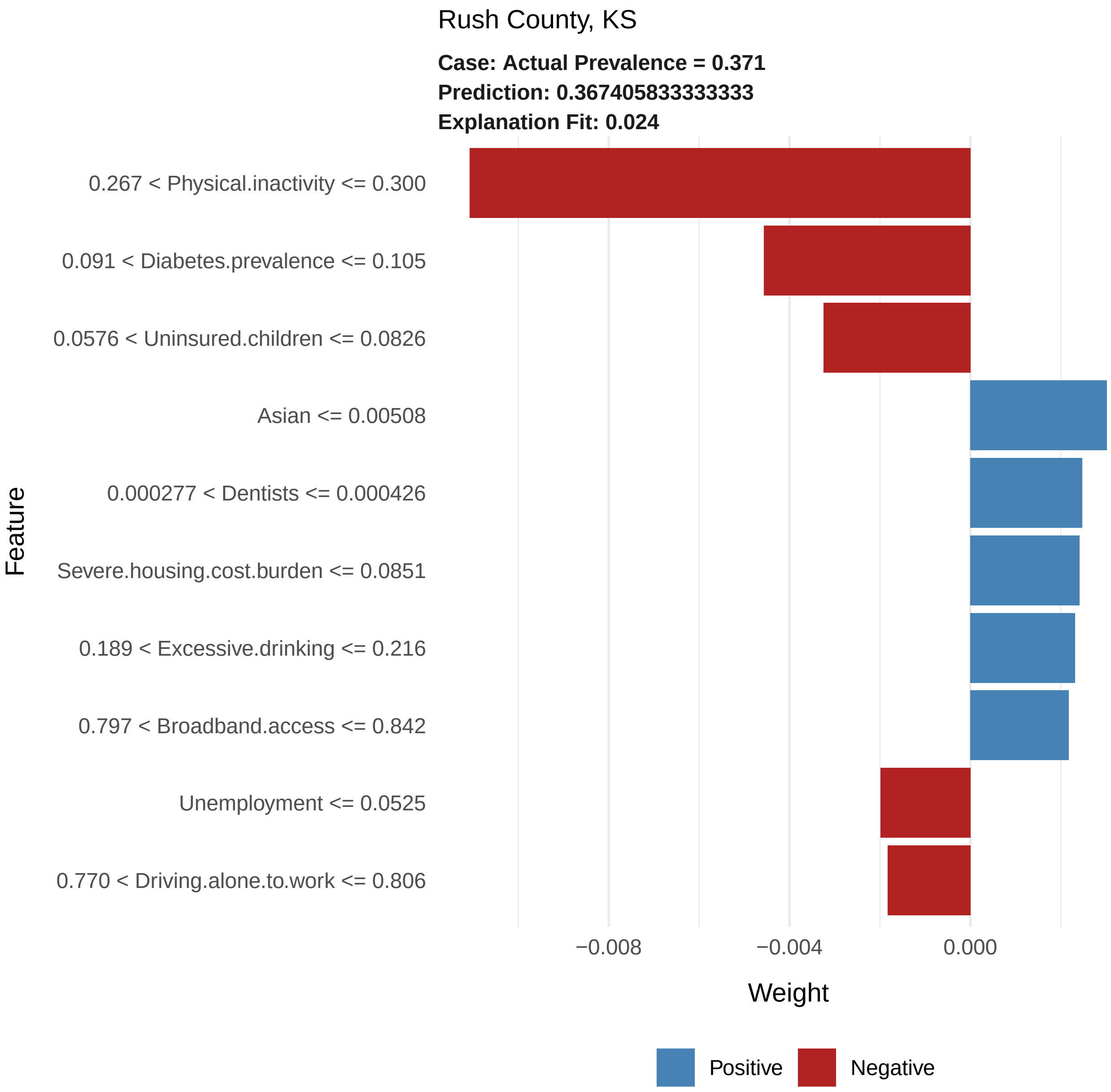

Rush County, KS
Case: Actual Prevalence = 0.371
Prediction: 0.367405833333333
Explanation Fit: 0.024
0.267 < Physical.inactivity <= 0.300
0.091 < Diabetes.prevalence <= 0.105
0.0576 < Uninsured.children <= 0.0826
Asian <= 0.00508
0.000277 < Dentists <= 0.000426
Severe.housing.cost.burden <= 0.0851
0.189 < Excessive.drinking <= 0.216
0.797 < Broadband.access <= 0.842
Unemployment <= 0.0525
0.770 < Driving.alone.to.work <= 0.806
Feature
−0.008
−0.004
0.000
Weight
Positive
Negative

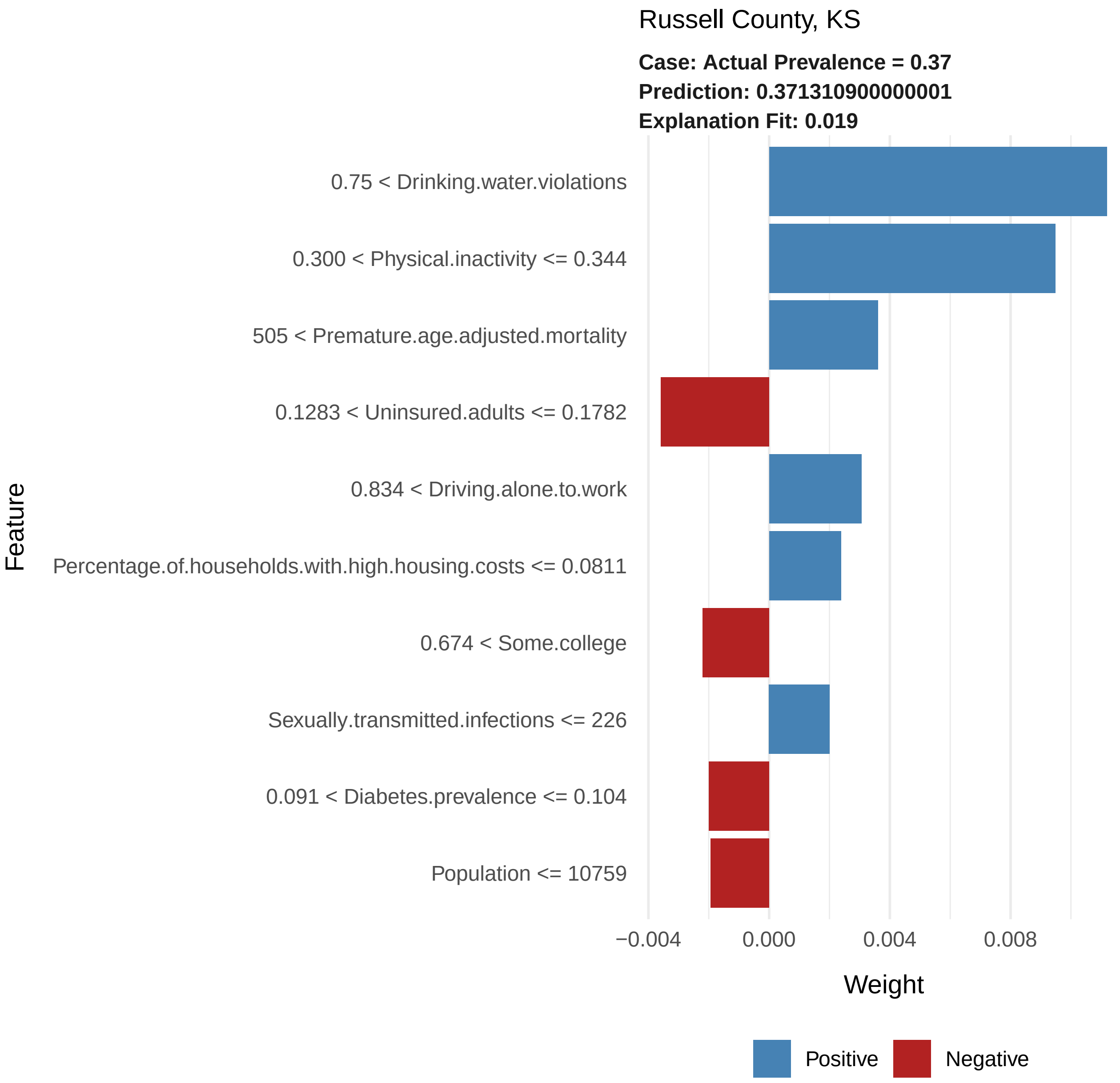

Russell County, KS
Case: Actual Prevalence = 0.37
Prediction: 0.371310900000001
Explanation Fit: 0.019
0.75 < Drinking.water.violations
0.300 < Physical.inactivity <= 0.344
505 < Premature.age.adjusted.mortality
0.1283 < Uninsured.adults <= 0.1782
0.834 < Driving.alone.to.work
Percentage.of.households.with.high.housing.costs <= 0.0811
0.674 < Some.college
Sexually.transmitted.infections <= 226
0.091 < Diabetes.prevalence <= 0.104
Population <= 10759
Feature
−0.004
0.000
0.004
0.008
Weight
Positive
Negative

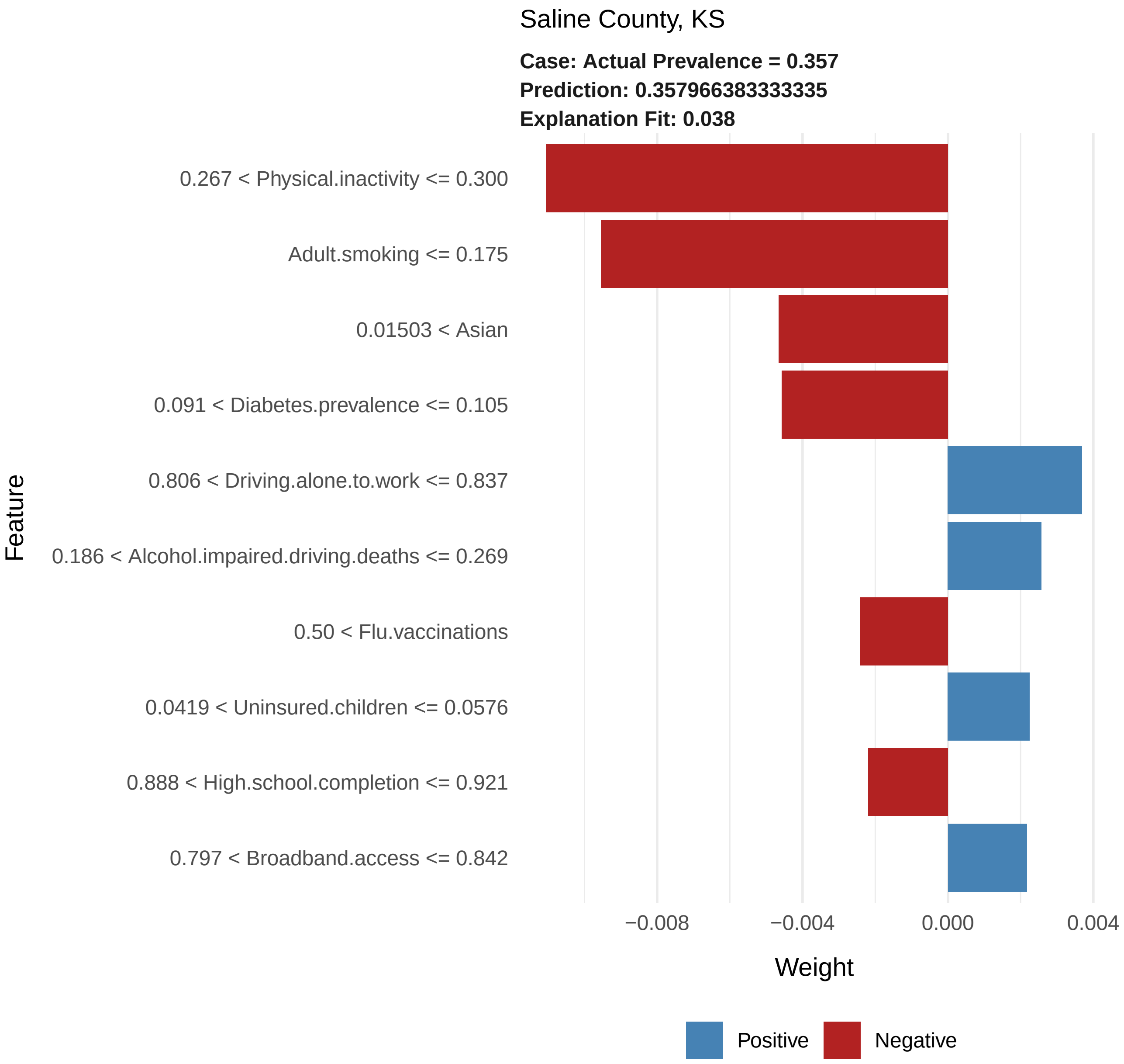

Saline County, KS
Case: Actual Prevalence = 0.357
Prediction: 0.357966383333335
Explanation Fit: 0.038
0.267 < Physical.inactivity <= 0.300
Adult.smoking <= 0.175
0.01503 < Asian
0.091 < Diabetes.prevalence <= 0.105
0.806 < Driving.alone.to.work <= 0.837
0.186 < Alcohol.impaired.driving.deaths <= 0.269
0.50 < Flu.vaccinations
0.0419 < Uninsured.children <= 0.0576
0.888 < High.school.completion <= 0.921
0.797 < Broadband.access <= 0.842
Feature
−0.008
−0.004
0.000
0.004
Weight
Positive
Negative

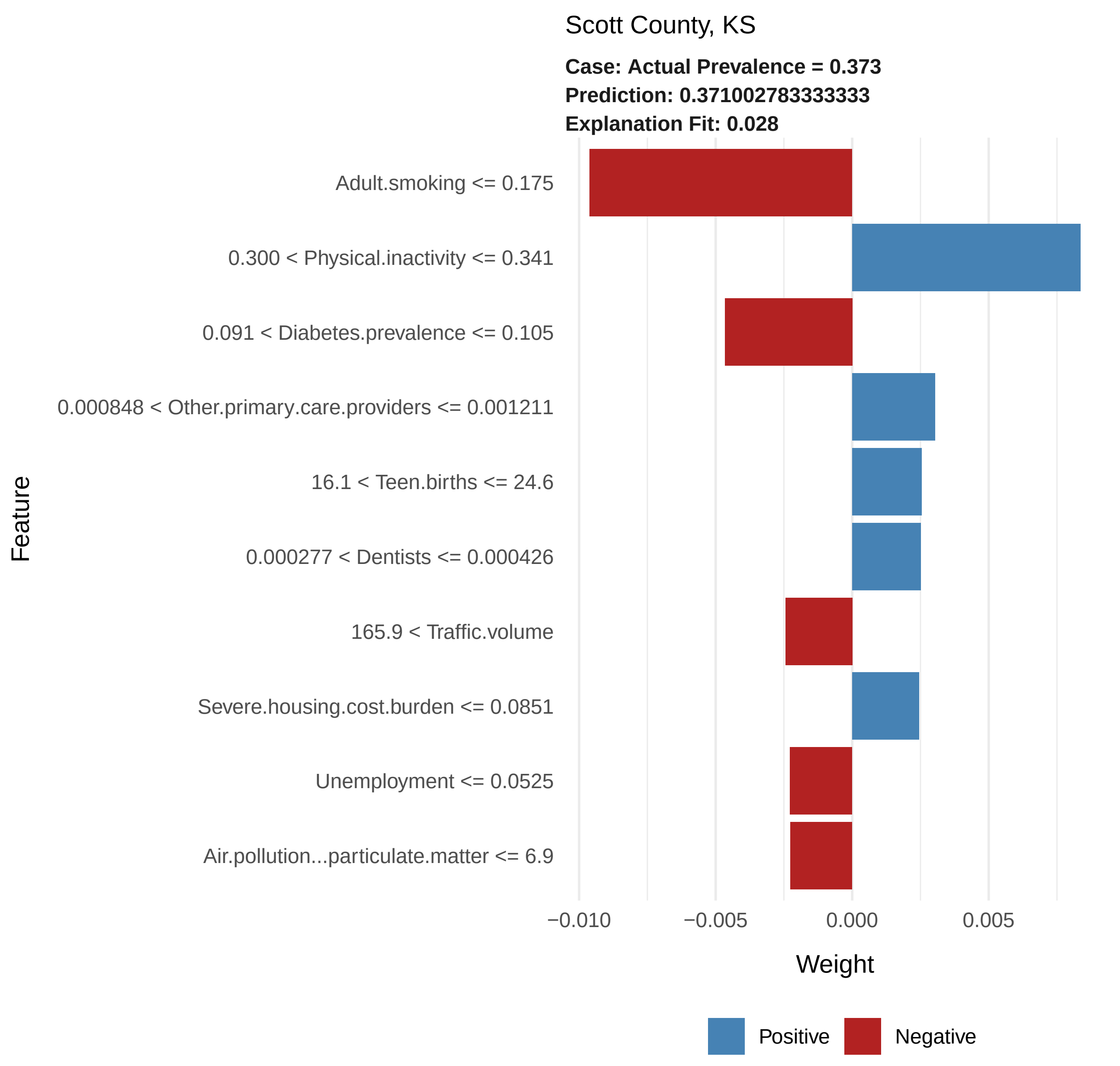
Scott County, KS
Case: Actual Prevalence = 0.373
Prediction: 0.371002783333333
Explanation Fit: 0.028
Adult.smoking <= 0.175
0.300 < Physical.inactivity <= 0.341
0.091 < Diabetes.prevalence <= 0.105
0.000848 < Other.primary.care.providers <= 0.001211
16.1 < Teen.births <= 24.6
0.000277 < Dentists <= 0.000426
165.9 < Traffic.volume
Severe.housing.cost.burden <= 0.0851
Unemployment <= 0.0525
Air.pollution...particulate.matter <= 6.9
Feature
−0.010
−0.005
0.000
0.005
Weight
Positive
Negative

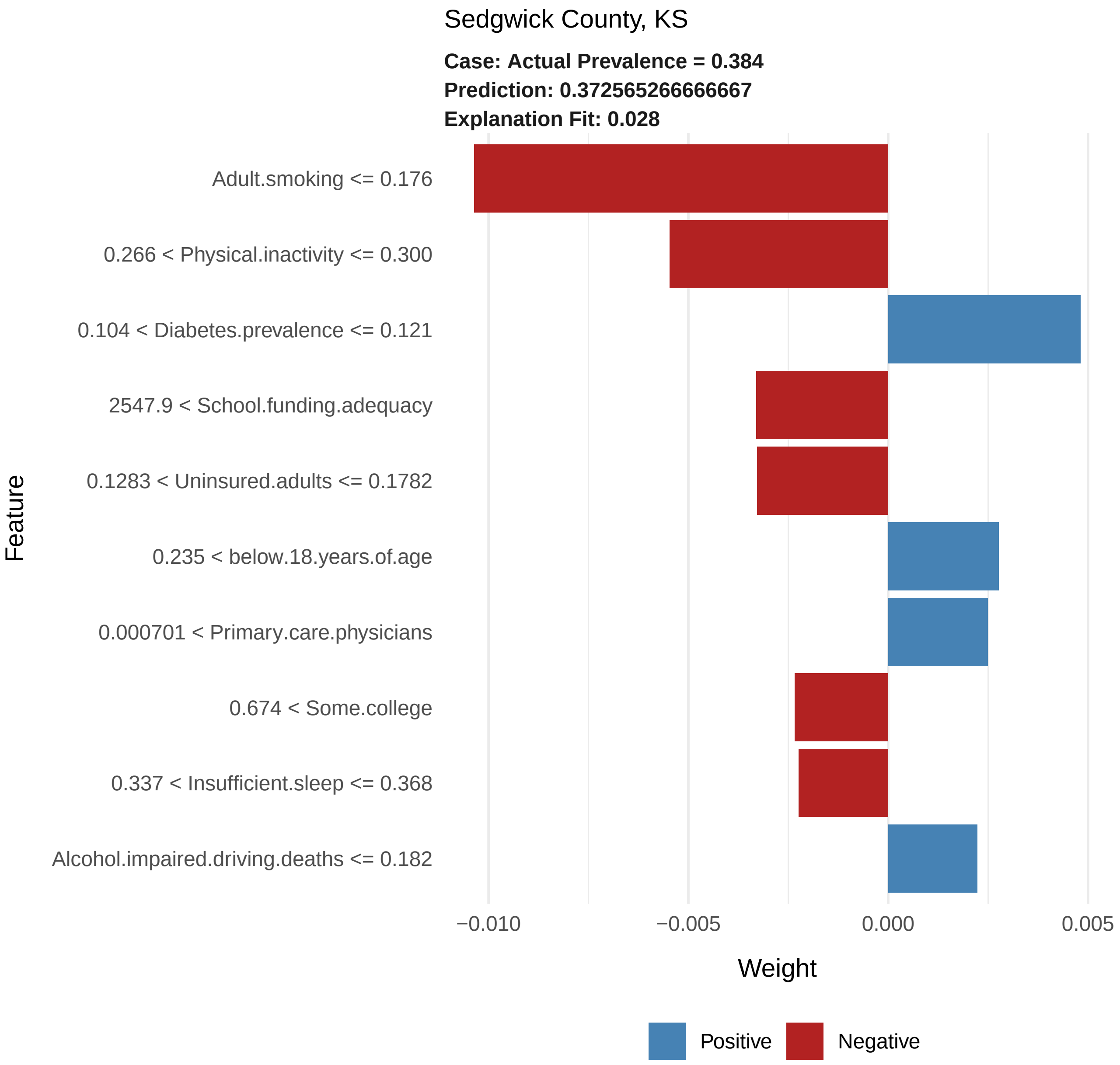

Sedgwick County, KS
Case: Actual Prevalence = 0.384
Prediction: 0.372565266666667
Explanation Fit: 0.028
Adult.smoking <= 0.176
0.266 < Physical.inactivity <= 0.300
0.104 < Diabetes.prevalence <= 0.121
2547.9 < School.funding.adequacy
0.1283 < Uninsured.adults <= 0.1782
0.235 < below.18.years.of.age
0.000701 < Primary.care.physicians
0.674 < Some.college
0.337 < Insufficient.sleep <= 0.368
Alcohol.impaired.driving.deaths <= 0.182
Feature
−0.010
−0.005
0.000
0.005
Weight
Positive
Negative

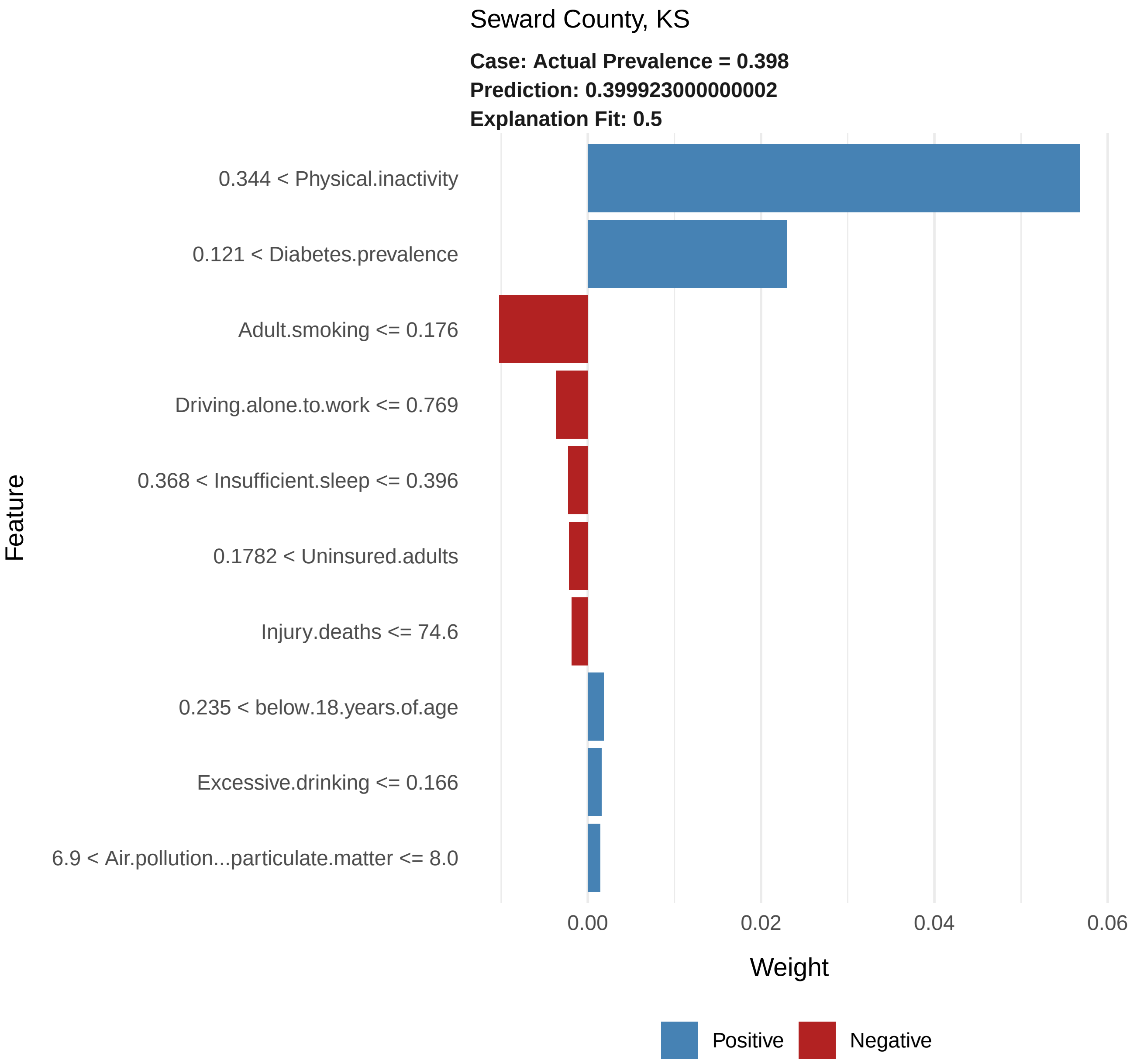

Seward County, KS
Case: Actual Prevalence = 0.398
Prediction: 0.399923000000002
Explanation Fit: 0.5
0.344 < Physical.inactivity
0.121 < Diabetes.prevalence
Adult.smoking <= 0.176
Driving.alone.to.work <= 0.769
0.368 < Insufficient.sleep <= 0.396
0.1782 < Uninsured.adults
Injury.deaths <= 74.6
0.235 < below.18.years.of.age
Excessive.drinking <= 0.166
6.9 < Air.pollution...particulate.matter <= 8.0
Feature
0.00
0.02
0.04
0.06
Weight
Positive
Negative

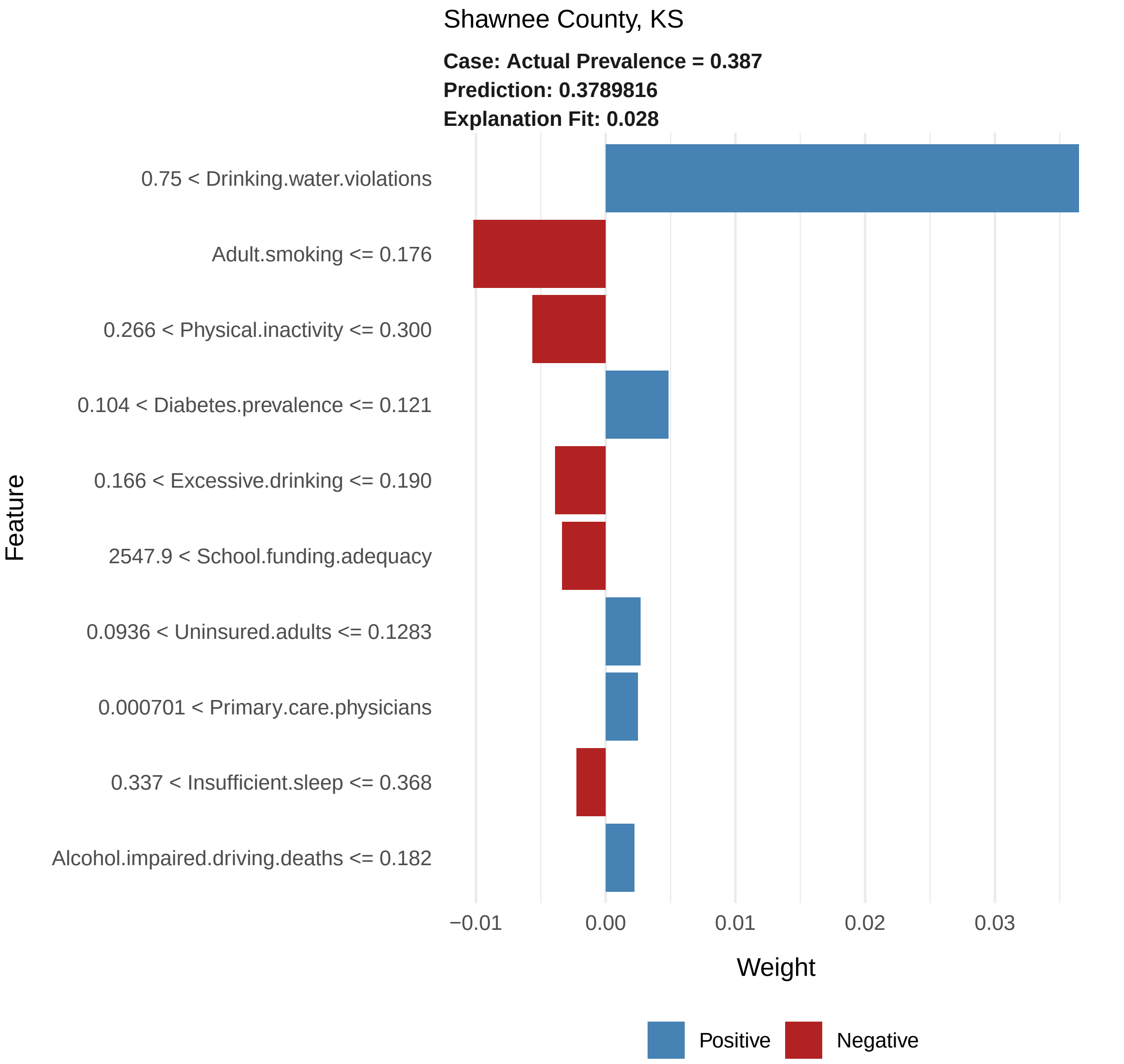
Shawnee County, KS
Case: Actual Prevalence = 0.387
Prediction: 0.3789816
Explanation Fit: 0.028
0.75 < Drinking.water.violations
Adult.smoking <= 0.176
0.266 < Physical.inactivity <= 0.300
0.104 < Diabetes.prevalence <= 0.121
0.166 < Excessive.drinking <= 0.190
2547.9 < School.funding.adequacy
0.0936 < Uninsured.adults <= 0.1283
0.000701 < Primary.care.physicians
0.337 < Insufficient.sleep <= 0.368
Alcohol.impaired.driving.deaths <= 0.182
Feature
−0.01
0.00
0.01
0.02
0.03
Weight
Positive
Negative

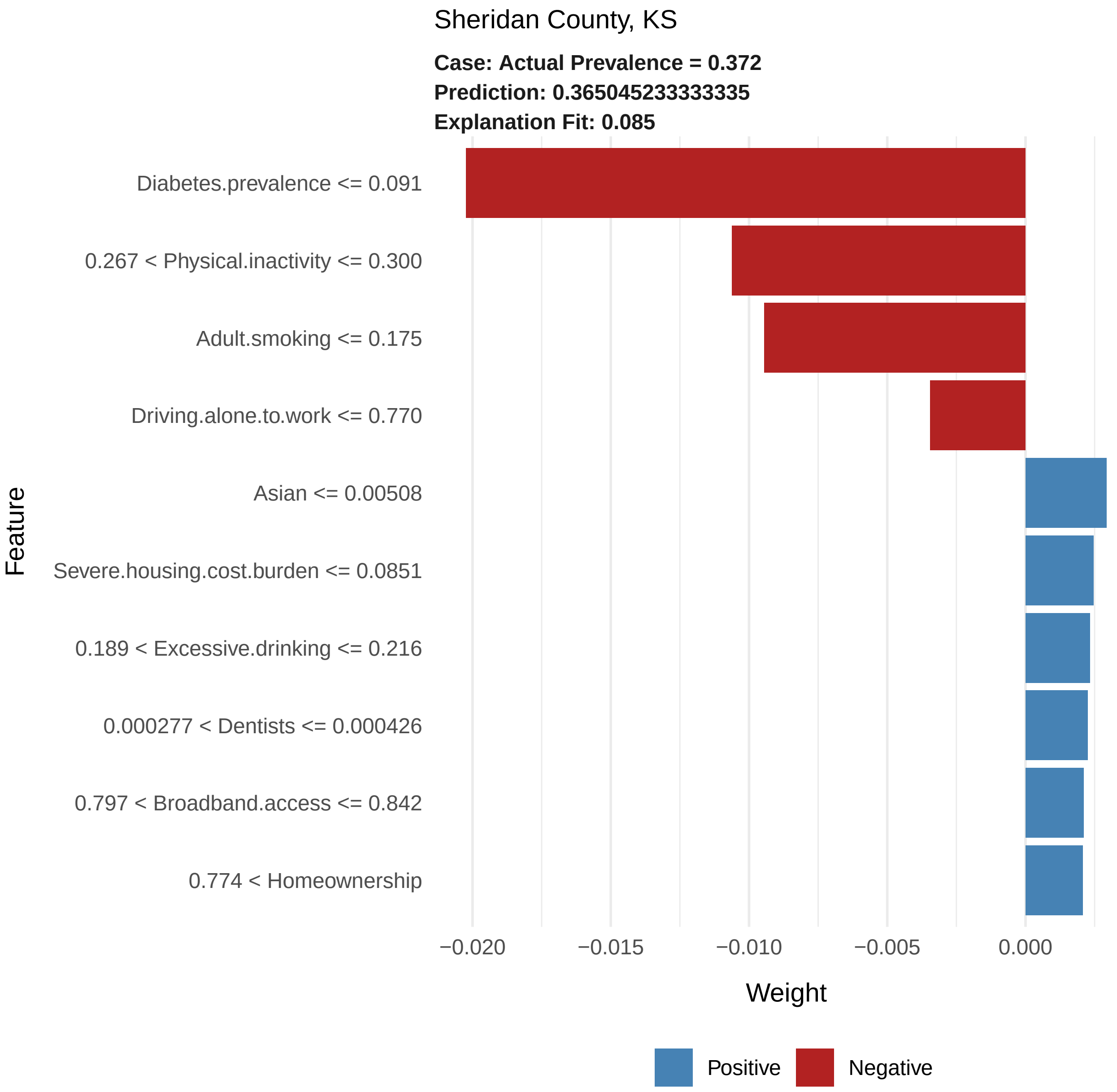

Sheridan County, KS
Case: Actual Prevalence = 0.372
Prediction: 0.365045233333335
Explanation Fit: 0.085
Diabetes.prevalence <= 0.091
0.267 < Physical.inactivity <= 0.300
Adult.smoking <= 0.175
Driving.alone.to.work <= 0.770
Asian <= 0.00508
Severe.housing.cost.burden <= 0.0851
0.189 < Excessive.drinking <= 0.216
0.000277 < Dentists <= 0.000426
0.797 < Broadband.access <= 0.842
0.774 < Homeownership
Feature
−0.020
−0.015
−0.010
−0.005
0.000
Weight
Positive
Negative

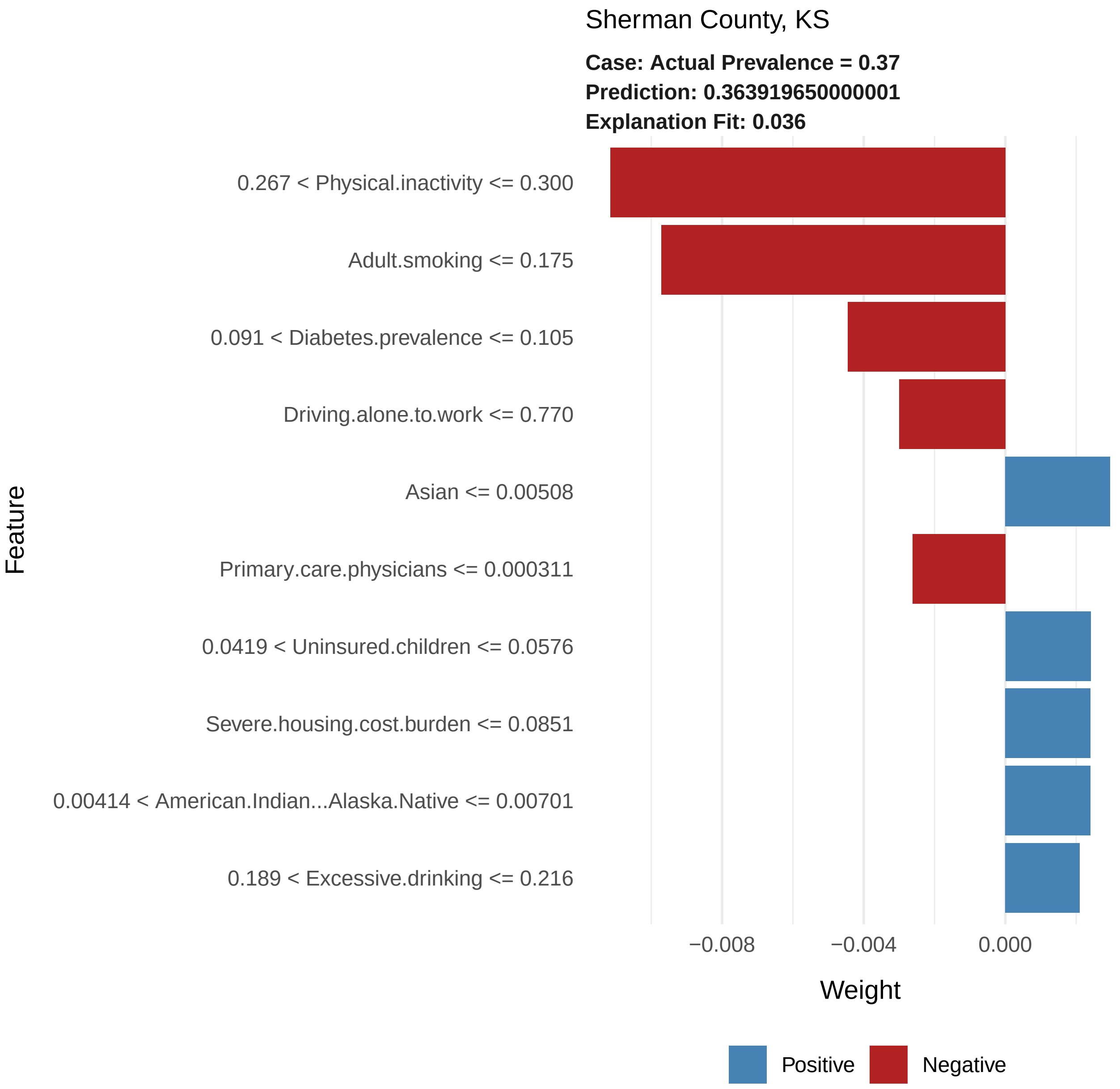
Sherman County, KS
Case: Actual Prevalence = 0.37
Prediction: 0.363919650000001
Explanation Fit: 0.036
0.267 < Physical.inactivity <= 0.300
Adult.smoking <= 0.175
0.091 < Diabetes.prevalence <= 0.105
Driving.alone.to.work <= 0.770
Asian <= 0.00508
Primary.care.physicians <= 0.000311
0.0419 < Uninsured.children <= 0.0576
Severe.housing.cost.burden <= 0.0851
0.00414 < American.Indian...Alaska.Native <= 0.00701
0.189 < Excessive.drinking <= 0.216
Feature
−0.008
−0.004
0.000
Weight
Positive
Negative

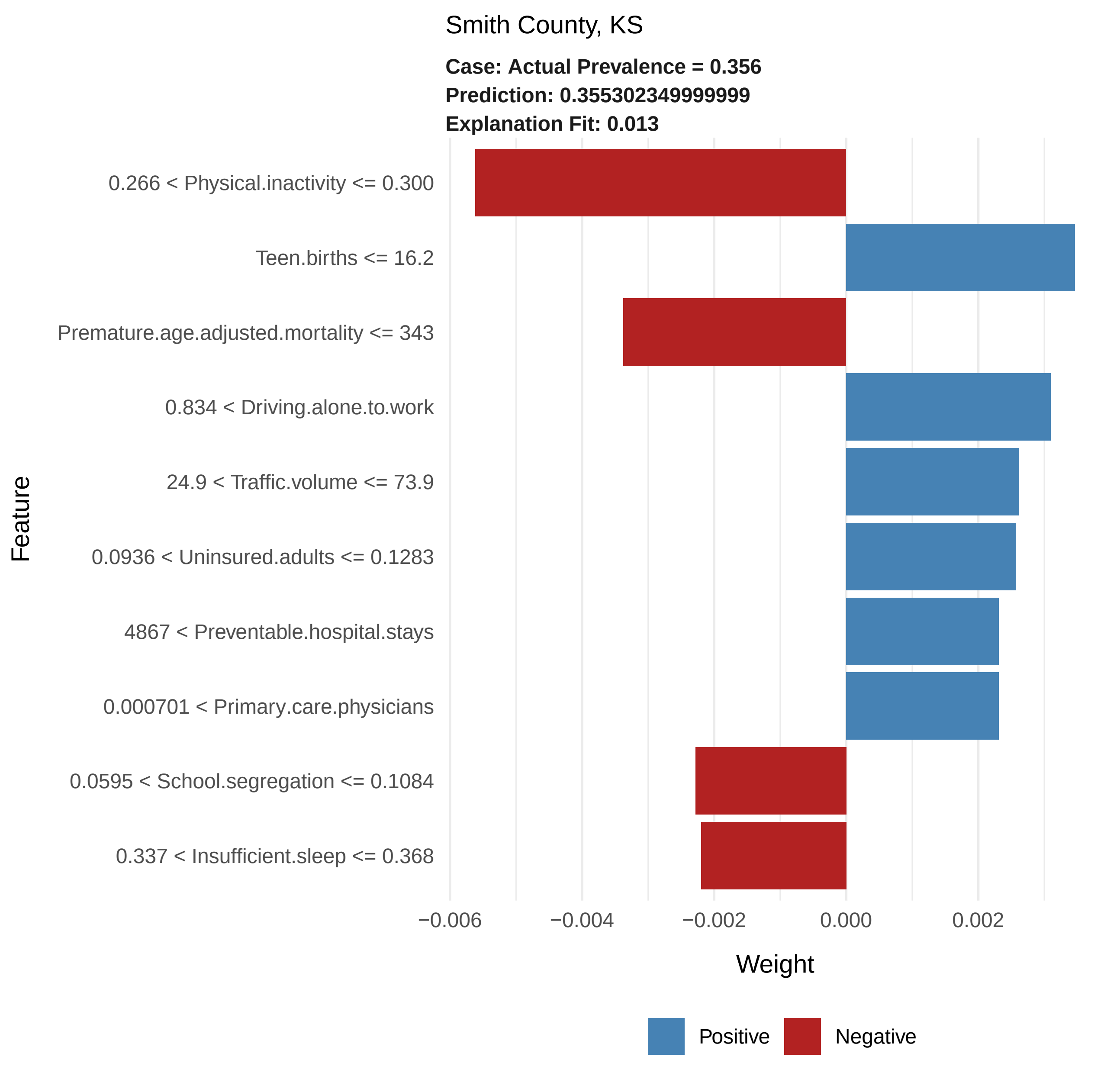
Smith County, KS
Case: Actual Prevalence = 0.356
Prediction: 0.355302349999999
Explanation Fit: 0.013
0.266 < Physical.inactivity <= 0.300
Teen.births <= 16.2
Premature.age.adjusted.mortality <= 343
0.834 < Driving.alone.to.work
24.9 < Traffic.volume <= 73.9
0.0936 < Uninsured.adults <= 0.1283
4867 < Preventable.hospital.stays
0.000701 < Primary.care.physicians
0.0595 < School.segregation <= 0.1084
0.337 < Insufficient.sleep <= 0.368
Feature
−0.006
−0.004
−0.002
0.000
0.002
Weight
Positive
Negative

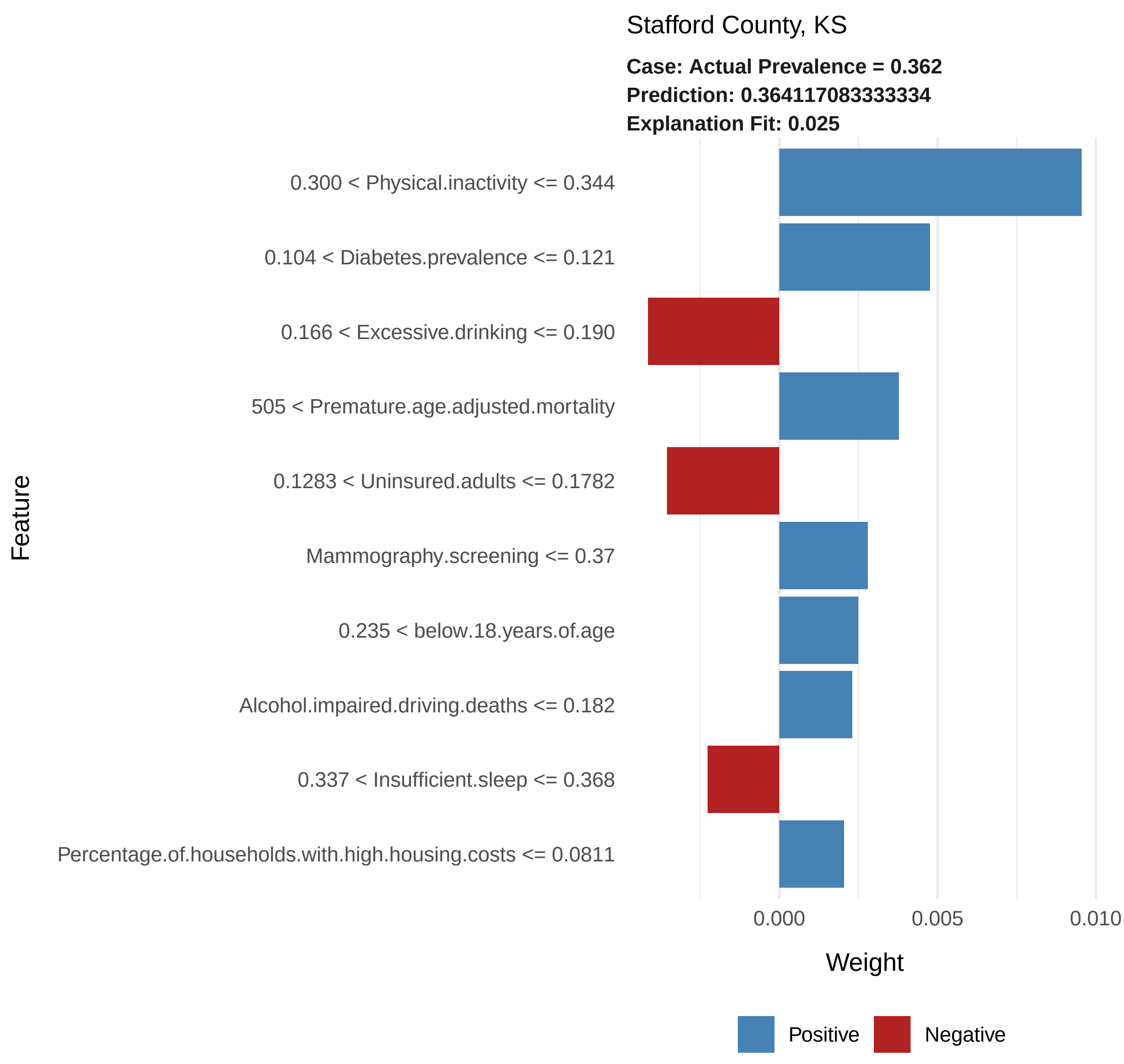

Stafford County, KS
Case: Actual Prevalence = 0.362
Prediction: 0.364117083333334
Explanation Fit: 0.025
0.300 < Physical.inactivity <= 0.344
0.104 < Diabetes.prevalence <= 0.121
0.166 < Excessive.drinking <= 0.190
505 < Premature.age.adjusted.mortality
0.1283 < Uninsured.adults <= 0.1782
Mammography.screening <= 0.37
0.235 < below.18.years.of.age
Alcohol.impaired.driving.deaths <= 0.182
0.337 < Insufficient.sleep <= 0.368
Percentage.of.households.with.high.housing.costs <= 0.0811
Feature
0.000
0.005
0.010
Weight
Positive
Negative

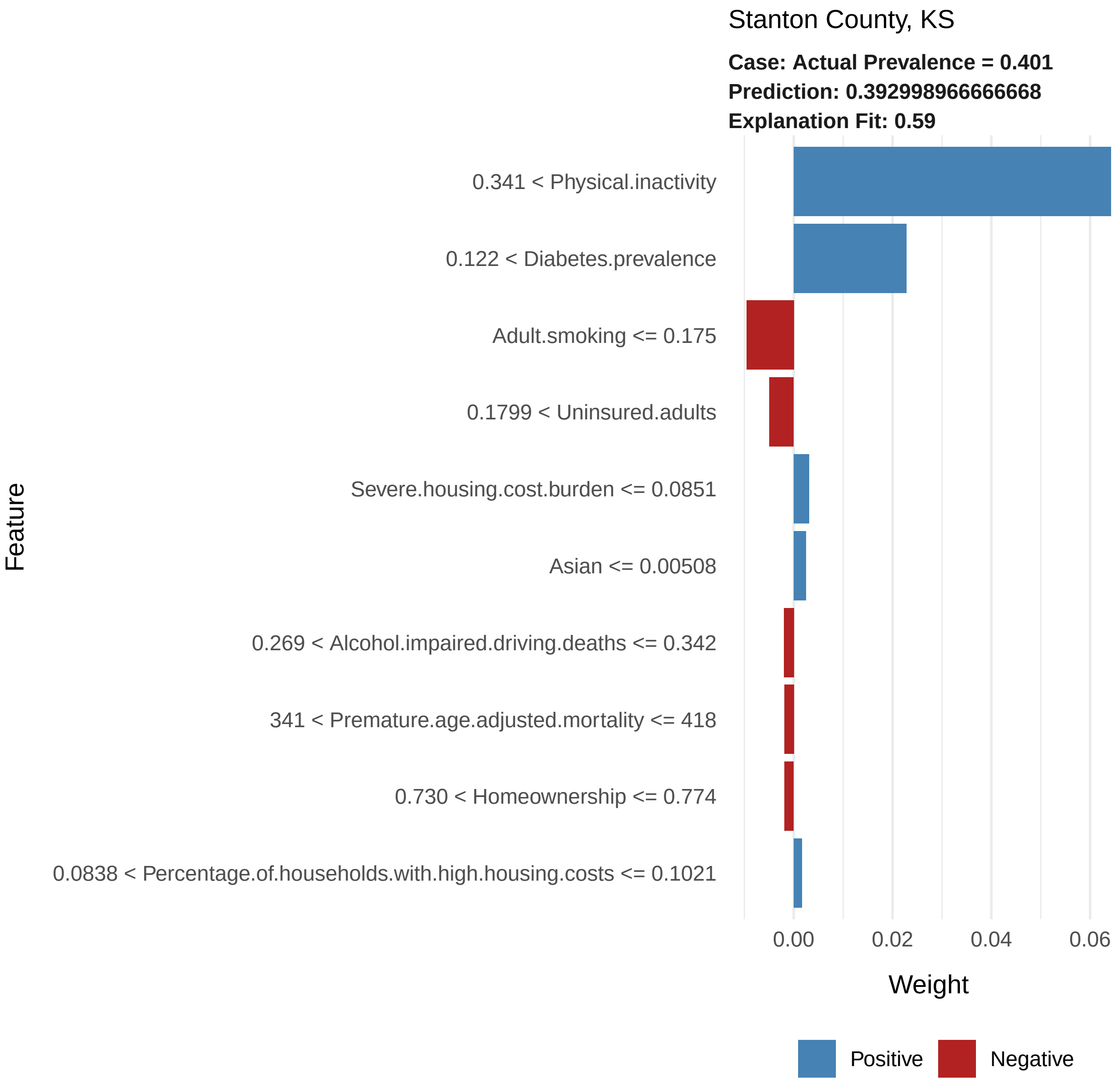
Stanton County, KS
Case: Actual Prevalence = 0.401
Prediction: 0.392998966666668
Explanation Fit: 0.59
0.341 < Physical.inactivity
0.122 < Diabetes.prevalence
Adult.smoking <= 0.175
0.1799 < Uninsured.adults
Severe.housing.cost.burden <= 0.0851
Asian <= 0.00508
0.269 < Alcohol.impaired.driving.deaths <= 0.342
341 < Premature.age.adjusted.mortality <= 418
0.730 < Homeownership <= 0.774
0.0838 < Percentage.of.households.with.high.housing.costs <= 0.1021
Feature
0.00
0.02
0.04
0.06
Weight
Positive
Negative

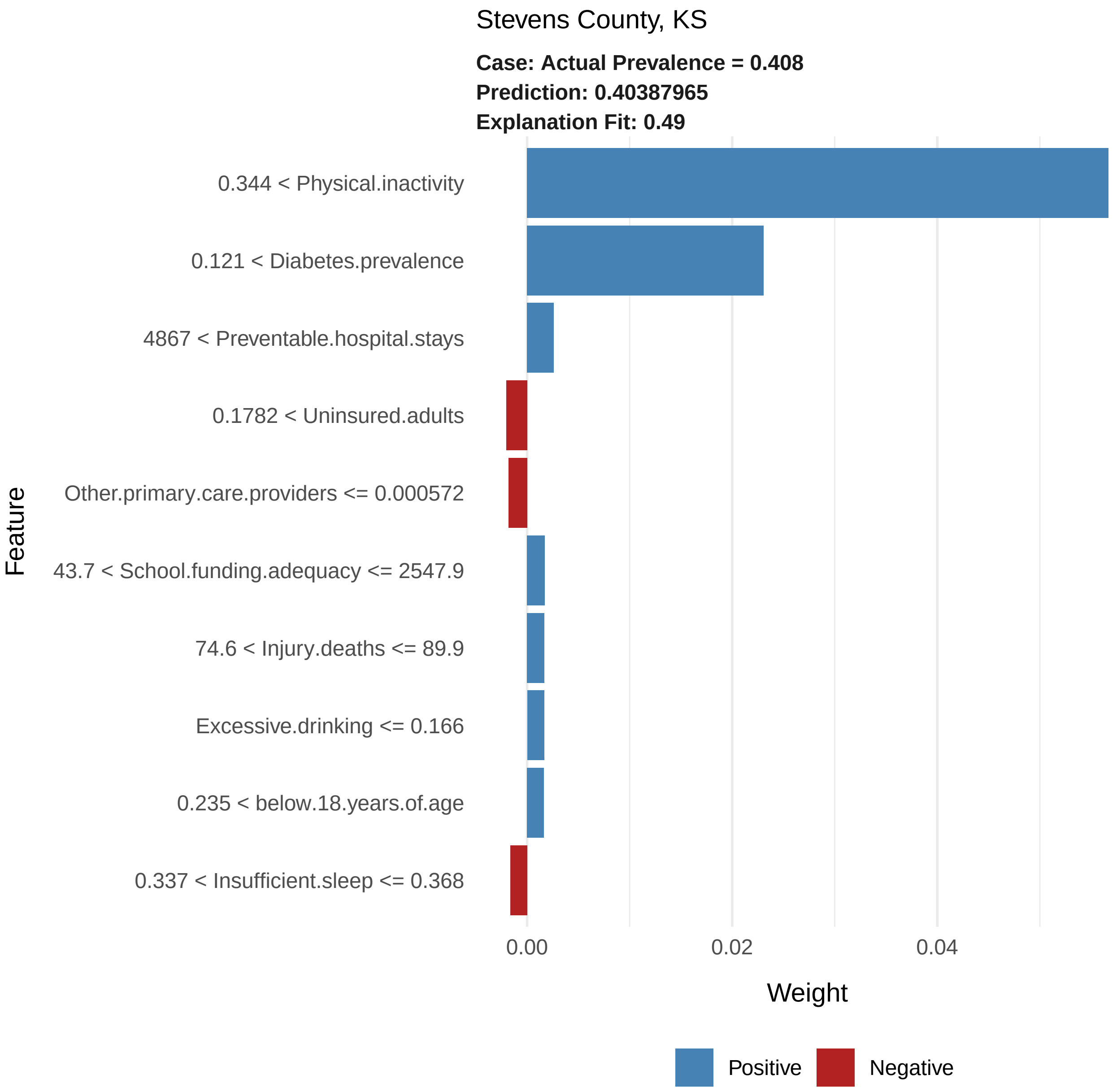

Stevens County, KS
Case: Actual Prevalence = 0.408
Prediction: 0.40387965
Explanation Fit: 0.49
0.344 < Physical.inactivity
0.121 < Diabetes.prevalence
4867 < Preventable.hospital.stays
0.1782 < Uninsured.adults
Other.primary.care.providers <= 0.000572
43.7 < School.funding.adequacy <= 2547.9
74.6 < Injury.deaths <= 89.9
Excessive.drinking <= 0.166
0.235 < below.18.years.of.age
0.337 < Insufficient.sleep <= 0.368
Feature
0.00
0.02
0.04
Weight
Positive
Negative

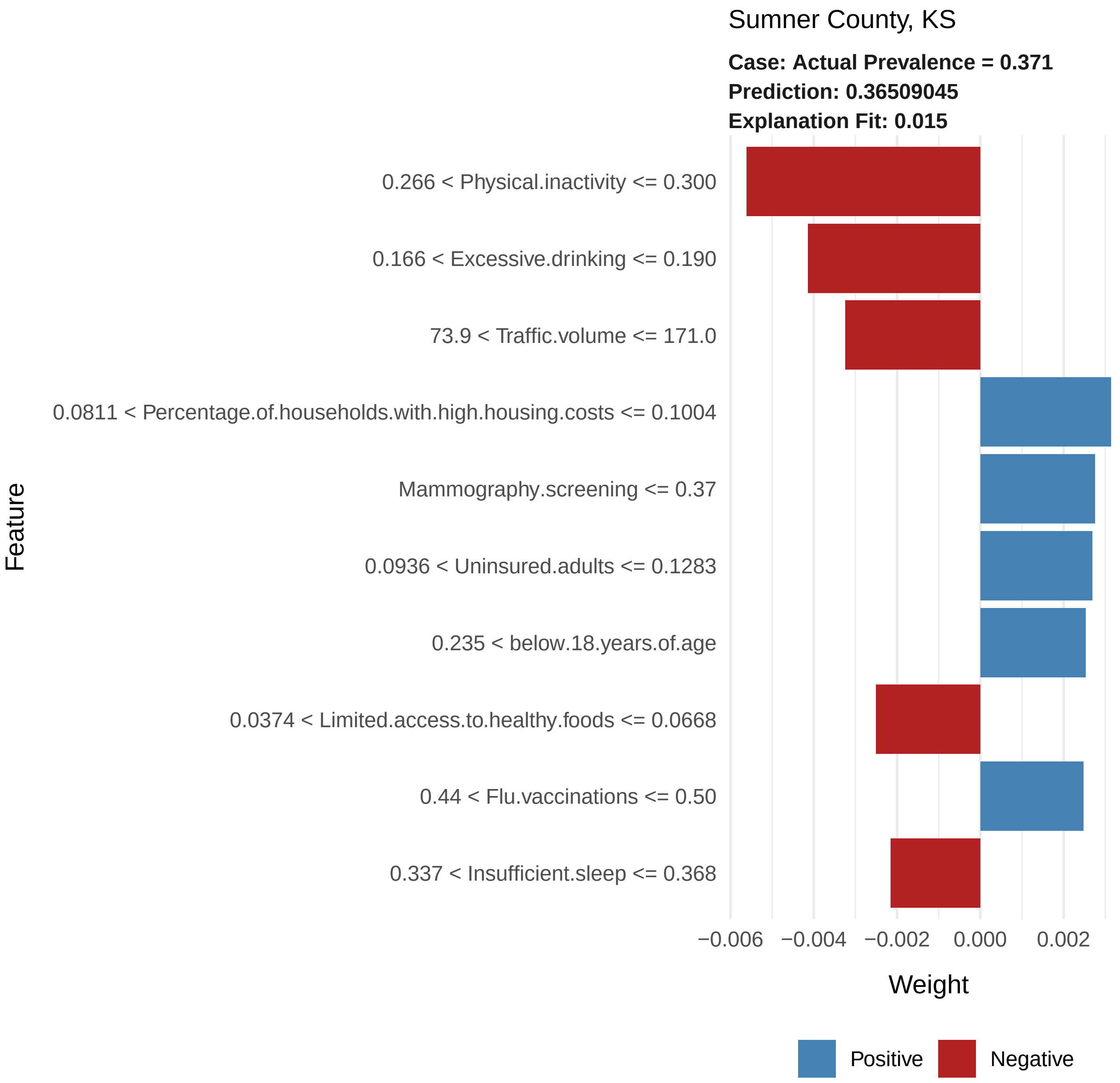

Sumner County, KS
Case: Actual Prevalence = 0.371
Prediction: 0.36509045
Explanation Fit: 0.015
0.266 < Physical.inactivity <= 0.300
0.166 < Excessive.drinking <= 0.190
73.9 < Traffic.volume <= 171.0
0.0811 < Percentage.of.households.with.high.housing.costs <= 0.1004
Mammography.screening <= 0.37
0.0936 < Uninsured.adults <= 0.1283
0.235 < below.18.years.of.age
0.0374 < Limited.access.to.healthy.foods <= 0.0668
0.44 < Flu.vaccinations <= 0.50
0.337 < Insufficient.sleep <= 0.368
Feature
−0.006
−0.004
−0.002
0.000
0.002
Weight
Positive
Negative

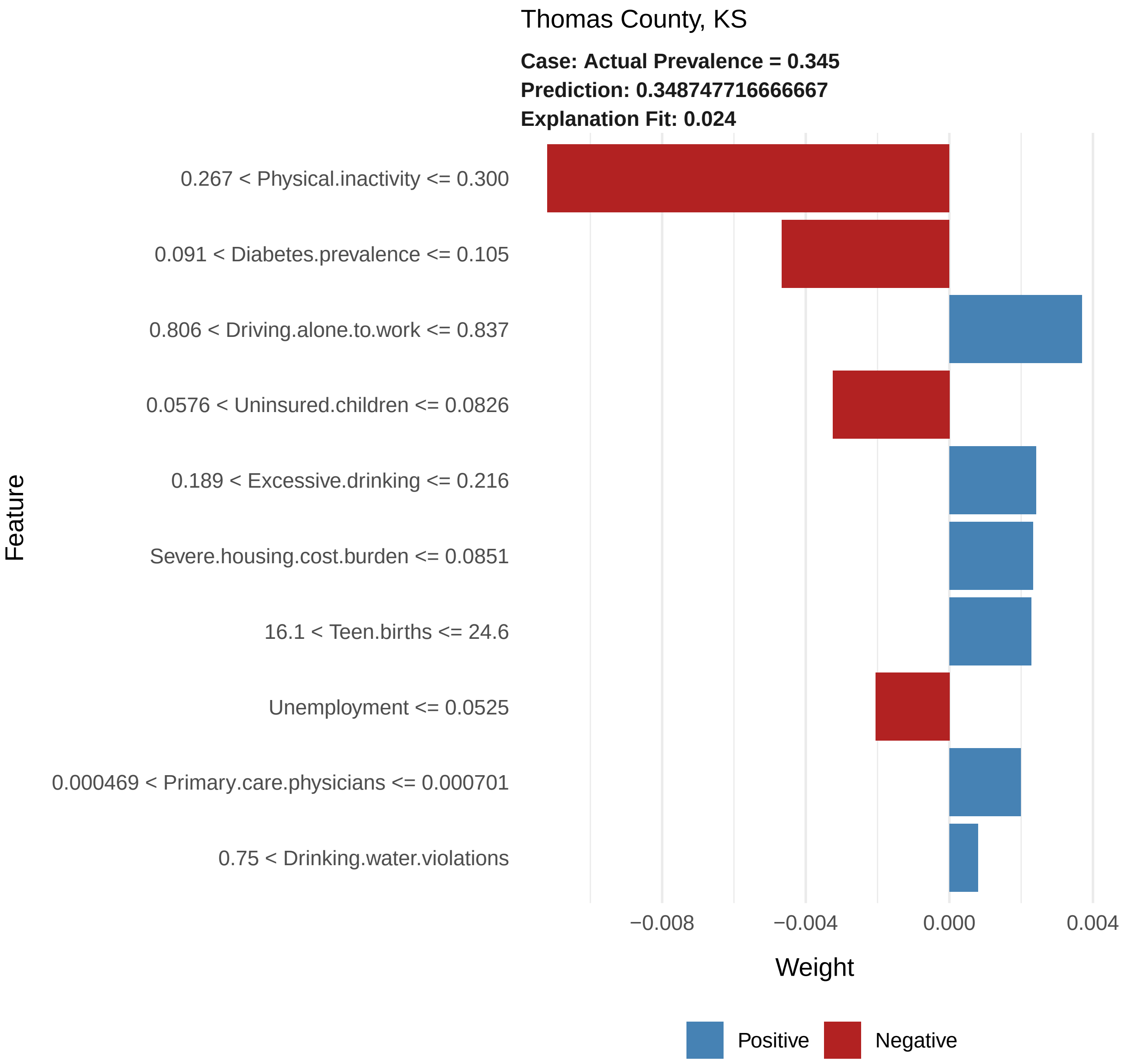

Thomas County, KS
Case: Actual Prevalence = 0.345
Prediction: 0.348747716666667
Explanation Fit: 0.024
0.267 < Physical.inactivity <= 0.300
0.091 < Diabetes.prevalence <= 0.105
0.806 < Driving.alone.to.work <= 0.837
0.0576 < Uninsured.children <= 0.0826
0.189 < Excessive.drinking <= 0.216
Severe.housing.cost.burden <= 0.0851
16.1 < Teen.births <= 24.6
Unemployment <= 0.0525
0.000469 < Primary.care.physicians <= 0.000701
0.75 < Drinking.water.violations
Feature
−0.008
−0.004
0.000
0.004
Weight
Positive
Negative

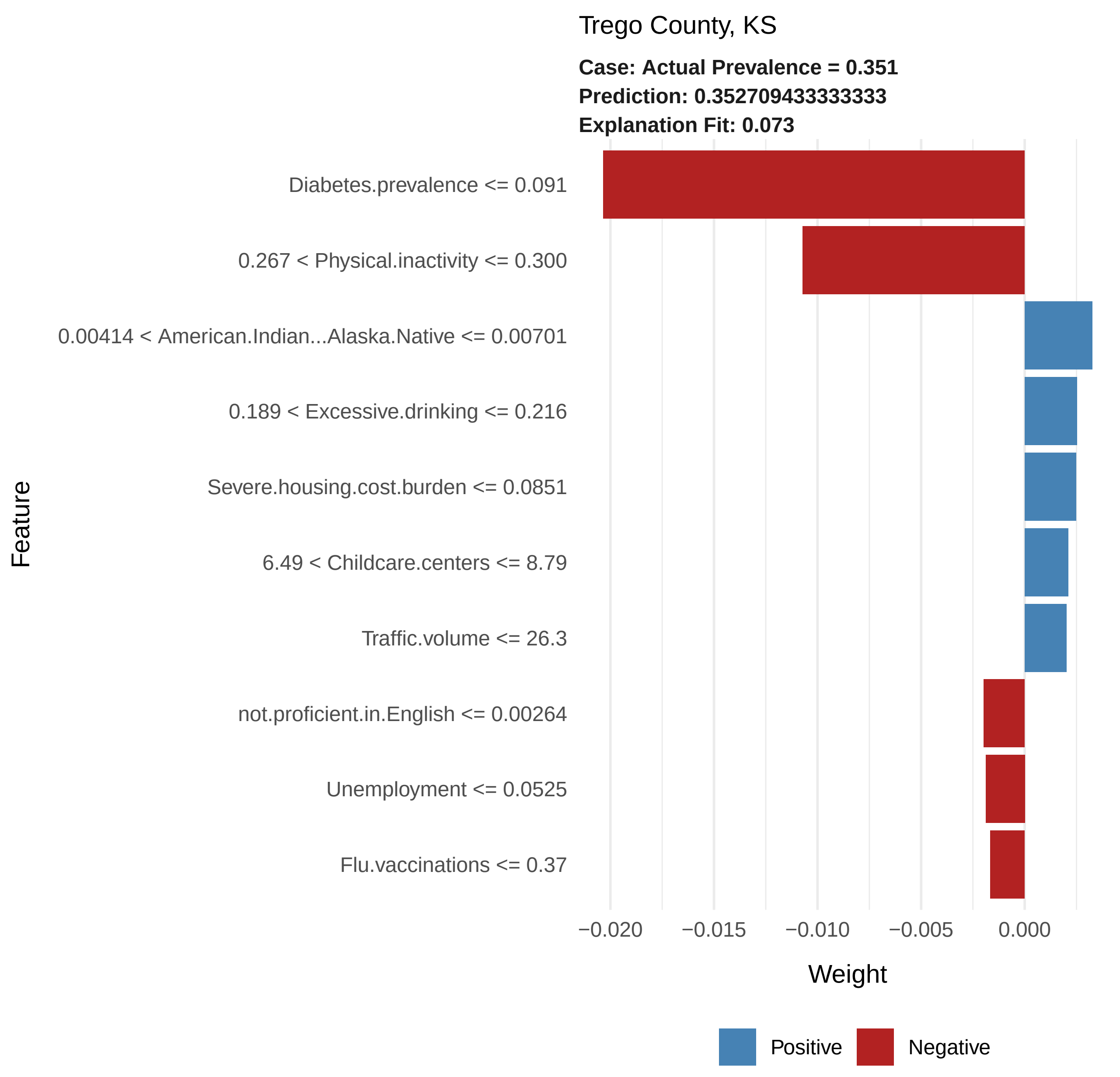

Trego County, KS
Case: Actual Prevalence = 0.351
Prediction: 0.352709433333333
Explanation Fit: 0.073
Diabetes.prevalence <= 0.091
0.267 < Physical.inactivity <= 0.300
0.00414 < American.Indian...Alaska.Native <= 0.00701
0.189 < Excessive.drinking <= 0.216
Severe.housing.cost.burden <= 0.0851
6.49 < Childcare.centers <= 8.79
Traffic.volume <= 26.3
not.proficient.in.English <= 0.00264
Unemployment <= 0.0525
Flu.vaccinations <= 0.37
Feature
−0.020
−0.015
−0.010
−0.005
0.000
Weight
Positive
Negative

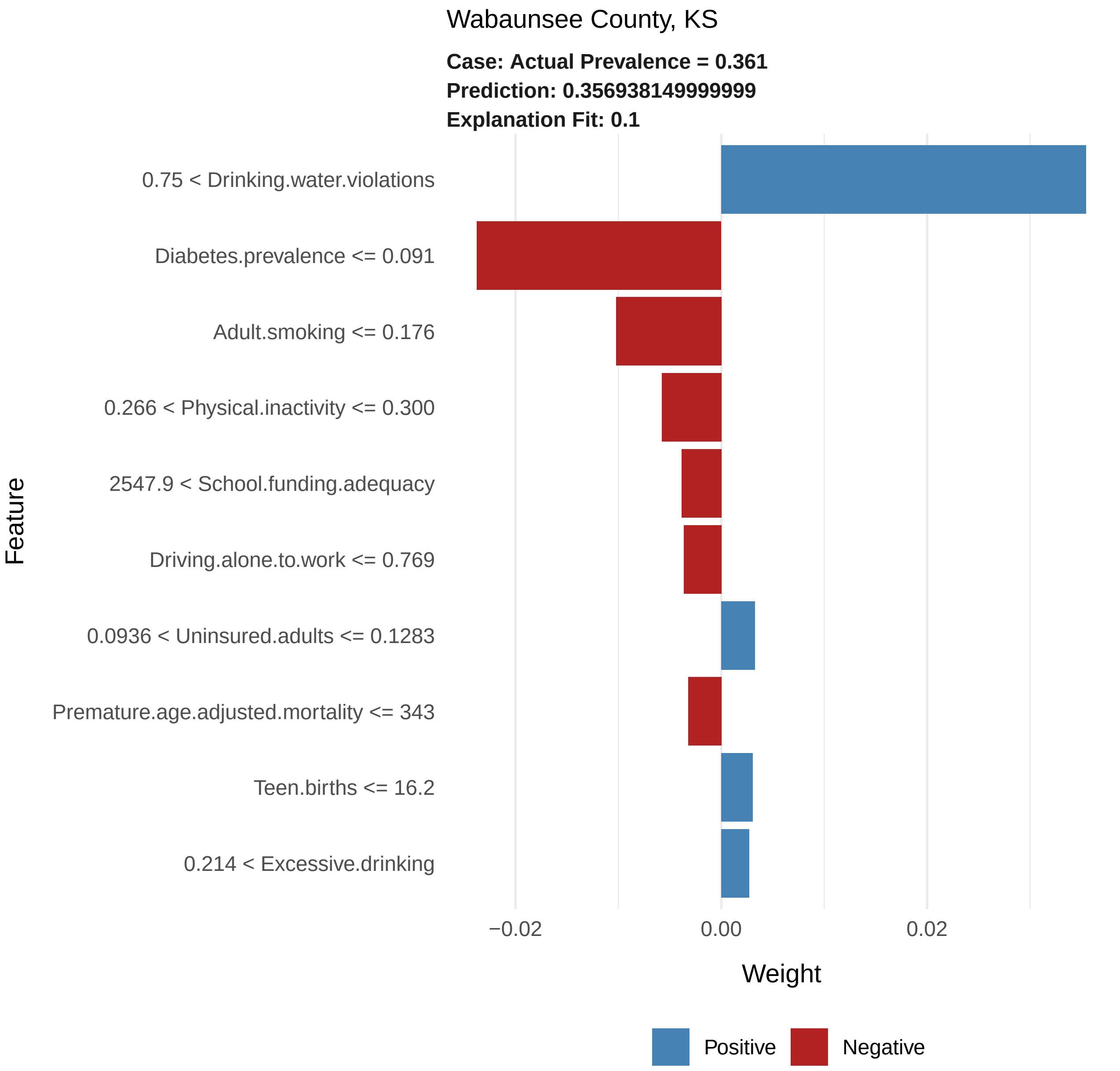

Wabaunsee County, KS
Case: Actual Prevalence = 0.361
Prediction: 0.356938149999999
Explanation Fit: 0.1
0.75 < Drinking.water.violations
Diabetes.prevalence <= 0.091
Adult.smoking <= 0.176
0.266 < Physical.inactivity <= 0.300
2547.9 < School.funding.adequacy
Driving.alone.to.work <= 0.769
0.0936 < Uninsured.adults <= 0.1283
Premature.age.adjusted.mortality <= 343
Teen.births <= 16.2
0.214 < Excessive.drinking
Feature
−0.02
0.00
0.02
Weight
Positive
Negative

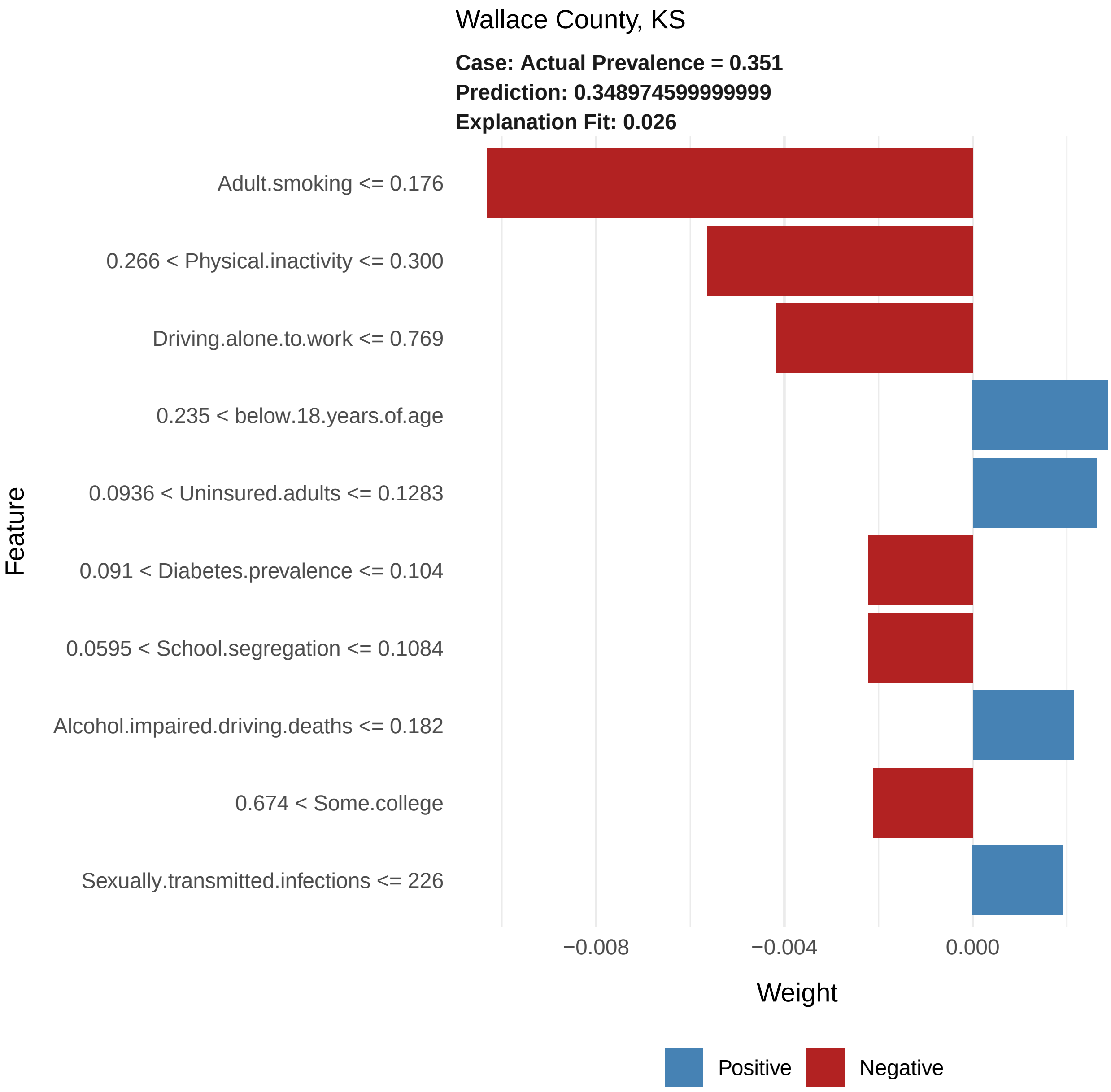

Wallace County, KS
Case: Actual Prevalence = 0.351
Prediction: 0.348974599999999
Explanation Fit: 0.026
Adult.smoking <= 0.176
0.266 < Physical.inactivity <= 0.300
Driving.alone.to.work <= 0.769
0.235 < below.18.years.of.age
0.0936 < Uninsured.adults <= 0.1283
0.091 < Diabetes.prevalence <= 0.104
0.0595 < School.segregation <= 0.1084
Alcohol.impaired.driving.deaths <= 0.182
0.674 < Some.college
Sexually.transmitted.infections <= 226
Feature
−0.008
−0.004
0.000
Weight
Positive
Negative

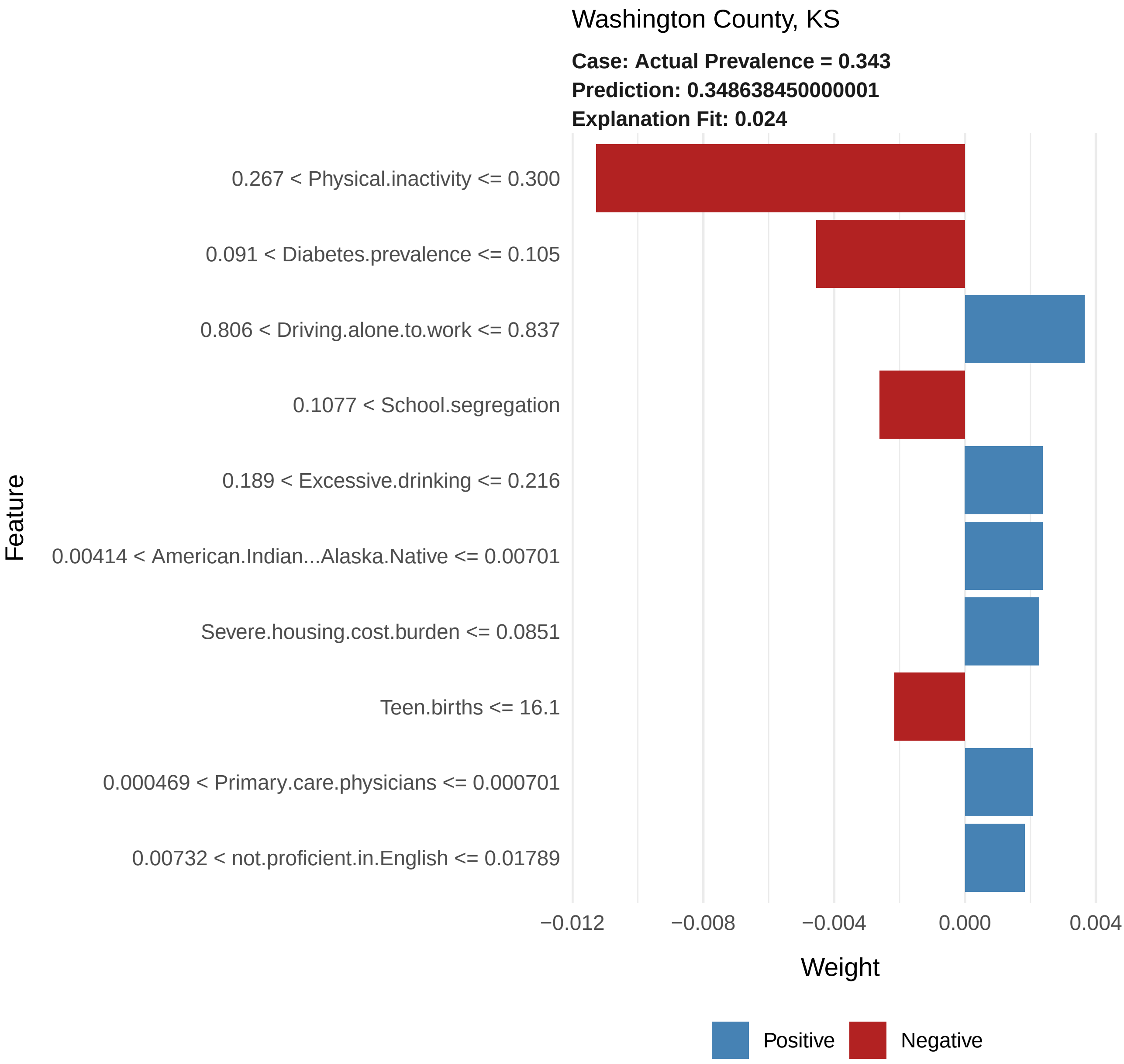
Washington County, KS
Case: Actual Prevalence = 0.343
Prediction: 0.348638450000001
Explanation Fit: 0.024
0.267 < Physical.inactivity <= 0.300
0.091 < Diabetes.prevalence <= 0.105
0.806 < Driving.alone.to.work <= 0.837
0.1077 < School.segregation
0.189 < Excessive.drinking <= 0.216
0.00414 < American.Indian...Alaska.Native <= 0.00701
Severe.housing.cost.burden <= 0.0851
Teen.births <= 16.1
0.000469 < Primary.care.physicians <= 0.000701
0.00732 < not.proficient.in.English <= 0.01789
Feature
−0.012
−0.008
−0.004
0.000
0.004
Weight
Positive
Negative

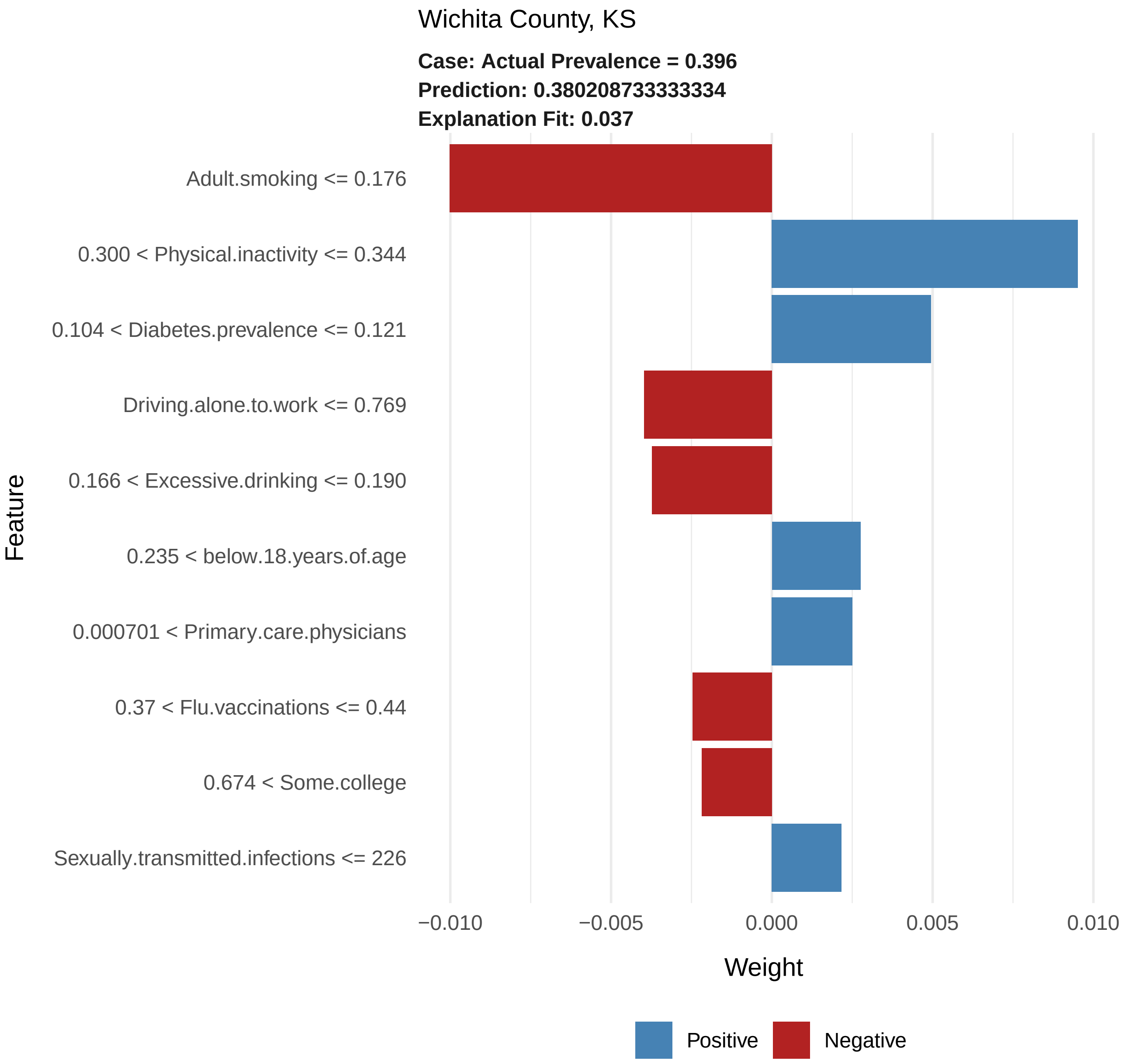

Wichita County, KS
Case: Actual Prevalence = 0.396
Prediction: 0.380208733333334
Explanation Fit: 0.037
Adult.smoking <= 0.176
0.300 < Physical.inactivity <= 0.344
0.104 < Diabetes.prevalence <= 0.121
Driving.alone.to.work <= 0.769
0.166 < Excessive.drinking <= 0.190
0.235 < below.18.years.of.age
0.000701 < Primary.care.physicians
0.37 < Flu.vaccinations <= 0.44
0.674 < Some.college
Sexually.transmitted.infections <= 226
Feature
−0.010
−0.005
0.000
0.005
0.010
Weight
Positive
Negative

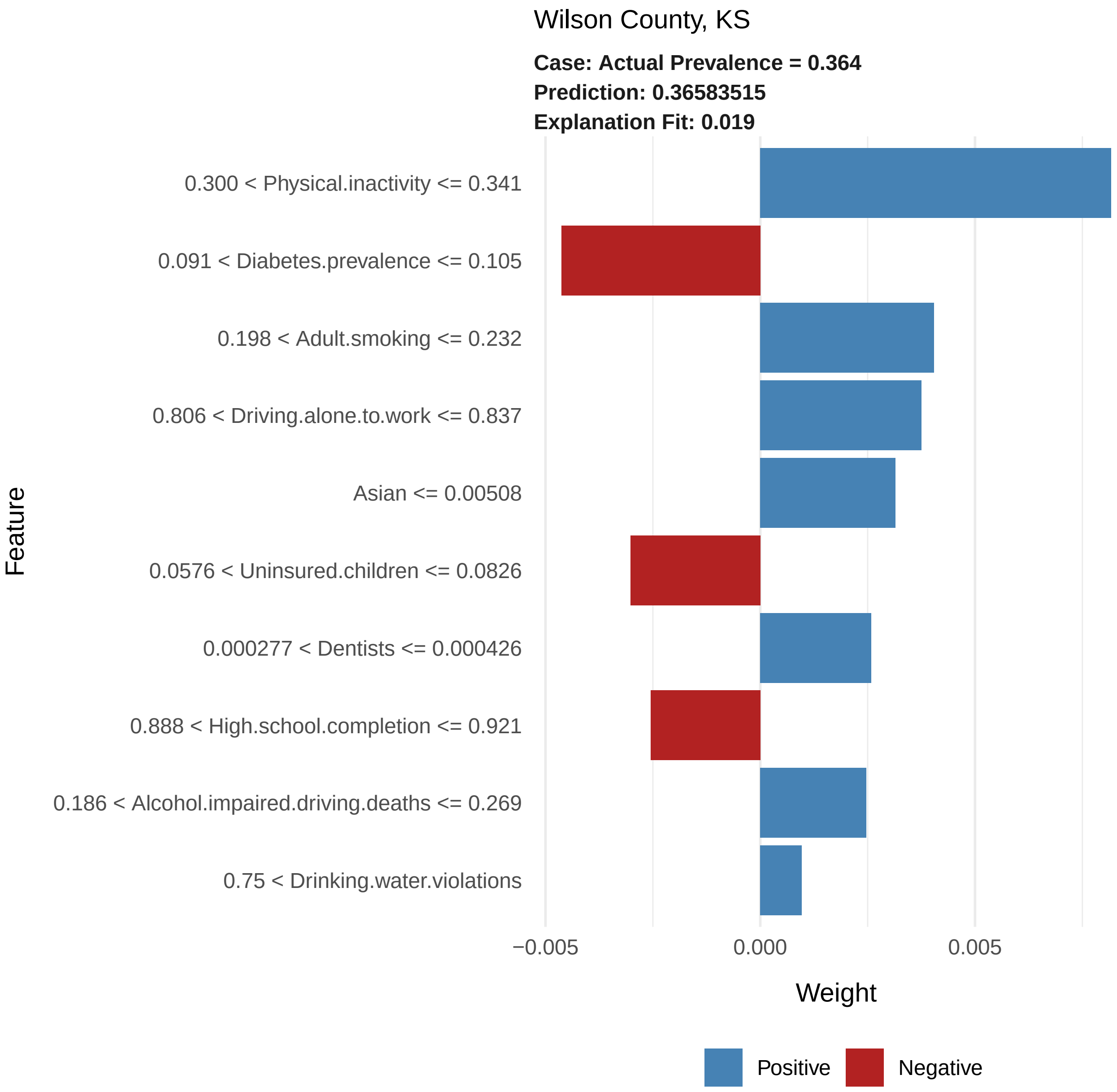
Wilson County, KS
Case: Actual Prevalence = 0.364
Prediction: 0.36583515
Explanation Fit: 0.019
Feature
0.300 < Physical.inactivity <= 0.341
0.091 < Diabetes.prevalence <= 0.105
0.198 < Adult.smoking <= 0.232
0.806 < Driving.alone.to.work <= 0.837
Asian <= 0.00508
0.0576 < Uninsured.children <= 0.0826
0.000277 < Dentists <= 0.000426
0.888 < High.school.completion <= 0.921
0.186 < Alcohol.impaired.driving.deaths <= 0.269
0.75 < Drinking.water.violations
−0.005
0.000
0.005
Weight
Positive
Negative

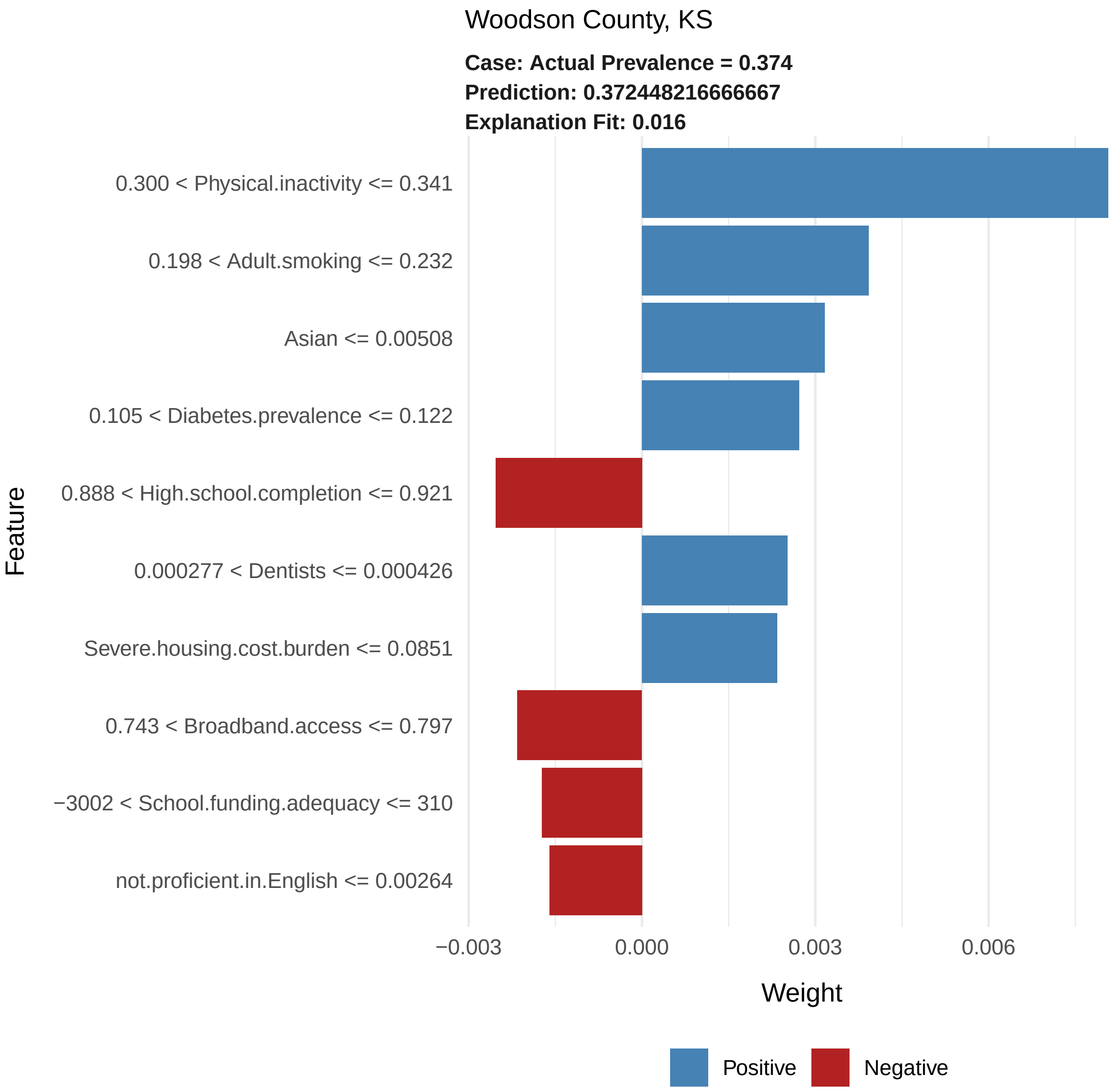

Woodson County, KS
Case: Actual Prevalence = 0.374
Prediction: 0.372448216666667
Explanation Fit: 0.016
0.300 < Physical.inactivity <= 0.341
0.198 < Adult.smoking <= 0.232
Asian <= 0.00508
0.105 < Diabetes.prevalence <= 0.122
0.888 < High.school.completion <= 0.921
0.000277 < Dentists <= 0.000426
Severe.housing.cost.burden <= 0.0851
0.743 < Broadband.access <= 0.797
−3002 < School.funding.adequacy <= 310
not.proficient.in.English <= 0.00264
Feature
−0.003
0.000
0.003
0.006
Weight
Positive
Negative

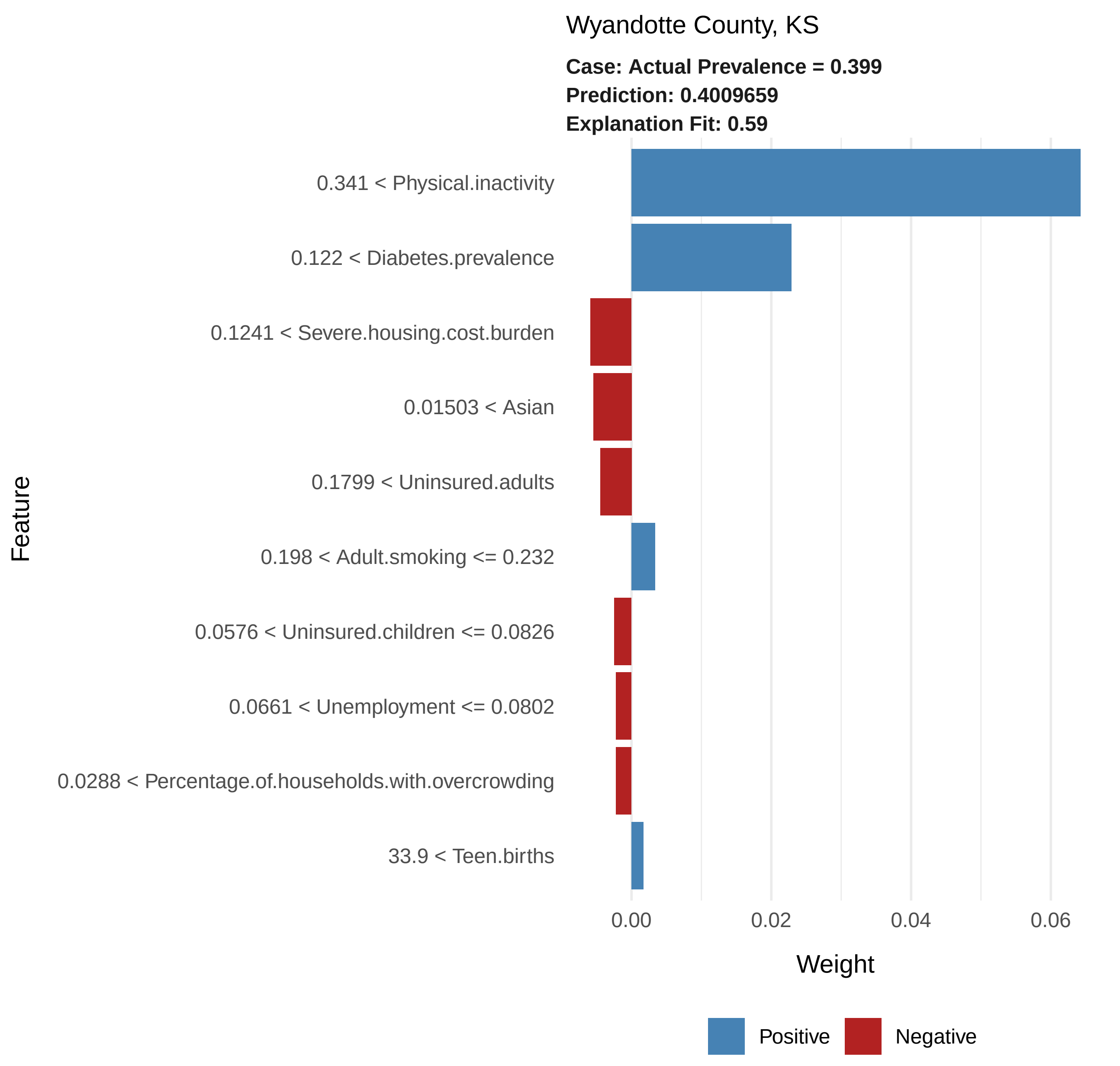

Wyandotte County, KS
Case: Actual Prevalence = 0.399
Prediction: 0.4009659
Explanation Fit: 0.59
0.341 < Physical.inactivity
0.122 < Diabetes.prevalence
0.1241 < Severe.housing.cost.burden
0.01503 < Asian
0.1799 < Uninsured.adults
0.198 < Adult.smoking <= 0.232
0.0576 < Uninsured.children <= 0.0826
0.0661 < Unemployment <= 0.0802
0.0288 < Percentage.of.households.with.overcrowding
33.9 < Teen.births
Feature
0.00
0.02
0.04
0.06
Weight
Positive
Negative

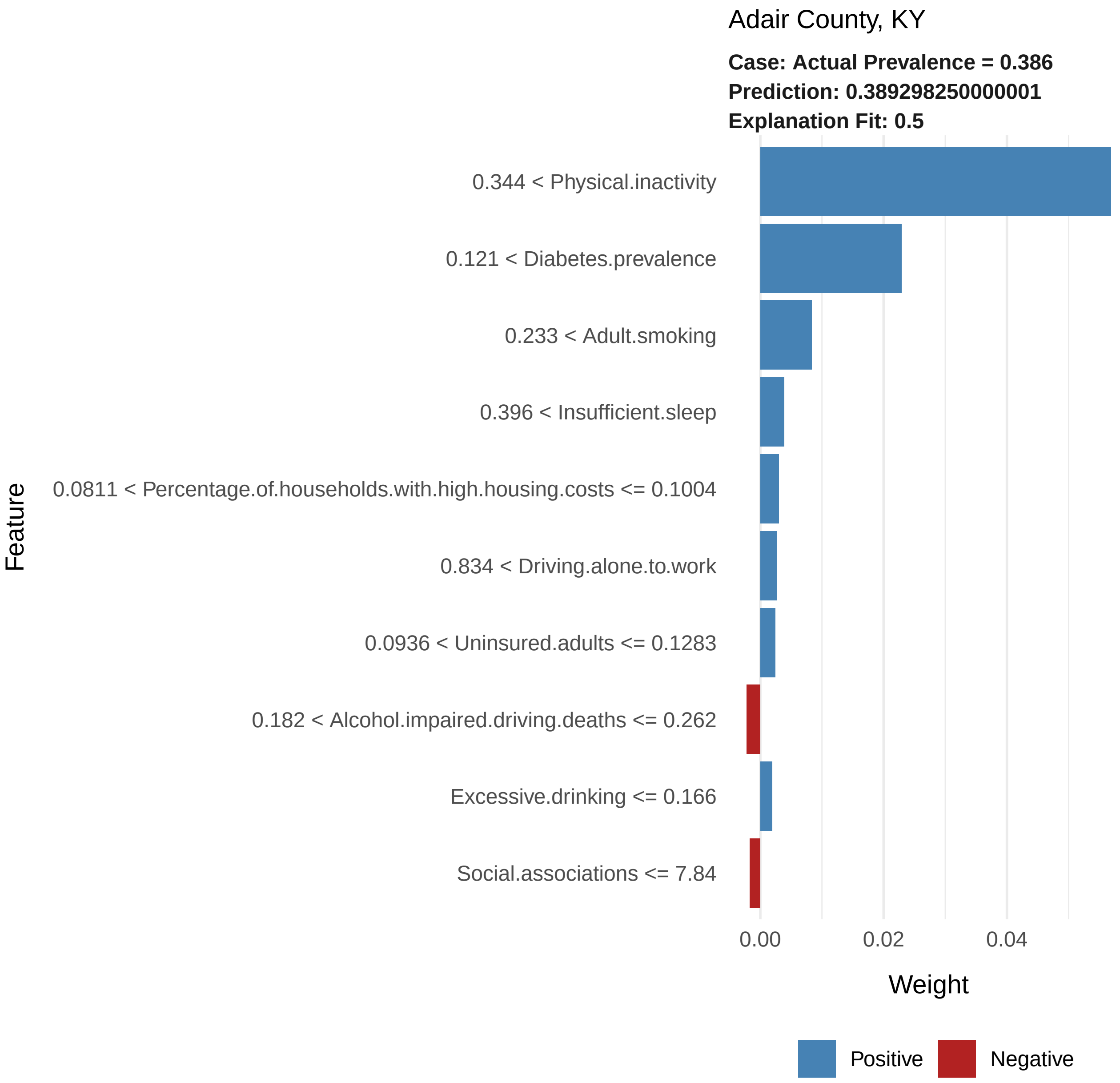

Adair County, KY
Case: Actual Prevalence = 0.386
Prediction: 0.389298250000001
Explanation Fit: 0.5
0.344 < Physical.inactivity
0.121 < Diabetes.prevalence
0.233 < Adult.smoking
0.396 < Insufficient.sleep
0.0811 < Percentage.of.households.with.high.housing.costs <= 0.1004
0.834 < Driving.alone.to.work
0.0936 < Uninsured.adults <= 0.1283
0.182 < Alcohol.impaired.driving.deaths <= 0.262
Excessive.drinking <= 0.166
Social.associations <= 7.84
Feature
0.00
0.02
0.04
Weight
Positive
Negative

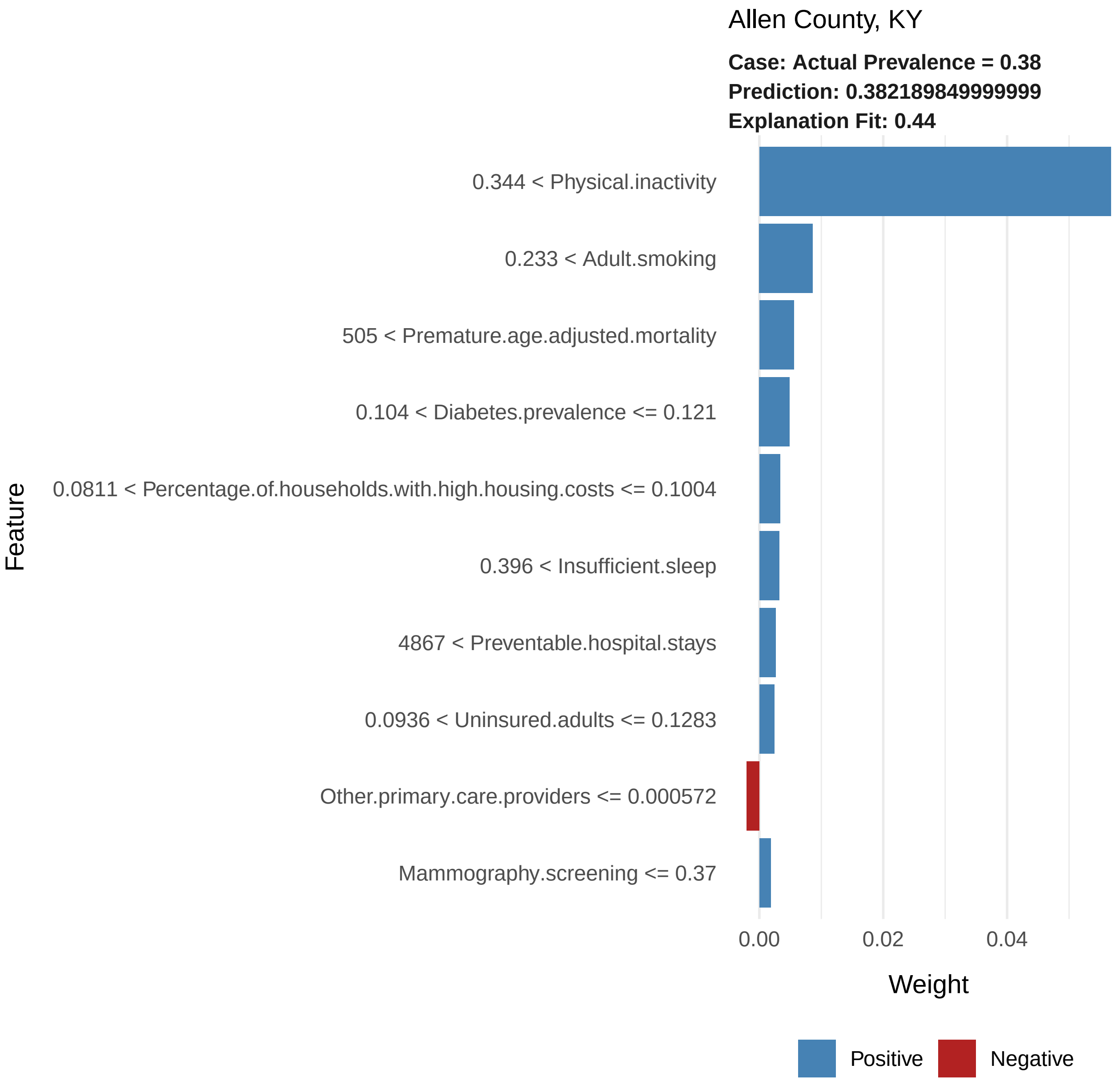

Allen County, KY
Case: Actual Prevalence = 0.38
Prediction: 0.382189849999999
Explanation Fit: 0.44
0.344 < Physical.inactivity
0.233 < Adult.smoking
505 < Premature.age.adjusted.mortality
0.104 < Diabetes.prevalence <= 0.121
0.0811 < Percentage.of.households.with.high.housing.costs <= 0.1004
0.396 < Insufficient.sleep
4867 < Preventable.hospital.stays
0.0936 < Uninsured.adults <= 0.1283
Other.primary.care.providers <= 0.000572
Mammography.screening <= 0.37
Feature
0.00
0.02
0.04
Weight
Positive
Negative

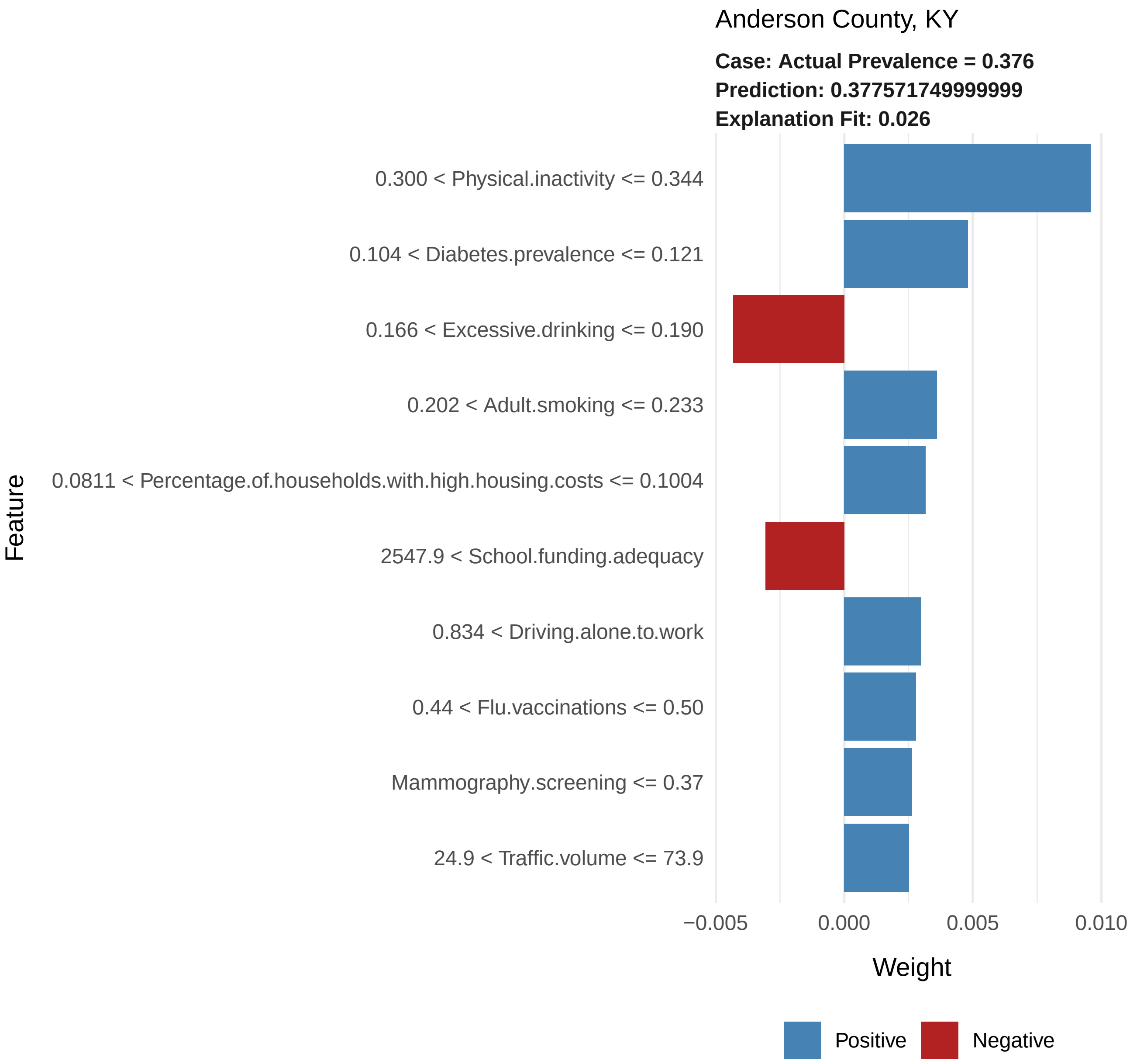

Anderson County, KY
Case: Actual Prevalence = 0.376
Prediction: 0.377571749999999
Explanation Fit: 0.026
0.300 < Physical.inactivity <= 0.344
0.104 < Diabetes.prevalence <= 0.121
0.166 < Excessive.drinking <= 0.190
0.202 < Adult.smoking <= 0.233
0.0811 < Percentage.of.households.with.high.housing.costs <= 0.1004
2547.9 < School.funding.adequacy
0.834 < Driving.alone.to.work
0.44 < Flu.vaccinations <= 0.50
Mammography.screening <= 0.37
24.9 < Traffic.volume <= 73.9
Feature
−0.005
0.000
0.005
0.010
Weight
Positive
Negative

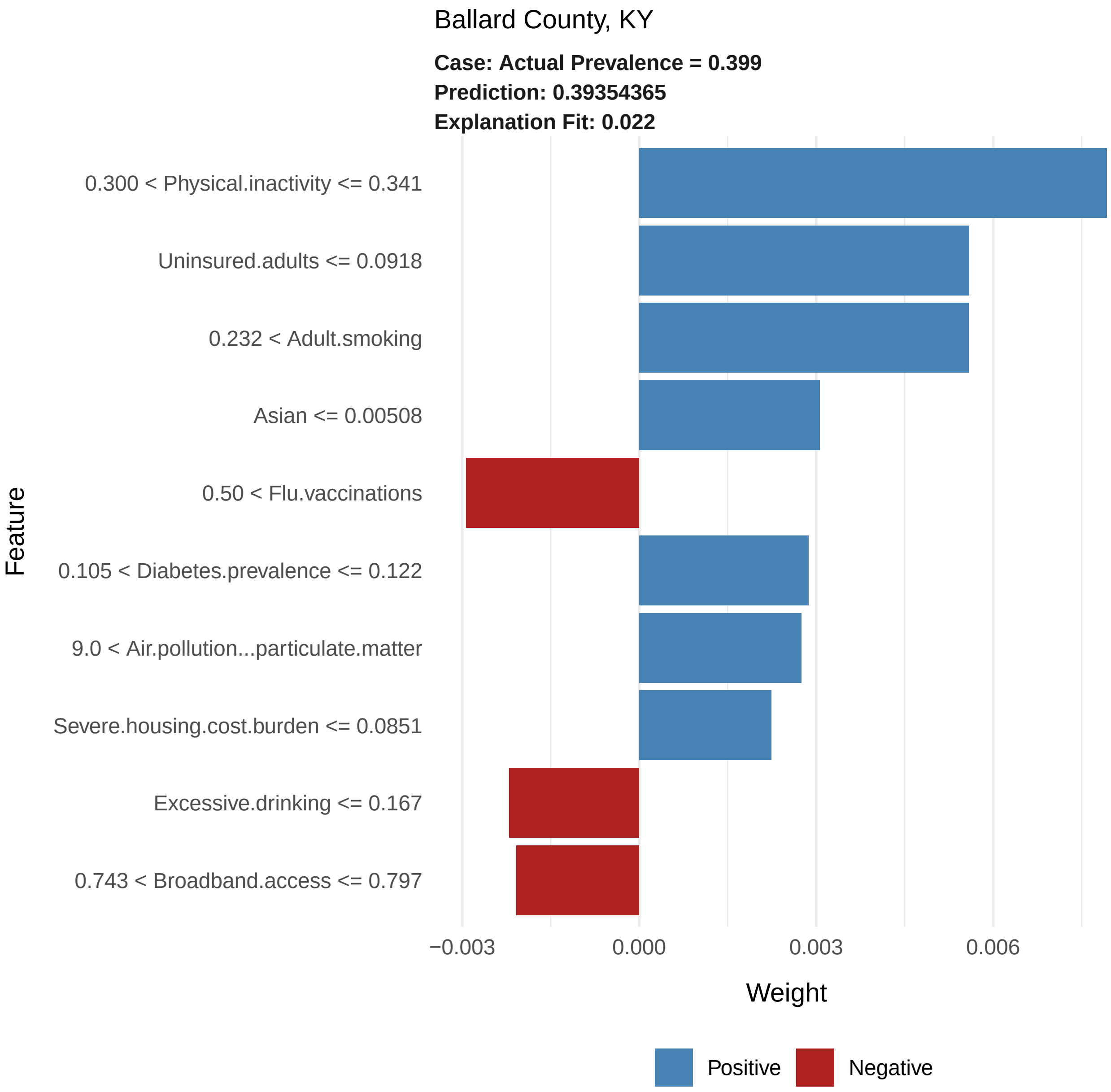
Ballard County, KY
Case: Actual Prevalence = 0.399
Prediction: 0.39354365
Explanation Fit: 0.022
0.300 < Physical.inactivity <= 0.341
Uninsured.adults <= 0.0918
0.232 < Adult.smoking
Asian <= 0.00508
0.50 < Flu.vaccinations
0.105 < Diabetes.prevalence <= 0.122
9.0 < Air.pollution...particulate.matter
Severe.housing.cost.burden <= 0.0851
Excessive.drinking <= 0.167
0.743 < Broadband.access <= 0.797
Feature
−0.003
0.000
0.003
0.006
Weight
Positive
Negative

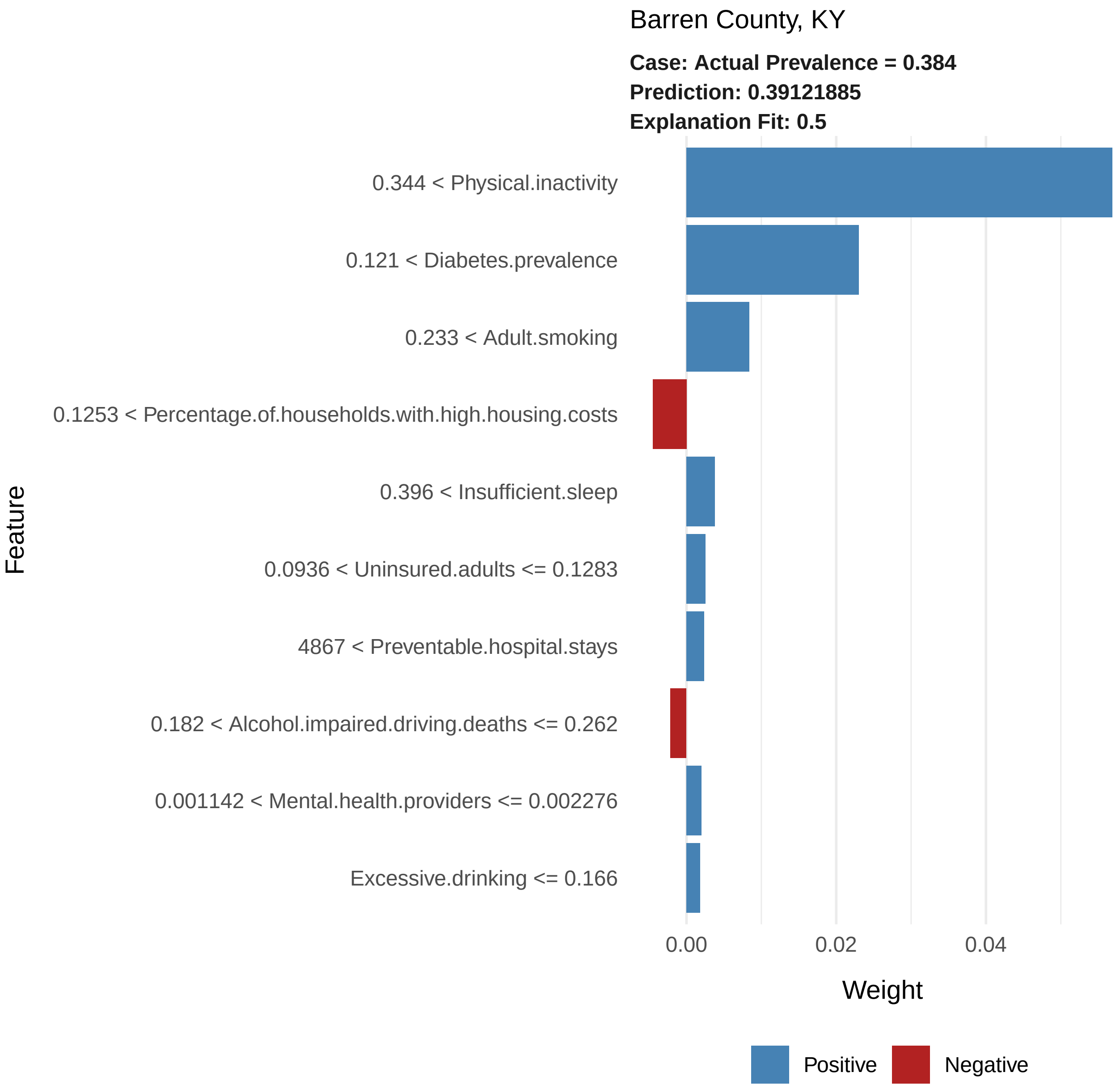
Barren County, KY
Case: Actual Prevalence = 0.384
Prediction: 0.39121885
Explanation Fit: 0.5
0.344 < Physical.inactivity
0.121 < Diabetes.prevalence
0.233 < Adult.smoking
0.1253 < Percentage.of.households.with.high.housing.costs
0.396 < Insufficient.sleep
0.0936 < Uninsured.adults <= 0.1283
4867 < Preventable.hospital.stays
0.182 < Alcohol.impaired.driving.deaths <= 0.262
0.001142 < Mental.health.providers <= 0.002276
Excessive.drinking <= 0.166
Feature
0.00
0.02
0.04
Weight
Positive
Negative

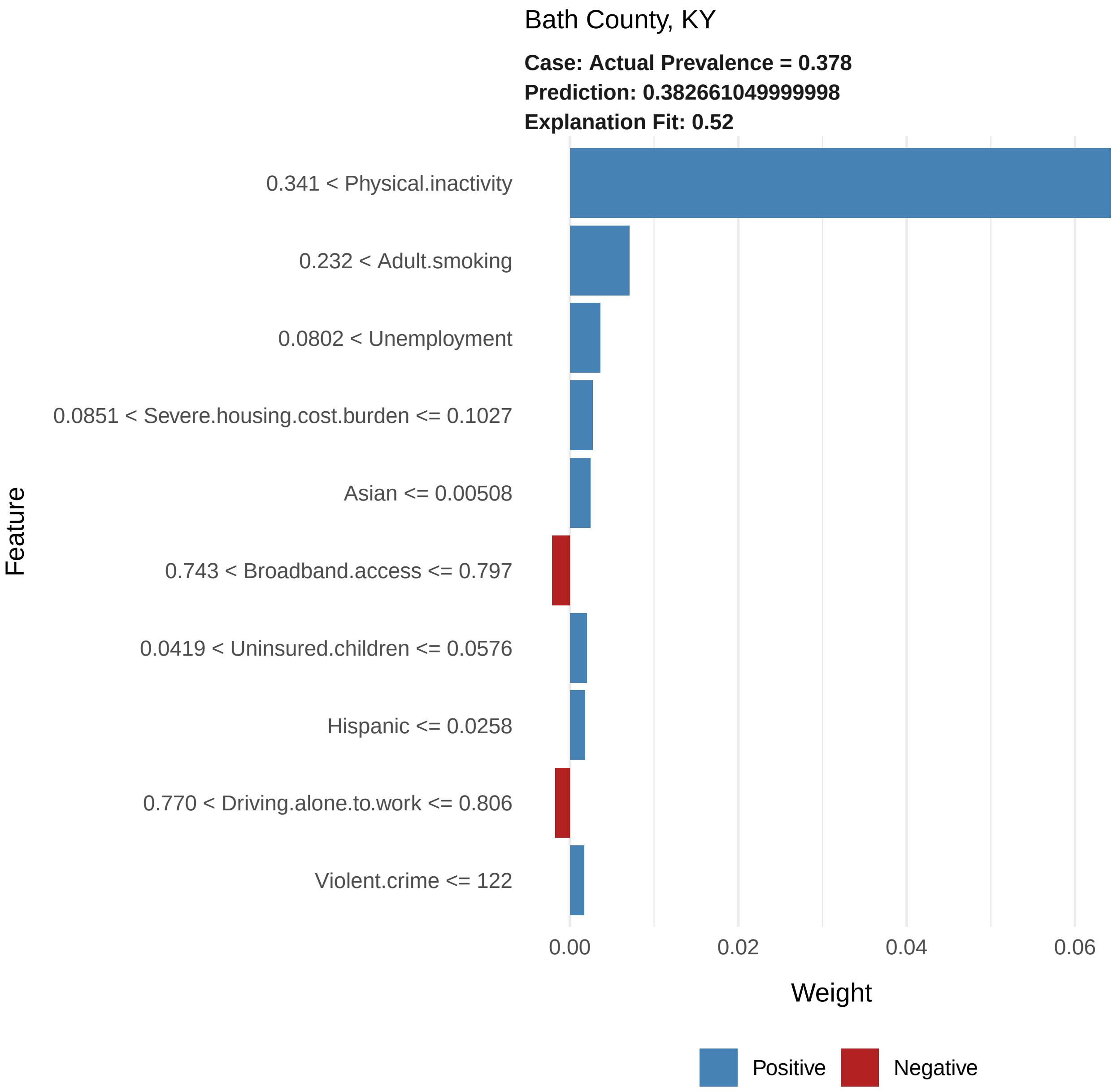

Bath County, KY
Case: Actual Prevalence = 0.378
Prediction: 0.382661049999998
Explanation Fit: 0.52
0.341 < Physical.inactivity
0.232 < Adult.smoking
0.0802 < Unemployment
0.0851 < Severe.housing.cost.burden <= 0.1027
Asian <= 0.00508
0.743 < Broadband.access <= 0.797
0.0419 < Uninsured.children <= 0.0576
Hispanic <= 0.0258
0.770 < Driving.alone.to.work <= 0.806
Violent.crime <= 122
Feature
0.00
0.02
0.04
0.06
Weight
Positive
Negative

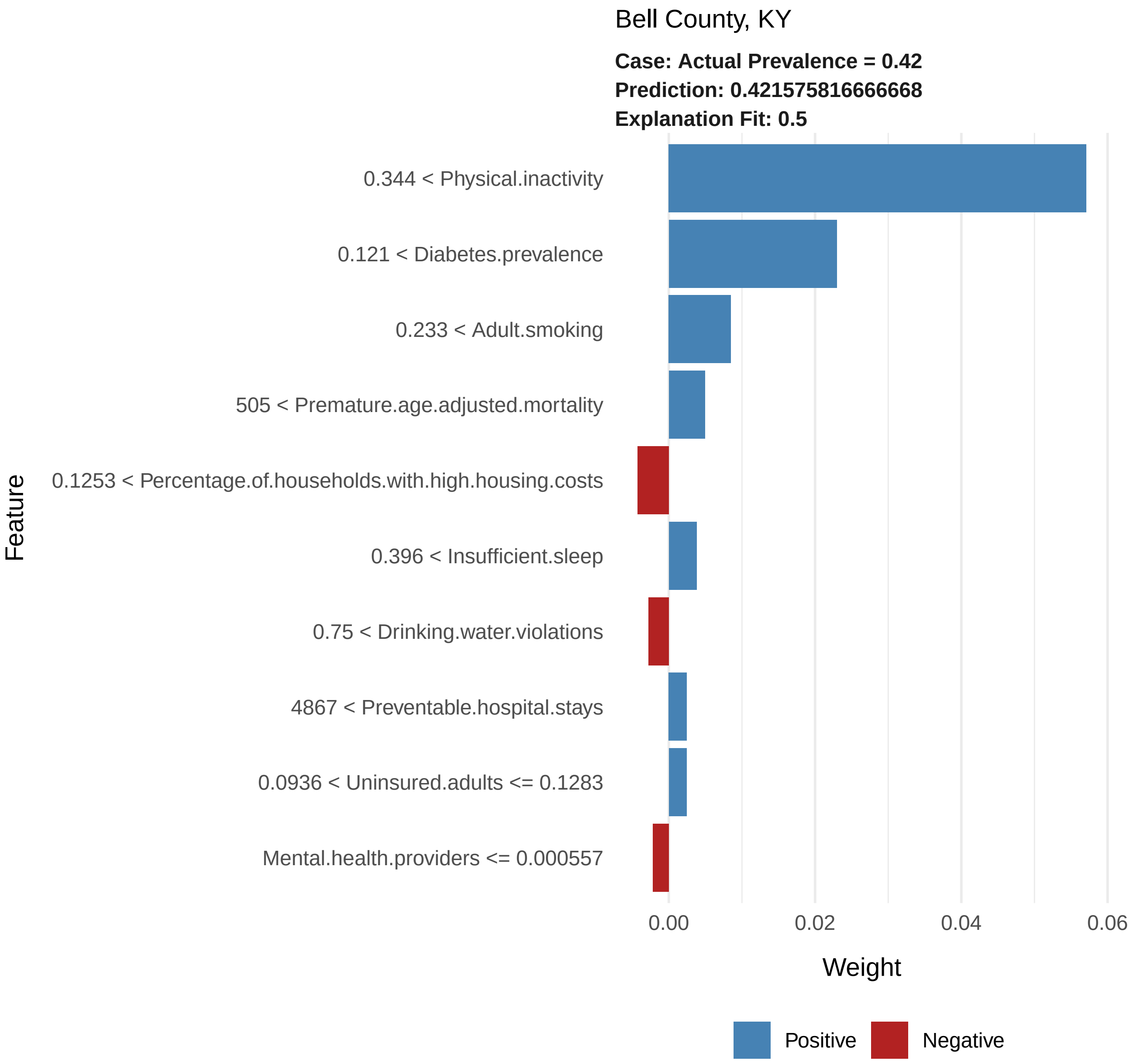
Bell County, KY
Case: Actual Prevalence = 0.42
Prediction: 0.421575816666668
Explanation Fit: 0.5
0.344 < Physical.inactivity
0.121 < Diabetes.prevalence
0.233 < Adult.smoking
505 < Premature.age.adjusted.mortality
0.1253 < Percentage.of.households.with.high.housing.costs
0.396 < Insufficient.sleep
0.75 < Drinking.water.violations
4867 < Preventable.hospital.stays
0.0936 < Uninsured.adults <= 0.1283
Mental.health.providers <= 0.000557
Feature
0.00
0.02
0.04
0.06
Weight
Positive
Negative

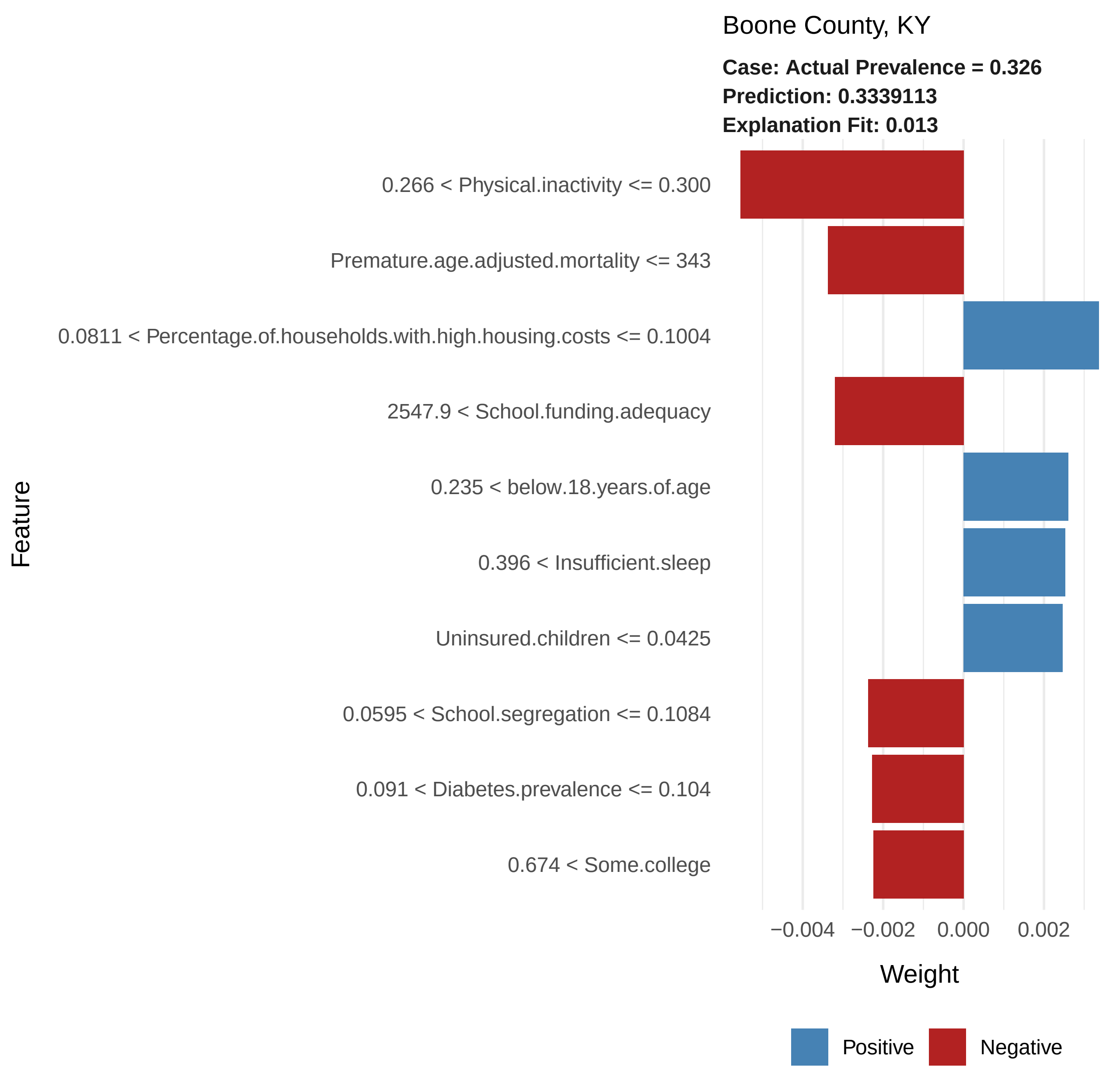
Boone County, KY
Case: Actual Prevalence = 0.326
Prediction: 0.3339113
Explanation Fit: 0.013
0.266 < Physical.inactivity <= 0.300
Premature.age.adjusted.mortality <= 343
0.0811 < Percentage.of.households.with.high.housing.costs <= 0.1004
2547.9 < School.funding.adequacy
0.235 < below.18.years.of.age
0.396 < Insufficient.sleep
Uninsured.children <= 0.0425
0.0595 < School.segregation <= 0.1084
0.091 < Diabetes.prevalence <= 0.104
0.674 < Some.college
Feature
−0.004
−0.002
0.000
0.002
Weight
Positive
Negative

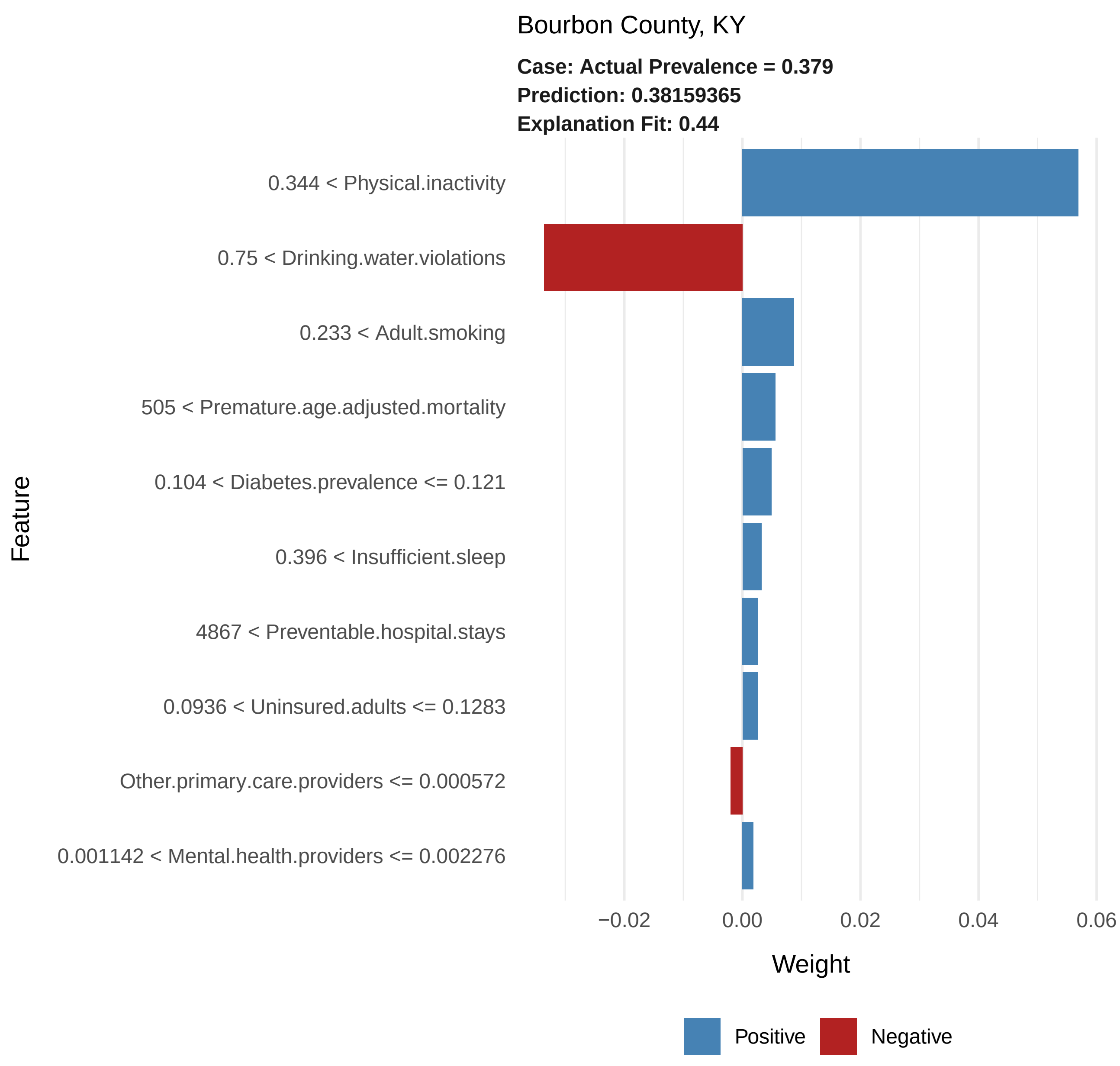

Bourbon County, KY
Case: Actual Prevalence = 0.379
Prediction: 0.38159365
Explanation Fit: 0.44
0.344 < Physical.inactivity
0.75 < Drinking.water.violations
0.233 < Adult.smoking
505 < Premature.age.adjusted.mortality
0.104 < Diabetes.prevalence <= 0.121
0.396 < Insufficient.sleep
4867 < Preventable.hospital.stays
0.0936 < Uninsured.adults <= 0.1283
Other.primary.care.providers <= 0.000572
0.001142 < Mental.health.providers <= 0.002276
Feature
−0.02
0.00
0.02
0.04
0.06
Weight
Positive
Negative

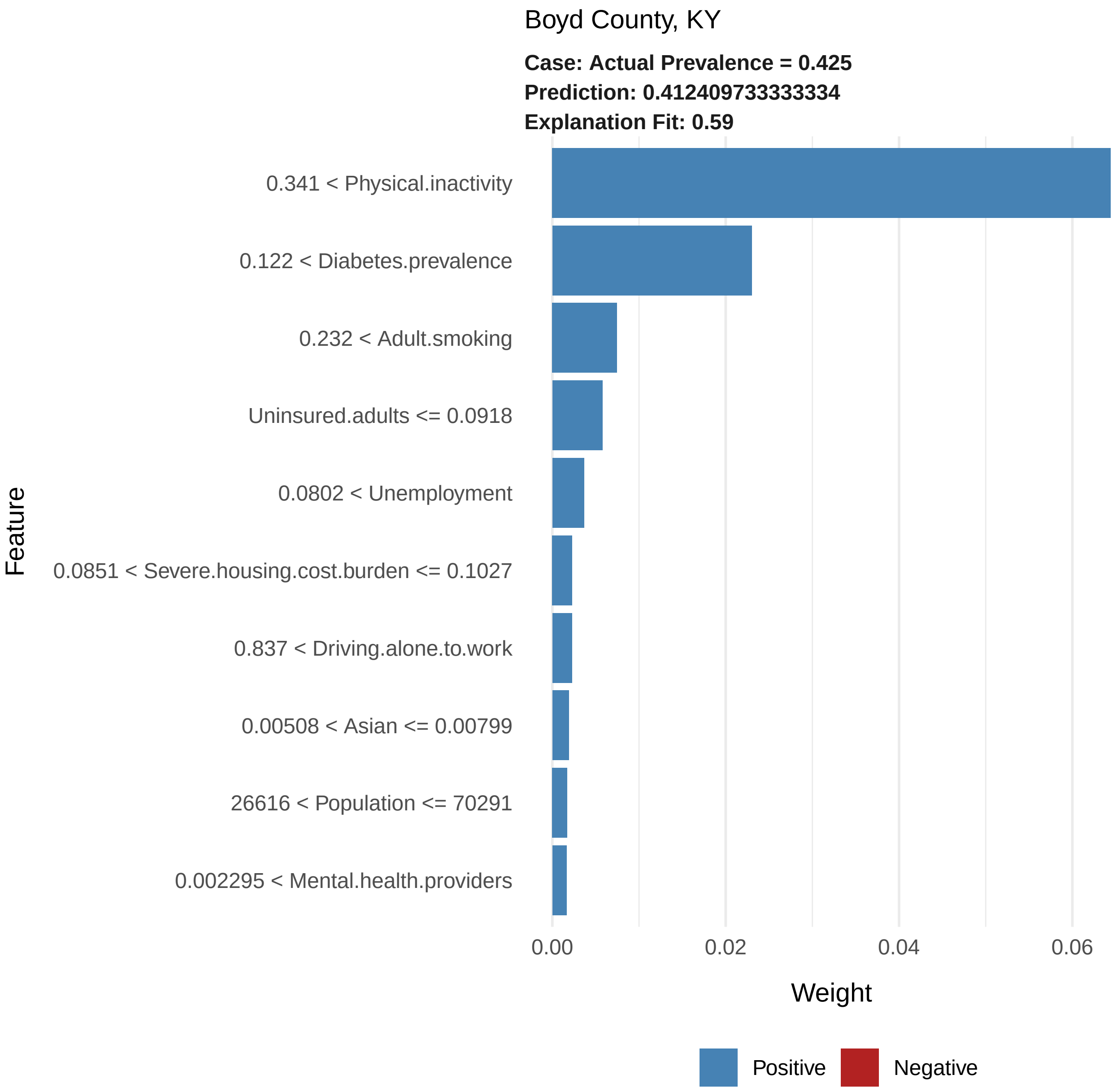
Boyd County, KY
Case: Actual Prevalence = 0.425
Prediction: 0.412409733333334
Explanation Fit: 0.59
0.341 < Physical.inactivity
0.122 < Diabetes.prevalence
0.232 < Adult.smoking
Uninsured.adults <= 0.0918
0.0802 < Unemployment
0.0851 < Severe.housing.cost.burden <= 0.1027
0.837 < Driving.alone.to.work
0.00508 < Asian <= 0.00799
26616 < Population <= 70291
0.002295 < Mental.health.providers
Feature
0.00
0.02
0.04
0.06
Weight
Positive
Negative

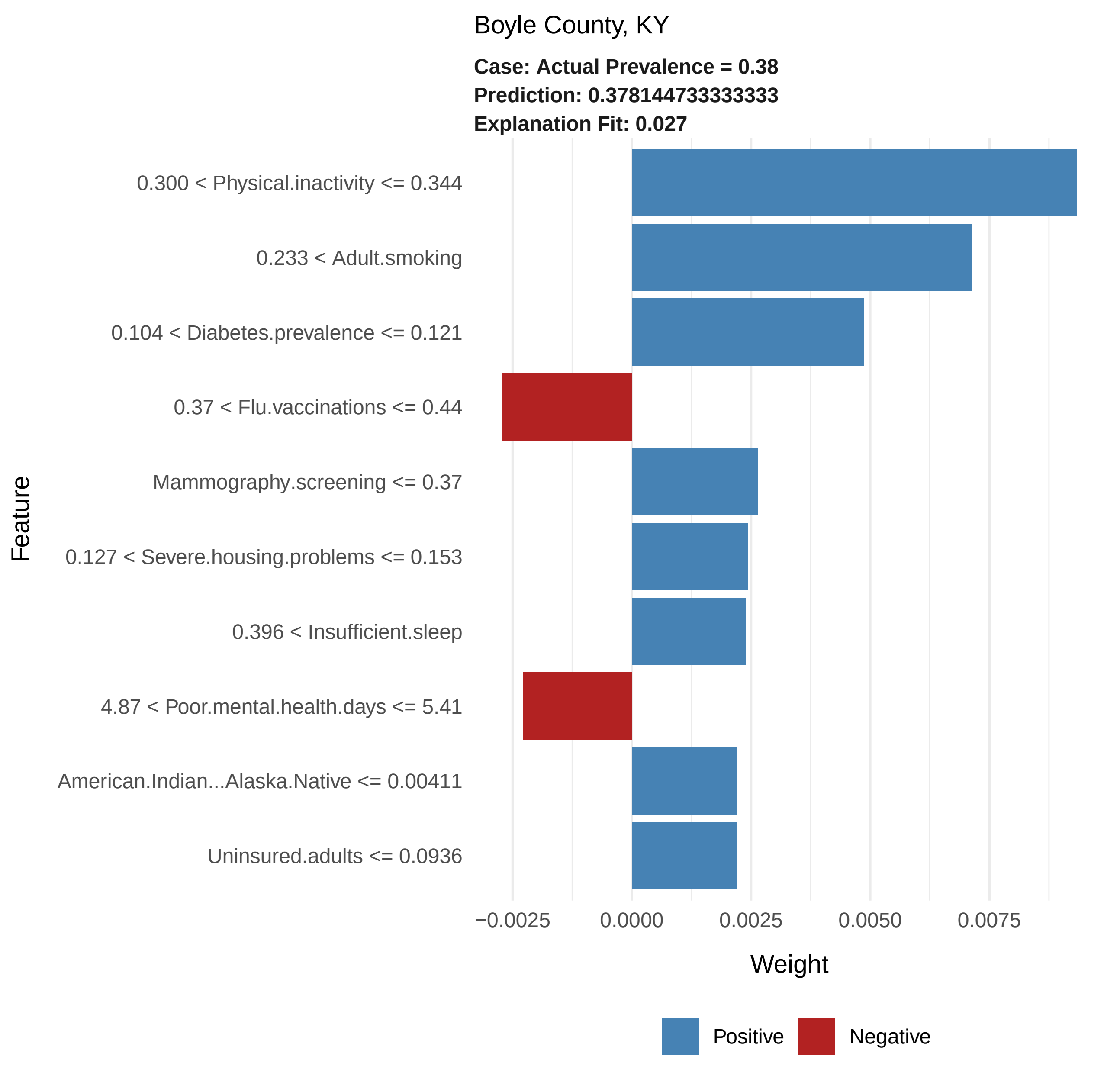

Boyle County, KY
Case: Actual Prevalence = 0.38
Prediction: 0.378144733333333
Explanation Fit: 0.027
0.300 < Physical.inactivity <= 0.344
0.233 < Adult.smoking
0.104 < Diabetes.prevalence <= 0.121
0.37 < Flu.vaccinations <= 0.44
Mammography.screening <= 0.37
0.127 < Severe.housing.problems <= 0.153
0.396 < Insufficient.sleep
4.87 < Poor.mental.health.days <= 5.41
American.Indian...Alaska.Native <= 0.00411
Uninsured.adults <= 0.0936
Feature
−0.0025
0.0000
0.0025
0.0050
0.0075
Weight
Positive
Negative

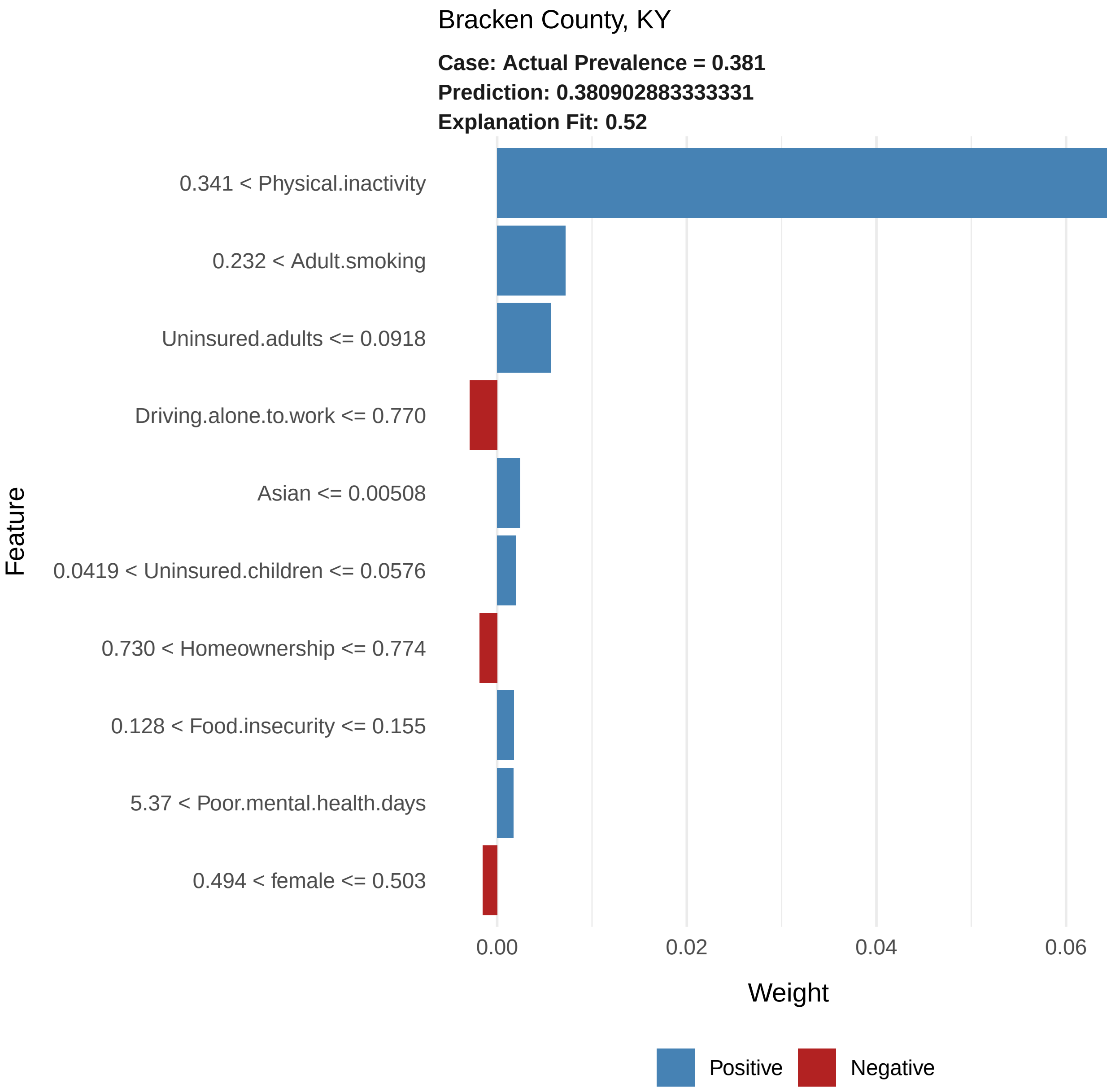

Bracken County, KY
Case: Actual Prevalence = 0.381
Prediction: 0.380902883333331
Explanation Fit: 0.52
0.341 < Physical.inactivity
0.232 < Adult.smoking
Uninsured.adults <= 0.0918
Driving.alone.to.work <= 0.770
Asian <= 0.00508
0.0419 < Uninsured.children <= 0.0576
0.730 < Homeownership <= 0.774
0.128 < Food.insecurity <= 0.155
5.37 < Poor.mental.health.days
0.494 < female <= 0.503
Feature
0.00
0.02
0.04
0.06
Weight
Positive
Negative

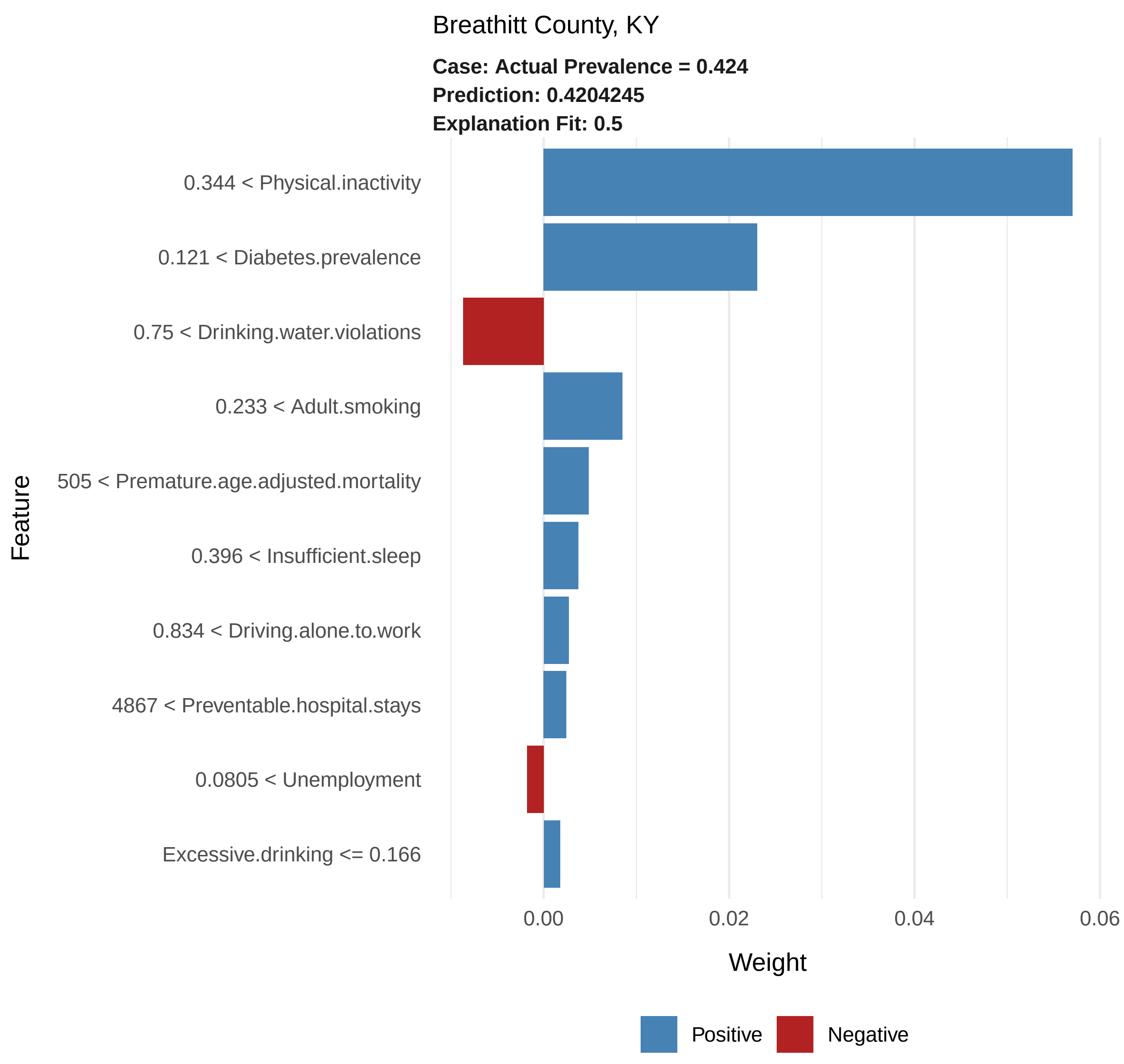
Breathitt County, KY
Case: Actual Prevalence = 0.424
Prediction: 0.4204245
Explanation Fit: 0.5
0.344 < Physical.inactivity
0.121 < Diabetes.prevalence
0.75 < Drinking.water.violations
0.233 < Adult.smoking
505 < Premature.age.adjusted.mortality
0.396 < Insufficient.sleep
0.834 < Driving.alone.to.work
4867 < Preventable.hospital.stays
0.0805 < Unemployment
Excessive.drinking <= 0.166
Feature
0.00
0.02
0.04
0.06
Weight
Positive
Negative

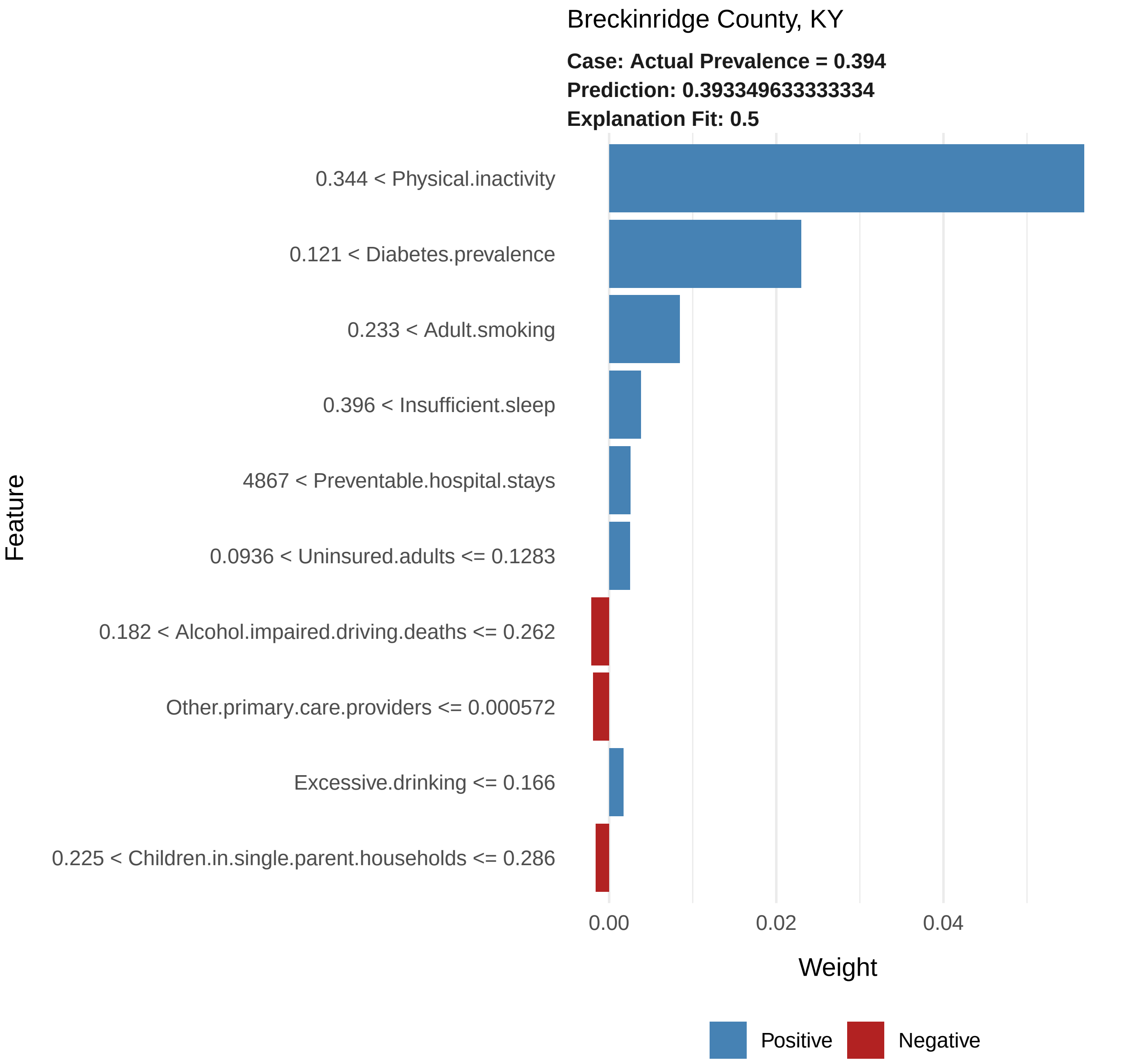

Breckinridge County, KY
Case: Actual Prevalence = 0.394
Prediction: 0.393349633333334
Explanation Fit: 0.5
0.344 < Physical.inactivity
0.121 < Diabetes.prevalence
0.233 < Adult.smoking
0.396 < Insufficient.sleep
4867 < Preventable.hospital.stays
0.0936 < Uninsured.adults <= 0.1283
0.182 < Alcohol.impaired.driving.deaths <= 0.262
Other.primary.care.providers <= 0.000572
Excessive.drinking <= 0.166
0.225 < Children.in.single.parent.households <= 0.286
Feature
0.00
0.02
0.04
Weight
Positive
Negative

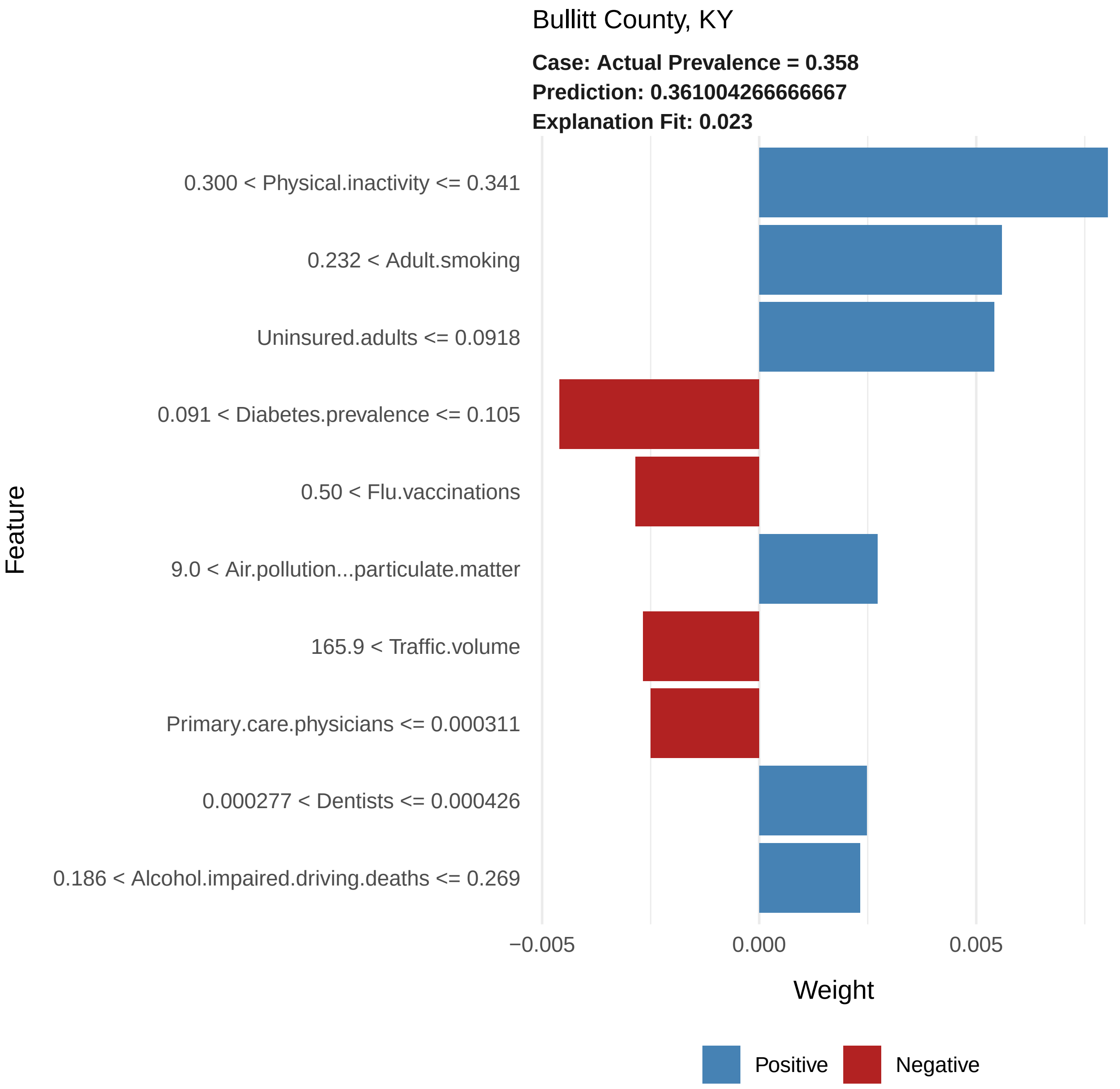
Bullitt County, KY
Case: Actual Prevalence = 0.358
Prediction: 0.361004266666667
Explanation Fit: 0.023
0.300 < Physical.inactivity <= 0.341
0.232 < Adult.smoking
Uninsured.adults <= 0.0918
0.091 < Diabetes.prevalence <= 0.105
0.50 < Flu.vaccinations
9.0 < Air.pollution...particulate.matter
165.9 < Traffic.volume
Primary.care.physicians <= 0.000311
0.000277 < Dentists <= 0.000426
0.186 < Alcohol.impaired.driving.deaths <= 0.269
Feature
−0.005
0.000
0.005
Weight
Positive
Negative

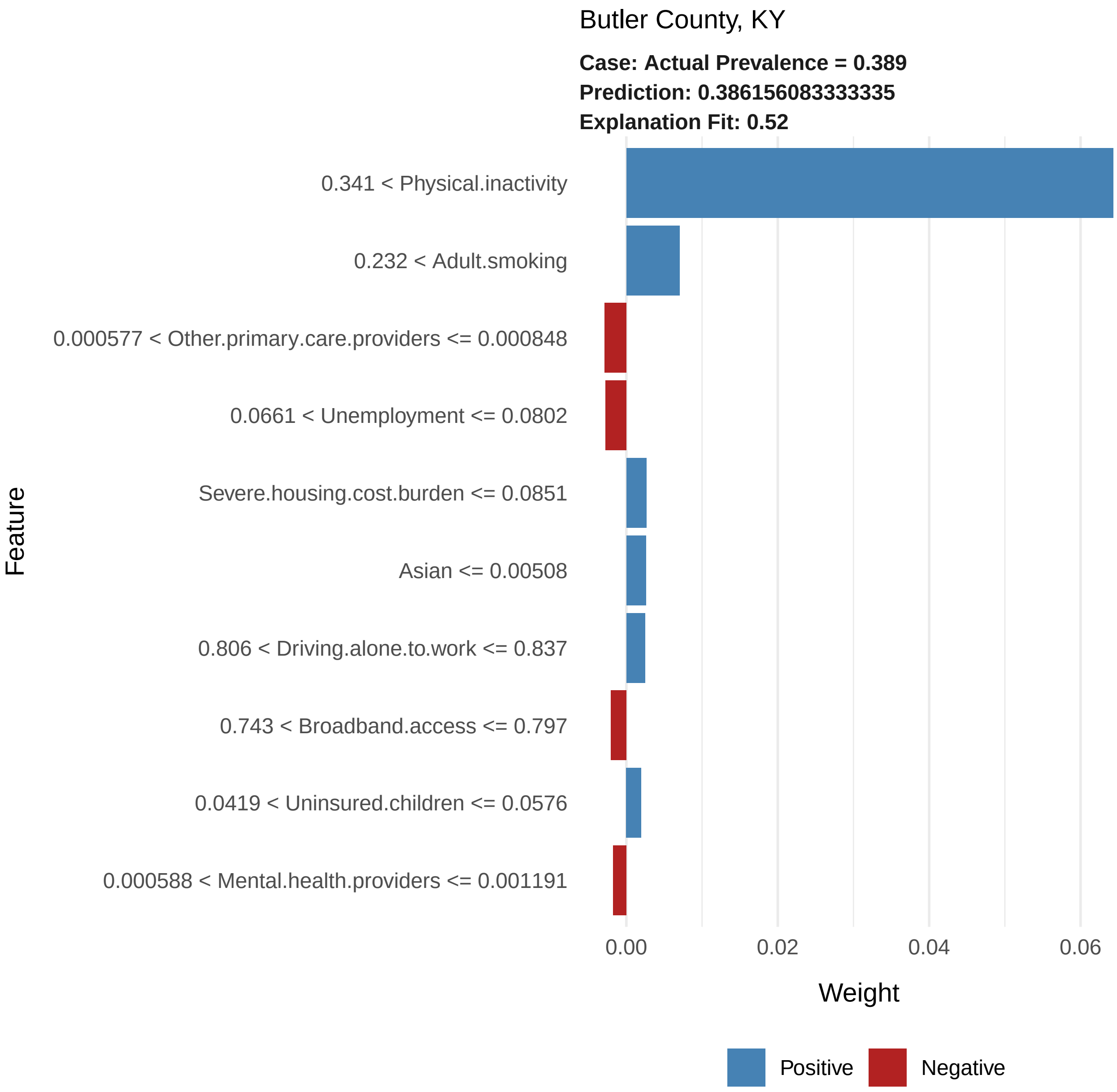
Butler County, KY
Case: Actual Prevalence = 0.389
Prediction: 0.386156083333335
Explanation Fit: 0.52
0.341 < Physical.inactivity
0.232 < Adult.smoking
0.000577 < Other.primary.care.providers <= 0.000848
0.0661 < Unemployment <= 0.0802
Severe.housing.cost.burden <= 0.0851
Asian <= 0.00508
0.806 < Driving.alone.to.work <= 0.837
0.743 < Broadband.access <= 0.797
0.0419 < Uninsured.children <= 0.0576
0.000588 < Mental.health.providers <= 0.001191
Feature
0.00
0.02
0.04
0.06
Weight
Positive
Negative

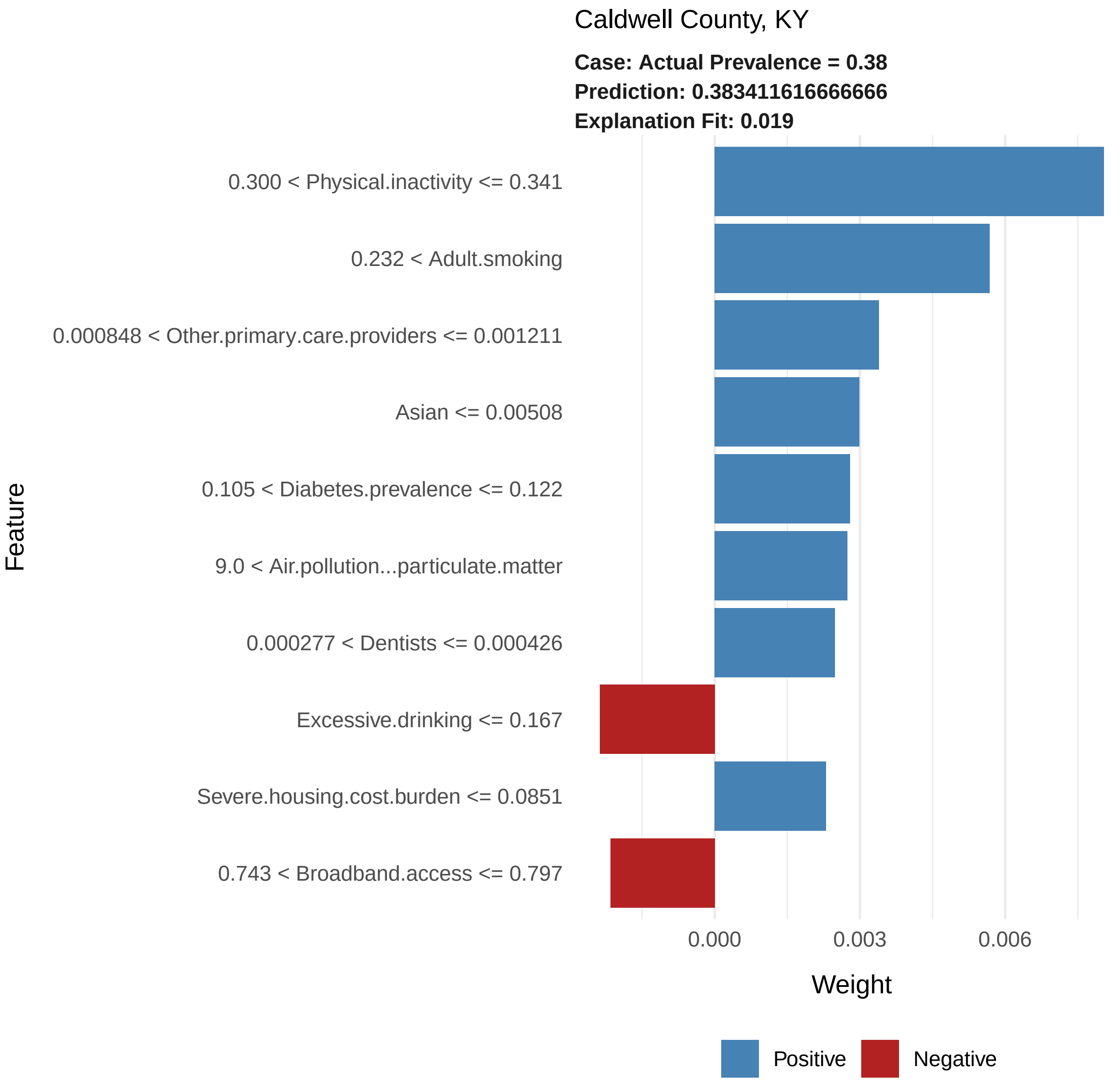

Caldwell County, KY
Case: Actual Prevalence = 0.38
Prediction: 0.383411616666666
Explanation Fit: 0.019
0.300 < Physical.inactivity <= 0.341
0.232 < Adult.smoking
0.000848 < Other.primary.care.providers <= 0.001211
Asian <= 0.00508
0.105 < Diabetes.prevalence <= 0.122
9.0 < Air.pollution...particulate.matter
0.000277 < Dentists <= 0.000426
Excessive.drinking <= 0.167
Severe.housing.cost.burden <= 0.0851
0.743 < Broadband.access <= 0.797
Feature
0.000
0.003
0.006
Weight
Positive
Negative

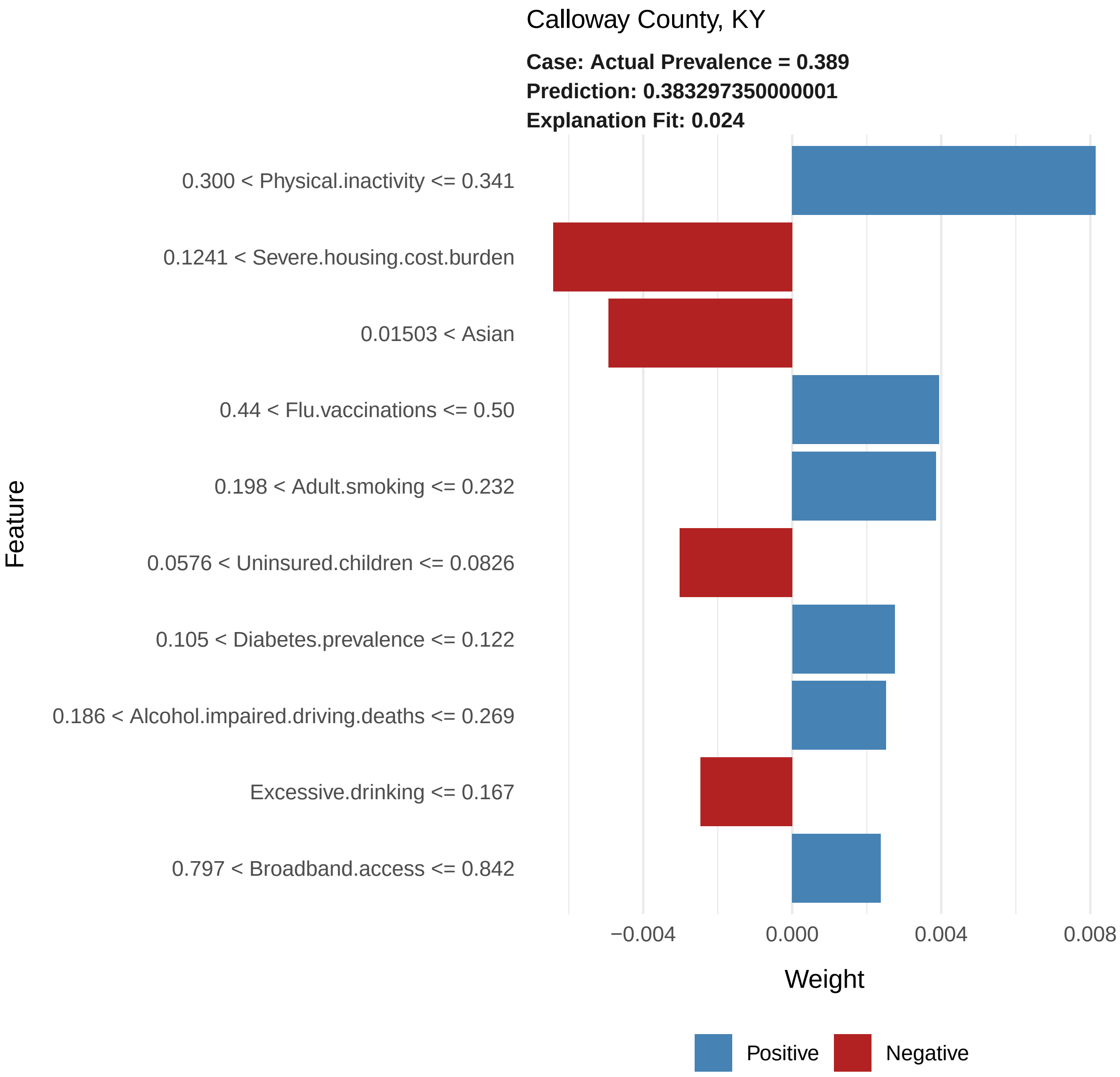

Calloway County, KY
Case: Actual Prevalence = 0.389
Prediction: 0.383297350000001
Explanation Fit: 0.024
0.300 < Physical.inactivity <= 0.341
0.1241 < Severe.housing.cost.burden
0.01503 < Asian
0.44 < Flu.vaccinations <= 0.50
0.198 < Adult.smoking <= 0.232
0.0576 < Uninsured.children <= 0.0826
0.105 < Diabetes.prevalence <= 0.122
0.186 < Alcohol.impaired.driving.deaths <= 0.269
Excessive.drinking <= 0.167
0.797 < Broadband.access <= 0.842
Feature
−0.004
0.000
0.004
0.008
Weight
Positive
Negative

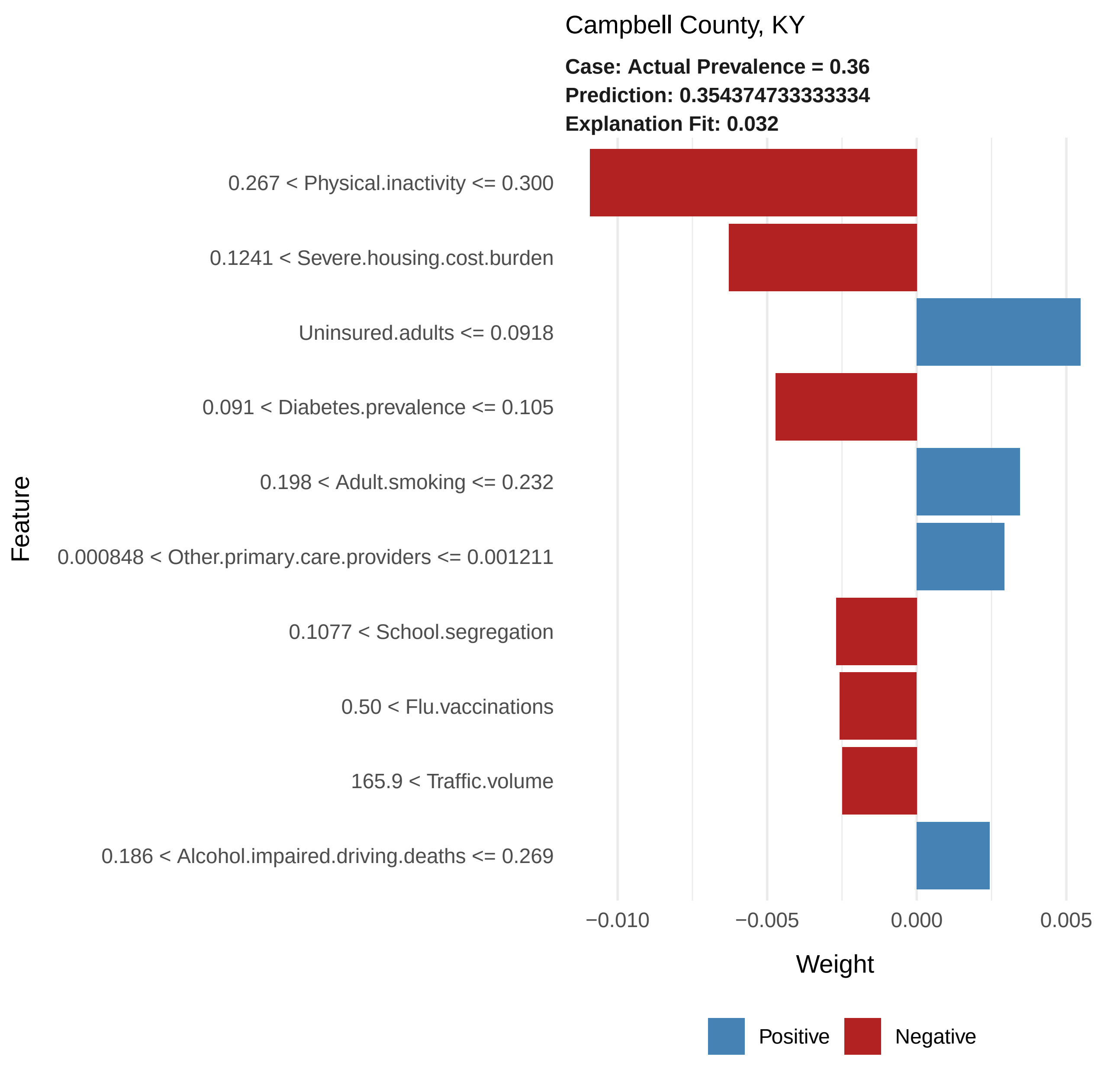

Campbell County, KY
Case: Actual Prevalence = 0.36
Prediction: 0.354374733333334
Explanation Fit: 0.032
0.267 < Physical.inactivity <= 0.300
0.1241 < Severe.housing.cost.burden
Uninsured.adults <= 0.0918
0.091 < Diabetes.prevalence <= 0.105
0.198 < Adult.smoking <= 0.232
0.000848 < Other.primary.care.providers <= 0.001211
0.1077 < School.segregation
0.50 < Flu.vaccinations
165.9 < Traffic.volume
0.186 < Alcohol.impaired.driving.deaths <= 0.269
Feature
−0.010
−0.005
0.000
0.005
Weight
Positive
Negative

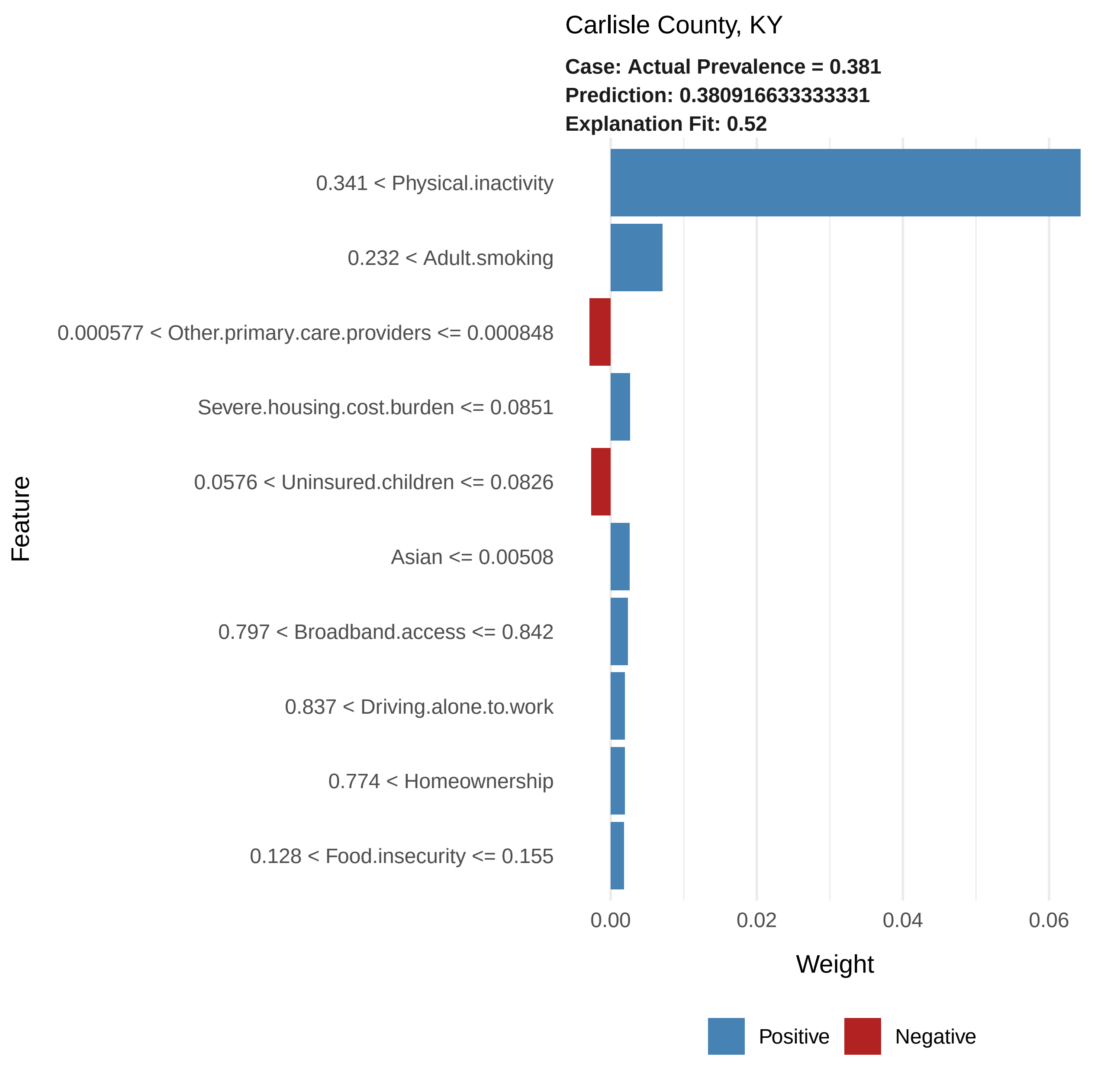

Carlisle County, KY
Case: Actual Prevalence = 0.381
Prediction: 0.380916633333331
Explanation Fit: 0.52
0.341 < Physical.inactivity
0.232 < Adult.smoking
0.000577 < Other.primary.care.providers <= 0.000848
Severe.housing.cost.burden <= 0.0851
0.0576 < Uninsured.children <= 0.0826
Asian <= 0.00508
0.797 < Broadband.access <= 0.842
0.837 < Driving.alone.to.work
0.774 < Homeownership
0.128 < Food.insecurity <= 0.155
Feature
0.00
0.02
0.04
0.06
Weight
Positive
Negative

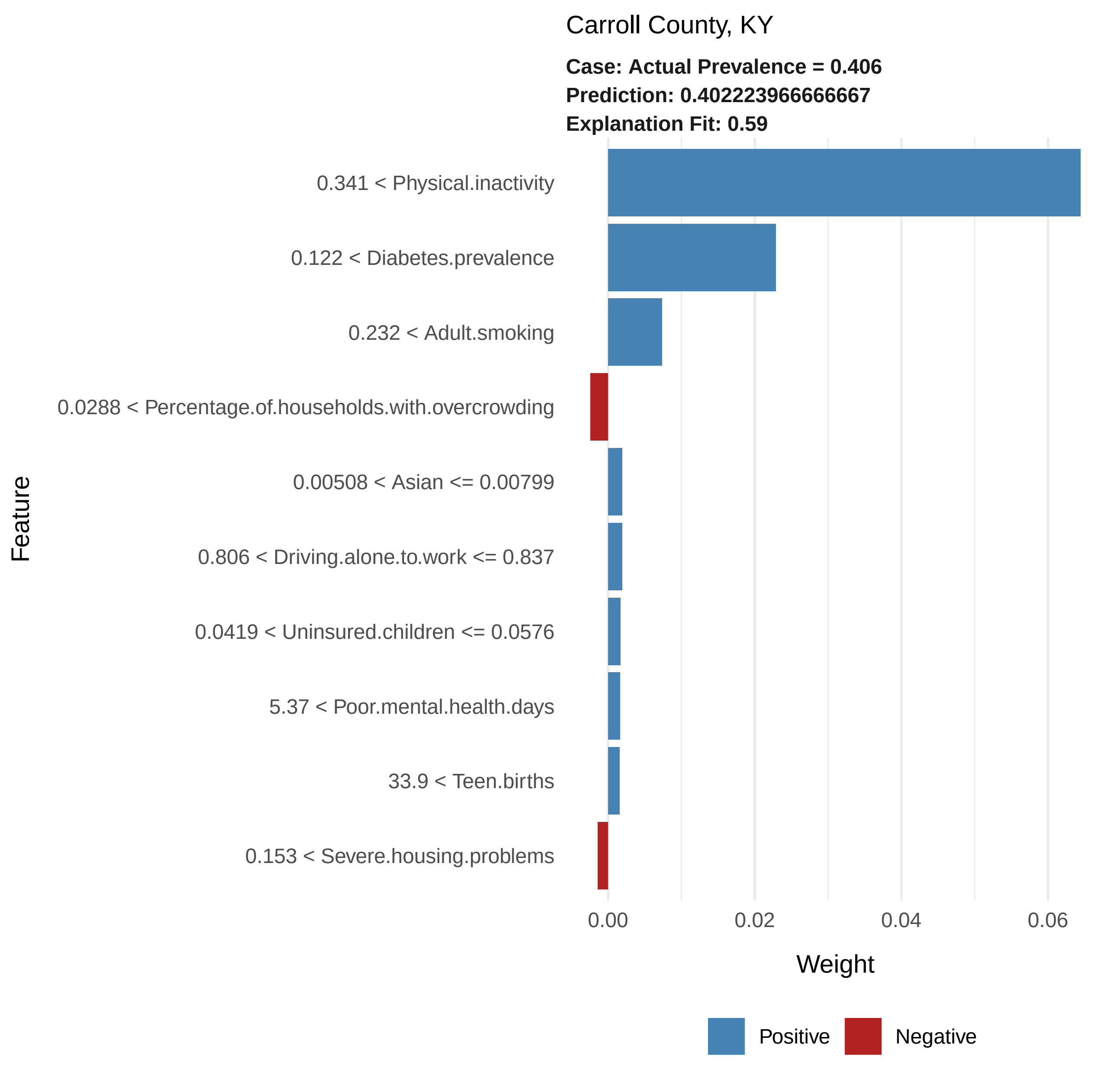

Carroll County, KY
Case: Actual Prevalence = 0.406
Prediction: 0.402223966666667
Explanation Fit: 0.59
0.341 < Physical.inactivity
0.122 < Diabetes.prevalence
0.232 < Adult.smoking
0.0288 < Percentage.of.households.with.overcrowding
0.00508 < Asian <= 0.00799
0.806 < Driving.alone.to.work <= 0.837
0.0419 < Uninsured.children <= 0.0576
5.37 < Poor.mental.health.days
33.9 < Teen.births
0.153 < Severe.housing.problems
Feature
0.00
0.02
0.04
0.06
Weight
Positive
Negative

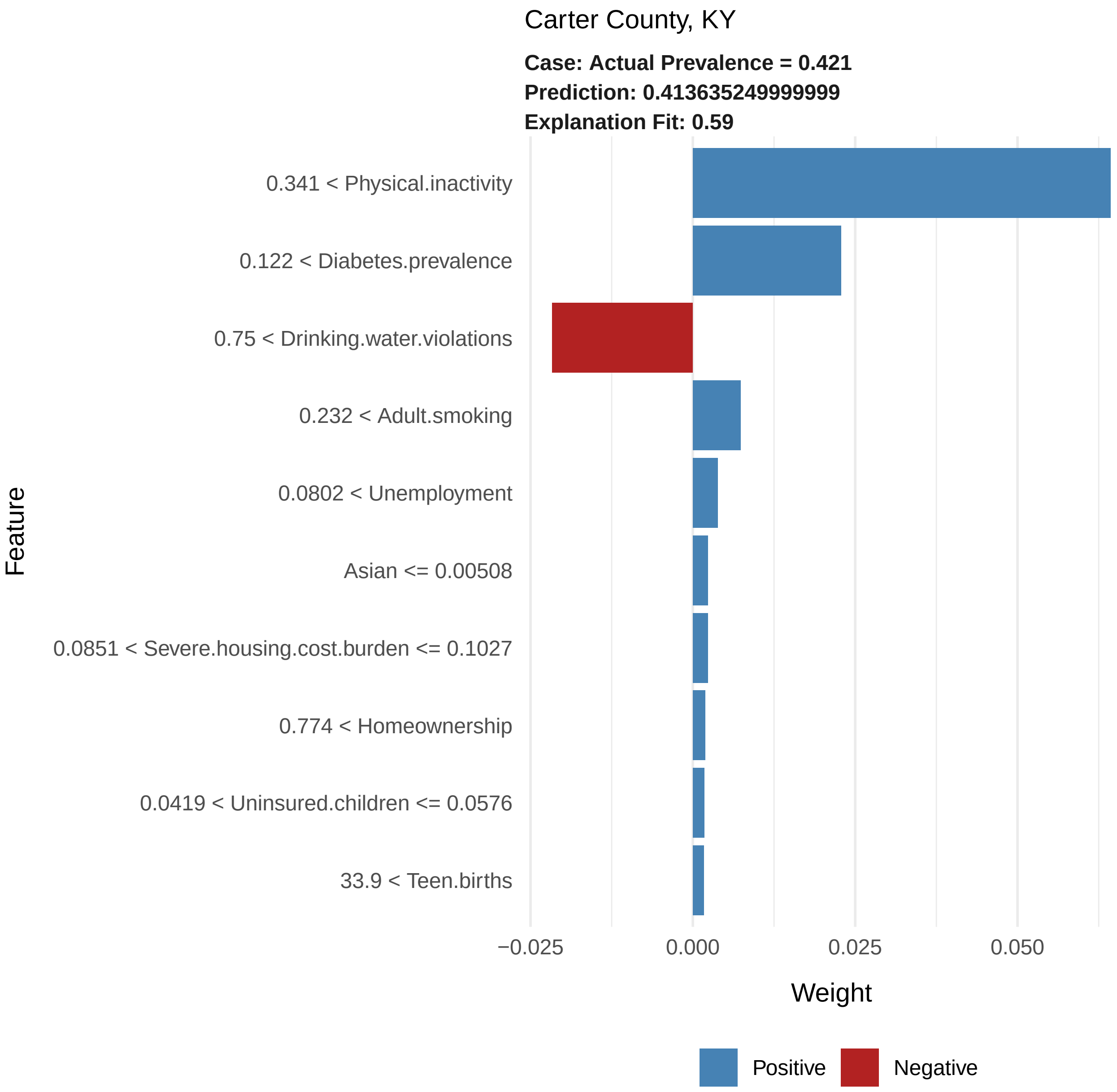
Carter County, KY
Case: Actual Prevalence = 0.421
Prediction: 0.413635249999999
Explanation Fit: 0.59
0.341 < Physical.inactivity
0.122 < Diabetes.prevalence
0.75 < Drinking.water.violations
0.232 < Adult.smoking
0.0802 < Unemployment
Asian <= 0.00508
0.0851 < Severe.housing.cost.burden <= 0.1027
0.774 < Homeownership
0.0419 < Uninsured.children <= 0.0576
33.9 < Teen.births
Feature
−0.025
0.000
0.025
0.050
Weight
Positive
Negative

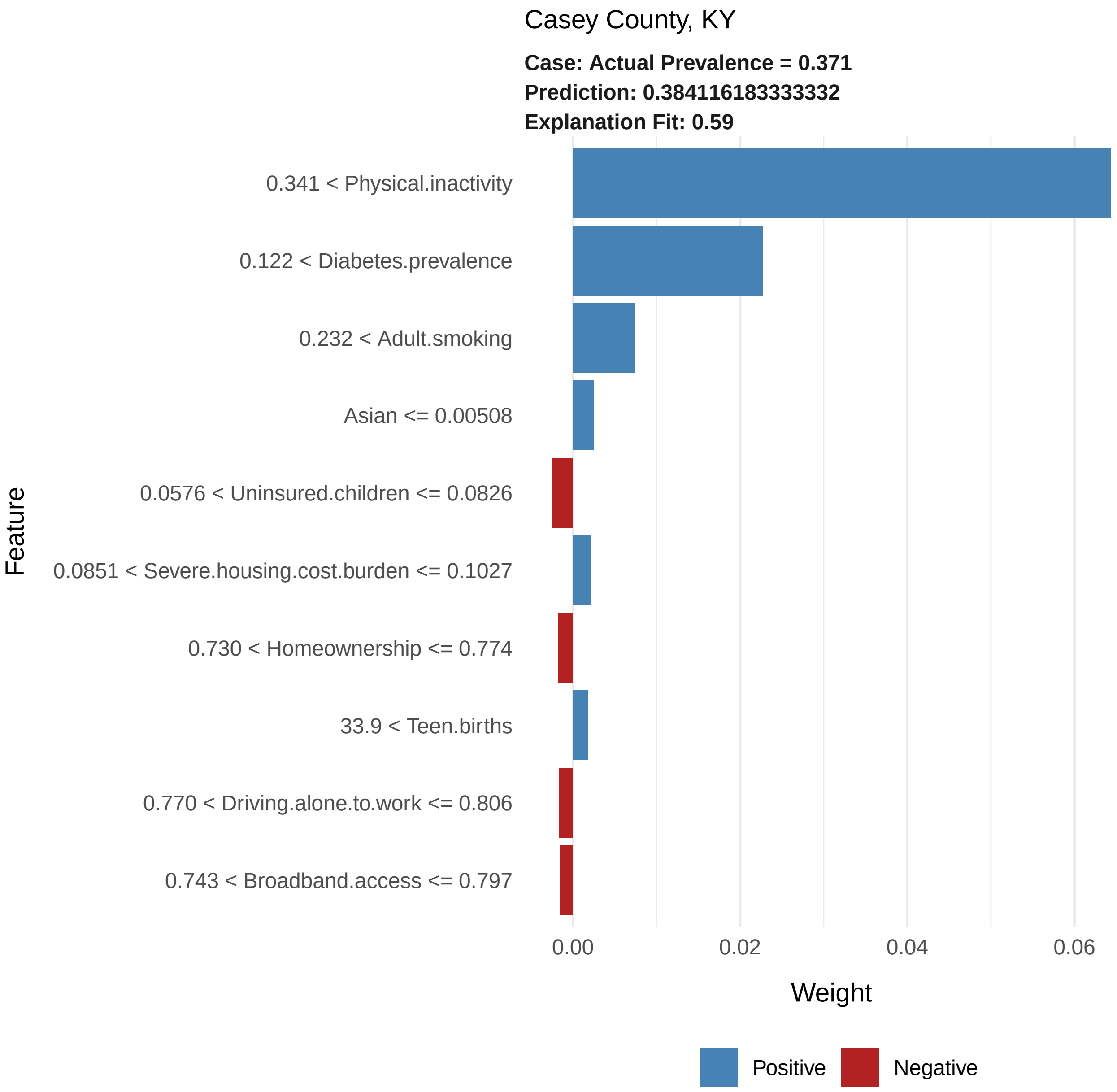
Casey County, KY
Case: Actual Prevalence = 0.371
Prediction: 0.384116183333332
Explanation Fit: 0.59
0.341 < Physical.inactivity
0.122 < Diabetes.prevalence
0.232 < Adult.smoking
Asian <= 0.00508
0.0576 < Uninsured.children <= 0.0826
0.0851 < Severe.housing.cost.burden <= 0.1027
0.730 < Homeownership <= 0.774
33.9 < Teen.births
0.770 < Driving.alone.to.work <= 0.806
0.743 < Broadband.access <= 0.797
Feature
0.00
0.02
0.04
0.06
Weight
Positive
Negative

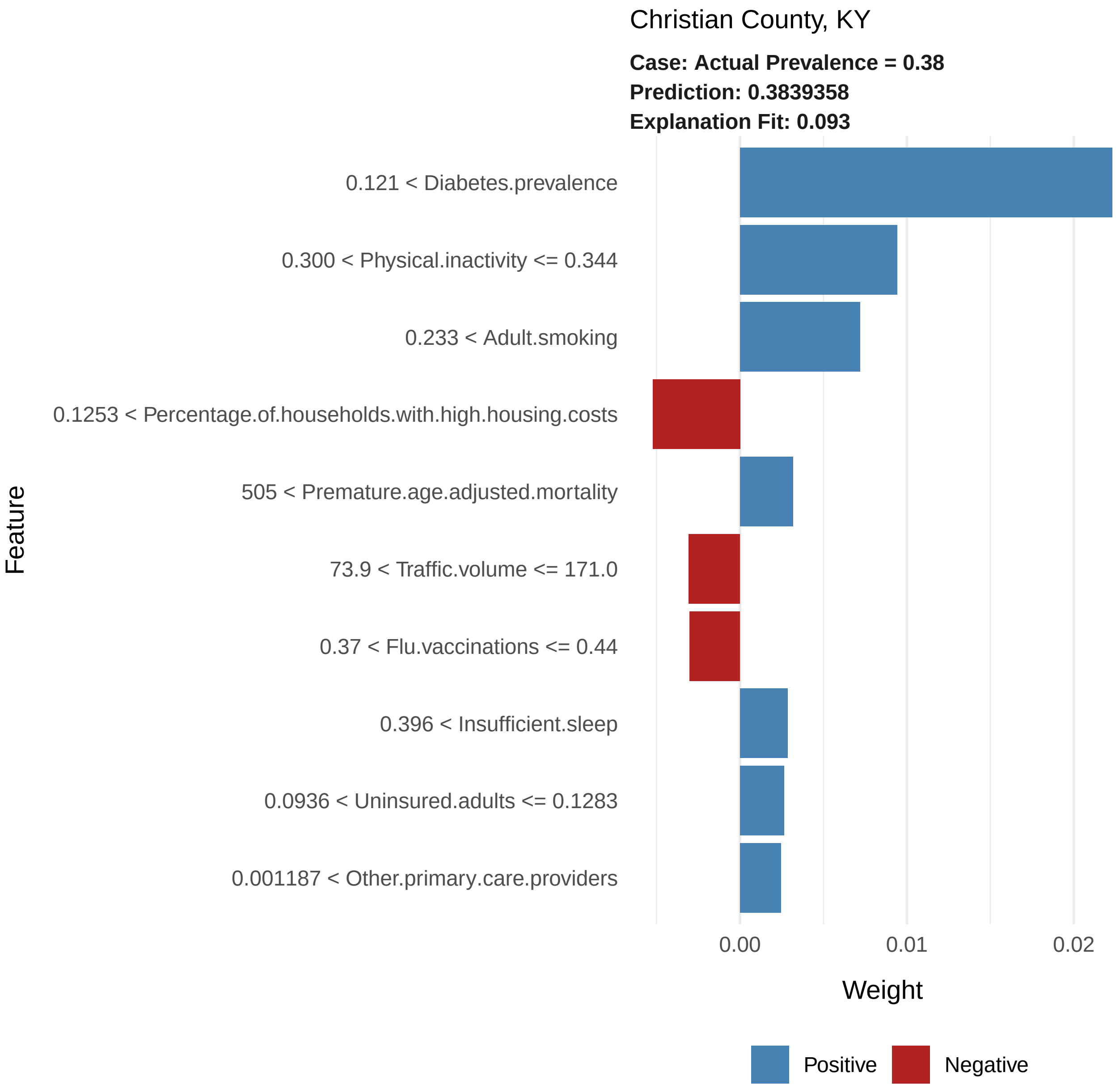

Christian County, KY
Case: Actual Prevalence = 0.38
Prediction: 0.3839358
Explanation Fit: 0.093
0.121 < Diabetes.prevalence
0.300 < Physical.inactivity <= 0.344
0.233 < Adult.smoking
0.1253 < Percentage.of.households.with.high.housing.costs
505 < Premature.age.adjusted.mortality
73.9 < Traffic.volume <= 171.0
0.37 < Flu.vaccinations <= 0.44
0.396 < Insufficient.sleep
0.0936 < Uninsured.adults <= 0.1283
0.001187 < Other.primary.care.providers
Feature
0.00
0.01
0.02
Weight
Positive
Negative

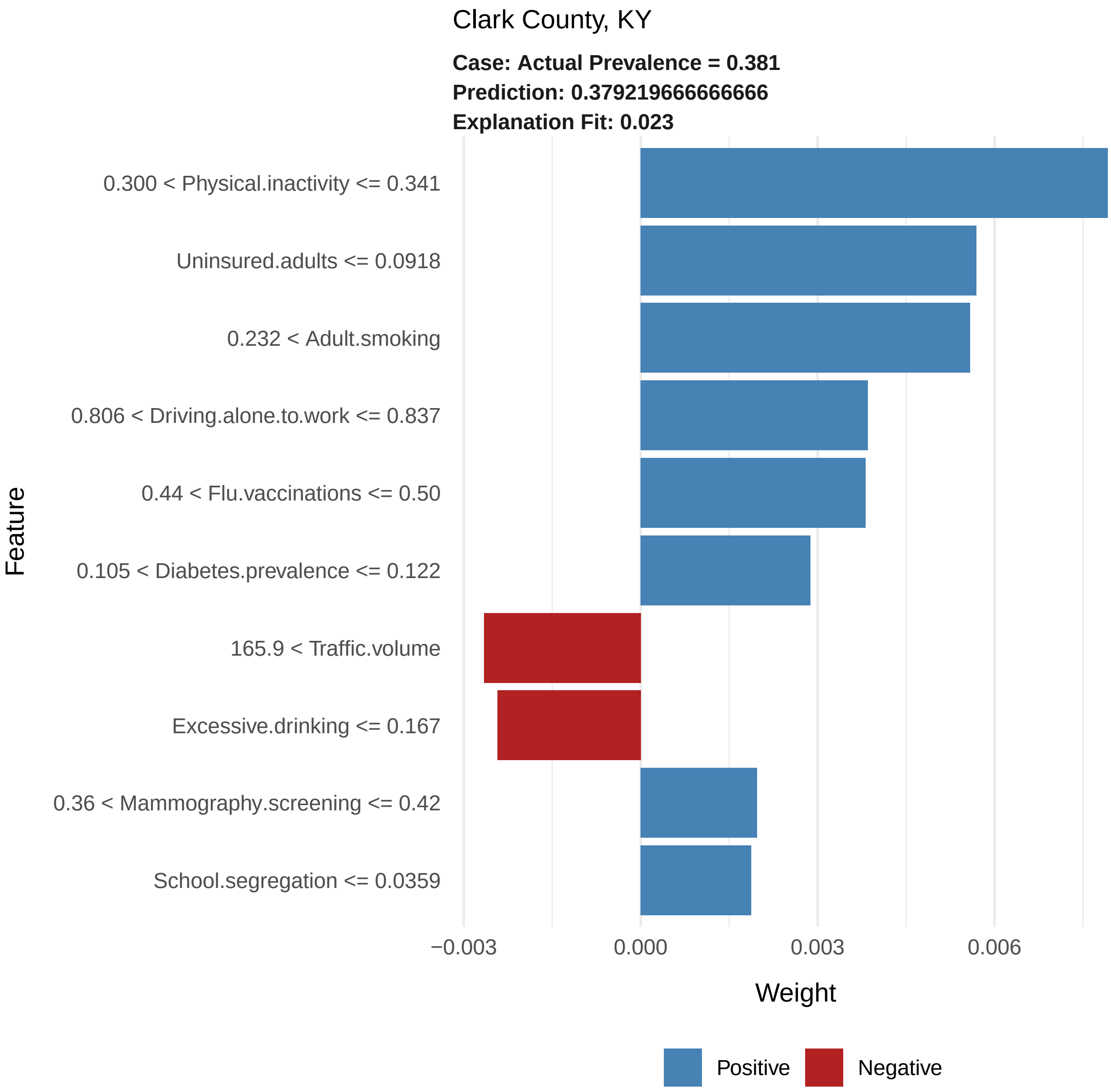

Clark County, KY
Case: Actual Prevalence = 0.381
Prediction: 0.379219666666666
Explanation Fit: 0.023
0.300 < Physical.inactivity <= 0.341
Uninsured.adults <= 0.0918
0.232 < Adult.smoking
0.806 < Driving.alone.to.work <= 0.837
0.44 < Flu.vaccinations <= 0.50
0.105 < Diabetes.prevalence <= 0.122
165.9 < Traffic.volume
Excessive.drinking <= 0.167
0.36 < Mammography.screening <= 0.42
School.segregation <= 0.0359
Feature
−0.003
0.000
0.003
0.006
Weight
Positive
Negative

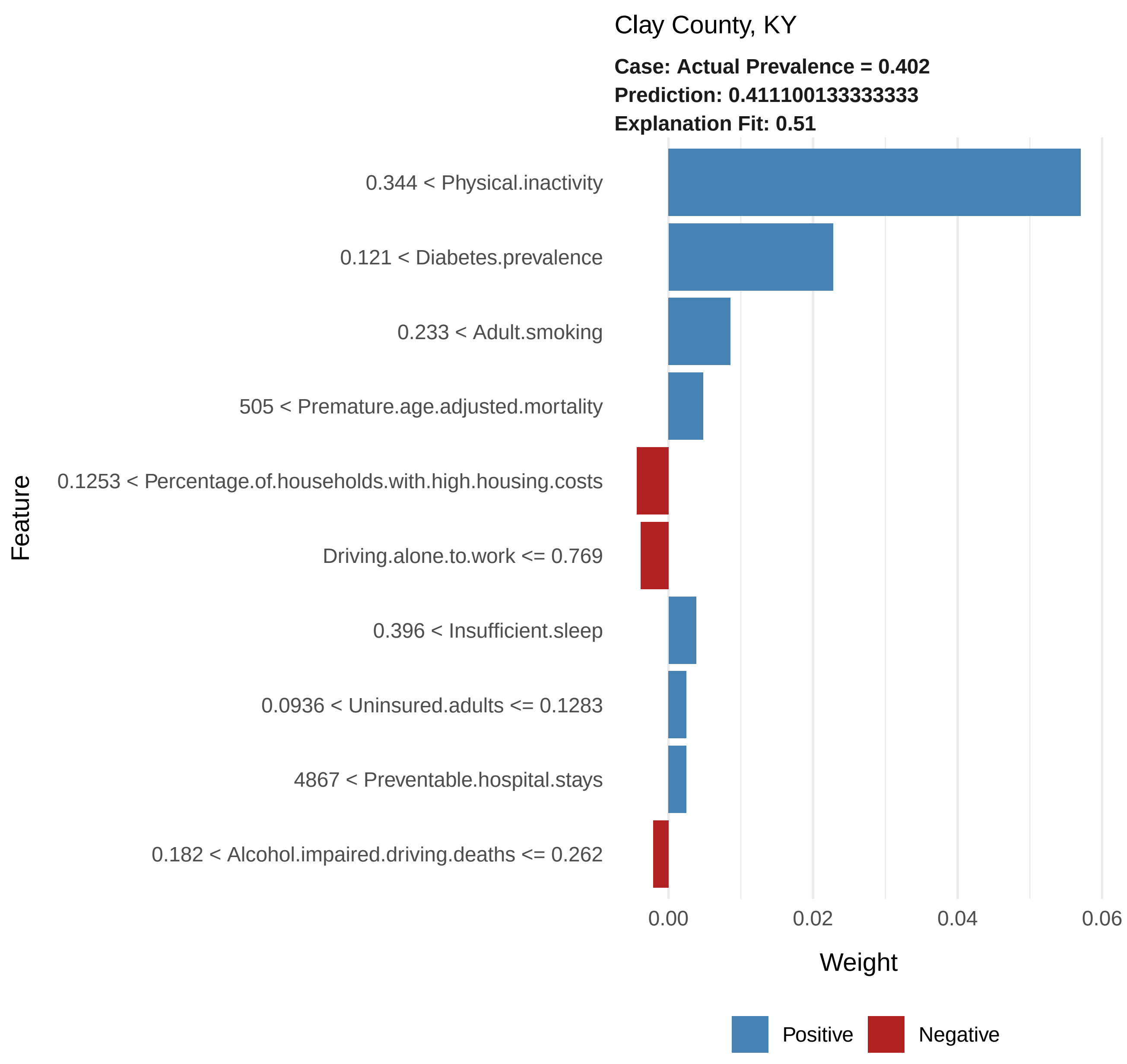

Clay County, KY
Case: Actual Prevalence = 0.402
Prediction: 0.411100133333333
Explanation Fit: 0.51
0.344 < Physical.inactivity
0.121 < Diabetes.prevalence
0.233 < Adult.smoking
505 < Premature.age.adjusted.mortality
0.1253 < Percentage.of.households.with.high.housing.costs
Driving.alone.to.work <= 0.769
0.396 < Insufficient.sleep
0.0936 < Uninsured.adults <= 0.1283
4867 < Preventable.hospital.stays
0.182 < Alcohol.impaired.driving.deaths <= 0.262
Feature
0.00
0.02
0.04
0.06
Weight
Positive
Negative

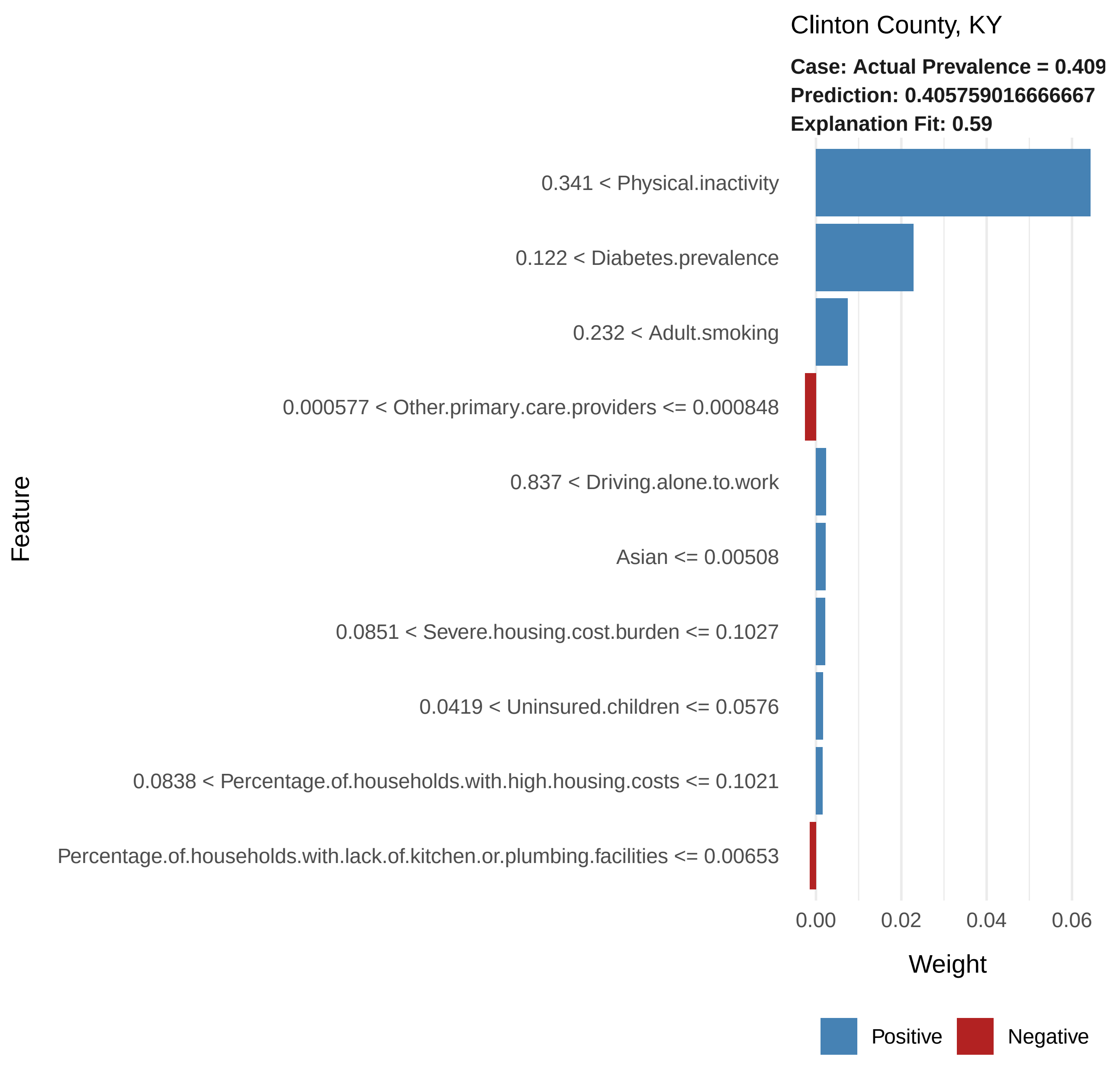
Clinton County, KY
Case: Actual Prevalence = 0.409
Prediction: 0.405759016666667
Explanation Fit: 0.59
0.341 < Physical.inactivity
0.122 < Diabetes.prevalence
0.232 < Adult.smoking
0.000577 < Other.primary.care.providers <= 0.000848
0.837 < Driving.alone.to.work
Asian <= 0.00508
0.0851 < Severe.housing.cost.burden <= 0.1027
0.0419 < Uninsured.children <= 0.0576
0.0838 < Percentage.of.households.with.high.housing.costs <= 0.1021
Percentage.of.households.with.lack.of.kitchen.or.plumbing.facilities <= 0.00653
Feature
0.00
0.02
0.04
0.06
Weight
Positive
Negative

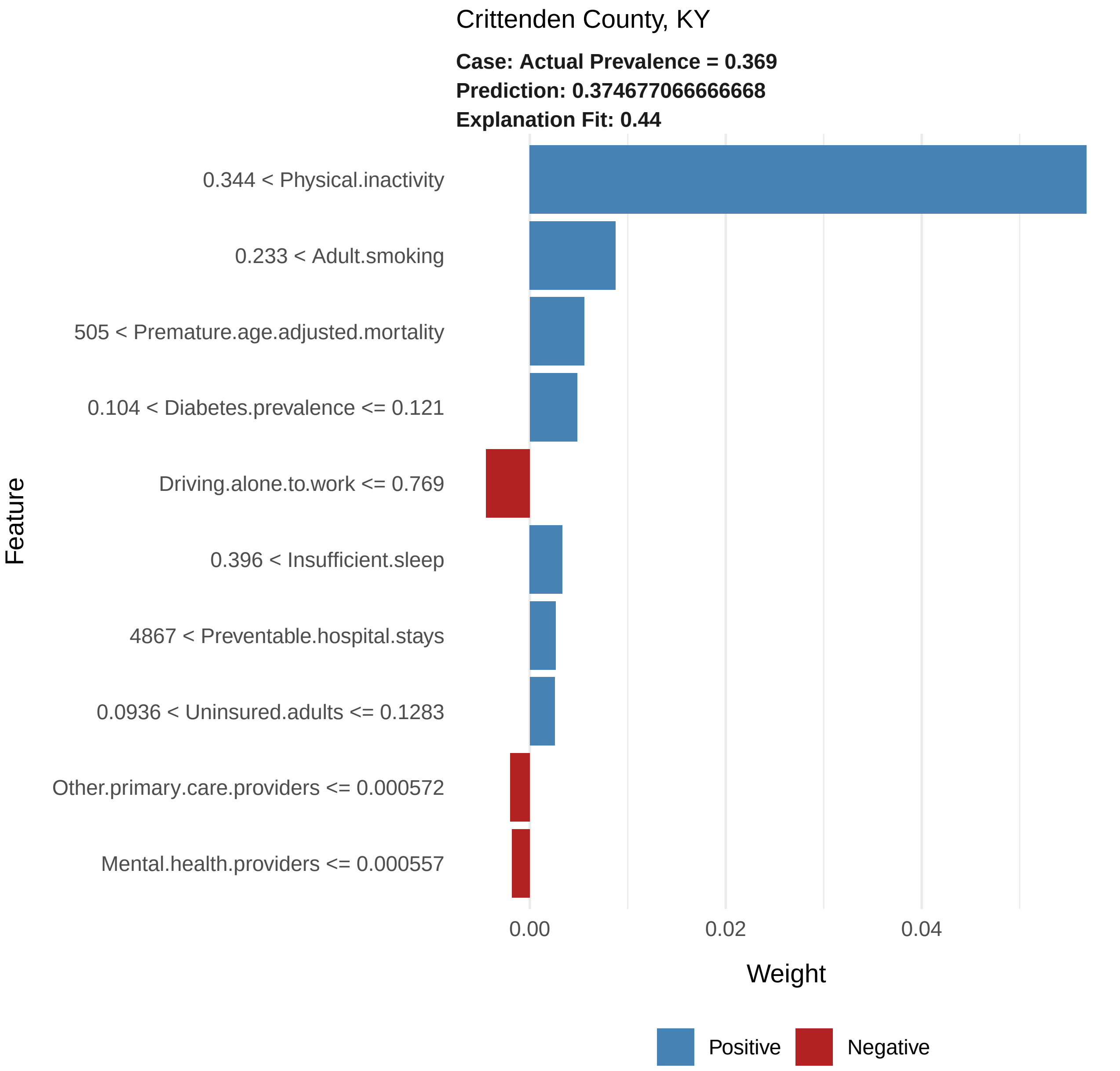

Crittenden County, KY
Case: Actual Prevalence = 0.369
Prediction: 0.374677066666668
Explanation Fit: 0.44
0.344 < Physical.inactivity
0.233 < Adult.smoking
505 < Premature.age.adjusted.mortality
0.104 < Diabetes.prevalence <= 0.121
Driving.alone.to.work <= 0.769
0.396 < Insufficient.sleep
4867 < Preventable.hospital.stays
0.0936 < Uninsured.adults <= 0.1283
Other.primary.care.providers <= 0.000572
Mental.health.providers <= 0.000557
Feature
0.00
0.02
0.04
Weight
Positive
Negative

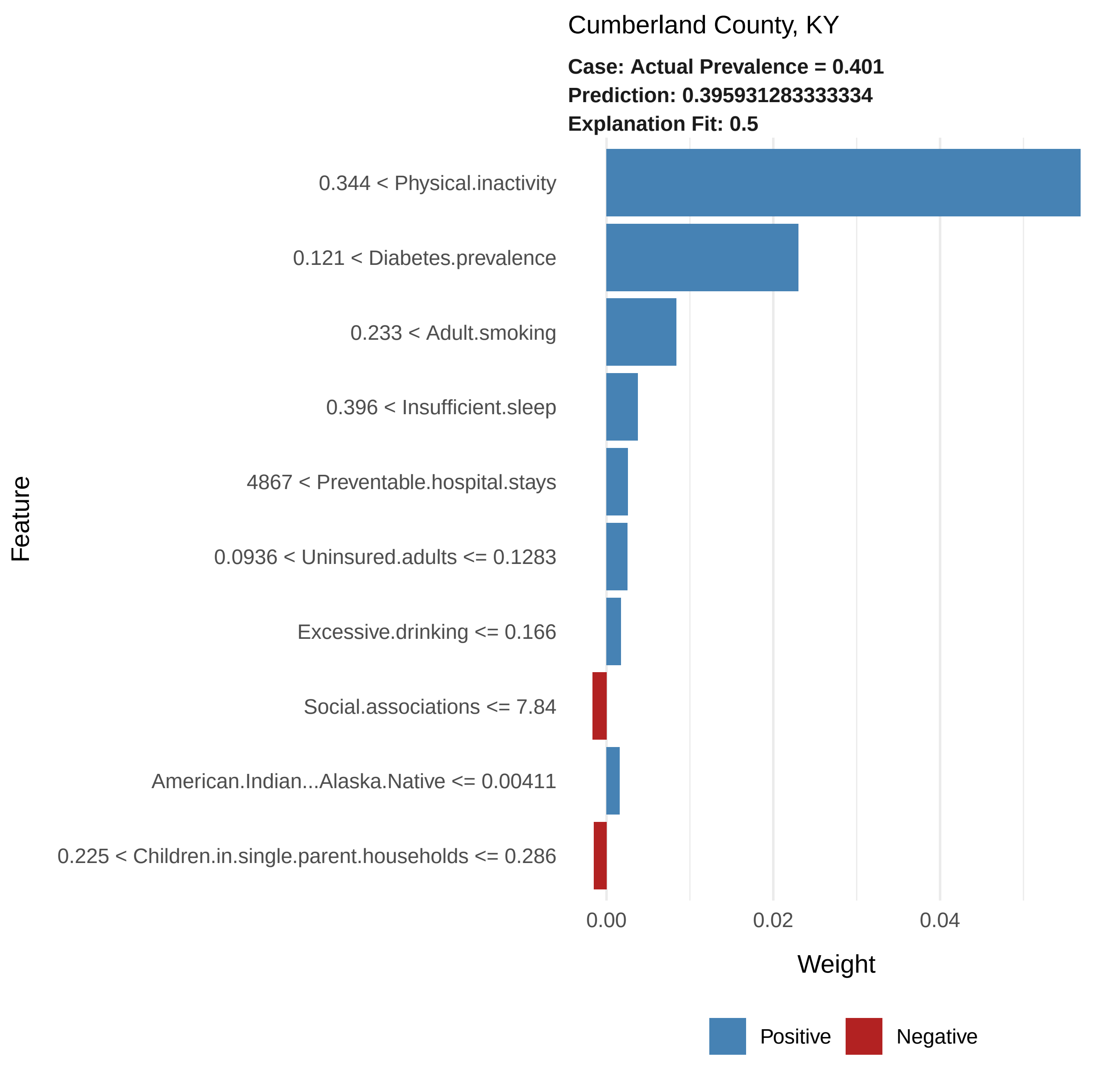

Cumberland County, KY
Case: Actual Prevalence = 0.401
Prediction: 0.395931283333334
Explanation Fit: 0.5
0.344 < Physical.inactivity
0.121 < Diabetes.prevalence
0.233 < Adult.smoking
0.396 < Insufficient.sleep
4867 < Preventable.hospital.stays
0.0936 < Uninsured.adults <= 0.1283
Excessive.drinking <= 0.166
Social.associations <= 7.84
American.Indian...Alaska.Native <= 0.00411
0.225 < Children.in.single.parent.households <= 0.286
Feature
0.00
0.02
0.04
Weight
Positive
Negative

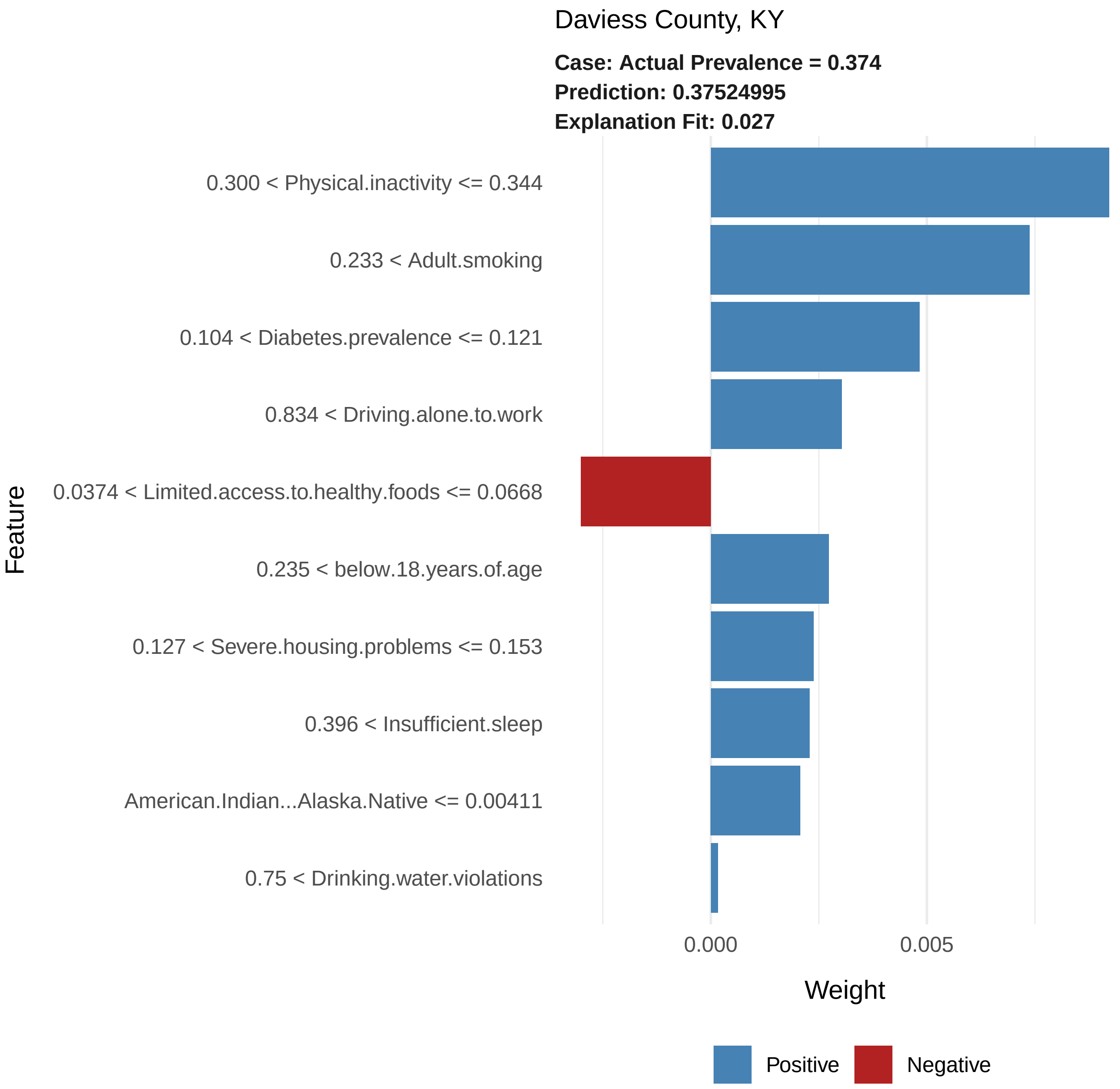
Daviess County, KY
Case: Actual Prevalence = 0.374
Prediction: 0.37524995
Explanation Fit: 0.027
0.300 < Physical.inactivity <= 0.344
0.233 < Adult.smoking
0.104 < Diabetes.prevalence <= 0.121
0.834 < Driving.alone.to.work
0.0374 < Limited.access.to.healthy.foods <= 0.0668
0.235 < below.18.years.of.age
0.127 < Severe.housing.problems <= 0.153
0.396 < Insufficient.sleep
American.Indian...Alaska.Native <= 0.00411
0.75 < Drinking.water.violations
Feature
0.000
0.005
Weight
Positive
Negative

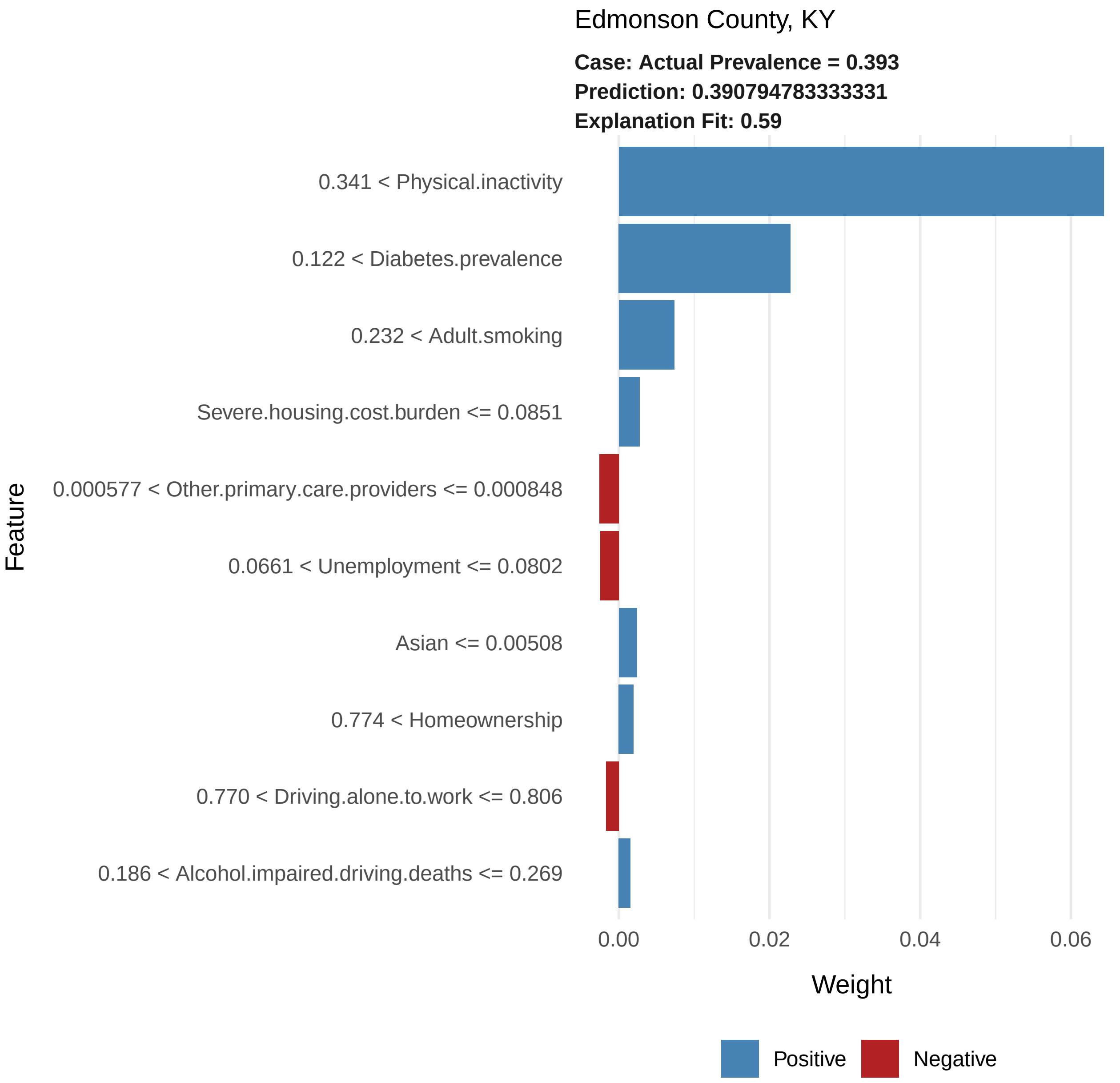

Edmonson County, KY
Case: Actual Prevalence = 0.393
Prediction: 0.390794783333331
Explanation Fit: 0.59
0.341 < Physical.inactivity
0.122 < Diabetes.prevalence
0.232 < Adult.smoking
Severe.housing.cost.burden <= 0.0851
0.000577 < Other.primary.care.providers <= 0.000848
0.0661 < Unemployment <= 0.0802
Asian <= 0.00508
0.774 < Homeownership
0.770 < Driving.alone.to.work <= 0.806
0.186 < Alcohol.impaired.driving.deaths <= 0.269
Feature
0.00
0.02
0.04
0.06
Weight
Positive
Negative

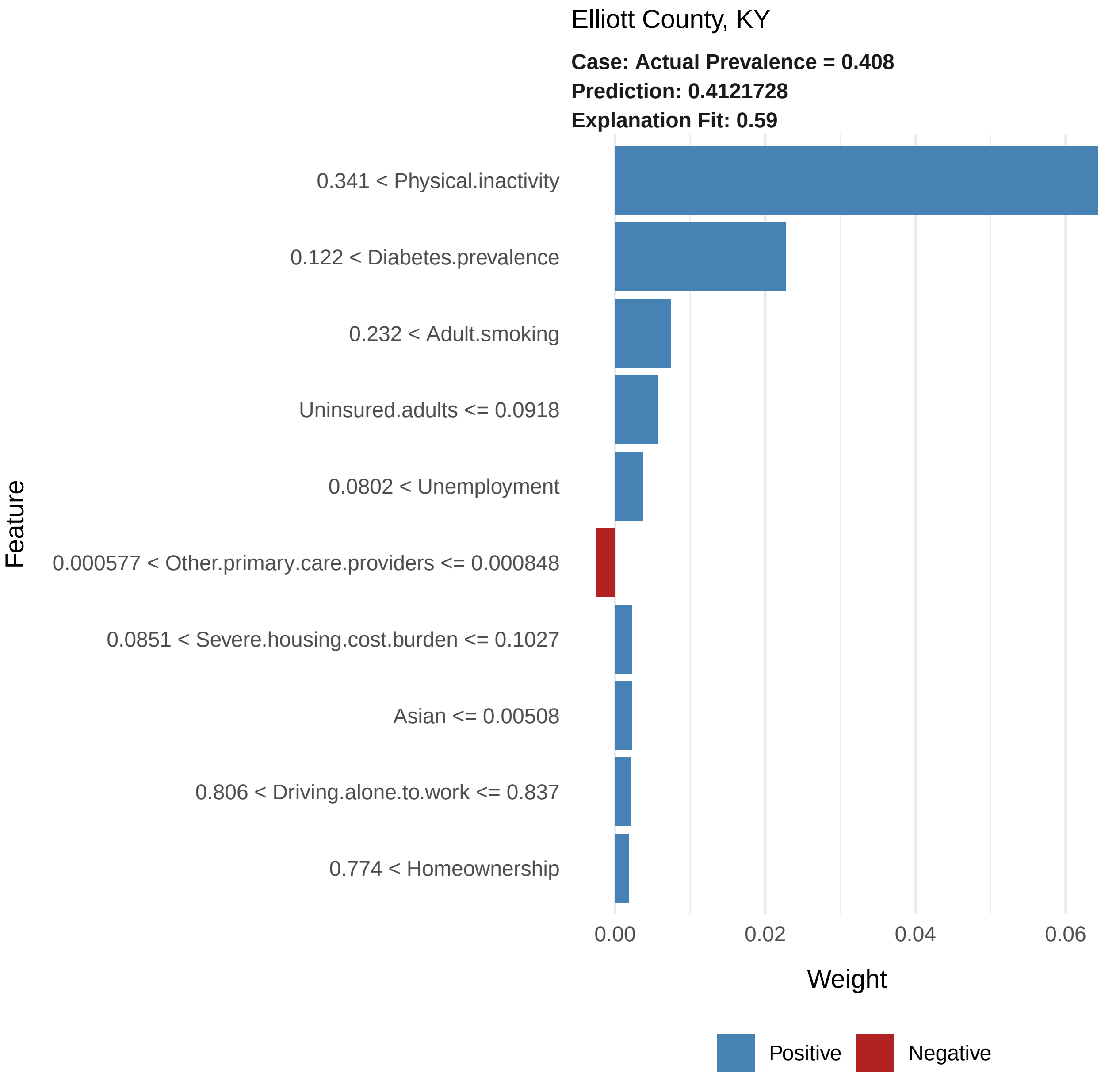

Elliott County, KY
Case: Actual Prevalence = 0.408
Prediction: 0.4121728
Explanation Fit: 0.59
0.341 < Physical.inactivity
0.122 < Diabetes.prevalence
0.232 < Adult.smoking
Uninsured.adults <= 0.0918
0.0802 < Unemployment
0.000577 < Other.primary.care.providers <= 0.000848
0.0851 < Severe.housing.cost.burden <= 0.1027
Asian <= 0.00508
0.806 < Driving.alone.to.work <= 0.837
0.774 < Homeownership
Feature
0.00
0.02
0.04
0.06
Weight
Positive
Negative

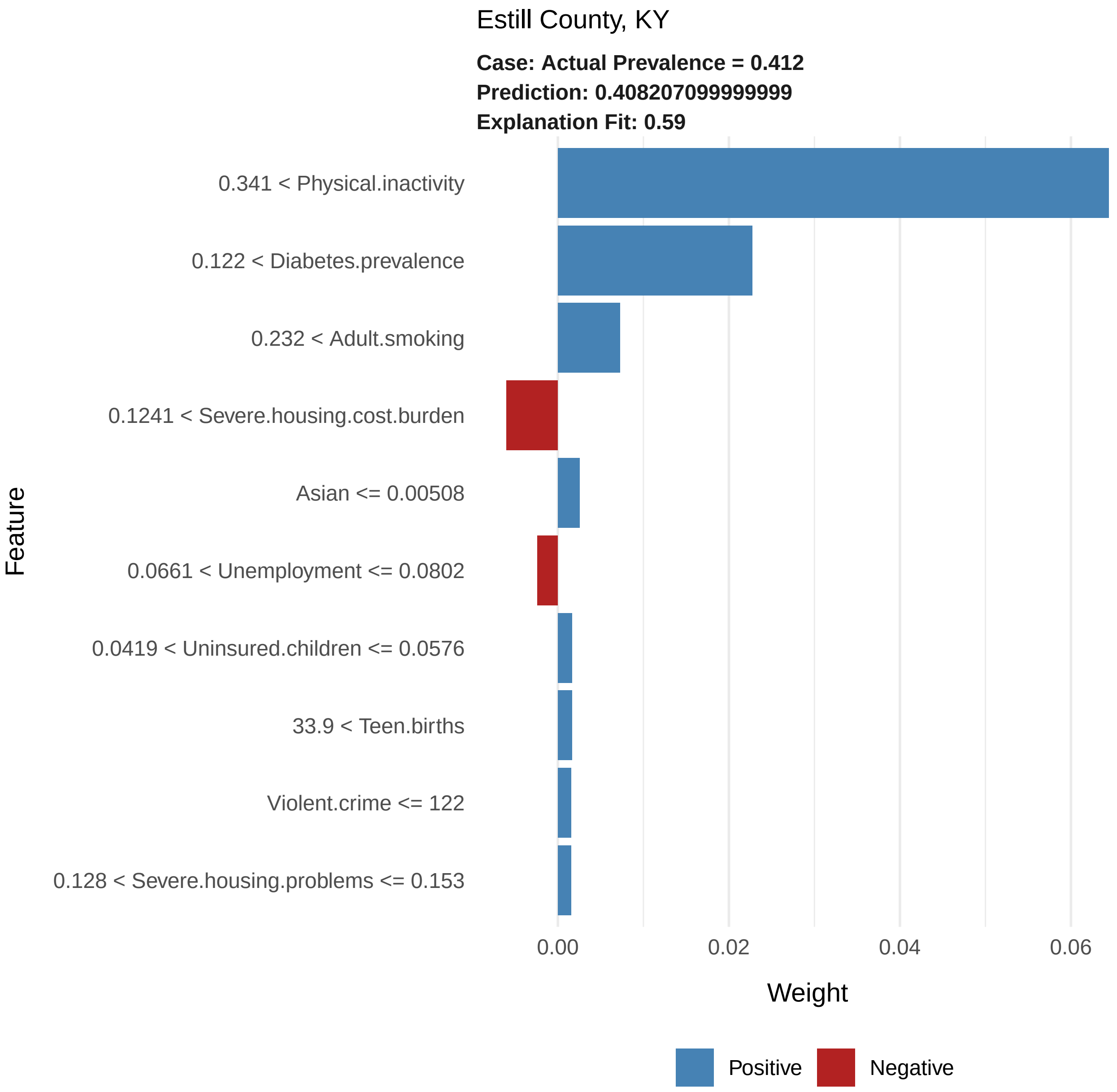

Estill County, KY
Case: Actual Prevalence = 0.412
Prediction: 0.408207099999999
Explanation Fit: 0.59
0.341 < Physical.inactivity
0.122 < Diabetes.prevalence
0.232 < Adult.smoking
0.1241 < Severe.housing.cost.burden
Asian <= 0.00508
0.0661 < Unemployment <= 0.0802
0.0419 < Uninsured.children <= 0.0576
33.9 < Teen.births
Violent.crime <= 122
0.128 < Severe.housing.problems <= 0.153
Feature
0.00
0.02
0.04
0.06
Weight
Positive
Negative

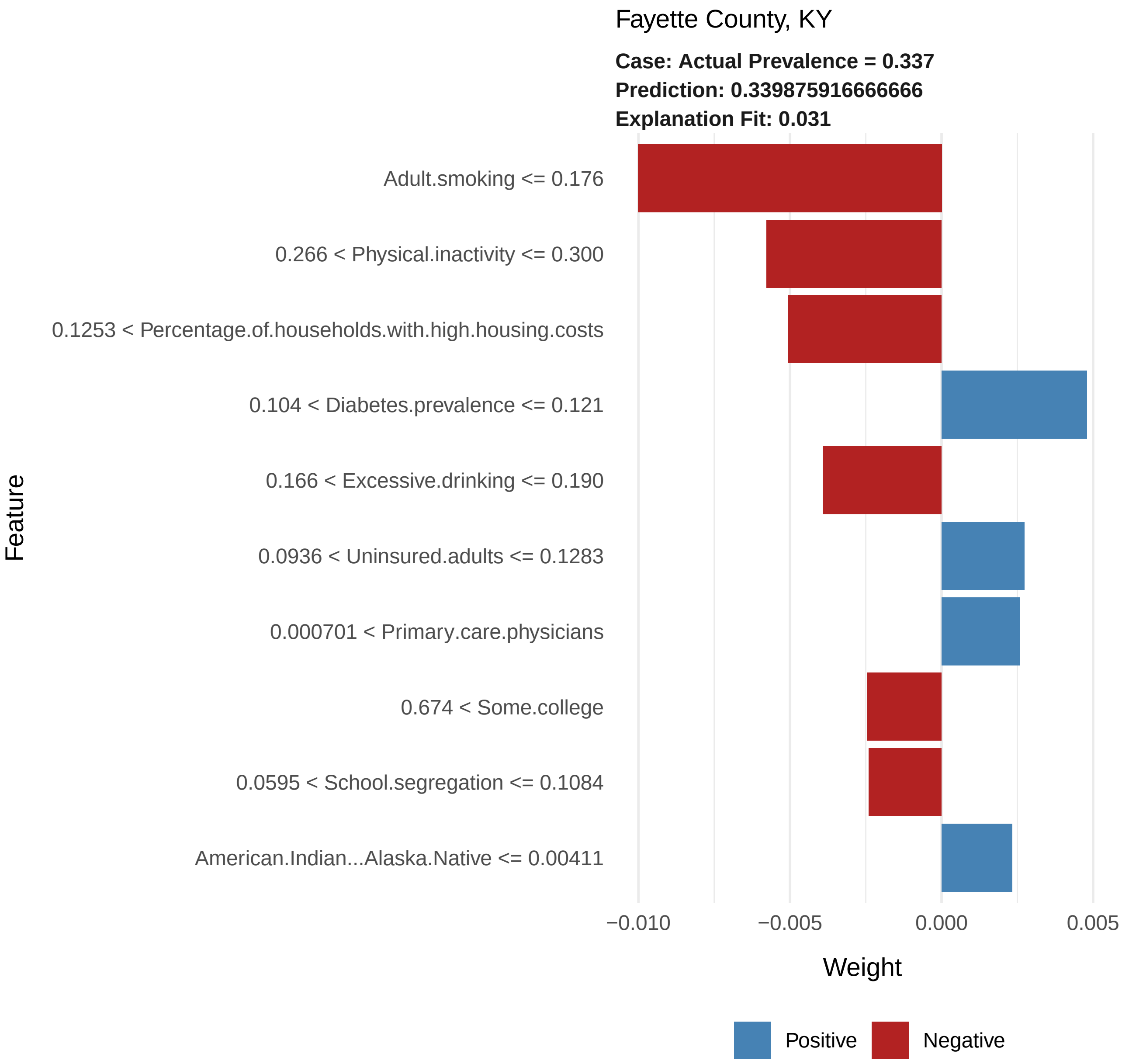

Fayette County, KY
Case: Actual Prevalence = 0.337
Prediction: 0.339875916666666
Explanation Fit: 0.031
Adult.smoking <= 0.176
0.266 < Physical.inactivity <= 0.300
0.1253 < Percentage.of.households.with.high.housing.costs
0.104 < Diabetes.prevalence <= 0.121
0.166 < Excessive.drinking <= 0.190
0.0936 < Uninsured.adults <= 0.1283
0.000701 < Primary.care.physicians
0.674 < Some.college
0.0595 < School.segregation <= 0.1084
American.Indian...Alaska.Native <= 0.00411
Feature
−0.010
−0.005
0.000
0.005
Weight
Positive
Negative

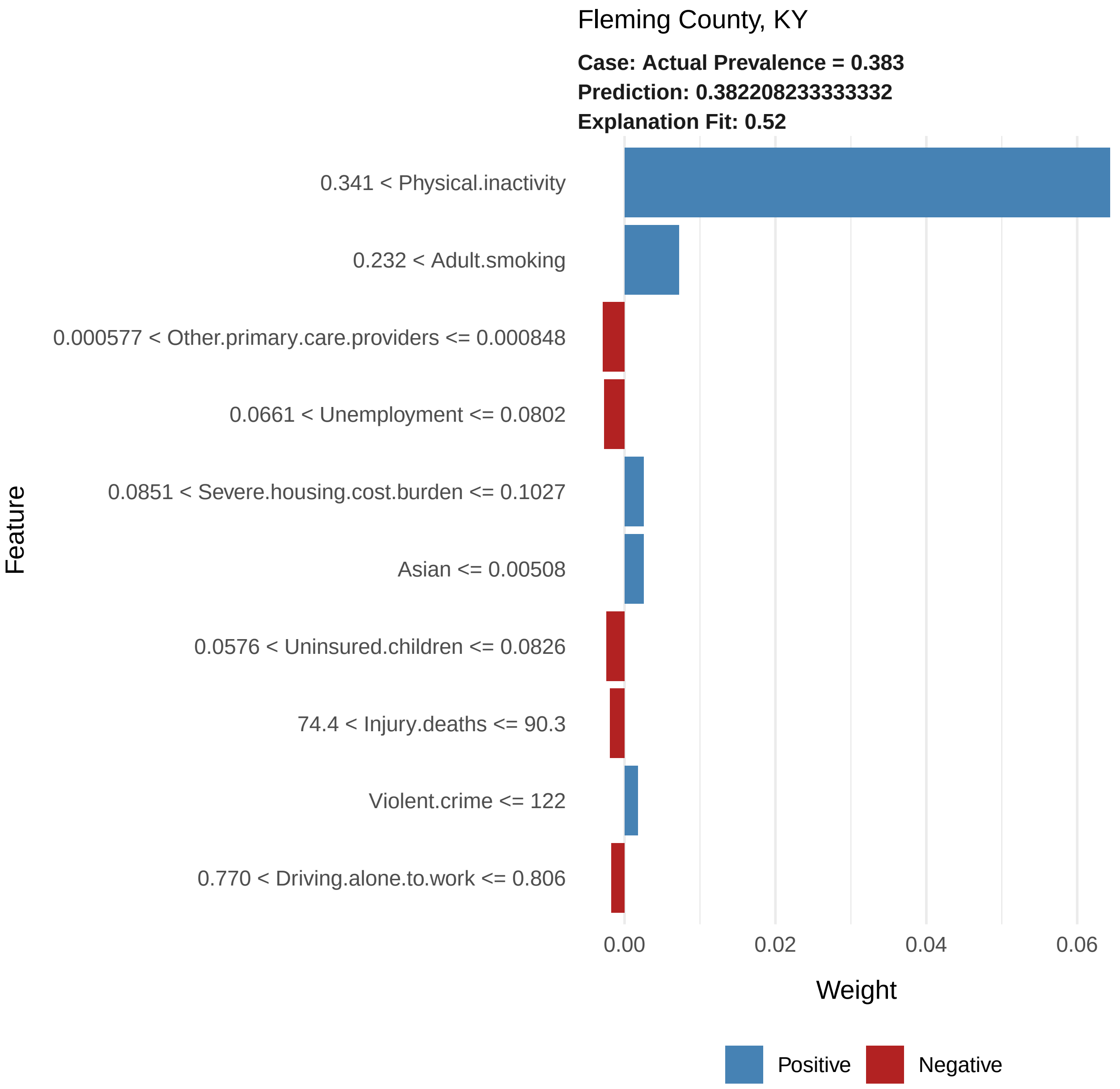

Fleming County, KY
Case: Actual Prevalence = 0.383
Prediction: 0.382208233333332
Explanation Fit: 0.52
0.341 < Physical.inactivity
0.232 < Adult.smoking
0.000577 < Other.primary.care.providers <= 0.000848
0.0661 < Unemployment <= 0.0802
0.0851 < Severe.housing.cost.burden <= 0.1027
Asian <= 0.00508
0.0576 < Uninsured.children <= 0.0826
74.4 < Injury.deaths <= 90.3
Violent.crime <= 122
0.770 < Driving.alone.to.work <= 0.806
Feature
0.00
0.02
0.04
0.06
Weight
Positive
Negative

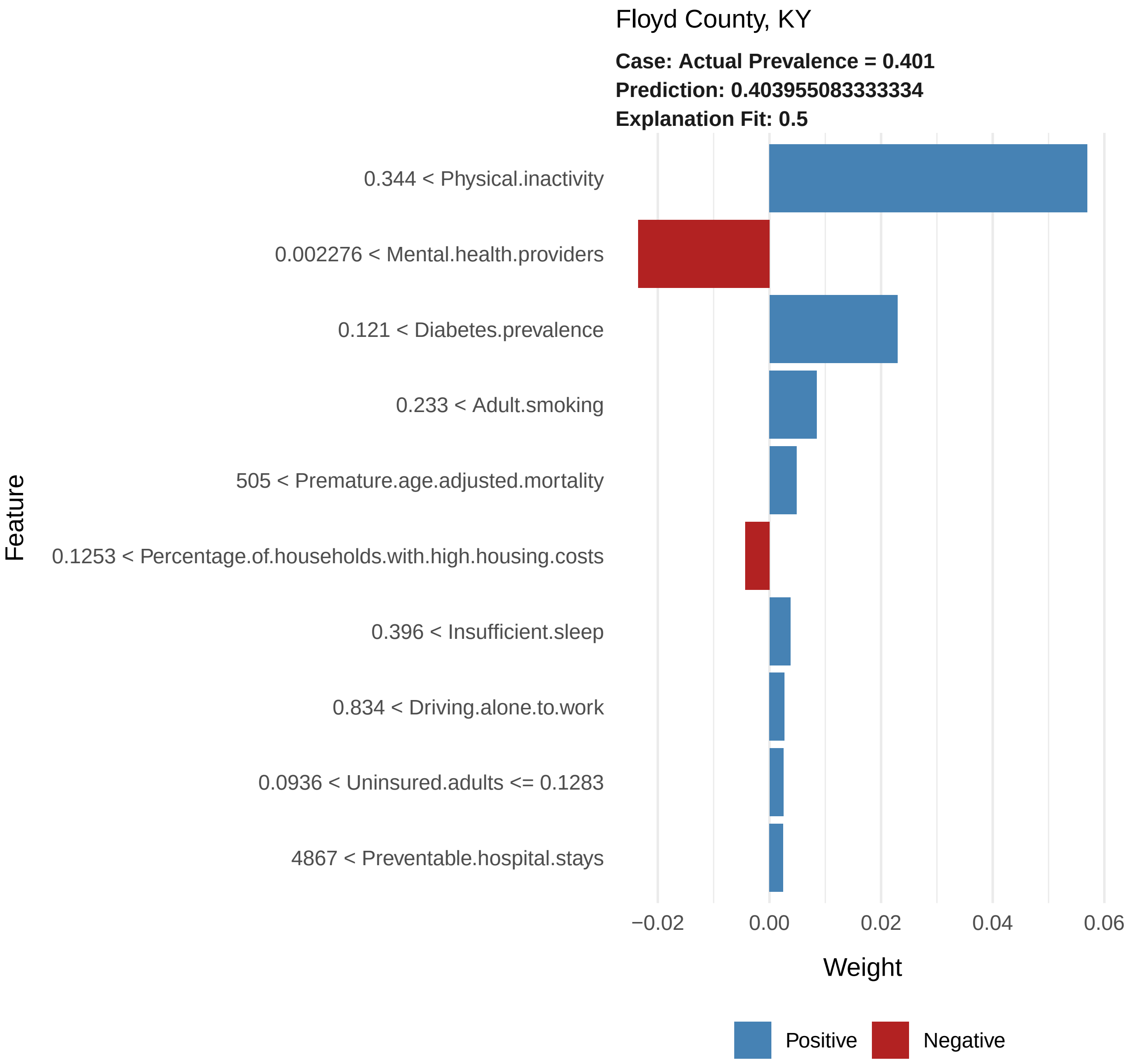
Floyd County, KY
Case: Actual Prevalence = 0.401
Prediction: 0.403955083333334
Explanation Fit: 0.5
0.344 < Physical.inactivity
0.002276 < Mental.health.providers
0.121 < Diabetes.prevalence
0.233 < Adult.smoking
505 < Premature.age.adjusted.mortality
0.1253 < Percentage.of.households.with.high.housing.costs
0.396 < Insufficient.sleep
0.834 < Driving.alone.to.work
0.0936 < Uninsured.adults <= 0.1283
4867 < Preventable.hospital.stays
Feature
−0.02
0.00
0.02
0.04
0.06
Weight
Positive
Negative

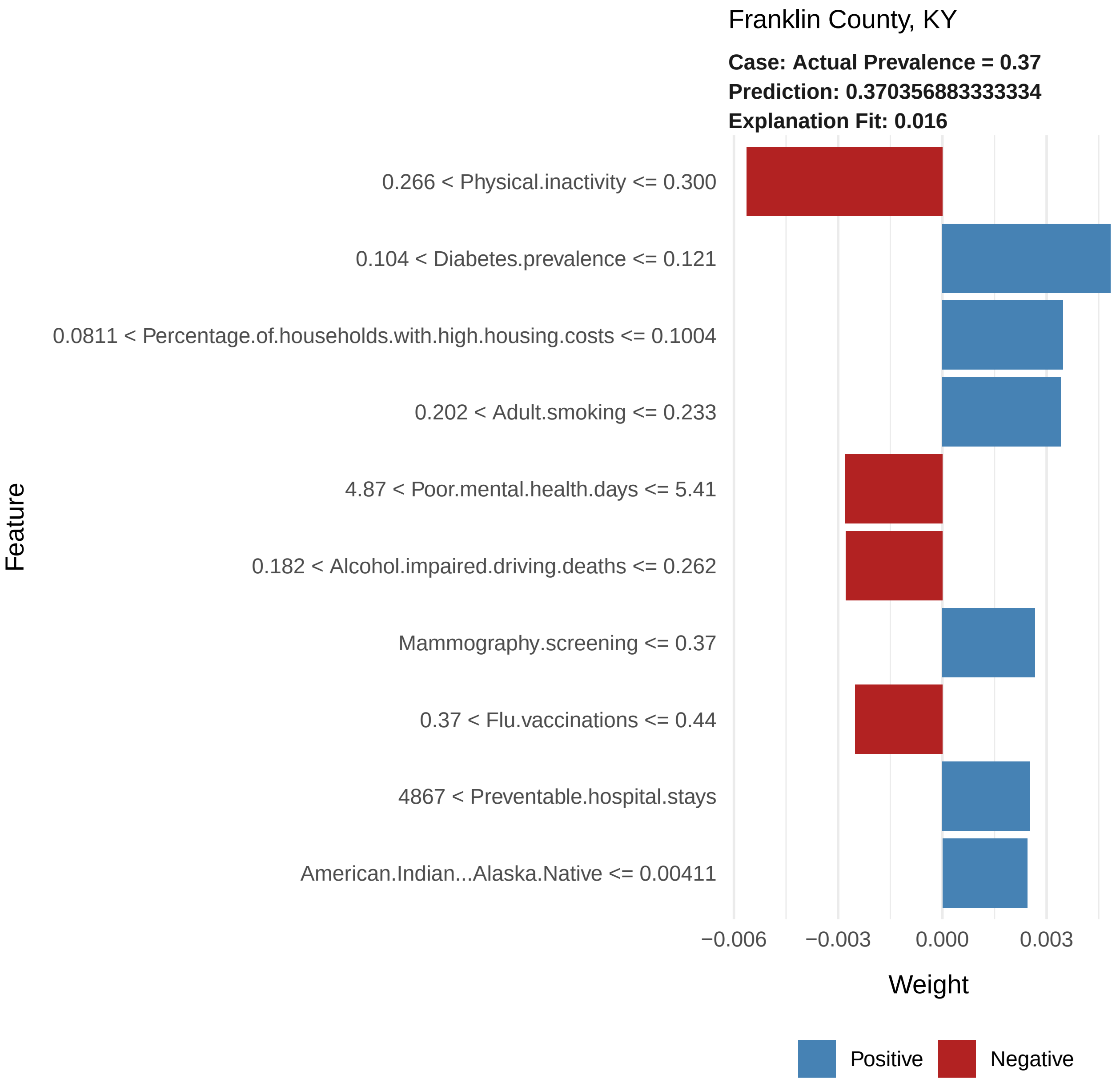

Franklin County, KY
Case: Actual Prevalence = 0.37
Prediction: 0.370356883333334
Explanation Fit: 0.016
0.266 < Physical.inactivity <= 0.300
0.104 < Diabetes.prevalence <= 0.121
0.0811 < Percentage.of.households.with.high.housing.costs <= 0.1004
0.202 < Adult.smoking <= 0.233
4.87 < Poor.mental.health.days <= 5.41
0.182 < Alcohol.impaired.driving.deaths <= 0.262
Mammography.screening <= 0.37
0.37 < Flu.vaccinations <= 0.44
4867 < Preventable.hospital.stays
American.Indian...Alaska.Native <= 0.00411
Feature
−0.006
−0.003
0.000
0.003
Weight
Positive
Negative

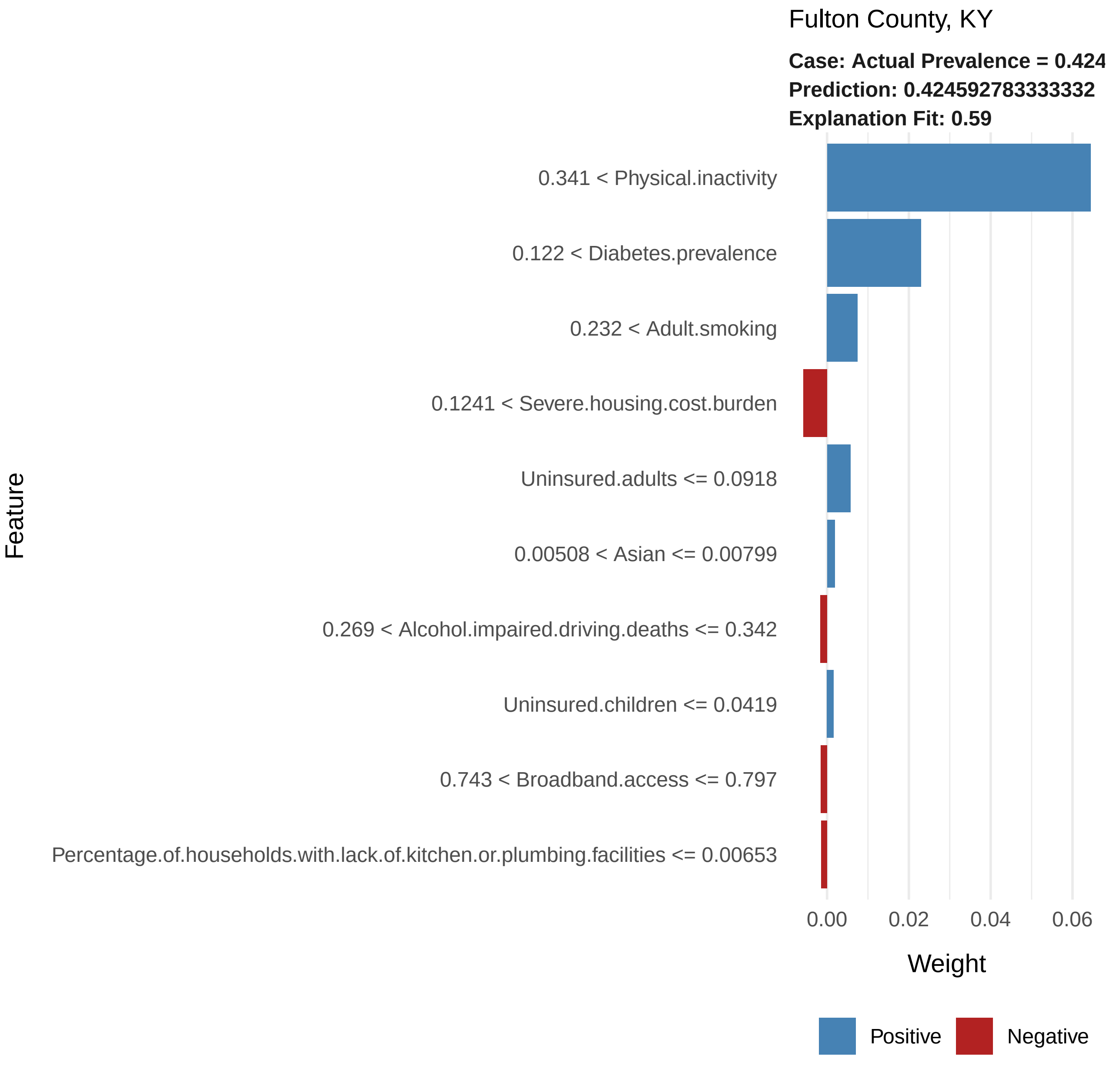

Fulton County, KY
Case: Actual Prevalence = 0.424
Prediction: 0.424592783333332
Explanation Fit: 0.59
0.341 < Physical.inactivity
0.122 < Diabetes.prevalence
0.232 < Adult.smoking
0.1241 < Severe.housing.cost.burden
Uninsured.adults <= 0.0918
0.00508 < Asian <= 0.00799
0.269 < Alcohol.impaired.driving.deaths <= 0.342
Uninsured.children <= 0.0419
0.743 < Broadband.access <= 0.797
Percentage.of.households.with.lack.of.kitchen.or.plumbing.facilities <= 0.00653
Feature
0.00
0.02
0.04
0.06
Weight
Positive
Negative

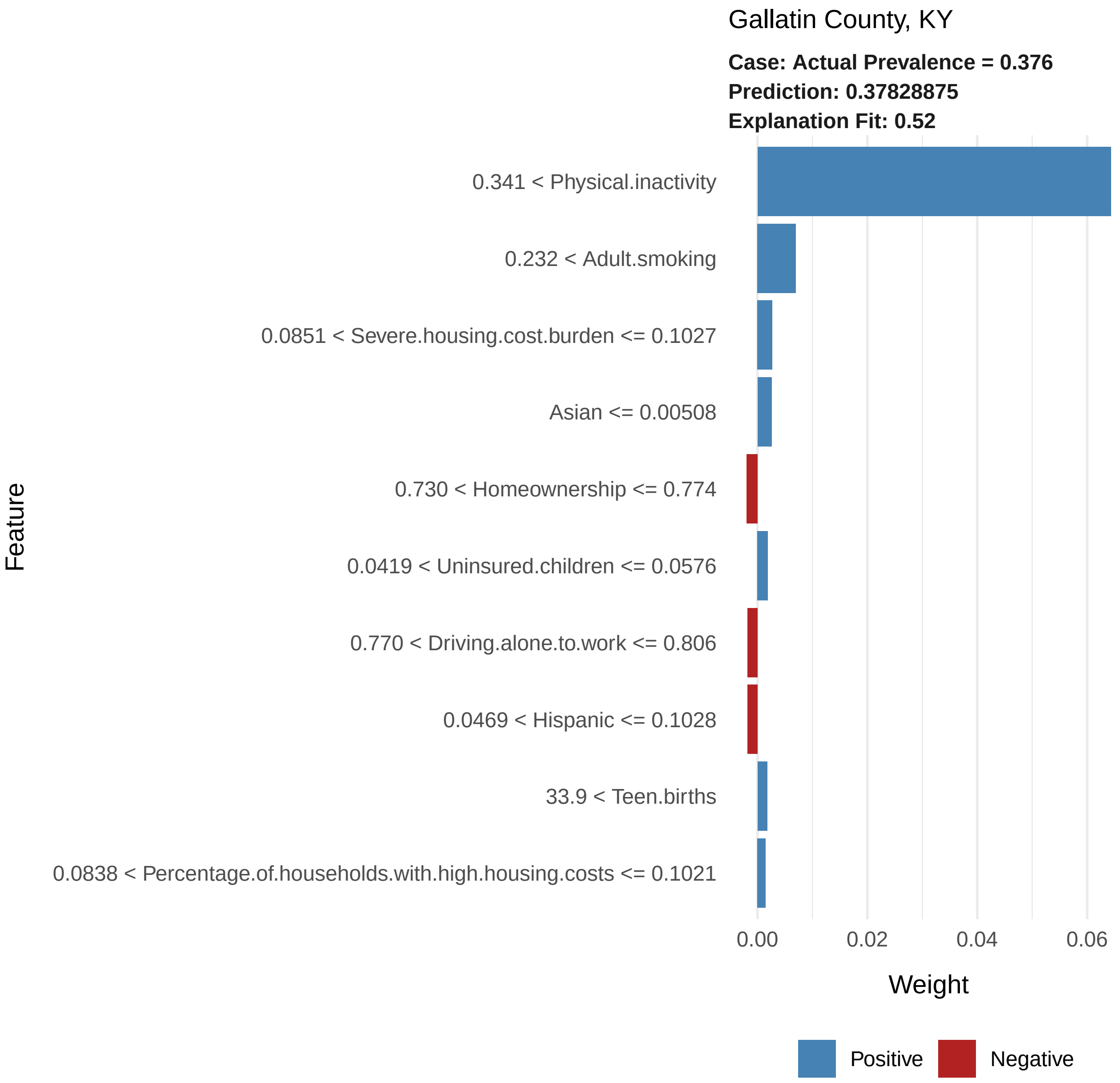

Gallatin County, KY
Case: Actual Prevalence = 0.376
Prediction: 0.37828875
Explanation Fit: 0.52
0.341 < Physical.inactivity
0.232 < Adult.smoking
0.0851 < Severe.housing.cost.burden <= 0.1027
Asian <= 0.00508
0.730 < Homeownership <= 0.774
0.0419 < Uninsured.children <= 0.0576
0.770 < Driving.alone.to.work <= 0.806
0.0469 < Hispanic <= 0.1028
33.9 < Teen.births
0.0838 < Percentage.of.households.with.high.housing.costs <= 0.1021
Feature
0.00
0.02
0.04
0.06
Weight
Positive
Negative

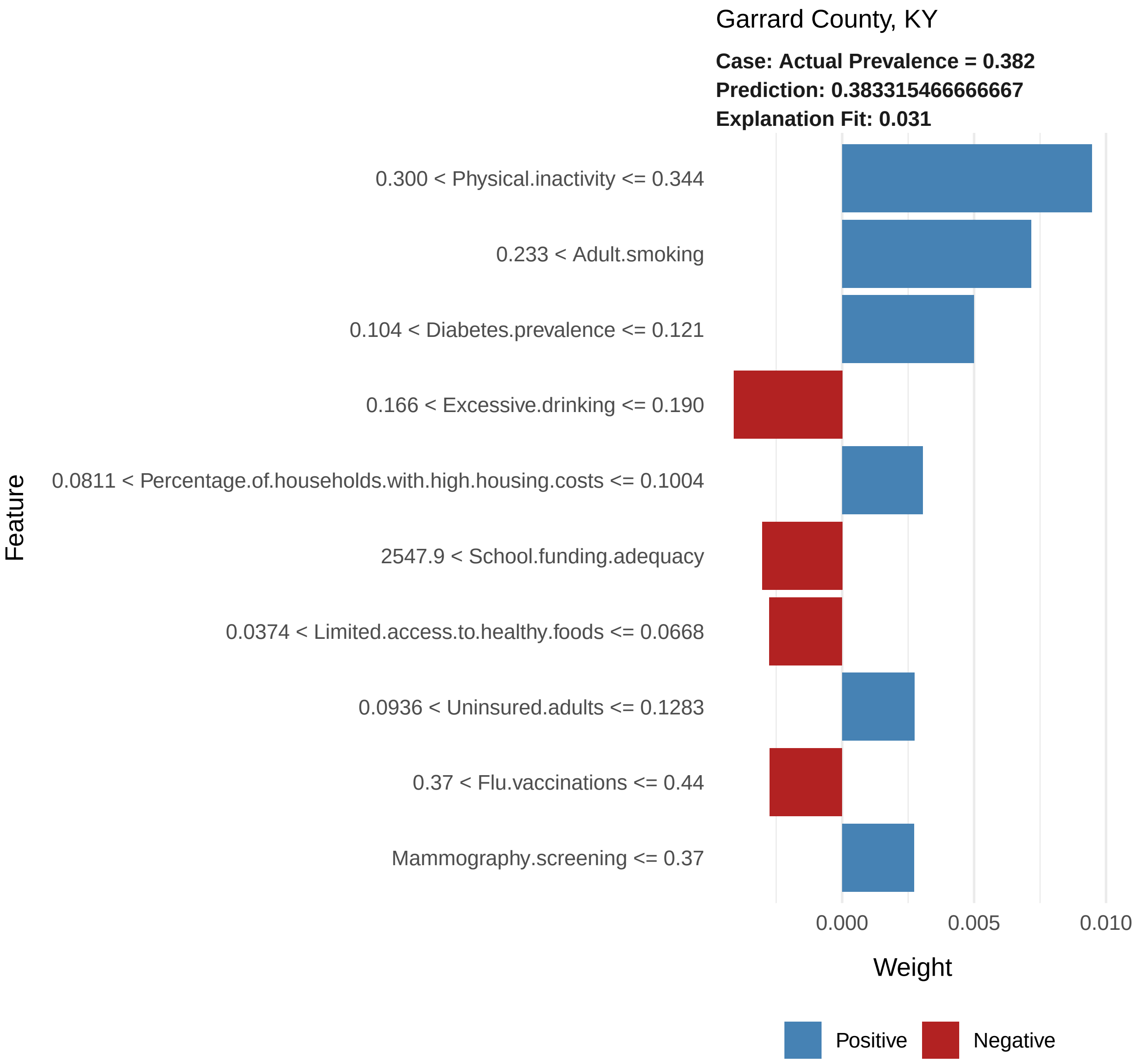

Garrard County, KY
Case: Actual Prevalence = 0.382
Prediction: 0.383315466666667
Explanation Fit: 0.031
0.300 < Physical.inactivity <= 0.344
0.233 < Adult.smoking
0.104 < Diabetes.prevalence <= 0.121
0.166 < Excessive.drinking <= 0.190
0.0811 < Percentage.of.households.with.high.housing.costs <= 0.1004
2547.9 < School.funding.adequacy
0.0374 < Limited.access.to.healthy.foods <= 0.0668
0.0936 < Uninsured.adults <= 0.1283
0.37 < Flu.vaccinations <= 0.44
Mammography.screening <= 0.37
Feature
0.000
0.005
0.010
Weight
Positive
Negative

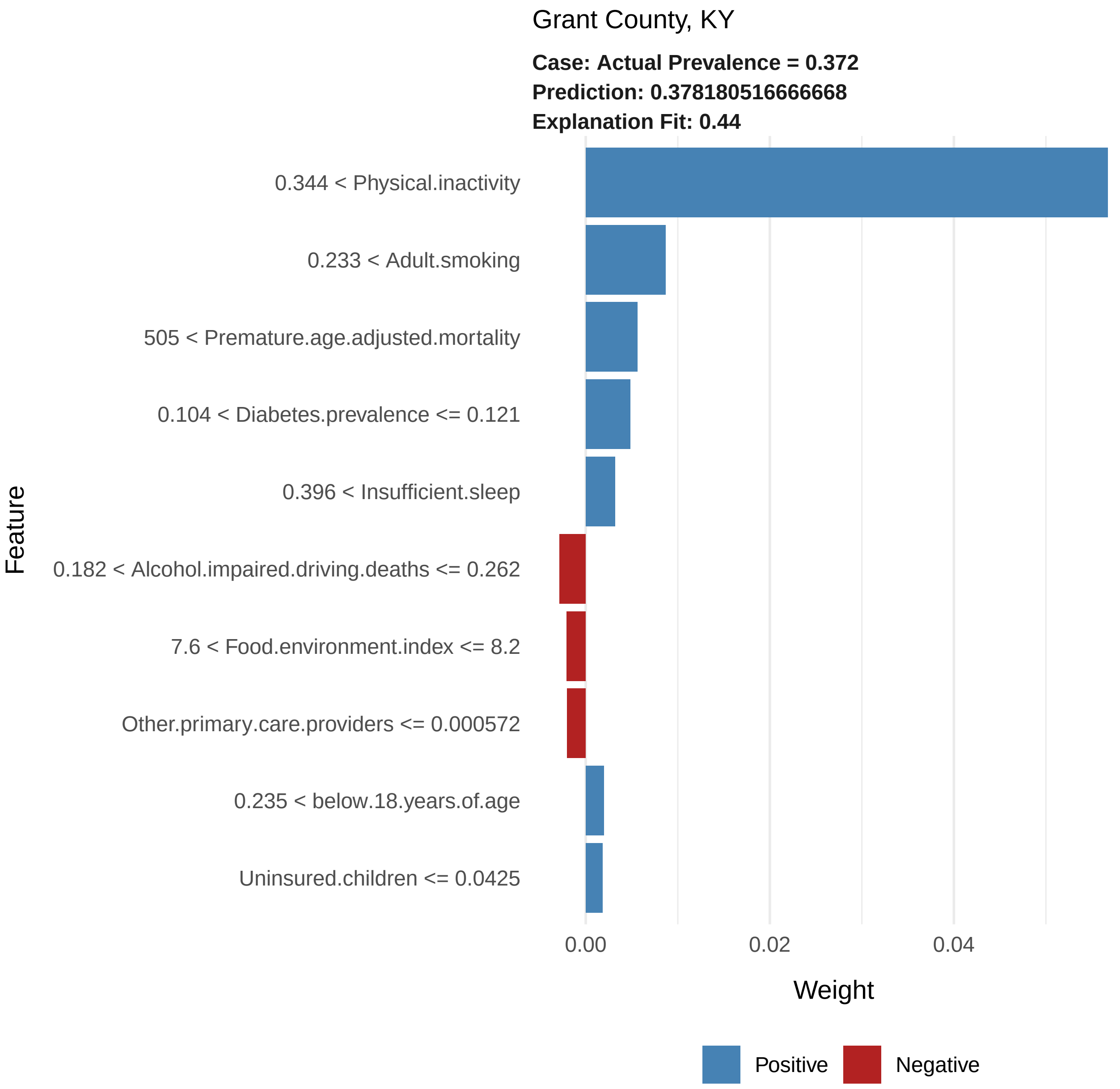

Grant County, KY
Case: Actual Prevalence = 0.372
Prediction: 0.378180516666668
Explanation Fit: 0.44
0.344 < Physical.inactivity
0.233 < Adult.smoking
505 < Premature.age.adjusted.mortality
0.104 < Diabetes.prevalence <= 0.121
0.396 < Insufficient.sleep
0.182 < Alcohol.impaired.driving.deaths <= 0.262
7.6 < Food.environment.index <= 8.2
Other.primary.care.providers <= 0.000572
0.235 < below.18.years.of.age
Uninsured.children <= 0.0425
Feature
0.00
0.02
0.04
Weight
Positive
Negative

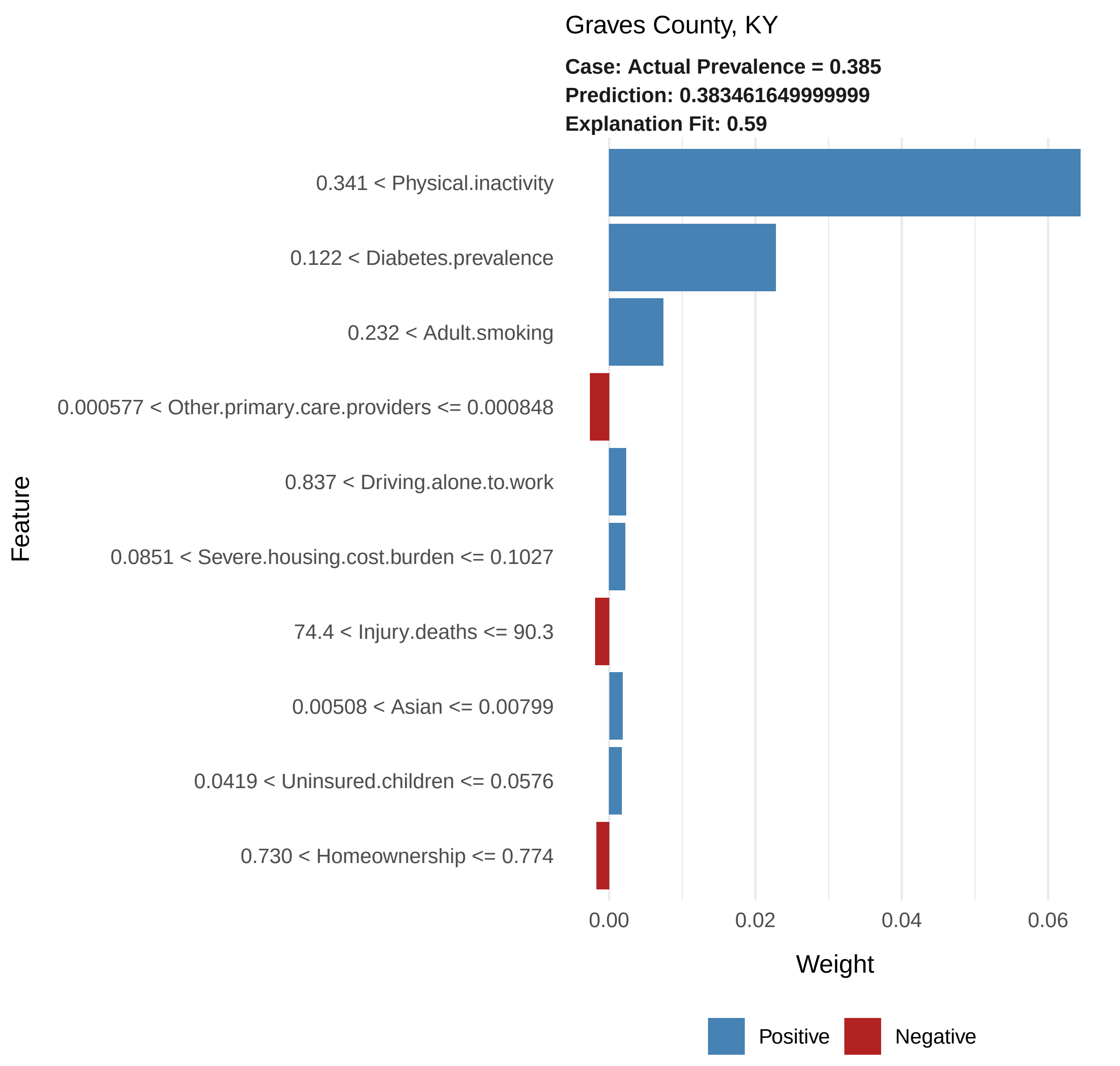

Graves County, KY
Case: Actual Prevalence = 0.385
Prediction: 0.383461649999999
Explanation Fit: 0.59
0.341 < Physical.inactivity
0.122 < Diabetes.prevalence
0.232 < Adult.smoking
0.000577 < Other.primary.care.providers <= 0.000848
0.837 < Driving.alone.to.work
0.0851 < Severe.housing.cost.burden <= 0.1027
74.4 < Injury.deaths <= 90.3
0.00508 < Asian <= 0.00799
0.0419 < Uninsured.children <= 0.0576
0.730 < Homeownership <= 0.774
Feature
0.00
0.02
0.04
0.06
Weight
Positive
Negative

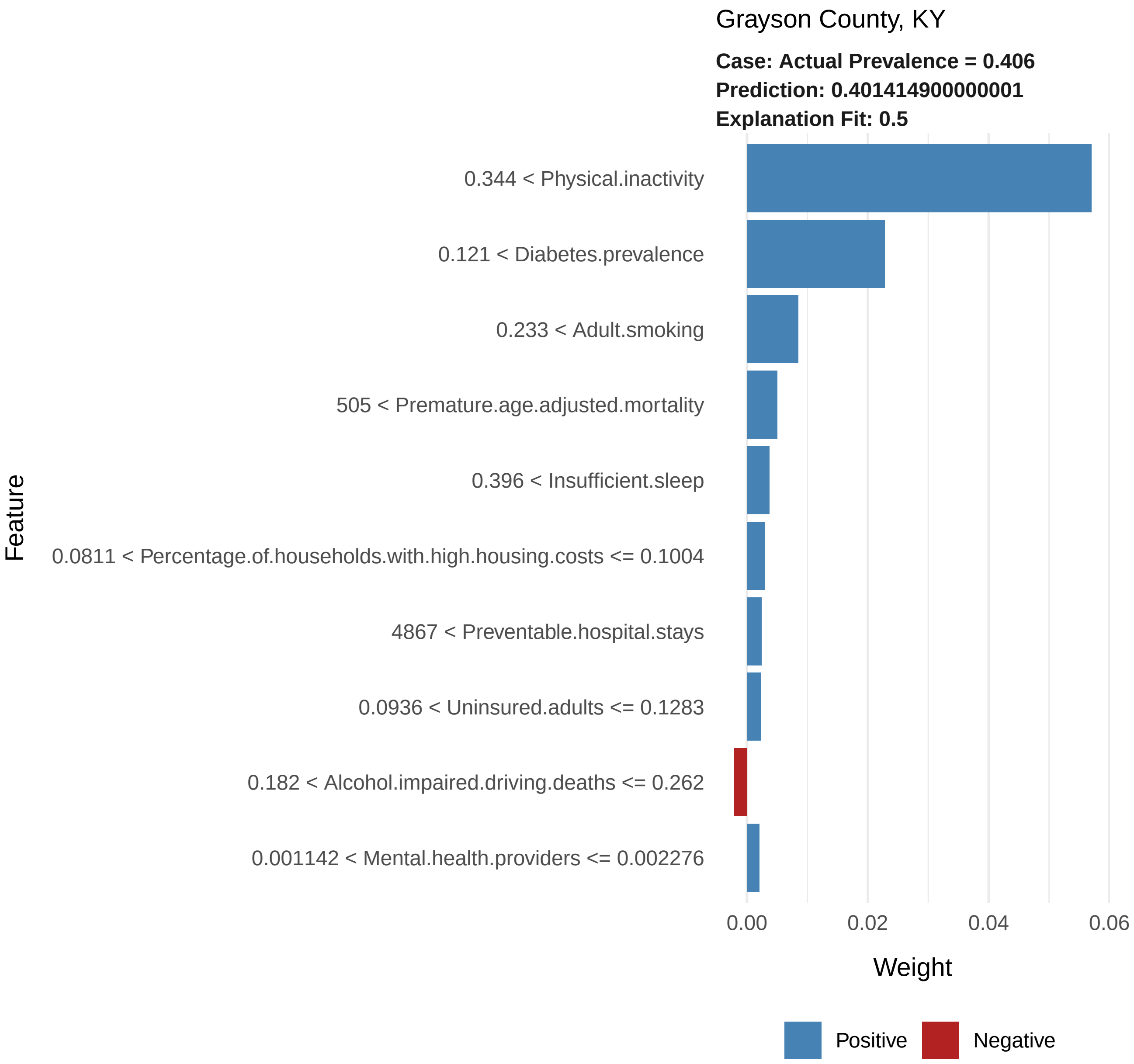
Grayson County, KY
Case: Actual Prevalence = 0.406
Prediction: 0.401414900000001
Explanation Fit: 0.5
0.344 < Physical.inactivity
0.121 < Diabetes.prevalence
0.233 < Adult.smoking
505 < Premature.age.adjusted.mortality
0.396 < Insufficient.sleep
0.0811 < Percentage.of.households.with.high.housing.costs <= 0.1004
4867 < Preventable.hospital.stays
0.0936 < Uninsured.adults <= 0.1283
0.182 < Alcohol.impaired.driving.deaths <= 0.262
0.001142 < Mental.health.providers <= 0.002276
Feature
0.00
0.02
0.04
0.06
Weight
Positive
Negative

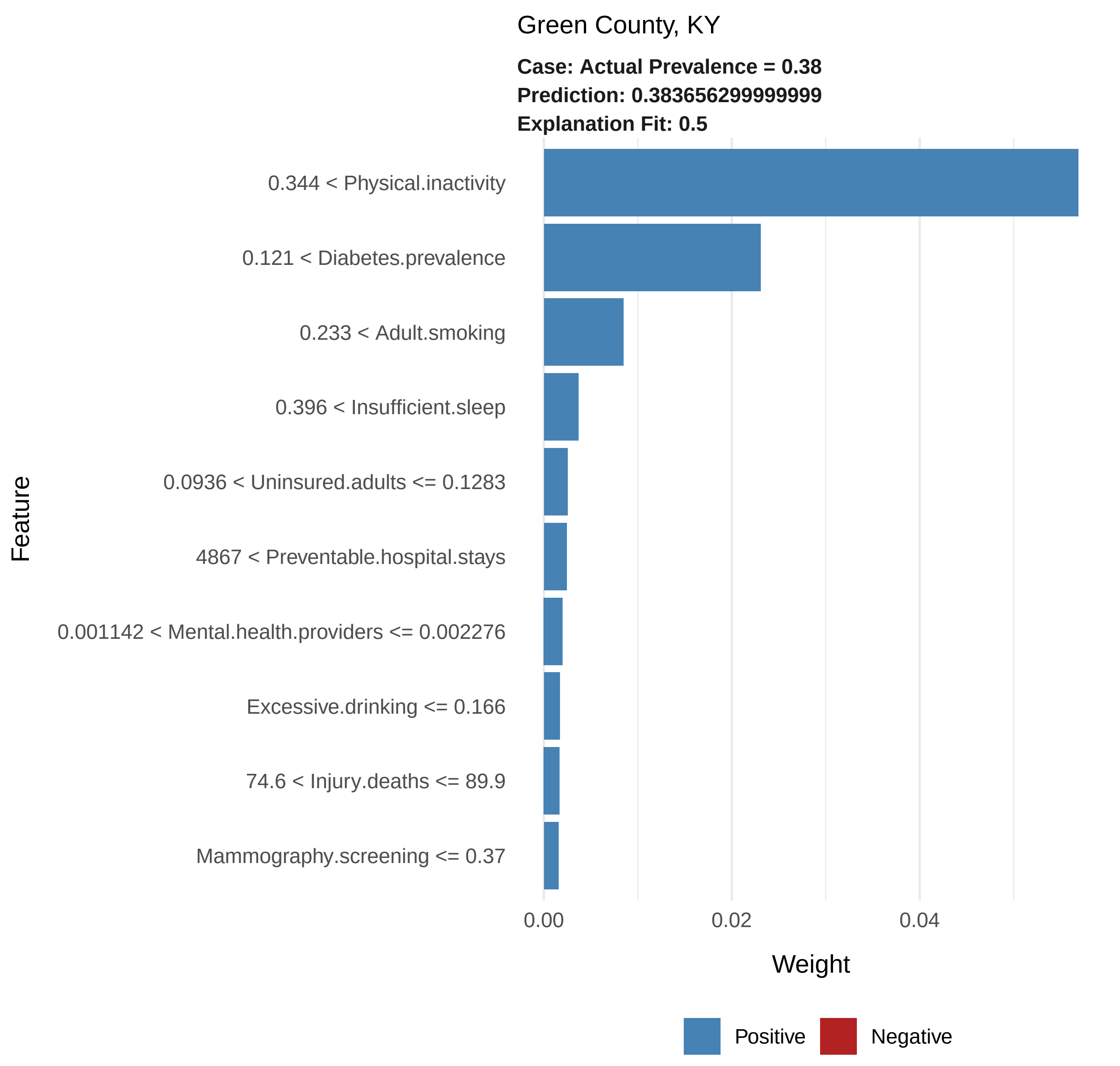

Green County, KY
Case: Actual Prevalence = 0.38
Prediction: 0.383656299999999
Explanation Fit: 0.5
0.344 < Physical.inactivity
0.121 < Diabetes.prevalence
0.233 < Adult.smoking
0.396 < Insufficient.sleep
0.0936 < Uninsured.adults <= 0.1283
4867 < Preventable.hospital.stays
0.001142 < Mental.health.providers <= 0.002276
Excessive.drinking <= 0.166
74.6 < Injury.deaths <= 89.9
Mammography.screening <= 0.37
Feature
0.00
0.02
0.04
Weight
Positive
Negative

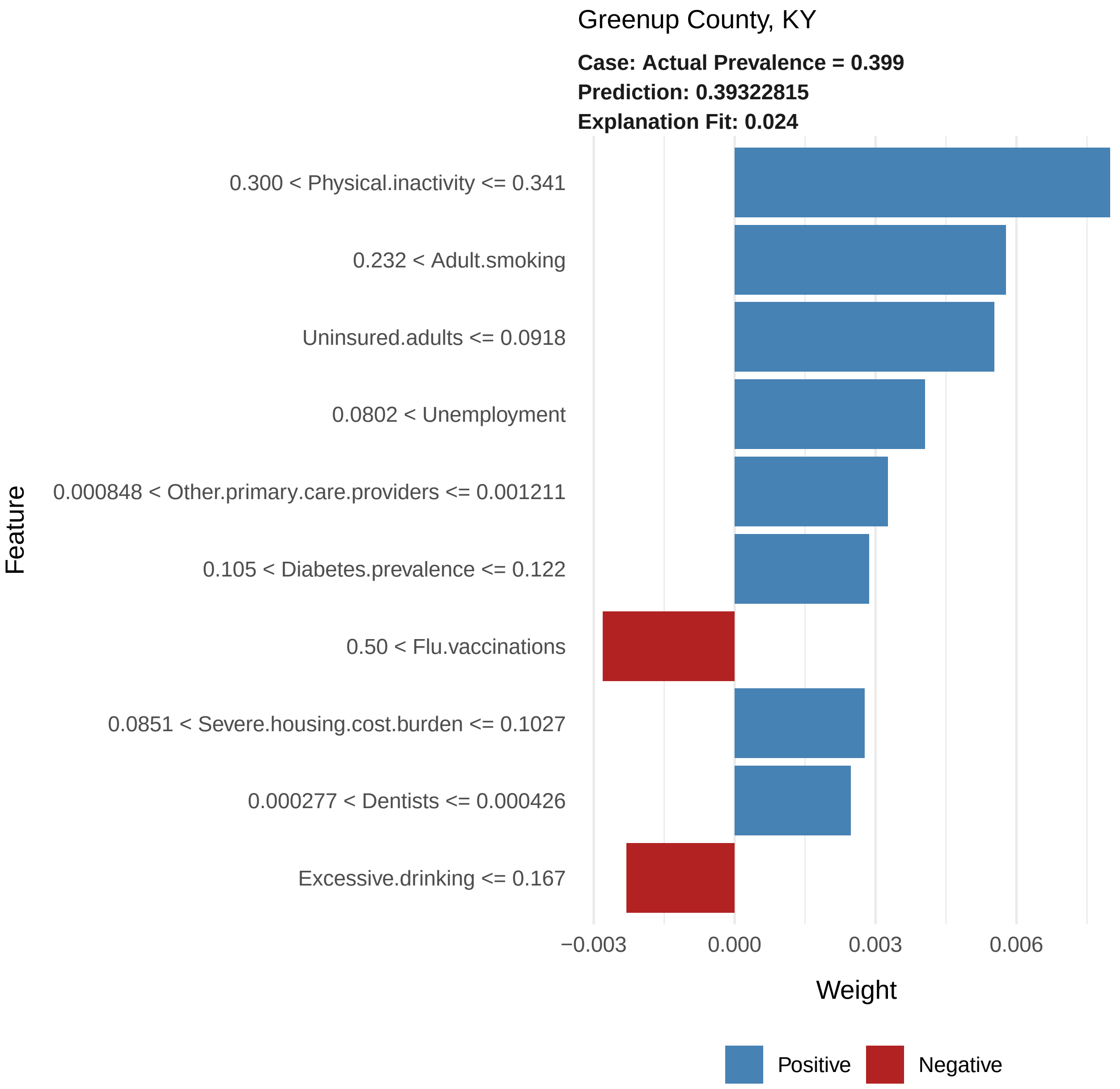
Greenup County, KY
Case: Actual Prevalence = 0.399
Prediction: 0.39322815
Explanation Fit: 0.024
0.300 < Physical.inactivity <= 0.341
0.232 < Adult.smoking
Uninsured.adults <= 0.0918
0.0802 < Unemployment
0.000848 < Other.primary.care.providers <= 0.001211
0.105 < Diabetes.prevalence <= 0.122
0.50 < Flu.vaccinations
0.0851 < Severe.housing.cost.burden <= 0.1027
0.000277 < Dentists <= 0.000426
Excessive.drinking <= 0.167
Feature
−0.003
0.000
0.003
0.006
Weight
Positive
Negative

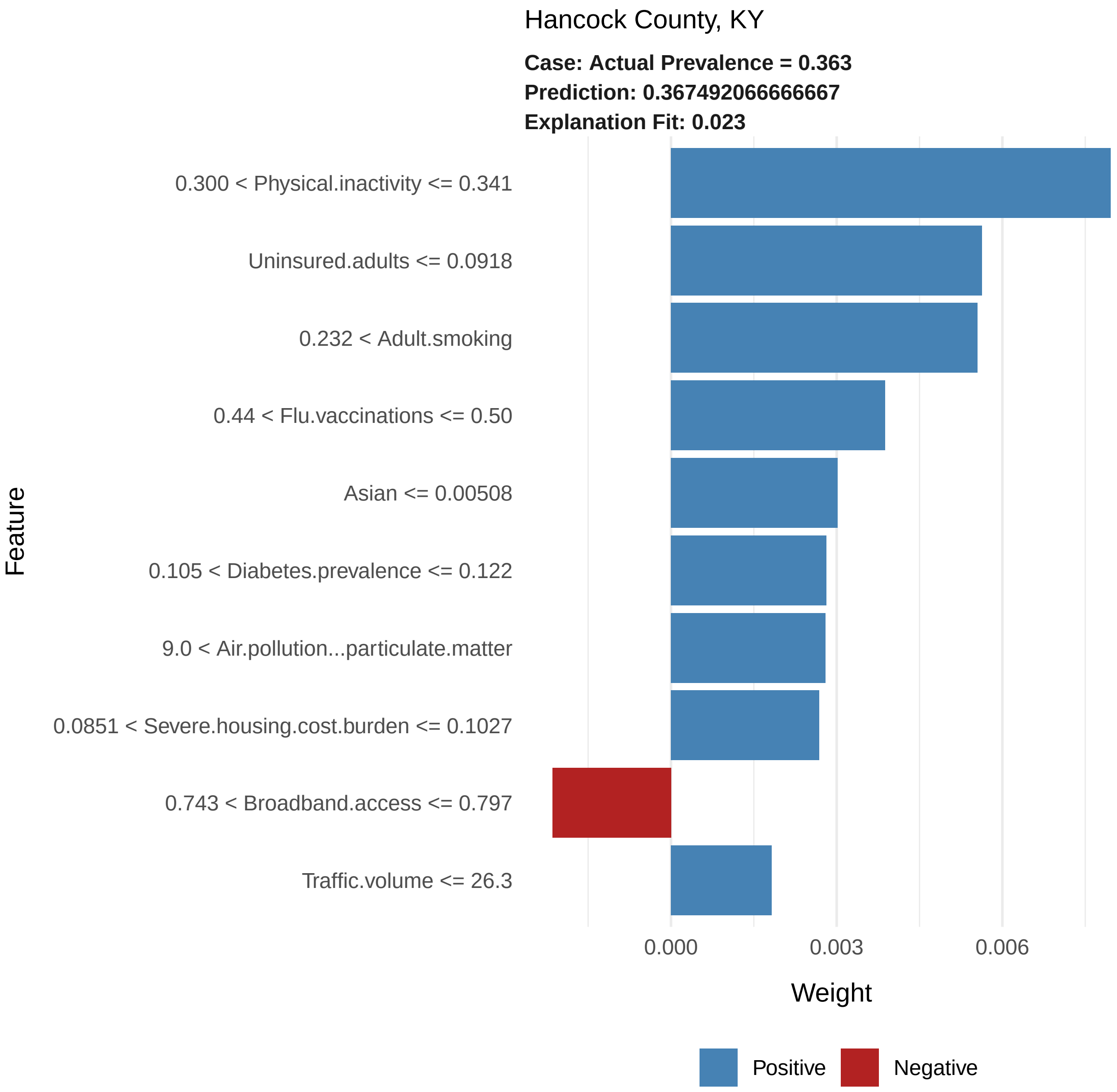
Hancock County, KY
Case: Actual Prevalence = 0.363
Prediction: 0.367492066666667
Explanation Fit: 0.023
0.300 < Physical.inactivity <= 0.341
Uninsured.adults <= 0.0918
0.232 < Adult.smoking
0.44 < Flu.vaccinations <= 0.50
Asian <= 0.00508
0.105 < Diabetes.prevalence <= 0.122
9.0 < Air.pollution...particulate.matter
0.0851 < Severe.housing.cost.burden <= 0.1027
0.743 < Broadband.access <= 0.797
Traffic.volume <= 26.3
Feature
0.000
0.003
0.006
Weight
Positive
Negative

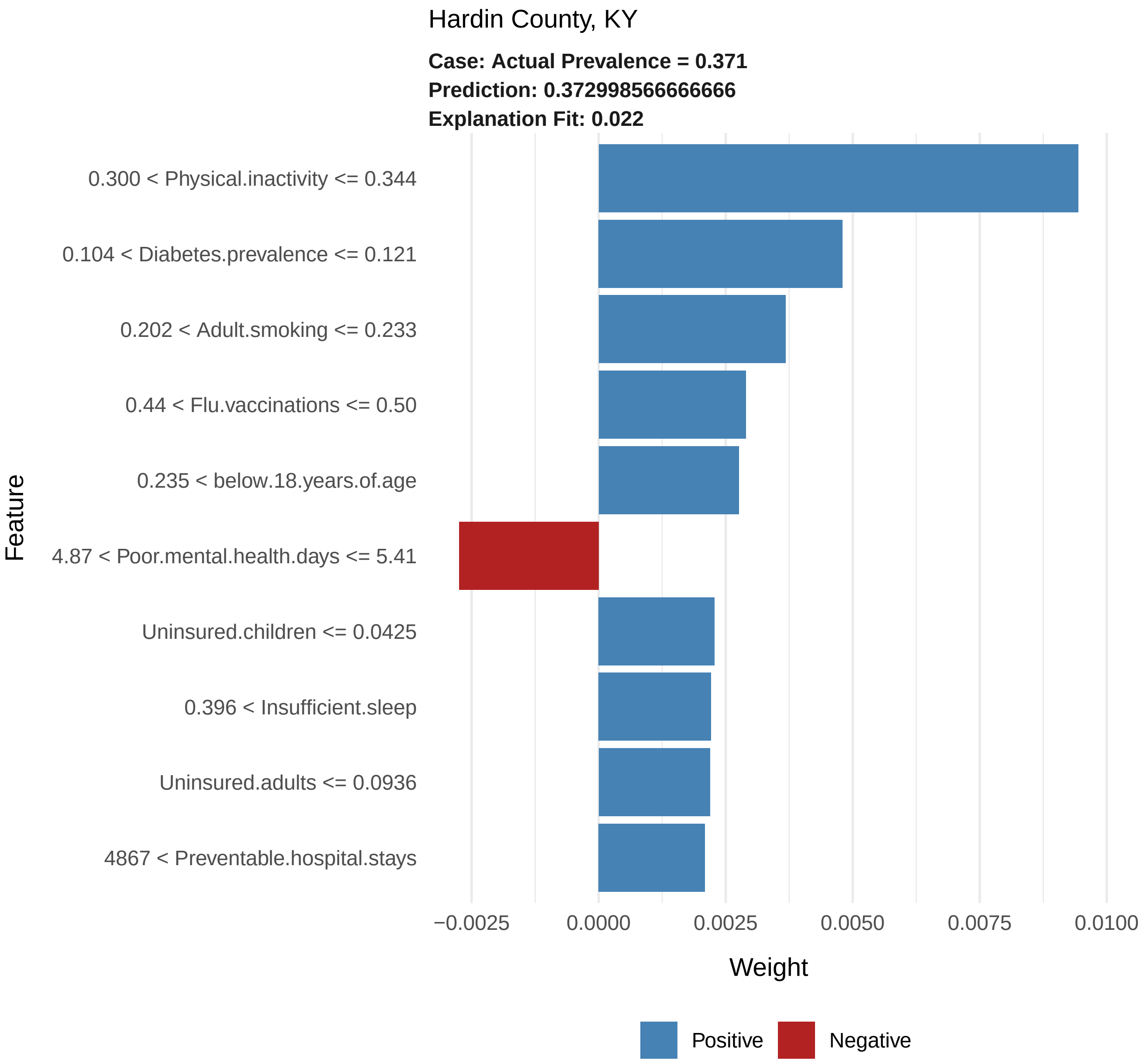
Hardin County, KY
Case: Actual Prevalence = 0.371
Prediction: 0.372998566666666
Explanation Fit: 0.022
0.300 < Physical.inactivity <= 0.344
0.104 < Diabetes.prevalence <= 0.121
0.202 < Adult.smoking <= 0.233
0.44 < Flu.vaccinations <= 0.50
0.235 < below.18.years.of.age
4.87 < Poor.mental.health.days <= 5.41
Uninsured.children <= 0.0425
0.396 < Insufficient.sleep
Uninsured.adults <= 0.0936
4867 < Preventable.hospital.stays
Feature
−0.0025
0.0000
0.0025
0.0050
0.0075
0.0100
Weight
Positive
Negative

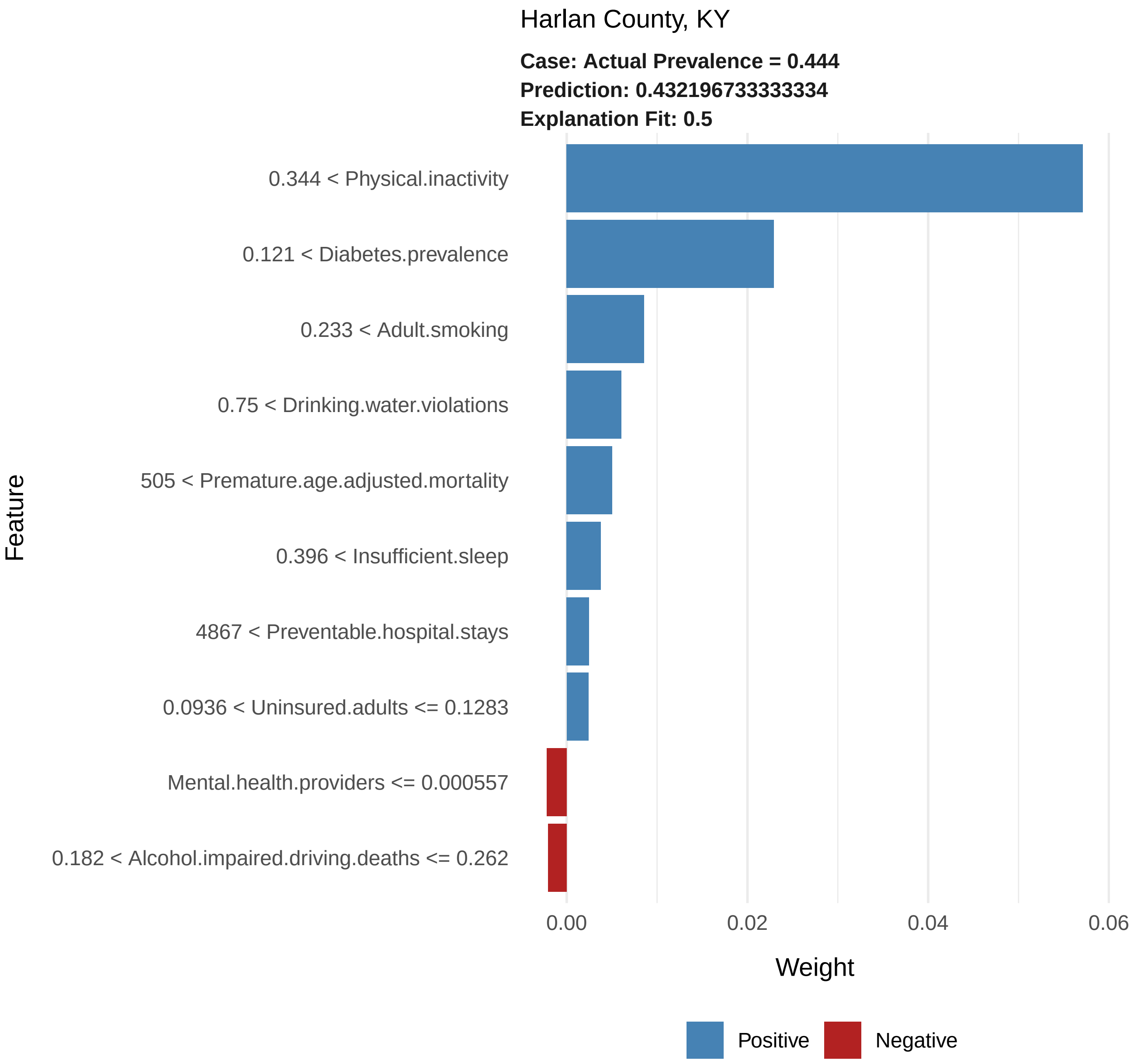
Harlan County, KY
Case: Actual Prevalence = 0.444
Prediction: 0.432196733333334
Explanation Fit: 0.5
0.344 < Physical.inactivity
0.121 < Diabetes.prevalence
0.233 < Adult.smoking
0.75 < Drinking.water.violations
505 < Premature.age.adjusted.mortality
0.396 < Insufficient.sleep
4867 < Preventable.hospital.stays
0.0936 < Uninsured.adults <= 0.1283
Mental.health.providers <= 0.000557
0.182 < Alcohol.impaired.driving.deaths <= 0.262
Feature
0.00
0.02
0.04
0.06
Weight
Positive
Negative

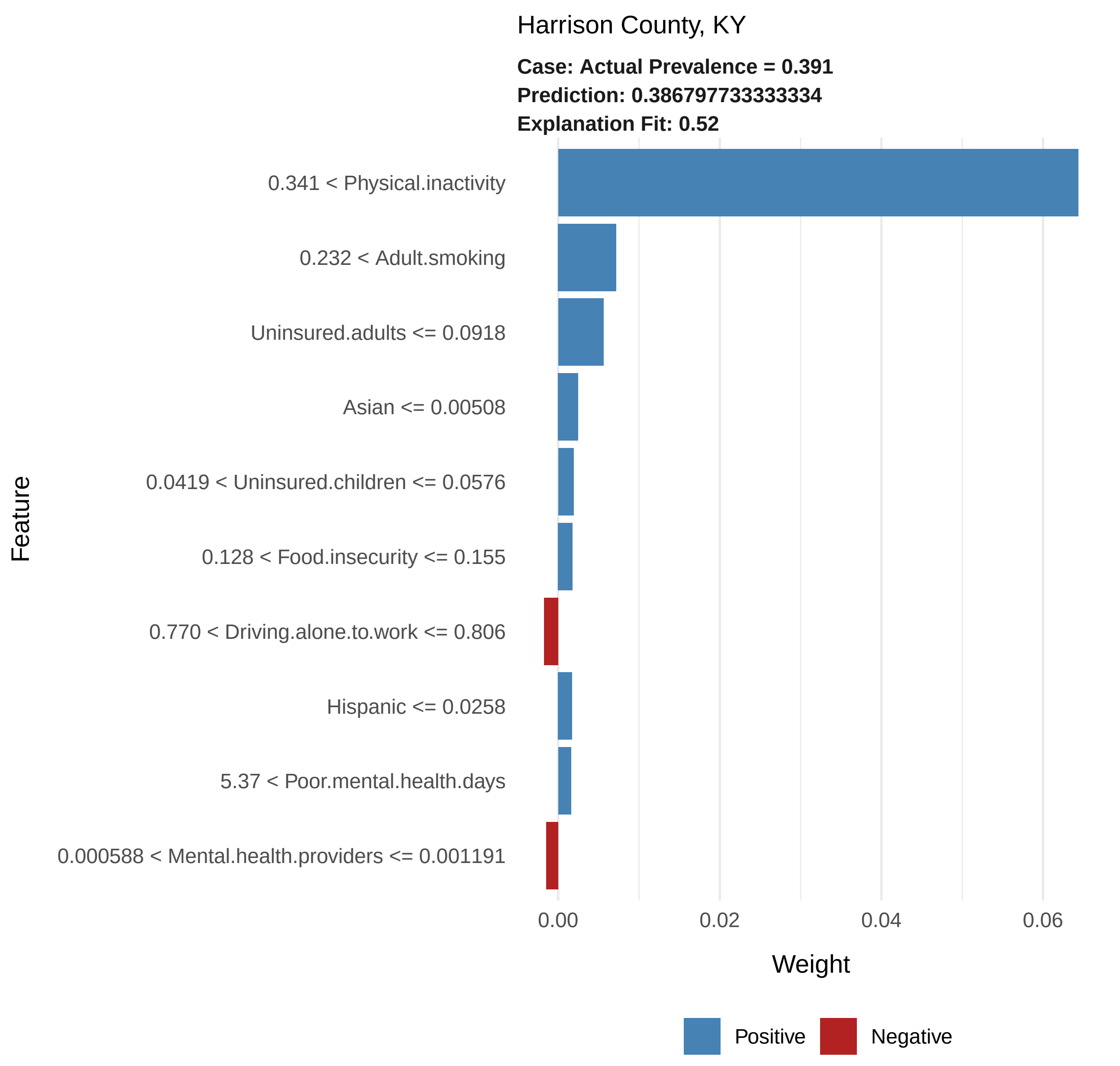

Harrison County, KY
Case: Actual Prevalence = 0.391
Prediction: 0.386797733333334
Explanation Fit: 0.52
0.341 < Physical.inactivity
0.232 < Adult.smoking
Uninsured.adults <= 0.0918
Asian <= 0.00508
0.0419 < Uninsured.children <= 0.0576
0.128 < Food.insecurity <= 0.155
0.770 < Driving.alone.to.work <= 0.806
Hispanic <= 0.0258
5.37 < Poor.mental.health.days
0.000588 < Mental.health.providers <= 0.001191
Feature
0.00
0.02
0.04
0.06
Weight
Positive
Negative

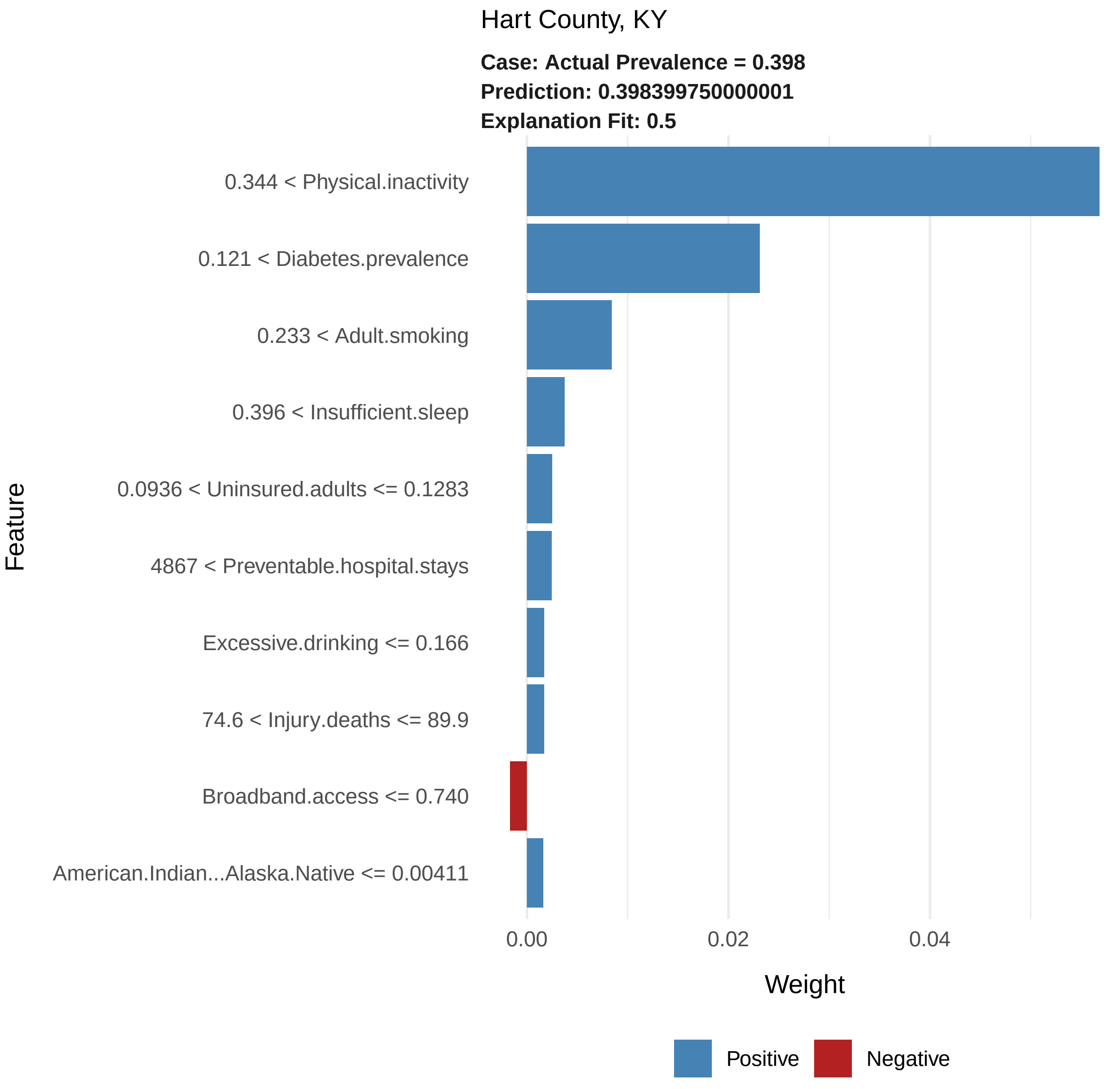

Hart County, KY
Case: Actual Prevalence = 0.398
Prediction: 0.398399750000001
Explanation Fit: 0.5
0.344 < Physical.inactivity
0.121 < Diabetes.prevalence
0.233 < Adult.smoking
0.396 < Insufficient.sleep
0.0936 < Uninsured.adults <= 0.1283
4867 < Preventable.hospital.stays
Excessive.drinking <= 0.166
74.6 < Injury.deaths <= 89.9
Broadband.access <= 0.740
American.Indian...Alaska.Native <= 0.00411
Feature
0.00
0.02
0.04
Weight
Positive
Negative

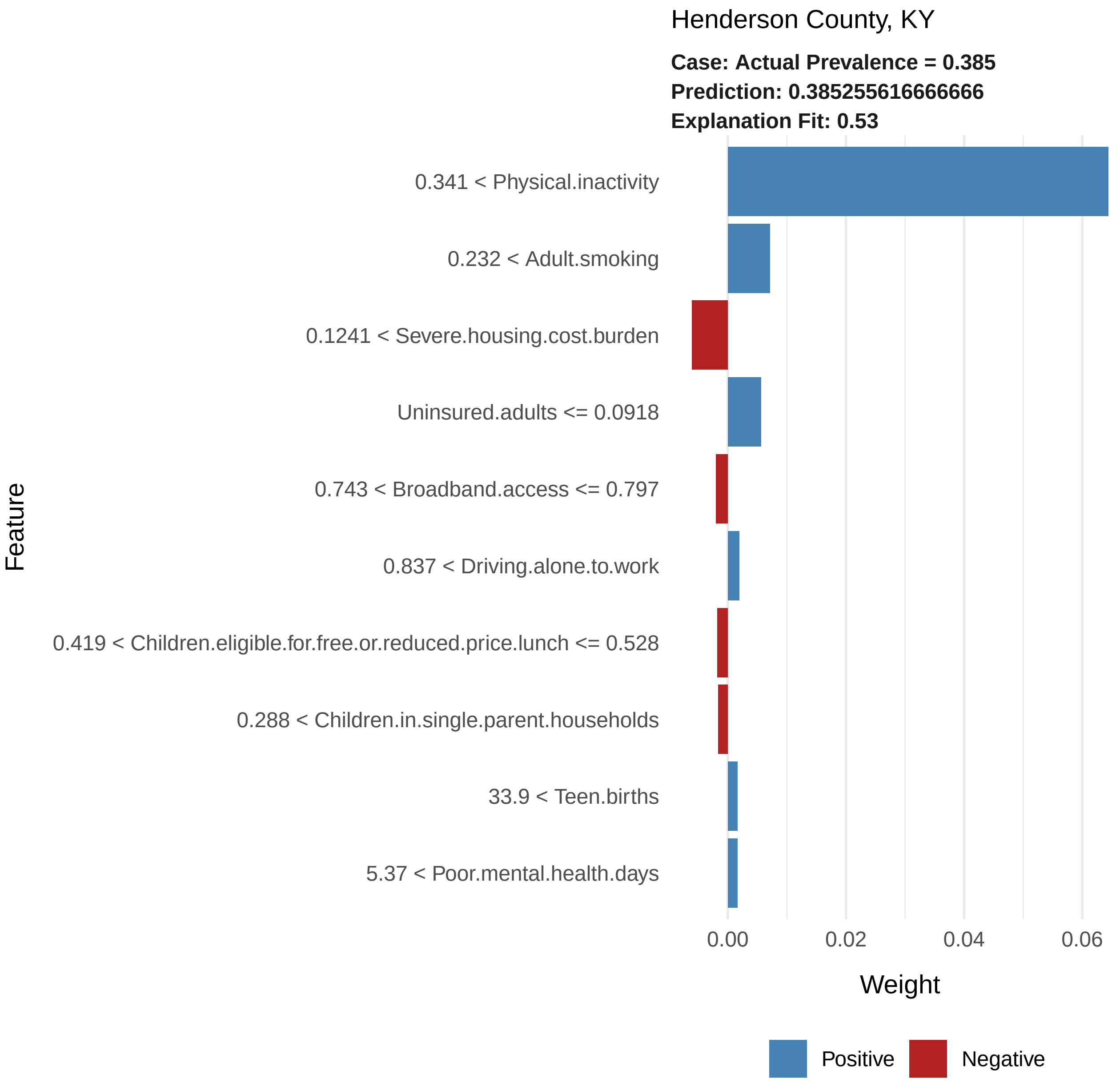

Henderson County, KY
Case: Actual Prevalence = 0.385
Prediction: 0.385255616666666
Explanation Fit: 0.53
0.341 < Physical.inactivity
0.232 < Adult.smoking
0.1241 < Severe.housing.cost.burden
Uninsured.adults <= 0.0918
0.743 < Broadband.access <= 0.797
0.837 < Driving.alone.to.work
0.419 < Children.eligible.for.free.or.reduced.price.lunch <= 0.528
0.288 < Children.in.single.parent.households
33.9 < Teen.births
5.37 < Poor.mental.health.days
Feature
0.00
0.02
0.04
0.06
Weight
Positive
Negative

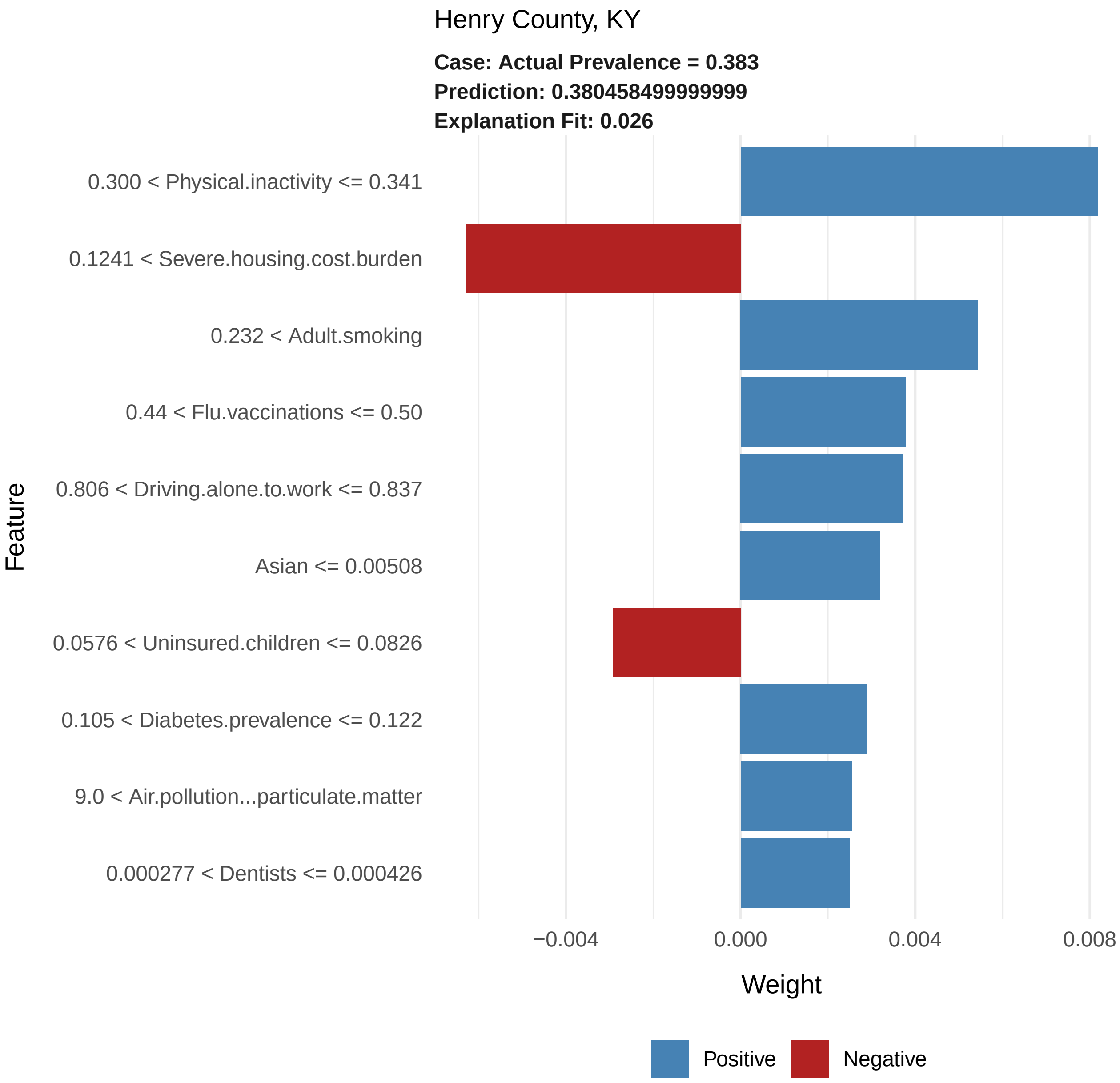

Henry County, KY
Case: Actual Prevalence = 0.383
Prediction: 0.380458499999999
Explanation Fit: 0.026
0.300 < Physical.inactivity <= 0.341
0.1241 < Severe.housing.cost.burden
0.232 < Adult.smoking
0.44 < Flu.vaccinations <= 0.50
0.806 < Driving.alone.to.work <= 0.837
Asian <= 0.00508
0.0576 < Uninsured.children <= 0.0826
0.105 < Diabetes.prevalence <= 0.122
9.0 < Air.pollution...particulate.matter
0.000277 < Dentists <= 0.000426
Feature
−0.004
0.000
0.004
0.008
Weight
Positive
Negative

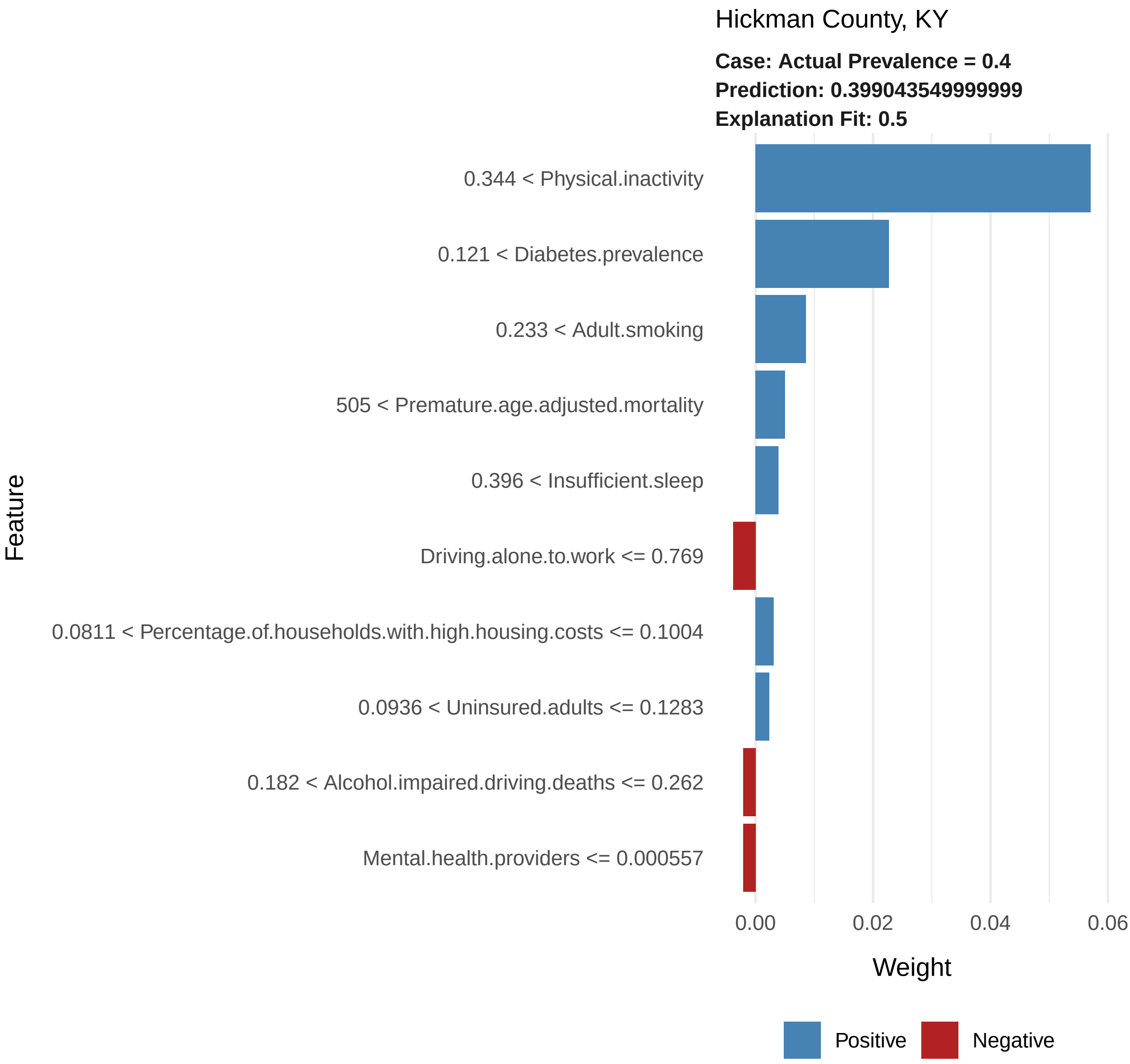

Hickman County, KY
Case: Actual Prevalence = 0.4
Prediction: 0.399043549999999
Explanation Fit: 0.5
0.344 < Physical.inactivity
0.121 < Diabetes.prevalence
0.233 < Adult.smoking
505 < Premature.age.adjusted.mortality
0.396 < Insufficient.sleep
Driving.alone.to.work <= 0.769
0.0811 < Percentage.of.households.with.high.housing.costs <= 0.1004
0.0936 < Uninsured.adults <= 0.1283
0.182 < Alcohol.impaired.driving.deaths <= 0.262
Mental.health.providers <= 0.000557
Feature
0.00
0.02
0.04
0.06
Weight
Positive
Negative

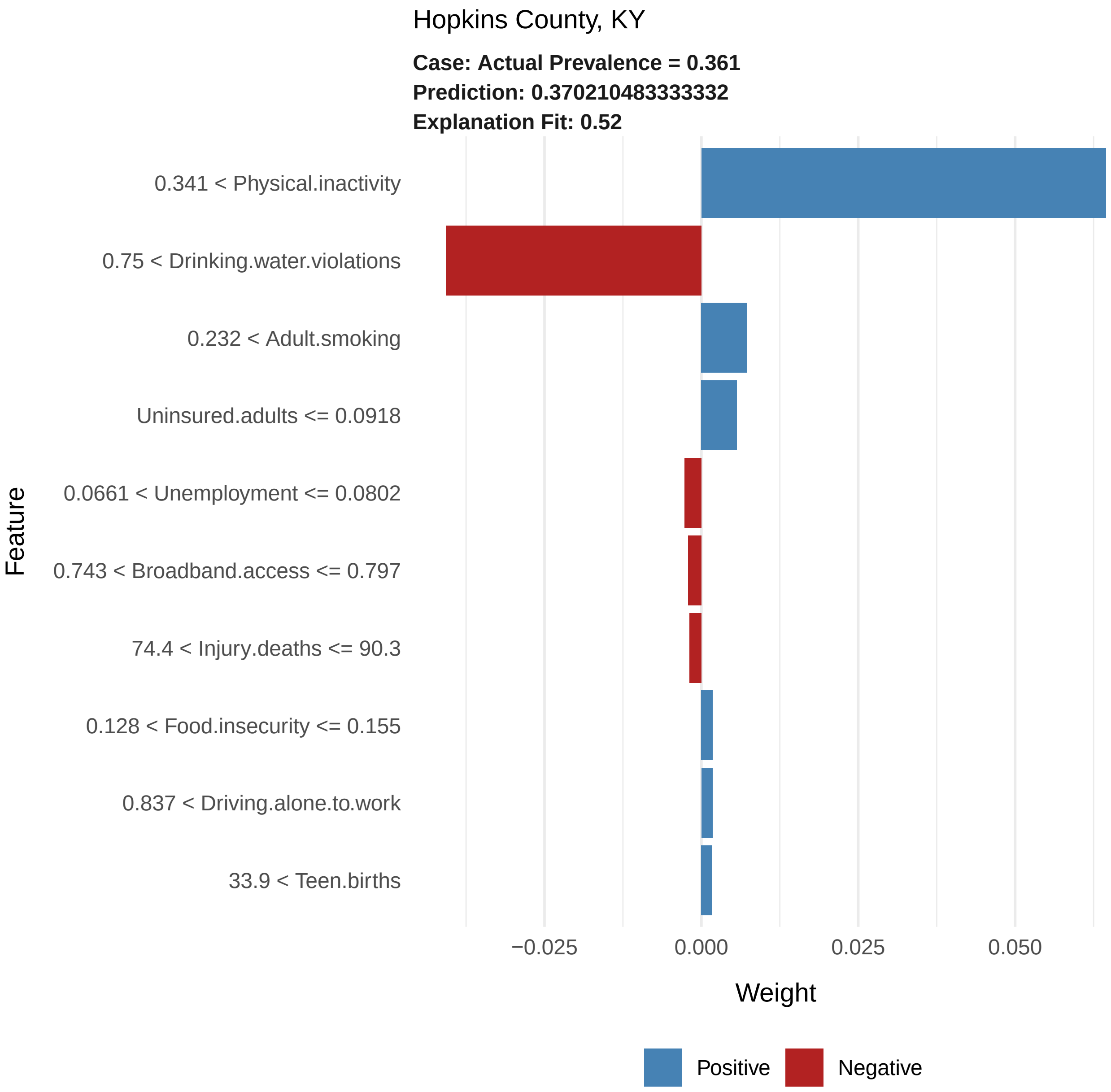

Hopkins County, KY
Case: Actual Prevalence = 0.361
Prediction: 0.370210483333332
Explanation Fit: 0.52
0.341 < Physical.inactivity
0.75 < Drinking.water.violations
0.232 < Adult.smoking
Uninsured.adults <= 0.0918
0.0661 < Unemployment <= 0.0802
0.743 < Broadband.access <= 0.797
74.4 < Injury.deaths <= 90.3
0.128 < Food.insecurity <= 0.155
0.837 < Driving.alone.to.work
33.9 < Teen.births
Feature
−0.025
0.000
0.025
0.050
Weight
Positive
Negative

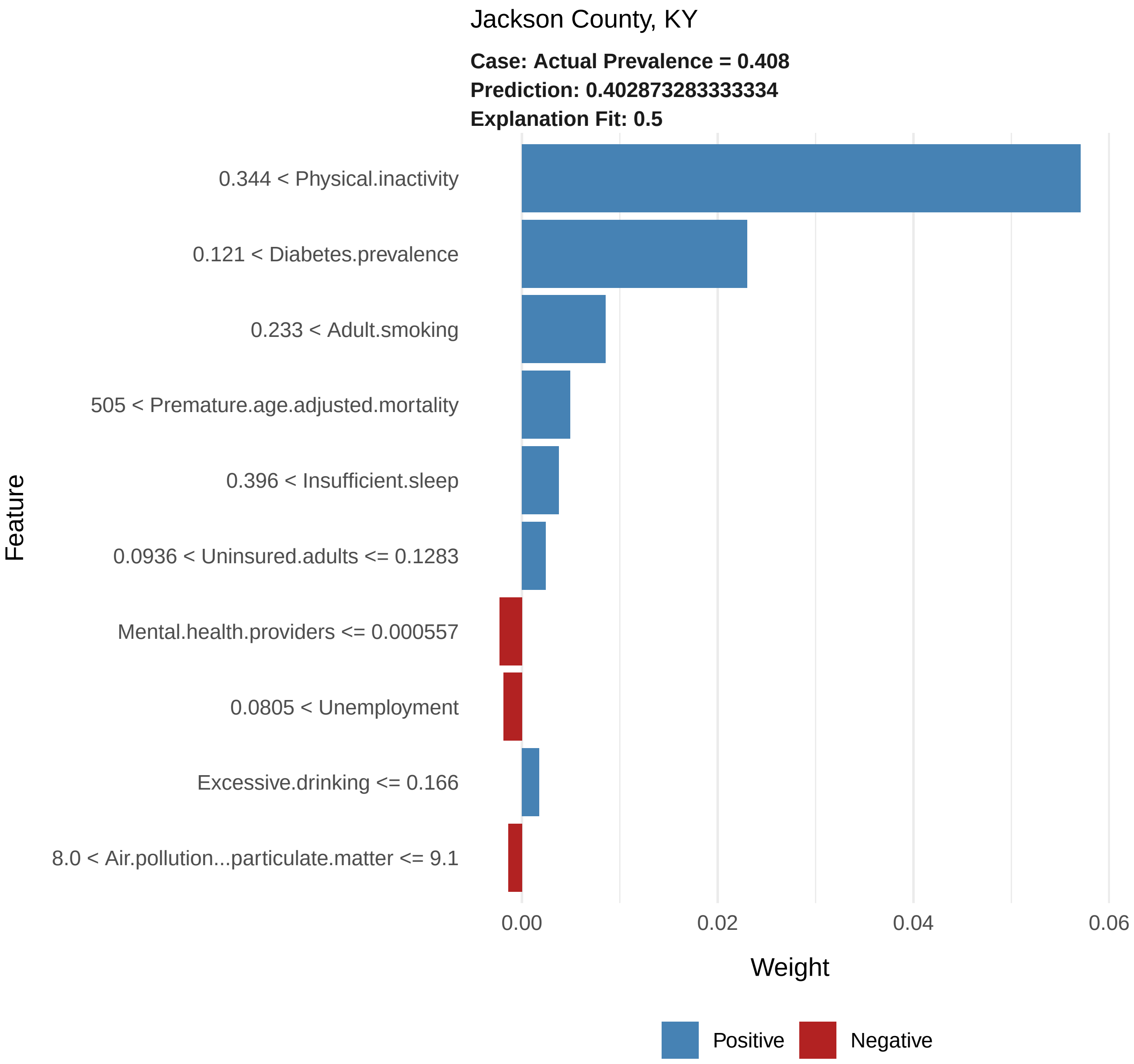

Jackson County, KY
Case: Actual Prevalence = 0.408
Prediction: 0.402873283333334
Explanation Fit: 0.5
0.344 < Physical.inactivity
0.121 < Diabetes.prevalence
0.233 < Adult.smoking
505 < Premature.age.adjusted.mortality
0.396 < Insufficient.sleep
0.0936 < Uninsured.adults <= 0.1283
Mental.health.providers <= 0.000557
0.0805 < Unemployment
Excessive.drinking <= 0.166
8.0 < Air.pollution...particulate.matter <= 9.1
Feature
0.00
0.02
0.04
0.06
Weight
Positive
Negative

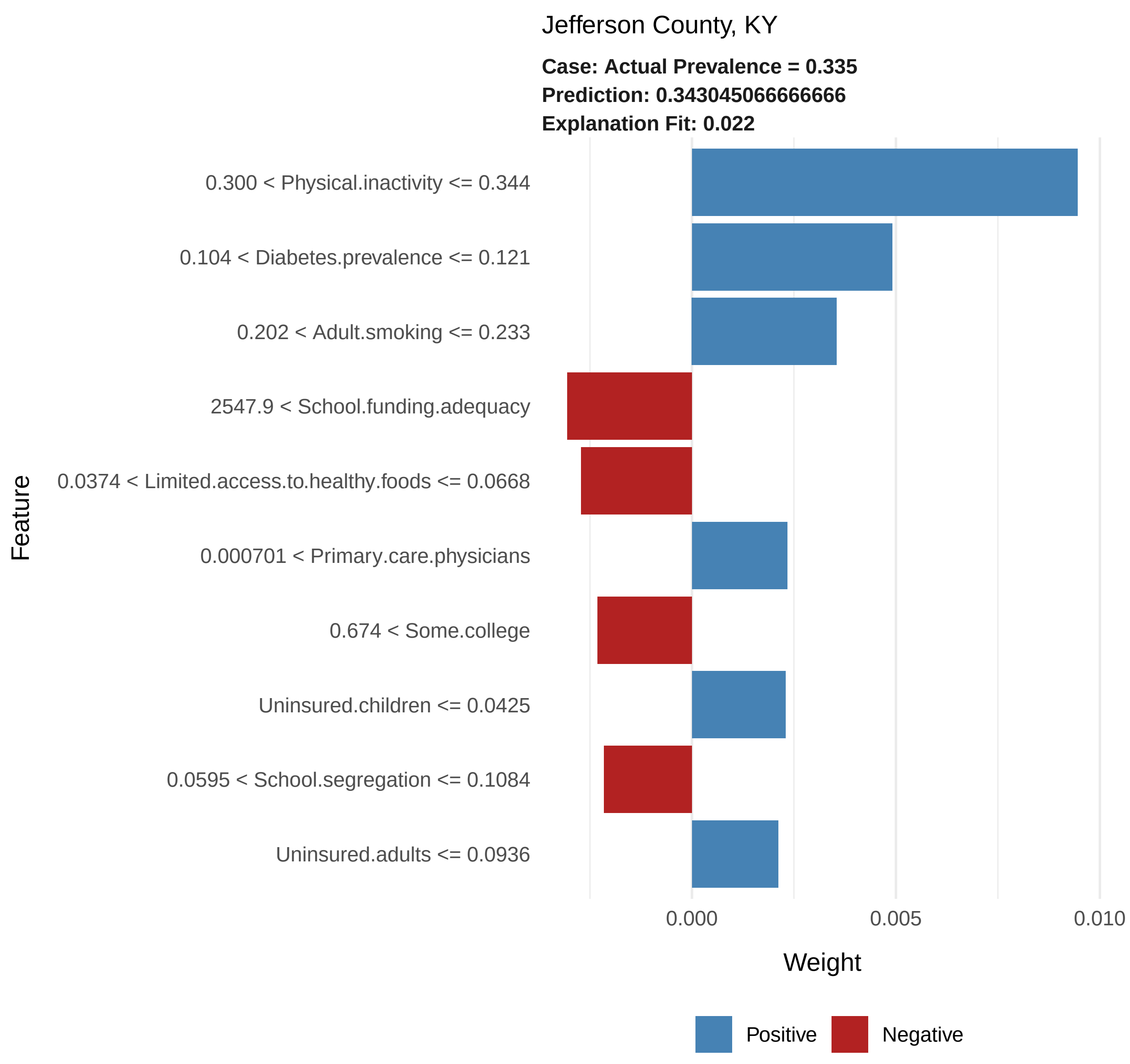
Jefferson County, KY
Case: Actual Prevalence = 0.335
Prediction: 0.343045066666666
Explanation Fit: 0.022
0.300 < Physical.inactivity <= 0.344
0.104 < Diabetes.prevalence <= 0.121
0.202 < Adult.smoking <= 0.233
2547.9 < School.funding.adequacy
0.0374 < Limited.access.to.healthy.foods <= 0.0668
0.000701 < Primary.care.physicians
0.674 < Some.college
Uninsured.children <= 0.0425
0.0595 < School.segregation <= 0.1084
Uninsured.adults <= 0.0936
Feature
0.000
0.005
0.010
Weight
Positive
Negative

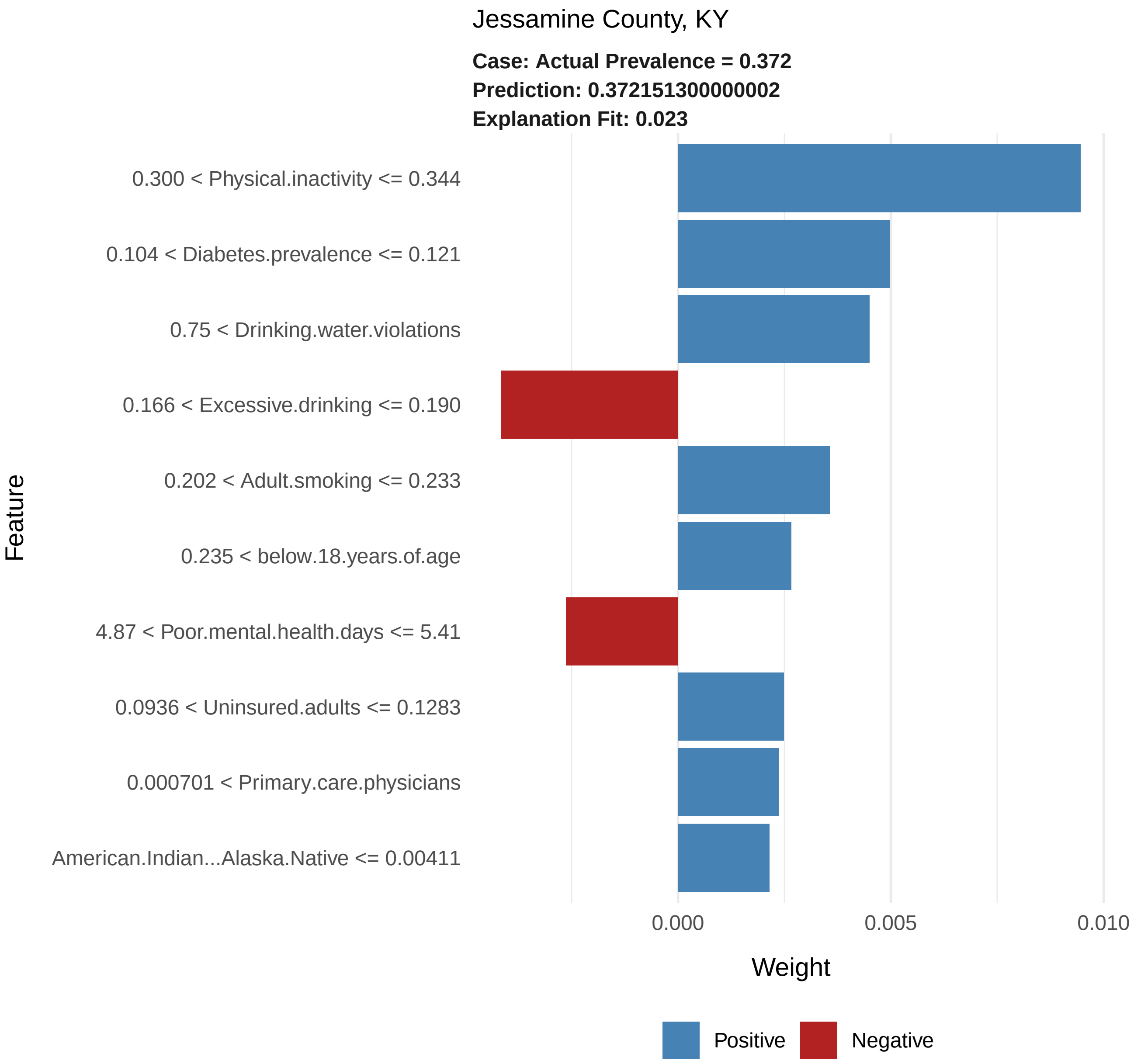

Jessamine County, KY
Case: Actual Prevalence = 0.372
Prediction: 0.372151300000002
Explanation Fit: 0.023
0.300 < Physical.inactivity <= 0.344
0.104 < Diabetes.prevalence <= 0.121
0.75 < Drinking.water.violations
0.166 < Excessive.drinking <= 0.190
0.202 < Adult.smoking <= 0.233
0.235 < below.18.years.of.age
4.87 < Poor.mental.health.days <= 5.41
0.0936 < Uninsured.adults <= 0.1283
0.000701 < Primary.care.physicians
American.Indian...Alaska.Native <= 0.00411
Feature
0.000
0.005
0.010
Weight
Positive
Negative

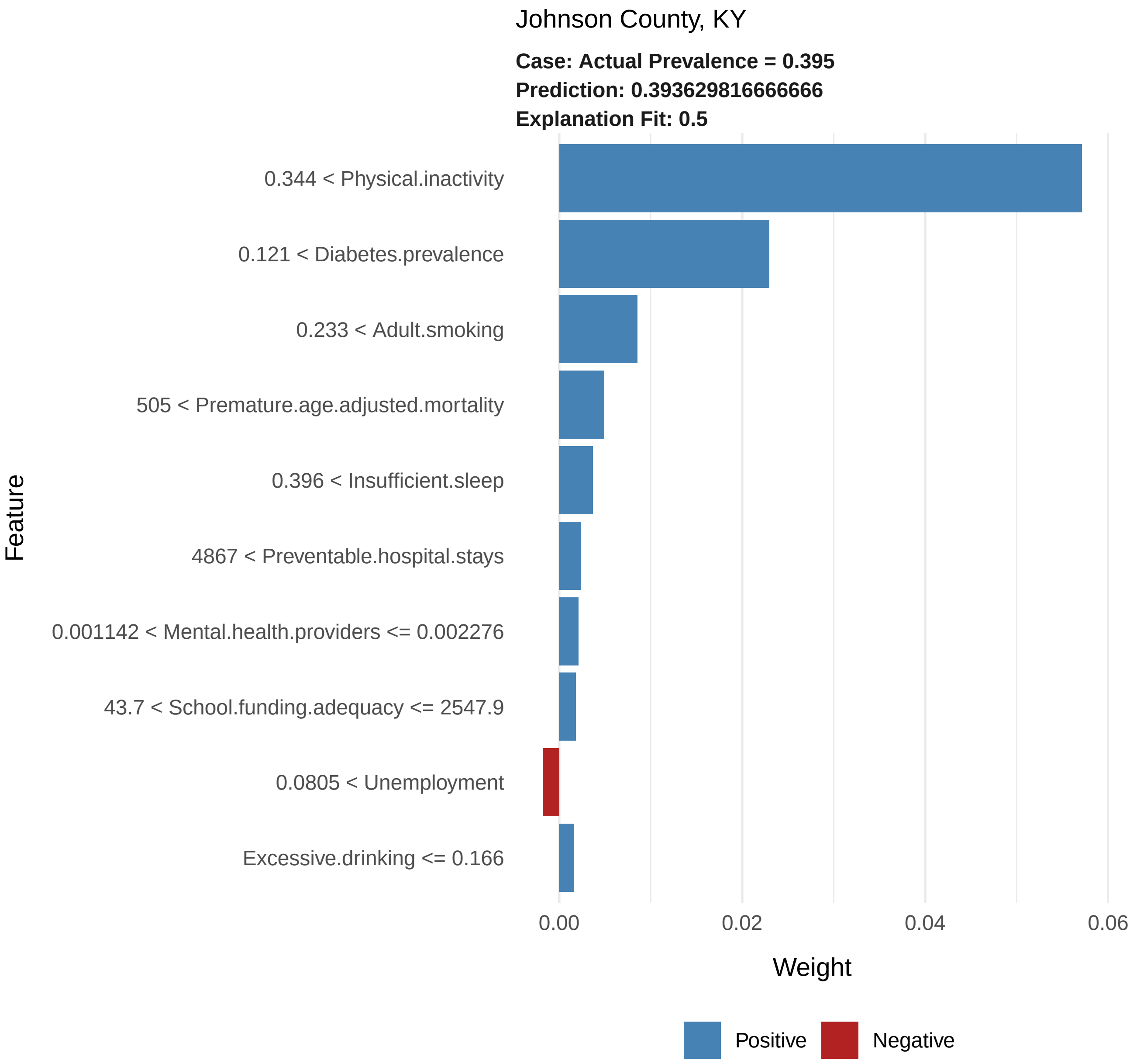
Johnson County, KY
Case: Actual Prevalence = 0.395
Prediction: 0.393629816666666
Explanation Fit: 0.5
0.344 < Physical.inactivity
0.121 < Diabetes.prevalence
0.233 < Adult.smoking
505 < Premature.age.adjusted.mortality
0.396 < Insufficient.sleep
4867 < Preventable.hospital.stays
0.001142 < Mental.health.providers <= 0.002276
43.7 < School.funding.adequacy <= 2547.9
0.0805 < Unemployment
Excessive.drinking <= 0.166
Feature
0.00
0.02
0.04
0.06
Weight
Positive
Negative

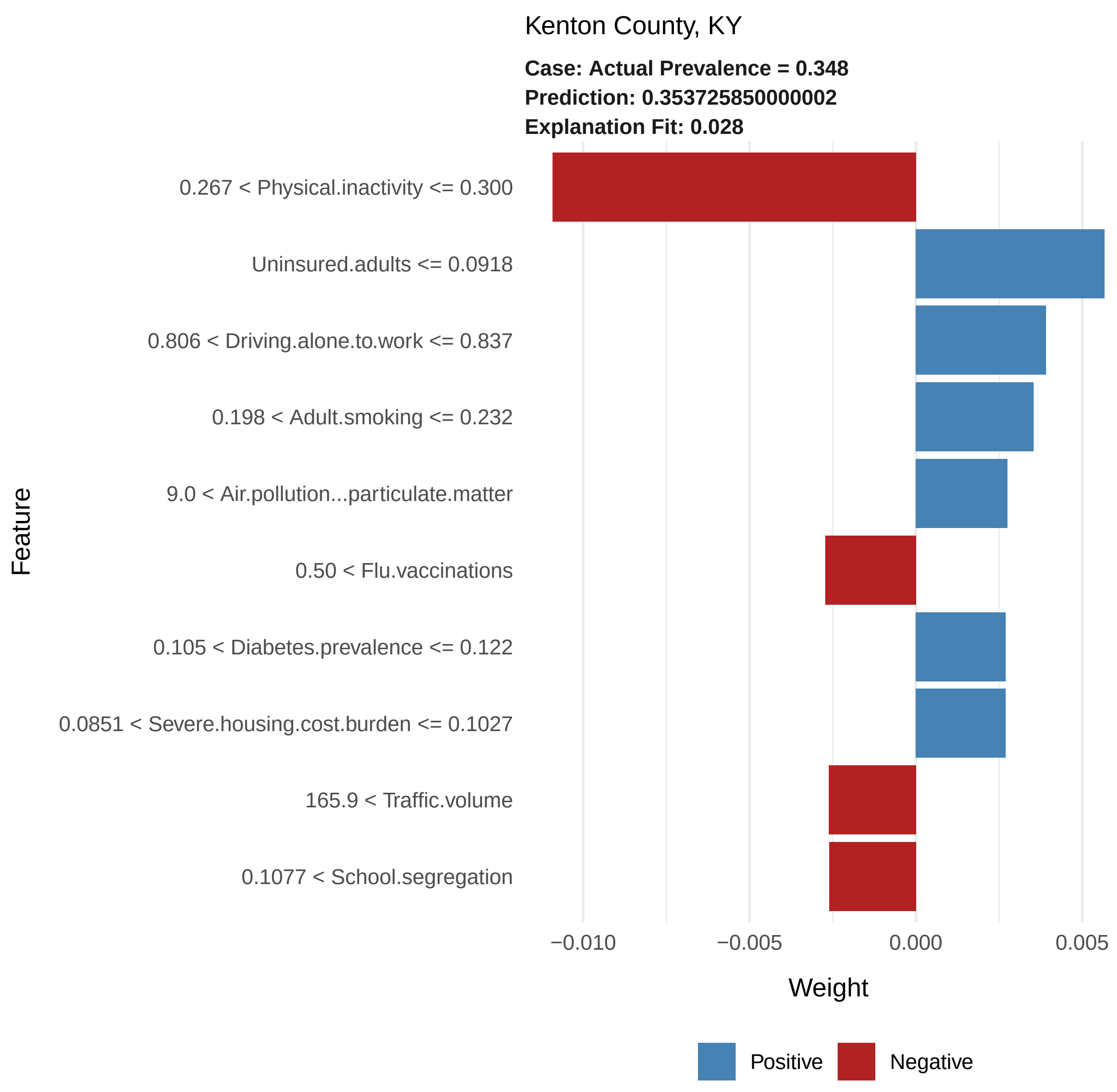
Kenton County, KY
Case: Actual Prevalence = 0.348
Prediction: 0.353725850000002
Explanation Fit: 0.028
0.267 < Physical.inactivity <= 0.300
Uninsured.adults <= 0.0918
0.806 < Driving.alone.to.work <= 0.837
0.198 < Adult.smoking <= 0.232
9.0 < Air.pollution...particulate.matter
0.50 < Flu.vaccinations
0.105 < Diabetes.prevalence <= 0.122
0.0851 < Severe.housing.cost.burden <= 0.1027
165.9 < Traffic.volume
0.1077 < School.segregation
Feature
−0.010
−0.005
0.000
0.005
Weight
Positive
Negative

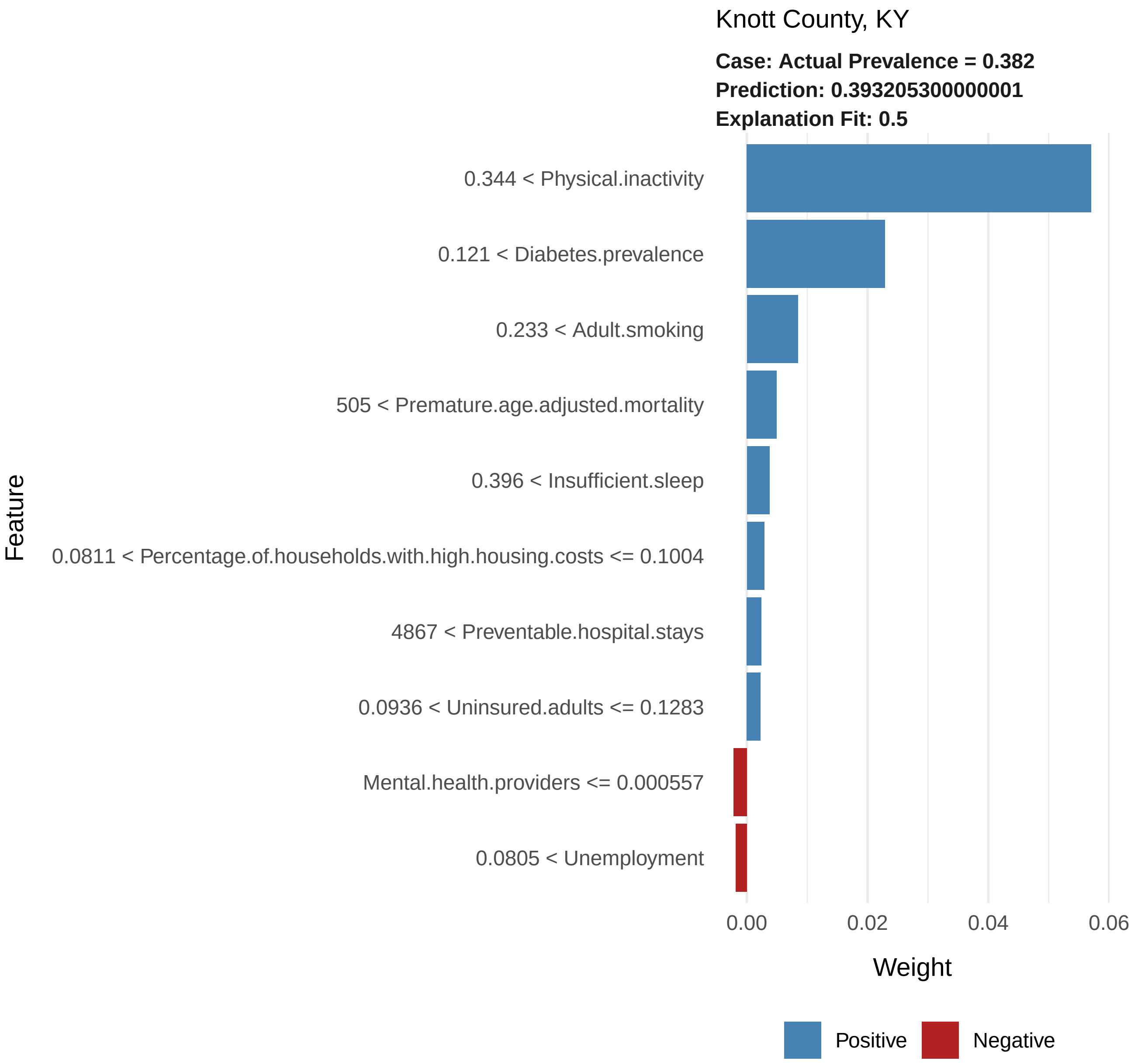
Knott County, KY
Case: Actual Prevalence = 0.382
Prediction: 0.393205300000001
Explanation Fit: 0.5
0.344 < Physical.inactivity
0.121 < Diabetes.prevalence
0.233 < Adult.smoking
505 < Premature.age.adjusted.mortality
0.396 < Insufficient.sleep
0.0811 < Percentage.of.households.with.high.housing.costs <= 0.1004
4867 < Preventable.hospital.stays
0.0936 < Uninsured.adults <= 0.1283
Mental.health.providers <= 0.000557
0.0805 < Unemployment
Feature
0.00
0.02
0.04
0.06
Weight
Positive
Negative

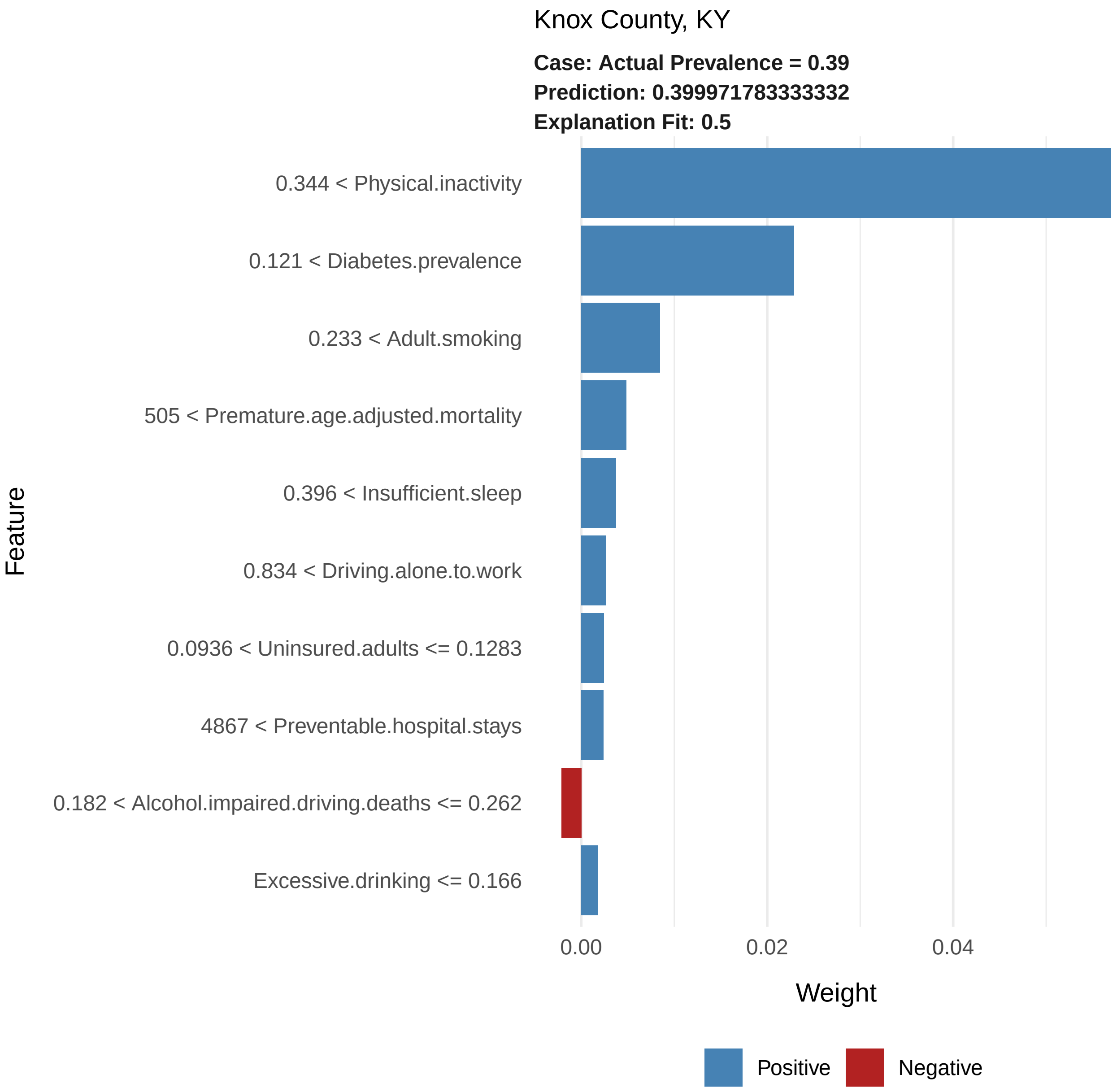
Knox County, KY
Case: Actual Prevalence = 0.39
Prediction: 0.399971783333332
Explanation Fit: 0.5
0.344 < Physical.inactivity
0.121 < Diabetes.prevalence
0.233 < Adult.smoking
505 < Premature.age.adjusted.mortality
0.396 < Insufficient.sleep
0.834 < Driving.alone.to.work
0.0936 < Uninsured.adults <= 0.1283
4867 < Preventable.hospital.stays
0.182 < Alcohol.impaired.driving.deaths <= 0.262
Excessive.drinking <= 0.166
Feature
0.00
0.02
0.04
Weight
Positive
Negative

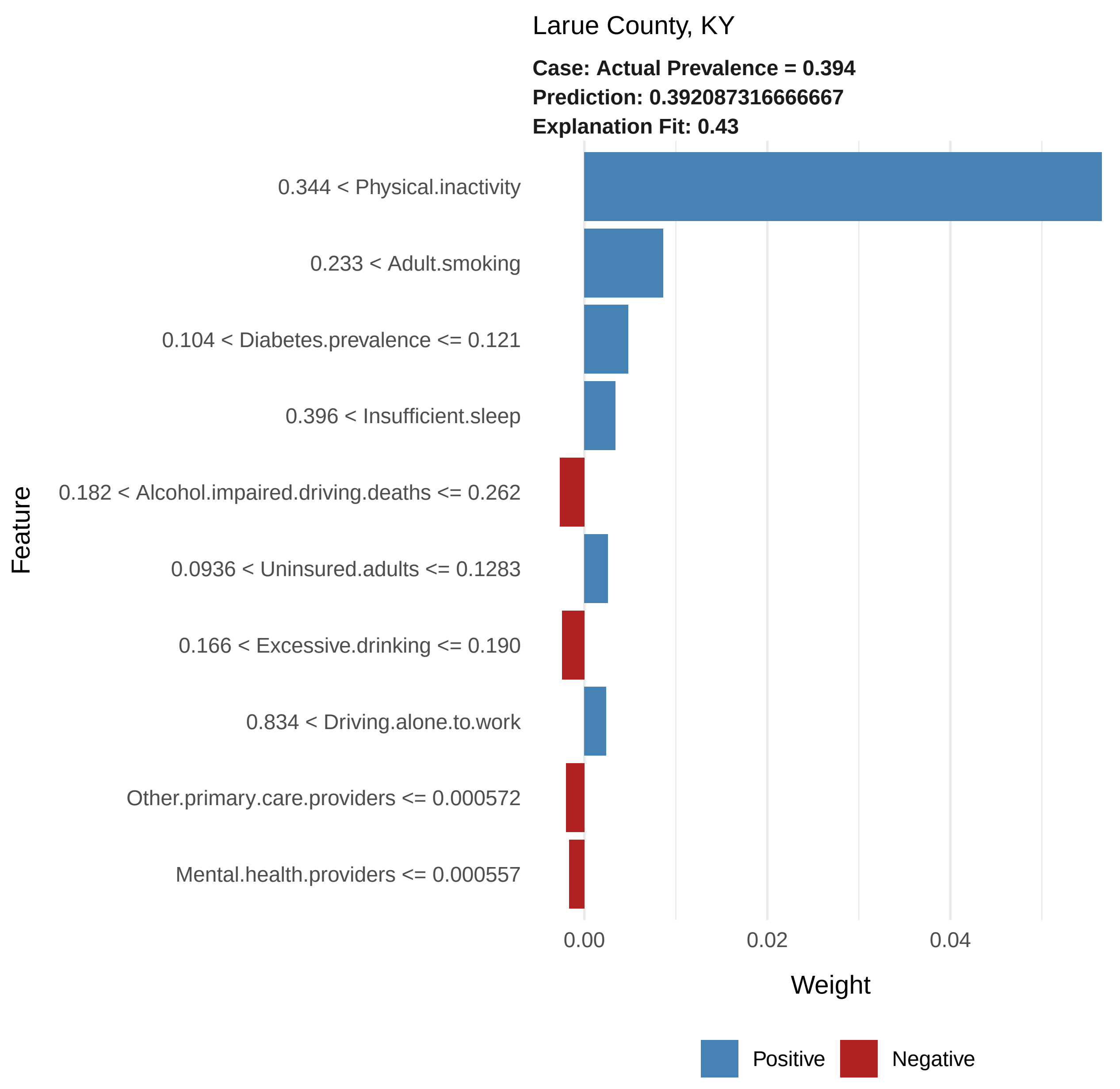

Larue County, KY
Case: Actual Prevalence = 0.394
Prediction: 0.392087316666667
Explanation Fit: 0.43
0.344 < Physical.inactivity
0.233 < Adult.smoking
0.104 < Diabetes.prevalence <= 0.121
0.396 < Insufficient.sleep
0.182 < Alcohol.impaired.driving.deaths <= 0.262
0.0936 < Uninsured.adults <= 0.1283
0.166 < Excessive.drinking <= 0.190
0.834 < Driving.alone.to.work
Other.primary.care.providers <= 0.000572
Mental.health.providers <= 0.000557
Feature
0.00
0.02
0.04
Weight
Positive
Negative

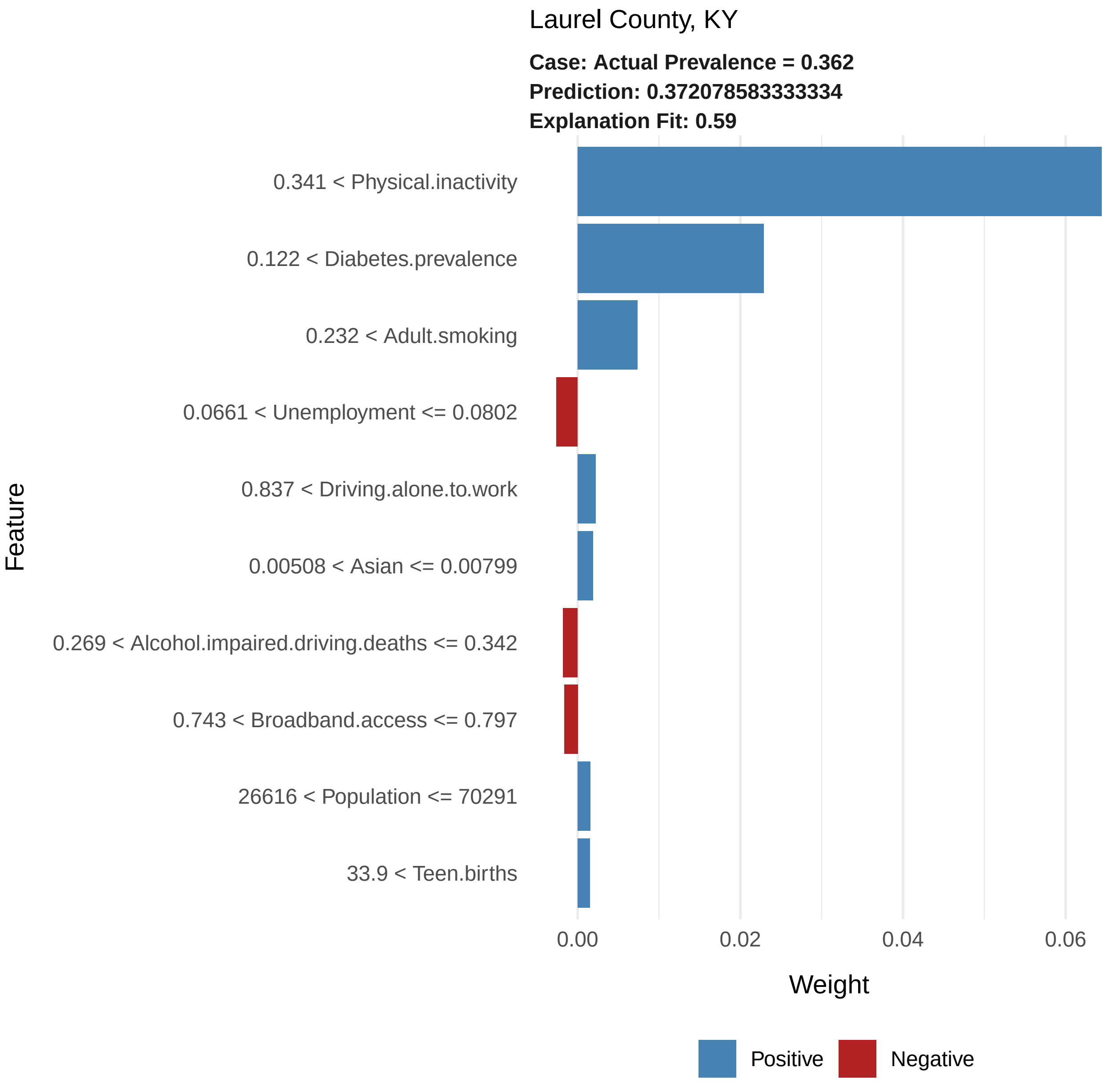

Laurel County, KY
Case: Actual Prevalence = 0.362
Prediction: 0.372078583333334
Explanation Fit: 0.59
0.341 < Physical.inactivity
0.122 < Diabetes.prevalence
0.232 < Adult.smoking
0.0661 < Unemployment <= 0.0802
0.837 < Driving.alone.to.work
0.00508 < Asian <= 0.00799
0.269 < Alcohol.impaired.driving.deaths <= 0.342
0.743 < Broadband.access <= 0.797
26616 < Population <= 70291
33.9 < Teen.births
Feature
0.00
0.02
0.04
0.06
Weight
Positive
Negative

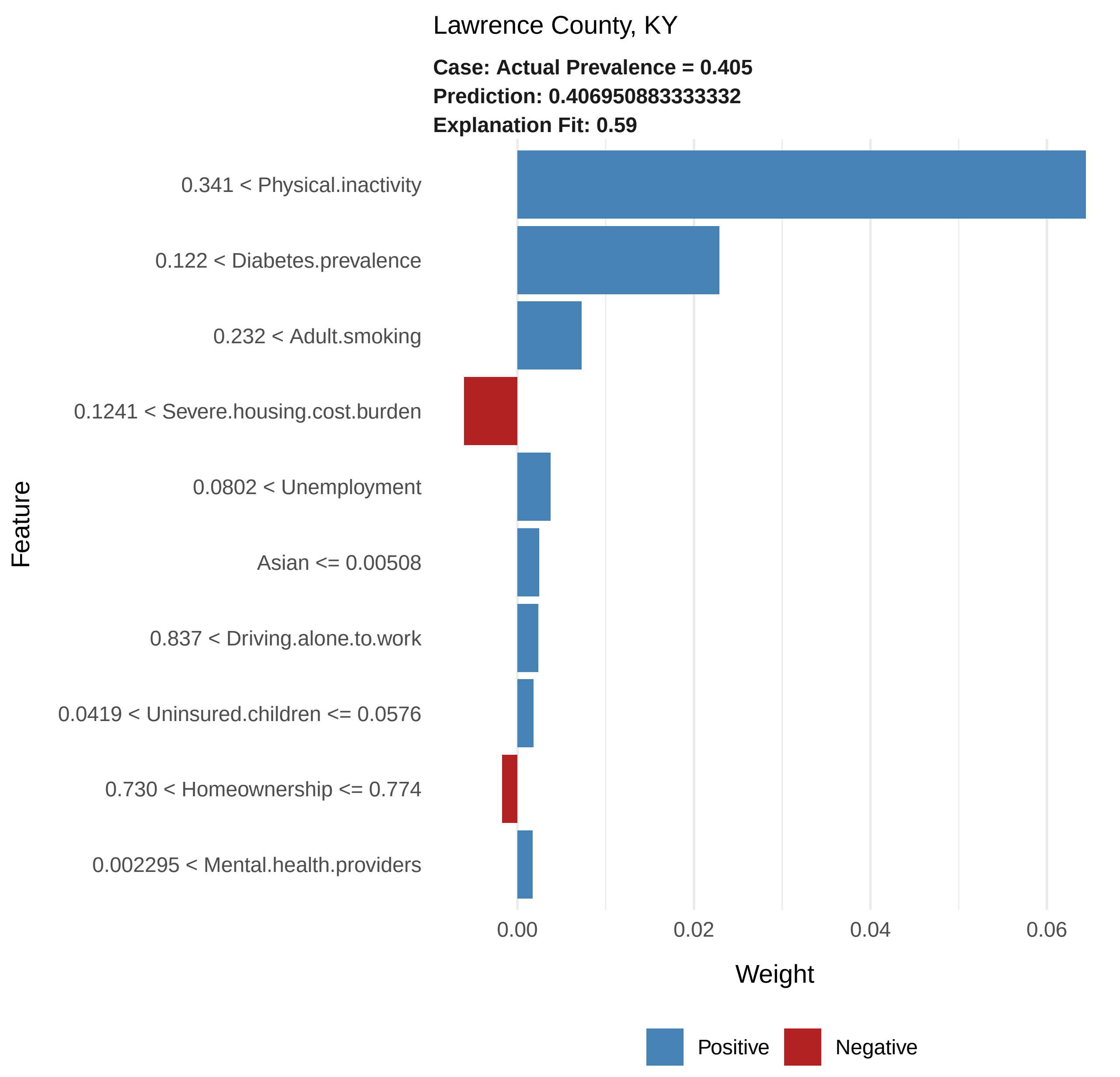
Lawrence County, KY
Case: Actual Prevalence = 0.405
Prediction: 0.406950883333332
Explanation Fit: 0.59
0.341 < Physical.inactivity
0.122 < Diabetes.prevalence
0.232 < Adult.smoking
0.1241 < Severe.housing.cost.burden
0.0802 < Unemployment
Asian <= 0.00508
0.837 < Driving.alone.to.work
0.0419 < Uninsured.children <= 0.0576
0.730 < Homeownership <= 0.774
0.002295 < Mental.health.providers
Feature
0.00
0.02
0.04
0.06
Weight
Positive
Negative

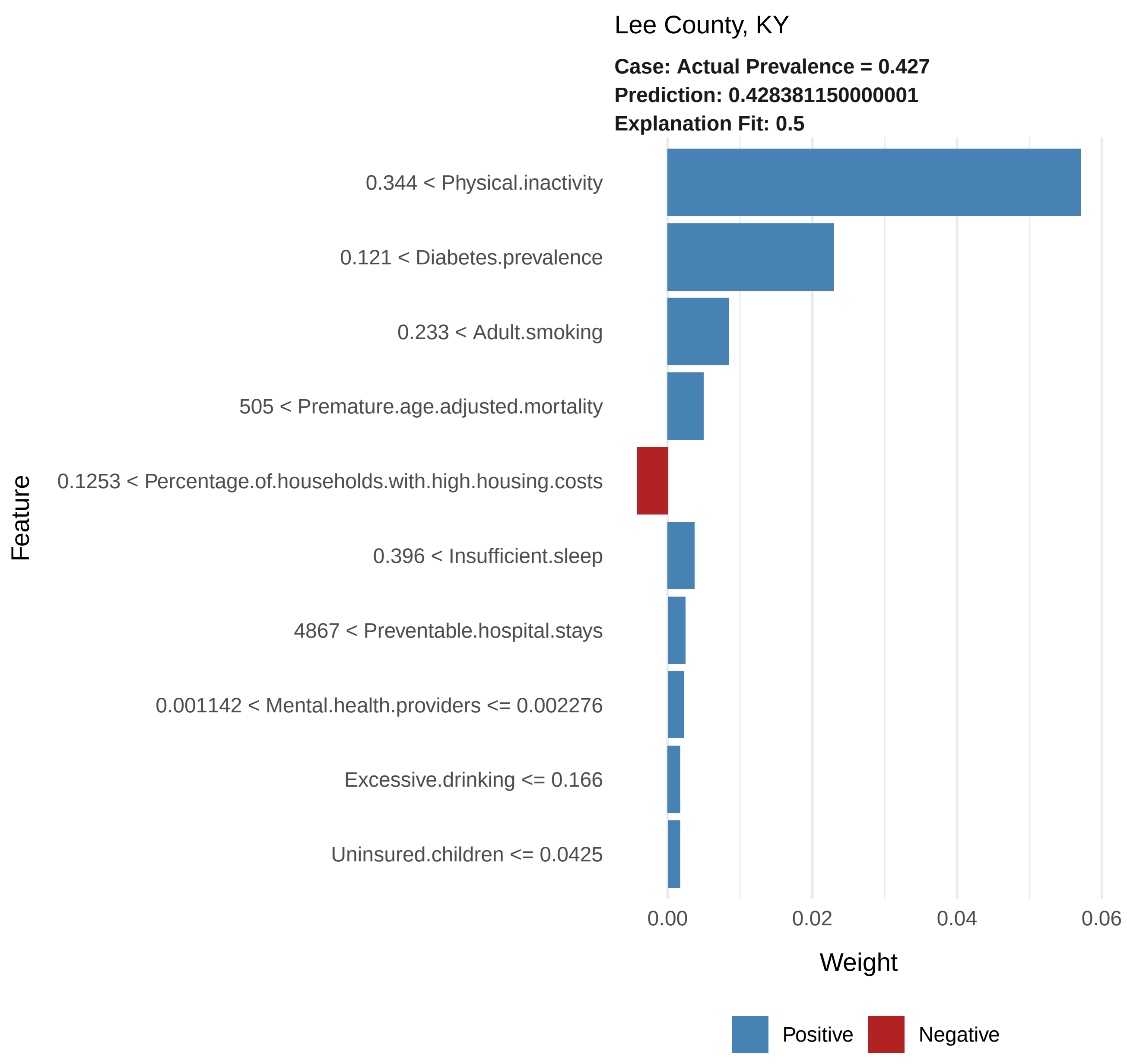
Lee County, KY
Case: Actual Prevalence = 0.427
Prediction: 0.428381150000001
Explanation Fit: 0.5
0.344 < Physical.inactivity
0.121 < Diabetes.prevalence
0.233 < Adult.smoking
505 < Premature.age.adjusted.mortality
0.1253 < Percentage.of.households.with.high.housing.costs
0.396 < Insufficient.sleep
4867 < Preventable.hospital.stays
0.001142 < Mental.health.providers <= 0.002276
Excessive.drinking <= 0.166
Uninsured.children <= 0.0425
Feature
0.00
0.02
0.04
0.06
Weight
Positive
Negative

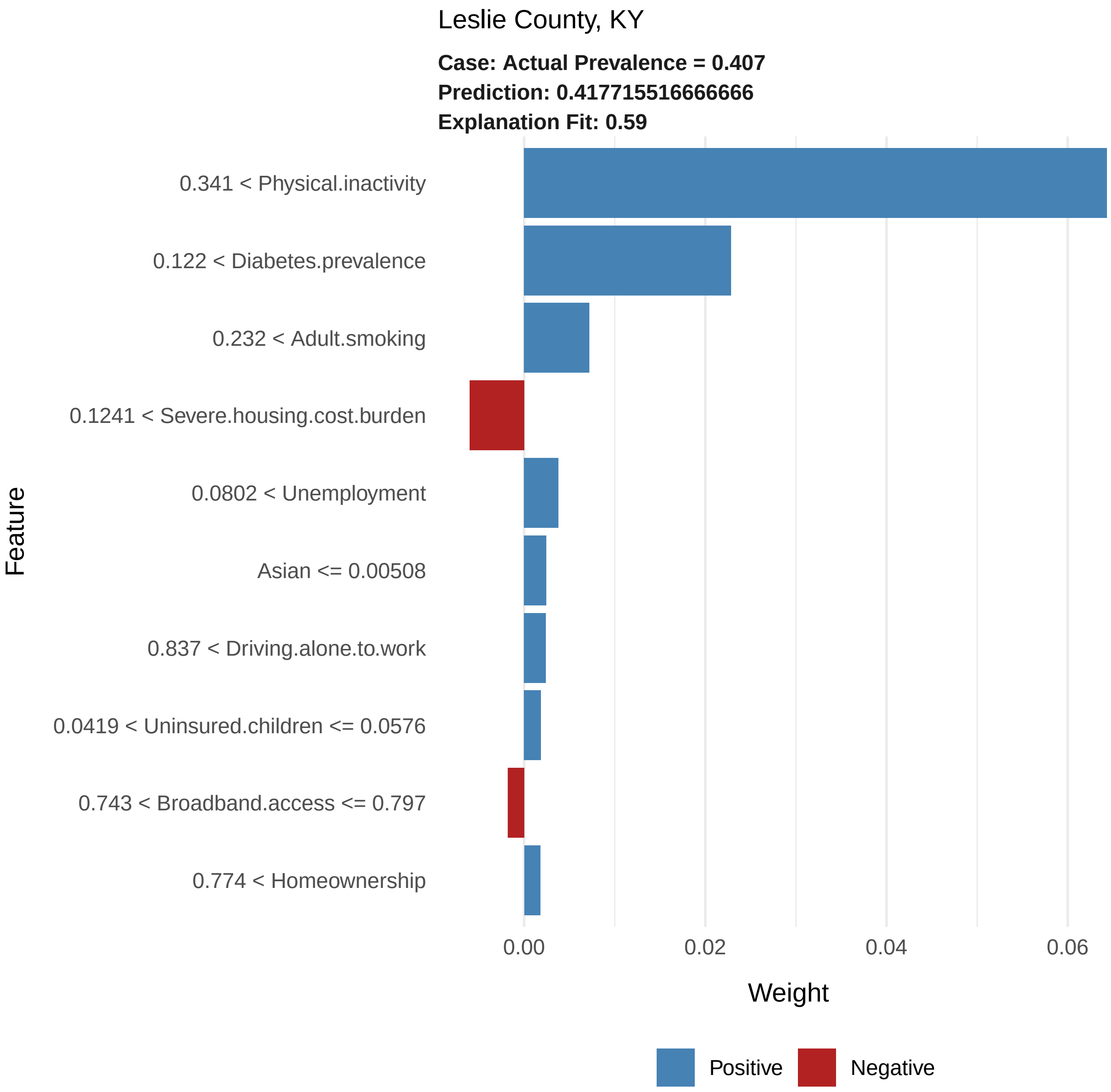
Leslie County, KY
Case: Actual Prevalence = 0.407
Prediction: 0.417715516666666
Explanation Fit: 0.59
0.341 < Physical.inactivity
0.122 < Diabetes.prevalence
0.232 < Adult.smoking
0.1241 < Severe.housing.cost.burden
0.0802 < Unemployment
Asian <= 0.00508
0.837 < Driving.alone.to.work
0.0419 < Uninsured.children <= 0.0576
0.743 < Broadband.access <= 0.797
0.774 < Homeownership
Feature
0.00
0.02
0.04
0.06
Weight
Positive
Negative

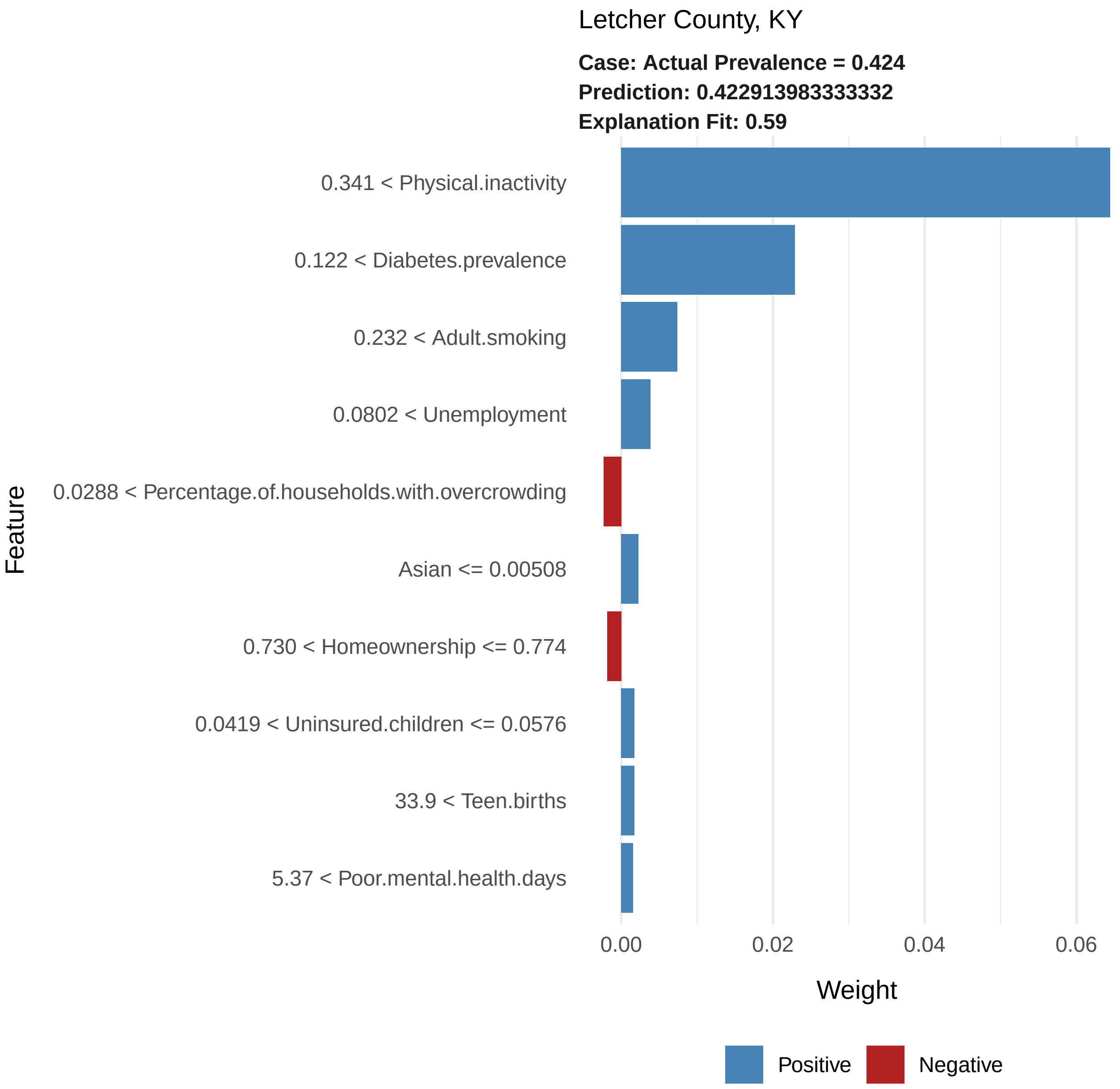

Letcher County, KY
Case: Actual Prevalence = 0.424
Prediction: 0.422913983333332
Explanation Fit: 0.59
0.341 < Physical.inactivity
0.122 < Diabetes.prevalence
0.232 < Adult.smoking
0.0802 < Unemployment
0.0288 < Percentage.of.households.with.overcrowding
Asian <= 0.00508
0.730 < Homeownership <= 0.774
0.0419 < Uninsured.children <= 0.0576
33.9 < Teen.births
5.37 < Poor.mental.health.days
Feature
0.00
0.02
0.04
0.06
Weight
Positive
Negative

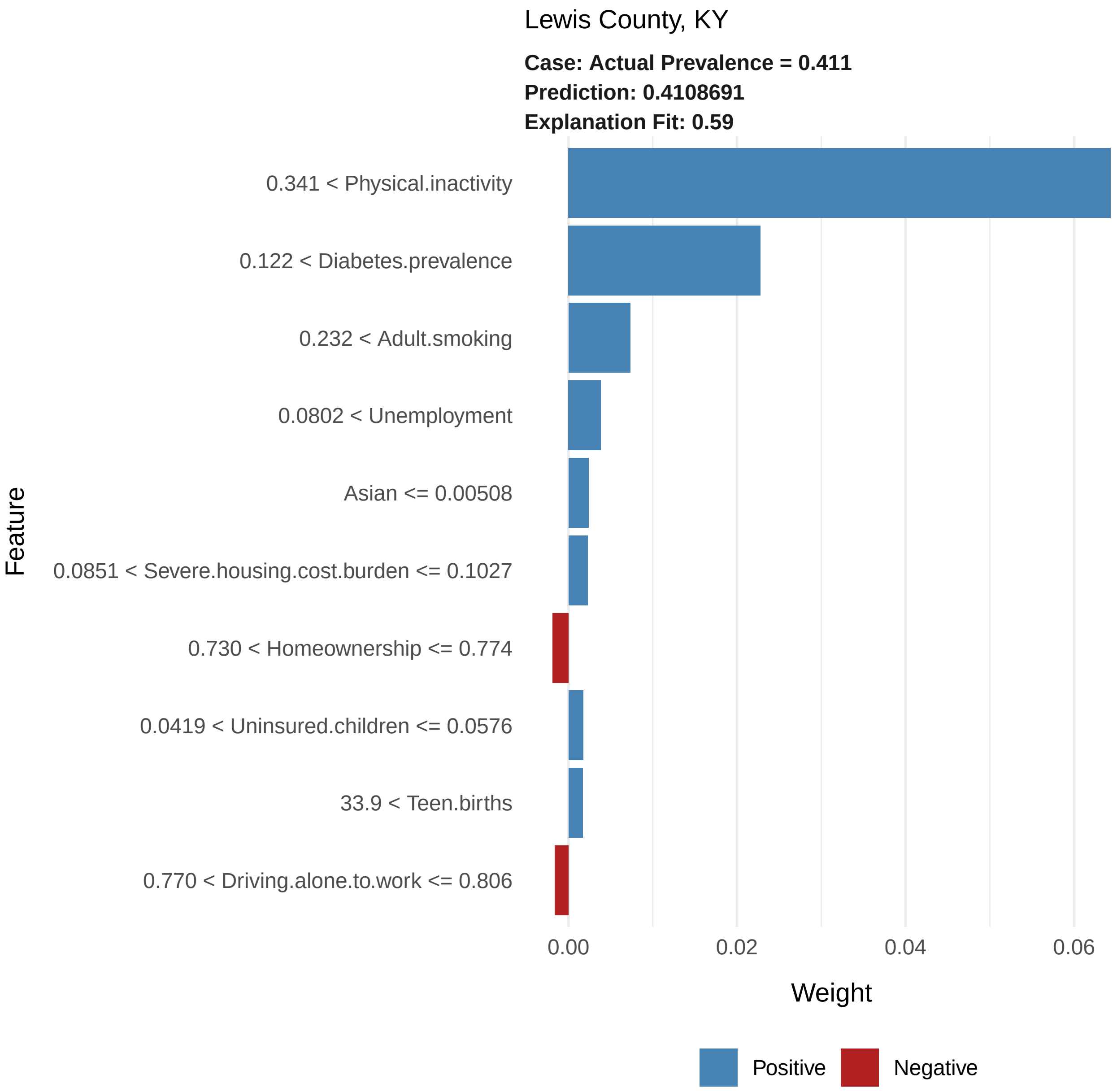
Lewis County, KY
Case: Actual Prevalence = 0.411
Prediction: 0.4108691
Explanation Fit: 0.59
0.341 < Physical.inactivity
0.122 < Diabetes.prevalence
0.232 < Adult.smoking
0.0802 < Unemployment
Asian <= 0.00508
0.0851 < Severe.housing.cost.burden <= 0.1027
0.730 < Homeownership <= 0.774
0.0419 < Uninsured.children <= 0.0576
33.9 < Teen.births
0.770 < Driving.alone.to.work <= 0.806
Feature
0.00
0.02
0.04
0.06
Weight
Positive
Negative

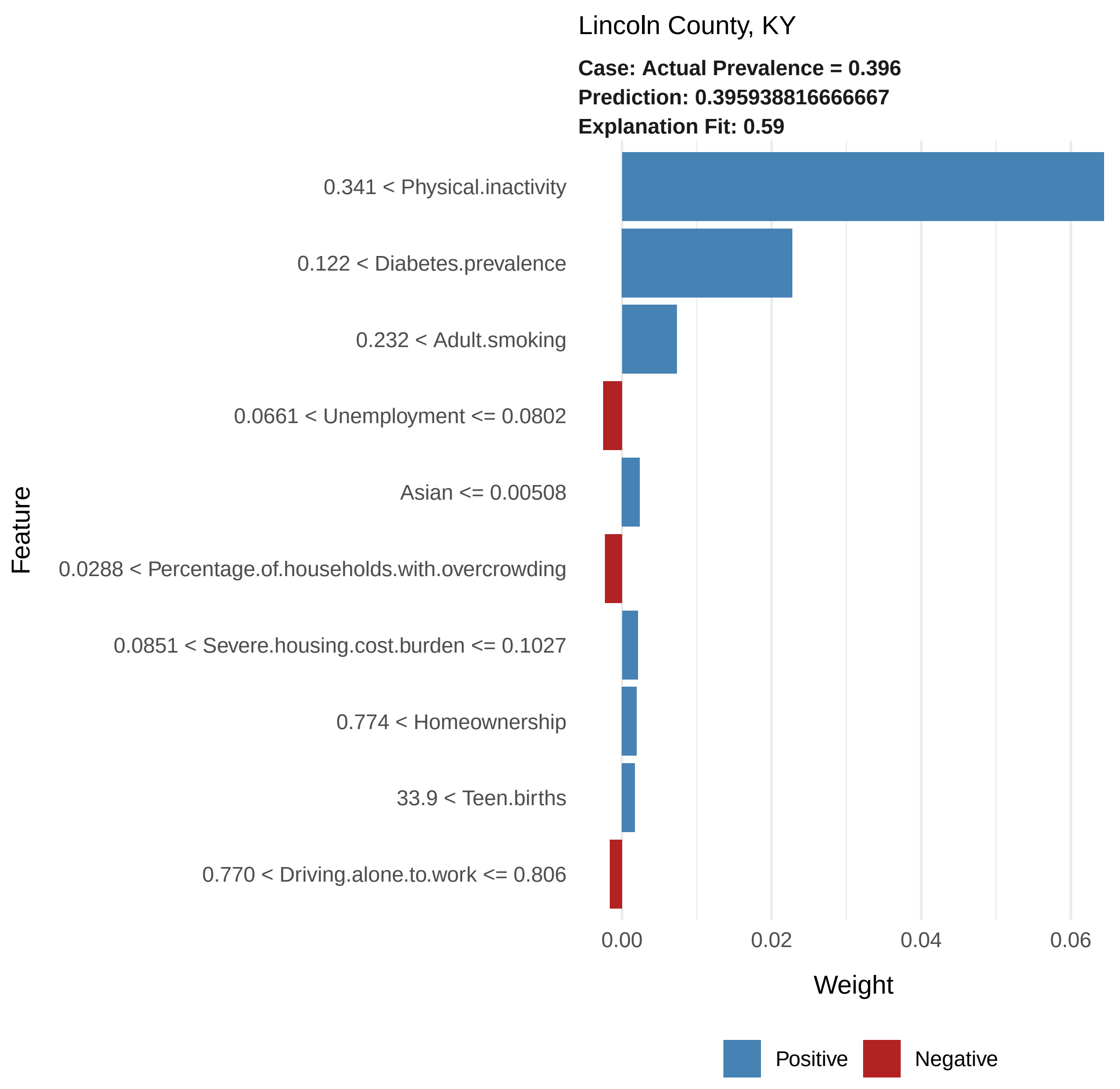

Lincoln County, KY
Case: Actual Prevalence = 0.396
Prediction: 0.395938816666667
Explanation Fit: 0.59
0.341 < Physical.inactivity
0.122 < Diabetes.prevalence
0.232 < Adult.smoking
0.0661 < Unemployment <= 0.0802
Asian <= 0.00508
0.0288 < Percentage.of.households.with.overcrowding
0.0851 < Severe.housing.cost.burden <= 0.1027
0.774 < Homeownership
33.9 < Teen.births
0.770 < Driving.alone.to.work <= 0.806
Feature
0.00
0.02
0.04
0.06
Weight
Positive
Negative

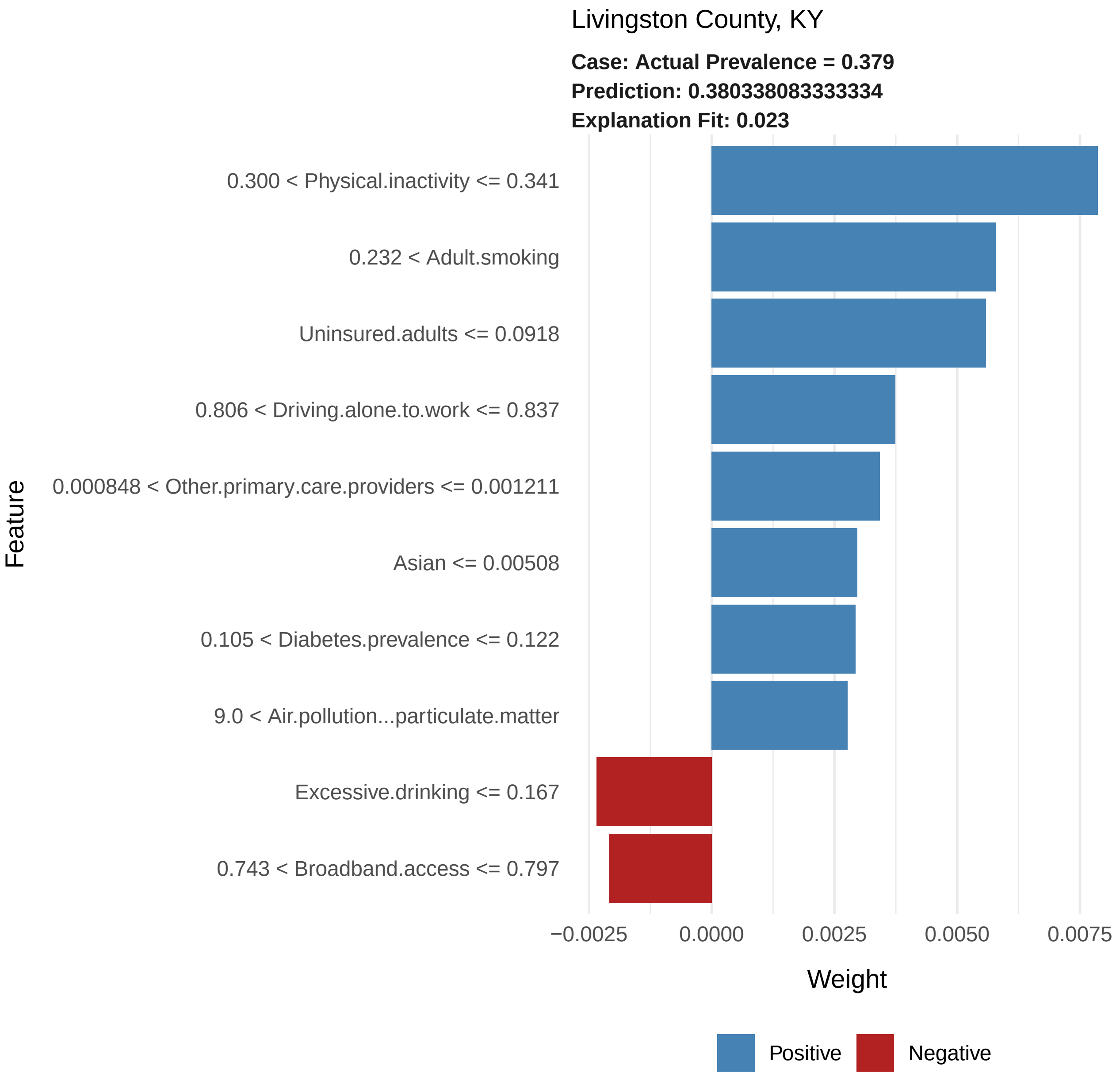

Livingston County, KY
Case: Actual Prevalence = 0.379
Prediction: 0.380338083333334
Explanation Fit: 0.023
0.300 < Physical.inactivity <= 0.341
0.232 < Adult.smoking
Uninsured.adults <= 0.0918
0.806 < Driving.alone.to.work <= 0.837
0.000848 < Other.primary.care.providers <= 0.001211
Asian <= 0.00508
0.105 < Diabetes.prevalence <= 0.122
9.0 < Air.pollution...particulate.matter
Excessive.drinking <= 0.167
0.743 < Broadband.access <= 0.797
Feature
−0.0025
0.0000
0.0025
0.0050
0.0075
Weight
Positive
Negative

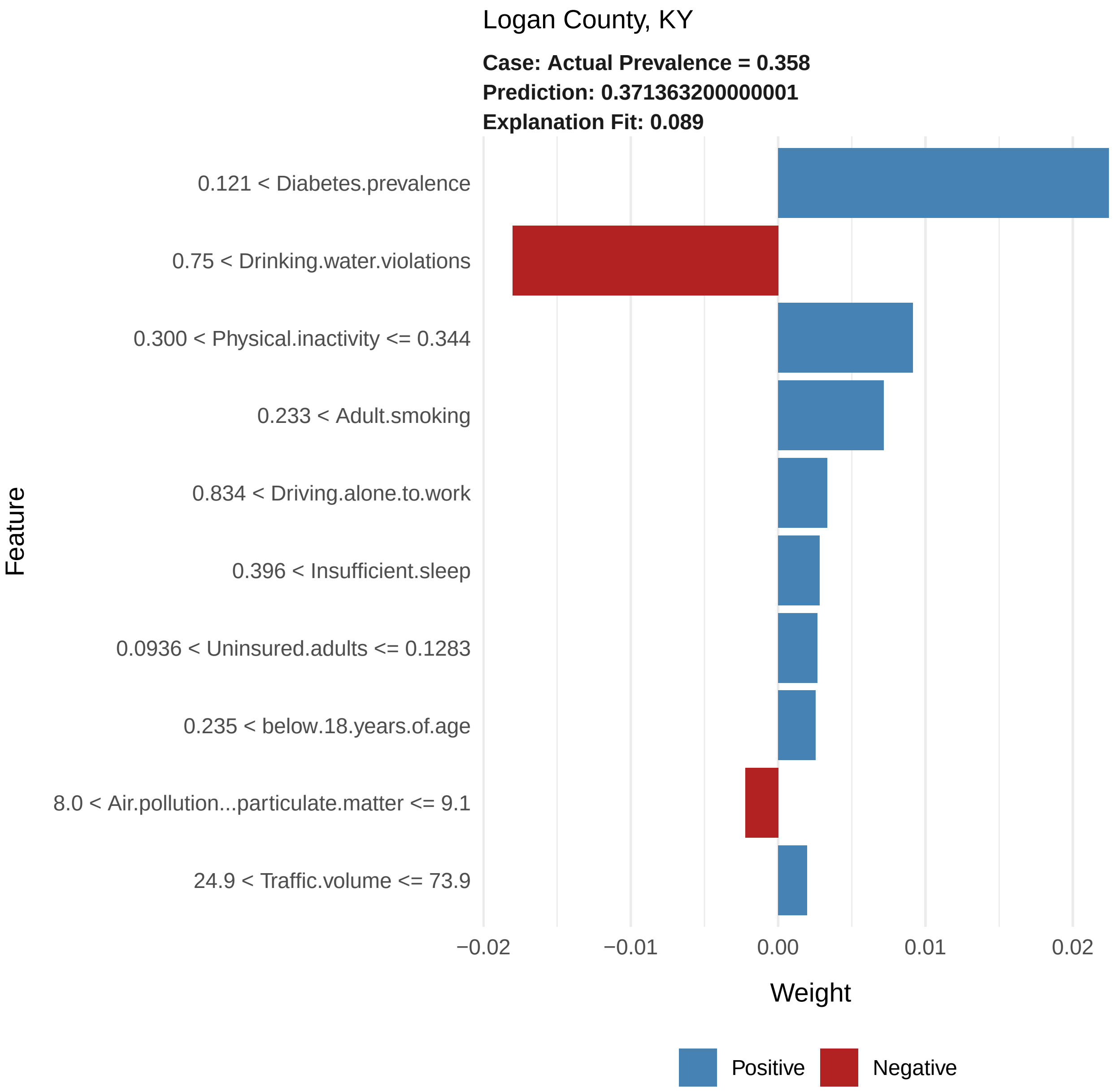

Logan County, KY
Case: Actual Prevalence = 0.358
Prediction: 0.371363200000001
Explanation Fit: 0.089
0.121 < Diabetes.prevalence
0.75 < Drinking.water.violations
0.300 < Physical.inactivity <= 0.344
0.233 < Adult.smoking
0.834 < Driving.alone.to.work
0.396 < Insufficient.sleep
0.0936 < Uninsured.adults <= 0.1283
0.235 < below.18.years.of.age
8.0 < Air.pollution...particulate.matter <= 9.1
24.9 < Traffic.volume <= 73.9
Feature
−0.02
−0.01
0.00
0.01
0.02
Weight
Positive
Negative

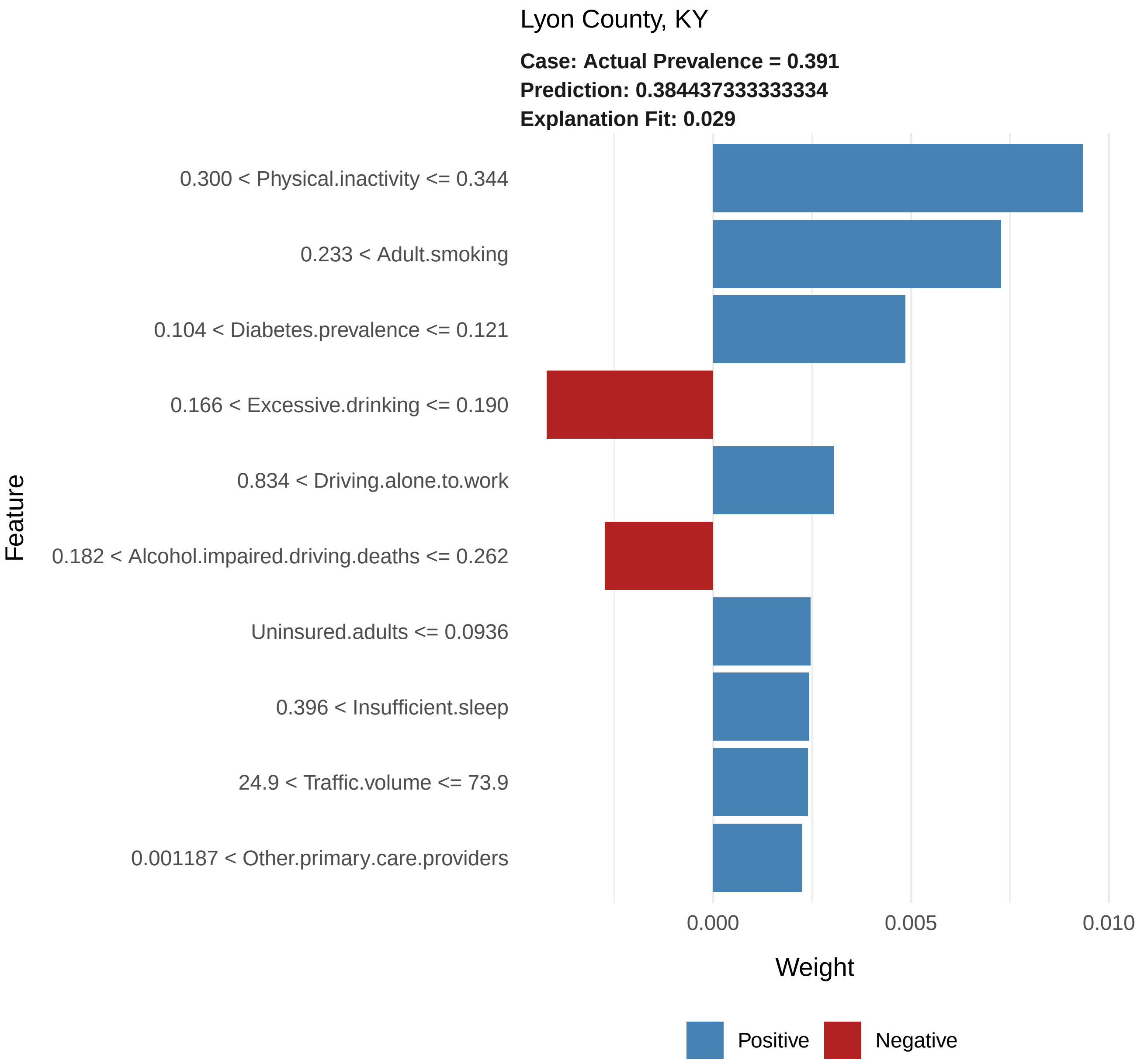

Lyon County, KY
Case: Actual Prevalence = 0.391
Prediction: 0.384437333333334
Explanation Fit: 0.029
0.300 < Physical.inactivity <= 0.344
0.233 < Adult.smoking
0.104 < Diabetes.prevalence <= 0.121
0.166 < Excessive.drinking <= 0.190
0.834 < Driving.alone.to.work
0.182 < Alcohol.impaired.driving.deaths <= 0.262
Uninsured.adults <= 0.0936
0.396 < Insufficient.sleep
24.9 < Traffic.volume <= 73.9
0.001187 < Other.primary.care.providers
Feature
0.000
0.005
0.010
Weight
Positive
Negative

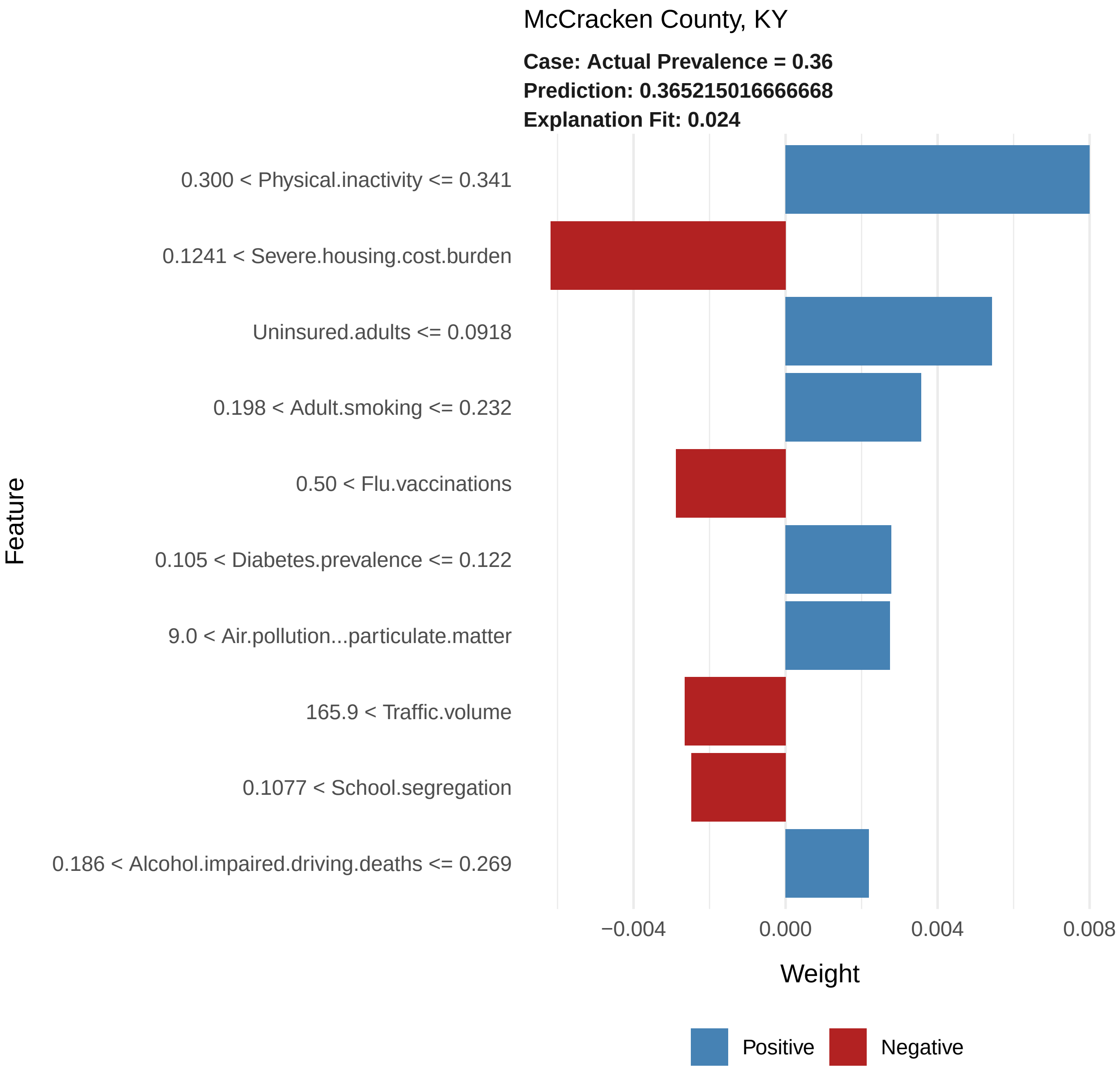
McCracken County, KY
Case: Actual Prevalence = 0.36
Prediction: 0.365215016666668
Explanation Fit: 0.024
0.300 < Physical.inactivity <= 0.341
0.1241 < Severe.housing.cost.burden
Uninsured.adults <= 0.0918
0.198 < Adult.smoking <= 0.232
0.50 < Flu.vaccinations
0.105 < Diabetes.prevalence <= 0.122
9.0 < Air.pollution...particulate.matter
165.9 < Traffic.volume
0.1077 < School.segregation
0.186 < Alcohol.impaired.driving.deaths <= 0.269
Feature
−0.004
0.000
0.004
0.008
Weight
Positive
Negative

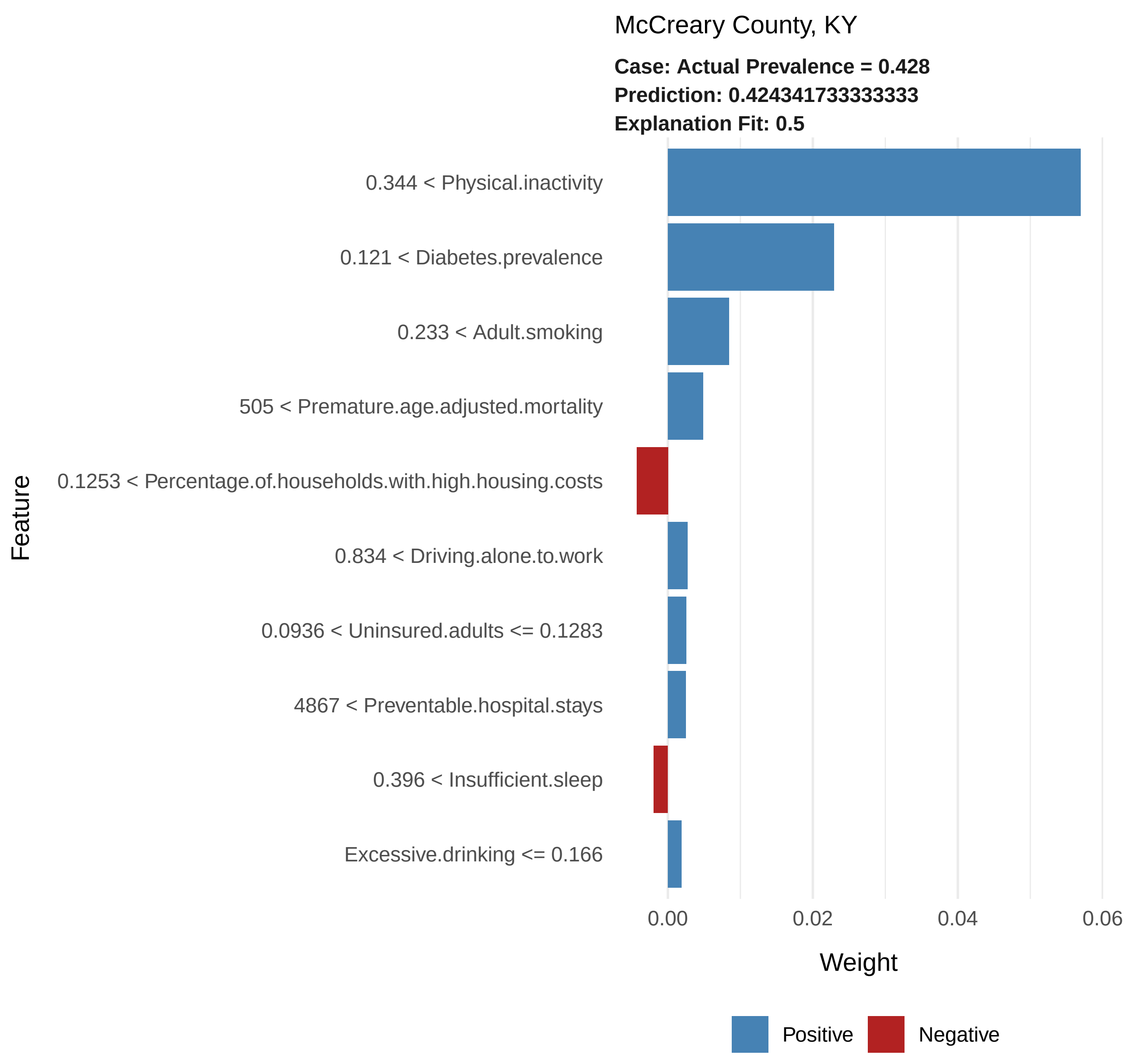
McCreary County, KY
Case: Actual Prevalence = 0.428
Prediction: 0.424341733333333
Explanation Fit: 0.5
0.344 < Physical.inactivity
0.121 < Diabetes.prevalence
0.233 < Adult.smoking
505 < Premature.age.adjusted.mortality
0.1253 < Percentage.of.households.with.high.housing.costs
0.834 < Driving.alone.to.work
0.0936 < Uninsured.adults <= 0.1283
4867 < Preventable.hospital.stays
0.396 < Insufficient.sleep
Excessive.drinking <= 0.166
Feature
0.00
0.02
0.04
0.06
Weight
Positive
Negative

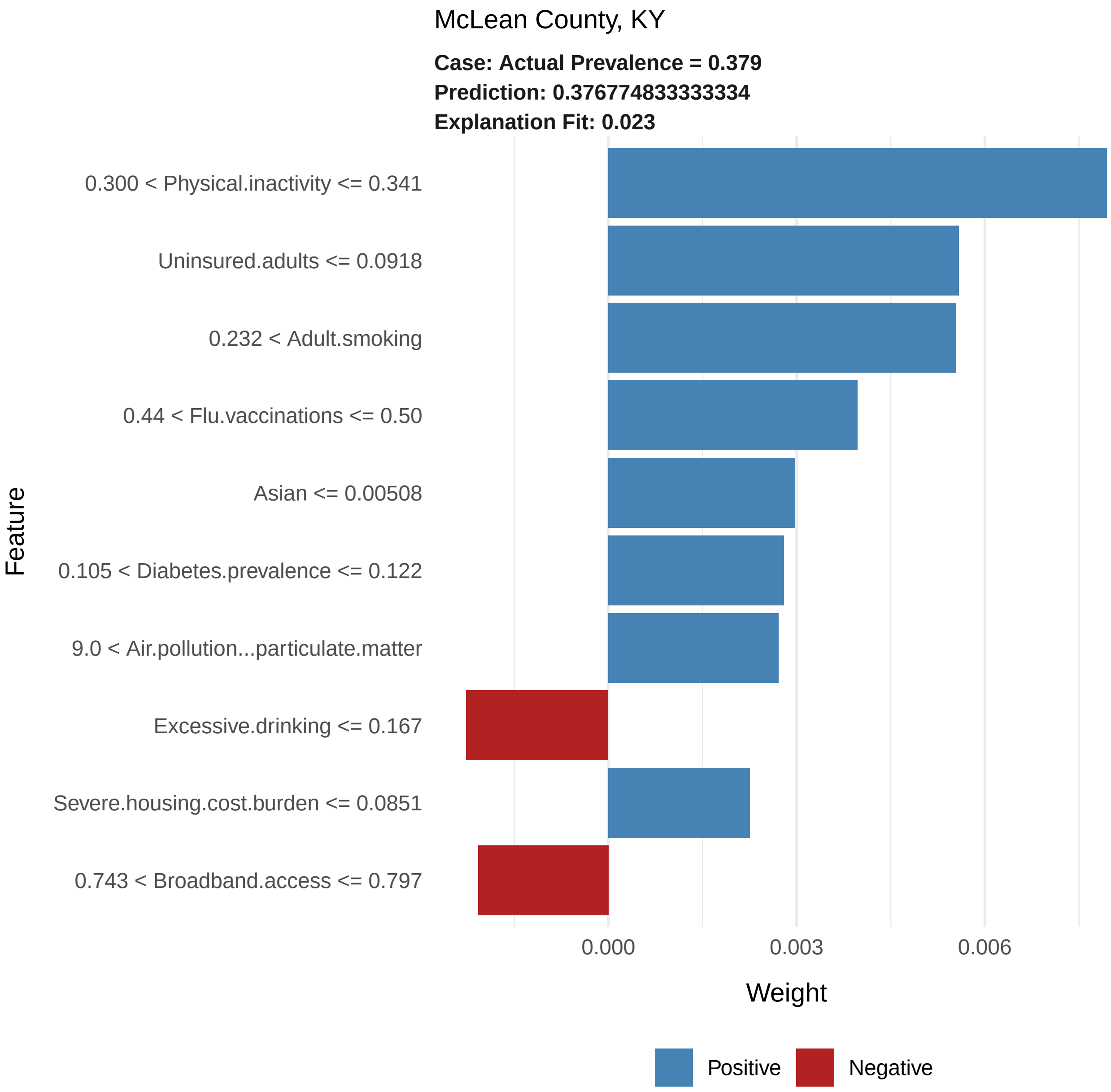

McLean County, KY
Case: Actual Prevalence = 0.379
Prediction: 0.376774833333334
Explanation Fit: 0.023
0.300 < Physical.inactivity <= 0.341
Uninsured.adults <= 0.0918
0.232 < Adult.smoking
0.44 < Flu.vaccinations <= 0.50
Asian <= 0.00508
0.105 < Diabetes.prevalence <= 0.122
9.0 < Air.pollution...particulate.matter
Excessive.drinking <= 0.167
Severe.housing.cost.burden <= 0.0851
0.743 < Broadband.access <= 0.797
Feature
0.000
0.003
0.006
Weight
Positive
Negative

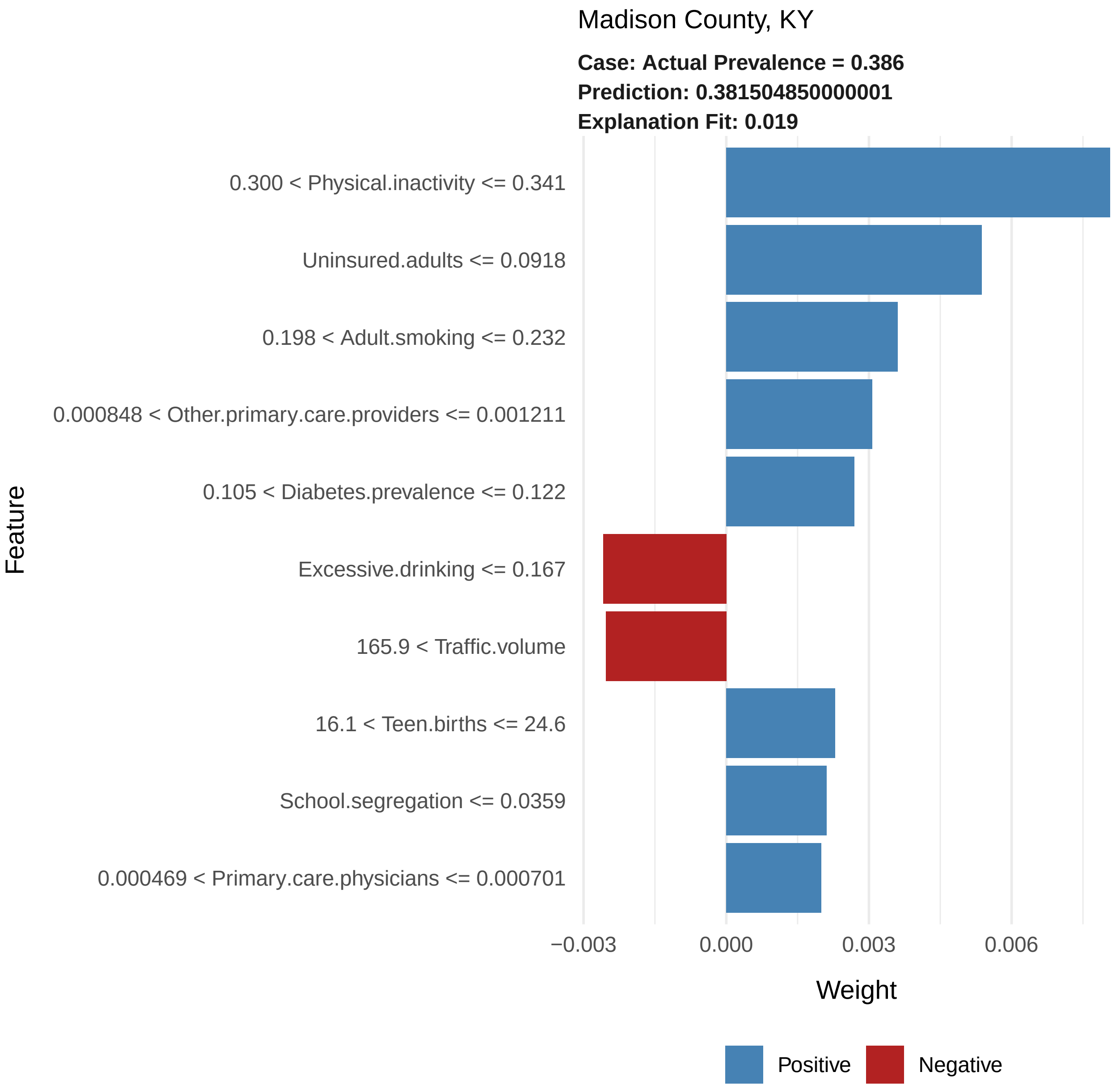

Madison County, KY
Case: Actual Prevalence = 0.386
Prediction: 0.381504850000001
Explanation Fit: 0.019
0.300 < Physical.inactivity <= 0.341
Uninsured.adults <= 0.0918
0.198 < Adult.smoking <= 0.232
0.000848 < Other.primary.care.providers <= 0.001211
0.105 < Diabetes.prevalence <= 0.122
Excessive.drinking <= 0.167
165.9 < Traffic.volume
16.1 < Teen.births <= 24.6
School.segregation <= 0.0359
0.000469 < Primary.care.physicians <= 0.000701
Feature
−0.003
0.000
0.003
0.006
Weight
Positive
Negative

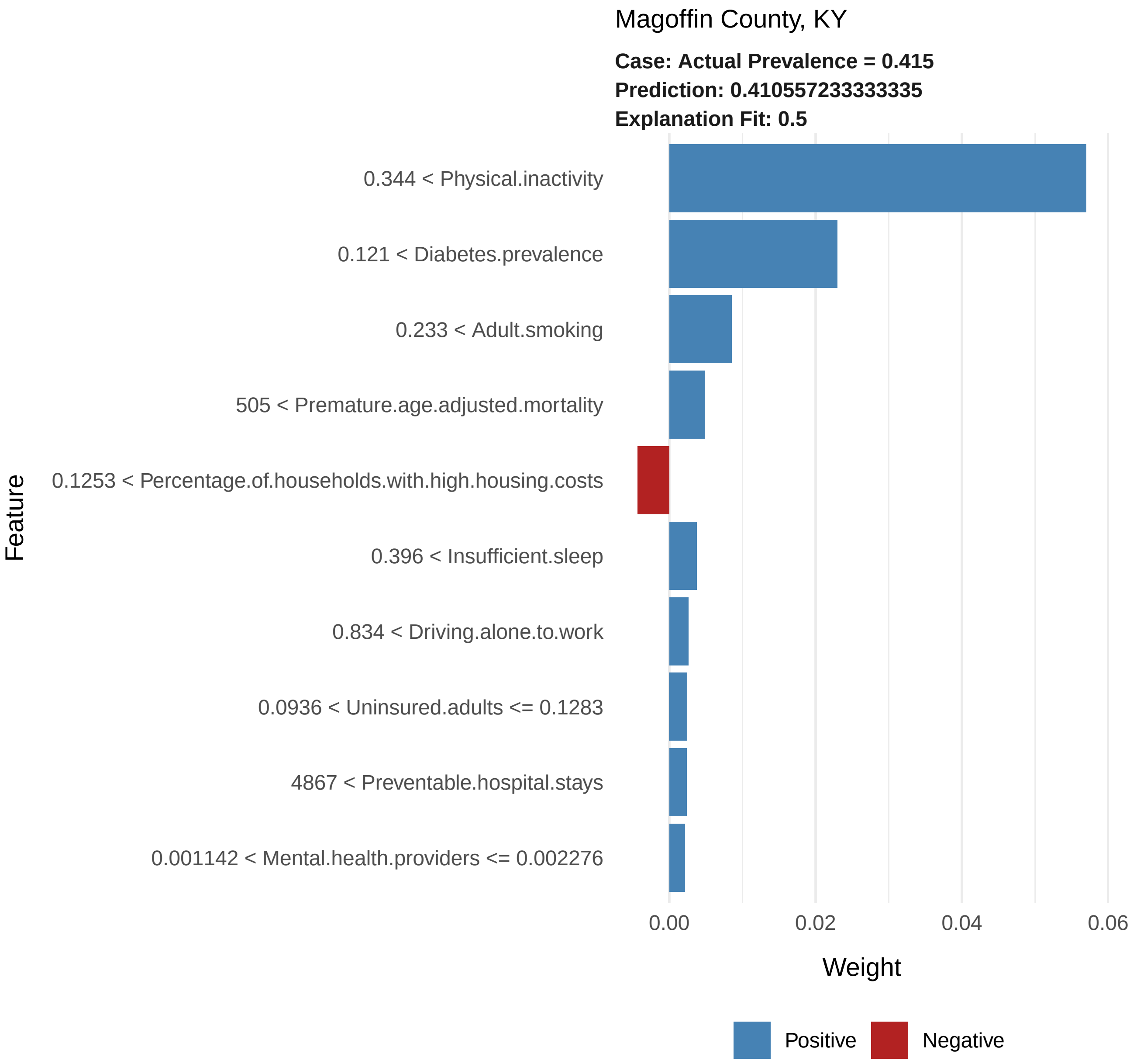

Magoffin County, KY
Case: Actual Prevalence = 0.415
Prediction: 0.410557233333335
Explanation Fit: 0.5
0.344 < Physical.inactivity
0.121 < Diabetes.prevalence
0.233 < Adult.smoking
505 < Premature.age.adjusted.mortality
0.1253 < Percentage.of.households.with.high.housing.costs
0.396 < Insufficient.sleep
0.834 < Driving.alone.to.work
0.0936 < Uninsured.adults <= 0.1283
4867 < Preventable.hospital.stays
0.001142 < Mental.health.providers <= 0.002276
Feature
0.00
0.02
0.04
0.06
Weight
Positive
Negative

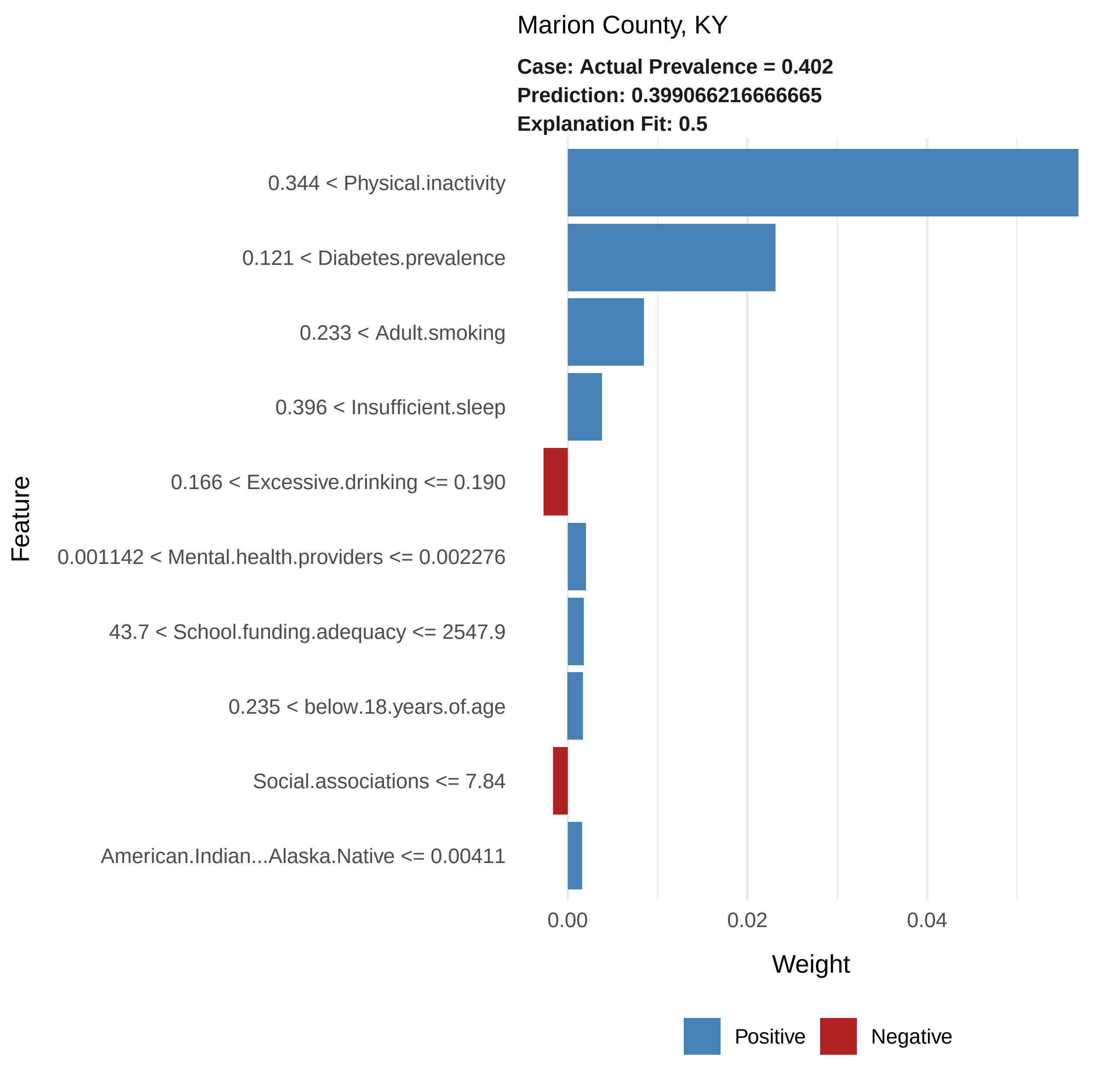

Marion County, KY
Case: Actual Prevalence = 0.402
Prediction: 0.399066216666665
Explanation Fit: 0.5
0.344 < Physical.inactivity
0.121 < Diabetes.prevalence
0.233 < Adult.smoking
0.396 < Insufficient.sleep
0.166 < Excessive.drinking <= 0.190
0.001142 < Mental.health.providers <= 0.002276
43.7 < School.funding.adequacy <= 2547.9
0.235 < below.18.years.of.age
Social.associations <= 7.84
American.Indian...Alaska.Native <= 0.00411
Feature
0.00
0.02
0.04
Weight
Positive
Negative

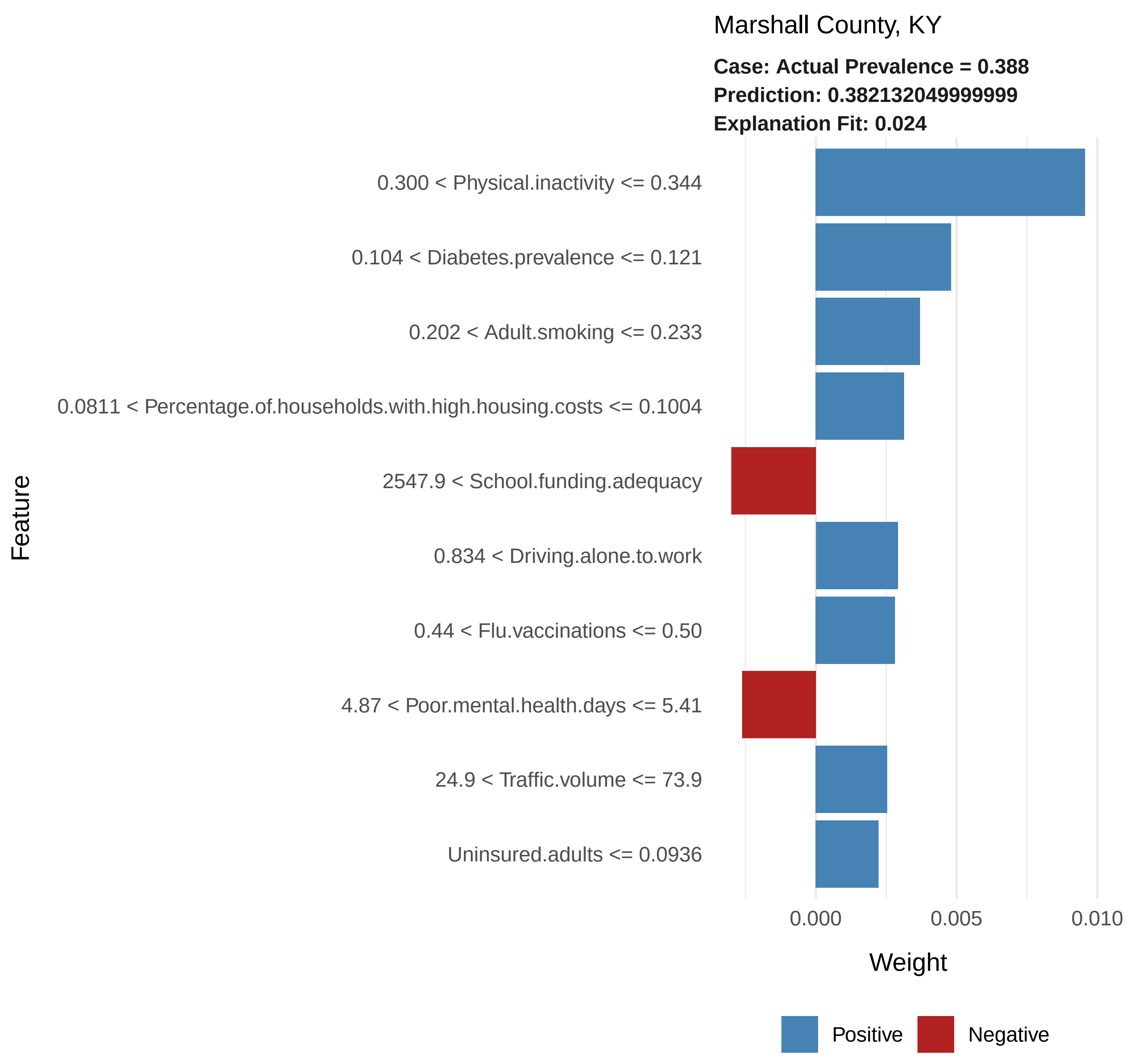
Marshall County, KY
Case: Actual Prevalence = 0.388
Prediction: 0.382132049999999
Explanation Fit: 0.024
0.300 < Physical.inactivity <= 0.344
0.104 < Diabetes.prevalence <= 0.121
0.202 < Adult.smoking <= 0.233
0.0811 < Percentage.of.households.with.high.housing.costs <= 0.1004
2547.9 < School.funding.adequacy
0.834 < Driving.alone.to.work
0.44 < Flu.vaccinations <= 0.50
4.87 < Poor.mental.health.days <= 5.41
24.9 < Traffic.volume <= 73.9
Uninsured.adults <= 0.0936
Feature
0.000
0.005
0.010
Weight
Positive
Negative

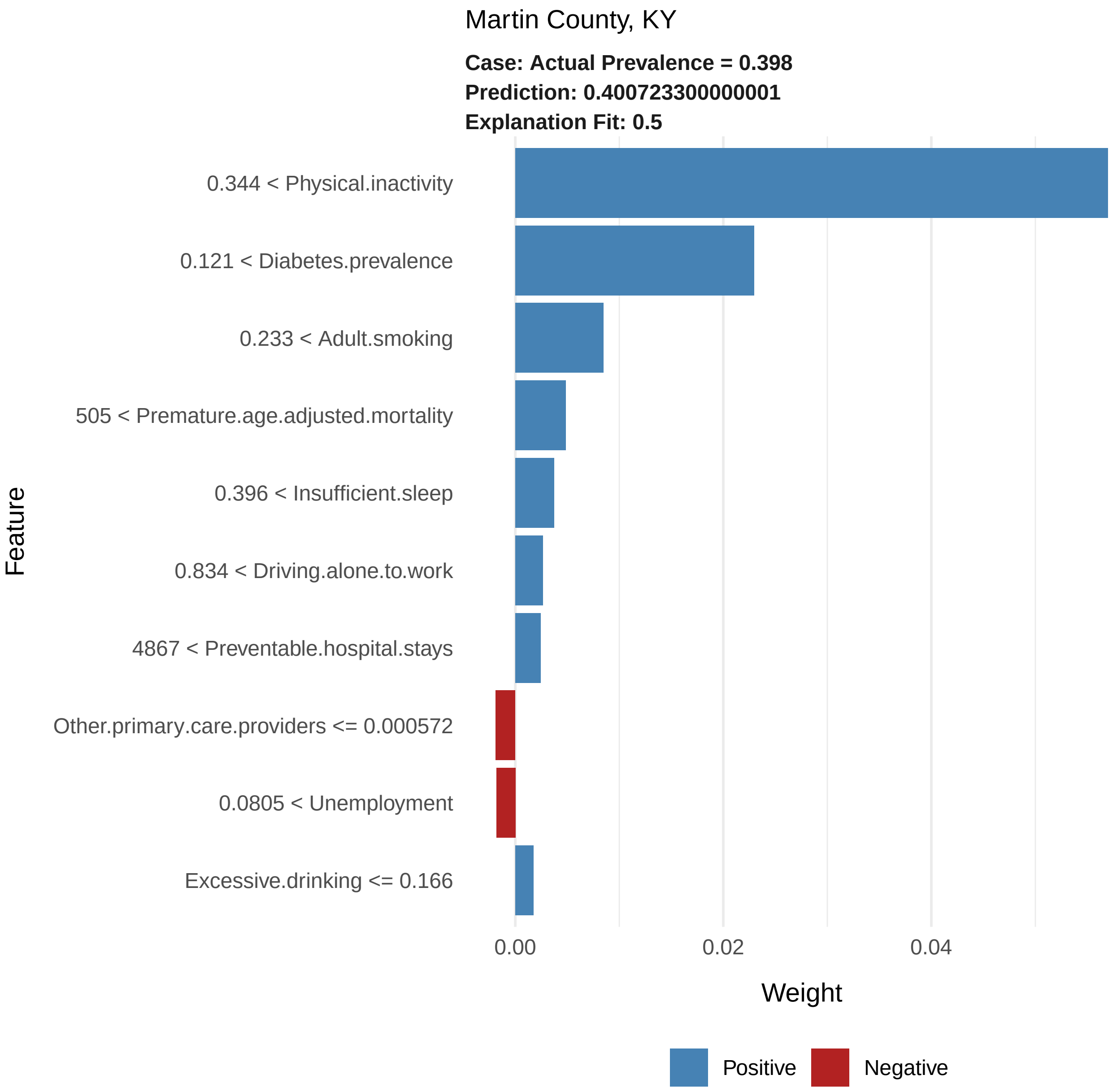

Martin County, KY
Case: Actual Prevalence = 0.398
Prediction: 0.400723300000001
Explanation Fit: 0.5
0.344 < Physical.inactivity
0.121 < Diabetes.prevalence
0.233 < Adult.smoking
505 < Premature.age.adjusted.mortality
0.396 < Insufficient.sleep
0.834 < Driving.alone.to.work
4867 < Preventable.hospital.stays
Other.primary.care.providers <= 0.000572
0.0805 < Unemployment
Excessive.drinking <= 0.166
Feature
0.00
0.02
0.04
Weight
Positive
Negative

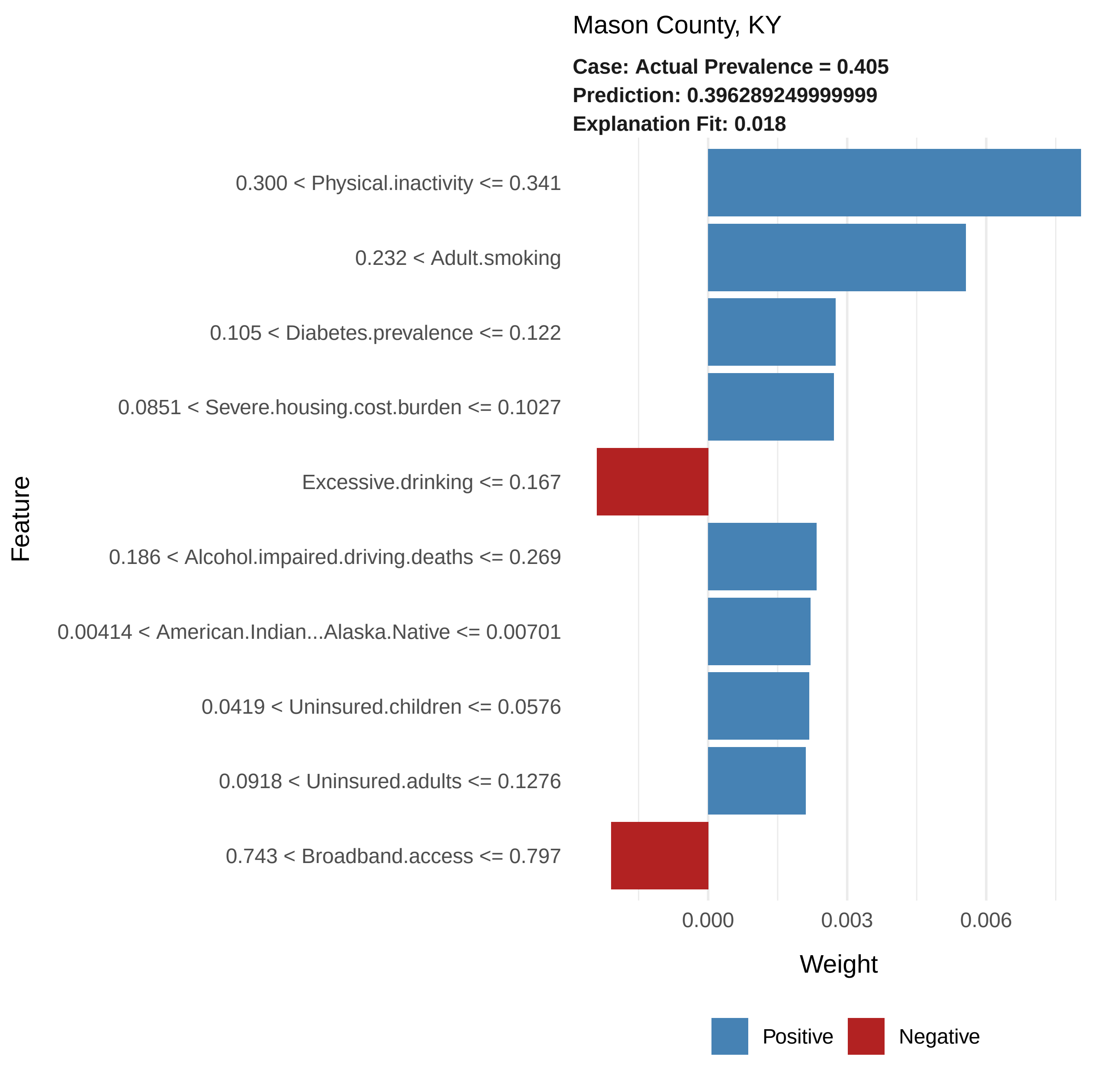
Mason County, KY
Case: Actual Prevalence = 0.405
Prediction: 0.396289249999999
Explanation Fit: 0.018
0.300 < Physical.inactivity <= 0.341
0.232 < Adult.smoking
0.105 < Diabetes.prevalence <= 0.122
0.0851 < Severe.housing.cost.burden <= 0.1027
Excessive.drinking <= 0.167
0.186 < Alcohol.impaired.driving.deaths <= 0.269
0.00414 < American.Indian...Alaska.Native <= 0.00701
0.0419 < Uninsured.children <= 0.0576
0.0918 < Uninsured.adults <= 0.1276
0.743 < Broadband.access <= 0.797
Feature
0.000
0.003
0.006
Weight
Positive
Negative

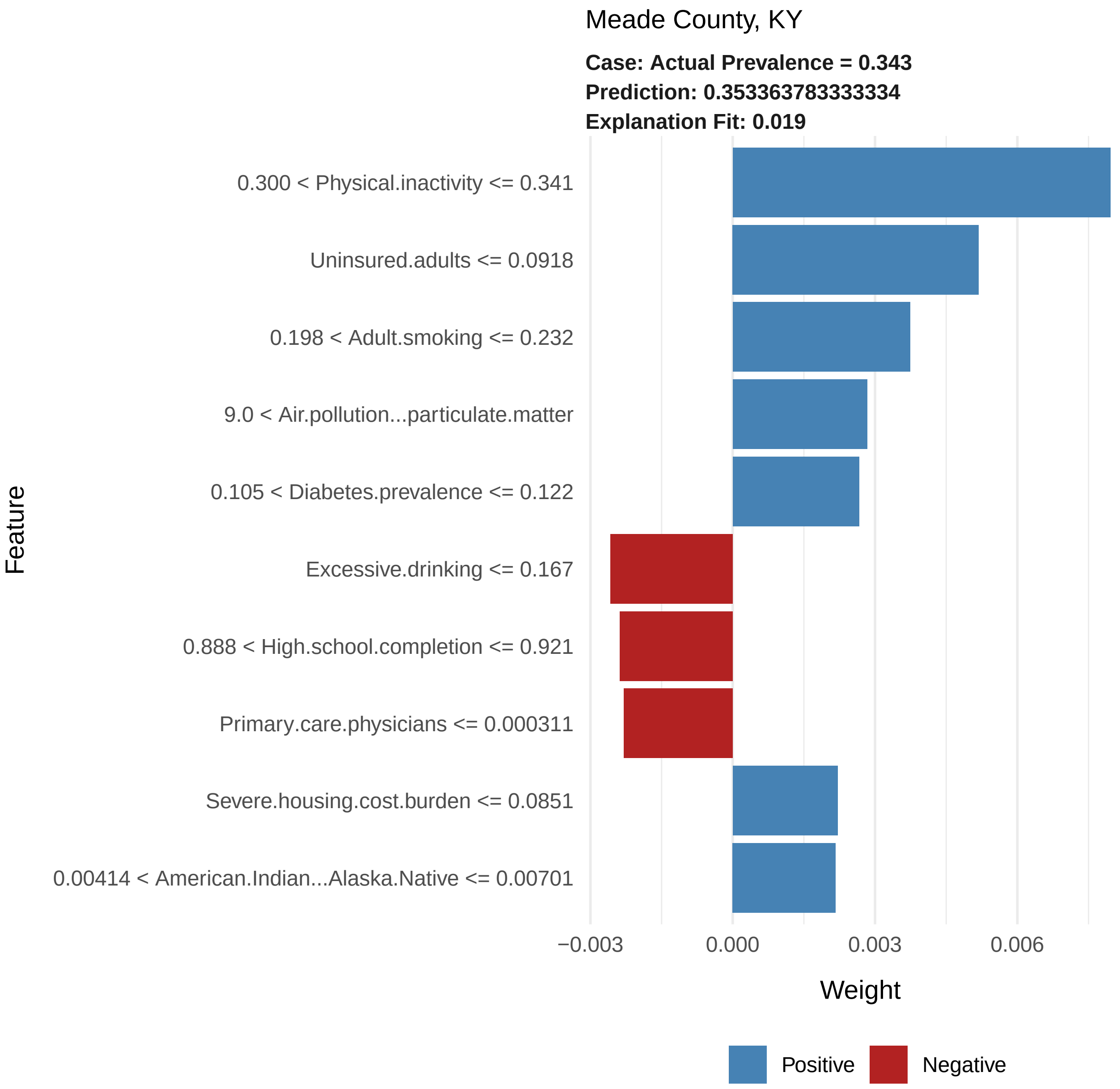
Meade County, KY
Case: Actual Prevalence = 0.343
Prediction: 0.353363783333334
Explanation Fit: 0.019
0.300 < Physical.inactivity <= 0.341
Uninsured.adults <= 0.0918
0.198 < Adult.smoking <= 0.232
9.0 < Air.pollution...particulate.matter
0.105 < Diabetes.prevalence <= 0.122
Excessive.drinking <= 0.167
0.888 < High.school.completion <= 0.921
Primary.care.physicians <= 0.000311
Severe.housing.cost.burden <= 0.0851
0.00414 < American.Indian...Alaska.Native <= 0.00701
Feature
−0.003
0.000
0.003
0.006
Weight
Positive
Negative

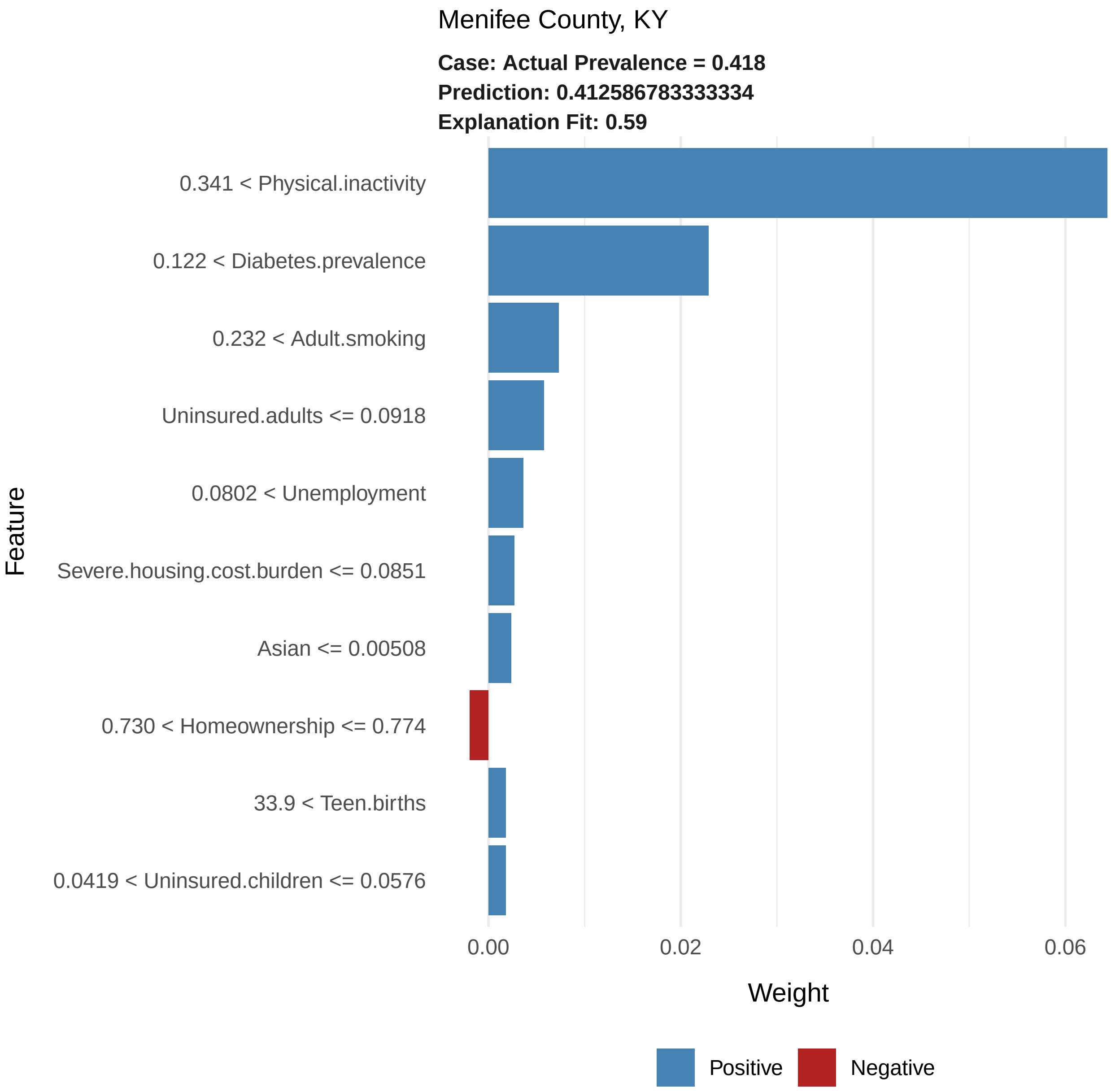
Menifee County, KY
Case: Actual Prevalence = 0.418
Prediction: 0.412586783333334
Explanation Fit: 0.59
0.341 < Physical.inactivity
0.122 < Diabetes.prevalence
0.232 < Adult.smoking
Uninsured.adults <= 0.0918
0.0802 < Unemployment
Severe.housing.cost.burden <= 0.0851
Asian <= 0.00508
0.730 < Homeownership <= 0.774
33.9 < Teen.births
0.0419 < Uninsured.children <= 0.0576
Feature
0.00
0.02
0.04
0.06
Weight
Positive
Negative

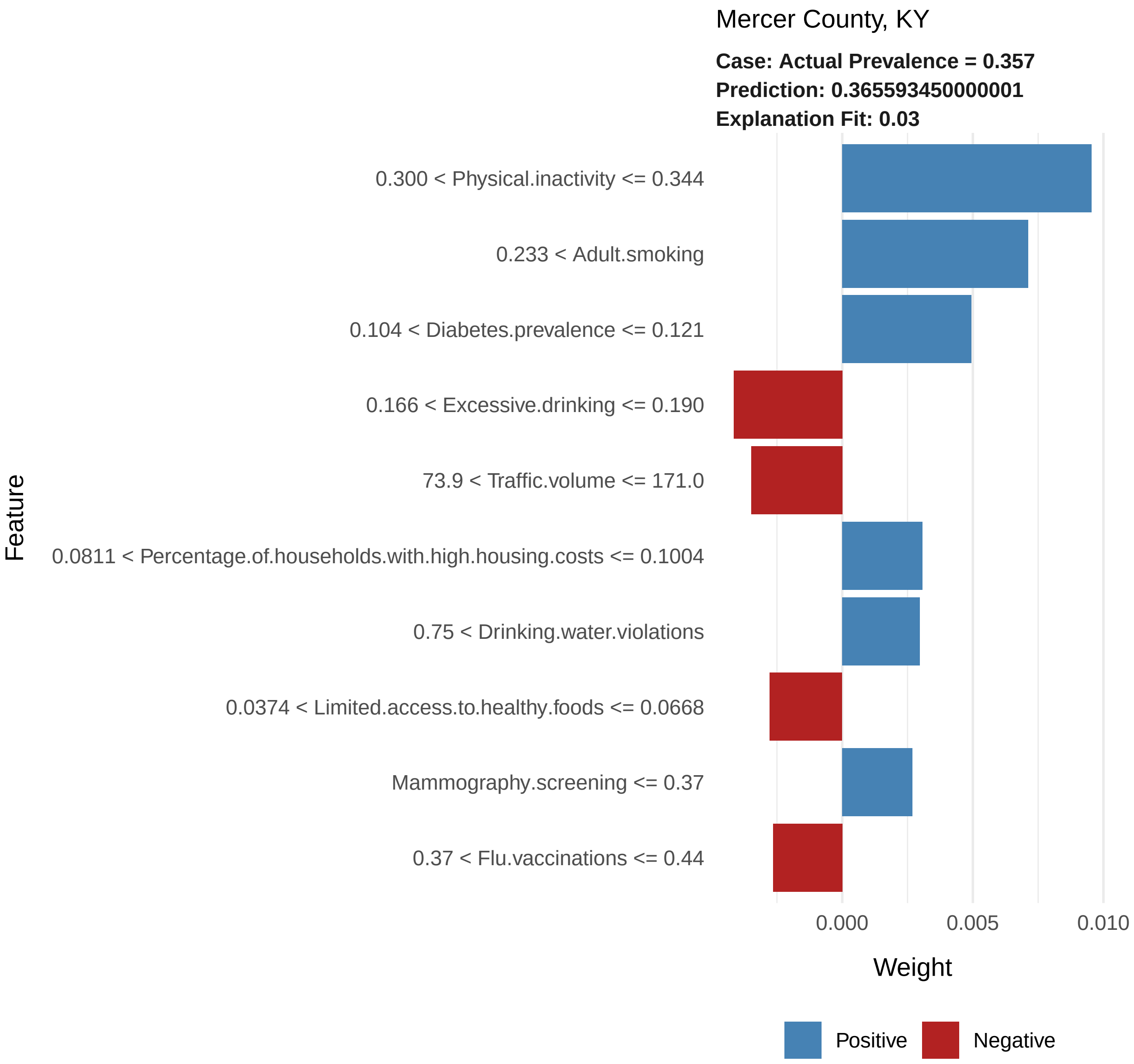
Mercer County, KY
Case: Actual Prevalence = 0.357
Prediction: 0.365593450000001
Explanation Fit: 0.03
0.300 < Physical.inactivity <= 0.344
0.233 < Adult.smoking
0.104 < Diabetes.prevalence <= 0.121
0.166 < Excessive.drinking <= 0.190
73.9 < Traffic.volume <= 171.0
0.0811 < Percentage.of.households.with.high.housing.costs <= 0.1004
0.75 < Drinking.water.violations
0.0374 < Limited.access.to.healthy.foods <= 0.0668
Mammography.screening <= 0.37
0.37 < Flu.vaccinations <= 0.44
Feature
0.000
0.005
0.010
Weight
Positive
Negative

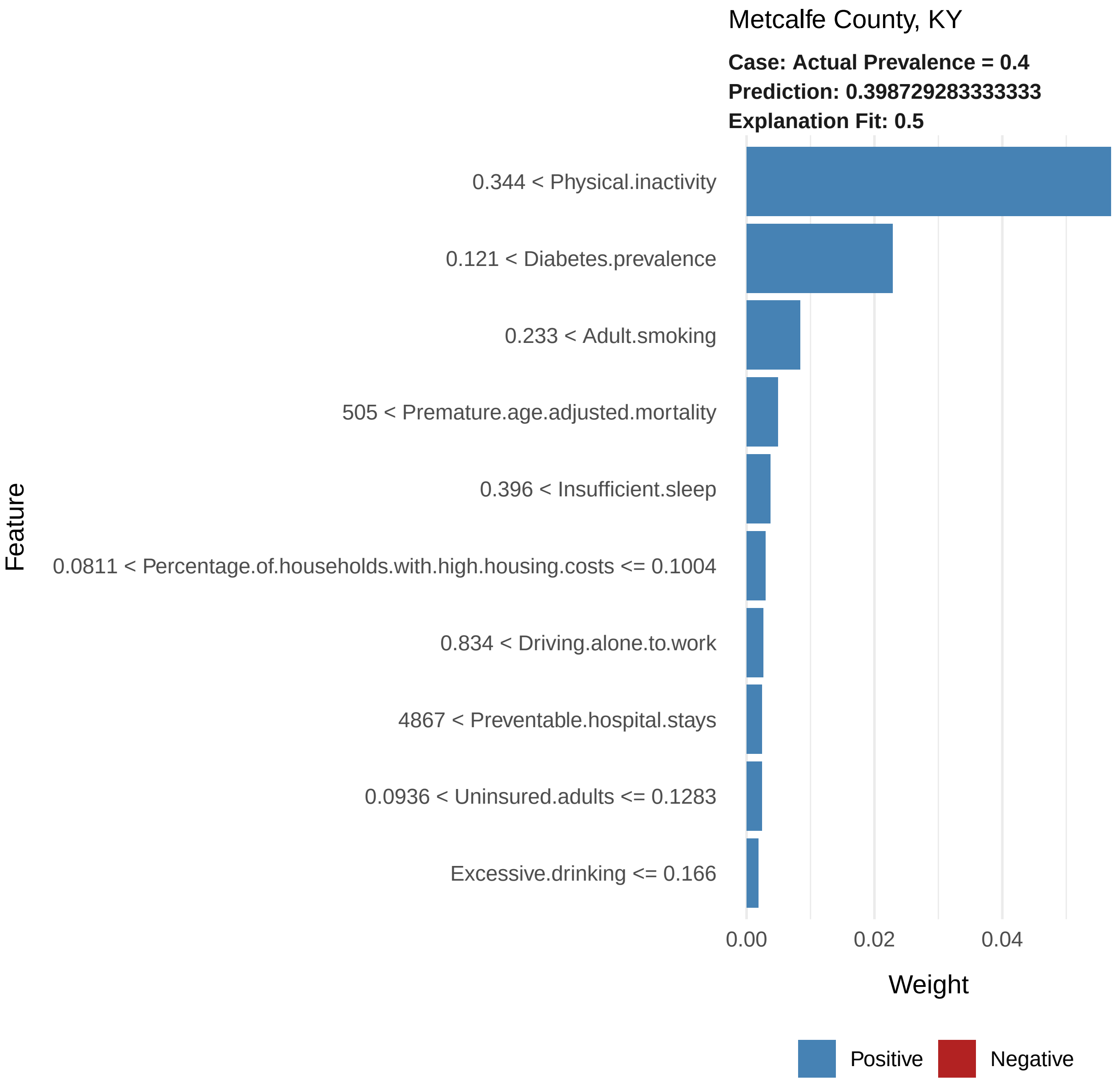

Metcalfe County, KY
Case: Actual Prevalence = 0.4
Prediction: 0.398729283333333
Explanation Fit: 0.5
0.344 < Physical.inactivity
0.121 < Diabetes.prevalence
0.233 < Adult.smoking
505 < Premature.age.adjusted.mortality
0.396 < Insufficient.sleep
0.0811 < Percentage.of.households.with.high.housing.costs <= 0.1004
0.834 < Driving.alone.to.work
4867 < Preventable.hospital.stays
0.0936 < Uninsured.adults <= 0.1283
Excessive.drinking <= 0.166
Feature
0.00
0.02
0.04
Weight
Positive
Negative

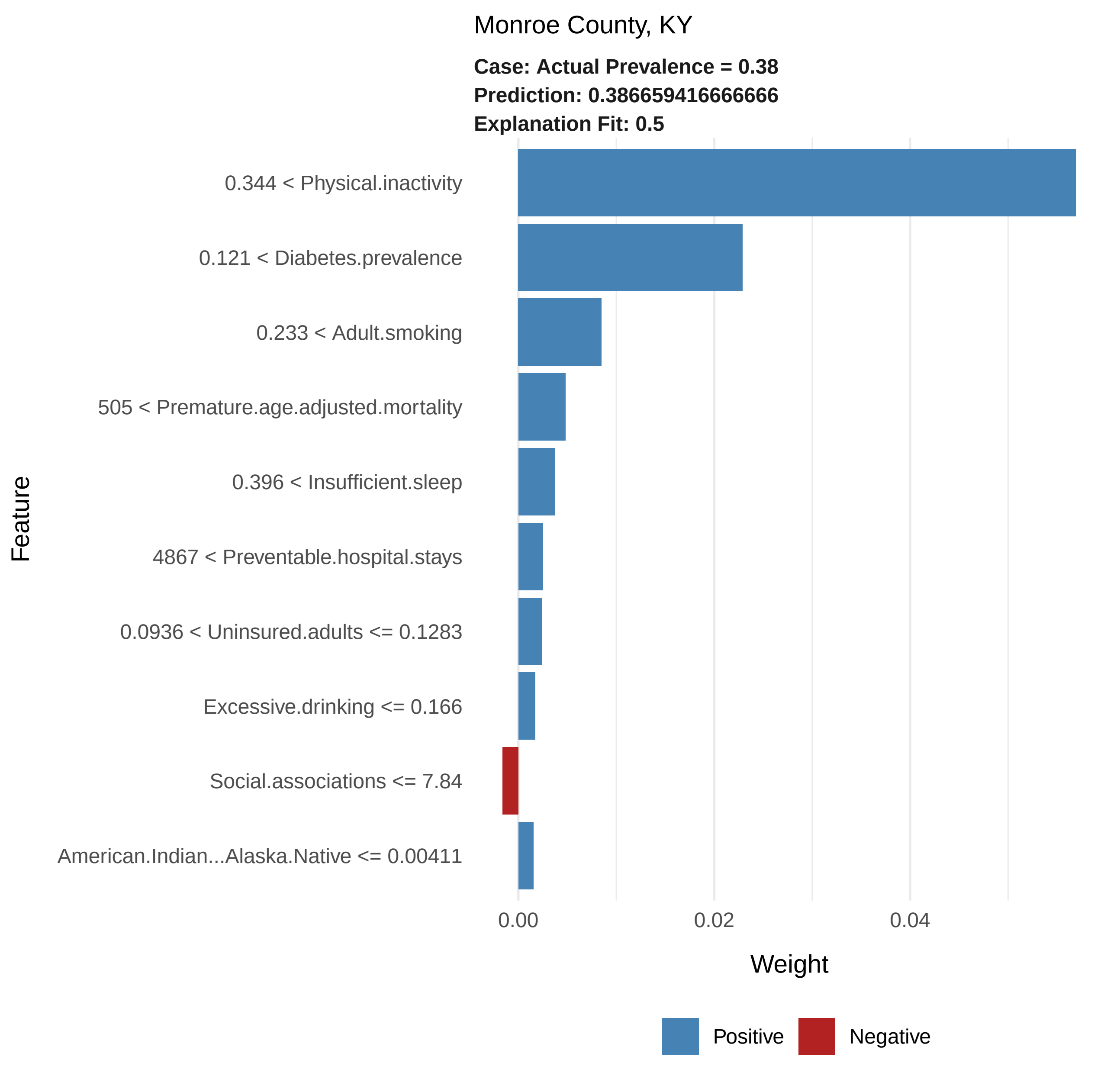
Monroe County, KY
Case: Actual Prevalence = 0.38
Prediction: 0.386659416666666
Explanation Fit: 0.5
0.344 < Physical.inactivity
0.121 < Diabetes.prevalence
0.233 < Adult.smoking
505 < Premature.age.adjusted.mortality
0.396 < Insufficient.sleep
4867 < Preventable.hospital.stays
0.0936 < Uninsured.adults <= 0.1283
Excessive.drinking <= 0.166
Social.associations <= 7.84
American.Indian...Alaska.Native <= 0.00411
Feature
0.00
0.02
0.04
Weight
Positive
Negative

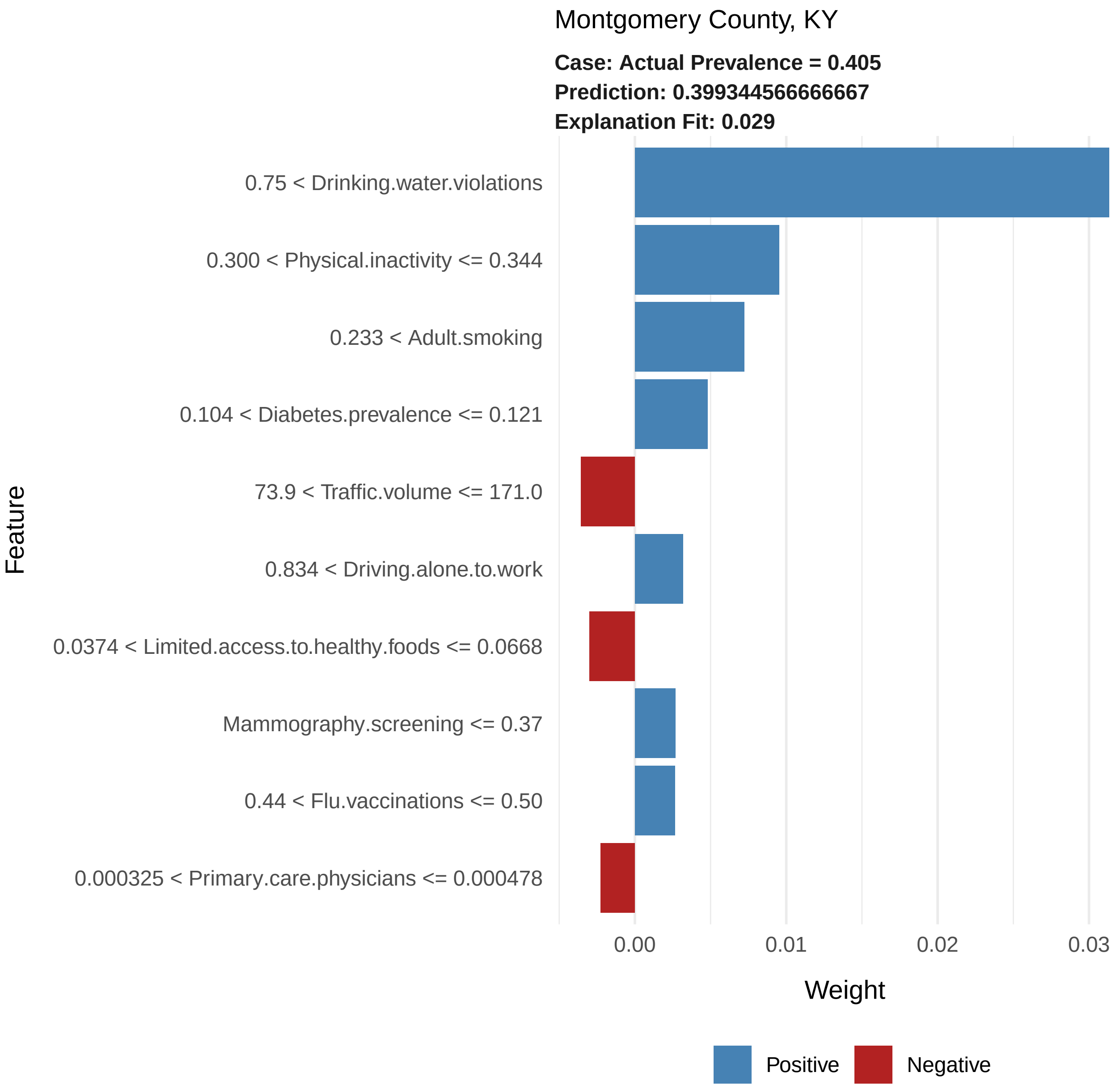

Montgomery County, KY
Case: Actual Prevalence = 0.405
Prediction: 0.399344566666667
Explanation Fit: 0.029
0.75 < Drinking.water.violations
0.300 < Physical.inactivity <= 0.344
0.233 < Adult.smoking
0.104 < Diabetes.prevalence <= 0.121
73.9 < Traffic.volume <= 171.0
0.834 < Driving.alone.to.work
0.0374 < Limited.access.to.healthy.foods <= 0.0668
Mammography.screening <= 0.37
0.44 < Flu.vaccinations <= 0.50
0.000325 < Primary.care.physicians <= 0.000478
Feature
0.00
0.01
0.02
0.03
Weight
Positive
Negative

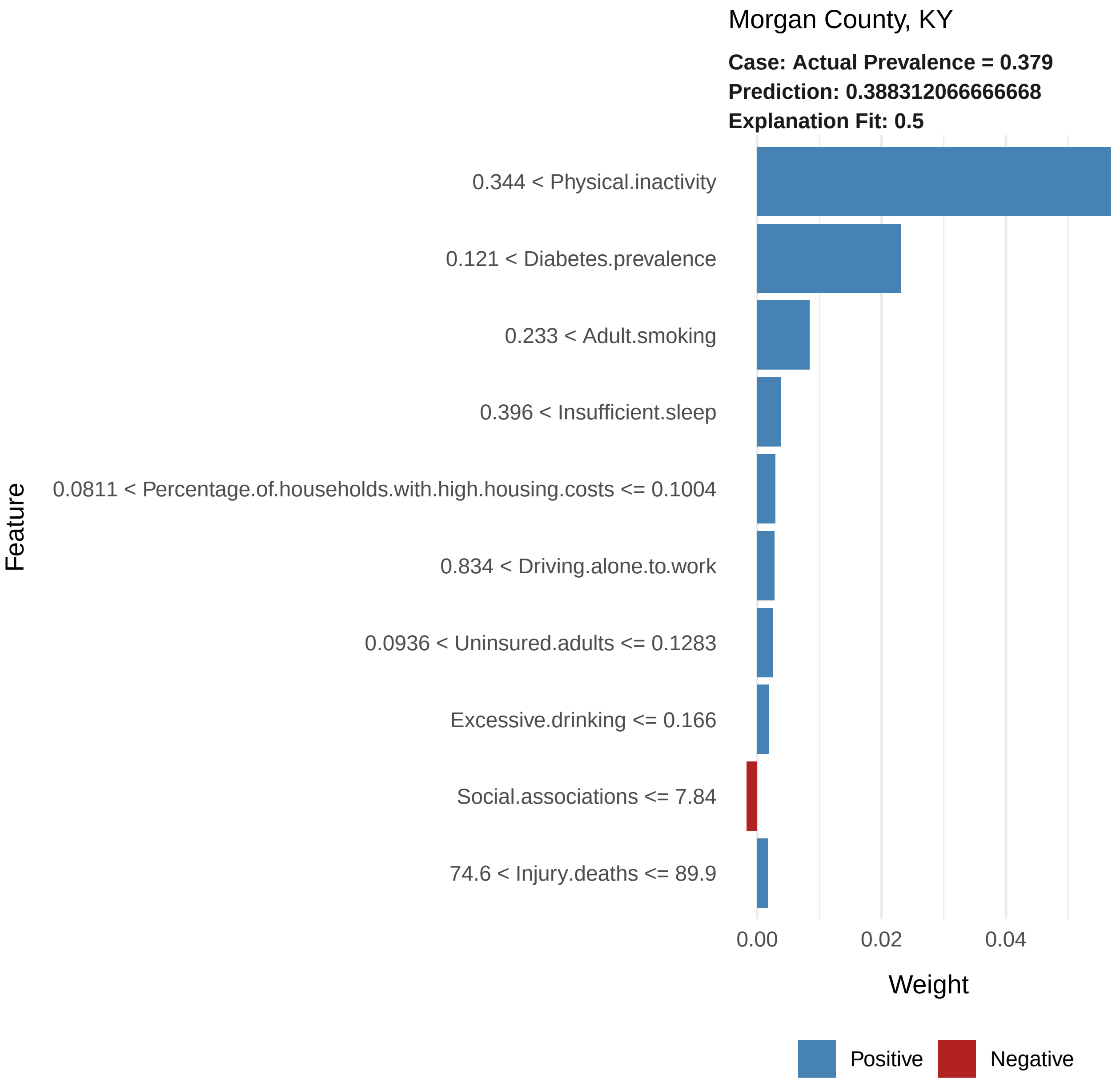
Morgan County, KY
Case: Actual Prevalence = 0.379
Prediction: 0.388312066666668
Explanation Fit: 0.5
0.344 < Physical.inactivity
0.121 < Diabetes.prevalence
0.233 < Adult.smoking
0.396 < Insufficient.sleep
0.0811 < Percentage.of.households.with.high.housing.costs <= 0.1004
0.834 < Driving.alone.to.work
0.0936 < Uninsured.adults <= 0.1283
Excessive.drinking <= 0.166
Social.associations <= 7.84
74.6 < Injury.deaths <= 89.9
Feature
0.00
0.02
0.04
Weight
Positive
Negative

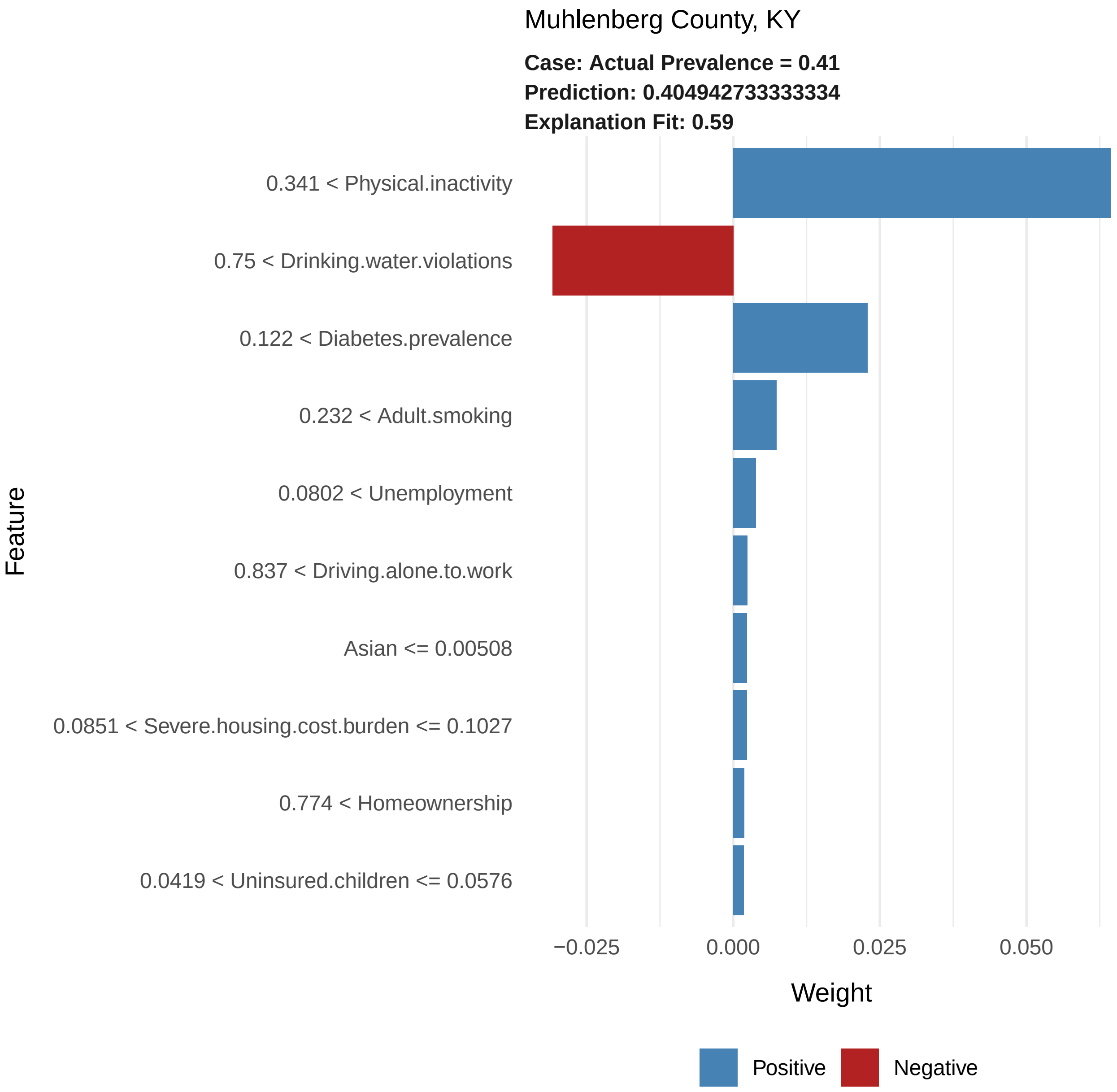

Muhlenberg County, KY
Case: Actual Prevalence = 0.41
Prediction: 0.404942733333334
Explanation Fit: 0.59
0.341 < Physical.inactivity
0.75 < Drinking.water.violations
0.122 < Diabetes.prevalence
0.232 < Adult.smoking
0.0802 < Unemployment
0.837 < Driving.alone.to.work
Asian <= 0.00508
0.0851 < Severe.housing.cost.burden <= 0.1027
0.774 < Homeownership
0.0419 < Uninsured.children <= 0.0576
Feature
−0.025
0.000
0.025
0.050
Weight
Positive
Negative

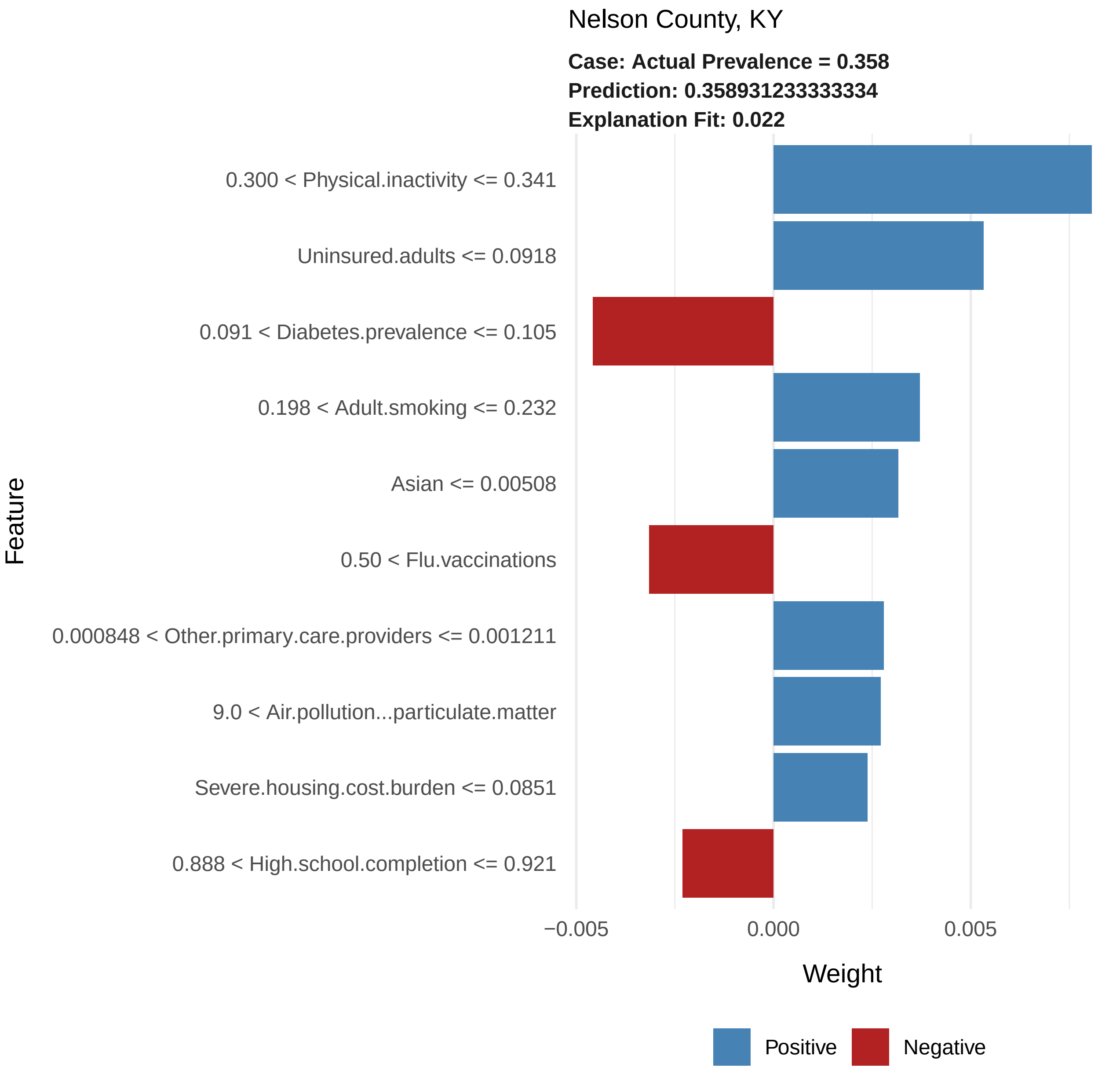

Nelson County, KY
Case: Actual Prevalence = 0.358
Prediction: 0.358931233333334
Explanation Fit: 0.022
0.300 < Physical.inactivity <= 0.341
Uninsured.adults <= 0.0918
0.091 < Diabetes.prevalence <= 0.105
0.198 < Adult.smoking <= 0.232
Asian <= 0.00508
0.50 < Flu.vaccinations
0.000848 < Other.primary.care.providers <= 0.001211
9.0 < Air.pollution...particulate.matter
Severe.housing.cost.burden <= 0.0851
0.888 < High.school.completion <= 0.921
Feature
−0.005
0.000
0.005
Weight
Positive
Negative

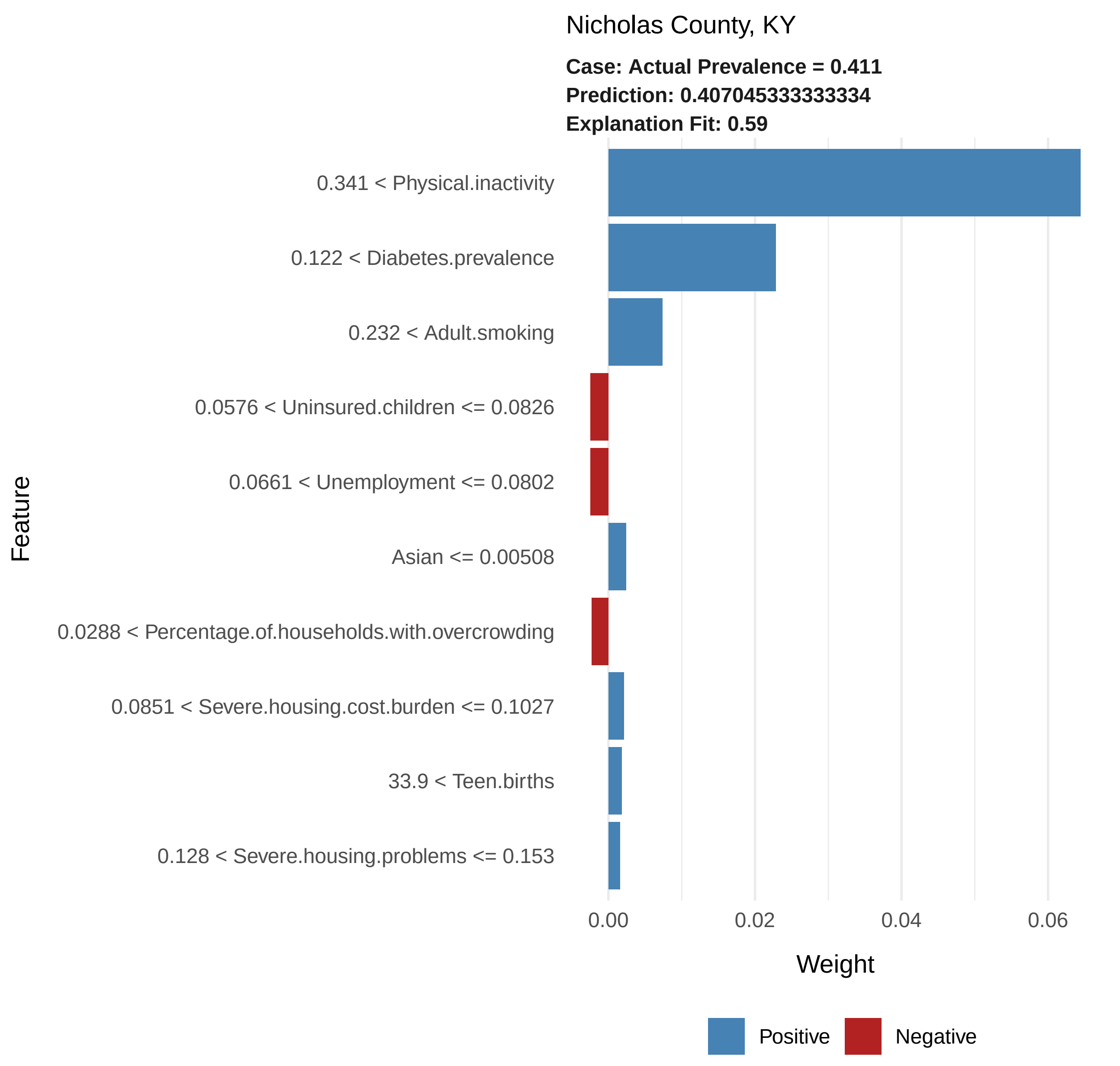
Nicholas County, KY
Case: Actual Prevalence = 0.411
Prediction: 0.407045333333334
Explanation Fit: 0.59
0.341 < Physical.inactivity
0.122 < Diabetes.prevalence
0.232 < Adult.smoking
0.0576 < Uninsured.children <= 0.0826
0.0661 < Unemployment <= 0.0802
Asian <= 0.00508
0.0288 < Percentage.of.households.with.overcrowding
0.0851 < Severe.housing.cost.burden <= 0.1027
33.9 < Teen.births
0.128 < Severe.housing.problems <= 0.153
Feature
0.00
0.02
0.04
0.06
Weight
Positive
Negative

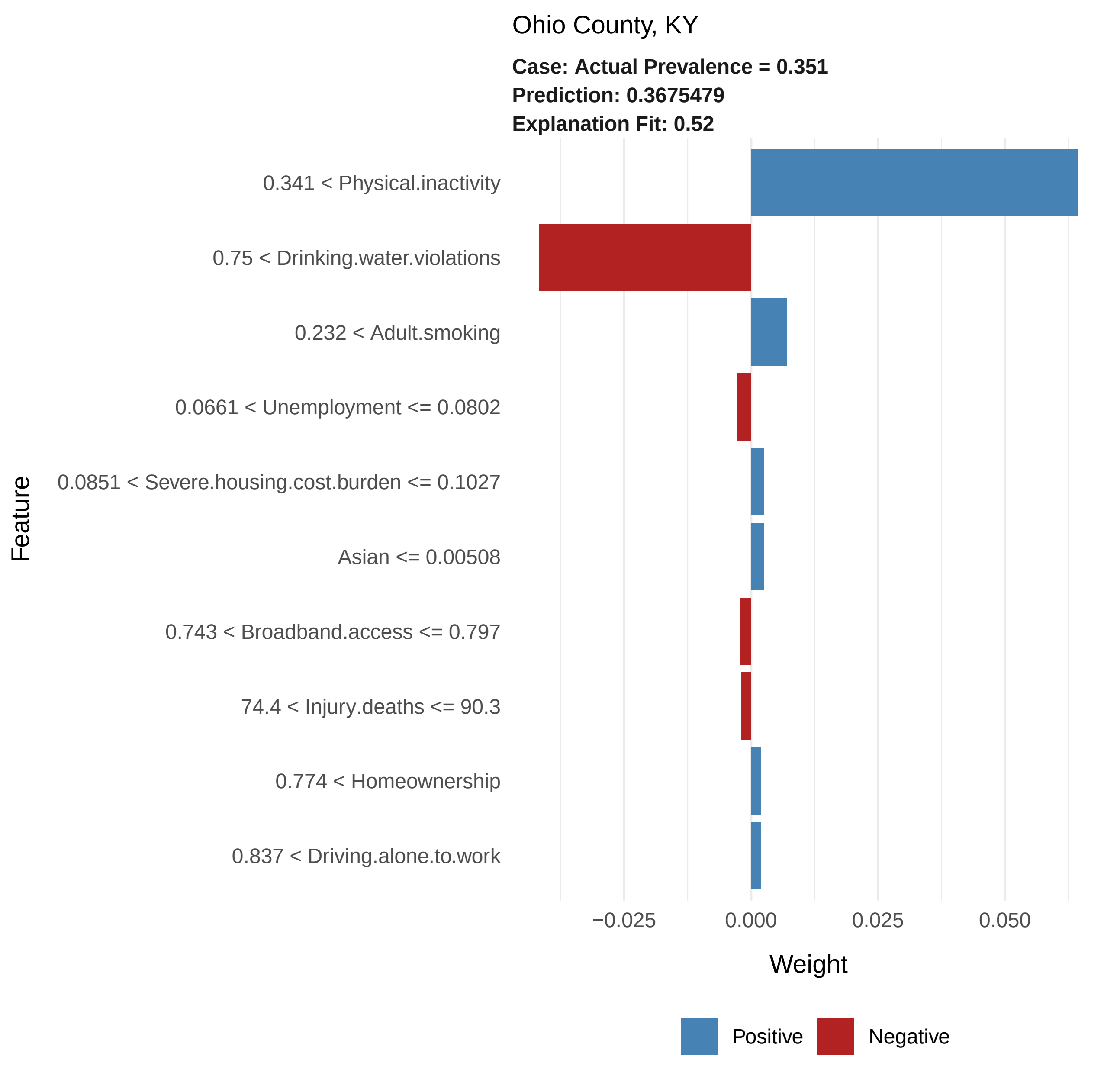

Ohio County, KY
Case: Actual Prevalence = 0.351
Prediction: 0.3675479
Explanation Fit: 0.52
0.341 < Physical.inactivity
0.75 < Drinking.water.violations
0.232 < Adult.smoking
0.0661 < Unemployment <= 0.0802
0.0851 < Severe.housing.cost.burden <= 0.1027
Asian <= 0.00508
0.743 < Broadband.access <= 0.797
74.4 < Injury.deaths <= 90.3
0.774 < Homeownership
0.837 < Driving.alone.to.work
Feature
−0.025
0.000
0.025
0.050
Weight
Positive
Negative

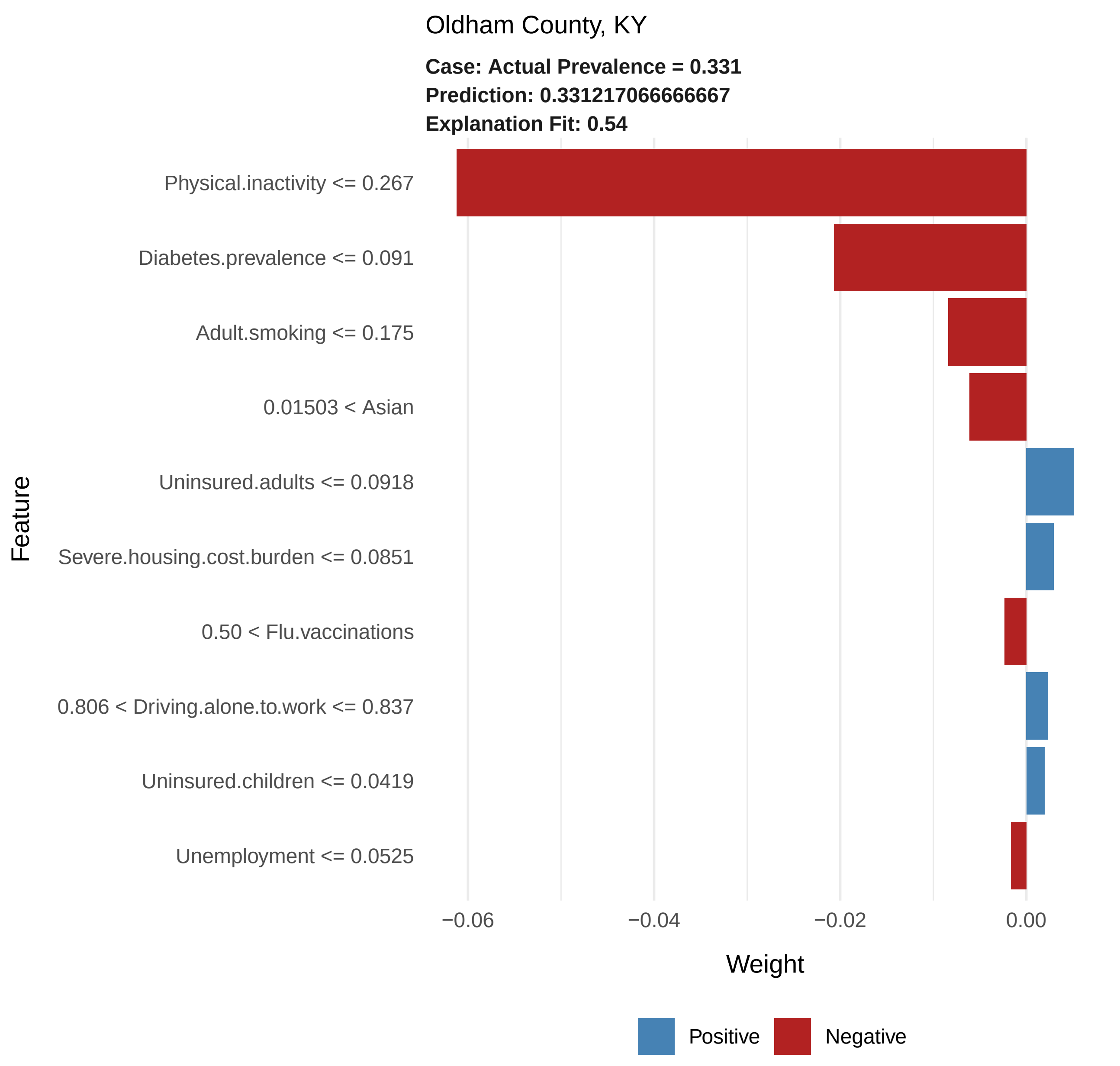

Oldham County, KY
Case: Actual Prevalence = 0.331
Prediction: 0.331217066666667
Explanation Fit: 0.54
Physical.inactivity <= 0.267
Diabetes.prevalence <= 0.091
Adult.smoking <= 0.175
0.01503 < Asian
Uninsured.adults <= 0.0918
Severe.housing.cost.burden <= 0.0851
0.50 < Flu.vaccinations
0.806 < Driving.alone.to.work <= 0.837
Uninsured.children <= 0.0419
Unemployment <= 0.0525
Feature
−0.06
−0.04
−0.02
0.00
Weight
Positive
Negative

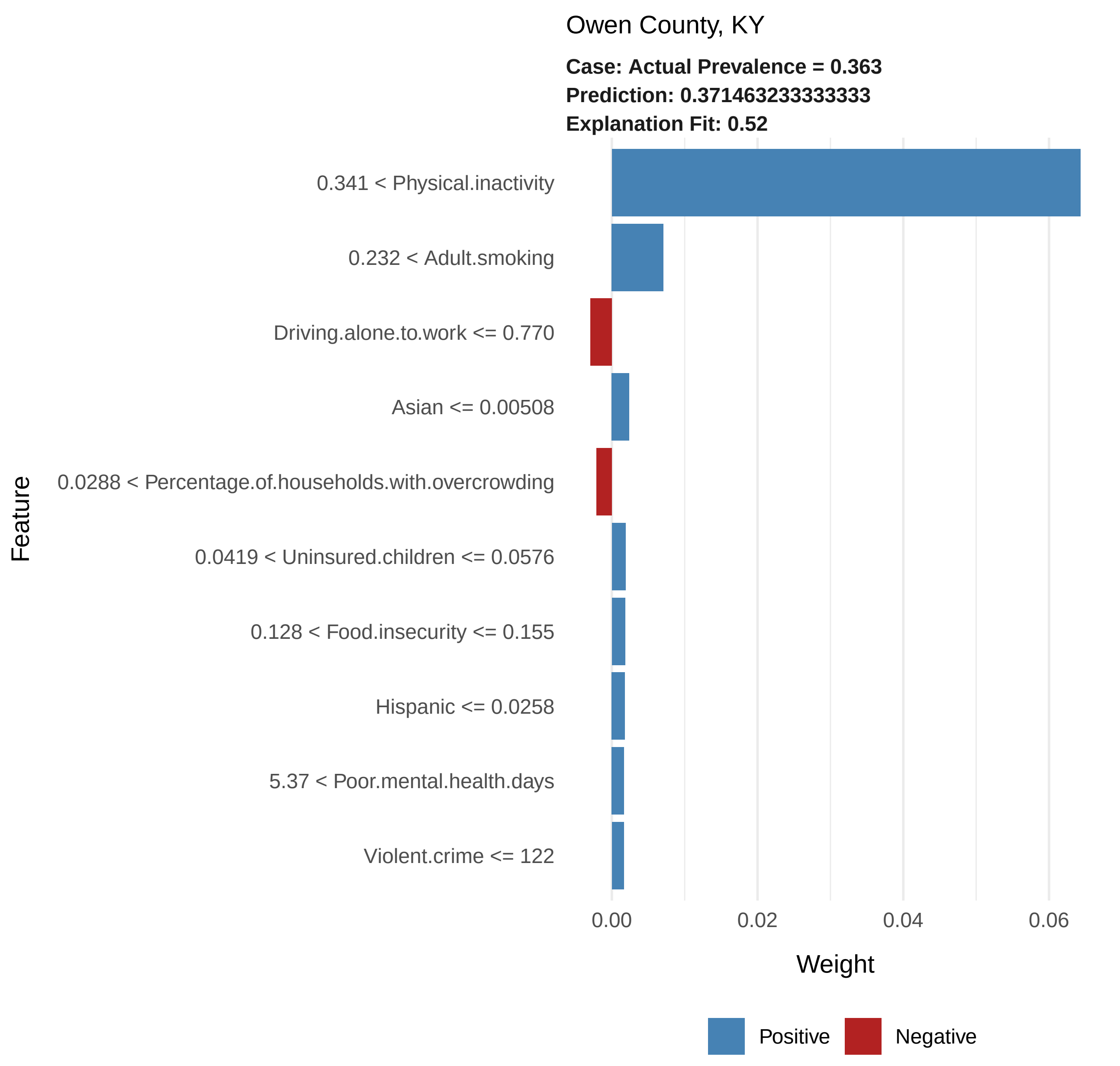

Owen County, KY
Case: Actual Prevalence = 0.363
Prediction: 0.371463233333333
Explanation Fit: 0.52
0.341 < Physical.inactivity
0.232 < Adult.smoking
Driving.alone.to.work <= 0.770
Asian <= 0.00508
0.0288 < Percentage.of.households.with.overcrowding
0.0419 < Uninsured.children <= 0.0576
0.128 < Food.insecurity <= 0.155
Hispanic <= 0.0258
5.37 < Poor.mental.health.days
Violent.crime <= 122
Feature
0.00
0.02
0.04
0.06
Weight
Positive
Negative

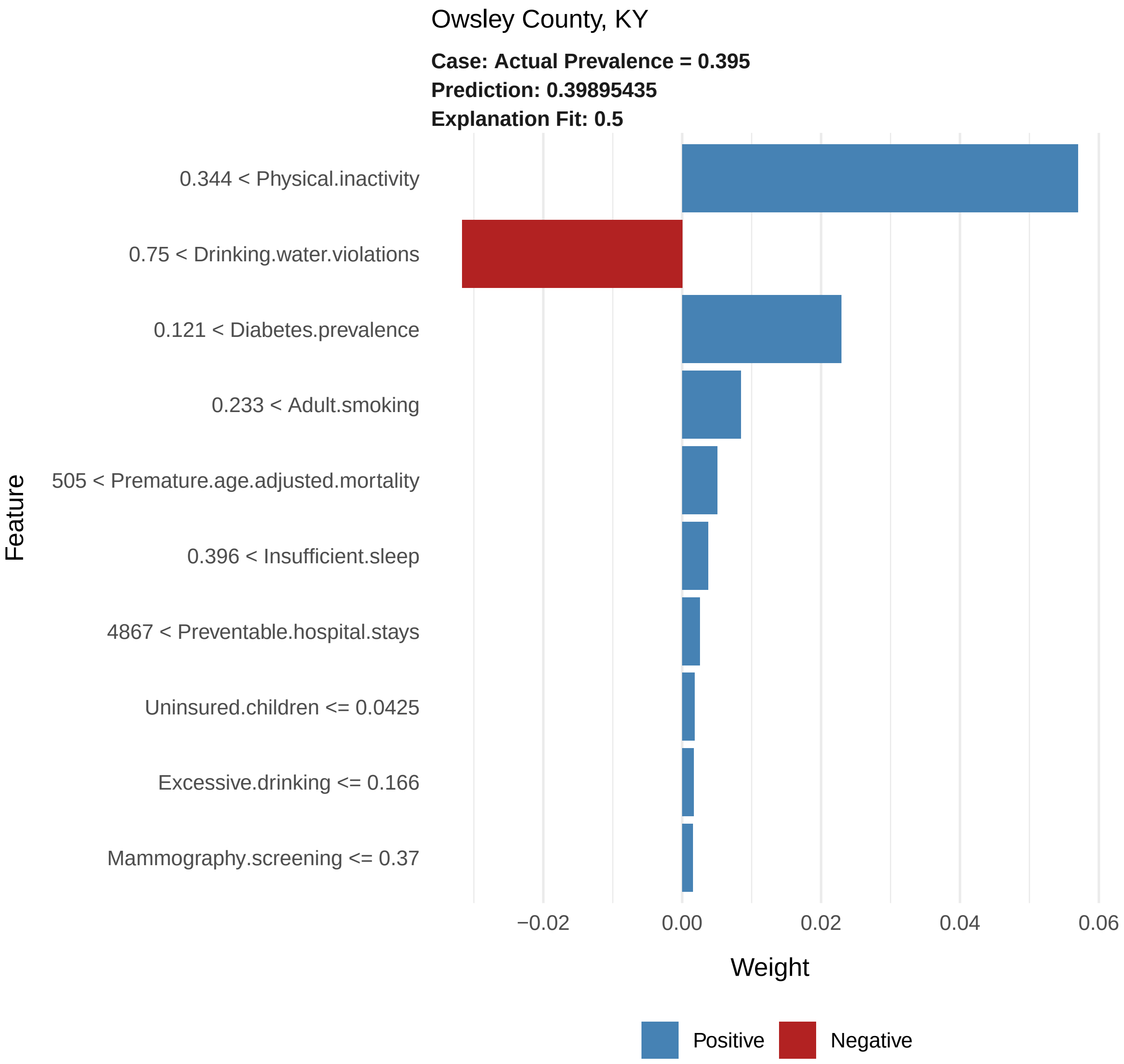
Owsley County, KY
Case: Actual Prevalence = 0.395
Prediction: 0.39895435
Explanation Fit: 0.5
0.344 < Physical.inactivity
0.75 < Drinking.water.violations
0.121 < Diabetes.prevalence
0.233 < Adult.smoking
505 < Premature.age.adjusted.mortality
0.396 < Insufficient.sleep
4867 < Preventable.hospital.stays
Uninsured.children <= 0.0425
Excessive.drinking <= 0.166
Mammography.screening <= 0.37
Feature
−0.02
0.00
0.02
0.04
0.06
Weight
Positive
Negative

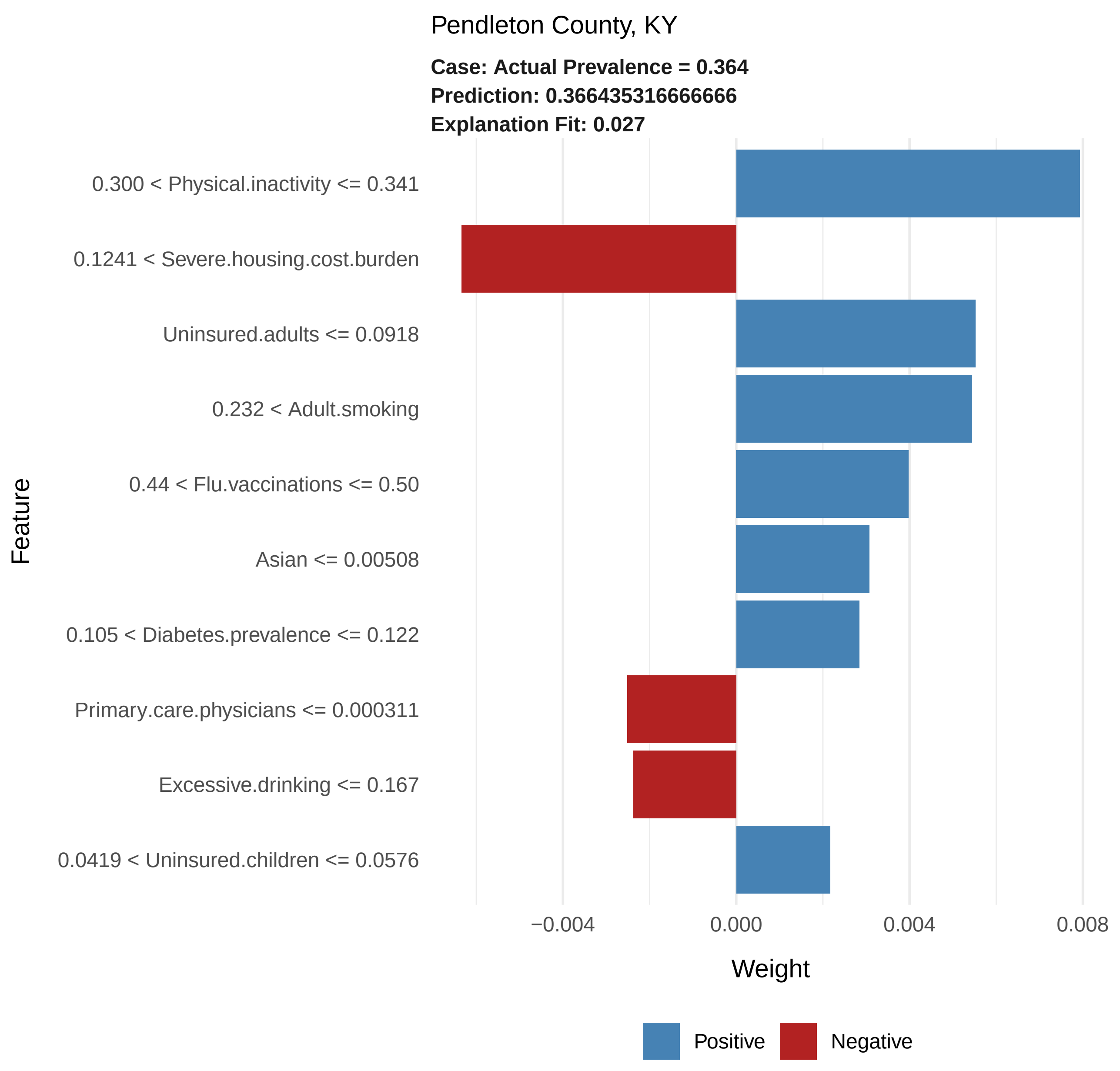
Pendleton County, KY
Case: Actual Prevalence = 0.364
Prediction: 0.366435316666666
Explanation Fit: 0.027
0.300 < Physical.inactivity <= 0.341
0.1241 < Severe.housing.cost.burden
Uninsured.adults <= 0.0918
0.232 < Adult.smoking
0.44 < Flu.vaccinations <= 0.50
Asian <= 0.00508
0.105 < Diabetes.prevalence <= 0.122
Primary.care.physicians <= 0.000311
Excessive.drinking <= 0.167
0.0419 < Uninsured.children <= 0.0576
Feature
−0.004
0.000
0.004
0.008
Weight
Positive
Negative

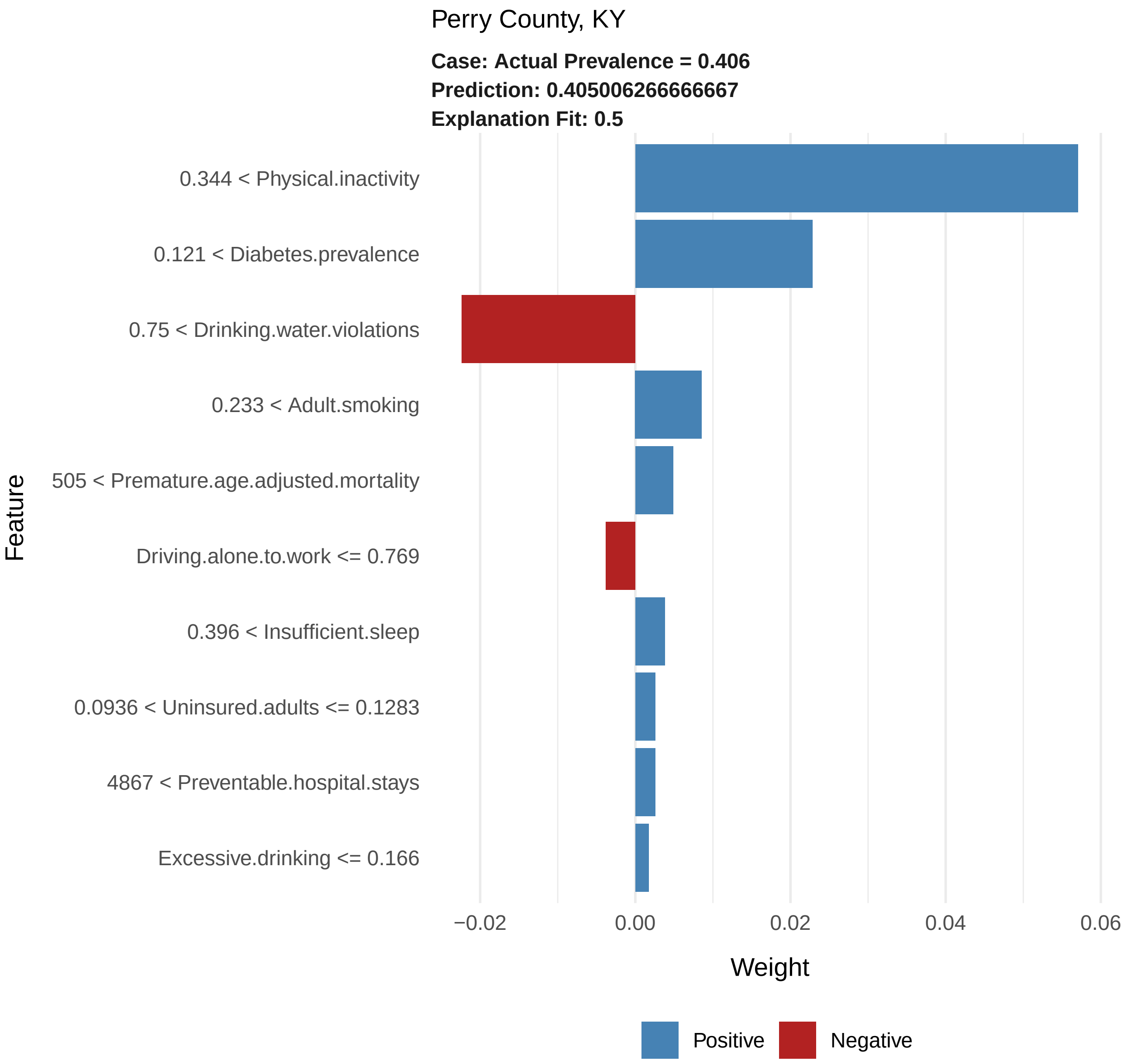
Perry County, KY
Case: Actual Prevalence = 0.406
Prediction: 0.405006266666667
Explanation Fit: 0.5
0.344 < Physical.inactivity
0.121 < Diabetes.prevalence
0.75 < Drinking.water.violations
0.233 < Adult.smoking
505 < Premature.age.adjusted.mortality
Driving.alone.to.work <= 0.769
0.396 < Insufficient.sleep
0.0936 < Uninsured.adults <= 0.1283
4867 < Preventable.hospital.stays
Excessive.drinking <= 0.166
Feature
−0.02
0.00
0.02
0.04
0.06
Weight
Positive
Negative

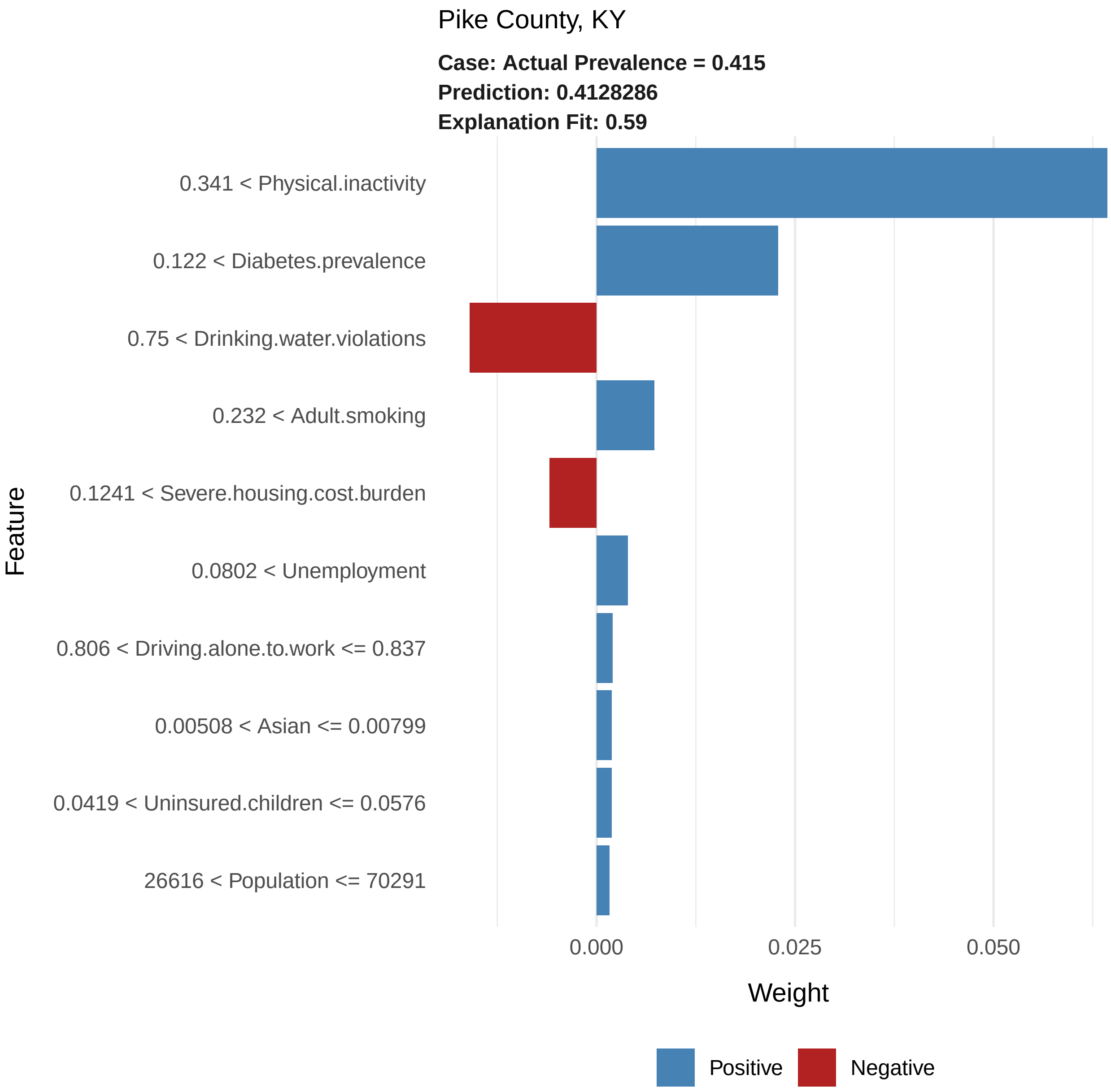
Pike County, KY
Case: Actual Prevalence = 0.415
Prediction: 0.4128286
Explanation Fit: 0.59
0.341 < Physical.inactivity
0.122 < Diabetes.prevalence
0.75 < Drinking.water.violations
0.232 < Adult.smoking
0.1241 < Severe.housing.cost.burden
0.0802 < Unemployment
0.806 < Driving.alone.to.work <= 0.837
0.00508 < Asian <= 0.00799
0.0419 < Uninsured.children <= 0.0576
26616 < Population <= 70291
Feature
0.000
0.025
0.050
Weight
Positive
Negative

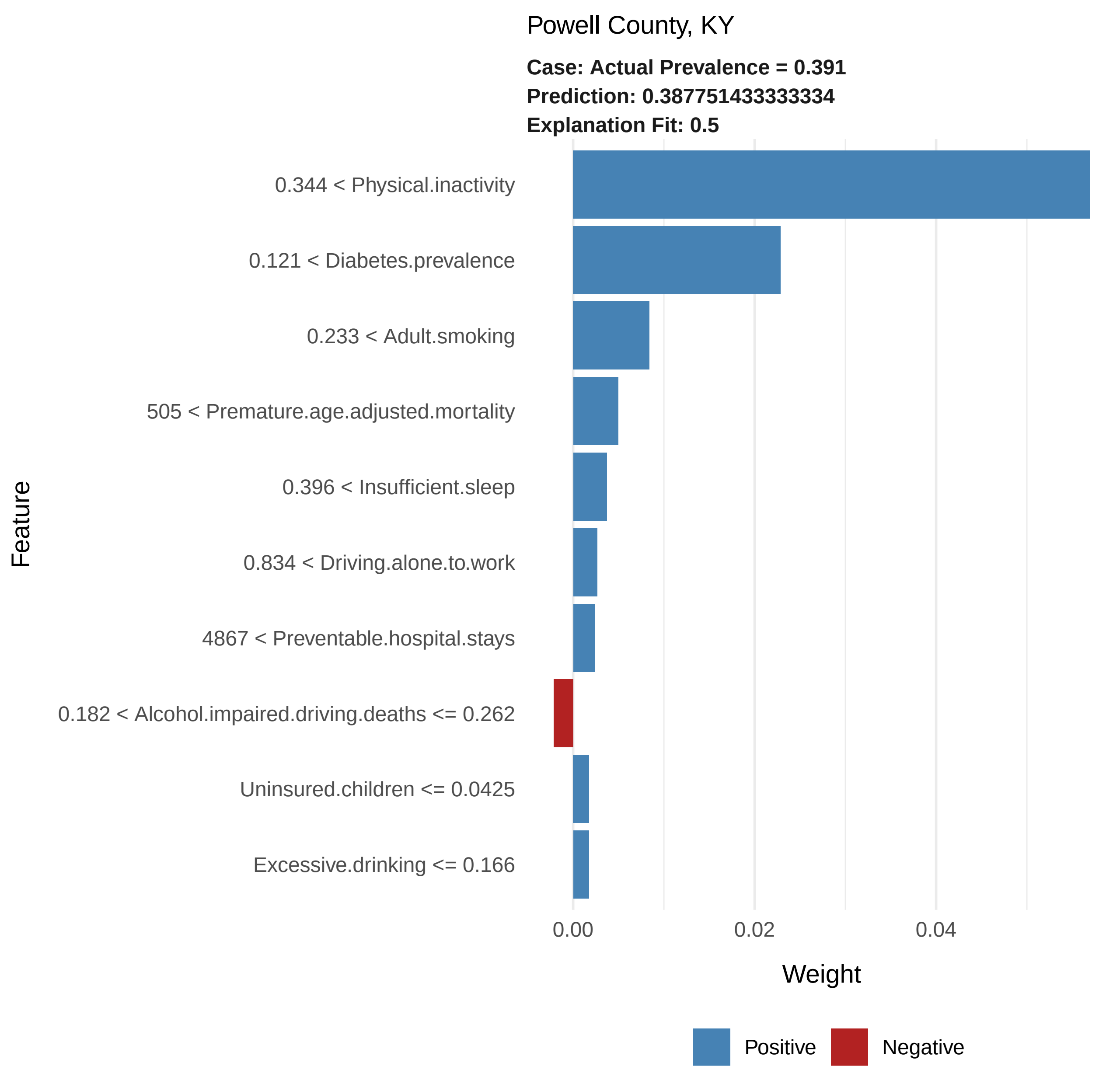

Powell County, KY
Case: Actual Prevalence = 0.391
Prediction: 0.387751433333334
Explanation Fit: 0.5
0.344 < Physical.inactivity
0.121 < Diabetes.prevalence
0.233 < Adult.smoking
505 < Premature.age.adjusted.mortality
0.396 < Insufficient.sleep
0.834 < Driving.alone.to.work
4867 < Preventable.hospital.stays
0.182 < Alcohol.impaired.driving.deaths <= 0.262
Uninsured.children <= 0.0425
Excessive.drinking <= 0.166
Feature
0.00
0.02
0.04
Weight
Positive
Negative

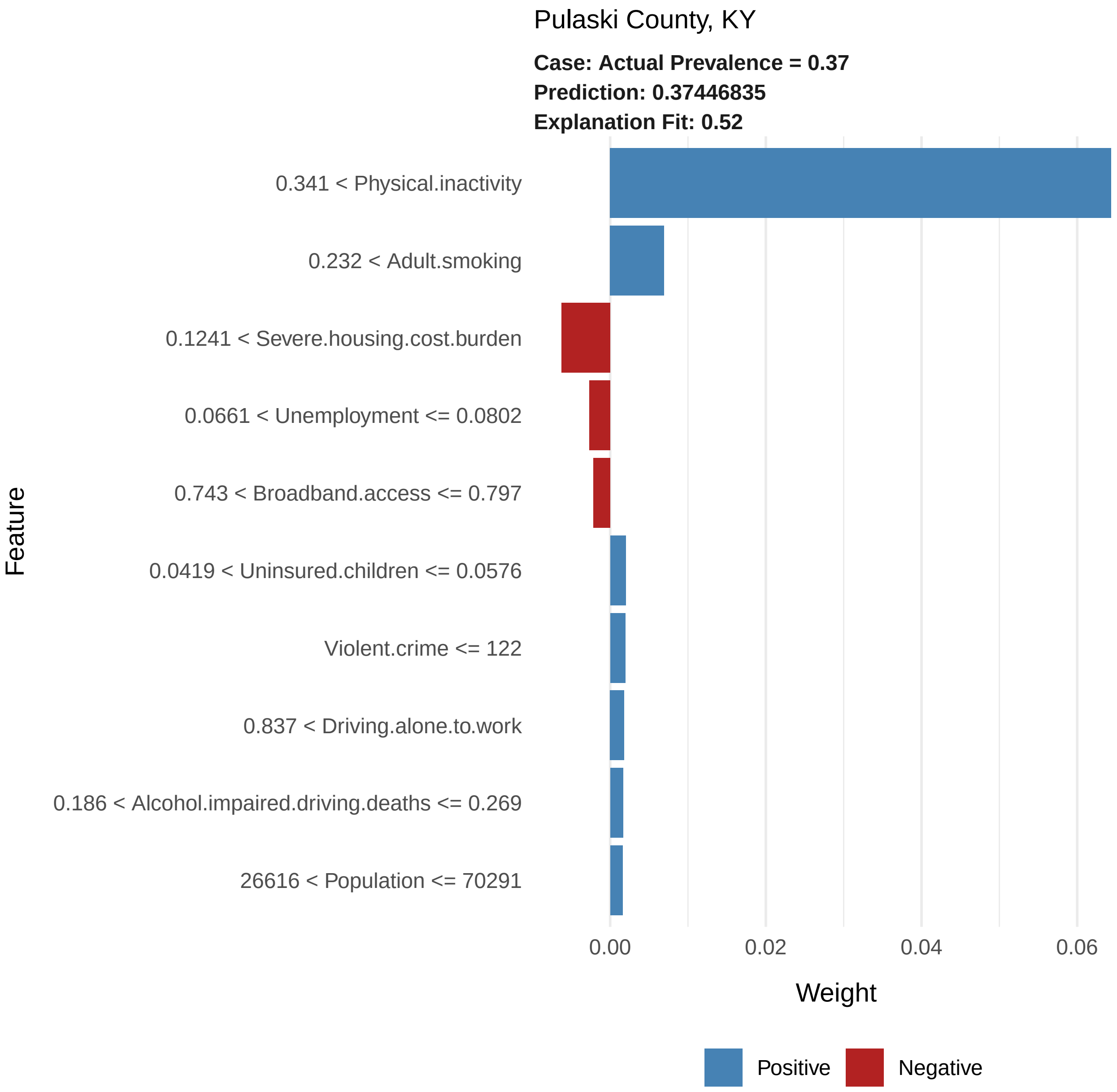

Pulaski County, KY
Case: Actual Prevalence = 0.37
Prediction: 0.37446835
Explanation Fit: 0.52
0.341 < Physical.inactivity
0.232 < Adult.smoking
0.1241 < Severe.housing.cost.burden
0.0661 < Unemployment <= 0.0802
0.743 < Broadband.access <= 0.797
0.0419 < Uninsured.children <= 0.0576
Violent.crime <= 122
0.837 < Driving.alone.to.work
0.186 < Alcohol.impaired.driving.deaths <= 0.269
26616 < Population <= 70291
Feature
0.00
0.02
0.04
0.06
Weight
Positive
Negative

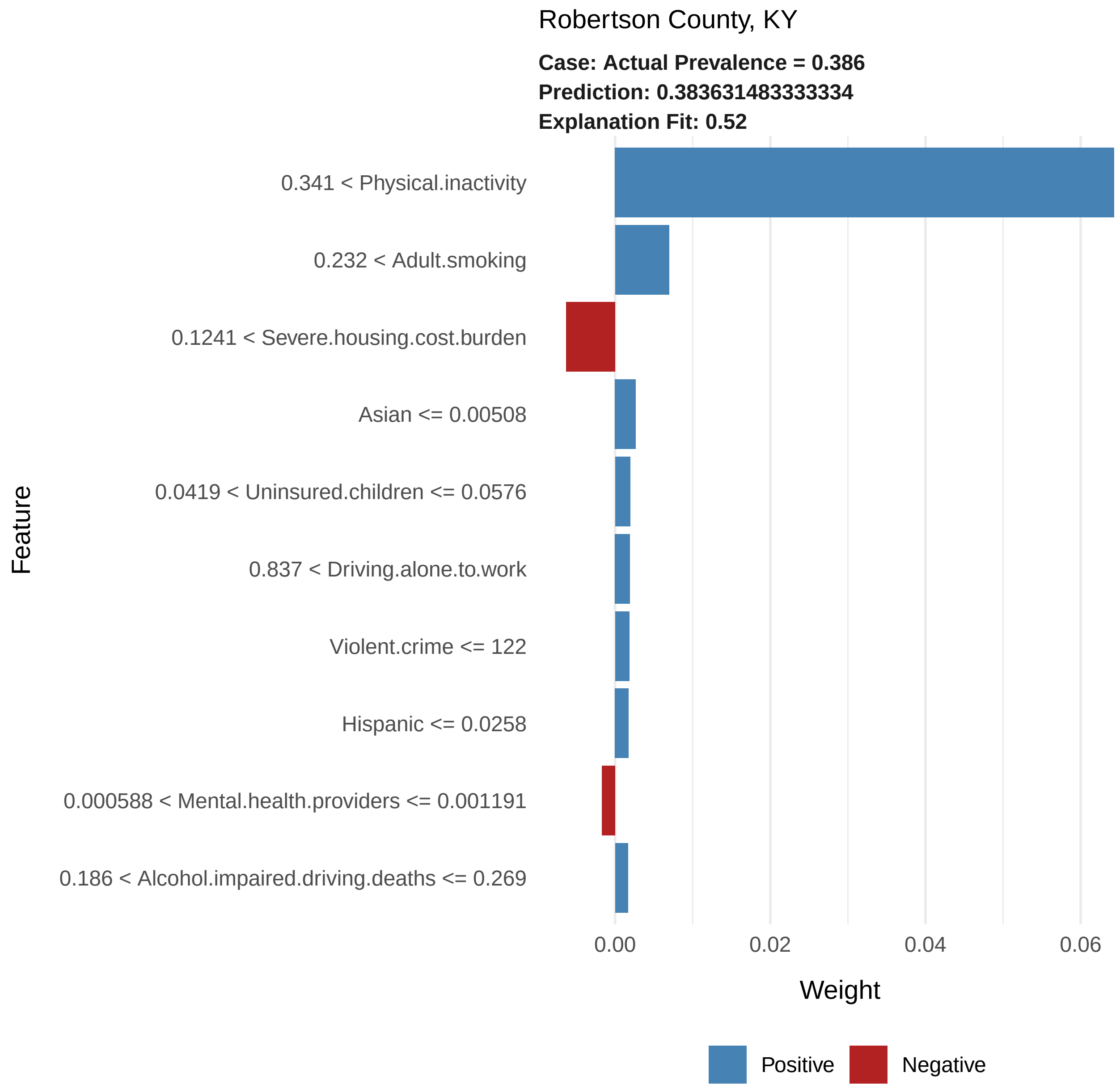

Robertson County, KY
Case: Actual Prevalence = 0.386
Prediction: 0.383631483333334
Explanation Fit: 0.52
0.341 < Physical.inactivity
0.232 < Adult.smoking
0.1241 < Severe.housing.cost.burden
Asian <= 0.00508
0.0419 < Uninsured.children <= 0.0576
0.837 < Driving.alone.to.work
Violent.crime <= 122
Hispanic <= 0.0258
0.000588 < Mental.health.providers <= 0.001191
0.186 < Alcohol.impaired.driving.deaths <= 0.269
Feature
0.00
0.02
0.04
0.06
Weight
Positive
Negative

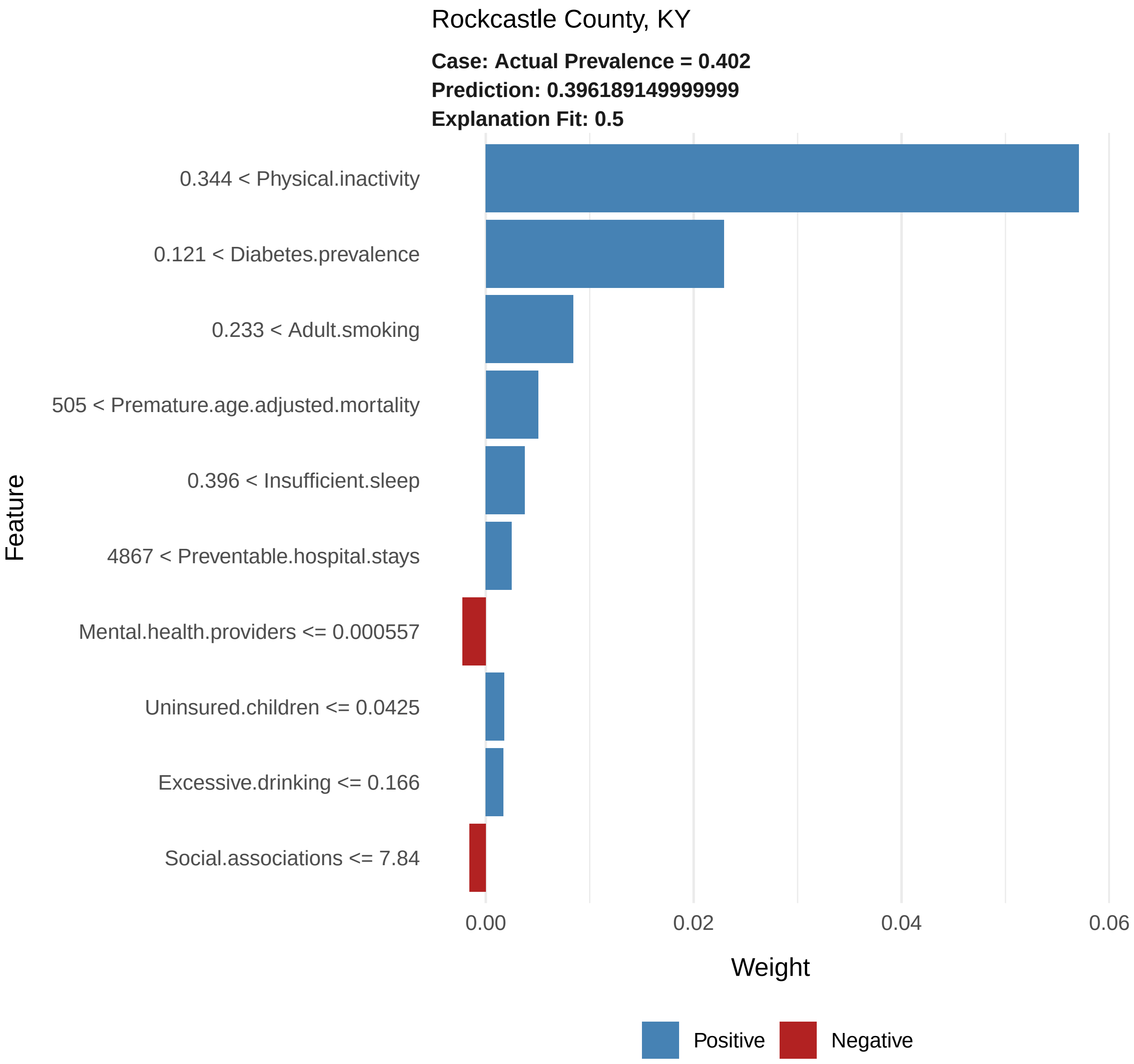

Rockcastle County, KY
Case: Actual Prevalence = 0.402
Prediction: 0.396189149999999
Explanation Fit: 0.5
0.344 < Physical.inactivity
0.121 < Diabetes.prevalence
0.233 < Adult.smoking
505 < Premature.age.adjusted.mortality
0.396 < Insufficient.sleep
4867 < Preventable.hospital.stays
Mental.health.providers <= 0.000557
Uninsured.children <= 0.0425
Excessive.drinking <= 0.166
Social.associations <= 7.84
Feature
0.00
0.02
0.04
0.06
Weight
Positive
Negative

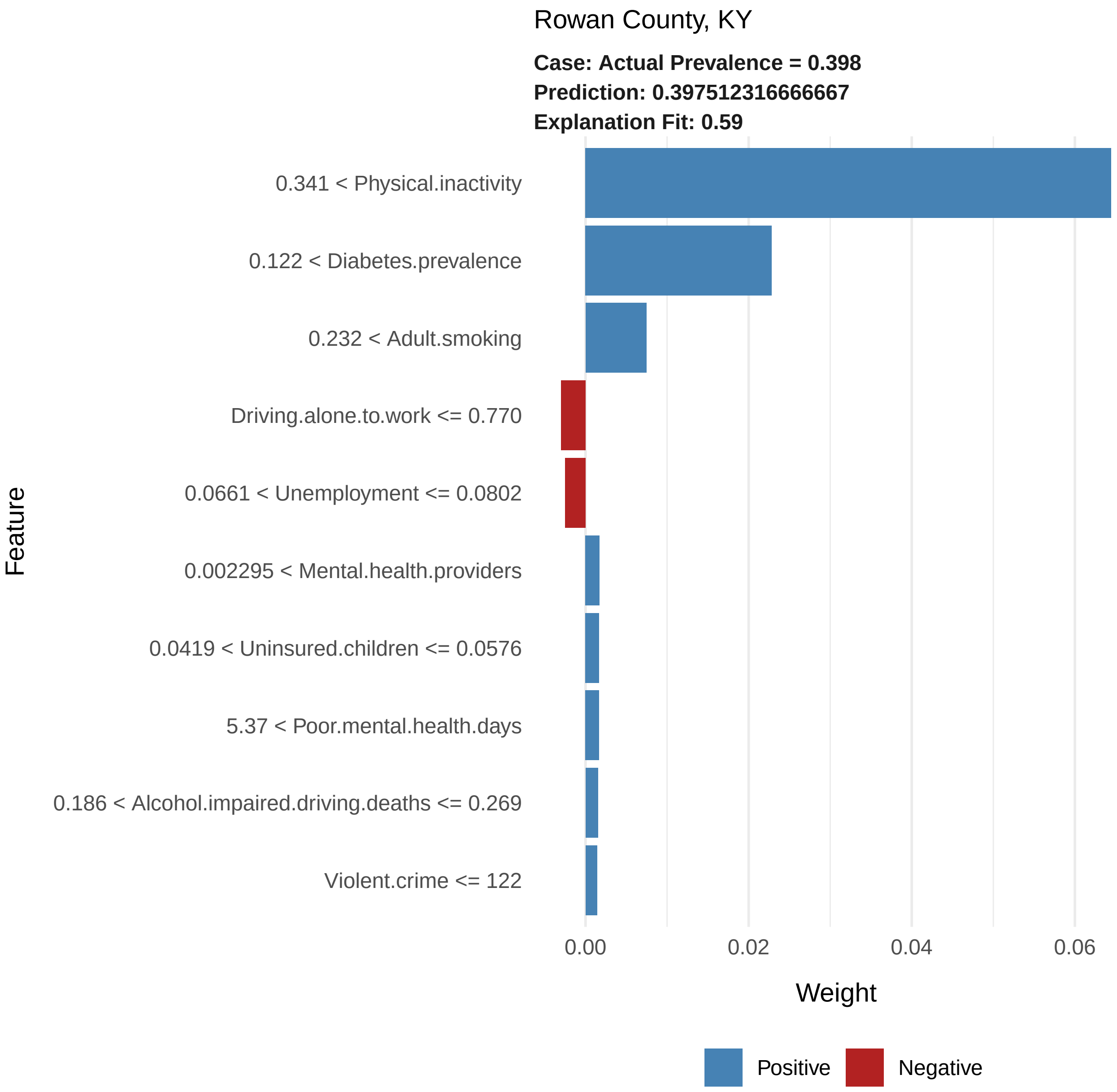
Rowan County, KY
Case: Actual Prevalence = 0.398
Prediction: 0.397512316666667
Explanation Fit: 0.59
0.341 < Physical.inactivity
0.122 < Diabetes.prevalence
0.232 < Adult.smoking
Driving.alone.to.work <= 0.770
0.0661 < Unemployment <= 0.0802
0.002295 < Mental.health.providers
0.0419 < Uninsured.children <= 0.0576
5.37 < Poor.mental.health.days
0.186 < Alcohol.impaired.driving.deaths <= 0.269
Violent.crime <= 122
Feature
0.00
0.02
0.04
0.06
Weight
Positive
Negative

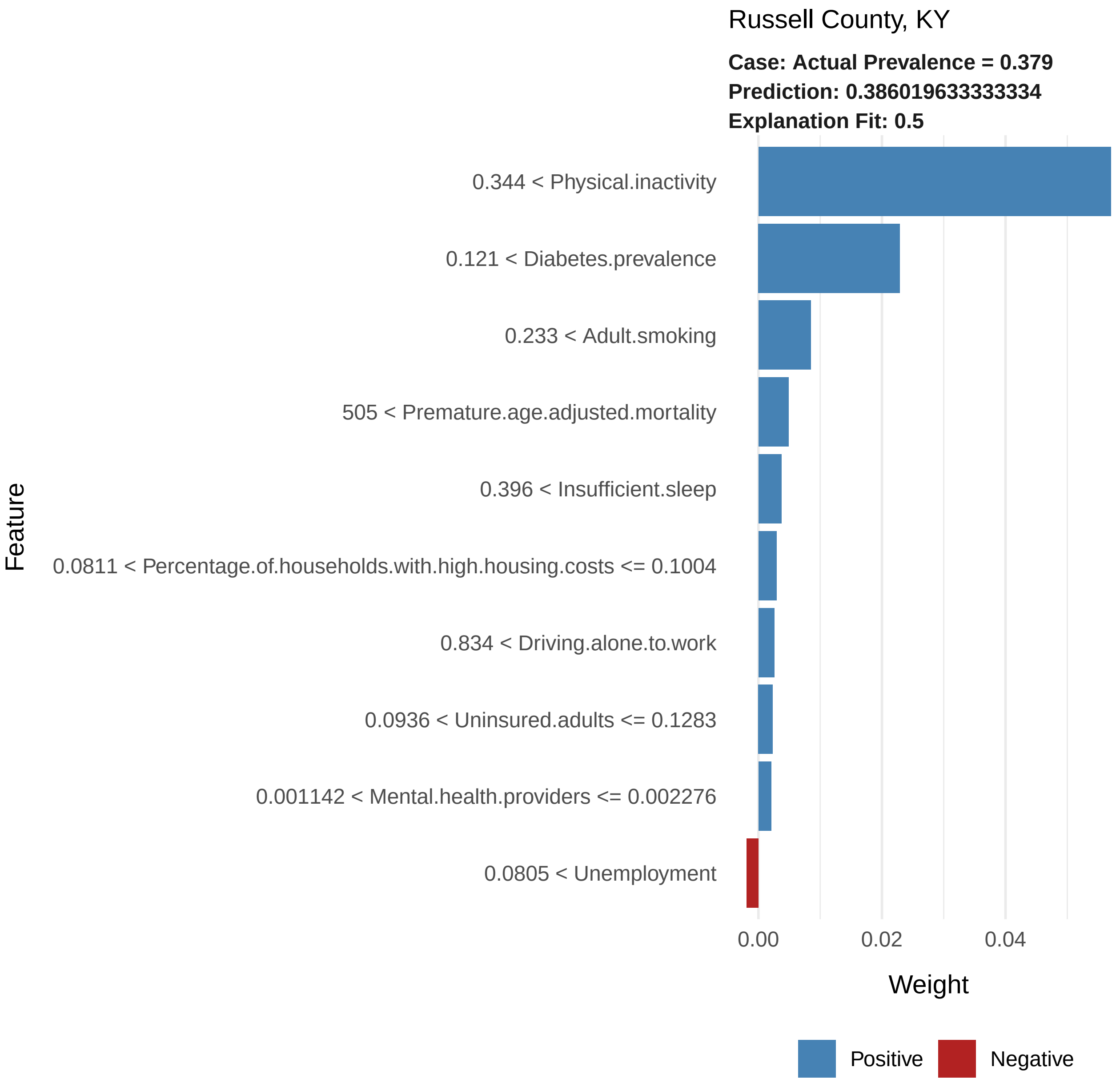

Russell County, KY
Case: Actual Prevalence = 0.379
Prediction: 0.386019633333334
Explanation Fit: 0.5
0.344 < Physical.inactivity
0.121 < Diabetes.prevalence
0.233 < Adult.smoking
505 < Premature.age.adjusted.mortality
0.396 < Insufficient.sleep
0.0811 < Percentage.of.households.with.high.housing.costs <= 0.1004
0.834 < Driving.alone.to.work
0.0936 < Uninsured.adults <= 0.1283
0.001142 < Mental.health.providers <= 0.002276
0.0805 < Unemployment
Feature
0.00
0.02
0.04
Weight
Positive
Negative

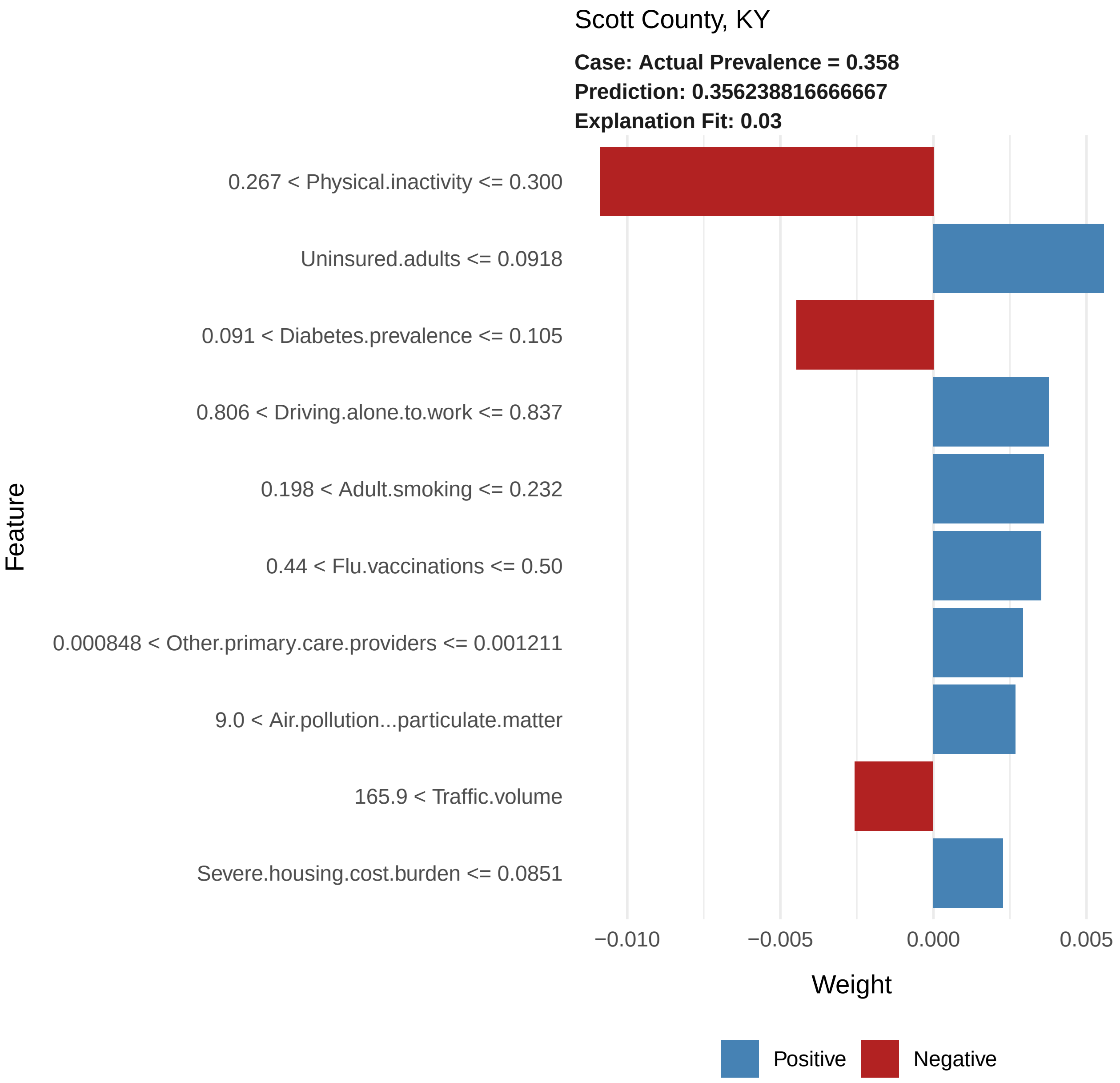

Scott County, KY
Case: Actual Prevalence = 0.358
Prediction: 0.356238816666667
Explanation Fit: 0.03
0.267 < Physical.inactivity <= 0.300
Uninsured.adults <= 0.0918
0.091 < Diabetes.prevalence <= 0.105
0.806 < Driving.alone.to.work <= 0.837
0.198 < Adult.smoking <= 0.232
0.44 < Flu.vaccinations <= 0.50
0.000848 < Other.primary.care.providers <= 0.001211
9.0 < Air.pollution...particulate.matter
165.9 < Traffic.volume
Severe.housing.cost.burden <= 0.0851
Feature
−0.010
−0.005
0.000
0.005
Weight
Positive
Negative

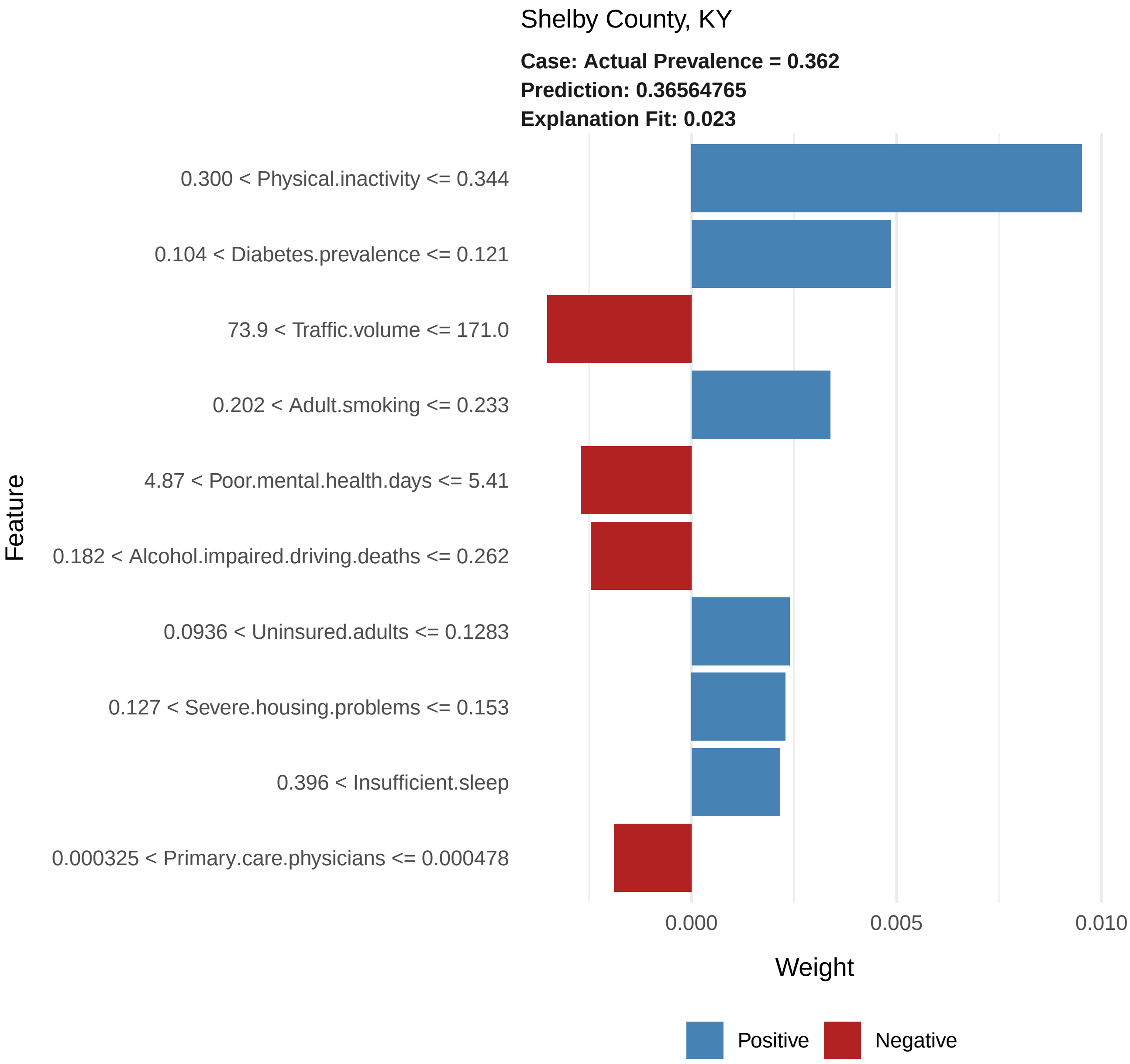
Shelby County, KY
Case: Actual Prevalence = 0.362
Prediction: 0.36564765
Explanation Fit: 0.023
0.300 < Physical.inactivity <= 0.344
0.104 < Diabetes.prevalence <= 0.121
73.9 < Traffic.volume <= 171.0
0.202 < Adult.smoking <= 0.233
4.87 < Poor.mental.health.days <= 5.41
0.182 < Alcohol.impaired.driving.deaths <= 0.262
0.0936 < Uninsured.adults <= 0.1283
0.127 < Severe.housing.problems <= 0.153
0.396 < Insufficient.sleep
0.000325 < Primary.care.physicians <= 0.000478
Feature
0.000
0.005
0.010
Weight
Positive
Negative

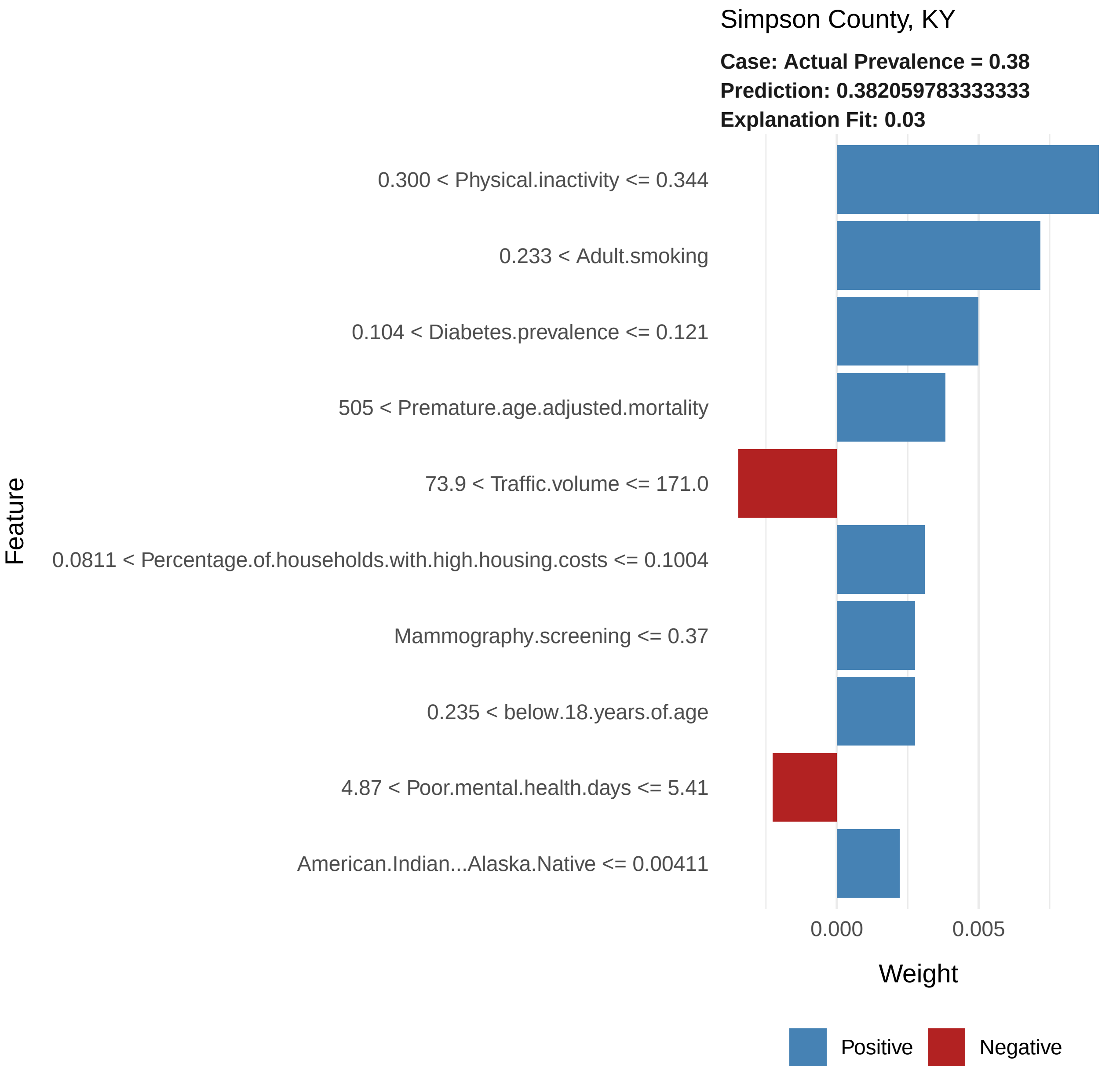
Simpson County, KY
Case: Actual Prevalence = 0.38
Prediction: 0.382059783333333
Explanation Fit: 0.03
0.300 < Physical.inactivity <= 0.344
0.233 < Adult.smoking
0.104 < Diabetes.prevalence <= 0.121
505 < Premature.age.adjusted.mortality
73.9 < Traffic.volume <= 171.0
0.0811 < Percentage.of.households.with.high.housing.costs <= 0.1004
Mammography.screening <= 0.37
0.235 < below.18.years.of.age
4.87 < Poor.mental.health.days <= 5.41
American.Indian...Alaska.Native <= 0.00411
Feature
0.000
0.005
Weight
Positive
Negative

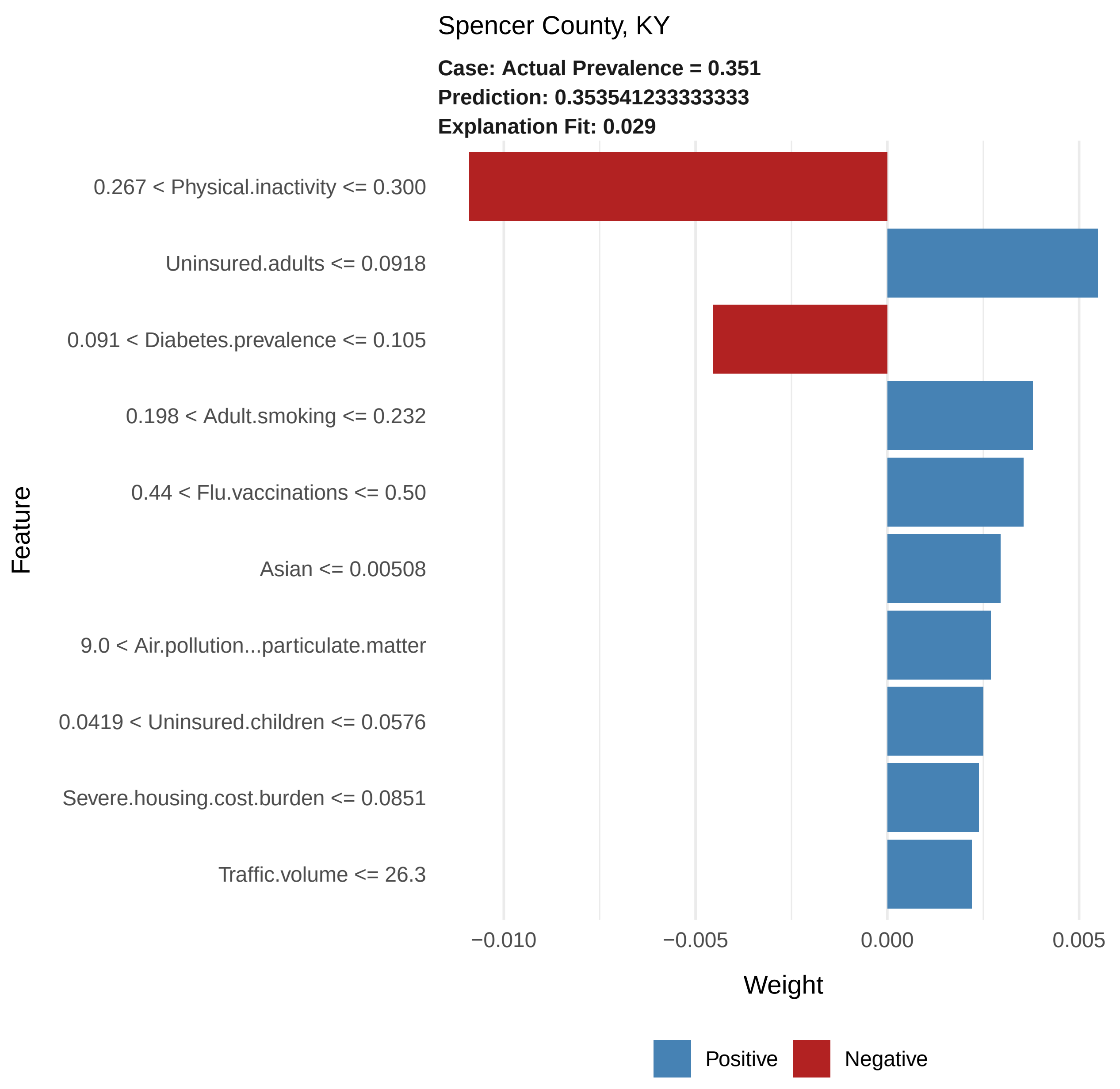

Spencer County, KY
Case: Actual Prevalence = 0.351
Prediction: 0.353541233333333
Explanation Fit: 0.029
0.267 < Physical.inactivity <= 0.300
Uninsured.adults <= 0.0918
0.091 < Diabetes.prevalence <= 0.105
0.198 < Adult.smoking <= 0.232
0.44 < Flu.vaccinations <= 0.50
Asian <= 0.00508
9.0 < Air.pollution...particulate.matter
0.0419 < Uninsured.children <= 0.0576
Severe.housing.cost.burden <= 0.0851
Traffic.volume <= 26.3
Feature
−0.010
−0.005
0.000
0.005
Weight
Positive
Negative

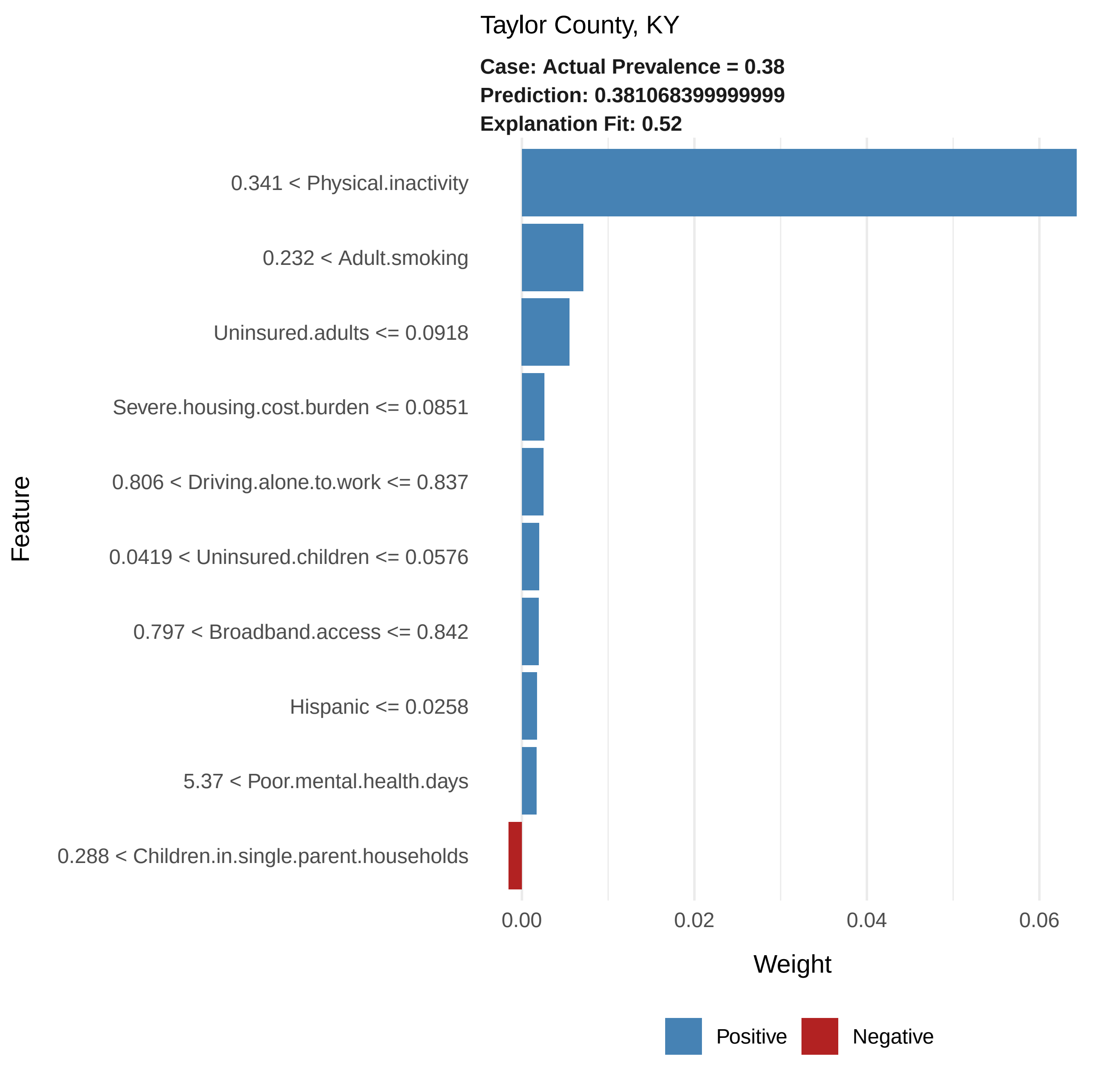

Taylor County, KY
Case: Actual Prevalence = 0.38
Prediction: 0.381068399999999
Explanation Fit: 0.52
0.341 < Physical.inactivity
0.232 < Adult.smoking
Uninsured.adults <= 0.0918
Severe.housing.cost.burden <= 0.0851
0.806 < Driving.alone.to.work <= 0.837
0.0419 < Uninsured.children <= 0.0576
0.797 < Broadband.access <= 0.842
Hispanic <= 0.0258
5.37 < Poor.mental.health.days
0.288 < Children.in.single.parent.households
Feature
0.00
0.02
0.04
0.06
Weight
Positive
Negative

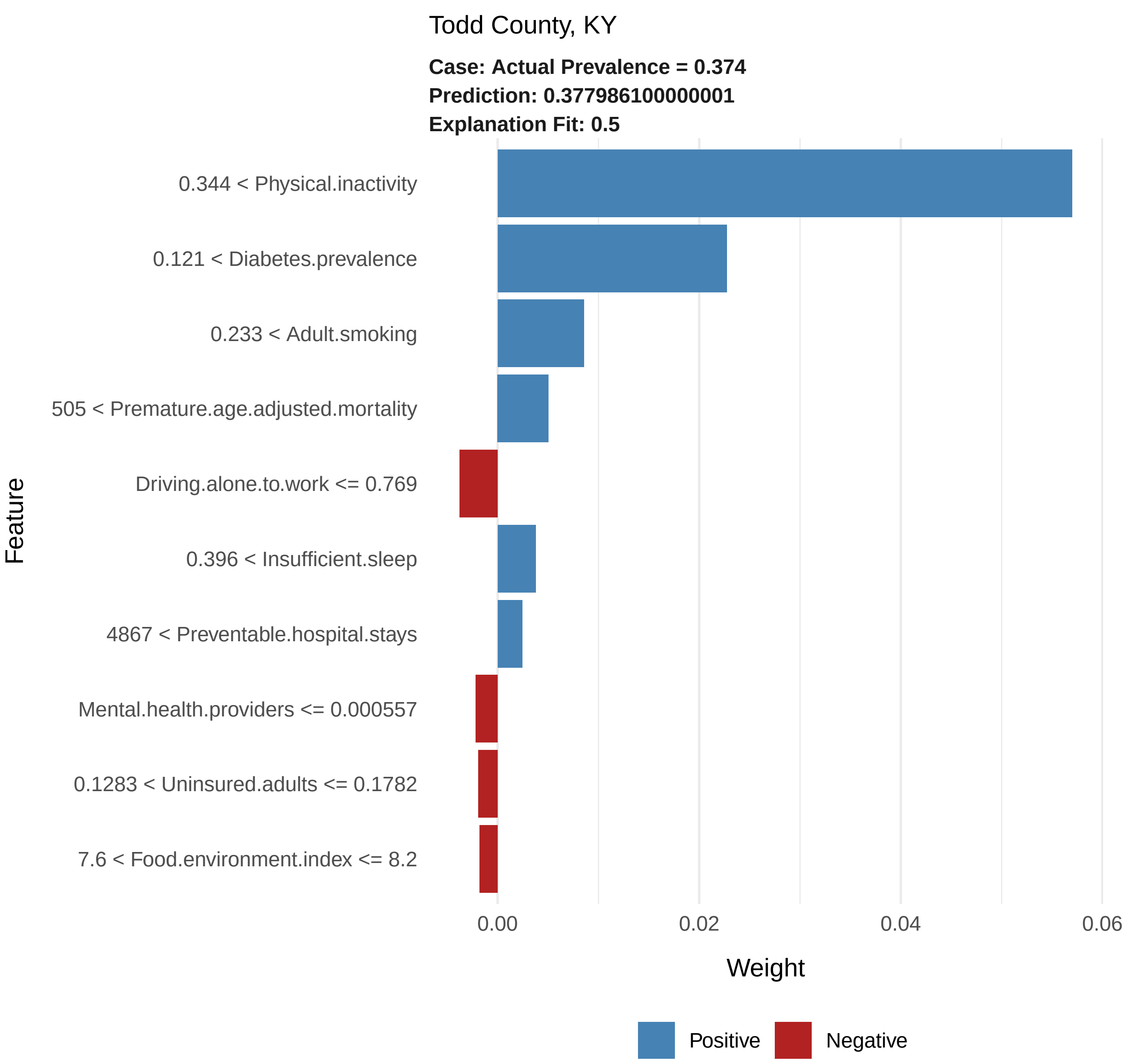
Todd County, KY
Case: Actual Prevalence = 0.374
Prediction: 0.377986100000001
Explanation Fit: 0.5
0.344 < Physical.inactivity
0.121 < Diabetes.prevalence
0.233 < Adult.smoking
505 < Premature.age.adjusted.mortality
Driving.alone.to.work <= 0.769
0.396 < Insufficient.sleep
4867 < Preventable.hospital.stays
Mental.health.providers <= 0.000557
0.1283 < Uninsured.adults <= 0.1782
7.6 < Food.environment.index <= 8.2
Feature
0.00
0.02
0.04
0.06
Weight
Positive
Negative

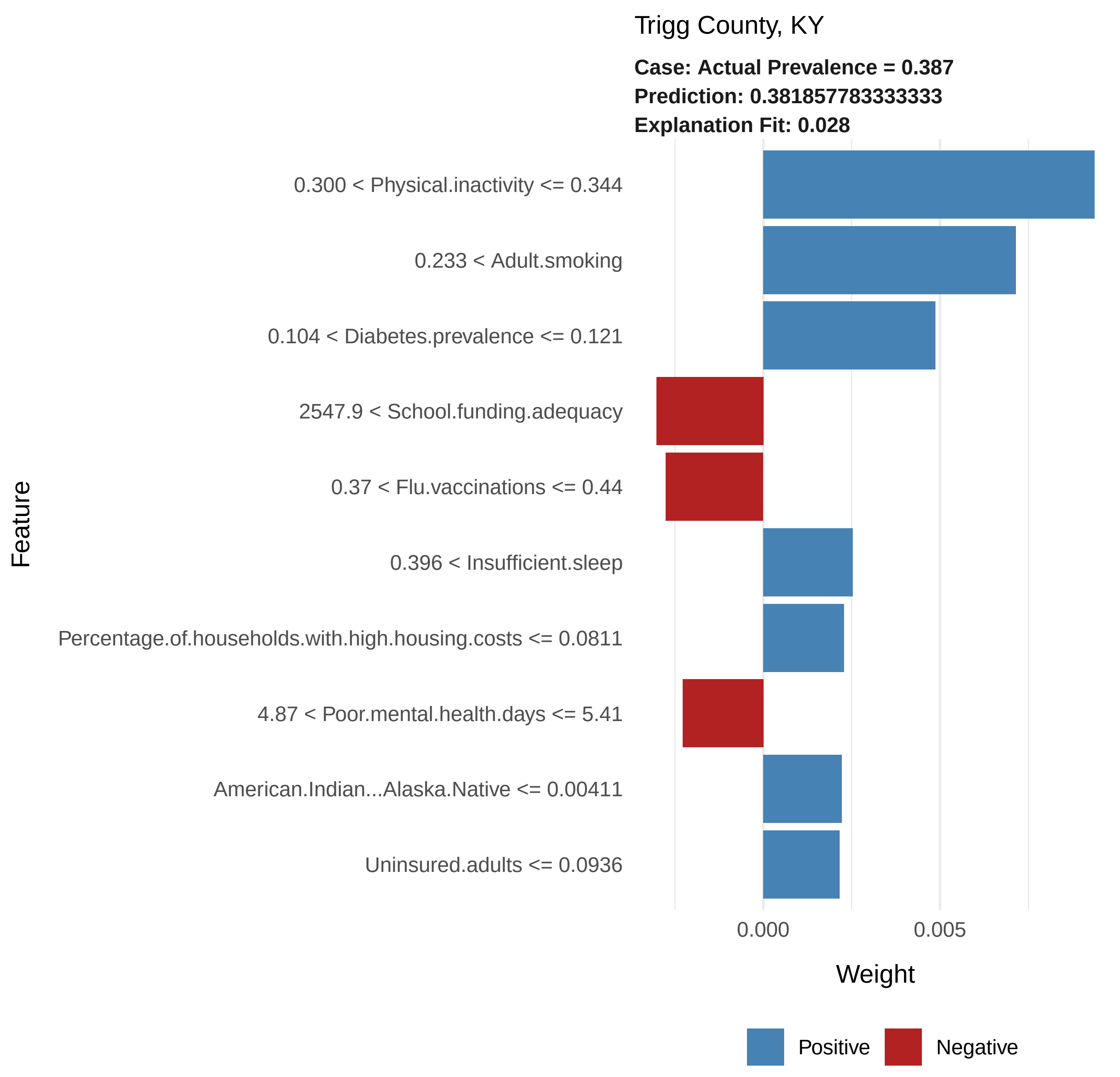

Trigg County, KY
Case: Actual Prevalence = 0.387
Prediction: 0.381857783333333
Explanation Fit: 0.028
0.300 < Physical.inactivity <= 0.344
0.233 < Adult.smoking
0.104 < Diabetes.prevalence <= 0.121
2547.9 < School.funding.adequacy
0.37 < Flu.vaccinations <= 0.44
0.396 < Insufficient.sleep
Percentage.of.households.with.high.housing.costs <= 0.0811
4.87 < Poor.mental.health.days <= 5.41
American.Indian...Alaska.Native <= 0.00411
Uninsured.adults <= 0.0936
Feature
0.000
0.005
Weight
Positive
Negative

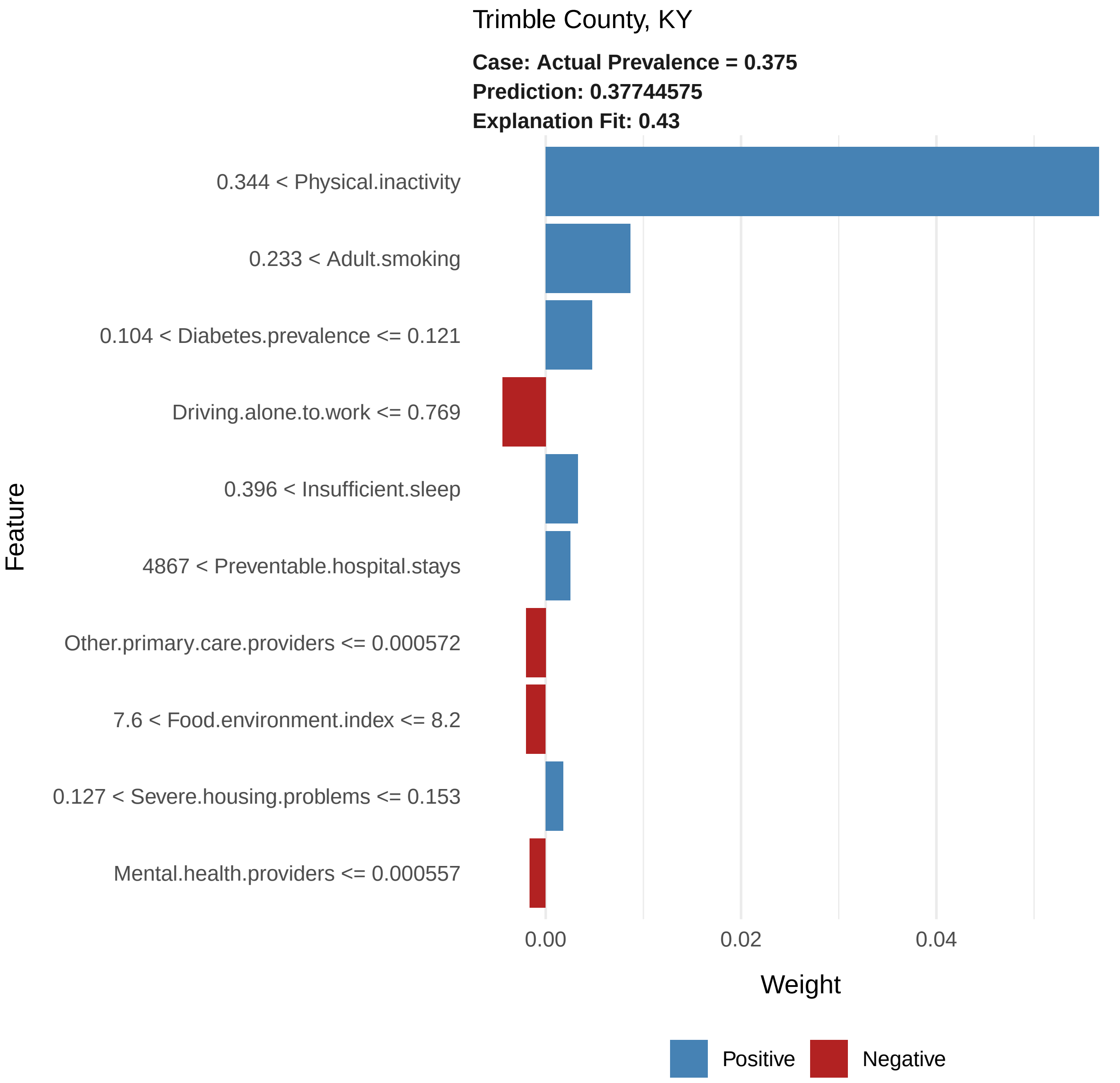
Trimble County, KY
Case: Actual Prevalence = 0.375
Prediction: 0.37744575
Explanation Fit: 0.43
0.344 < Physical.inactivity
0.233 < Adult.smoking
0.104 < Diabetes.prevalence <= 0.121
Driving.alone.to.work <= 0.769
0.396 < Insufficient.sleep
4867 < Preventable.hospital.stays
Other.primary.care.providers <= 0.000572
7.6 < Food.environment.index <= 8.2
0.127 < Severe.housing.problems <= 0.153
Mental.health.providers <= 0.000557
Feature
0.00
0.02
0.04
Weight
Positive
Negative

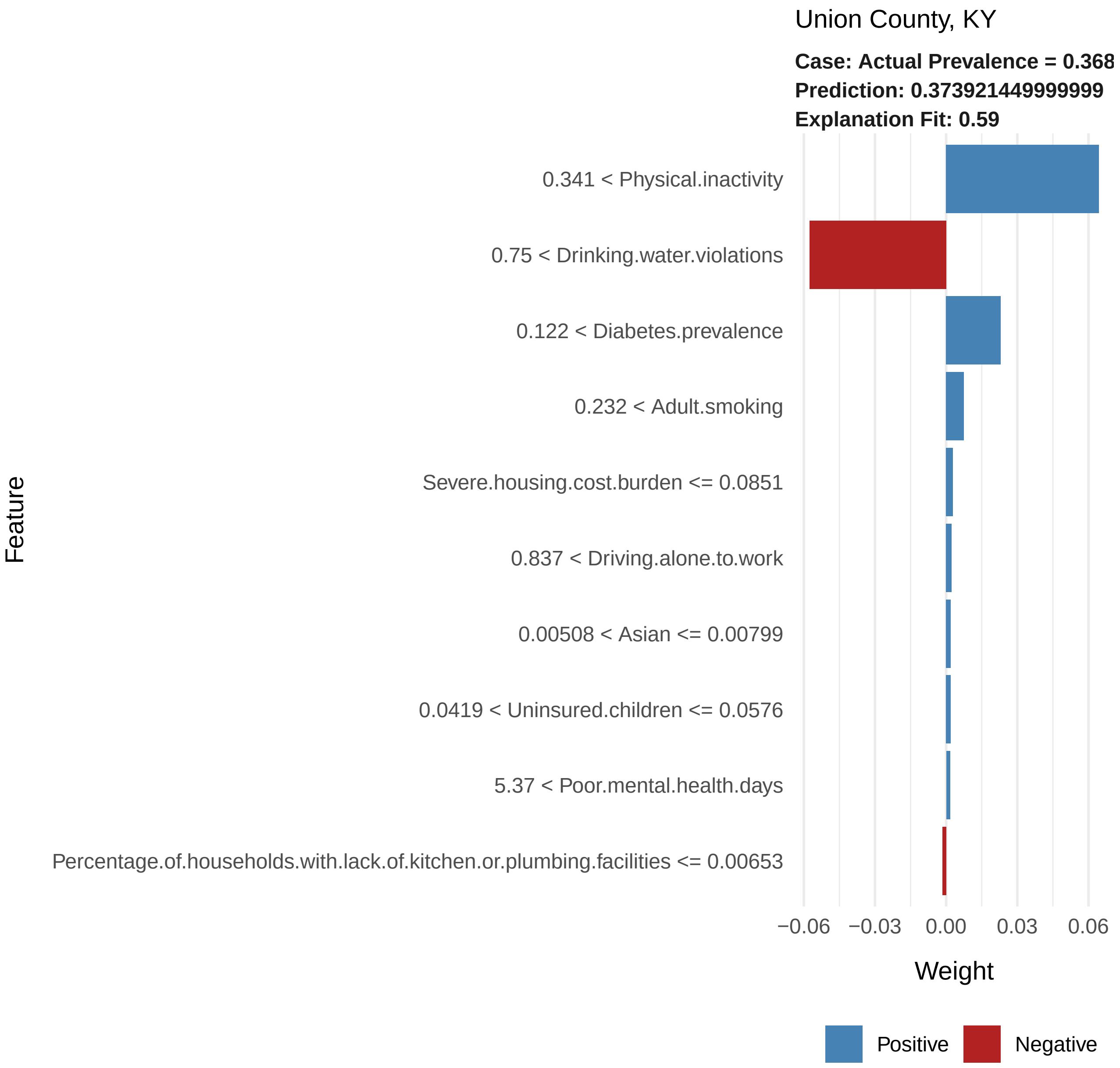

Union County, KY
Case: Actual Prevalence = 0.368
Prediction: 0.373921449999999
Explanation Fit: 0.59
0.341 < Physical.inactivity
0.75 < Drinking.water.violations
0.122 < Diabetes.prevalence
0.232 < Adult.smoking
Severe.housing.cost.burden <= 0.0851
0.837 < Driving.alone.to.work
0.00508 < Asian <= 0.00799
0.0419 < Uninsured.children <= 0.0576
5.37 < Poor.mental.health.days
Percentage.of.households.with.lack.of.kitchen.or.plumbing.facilities <= 0.00653
Feature
−0.06
−0.03
0.00
0.03
0.06
Weight
Positive
Negative

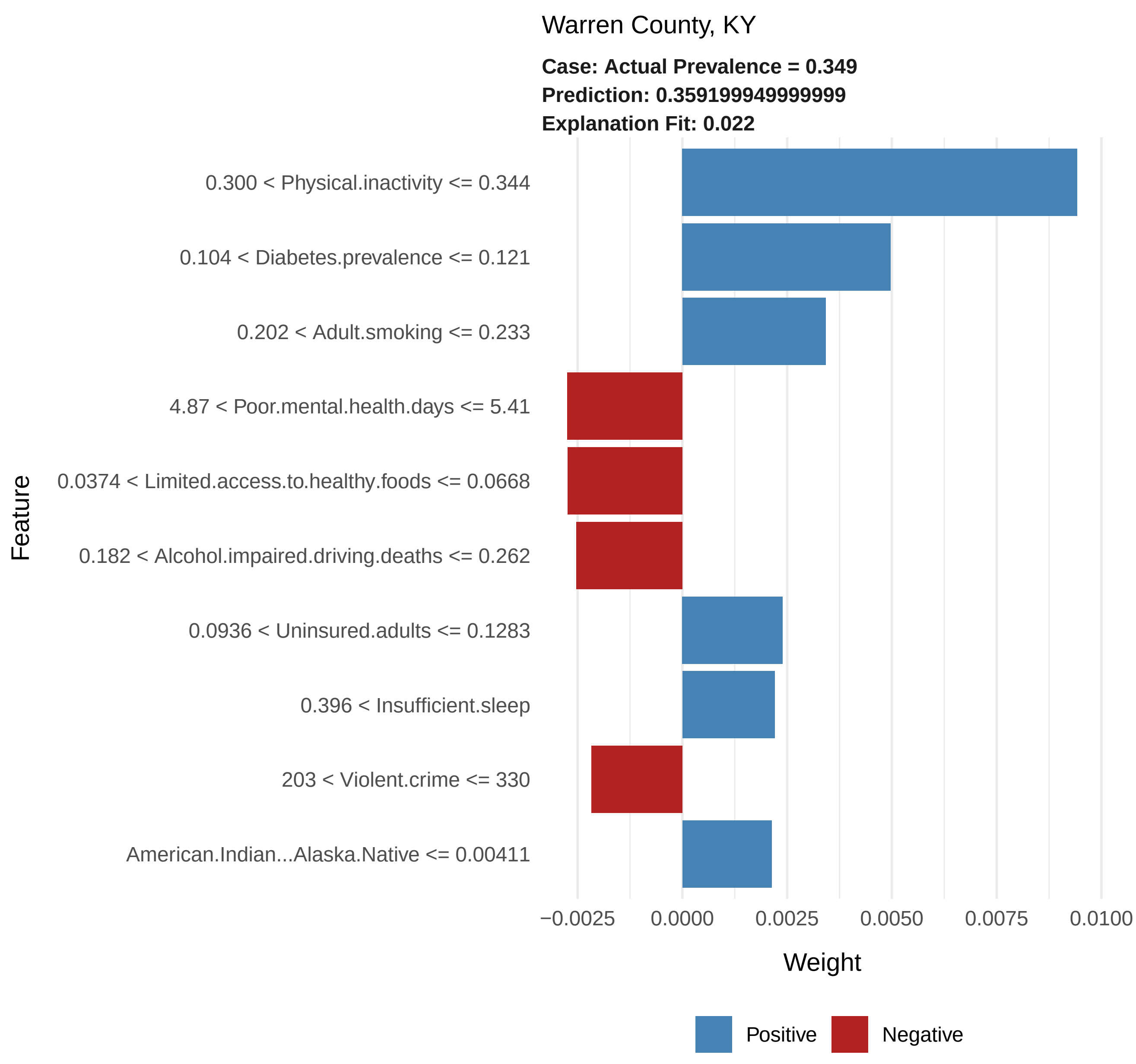
Warren County, KY
Case: Actual Prevalence = 0.349
Prediction: 0.359199949999999
Explanation Fit: 0.022
Feature
0.300 < Physical.inactivity <= 0.344
0.104 < Diabetes.prevalence <= 0.121
0.202 < Adult.smoking <= 0.233
4.87 < Poor.mental.health.days <= 5.41
0.0374 < Limited.access.to.healthy.foods <= 0.0668
0.182 < Alcohol.impaired.driving.deaths <= 0.262
0.0936 < Uninsured.adults <= 0.1283
0.396 < Insufficient.sleep
203 < Violent.crime <= 330
American.Indian...Alaska.Native <= 0.00411
−0.0025
0.0000
0.0025
0.0050
0.0075
0.0100
Weight
Positive
Negative

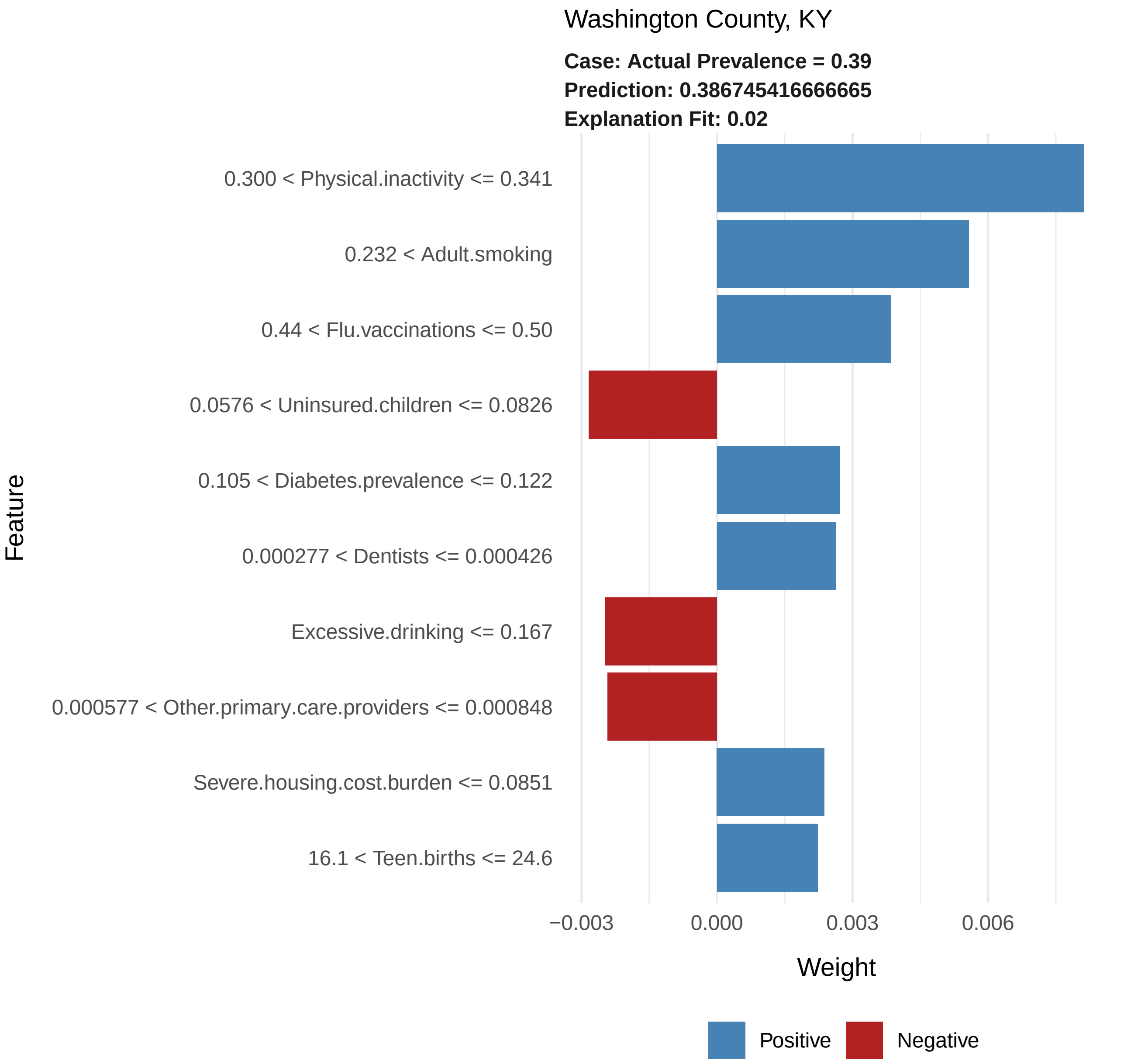
Washington County, KY
Case: Actual Prevalence = 0.39
Prediction: 0.386745416666665
Explanation Fit: 0.02
0.300 < Physical.inactivity <= 0.341
0.232 < Adult.smoking
0.44 < Flu.vaccinations <= 0.50
0.0576 < Uninsured.children <= 0.0826
0.105 < Diabetes.prevalence <= 0.122
0.000277 < Dentists <= 0.000426
Excessive.drinking <= 0.167
0.000577 < Other.primary.care.providers <= 0.000848
Severe.housing.cost.burden <= 0.0851
16.1 < Teen.births <= 24.6
Feature
−0.003
0.000
0.003
0.006
Weight
Positive
Negative

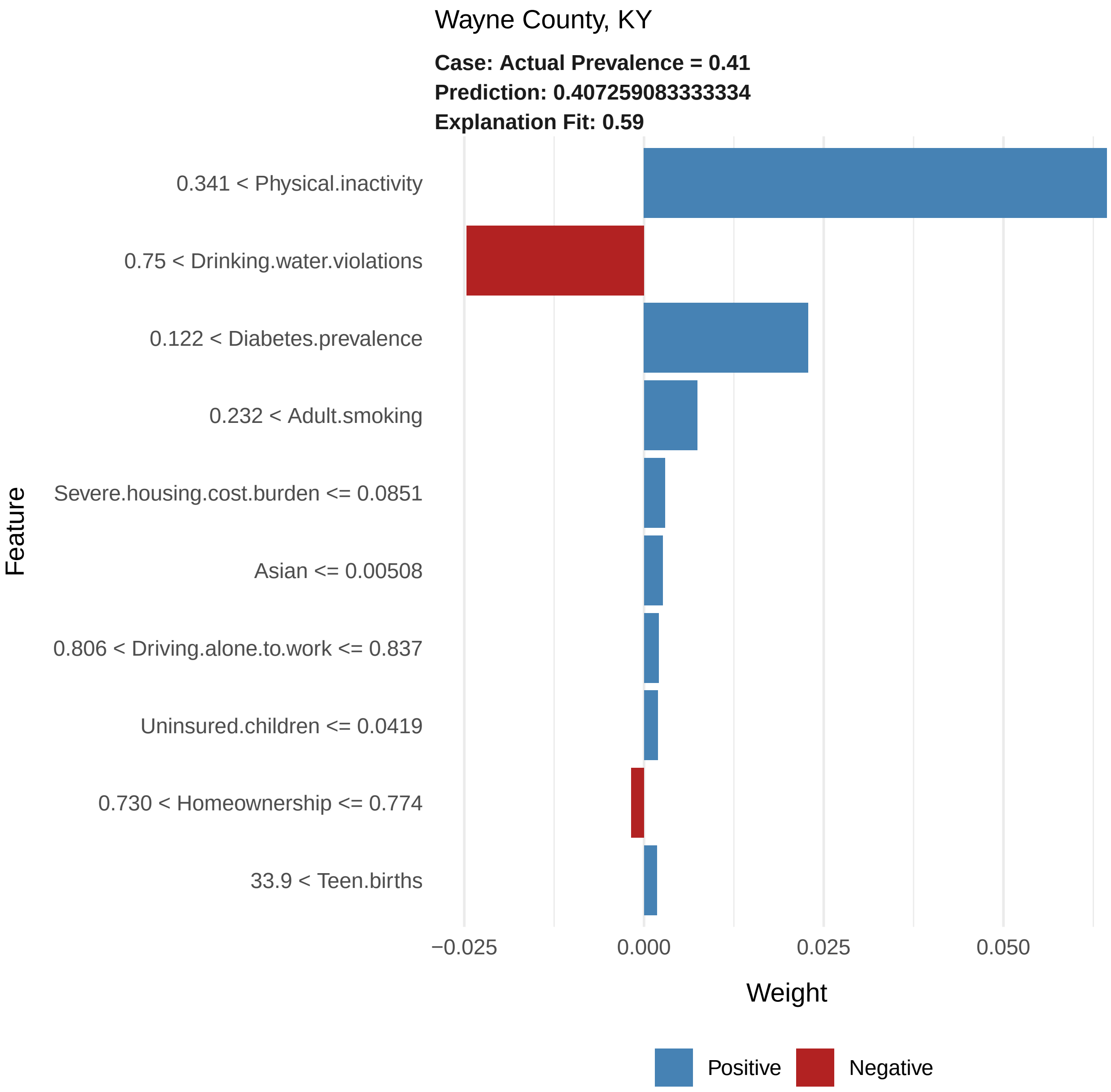
Wayne County, KY
Case: Actual Prevalence = 0.41
Prediction: 0.407259083333334
Explanation Fit: 0.59
0.341 < Physical.inactivity
0.75 < Drinking.water.violations
0.122 < Diabetes.prevalence
0.232 < Adult.smoking
Severe.housing.cost.burden <= 0.0851
Asian <= 0.00508
0.806 < Driving.alone.to.work <= 0.837
Uninsured.children <= 0.0419
0.730 < Homeownership <= 0.774
33.9 < Teen.births
Feature
−0.025
0.000
0.025
0.050
Weight
Positive
Negative

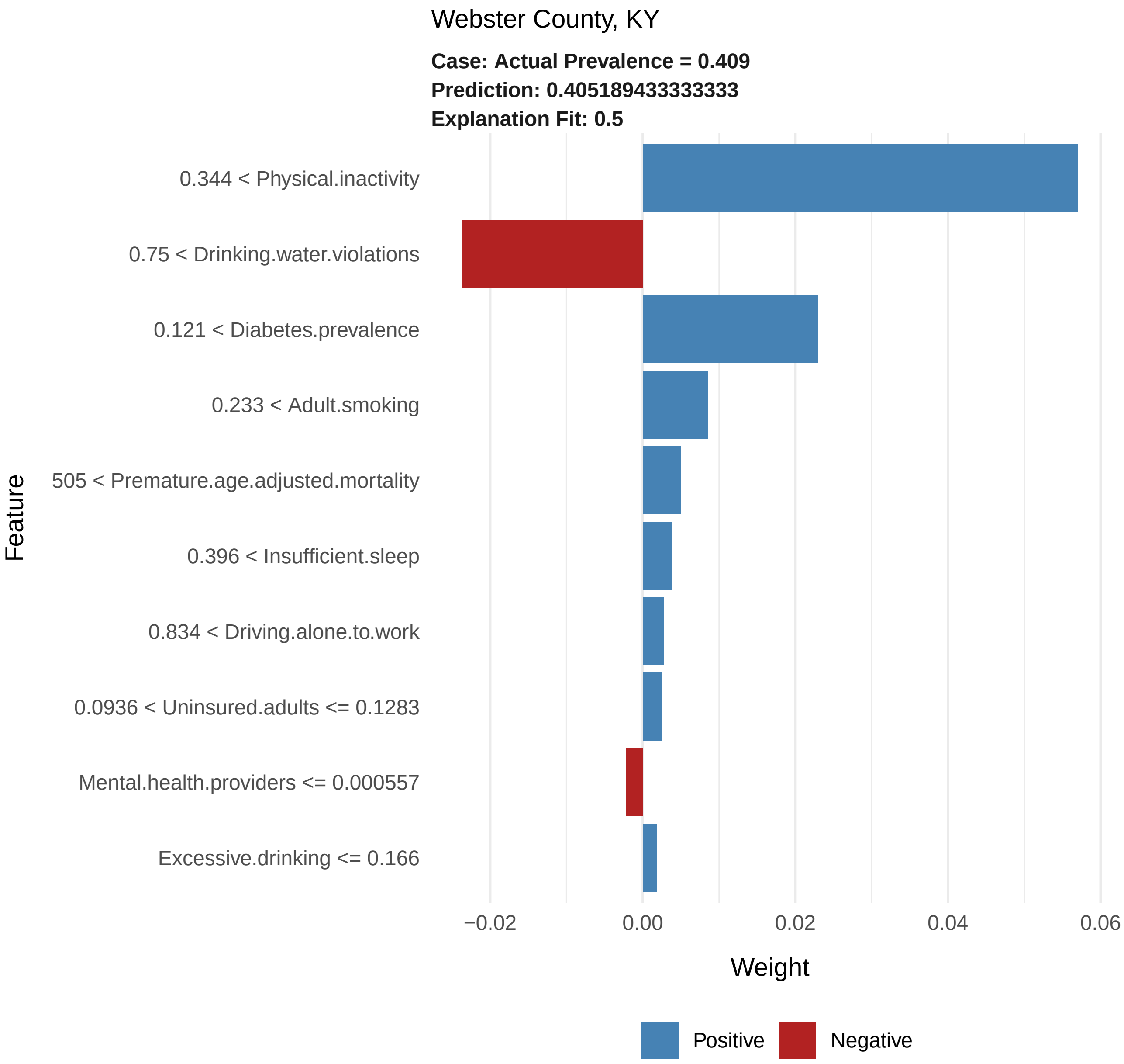

Webster County, KY
Case: Actual Prevalence = 0.409
Prediction: 0.405189433333333
Explanation Fit: 0.5
0.344 < Physical.inactivity
0.75 < Drinking.water.violations
0.121 < Diabetes.prevalence
0.233 < Adult.smoking
505 < Premature.age.adjusted.mortality
0.396 < Insufficient.sleep
0.834 < Driving.alone.to.work
0.0936 < Uninsured.adults <= 0.1283
Mental.health.providers <= 0.000557
Excessive.drinking <= 0.166
Feature
−0.02
0.00
0.02
0.04
0.06
Weight
Positive
Negative

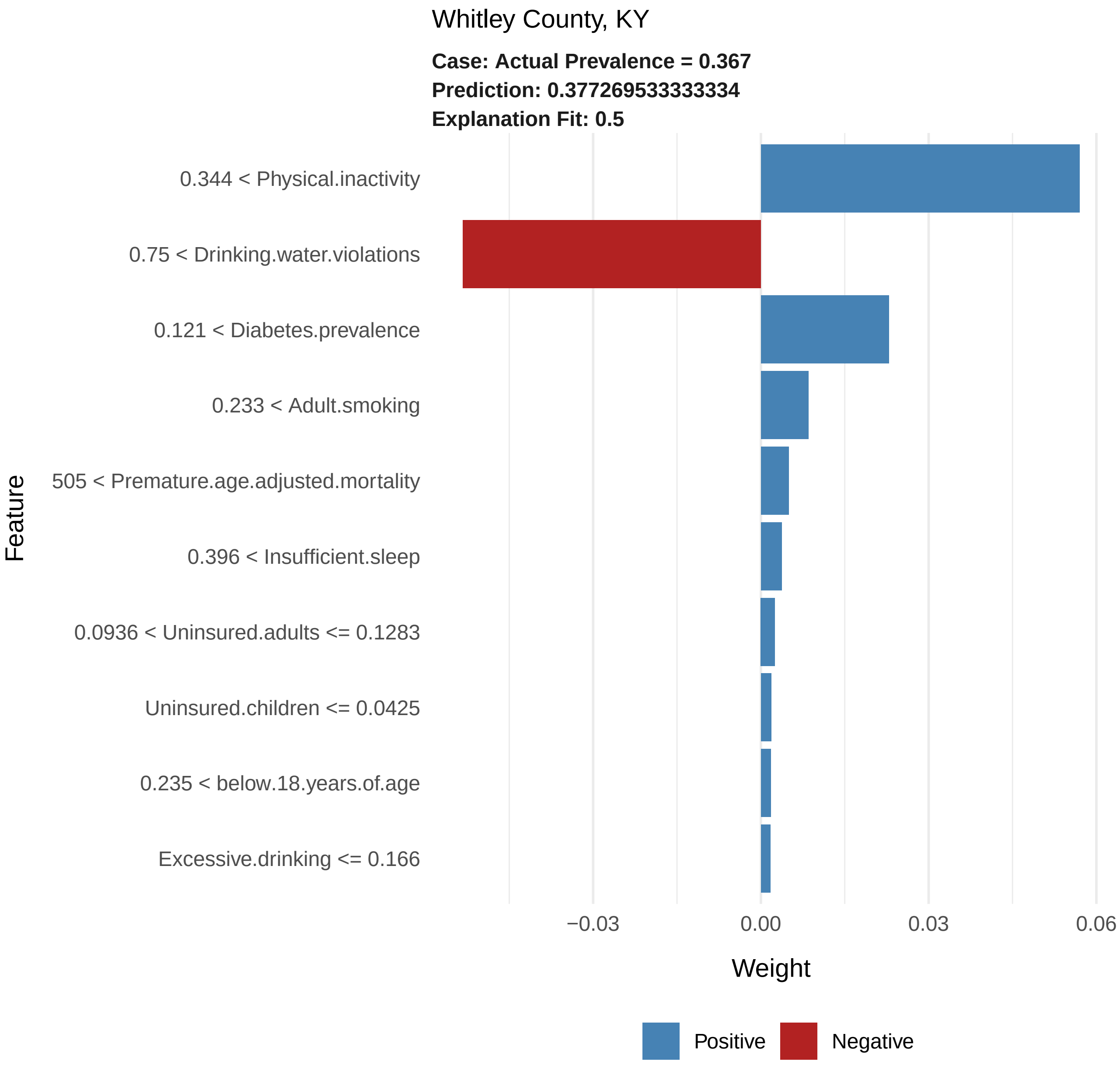

Whitley County, KY
Case: Actual Prevalence = 0.367
Prediction: 0.377269533333334
Explanation Fit: 0.5
0.344 < Physical.inactivity
0.75 < Drinking.water.violations
0.121 < Diabetes.prevalence
0.233 < Adult.smoking
505 < Premature.age.adjusted.mortality
0.396 < Insufficient.sleep
0.0936 < Uninsured.adults <= 0.1283
Uninsured.children <= 0.0425
0.235 < below.18.years.of.age
Excessive.drinking <= 0.166
Feature
−0.03
0.00
0.03
0.06
Weight
Positive
Negative

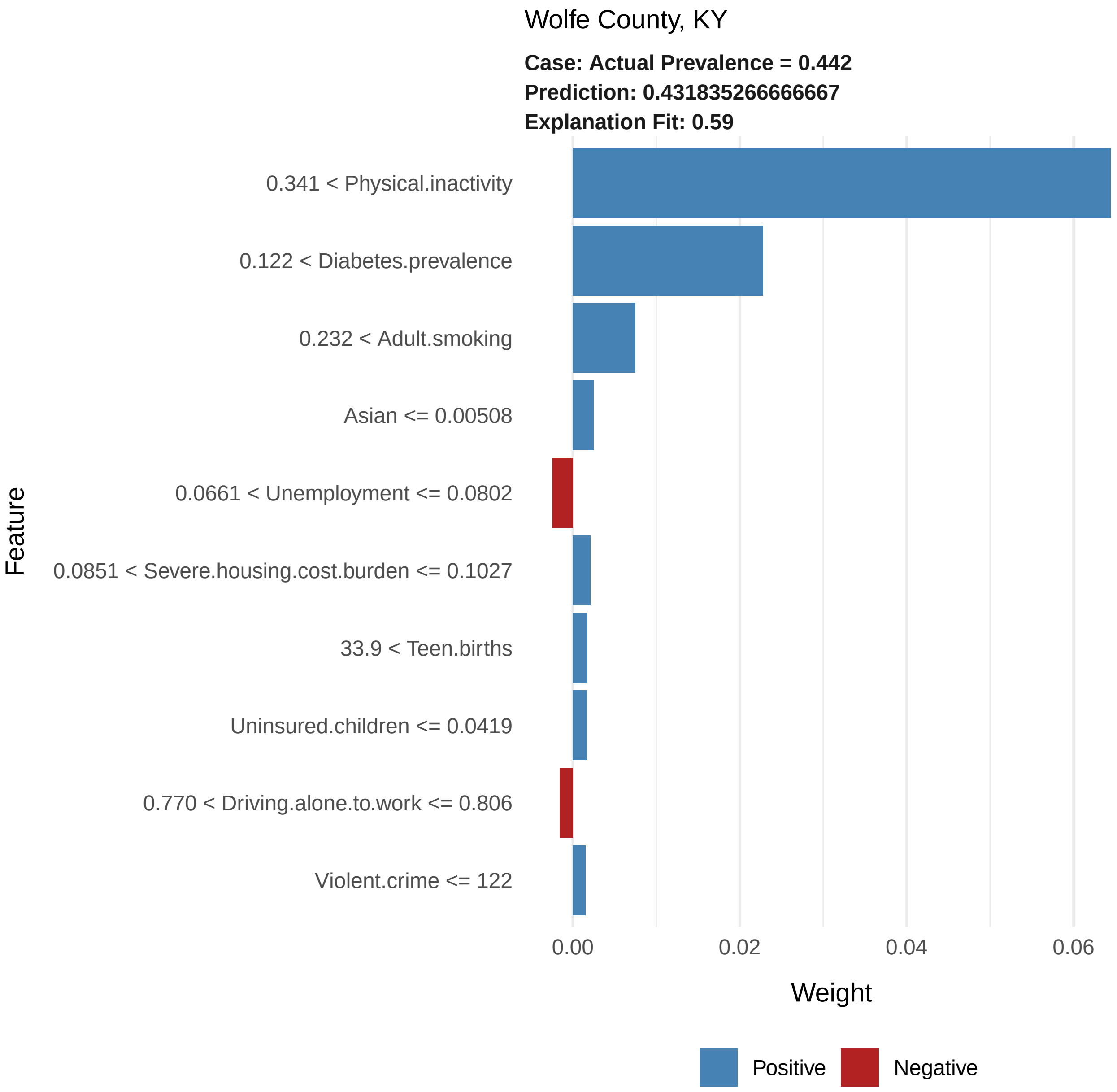

Wolfe County, KY
Case: Actual Prevalence = 0.442
Prediction: 0.431835266666667
Explanation Fit: 0.59
0.341 < Physical.inactivity
0.122 < Diabetes.prevalence
0.232 < Adult.smoking
Asian <= 0.00508
0.0661 < Unemployment <= 0.0802
0.0851 < Severe.housing.cost.burden <= 0.1027
33.9 < Teen.births
Uninsured.children <= 0.0419
0.770 < Driving.alone.to.work <= 0.806
Violent.crime <= 122
Feature
0.00
0.02
0.04
0.06
Weight
Positive
Negative

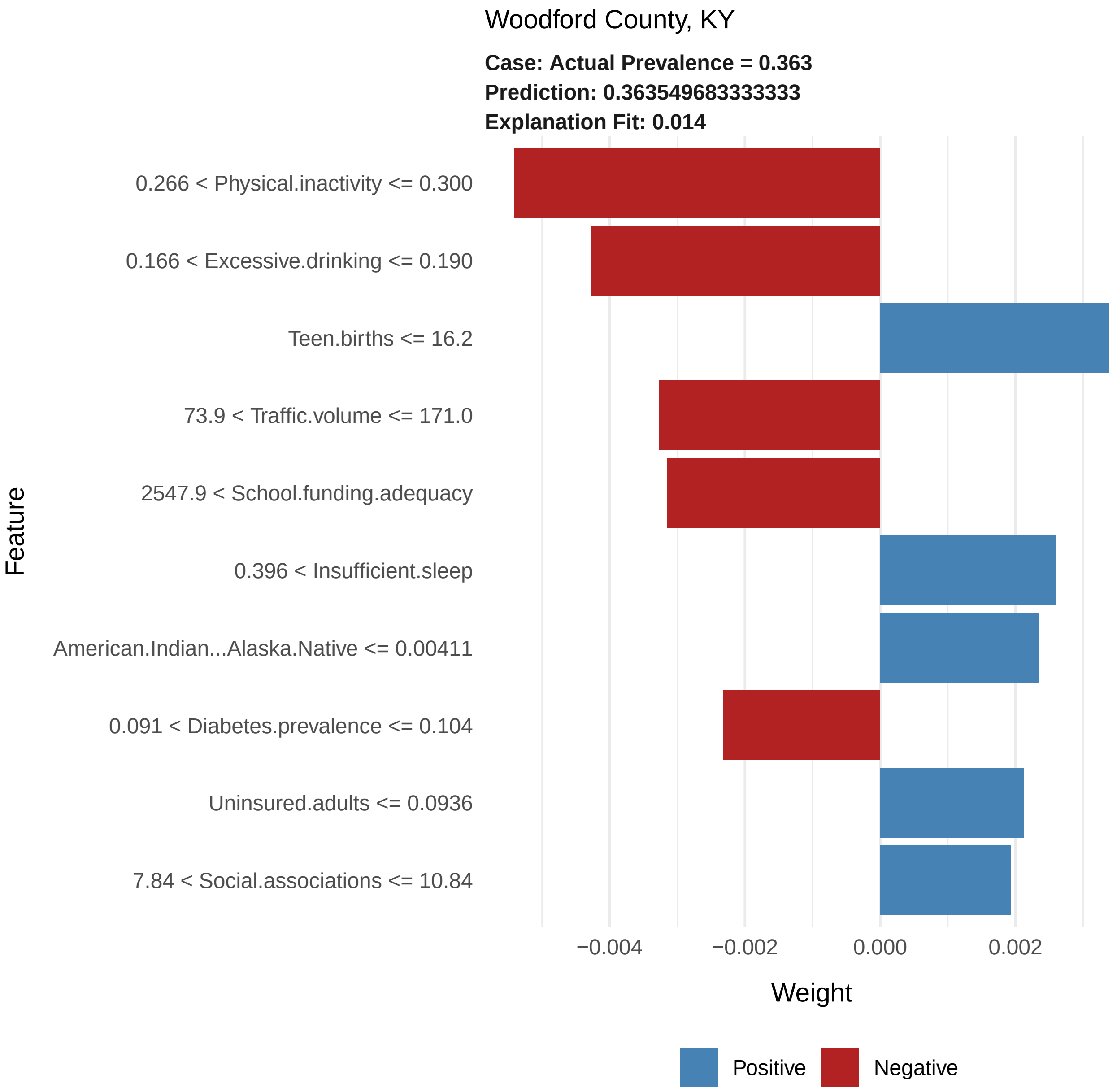
Woodford County, KY
Case: Actual Prevalence = 0.363
Prediction: 0.363549683333333
Explanation Fit: 0.014
0.266 < Physical.inactivity <= 0.300
0.166 < Excessive.drinking <= 0.190
Teen.births <= 16.2
73.9 < Traffic.volume <= 171.0
2547.9 < School.funding.adequacy
0.396 < Insufficient.sleep
American.Indian...Alaska.Native <= 0.00411
0.091 < Diabetes.prevalence <= 0.104
Uninsured.adults <= 0.0936
7.84 < Social.associations <= 10.84
Feature
−0.004
−0.002
0.000
0.002
Weight
Positive
Negative

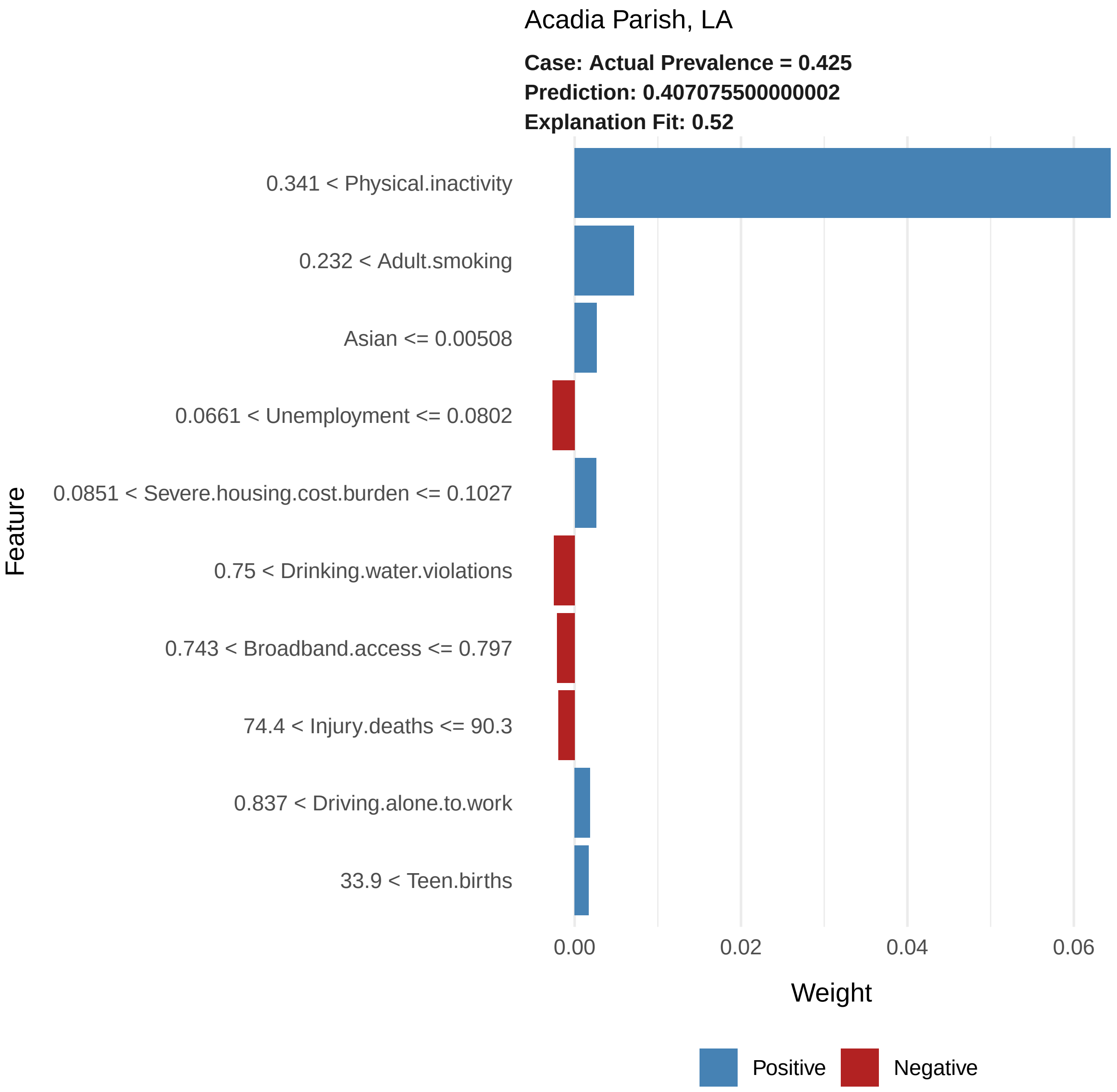

Acadia Parish, LA
Case: Actual Prevalence = 0.425
Prediction: 0.407075500000002
Explanation Fit: 0.52
0.341 < Physical.inactivity
0.232 < Adult.smoking
Asian <= 0.00508
0.0661 < Unemployment <= 0.0802
0.0851 < Severe.housing.cost.burden <= 0.1027
0.75 < Drinking.water.violations
0.743 < Broadband.access <= 0.797
74.4 < Injury.deaths <= 90.3
0.837 < Driving.alone.to.work
33.9 < Teen.births
Feature
0.00
0.02
0.04
0.06
Weight
Positive
Negative

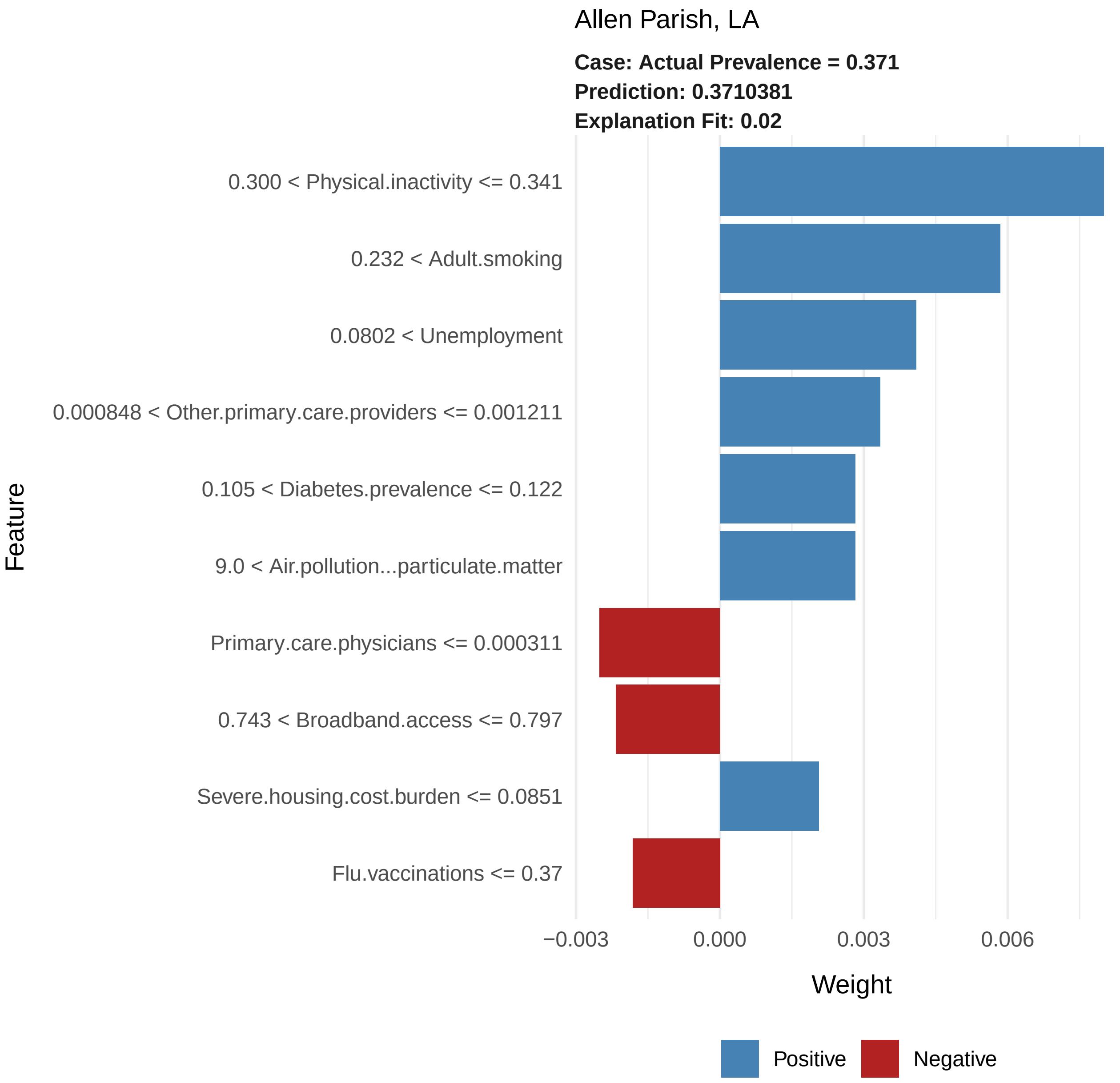

Allen Parish, LA
Case: Actual Prevalence = 0.371
Prediction: 0.3710381
Explanation Fit: 0.02
0.300 < Physical.inactivity <= 0.341
0.232 < Adult.smoking
0.0802 < Unemployment
0.000848 < Other.primary.care.providers <= 0.001211
0.105 < Diabetes.prevalence <= 0.122
9.0 < Air.pollution...particulate.matter
Primary.care.physicians <= 0.000311
0.743 < Broadband.access <= 0.797
Severe.housing.cost.burden <= 0.0851
Flu.vaccinations <= 0.37
Feature
−0.003
0.000
0.003
0.006
Weight
Positive
Negative

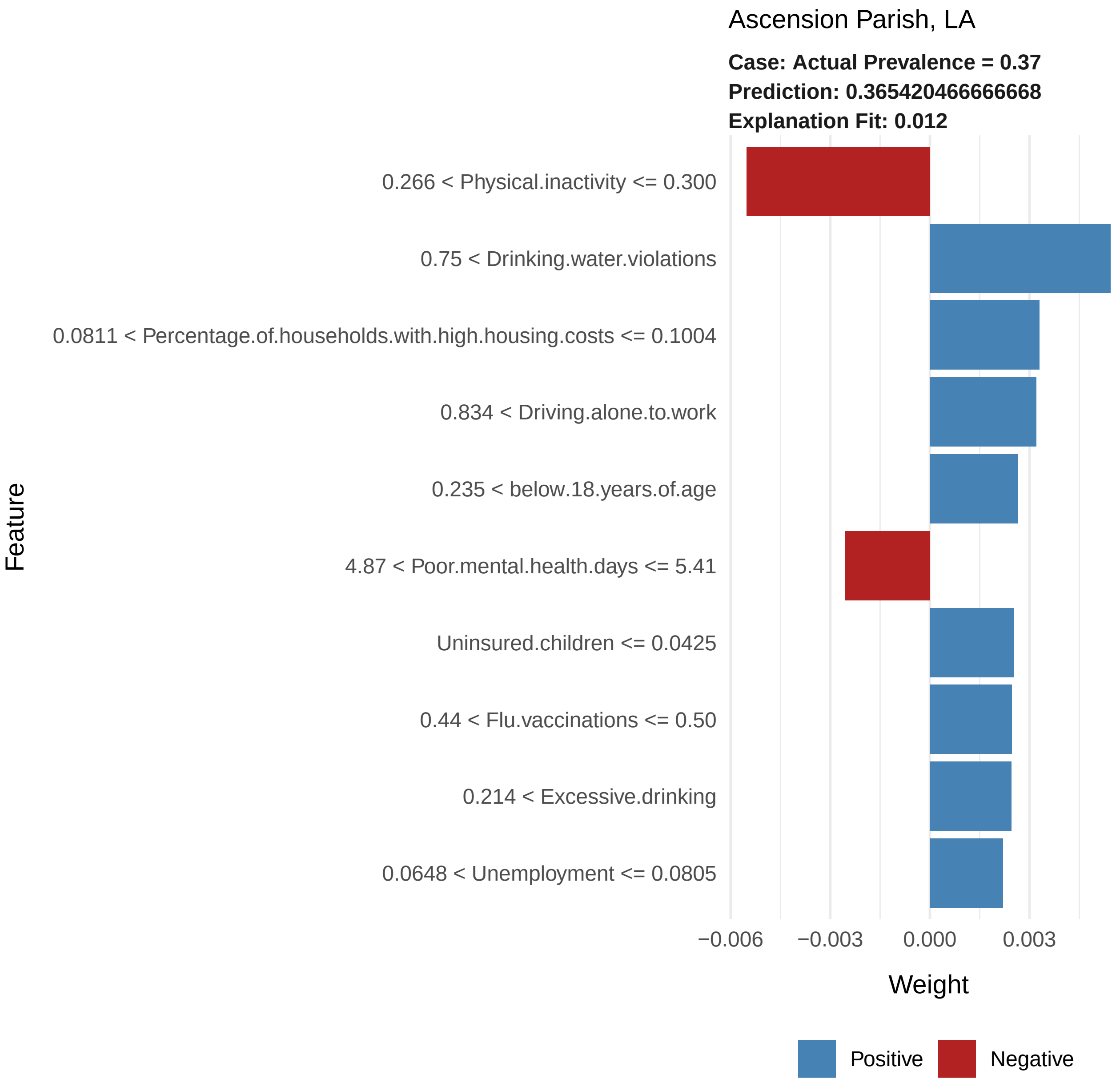

Ascension Parish, LA
Case: Actual Prevalence = 0.37
Prediction: 0.365420466666668
Explanation Fit: 0.012
0.266 < Physical.inactivity <= 0.300
0.75 < Drinking.water.violations
0.0811 < Percentage.of.households.with.high.housing.costs <= 0.1004
0.834 < Driving.alone.to.work
0.235 < below.18.years.of.age
4.87 < Poor.mental.health.days <= 5.41
Uninsured.children <= 0.0425
0.44 < Flu.vaccinations <= 0.50
0.214 < Excessive.drinking
0.0648 < Unemployment <= 0.0805
Feature
−0.006
−0.003
0.000
0.003
Weight
Positive
Negative

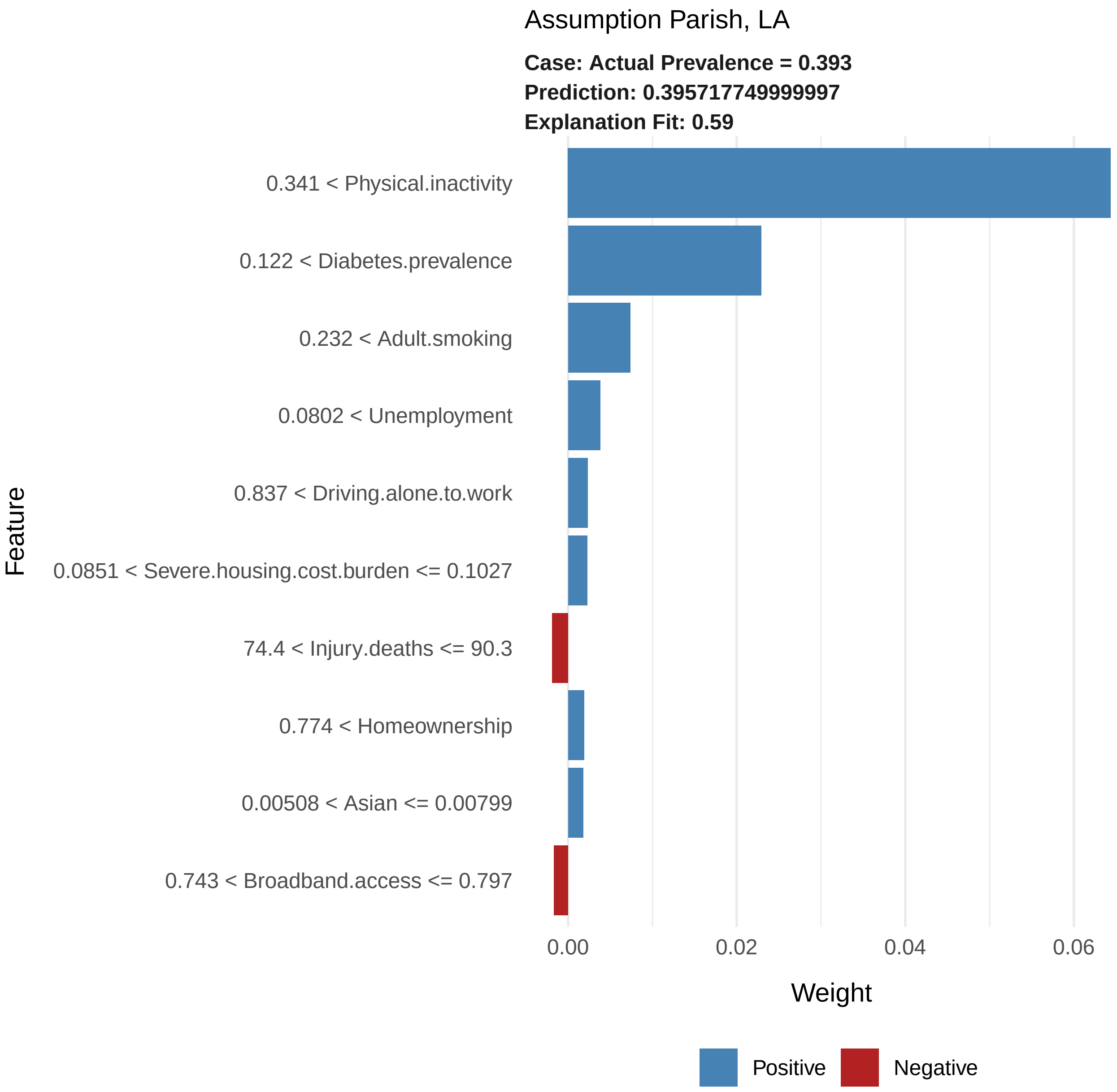

Assumption Parish, LA
Case: Actual Prevalence = 0.393
Prediction: 0.395717749999997
Explanation Fit: 0.59
0.341 < Physical.inactivity
0.122 < Diabetes.prevalence
0.232 < Adult.smoking
0.0802 < Unemployment
0.837 < Driving.alone.to.work
0.0851 < Severe.housing.cost.burden <= 0.1027
74.4 < Injury.deaths <= 90.3
0.774 < Homeownership
0.00508 < Asian <= 0.00799
0.743 < Broadband.access <= 0.797
Feature
0.00
0.02
0.04
0.06
Weight
Positive
Negative

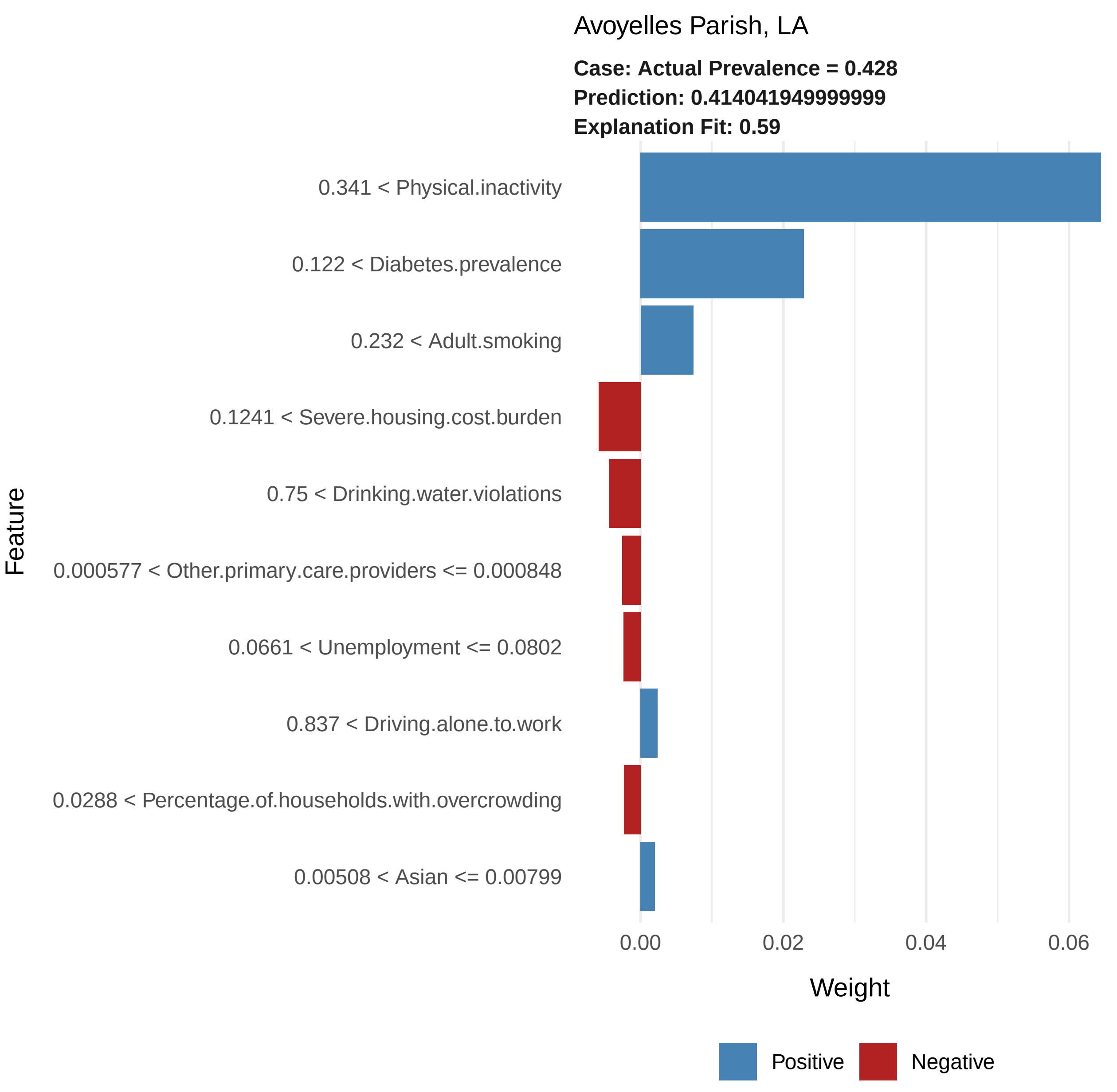

Avoyelles Parish, LA
Case: Actual Prevalence = 0.428
Prediction: 0.414041949999999
Explanation Fit: 0.59
0.341 < Physical.inactivity
0.122 < Diabetes.prevalence
0.232 < Adult.smoking
0.1241 < Severe.housing.cost.burden
0.75 < Drinking.water.violations
0.000577 < Other.primary.care.providers <= 0.000848
0.0661 < Unemployment <= 0.0802
0.837 < Driving.alone.to.work
0.0288 < Percentage.of.households.with.overcrowding
0.00508 < Asian <= 0.00799
Feature
0.00
0.02
0.04
0.06
Weight
Positive
Negative

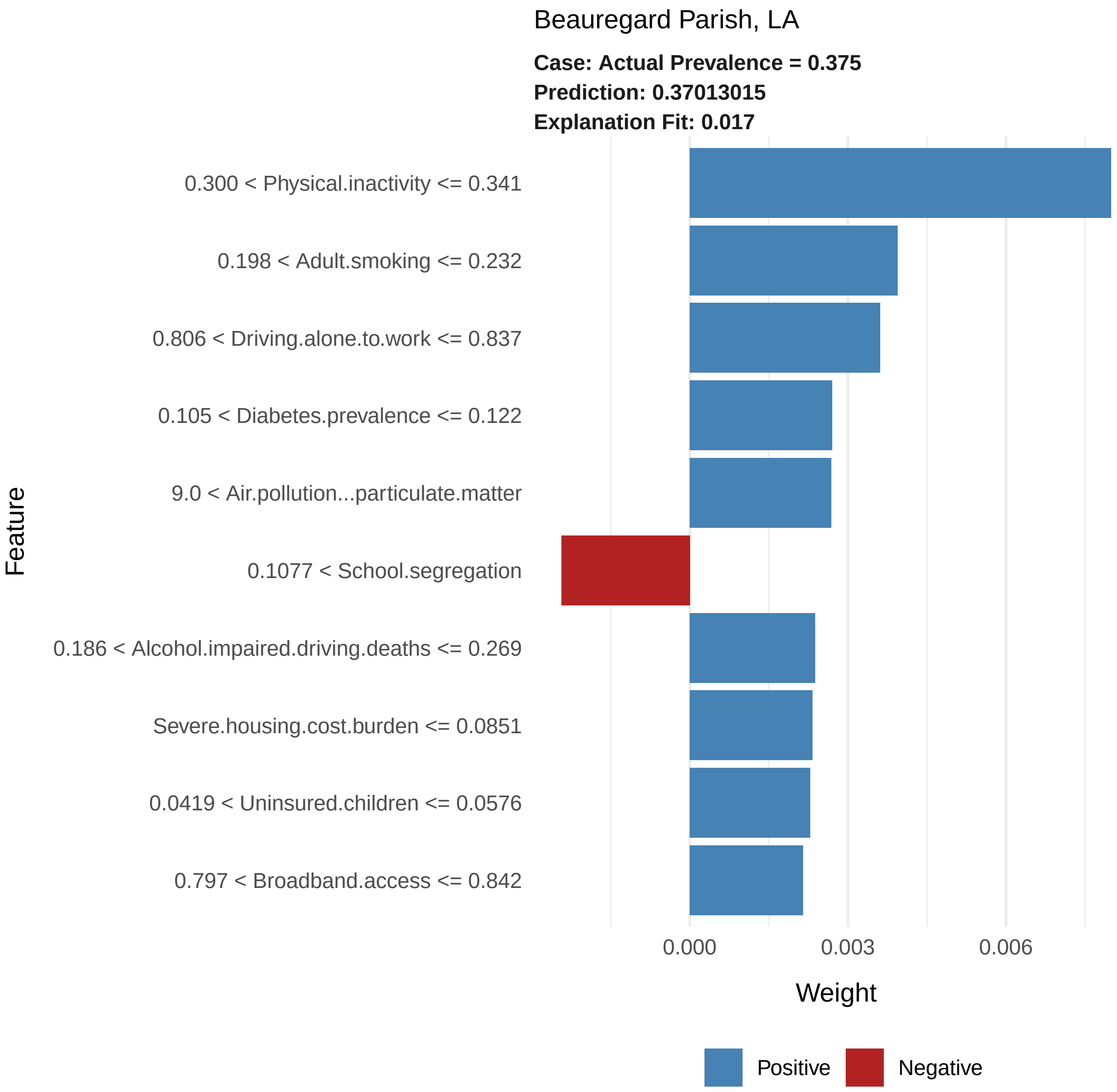

Beauregard Parish, LA
Case: Actual Prevalence = 0.375
Prediction: 0.37013015
Explanation Fit: 0.017
0.300 < Physical.inactivity <= 0.341
0.198 < Adult.smoking <= 0.232
0.806 < Driving.alone.to.work <= 0.837
0.105 < Diabetes.prevalence <= 0.122
9.0 < Air.pollution...particulate.matter
0.1077 < School.segregation
0.186 < Alcohol.impaired.driving.deaths <= 0.269
Severe.housing.cost.burden <= 0.0851
0.0419 < Uninsured.children <= 0.0576
0.797 < Broadband.access <= 0.842
Feature
0.000
0.003
0.006
Weight
Positive
Negative

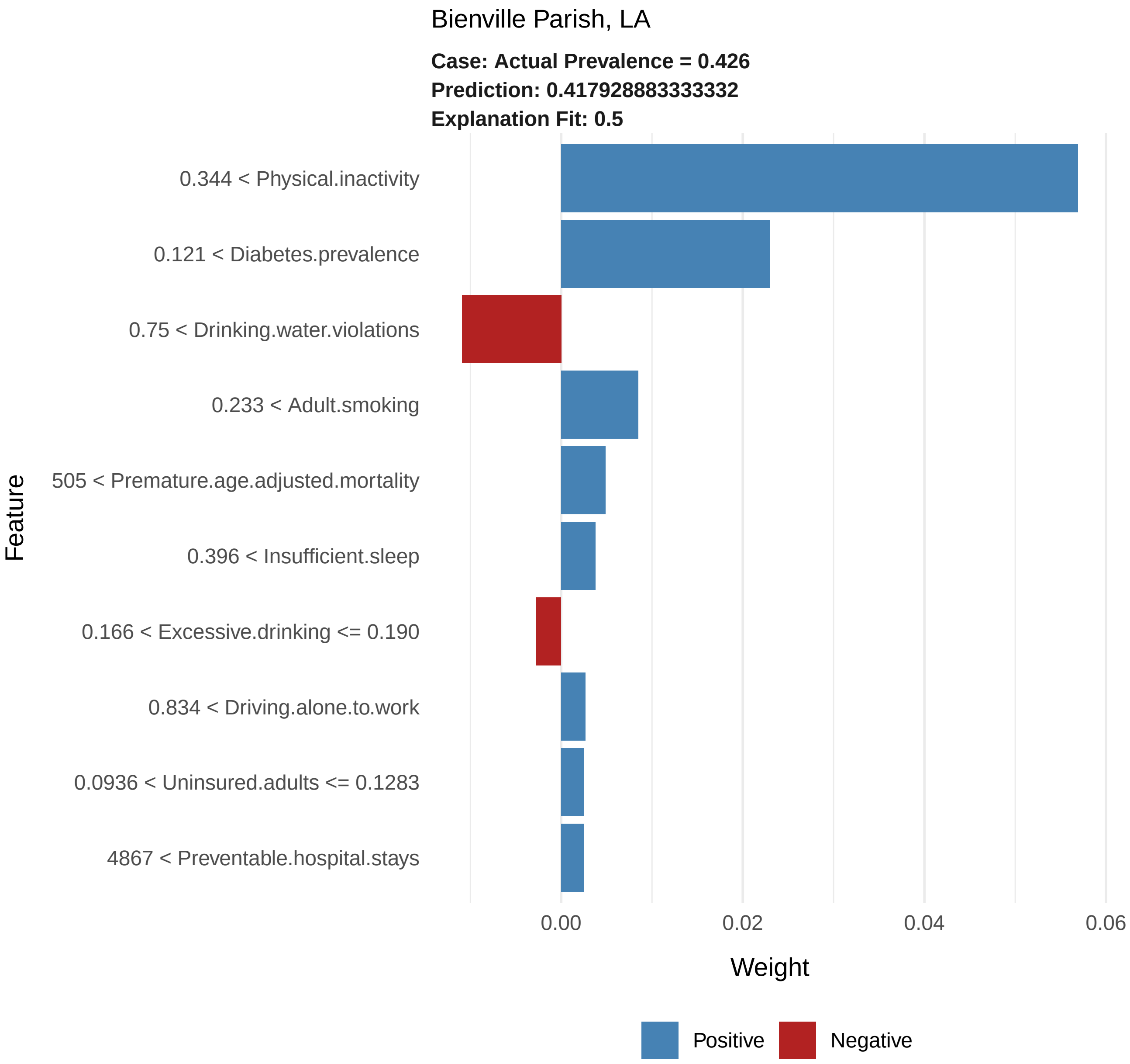

Bienville Parish, LA
Case: Actual Prevalence = 0.426
Prediction: 0.417928883333332
Explanation Fit: 0.5
0.344 < Physical.inactivity
0.121 < Diabetes.prevalence
0.75 < Drinking.water.violations
0.233 < Adult.smoking
505 < Premature.age.adjusted.mortality
0.396 < Insufficient.sleep
0.166 < Excessive.drinking <= 0.190
0.834 < Driving.alone.to.work
0.0936 < Uninsured.adults <= 0.1283
4867 < Preventable.hospital.stays
Feature
0.00
0.02
0.04
0.06
Weight
Positive
Negative

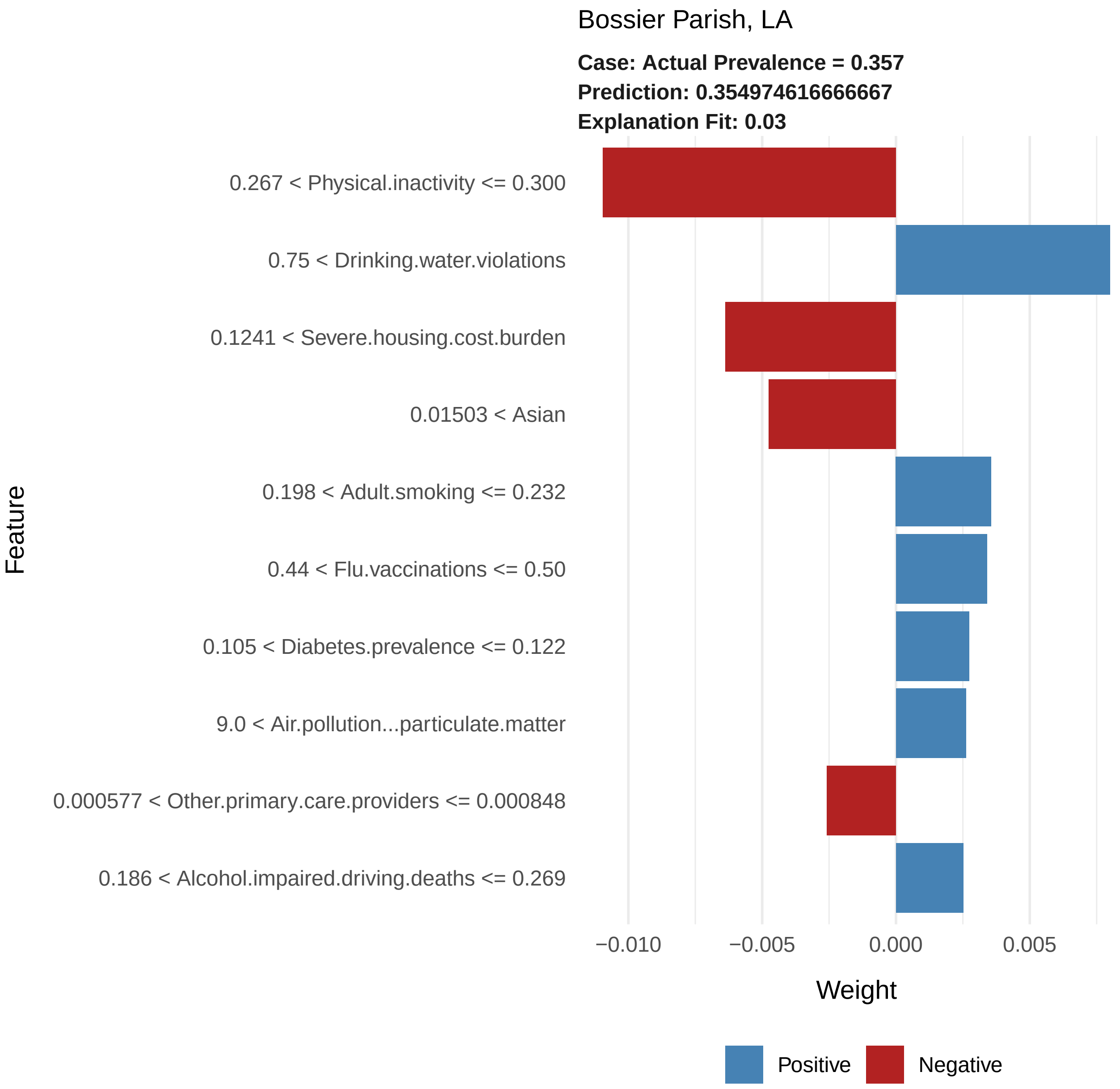
Bossier Parish, LA
Case: Actual Prevalence = 0.357
Prediction: 0.354974616666667
Explanation Fit: 0.03
0.267 < Physical.inactivity <= 0.300
0.75 < Drinking.water.violations
0.1241 < Severe.housing.cost.burden
0.01503 < Asian
0.198 < Adult.smoking <= 0.232
0.44 < Flu.vaccinations <= 0.50
0.105 < Diabetes.prevalence <= 0.122
9.0 < Air.pollution...particulate.matter
0.000577 < Other.primary.care.providers <= 0.000848
0.186 < Alcohol.impaired.driving.deaths <= 0.269
Feature
−0.010
−0.005
0.000
0.005
Weight
Positive
Negative

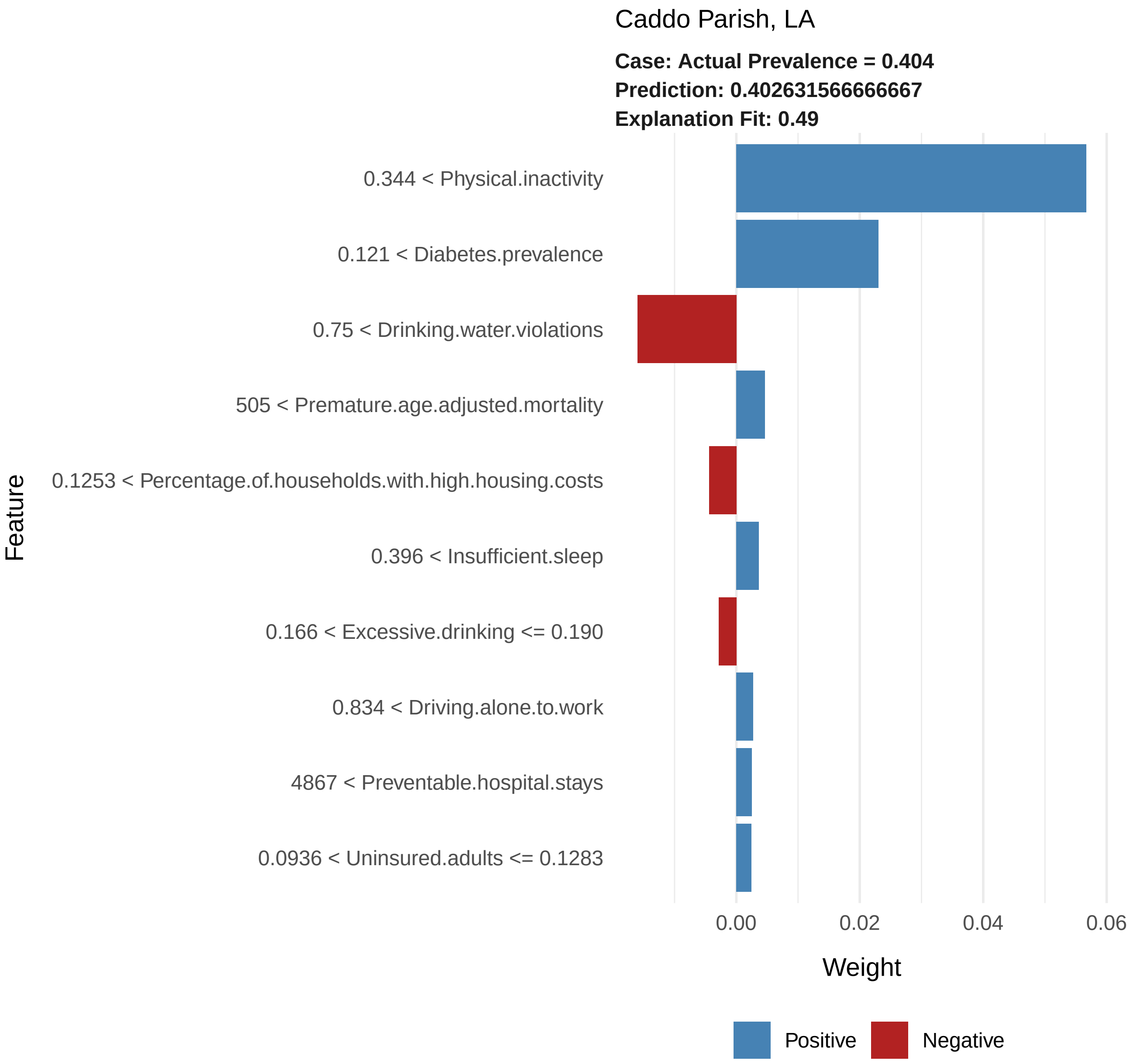

Caddo Parish, LA
Case: Actual Prevalence = 0.404
Prediction: 0.402631566666667
Explanation Fit: 0.49
0.344 < Physical.inactivity
0.121 < Diabetes.prevalence
0.75 < Drinking.water.violations
505 < Premature.age.adjusted.mortality
0.1253 < Percentage.of.households.with.high.housing.costs
0.396 < Insufficient.sleep
0.166 < Excessive.drinking <= 0.190
0.834 < Driving.alone.to.work
4867 < Preventable.hospital.stays
0.0936 < Uninsured.adults <= 0.1283
Feature
0.00
0.02
0.04
0.06
Weight
Positive
Negative

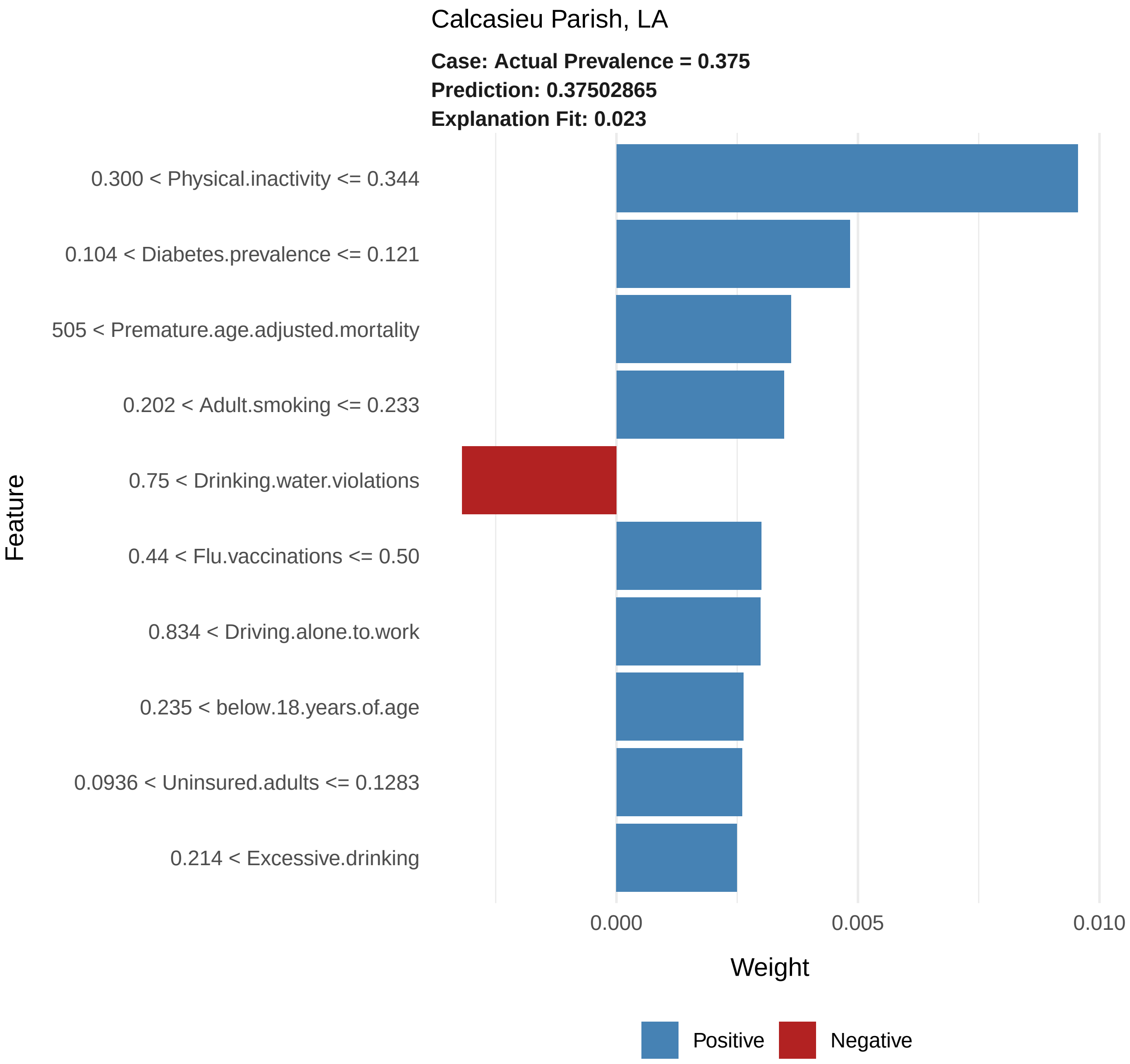

Calcasieu Parish, LA
Case: Actual Prevalence = 0.375
Prediction: 0.37502865
Explanation Fit: 0.023
0.300 < Physical.inactivity <= 0.344
0.104 < Diabetes.prevalence <= 0.121
505 < Premature.age.adjusted.mortality
0.202 < Adult.smoking <= 0.233
0.75 < Drinking.water.violations
0.44 < Flu.vaccinations <= 0.50
0.834 < Driving.alone.to.work
0.235 < below.18.years.of.age
0.0936 < Uninsured.adults <= 0.1283
0.214 < Excessive.drinking
Feature
0.000
0.005
0.010
Weight
Positive
Negative

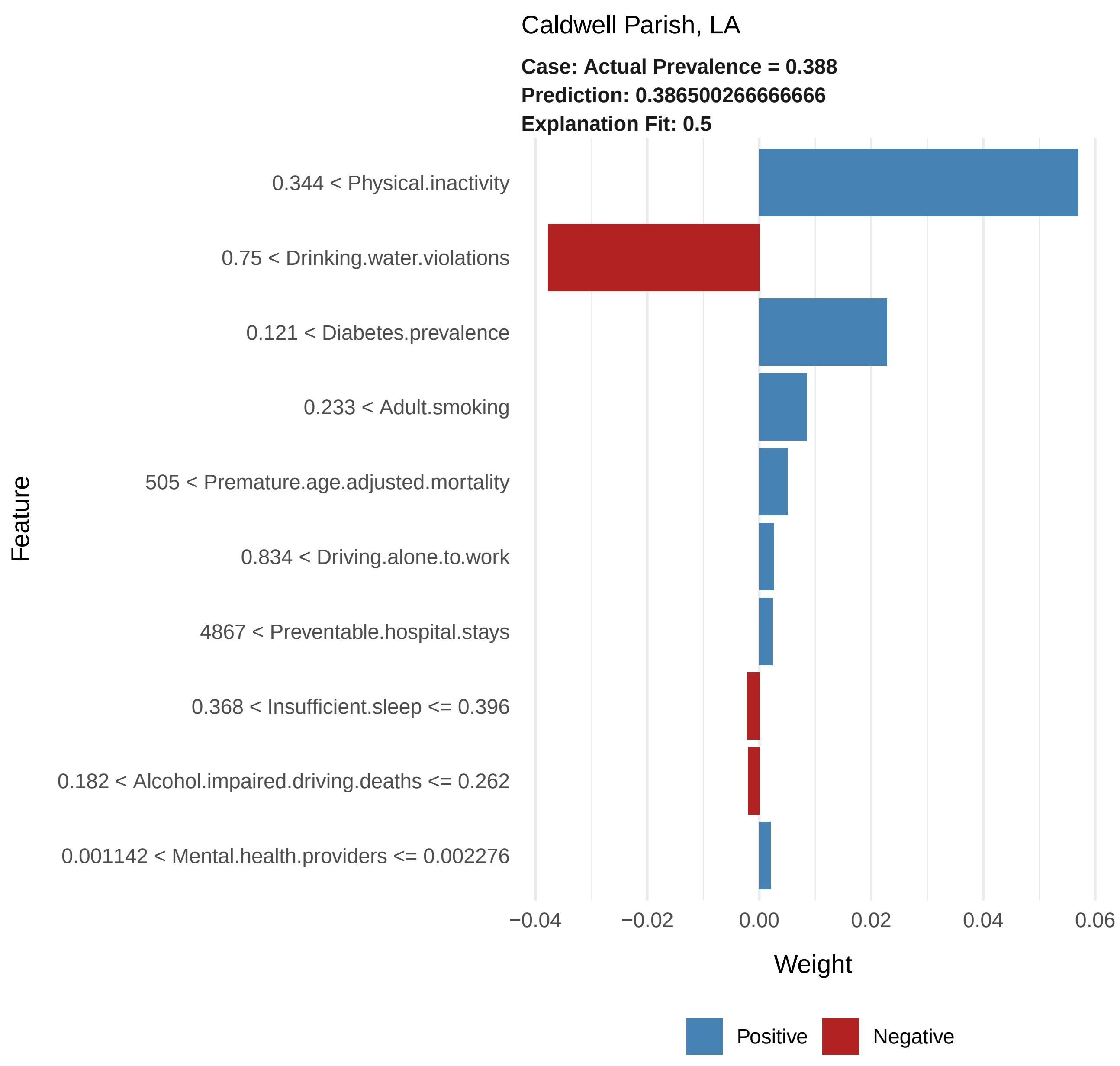

Caldwell Parish, LA
Case: Actual Prevalence = 0.388
Prediction: 0.386500266666666
Explanation Fit: 0.5
0.344 < Physical.inactivity
0.75 < Drinking.water.violations
0.121 < Diabetes.prevalence
0.233 < Adult.smoking
505 < Premature.age.adjusted.mortality
0.834 < Driving.alone.to.work
4867 < Preventable.hospital.stays
0.368 < Insufficient.sleep <= 0.396
0.182 < Alcohol.impaired.driving.deaths <= 0.262
0.001142 < Mental.health.providers <= 0.002276
Feature
−0.04
−0.02
0.00
0.02
0.04
0.06
Weight
Positive
Negative

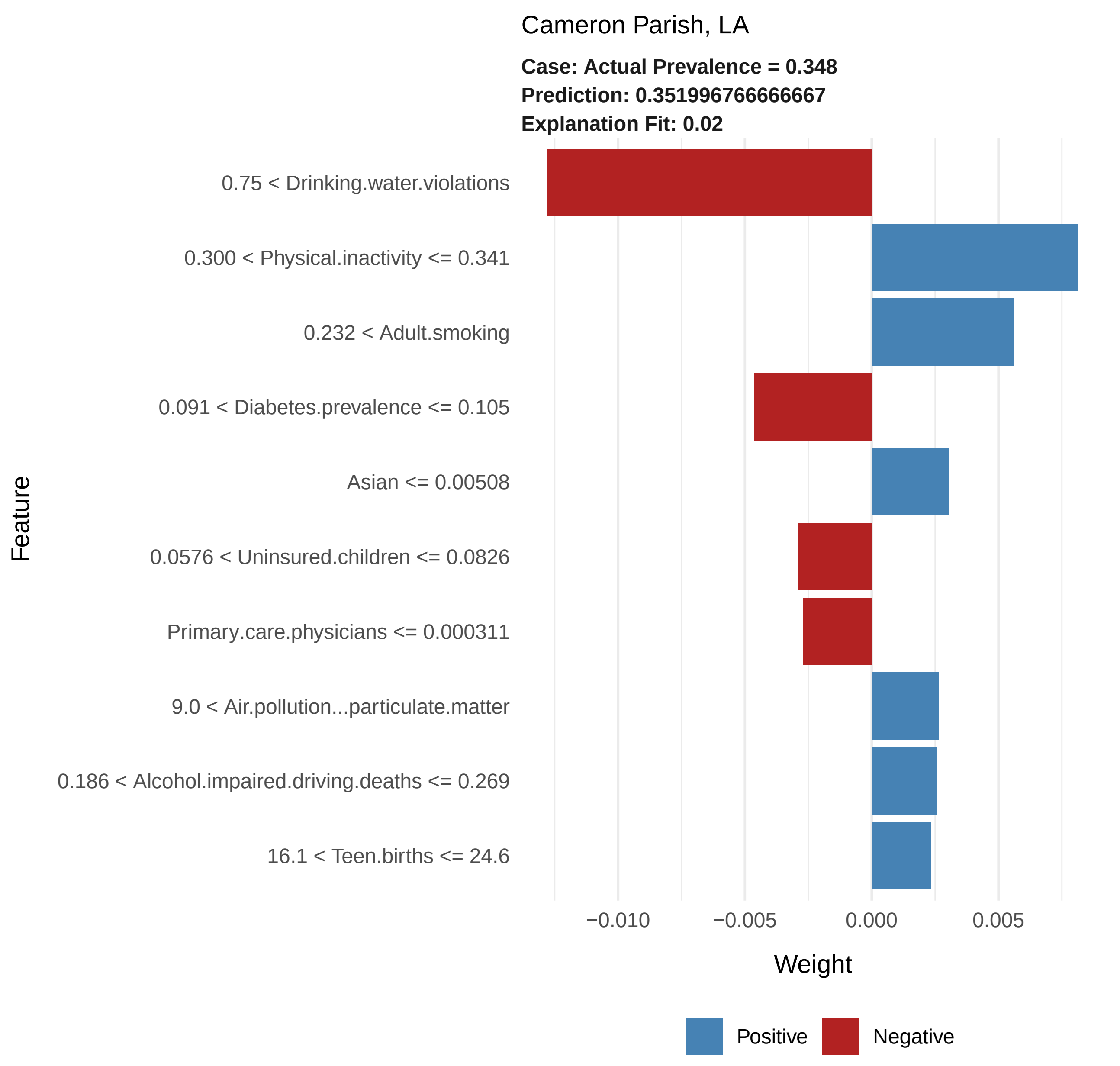
Cameron Parish, LA
Case: Actual Prevalence = 0.348
Prediction: 0.351996766666667
Explanation Fit: 0.02
0.75 < Drinking.water.violations
0.300 < Physical.inactivity <= 0.341
0.232 < Adult.smoking
0.091 < Diabetes.prevalence <= 0.105
Asian <= 0.00508
0.0576 < Uninsured.children <= 0.0826
Primary.care.physicians <= 0.000311
9.0 < Air.pollution...particulate.matter
0.186 < Alcohol.impaired.driving.deaths <= 0.269
16.1 < Teen.births <= 24.6
Feature
−0.010
−0.005
0.000
0.005
Weight
Positive
Negative

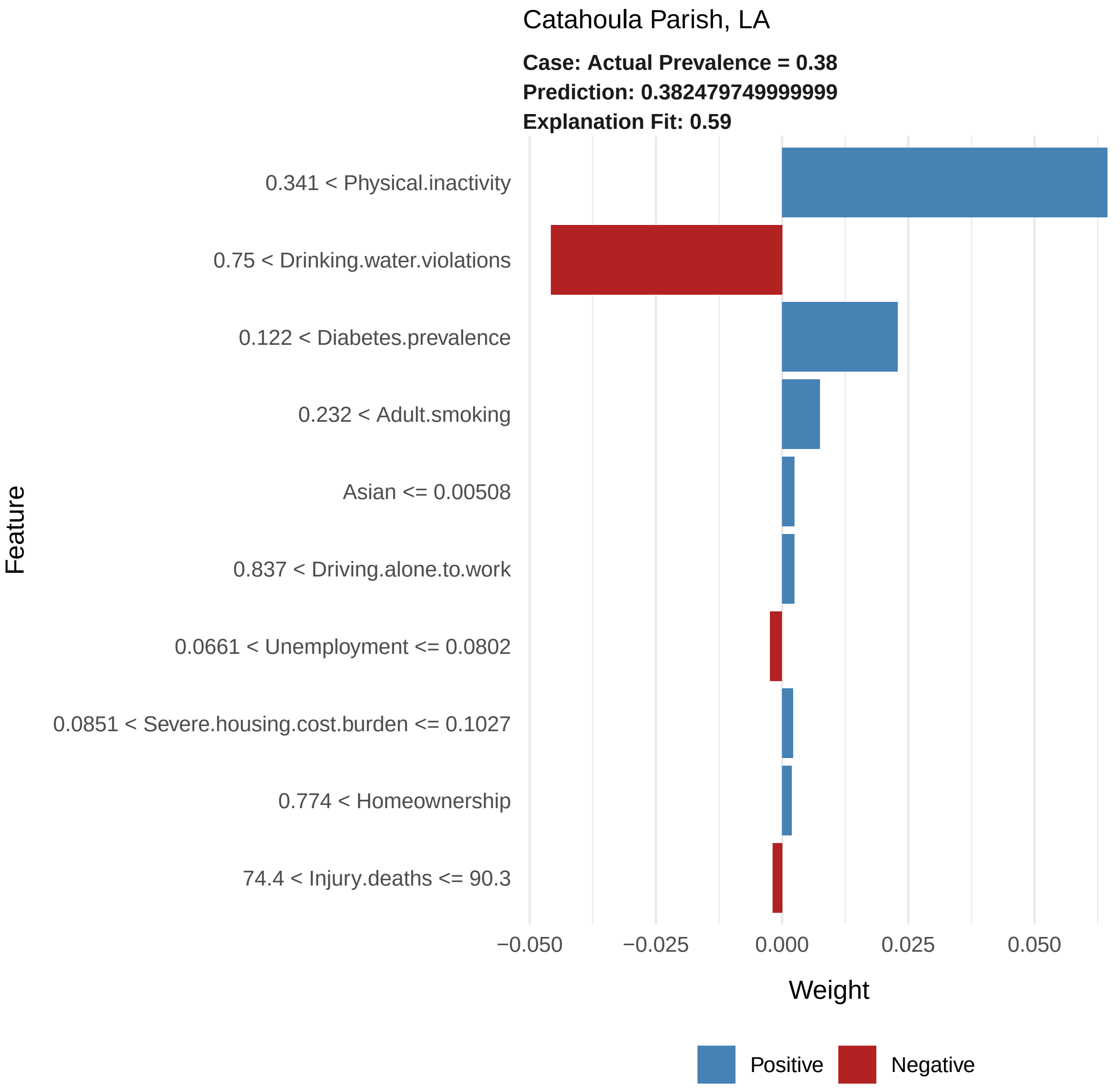
Catahoula Parish, LA
Case: Actual Prevalence = 0.38
Prediction: 0.382479749999999
Explanation Fit: 0.59
0.341 < Physical.inactivity
0.75 < Drinking.water.violations
0.122 < Diabetes.prevalence
0.232 < Adult.smoking
Asian <= 0.00508
0.837 < Driving.alone.to.work
0.0661 < Unemployment <= 0.0802
0.0851 < Severe.housing.cost.burden <= 0.1027
0.774 < Homeownership
74.4 < Injury.deaths <= 90.3
Feature
−0.050
−0.025
0.000
0.025
0.050
Weight
Positive
Negative

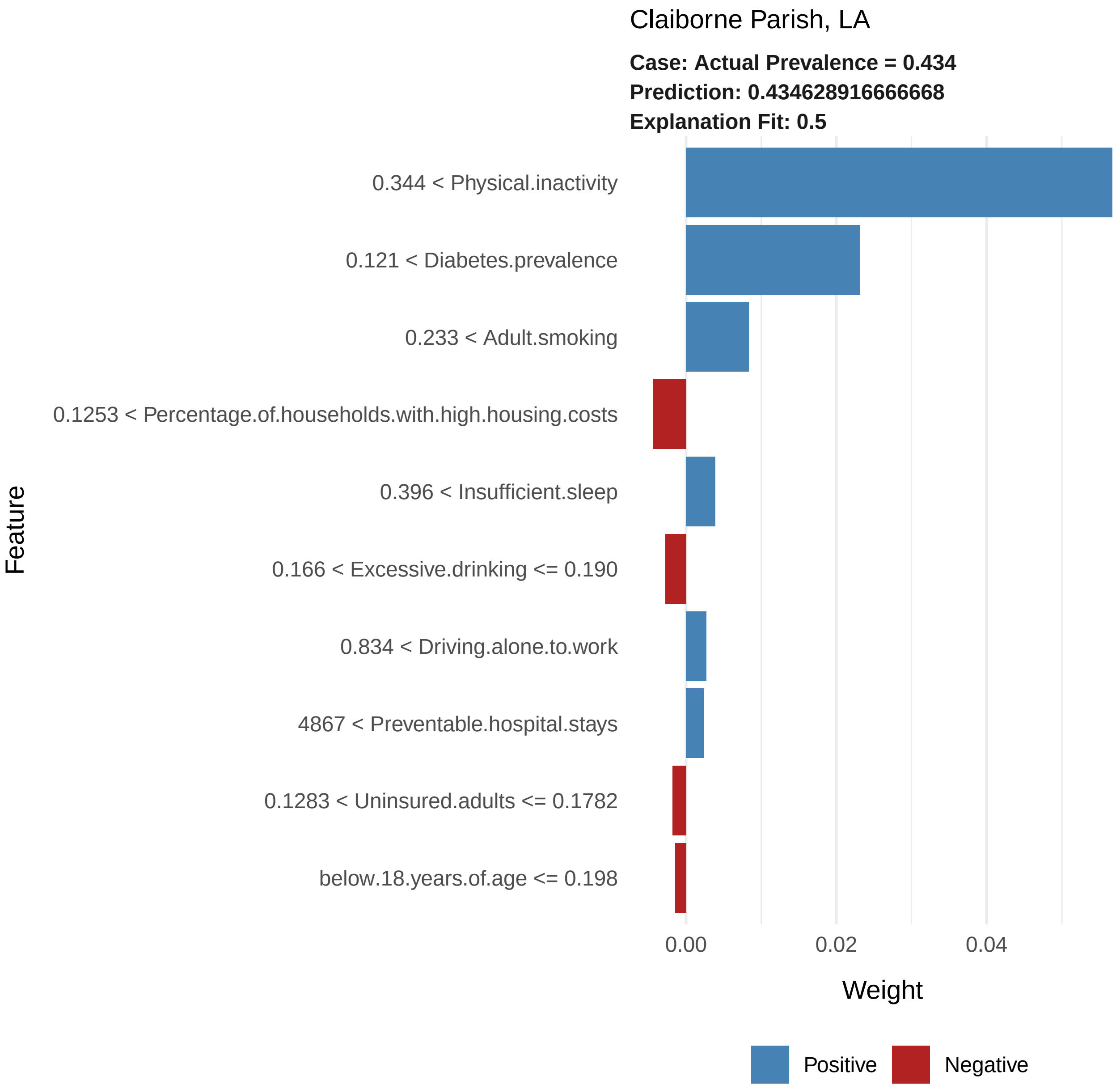

Claiborne Parish, LA
Case: Actual Prevalence = 0.434
Prediction: 0.434628916666668
Explanation Fit: 0.5
0.344 < Physical.inactivity
0.121 < Diabetes.prevalence
0.233 < Adult.smoking
0.1253 < Percentage.of.households.with.high.housing.costs
0.396 < Insufficient.sleep
0.166 < Excessive.drinking <= 0.190
0.834 < Driving.alone.to.work
4867 < Preventable.hospital.stays
0.1283 < Uninsured.adults <= 0.1782
below.18.years.of.age <= 0.198
Feature
0.00
0.02
0.04
Weight
Positive
Negative

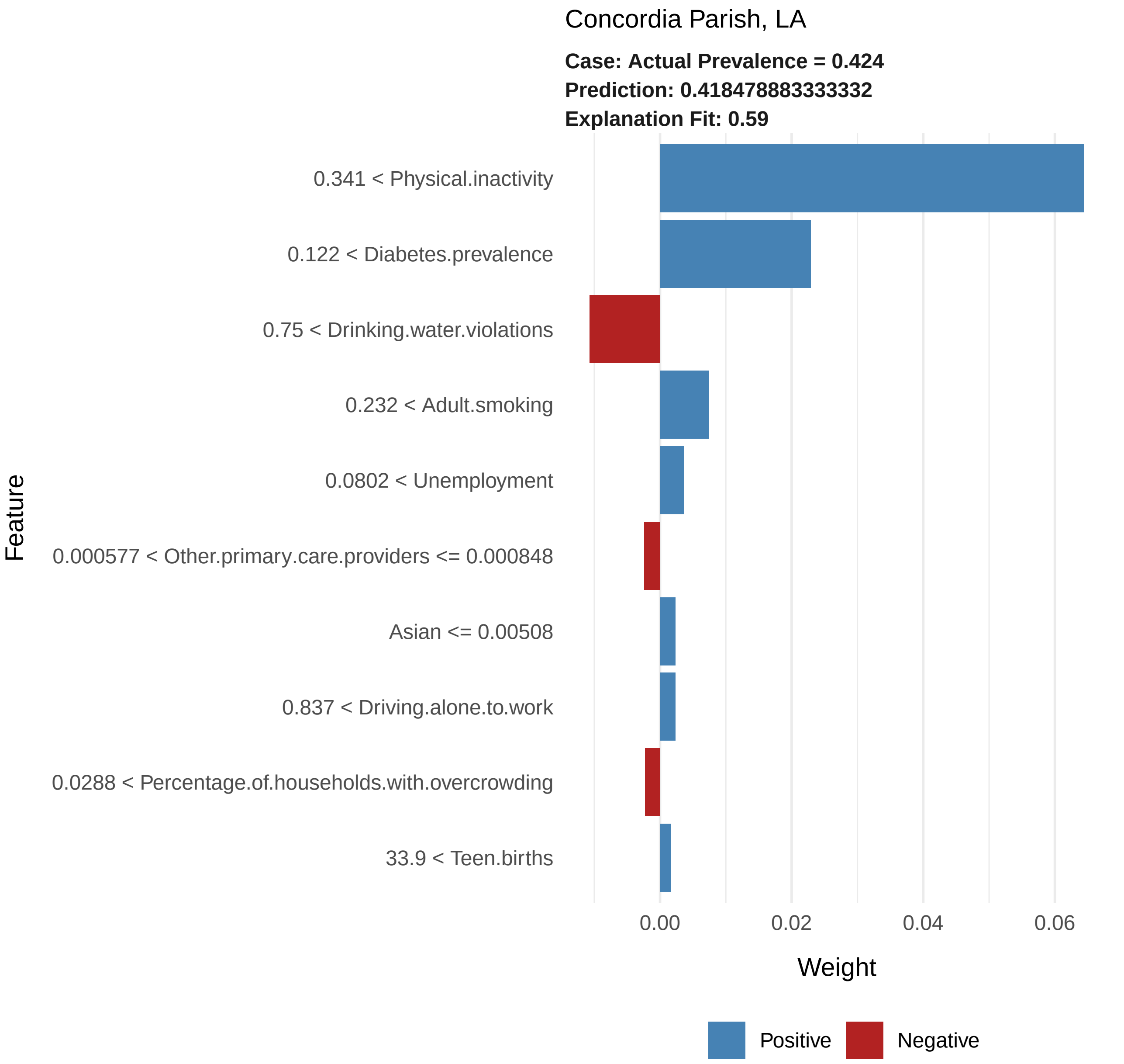

Concordia Parish, LA
Case: Actual Prevalence = 0.424
Prediction: 0.418478883333332
Explanation Fit: 0.59
0.341 < Physical.inactivity
0.122 < Diabetes.prevalence
0.75 < Drinking.water.violations
0.232 < Adult.smoking
0.0802 < Unemployment
0.000577 < Other.primary.care.providers <= 0.000848
Asian <= 0.00508
0.837 < Driving.alone.to.work
0.0288 < Percentage.of.households.with.overcrowding
33.9 < Teen.births
Feature
0.00
0.02
0.04
0.06
Weight
Positive
Negative

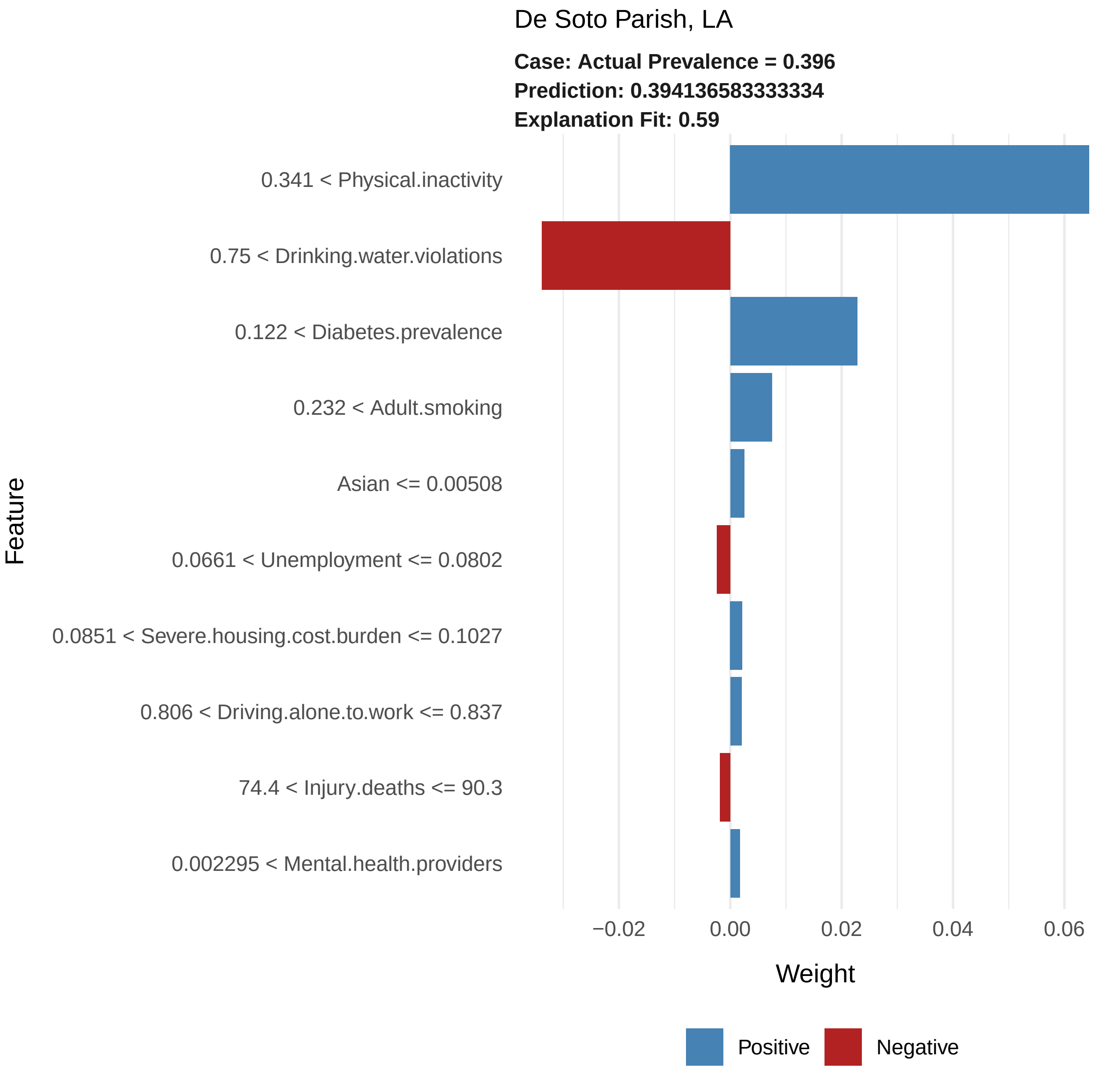

De Soto Parish, LA
Case: Actual Prevalence = 0.396
Prediction: 0.394136583333334
Explanation Fit: 0.59
0.341 < Physical.inactivity
0.75 < Drinking.water.violations
0.122 < Diabetes.prevalence
0.232 < Adult.smoking
Asian <= 0.00508
0.0661 < Unemployment <= 0.0802
0.0851 < Severe.housing.cost.burden <= 0.1027
0.806 < Driving.alone.to.work <= 0.837
74.4 < Injury.deaths <= 90.3
0.002295 < Mental.health.providers
Feature
−0.02
0.00
0.02
0.04
0.06
Weight
Positive
Negative

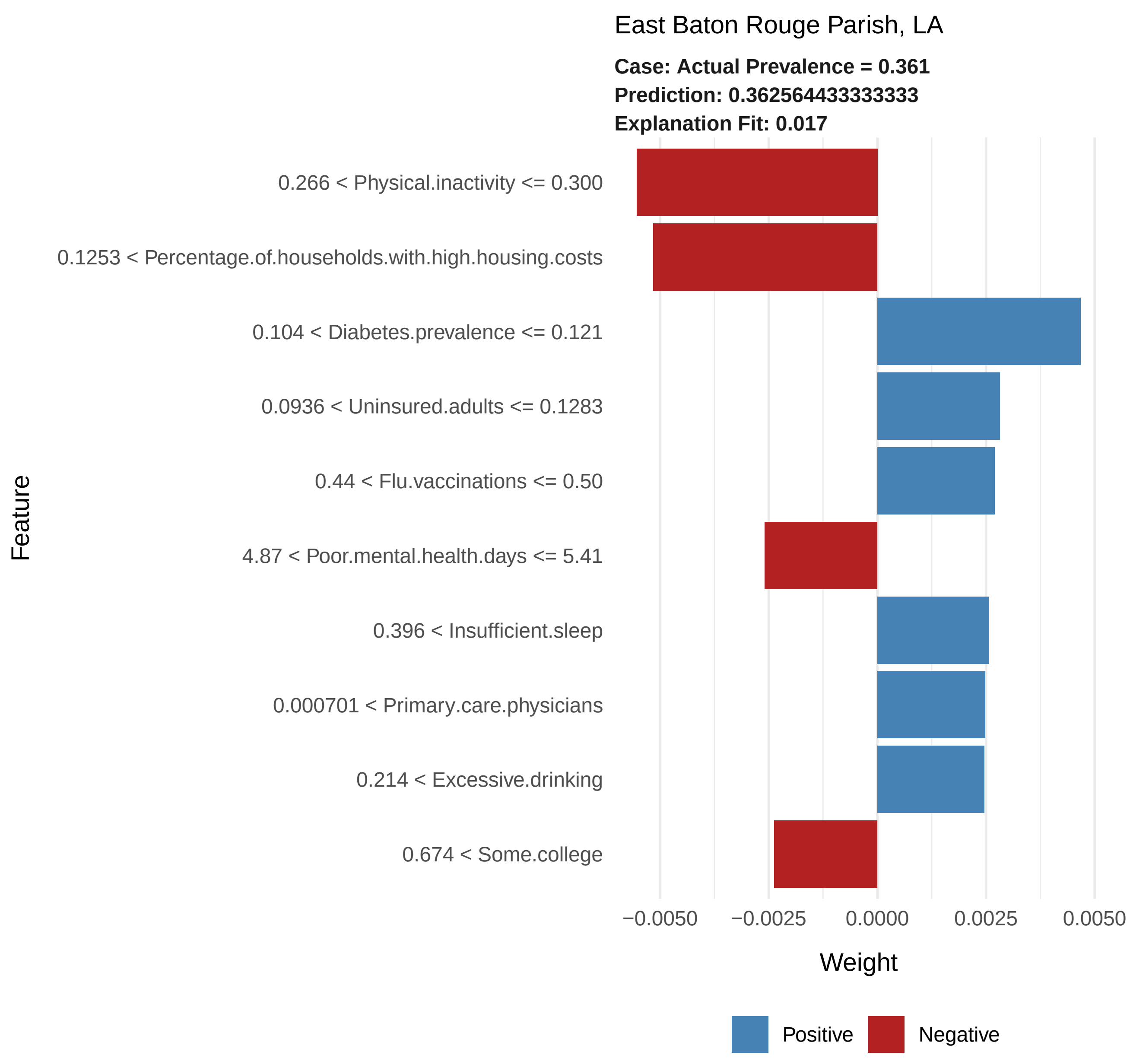

East Baton Rouge Parish, LA
Case: Actual Prevalence = 0.361
Prediction: 0.362564433333333
Explanation Fit: 0.017
0.266 < Physical.inactivity <= 0.300
0.1253 < Percentage.of.households.with.high.housing.costs
0.104 < Diabetes.prevalence <= 0.121
0.0936 < Uninsured.adults <= 0.1283
0.44 < Flu.vaccinations <= 0.50
4.87 < Poor.mental.health.days <= 5.41
0.396 < Insufficient.sleep
0.000701 < Primary.care.physicians
0.214 < Excessive.drinking
0.674 < Some.college
Feature
−0.0050
−0.0025
0.0000
0.0025
0.0050
Weight
Positive
Negative

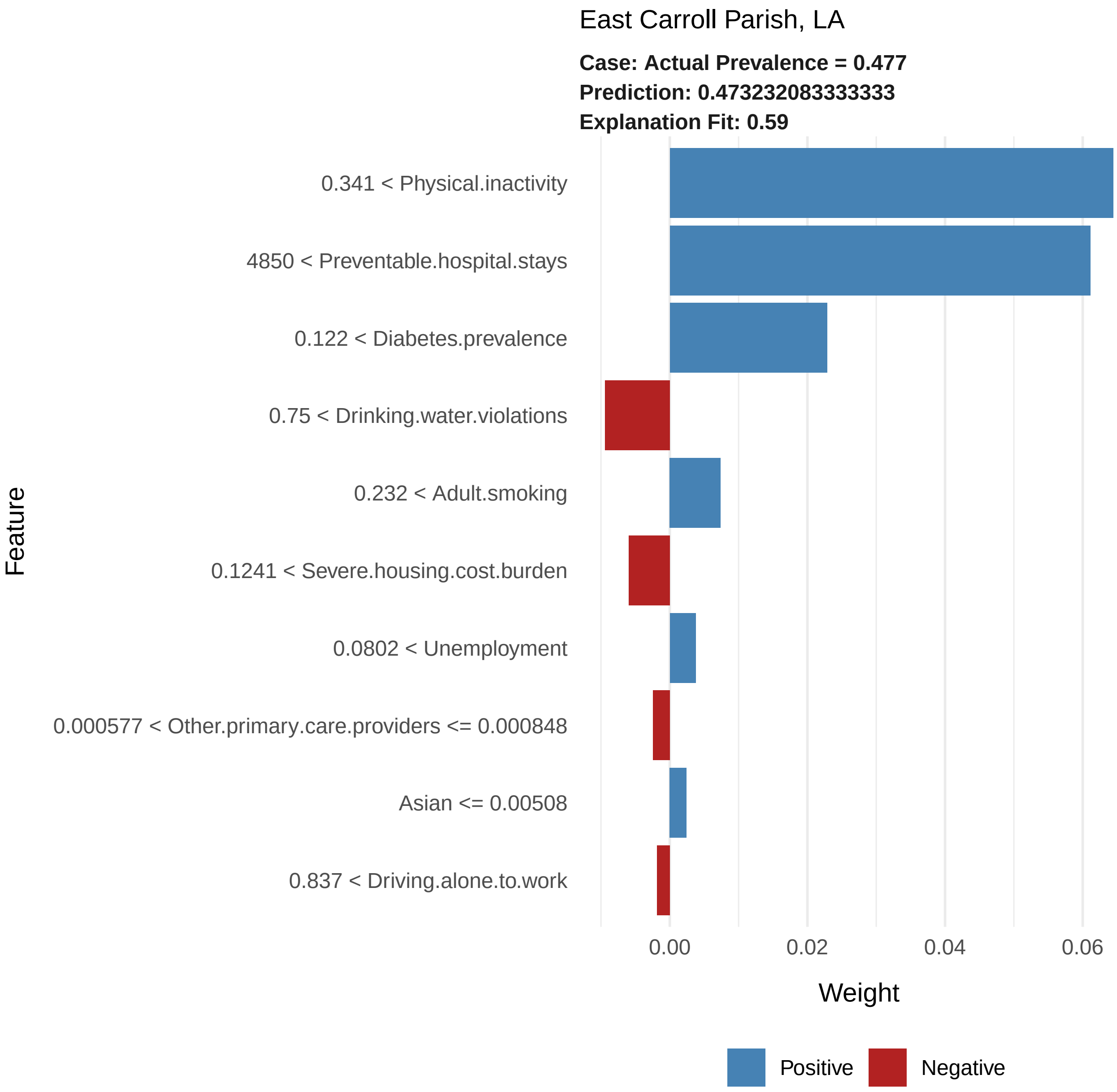

East Carroll Parish, LA
Case: Actual Prevalence = 0.477
Prediction: 0.473232083333333
Explanation Fit: 0.59
0.341 < Physical.inactivity
4850 < Preventable.hospital.stays
0.122 < Diabetes.prevalence
0.75 < Drinking.water.violations
0.232 < Adult.smoking
0.1241 < Severe.housing.cost.burden
0.0802 < Unemployment
0.000577 < Other.primary.care.providers <= 0.000848
Asian <= 0.00508
0.837 < Driving.alone.to.work
Feature
0.00
0.02
0.04
0.06
Weight
Positive
Negative

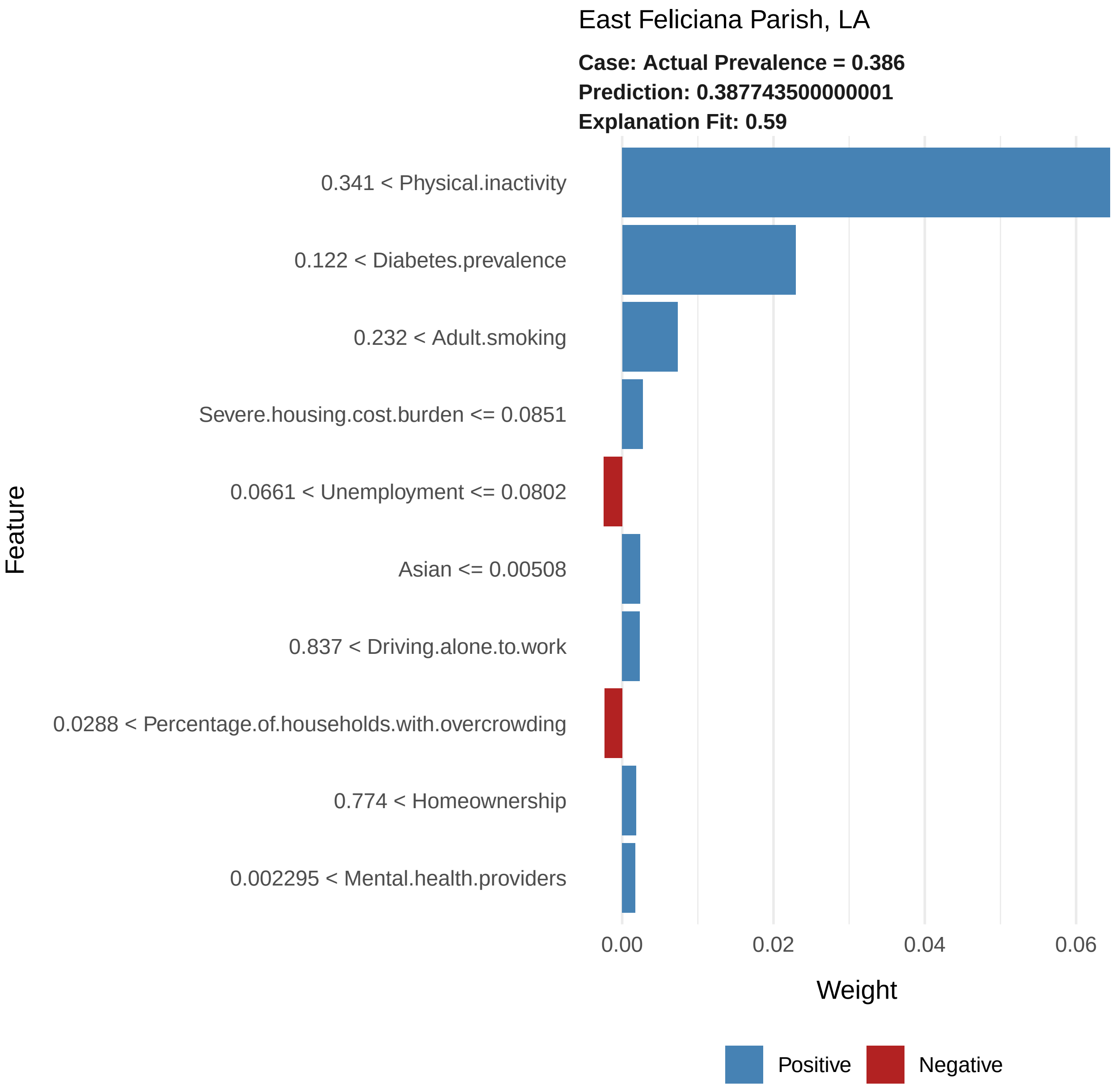
East Feliciana Parish, LA
Case: Actual Prevalence = 0.386
Prediction: 0.387743500000001
Explanation Fit: 0.59
0.341 < Physical.inactivity
0.122 < Diabetes.prevalence
0.232 < Adult.smoking
Severe.housing.cost.burden <= 0.0851
0.0661 < Unemployment <= 0.0802
Asian <= 0.00508
0.837 < Driving.alone.to.work
0.0288 < Percentage.of.households.with.overcrowding
0.774 < Homeownership
0.002295 < Mental.health.providers
Feature
0.00
0.02
0.04
0.06
Weight
Positive
Negative

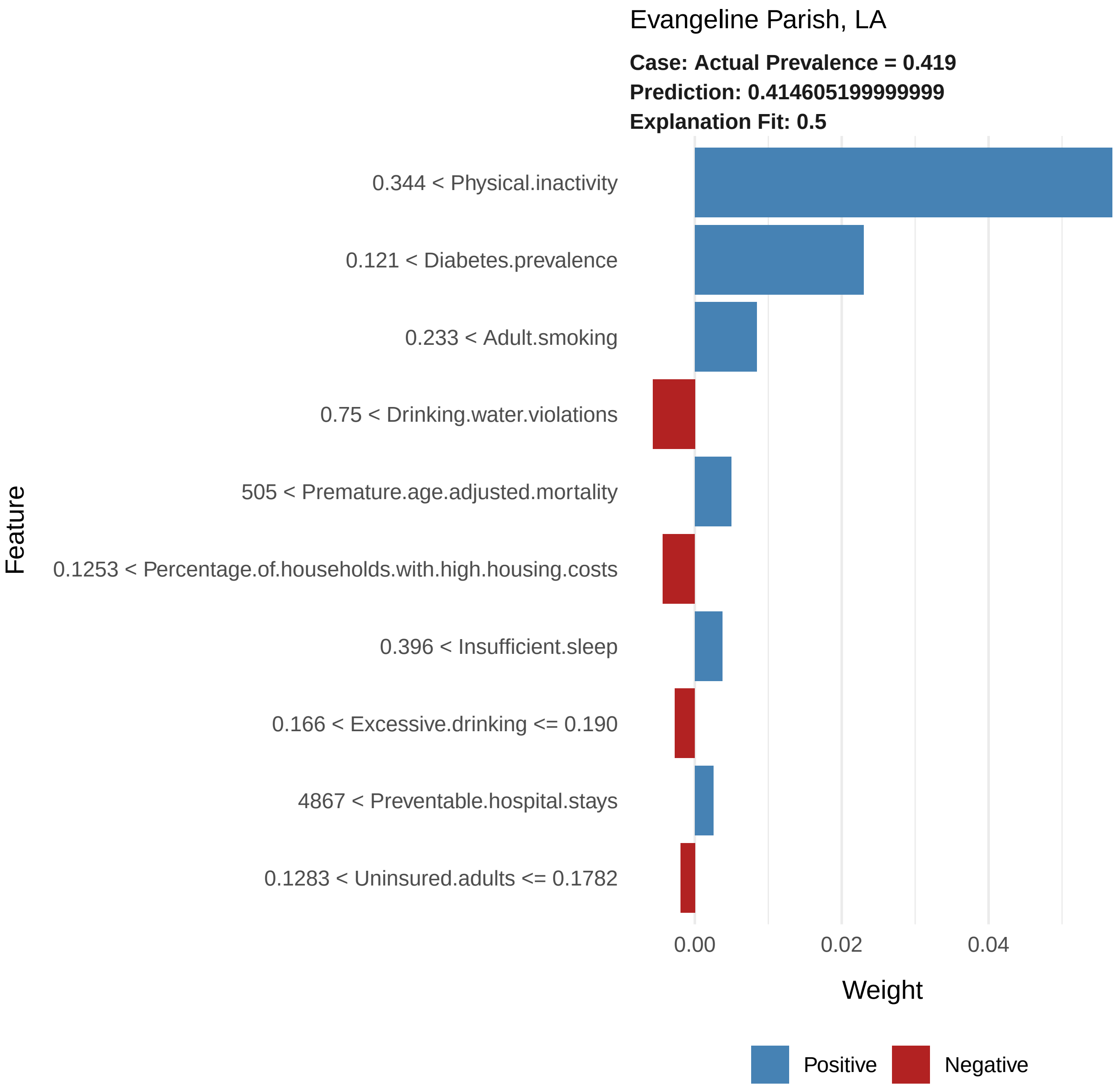
Evangeline Parish, LA
Case: Actual Prevalence = 0.419
Prediction: 0.414605199999999
Explanation Fit: 0.5
0.344 < Physical.inactivity
0.121 < Diabetes.prevalence
0.233 < Adult.smoking
0.75 < Drinking.water.violations
505 < Premature.age.adjusted.mortality
0.1253 < Percentage.of.households.with.high.housing.costs
0.396 < Insufficient.sleep
0.166 < Excessive.drinking <= 0.190
4867 < Preventable.hospital.stays
0.1283 < Uninsured.adults <= 0.1782
Feature
0.00
0.02
0.04
Weight
Positive
Negative

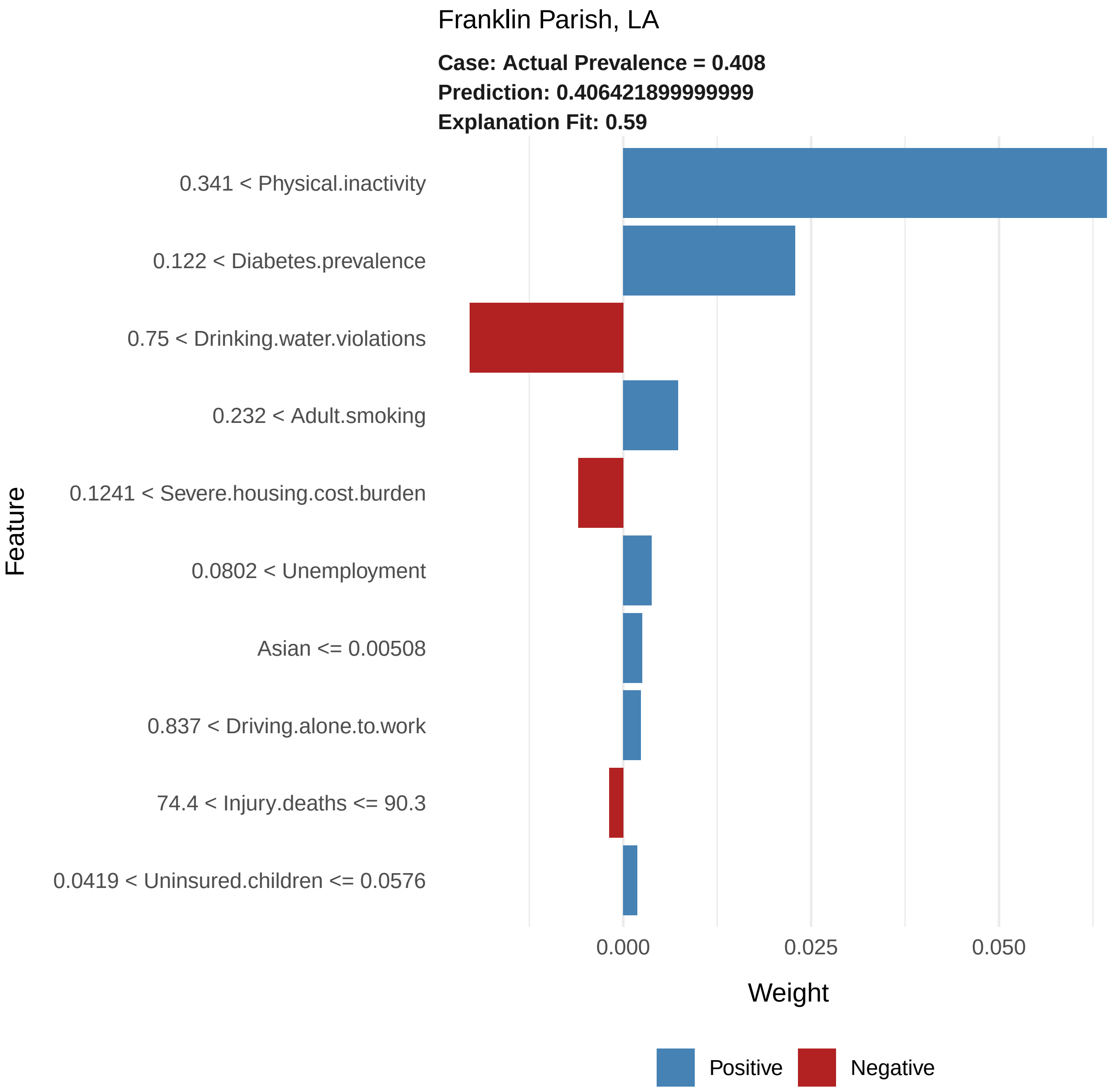

Franklin Parish, LA
Case: Actual Prevalence = 0.408
Prediction: 0.406421899999999
Explanation Fit: 0.59
0.341 < Physical.inactivity
0.122 < Diabetes.prevalence
0.75 < Drinking.water.violations
0.232 < Adult.smoking
0.1241 < Severe.housing.cost.burden
0.0802 < Unemployment
Asian <= 0.00508
0.837 < Driving.alone.to.work
74.4 < Injury.deaths <= 90.3
0.0419 < Uninsured.children <= 0.0576
Feature
0.000
0.025
0.050
Weight
Positive
Negative

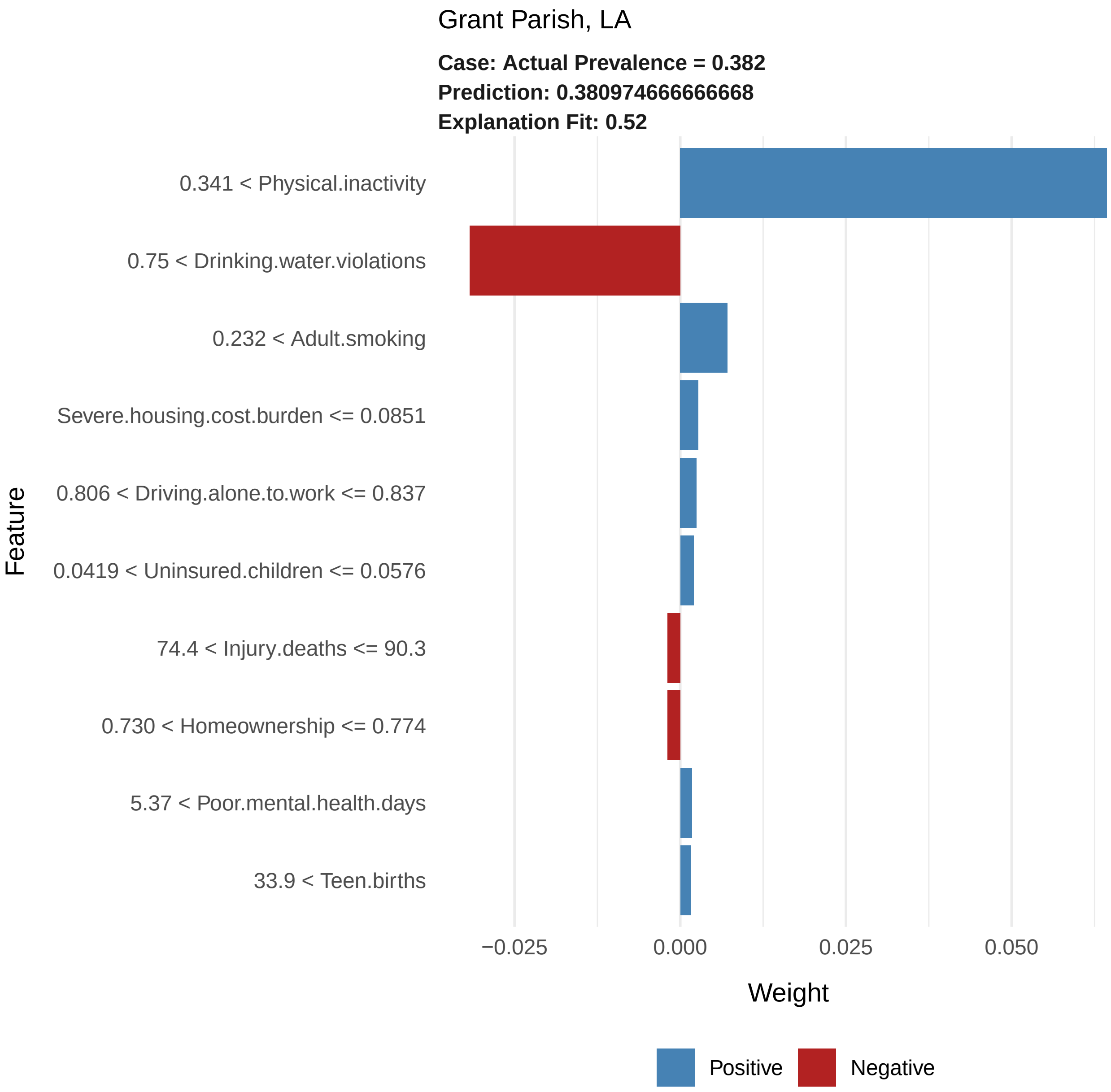

Grant Parish, LA
Case: Actual Prevalence = 0.382
Prediction: 0.380974666666668
Explanation Fit: 0.52
0.341 < Physical.inactivity
0.75 < Drinking.water.violations
0.232 < Adult.smoking
Severe.housing.cost.burden <= 0.0851
0.806 < Driving.alone.to.work <= 0.837
0.0419 < Uninsured.children <= 0.0576
74.4 < Injury.deaths <= 90.3
0.730 < Homeownership <= 0.774
5.37 < Poor.mental.health.days
33.9 < Teen.births
Feature
−0.025
0.000
0.025
0.050
Weight
Positive
Negative

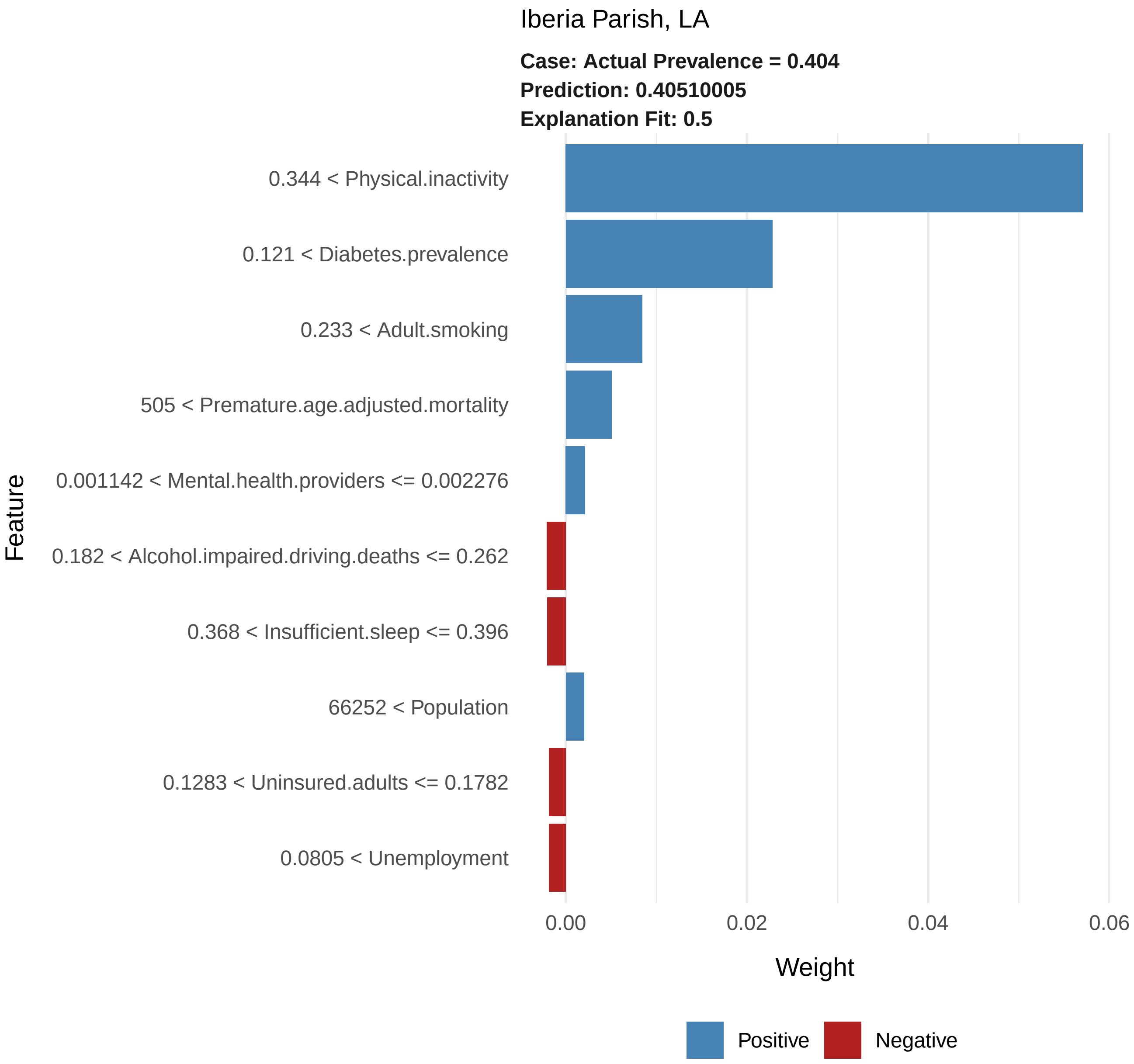
Iberia Parish, LA
Case: Actual Prevalence = 0.404
Prediction: 0.40510005
Explanation Fit: 0.5
0.344 < Physical.inactivity
0.121 < Diabetes.prevalence
0.233 < Adult.smoking
505 < Premature.age.adjusted.mortality
0.001142 < Mental.health.providers <= 0.002276
0.182 < Alcohol.impaired.driving.deaths <= 0.262
0.368 < Insufficient.sleep <= 0.396
66252 < Population
0.1283 < Uninsured.adults <= 0.1782
0.0805 < Unemployment
Feature
0.00
0.02
0.04
0.06
Weight
Positive
Negative

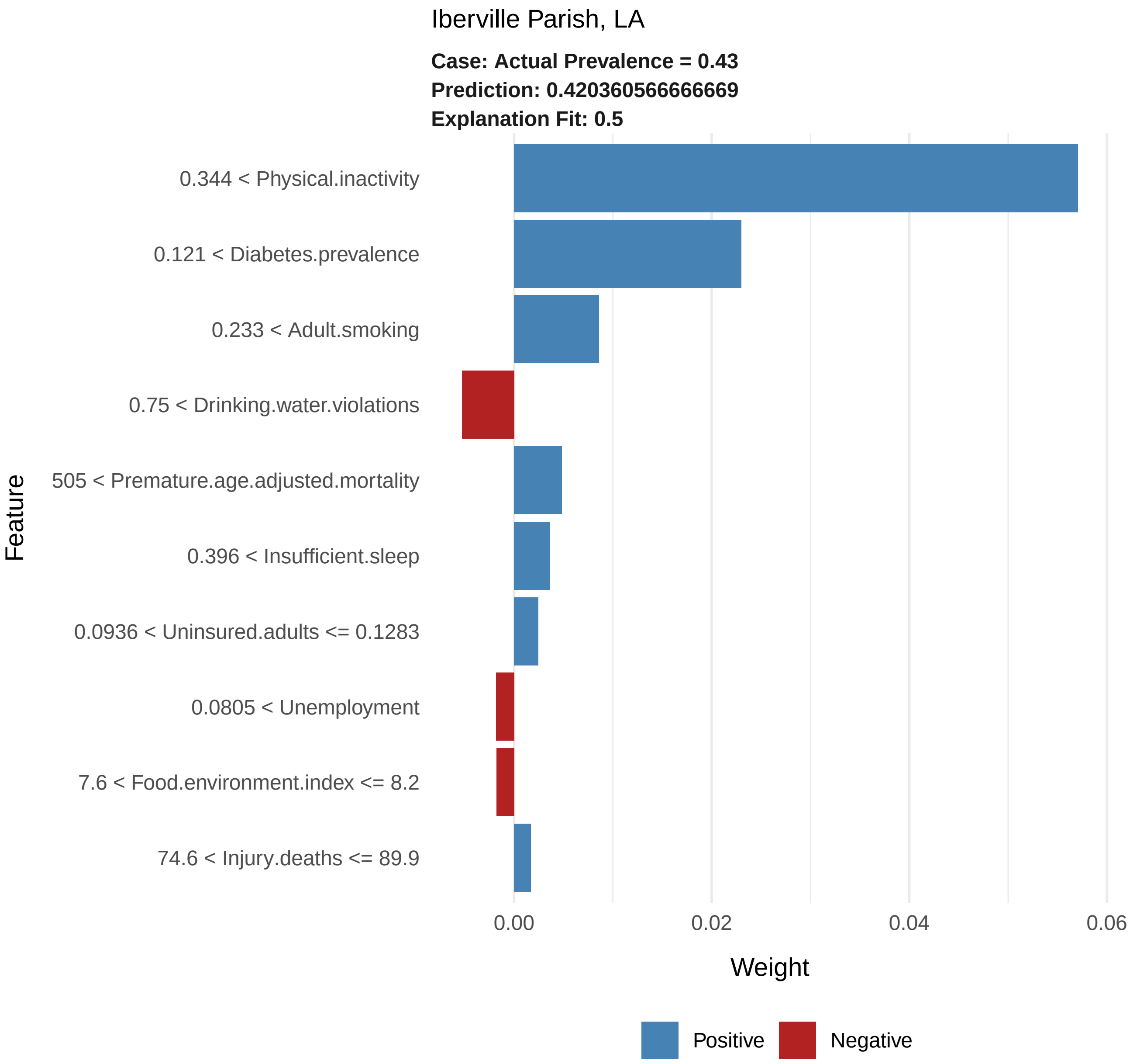
Iberville Parish, LA
Case: Actual Prevalence = 0.43
Prediction: 0.420360566666669
Explanation Fit: 0.5
0.344 < Physical.inactivity
0.121 < Diabetes.prevalence
0.233 < Adult.smoking
0.75 < Drinking.water.violations
505 < Premature.age.adjusted.mortality
0.396 < Insufficient.sleep
0.0936 < Uninsured.adults <= 0.1283
0.0805 < Unemployment
7.6 < Food.environment.index <= 8.2
74.6 < Injury.deaths <= 89.9
Feature
0.00
0.02
0.04
0.06
Weight
Positive
Negative

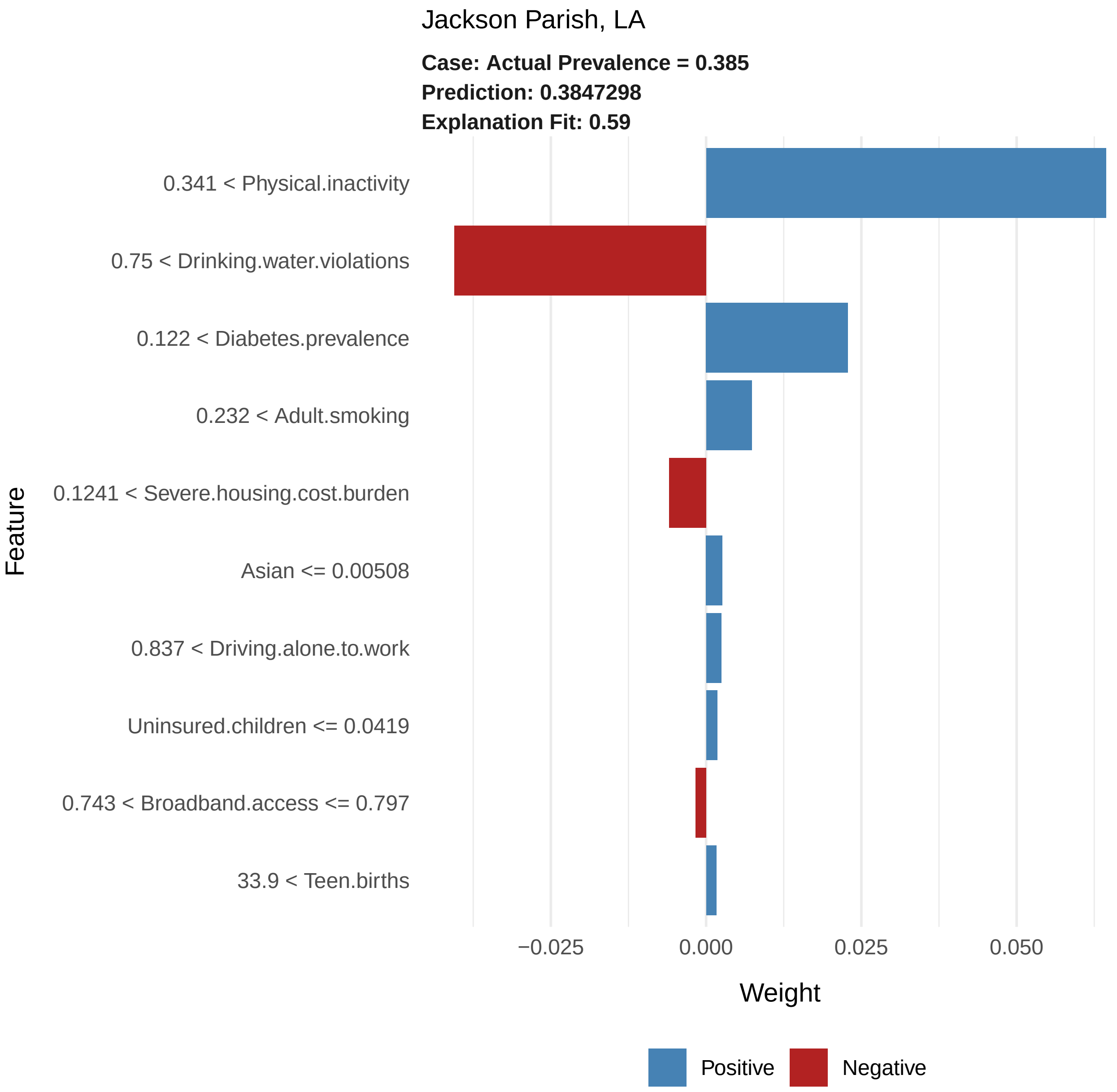

Jackson Parish, LA
Case: Actual Prevalence = 0.385
Prediction: 0.3847298
Explanation Fit: 0.59
0.341 < Physical.inactivity
0.75 < Drinking.water.violations
0.122 < Diabetes.prevalence
0.232 < Adult.smoking
0.1241 < Severe.housing.cost.burden
Asian <= 0.00508
0.837 < Driving.alone.to.work
Uninsured.children <= 0.0419
0.743 < Broadband.access <= 0.797
33.9 < Teen.births
Feature
−0.025
0.000
0.025
0.050
Weight
Positive
Negative

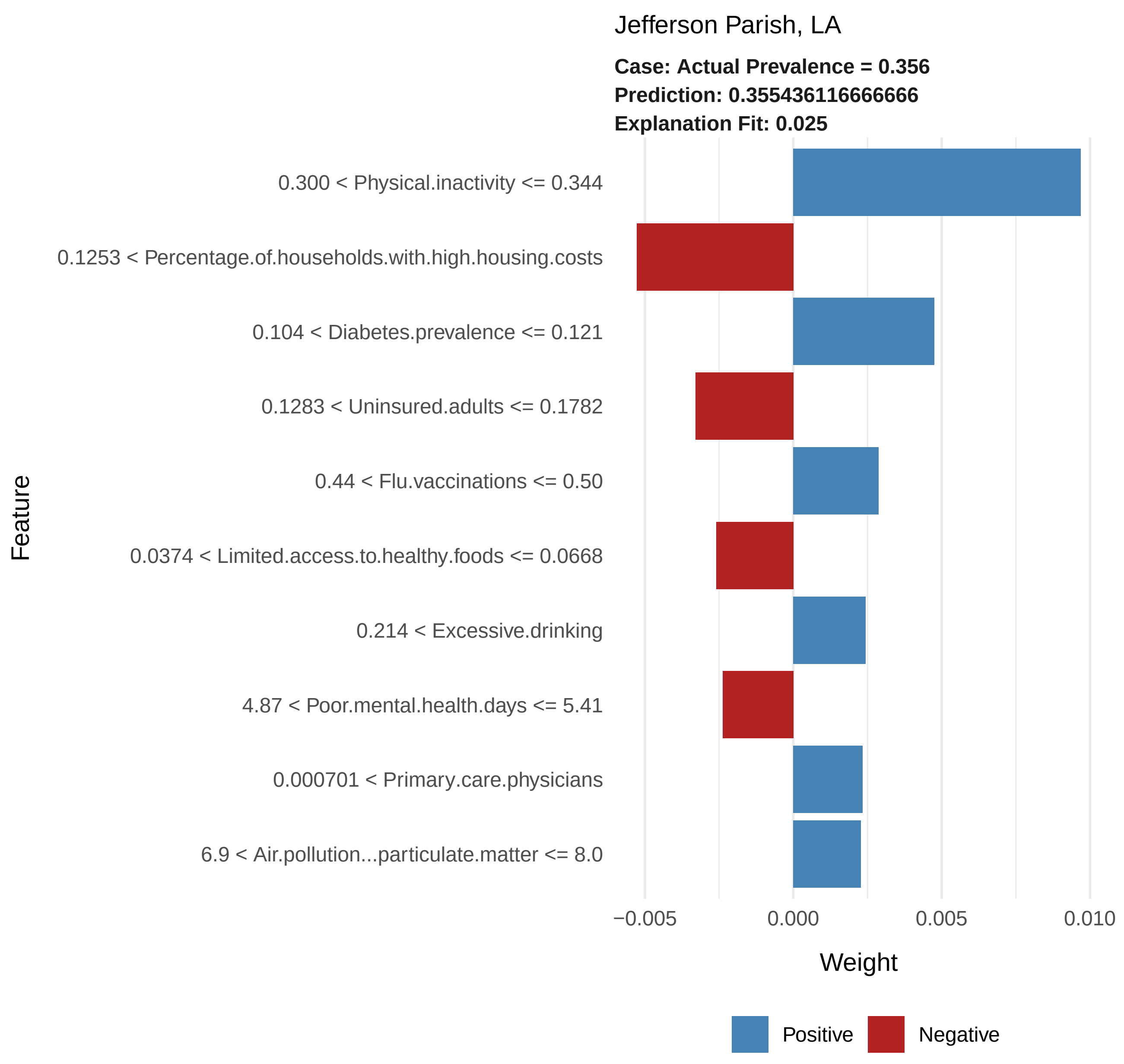
Jefferson Parish, LA
Case: Actual Prevalence = 0.356
Prediction: 0.355436116666666
Explanation Fit: 0.025
0.300 < Physical.inactivity <= 0.344
0.1253 < Percentage.of.households.with.high.housing.costs
0.104 < Diabetes.prevalence <= 0.121
0.1283 < Uninsured.adults <= 0.1782
0.44 < Flu.vaccinations <= 0.50
0.0374 < Limited.access.to.healthy.foods <= 0.0668
0.214 < Excessive.drinking
4.87 < Poor.mental.health.days <= 5.41
0.000701 < Primary.care.physicians
6.9 < Air.pollution...particulate.matter <= 8.0
Feature
−0.005
0.000
0.005
0.010
Weight
Positive
Negative

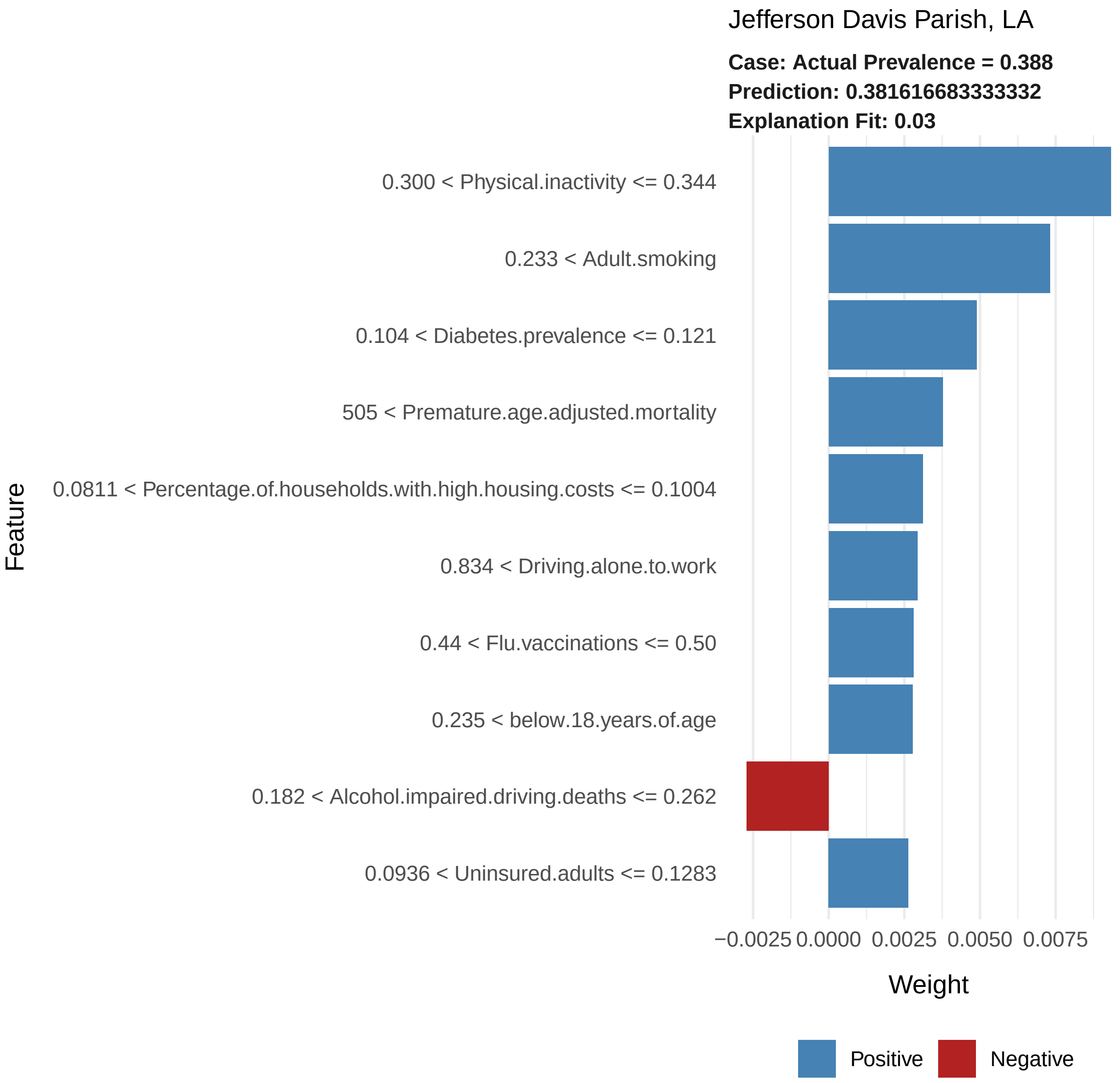

Jefferson Davis Parish, LA
Case: Actual Prevalence = 0.388
Prediction: 0.381616683333332
Explanation Fit: 0.03
0.300 < Physical.inactivity <= 0.344
0.233 < Adult.smoking
0.104 < Diabetes.prevalence <= 0.121
505 < Premature.age.adjusted.mortality
0.0811 < Percentage.of.households.with.high.housing.costs <= 0.1004
0.834 < Driving.alone.to.work
0.44 < Flu.vaccinations <= 0.50
0.235 < below.18.years.of.age
0.182 < Alcohol.impaired.driving.deaths <= 0.262
0.0936 < Uninsured.adults <= 0.1283
Feature
−0.0025 0.0000 0.0025 0.0050 0.0075
Weight
Positive
Negative

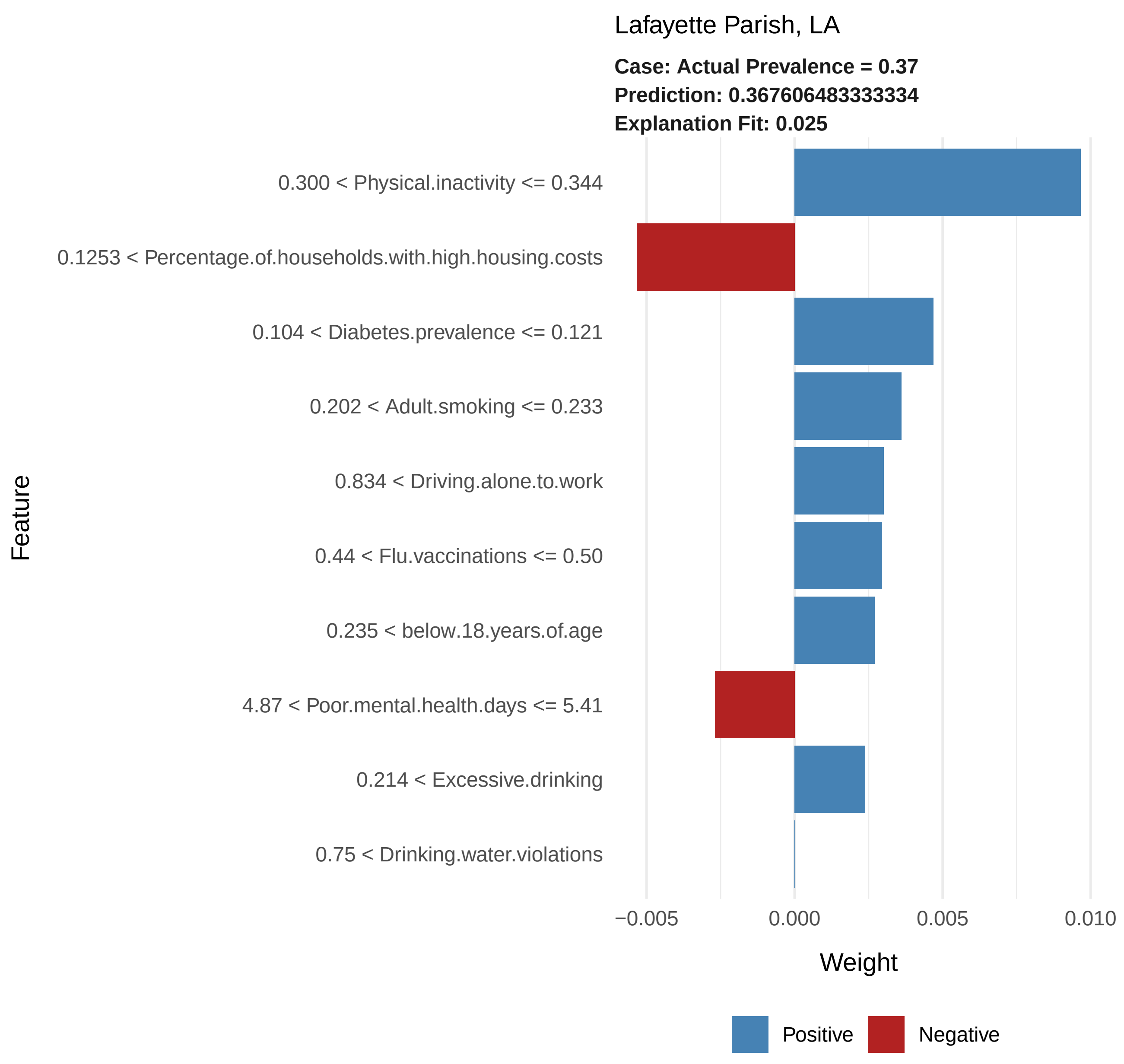

Lafayette Parish, LA
Case: Actual Prevalence = 0.37
Prediction: 0.367606483333334
Explanation Fit: 0.025
Feature
0.300 < Physical.inactivity <= 0.344
0.1253 < Percentage.of.households.with.high.housing.costs
0.104 < Diabetes.prevalence <= 0.121
0.202 < Adult.smoking <= 0.233
0.834 < Driving.alone.to.work
0.44 < Flu.vaccinations <= 0.50
0.235 < below.18.years.of.age
4.87 < Poor.mental.health.days <= 5.41
0.214 < Excessive.drinking
0.75 < Drinking.water.violations
−0.005
0.000
0.005
0.010
Weight
Positive
Negative

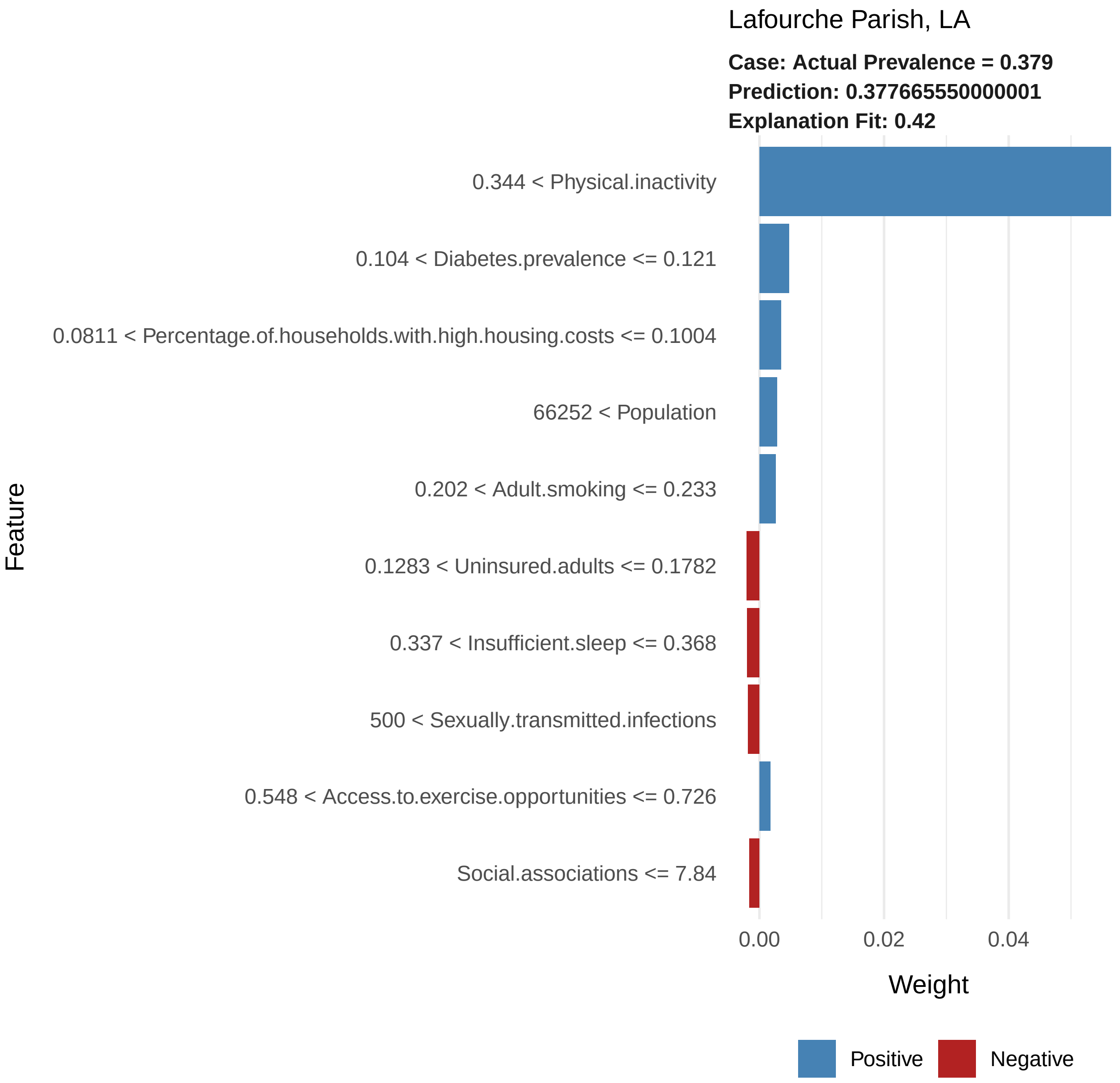

Lafourche Parish, LA
Case: Actual Prevalence = 0.379
Prediction: 0.377665550000001
Explanation Fit: 0.42
0.344 < Physical.inactivity
0.104 < Diabetes.prevalence <= 0.121
0.0811 < Percentage.of.households.with.high.housing.costs <= 0.1004
66252 < Population
0.202 < Adult.smoking <= 0.233
0.1283 < Uninsured.adults <= 0.1782
0.337 < Insufficient.sleep <= 0.368
500 < Sexually.transmitted.infections
0.548 < Access.to.exercise.opportunities <= 0.726
Social.associations <= 7.84
Feature
0.00
0.02
0.04
Weight
Positive
Negative

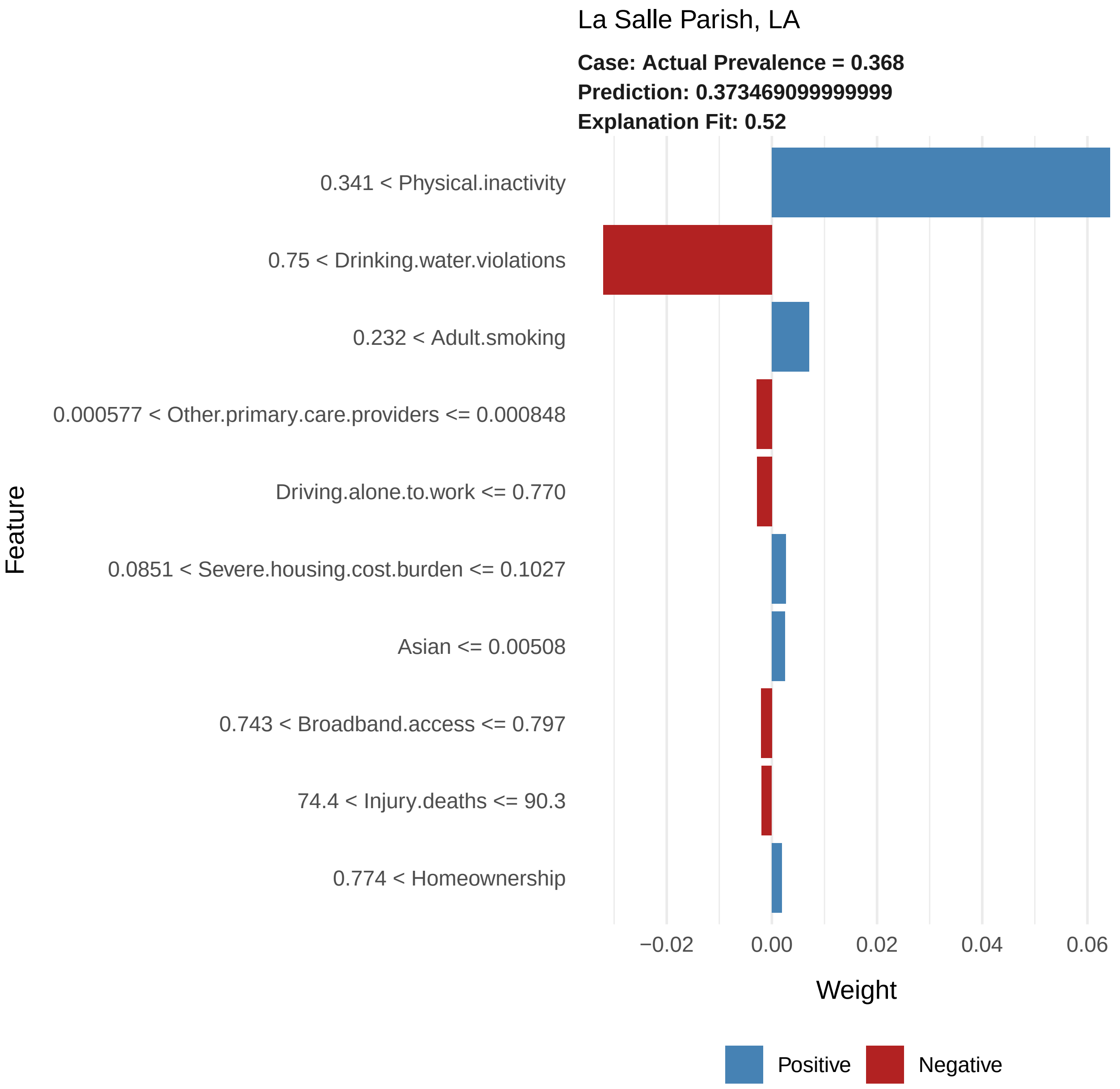
La Salle Parish, LA
Case: Actual Prevalence = 0.368
Prediction: 0.373469099999999
Explanation Fit: 0.52
0.341 < Physical.inactivity
0.75 < Drinking.water.violations
0.232 < Adult.smoking
0.000577 < Other.primary.care.providers <= 0.000848
Driving.alone.to.work <= 0.770
0.0851 < Severe.housing.cost.burden <= 0.1027
Asian <= 0.00508
0.743 < Broadband.access <= 0.797
74.4 < Injury.deaths <= 90.3
0.774 < Homeownership
Feature
−0.02
0.00
0.02
0.04
0.06
Weight
Positive
Negative

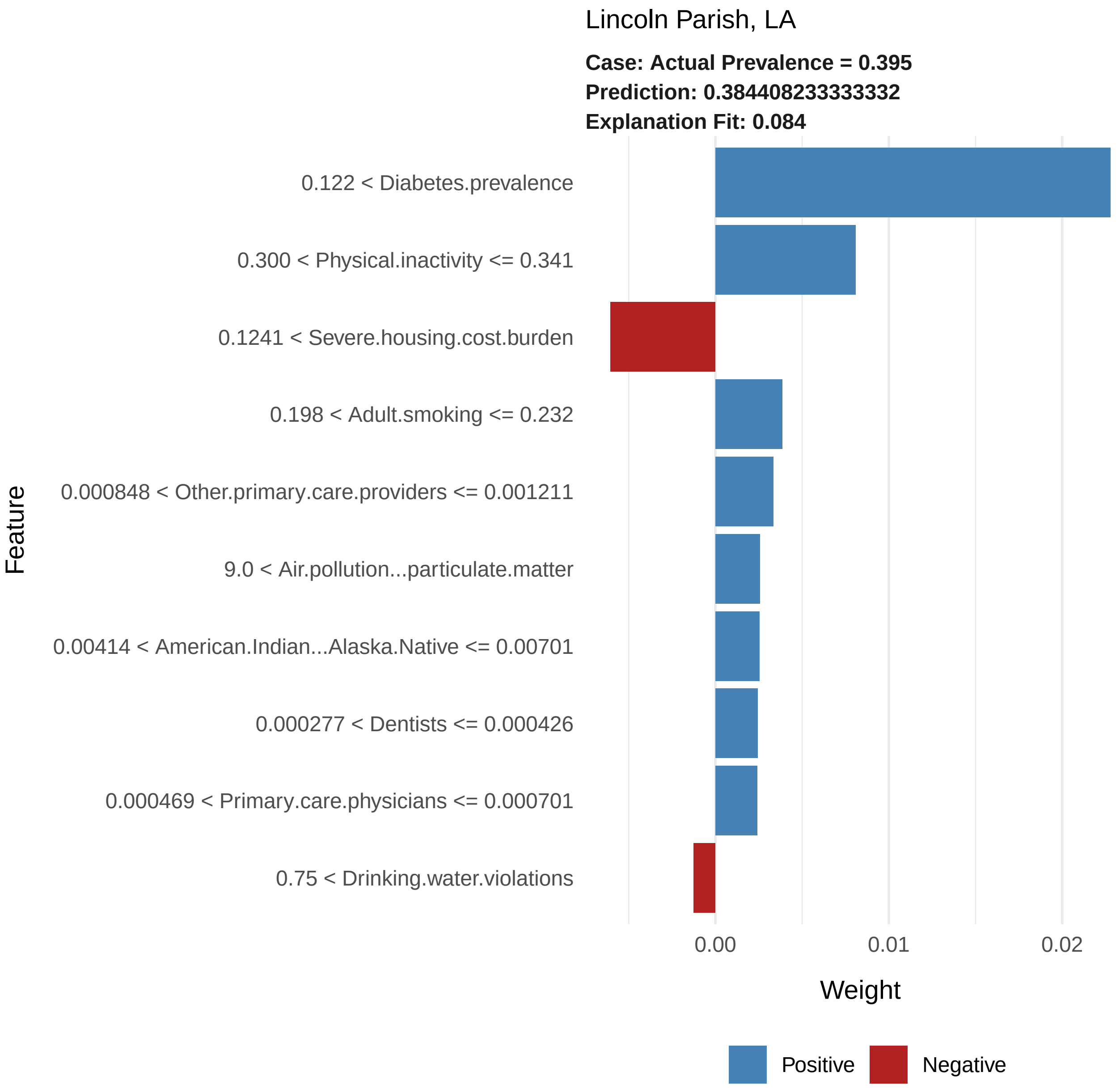
Lincoln Parish, LA
Case: Actual Prevalence = 0.395
Prediction: 0.384408233333332
Explanation Fit: 0.084
0.122 < Diabetes.prevalence
0.300 < Physical.inactivity <= 0.341
0.1241 < Severe.housing.cost.burden
0.198 < Adult.smoking <= 0.232
0.000848 < Other.primary.care.providers <= 0.001211
9.0 < Air.pollution...particulate.matter
0.00414 < American.Indian...Alaska.Native <= 0.00701
0.000277 < Dentists <= 0.000426
0.000469 < Primary.care.physicians <= 0.000701
0.75 < Drinking.water.violations
Feature
0.00
0.01
0.02
Weight
Positive
Negative

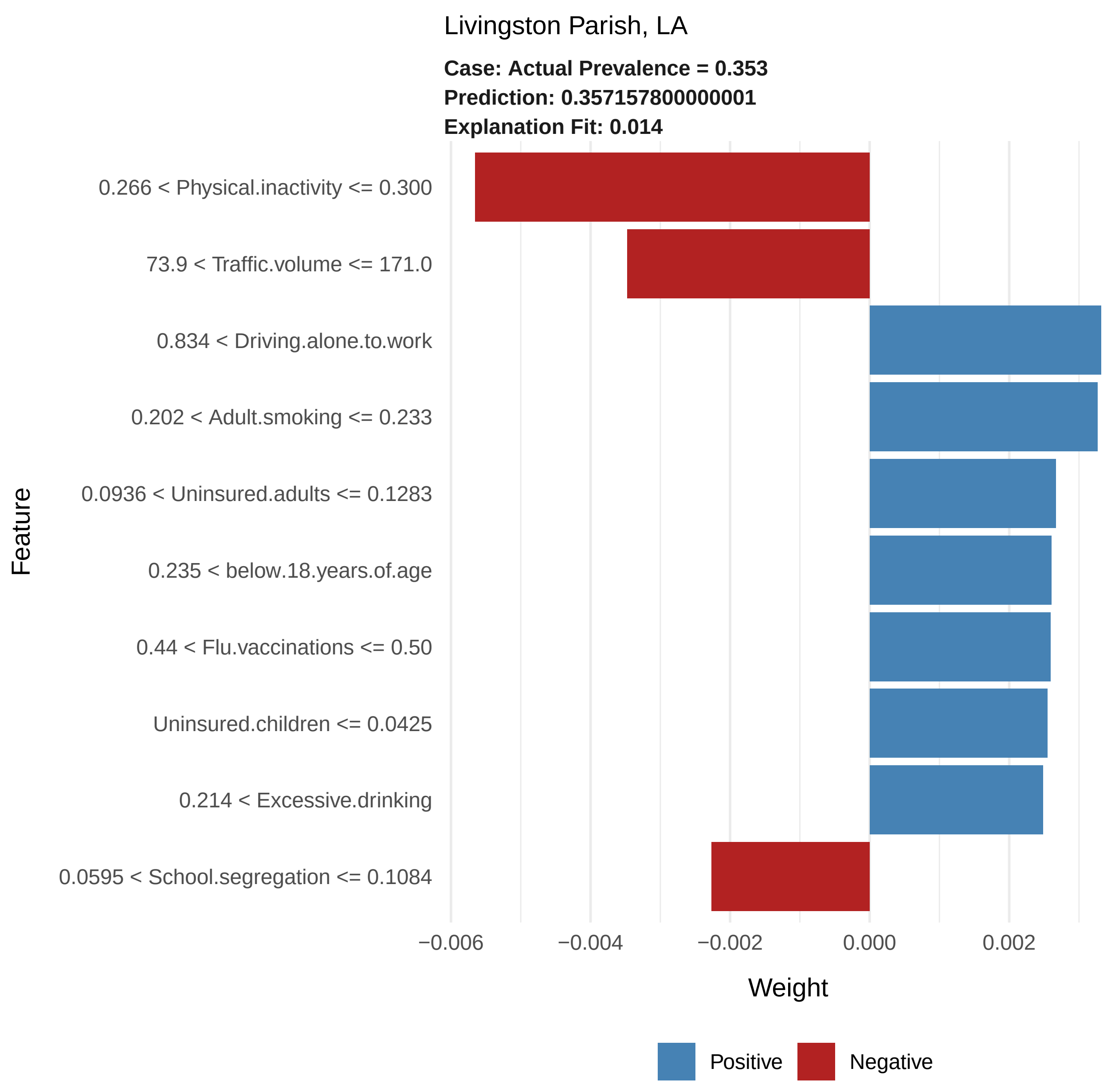
Livingston Parish, LA
Case: Actual Prevalence = 0.353
Prediction: 0.357157800000001
Explanation Fit: 0.014
0.266 < Physical.inactivity <= 0.300
73.9 < Traffic.volume <= 171.0
0.834 < Driving.alone.to.work
0.202 < Adult.smoking <= 0.233
0.0936 < Uninsured.adults <= 0.1283
0.235 < below.18.years.of.age
0.44 < Flu.vaccinations <= 0.50
Uninsured.children <= 0.0425
0.214 < Excessive.drinking
0.0595 < School.segregation <= 0.1084
Feature
−0.006
−0.004
−0.002
0.000
0.002
Weight
Positive
Negative

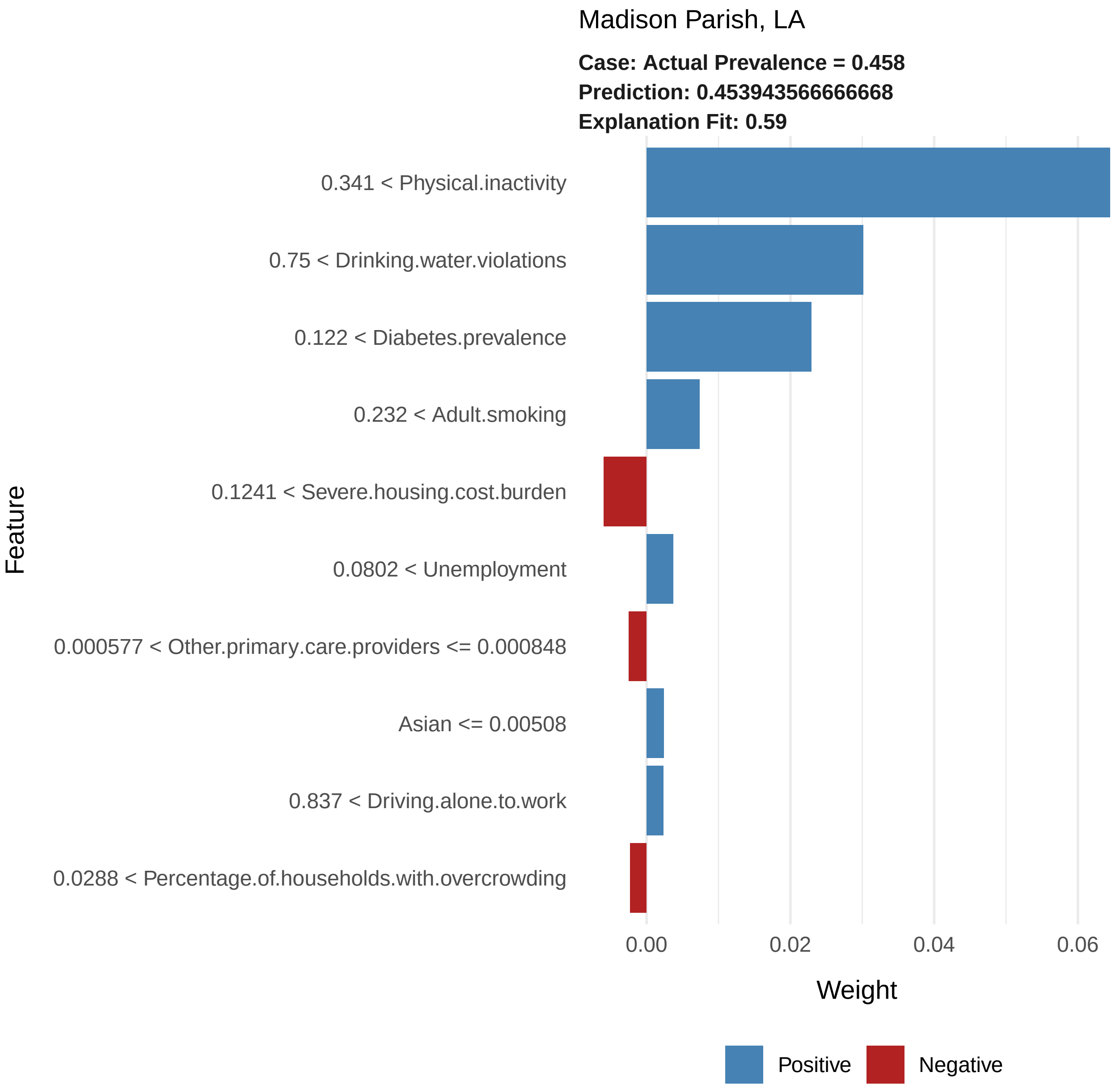

Madison Parish, LA
Case: Actual Prevalence = 0.458
Prediction: 0.453943566666668
Explanation Fit: 0.59
0.341 < Physical.inactivity
0.75 < Drinking.water.violations
0.122 < Diabetes.prevalence
0.232 < Adult.smoking
0.1241 < Severe.housing.cost.burden
0.0802 < Unemployment
0.000577 < Other.primary.care.providers <= 0.000848
Asian <= 0.00508
0.837 < Driving.alone.to.work
0.0288 < Percentage.of.households.with.overcrowding
Feature
0.00
0.02
0.04
0.06
Weight
Positive
Negative

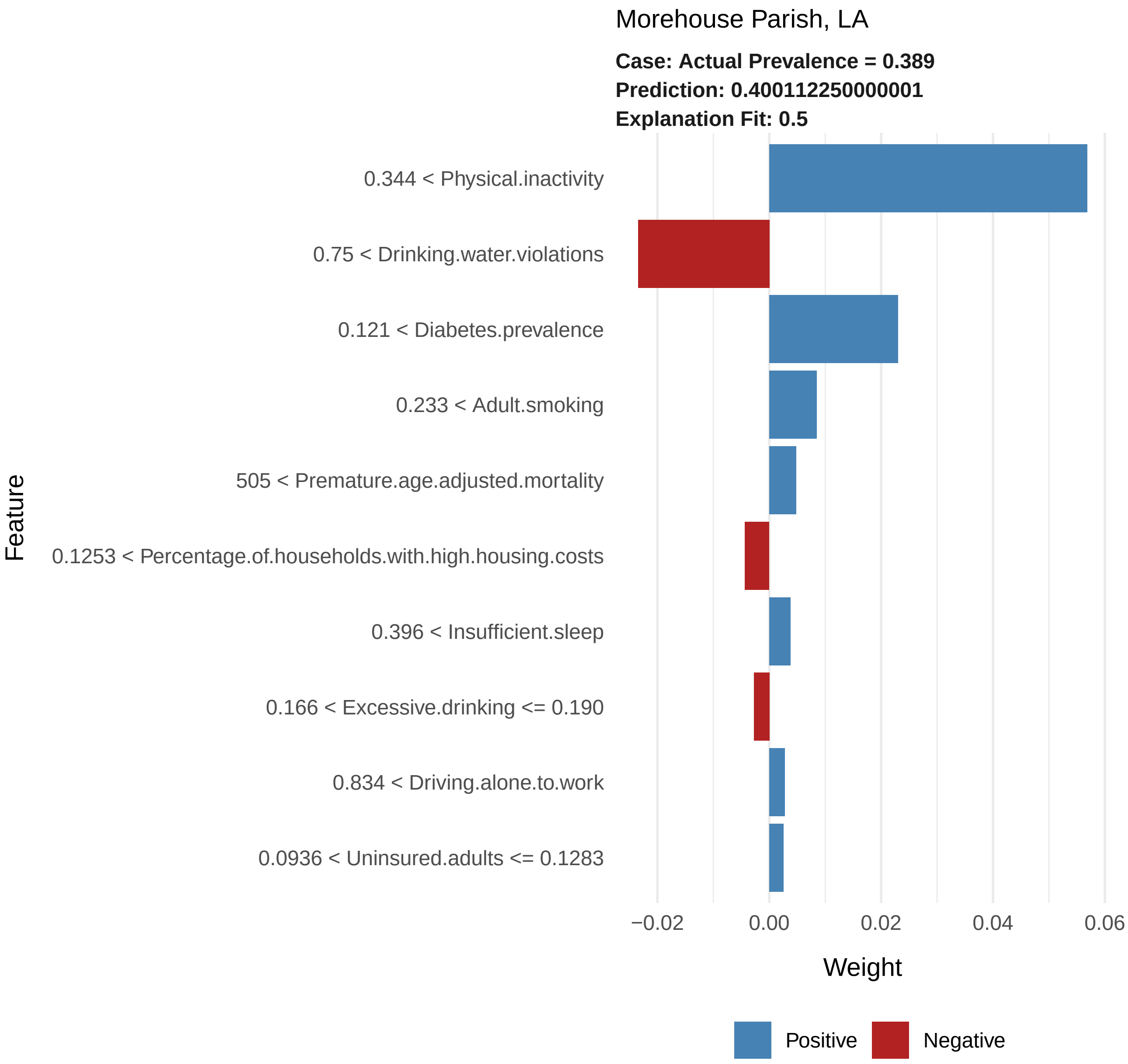

Morehouse Parish, LA
Case: Actual Prevalence = 0.389
Prediction: 0.400112250000001
Explanation Fit: 0.5
0.344 < Physical.inactivity
0.75 < Drinking.water.violations
0.121 < Diabetes.prevalence
0.233 < Adult.smoking
505 < Premature.age.adjusted.mortality
0.1253 < Percentage.of.households.with.high.housing.costs
0.396 < Insufficient.sleep
0.166 < Excessive.drinking <= 0.190
0.834 < Driving.alone.to.work
0.0936 < Uninsured.adults <= 0.1283
Feature
−0.02
0.00
0.02
0.04
0.06
Weight
Positive
Negative

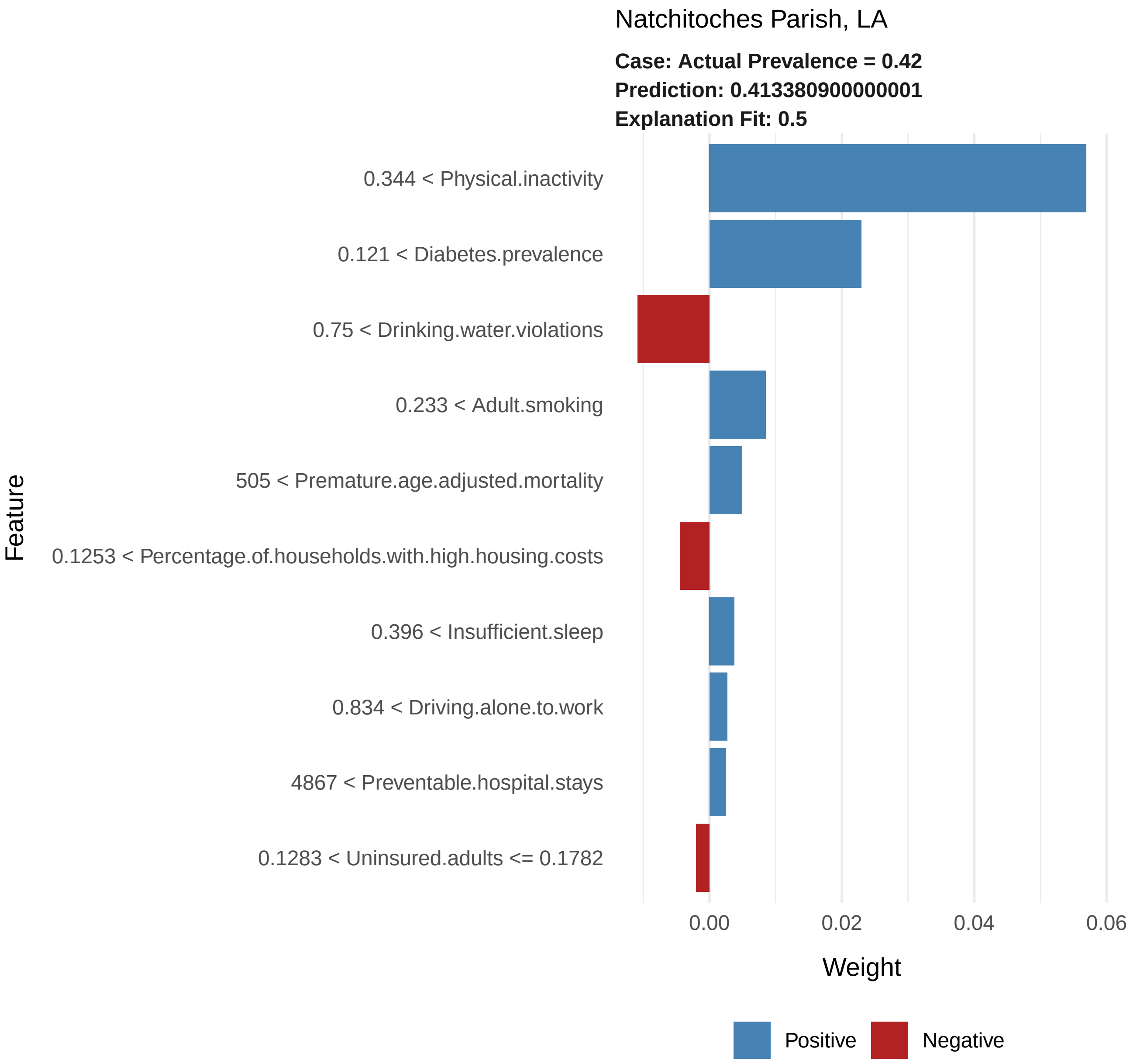

Natchitoches Parish, LA
Case: Actual Prevalence = 0.42
Prediction: 0.413380900000001
Explanation Fit: 0.5
0.344 < Physical.inactivity
0.121 < Diabetes.prevalence
0.75 < Drinking.water.violations
0.233 < Adult.smoking
505 < Premature.age.adjusted.mortality
0.1253 < Percentage.of.households.with.high.housing.costs
0.396 < Insufficient.sleep
0.834 < Driving.alone.to.work
4867 < Preventable.hospital.stays
0.1283 < Uninsured.adults <= 0.1782
Feature
0.00
0.02
0.04
0.06
Weight
Positive
Negative

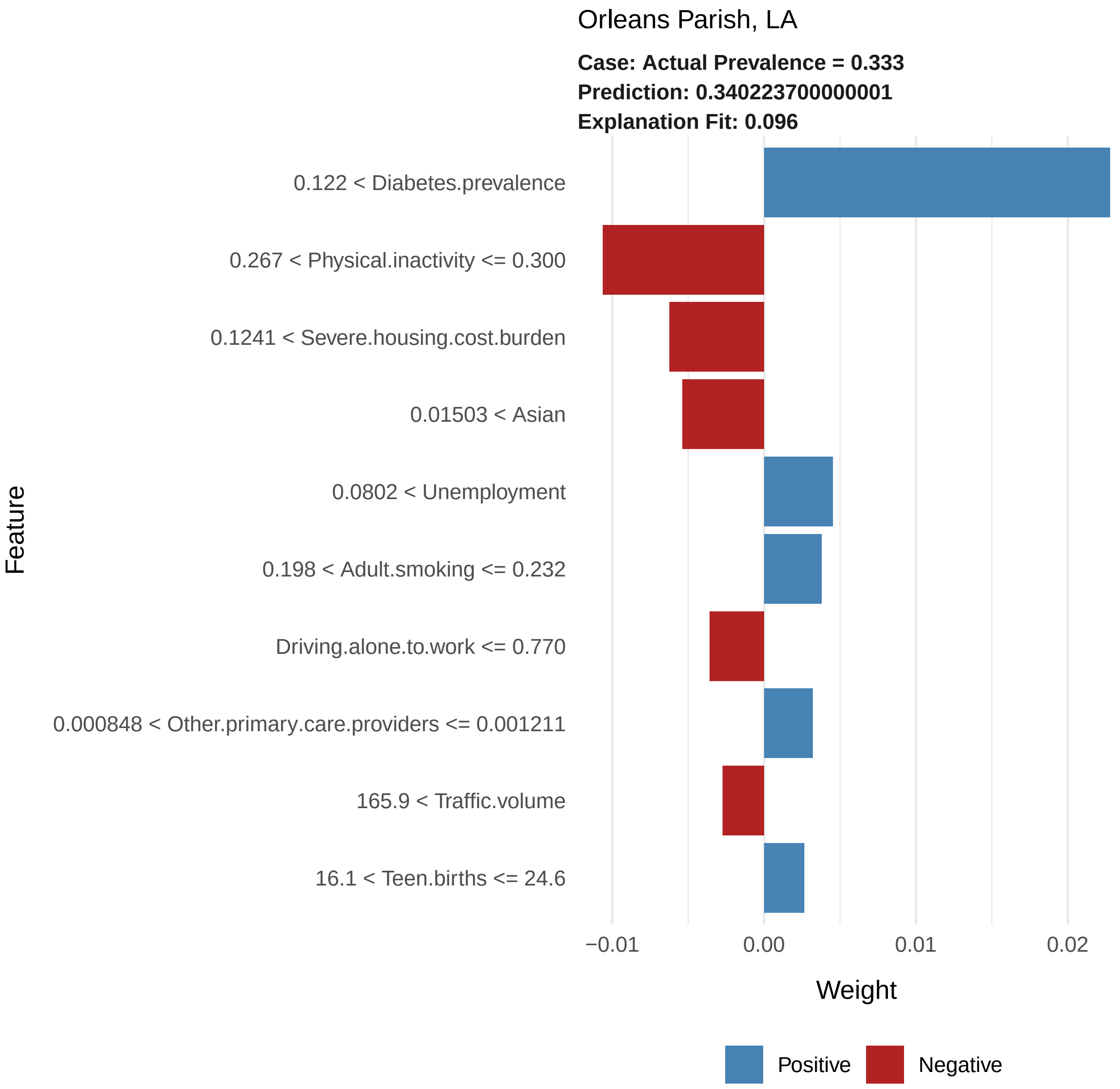

Orleans Parish, LA
Case: Actual Prevalence = 0.333
Prediction: 0.340223700000001
Explanation Fit: 0.096
0.122 < Diabetes.prevalence
0.267 < Physical.inactivity <= 0.300
0.1241 < Severe.housing.cost.burden
0.01503 < Asian
0.0802 < Unemployment
0.198 < Adult.smoking <= 0.232
Driving.alone.to.work <= 0.770
0.000848 < Other.primary.care.providers <= 0.001211
165.9 < Traffic.volume
16.1 < Teen.births <= 24.6
Feature
−0.01
0.00
0.01
0.02
Weight
Positive
Negative

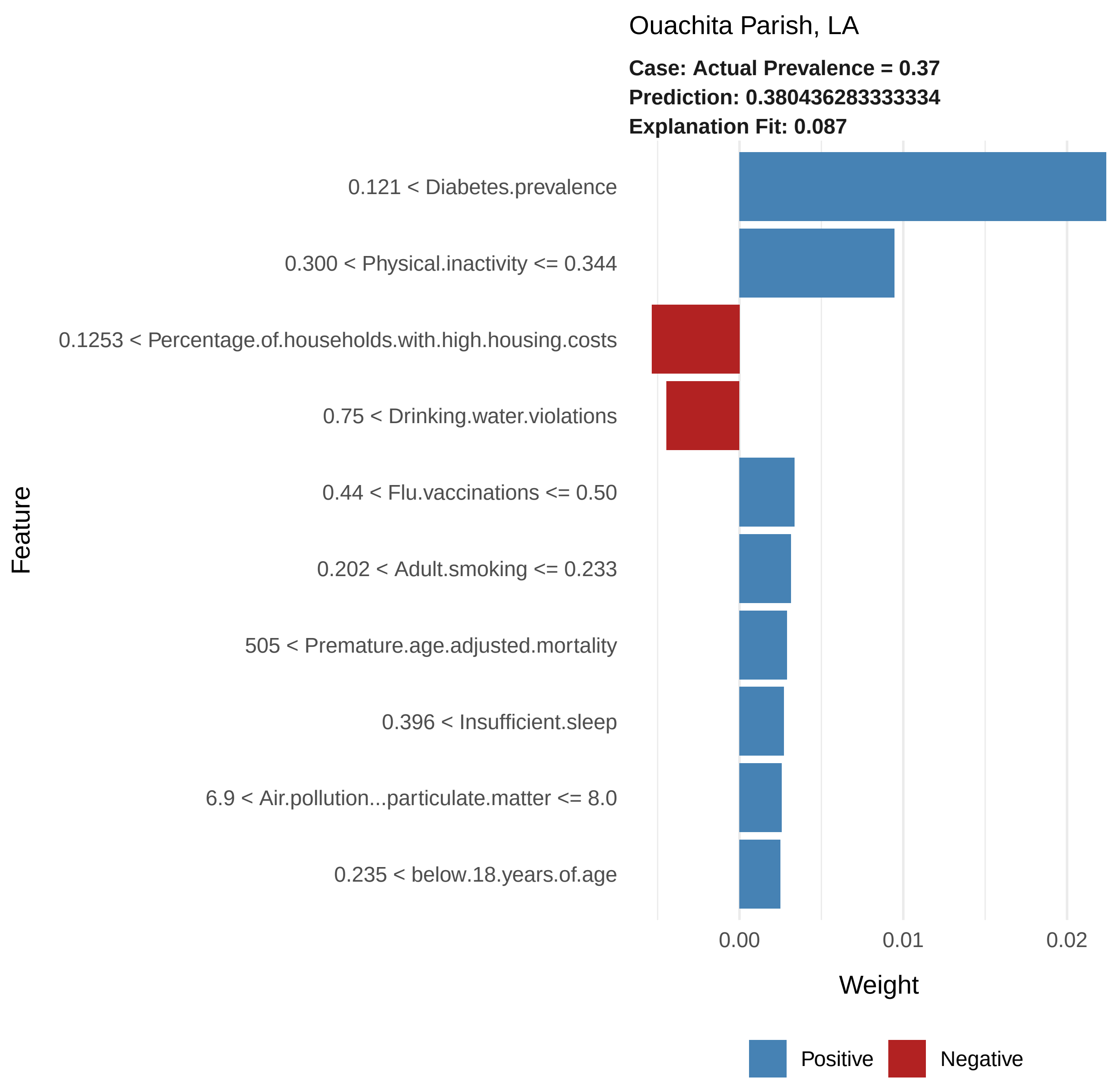
Ouachita Parish, LA
Case: Actual Prevalence = 0.37
Prediction: 0.380436283333334
Explanation Fit: 0.087
0.121 < Diabetes.prevalence
0.300 < Physical.inactivity <= 0.344
0.1253 < Percentage.of.households.with.high.housing.costs
0.75 < Drinking.water.violations
0.44 < Flu.vaccinations <= 0.50
0.202 < Adult.smoking <= 0.233
505 < Premature.age.adjusted.mortality
0.396 < Insufficient.sleep
6.9 < Air.pollution...particulate.matter <= 8.0
0.235 < below.18.years.of.age
Feature
0.00
0.01
0.02
Weight
Positive
Negative

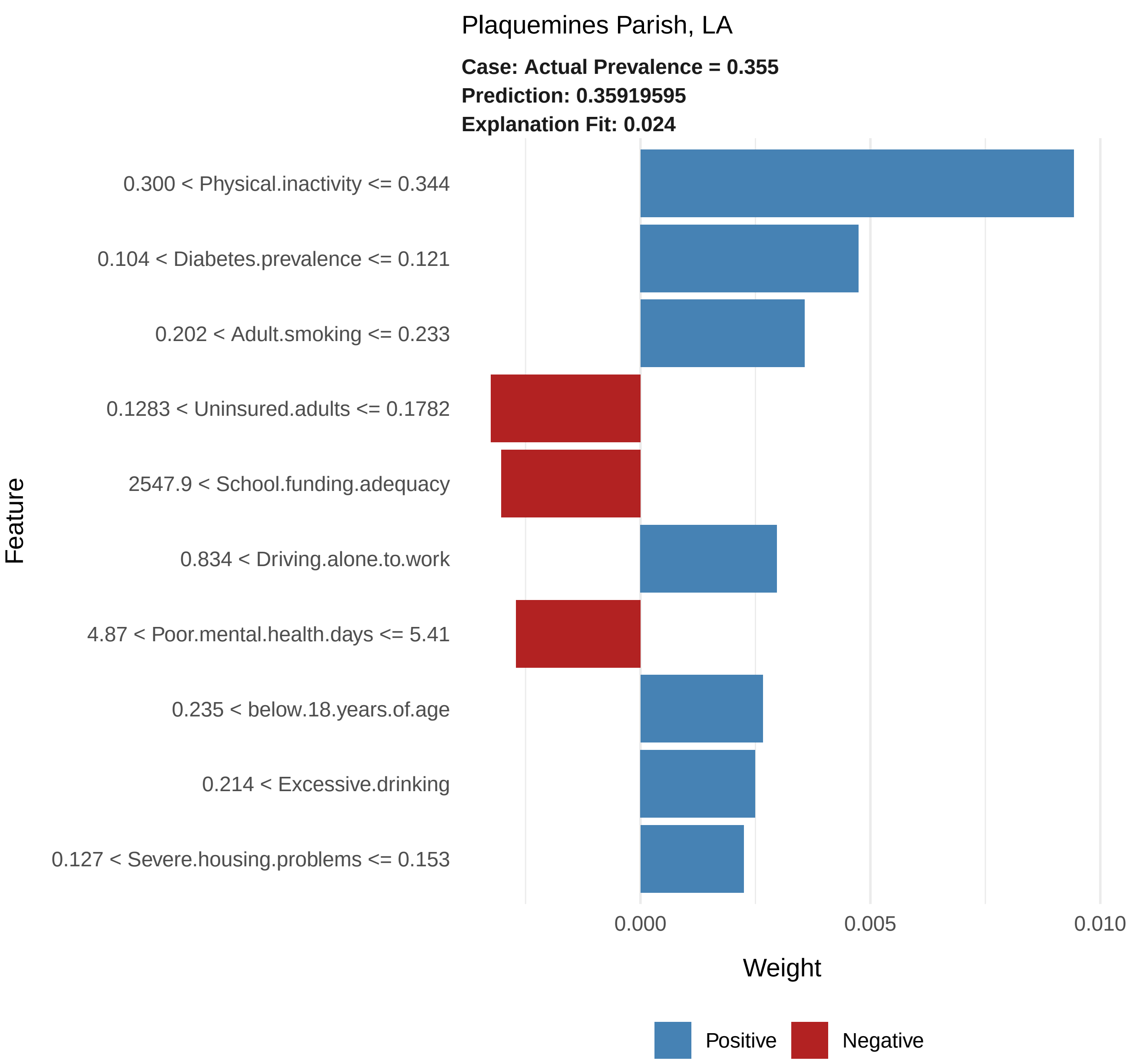

Plaquemines Parish, LA
Case: Actual Prevalence = 0.355
Prediction: 0.35919595
Explanation Fit: 0.024
0.300 < Physical.inactivity <= 0.344
0.104 < Diabetes.prevalence <= 0.121
0.202 < Adult.smoking <= 0.233
0.1283 < Uninsured.adults <= 0.1782
2547.9 < School.funding.adequacy
0.834 < Driving.alone.to.work
4.87 < Poor.mental.health.days <= 5.41
0.235 < below.18.years.of.age
0.214 < Excessive.drinking
0.127 < Severe.housing.problems <= 0.153
Feature
0.000
0.005
0.010
Weight
Positive
Negative

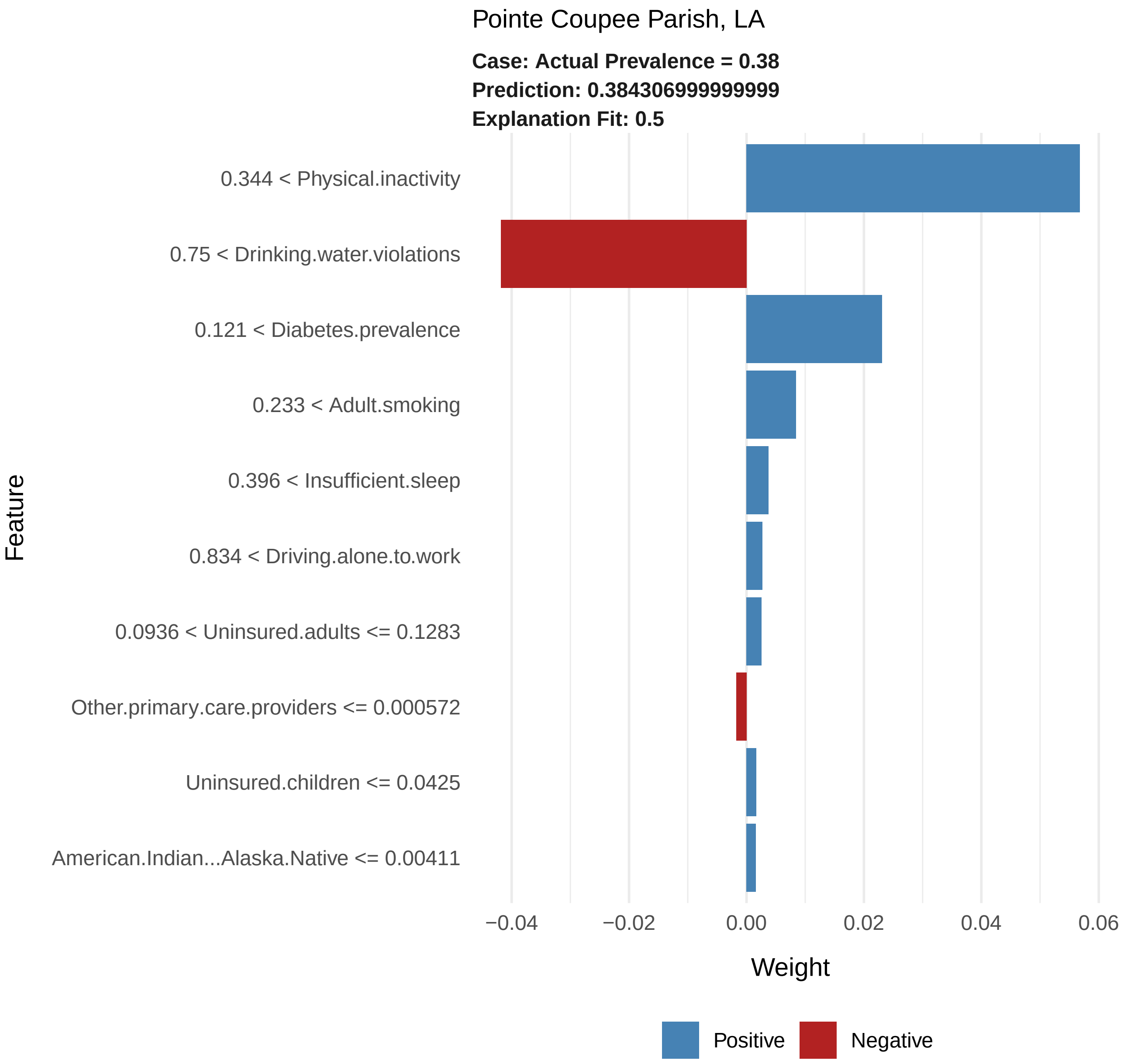

Pointe Coupee Parish, LA
Case: Actual Prevalence = 0.38
Prediction: 0.384306999999999
Explanation Fit: 0.5
0.344 < Physical.inactivity
0.75 < Drinking.water.violations
0.121 < Diabetes.prevalence
0.233 < Adult.smoking
0.396 < Insufficient.sleep
0.834 < Driving.alone.to.work
0.0936 < Uninsured.adults <= 0.1283
Other.primary.care.providers <= 0.000572
Uninsured.children <= 0.0425
American.Indian...Alaska.Native <= 0.00411
Feature
−0.04
−0.02
0.00
0.02
0.04
0.06
Weight
Positive
Negative

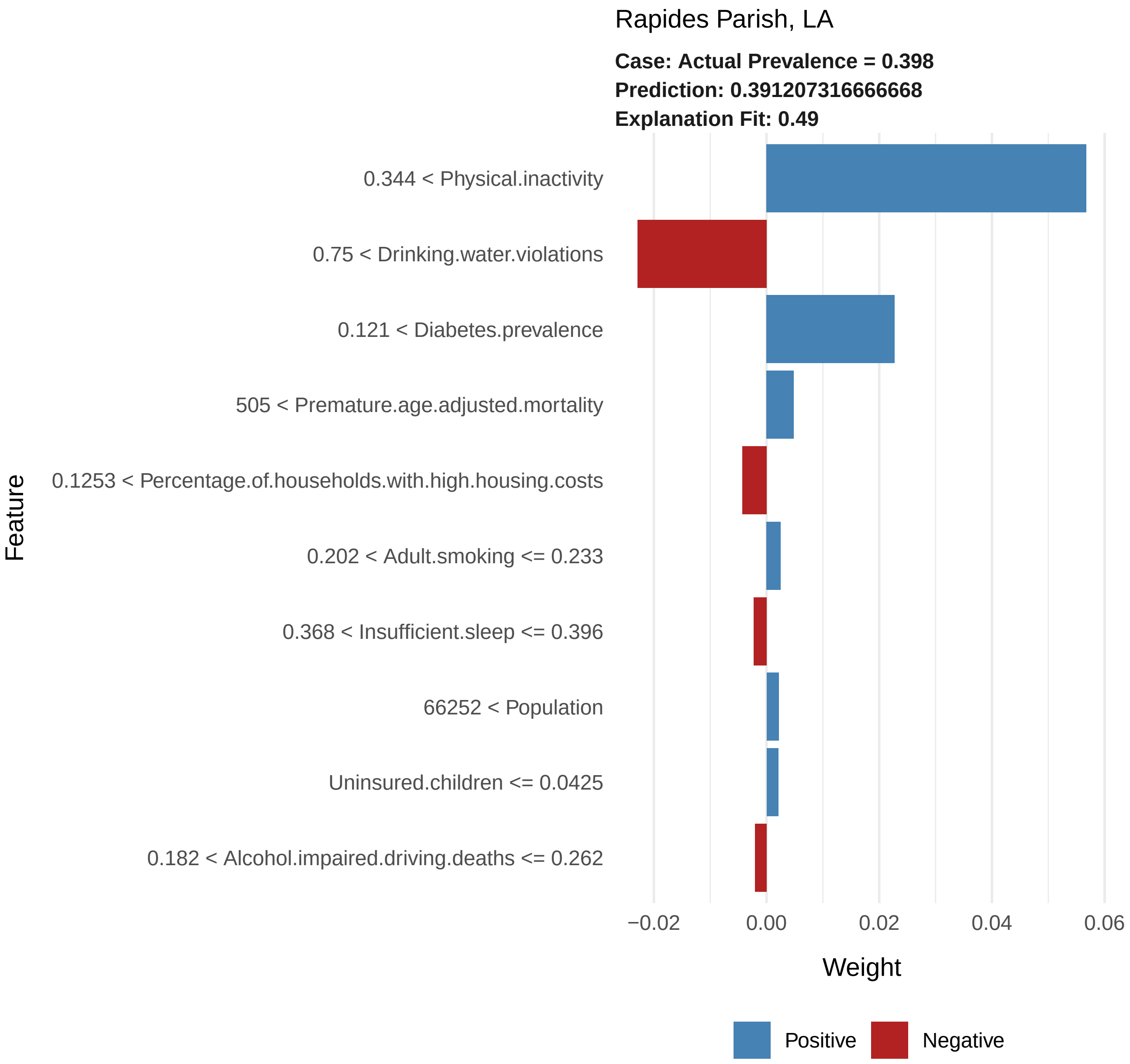

Rapides Parish, LA
Case: Actual Prevalence = 0.398
Prediction: 0.391207316666668
Explanation Fit: 0.49
0.344 < Physical.inactivity
0.75 < Drinking.water.violations
0.121 < Diabetes.prevalence
505 < Premature.age.adjusted.mortality
0.1253 < Percentage.of.households.with.high.housing.costs
0.202 < Adult.smoking <= 0.233
0.368 < Insufficient.sleep <= 0.396
66252 < Population
Uninsured.children <= 0.0425
0.182 < Alcohol.impaired.driving.deaths <= 0.262
Feature
−0.02
0.00
0.02
0.04
0.06
Weight
Positive
Negative

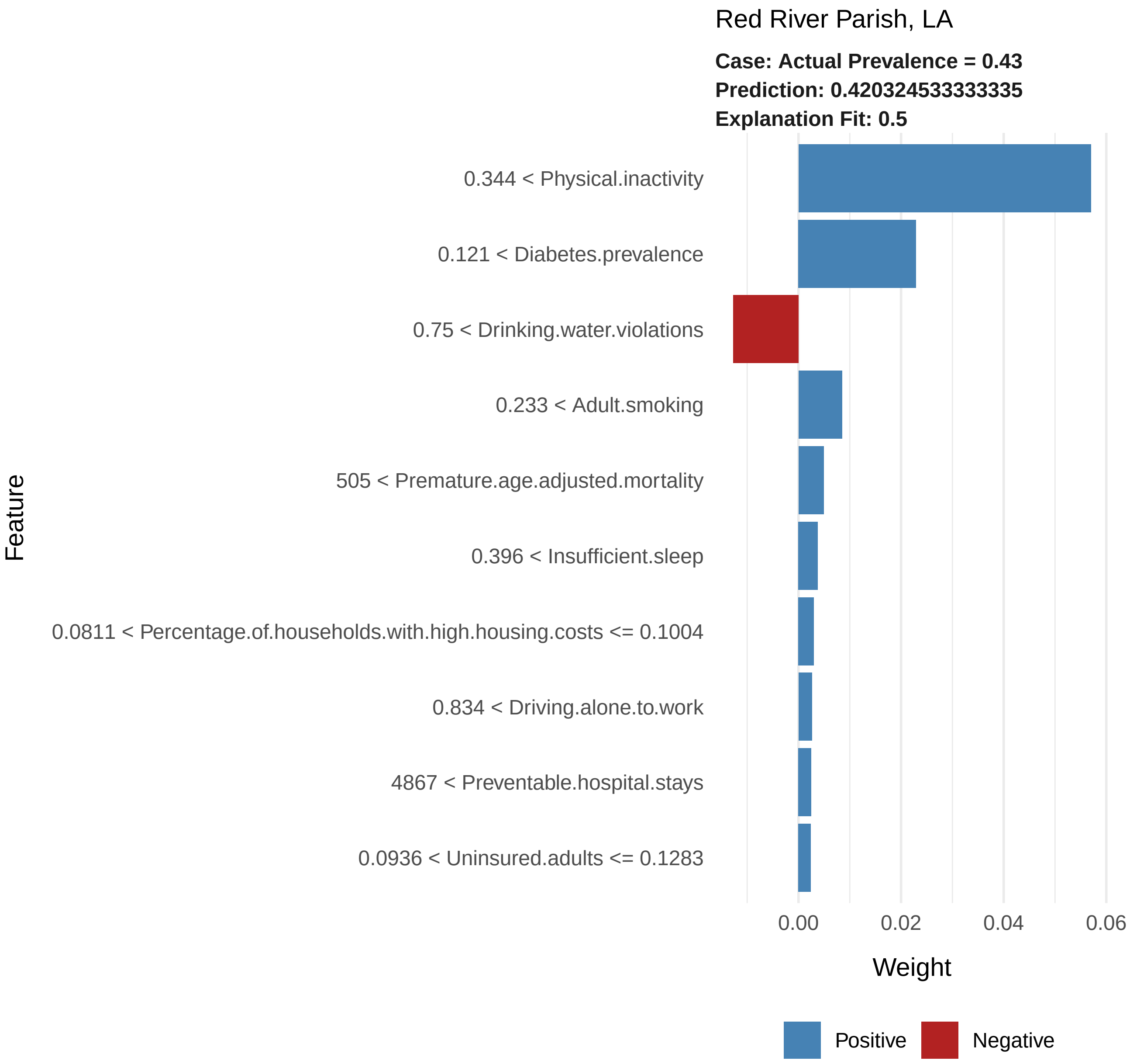

Red River Parish, LA
Case: Actual Prevalence = 0.43
Prediction: 0.420324533333335
Explanation Fit: 0.5
0.344 < Physical.inactivity
0.121 < Diabetes.prevalence
0.75 < Drinking.water.violations
0.233 < Adult.smoking
505 < Premature.age.adjusted.mortality
0.396 < Insufficient.sleep
0.0811 < Percentage.of.households.with.high.housing.costs <= 0.1004
0.834 < Driving.alone.to.work
4867 < Preventable.hospital.stays
0.0936 < Uninsured.adults <= 0.1283
Feature
0.00
0.02
0.04
0.06
Weight
Positive
Negative

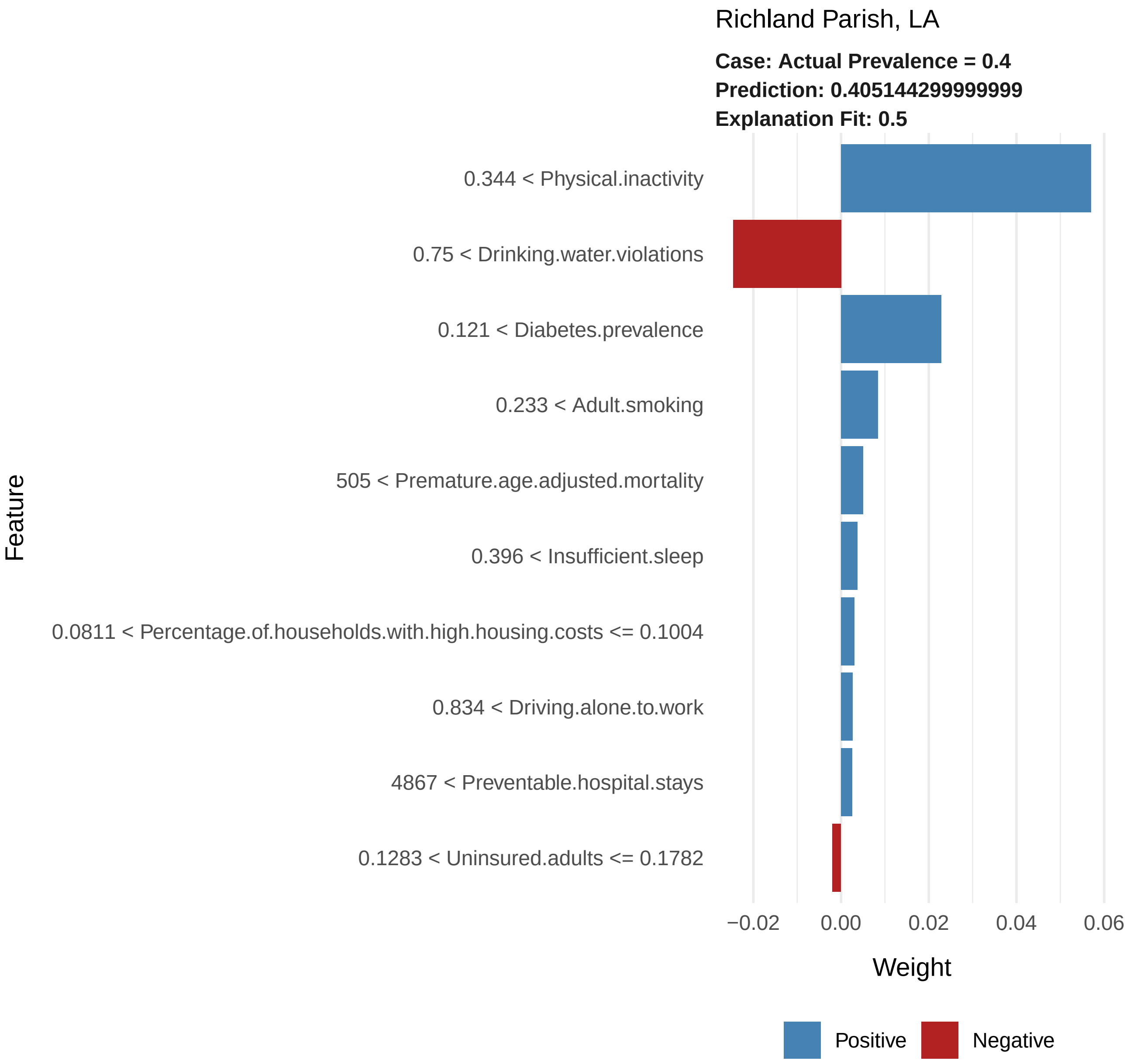
Richland Parish, LA
Case: Actual Prevalence = 0.4
Prediction: 0.405144299999999
Explanation Fit: 0.5
0.344 < Physical.inactivity
0.75 < Drinking.water.violations
0.121 < Diabetes.prevalence
0.233 < Adult.smoking
505 < Premature.age.adjusted.mortality
0.396 < Insufficient.sleep
0.0811 < Percentage.of.households.with.high.housing.costs <= 0.1004
0.834 < Driving.alone.to.work
4867 < Preventable.hospital.stays
0.1283 < Uninsured.adults <= 0.1782
Feature
−0.02
0.00
0.02
0.04
0.06
Weight
Positive
Negative

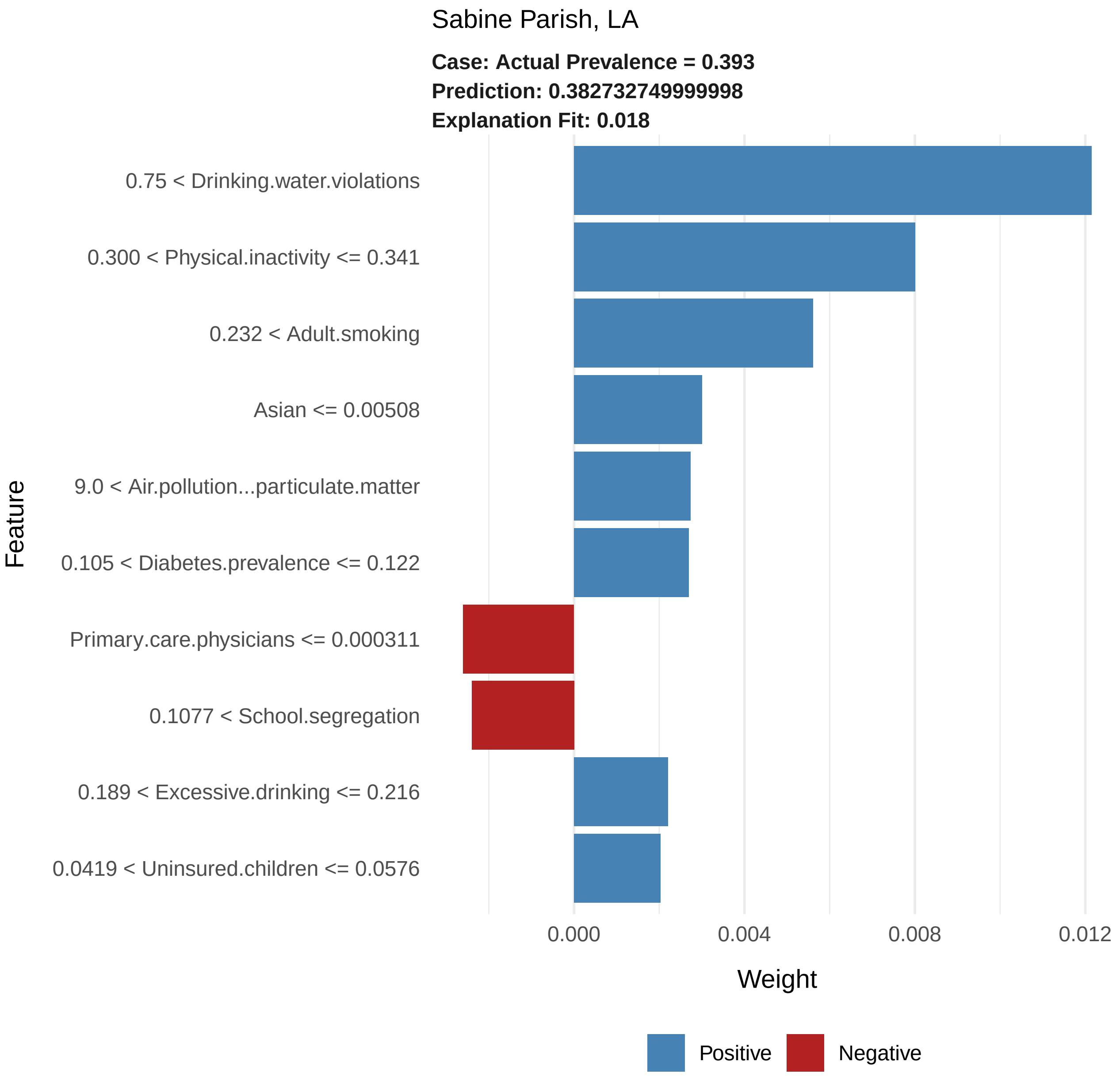
Sabine Parish, LA
Case: Actual Prevalence = 0.393
Prediction: 0.382732749999998
Explanation Fit: 0.018
0.75 < Drinking.water.violations
0.300 < Physical.inactivity <= 0.341
0.232 < Adult.smoking
Asian <= 0.00508
9.0 < Air.pollution...particulate.matter
0.105 < Diabetes.prevalence <= 0.122
Primary.care.physicians <= 0.000311
0.1077 < School.segregation
0.189 < Excessive.drinking <= 0.216
0.0419 < Uninsured.children <= 0.0576
Feature
0.000
0.004
0.008
0.012
Weight
Positive
Negative

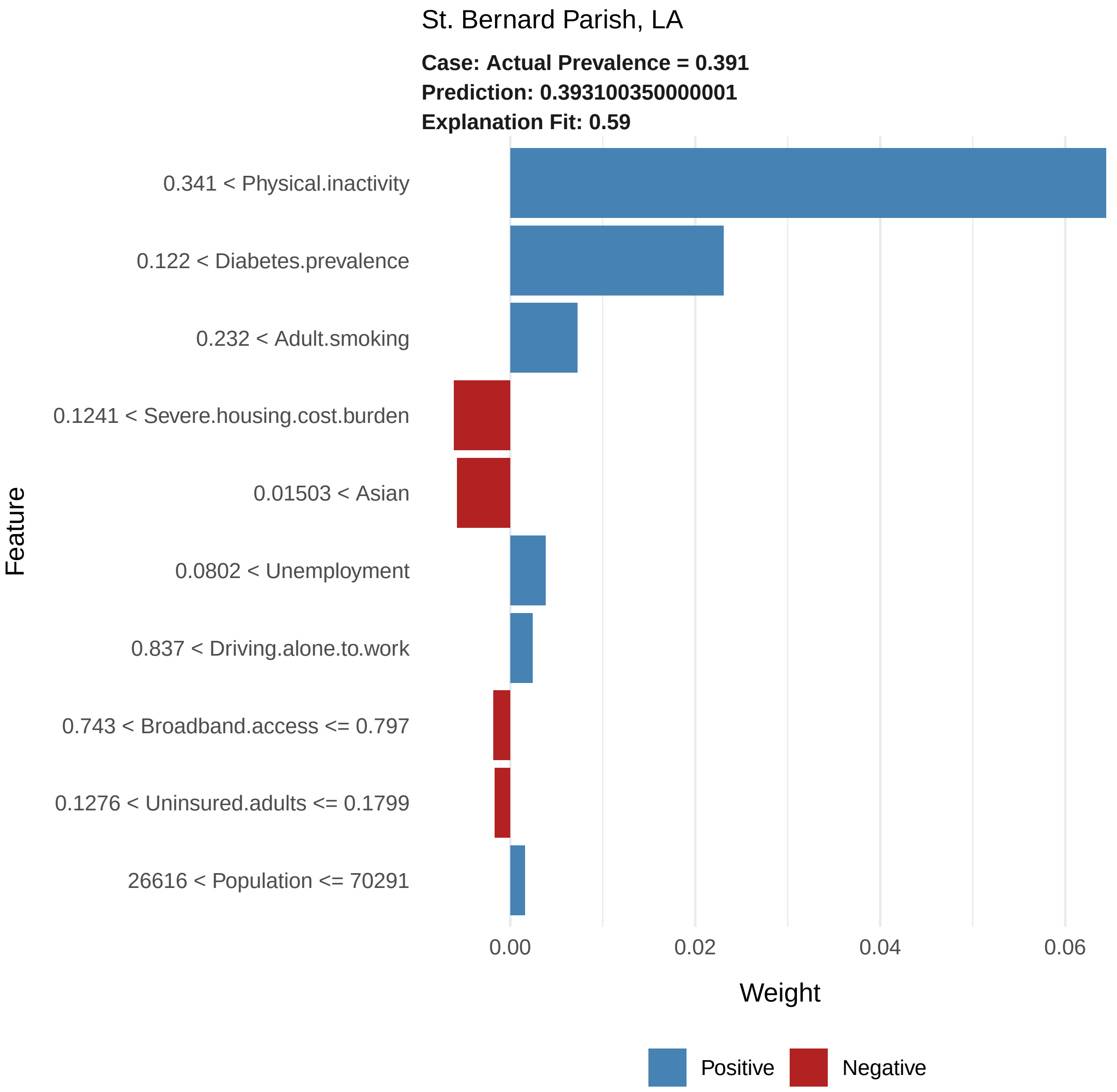
St. Bernard Parish, LA
Case: Actual Prevalence = 0.391
Prediction: 0.393100350000001
Explanation Fit: 0.59
Feature
0.341 < Physical.inactivity
0.122 < Diabetes.prevalence
0.232 < Adult.smoking
0.1241 < Severe.housing.cost.burden
0.01503 < Asian
0.0802 < Unemployment
0.837 < Driving.alone.to.work
0.743 < Broadband.access <= 0.797
0.1276 < Uninsured.adults <= 0.1799
26616 < Population <= 70291
0.00
0.02
0.04
0.06
Weight
Positive
Negative

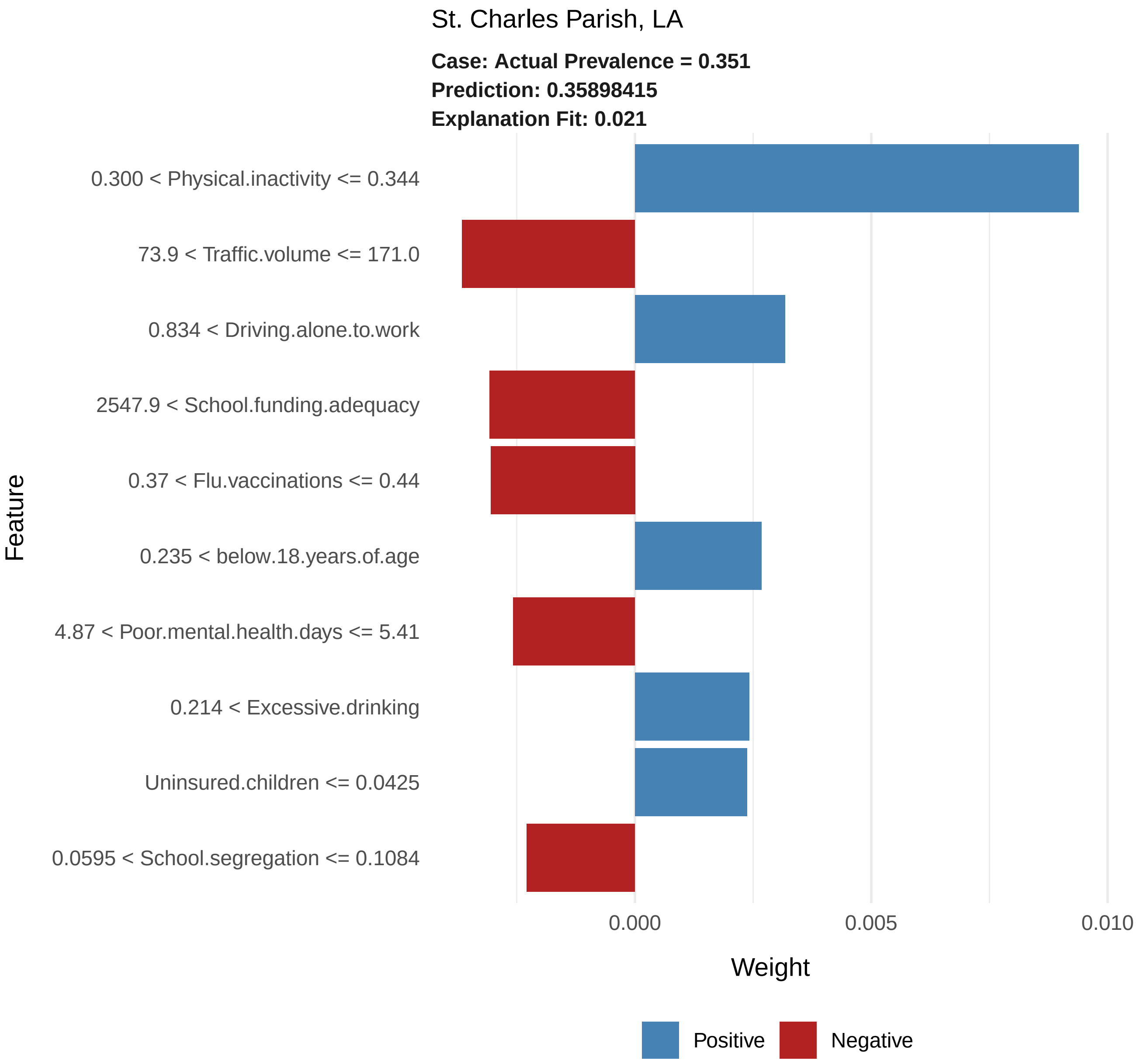
St. Charles Parish, LA
Case: Actual Prevalence = 0.351
Prediction: 0.35898415
Explanation Fit: 0.021
0.300 < Physical.inactivity <= 0.344
73.9 < Traffic.volume <= 171.0
0.834 < Driving.alone.to.work
2547.9 < School.funding.adequacy
0.37 < Flu.vaccinations <= 0.44
0.235 < below.18.years.of.age
4.87 < Poor.mental.health.days <= 5.41
0.214 < Excessive.drinking
Uninsured.children <= 0.0425
0.0595 < School.segregation <= 0.1084
Feature
0.000
0.005
0.010
Weight
Positive
Negative

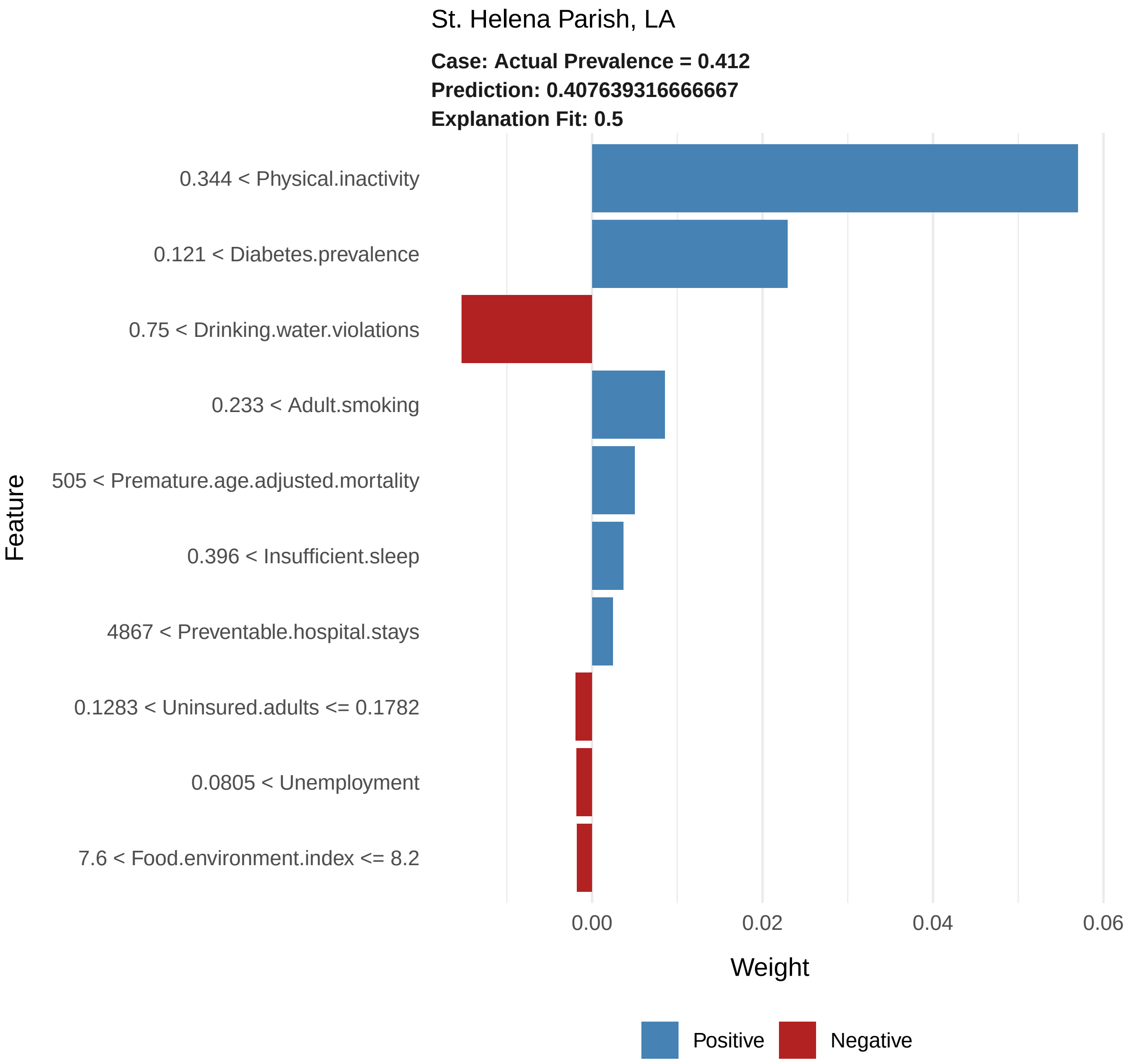
St. Helena Parish, LA
Case: Actual Prevalence = 0.412
Prediction: 0.407639316666667
Explanation Fit: 0.5
0.344 < Physical.inactivity
0.121 < Diabetes.prevalence
0.75 < Drinking.water.violations
0.233 < Adult.smoking
505 < Premature.age.adjusted.mortality
0.396 < Insufficient.sleep
4867 < Preventable.hospital.stays
0.1283 < Uninsured.adults <= 0.1782
0.0805 < Unemployment
7.6 < Food.environment.index <= 8.2
Feature
0.00
0.02
0.04
0.06
Weight
Positive
Negative

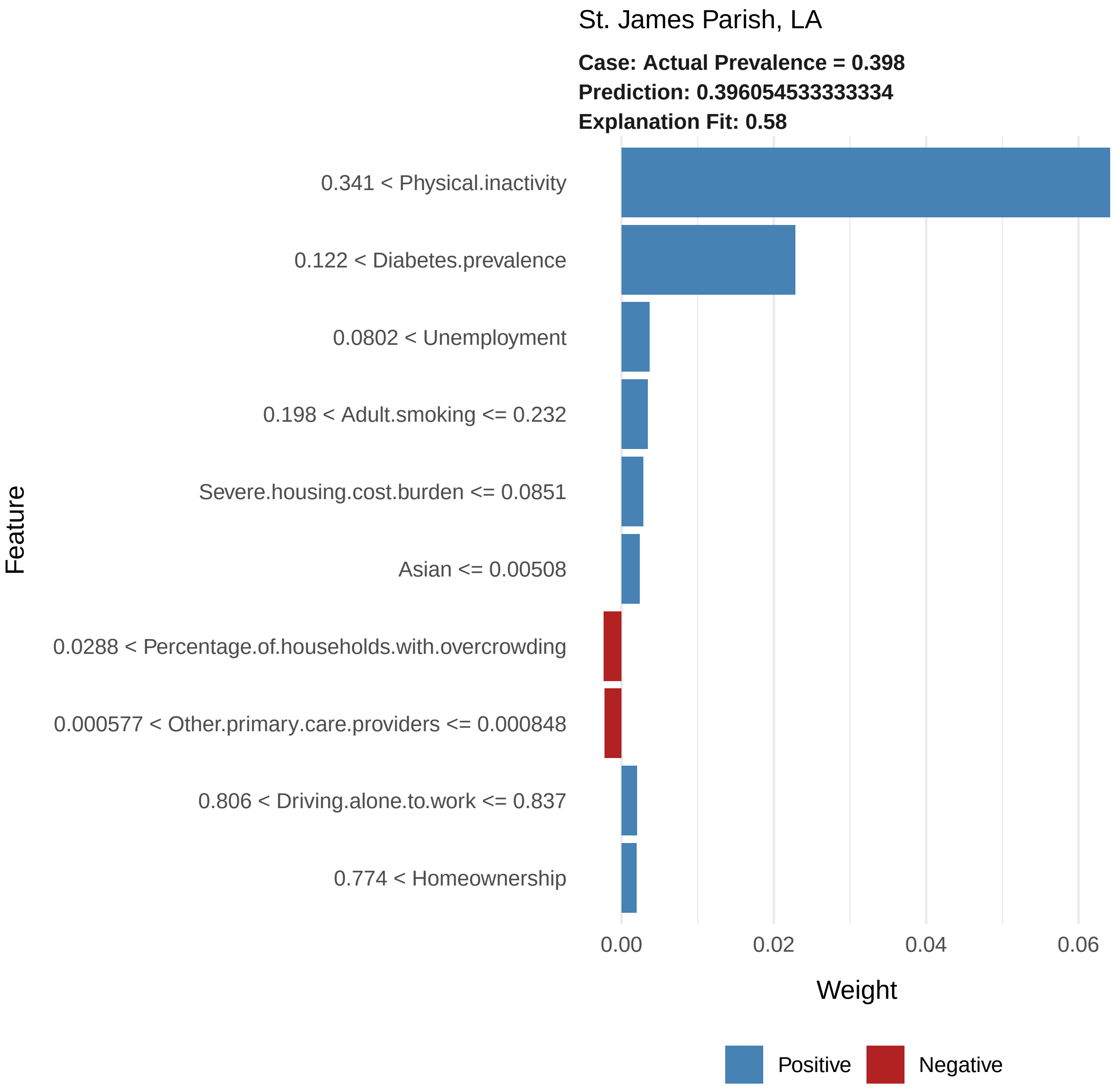
St. James Parish, LA
Case: Actual Prevalence = 0.398
Prediction: 0.396054533333334
Explanation Fit: 0.58
0.341 < Physical.inactivity
0.122 < Diabetes.prevalence
0.0802 < Unemployment
0.198 < Adult.smoking <= 0.232
Severe.housing.cost.burden <= 0.0851
Asian <= 0.00508
0.0288 < Percentage.of.households.with.overcrowding
0.000577 < Other.primary.care.providers <= 0.000848
0.806 < Driving.alone.to.work <= 0.837
0.774 < Homeownership
Feature
0.00
0.02
0.04
0.06
Weight
Positive
Negative

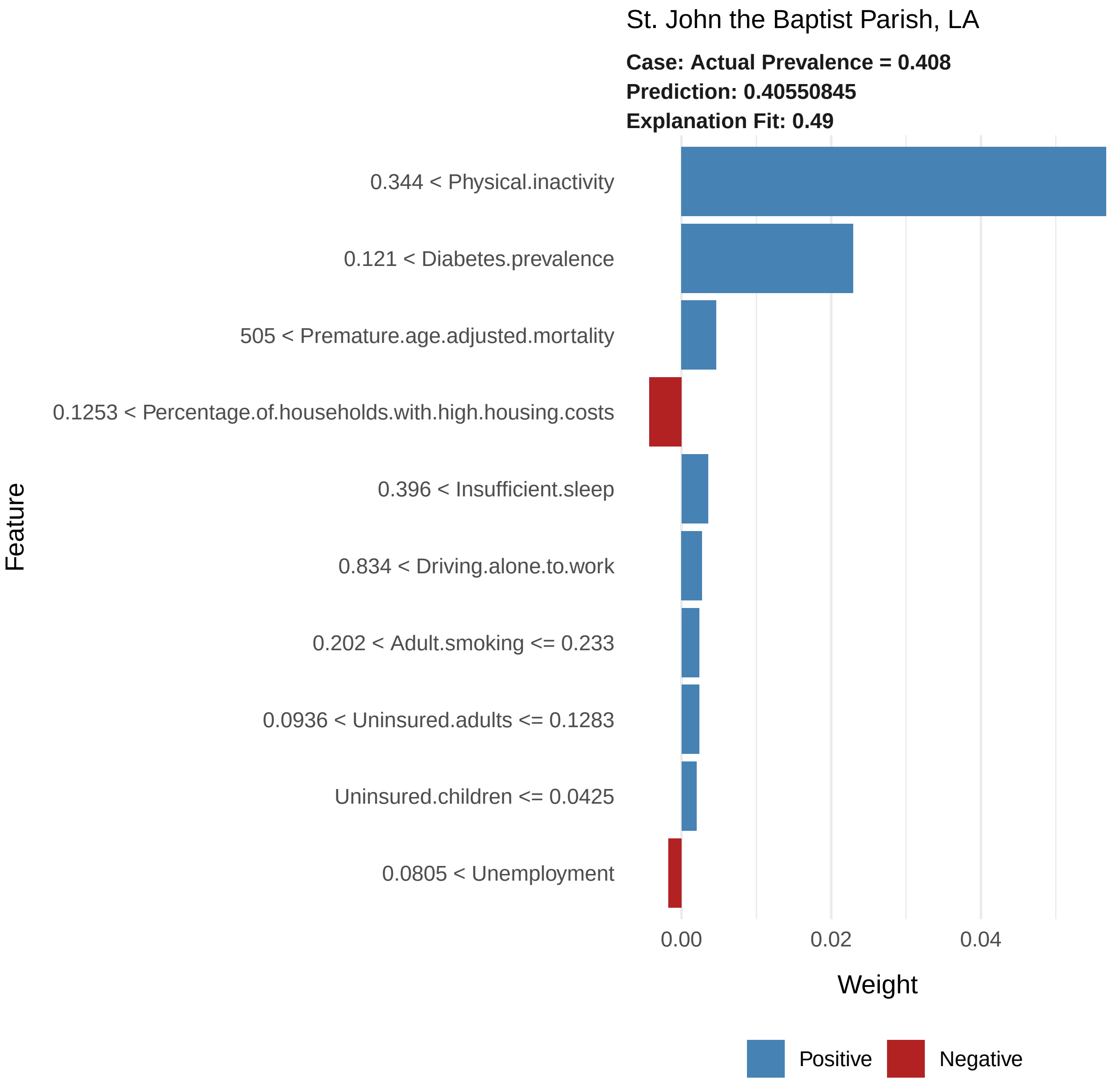
St. John the Baptist Parish, LA
Case: Actual Prevalence = 0.408
Prediction: 0.40550845
Explanation Fit: 0.49
0.344 < Physical.inactivity
0.121 < Diabetes.prevalence
505 < Premature.age.adjusted.mortality
0.1253 < Percentage.of.households.with.high.housing.costs
0.396 < Insufficient.sleep
0.834 < Driving.alone.to.work
0.202 < Adult.smoking <= 0.233
0.0936 < Uninsured.adults <= 0.1283
Uninsured.children <= 0.0425
0.0805 < Unemployment
Feature
0.00
0.02
0.04
Weight
Positive
Negative

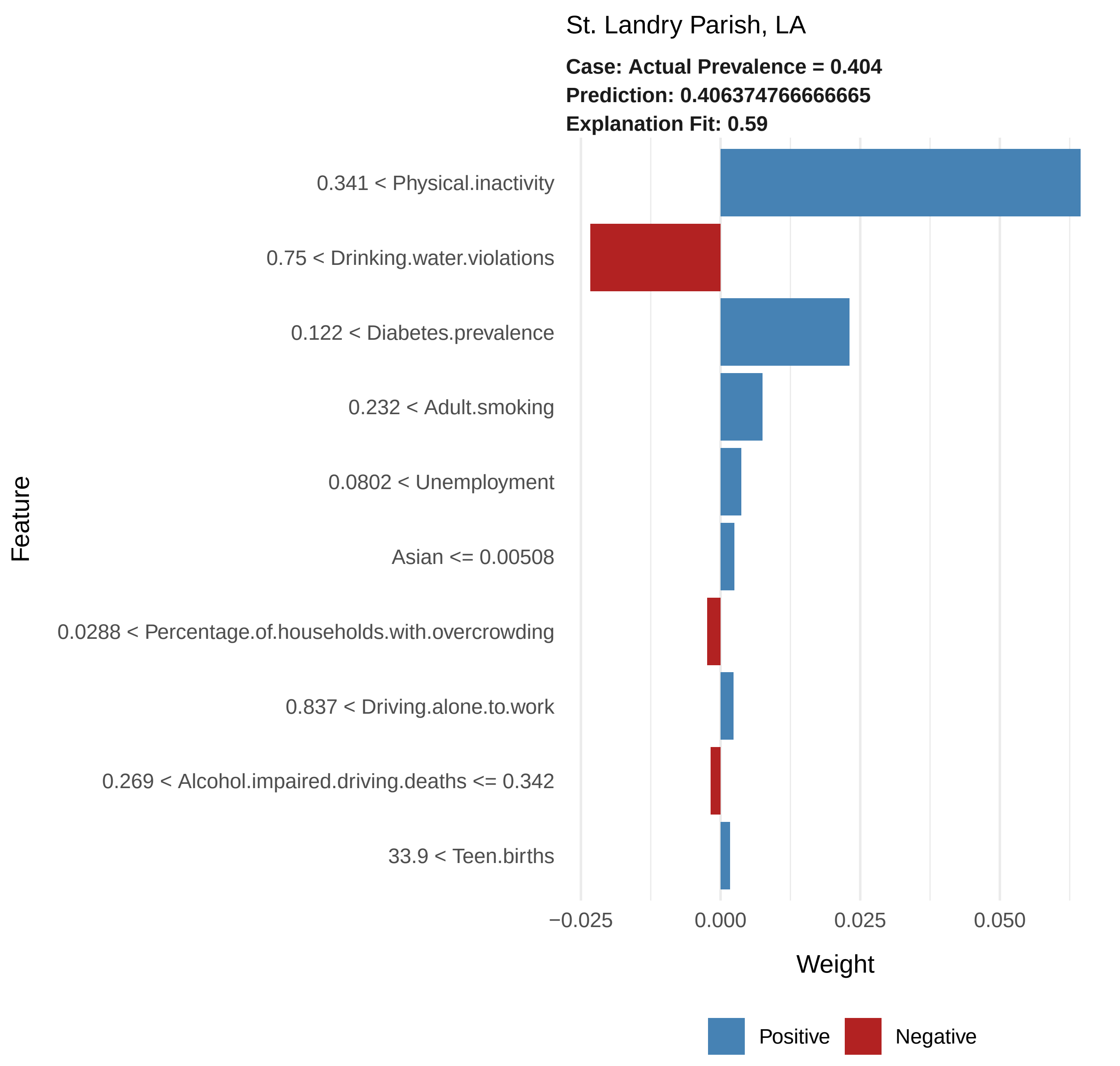

St. Landry Parish, LA
Case: Actual Prevalence = 0.404
Prediction: 0.406374766666665
Explanation Fit: 0.59
0.341 < Physical.inactivity
0.75 < Drinking.water.violations
0.122 < Diabetes.prevalence
0.232 < Adult.smoking
0.0802 < Unemployment
Asian <= 0.00508
0.0288 < Percentage.of.households.with.overcrowding
0.837 < Driving.alone.to.work
0.269 < Alcohol.impaired.driving.deaths <= 0.342
33.9 < Teen.births
Feature
−0.025
0.000
0.025
0.050
Weight
Positive
Negative

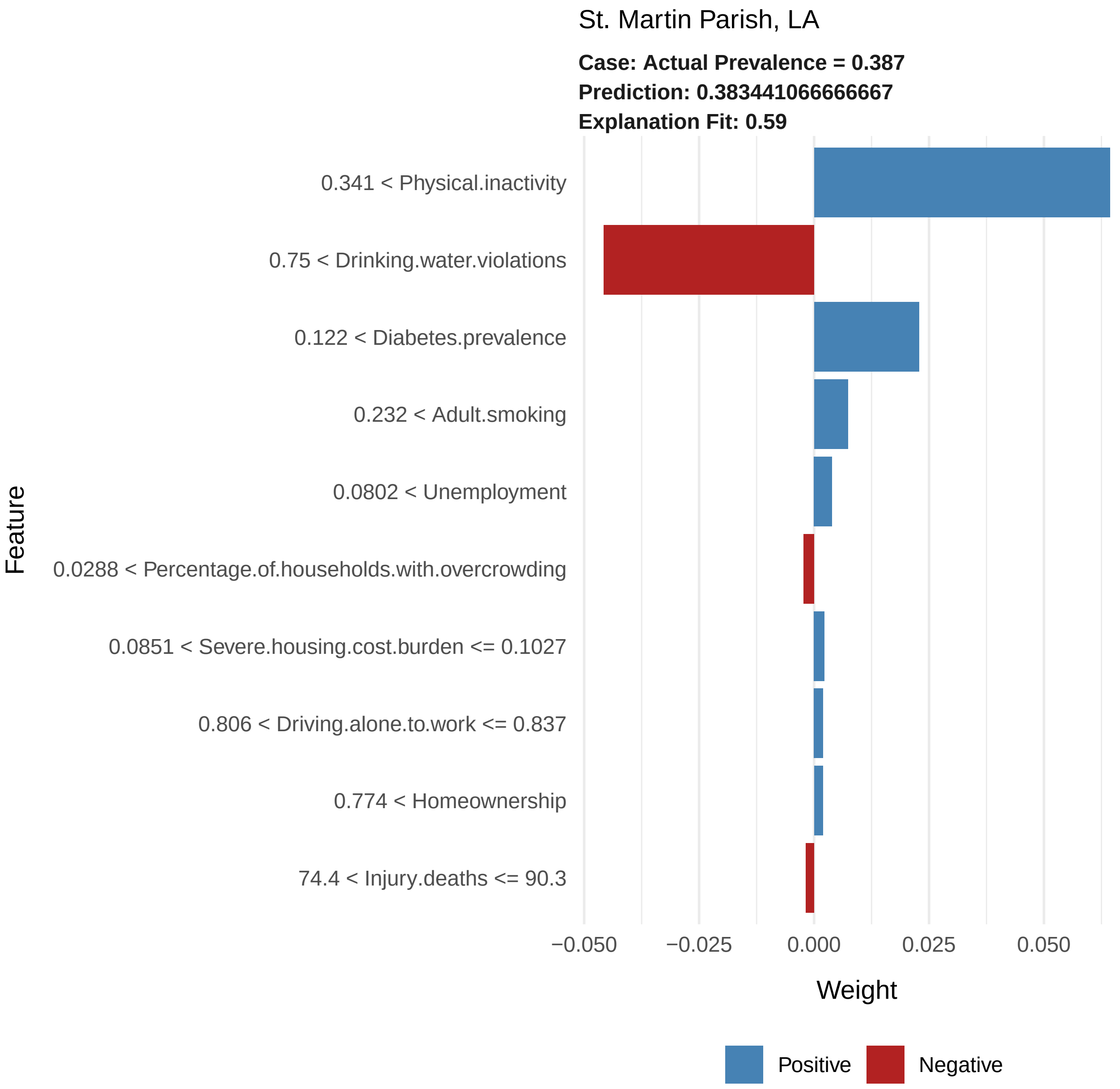

St. Martin Parish, LA
Case: Actual Prevalence = 0.387
Prediction: 0.383441066666667
Explanation Fit: 0.59
0.341 < Physical.inactivity
0.75 < Drinking.water.violations
0.122 < Diabetes.prevalence
0.232 < Adult.smoking
0.0802 < Unemployment
0.0288 < Percentage.of.households.with.overcrowding
0.0851 < Severe.housing.cost.burden <= 0.1027
0.806 < Driving.alone.to.work <= 0.837
0.774 < Homeownership
74.4 < Injury.deaths <= 90.3
Feature
−0.050
−0.025
0.000
0.025
0.050
Weight
Positive
Negative

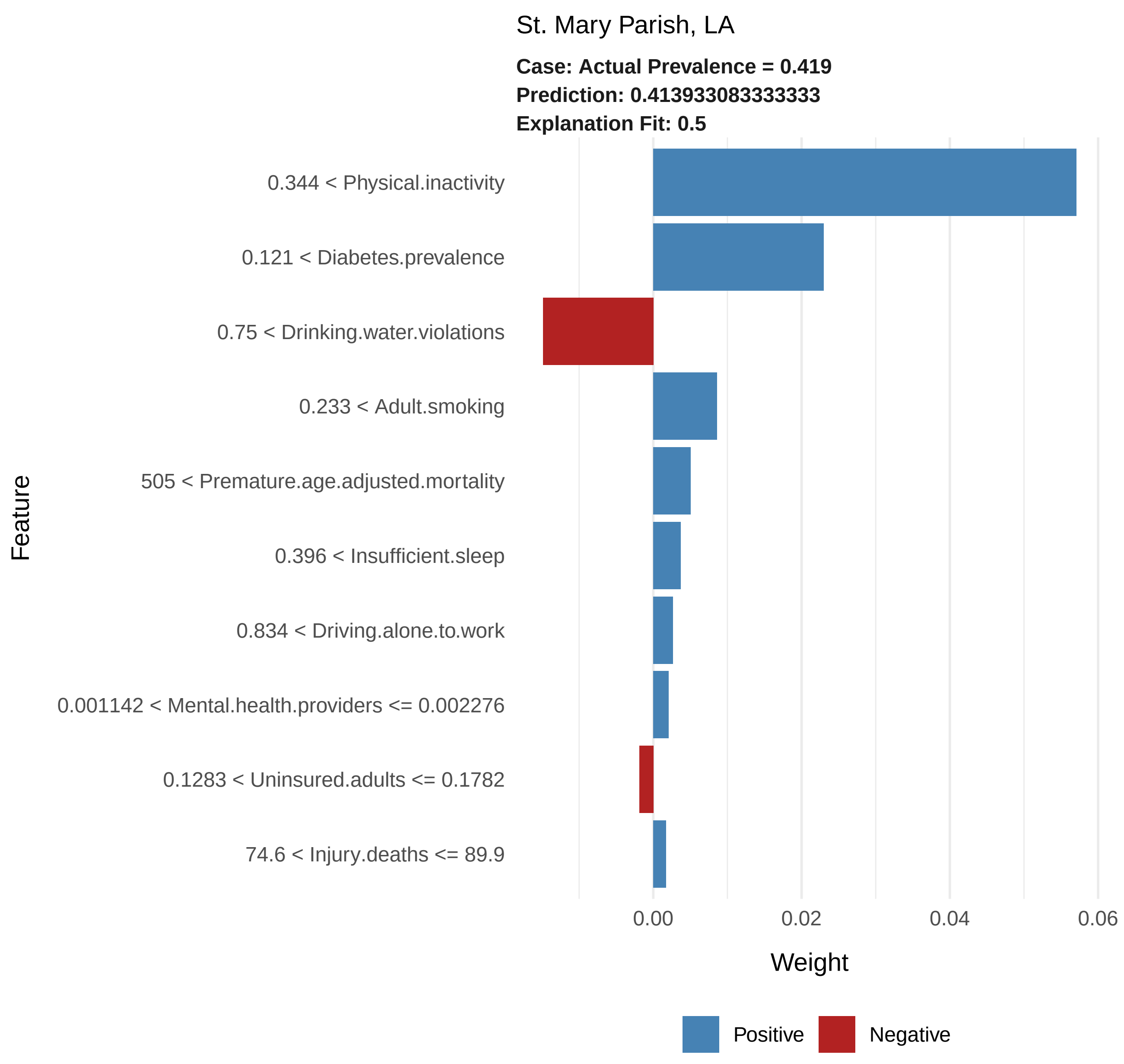
St. Mary Parish, LA
Case: Actual Prevalence = 0.419
Prediction: 0.413933083333333
Explanation Fit: 0.5
0.344 < Physical.inactivity
0.121 < Diabetes.prevalence
0.75 < Drinking.water.violations
0.233 < Adult.smoking
505 < Premature.age.adjusted.mortality
0.396 < Insufficient.sleep
0.834 < Driving.alone.to.work
0.001142 < Mental.health.providers <= 0.002276
0.1283 < Uninsured.adults <= 0.1782
74.6 < Injury.deaths <= 89.9
Feature
0.00
0.02
0.04
0.06
Weight
Positive
Negative

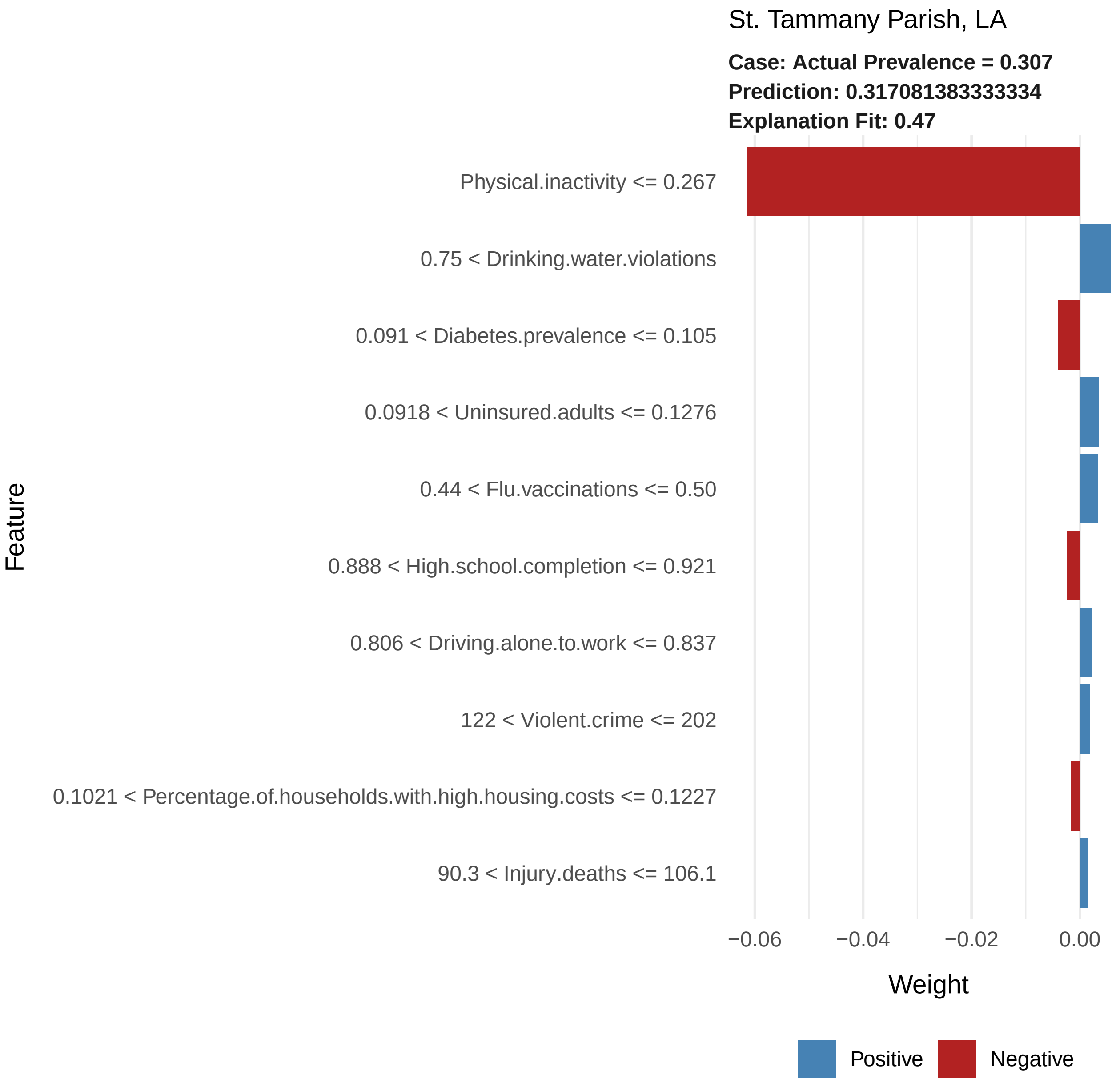
St. Tammany Parish, LA
Case: Actual Prevalence = 0.307
Prediction: 0.317081383333334
Explanation Fit: 0.47
Feature
Physical.inactivity <= 0.267
0.75 < Drinking.water.violations
0.091 < Diabetes.prevalence <= 0.105
0.0918 < Uninsured.adults <= 0.1276
0.44 < Flu.vaccinations <= 0.50
0.888 < High.school.completion <= 0.921
0.806 < Driving.alone.to.work <= 0.837
122 < Violent.crime <= 202
0.1021 < Percentage.of.households.with.high.housing.costs <= 0.1227
90.3 < Injury.deaths <= 106.1
−0.06
−0.04
−0.02
0.00
Weight
Positive
Negative

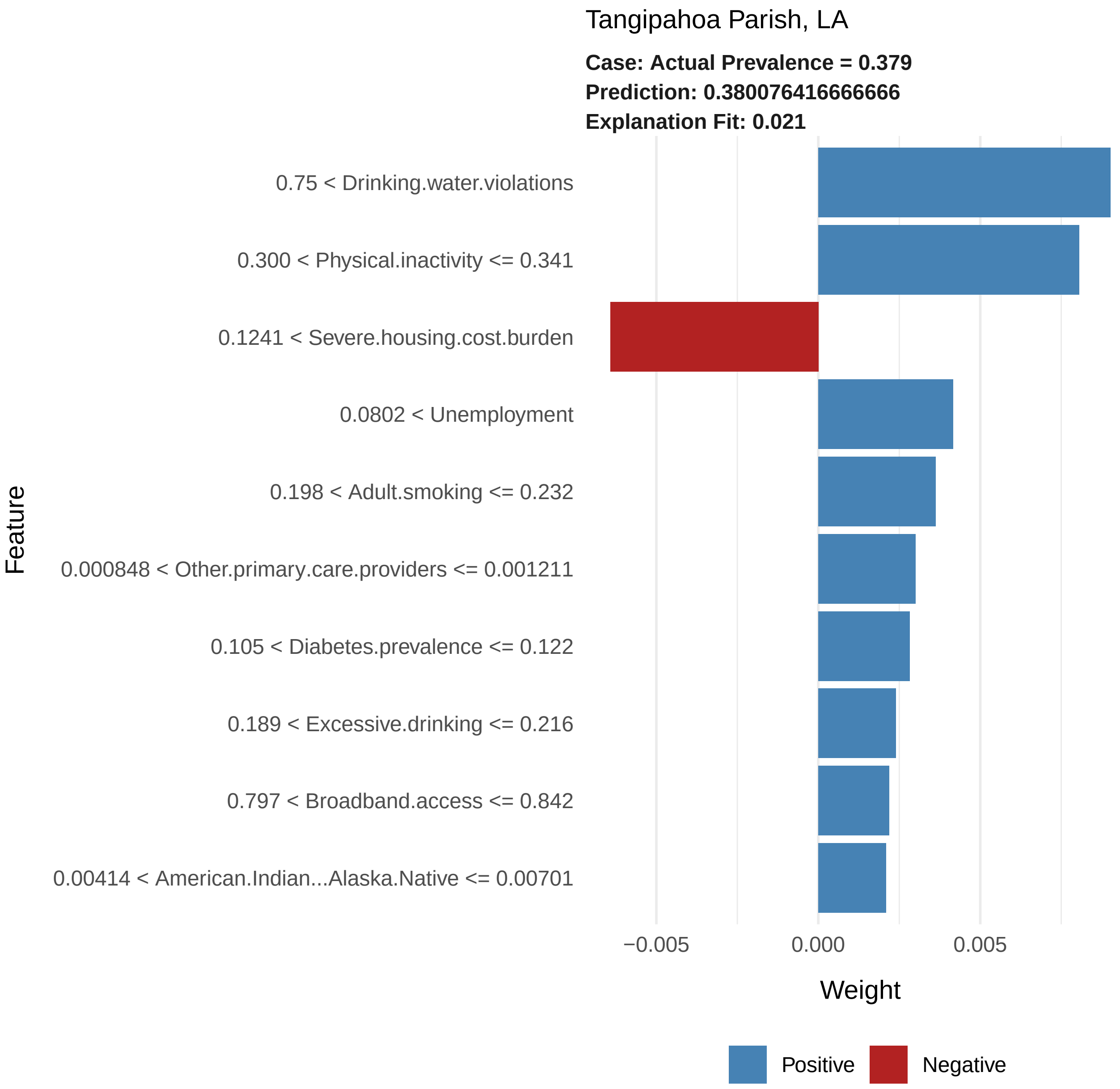

Tangipahoa Parish, LA
Case: Actual Prevalence = 0.379
Prediction: 0.380076416666666
Explanation Fit: 0.021
0.75 < Drinking.water.violations
0.300 < Physical.inactivity <= 0.341
0.1241 < Severe.housing.cost.burden
0.0802 < Unemployment
0.198 < Adult.smoking <= 0.232
0.000848 < Other.primary.care.providers <= 0.001211
0.105 < Diabetes.prevalence <= 0.122
0.189 < Excessive.drinking <= 0.216
0.797 < Broadband.access <= 0.842
0.00414 < American.Indian...Alaska.Native <= 0.00701
Feature
−0.005
0.000
0.005
Weight
Positive
Negative

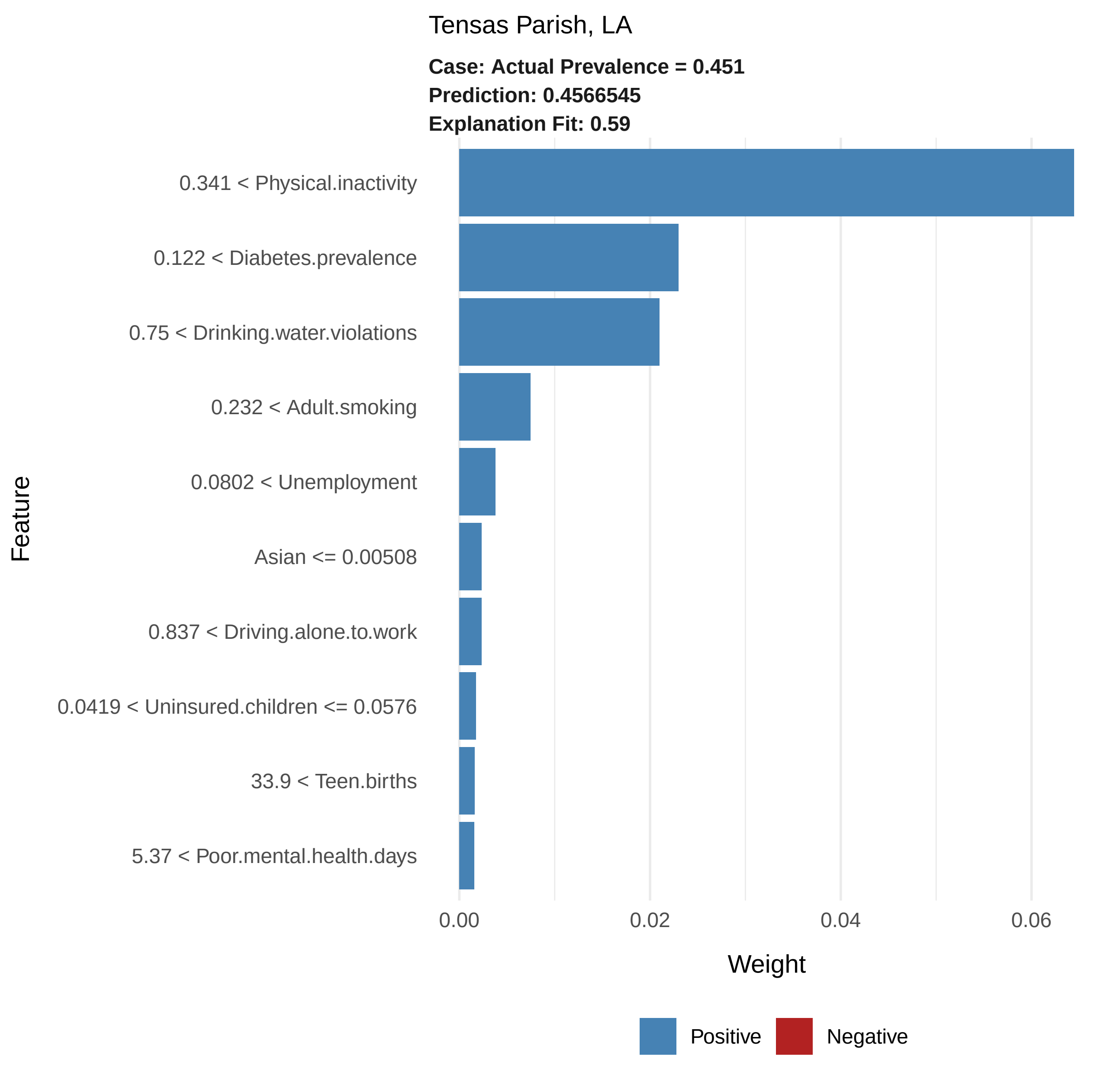
Tensas Parish, LA
Case: Actual Prevalence = 0.451
Prediction: 0.4566545
Explanation Fit: 0.59
0.341 < Physical.inactivity
0.122 < Diabetes.prevalence
0.75 < Drinking.water.violations
0.232 < Adult.smoking
0.0802 < Unemployment
Asian <= 0.00508
0.837 < Driving.alone.to.work
0.0419 < Uninsured.children <= 0.0576
33.9 < Teen.births
5.37 < Poor.mental.health.days
Feature
0.00
0.02
0.04
0.06
Weight
Positive
Negative

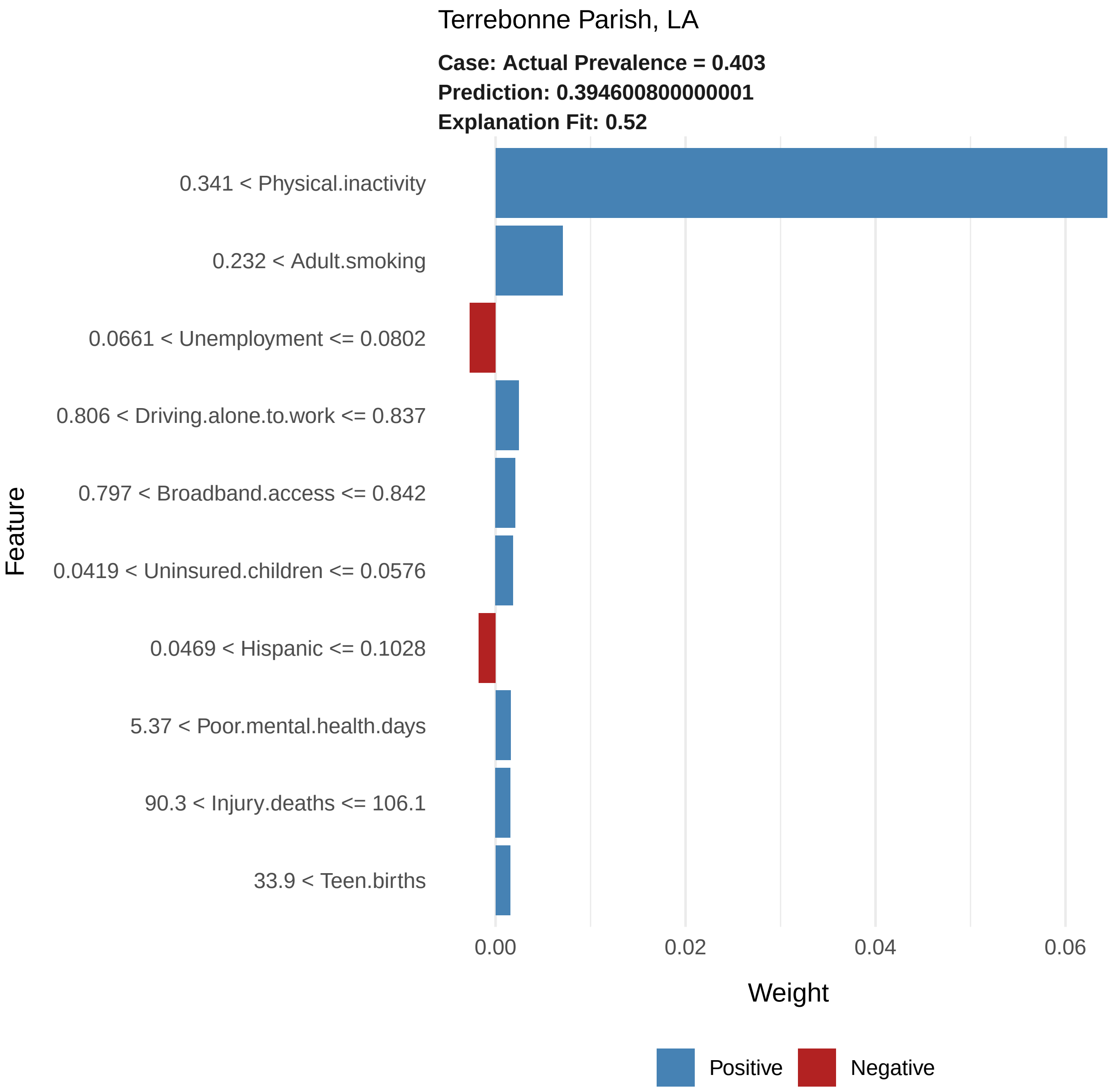
Terrebonne Parish, LA
Case: Actual Prevalence = 0.403
Prediction: 0.394600800000001
Explanation Fit: 0.52
0.341 < Physical.inactivity
0.232 < Adult.smoking
0.0661 < Unemployment <= 0.0802
0.806 < Driving.alone.to.work <= 0.837
0.797 < Broadband.access <= 0.842
0.0419 < Uninsured.children <= 0.0576
0.0469 < Hispanic <= 0.1028
5.37 < Poor.mental.health.days
90.3 < Injury.deaths <= 106.1
33.9 < Teen.births
Feature
0.00
0.02
0.04
0.06
Weight
Positive
Negative

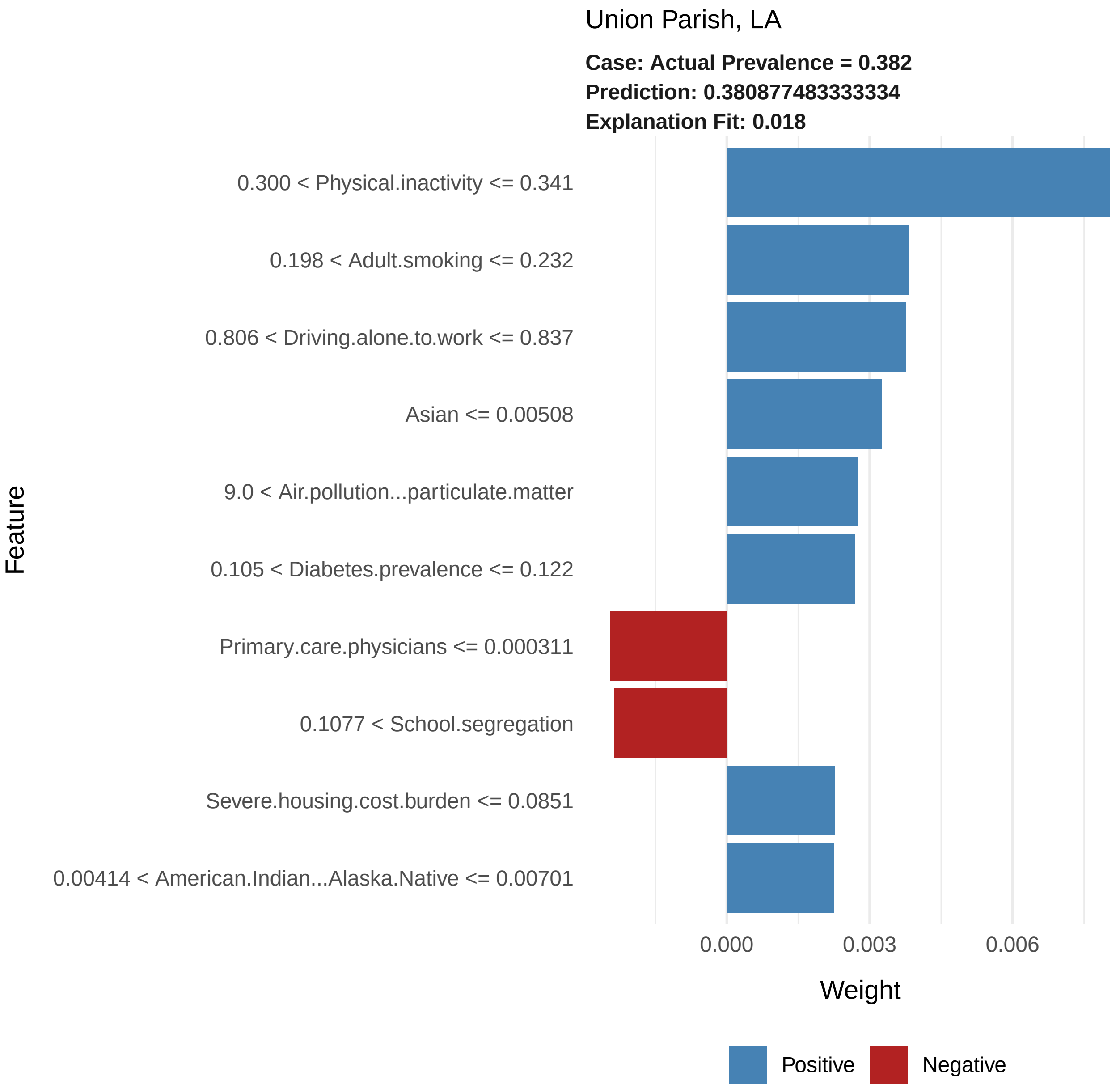

Union Parish, LA
Case: Actual Prevalence = 0.382
Prediction: 0.380877483333334
Explanation Fit: 0.018
0.300 < Physical.inactivity <= 0.341
0.198 < Adult.smoking <= 0.232
0.806 < Driving.alone.to.work <= 0.837
Asian <= 0.00508
9.0 < Air.pollution...particulate.matter
0.105 < Diabetes.prevalence <= 0.122
Primary.care.physicians <= 0.000311
0.1077 < School.segregation
Severe.housing.cost.burden <= 0.0851
0.00414 < American.Indian...Alaska.Native <= 0.00701
Feature
0.000
0.003
0.006
Weight
Positive
Negative

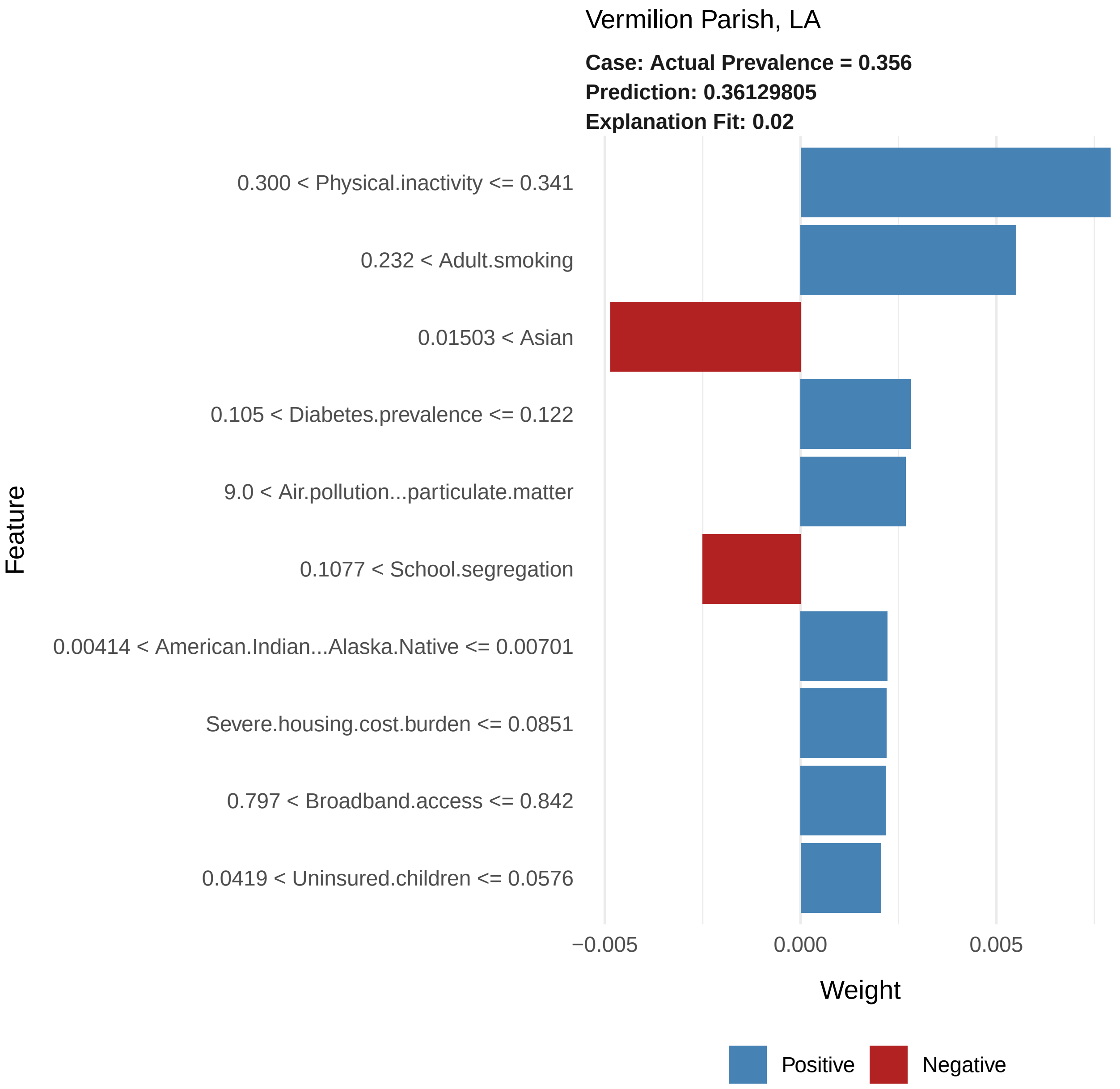

Vermilion Parish, LA
Case: Actual Prevalence = 0.356
Prediction: 0.36129805
Explanation Fit: 0.02
0.300 < Physical.inactivity <= 0.341
0.232 < Adult.smoking
0.01503 < Asian
0.105 < Diabetes.prevalence <= 0.122
9.0 < Air.pollution...particulate.matter
0.1077 < School.segregation
0.00414 < American.Indian...Alaska.Native <= 0.00701
Severe.housing.cost.burden <= 0.0851
0.797 < Broadband.access <= 0.842
0.0419 < Uninsured.children <= 0.0576
Feature
−0.005
0.000
0.005
Weight
Positive
Negative

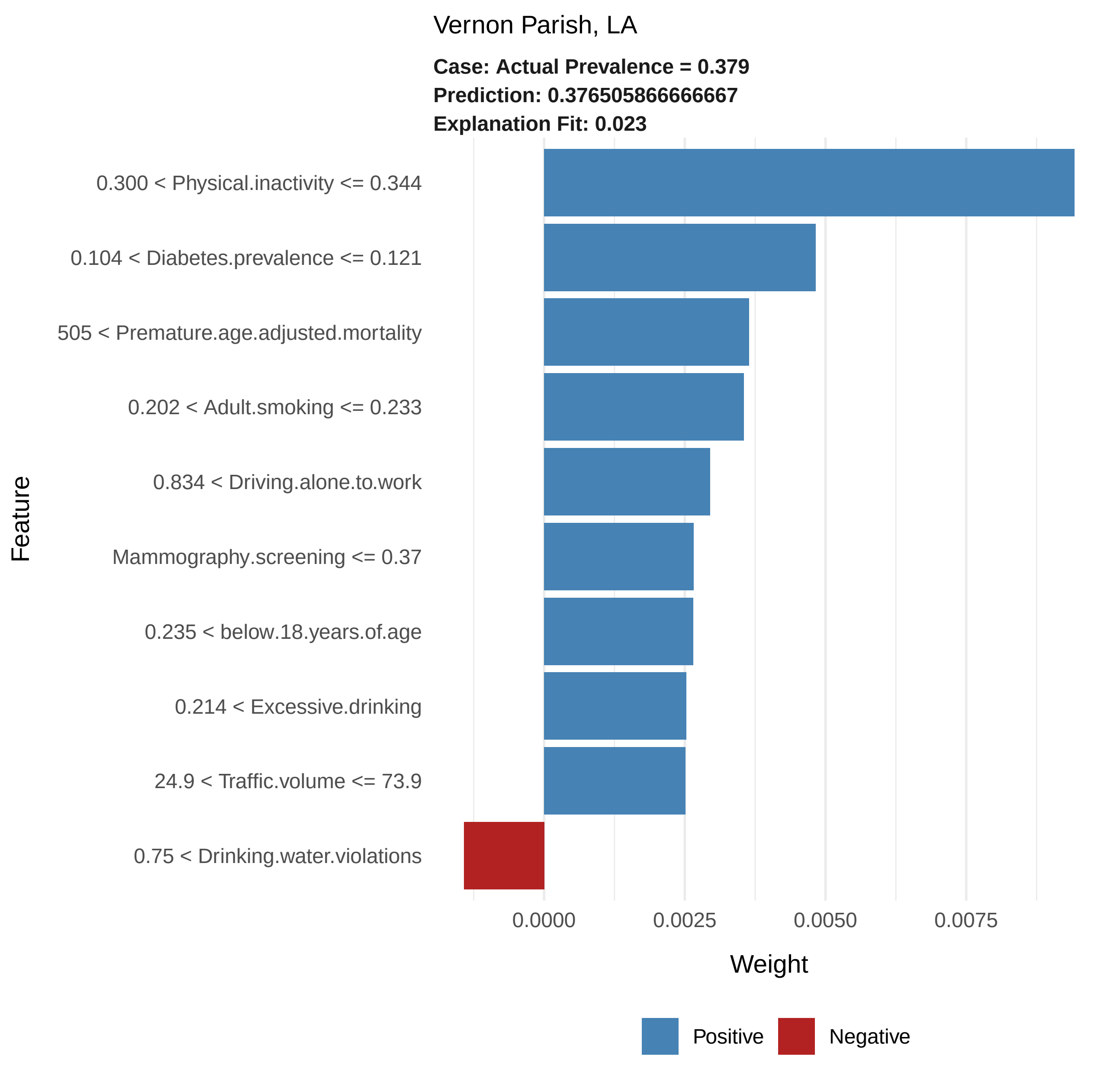
Vernon Parish, LA
Case: Actual Prevalence = 0.379
Prediction: 0.376505866666667
Explanation Fit: 0.023
0.300 < Physical.inactivity <= 0.344
0.104 < Diabetes.prevalence <= 0.121
505 < Premature.age.adjusted.mortality
0.202 < Adult.smoking <= 0.233
0.834 < Driving.alone.to.work
Mammography.screening <= 0.37
0.235 < below.18.years.of.age
0.214 < Excessive.drinking
24.9 < Traffic.volume <= 73.9
0.75 < Drinking.water.violations
Feature
0.0000
0.0025
0.0050
0.0075
Weight
Positive
Negative

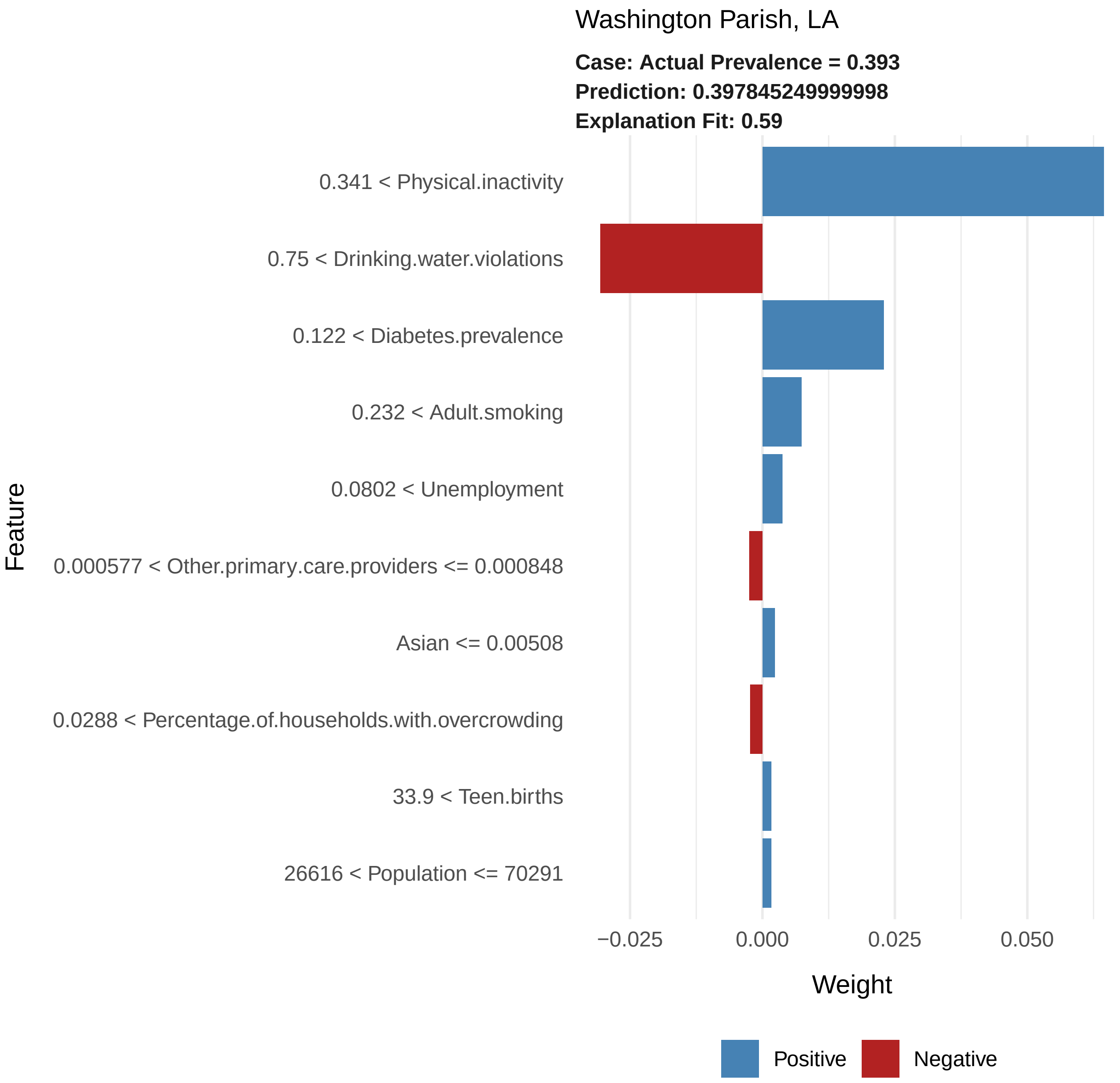

Washington Parish, LA
Case: Actual Prevalence = 0.393
Prediction: 0.397845249999998
Explanation Fit: 0.59
0.341 < Physical.inactivity
0.75 < Drinking.water.violations
0.122 < Diabetes.prevalence
0.232 < Adult.smoking
0.0802 < Unemployment
0.000577 < Other.primary.care.providers <= 0.000848
Asian <= 0.00508
0.0288 < Percentage.of.households.with.overcrowding
33.9 < Teen.births
26616 < Population <= 70291
Feature
−0.025
0.000
0.025
0.050
Weight
Positive
Negative

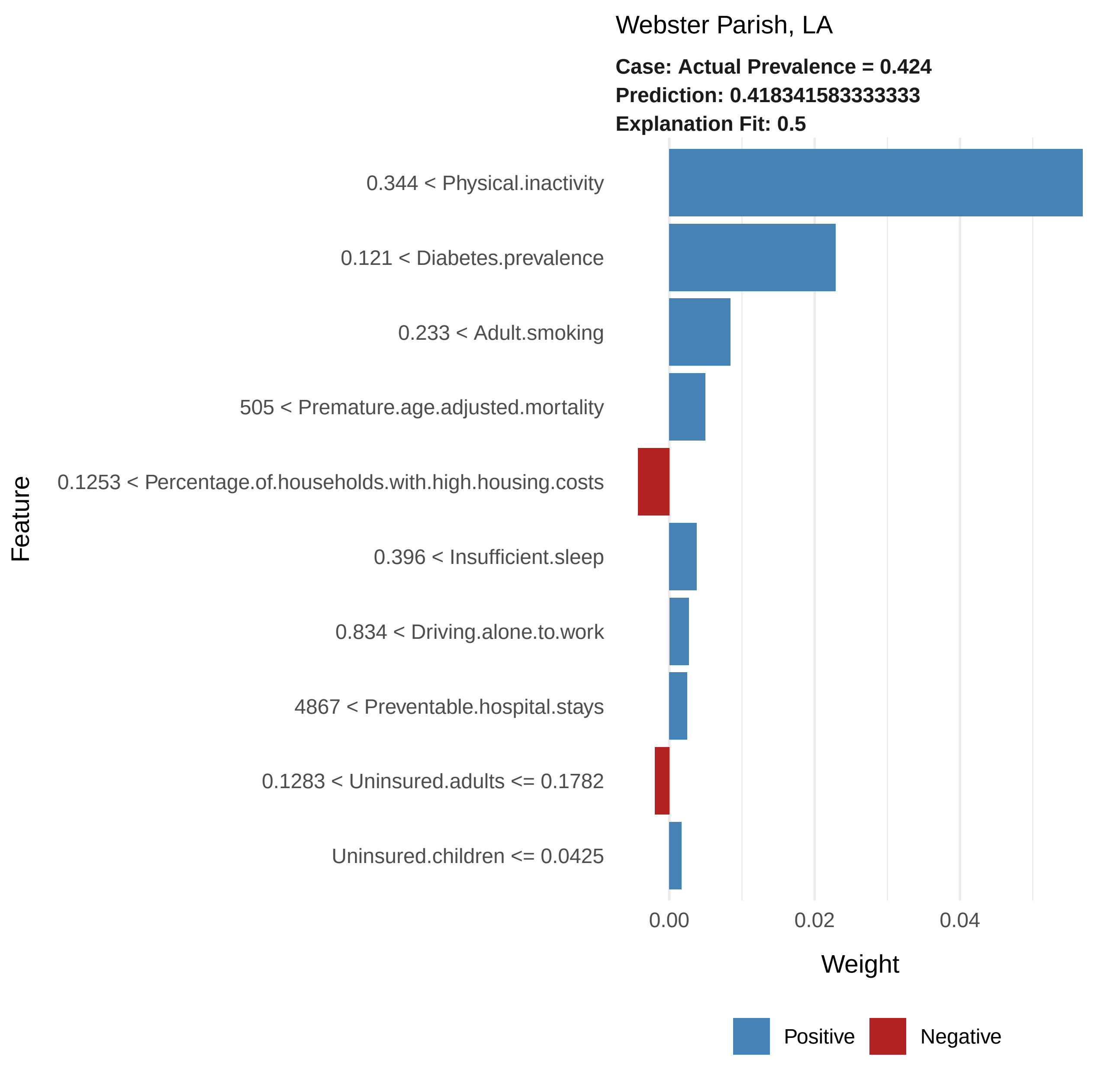

Webster Parish, LA
Case: Actual Prevalence = 0.424
Prediction: 0.418341583333333
Explanation Fit: 0.5
0.344 < Physical.inactivity
0.121 < Diabetes.prevalence
0.233 < Adult.smoking
505 < Premature.age.adjusted.mortality
0.1253 < Percentage.of.households.with.high.housing.costs
0.396 < Insufficient.sleep
0.834 < Driving.alone.to.work
4867 < Preventable.hospital.stays
0.1283 < Uninsured.adults <= 0.1782
Uninsured.children <= 0.0425
Feature
0.00
0.02
0.04
Weight
Positive
Negative

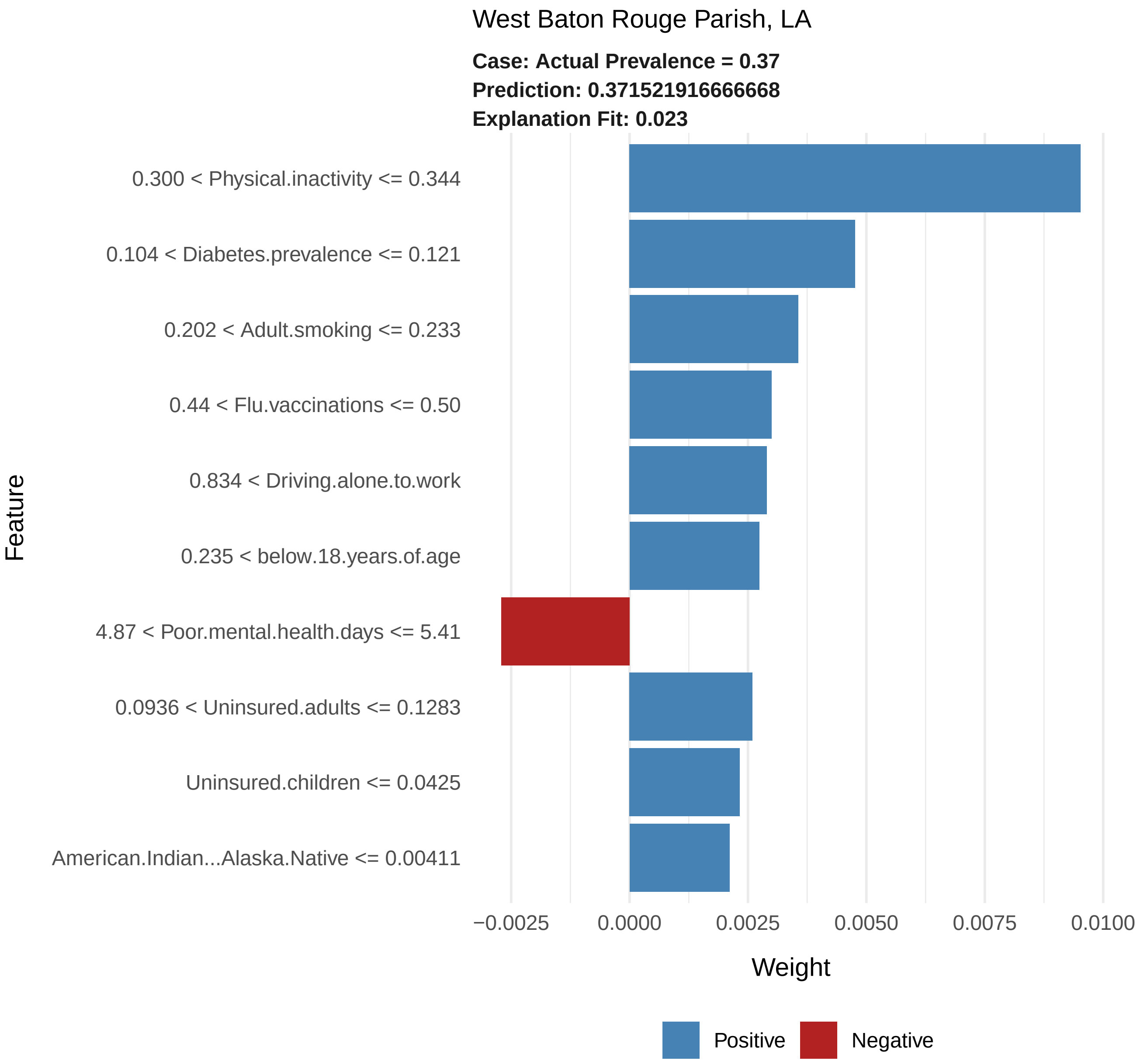
West Baton Rouge Parish, LA
Case: Actual Prevalence = 0.37
Prediction: 0.371521916666668
Explanation Fit: 0.023
0.300 < Physical.inactivity <= 0.344
0.104 < Diabetes.prevalence <= 0.121
0.202 < Adult.smoking <= 0.233
0.44 < Flu.vaccinations <= 0.50
0.834 < Driving.alone.to.work
0.235 < below.18.years.of.age
4.87 < Poor.mental.health.days <= 5.41
0.0936 < Uninsured.adults <= 0.1283
Uninsured.children <= 0.0425
American.Indian...Alaska.Native <= 0.00411
Feature
−0.0025
0.0000
0.0025
0.0050
0.0075
0.0100
Weight
Positive
Negative

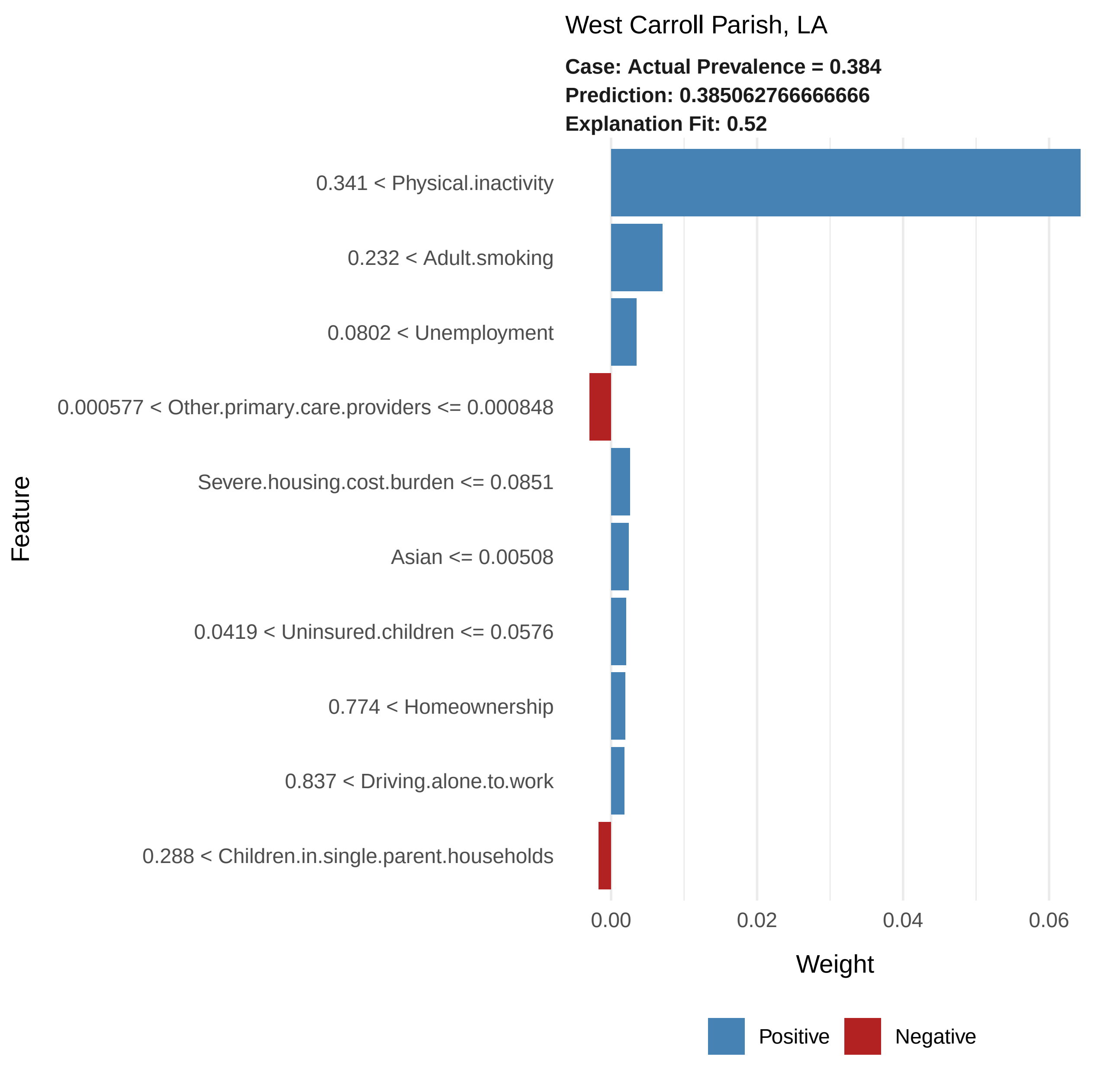

West Carroll Parish, LA
Case: Actual Prevalence = 0.384
Prediction: 0.385062766666666
Explanation Fit: 0.52
0.341 < Physical.inactivity
0.232 < Adult.smoking
0.0802 < Unemployment
0.000577 < Other.primary.care.providers <= 0.000848
Severe.housing.cost.burden <= 0.0851
Asian <= 0.00508
0.0419 < Uninsured.children <= 0.0576
0.774 < Homeownership
0.837 < Driving.alone.to.work
0.288 < Children.in.single.parent.households
Feature
0.00
0.02
0.04
0.06
Weight
Positive
Negative

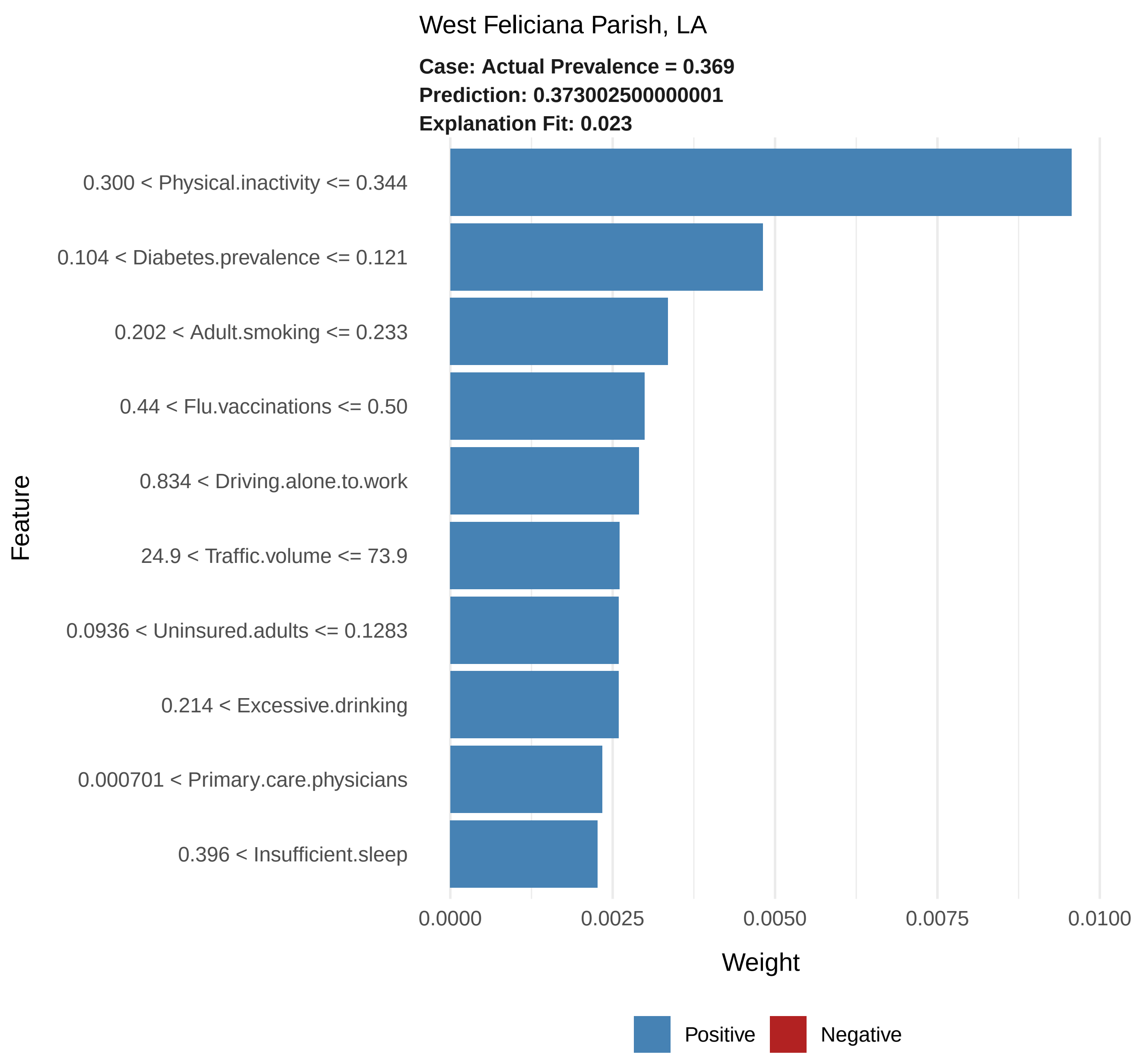
West Feliciana Parish, LA
Case: Actual Prevalence = 0.369
Prediction: 0.373002500000001
Explanation Fit: 0.023
0.300 < Physical.inactivity <= 0.344
0.104 < Diabetes.prevalence <= 0.121
0.202 < Adult.smoking <= 0.233
0.44 < Flu.vaccinations <= 0.50
0.834 < Driving.alone.to.work
24.9 < Traffic.volume <= 73.9
0.0936 < Uninsured.adults <= 0.1283
0.214 < Excessive.drinking
0.000701 < Primary.care.physicians
0.396 < Insufficient.sleep
Feature
0.0000
0.0025
0.0050
0.0075
0.0100
Weight
Positive
Negative

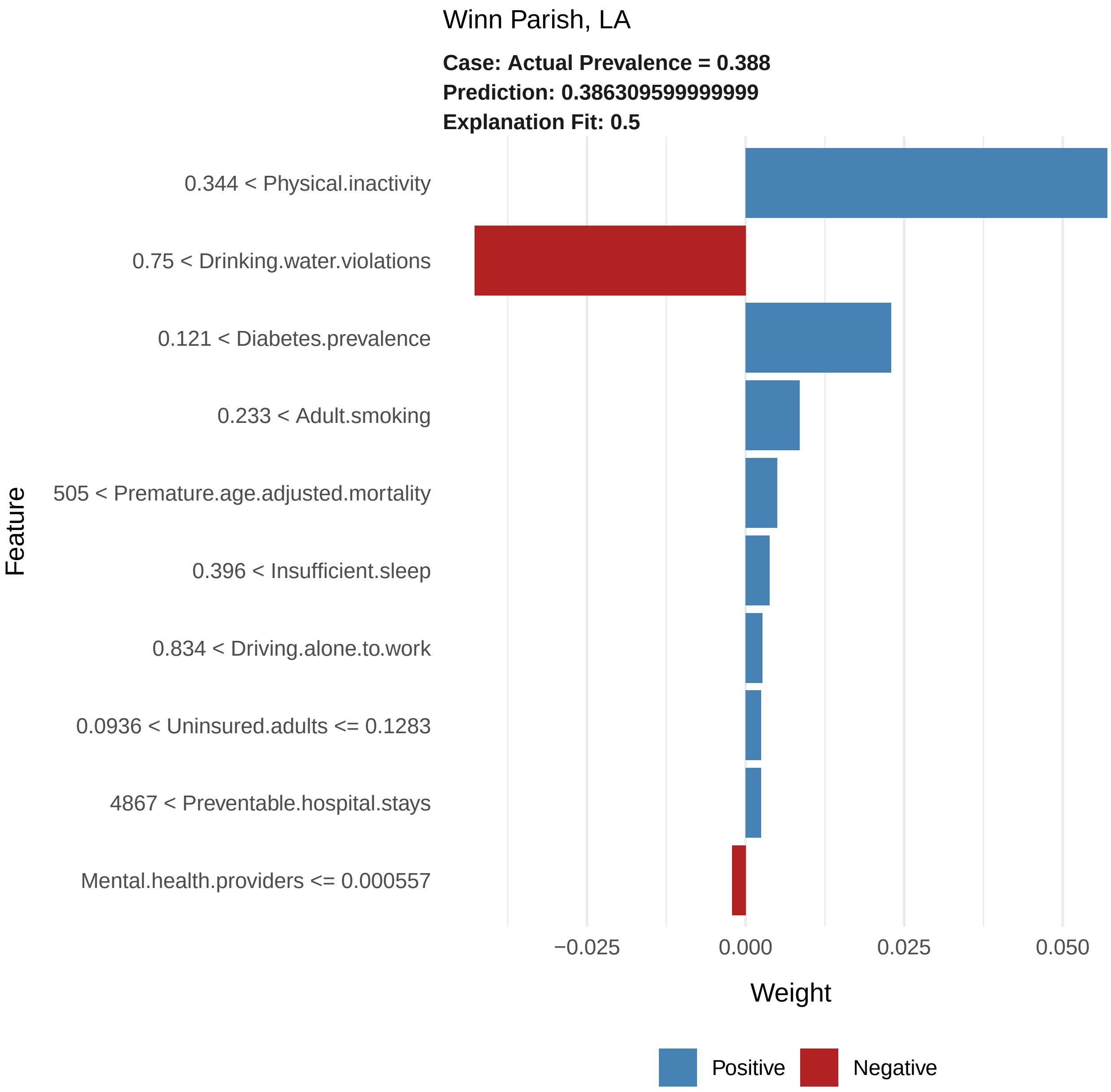

Winn Parish, LA
Case: Actual Prevalence = 0.388
Prediction: 0.386309599999999
Explanation Fit: 0.5
0.344 < Physical.inactivity
0.75 < Drinking.water.violations
0.121 < Diabetes.prevalence
0.233 < Adult.smoking
505 < Premature.age.adjusted.mortality
0.396 < Insufficient.sleep
0.834 < Driving.alone.to.work
0.0936 < Uninsured.adults <= 0.1283
4867 < Preventable.hospital.stays
Mental.health.providers <= 0.000557
Feature
−0.025
0.000
0.025
0.050
Weight
Positive
Negative

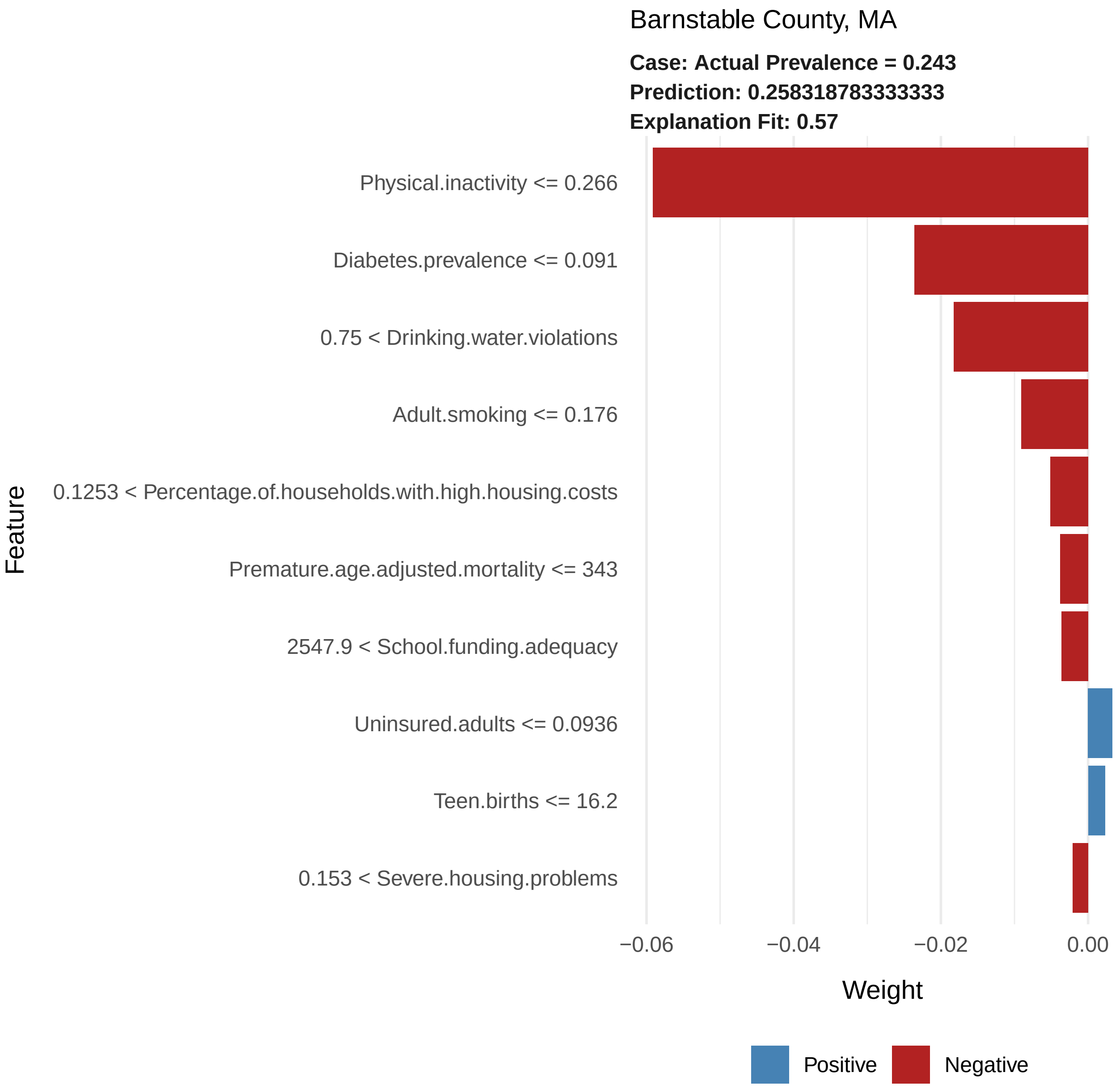

Barnstable County, MA
Case: Actual Prevalence = 0.243
Prediction: 0.258318783333333
Explanation Fit: 0.57
Physical.inactivity <= 0.266
Diabetes.prevalence <= 0.091
0.75 < Drinking.water.violations
Adult.smoking <= 0.176
0.1253 < Percentage.of.households.with.high.housing.costs
Premature.age.adjusted.mortality <= 343
2547.9 < School.funding.adequacy
Uninsured.adults <= 0.0936
Teen.births <= 16.2
0.153 < Severe.housing.problems
Feature
−0.06
−0.04
−0.02
0.00
Weight
Positive
Negative

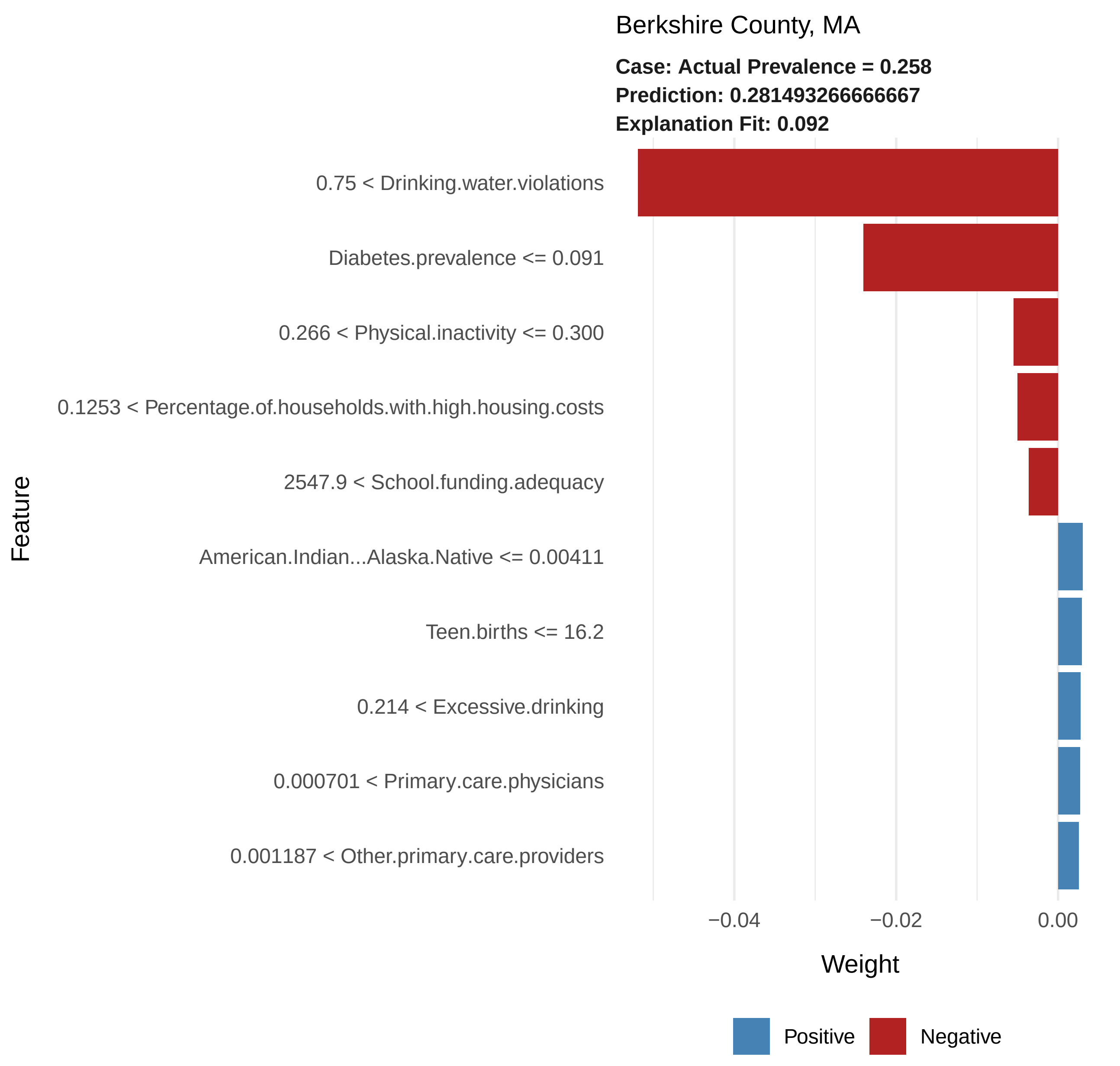
Berkshire County, MA
Case: Actual Prevalence = 0.258
Prediction: 0.281493266666667
Explanation Fit: 0.092
0.75 < Drinking.water.violations
Diabetes.prevalence <= 0.091
0.266 < Physical.inactivity <= 0.300
0.1253 < Percentage.of.households.with.high.housing.costs
2547.9 < School.funding.adequacy
American.Indian...Alaska.Native <= 0.00411
Teen.births <= 16.2
0.214 < Excessive.drinking
0.000701 < Primary.care.physicians
0.001187 < Other.primary.care.providers
Feature
−0.04
−0.02
0.00
Weight
Positive
Negative

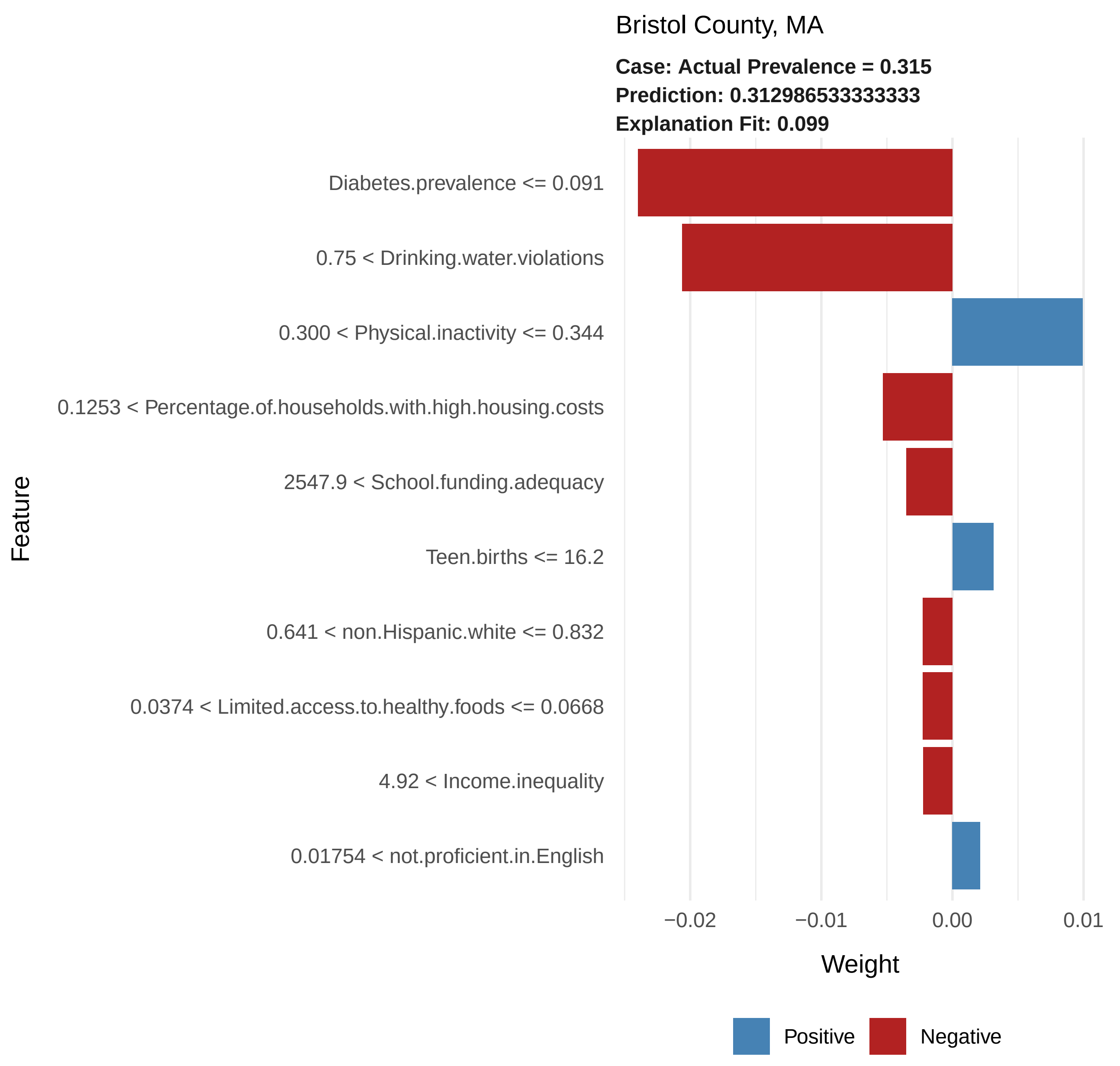

Bristol County, MA
Case: Actual Prevalence = 0.315
Prediction: 0.312986533333333
Explanation Fit: 0.099
Diabetes.prevalence <= 0.091
0.75 < Drinking.water.violations
0.300 < Physical.inactivity <= 0.344
0.1253 < Percentage.of.households.with.high.housing.costs
2547.9 < School.funding.adequacy
Teen.births <= 16.2
0.641 < non.Hispanic.white <= 0.832
0.0374 < Limited.access.to.healthy.foods <= 0.0668
4.92 < Income.inequality
0.01754 < not.proficient.in.English
Feature
−0.02
−0.01
0.00
0.01
Weight
Positive
Negative

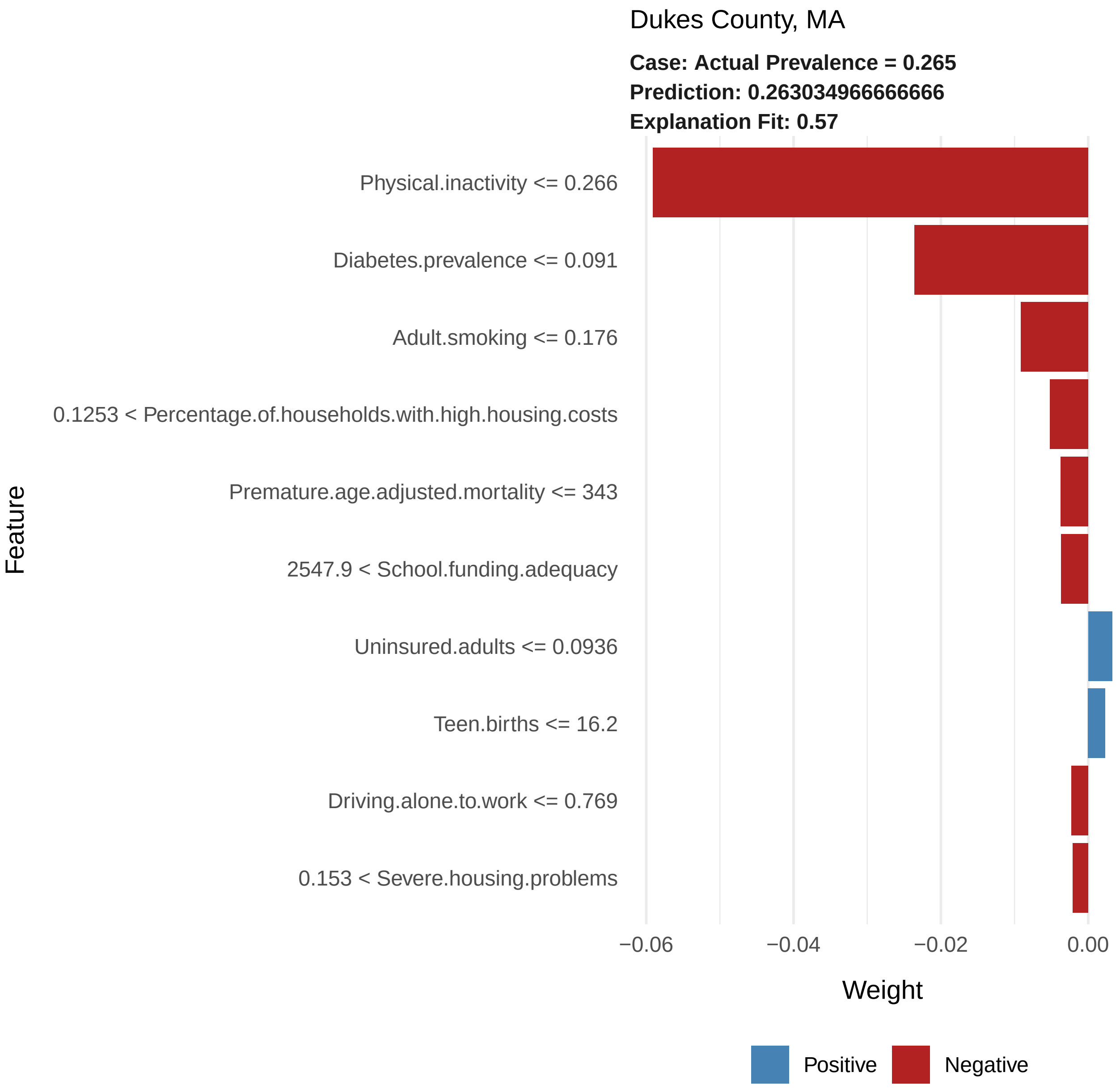

Dukes County, MA
Case: Actual Prevalence = 0.265
Prediction: 0.263034966666666
Explanation Fit: 0.57
Physical.inactivity <= 0.266
Diabetes.prevalence <= 0.091
Adult.smoking <= 0.176
0.1253 < Percentage.of.households.with.high.housing.costs
Premature.age.adjusted.mortality <= 343
2547.9 < School.funding.adequacy
Uninsured.adults <= 0.0936
Teen.births <= 16.2
Driving.alone.to.work <= 0.769
0.153 < Severe.housing.problems
Feature
−0.06
−0.04
−0.02
0.00
Weight
Positive
Negative

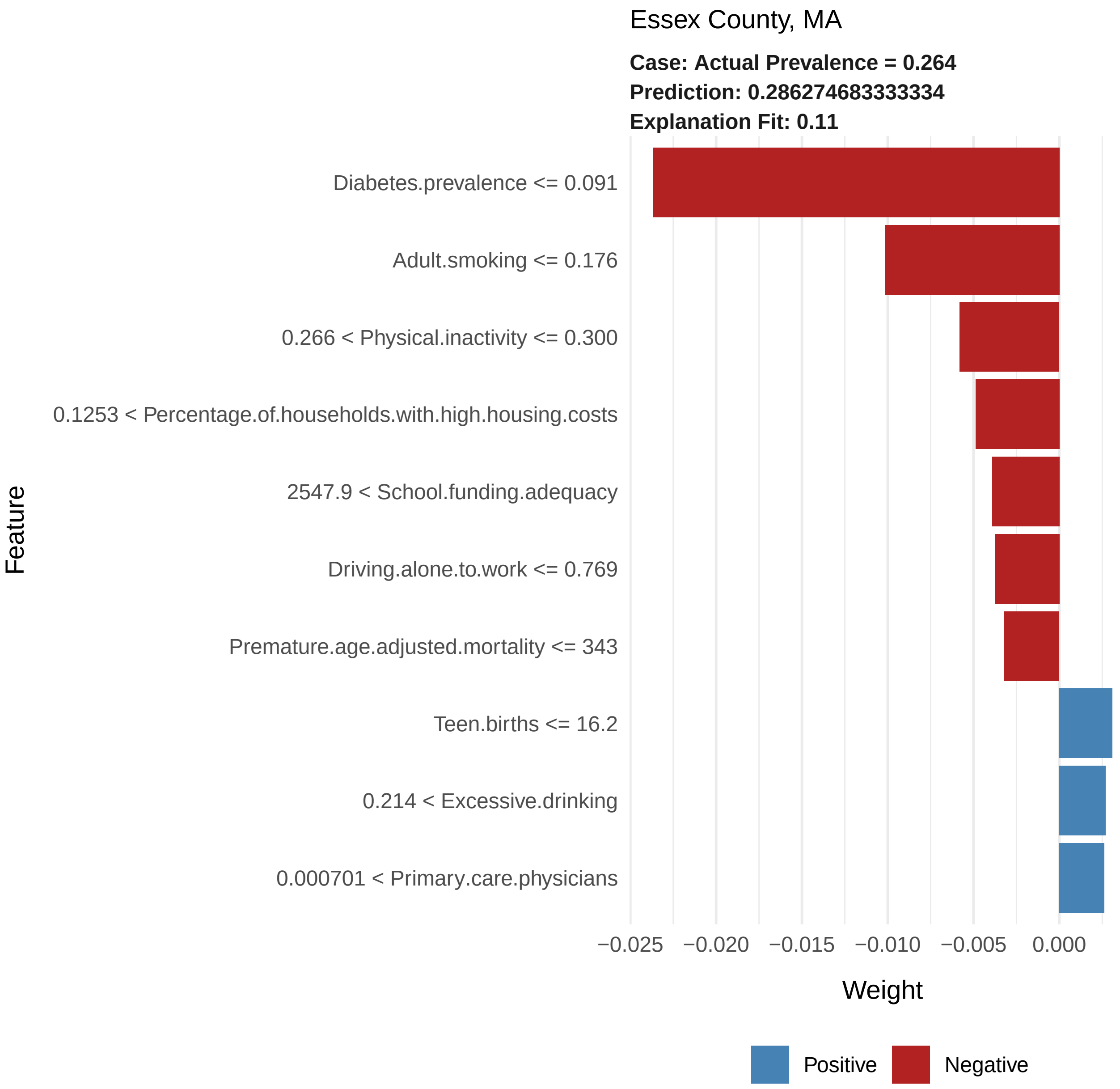
Essex County, MA
Case: Actual Prevalence = 0.264
Prediction: 0.286274683333334
Explanation Fit: 0.11
Diabetes.prevalence <= 0.091
Adult.smoking <= 0.176
0.266 < Physical.inactivity <= 0.300
0.1253 < Percentage.of.households.with.high.housing.costs
2547.9 < School.funding.adequacy
Driving.alone.to.work <= 0.769
Premature.age.adjusted.mortality <= 343
Teen.births <= 16.2
0.214 < Excessive.drinking
0.000701 < Primary.care.physicians
Feature
−0.025
−0.020
−0.015
−0.010
−0.005
0.000
Weight
Positive
Negative

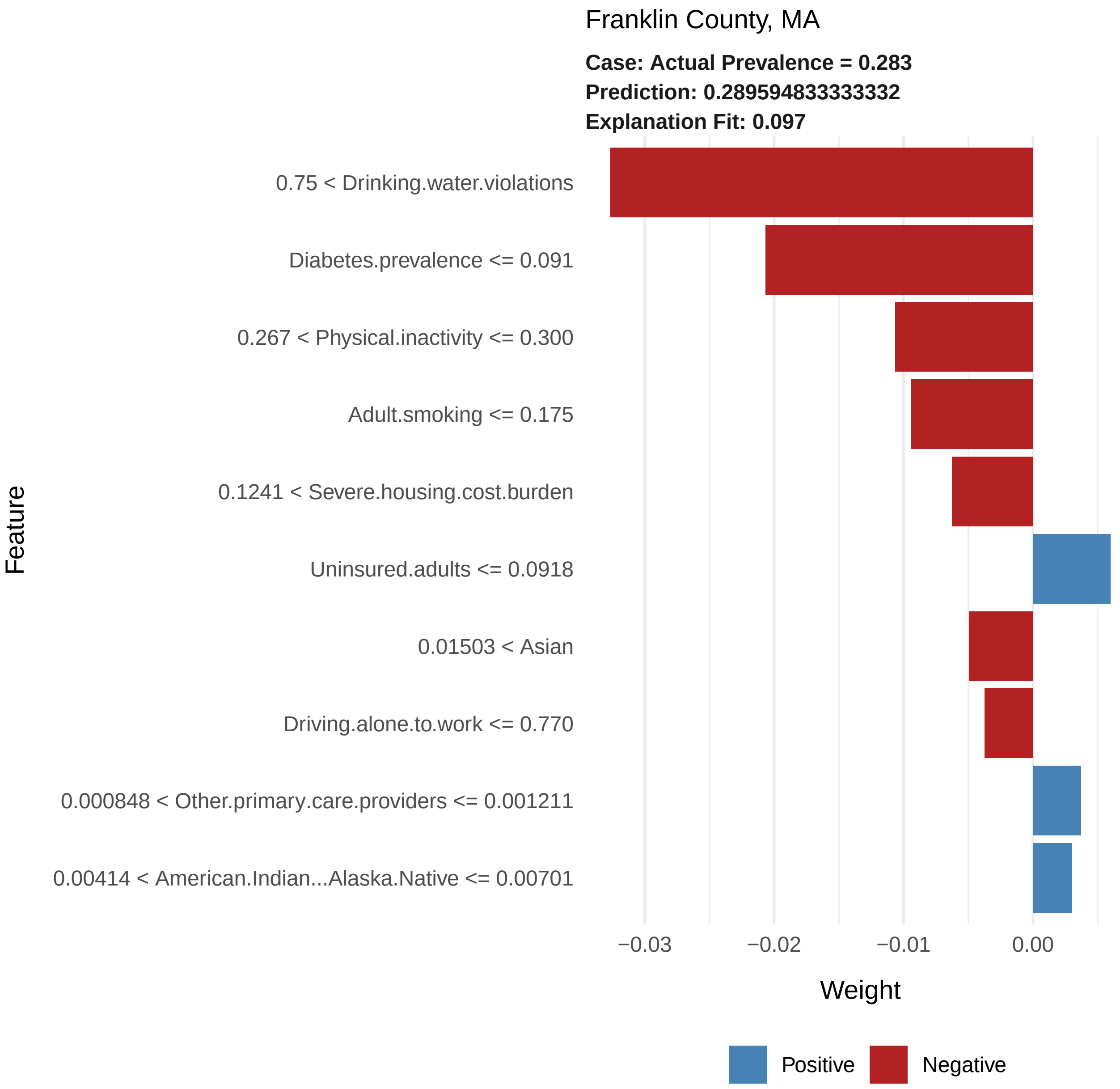
Franklin County, MA
Case: Actual Prevalence = 0.283
Prediction: 0.289594833333332
Explanation Fit: 0.097
0.75 < Drinking.water.violations
Diabetes.prevalence <= 0.091
0.267 < Physical.inactivity <= 0.300
Adult.smoking <= 0.175
0.1241 < Severe.housing.cost.burden
Uninsured.adults <= 0.0918
0.01503 < Asian
Driving.alone.to.work <= 0.770
0.000848 < Other.primary.care.providers <= 0.001211
0.00414 < American.Indian...Alaska.Native <= 0.00701
Feature
−0.03
−0.02
−0.01
0.00
Weight
Positive
Negative

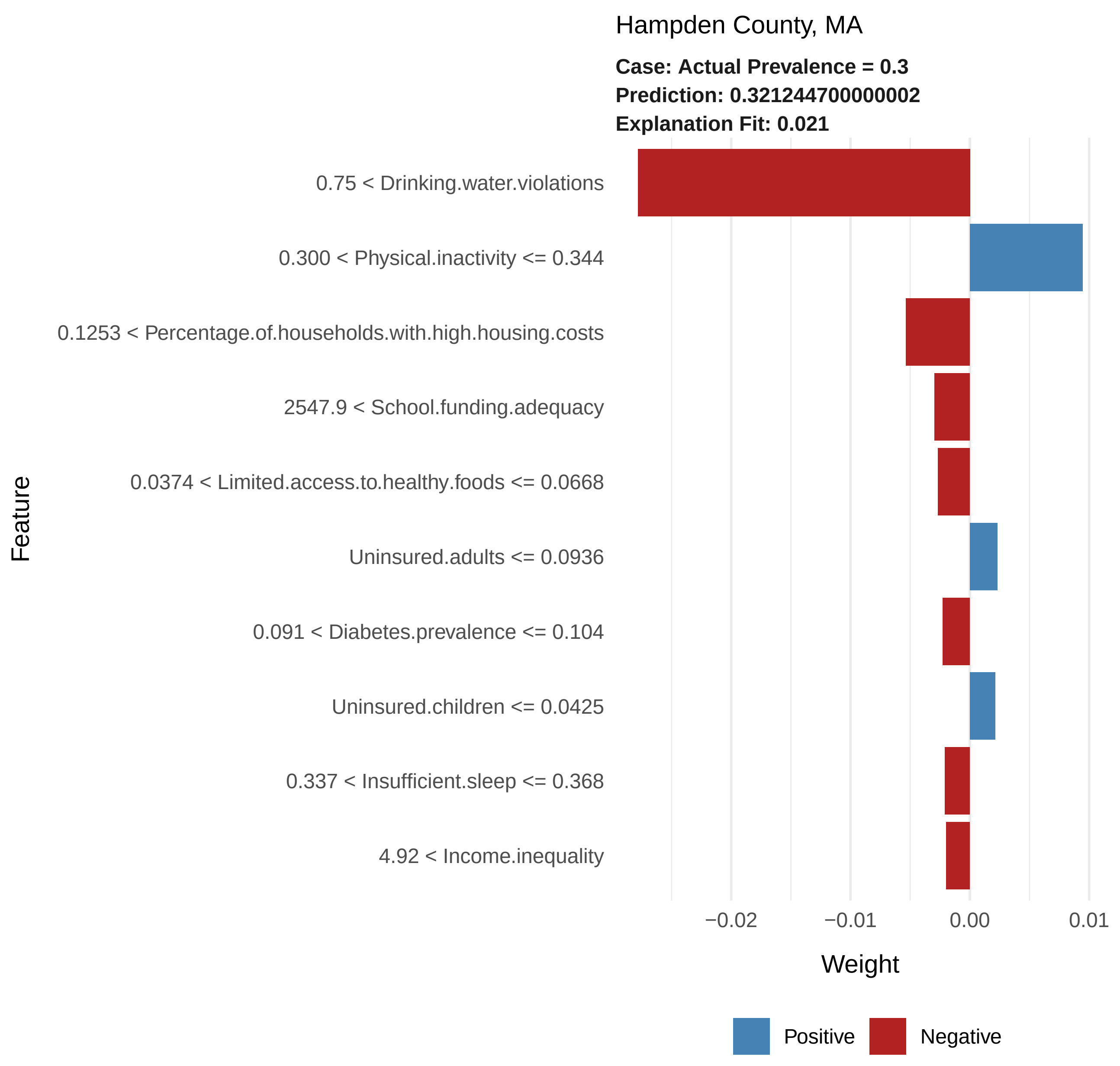
Hampden County, MA
Case: Actual Prevalence = 0.3
Prediction: 0.321244700000002
Explanation Fit: 0.021
0.75 < Drinking.water.violations
0.300 < Physical.inactivity <= 0.344
0.1253 < Percentage.of.households.with.high.housing.costs
2547.9 < School.funding.adequacy
0.0374 < Limited.access.to.healthy.foods <= 0.0668
Uninsured.adults <= 0.0936
0.091 < Diabetes.prevalence <= 0.104
Uninsured.children <= 0.0425
0.337 < Insufficient.sleep <= 0.368
4.92 < Income.inequality
Feature
−0.02
−0.01
0.00
0.01
Weight
Positive
Negative

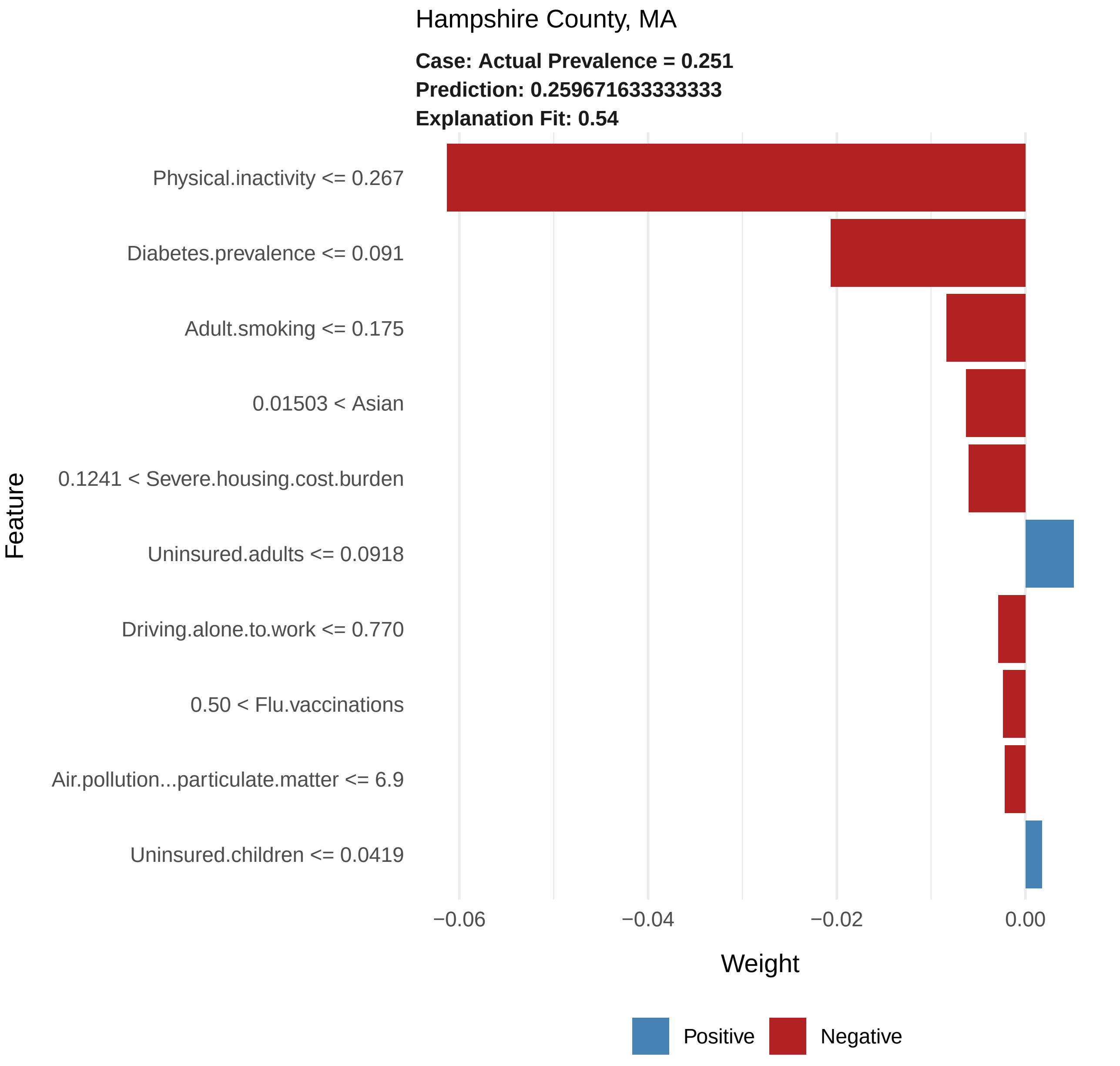
Hampshire County, MA
Case: Actual Prevalence = 0.251
Prediction: 0.259671633333333
Explanation Fit: 0.54
Feature
Physical.inactivity <= 0.267
Diabetes.prevalence <= 0.091
Adult.smoking <= 0.175
0.01503 < Asian
0.1241 < Severe.housing.cost.burden
Uninsured.adults <= 0.0918
Driving.alone.to.work <= 0.770
0.50 < Flu.vaccinations
Air.pollution...particulate.matter <= 6.9
Uninsured.children <= 0.0419
−0.06
−0.04
−0.02
0.00
Weight
Positive
Negative

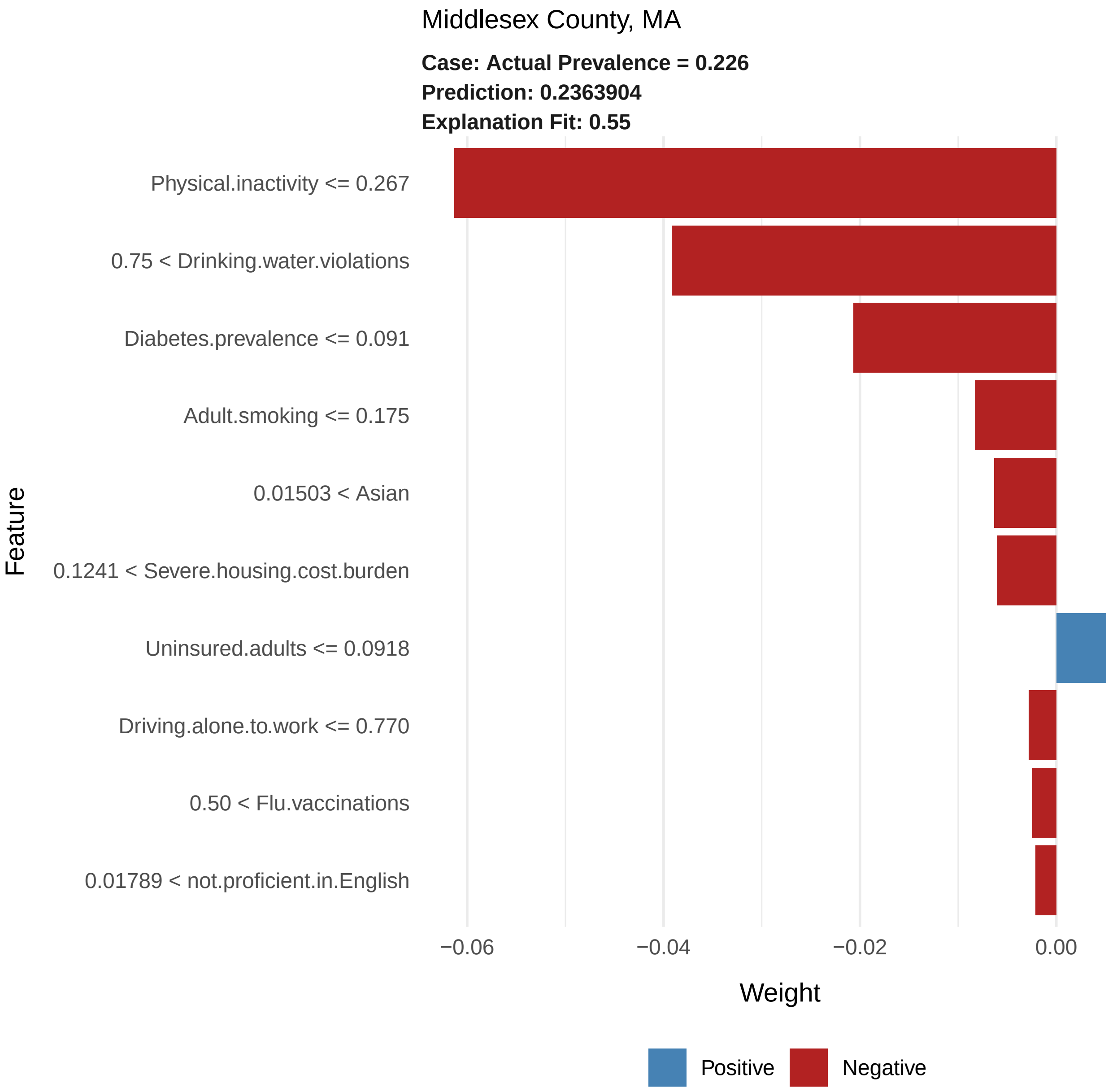
Middlesex County, MA
Case: Actual Prevalence = 0.226
Prediction: 0.2363904
Explanation Fit: 0.55
Physical.inactivity <= 0.267
0.75 < Drinking.water.violations
Diabetes.prevalence <= 0.091
Adult.smoking <= 0.175
0.01503 < Asian
0.1241 < Severe.housing.cost.burden
Uninsured.adults <= 0.0918
Driving.alone.to.work <= 0.770
0.50 < Flu.vaccinations
0.01789 < not.proficient.in.English
Feature
−0.06
−0.04
−0.02
0.00
Weight
Positive
Negative

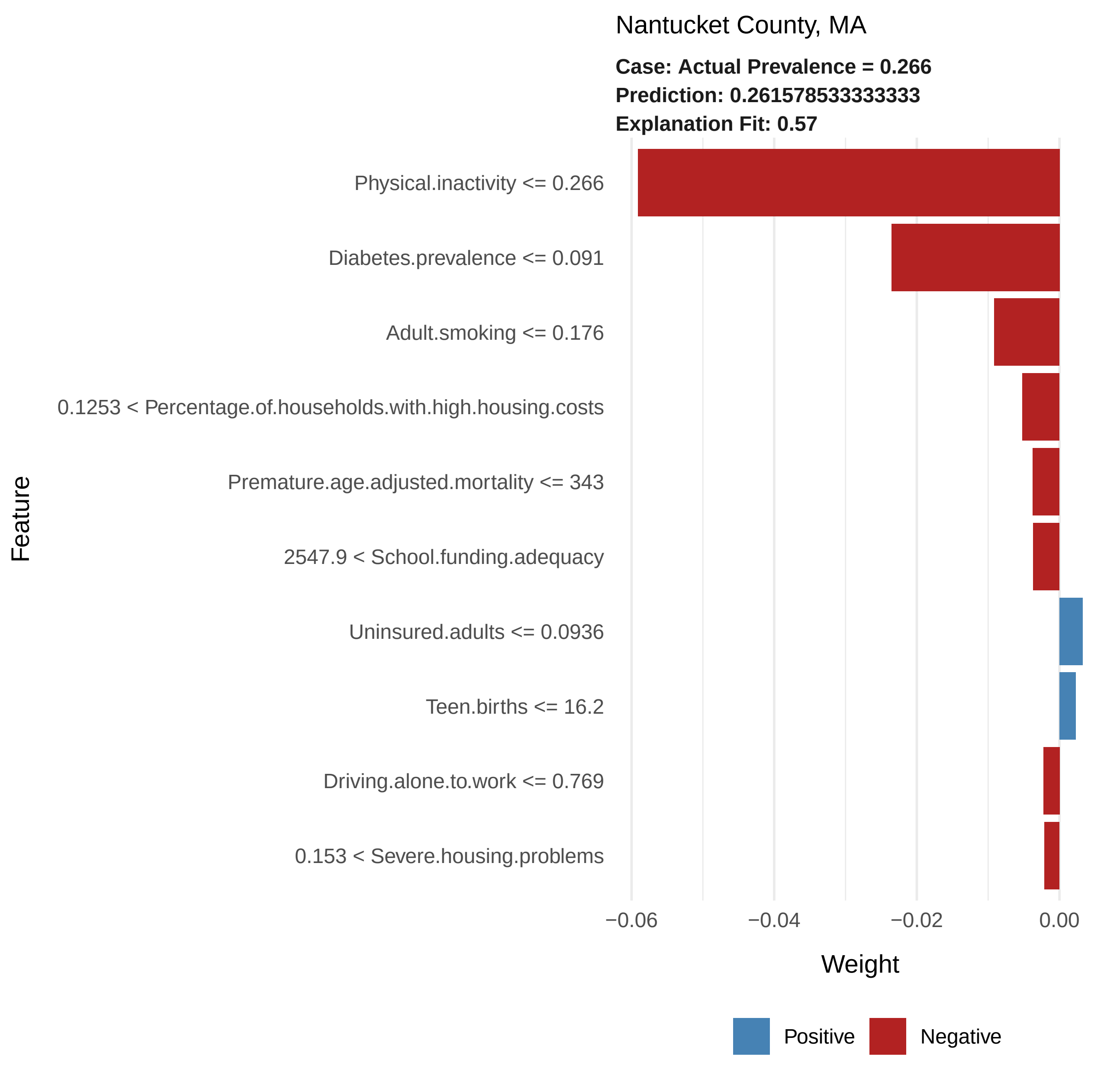

Nantucket County, MA
Case: Actual Prevalence = 0.266
Prediction: 0.261578533333333
Explanation Fit: 0.57
Physical.inactivity <= 0.266
Diabetes.prevalence <= 0.091
Adult.smoking <= 0.176
0.1253 < Percentage.of.households.with.high.housing.costs
Premature.age.adjusted.mortality <= 343
2547.9 < School.funding.adequacy
Uninsured.adults <= 0.0936
Teen.births <= 16.2
Driving.alone.to.work <= 0.769
0.153 < Severe.housing.problems
Feature
−0.06
−0.04
−0.02
0.00
Weight
Positive
Negative

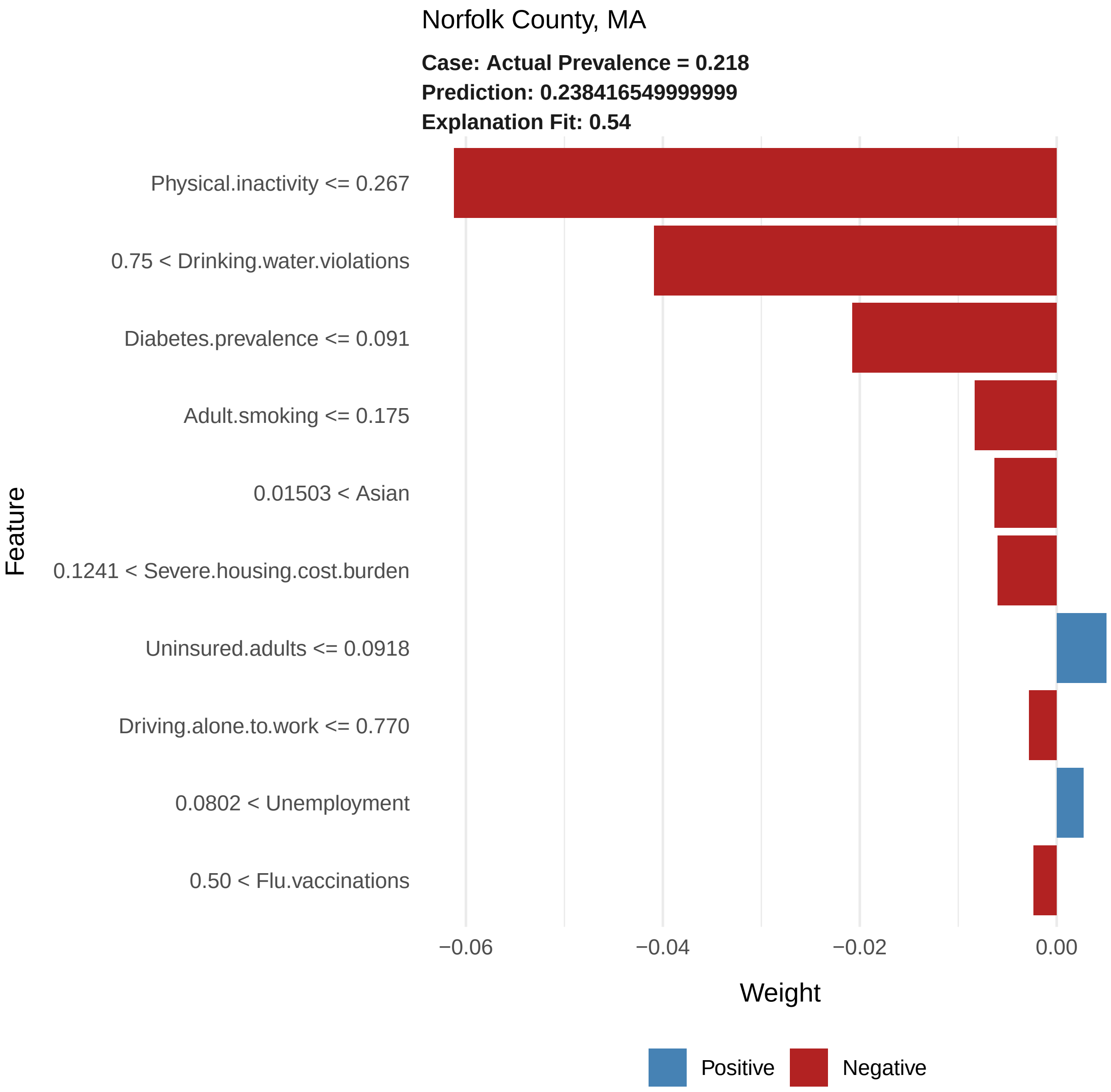

Norfolk County, MA
Case: Actual Prevalence = 0.218
Prediction: 0.238416549999999
Explanation Fit: 0.54
Physical.inactivity <= 0.267
0.75 < Drinking.water.violations
Diabetes.prevalence <= 0.091
Adult.smoking <= 0.175
0.01503 < Asian
0.1241 < Severe.housing.cost.burden
Uninsured.adults <= 0.0918
Driving.alone.to.work <= 0.770
0.0802 < Unemployment
0.50 < Flu.vaccinations
Feature
−0.06
−0.04
−0.02
0.00
Weight
Positive
Negative

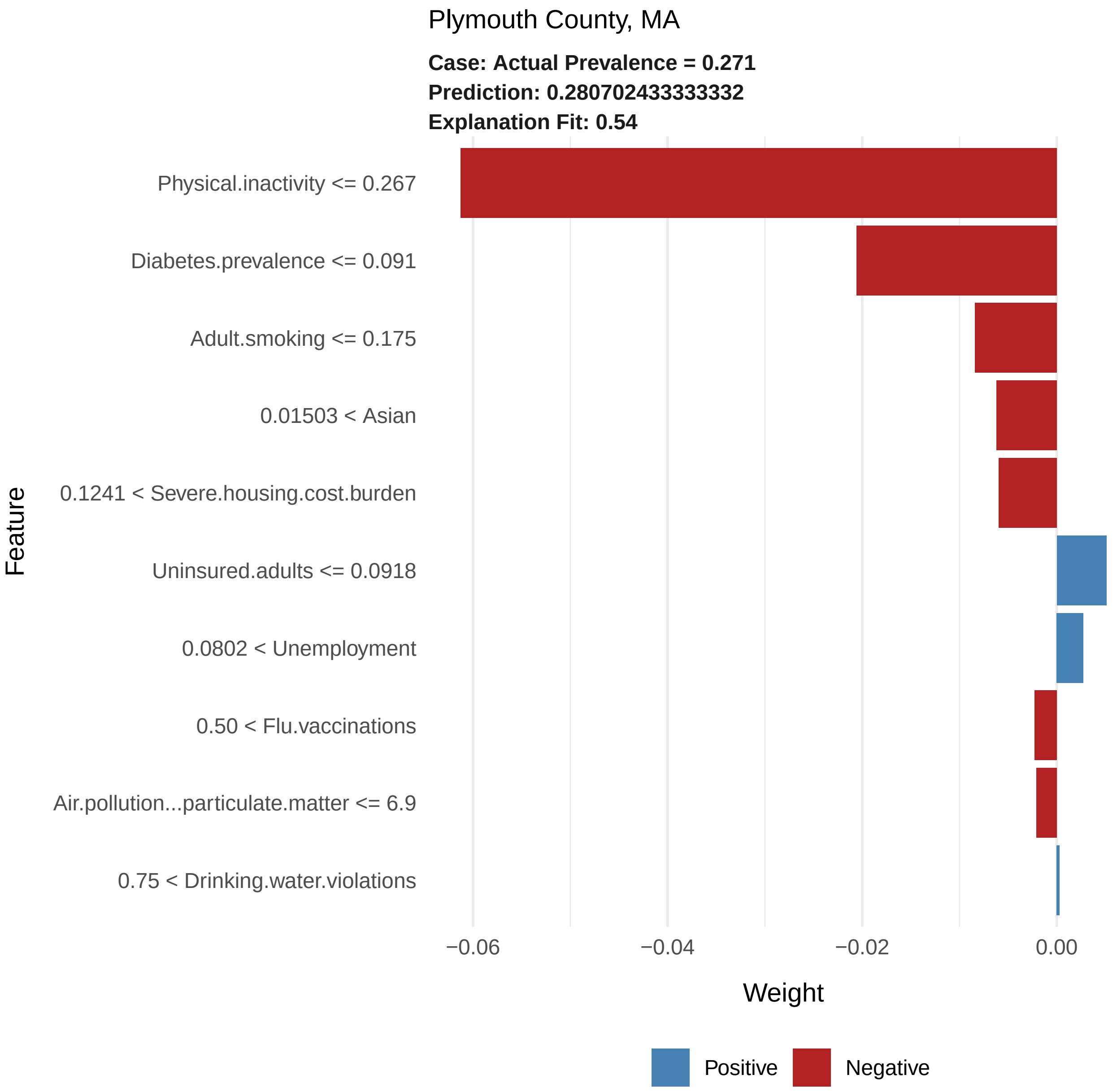

Plymouth County, MA
Case: Actual Prevalence = 0.271
Prediction: 0.280702433333332
Explanation Fit: 0.54
Physical.inactivity <= 0.267
Diabetes.prevalence <= 0.091
Adult.smoking <= 0.175
0.01503 < Asian
0.1241 < Severe.housing.cost.burden
Uninsured.adults <= 0.0918
0.0802 < Unemployment
0.50 < Flu.vaccinations
Air.pollution...particulate.matter <= 6.9
0.75 < Drinking.water.violations
Feature
−0.06
−0.04
−0.02
0.00
Weight
Positive
Negative

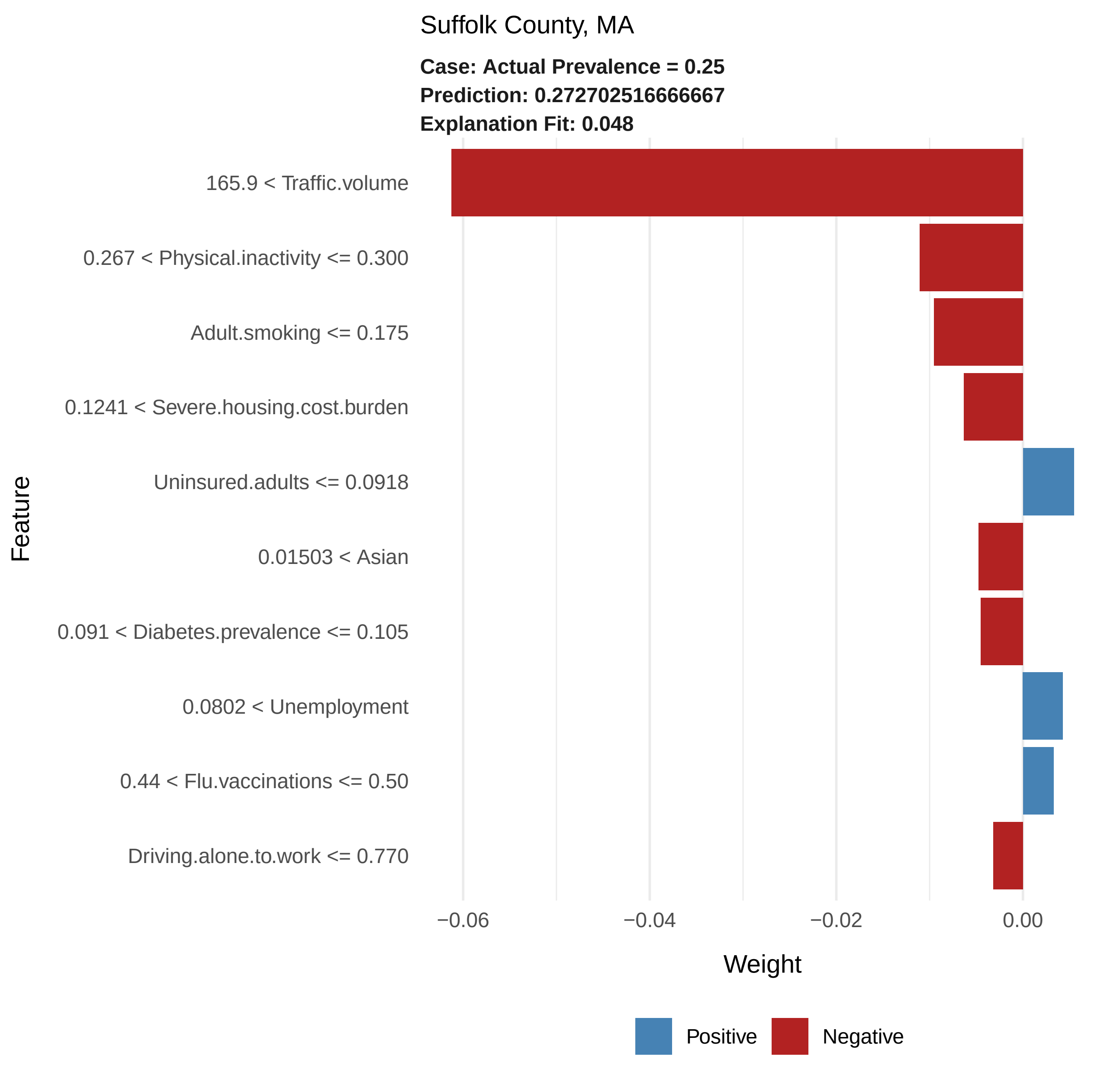

Suffolk County, MA
Case: Actual Prevalence = 0.25
Prediction: 0.272702516666667
Explanation Fit: 0.048
165.9 < Traffic.volume
0.267 < Physical.inactivity <= 0.300
Adult.smoking <= 0.175
0.1241 < Severe.housing.cost.burden
Uninsured.adults <= 0.0918
0.01503 < Asian
0.091 < Diabetes.prevalence <= 0.105
0.0802 < Unemployment
0.44 < Flu.vaccinations <= 0.50
Driving.alone.to.work <= 0.770
Feature
−0.06
−0.04
−0.02
0.00
Weight
Positive
Negative

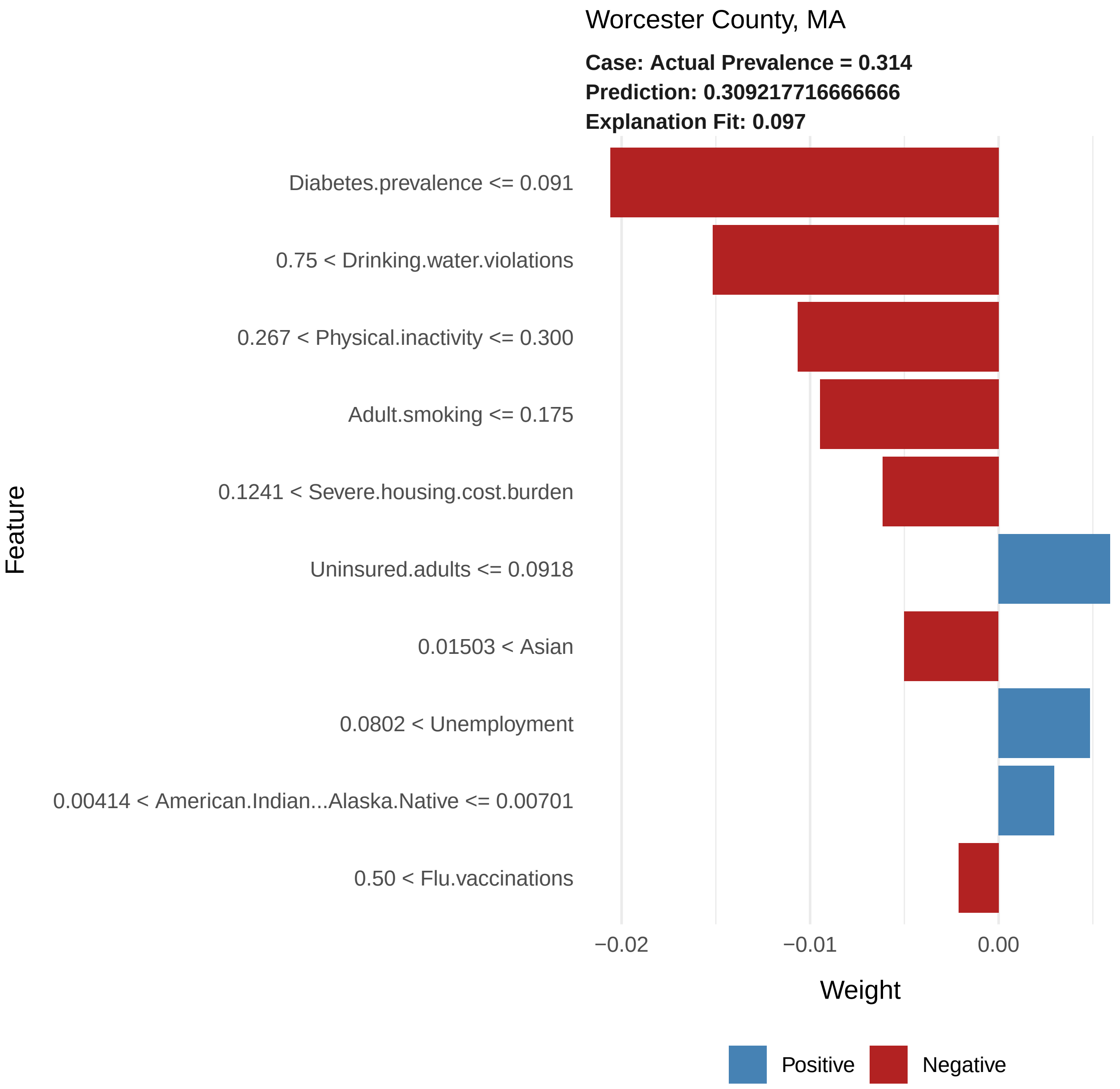
Worcester County, MA
Case: Actual Prevalence = 0.314
Prediction: 0.309217716666666
Explanation Fit: 0.097
Feature
Diabetes.prevalence <= 0.091
0.75 < Drinking.water.violations
0.267 < Physical.inactivity <= 0.300
Adult.smoking <= 0.175
0.1241 < Severe.housing.cost.burden
Uninsured.adults <= 0.0918
0.01503 < Asian
0.0802 < Unemployment
0.00414 < American.Indian...Alaska.Native <= 0.00701
0.50 < Flu.vaccinations
−0.02
−0.01
0.00
Weight
Positive
Negative

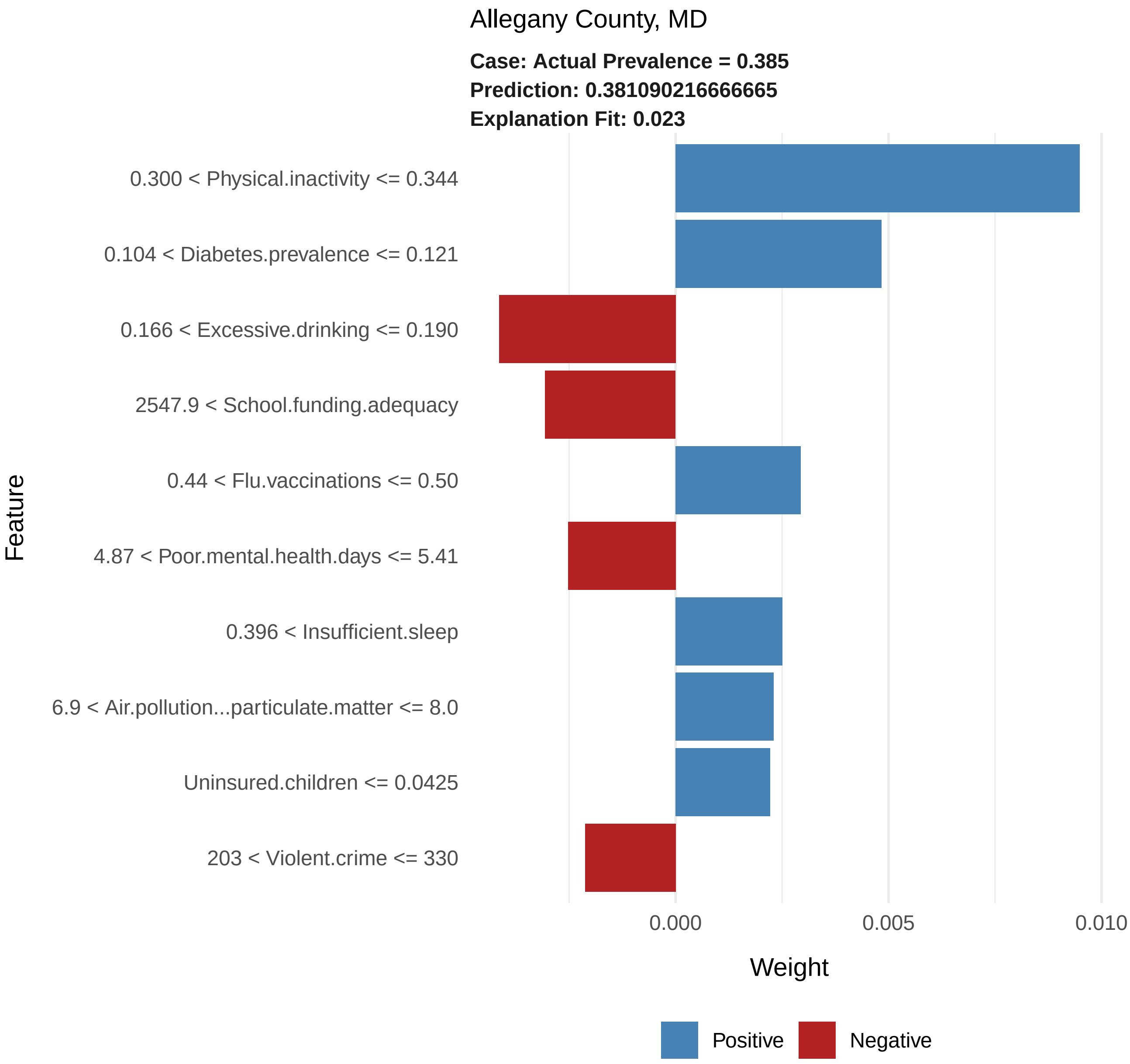

Allegany County, MD
Case: Actual Prevalence = 0.385
Prediction: 0.381090216666665
Explanation Fit: 0.023
0.300 < Physical.inactivity <= 0.344
0.104 < Diabetes.prevalence <= 0.121
0.166 < Excessive.drinking <= 0.190
2547.9 < School.funding.adequacy
0.44 < Flu.vaccinations <= 0.50
4.87 < Poor.mental.health.days <= 5.41
0.396 < Insufficient.sleep
6.9 < Air.pollution...particulate.matter <= 8.0
Uninsured.children <= 0.0425
203 < Violent.crime <= 330
Feature
0.000
0.005
0.010
Weight
Positive
Negative

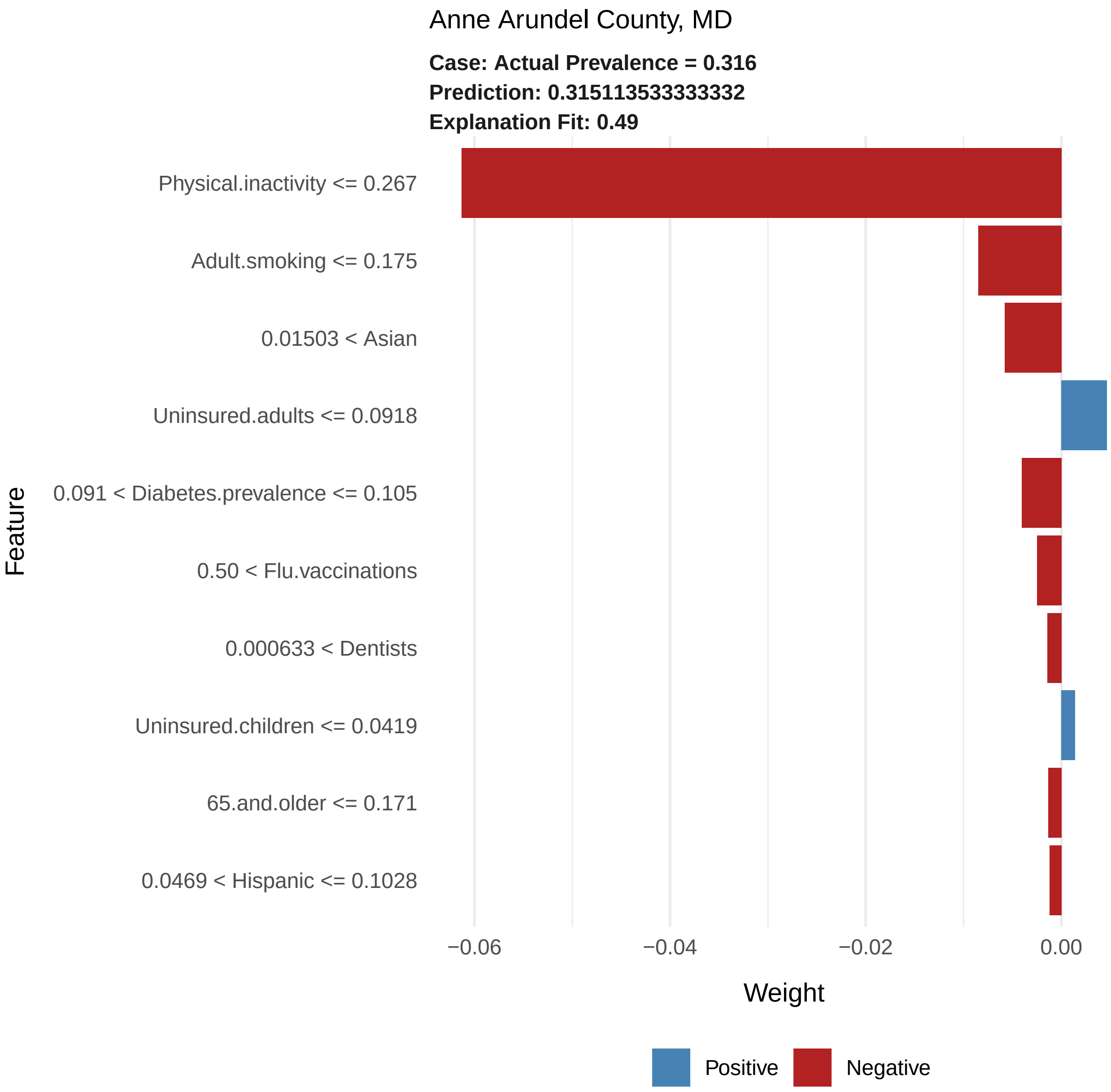

Anne Arundel County, MD
Case: Actual Prevalence = 0.316
Prediction: 0.315113533333332
Explanation Fit: 0.49
Physical.inactivity <= 0.267
Adult.smoking <= 0.175
0.01503 < Asian
Uninsured.adults <= 0.0918
0.091 < Diabetes.prevalence <= 0.105
0.50 < Flu.vaccinations
0.000633 < Dentists
Uninsured.children <= 0.0419
65.and.older <= 0.171
0.0469 < Hispanic <= 0.1028
Feature
−0.06
−0.04
−0.02
0.00
Weight
Positive
Negative

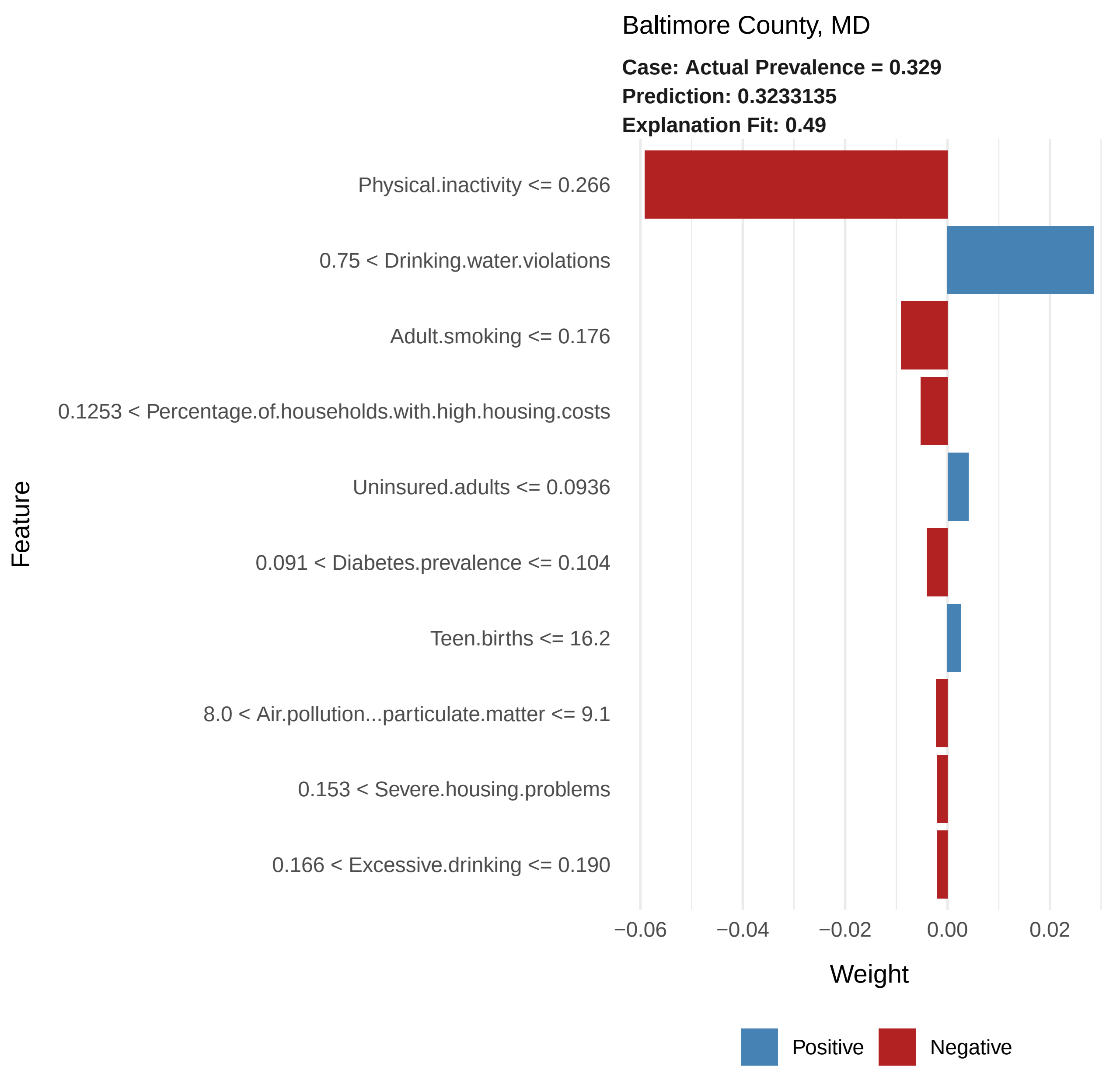

Baltimore County, MD
Case: Actual Prevalence = 0.329
Prediction: 0.3233135
Explanation Fit: 0.49
Physical.inactivity <= 0.266
0.75 < Drinking.water.violations
Adult.smoking <= 0.176
0.1253 < Percentage.of.households.with.high.housing.costs
Uninsured.adults <= 0.0936
0.091 < Diabetes.prevalence <= 0.104
Teen.births <= 16.2
8.0 < Air.pollution...particulate.matter <= 9.1
0.153 < Severe.housing.problems
0.166 < Excessive.drinking <= 0.190
Feature
−0.06
−0.04
−0.02
0.00
0.02
Weight
Positive
Negative

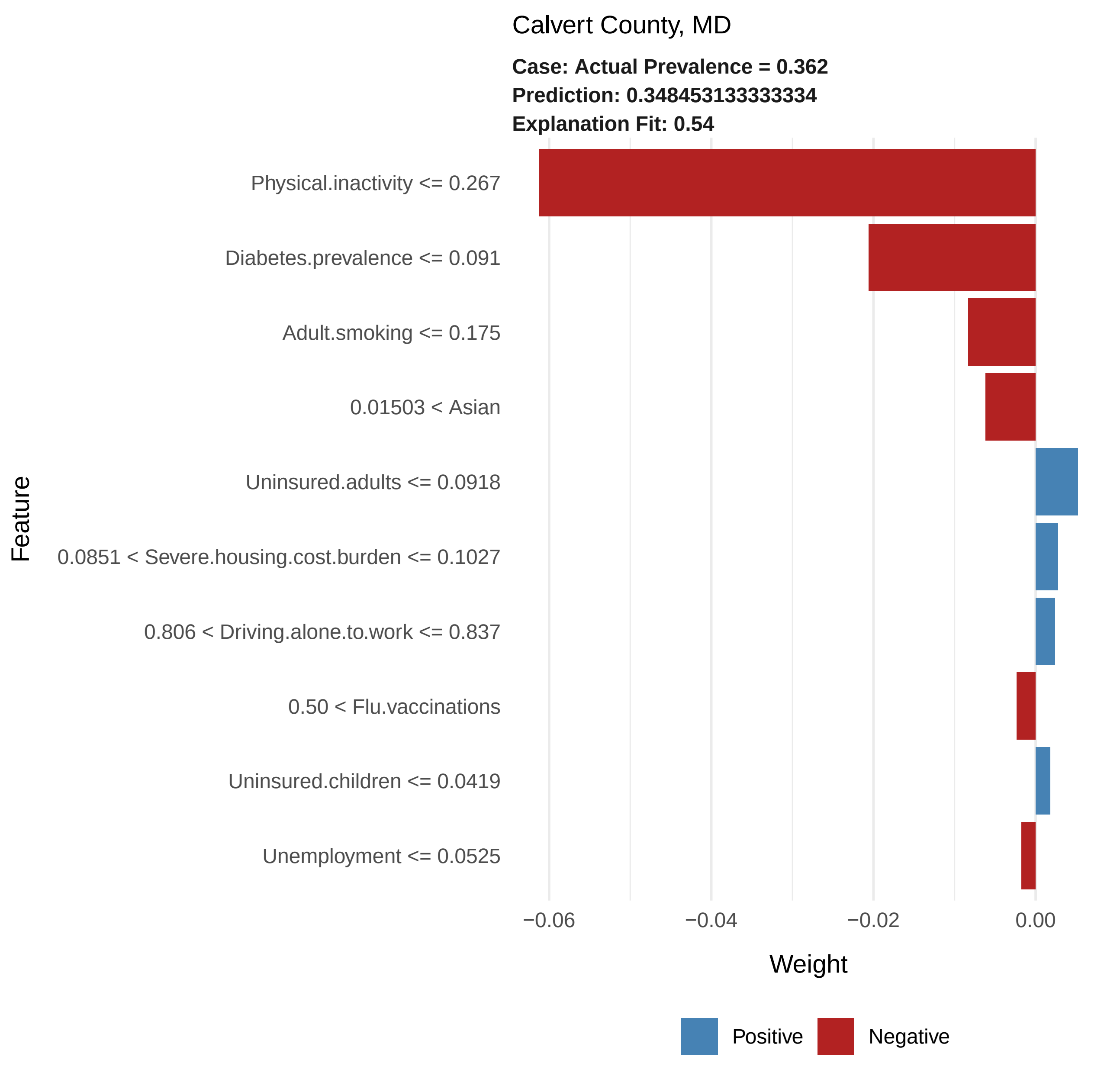
Calvert County, MD
Case: Actual Prevalence = 0.362
Prediction: 0.348453133333334
Explanation Fit: 0.54
Physical.inactivity <= 0.267
Diabetes.prevalence <= 0.091
Adult.smoking <= 0.175
0.01503 < Asian
Uninsured.adults <= 0.0918
0.0851 < Severe.housing.cost.burden <= 0.1027
0.806 < Driving.alone.to.work <= 0.837
0.50 < Flu.vaccinations
Uninsured.children <= 0.0419
Unemployment <= 0.0525
Feature
−0.06
−0.04
−0.02
0.00
Weight
Positive
Negative

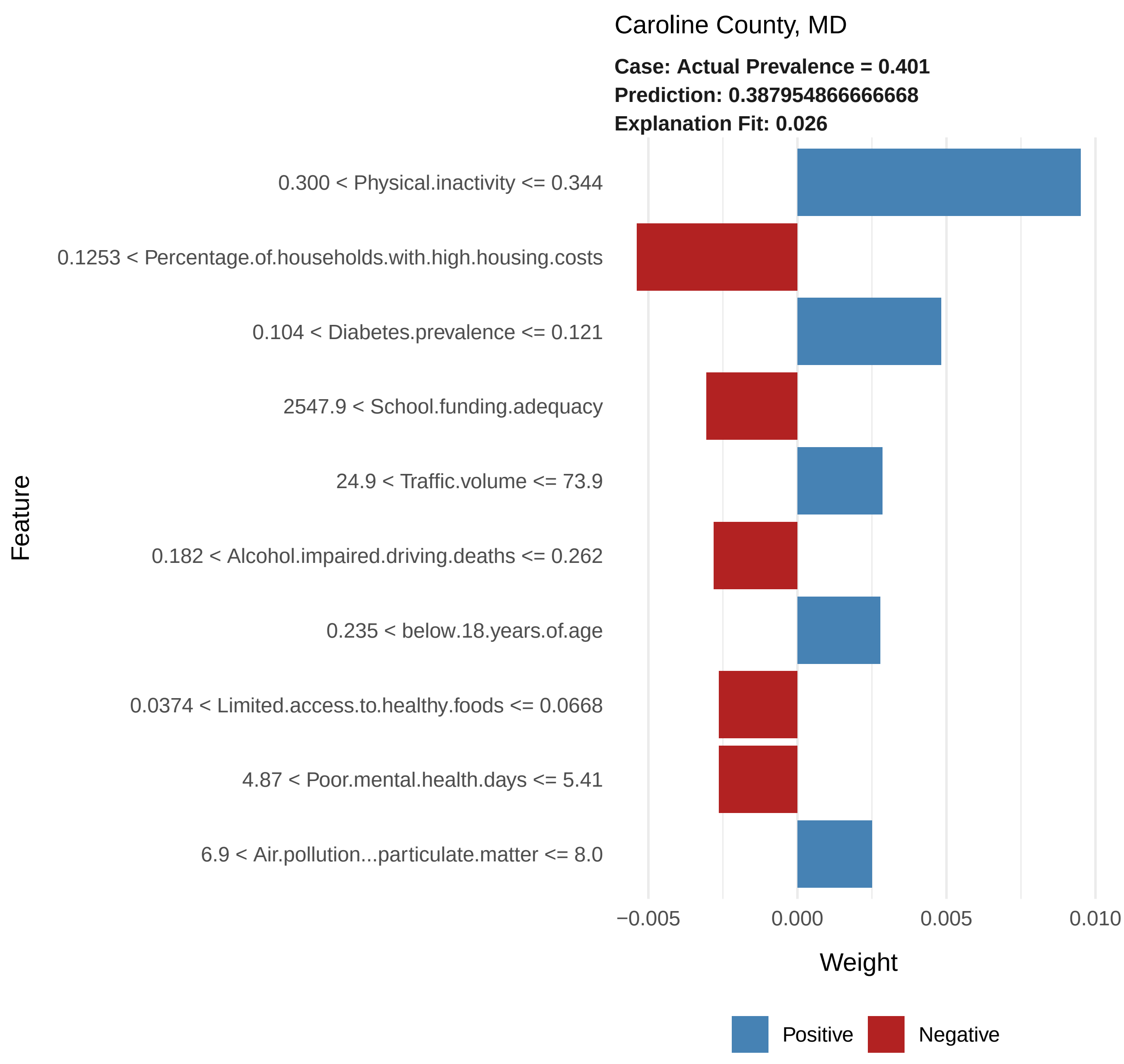
Caroline County, MD
Case: Actual Prevalence = 0.401
Prediction: 0.387954866666668
Explanation Fit: 0.026
0.300 < Physical.inactivity <= 0.344
0.1253 < Percentage.of.households.with.high.housing.costs
0.104 < Diabetes.prevalence <= 0.121
2547.9 < School.funding.adequacy
24.9 < Traffic.volume <= 73.9
0.182 < Alcohol.impaired.driving.deaths <= 0.262
0.235 < below.18.years.of.age
0.0374 < Limited.access.to.healthy.foods <= 0.0668
4.87 < Poor.mental.health.days <= 5.41
6.9 < Air.pollution...particulate.matter <= 8.0
Feature
−0.005
0.000
0.005
0.010
Weight
Positive
Negative

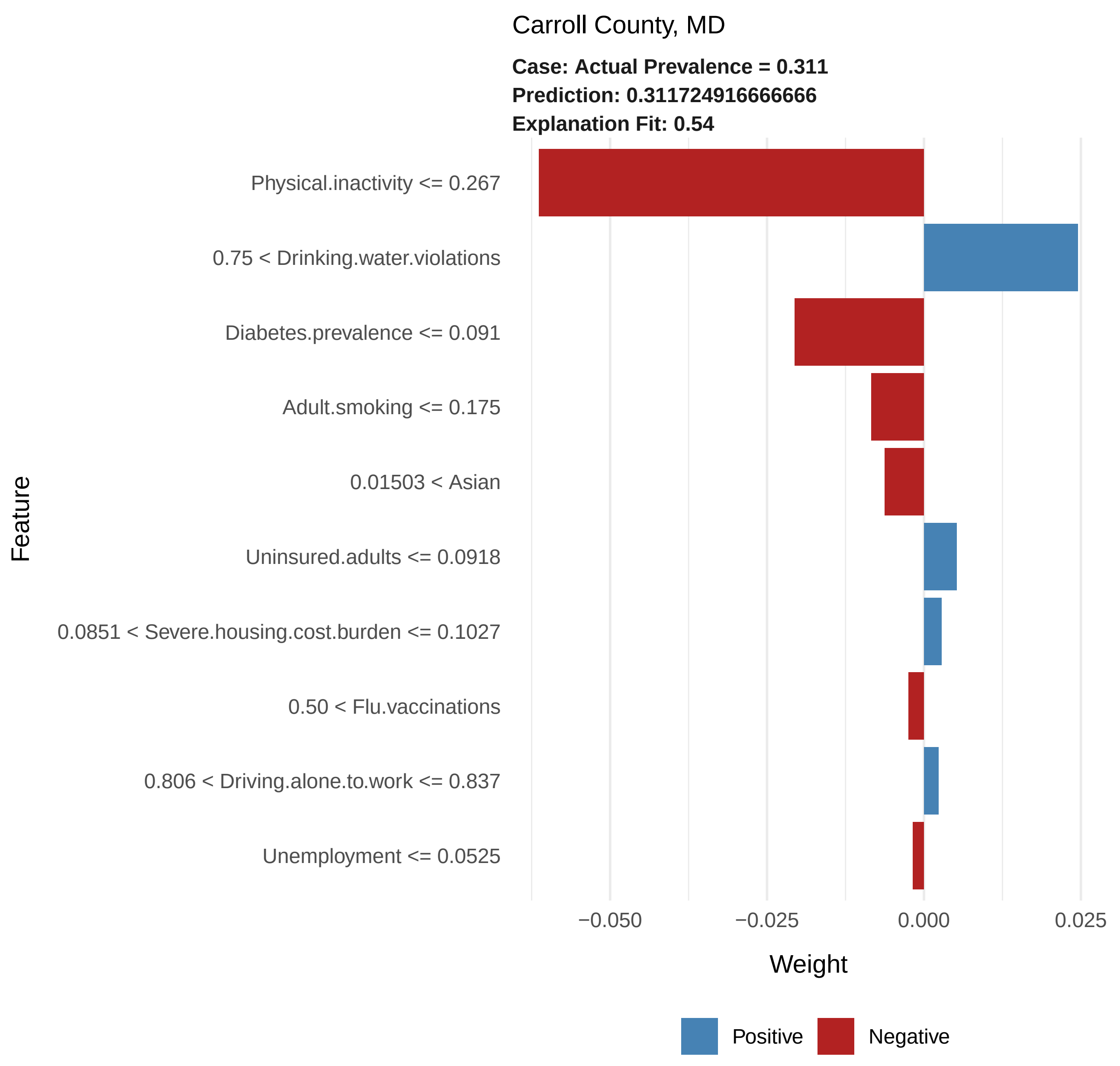
Carroll County, MD
Case: Actual Prevalence = 0.311
Prediction: 0.311724916666666
Explanation Fit: 0.54
Physical.inactivity <= 0.267
0.75 < Drinking.water.violations
Diabetes.prevalence <= 0.091
Adult.smoking <= 0.175
0.01503 < Asian
Uninsured.adults <= 0.0918
0.0851 < Severe.housing.cost.burden <= 0.1027
0.50 < Flu.vaccinations
0.806 < Driving.alone.to.work <= 0.837
Unemployment <= 0.0525
Feature
−0.050
−0.025
0.000
0.025
Weight
Positive
Negative

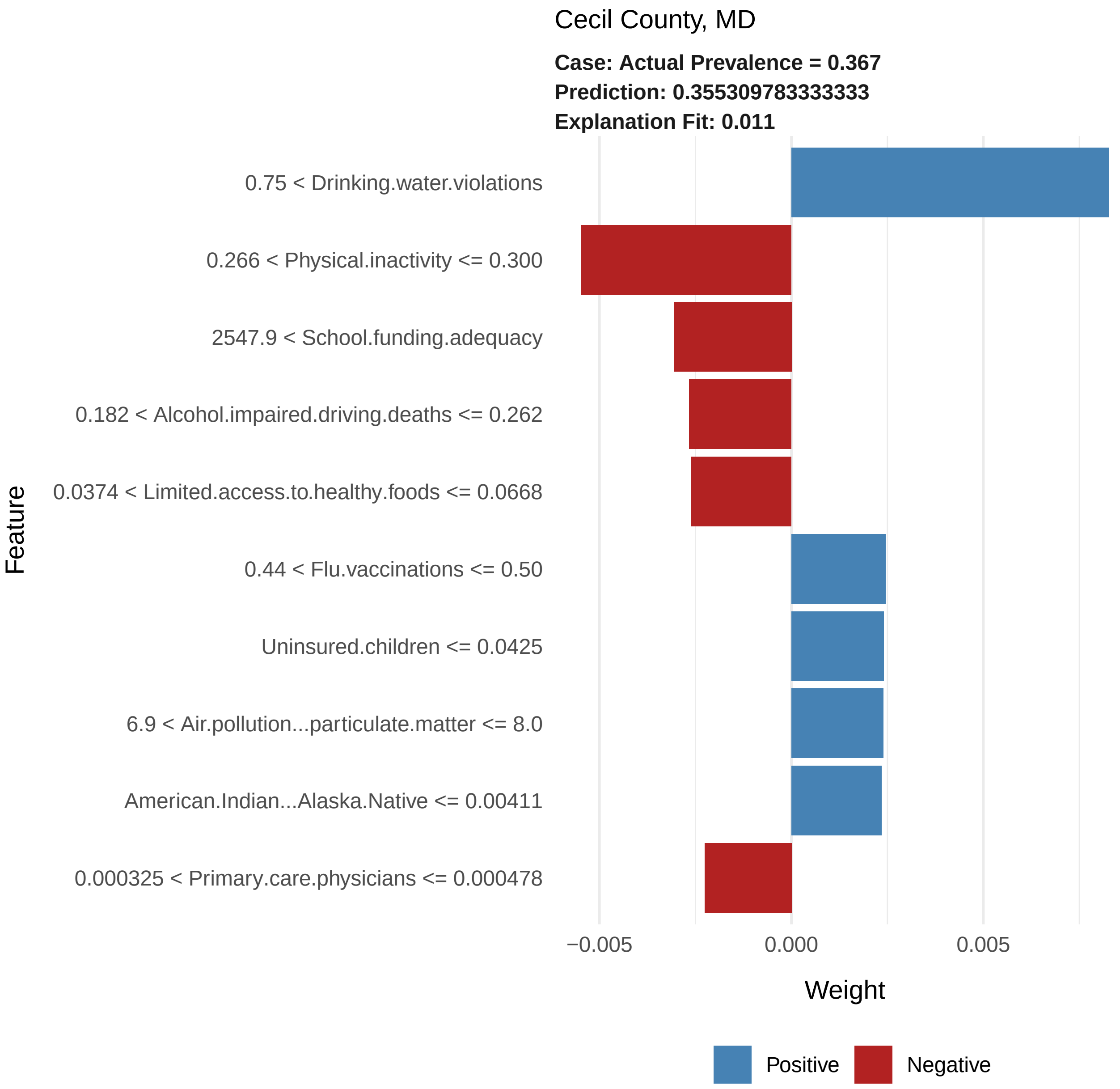

Cecil County, MD
Case: Actual Prevalence = 0.367
Prediction: 0.355309783333333
Explanation Fit: 0.011
0.75 < Drinking.water.violations
0.266 < Physical.inactivity <= 0.300
2547.9 < School.funding.adequacy
0.182 < Alcohol.impaired.driving.deaths <= 0.262
0.0374 < Limited.access.to.healthy.foods <= 0.0668
0.44 < Flu.vaccinations <= 0.50
Uninsured.children <= 0.0425
6.9 < Air.pollution...particulate.matter <= 8.0
American.Indian...Alaska.Native <= 0.00411
0.000325 < Primary.care.physicians <= 0.000478
Feature
−0.005
0.000
0.005
Weight
Positive
Negative

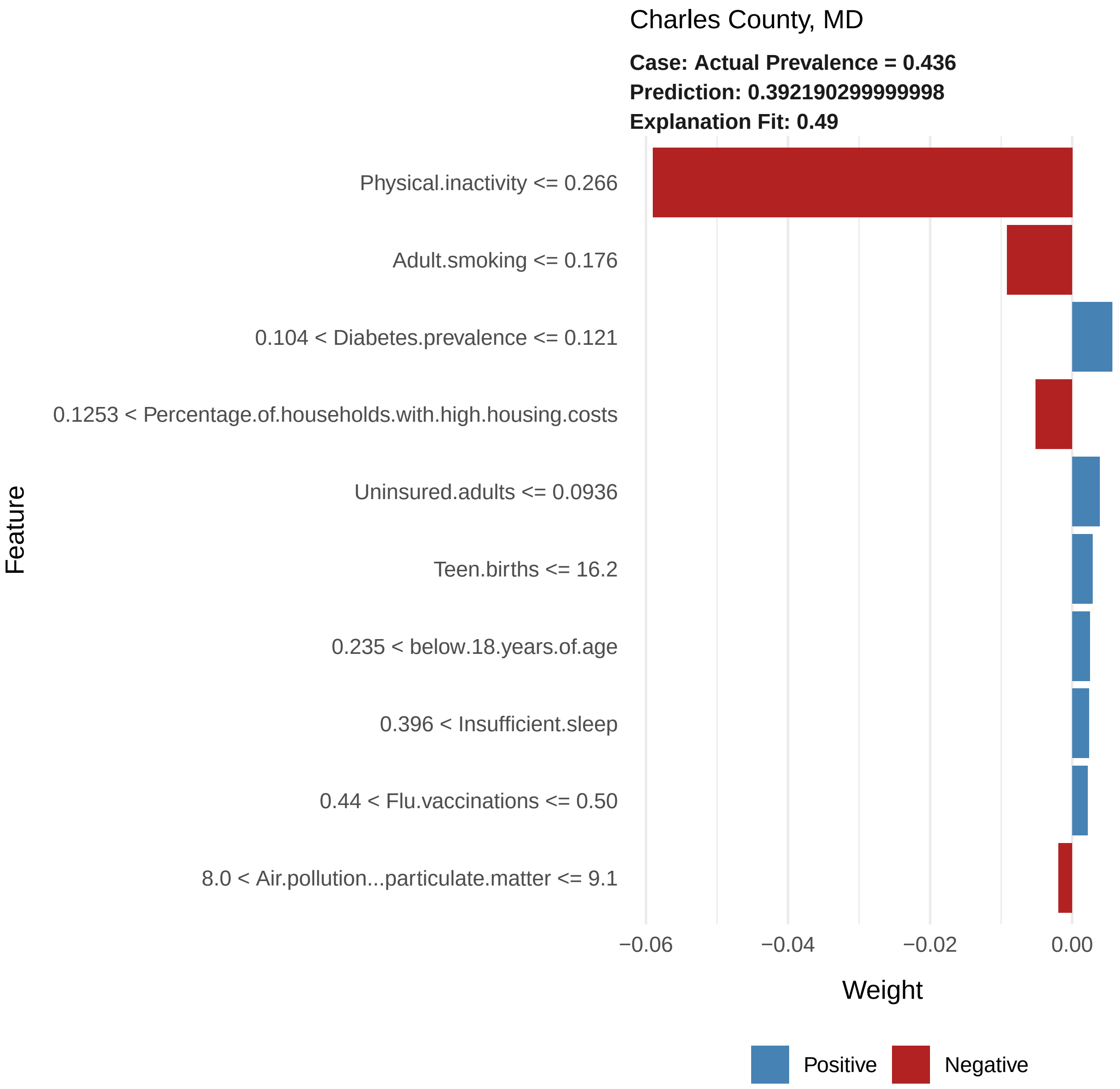

Charles County, MD
Case: Actual Prevalence = 0.436
Prediction: 0.392190299999998
Explanation Fit: 0.49
Physical.inactivity <= 0.266
Adult.smoking <= 0.176
0.104 < Diabetes.prevalence <= 0.121
0.1253 < Percentage.of.households.with.high.housing.costs
Uninsured.adults <= 0.0936
Teen.births <= 16.2
0.235 < below.18.years.of.age
0.396 < Insufficient.sleep
0.44 < Flu.vaccinations <= 0.50
8.0 < Air.pollution...particulate.matter <= 9.1
Feature
−0.06
−0.04
−0.02
0.00
Weight
Positive
Negative

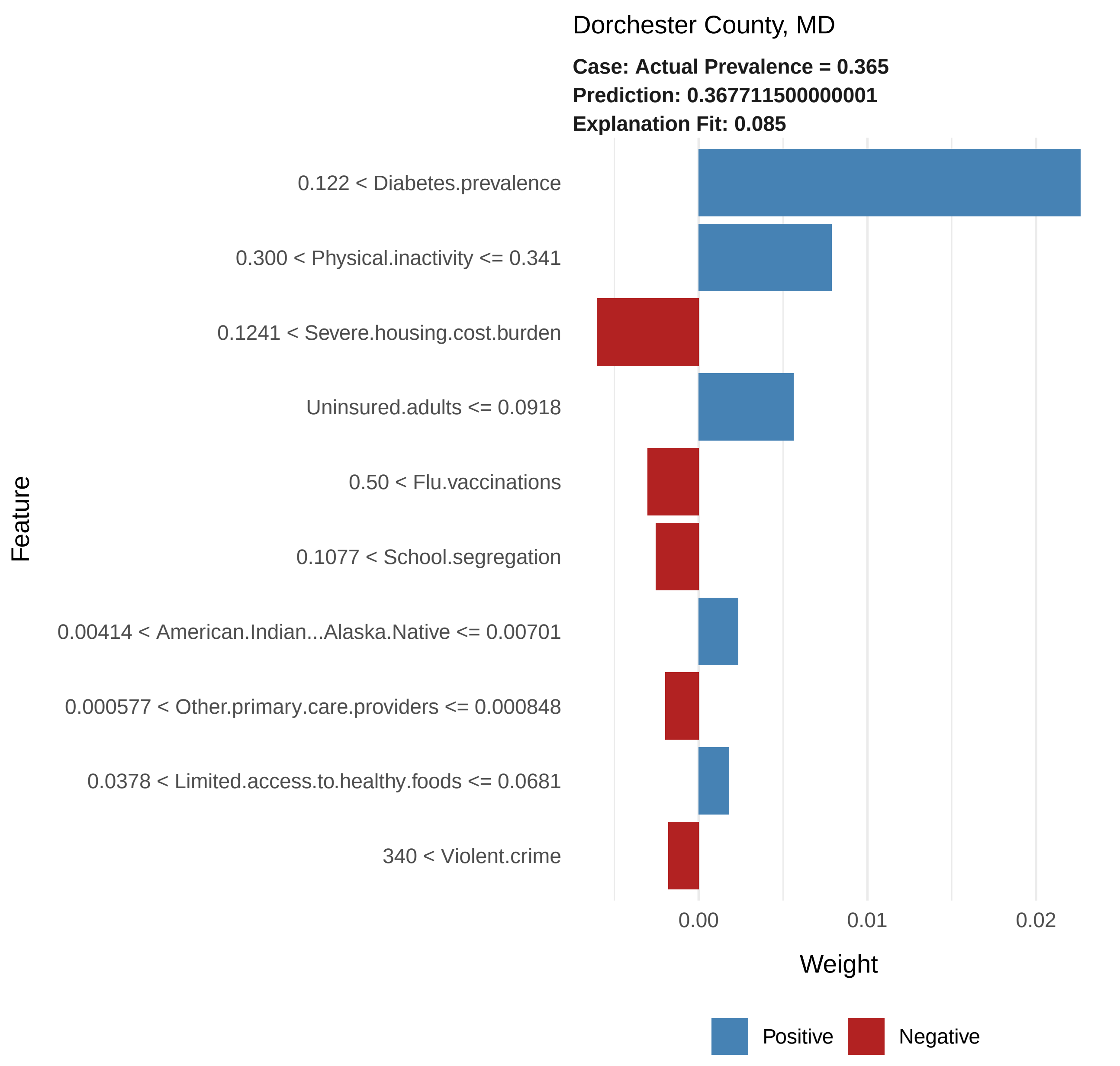

Dorchester County, MD
Case: Actual Prevalence = 0.365
Prediction: 0.367711500000001
Explanation Fit: 0.085
0.122 < Diabetes.prevalence
0.300 < Physical.inactivity <= 0.341
0.1241 < Severe.housing.cost.burden
Uninsured.adults <= 0.0918
0.50 < Flu.vaccinations
0.1077 < School.segregation
0.00414 < American.Indian...Alaska.Native <= 0.00701
0.000577 < Other.primary.care.providers <= 0.000848
0.0378 < Limited.access.to.healthy.foods <= 0.0681
340 < Violent.crime
Feature
0.00
0.01
0.02
Weight
Positive
Negative

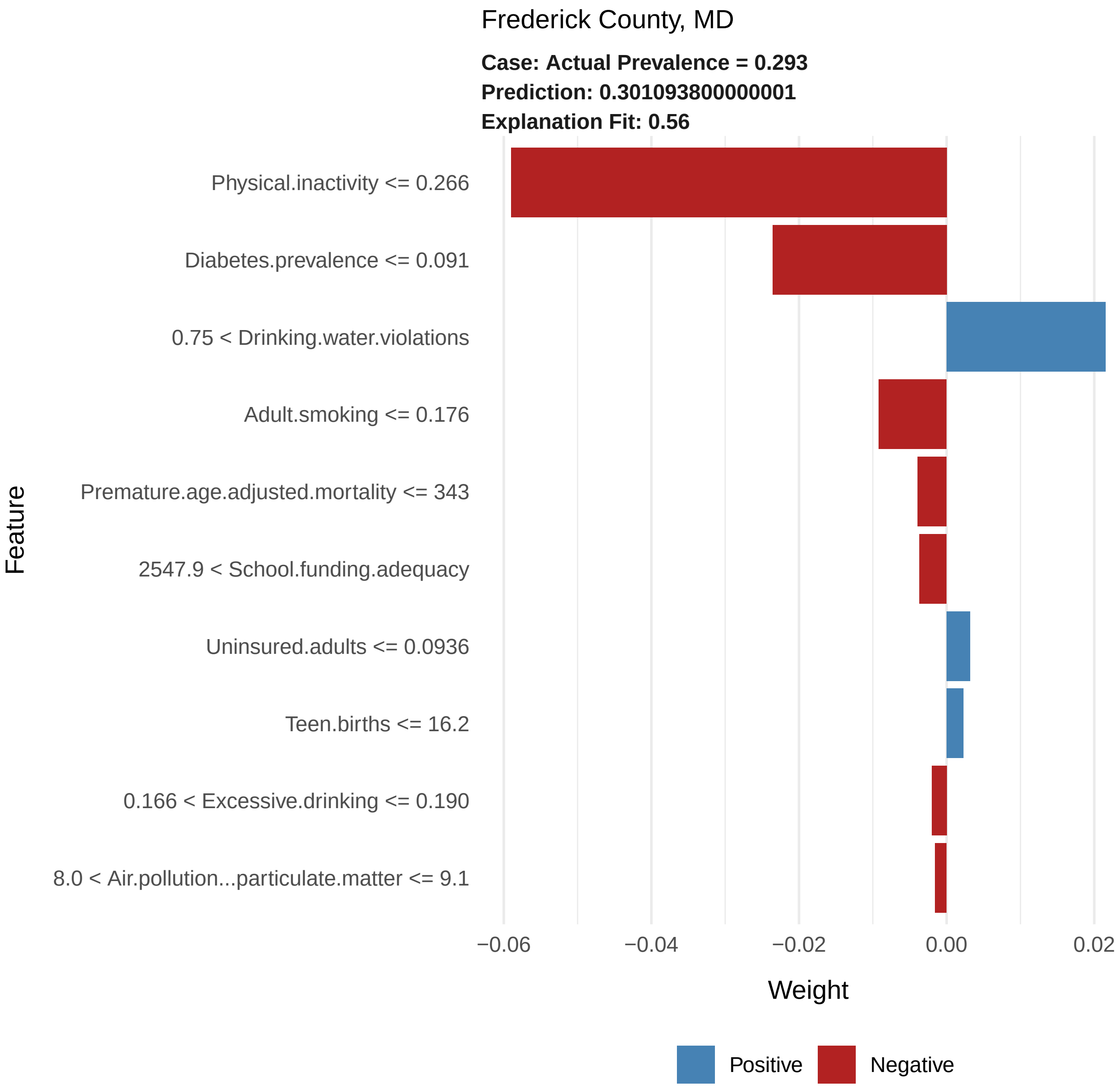

Frederick County, MD
Case: Actual Prevalence = 0.293
Prediction: 0.301093800000001
Explanation Fit: 0.56
Physical.inactivity <= 0.266
Diabetes.prevalence <= 0.091
0.75 < Drinking.water.violations
Adult.smoking <= 0.176
Premature.age.adjusted.mortality <= 343
2547.9 < School.funding.adequacy
Uninsured.adults <= 0.0936
Teen.births <= 16.2
0.166 < Excessive.drinking <= 0.190
8.0 < Air.pollution...particulate.matter <= 9.1
Feature
−0.06
−0.04
−0.02
0.00
0.02
Weight
Positive
Negative

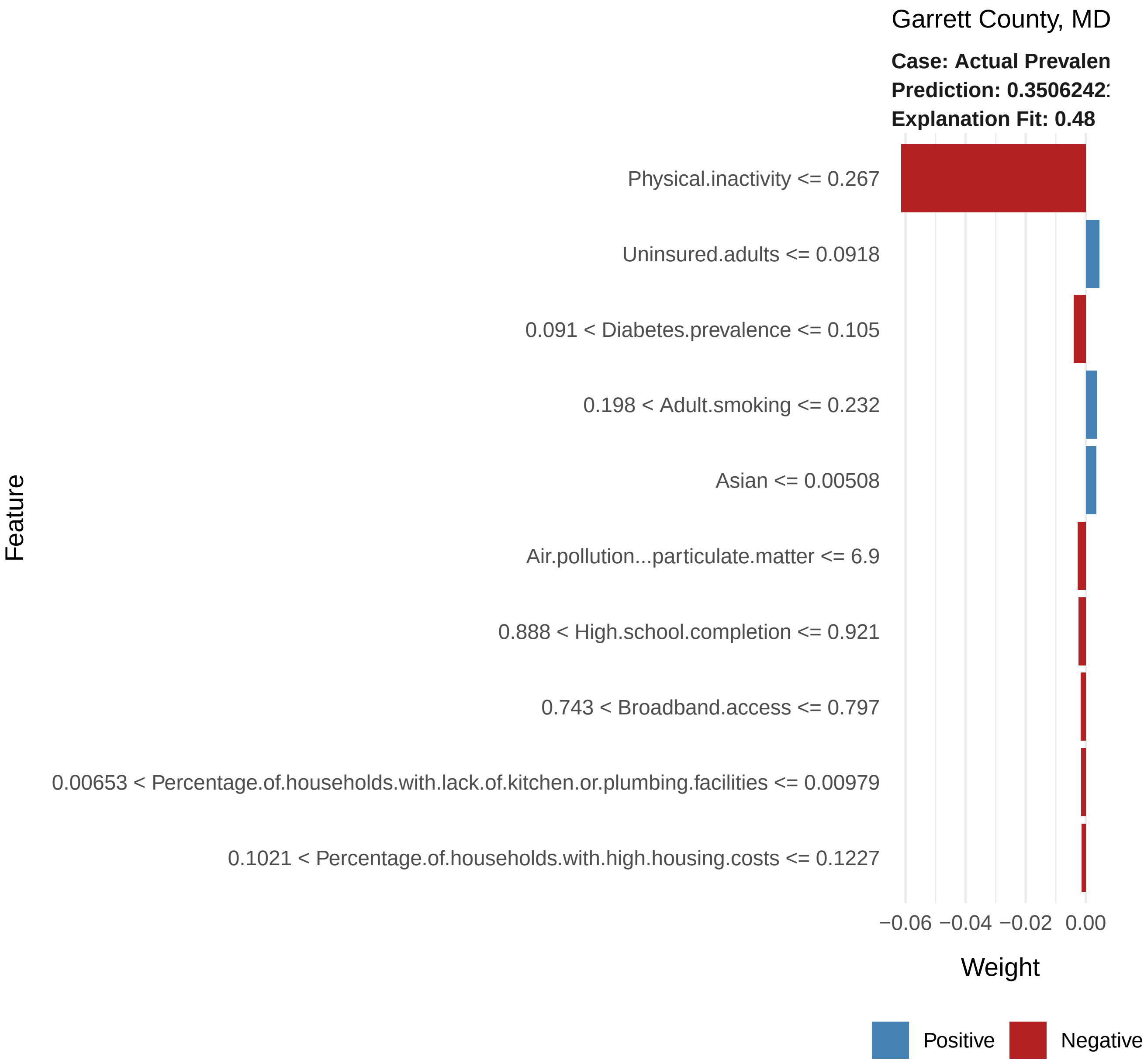
Garrett County, MD
Case: Actual Prevalen
Prediction: 0.3506242
Explanation Fit: 0.48
Physical.inactivity <= 0.267
Uninsured.adults <= 0.0918
0.091 < Diabetes.prevalence <= 0.105
0.198 < Adult.smoking <= 0.232
Asian <= 0.00508
Air.pollution...particulate.matter <= 6.9
0.888 < High.school.completion <= 0.921
0.743 < Broadband.access <= 0.797
0.00653 < Percentage.of.households.with.lack.of.kitchen.or.plumbing.facilities <= 0.00979
0.1021 < Percentage.of.households.with.high.housing.costs <= 0.1227
Feature
−0.06 −0.04 −0.02 0.00
Weight
Positive
Negative

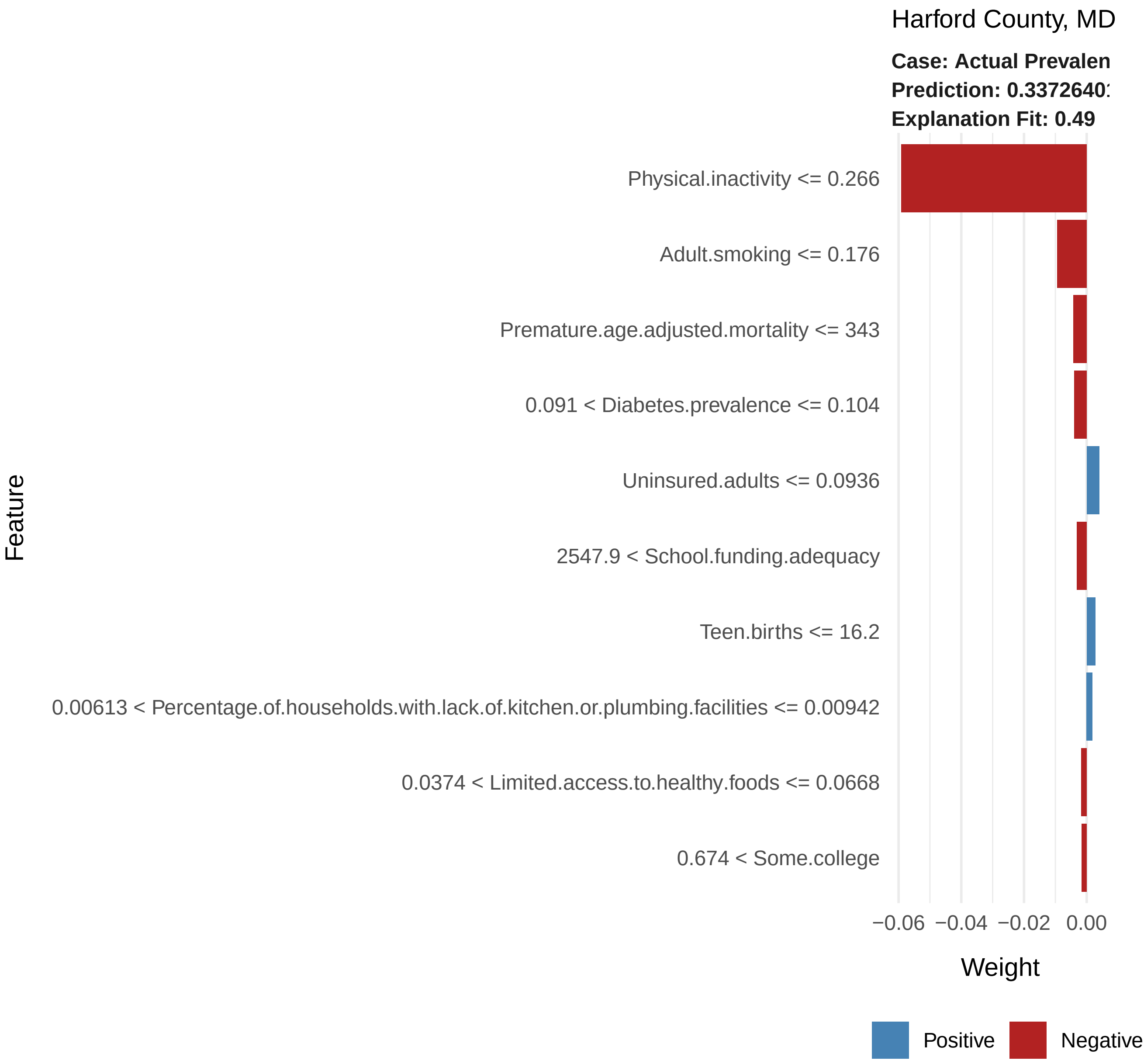

Harford County, MD
Case: Actual Prevalen
Prediction: 0.3372640
Explanation Fit: 0.49
Physical.inactivity <= 0.266
Adult.smoking <= 0.176
Premature.age.adjusted.mortality <= 343
0.091 < Diabetes.prevalence <= 0.104
Uninsured.adults <= 0.0936
2547.9 < School.funding.adequacy
Teen.births <= 16.2
0.00613 < Percentage.of.households.with.lack.of.kitchen.or.plumbing.facilities <= 0.00942
0.0374 < Limited.access.to.healthy.foods <= 0.0668
0.674 < Some.college
Feature
−0.06 −0.04 −0.02 0.00
Weight
Positive
Negative

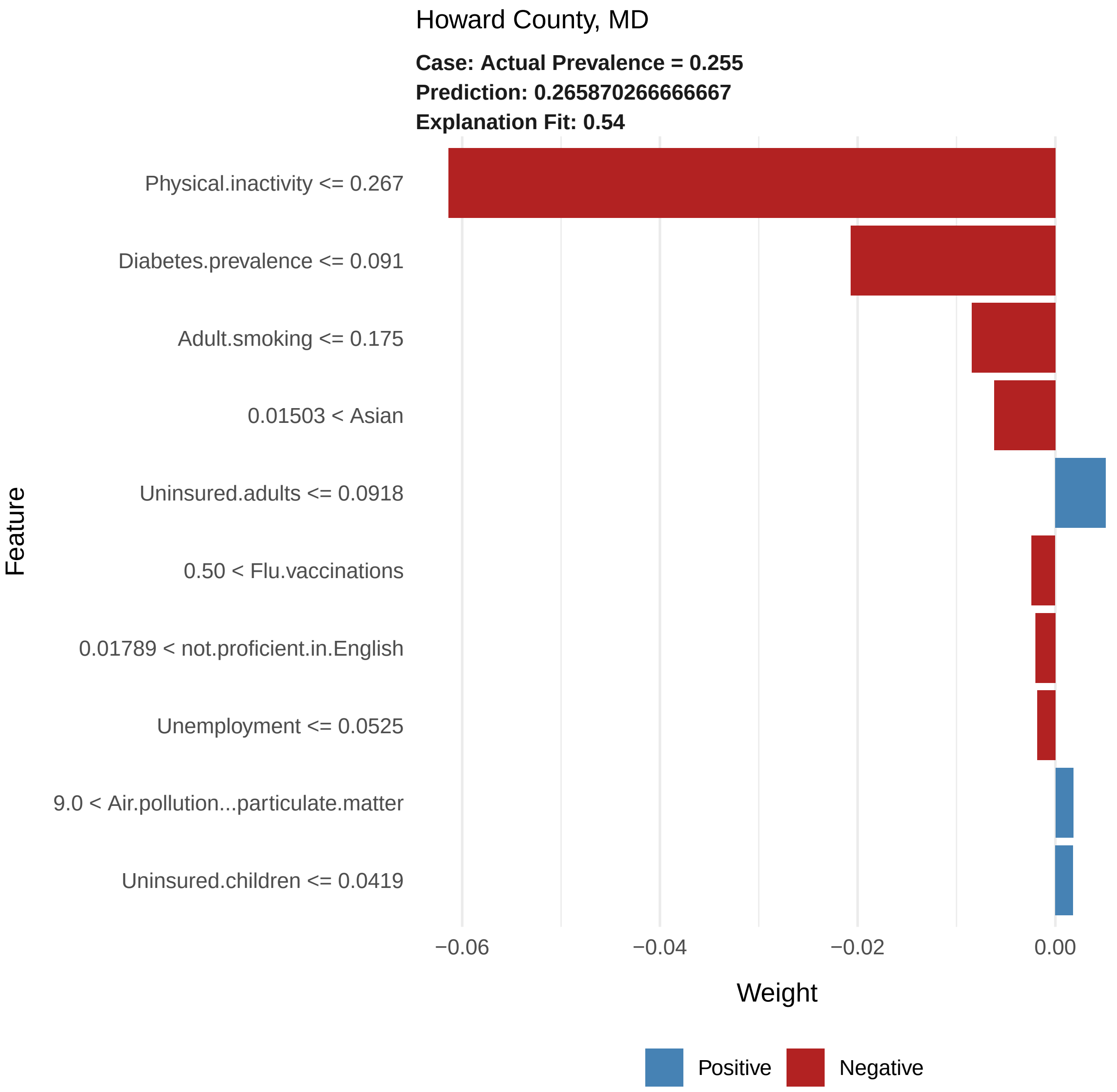
Howard County, MD
Case: Actual Prevalence = 0.255
Prediction: 0.265870266666667
Explanation Fit: 0.54
Physical.inactivity <= 0.267
Diabetes.prevalence <= 0.091
Adult.smoking <= 0.175
0.01503 < Asian
Uninsured.adults <= 0.0918
0.50 < Flu.vaccinations
0.01789 < not.proficient.in.English
Unemployment <= 0.0525
9.0 < Air.pollution...particulate.matter
Uninsured.children <= 0.0419
Feature
−0.06
−0.04
−0.02
0.00
Weight
Positive
Negative

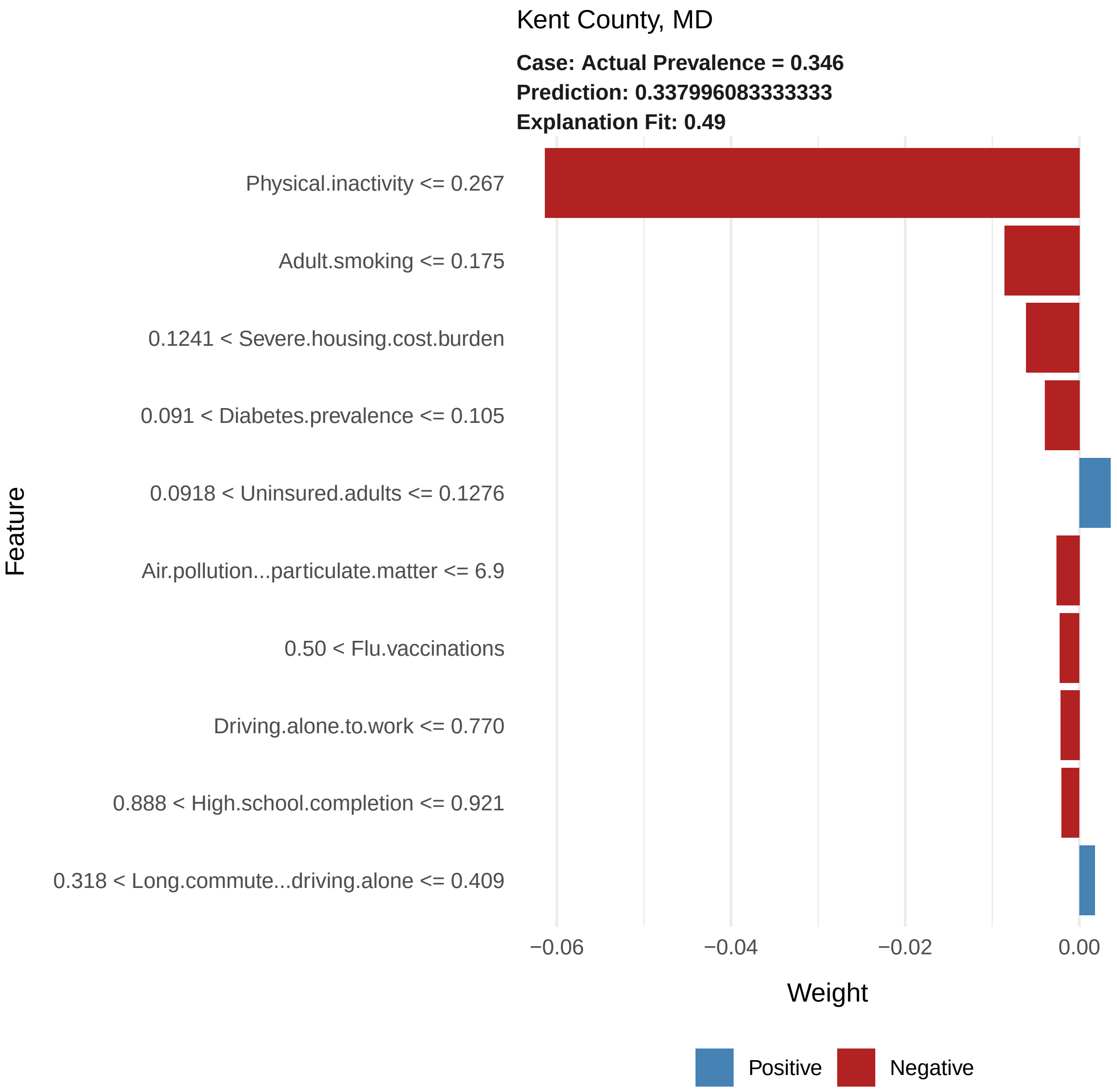
Kent County, MD
Case: Actual Prevalence = 0.346
Prediction: 0.337996083333333
Explanation Fit: 0.49
Physical.inactivity <= 0.267
Adult.smoking <= 0.175
0.1241 < Severe.housing.cost.burden
0.091 < Diabetes.prevalence <= 0.105
0.0918 < Uninsured.adults <= 0.1276
Air.pollution...particulate.matter <= 6.9
0.50 < Flu.vaccinations
Driving.alone.to.work <= 0.770
0.888 < High.school.completion <= 0.921
0.318 < Long.commute...driving.alone <= 0.409
Feature
−0.06
−0.04
−0.02
0.00
Weight
Positive
Negative

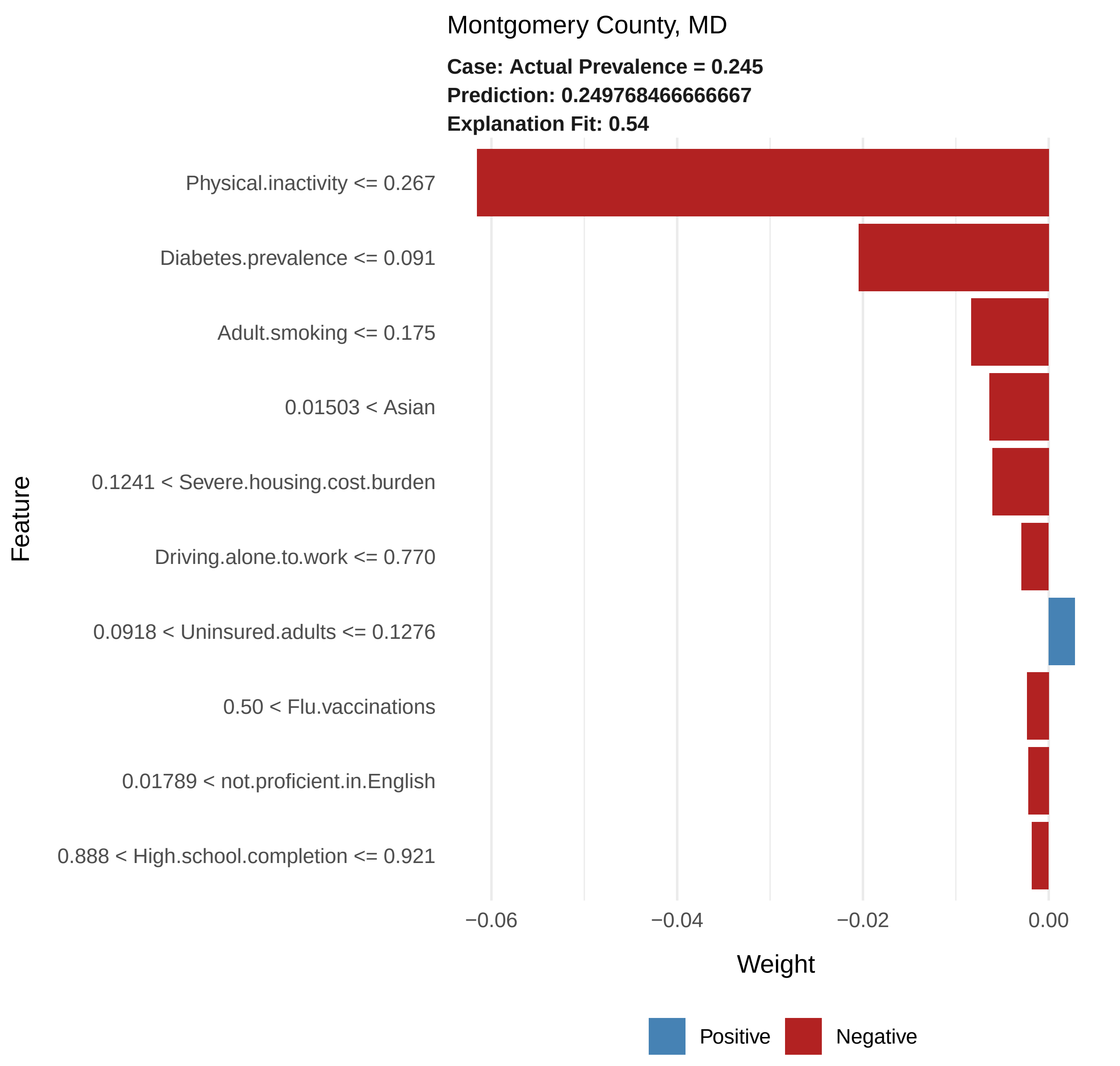

Montgomery County, MD
Case: Actual Prevalence = 0.245
Prediction: 0.249768466666667
Explanation Fit: 0.54
Physical.inactivity <= 0.267
Diabetes.prevalence <= 0.091
Adult.smoking <= 0.175
0.01503 < Asian
0.1241 < Severe.housing.cost.burden
Driving.alone.to.work <= 0.770
0.0918 < Uninsured.adults <= 0.1276
0.50 < Flu.vaccinations
0.01789 < not.proficient.in.English
0.888 < High.school.completion <= 0.921
Feature
−0.06
−0.04
−0.02
0.00
Weight
Positive
Negative

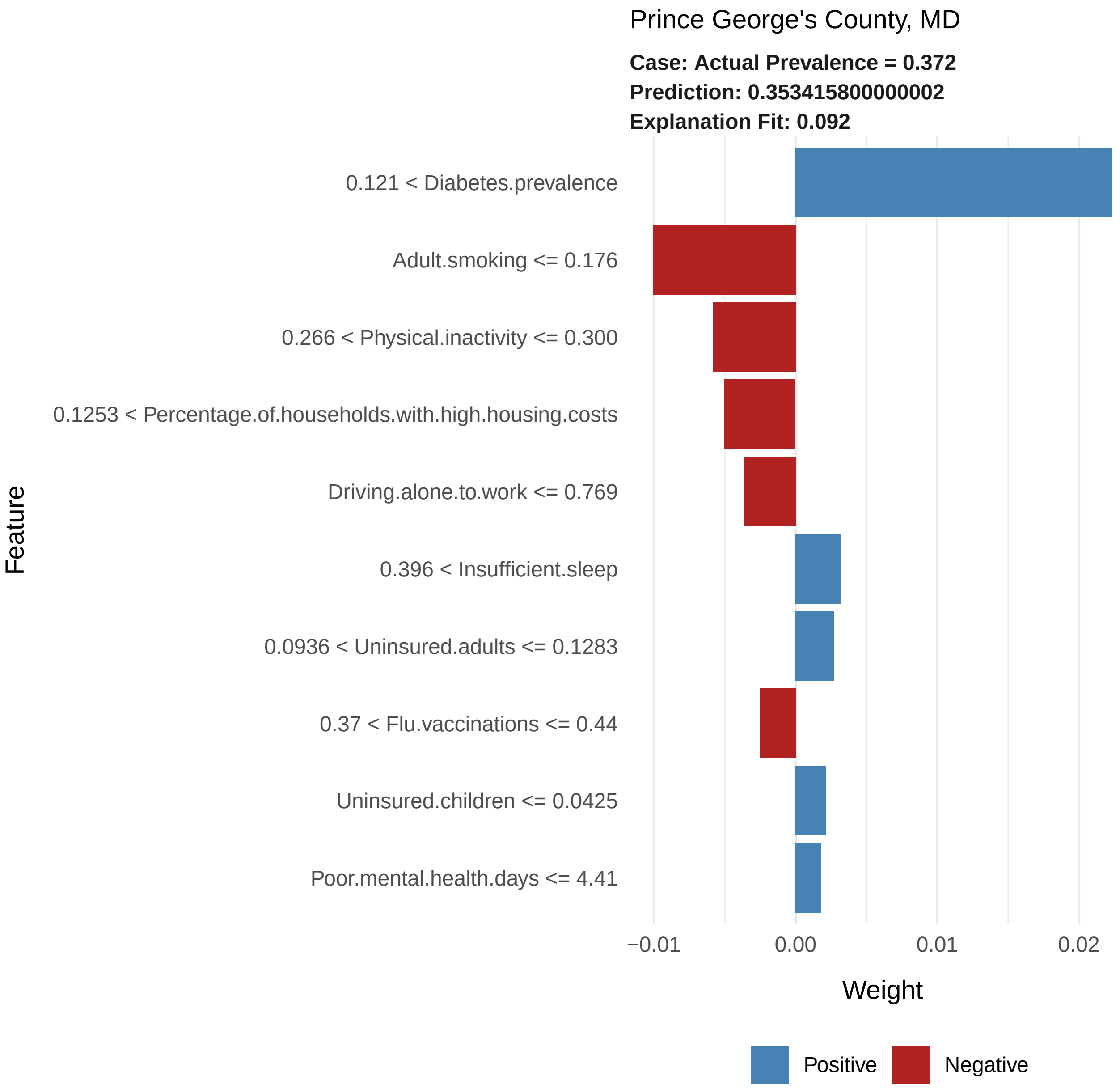
Prince George's County, MD
Case: Actual Prevalence = 0.372
Prediction: 0.353415800000002
Explanation Fit: 0.092
0.121 < Diabetes.prevalence
Adult.smoking <= 0.176
0.266 < Physical.inactivity <= 0.300
0.1253 < Percentage.of.households.with.high.housing.costs
Driving.alone.to.work <= 0.769
0.396 < Insufficient.sleep
0.0936 < Uninsured.adults <= 0.1283
0.37 < Flu.vaccinations <= 0.44
Uninsured.children <= 0.0425
Poor.mental.health.days <= 4.41
Feature
−0.01
0.00
0.01
0.02
Weight
Positive
Negative

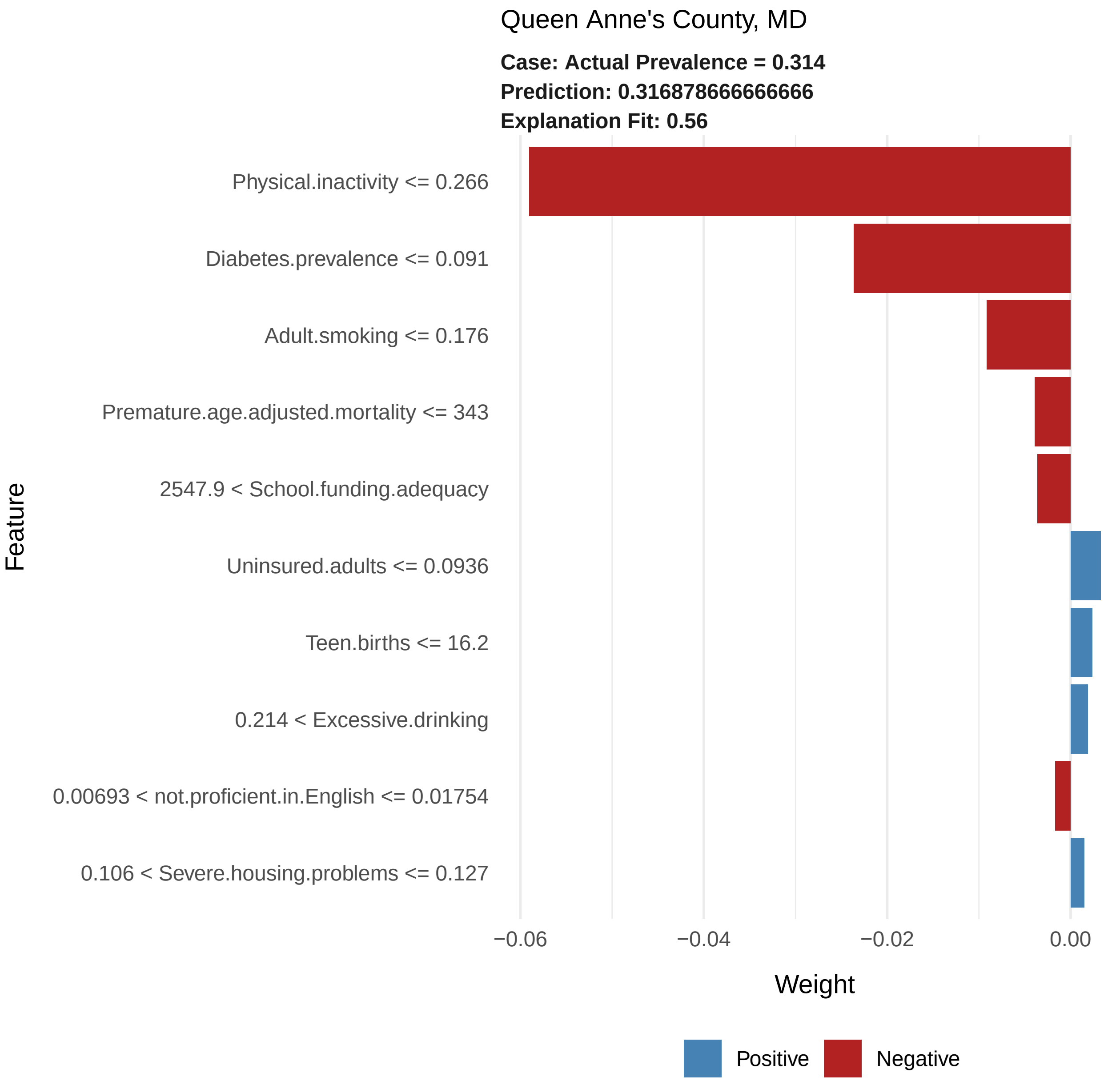

Queen Anne's County, MD
Case: Actual Prevalence = 0.314
Prediction: 0.316878666666666
Explanation Fit: 0.56
Physical.inactivity <= 0.266
Diabetes.prevalence <= 0.091
Adult.smoking <= 0.176
Premature.age.adjusted.mortality <= 343
2547.9 < School.funding.adequacy
Uninsured.adults <= 0.0936
Teen.births <= 16.2
0.214 < Excessive.drinking
0.00693 < not.proficient.in.English <= 0.01754
0.106 < Severe.housing.problems <= 0.127
Feature
−0.06
−0.04
−0.02
0.00
Weight
Positive
Negative

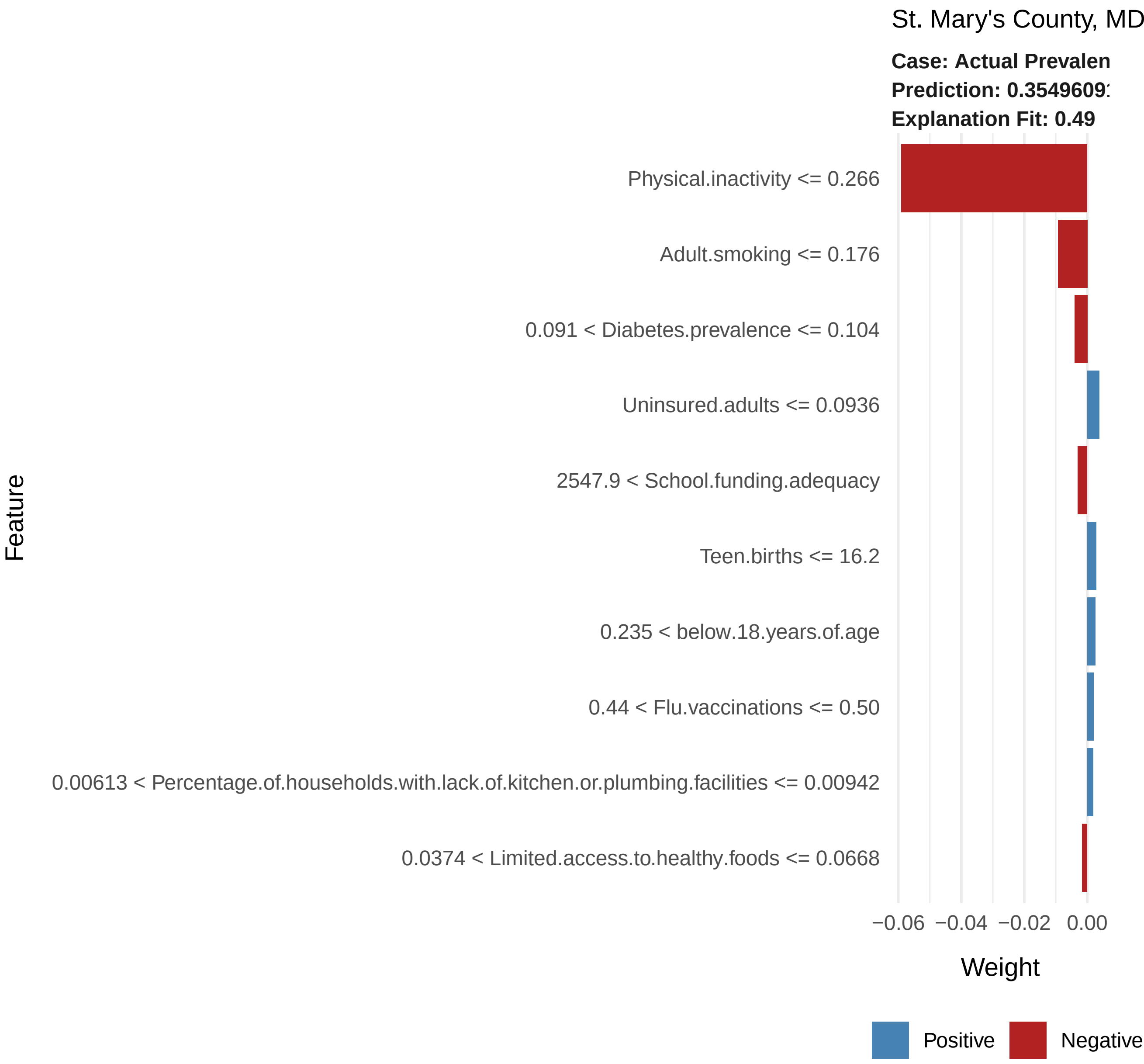

St. Mary's County, MD
Case: Actual Prevalen
Prediction: 0.3549609
Explanation Fit: 0.49
Physical.inactivity <= 0.266
Adult.smoking <= 0.176
0.091 < Diabetes.prevalence <= 0.104
Uninsured.adults <= 0.0936
2547.9 < School.funding.adequacy
Teen.births <= 16.2
0.235 < below.18.years.of.age
0.44 < Flu.vaccinations <= 0.50
0.00613 < Percentage.of.households.with.lack.of.kitchen.or.plumbing.facilities <= 0.00942
0.0374 < Limited.access.to.healthy.foods <= 0.0668
Feature
−0.06 −0.04 −0.02 0.00
Weight
Positive
Negative

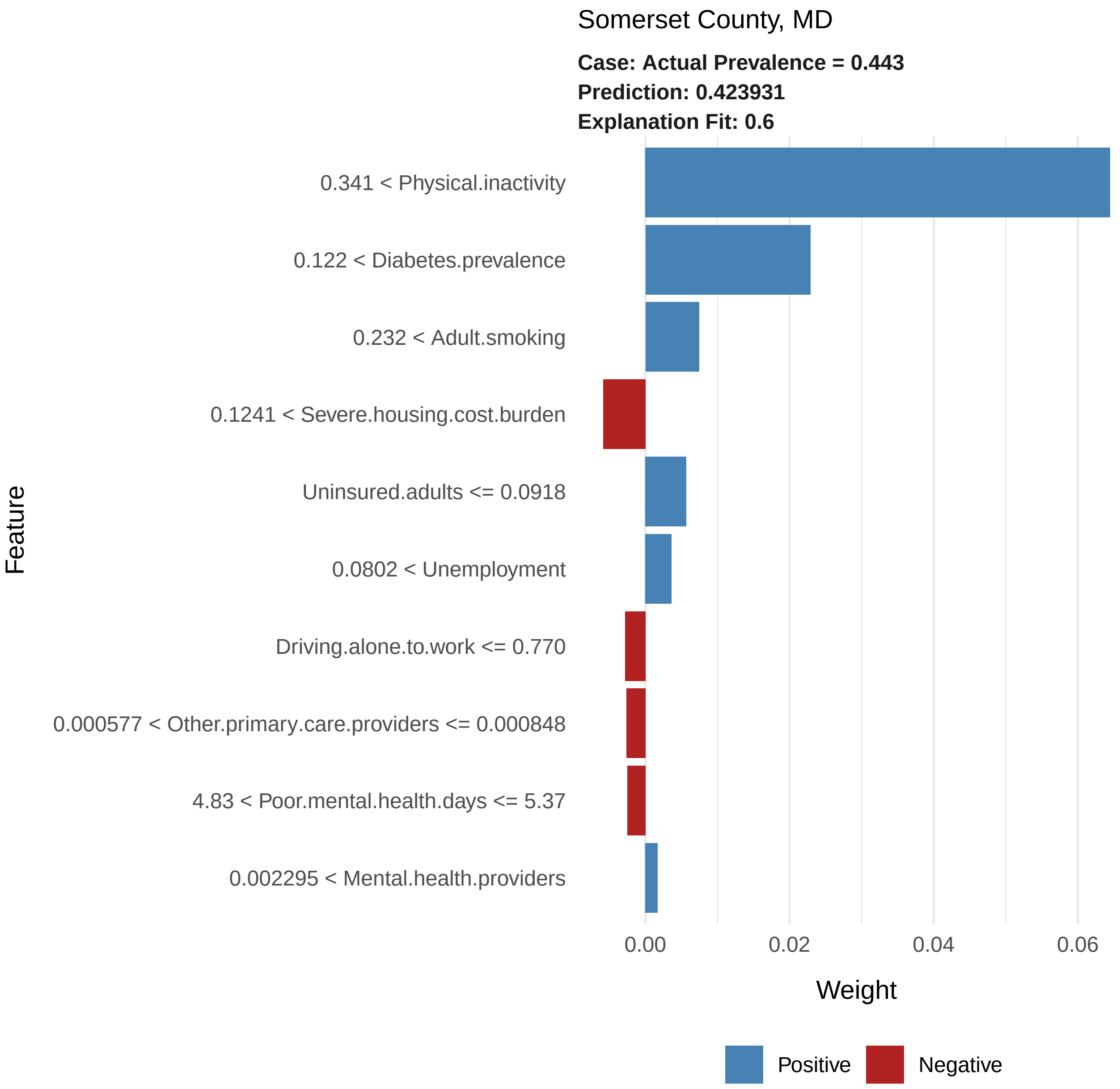
Somerset County, MD
Case: Actual Prevalence = 0.443
Prediction: 0.423931
Explanation Fit: 0.6
0.341 < Physical.inactivity
0.122 < Diabetes.prevalence
0.232 < Adult.smoking
0.1241 < Severe.housing.cost.burden
Uninsured.adults <= 0.0918
0.0802 < Unemployment
Driving.alone.to.work <= 0.770
0.000577 < Other.primary.care.providers <= 0.000848
4.83 < Poor.mental.health.days <= 5.37
0.002295 < Mental.health.providers
Feature
0.00
0.02
0.04
0.06
Weight
Positive
Negative

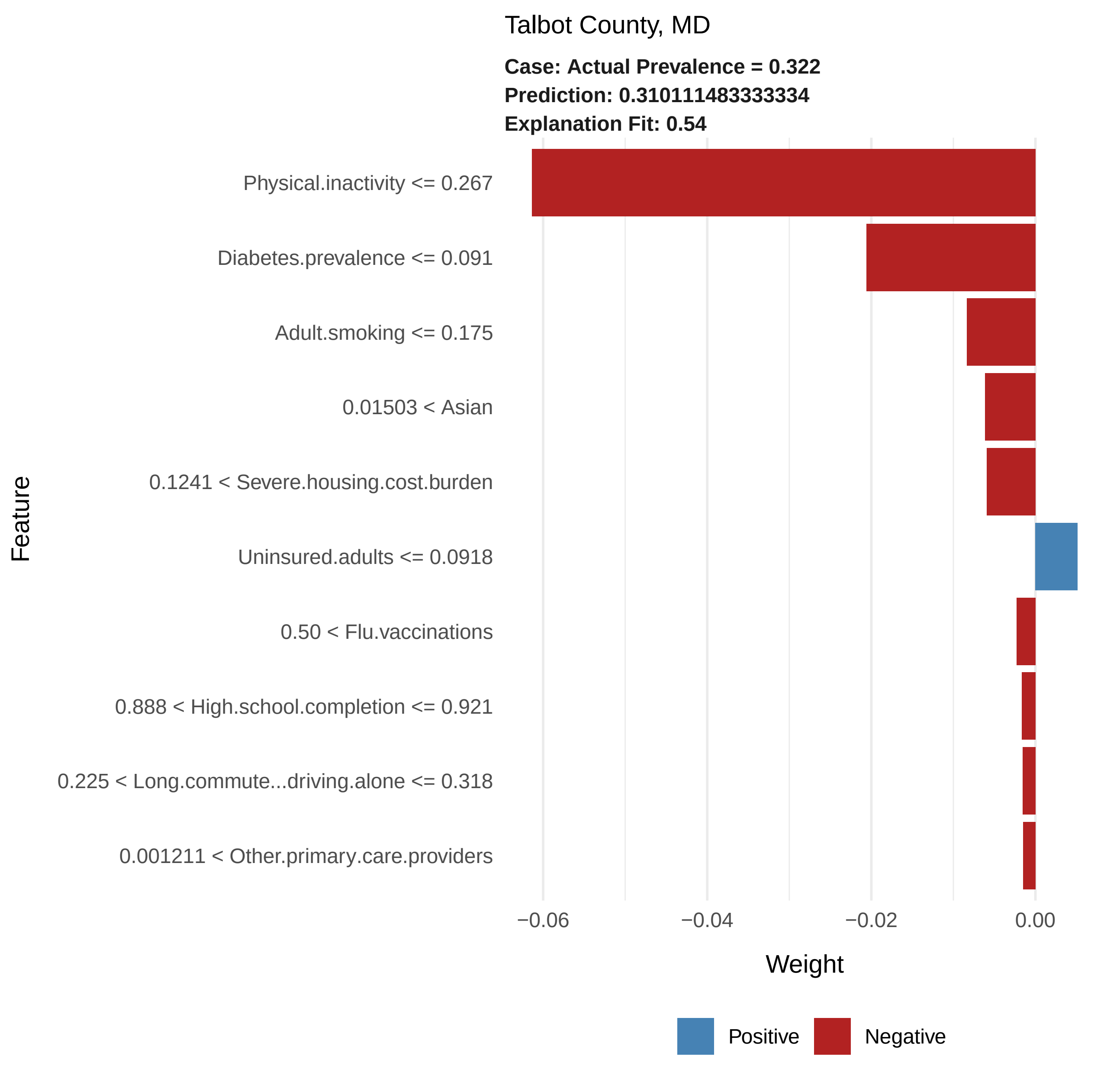

Talbot County, MD
Case: Actual Prevalence = 0.322
Prediction: 0.310111483333334
Explanation Fit: 0.54
Physical.inactivity <= 0.267
Diabetes.prevalence <= 0.091
Adult.smoking <= 0.175
0.01503 < Asian
0.1241 < Severe.housing.cost.burden
Uninsured.adults <= 0.0918
0.50 < Flu.vaccinations
0.888 < High.school.completion <= 0.921
0.225 < Long.commute...driving.alone <= 0.318
0.001211 < Other.primary.care.providers
Feature
−0.06
−0.04
−0.02
0.00
Weight
Positive
Negative

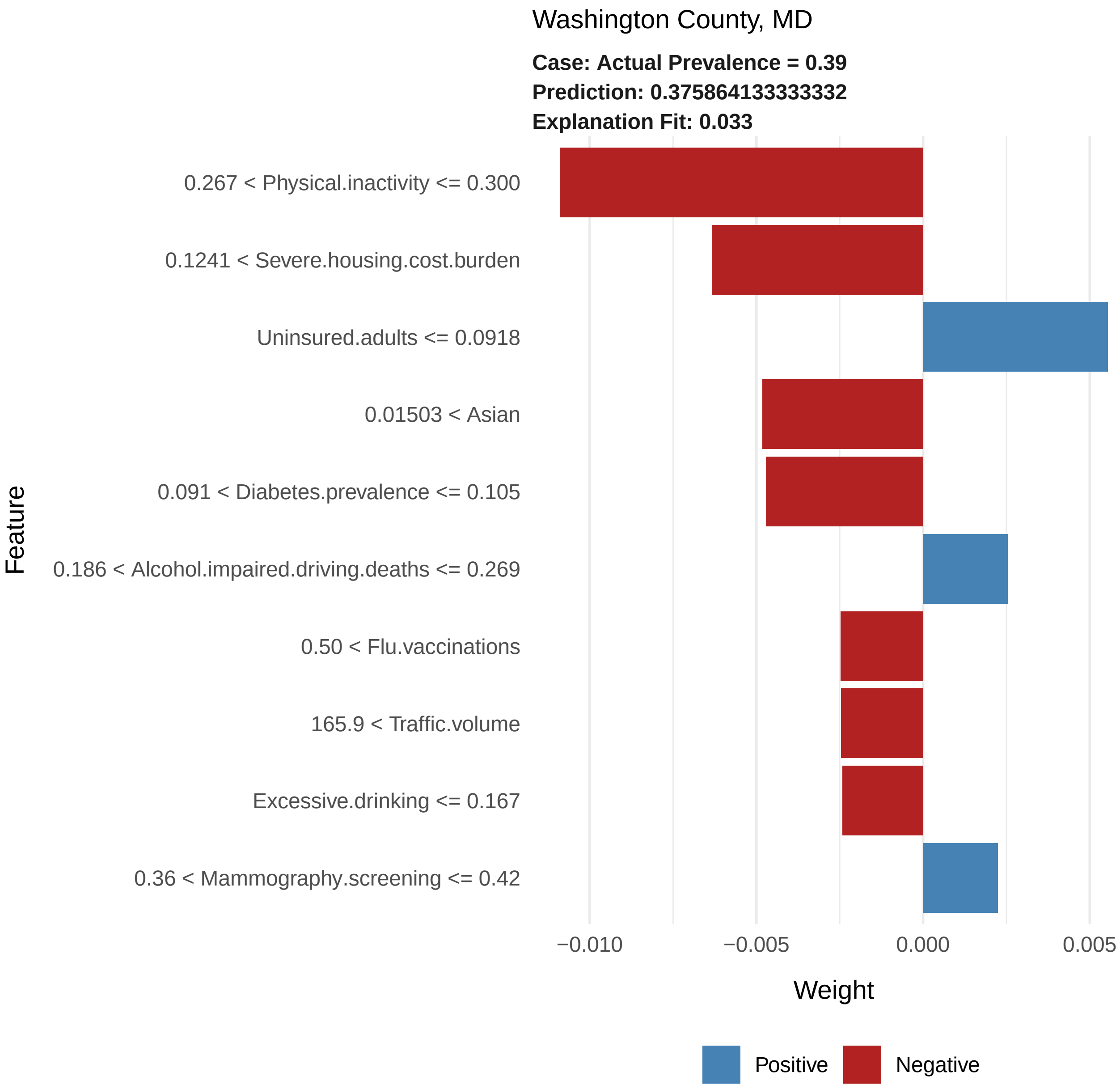

Washington County, MD
Case: Actual Prevalence = 0.39
Prediction: 0.375864133333332
Explanation Fit: 0.033
0.267 < Physical.inactivity <= 0.300
0.1241 < Severe.housing.cost.burden
Uninsured.adults <= 0.0918
0.01503 < Asian
0.091 < Diabetes.prevalence <= 0.105
0.186 < Alcohol.impaired.driving.deaths <= 0.269
0.50 < Flu.vaccinations
165.9 < Traffic.volume
Excessive.drinking <= 0.167
0.36 < Mammography.screening <= 0.42
Feature
−0.010
−0.005
0.000
0.005
Weight
Positive
Negative

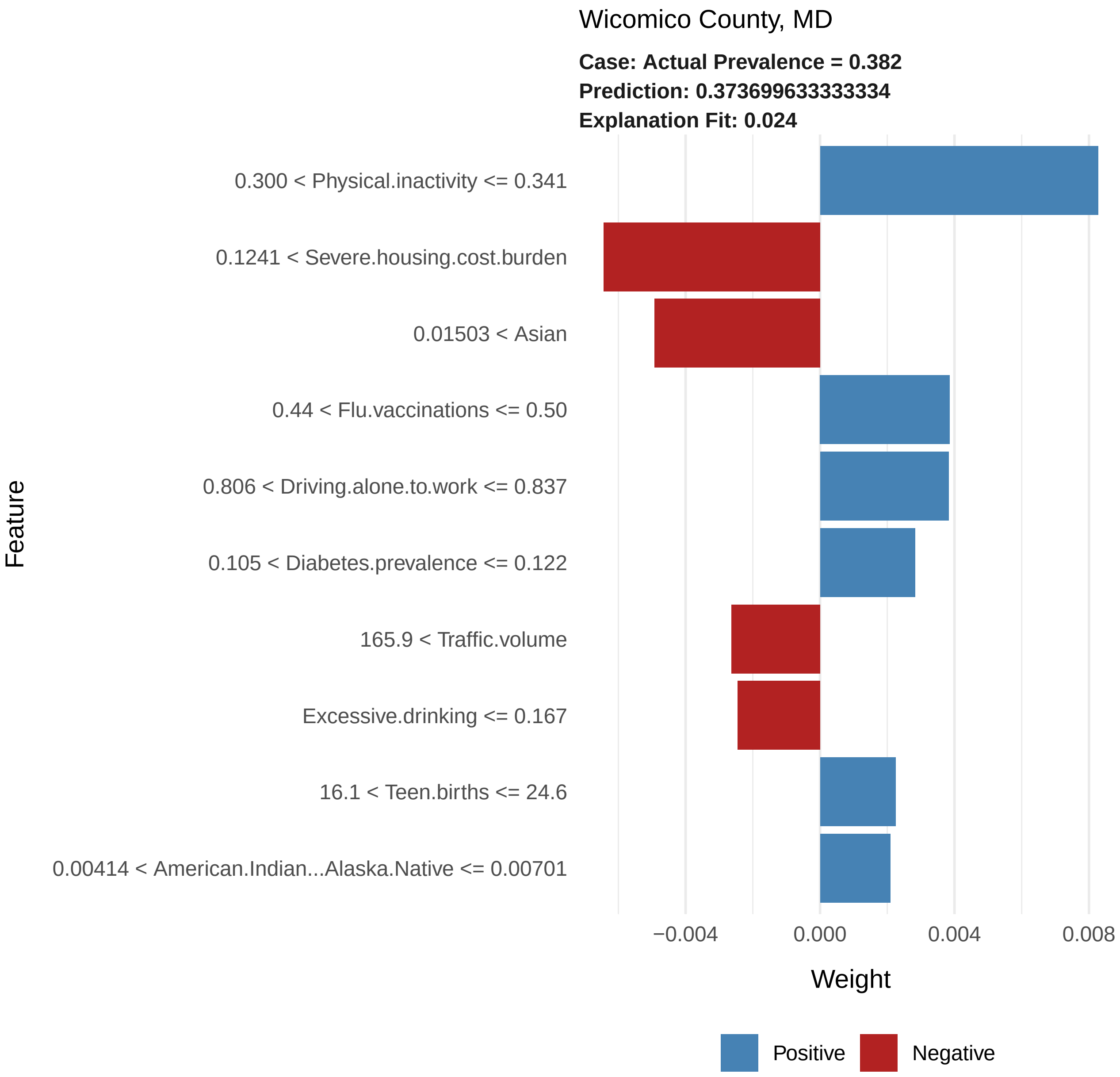

Wicomico County, MD
Case: Actual Prevalence = 0.382
Prediction: 0.373699633333334
Explanation Fit: 0.024
0.300 < Physical.inactivity <= 0.341
0.1241 < Severe.housing.cost.burden
0.01503 < Asian
0.44 < Flu.vaccinations <= 0.50
0.806 < Driving.alone.to.work <= 0.837
0.105 < Diabetes.prevalence <= 0.122
165.9 < Traffic.volume
Excessive.drinking <= 0.167
16.1 < Teen.births <= 24.6
0.00414 < American.Indian...Alaska.Native <= 0.00701
Feature
−0.004
0.000
0.004
0.008
Weight
Positive
Negative

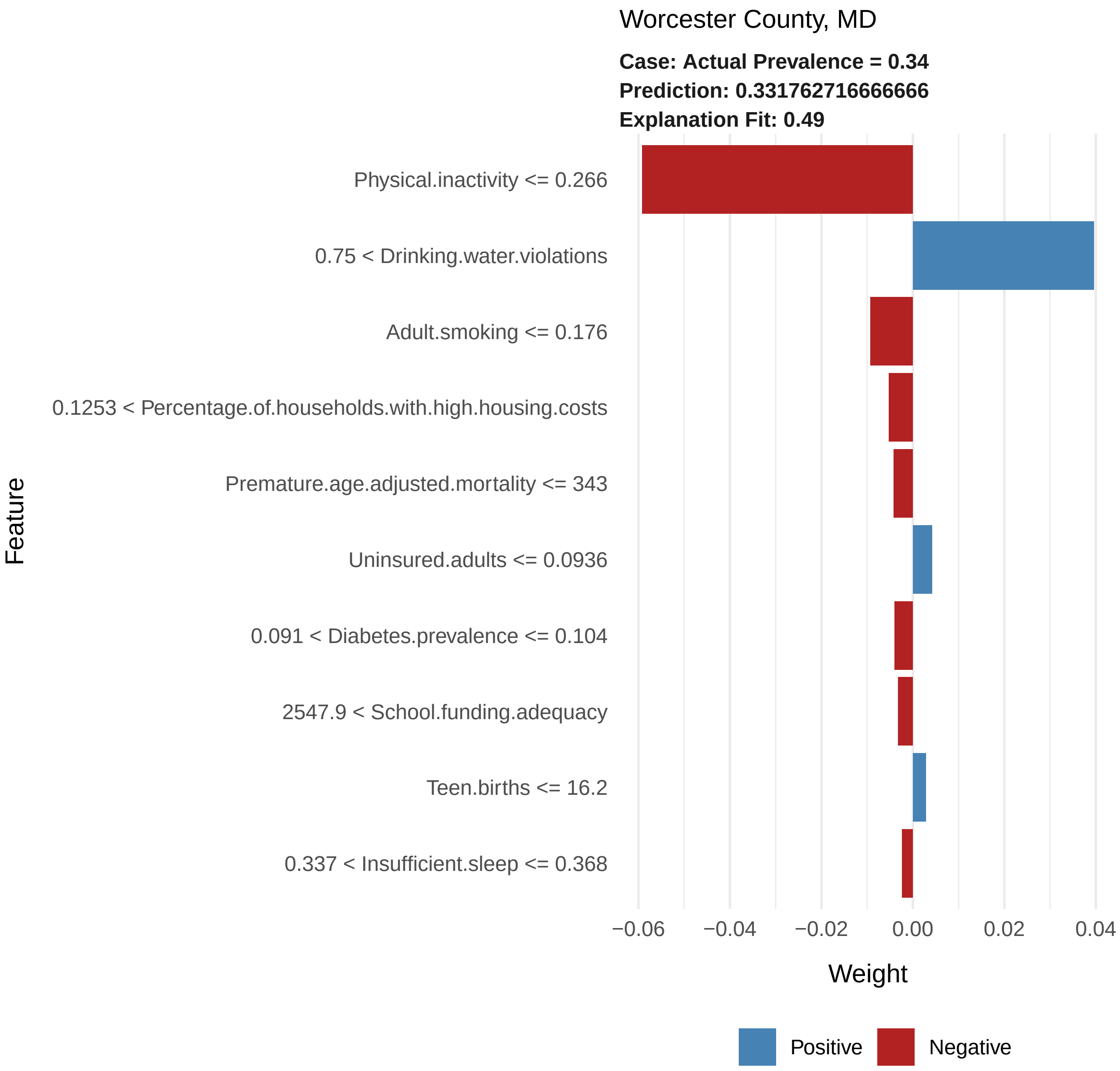

Worcester County, MD
Case: Actual Prevalence = 0.34
Prediction: 0.331762716666666
Explanation Fit: 0.49
Physical.inactivity <= 0.266
0.75 < Drinking.water.violations
Adult.smoking <= 0.176
0.1253 < Percentage.of.households.with.high.housing.costs
Premature.age.adjusted.mortality <= 343
Uninsured.adults <= 0.0936
0.091 < Diabetes.prevalence <= 0.104
2547.9 < School.funding.adequacy
Teen.births <= 16.2
0.337 < Insufficient.sleep <= 0.368
Feature
−0.06
−0.04
−0.02
0.00
0.02
0.04
Weight
Positive
Negative

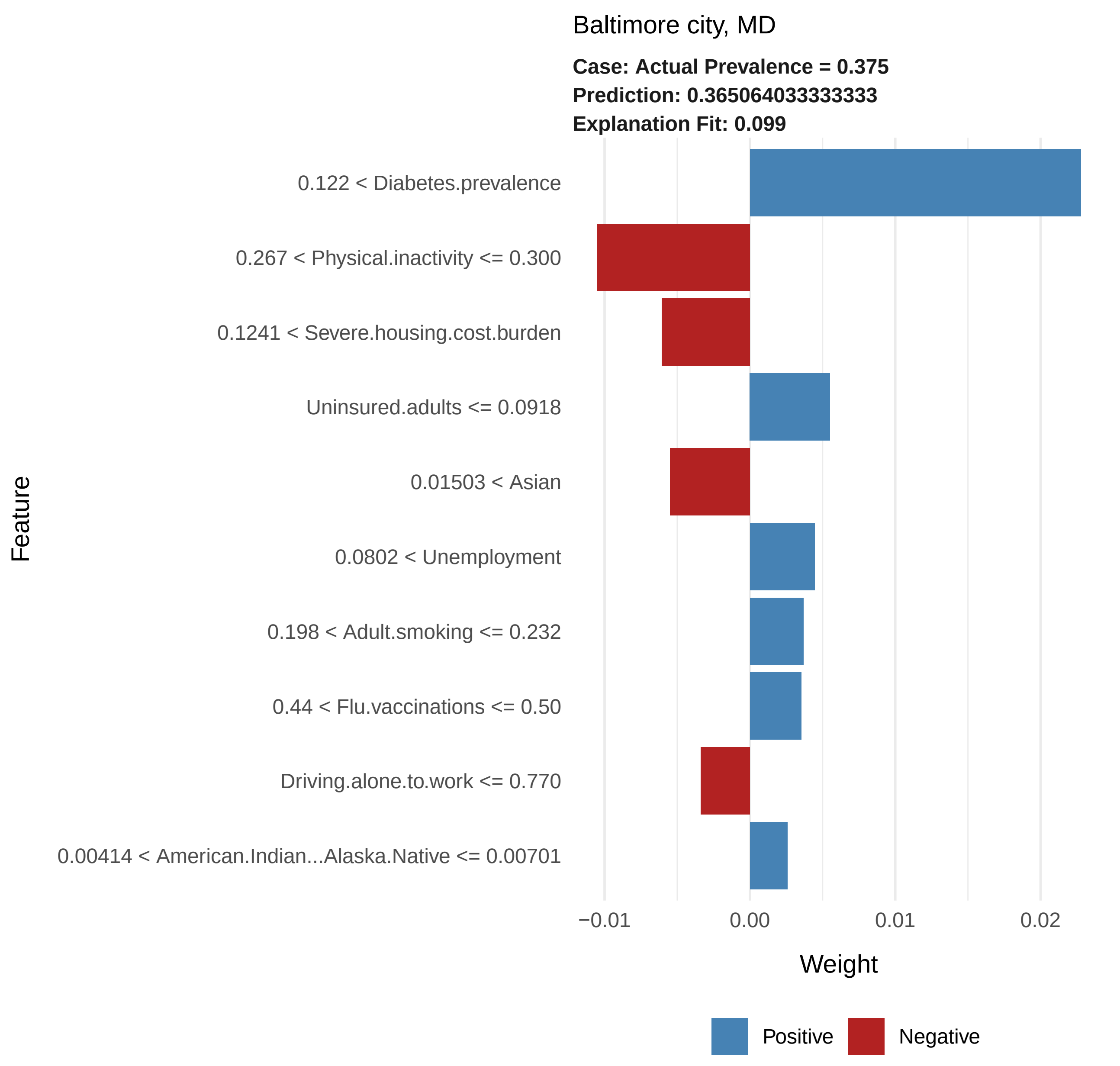
Baltimore city, MD
Case: Actual Prevalence = 0.375
Prediction: 0.365064033333333
Explanation Fit: 0.099
0.122 < Diabetes.prevalence
0.267 < Physical.inactivity <= 0.300
0.1241 < Severe.housing.cost.burden
Uninsured.adults <= 0.0918
0.01503 < Asian
0.0802 < Unemployment
0.198 < Adult.smoking <= 0.232
0.44 < Flu.vaccinations <= 0.50
Driving.alone.to.work <= 0.770
0.00414 < American.Indian...Alaska.Native <= 0.00701
Feature
−0.01
0.00
0.01
0.02
Weight
Positive
Negative

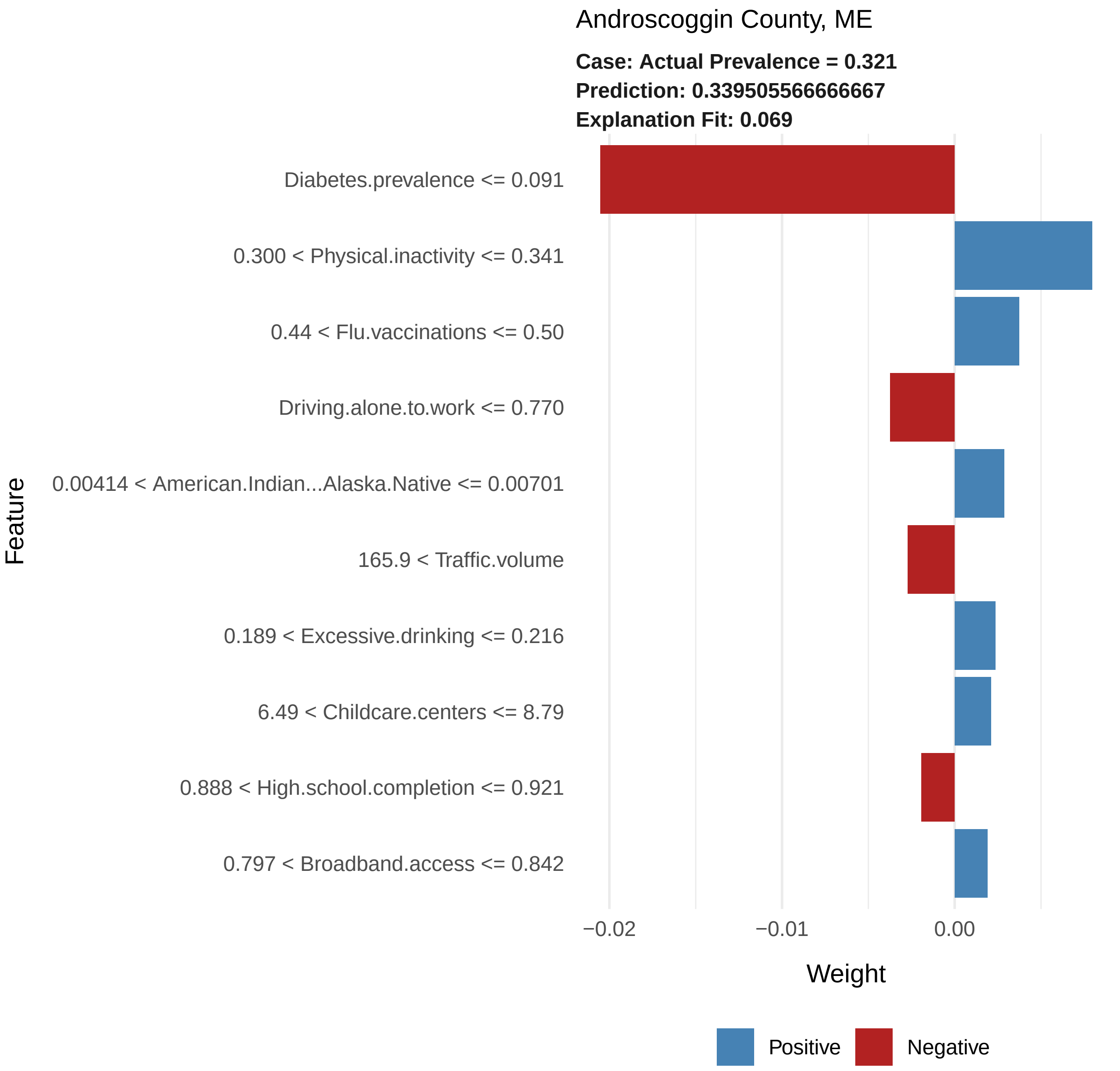
Androscoggin County, ME
Case: Actual Prevalence = 0.321
Prediction: 0.339505566666667
Explanation Fit: 0.069
Diabetes.prevalence <= 0.091
0.300 < Physical.inactivity <= 0.341
0.44 < Flu.vaccinations <= 0.50
Driving.alone.to.work <= 0.770
0.00414 < American.Indian...Alaska.Native <= 0.00701
165.9 < Traffic.volume
0.189 < Excessive.drinking <= 0.216
6.49 < Childcare.centers <= 8.79
0.888 < High.school.completion <= 0.921
0.797 < Broadband.access <= 0.842
Feature
−0.02
−0.01
0.00
Weight
Positive
Negative

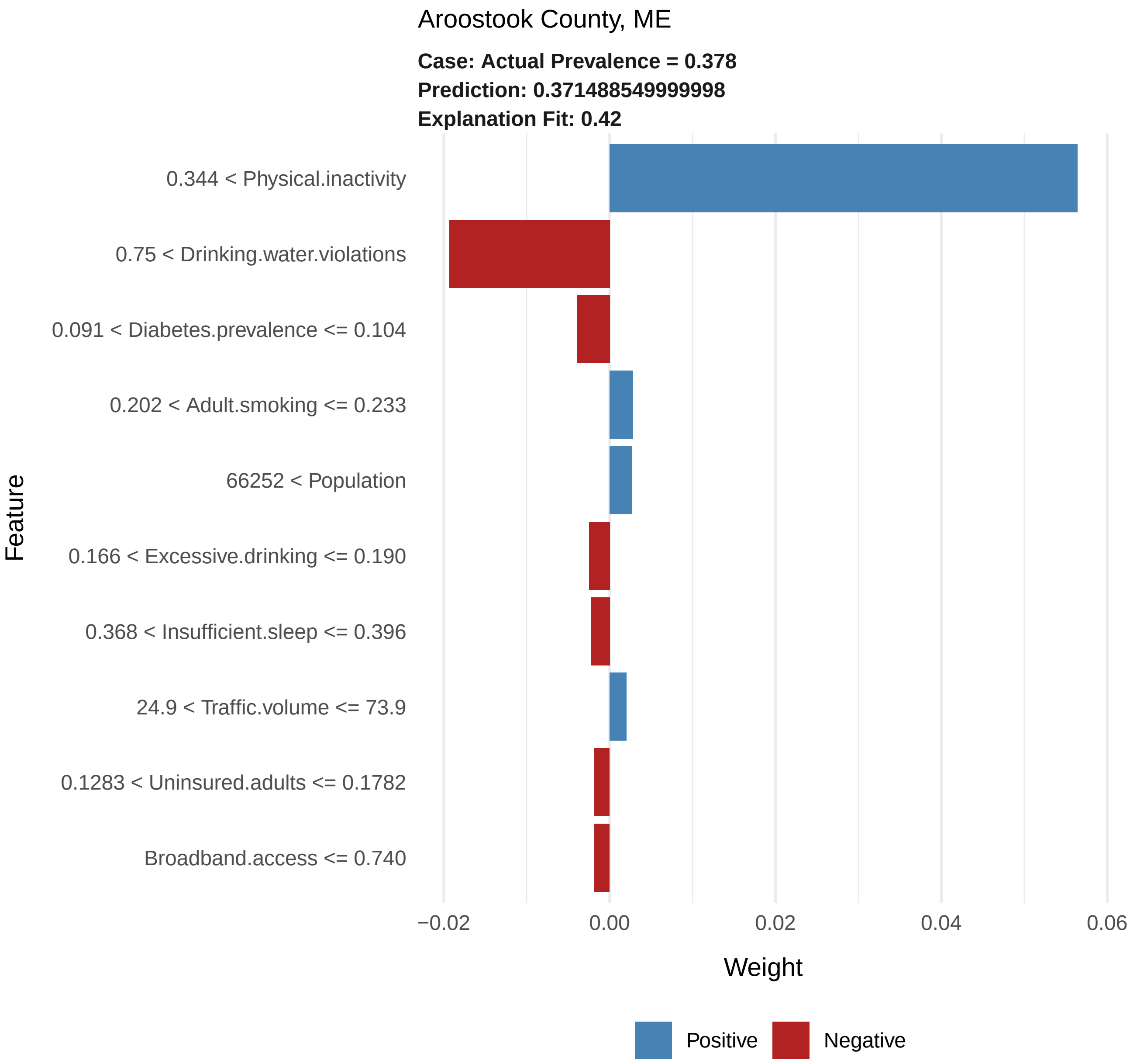

Aroostook County, ME
Case: Actual Prevalence = 0.378
Prediction: 0.371488549999998
Explanation Fit: 0.42
0.344 < Physical.inactivity
0.75 < Drinking.water.violations
0.091 < Diabetes.prevalence <= 0.104
0.202 < Adult.smoking <= 0.233
66252 < Population
0.166 < Excessive.drinking <= 0.190
0.368 < Insufficient.sleep <= 0.396
24.9 < Traffic.volume <= 73.9
0.1283 < Uninsured.adults <= 0.1782
Broadband.access <= 0.740
Feature
−0.02
0.00
0.02
0.04
0.06
Weight
Positive
Negative

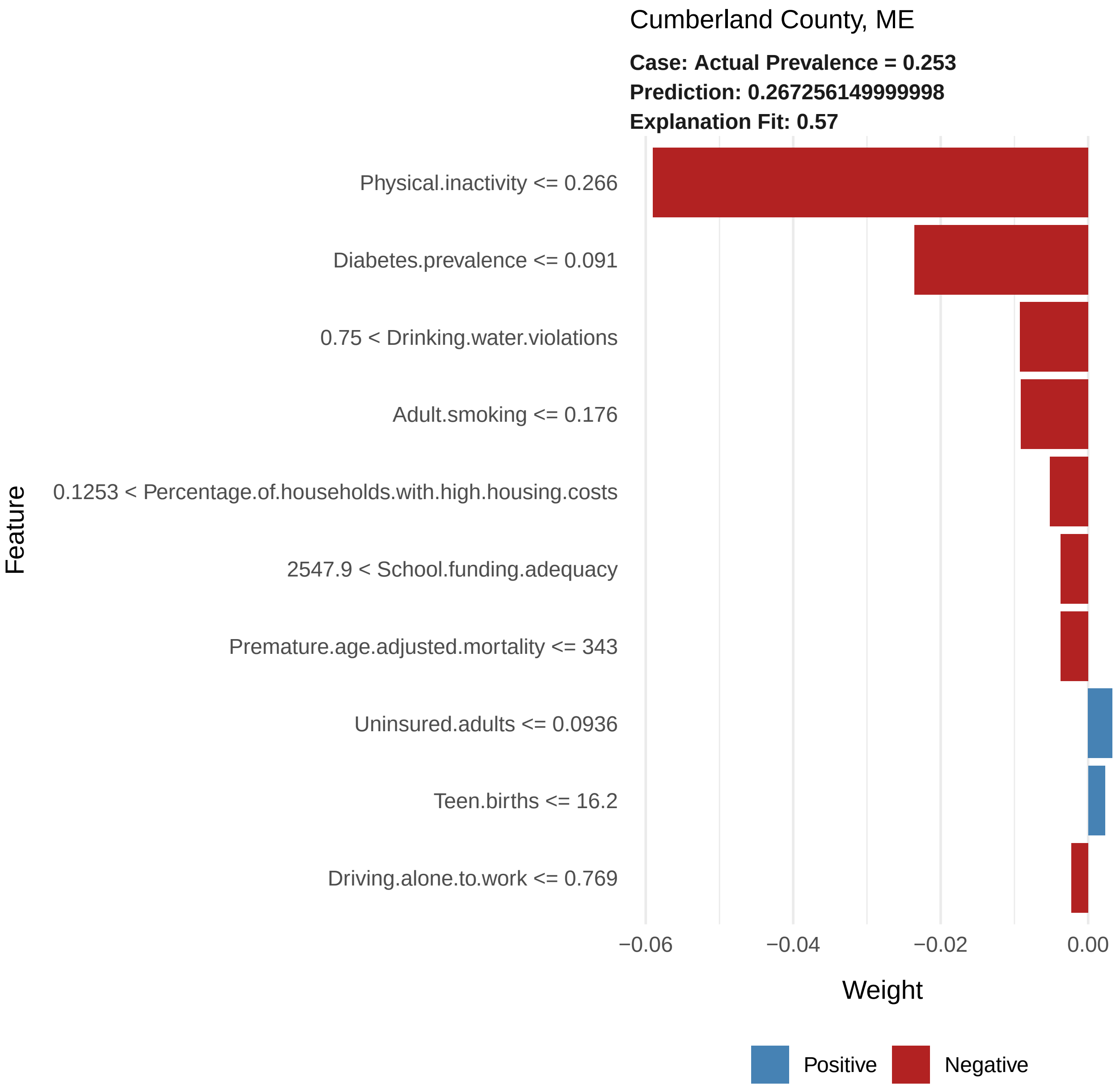
Cumberland County, ME
Case: Actual Prevalence = 0.253
Prediction: 0.267256149999998
Explanation Fit: 0.57
Physical.inactivity <= 0.266
Diabetes.prevalence <= 0.091
0.75 < Drinking.water.violations
Adult.smoking <= 0.176
0.1253 < Percentage.of.households.with.high.housing.costs
2547.9 < School.funding.adequacy
Premature.age.adjusted.mortality <= 343
Uninsured.adults <= 0.0936
Teen.births <= 16.2
Driving.alone.to.work <= 0.769
Feature
−0.06
−0.04
−0.02
0.00
Weight
Positive
Negative

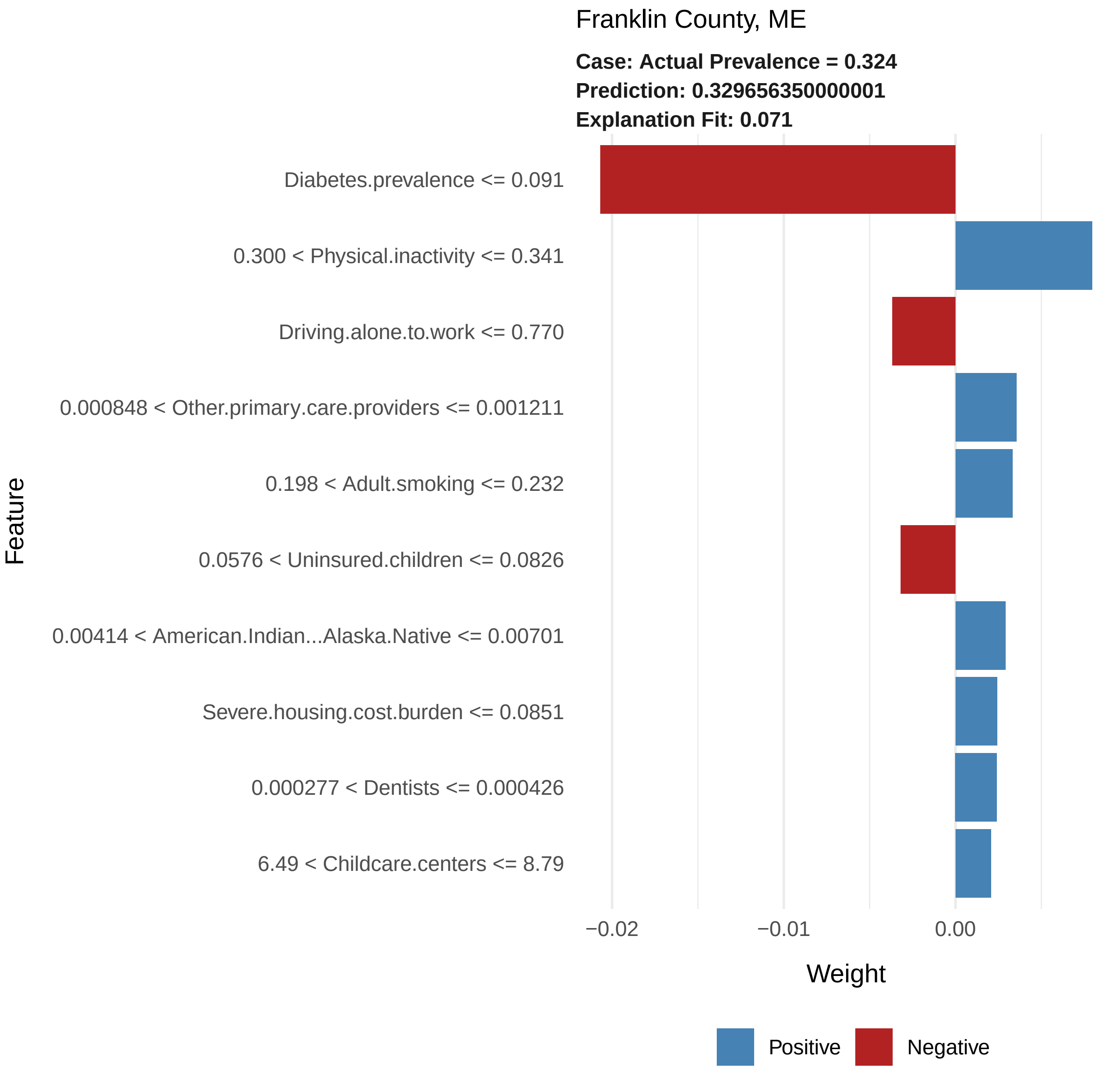

Franklin County, ME
Case: Actual Prevalence = 0.324
Prediction: 0.329656350000001
Explanation Fit: 0.071
Diabetes.prevalence <= 0.091
0.300 < Physical.inactivity <= 0.341
Driving.alone.to.work <= 0.770
0.000848 < Other.primary.care.providers <= 0.001211
0.198 < Adult.smoking <= 0.232
0.0576 < Uninsured.children <= 0.0826
0.00414 < American.Indian...Alaska.Native <= 0.00701
Severe.housing.cost.burden <= 0.0851
0.000277 < Dentists <= 0.000426
6.49 < Childcare.centers <= 8.79
Feature
−0.02
−0.01
0.00
Weight
Positive
Negative

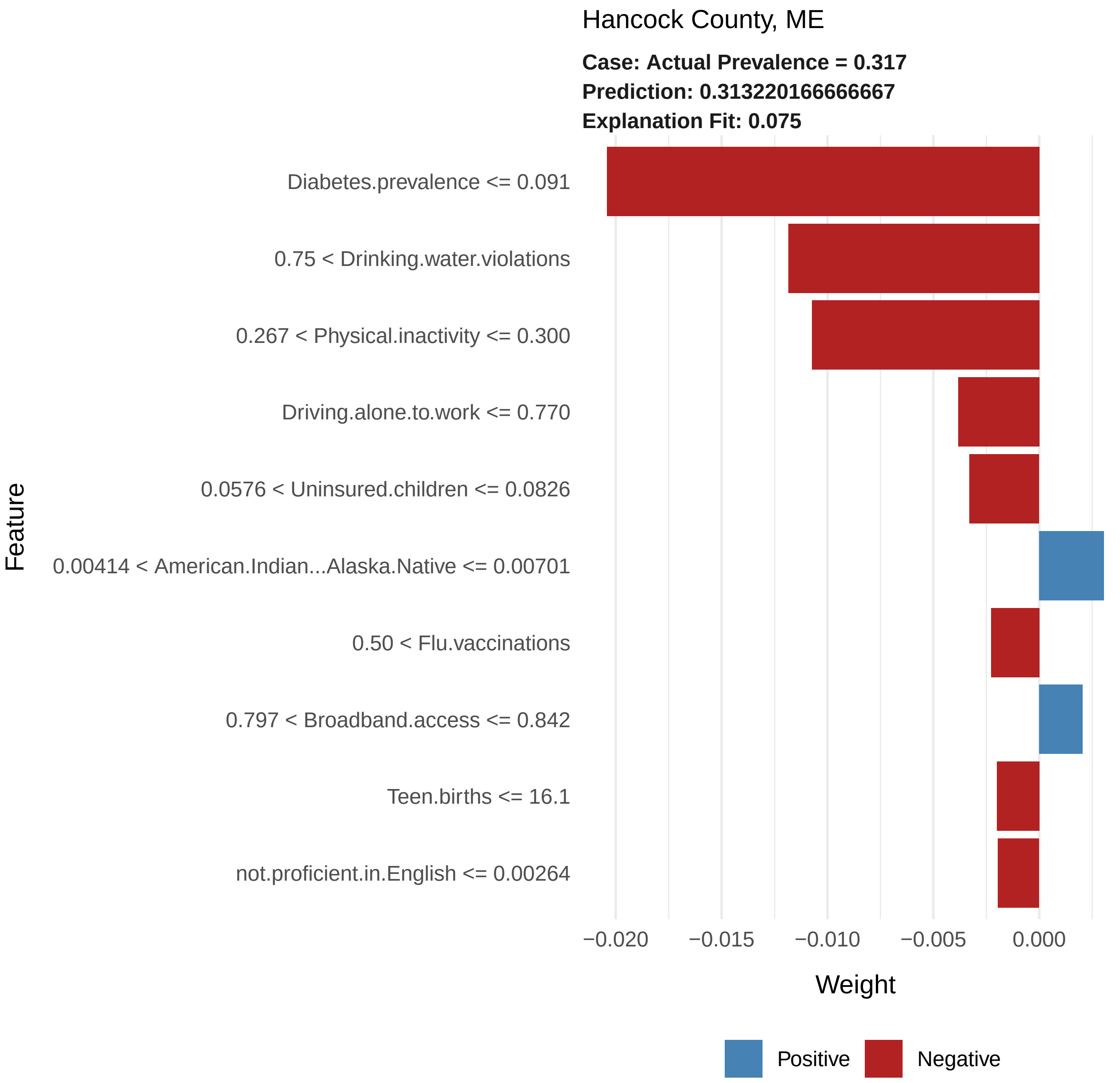

Hancock County, ME
Case: Actual Prevalence = 0.317
Prediction: 0.313220166666667
Explanation Fit: 0.075
Diabetes.prevalence <= 0.091
0.75 < Drinking.water.violations
0.267 < Physical.inactivity <= 0.300
Driving.alone.to.work <= 0.770
0.0576 < Uninsured.children <= 0.0826
0.00414 < American.Indian...Alaska.Native <= 0.00701
0.50 < Flu.vaccinations
0.797 < Broadband.access <= 0.842
Teen.births <= 16.1
not.proficient.in.English <= 0.00264
Feature
−0.020
−0.015
−0.010
−0.005
0.000
Weight
Positive
Negative

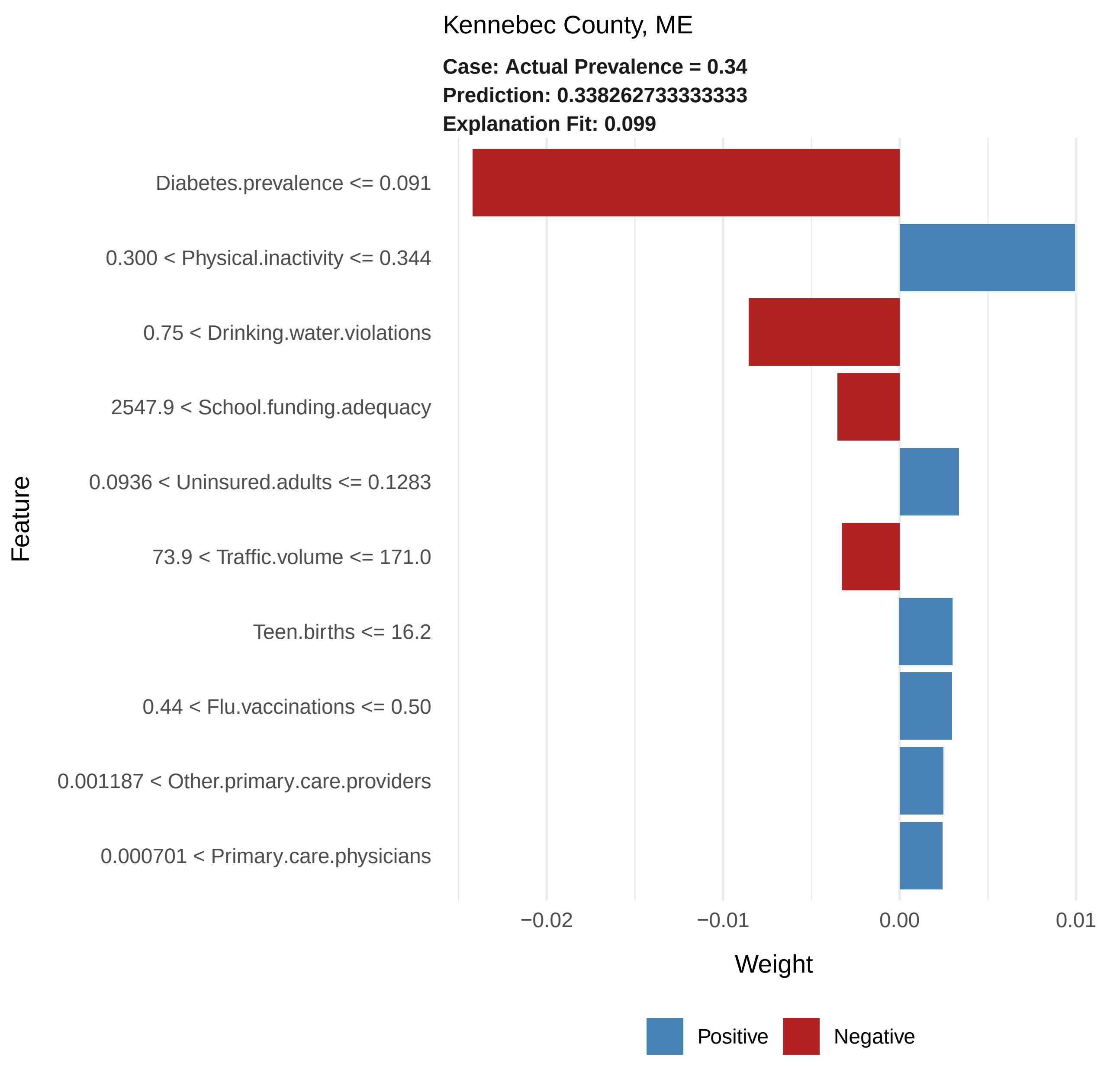

Kennebec County, ME
Case: Actual Prevalence = 0.34
Prediction: 0.338262733333333
Explanation Fit: 0.099
Diabetes.prevalence <= 0.091
0.300 < Physical.inactivity <= 0.344
0.75 < Drinking.water.violations
2547.9 < School.funding.adequacy
0.0936 < Uninsured.adults <= 0.1283
73.9 < Traffic.volume <= 171.0
Teen.births <= 16.2
0.44 < Flu.vaccinations <= 0.50
0.001187 < Other.primary.care.providers
0.000701 < Primary.care.physicians
Feature
−0.02
−0.01
0.00
0.01
Weight
Positive
Negative

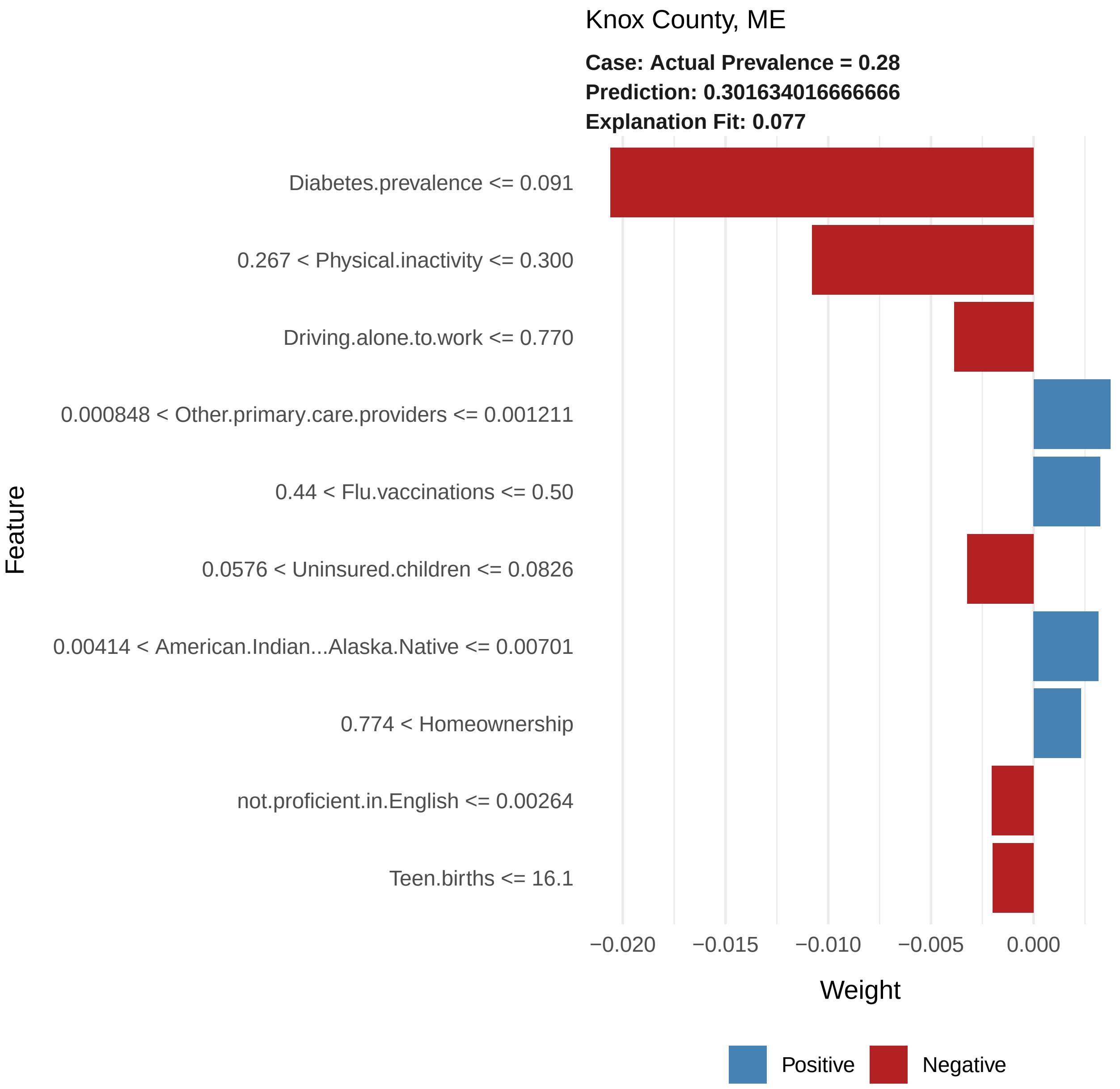

Knox County, ME
Case: Actual Prevalence = 0.28
Prediction: 0.301634016666666
Explanation Fit: 0.077
Diabetes.prevalence <= 0.091
0.267 < Physical.inactivity <= 0.300
Driving.alone.to.work <= 0.770
0.000848 < Other.primary.care.providers <= 0.001211
0.44 < Flu.vaccinations <= 0.50
0.0576 < Uninsured.children <= 0.0826
0.00414 < American.Indian...Alaska.Native <= 0.00701
0.774 < Homeownership
not.proficient.in.English <= 0.00264
Teen.births <= 16.1
Feature
−0.020
−0.015
−0.010
−0.005
0.000
Weight
Positive
Negative

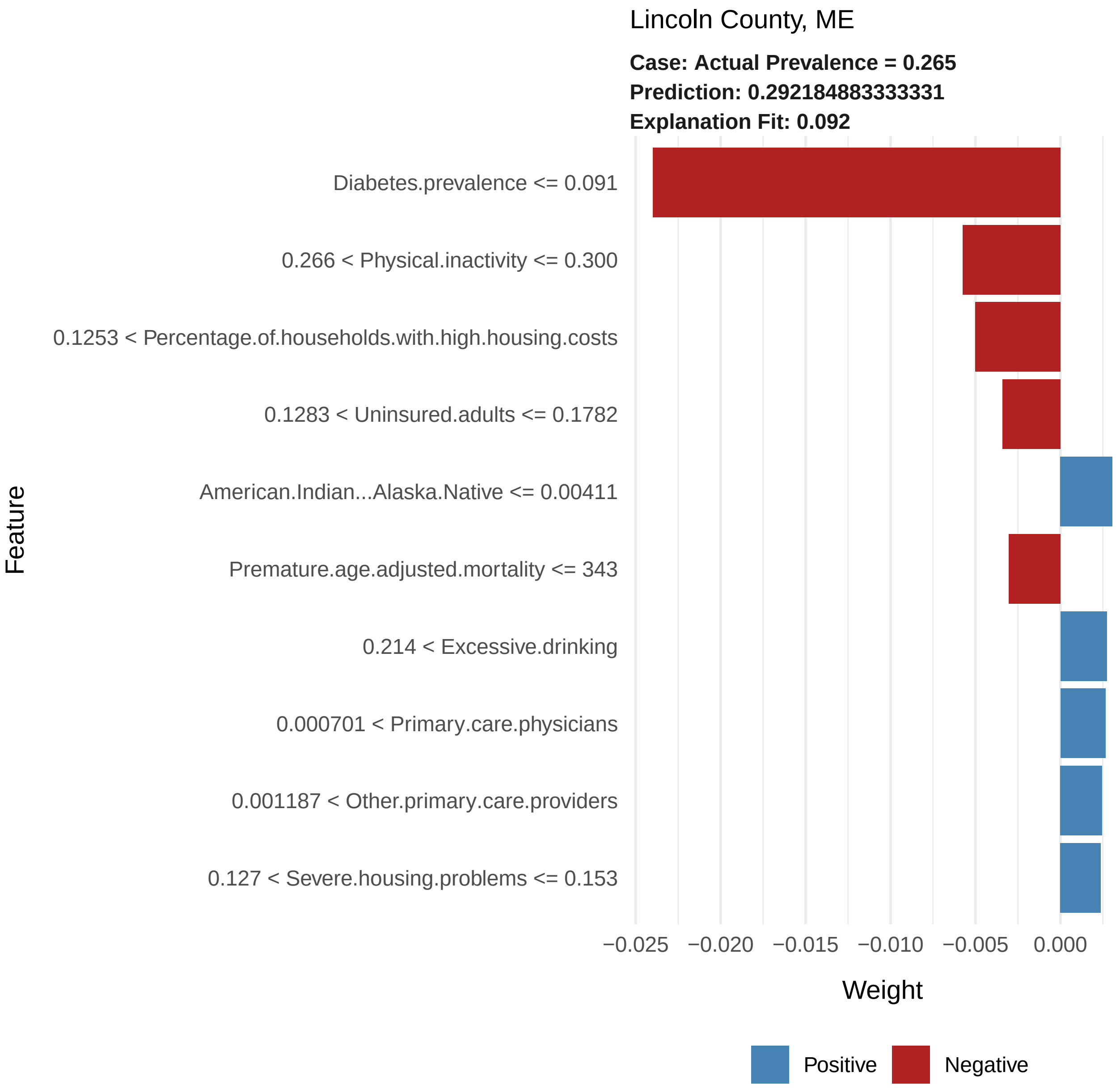
Lincoln County, ME
Case: Actual Prevalence = 0.265
Prediction: 0.292184883333331
Explanation Fit: 0.092
Diabetes.prevalence <= 0.091
0.266 < Physical.inactivity <= 0.300
0.1253 < Percentage.of.households.with.high.housing.costs
0.1283 < Uninsured.adults <= 0.1782
American.Indian...Alaska.Native <= 0.00411
Premature.age.adjusted.mortality <= 343
0.214 < Excessive.drinking
0.000701 < Primary.care.physicians
0.001187 < Other.primary.care.providers
0.127 < Severe.housing.problems <= 0.153
Feature
−0.025
−0.020
−0.015
−0.010
−0.005
0.000
Weight
Positive
Negative

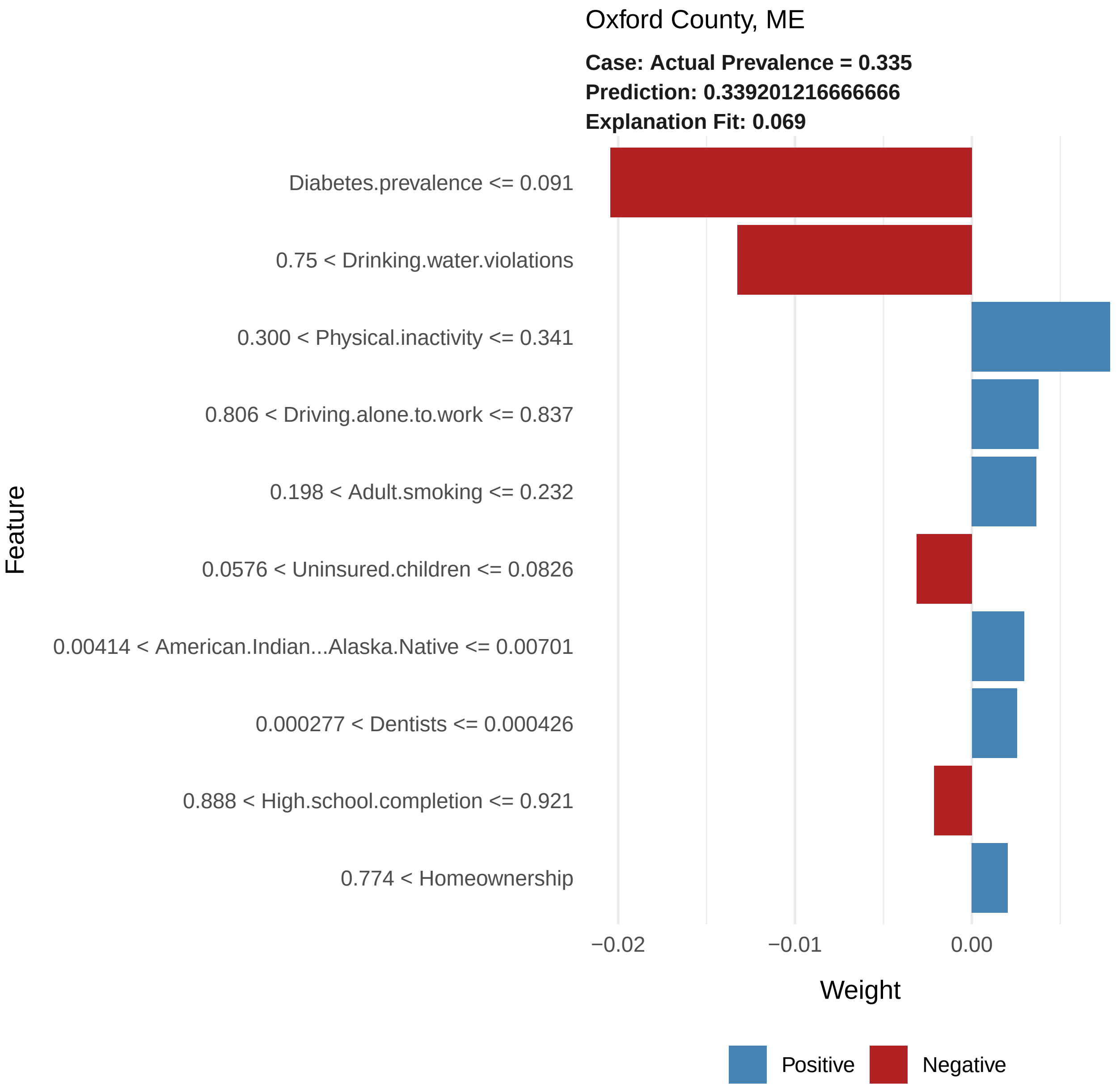

Oxford County, ME
Case: Actual Prevalence = 0.335
Prediction: 0.339201216666666
Explanation Fit: 0.069
Diabetes.prevalence <= 0.091
0.75 < Drinking.water.violations
0.300 < Physical.inactivity <= 0.341
0.806 < Driving.alone.to.work <= 0.837
0.198 < Adult.smoking <= 0.232
0.0576 < Uninsured.children <= 0.0826
0.00414 < American.Indian...Alaska.Native <= 0.00701
0.000277 < Dentists <= 0.000426
0.888 < High.school.completion <= 0.921
0.774 < Homeownership
Feature
−0.02
−0.01
0.00
Weight
Positive
Negative

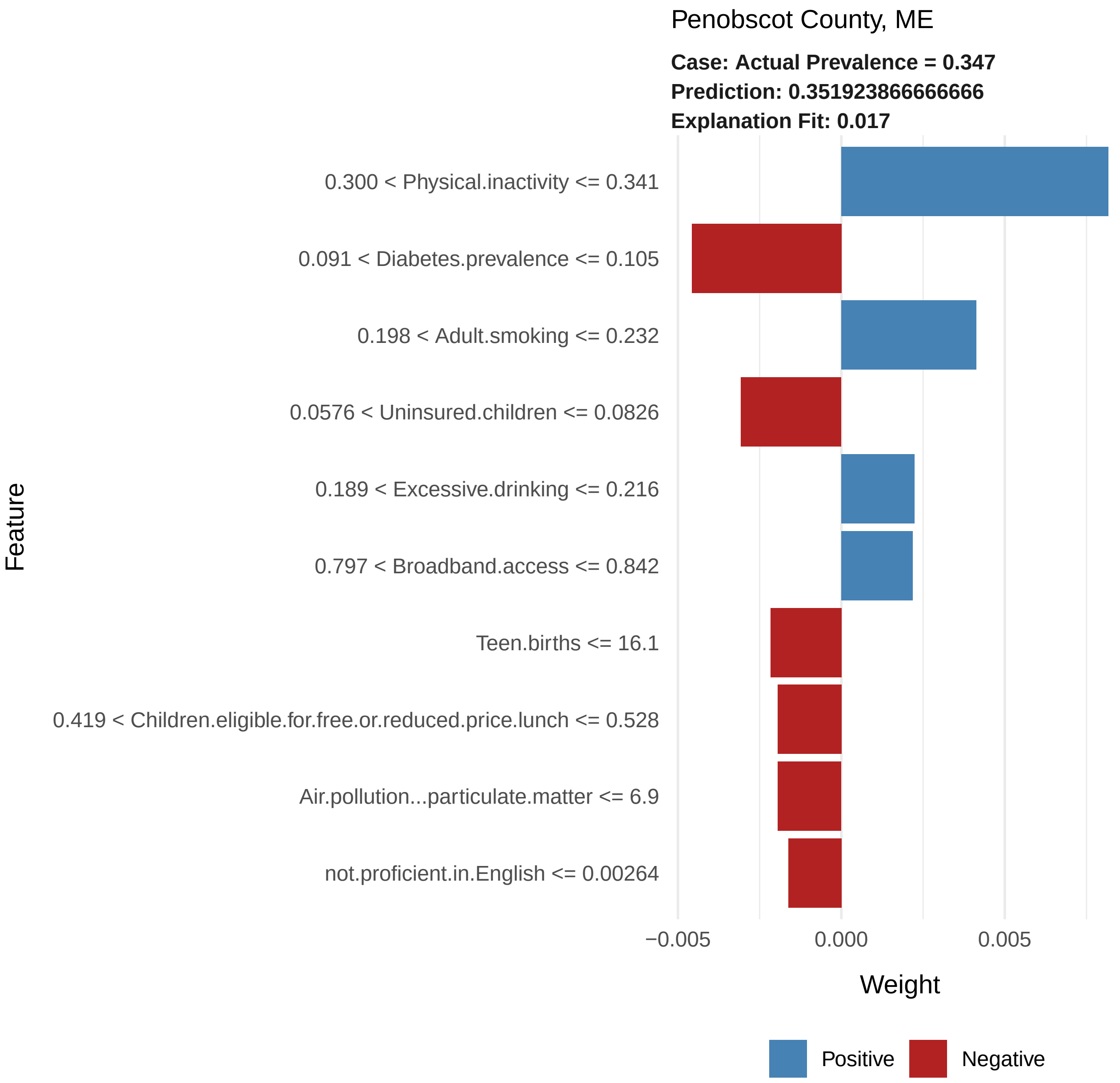
Penobscot County, ME
Case: Actual Prevalence = 0.347
Prediction: 0.351923866666666
Explanation Fit: 0.017
0.300 < Physical.inactivity <= 0.341
0.091 < Diabetes.prevalence <= 0.105
0.198 < Adult.smoking <= 0.232
0.0576 < Uninsured.children <= 0.0826
0.189 < Excessive.drinking <= 0.216
0.797 < Broadband.access <= 0.842
Teen.births <= 16.1
0.419 < Children.eligible.for.free.or.reduced.price.lunch <= 0.528
Air.pollution...particulate.matter <= 6.9
not.proficient.in.English <= 0.00264
Feature
−0.005
0.000
0.005
Weight
Positive
Negative

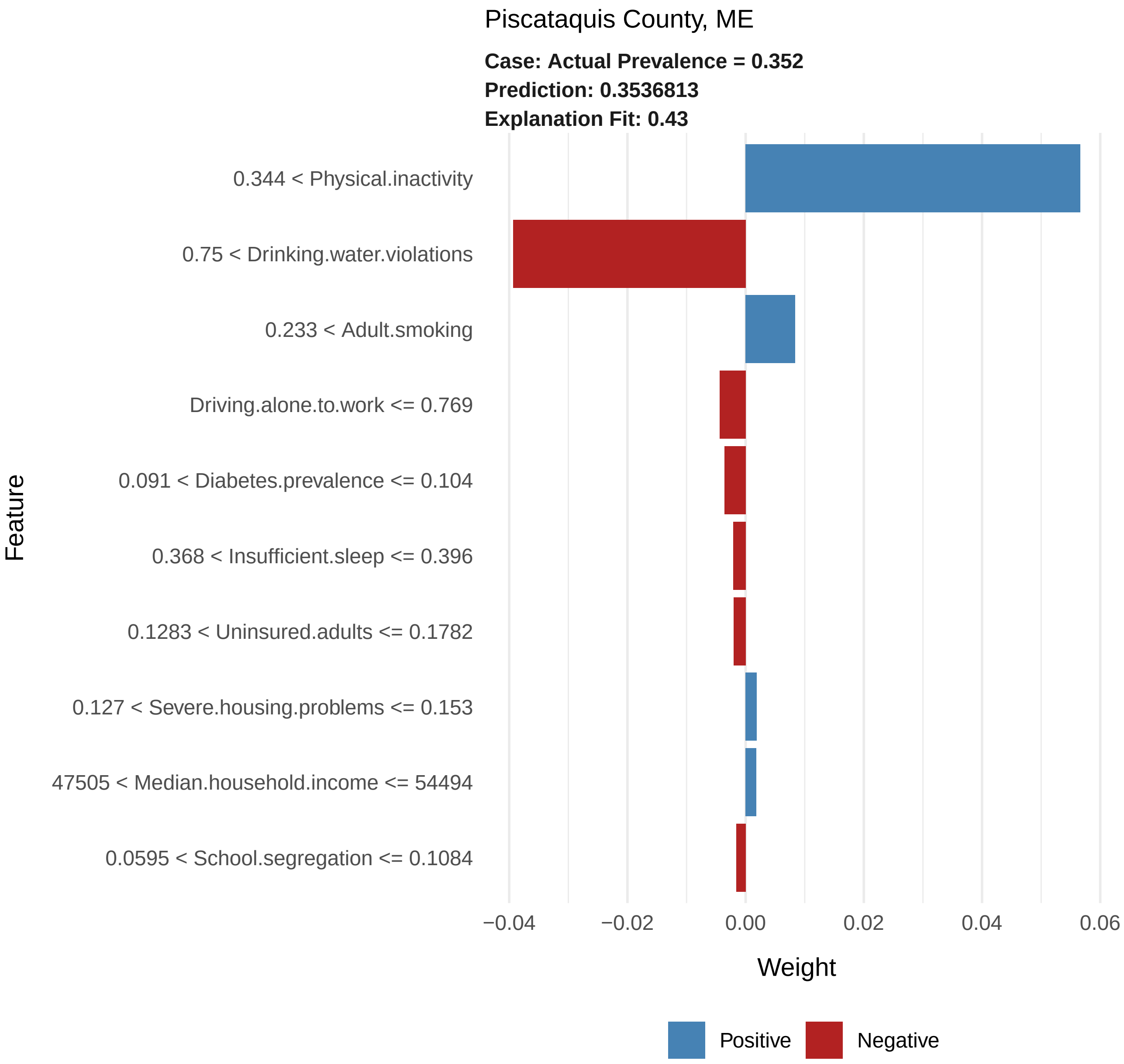

Piscataquis County, ME
Case: Actual Prevalence = 0.352
Prediction: 0.3536813
Explanation Fit: 0.43
0.344 < Physical.inactivity
0.75 < Drinking.water.violations
0.233 < Adult.smoking
Driving.alone.to.work <= 0.769
0.091 < Diabetes.prevalence <= 0.104
0.368 < Insufficient.sleep <= 0.396
0.1283 < Uninsured.adults <= 0.1782
0.127 < Severe.housing.problems <= 0.153
47505 < Median.household.income <= 54494
0.0595 < School.segregation <= 0.1084
Feature
−0.04
−0.02
0.00
0.02
0.04
0.06
Weight
Positive
Negative

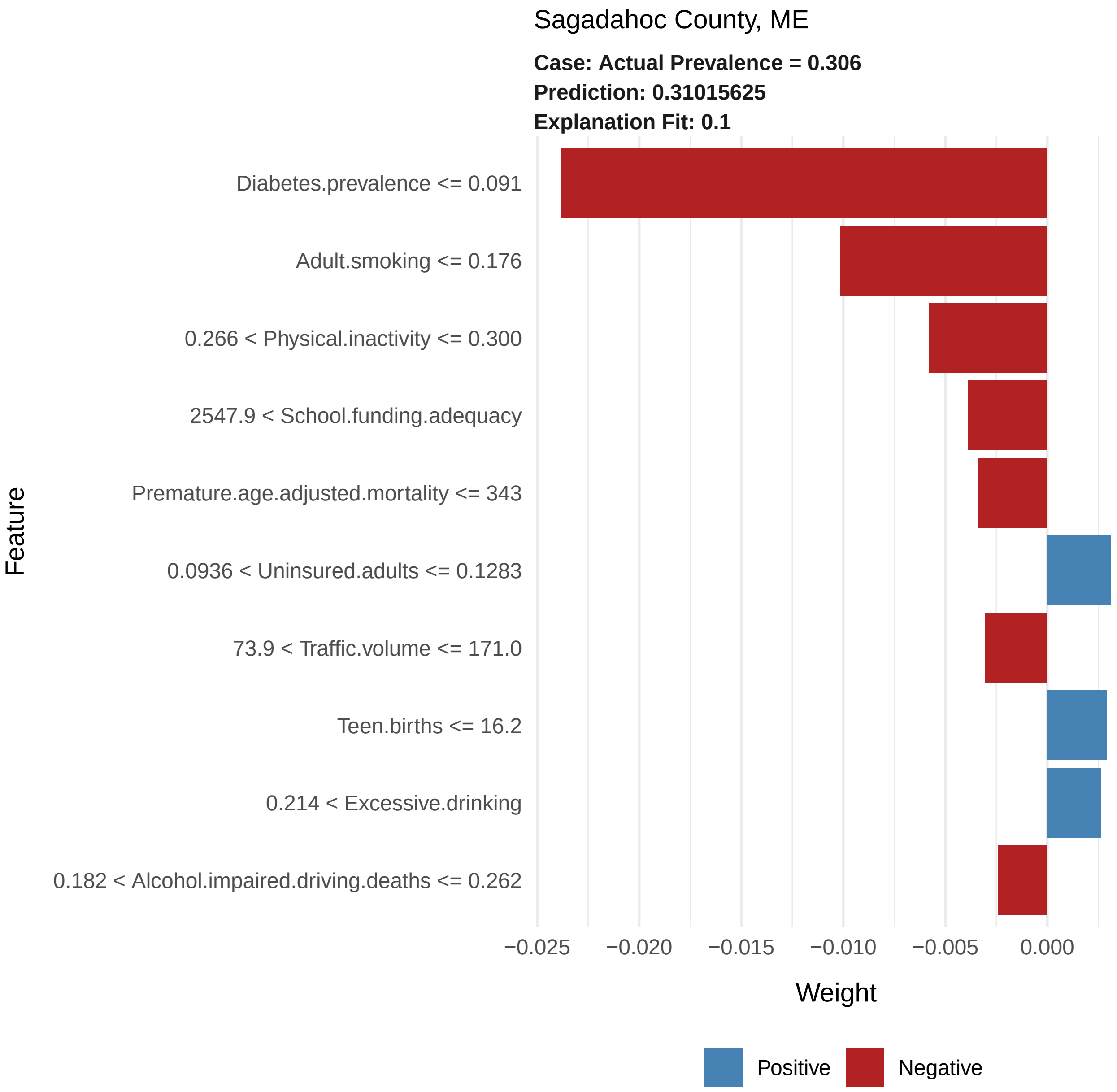

Sagadahoc County, ME
Case: Actual Prevalence = 0.306
Prediction: 0.31015625
Explanation Fit: 0.1
Diabetes.prevalence <= 0.091
Adult.smoking <= 0.176
0.266 < Physical.inactivity <= 0.300
2547.9 < School.funding.adequacy
Premature.age.adjusted.mortality <= 343
0.0936 < Uninsured.adults <= 0.1283
73.9 < Traffic.volume <= 171.0
Teen.births <= 16.2
0.214 < Excessive.drinking
0.182 < Alcohol.impaired.driving.deaths <= 0.262
Feature
−0.025
−0.020
−0.015
−0.010
−0.005
0.000
Weight
Positive
Negative

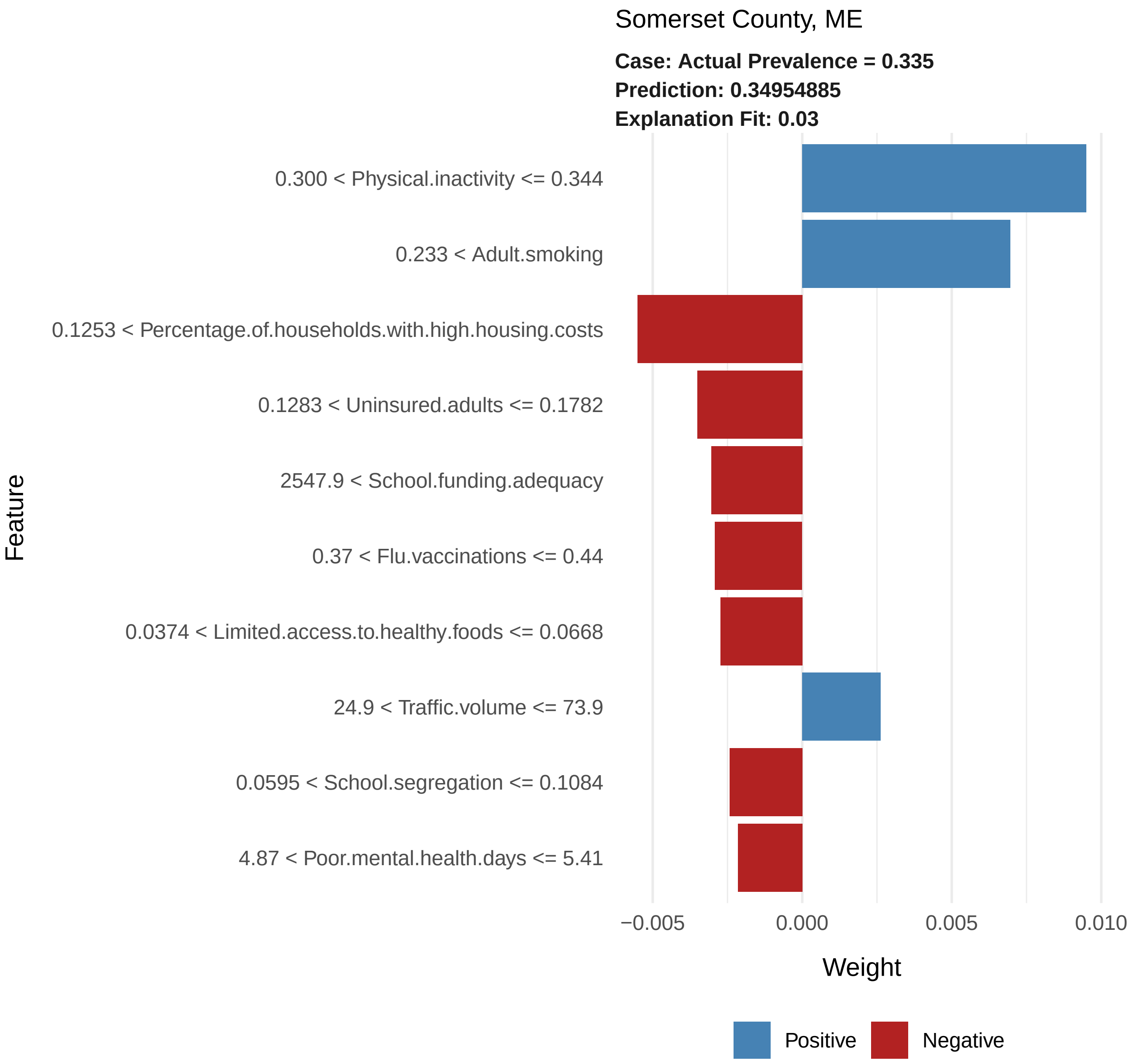

Somerset County, ME
Case: Actual Prevalence = 0.335
Prediction: 0.34954885
Explanation Fit: 0.03
0.300 < Physical.inactivity <= 0.344
0.233 < Adult.smoking
0.1253 < Percentage.of.households.with.high.housing.costs
0.1283 < Uninsured.adults <= 0.1782
2547.9 < School.funding.adequacy
0.37 < Flu.vaccinations <= 0.44
0.0374 < Limited.access.to.healthy.foods <= 0.0668
24.9 < Traffic.volume <= 73.9
0.0595 < School.segregation <= 0.1084
4.87 < Poor.mental.health.days <= 5.41
Feature
−0.005
0.000
0.005
0.010
Weight
Positive
Negative

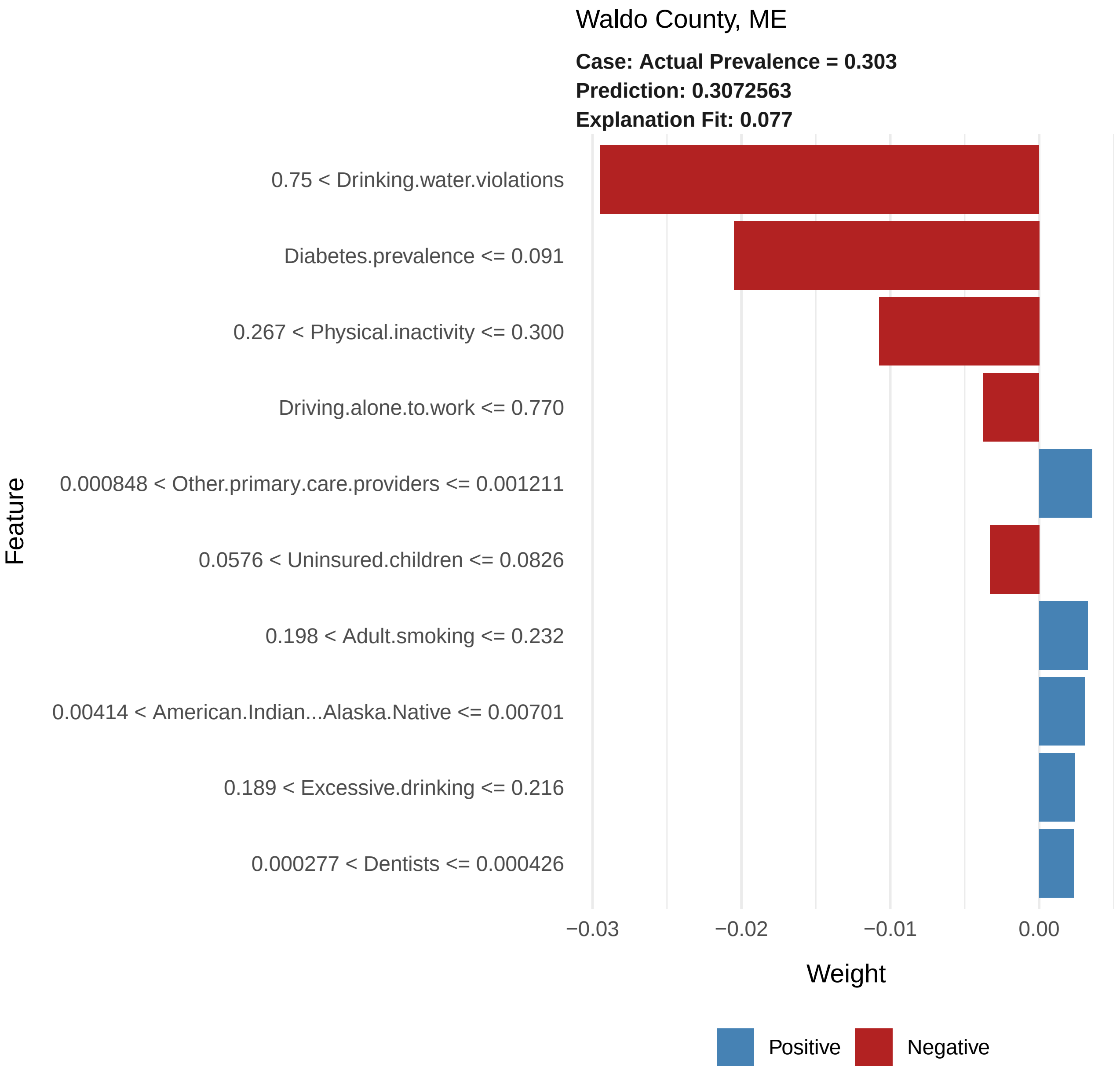

Waldo County, ME
Case: Actual Prevalence = 0.303
Prediction: 0.3072563
Explanation Fit: 0.077
0.75 < Drinking.water.violations
Diabetes.prevalence <= 0.091
0.267 < Physical.inactivity <= 0.300
Driving.alone.to.work <= 0.770
0.000848 < Other.primary.care.providers <= 0.001211
0.0576 < Uninsured.children <= 0.0826
0.198 < Adult.smoking <= 0.232
0.00414 < American.Indian...Alaska.Native <= 0.00701
0.189 < Excessive.drinking <= 0.216
0.000277 < Dentists <= 0.000426
Feature
−0.03
−0.02
−0.01
0.00
Weight
Positive
Negative

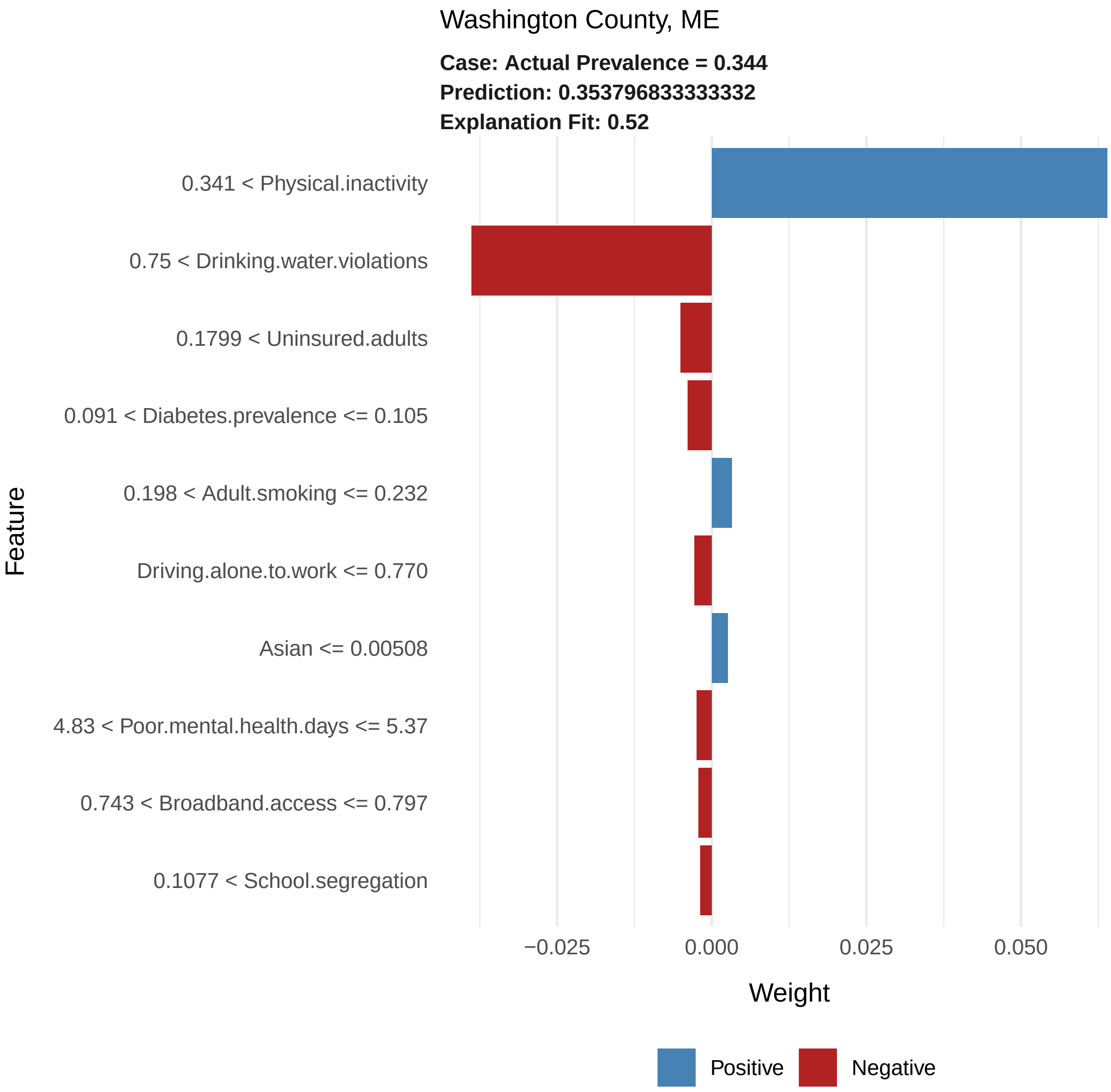
Washington County, ME
Case: Actual Prevalence = 0.344
Prediction: 0.3537968333333332
Explanation Fit: 0.52
0.341 < Physical.inactivity
0.75 < Drinking.water.violations
0.1799 < Uninsured.adults
0.091 < Diabetes.prevalence <= 0.105
0.198 < Adult.smoking <= 0.232
Driving.alone.to.work <= 0.770
Asian <= 0.00508
4.83 < Poor.mental.health.days <= 5.37
0.743 < Broadband.access <= 0.797
0.1077 < School.segregation
Feature
−0.025
0.000
0.025
0.050
Weight
Positive
Negative

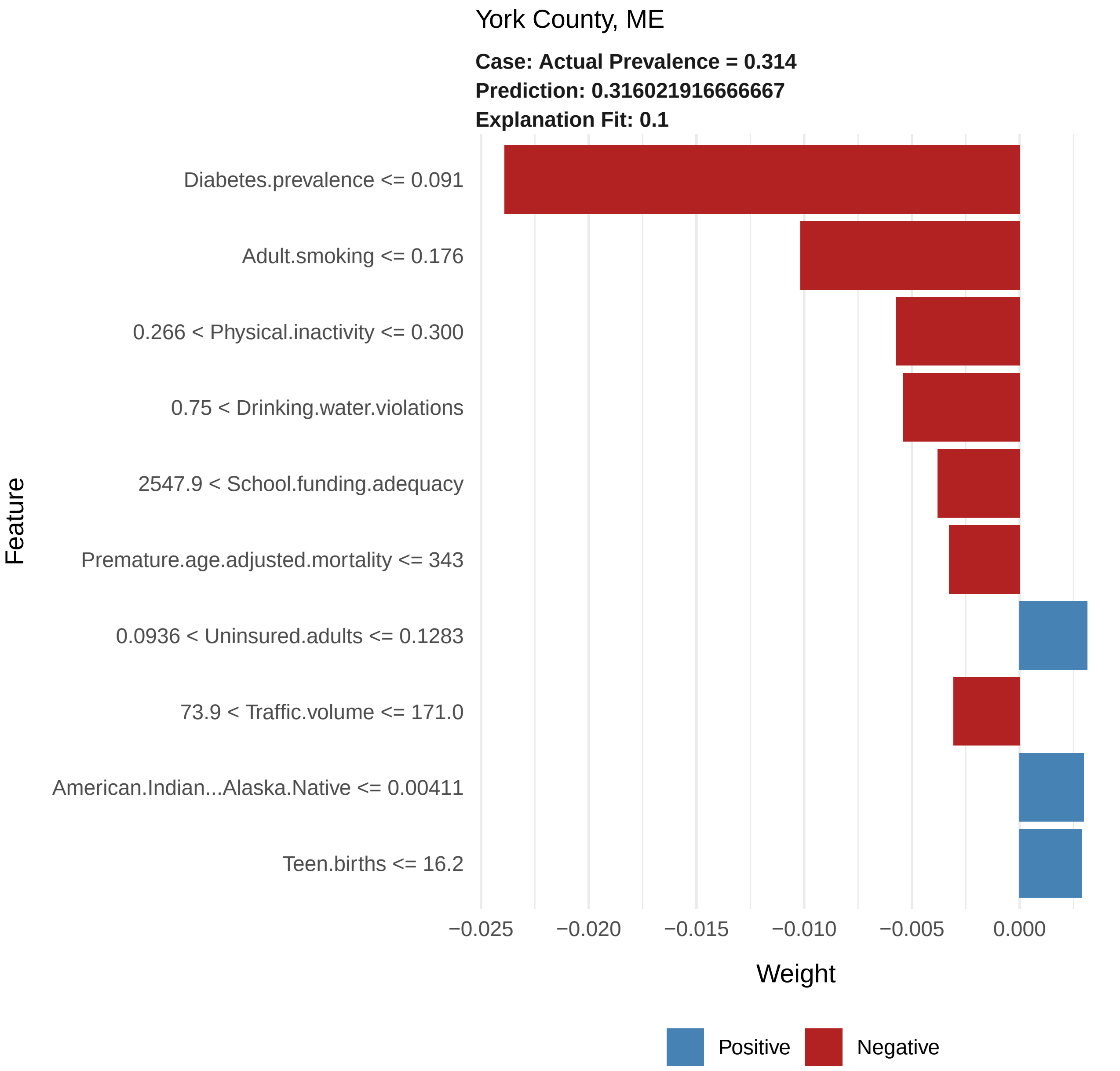

York County, ME
Case: Actual Prevalence = 0.314
Prediction: 0.316021916666667
Explanation Fit: 0.1
Diabetes.prevalence <= 0.091
Adult.smoking <= 0.176
0.266 < Physical.inactivity <= 0.300
0.75 < Drinking.water.violations
2547.9 < School.funding.adequacy
Premature.age.adjusted.mortality <= 343
0.0936 < Uninsured.adults <= 0.1283
73.9 < Traffic.volume <= 171.0
American.Indian...Alaska.Native <= 0.00411
Teen.births <= 16.2
Feature
−0.025
−0.020
−0.015
−0.010
−0.005
0.000
Weight
Positive
Negative

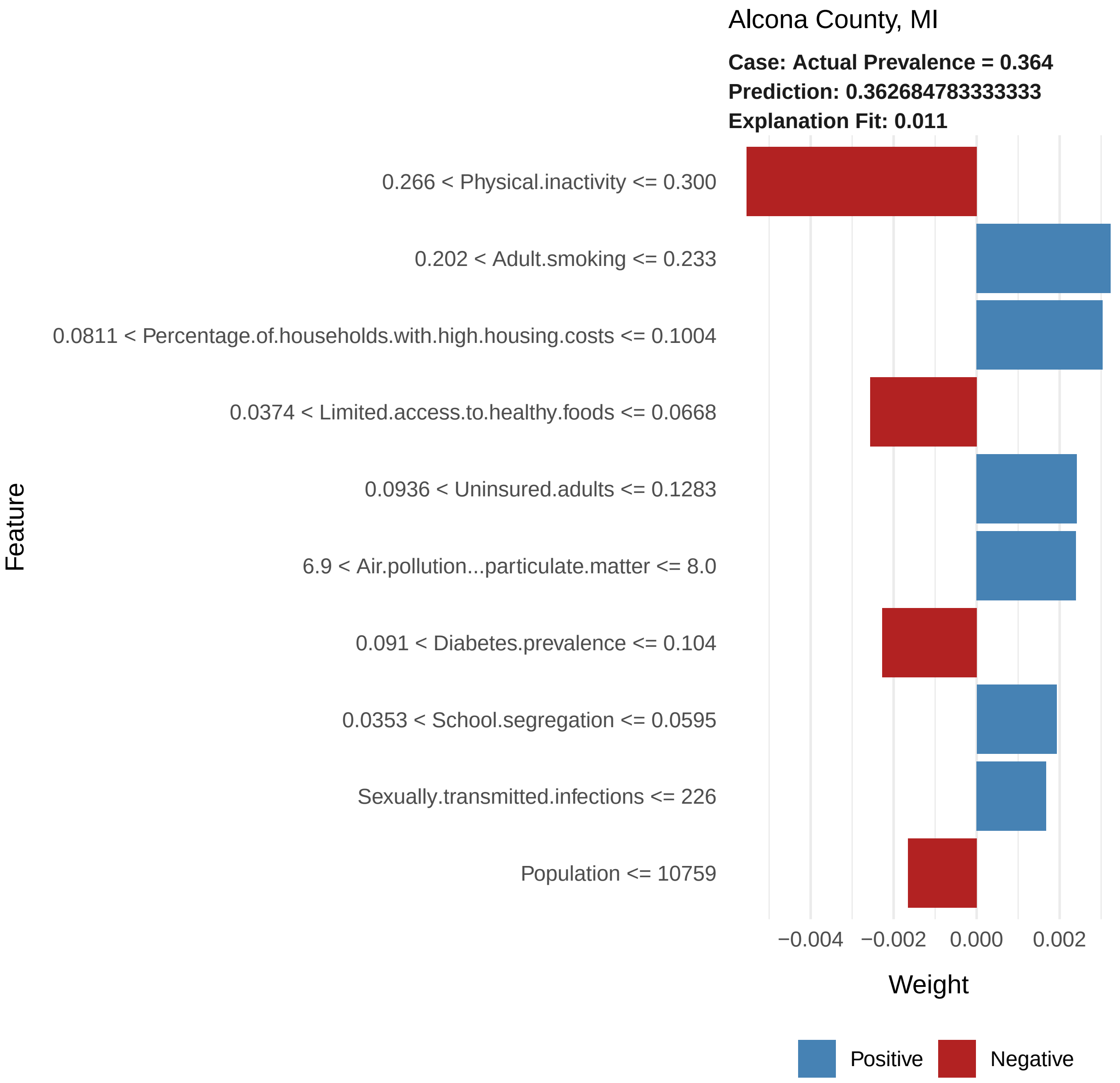

Alcona County, MI
Case: Actual Prevalence = 0.364
Prediction: 0.362684783333333
Explanation Fit: 0.011
0.266 < Physical.inactivity <= 0.300
0.202 < Adult.smoking <= 0.233
0.0811 < Percentage.of.households.with.high.housing.costs <= 0.1004
0.0374 < Limited.access.to.healthy.foods <= 0.0668
0.0936 < Uninsured.adults <= 0.1283
6.9 < Air.pollution...particulate.matter <= 8.0
0.091 < Diabetes.prevalence <= 0.104
0.0353 < School.segregation <= 0.0595
Sexually.transmitted.infections <= 226
Population <= 10759
Feature
−0.004
−0.002
0.000
0.002
Weight
Positive
Negative

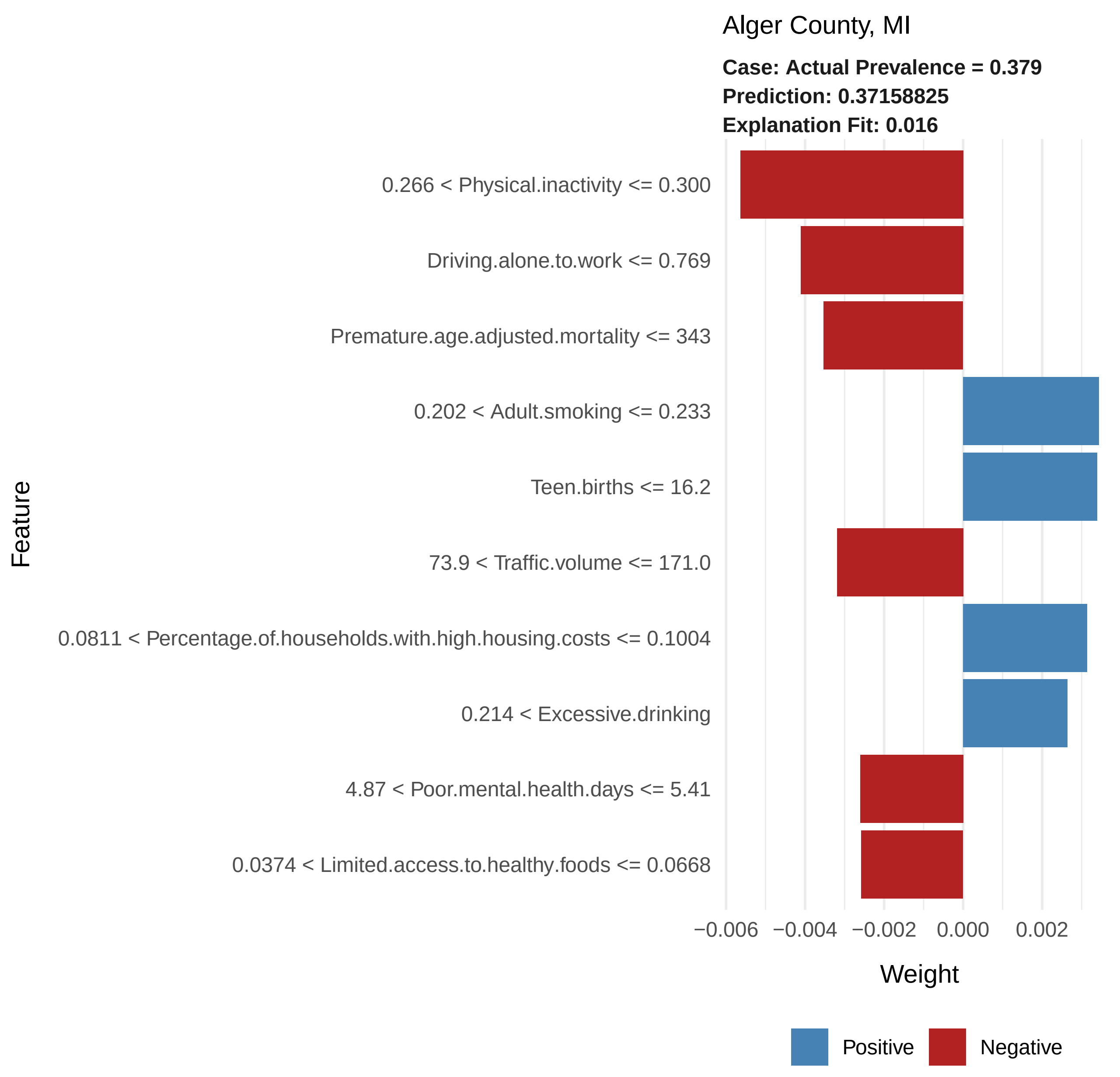

Alger County, MI
Case: Actual Prevalence = 0.379
Prediction: 0.37158825
Explanation Fit: 0.016
0.266 < Physical.inactivity <= 0.300
Driving.alone.to.work <= 0.769
Premature.age.adjusted.mortality <= 343
0.202 < Adult.smoking <= 0.233
Teen.births <= 16.2
73.9 < Traffic.volume <= 171.0
0.0811 < Percentage.of.households.with.high.housing.costs <= 0.1004
0.214 < Excessive.drinking
4.87 < Poor.mental.health.days <= 5.41
0.0374 < Limited.access.to.healthy.foods <= 0.0668
Feature
−0.006
−0.004
−0.002
0.000
0.002
Weight
Positive
Negative

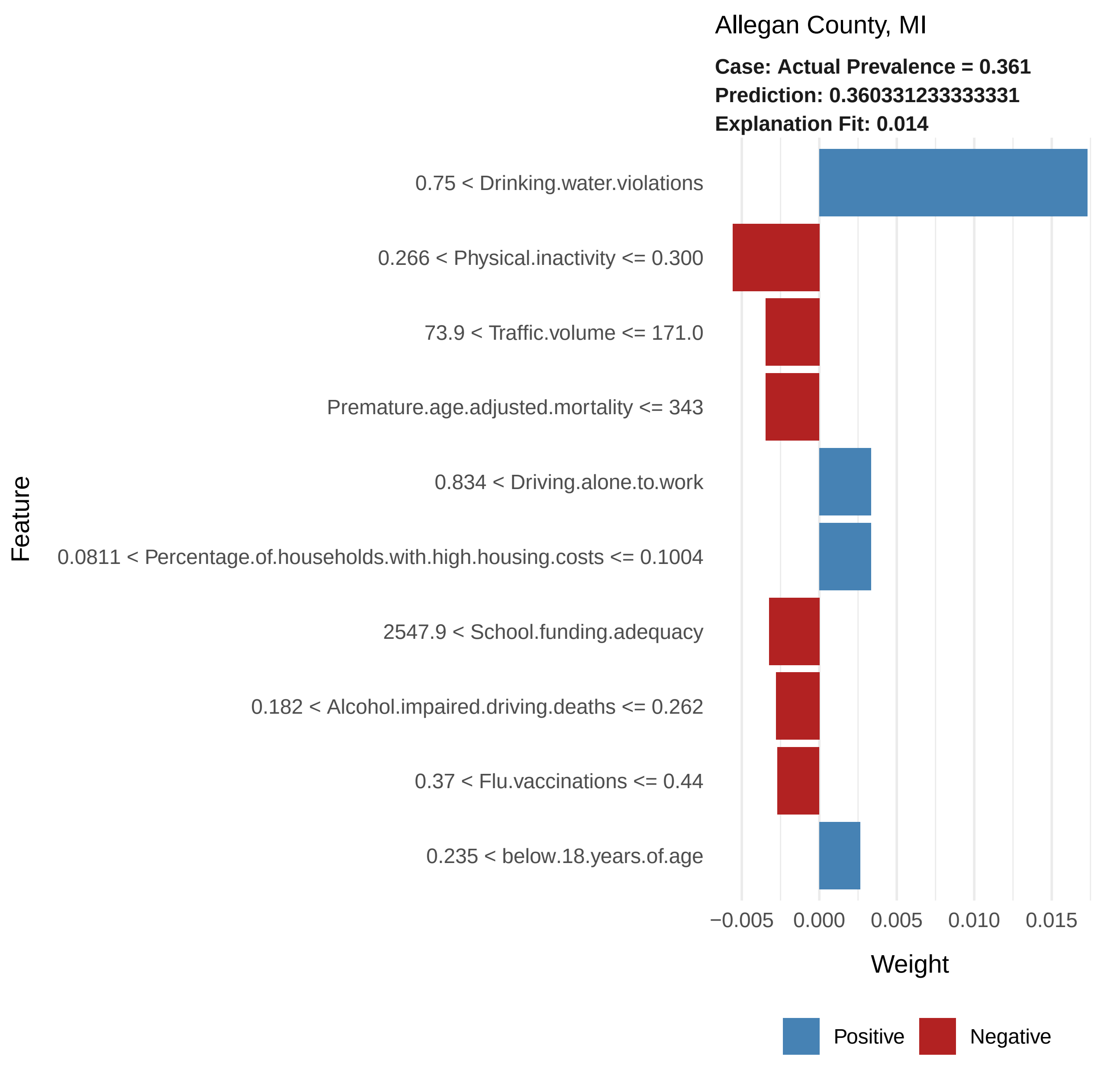
Allegan County, MI
Case: Actual Prevalence = 0.361
Prediction: 0.360331233333331
Explanation Fit: 0.014
0.75 < Drinking.water.violations
0.266 < Physical.inactivity <= 0.300
73.9 < Traffic.volume <= 171.0
Premature.age.adjusted.mortality <= 343
0.834 < Driving.alone.to.work
0.0811 < Percentage.of.households.with.high.housing.costs <= 0.1004
2547.9 < School.funding.adequacy
0.182 < Alcohol.impaired.driving.deaths <= 0.262
0.37 < Flu.vaccinations <= 0.44
0.235 < below.18.years.of.age
Feature
−0.005
0.000
0.005
0.010
0.015
Weight
Positive
Negative

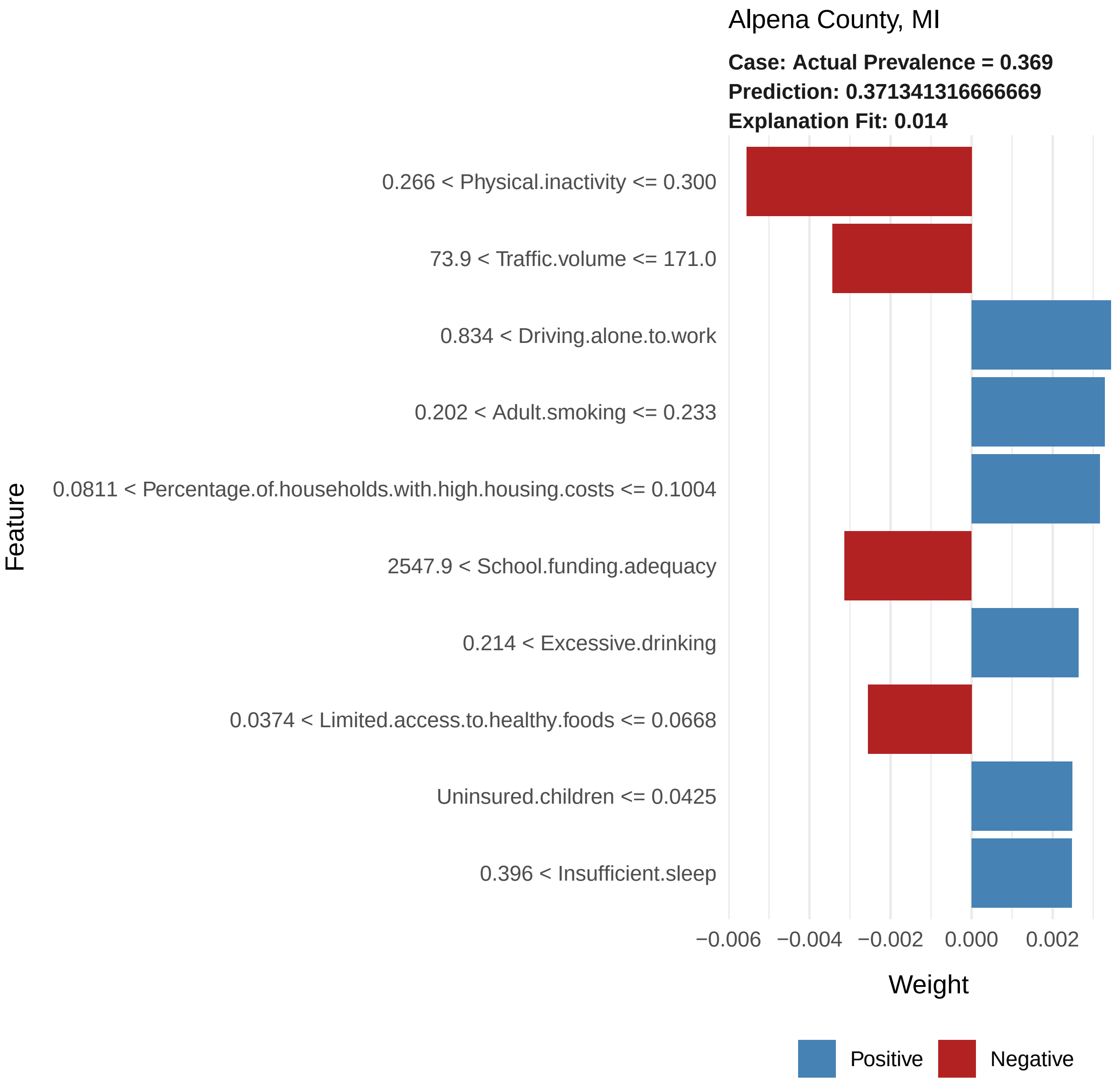

Alpena County, MI
Case: Actual Prevalence = 0.369
Prediction: 0.371341316666669
Explanation Fit: 0.014
0.266 < Physical.inactivity <= 0.300
73.9 < Traffic.volume <= 171.0
0.834 < Driving.alone.to.work
0.202 < Adult.smoking <= 0.233
0.0811 < Percentage.of.households.with.high.housing.costs <= 0.1004
2547.9 < School.funding.adequacy
0.214 < Excessive.drinking
0.0374 < Limited.access.to.healthy.foods <= 0.0668
Uninsured.children <= 0.0425
0.396 < Insufficient.sleep
Feature
−0.006
−0.004
−0.002
0.000
0.002
Weight
Positive
Negative

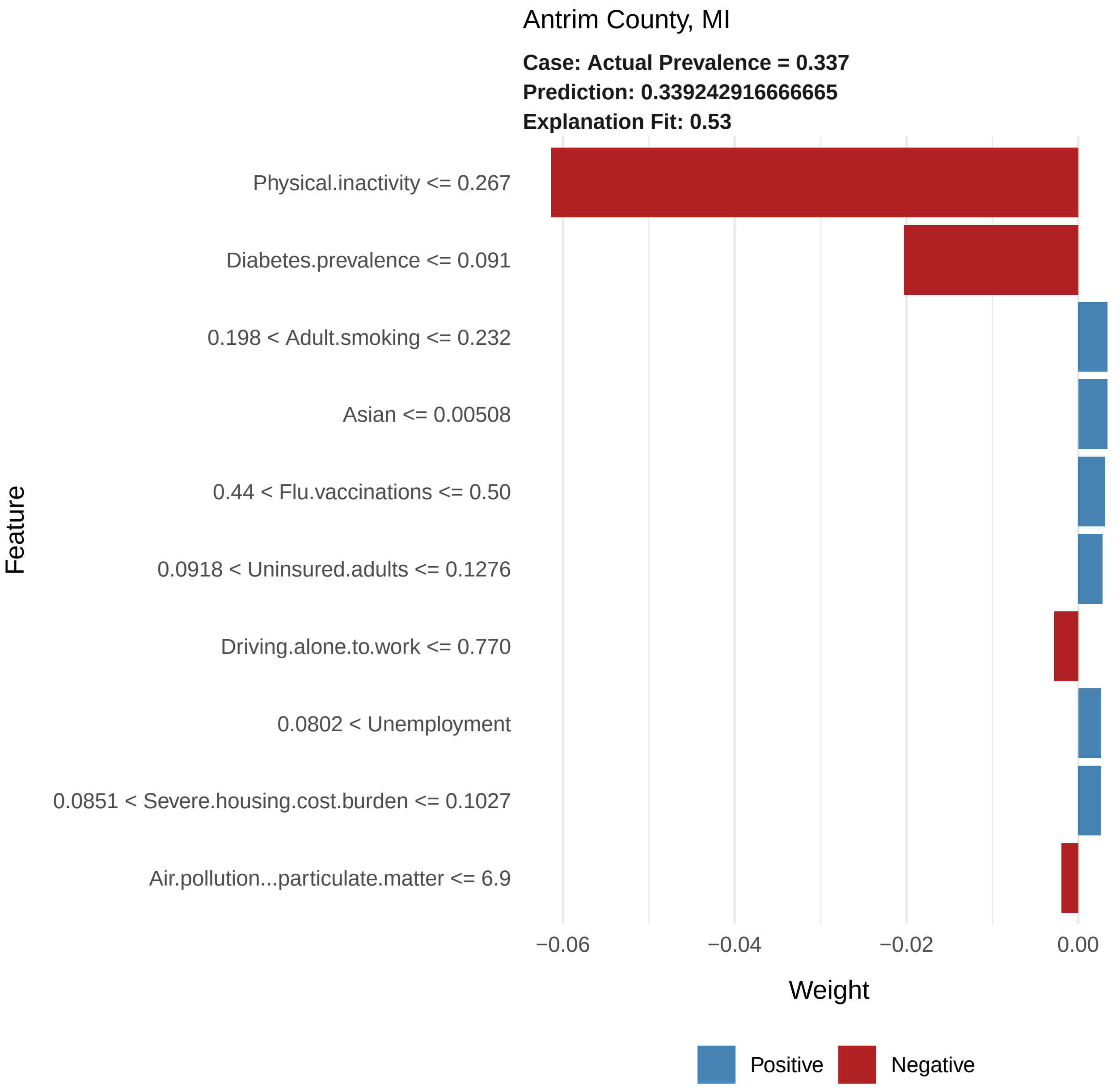

Antrim County, MI
Case: Actual Prevalence = 0.337
Prediction: 0.339242916666665
Explanation Fit: 0.53
Physical.inactivity <= 0.267
Diabetes.prevalence <= 0.091
0.198 < Adult.smoking <= 0.232
Asian <= 0.00508
0.44 < Flu.vaccinations <= 0.50
0.0918 < Uninsured.adults <= 0.1276
Driving.alone.to.work <= 0.770
0.0802 < Unemployment
0.0851 < Severe.housing.cost.burden <= 0.1027
Air.pollution...particulate.matter <= 6.9
Feature
−0.06
−0.04
−0.02
0.00
Weight
Positive
Negative

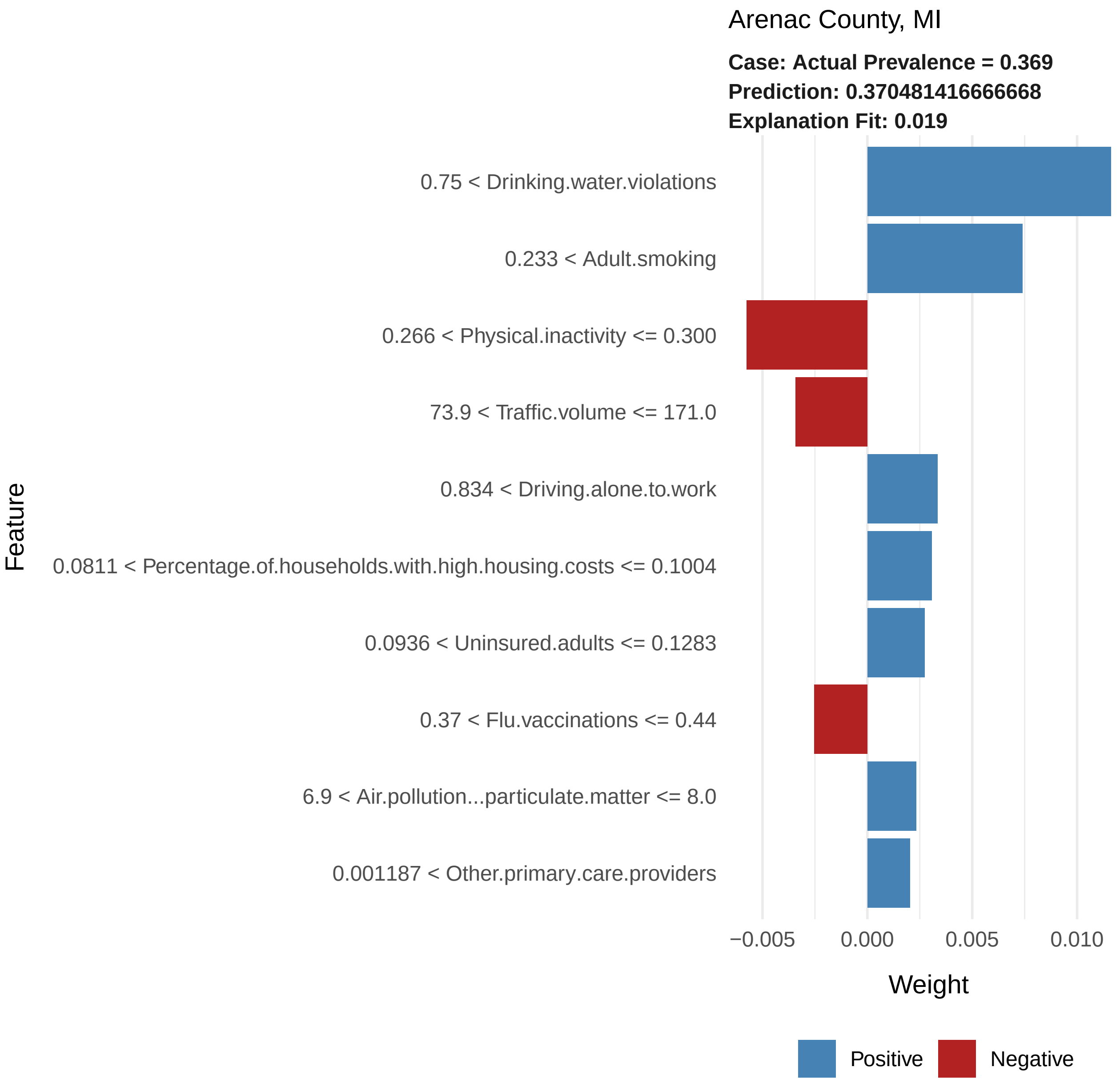
Arenac County, MI
Case: Actual Prevalence = 0.369
Prediction: 0.370481416666668
Explanation Fit: 0.019
0.75 < Drinking.water.violations
0.233 < Adult.smoking
0.266 < Physical.inactivity <= 0.300
73.9 < Traffic.volume <= 171.0
0.834 < Driving.alone.to.work
0.0811 < Percentage.of.households.with.high.housing.costs <= 0.1004
0.0936 < Uninsured.adults <= 0.1283
0.37 < Flu.vaccinations <= 0.44
6.9 < Air.pollution...particulate.matter <= 8.0
0.001187 < Other.primary.care.providers
Feature
−0.005
0.000
0.005
0.010
Weight
Positive
Negative

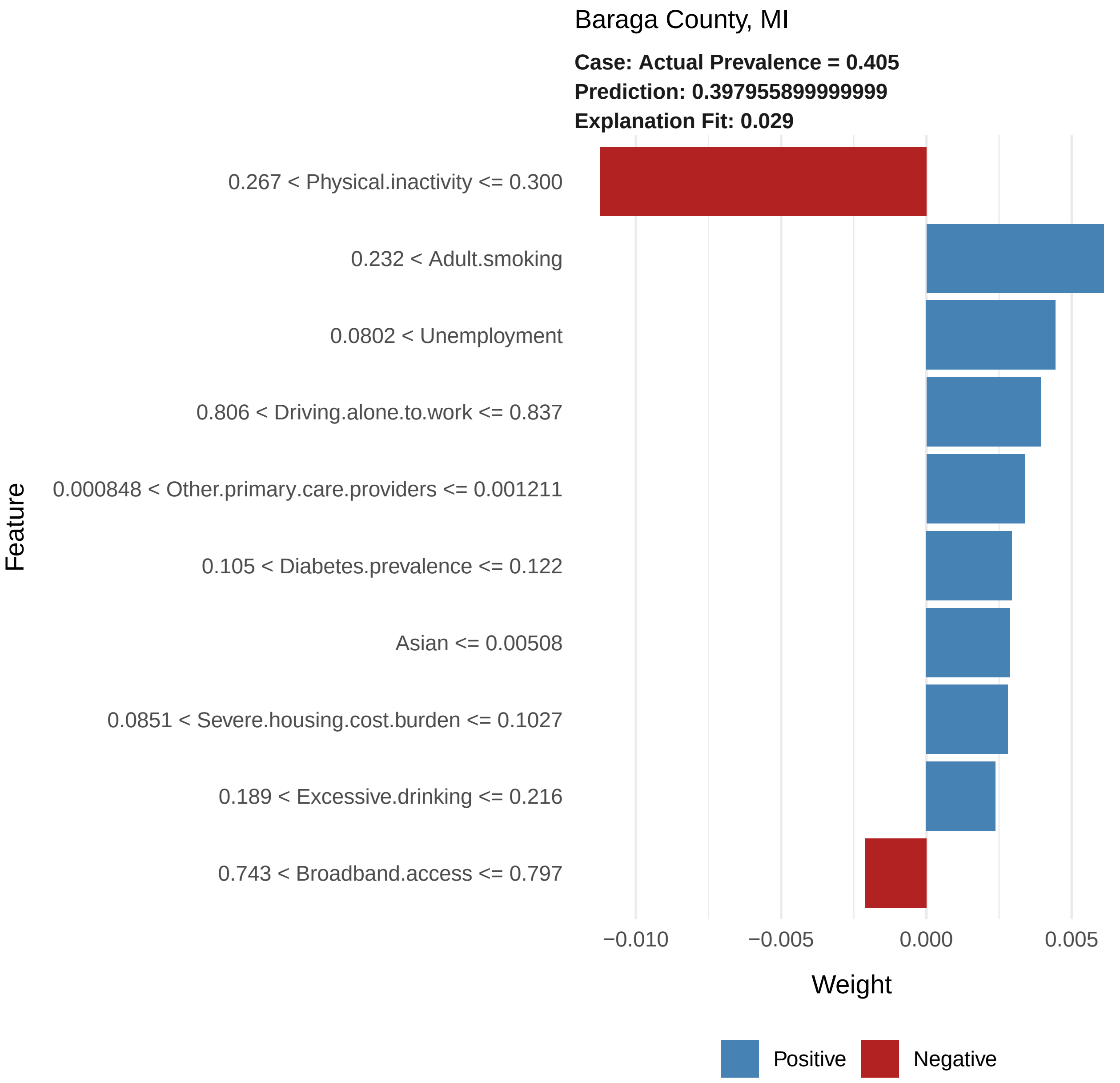

Baraga County, MI
Case: Actual Prevalence = 0.405
Prediction: 0.397955899999999
Explanation Fit: 0.029
0.267 < Physical.inactivity <= 0.300
0.232 < Adult.smoking
0.0802 < Unemployment
0.806 < Driving.alone.to.work <= 0.837
0.000848 < Other.primary.care.providers <= 0.001211
0.105 < Diabetes.prevalence <= 0.122
Asian <= 0.00508
0.0851 < Severe.housing.cost.burden <= 0.1027
0.189 < Excessive.drinking <= 0.216
0.743 < Broadband.access <= 0.797
Feature
−0.010
−0.005
0.000
0.005
Weight
Positive
Negative

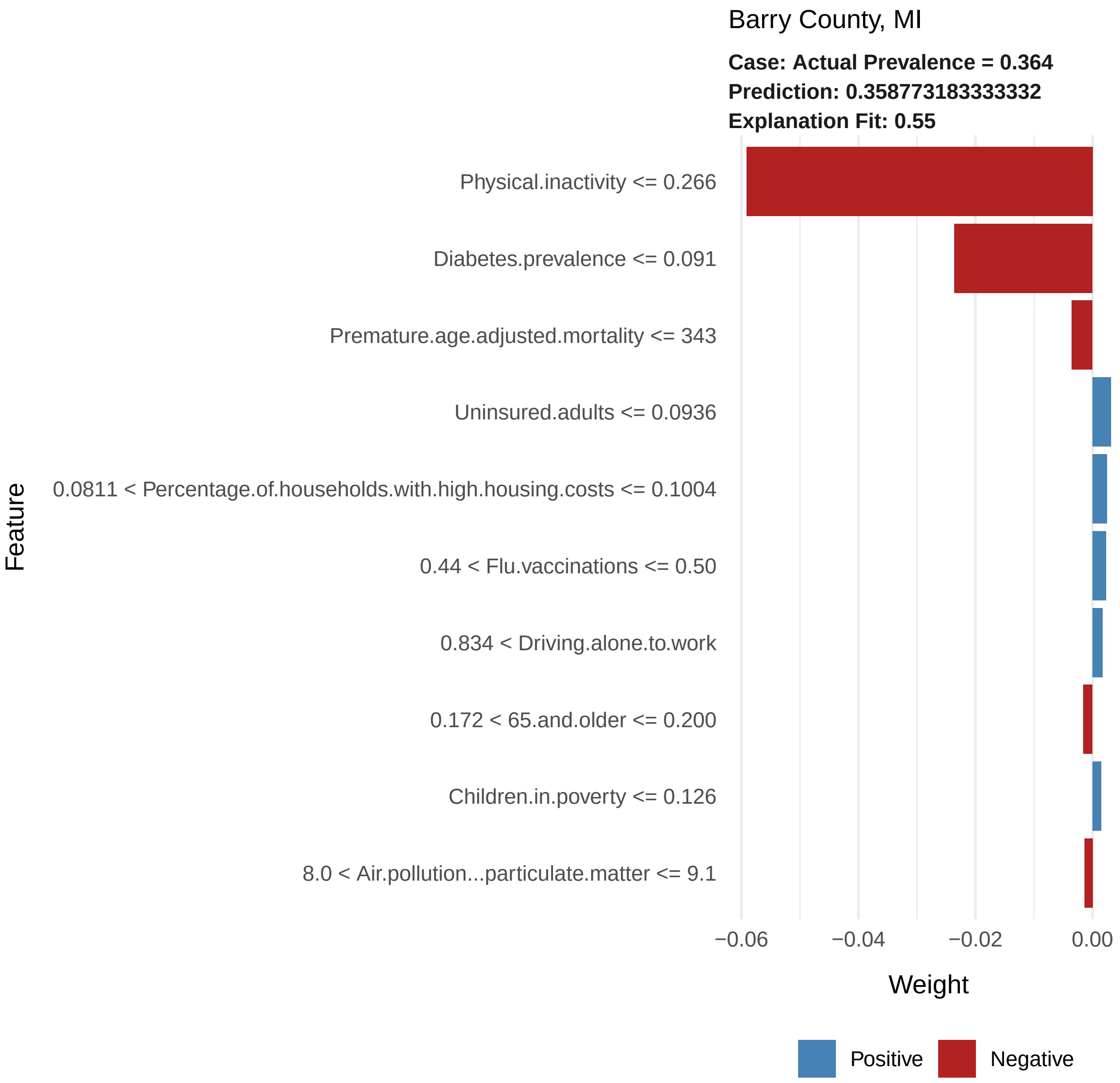

Barry County, MI
Case: Actual Prevalence = 0.364
Prediction: 0.358773183333332
Explanation Fit: 0.55
Physical.inactivity <= 0.266
Diabetes.prevalence <= 0.091
Premature.age.adjusted.mortality <= 343
Uninsured.adults <= 0.0936
0.0811 < Percentage.of.households.with.high.housing.costs <= 0.1004
0.44 < Flu.vaccinations <= 0.50
0.834 < Driving.alone.to.work
0.172 < 65.and.older <= 0.200
Children.in.poverty <= 0.126
8.0 < Air.pollution...particulate.matter <= 9.1
Feature
−0.06
−0.04
−0.02
0.00
Weight
Positive
Negative

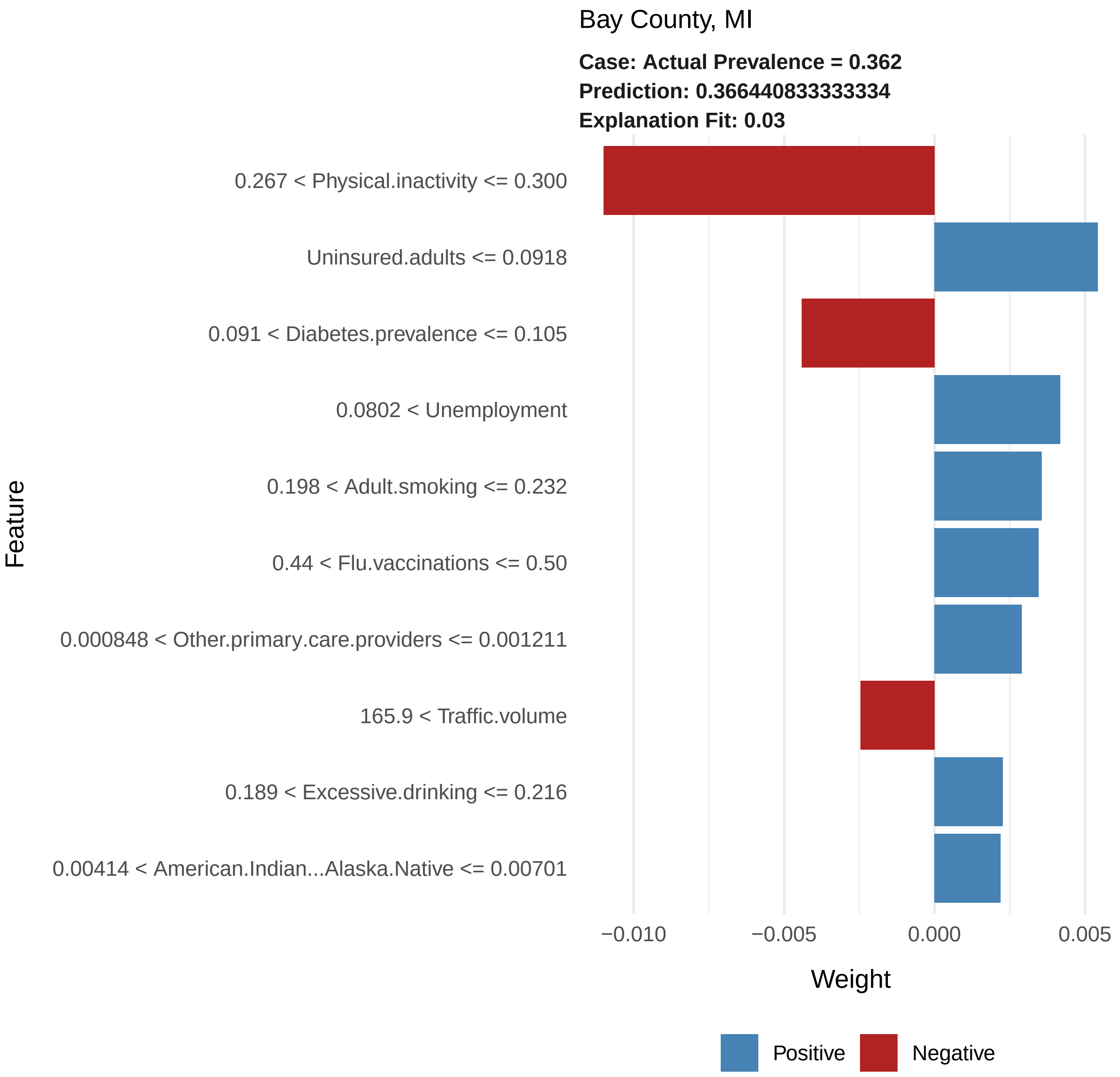

Bay County, MI
Case: Actual Prevalence = 0.362
Prediction: 0.366440833333334
Explanation Fit: 0.03
0.267 < Physical.inactivity <= 0.300
Uninsured.adults <= 0.0918
0.091 < Diabetes.prevalence <= 0.105
0.0802 < Unemployment
0.198 < Adult.smoking <= 0.232
0.44 < Flu.vaccinations <= 0.50
0.000848 < Other.primary.care.providers <= 0.001211
165.9 < Traffic.volume
0.189 < Excessive.drinking <= 0.216
0.00414 < American.Indian...Alaska.Native <= 0.00701
Feature
−0.010
−0.005
0.000
0.005
Weight
Positive
Negative

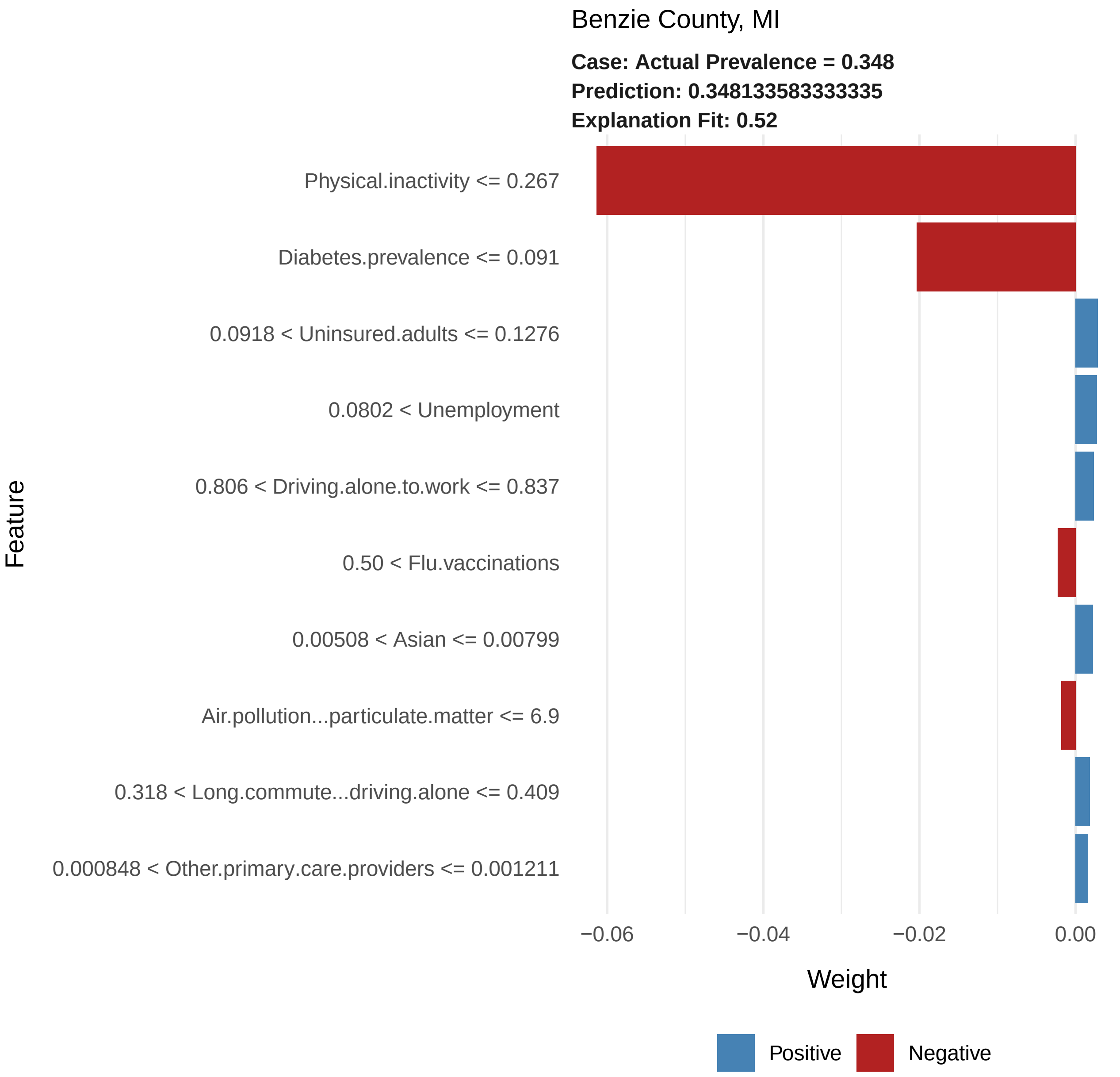
Benzie County, MI
Case: Actual Prevalence = 0.348
Prediction: 0.348133583333335
Explanation Fit: 0.52
Physical.inactivity <= 0.267
Diabetes.prevalence <= 0.091
0.0918 < Uninsured.adults <= 0.1276
0.0802 < Unemployment
0.806 < Driving.alone.to.work <= 0.837
0.50 < Flu.vaccinations
0.00508 < Asian <= 0.00799
Air.pollution...particulate.matter <= 6.9
0.318 < Long.commute...driving.alone <= 0.409
0.000848 < Other.primary.care.providers <= 0.001211
Feature
−0.06
−0.04
−0.02
0.00
Weight
Positive
Negative

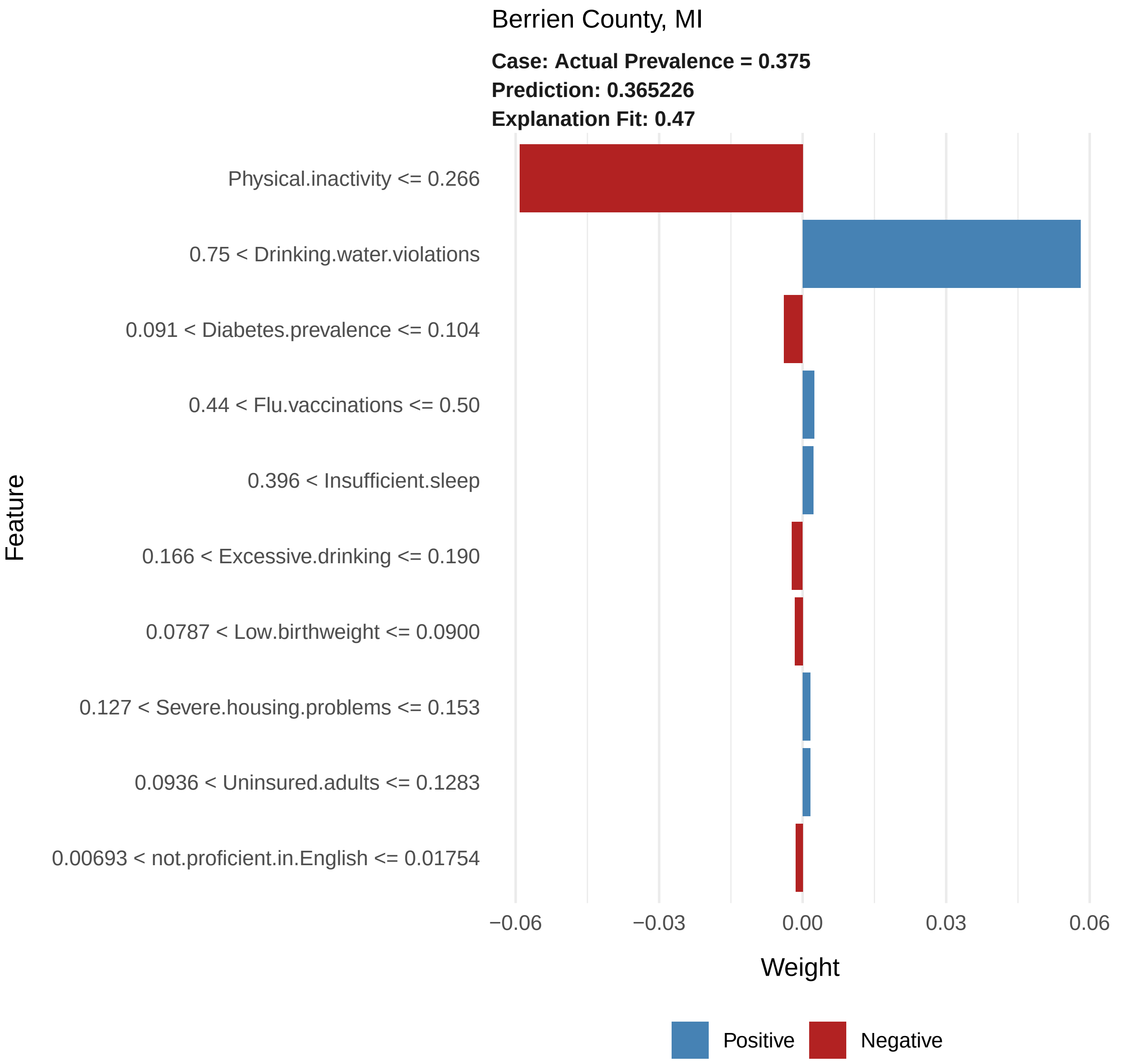

Berrien County, MI
Case: Actual Prevalence = 0.375
Prediction: 0.365226
Explanation Fit: 0.47
Physical.inactivity <= 0.266
0.75 < Drinking.water.violations
0.091 < Diabetes.prevalence <= 0.104
0.44 < Flu.vaccinations <= 0.50
0.396 < Insufficient.sleep
0.166 < Excessive.drinking <= 0.190
0.0787 < Low.birthweight <= 0.0900
0.127 < Severe.housing.problems <= 0.153
0.0936 < Uninsured.adults <= 0.1283
0.00693 < not.proficient.in.English <= 0.01754
Feature
−0.06
−0.03
0.00
0.03
0.06
Weight
Positive
Negative

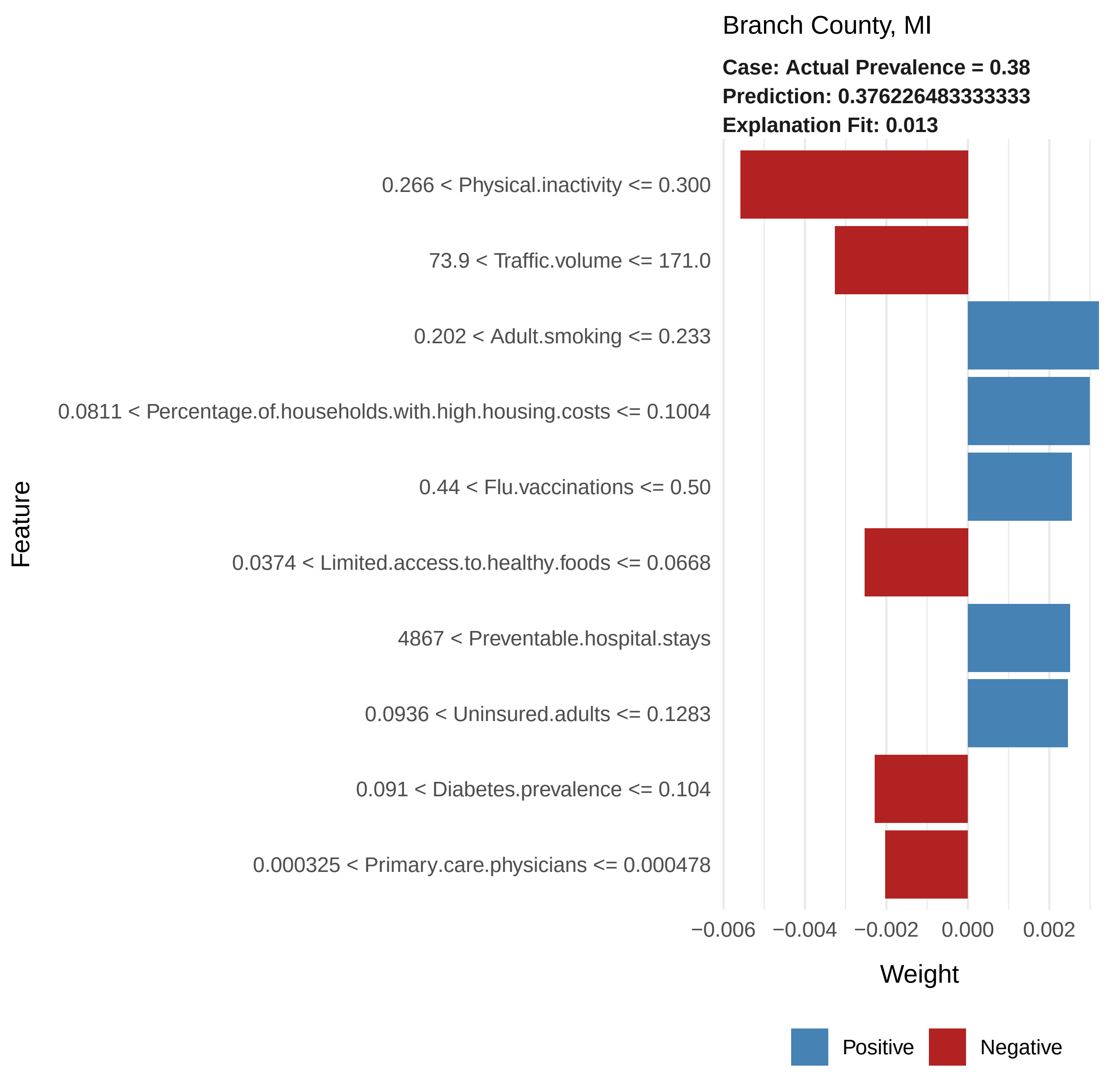
Branch County, MI
Case: Actual Prevalence = 0.38
Prediction: 0.376226483333333
Explanation Fit: 0.013
0.266 < Physical.inactivity <= 0.300
73.9 < Traffic.volume <= 171.0
0.202 < Adult.smoking <= 0.233
0.0811 < Percentage.of.households.with.high.housing.costs <= 0.1004
0.44 < Flu.vaccinations <= 0.50
0.0374 < Limited.access.to.healthy.foods <= 0.0668
4867 < Preventable.hospital.stays
0.0936 < Uninsured.adults <= 0.1283
0.091 < Diabetes.prevalence <= 0.104
0.000325 < Primary.care.physicians <= 0.000478
Feature
−0.006
−0.004
−0.002
0.000
0.002
Weight
Positive
Negative

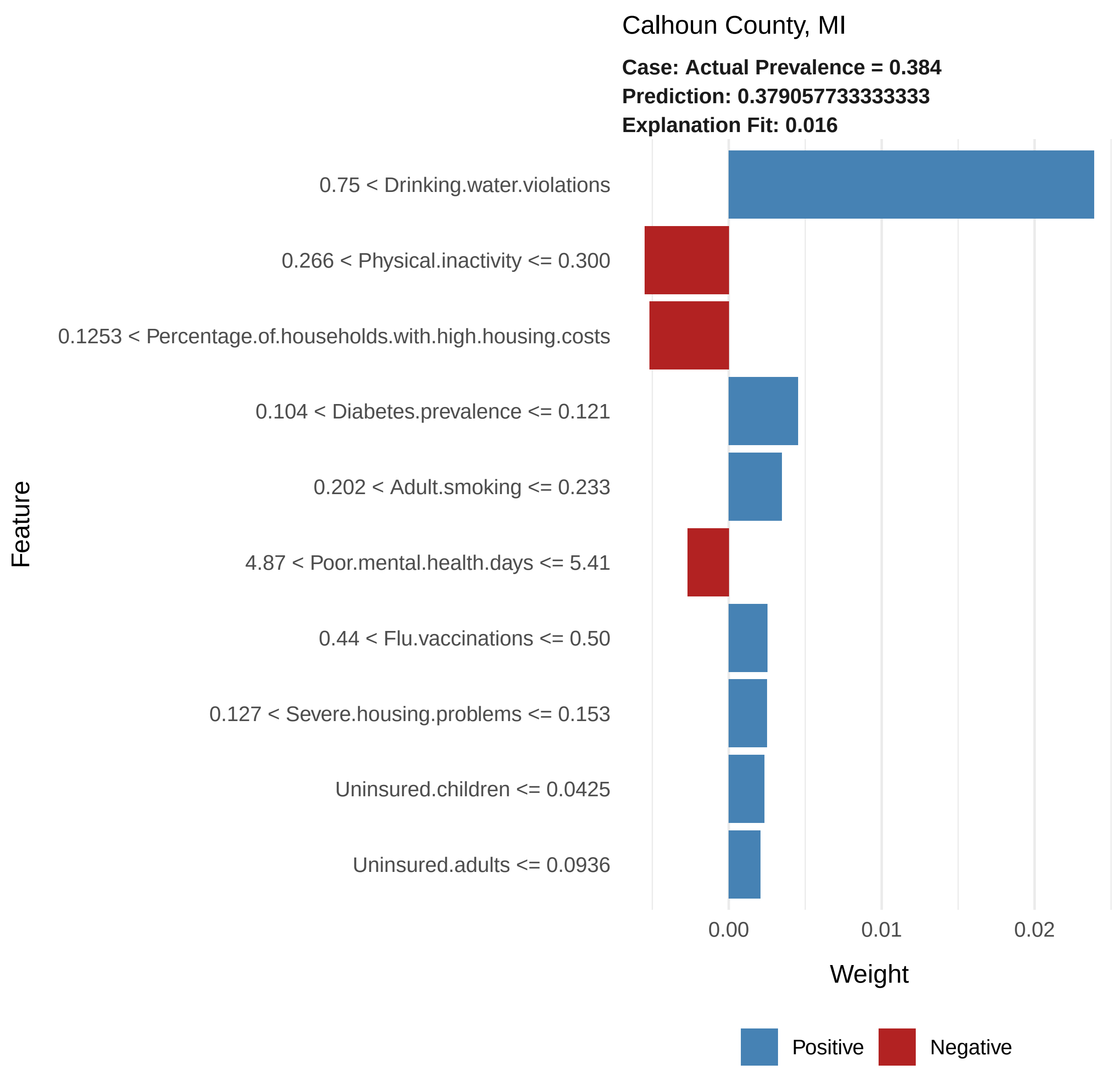

Calhoun County, MI
Case: Actual Prevalence = 0.384
Prediction: 0.379057733333333
Explanation Fit: 0.016
0.75 < Drinking.water.violations
0.266 < Physical.inactivity <= 0.300
0.1253 < Percentage.of.households.with.high.housing.costs
0.104 < Diabetes.prevalence <= 0.121
0.202 < Adult.smoking <= 0.233
4.87 < Poor.mental.health.days <= 5.41
0.44 < Flu.vaccinations <= 0.50
0.127 < Severe.housing.problems <= 0.153
Uninsured.children <= 0.0425
Uninsured.adults <= 0.0936
Feature
0.00
0.01
0.02
Weight
Positive
Negative

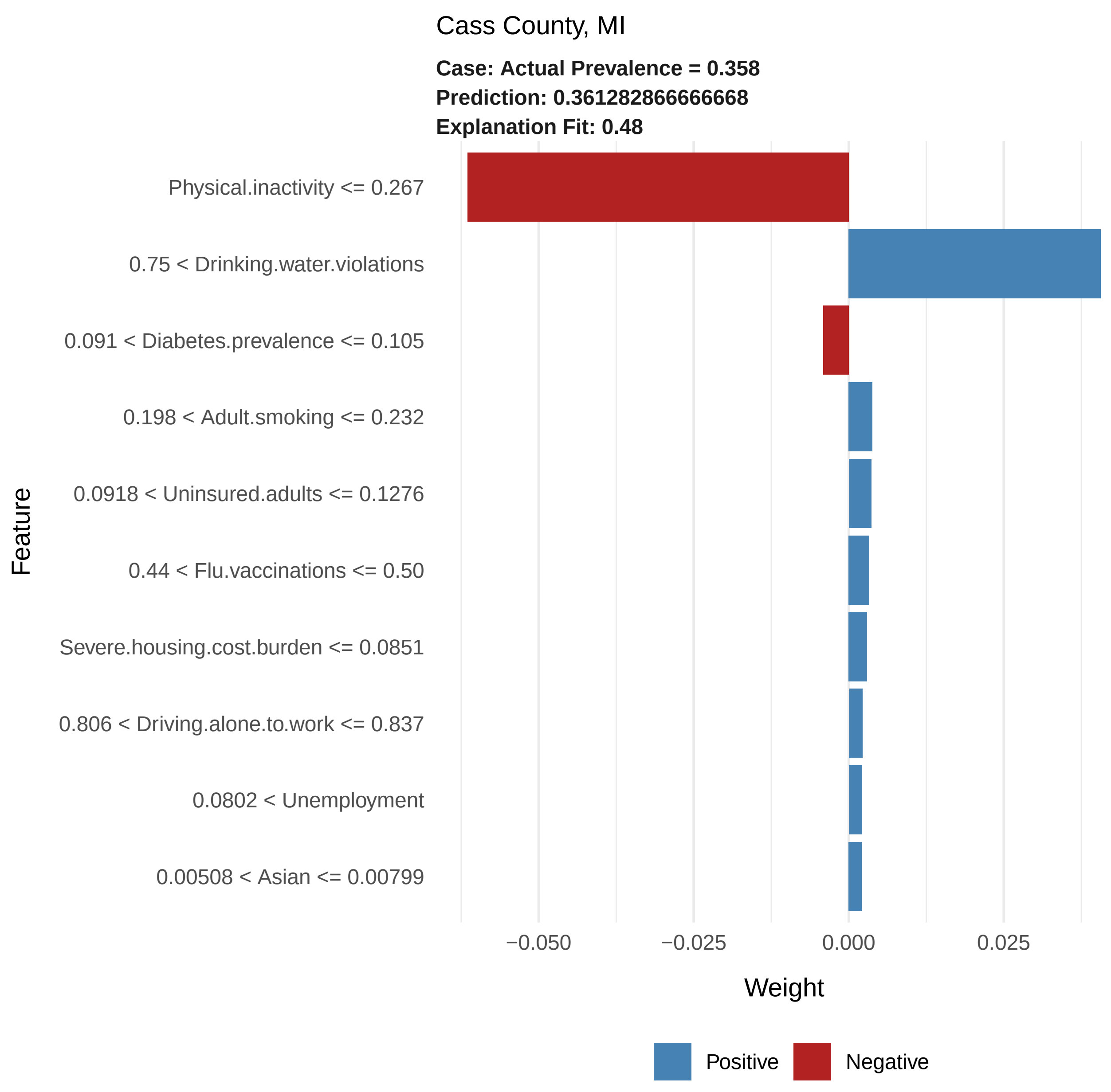
Cass County, MI
Case: Actual Prevalence = 0.358
Prediction: 0.361282866666668
Explanation Fit: 0.48
Physical.inactivity <= 0.267
0.75 < Drinking.water.violations
0.091 < Diabetes.prevalence <= 0.105
0.198 < Adult.smoking <= 0.232
0.0918 < Uninsured.adults <= 0.1276
0.44 < Flu.vaccinations <= 0.50
Severe.housing.cost.burden <= 0.0851
0.806 < Driving.alone.to.work <= 0.837
0.0802 < Unemployment
0.00508 < Asian <= 0.00799
Feature
−0.050
−0.025
0.000
0.025
Weight
Positive
Negative

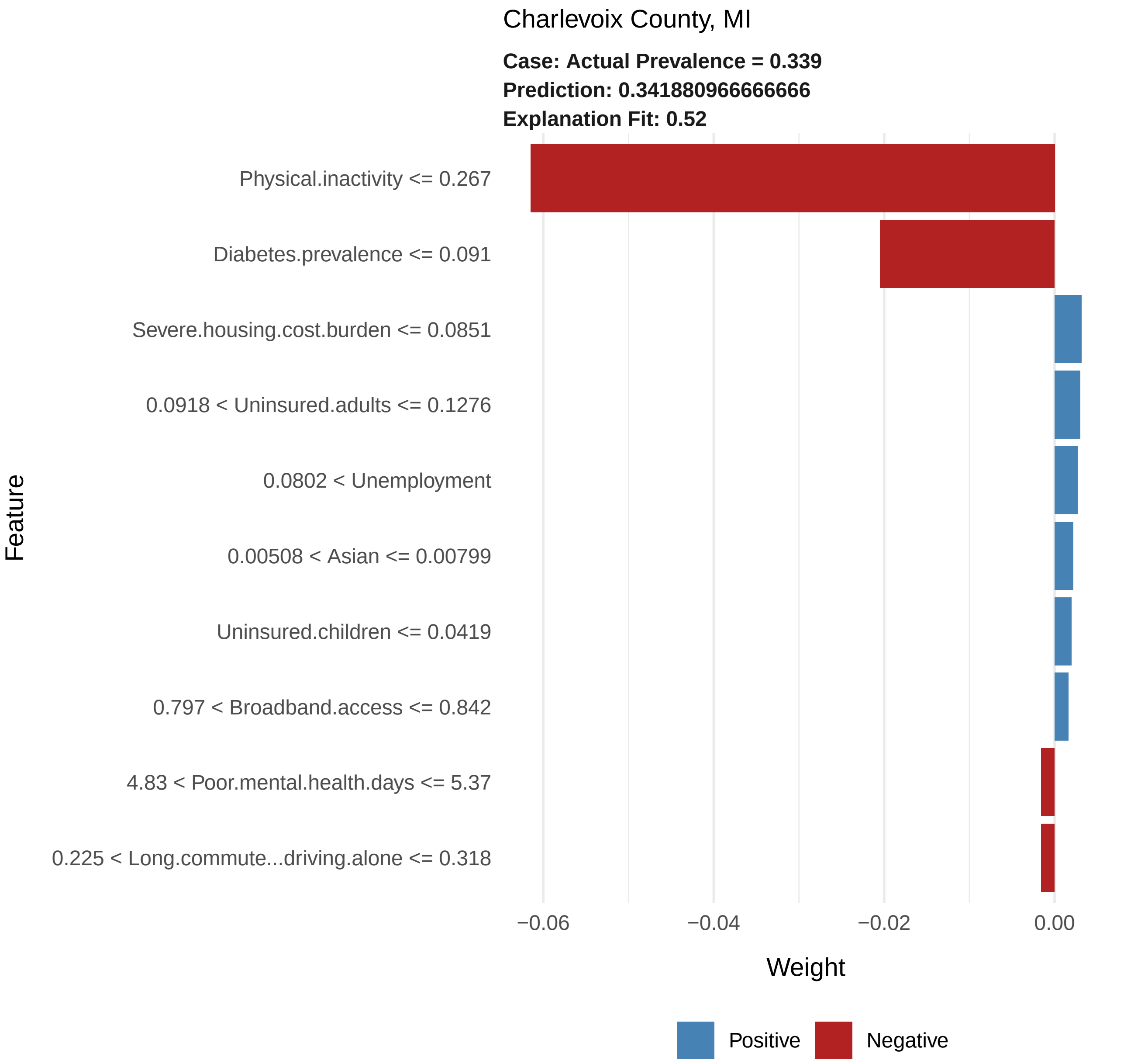
Charlevoix County, MI
Case: Actual Prevalence = 0.339
Prediction: 0.341880966666666
Explanation Fit: 0.52
Physical.inactivity <= 0.267
Diabetes.prevalence <= 0.091
Severe.housing.cost.burden <= 0.0851
0.0918 < Uninsured.adults <= 0.1276
0.0802 < Unemployment
0.00508 < Asian <= 0.00799
Uninsured.children <= 0.0419
0.797 < Broadband.access <= 0.842
4.83 < Poor.mental.health.days <= 5.37
0.225 < Long.commute...driving.alone <= 0.318
Feature
−0.06
−0.04
−0.02
0.00
Weight
Positive
Negative

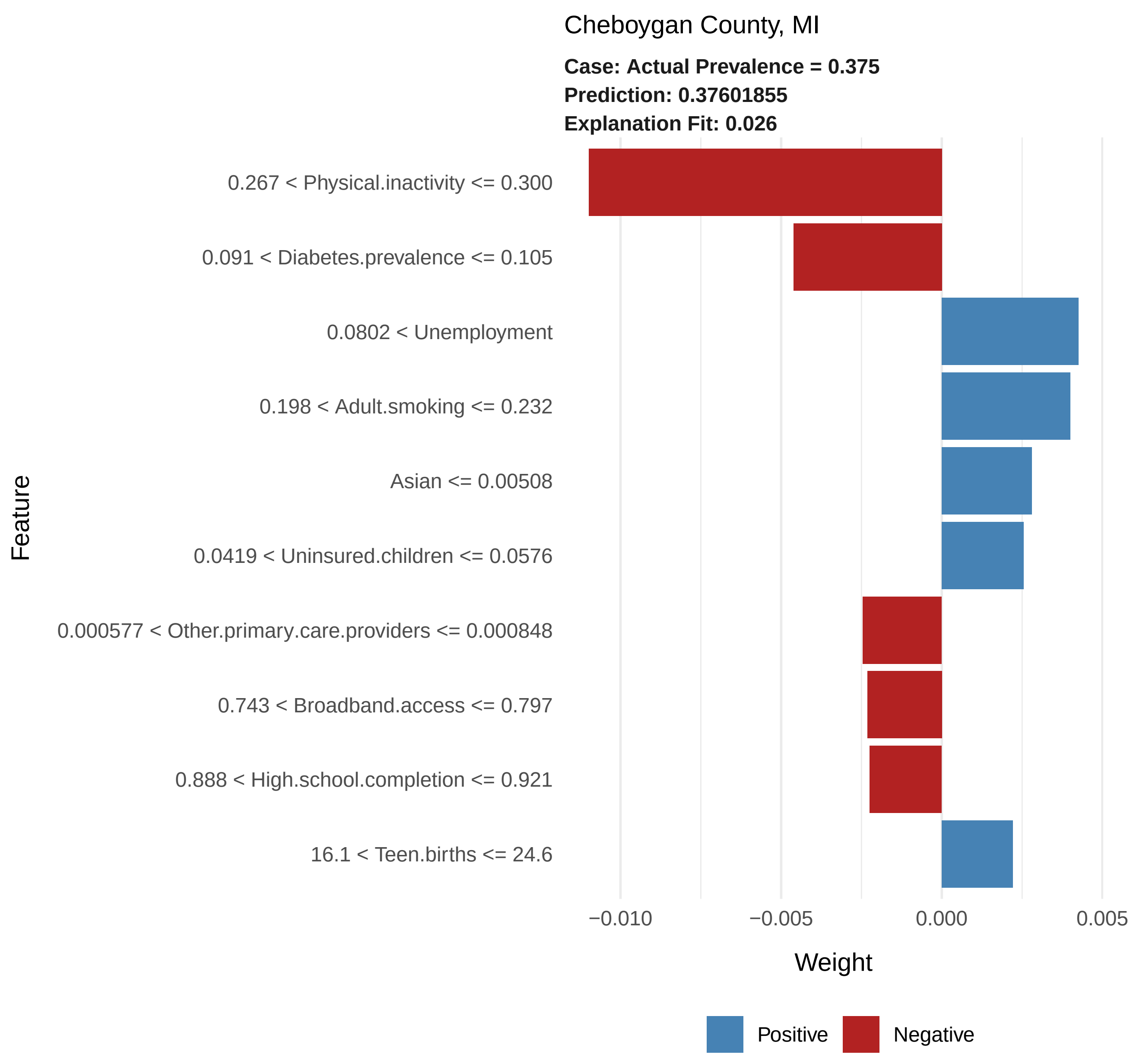
Cheboygan County, MI
Case: Actual Prevalence = 0.375
Prediction: 0.37601855
Explanation Fit: 0.026
0.267 < Physical.inactivity <= 0.300
0.091 < Diabetes.prevalence <= 0.105
0.0802 < Unemployment
0.198 < Adult.smoking <= 0.232
Asian <= 0.00508
0.0419 < Uninsured.children <= 0.0576
0.000577 < Other.primary.care.providers <= 0.000848
0.743 < Broadband.access <= 0.797
0.888 < High.school.completion <= 0.921
16.1 < Teen.births <= 24.6
Feature
−0.010
−0.005
0.000
0.005
Weight
Positive
Negative

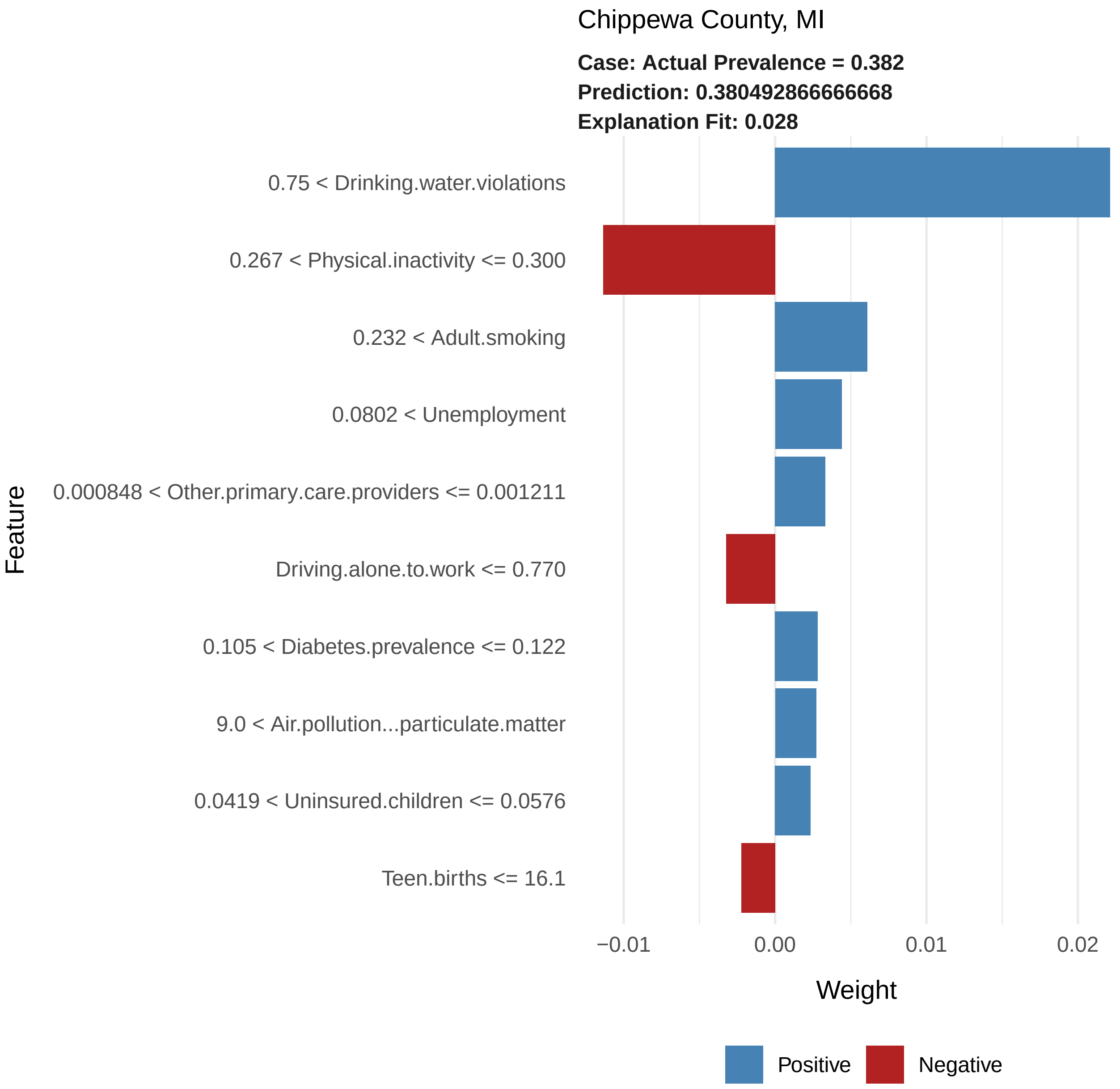

Chippewa County, MI
Case: Actual Prevalence = 0.382
Prediction: 0.380492866666668
Explanation Fit: 0.028
0.75 < Drinking.water.violations
0.267 < Physical.inactivity <= 0.300
0.232 < Adult.smoking
0.0802 < Unemployment
0.000848 < Other.primary.care.providers <= 0.001211
Driving.alone.to.work <= 0.770
0.105 < Diabetes.prevalence <= 0.122
9.0 < Air.pollution...particulate.matter
0.0419 < Uninsured.children <= 0.0576
Teen.births <= 16.1
Feature
−0.01
0.00
0.01
0.02
Weight
Positive
Negative

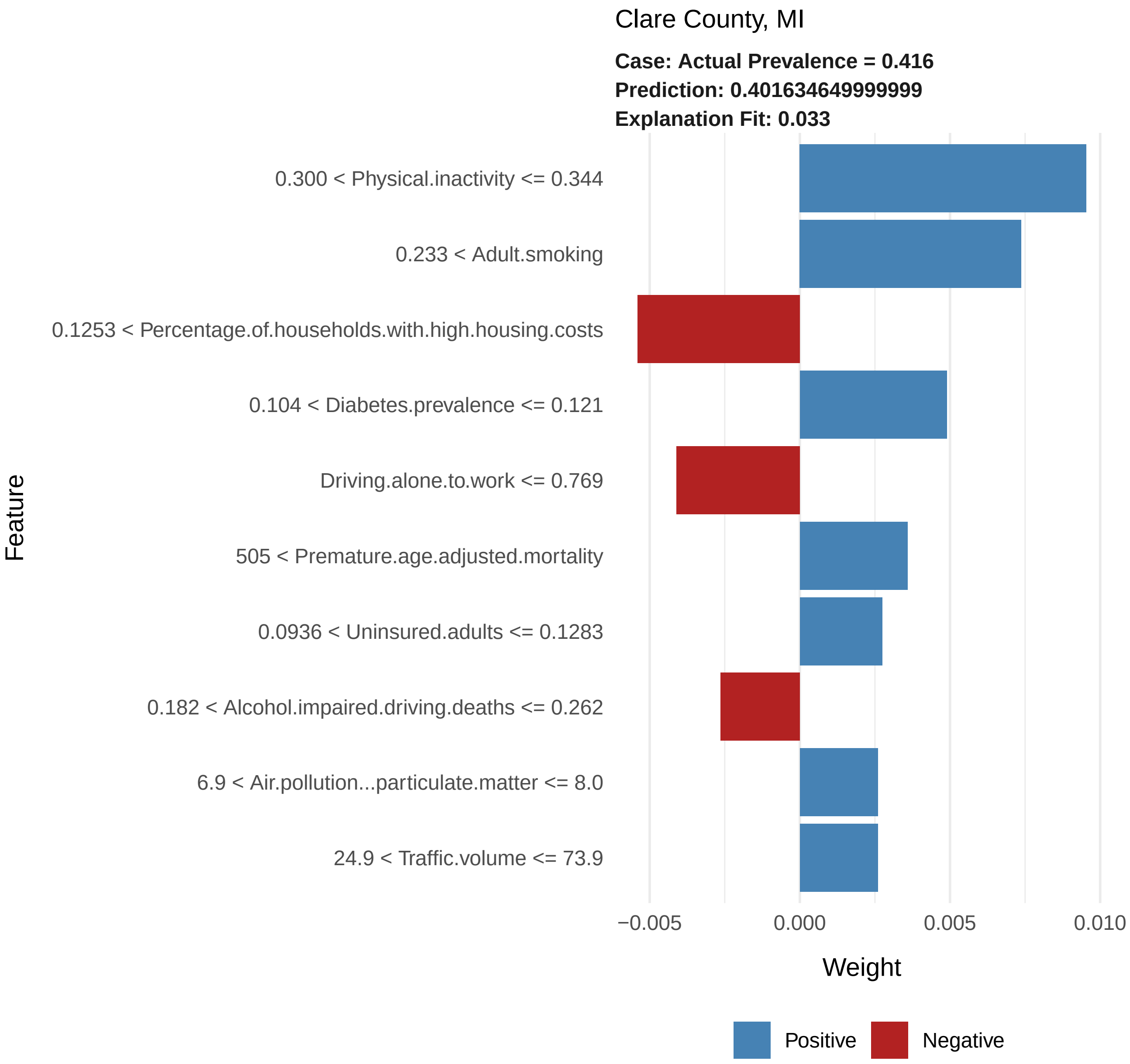
Clare County, MI
Case: Actual Prevalence = 0.416
Prediction: 0.401634649999999
Explanation Fit: 0.033
0.300 < Physical.inactivity <= 0.344
0.233 < Adult.smoking
0.1253 < Percentage.of.households.with.high.housing.costs
0.104 < Diabetes.prevalence <= 0.121
Driving.alone.to.work <= 0.769
505 < Premature.age.adjusted.mortality
0.0936 < Uninsured.adults <= 0.1283
0.182 < Alcohol.impaired.driving.deaths <= 0.262
6.9 < Air.pollution...particulate.matter <= 8.0
24.9 < Traffic.volume <= 73.9
Feature
−0.005
0.000
0.005
0.010
Weight
Positive
Negative

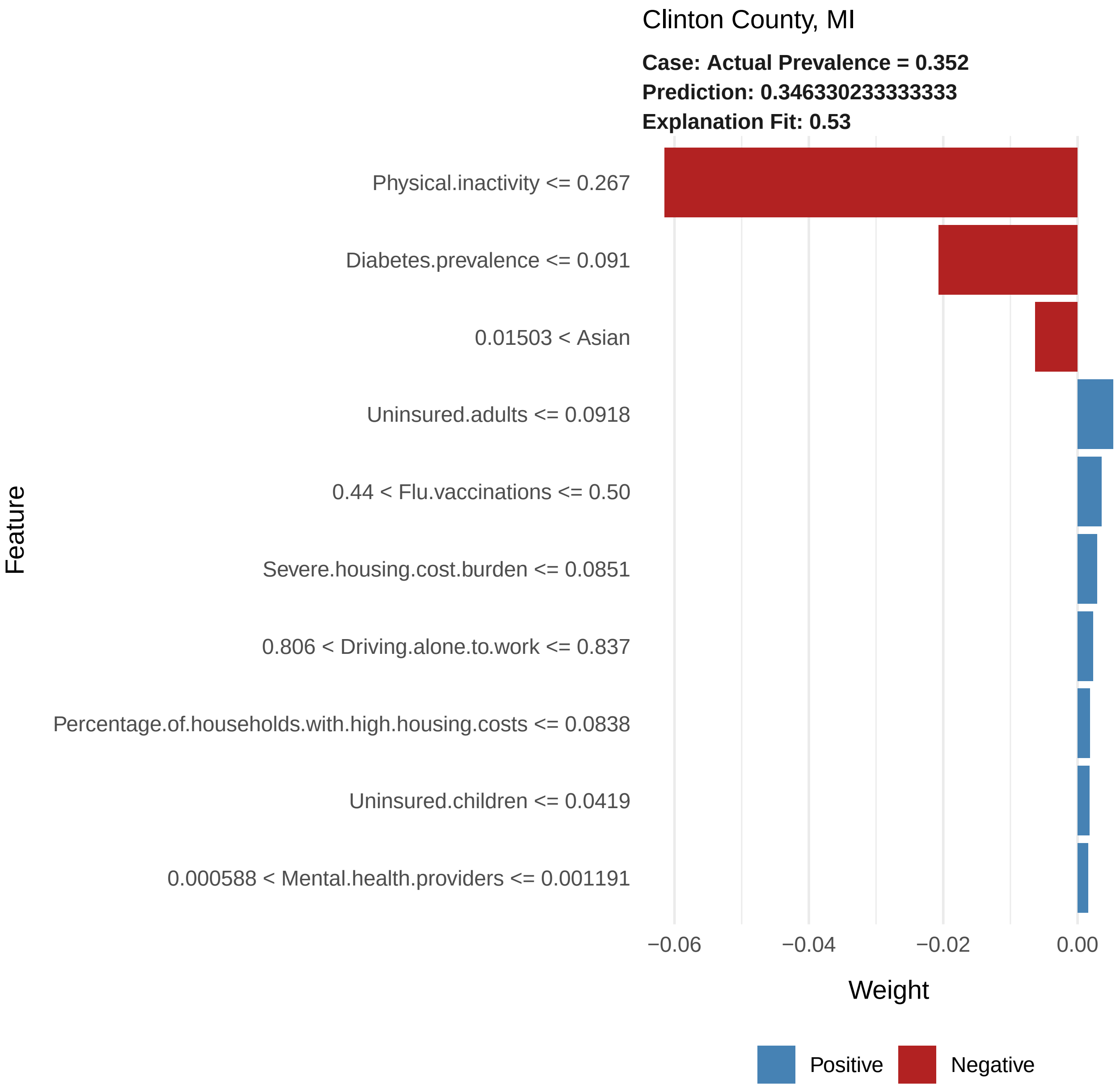
Clinton County, MI
Case: Actual Prevalence = 0.352
Prediction: 0.346330233333333
Explanation Fit: 0.53
Physical.inactivity <= 0.267
Diabetes.prevalence <= 0.091
0.01503 < Asian
Uninsured.adults <= 0.0918
0.44 < Flu.vaccinations <= 0.50
Severe.housing.cost.burden <= 0.0851
0.806 < Driving.alone.to.work <= 0.837
Percentage.of.households.with.high.housing.costs <= 0.0838
Uninsured.children <= 0.0419
0.000588 < Mental.health.providers <= 0.001191
Feature
−0.06
−0.04
−0.02
0.00
Weight
Positive
Negative

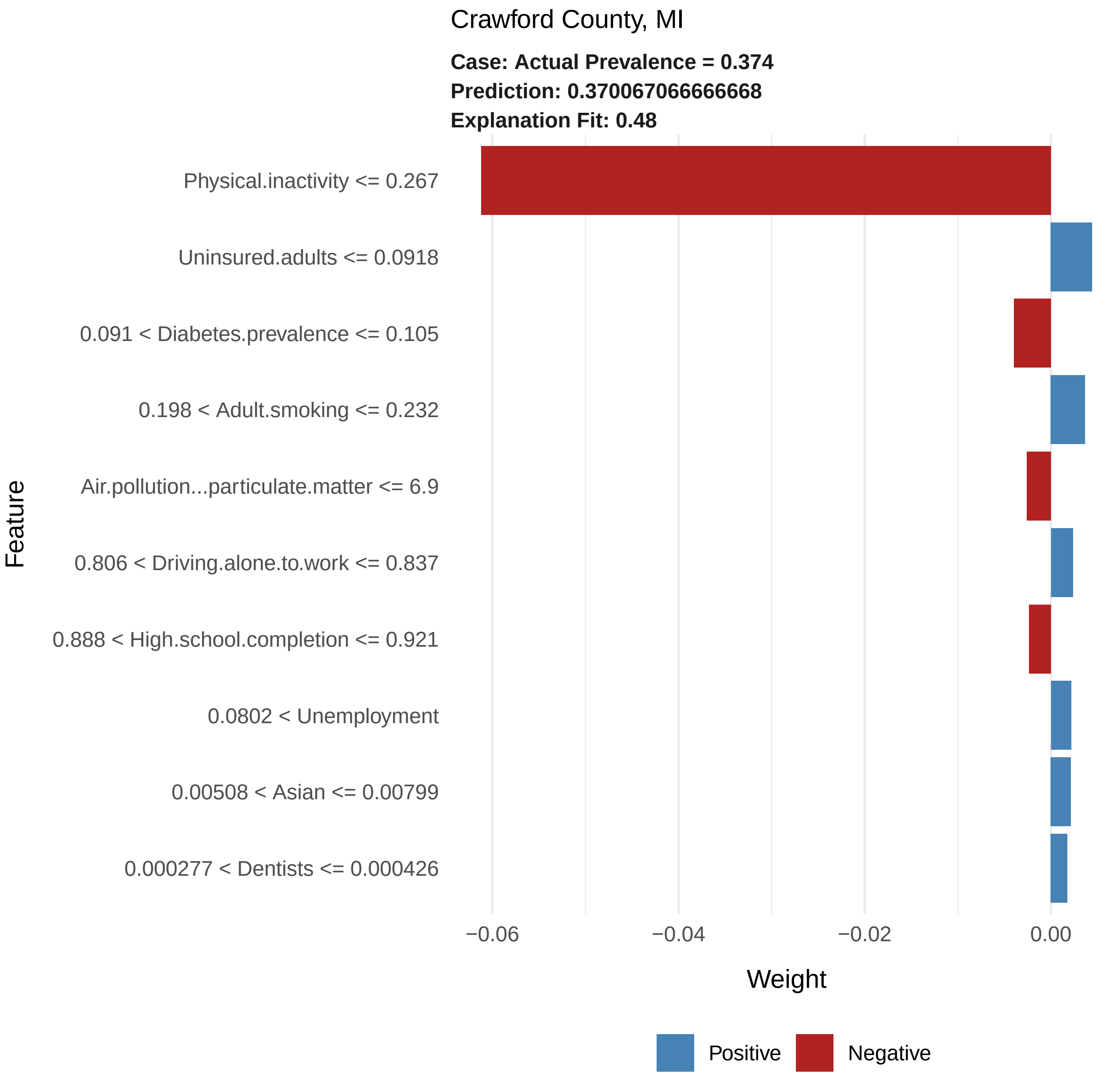
Crawford County, MI
Case: Actual Prevalence = 0.374
Prediction: 0.370067066666668
Explanation Fit: 0.48
Physical.inactivity <= 0.267
Uninsured.adults <= 0.0918
0.091 < Diabetes.prevalence <= 0.105
0.198 < Adult.smoking <= 0.232
Air.pollution...particulate.matter <= 6.9
0.806 < Driving.alone.to.work <= 0.837
0.888 < High.school.completion <= 0.921
0.0802 < Unemployment
0.00508 < Asian <= 0.00799
0.000277 < Dentists <= 0.000426
Feature
−0.06
−0.04
−0.02
0.00
Weight
Positive
Negative

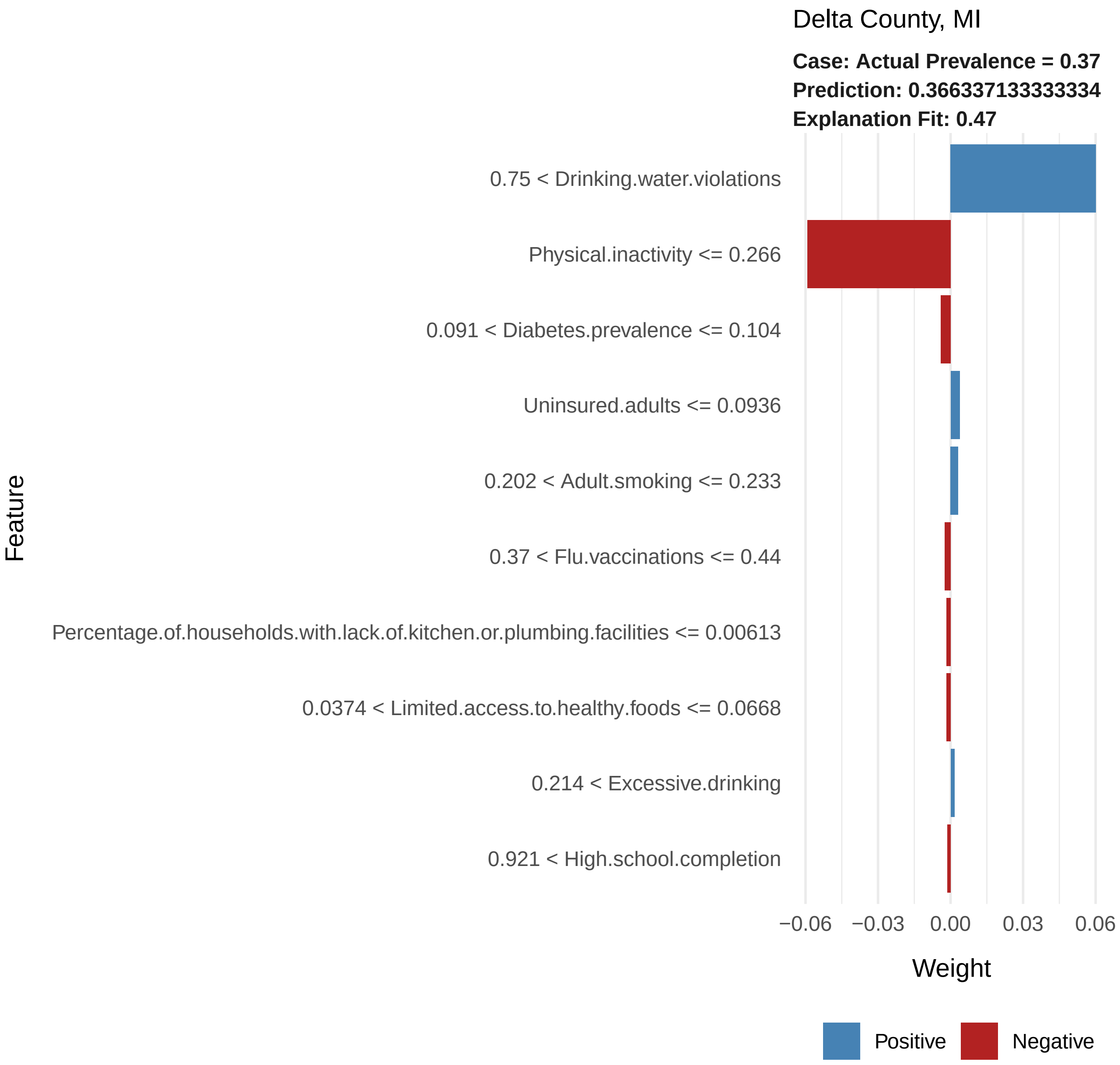

Delta County, MI
Case: Actual Prevalence = 0.37
Prediction: 0.366337133333334
Explanation Fit: 0.47
0.75 < Drinking.water.violations
Physical.inactivity <= 0.266
0.091 < Diabetes.prevalence <= 0.104
Uninsured.adults <= 0.0936
0.202 < Adult.smoking <= 0.233
0.37 < Flu.vaccinations <= 0.44
Percentage.of.households.with.lack.of.kitchen.or.plumbing.facilities <= 0.00613
0.0374 < Limited.access.to.healthy.foods <= 0.0668
0.214 < Excessive.drinking
0.921 < High.school.completion
Feature
−0.06
−0.03
0.00
0.03
0.06
Weight
Positive
Negative

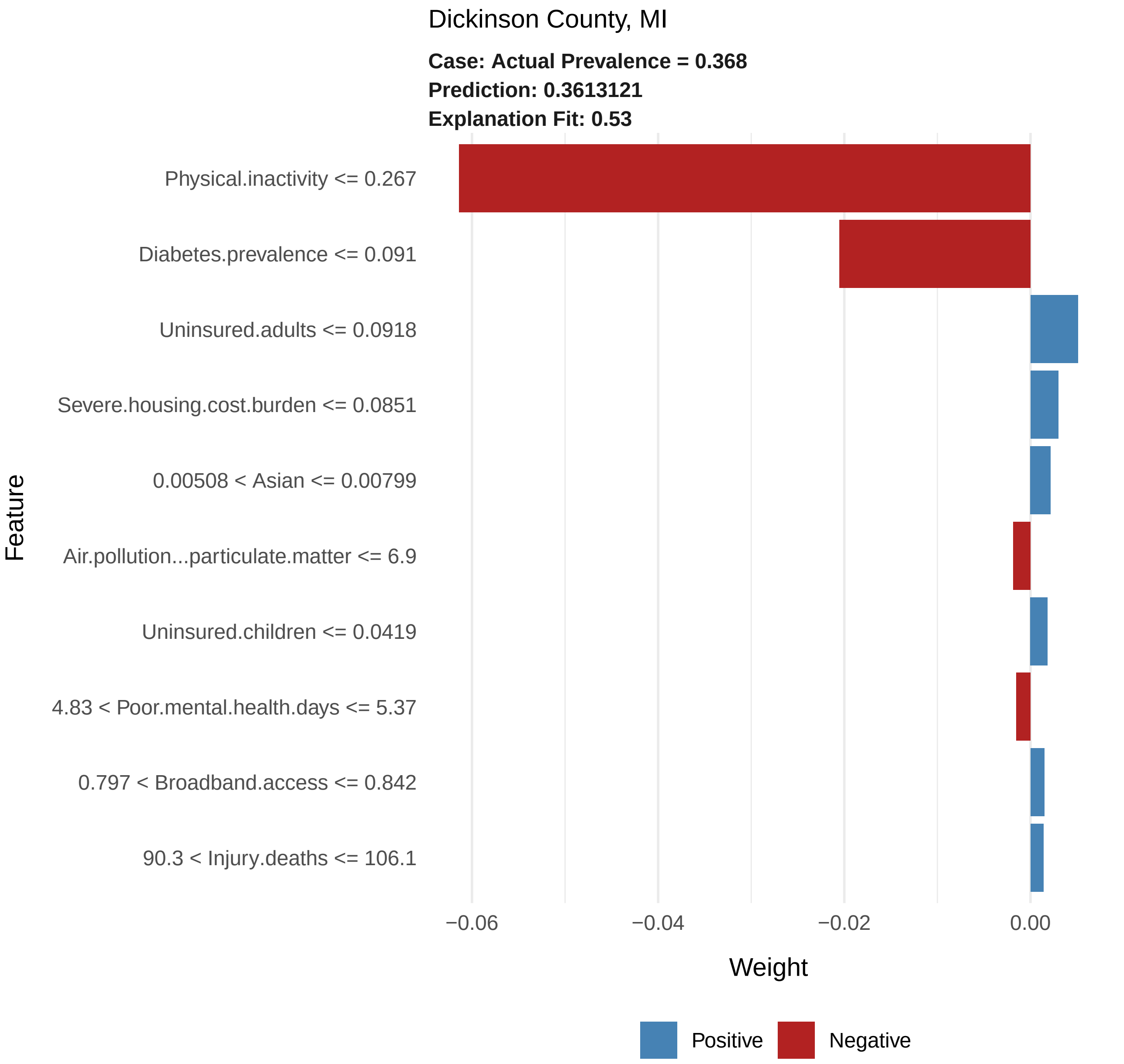

Dickinson County, MI
Case: Actual Prevalence = 0.368
Prediction: 0.3613121
Explanation Fit: 0.53
Physical.inactivity <= 0.267
Diabetes.prevalence <= 0.091
Uninsured.adults <= 0.0918
Severe.housing.cost.burden <= 0.0851
0.00508 < Asian <= 0.00799
Air.pollution...particulate.matter <= 6.9
Uninsured.children <= 0.0419
4.83 < Poor.mental.health.days <= 5.37
0.797 < Broadband.access <= 0.842
90.3 < Injury.deaths <= 106.1
Feature
−0.06
−0.04
−0.02
0.00
Weight
Positive
Negative

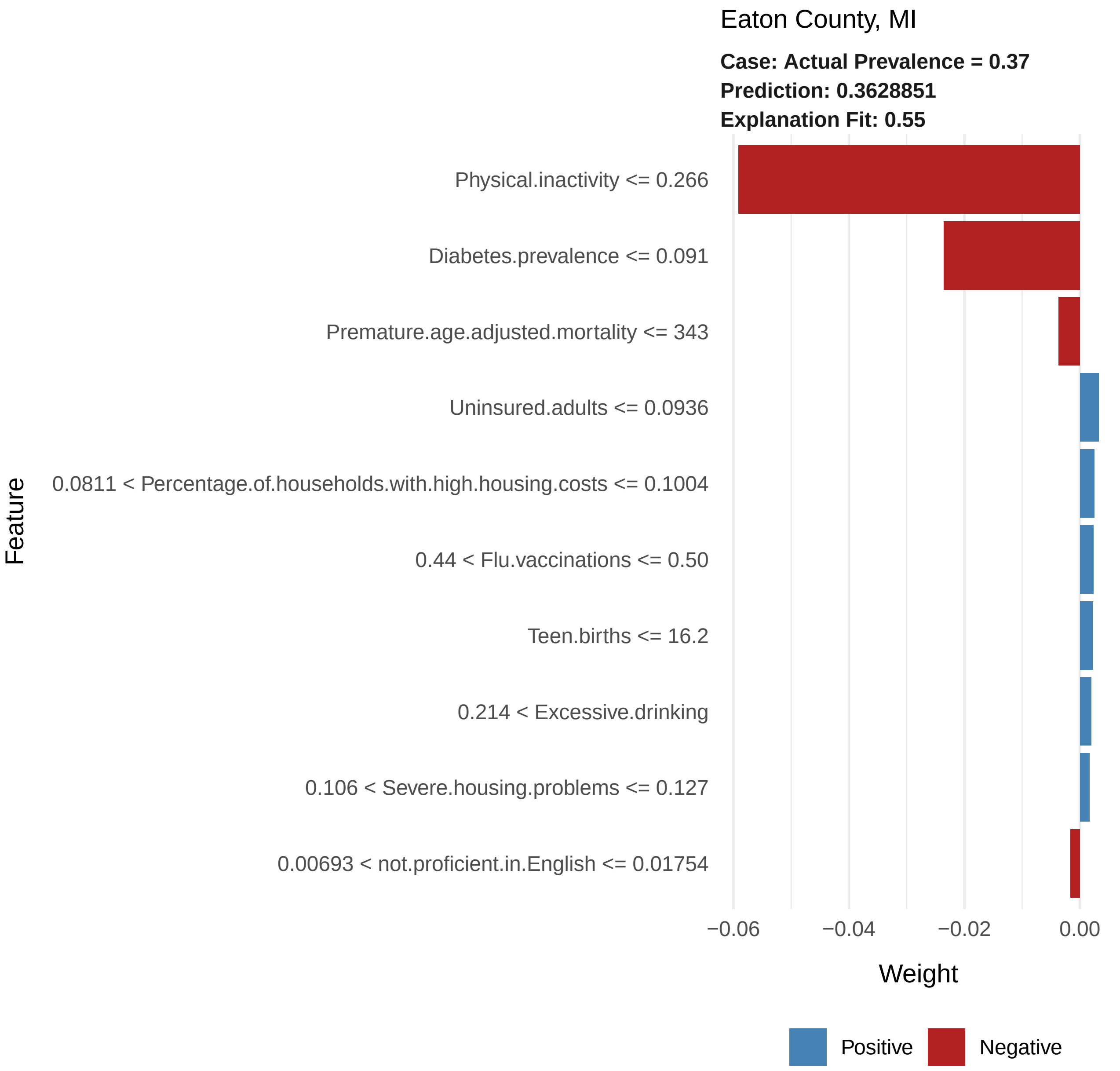

Eaton County, MI
Case: Actual Prevalence = 0.37
Prediction: 0.3628851
Explanation Fit: 0.55
Physical.inactivity <= 0.266
Diabetes.prevalence <= 0.091
Premature.age.adjusted.mortality <= 343
Uninsured.adults <= 0.0936
0.0811 < Percentage.of.households.with.high.housing.costs <= 0.1004
0.44 < Flu.vaccinations <= 0.50
Teen.births <= 16.2
0.214 < Excessive.drinking
0.106 < Severe.housing.problems <= 0.127
0.00693 < not.proficient.in.English <= 0.01754
Feature
−0.06
−0.04
−0.02
0.00
Weight
Positive
Negative

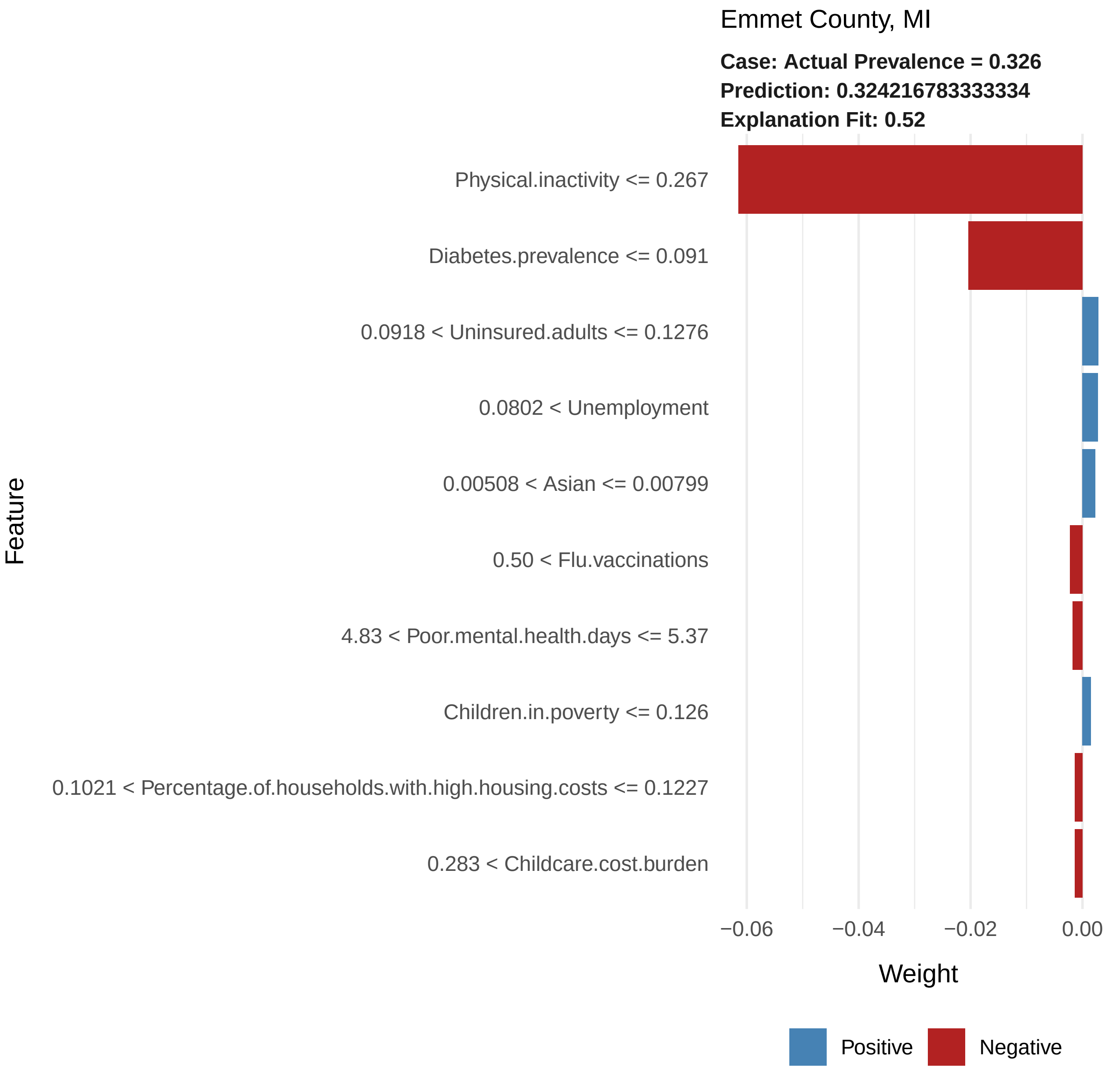

Emmet County, MI
Case: Actual Prevalence = 0.326
Prediction: 0.324216783333334
Explanation Fit: 0.52
Physical.inactivity <= 0.267
Diabetes.prevalence <= 0.091
0.0918 < Uninsured.adults <= 0.1276
0.0802 < Unemployment
0.00508 < Asian <= 0.00799
0.50 < Flu.vaccinations
4.83 < Poor.mental.health.days <= 5.37
Children.in.poverty <= 0.126
0.1021 < Percentage.of.households.with.high.housing.costs <= 0.1227
0.283 < Childcare.cost.burden
Feature
−0.06
−0.04
−0.02
0.00
Weight
Positive
Negative

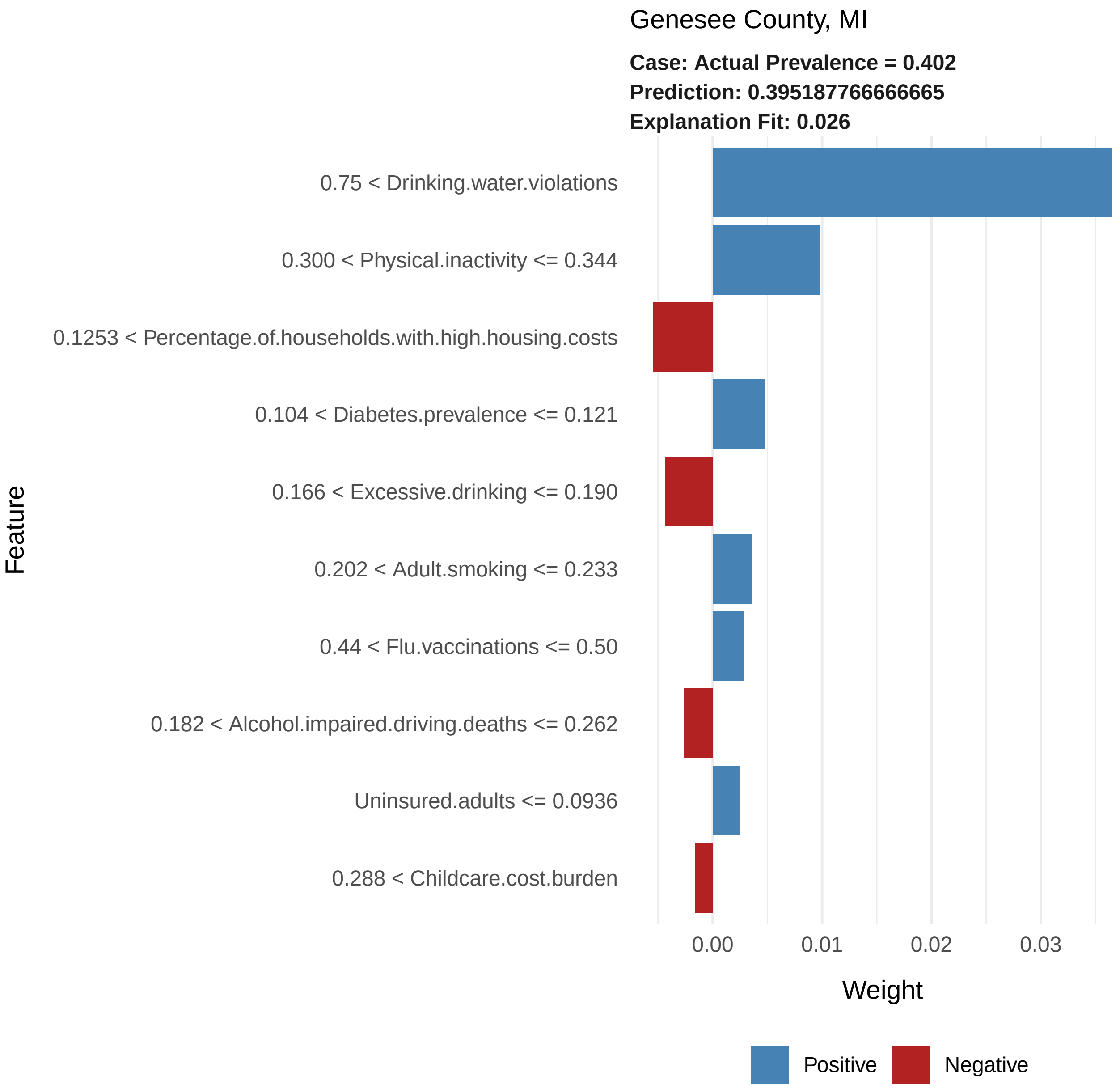
Genesee County, MI
Case: Actual Prevalence = 0.402
Prediction: 0.395187766666665
Explanation Fit: 0.026
0.75 < Drinking.water.violations
0.300 < Physical.inactivity <= 0.344
0.1253 < Percentage.of.households.with.high.housing.costs
0.104 < Diabetes.prevalence <= 0.121
0.166 < Excessive.drinking <= 0.190
0.202 < Adult.smoking <= 0.233
0.44 < Flu.vaccinations <= 0.50
0.182 < Alcohol.impaired.driving.deaths <= 0.262
Uninsured.adults <= 0.0936
0.288 < Childcare.cost.burden
Feature
0.00
0.01
0.02
0.03
Weight
Positive
Negative

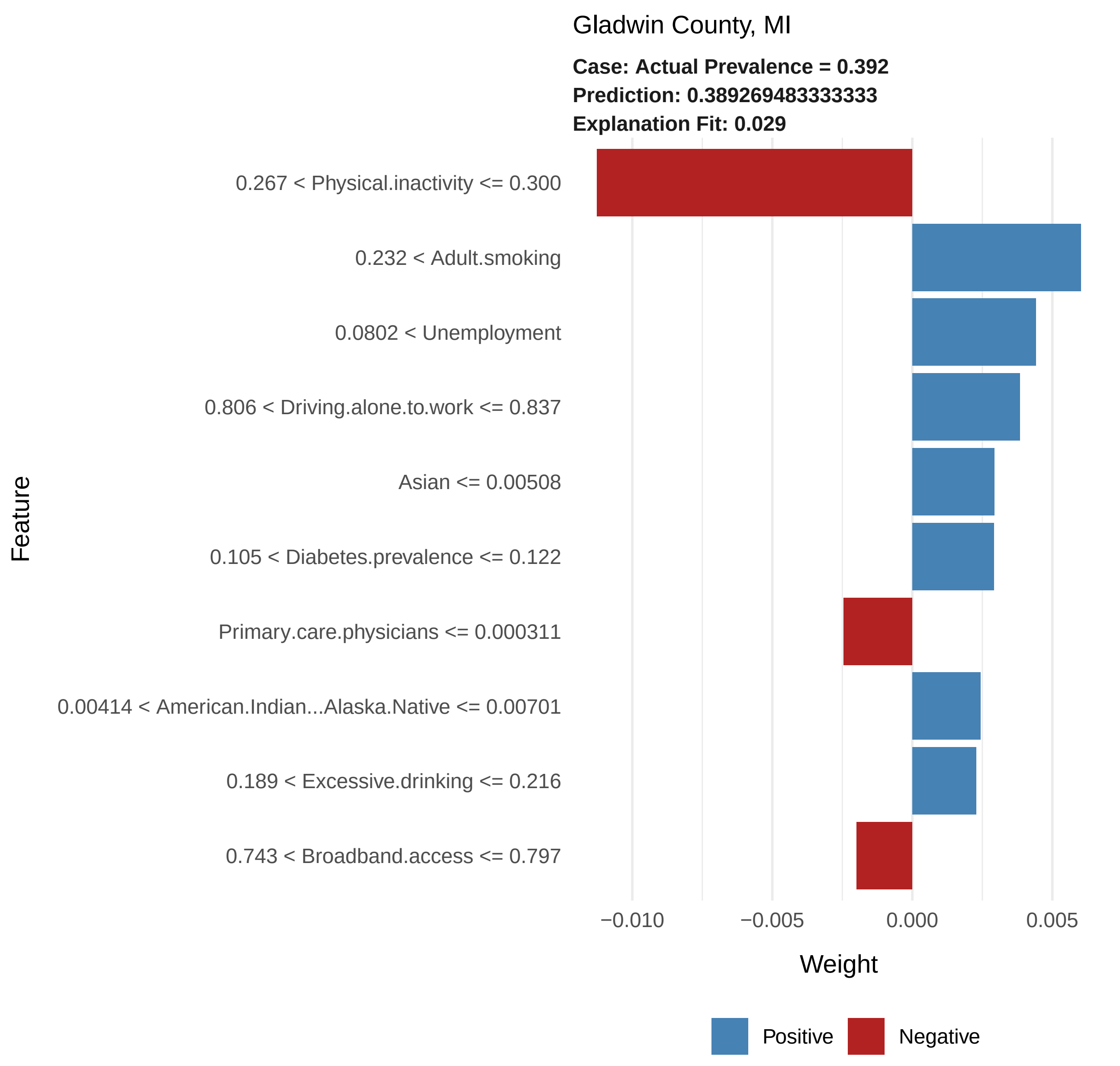
Gladwin County, MI
Case: Actual Prevalence = 0.392
Prediction: 0.389269483333333
Explanation Fit: 0.029
0.267 < Physical.inactivity <= 0.300
0.232 < Adult.smoking
0.0802 < Unemployment
0.806 < Driving.alone.to.work <= 0.837
Asian <= 0.00508
0.105 < Diabetes.prevalence <= 0.122
Primary.care.physicians <= 0.000311
0.00414 < American.Indian...Alaska.Native <= 0.00701
0.189 < Excessive.drinking <= 0.216
0.743 < Broadband.access <= 0.797
Feature
−0.010
−0.005
0.000
0.005
Weight
Positive
Negative

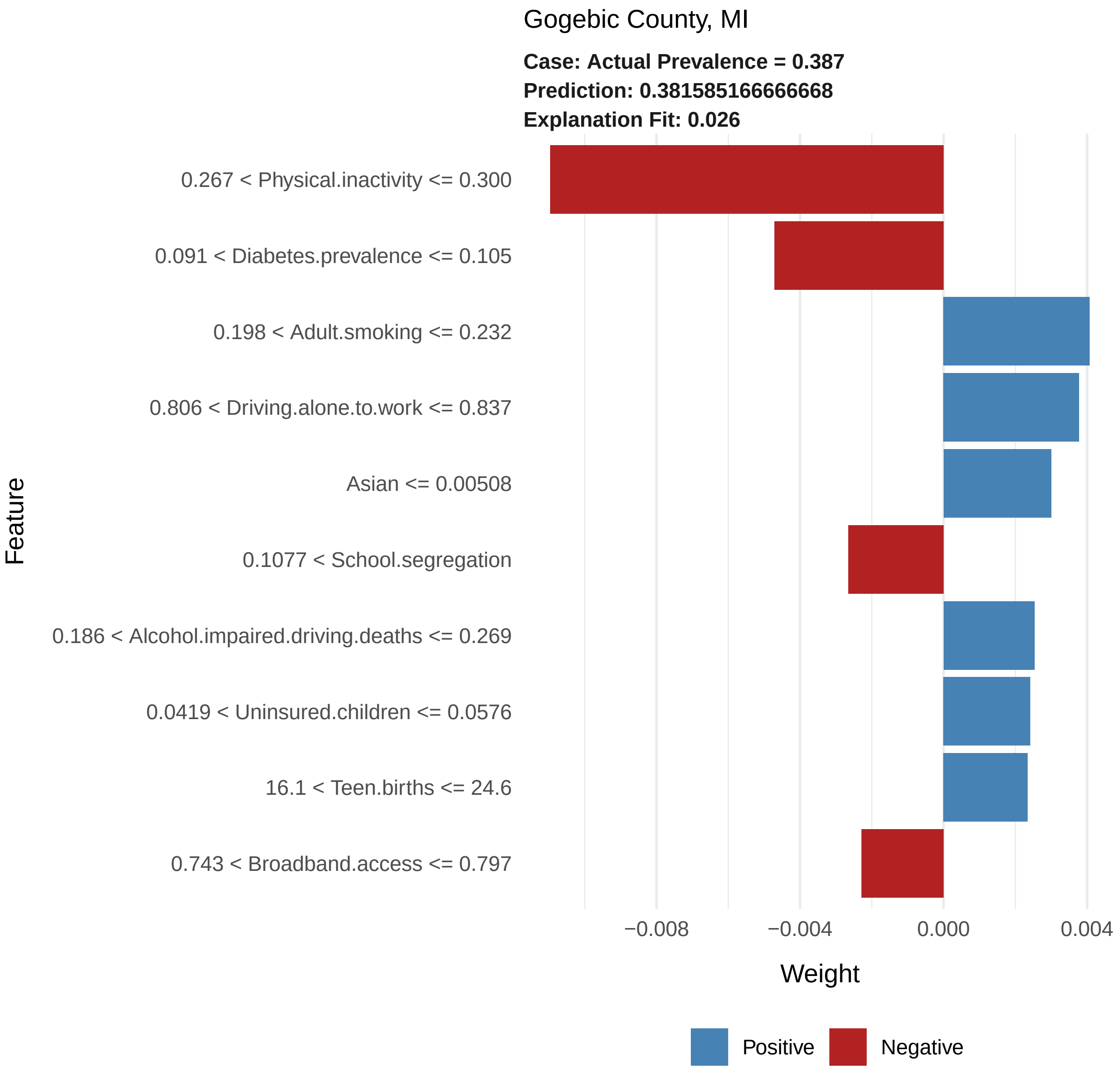
Gogebic County, MI
Case: Actual Prevalence = 0.387
Prediction: 0.381585166666668
Explanation Fit: 0.026
Feature
0.267 < Physical.inactivity <= 0.300
0.091 < Diabetes.prevalence <= 0.105
0.198 < Adult.smoking <= 0.232
0.806 < Driving.alone.to.work <= 0.837
Asian <= 0.00508
0.1077 < School.segregation
0.186 < Alcohol.impaired.driving.deaths <= 0.269
0.0419 < Uninsured.children <= 0.0576
16.1 < Teen.births <= 24.6
0.743 < Broadband.access <= 0.797
−0.008
−0.004
0.000
0.004
Weight
Positive
Negative

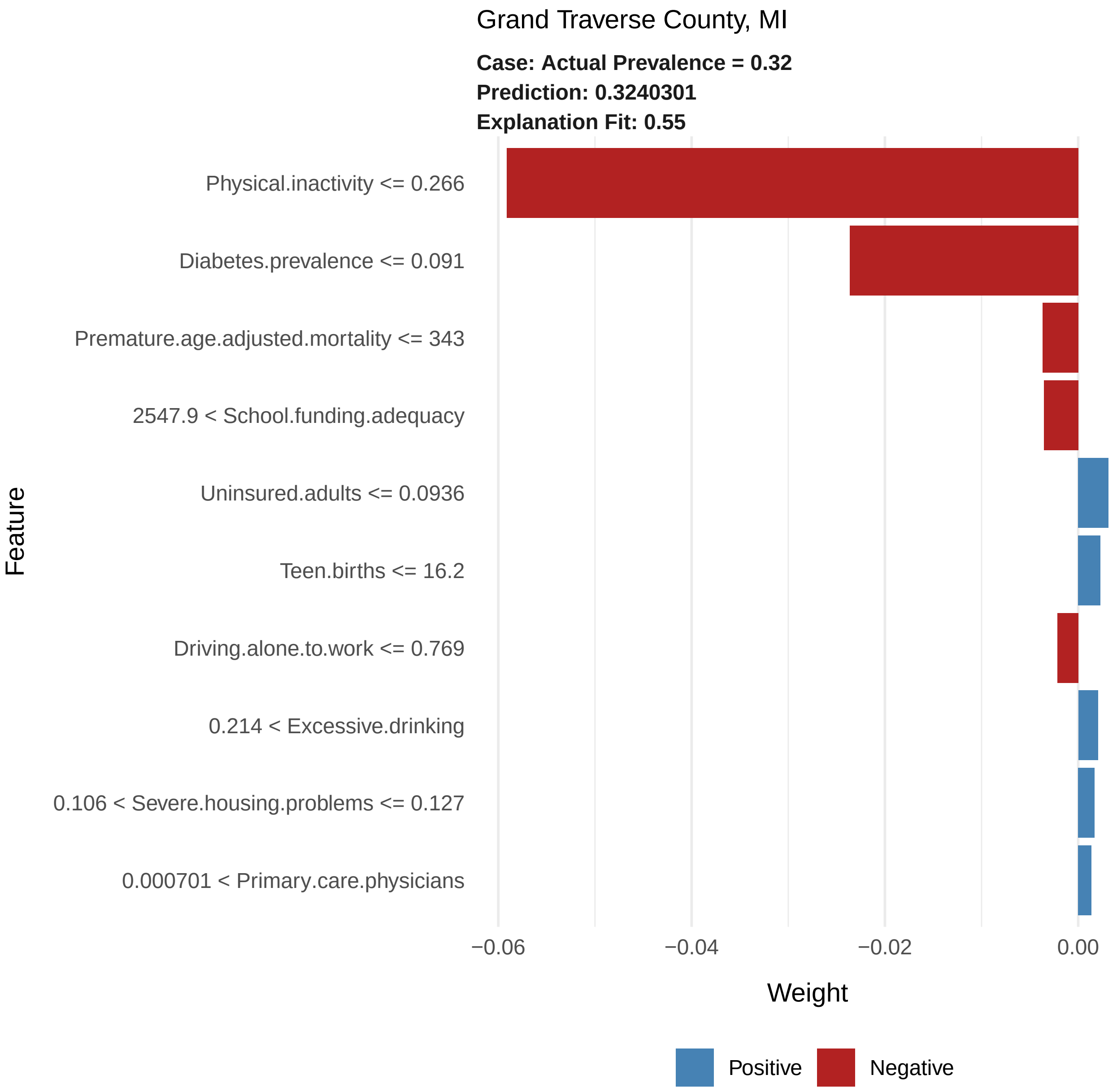

Grand Traverse County, MI
Case: Actual Prevalence = 0.32
Prediction: 0.3240301
Explanation Fit: 0.55
Physical.inactivity <= 0.266
Diabetes.prevalence <= 0.091
Premature.age.adjusted.mortality <= 343
2547.9 < School.funding.adequacy
Uninsured.adults <= 0.0936
Teen.births <= 16.2
Driving.alone.to.work <= 0.769
0.214 < Excessive.drinking
0.106 < Severe.housing.problems <= 0.127
0.000701 < Primary.care.physicians
Feature
−0.06
−0.04
−0.02
0.00
Weight
Positive
Negative

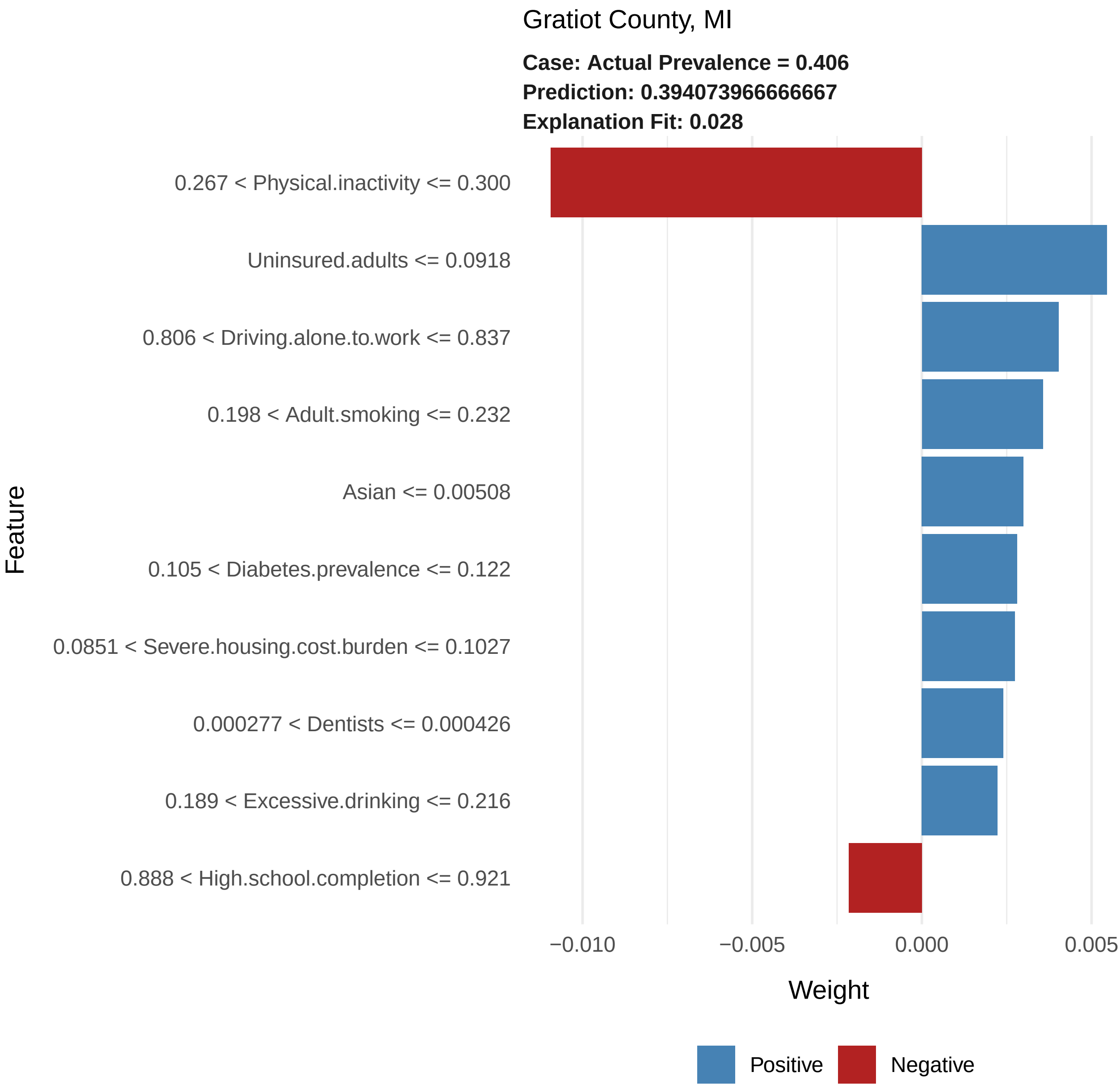
Gratiot County, MI
Case: Actual Prevalence = 0.406
Prediction: 0.394073966666667
Explanation Fit: 0.028
0.267 < Physical.inactivity <= 0.300
Uninsured.adults <= 0.0918
0.806 < Driving.alone.to.work <= 0.837
0.198 < Adult.smoking <= 0.232
Asian <= 0.00508
0.105 < Diabetes.prevalence <= 0.122
0.0851 < Severe.housing.cost.burden <= 0.1027
0.000277 < Dentists <= 0.000426
0.189 < Excessive.drinking <= 0.216
0.888 < High.school.completion <= 0.921
Feature
−0.010
−0.005
0.000
0.005
Weight
Positive
Negative

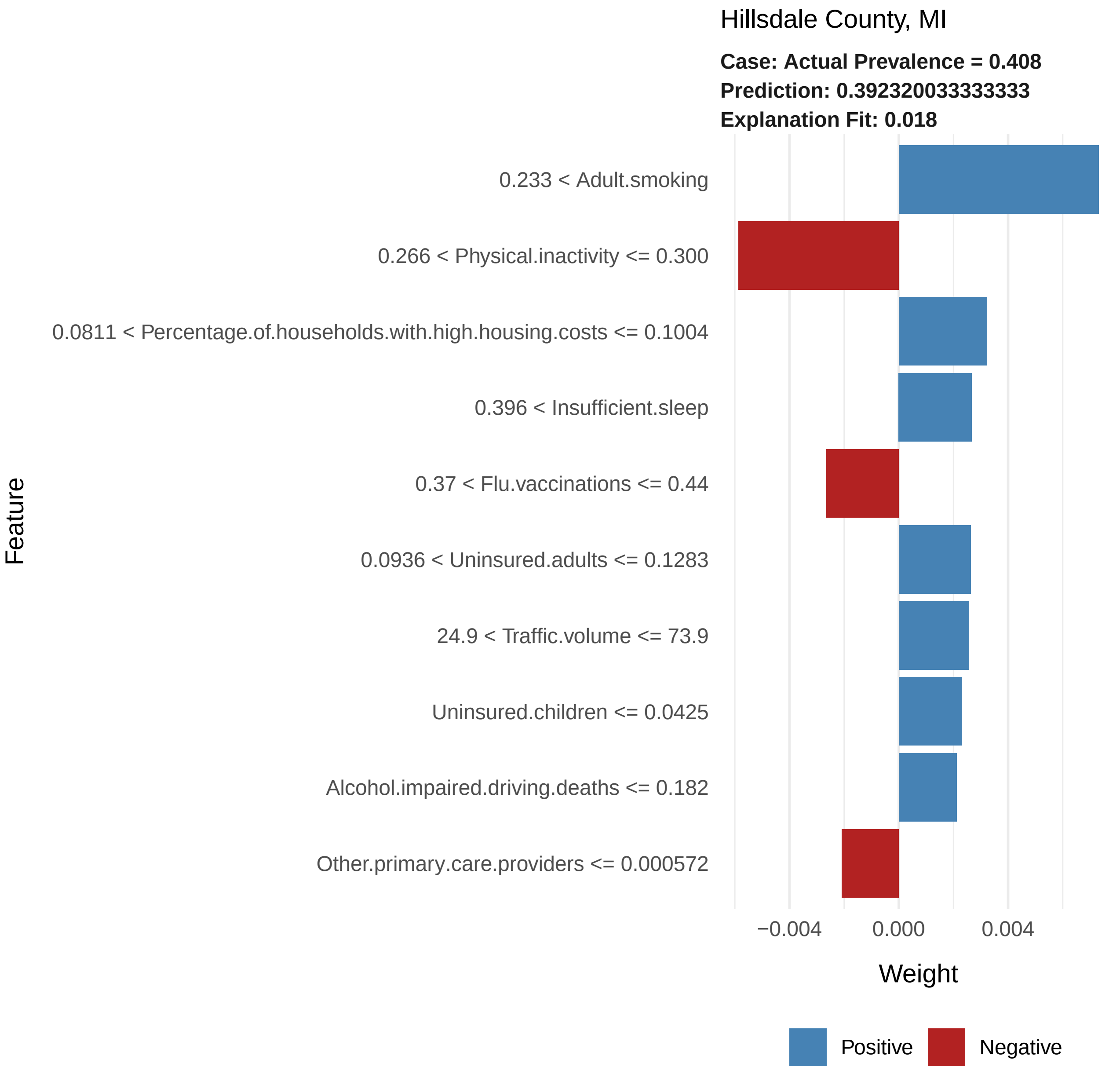
Hillsdale County, MI
Case: Actual Prevalence = 0.408
Prediction: 0.392320033333333
Explanation Fit: 0.018
0.233 < Adult.smoking
0.266 < Physical.inactivity <= 0.300
0.0811 < Percentage.of.households.with.high.housing.costs <= 0.1004
0.396 < Insufficient.sleep
0.37 < Flu.vaccinations <= 0.44
0.0936 < Uninsured.adults <= 0.1283
24.9 < Traffic.volume <= 73.9
Uninsured.children <= 0.0425
Alcohol.impaired.driving.deaths <= 0.182
Other.primary.care.providers <= 0.000572
Feature
−0.004
0.000
0.004
Weight
Positive
Negative

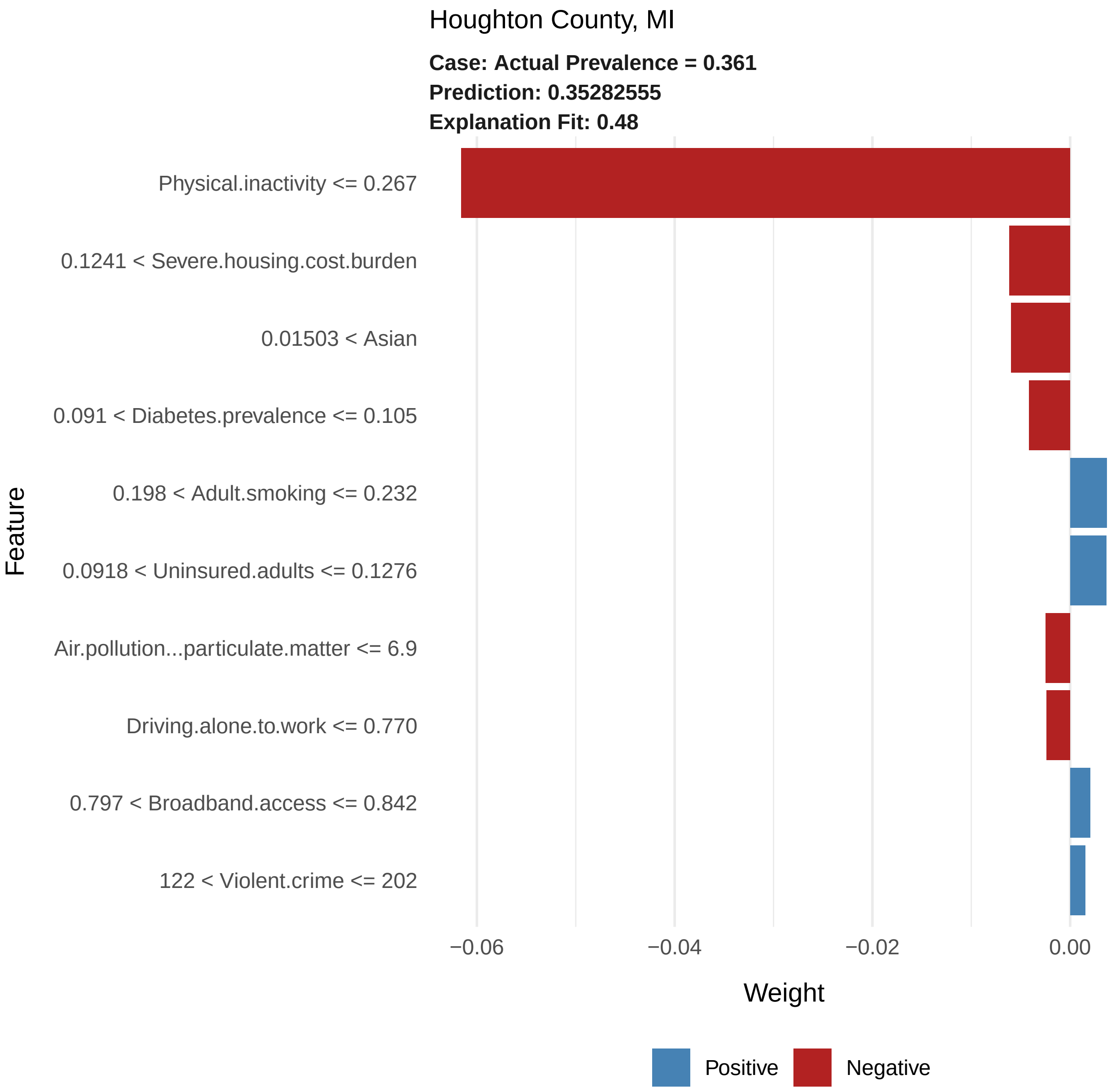

Houghton County, MI
Case: Actual Prevalence = 0.361
Prediction: 0.35282555
Explanation Fit: 0.48
Physical.inactivity <= 0.267
0.1241 < Severe.housing.cost.burden
0.01503 < Asian
0.091 < Diabetes.prevalence <= 0.105
0.198 < Adult.smoking <= 0.232
0.0918 < Uninsured.adults <= 0.1276
Air.pollution...particulate.matter <= 6.9
Driving.alone.to.work <= 0.770
0.797 < Broadband.access <= 0.842
122 < Violent.crime <= 202
Feature
−0.06
−0.04
−0.02
0.00
Weight
Positive
Negative

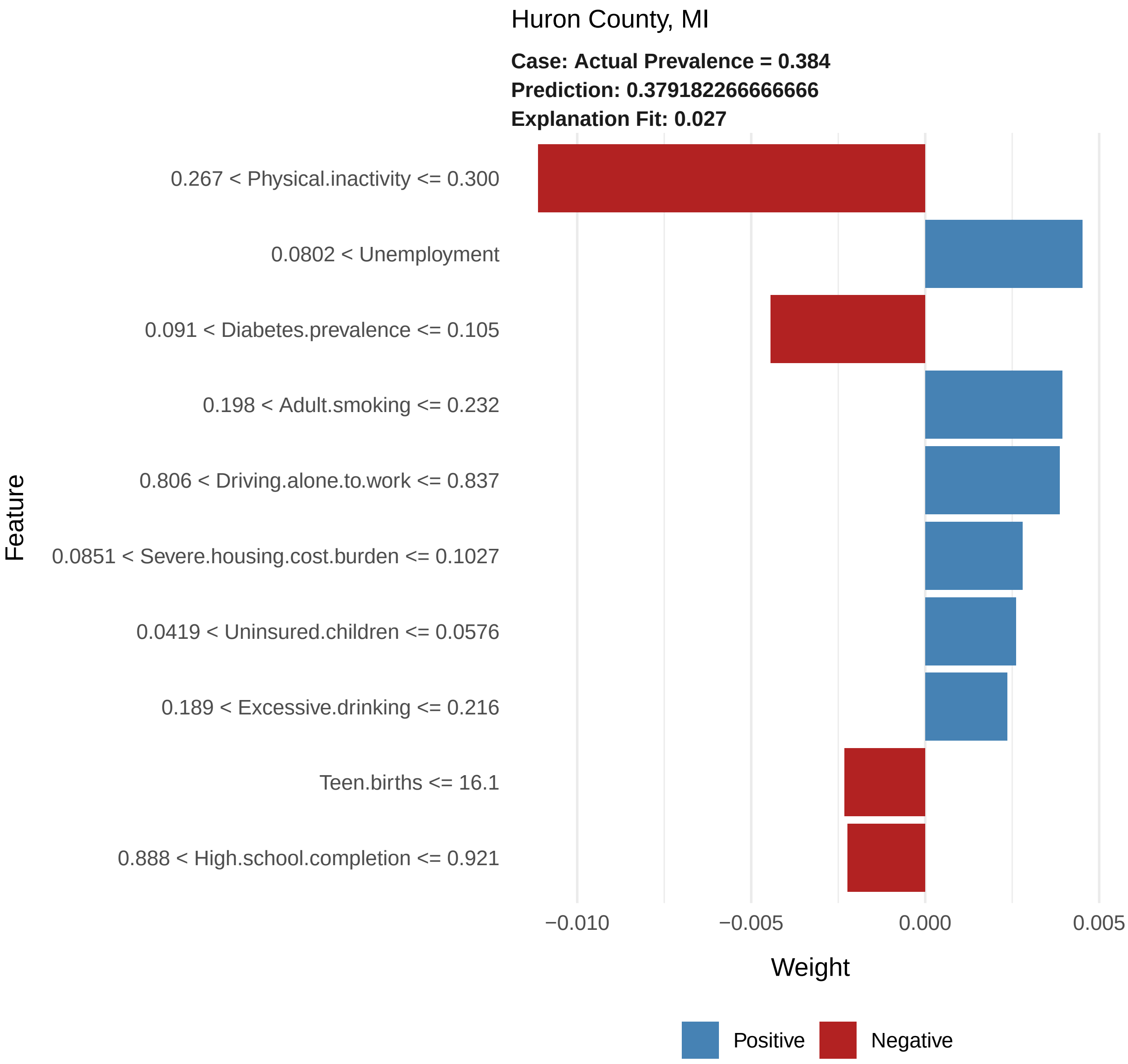

Huron County, MI
Case: Actual Prevalence = 0.384
Prediction: 0.379182266666666
Explanation Fit: 0.027
0.267 < Physical.inactivity <= 0.300
0.0802 < Unemployment
0.091 < Diabetes.prevalence <= 0.105
0.198 < Adult.smoking <= 0.232
0.806 < Driving.alone.to.work <= 0.837
0.0851 < Severe.housing.cost.burden <= 0.1027
0.0419 < Uninsured.children <= 0.0576
0.189 < Excessive.drinking <= 0.216
Teen.births <= 16.1
0.888 < High.school.completion <= 0.921
Feature
−0.010
−0.005
0.000
0.005
Weight
Positive
Negative

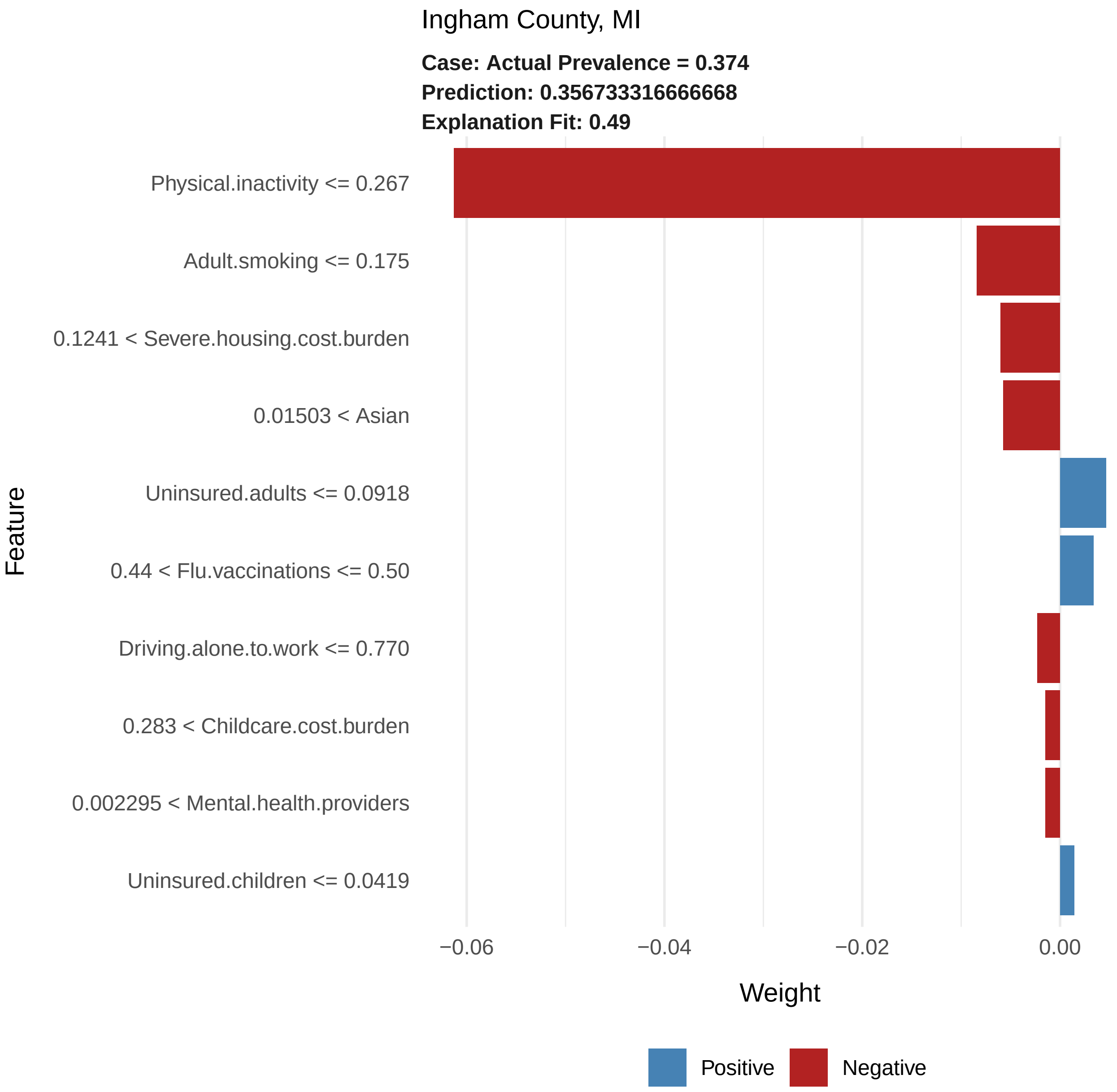
Ingham County, MI
Case: Actual Prevalence = 0.374
Prediction: 0.356733316666668
Explanation Fit: 0.49
Physical.inactivity <= 0.267
Adult.smoking <= 0.175
0.1241 < Severe.housing.cost.burden
0.01503 < Asian
Uninsured.adults <= 0.0918
0.44 < Flu.vaccinations <= 0.50
Driving.alone.to.work <= 0.770
0.283 < Childcare.cost.burden
0.002295 < Mental.health.providers
Uninsured.children <= 0.0419
Feature
−0.06
−0.04
−0.02
0.00
Weight
Positive
Negative

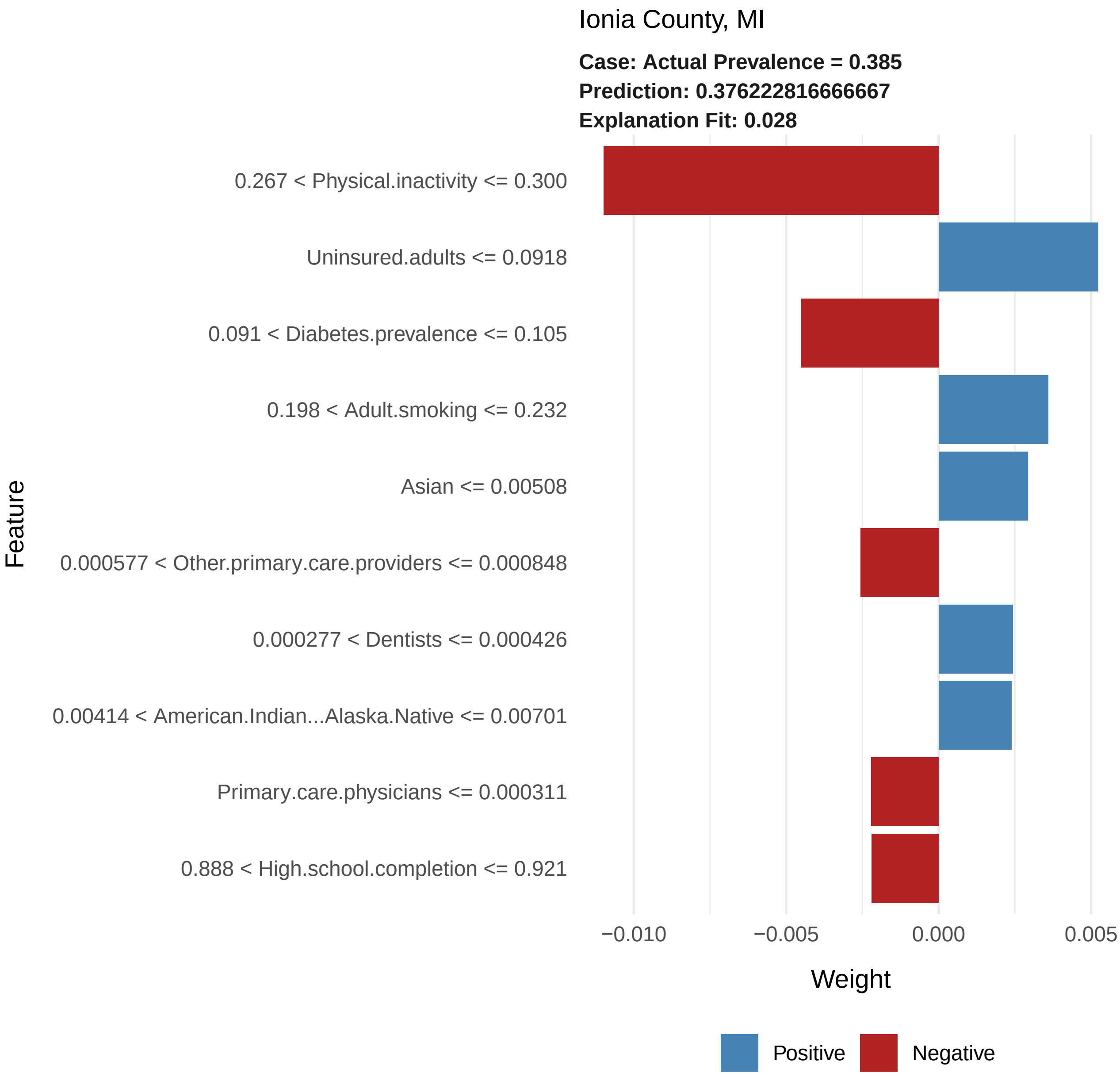

Ionia County, MI
Case: Actual Prevalence = 0.385
Prediction: 0.376222816666667
Explanation Fit: 0.028
0.267 < Physical.inactivity <= 0.300
Uninsured.adults <= 0.0918
0.091 < Diabetes.prevalence <= 0.105
0.198 < Adult.smoking <= 0.232
Asian <= 0.00508
0.000577 < Other.primary.care.providers <= 0.000848
0.000277 < Dentists <= 0.000426
0.00414 < American.Indian...Alaska.Native <= 0.00701
Primary.care.physicians <= 0.000311
0.888 < High.school.completion <= 0.921
Feature
−0.010
−0.005
0.000
0.005
Weight
Positive
Negative

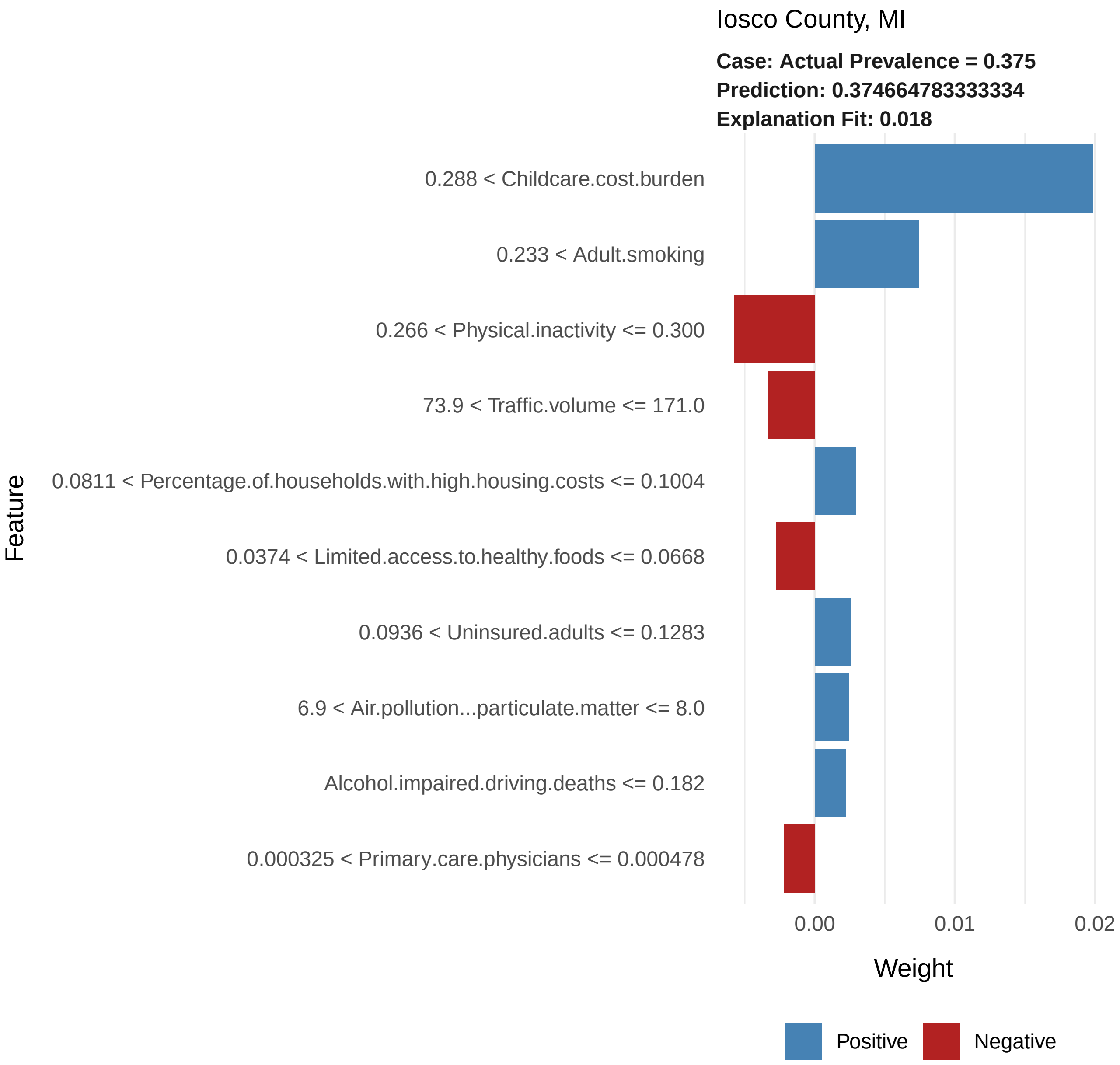
Iosco County, MI
Case: Actual Prevalence = 0.375
Prediction: 0.374664783333334
Explanation Fit: 0.018
0.288 < Childcare.cost.burden
0.233 < Adult.smoking
0.266 < Physical.inactivity <= 0.300
73.9 < Traffic.volume <= 171.0
0.0811 < Percentage.of.households.with.high.housing.costs <= 0.1004
0.0374 < Limited.access.to.healthy.foods <= 0.0668
0.0936 < Uninsured.adults <= 0.1283
6.9 < Air.pollution...particulate.matter <= 8.0
Alcohol.impaired.driving.deaths <= 0.182
0.000325 < Primary.care.physicians <= 0.000478
Feature
0.00
0.01
0.02
Weight
Positive
Negative

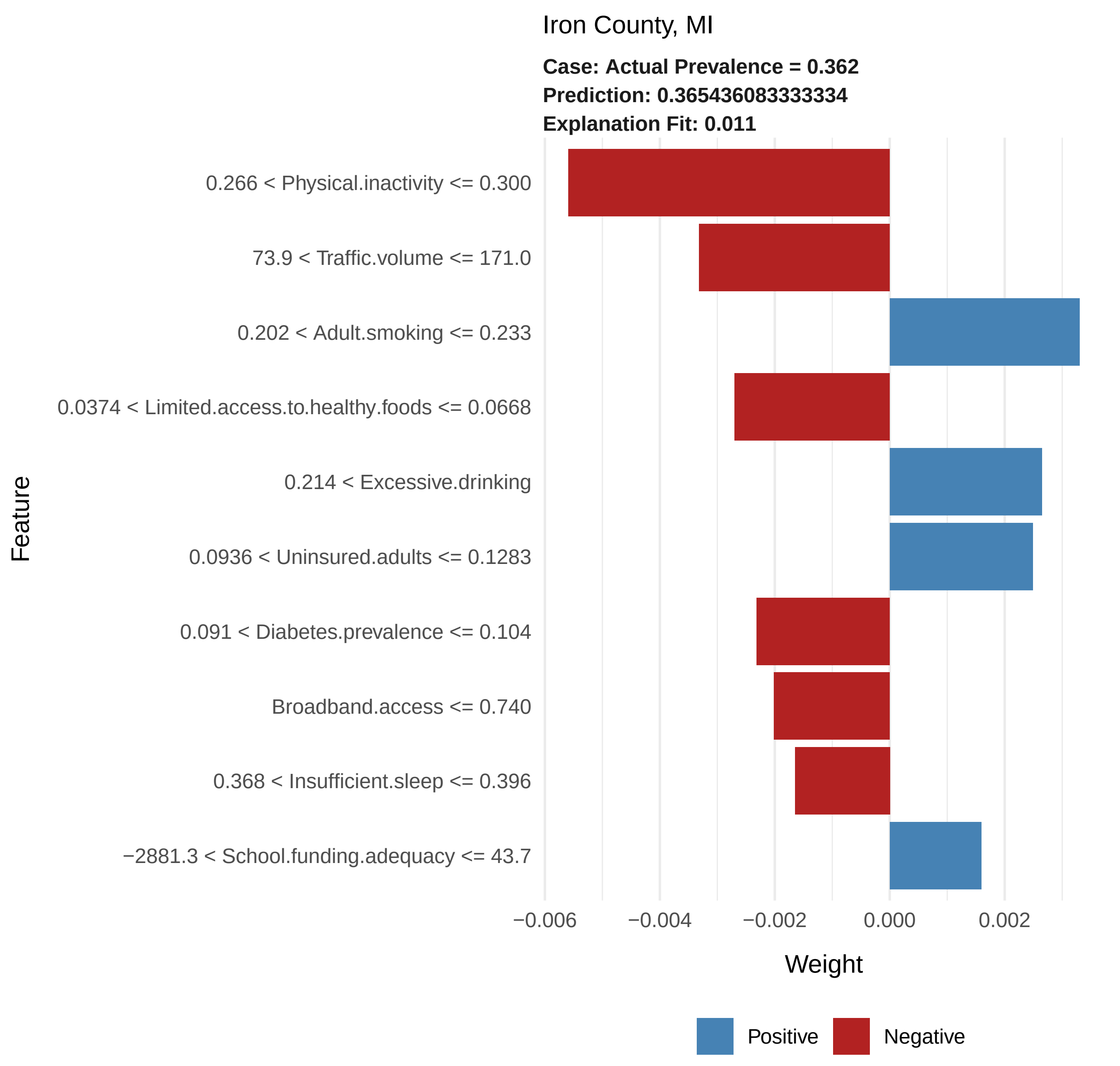

Iron County, MI
Case: Actual Prevalence = 0.362
Prediction: 0.365436083333334
Explanation Fit: 0.011
0.266 < Physical.inactivity <= 0.300
73.9 < Traffic.volume <= 171.0
0.202 < Adult.smoking <= 0.233
0.0374 < Limited.access.to.healthy.foods <= 0.0668
0.214 < Excessive.drinking
0.0936 < Uninsured.adults <= 0.1283
0.091 < Diabetes.prevalence <= 0.104
Broadband.access <= 0.740
0.368 < Insufficient.sleep <= 0.396
−2881.3 < School.funding.adequacy <= 43.7
Feature
−0.006
−0.004
−0.002
0.000
0.002
Weight
Positive
Negative

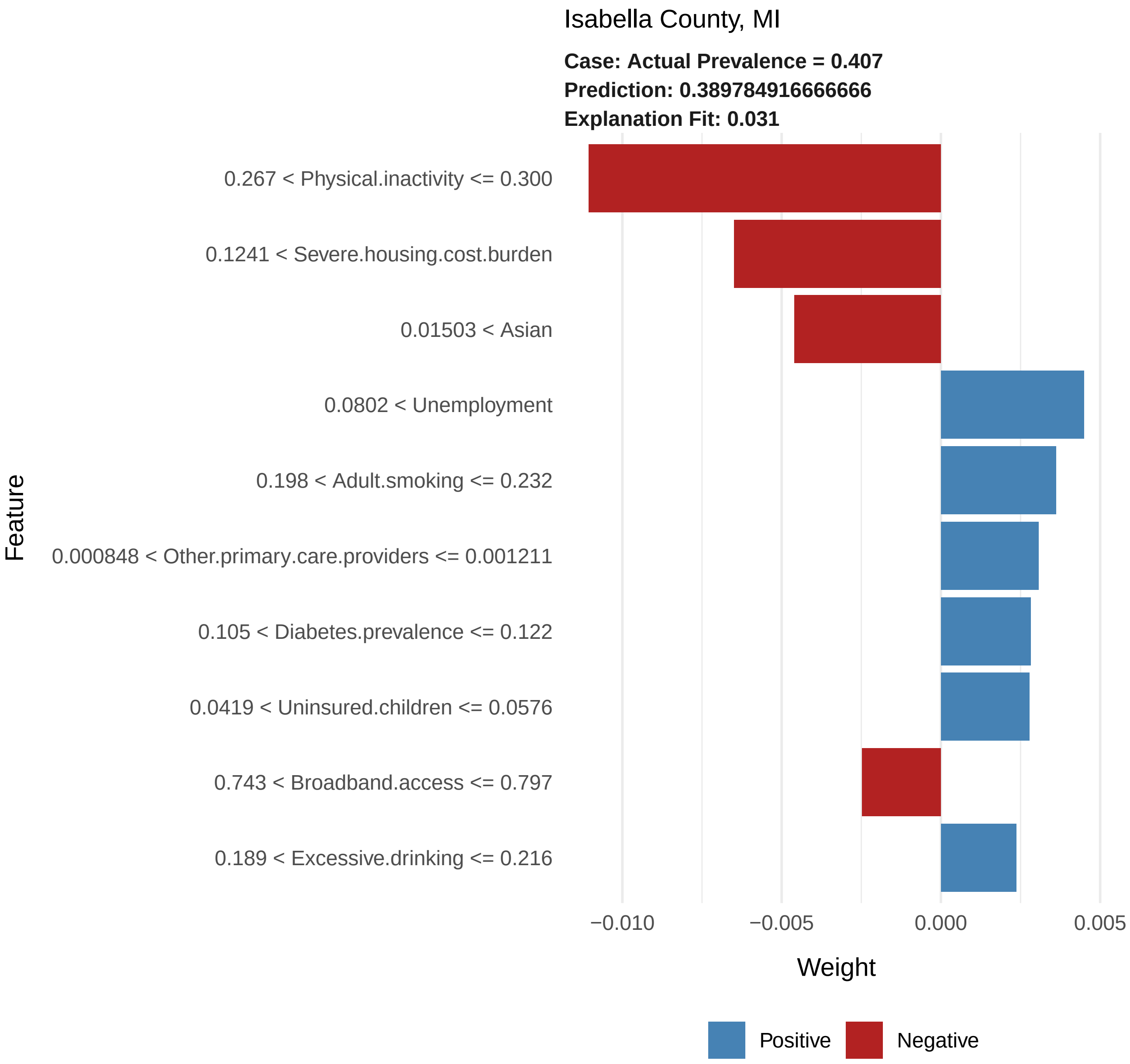

Isabella County, MI
Case: Actual Prevalence = 0.407
Prediction: 0.389784916666666
Explanation Fit: 0.031
Feature
0.267 < Physical.inactivity <= 0.300
0.1241 < Severe.housing.cost.burden
0.01503 < Asian
0.0802 < Unemployment
0.198 < Adult.smoking <= 0.232
0.000848 < Other.primary.care.providers <= 0.001211
0.105 < Diabetes.prevalence <= 0.122
0.0419 < Uninsured.children <= 0.0576
0.743 < Broadband.access <= 0.797
0.189 < Excessive.drinking <= 0.216
−0.010
−0.005
0.000
0.005
Weight
Positive
Negative

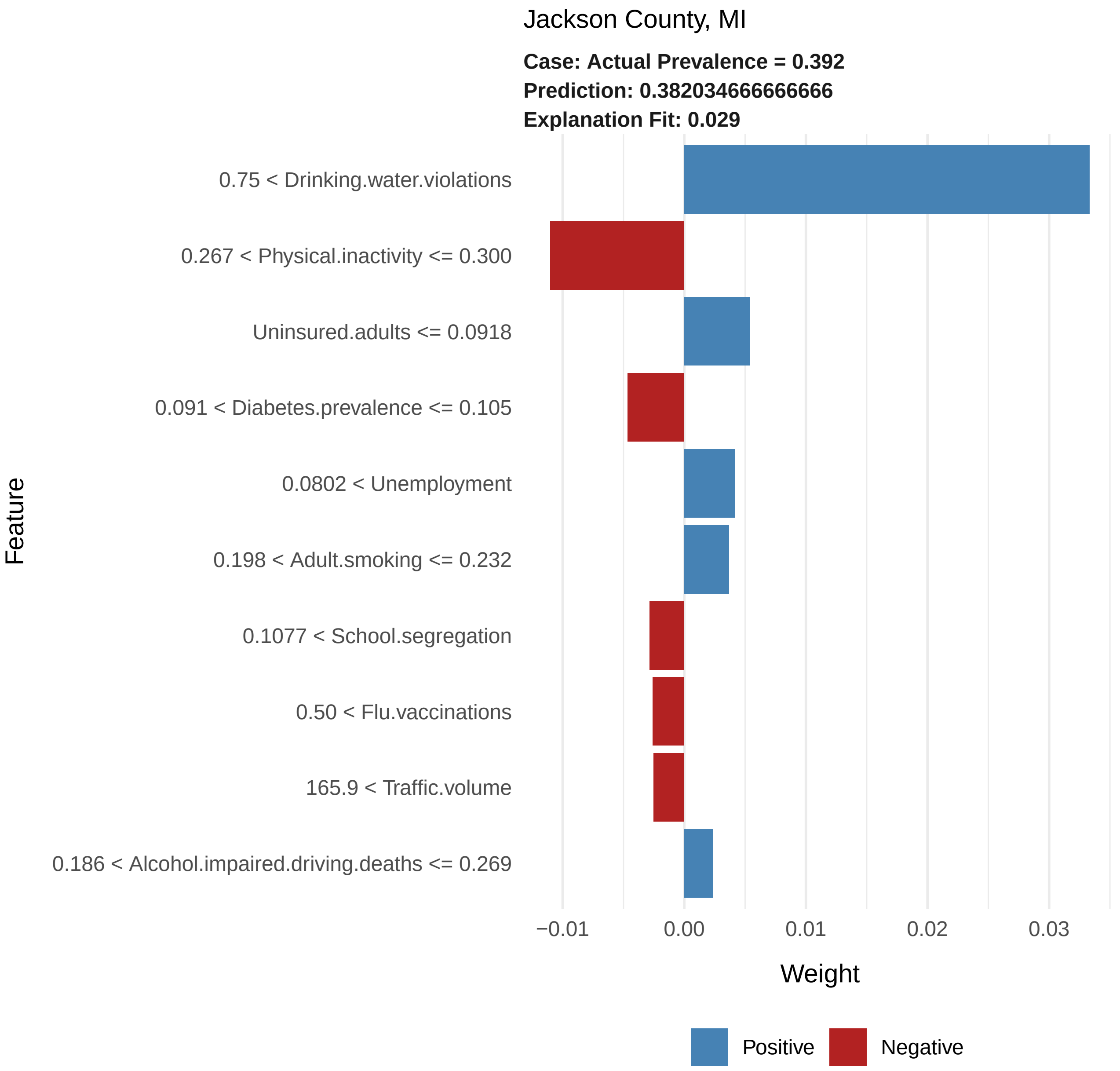
Jackson County, MI
Case: Actual Prevalence = 0.392
Prediction: 0.382034666666666
Explanation Fit: 0.029
0.75 < Drinking.water.violations
0.267 < Physical.inactivity <= 0.300
Uninsured.adults <= 0.0918
0.091 < Diabetes.prevalence <= 0.105
0.0802 < Unemployment
0.198 < Adult.smoking <= 0.232
0.1077 < School.segregation
0.50 < Flu.vaccinations
165.9 < Traffic.volume
0.186 < Alcohol.impaired.driving.deaths <= 0.269
Feature
−0.01
0.00
0.01
0.02
0.03
Weight
Positive
Negative

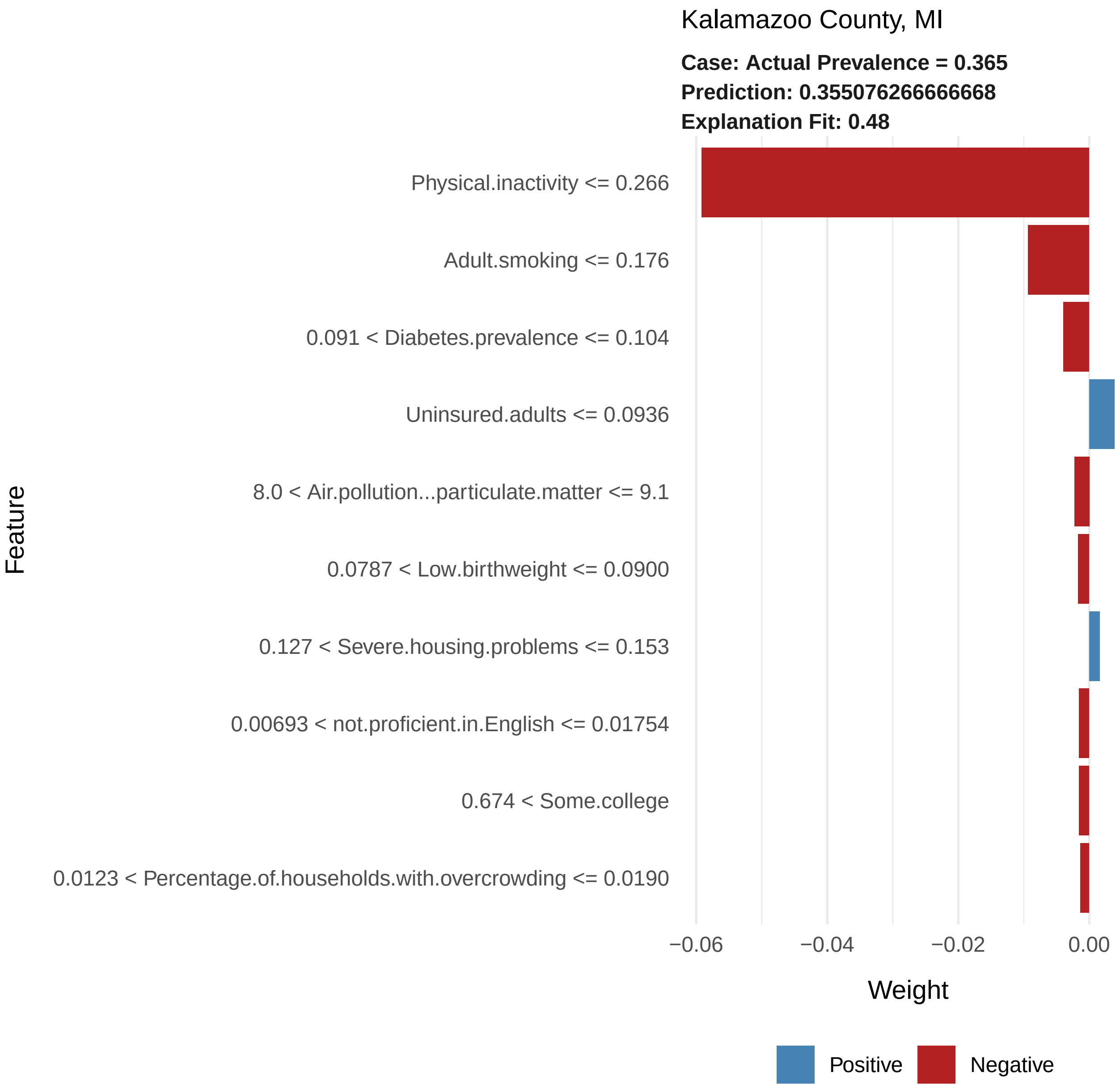
Kalamazoo County, MI
Case: Actual Prevalence = 0.365
Prediction: 0.355076266666668
Explanation Fit: 0.48
Physical.inactivity <= 0.266
Adult.smoking <= 0.176
0.091 < Diabetes.prevalence <= 0.104
Uninsured.adults <= 0.0936
8.0 < Air.pollution...particulate.matter <= 9.1
0.0787 < Low.birthweight <= 0.0900
0.127 < Severe.housing.problems <= 0.153
0.00693 < not.proficient.in.English <= 0.01754
0.674 < Some.college
0.0123 < Percentage.of.households.with.overcrowding <= 0.0190
Feature
−0.06
−0.04
−0.02
0.00
Weight
Positive
Negative

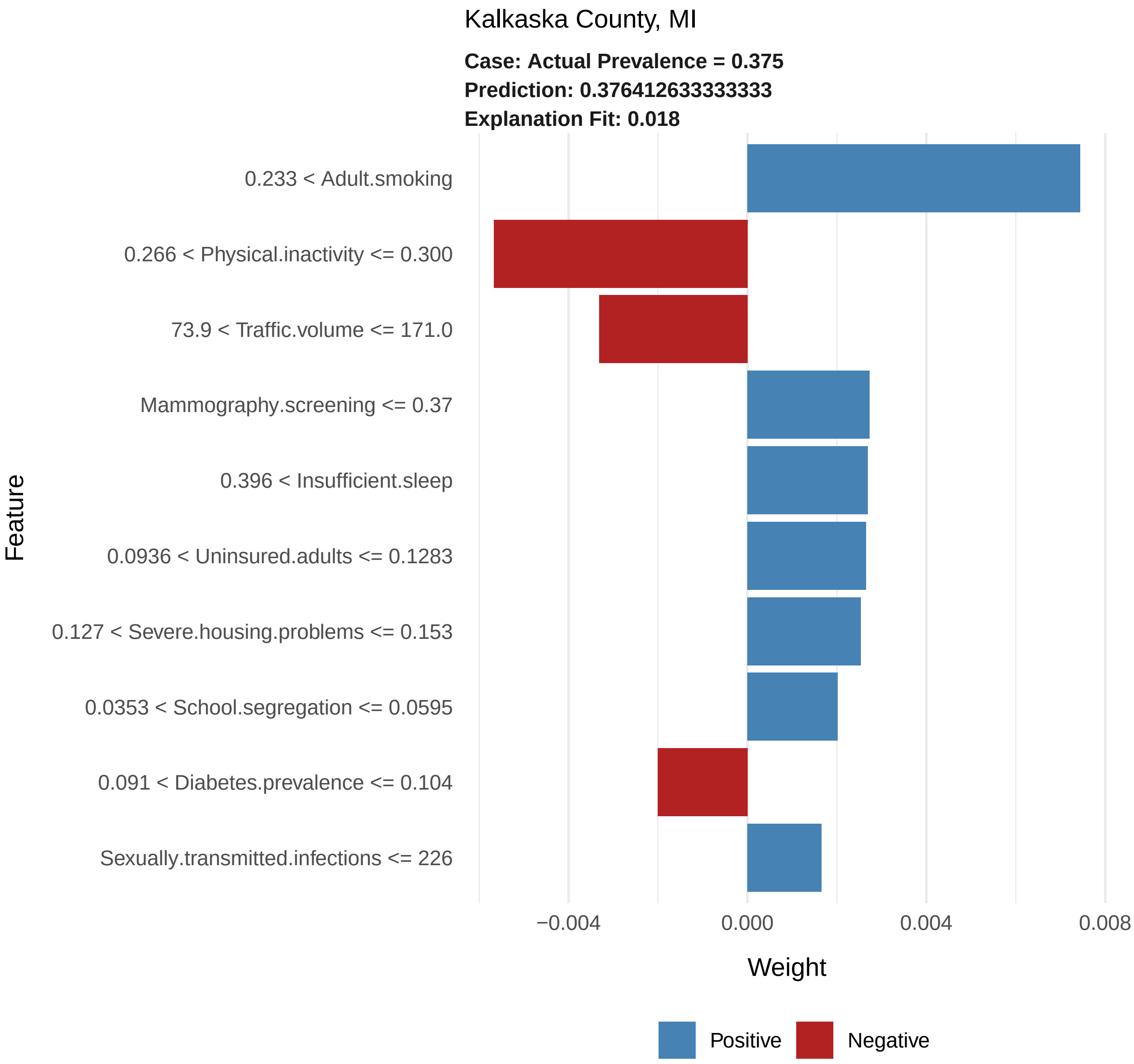

Kalkaska County, MI
Case: Actual Prevalence = 0.375
Prediction: 0.376412633333333
Explanation Fit: 0.018
0.233 < Adult.smoking
0.266 < Physical.inactivity <= 0.300
73.9 < Traffic.volume <= 171.0
Mammography.screening <= 0.37
0.396 < Insufficient.sleep
0.0936 < Uninsured.adults <= 0.1283
0.127 < Severe.housing.problems <= 0.153
0.0353 < School.segregation <= 0.0595
0.091 < Diabetes.prevalence <= 0.104
Sexually.transmitted.infections <= 226
Feature
−0.004
0.000
0.004
0.008
Weight
Positive
Negative

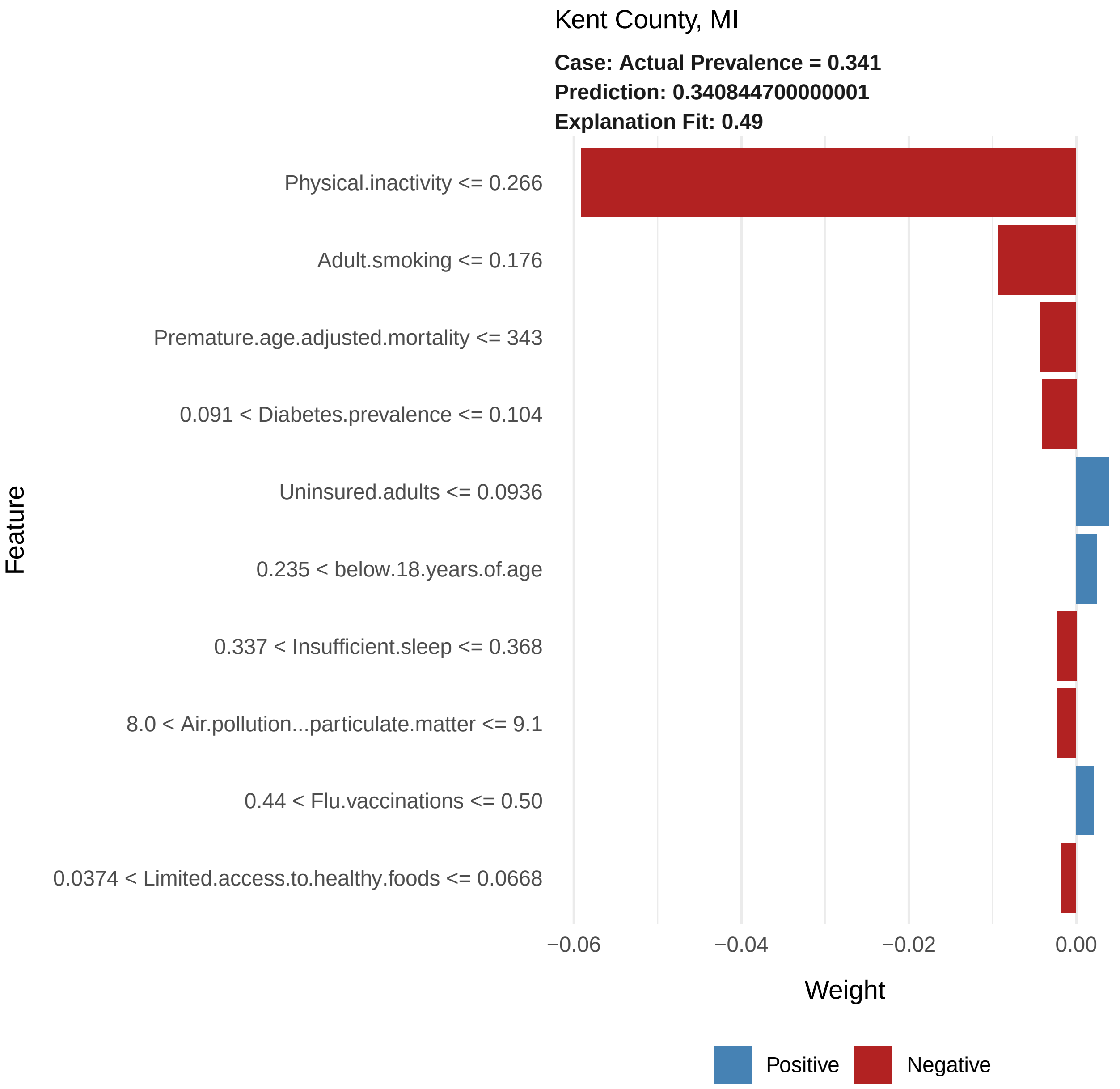

Kent County, MI
Case: Actual Prevalence = 0.341
Prediction: 0.340844700000001
Explanation Fit: 0.49
Physical.inactivity <= 0.266
Adult.smoking <= 0.176
Premature.age.adjusted.mortality <= 343
0.091 < Diabetes.prevalence <= 0.104
Uninsured.adults <= 0.0936
0.235 < below.18.years.of.age
0.337 < Insufficient.sleep <= 0.368
8.0 < Air.pollution...particulate.matter <= 9.1
0.44 < Flu.vaccinations <= 0.50
0.0374 < Limited.access.to.healthy.foods <= 0.0668
Feature
−0.06
−0.04
−0.02
0.00
Weight
Positive
Negative

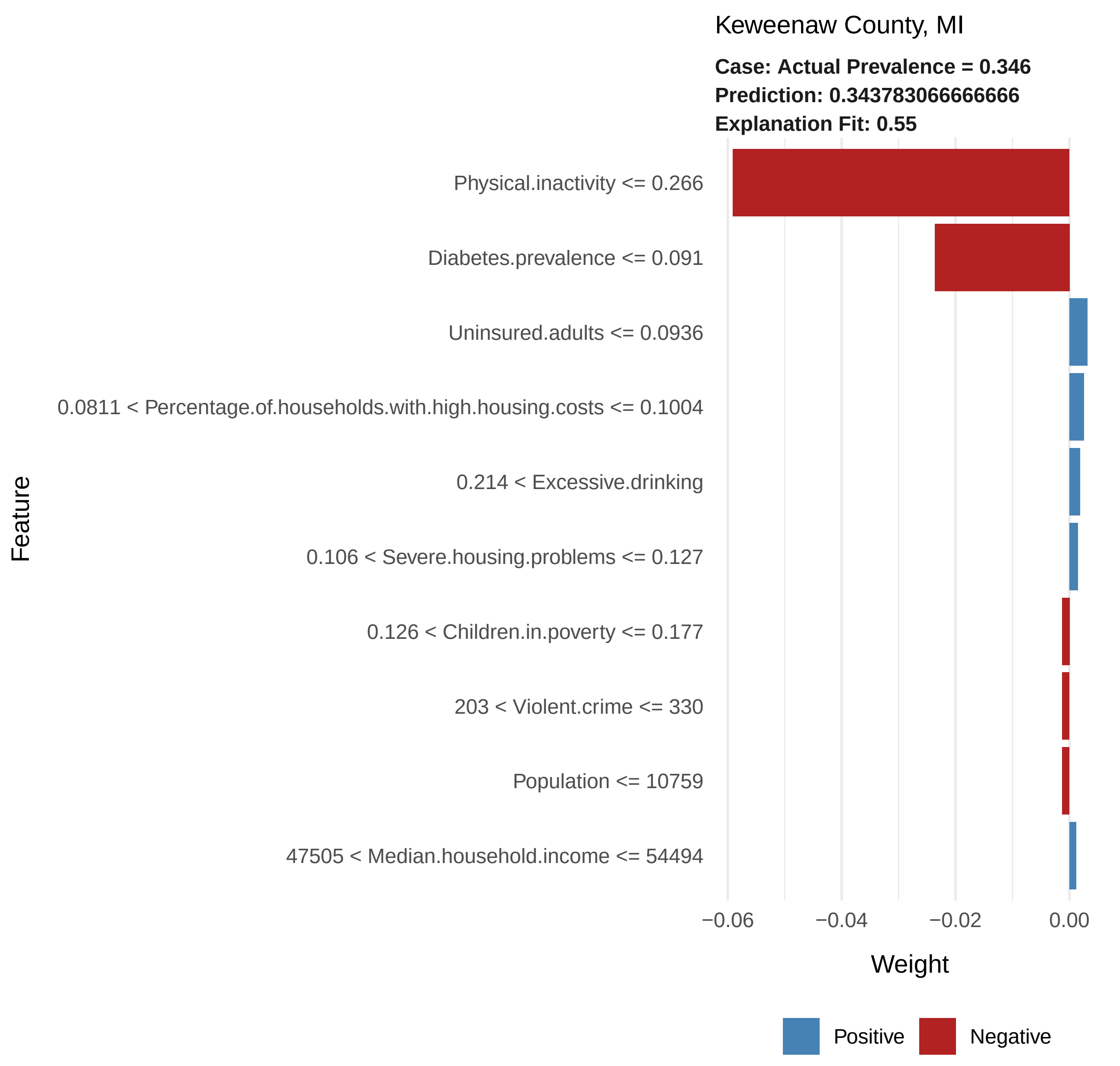
Keweenaw County, MI
Case: Actual Prevalence = 0.346
Prediction: 0.343783066666666
Explanation Fit: 0.55
Physical.inactivity <= 0.266
Diabetes.prevalence <= 0.091
Uninsured.adults <= 0.0936
0.0811 < Percentage.of.households.with.high.housing.costs <= 0.1004
0.214 < Excessive.drinking
0.106 < Severe.housing.problems <= 0.127
0.126 < Children.in.poverty <= 0.177
203 < Violent.crime <= 330
Population <= 10759
47505 < Median.household.income <= 54494
Feature
−0.06
−0.04
−0.02
0.00
Weight
Positive
Negative

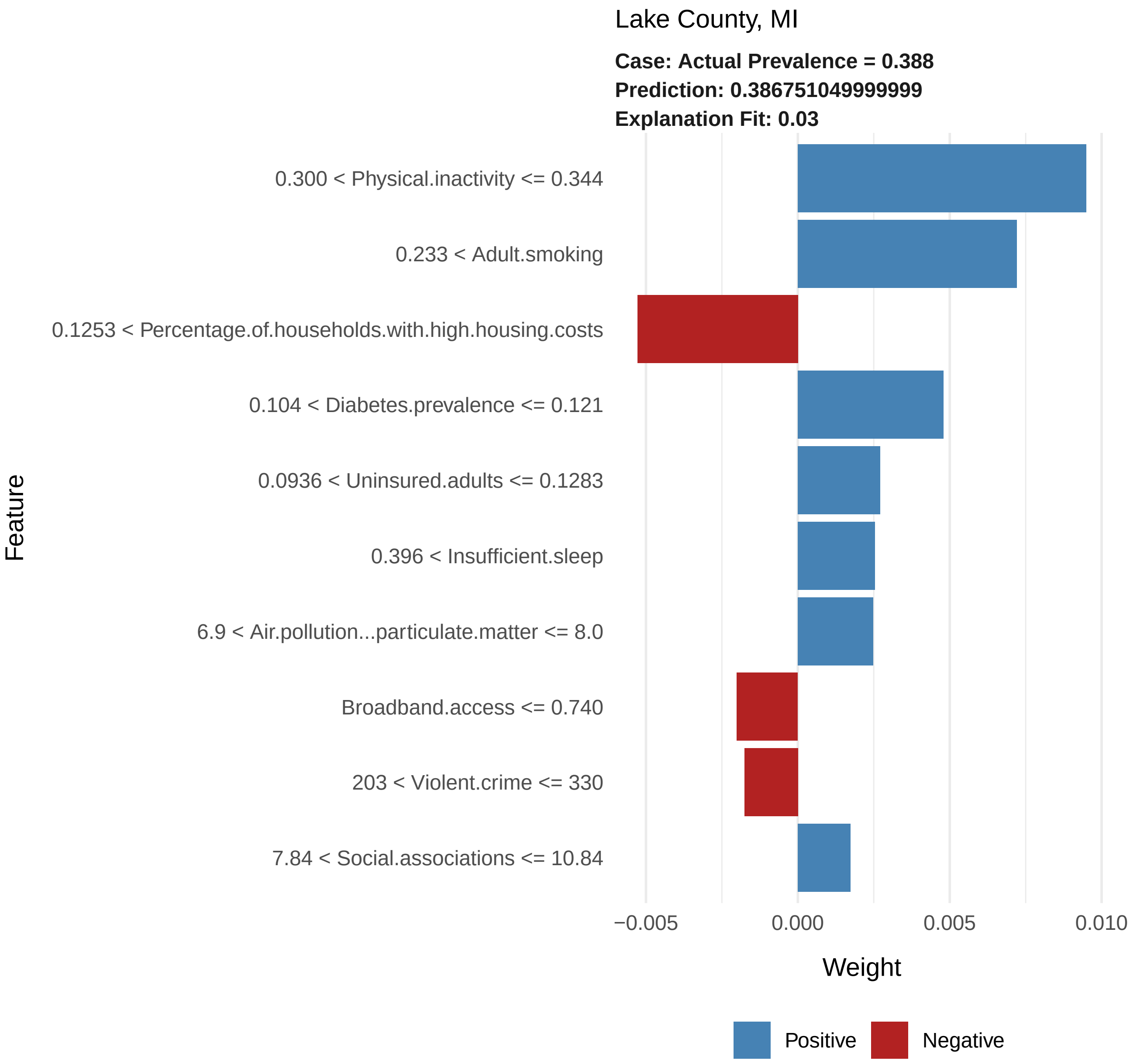

Lake County, MI
Case: Actual Prevalence = 0.388
Prediction: 0.386751049999999
Explanation Fit: 0.03
0.300 < Physical.inactivity <= 0.344
0.233 < Adult.smoking
0.1253 < Percentage.of.households.with.high.housing.costs
0.104 < Diabetes.prevalence <= 0.121
0.0936 < Uninsured.adults <= 0.1283
0.396 < Insufficient.sleep
6.9 < Air.pollution...particulate.matter <= 8.0
Broadband.access <= 0.740
203 < Violent.crime <= 330
7.84 < Social.associations <= 10.84
Feature
−0.005
0.000
0.005
0.010
Weight
Positive
Negative

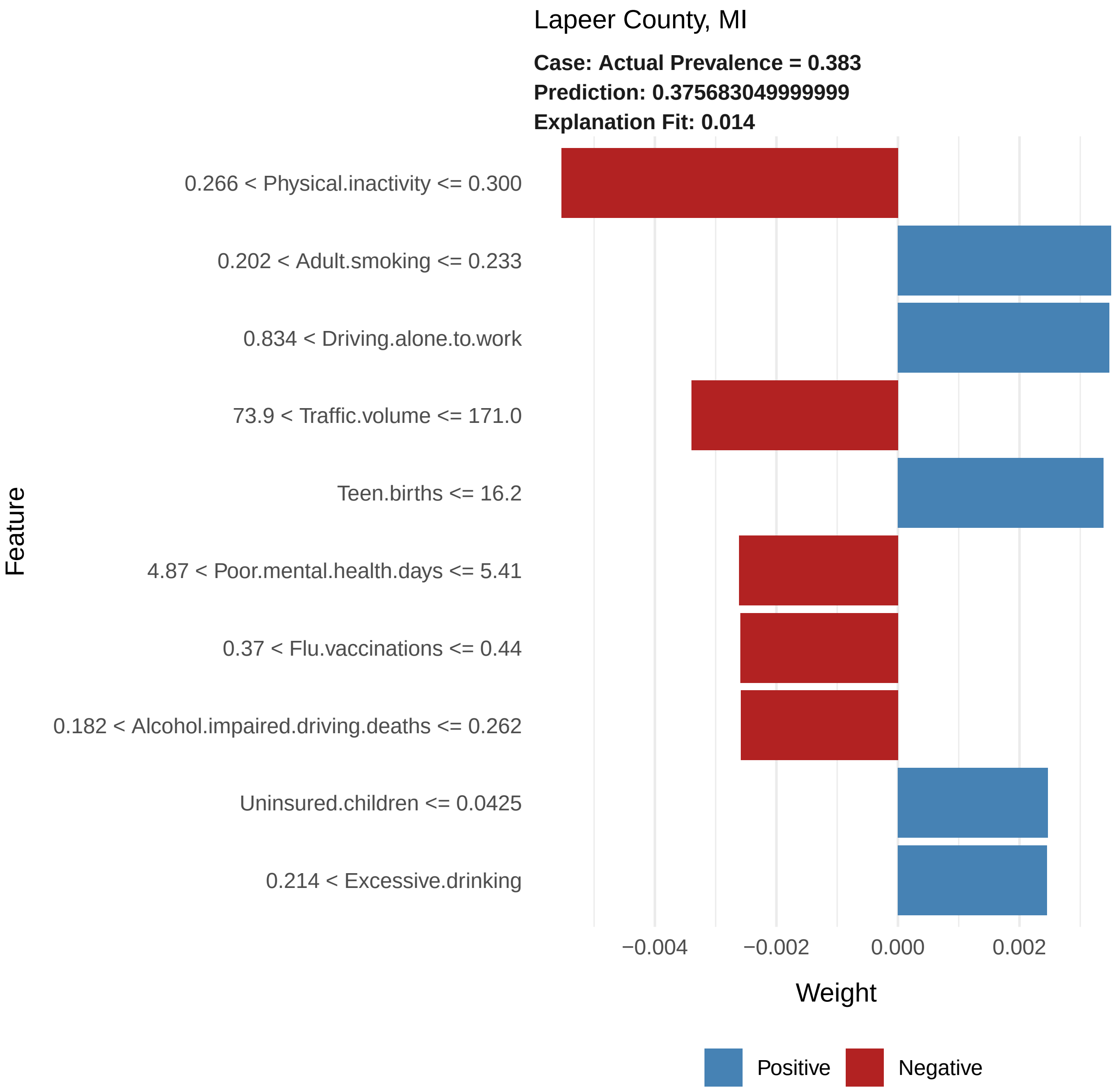
Lapeer County, MI
Case: Actual Prevalence = 0.383
Prediction: 0.375683049999999
Explanation Fit: 0.014
0.266 < Physical.inactivity <= 0.300
0.202 < Adult.smoking <= 0.233
0.834 < Driving.alone.to.work
73.9 < Traffic.volume <= 171.0
Teen.births <= 16.2
4.87 < Poor.mental.health.days <= 5.41
0.37 < Flu.vaccinations <= 0.44
0.182 < Alcohol.impaired.driving.deaths <= 0.262
Uninsured.children <= 0.0425
0.214 < Excessive.drinking
Feature
−0.004
−0.002
0.000
0.002
Weight
Positive
Negative

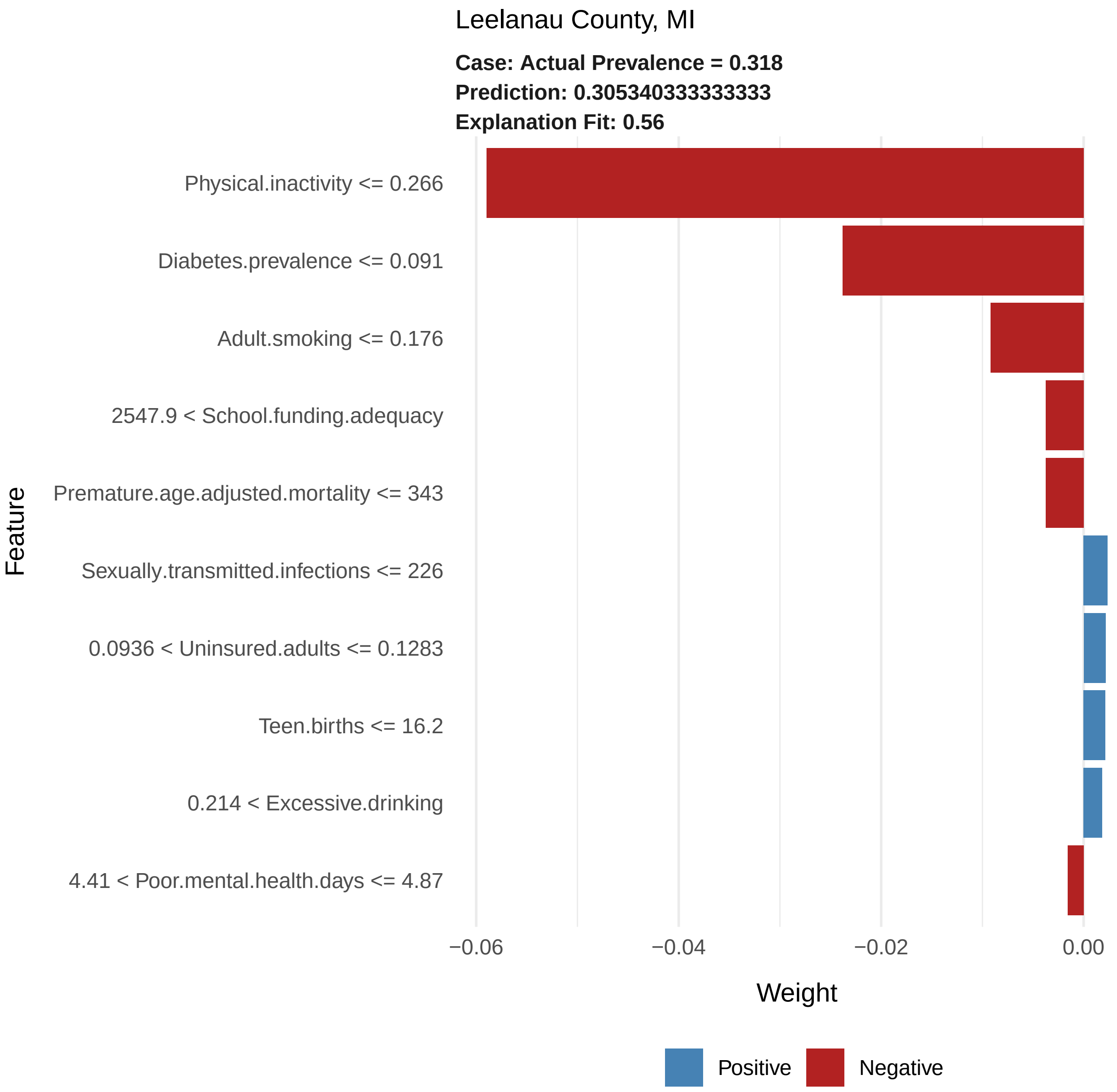

Leelanau County, MI
Case: Actual Prevalence = 0.318
Prediction: 0.305340333333333
Explanation Fit: 0.56
Physical.inactivity <= 0.266
Diabetes.prevalence <= 0.091
Adult.smoking <= 0.176
2547.9 < School.funding.adequacy
Premature.age.adjusted.mortality <= 343
Sexually.transmitted.infections <= 226
0.0936 < Uninsured.adults <= 0.1283
Teen.births <= 16.2
0.214 < Excessive.drinking
4.41 < Poor.mental.health.days <= 4.87
Feature
−0.06
−0.04
−0.02
0.00
Weight
Positive
Negative

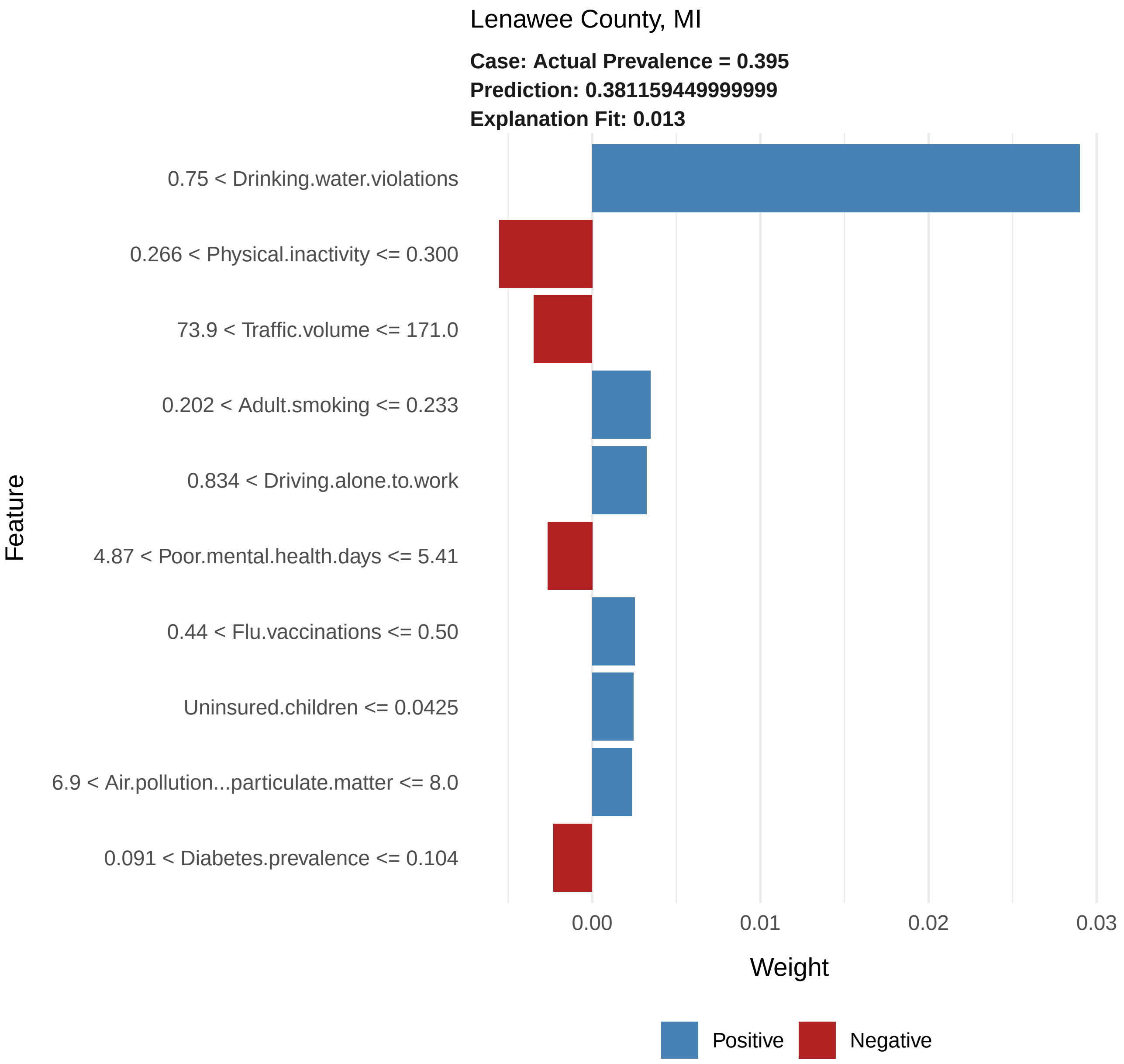
Lenawee County, MI
Case: Actual Prevalence = 0.395
Prediction: 0.381159449999999
Explanation Fit: 0.013
0.75 < Drinking.water.violations
0.266 < Physical.inactivity <= 0.300
73.9 < Traffic.volume <= 171.0
0.202 < Adult.smoking <= 0.233
0.834 < Driving.alone.to.work
4.87 < Poor.mental.health.days <= 5.41
0.44 < Flu.vaccinations <= 0.50
Uninsured.children <= 0.0425
6.9 < Air.pollution...particulate.matter <= 8.0
0.091 < Diabetes.prevalence <= 0.104
Feature
0.00
0.01
0.02
0.03
Weight
Positive
Negative

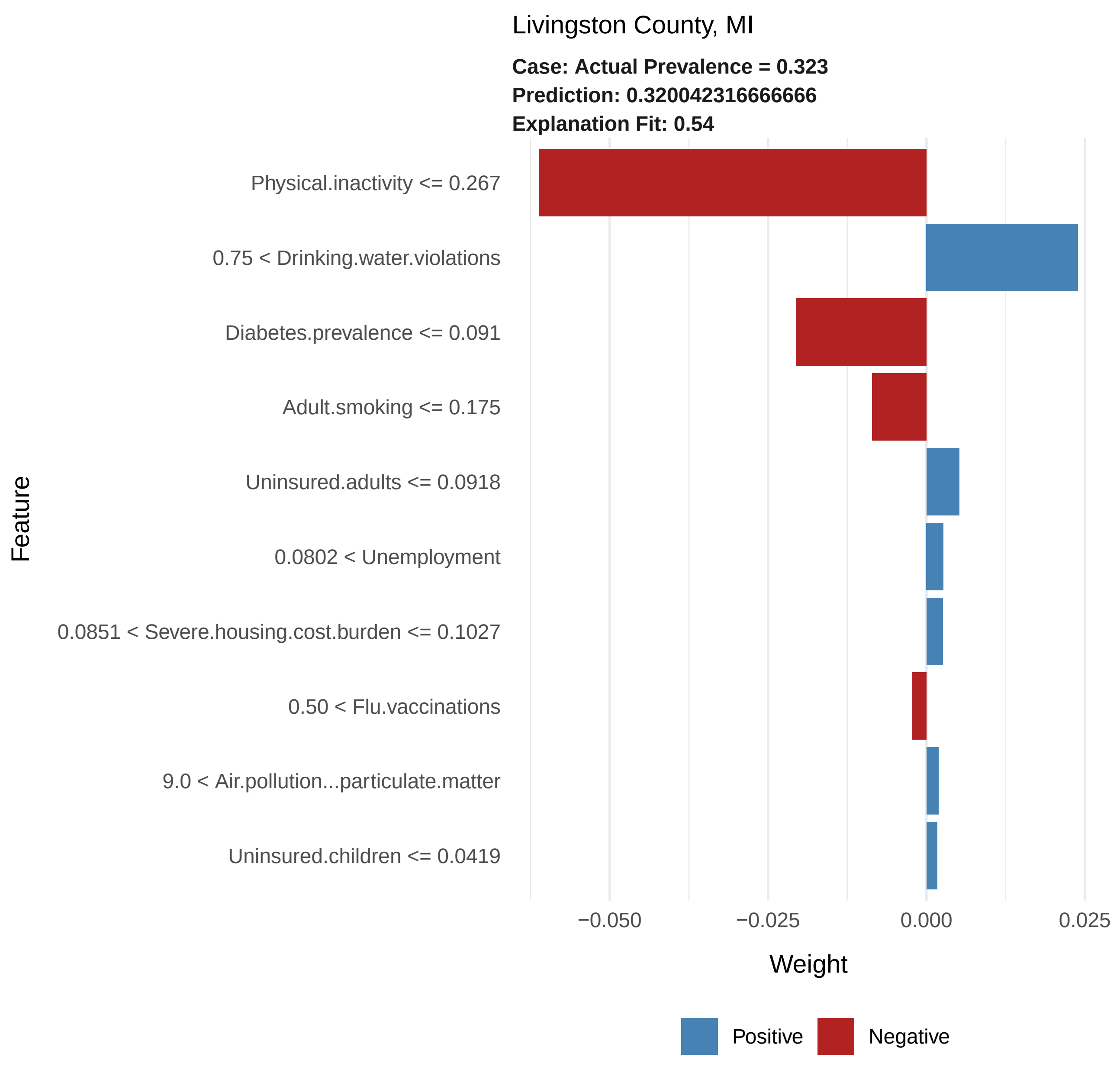

Livingston County, MI
Case: Actual Prevalence = 0.323
Prediction: 0.320042316666666
Explanation Fit: 0.54
Physical.inactivity <= 0.267
0.75 < Drinking.water.violations
Diabetes.prevalence <= 0.091
Adult.smoking <= 0.175
Uninsured.adults <= 0.0918
0.0802 < Unemployment
0.0851 < Severe.housing.cost.burden <= 0.1027
0.50 < Flu.vaccinations
9.0 < Air.pollution...particulate.matter
Uninsured.children <= 0.0419
Feature
−0.050
−0.025
0.000
0.025
Weight
Positive
Negative

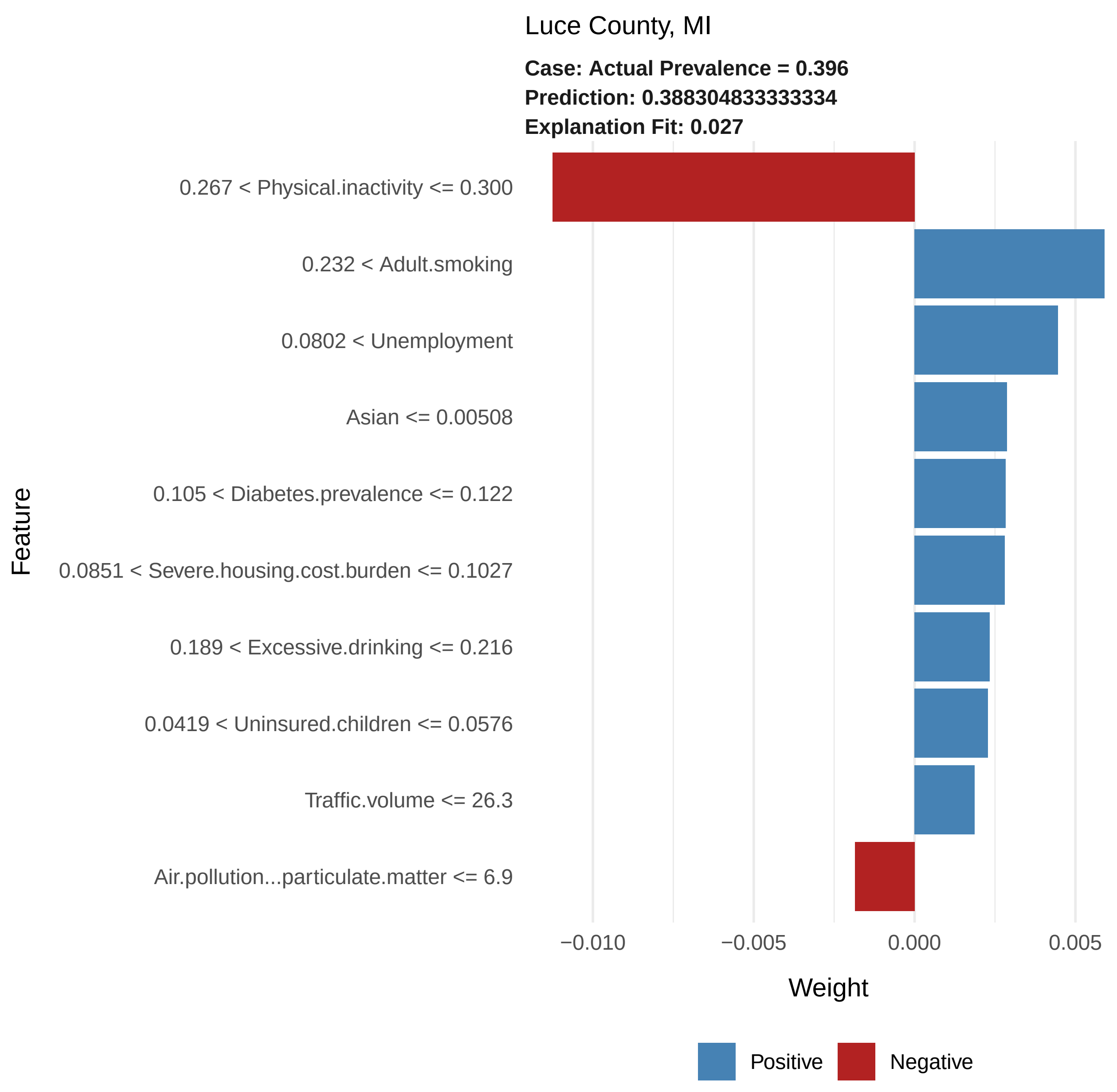
Luce County, MI
Case: Actual Prevalence = 0.396
Prediction: 0.388304833333334
Explanation Fit: 0.027
0.267 < Physical.inactivity <= 0.300
0.232 < Adult.smoking
0.0802 < Unemployment
Asian <= 0.00508
0.105 < Diabetes.prevalence <= 0.122
0.0851 < Severe.housing.cost.burden <= 0.1027
0.189 < Excessive.drinking <= 0.216
0.0419 < Uninsured.children <= 0.0576
Traffic.volume <= 26.3
Air.pollution...particulate.matter <= 6.9
Feature
−0.010
−0.005
0.000
0.005
Weight
Positive
Negative

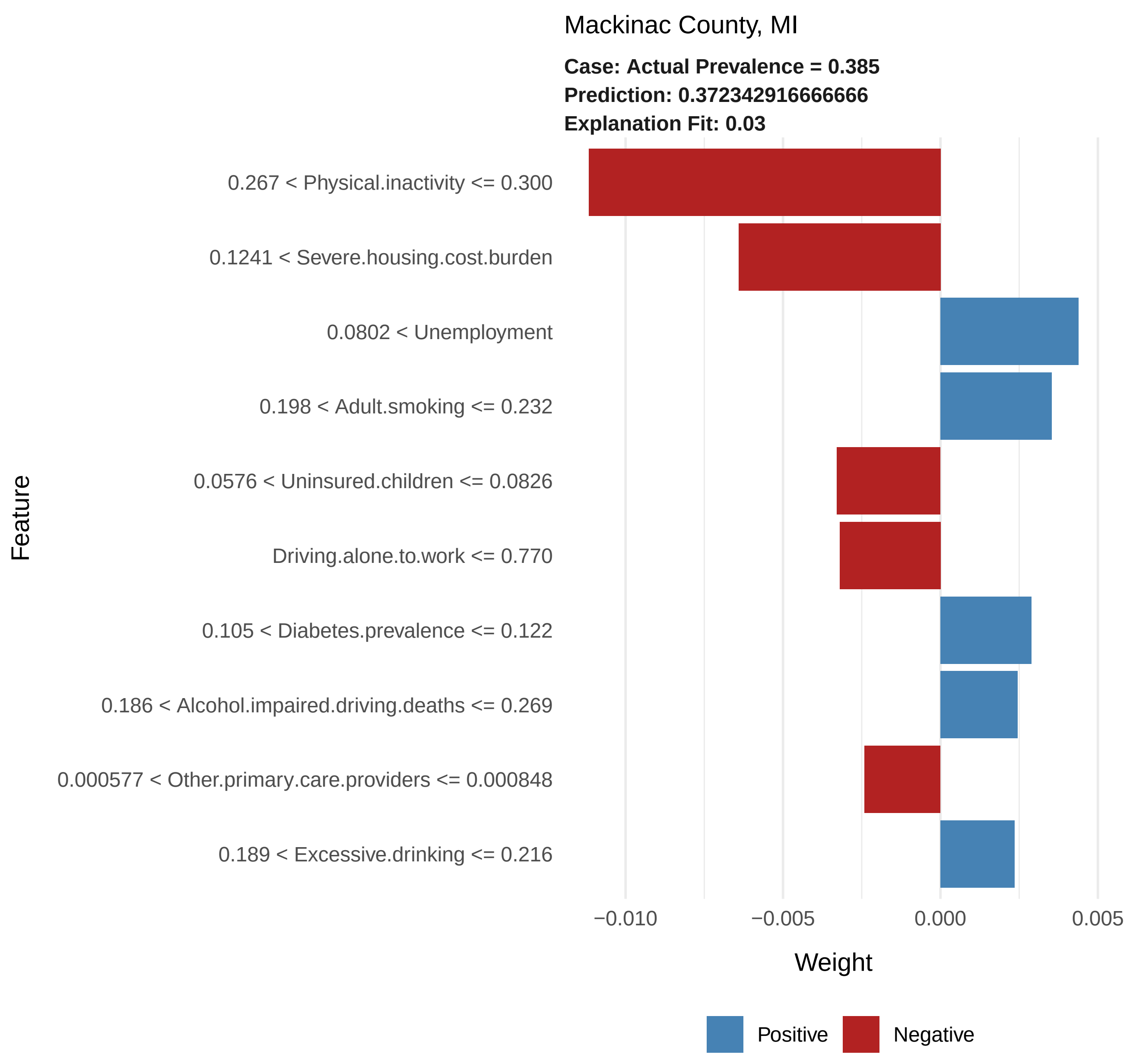
Mackinac County, MI
Case: Actual Prevalence = 0.385
Prediction: 0.372342916666666
Explanation Fit: 0.03
0.267 < Physical.inactivity <= 0.300
0.1241 < Severe.housing.cost.burden
0.0802 < Unemployment
0.198 < Adult.smoking <= 0.232
0.0576 < Uninsured.children <= 0.0826
Driving.alone.to.work <= 0.770
0.105 < Diabetes.prevalence <= 0.122
0.186 < Alcohol.impaired.driving.deaths <= 0.269
0.000577 < Other.primary.care.providers <= 0.000848
0.189 < Excessive.drinking <= 0.216
Feature
−0.010
−0.005
0.000
0.005
Weight
Positive
Negative

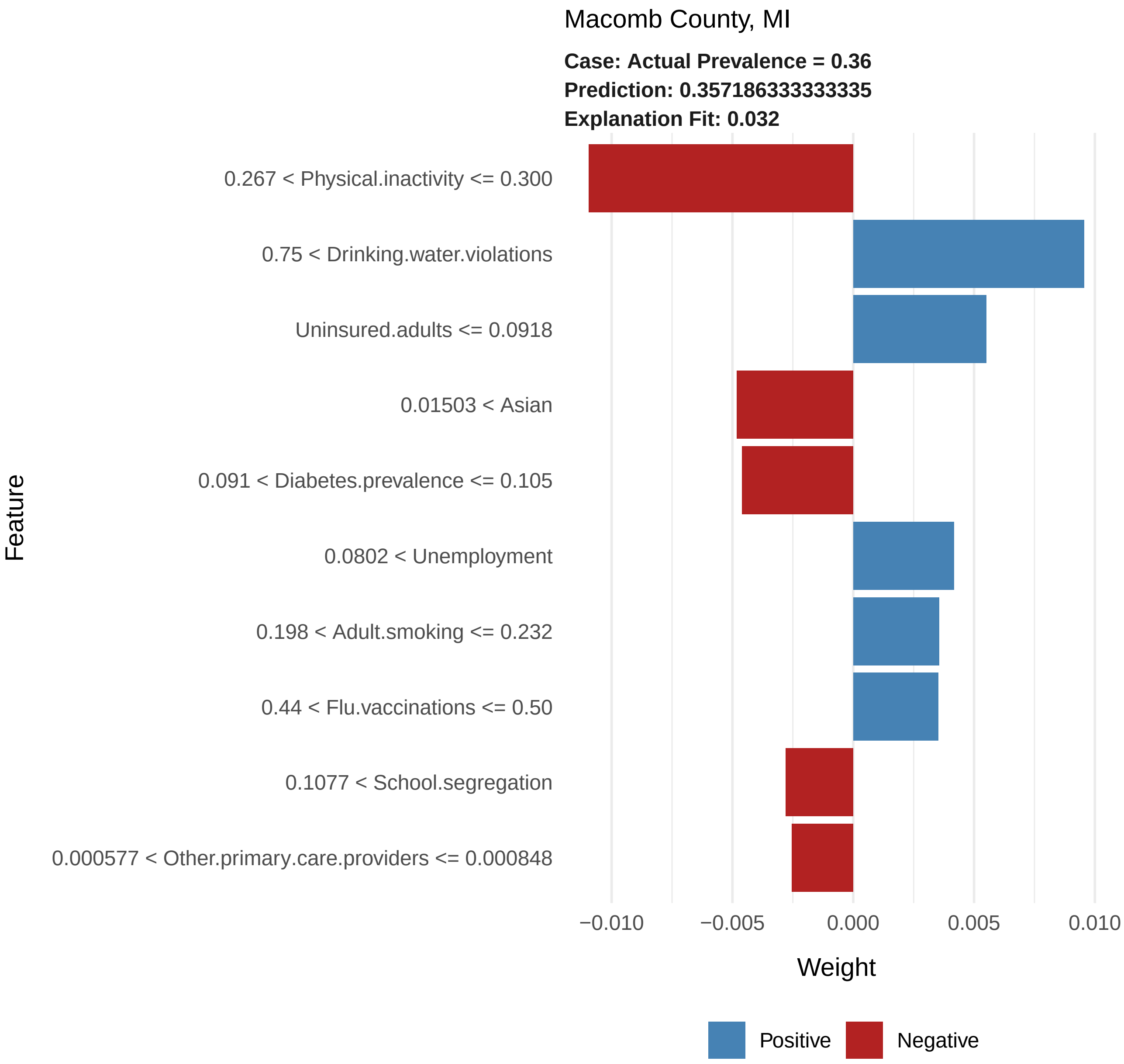

Macomb County, MI
Case: Actual Prevalence = 0.36
Prediction: 0.357186333333335
Explanation Fit: 0.032
0.267 < Physical.inactivity <= 0.300
0.75 < Drinking.water.violations
Uninsured.adults <= 0.0918
0.01503 < Asian
0.091 < Diabetes.prevalence <= 0.105
0.0802 < Unemployment
0.198 < Adult.smoking <= 0.232
0.44 < Flu.vaccinations <= 0.50
0.1077 < School.segregation
0.000577 < Other.primary.care.providers <= 0.000848
Feature
−0.010
−0.005
0.000
0.005
0.010
Weight
Positive
Negative

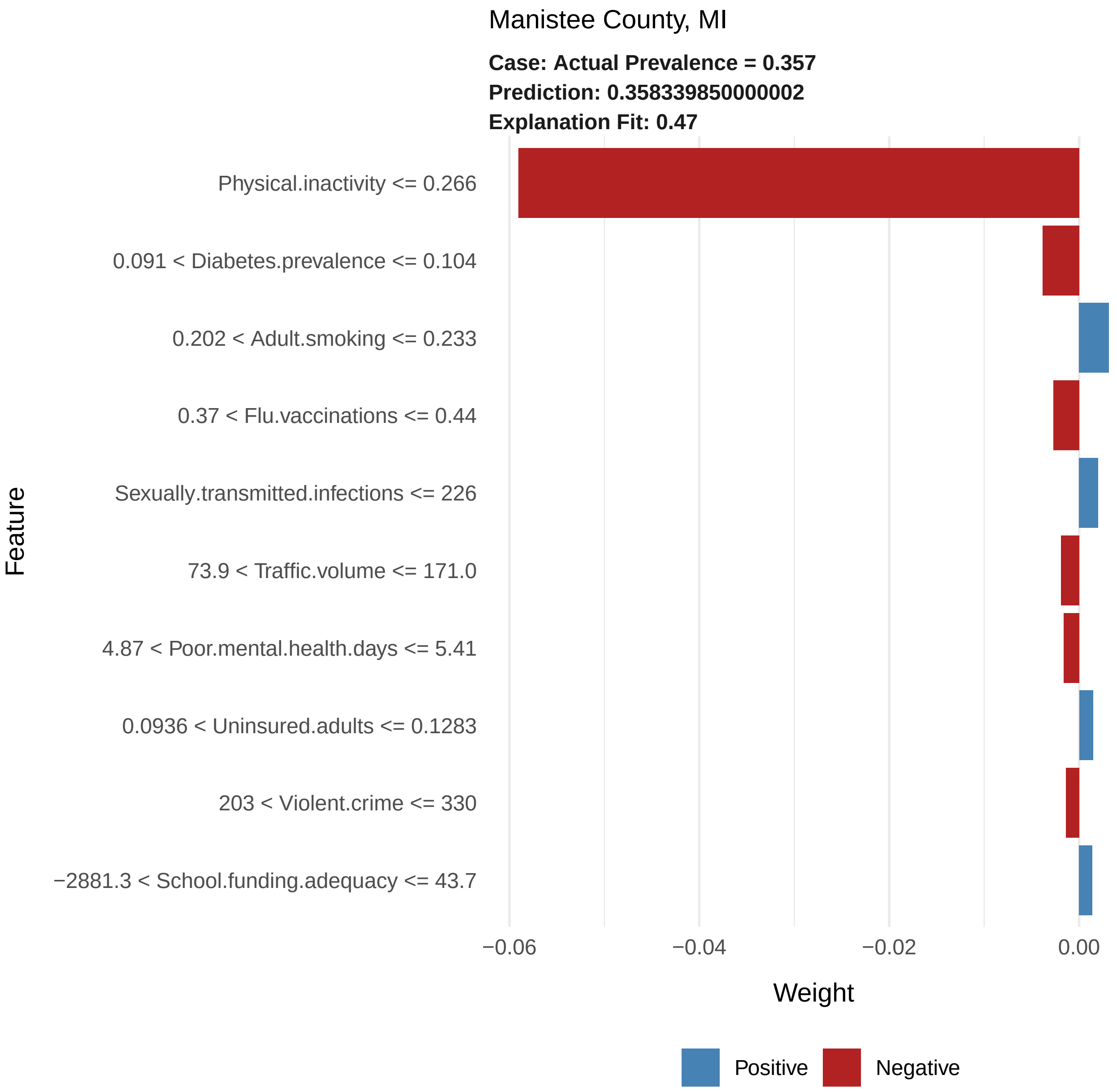

Manistee County, MI
Case: Actual Prevalence = 0.357
Prediction: 0.358339850000002
Explanation Fit: 0.47
Physical.inactivity <= 0.266
0.091 < Diabetes.prevalence <= 0.104
0.202 < Adult.smoking <= 0.233
0.37 < Flu.vaccinations <= 0.44
Sexually.transmitted.infections <= 226
73.9 < Traffic.volume <= 171.0
4.87 < Poor.mental.health.days <= 5.41
0.0936 < Uninsured.adults <= 0.1283
203 < Violent.crime <= 330
−2881.3 < School.funding.adequacy <= 43.7
Feature
−0.06
−0.04
−0.02
0.00
Weight
Positive
Negative

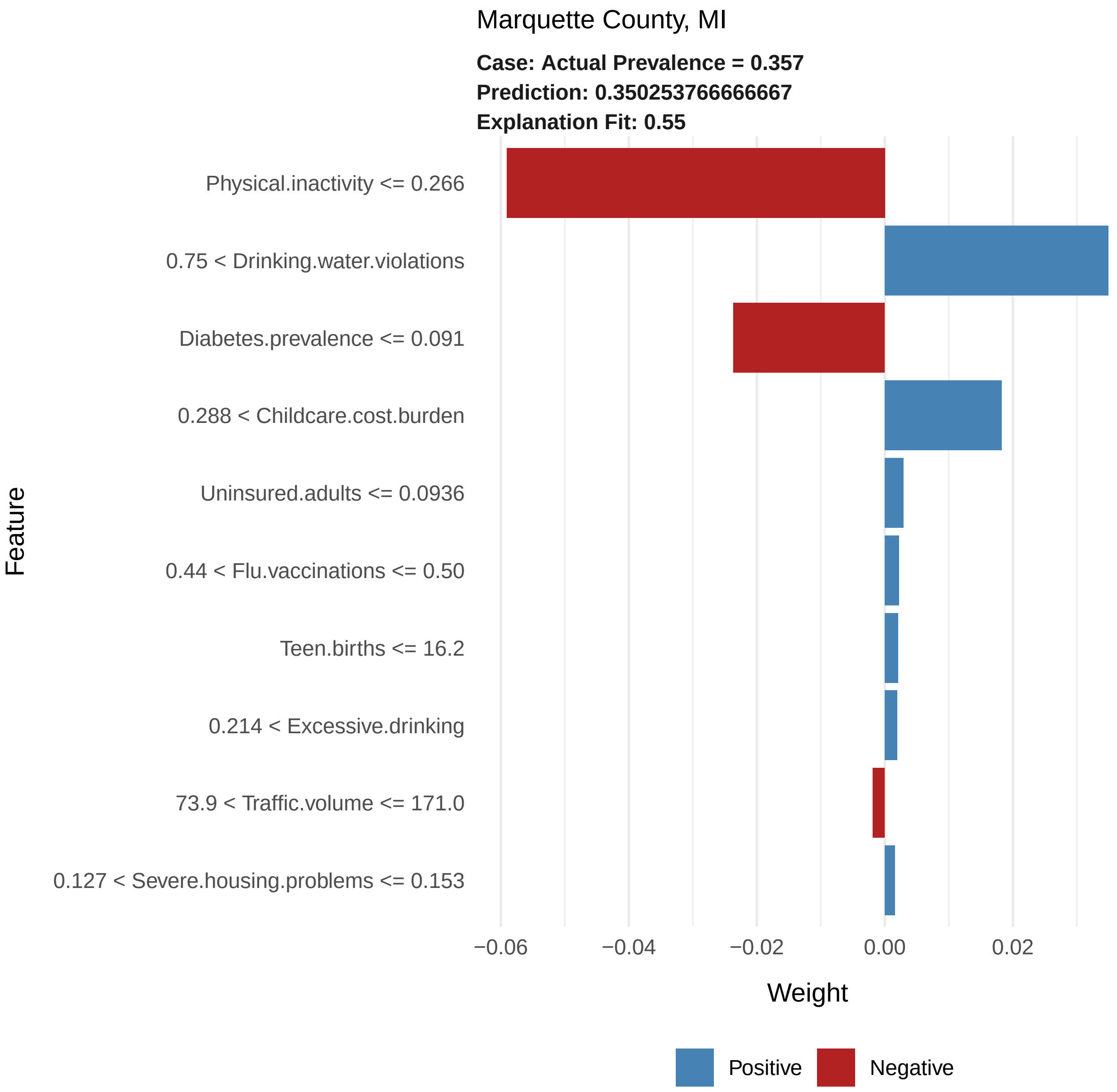

Marquette County, MI
Case: Actual Prevalence = 0.357
Prediction: 0.350253766666667
Explanation Fit: 0.55
Physical.inactivity <= 0.266
0.75 < Drinking.water.violations
Diabetes.prevalence <= 0.091
0.288 < Childcare.cost.burden
Uninsured.adults <= 0.0936
0.44 < Flu.vaccinations <= 0.50
Teen.births <= 16.2
0.214 < Excessive.drinking
73.9 < Traffic.volume <= 171.0
0.127 < Severe.housing.problems <= 0.153
Feature
−0.06
−0.04
−0.02
0.00
0.02
Weight
Positive
Negative

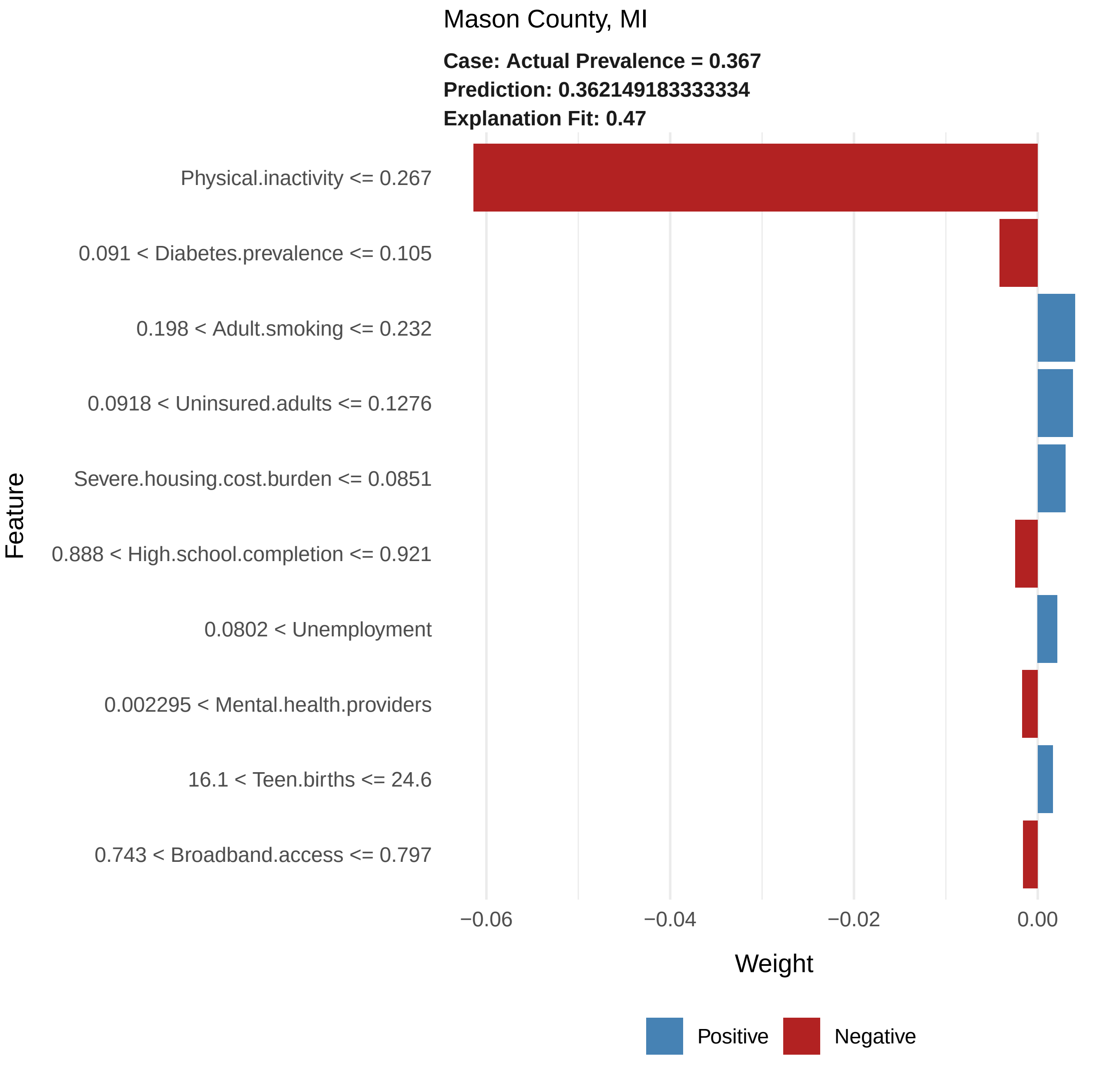

Mason County, MI
Case: Actual Prevalence = 0.367
Prediction: 0.362149183333334
Explanation Fit: 0.47
Physical.inactivity <= 0.267
0.091 < Diabetes.prevalence <= 0.105
0.198 < Adult.smoking <= 0.232
0.0918 < Uninsured.adults <= 0.1276
Severe.housing.cost.burden <= 0.0851
0.888 < High.school.completion <= 0.921
0.0802 < Unemployment
0.002295 < Mental.health.providers
16.1 < Teen.births <= 24.6
0.743 < Broadband.access <= 0.797
Feature
−0.06
−0.04
−0.02
0.00
Weight
Positive
Negative

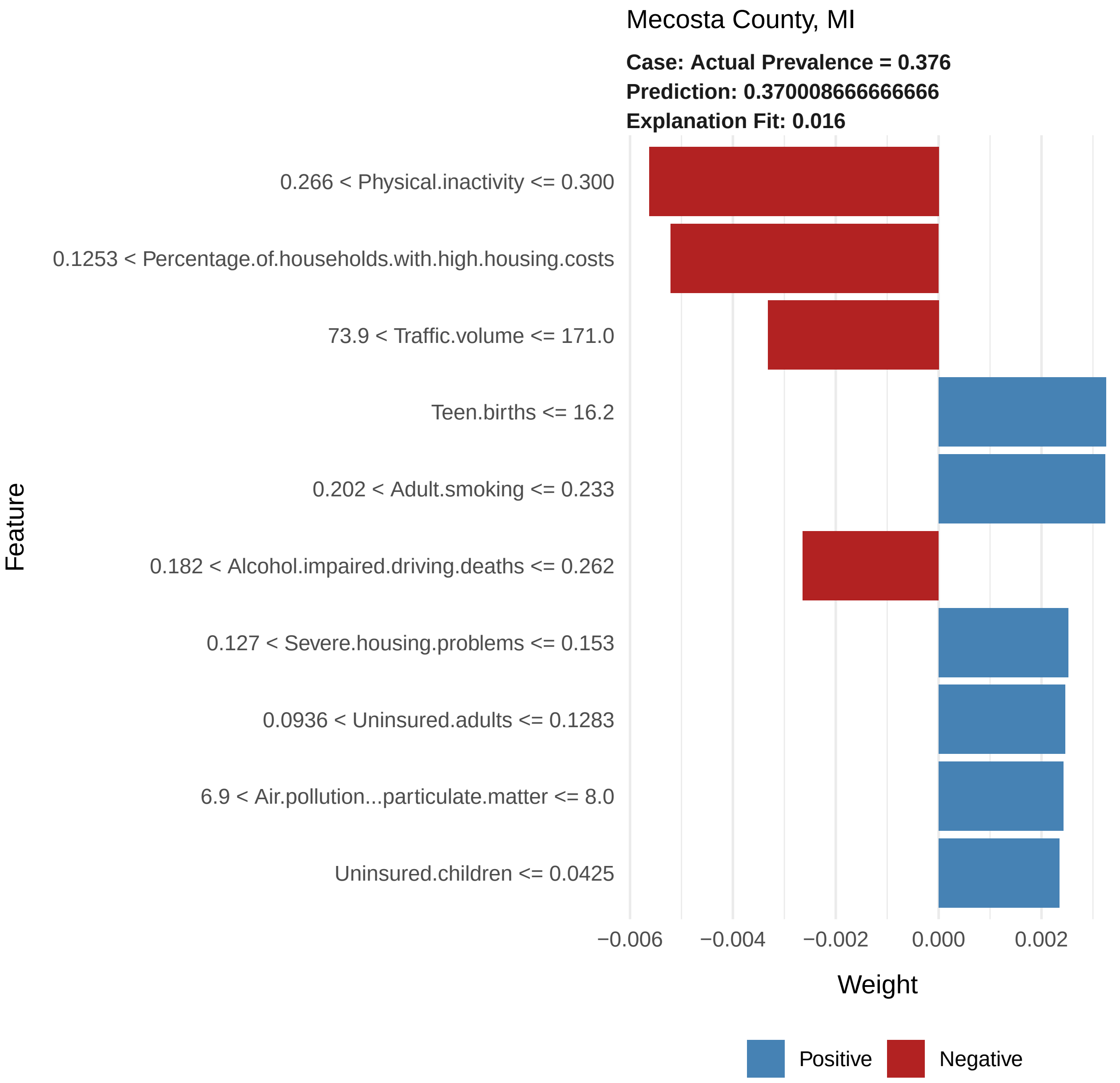

Mecosta County, MI
Case: Actual Prevalence = 0.376
Prediction: 0.370008666666666
Explanation Fit: 0.016
0.266 < Physical.inactivity <= 0.300
0.1253 < Percentage.of.households.with.high.housing.costs
73.9 < Traffic.volume <= 171.0
Teen.births <= 16.2
0.202 < Adult.smoking <= 0.233
0.182 < Alcohol.impaired.driving.deaths <= 0.262
0.127 < Severe.housing.problems <= 0.153
0.0936 < Uninsured.adults <= 0.1283
6.9 < Air.pollution...particulate.matter <= 8.0
Uninsured.children <= 0.0425
Feature
−0.006
−0.004
−0.002
0.000
0.002
Weight
Positive
Negative

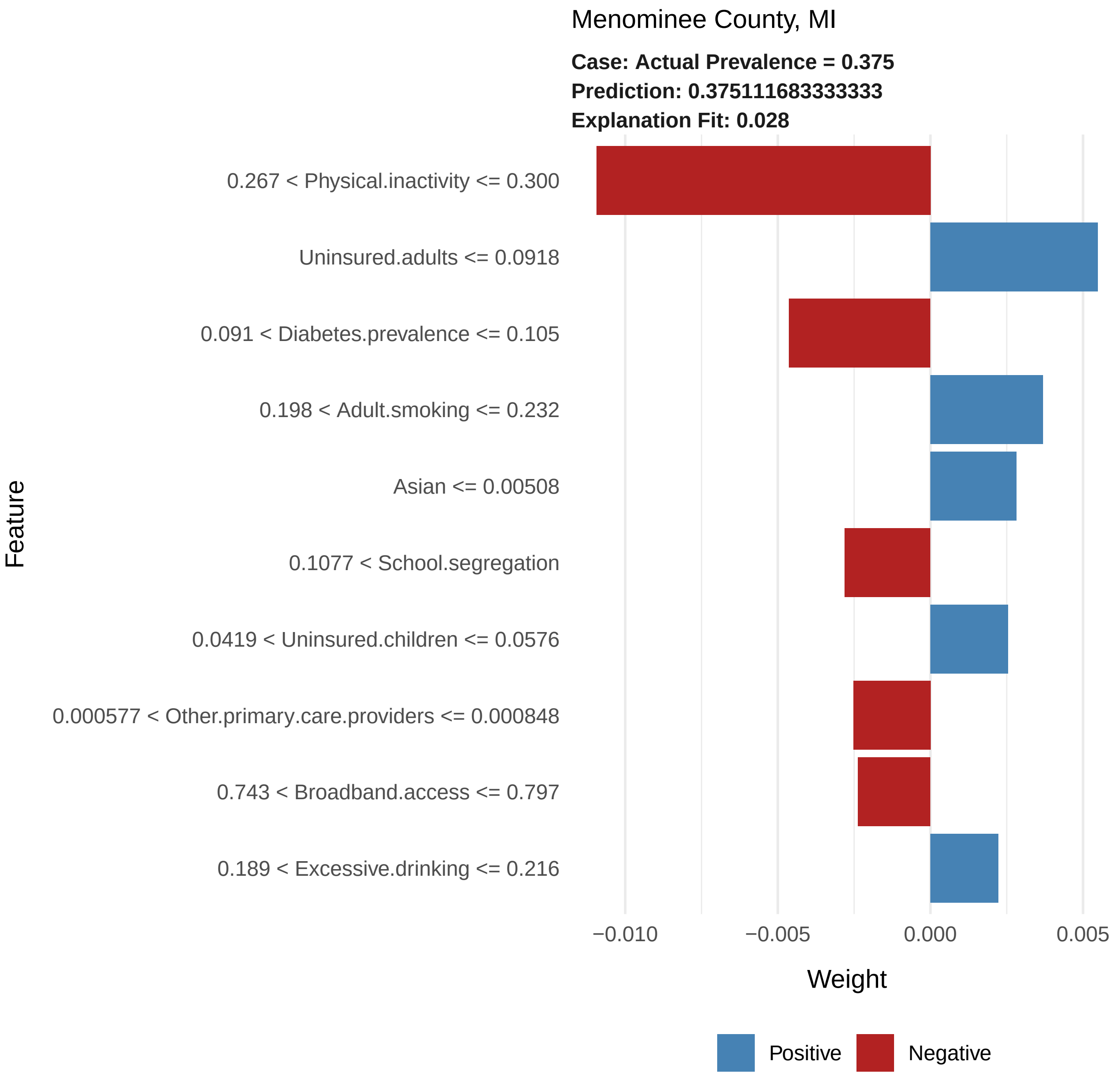

Menominee County, MI
Case: Actual Prevalence = 0.375
Prediction: 0.375111683333333
Explanation Fit: 0.028
0.267 < Physical.inactivity <= 0.300
Uninsured.adults <= 0.0918
0.091 < Diabetes.prevalence <= 0.105
0.198 < Adult.smoking <= 0.232
Asian <= 0.00508
0.1077 < School.segregation
0.0419 < Uninsured.children <= 0.0576
0.000577 < Other.primary.care.providers <= 0.000848
0.743 < Broadband.access <= 0.797
0.189 < Excessive.drinking <= 0.216
Feature
−0.010
−0.005
0.000
0.005
Weight
Positive
Negative

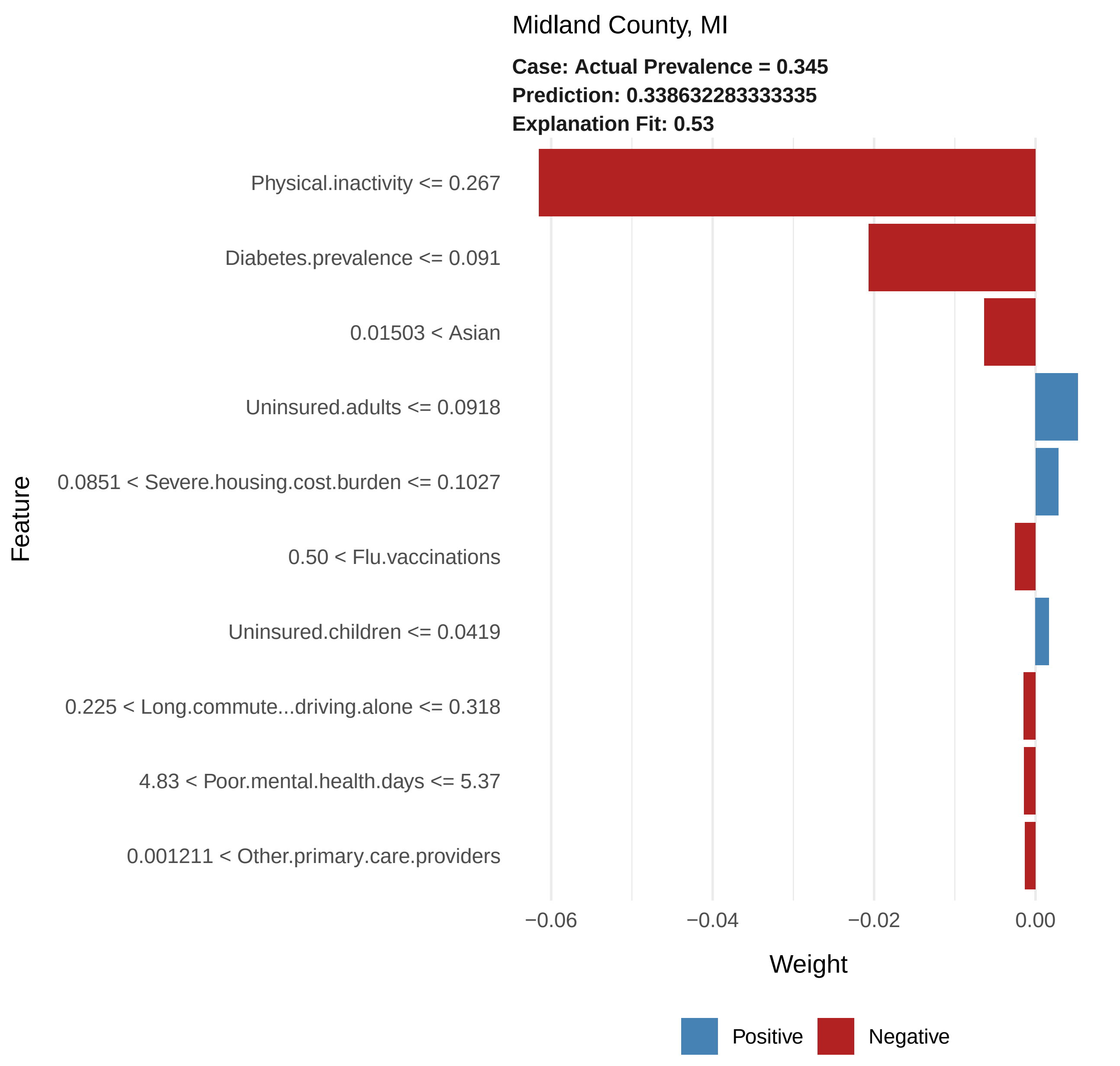
Midland County, MI
Case: Actual Prevalence = 0.345
Prediction: 0.338632283333335
Explanation Fit: 0.53
Physical.inactivity <= 0.267
Diabetes.prevalence <= 0.091
0.01503 < Asian
Uninsured.adults <= 0.0918
0.0851 < Severe.housing.cost.burden <= 0.1027
0.50 < Flu.vaccinations
Uninsured.children <= 0.0419
0.225 < Long.commute...driving.alone <= 0.318
4.83 < Poor.mental.health.days <= 5.37
0.001211 < Other.primary.care.providers
Feature
−0.06
−0.04
−0.02
0.00
Weight
Positive
Negative

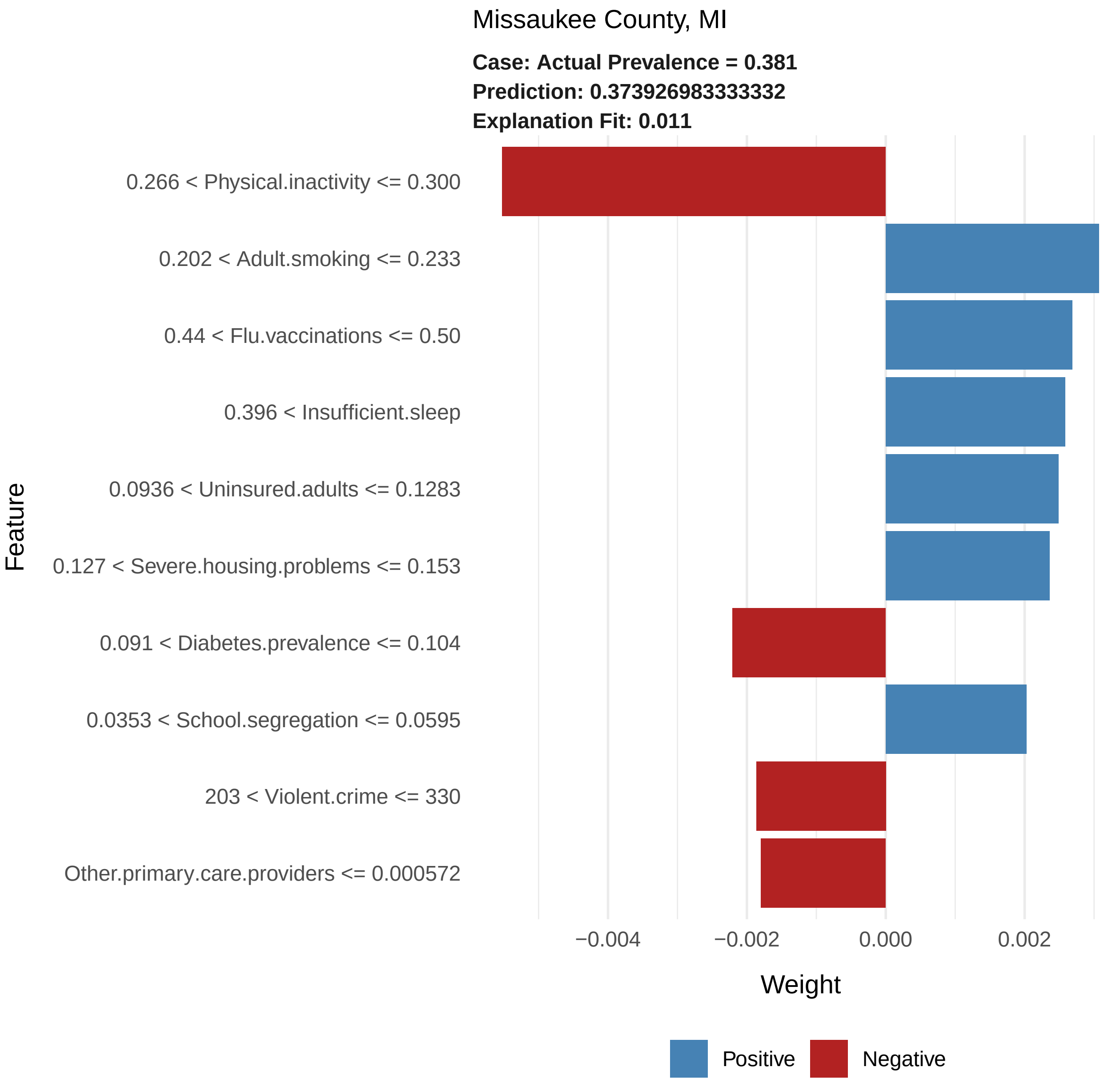

Missaukee County, MI
Case: Actual Prevalence = 0.381
Prediction: 0.373926983333332
Explanation Fit: 0.011
0.266 < Physical.inactivity <= 0.300
0.202 < Adult.smoking <= 0.233
0.44 < Flu.vaccinations <= 0.50
0.396 < Insufficient.sleep
0.0936 < Uninsured.adults <= 0.1283
0.127 < Severe.housing.problems <= 0.153
0.091 < Diabetes.prevalence <= 0.104
0.0353 < School.segregation <= 0.0595
203 < Violent.crime <= 330
Other.primary.care.providers <= 0.000572
Feature
−0.004
−0.002
0.000
0.002
Weight
Positive
Negative

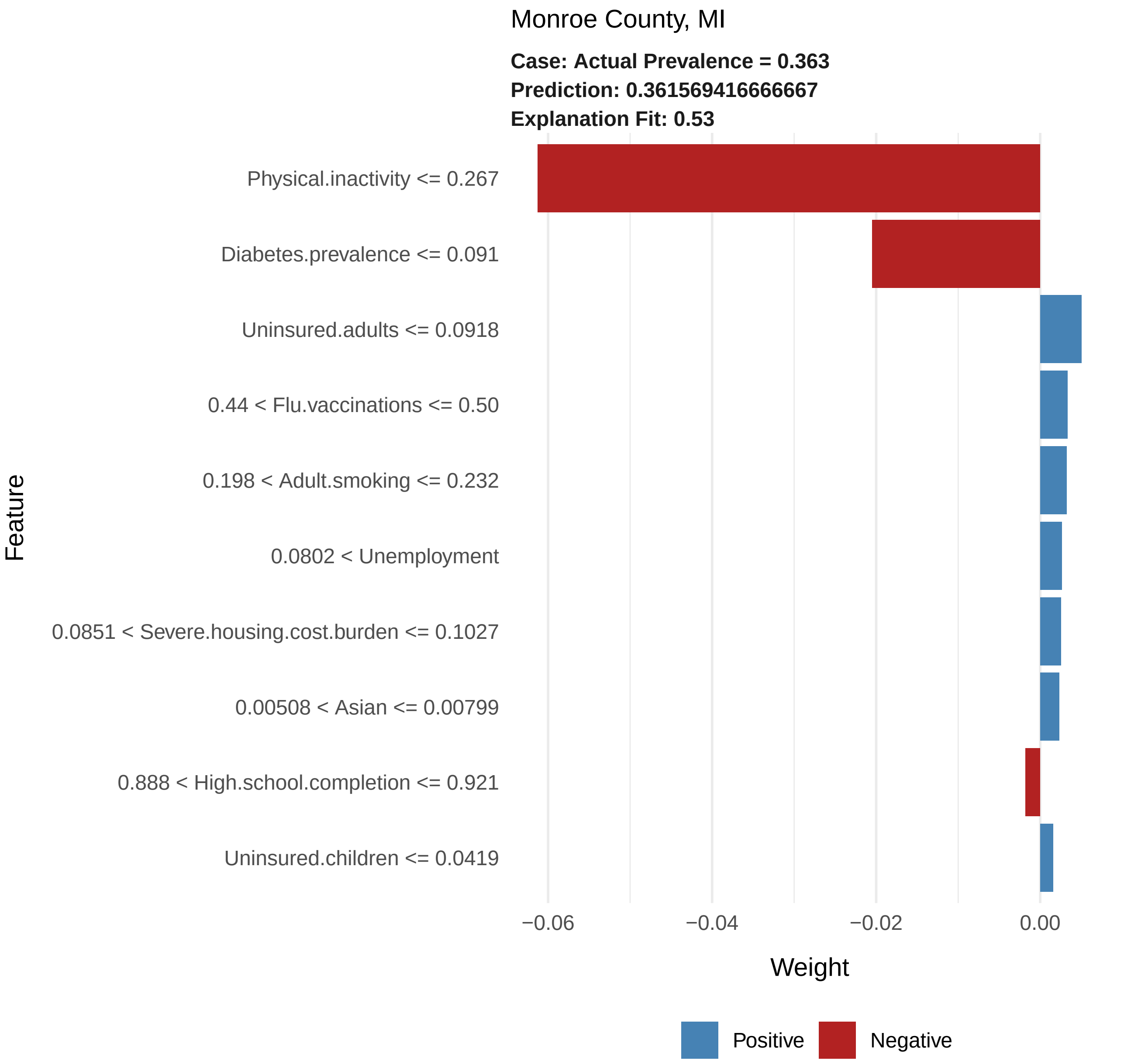
Monroe County, MI
Case: Actual Prevalence = 0.363
Prediction: 0.361569416666667
Explanation Fit: 0.53
Physical.inactivity <= 0.267
Diabetes.prevalence <= 0.091
Uninsured.adults <= 0.0918
0.44 < Flu.vaccinations <= 0.50
0.198 < Adult.smoking <= 0.232
0.0802 < Unemployment
0.0851 < Severe.housing.cost.burden <= 0.1027
0.00508 < Asian <= 0.00799
0.888 < High.school.completion <= 0.921
Uninsured.children <= 0.0419
Feature
−0.06
−0.04
−0.02
0.00
Weight
Positive
Negative

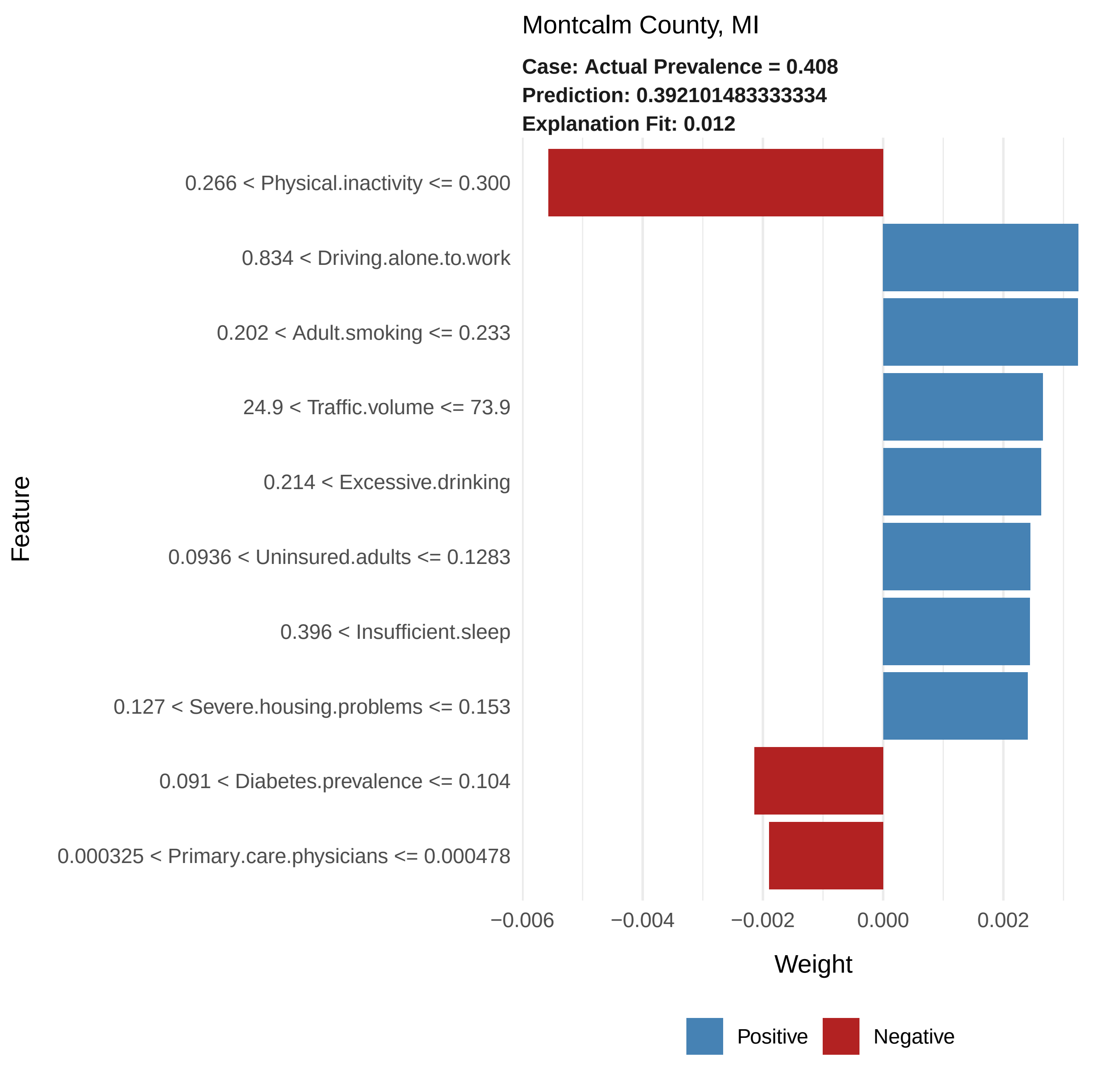

Montcalm County, MI
Case: Actual Prevalence = 0.408
Prediction: 0.392101483333334
Explanation Fit: 0.012
0.266 < Physical.inactivity <= 0.300
0.834 < Driving.alone.to.work
0.202 < Adult.smoking <= 0.233
24.9 < Traffic.volume <= 73.9
0.214 < Excessive.drinking
0.0936 < Uninsured.adults <= 0.1283
0.396 < Insufficient.sleep
0.127 < Severe.housing.problems <= 0.153
0.091 < Diabetes.prevalence <= 0.104
0.000325 < Primary.care.physicians <= 0.000478
Feature
−0.006
−0.004
−0.002
0.000
0.002
Weight
Positive
Negative

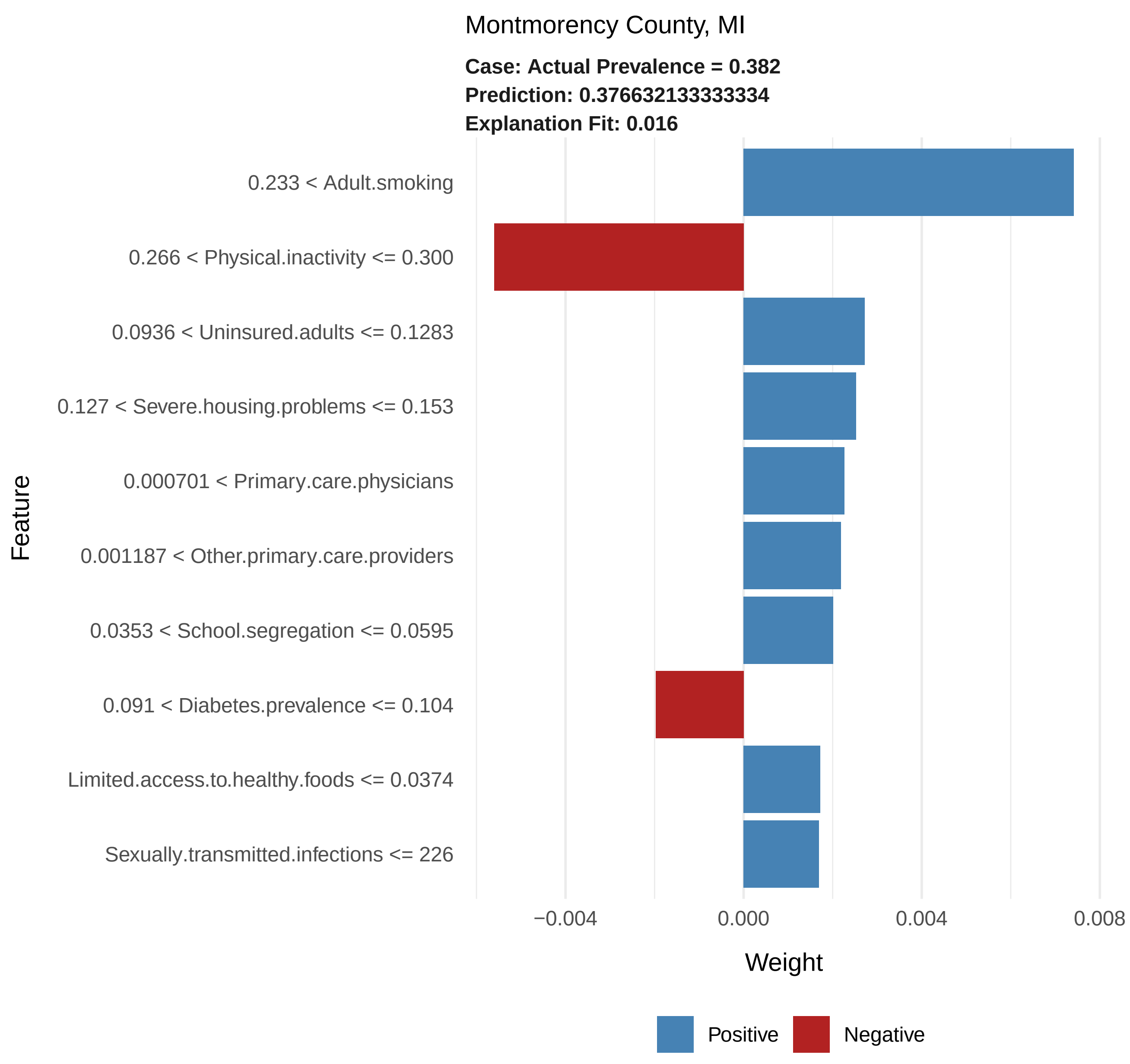
Montmorency County, MI
Case: Actual Prevalence = 0.382
Prediction: 0.376632133333334
Explanation Fit: 0.016
0.233 < Adult.smoking
0.266 < Physical.inactivity <= 0.300
0.0936 < Uninsured.adults <= 0.1283
0.127 < Severe.housing.problems <= 0.153
0.000701 < Primary.care.physicians
0.001187 < Other.primary.care.providers
0.0353 < School.segregation <= 0.0595
0.091 < Diabetes.prevalence <= 0.104
Limited.access.to.healthy.foods <= 0.0374
Sexually.transmitted.infections <= 226
Feature
−0.004
0.000
0.004
0.008
Weight
Positive
Negative

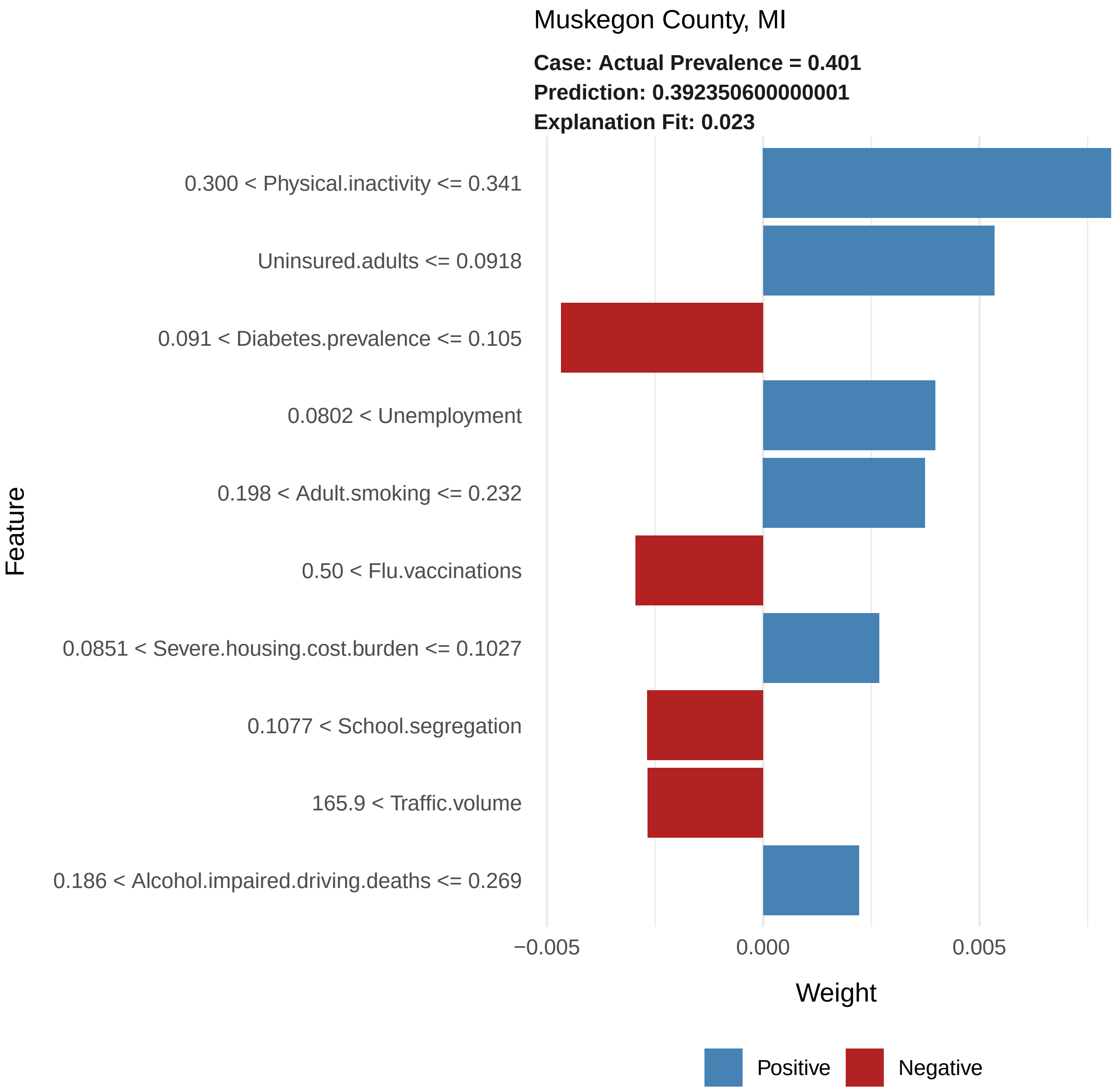

Muskegon County, MI
Case: Actual Prevalence = 0.401
Prediction: 0.392350600000001
Explanation Fit: 0.023
0.300 < Physical.inactivity <= 0.341
Uninsured.adults <= 0.0918
0.091 < Diabetes.prevalence <= 0.105
0.0802 < Unemployment
0.198 < Adult.smoking <= 0.232
0.50 < Flu.vaccinations
0.0851 < Severe.housing.cost.burden <= 0.1027
0.1077 < School.segregation
165.9 < Traffic.volume
0.186 < Alcohol.impaired.driving.deaths <= 0.269
Feature
−0.005
0.000
0.005
Weight
Positive
Negative

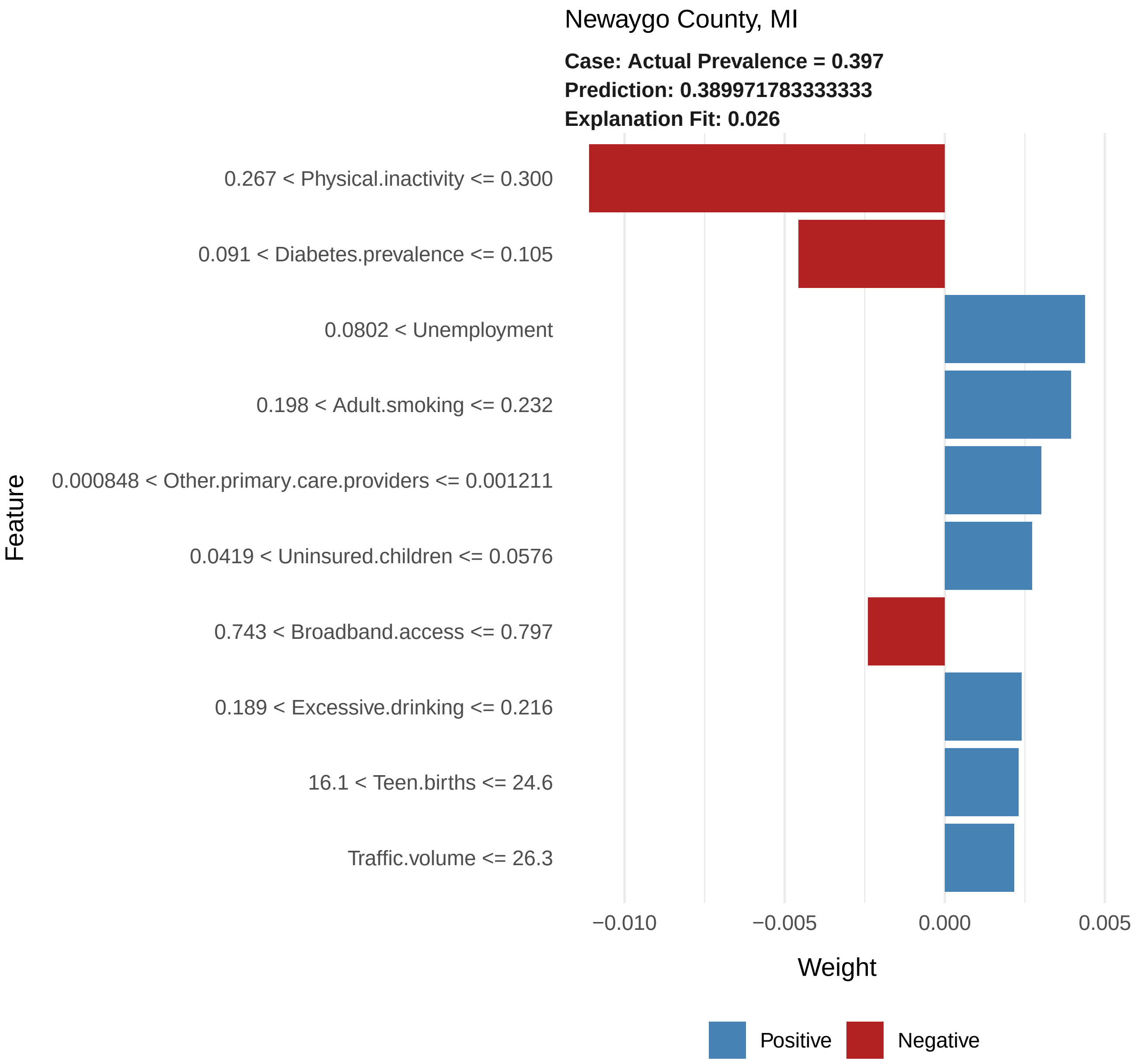

Newaygo County, MI
Case: Actual Prevalence = 0.397
Prediction: 0.389971783333333
Explanation Fit: 0.026
0.267 < Physical.inactivity <= 0.300
0.091 < Diabetes.prevalence <= 0.105
0.0802 < Unemployment
0.198 < Adult.smoking <= 0.232
0.000848 < Other.primary.care.providers <= 0.001211
0.0419 < Uninsured.children <= 0.0576
0.743 < Broadband.access <= 0.797
0.189 < Excessive.drinking <= 0.216
16.1 < Teen.births <= 24.6
Traffic.volume <= 26.3
Feature
−0.010
−0.005
0.000
0.005
Weight
Positive
Negative

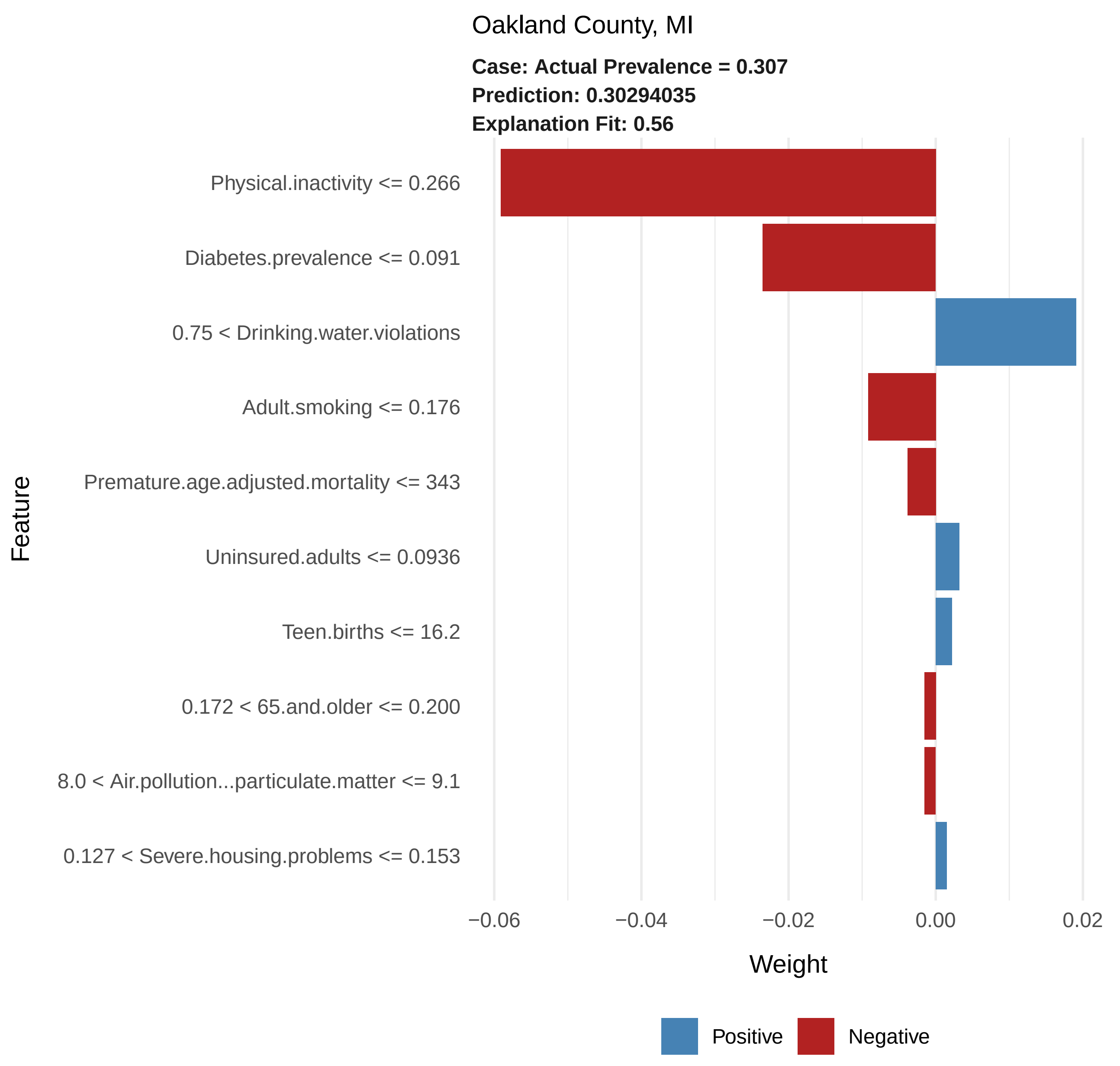

Oakland County, MI
Case: Actual Prevalence = 0.307
Prediction: 0.30294035
Explanation Fit: 0.56
Physical.inactivity <= 0.266
Diabetes.prevalence <= 0.091
0.75 < Drinking.water.violations
Adult.smoking <= 0.176
Premature.age.adjusted.mortality <= 343
Uninsured.adults <= 0.0936
Teen.births <= 16.2
0.172 < 65.and.older <= 0.200
8.0 < Air.pollution...particulate.matter <= 9.1
0.127 < Severe.housing.problems <= 0.153
Feature
−0.06
−0.04
−0.02
0.00
0.02
Weight
Positive
Negative

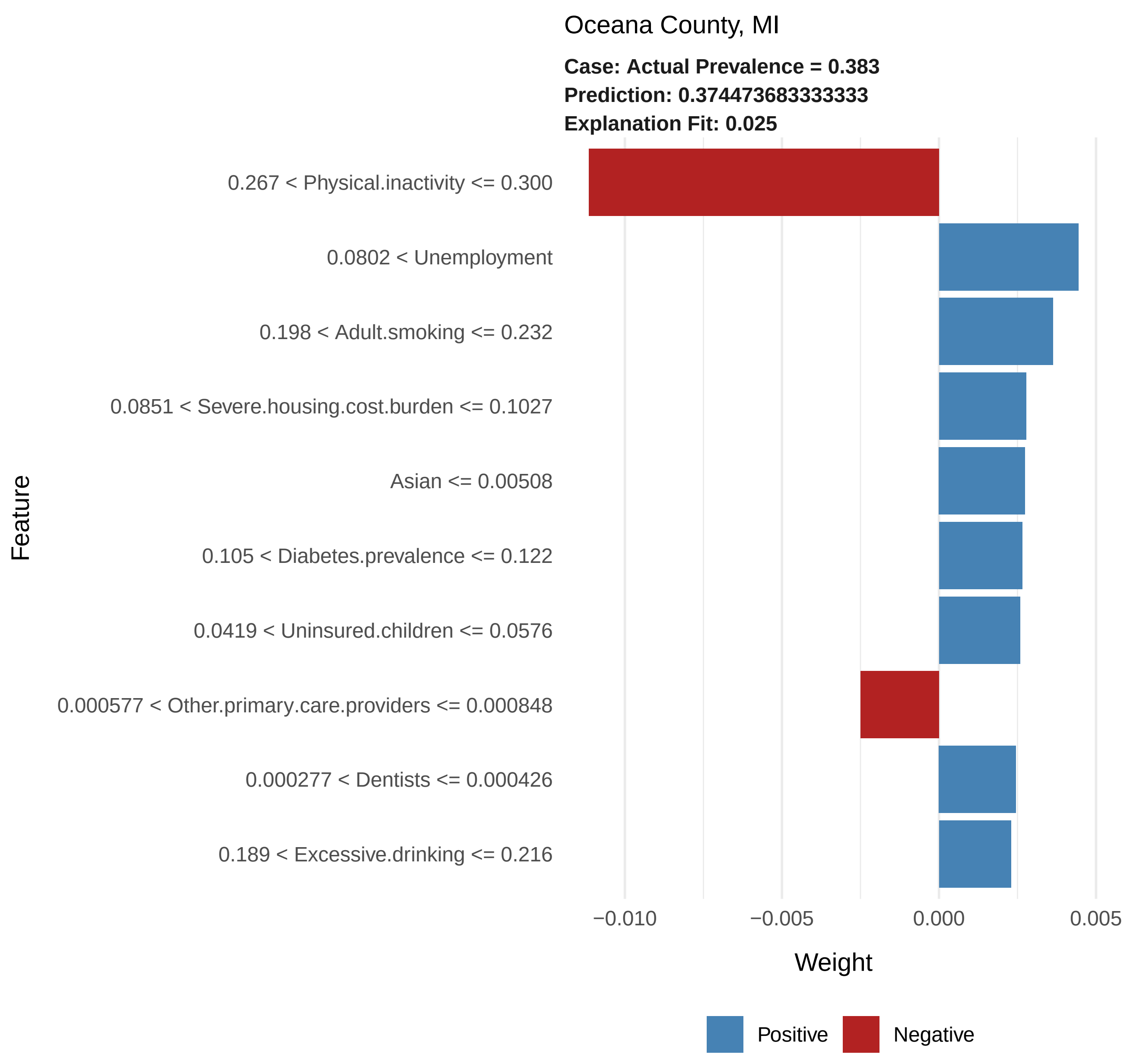

Oceana County, MI
Case: Actual Prevalence = 0.383
Prediction: 0.374473683333333
Explanation Fit: 0.025
0.267 < Physical.inactivity <= 0.300
0.0802 < Unemployment
0.198 < Adult.smoking <= 0.232
0.0851 < Severe.housing.cost.burden <= 0.1027
Asian <= 0.00508
0.105 < Diabetes.prevalence <= 0.122
0.0419 < Uninsured.children <= 0.0576
0.000577 < Other.primary.care.providers <= 0.000848
0.000277 < Dentists <= 0.000426
0.189 < Excessive.drinking <= 0.216
Feature
−0.010
−0.005
0.000
0.005
Weight
Positive
Negative

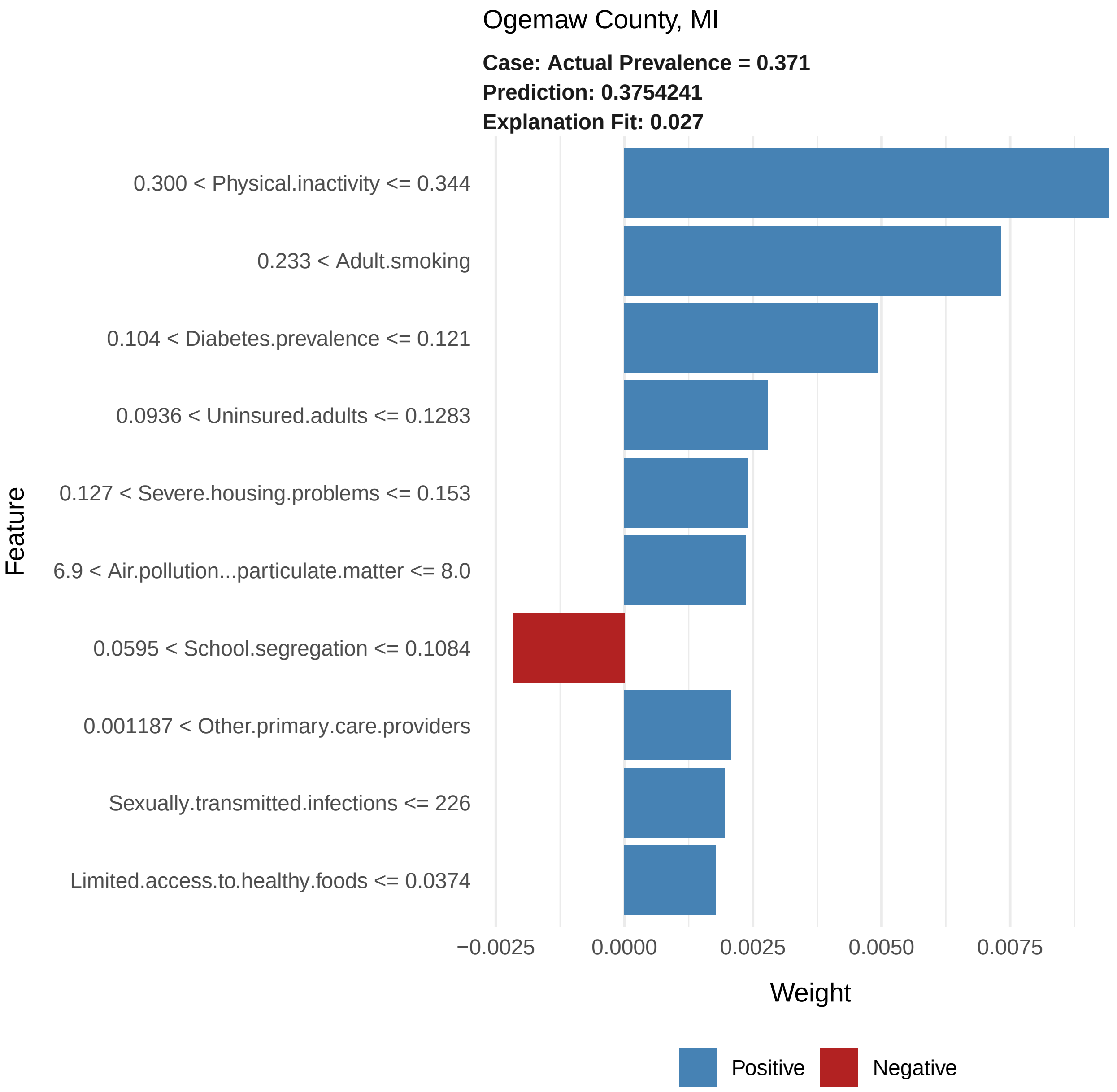

Ogemaw County, MI
Case: Actual Prevalence = 0.371
Prediction: 0.3754241
Explanation Fit: 0.027
0.300 < Physical.inactivity <= 0.344
0.233 < Adult.smoking
0.104 < Diabetes.prevalence <= 0.121
0.0936 < Uninsured.adults <= 0.1283
0.127 < Severe.housing.problems <= 0.153
6.9 < Air.pollution...particulate.matter <= 8.0
0.0595 < School.segregation <= 0.1084
0.001187 < Other.primary.care.providers
Sexually.transmitted.infections <= 226
Limited.access.to.healthy.foods <= 0.0374
Feature
−0.0025
0.0000
0.0025
0.0050
0.0075
Weight
Positive
Negative

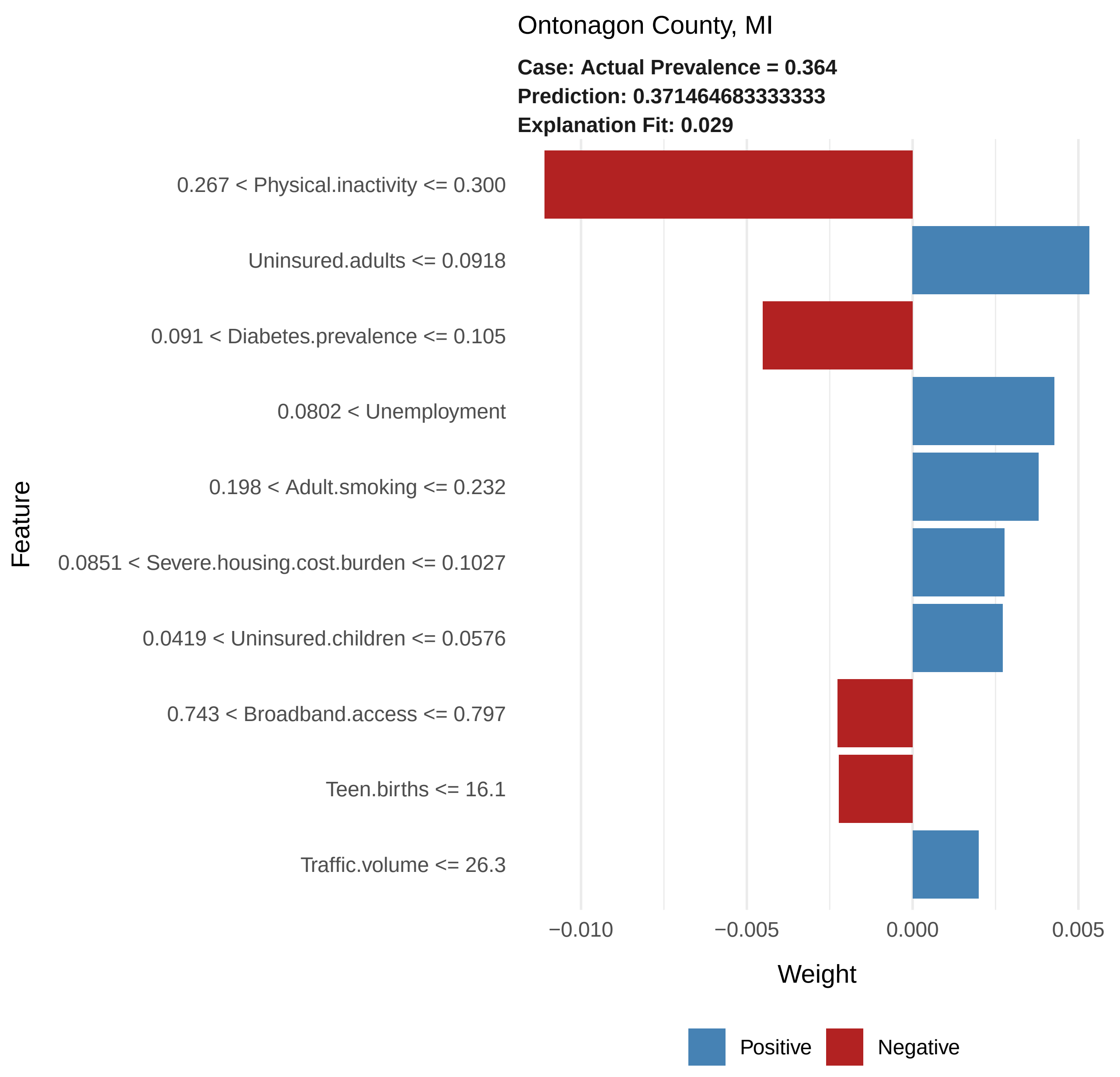
Ontonagon County, MI
Case: Actual Prevalence = 0.364
Prediction: 0.371464683333333
Explanation Fit: 0.029
0.267 < Physical.inactivity <= 0.300
Uninsured.adults <= 0.0918
0.091 < Diabetes.prevalence <= 0.105
0.0802 < Unemployment
0.198 < Adult.smoking <= 0.232
0.0851 < Severe.housing.cost.burden <= 0.1027
0.0419 < Uninsured.children <= 0.0576
0.743 < Broadband.access <= 0.797
Teen.births <= 16.1
Traffic.volume <= 26.3
Feature
−0.010
−0.005
0.000
0.005
Weight
Positive
Negative

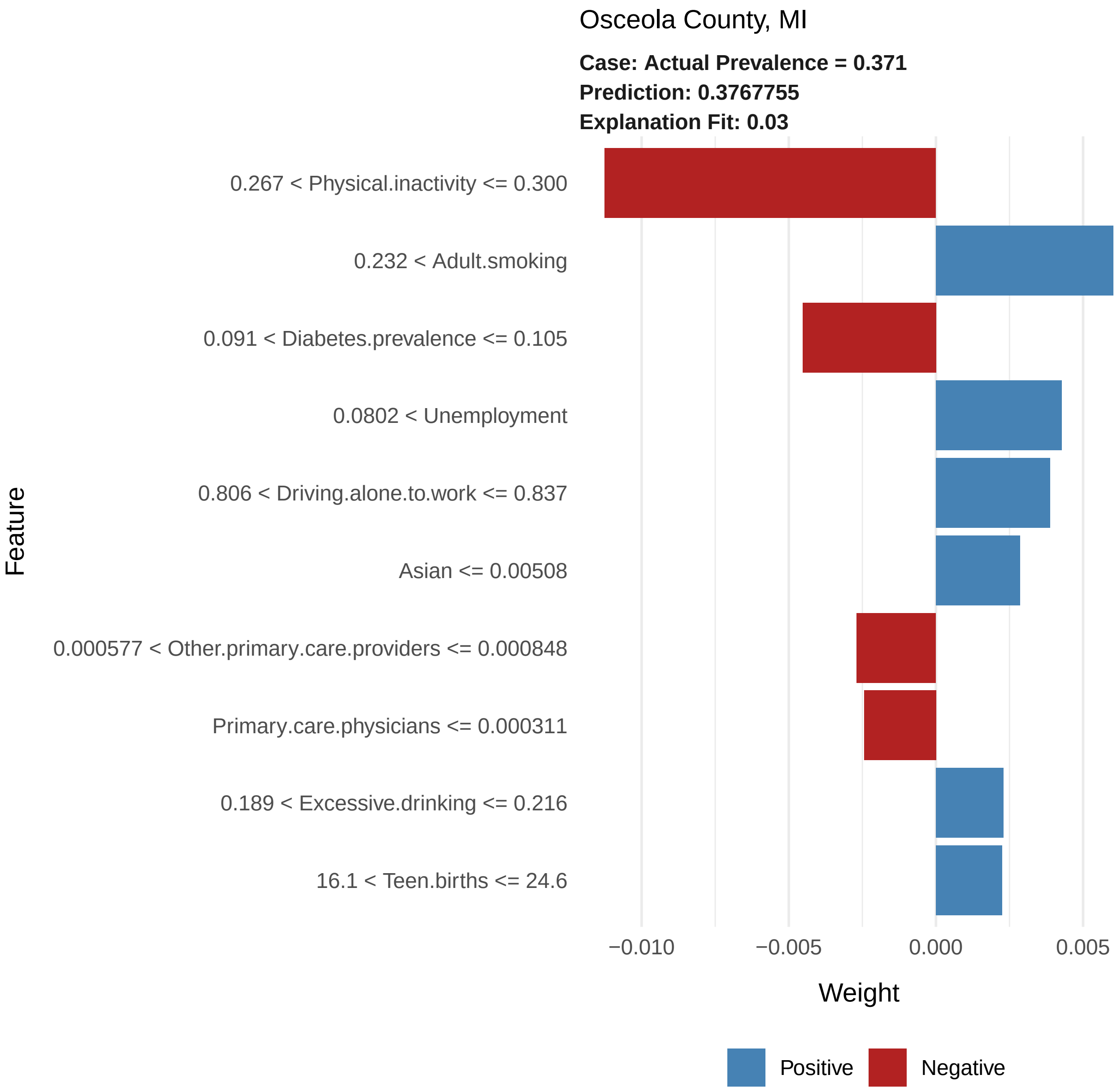

Osceola County, MI
Case: Actual Prevalence = 0.371
Prediction: 0.3767755
Explanation Fit: 0.03
0.267 < Physical.inactivity <= 0.300
0.232 < Adult.smoking
0.091 < Diabetes.prevalence <= 0.105
0.0802 < Unemployment
0.806 < Driving.alone.to.work <= 0.837
Asian <= 0.00508
0.000577 < Other.primary.care.providers <= 0.000848
Primary.care.physicians <= 0.000311
0.189 < Excessive.drinking <= 0.216
16.1 < Teen.births <= 24.6
Feature
−0.010
−0.005
0.000
0.005
Weight
Positive
Negative

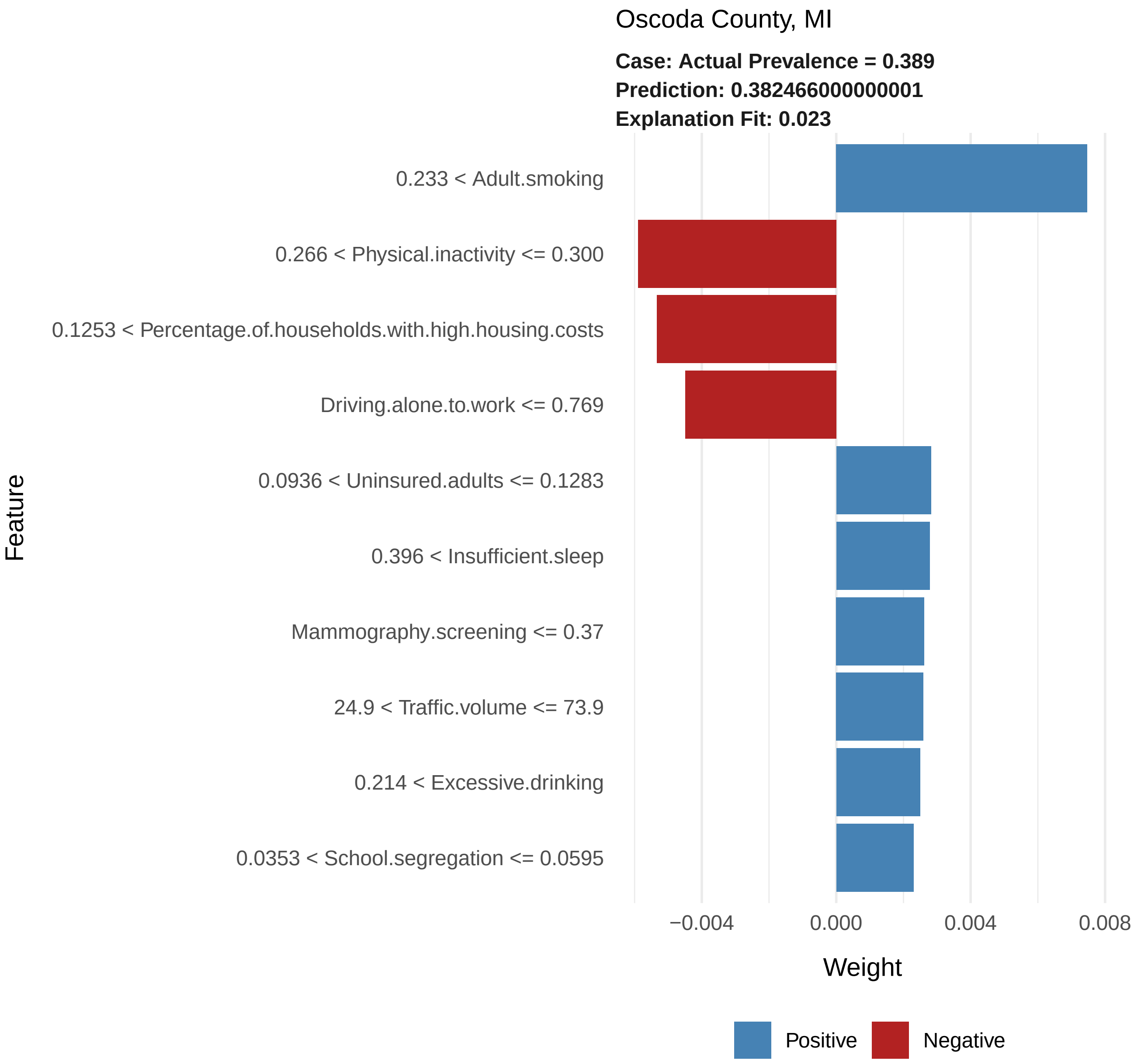

Oscoda County, MI
Case: Actual Prevalence = 0.389
Prediction: 0.382466000000001
Explanation Fit: 0.023
0.233 < Adult.smoking
0.266 < Physical.inactivity <= 0.300
0.1253 < Percentage.of.households.with.high.housing.costs
Driving.alone.to.work <= 0.769
0.0936 < Uninsured.adults <= 0.1283
0.396 < Insufficient.sleep
Mammography.screening <= 0.37
24.9 < Traffic.volume <= 73.9
0.214 < Excessive.drinking
0.0353 < School.segregation <= 0.0595
Feature
−0.004
0.000
0.004
0.008
Weight
Positive
Negative

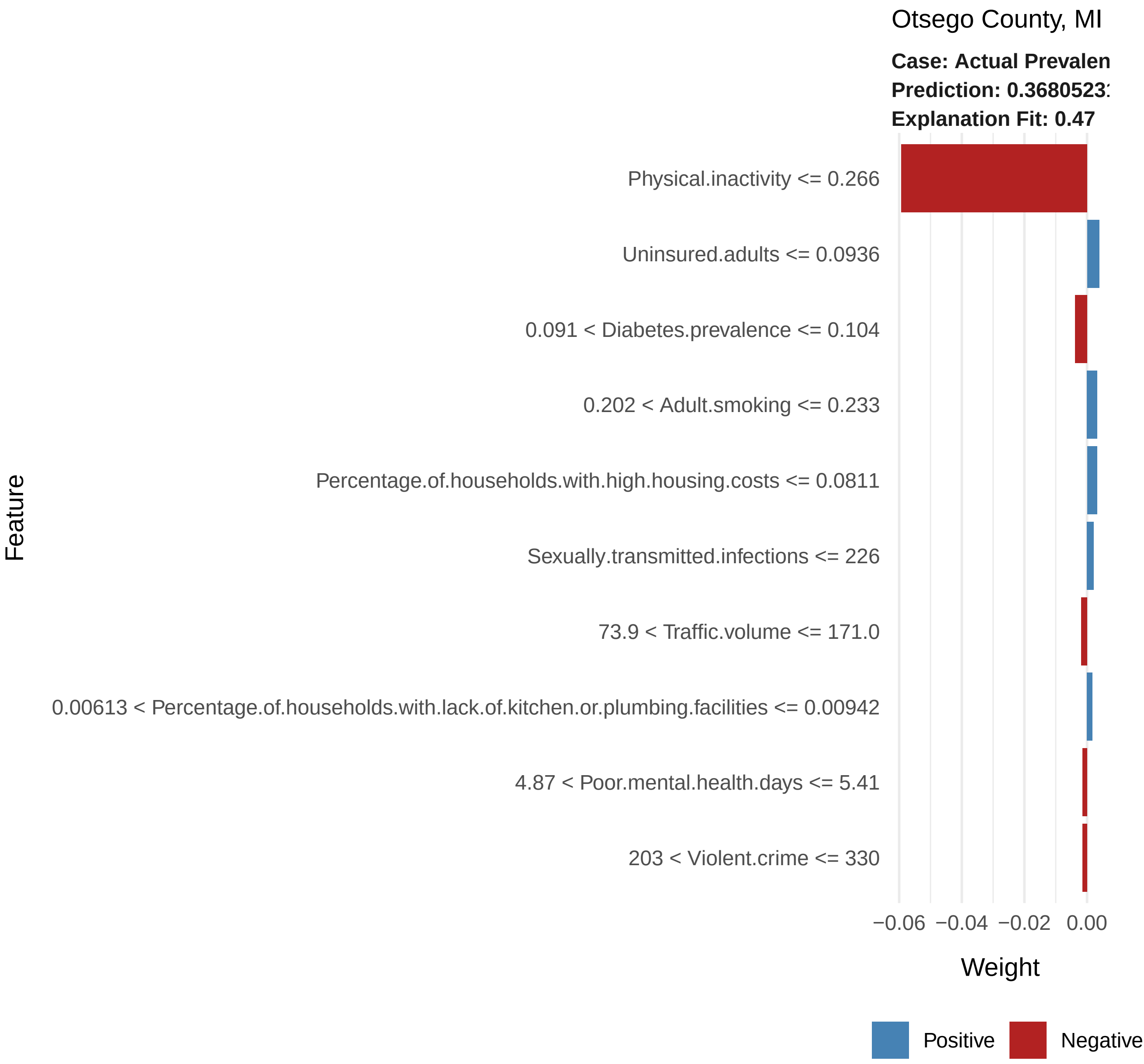

Otsego County, MI
Case: Actual Prevalen
Prediction: 0.3680523
Explanation Fit: 0.47
Physical.inactivity <= 0.266
Uninsured.adults <= 0.0936
0.091 < Diabetes.prevalence <= 0.104
0.202 < Adult.smoking <= 0.233
Percentage.of.households.with.high.housing.costs <= 0.0811
Sexually.transmitted.infections <= 226
73.9 < Traffic.volume <= 171.0
0.00613 < Percentage.of.households.with.lack.of.kitchen.or.plumbing.facilities <= 0.00942
4.87 < Poor.mental.health.days <= 5.41
203 < Violent.crime <= 330
Feature
−0.06
−0.04
−0.02
0.00
Weight
Positive
Negative

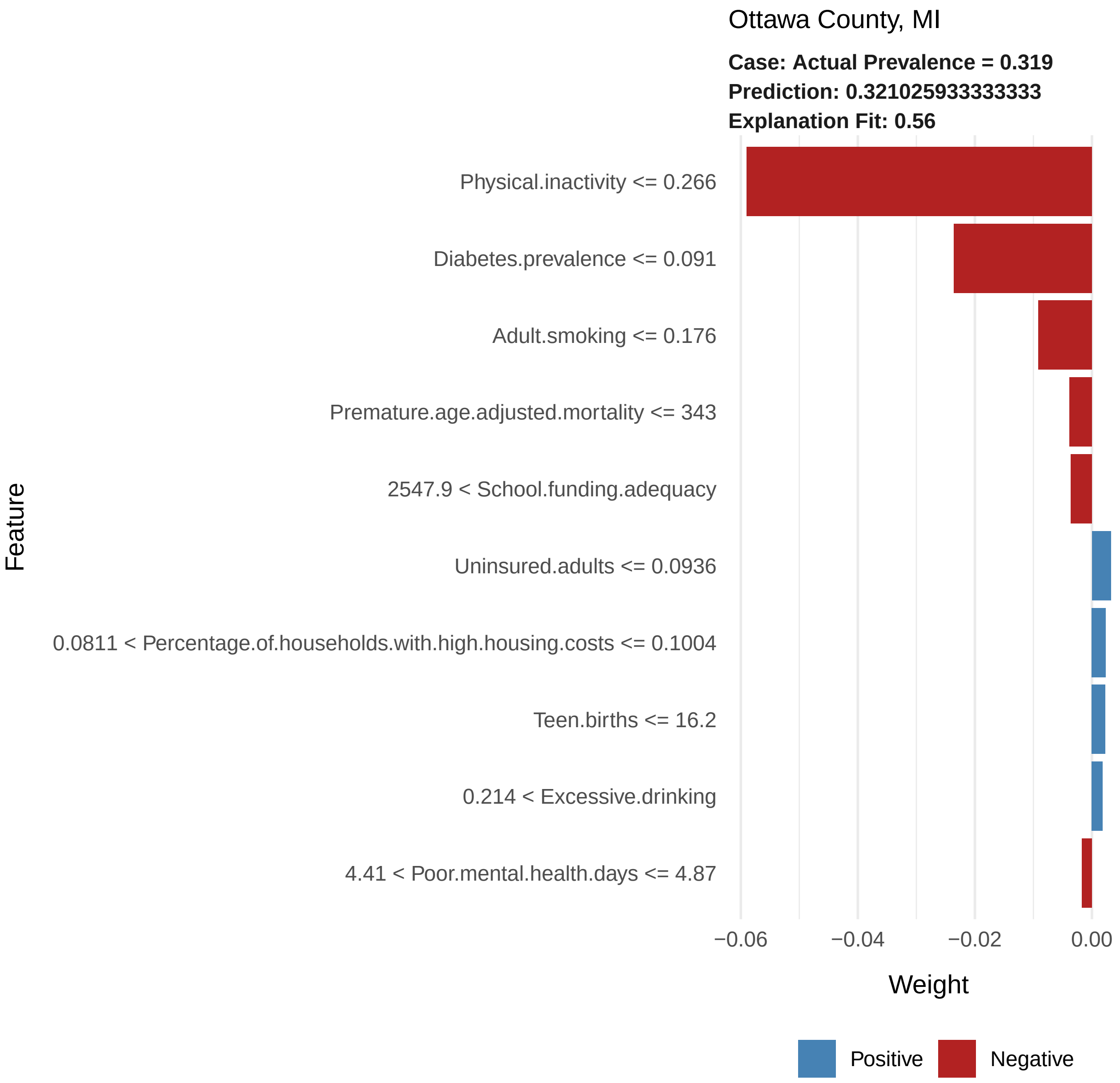
Ottawa County, MI
Case: Actual Prevalence = 0.319
Prediction: 0.321025933333333
Explanation Fit: 0.56
Physical.inactivity <= 0.266
Diabetes.prevalence <= 0.091
Adult.smoking <= 0.176
Premature.age.adjusted.mortality <= 343
2547.9 < School.funding.adequacy
Uninsured.adults <= 0.0936
0.0811 < Percentage.of.households.with.high.housing.costs <= 0.1004
Teen.births <= 16.2
0.214 < Excessive.drinking
4.41 < Poor.mental.health.days <= 4.87
Feature
−0.06
−0.04
−0.02
0.00
Weight
Positive
Negative

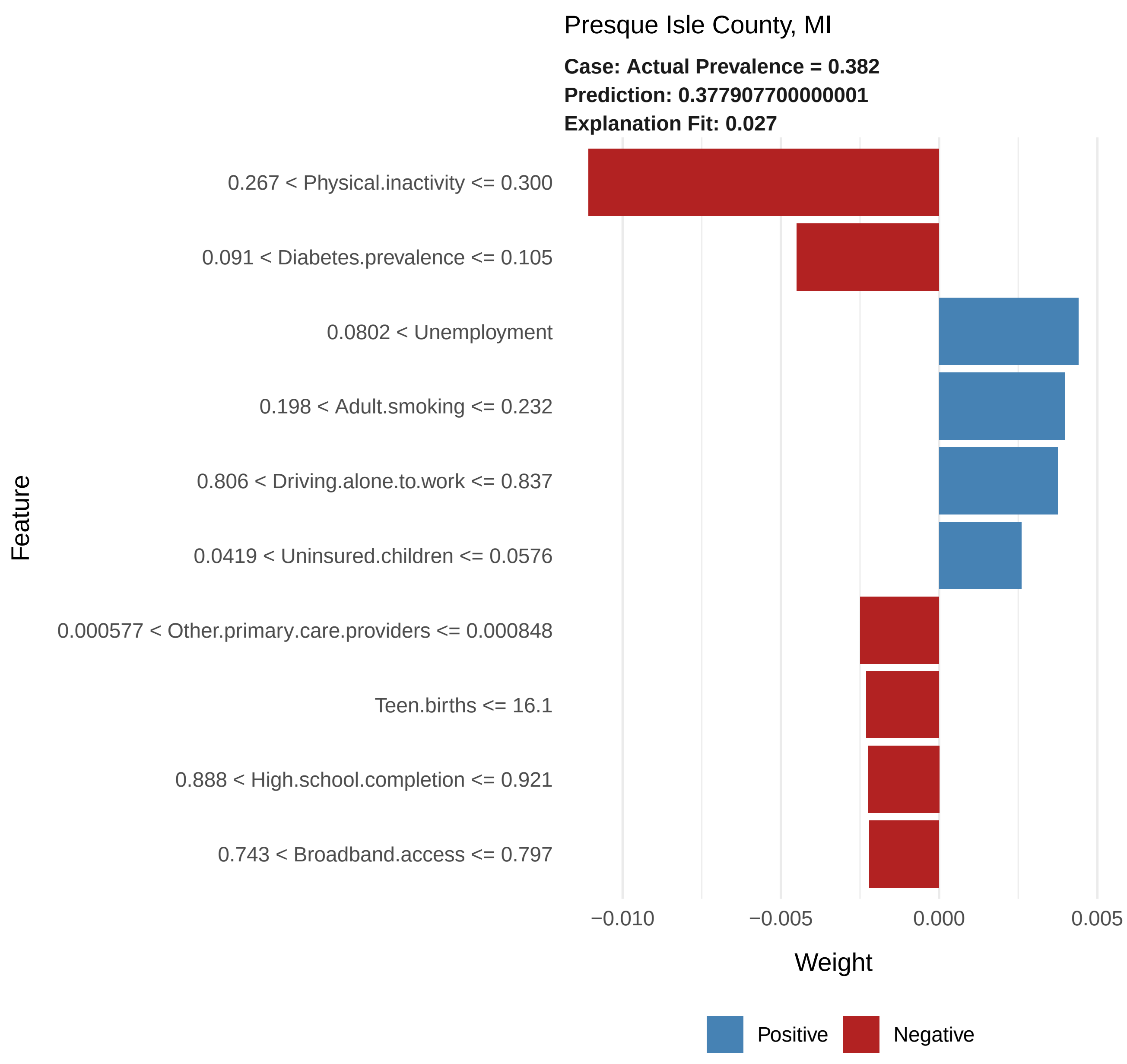

Presque Isle County, MI
Case: Actual Prevalence = 0.382
Prediction: 0.377907700000001
Explanation Fit: 0.027
0.267 < Physical.inactivity <= 0.300
0.091 < Diabetes.prevalence <= 0.105
0.0802 < Unemployment
0.198 < Adult.smoking <= 0.232
0.806 < Driving.alone.to.work <= 0.837
0.0419 < Uninsured.children <= 0.0576
0.000577 < Other.primary.care.providers <= 0.000848
Teen.births <= 16.1
0.888 < High.school.completion <= 0.921
0.743 < Broadband.access <= 0.797
Feature
−0.010
−0.005
0.000
0.005
Weight
Positive
Negative

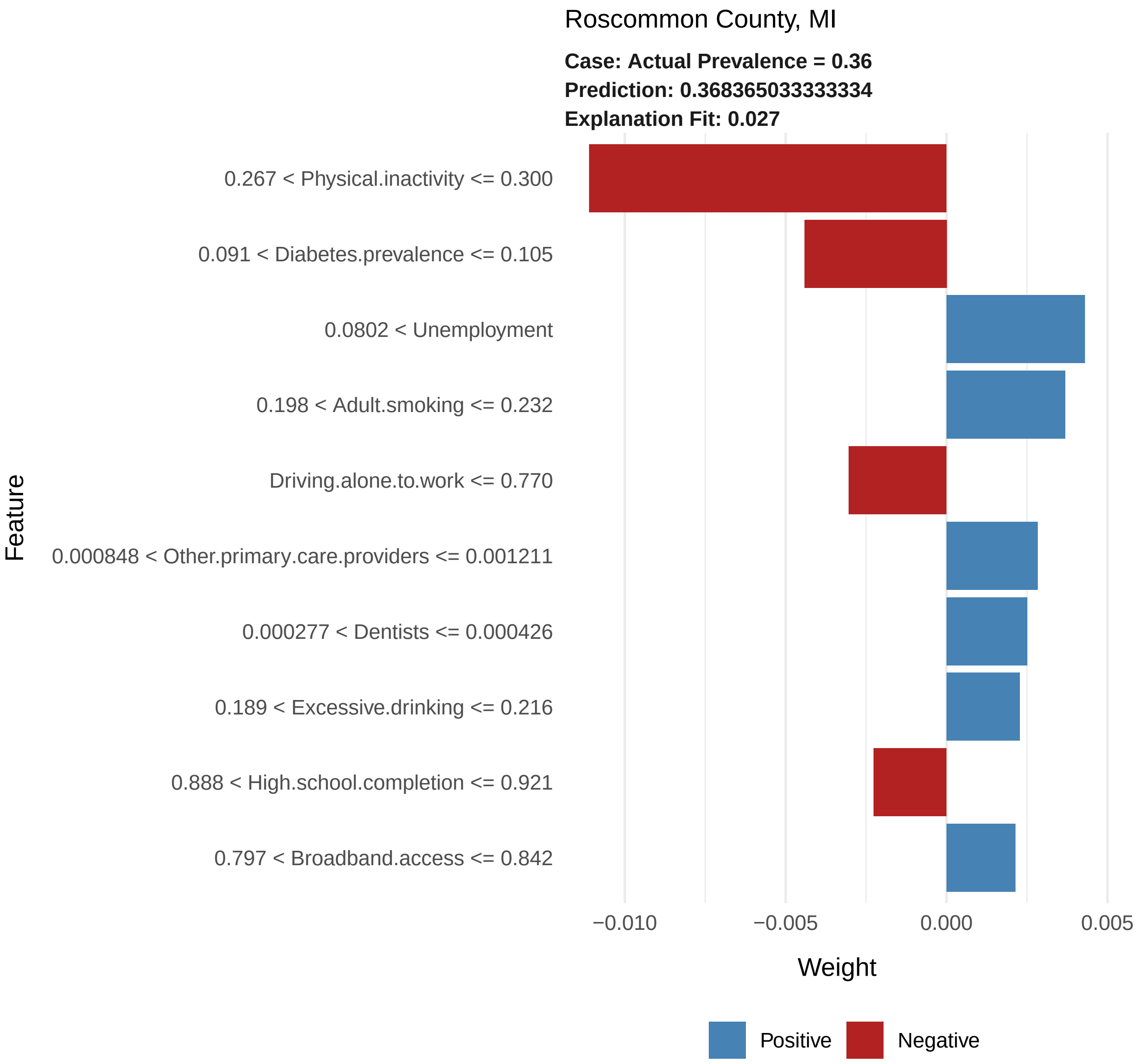

Roscommon County, MI
Case: Actual Prevalence = 0.36
Prediction: 0.368365033333334
Explanation Fit: 0.027
0.267 < Physical.inactivity <= 0.300
0.091 < Diabetes.prevalence <= 0.105
0.0802 < Unemployment
0.198 < Adult.smoking <= 0.232
Driving.alone.to.work <= 0.770
0.000848 < Other.primary.care.providers <= 0.001211
0.000277 < Dentists <= 0.000426
0.189 < Excessive.drinking <= 0.216
0.888 < High.school.completion <= 0.921
0.797 < Broadband.access <= 0.842
Feature
−0.010
−0.005
0.000
0.005
Weight
Positive
Negative

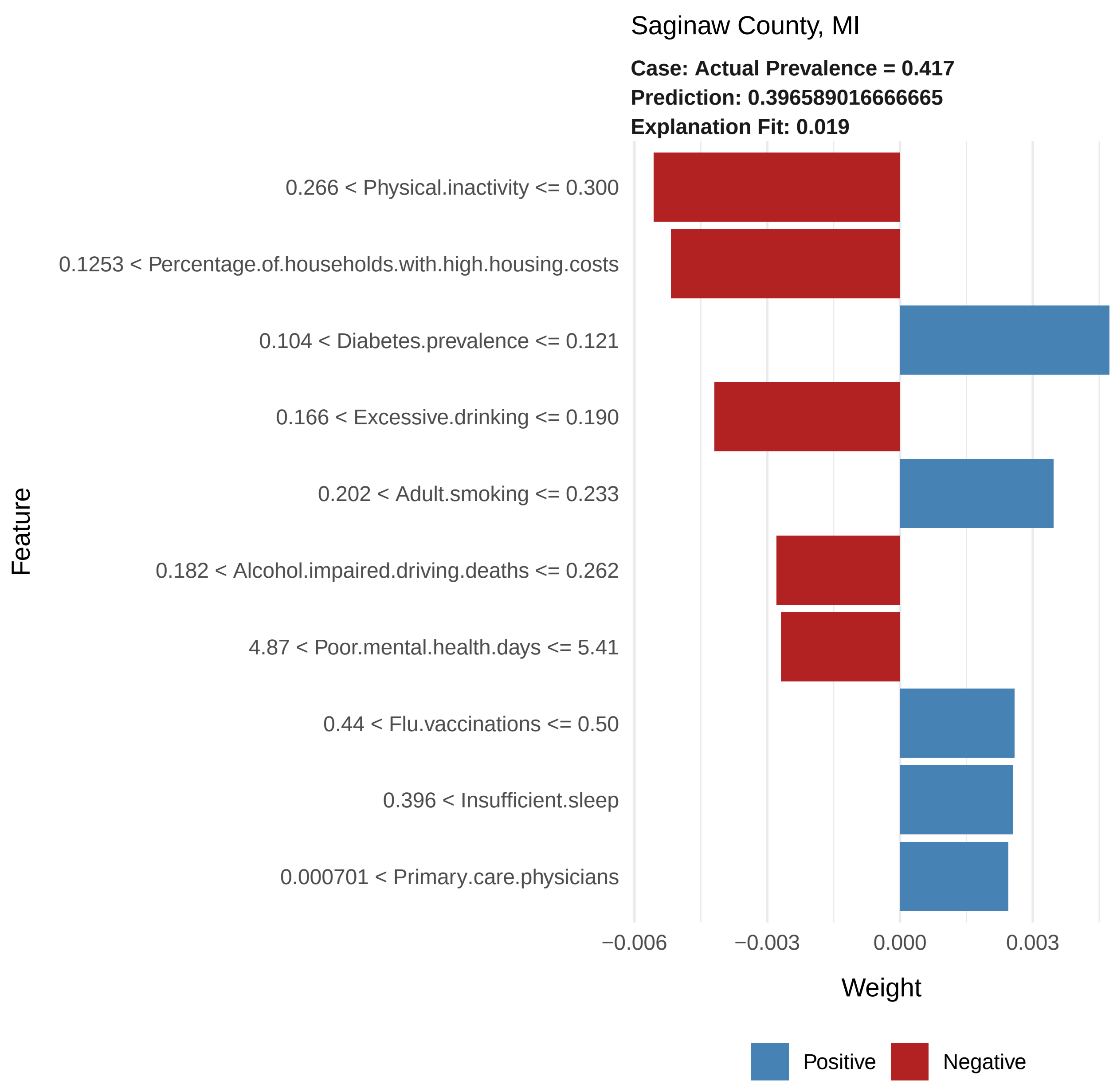
Saginaw County, MI
Case: Actual Prevalence = 0.417
Prediction: 0.396589016666665
Explanation Fit: 0.019
0.266 < Physical.inactivity <= 0.300
0.1253 < Percentage.of.households.with.high.housing.costs
0.104 < Diabetes.prevalence <= 0.121
0.166 < Excessive.drinking <= 0.190
0.202 < Adult.smoking <= 0.233
0.182 < Alcohol.impaired.driving.deaths <= 0.262
4.87 < Poor.mental.health.days <= 5.41
0.44 < Flu.vaccinations <= 0.50
0.396 < Insufficient.sleep
0.000701 < Primary.care.physicians
Feature
−0.006
−0.003
0.000
0.003
Weight
Positive
Negative

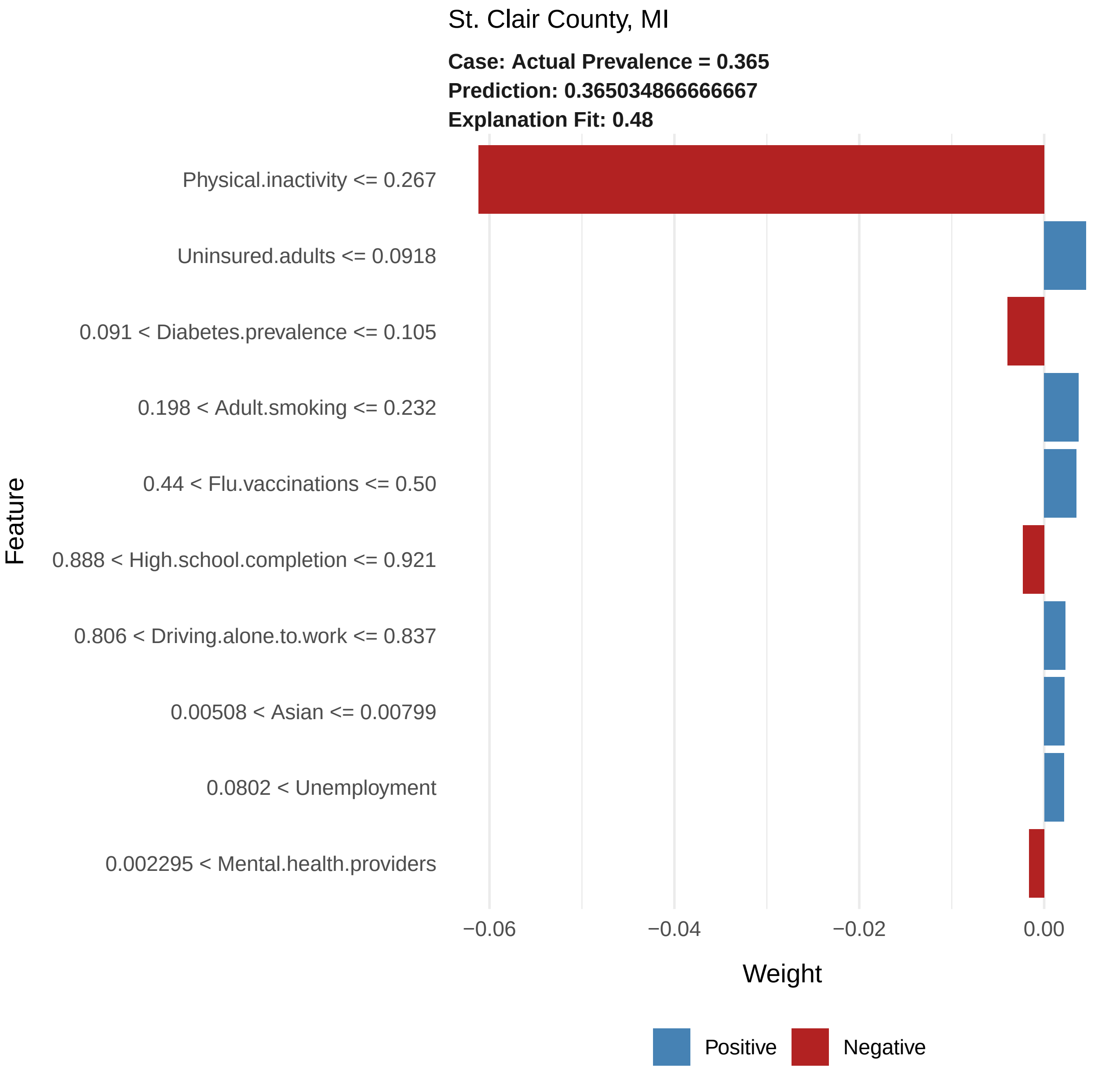
St. Clair County, MI
Case: Actual Prevalence = 0.365
Prediction: 0.365034866666667
Explanation Fit: 0.48
Physical.inactivity <= 0.267
Uninsured.adults <= 0.0918
0.091 < Diabetes.prevalence <= 0.105
0.198 < Adult.smoking <= 0.232
0.44 < Flu.vaccinations <= 0.50
0.888 < High.school.completion <= 0.921
0.806 < Driving.alone.to.work <= 0.837
0.00508 < Asian <= 0.00799
0.0802 < Unemployment
0.002295 < Mental.health.providers
Feature
−0.06
−0.04
−0.02
0.00
Weight
Positive
Negative

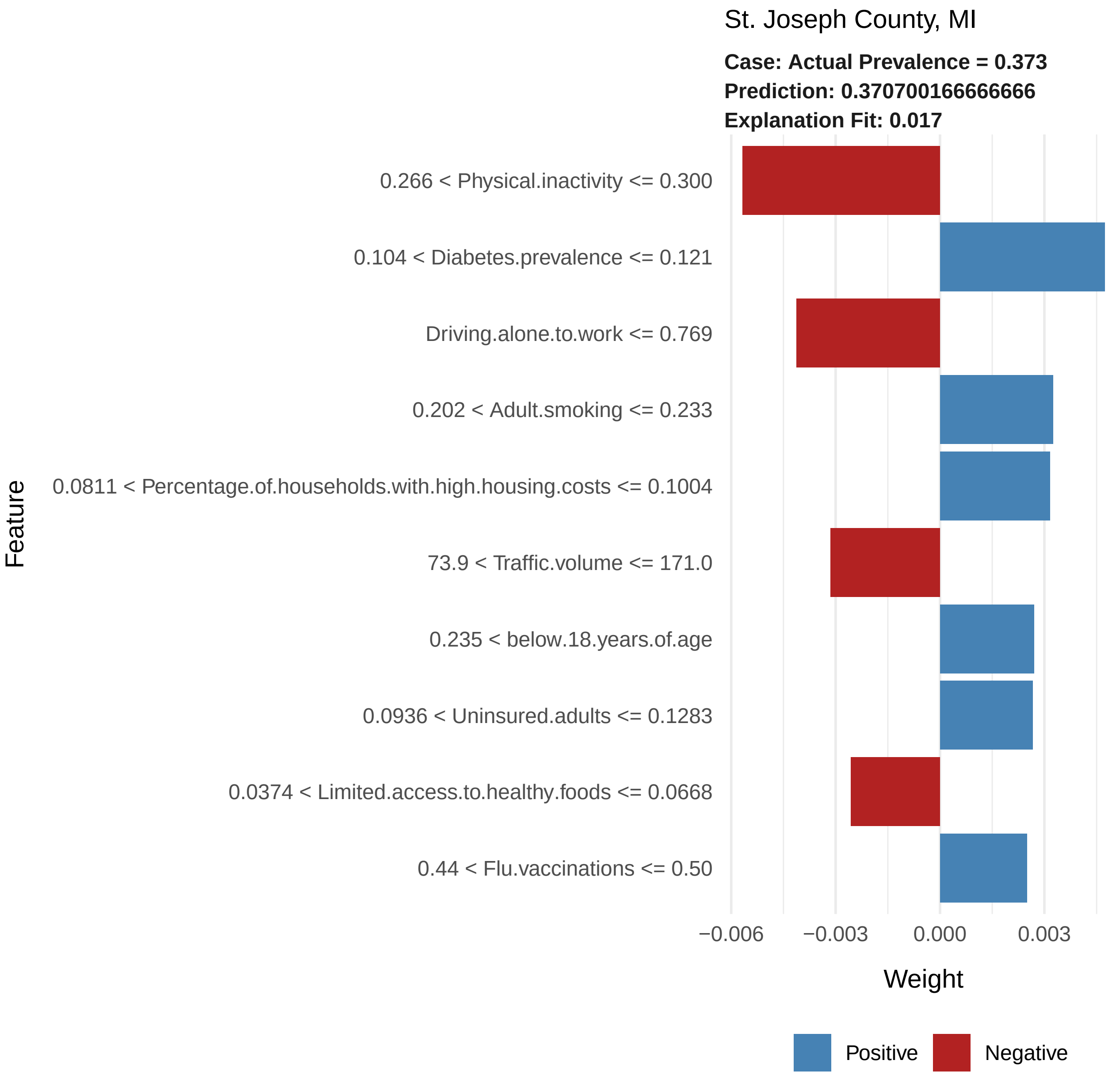
St. Joseph County, MI
Case: Actual Prevalence = 0.373
Prediction: 0.370700166666666
Explanation Fit: 0.017
0.266 < Physical.inactivity <= 0.300
0.104 < Diabetes.prevalence <= 0.121
Driving.alone.to.work <= 0.769
0.202 < Adult.smoking <= 0.233
0.0811 < Percentage.of.households.with.high.housing.costs <= 0.1004
73.9 < Traffic.volume <= 171.0
0.235 < below.18.years.of.age
0.0936 < Uninsured.adults <= 0.1283
0.0374 < Limited.access.to.healthy.foods <= 0.0668
0.44 < Flu.vaccinations <= 0.50
Feature
−0.006
−0.003
0.000
0.003
Weight
Positive
Negative

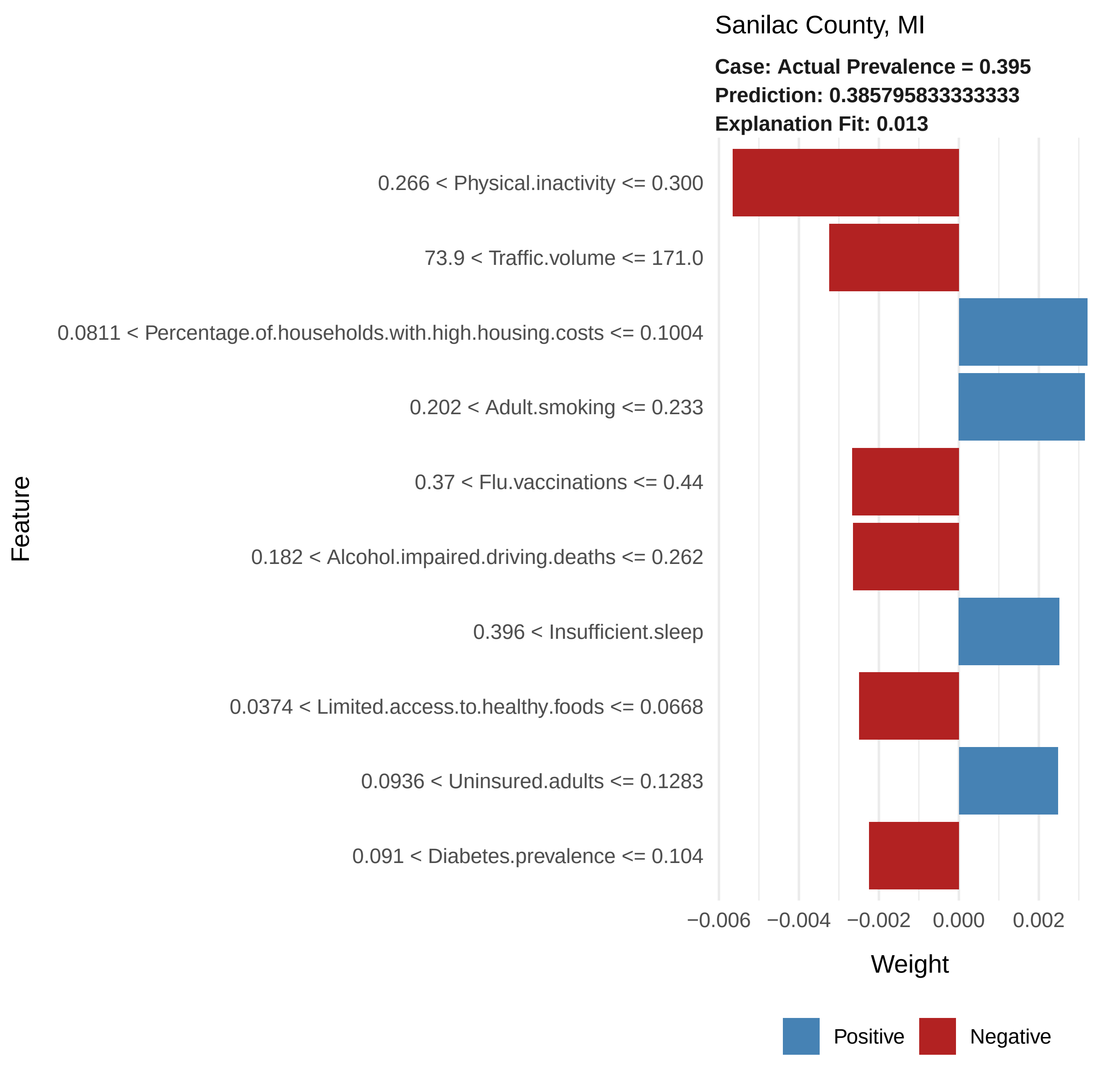
Sanilac County, MI
Case: Actual Prevalence = 0.395
Prediction: 0.385795833333333
Explanation Fit: 0.013
0.266 < Physical.inactivity <= 0.300
73.9 < Traffic.volume <= 171.0
0.0811 < Percentage.of.households.with.high.housing.costs <= 0.1004
0.202 < Adult.smoking <= 0.233
0.37 < Flu.vaccinations <= 0.44
0.182 < Alcohol.impaired.driving.deaths <= 0.262
0.396 < Insufficient.sleep
0.0374 < Limited.access.to.healthy.foods <= 0.0668
0.0936 < Uninsured.adults <= 0.1283
0.091 < Diabetes.prevalence <= 0.104
Feature
−0.006
−0.004
−0.002
0.000
0.002
Weight
Positive
Negative

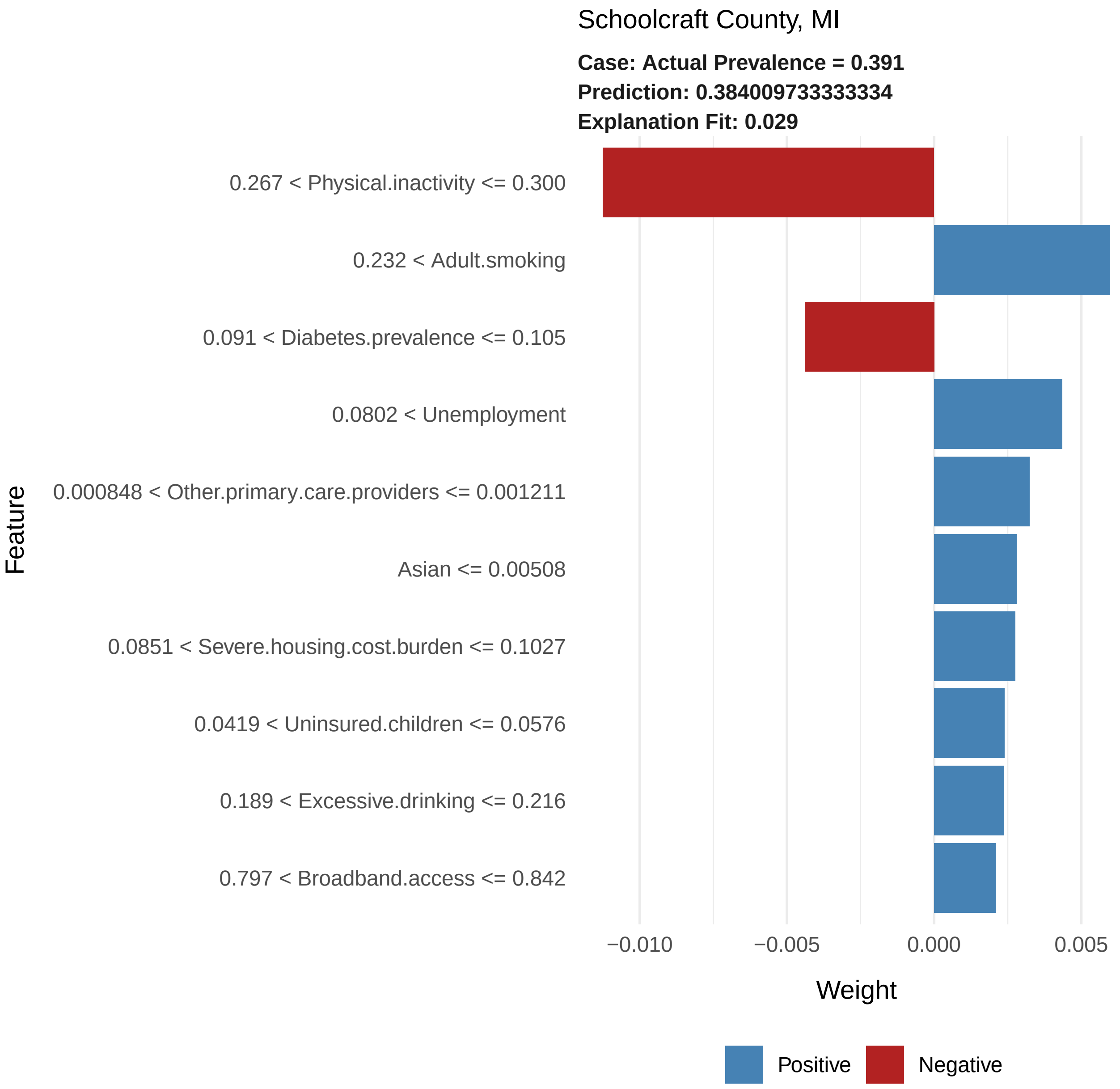

Schoolcraft County, MI
Case: Actual Prevalence = 0.391
Prediction: 0.384009733333334
Explanation Fit: 0.029
0.267 < Physical.inactivity <= 0.300
0.232 < Adult.smoking
0.091 < Diabetes.prevalence <= 0.105
0.0802 < Unemployment
0.000848 < Other.primary.care.providers <= 0.001211
Asian <= 0.00508
0.0851 < Severe.housing.cost.burden <= 0.1027
0.0419 < Uninsured.children <= 0.0576
0.189 < Excessive.drinking <= 0.216
0.797 < Broadband.access <= 0.842
Feature
−0.010
−0.005
0.000
0.005
Weight
Positive
Negative

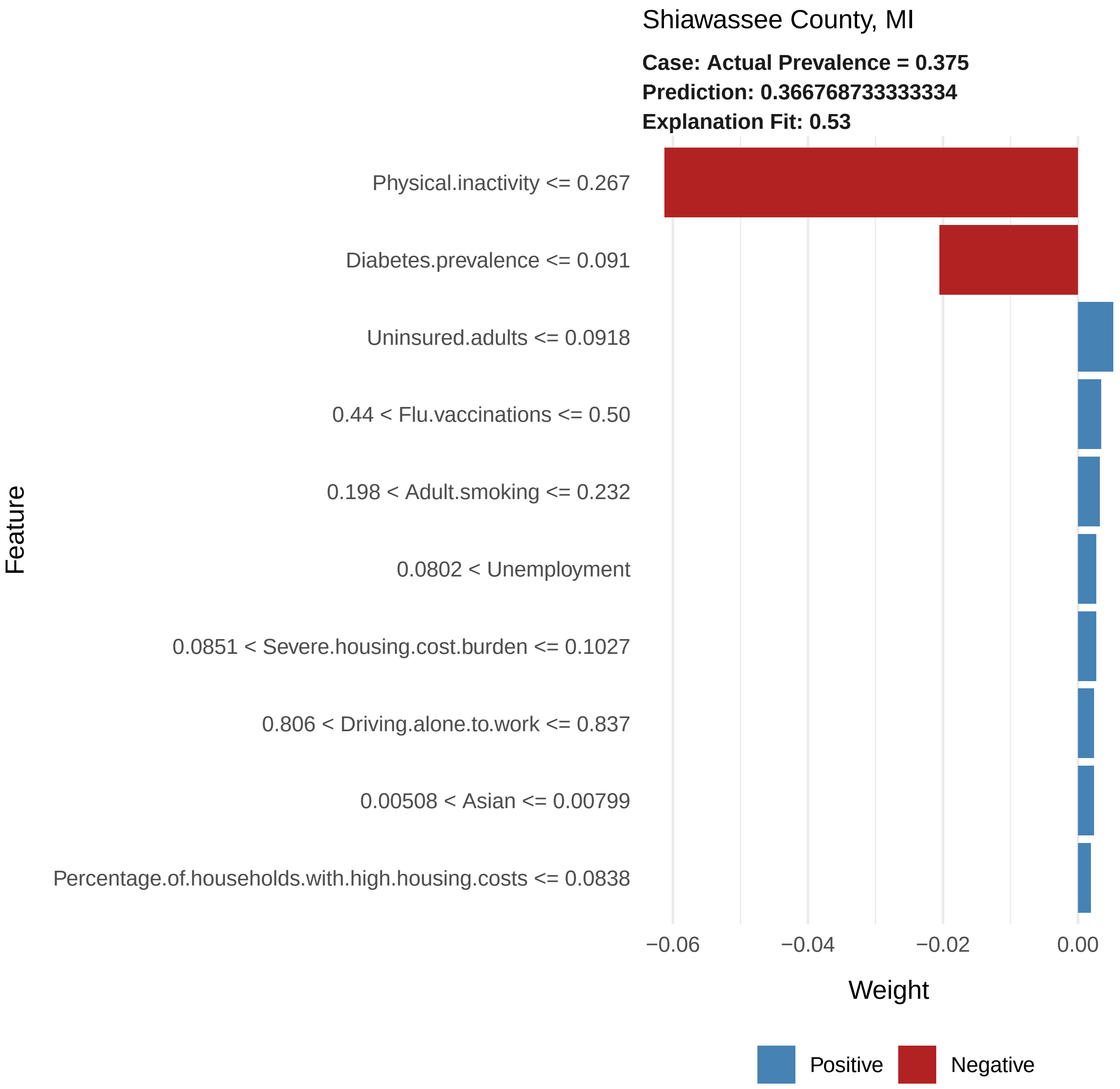

Shiawassee County, MI
Case: Actual Prevalence = 0.375
Prediction: 0.366768733333334
Explanation Fit: 0.53
Physical.inactivity <= 0.267
Diabetes.prevalence <= 0.091
Uninsured.adults <= 0.0918
0.44 < Flu.vaccinations <= 0.50
0.198 < Adult.smoking <= 0.232
0.0802 < Unemployment
0.0851 < Severe.housing.cost.burden <= 0.1027
0.806 < Driving.alone.to.work <= 0.837
0.00508 < Asian <= 0.00799
Percentage.of.households.with.high.housing.costs <= 0.0838
Feature
−0.06
−0.04
−0.02
0.00
Weight
Positive
Negative

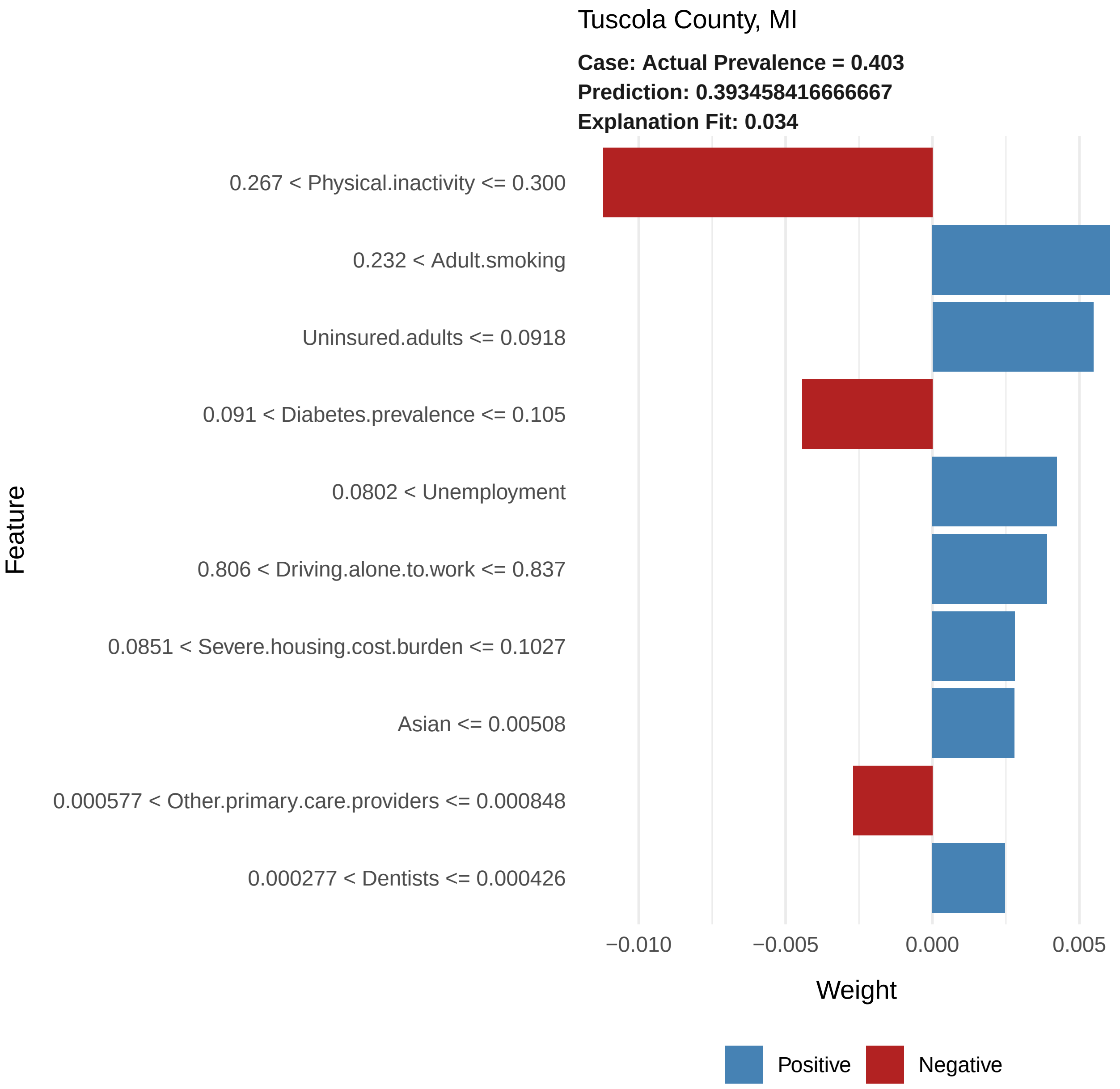
Tuscola County, MI
Case: Actual Prevalence = 0.403
Prediction: 0.393458416666667
Explanation Fit: 0.034
0.267 < Physical.inactivity <= 0.300
0.232 < Adult.smoking
Uninsured.adults <= 0.0918
0.091 < Diabetes.prevalence <= 0.105
0.0802 < Unemployment
0.806 < Driving.alone.to.work <= 0.837
0.0851 < Severe.housing.cost.burden <= 0.1027
Asian <= 0.00508
0.000577 < Other.primary.care.providers <= 0.000848
0.000277 < Dentists <= 0.000426
Feature
−0.010
−0.005
0.000
0.005
Weight
Positive
Negative

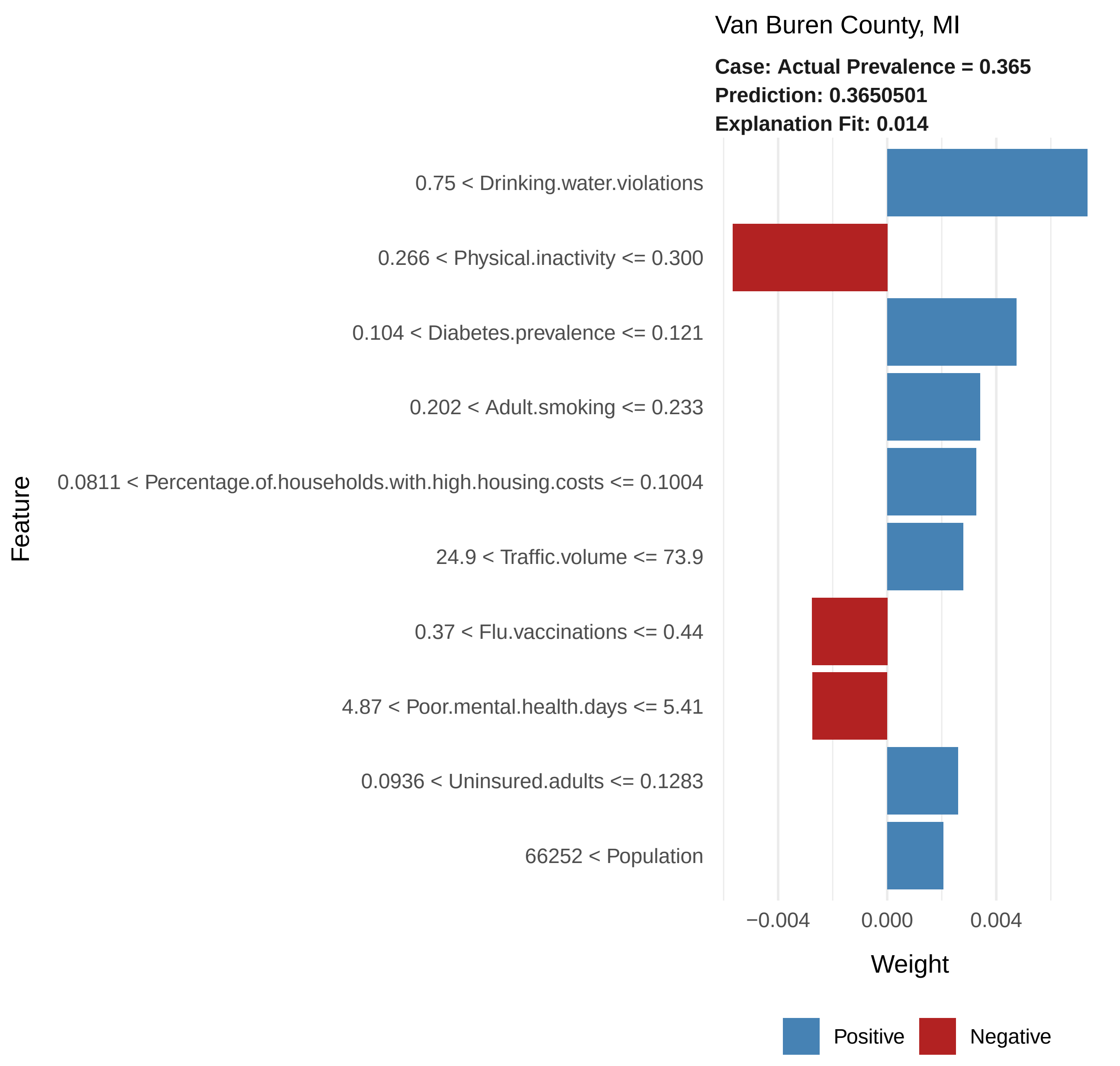
Van Buren County, MI
Case: Actual Prevalence = 0.365
Prediction: 0.3650501
Explanation Fit: 0.014
0.75 < Drinking.water.violations
0.266 < Physical.inactivity <= 0.300
0.104 < Diabetes.prevalence <= 0.121
0.202 < Adult.smoking <= 0.233
0.0811 < Percentage.of.households.with.high.housing.costs <= 0.1004
24.9 < Traffic.volume <= 73.9
0.37 < Flu.vaccinations <= 0.44
4.87 < Poor.mental.health.days <= 5.41
0.0936 < Uninsured.adults <= 0.1283
66252 < Population
Feature
−0.004
0.000
0.004
Weight
Positive
Negative

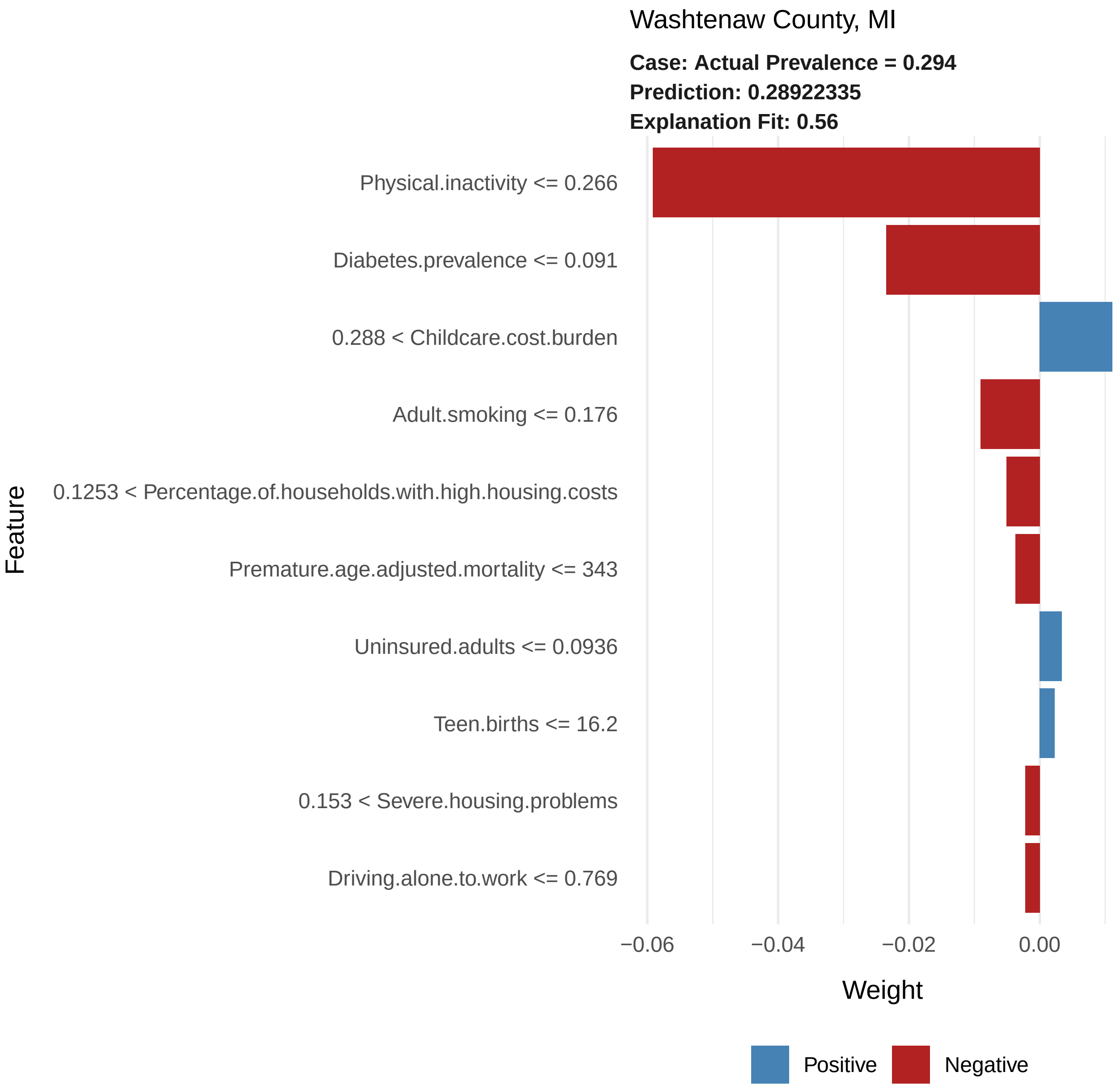

Washtenaw County, MI
Case: Actual Prevalence = 0.294
Prediction: 0.28922335
Explanation Fit: 0.56
Physical.inactivity <= 0.266
Diabetes.prevalence <= 0.091
0.288 < Childcare.cost.burden
Adult.smoking <= 0.176
0.1253 < Percentage.of.households.with.high.housing.costs
Premature.age.adjusted.mortality <= 343
Uninsured.adults <= 0.0936
Teen.births <= 16.2
0.153 < Severe.housing.problems
Driving.alone.to.work <= 0.769
Feature
−0.06
−0.04
−0.02
0.00
Weight
Positive
Negative

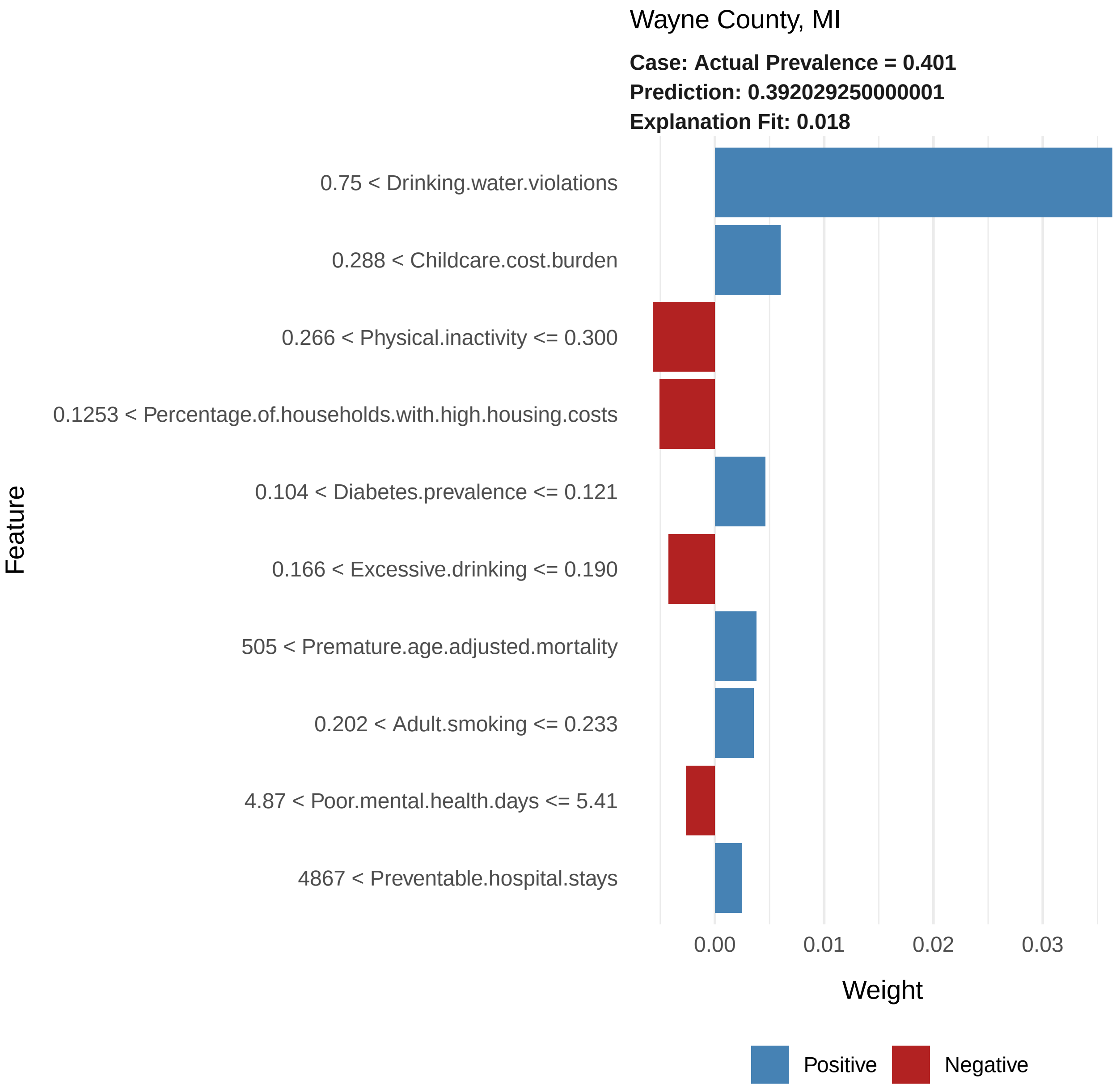

Wayne County, MI
Case: Actual Prevalence = 0.401
Prediction: 0.392029250000001
Explanation Fit: 0.018
0.75 < Drinking.water.violations
0.288 < Childcare.cost.burden
0.266 < Physical.inactivity <= 0.300
0.1253 < Percentage.of.households.with.high.housing.costs
0.104 < Diabetes.prevalence <= 0.121
0.166 < Excessive.drinking <= 0.190
505 < Premature.age.adjusted.mortality
0.202 < Adult.smoking <= 0.233
4.87 < Poor.mental.health.days <= 5.41
4867 < Preventable.hospital.stays
Feature
0.00
0.01
0.02
0.03
Weight
Positive
Negative

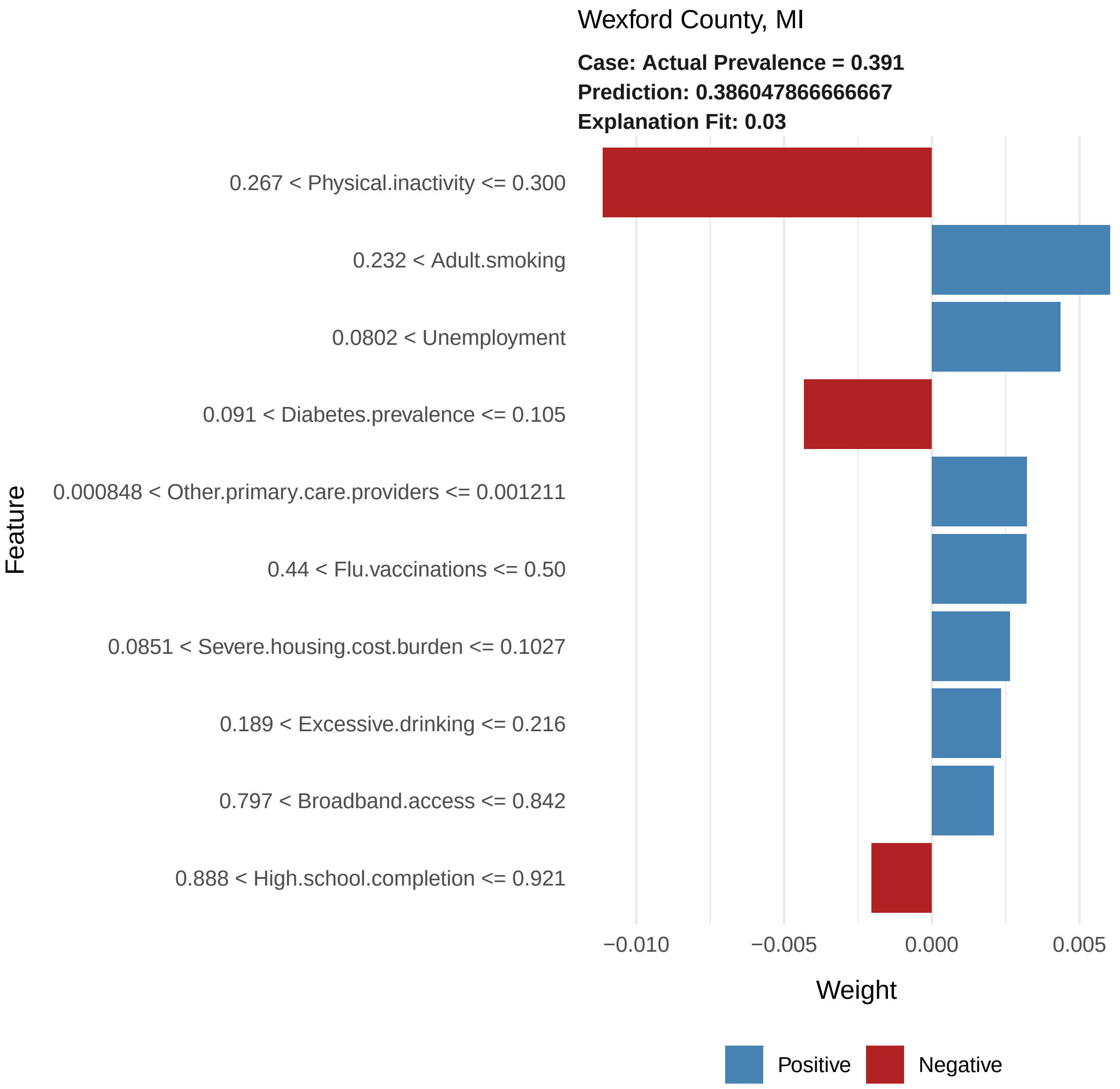
Wexford County, MI
Case: Actual Prevalence = 0.391
Prediction: 0.386047866666667
Explanation Fit: 0.03
0.267 < Physical.inactivity <= 0.300
0.232 < Adult.smoking
0.0802 < Unemployment
0.091 < Diabetes.prevalence <= 0.105
0.000848 < Other.primary.care.providers <= 0.001211
0.44 < Flu.vaccinations <= 0.50
0.0851 < Severe.housing.cost.burden <= 0.1027
0.189 < Excessive.drinking <= 0.216
0.797 < Broadband.access <= 0.842
0.888 < High.school.completion <= 0.921
Feature
−0.010
−0.005
0.000
0.005
Weight
Positive
Negative

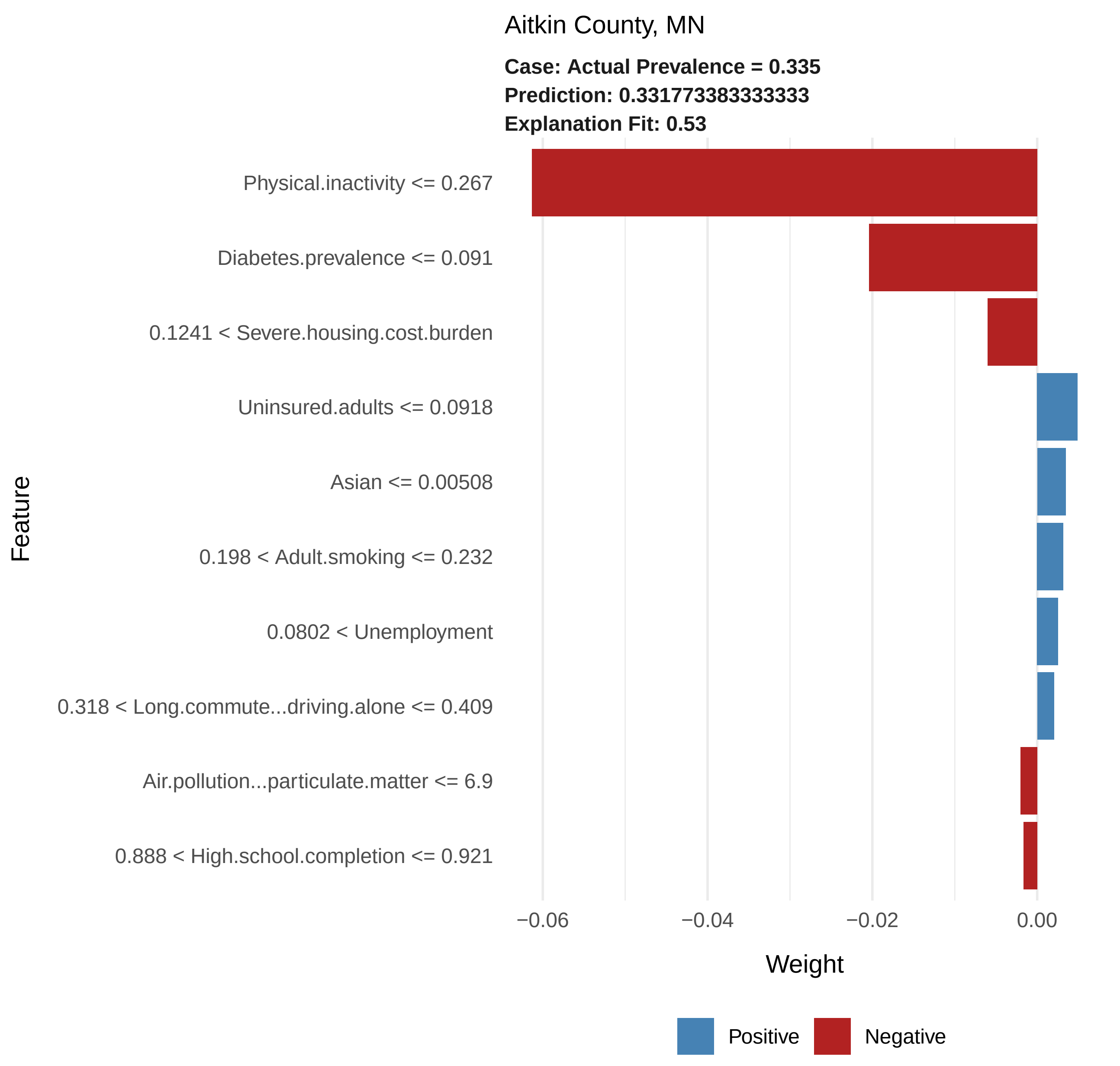

Aitkin County, MN
Case: Actual Prevalence = 0.335
Prediction: 0.331773383333333
Explanation Fit: 0.53
Physical.inactivity <= 0.267
Diabetes.prevalence <= 0.091
0.1241 < Severe.housing.cost.burden
Uninsured.adults <= 0.0918
Asian <= 0.00508
0.198 < Adult.smoking <= 0.232
0.0802 < Unemployment
0.318 < Long.commute...driving.alone <= 0.409
Air.pollution...particulate.matter <= 6.9
0.888 < High.school.completion <= 0.921
Feature
−0.06
−0.04
−0.02
0.00
Weight
Positive
Negative

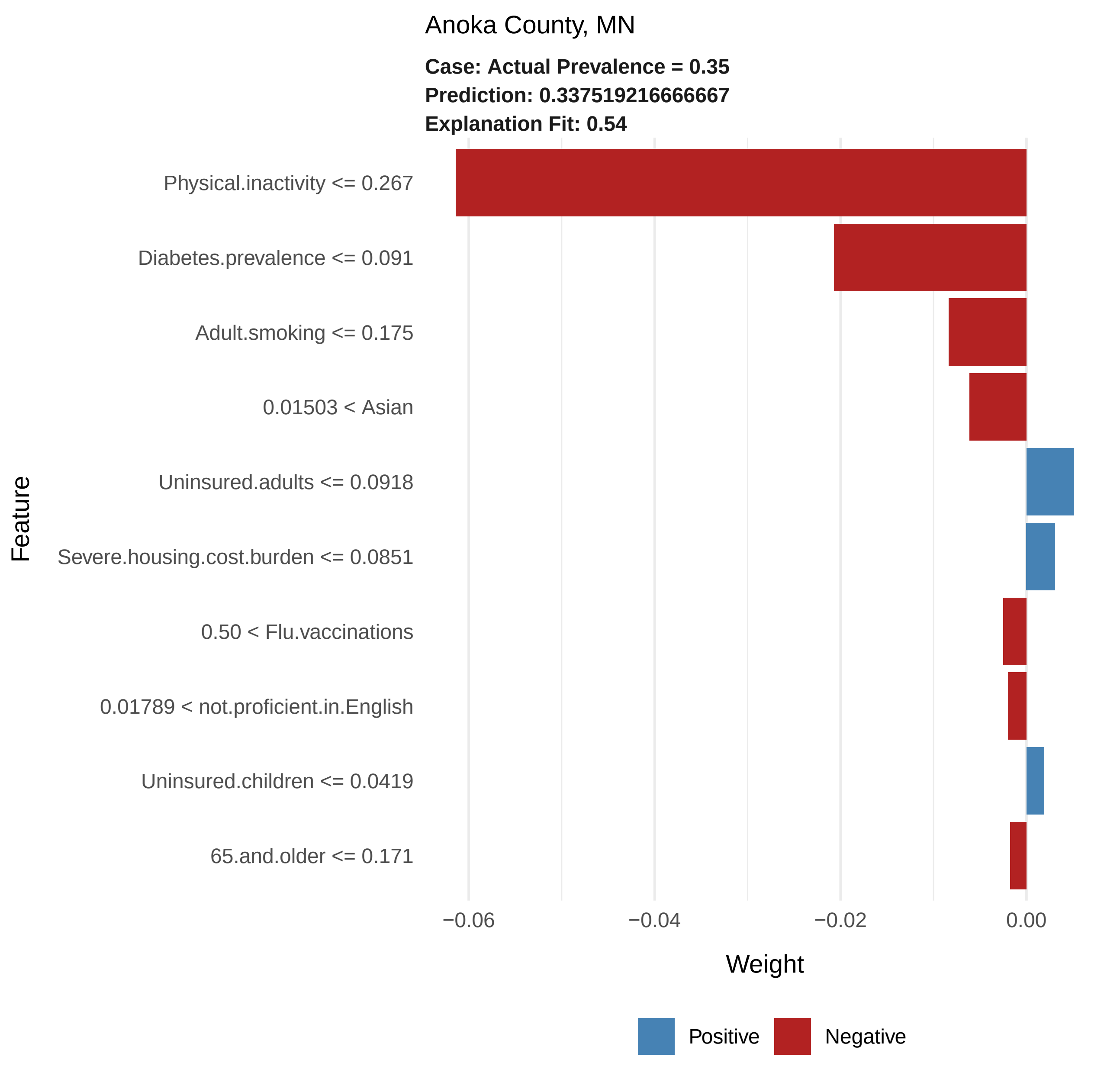

Anoka County, MN
Case: Actual Prevalence = 0.35
Prediction: 0.337519216666667
Explanation Fit: 0.54
Physical.inactivity <= 0.267
Diabetes.prevalence <= 0.091
Adult.smoking <= 0.175
0.01503 < Asian
Uninsured.adults <= 0.0918
Severe.housing.cost.burden <= 0.0851
0.50 < Flu.vaccinations
0.01789 < not.proficient.in.English
Uninsured.children <= 0.0419
65.and.older <= 0.171
Feature
−0.06
−0.04
−0.02
0.00
Weight
Positive
Negative

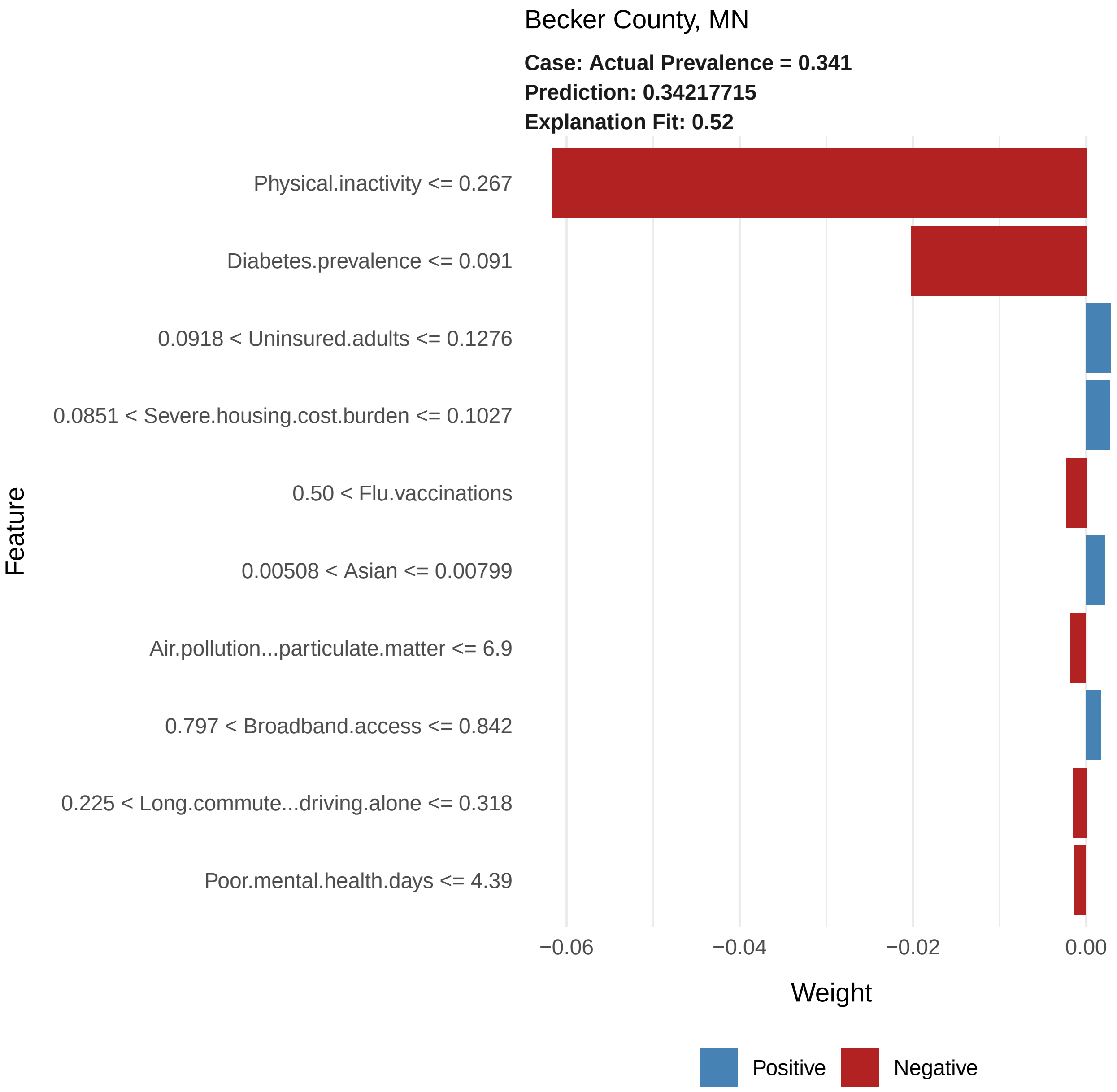

Becker County, MN
Case: Actual Prevalence = 0.341
Prediction: 0.34217715
Explanation Fit: 0.52
Physical.inactivity <= 0.267
Diabetes.prevalence <= 0.091
0.0918 < Uninsured.adults <= 0.1276
0.0851 < Severe.housing.cost.burden <= 0.1027
0.50 < Flu.vaccinations
0.00508 < Asian <= 0.00799
Air.pollution...particulate.matter <= 6.9
0.797 < Broadband.access <= 0.842
0.225 < Long.commute...driving.alone <= 0.318
Poor.mental.health.days <= 4.39
Feature
−0.06
−0.04
−0.02
0.00
Weight
Positive
Negative

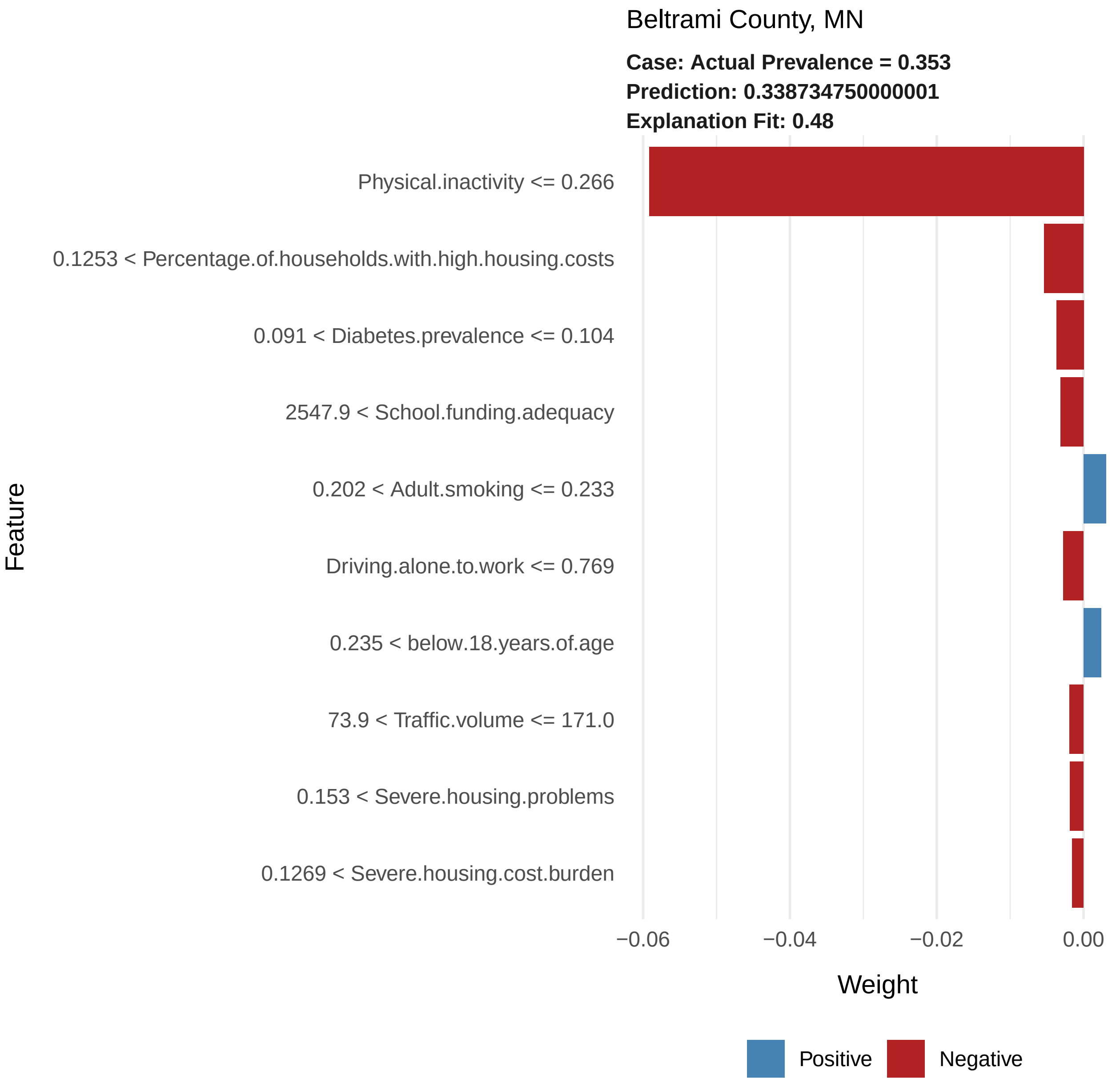

Beltrami County, MN
Case: Actual Prevalence = 0.353
Prediction: 0.338734750000001
Explanation Fit: 0.48
Physical.inactivity <= 0.266
0.1253 < Percentage.of.households.with.high.housing.costs
0.091 < Diabetes.prevalence <= 0.104
2547.9 < School.funding.adequacy
0.202 < Adult.smoking <= 0.233
Driving.alone.to.work <= 0.769
0.235 < below.18.years.of.age
73.9 < Traffic.volume <= 171.0
0.153 < Severe.housing.problems
0.1269 < Severe.housing.cost.burden
Feature
−0.06
−0.04
−0.02
0.00
Weight
Positive
Negative

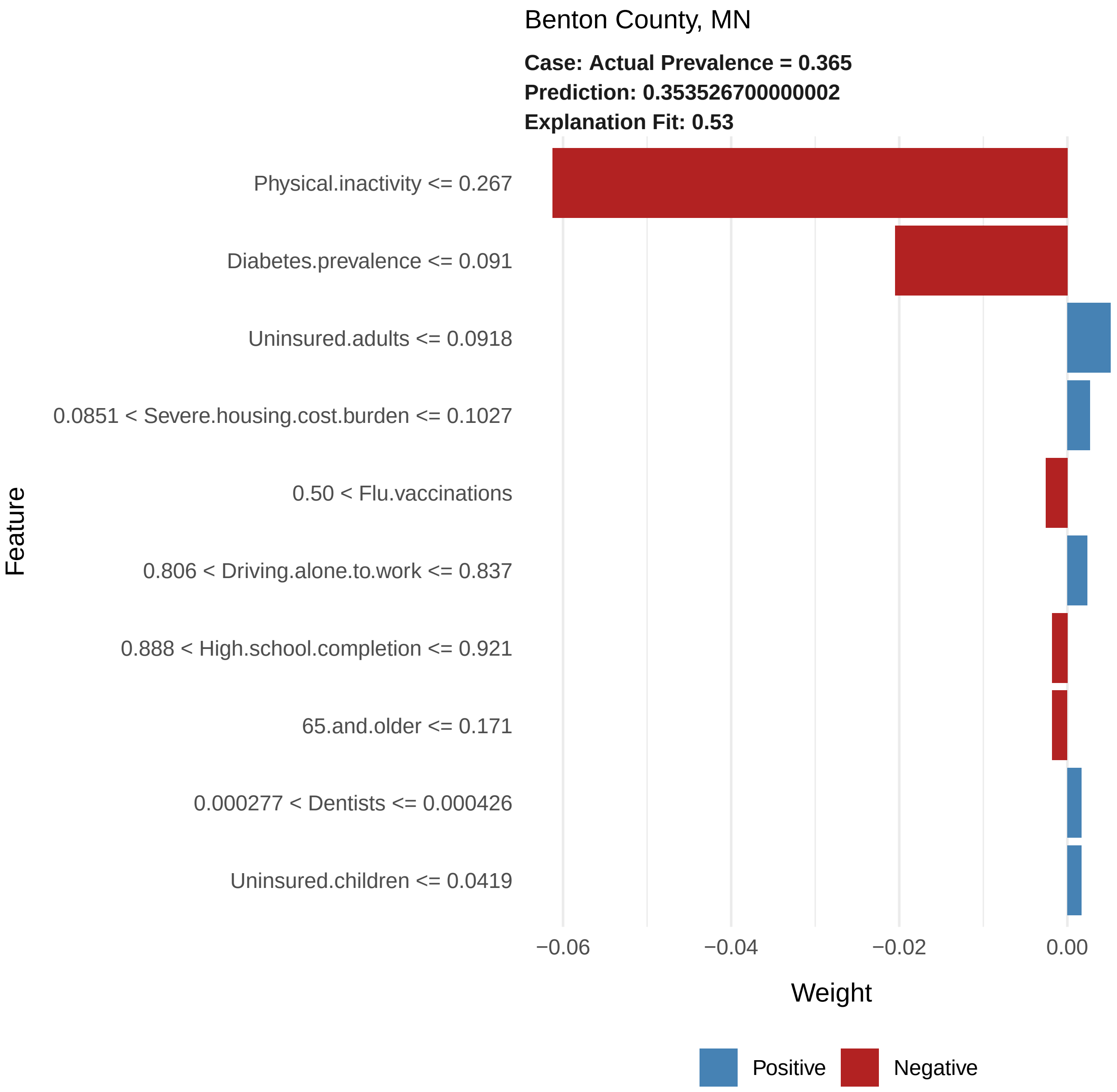
Benton County, MN
Case: Actual Prevalence = 0.365
Prediction: 0.353526700000002
Explanation Fit: 0.53
Physical.inactivity <= 0.267
Diabetes.prevalence <= 0.091
Uninsured.adults <= 0.0918
0.0851 < Severe.housing.cost.burden <= 0.1027
0.50 < Flu.vaccinations
0.806 < Driving.alone.to.work <= 0.837
0.888 < High.school.completion <= 0.921
65.and.older <= 0.171
0.000277 < Dentists <= 0.000426
Uninsured.children <= 0.0419
Feature
−0.06
−0.04
−0.02
0.00
Weight
Positive
Negative

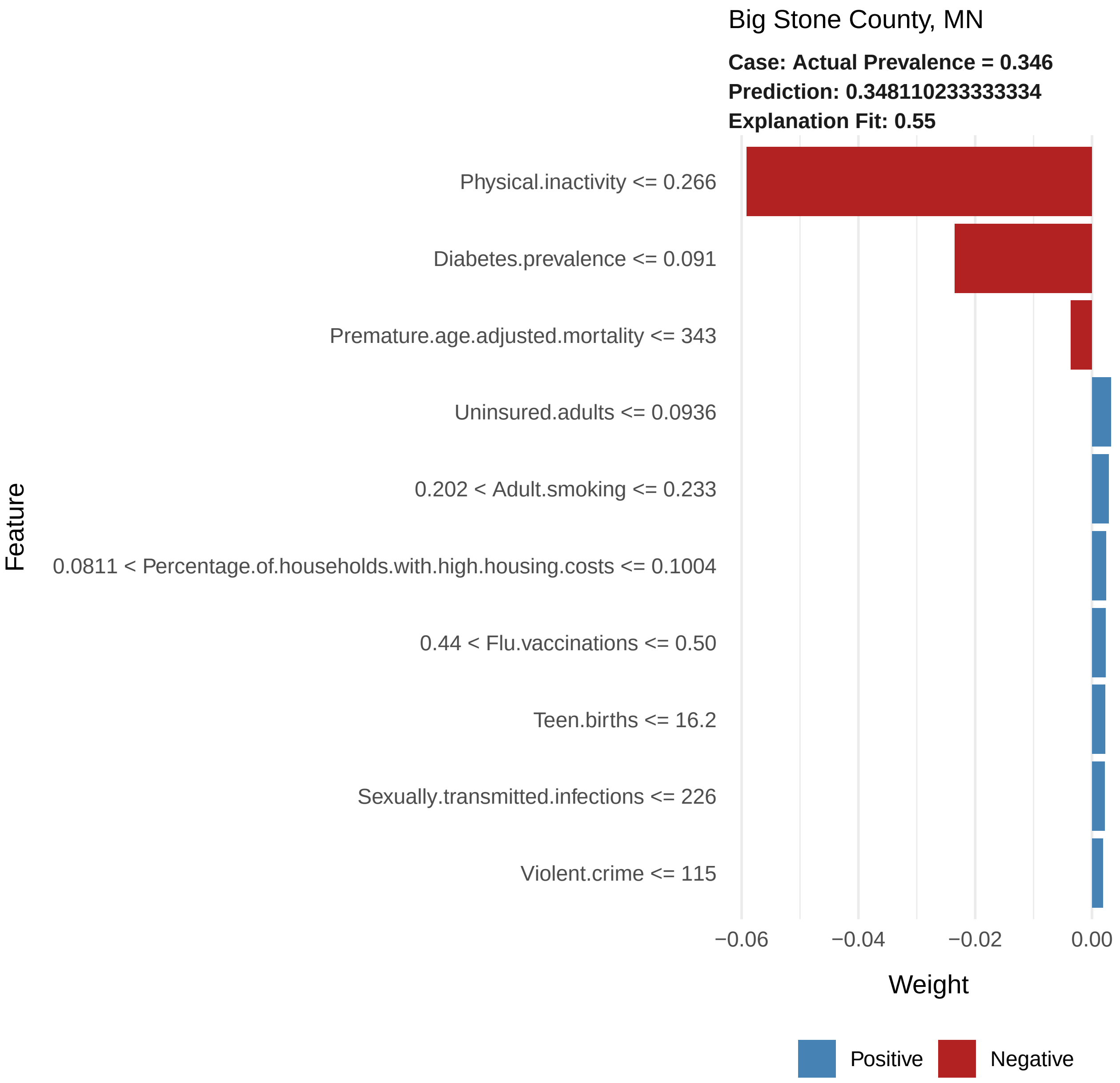
Big Stone County, MN
Case: Actual Prevalence = 0.346
Prediction: 0.348110233333334
Explanation Fit: 0.55
Physical.inactivity <= 0.266
Diabetes.prevalence <= 0.091
Premature.age.adjusted.mortality <= 343
Uninsured.adults <= 0.0936
0.202 < Adult.smoking <= 0.233
0.0811 < Percentage.of.households.with.high.housing.costs <= 0.1004
0.44 < Flu.vaccinations <= 0.50
Teen.births <= 16.2
Sexually.transmitted.infections <= 226
Violent.crime <= 115
Feature
−0.06
−0.04
−0.02
0.00
Weight
Positive
Negative

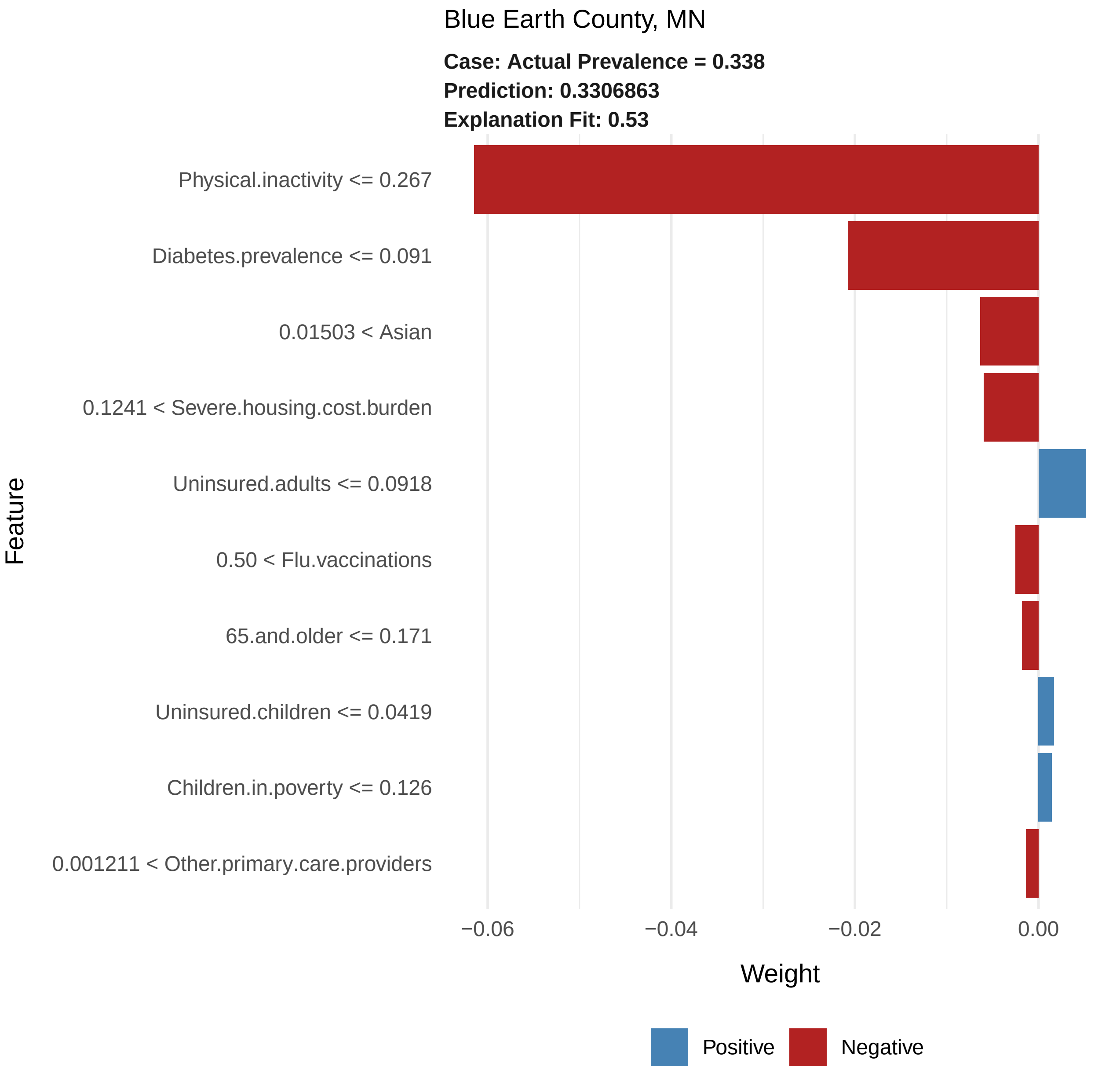

Blue Earth County, MN
Case: Actual Prevalence = 0.338
Prediction: 0.3306863
Explanation Fit: 0.53
Physical.inactivity <= 0.267
Diabetes.prevalence <= 0.091
0.01503 < Asian
0.1241 < Severe.housing.cost.burden
Uninsured.adults <= 0.0918
0.50 < Flu.vaccinations
65.and.older <= 0.171
Uninsured.children <= 0.0419
Children.in.poverty <= 0.126
0.001211 < Other.primary.care.providers
Feature
−0.06
−0.04
−0.02
0.00
Weight
Positive
Negative

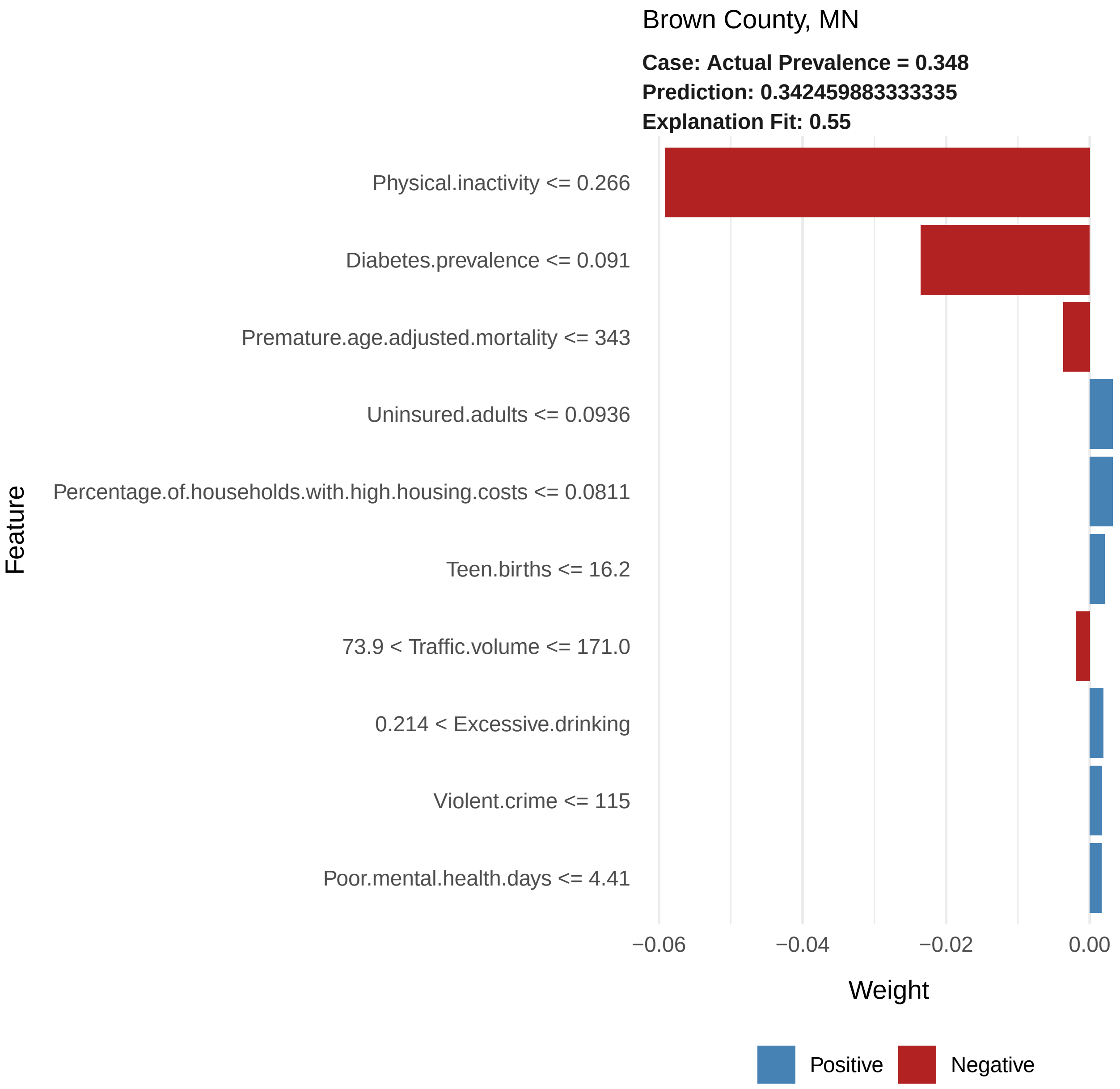
Brown County, MN
Case: Actual Prevalence = 0.348
Prediction: 0.342459883333335
Explanation Fit: 0.55
Physical.inactivity <= 0.266
Diabetes.prevalence <= 0.091
Premature.age.adjusted.mortality <= 343
Uninsured.adults <= 0.0936
Percentage.of.households.with.high.housing.costs <= 0.0811
Teen.births <= 16.2
73.9 < Traffic.volume <= 171.0
0.214 < Excessive.drinking
Violent.crime <= 115
Poor.mental.health.days <= 4.41
Feature
−0.06
−0.04
−0.02
0.00
Weight
Positive
Negative

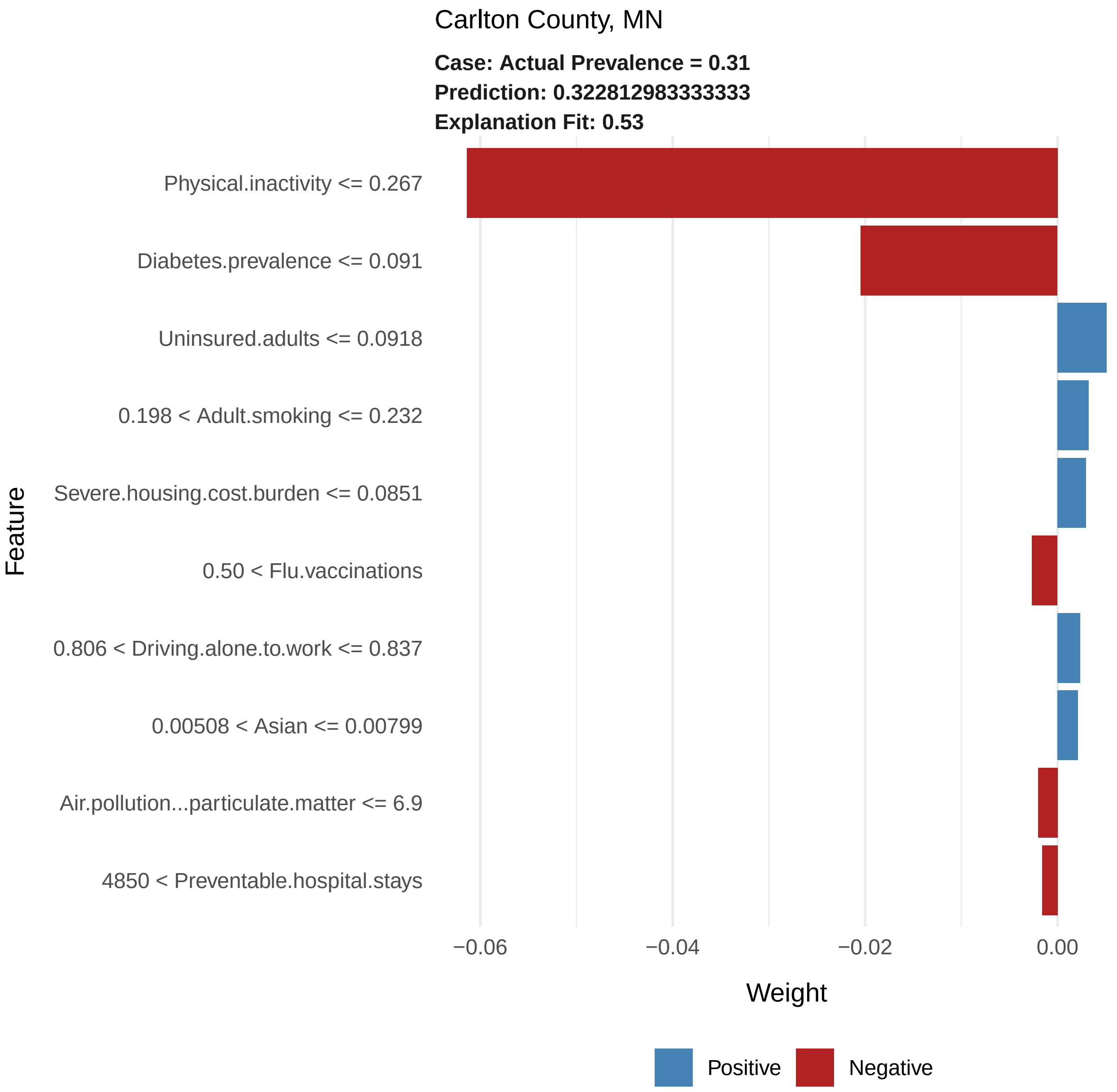

Carlton County, MN
Case: Actual Prevalence = 0.31
Prediction: 0.322812983333333
Explanation Fit: 0.53
Physical.inactivity <= 0.267
Diabetes.prevalence <= 0.091
Uninsured.adults <= 0.0918
0.198 < Adult.smoking <= 0.232
Severe.housing.cost.burden <= 0.0851
0.50 < Flu.vaccinations
0.806 < Driving.alone.to.work <= 0.837
0.00508 < Asian <= 0.00799
Air.pollution...particulate.matter <= 6.9
4850 < Preventable.hospital.stays
Feature
−0.06
−0.04
−0.02
0.00
Weight
Positive
Negative

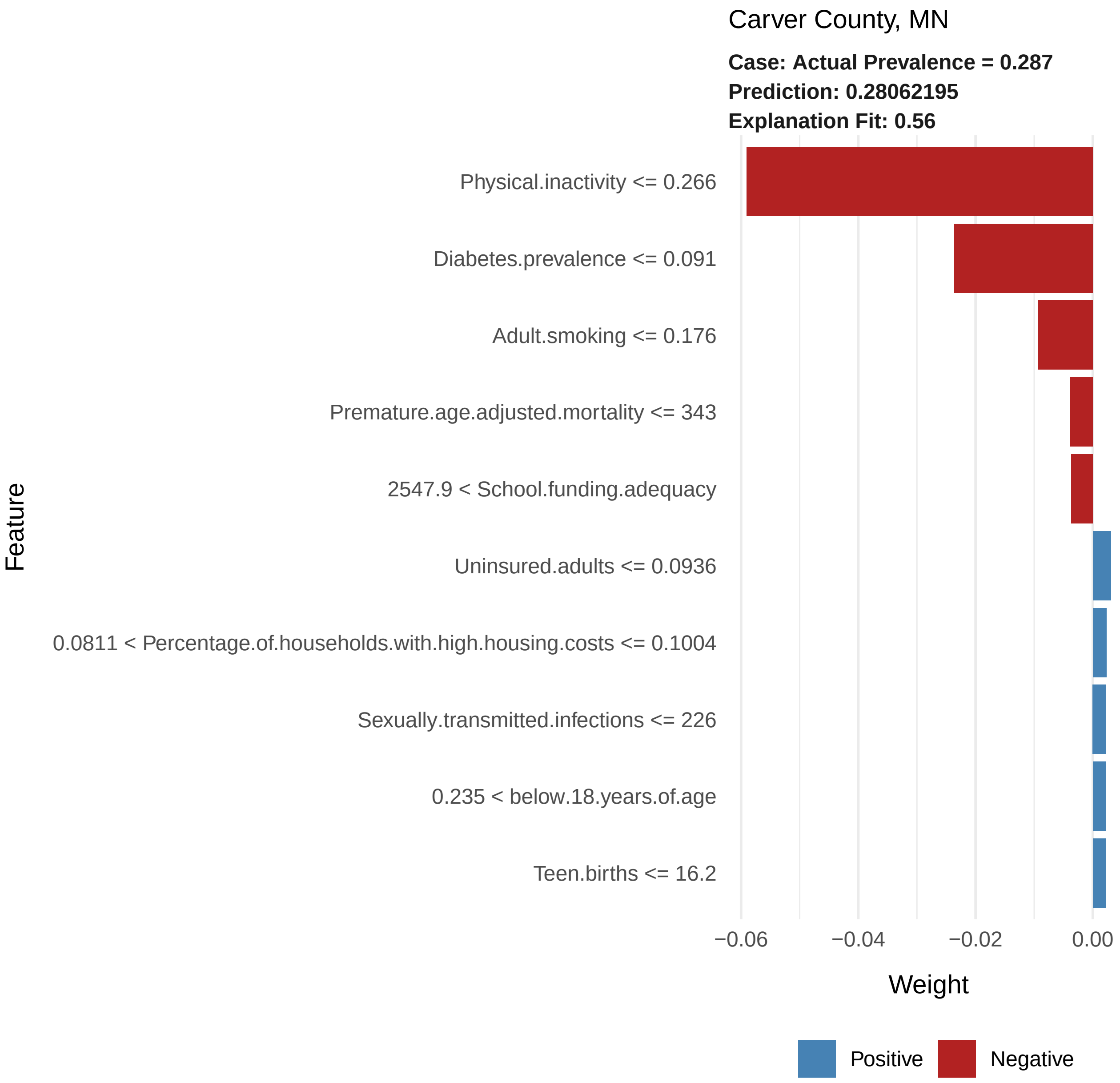

Carver County, MN
Case: Actual Prevalence = 0.287
Prediction: 0.28062195
Explanation Fit: 0.56
Physical.inactivity <= 0.266
Diabetes.prevalence <= 0.091
Adult.smoking <= 0.176
Premature.age.adjusted.mortality <= 343
2547.9 < School.funding.adequacy
Uninsured.adults <= 0.0936
0.0811 < Percentage.of.households.with.high.housing.costs <= 0.1004
Sexually.transmitted.infections <= 226
0.235 < below.18.years.of.age
Teen.births <= 16.2
Feature
−0.06
−0.04
−0.02
0.00
Weight
Positive
Negative

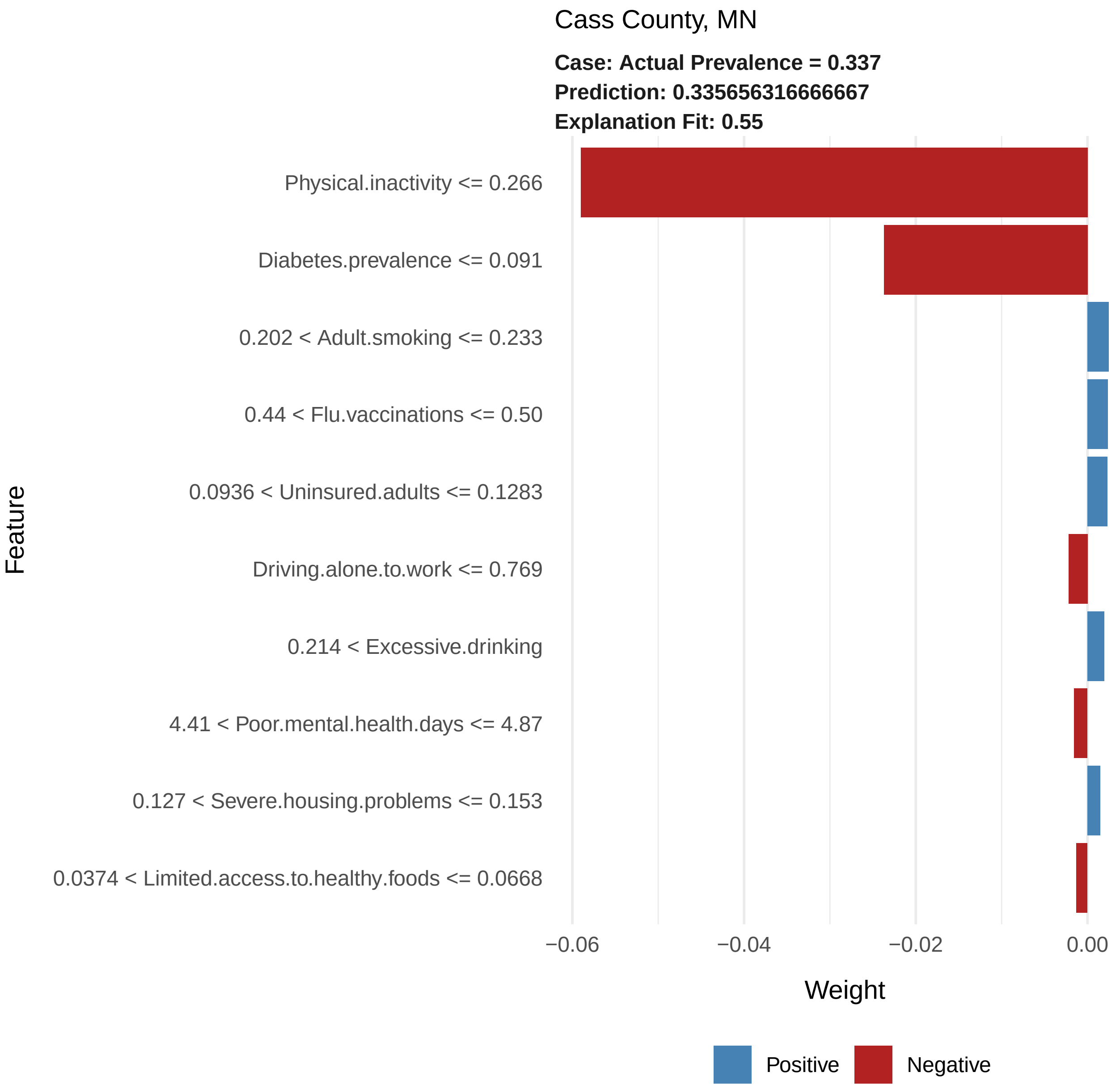
Cass County, MN
Case: Actual Prevalence = 0.337
Prediction: 0.335656316666667
Explanation Fit: 0.55
Physical.inactivity <= 0.266
Diabetes.prevalence <= 0.091
0.202 < Adult.smoking <= 0.233
0.44 < Flu.vaccinations <= 0.50
0.0936 < Uninsured.adults <= 0.1283
Driving.alone.to.work <= 0.769
0.214 < Excessive.drinking
4.41 < Poor.mental.health.days <= 4.87
0.127 < Severe.housing.problems <= 0.153
0.0374 < Limited.access.to.healthy.foods <= 0.0668
Feature
−0.06
−0.04
−0.02
0.00
Weight
Positive
Negative

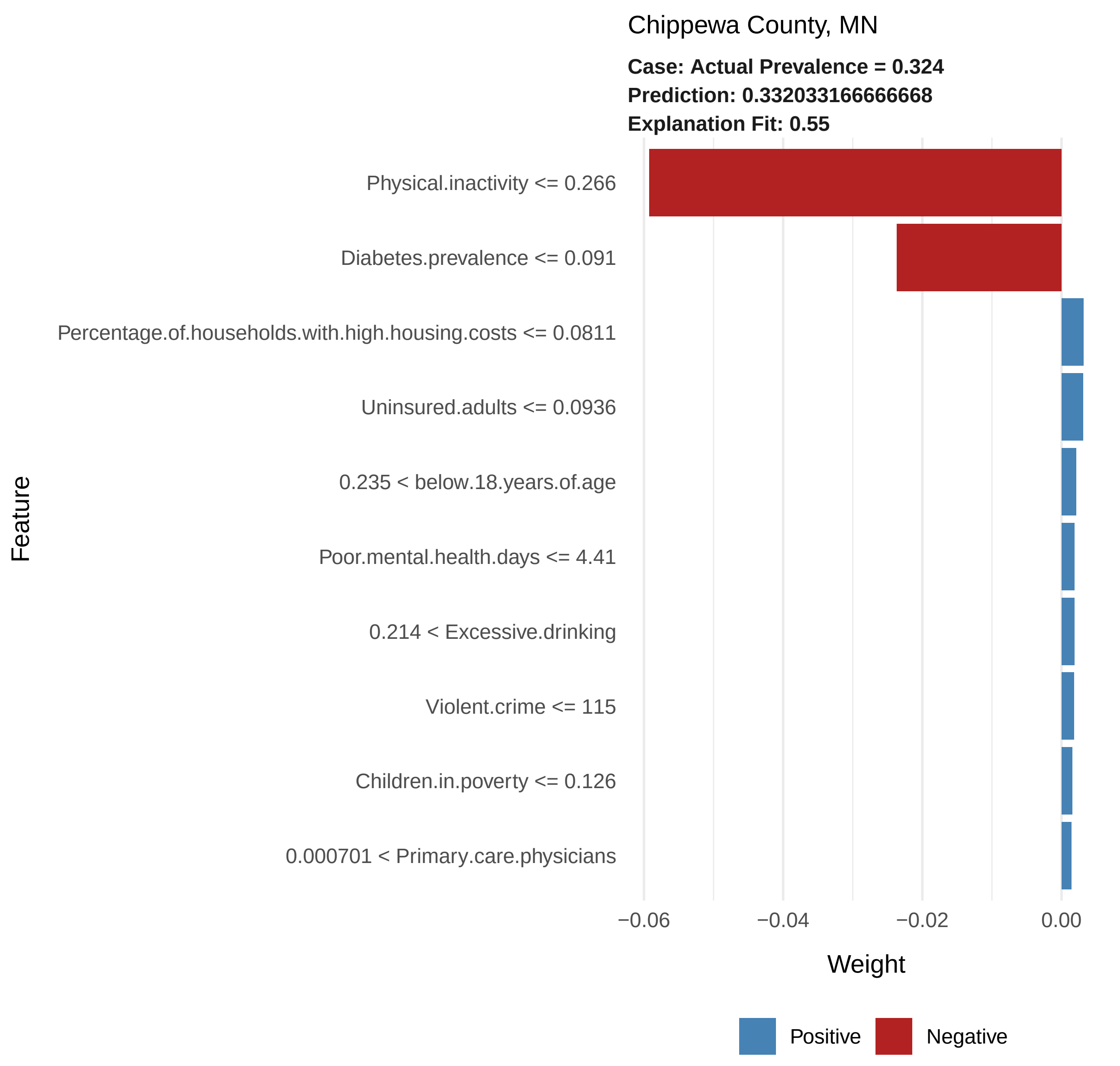

Chippewa County, MN
Case: Actual Prevalence = 0.324
Prediction: 0.332033166666668
Explanation Fit: 0.55
Physical.inactivity <= 0.266
Diabetes.prevalence <= 0.091
Percentage.of.households.with.high.housing.costs <= 0.0811
Uninsured.adults <= 0.0936
0.235 < below.18.years.of.age
Poor.mental.health.days <= 4.41
0.214 < Excessive.drinking
Violent.crime <= 115
Children.in.poverty <= 0.126
0.000701 < Primary.care.physicians
Feature
−0.06
−0.04
−0.02
0.00
Weight
Positive
Negative

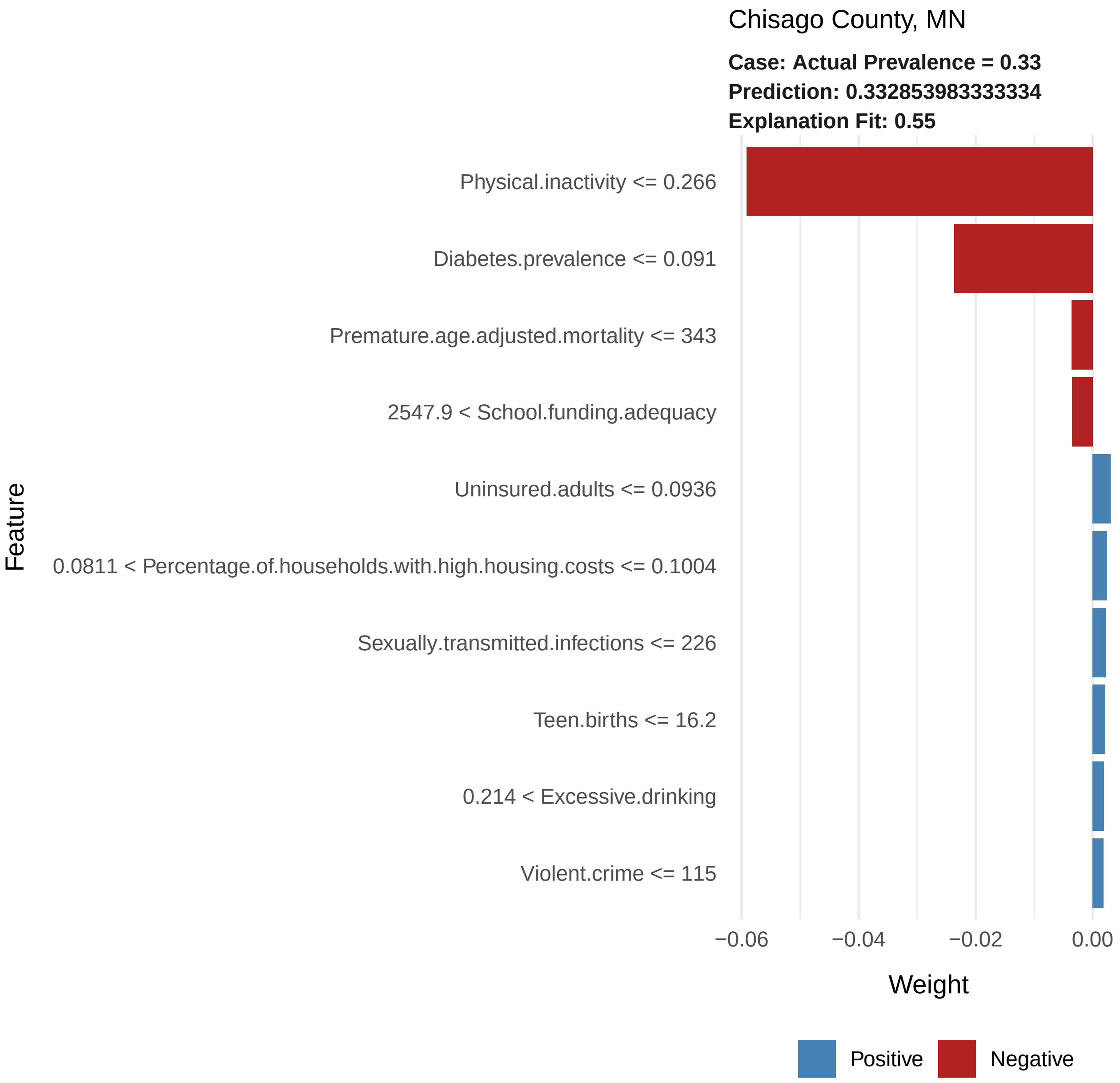

Chisago County, MN
Case: Actual Prevalence = 0.33
Prediction: 0.332853983333334
Explanation Fit: 0.55
Physical.inactivity <= 0.266
Diabetes.prevalence <= 0.091
Premature.age.adjusted.mortality <= 343
2547.9 < School.funding.adequacy
Uninsured.adults <= 0.0936
0.0811 < Percentage.of.households.with.high.housing.costs <= 0.1004
Sexually.transmitted.infections <= 226
Teen.births <= 16.2
0.214 < Excessive.drinking
Violent.crime <= 115
Feature
−0.06
−0.04
−0.02
0.00
Weight
Positive
Negative

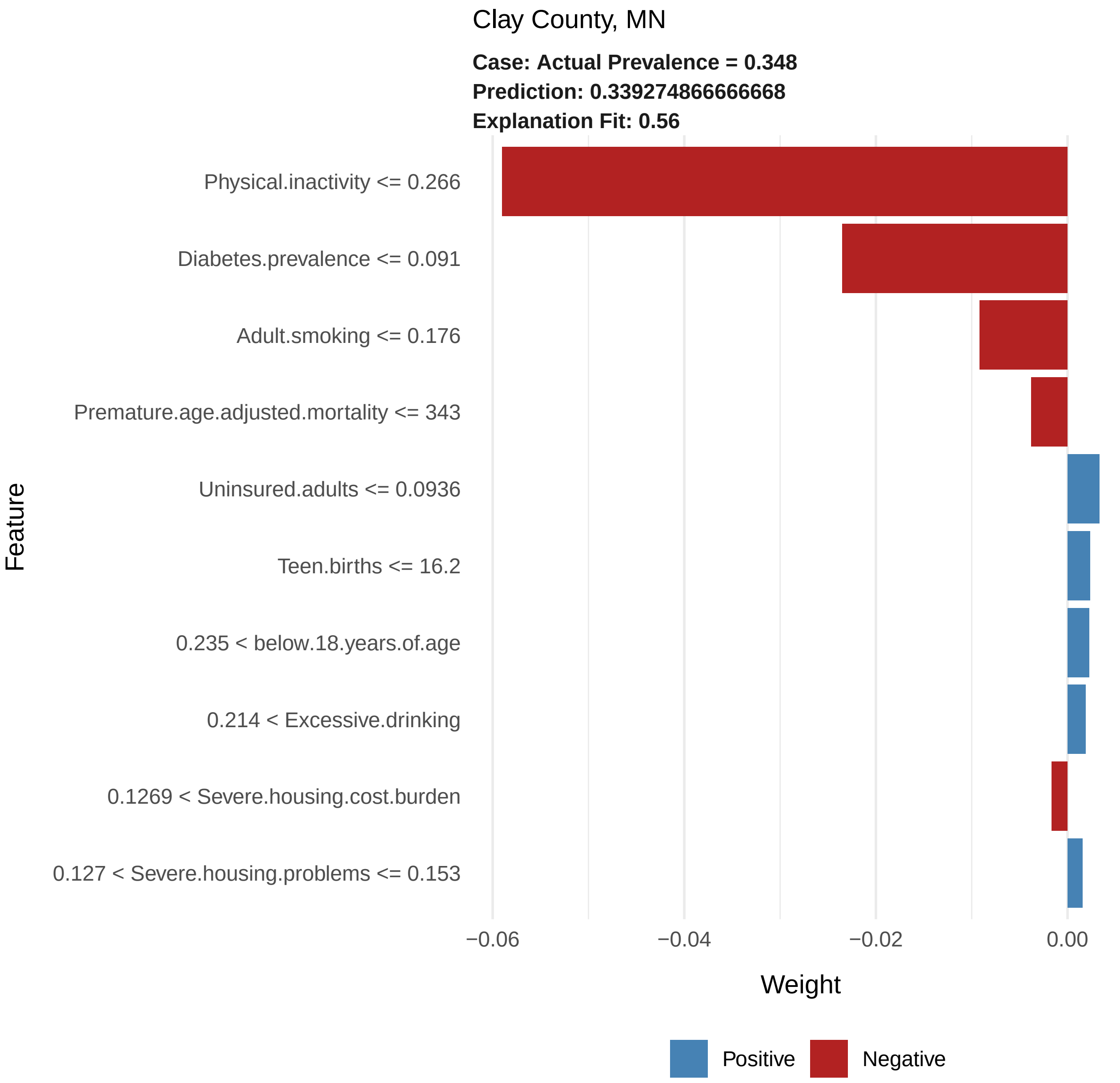

Clay County, MN
Case: Actual Prevalence = 0.348
Prediction: 0.339274866666668
Explanation Fit: 0.56
Physical.inactivity <= 0.266
Diabetes.prevalence <= 0.091
Adult.smoking <= 0.176
Premature.age.adjusted.mortality <= 343
Uninsured.adults <= 0.0936
Teen.births <= 16.2
0.235 < below.18.years.of.age
0.214 < Excessive.drinking
0.1269 < Severe.housing.cost.burden
0.127 < Severe.housing.problems <= 0.153
Feature
−0.06
−0.04
−0.02
0.00
Weight
Positive
Negative

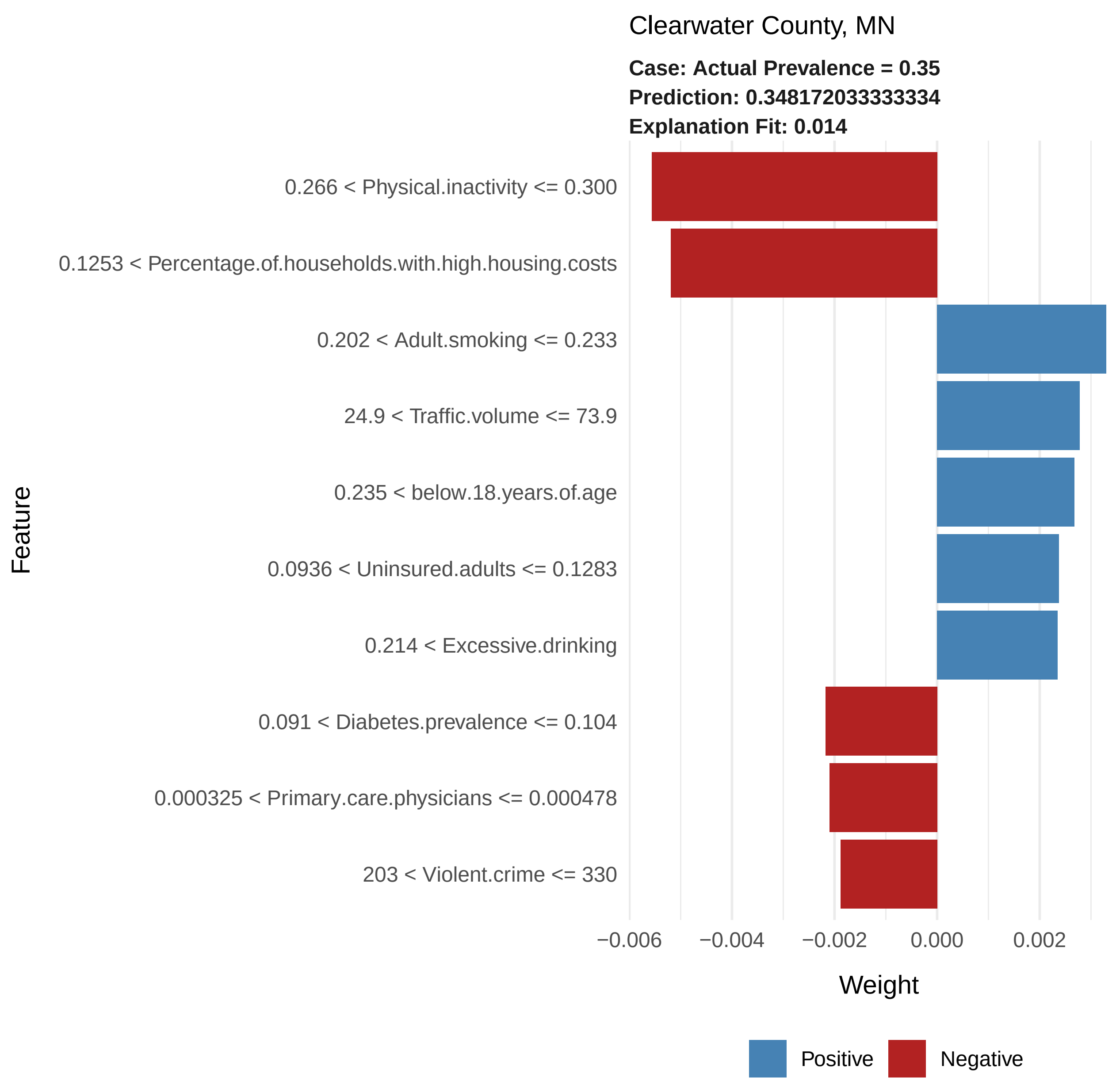

Clearwater County, MN
Case: Actual Prevalence = 0.35
Prediction: 0.348172033333334
Explanation Fit: 0.014
0.266 < Physical.inactivity <= 0.300
0.1253 < Percentage.of.households.with.high.housing.costs
0.202 < Adult.smoking <= 0.233
24.9 < Traffic.volume <= 73.9
0.235 < below.18.years.of.age
0.0936 < Uninsured.adults <= 0.1283
0.214 < Excessive.drinking
0.091 < Diabetes.prevalence <= 0.104
0.000325 < Primary.care.physicians <= 0.000478
203 < Violent.crime <= 330
Feature
−0.006
−0.004
−0.002
0.000
0.002
Weight
Positive
Negative

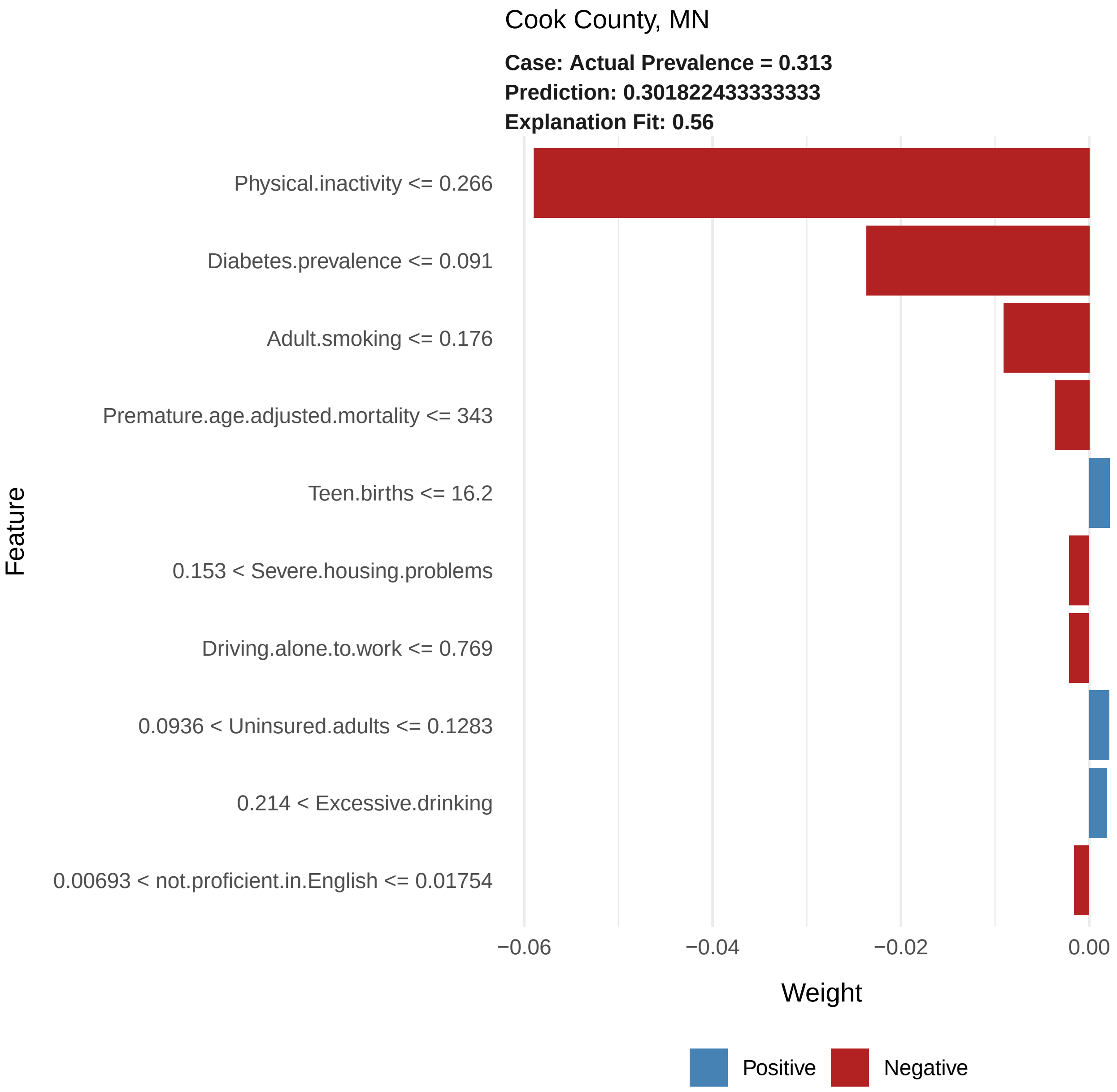
Cook County, MN
Case: Actual Prevalence = 0.313
Prediction: 0.301822433333333
Explanation Fit: 0.56
Physical.inactivity <= 0.266
Diabetes.prevalence <= 0.091
Adult.smoking <= 0.176
Premature.age.adjusted.mortality <= 343
Teen.births <= 16.2
0.153 < Severe.housing.problems
Driving.alone.to.work <= 0.769
0.0936 < Uninsured.adults <= 0.1283
0.214 < Excessive.drinking
0.00693 < not.proficient.in.English <= 0.01754
Feature
−0.06
−0.04
−0.02
0.00
Weight
Positive
Negative

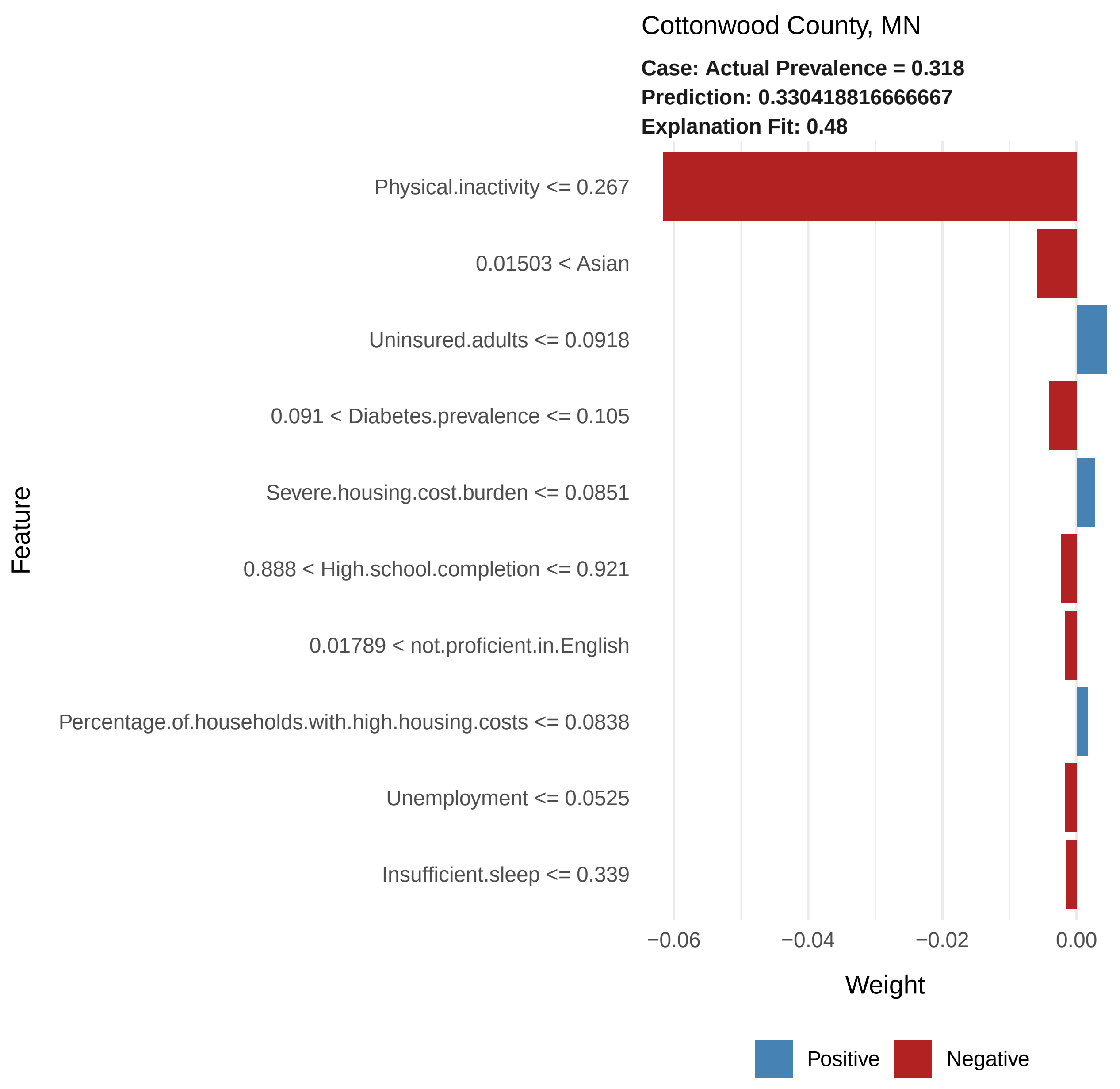

Cottonwood County, MN
Case: Actual Prevalence = 0.318
Prediction: 0.330418816666667
Explanation Fit: 0.48
Physical.inactivity <= 0.267
0.01503 < Asian
Uninsured.adults <= 0.0918
0.091 < Diabetes.prevalence <= 0.105
Severe.housing.cost.burden <= 0.0851
0.888 < High.school.completion <= 0.921
0.01789 < not.proficient.in.English
Percentage.of.households.with.high.housing.costs <= 0.0838
Unemployment <= 0.0525
Insufficient.sleep <= 0.339
Feature
−0.06
−0.04
−0.02
0.00
Weight
Positive
Negative

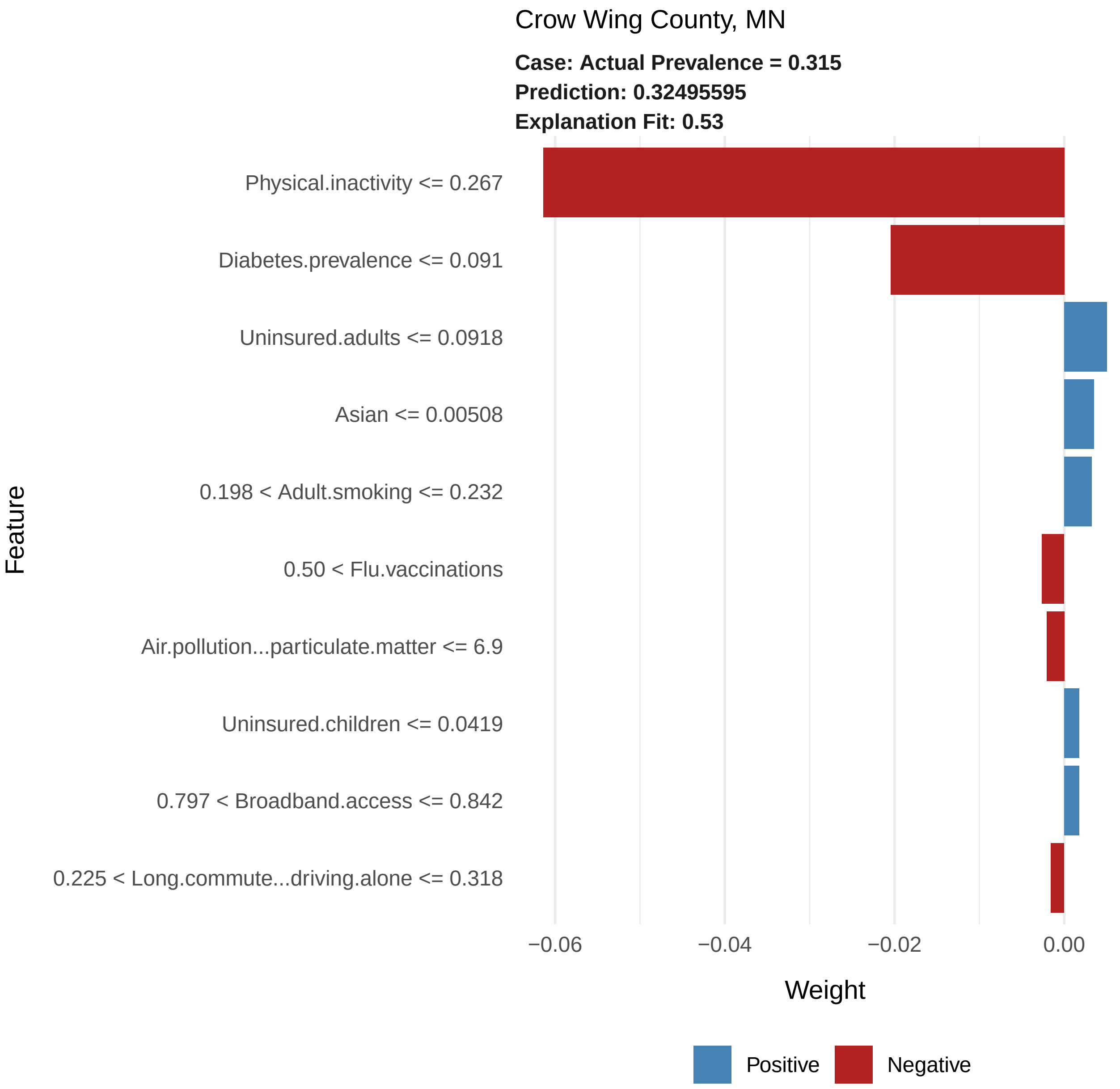
Crow Wing County, MN
Case: Actual Prevalence = 0.315
Prediction: 0.32495595
Explanation Fit: 0.53
Physical.inactivity <= 0.267
Diabetes.prevalence <= 0.091
Uninsured.adults <= 0.0918
Asian <= 0.00508
0.198 < Adult.smoking <= 0.232
0.50 < Flu.vaccinations
Air.pollution...particulate.matter <= 6.9
Uninsured.children <= 0.0419
0.797 < Broadband.access <= 0.842
0.225 < Long.commute...driving.alone <= 0.318
Feature
−0.06
−0.04
−0.02
0.00
Weight
Positive
Negative

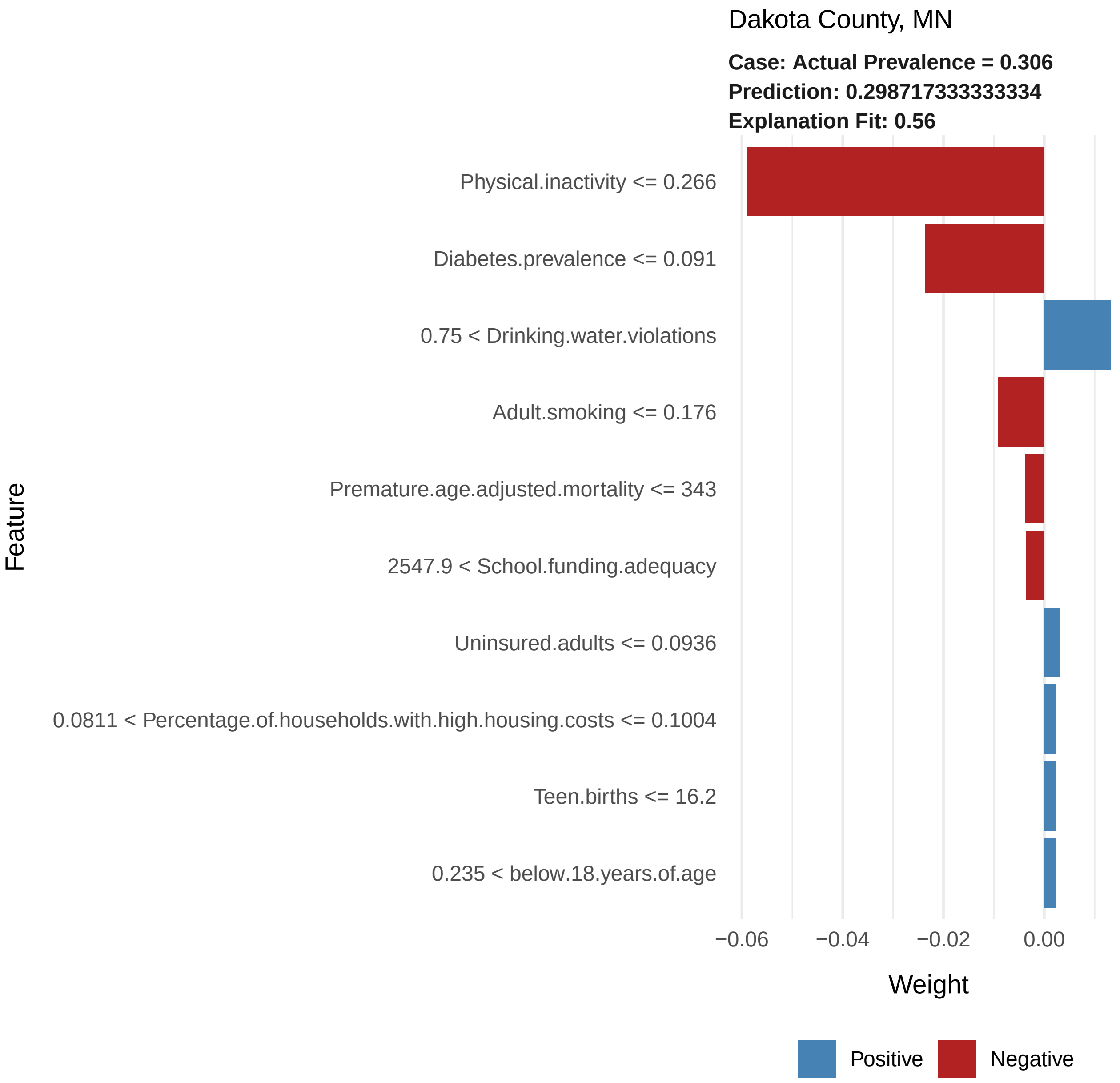

Dakota County, MN
Case: Actual Prevalence = 0.306
Prediction: 0.298717333333334
Explanation Fit: 0.56
Physical.inactivity <= 0.266
Diabetes.prevalence <= 0.091
0.75 < Drinking.water.violations
Adult.smoking <= 0.176
Premature.age.adjusted.mortality <= 343
2547.9 < School.funding.adequacy
Uninsured.adults <= 0.0936
0.0811 < Percentage.of.households.with.high.housing.costs <= 0.1004
Teen.births <= 16.2
0.235 < below.18.years.of.age
Feature
−0.06
−0.04
−0.02
0.00
Weight
Positive
Negative

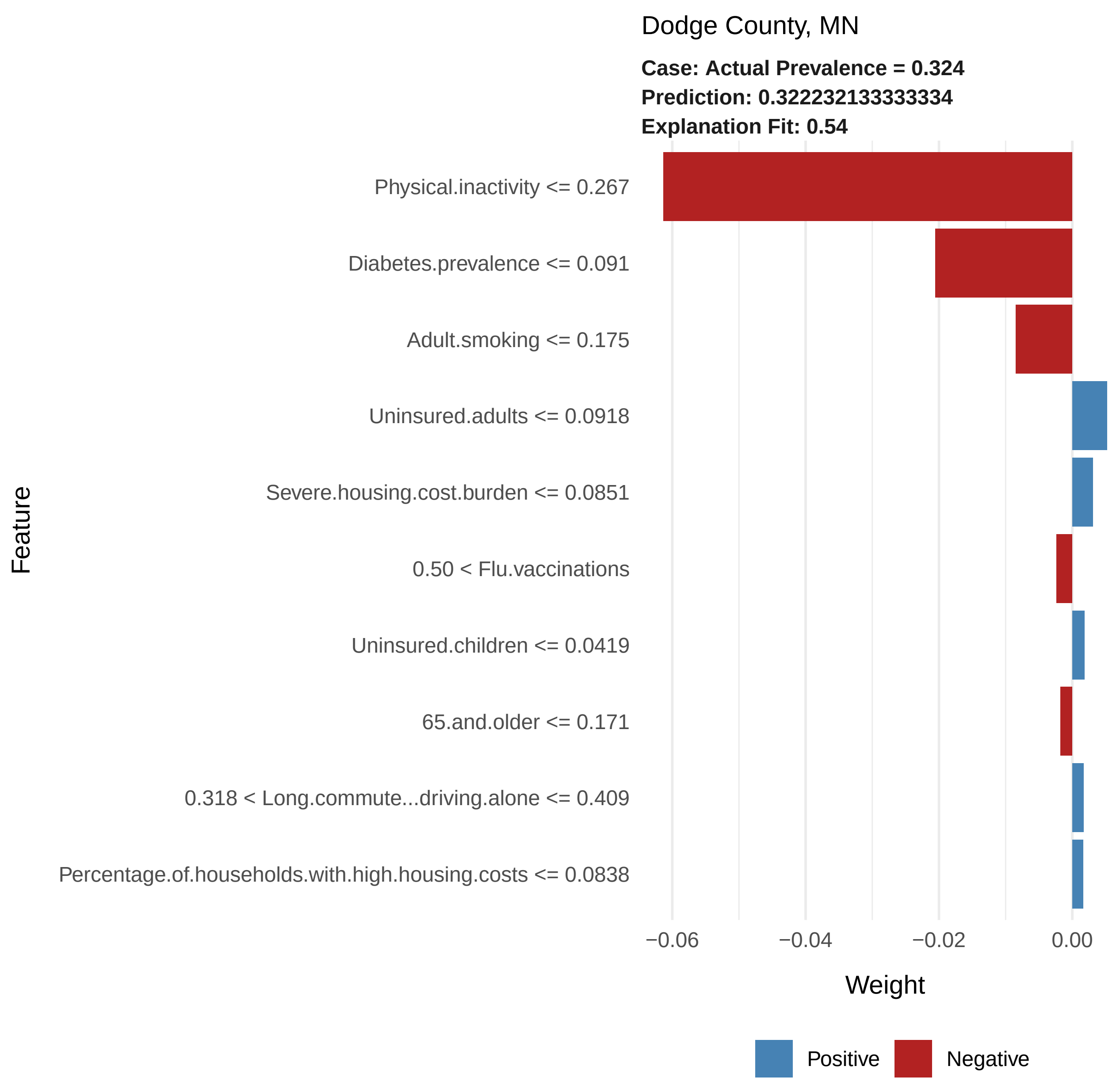
Dodge County, MN
Case: Actual Prevalence = 0.324
Prediction: 0.322232133333334
Explanation Fit: 0.54
Physical.inactivity <= 0.267
Diabetes.prevalence <= 0.091
Adult.smoking <= 0.175
Uninsured.adults <= 0.0918
Severe.housing.cost.burden <= 0.0851
0.50 < Flu.vaccinations
Uninsured.children <= 0.0419
65.and.older <= 0.171
0.318 < Long.commute...driving.alone <= 0.409
Percentage.of.households.with.high.housing.costs <= 0.0838
Feature
−0.06
−0.04
−0.02
0.00
Weight
Positive
Negative

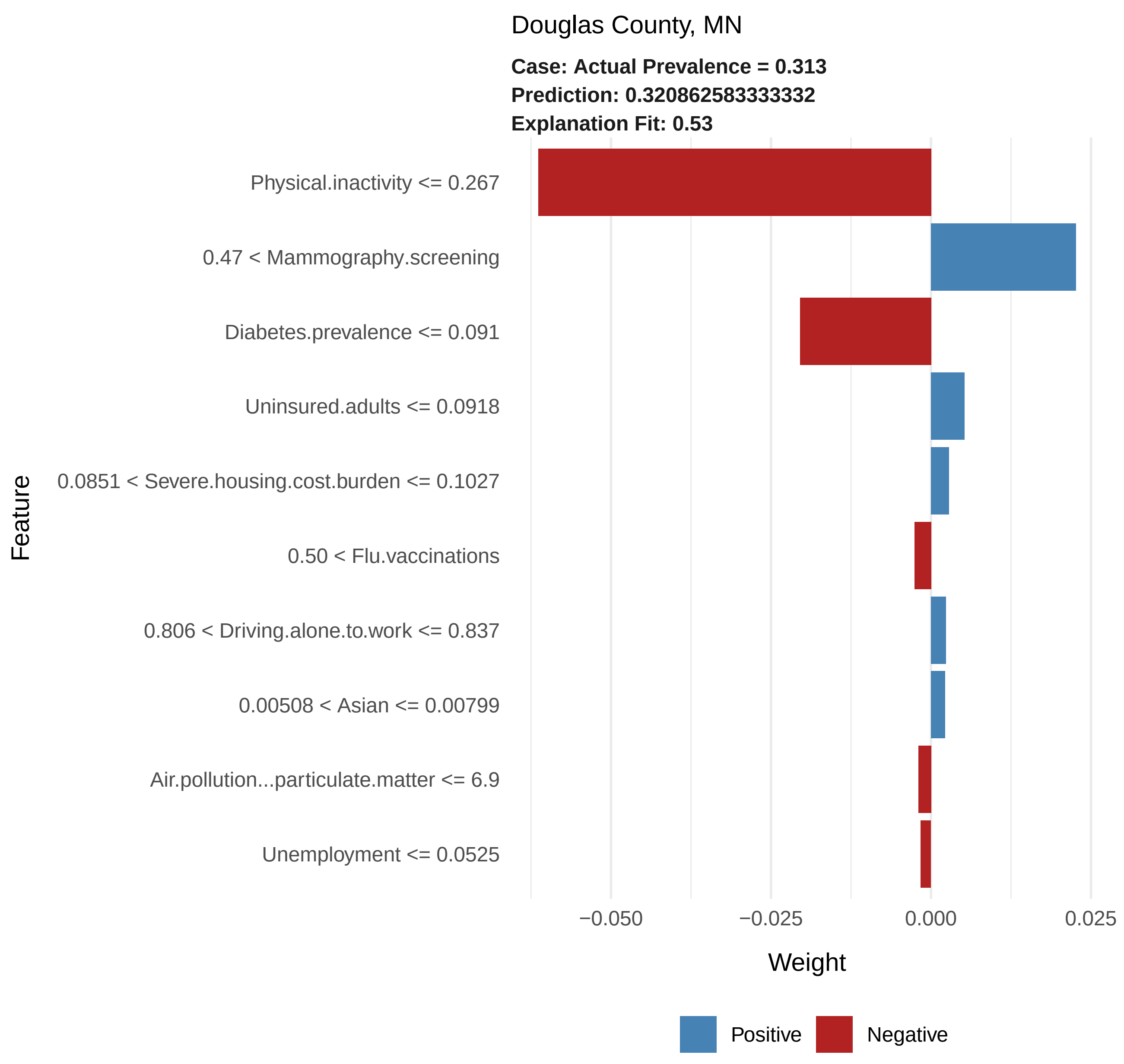

Douglas County, MN
Case: Actual Prevalence = 0.313
Prediction: 0.320862583333332
Explanation Fit: 0.53
Physical.inactivity <= 0.267
0.47 < Mammography.screening
Diabetes.prevalence <= 0.091
Uninsured.adults <= 0.0918
0.0851 < Severe.housing.cost.burden <= 0.1027
0.50 < Flu.vaccinations
0.806 < Driving.alone.to.work <= 0.837
0.00508 < Asian <= 0.00799
Air.pollution...particulate.matter <= 6.9
Unemployment <= 0.0525
Feature
−0.050
−0.025
0.000
0.025
Weight
Positive
Negative

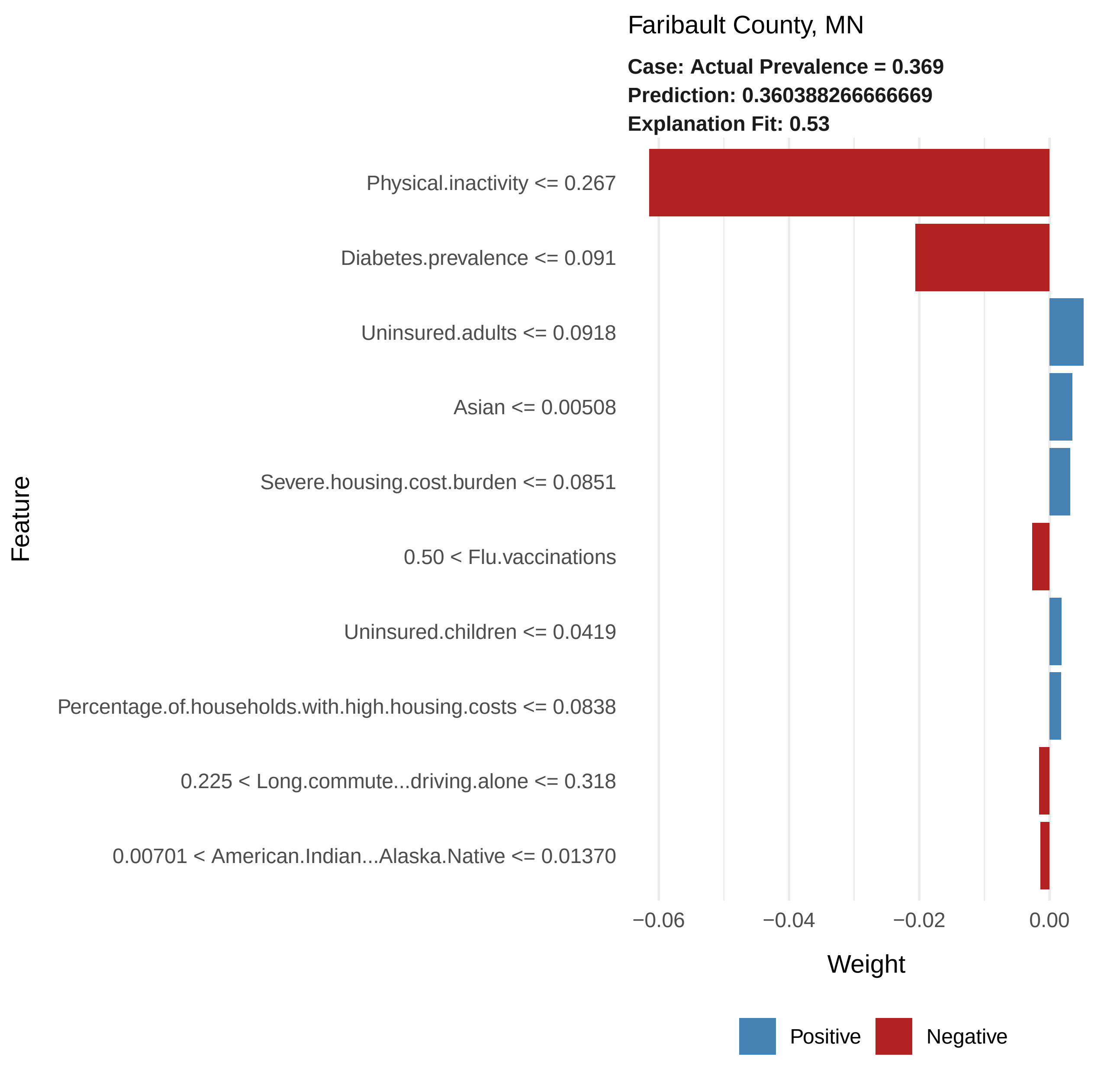

Faribault County, MN
Case: Actual Prevalence = 0.369
Prediction: 0.360388266666669
Explanation Fit: 0.53
Physical.inactivity <= 0.267
Diabetes.prevalence <= 0.091
Uninsured.adults <= 0.0918
Asian <= 0.00508
Severe.housing.cost.burden <= 0.0851
0.50 < Flu.vaccinations
Uninsured.children <= 0.0419
Percentage.of.households.with.high.housing.costs <= 0.0838
0.225 < Long.commute...driving.alone <= 0.318
0.00701 < American.Indian...Alaska.Native <= 0.01370
Feature
−0.06
−0.04
−0.02
0.00
Weight
Positive
Negative

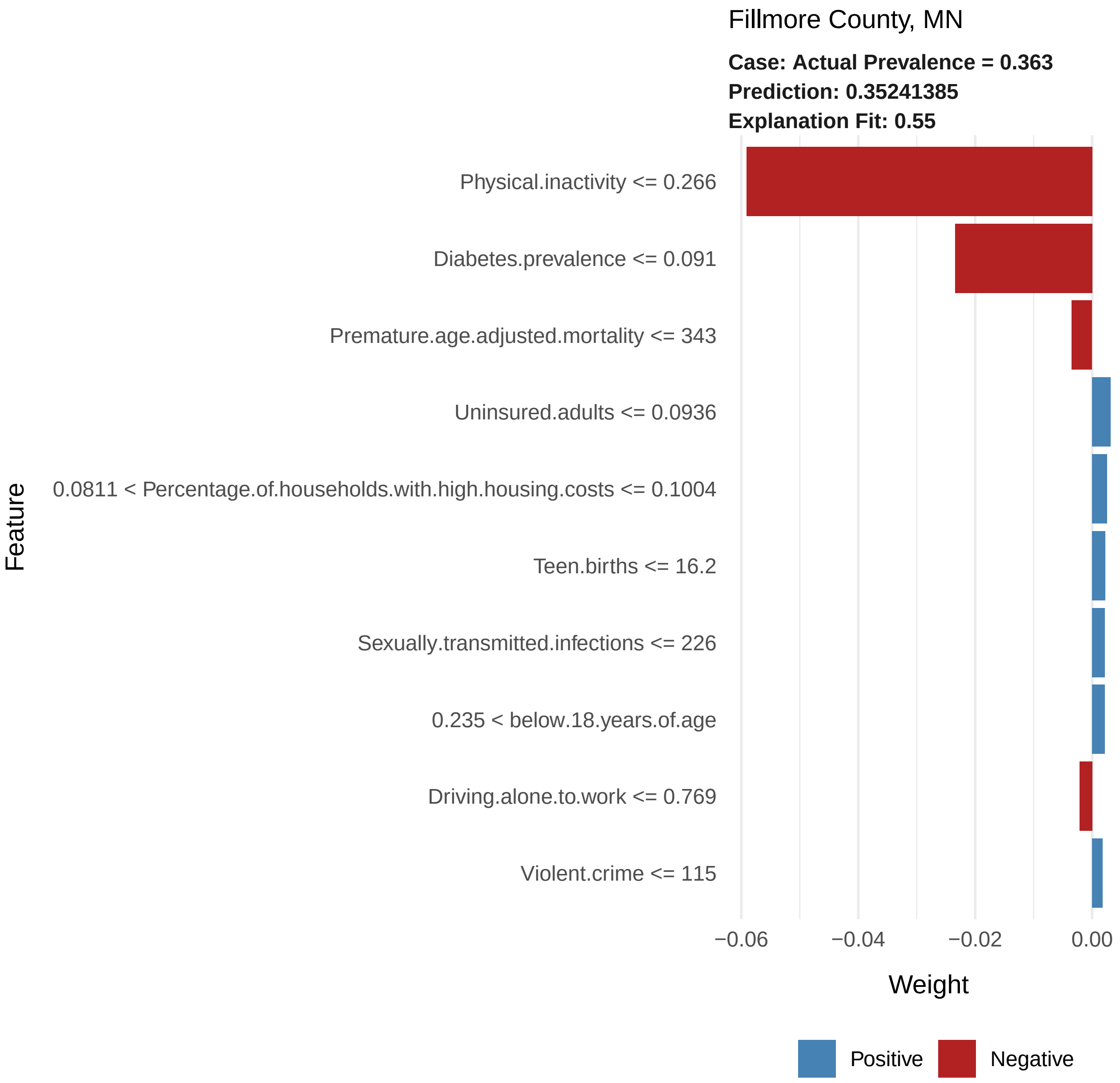

Fillmore County, MN
Case: Actual Prevalence = 0.363
Prediction: 0.35241385
Explanation Fit: 0.55
Physical.inactivity <= 0.266
Diabetes.prevalence <= 0.091
Premature.age.adjusted.mortality <= 343
Uninsured.adults <= 0.0936
0.0811 < Percentage.of.households.with.high.housing.costs <= 0.1004
Teen.births <= 16.2
Sexually.transmitted.infections <= 226
0.235 < below.18.years.of.age
Driving.alone.to.work <= 0.769
Violent.crime <= 115
Feature
−0.06
−0.04
−0.02
0.00
Weight
Positive
Negative

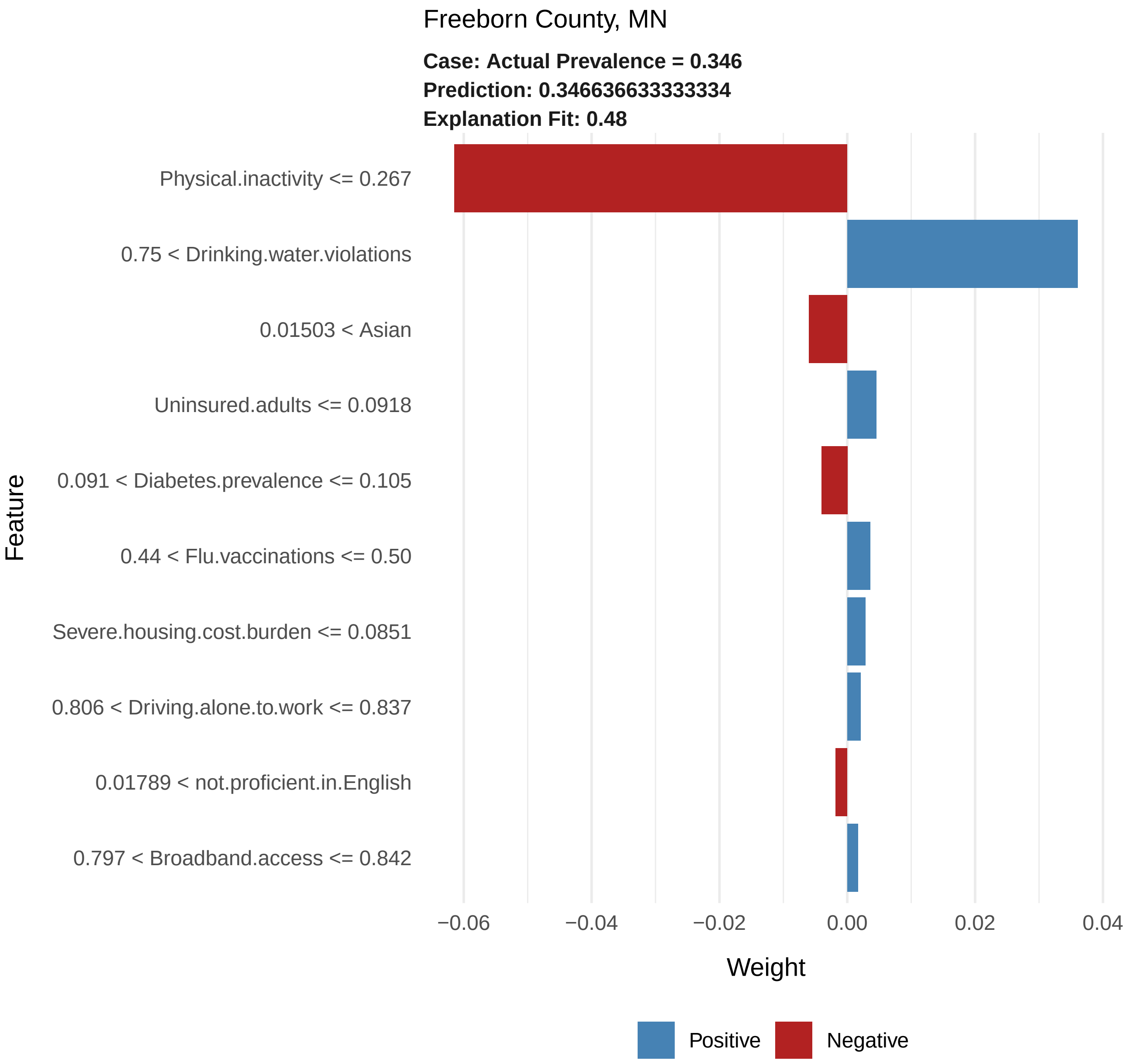
Freeborn County, MN
Case: Actual Prevalence = 0.346
Prediction: 0.346636633333334
Explanation Fit: 0.48
Physical.inactivity <= 0.267
0.75 < Drinking.water.violations
0.01503 < Asian
Uninsured.adults <= 0.0918
0.091 < Diabetes.prevalence <= 0.105
0.44 < Flu.vaccinations <= 0.50
Severe.housing.cost.burden <= 0.0851
0.806 < Driving.alone.to.work <= 0.837
0.01789 < not.proficient.in.English
0.797 < Broadband.access <= 0.842
Feature
−0.06
−0.04
−0.02
0.00
0.02
0.04
Weight
Positive
Negative

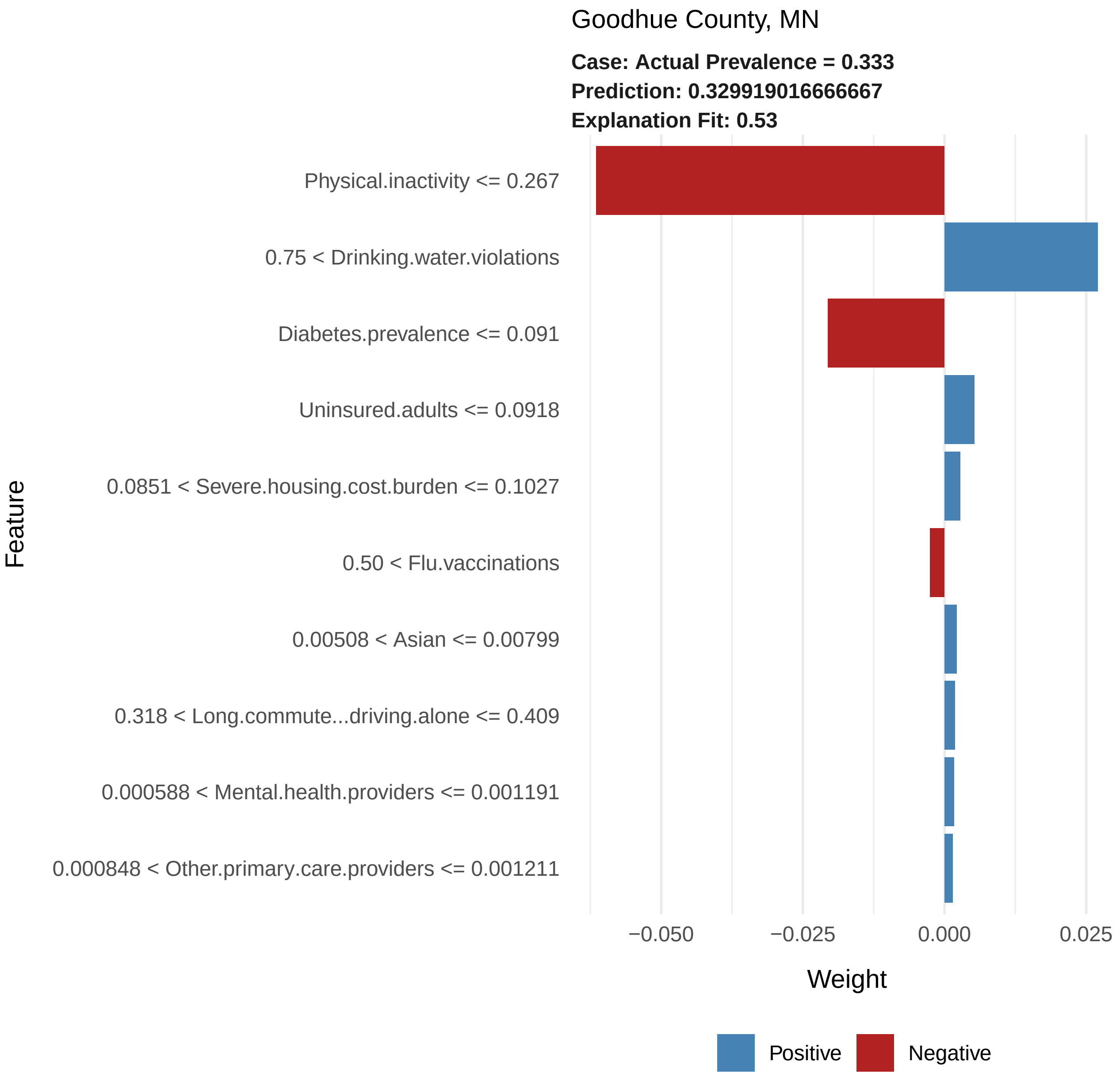
Goodhue County, MN
Case: Actual Prevalence = 0.333
Prediction: 0.329919016666667
Explanation Fit: 0.53
Physical.inactivity <= 0.267
0.75 < Drinking.water.violations
Diabetes.prevalence <= 0.091
Uninsured.adults <= 0.0918
0.0851 < Severe.housing.cost.burden <= 0.1027
0.50 < Flu.vaccinations
0.00508 < Asian <= 0.00799
0.318 < Long.commute...driving.alone <= 0.409
0.000588 < Mental.health.providers <= 0.001191
0.000848 < Other.primary.care.providers <= 0.001211
Feature
−0.050
−0.025
0.000
0.025
Weight
Positive
Negative

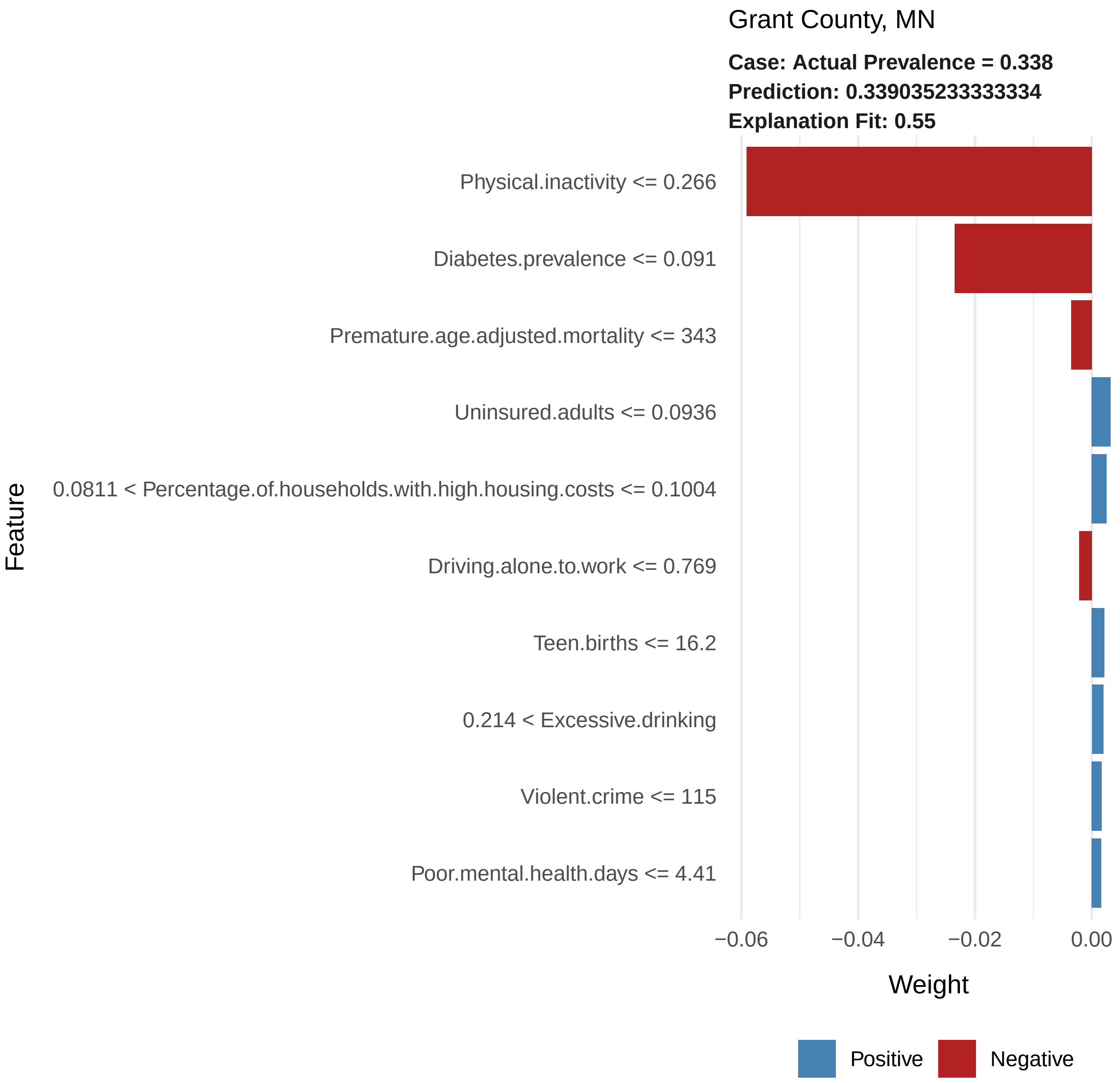

Grant County, MN
Case: Actual Prevalence = 0.338
Prediction: 0.339035233333334
Explanation Fit: 0.55
Physical.inactivity <= 0.266
Diabetes.prevalence <= 0.091
Premature.age.adjusted.mortality <= 343
Uninsured.adults <= 0.0936
0.0811 < Percentage.of.households.with.high.housing.costs <= 0.1004
Driving.alone.to.work <= 0.769
Teen.births <= 16.2
0.214 < Excessive.drinking
Violent.crime <= 115
Poor.mental.health.days <= 4.41
Feature
−0.06
−0.04
−0.02
0.00
Weight
Positive
Negative

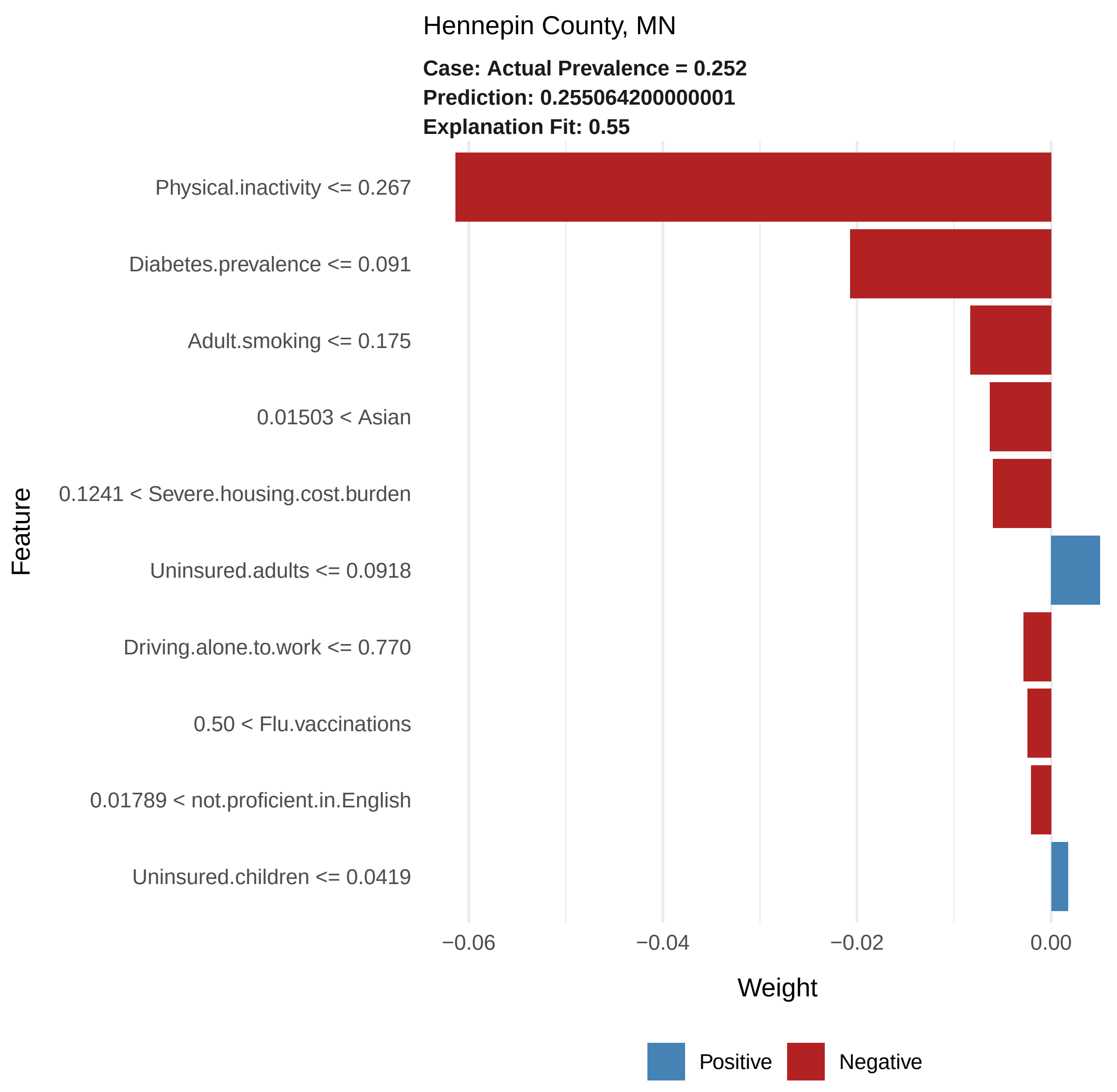

Hennepin County, MN
Case: Actual Prevalence = 0.252
Prediction: 0.255064200000001
Explanation Fit: 0.55
Physical.inactivity <= 0.267
Diabetes.prevalence <= 0.091
Adult.smoking <= 0.175
0.01503 < Asian
0.1241 < Severe.housing.cost.burden
Uninsured.adults <= 0.0918
Driving.alone.to.work <= 0.770
0.50 < Flu.vaccinations
0.01789 < not.proficient.in.English
Uninsured.children <= 0.0419
Feature
−0.06
−0.04
−0.02
0.00
Weight
Positive
Negative

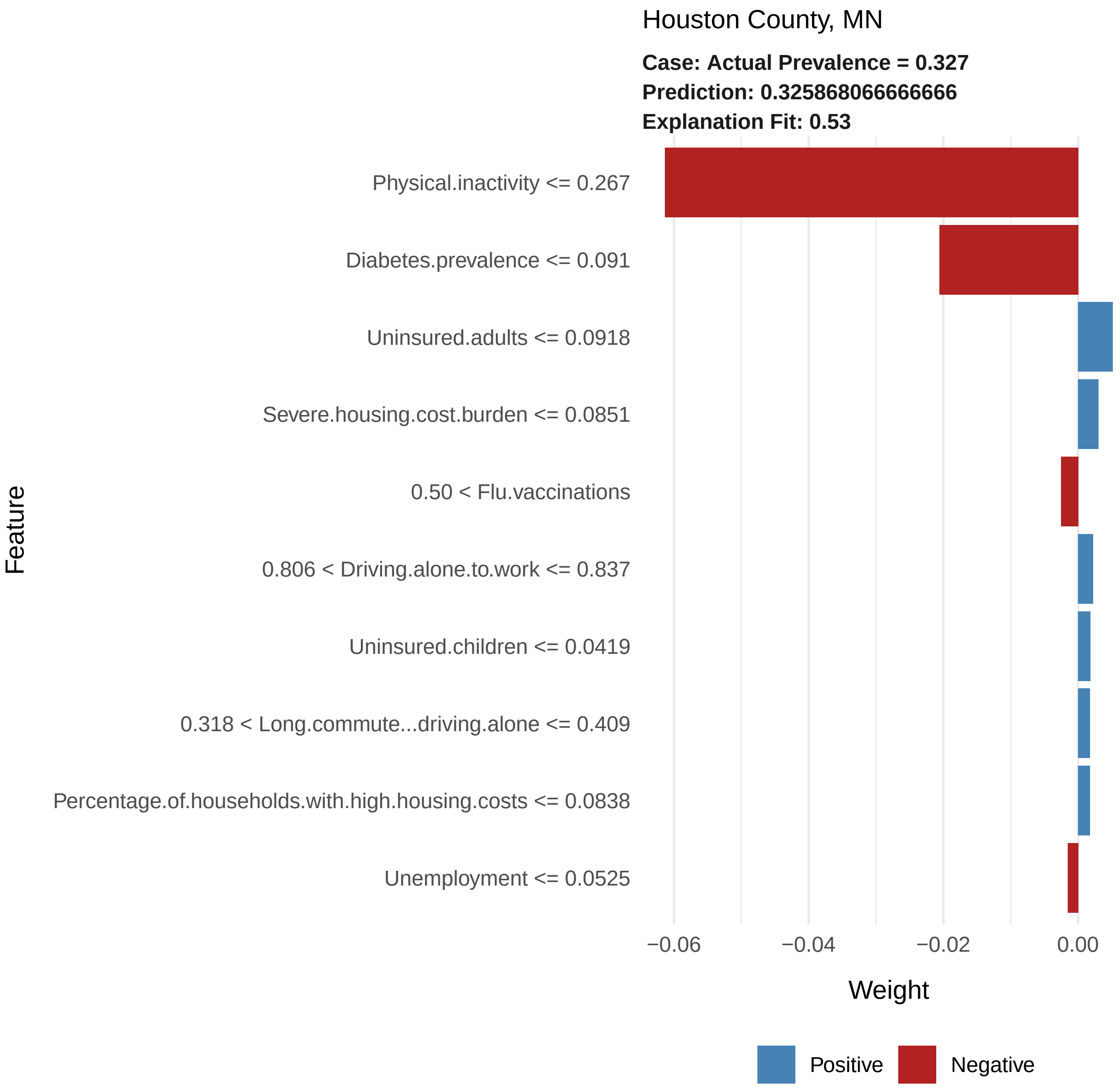
Houston County, MN
Case: Actual Prevalence = 0.327
Prediction: 0.325868066666666
Explanation Fit: 0.53
Feature
Physical.inactivity <= 0.267
Diabetes.prevalence <= 0.091
Uninsured.adults <= 0.0918
Severe.housing.cost.burden <= 0.0851
0.50 < Flu.vaccinations
0.806 < Driving.alone.to.work <= 0.837
Uninsured.children <= 0.0419
0.318 < Long.commute...driving.alone <= 0.409
Percentage.of.households.with.high.housing.costs <= 0.0838
Unemployment <= 0.0525
−0.06
−0.04
−0.02
0.00
Weight
Positive
Negative

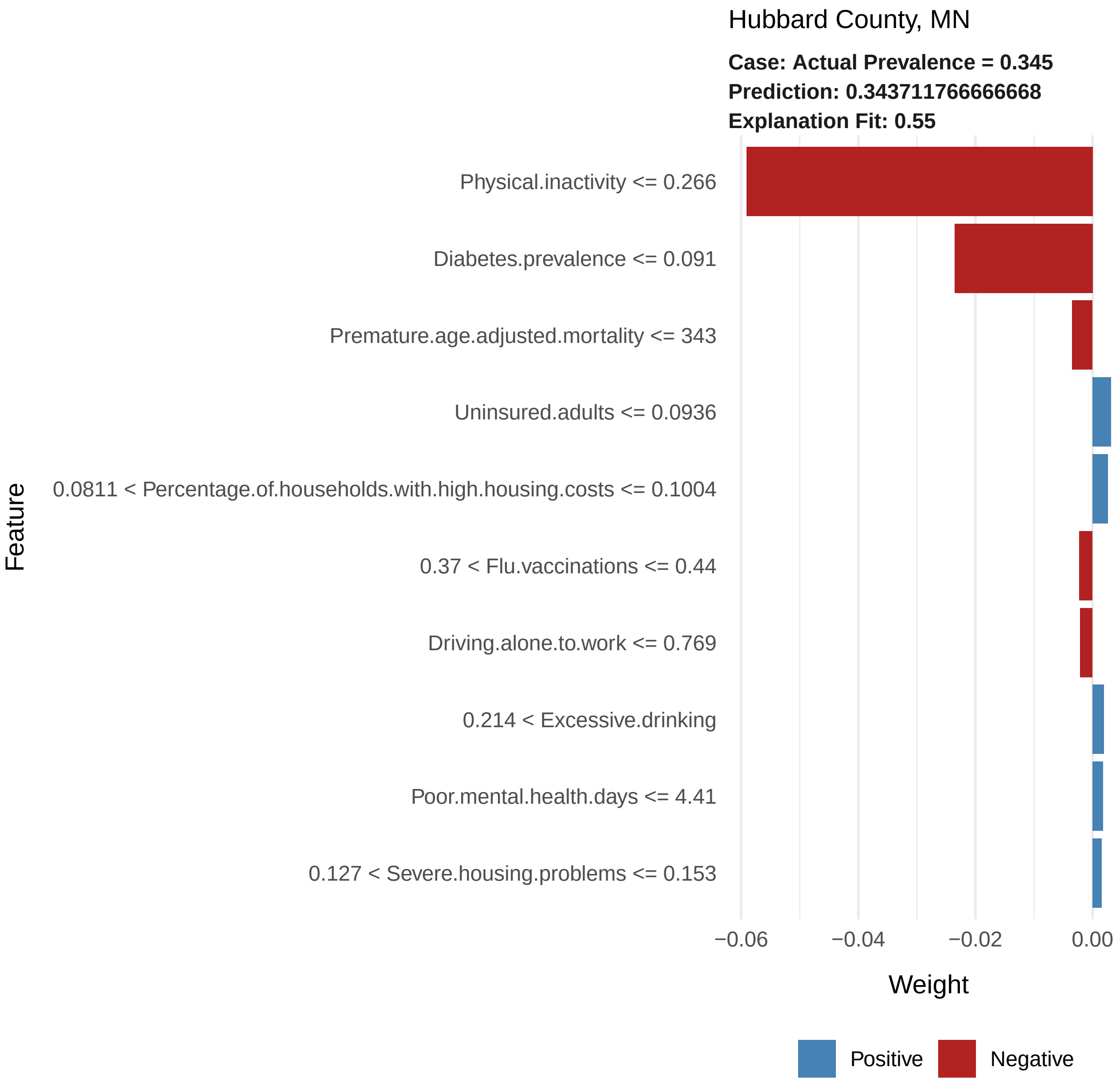
Hubbard County, MN
Case: Actual Prevalence = 0.345
Prediction: 0.343711766666668
Explanation Fit: 0.55
Physical.inactivity <= 0.266
Diabetes.prevalence <= 0.091
Premature.age.adjusted.mortality <= 343
Uninsured.adults <= 0.0936
0.0811 < Percentage.of.households.with.high.housing.costs <= 0.1004
0.37 < Flu.vaccinations <= 0.44
Driving.alone.to.work <= 0.769
0.214 < Excessive.drinking
Poor.mental.health.days <= 4.41
0.127 < Severe.housing.problems <= 0.153
Feature
−0.06
−0.04
−0.02
0.00
Weight
Positive
Negative

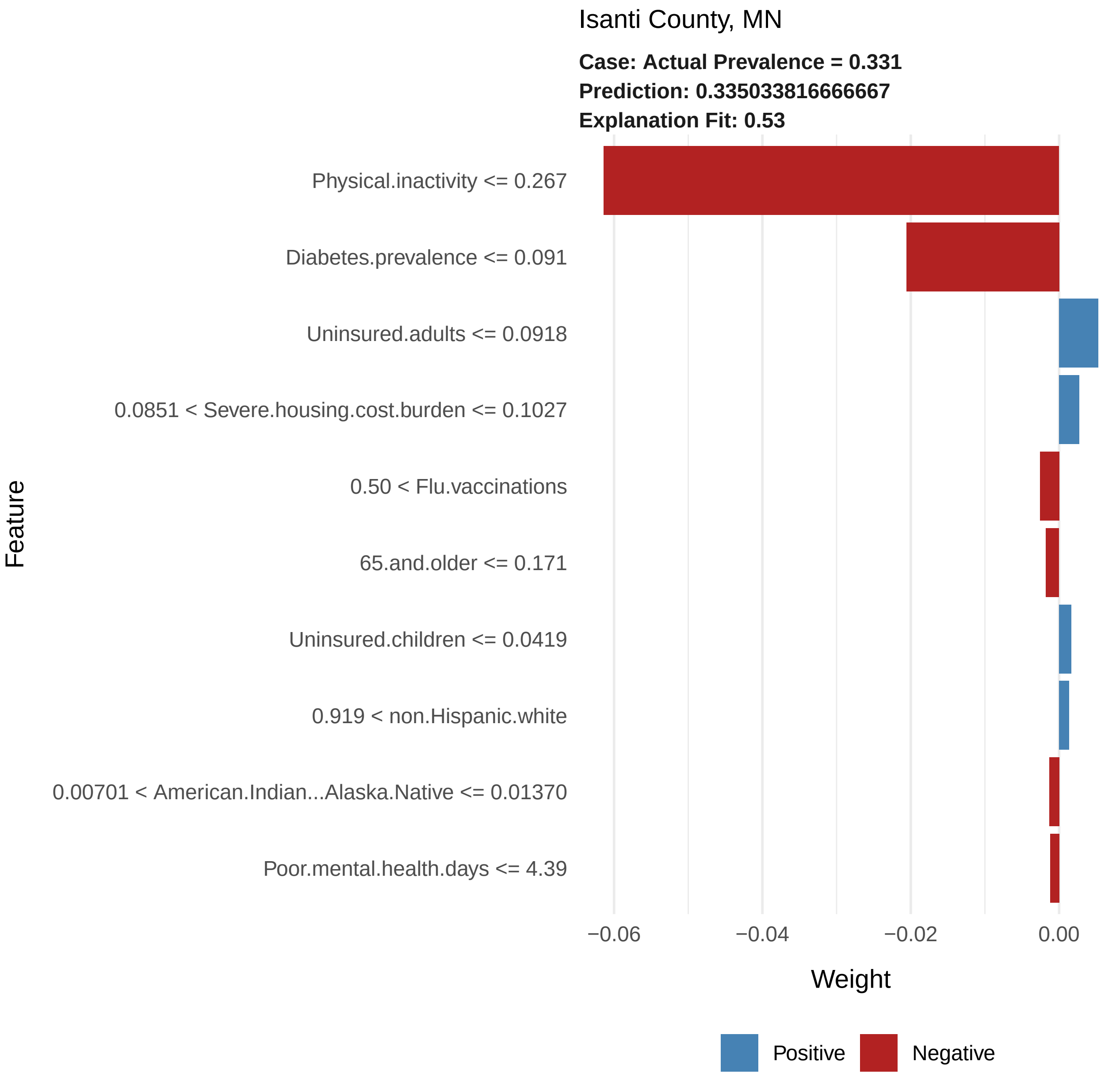
Isanti County, MN
Case: Actual Prevalence = 0.331
Prediction: 0.335033816666667
Explanation Fit: 0.53
Physical.inactivity <= 0.267
Diabetes.prevalence <= 0.091
Uninsured.adults <= 0.0918
0.0851 < Severe.housing.cost.burden <= 0.1027
0.50 < Flu.vaccinations
65.and.older <= 0.171
Uninsured.children <= 0.0419
0.919 < non.Hispanic.white
0.00701 < American.Indian...Alaska.Native <= 0.01370
Poor.mental.health.days <= 4.39
Feature
−0.06
−0.04
−0.02
0.00
Weight
Positive
Negative

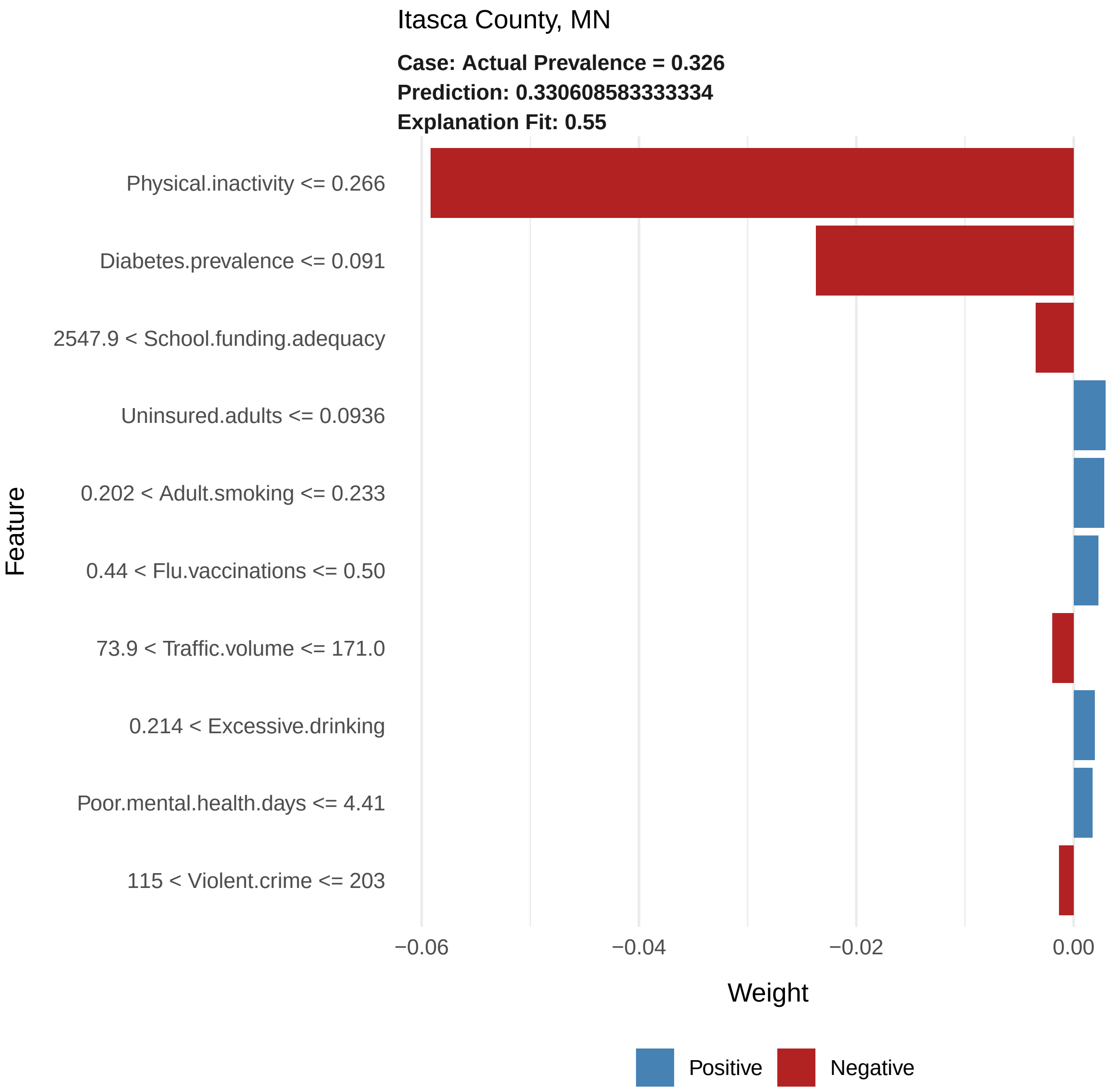

Itasca County, MN
Case: Actual Prevalence = 0.326
Prediction: 0.330608583333334
Explanation Fit: 0.55
Physical.inactivity <= 0.266
Diabetes.prevalence <= 0.091
2547.9 < School.funding.adequacy
Uninsured.adults <= 0.0936
0.202 < Adult.smoking <= 0.233
0.44 < Flu.vaccinations <= 0.50
73.9 < Traffic.volume <= 171.0
0.214 < Excessive.drinking
Poor.mental.health.days <= 4.41
115 < Violent.crime <= 203
Feature
−0.06
−0.04
−0.02
0.00
Weight
Positive
Negative

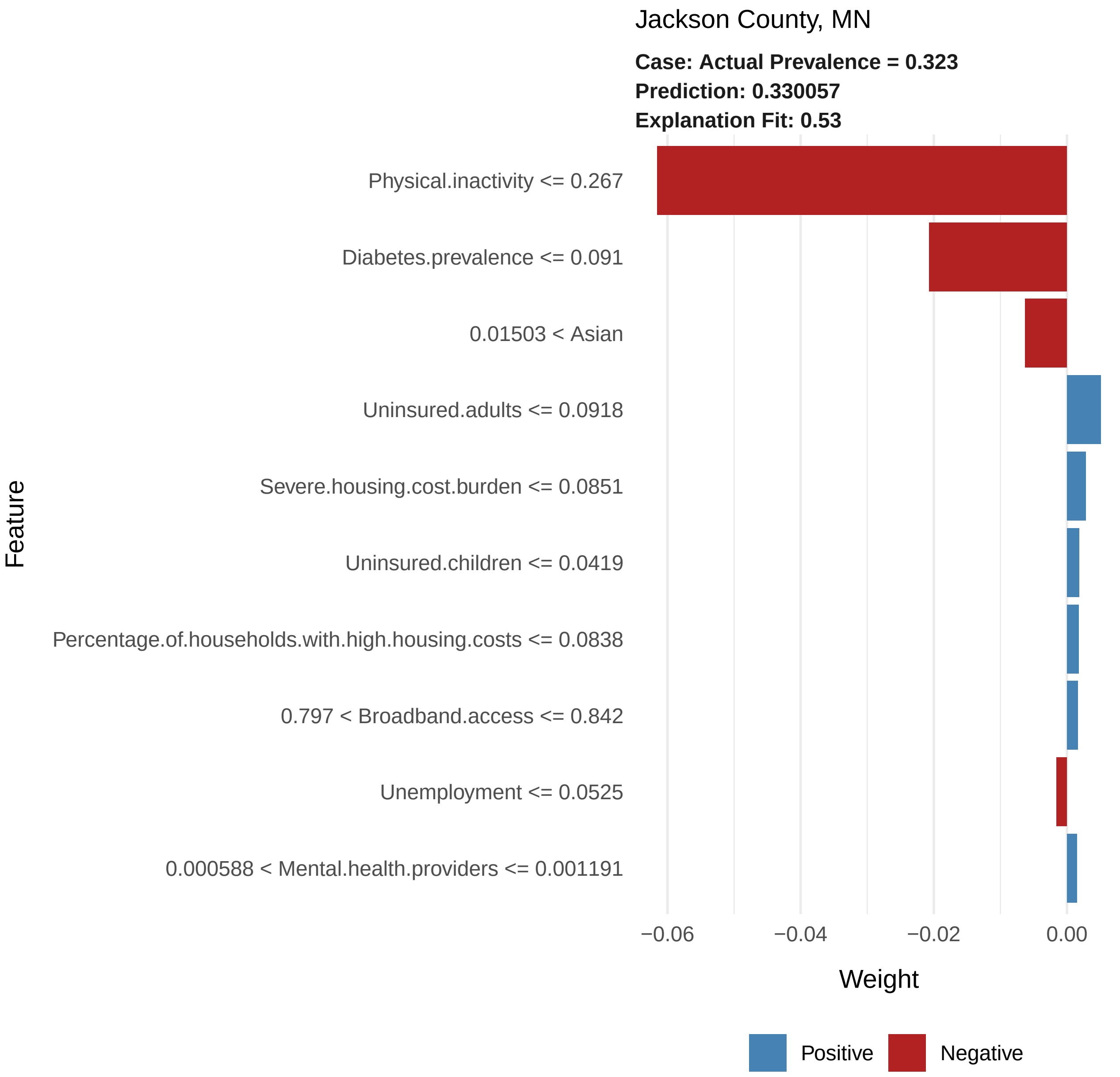

Jackson County, MN
Case: Actual Prevalence = 0.323
Prediction: 0.330057
Explanation Fit: 0.53
Physical.inactivity <= 0.267
Diabetes.prevalence <= 0.091
0.01503 < Asian
Uninsured.adults <= 0.0918
Severe.housing.cost.burden <= 0.0851
Uninsured.children <= 0.0419
Percentage.of.households.with.high.housing.costs <= 0.0838
0.797 < Broadband.access <= 0.842
Unemployment <= 0.0525
0.000588 < Mental.health.providers <= 0.001191
Feature
−0.06
−0.04
−0.02
0.00
Weight
Positive
Negative

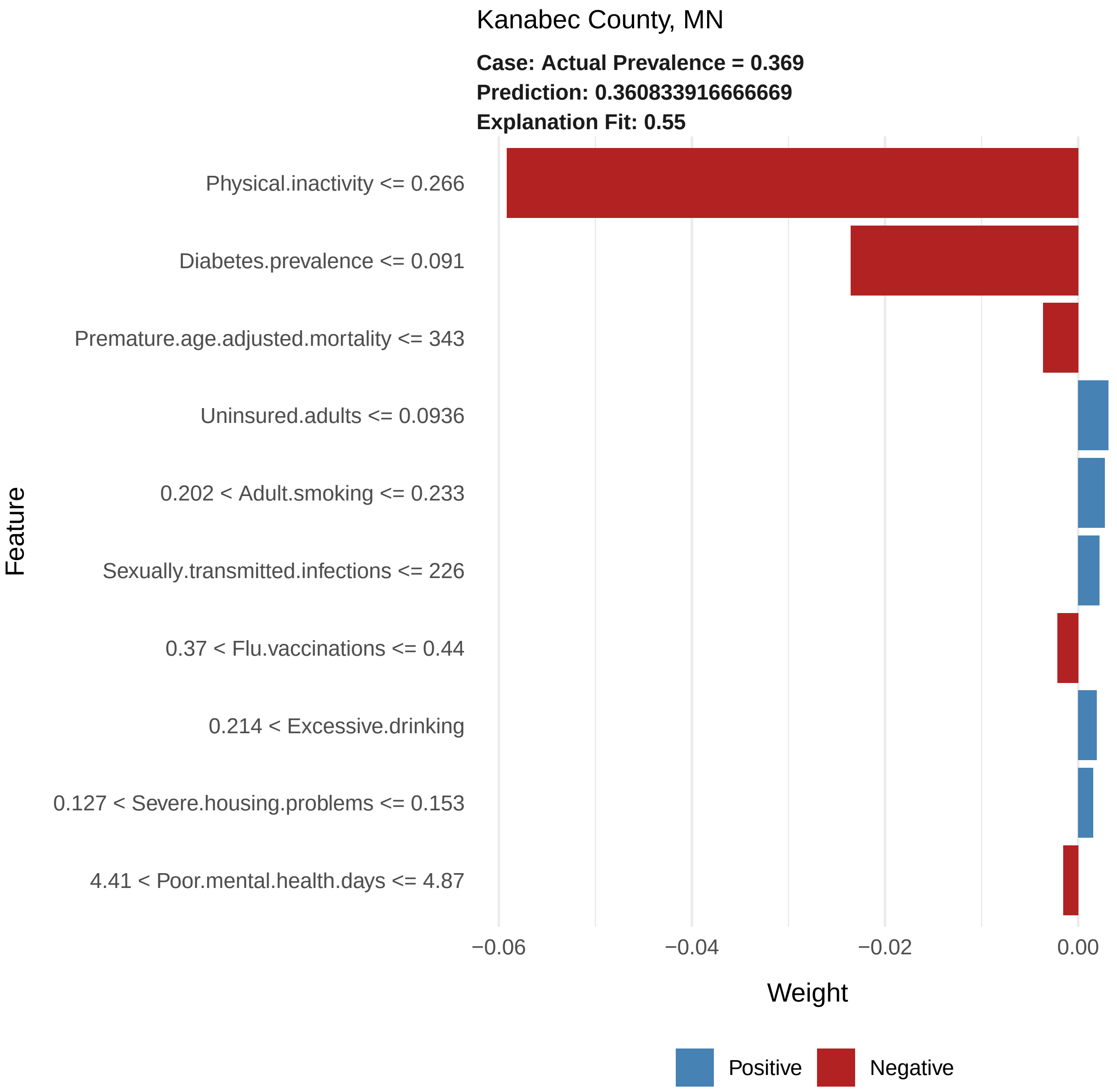
Kanabec County, MN
Case: Actual Prevalence = 0.369
Prediction: 0.360833916666669
Explanation Fit: 0.55
Physical.inactivity <= 0.266
Diabetes.prevalence <= 0.091
Premature.age.adjusted.mortality <= 343
Uninsured.adults <= 0.0936
0.202 < Adult.smoking <= 0.233
Sexually.transmitted.infections <= 226
0.37 < Flu.vaccinations <= 0.44
0.214 < Excessive.drinking
0.127 < Severe.housing.problems <= 0.153
4.41 < Poor.mental.health.days <= 4.87
Feature
−0.06
−0.04
−0.02
0.00
Weight
Positive
Negative

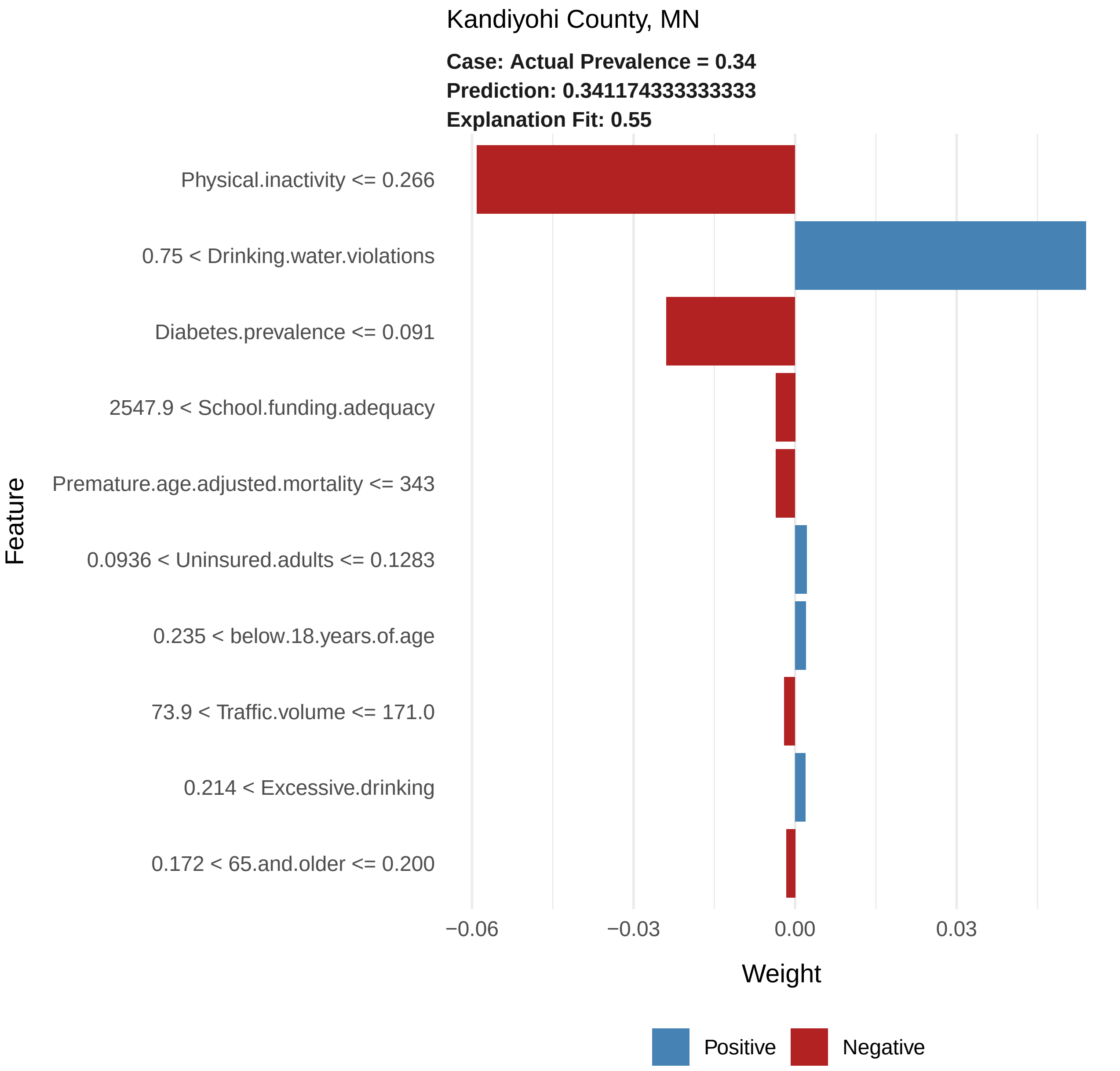

Kandiyohi County, MN
Case: Actual Prevalence = 0.34
Prediction: 0.341174333333333
Explanation Fit: 0.55
Physical.inactivity <= 0.266
0.75 < Drinking.water.violations
Diabetes.prevalence <= 0.091
2547.9 < School.funding.adequacy
Premature.age.adjusted.mortality <= 343
0.0936 < Uninsured.adults <= 0.1283
0.235 < below.18.years.of.age
73.9 < Traffic.volume <= 171.0
0.214 < Excessive.drinking
0.172 < 65.and.older <= 0.200
Feature
−0.06
−0.03
0.00
0.03
Weight
Positive
Negative

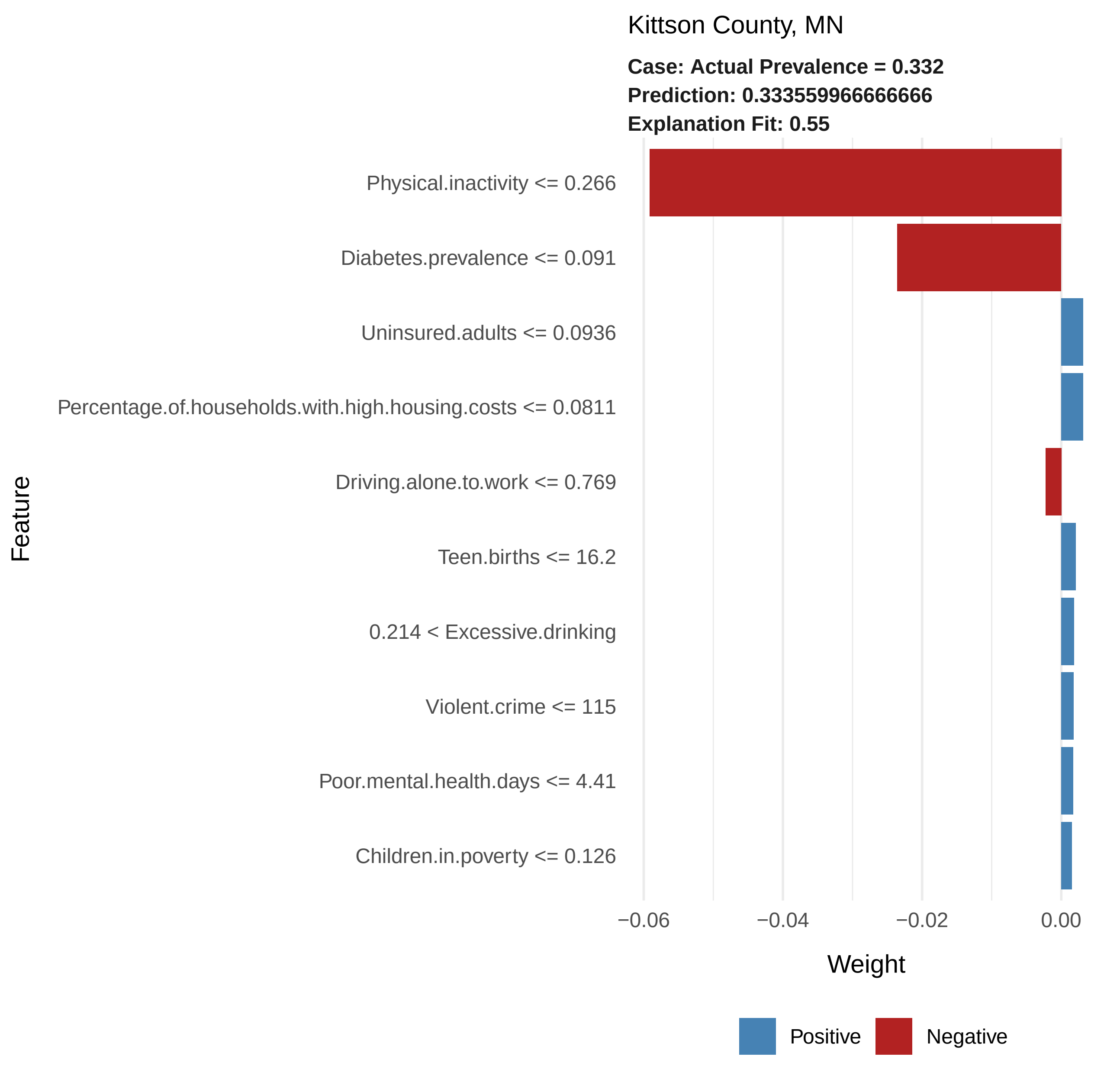
Kittson County, MN
Case: Actual Prevalence = 0.332
Prediction: 0.333559966666666
Explanation Fit: 0.55
Physical.inactivity <= 0.266
Diabetes.prevalence <= 0.091
Uninsured.adults <= 0.0936
Percentage.of.households.with.high.housing.costs <= 0.0811
Driving.alone.to.work <= 0.769
Teen.births <= 16.2
0.214 < Excessive.drinking
Violent.crime <= 115
Poor.mental.health.days <= 4.41
Children.in.poverty <= 0.126
Feature
−0.06
−0.04
−0.02
0.00
Weight
Positive
Negative

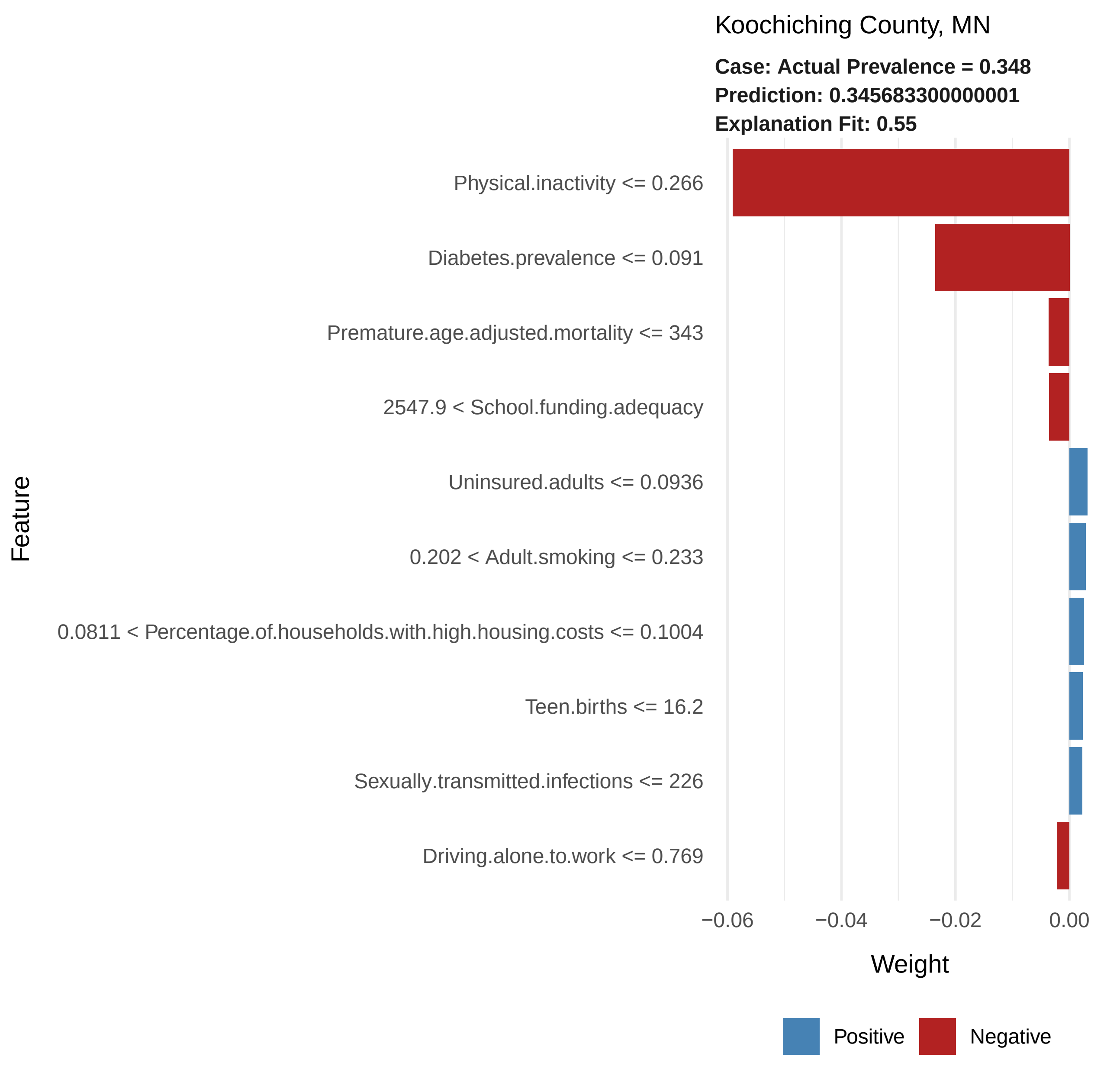
Koochiching County, MN
Case: Actual Prevalence = 0.348
Prediction: 0.345683300000001
Explanation Fit: 0.55
Physical.inactivity <= 0.266
Diabetes.prevalence <= 0.091
Premature.age.adjusted.mortality <= 343
2547.9 < School.funding.adequacy
Uninsured.adults <= 0.0936
0.202 < Adult.smoking <= 0.233
0.0811 < Percentage.of.households.with.high.housing.costs <= 0.1004
Teen.births <= 16.2
Sexually.transmitted.infections <= 226
Driving.alone.to.work <= 0.769
Feature
−0.06
−0.04
−0.02
0.00
Weight
Positive
Negative

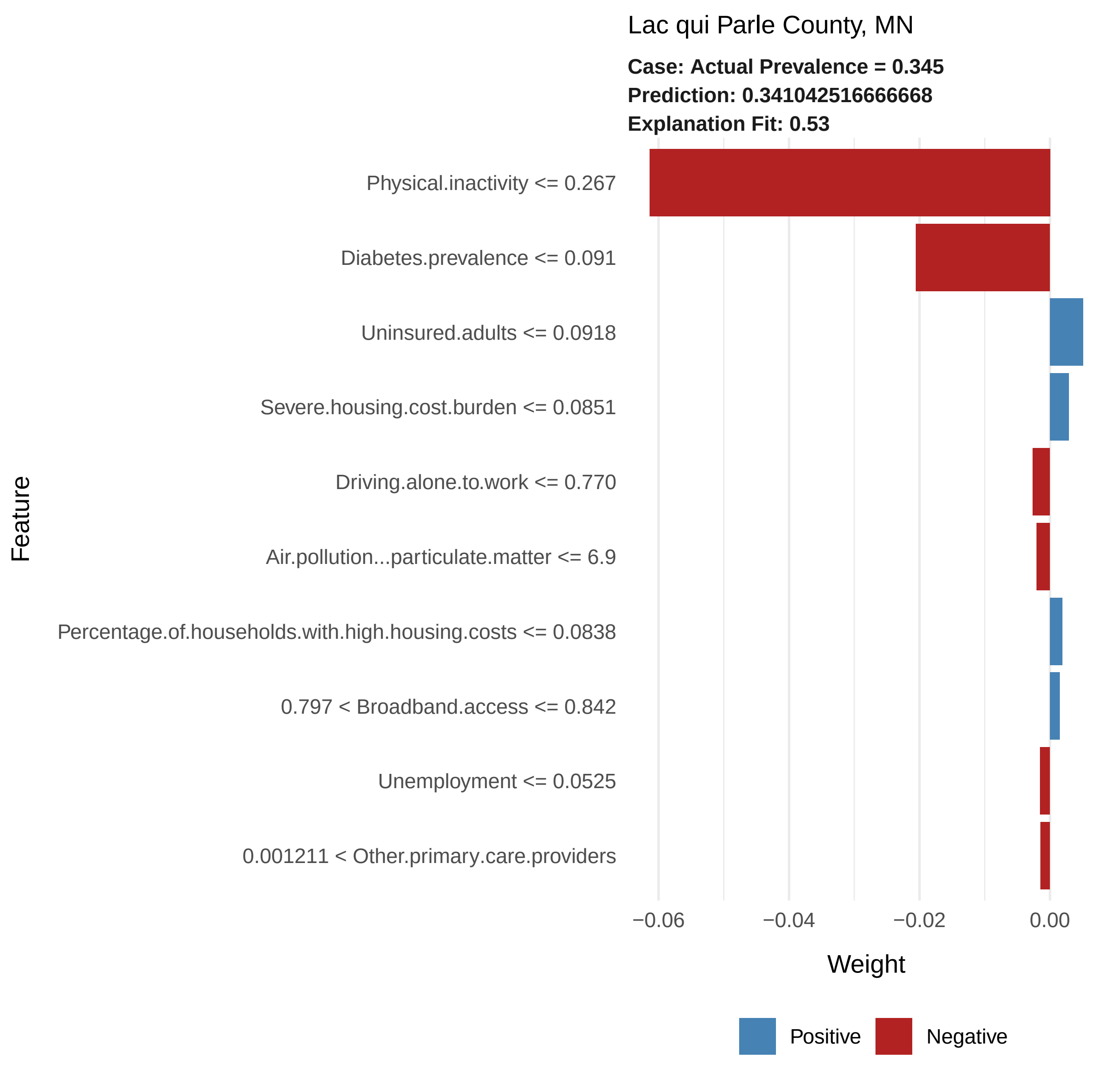

Lac qui Parle County, MN
Case: Actual Prevalence = 0.345
Prediction: 0.341042516666668
Explanation Fit: 0.53
Physical.inactivity <= 0.267
Diabetes.prevalence <= 0.091
Uninsured.adults <= 0.0918
Severe.housing.cost.burden <= 0.0851
Driving.alone.to.work <= 0.770
Air.pollution...particulate.matter <= 6.9
Percentage.of.households.with.high.housing.costs <= 0.0838
0.797 < Broadband.access <= 0.842
Unemployment <= 0.0525
0.001211 < Other.primary.care.providers
Feature
−0.06
−0.04
−0.02
0.00
Weight
Positive
Negative

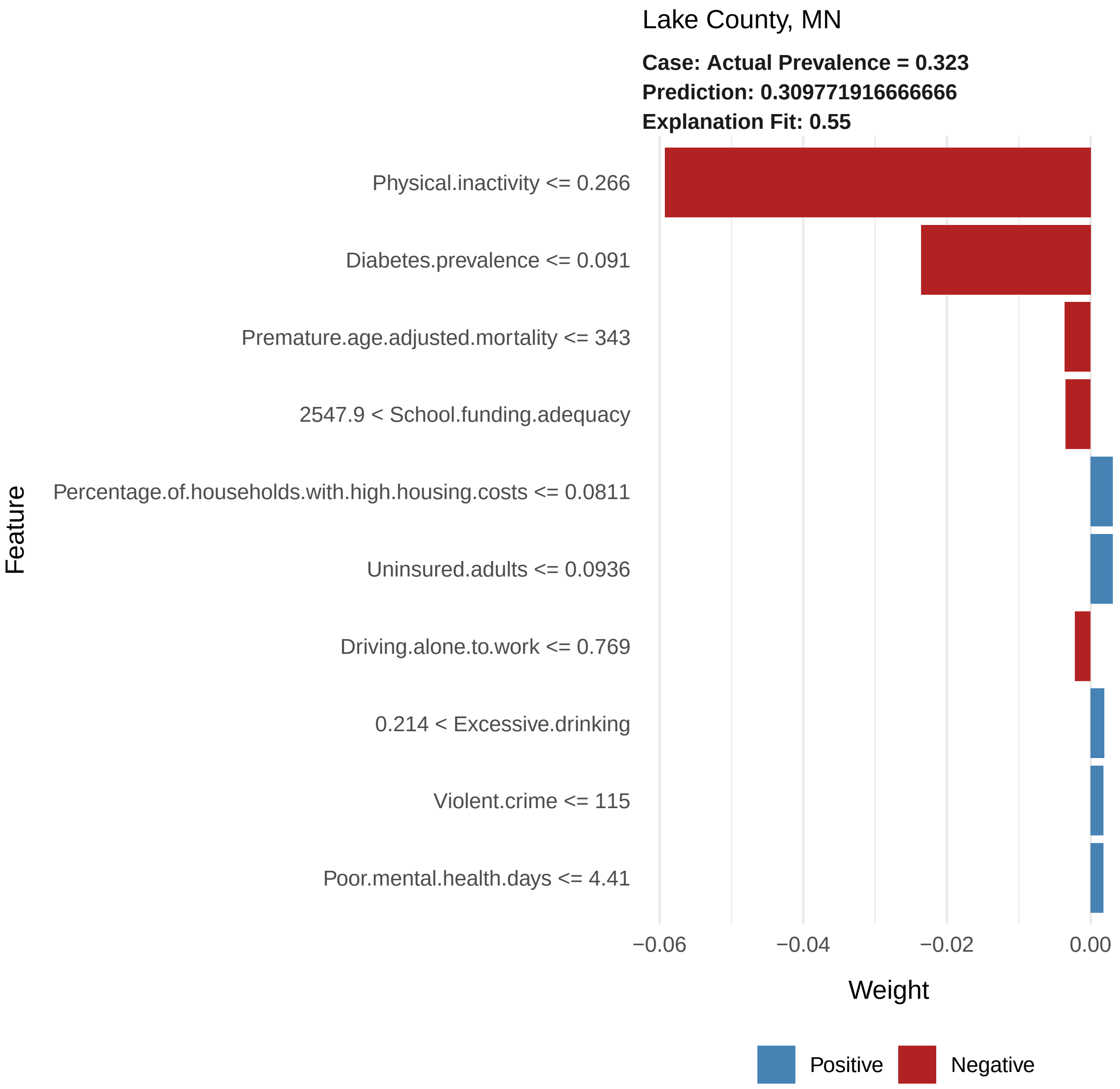

Lake County, MN
Case: Actual Prevalence = 0.323
Prediction: 0.309771916666666
Explanation Fit: 0.55
Physical.inactivity <= 0.266
Diabetes.prevalence <= 0.091
Premature.age.adjusted.mortality <= 343
2547.9 < School.funding.adequacy
Percentage.of.households.with.high.housing.costs <= 0.0811
Uninsured.adults <= 0.0936
Driving.alone.to.work <= 0.769
0.214 < Excessive.drinking
Violent.crime <= 115
Poor.mental.health.days <= 4.41
Feature
−0.06
−0.04
−0.02
0.00
Weight
Positive
Negative

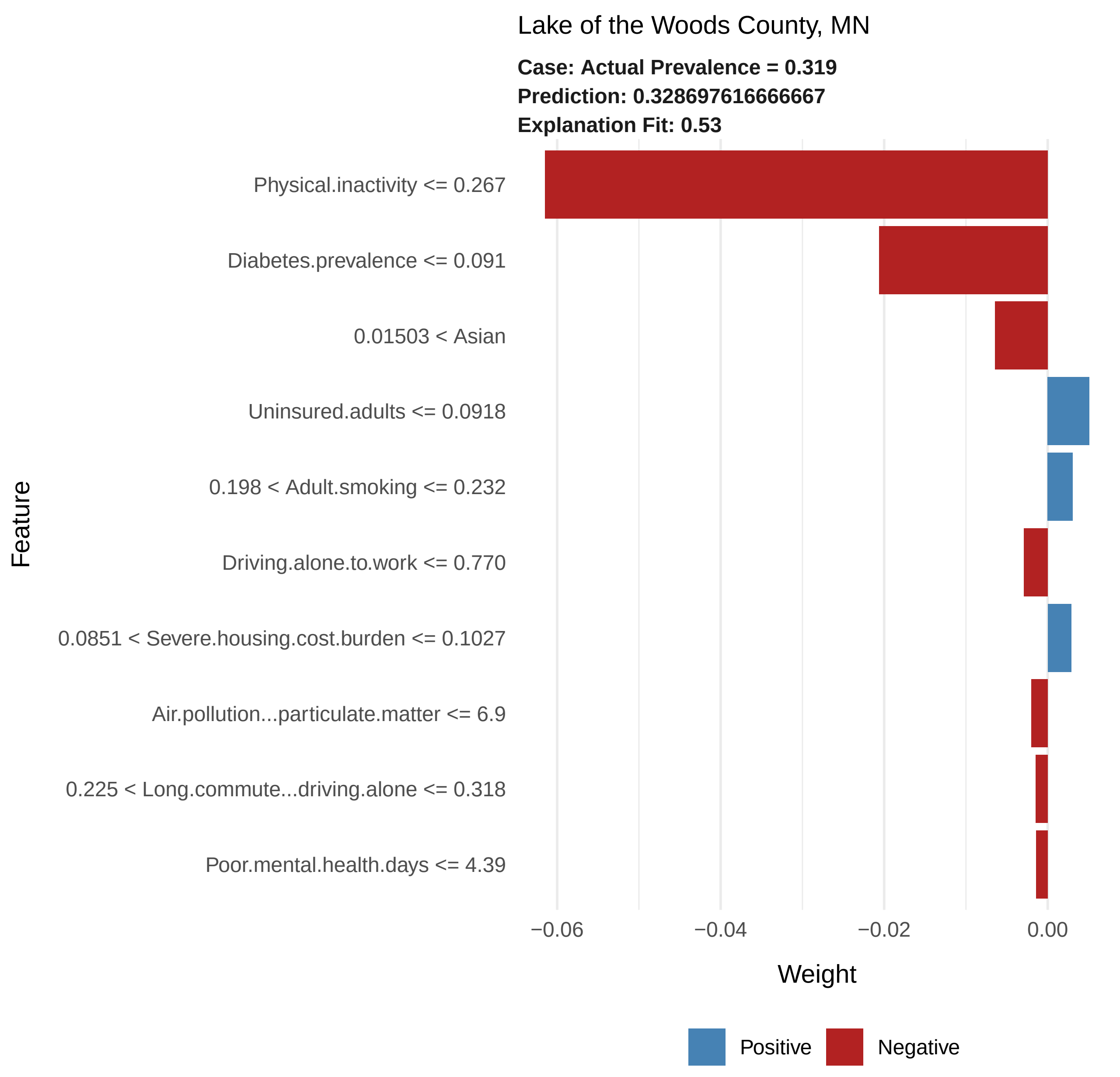

Lake of the Woods County, MN
Case: Actual Prevalence = 0.319
Prediction: 0.328697616666667
Explanation Fit: 0.53
Physical.inactivity <= 0.267
Diabetes.prevalence <= 0.091
0.01503 < Asian
Uninsured.adults <= 0.0918
0.198 < Adult.smoking <= 0.232
Driving.alone.to.work <= 0.770
0.0851 < Severe.housing.cost.burden <= 0.1027
Air.pollution...particulate.matter <= 6.9
0.225 < Long.commute...driving.alone <= 0.318
Poor.mental.health.days <= 4.39
Feature
−0.06
−0.04
−0.02
0.00
Weight
Positive
Negative

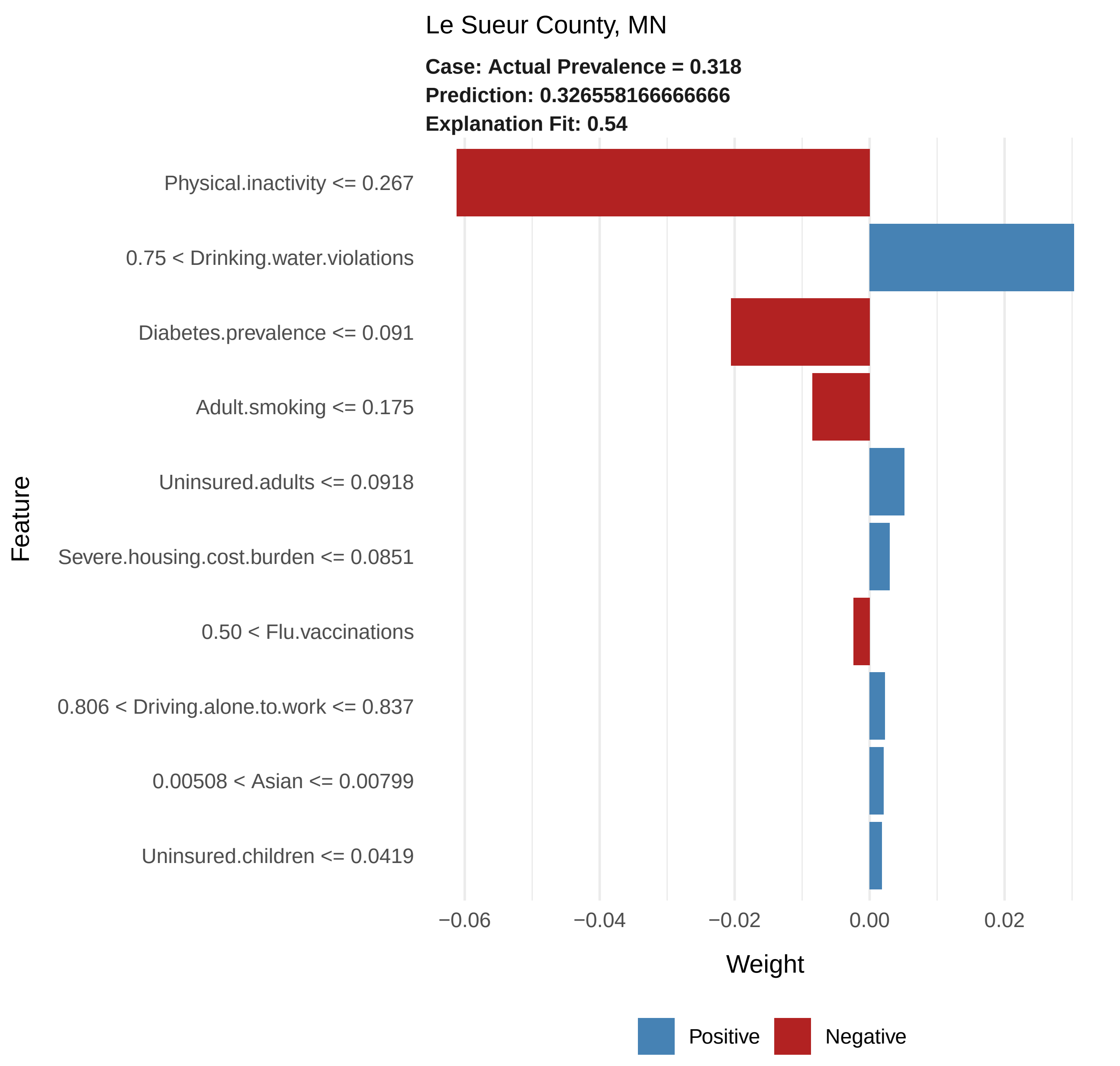

Le Sueur County, MN
Case: Actual Prevalence = 0.318
Prediction: 0.326558166666666
Explanation Fit: 0.54
Physical.inactivity <= 0.267
0.75 < Drinking.water.violations
Diabetes.prevalence <= 0.091
Adult.smoking <= 0.175
Uninsured.adults <= 0.0918
Severe.housing.cost.burden <= 0.0851
0.50 < Flu.vaccinations
0.806 < Driving.alone.to.work <= 0.837
0.00508 < Asian <= 0.00799
Uninsured.children <= 0.0419
Feature
−0.06
−0.04
−0.02
0.00
0.02
Weight
Positive
Negative

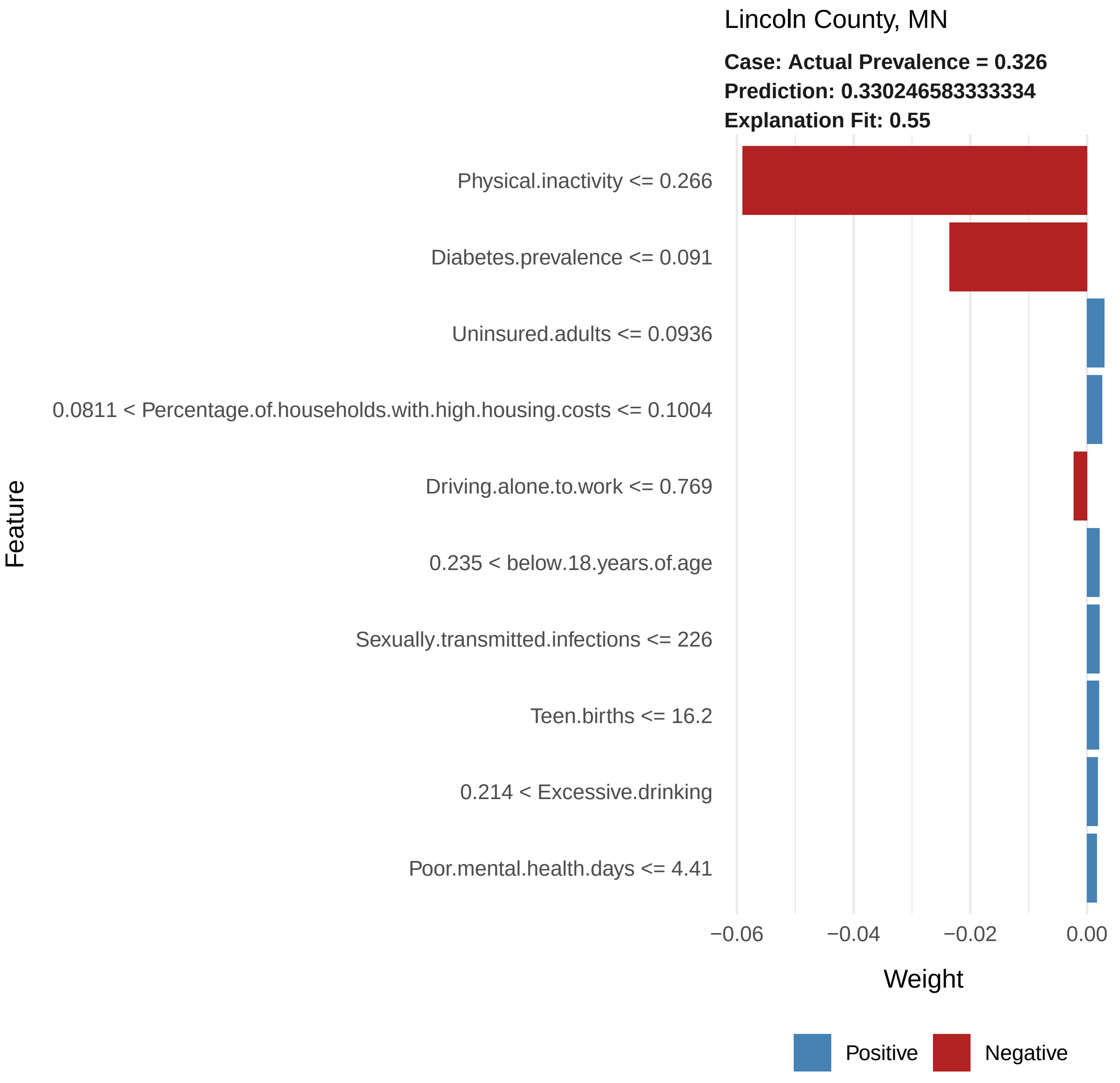

Lincoln County, MN
Case: Actual Prevalence = 0.326
Prediction: 0.330246583333334
Explanation Fit: 0.55
Physical.inactivity <= 0.266
Diabetes.prevalence <= 0.091
Uninsured.adults <= 0.0936
0.0811 < Percentage.of.households.with.high.housing.costs <= 0.1004
Driving.alone.to.work <= 0.769
0.235 < below.18.years.of.age
Sexually.transmitted.infections <= 226
Teen.births <= 16.2
0.214 < Excessive.drinking
Poor.mental.health.days <= 4.41
Feature
−0.06
−0.04
−0.02
0.00
Weight
Positive
Negative

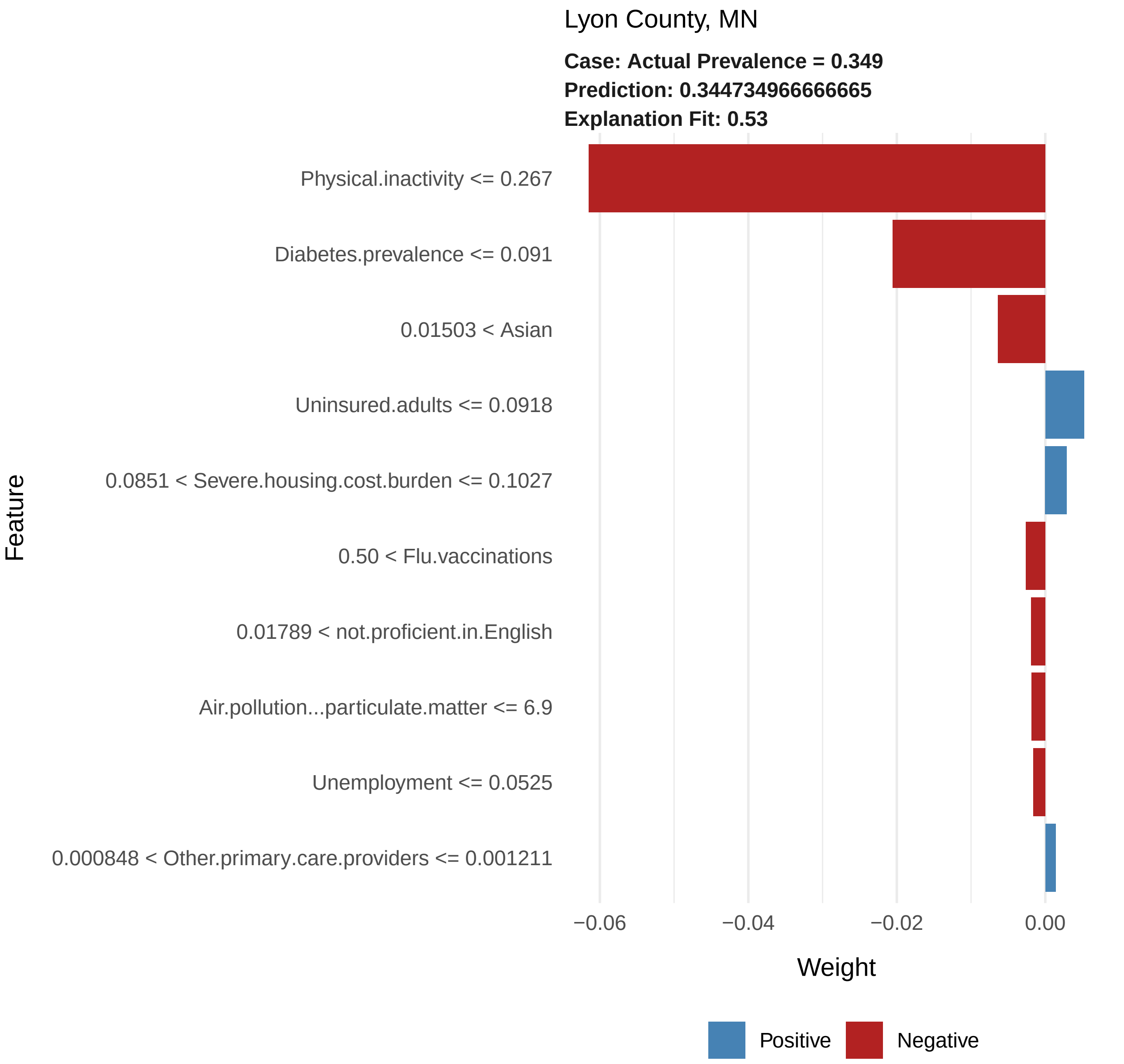
Lyon County, MN
Case: Actual Prevalence = 0.349
Prediction: 0.344734966666665
Explanation Fit: 0.53
Physical.inactivity <= 0.267
Diabetes.prevalence <= 0.091
0.01503 < Asian
Uninsured.adults <= 0.0918
0.0851 < Severe.housing.cost.burden <= 0.1027
0.50 < Flu.vaccinations
0.01789 < not.proficient.in.English
Air.pollution...particulate.matter <= 6.9
Unemployment <= 0.0525
0.000848 < Other.primary.care.providers <= 0.001211
Feature
−0.06
−0.04
−0.02
0.00
Weight
Positive
Negative

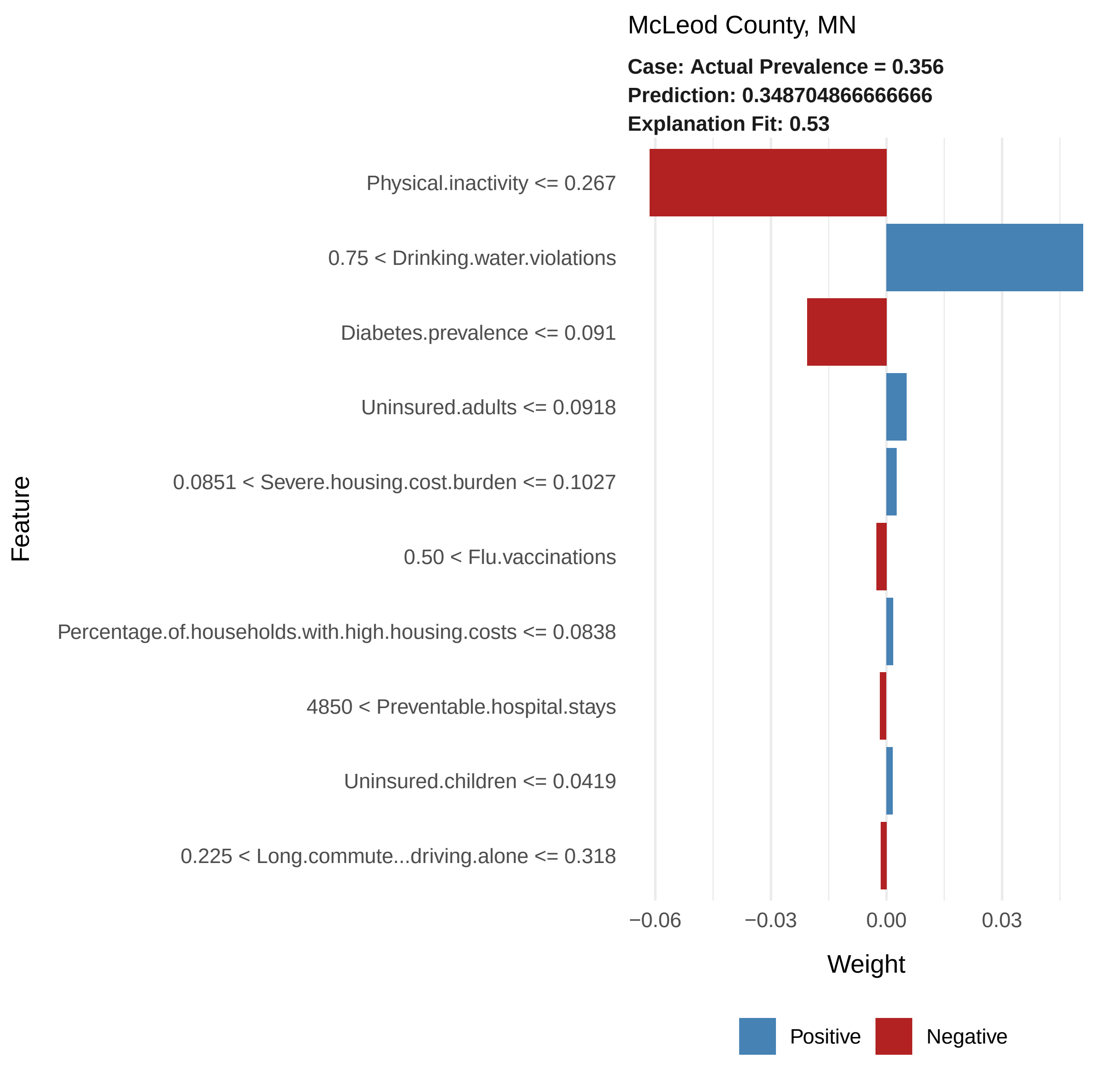
McLeod County, MN
Case: Actual Prevalence = 0.356
Prediction: 0.348704866666666
Explanation Fit: 0.53
Physical.inactivity <= 0.267
0.75 < Drinking.water.violations
Diabetes.prevalence <= 0.091
Uninsured.adults <= 0.0918
0.0851 < Severe.housing.cost.burden <= 0.1027
0.50 < Flu.vaccinations
Percentage.of.households.with.high.housing.costs <= 0.0838
4850 < Preventable.hospital.stays
Uninsured.children <= 0.0419
0.225 < Long.commute...driving.alone <= 0.318
Feature
−0.06
−0.03
0.00
0.03
Weight
Positive
Negative

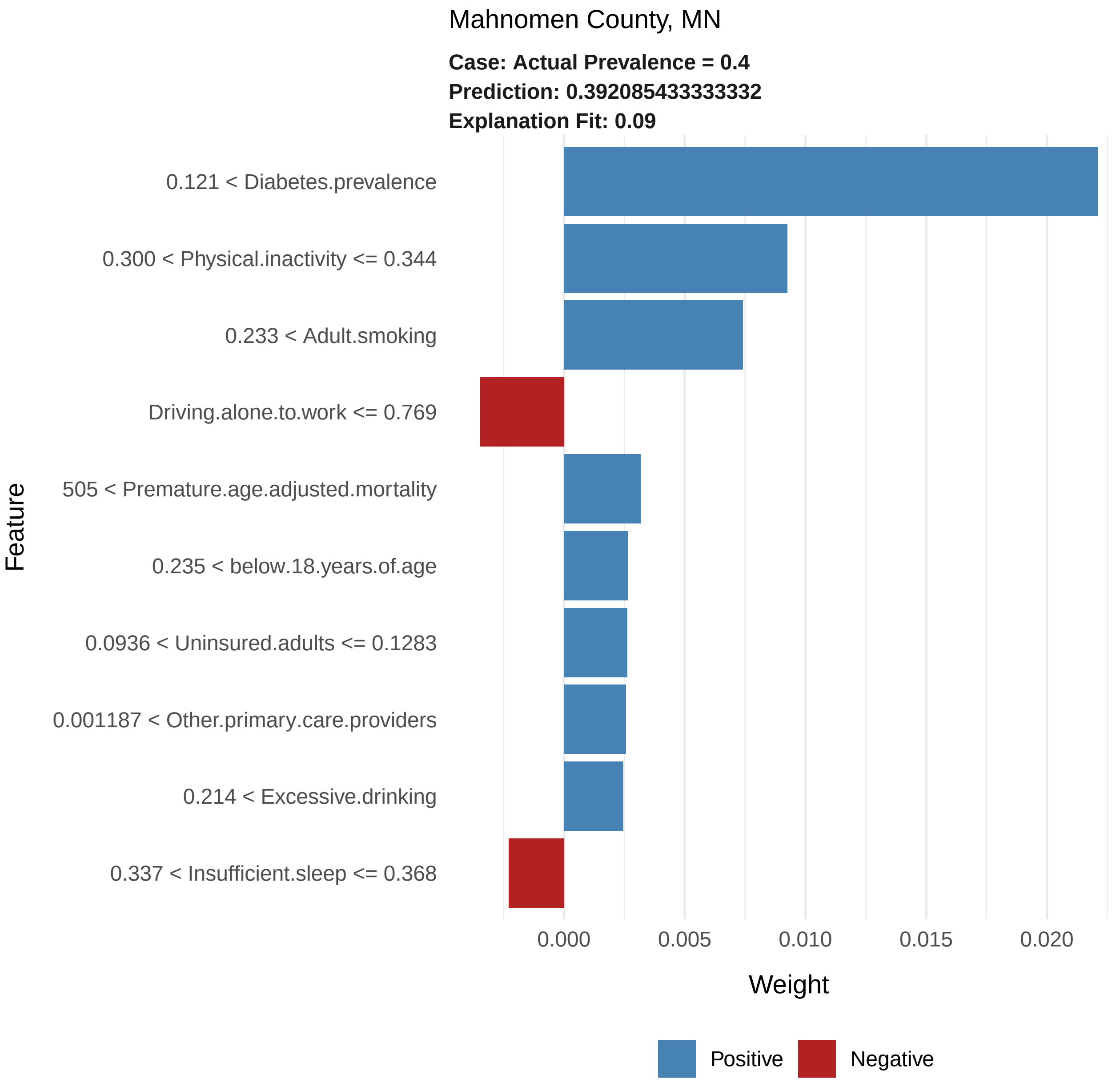
Mahnomen County, MN
Case: Actual Prevalence = 0.4
Prediction: 0.392085433333332
Explanation Fit: 0.09
Feature
0.121 < Diabetes.prevalence
0.300 < Physical.inactivity <= 0.344
0.233 < Adult.smoking
Driving.alone.to.work <= 0.769
505 < Premature.age.adjusted.mortality
0.235 < below.18.years.of.age
0.0936 < Uninsured.adults <= 0.1283
0.001187 < Other.primary.care.providers
0.214 < Excessive.drinking
0.337 < Insufficient.sleep <= 0.368
0.000
0.005
0.010
0.015
0.020
Weight
Positive
Negative

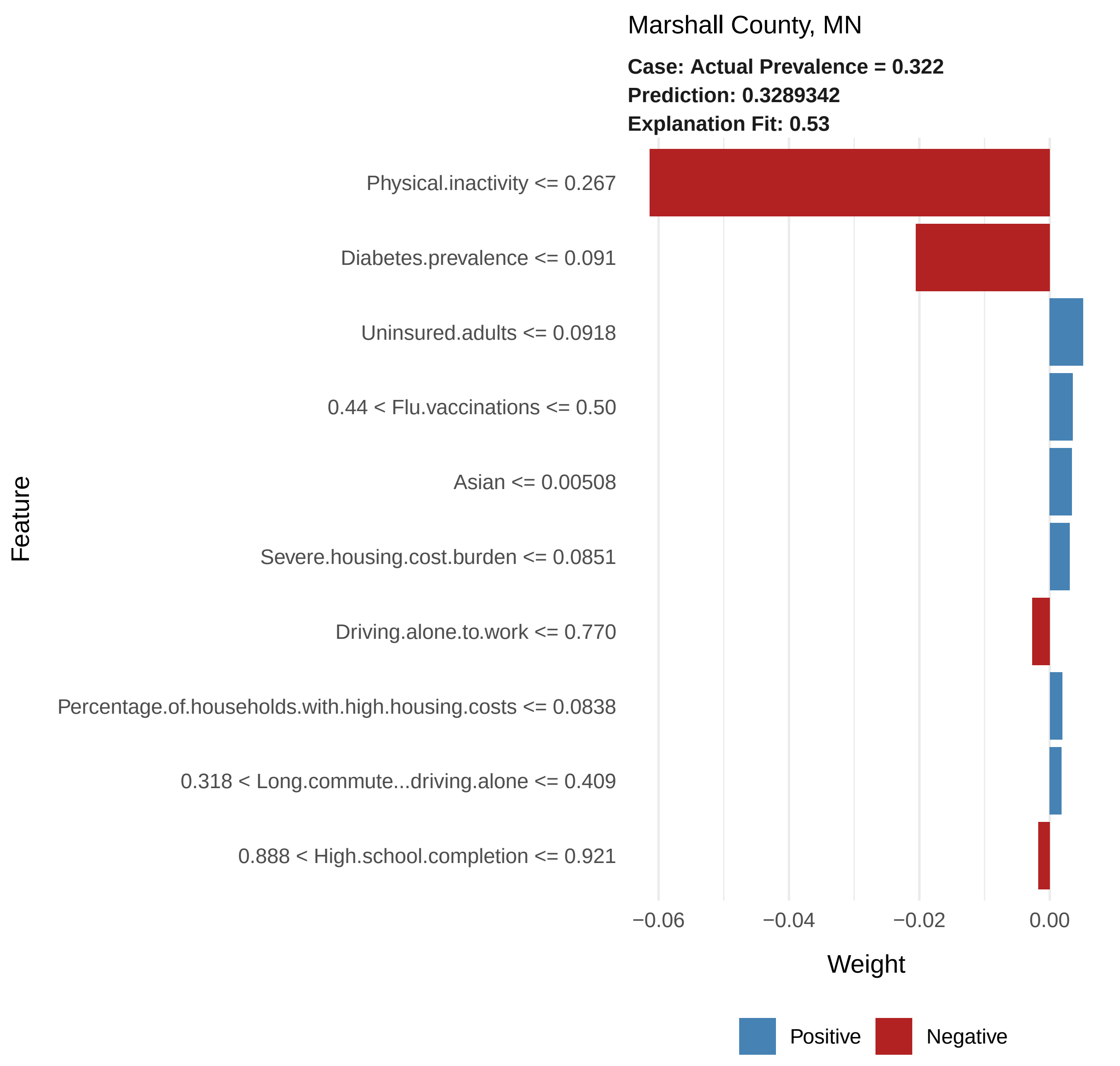

Marshall County, MN
Case: Actual Prevalence = 0.322
Prediction: 0.3289342
Explanation Fit: 0.53
Physical.inactivity <= 0.267
Diabetes.prevalence <= 0.091
Uninsured.adults <= 0.0918
0.44 < Flu.vaccinations <= 0.50
Asian <= 0.00508
Severe.housing.cost.burden <= 0.0851
Driving.alone.to.work <= 0.770
Percentage.of.households.with.high.housing.costs <= 0.0838
0.318 < Long.commute...driving.alone <= 0.409
0.888 < High.school.completion <= 0.921
Feature
−0.06
−0.04
−0.02
0.00
Weight
Positive
Negative

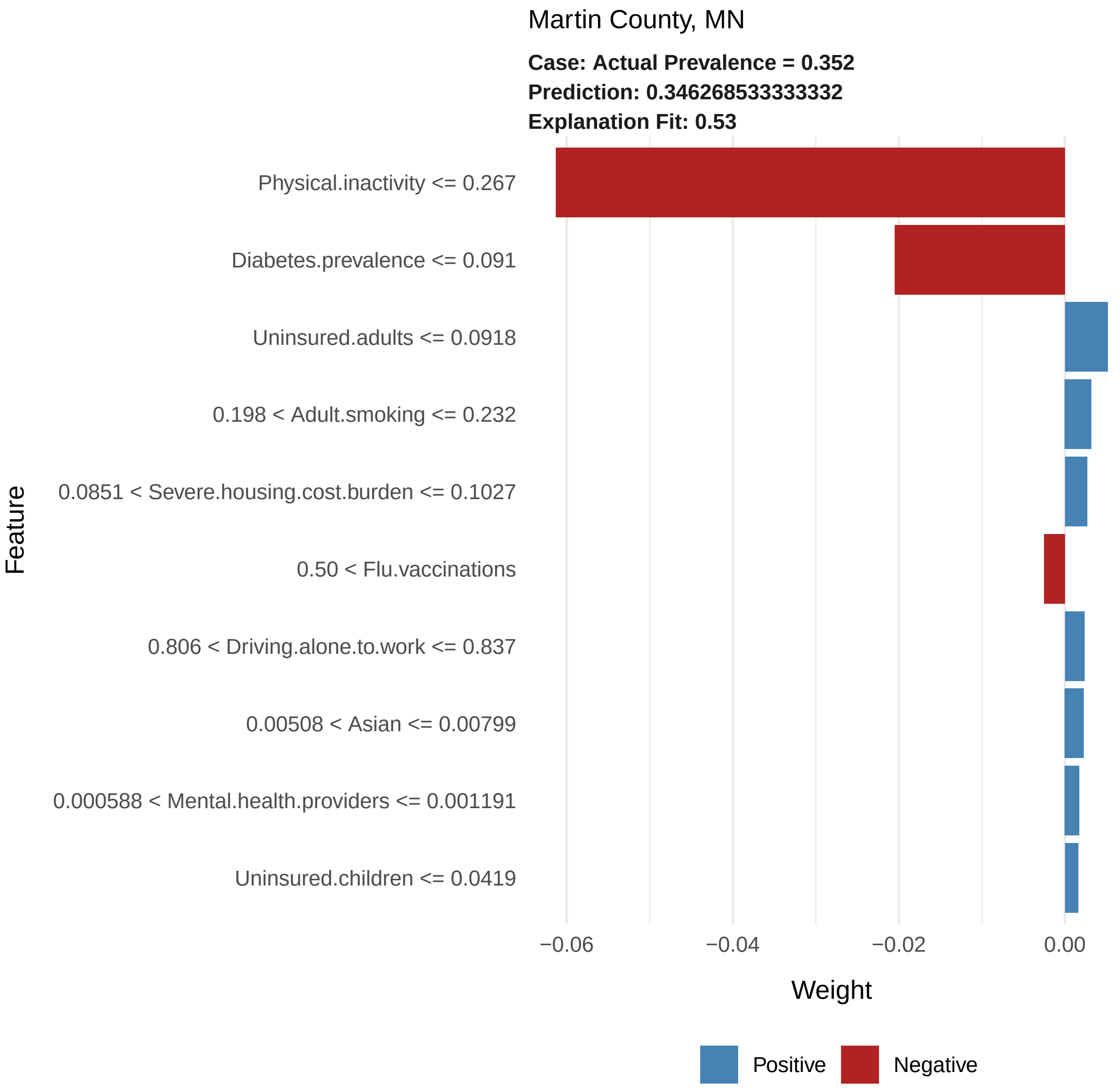

Martin County, MN
Case: Actual Prevalence = 0.352
Prediction: 0.346268533333332
Explanation Fit: 0.53
Physical.inactivity <= 0.267
Diabetes.prevalence <= 0.091
Uninsured.adults <= 0.0918
0.198 < Adult.smoking <= 0.232
0.0851 < Severe.housing.cost.burden <= 0.1027
0.50 < Flu.vaccinations
0.806 < Driving.alone.to.work <= 0.837
0.00508 < Asian <= 0.00799
0.000588 < Mental.health.providers <= 0.001191
Uninsured.children <= 0.0419
Feature
−0.06
−0.04
−0.02
0.00
Weight
Positive
Negative

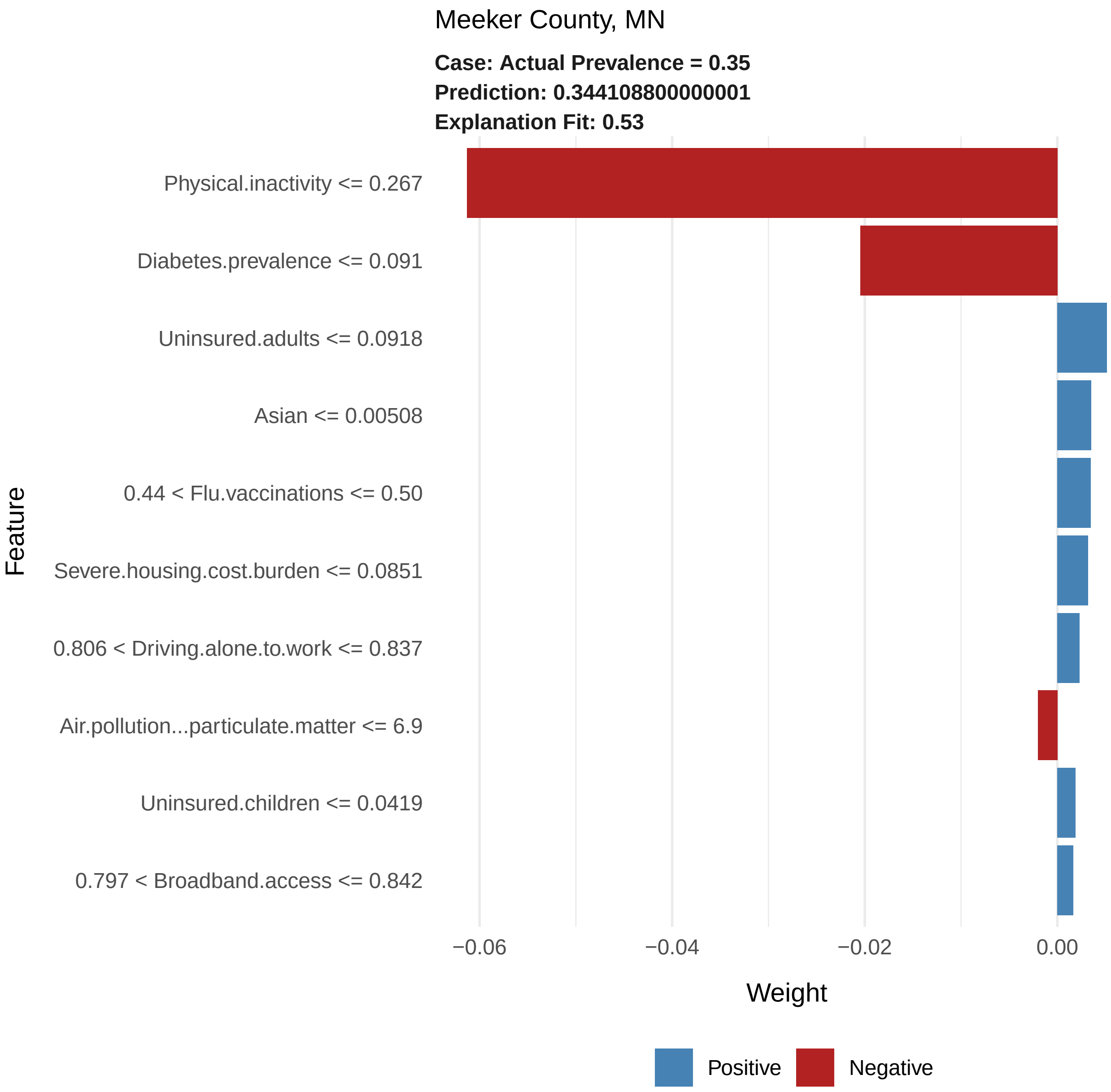

Meeker County, MN
Case: Actual Prevalence = 0.35
Prediction: 0.344108800000001
Explanation Fit: 0.53
Physical.inactivity <= 0.267
Diabetes.prevalence <= 0.091
Uninsured.adults <= 0.0918
Asian <= 0.00508
0.44 < Flu.vaccinations <= 0.50
Severe.housing.cost.burden <= 0.0851
0.806 < Driving.alone.to.work <= 0.837
Air.pollution...particulate.matter <= 6.9
Uninsured.children <= 0.0419
0.797 < Broadband.access <= 0.842
Feature
−0.06
−0.04
−0.02
0.00
Weight
Positive
Negative

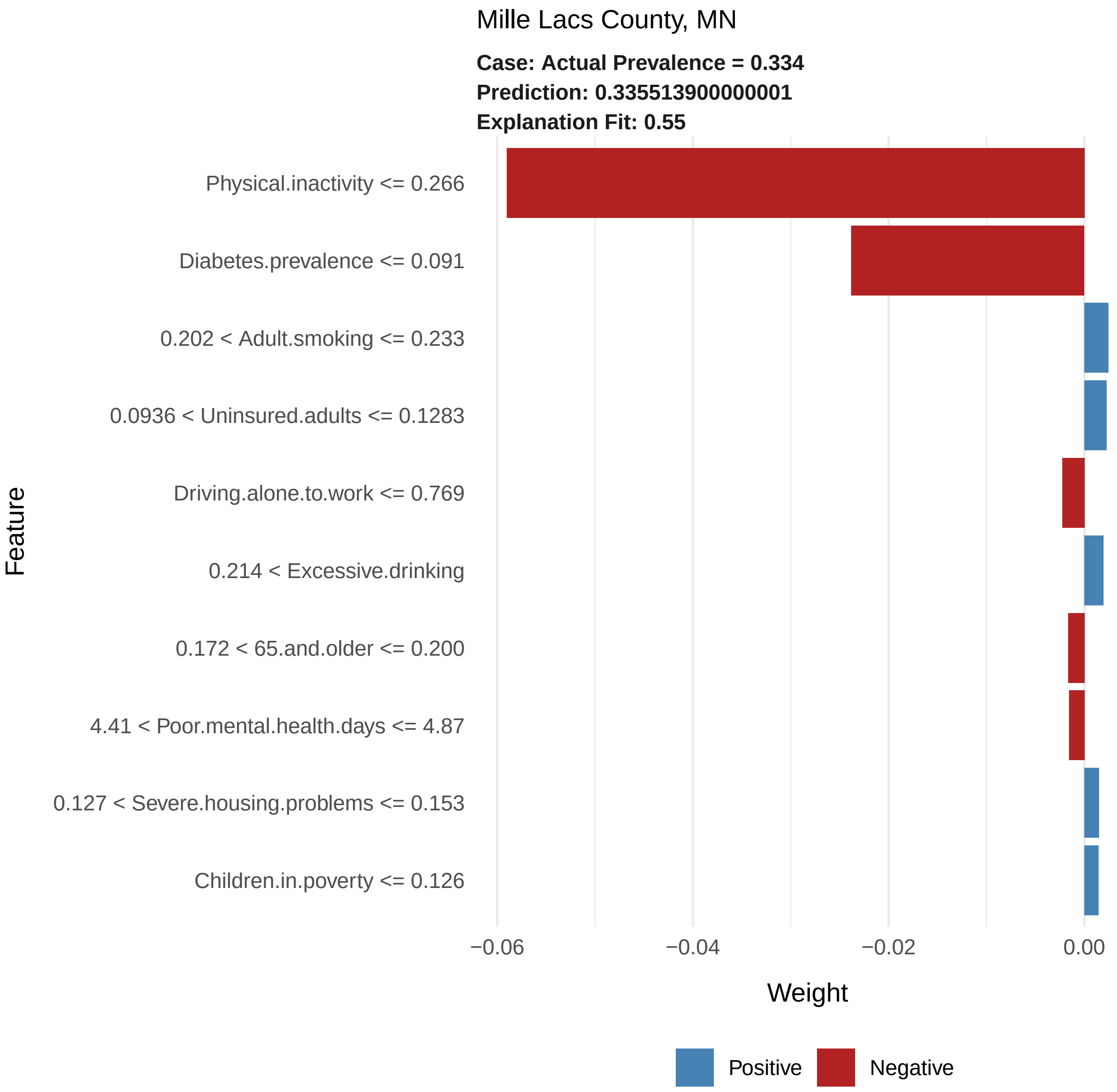
Mille Lacs County, MN
Case: Actual Prevalence = 0.334
Prediction: 0.335513900000001
Explanation Fit: 0.55
Physical.inactivity <= 0.266
Diabetes.prevalence <= 0.091
0.202 < Adult.smoking <= 0.233
0.0936 < Uninsured.adults <= 0.1283
Driving.alone.to.work <= 0.769
0.214 < Excessive.drinking
0.172 < 65.and.older <= 0.200
4.41 < Poor.mental.health.days <= 4.87
0.127 < Severe.housing.problems <= 0.153
Children.in.poverty <= 0.126
Feature
−0.06
−0.04
−0.02
0.00
Weight
Positive
Negative

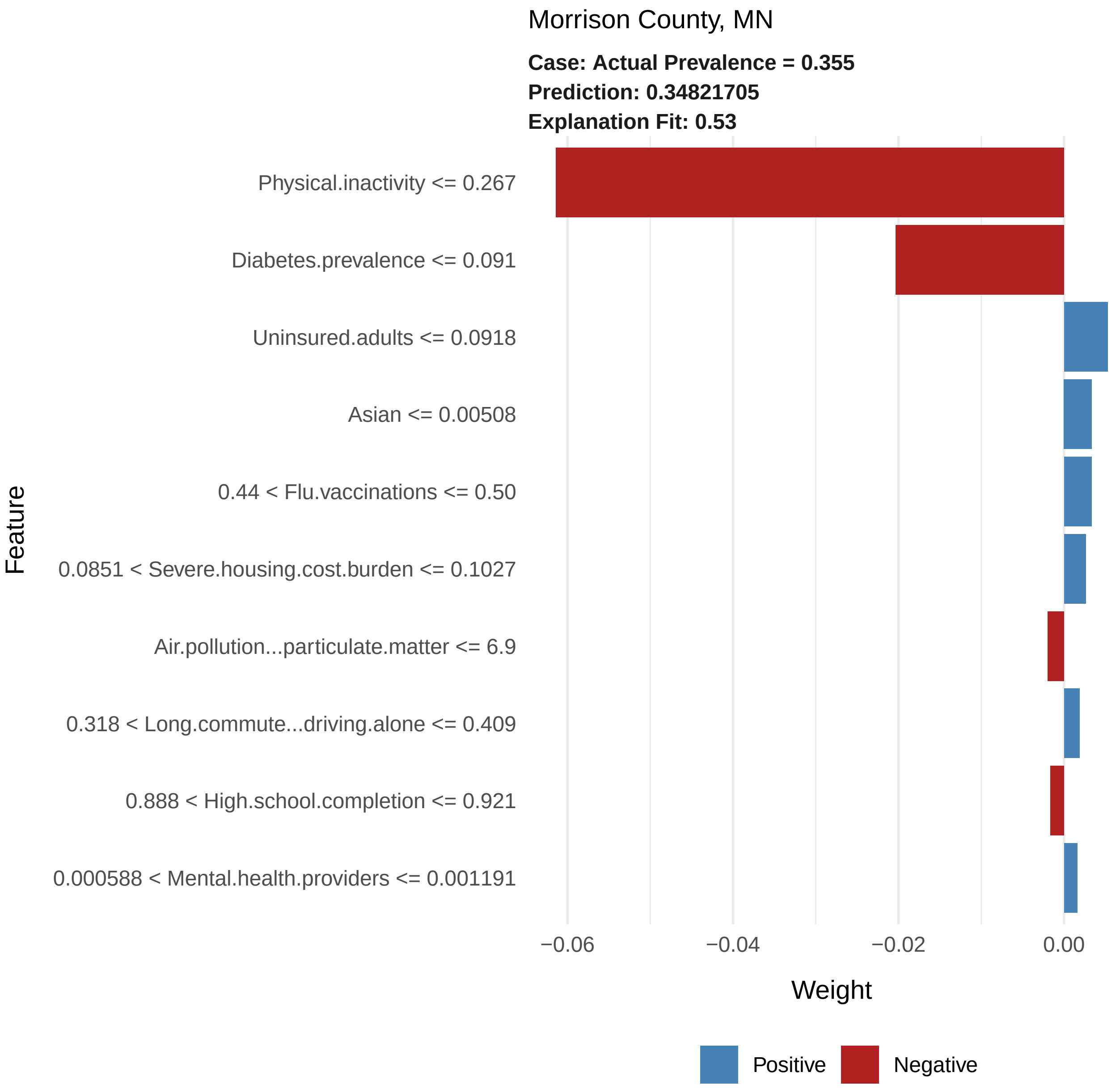
Morrison County, MN
Case: Actual Prevalence = 0.355
Prediction: 0.34821705
Explanation Fit: 0.53
Physical.inactivity <= 0.267
Diabetes.prevalence <= 0.091
Uninsured.adults <= 0.0918
Asian <= 0.00508
0.44 < Flu.vaccinations <= 0.50
0.0851 < Severe.housing.cost.burden <= 0.1027
Air.pollution...particulate.matter <= 6.9
0.318 < Long.commute...driving.alone <= 0.409
0.888 < High.school.completion <= 0.921
0.000588 < Mental.health.providers <= 0.001191
Feature
−0.06
−0.04
−0.02
0.00
Weight
Positive
Negative

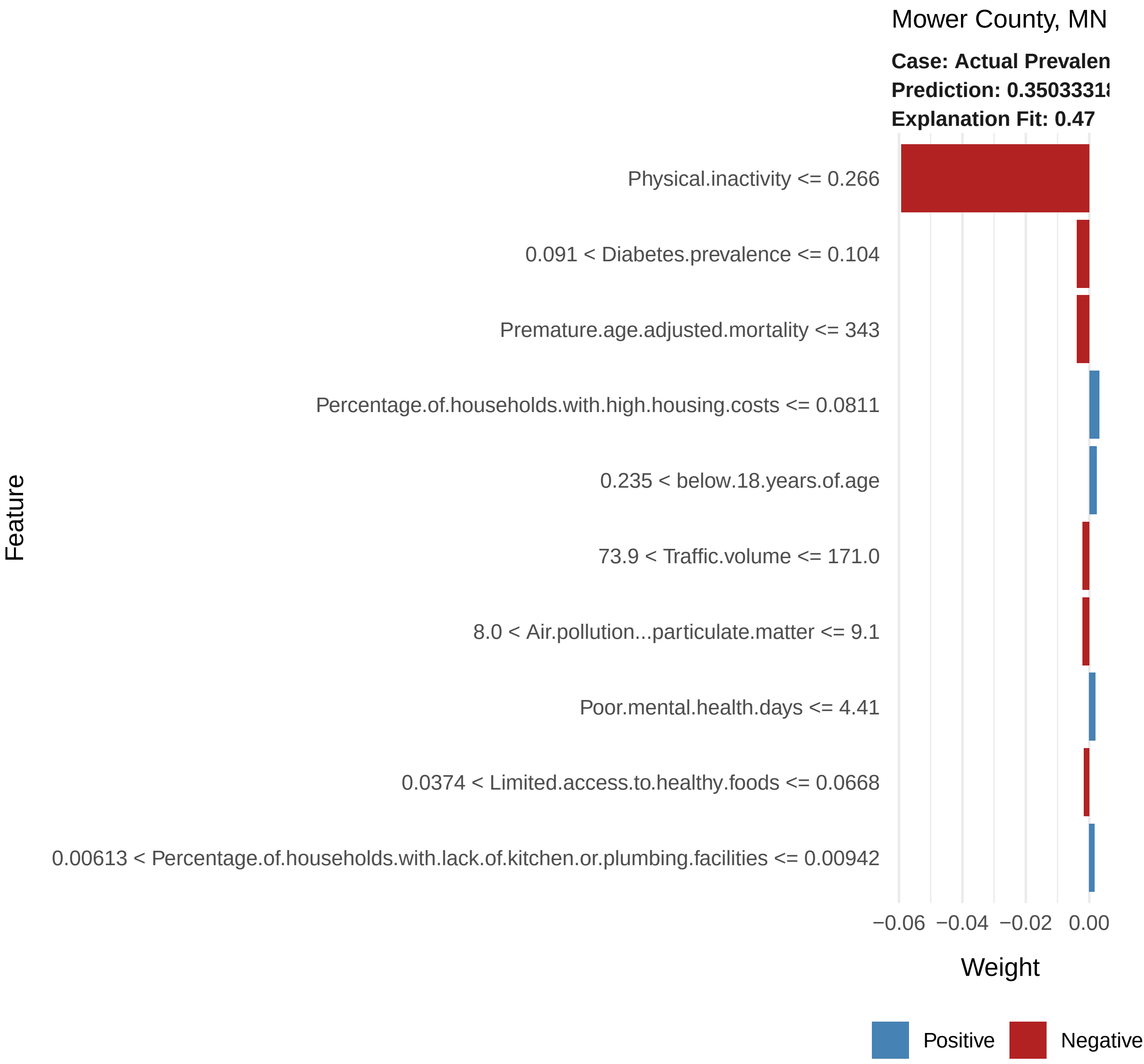

Mower County, MN
Case: Actual Prevalen
Prediction: 0.3503331
Explanation Fit: 0.47
Feature
Physical.inactivity <= 0.266
0.091 < Diabetes.prevalence <= 0.104
Premature.age.adjusted.mortality <= 343
Percentage.of.households.with.high.housing.costs <= 0.0811
0.235 < below.18.years.of.age
73.9 < Traffic.volume <= 171.0
8.0 < Air.pollution...particulate.matter <= 9.1
Poor.mental.health.days <= 4.41
0.0374 < Limited.access.to.healthy.foods <= 0.0668
0.00613 < Percentage.of.households.with.lack.of.kitchen.or.plumbing.facilities <= 0.00942
−0.06
−0.04
−0.02
0.00
Weight
Positive
Negative

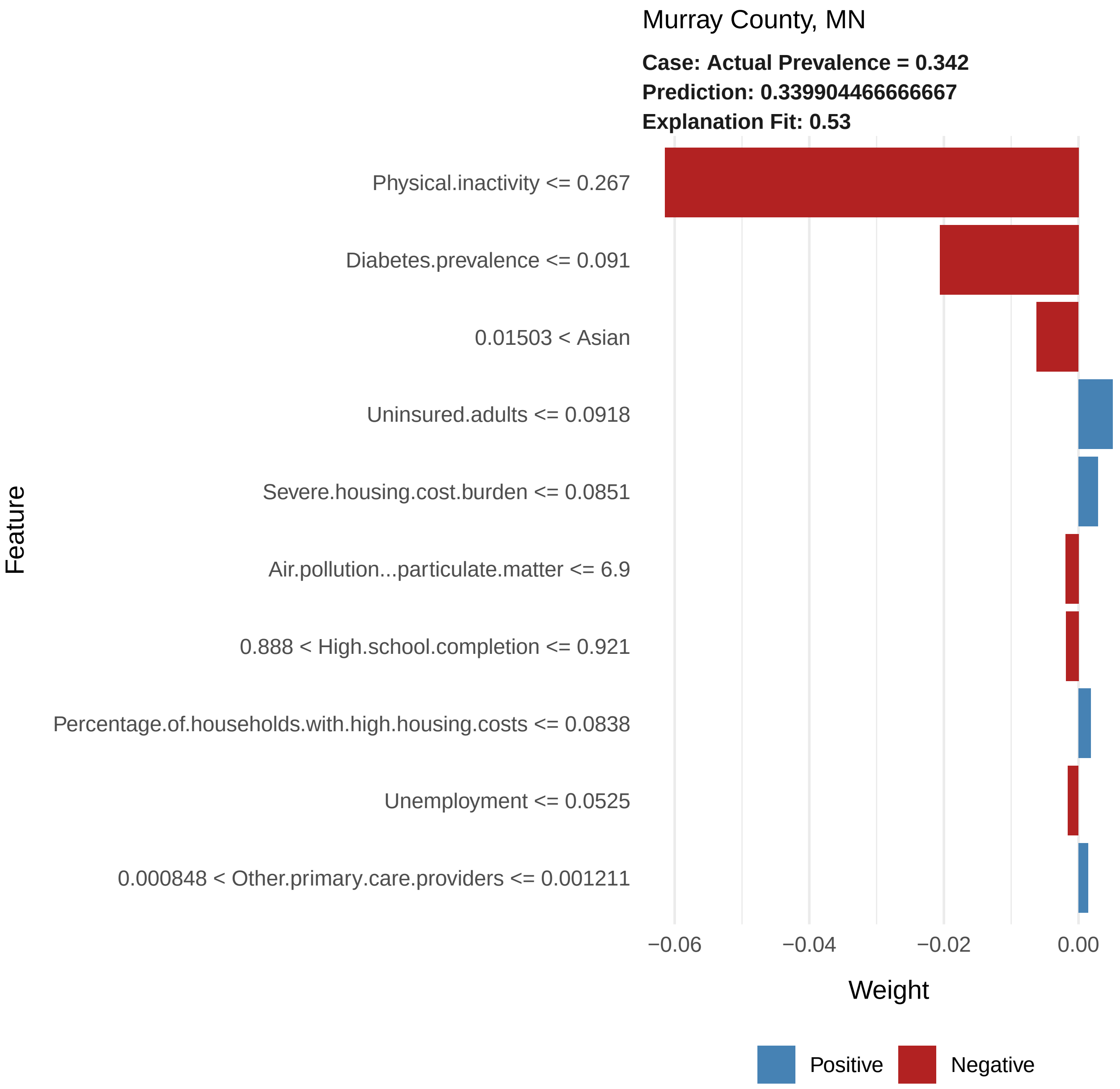

Murray County, MN
Case: Actual Prevalence = 0.342
Prediction: 0.339904466666667
Explanation Fit: 0.53
Physical.inactivity <= 0.267
Diabetes.prevalence <= 0.091
0.01503 < Asian
Uninsured.adults <= 0.0918
Severe.housing.cost.burden <= 0.0851
Air.pollution...particulate.matter <= 6.9
0.888 < High.school.completion <= 0.921
Percentage.of.households.with.high.housing.costs <= 0.0838
Unemployment <= 0.0525
0.000848 < Other.primary.care.providers <= 0.001211
Feature
−0.06
−0.04
−0.02
0.00
Weight
Positive
Negative

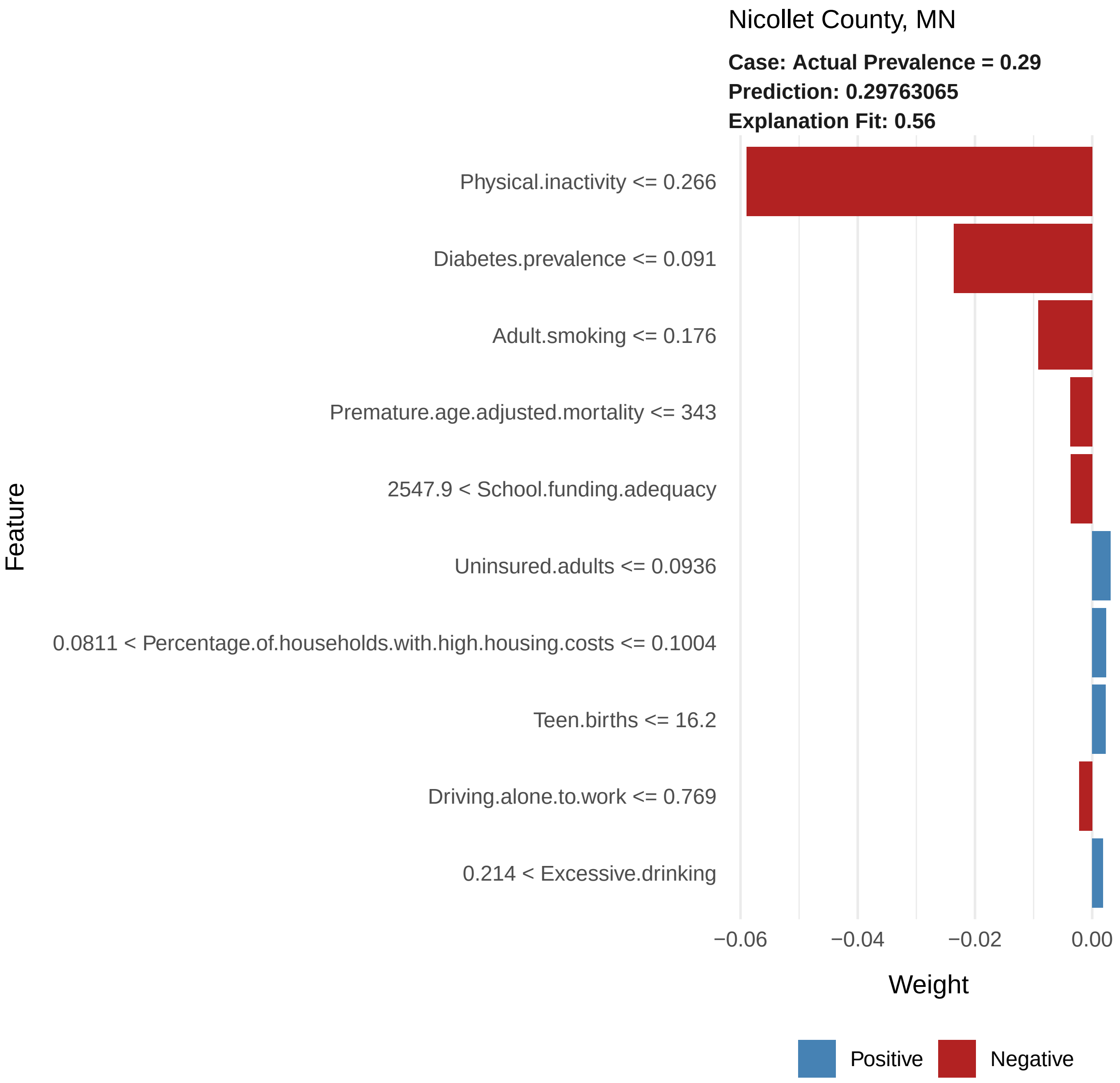

Nicollet County, MN
Case: Actual Prevalence = 0.29
Prediction: 0.29763065
Explanation Fit: 0.56
Physical.inactivity <= 0.266
Diabetes.prevalence <= 0.091
Adult.smoking <= 0.176
Premature.age.adjusted.mortality <= 343
2547.9 < School.funding.adequacy
Uninsured.adults <= 0.0936
0.0811 < Percentage.of.households.with.high.housing.costs <= 0.1004
Teen.births <= 16.2
Driving.alone.to.work <= 0.769
0.214 < Excessive.drinking
Feature
−0.06
−0.04
−0.02
0.00
Weight
Positive
Negative

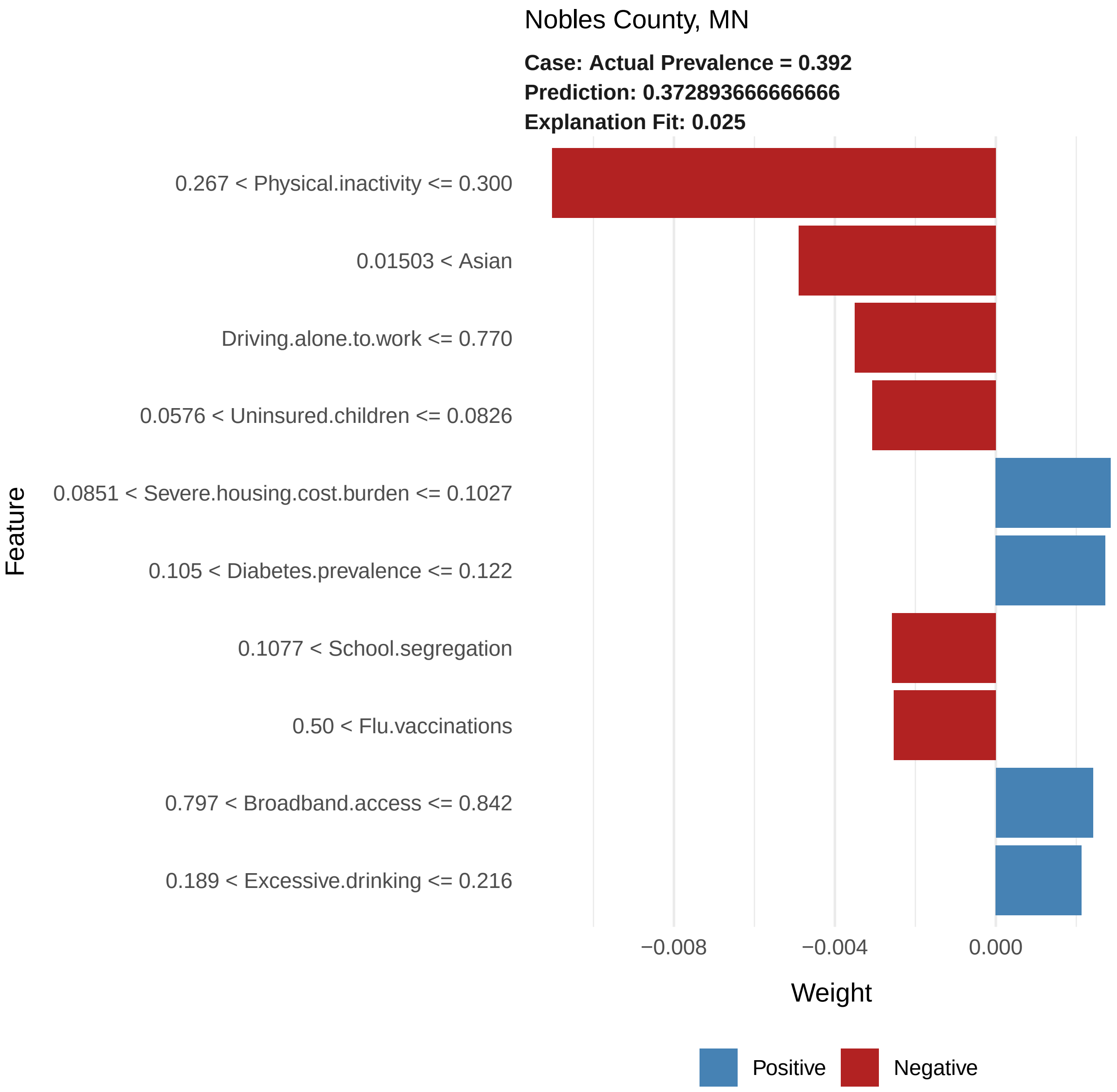
Nobles County, MN
Case: Actual Prevalence = 0.392
Prediction: 0.372893666666666
Explanation Fit: 0.025
0.267 < Physical.inactivity <= 0.300
0.01503 < Asian
Driving.alone.to.work <= 0.770
0.0576 < Uninsured.children <= 0.0826
0.0851 < Severe.housing.cost.burden <= 0.1027
0.105 < Diabetes.prevalence <= 0.122
0.1077 < School.segregation
0.50 < Flu.vaccinations
0.797 < Broadband.access <= 0.842
0.189 < Excessive.drinking <= 0.216
Feature
−0.008
−0.004
0.000
Weight
Positive
Negative

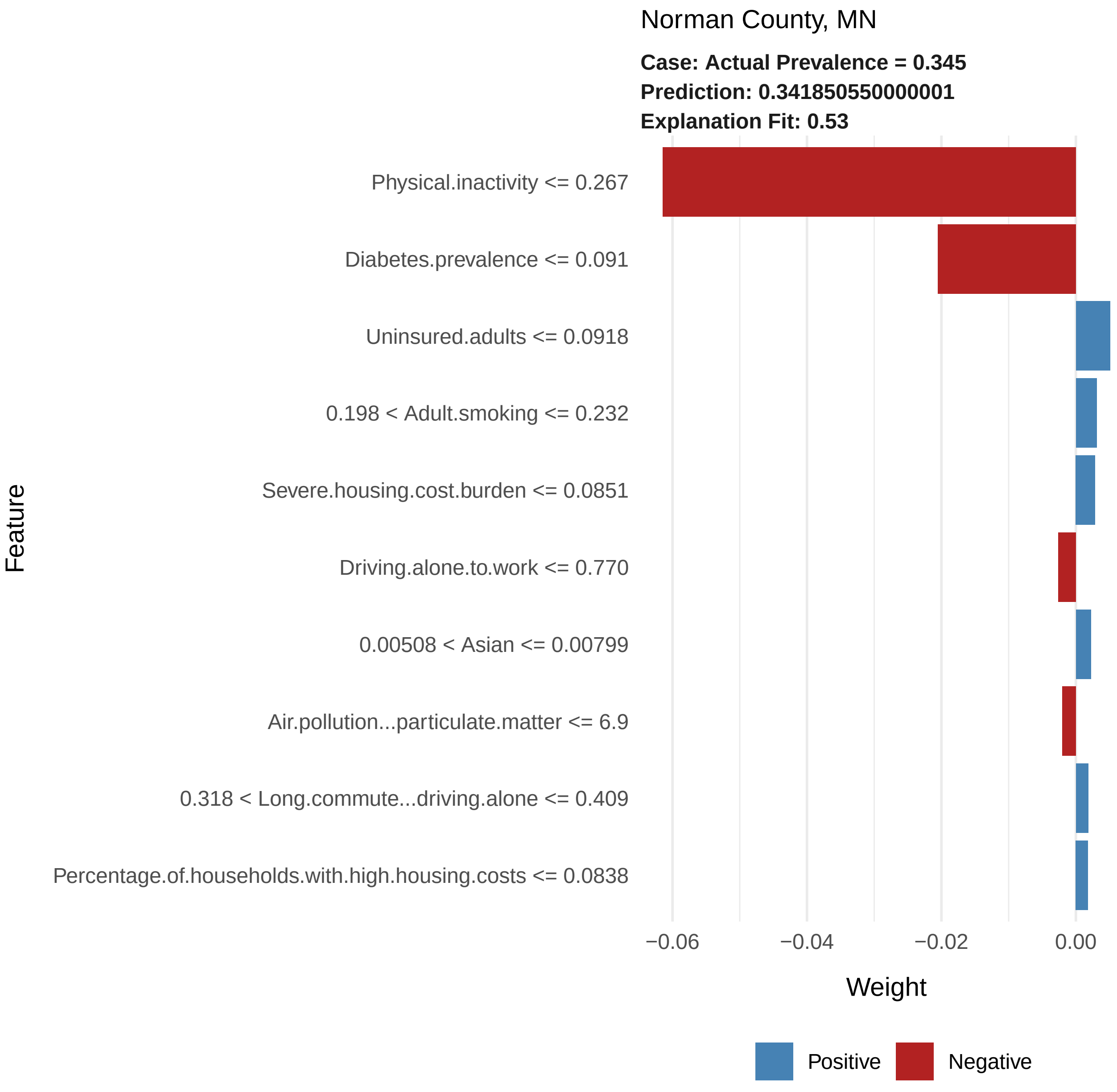

Norman County, MN
Case: Actual Prevalence = 0.345
Prediction: 0.341850550000001
Explanation Fit: 0.53
Physical.inactivity <= 0.267
Diabetes.prevalence <= 0.091
Uninsured.adults <= 0.0918
0.198 < Adult.smoking <= 0.232
Severe.housing.cost.burden <= 0.0851
Driving.alone.to.work <= 0.770
0.00508 < Asian <= 0.00799
Air.pollution...particulate.matter <= 6.9
0.318 < Long.commute...driving.alone <= 0.409
Percentage.of.households.with.high.housing.costs <= 0.0838
Feature
−0.06
−0.04
−0.02
0.00
Weight
Positive
Negative

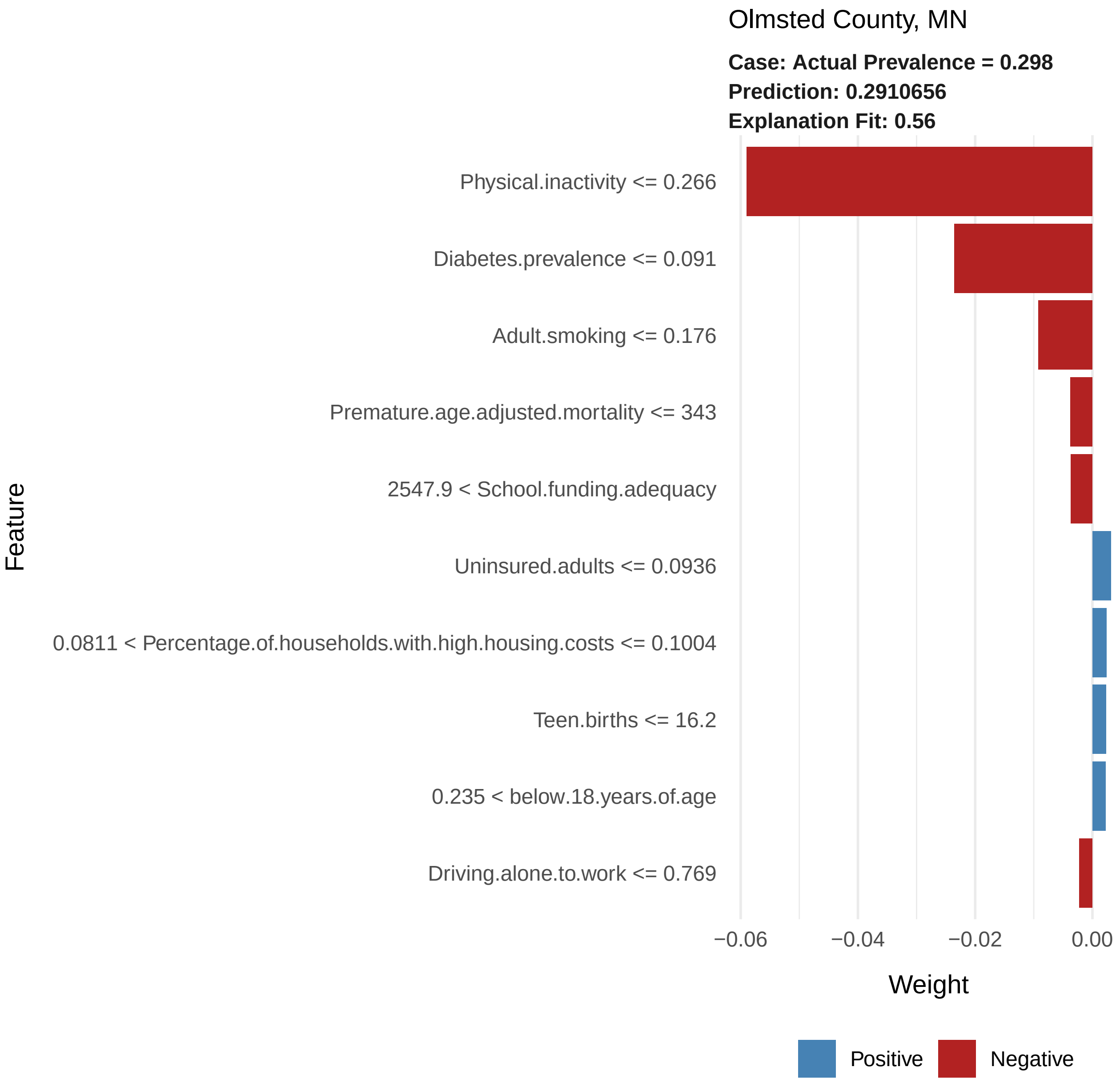

Olmsted County, MN
Case: Actual Prevalence = 0.298
Prediction: 0.2910656
Explanation Fit: 0.56
Physical.inactivity <= 0.266
Diabetes.prevalence <= 0.091
Adult.smoking <= 0.176
Premature.age.adjusted.mortality <= 343
2547.9 < School.funding.adequacy
Uninsured.adults <= 0.0936
0.0811 < Percentage.of.households.with.high.housing.costs <= 0.1004
Teen.births <= 16.2
0.235 < below.18.years.of.age
Driving.alone.to.work <= 0.769
Feature
−0.06
−0.04
−0.02
0.00
Weight
Positive
Negative

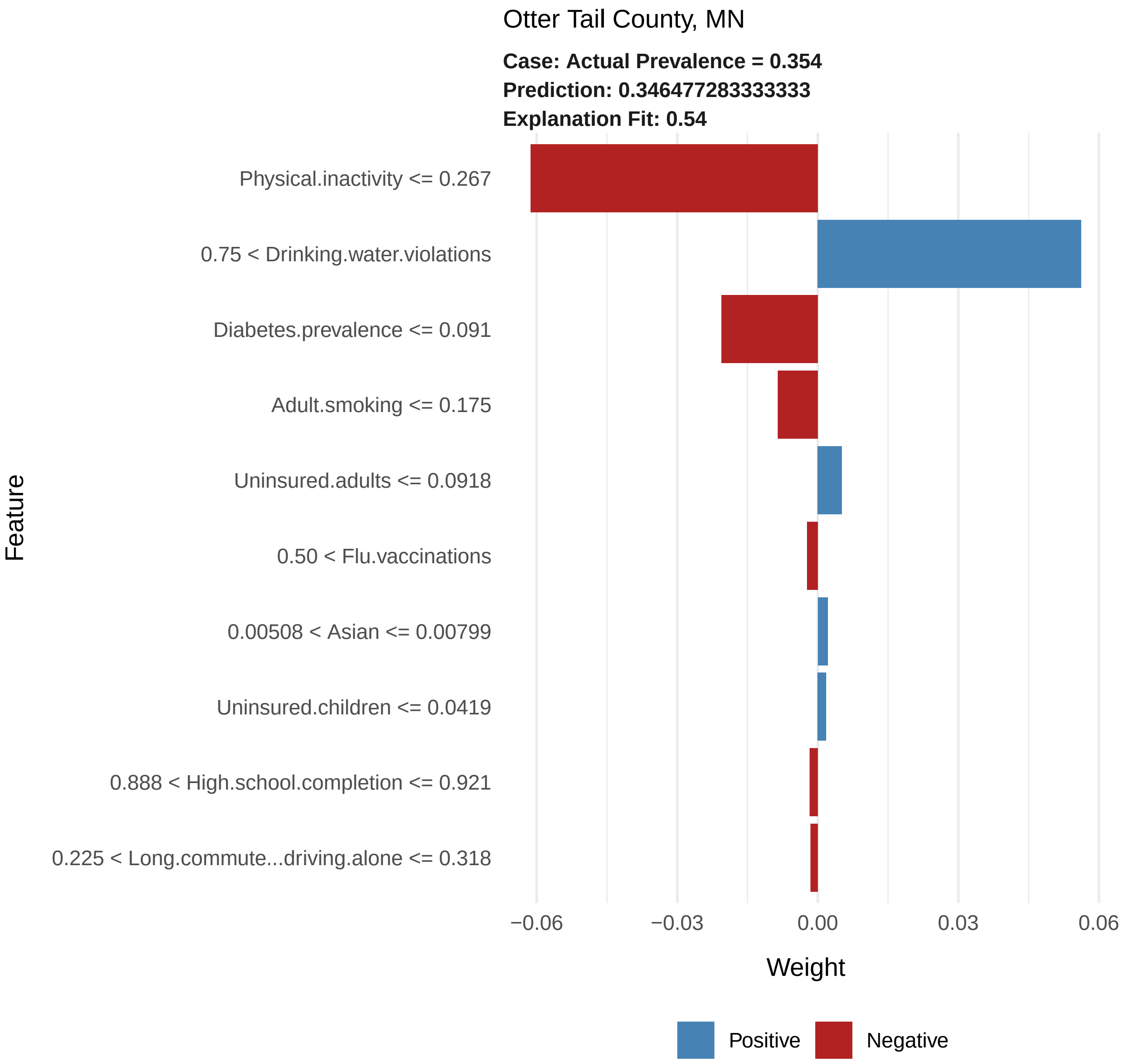

Otter Tail County, MN
Case: Actual Prevalence = 0.354
Prediction: 0.346477283333333
Explanation Fit: 0.54
Physical.inactivity <= 0.267
0.75 < Drinking.water.violations
Diabetes.prevalence <= 0.091
Adult.smoking <= 0.175
Uninsured.adults <= 0.0918
0.50 < Flu.vaccinations
0.00508 < Asian <= 0.00799
Uninsured.children <= 0.0419
0.888 < High.school.completion <= 0.921
0.225 < Long.commute...driving.alone <= 0.318
Feature
−0.06
−0.03
0.00
0.03
0.06
Weight
Positive
Negative

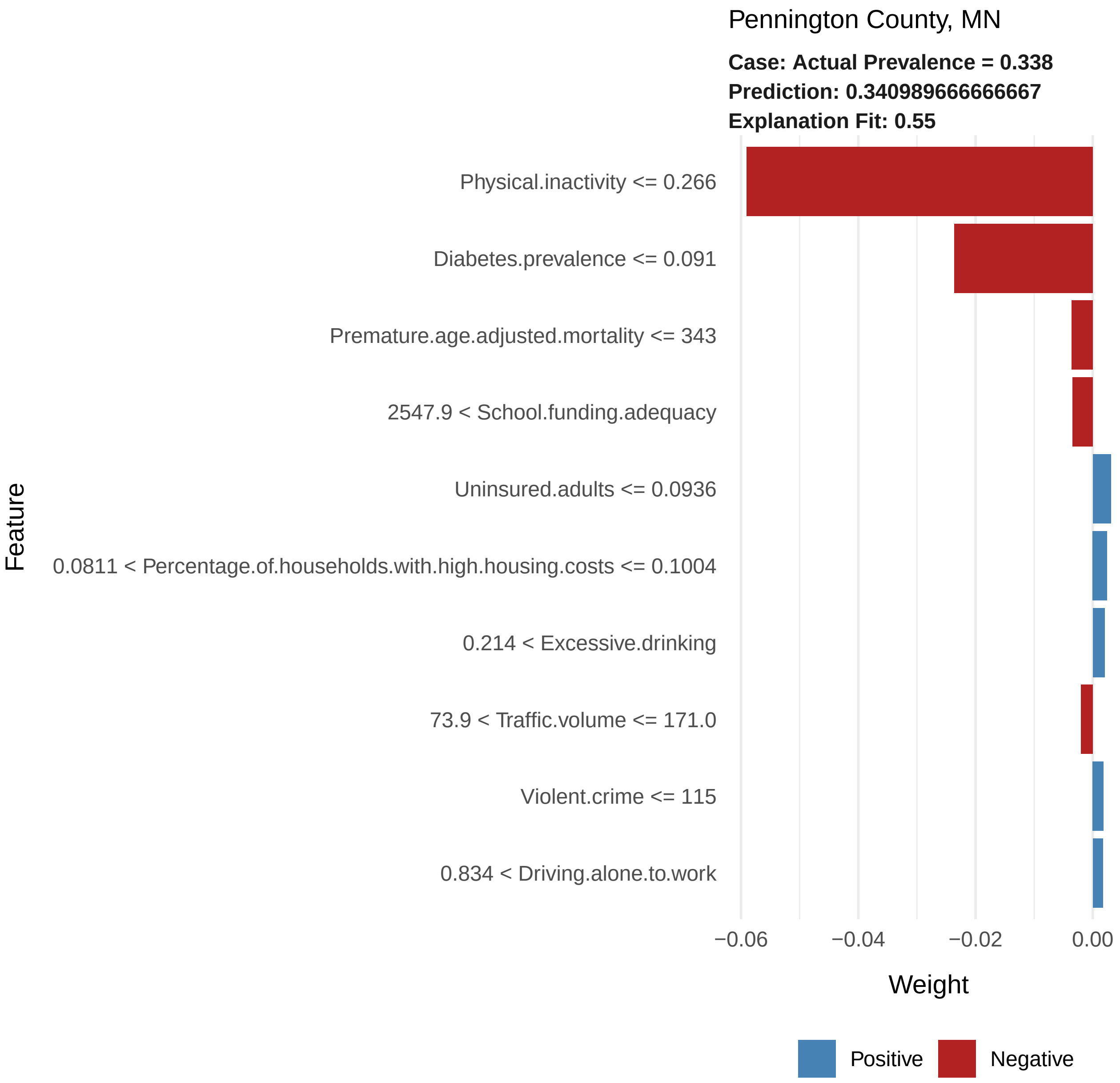

Pennington County, MN
Case: Actual Prevalence = 0.338
Prediction: 0.340989666666667
Explanation Fit: 0.55
Physical.inactivity <= 0.266
Diabetes.prevalence <= 0.091
Premature.age.adjusted.mortality <= 343
2547.9 < School.funding.adequacy
Uninsured.adults <= 0.0936
0.0811 < Percentage.of.households.with.high.housing.costs <= 0.1004
0.214 < Excessive.drinking
73.9 < Traffic.volume <= 171.0
Violent.crime <= 115
0.834 < Driving.alone.to.work
Feature
−0.06
−0.04
−0.02
0.00
Weight
Positive
Negative

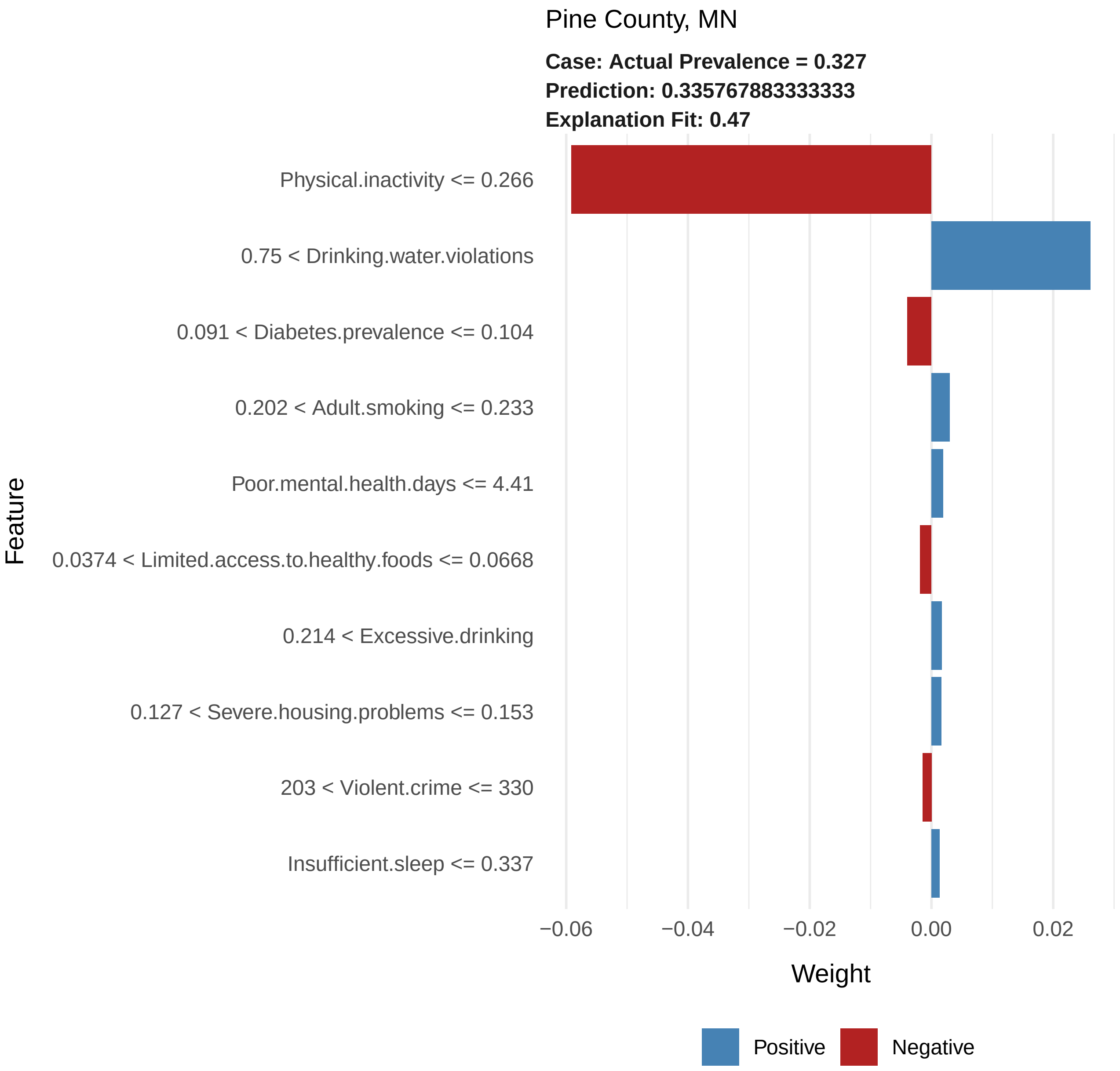

Pine County, MN
Case: Actual Prevalence = 0.327
Prediction: 0.335767883333333
Explanation Fit: 0.47
Physical.inactivity <= 0.266
0.75 < Drinking.water.violations
0.091 < Diabetes.prevalence <= 0.104
0.202 < Adult.smoking <= 0.233
Poor.mental.health.days <= 4.41
0.0374 < Limited.access.to.healthy.foods <= 0.0668
0.214 < Excessive.drinking
0.127 < Severe.housing.problems <= 0.153
203 < Violent.crime <= 330
Insufficient.sleep <= 0.337
Feature
−0.06
−0.04
−0.02
0.00
0.02
Weight
Positive
Negative

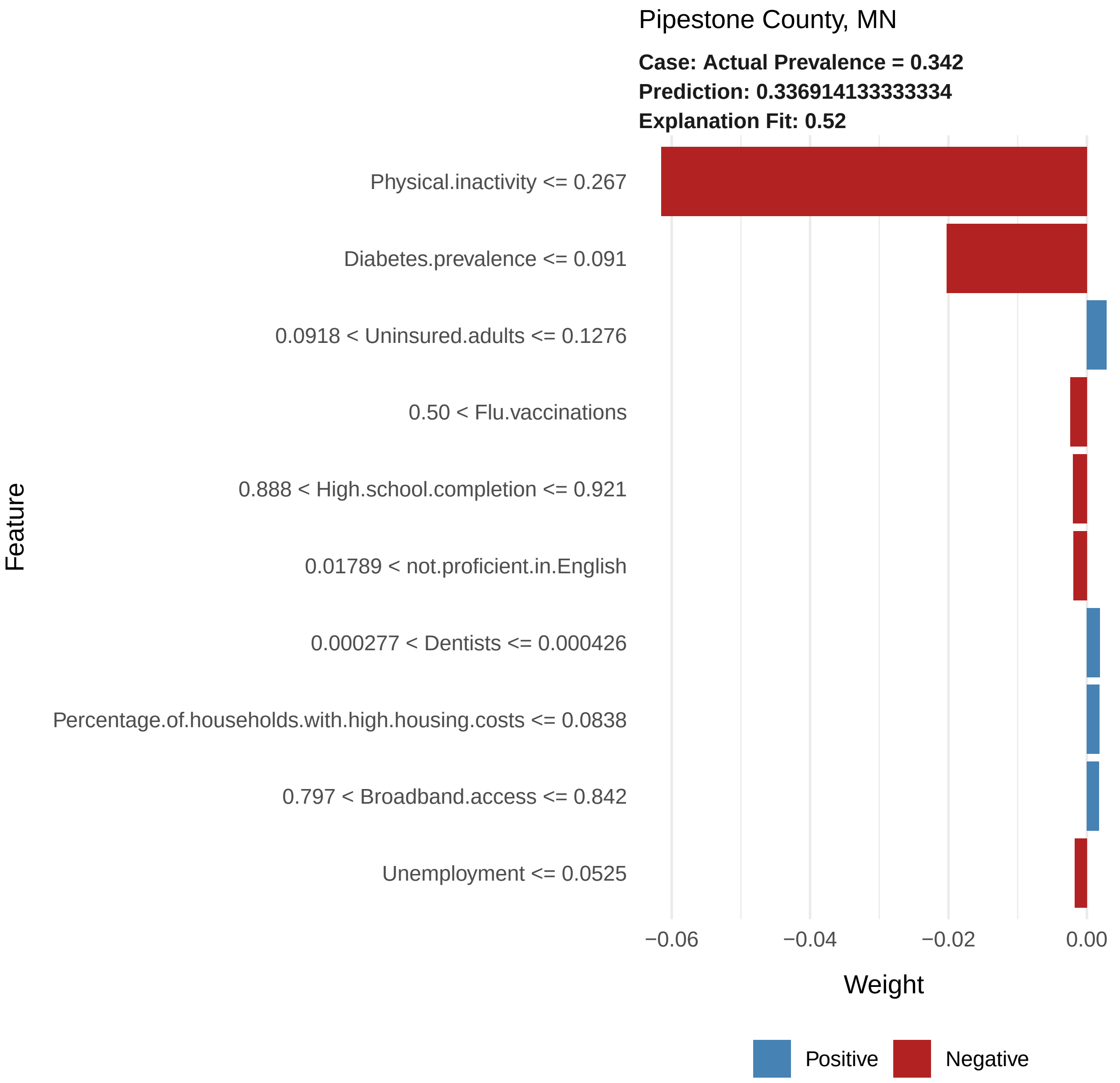
Pipestone County, MN
Case: Actual Prevalence = 0.342
Prediction: 0.336914133333334
Explanation Fit: 0.52
Physical.inactivity <= 0.267
Diabetes.prevalence <= 0.091
0.0918 < Uninsured.adults <= 0.1276
0.50 < Flu.vaccinations
0.888 < High.school.completion <= 0.921
0.01789 < not.proficient.in.English
0.000277 < Dentists <= 0.000426
Percentage.of.households.with.high.housing.costs <= 0.0838
0.797 < Broadband.access <= 0.842
Unemployment <= 0.0525
Feature
−0.06
−0.04
−0.02
0.00
Weight
Positive
Negative

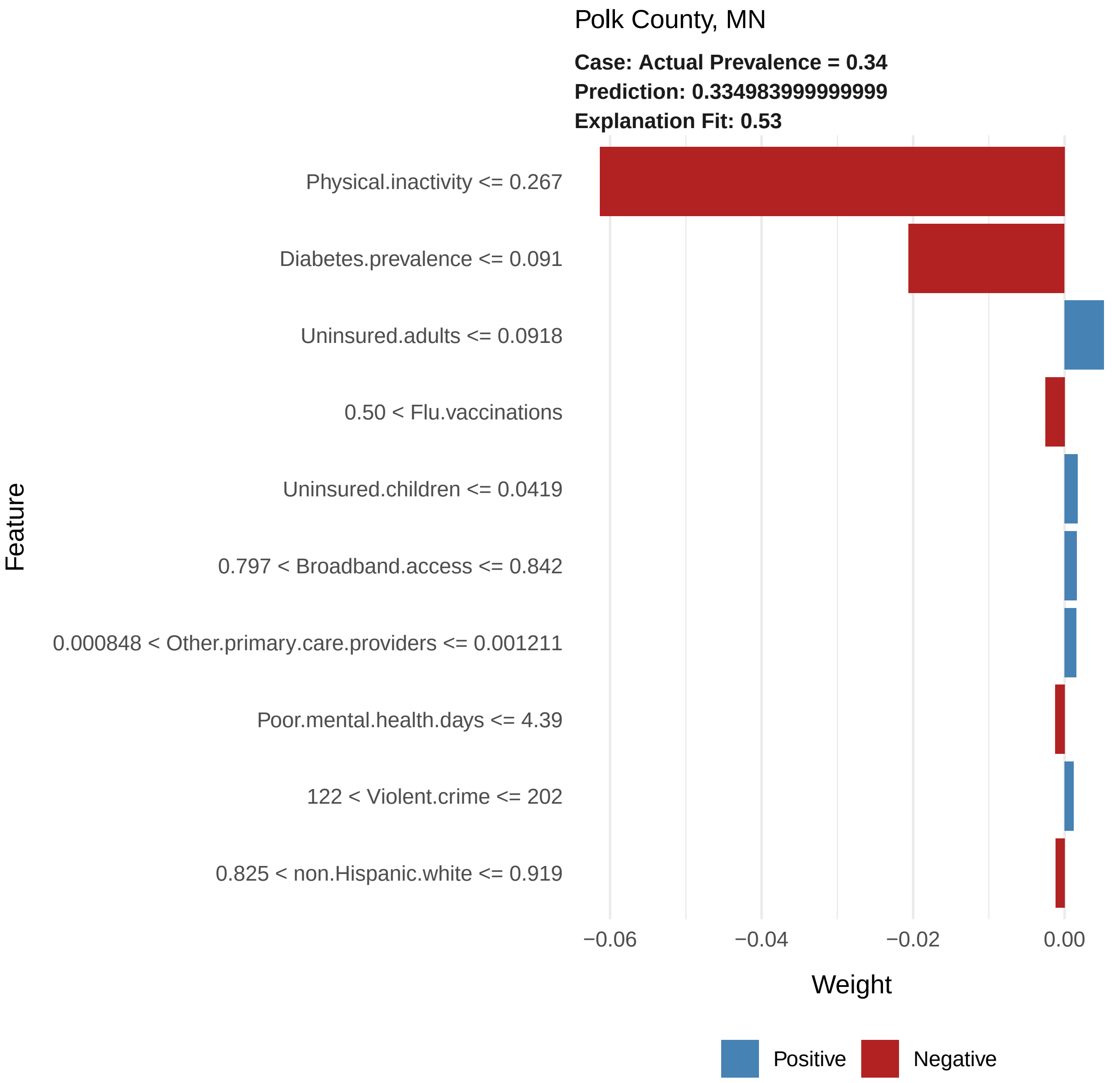
Polk County, MN
Case: Actual Prevalence = 0.34
Prediction: 0.334983999999999
Explanation Fit: 0.53
Physical.inactivity <= 0.267
Diabetes.prevalence <= 0.091
Uninsured.adults <= 0.0918
0.50 < Flu.vaccinations
Uninsured.children <= 0.0419
0.797 < Broadband.access <= 0.842
0.000848 < Other.primary.care.providers <= 0.001211
Poor.mental.health.days <= 4.39
122 < Violent.crime <= 202
0.825 < non.Hispanic.white <= 0.919
Feature
−0.06
−0.04
−0.02
0.00
Weight
Positive
Negative

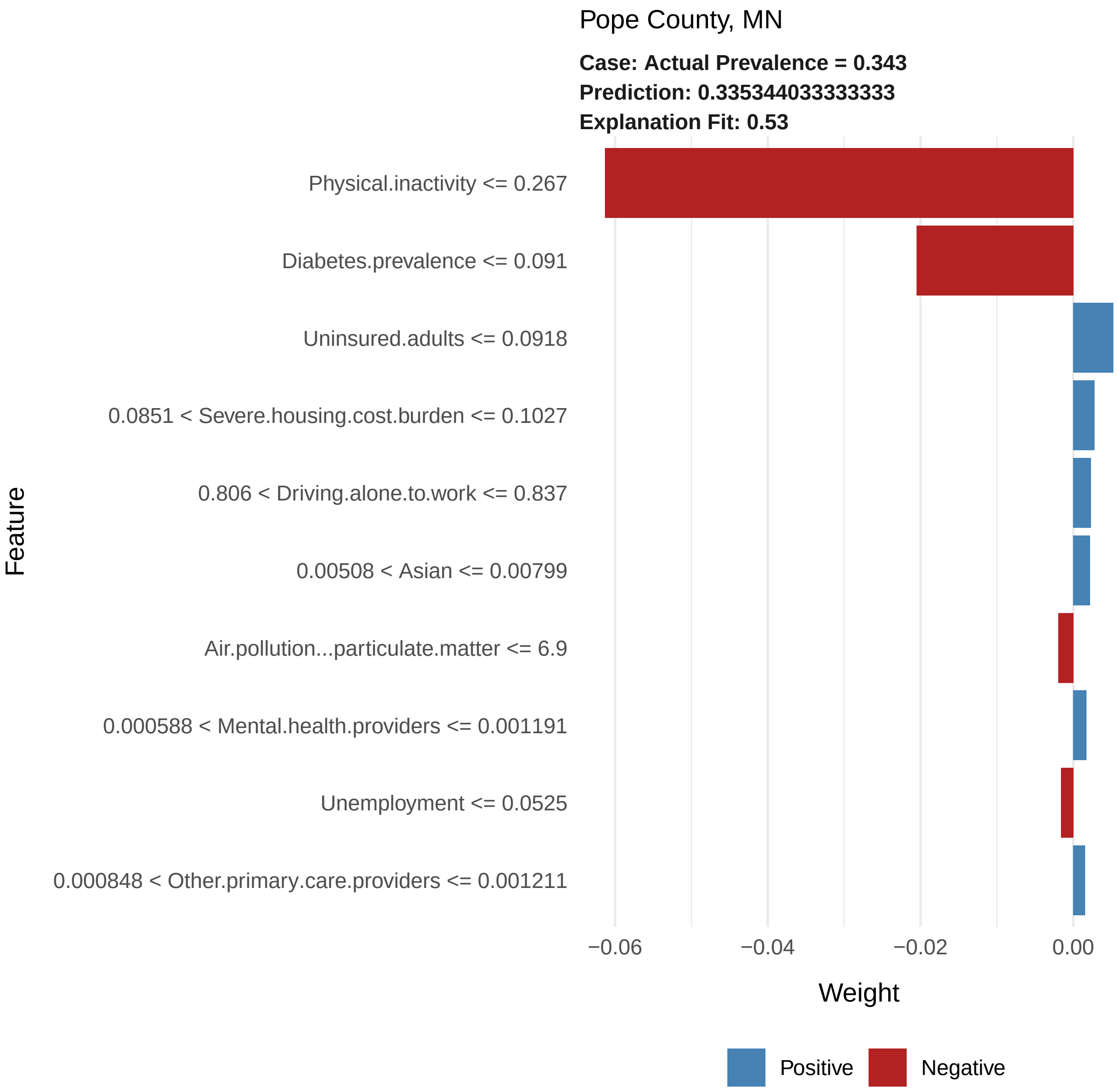
Pope County, MN
Case: Actual Prevalence = 0.343
Prediction: 0.335344033333333
Explanation Fit: 0.53
Physical.inactivity <= 0.267
Diabetes.prevalence <= 0.091
Uninsured.adults <= 0.0918
0.0851 < Severe.housing.cost.burden <= 0.1027
0.806 < Driving.alone.to.work <= 0.837
0.00508 < Asian <= 0.00799
Air.pollution...particulate.matter <= 6.9
0.000588 < Mental.health.providers <= 0.001191
Unemployment <= 0.0525
0.000848 < Other.primary.care.providers <= 0.001211
Feature
−0.06
−0.04
−0.02
0.00
Weight
Positive
Negative

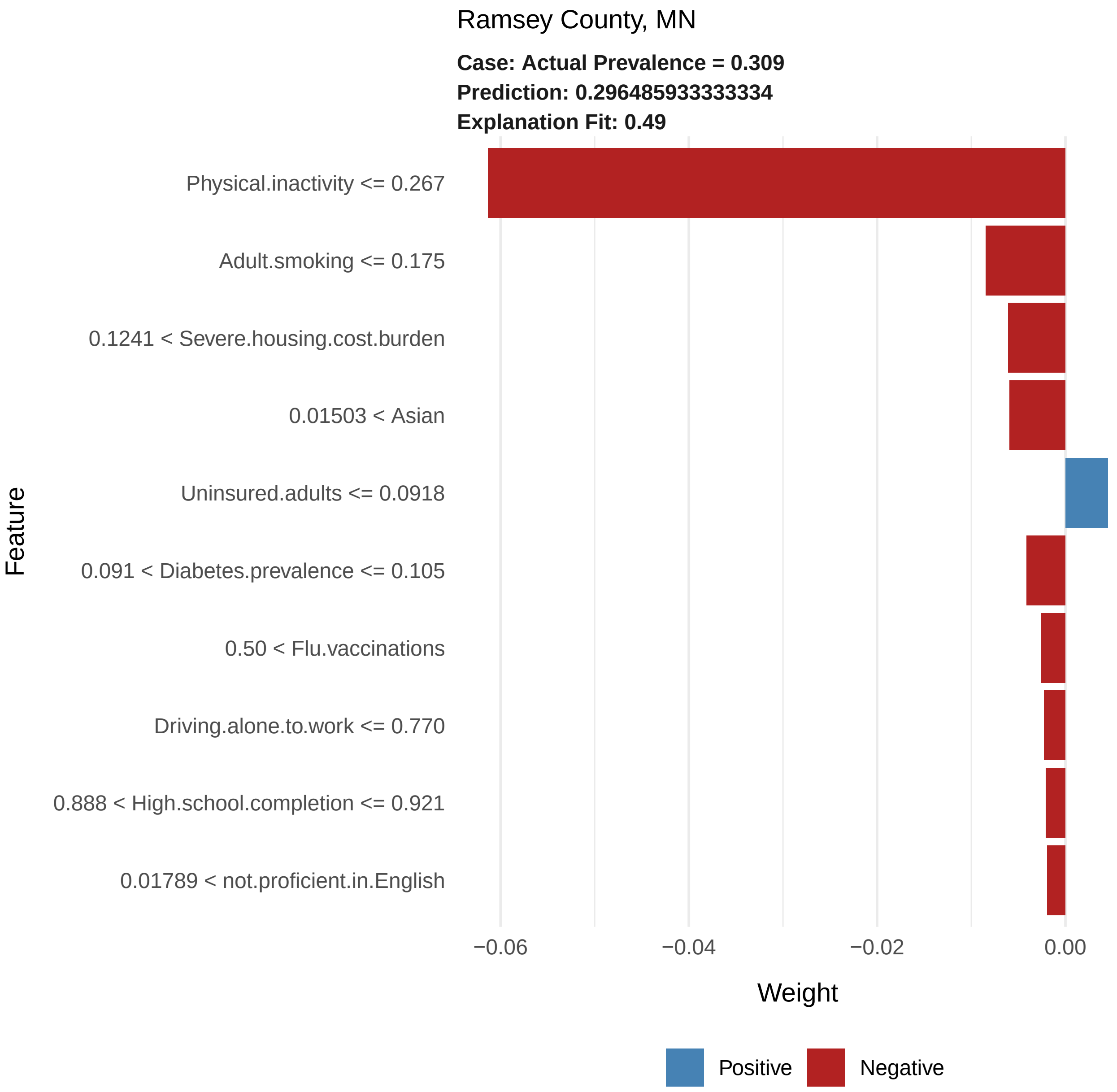
Ramsey County, MN
Case: Actual Prevalence = 0.309
Prediction: 0.296485933333334
Explanation Fit: 0.49
Physical.inactivity <= 0.267
Adult.smoking <= 0.175
0.1241 < Severe.housing.cost.burden
0.01503 < Asian
Uninsured.adults <= 0.0918
0.091 < Diabetes.prevalence <= 0.105
0.50 < Flu.vaccinations
Driving.alone.to.work <= 0.770
0.888 < High.school.completion <= 0.921
0.01789 < not.proficient.in.English
Feature
−0.06
−0.04
−0.02
0.00
Weight
Positive
Negative

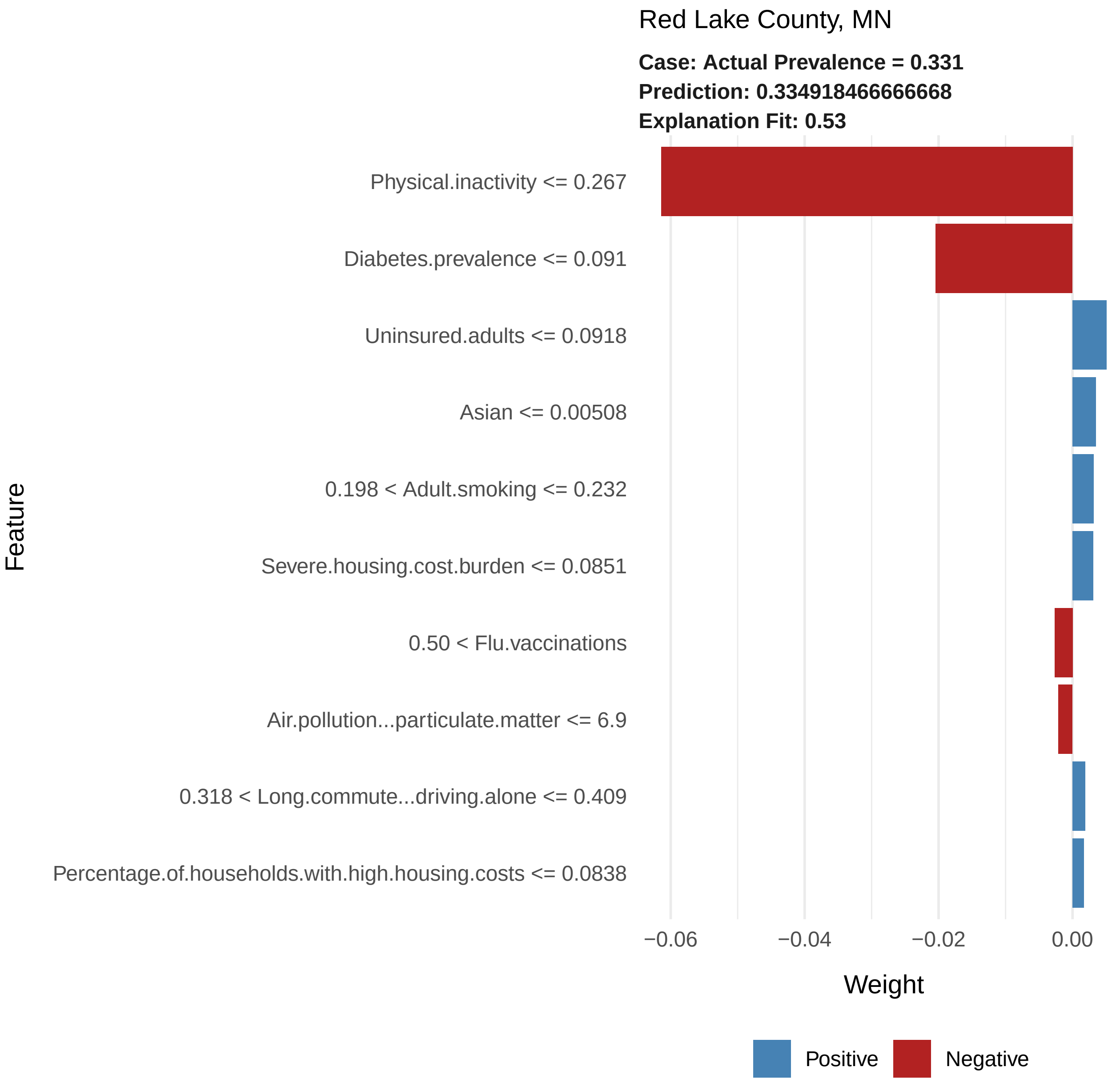
Red Lake County, MN
Case: Actual Prevalence = 0.331
Prediction: 0.334918466666668
Explanation Fit: 0.53
Physical.inactivity <= 0.267
Diabetes.prevalence <= 0.091
Uninsured.adults <= 0.0918
Asian <= 0.00508
0.198 < Adult.smoking <= 0.232
Severe.housing.cost.burden <= 0.0851
0.50 < Flu.vaccinations
Air.pollution...particulate.matter <= 6.9
0.318 < Long.commute...driving.alone <= 0.409
Percentage.of.households.with.high.housing.costs <= 0.0838
Feature
−0.06
−0.04
−0.02
0.00
Weight
Positive
Negative

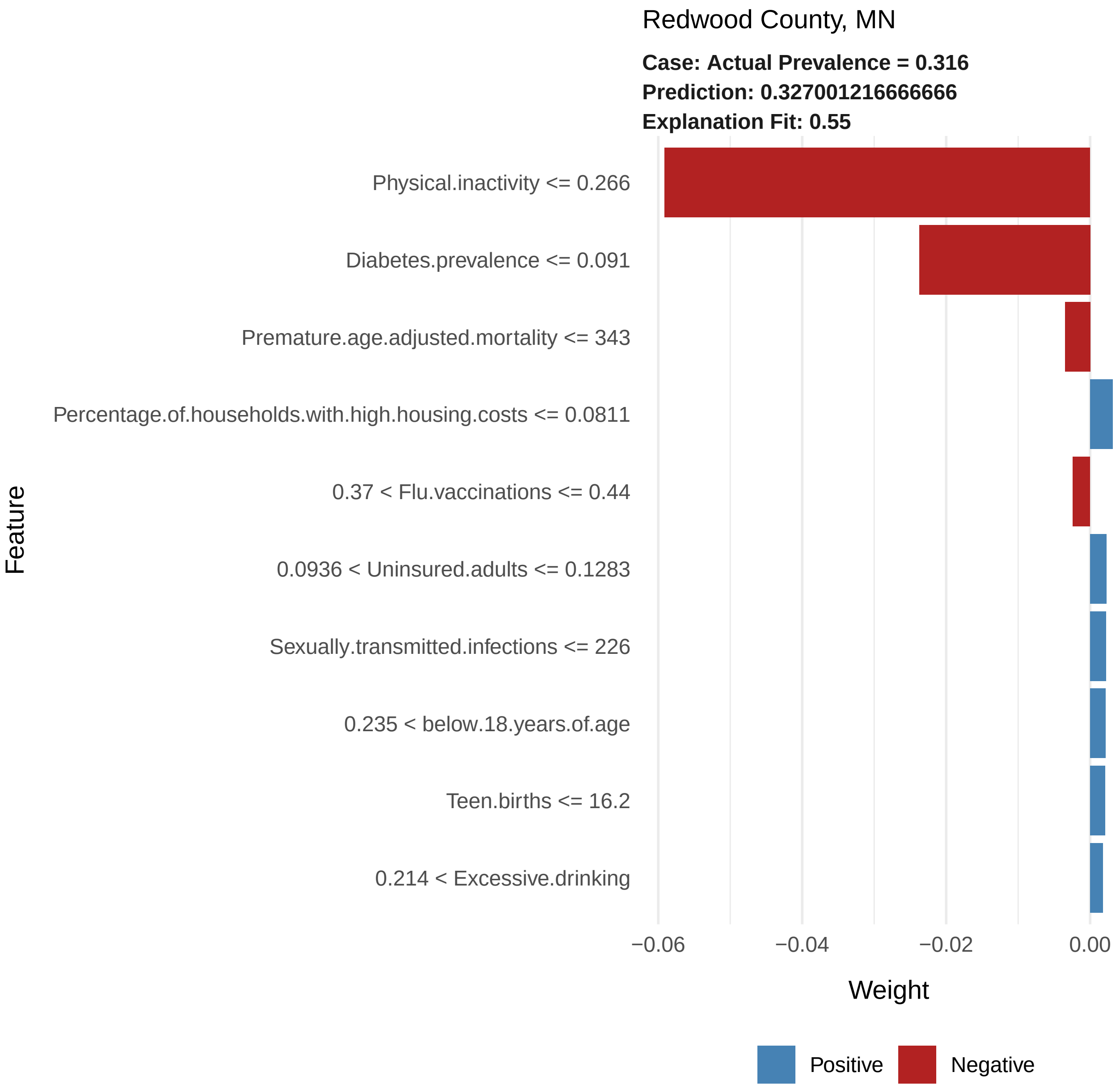
Redwood County, MN
Case: Actual Prevalence = 0.316
Prediction: 0.327001216666666
Explanation Fit: 0.55
Physical.inactivity <= 0.266
Diabetes.prevalence <= 0.091
Premature.age.adjusted.mortality <= 343
Percentage.of.households.with.high.housing.costs <= 0.0811
0.37 < Flu.vaccinations <= 0.44
0.0936 < Uninsured.adults <= 0.1283
Sexually.transmitted.infections <= 226
0.235 < below.18.years.of.age
Teen.births <= 16.2
0.214 < Excessive.drinking
Feature
−0.06
−0.04
−0.02
0.00
Weight
Positive
Negative

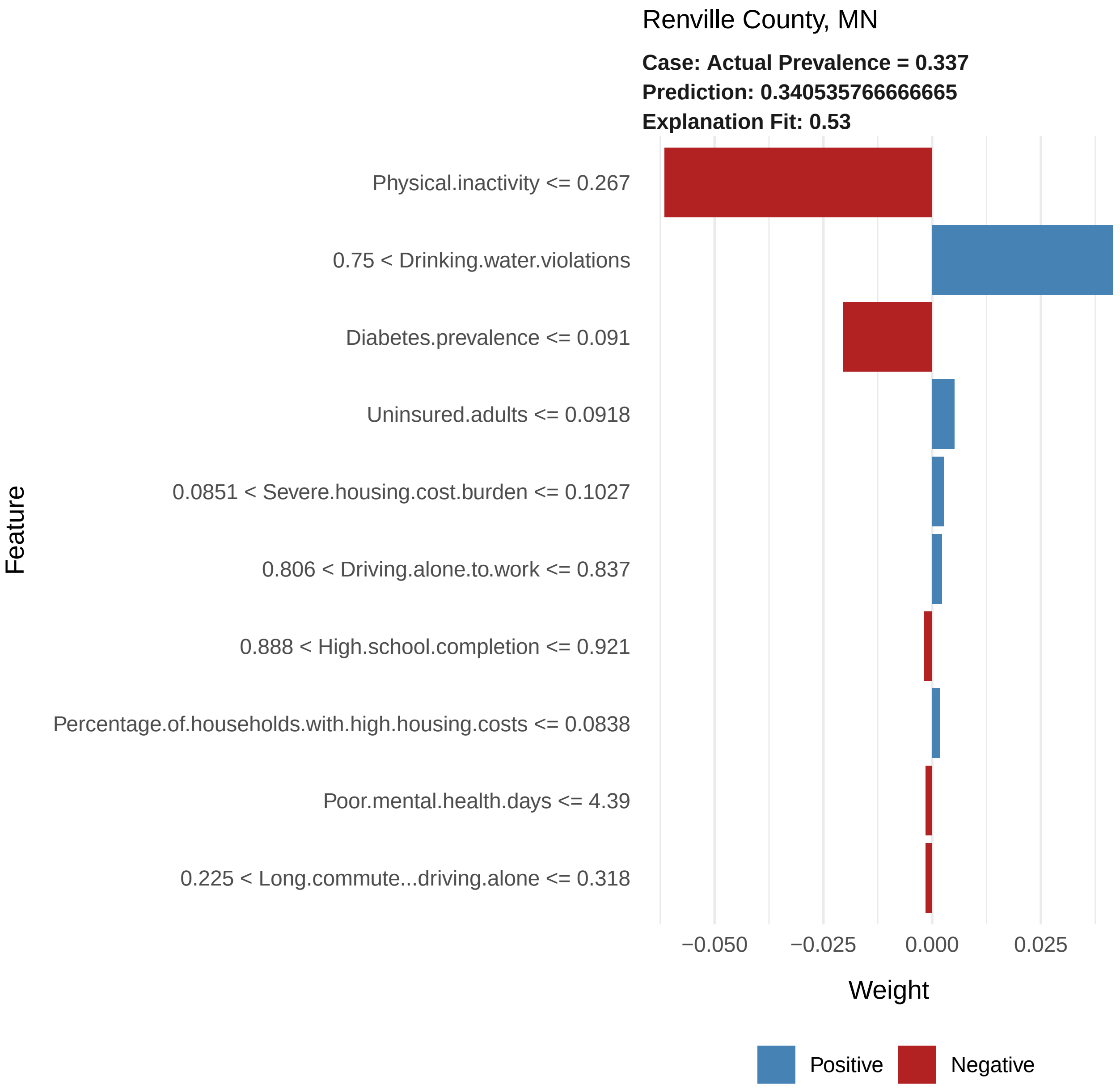
Renville County, MN
Case: Actual Prevalence = 0.337
Prediction: 0.340535766666665
Explanation Fit: 0.53
Physical.inactivity <= 0.267
0.75 < Drinking.water.violations
Diabetes.prevalence <= 0.091
Uninsured.adults <= 0.0918
0.0851 < Severe.housing.cost.burden <= 0.1027
0.806 < Driving.alone.to.work <= 0.837
0.888 < High.school.completion <= 0.921
Percentage.of.households.with.high.housing.costs <= 0.0838
Poor.mental.health.days <= 4.39
0.225 < Long.commute...driving.alone <= 0.318
Feature
−0.050
−0.025
0.000
0.025
Weight
Positive
Negative

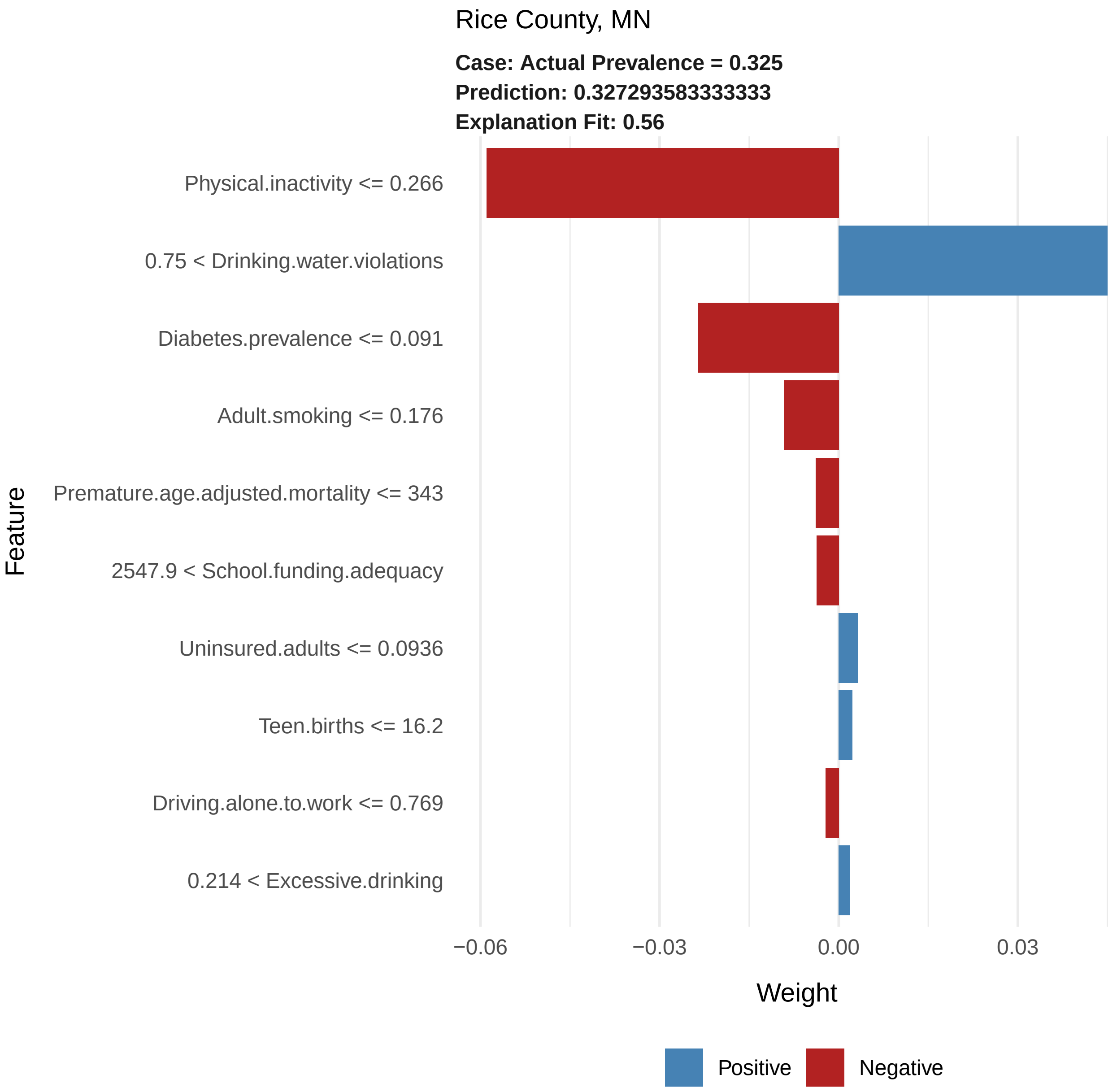

Rice County, MN
Case: Actual Prevalence = 0.325
Prediction: 0.327293583333333
Explanation Fit: 0.56
Physical.inactivity <= 0.266
0.75 < Drinking.water.violations
Diabetes.prevalence <= 0.091
Adult.smoking <= 0.176
Premature.age.adjusted.mortality <= 343
2547.9 < School.funding.adequacy
Uninsured.adults <= 0.0936
Teen.births <= 16.2
Driving.alone.to.work <= 0.769
0.214 < Excessive.drinking
Feature
−0.06
−0.03
0.00
0.03
Weight
Positive
Negative

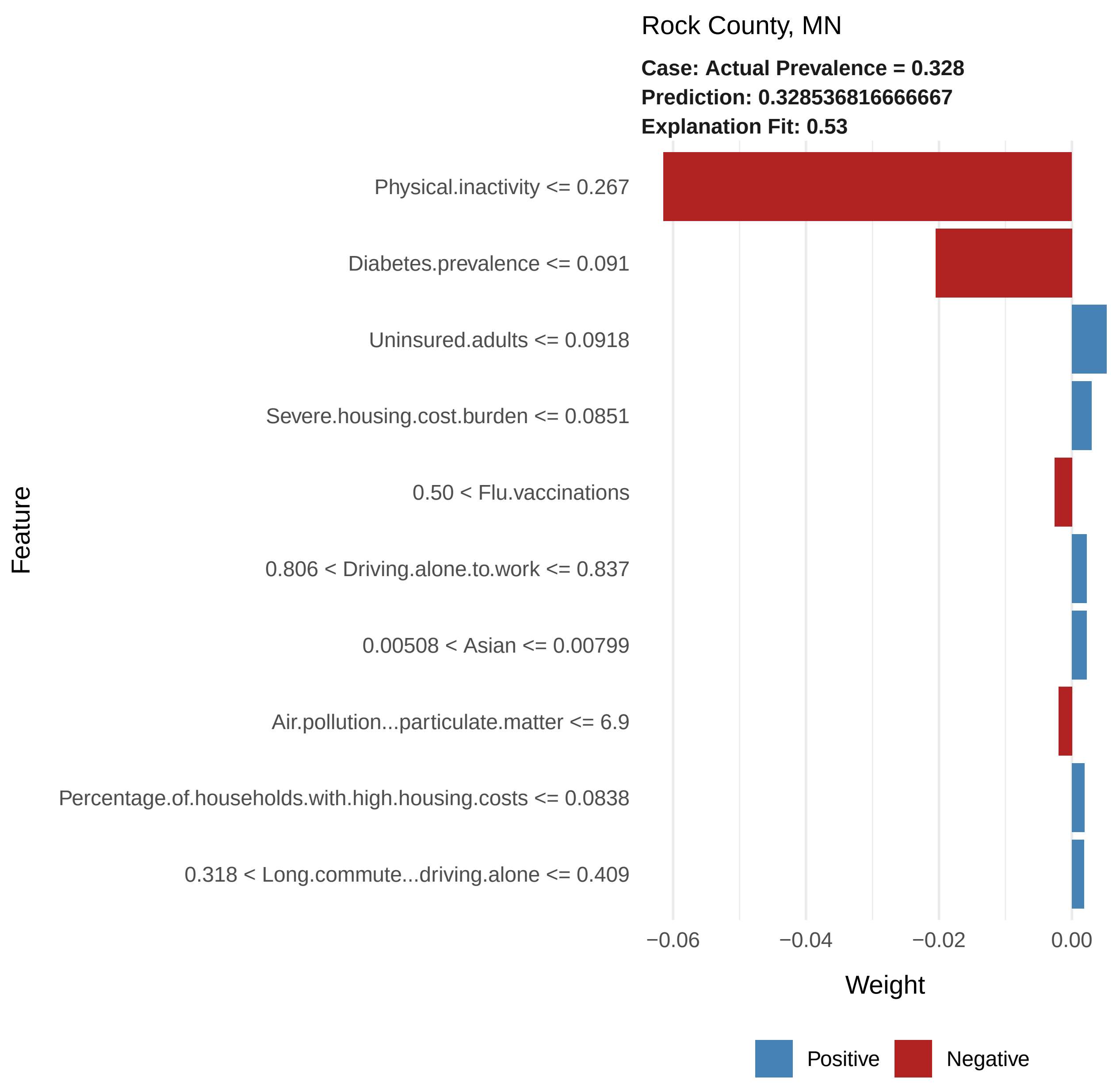
Rock County, MN
Case: Actual Prevalence = 0.328
Prediction: 0.328536816666667
Explanation Fit: 0.53
Physical.inactivity <= 0.267
Diabetes.prevalence <= 0.091
Uninsured.adults <= 0.0918
Severe.housing.cost.burden <= 0.0851
0.50 < Flu.vaccinations
0.806 < Driving.alone.to.work <= 0.837
0.00508 < Asian <= 0.00799
Air.pollution...particulate.matter <= 6.9
Percentage.of.households.with.high.housing.costs <= 0.0838
0.318 < Long.commute...driving.alone <= 0.409
Feature
−0.06
−0.04
−0.02
0.00
Weight
Positive
Negative

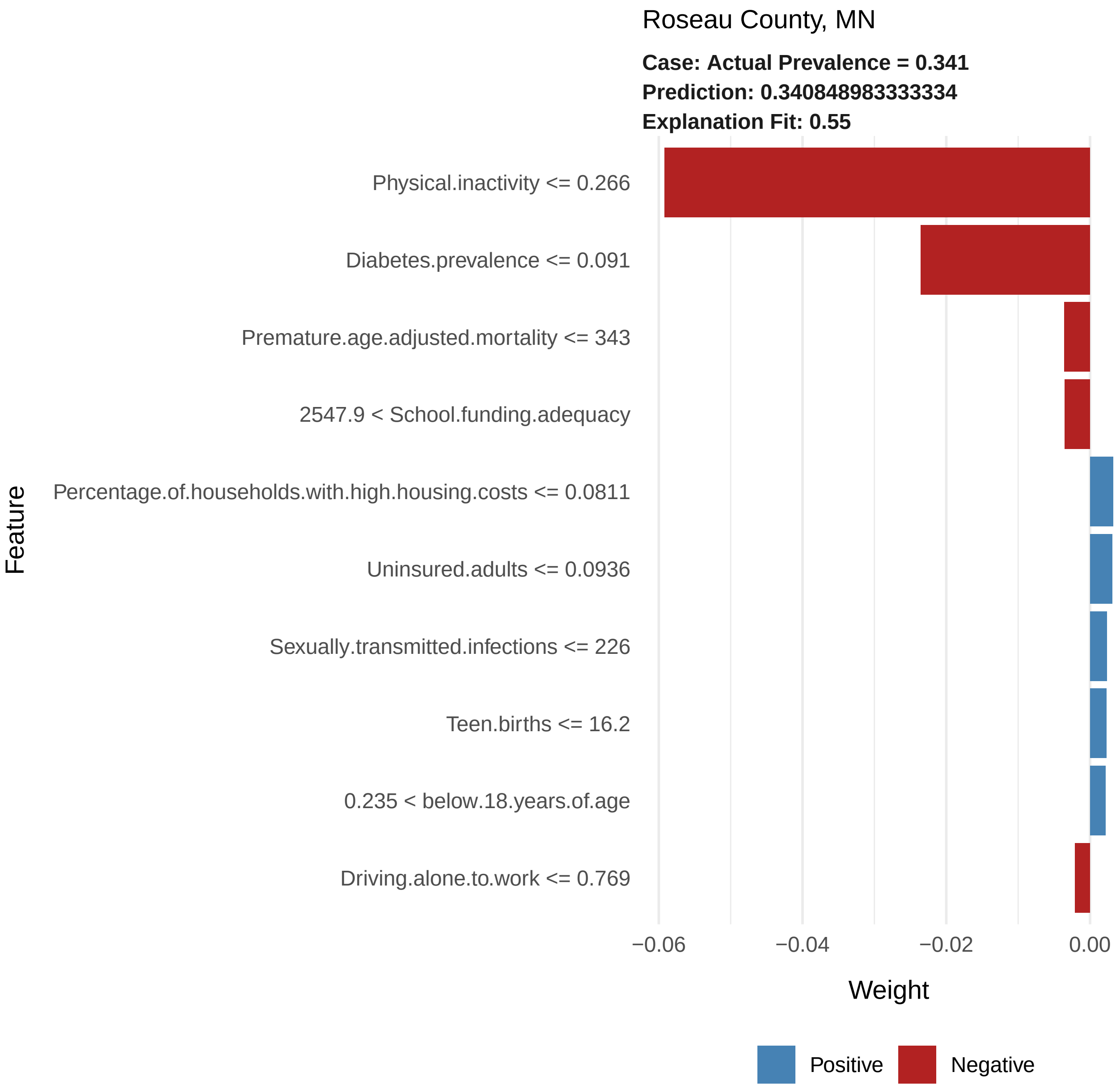

Roseau County, MN
Case: Actual Prevalence = 0.341
Prediction: 0.340848983333334
Explanation Fit: 0.55
Physical.inactivity <= 0.266
Diabetes.prevalence <= 0.091
Premature.age.adjusted.mortality <= 343
2547.9 < School.funding.adequacy
Percentage.of.households.with.high.housing.costs <= 0.0811
Uninsured.adults <= 0.0936
Sexually.transmitted.infections <= 226
Teen.births <= 16.2
0.235 < below.18.years.of.age
Driving.alone.to.work <= 0.769
Feature
−0.06
−0.04
−0.02
0.00
Weight
Positive
Negative

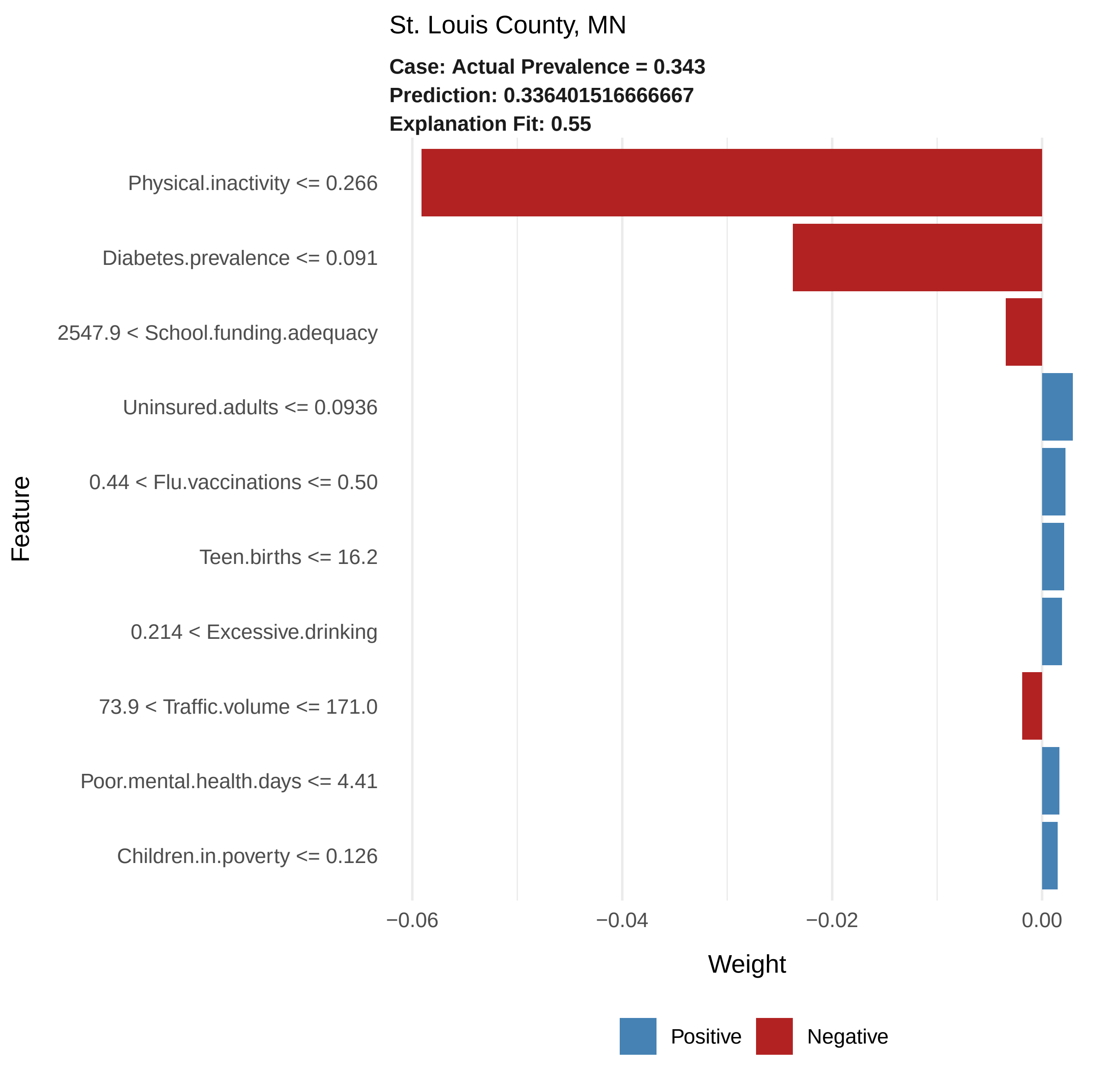
St. Louis County, MN
Case: Actual Prevalence = 0.343
Prediction: 0.336401516666667
Explanation Fit: 0.55
Physical.inactivity <= 0.266
Diabetes.prevalence <= 0.091
2547.9 < School.funding.adequacy
Uninsured.adults <= 0.0936
0.44 < Flu.vaccinations <= 0.50
Teen.births <= 16.2
0.214 < Excessive.drinking
73.9 < Traffic.volume <= 171.0
Poor.mental.health.days <= 4.41
Children.in.poverty <= 0.126
Feature
−0.06
−0.04
−0.02
0.00
Weight
Positive
Negative

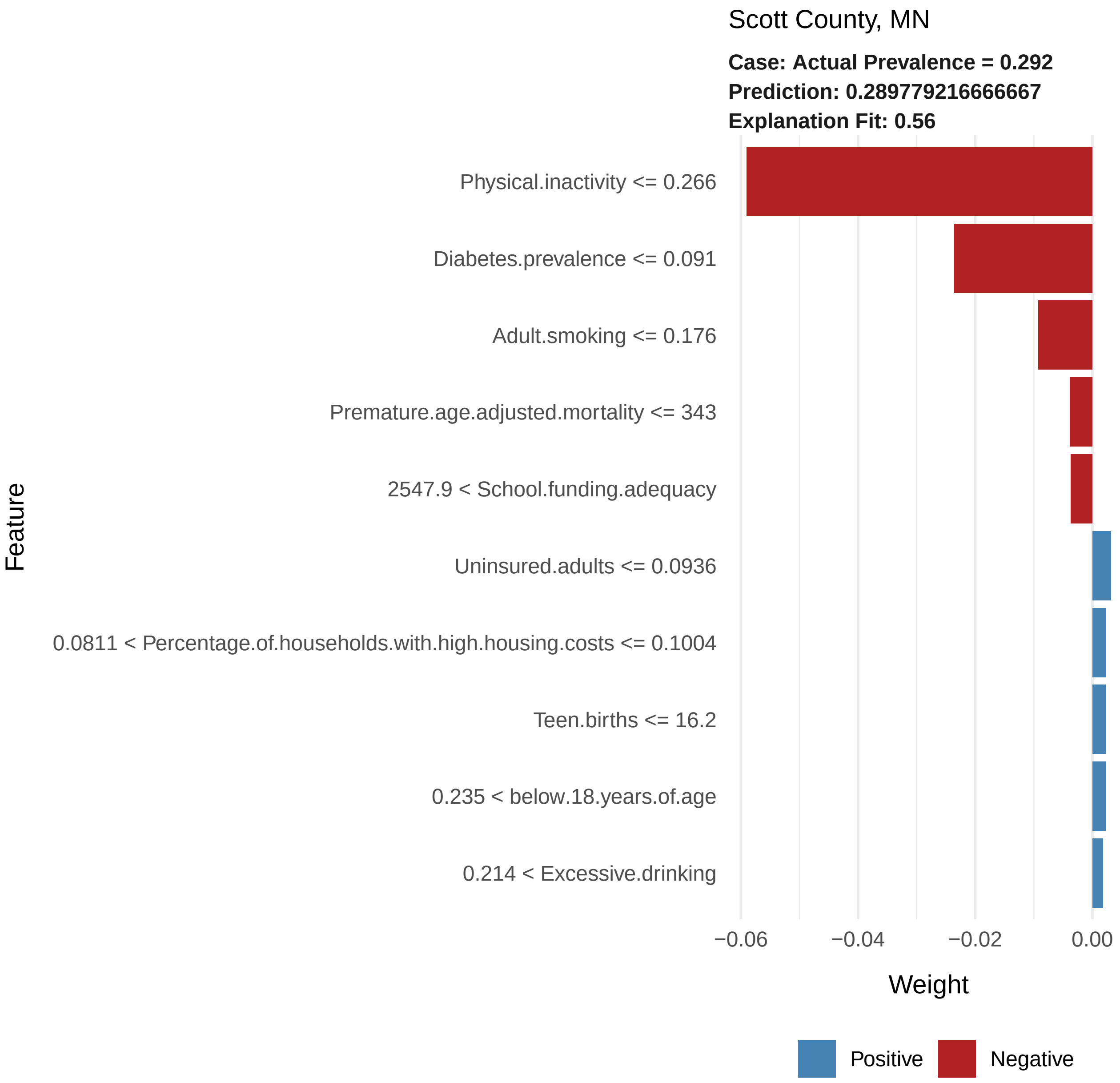

Scott County, MN
Case: Actual Prevalence = 0.292
Prediction: 0.289779216666667
Explanation Fit: 0.56
Physical.inactivity <= 0.266
Diabetes.prevalence <= 0.091
Adult.smoking <= 0.176
Premature.age.adjusted.mortality <= 343
2547.9 < School.funding.adequacy
Uninsured.adults <= 0.0936
0.0811 < Percentage.of.households.with.high.housing.costs <= 0.1004
Teen.births <= 16.2
0.235 < below.18.years.of.age
0.214 < Excessive.drinking
Feature
−0.06
−0.04
−0.02
0.00
Weight
Positive
Negative

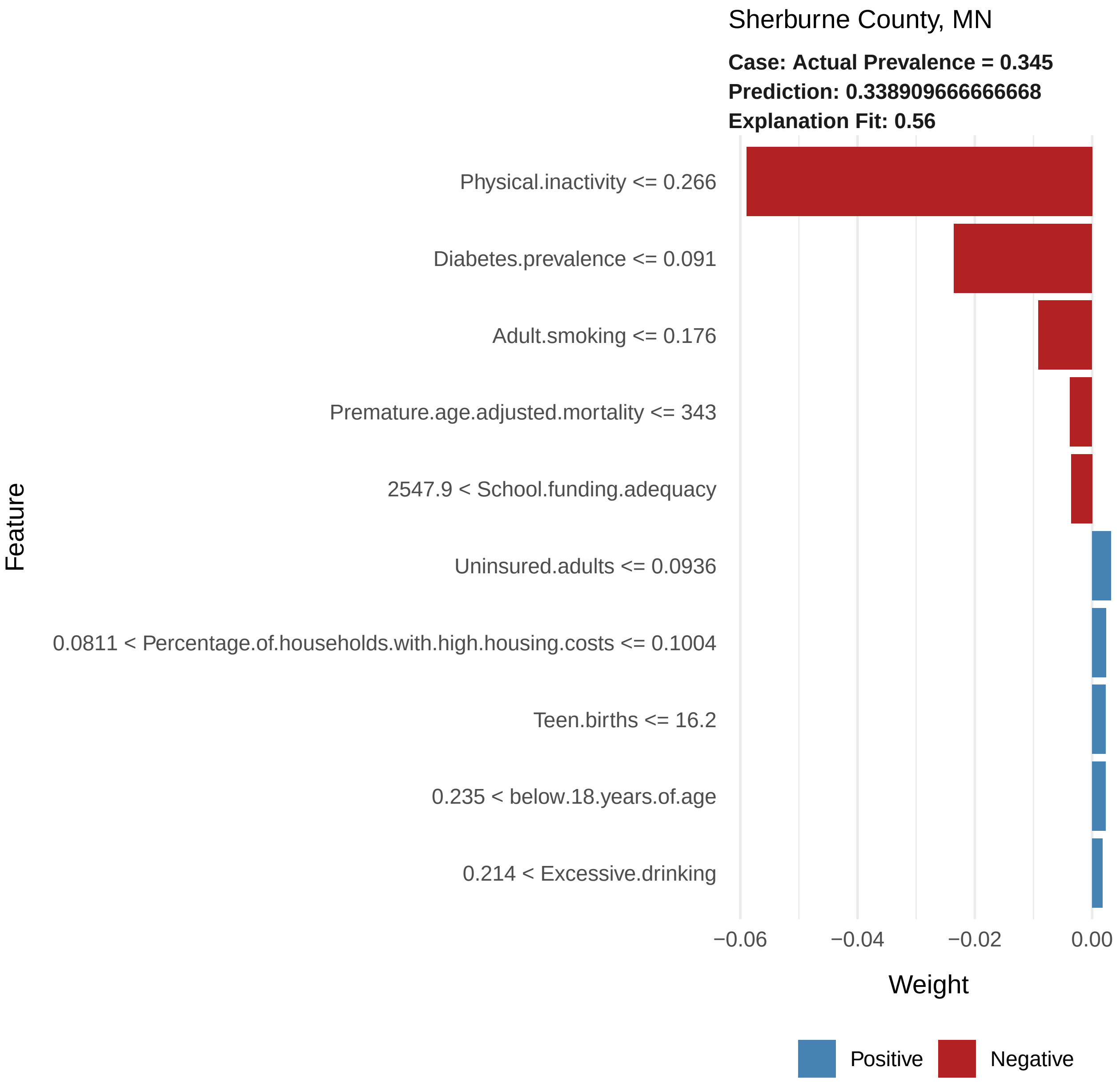
Sherburne County, MN
Case: Actual Prevalence = 0.345
Prediction: 0.338909666666668
Explanation Fit: 0.56
Physical.inactivity <= 0.266
Diabetes.prevalence <= 0.091
Adult.smoking <= 0.176
Premature.age.adjusted.mortality <= 343
2547.9 < School.funding.adequacy
Uninsured.adults <= 0.0936
0.0811 < Percentage.of.households.with.high.housing.costs <= 0.1004
Teen.births <= 16.2
0.235 < below.18.years.of.age
0.214 < Excessive.drinking
Feature
−0.06
−0.04
−0.02
0.00
Weight
Positive
Negative

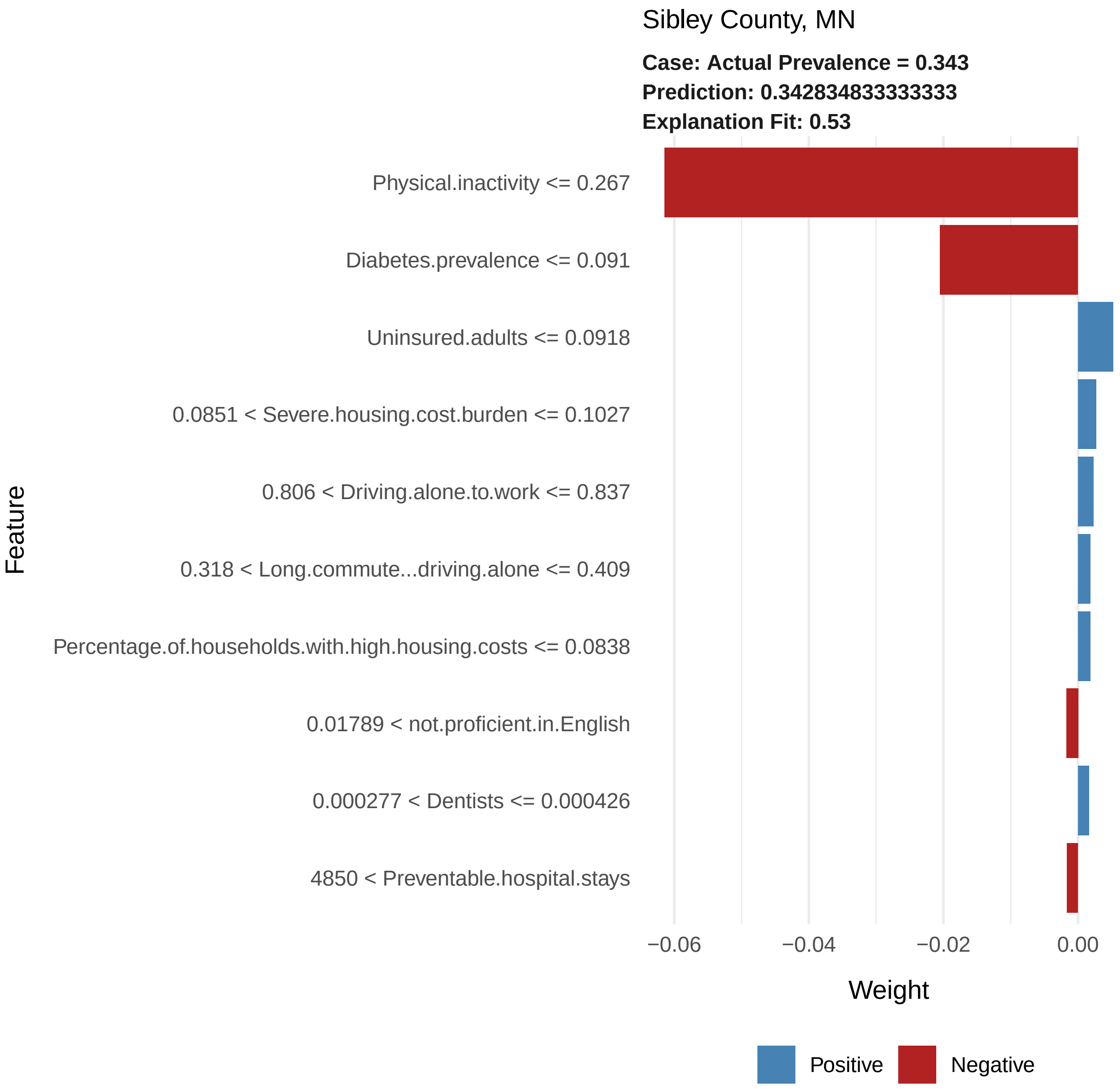

Sibley County, MN
Case: Actual Prevalence = 0.343
Prediction: 0.342834833333333
Explanation Fit: 0.53
Physical.inactivity <= 0.267
Diabetes.prevalence <= 0.091
Uninsured.adults <= 0.0918
0.0851 < Severe.housing.cost.burden <= 0.1027
0.806 < Driving.alone.to.work <= 0.837
0.318 < Long.commute...driving.alone <= 0.409
Percentage.of.households.with.high.housing.costs <= 0.0838
0.01789 < not.proficient.in.English
0.000277 < Dentists <= 0.000426
4850 < Preventable.hospital.stays
Feature
−0.06
−0.04
−0.02
0.00
Weight
Positive
Negative

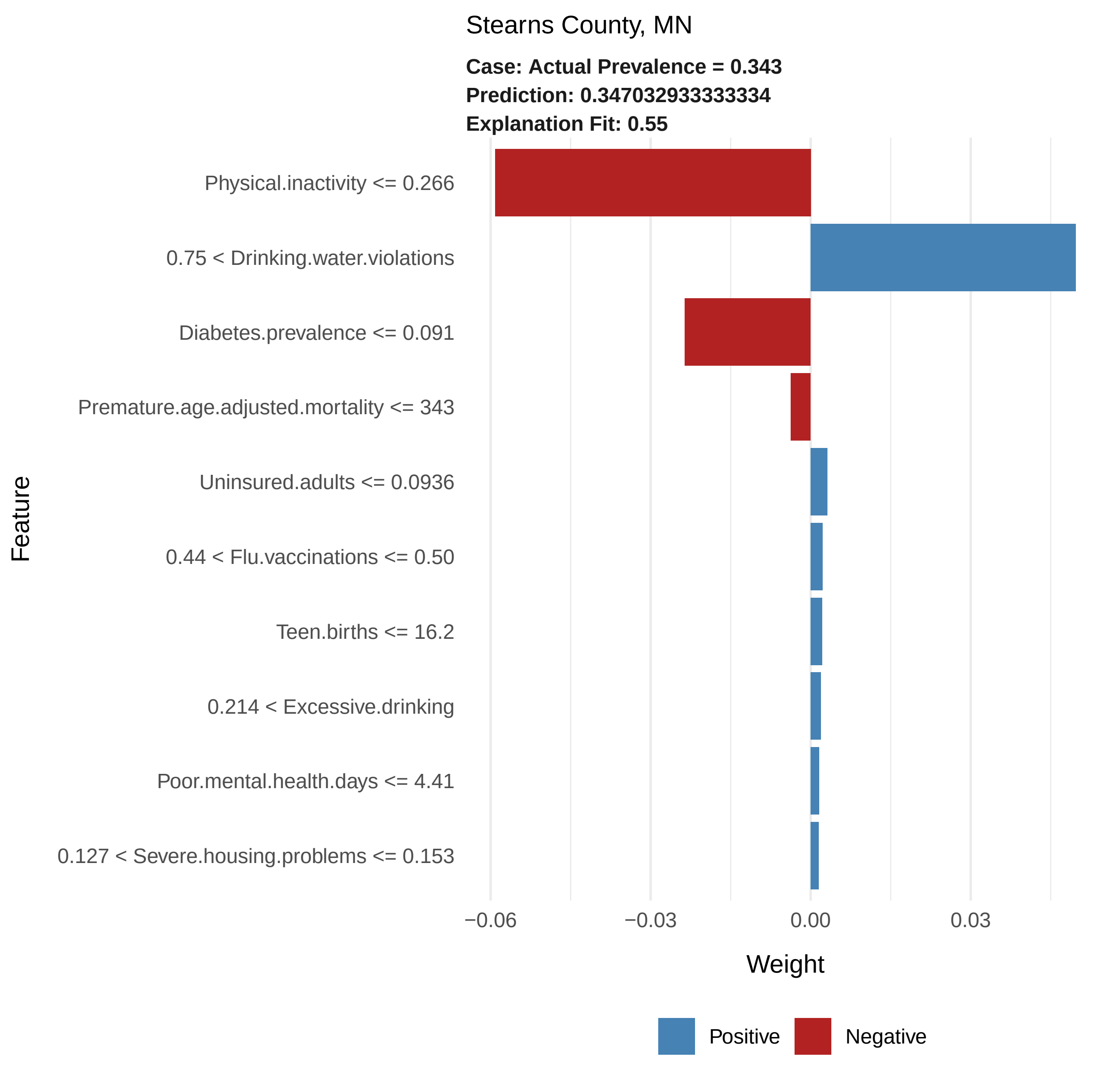

Stearns County, MN
Case: Actual Prevalence = 0.343
Prediction: 0.347032933333334
Explanation Fit: 0.55
Physical.inactivity <= 0.266
0.75 < Drinking.water.violations
Diabetes.prevalence <= 0.091
Premature.age.adjusted.mortality <= 343
Uninsured.adults <= 0.0936
0.44 < Flu.vaccinations <= 0.50
Teen.births <= 16.2
0.214 < Excessive.drinking
Poor.mental.health.days <= 4.41
0.127 < Severe.housing.problems <= 0.153
Feature
−0.06
−0.03
0.00
0.03
Weight
Positive
Negative

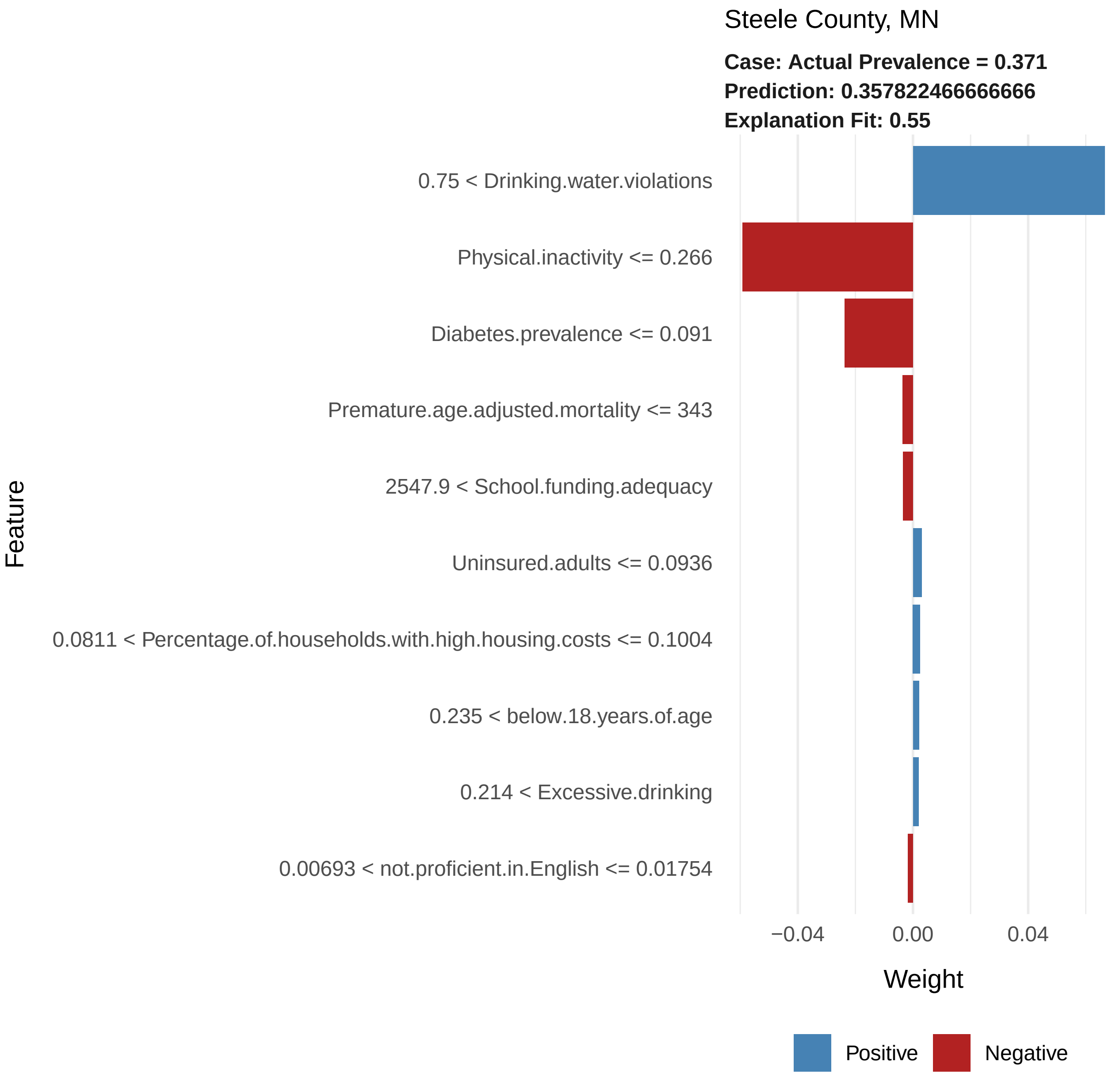
Steele County, MN
Case: Actual Prevalence = 0.371
Prediction: 0.357822466666666
Explanation Fit: 0.55
0.75 < Drinking.water.violations
Physical.inactivity <= 0.266
Diabetes.prevalence <= 0.091
Premature.age.adjusted.mortality <= 343
2547.9 < School.funding.adequacy
Uninsured.adults <= 0.0936
0.0811 < Percentage.of.households.with.high.housing.costs <= 0.1004
0.235 < below.18.years.of.age
0.214 < Excessive.drinking
0.00693 < not.proficient.in.English <= 0.01754
Feature
−0.04
0.00
0.04
Weight
Positive
Negative

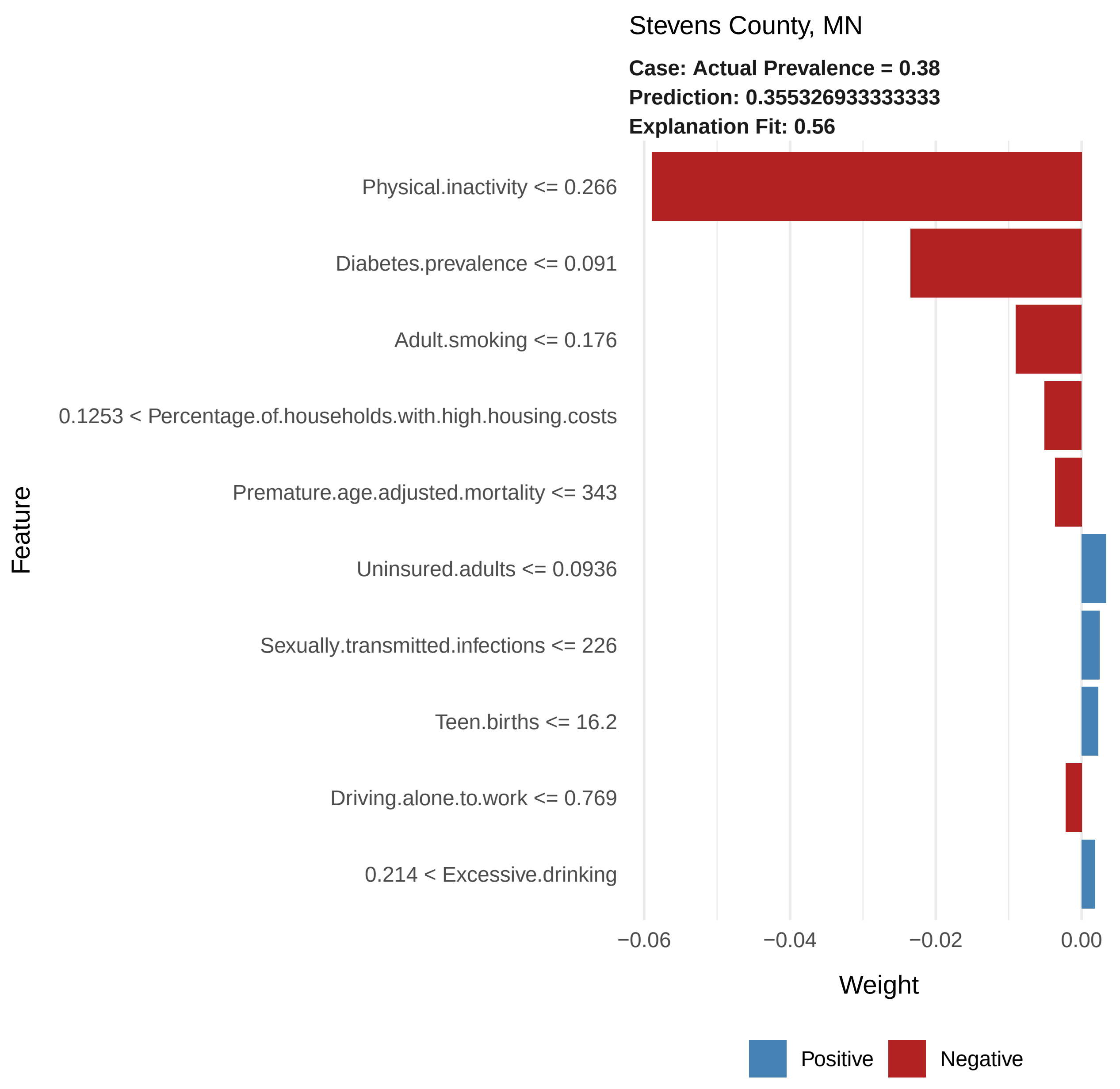
Stevens County, MN
Case: Actual Prevalence = 0.38
Prediction: 0.355326933333333
Explanation Fit: 0.56
Physical.inactivity <= 0.266
Diabetes.prevalence <= 0.091
Adult.smoking <= 0.176
0.1253 < Percentage.of.households.with.high.housing.costs
Premature.age.adjusted.mortality <= 343
Uninsured.adults <= 0.0936
Sexually.transmitted.infections <= 226
Teen.births <= 16.2
Driving.alone.to.work <= 0.769
0.214 < Excessive.drinking
Feature
−0.06
−0.04
−0.02
0.00
Weight
Positive
Negative

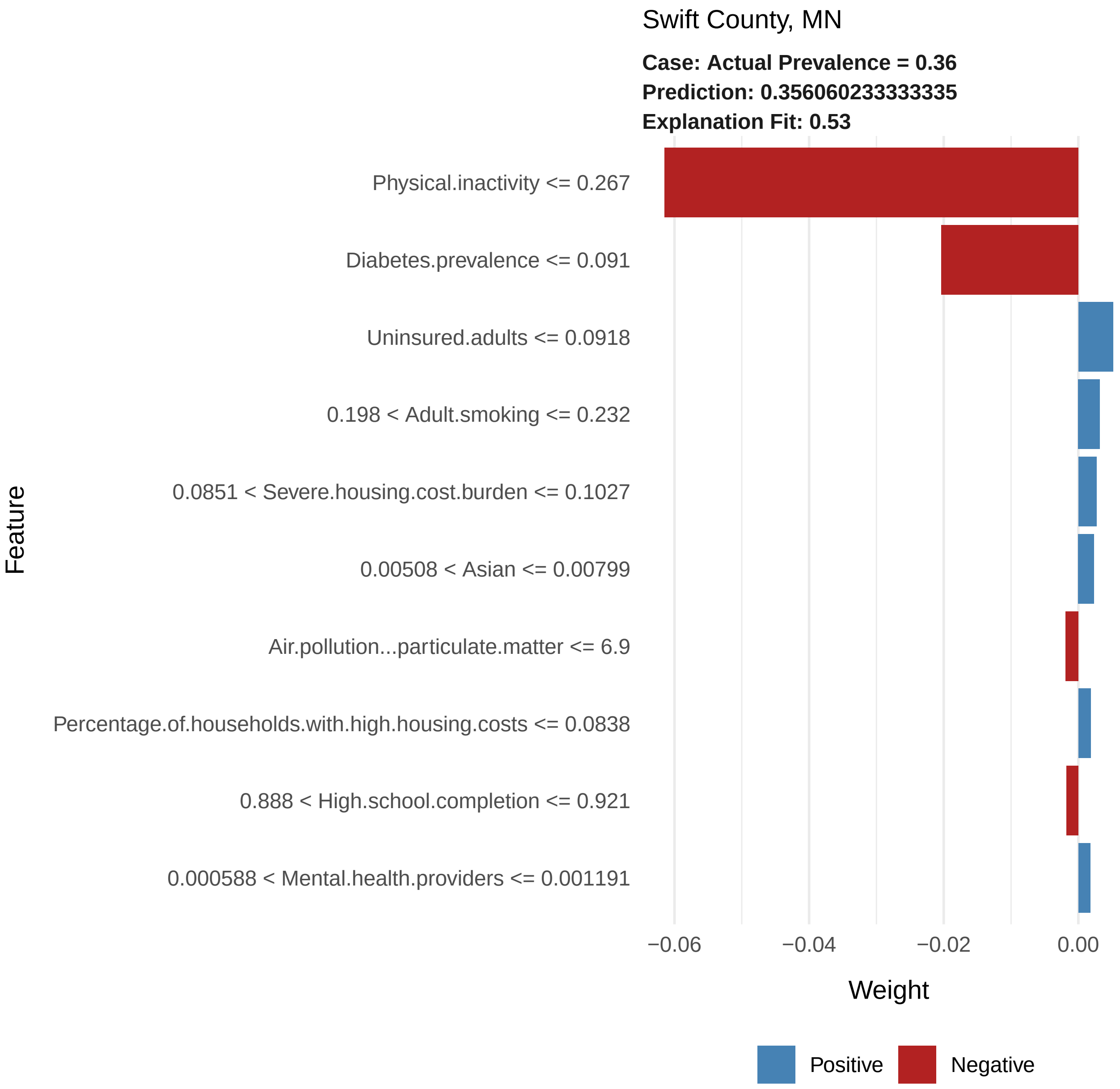
Swift County, MN
Case: Actual Prevalence = 0.36
Prediction: 0.356060233333335
Explanation Fit: 0.53
Physical.inactivity <= 0.267
Diabetes.prevalence <= 0.091
Uninsured.adults <= 0.0918
0.198 < Adult.smoking <= 0.232
0.0851 < Severe.housing.cost.burden <= 0.1027
0.00508 < Asian <= 0.00799
Air.pollution...particulate.matter <= 6.9
Percentage.of.households.with.high.housing.costs <= 0.0838
0.888 < High.school.completion <= 0.921
0.000588 < Mental.health.providers <= 0.001191
Feature
−0.06
−0.04
−0.02
0.00
Weight
Positive
Negative

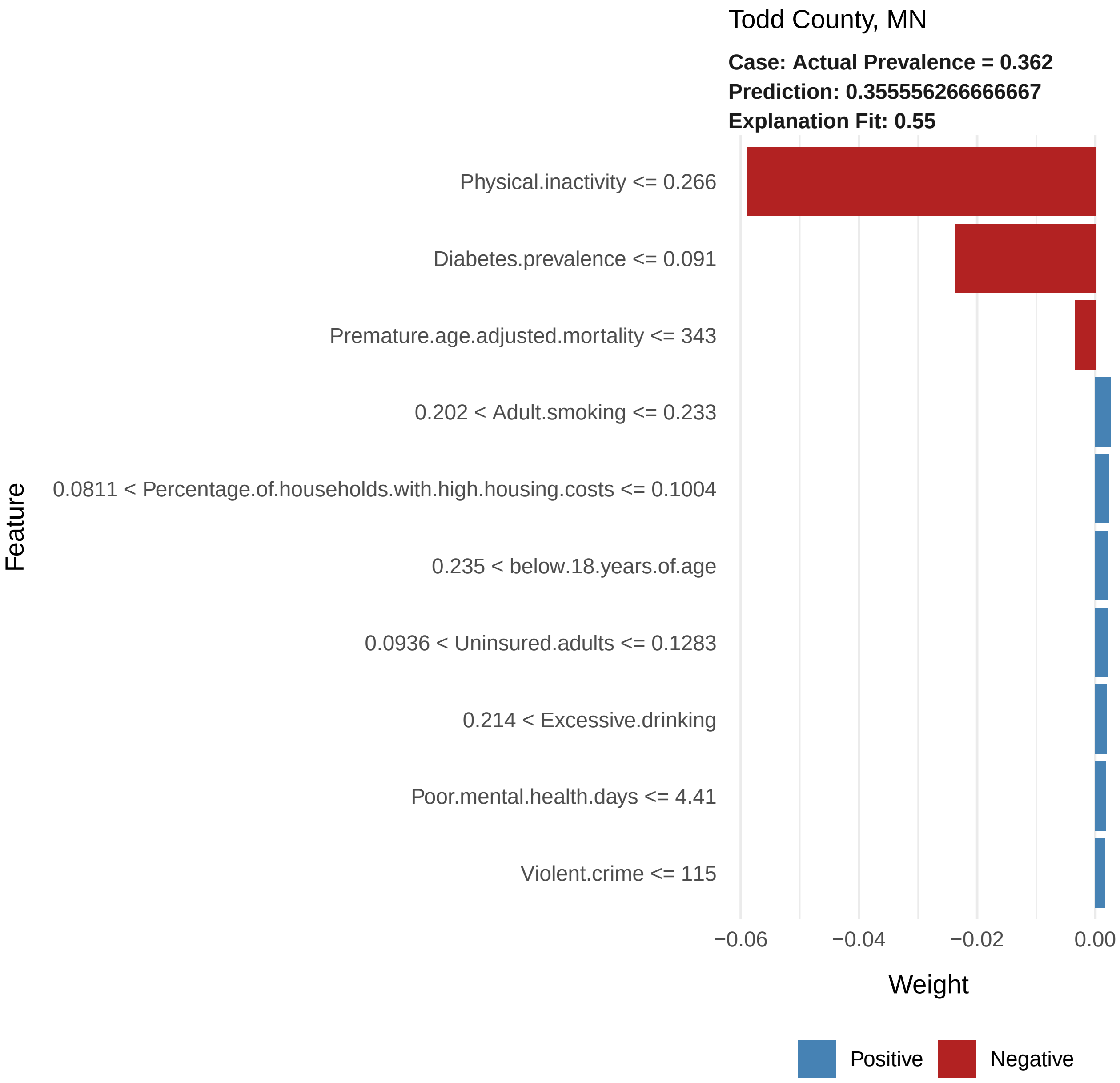

Todd County, MN
Case: Actual Prevalence = 0.362
Prediction: 0.355556266666667
Explanation Fit: 0.55
Physical.inactivity <= 0.266
Diabetes.prevalence <= 0.091
Premature.age.adjusted.mortality <= 343
0.202 < Adult.smoking <= 0.233
0.0811 < Percentage.of.households.with.high.housing.costs <= 0.1004
0.235 < below.18.years.of.age
0.0936 < Uninsured.adults <= 0.1283
0.214 < Excessive.drinking
Poor.mental.health.days <= 4.41
Violent.crime <= 115
Feature
−0.06
−0.04
−0.02
0.00
Weight
Positive
Negative

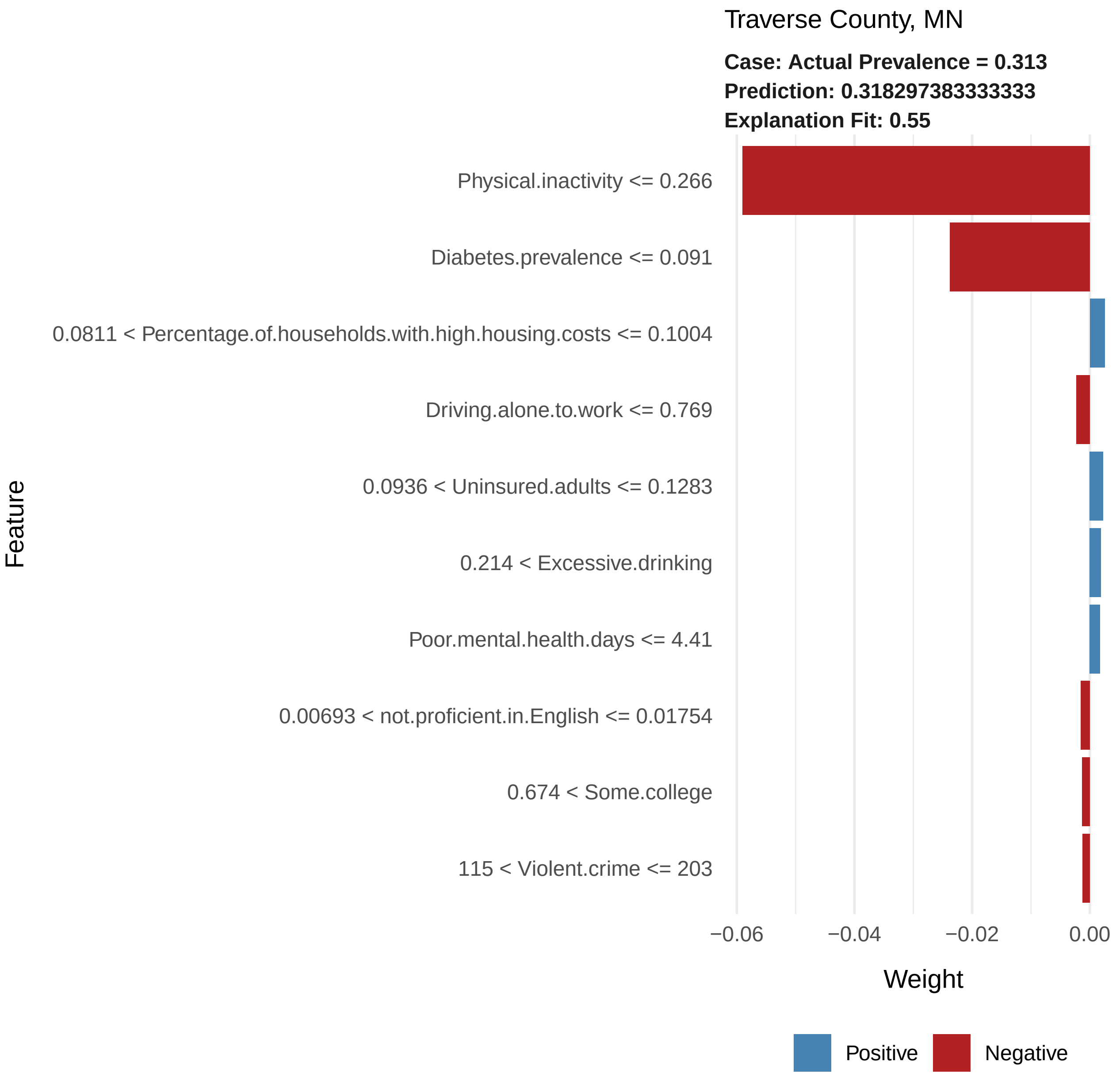

Traverse County, MN
Case: Actual Prevalence = 0.313
Prediction: 0.318297383333333
Explanation Fit: 0.55
Physical.inactivity <= 0.266
Diabetes.prevalence <= 0.091
0.0811 < Percentage.of.households.with.high.housing.costs <= 0.1004
Driving.alone.to.work <= 0.769
0.0936 < Uninsured.adults <= 0.1283
0.214 < Excessive.drinking
Poor.mental.health.days <= 4.41
0.00693 < not.proficient.in.English <= 0.01754
0.674 < Some.college
115 < Violent.crime <= 203
Feature
−0.06
−0.04
−0.02
0.00
Weight
Positive
Negative

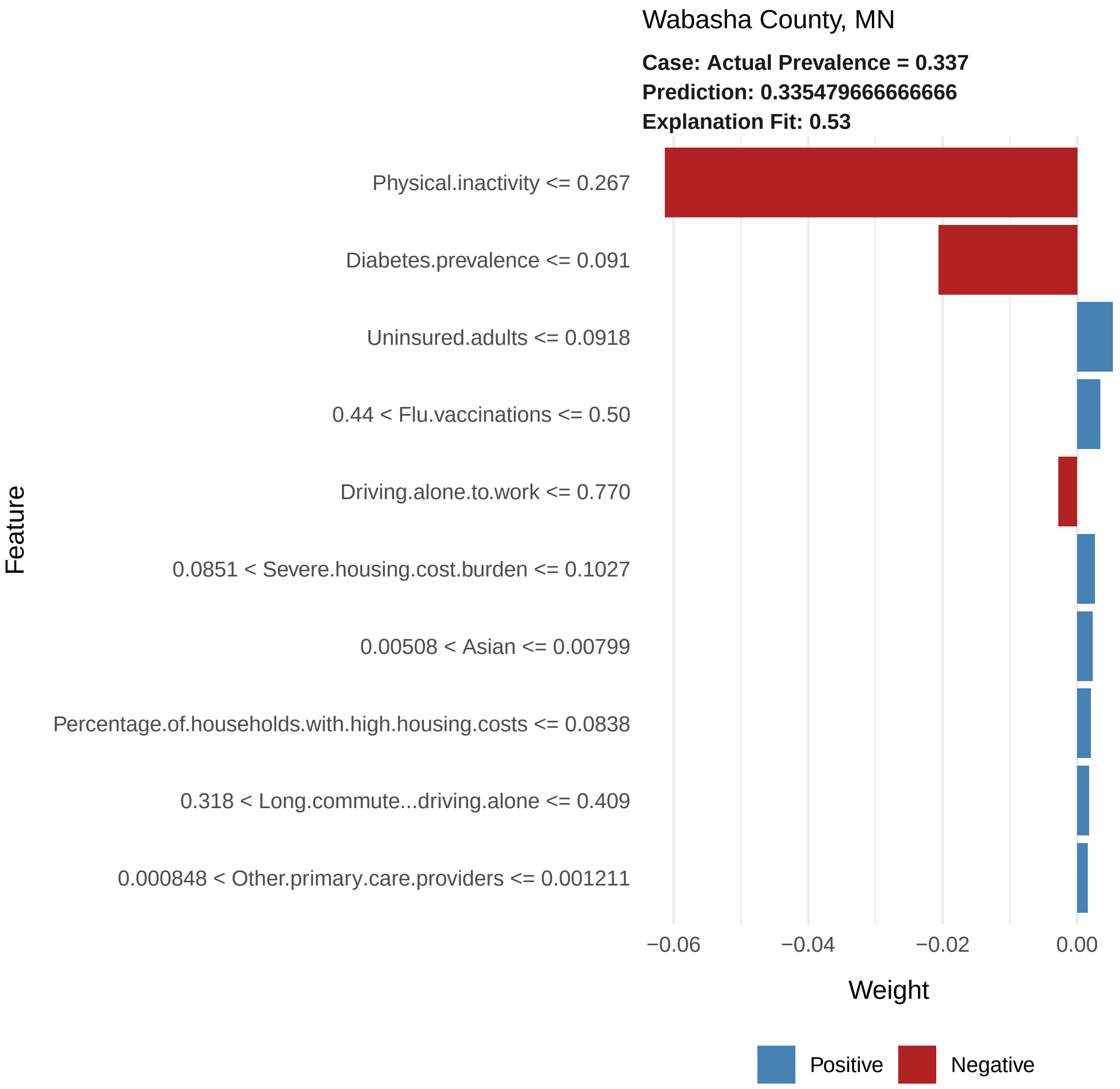

Wabasha County, MN
Case: Actual Prevalence = 0.337
Prediction: 0.335479666666666
Explanation Fit: 0.53
Physical.inactivity <= 0.267
Diabetes.prevalence <= 0.091
Uninsured.adults <= 0.0918
0.44 < Flu.vaccinations <= 0.50
Driving.alone.to.work <= 0.770
0.0851 < Severe.housing.cost.burden <= 0.1027
0.00508 < Asian <= 0.00799
Percentage.of.households.with.high.housing.costs <= 0.0838
0.318 < Long.commute...driving.alone <= 0.409
0.000848 < Other.primary.care.providers <= 0.001211
Feature
−0.06
−0.04
−0.02
0.00
Weight
Positive
Negative

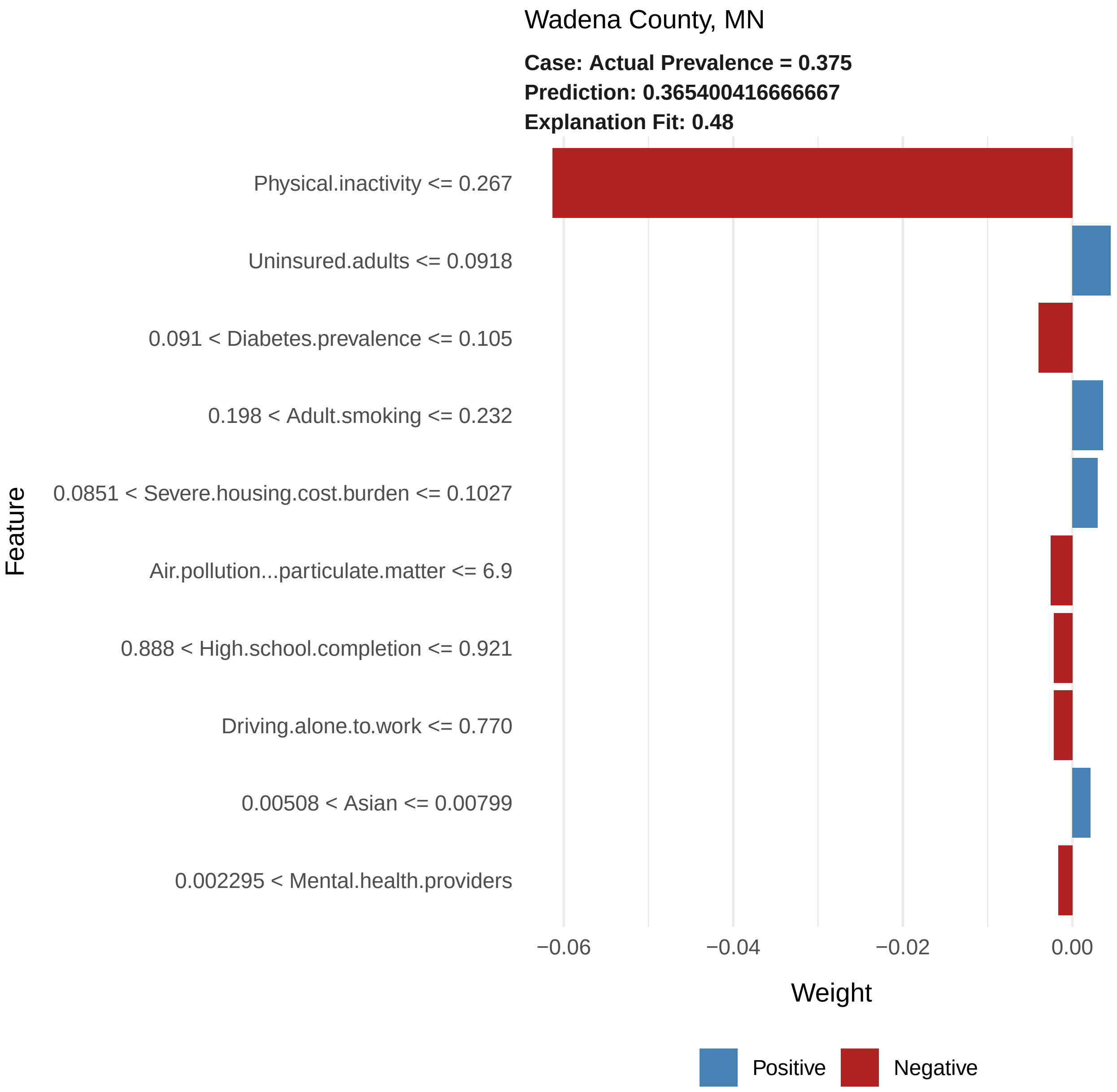

Wadena County, MN
Case: Actual Prevalence = 0.375
Prediction: 0.365400416666667
Explanation Fit: 0.48
Physical.inactivity <= 0.267
Uninsured.adults <= 0.0918
0.091 < Diabetes.prevalence <= 0.105
0.198 < Adult.smoking <= 0.232
0.0851 < Severe.housing.cost.burden <= 0.1027
Air.pollution...particulate.matter <= 6.9
0.888 < High.school.completion <= 0.921
Driving.alone.to.work <= 0.770
0.00508 < Asian <= 0.00799
0.002295 < Mental.health.providers
Feature
−0.06
−0.04
−0.02
0.00
Weight
Positive
Negative

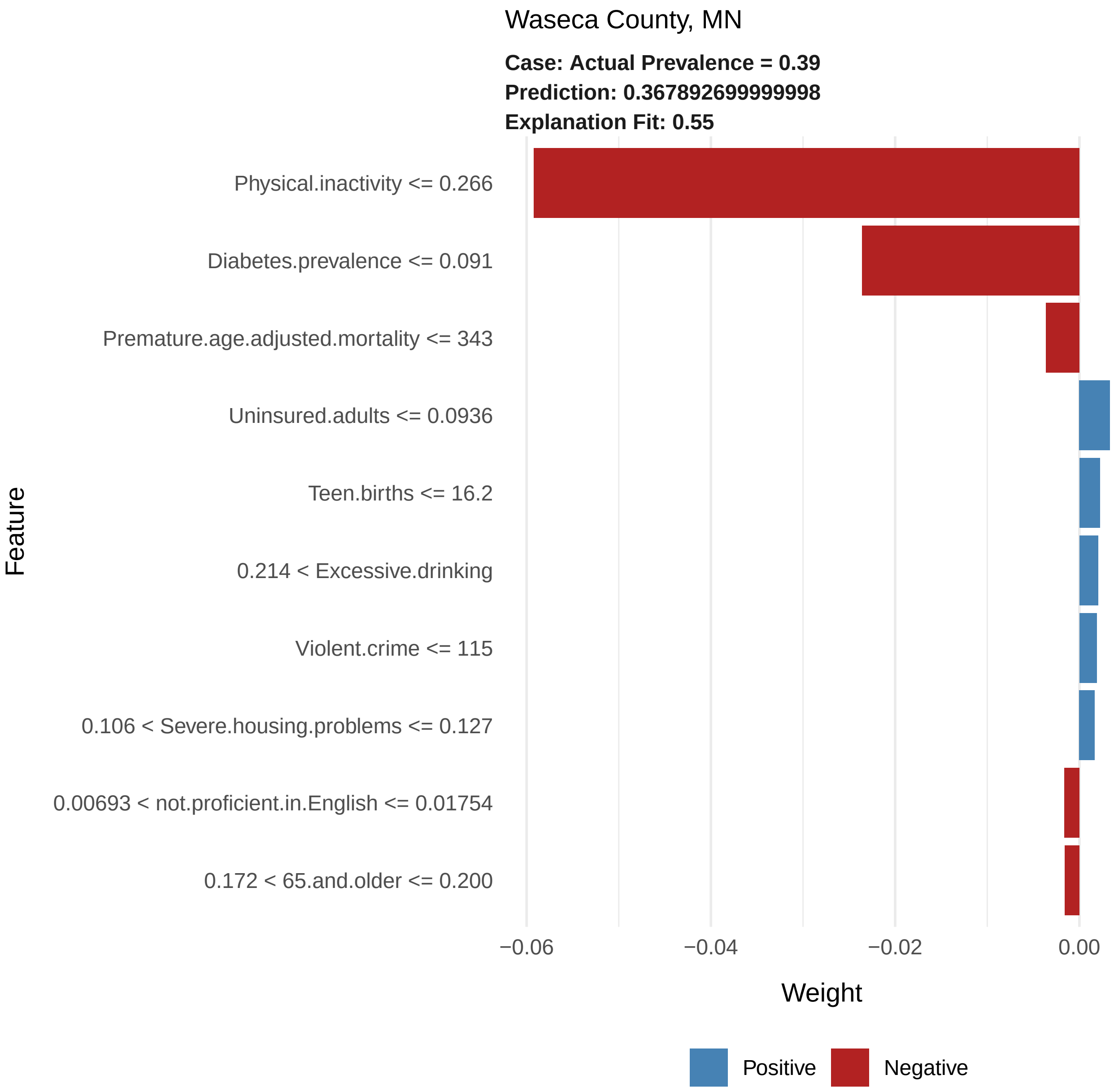

Waseca County, MN
Case: Actual Prevalence = 0.39
Prediction: 0.367892699999998
Explanation Fit: 0.55
Physical.inactivity <= 0.266
Diabetes.prevalence <= 0.091
Premature.age.adjusted.mortality <= 343
Uninsured.adults <= 0.0936
Teen.births <= 16.2
0.214 < Excessive.drinking
Violent.crime <= 115
0.106 < Severe.housing.problems <= 0.127
0.00693 < not.proficient.in.English <= 0.01754
0.172 < 65.and.older <= 0.200
Feature
−0.06
−0.04
−0.02
0.00
Weight
Positive
Negative

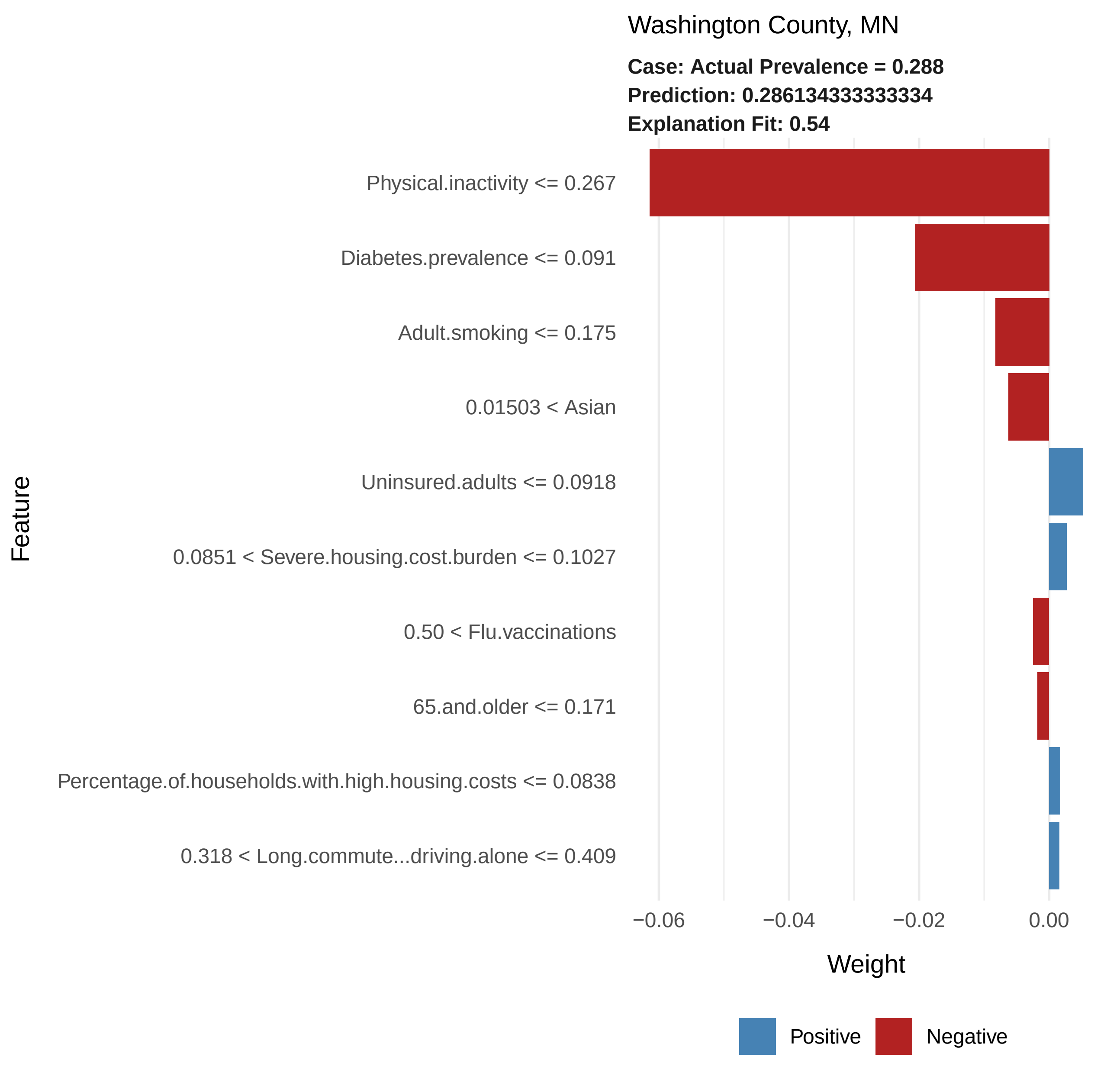

Washington County, MN
Case: Actual Prevalence = 0.288
Prediction: 0.286134333333334
Explanation Fit: 0.54
Physical.inactivity <= 0.267
Diabetes.prevalence <= 0.091
Adult.smoking <= 0.175
0.01503 < Asian
Uninsured.adults <= 0.0918
0.0851 < Severe.housing.cost.burden <= 0.1027
0.50 < Flu.vaccinations
65.and.older <= 0.171
Percentage.of.households.with.high.housing.costs <= 0.0838
0.318 < Long.commute...driving.alone <= 0.409
Feature
−0.06
−0.04
−0.02
0.00
Weight
Positive
Negative

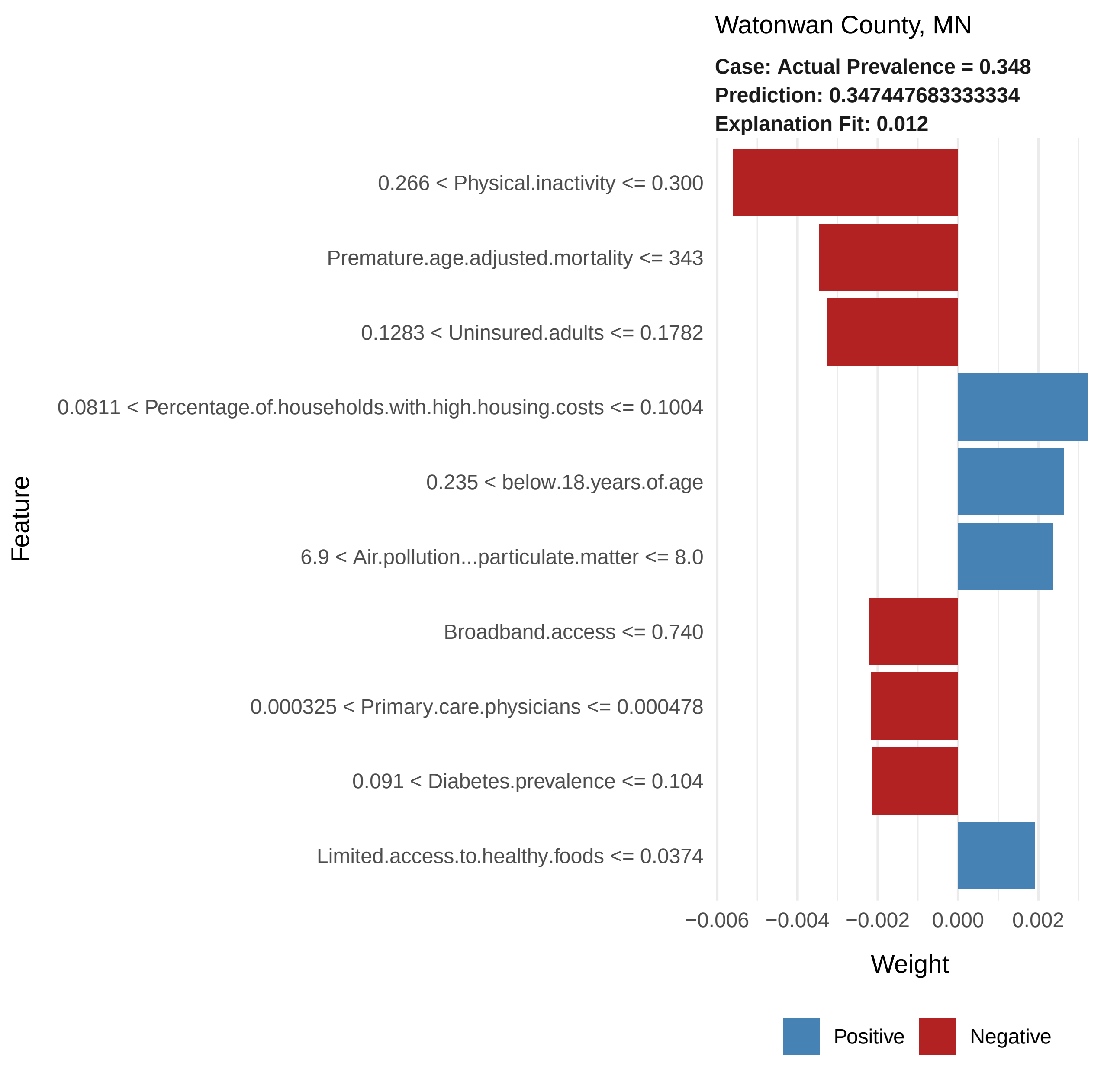

Watonwan County, MN
Case: Actual Prevalence = 0.348
Prediction: 0.347447683333334
Explanation Fit: 0.012
0.266 < Physical.inactivity <= 0.300
Premature.age.adjusted.mortality <= 343
0.1283 < Uninsured.adults <= 0.1782
0.0811 < Percentage.of.households.with.high.housing.costs <= 0.1004
0.235 < below.18.years.of.age
6.9 < Air.pollution...particulate.matter <= 8.0
Broadband.access <= 0.740
0.000325 < Primary.care.physicians <= 0.000478
0.091 < Diabetes.prevalence <= 0.104
Limited.access.to.healthy.foods <= 0.0374
Feature
−0.006
−0.004
−0.002
0.000
0.002
Weight
Positive
Negative

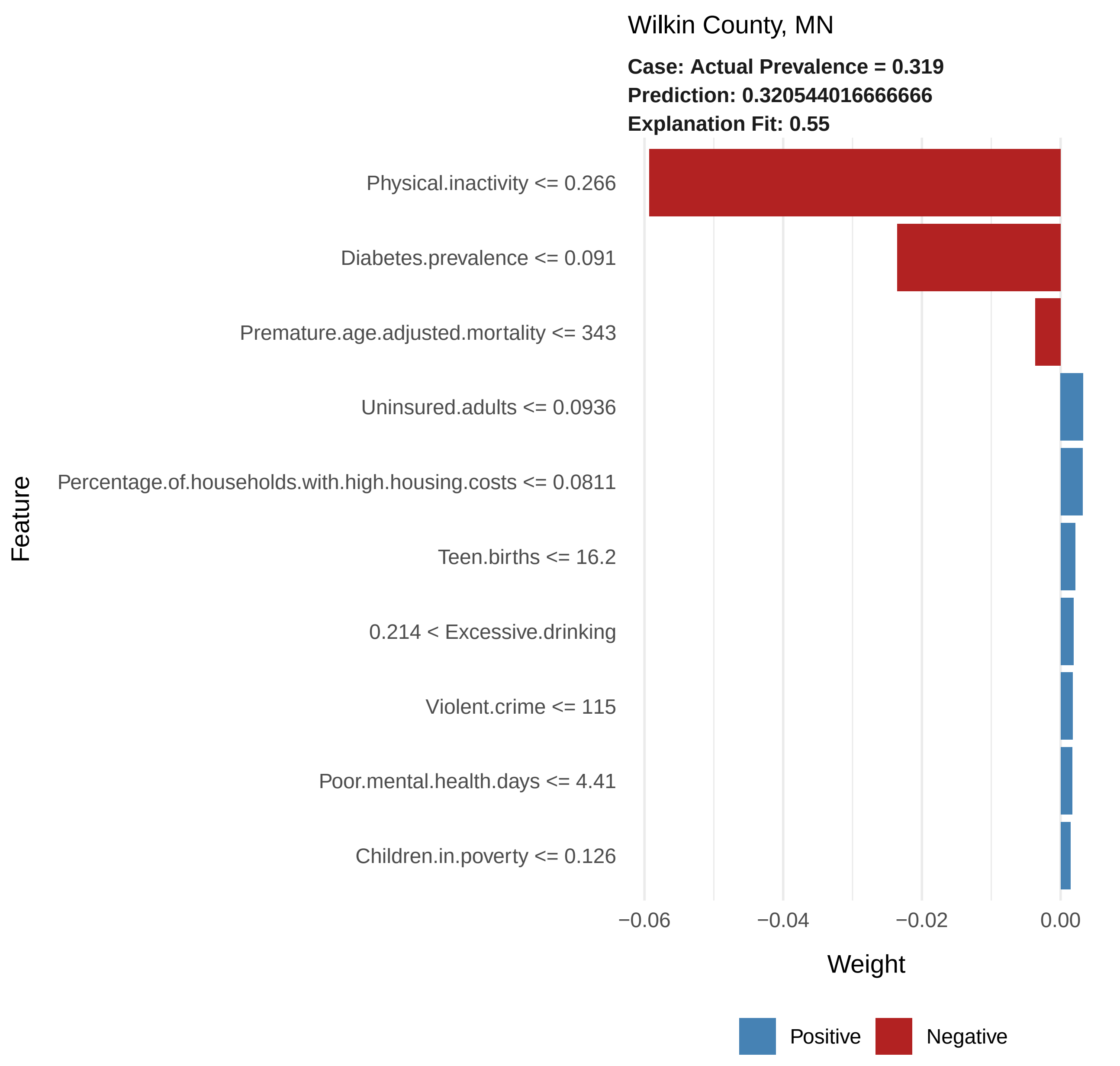

Wilkin County, MN
Case: Actual Prevalence = 0.319
Prediction: 0.320544016666666
Explanation Fit: 0.55
Physical.inactivity <= 0.266
Diabetes.prevalence <= 0.091
Premature.age.adjusted.mortality <= 343
Uninsured.adults <= 0.0936
Percentage.of.households.with.high.housing.costs <= 0.0811
Teen.births <= 16.2
0.214 < Excessive.drinking
Violent.crime <= 115
Poor.mental.health.days <= 4.41
Children.in.poverty <= 0.126
Feature
−0.06
−0.04
−0.02
0.00
Weight
Positive
Negative

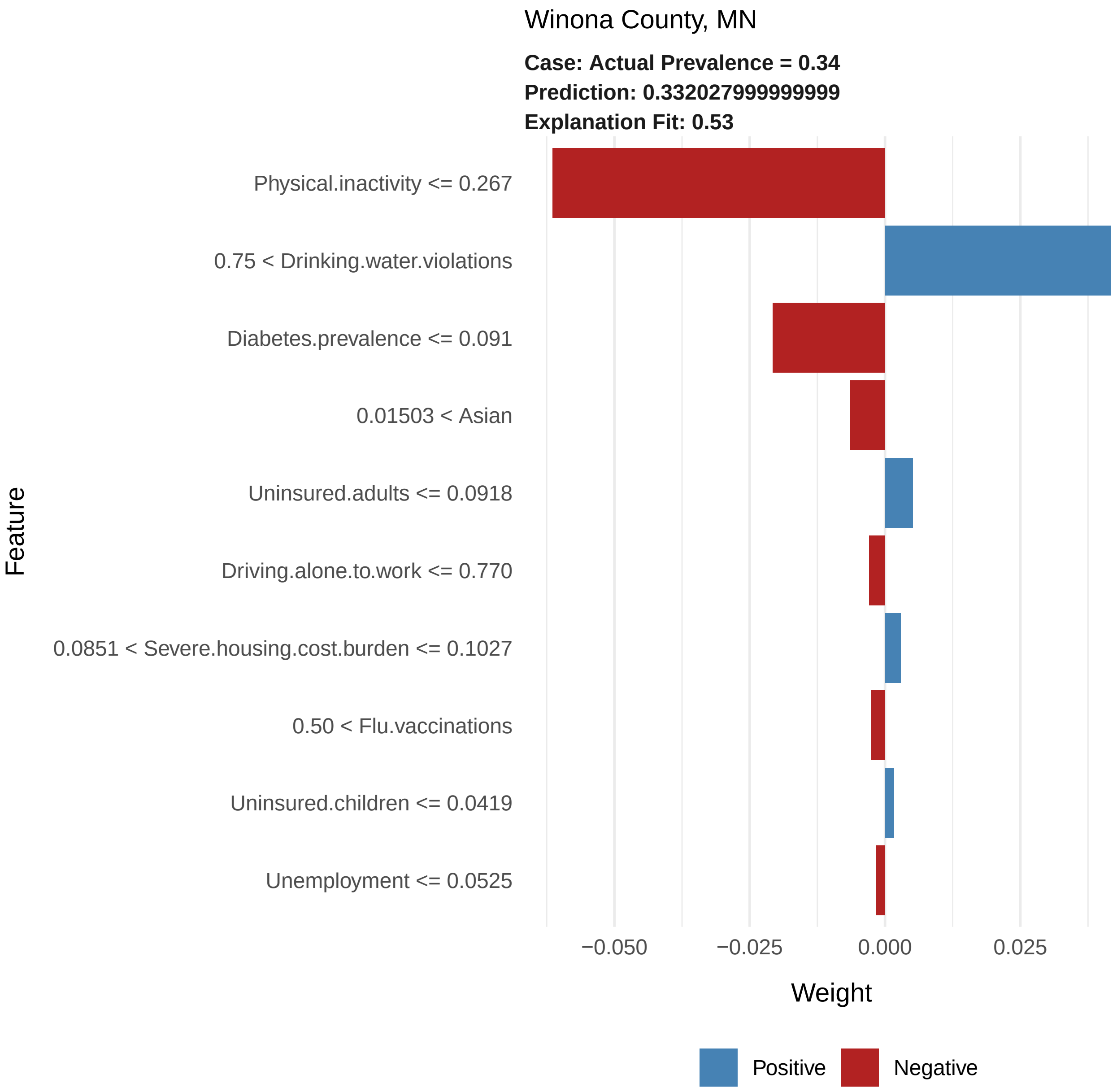

Winona County, MN
Case: Actual Prevalence = 0.34
Prediction: 0.332027999999999
Explanation Fit: 0.53
Physical.inactivity <= 0.267
0.75 < Drinking.water.violations
Diabetes.prevalence <= 0.091
0.01503 < Asian
Uninsured.adults <= 0.0918
Driving.alone.to.work <= 0.770
0.0851 < Severe.housing.cost.burden <= 0.1027
0.50 < Flu.vaccinations
Uninsured.children <= 0.0419
Unemployment <= 0.0525
Feature
−0.050
−0.025
0.000
0.025
Weight
Positive
Negative

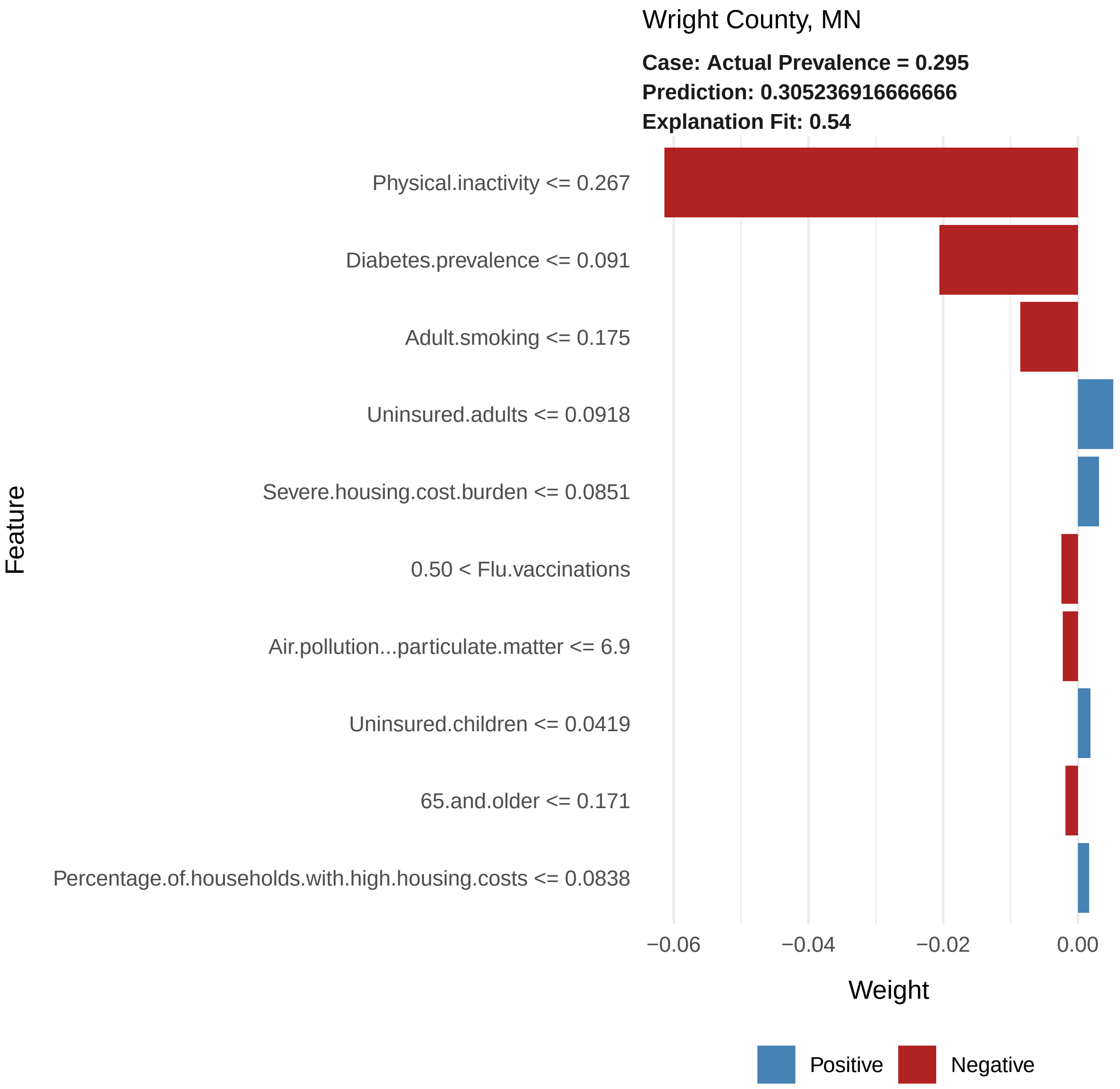

Wright County, MN
Case: Actual Prevalence = 0.295
Prediction: 0.305236916666666
Explanation Fit: 0.54
Physical.inactivity <= 0.267
Diabetes.prevalence <= 0.091
Adult.smoking <= 0.175
Uninsured.adults <= 0.0918
Severe.housing.cost.burden <= 0.0851
0.50 < Flu.vaccinations
Air.pollution...particulate.matter <= 6.9
Uninsured.children <= 0.0419
65.and.older <= 0.171
Percentage.of.households.with.high.housing.costs <= 0.0838
Feature
−0.06
−0.04
−0.02
0.00
Weight
Positive
Negative

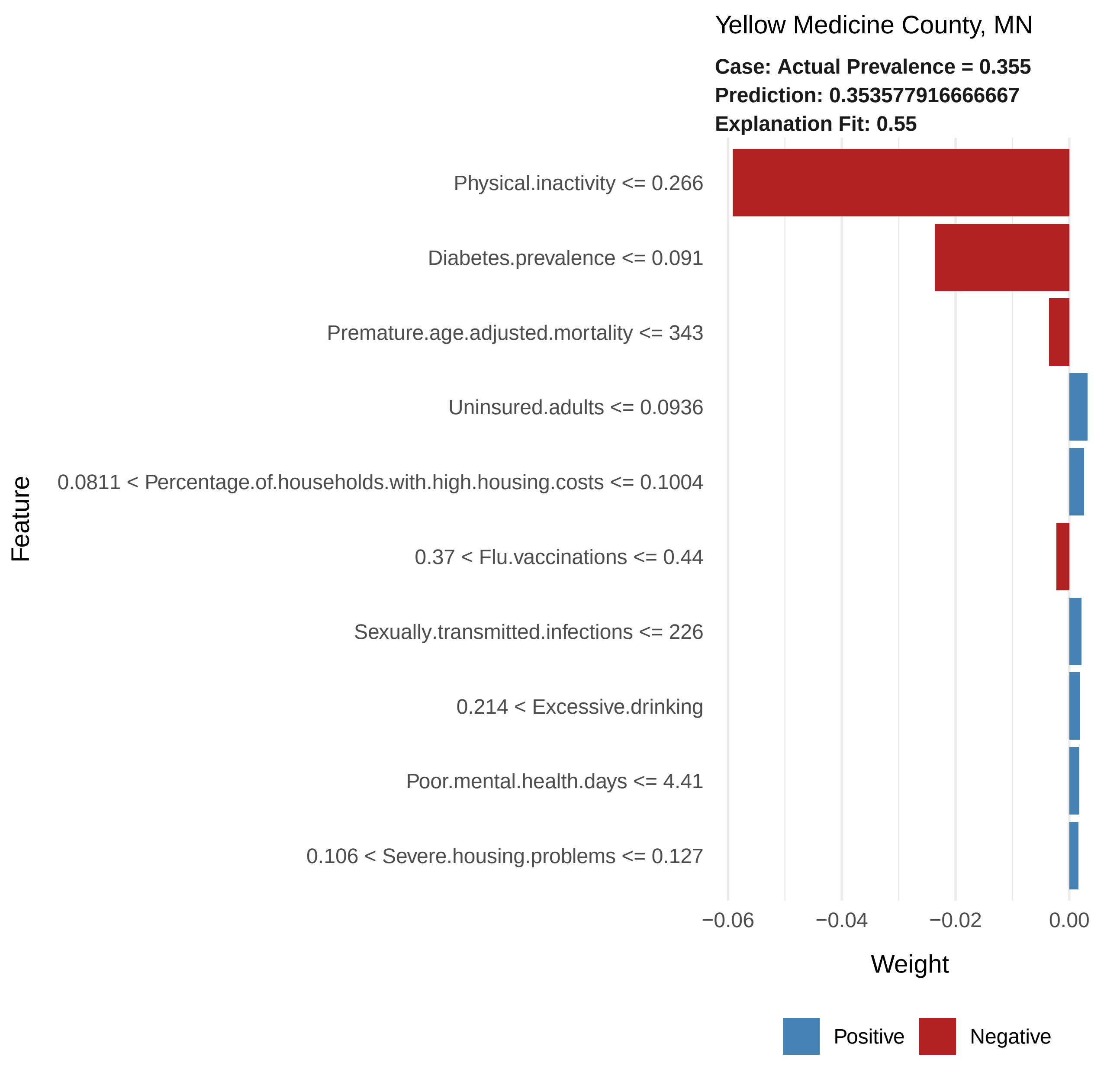
Yellow Medicine County, MN
Case: Actual Prevalence = 0.355
Prediction: 0.353577916666667
Explanation Fit: 0.55
Physical.inactivity <= 0.266
Diabetes.prevalence <= 0.091
Premature.age.adjusted.mortality <= 343
Uninsured.adults <= 0.0936
0.0811 < Percentage.of.households.with.high.housing.costs <= 0.1004
0.37 < Flu.vaccinations <= 0.44
Sexually.transmitted.infections <= 226
0.214 < Excessive.drinking
Poor.mental.health.days <= 4.41
0.106 < Severe.housing.problems <= 0.127
Feature
−0.06
−0.04
−0.02
0.00
Weight
Positive
Negative

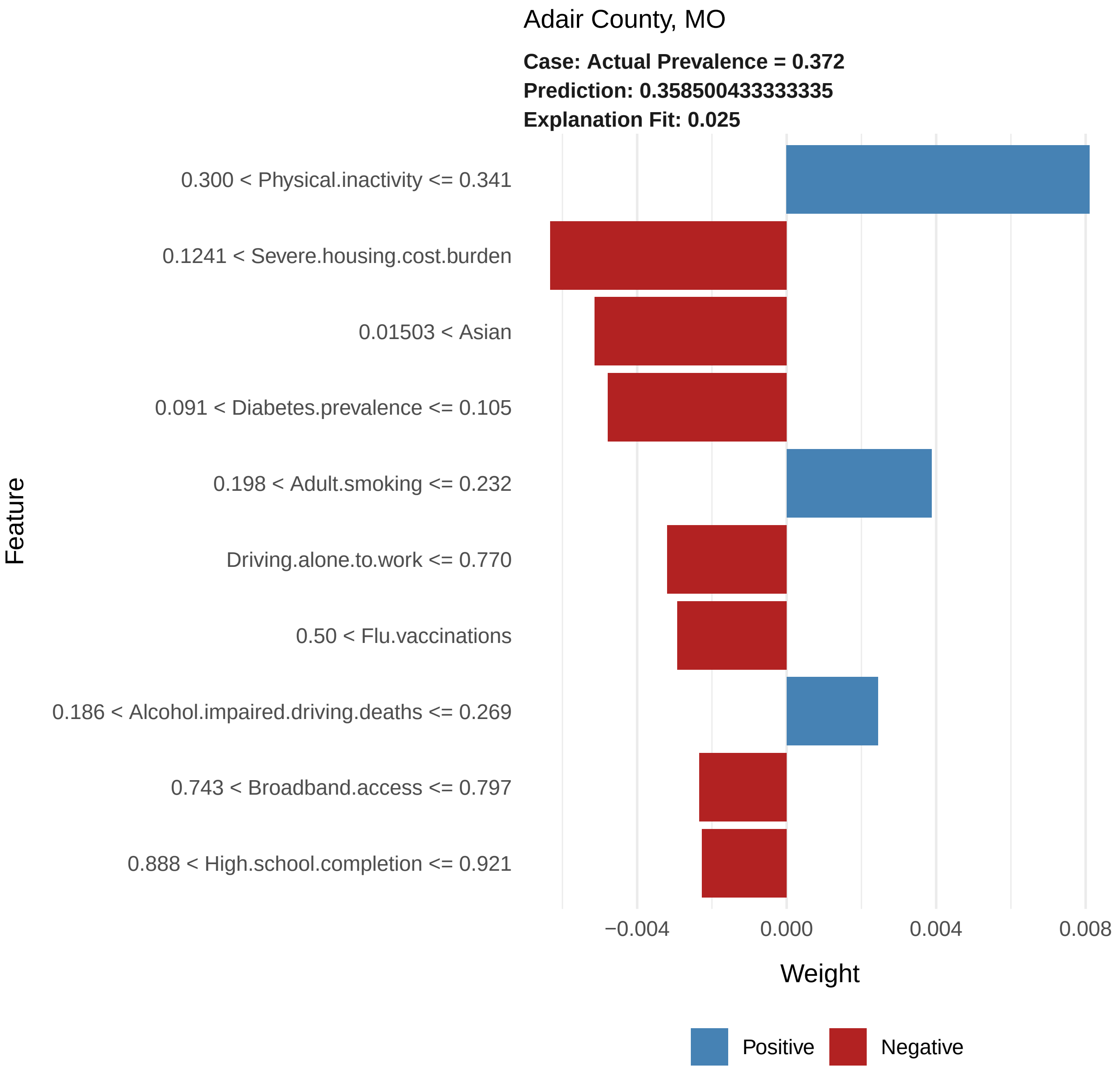

Adair County, MO
Case: Actual Prevalence = 0.372
Prediction: 0.358500433333335
Explanation Fit: 0.025
0.300 < Physical.inactivity <= 0.341
0.1241 < Severe.housing.cost.burden
0.01503 < Asian
0.091 < Diabetes.prevalence <= 0.105
0.198 < Adult.smoking <= 0.232
Driving.alone.to.work <= 0.770
0.50 < Flu.vaccinations
0.186 < Alcohol.impaired.driving.deaths <= 0.269
0.743 < Broadband.access <= 0.797
0.888 < High.school.completion <= 0.921
Feature
−0.004
0.000
0.004
0.008
Weight
Positive
Negative

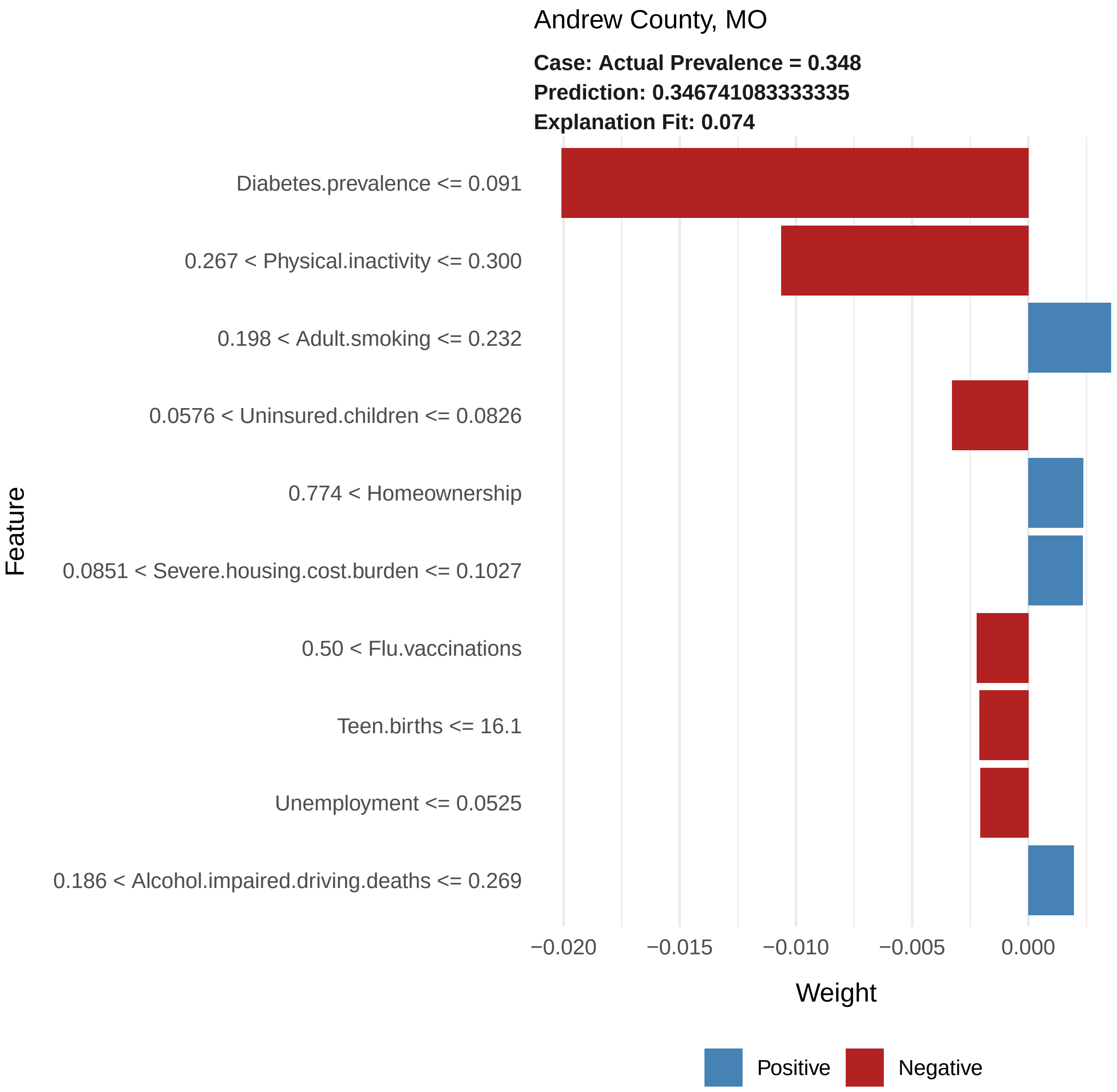
Andrew County, MO
Case: Actual Prevalence = 0.348
Prediction: 0.346741083333335
Explanation Fit: 0.074
Diabetes.prevalence <= 0.091
0.267 < Physical.inactivity <= 0.300
0.198 < Adult.smoking <= 0.232
0.0576 < Uninsured.children <= 0.0826
0.774 < Homeownership
0.0851 < Severe.housing.cost.burden <= 0.1027
0.50 < Flu.vaccinations
Teen.births <= 16.1
Unemployment <= 0.0525
0.186 < Alcohol.impaired.driving.deaths <= 0.269
Feature
−0.020
−0.015
−0.010
−0.005
0.000
Weight
Positive
Negative

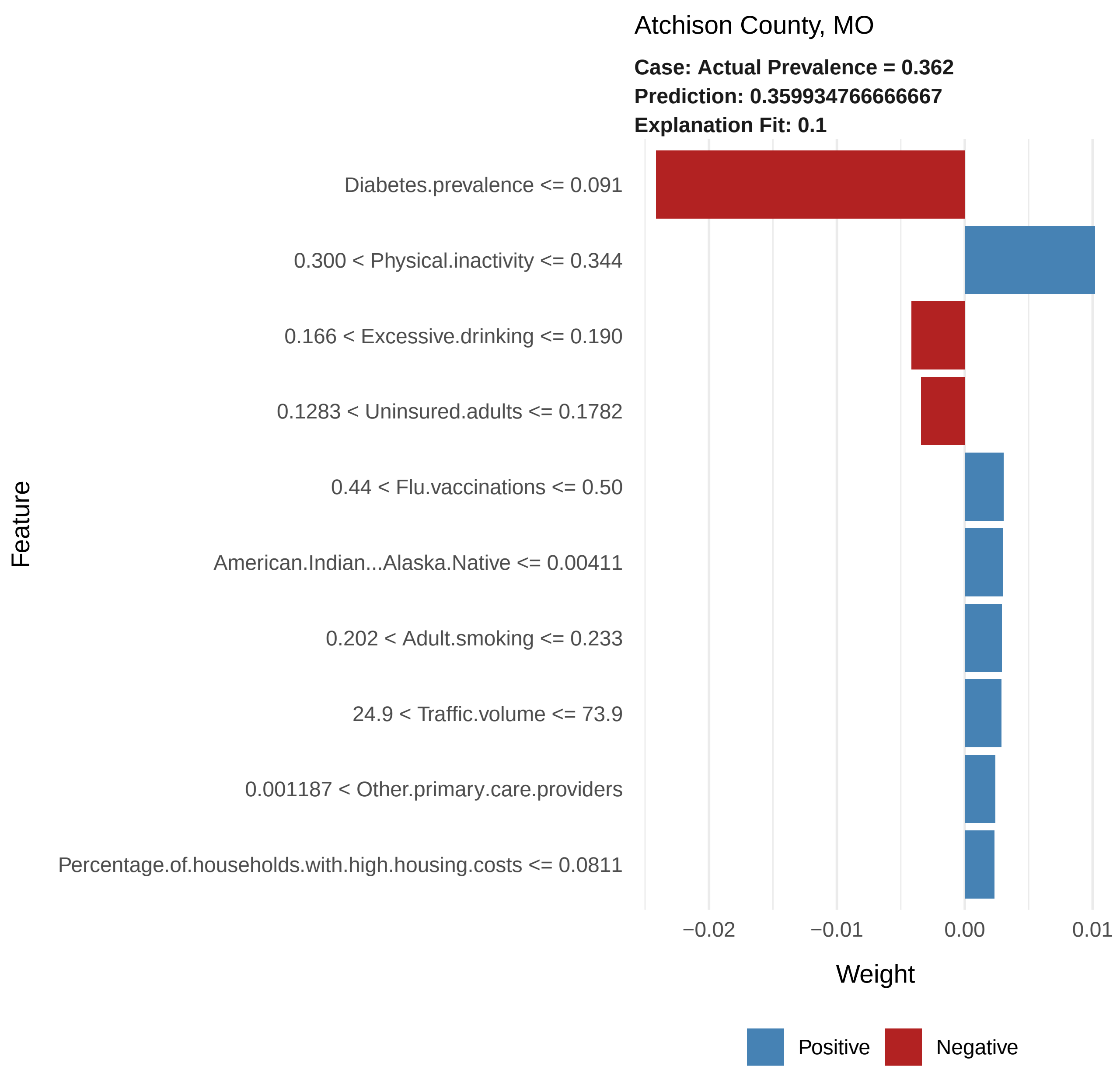
Atchison County, MO
Case: Actual Prevalence = 0.362
Prediction: 0.359934766666667
Explanation Fit: 0.1
Diabetes.prevalence <= 0.091
0.300 < Physical.inactivity <= 0.344
0.166 < Excessive.drinking <= 0.190
0.1283 < Uninsured.adults <= 0.1782
0.44 < Flu.vaccinations <= 0.50
American.Indian...Alaska.Native <= 0.00411
0.202 < Adult.smoking <= 0.233
24.9 < Traffic.volume <= 73.9
0.001187 < Other.primary.care.providers
Percentage.of.households.with.high.housing.costs <= 0.0811
Feature
−0.02
−0.01
0.00
0.01
Weight
Positive
Negative

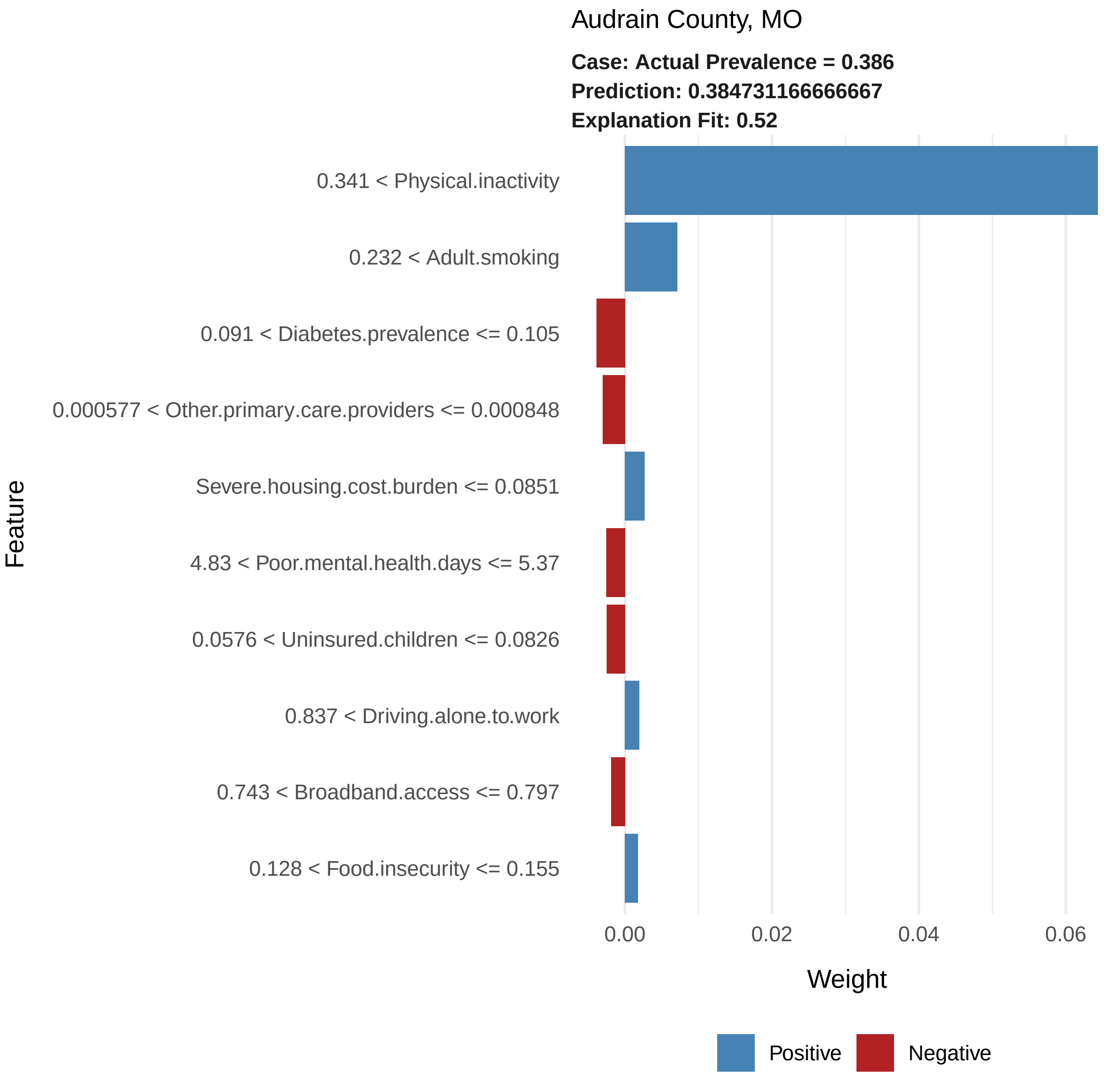

Audrain County, MO
Case: Actual Prevalence = 0.386
Prediction: 0.384731166666667
Explanation Fit: 0.52
0.341 < Physical.inactivity
0.232 < Adult.smoking
0.091 < Diabetes.prevalence <= 0.105
0.000577 < Other.primary.care.providers <= 0.000848
Severe.housing.cost.burden <= 0.0851
4.83 < Poor.mental.health.days <= 5.37
0.0576 < Uninsured.children <= 0.0826
0.837 < Driving.alone.to.work
0.743 < Broadband.access <= 0.797
0.128 < Food.insecurity <= 0.155
Feature
0.00
0.02
0.04
0.06
Weight
Positive
Negative

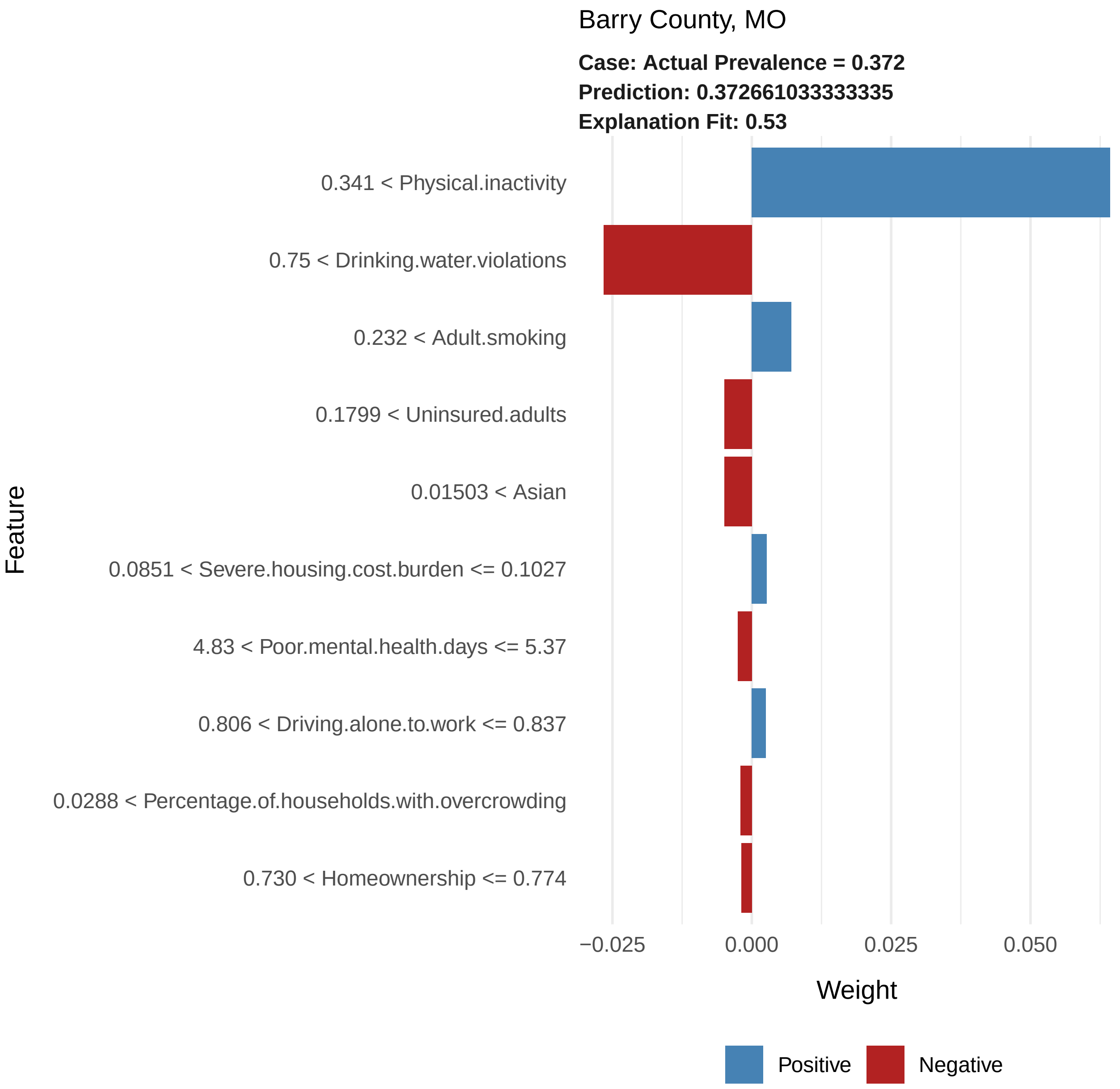

Barry County, MO
Case: Actual Prevalence = 0.372
Prediction: 0.372661033333335
Explanation Fit: 0.53
0.341 < Physical.inactivity
0.75 < Drinking.water.violations
0.232 < Adult.smoking
0.1799 < Uninsured.adults
0.01503 < Asian
0.0851 < Severe.housing.cost.burden <= 0.1027
4.83 < Poor.mental.health.days <= 5.37
0.806 < Driving.alone.to.work <= 0.837
0.0288 < Percentage.of.households.with.overcrowding
0.730 < Homeownership <= 0.774
Feature
−0.025
0.000
0.025
0.050
Weight
Positive
Negative

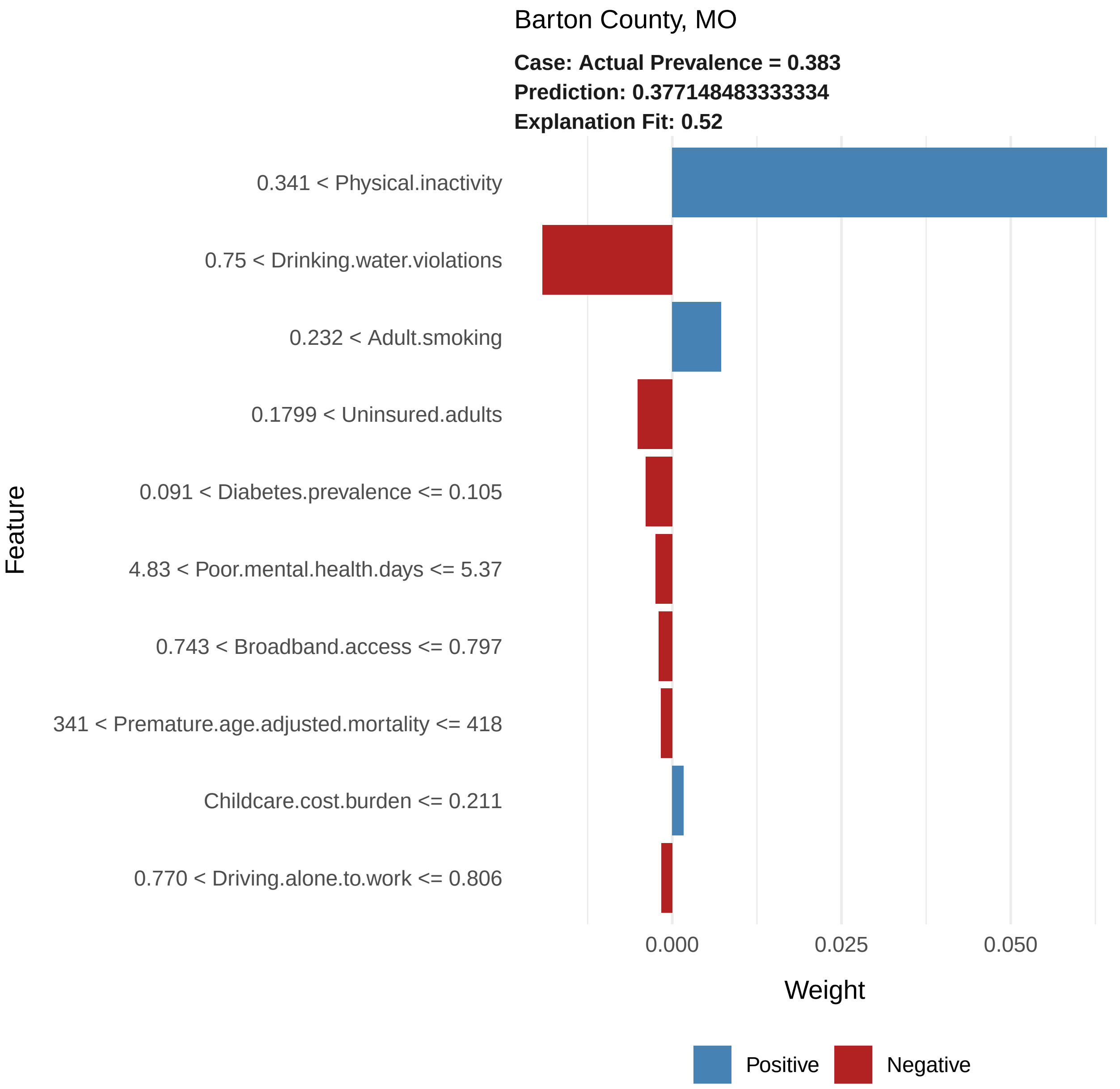
Barton County, MO
Case: Actual Prevalence = 0.383
Prediction: 0.377148483333334
Explanation Fit: 0.52
0.341 < Physical.inactivity
0.75 < Drinking.water.violations
0.232 < Adult.smoking
0.1799 < Uninsured.adults
0.091 < Diabetes.prevalence <= 0.105
4.83 < Poor.mental.health.days <= 5.37
0.743 < Broadband.access <= 0.797
341 < Premature.age.adjusted.mortality <= 418
Childcare.cost.burden <= 0.211
0.770 < Driving.alone.to.work <= 0.806
Feature
0.000
0.025
0.050
Weight
Positive
Negative

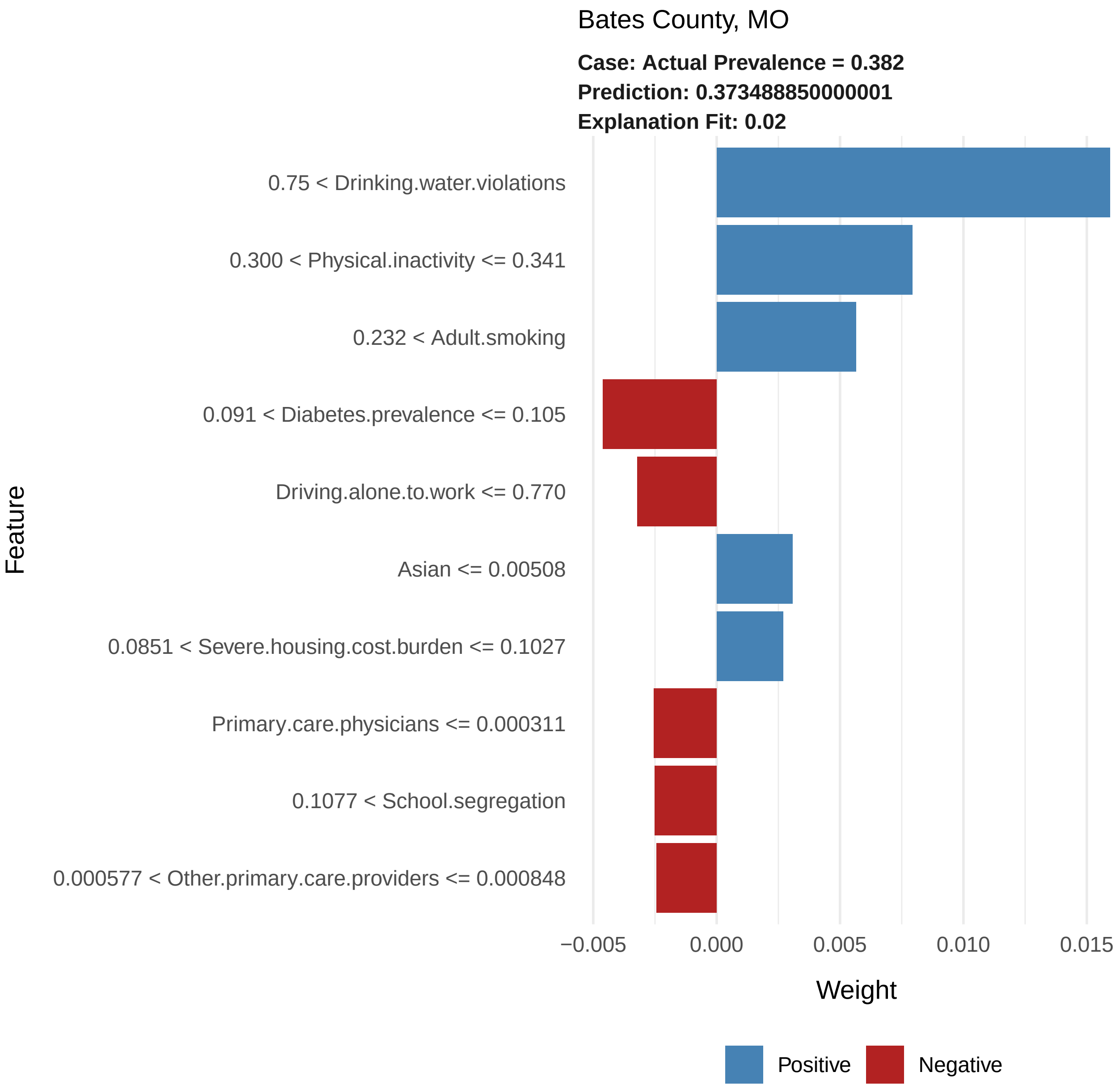
Bates County, MO
Case: Actual Prevalence = 0.382
Prediction: 0.373488850000001
Explanation Fit: 0.02
0.75 < Drinking.water.violations
0.300 < Physical.inactivity <= 0.341
0.232 < Adult.smoking
0.091 < Diabetes.prevalence <= 0.105
Driving.alone.to.work <= 0.770
Asian <= 0.00508
0.0851 < Severe.housing.cost.burden <= 0.1027
Primary.care.physicians <= 0.000311
0.1077 < School.segregation
0.000577 < Other.primary.care.providers <= 0.000848
Feature
−0.005
0.000
0.005
0.010
0.015
Weight
Positive
Negative

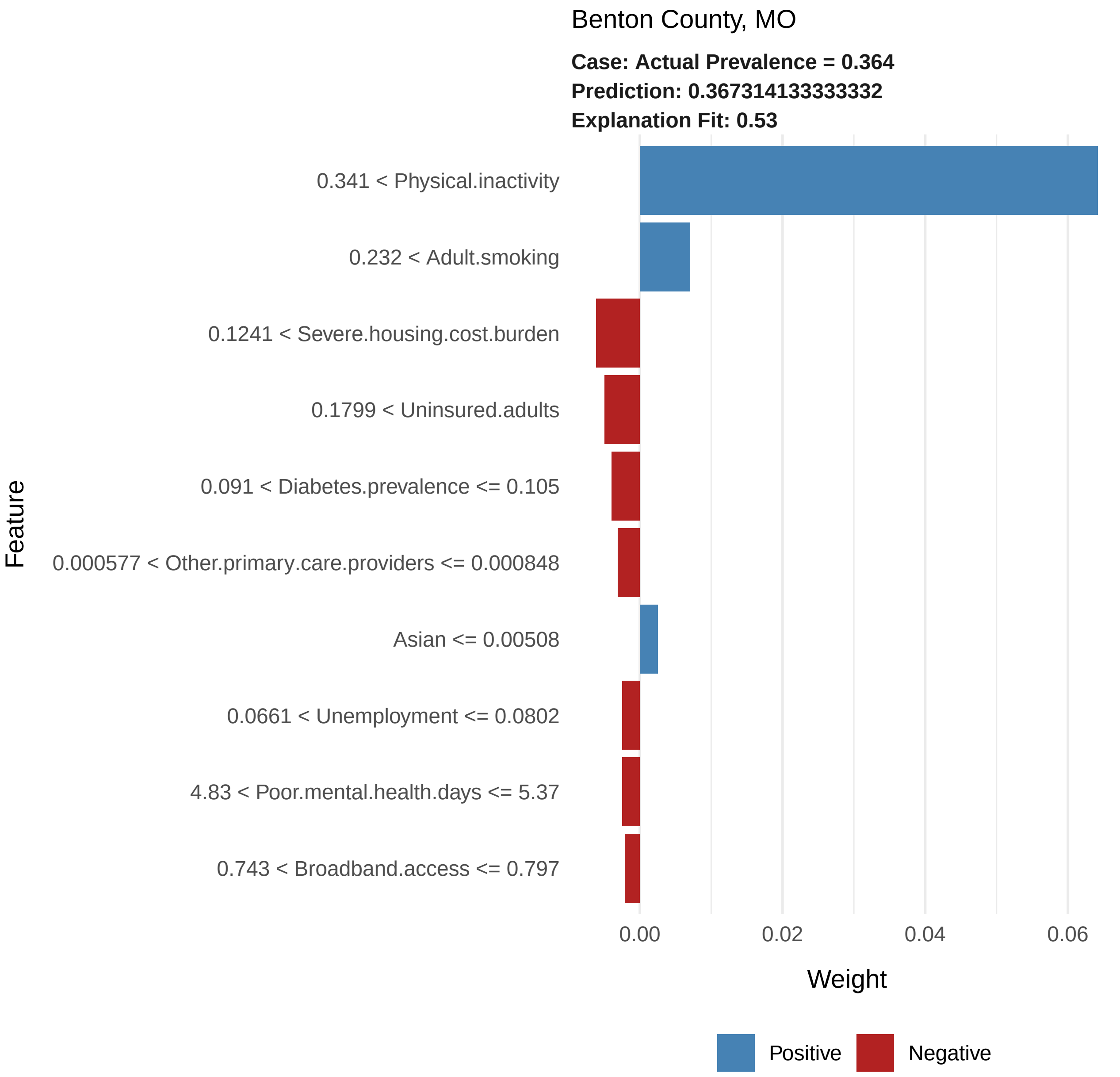

Benton County, MO
Case: Actual Prevalence = 0.364
Prediction: 0.367314133333332
Explanation Fit: 0.53
0.341 < Physical.inactivity
0.232 < Adult.smoking
0.1241 < Severe.housing.cost.burden
0.1799 < Uninsured.adults
0.091 < Diabetes.prevalence <= 0.105
0.000577 < Other.primary.care.providers <= 0.000848
Asian <= 0.00508
0.0661 < Unemployment <= 0.0802
4.83 < Poor.mental.health.days <= 5.37
0.743 < Broadband.access <= 0.797
Feature
0.00
0.02
0.04
0.06
Weight
Positive
Negative

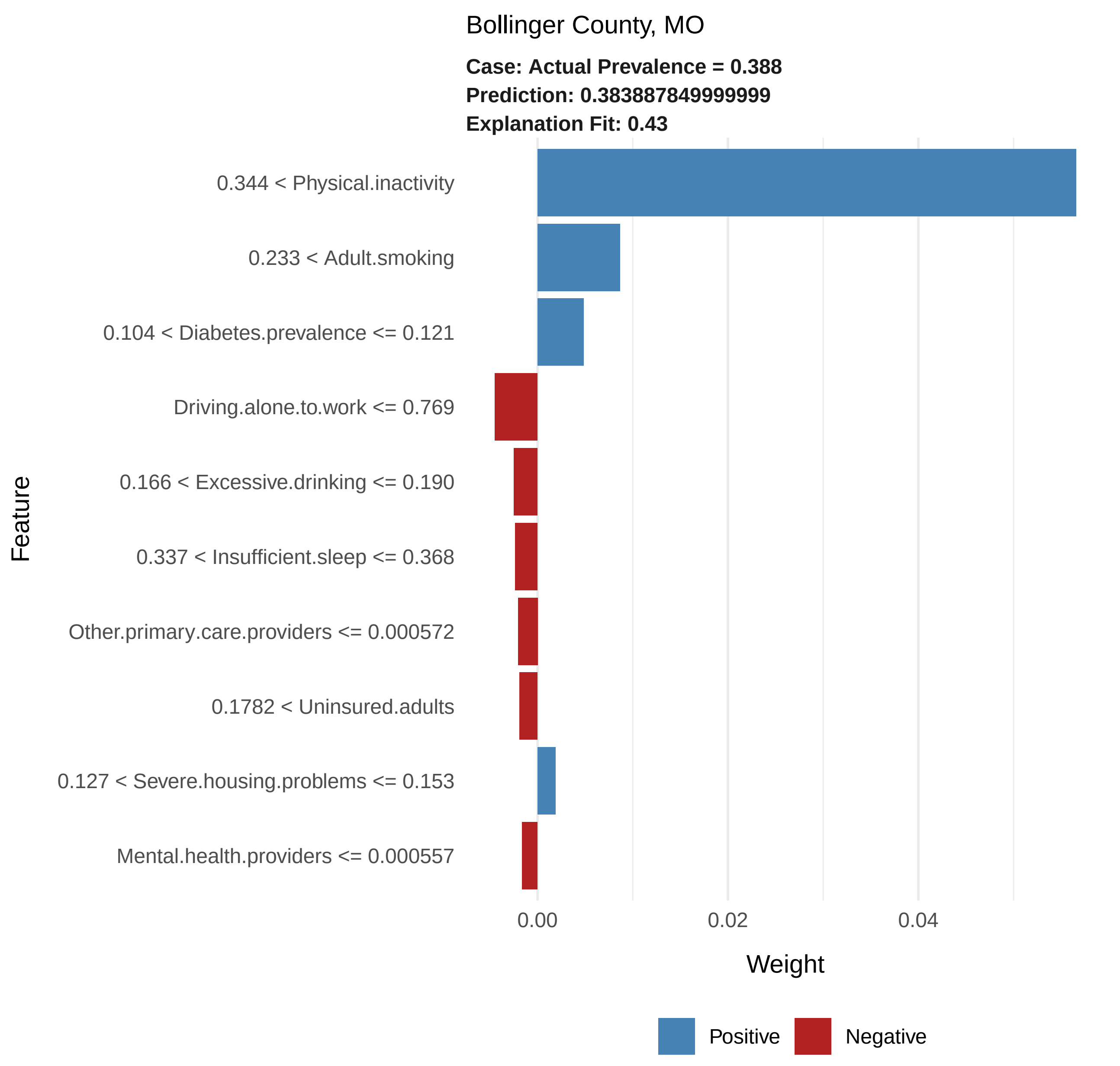

Bollinger County, MO
Case: Actual Prevalence = 0.388
Prediction: 0.383887849999999
Explanation Fit: 0.43
0.344 < Physical.inactivity
0.233 < Adult.smoking
0.104 < Diabetes.prevalence <= 0.121
Driving.alone.to.work <= 0.769
0.166 < Excessive.drinking <= 0.190
0.337 < Insufficient.sleep <= 0.368
Other.primary.care.providers <= 0.000572
0.1782 < Uninsured.adults
0.127 < Severe.housing.problems <= 0.153
Mental.health.providers <= 0.000557
Feature
0.00
0.02
0.04
Weight
Positive
Negative

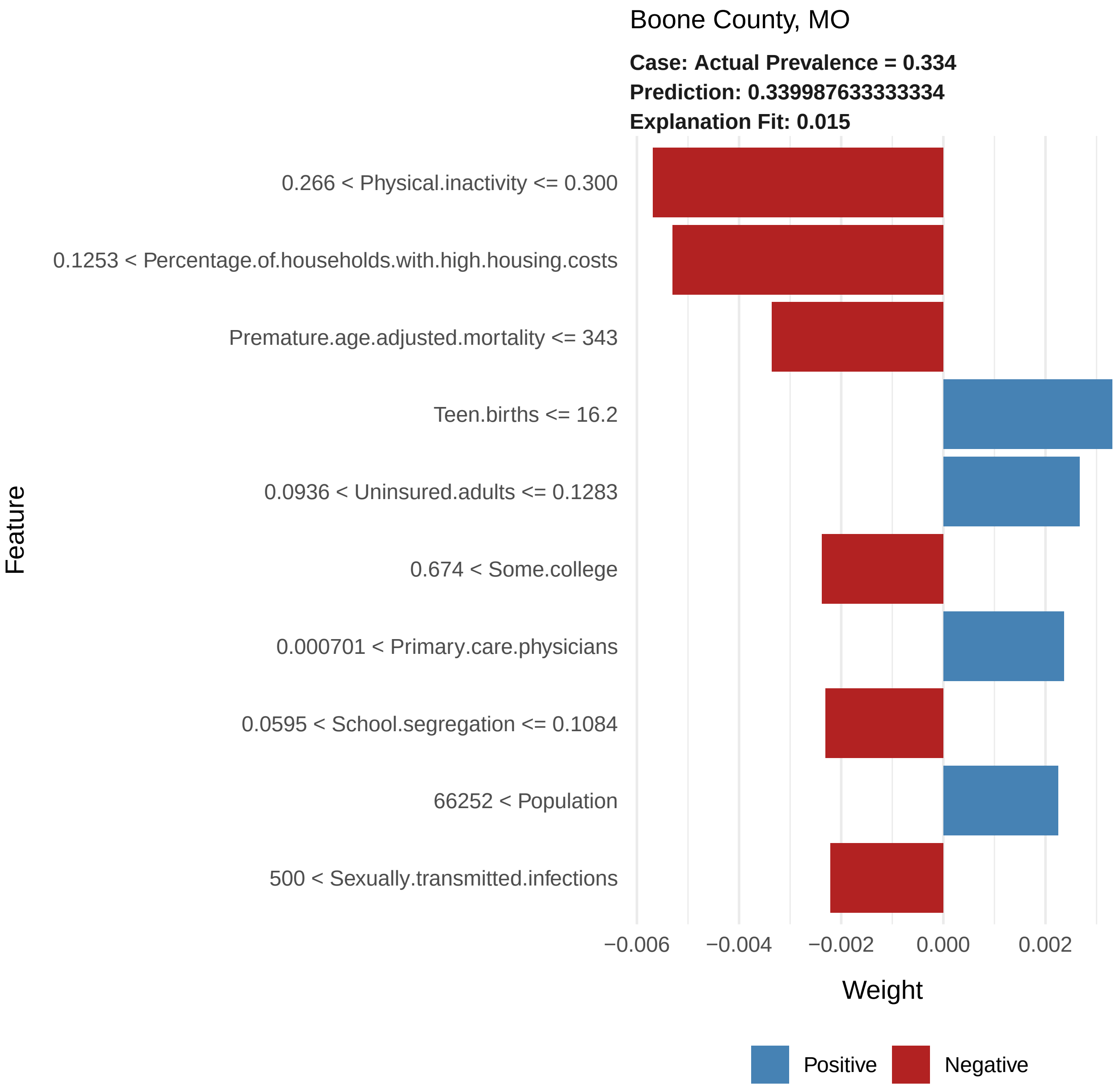

Boone County, MO
Case: Actual Prevalence = 0.334
Prediction: 0.339987633333334
Explanation Fit: 0.015
0.266 < Physical.inactivity <= 0.300
0.1253 < Percentage.of.households.with.high.housing.costs
Premature.age.adjusted.mortality <= 343
Teen.births <= 16.2
0.0936 < Uninsured.adults <= 0.1283
0.674 < Some.college
0.000701 < Primary.care.physicians
0.0595 < School.segregation <= 0.1084
66252 < Population
500 < Sexually.transmitted.infections
Feature
−0.006
−0.004
−0.002
0.000
0.002
Weight
Positive
Negative

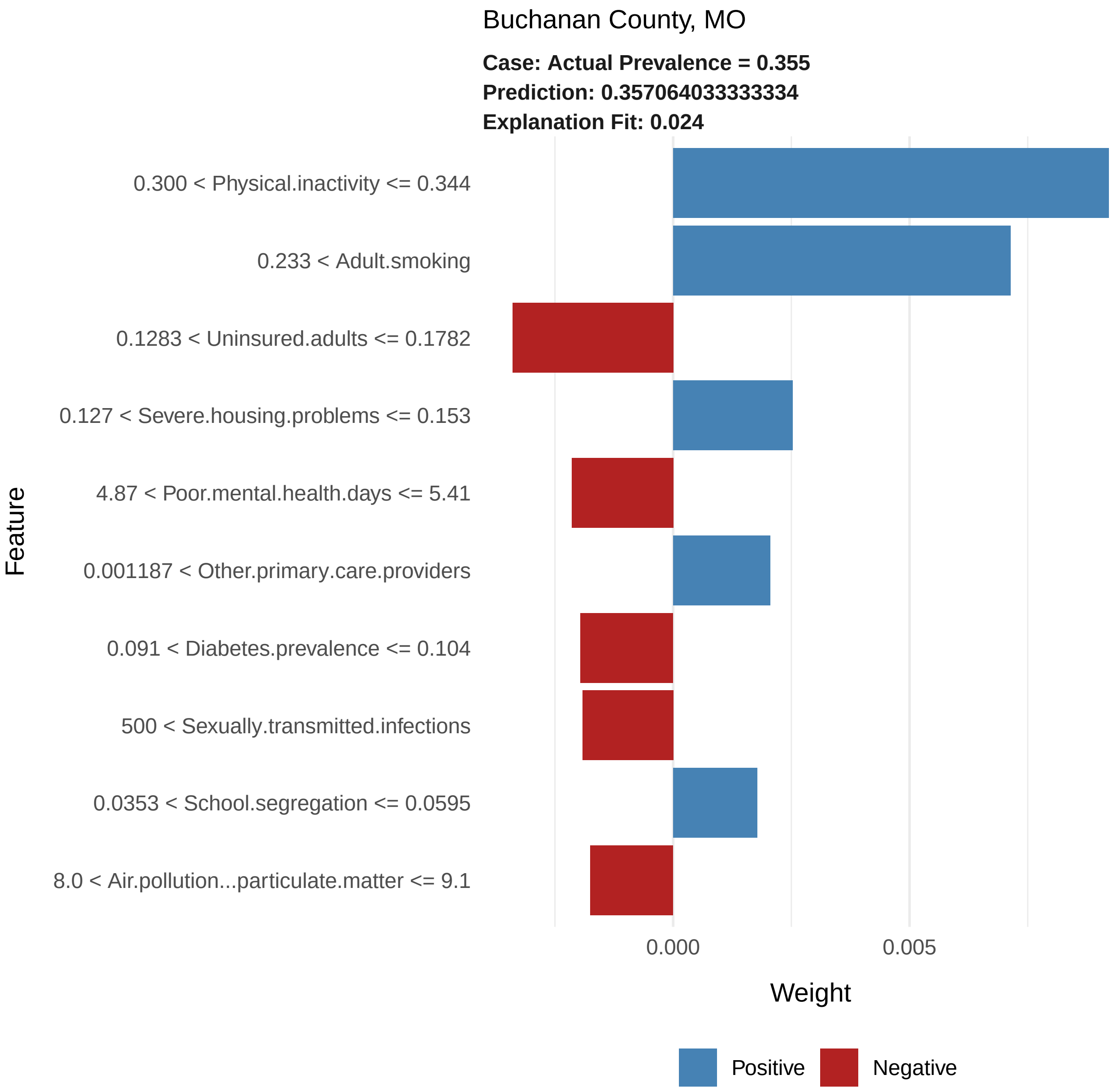

Buchanan County, MO
Case: Actual Prevalence = 0.355
Prediction: 0.357064033333334
Explanation Fit: 0.024
0.300 < Physical.inactivity <= 0.344
0.233 < Adult.smoking
0.1283 < Uninsured.adults <= 0.1782
0.127 < Severe.housing.problems <= 0.153
4.87 < Poor.mental.health.days <= 5.41
0.001187 < Other.primary.care.providers
0.091 < Diabetes.prevalence <= 0.104
500 < Sexually.transmitted.infections
0.0353 < School.segregation <= 0.0595
8.0 < Air.pollution...particulate.matter <= 9.1
Feature
0.000
0.005
Weight
Positive
Negative

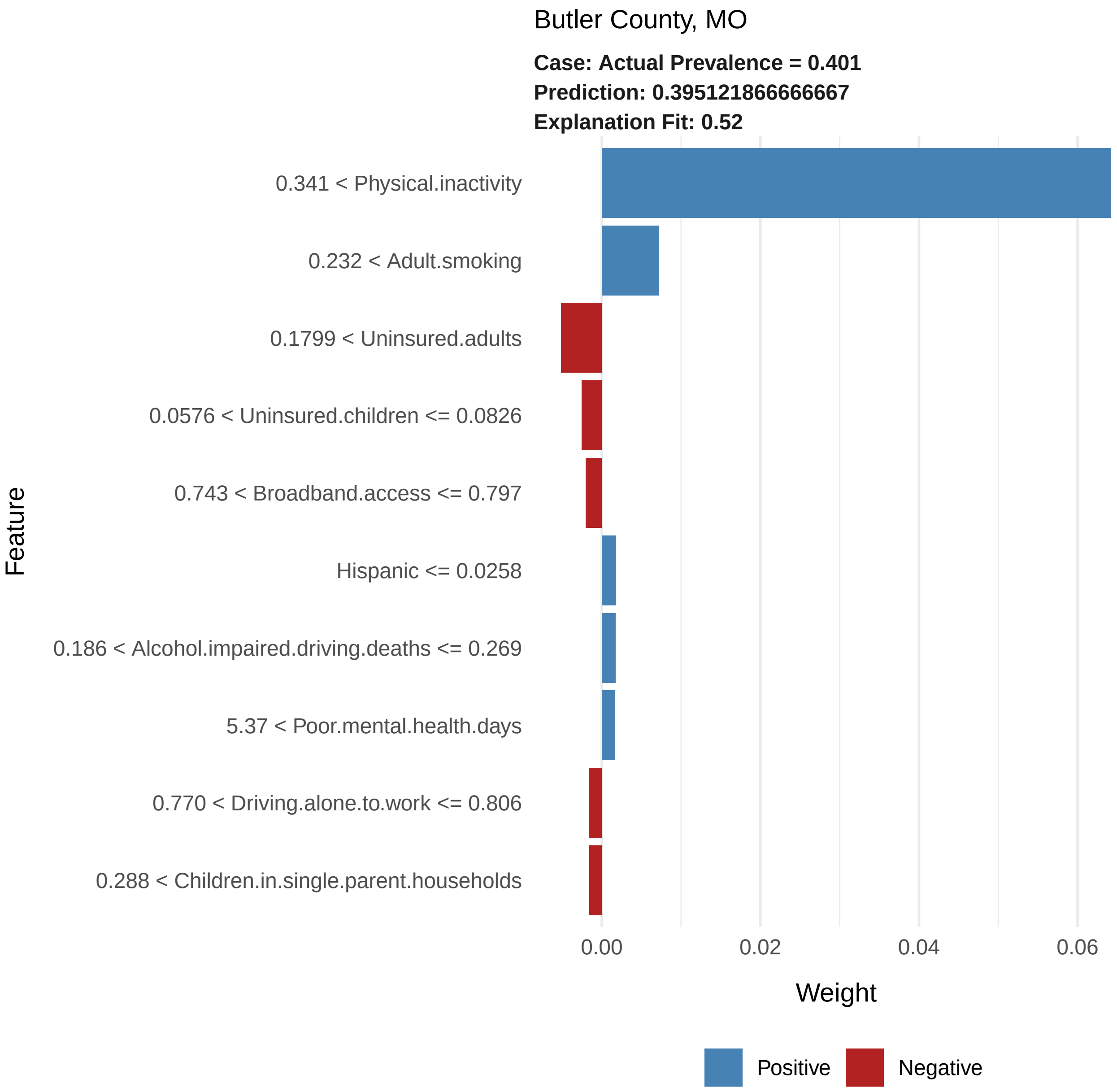
Butler County, MO
Case: Actual Prevalence = 0.401
Prediction: 0.395121866666667
Explanation Fit: 0.52
0.341 < Physical.inactivity
0.232 < Adult.smoking
0.1799 < Uninsured.adults
0.0576 < Uninsured.children <= 0.0826
0.743 < Broadband.access <= 0.797
Hispanic <= 0.0258
0.186 < Alcohol.impaired.driving.deaths <= 0.269
5.37 < Poor.mental.health.days
0.770 < Driving.alone.to.work <= 0.806
0.288 < Children.in.single.parent.households
Feature
0.00
0.02
0.04
0.06
Weight
Positive
Negative

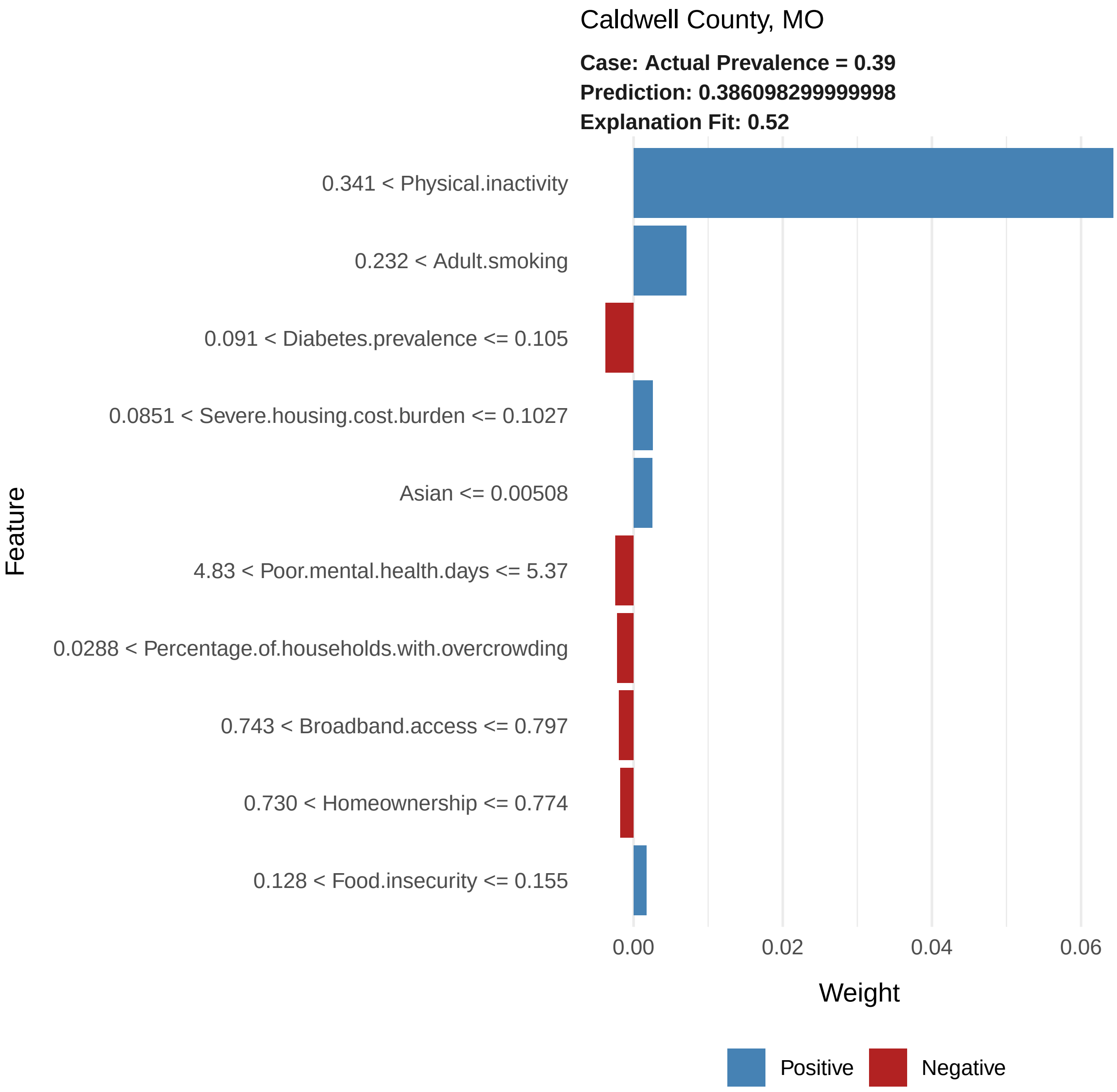
Caldwell County, MO
Case: Actual Prevalence = 0.39
Prediction: 0.386098299999998
Explanation Fit: 0.52
0.341 < Physical.inactivity
0.232 < Adult.smoking
0.091 < Diabetes.prevalence <= 0.105
0.0851 < Severe.housing.cost.burden <= 0.1027
Asian <= 0.00508
4.83 < Poor.mental.health.days <= 5.37
0.0288 < Percentage.of.households.with.overcrowding
0.743 < Broadband.access <= 0.797
0.730 < Homeownership <= 0.774
0.128 < Food.insecurity <= 0.155
Feature
0.00
0.02
0.04
0.06
Weight
Positive
Negative

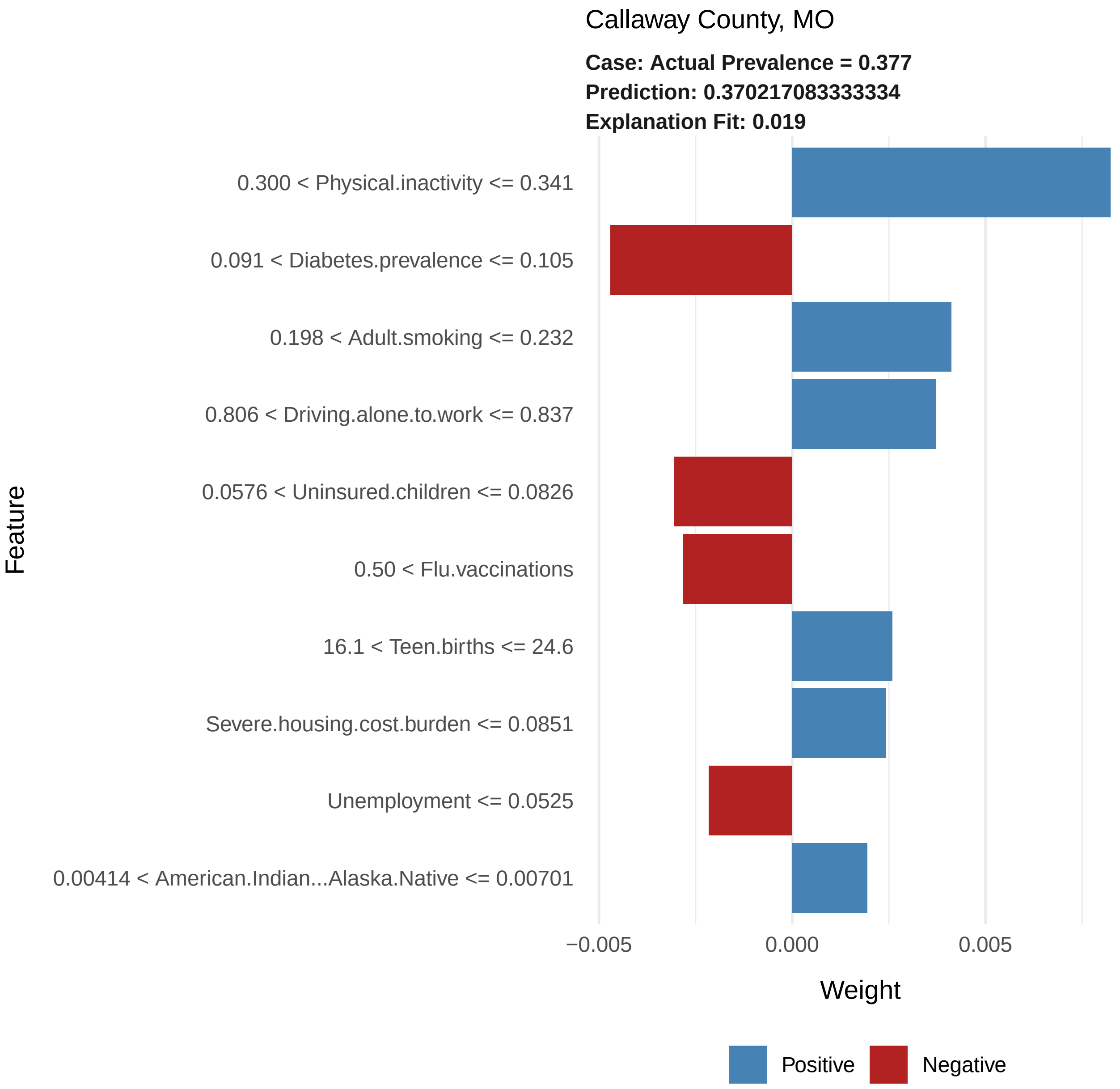
Callaway County, MO
Case: Actual Prevalence = 0.377
Prediction: 0.370217083333334
Explanation Fit: 0.019
0.300 < Physical.inactivity <= 0.341
0.091 < Diabetes.prevalence <= 0.105
0.198 < Adult.smoking <= 0.232
0.806 < Driving.alone.to.work <= 0.837
0.0576 < Uninsured.children <= 0.0826
0.50 < Flu.vaccinations
16.1 < Teen.births <= 24.6
Severe.housing.cost.burden <= 0.0851
Unemployment <= 0.0525
0.00414 < American.Indian...Alaska.Native <= 0.00701
Feature
−0.005
0.000
0.005
Weight
Positive
Negative

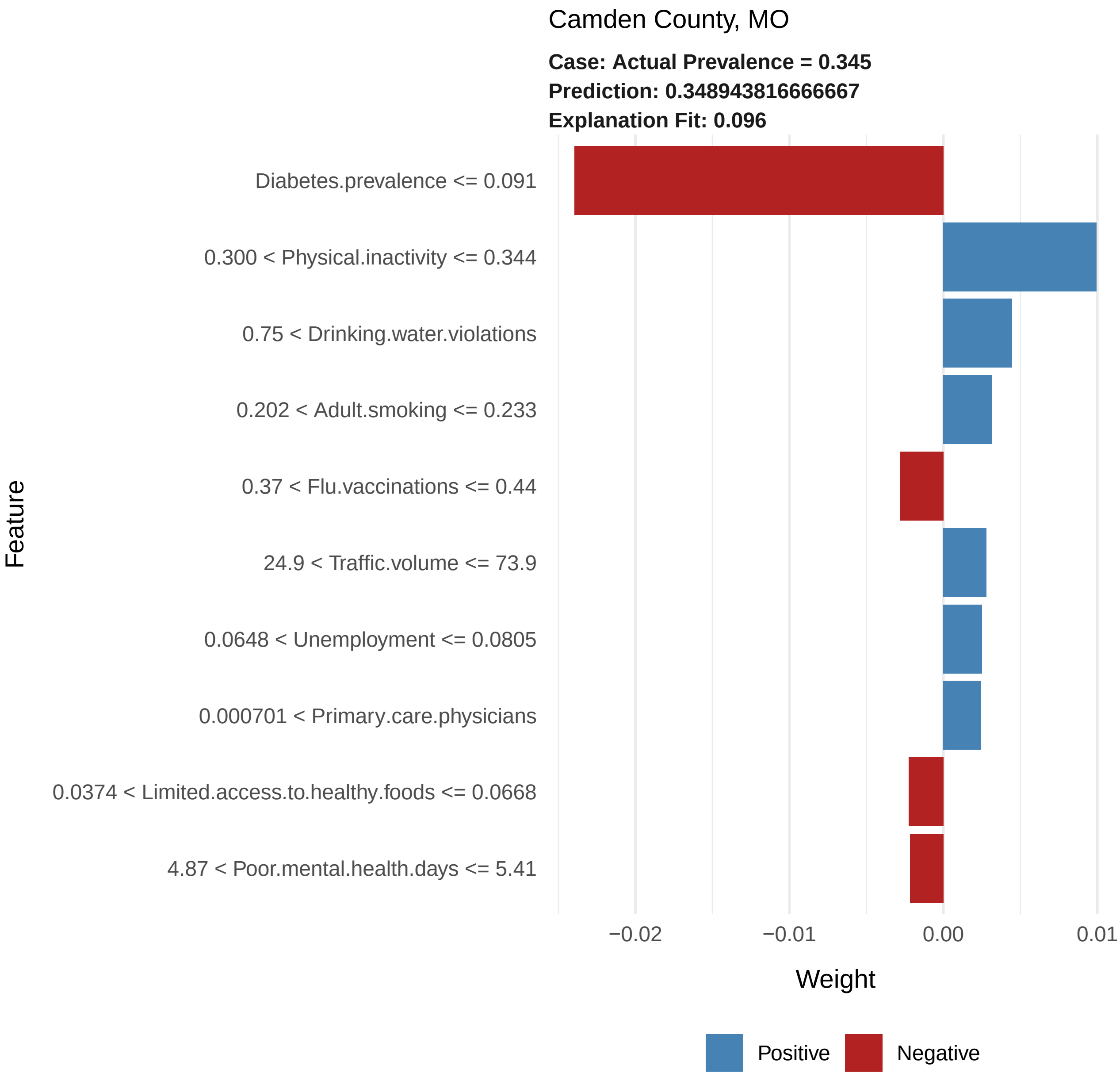

Camden County, MO
Case: Actual Prevalence = 0.345
Prediction: 0.348943816666667
Explanation Fit: 0.096
Diabetes.prevalence <= 0.091
0.300 < Physical.inactivity <= 0.344
0.75 < Drinking.water.violations
0.202 < Adult.smoking <= 0.233
0.37 < Flu.vaccinations <= 0.44
24.9 < Traffic.volume <= 73.9
0.0648 < Unemployment <= 0.0805
0.000701 < Primary.care.physicians
0.0374 < Limited.access.to.healthy.foods <= 0.0668
4.87 < Poor.mental.health.days <= 5.41
Feature
−0.02
−0.01
0.00
0.01
Weight
Positive
Negative

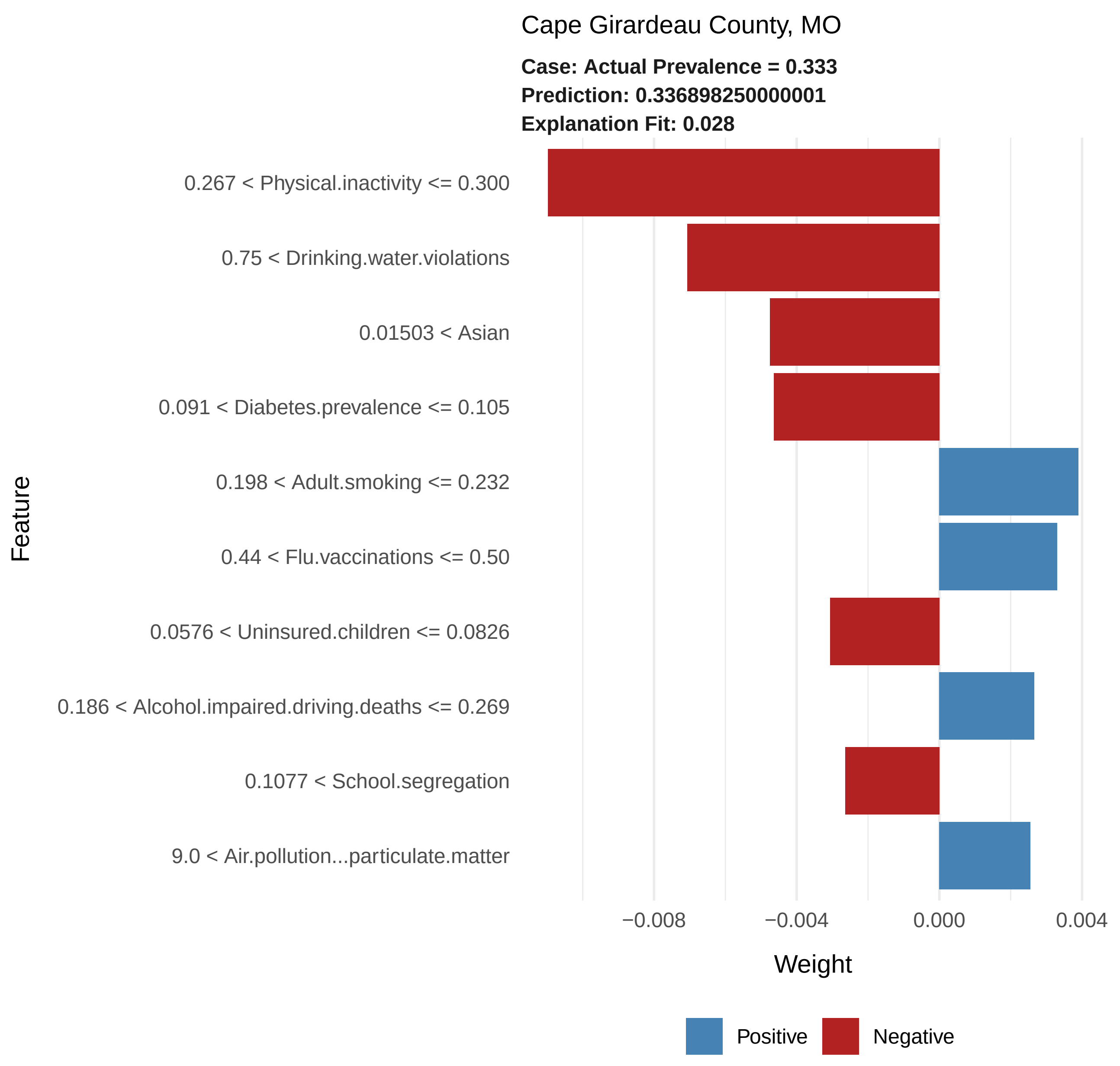
Cape Girardeau County, MO
Case: Actual Prevalence = 0.333
Prediction: 0.336898250000001
Explanation Fit: 0.028
0.267 < Physical.inactivity <= 0.300
0.75 < Drinking.water.violations
0.01503 < Asian
0.091 < Diabetes.prevalence <= 0.105
0.198 < Adult.smoking <= 0.232
0.44 < Flu.vaccinations <= 0.50
0.0576 < Uninsured.children <= 0.0826
0.186 < Alcohol.impaired.driving.deaths <= 0.269
0.1077 < School.segregation
9.0 < Air.pollution...particulate.matter
Feature
−0.008
−0.004
0.000
0.004
Weight
Positive
Negative

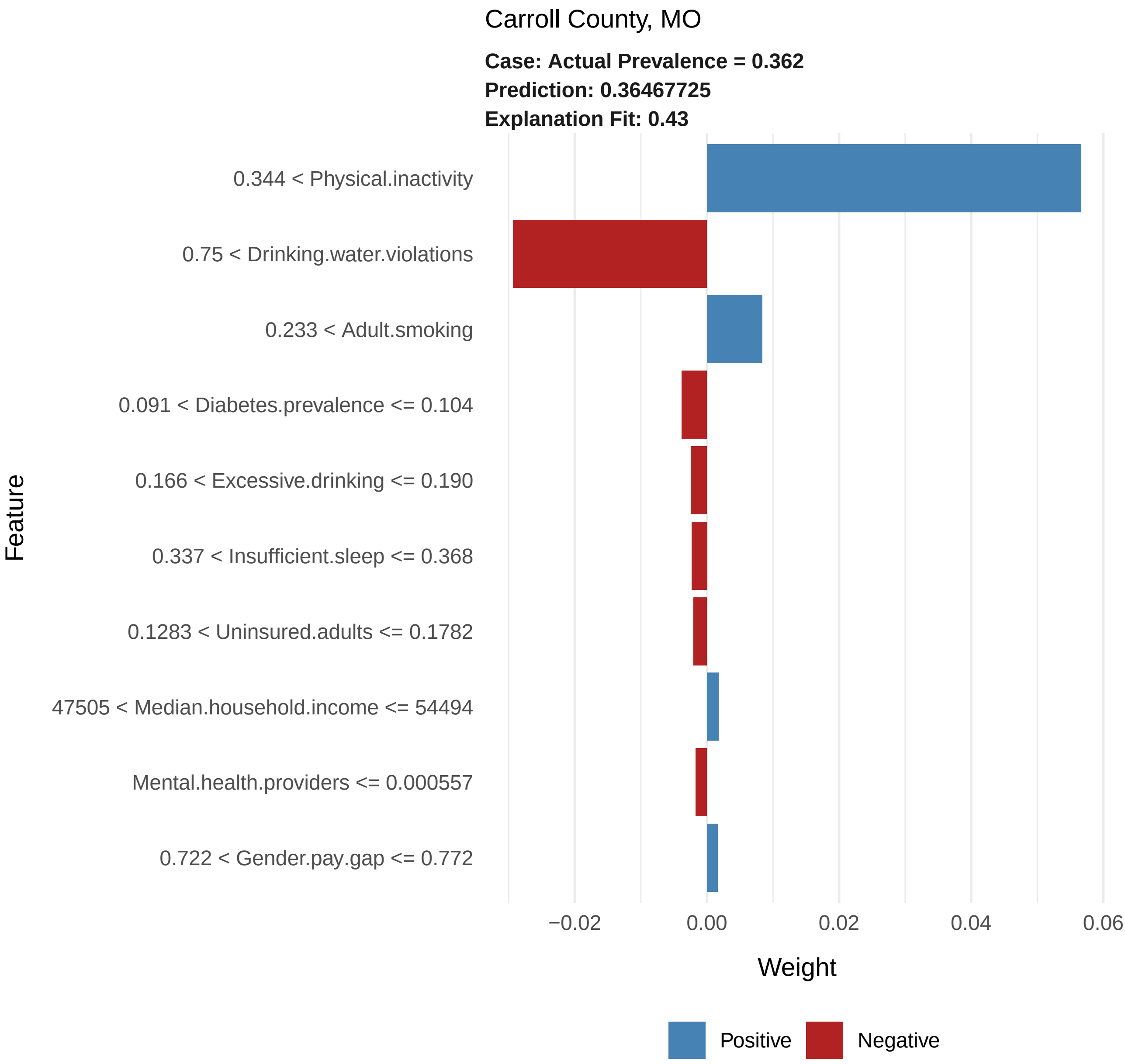

Carroll County, MO
Case: Actual Prevalence = 0.362
Prediction: 0.36467725
Explanation Fit: 0.43
0.344 < Physical.inactivity
0.75 < Drinking.water.violations
0.233 < Adult.smoking
0.091 < Diabetes.prevalence <= 0.104
0.166 < Excessive.drinking <= 0.190
0.337 < Insufficient.sleep <= 0.368
0.1283 < Uninsured.adults <= 0.1782
47505 < Median.household.income <= 54494
Mental.health.providers <= 0.000557
0.722 < Gender.pay.gap <= 0.772
Feature
−0.02
0.00
0.02
0.04
0.06
Weight
Positive
Negative

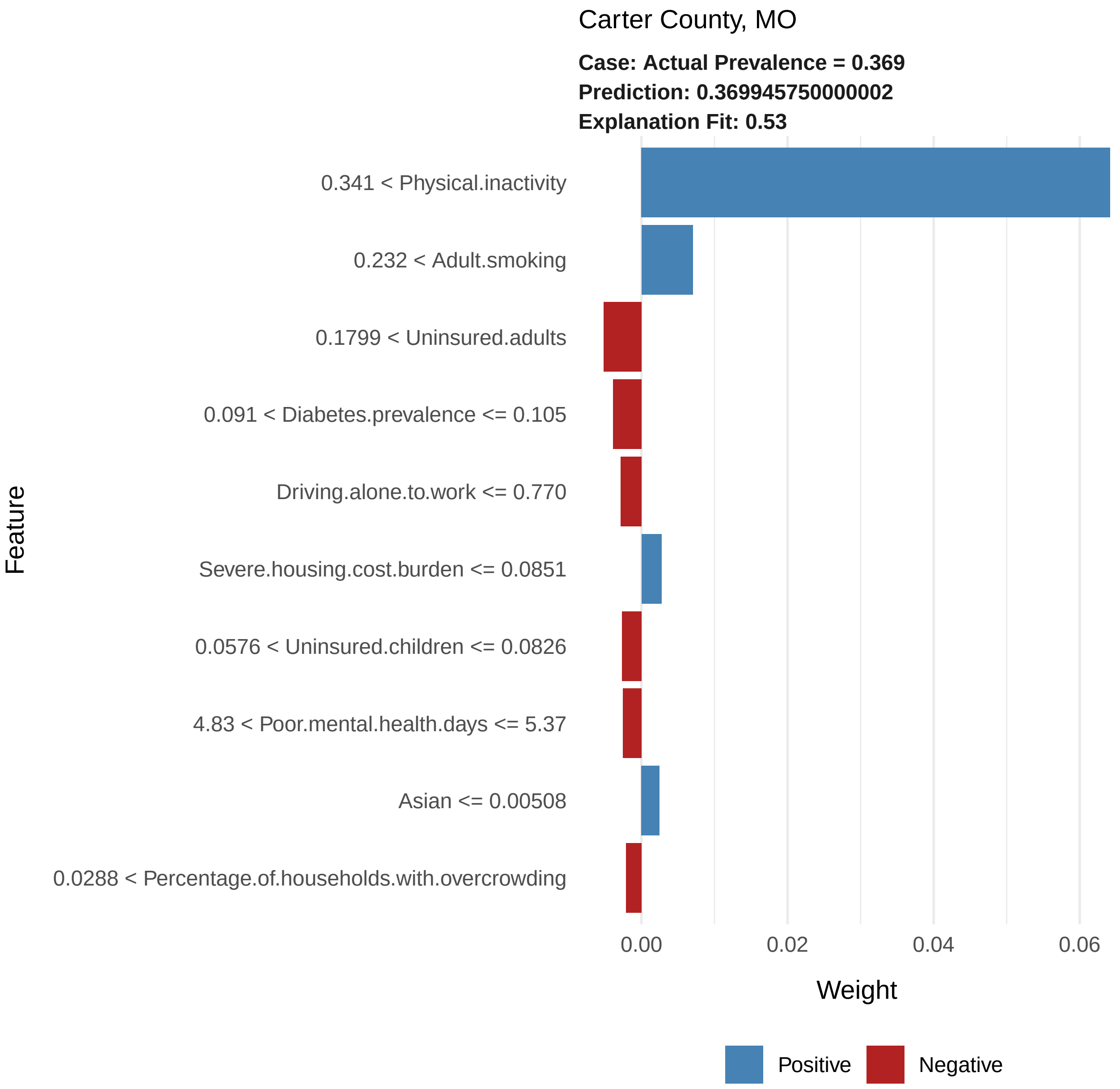

Carter County, MO
Case: Actual Prevalence = 0.369
Prediction: 0.369945750000002
Explanation Fit: 0.53
0.341 < Physical.inactivity
0.232 < Adult.smoking
0.1799 < Uninsured.adults
0.091 < Diabetes.prevalence <= 0.105
Driving.alone.to.work <= 0.770
Severe.housing.cost.burden <= 0.0851
0.0576 < Uninsured.children <= 0.0826
4.83 < Poor.mental.health.days <= 5.37
Asian <= 0.00508
0.0288 < Percentage.of.households.with.overcrowding
Feature
0.00
0.02
0.04
0.06
Weight
Positive
Negative

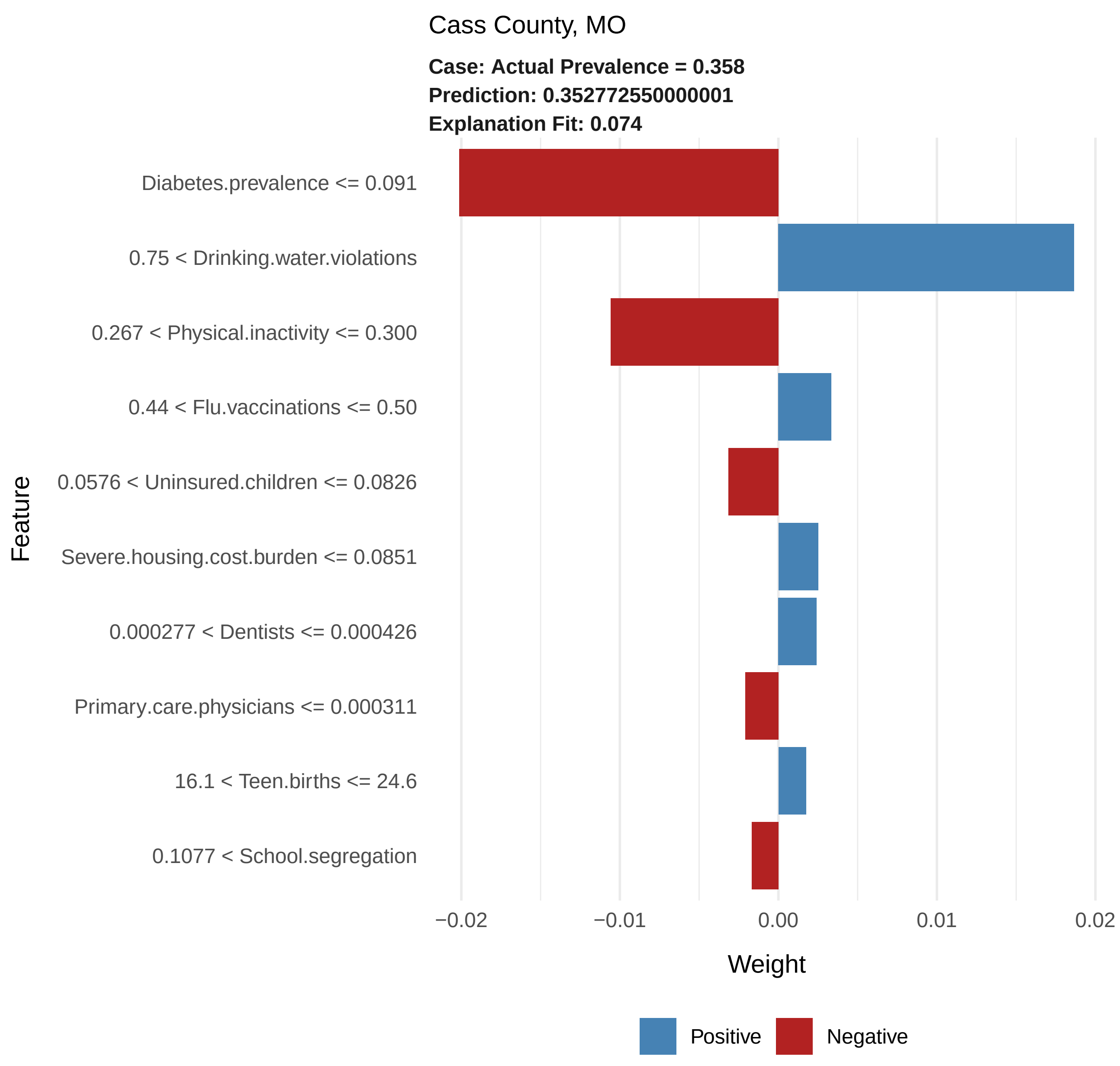
Cass County, MO
Case: Actual Prevalence = 0.358
Prediction: 0.352772550000001
Explanation Fit: 0.074
Diabetes.prevalence <= 0.091
0.75 < Drinking.water.violations
0.267 < Physical.inactivity <= 0.300
0.44 < Flu.vaccinations <= 0.50
0.0576 < Uninsured.children <= 0.0826
Severe.housing.cost.burden <= 0.0851
0.000277 < Dentists <= 0.000426
Primary.care.physicians <= 0.000311
16.1 < Teen.births <= 24.6
0.1077 < School.segregation
Feature
−0.02
−0.01
0.00
0.01
0.02
Weight
Positive
Negative

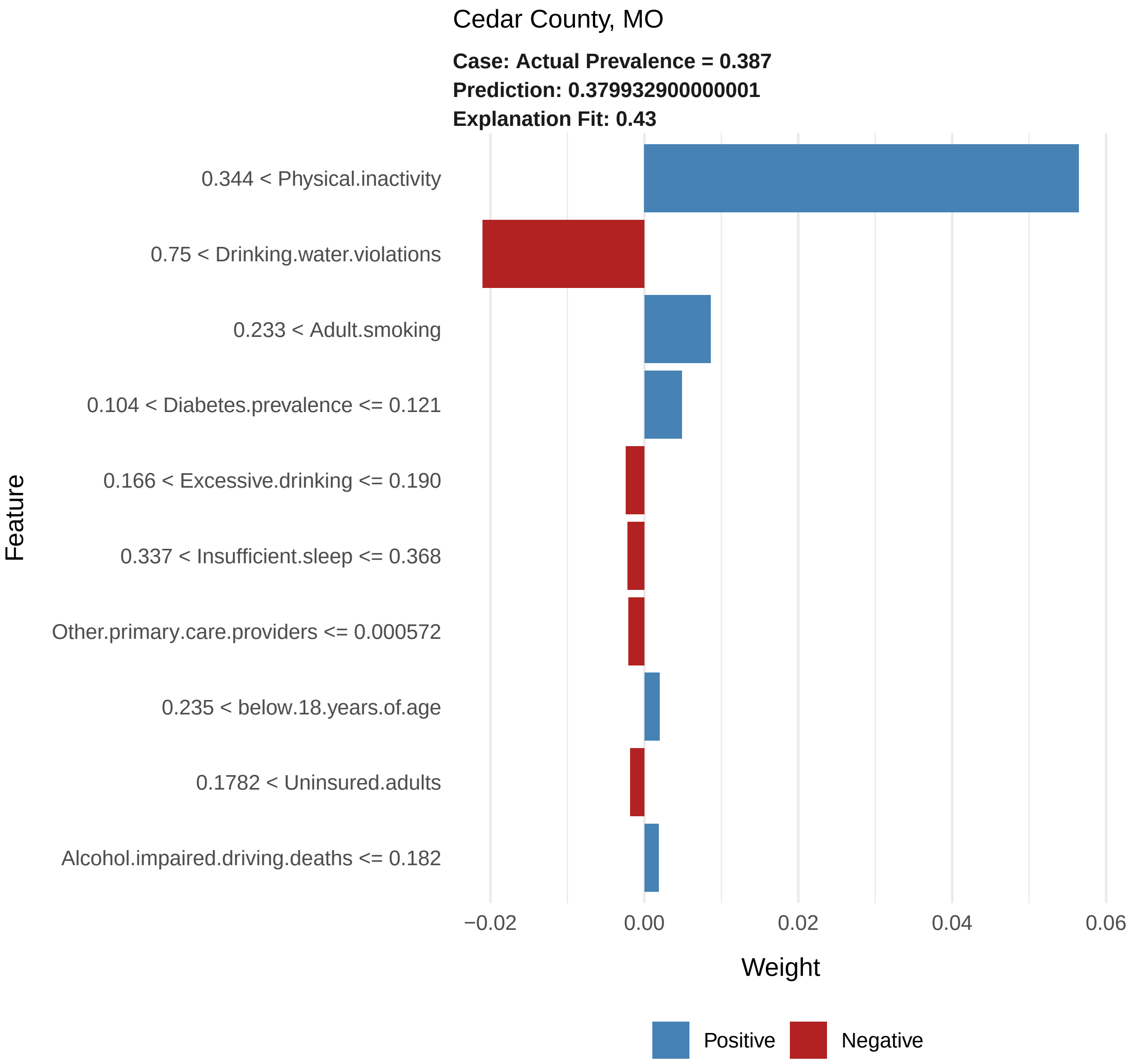
Cedar County, MO
Case: Actual Prevalence = 0.387
Prediction: 0.379932900000001
Explanation Fit: 0.43
0.344 < Physical.inactivity
0.75 < Drinking.water.violations
0.233 < Adult.smoking
0.104 < Diabetes.prevalence <= 0.121
0.166 < Excessive.drinking <= 0.190
0.337 < Insufficient.sleep <= 0.368
Other.primary.care.providers <= 0.000572
0.235 < below.18.years.of.age
0.1782 < Uninsured.adults
Alcohol.impaired.driving.deaths <= 0.182
Feature
−0.02
0.00
0.02
0.04
0.06
Weight
Positive
Negative

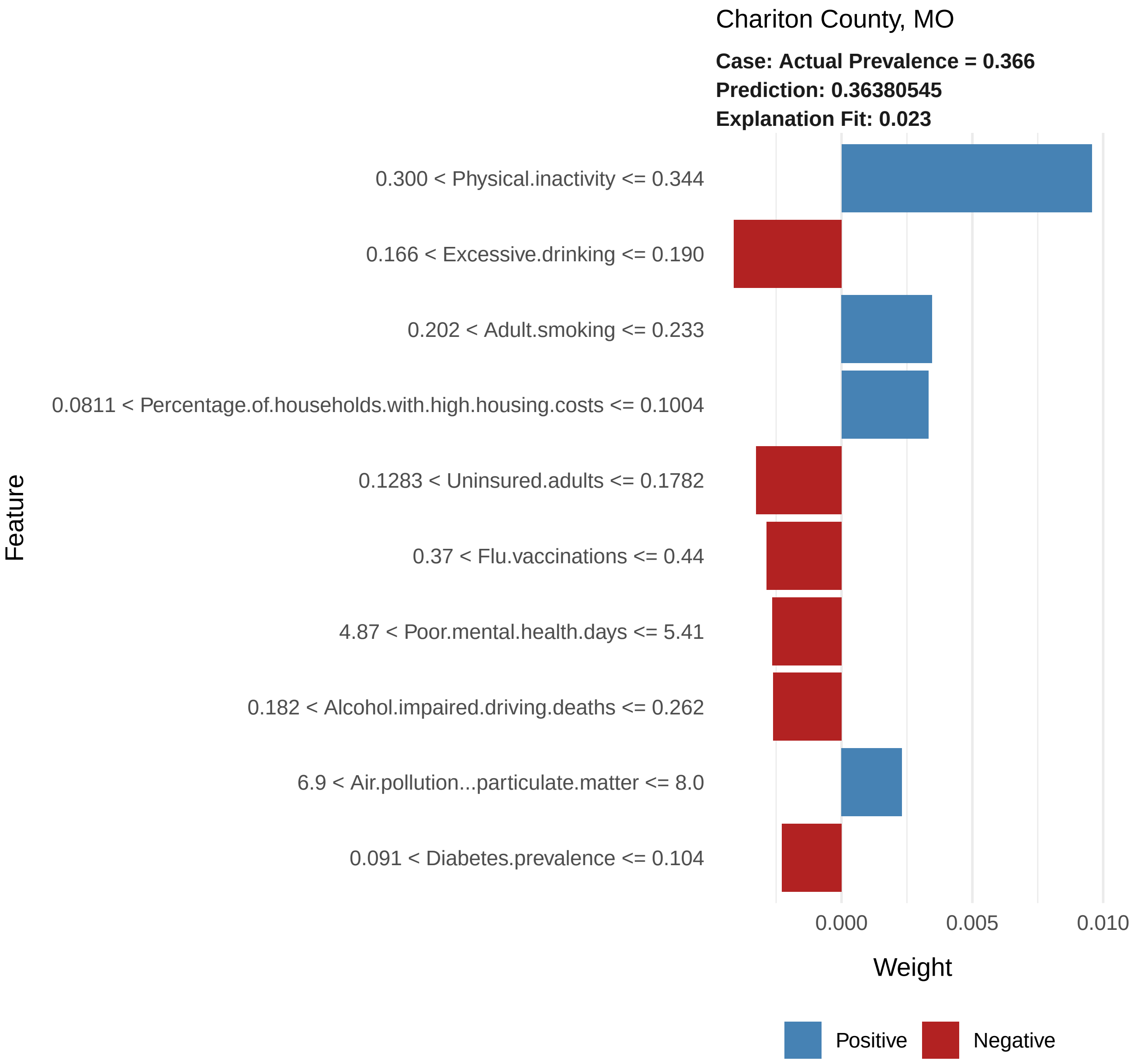
Chariton County, MO
Case: Actual Prevalence = 0.366
Prediction: 0.36380545
Explanation Fit: 0.023
0.300 < Physical.inactivity <= 0.344
0.166 < Excessive.drinking <= 0.190
0.202 < Adult.smoking <= 0.233
0.0811 < Percentage.of.households.with.high.housing.costs <= 0.1004
0.1283 < Uninsured.adults <= 0.1782
0.37 < Flu.vaccinations <= 0.44
4.87 < Poor.mental.health.days <= 5.41
0.182 < Alcohol.impaired.driving.deaths <= 0.262
6.9 < Air.pollution...particulate.matter <= 8.0
0.091 < Diabetes.prevalence <= 0.104
Feature
0.000
0.005
0.010
Weight
Positive
Negative

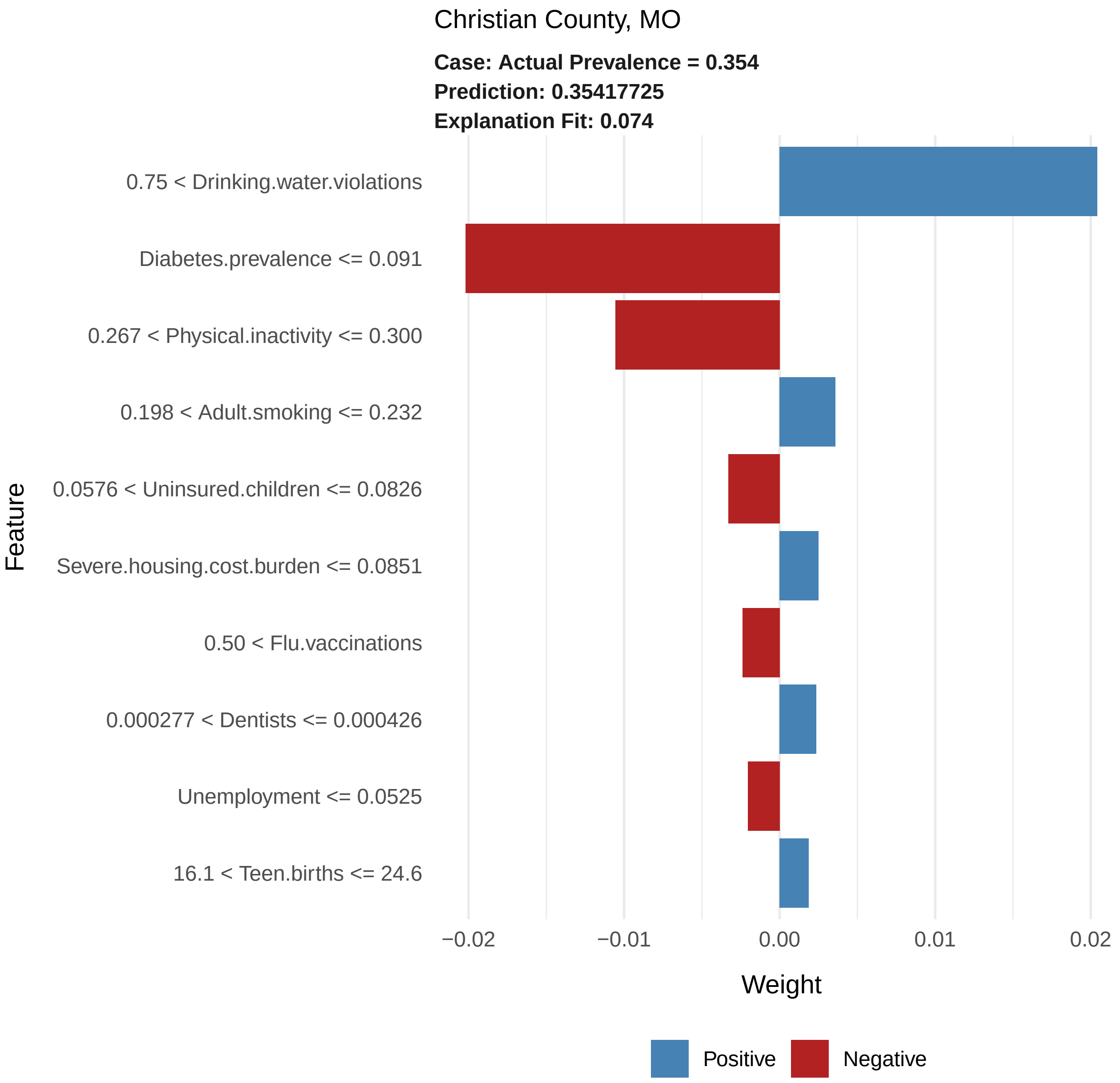
Christian County, MO
Case: Actual Prevalence = 0.354
Prediction: 0.35417725
Explanation Fit: 0.074
0.75 < Drinking.water.violations
Diabetes.prevalence <= 0.091
0.267 < Physical.inactivity <= 0.300
0.198 < Adult.smoking <= 0.232
0.0576 < Uninsured.children <= 0.0826
Severe.housing.cost.burden <= 0.0851
0.50 < Flu.vaccinations
0.000277 < Dentists <= 0.000426
Unemployment <= 0.0525
16.1 < Teen.births <= 24.6
Feature
−0.02
−0.01
0.00
0.01
0.02
Weight
Positive
Negative

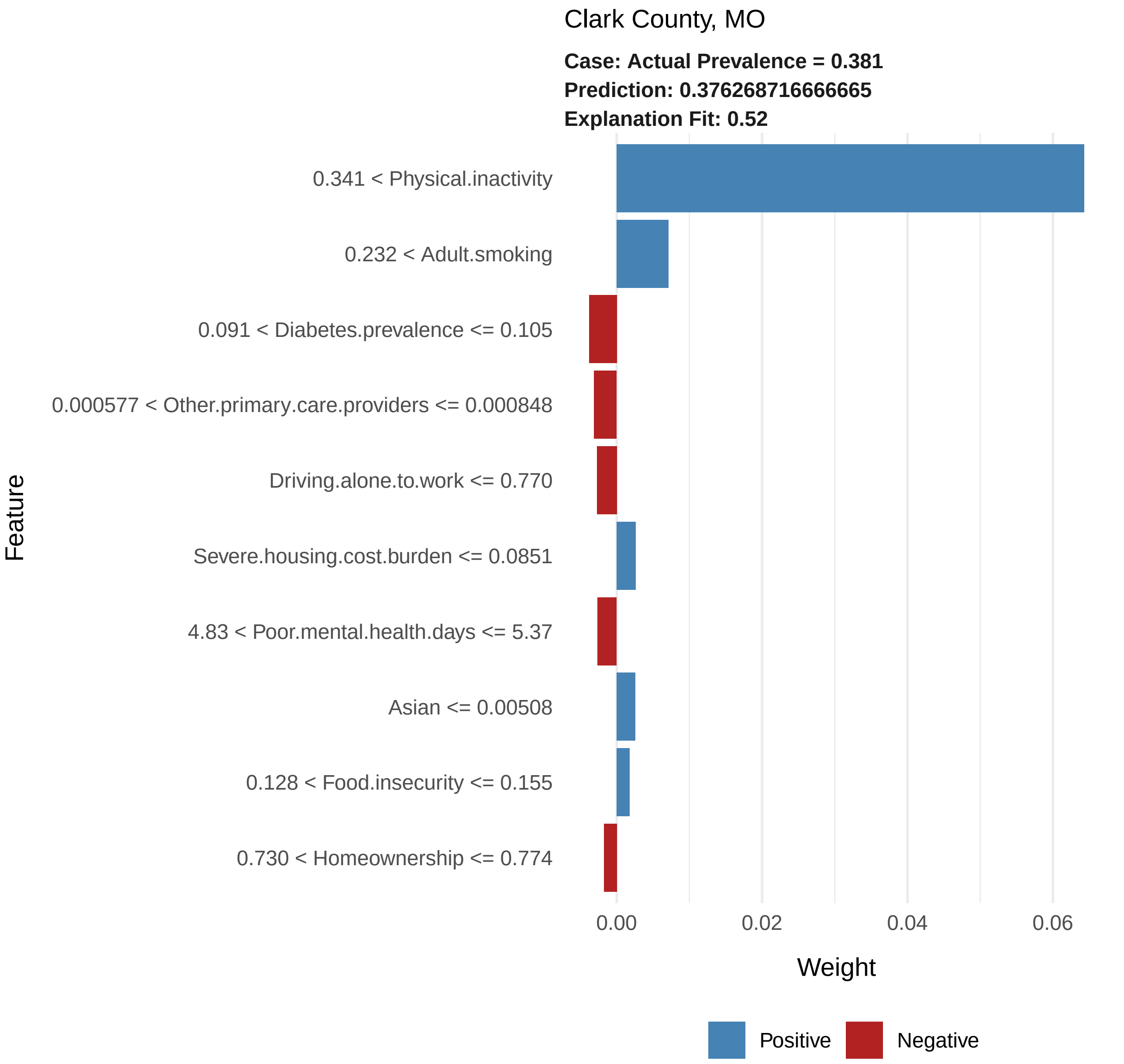

Clark County, MO
Case: Actual Prevalence = 0.381
Prediction: 0.376268716666665
Explanation Fit: 0.52
0.341 < Physical.inactivity
0.232 < Adult.smoking
0.091 < Diabetes.prevalence <= 0.105
0.000577 < Other.primary.care.providers <= 0.000848
Driving.alone.to.work <= 0.770
Severe.housing.cost.burden <= 0.0851
4.83 < Poor.mental.health.days <= 5.37
Asian <= 0.00508
0.128 < Food.insecurity <= 0.155
0.730 < Homeownership <= 0.774
Feature
0.00
0.02
0.04
0.06
Weight
Positive
Negative

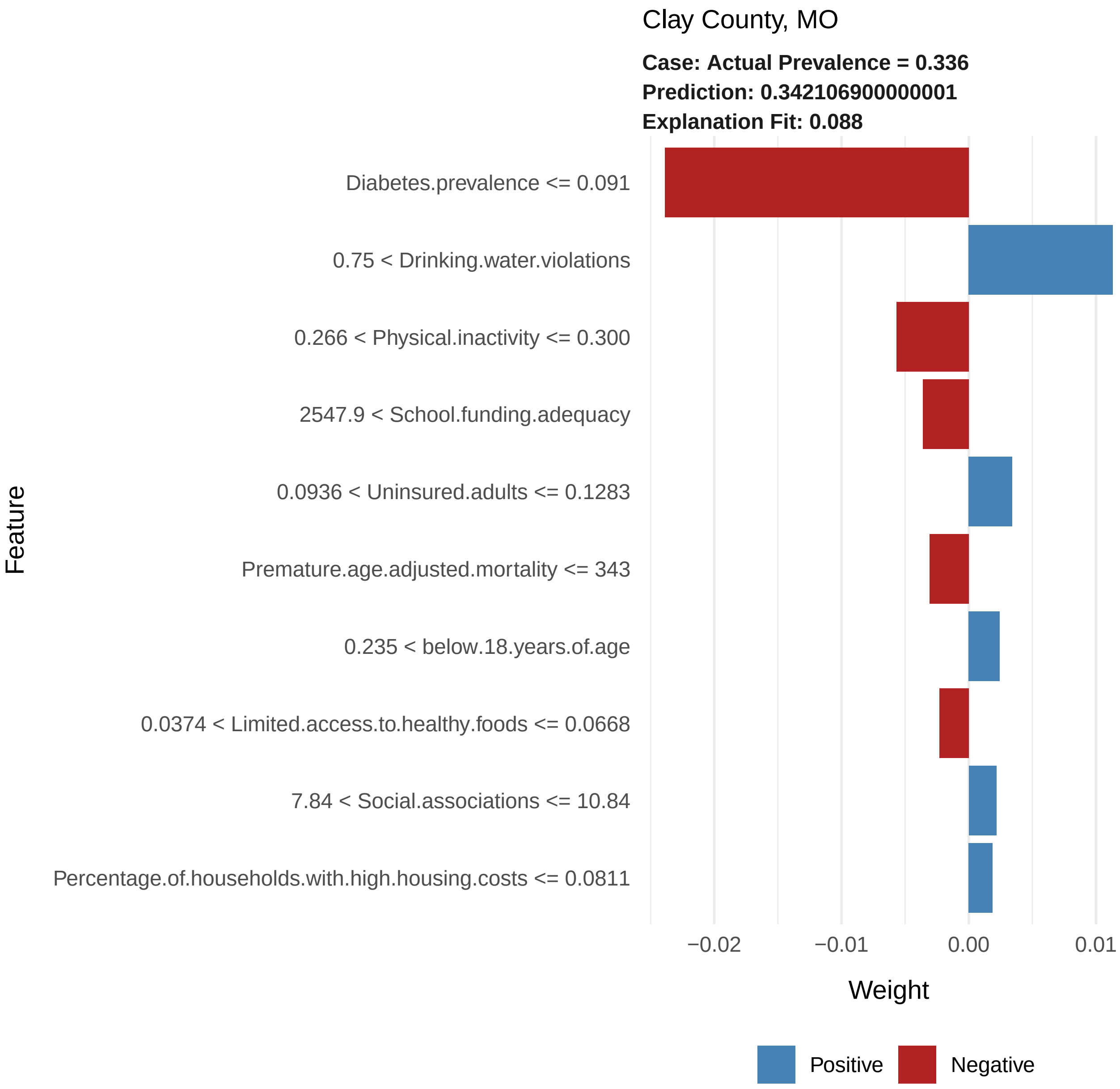
Clay County, MO
Case: Actual Prevalence = 0.336
Prediction: 0.342106900000001
Explanation Fit: 0.088
Diabetes.prevalence <= 0.091
0.75 < Drinking.water.violations
0.266 < Physical.inactivity <= 0.300
2547.9 < School.funding.adequacy
0.0936 < Uninsured.adults <= 0.1283
Premature.age.adjusted.mortality <= 343
0.235 < below.18.years.of.age
0.0374 < Limited.access.to.healthy.foods <= 0.0668
7.84 < Social.associations <= 10.84
Percentage.of.households.with.high.housing.costs <= 0.0811
Feature
−0.02
−0.01
0.00
0.01
Weight
Positive
Negative

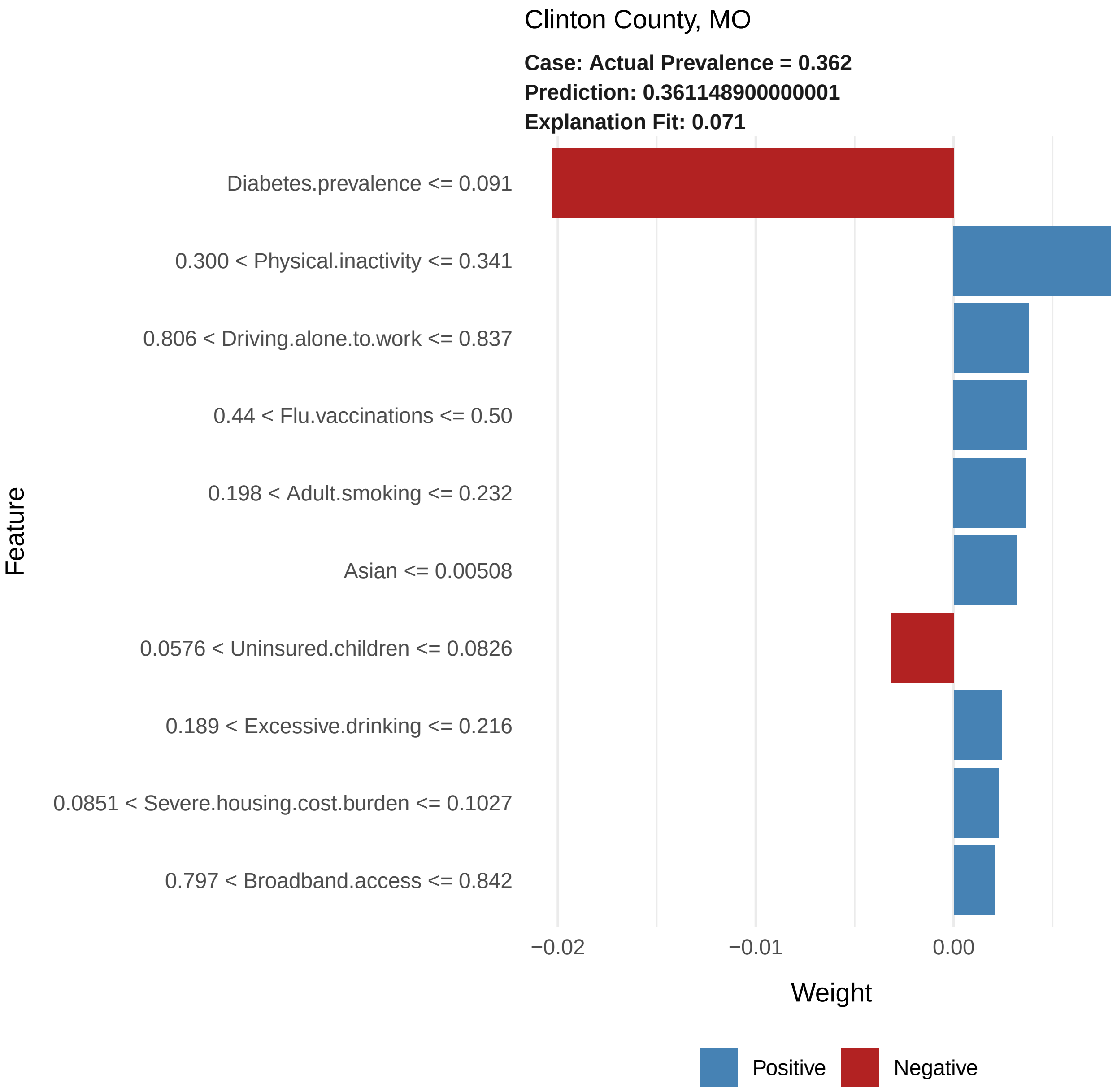

Clinton County, MO
Case: Actual Prevalence = 0.362
Prediction: 0.361148900000001
Explanation Fit: 0.071
Diabetes.prevalence <= 0.091
0.300 < Physical.inactivity <= 0.341
0.806 < Driving.alone.to.work <= 0.837
0.44 < Flu.vaccinations <= 0.50
0.198 < Adult.smoking <= 0.232
Asian <= 0.00508
0.0576 < Uninsured.children <= 0.0826
0.189 < Excessive.drinking <= 0.216
0.0851 < Severe.housing.cost.burden <= 0.1027
0.797 < Broadband.access <= 0.842
Feature
−0.02
−0.01
0.00
Weight
Positive
Negative

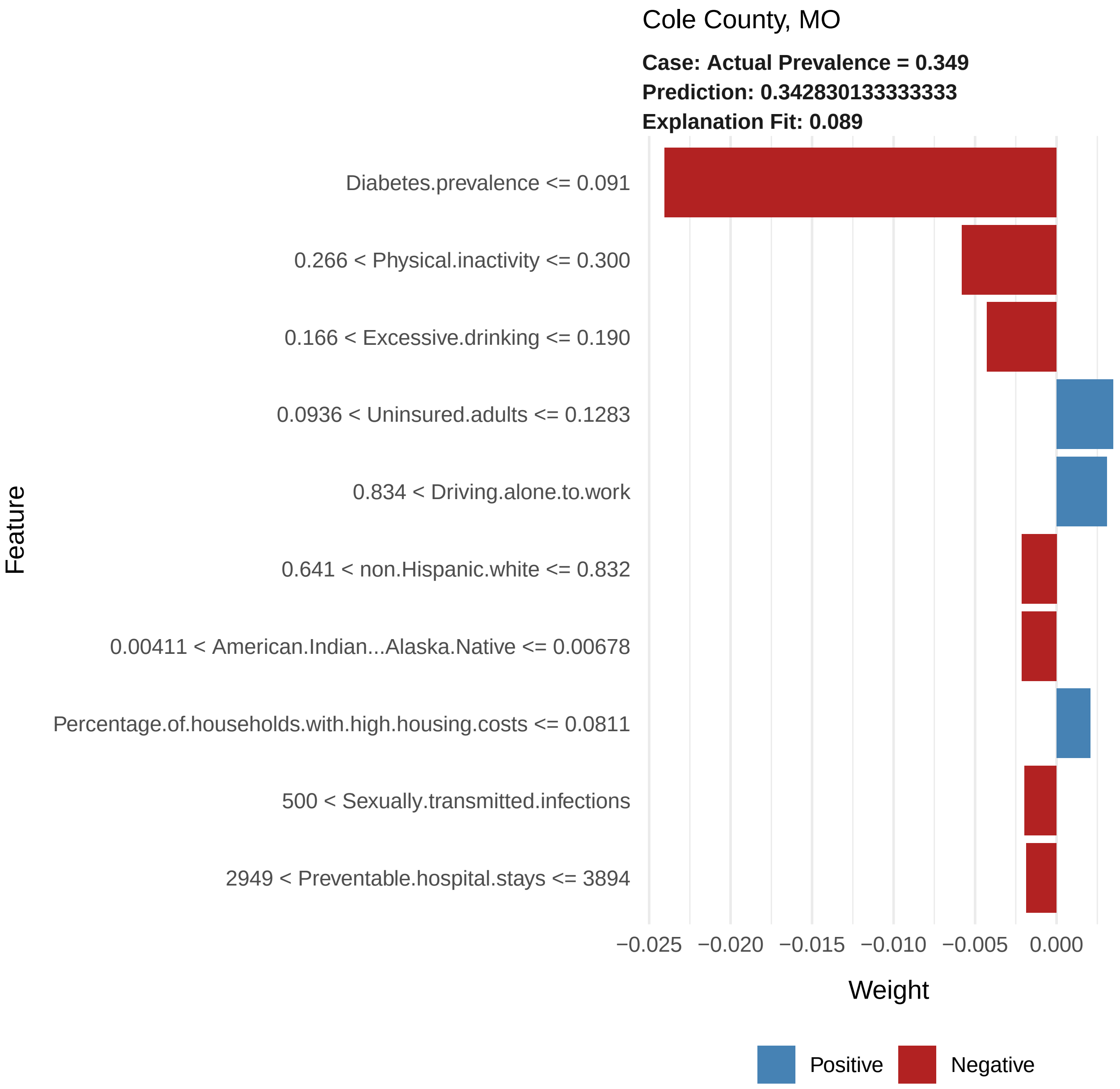

Cole County, MO
Case: Actual Prevalence = 0.349
Prediction: 0.342830133333333
Explanation Fit: 0.089
Diabetes.prevalence <= 0.091
0.266 < Physical.inactivity <= 0.300
0.166 < Excessive.drinking <= 0.190
0.0936 < Uninsured.adults <= 0.1283
0.834 < Driving.alone.to.work
0.641 < non.Hispanic.white <= 0.832
0.00411 < American.Indian...Alaska.Native <= 0.00678
Percentage.of.households.with.high.housing.costs <= 0.0811
500 < Sexually.transmitted.infections
2949 < Preventable.hospital.stays <= 3894
Feature
−0.025
−0.020
−0.015
−0.010
−0.005
0.000
Weight
Positive
Negative

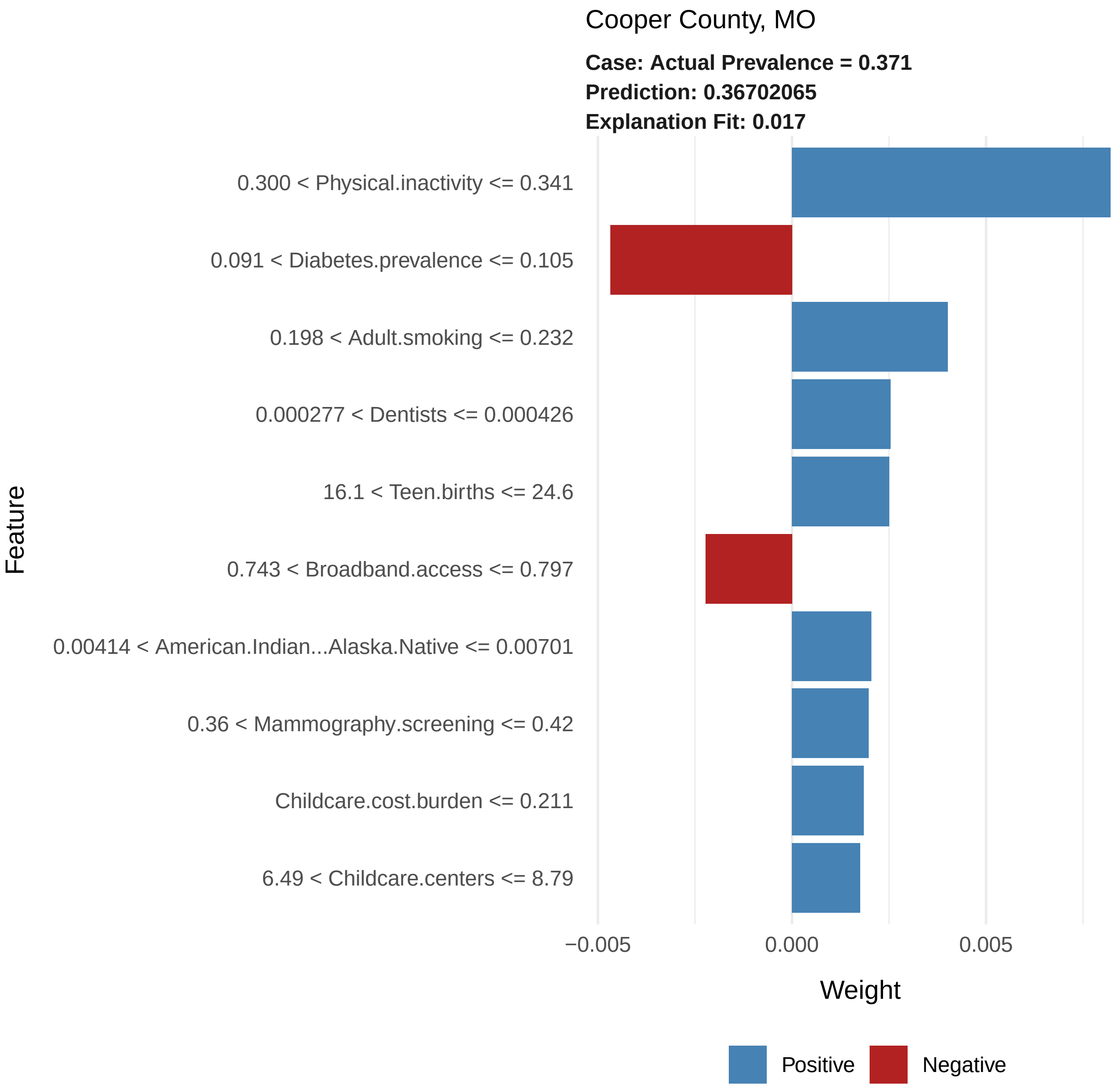

Cooper County, MO
Case: Actual Prevalence = 0.371
Prediction: 0.36702065
Explanation Fit: 0.017
0.300 < Physical.inactivity <= 0.341
0.091 < Diabetes.prevalence <= 0.105
0.198 < Adult.smoking <= 0.232
0.000277 < Dentists <= 0.000426
16.1 < Teen.births <= 24.6
0.743 < Broadband.access <= 0.797
0.00414 < American.Indian...Alaska.Native <= 0.00701
0.36 < Mammography.screening <= 0.42
Childcare.cost.burden <= 0.211
6.49 < Childcare.centers <= 8.79
Feature
−0.005
0.000
0.005
Weight
Positive
Negative

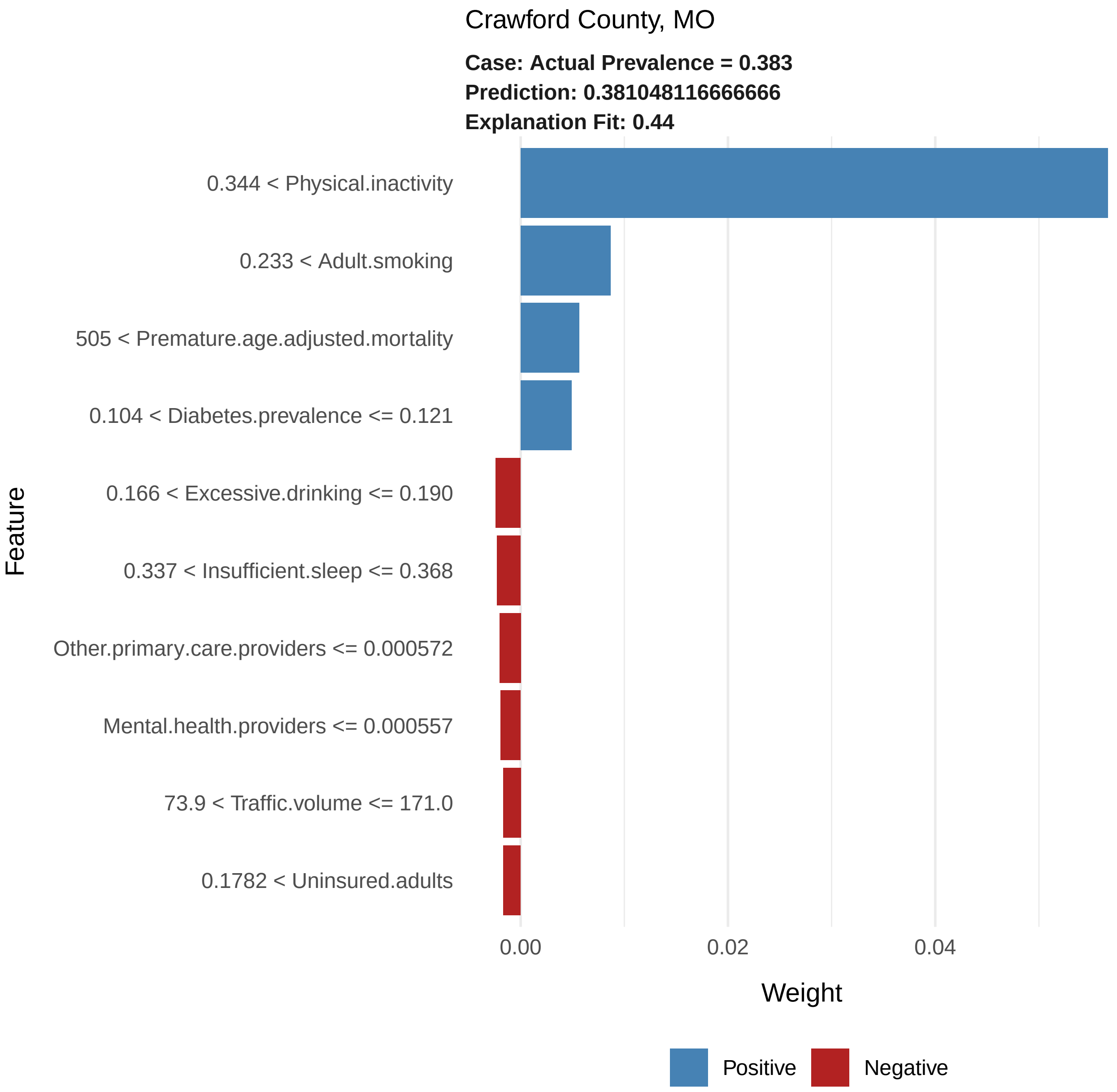
Crawford County, MO
Case: Actual Prevalence = 0.383
Prediction: 0.381048116666666
Explanation Fit: 0.44
0.344 < Physical.inactivity
0.233 < Adult.smoking
505 < Premature.age.adjusted.mortality
0.104 < Diabetes.prevalence <= 0.121
0.166 < Excessive.drinking <= 0.190
0.337 < Insufficient.sleep <= 0.368
Other.primary.care.providers <= 0.000572
Mental.health.providers <= 0.000557
73.9 < Traffic.volume <= 171.0
0.1782 < Uninsured.adults
Feature
0.00
0.02
0.04
Weight
Positive
Negative

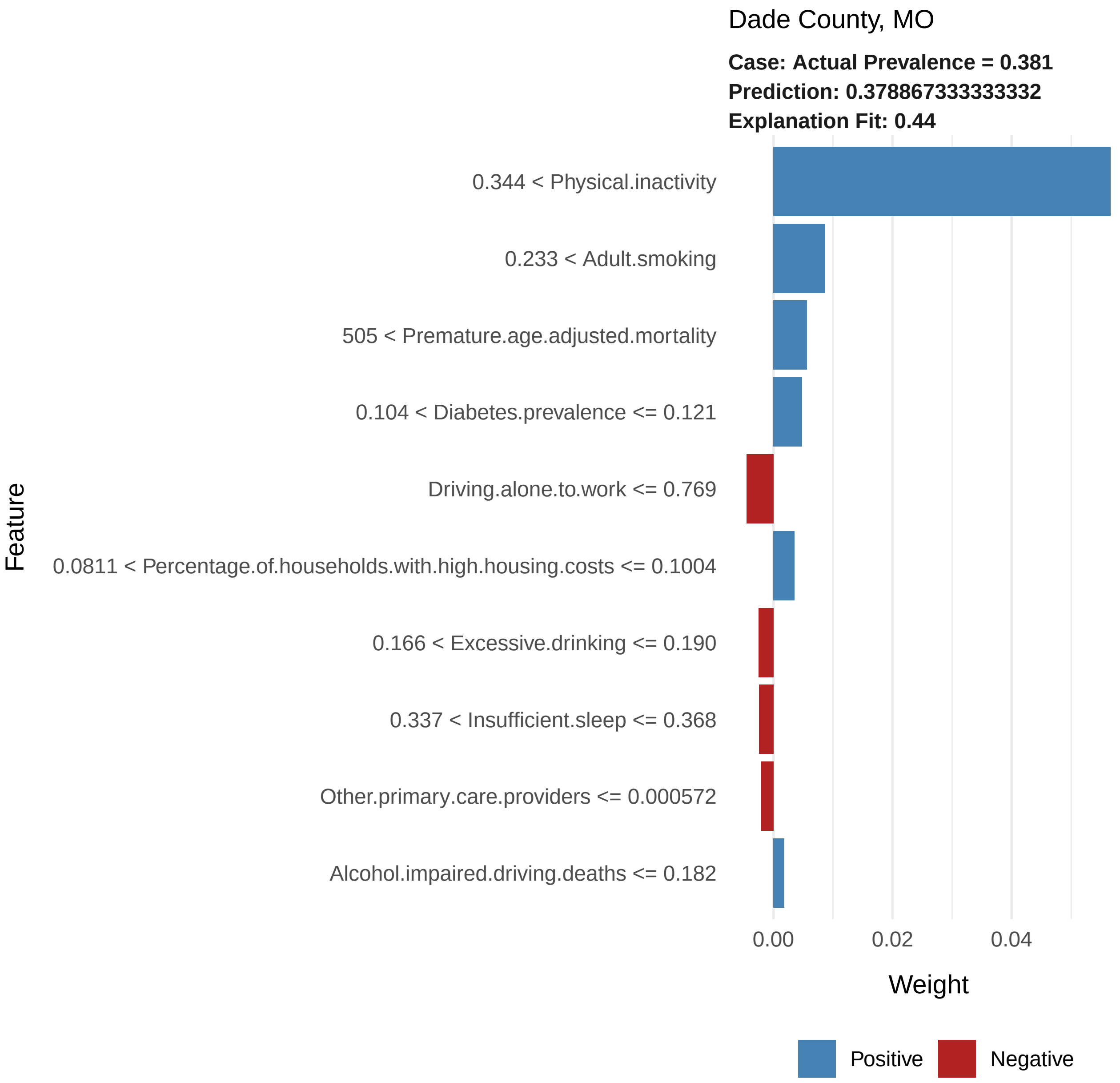
Dade County, MO
Case: Actual Prevalence = 0.381
Prediction: 0.378867333333332
Explanation Fit: 0.44
0.344 < Physical.inactivity
0.233 < Adult.smoking
505 < Premature.age.adjusted.mortality
0.104 < Diabetes.prevalence <= 0.121
Driving.alone.to.work <= 0.769
0.0811 < Percentage.of.households.with.high.housing.costs <= 0.1004
0.166 < Excessive.drinking <= 0.190
0.337 < Insufficient.sleep <= 0.368
Other.primary.care.providers <= 0.000572
Alcohol.impaired.driving.deaths <= 0.182
Feature
0.00
0.02
0.04
Weight
Positive
Negative

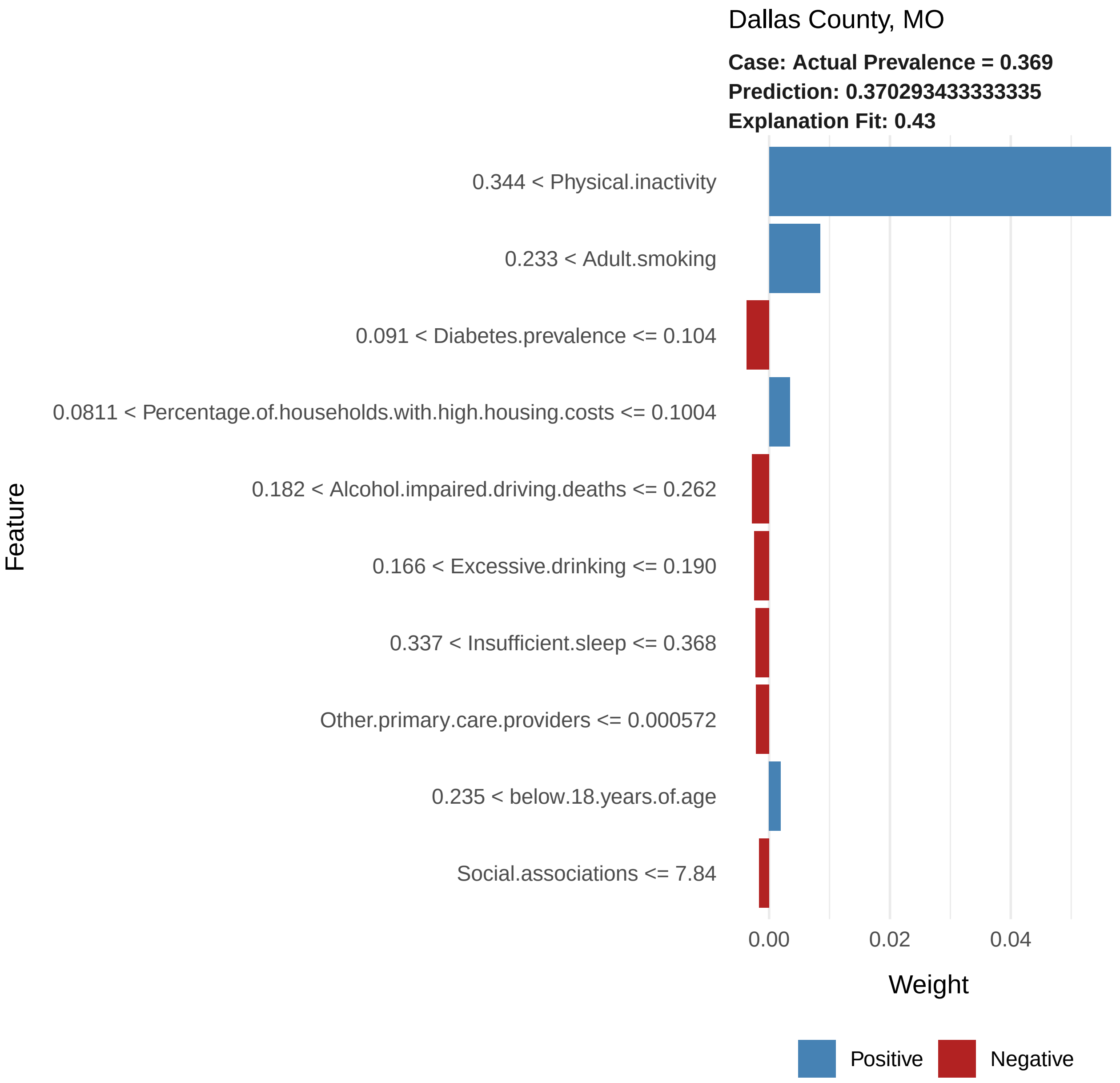
Dallas County, MO
Case: Actual Prevalence = 0.369
Prediction: 0.370293433333335
Explanation Fit: 0.43
0.344 < Physical.inactivity
0.233 < Adult.smoking
0.091 < Diabetes.prevalence <= 0.104
0.0811 < Percentage.of.households.with.high.housing.costs <= 0.1004
0.182 < Alcohol.impaired.driving.deaths <= 0.262
0.166 < Excessive.drinking <= 0.190
0.337 < Insufficient.sleep <= 0.368
Other.primary.care.providers <= 0.000572
0.235 < below.18.years.of.age
Social.associations <= 7.84
Feature
0.00
0.02
0.04
Weight
Positive
Negative

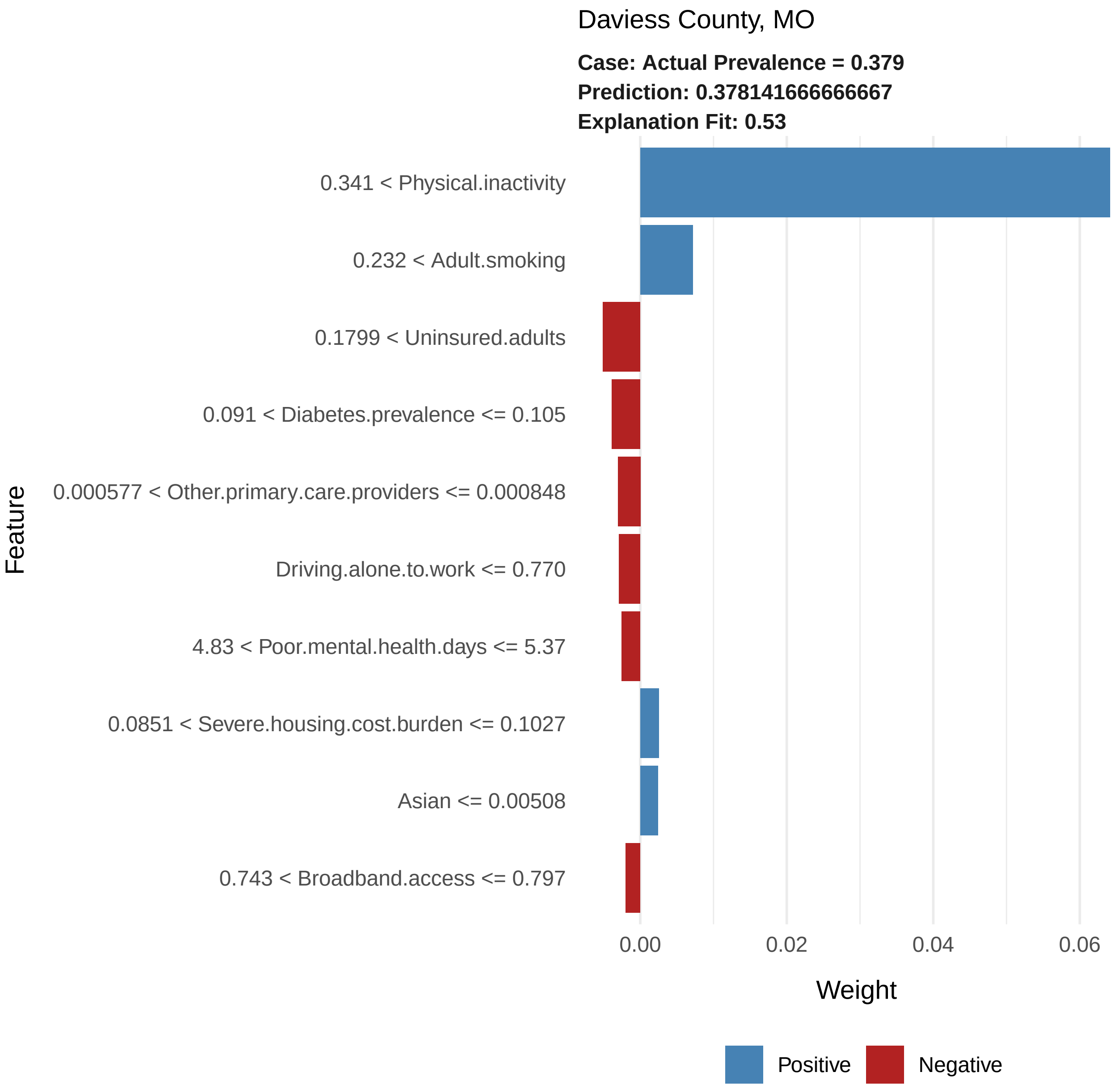
Daviess County, MO
Case: Actual Prevalence = 0.379
Prediction: 0.378141666666667
Explanation Fit: 0.53
0.341 < Physical.inactivity
0.232 < Adult.smoking
0.1799 < Uninsured.adults
0.091 < Diabetes.prevalence <= 0.105
0.000577 < Other.primary.care.providers <= 0.000848
Driving.alone.to.work <= 0.770
4.83 < Poor.mental.health.days <= 5.37
0.0851 < Severe.housing.cost.burden <= 0.1027
Asian <= 0.00508
0.743 < Broadband.access <= 0.797
Feature
0.00
0.02
0.04
0.06
Weight
Positive
Negative

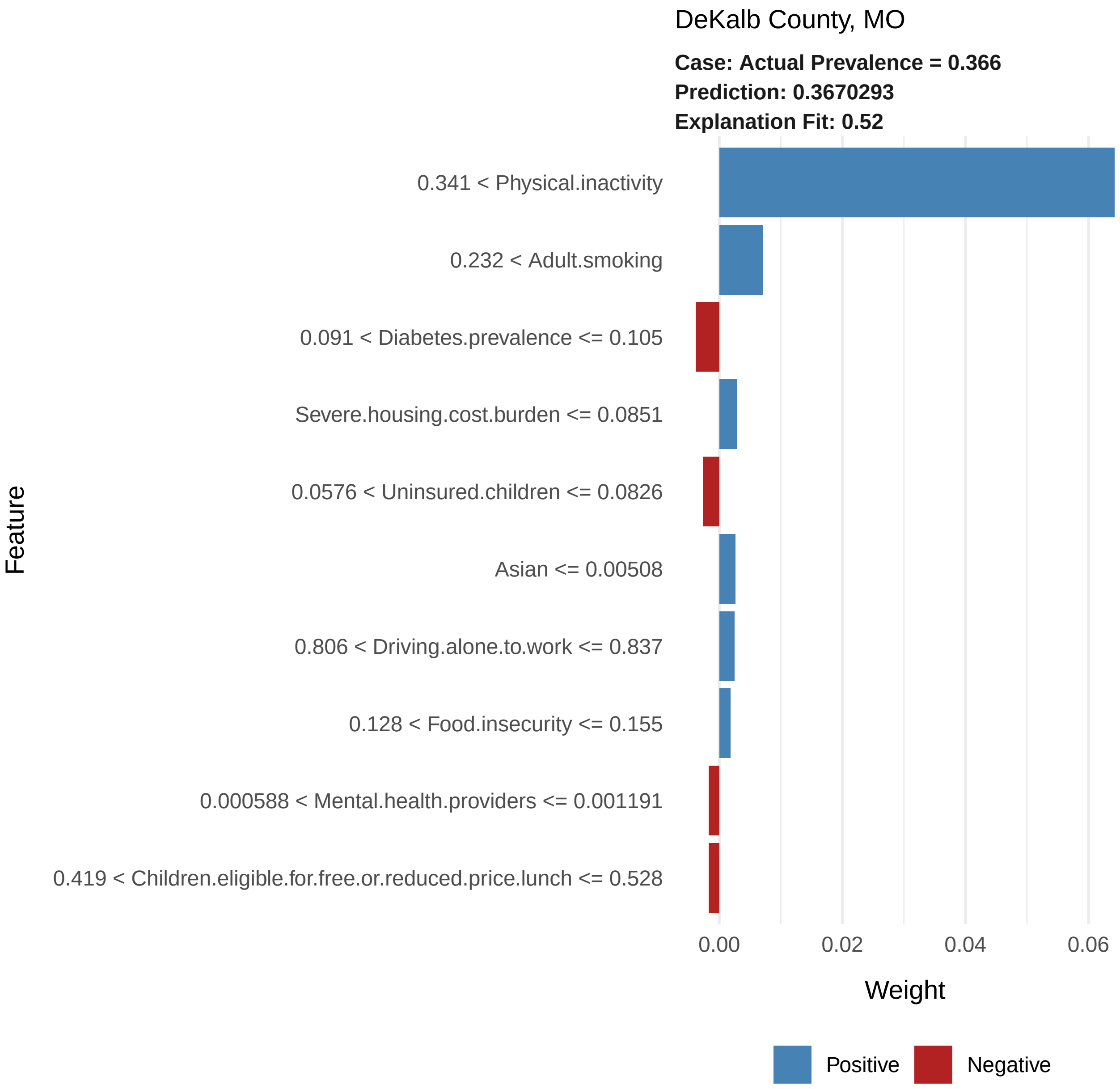
DeKalb County, MO
Case: Actual Prevalence = 0.366
Prediction: 0.3670293
Explanation Fit: 0.52
0.341 < Physical.inactivity
0.232 < Adult.smoking
0.091 < Diabetes.prevalence <= 0.105
Severe.housing.cost.burden <= 0.0851
0.0576 < Uninsured.children <= 0.0826
Asian <= 0.00508
0.806 < Driving.alone.to.work <= 0.837
0.128 < Food.insecurity <= 0.155
0.000588 < Mental.health.providers <= 0.001191
0.419 < Children.eligible.for.free.or.reduced.price.lunch <= 0.528
Feature
0.00
0.02
0.04
0.06
Weight
Positive
Negative

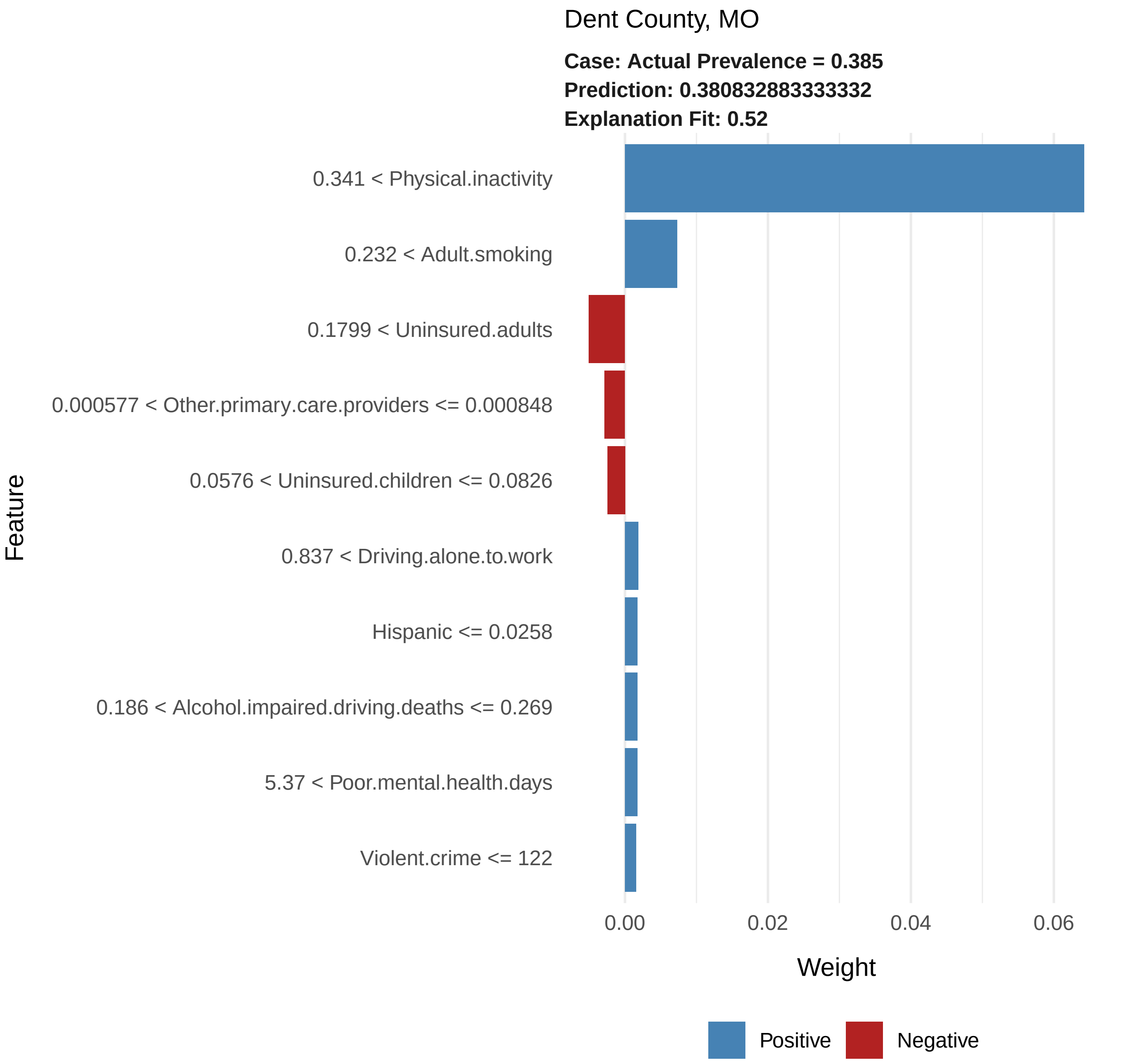

Dent County, MO
Case: Actual Prevalence = 0.385
Prediction: 0.380832883333332
Explanation Fit: 0.52
0.341 < Physical.inactivity
0.232 < Adult.smoking
0.1799 < Uninsured.adults
0.000577 < Other.primary.care.providers <= 0.000848
0.0576 < Uninsured.children <= 0.0826
0.837 < Driving.alone.to.work
Hispanic <= 0.0258
0.186 < Alcohol.impaired.driving.deaths <= 0.269
5.37 < Poor.mental.health.days
Violent.crime <= 122
Feature
0.00
0.02
0.04
0.06
Weight
Positive
Negative

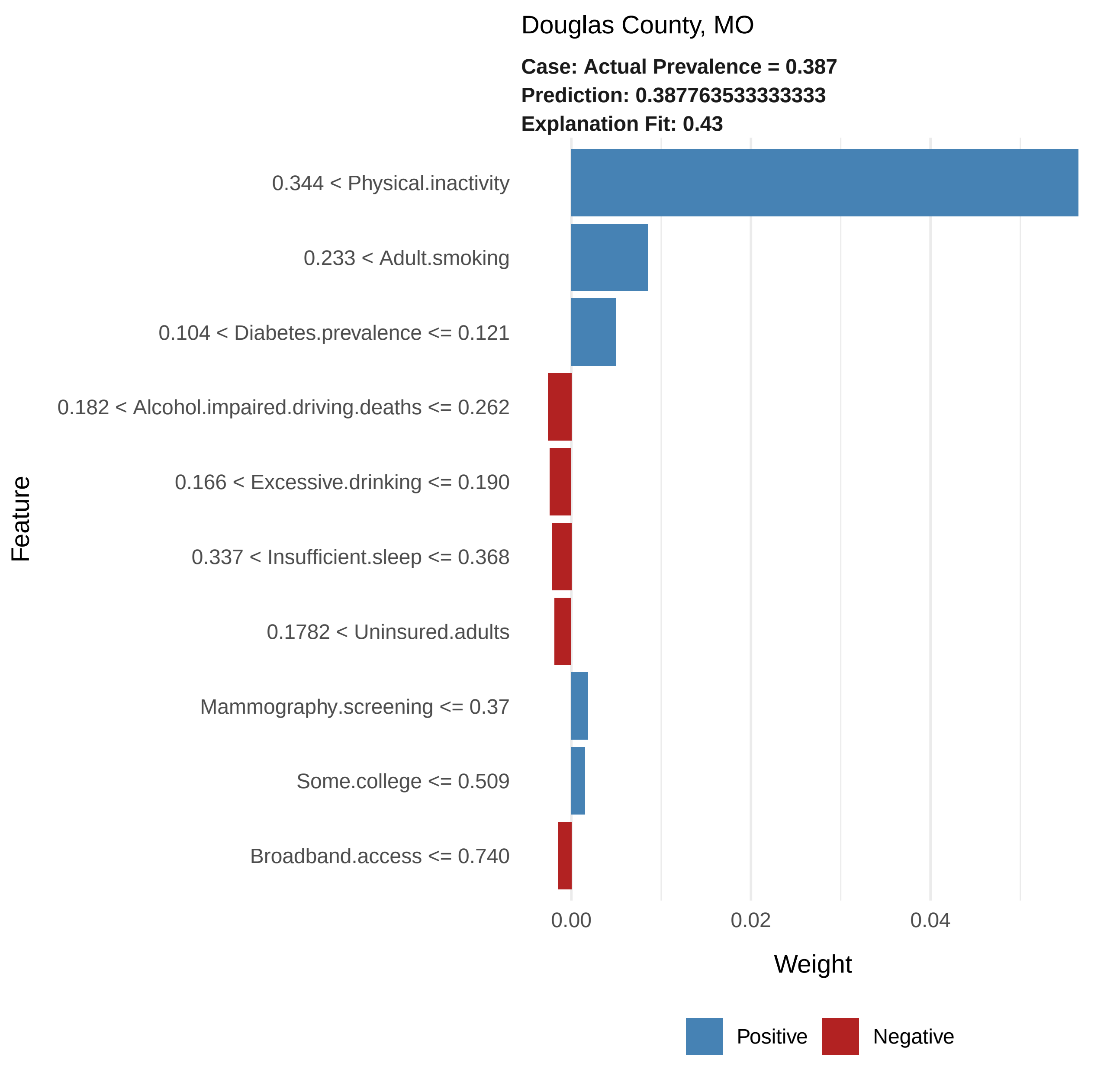
Douglas County, MO
Case: Actual Prevalence = 0.387
Prediction: 0.387763533333333
Explanation Fit: 0.43
0.344 < Physical.inactivity
0.233 < Adult.smoking
0.104 < Diabetes.prevalence <= 0.121
0.182 < Alcohol.impaired.driving.deaths <= 0.262
0.166 < Excessive.drinking <= 0.190
0.337 < Insufficient.sleep <= 0.368
0.1782 < Uninsured.adults
Mammography.screening <= 0.37
Some.college <= 0.509
Broadband.access <= 0.740
Feature
0.00
0.02
0.04
Weight
Positive
Negative

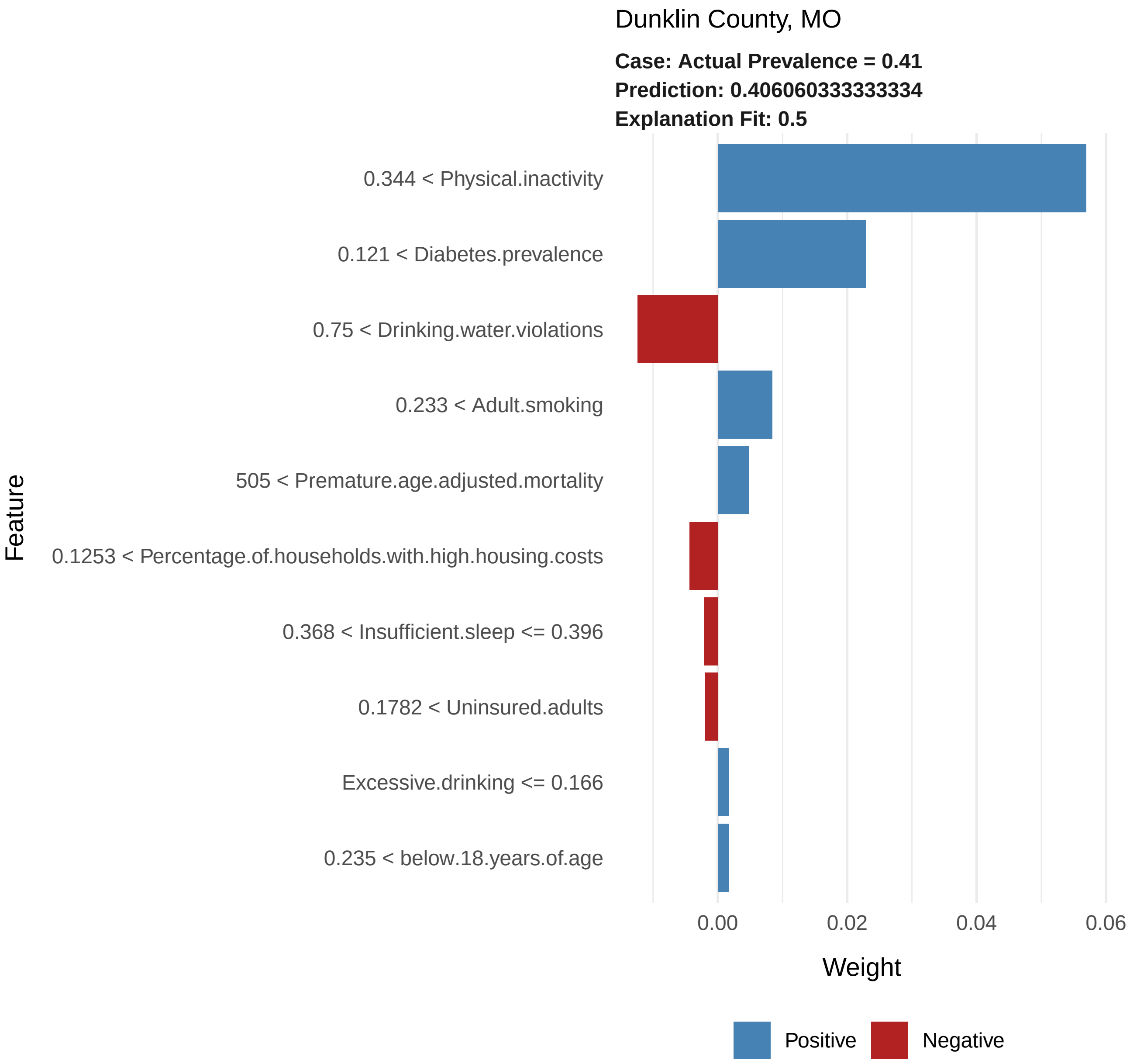
Dunklin County, MO
Case: Actual Prevalence = 0.41
Prediction: 0.406060333333334
Explanation Fit: 0.5
0.344 < Physical.inactivity
0.121 < Diabetes.prevalence
0.75 < Drinking.water.violations
0.233 < Adult.smoking
505 < Premature.age.adjusted.mortality
0.1253 < Percentage.of.households.with.high.housing.costs
0.368 < Insufficient.sleep <= 0.396
0.1782 < Uninsured.adults
Excessive.drinking <= 0.166
0.235 < below.18.years.of.age
Feature
0.00
0.02
0.04
0.06
Weight
Positive
Negative

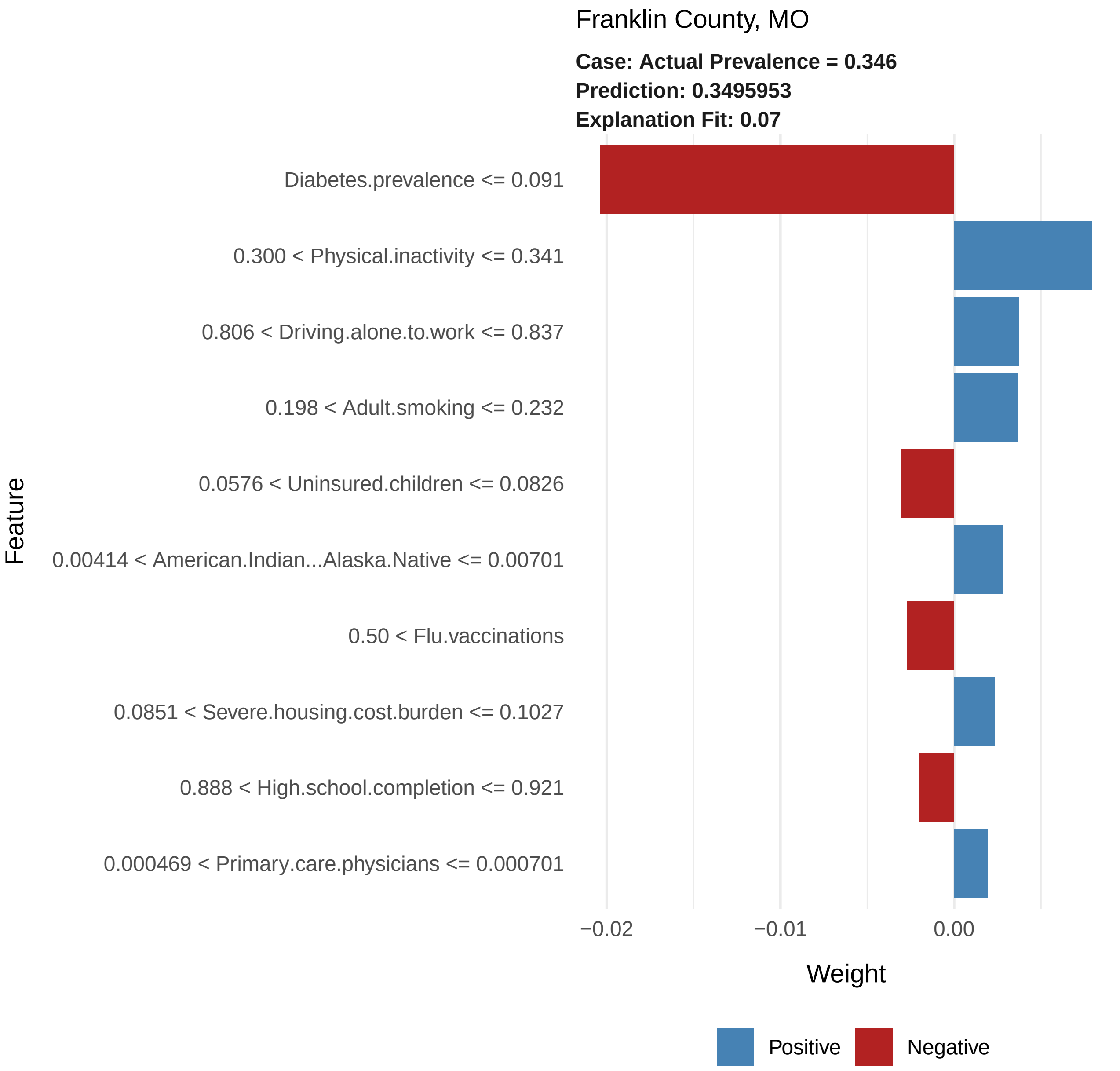
Franklin County, MO
Case: Actual Prevalence = 0.346
Prediction: 0.3495953
Explanation Fit: 0.07
Feature
Diabetes.prevalence <= 0.091
0.300 < Physical.inactivity <= 0.341
0.806 < Driving.alone.to.work <= 0.837
0.198 < Adult.smoking <= 0.232
0.0576 < Uninsured.children <= 0.0826
0.00414 < American.Indian...Alaska.Native <= 0.00701
0.50 < Flu.vaccinations
0.0851 < Severe.housing.cost.burden <= 0.1027
0.888 < High.school.completion <= 0.921
0.000469 < Primary.care.physicians <= 0.000701
−0.02
−0.01
0.00
Weight
Positive
Negative

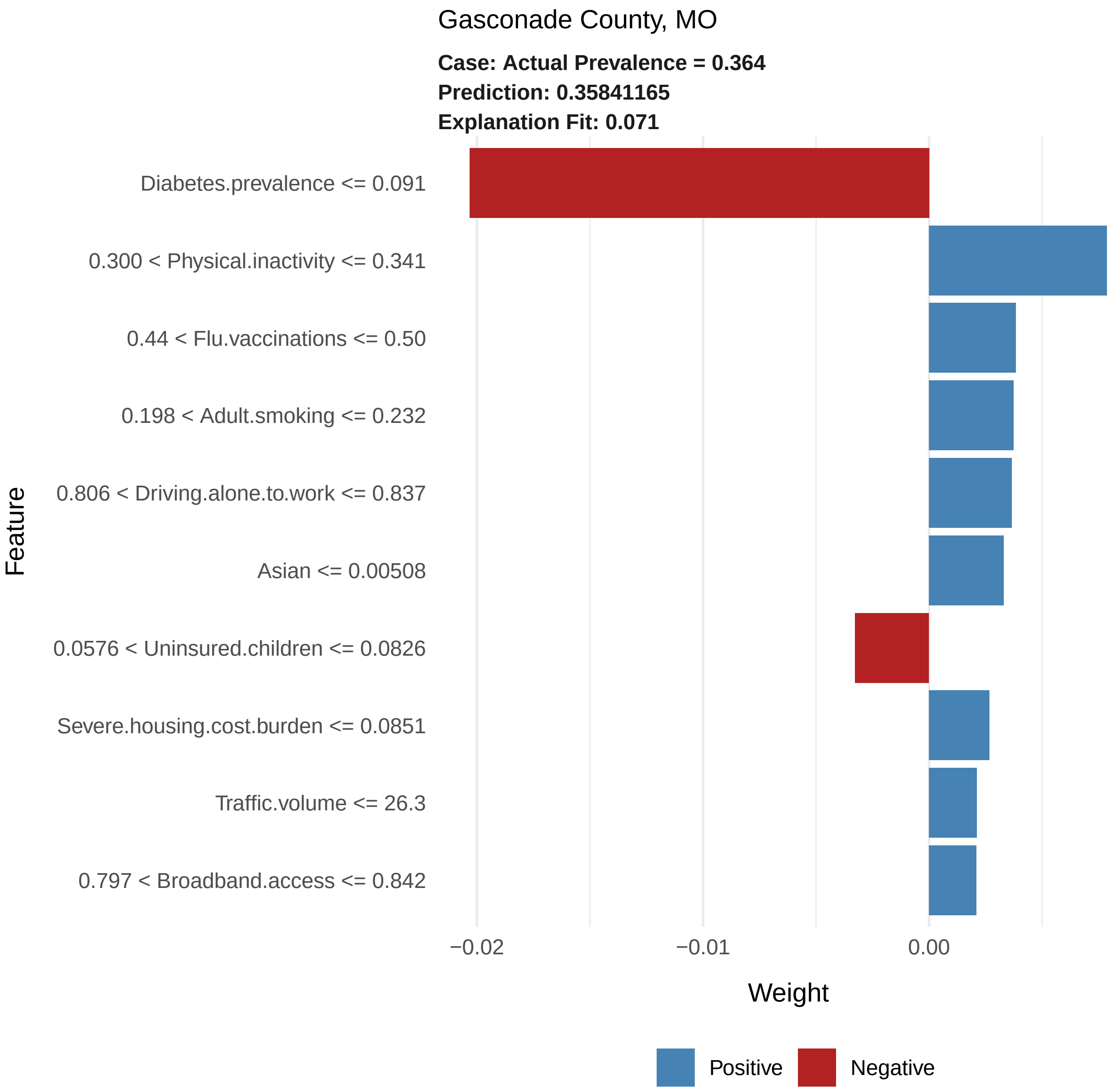
Gasconade County, MO
Case: Actual Prevalence = 0.364
Prediction: 0.35841165
Explanation Fit: 0.071
Diabetes.prevalence <= 0.091
0.300 < Physical.inactivity <= 0.341
0.44 < Flu.vaccinations <= 0.50
0.198 < Adult.smoking <= 0.232
0.806 < Driving.alone.to.work <= 0.837
Asian <= 0.00508
0.0576 < Uninsured.children <= 0.0826
Severe.housing.cost.burden <= 0.0851
Traffic.volume <= 26.3
0.797 < Broadband.access <= 0.842
Feature
−0.02
−0.01
0.00
Weight
Positive
Negative

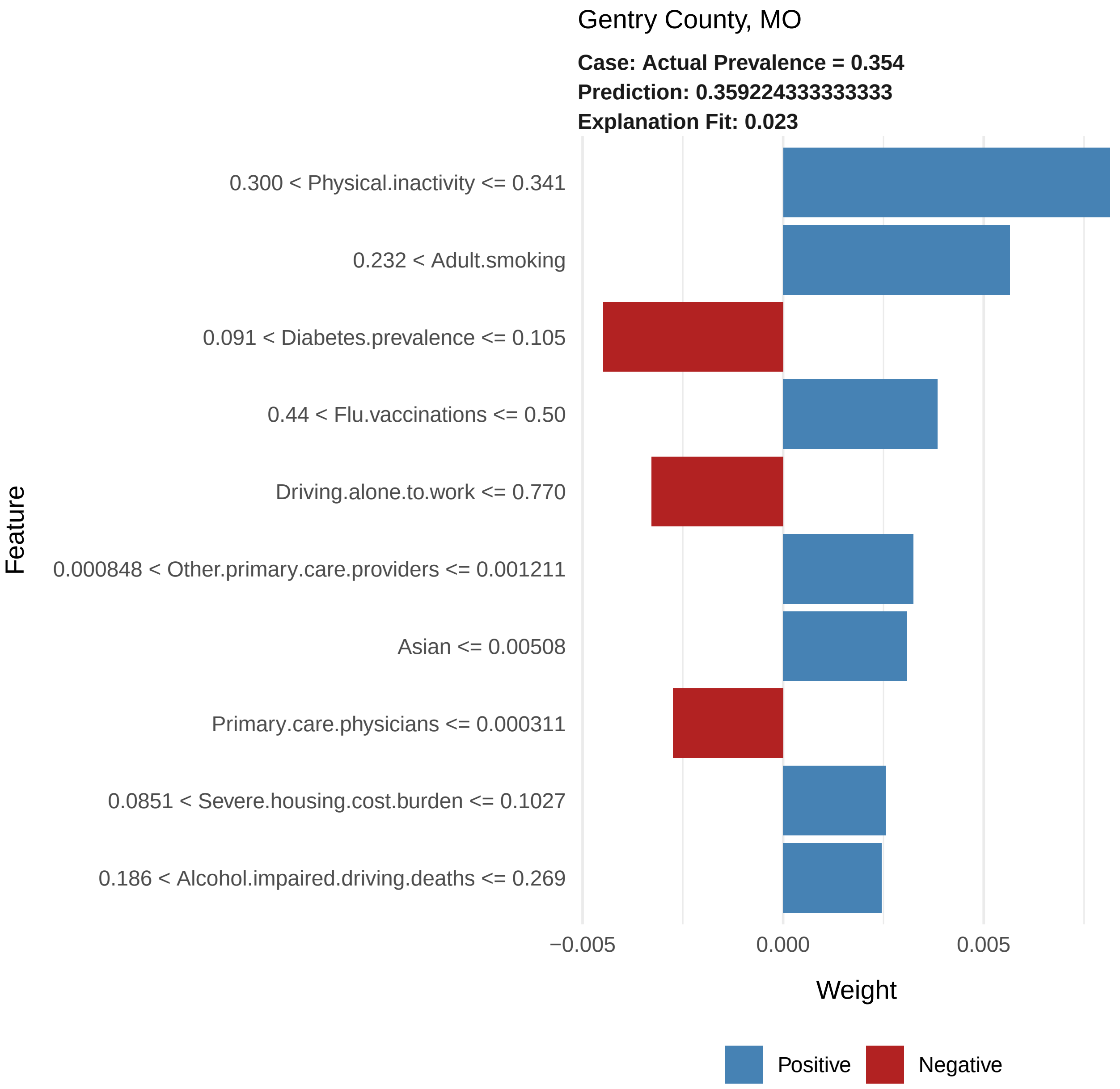

Gentry County, MO
Case: Actual Prevalence = 0.354
Prediction: 0.359224333333333
Explanation Fit: 0.023
0.300 < Physical.inactivity <= 0.341
0.232 < Adult.smoking
0.091 < Diabetes.prevalence <= 0.105
0.44 < Flu.vaccinations <= 0.50
Driving.alone.to.work <= 0.770
0.000848 < Other.primary.care.providers <= 0.001211
Asian <= 0.00508
Primary.care.physicians <= 0.000311
0.0851 < Severe.housing.cost.burden <= 0.1027
0.186 < Alcohol.impaired.driving.deaths <= 0.269
Feature
−0.005
0.000
0.005
Weight
Positive
Negative

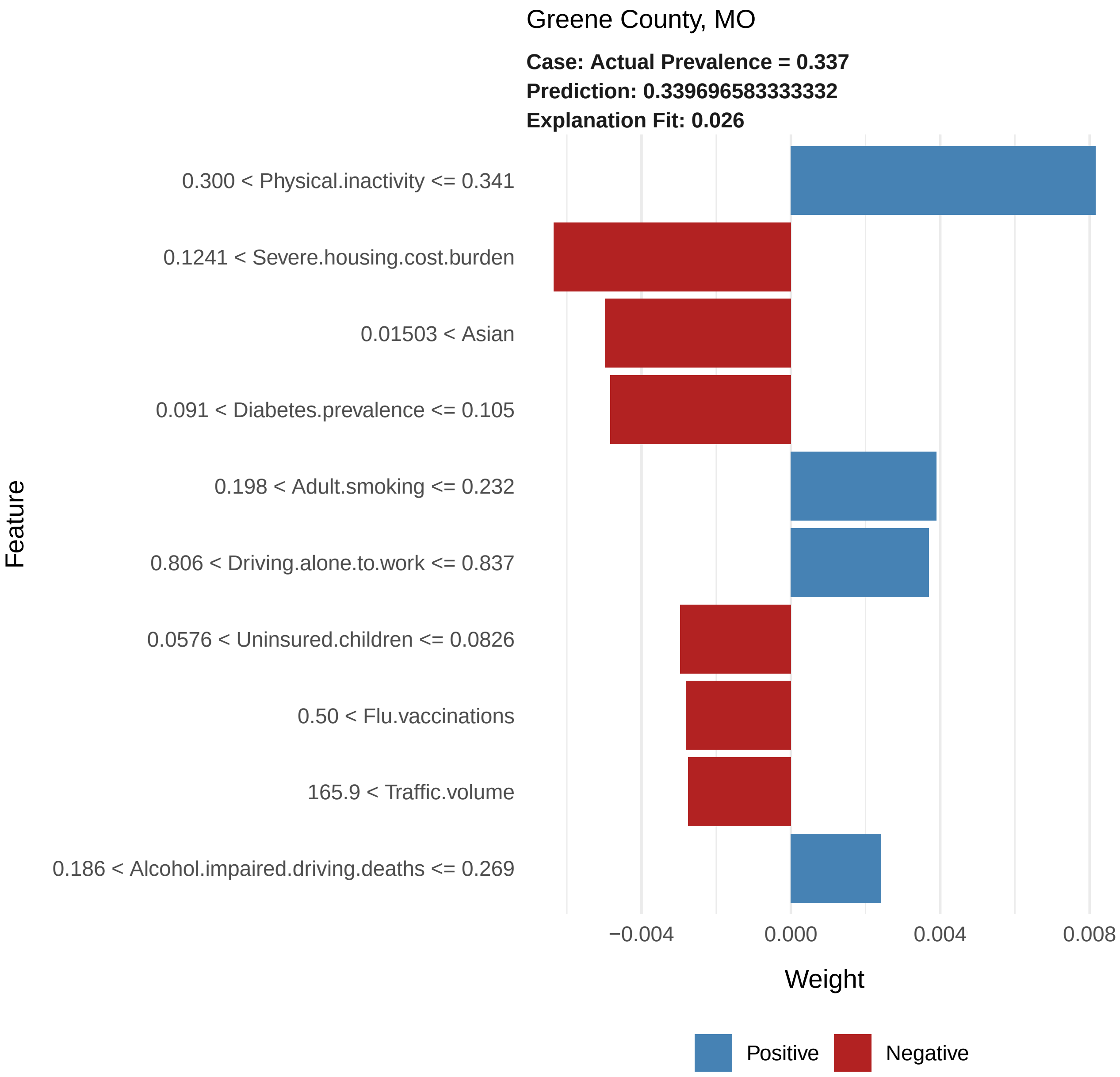
Greene County, MO
Case: Actual Prevalence = 0.337
Prediction: 0.339696583333332
Explanation Fit: 0.026
0.300 < Physical.inactivity <= 0.341
0.1241 < Severe.housing.cost.burden
0.01503 < Asian
0.091 < Diabetes.prevalence <= 0.105
0.198 < Adult.smoking <= 0.232
0.806 < Driving.alone.to.work <= 0.837
0.0576 < Uninsured.children <= 0.0826
0.50 < Flu.vaccinations
165.9 < Traffic.volume
0.186 < Alcohol.impaired.driving.deaths <= 0.269
Feature
−0.004
0.000
0.004
0.008
Weight
Positive
Negative

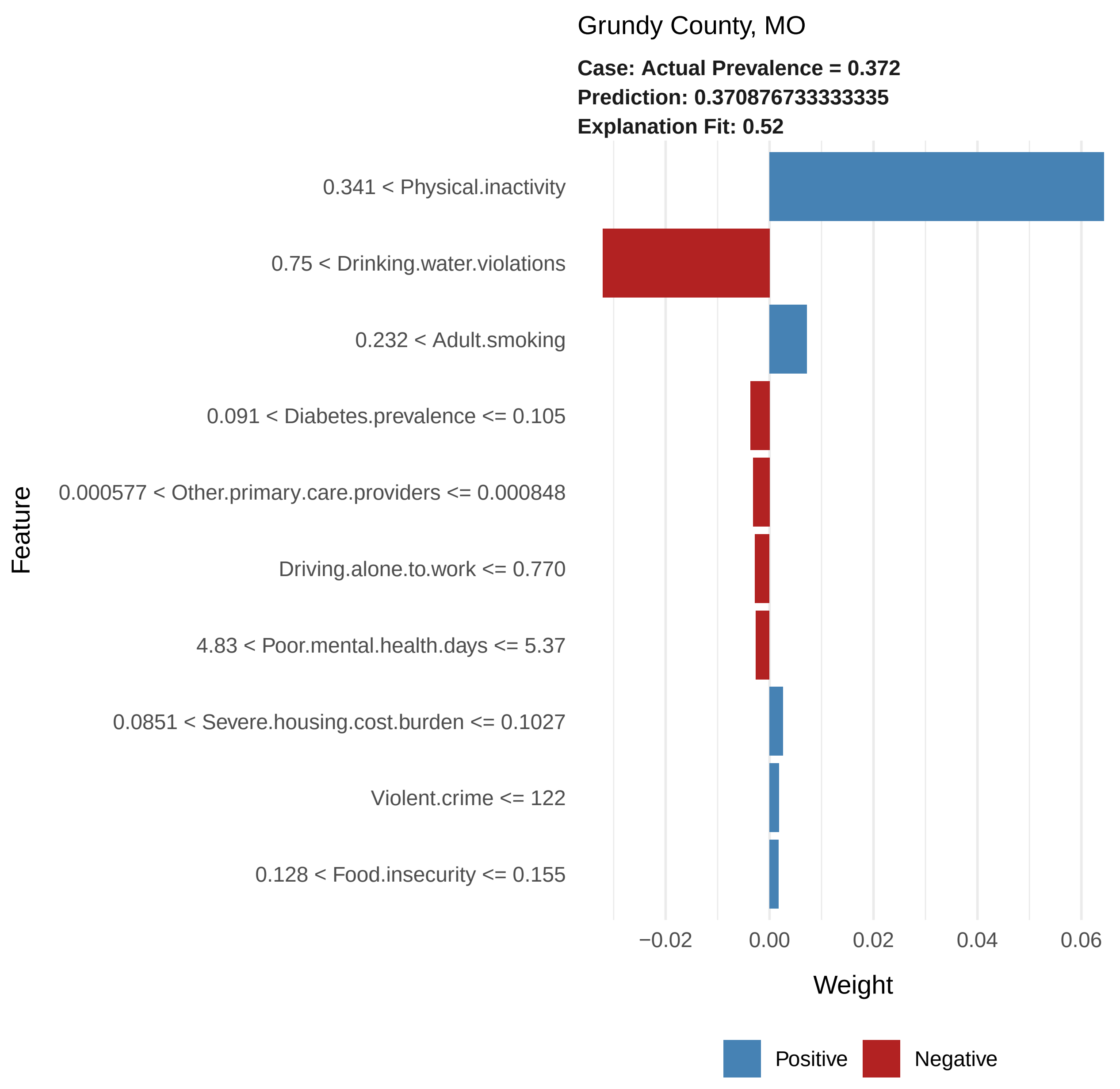

Grundy County, MO
Case: Actual Prevalence = 0.372
Prediction: 0.370876733333335
Explanation Fit: 0.52
0.341 < Physical.inactivity
0.75 < Drinking.water.violations
0.232 < Adult.smoking
0.091 < Diabetes.prevalence <= 0.105
0.000577 < Other.primary.care.providers <= 0.000848
Driving.alone.to.work <= 0.770
4.83 < Poor.mental.health.days <= 5.37
0.0851 < Severe.housing.cost.burden <= 0.1027
Violent.crime <= 122
0.128 < Food.insecurity <= 0.155
Feature
−0.02
0.00
0.02
0.04
0.06
Weight
Positive
Negative

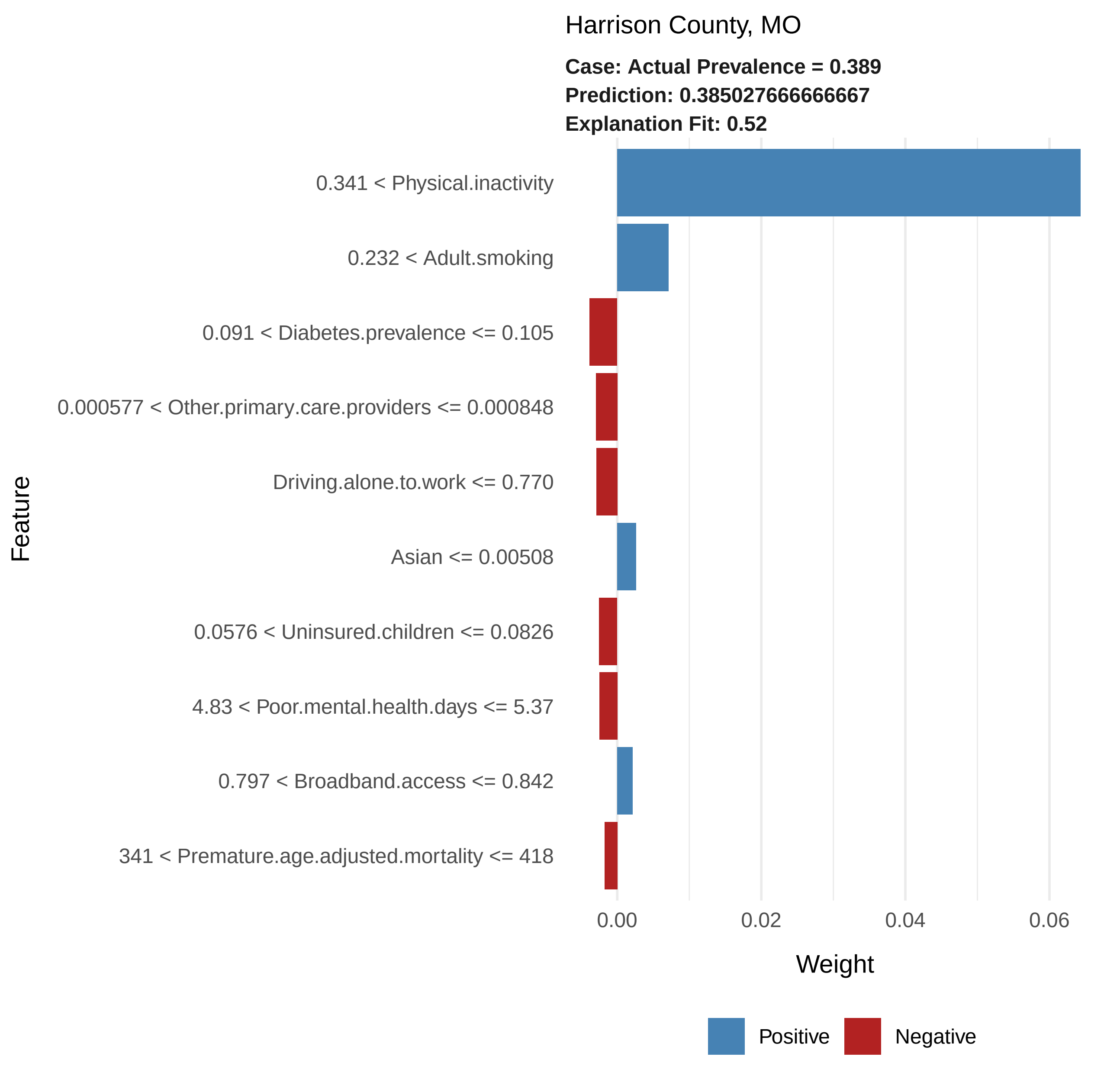
Harrison County, MO
Case: Actual Prevalence = 0.389
Prediction: 0.385027666666667
Explanation Fit: 0.52
Feature
0.341 < Physical.inactivity
0.232 < Adult.smoking
0.091 < Diabetes.prevalence <= 0.105
0.000577 < Other.primary.care.providers <= 0.000848
Driving.alone.to.work <= 0.770
Asian <= 0.00508
0.0576 < Uninsured.children <= 0.0826
4.83 < Poor.mental.health.days <= 5.37
0.797 < Broadband.access <= 0.842
341 < Premature.age.adjusted.mortality <= 418
0.00
0.02
0.04
0.06
Weight
Positive
Negative

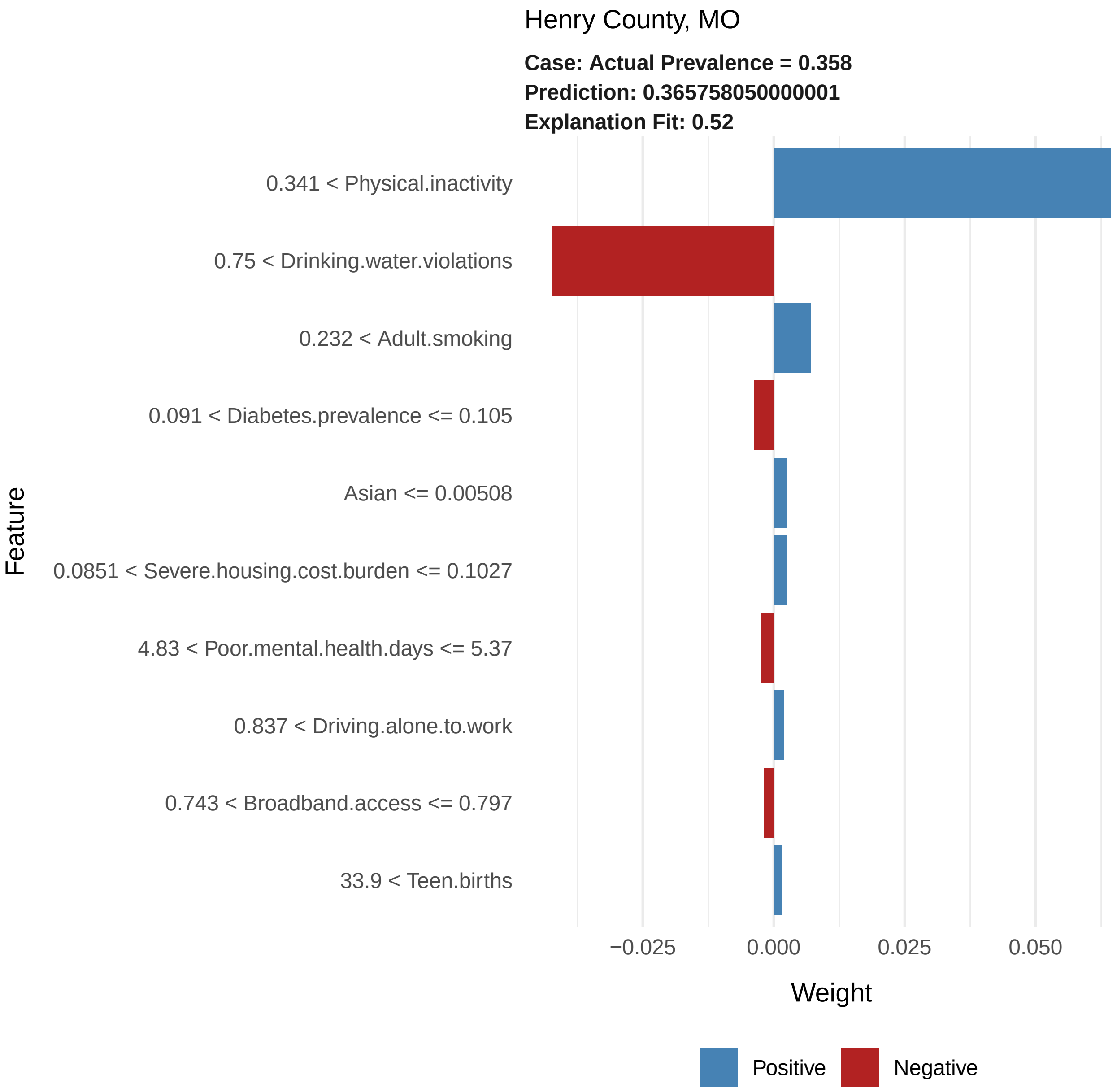

Henry County, MO
Case: Actual Prevalence = 0.358
Prediction: 0.365758050000001
Explanation Fit: 0.52
0.341 < Physical.inactivity
0.75 < Drinking.water.violations
0.232 < Adult.smoking
0.091 < Diabetes.prevalence <= 0.105
Asian <= 0.00508
0.0851 < Severe.housing.cost.burden <= 0.1027
4.83 < Poor.mental.health.days <= 5.37
0.837 < Driving.alone.to.work
0.743 < Broadband.access <= 0.797
33.9 < Teen.births
Feature
−0.025
0.000
0.025
0.050
Weight
Positive
Negative

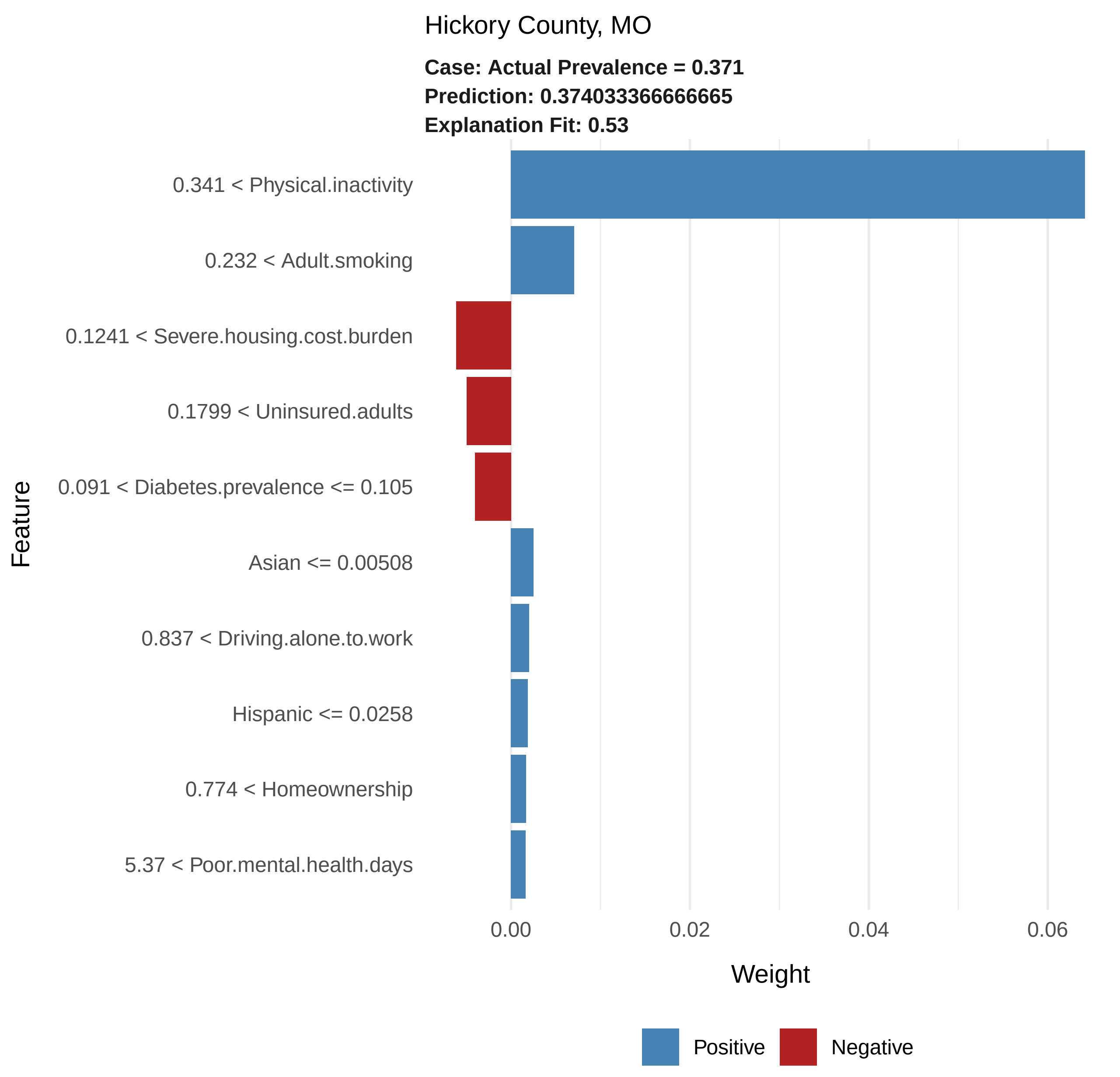

Hickory County, MO
Case: Actual Prevalence = 0.371
Prediction: 0.374033366666665
Explanation Fit: 0.53
0.341 < Physical.inactivity
0.232 < Adult.smoking
0.1241 < Severe.housing.cost.burden
0.1799 < Uninsured.adults
0.091 < Diabetes.prevalence <= 0.105
Asian <= 0.00508
0.837 < Driving.alone.to.work
Hispanic <= 0.0258
0.774 < Homeownership
5.37 < Poor.mental.health.days
Feature
0.00
0.02
0.04
0.06
Weight
Positive
Negative

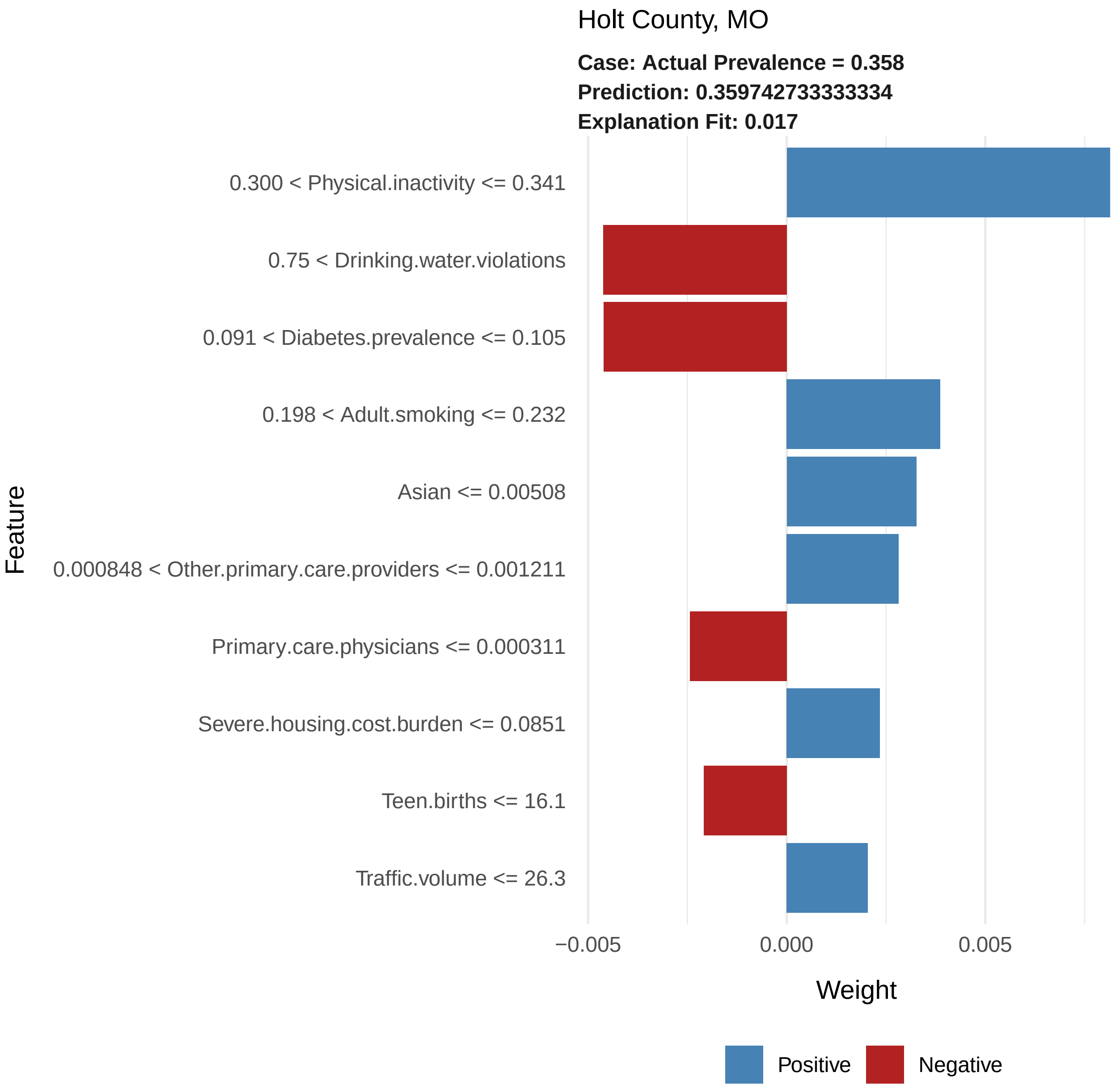
Holt County, MO
Case: Actual Prevalence = 0.358
Prediction: 0.359742733333334
Explanation Fit: 0.017
0.300 < Physical.inactivity <= 0.341
0.75 < Drinking.water.violations
0.091 < Diabetes.prevalence <= 0.105
0.198 < Adult.smoking <= 0.232
Asian <= 0.00508
0.000848 < Other.primary.care.providers <= 0.001211
Primary.care.physicians <= 0.000311
Severe.housing.cost.burden <= 0.0851
Teen.births <= 16.1
Traffic.volume <= 26.3
Feature
−0.005
0.000
0.005
Weight
Positive
Negative

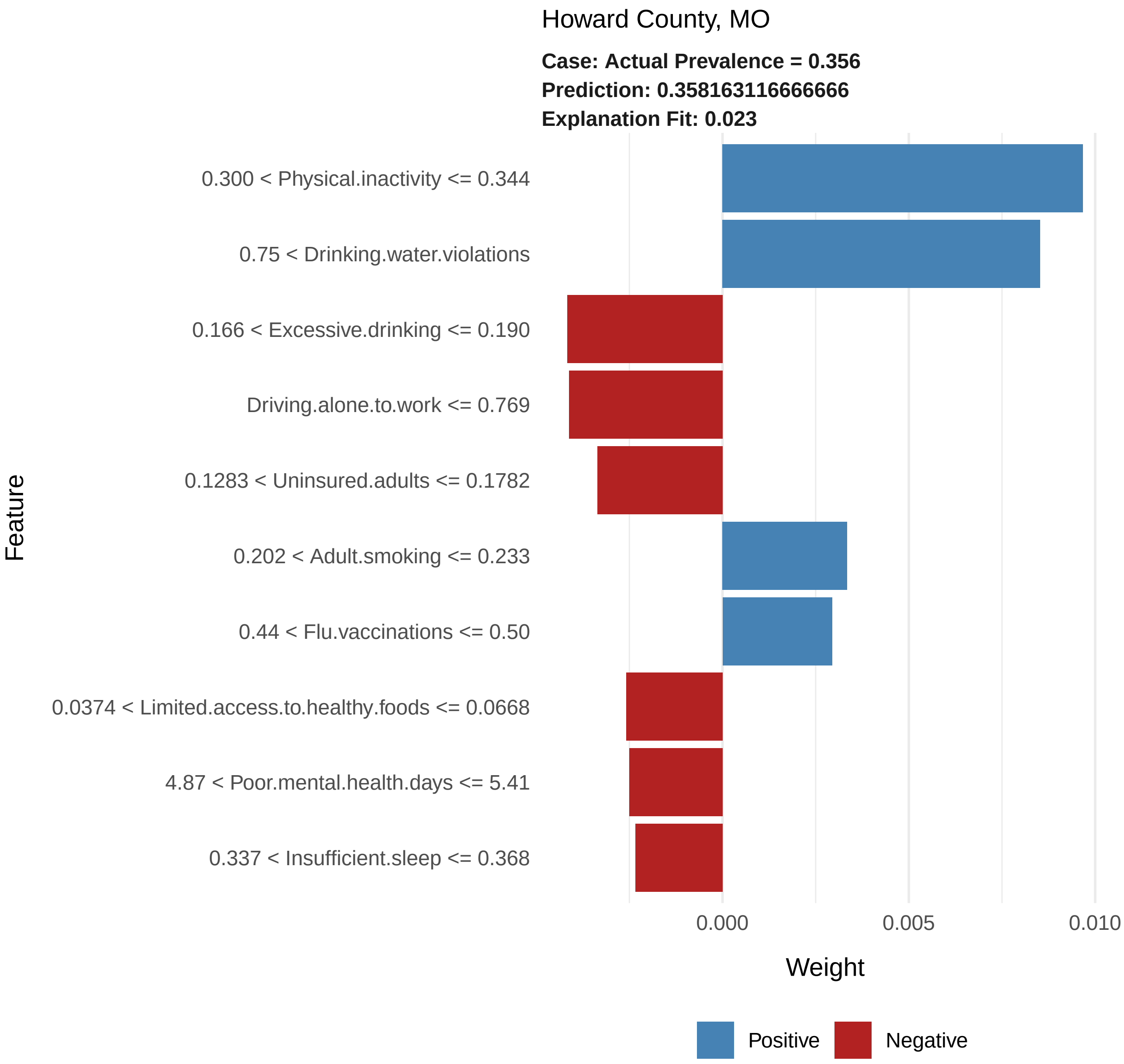

Howard County, MO
Case: Actual Prevalence = 0.356
Prediction: 0.358163116666666
Explanation Fit: 0.023
0.300 < Physical.inactivity <= 0.344
0.75 < Drinking.water.violations
0.166 < Excessive.drinking <= 0.190
Driving.alone.to.work <= 0.769
0.1283 < Uninsured.adults <= 0.1782
0.202 < Adult.smoking <= 0.233
0.44 < Flu.vaccinations <= 0.50
0.0374 < Limited.access.to.healthy.foods <= 0.0668
4.87 < Poor.mental.health.days <= 5.41
0.337 < Insufficient.sleep <= 0.368
Feature
0.000
0.005
0.010
Weight
Positive
Negative

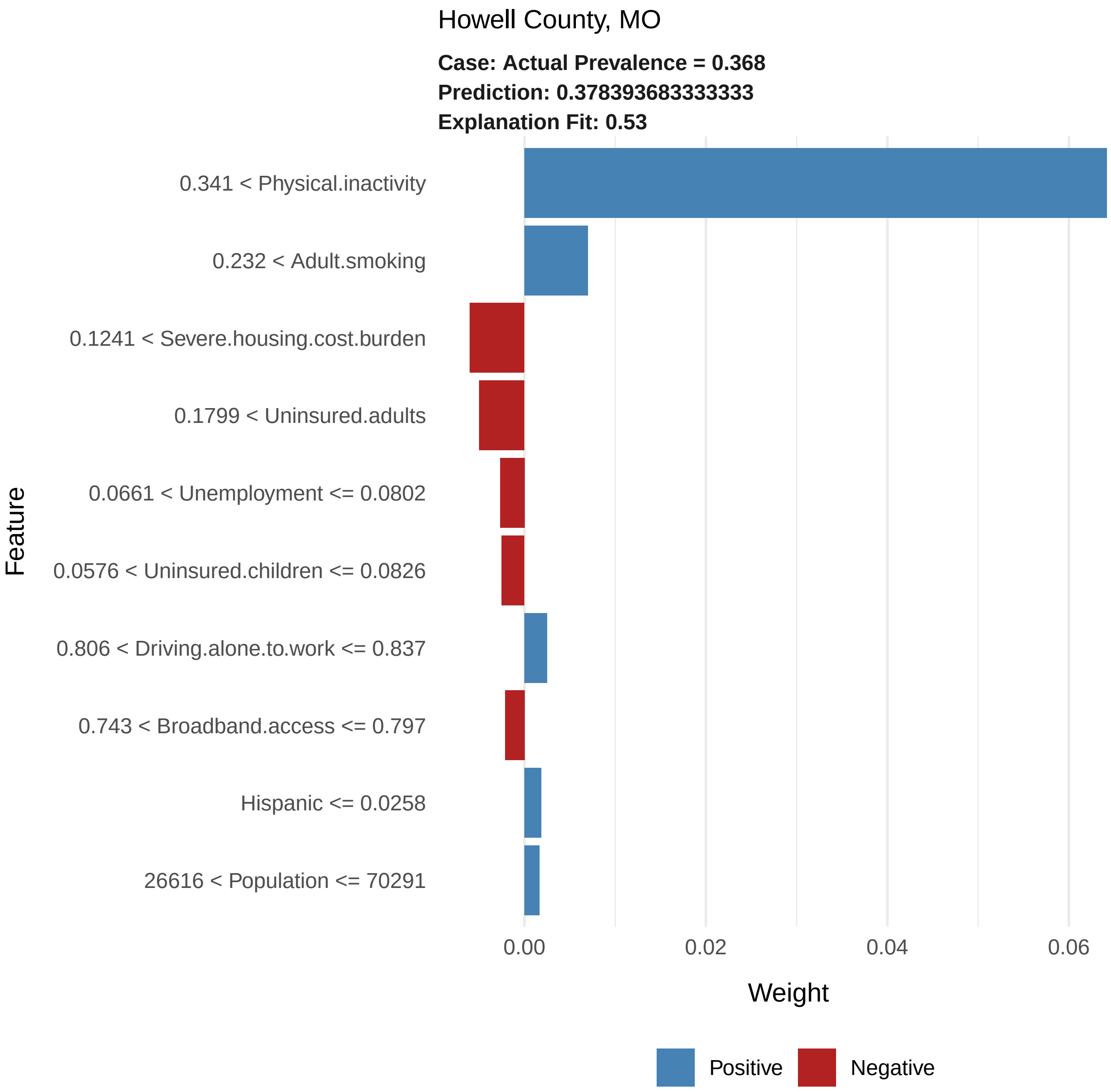

Howell County, MO
Case: Actual Prevalence = 0.368
Prediction: 0.378393683333333
Explanation Fit: 0.53
0.341 < Physical.inactivity
0.232 < Adult.smoking
0.1241 < Severe.housing.cost.burden
0.1799 < Uninsured.adults
0.0661 < Unemployment <= 0.0802
0.0576 < Uninsured.children <= 0.0826
0.806 < Driving.alone.to.work <= 0.837
0.743 < Broadband.access <= 0.797
Hispanic <= 0.0258
26616 < Population <= 70291
Feature
0.00
0.02
0.04
0.06
Weight
Positive
Negative

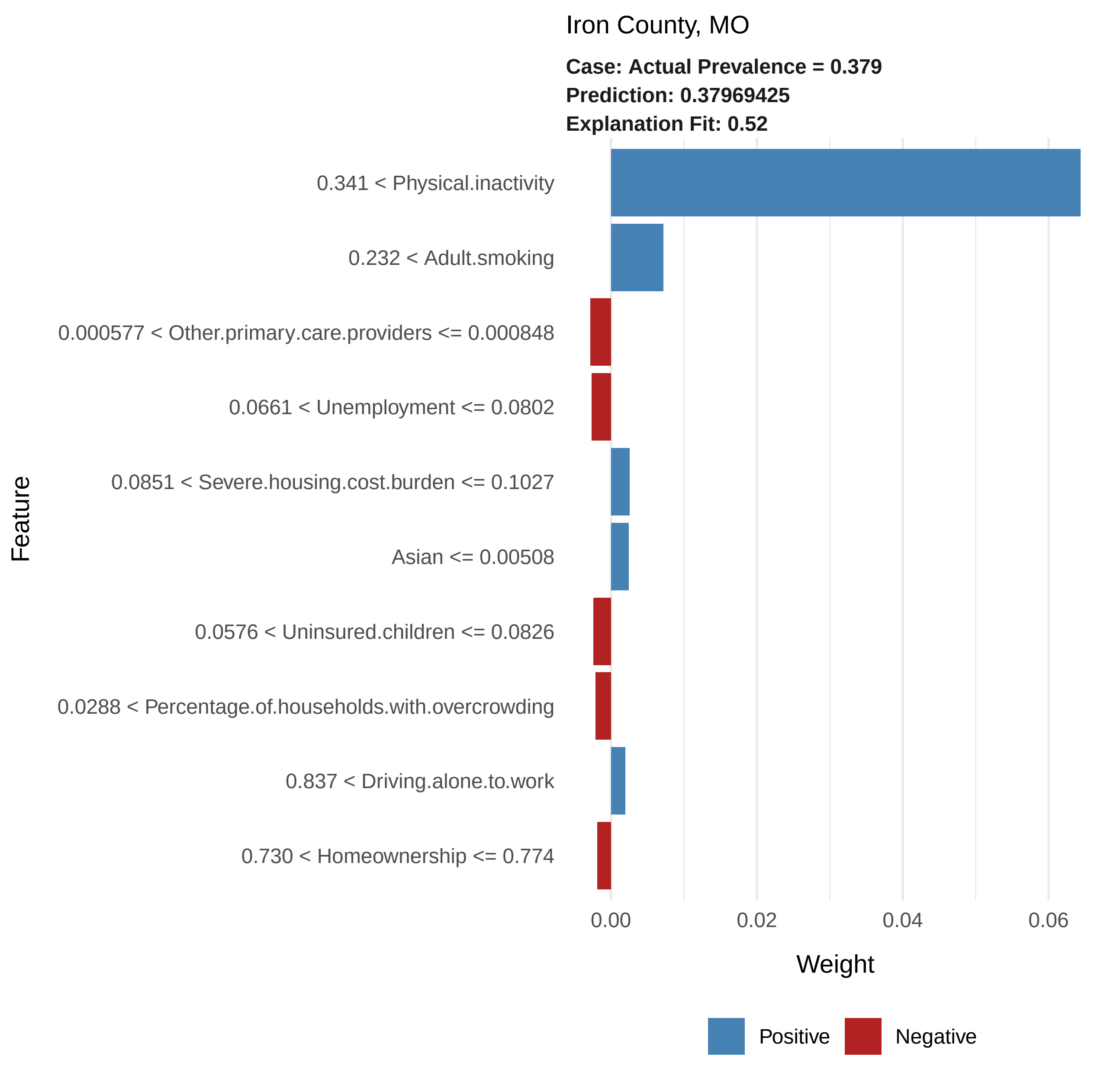

Iron County, MO
Case: Actual Prevalence = 0.379
Prediction: 0.37969425
Explanation Fit: 0.52
0.341 < Physical.inactivity
0.232 < Adult.smoking
0.000577 < Other.primary.care.providers <= 0.000848
0.0661 < Unemployment <= 0.0802
0.0851 < Severe.housing.cost.burden <= 0.1027
Asian <= 0.00508
0.0576 < Uninsured.children <= 0.0826
0.0288 < Percentage.of.households.with.overcrowding
0.837 < Driving.alone.to.work
0.730 < Homeownership <= 0.774
Feature
0.00
0.02
0.04
0.06
Weight
Positive
Negative

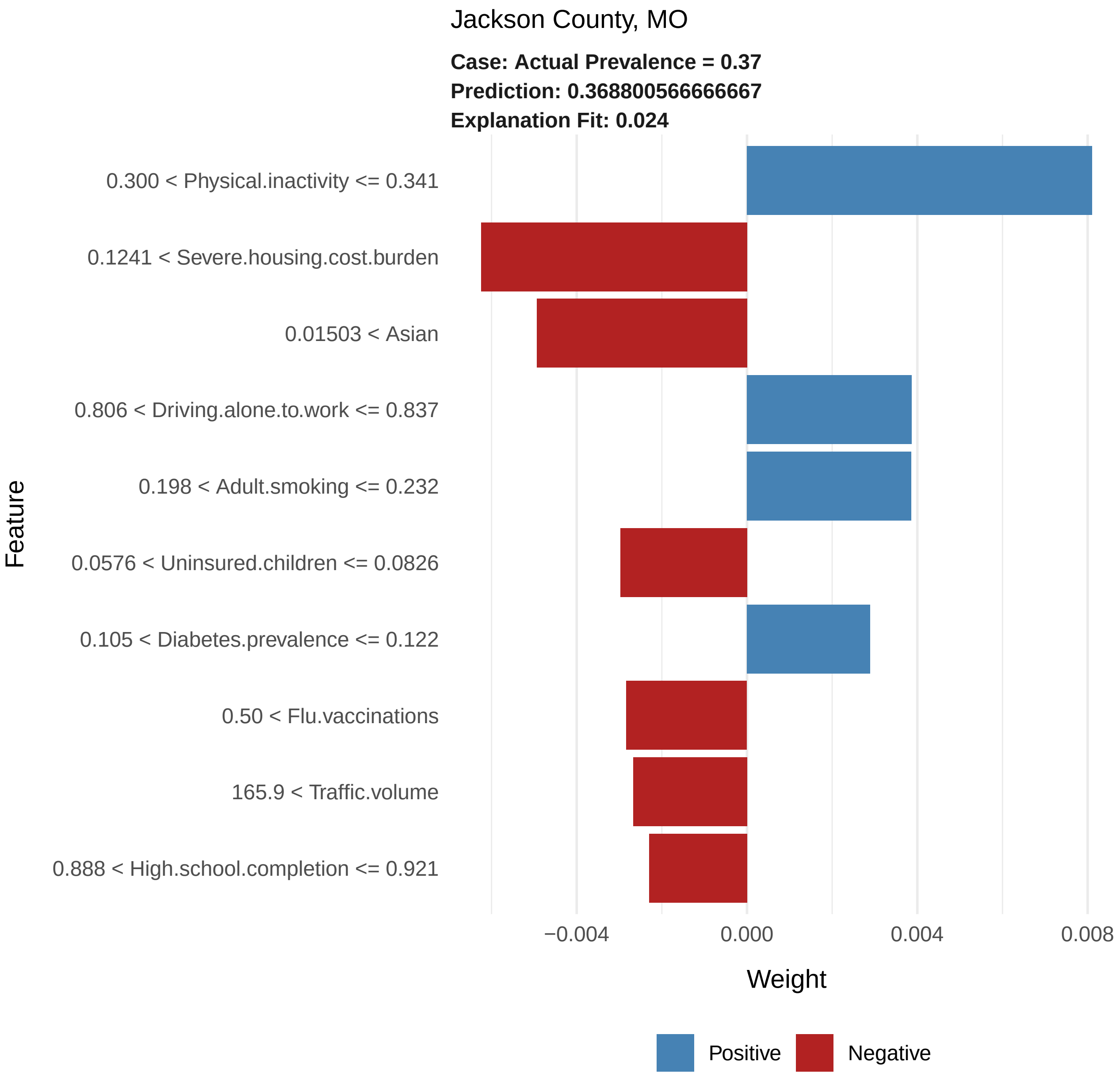

Jackson County, MO
Case: Actual Prevalence = 0.37
Prediction: 0.368800566666667
Explanation Fit: 0.024
0.300 < Physical.inactivity <= 0.341
0.1241 < Severe.housing.cost.burden
0.01503 < Asian
0.806 < Driving.alone.to.work <= 0.837
0.198 < Adult.smoking <= 0.232
0.0576 < Uninsured.children <= 0.0826
0.105 < Diabetes.prevalence <= 0.122
0.50 < Flu.vaccinations
165.9 < Traffic.volume
0.888 < High.school.completion <= 0.921
Feature
−0.004
0.000
0.004
0.008
Weight
Positive
Negative

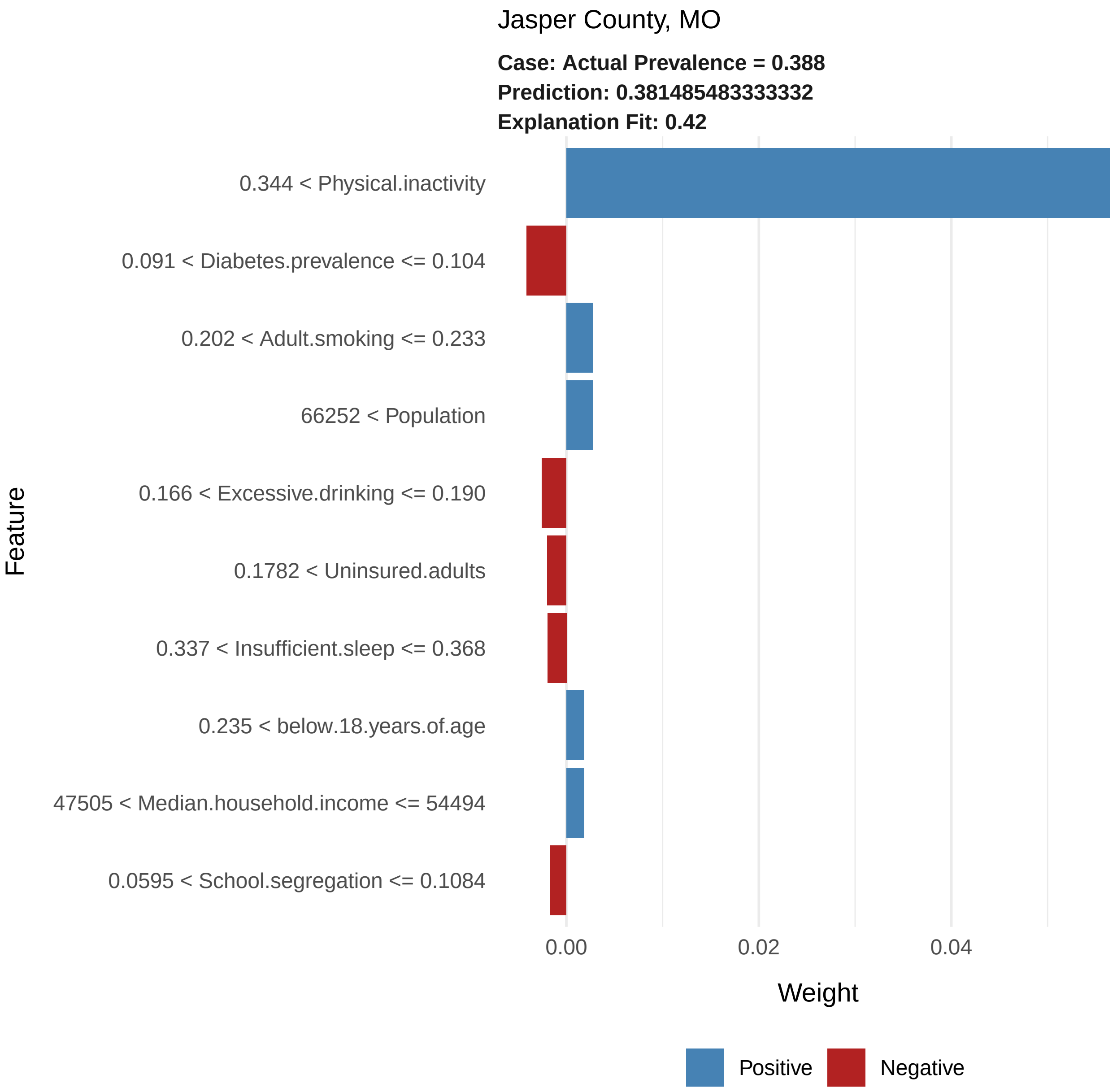
Jasper County, MO
Case: Actual Prevalence = 0.388
Prediction: 0.381485483333332
Explanation Fit: 0.42
0.344 < Physical.inactivity
0.091 < Diabetes.prevalence <= 0.104
0.202 < Adult.smoking <= 0.233
66252 < Population
0.166 < Excessive.drinking <= 0.190
0.1782 < Uninsured.adults
0.337 < Insufficient.sleep <= 0.368
0.235 < below.18.years.of.age
47505 < Median.household.income <= 54494
0.0595 < School.segregation <= 0.1084
Feature
0.00
0.02
0.04
Weight
Positive
Negative

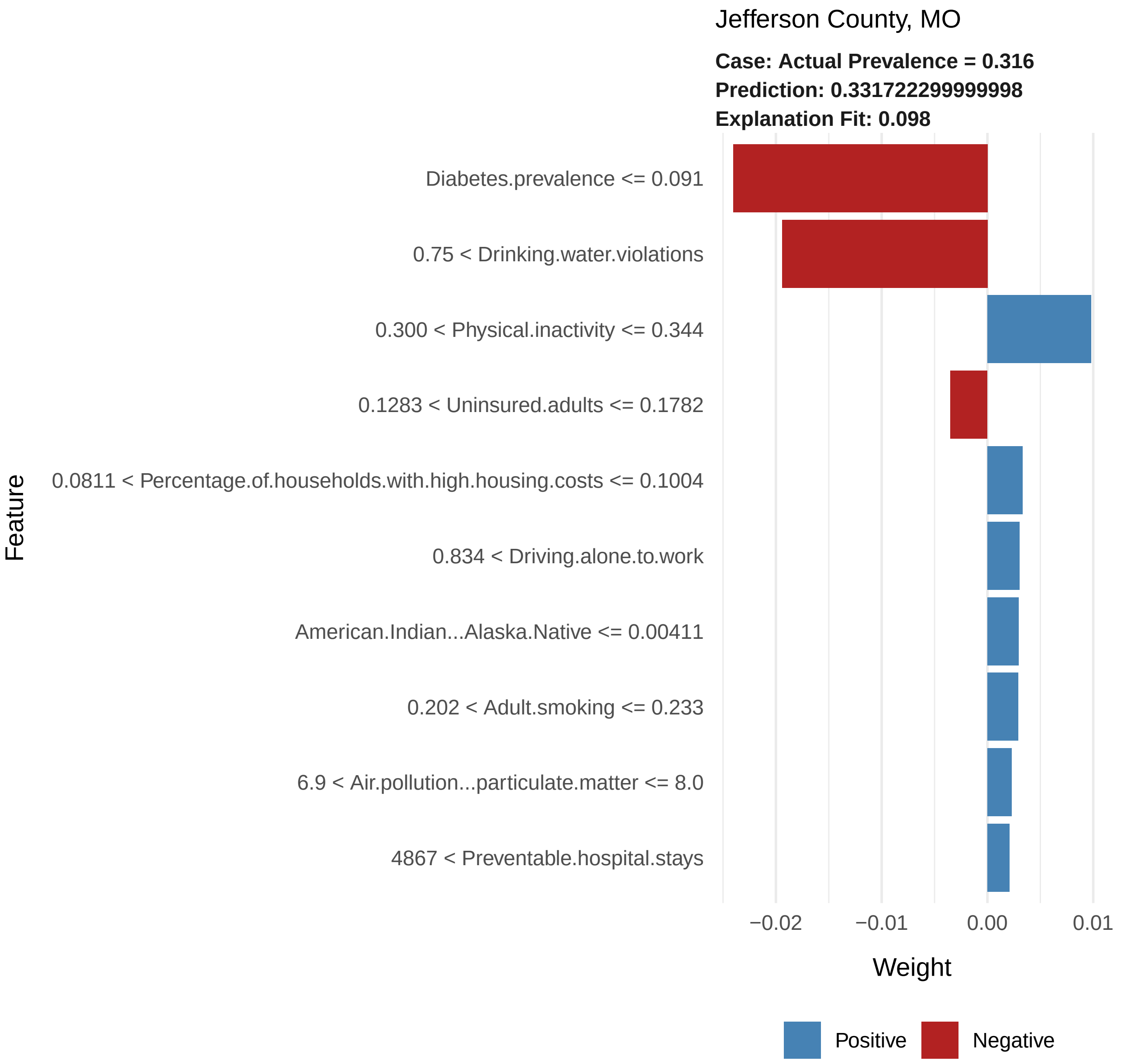

Jefferson County, MO
Case: Actual Prevalence = 0.316
Prediction: 0.331722299999998
Explanation Fit: 0.098
Diabetes.prevalence <= 0.091
0.75 < Drinking.water.violations
0.300 < Physical.inactivity <= 0.344
0.1283 < Uninsured.adults <= 0.1782
0.0811 < Percentage.of.households.with.high.housing.costs <= 0.1004
0.834 < Driving.alone.to.work
American.Indian...Alaska.Native <= 0.00411
0.202 < Adult.smoking <= 0.233
6.9 < Air.pollution...particulate.matter <= 8.0
4867 < Preventable.hospital.stays
Feature
−0.02
−0.01
0.00
0.01
Weight
Positive
Negative

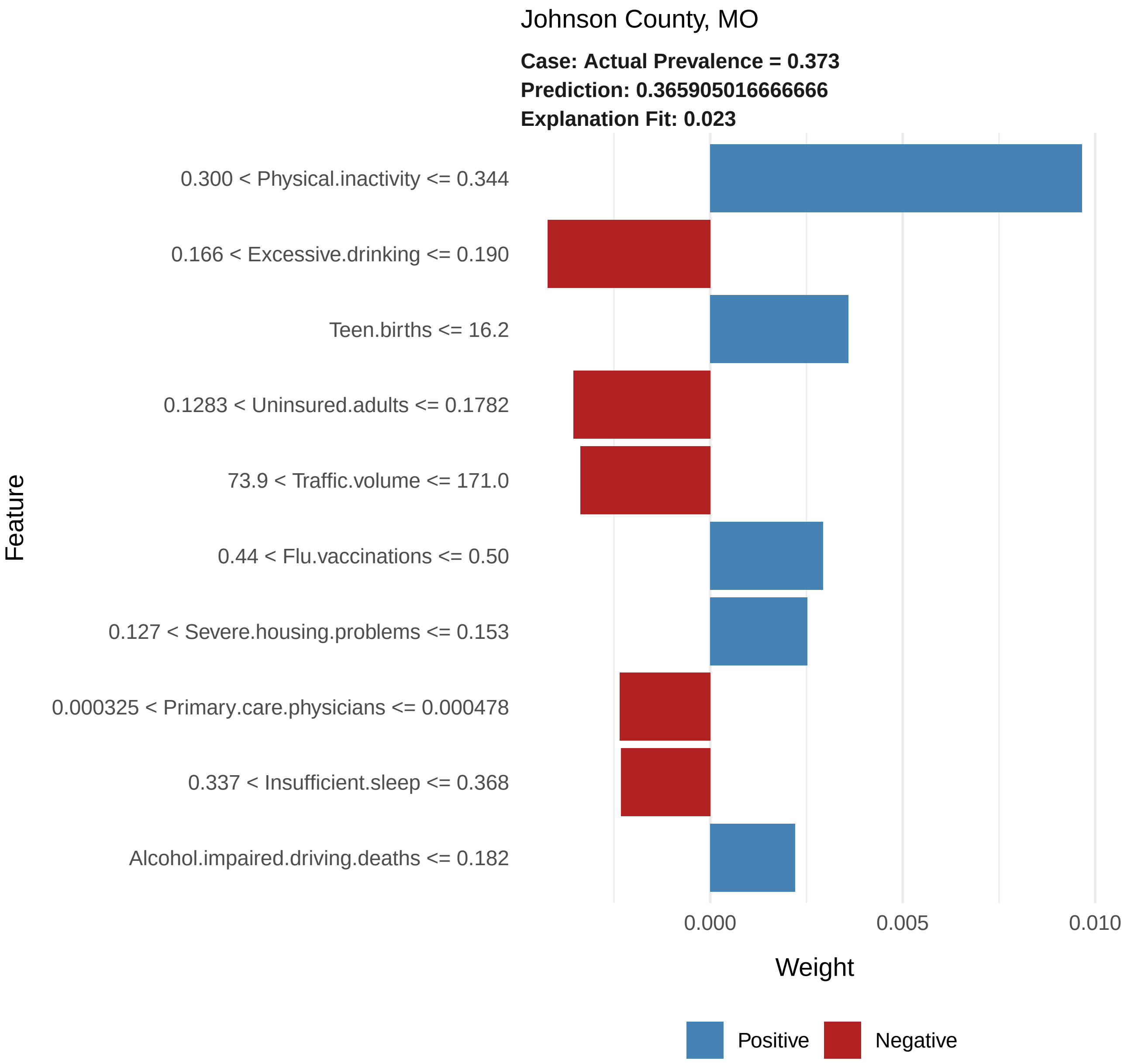
Johnson County, MO
Case: Actual Prevalence = 0.373
Prediction: 0.365905016666666
Explanation Fit: 0.023
0.300 < Physical.inactivity <= 0.344
0.166 < Excessive.drinking <= 0.190
Teen.births <= 16.2
0.1283 < Uninsured.adults <= 0.1782
73.9 < Traffic.volume <= 171.0
0.44 < Flu.vaccinations <= 0.50
0.127 < Severe.housing.problems <= 0.153
0.000325 < Primary.care.physicians <= 0.000478
0.337 < Insufficient.sleep <= 0.368
Alcohol.impaired.driving.deaths <= 0.182
Feature
0.000
0.005
0.010
Weight
Positive
Negative

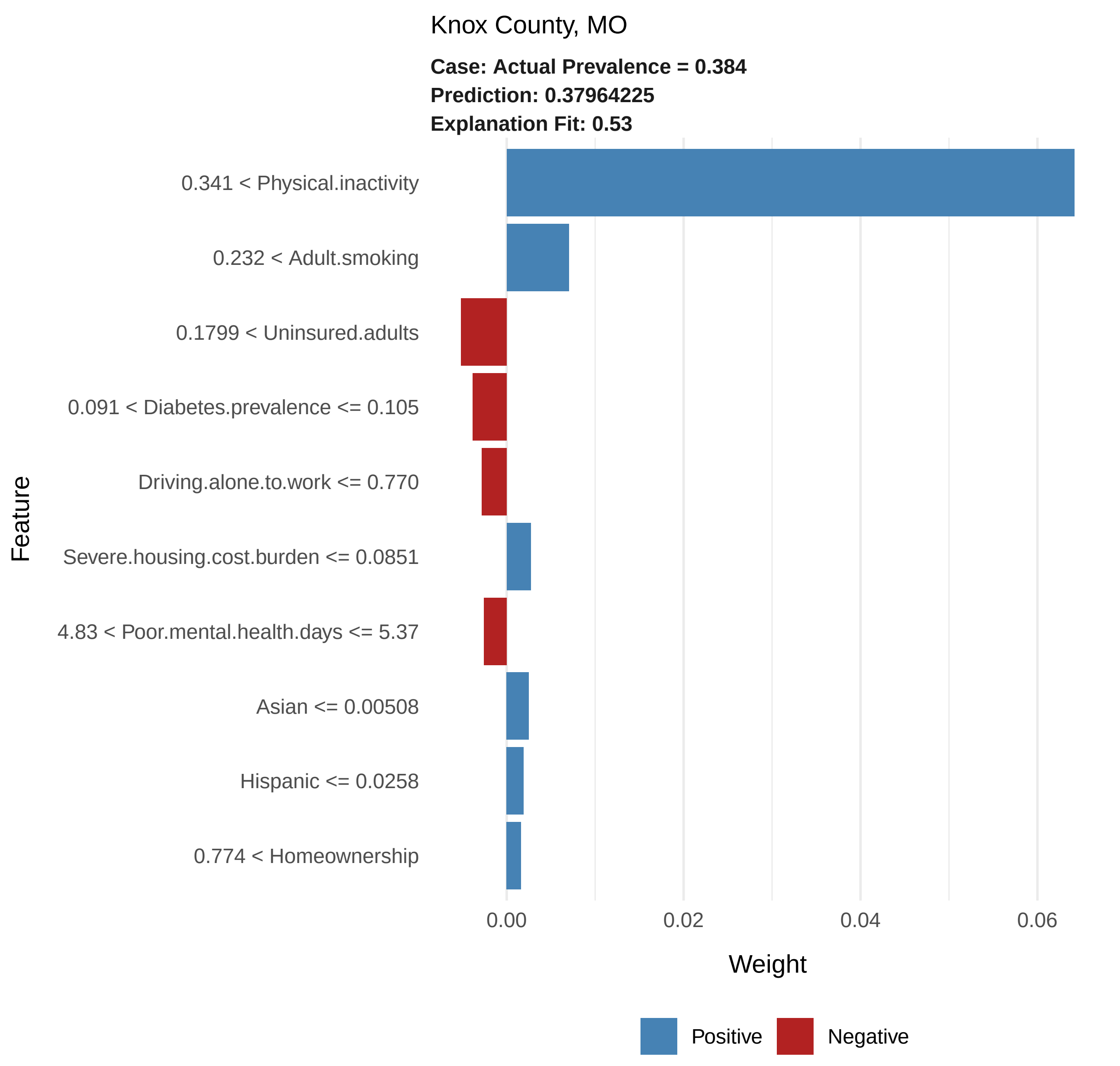

Knox County, MO
Case: Actual Prevalence = 0.384
Prediction: 0.37964225
Explanation Fit: 0.53
0.341 < Physical.inactivity
0.232 < Adult.smoking
0.1799 < Uninsured.adults
0.091 < Diabetes.prevalence <= 0.105
Driving.alone.to.work <= 0.770
Severe.housing.cost.burden <= 0.0851
4.83 < Poor.mental.health.days <= 5.37
Asian <= 0.00508
Hispanic <= 0.0258
0.774 < Homeownership
Feature
0.00
0.02
0.04
0.06
Weight
Positive
Negative

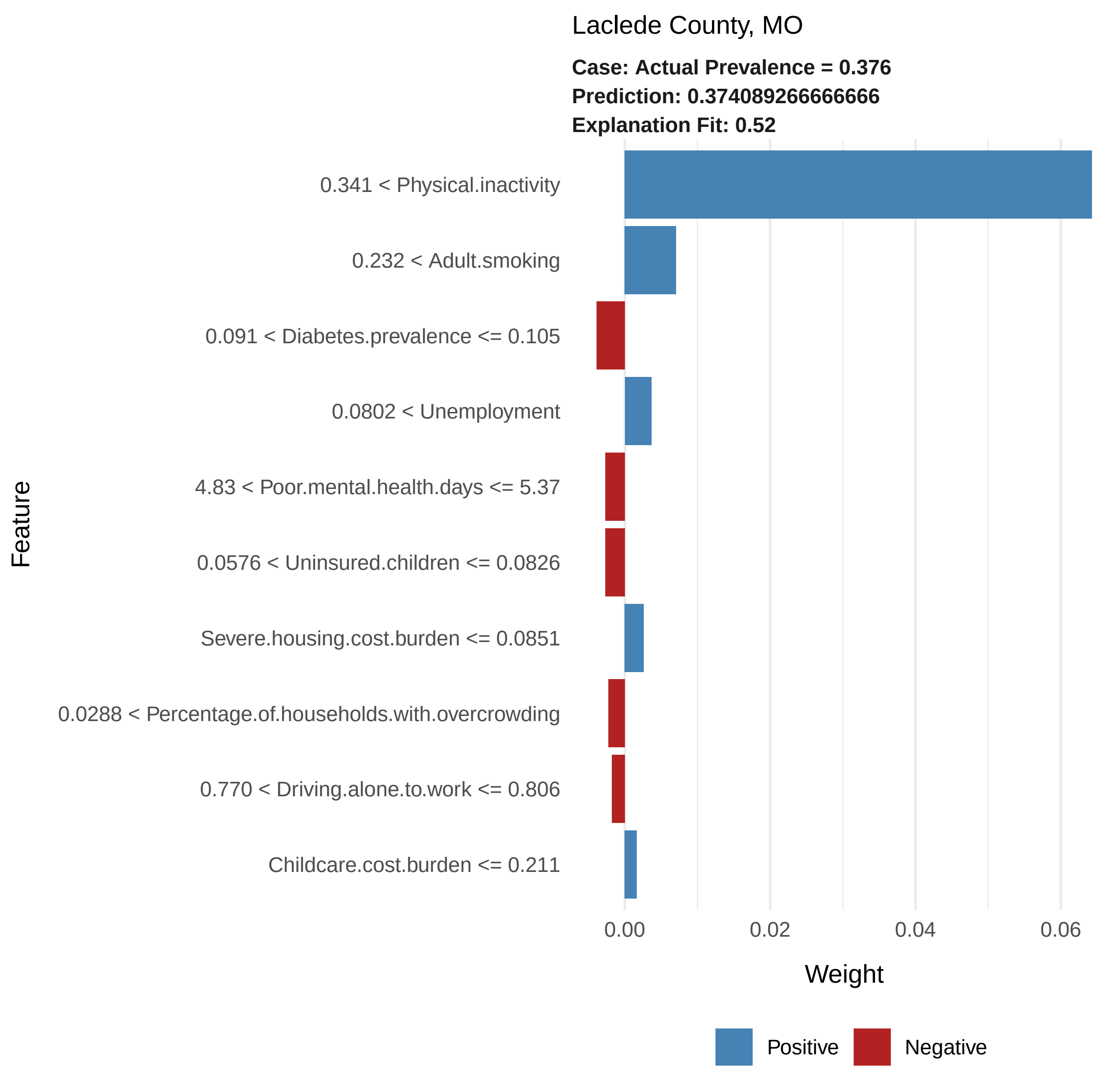
Laclede County, MO
Case: Actual Prevalence = 0.376
Prediction: 0.374089266666666
Explanation Fit: 0.52
0.341 < Physical.inactivity
0.232 < Adult.smoking
0.091 < Diabetes.prevalence <= 0.105
0.0802 < Unemployment
4.83 < Poor.mental.health.days <= 5.37
0.0576 < Uninsured.children <= 0.0826
Severe.housing.cost.burden <= 0.0851
0.0288 < Percentage.of.households.with.overcrowding
0.770 < Driving.alone.to.work <= 0.806
Childcare.cost.burden <= 0.211
Feature
0.00
0.02
0.04
0.06
Weight
Positive
Negative

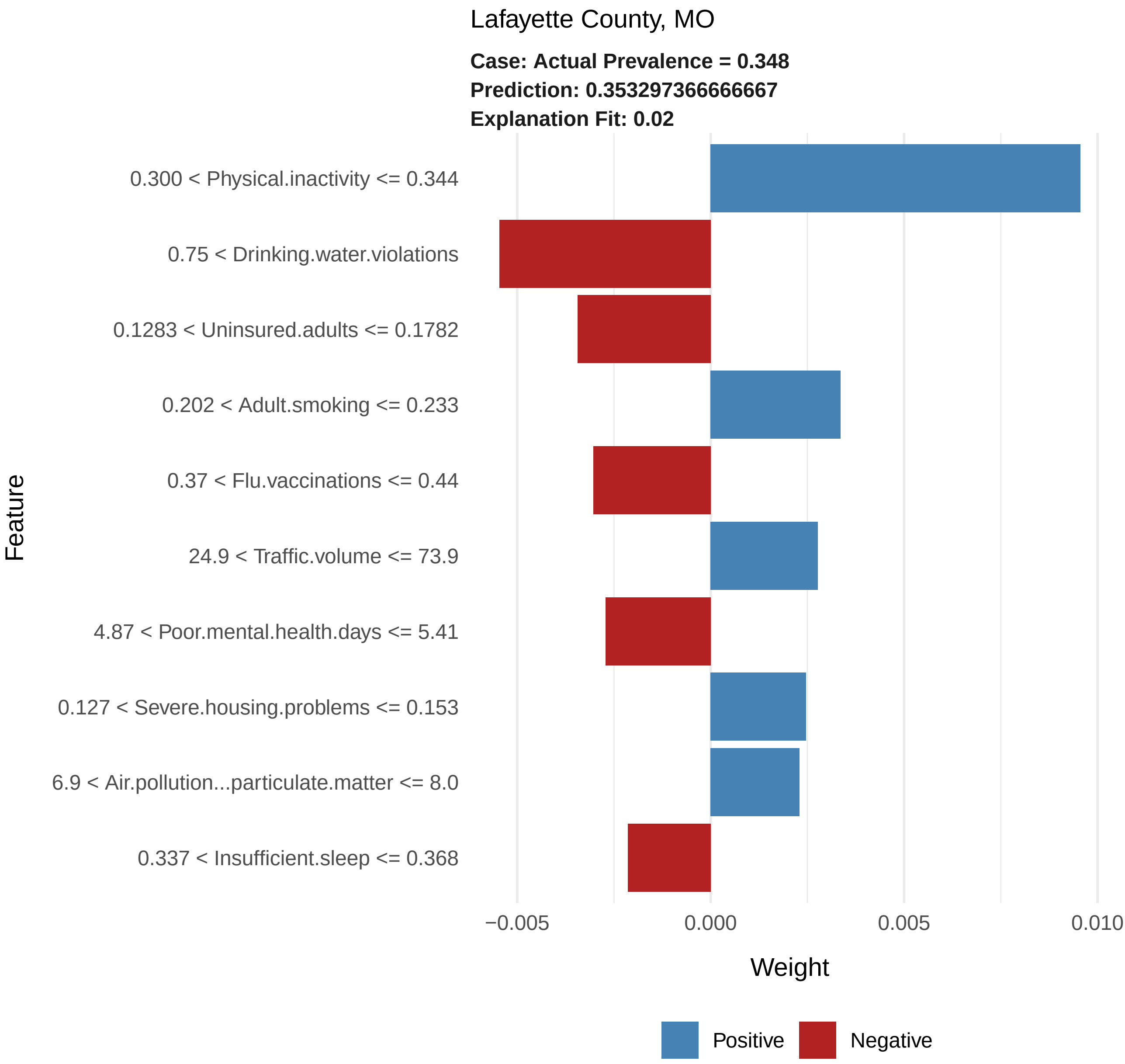

Lafayette County, MO
Case: Actual Prevalence = 0.348
Prediction: 0.353297366666667
Explanation Fit: 0.02
0.300 < Physical.inactivity <= 0.344
0.75 < Drinking.water.violations
0.1283 < Uninsured.adults <= 0.1782
0.202 < Adult.smoking <= 0.233
0.37 < Flu.vaccinations <= 0.44
24.9 < Traffic.volume <= 73.9
4.87 < Poor.mental.health.days <= 5.41
0.127 < Severe.housing.problems <= 0.153
6.9 < Air.pollution...particulate.matter <= 8.0
0.337 < Insufficient.sleep <= 0.368
Feature
−0.005
0.000
0.005
0.010
Weight
Positive
Negative

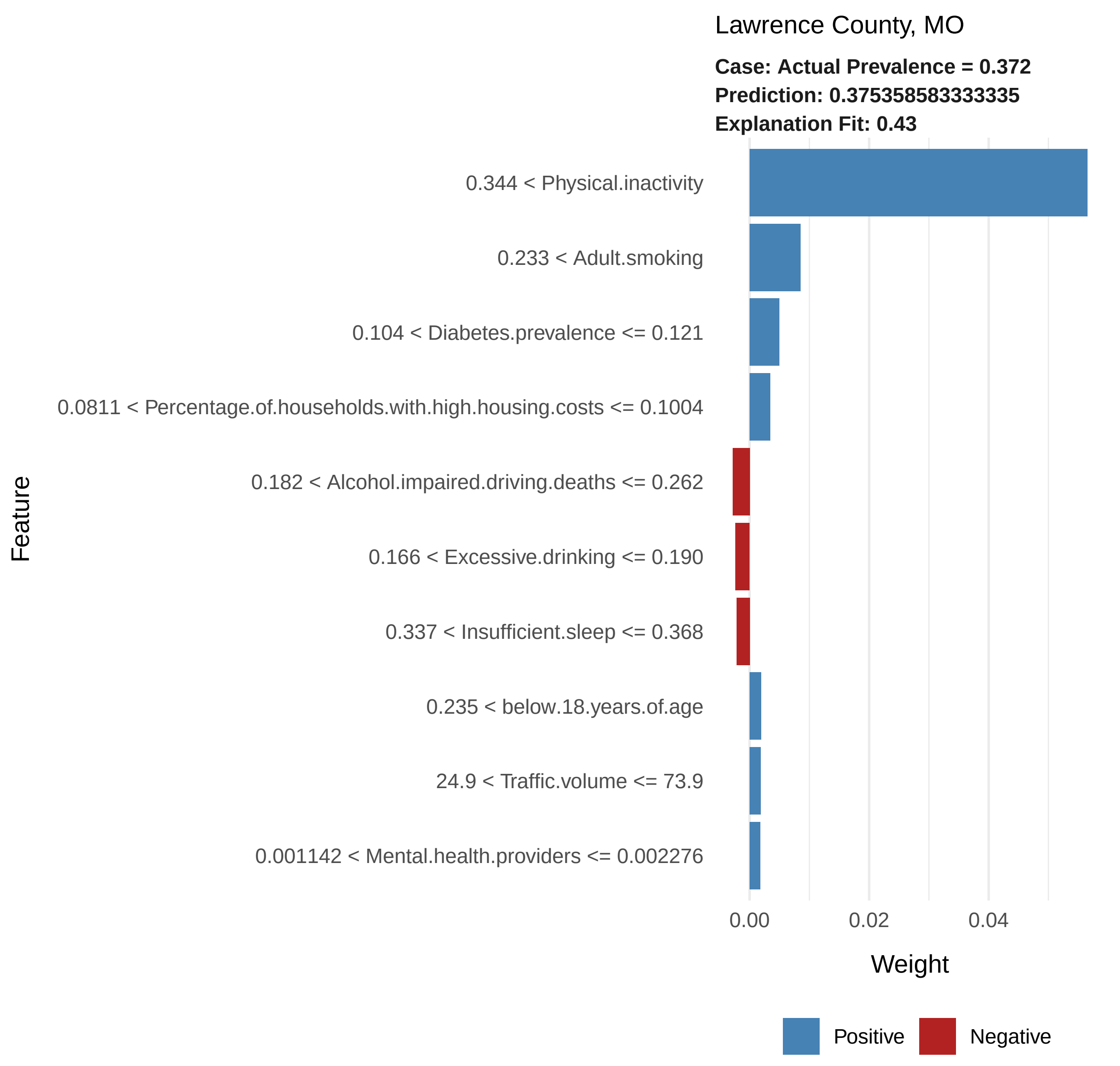

Lawrence County, MO
Case: Actual Prevalence = 0.372
Prediction: 0.375358583333335
Explanation Fit: 0.43
0.344 < Physical.inactivity
0.233 < Adult.smoking
0.104 < Diabetes.prevalence <= 0.121
0.0811 < Percentage.of.households.with.high.housing.costs <= 0.1004
0.182 < Alcohol.impaired.driving.deaths <= 0.262
0.166 < Excessive.drinking <= 0.190
0.337 < Insufficient.sleep <= 0.368
0.235 < below.18.years.of.age
24.9 < Traffic.volume <= 73.9
0.001142 < Mental.health.providers <= 0.002276
Feature
0.00
0.02
0.04
Weight
Positive
Negative

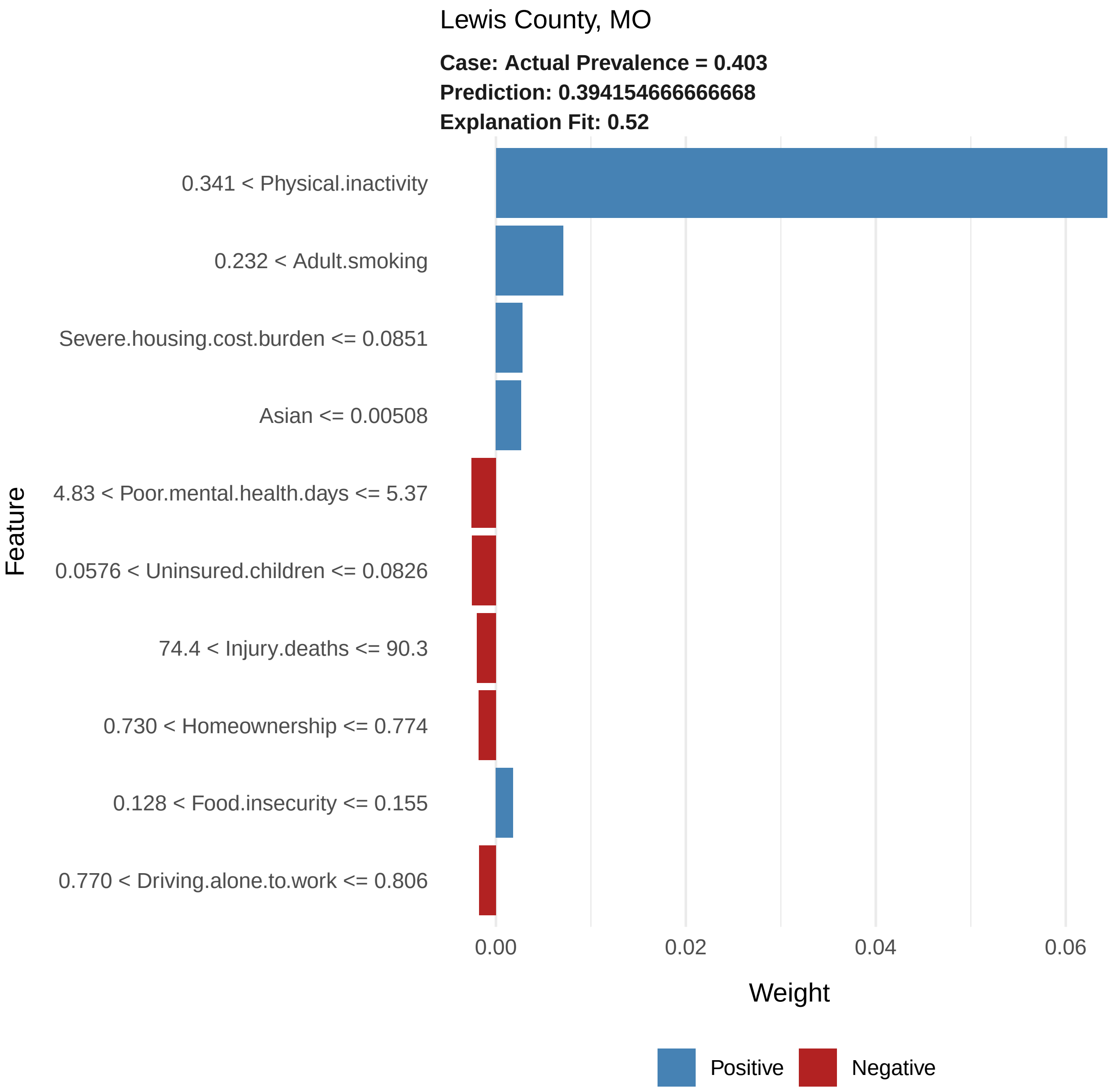
Lewis County, MO
Case: Actual Prevalence = 0.403
Prediction: 0.394154666666668
Explanation Fit: 0.52
0.341 < Physical.inactivity
0.232 < Adult.smoking
Severe.housing.cost.burden <= 0.0851
Asian <= 0.00508
4.83 < Poor.mental.health.days <= 5.37
0.0576 < Uninsured.children <= 0.0826
74.4 < Injury.deaths <= 90.3
0.730 < Homeownership <= 0.774
0.128 < Food.insecurity <= 0.155
0.770 < Driving.alone.to.work <= 0.806
Feature
0.00
0.02
0.04
0.06
Weight
Positive
Negative

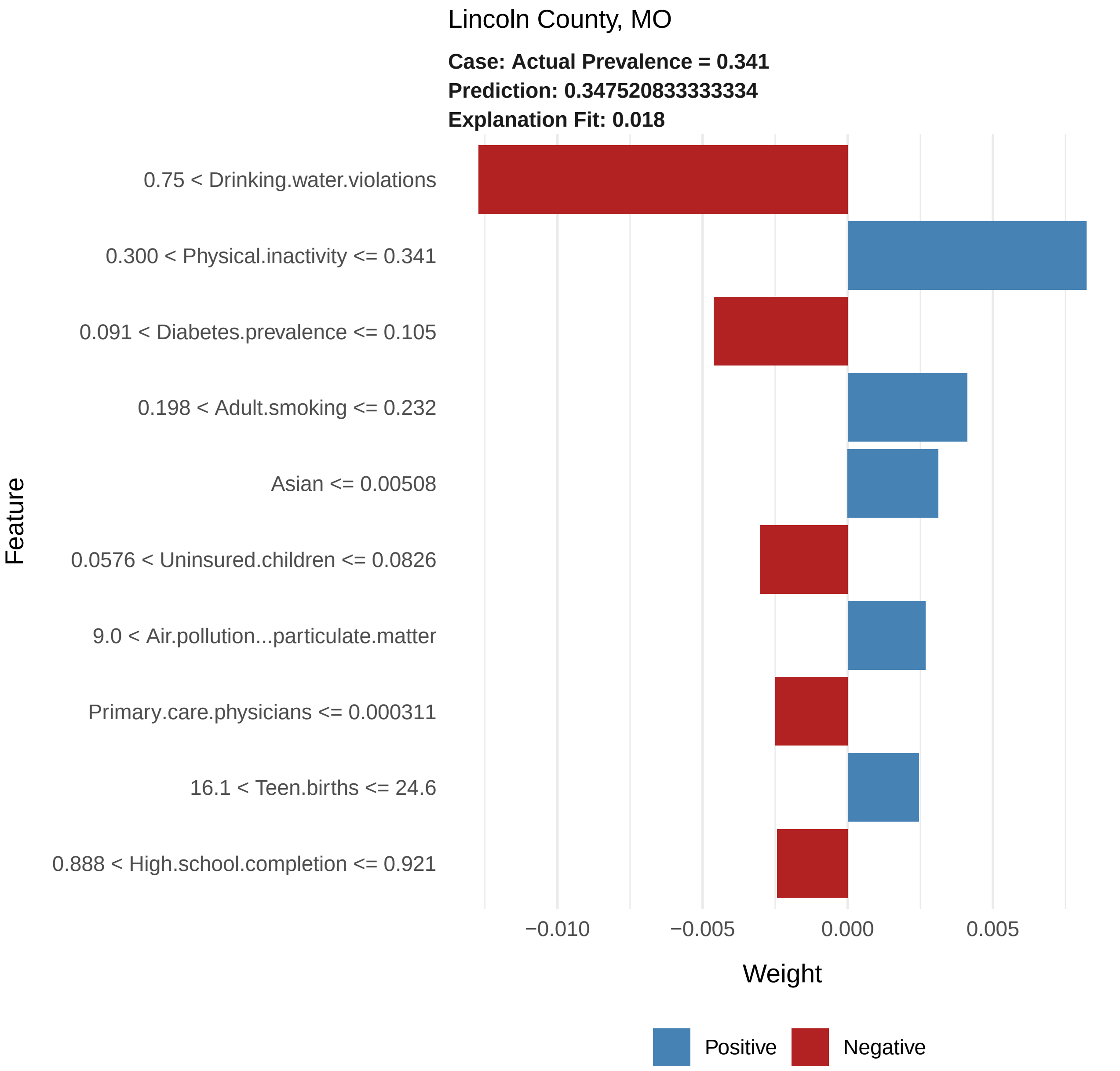

Lincoln County, MO
Case: Actual Prevalence = 0.341
Prediction: 0.347520833333334
Explanation Fit: 0.018
Feature
0.75 < Drinking.water.violations
0.300 < Physical.inactivity <= 0.341
0.091 < Diabetes.prevalence <= 0.105
0.198 < Adult.smoking <= 0.232
Asian <= 0.00508
0.0576 < Uninsured.children <= 0.0826
9.0 < Air.pollution...particulate.matter
Primary.care.physicians <= 0.000311
16.1 < Teen.births <= 24.6
0.888 < High.school.completion <= 0.921
−0.010
−0.005
0.000
0.005
Weight
Positive
Negative

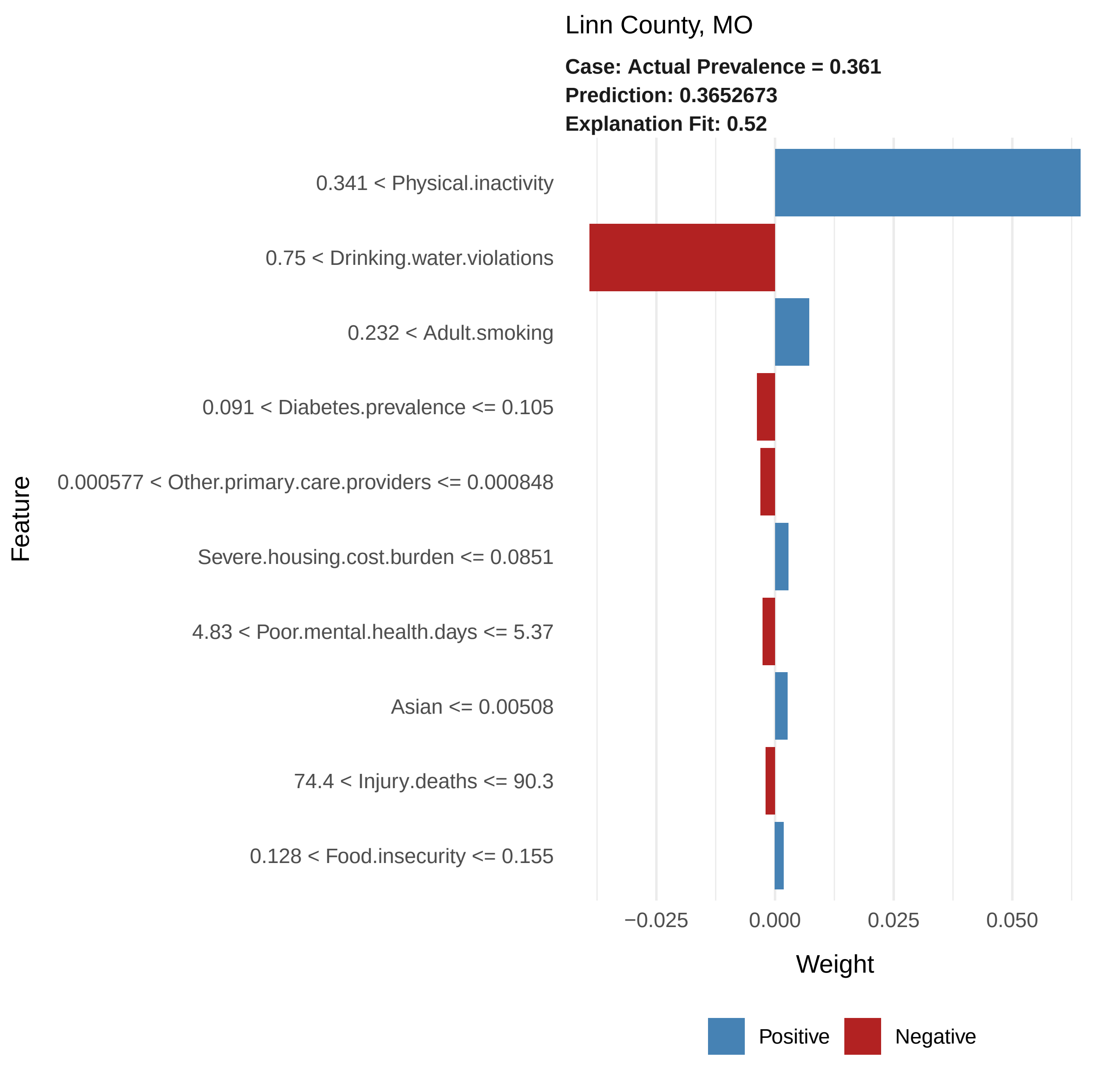
Linn County, MO
Case: Actual Prevalence = 0.361
Prediction: 0.3652673
Explanation Fit: 0.52
0.341 < Physical.inactivity
0.75 < Drinking.water.violations
0.232 < Adult.smoking
0.091 < Diabetes.prevalence <= 0.105
0.000577 < Other.primary.care.providers <= 0.000848
Severe.housing.cost.burden <= 0.0851
4.83 < Poor.mental.health.days <= 5.37
Asian <= 0.00508
74.4 < Injury.deaths <= 90.3
0.128 < Food.insecurity <= 0.155
Feature
−0.025
0.000
0.025
0.050
Weight
Positive
Negative

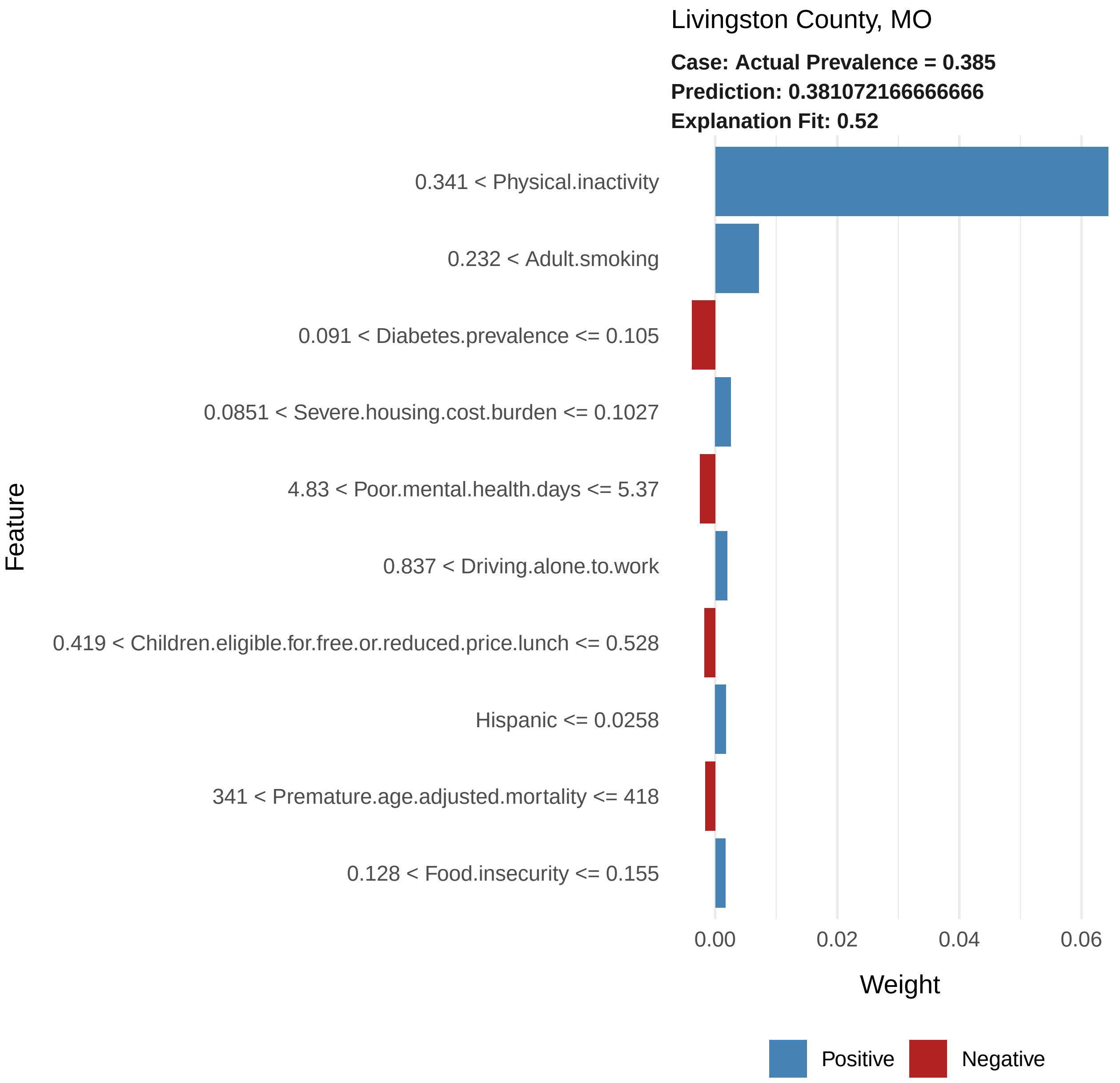

Livingston County, MO
Case: Actual Prevalence = 0.385
Prediction: 0.381072166666666
Explanation Fit: 0.52
0.341 < Physical.inactivity
0.232 < Adult.smoking
0.091 < Diabetes.prevalence <= 0.105
0.0851 < Severe.housing.cost.burden <= 0.1027
4.83 < Poor.mental.health.days <= 5.37
0.837 < Driving.alone.to.work
0.419 < Children.eligible.for.free.or.reduced.price.lunch <= 0.528
Hispanic <= 0.0258
341 < Premature.age.adjusted.mortality <= 418
0.128 < Food.insecurity <= 0.155
Feature
0.00
0.02
0.04
0.06
Weight
Positive
Negative

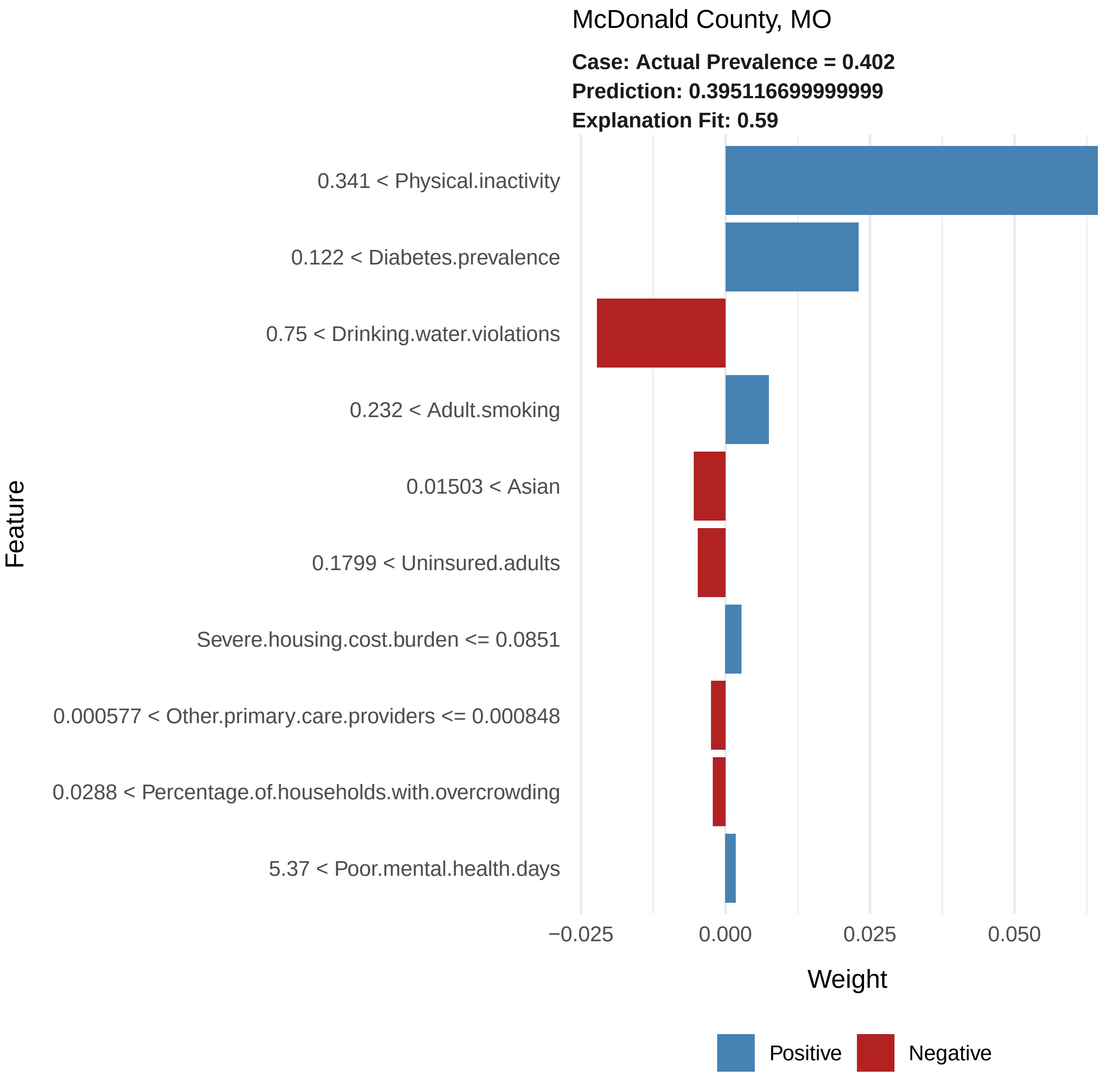

McDonald County, MO
Case: Actual Prevalence = 0.402
Prediction: 0.395116699999999
Explanation Fit: 0.59
0.341 < Physical.inactivity
0.122 < Diabetes.prevalence
0.75 < Drinking.water.violations
0.232 < Adult.smoking
0.01503 < Asian
0.1799 < Uninsured.adults
Severe.housing.cost.burden <= 0.0851
0.000577 < Other.primary.care.providers <= 0.000848
0.0288 < Percentage.of.households.with.overcrowding
5.37 < Poor.mental.health.days
Feature
−0.025
0.000
0.025
0.050
Weight
Positive
Negative

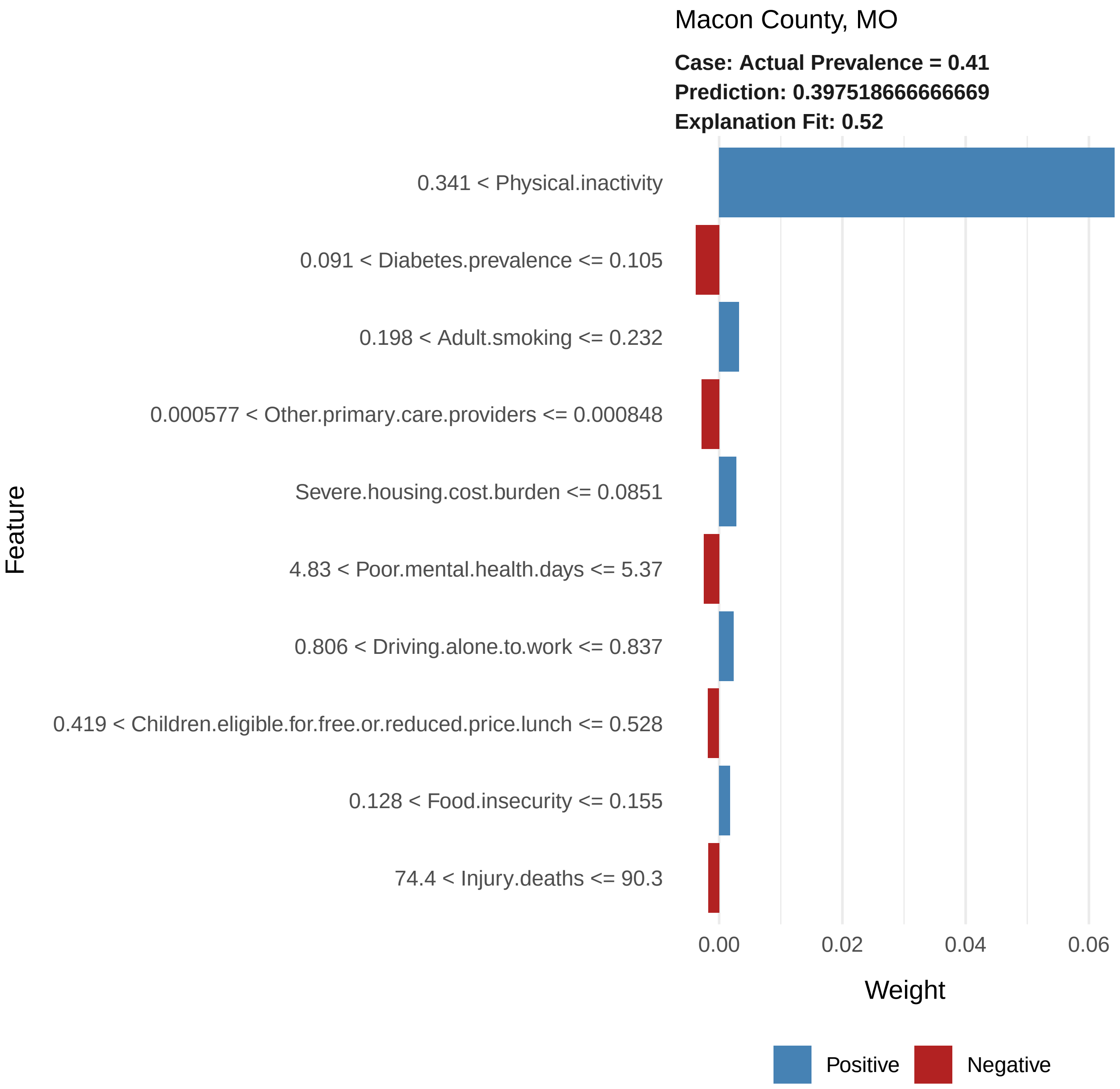
Macon County, MO
Case: Actual Prevalence = 0.41
Prediction: 0.397518666666669
Explanation Fit: 0.52
0.341 < Physical.inactivity
0.091 < Diabetes.prevalence <= 0.105
0.198 < Adult.smoking <= 0.232
0.000577 < Other.primary.care.providers <= 0.000848
Severe.housing.cost.burden <= 0.0851
4.83 < Poor.mental.health.days <= 5.37
0.806 < Driving.alone.to.work <= 0.837
0.419 < Children.eligible.for.free.or.reduced.price.lunch <= 0.528
0.128 < Food.insecurity <= 0.155
74.4 < Injury.deaths <= 90.3
Feature
0.00
0.02
0.04
0.06
Weight
Positive
Negative

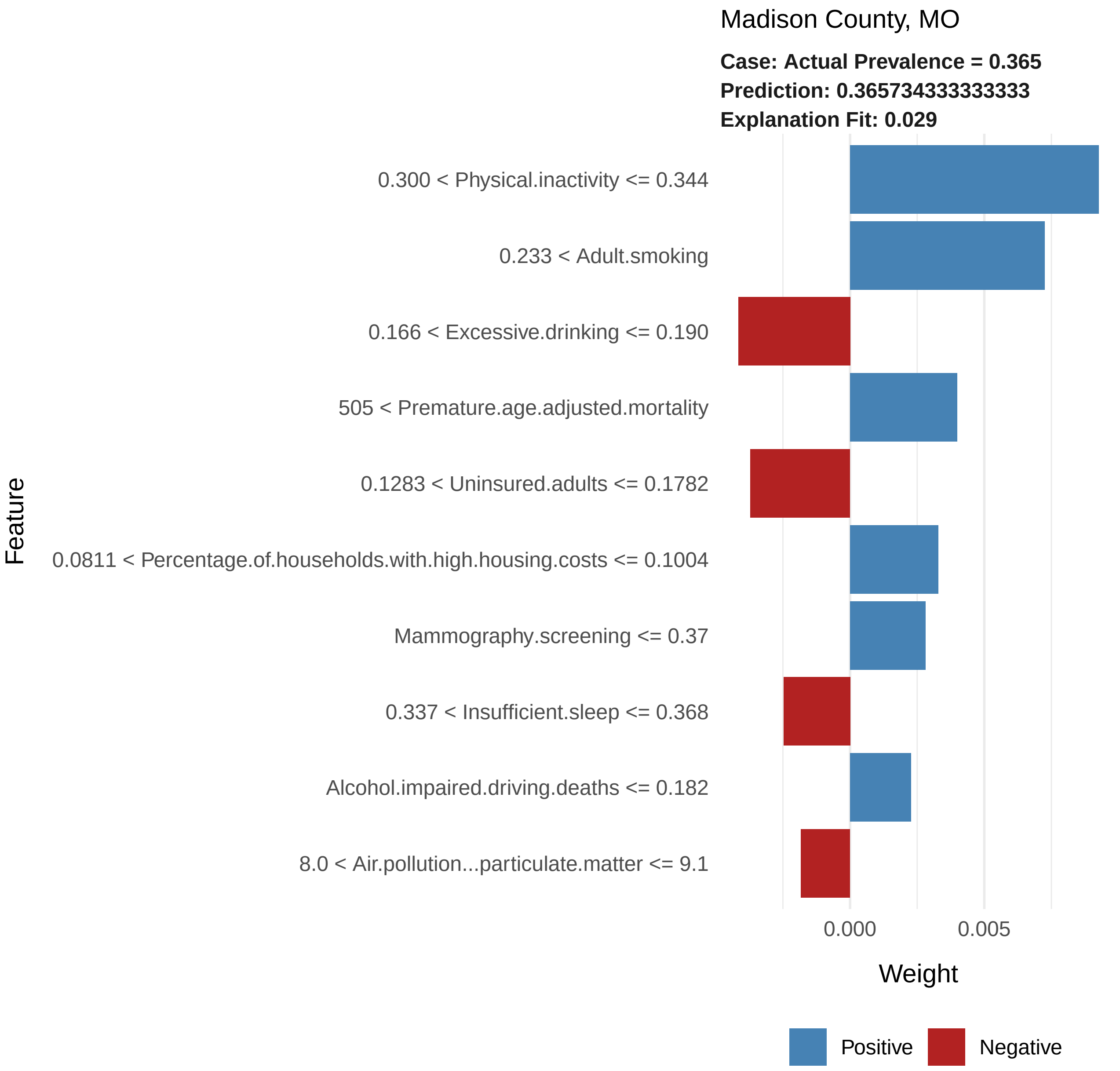

Madison County, MO
Case: Actual Prevalence = 0.365
Prediction: 0.365734333333333
Explanation Fit: 0.029
0.300 < Physical.inactivity <= 0.344
0.233 < Adult.smoking
0.166 < Excessive.drinking <= 0.190
505 < Premature.age.adjusted.mortality
0.1283 < Uninsured.adults <= 0.1782
0.0811 < Percentage.of.households.with.high.housing.costs <= 0.1004
Mammography.screening <= 0.37
0.337 < Insufficient.sleep <= 0.368
Alcohol.impaired.driving.deaths <= 0.182
8.0 < Air.pollution...particulate.matter <= 9.1
Feature
0.000
0.005
Weight
Positive
Negative

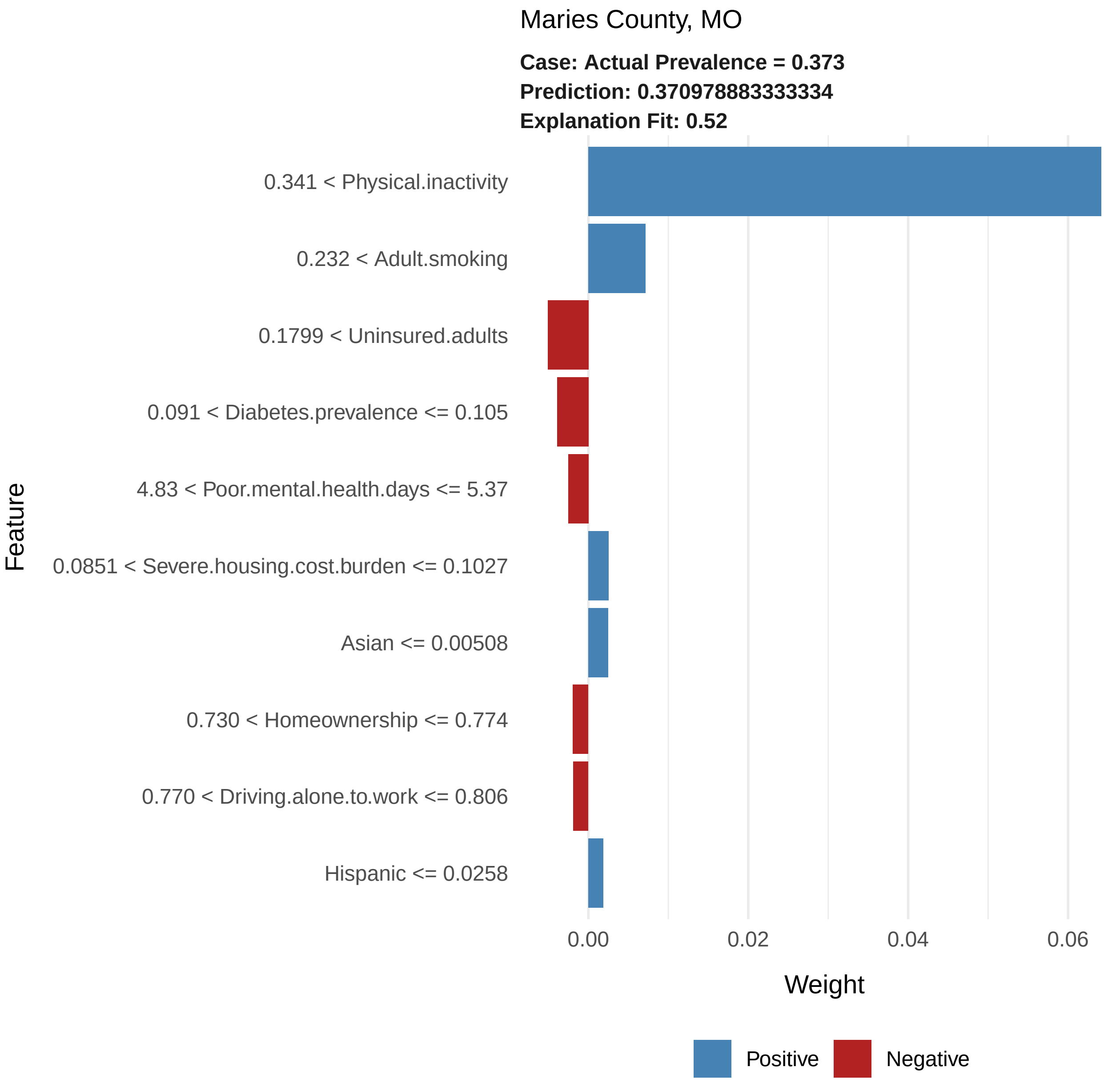

Maries County, MO
Case: Actual Prevalence = 0.373
Prediction: 0.370978883333334
Explanation Fit: 0.52
0.341 < Physical.inactivity
0.232 < Adult.smoking
0.1799 < Uninsured.adults
0.091 < Diabetes.prevalence <= 0.105
4.83 < Poor.mental.health.days <= 5.37
0.0851 < Severe.housing.cost.burden <= 0.1027
Asian <= 0.00508
0.730 < Homeownership <= 0.774
0.770 < Driving.alone.to.work <= 0.806
Hispanic <= 0.0258
Feature
0.00
0.02
0.04
0.06
Weight
Positive
Negative

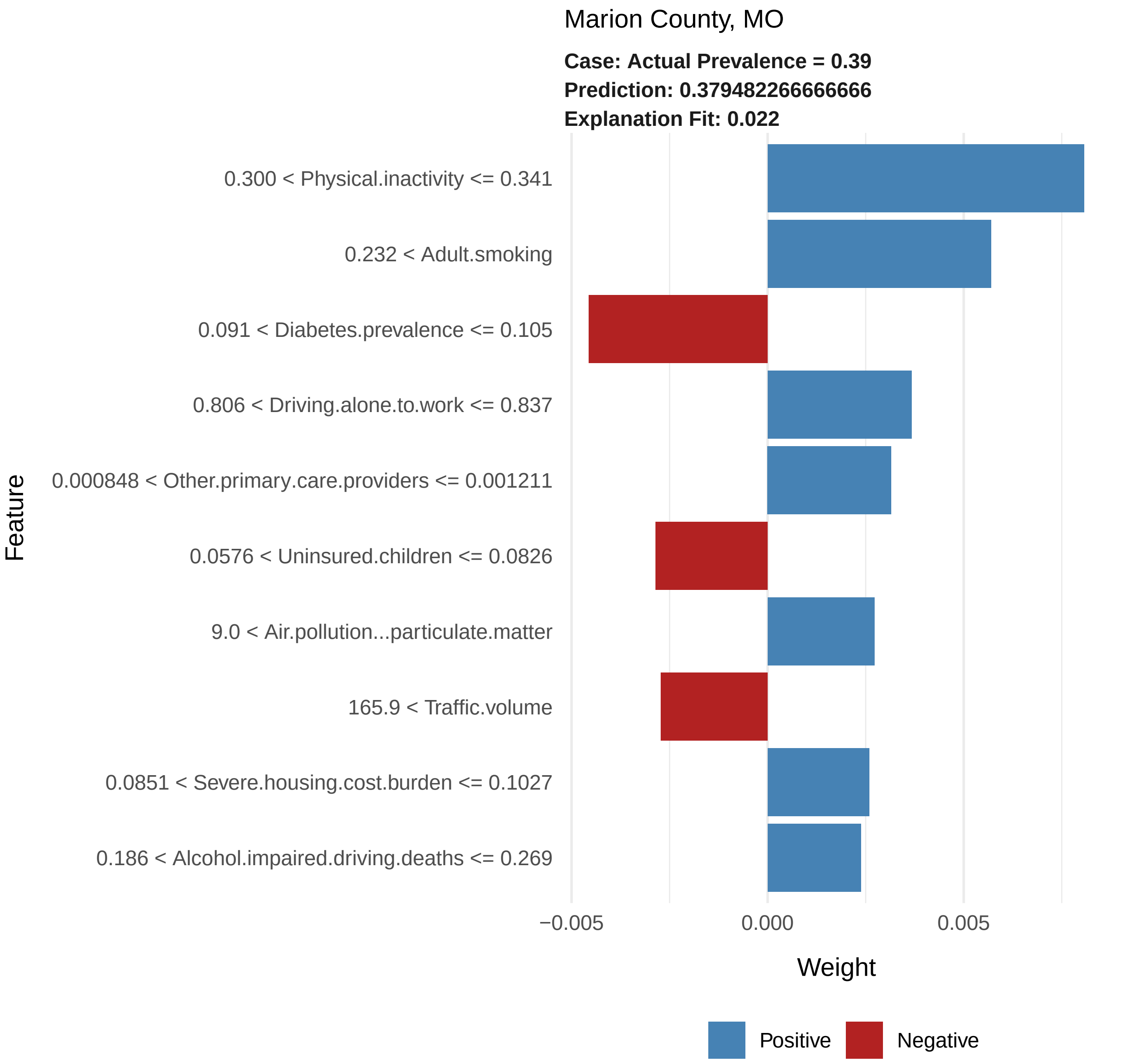
Marion County, MO
Case: Actual Prevalence = 0.39
Prediction: 0.379482266666666
Explanation Fit: 0.022
0.300 < Physical.inactivity <= 0.341
0.232 < Adult.smoking
0.091 < Diabetes.prevalence <= 0.105
0.806 < Driving.alone.to.work <= 0.837
0.000848 < Other.primary.care.providers <= 0.001211
0.0576 < Uninsured.children <= 0.0826
9.0 < Air.pollution...particulate.matter
165.9 < Traffic.volume
0.0851 < Severe.housing.cost.burden <= 0.1027
0.186 < Alcohol.impaired.driving.deaths <= 0.269
Feature
−0.005
0.000
0.005
Weight
Positive
Negative

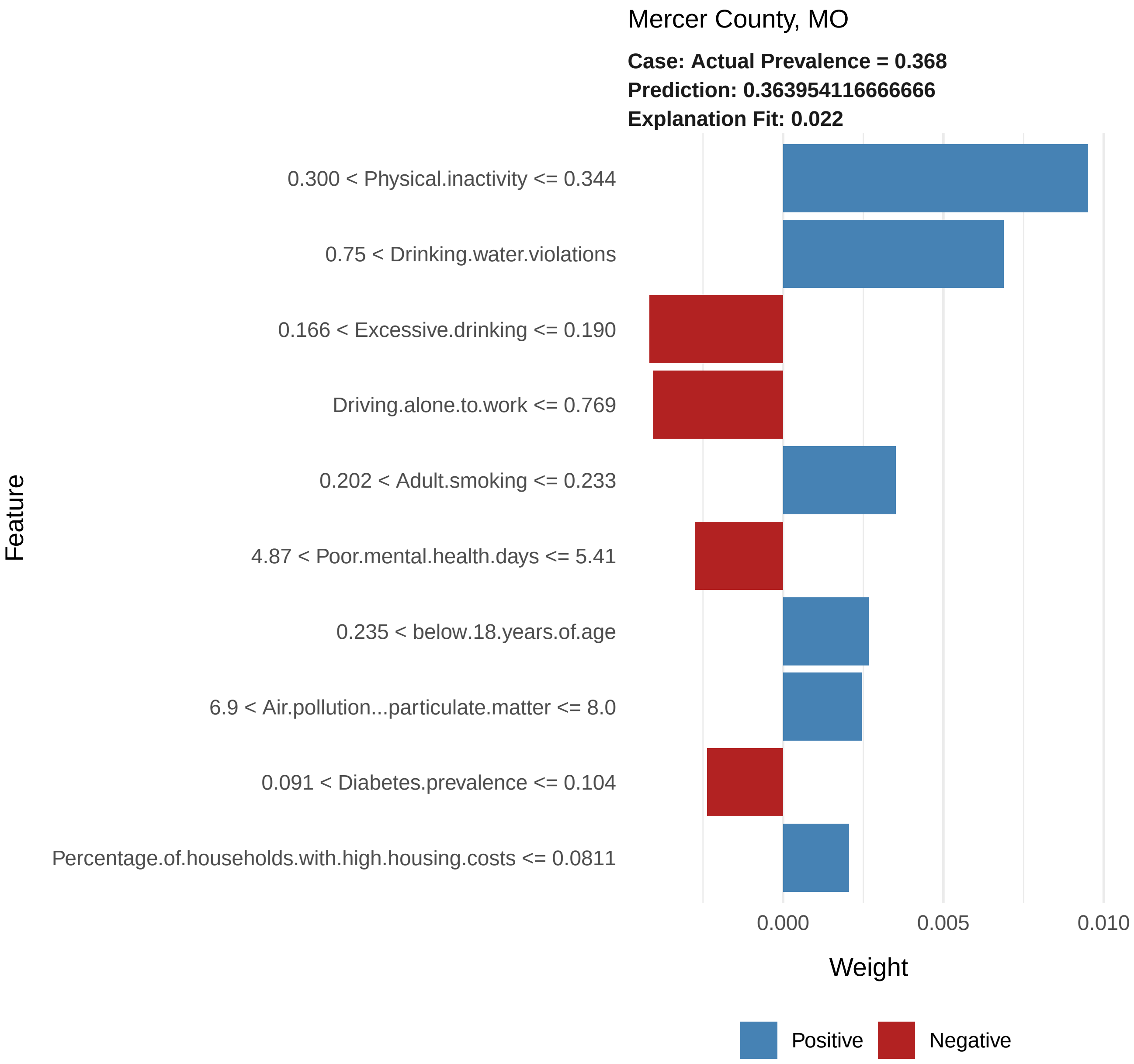
Mercer County, MO
Case: Actual Prevalence = 0.368
Prediction: 0.363954116666666
Explanation Fit: 0.022
0.300 < Physical.inactivity <= 0.344
0.75 < Drinking.water.violations
0.166 < Excessive.drinking <= 0.190
Driving.alone.to.work <= 0.769
0.202 < Adult.smoking <= 0.233
4.87 < Poor.mental.health.days <= 5.41
0.235 < below.18.years.of.age
6.9 < Air.pollution...particulate.matter <= 8.0
0.091 < Diabetes.prevalence <= 0.104
Percentage.of.households.with.high.housing.costs <= 0.0811
Feature
0.000
0.005
0.010
Weight
Positive
Negative

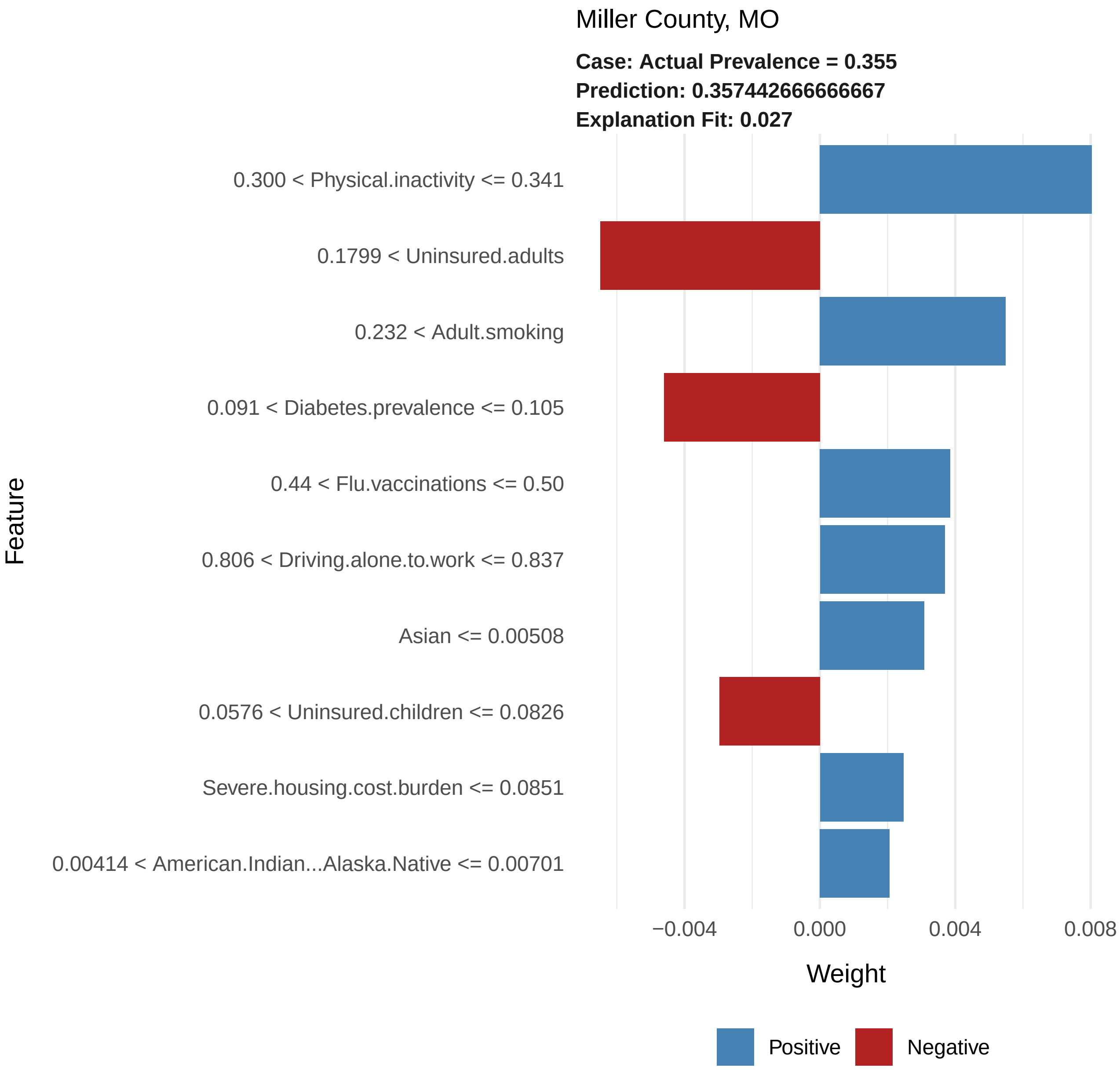

Miller County, MO
Case: Actual Prevalence = 0.355
Prediction: 0.357442666666667
Explanation Fit: 0.027
0.300 < Physical.inactivity <= 0.341
0.1799 < Uninsured.adults
0.232 < Adult.smoking
0.091 < Diabetes.prevalence <= 0.105
0.44 < Flu.vaccinations <= 0.50
0.806 < Driving.alone.to.work <= 0.837
Asian <= 0.00508
0.0576 < Uninsured.children <= 0.0826
Severe.housing.cost.burden <= 0.0851
0.00414 < American.Indian...Alaska.Native <= 0.00701
Feature
−0.004
0.000
0.004
0.008
Weight
Positive
Negative

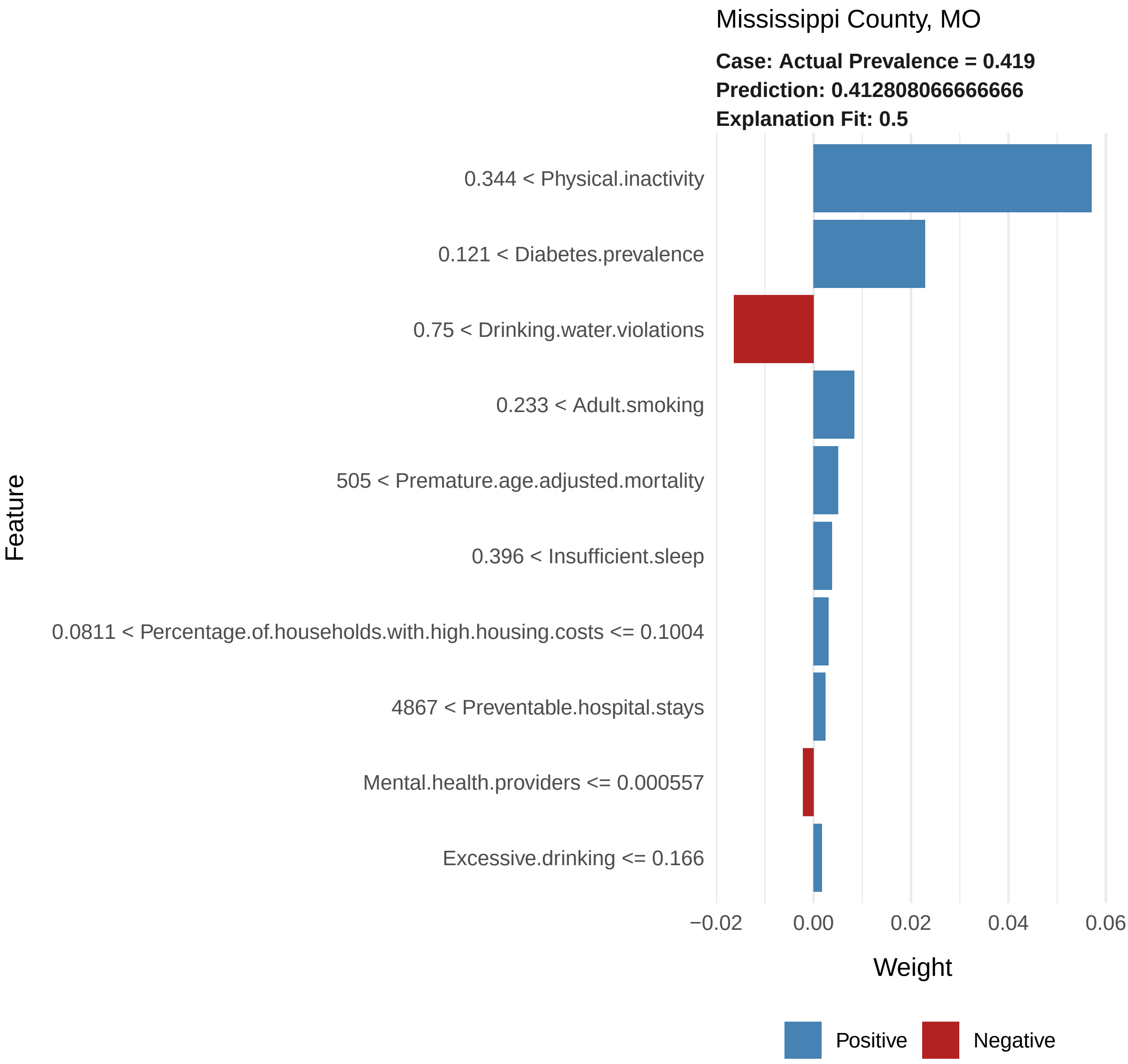
Mississippi County, MO
Case: Actual Prevalence = 0.419
Prediction: 0.412808066666666
Explanation Fit: 0.5
0.344 < Physical.inactivity
0.121 < Diabetes.prevalence
0.75 < Drinking.water.violations
0.233 < Adult.smoking
505 < Premature.age.adjusted.mortality
0.396 < Insufficient.sleep
0.0811 < Percentage.of.households.with.high.housing.costs <= 0.1004
4867 < Preventable.hospital.stays
Mental.health.providers <= 0.000557
Excessive.drinking <= 0.166
Feature
−0.02
0.00
0.02
0.04
0.06
Weight
Positive
Negative

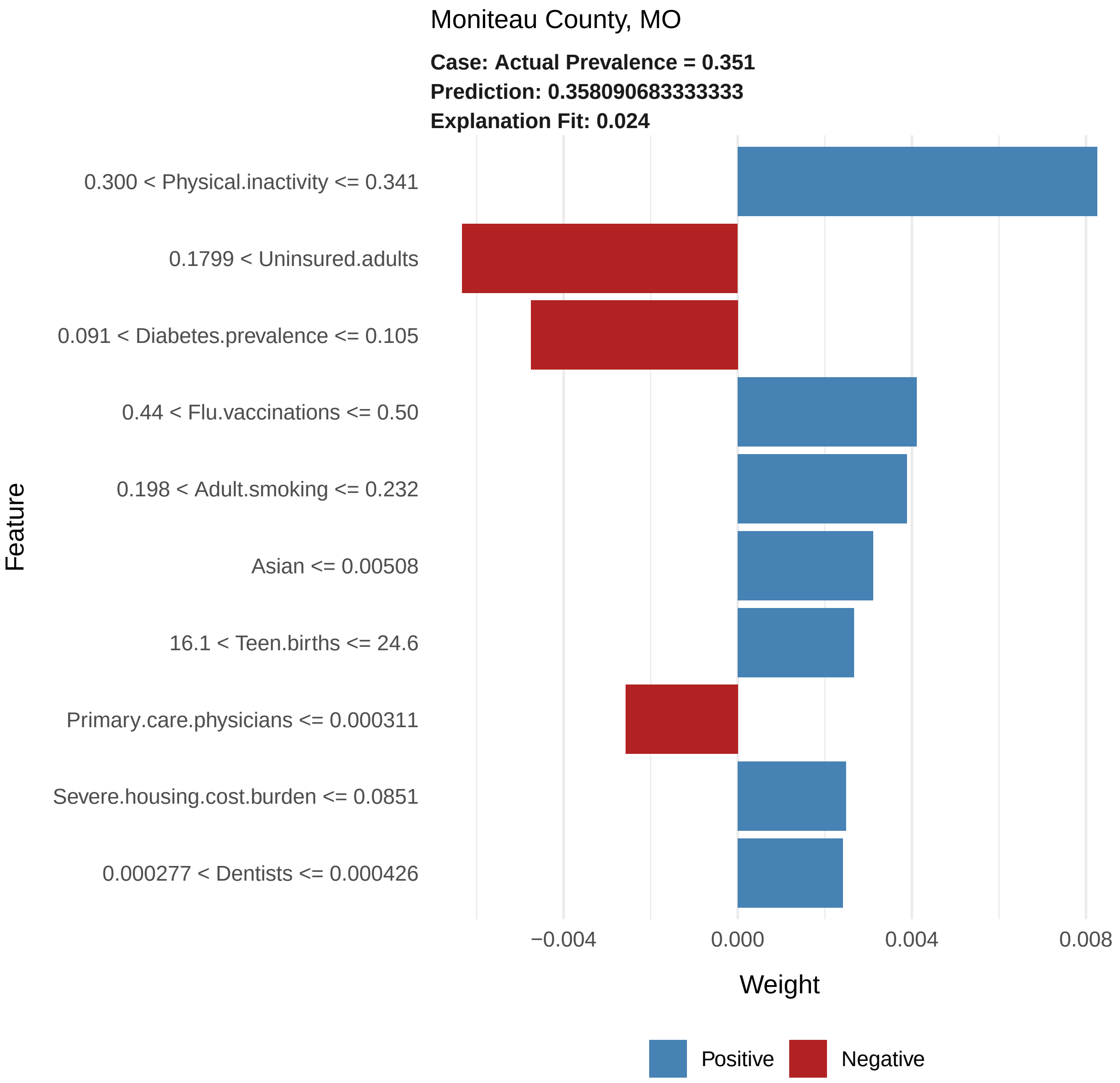

Moniteau County, MO
Case: Actual Prevalence = 0.351
Prediction: 0.358090683333333
Explanation Fit: 0.024
0.300 < Physical.inactivity <= 0.341
0.1799 < Uninsured.adults
0.091 < Diabetes.prevalence <= 0.105
0.44 < Flu.vaccinations <= 0.50
0.198 < Adult.smoking <= 0.232
Asian <= 0.00508
16.1 < Teen.births <= 24.6
Primary.care.physicians <= 0.000311
Severe.housing.cost.burden <= 0.0851
0.000277 < Dentists <= 0.000426
Feature
−0.004
0.000
0.004
0.008
Weight
Positive
Negative

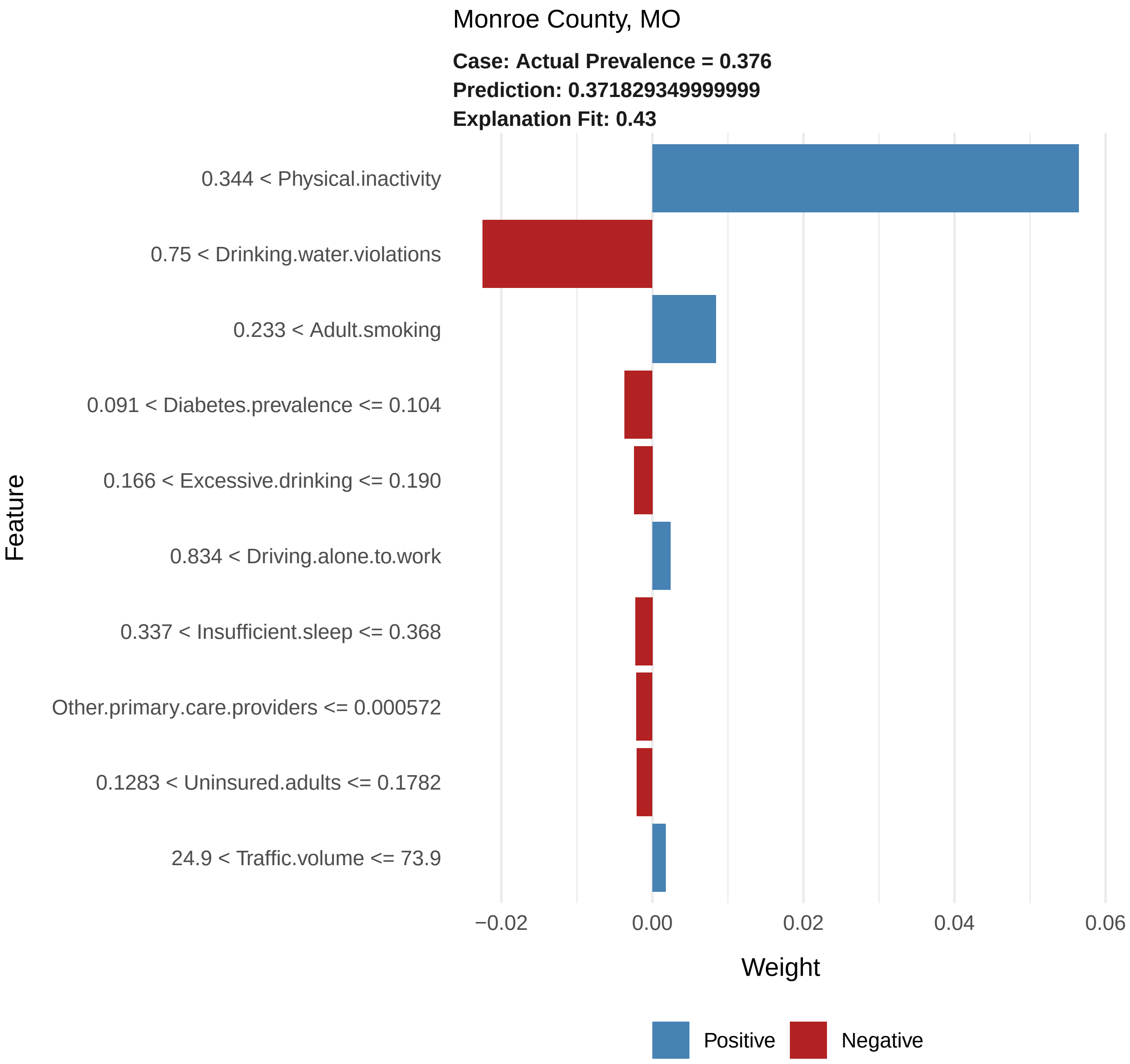

Monroe County, MO
Case: Actual Prevalence = 0.376
Prediction: 0.371829349999999
Explanation Fit: 0.43
0.344 < Physical.inactivity
0.75 < Drinking.water.violations
0.233 < Adult.smoking
0.091 < Diabetes.prevalence <= 0.104
0.166 < Excessive.drinking <= 0.190
0.834 < Driving.alone.to.work
0.337 < Insufficient.sleep <= 0.368
Other.primary.care.providers <= 0.000572
0.1283 < Uninsured.adults <= 0.1782
24.9 < Traffic.volume <= 73.9
Feature
−0.02
0.00
0.02
0.04
0.06
Weight
Positive
Negative

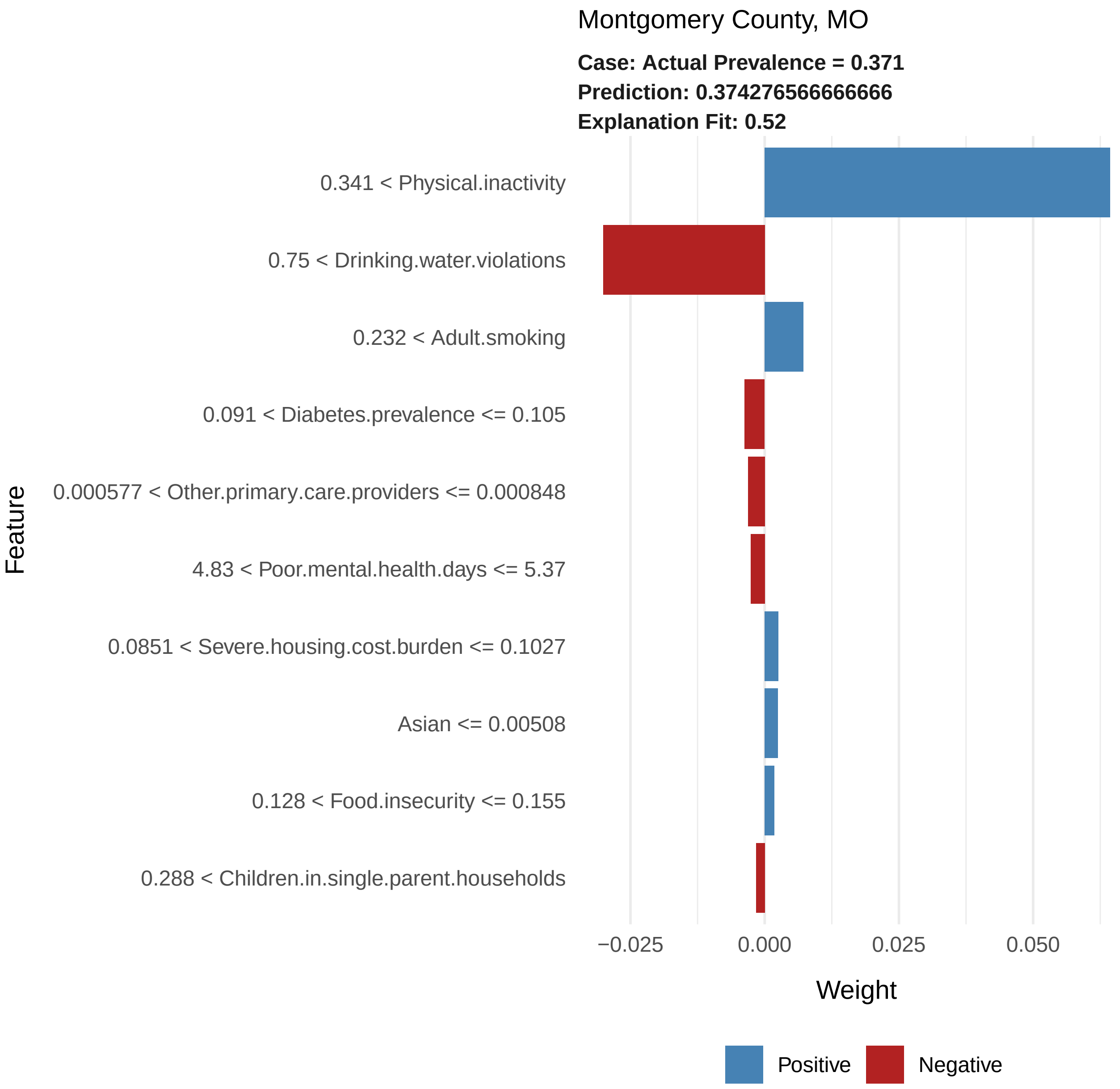
Montgomery County, MO
Case: Actual Prevalence = 0.371
Prediction: 0.374276566666666
Explanation Fit: 0.52
0.341 < Physical.inactivity
0.75 < Drinking.water.violations
0.232 < Adult.smoking
0.091 < Diabetes.prevalence <= 0.105
0.000577 < Other.primary.care.providers <= 0.000848
4.83 < Poor.mental.health.days <= 5.37
0.0851 < Severe.housing.cost.burden <= 0.1027
Asian <= 0.00508
0.128 < Food.insecurity <= 0.155
0.288 < Children.in.single.parent.households
Feature
−0.025
0.000
0.025
0.050
Weight
Positive
Negative

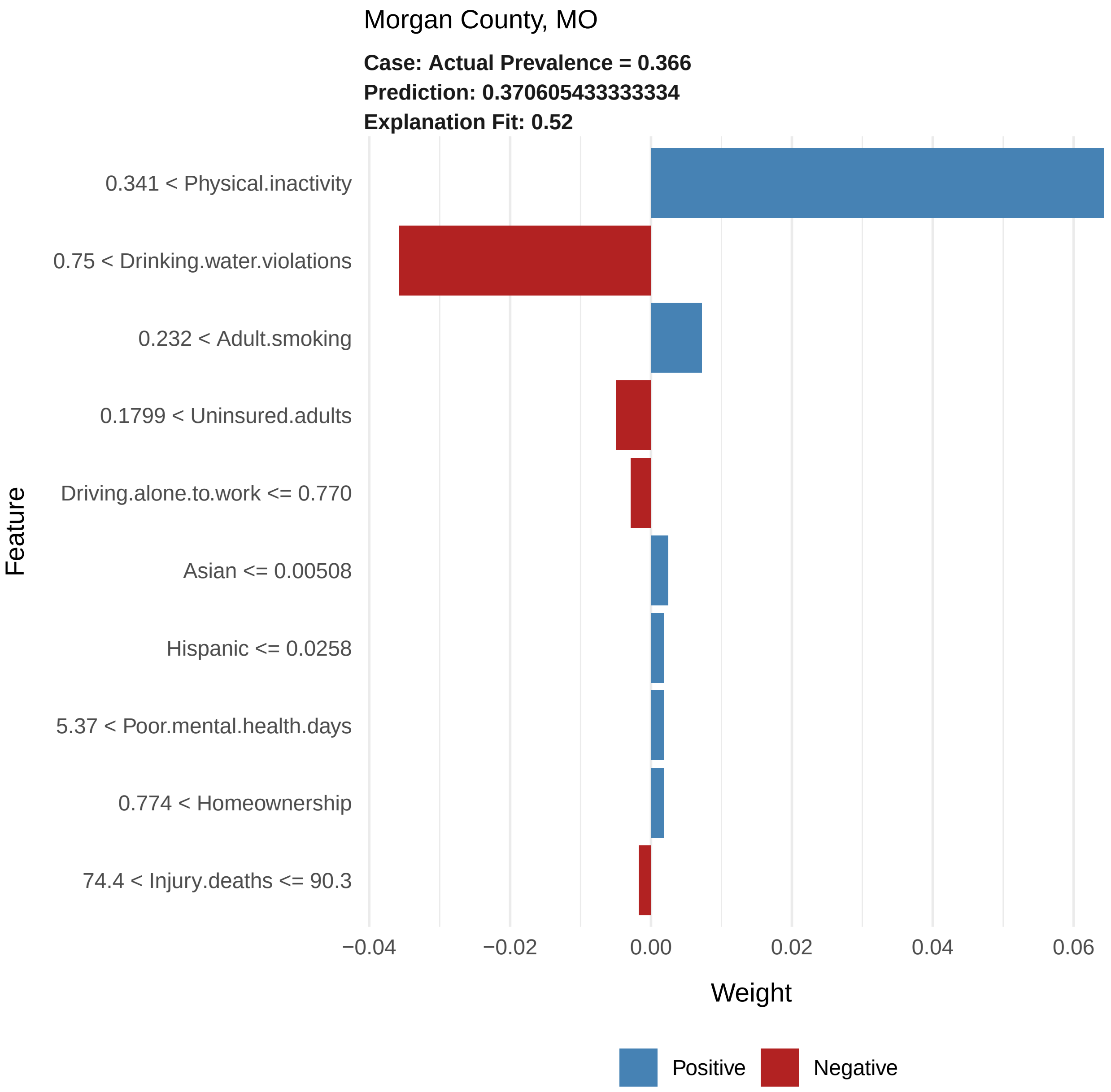
Morgan County, MO
Case: Actual Prevalence = 0.366
Prediction: 0.370605433333334
Explanation Fit: 0.52
0.341 < Physical.inactivity
0.75 < Drinking.water.violations
0.232 < Adult.smoking
0.1799 < Uninsured.adults
Driving.alone.to.work <= 0.770
Asian <= 0.00508
Hispanic <= 0.0258
5.37 < Poor.mental.health.days
0.774 < Homeownership
74.4 < Injury.deaths <= 90.3
Feature
−0.04
−0.02
0.00
0.02
0.04
0.06
Weight
Positive
Negative

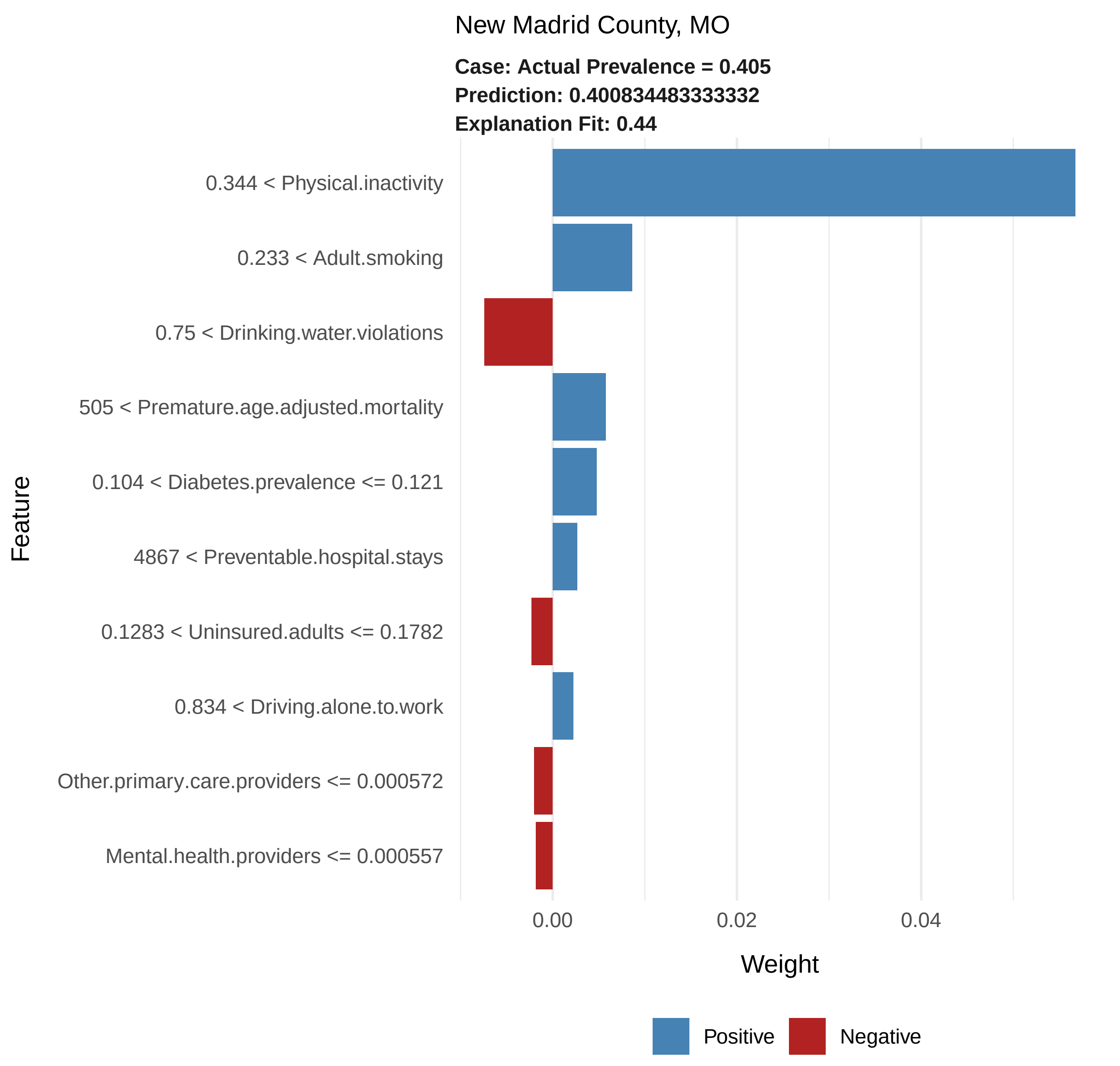

New Madrid County, MO
Case: Actual Prevalence = 0.405
Prediction: 0.400834483333332
Explanation Fit: 0.44
0.344 < Physical.inactivity
0.233 < Adult.smoking
0.75 < Drinking.water.violations
505 < Premature.age.adjusted.mortality
0.104 < Diabetes.prevalence <= 0.121
4867 < Preventable.hospital.stays
0.1283 < Uninsured.adults <= 0.1782
0.834 < Driving.alone.to.work
Other.primary.care.providers <= 0.000572
Mental.health.providers <= 0.000557
Feature
0.00
0.02
0.04
Weight
Positive
Negative

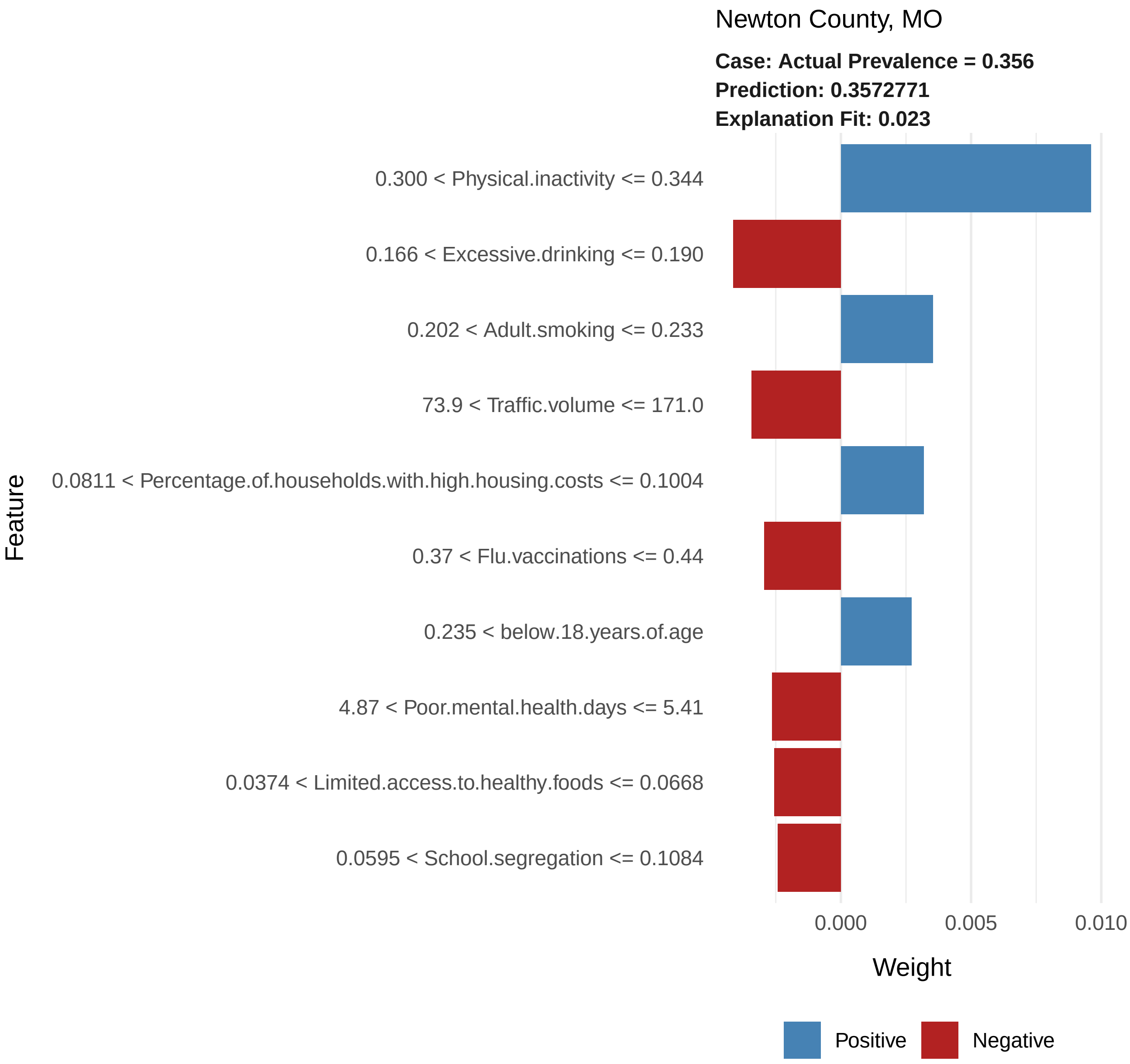
Newton County, MO
Case: Actual Prevalence = 0.356
Prediction: 0.3572771
Explanation Fit: 0.023
0.300 < Physical.inactivity <= 0.344
0.166 < Excessive.drinking <= 0.190
0.202 < Adult.smoking <= 0.233
73.9 < Traffic.volume <= 171.0
0.0811 < Percentage.of.households.with.high.housing.costs <= 0.1004
0.37 < Flu.vaccinations <= 0.44
0.235 < below.18.years.of.age
4.87 < Poor.mental.health.days <= 5.41
0.0374 < Limited.access.to.healthy.foods <= 0.0668
0.0595 < School.segregation <= 0.1084
Feature
0.000
0.005
0.010
Weight
Positive
Negative

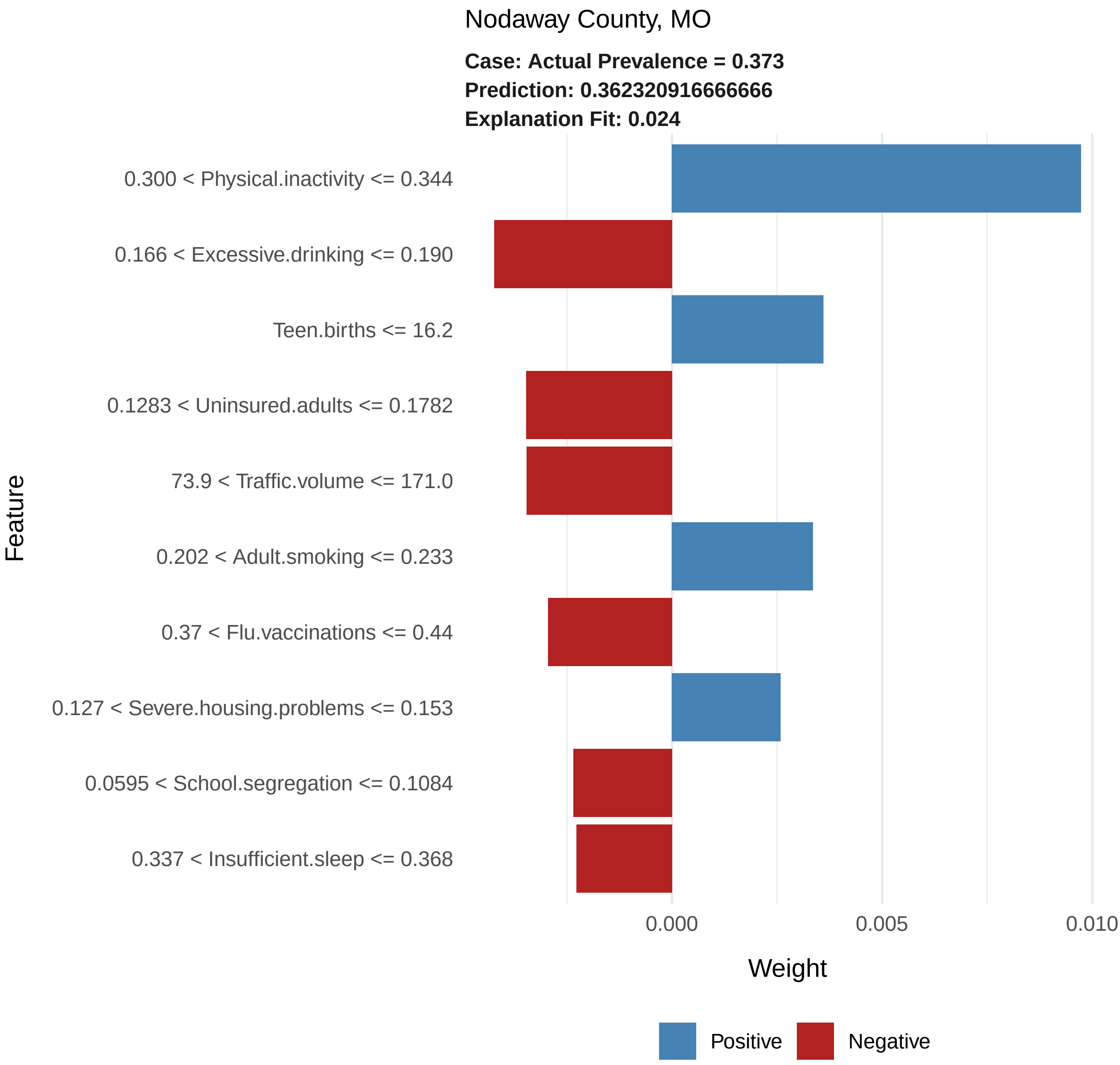
Nodaway County, MO
Case: Actual Prevalence = 0.373
Prediction: 0.362320916666666
Explanation Fit: 0.024
0.300 < Physical.inactivity <= 0.344
0.166 < Excessive.drinking <= 0.190
Teen.births <= 16.2
0.1283 < Uninsured.adults <= 0.1782
73.9 < Traffic.volume <= 171.0
0.202 < Adult.smoking <= 0.233
0.37 < Flu.vaccinations <= 0.44
0.127 < Severe.housing.problems <= 0.153
0.0595 < School.segregation <= 0.1084
0.337 < Insufficient.sleep <= 0.368
Feature
0.000
0.005
0.010
Weight
Positive
Negative

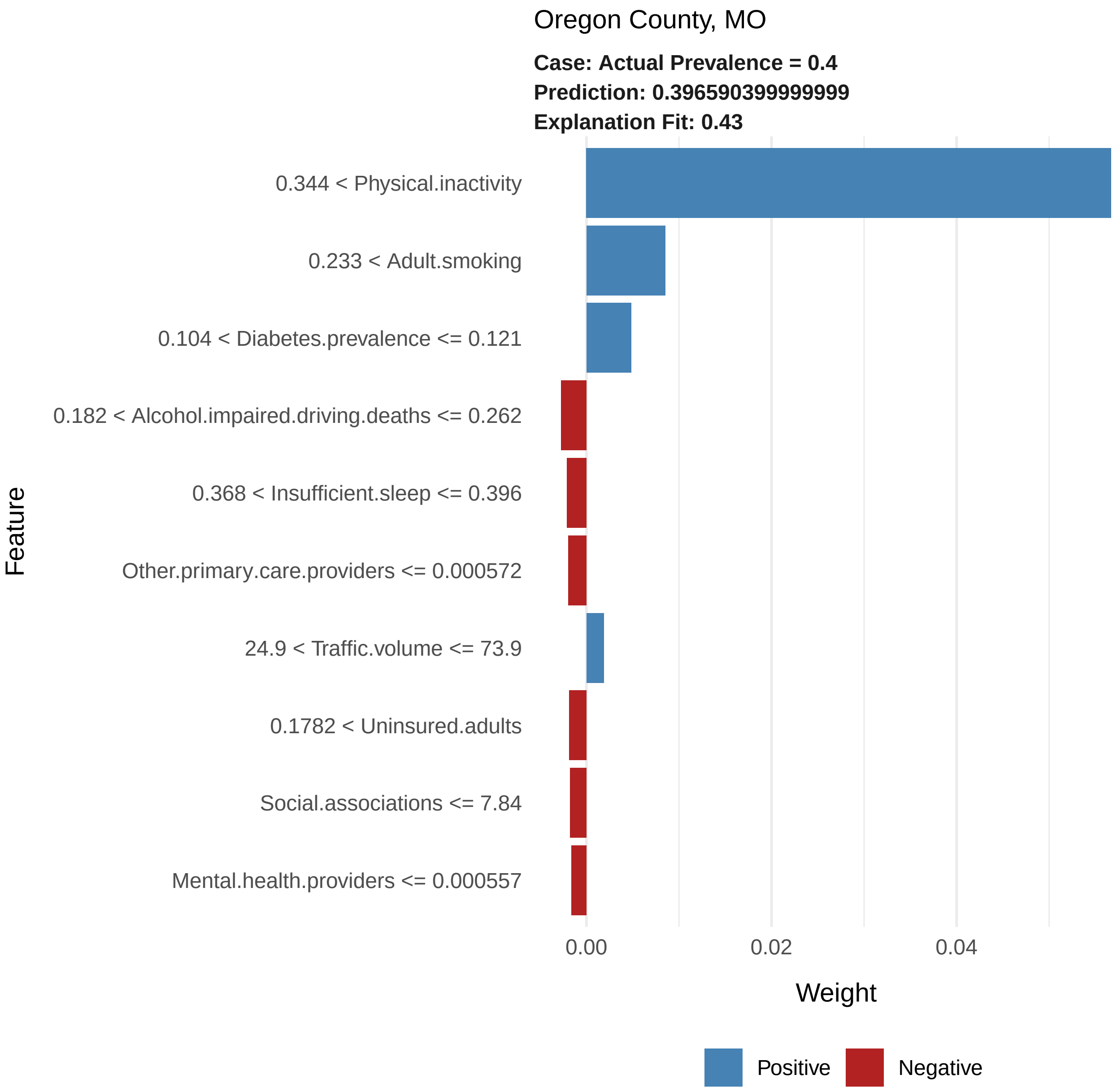

Oregon County, MO
Case: Actual Prevalence = 0.4
Prediction: 0.396590399999999
Explanation Fit: 0.43
0.344 < Physical.inactivity
0.233 < Adult.smoking
0.104 < Diabetes.prevalence <= 0.121
0.182 < Alcohol.impaired.driving.deaths <= 0.262
0.368 < Insufficient.sleep <= 0.396
Other.primary.care.providers <= 0.000572
24.9 < Traffic.volume <= 73.9
0.1782 < Uninsured.adults
Social.associations <= 7.84
Mental.health.providers <= 0.000557
Feature
0.00
0.02
0.04
Weight
Positive
Negative

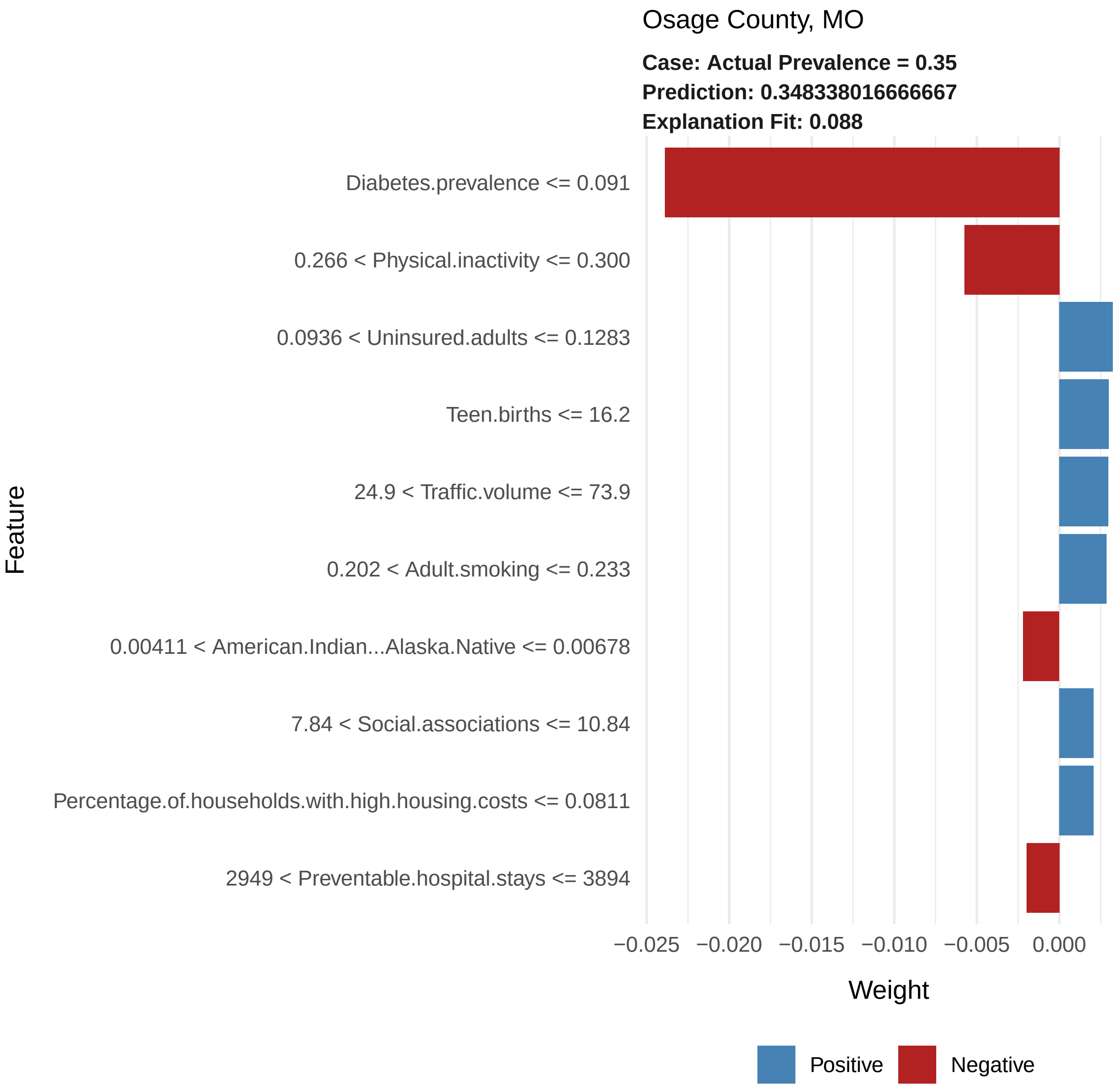

Osage County, MO
Case: Actual Prevalence = 0.35
Prediction: 0.348338016666667
Explanation Fit: 0.088
Diabetes.prevalence <= 0.091
0.266 < Physical.inactivity <= 0.300
0.0936 < Uninsured.adults <= 0.1283
Teen.births <= 16.2
24.9 < Traffic.volume <= 73.9
0.202 < Adult.smoking <= 0.233
0.00411 < American.Indian...Alaska.Native <= 0.00678
7.84 < Social.associations <= 10.84
Percentage.of.households.with.high.housing.costs <= 0.0811
2949 < Preventable.hospital.stays <= 3894
Feature
−0.025
−0.020
−0.015
−0.010
−0.005
0.000
Weight
Positive
Negative

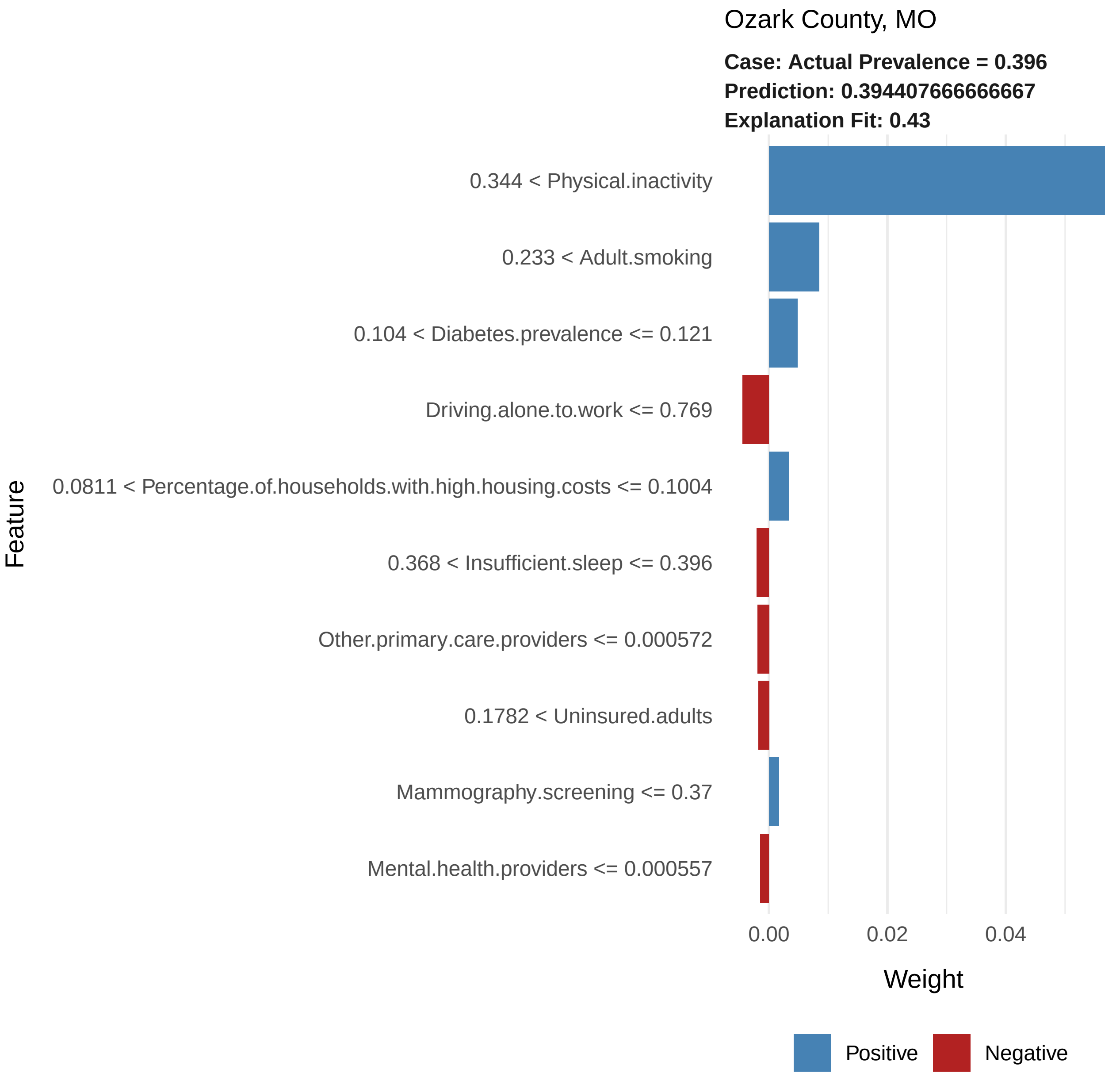
Ozark County, MO
Case: Actual Prevalence = 0.396
Prediction: 0.394407666666667
Explanation Fit: 0.43
0.344 < Physical.inactivity
0.233 < Adult.smoking
0.104 < Diabetes.prevalence <= 0.121
Driving.alone.to.work <= 0.769
0.0811 < Percentage.of.households.with.high.housing.costs <= 0.1004
0.368 < Insufficient.sleep <= 0.396
Other.primary.care.providers <= 0.000572
0.1782 < Uninsured.adults
Mammography.screening <= 0.37
Mental.health.providers <= 0.000557
Feature
0.00
0.02
0.04
Weight
Positive
Negative

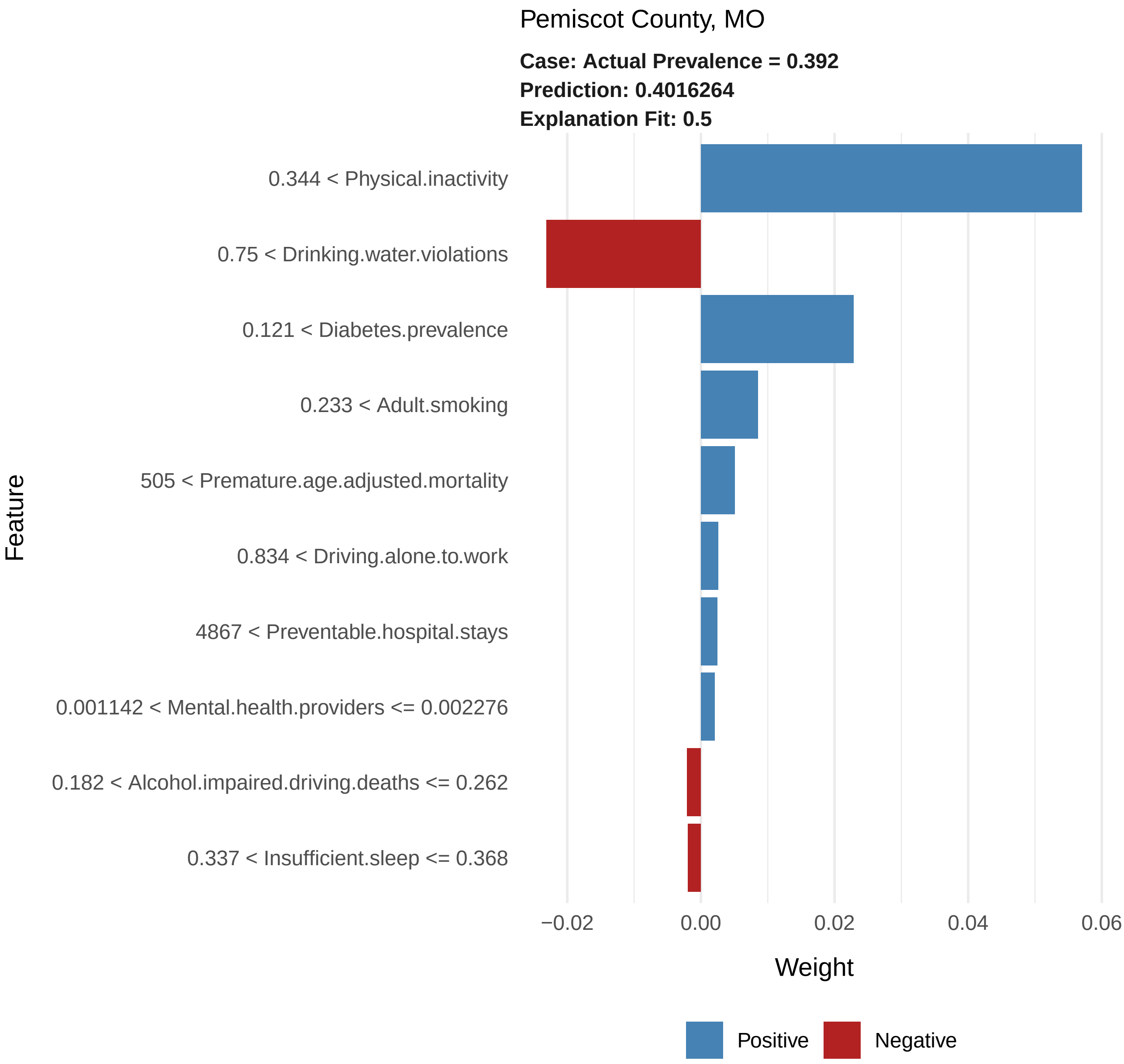
Pemiscot County, MO
Case: Actual Prevalence = 0.392
Prediction: 0.4016264
Explanation Fit: 0.5
0.344 < Physical.inactivity
0.75 < Drinking.water.violations
0.121 < Diabetes.prevalence
0.233 < Adult.smoking
505 < Premature.age.adjusted.mortality
0.834 < Driving.alone.to.work
4867 < Preventable.hospital.stays
0.001142 < Mental.health.providers <= 0.002276
0.182 < Alcohol.impaired.driving.deaths <= 0.262
0.337 < Insufficient.sleep <= 0.368
Feature
−0.02
0.00
0.02
0.04
0.06
Weight
Positive
Negative

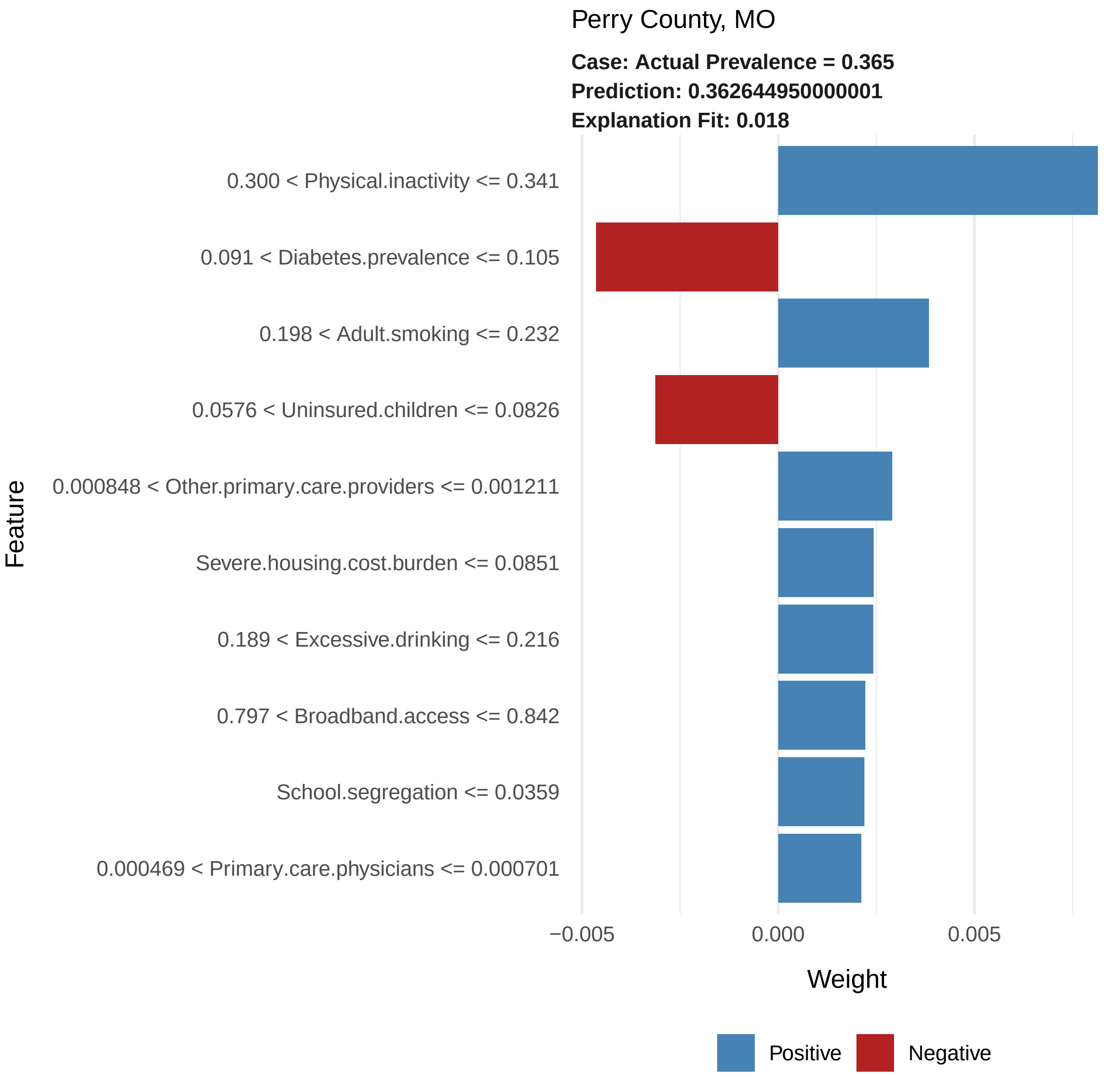
Perry County, MO
Case: Actual Prevalence = 0.365
Prediction: 0.362644950000001
Explanation Fit: 0.018
0.300 < Physical.inactivity <= 0.341
0.091 < Diabetes.prevalence <= 0.105
0.198 < Adult.smoking <= 0.232
0.0576 < Uninsured.children <= 0.0826
0.000848 < Other.primary.care.providers <= 0.001211
Severe.housing.cost.burden <= 0.0851
0.189 < Excessive.drinking <= 0.216
0.797 < Broadband.access <= 0.842
School.segregation <= 0.0359
0.000469 < Primary.care.physicians <= 0.000701
Feature
−0.005
0.000
0.005
Weight
Positive
Negative

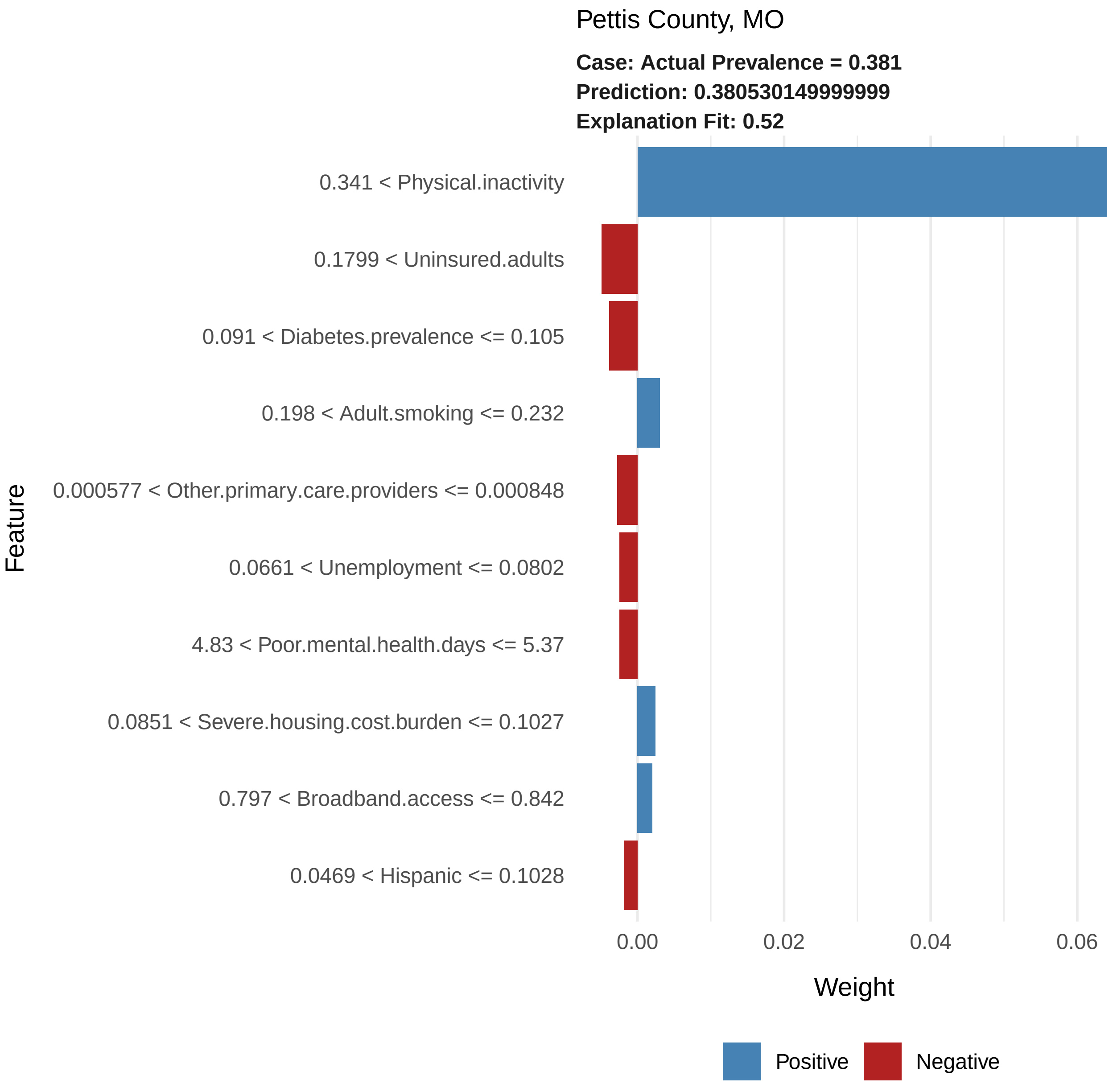
Pettis County, MO
Case: Actual Prevalence = 0.381
Prediction: 0.380530149999999
Explanation Fit: 0.52
0.341 < Physical.inactivity
0.1799 < Uninsured.adults
0.091 < Diabetes.prevalence <= 0.105
0.198 < Adult.smoking <= 0.232
0.000577 < Other.primary.care.providers <= 0.000848
0.0661 < Unemployment <= 0.0802
4.83 < Poor.mental.health.days <= 5.37
0.0851 < Severe.housing.cost.burden <= 0.1027
0.797 < Broadband.access <= 0.842
0.0469 < Hispanic <= 0.1028
Feature
0.00
0.02
0.04
0.06
Weight
Positive
Negative

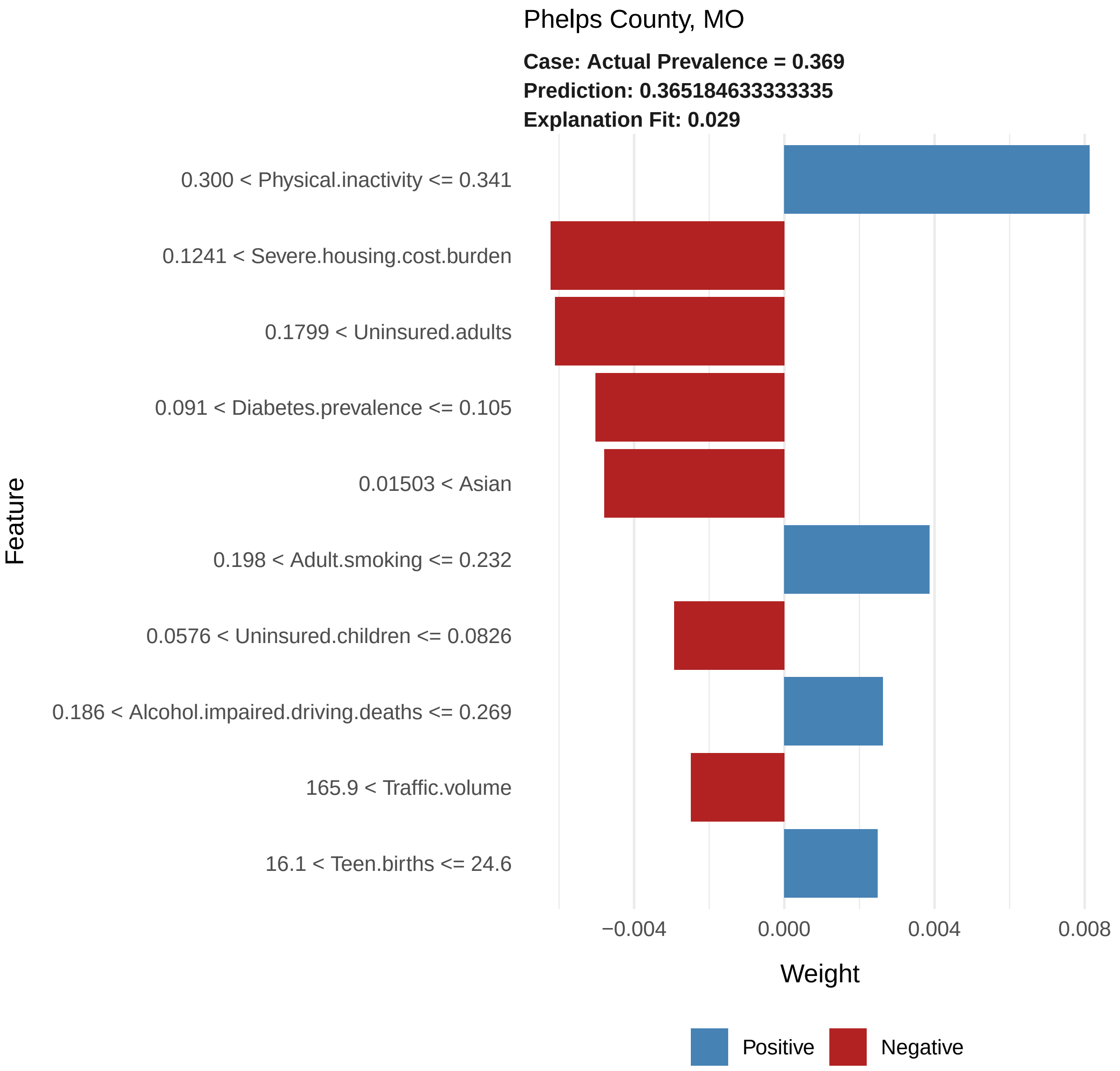
Phelps County, MO
Case: Actual Prevalence = 0.369
Prediction: 0.365184633333335
Explanation Fit: 0.029
0.300 < Physical.inactivity <= 0.341
0.1241 < Severe.housing.cost.burden
0.1799 < Uninsured.adults
0.091 < Diabetes.prevalence <= 0.105
0.01503 < Asian
0.198 < Adult.smoking <= 0.232
0.0576 < Uninsured.children <= 0.0826
0.186 < Alcohol.impaired.driving.deaths <= 0.269
165.9 < Traffic.volume
16.1 < Teen.births <= 24.6
Feature
−0.004
0.000
0.004
0.008
Weight
Positive
Negative

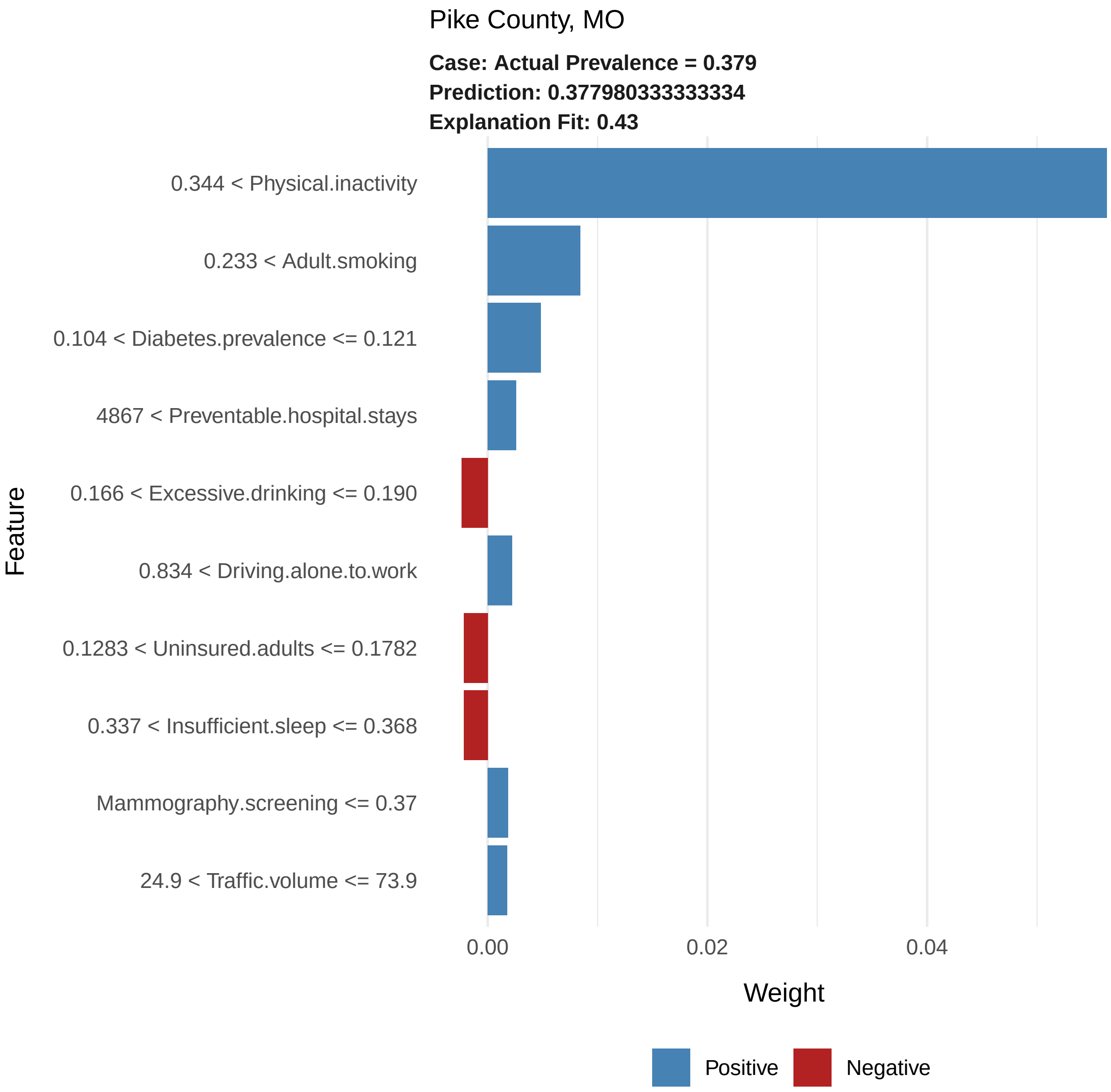

Pike County, MO
Case: Actual Prevalence = 0.379
Prediction: 0.377980333333334
Explanation Fit: 0.43
0.344 < Physical.inactivity
0.233 < Adult.smoking
0.104 < Diabetes.prevalence <= 0.121
4867 < Preventable.hospital.stays
0.166 < Excessive.drinking <= 0.190
0.834 < Driving.alone.to.work
0.1283 < Uninsured.adults <= 0.1782
0.337 < Insufficient.sleep <= 0.368
Mammography.screening <= 0.37
24.9 < Traffic.volume <= 73.9
Feature
0.00
0.02
0.04
Weight
Positive
Negative

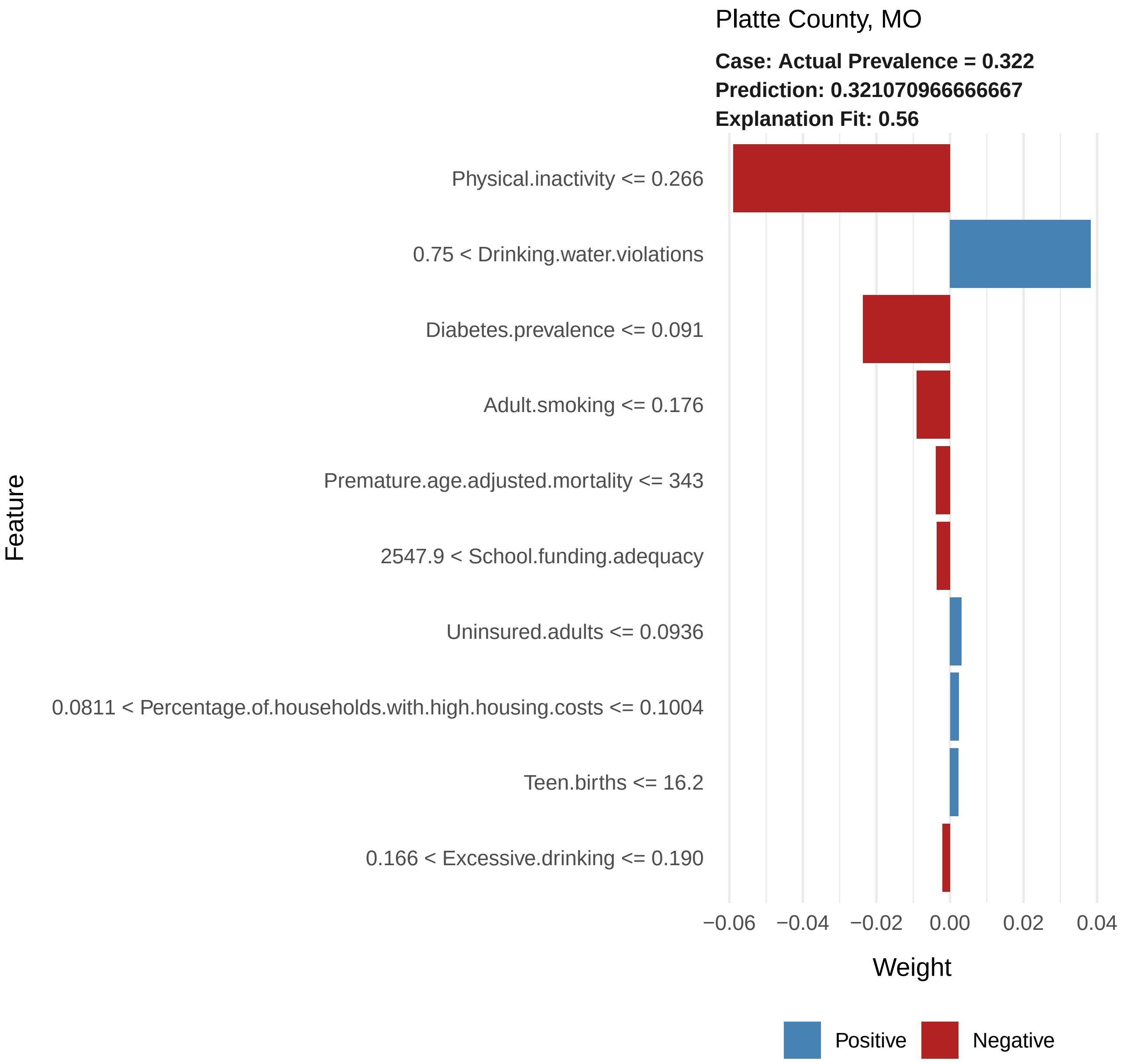
Platte County, MO
Case: Actual Prevalence = 0.322
Prediction: 0.321070966666667
Explanation Fit: 0.56
Physical.inactivity <= 0.266
0.75 < Drinking.water.violations
Diabetes.prevalence <= 0.091
Adult.smoking <= 0.176
Premature.age.adjusted.mortality <= 343
2547.9 < School.funding.adequacy
Uninsured.adults <= 0.0936
0.0811 < Percentage.of.households.with.high.housing.costs <= 0.1004
Teen.births <= 16.2
0.166 < Excessive.drinking <= 0.190
Feature
−0.06
−0.04
−0.02
0.00
0.02
0.04
Weight
Positive
Negative

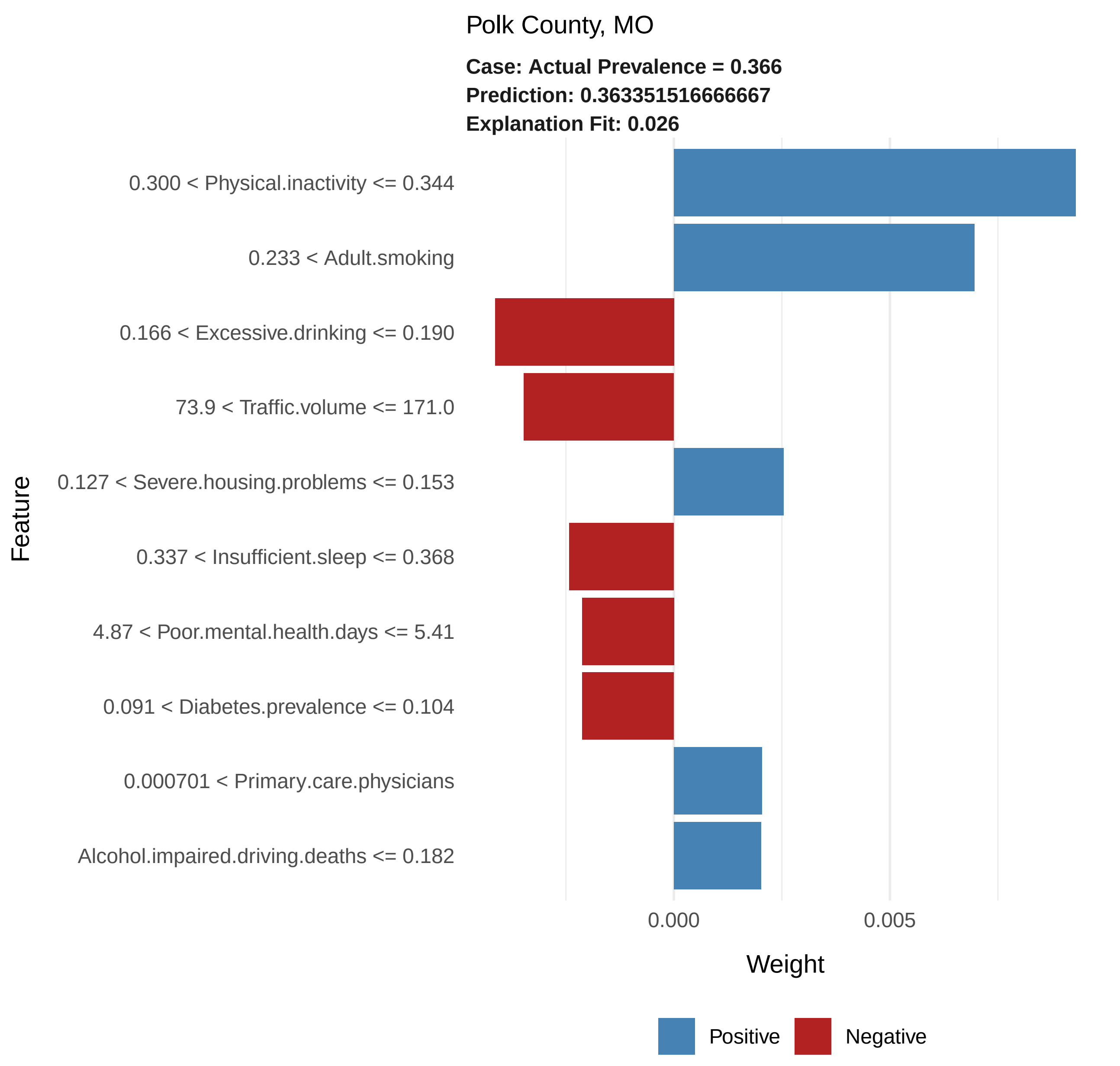
Polk County, MO
Case: Actual Prevalence = 0.366
Prediction: 0.363351516666667
Explanation Fit: 0.026
0.300 < Physical.inactivity <= 0.344
0.233 < Adult.smoking
0.166 < Excessive.drinking <= 0.190
73.9 < Traffic.volume <= 171.0
0.127 < Severe.housing.problems <= 0.153
0.337 < Insufficient.sleep <= 0.368
4.87 < Poor.mental.health.days <= 5.41
0.091 < Diabetes.prevalence <= 0.104
0.000701 < Primary.care.physicians
Alcohol.impaired.driving.deaths <= 0.182
Feature
0.000
0.005
Weight
Positive
Negative

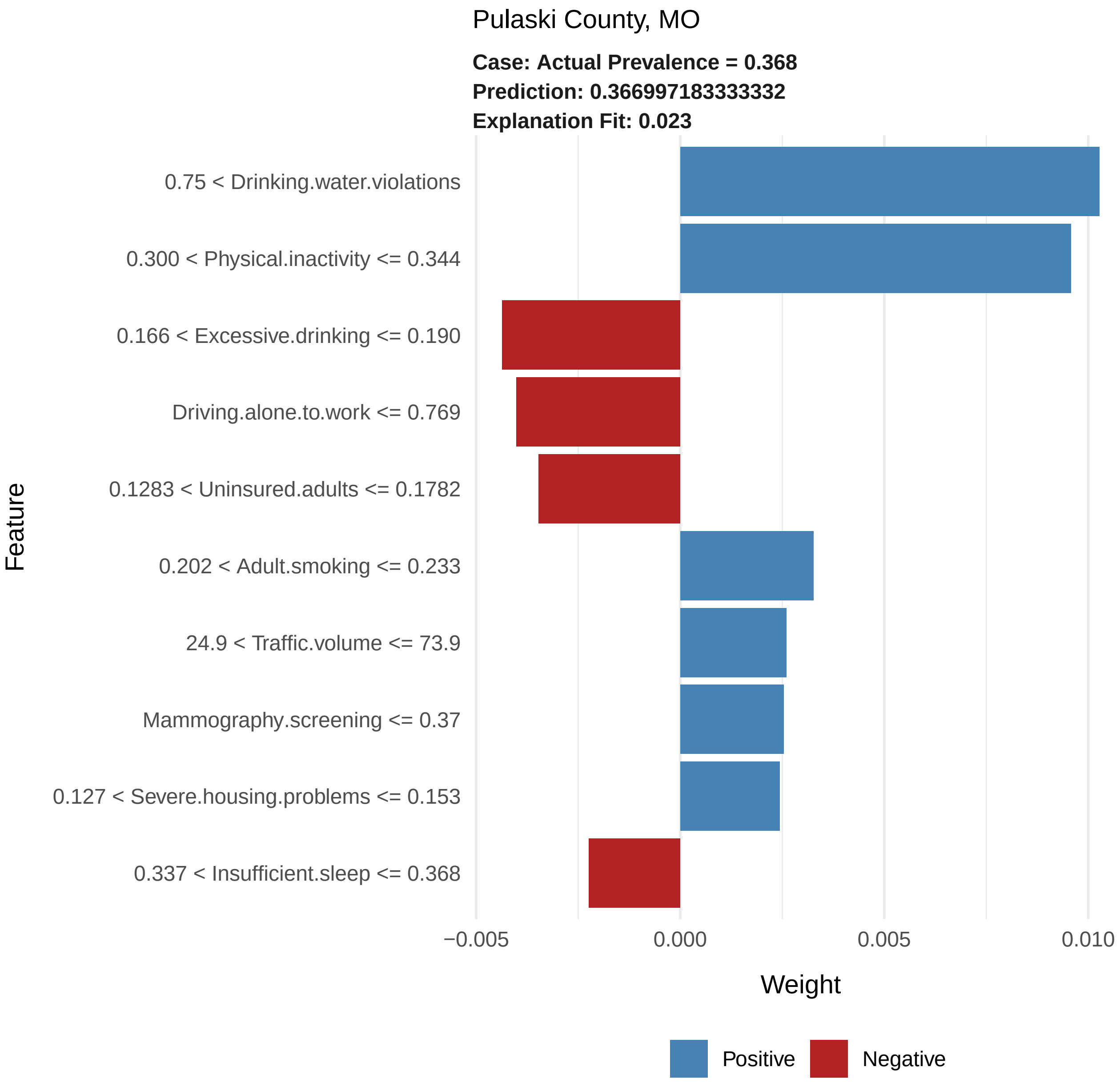

Pulaski County, MO
Case: Actual Prevalence = 0.368
Prediction: 0.366997183333332
Explanation Fit: 0.023
0.75 < Drinking.water.violations
0.300 < Physical.inactivity <= 0.344
0.166 < Excessive.drinking <= 0.190
Driving.alone.to.work <= 0.769
0.1283 < Uninsured.adults <= 0.1782
0.202 < Adult.smoking <= 0.233
24.9 < Traffic.volume <= 73.9
Mammography.screening <= 0.37
0.127 < Severe.housing.problems <= 0.153
0.337 < Insufficient.sleep <= 0.368
Feature
−0.005
0.000
0.005
0.010
Weight
Positive
Negative

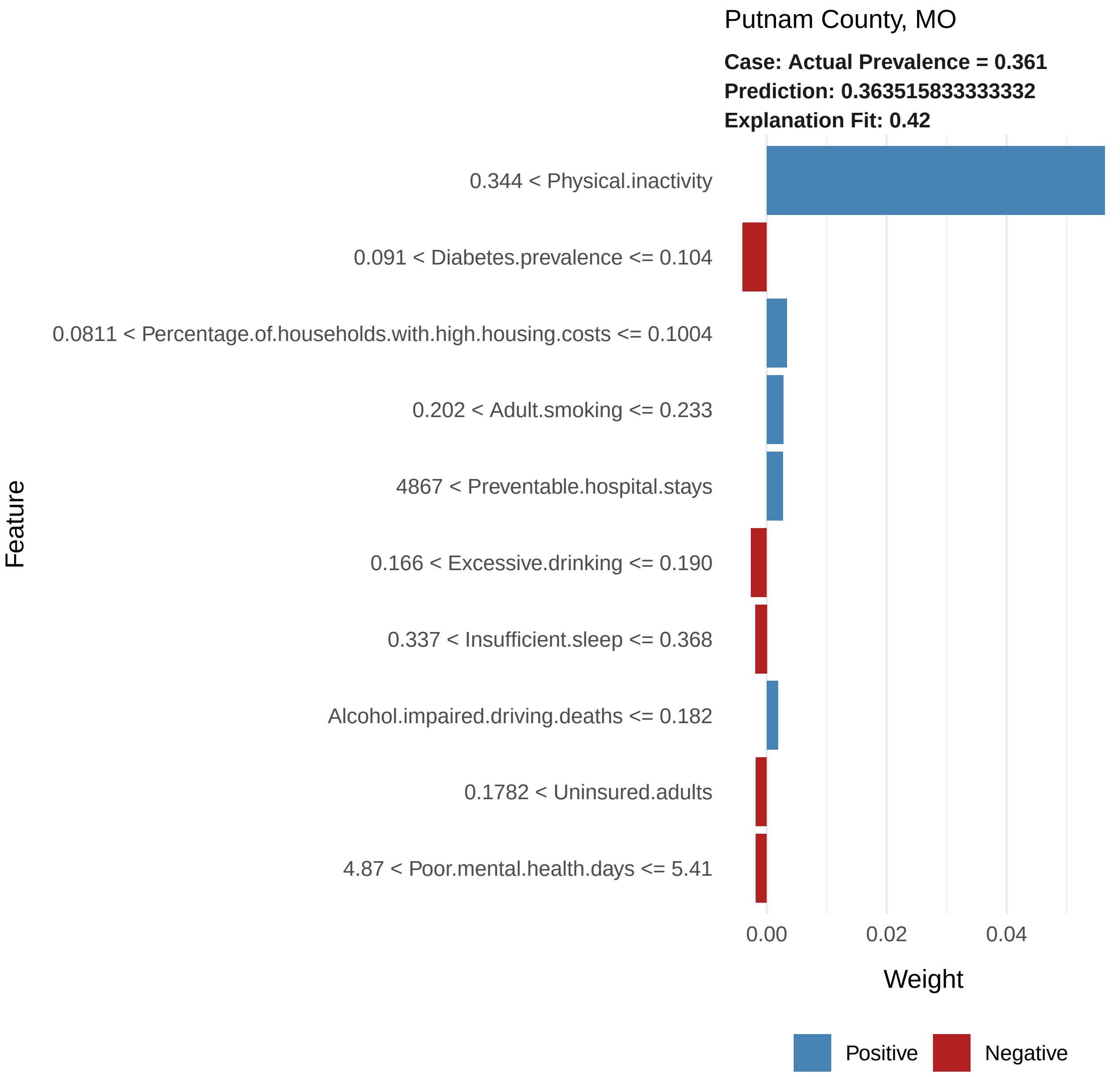
Putnam County, MO
Case: Actual Prevalence = 0.361
Prediction: 0.363515833333332
Explanation Fit: 0.42
0.344 < Physical.inactivity
0.091 < Diabetes.prevalence <= 0.104
0.0811 < Percentage.of.households.with.high.housing.costs <= 0.1004
0.202 < Adult.smoking <= 0.233
4867 < Preventable.hospital.stays
0.166 < Excessive.drinking <= 0.190
0.337 < Insufficient.sleep <= 0.368
Alcohol.impaired.driving.deaths <= 0.182
0.1782 < Uninsured.adults
4.87 < Poor.mental.health.days <= 5.41
Feature
0.00
0.02
0.04
Weight
Positive
Negative

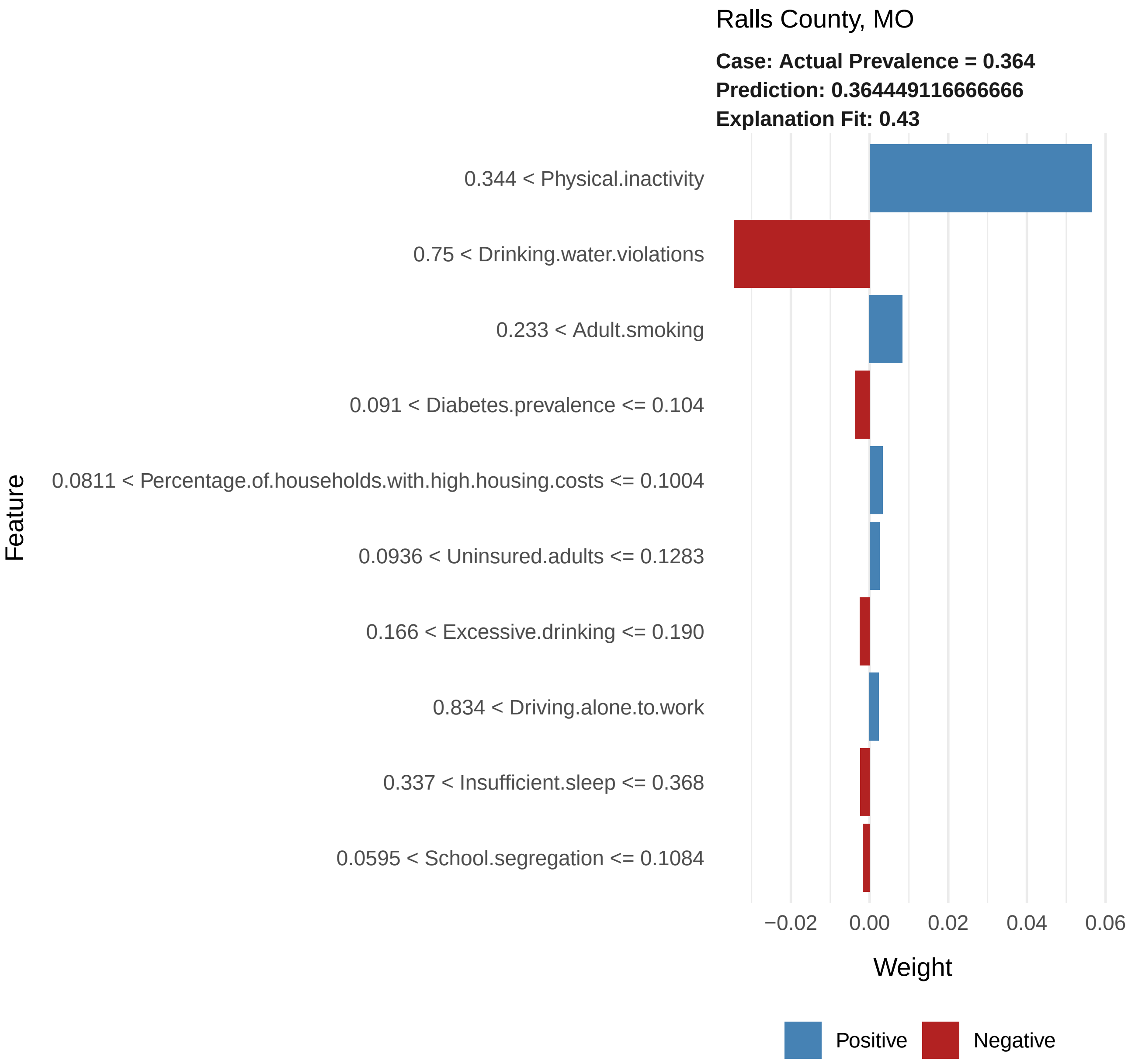
Ralls County, MO
Case: Actual Prevalence = 0.364
Prediction: 0.364449116666666
Explanation Fit: 0.43
0.344 < Physical.inactivity
0.75 < Drinking.water.violations
0.233 < Adult.smoking
0.091 < Diabetes.prevalence <= 0.104
0.0811 < Percentage.of.households.with.high.housing.costs <= 0.1004
0.0936 < Uninsured.adults <= 0.1283
0.166 < Excessive.drinking <= 0.190
0.834 < Driving.alone.to.work
0.337 < Insufficient.sleep <= 0.368
0.0595 < School.segregation <= 0.1084
Feature
−0.02
0.00
0.02
0.04
0.06
Weight
Positive
Negative

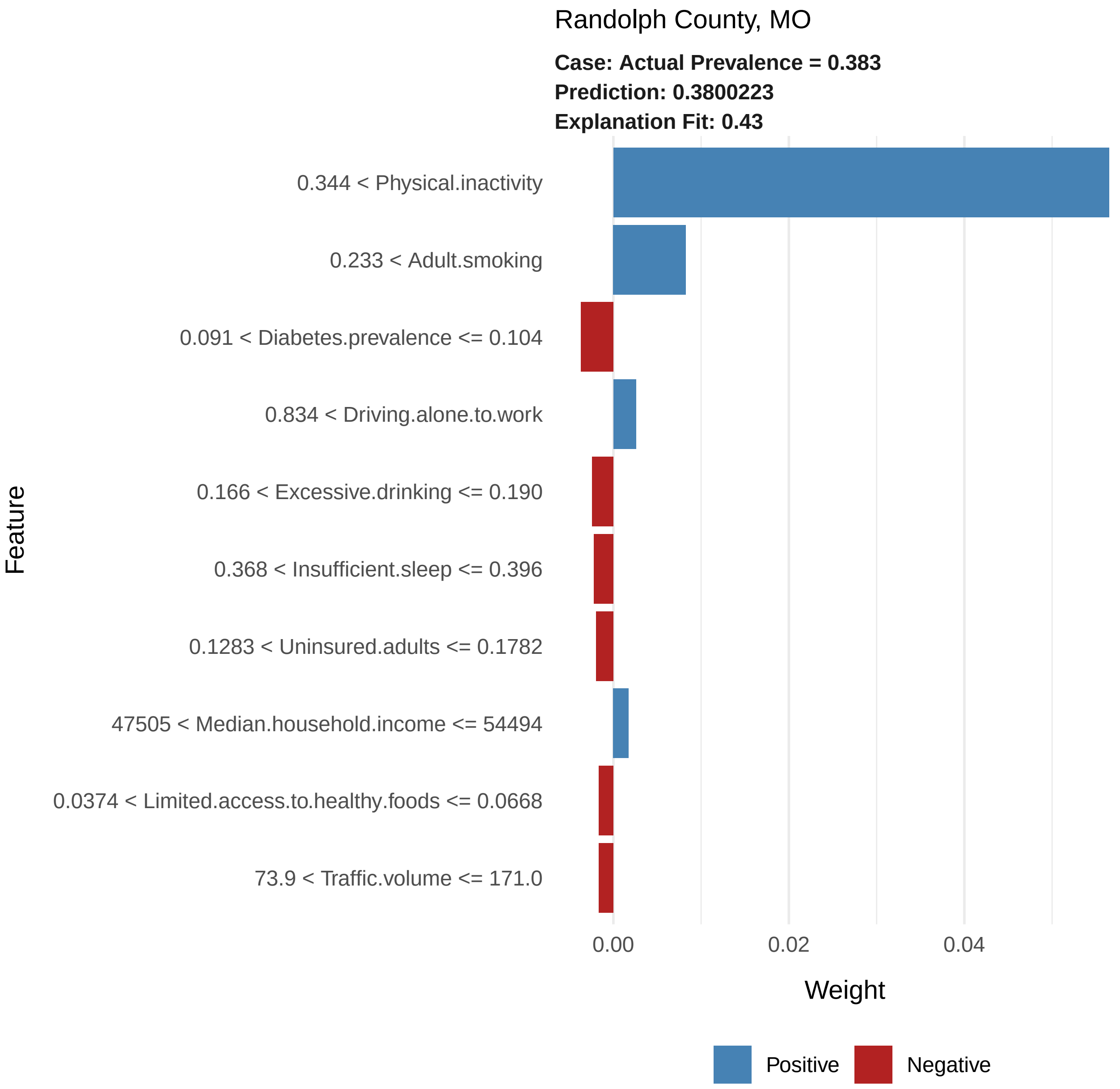
Randolph County, MO
Case: Actual Prevalence = 0.383
Prediction: 0.3800223
Explanation Fit: 0.43
0.344 < Physical.inactivity
0.233 < Adult.smoking
0.091 < Diabetes.prevalence <= 0.104
0.834 < Driving.alone.to.work
0.166 < Excessive.drinking <= 0.190
0.368 < Insufficient.sleep <= 0.396
0.1283 < Uninsured.adults <= 0.1782
47505 < Median.household.income <= 54494
0.0374 < Limited.access.to.healthy.foods <= 0.0668
73.9 < Traffic.volume <= 171.0
Feature
0.00
0.02
0.04
Weight
Positive
Negative

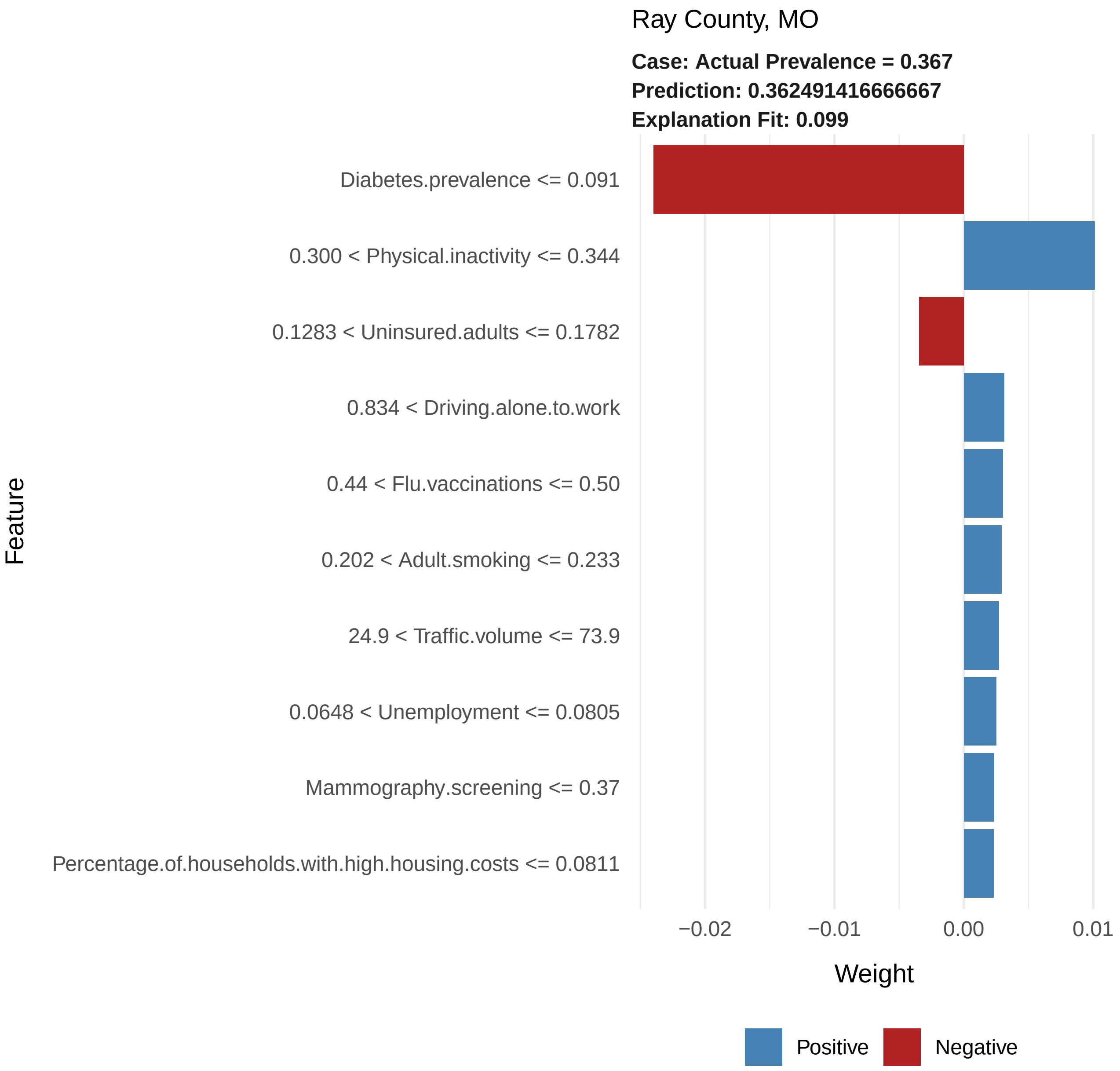

Ray County, MO
Case: Actual Prevalence = 0.367
Prediction: 0.362491416666667
Explanation Fit: 0.099
Diabetes.prevalence <= 0.091
0.300 < Physical.inactivity <= 0.344
0.1283 < Uninsured.adults <= 0.1782
0.834 < Driving.alone.to.work
0.44 < Flu.vaccinations <= 0.50
0.202 < Adult.smoking <= 0.233
24.9 < Traffic.volume <= 73.9
0.0648 < Unemployment <= 0.0805
Mammography.screening <= 0.37
Percentage.of.households.with.high.housing.costs <= 0.0811
Feature
−0.02
−0.01
0.00
0.01
Weight
Positive
Negative

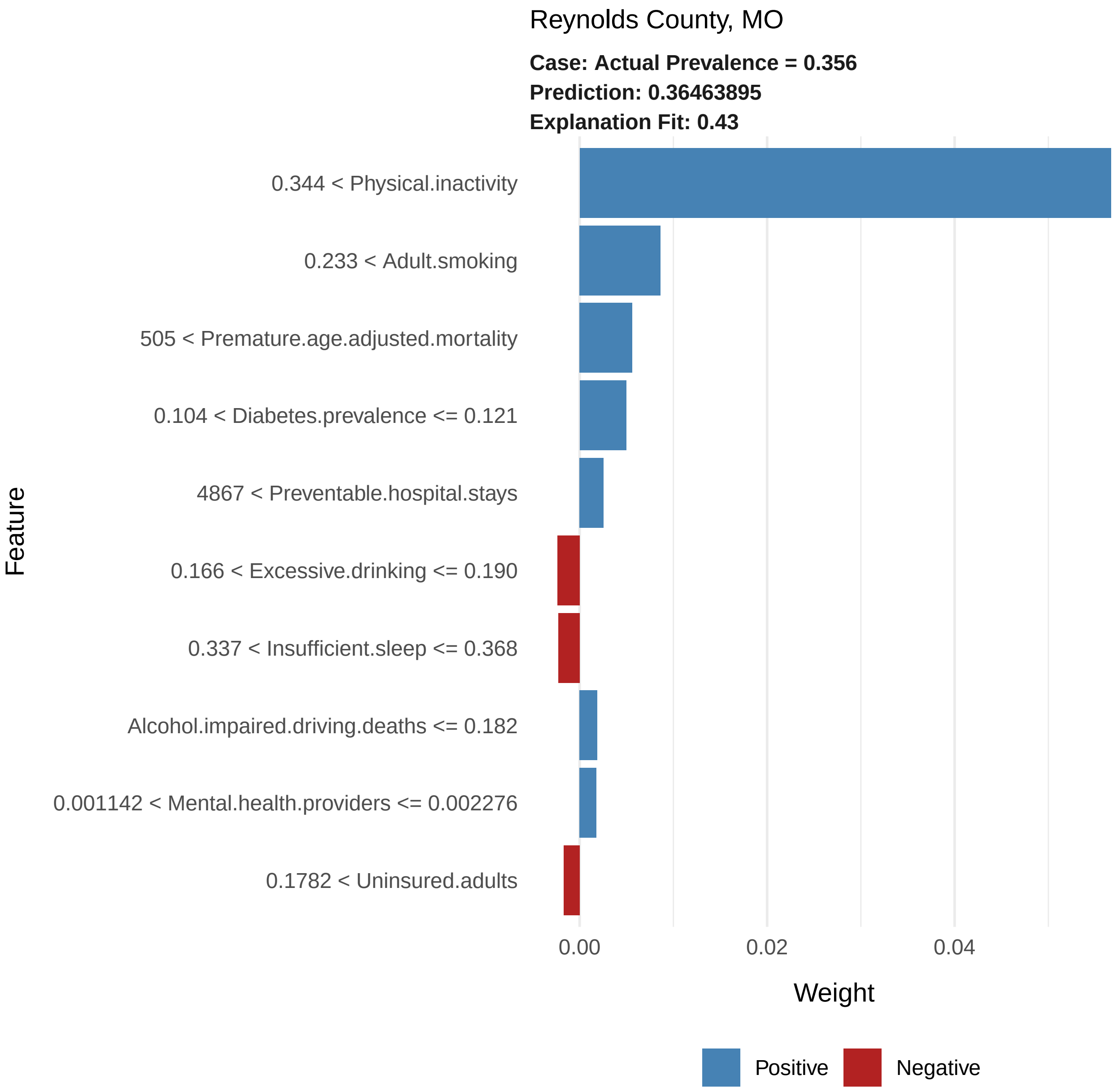
Reynolds County, MO
Case: Actual Prevalence = 0.356
Prediction: 0.36463895
Explanation Fit: 0.43
0.344 < Physical.inactivity
0.233 < Adult.smoking
505 < Premature.age.adjusted.mortality
0.104 < Diabetes.prevalence <= 0.121
4867 < Preventable.hospital.stays
0.166 < Excessive.drinking <= 0.190
0.337 < Insufficient.sleep <= 0.368
Alcohol.impaired.driving.deaths <= 0.182
0.001142 < Mental.health.providers <= 0.002276
0.1782 < Uninsured.adults
Feature
0.00
0.02
0.04
Weight
Positive
Negative

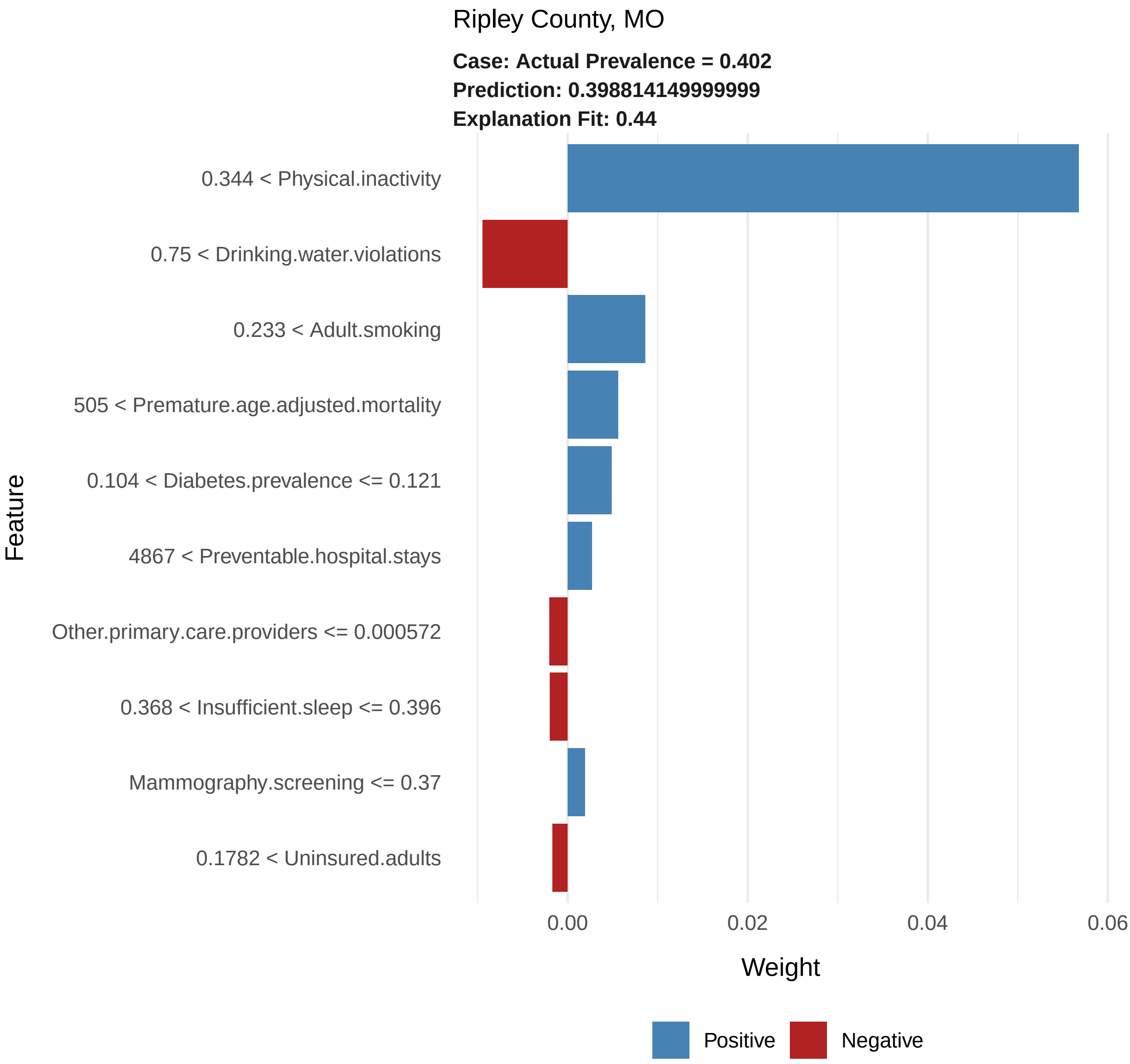
Ripley County, MO
Case: Actual Prevalence = 0.402
Prediction: 0.398814149999999
Explanation Fit: 0.44
0.344 < Physical.inactivity
0.75 < Drinking.water.violations
0.233 < Adult.smoking
505 < Premature.age.adjusted.mortality
0.104 < Diabetes.prevalence <= 0.121
4867 < Preventable.hospital.stays
Other.primary.care.providers <= 0.000572
0.368 < Insufficient.sleep <= 0.396
Mammography.screening <= 0.37
0.1782 < Uninsured.adults
Feature
0.00
0.02
0.04
0.06
Weight
Positive
Negative

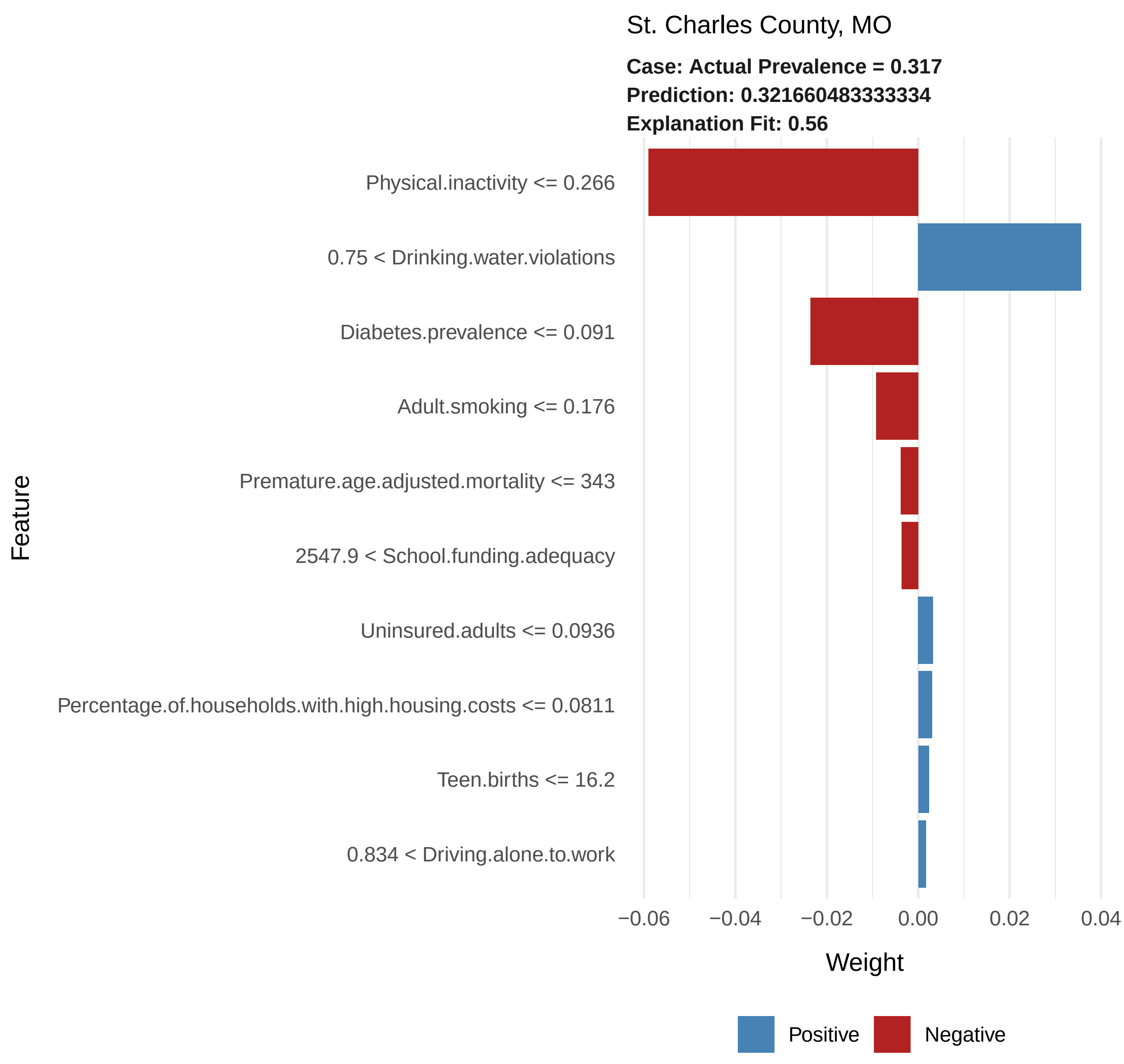

St. Charles County, MO
Case: Actual Prevalence = 0.317
Prediction: 0.321660483333334
Explanation Fit: 0.56
Physical.inactivity <= 0.266
0.75 < Drinking.water.violations
Diabetes.prevalence <= 0.091
Adult.smoking <= 0.176
Premature.age.adjusted.mortality <= 343
2547.9 < School.funding.adequacy
Uninsured.adults <= 0.0936
Percentage.of.households.with.high.housing.costs <= 0.0811
Teen.births <= 16.2
0.834 < Driving.alone.to.work
Feature
−0.06
−0.04
−0.02
0.00
0.02
0.04
Weight
Positive
Negative

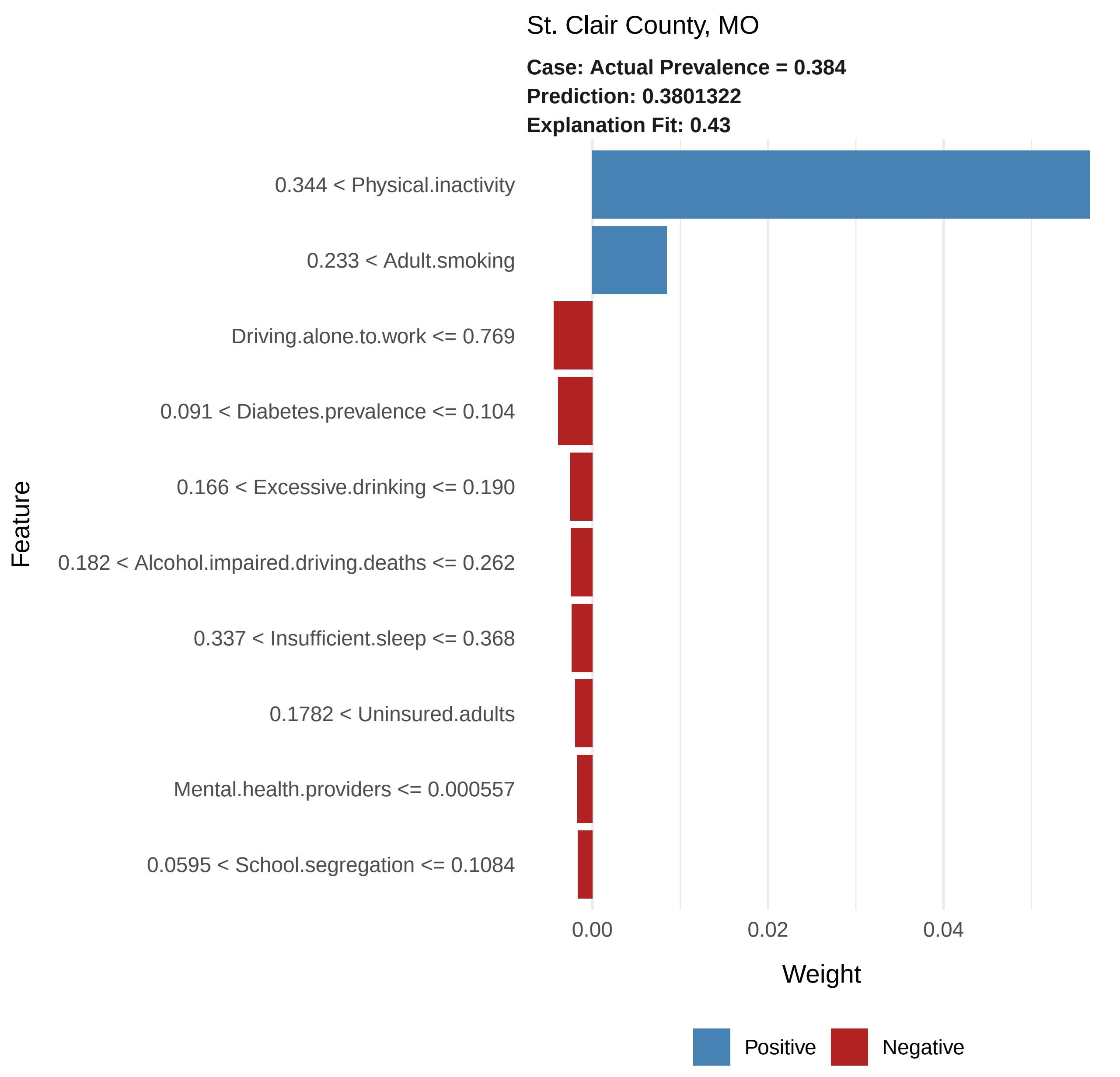
St. Clair County, MO
Case: Actual Prevalence = 0.384
Prediction: 0.3801322
Explanation Fit: 0.43
0.344 < Physical.inactivity
0.233 < Adult.smoking
Driving.alone.to.work <= 0.769
0.091 < Diabetes.prevalence <= 0.104
0.166 < Excessive.drinking <= 0.190
0.182 < Alcohol.impaired.driving.deaths <= 0.262
0.337 < Insufficient.sleep <= 0.368
0.1782 < Uninsured.adults
Mental.health.providers <= 0.000557
0.0595 < School.segregation <= 0.1084
Feature
0.00
0.02
0.04
Weight
Positive
Negative

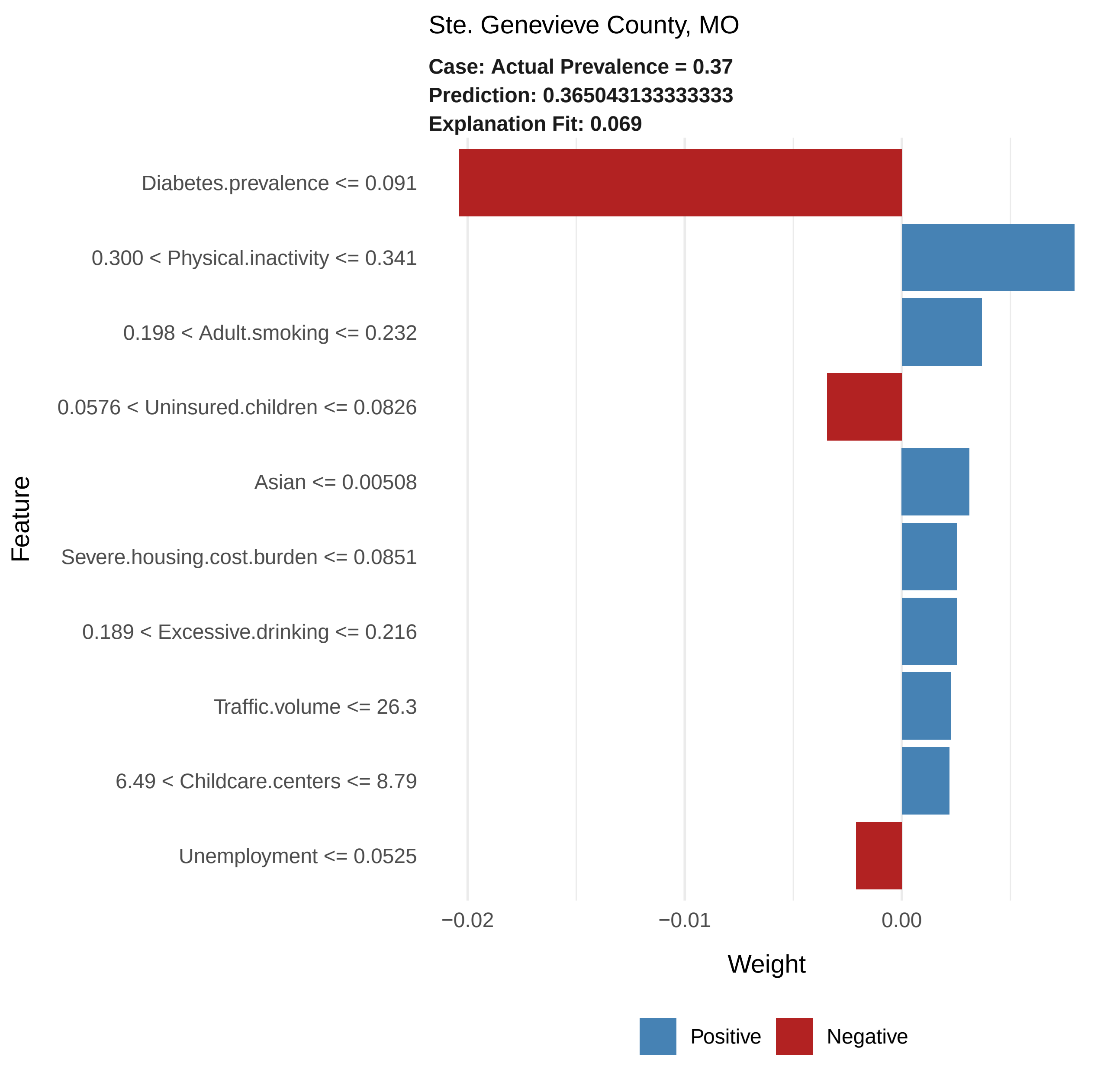

Ste. Genevieve County, MO
Case: Actual Prevalence = 0.37
Prediction: 0.365043133333333
Explanation Fit: 0.069
Diabetes.prevalence <= 0.091
0.300 < Physical.inactivity <= 0.341
0.198 < Adult.smoking <= 0.232
0.0576 < Uninsured.children <= 0.0826
Asian <= 0.00508
Severe.housing.cost.burden <= 0.0851
0.189 < Excessive.drinking <= 0.216
Traffic.volume <= 26.3
6.49 < Childcare.centers <= 8.79
Unemployment <= 0.0525
Feature
−0.02
−0.01
0.00
Weight
Positive
Negative

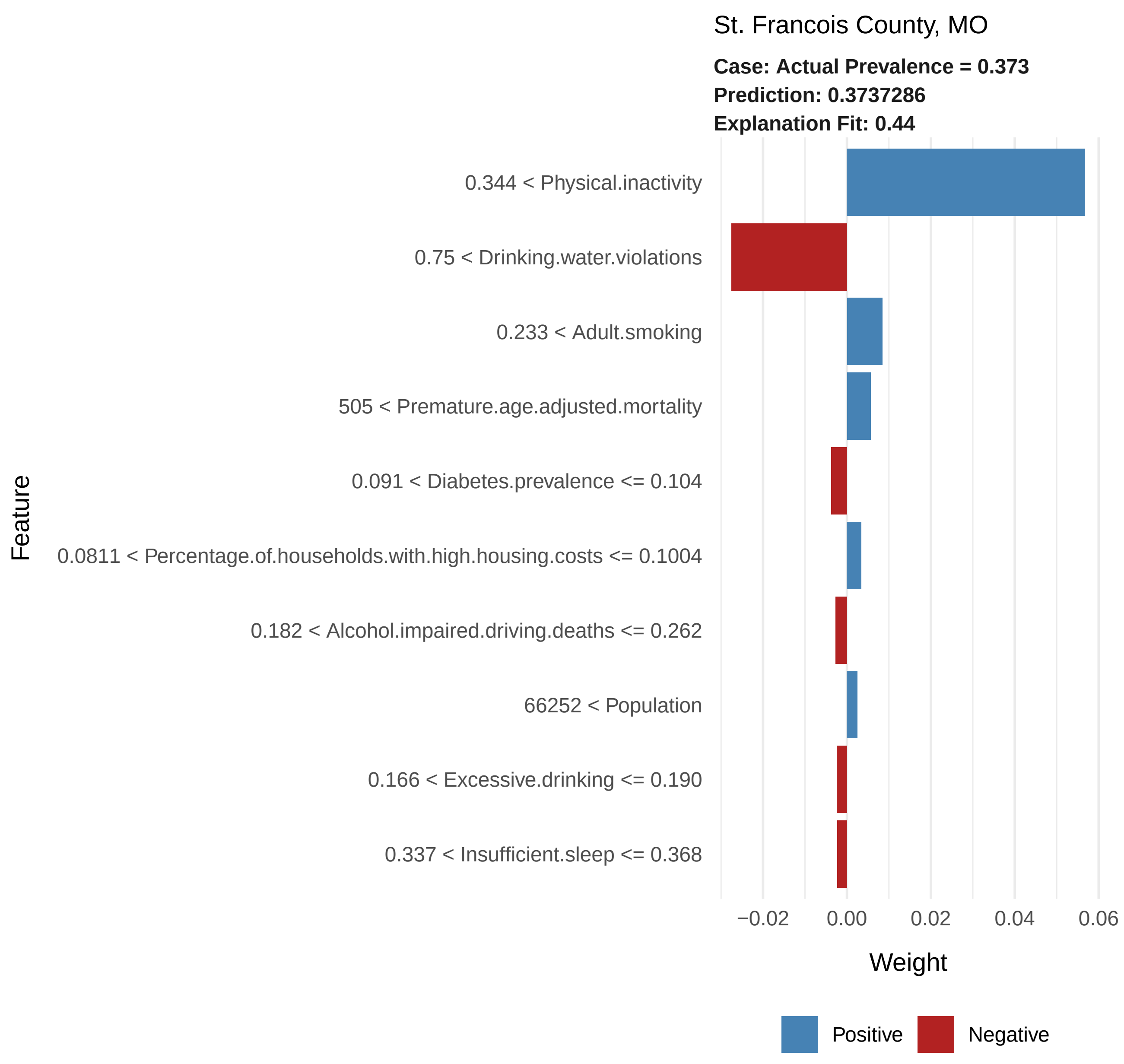
St. Francois County, MO
Case: Actual Prevalence = 0.373
Prediction: 0.3737286
Explanation Fit: 0.44
0.344 < Physical.inactivity
0.75 < Drinking.water.violations
0.233 < Adult.smoking
505 < Premature.age.adjusted.mortality
0.091 < Diabetes.prevalence <= 0.104
0.0811 < Percentage.of.households.with.high.housing.costs <= 0.1004
0.182 < Alcohol.impaired.driving.deaths <= 0.262
66252 < Population
0.166 < Excessive.drinking <= 0.190
0.337 < Insufficient.sleep <= 0.368
Feature
−0.02
0.00
0.02
0.04
0.06
Weight
Positive
Negative

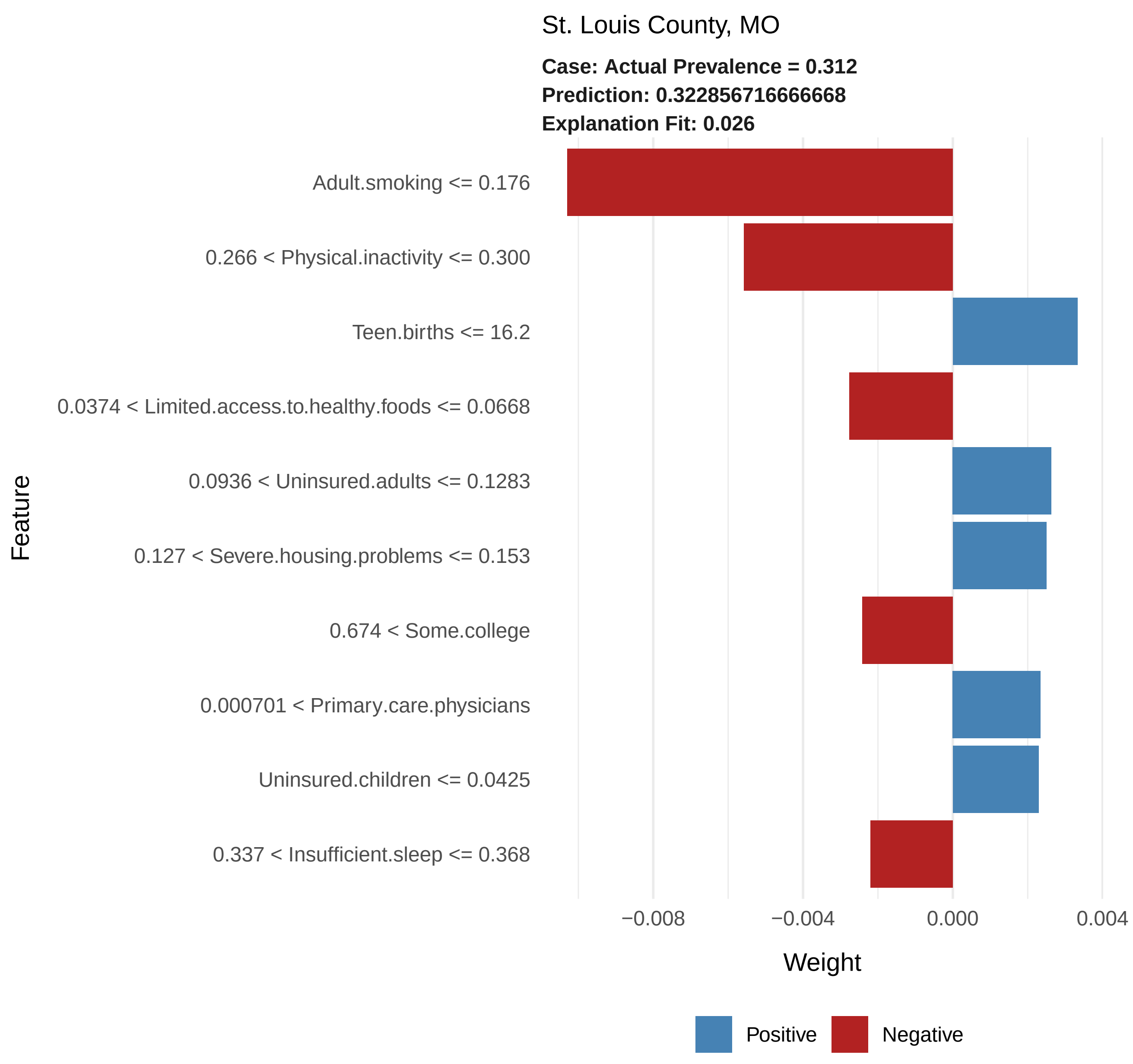

St. Louis County, MO
Case: Actual Prevalence = 0.312
Prediction: 0.322856716666668
Explanation Fit: 0.026
Feature
Adult.smoking <= 0.176
0.266 < Physical.inactivity <= 0.300
Teen.births <= 16.2
0.0374 < Limited.access.to.healthy.foods <= 0.0668
0.0936 < Uninsured.adults <= 0.1283
0.127 < Severe.housing.problems <= 0.153
0.674 < Some.college
0.000701 < Primary.care.physicians
Uninsured.children <= 0.0425
0.337 < Insufficient.sleep <= 0.368
−0.008
−0.004
0.000
0.004
Weight
Positive
Negative

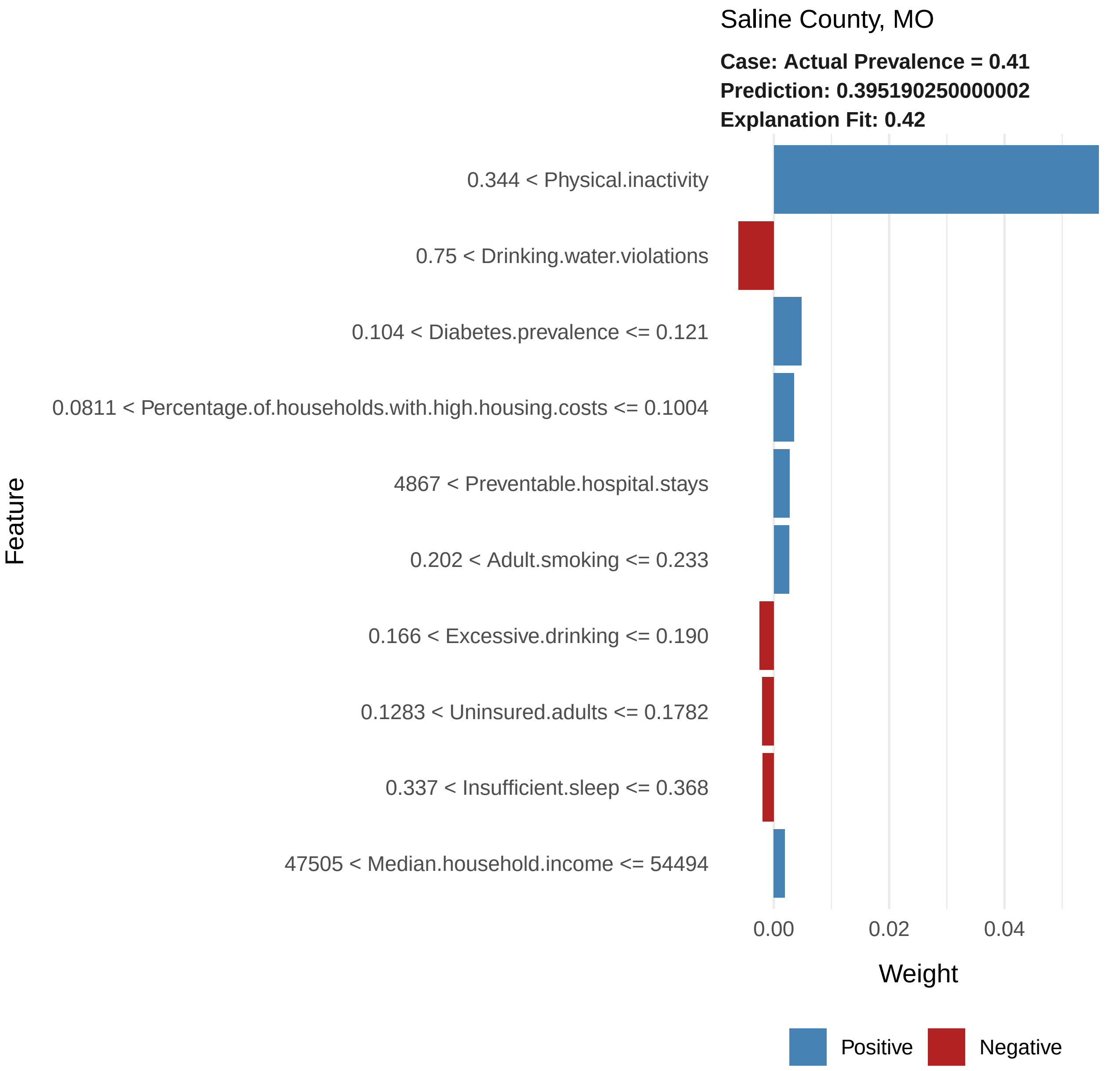
Saline County, MO
Case: Actual Prevalence = 0.41
Prediction: 0.395190250000002
Explanation Fit: 0.42
0.344 < Physical.inactivity
0.75 < Drinking.water.violations
0.104 < Diabetes.prevalence <= 0.121
0.0811 < Percentage.of.households.with.high.housing.costs <= 0.1004
4867 < Preventable.hospital.stays
0.202 < Adult.smoking <= 0.233
0.166 < Excessive.drinking <= 0.190
0.1283 < Uninsured.adults <= 0.1782
0.337 < Insufficient.sleep <= 0.368
47505 < Median.household.income <= 54494
Feature
0.00
0.02
0.04
Weight
Positive
Negative

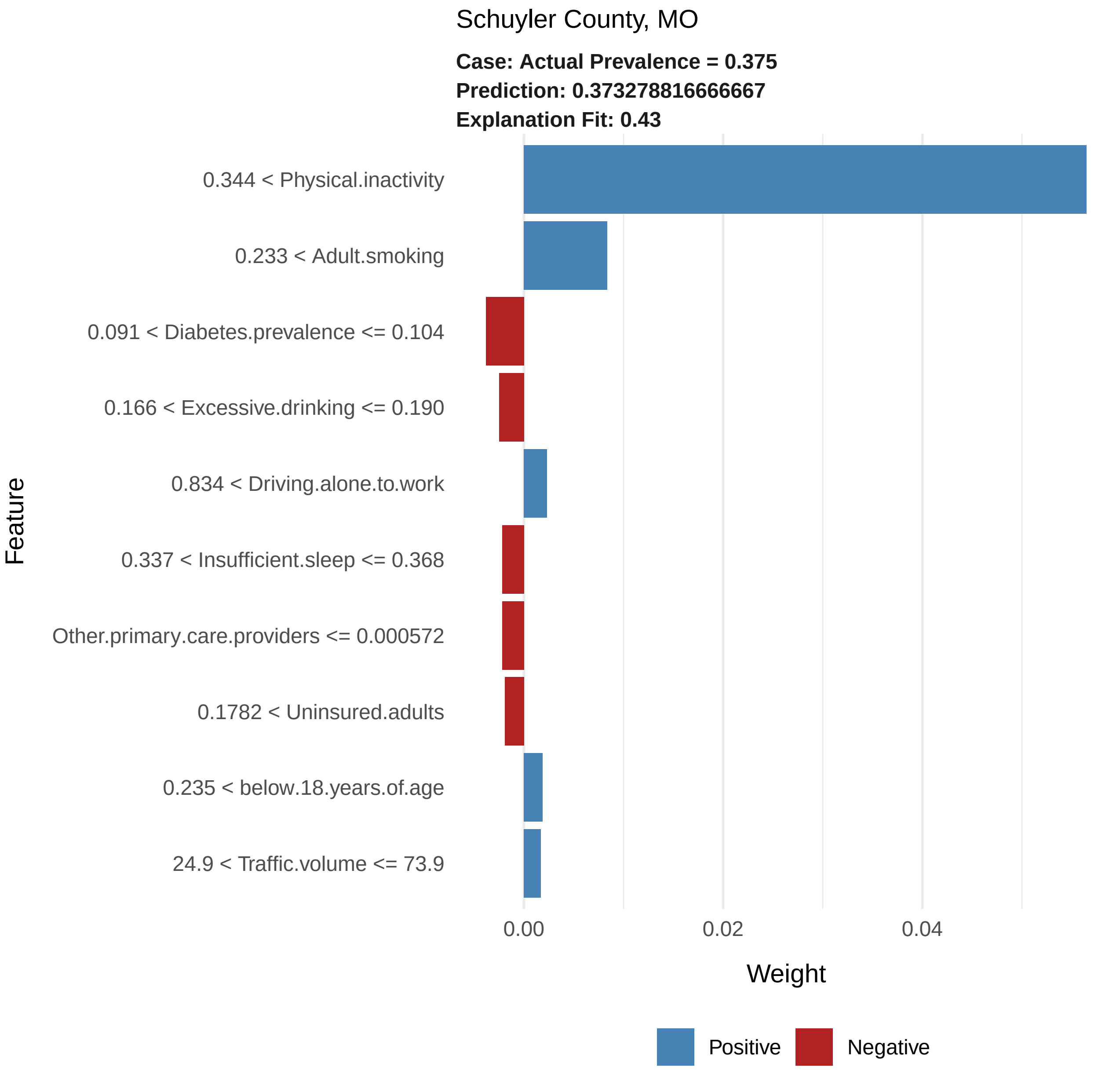

Schuyler County, MO
Case: Actual Prevalence = 0.375
Prediction: 0.373278816666667
Explanation Fit: 0.43
0.344 < Physical.inactivity
0.233 < Adult.smoking
0.091 < Diabetes.prevalence <= 0.104
0.166 < Excessive.drinking <= 0.190
0.834 < Driving.alone.to.work
0.337 < Insufficient.sleep <= 0.368
Other.primary.care.providers <= 0.000572
0.1782 < Uninsured.adults
0.235 < below.18.years.of.age
24.9 < Traffic.volume <= 73.9
Feature
0.00
0.02
0.04
Weight
Positive
Negative

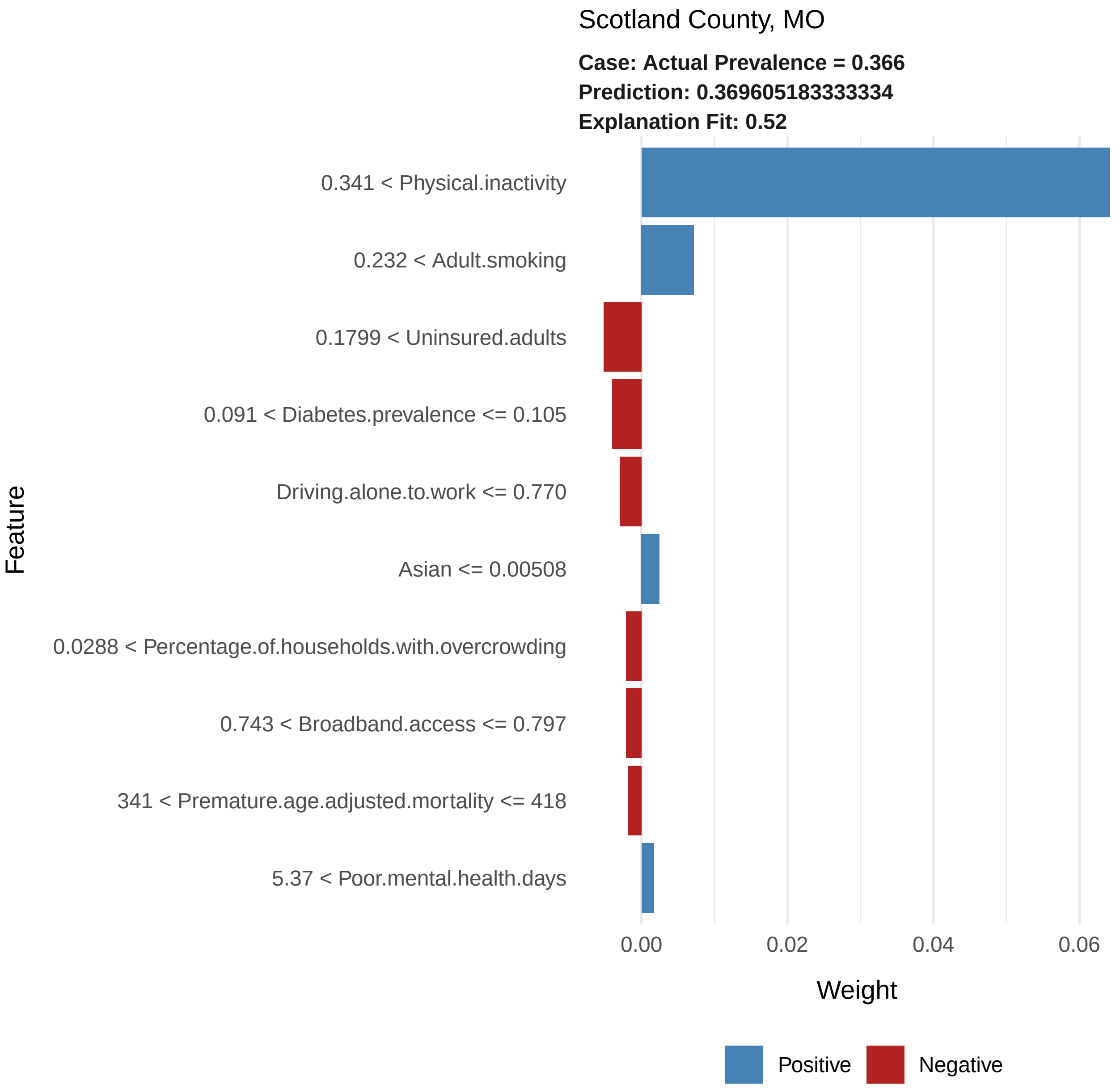
Scotland County, MO
Case: Actual Prevalence = 0.366
Prediction: 0.369605183333334
Explanation Fit: 0.52
0.341 < Physical.inactivity
0.232 < Adult.smoking
0.1799 < Uninsured.adults
0.091 < Diabetes.prevalence <= 0.105
Driving.alone.to.work <= 0.770
Asian <= 0.00508
0.0288 < Percentage.of.households.with.overcrowding
0.743 < Broadband.access <= 0.797
341 < Premature.age.adjusted.mortality <= 418
5.37 < Poor.mental.health.days
Feature
0.00
0.02
0.04
0.06
Weight
Positive
Negative

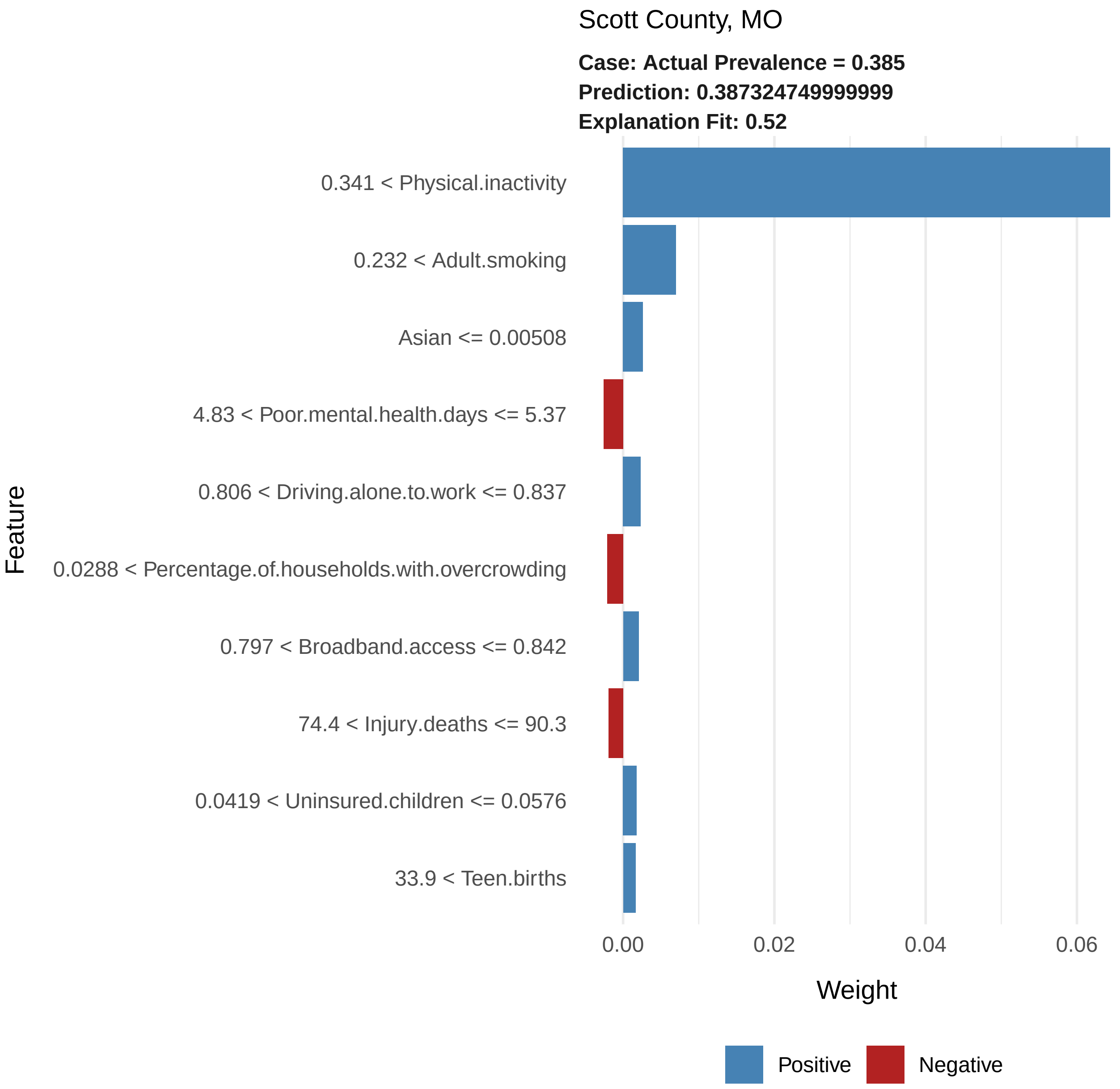

Scott County, MO
Case: Actual Prevalence = 0.385
Prediction: 0.387324749999999
Explanation Fit: 0.52
0.341 < Physical.inactivity
0.232 < Adult.smoking
Asian <= 0.00508
4.83 < Poor.mental.health.days <= 5.37
0.806 < Driving.alone.to.work <= 0.837
0.0288 < Percentage.of.households.with.overcrowding
0.797 < Broadband.access <= 0.842
74.4 < Injury.deaths <= 90.3
0.0419 < Uninsured.children <= 0.0576
33.9 < Teen.births
Feature
0.00
0.02
0.04
0.06
Weight
Positive
Negative

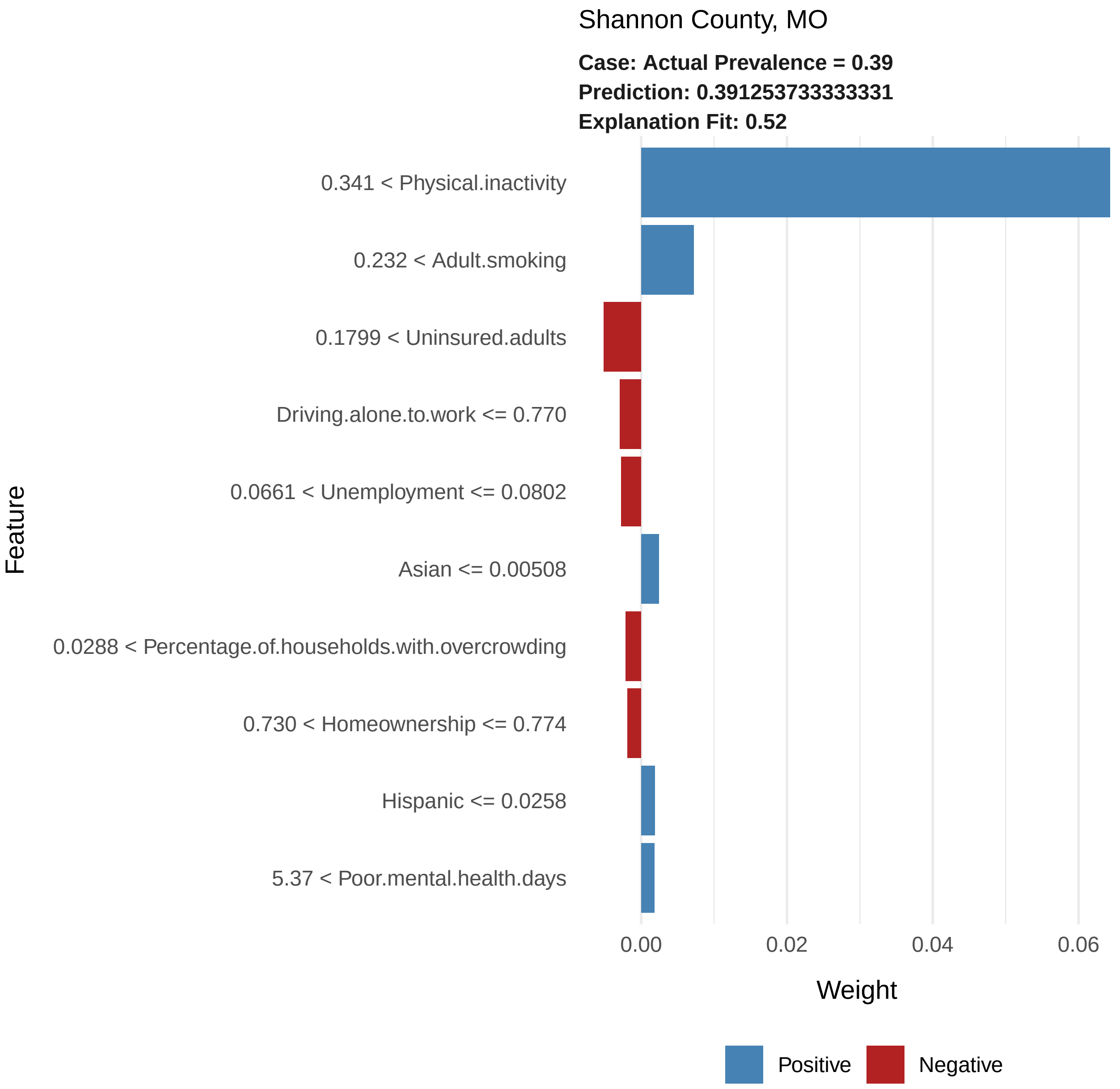
Shannon County, MO
Case: Actual Prevalence = 0.39
Prediction: 0.391253733333331
Explanation Fit: 0.52
0.341 < Physical.inactivity
0.232 < Adult.smoking
0.1799 < Uninsured.adults
Driving.alone.to.work <= 0.770
0.0661 < Unemployment <= 0.0802
Asian <= 0.00508
0.0288 < Percentage.of.households.with.overcrowding
0.730 < Homeownership <= 0.774
Hispanic <= 0.0258
5.37 < Poor.mental.health.days
Feature
0.00
0.02
0.04
0.06
Weight
Positive
Negative

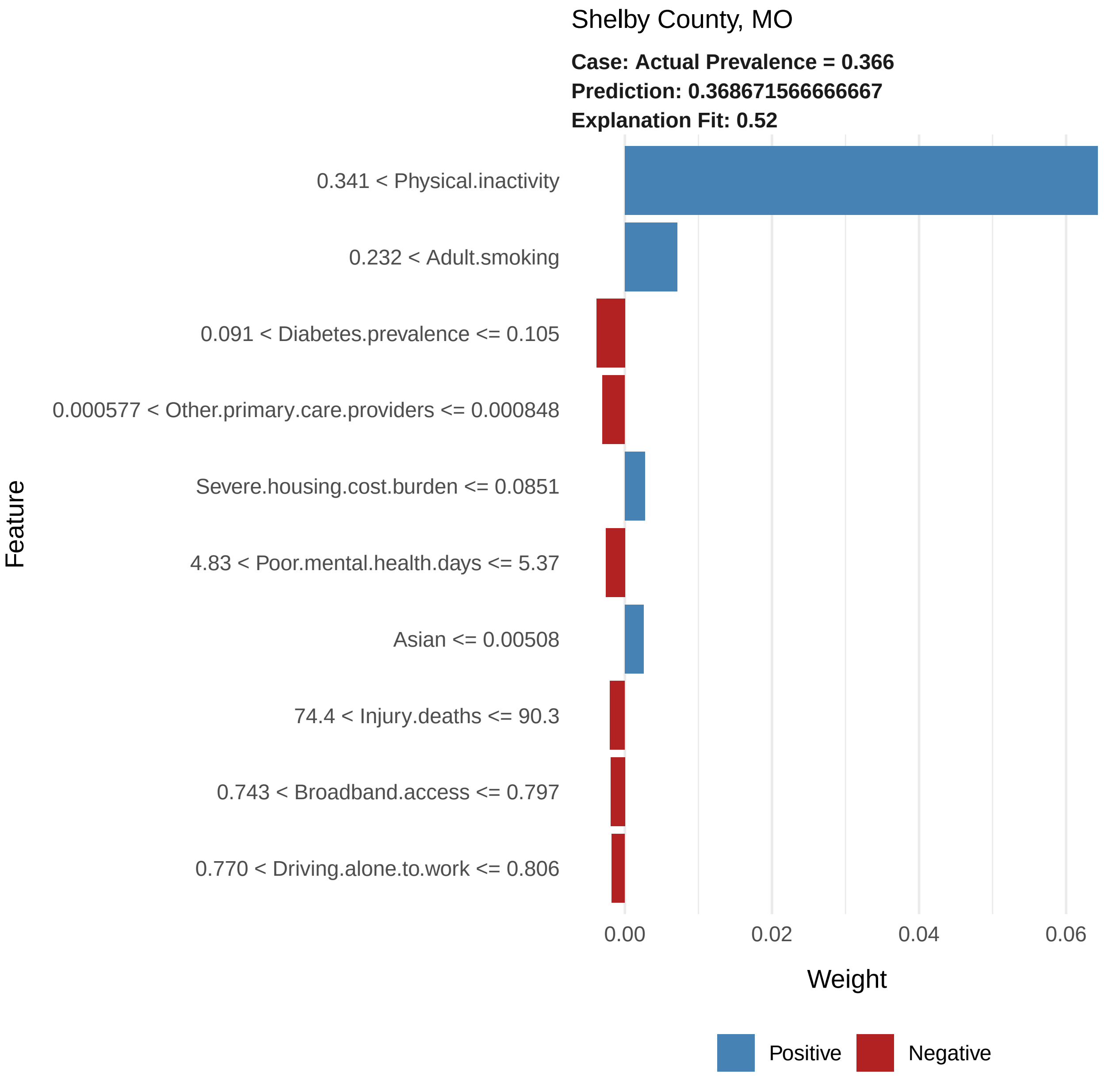

Shelby County, MO
Case: Actual Prevalence = 0.366
Prediction: 0.368671566666667
Explanation Fit: 0.52
0.341 < Physical.inactivity
0.232 < Adult.smoking
0.091 < Diabetes.prevalence <= 0.105
0.000577 < Other.primary.care.providers <= 0.000848
Severe.housing.cost.burden <= 0.0851
4.83 < Poor.mental.health.days <= 5.37
Asian <= 0.00508
74.4 < Injury.deaths <= 90.3
0.743 < Broadband.access <= 0.797
0.770 < Driving.alone.to.work <= 0.806
Feature
0.00
0.02
0.04
0.06
Weight
Positive
Negative

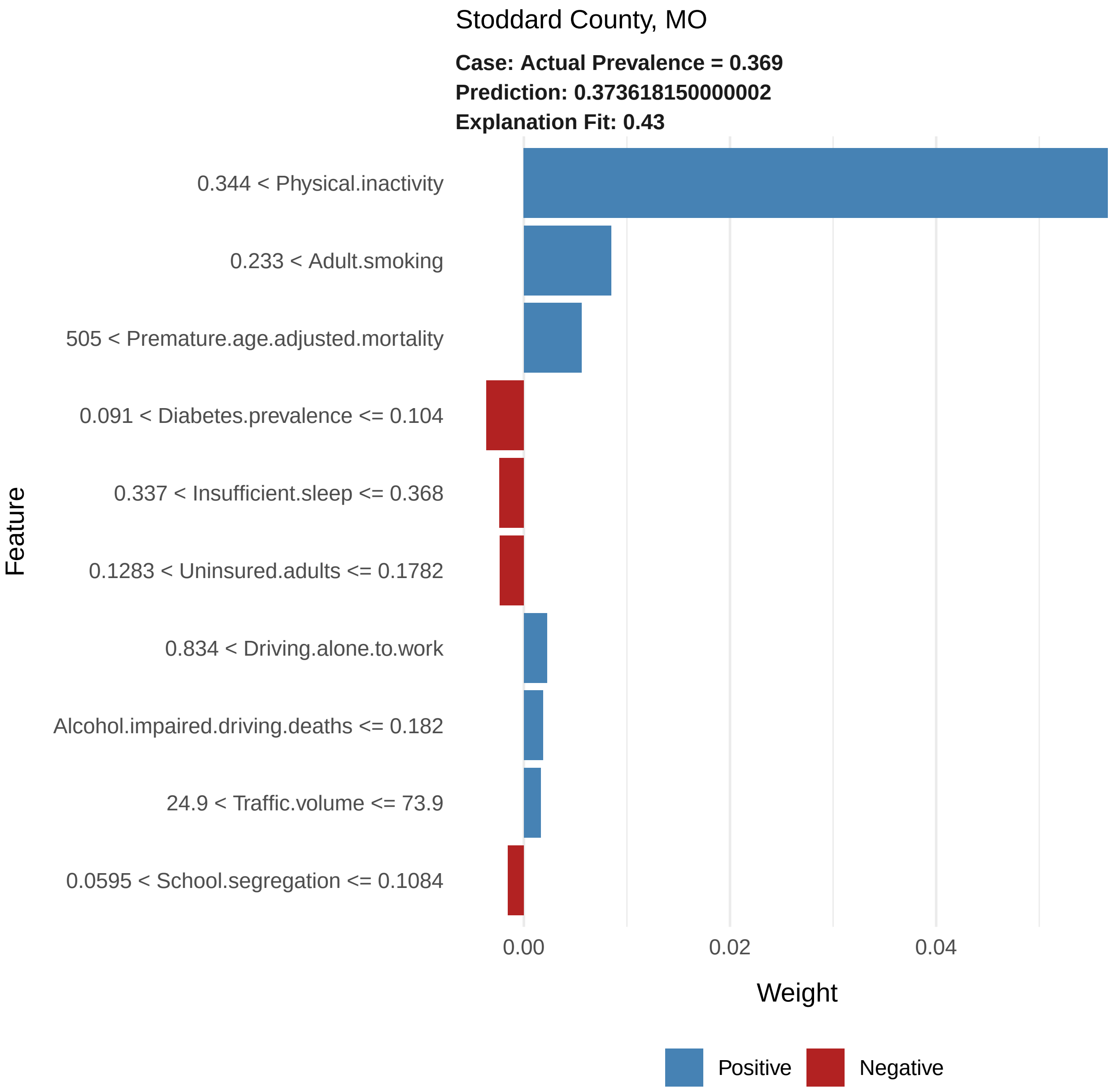
Stoddard County, MO
Case: Actual Prevalence = 0.369
Prediction: 0.373618150000002
Explanation Fit: 0.43
0.344 < Physical.inactivity
0.233 < Adult.smoking
505 < Premature.age.adjusted.mortality
0.091 < Diabetes.prevalence <= 0.104
0.337 < Insufficient.sleep <= 0.368
0.1283 < Uninsured.adults <= 0.1782
0.834 < Driving.alone.to.work
Alcohol.impaired.driving.deaths <= 0.182
24.9 < Traffic.volume <= 73.9
0.0595 < School.segregation <= 0.1084
Feature
0.00
0.02
0.04
Weight
Positive
Negative

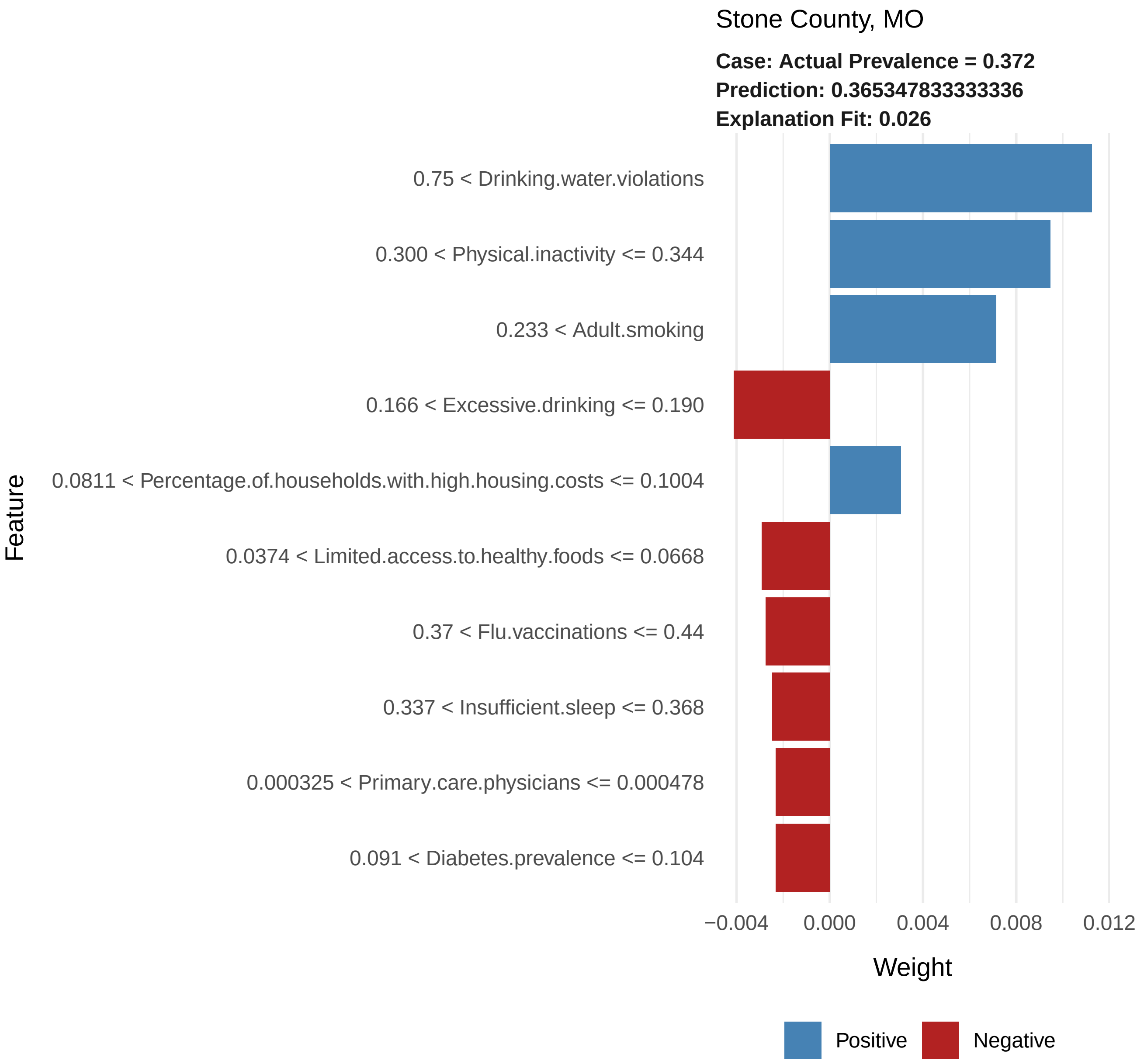
Stone County, MO
Case: Actual Prevalence = 0.372
Prediction: 0.365347833333336
Explanation Fit: 0.026
0.75 < Drinking.water.violations
0.300 < Physical.inactivity <= 0.344
0.233 < Adult.smoking
0.166 < Excessive.drinking <= 0.190
0.0811 < Percentage.of.households.with.high.housing.costs <= 0.1004
0.0374 < Limited.access.to.healthy.foods <= 0.0668
0.37 < Flu.vaccinations <= 0.44
0.337 < Insufficient.sleep <= 0.368
0.000325 < Primary.care.physicians <= 0.000478
0.091 < Diabetes.prevalence <= 0.104
Feature
−0.004
0.000
0.004
0.008
0.012
Weight
Positive
Negative

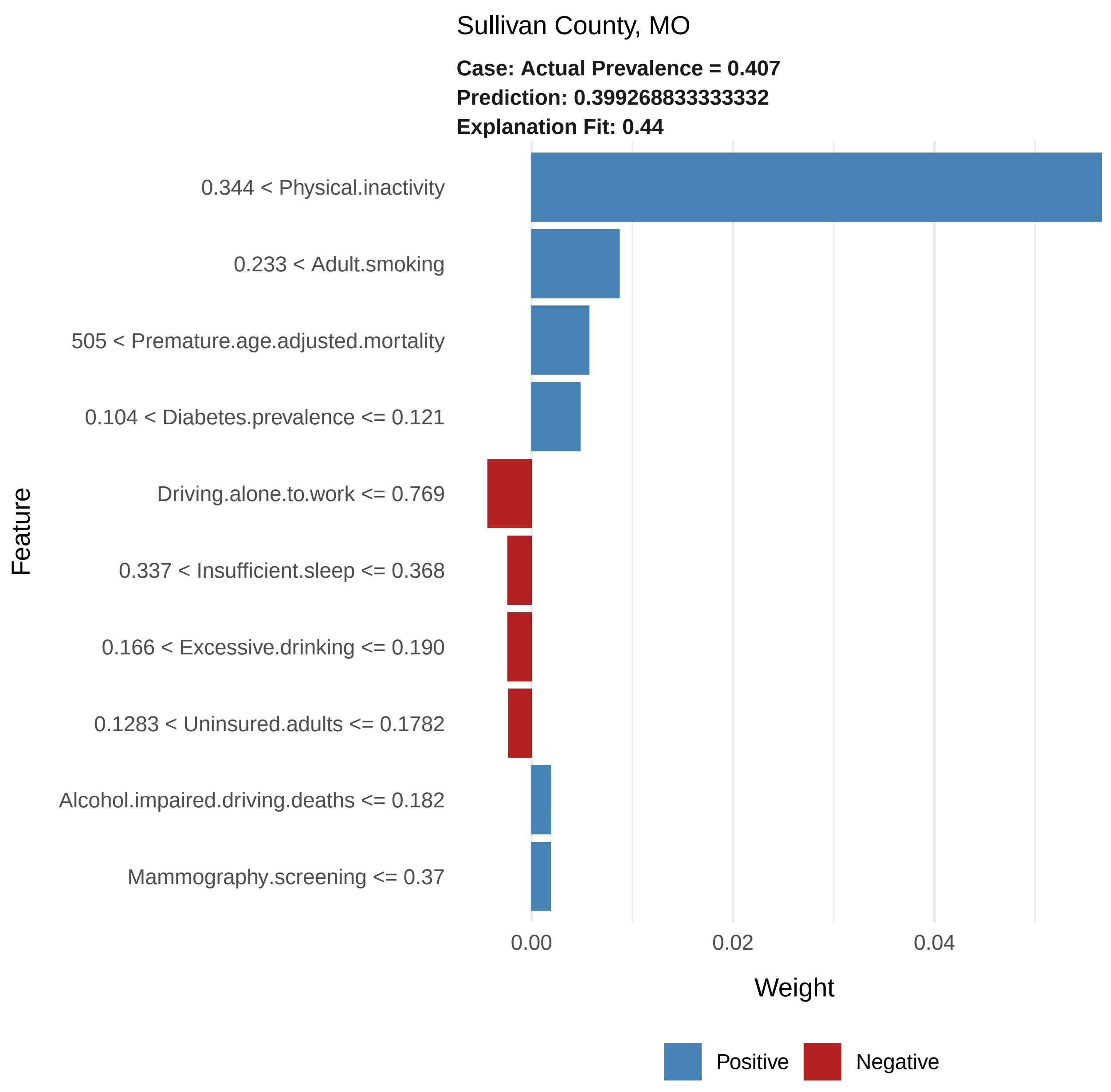

Sullivan County, MO
Case: Actual Prevalence = 0.407
Prediction: 0.399268833333332
Explanation Fit: 0.44
0.344 < Physical.inactivity
0.233 < Adult.smoking
505 < Premature.age.adjusted.mortality
0.104 < Diabetes.prevalence <= 0.121
Driving.alone.to.work <= 0.769
0.337 < Insufficient.sleep <= 0.368
0.166 < Excessive.drinking <= 0.190
0.1283 < Uninsured.adults <= 0.1782
Alcohol.impaired.driving.deaths <= 0.182
Mammography.screening <= 0.37
Feature
0.00
0.02
0.04
Weight
Positive
Negative

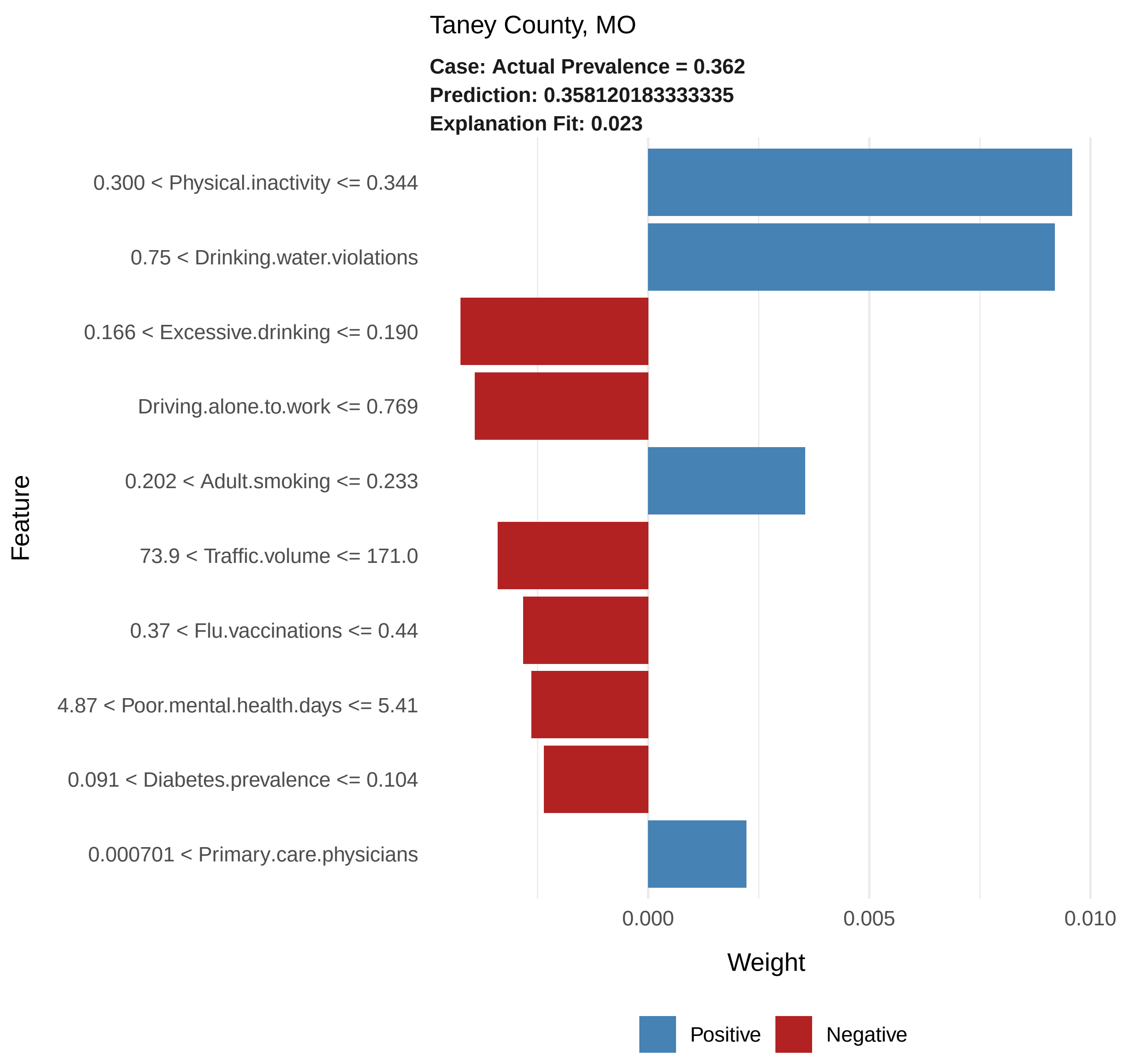

Taney County, MO
Case: Actual Prevalence = 0.362
Prediction: 0.358120183333335
Explanation Fit: 0.023
0.300 < Physical.inactivity <= 0.344
0.75 < Drinking.water.violations
0.166 < Excessive.drinking <= 0.190
Driving.alone.to.work <= 0.769
0.202 < Adult.smoking <= 0.233
73.9 < Traffic.volume <= 171.0
0.37 < Flu.vaccinations <= 0.44
4.87 < Poor.mental.health.days <= 5.41
0.091 < Diabetes.prevalence <= 0.104
0.000701 < Primary.care.physicians
Feature
0.000
0.005
0.010
Weight
Positive
Negative

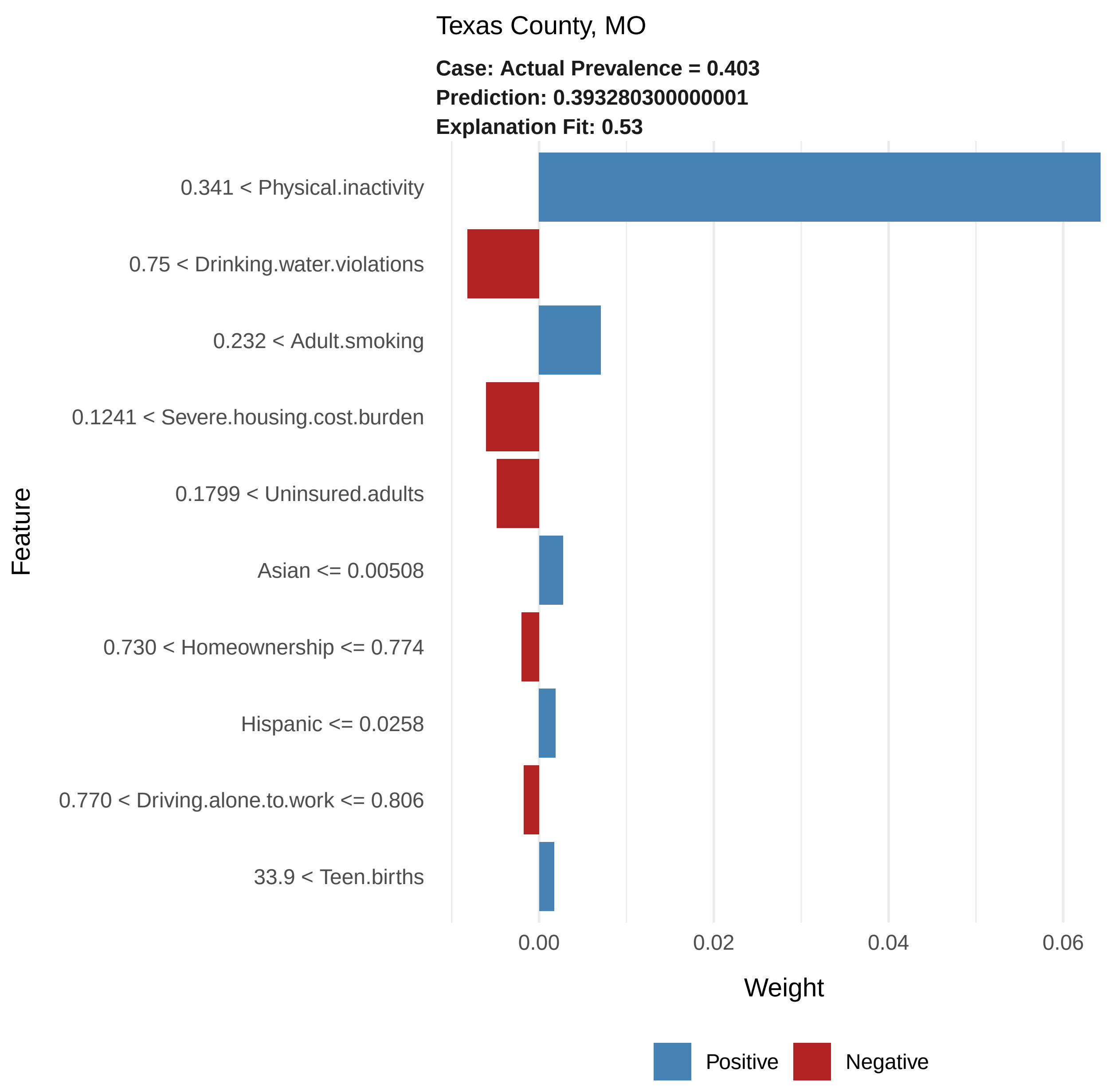
Texas County, MO
Case: Actual Prevalence = 0.403
Prediction: 0.393280300000001
Explanation Fit: 0.53
0.341 < Physical.inactivity
0.75 < Drinking.water.violations
0.232 < Adult.smoking
0.1241 < Severe.housing.cost.burden
0.1799 < Uninsured.adults
Asian <= 0.00508
0.730 < Homeownership <= 0.774
Hispanic <= 0.0258
0.770 < Driving.alone.to.work <= 0.806
33.9 < Teen.births
Feature
0.00
0.02
0.04
0.06
Weight
Positive
Negative

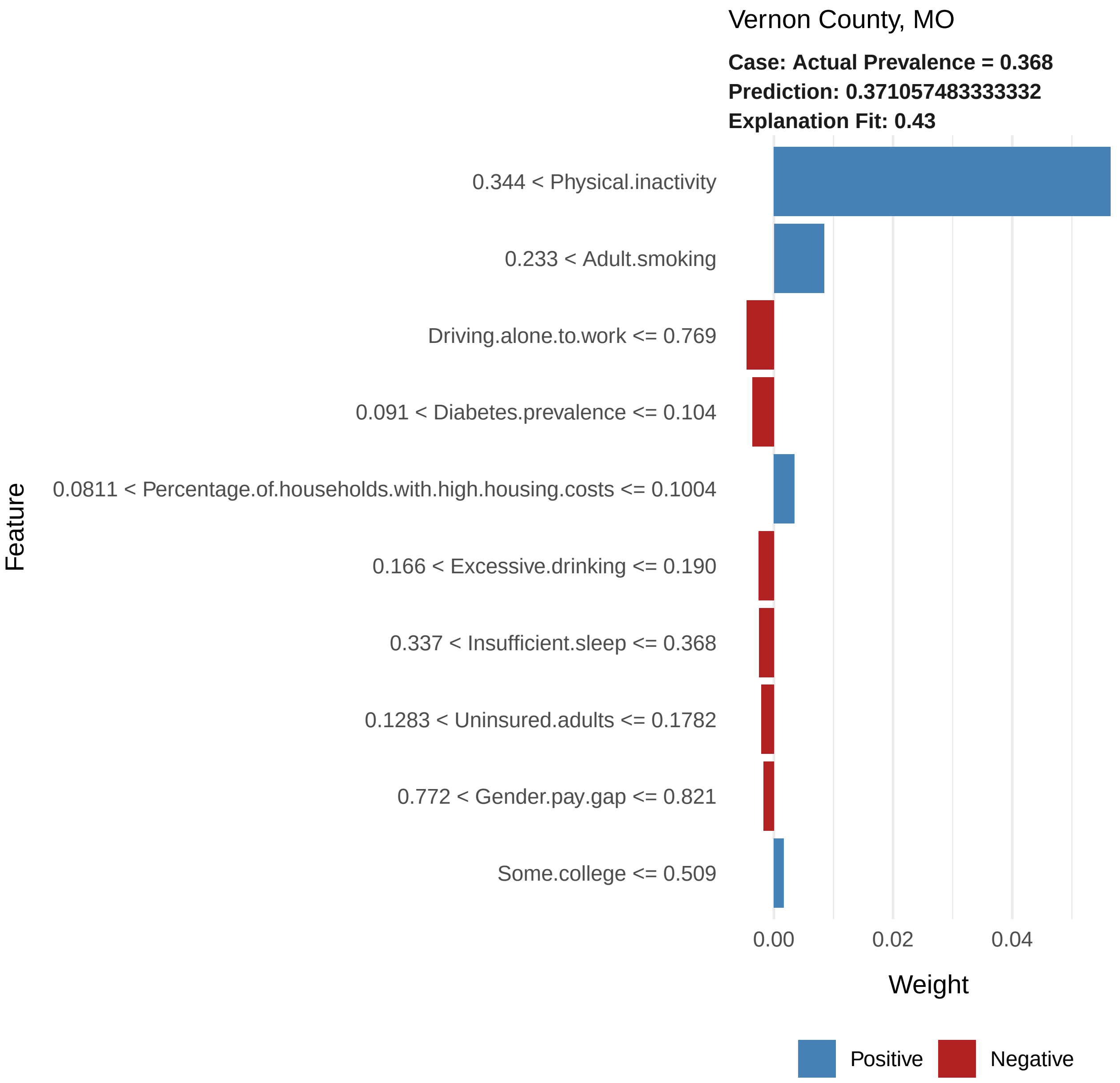

Vernon County, MO
Case: Actual Prevalence = 0.368
Prediction: 0.371057483333332
Explanation Fit: 0.43
0.344 < Physical.inactivity
0.233 < Adult.smoking
Driving.alone.to.work <= 0.769
0.091 < Diabetes.prevalence <= 0.104
0.0811 < Percentage.of.households.with.high.housing.costs <= 0.1004
0.166 < Excessive.drinking <= 0.190
0.337 < Insufficient.sleep <= 0.368
0.1283 < Uninsured.adults <= 0.1782
0.772 < Gender.pay.gap <= 0.821
Some.college <= 0.509
Feature
0.00
0.02
0.04
Weight
Positive
Negative

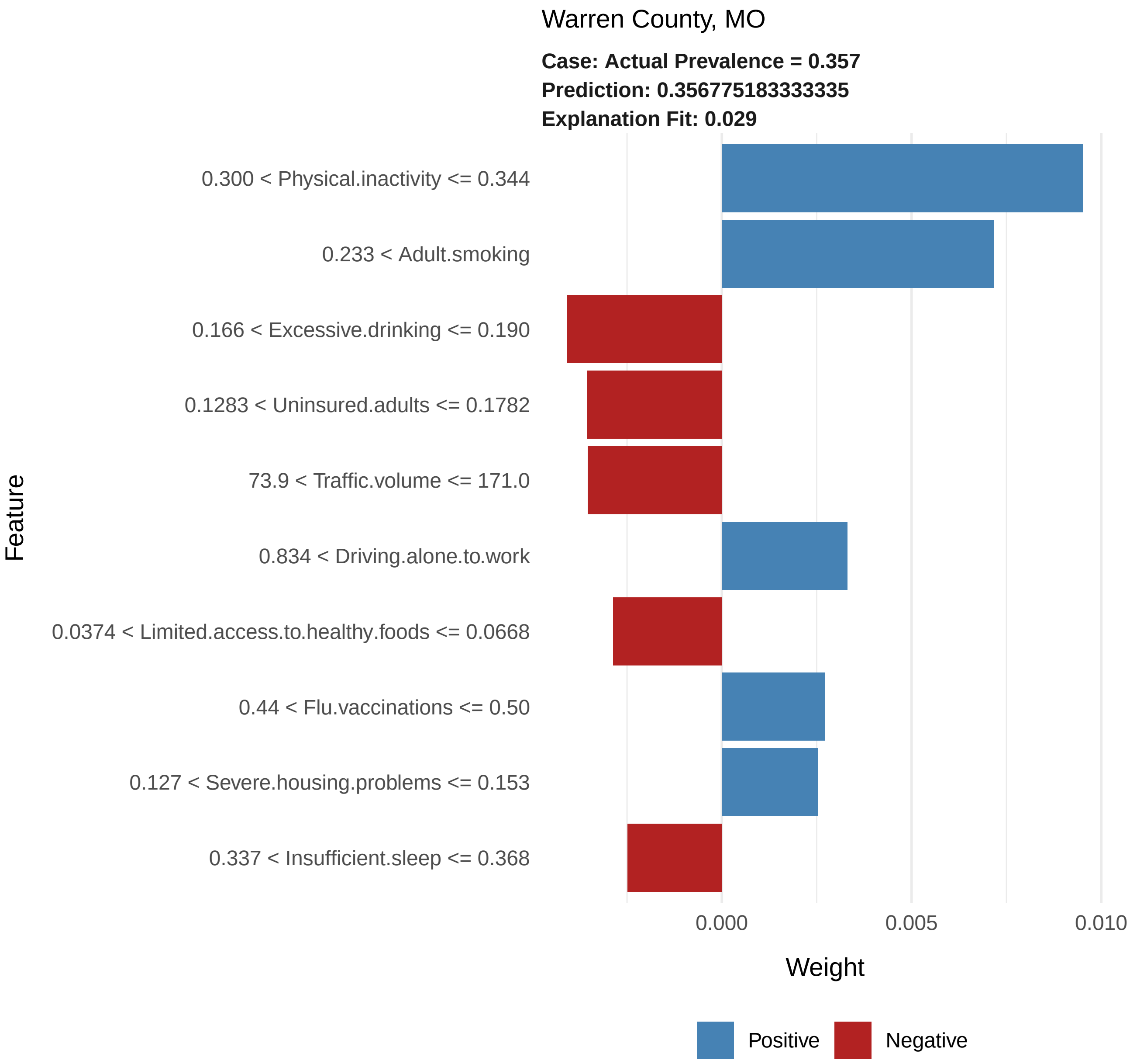

Warren County, MO
Case: Actual Prevalence = 0.357
Prediction: 0.356775183333335
Explanation Fit: 0.029
0.300 < Physical.inactivity <= 0.344
0.233 < Adult.smoking
0.166 < Excessive.drinking <= 0.190
0.1283 < Uninsured.adults <= 0.1782
73.9 < Traffic.volume <= 171.0
0.834 < Driving.alone.to.work
0.0374 < Limited.access.to.healthy.foods <= 0.0668
0.44 < Flu.vaccinations <= 0.50
0.127 < Severe.housing.problems <= 0.153
0.337 < Insufficient.sleep <= 0.368
Feature
0.000
0.005
0.010
Weight
Positive
Negative

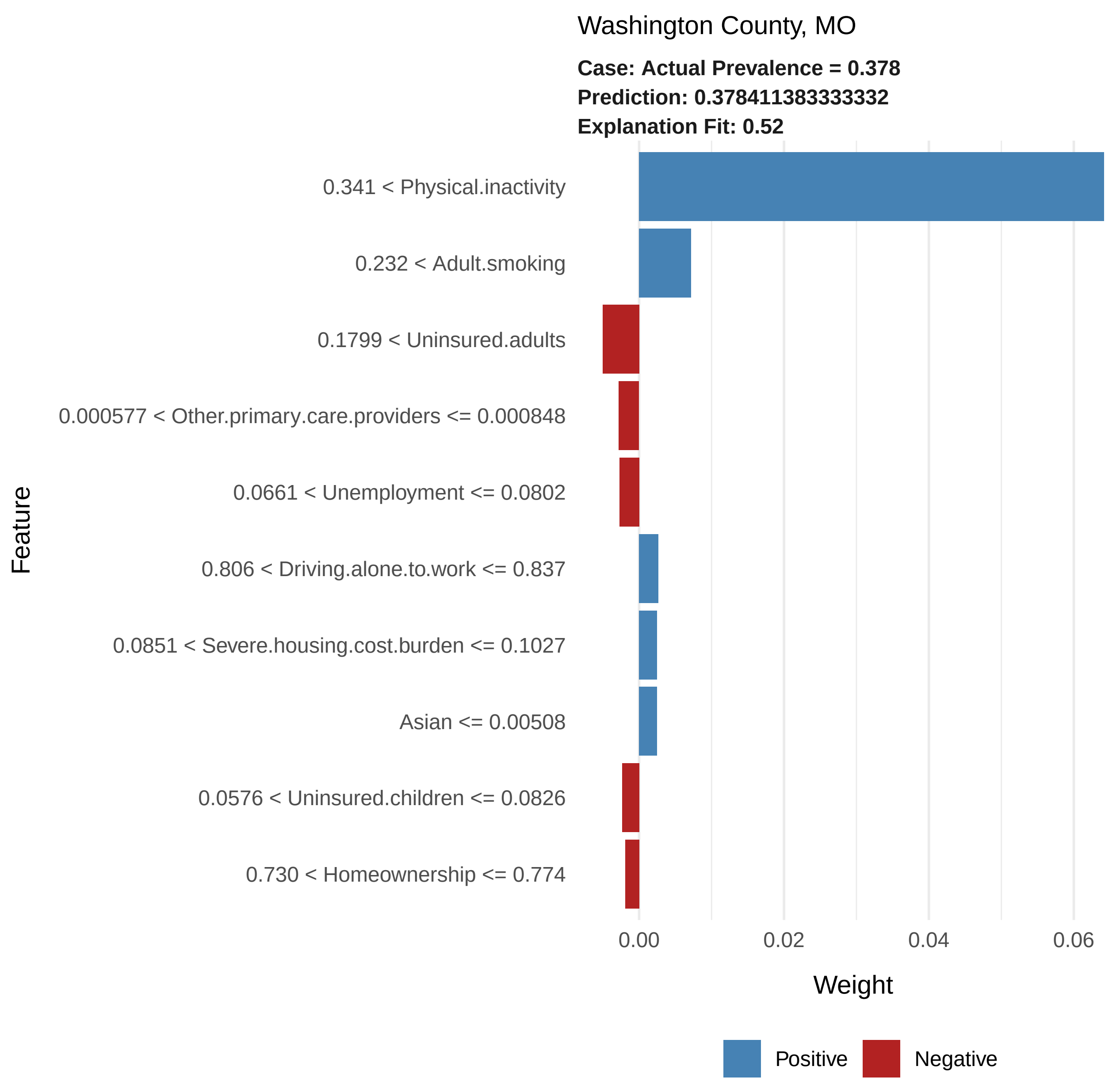

Washington County, MO
Case: Actual Prevalence = 0.378
Prediction: 0.378411383333332
Explanation Fit: 0.52
0.341 < Physical.inactivity
0.232 < Adult.smoking
0.1799 < Uninsured.adults
0.000577 < Other.primary.care.providers <= 0.000848
0.0661 < Unemployment <= 0.0802
0.806 < Driving.alone.to.work <= 0.837
0.0851 < Severe.housing.cost.burden <= 0.1027
Asian <= 0.00508
0.0576 < Uninsured.children <= 0.0826
0.730 < Homeownership <= 0.774
Feature
0.00
0.02
0.04
0.06
Weight
Positive
Negative

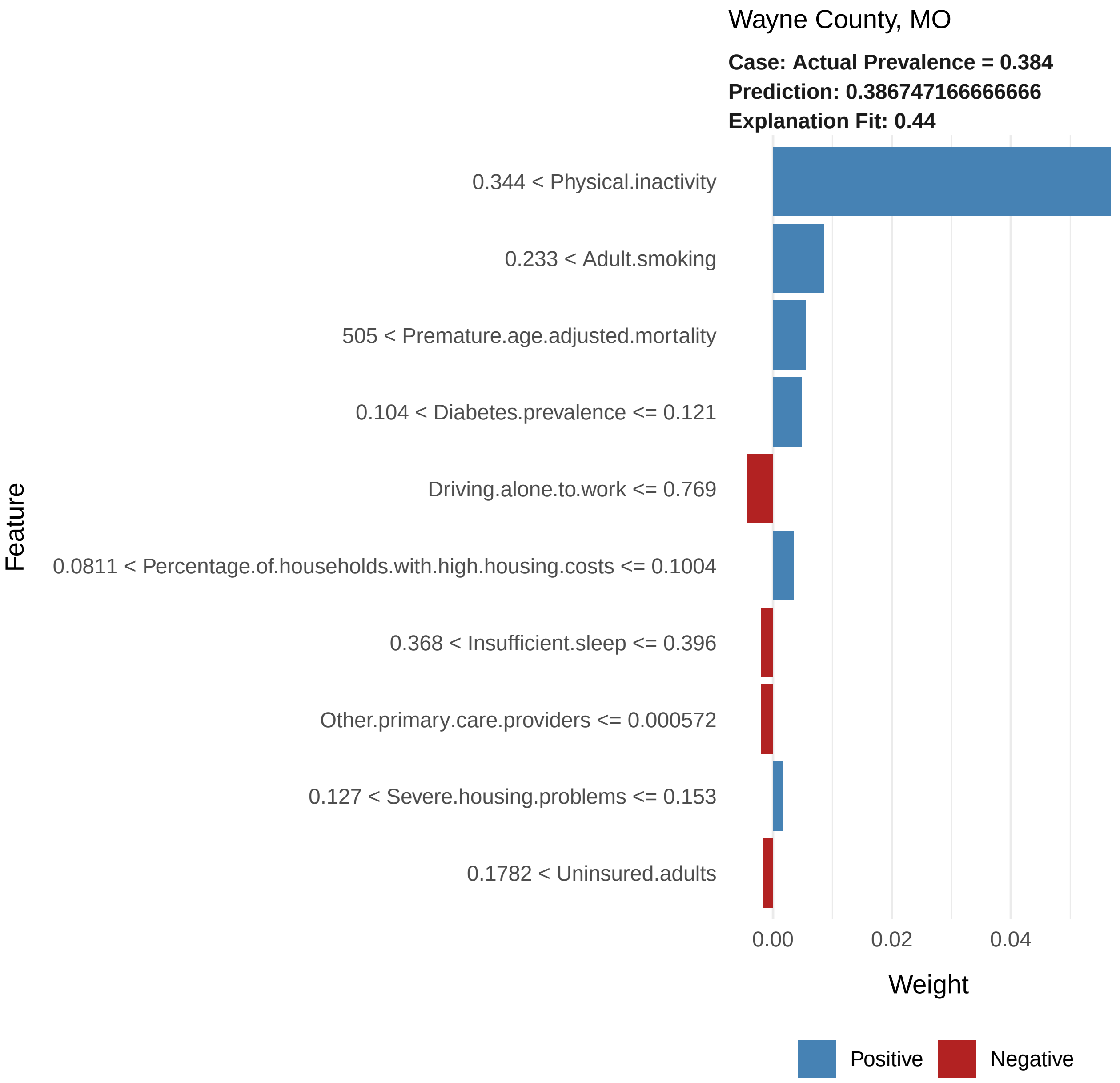

Wayne County, MO
Case: Actual Prevalence = 0.384
Prediction: 0.386747166666666
Explanation Fit: 0.44
0.344 < Physical.inactivity
0.233 < Adult.smoking
505 < Premature.age.adjusted.mortality
0.104 < Diabetes.prevalence <= 0.121
Driving.alone.to.work <= 0.769
0.0811 < Percentage.of.households.with.high.housing.costs <= 0.1004
0.368 < Insufficient.sleep <= 0.396
Other.primary.care.providers <= 0.000572
0.127 < Severe.housing.problems <= 0.153
0.1782 < Uninsured.adults
Feature
0.00
0.02
0.04
Weight
Positive
Negative

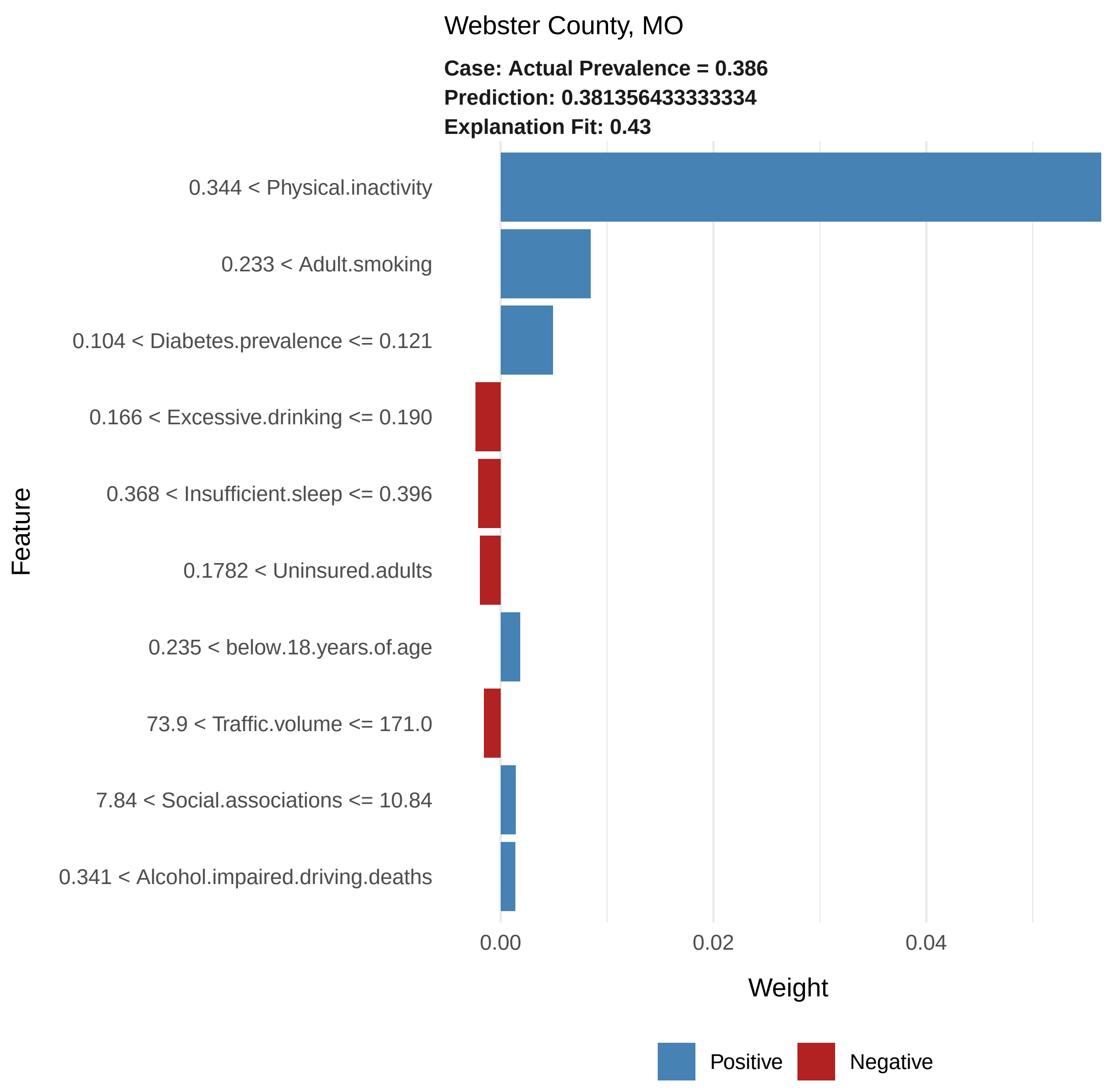
Webster County, MO
Case: Actual Prevalence = 0.386
Prediction: 0.381356433333334
Explanation Fit: 0.43
0.344 < Physical.inactivity
0.233 < Adult.smoking
0.104 < Diabetes.prevalence <= 0.121
0.166 < Excessive.drinking <= 0.190
0.368 < Insufficient.sleep <= 0.396
0.1782 < Uninsured.adults
0.235 < below.18.years.of.age
73.9 < Traffic.volume <= 171.0
7.84 < Social.associations <= 10.84
0.341 < Alcohol.impaired.driving.deaths
Feature
0.00
0.02
0.04
Weight
Positive
Negative

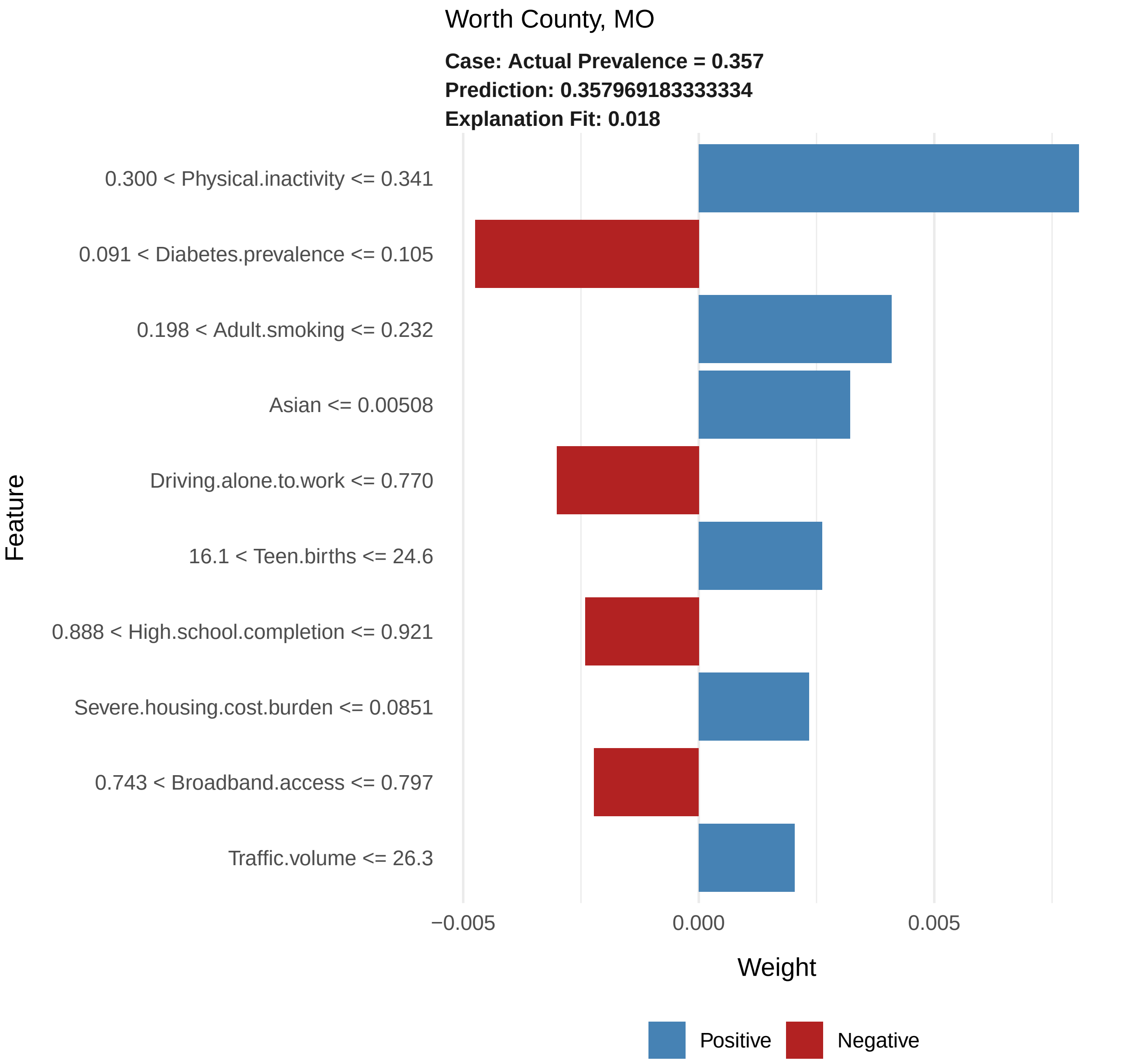
Worth County, MO
Case: Actual Prevalence = 0.357
Prediction: 0.357969183333334
Explanation Fit: 0.018
0.300 < Physical.inactivity <= 0.341
0.091 < Diabetes.prevalence <= 0.105
0.198 < Adult.smoking <= 0.232
Asian <= 0.00508
Driving.alone.to.work <= 0.770
16.1 < Teen.births <= 24.6
0.888 < High.school.completion <= 0.921
Severe.housing.cost.burden <= 0.0851
0.743 < Broadband.access <= 0.797
Traffic.volume <= 26.3
Feature
−0.005
0.000
0.005
Weight
Positive
Negative

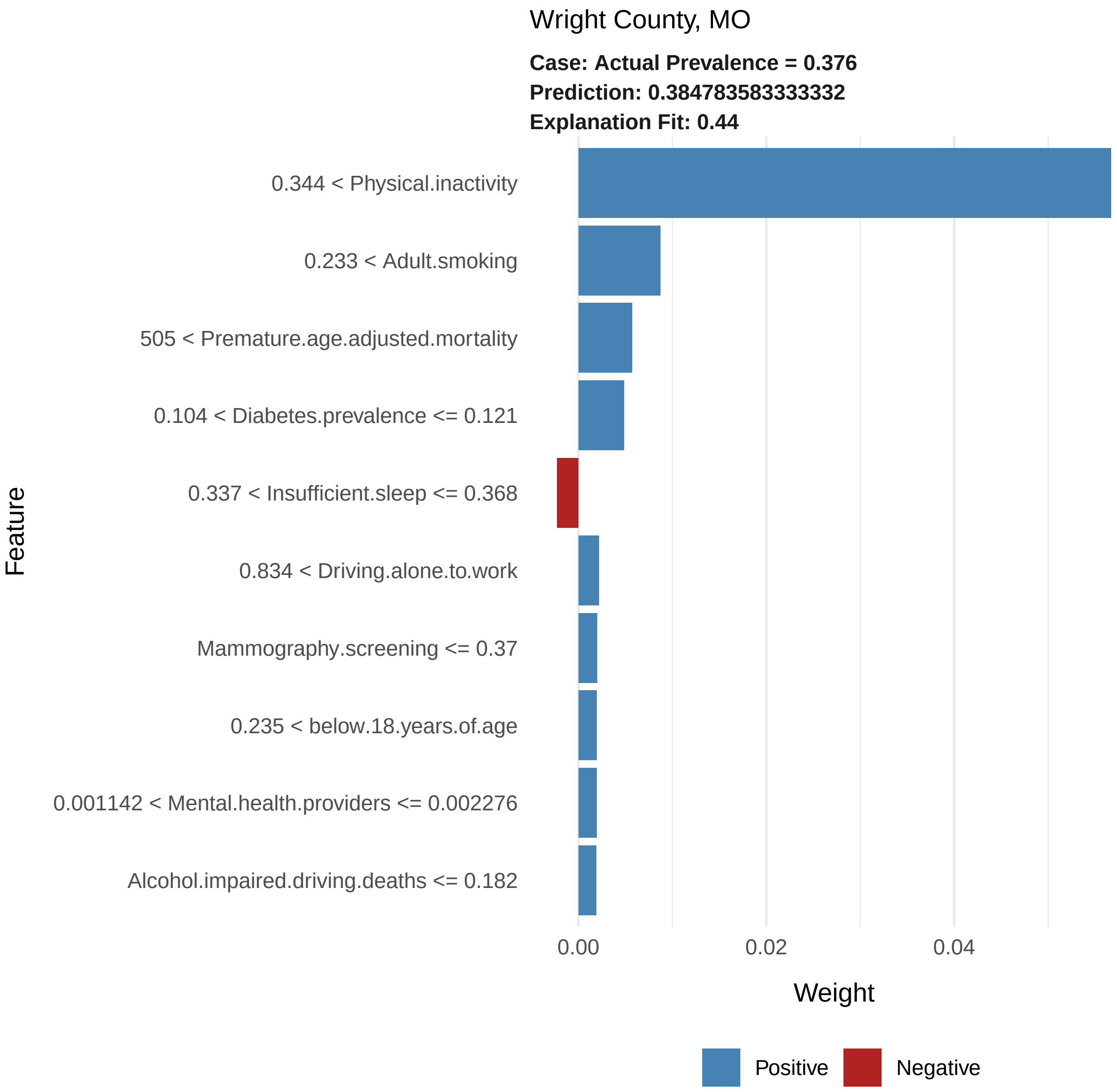

Wright County, MO
Case: Actual Prevalence = 0.376
Prediction: 0.384783583333332
Explanation Fit: 0.44
0.344 < Physical.inactivity
0.233 < Adult.smoking
505 < Premature.age.adjusted.mortality
0.104 < Diabetes.prevalence <= 0.121
0.337 < Insufficient.sleep <= 0.368
0.834 < Driving.alone.to.work
Mammography.screening <= 0.37
0.235 < below.18.years.of.age
0.001142 < Mental.health.providers <= 0.002276
Alcohol.impaired.driving.deaths <= 0.182
Feature
0.00
0.02
0.04
Weight
Positive
Negative

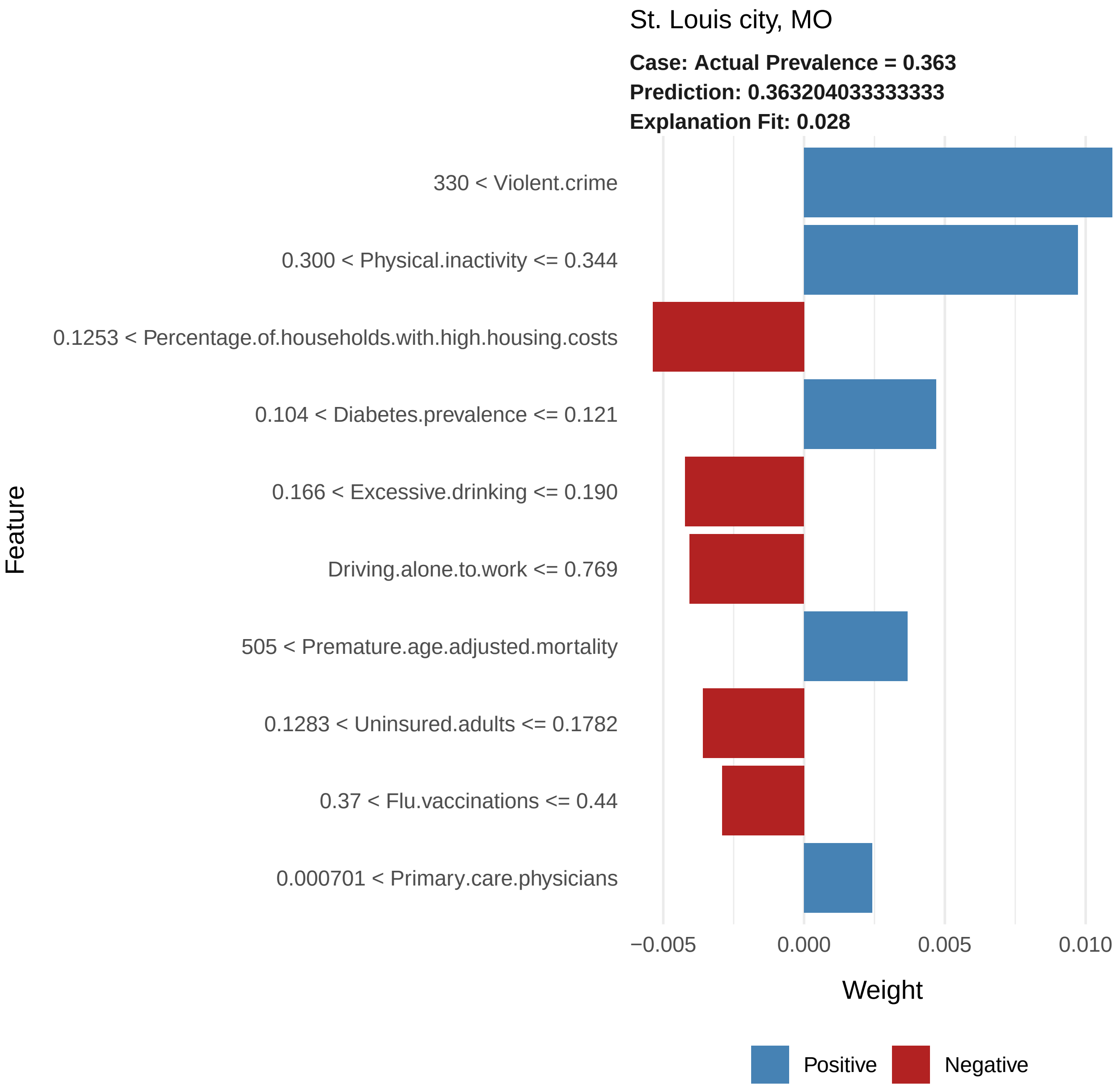
St. Louis city, MO
Case: Actual Prevalence = 0.363
Prediction: 0.363204033333333
Explanation Fit: 0.028
330 < Violent.crime
0.300 < Physical.inactivity <= 0.344
0.1253 < Percentage.of.households.with.high.housing.costs
0.104 < Diabetes.prevalence <= 0.121
0.166 < Excessive.drinking <= 0.190
Driving.alone.to.work <= 0.769
505 < Premature.age.adjusted.mortality
0.1283 < Uninsured.adults <= 0.1782
0.37 < Flu.vaccinations <= 0.44
0.000701 < Primary.care.physicians
Feature
−0.005
0.000
0.005
0.010
Weight
Positive
Negative

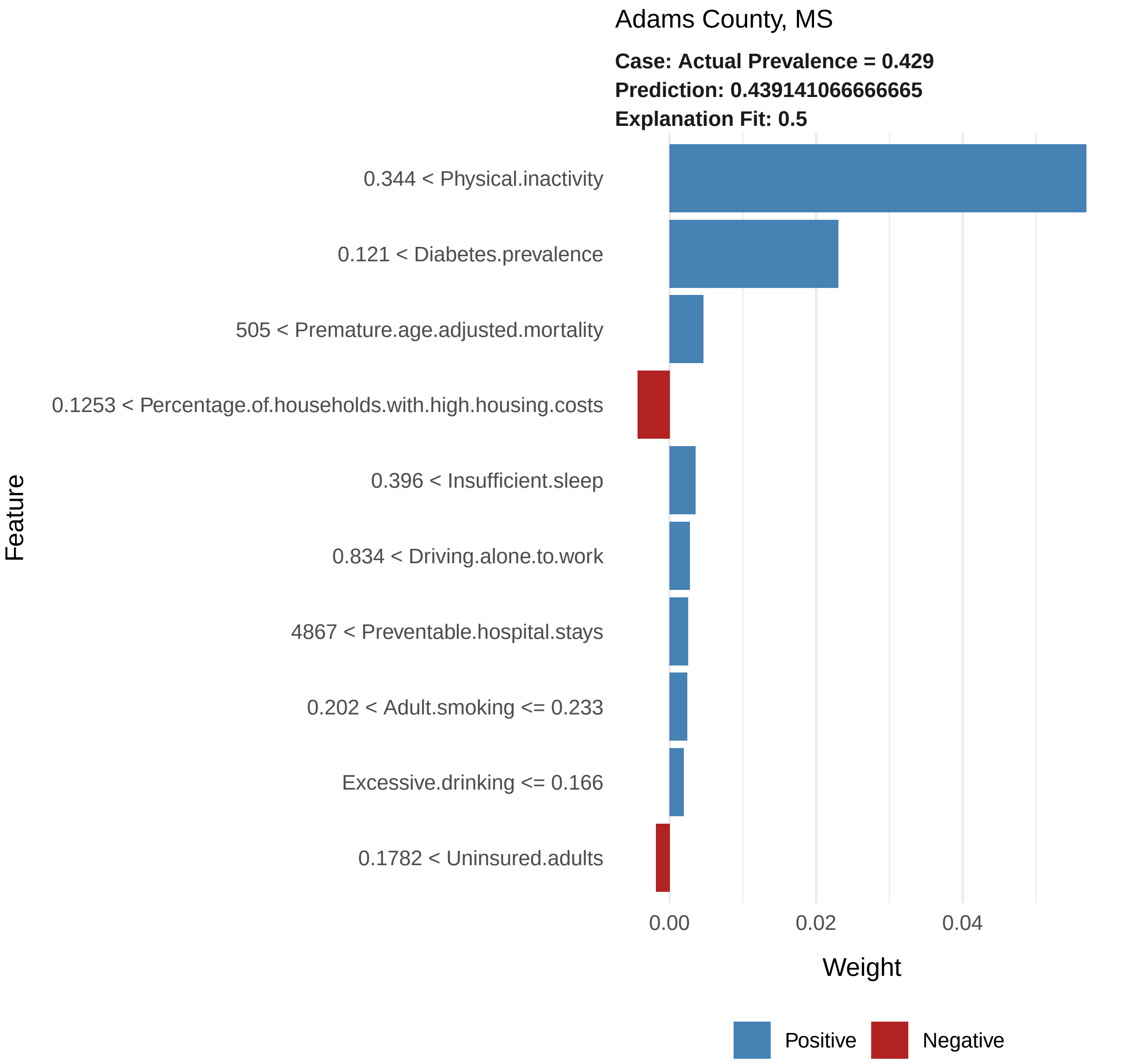
Adams County, MS
Case: Actual Prevalence = 0.429
Prediction: 0.439141066666665
Explanation Fit: 0.5
0.344 < Physical.inactivity
0.121 < Diabetes.prevalence
505 < Premature.age.adjusted.mortality
0.1253 < Percentage.of.households.with.high.housing.costs
0.396 < Insufficient.sleep
0.834 < Driving.alone.to.work
4867 < Preventable.hospital.stays
0.202 < Adult.smoking <= 0.233
Excessive.drinking <= 0.166
0.1782 < Uninsured.adults
Feature
0.00
0.02
0.04
Weight
Positive
Negative

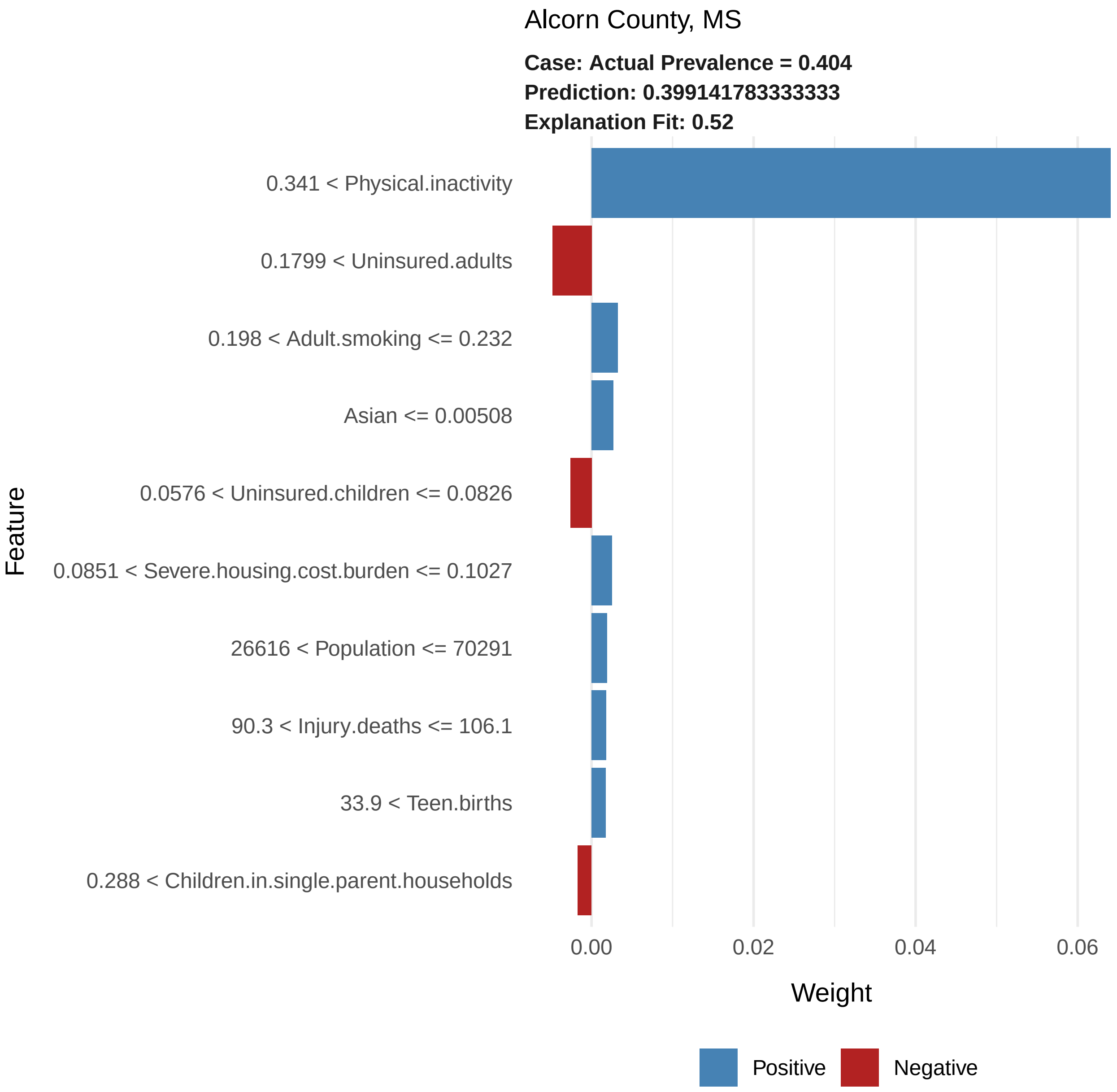

Alcorn County, MS
Case: Actual Prevalence = 0.404
Prediction: 0.399141783333333
Explanation Fit: 0.52
0.341 < Physical.inactivity
0.1799 < Uninsured.adults
0.198 < Adult.smoking <= 0.232
Asian <= 0.00508
0.0576 < Uninsured.children <= 0.0826
0.0851 < Severe.housing.cost.burden <= 0.1027
26616 < Population <= 70291
90.3 < Injury.deaths <= 106.1
33.9 < Teen.births
0.288 < Children.in.single.parent.households
Feature
0.00
0.02
0.04
0.06
Weight
Positive
Negative

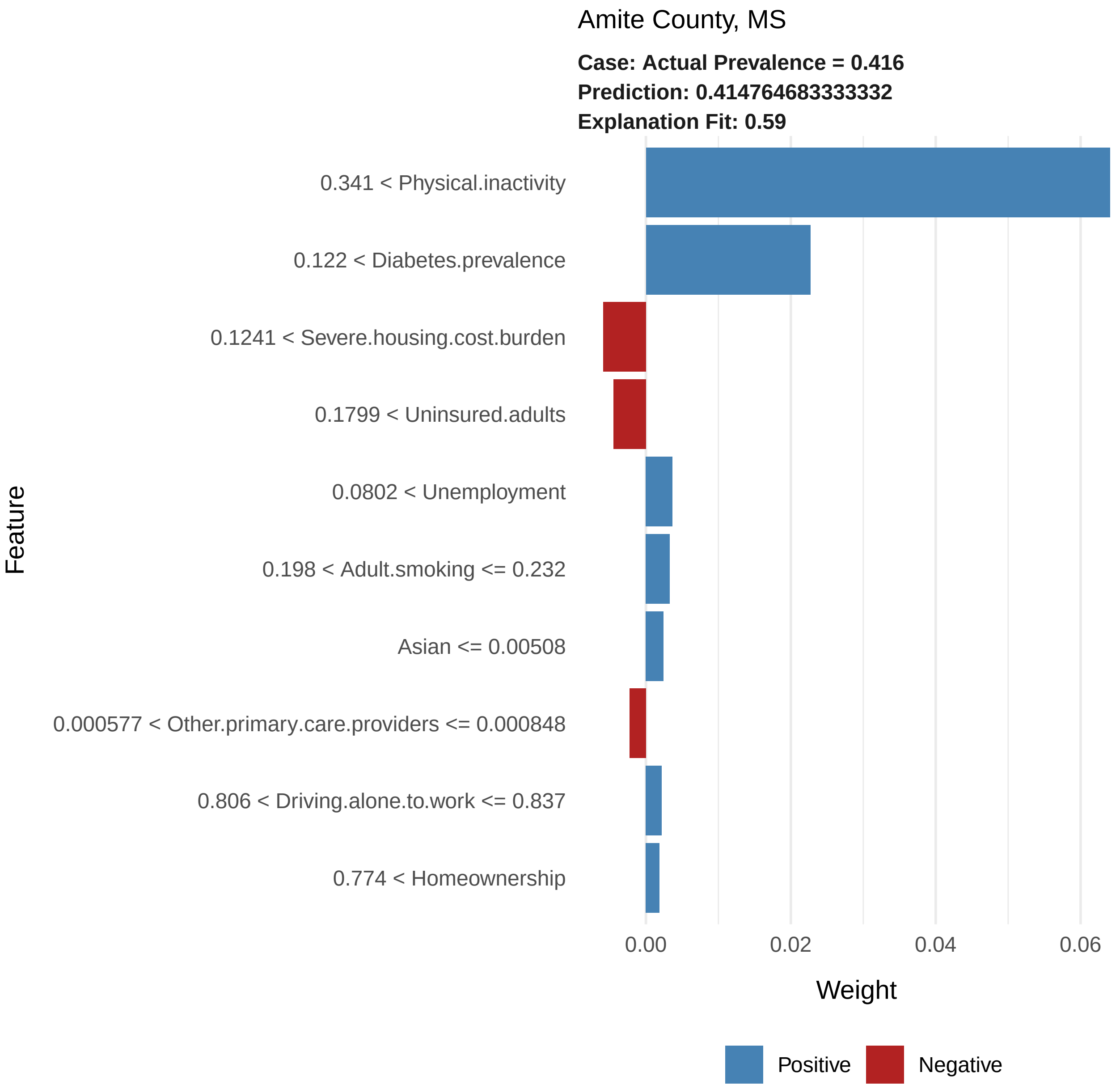
Amite County, MS
Case: Actual Prevalence = 0.416
Prediction: 0.414764683333332
Explanation Fit: 0.59
0.341 < Physical.inactivity
0.122 < Diabetes.prevalence
0.1241 < Severe.housing.cost.burden
0.1799 < Uninsured.adults
0.0802 < Unemployment
0.198 < Adult.smoking <= 0.232
Asian <= 0.00508
0.000577 < Other.primary.care.providers <= 0.000848
0.806 < Driving.alone.to.work <= 0.837
0.774 < Homeownership
Feature
0.00
0.02
0.04
0.06
Weight
Positive
Negative

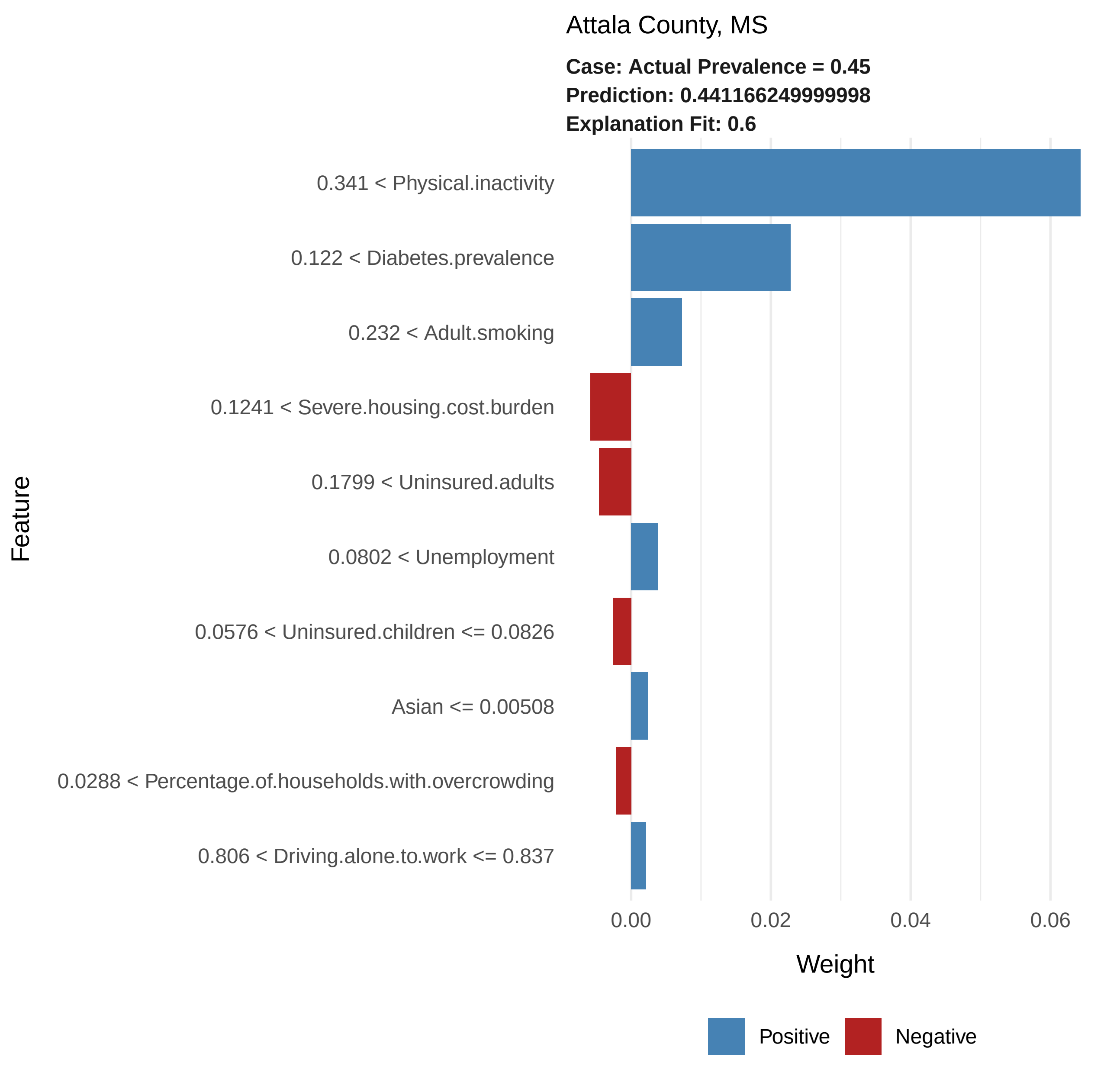

Attala County, MS
Case: Actual Prevalence = 0.45
Prediction: 0.441166249999998
Explanation Fit: 0.6
0.341 < Physical.inactivity
0.122 < Diabetes.prevalence
0.232 < Adult.smoking
0.1241 < Severe.housing.cost.burden
0.1799 < Uninsured.adults
0.0802 < Unemployment
0.0576 < Uninsured.children <= 0.0826
Asian <= 0.00508
0.0288 < Percentage.of.households.with.overcrowding
0.806 < Driving.alone.to.work <= 0.837
Feature
0.00
0.02
0.04
0.06
Weight
Positive
Negative

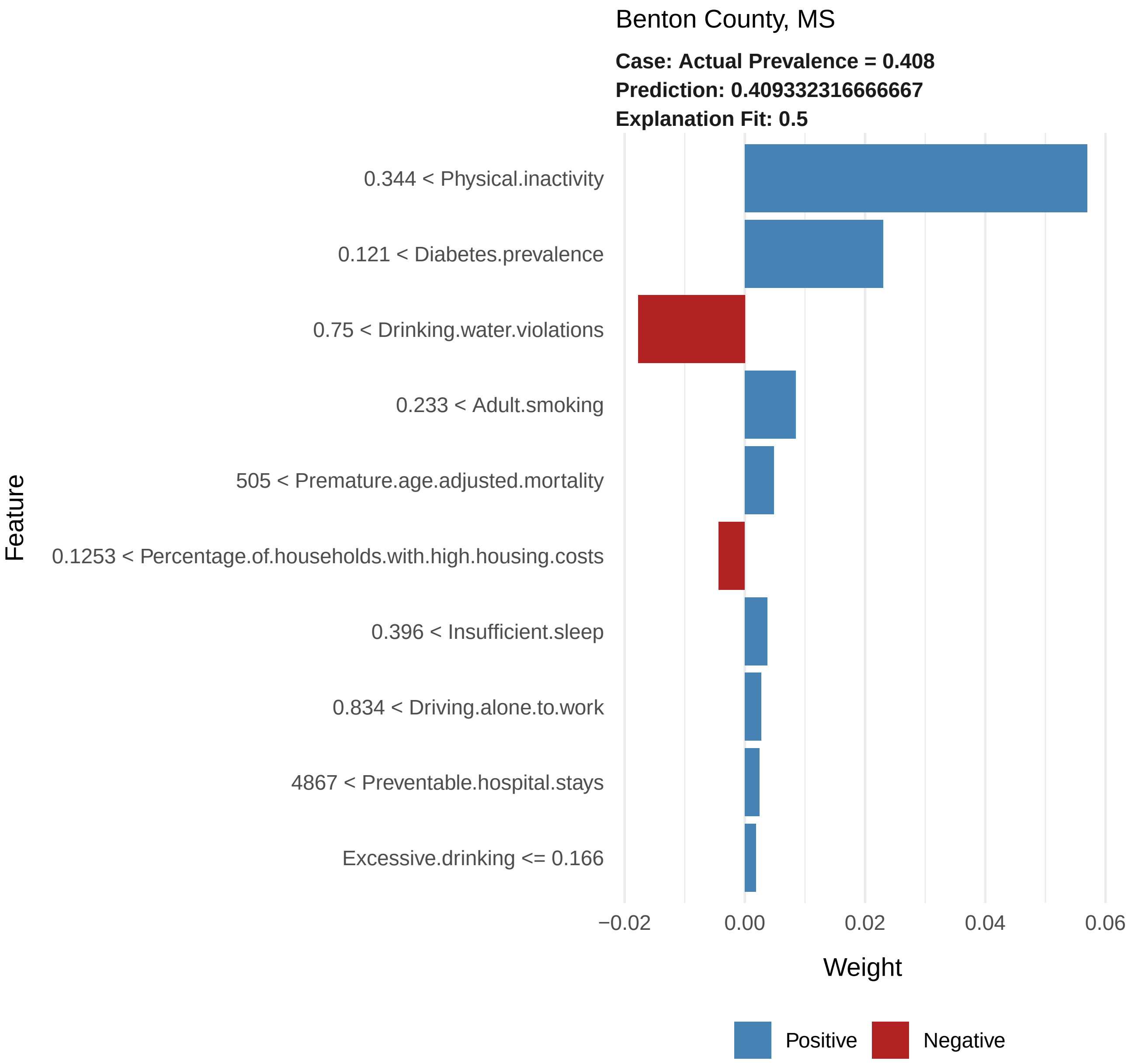

Benton County, MS
Case: Actual Prevalence = 0.408
Prediction: 0.409332316666667
Explanation Fit: 0.5
0.344 < Physical.inactivity
0.121 < Diabetes.prevalence
0.75 < Drinking.water.violations
0.233 < Adult.smoking
505 < Premature.age.adjusted.mortality
0.1253 < Percentage.of.households.with.high.housing.costs
0.396 < Insufficient.sleep
0.834 < Driving.alone.to.work
4867 < Preventable.hospital.stays
Excessive.drinking <= 0.166
Feature
−0.02
0.00
0.02
0.04
0.06
Weight
Positive
Negative

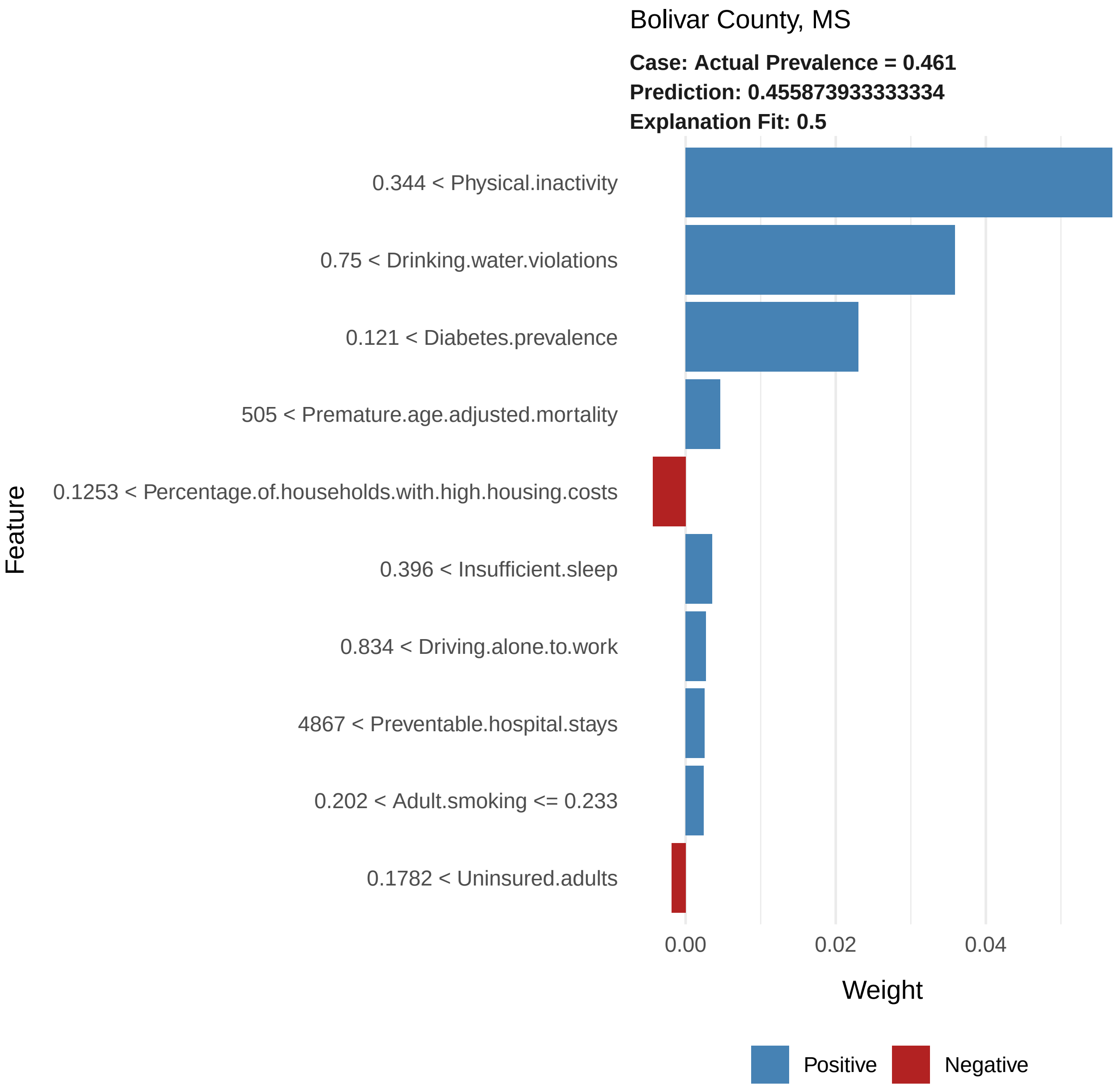

Bolivar County, MS
Case: Actual Prevalence = 0.461
Prediction: 0.455873933333334
Explanation Fit: 0.5
0.344 < Physical.inactivity
0.75 < Drinking.water.violations
0.121 < Diabetes.prevalence
505 < Premature.age.adjusted.mortality
0.1253 < Percentage.of.households.with.high.housing.costs
0.396 < Insufficient.sleep
0.834 < Driving.alone.to.work
4867 < Preventable.hospital.stays
0.202 < Adult.smoking <= 0.233
0.1782 < Uninsured.adults
Feature
0.00
0.02
0.04
Weight
Positive
Negative

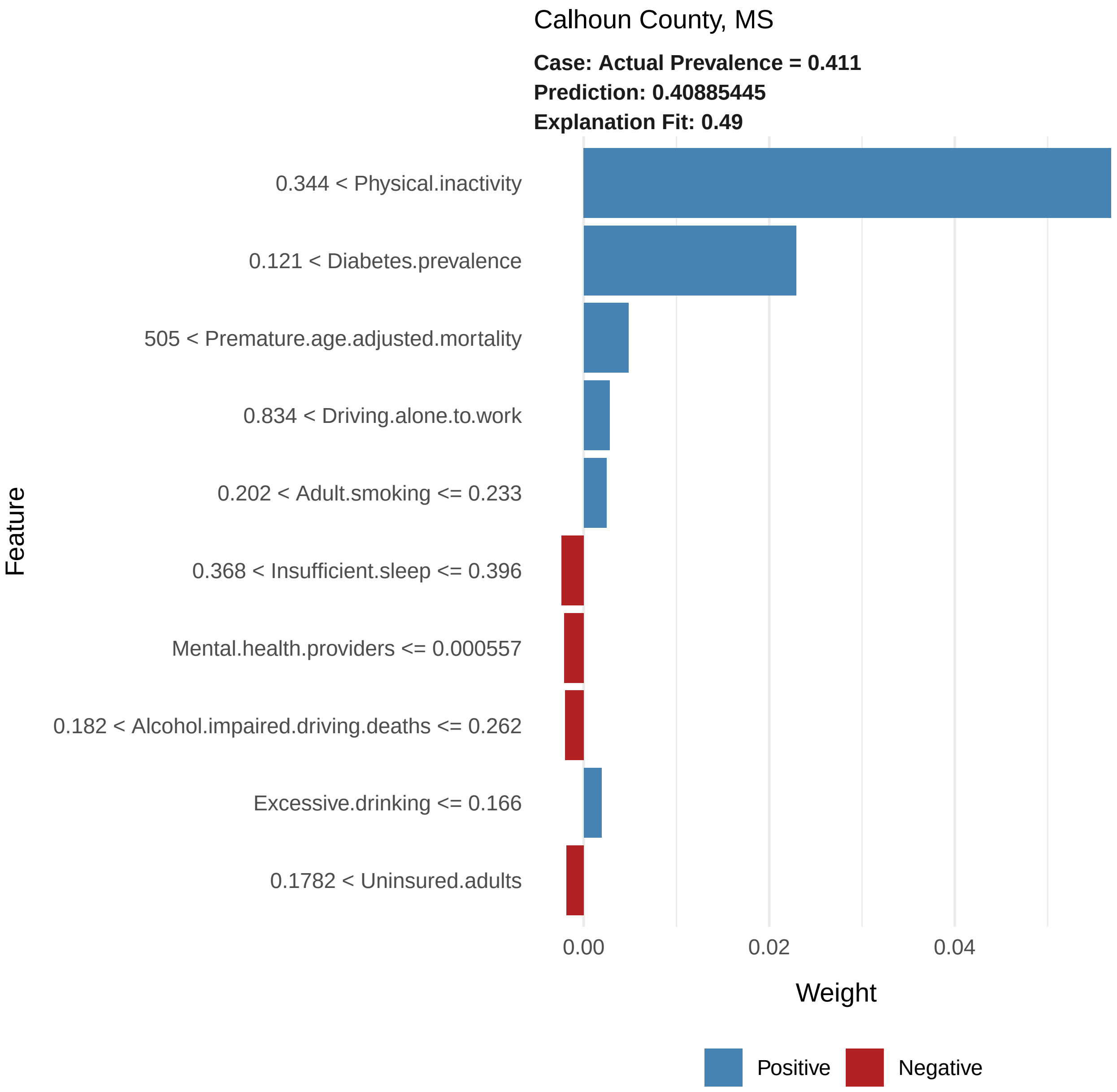

Calhoun County, MS
Case: Actual Prevalence = 0.411
Prediction: 0.40885445
Explanation Fit: 0.49
0.344 < Physical.inactivity
0.121 < Diabetes.prevalence
505 < Premature.age.adjusted.mortality
0.834 < Driving.alone.to.work
0.202 < Adult.smoking <= 0.233
0.368 < Insufficient.sleep <= 0.396
Mental.health.providers <= 0.000557
0.182 < Alcohol.impaired.driving.deaths <= 0.262
Excessive.drinking <= 0.166
0.1782 < Uninsured.adults
Feature
0.00
0.02
0.04
Weight
Positive
Negative

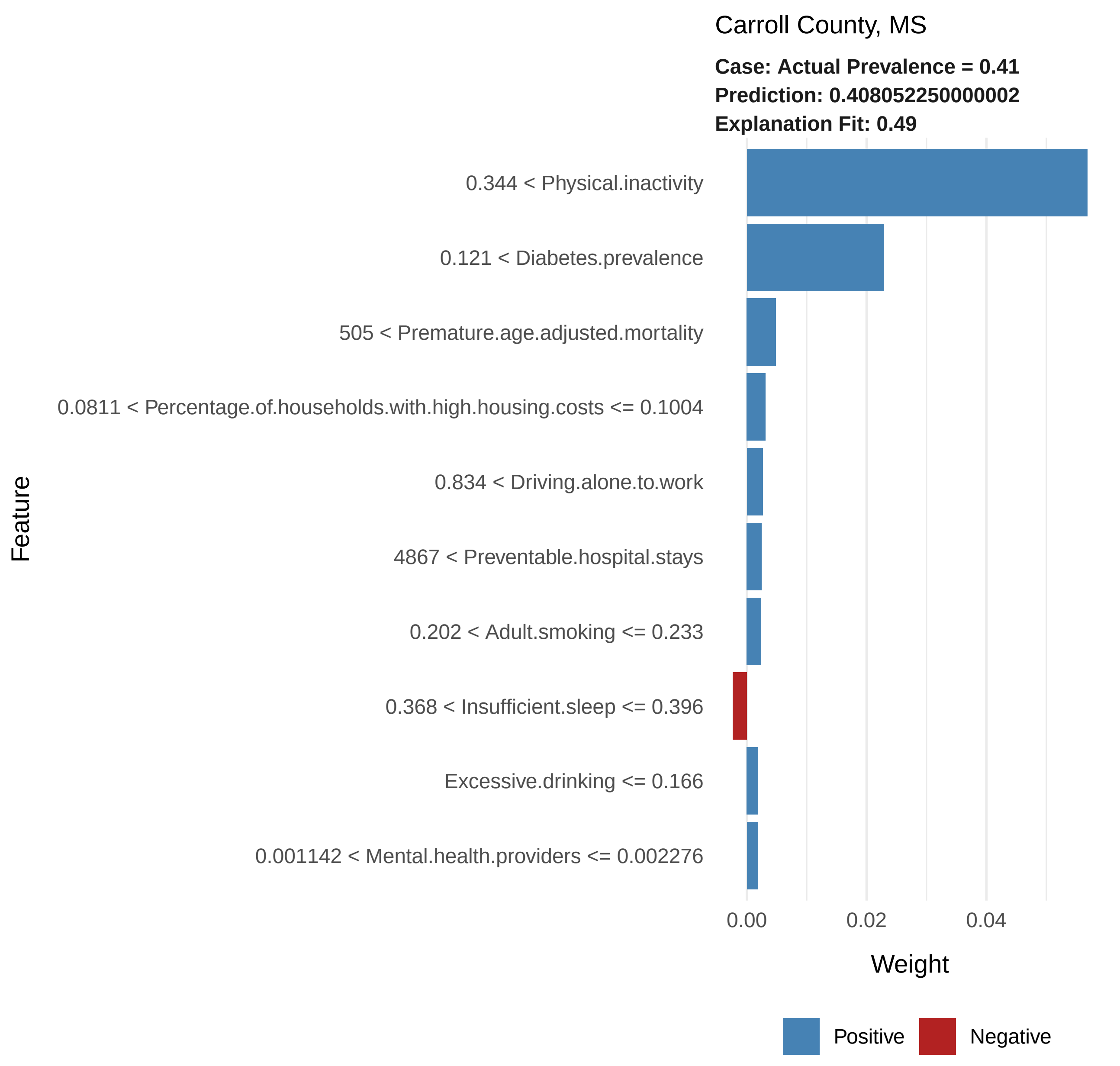

Carroll County, MS
Case: Actual Prevalence = 0.41
Prediction: 0.408052250000002
Explanation Fit: 0.49
0.344 < Physical.inactivity
0.121 < Diabetes.prevalence
505 < Premature.age.adjusted.mortality
0.0811 < Percentage.of.households.with.high.housing.costs <= 0.1004
0.834 < Driving.alone.to.work
4867 < Preventable.hospital.stays
0.202 < Adult.smoking <= 0.233
0.368 < Insufficient.sleep <= 0.396
Excessive.drinking <= 0.166
0.001142 < Mental.health.providers <= 0.002276
Feature
0.00
0.02
0.04
Weight
Positive
Negative

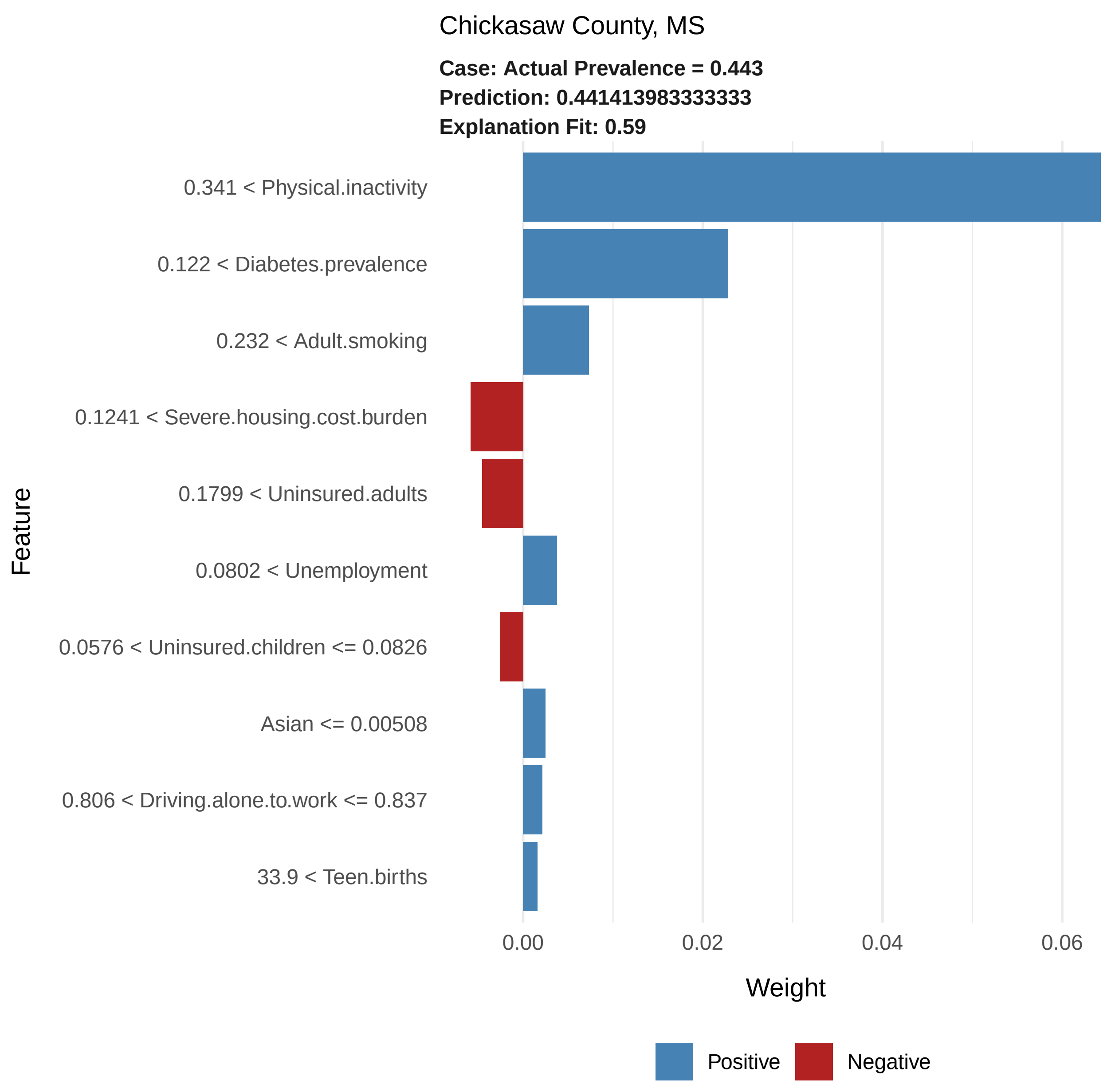
Chickasaw County, MS
Case: Actual Prevalence = 0.443
Prediction: 0.441413983333333
Explanation Fit: 0.59
0.341 < Physical.inactivity
0.122 < Diabetes.prevalence
0.232 < Adult.smoking
0.1241 < Severe.housing.cost.burden
0.1799 < Uninsured.adults
0.0802 < Unemployment
0.0576 < Uninsured.children <= 0.0826
Asian <= 0.00508
0.806 < Driving.alone.to.work <= 0.837
33.9 < Teen.births
Feature
0.00
0.02
0.04
0.06
Weight
Positive
Negative

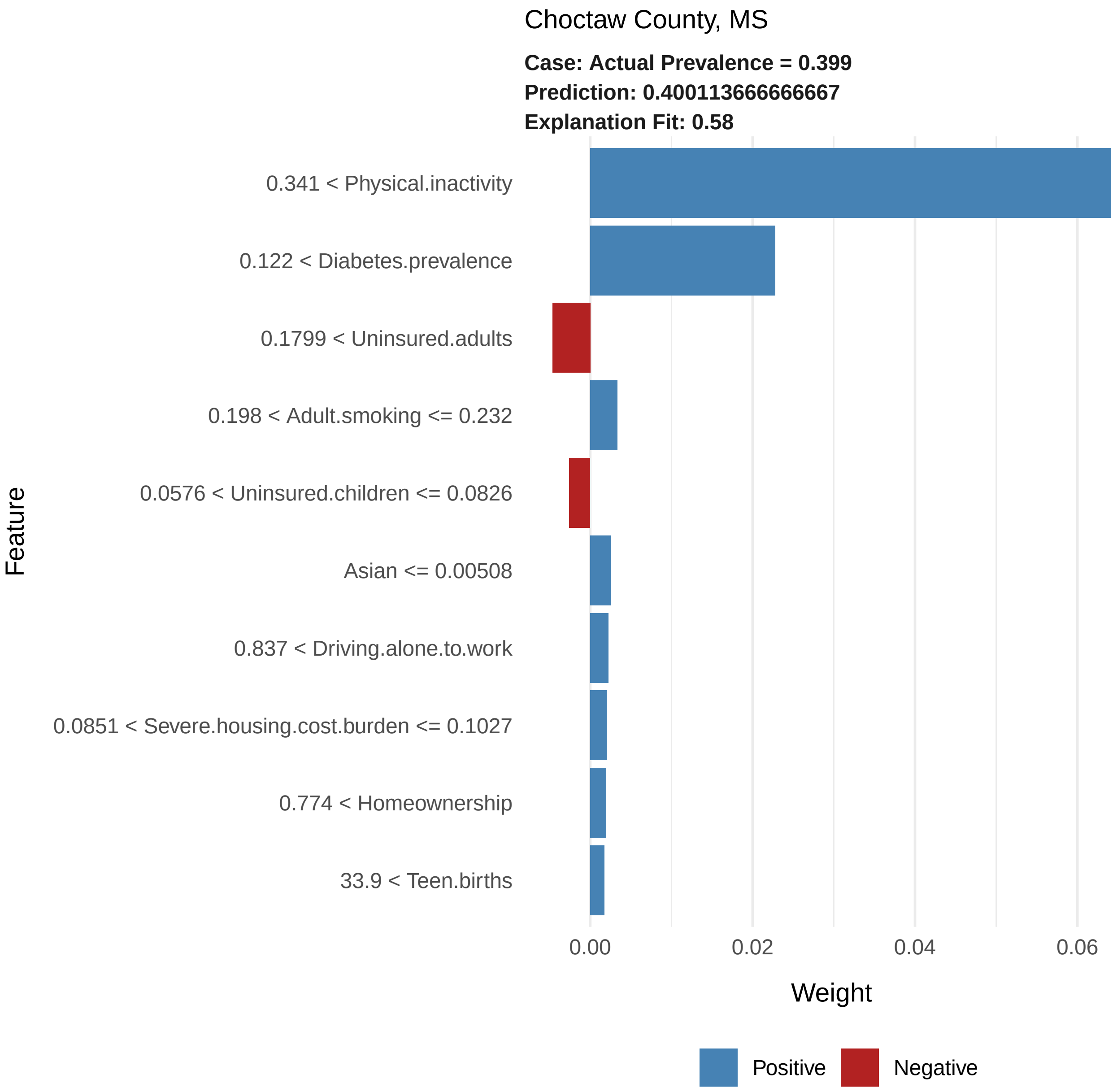

Choctaw County, MS
Case: Actual Prevalence = 0.399
Prediction: 0.400113666666667
Explanation Fit: 0.58
0.341 < Physical.inactivity
0.122 < Diabetes.prevalence
0.1799 < Uninsured.adults
0.198 < Adult.smoking <= 0.232
0.0576 < Uninsured.children <= 0.0826
Asian <= 0.00508
0.837 < Driving.alone.to.work
0.0851 < Severe.housing.cost.burden <= 0.1027
0.774 < Homeownership
33.9 < Teen.births
Feature
0.00
0.02
0.04
0.06
Weight
Positive
Negative

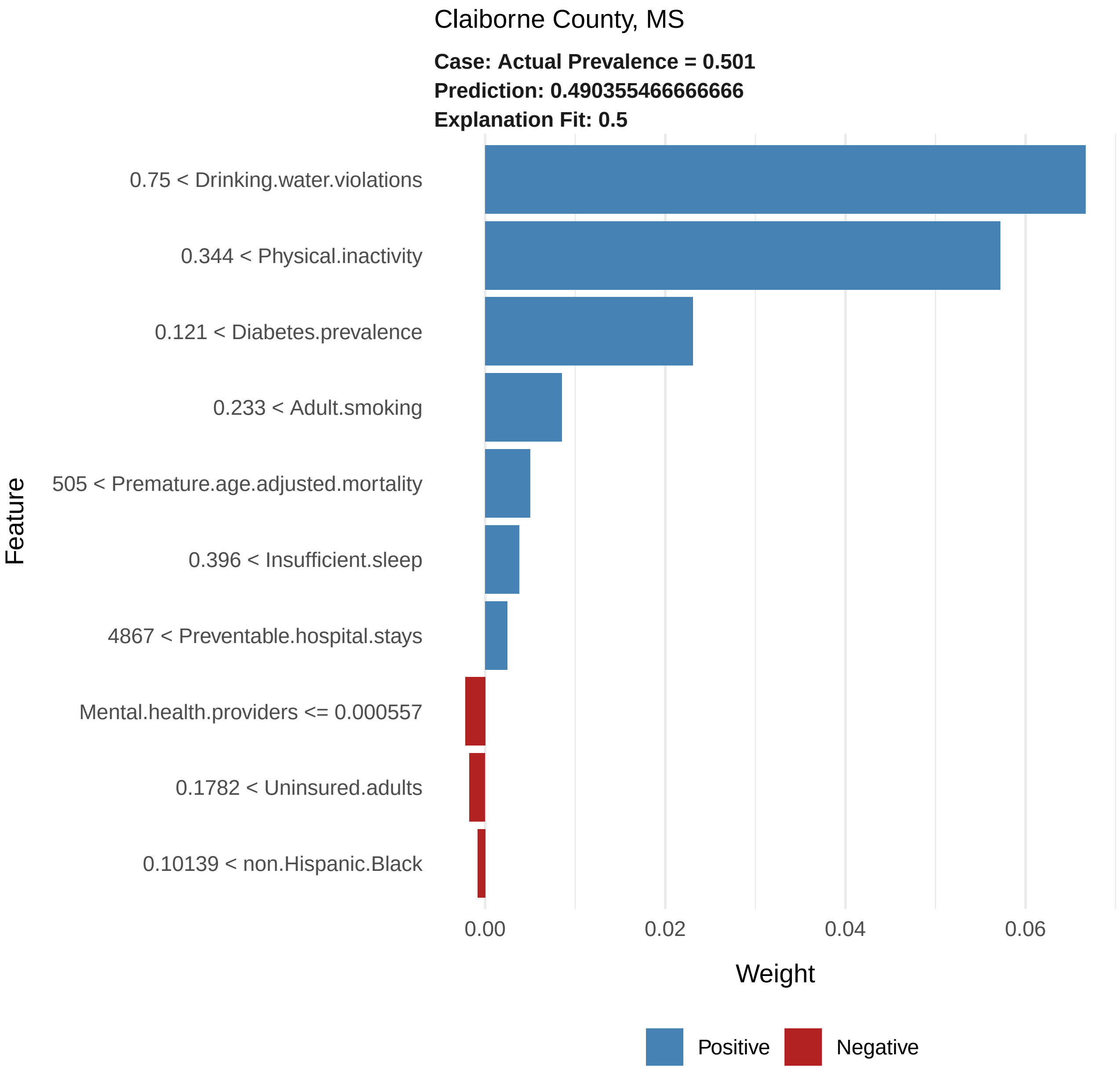
Claiborne County, MS
Case: Actual Prevalence = 0.501
Prediction: 0.490355466666666
Explanation Fit: 0.5
0.75 < Drinking.water.violations
0.344 < Physical.inactivity
0.121 < Diabetes.prevalence
0.233 < Adult.smoking
505 < Premature.age.adjusted.mortality
0.396 < Insufficient.sleep
4867 < Preventable.hospital.stays
Mental.health.providers <= 0.000557
0.1782 < Uninsured.adults
0.10139 < non.Hispanic.Black
Feature
0.00
0.02
0.04
0.06
Weight
Positive
Negative

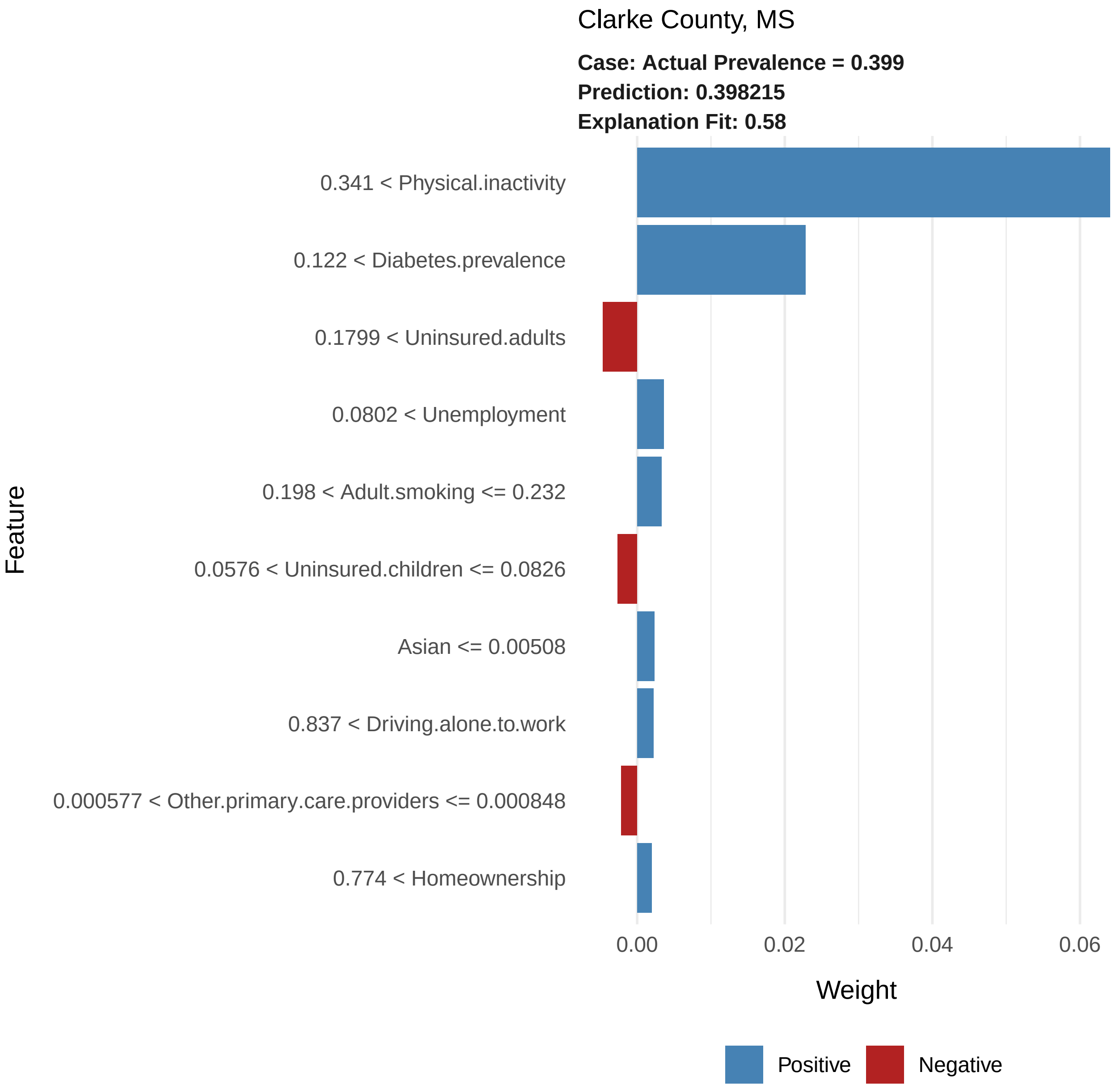

Clarke County, MS
Case: Actual Prevalence = 0.399
Prediction: 0.398215
Explanation Fit: 0.58
0.341 < Physical.inactivity
0.122 < Diabetes.prevalence
0.1799 < Uninsured.adults
0.0802 < Unemployment
0.198 < Adult.smoking <= 0.232
0.0576 < Uninsured.children <= 0.0826
Asian <= 0.00508
0.837 < Driving.alone.to.work
0.000577 < Other.primary.care.providers <= 0.000848
0.774 < Homeownership
Feature
0.00
0.02
0.04
0.06
Weight
Positive
Negative

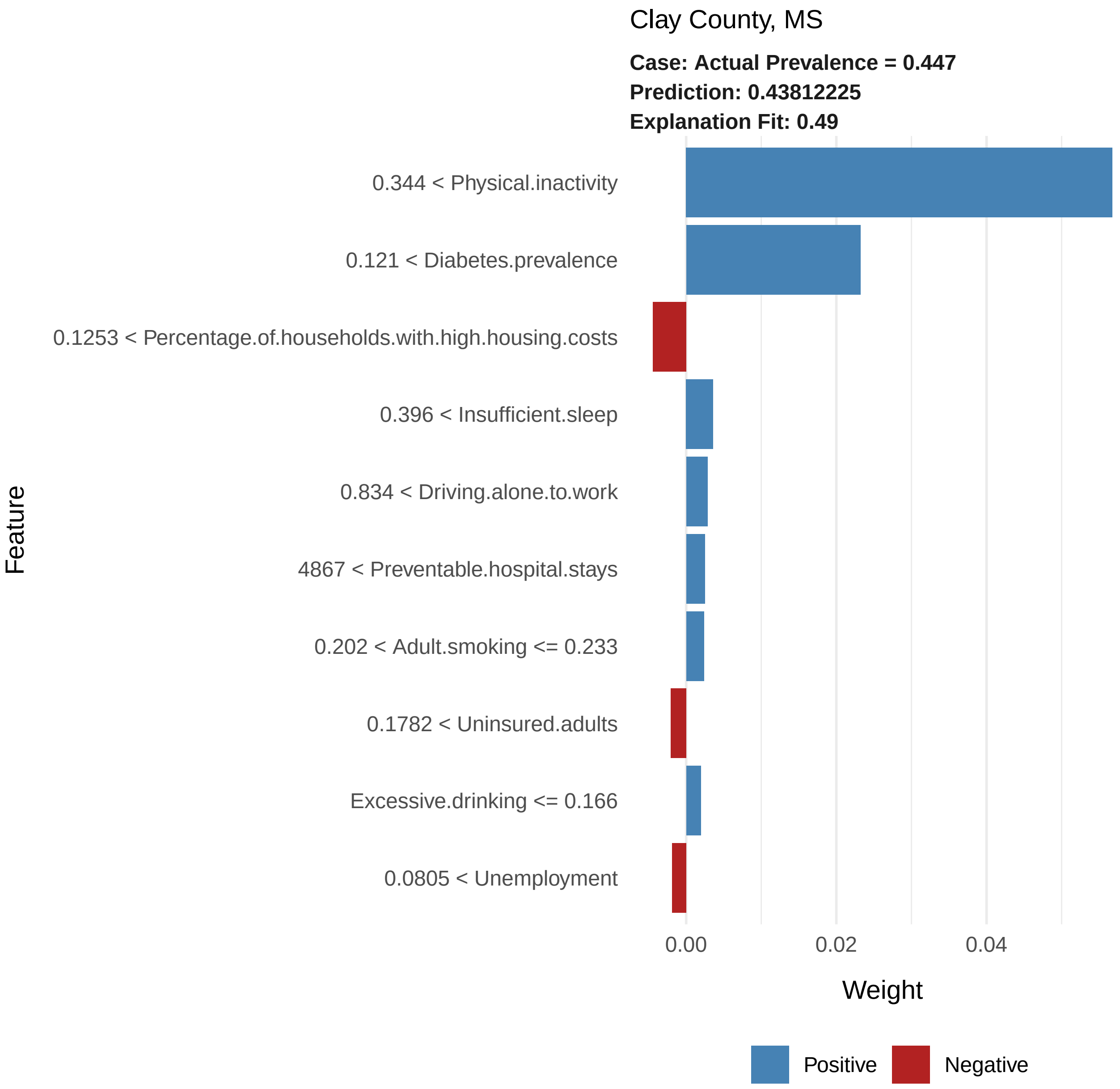

Clay County, MS
Case: Actual Prevalence = 0.447
Prediction: 0.43812225
Explanation Fit: 0.49
0.344 < Physical.inactivity
0.121 < Diabetes.prevalence
0.1253 < Percentage.of.households.with.high.housing.costs
0.396 < Insufficient.sleep
0.834 < Driving.alone.to.work
4867 < Preventable.hospital.stays
0.202 < Adult.smoking <= 0.233
0.1782 < Uninsured.adults
Excessive.drinking <= 0.166
0.0805 < Unemployment
Feature
0.00
0.02
0.04
Weight
Positive
Negative

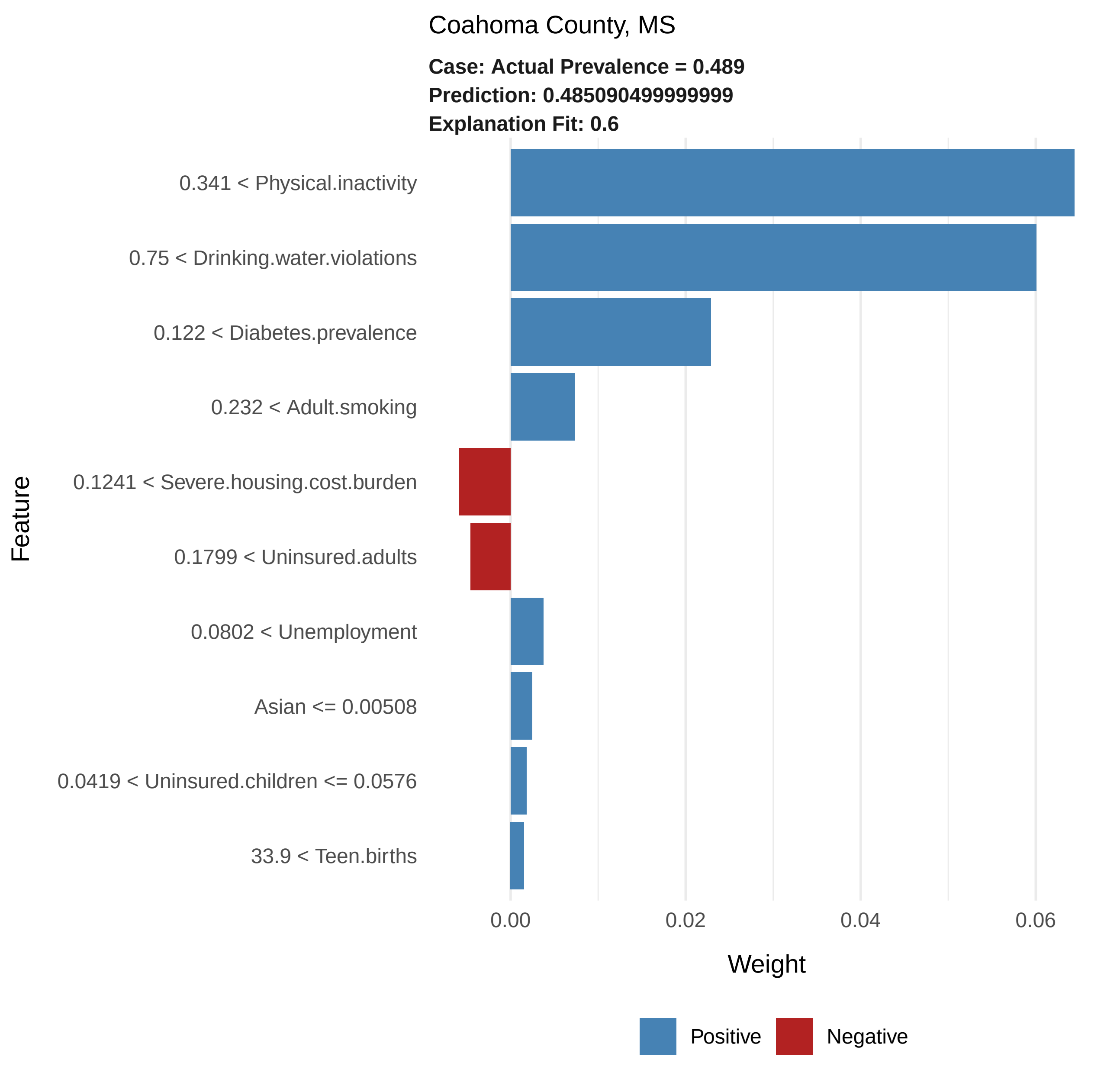

Coahoma County, MS
Case: Actual Prevalence = 0.489
Prediction: 0.485090499999999
Explanation Fit: 0.6
0.341 < Physical.inactivity
0.75 < Drinking.water.violations
0.122 < Diabetes.prevalence
0.232 < Adult.smoking
0.1241 < Severe.housing.cost.burden
0.1799 < Uninsured.adults
0.0802 < Unemployment
Asian <= 0.00508
0.0419 < Uninsured.children <= 0.0576
33.9 < Teen.births
Feature
0.00
0.02
0.04
0.06
Weight
Positive
Negative

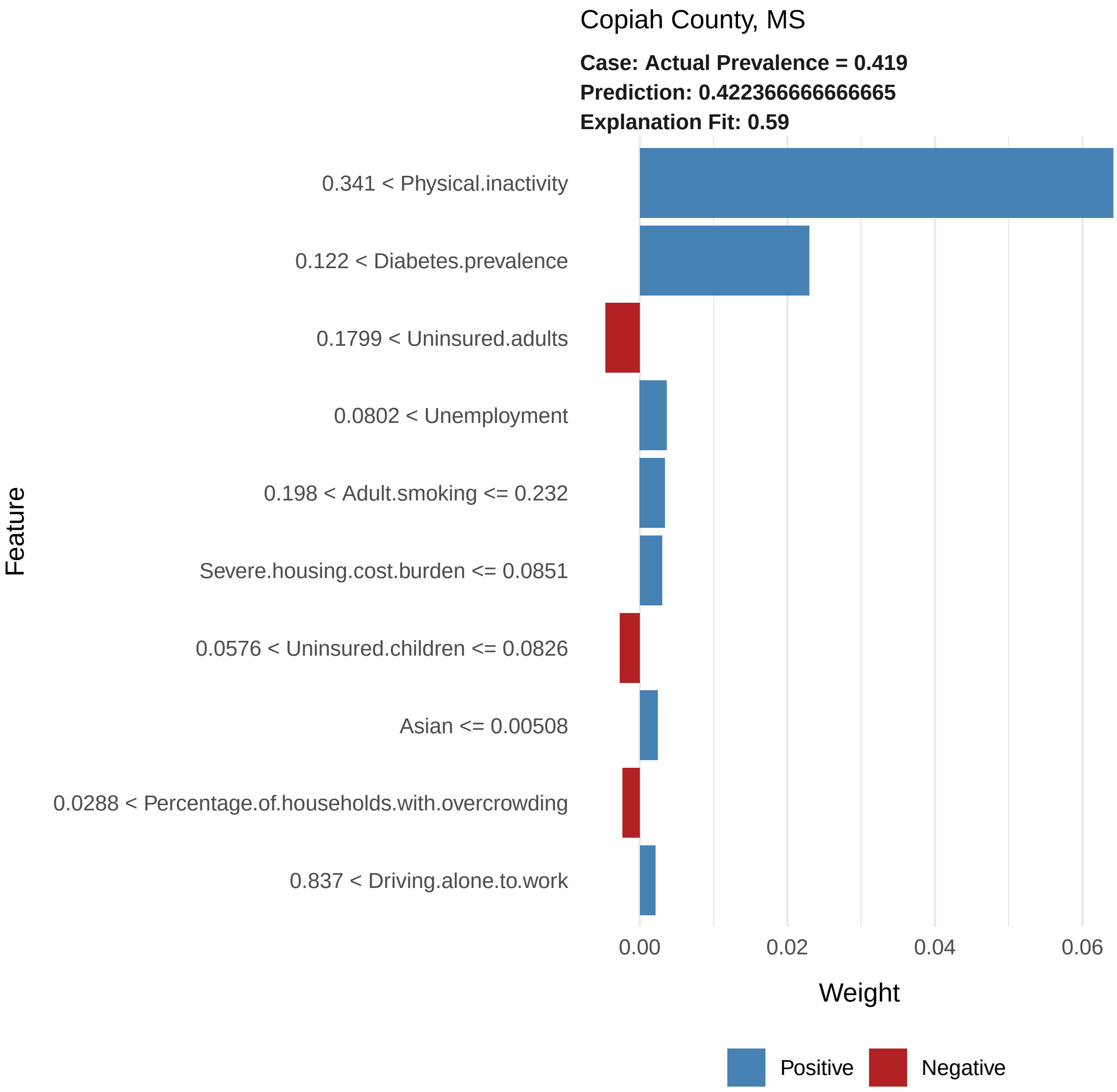
Copiah County, MS
Case: Actual Prevalence = 0.419
Prediction: 0.422366666666665
Explanation Fit: 0.59
0.341 < Physical.inactivity
0.122 < Diabetes.prevalence
0.1799 < Uninsured.adults
0.0802 < Unemployment
0.198 < Adult.smoking <= 0.232
Severe.housing.cost.burden <= 0.0851
0.0576 < Uninsured.children <= 0.0826
Asian <= 0.00508
0.0288 < Percentage.of.households.with.overcrowding
0.837 < Driving.alone.to.work
Feature
0.00
0.02
0.04
0.06
Weight
Positive
Negative

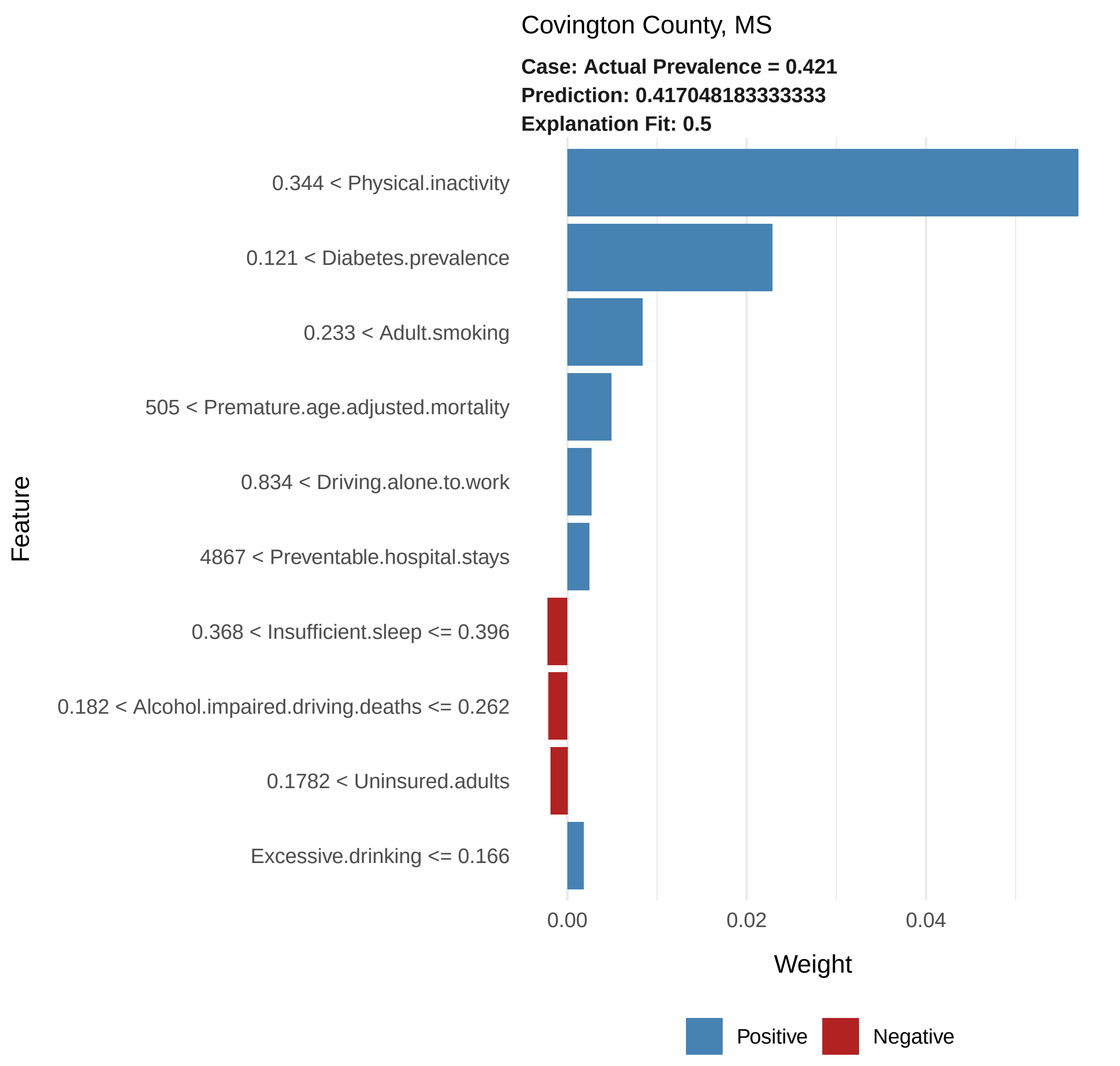

Covington County, MS
Case: Actual Prevalence = 0.421
Prediction: 0.417048183333333
Explanation Fit: 0.5
0.344 < Physical.inactivity
0.121 < Diabetes.prevalence
0.233 < Adult.smoking
505 < Premature.age.adjusted.mortality
0.834 < Driving.alone.to.work
4867 < Preventable.hospital.stays
0.368 < Insufficient.sleep <= 0.396
0.182 < Alcohol.impaired.driving.deaths <= 0.262
0.1782 < Uninsured.adults
Excessive.drinking <= 0.166
Feature
0.00
0.02
0.04
Weight
Positive
Negative

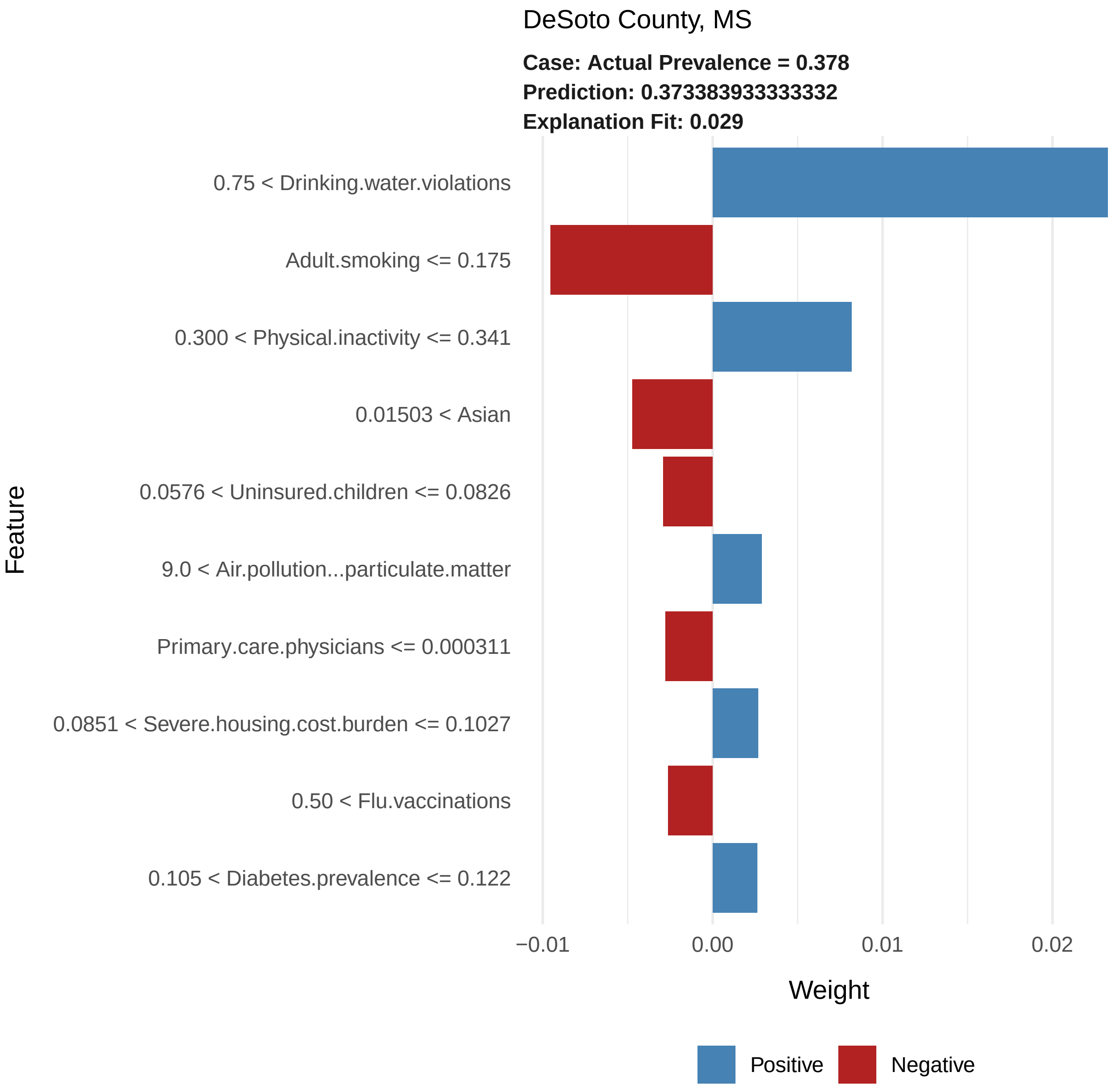

DeSoto County, MS
Case: Actual Prevalence = 0.378
Prediction: 0.373383933333332
Explanation Fit: 0.029
0.75 < Drinking.water.violations
Adult.smoking <= 0.175
0.300 < Physical.inactivity <= 0.341
0.01503 < Asian
0.0576 < Uninsured.children <= 0.0826
9.0 < Air.pollution...particulate.matter
Primary.care.physicians <= 0.000311
0.0851 < Severe.housing.cost.burden <= 0.1027
0.50 < Flu.vaccinations
0.105 < Diabetes.prevalence <= 0.122
Feature
−0.01
0.00
0.01
0.02
Weight
Positive
Negative

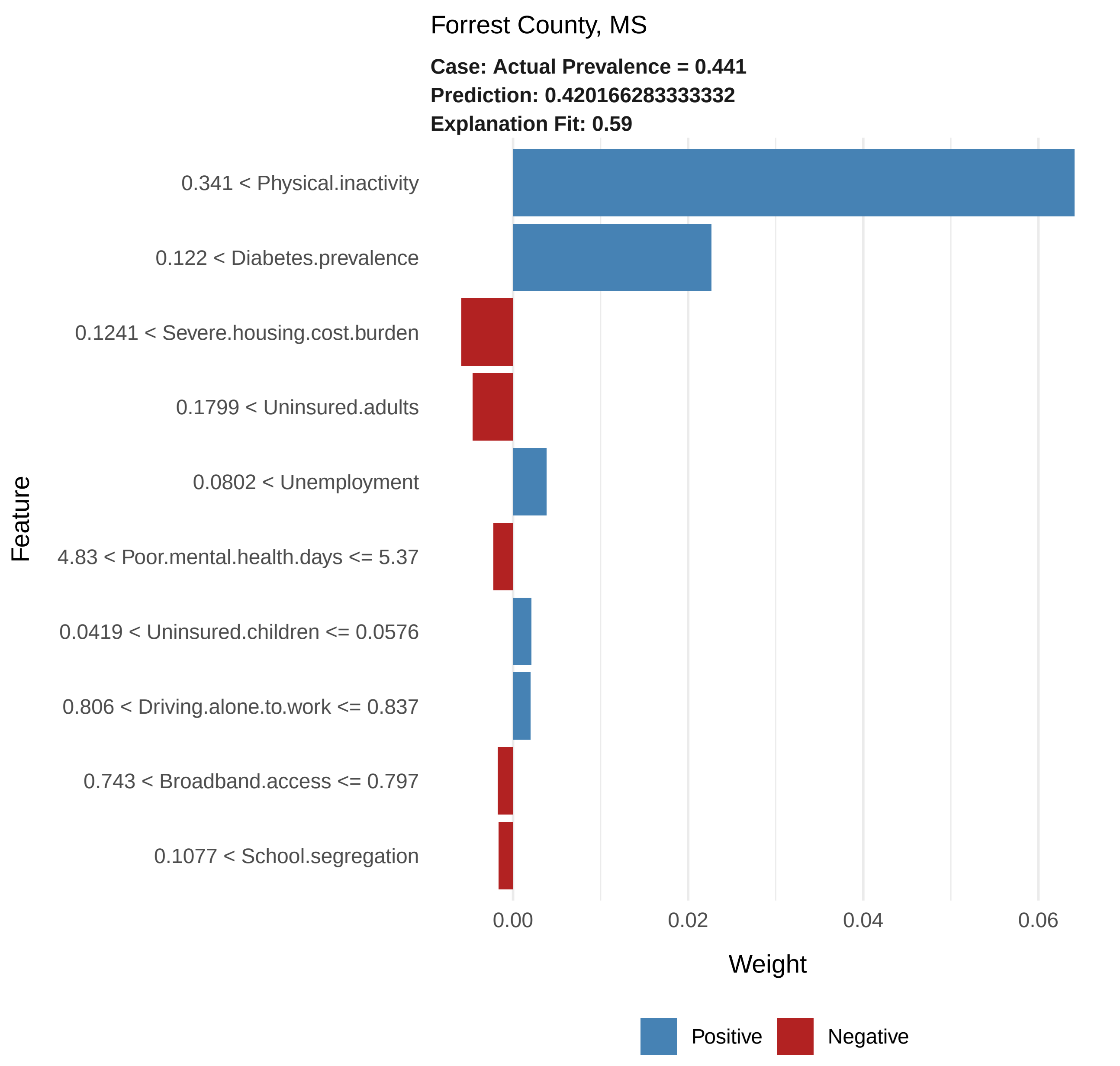

Forrest County, MS
Case: Actual Prevalence = 0.441
Prediction: 0.420166283333332
Explanation Fit: 0.59
0.341 < Physical.inactivity
0.122 < Diabetes.prevalence
0.1241 < Severe.housing.cost.burden
0.1799 < Uninsured.adults
0.0802 < Unemployment
4.83 < Poor.mental.health.days <= 5.37
0.0419 < Uninsured.children <= 0.0576
0.806 < Driving.alone.to.work <= 0.837
0.743 < Broadband.access <= 0.797
0.1077 < School.segregation
Feature
0.00
0.02
0.04
0.06
Weight
Positive
Negative

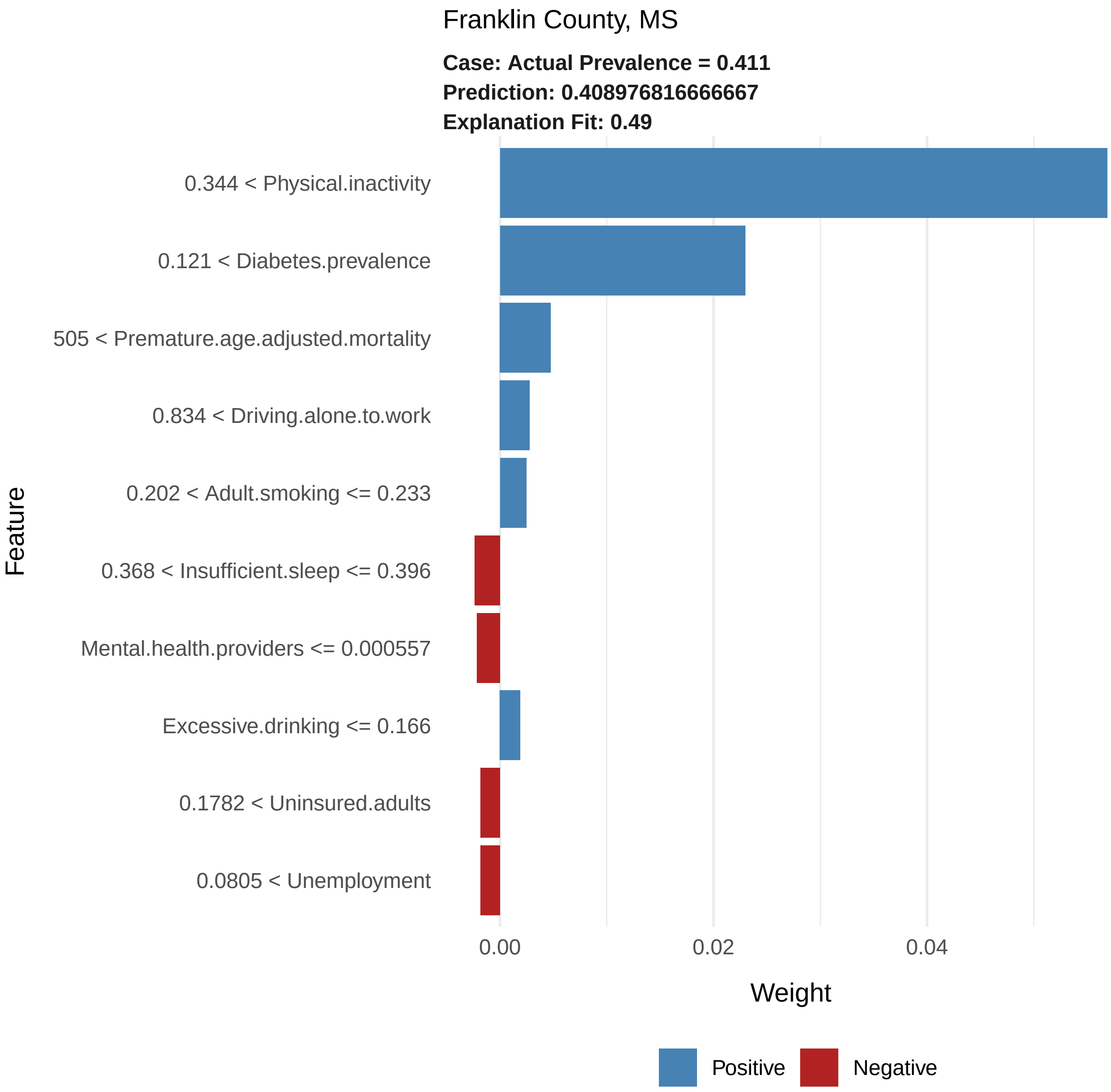

Franklin County, MS
Case: Actual Prevalence = 0.411
Prediction: 0.408976816666667
Explanation Fit: 0.49
0.344 < Physical.inactivity
0.121 < Diabetes.prevalence
505 < Premature.age.adjusted.mortality
0.834 < Driving.alone.to.work
0.202 < Adult.smoking <= 0.233
0.368 < Insufficient.sleep <= 0.396
Mental.health.providers <= 0.000557
Excessive.drinking <= 0.166
0.1782 < Uninsured.adults
0.0805 < Unemployment
Feature
0.00
0.02
0.04
Weight
Positive
Negative

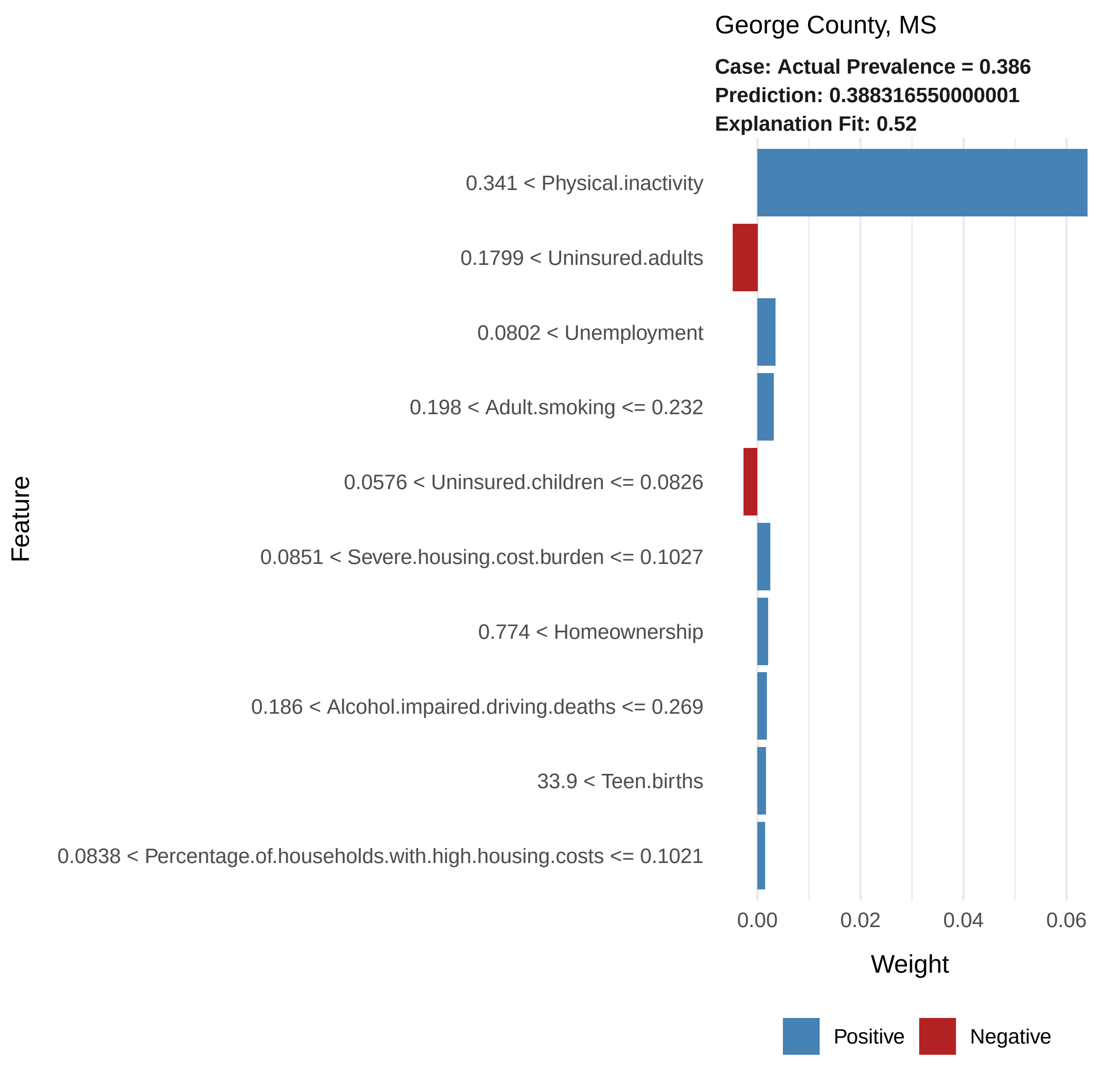
George County, MS
Case: Actual Prevalence = 0.386
Prediction: 0.388316550000001
Explanation Fit: 0.52
0.341 < Physical.inactivity
0.1799 < Uninsured.adults
0.0802 < Unemployment
0.198 < Adult.smoking <= 0.232
0.0576 < Uninsured.children <= 0.0826
0.0851 < Severe.housing.cost.burden <= 0.1027
0.774 < Homeownership
0.186 < Alcohol.impaired.driving.deaths <= 0.269
33.9 < Teen.births
0.0838 < Percentage.of.households.with.high.housing.costs <= 0.1021
Feature
0.00
0.02
0.04
0.06
Weight
Positive
Negative

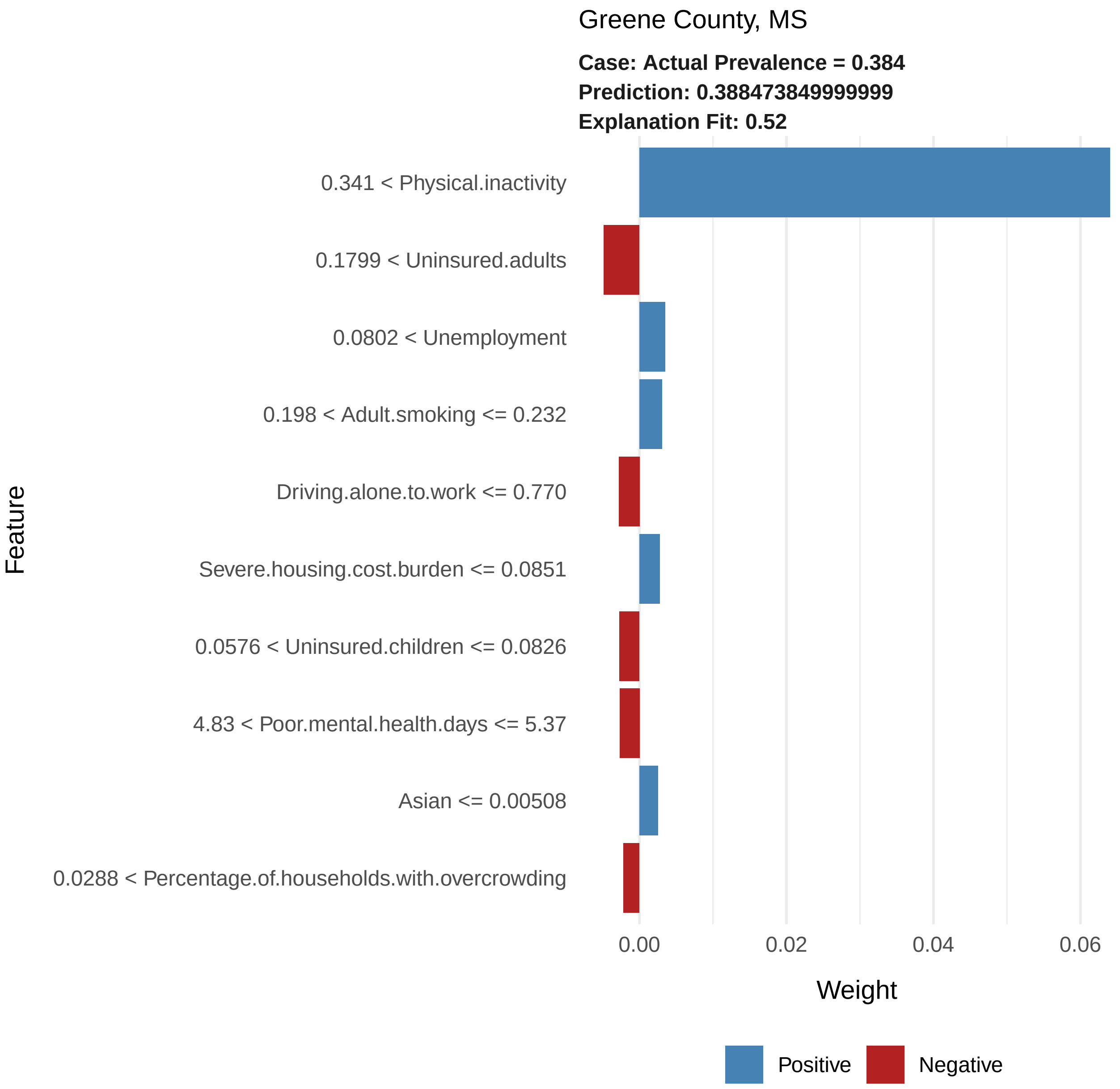
Greene County, MS
Case: Actual Prevalence = 0.384
Prediction: 0.388473849999999
Explanation Fit: 0.52
0.341 < Physical.inactivity
0.1799 < Uninsured.adults
0.0802 < Unemployment
0.198 < Adult.smoking <= 0.232
Driving.alone.to.work <= 0.770
Severe.housing.cost.burden <= 0.0851
0.0576 < Uninsured.children <= 0.0826
4.83 < Poor.mental.health.days <= 5.37
Asian <= 0.00508
0.0288 < Percentage.of.households.with.overcrowding
Feature
0.00
0.02
0.04
0.06
Weight
Positive
Negative

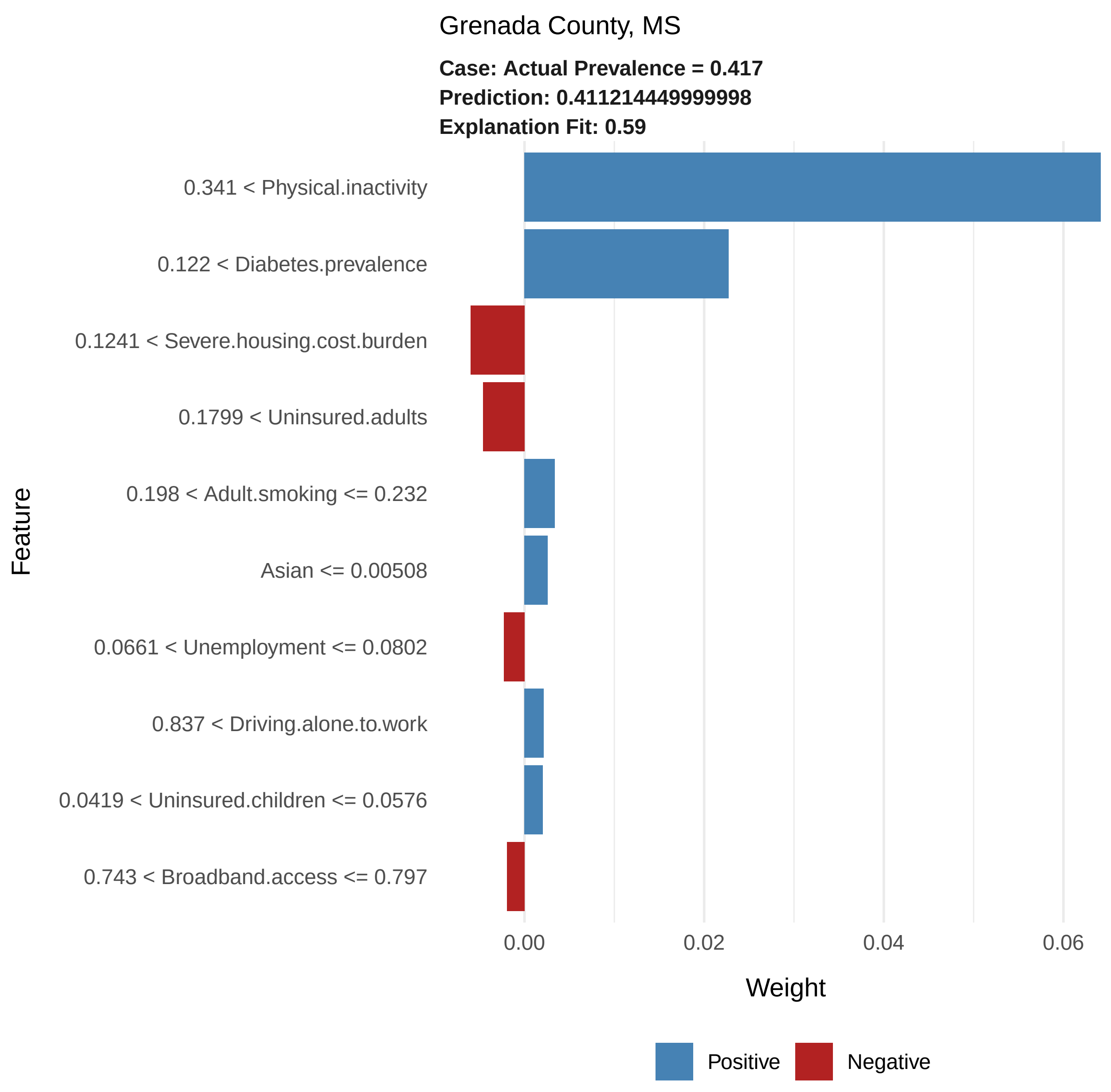
Grenada County, MS
Case: Actual Prevalence = 0.417
Prediction: 0.411214449999998
Explanation Fit: 0.59
0.341 < Physical.inactivity
0.122 < Diabetes.prevalence
0.1241 < Severe.housing.cost.burden
0.1799 < Uninsured.adults
0.198 < Adult.smoking <= 0.232
Asian <= 0.00508
0.0661 < Unemployment <= 0.0802
0.837 < Driving.alone.to.work
0.0419 < Uninsured.children <= 0.0576
0.743 < Broadband.access <= 0.797
Feature
0.00
0.02
0.04
0.06
Weight
Positive
Negative

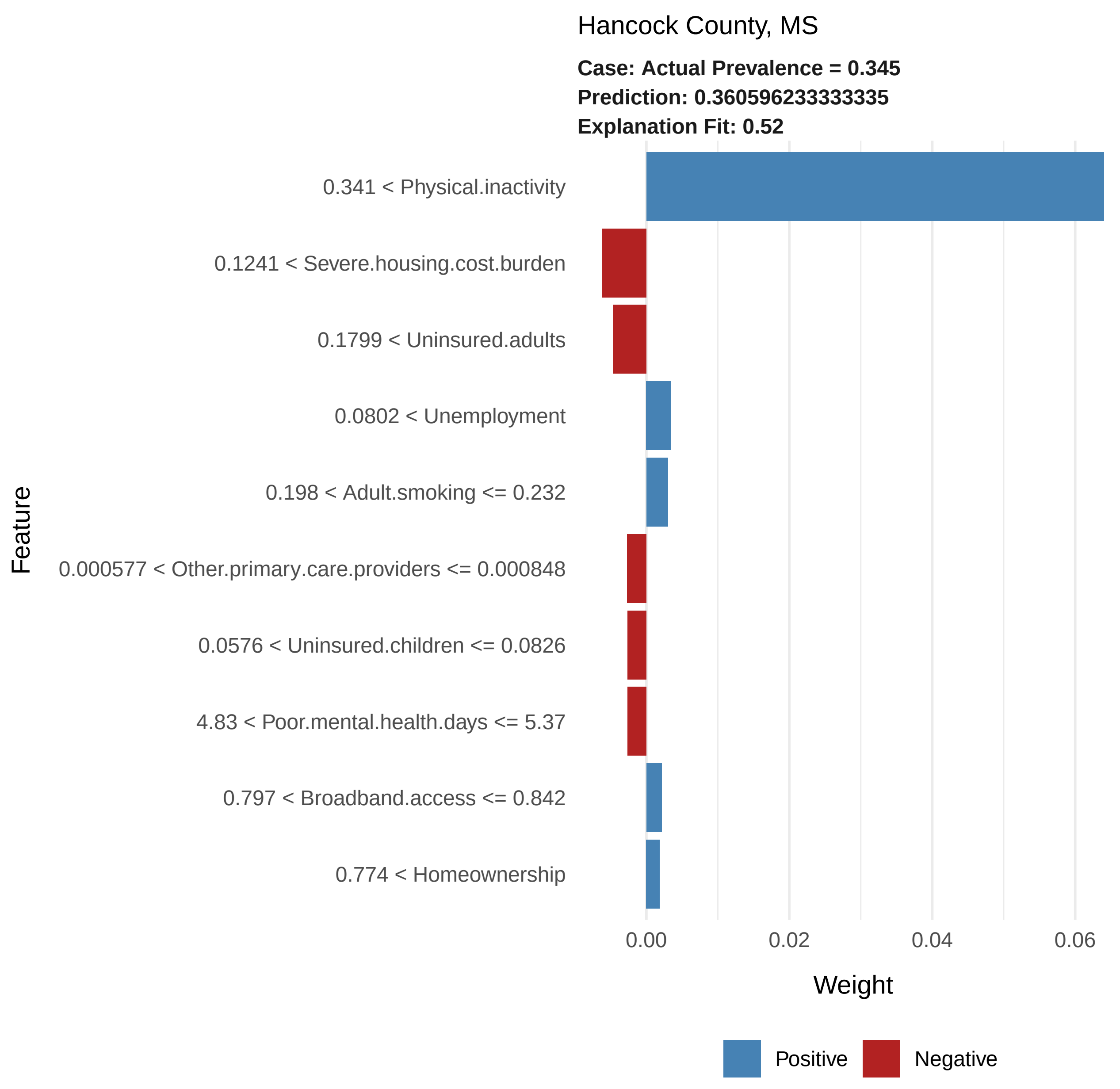

Hancock County, MS
Case: Actual Prevalence = 0.345
Prediction: 0.360596233333335
Explanation Fit: 0.52
0.341 < Physical.inactivity
0.1241 < Severe.housing.cost.burden
0.1799 < Uninsured.adults
0.0802 < Unemployment
0.198 < Adult.smoking <= 0.232
0.000577 < Other.primary.care.providers <= 0.000848
0.0576 < Uninsured.children <= 0.0826
4.83 < Poor.mental.health.days <= 5.37
0.797 < Broadband.access <= 0.842
0.774 < Homeownership
Feature
0.00
0.02
0.04
0.06
Weight
Positive
Negative

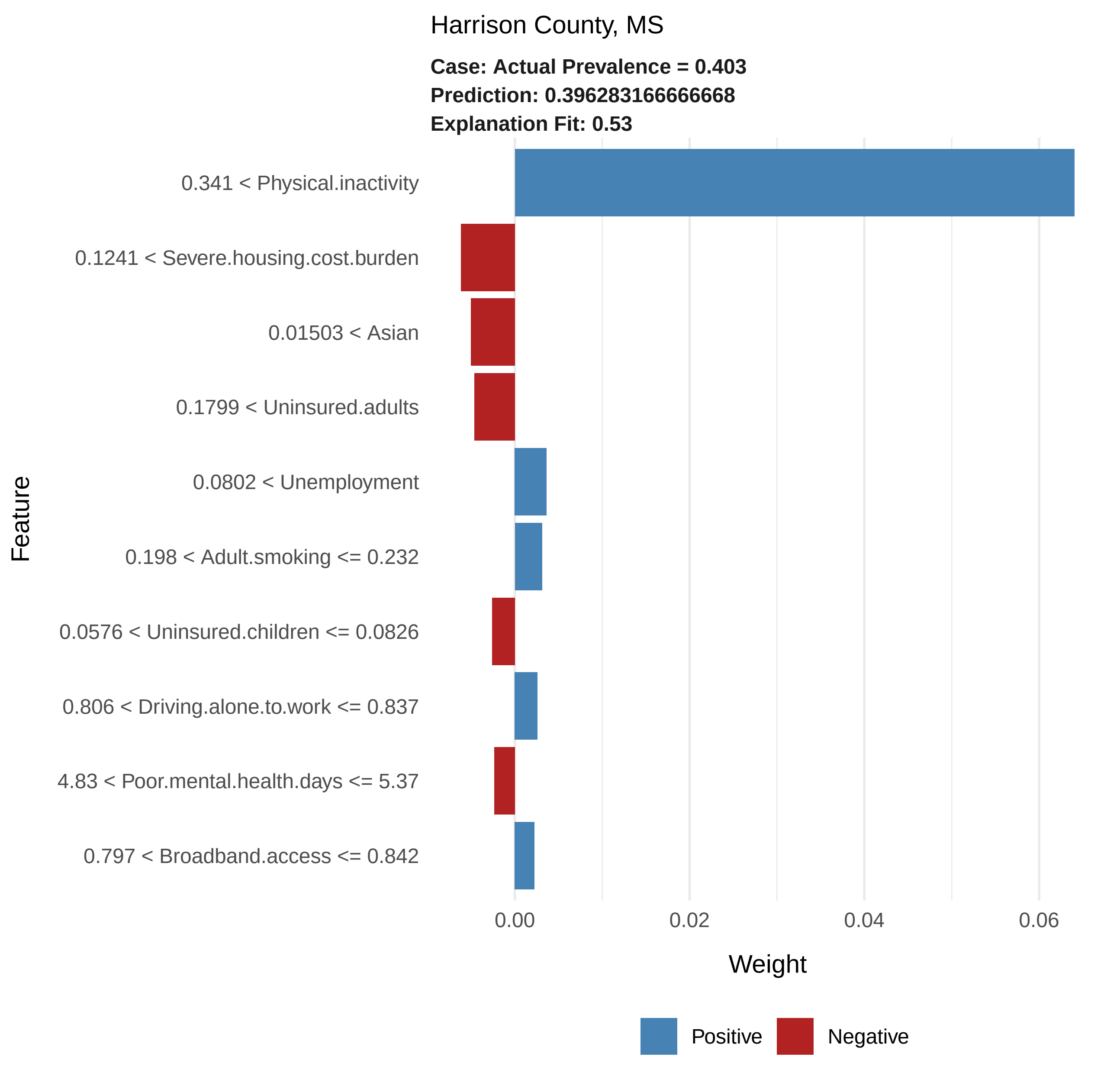

Harrison County, MS
Case: Actual Prevalence = 0.403
Prediction: 0.396283166666668
Explanation Fit: 0.53
0.341 < Physical.inactivity
0.1241 < Severe.housing.cost.burden
0.01503 < Asian
0.1799 < Uninsured.adults
0.0802 < Unemployment
0.198 < Adult.smoking <= 0.232
0.0576 < Uninsured.children <= 0.0826
0.806 < Driving.alone.to.work <= 0.837
4.83 < Poor.mental.health.days <= 5.37
0.797 < Broadband.access <= 0.842
Feature
0.00
0.02
0.04
0.06
Weight
Positive
Negative

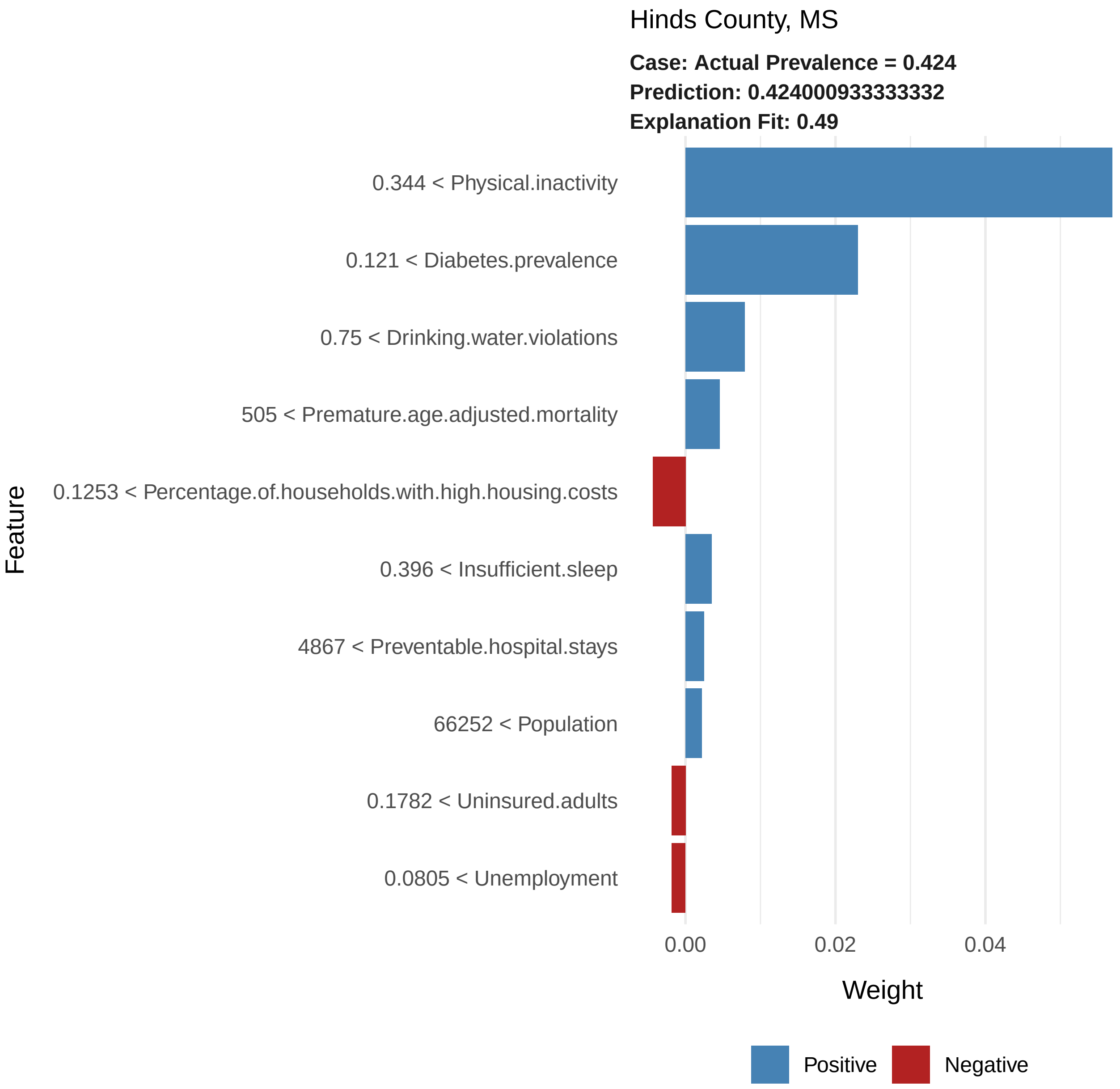
Hinds County, MS
Case: Actual Prevalence = 0.424
Prediction: 0.424000933333332
Explanation Fit: 0.49
0.344 < Physical.inactivity
0.121 < Diabetes.prevalence
0.75 < Drinking.water.violations
505 < Premature.age.adjusted.mortality
0.1253 < Percentage.of.households.with.high.housing.costs
0.396 < Insufficient.sleep
4867 < Preventable.hospital.stays
66252 < Population
0.1782 < Uninsured.adults
0.0805 < Unemployment
Feature
0.00
0.02
0.04
Weight
Positive
Negative

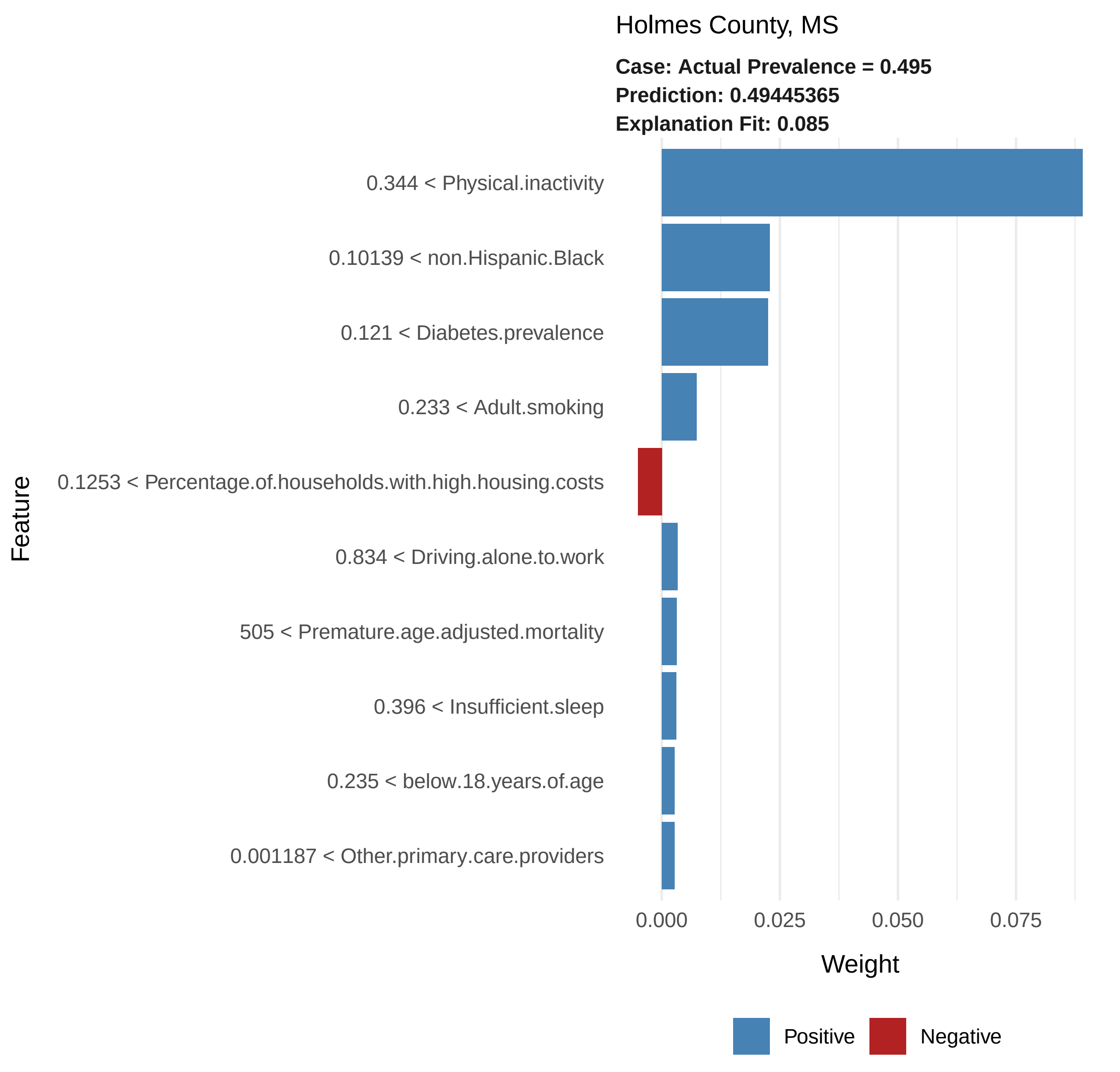
Holmes County, MS
Case: Actual Prevalence = 0.495
Prediction: 0.49445365
Explanation Fit: 0.085
0.344 < Physical.inactivity
0.10139 < non.Hispanic.Black
0.121 < Diabetes.prevalence
0.233 < Adult.smoking
0.1253 < Percentage.of.households.with.high.housing.costs
0.834 < Driving.alone.to.work
505 < Premature.age.adjusted.mortality
0.396 < Insufficient.sleep
0.235 < below.18.years.of.age
0.001187 < Other.primary.care.providers
Feature
0.000
0.025
0.050
0.075
Weight
Positive
Negative

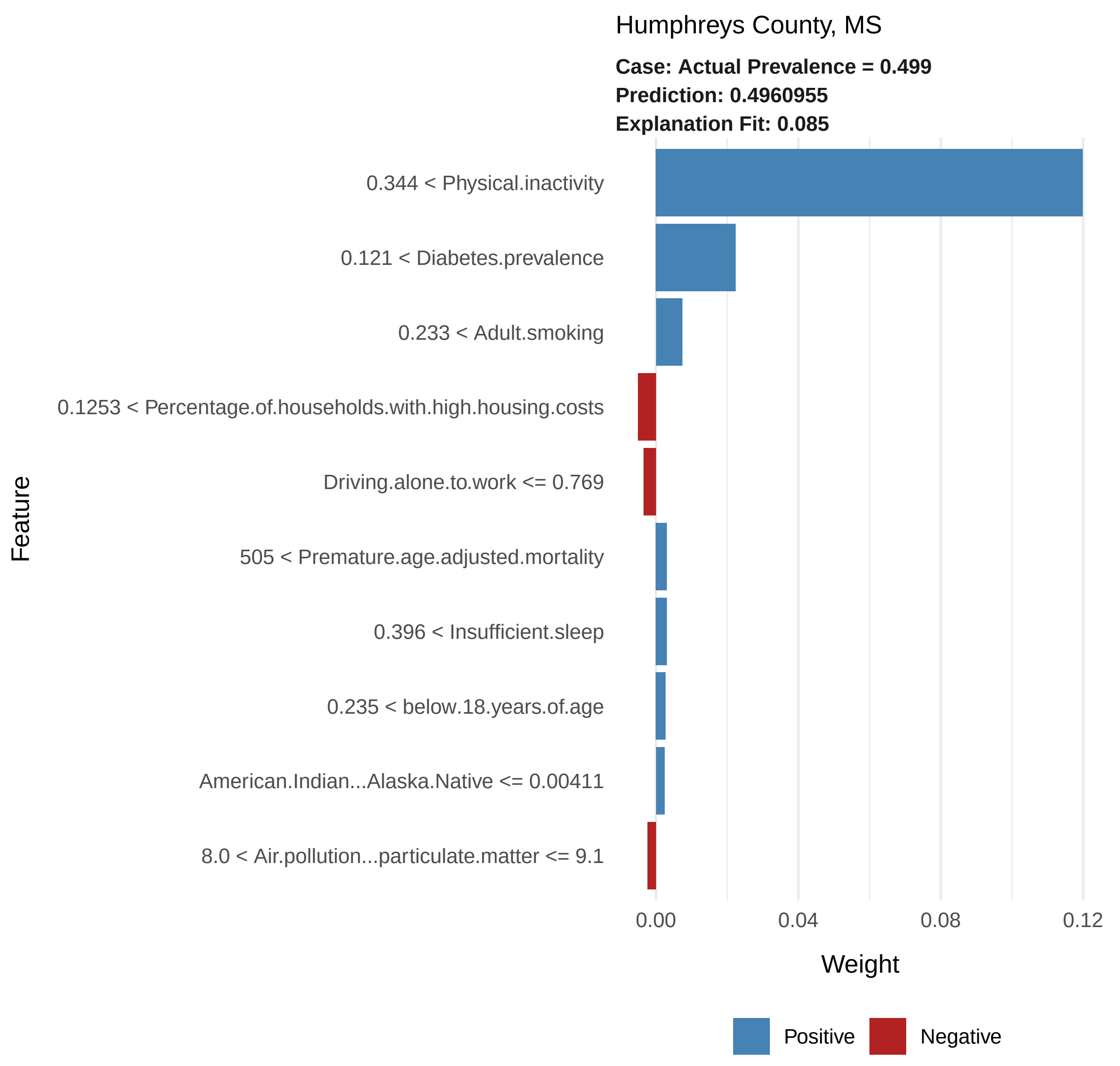

Humphreys County, MS
Case: Actual Prevalence = 0.499
Prediction: 0.4960955
Explanation Fit: 0.085
0.344 < Physical.inactivity
0.121 < Diabetes.prevalence
0.233 < Adult.smoking
0.1253 < Percentage.of.households.with.high.housing.costs
Driving.alone.to.work <= 0.769
505 < Premature.age.adjusted.mortality
0.396 < Insufficient.sleep
0.235 < below.18.years.of.age
American.Indian...Alaska.Native <= 0.00411
8.0 < Air.pollution...particulate.matter <= 9.1
Feature
0.00
0.04
0.08
0.12
Weight
Positive
Negative

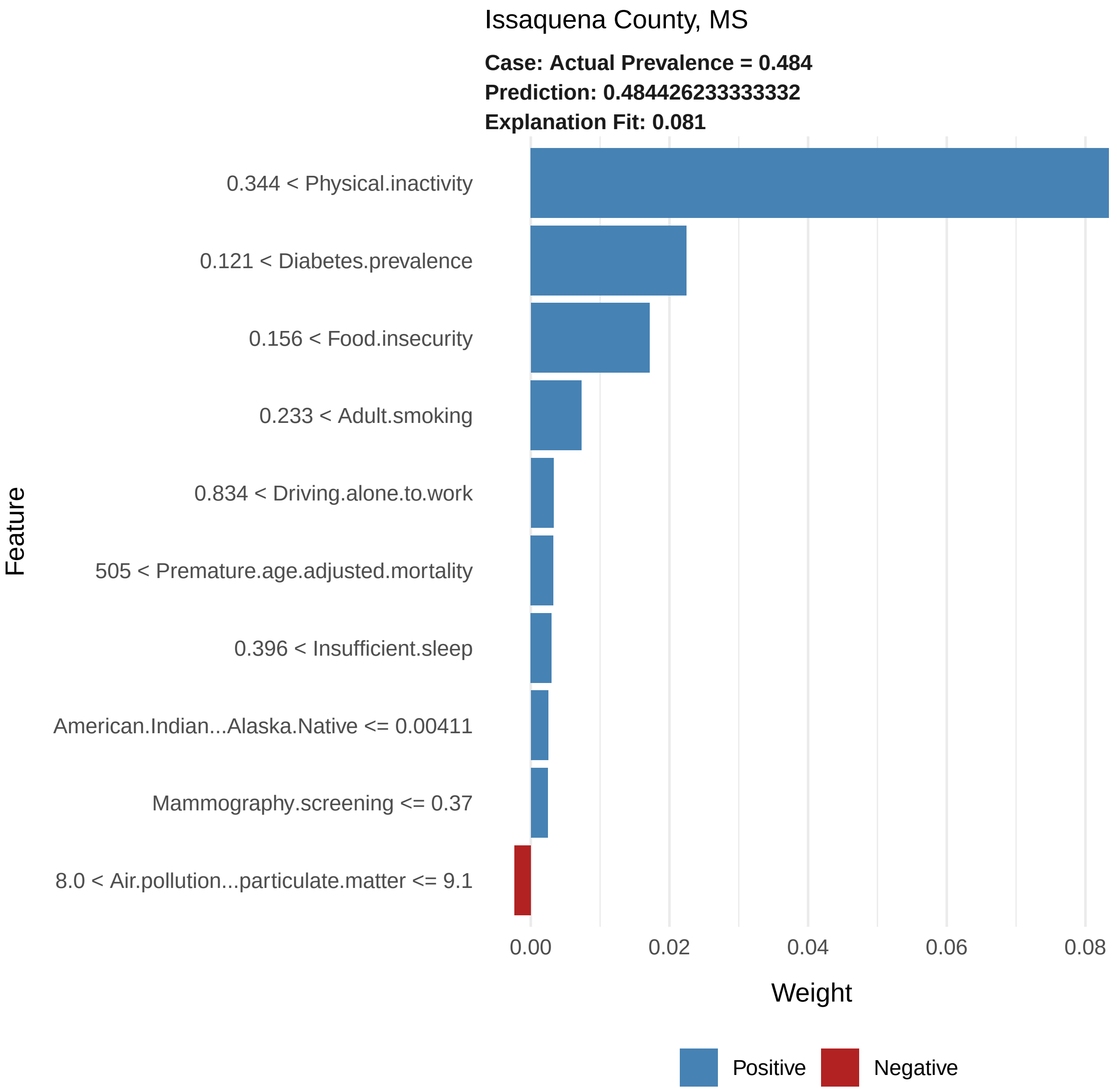
Issaquena County, MS
Case: Actual Prevalence = 0.484
Prediction: 0.484426233333332
Explanation Fit: 0.081
0.344 < Physical.inactivity
0.121 < Diabetes.prevalence
0.156 < Food.insecurity
0.233 < Adult.smoking
0.834 < Driving.alone.to.work
505 < Premature.age.adjusted.mortality
0.396 < Insufficient.sleep
American.Indian...Alaska.Native <= 0.00411
Mammography.screening <= 0.37
8.0 < Air.pollution...particulate.matter <= 9.1
Feature
0.00
0.02
0.04
0.06
0.08
Weight
Positive
Negative

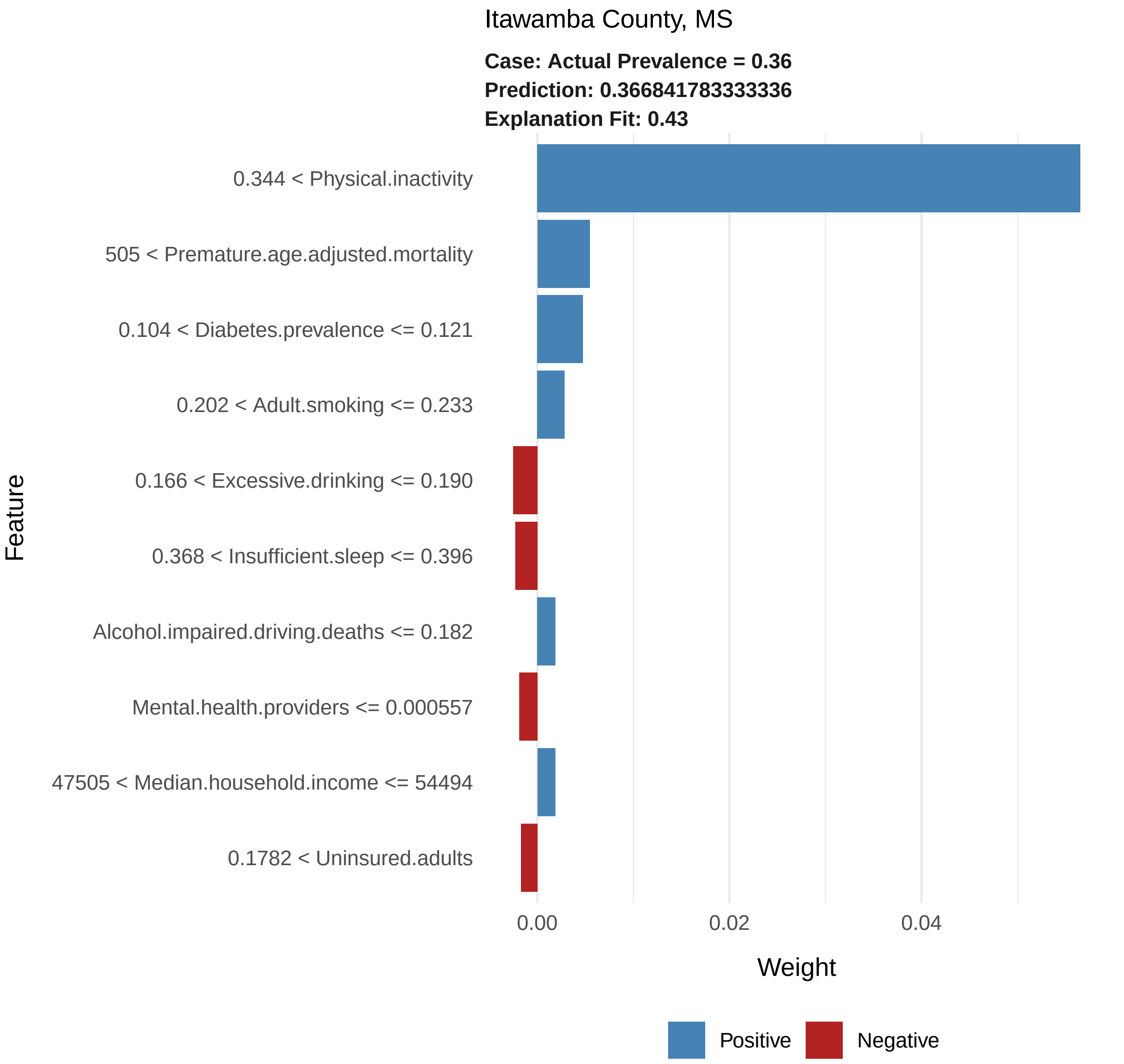

Itawamba County, MS
Case: Actual Prevalence = 0.36
Prediction: 0.366841783333336
Explanation Fit: 0.43
0.344 < Physical.inactivity
505 < Premature.age.adjusted.mortality
0.104 < Diabetes.prevalence <= 0.121
0.202 < Adult.smoking <= 0.233
0.166 < Excessive.drinking <= 0.190
0.368 < Insufficient.sleep <= 0.396
Alcohol.impaired.driving.deaths <= 0.182
Mental.health.providers <= 0.000557
47505 < Median.household.income <= 54494
0.1782 < Uninsured.adults
Feature
0.00
0.02
0.04
Weight
Positive
Negative

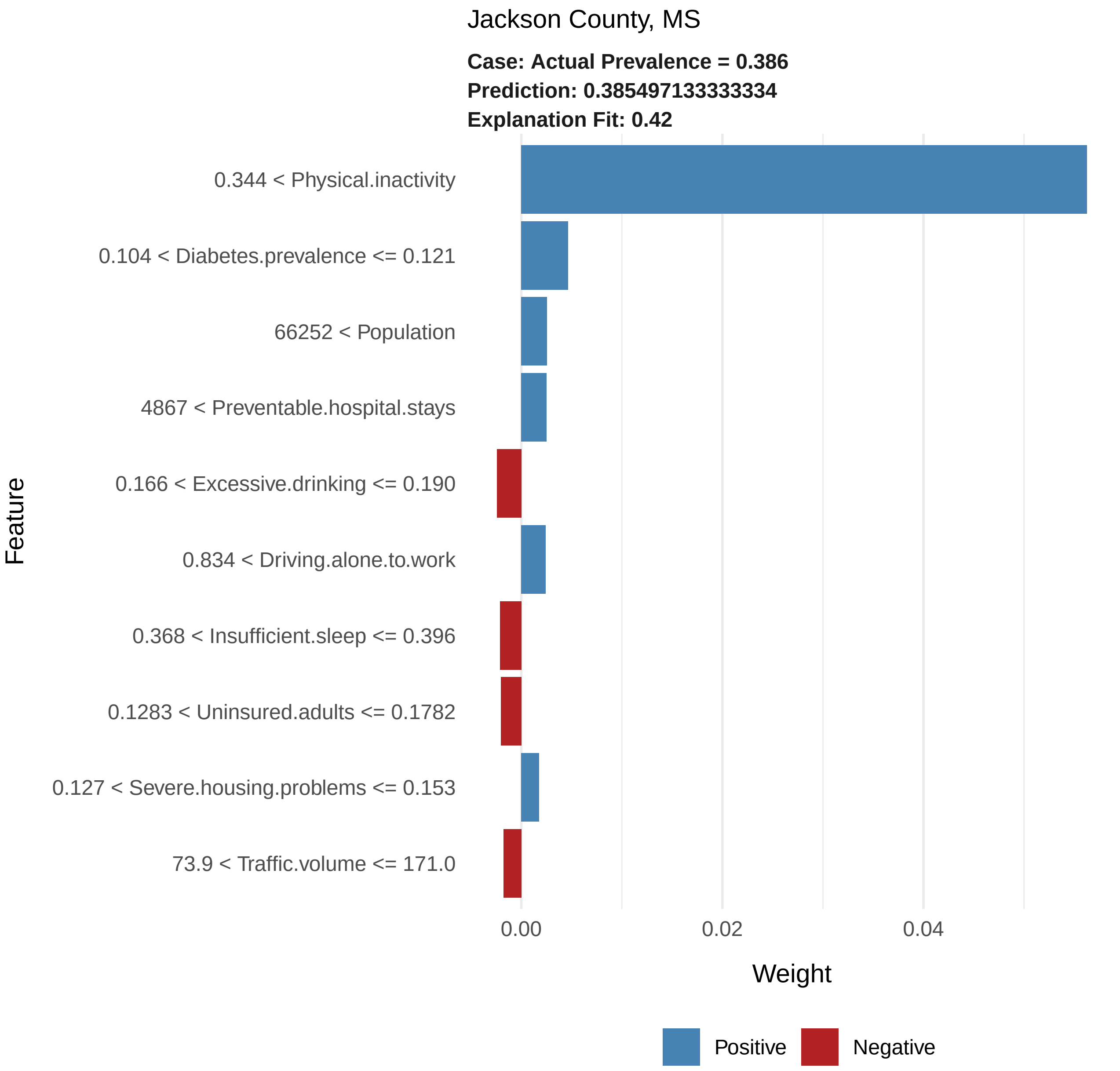

Jackson County, MS
Case: Actual Prevalence = 0.386
Prediction: 0.385497133333334
Explanation Fit: 0.42
0.344 < Physical.inactivity
0.104 < Diabetes.prevalence <= 0.121
66252 < Population
4867 < Preventable.hospital.stays
0.166 < Excessive.drinking <= 0.190
0.834 < Driving.alone.to.work
0.368 < Insufficient.sleep <= 0.396
0.1283 < Uninsured.adults <= 0.1782
0.127 < Severe.housing.problems <= 0.153
73.9 < Traffic.volume <= 171.0
Feature
0.00
0.02
0.04
Weight
Positive
Negative

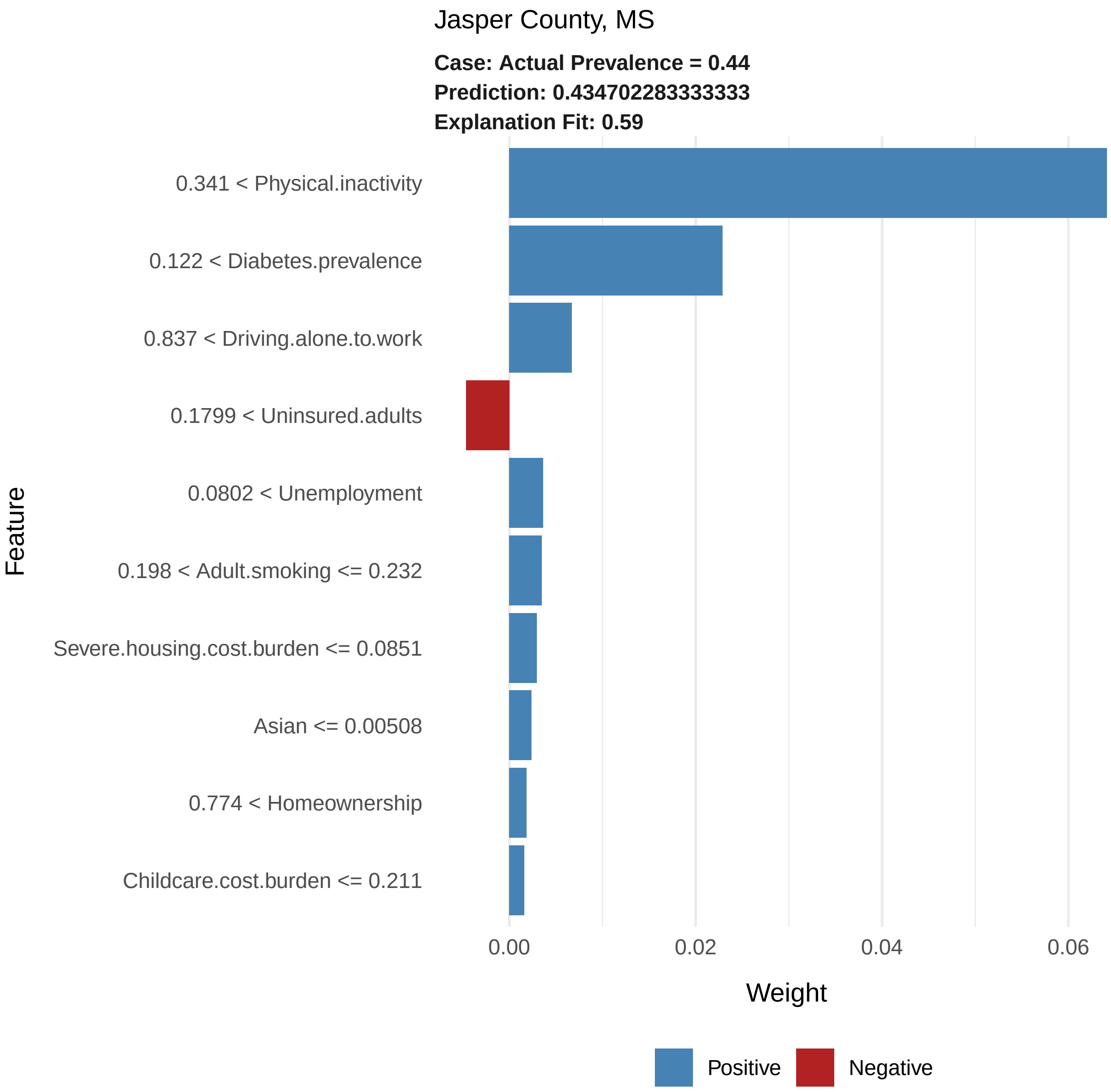

Jasper County, MS
Case: Actual Prevalence = 0.44
Prediction: 0.434702283333333
Explanation Fit: 0.59
0.341 < Physical.inactivity
0.122 < Diabetes.prevalence
0.837 < Driving.alone.to.work
0.1799 < Uninsured.adults
0.0802 < Unemployment
0.198 < Adult.smoking <= 0.232
Severe.housing.cost.burden <= 0.0851
Asian <= 0.00508
0.774 < Homeownership
Childcare.cost.burden <= 0.211
Feature
0.00
0.02
0.04
0.06
Weight
Positive
Negative

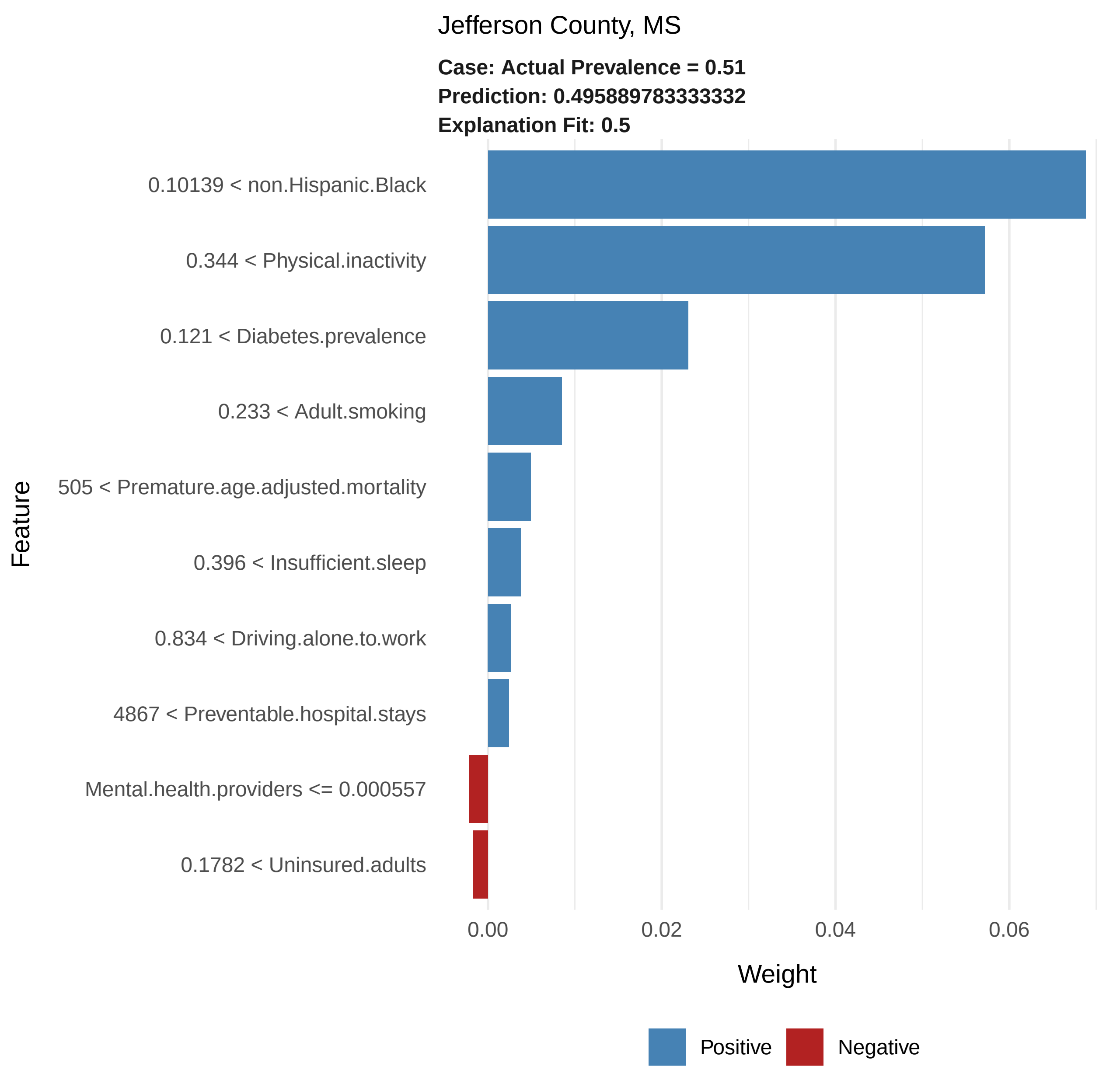

Jefferson County, MS
Case: Actual Prevalence = 0.51
Prediction: 0.495889783333332
Explanation Fit: 0.5
0.10139 < non.Hispanic.Black
0.344 < Physical.inactivity
0.121 < Diabetes.prevalence
0.233 < Adult.smoking
505 < Premature.age.adjusted.mortality
0.396 < Insufficient.sleep
0.834 < Driving.alone.to.work
4867 < Preventable.hospital.stays
Mental.health.providers <= 0.000557
0.1782 < Uninsured.adults
Feature
0.00
0.02
0.04
0.06
Weight
Positive
Negative

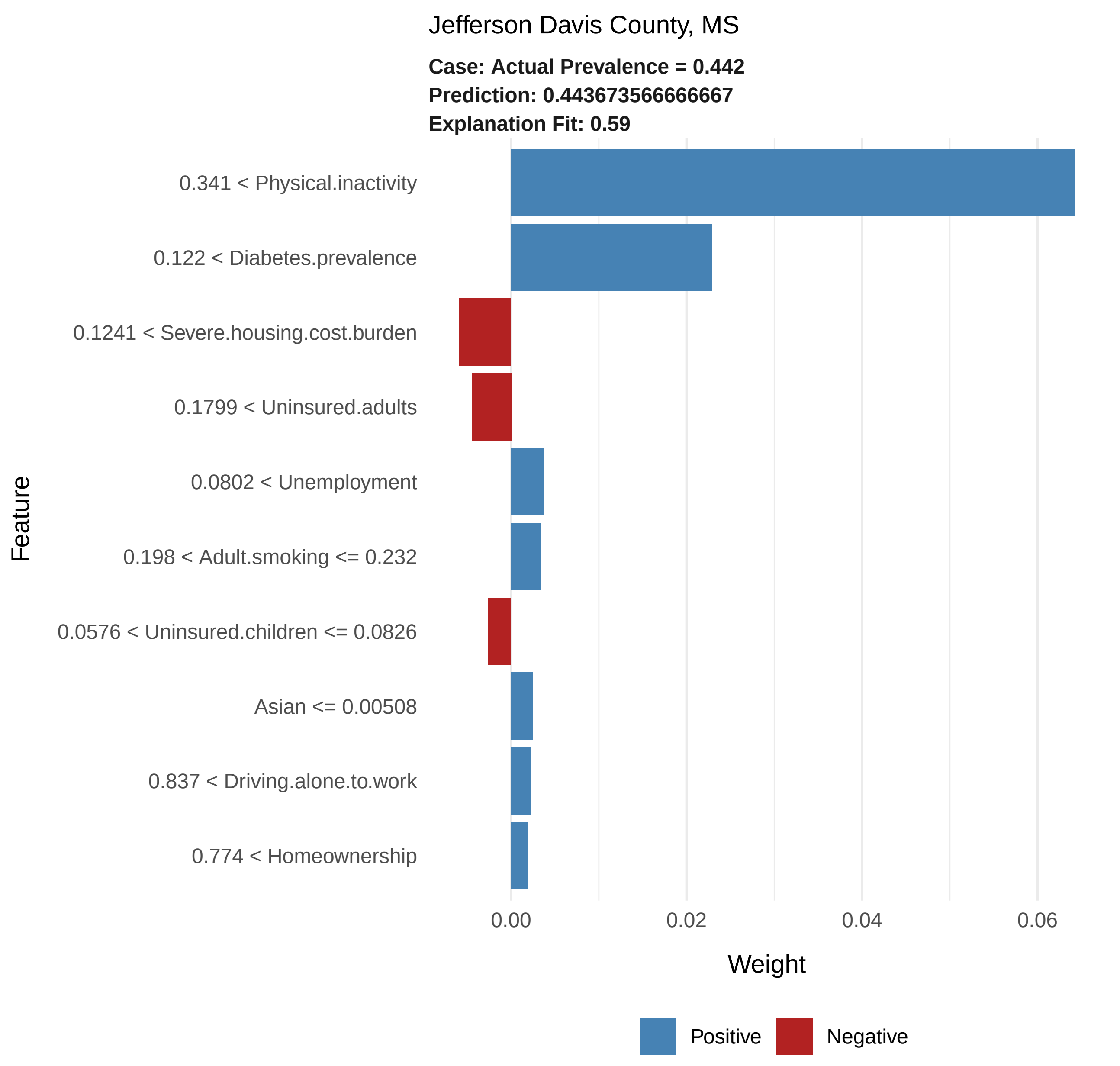
Jefferson Davis County, MS
Case: Actual Prevalence = 0.442
Prediction: 0.443673566666667
Explanation Fit: 0.59
0.341 < Physical.inactivity
0.122 < Diabetes.prevalence
0.1241 < Severe.housing.cost.burden
0.1799 < Uninsured.adults
0.0802 < Unemployment
0.198 < Adult.smoking <= 0.232
0.0576 < Uninsured.children <= 0.0826
Asian <= 0.00508
0.837 < Driving.alone.to.work
0.774 < Homeownership
Feature
0.00
0.02
0.04
0.06
Weight
Positive
Negative

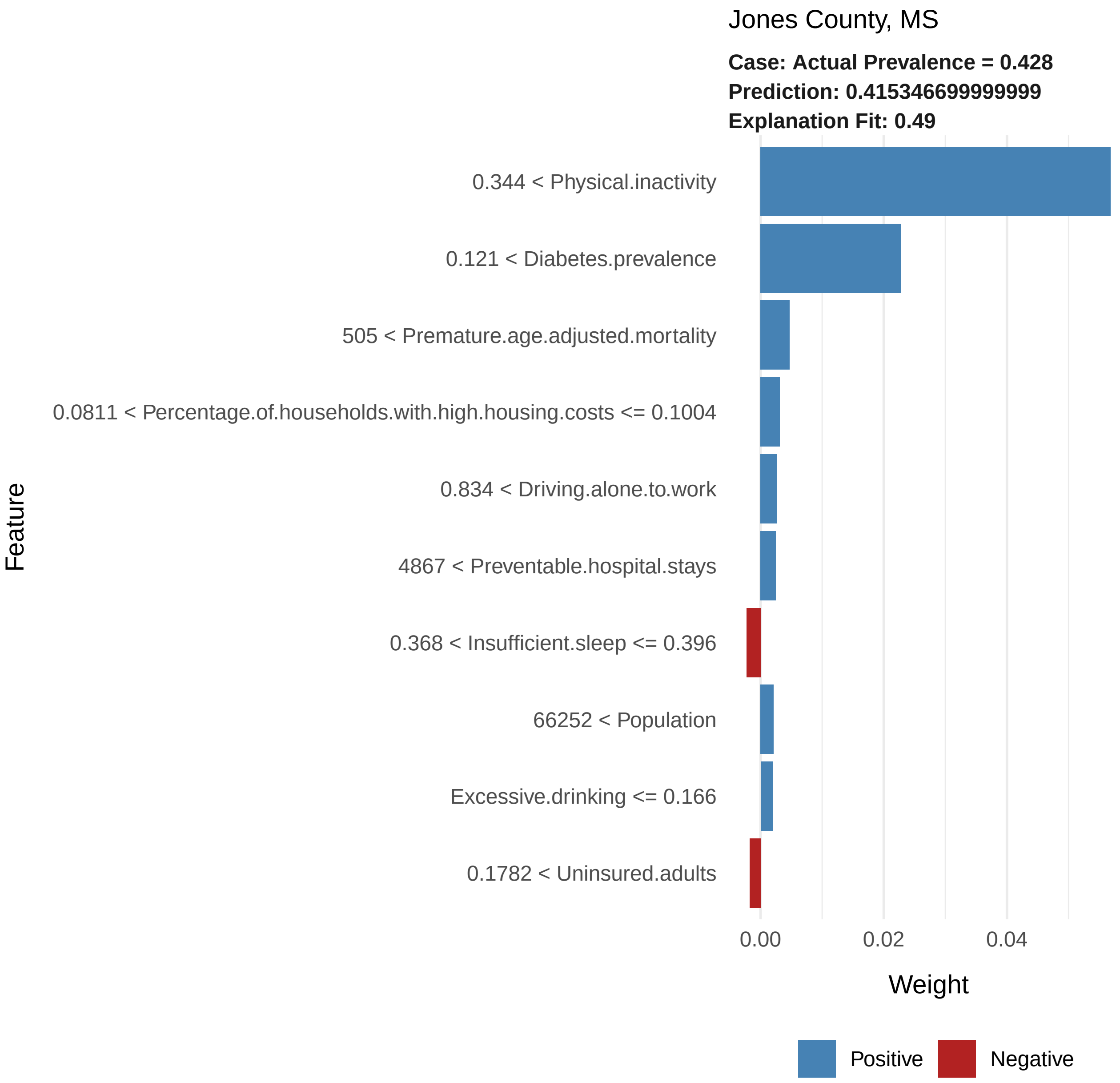
Jones County, MS
Case: Actual Prevalence = 0.428
Prediction: 0.415346699999999
Explanation Fit: 0.49
0.344 < Physical.inactivity
0.121 < Diabetes.prevalence
505 < Premature.age.adjusted.mortality
0.0811 < Percentage.of.households.with.high.housing.costs <= 0.1004
0.834 < Driving.alone.to.work
4867 < Preventable.hospital.stays
0.368 < Insufficient.sleep <= 0.396
66252 < Population
Excessive.drinking <= 0.166
0.1782 < Uninsured.adults
Feature
0.00
0.02
0.04
Weight
Positive
Negative

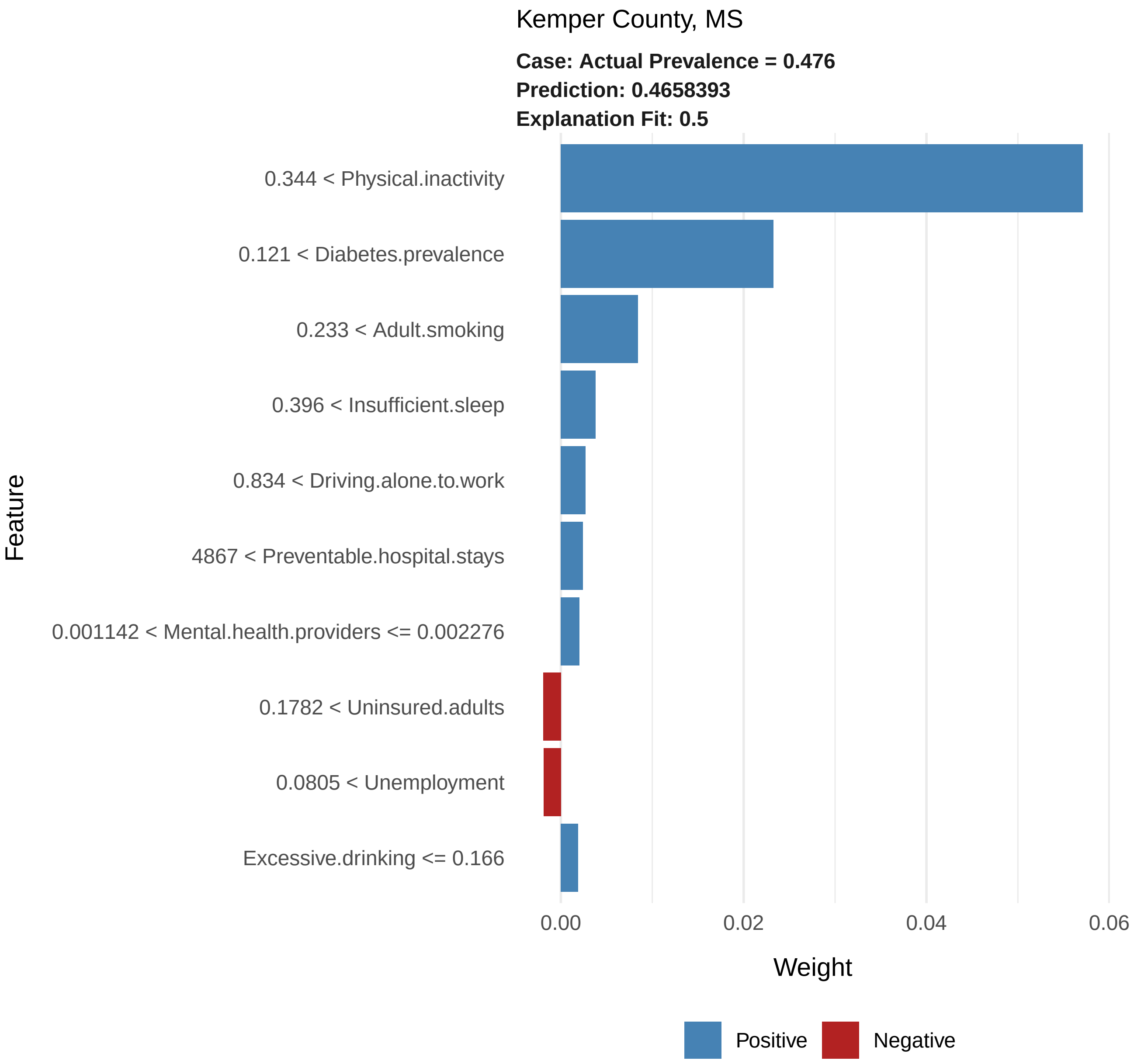

Kemper County, MS
Case: Actual Prevalence = 0.476
Prediction: 0.4658393
Explanation Fit: 0.5
0.344 < Physical.inactivity
0.121 < Diabetes.prevalence
0.233 < Adult.smoking
0.396 < Insufficient.sleep
0.834 < Driving.alone.to.work
4867 < Preventable.hospital.stays
0.001142 < Mental.health.providers <= 0.002276
0.1782 < Uninsured.adults
0.0805 < Unemployment
Excessive.drinking <= 0.166
Feature
0.00
0.02
0.04
0.06
Weight
Positive
Negative

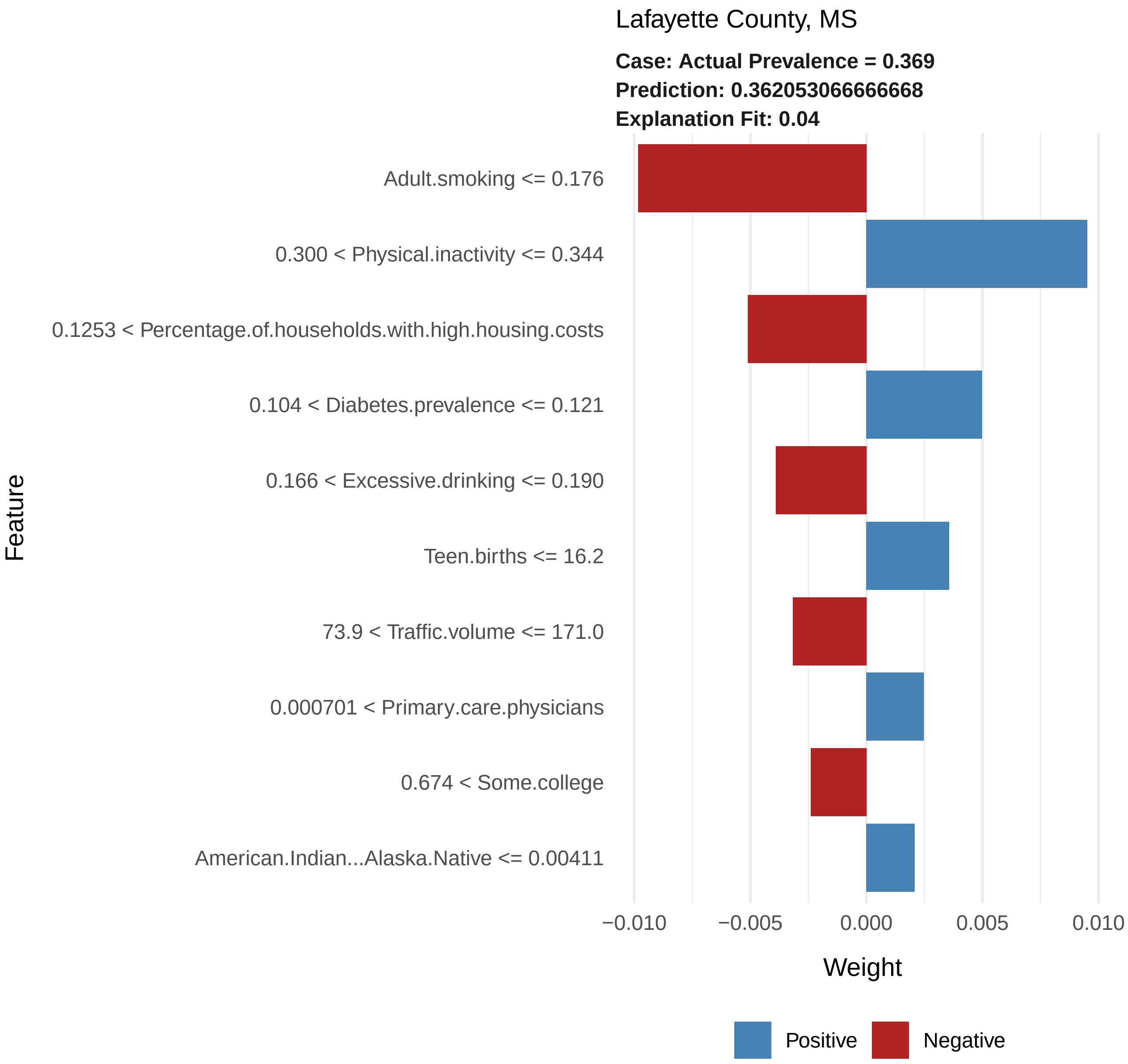

Lafayette County, MS
Case: Actual Prevalence = 0.369
Prediction: 0.362053066666668
Explanation Fit: 0.04
Adult.smoking <= 0.176
0.300 < Physical.inactivity <= 0.344
0.1253 < Percentage.of.households.with.high.housing.costs
0.104 < Diabetes.prevalence <= 0.121
0.166 < Excessive.drinking <= 0.190
Teen.births <= 16.2
73.9 < Traffic.volume <= 171.0
0.000701 < Primary.care.physicians
0.674 < Some.college
American.Indian...Alaska.Native <= 0.00411
Feature
−0.010
−0.005
0.000
0.005
0.010
Weight
Positive
Negative

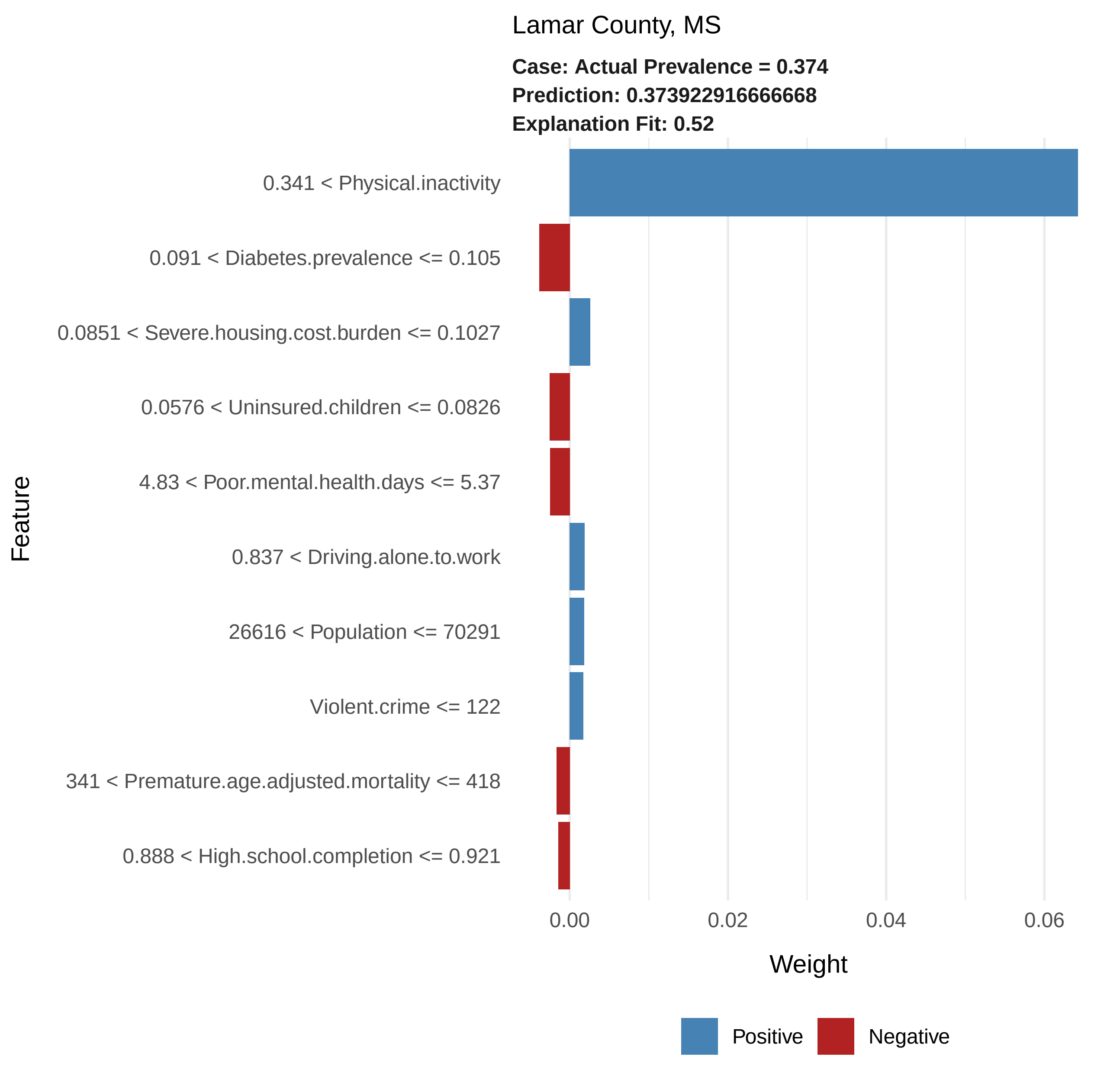

Lamar County, MS
Case: Actual Prevalence = 0.374
Prediction: 0.373922916666668
Explanation Fit: 0.52
0.341 < Physical.inactivity
0.091 < Diabetes.prevalence <= 0.105
0.0851 < Severe.housing.cost.burden <= 0.1027
0.0576 < Uninsured.children <= 0.0826
4.83 < Poor.mental.health.days <= 5.37
0.837 < Driving.alone.to.work
26616 < Population <= 70291
Violent.crime <= 122
341 < Premature.age.adjusted.mortality <= 418
0.888 < High.school.completion <= 0.921
Feature
0.00
0.02
0.04
0.06
Weight
Positive
Negative

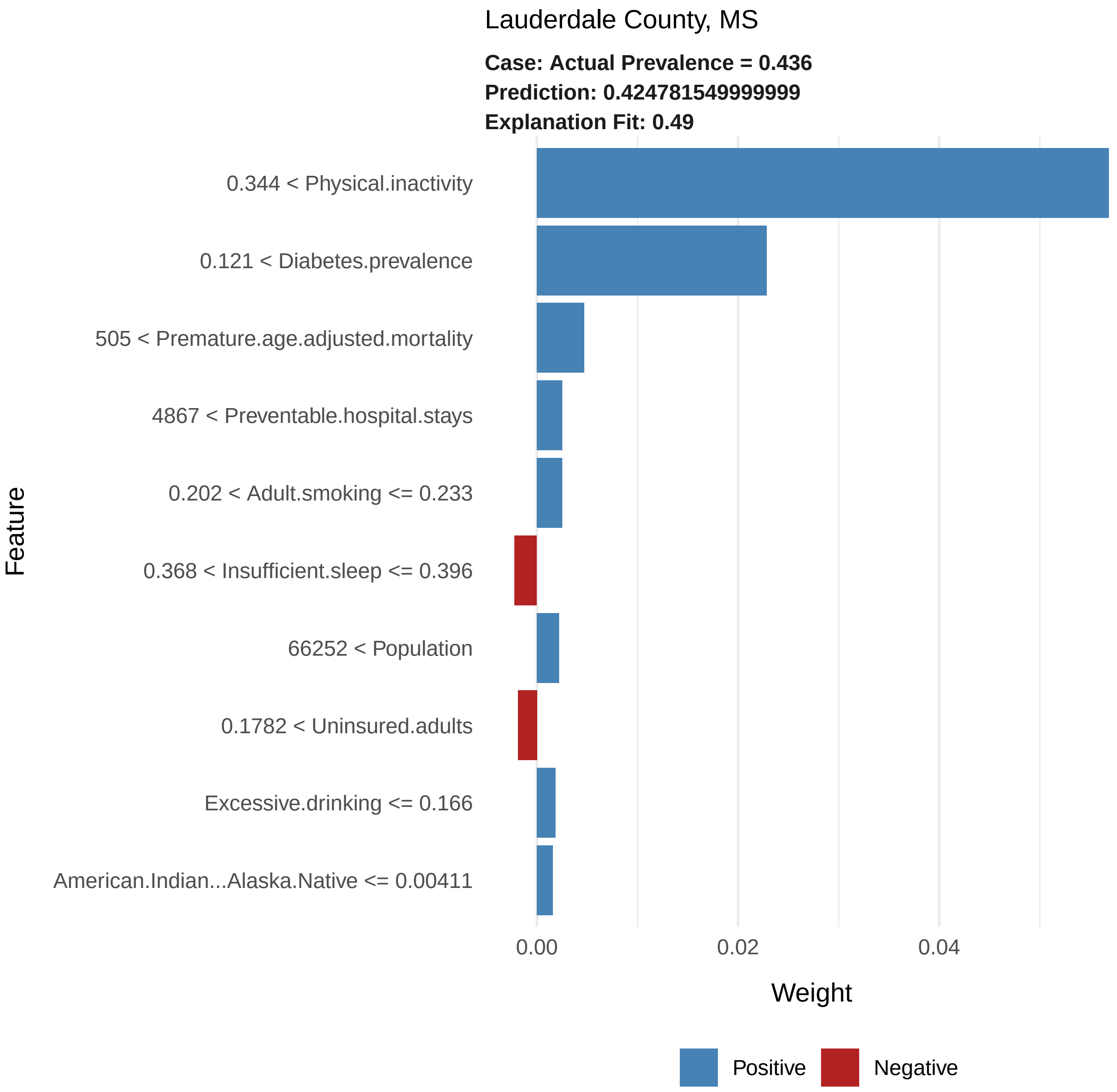
Lauderdale County, MS
Case: Actual Prevalence = 0.436
Prediction: 0.424781549999999
Explanation Fit: 0.49
0.344 < Physical.inactivity
0.121 < Diabetes.prevalence
505 < Premature.age.adjusted.mortality
4867 < Preventable.hospital.stays
0.202 < Adult.smoking <= 0.233
0.368 < Insufficient.sleep <= 0.396
66252 < Population
0.1782 < Uninsured.adults
Excessive.drinking <= 0.166
American.Indian...Alaska.Native <= 0.00411
Feature
0.00
0.02
0.04
Weight
Positive
Negative

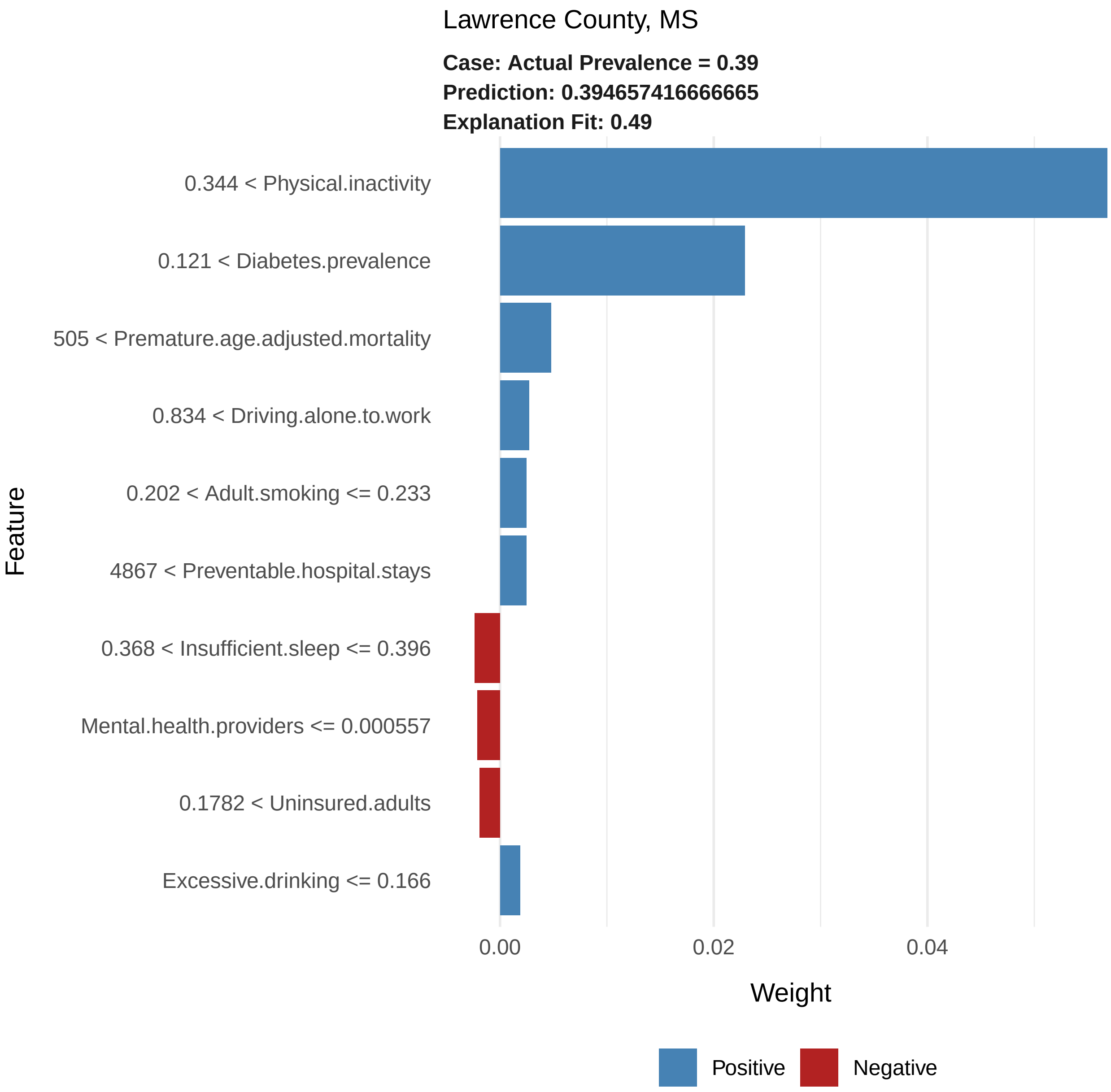
Lawrence County, MS
Case: Actual Prevalence = 0.39
Prediction: 0.394657416666665
Explanation Fit: 0.49
0.344 < Physical.inactivity
0.121 < Diabetes.prevalence
505 < Premature.age.adjusted.mortality
0.834 < Driving.alone.to.work
0.202 < Adult.smoking <= 0.233
4867 < Preventable.hospital.stays
0.368 < Insufficient.sleep <= 0.396
Mental.health.providers <= 0.000557
0.1782 < Uninsured.adults
Excessive.drinking <= 0.166
Feature
0.00
0.02
0.04
Weight
Positive
Negative

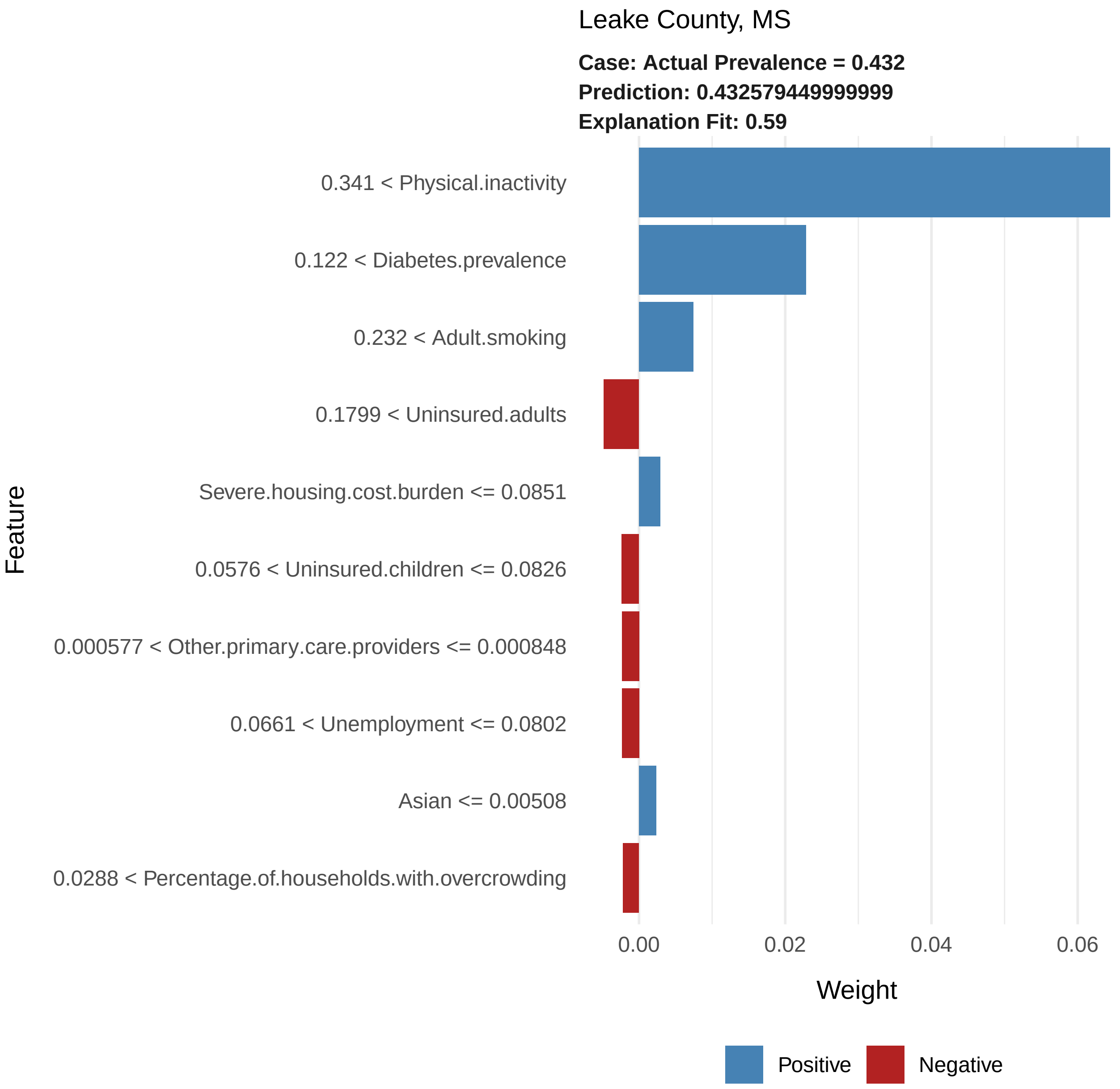

Leake County, MS
Case: Actual Prevalence = 0.432
Prediction: 0.432579449999999
Explanation Fit: 0.59
0.341 < Physical.inactivity
0.122 < Diabetes.prevalence
0.232 < Adult.smoking
0.1799 < Uninsured.adults
Severe.housing.cost.burden <= 0.0851
0.0576 < Uninsured.children <= 0.0826
0.000577 < Other.primary.care.providers <= 0.000848
0.0661 < Unemployment <= 0.0802
Asian <= 0.00508
0.0288 < Percentage.of.households.with.overcrowding
Feature
0.00
0.02
0.04
0.06
Weight
Positive
Negative

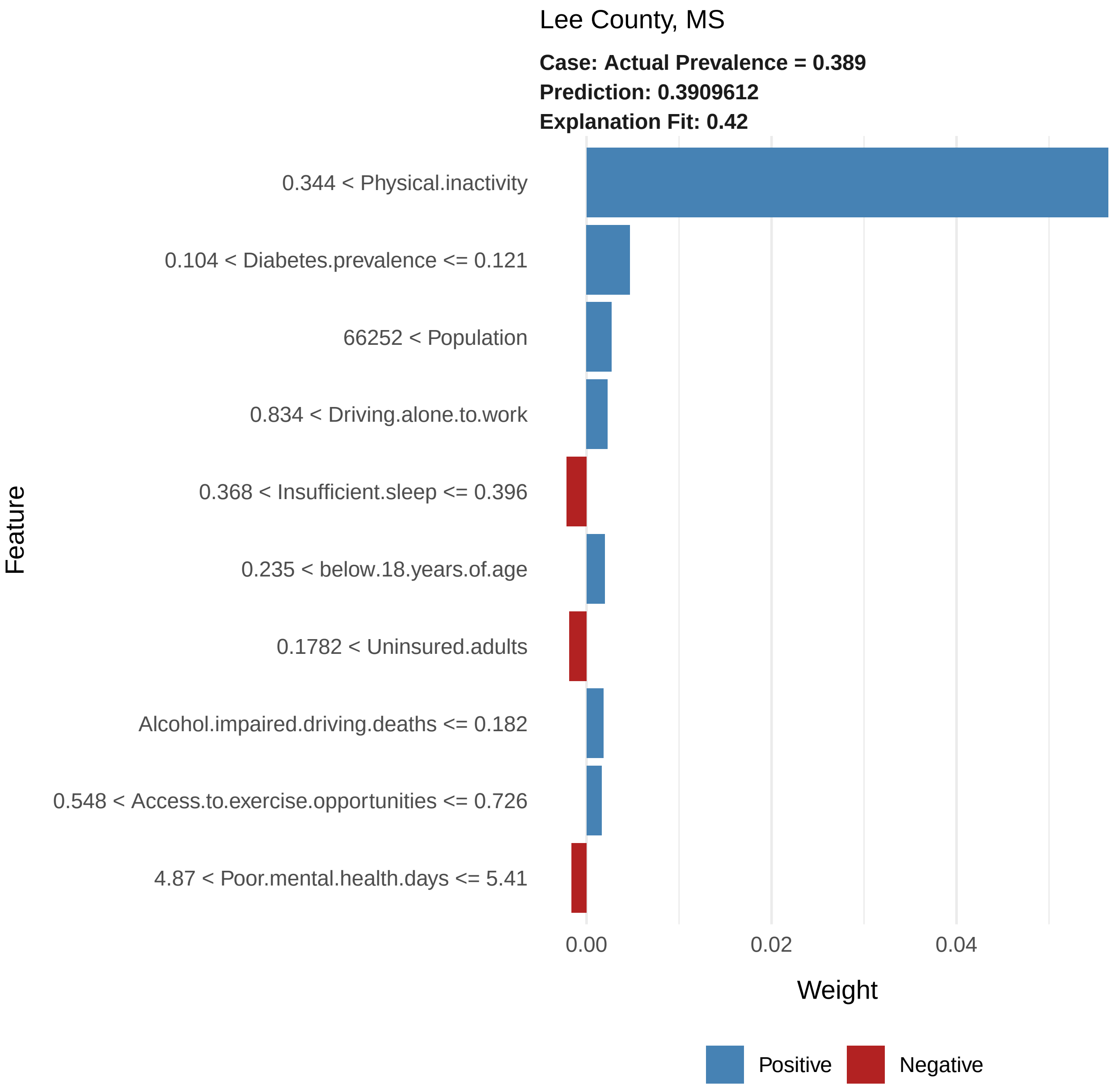
Lee County, MS
Case: Actual Prevalence = 0.389
Prediction: 0.3909612
Explanation Fit: 0.42
0.344 < Physical.inactivity
0.104 < Diabetes.prevalence <= 0.121
66252 < Population
0.834 < Driving.alone.to.work
0.368 < Insufficient.sleep <= 0.396
0.235 < below.18.years.of.age
0.1782 < Uninsured.adults
Alcohol.impaired.driving.deaths <= 0.182
0.548 < Access.to.exercise.opportunities <= 0.726
4.87 < Poor.mental.health.days <= 5.41
Feature
0.00
0.02
0.04
Weight
Positive
Negative

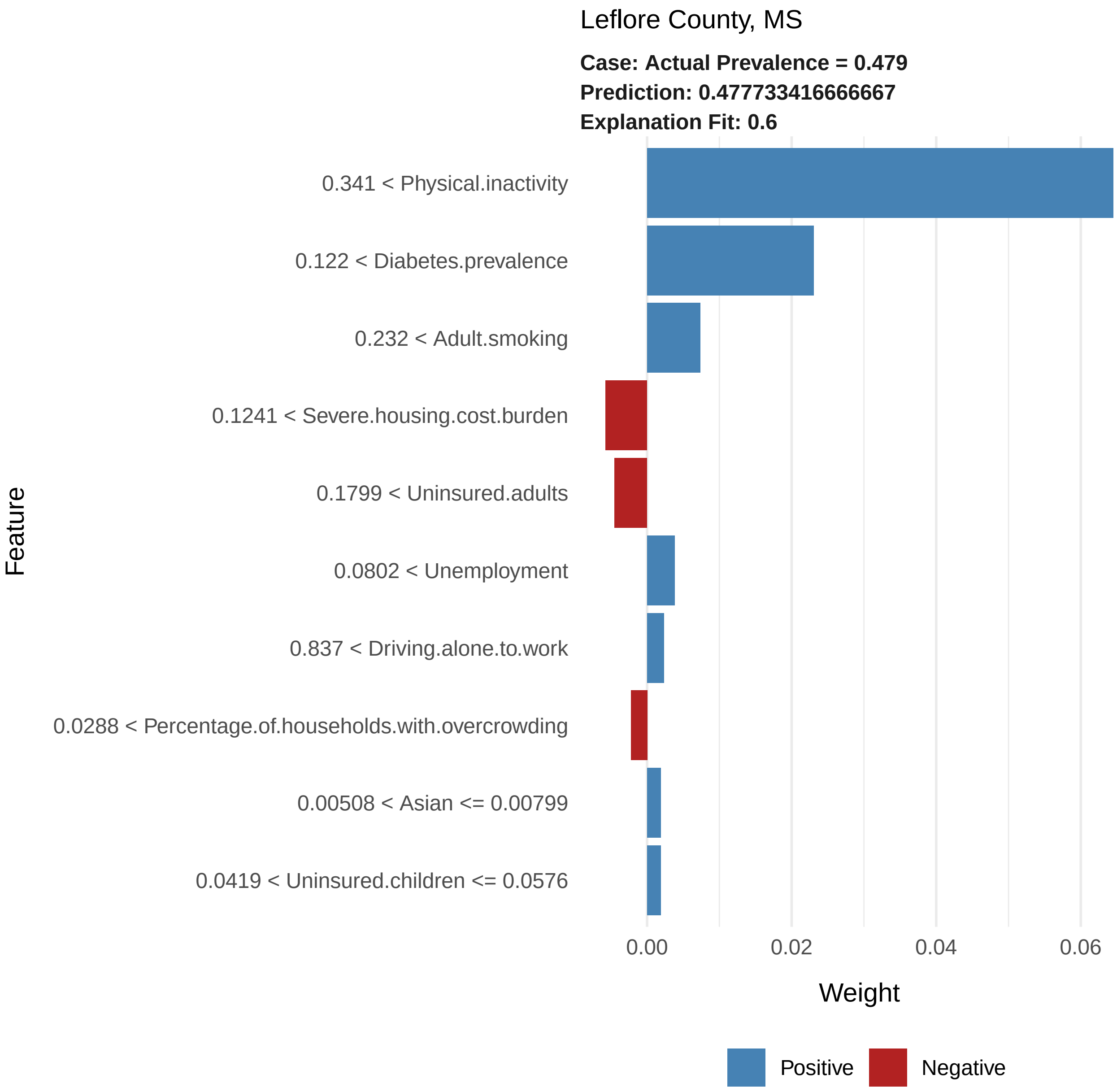
Leflore County, MS
Case: Actual Prevalence = 0.479
Prediction: 0.477733416666667
Explanation Fit: 0.6
0.341 < Physical.inactivity
0.122 < Diabetes.prevalence
0.232 < Adult.smoking
0.1241 < Severe.housing.cost.burden
0.1799 < Uninsured.adults
0.0802 < Unemployment
0.837 < Driving.alone.to.work
0.0288 < Percentage.of.households.with.overcrowding
0.00508 < Asian <= 0.00799
0.0419 < Uninsured.children <= 0.0576
Feature
0.00
0.02
0.04
0.06
Weight
Positive
Negative

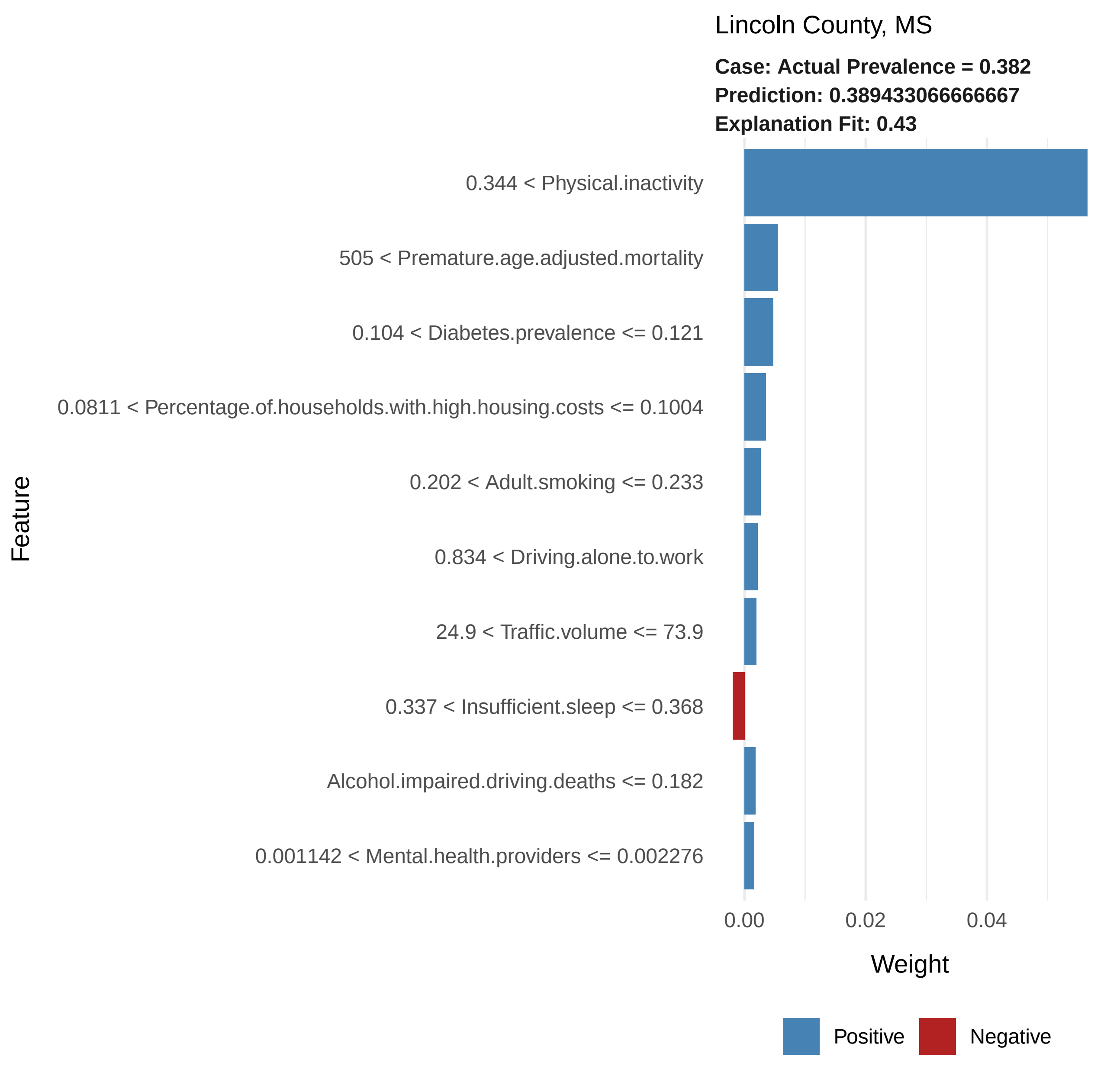
Lincoln County, MS
Case: Actual Prevalence = 0.382
Prediction: 0.389433066666667
Explanation Fit: 0.43
0.344 < Physical.inactivity
505 < Premature.age.adjusted.mortality
0.104 < Diabetes.prevalence <= 0.121
0.0811 < Percentage.of.households.with.high.housing.costs <= 0.1004
0.202 < Adult.smoking <= 0.233
0.834 < Driving.alone.to.work
24.9 < Traffic.volume <= 73.9
0.337 < Insufficient.sleep <= 0.368
Alcohol.impaired.driving.deaths <= 0.182
0.001142 < Mental.health.providers <= 0.002276
Feature
0.00
0.02
0.04
Weight
Positive
Negative

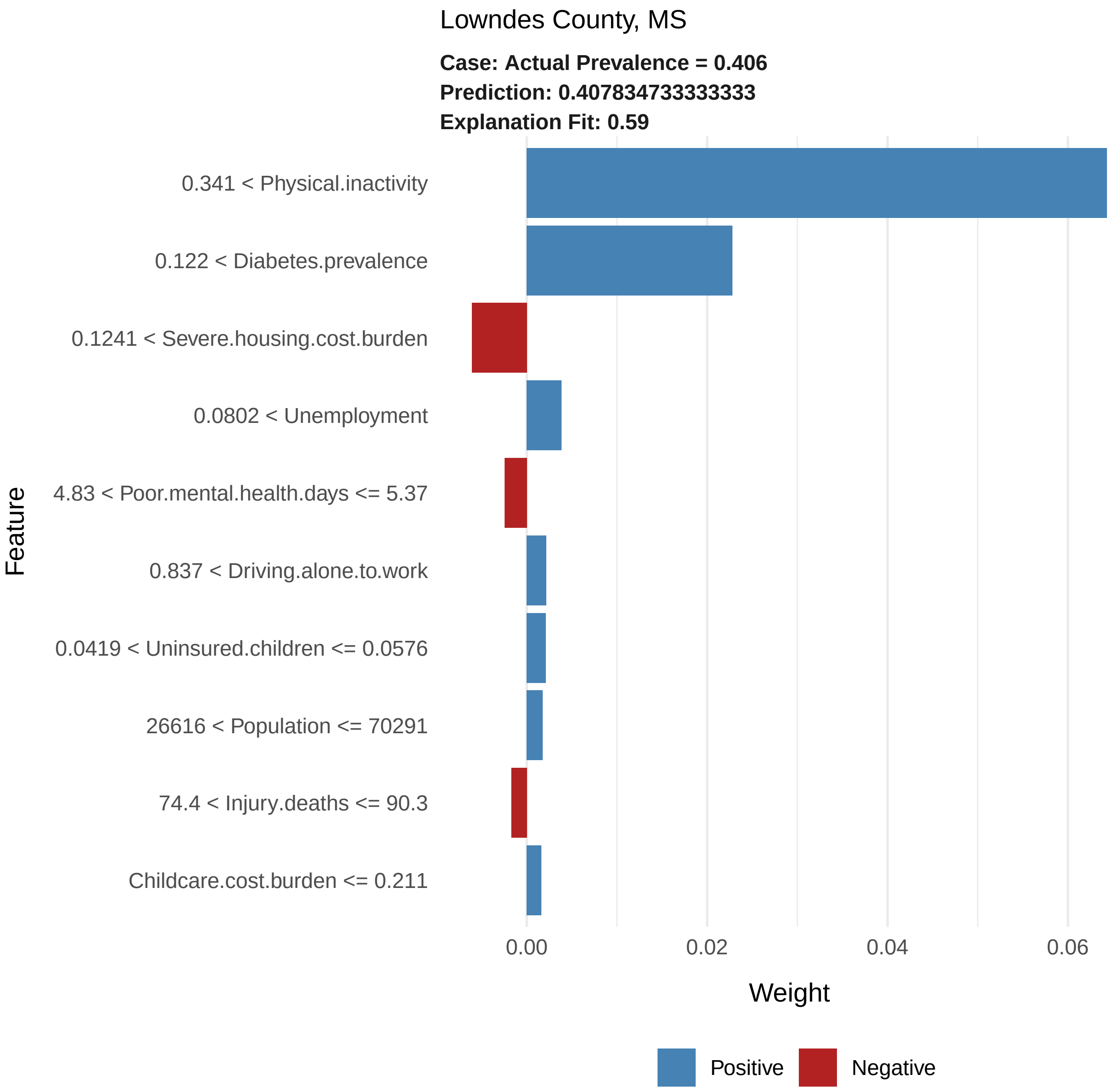
Lowndes County, MS
Case: Actual Prevalence = 0.406
Prediction: 0.407834733333333
Explanation Fit: 0.59
0.341 < Physical.inactivity
0.122 < Diabetes.prevalence
0.1241 < Severe.housing.cost.burden
0.0802 < Unemployment
4.83 < Poor.mental.health.days <= 5.37
0.837 < Driving.alone.to.work
0.0419 < Uninsured.children <= 0.0576
26616 < Population <= 70291
74.4 < Injury.deaths <= 90.3
Childcare.cost.burden <= 0.211
Feature
0.00
0.02
0.04
0.06
Weight
Positive
Negative

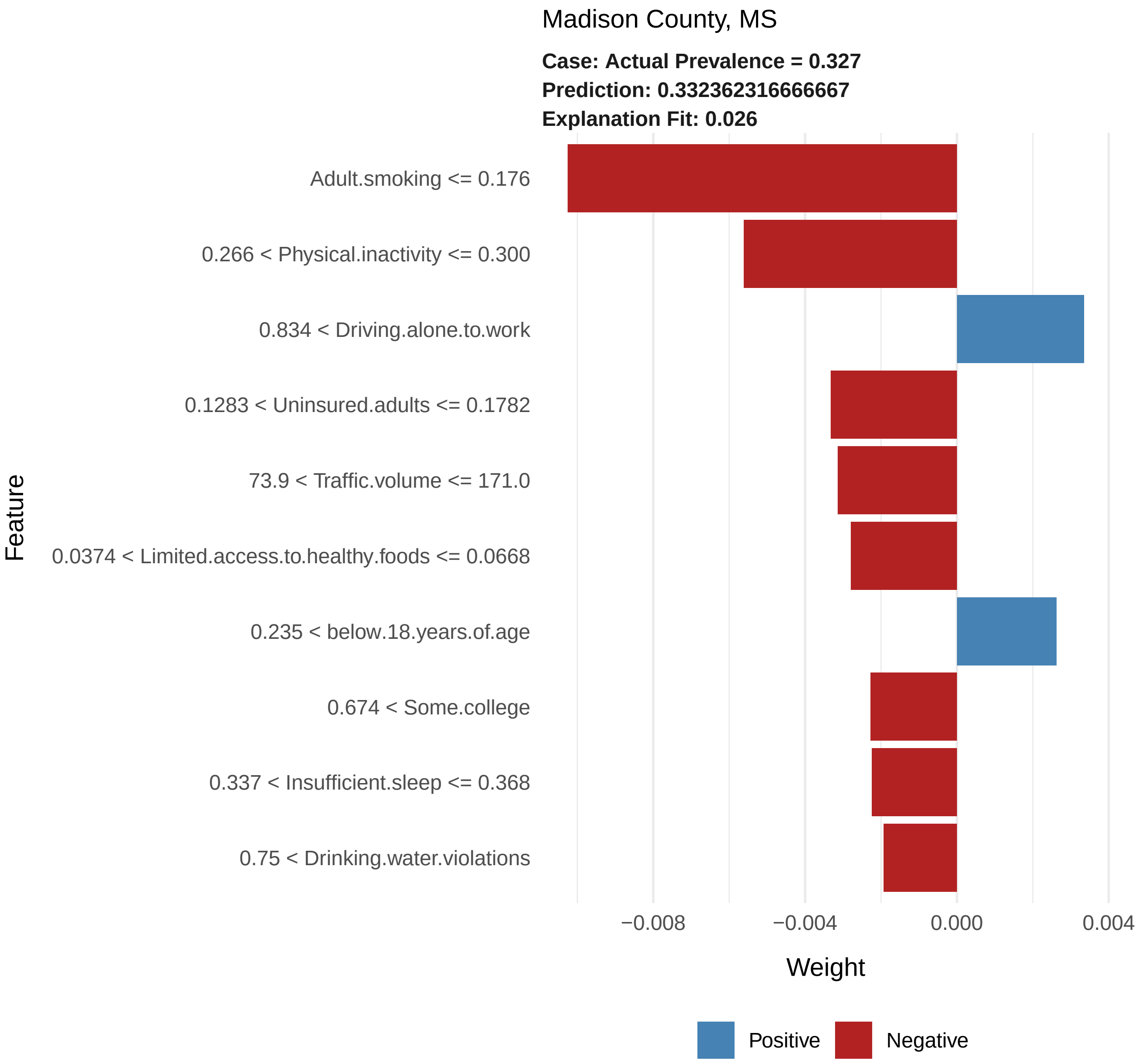

Madison County, MS
Case: Actual Prevalence = 0.327
Prediction: 0.332362316666667
Explanation Fit: 0.026
Adult.smoking <= 0.176
0.266 < Physical.inactivity <= 0.300
0.834 < Driving.alone.to.work
0.1283 < Uninsured.adults <= 0.1782
73.9 < Traffic.volume <= 171.0
0.0374 < Limited.access.to.healthy.foods <= 0.0668
0.235 < below.18.years.of.age
0.674 < Some.college
0.337 < Insufficient.sleep <= 0.368
0.75 < Drinking.water.violations
Feature
−0.008
−0.004
0.000
0.004
Weight
Positive
Negative

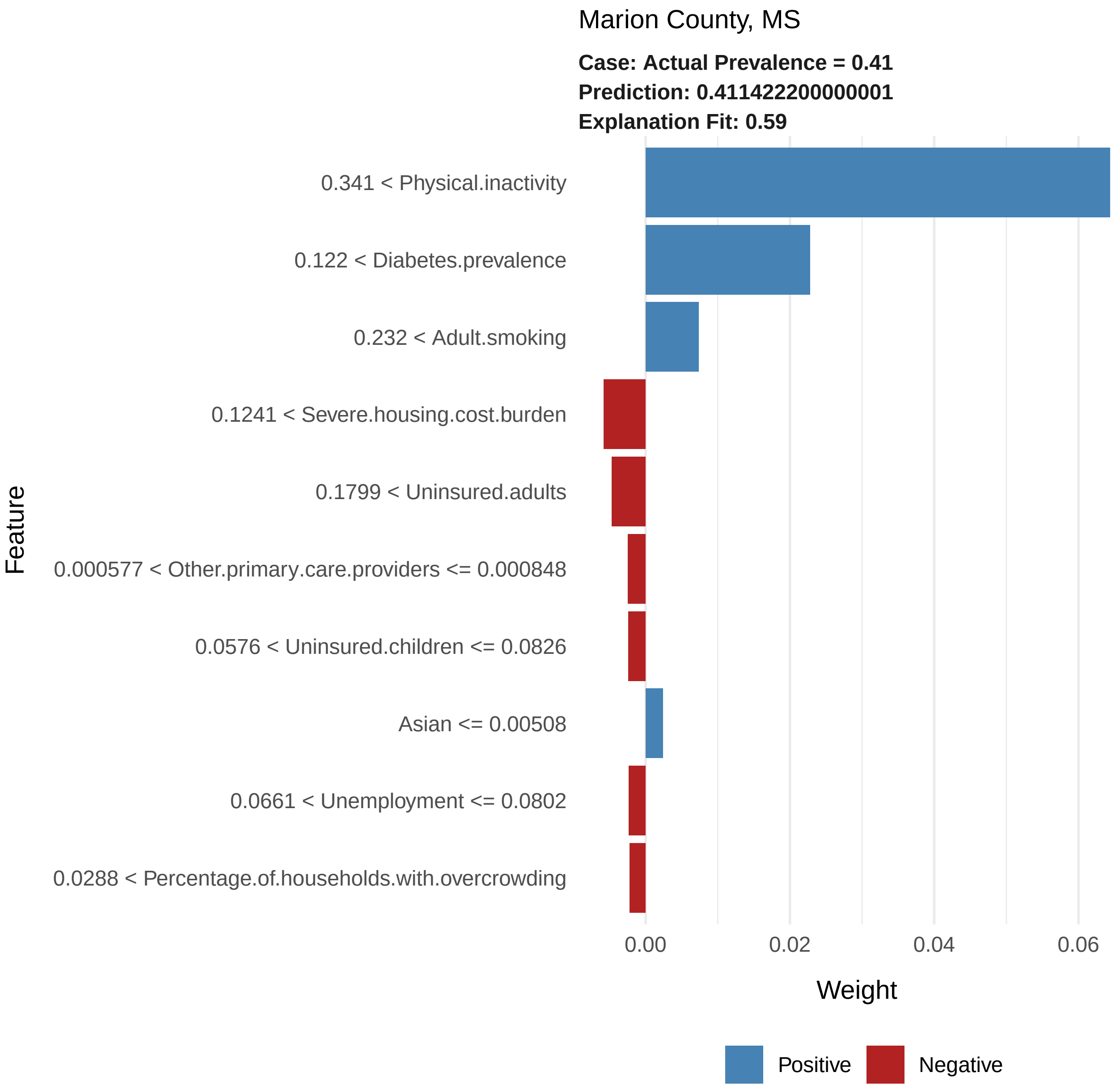
Marion County, MS
Case: Actual Prevalence = 0.41
Prediction: 0.411422200000001
Explanation Fit: 0.59
0.341 < Physical.inactivity
0.122 < Diabetes.prevalence
0.232 < Adult.smoking
0.1241 < Severe.housing.cost.burden
0.1799 < Uninsured.adults
0.000577 < Other.primary.care.providers <= 0.000848
0.0576 < Uninsured.children <= 0.0826
Asian <= 0.00508
0.0661 < Unemployment <= 0.0802
0.0288 < Percentage.of.households.with.overcrowding
Feature
0.00
0.02
0.04
0.06
Weight
Positive
Negative

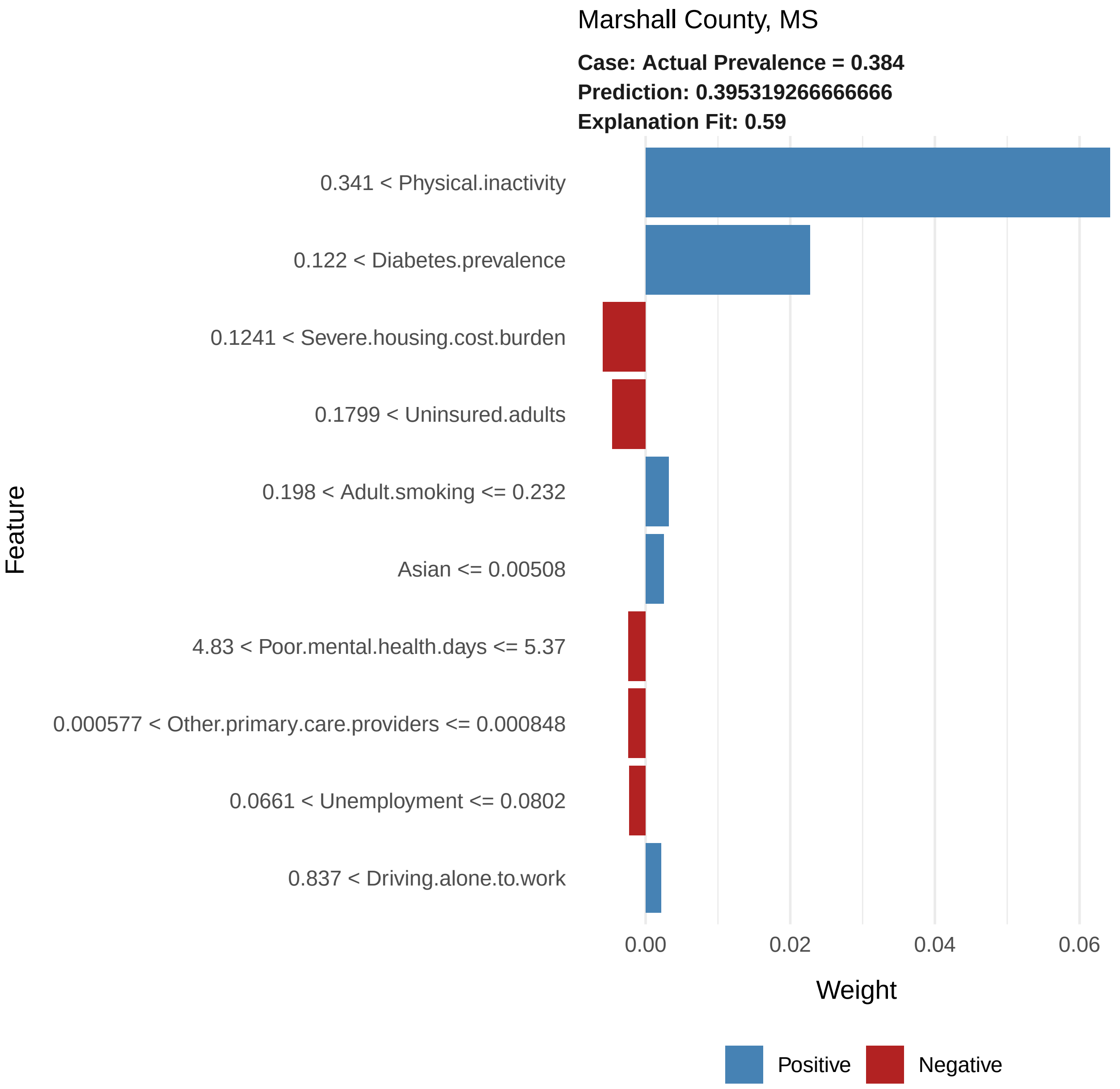

Marshall County, MS
Case: Actual Prevalence = 0.384
Prediction: 0.395319266666666
Explanation Fit: 0.59
Feature
0.341 < Physical.inactivity
0.122 < Diabetes.prevalence
0.1241 < Severe.housing.cost.burden
0.1799 < Uninsured.adults
0.198 < Adult.smoking <= 0.232
Asian <= 0.00508
4.83 < Poor.mental.health.days <= 5.37
0.000577 < Other.primary.care.providers <= 0.000848
0.0661 < Unemployment <= 0.0802
0.837 < Driving.alone.to.work
0.00
0.02
0.04
0.06
Weight
Positive
Negative

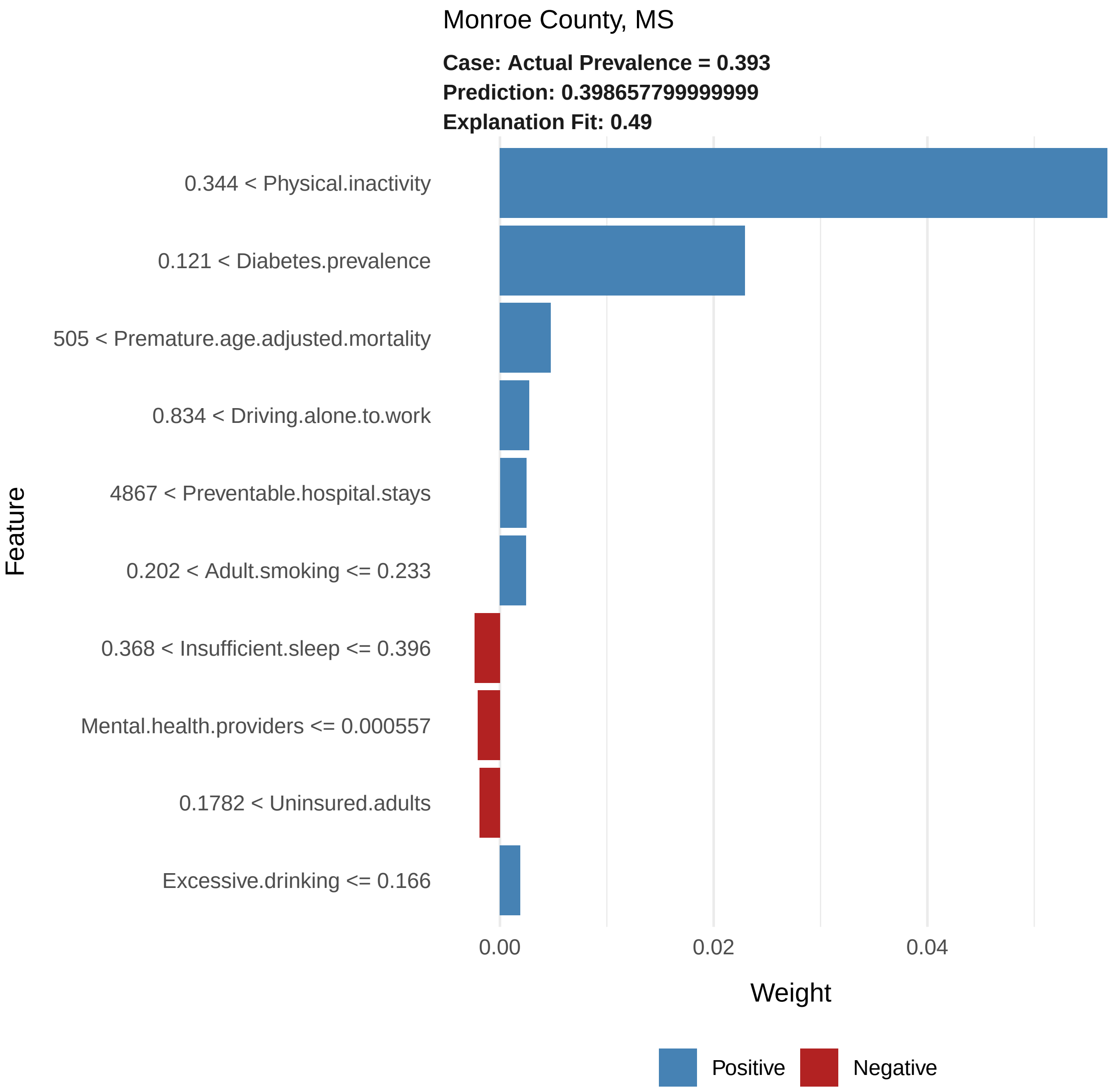

Monroe County, MS
Case: Actual Prevalence = 0.393
Prediction: 0.398657799999999
Explanation Fit: 0.49
0.344 < Physical.inactivity
0.121 < Diabetes.prevalence
505 < Premature.age.adjusted.mortality
0.834 < Driving.alone.to.work
4867 < Preventable.hospital.stays
0.202 < Adult.smoking <= 0.233
0.368 < Insufficient.sleep <= 0.396
Mental.health.providers <= 0.000557
0.1782 < Uninsured.adults
Excessive.drinking <= 0.166
Feature
0.00
0.02
0.04
Weight
Positive
Negative

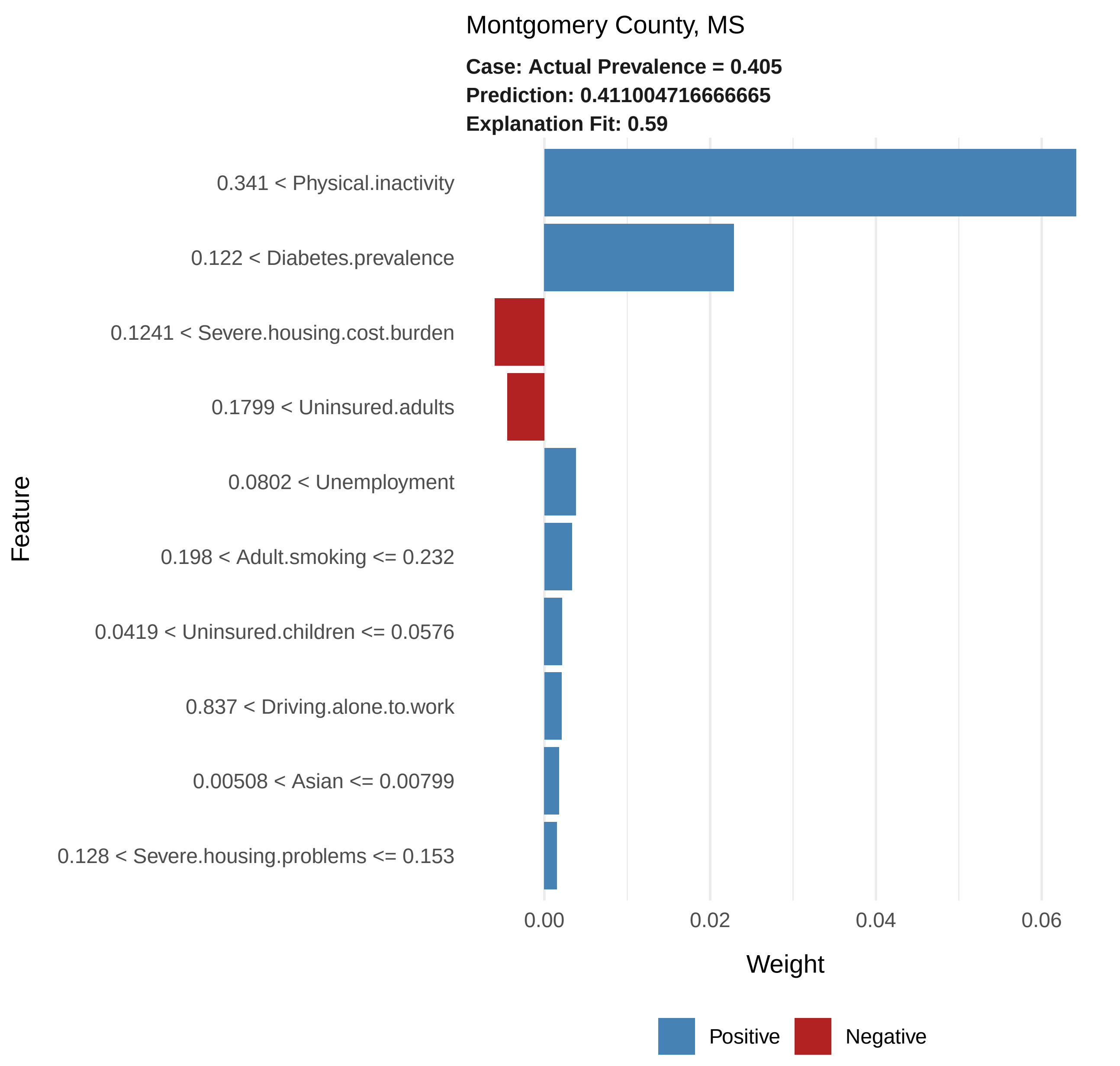
Montgomery County, MS
Case: Actual Prevalence = 0.405
Prediction: 0.411004716666665
Explanation Fit: 0.59
0.341 < Physical.inactivity
0.122 < Diabetes.prevalence
0.1241 < Severe.housing.cost.burden
0.1799 < Uninsured.adults
0.0802 < Unemployment
0.198 < Adult.smoking <= 0.232
0.0419 < Uninsured.children <= 0.0576
0.837 < Driving.alone.to.work
0.00508 < Asian <= 0.00799
0.128 < Severe.housing.problems <= 0.153
Feature
0.00
0.02
0.04
0.06
Weight
Positive
Negative

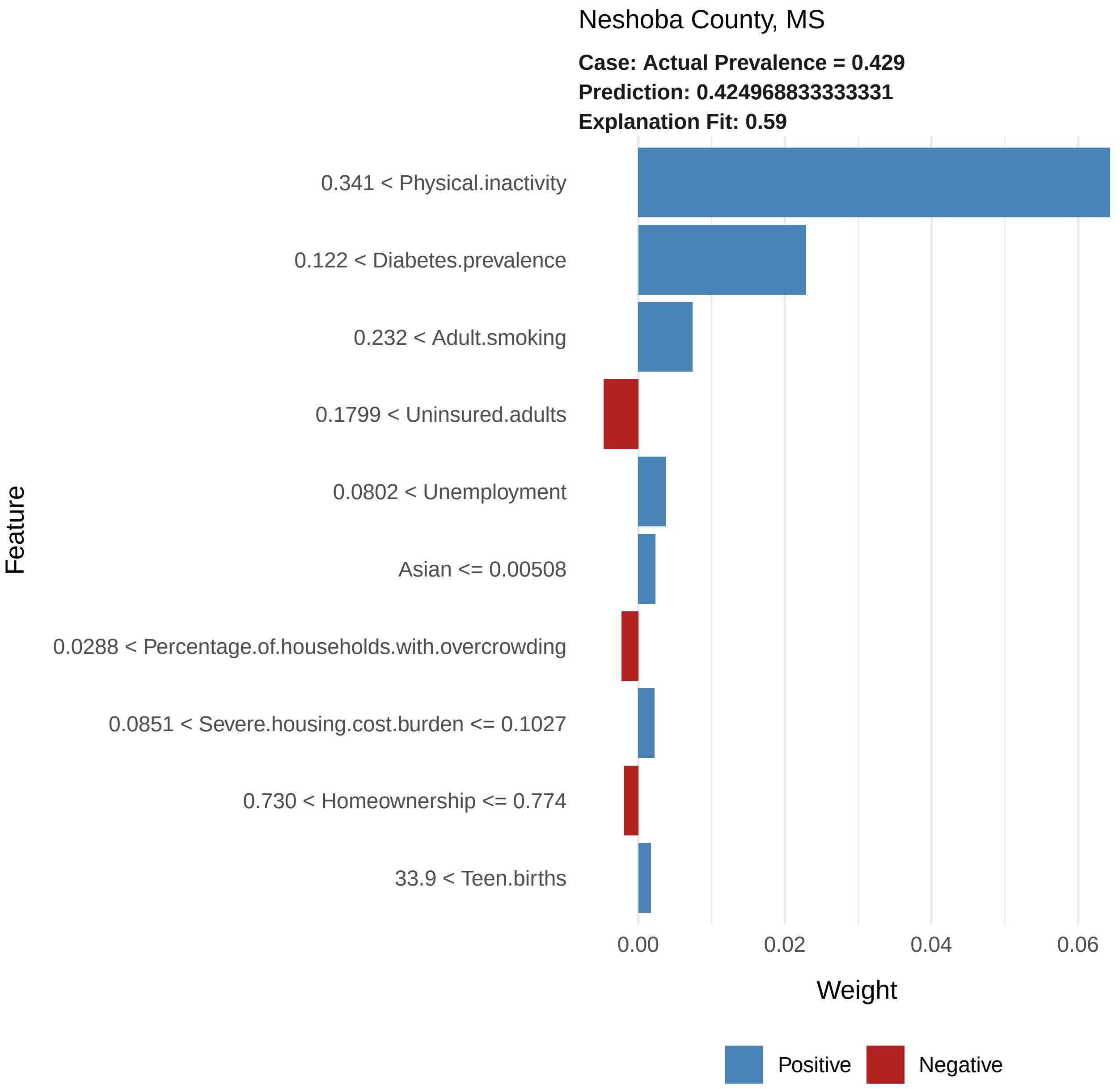

Neshoba County, MS
Case: Actual Prevalence = 0.429
Prediction: 0.424968833333331
Explanation Fit: 0.59
0.341 < Physical.inactivity
0.122 < Diabetes.prevalence
0.232 < Adult.smoking
0.1799 < Uninsured.adults
0.0802 < Unemployment
Asian <= 0.00508
0.0288 < Percentage.of.households.with.overcrowding
0.0851 < Severe.housing.cost.burden <= 0.1027
0.730 < Homeownership <= 0.774
33.9 < Teen.births
Feature
0.00
0.02
0.04
0.06
Weight
Positive
Negative

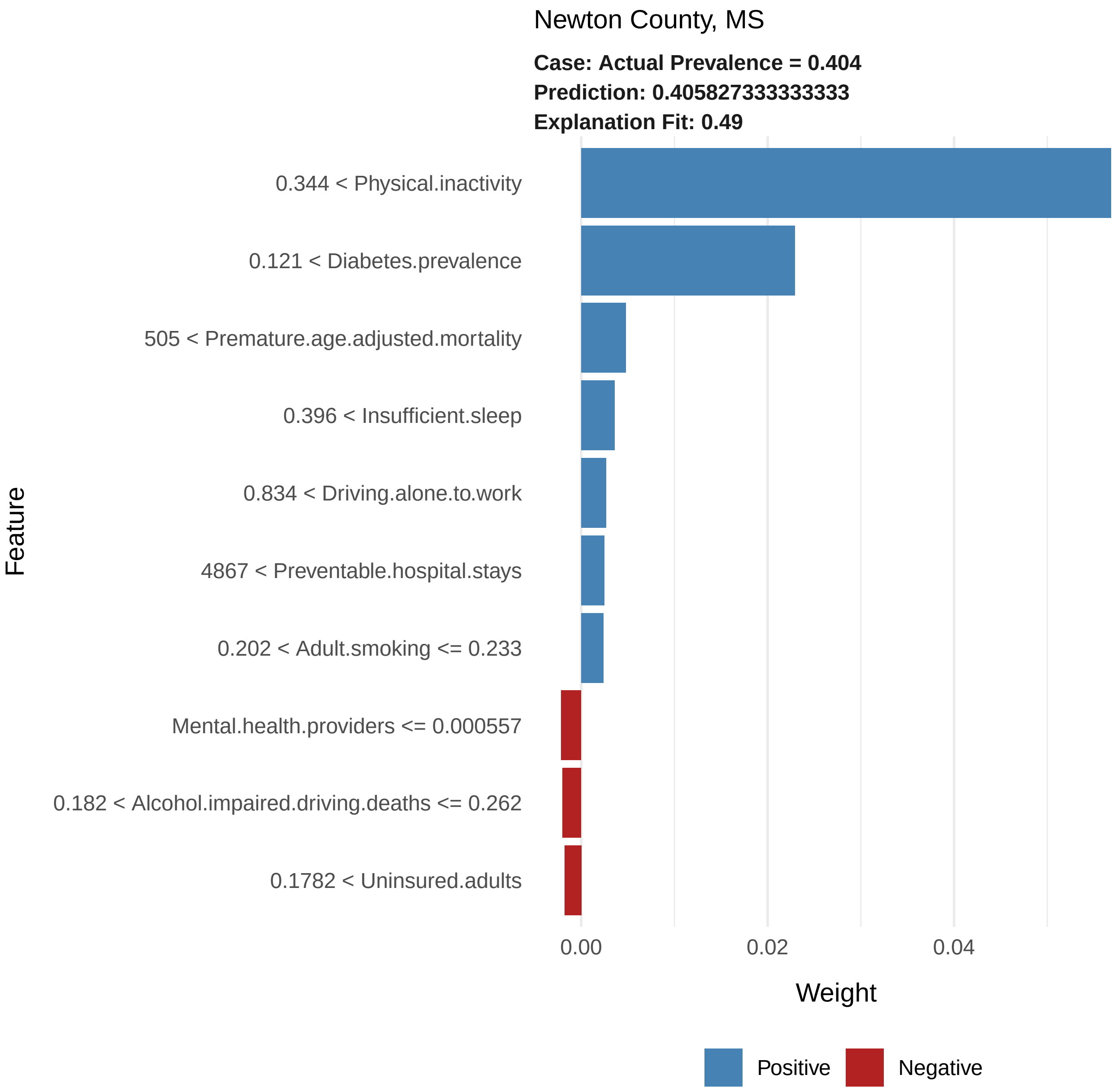

Newton County, MS
Case: Actual Prevalence = 0.404
Prediction: 0.405827333333333
Explanation Fit: 0.49
0.344 < Physical.inactivity
0.121 < Diabetes.prevalence
505 < Premature.age.adjusted.mortality
0.396 < Insufficient.sleep
0.834 < Driving.alone.to.work
4867 < Preventable.hospital.stays
0.202 < Adult.smoking <= 0.233
Mental.health.providers <= 0.000557
0.182 < Alcohol.impaired.driving.deaths <= 0.262
0.1782 < Uninsured.adults
Feature
0.00
0.02
0.04
Weight
Positive
Negative

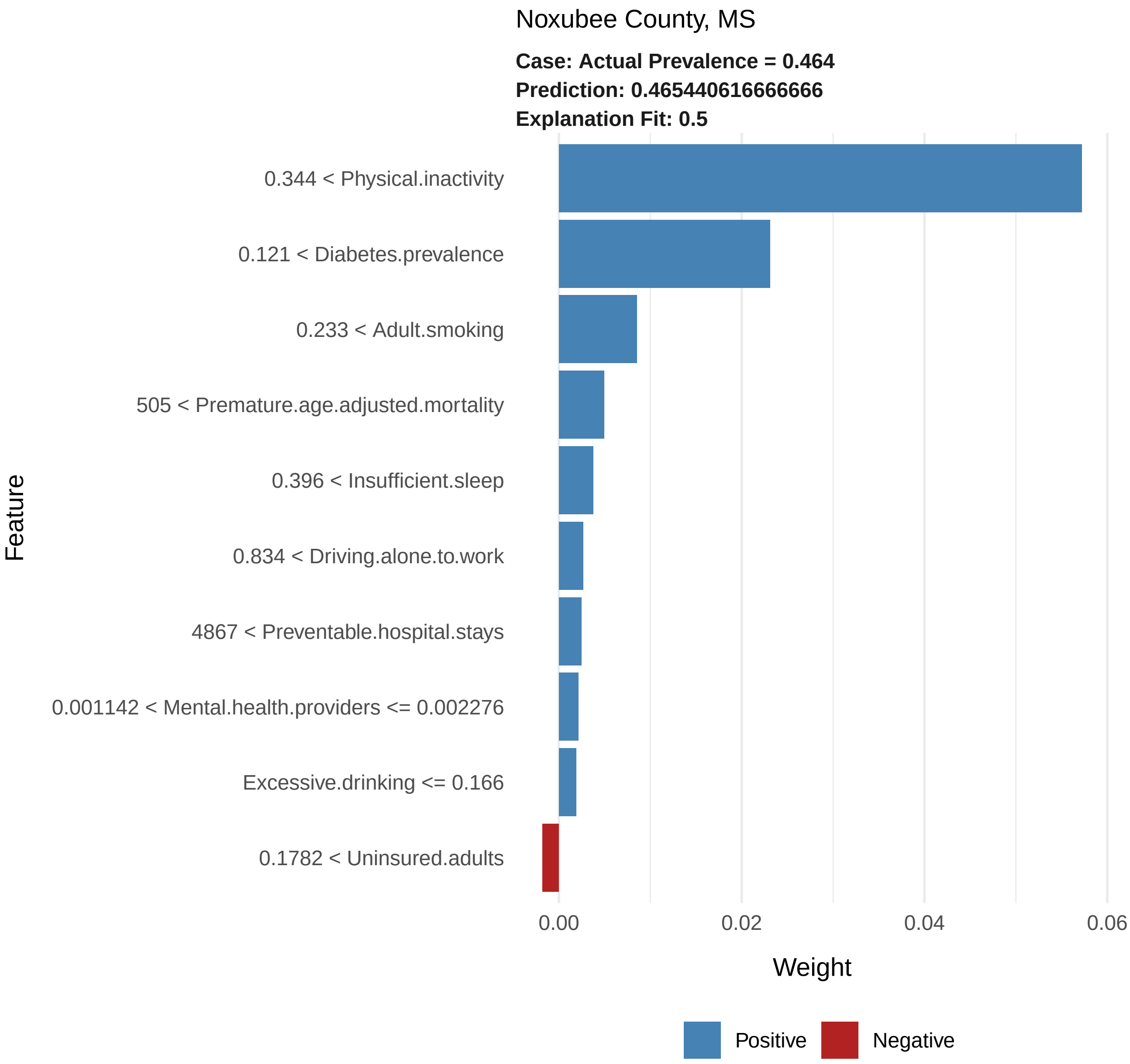

Noxubee County, MS
Case: Actual Prevalence = 0.464
Prediction: 0.465440616666666
Explanation Fit: 0.5
0.344 < Physical.inactivity
0.121 < Diabetes.prevalence
0.233 < Adult.smoking
505 < Premature.age.adjusted.mortality
0.396 < Insufficient.sleep
0.834 < Driving.alone.to.work
4867 < Preventable.hospital.stays
0.001142 < Mental.health.providers <= 0.002276
Excessive.drinking <= 0.166
0.1782 < Uninsured.adults
Feature
0.00
0.02
0.04
0.06
Weight
Positive
Negative

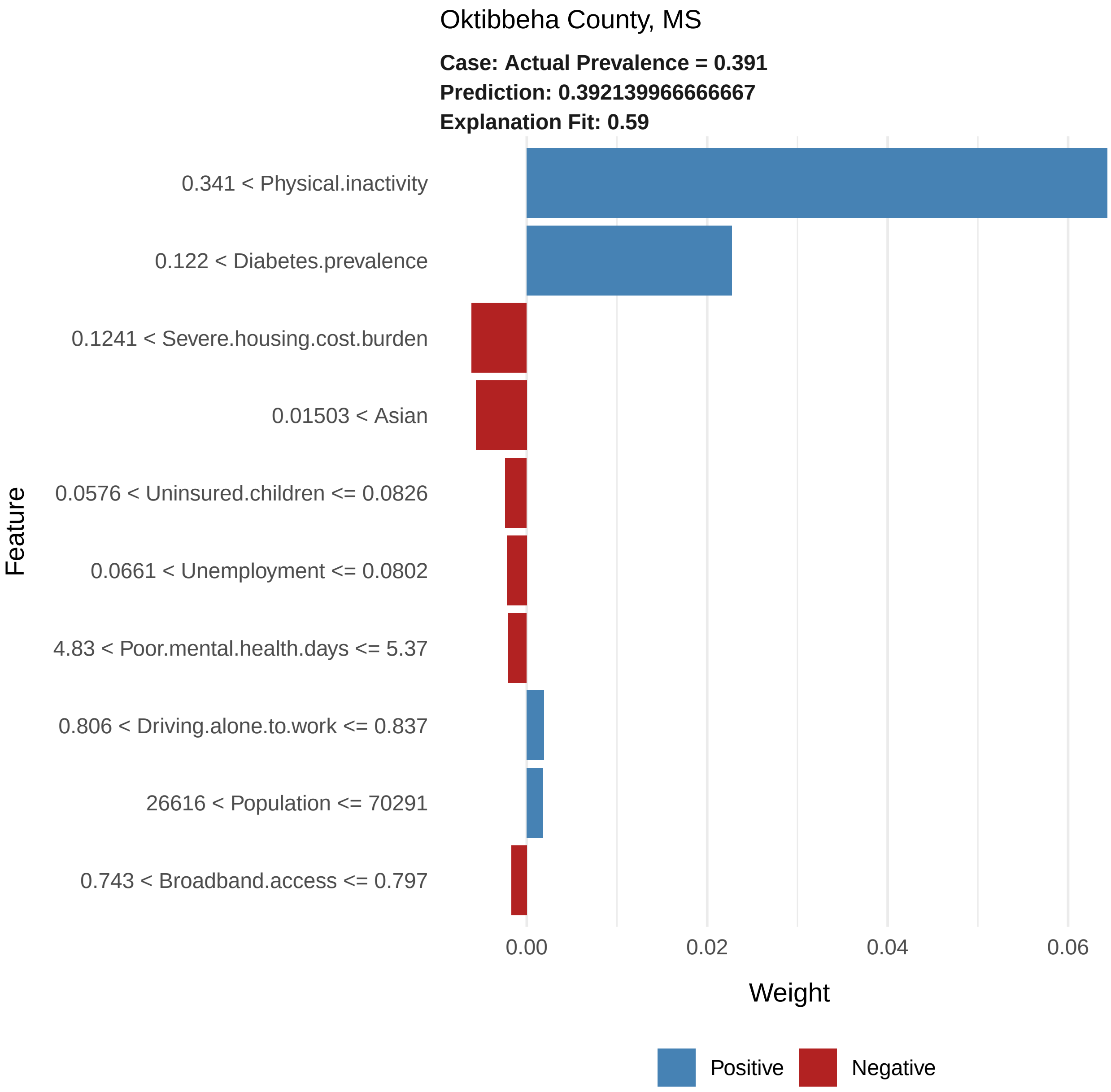

Oktibbeha County, MS
Case: Actual Prevalence = 0.391
Prediction: 0.392139966666667
Explanation Fit: 0.59
0.341 < Physical.inactivity
0.122 < Diabetes.prevalence
0.1241 < Severe.housing.cost.burden
0.01503 < Asian
0.0576 < Uninsured.children <= 0.0826
0.0661 < Unemployment <= 0.0802
4.83 < Poor.mental.health.days <= 5.37
0.806 < Driving.alone.to.work <= 0.837
26616 < Population <= 70291
0.743 < Broadband.access <= 0.797
Feature
0.00
0.02
0.04
0.06
Weight
Positive
Negative

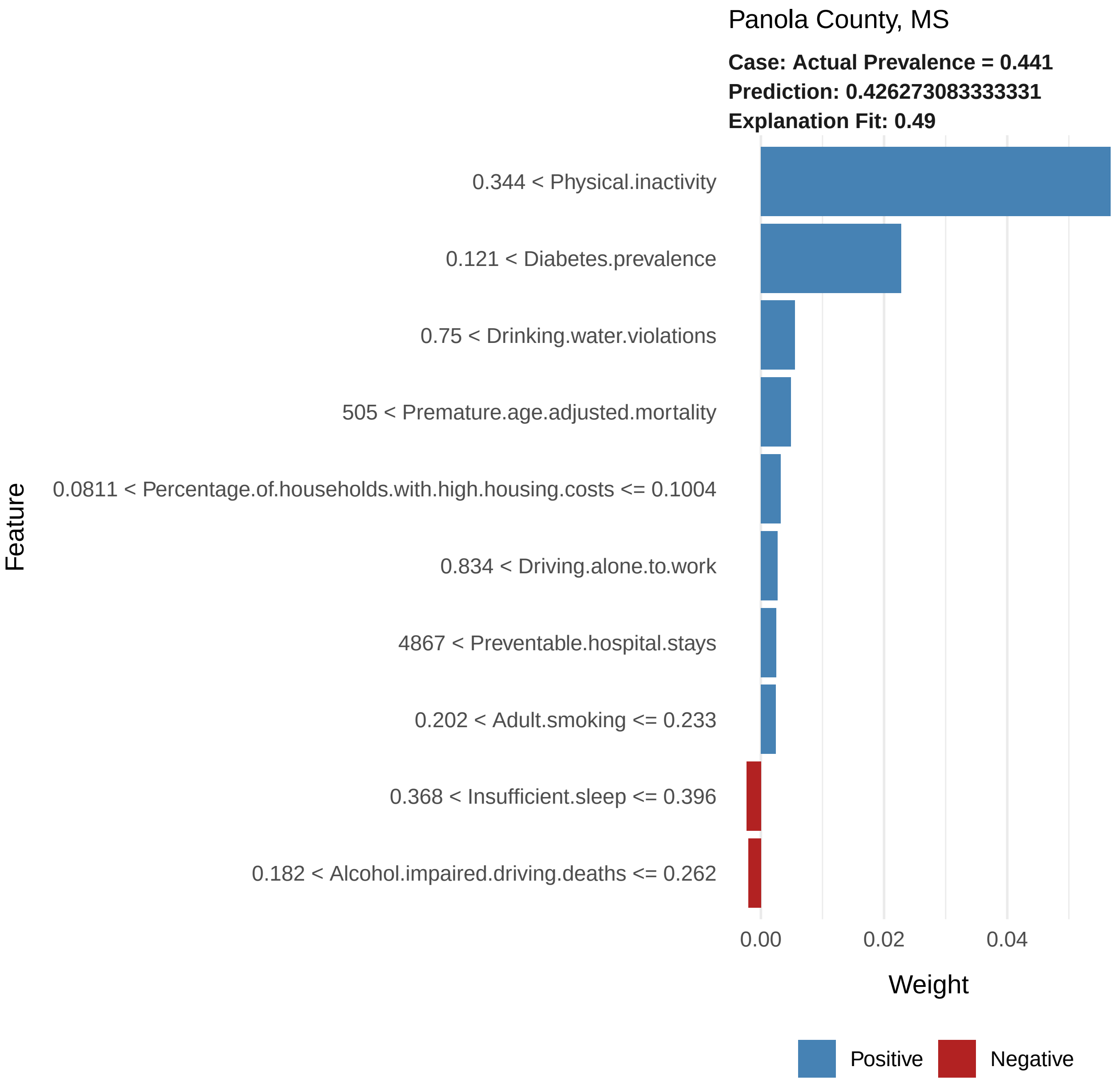

Panola County, MS
Case: Actual Prevalence = 0.441
Prediction: 0.426273083333331
Explanation Fit: 0.49
0.344 < Physical.inactivity
0.121 < Diabetes.prevalence
0.75 < Drinking.water.violations
505 < Premature.age.adjusted.mortality
0.0811 < Percentage.of.households.with.high.housing.costs <= 0.1004
0.834 < Driving.alone.to.work
4867 < Preventable.hospital.stays
0.202 < Adult.smoking <= 0.233
0.368 < Insufficient.sleep <= 0.396
0.182 < Alcohol.impaired.driving.deaths <= 0.262
Feature
0.00
0.02
0.04
Weight
Positive
Negative

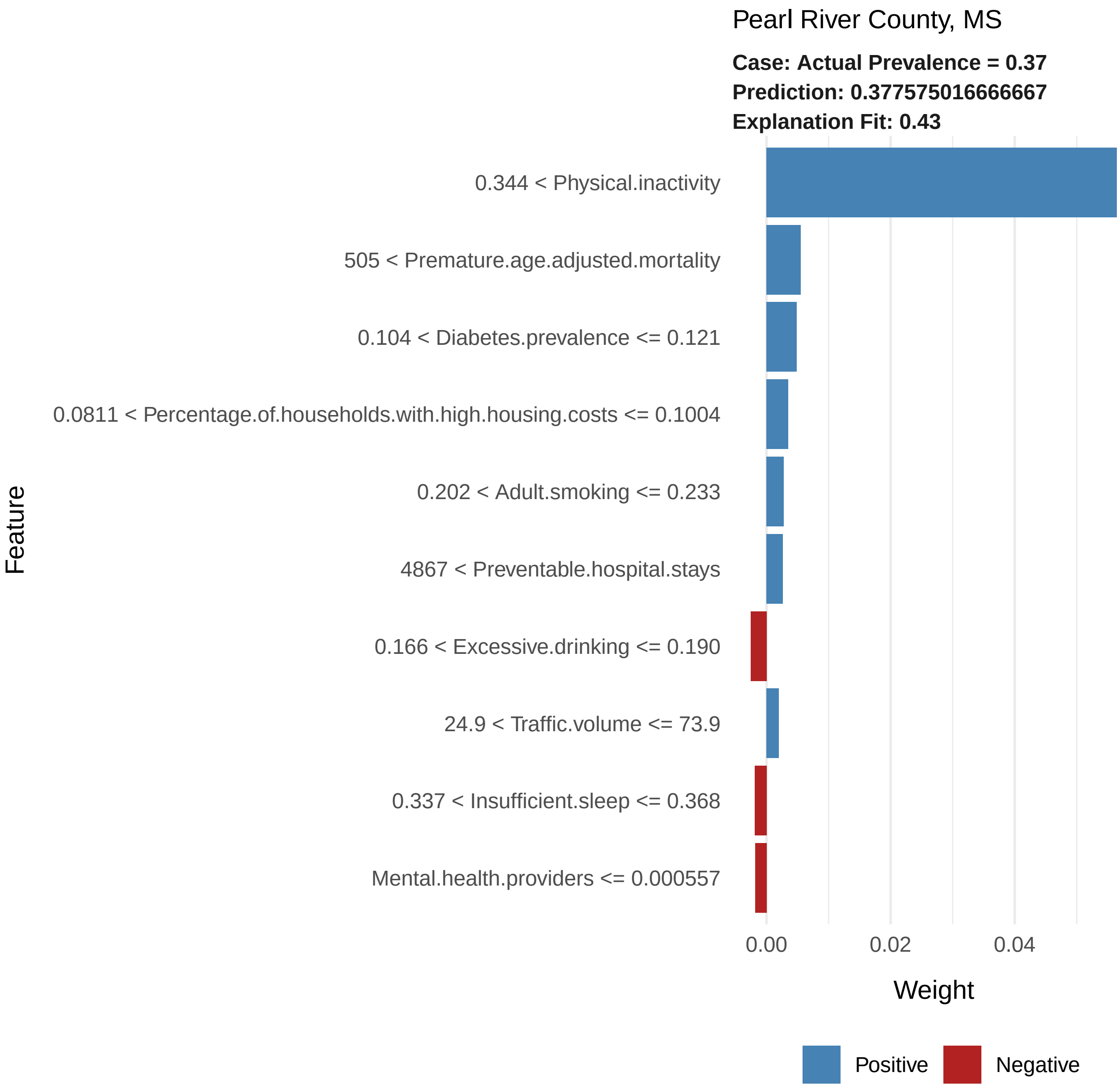
Pearl River County, MS
Case: Actual Prevalence = 0.37
Prediction: 0.377575016666667
Explanation Fit: 0.43
0.344 < Physical.inactivity
505 < Premature.age.adjusted.mortality
0.104 < Diabetes.prevalence <= 0.121
0.0811 < Percentage.of.households.with.high.housing.costs <= 0.1004
0.202 < Adult.smoking <= 0.233
4867 < Preventable.hospital.stays
0.166 < Excessive.drinking <= 0.190
24.9 < Traffic.volume <= 73.9
0.337 < Insufficient.sleep <= 0.368
Mental.health.providers <= 0.000557
Feature
0.00
0.02
0.04
Weight
Positive
Negative

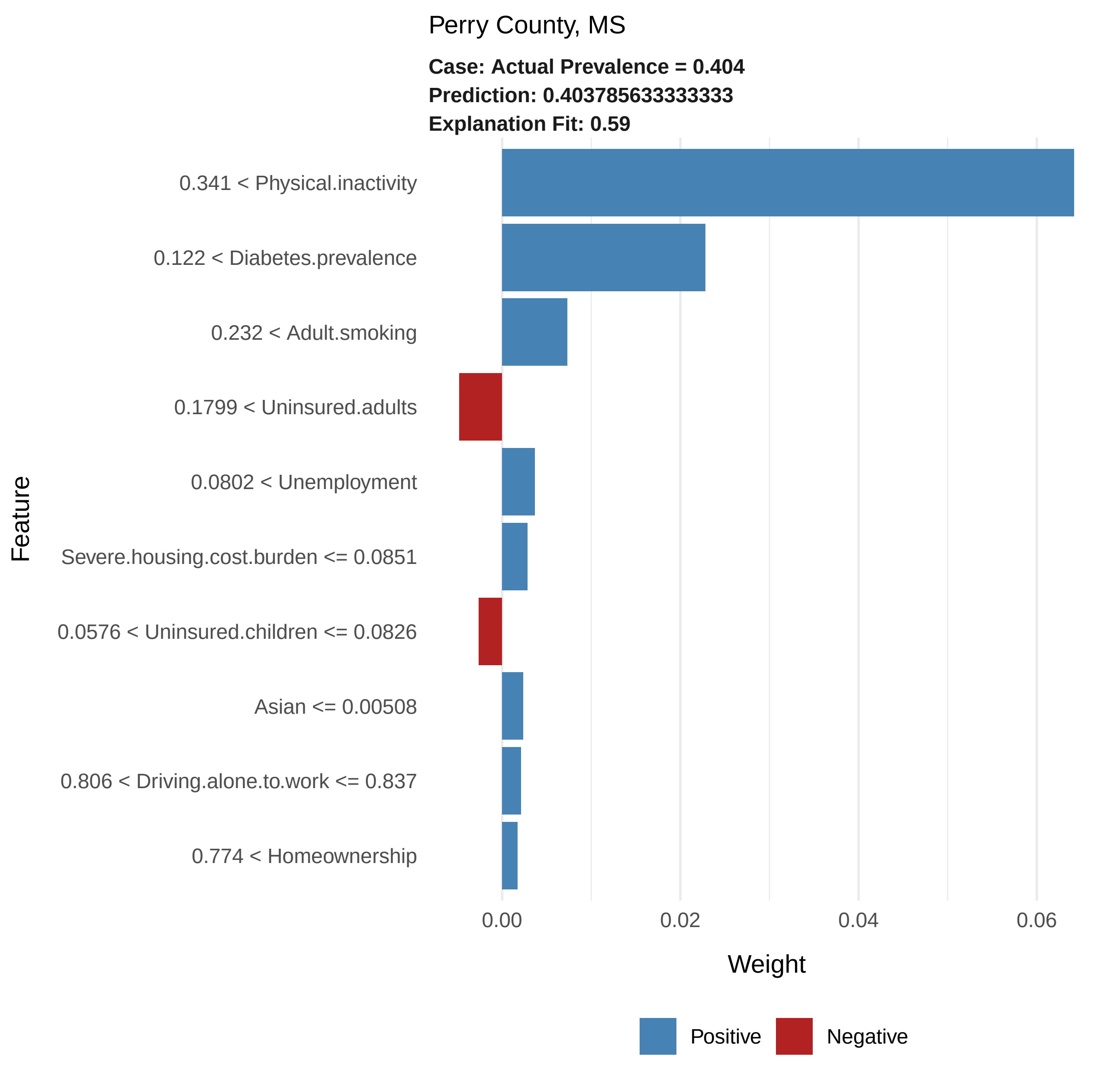

Perry County, MS
Case: Actual Prevalence = 0.404
Prediction: 0.403785633333333
Explanation Fit: 0.59
0.341 < Physical.inactivity
0.122 < Diabetes.prevalence
0.232 < Adult.smoking
0.1799 < Uninsured.adults
0.0802 < Unemployment
Severe.housing.cost.burden <= 0.0851
0.0576 < Uninsured.children <= 0.0826
Asian <= 0.00508
0.806 < Driving.alone.to.work <= 0.837
0.774 < Homeownership
Feature
0.00
0.02
0.04
0.06
Weight
Positive
Negative

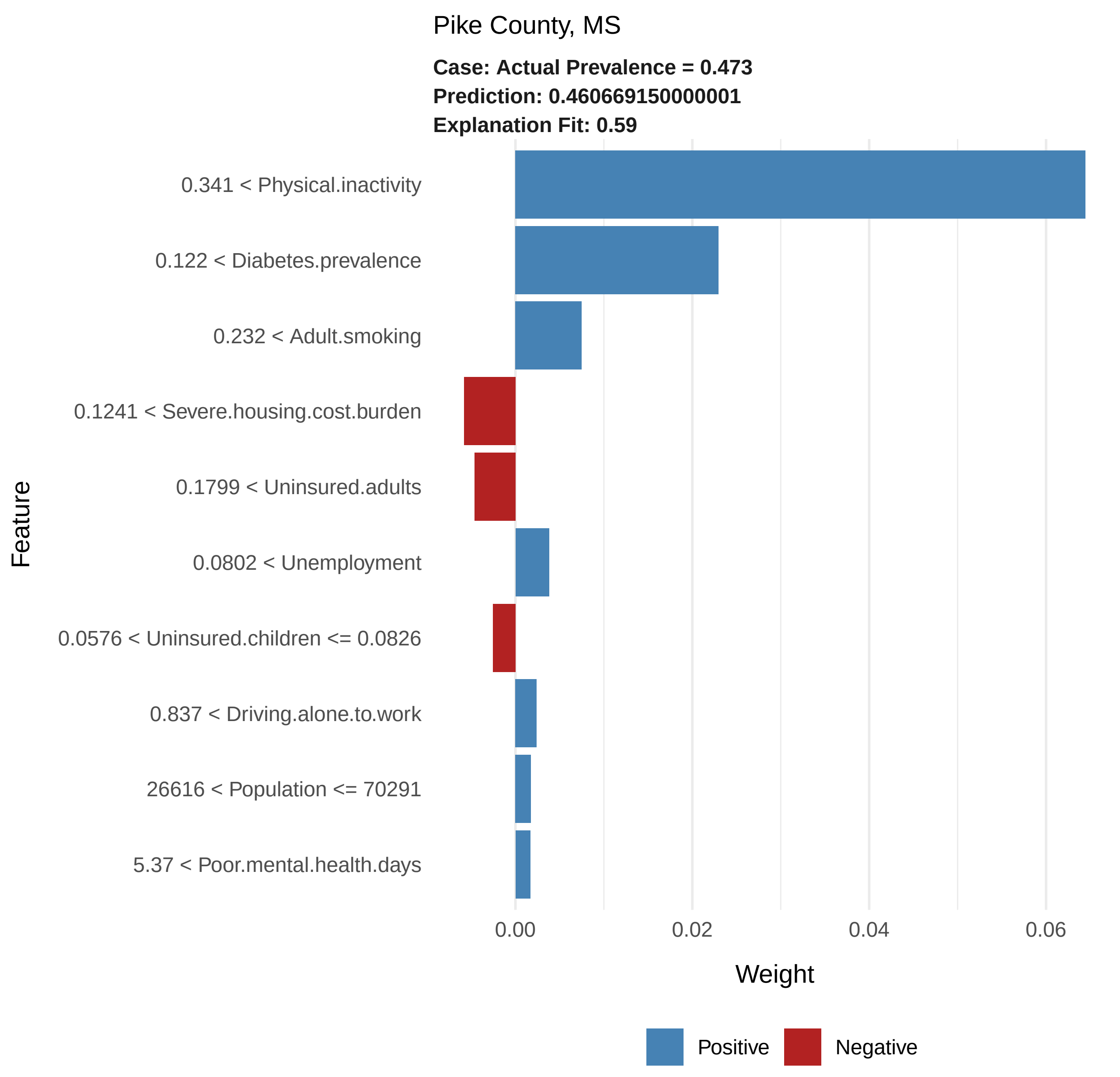
Pike County, MS
Case: Actual Prevalence = 0.473
Prediction: 0.460669150000001
Explanation Fit: 0.59
0.341 < Physical.inactivity
0.122 < Diabetes.prevalence
0.232 < Adult.smoking
0.1241 < Severe.housing.cost.burden
0.1799 < Uninsured.adults
0.0802 < Unemployment
0.0576 < Uninsured.children <= 0.0826
0.837 < Driving.alone.to.work
26616 < Population <= 70291
5.37 < Poor.mental.health.days
Feature
0.00
0.02
0.04
0.06
Weight
Positive
Negative

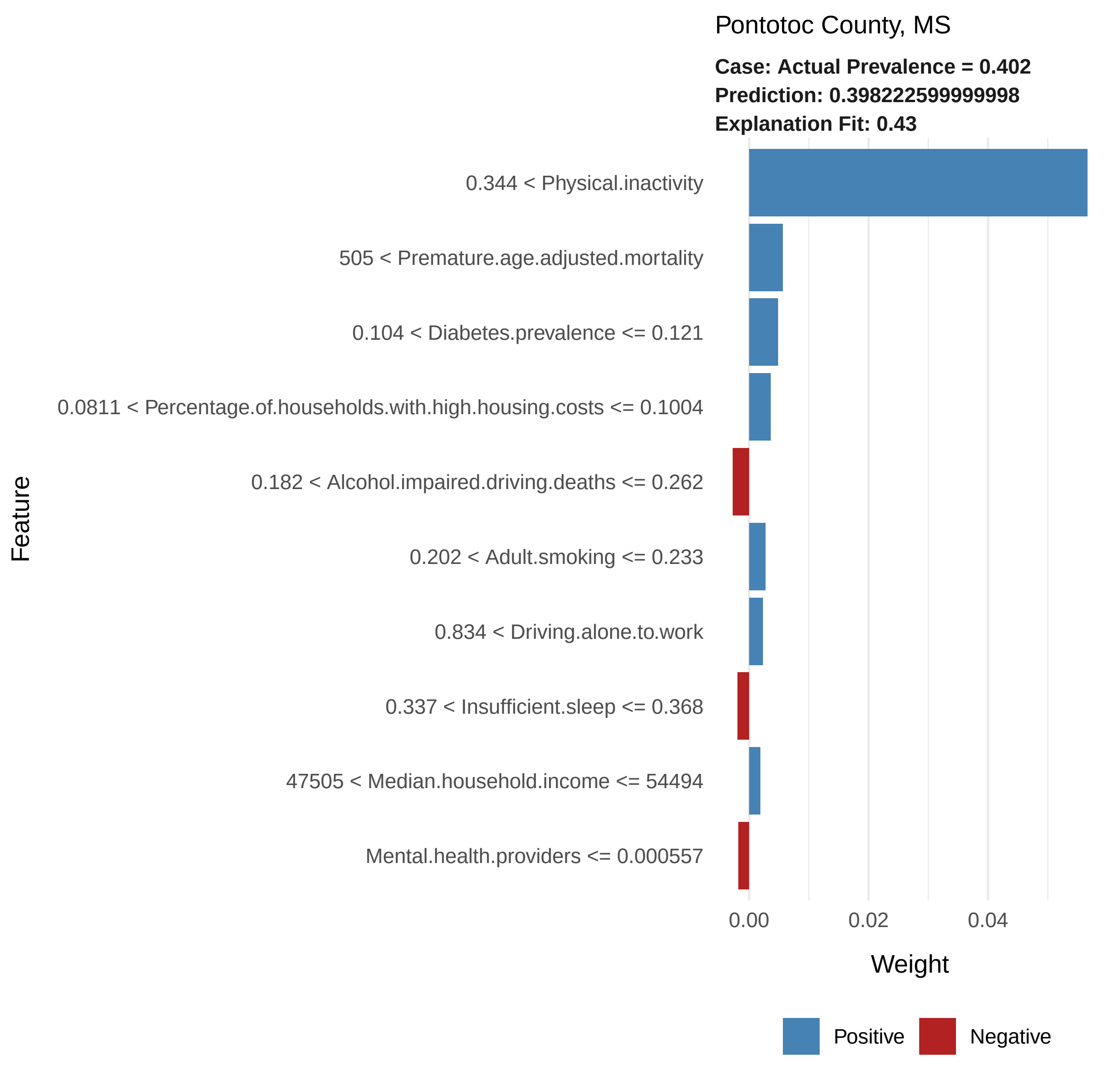

Pontotoc County, MS
Case: Actual Prevalence = 0.402
Prediction: 0.398222599999998
Explanation Fit: 0.43
0.344 < Physical.inactivity
505 < Premature.age.adjusted.mortality
0.104 < Diabetes.prevalence <= 0.121
0.0811 < Percentage.of.households.with.high.housing.costs <= 0.1004
0.182 < Alcohol.impaired.driving.deaths <= 0.262
0.202 < Adult.smoking <= 0.233
0.834 < Driving.alone.to.work
0.337 < Insufficient.sleep <= 0.368
47505 < Median.household.income <= 54494
Mental.health.providers <= 0.000557
Feature
0.00
0.02
0.04
Weight
Positive
Negative

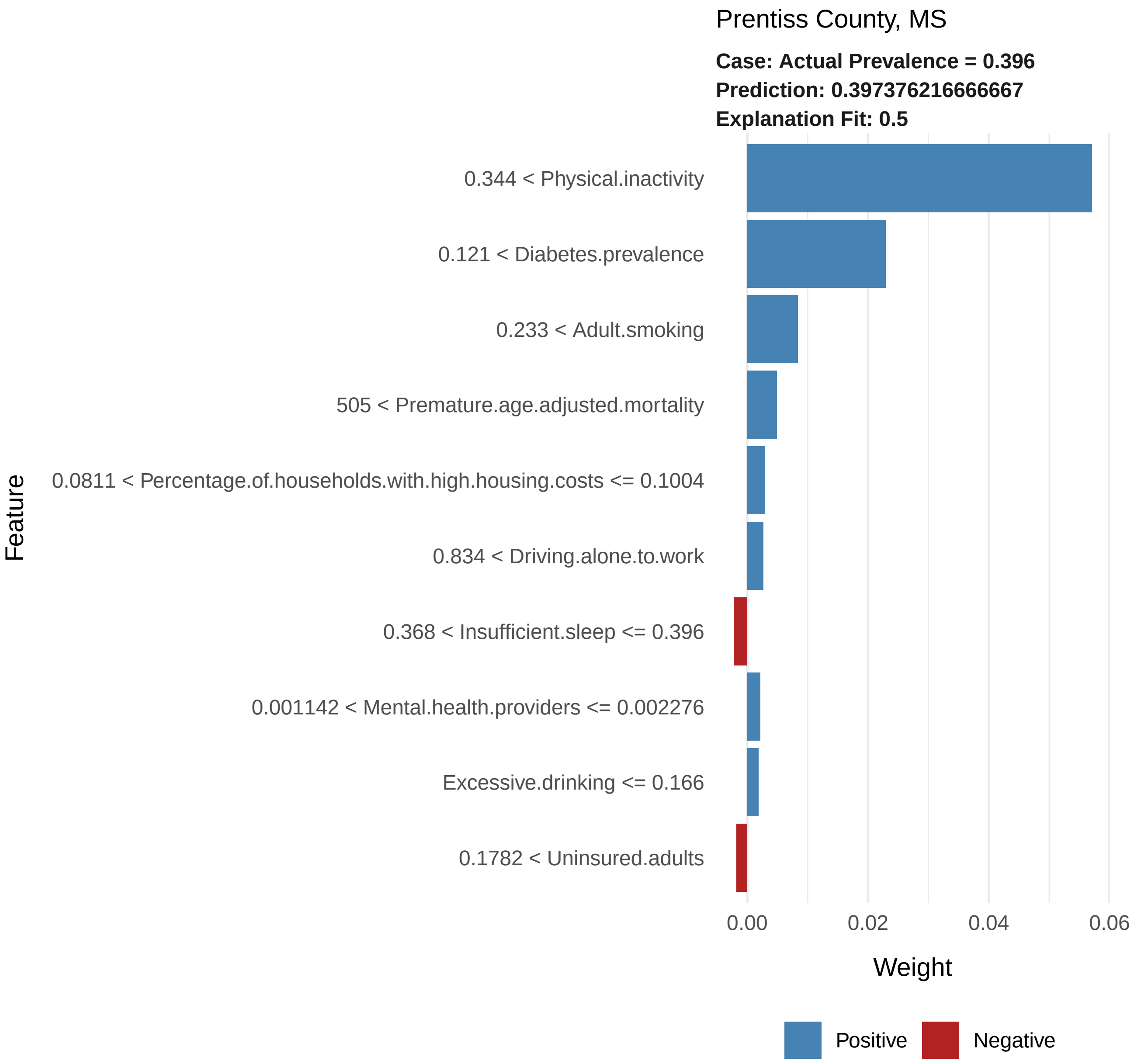
Prentiss County, MS
Case: Actual Prevalence = 0.396
Prediction: 0.397376216666667
Explanation Fit: 0.5
0.344 < Physical.inactivity
0.121 < Diabetes.prevalence
0.233 < Adult.smoking
505 < Premature.age.adjusted.mortality
0.0811 < Percentage.of.households.with.high.housing.costs <= 0.1004
0.834 < Driving.alone.to.work
0.368 < Insufficient.sleep <= 0.396
0.001142 < Mental.health.providers <= 0.002276
Excessive.drinking <= 0.166
0.1782 < Uninsured.adults
Feature
0.00
0.02
0.04
0.06
Weight
Positive
Negative

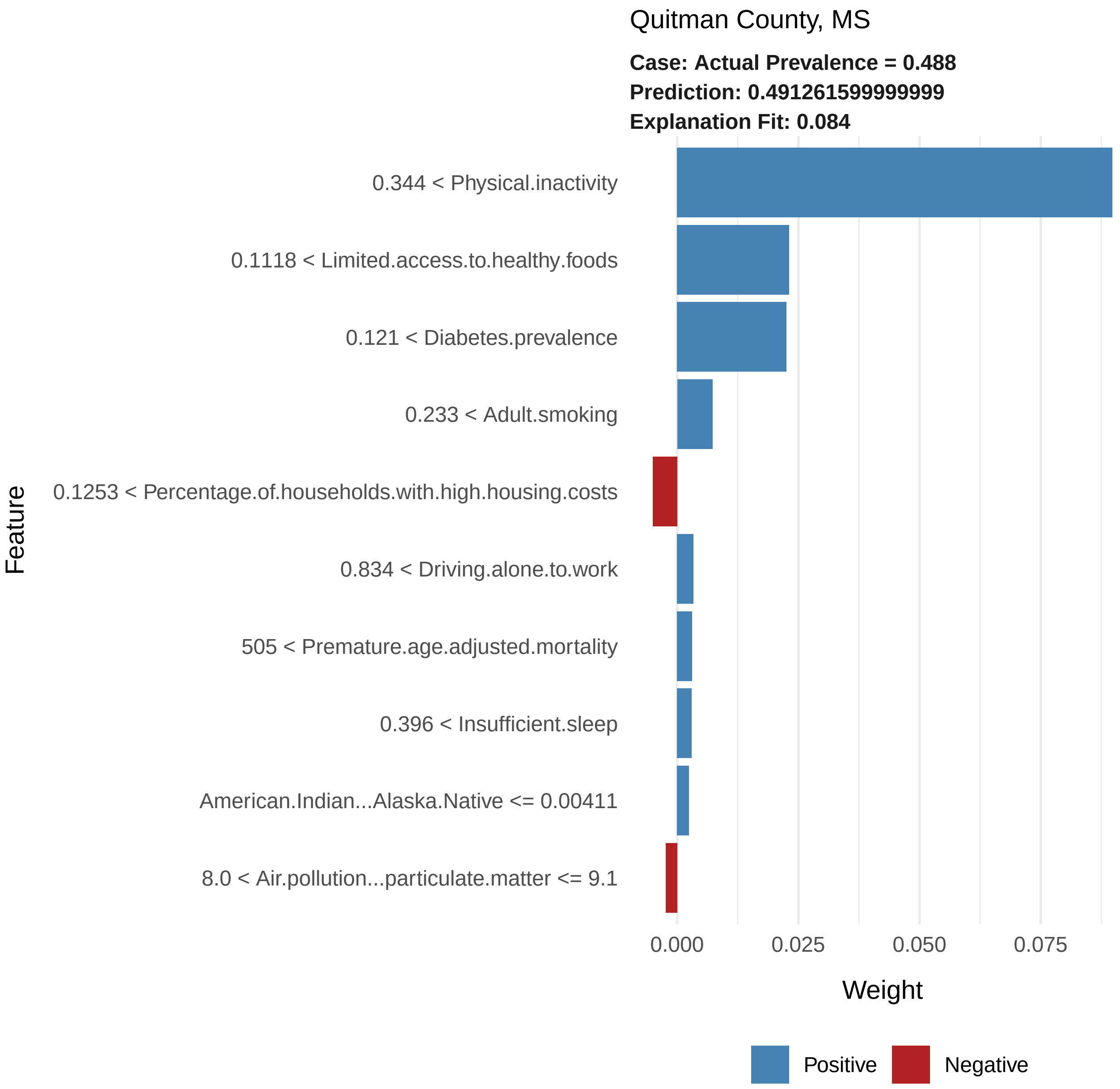

Quitman County, MS
Case: Actual Prevalence = 0.488
Prediction: 0.491261599999999
Explanation Fit: 0.084
0.344 < Physical.inactivity
0.1118 < Limited.access.to.healthy.foods
0.121 < Diabetes.prevalence
0.233 < Adult.smoking
0.1253 < Percentage.of.households.with.high.housing.costs
0.834 < Driving.alone.to.work
505 < Premature.age.adjusted.mortality
0.396 < Insufficient.sleep
American.Indian...Alaska.Native <= 0.00411
8.0 < Air.pollution...particulate.matter <= 9.1
Feature
0.000
0.025
0.050
0.075
Weight
Positive
Negative

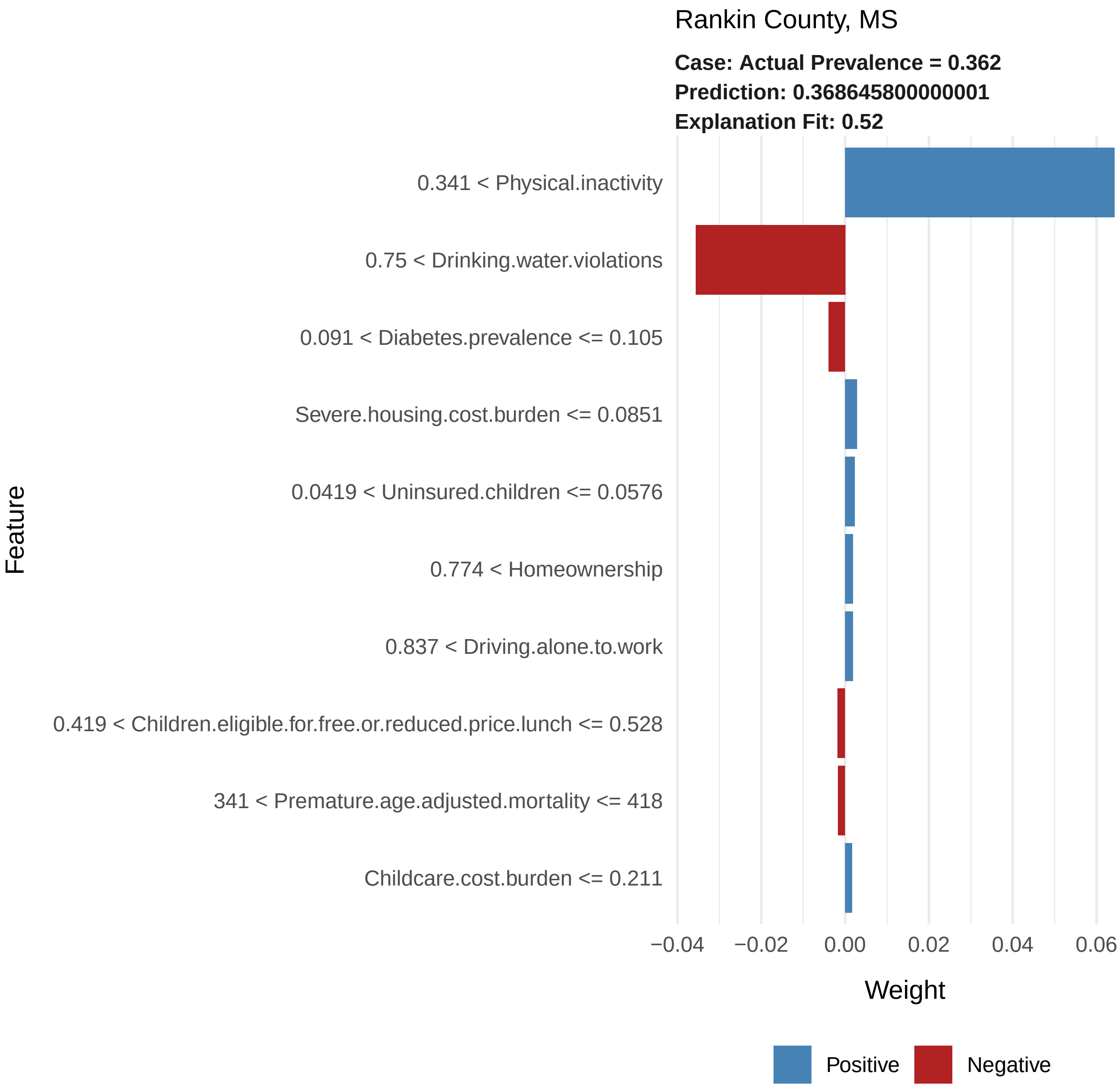

Rankin County, MS
Case: Actual Prevalence = 0.362
Prediction: 0.368645800000001
Explanation Fit: 0.52
0.341 < Physical.inactivity
0.75 < Drinking.water.violations
0.091 < Diabetes.prevalence <= 0.105
Severe.housing.cost.burden <= 0.0851
0.0419 < Uninsured.children <= 0.0576
0.774 < Homeownership
0.837 < Driving.alone.to.work
0.419 < Children.eligible.for.free.or.reduced.price.lunch <= 0.528
341 < Premature.age.adjusted.mortality <= 418
Childcare.cost.burden <= 0.211
Feature
−0.04
−0.02
0.00
0.02
0.04
0.06
Weight
Positive
Negative

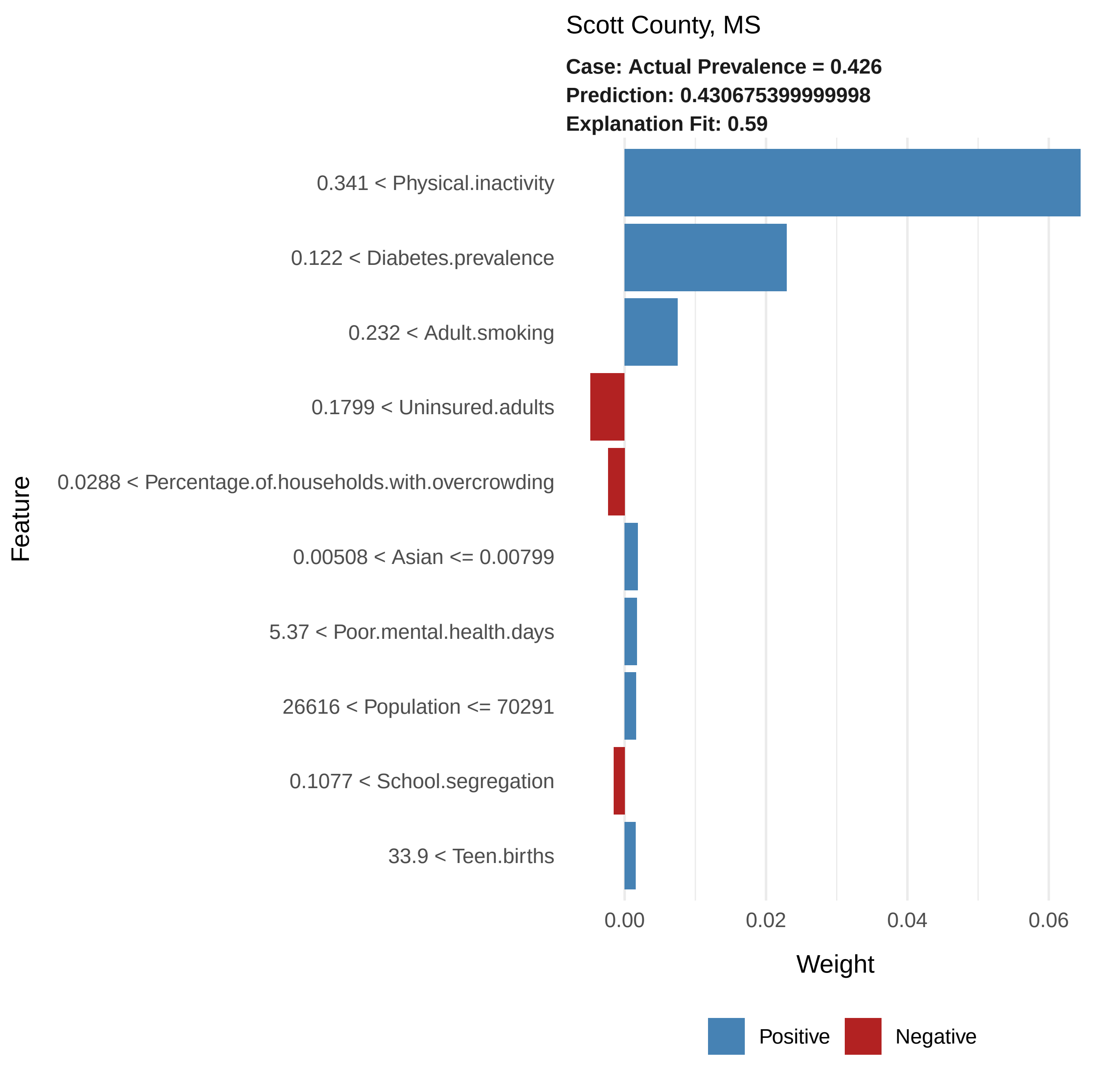
Scott County, MS
Case: Actual Prevalence = 0.426
Prediction: 0.430675399999998
Explanation Fit: 0.59
0.341 < Physical.inactivity
0.122 < Diabetes.prevalence
0.232 < Adult.smoking
0.1799 < Uninsured.adults
0.0288 < Percentage.of.households.with.overcrowding
0.00508 < Asian <= 0.00799
5.37 < Poor.mental.health.days
26616 < Population <= 70291
0.1077 < School.segregation
33.9 < Teen.births
Feature
0.00
0.02
0.04
0.06
Weight
Positive
Negative

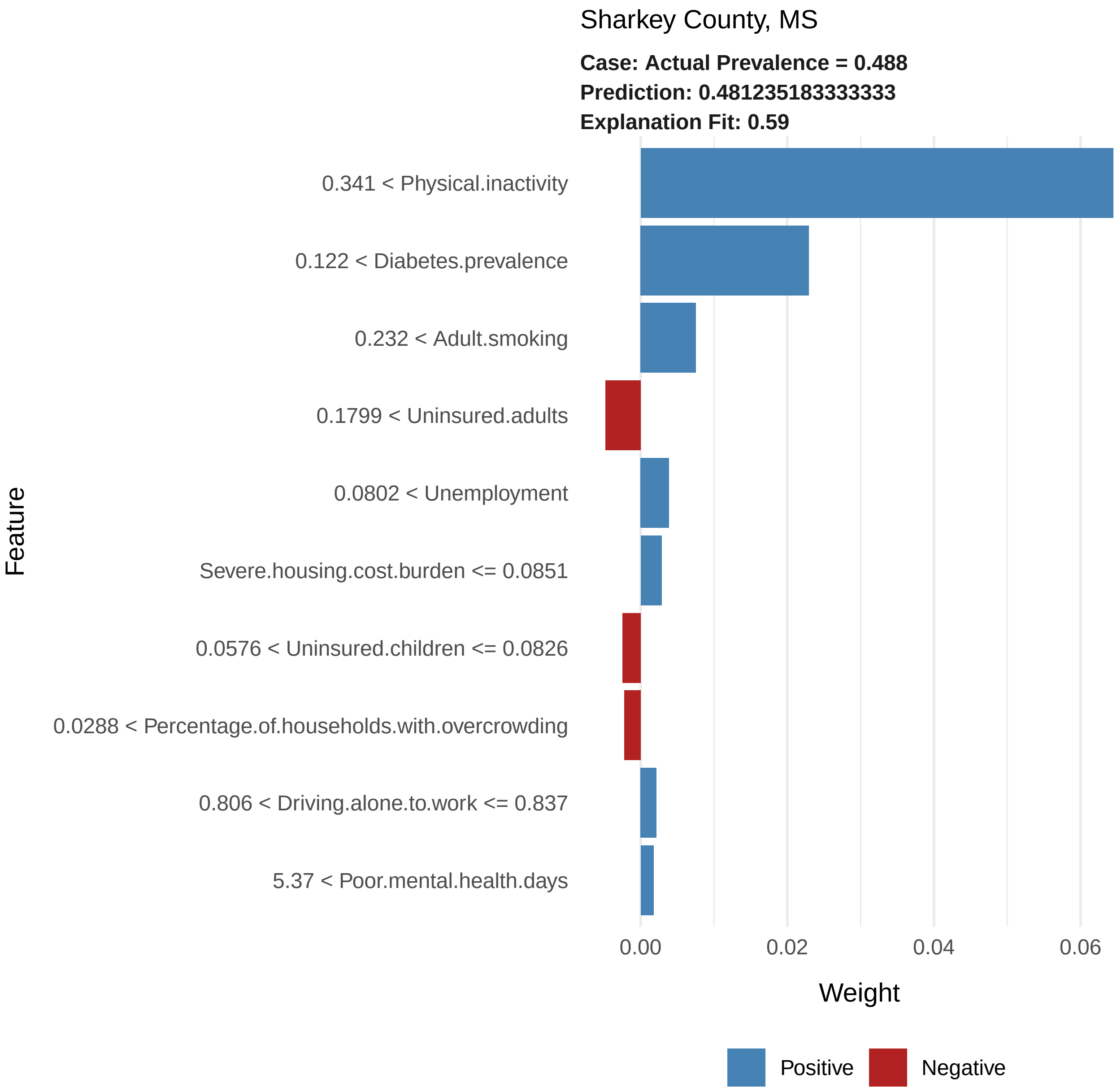
Sharkey County, MS
Case: Actual Prevalence = 0.488
Prediction: 0.481235183333333
Explanation Fit: 0.59
0.341 < Physical.inactivity
0.122 < Diabetes.prevalence
0.232 < Adult.smoking
0.1799 < Uninsured.adults
0.0802 < Unemployment
Severe.housing.cost.burden <= 0.0851
0.0576 < Uninsured.children <= 0.0826
0.0288 < Percentage.of.households.with.overcrowding
0.806 < Driving.alone.to.work <= 0.837
5.37 < Poor.mental.health.days
Feature
0.00
0.02
0.04
0.06
Weight
Positive
Negative

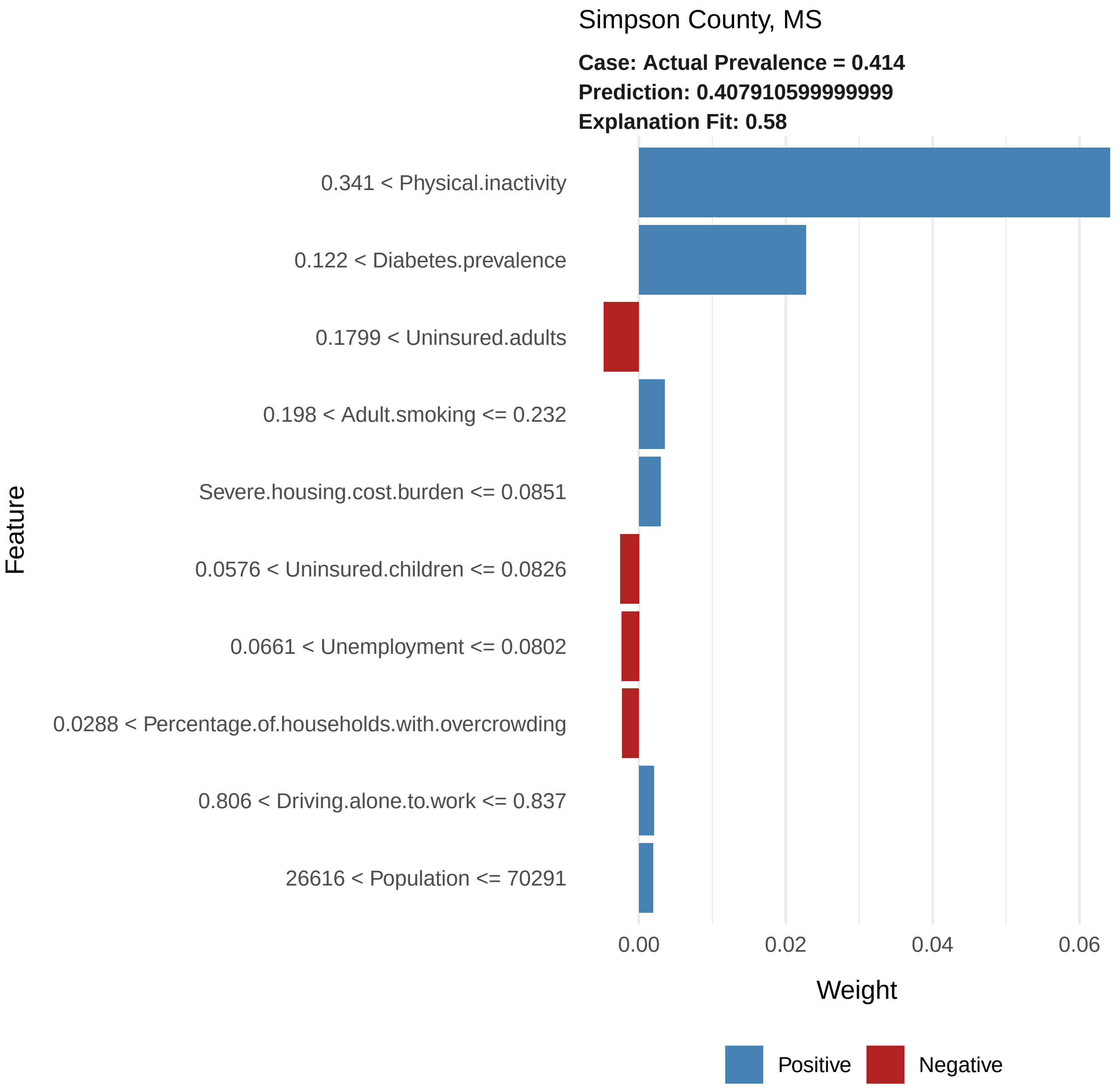

Simpson County, MS
Case: Actual Prevalence = 0.414
Prediction: 0.407910599999999
Explanation Fit: 0.58
0.341 < Physical.inactivity
0.122 < Diabetes.prevalence
0.1799 < Uninsured.adults
0.198 < Adult.smoking <= 0.232
Severe.housing.cost.burden <= 0.0851
0.0576 < Uninsured.children <= 0.0826
0.0661 < Unemployment <= 0.0802
0.0288 < Percentage.of.households.with.overcrowding
0.806 < Driving.alone.to.work <= 0.837
26616 < Population <= 70291
Feature
0.00
0.02
0.04
0.06
Weight
Positive
Negative

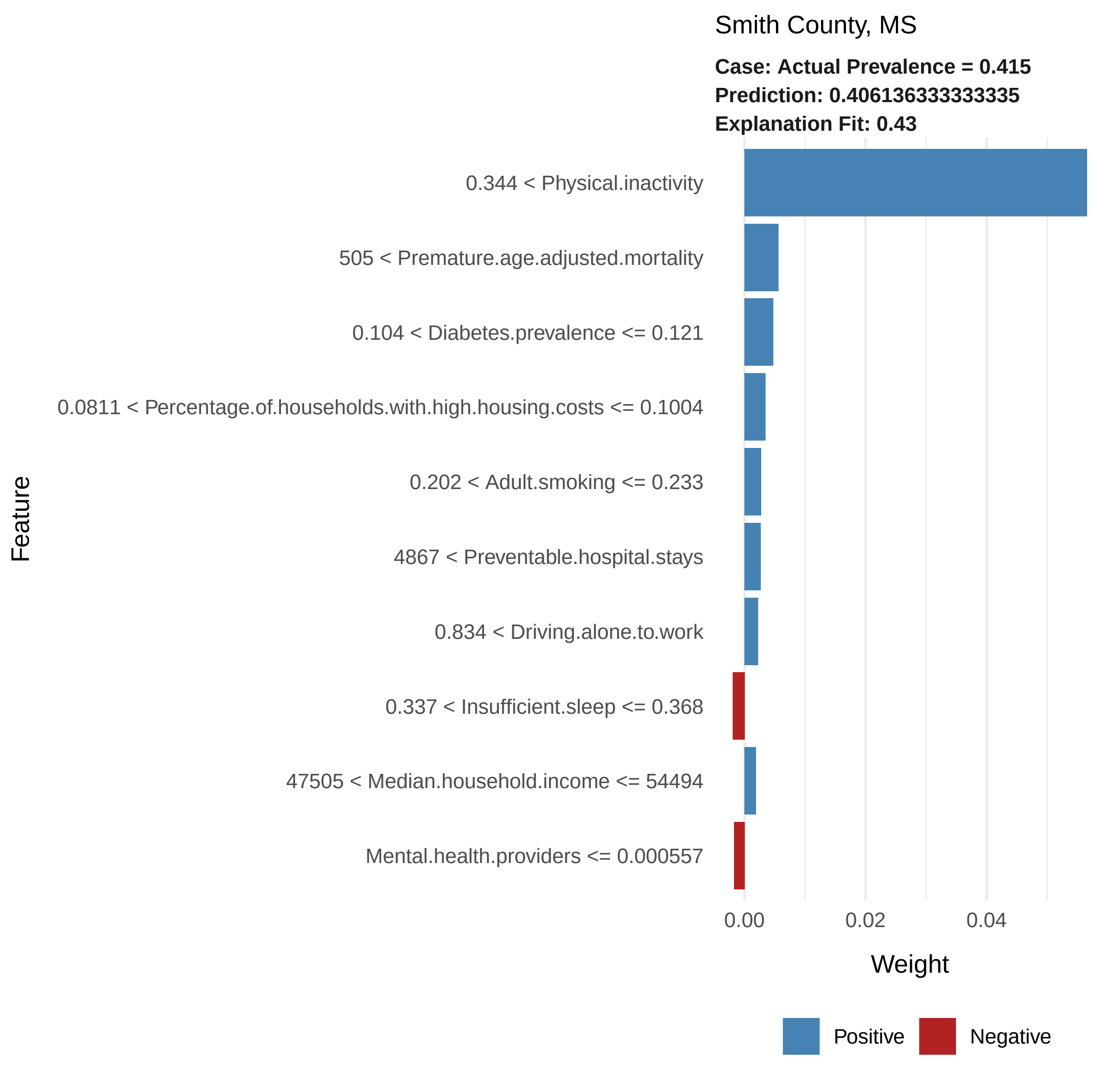

Smith County, MS
Case: Actual Prevalence = 0.415
Prediction: 0.406136333333335
Explanation Fit: 0.43
0.344 < Physical.inactivity
505 < Premature.age.adjusted.mortality
0.104 < Diabetes.prevalence <= 0.121
0.0811 < Percentage.of.households.with.high.housing.costs <= 0.1004
0.202 < Adult.smoking <= 0.233
4867 < Preventable.hospital.stays
0.834 < Driving.alone.to.work
0.337 < Insufficient.sleep <= 0.368
47505 < Median.household.income <= 54494
Mental.health.providers <= 0.000557
Feature
0.00
0.02
0.04
Weight
Positive
Negative

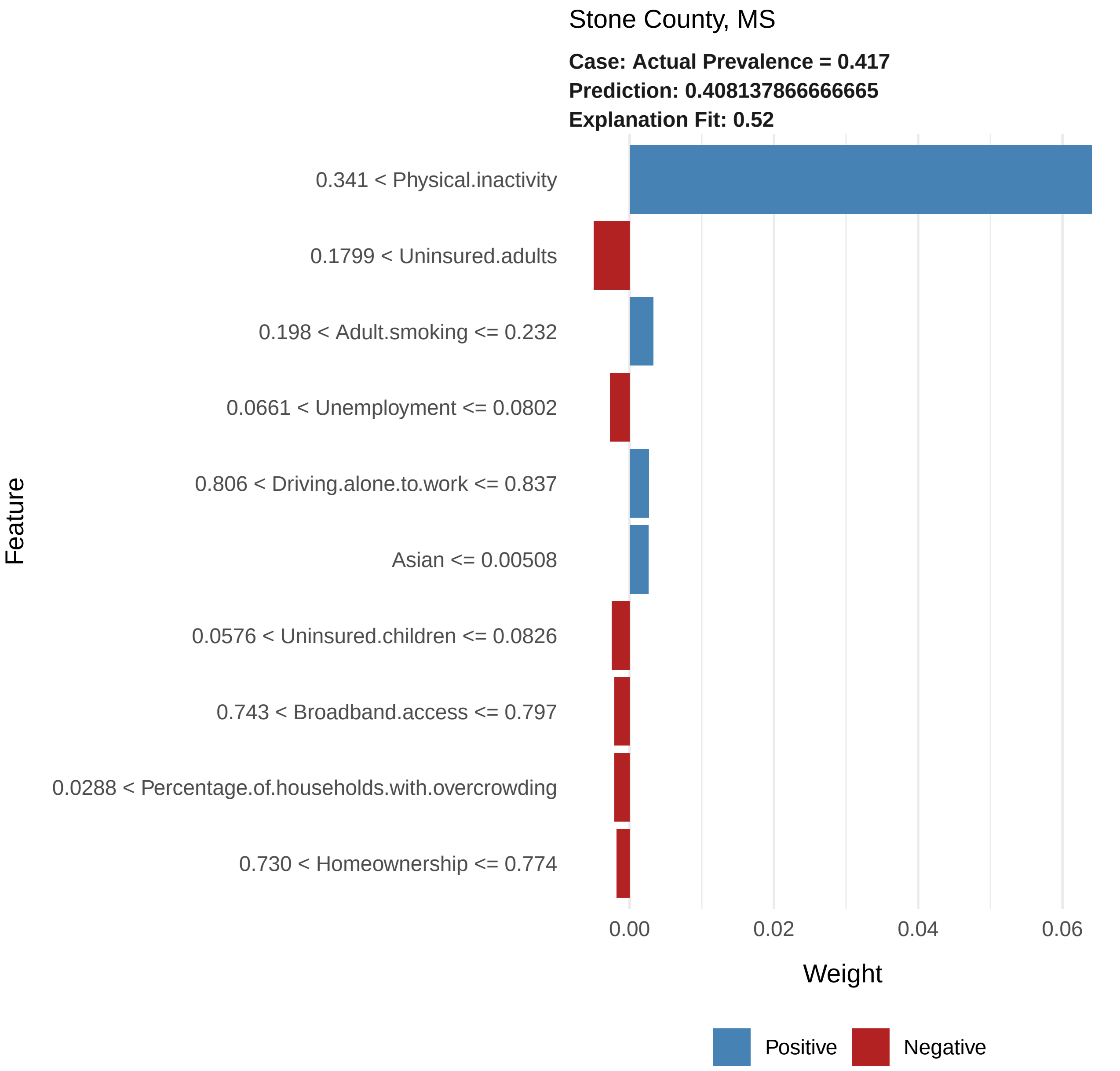

Stone County, MS
Case: Actual Prevalence = 0.417
Prediction: 0.408137866666665
Explanation Fit: 0.52
Feature
0.341 < Physical.inactivity
0.1799 < Uninsured.adults
0.198 < Adult.smoking <= 0.232
0.0661 < Unemployment <= 0.0802
0.806 < Driving.alone.to.work <= 0.837
Asian <= 0.00508
0.0576 < Uninsured.children <= 0.0826
0.743 < Broadband.access <= 0.797
0.0288 < Percentage.of.households.with.overcrowding
0.730 < Homeownership <= 0.774
0.00
0.02
0.04
0.06
Weight
Positive
Negative

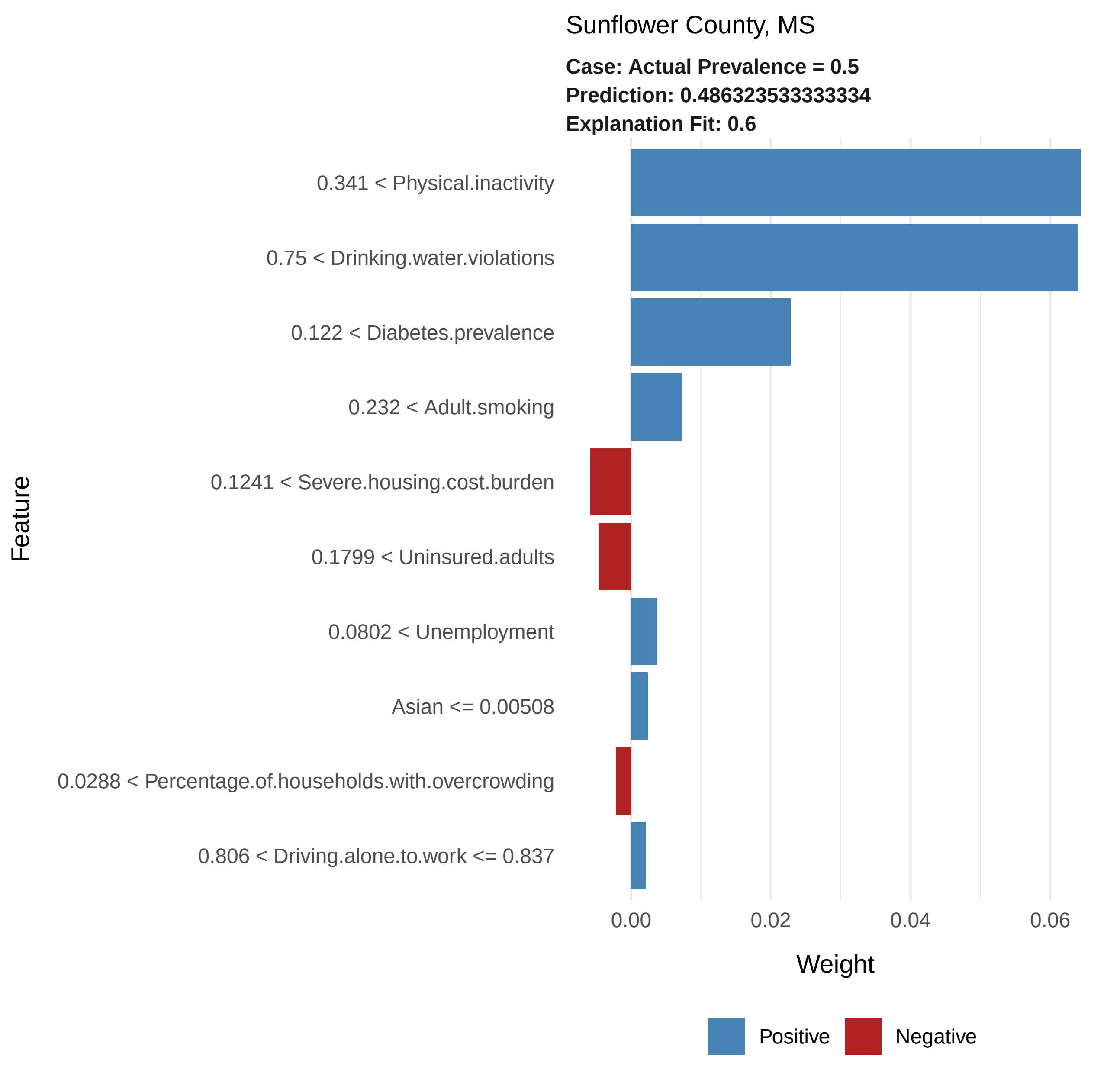
Sunflower County, MS
Case: Actual Prevalence = 0.5
Prediction: 0.486323533333334
Explanation Fit: 0.6
0.341 < Physical.inactivity
0.75 < Drinking.water.violations
0.122 < Diabetes.prevalence
0.232 < Adult.smoking
0.1241 < Severe.housing.cost.burden
0.1799 < Uninsured.adults
0.0802 < Unemployment
Asian <= 0.00508
0.0288 < Percentage.of.households.with.overcrowding
0.806 < Driving.alone.to.work <= 0.837
Feature
0.00
0.02
0.04
0.06
Weight
Positive
Negative

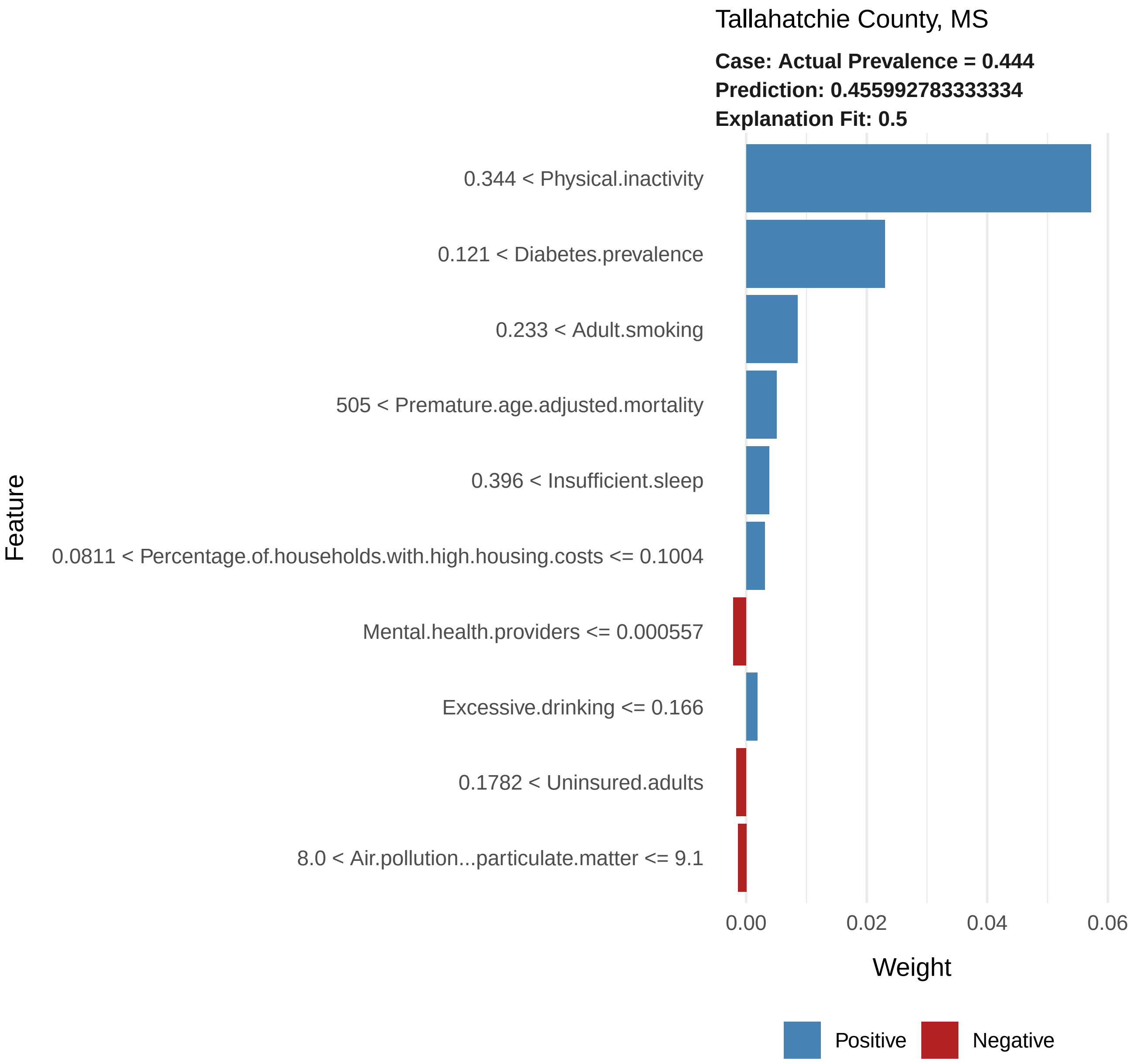
Tallahatchie County, MS
Case: Actual Prevalence = 0.444
Prediction: 0.455992783333334
Explanation Fit: 0.5
0.344 < Physical.inactivity
0.121 < Diabetes.prevalence
0.233 < Adult.smoking
505 < Premature.age.adjusted.mortality
0.396 < Insufficient.sleep
0.0811 < Percentage.of.households.with.high.housing.costs <= 0.1004
Mental.health.providers <= 0.000557
Excessive.drinking <= 0.166
0.1782 < Uninsured.adults
8.0 < Air.pollution...particulate.matter <= 9.1
Feature
0.00
0.02
0.04
0.06
Weight
Positive
Negative

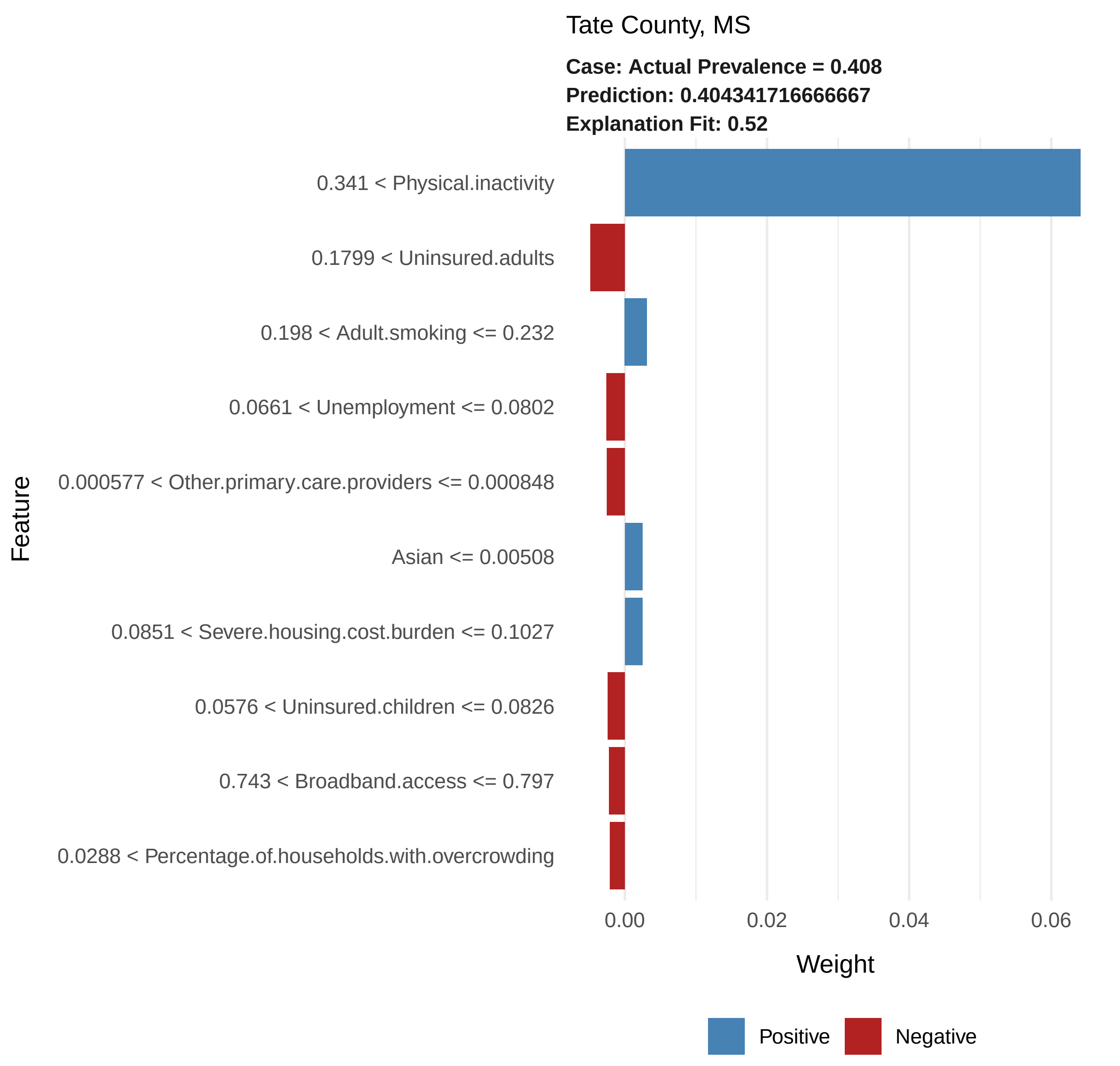

Tate County, MS
Case: Actual Prevalence = 0.408
Prediction: 0.404341716666667
Explanation Fit: 0.52
0.341 < Physical.inactivity
0.1799 < Uninsured.adults
0.198 < Adult.smoking <= 0.232
0.0661 < Unemployment <= 0.0802
0.000577 < Other.primary.care.providers <= 0.000848
Asian <= 0.00508
0.0851 < Severe.housing.cost.burden <= 0.1027
0.0576 < Uninsured.children <= 0.0826
0.743 < Broadband.access <= 0.797
0.0288 < Percentage.of.households.with.overcrowding
Feature
0.00
0.02
0.04
0.06
Weight
Positive
Negative

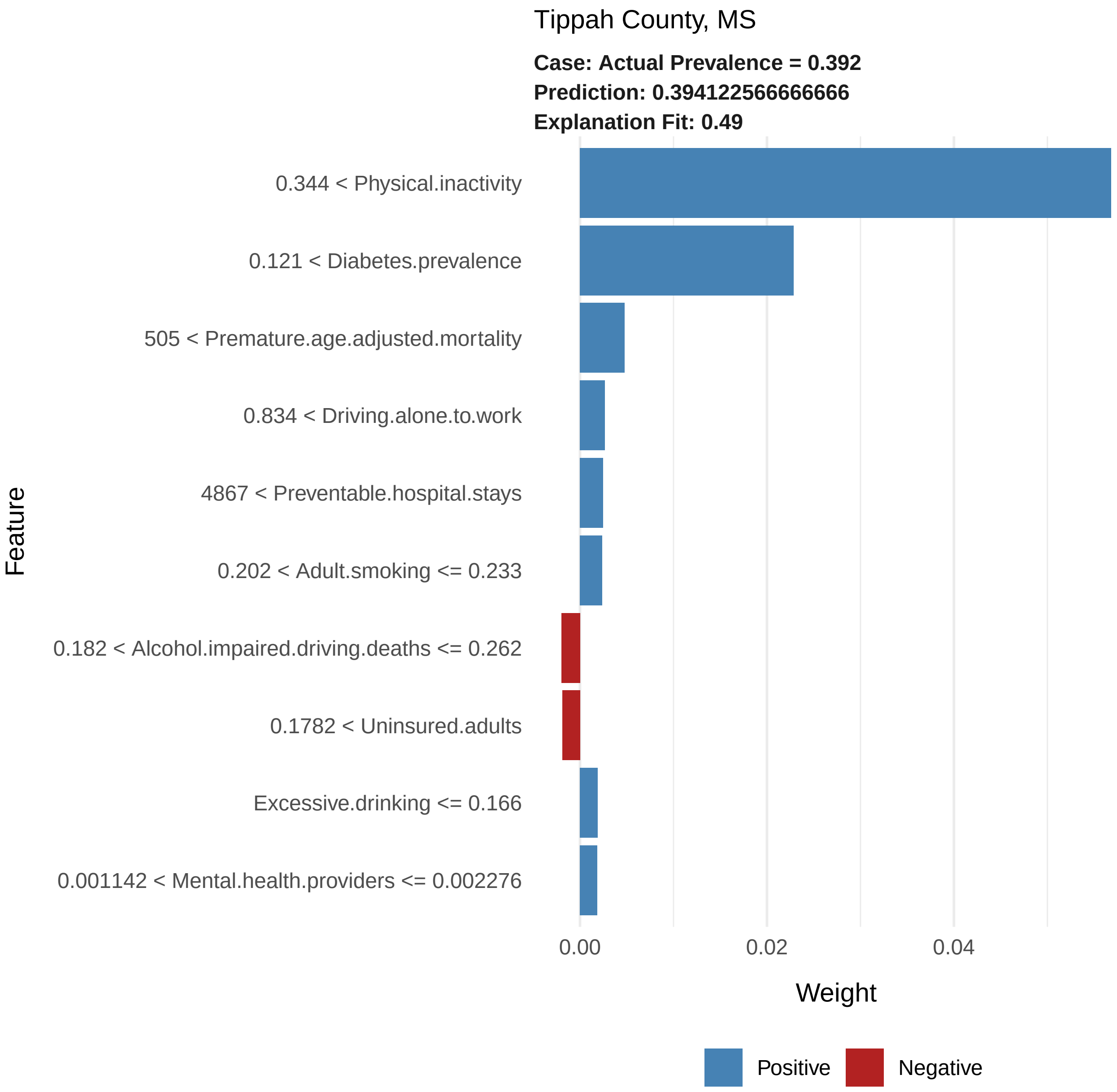

Tippah County, MS
Case: Actual Prevalence = 0.392
Prediction: 0.394122566666666
Explanation Fit: 0.49
0.344 < Physical.inactivity
0.121 < Diabetes.prevalence
505 < Premature.age.adjusted.mortality
0.834 < Driving.alone.to.work
4867 < Preventable.hospital.stays
0.202 < Adult.smoking <= 0.233
0.182 < Alcohol.impaired.driving.deaths <= 0.262
0.1782 < Uninsured.adults
Excessive.drinking <= 0.166
0.001142 < Mental.health.providers <= 0.002276
Feature
0.00
0.02
0.04
Weight
Positive
Negative

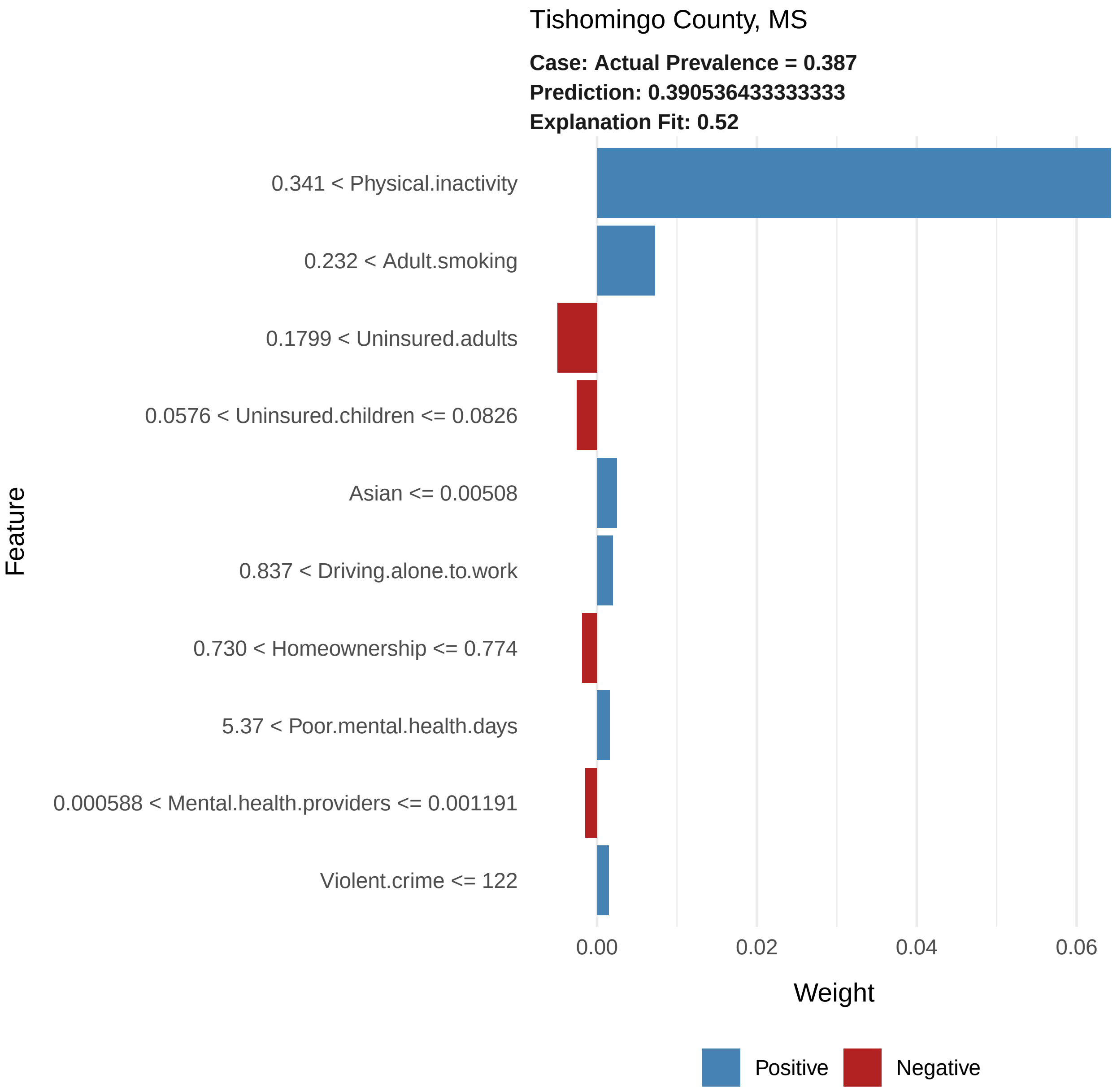
Tishomingo County, MS
Case: Actual Prevalence = 0.387
Prediction: 0.390536433333333
Explanation Fit: 0.52
0.341 < Physical.inactivity
0.232 < Adult.smoking
0.1799 < Uninsured.adults
0.0576 < Uninsured.children <= 0.0826
Asian <= 0.00508
0.837 < Driving.alone.to.work
0.730 < Homeownership <= 0.774
5.37 < Poor.mental.health.days
0.000588 < Mental.health.providers <= 0.001191
Violent.crime <= 122
Feature
0.00
0.02
0.04
0.06
Weight
Positive
Negative

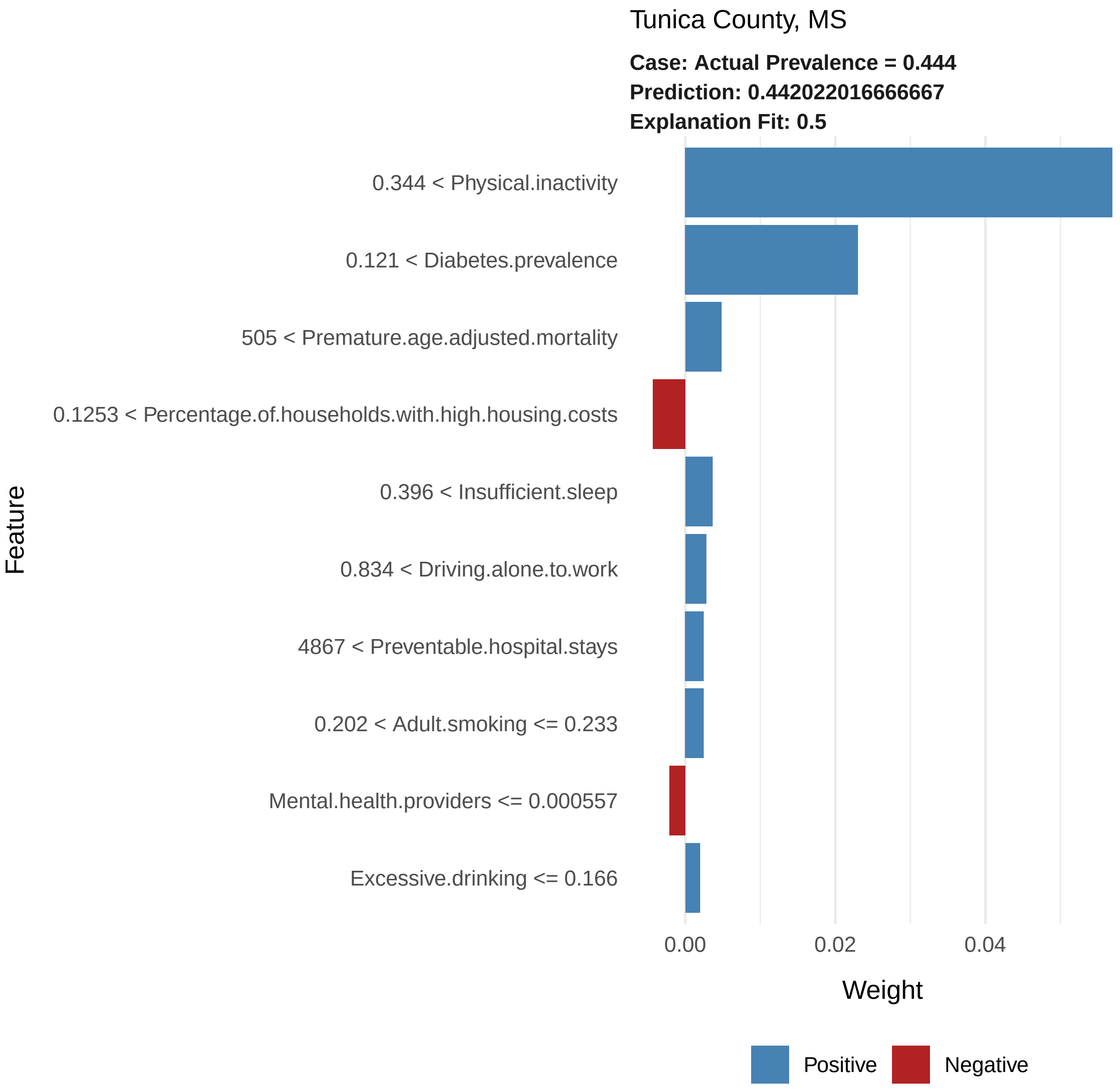
Tunica County, MS
Case: Actual Prevalence = 0.444
Prediction: 0.442022016666667
Explanation Fit: 0.5
0.344 < Physical.inactivity
0.121 < Diabetes.prevalence
505 < Premature.age.adjusted.mortality
0.1253 < Percentage.of.households.with.high.housing.costs
0.396 < Insufficient.sleep
0.834 < Driving.alone.to.work
4867 < Preventable.hospital.stays
0.202 < Adult.smoking <= 0.233
Mental.health.providers <= 0.000557
Excessive.drinking <= 0.166
Feature
0.00
0.02
0.04
Weight
Positive
Negative

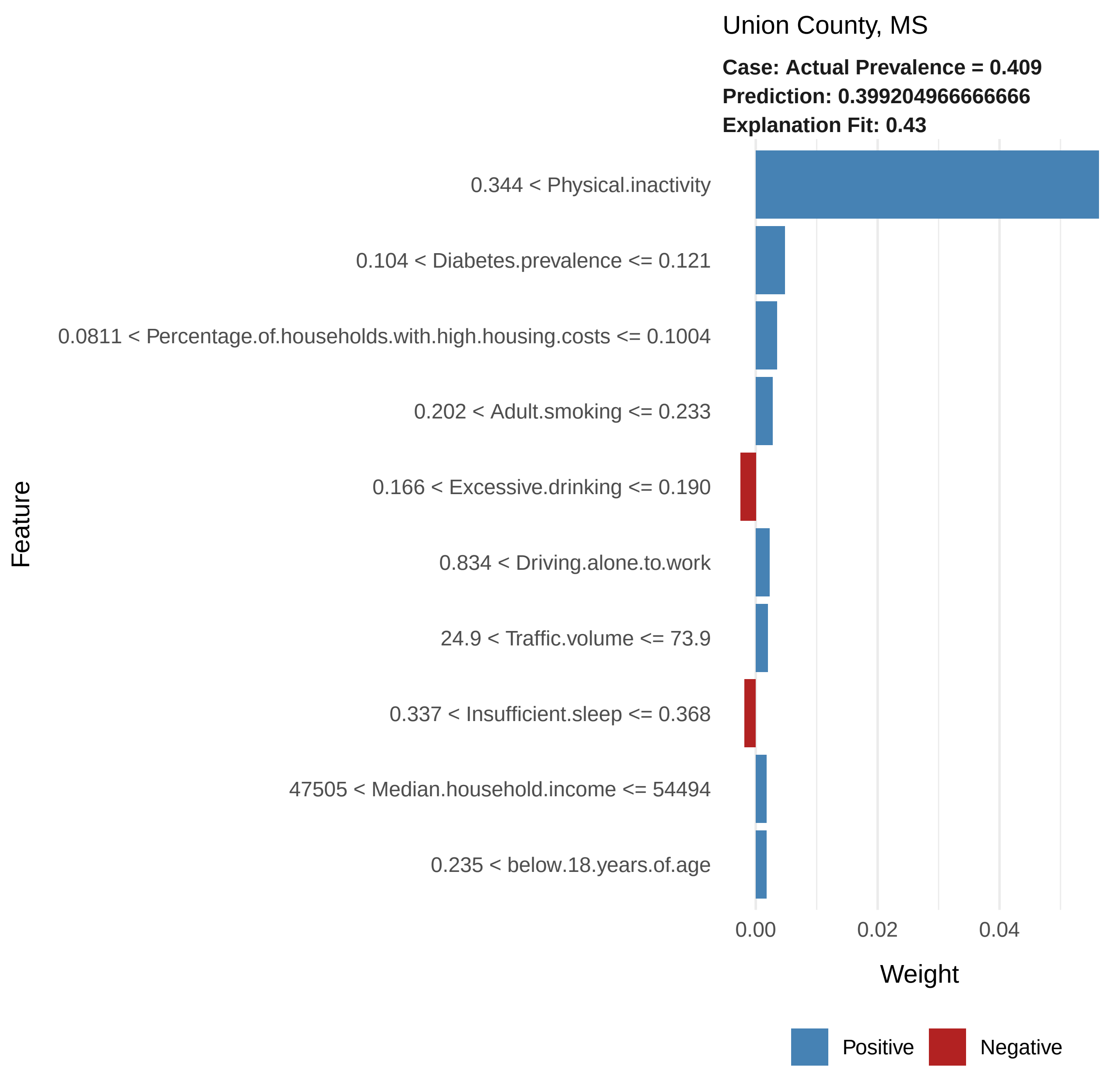

Union County, MS
Case: Actual Prevalence = 0.409
Prediction: 0.399204966666666
Explanation Fit: 0.43
0.344 < Physical.inactivity
0.104 < Diabetes.prevalence <= 0.121
0.0811 < Percentage.of.households.with.high.housing.costs <= 0.1004
0.202 < Adult.smoking <= 0.233
0.166 < Excessive.drinking <= 0.190
0.834 < Driving.alone.to.work
24.9 < Traffic.volume <= 73.9
0.337 < Insufficient.sleep <= 0.368
47505 < Median.household.income <= 54494
0.235 < below.18.years.of.age
Feature
0.00
0.02
0.04
Weight
Positive
Negative

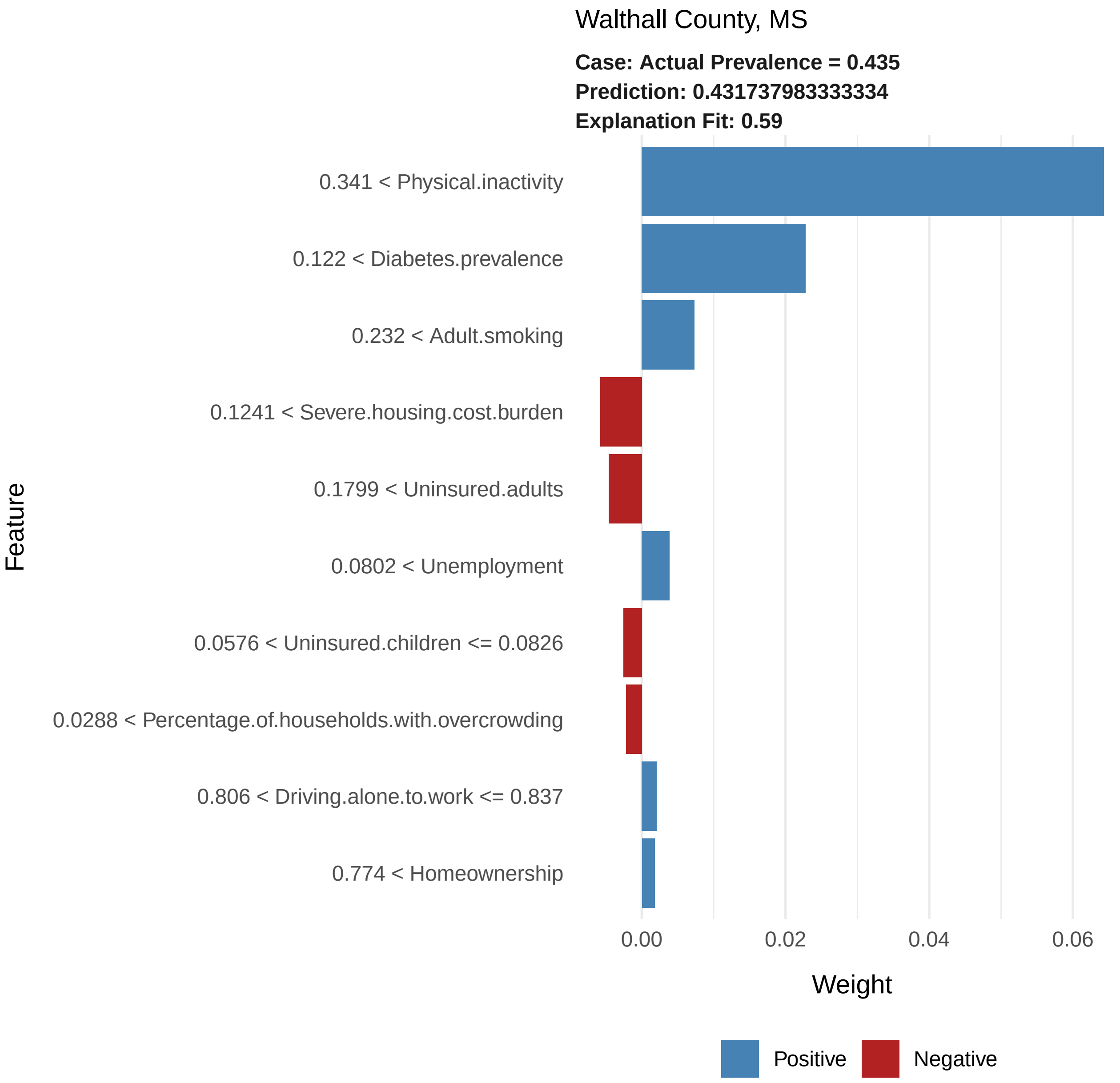

Walthall County, MS
Case: Actual Prevalence = 0.435
Prediction: 0.431737983333334
Explanation Fit: 0.59
0.341 < Physical.inactivity
0.122 < Diabetes.prevalence
0.232 < Adult.smoking
0.1241 < Severe.housing.cost.burden
0.1799 < Uninsured.adults
0.0802 < Unemployment
0.0576 < Uninsured.children <= 0.0826
0.0288 < Percentage.of.households.with.overcrowding
0.806 < Driving.alone.to.work <= 0.837
0.774 < Homeownership
Feature
0.00
0.02
0.04
0.06
Weight
Positive
Negative

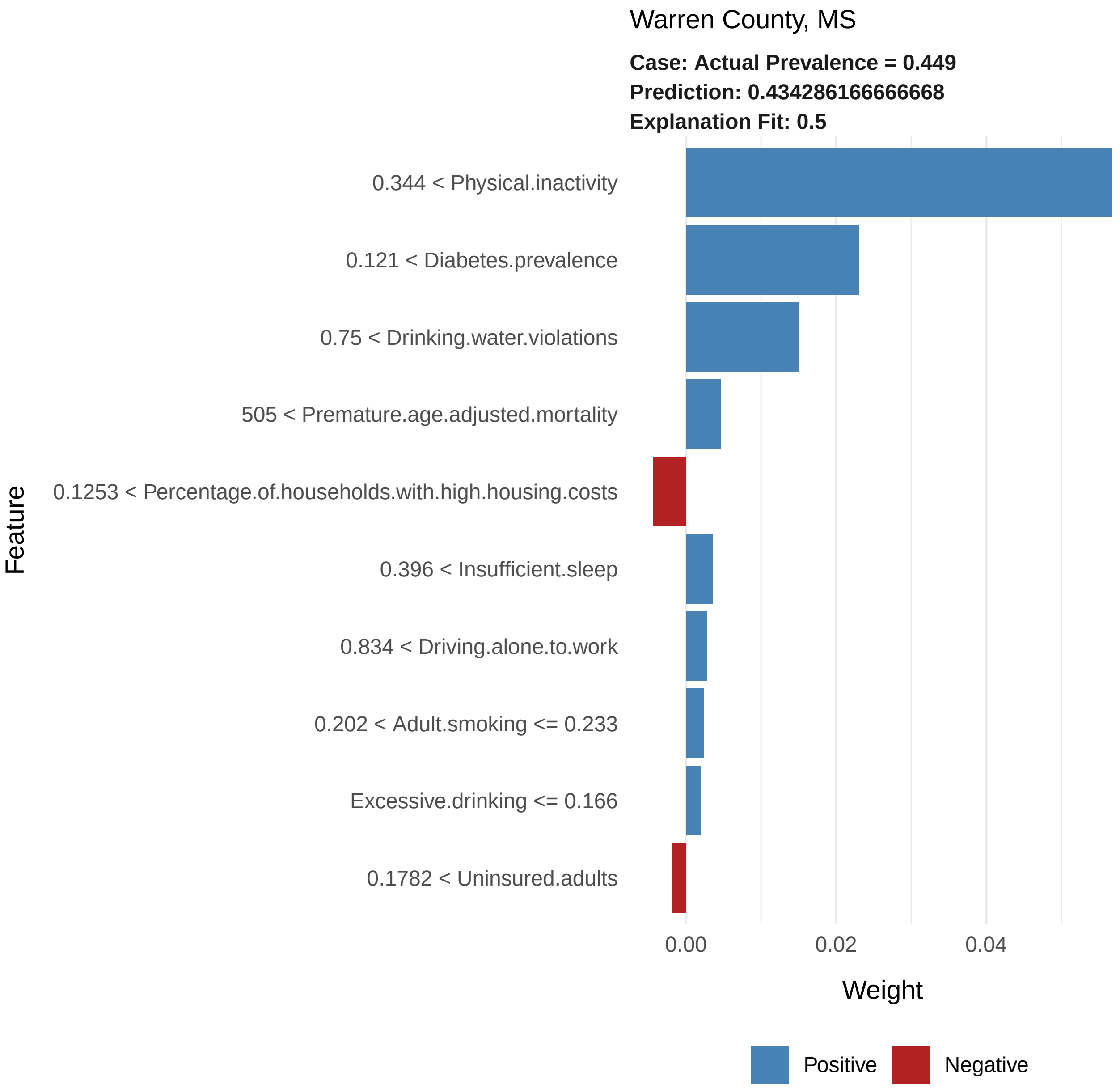

Warren County, MS
Case: Actual Prevalence = 0.449
Prediction: 0.434286166666668
Explanation Fit: 0.5
0.344 < Physical.inactivity
0.121 < Diabetes.prevalence
0.75 < Drinking.water.violations
505 < Premature.age.adjusted.mortality
0.1253 < Percentage.of.households.with.high.housing.costs
0.396 < Insufficient.sleep
0.834 < Driving.alone.to.work
0.202 < Adult.smoking <= 0.233
Excessive.drinking <= 0.166
0.1782 < Uninsured.adults
Feature
0.00
0.02
0.04
Weight
Positive
Negative

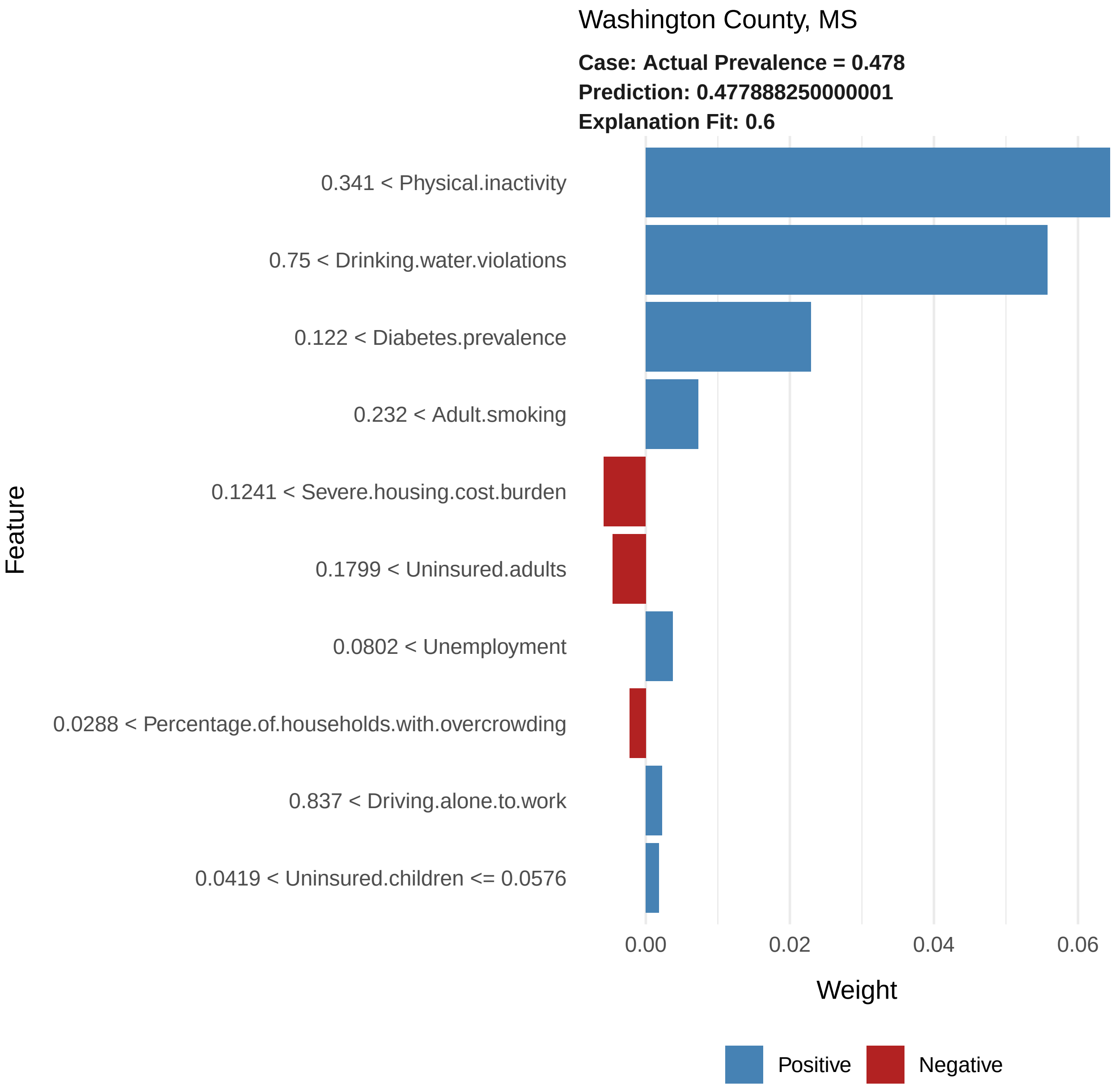
Washington County, MS
Case: Actual Prevalence = 0.478
Prediction: 0.477888250000001
Explanation Fit: 0.6
0.341 < Physical.inactivity
0.75 < Drinking.water.violations
0.122 < Diabetes.prevalence
0.232 < Adult.smoking
0.1241 < Severe.housing.cost.burden
0.1799 < Uninsured.adults
0.0802 < Unemployment
0.0288 < Percentage.of.households.with.overcrowding
0.837 < Driving.alone.to.work
0.0419 < Uninsured.children <= 0.0576
Feature
0.00
0.02
0.04
0.06
Weight
Positive
Negative

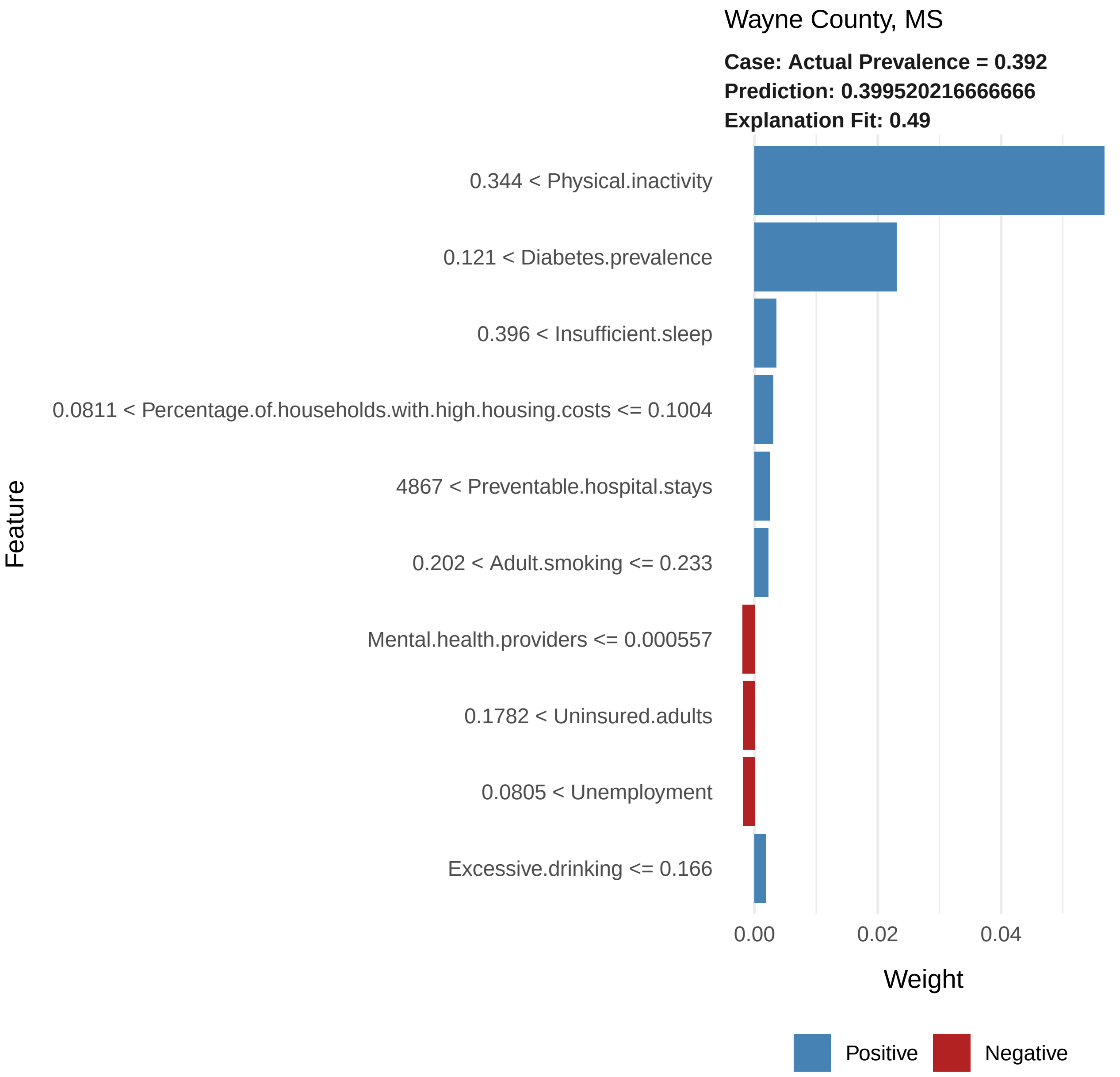

Wayne County, MS
Case: Actual Prevalence = 0.392
Prediction: 0.399520216666666
Explanation Fit: 0.49
0.344 < Physical.inactivity
0.121 < Diabetes.prevalence
0.396 < Insufficient.sleep
0.0811 < Percentage.of.households.with.high.housing.costs <= 0.1004
4867 < Preventable.hospital.stays
0.202 < Adult.smoking <= 0.233
Mental.health.providers <= 0.000557
0.1782 < Uninsured.adults
0.0805 < Unemployment
Excessive.drinking <= 0.166
Feature
0.00
0.02
0.04
Weight
Positive
Negative

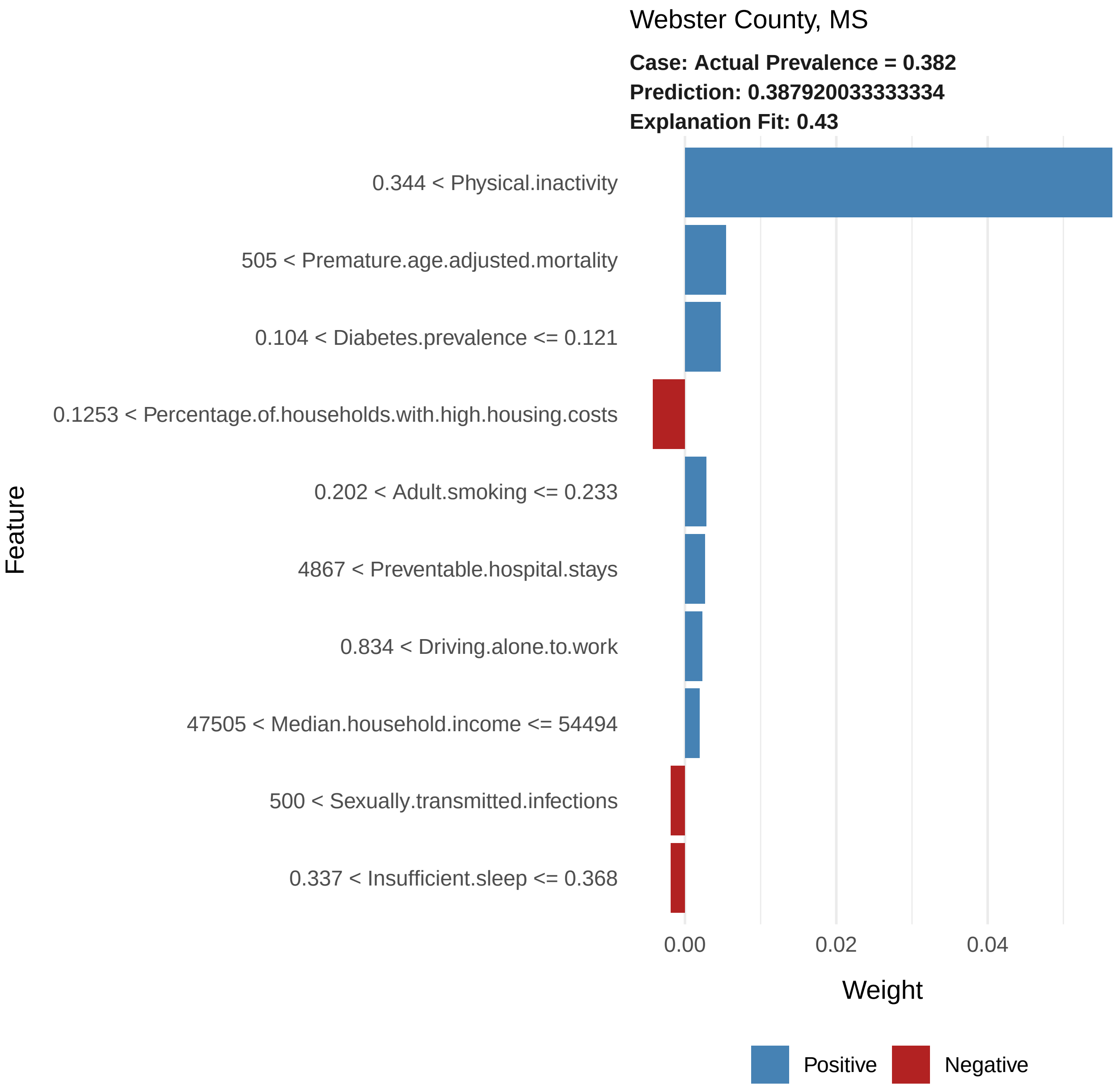
Webster County, MS
Case: Actual Prevalence = 0.382
Prediction: 0.387920033333334
Explanation Fit: 0.43
0.344 < Physical.inactivity
505 < Premature.age.adjusted.mortality
0.104 < Diabetes.prevalence <= 0.121
0.1253 < Percentage.of.households.with.high.housing.costs
0.202 < Adult.smoking <= 0.233
4867 < Preventable.hospital.stays
0.834 < Driving.alone.to.work
47505 < Median.household.income <= 54494
500 < Sexually.transmitted.infections
0.337 < Insufficient.sleep <= 0.368
Feature
0.00
0.02
0.04
Weight
Positive
Negative

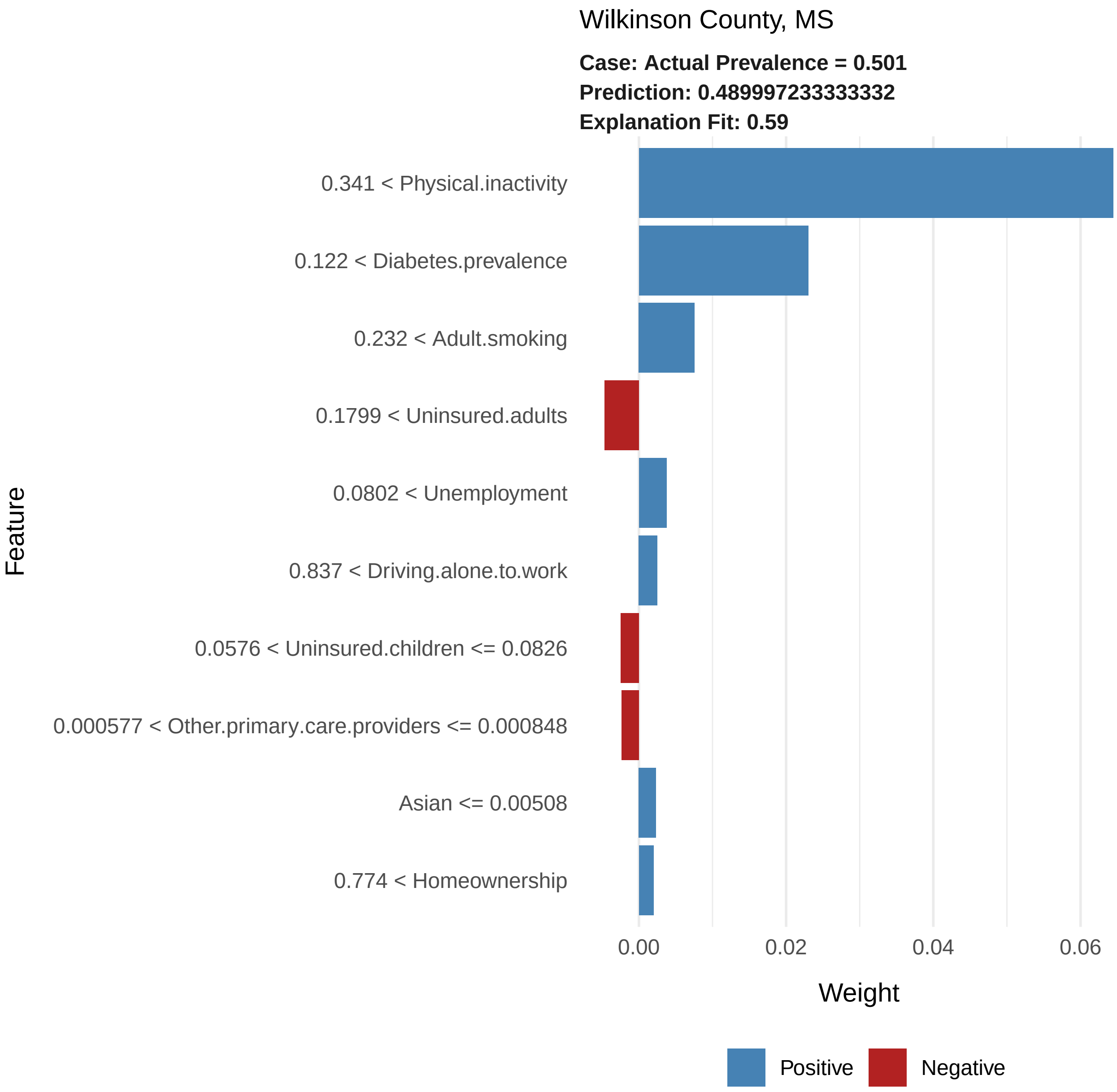

Wilkinson County, MS
Case: Actual Prevalence = 0.501
Prediction: 0.489997233333332
Explanation Fit: 0.59
0.341 < Physical.inactivity
0.122 < Diabetes.prevalence
0.232 < Adult.smoking
0.1799 < Uninsured.adults
0.0802 < Unemployment
0.837 < Driving.alone.to.work
0.0576 < Uninsured.children <= 0.0826
0.000577 < Other.primary.care.providers <= 0.000848
Asian <= 0.00508
0.774 < Homeownership
Feature
0.00
0.02
0.04
0.06
Weight
Positive
Negative

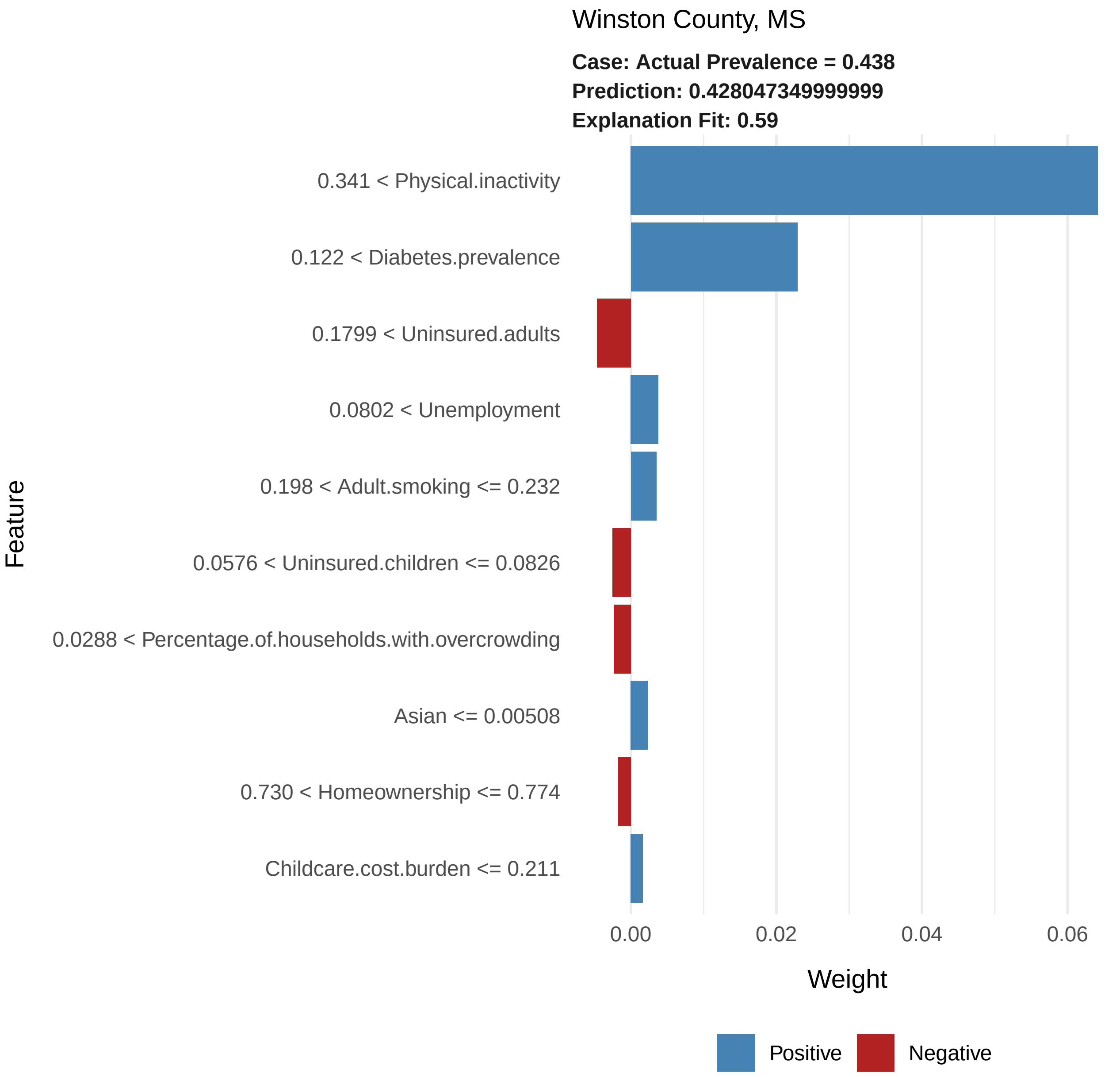

Winston County, MS
Case: Actual Prevalence = 0.438
Prediction: 0.428047349999999
Explanation Fit: 0.59
0.341 < Physical.inactivity
0.122 < Diabetes.prevalence
0.1799 < Uninsured.adults
0.0802 < Unemployment
0.198 < Adult.smoking <= 0.232
0.0576 < Uninsured.children <= 0.0826
0.0288 < Percentage.of.households.with.overcrowding
Asian <= 0.00508
0.730 < Homeownership <= 0.774
Childcare.cost.burden <= 0.211
Feature
0.00
0.02
0.04
0.06
Weight
Positive
Negative

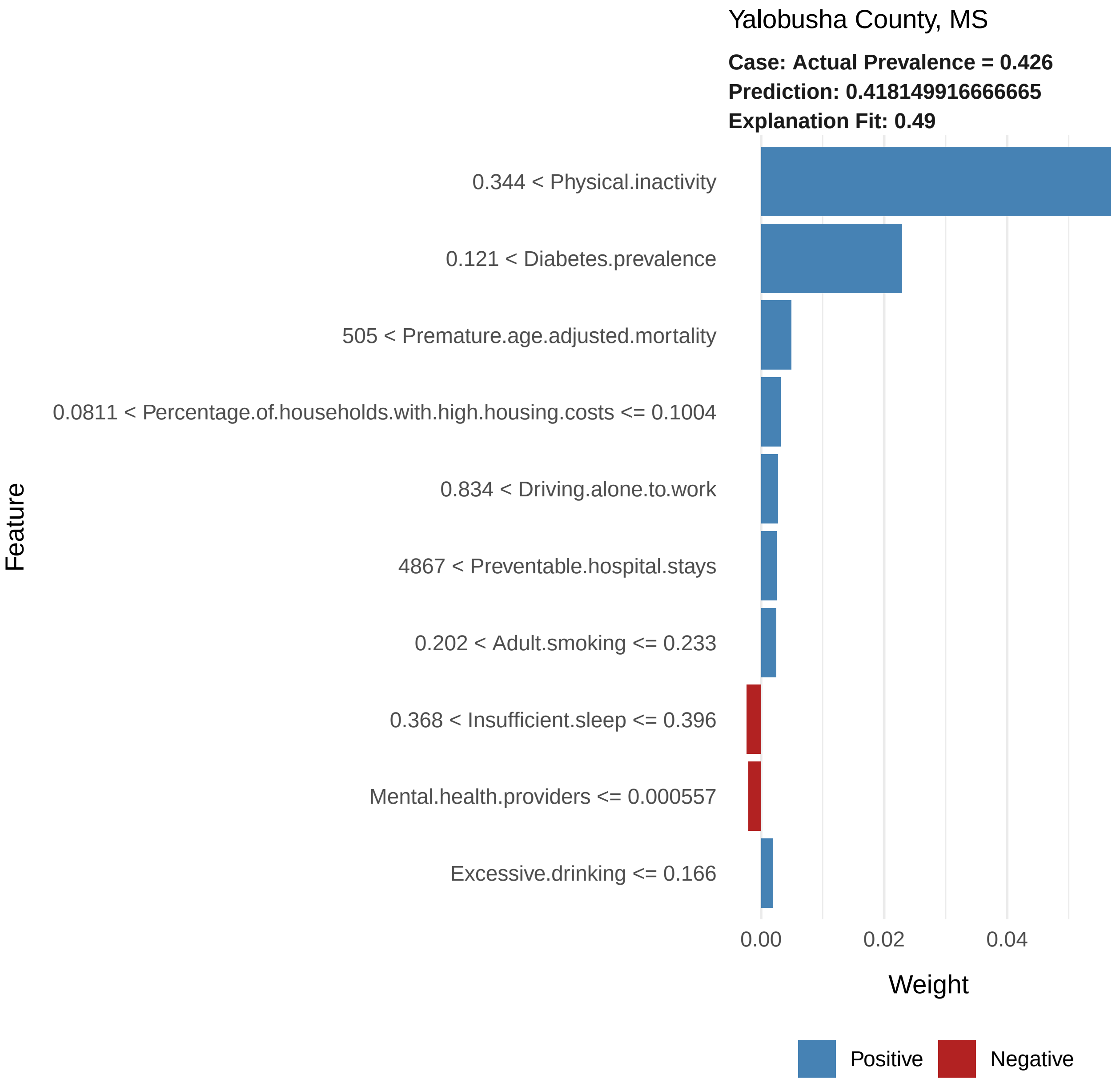

Yalobusha County, MS
Case: Actual Prevalence = 0.426
Prediction: 0.418149916666665
Explanation Fit: 0.49
0.344 < Physical.inactivity
0.121 < Diabetes.prevalence
505 < Premature.age.adjusted.mortality
0.0811 < Percentage.of.households.with.high.housing.costs <= 0.1004
0.834 < Driving.alone.to.work
4867 < Preventable.hospital.stays
0.202 < Adult.smoking <= 0.233
0.368 < Insufficient.sleep <= 0.396
Mental.health.providers <= 0.000557
Excessive.drinking <= 0.166
Feature
0.00
0.02
0.04
Weight
Positive
Negative

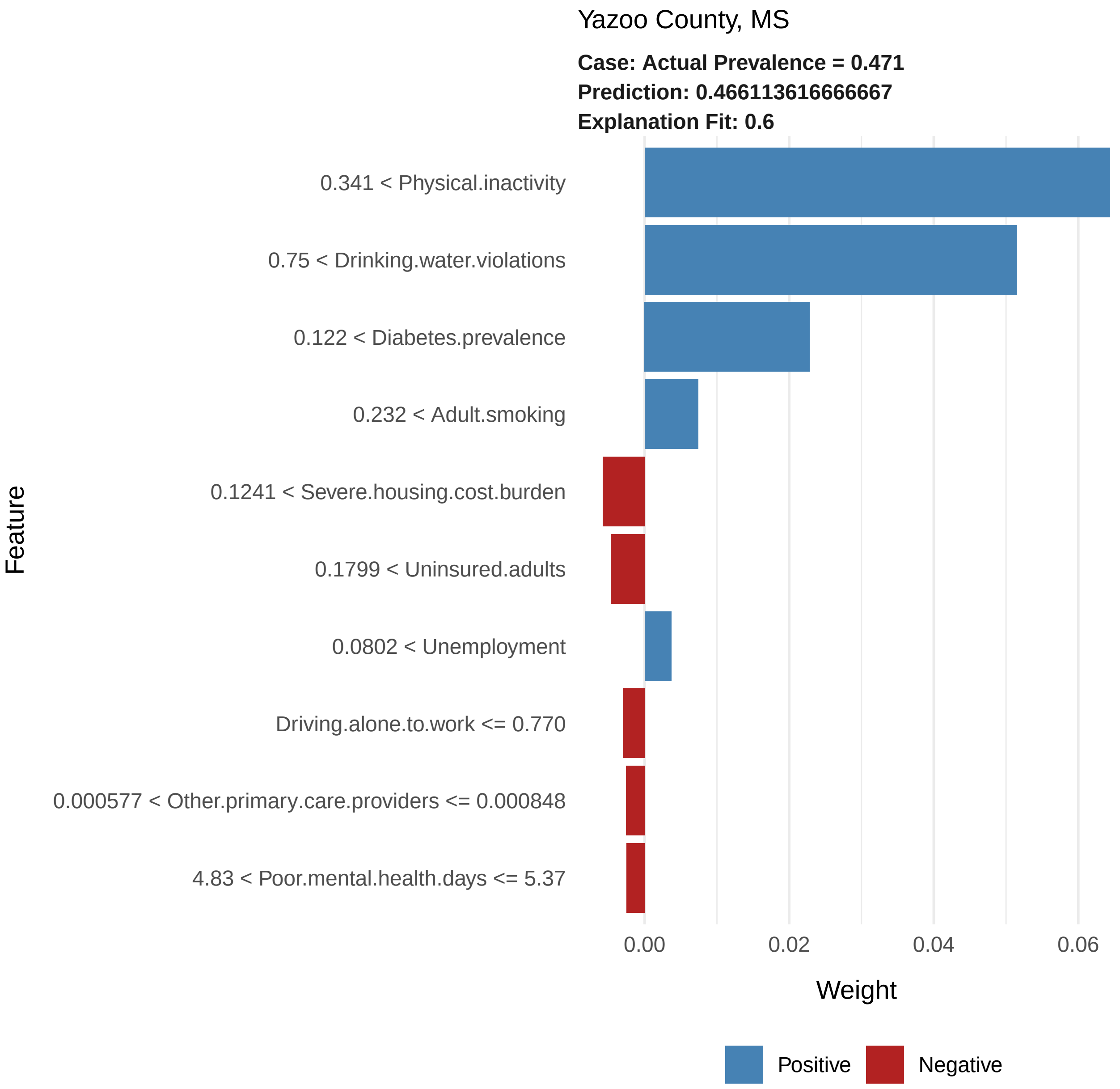

Yazoo County, MS
Case: Actual Prevalence = 0.471
Prediction: 0.466113616666667
Explanation Fit: 0.6
0.341 < Physical.inactivity
0.75 < Drinking.water.violations
0.122 < Diabetes.prevalence
0.232 < Adult.smoking
0.1241 < Severe.housing.cost.burden
0.1799 < Uninsured.adults
0.0802 < Unemployment
Driving.alone.to.work <= 0.770
0.000577 < Other.primary.care.providers <= 0.000848
4.83 < Poor.mental.health.days <= 5.37
Feature
0.00
0.02
0.04
0.06
Weight
Positive
Negative

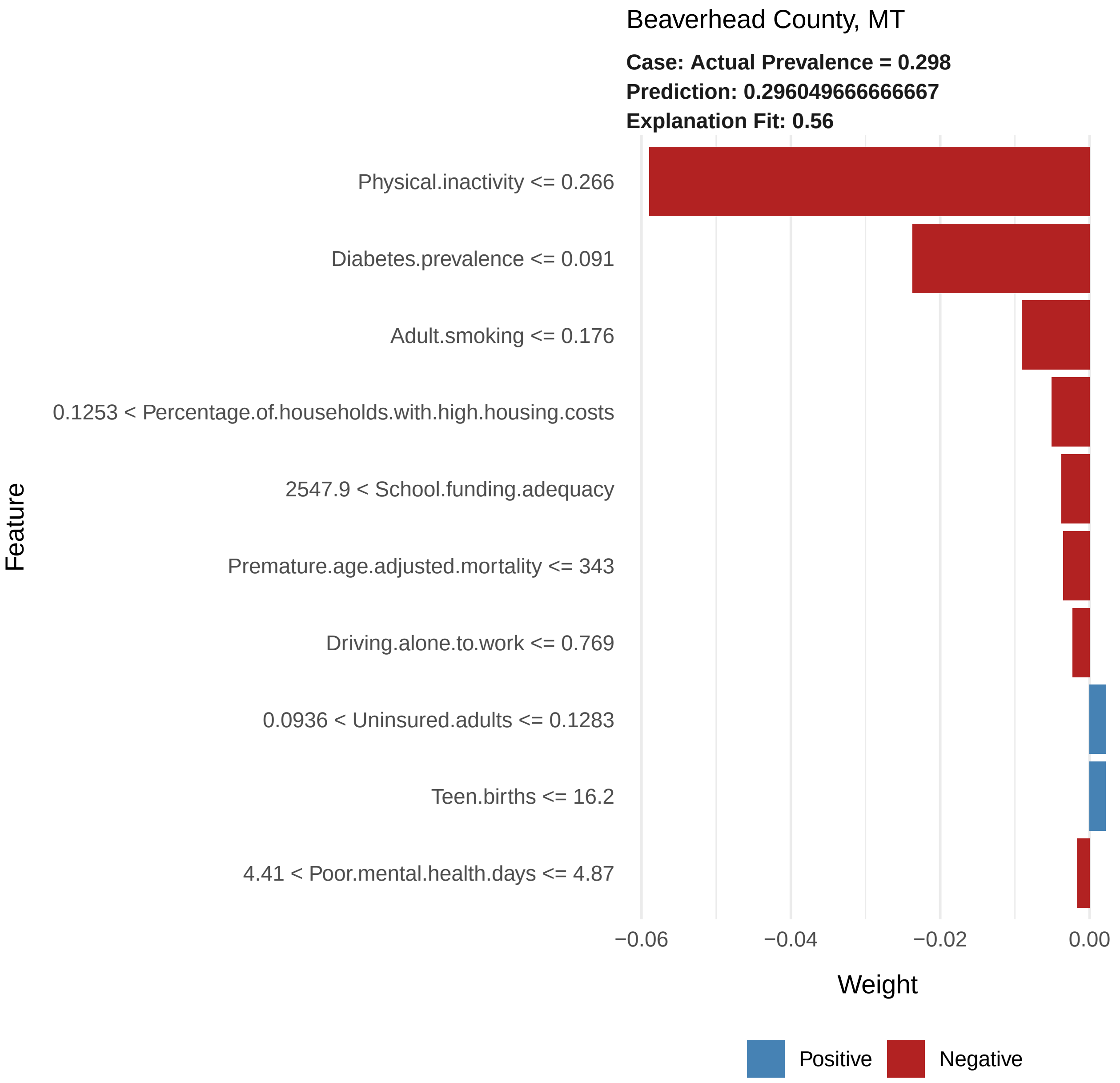

Beaverhead County, MT
Case: Actual Prevalence = 0.298
Prediction: 0.296049666666667
Explanation Fit: 0.56
Physical.inactivity <= 0.266
Diabetes.prevalence <= 0.091
Adult.smoking <= 0.176
0.1253 < Percentage.of.households.with.high.housing.costs
2547.9 < School.funding.adequacy
Premature.age.adjusted.mortality <= 343
Driving.alone.to.work <= 0.769
0.0936 < Uninsured.adults <= 0.1283
Teen.births <= 16.2
4.41 < Poor.mental.health.days <= 4.87
Feature
−0.06
−0.04
−0.02
0.00
Weight
Positive
Negative

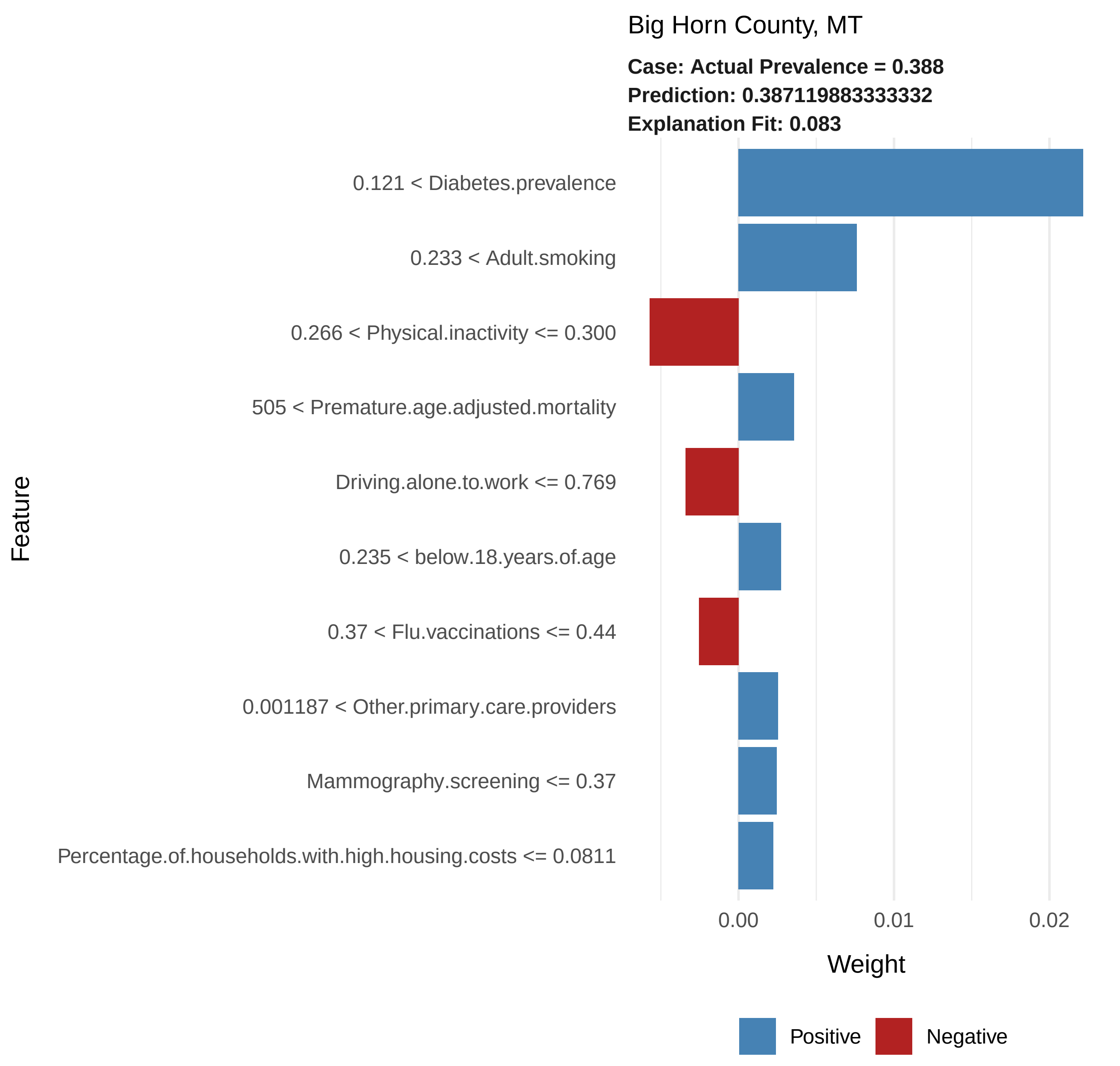

Big Horn County, MT
Case: Actual Prevalence = 0.388
Prediction: 0.3871198833333332
Explanation Fit: 0.083
Feature
0.121 < Diabetes.prevalence
0.233 < Adult.smoking
0.266 < Physical.inactivity <= 0.300
505 < Premature.age.adjusted.mortality
Driving.alone.to.work <= 0.769
0.235 < below.18.years.of.age
0.37 < Flu.vaccinations <= 0.44
0.001187 < Other.primary.care.providers
Mammography.screening <= 0.37
Percentage.of.households.with.high.housing.costs <= 0.0811
0.00
0.01
0.02
Weight
Positive
Negative

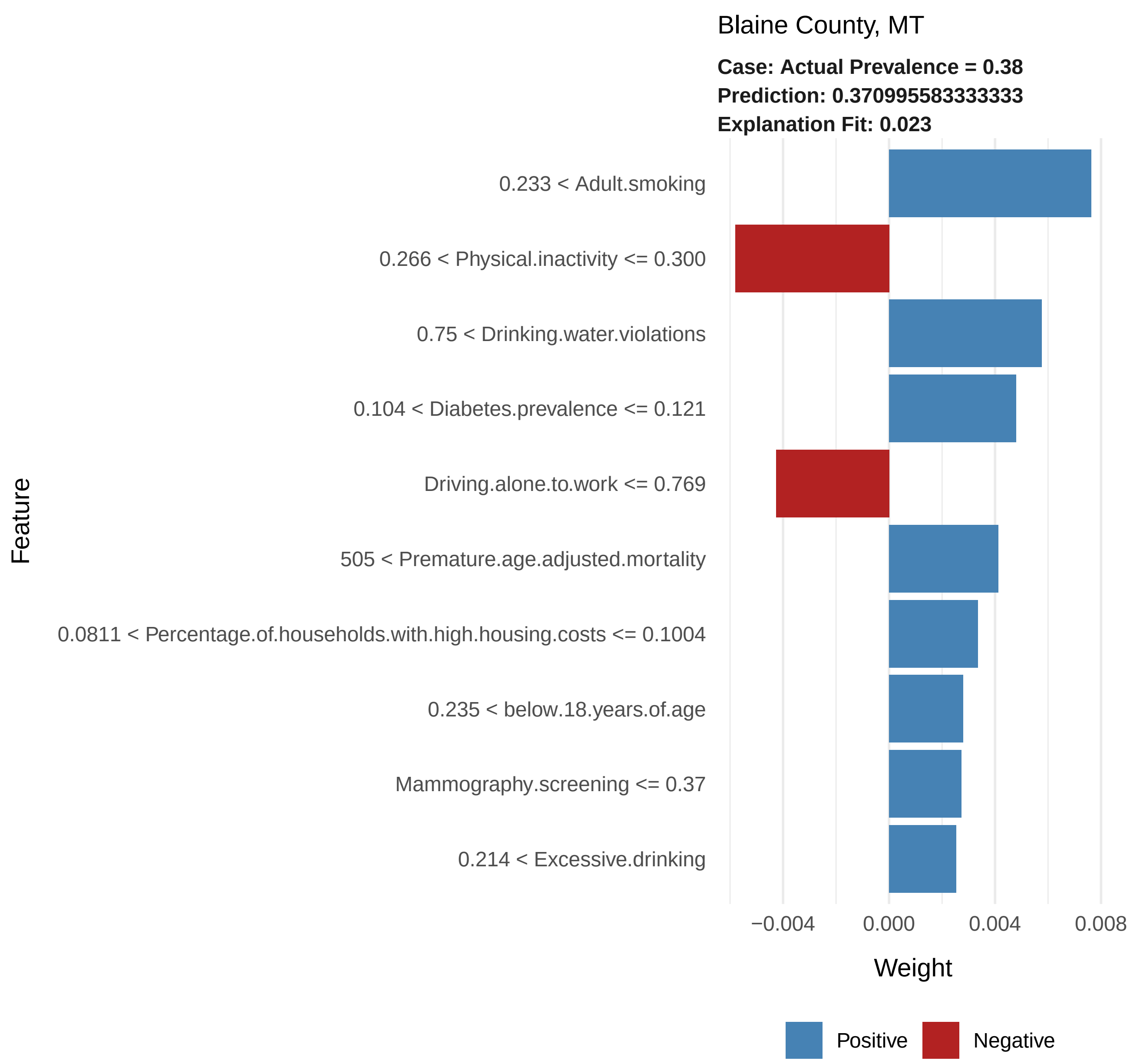

Blaine County, MT
Case: Actual Prevalence = 0.38
Prediction: 0.370995583333333
Explanation Fit: 0.023
0.233 < Adult.smoking
0.266 < Physical.inactivity <= 0.300
0.75 < Drinking.water.violations
0.104 < Diabetes.prevalence <= 0.121
Driving.alone.to.work <= 0.769
505 < Premature.age.adjusted.mortality
0.0811 < Percentage.of.households.with.high.housing.costs <= 0.1004
0.235 < below.18.years.of.age
Mammography.screening <= 0.37
0.214 < Excessive.drinking
Feature
−0.004
0.000
0.004
0.008
Weight
Positive
Negative

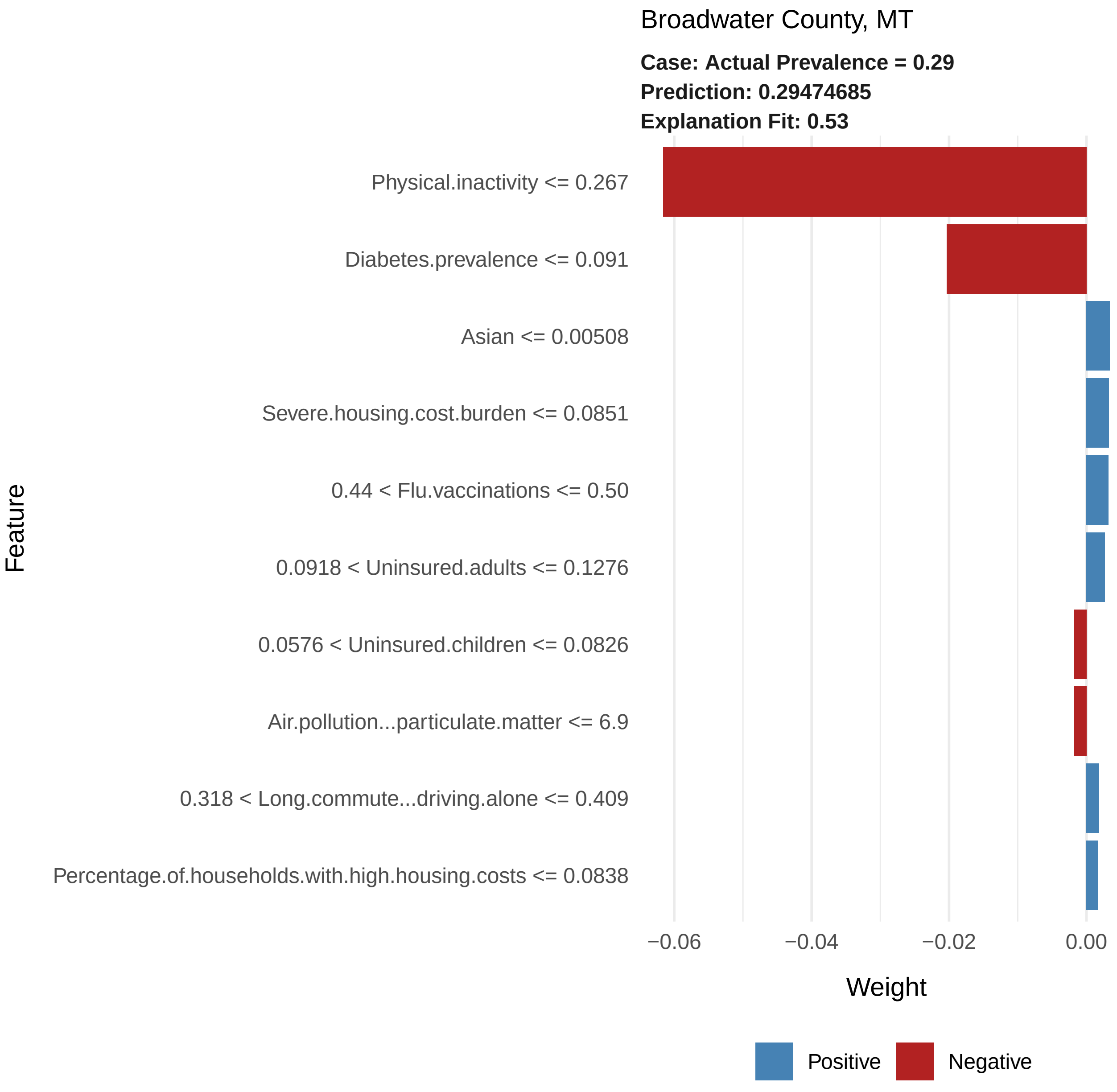
Broadwater County, MT
Case: Actual Prevalence = 0.29
Prediction: 0.29474685
Explanation Fit: 0.53
Physical.inactivity <= 0.267
Diabetes.prevalence <= 0.091
Asian <= 0.00508
Severe.housing.cost.burden <= 0.0851
0.44 < Flu.vaccinations <= 0.50
0.0918 < Uninsured.adults <= 0.1276
0.0576 < Uninsured.children <= 0.0826
Air.pollution...particulate.matter <= 6.9
0.318 < Long.commute...driving.alone <= 0.409
Percentage.of.households.with.high.housing.costs <= 0.0838
Feature
−0.06
−0.04
−0.02
0.00
Weight
Positive
Negative

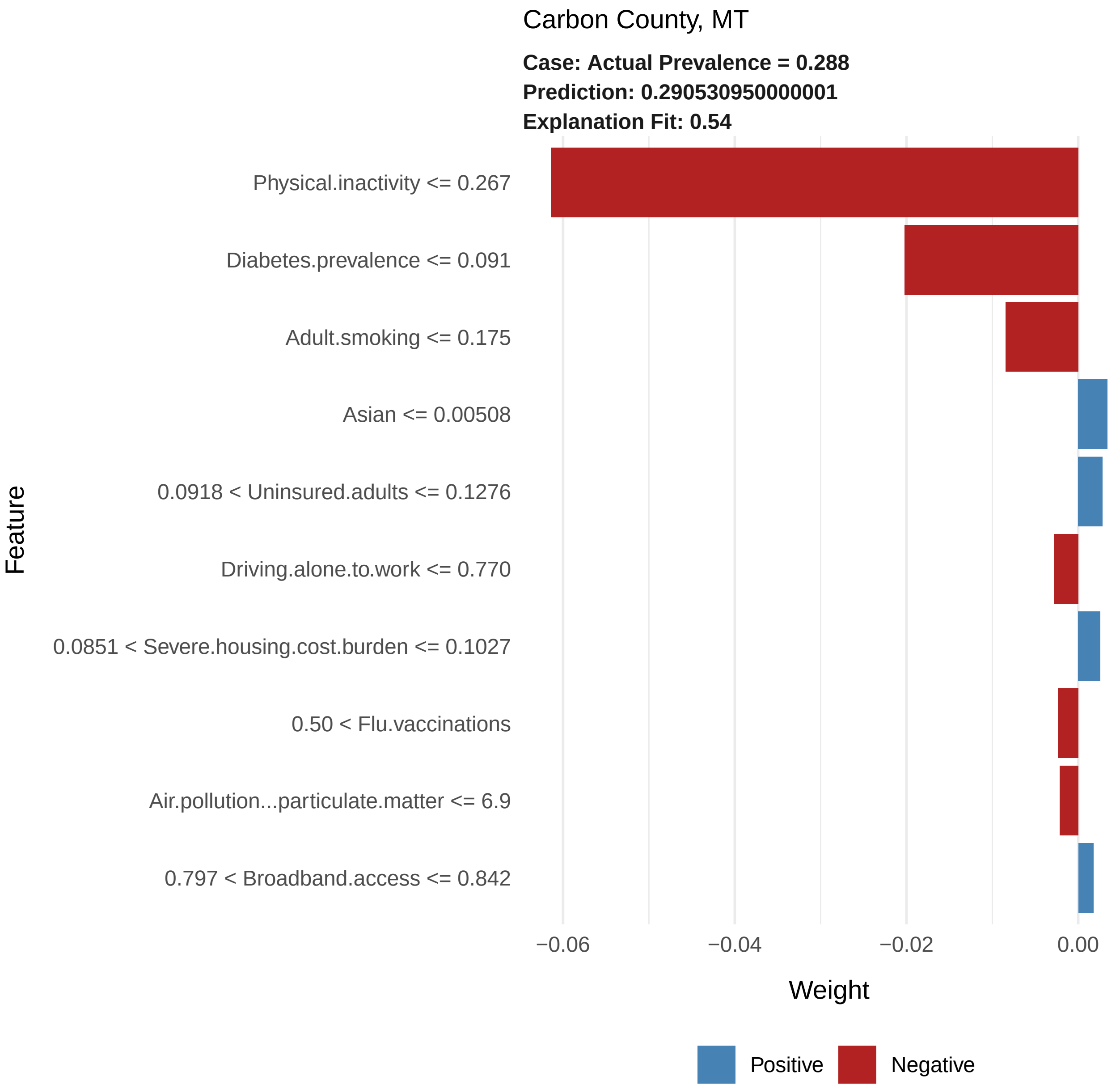
Carbon County, MT
Case: Actual Prevalence = 0.288
Prediction: 0.290530950000001
Explanation Fit: 0.54
Physical.inactivity <= 0.267
Diabetes.prevalence <= 0.091
Adult.smoking <= 0.175
Asian <= 0.00508
0.0918 < Uninsured.adults <= 0.1276
Driving.alone.to.work <= 0.770
0.0851 < Severe.housing.cost.burden <= 0.1027
0.50 < Flu.vaccinations
Air.pollution...particulate.matter <= 6.9
0.797 < Broadband.access <= 0.842
Feature
−0.06
−0.04
−0.02
0.00
Weight
Positive
Negative

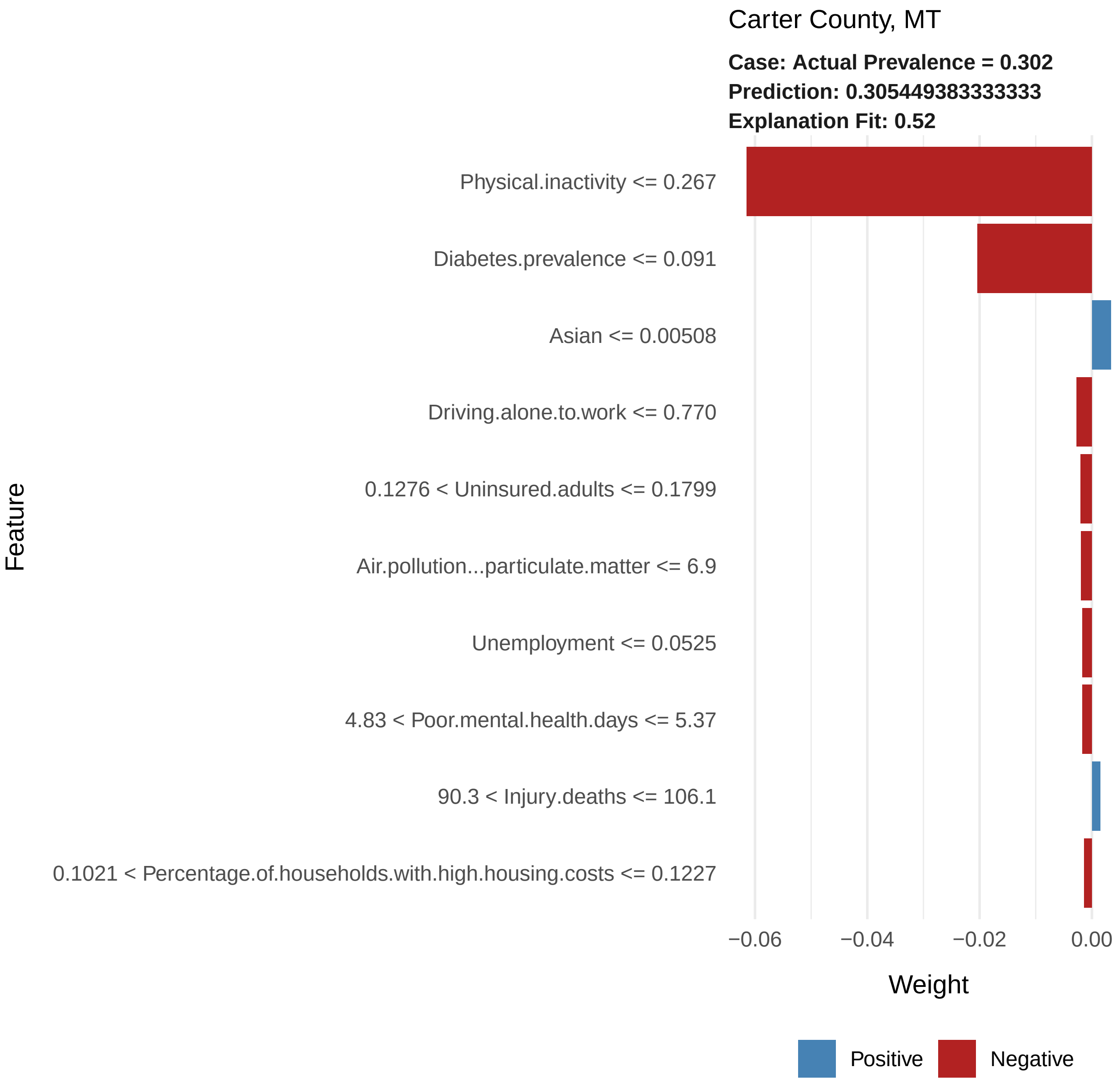

Carter County, MT
Case: Actual Prevalence = 0.302
Prediction: 0.305449383333333
Explanation Fit: 0.52
Physical.inactivity <= 0.267
Diabetes.prevalence <= 0.091
Asian <= 0.00508
Driving.alone.to.work <= 0.770
0.1276 < Uninsured.adults <= 0.1799
Air.pollution...particulate.matter <= 6.9
Unemployment <= 0.0525
4.83 < Poor.mental.health.days <= 5.37
90.3 < Injury.deaths <= 106.1
0.1021 < Percentage.of.households.with.high.housing.costs <= 0.1227
Feature
−0.06
−0.04
−0.02
0.00
Weight
Positive
Negative

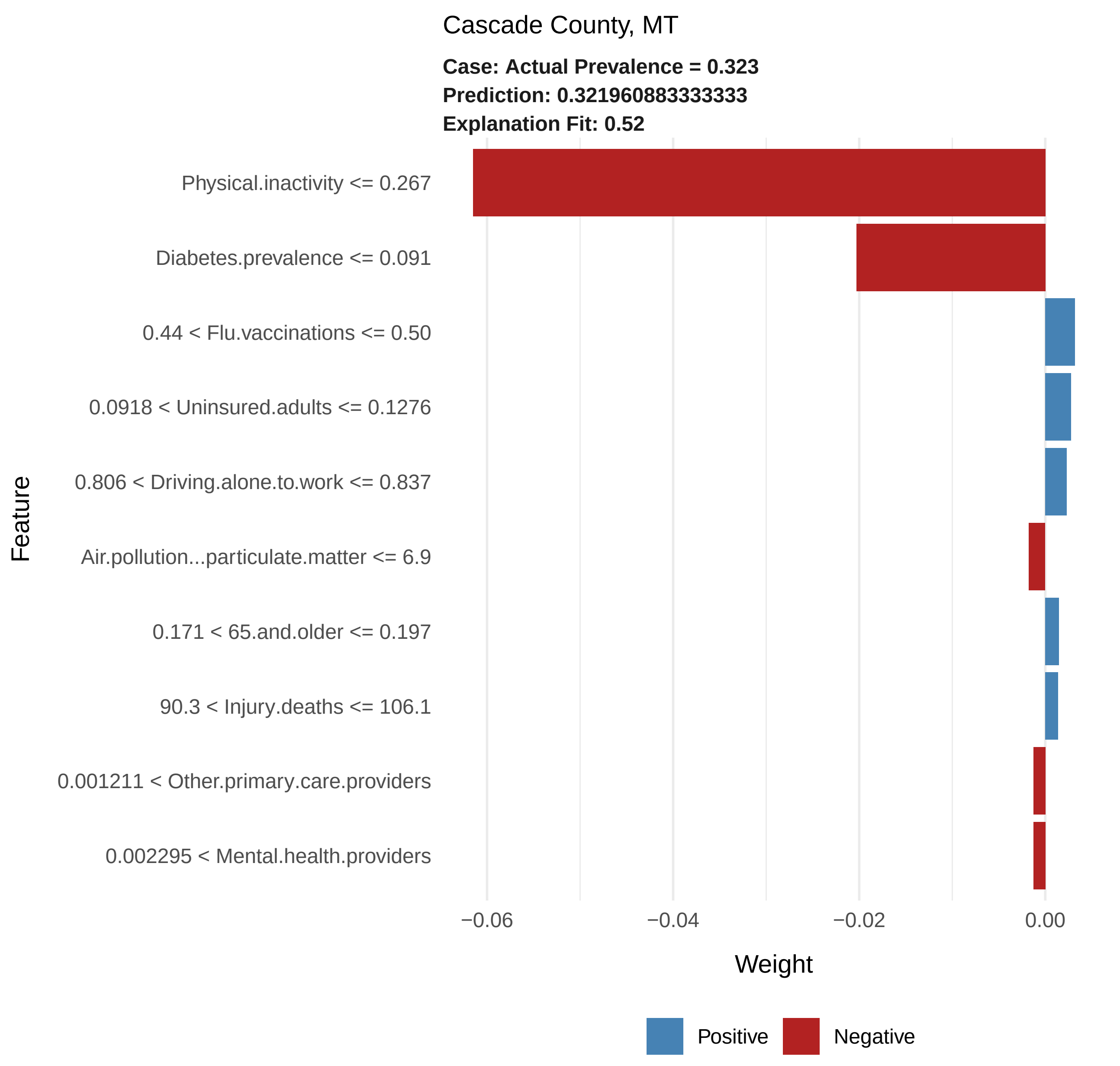

Cascade County, MT
Case: Actual Prevalence = 0.323
Prediction: 0.321960883333333
Explanation Fit: 0.52
Physical.inactivity <= 0.267
Diabetes.prevalence <= 0.091
0.44 < Flu.vaccinations <= 0.50
0.0918 < Uninsured.adults <= 0.1276
0.806 < Driving.alone.to.work <= 0.837
Air.pollution...particulate.matter <= 6.9
0.171 < 65.and.older <= 0.197
90.3 < Injury.deaths <= 106.1
0.001211 < Other.primary.care.providers
0.002295 < Mental.health.providers
Feature
−0.06
−0.04
−0.02
0.00
Weight
Positive
Negative

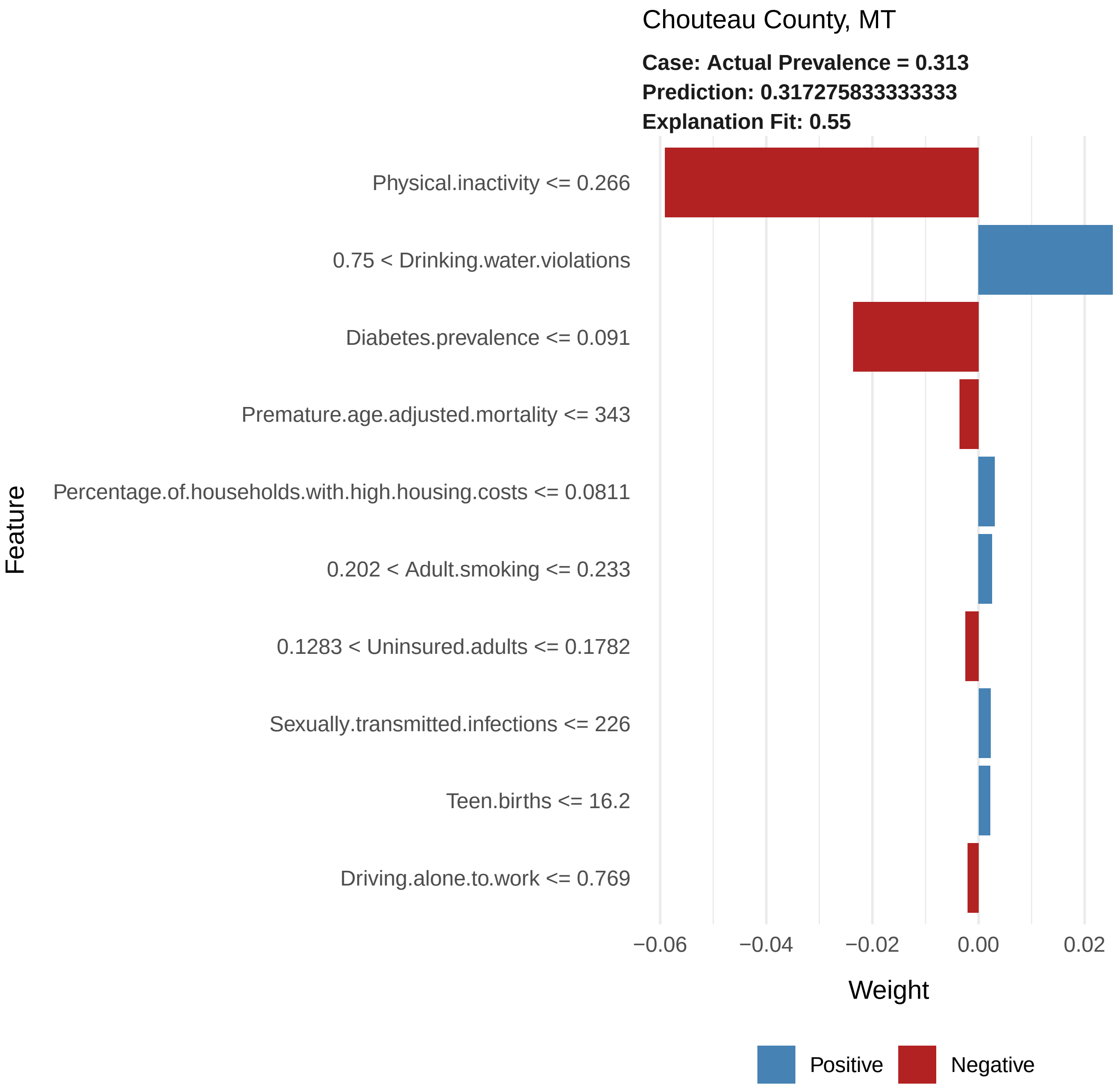
Chouteau County, MT
Case: Actual Prevalence = 0.313
Prediction: 0.317275833333333
Explanation Fit: 0.55
Physical.inactivity <= 0.266
0.75 < Drinking.water.violations
Diabetes.prevalence <= 0.091
Premature.age.adjusted.mortality <= 343
Percentage.of.households.with.high.housing.costs <= 0.0811
0.202 < Adult.smoking <= 0.233
0.1283 < Uninsured.adults <= 0.1782
Sexually.transmitted.infections <= 226
Teen.births <= 16.2
Driving.alone.to.work <= 0.769
Feature
−0.06
−0.04
−0.02
0.00
0.02
Weight
Positive
Negative

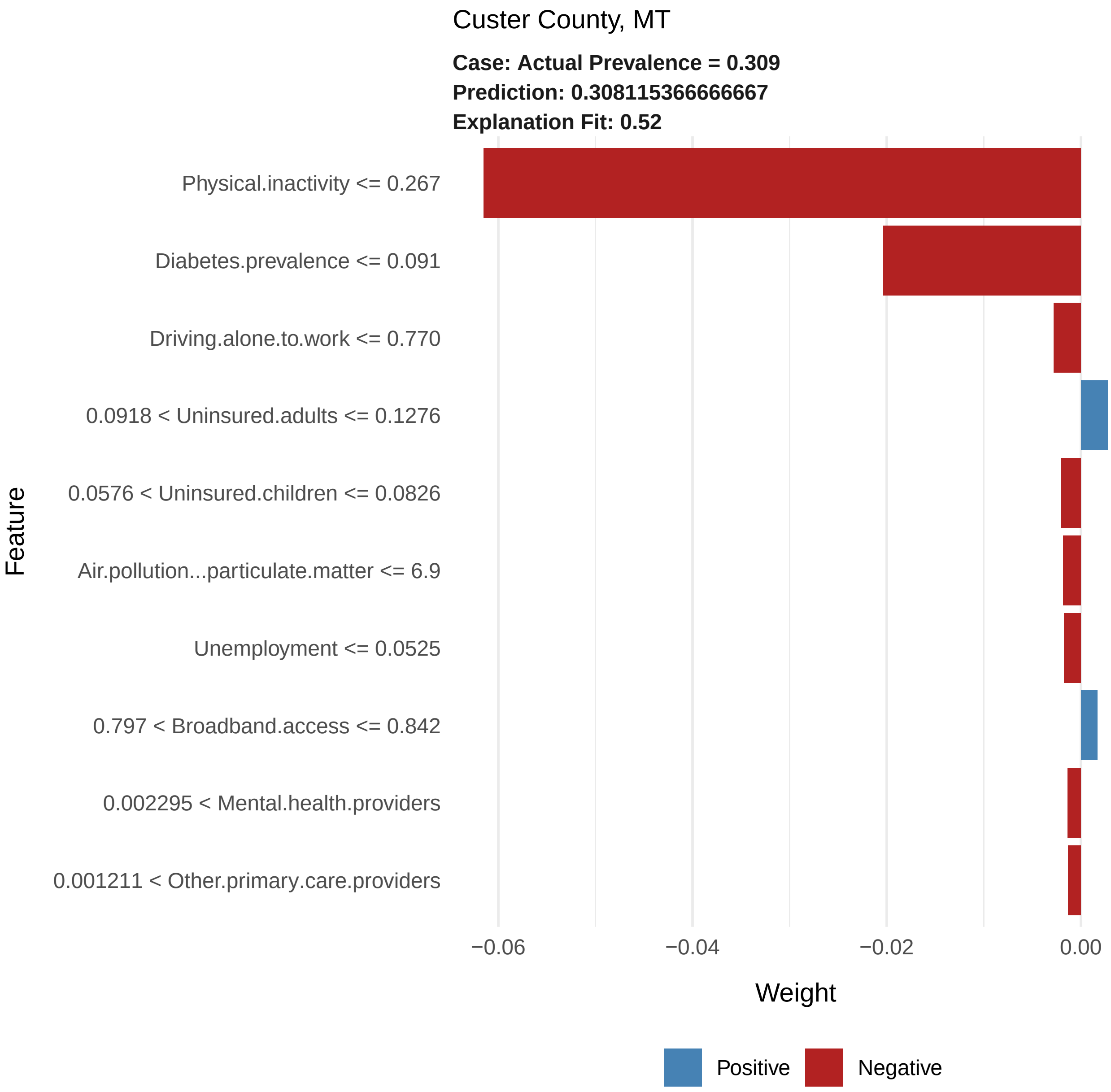

Custer County, MT
Case: Actual Prevalence = 0.309
Prediction: 0.308115366666667
Explanation Fit: 0.52
Physical.inactivity <= 0.267
Diabetes.prevalence <= 0.091
Driving.alone.to.work <= 0.770
0.0918 < Uninsured.adults <= 0.1276
0.0576 < Uninsured.children <= 0.0826
Air.pollution...particulate.matter <= 6.9
Unemployment <= 0.0525
0.797 < Broadband.access <= 0.842
0.002295 < Mental.health.providers
0.001211 < Other.primary.care.providers
Feature
−0.06
−0.04
−0.02
0.00
Weight
Positive
Negative

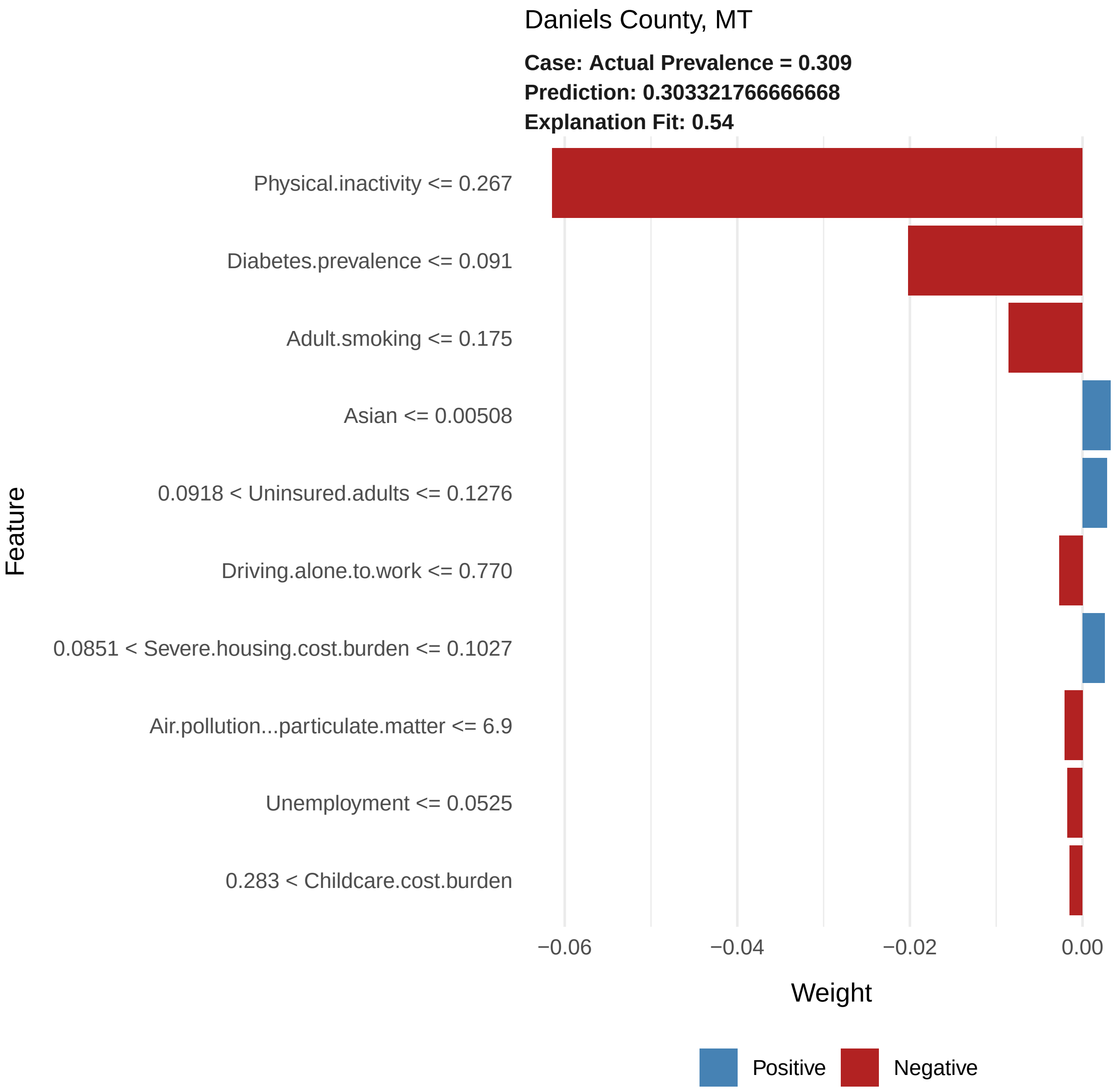
Daniels County, MT
Case: Actual Prevalence = 0.309
Prediction: 0.303321766666668
Explanation Fit: 0.54
Physical.inactivity <= 0.267
Diabetes.prevalence <= 0.091
Adult.smoking <= 0.175
Asian <= 0.00508
0.0918 < Uninsured.adults <= 0.1276
Driving.alone.to.work <= 0.770
0.0851 < Severe.housing.cost.burden <= 0.1027
Air.pollution...particulate.matter <= 6.9
Unemployment <= 0.0525
0.283 < Childcare.cost.burden
Feature
−0.06
−0.04
−0.02
0.00
Weight
Positive
Negative

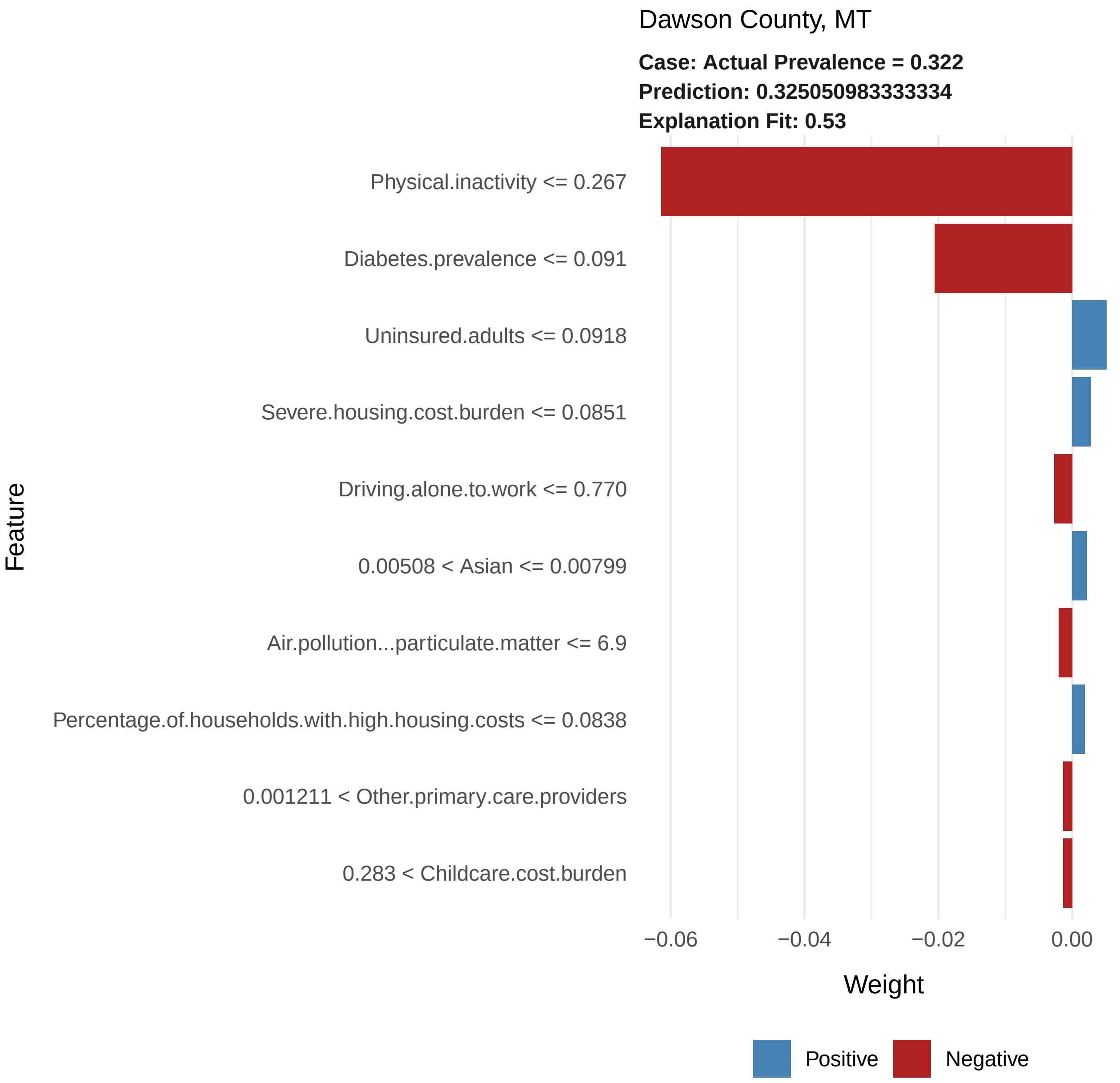
Dawson County, MT
Case: Actual Prevalence = 0.322
Prediction: 0.325050983333334
Explanation Fit: 0.53
Physical.inactivity <= 0.267
Diabetes.prevalence <= 0.091
Uninsured.adults <= 0.0918
Severe.housing.cost.burden <= 0.0851
Driving.alone.to.work <= 0.770
0.00508 < Asian <= 0.00799
Air.pollution...particulate.matter <= 6.9
Percentage.of.households.with.high.housing.costs <= 0.0838
0.001211 < Other.primary.care.providers
0.283 < Childcare.cost.burden
Feature
−0.06
−0.04
−0.02
0.00
Weight
Positive
Negative

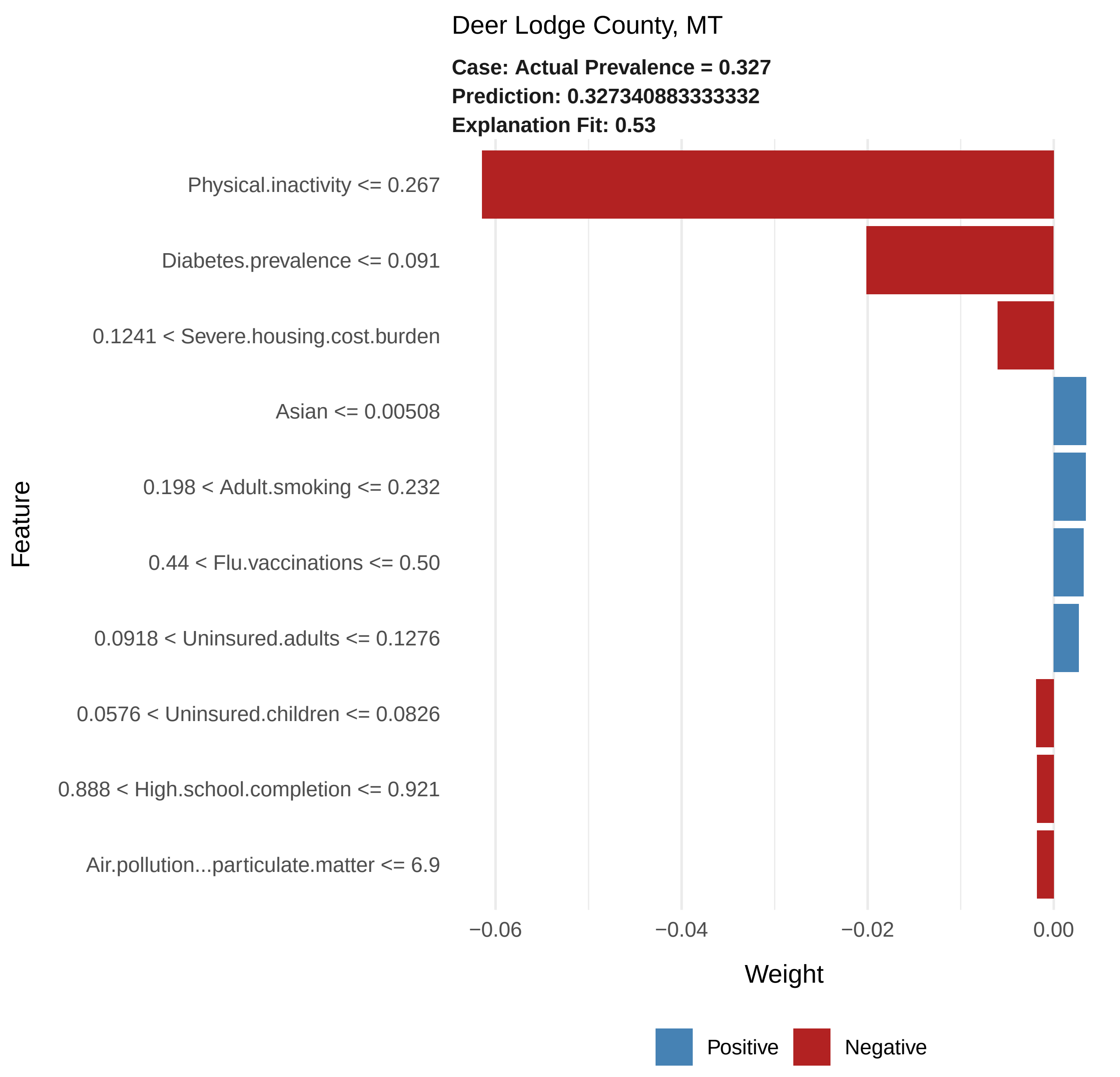

Deer Lodge County, MT
Case: Actual Prevalence = 0.327
Prediction: 0.327340883333332
Explanation Fit: 0.53
Physical.inactivity <= 0.267
Diabetes.prevalence <= 0.091
0.1241 < Severe.housing.cost.burden
Asian <= 0.00508
0.198 < Adult.smoking <= 0.232
0.44 < Flu.vaccinations <= 0.50
0.0918 < Uninsured.adults <= 0.1276
0.0576 < Uninsured.children <= 0.0826
0.888 < High.school.completion <= 0.921
Air.pollution...particulate.matter <= 6.9
Feature
−0.06
−0.04
−0.02
0.00
Weight
Positive
Negative

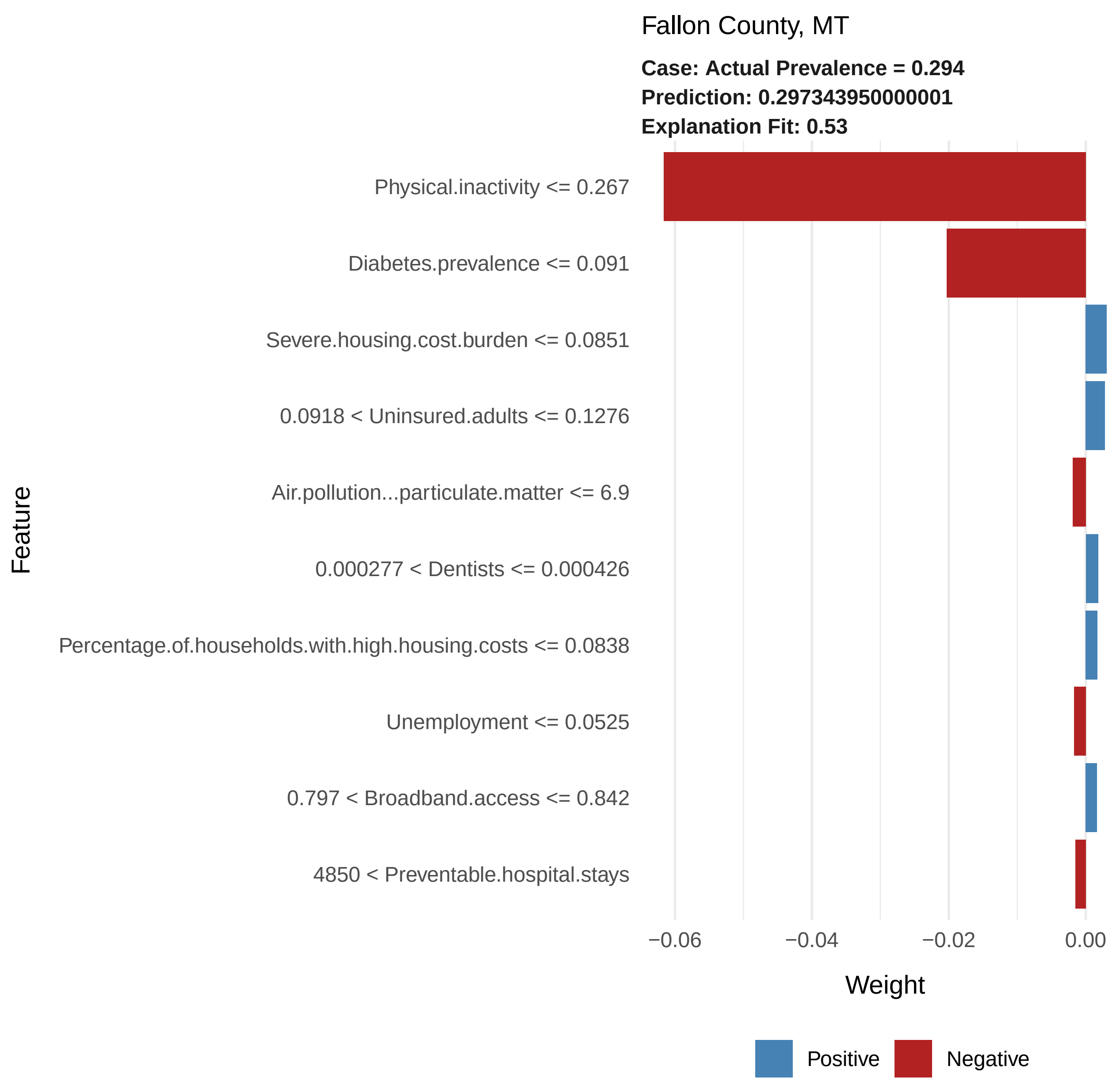
Fallon County, MT
Case: Actual Prevalence = 0.294
Prediction: 0.297343950000001
Explanation Fit: 0.53
Physical.inactivity <= 0.267
Diabetes.prevalence <= 0.091
Severe.housing.cost.burden <= 0.0851
0.0918 < Uninsured.adults <= 0.1276
Air.pollution...particulate.matter <= 6.9
0.000277 < Dentists <= 0.000426
Percentage.of.households.with.high.housing.costs <= 0.0838
Unemployment <= 0.0525
0.797 < Broadband.access <= 0.842
4850 < Preventable.hospital.stays
Feature
−0.06
−0.04
−0.02
0.00
Weight
Positive
Negative

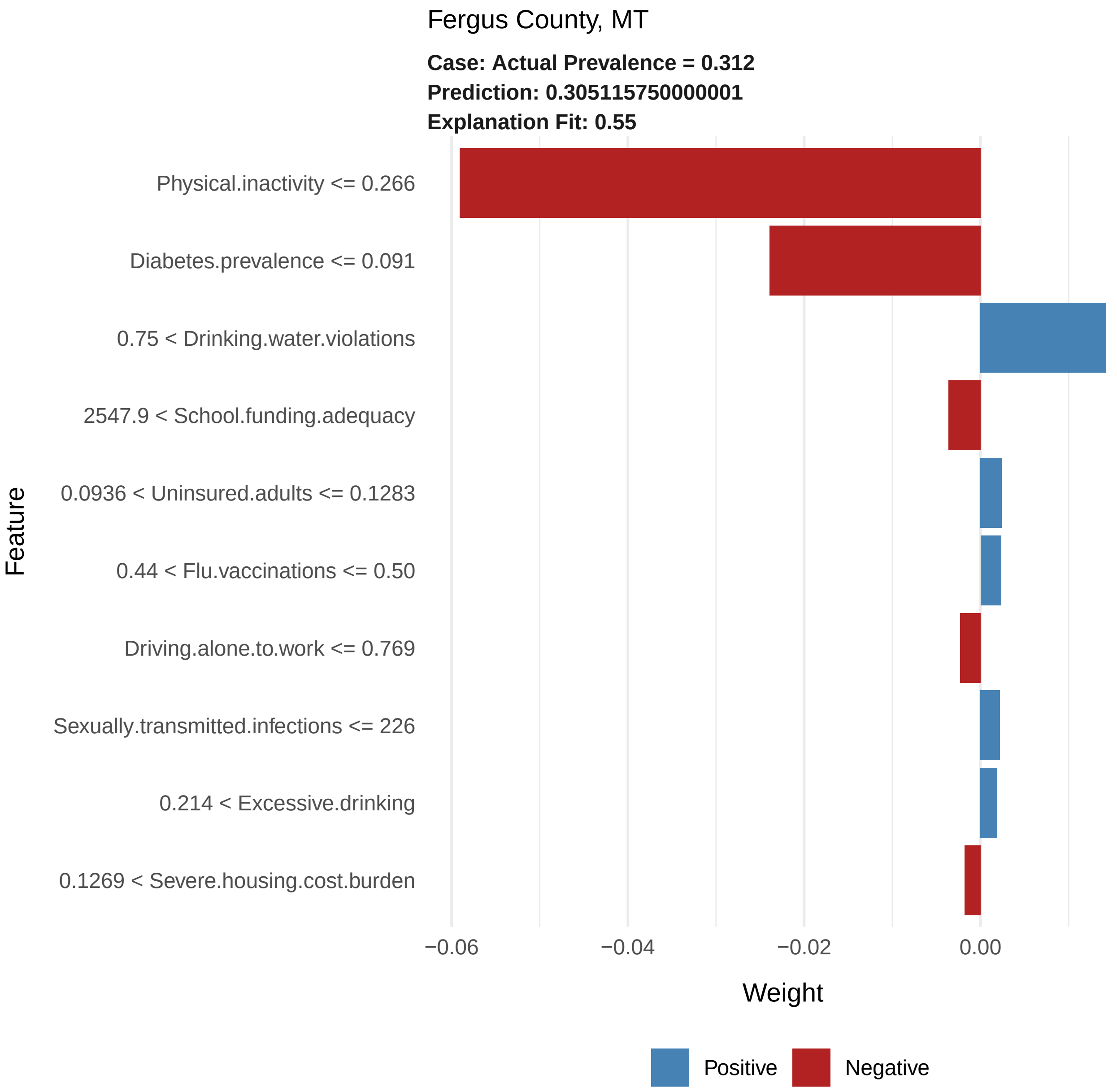

Fergus County, MT
Case: Actual Prevalence = 0.312
Prediction: 0.305115750000001
Explanation Fit: 0.55
Physical.inactivity <= 0.266
Diabetes.prevalence <= 0.091
0.75 < Drinking.water.violations
2547.9 < School.funding.adequacy
0.0936 < Uninsured.adults <= 0.1283
0.44 < Flu.vaccinations <= 0.50
Driving.alone.to.work <= 0.769
Sexually.transmitted.infections <= 226
0.214 < Excessive.drinking
0.1269 < Severe.housing.cost.burden
Feature
−0.06
−0.04
−0.02
0.00
Weight
Positive
Negative

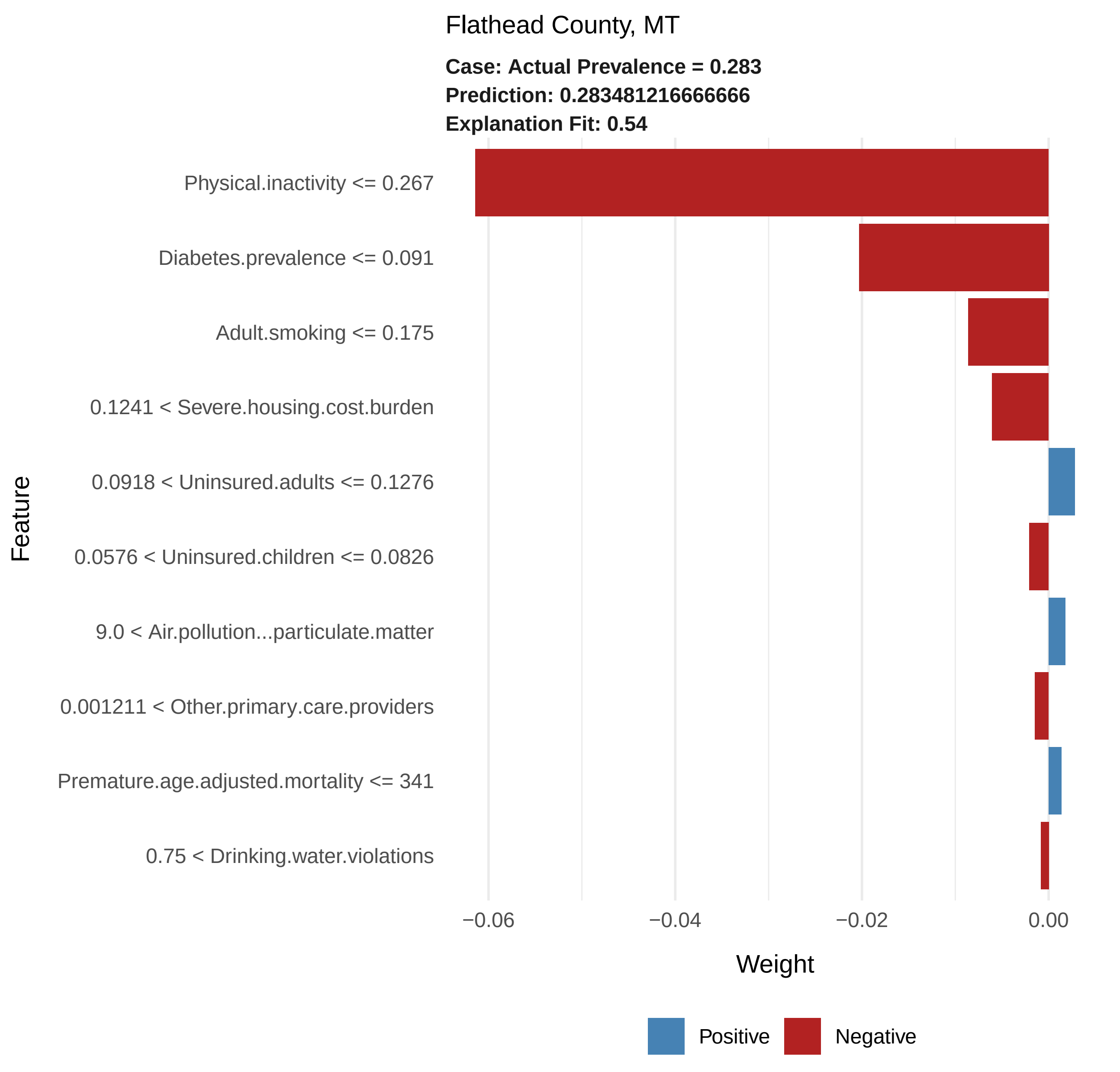

Flathead County, MT
Case: Actual Prevalence = 0.283
Prediction: 0.283481216666666
Explanation Fit: 0.54
Physical.inactivity <= 0.267
Diabetes.prevalence <= 0.091
Adult.smoking <= 0.175
0.1241 < Severe.housing.cost.burden
0.0918 < Uninsured.adults <= 0.1276
0.0576 < Uninsured.children <= 0.0826
9.0 < Air.pollution...particulate.matter
0.001211 < Other.primary.care.providers
Premature.age.adjusted.mortality <= 341
0.75 < Drinking.water.violations
Feature
−0.06
−0.04
−0.02
0.00
Weight
Positive
Negative

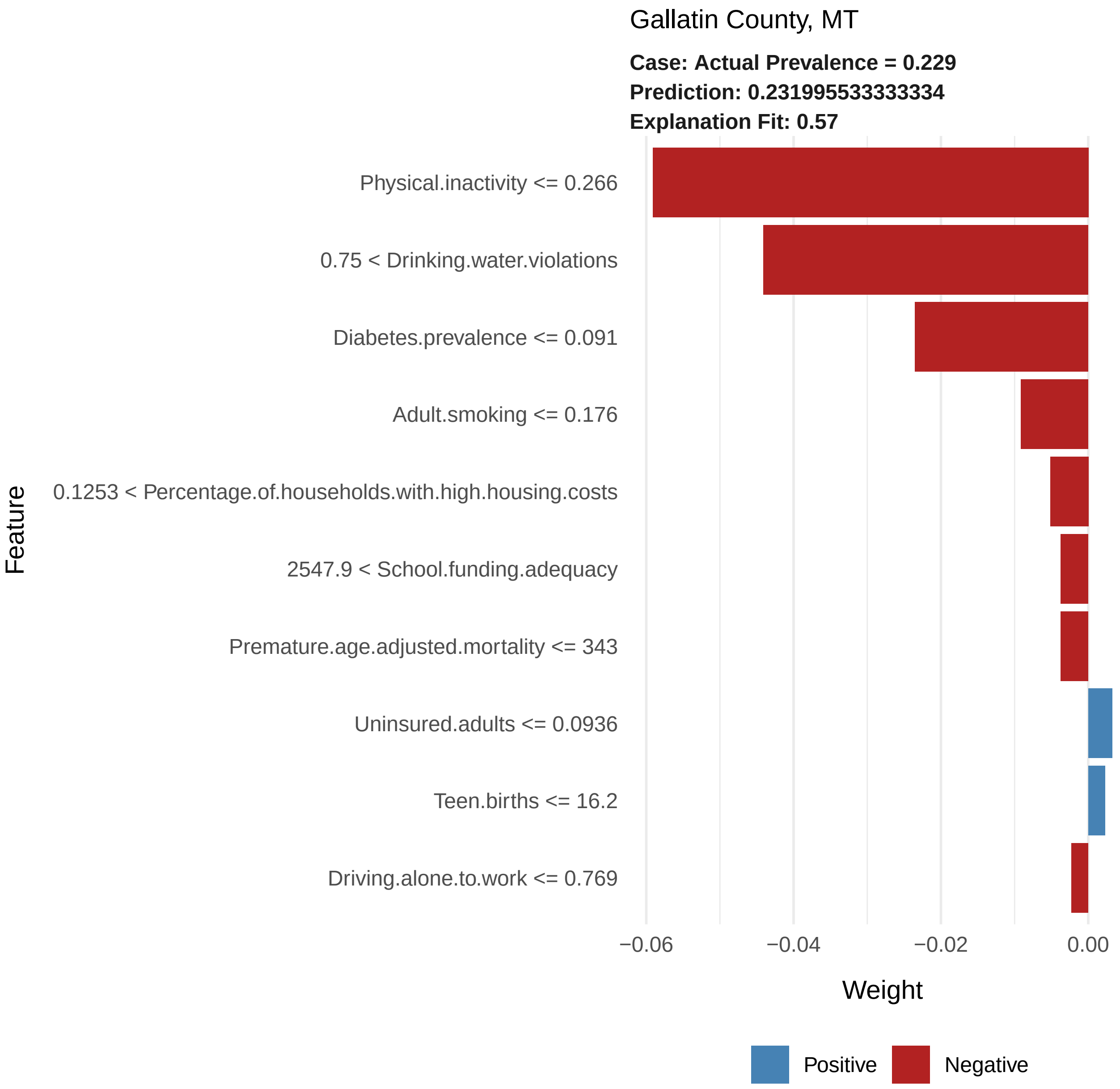

Gallatin County, MT
Case: Actual Prevalence = 0.229
Prediction: 0.231995533333334
Explanation Fit: 0.57
Feature
Physical.inactivity <= 0.266
0.75 < Drinking.water.violations
Diabetes.prevalence <= 0.091
Adult.smoking <= 0.176
0.1253 < Percentage.of.households.with.high.housing.costs
2547.9 < School.funding.adequacy
Premature.age.adjusted.mortality <= 343
Uninsured.adults <= 0.0936
Teen.births <= 16.2
Driving.alone.to.work <= 0.769
−0.06
−0.04
−0.02
0.00
Weight
Positive
Negative

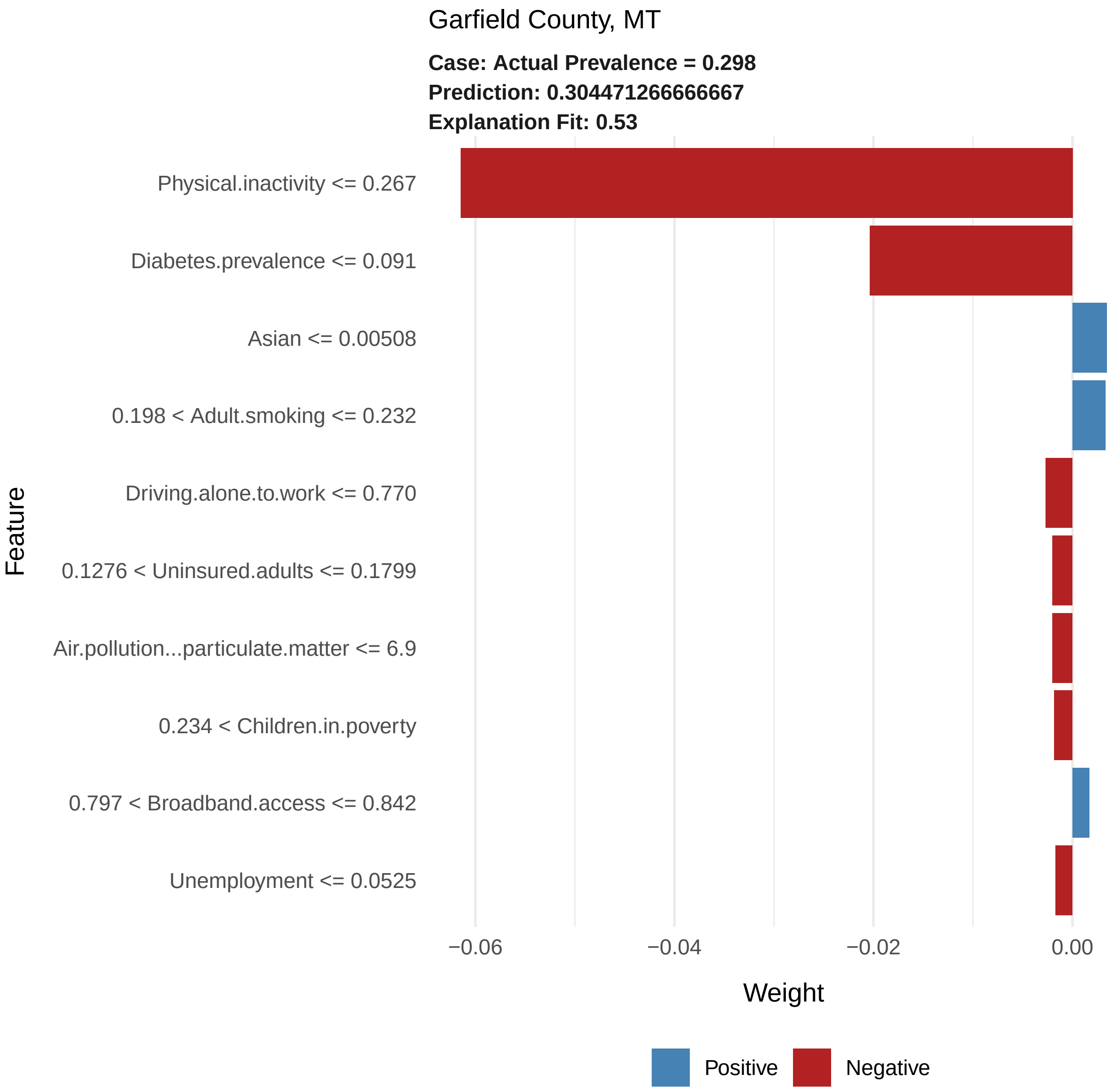
Garfield County, MT
Case: Actual Prevalence = 0.298
Prediction: 0.304471266666667
Explanation Fit: 0.53
Physical.inactivity <= 0.267
Diabetes.prevalence <= 0.091
Asian <= 0.00508
0.198 < Adult.smoking <= 0.232
Driving.alone.to.work <= 0.770
0.1276 < Uninsured.adults <= 0.1799
Air.pollution...particulate.matter <= 6.9
0.234 < Children.in.poverty
0.797 < Broadband.access <= 0.842
Unemployment <= 0.0525
Feature
−0.06
−0.04
−0.02
0.00
Weight
Positive
Negative

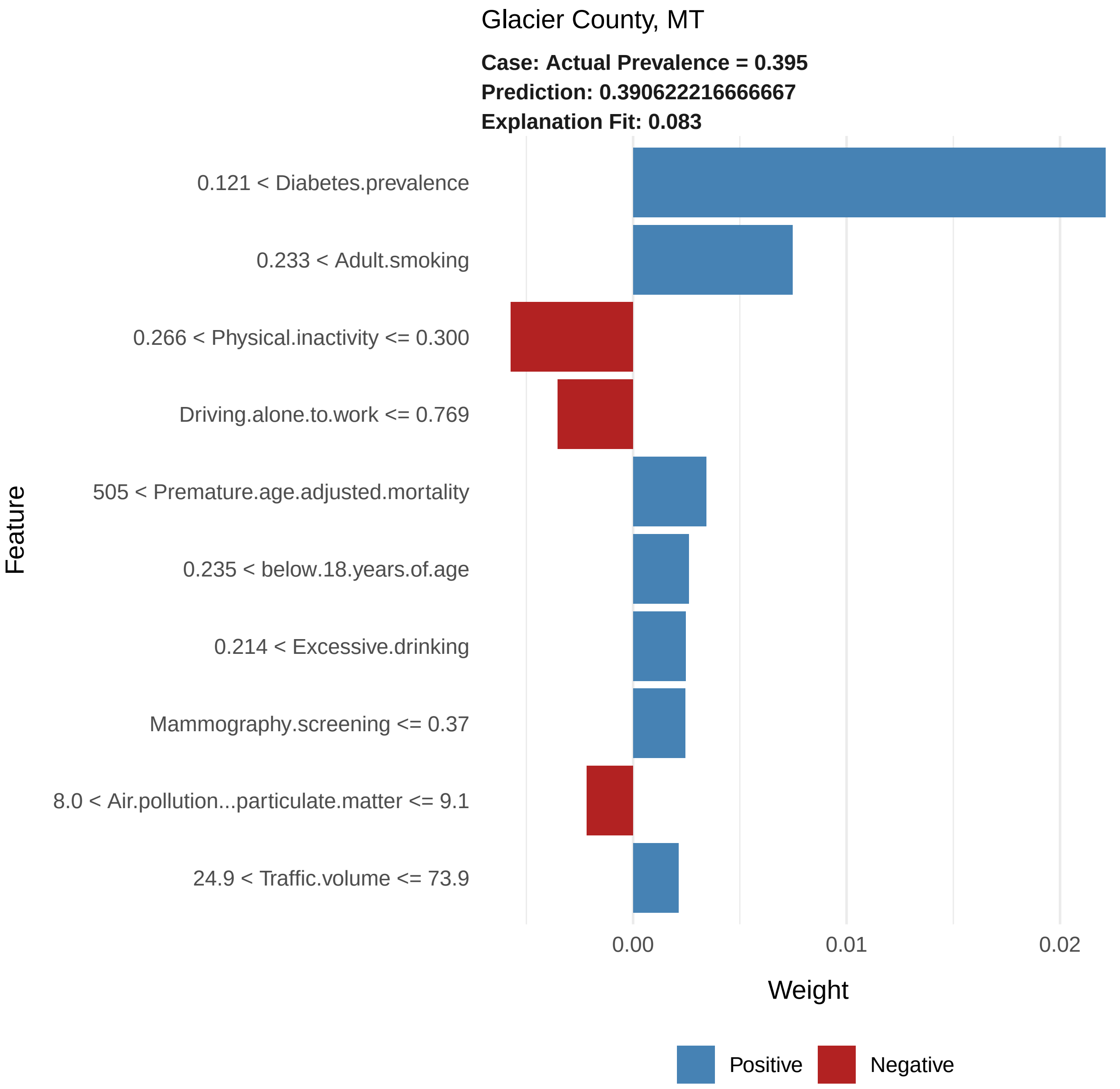

Glacier County, MT
Case: Actual Prevalence = 0.395
Prediction: 0.390622216666667
Explanation Fit: 0.083
0.121 < Diabetes.prevalence
0.233 < Adult.smoking
0.266 < Physical.inactivity <= 0.300
Driving.alone.to.work <= 0.769
505 < Premature.age.adjusted.mortality
0.235 < below.18.years.of.age
0.214 < Excessive.drinking
Mammography.screening <= 0.37
8.0 < Air.pollution...particulate.matter <= 9.1
24.9 < Traffic.volume <= 73.9
Feature
0.00
0.01
0.02
Weight
Positive
Negative

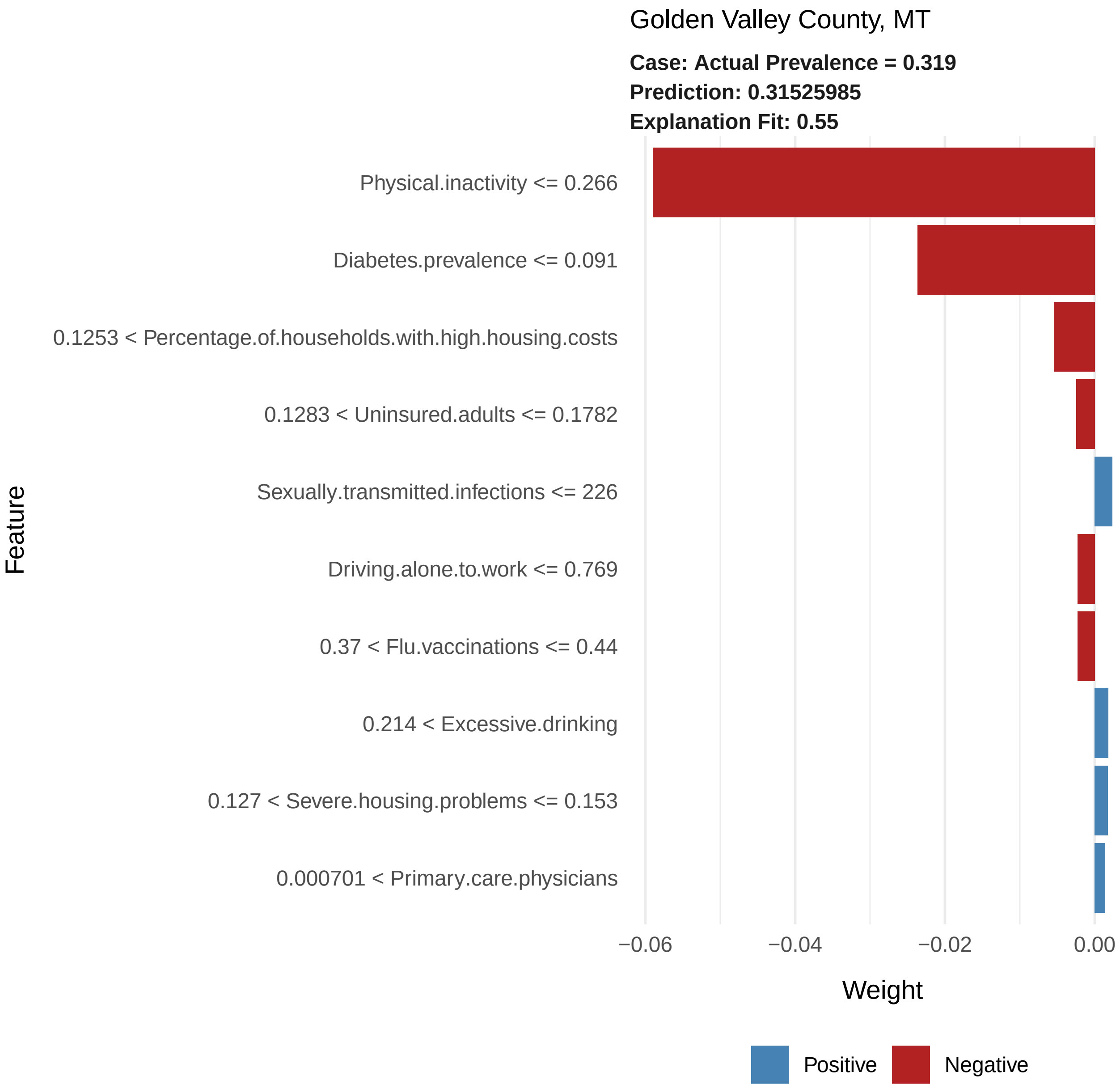

Golden Valley County, MT
Case: Actual Prevalence = 0.319
Prediction: 0.31525985
Explanation Fit: 0.55
Physical.inactivity <= 0.266
Diabetes.prevalence <= 0.091
0.1253 < Percentage.of.households.with.high.housing.costs
0.1283 < Uninsured.adults <= 0.1782
Sexually.transmitted.infections <= 226
Driving.alone.to.work <= 0.769
0.37 < Flu.vaccinations <= 0.44
0.214 < Excessive.drinking
0.127 < Severe.housing.problems <= 0.153
0.000701 < Primary.care.physicians
Feature
−0.06
−0.04
−0.02
0.00
Weight
Positive
Negative

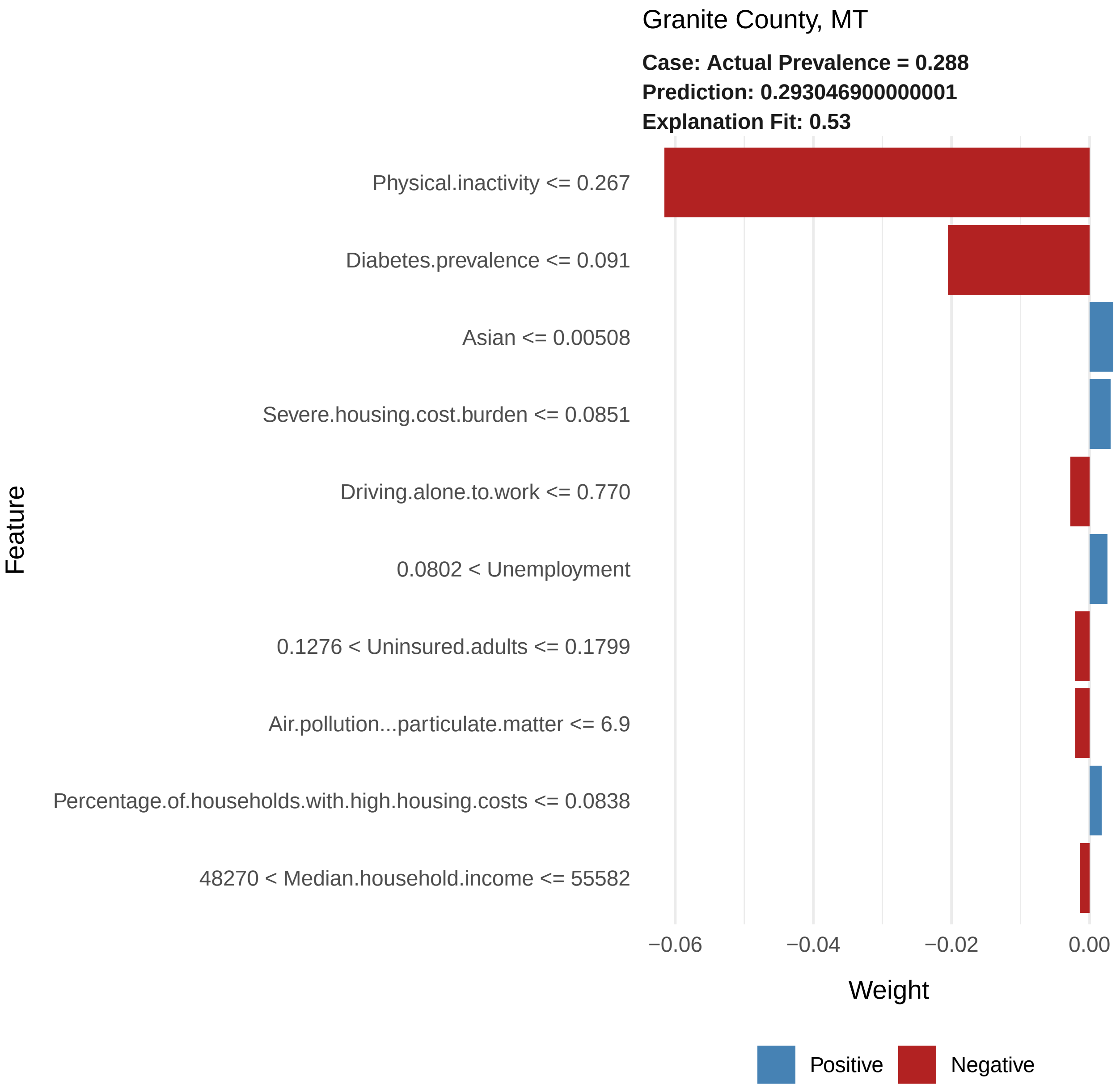

Granite County, MT
Case: Actual Prevalence = 0.288
Prediction: 0.293046900000001
Explanation Fit: 0.53
Physical.inactivity <= 0.267
Diabetes.prevalence <= 0.091
Asian <= 0.00508
Severe.housing.cost.burden <= 0.0851
Driving.alone.to.work <= 0.770
0.0802 < Unemployment
0.1276 < Uninsured.adults <= 0.1799
Air.pollution...particulate.matter <= 6.9
Percentage.of.households.with.high.housing.costs <= 0.0838
48270 < Median.household.income <= 55582
Feature
−0.06
−0.04
−0.02
0.00
Weight
Positive
Negative

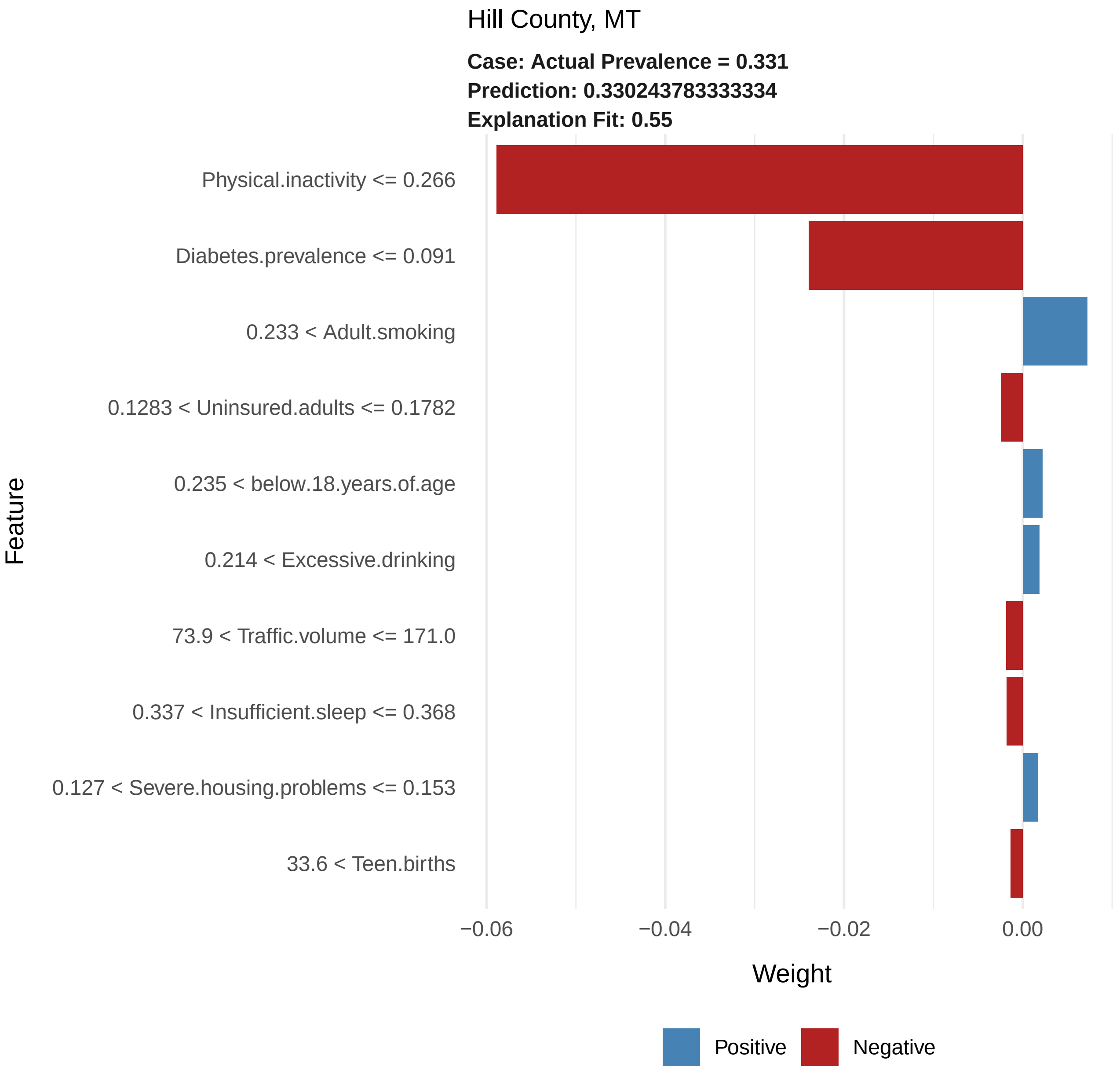
Hill County, MT
Case: Actual Prevalence = 0.331
Prediction: 0.330243783333334
Explanation Fit: 0.55
Physical.inactivity <= 0.266
Diabetes.prevalence <= 0.091
0.233 < Adult.smoking
0.1283 < Uninsured.adults <= 0.1782
0.235 < below.18.years.of.age
0.214 < Excessive.drinking
73.9 < Traffic.volume <= 171.0
0.337 < Insufficient.sleep <= 0.368
0.127 < Severe.housing.problems <= 0.153
33.6 < Teen.births
Feature
−0.06
−0.04
−0.02
0.00
Weight
Positive
Negative

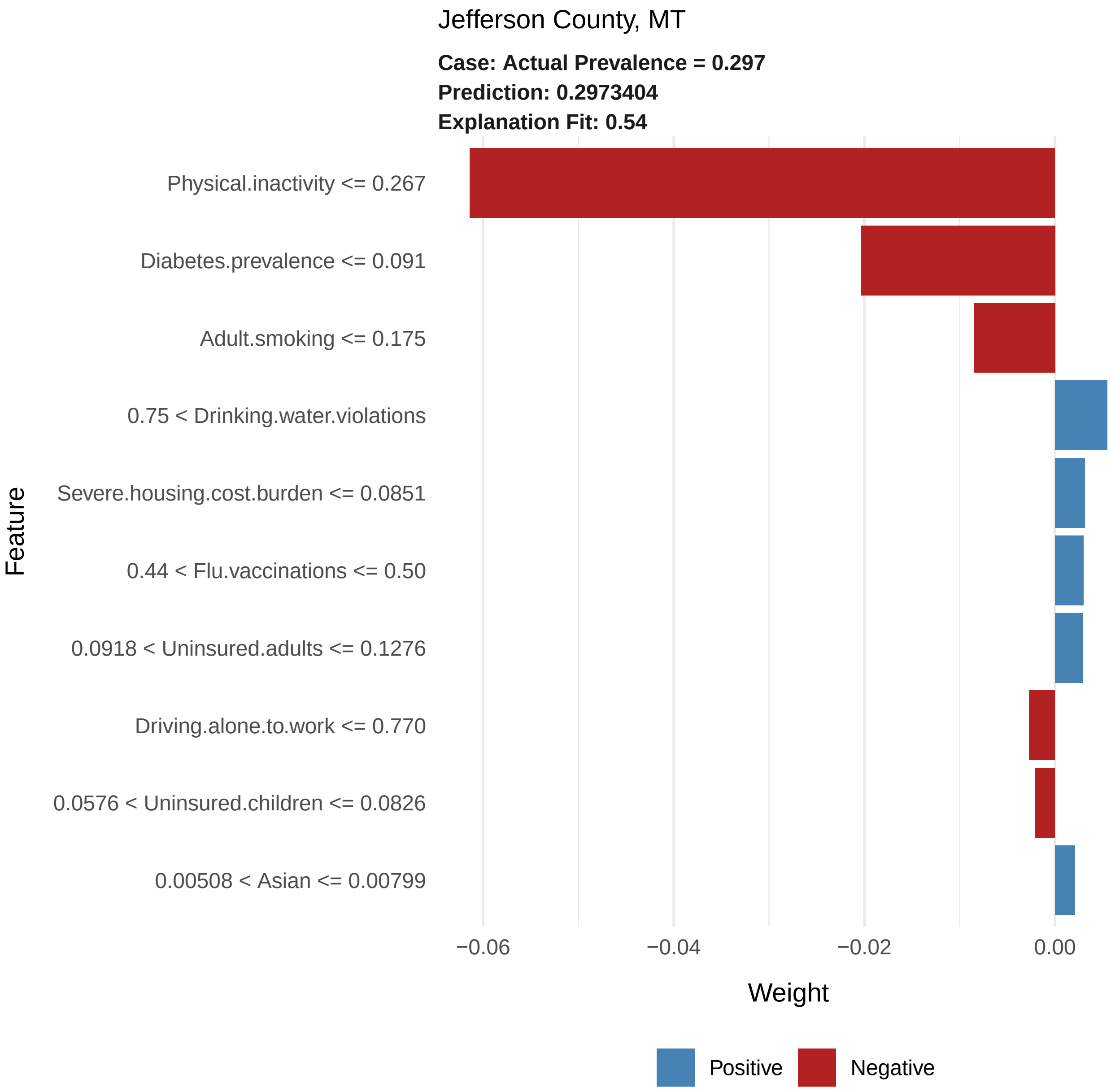

Jefferson County, MT
Case: Actual Prevalence = 0.297
Prediction: 0.2973404
Explanation Fit: 0.54
Physical.inactivity <= 0.267
Diabetes.prevalence <= 0.091
Adult.smoking <= 0.175
0.75 < Drinking.water.violations
Severe.housing.cost.burden <= 0.0851
0.44 < Flu.vaccinations <= 0.50
0.0918 < Uninsured.adults <= 0.1276
Driving.alone.to.work <= 0.770
0.0576 < Uninsured.children <= 0.0826
0.00508 < Asian <= 0.00799
Feature
−0.06
−0.04
−0.02
0.00
Weight
Positive
Negative

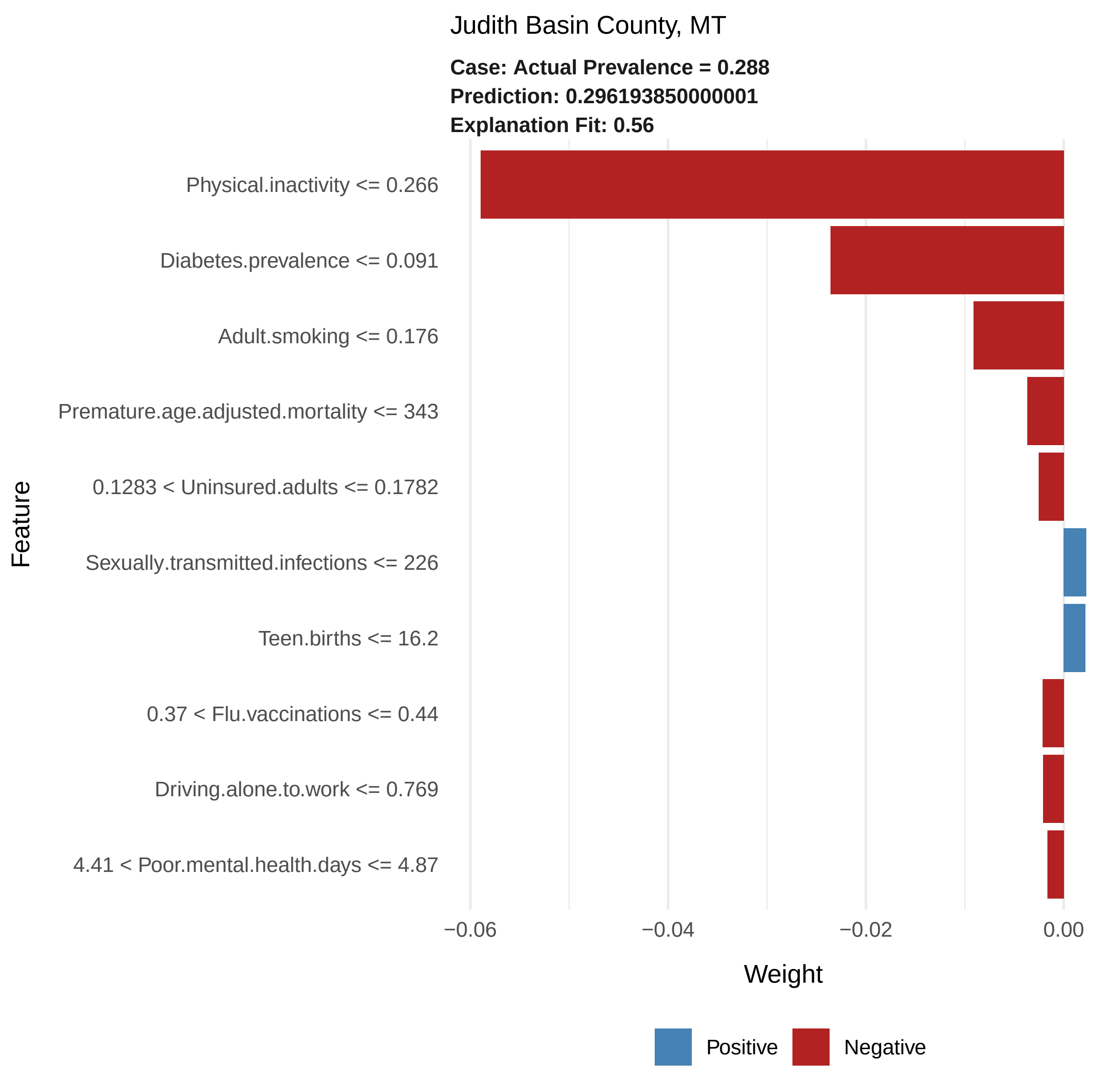

Judith Basin County, MT
Case: Actual Prevalence = 0.288
Prediction: 0.296193850000001
Explanation Fit: 0.56
Physical.inactivity <= 0.266
Diabetes.prevalence <= 0.091
Adult.smoking <= 0.176
Premature.age.adjusted.mortality <= 343
0.1283 < Uninsured.adults <= 0.1782
Sexually.transmitted.infections <= 226
Teen.births <= 16.2
0.37 < Flu.vaccinations <= 0.44
Driving.alone.to.work <= 0.769
4.41 < Poor.mental.health.days <= 4.87
Feature
−0.06
−0.04
−0.02
0.00
Weight
Positive
Negative

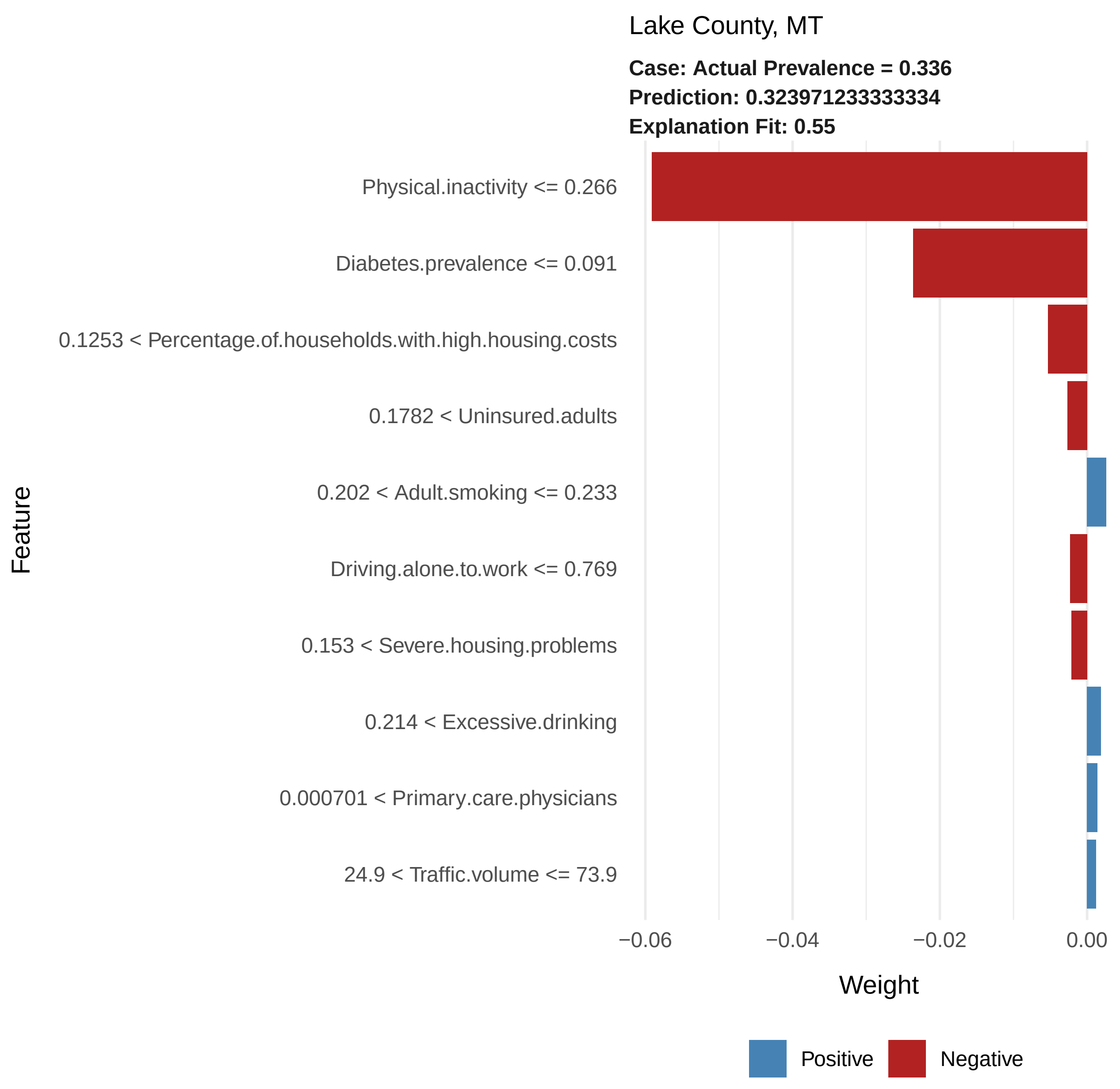

Lake County, MT
Case: Actual Prevalence = 0.336
Prediction: 0.323971233333334
Explanation Fit: 0.55
Physical.inactivity <= 0.266
Diabetes.prevalence <= 0.091
0.1253 < Percentage.of.households.with.high.housing.costs
0.1782 < Uninsured.adults
0.202 < Adult.smoking <= 0.233
Driving.alone.to.work <= 0.769
0.153 < Severe.housing.problems
0.214 < Excessive.drinking
0.000701 < Primary.care.physicians
24.9 < Traffic.volume <= 73.9
Feature
−0.06
−0.04
−0.02
0.00
Weight
Positive
Negative

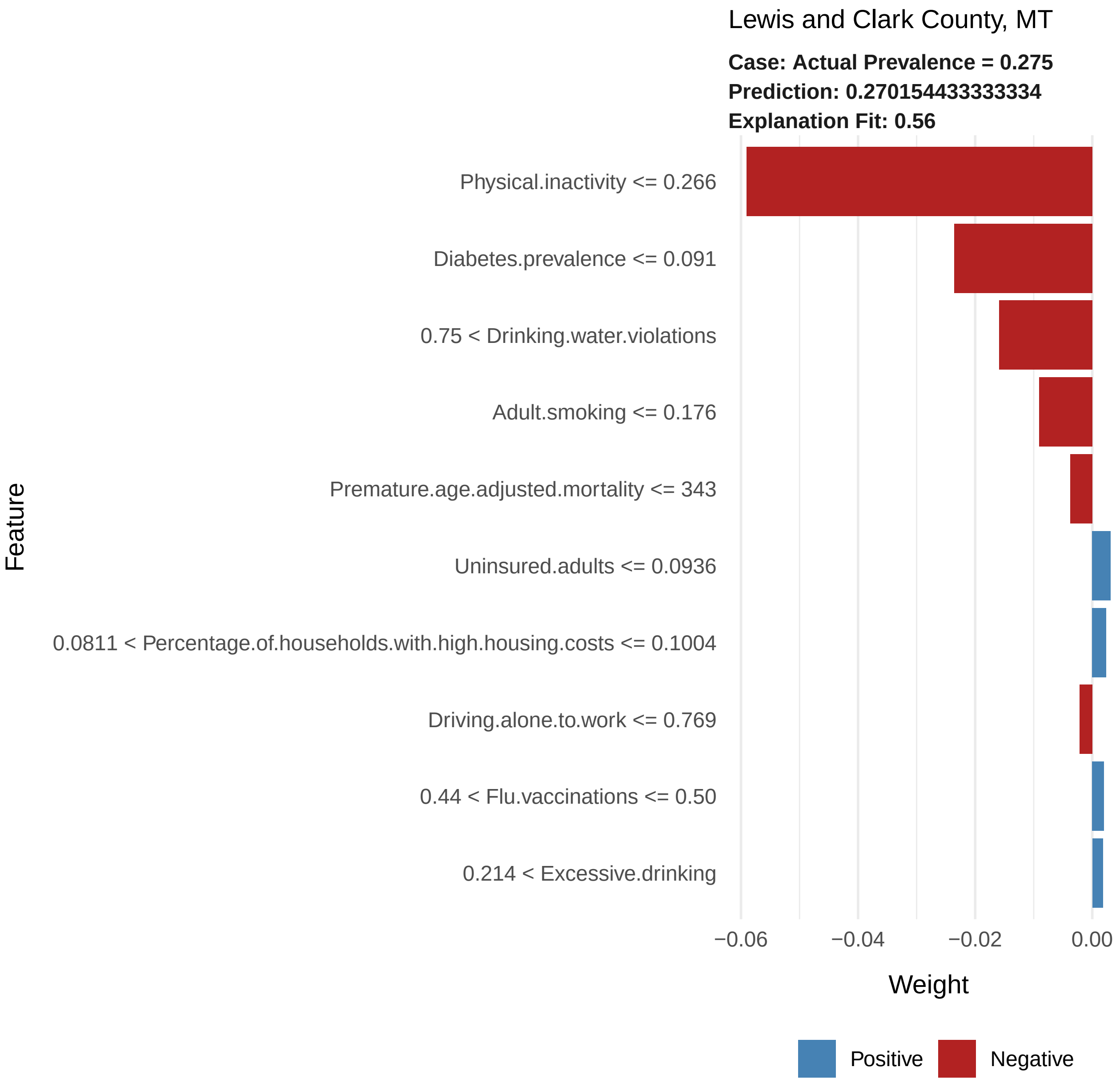

Lewis and Clark County, MT
Case: Actual Prevalence = 0.275
Prediction: 0.270154433333334
Explanation Fit: 0.56
Physical.inactivity <= 0.266
Diabetes.prevalence <= 0.091
0.75 < Drinking.water.violations
Adult.smoking <= 0.176
Premature.age.adjusted.mortality <= 343
Uninsured.adults <= 0.0936
0.0811 < Percentage.of.households.with.high.housing.costs <= 0.1004
Driving.alone.to.work <= 0.769
0.44 < Flu.vaccinations <= 0.50
0.214 < Excessive.drinking
Feature
−0.06
−0.04
−0.02
0.00
Weight
Positive
Negative

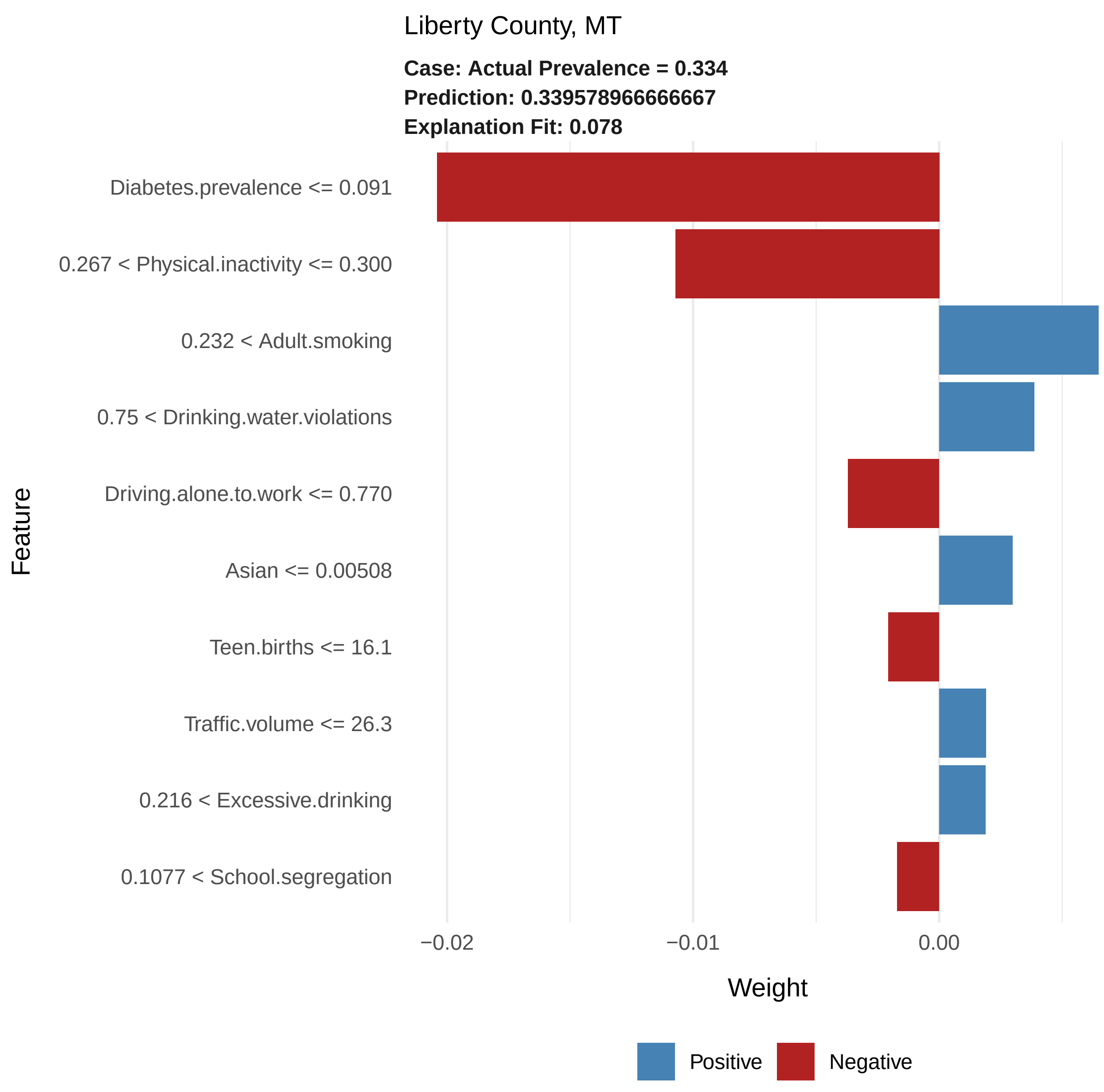

Liberty County, MT
Case: Actual Prevalence = 0.334
Prediction: 0.339578966666667
Explanation Fit: 0.078
Diabetes.prevalence <= 0.091
0.267 < Physical.inactivity <= 0.300
0.232 < Adult.smoking
0.75 < Drinking.water.violations
Driving.alone.to.work <= 0.770
Asian <= 0.00508
Teen.births <= 16.1
Traffic.volume <= 26.3
0.216 < Excessive.drinking
0.1077 < School.segregation
Feature
−0.02
−0.01
0.00
Weight
Positive
Negative

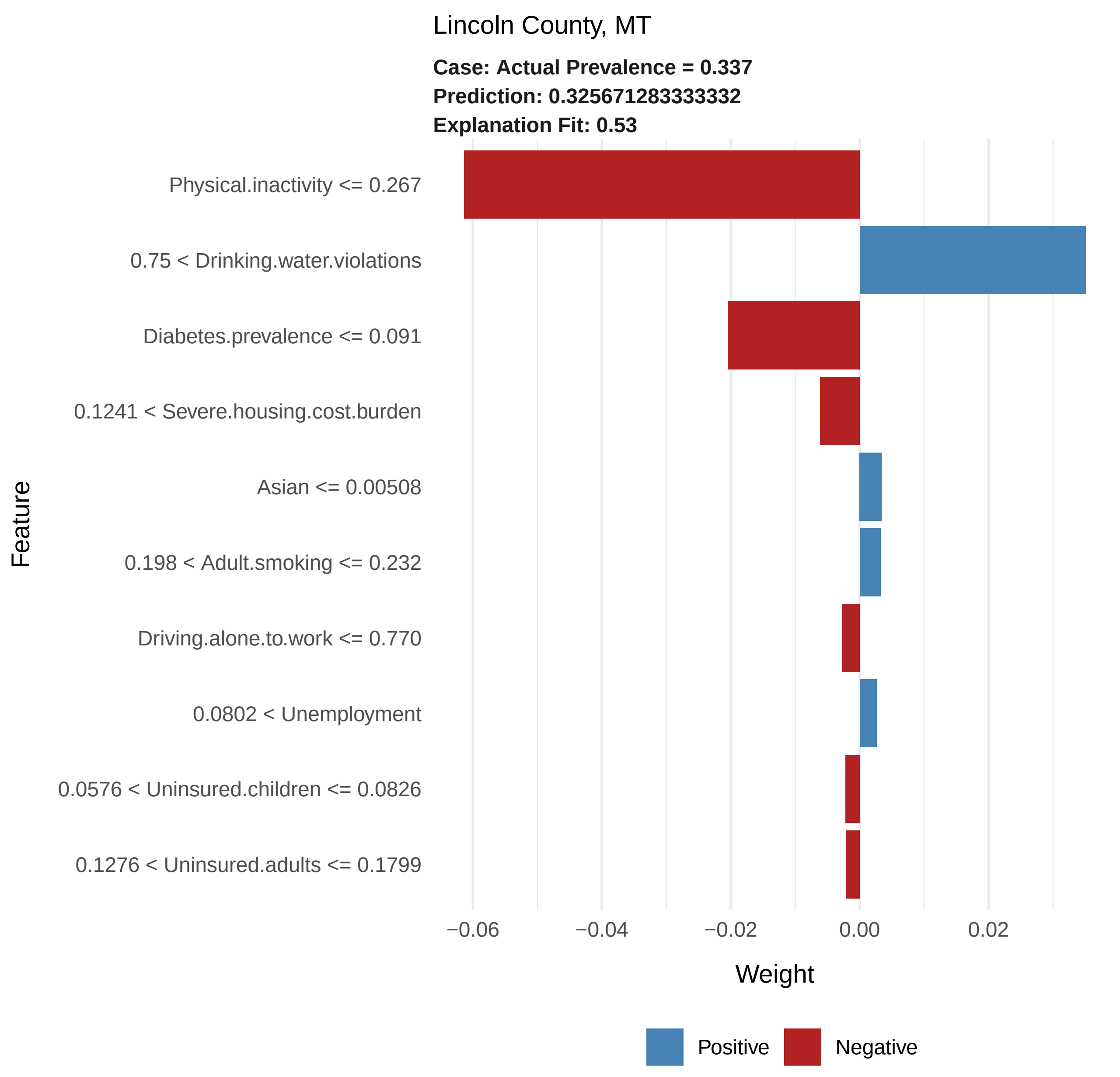
Lincoln County, MT
Case: Actual Prevalence = 0.337
Prediction: 0.325671283333332
Explanation Fit: 0.53
Physical.inactivity <= 0.267
0.75 < Drinking.water.violations
Diabetes.prevalence <= 0.091
0.1241 < Severe.housing.cost.burden
Asian <= 0.00508
0.198 < Adult.smoking <= 0.232
Driving.alone.to.work <= 0.770
0.0802 < Unemployment
0.0576 < Uninsured.children <= 0.0826
0.1276 < Uninsured.adults <= 0.1799
Feature
−0.06
−0.04
−0.02
0.00
0.02
Weight
Positive
Negative

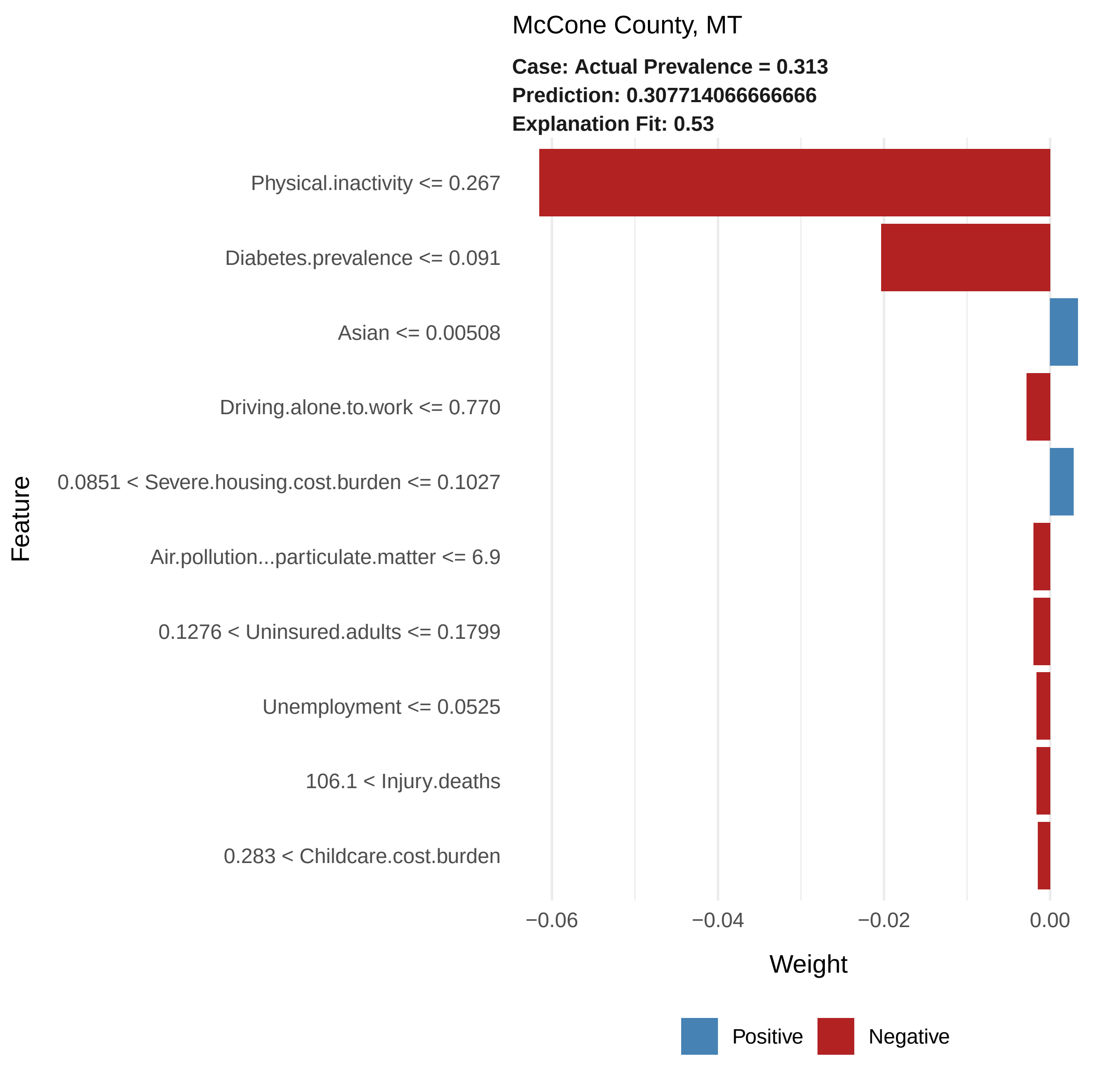

McCone County, MT
Case: Actual Prevalence = 0.313
Prediction: 0.307714066666666
Explanation Fit: 0.53
Physical.inactivity <= 0.267
Diabetes.prevalence <= 0.091
Asian <= 0.00508
Driving.alone.to.work <= 0.770
0.0851 < Severe.housing.cost.burden <= 0.1027
Air.pollution...particulate.matter <= 6.9
0.1276 < Uninsured.adults <= 0.1799
Unemployment <= 0.0525
106.1 < Injury.deaths
0.283 < Childcare.cost.burden
Feature
−0.06
−0.04
−0.02
0.00
Weight
Positive
Negative

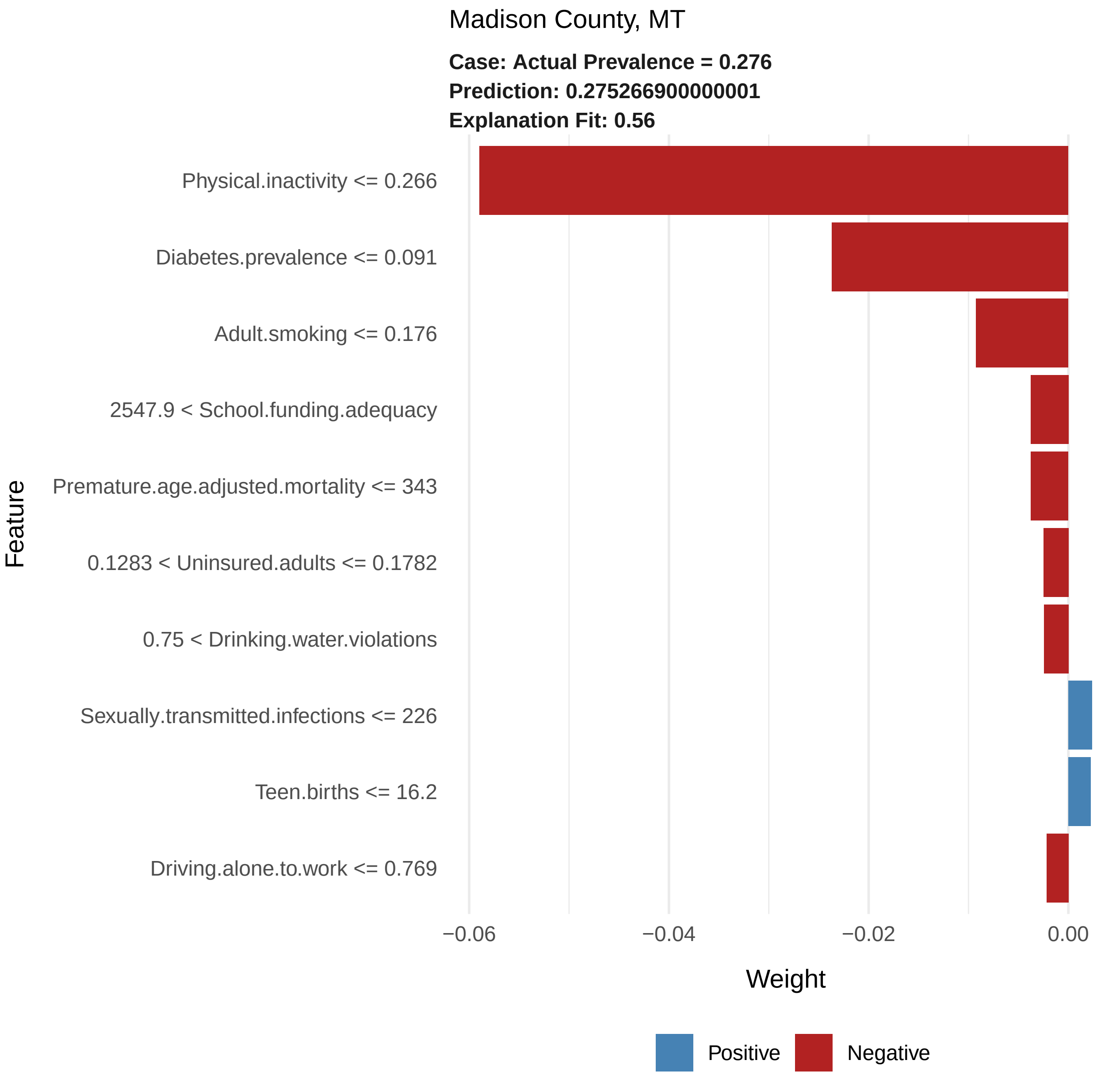

Madison County, MT
Case: Actual Prevalence = 0.276
Prediction: 0.275266900000001
Explanation Fit: 0.56
Physical.inactivity <= 0.266
Diabetes.prevalence <= 0.091
Adult.smoking <= 0.176
2547.9 < School.funding.adequacy
Premature.age.adjusted.mortality <= 343
0.1283 < Uninsured.adults <= 0.1782
0.75 < Drinking.water.violations
Sexually.transmitted.infections <= 226
Teen.births <= 16.2
Driving.alone.to.work <= 0.769
Feature
−0.06
−0.04
−0.02
0.00
Weight
Positive
Negative

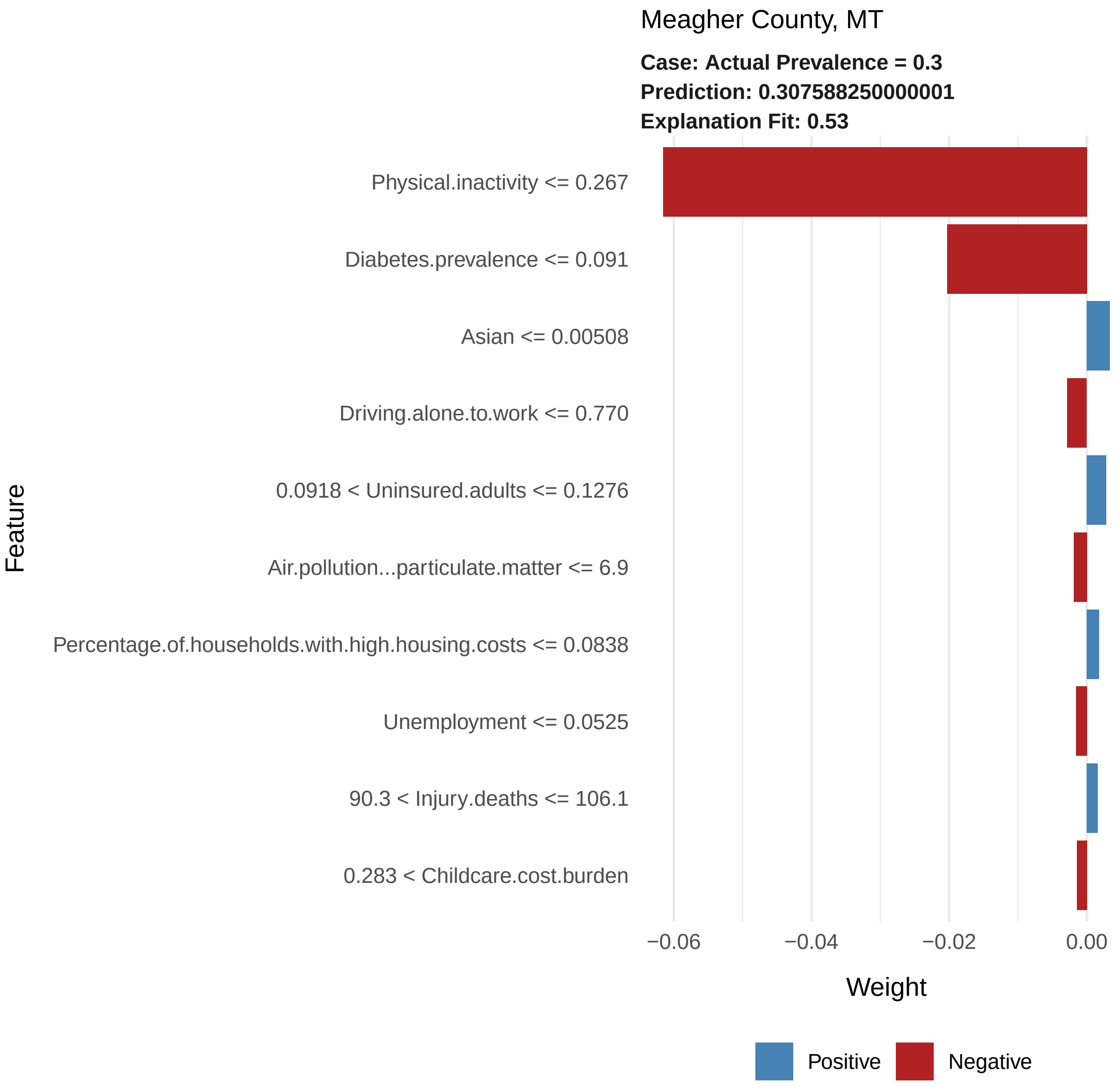

Meagher County, MT
Case: Actual Prevalence = 0.3
Prediction: 0.307588250000001
Explanation Fit: 0.53
Physical.inactivity <= 0.267
Diabetes.prevalence <= 0.091
Asian <= 0.00508
Driving.alone.to.work <= 0.770
0.0918 < Uninsured.adults <= 0.1276
Air.pollution...particulate.matter <= 6.9
Percentage.of.households.with.high.housing.costs <= 0.0838
Unemployment <= 0.0525
90.3 < Injury.deaths <= 106.1
0.283 < Childcare.cost.burden
Feature
−0.06
−0.04
−0.02
0.00
Weight
Positive
Negative

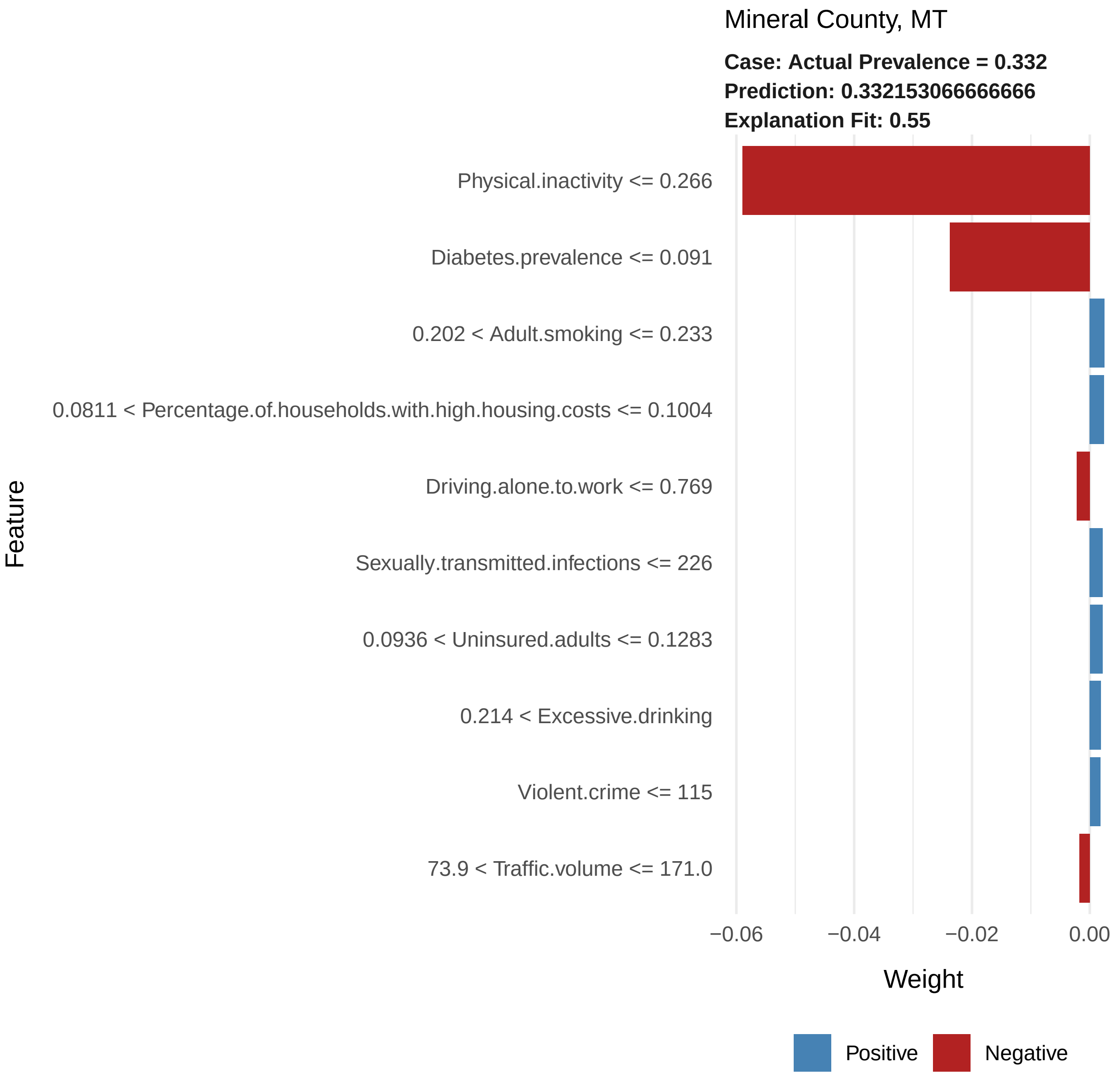

Mineral County, MT
Case: Actual Prevalence = 0.332
Prediction: 0.332153066666666
Explanation Fit: 0.55
Physical.inactivity <= 0.266
Diabetes.prevalence <= 0.091
0.202 < Adult.smoking <= 0.233
0.0811 < Percentage.of.households.with.high.housing.costs <= 0.1004
Driving.alone.to.work <= 0.769
Sexually.transmitted.infections <= 226
0.0936 < Uninsured.adults <= 0.1283
0.214 < Excessive.drinking
Violent.crime <= 115
73.9 < Traffic.volume <= 171.0
Feature
−0.06
−0.04
−0.02
0.00
Weight
Positive
Negative

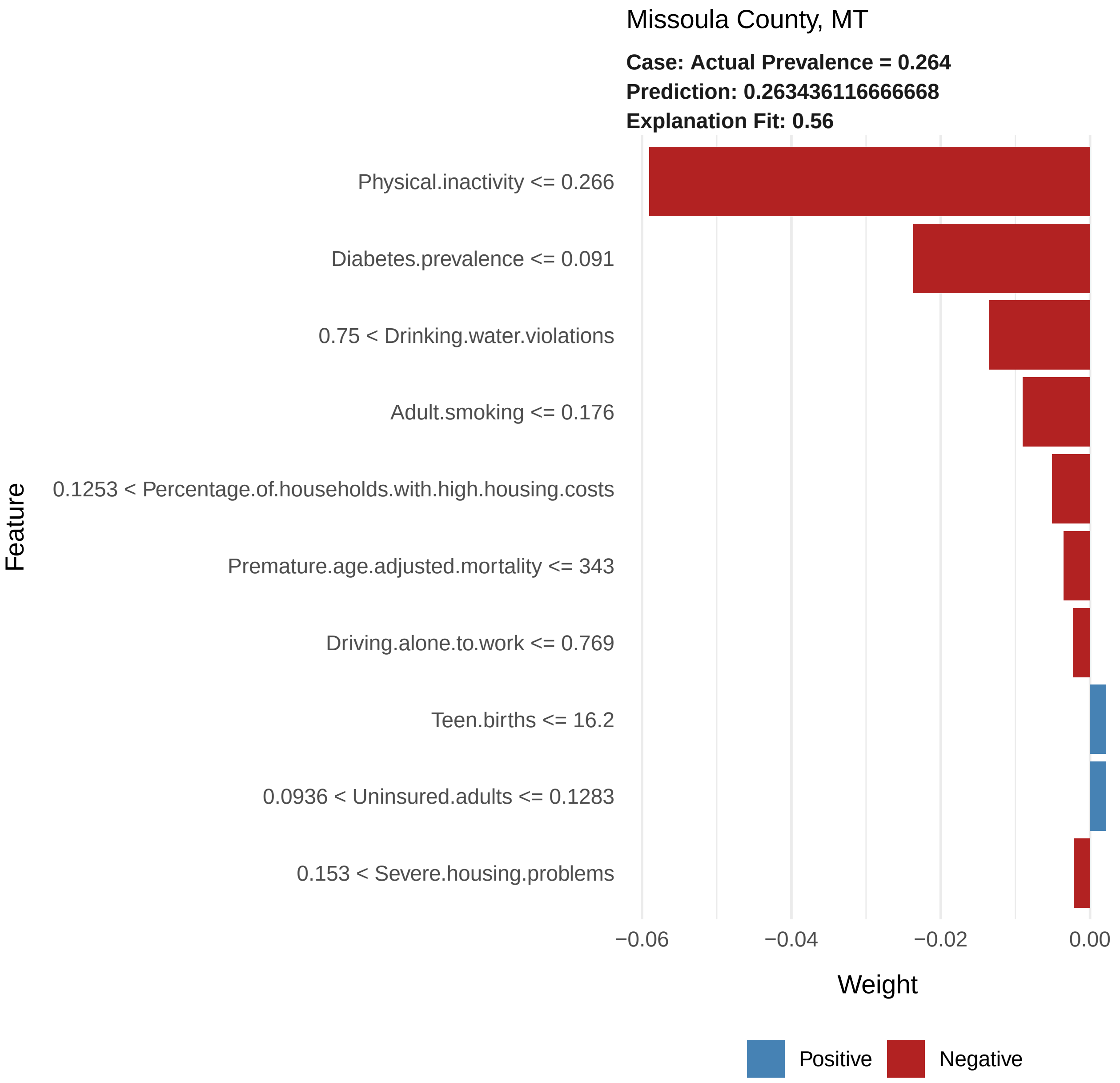
Missoula County, MT
Case: Actual Prevalence = 0.264
Prediction: 0.263436116666668
Explanation Fit: 0.56
Physical.inactivity <= 0.266
Diabetes.prevalence <= 0.091
0.75 < Drinking.water.violations
Adult.smoking <= 0.176
0.1253 < Percentage.of.households.with.high.housing.costs
Premature.age.adjusted.mortality <= 343
Driving.alone.to.work <= 0.769
Teen.births <= 16.2
0.0936 < Uninsured.adults <= 0.1283
0.153 < Severe.housing.problems
Feature
−0.06
−0.04
−0.02
0.00
Weight
Positive
Negative

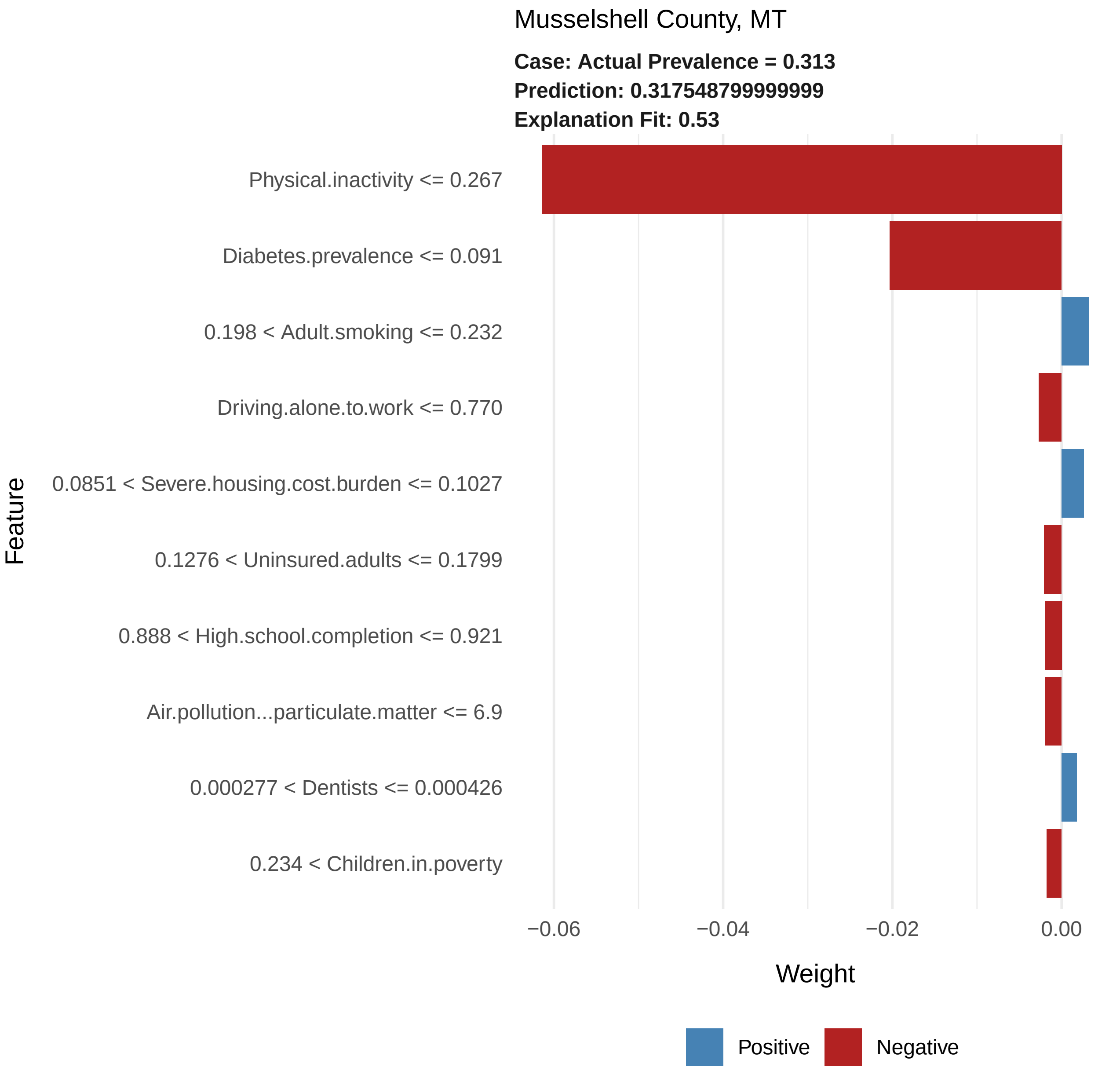
Musselshell County, MT
Case: Actual Prevalence = 0.313
Prediction: 0.317548799999999
Explanation Fit: 0.53
Physical.inactivity <= 0.267
Diabetes.prevalence <= 0.091
0.198 < Adult.smoking <= 0.232
Driving.alone.to.work <= 0.770
0.0851 < Severe.housing.cost.burden <= 0.1027
0.1276 < Uninsured.adults <= 0.1799
0.888 < High.school.completion <= 0.921
Air.pollution...particulate.matter <= 6.9
0.000277 < Dentists <= 0.000426
0.234 < Children.in.poverty
Feature
−0.06
−0.04
−0.02
0.00
Weight
Positive
Negative

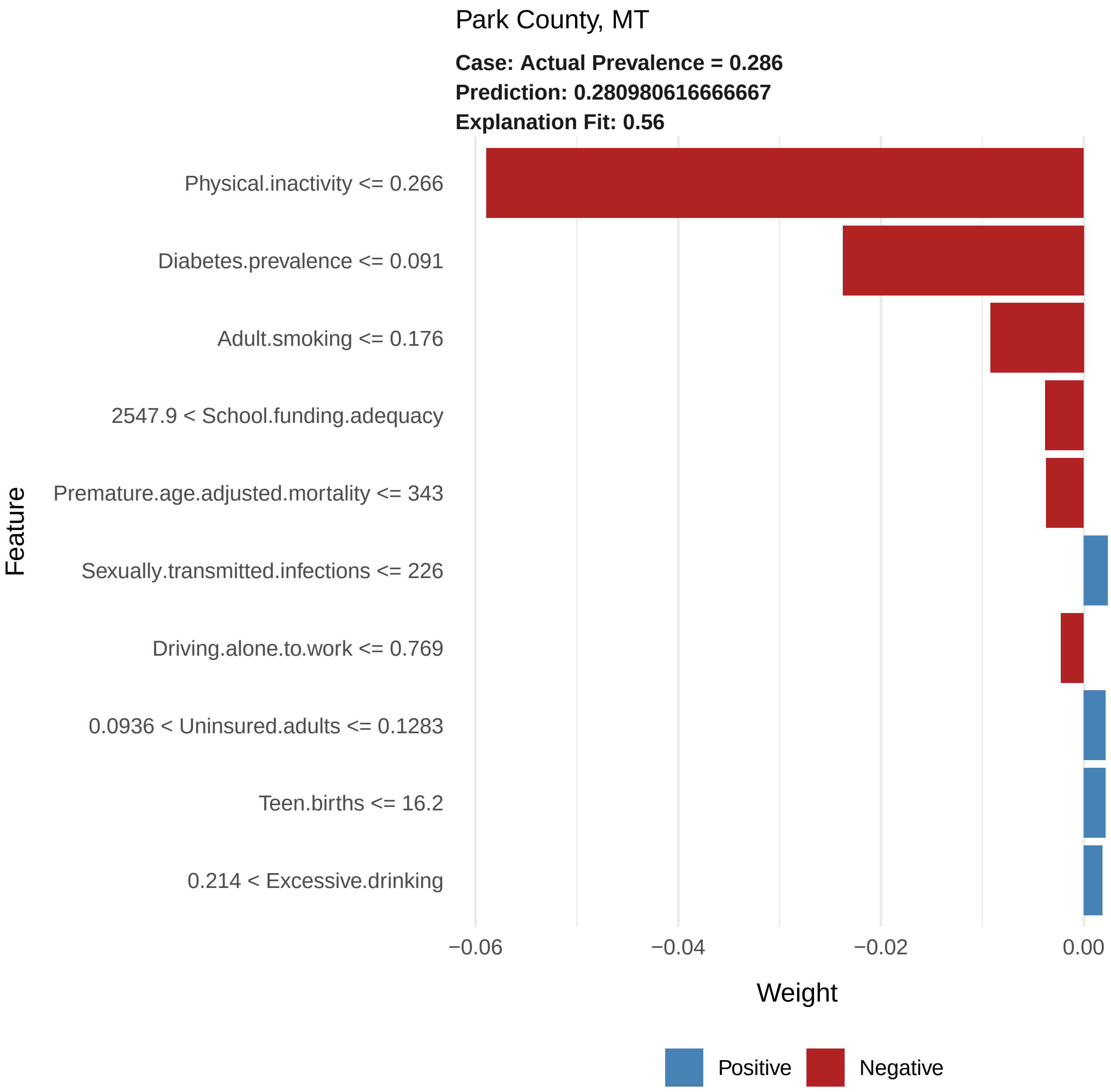

Park County, MT
Case: Actual Prevalence = 0.286
Prediction: 0.280980616666667
Explanation Fit: 0.56
Physical.inactivity <= 0.266
Diabetes.prevalence <= 0.091
Adult.smoking <= 0.176
2547.9 < School.funding.adequacy
Premature.age.adjusted.mortality <= 343
Sexually.transmitted.infections <= 226
Driving.alone.to.work <= 0.769
0.0936 < Uninsured.adults <= 0.1283
Teen.births <= 16.2
0.214 < Excessive.drinking
Feature
−0.06
−0.04
−0.02
0.00
Weight
Positive
Negative

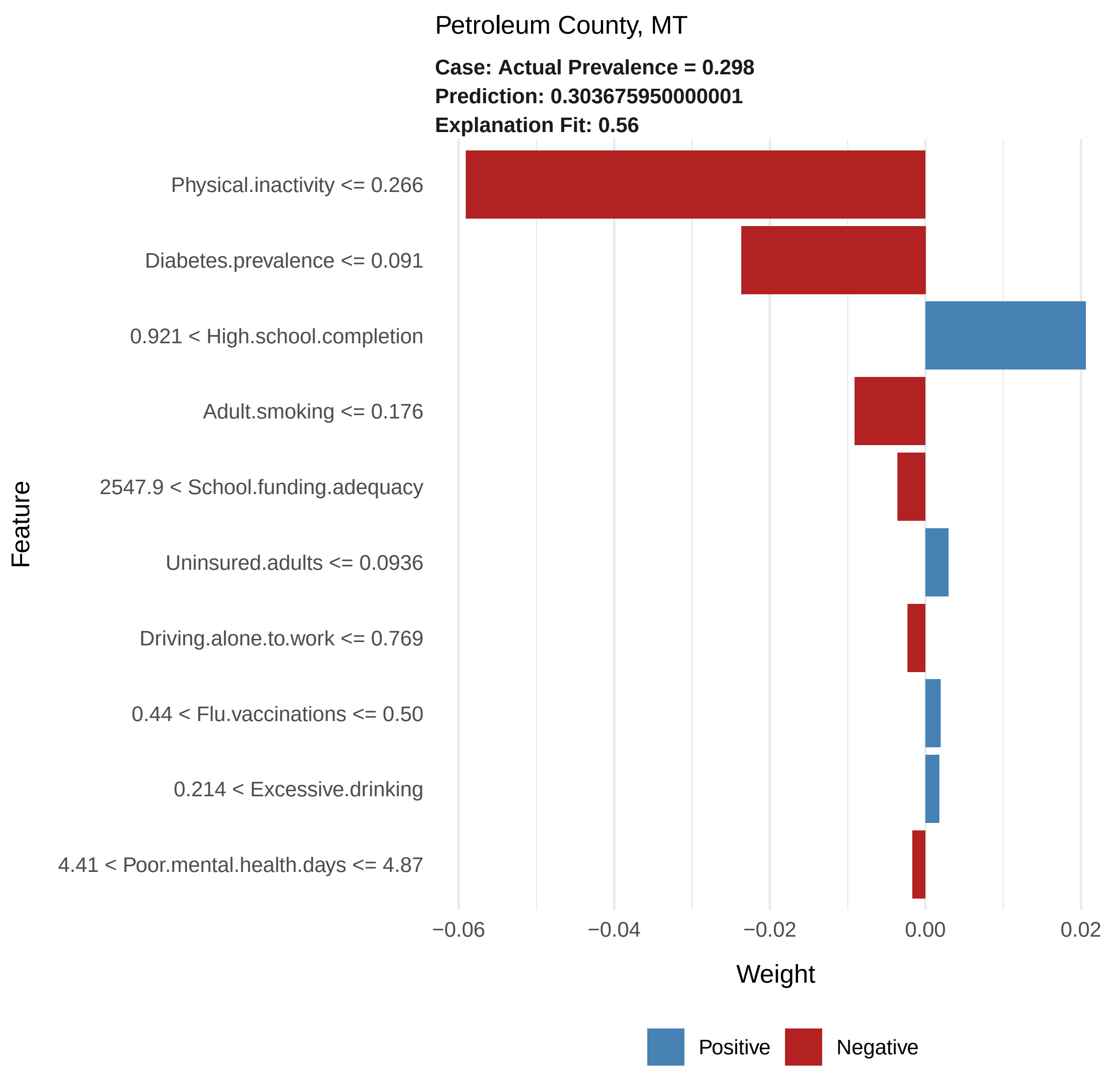
Petroleum County, MT
Case: Actual Prevalence = 0.298
Prediction: 0.303675950000001
Explanation Fit: 0.56
Physical.inactivity <= 0.266
Diabetes.prevalence <= 0.091
0.921 < High.school.completion
Adult.smoking <= 0.176
2547.9 < School.funding.adequacy
Uninsured.adults <= 0.0936
Driving.alone.to.work <= 0.769
0.44 < Flu.vaccinations <= 0.50
0.214 < Excessive.drinking
4.41 < Poor.mental.health.days <= 4.87
Feature
−0.06
−0.04
−0.02
0.00
0.02
Weight
Positive
Negative

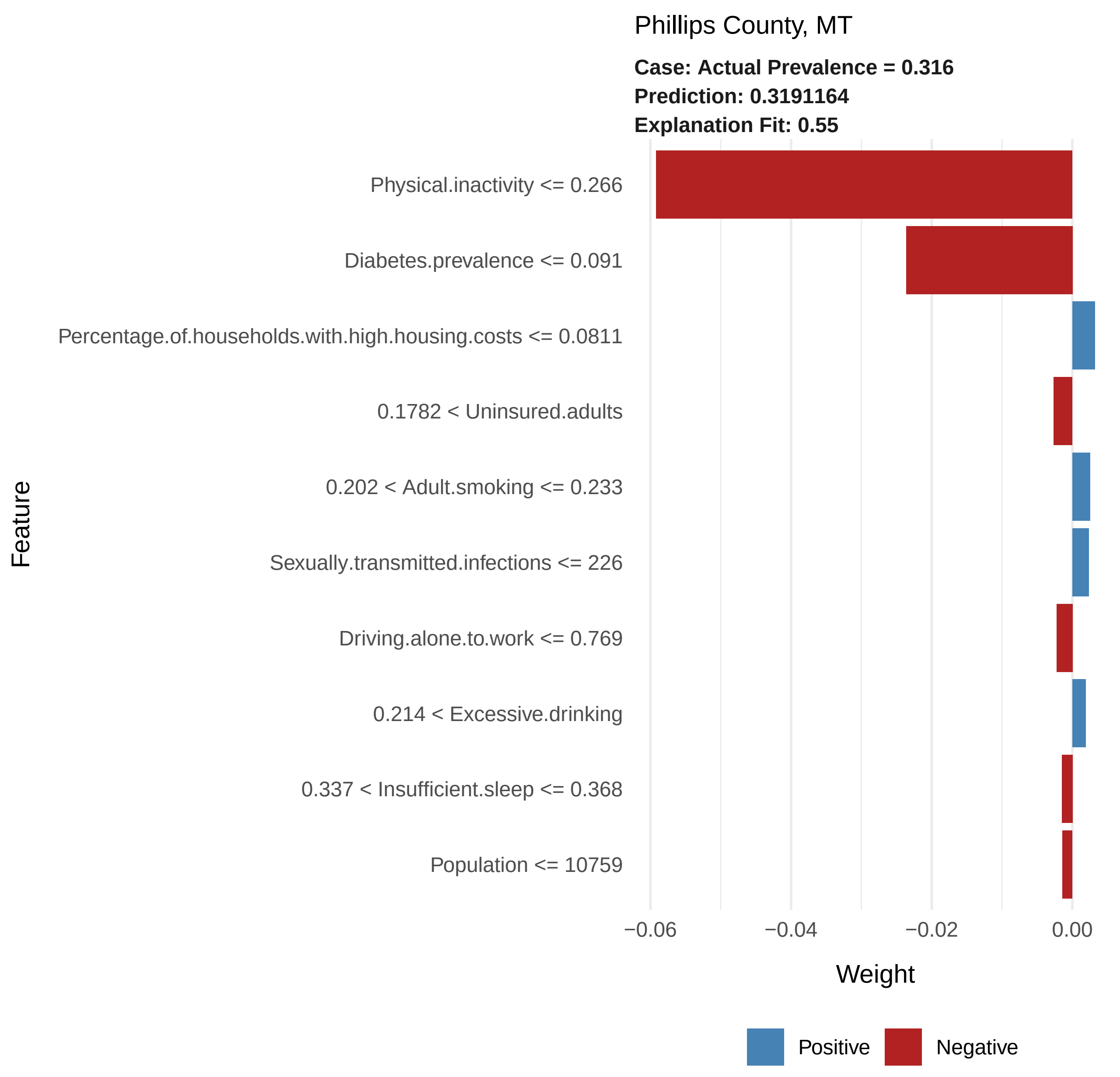

Phillips County, MT
Case: Actual Prevalence = 0.316
Prediction: 0.3191164
Explanation Fit: 0.55
Physical.inactivity <= 0.266
Diabetes.prevalence <= 0.091
Percentage.of.households.with.high.housing.costs <= 0.0811
0.1782 < Uninsured.adults
0.202 < Adult.smoking <= 0.233
Sexually.transmitted.infections <= 226
Driving.alone.to.work <= 0.769
0.214 < Excessive.drinking
0.337 < Insufficient.sleep <= 0.368
Population <= 10759
Feature
−0.06
−0.04
−0.02
0.00
Weight
Positive
Negative

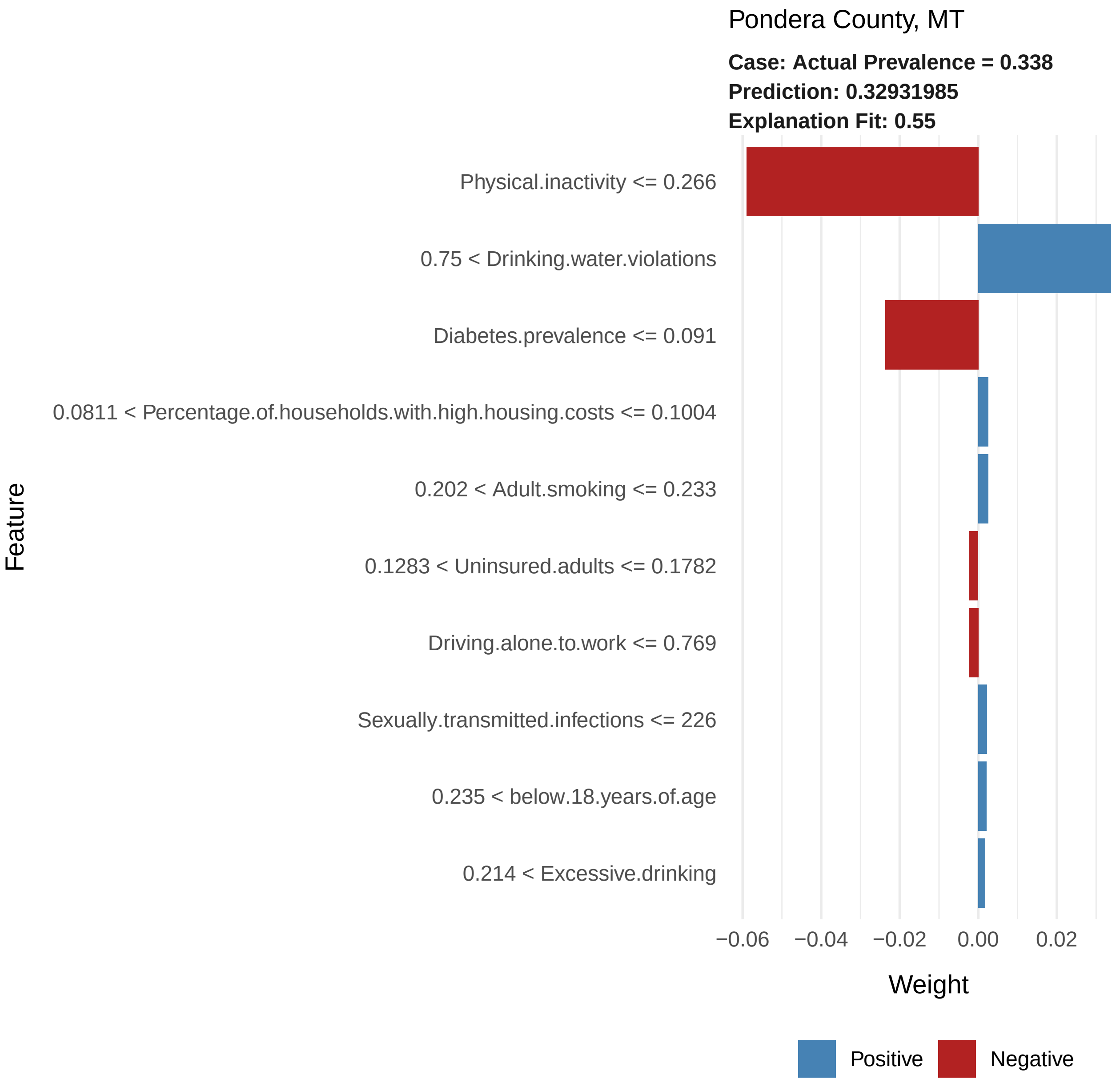

Pondera County, MT
Case: Actual Prevalence = 0.338
Prediction: 0.32931985
Explanation Fit: 0.55
Physical.inactivity <= 0.266
0.75 < Drinking.water.violations
Diabetes.prevalence <= 0.091
0.0811 < Percentage.of.households.with.high.housing.costs <= 0.1004
0.202 < Adult.smoking <= 0.233
0.1283 < Uninsured.adults <= 0.1782
Driving.alone.to.work <= 0.769
Sexually.transmitted.infections <= 226
0.235 < below.18.years.of.age
0.214 < Excessive.drinking
Feature
−0.06
−0.04
−0.02
0.00
0.02
Weight
Positive
Negative

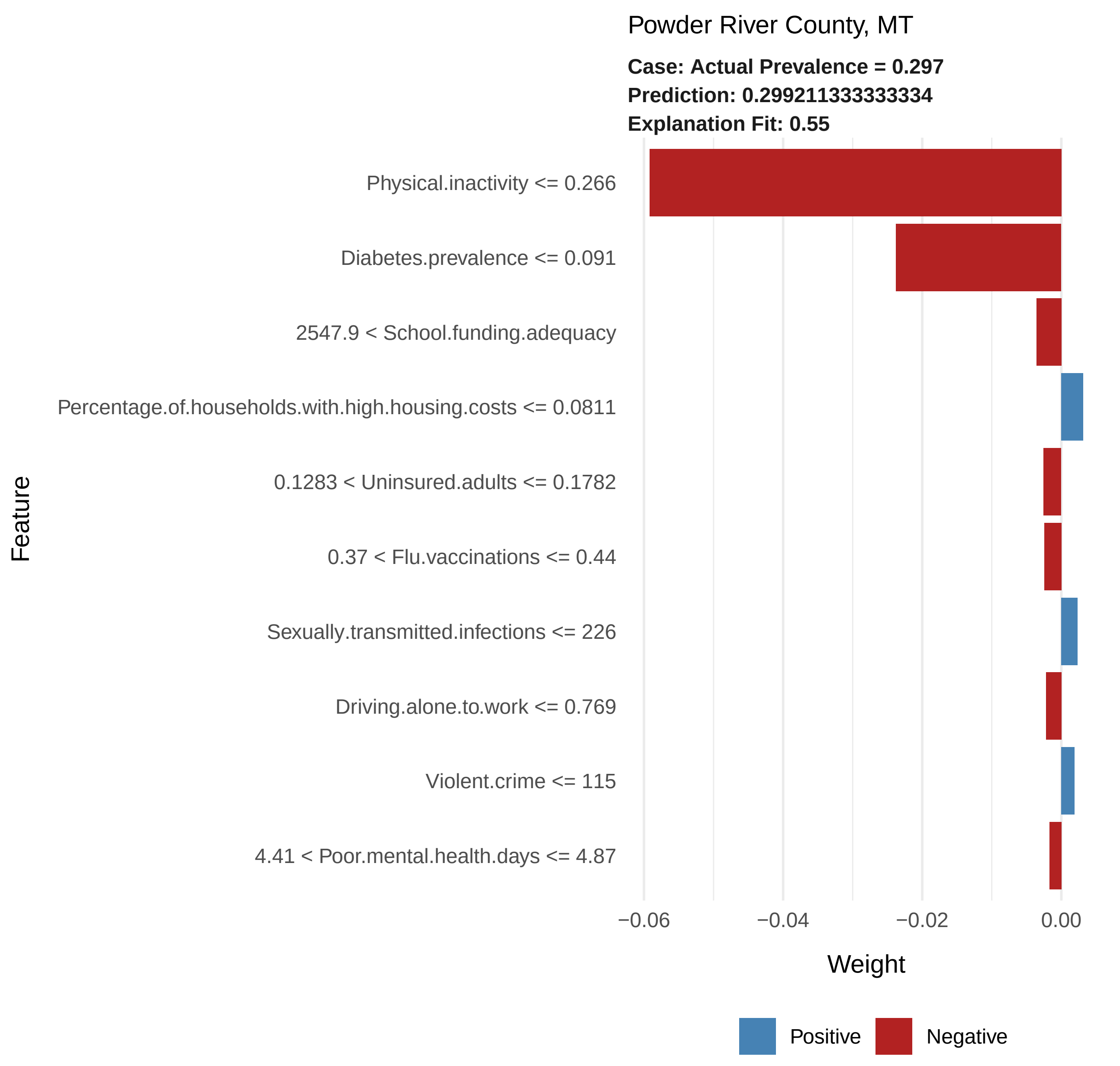
Powder River County, MT
Case: Actual Prevalence = 0.297
Prediction: 0.299211333333334
Explanation Fit: 0.55
Physical.inactivity <= 0.266
Diabetes.prevalence <= 0.091
2547.9 < School.funding.adequacy
Percentage.of.households.with.high.housing.costs <= 0.0811
0.1283 < Uninsured.adults <= 0.1782
0.37 < Flu.vaccinations <= 0.44
Sexually.transmitted.infections <= 226
Driving.alone.to.work <= 0.769
Violent.crime <= 115
4.41 < Poor.mental.health.days <= 4.87
Feature
−0.06
−0.04
−0.02
0.00
Weight
Positive
Negative

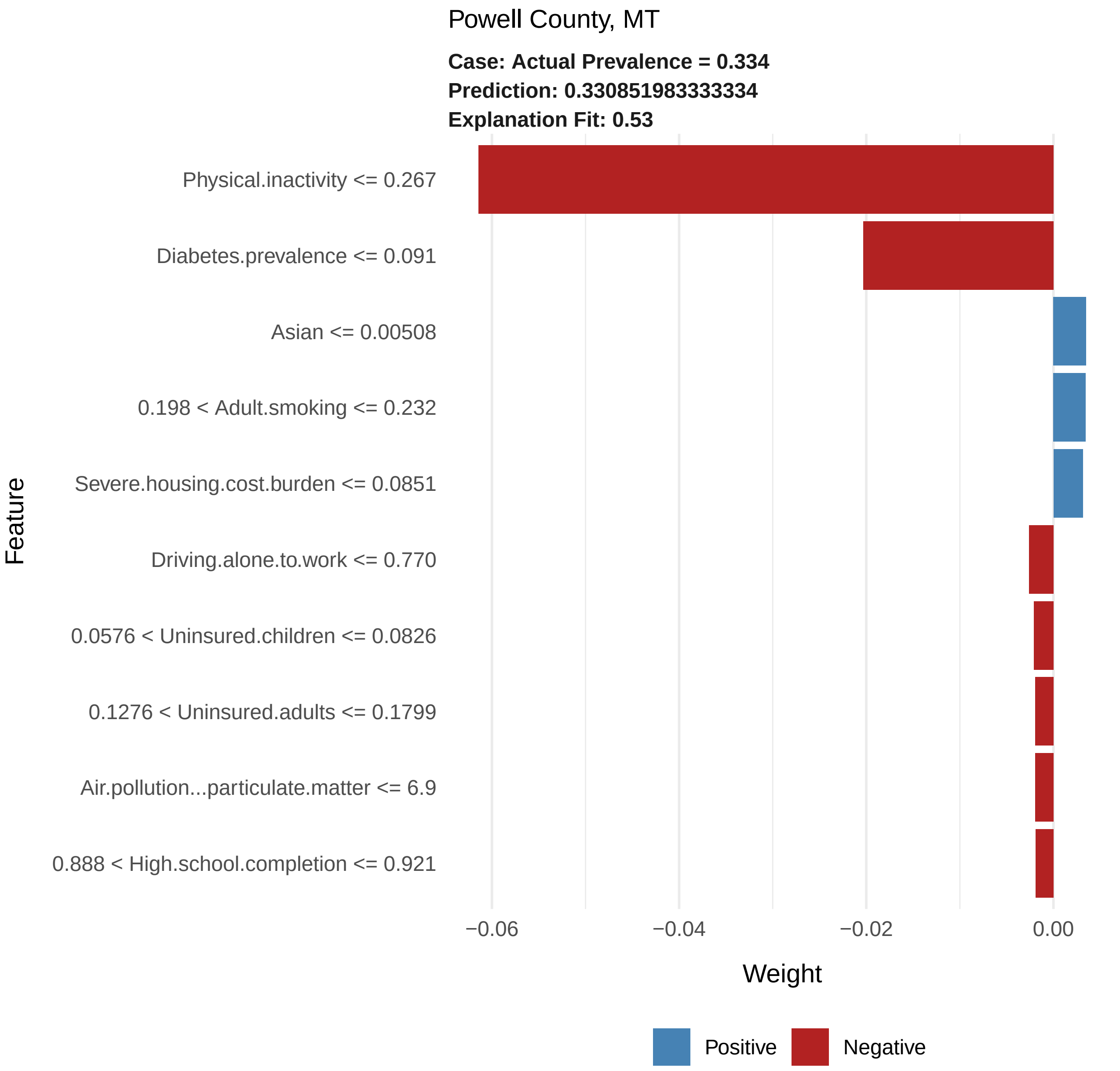
Powell County, MT
Case: Actual Prevalence = 0.334
Prediction: 0.330851983333334
Explanation Fit: 0.53
Physical.inactivity <= 0.267
Diabetes.prevalence <= 0.091
Asian <= 0.00508
0.198 < Adult.smoking <= 0.232
Severe.housing.cost.burden <= 0.0851
Driving.alone.to.work <= 0.770
0.0576 < Uninsured.children <= 0.0826
0.1276 < Uninsured.adults <= 0.1799
Air.pollution...particulate.matter <= 6.9
0.888 < High.school.completion <= 0.921
Feature
−0.06
−0.04
−0.02
0.00
Weight
Positive
Negative

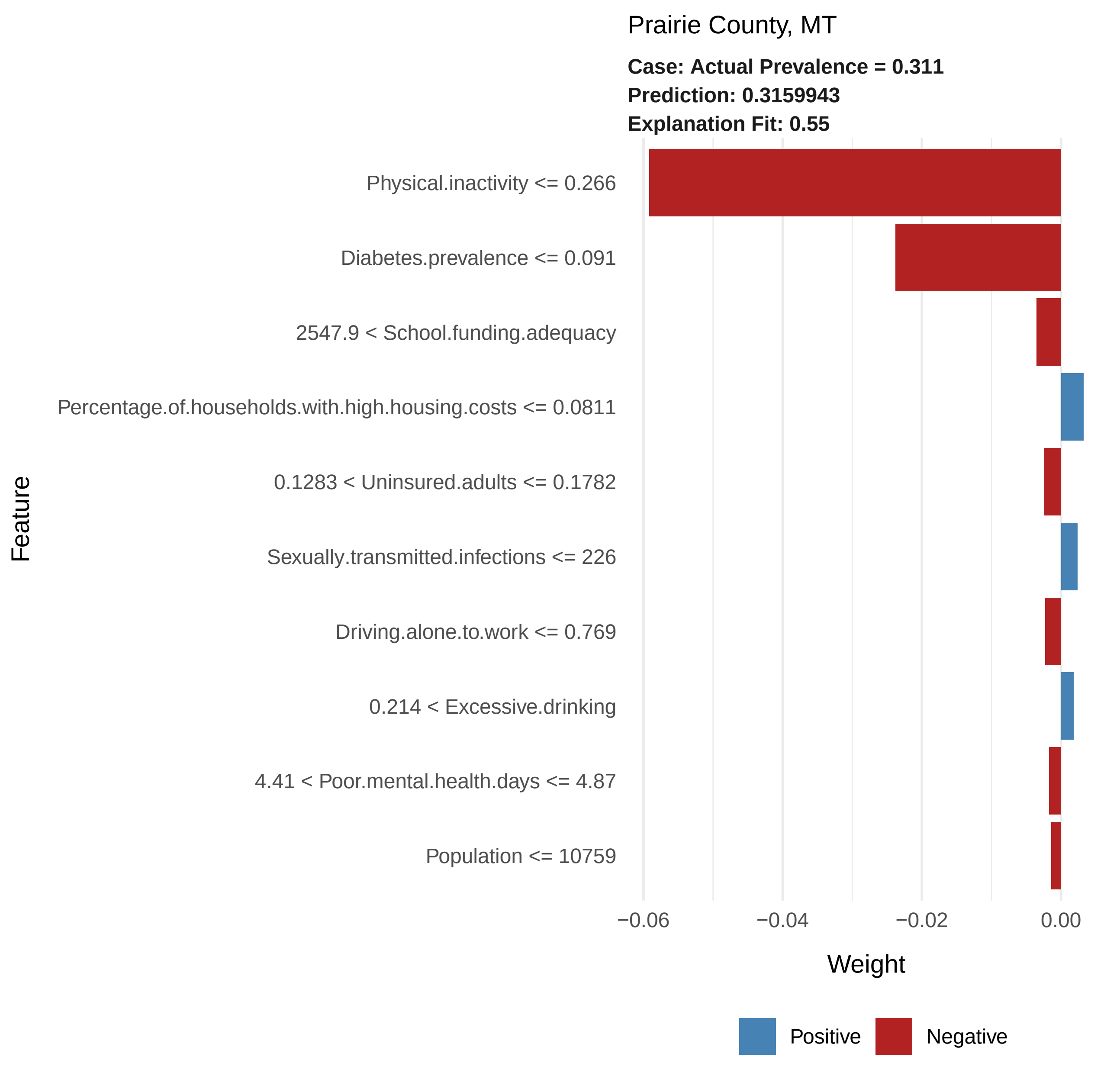
Prairie County, MT
Case: Actual Prevalence = 0.311
Prediction: 0.3159943
Explanation Fit: 0.55
Physical.inactivity <= 0.266
Diabetes.prevalence <= 0.091
2547.9 < School.funding.adequacy
Percentage.of.households.with.high.housing.costs <= 0.0811
0.1283 < Uninsured.adults <= 0.1782
Sexually.transmitted.infections <= 226
Driving.alone.to.work <= 0.769
0.214 < Excessive.drinking
4.41 < Poor.mental.health.days <= 4.87
Population <= 10759
Feature
−0.06
−0.04
−0.02
0.00
Weight
Positive
Negative

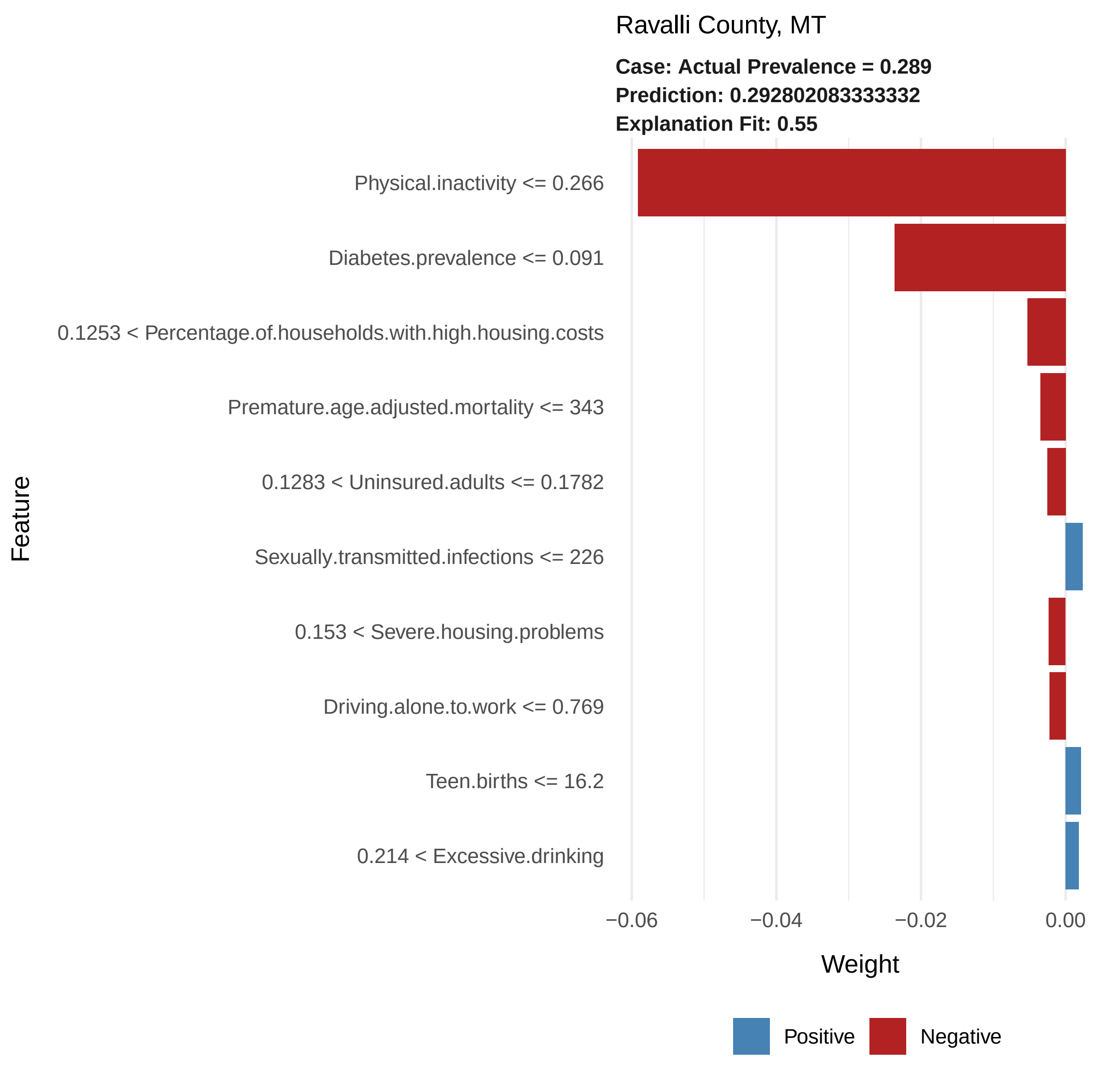

Ravalli County, MT
Case: Actual Prevalence = 0.289
Prediction: 0.292802083333332
Explanation Fit: 0.55
Physical.inactivity <= 0.266
Diabetes.prevalence <= 0.091
0.1253 < Percentage.of.households.with.high.housing.costs
Premature.age.adjusted.mortality <= 343
0.1283 < Uninsured.adults <= 0.1782
Sexually.transmitted.infections <= 226
0.153 < Severe.housing.problems
Driving.alone.to.work <= 0.769
Teen.births <= 16.2
0.214 < Excessive.drinking
Feature
−0.06
−0.04
−0.02
0.00
Weight
Positive
Negative

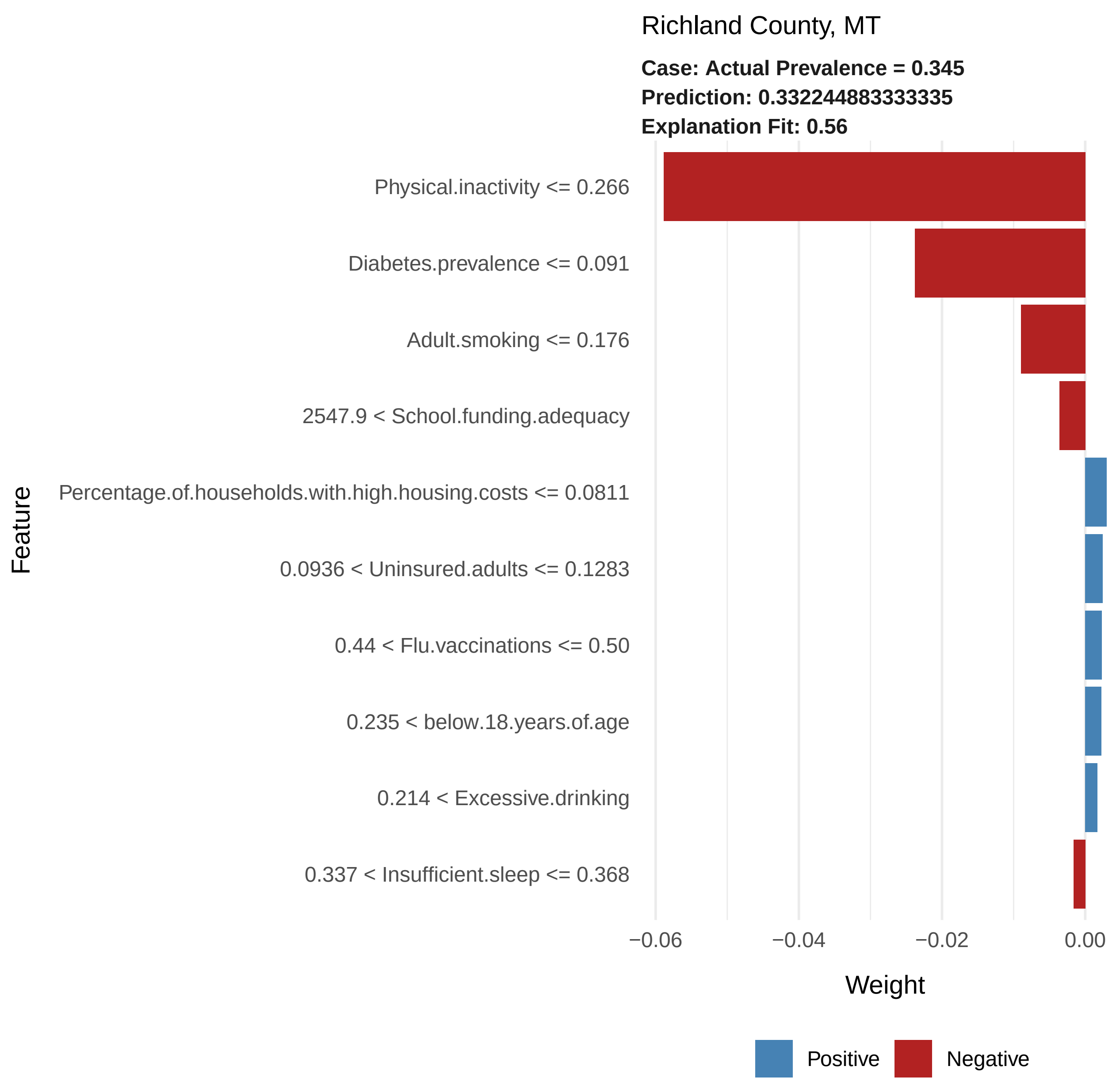
Richland County, MT
Case: Actual Prevalence = 0.345
Prediction: 0.332244883333335
Explanation Fit: 0.56
Physical.inactivity <= 0.266
Diabetes.prevalence <= 0.091
Adult.smoking <= 0.176
2547.9 < School.funding.adequacy
Percentage.of.households.with.high.housing.costs <= 0.0811
0.0936 < Uninsured.adults <= 0.1283
0.44 < Flu.vaccinations <= 0.50
0.235 < below.18.years.of.age
0.214 < Excessive.drinking
0.337 < Insufficient.sleep <= 0.368
Feature
−0.06
−0.04
−0.02
0.00
Weight
Positive
Negative

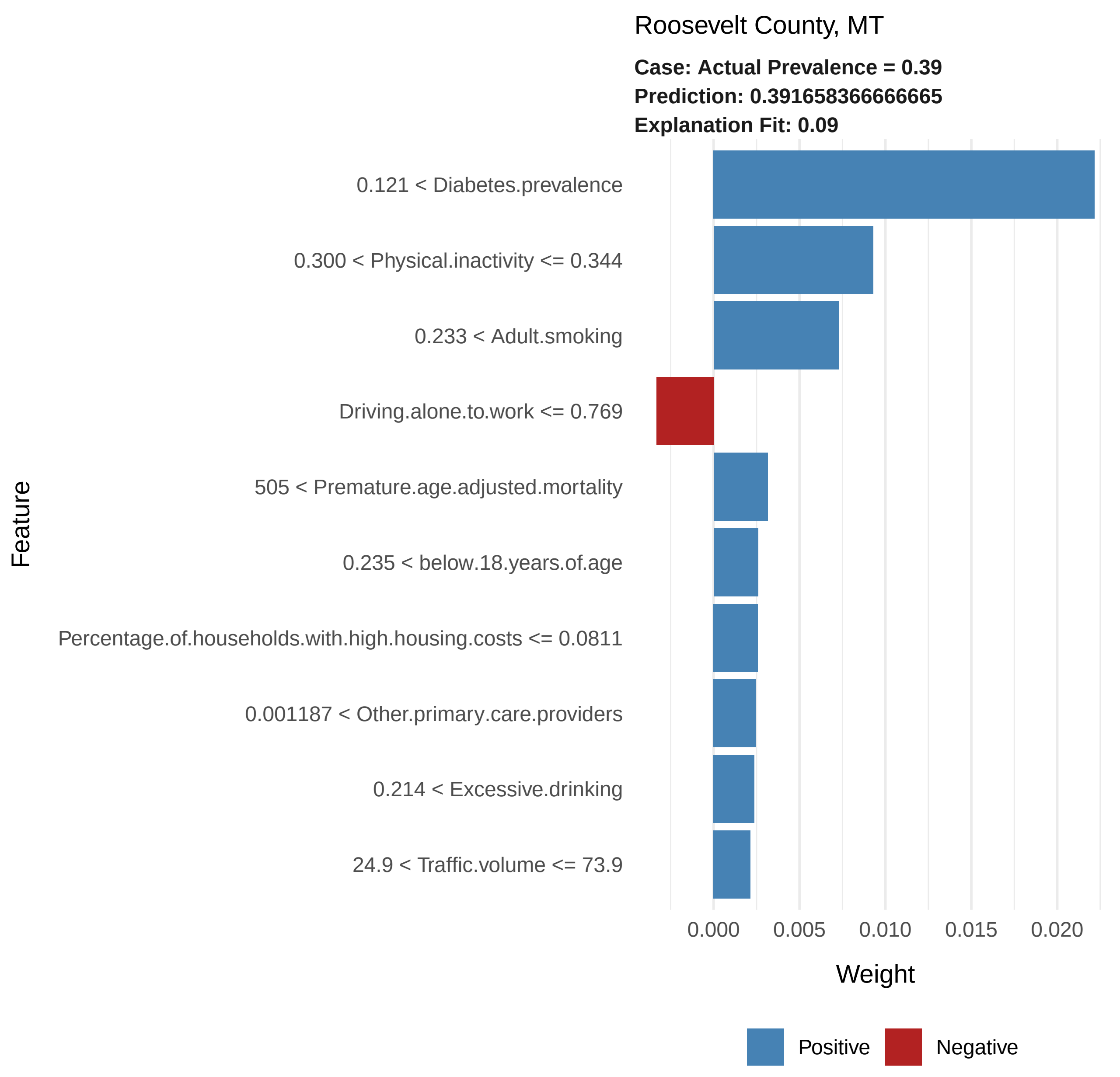

Roosevelt County, MT
Case: Actual Prevalence = 0.39
Prediction: 0.391658366666665
Explanation Fit: 0.09
0.121 < Diabetes.prevalence
0.300 < Physical.inactivity <= 0.344
0.233 < Adult.smoking
Driving.alone.to.work <= 0.769
505 < Premature.age.adjusted.mortality
0.235 < below.18.years.of.age
Percentage.of.households.with.high.housing.costs <= 0.0811
0.001187 < Other.primary.care.providers
0.214 < Excessive.drinking
24.9 < Traffic.volume <= 73.9
Feature
0.000
0.005
0.010
0.015
0.020
Weight
Positive
Negative

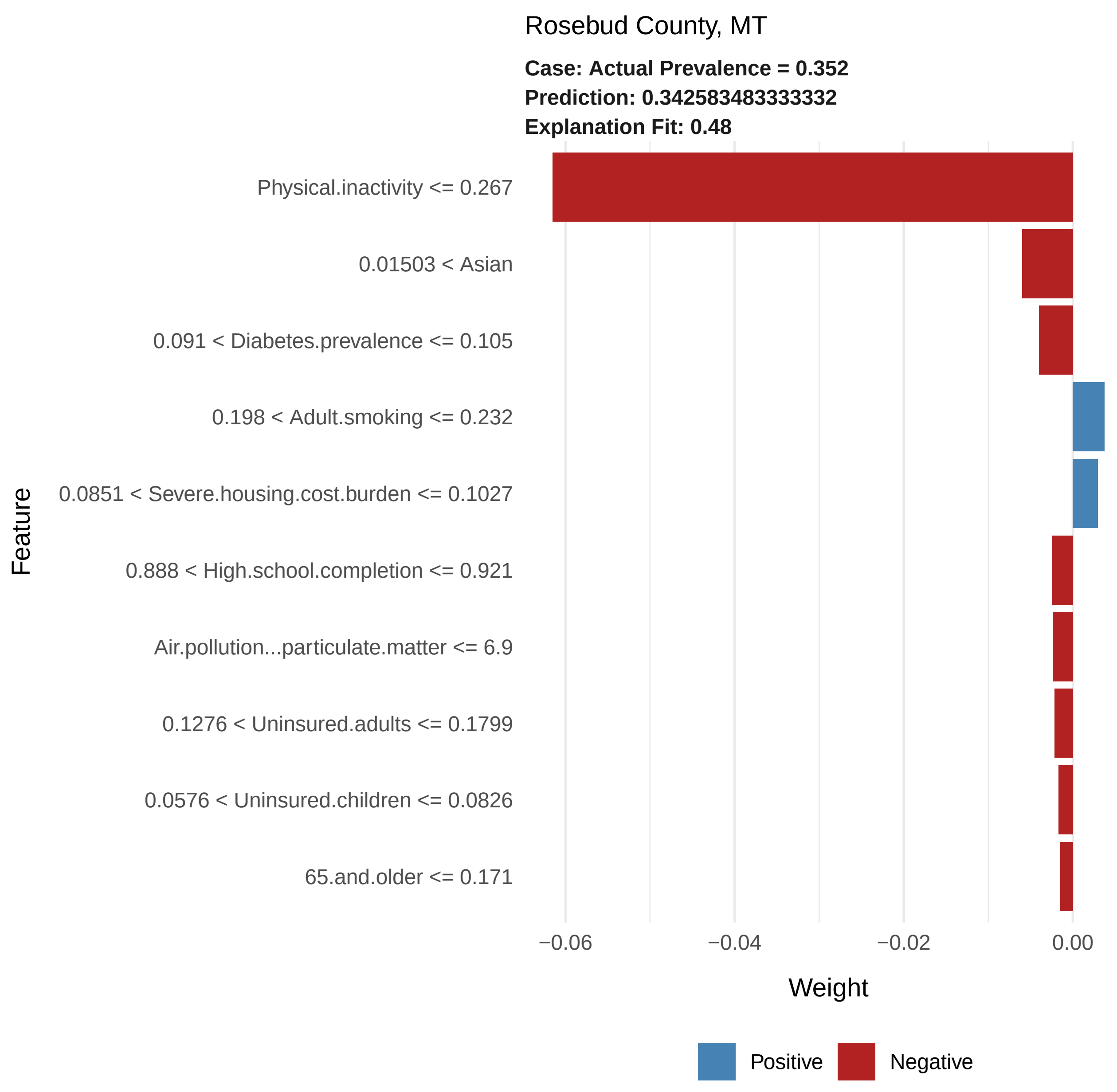
Rosebud County, MT
Case: Actual Prevalence = 0.352
Prediction: 0.342583483333332
Explanation Fit: 0.48
Physical.inactivity <= 0.267
0.01503 < Asian
0.091 < Diabetes.prevalence <= 0.105
0.198 < Adult.smoking <= 0.232
0.0851 < Severe.housing.cost.burden <= 0.1027
0.888 < High.school.completion <= 0.921
Air.pollution...particulate.matter <= 6.9
0.1276 < Uninsured.adults <= 0.1799
0.0576 < Uninsured.children <= 0.0826
65.and.older <= 0.171
Feature
−0.06
−0.04
−0.02
0.00
Weight
Positive
Negative

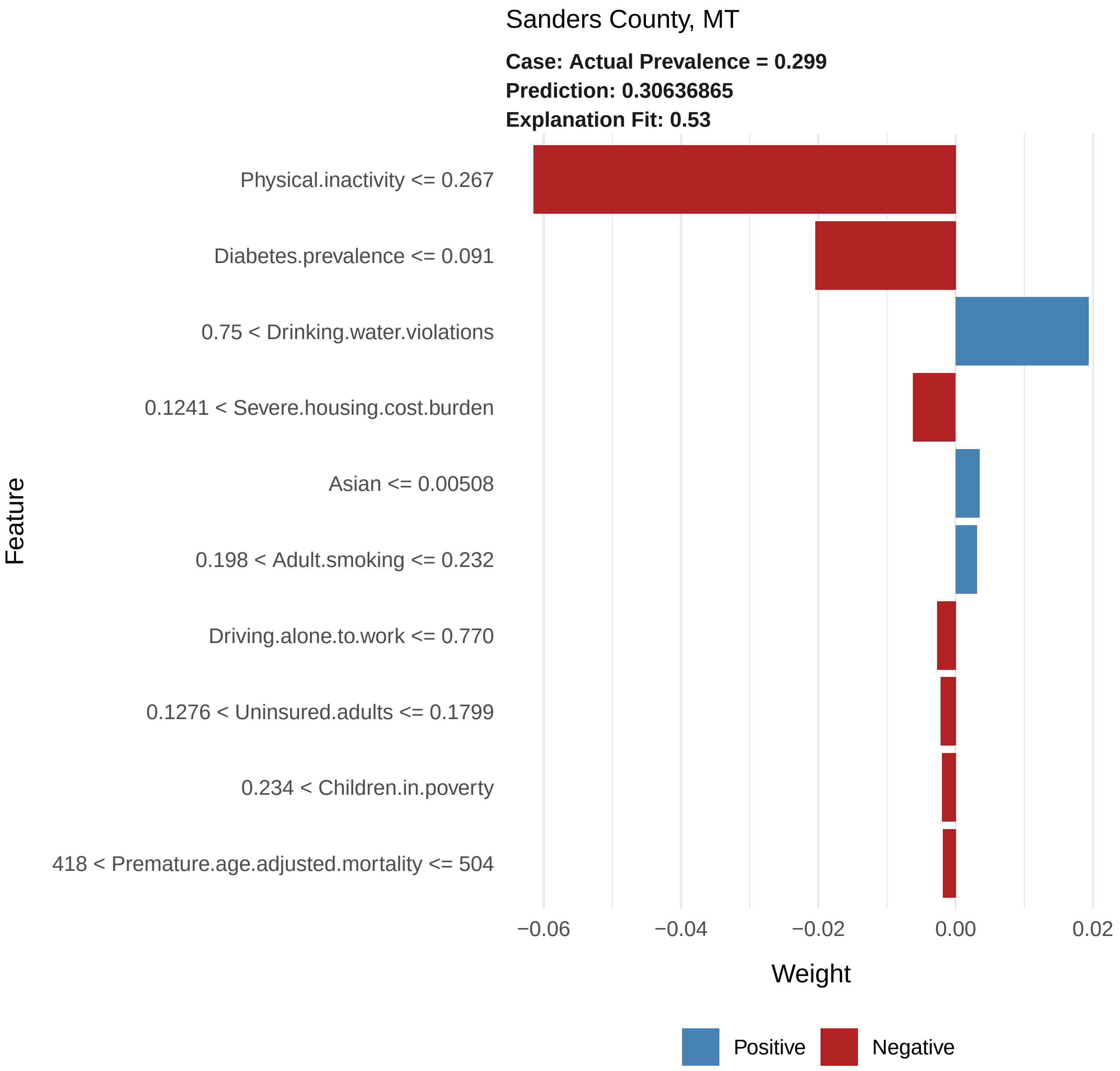

Sanders County, MT
Case: Actual Prevalence = 0.299
Prediction: 0.30636865
Explanation Fit: 0.53
Physical.inactivity <= 0.267
Diabetes.prevalence <= 0.091
0.75 < Drinking.water.violations
0.1241 < Severe.housing.cost.burden
Asian <= 0.00508
0.198 < Adult.smoking <= 0.232
Driving.alone.to.work <= 0.770
0.1276 < Uninsured.adults <= 0.1799
0.234 < Children.in.poverty
418 < Premature.age.adjusted.mortality <= 504
Feature
−0.06
−0.04
−0.02
0.00
0.02
Weight
Positive
Negative

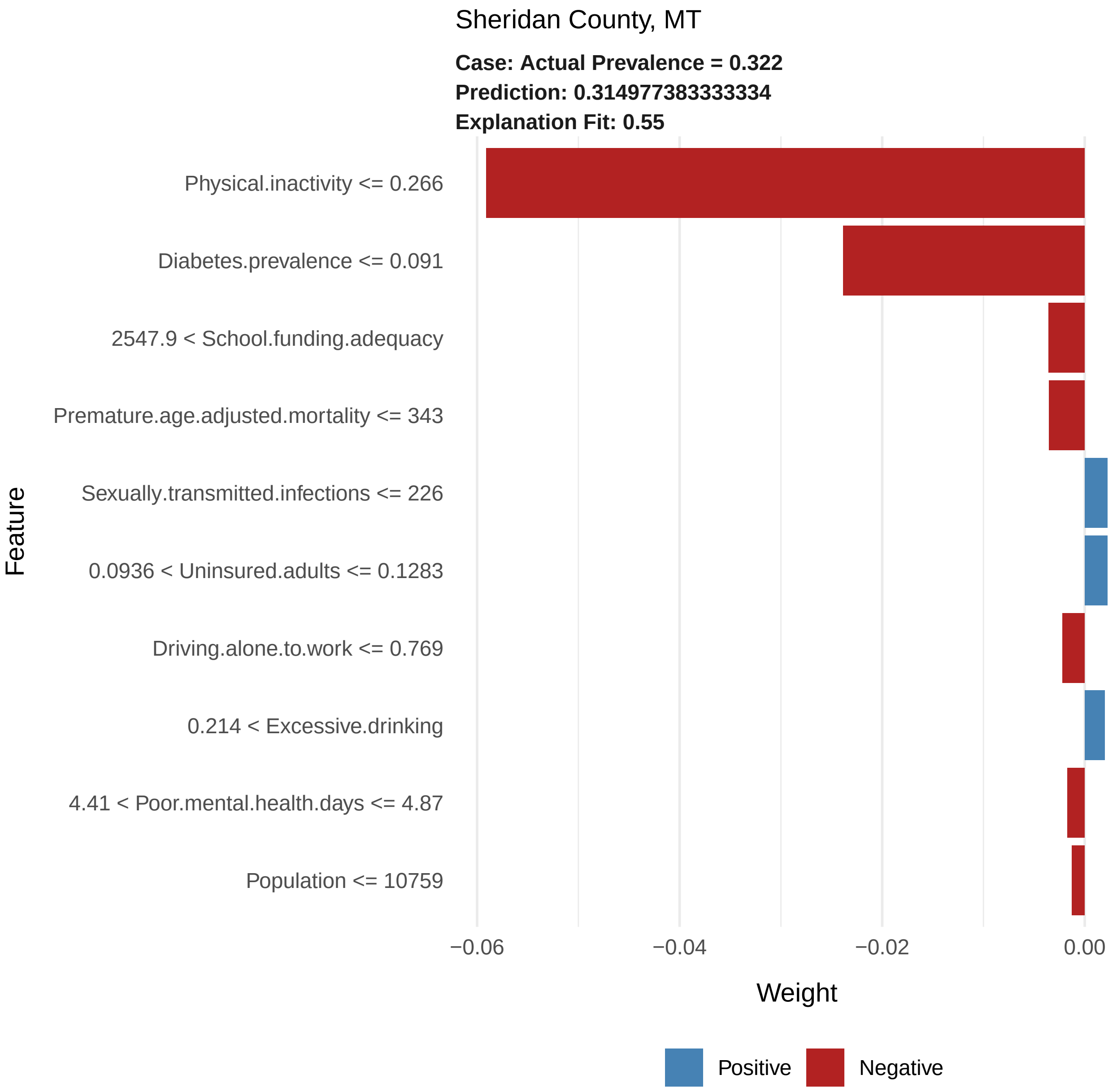
Sheridan County, MT
Case: Actual Prevalence = 0.322
Prediction: 0.314977383333334
Explanation Fit: 0.55
Physical.inactivity <= 0.266
Diabetes.prevalence <= 0.091
2547.9 < School.funding.adequacy
Premature.age.adjusted.mortality <= 343
Sexually.transmitted.infections <= 226
0.0936 < Uninsured.adults <= 0.1283
Driving.alone.to.work <= 0.769
0.214 < Excessive.drinking
4.41 < Poor.mental.health.days <= 4.87
Population <= 10759
Feature
−0.06
−0.04
−0.02
0.00
Weight
Positive
Negative

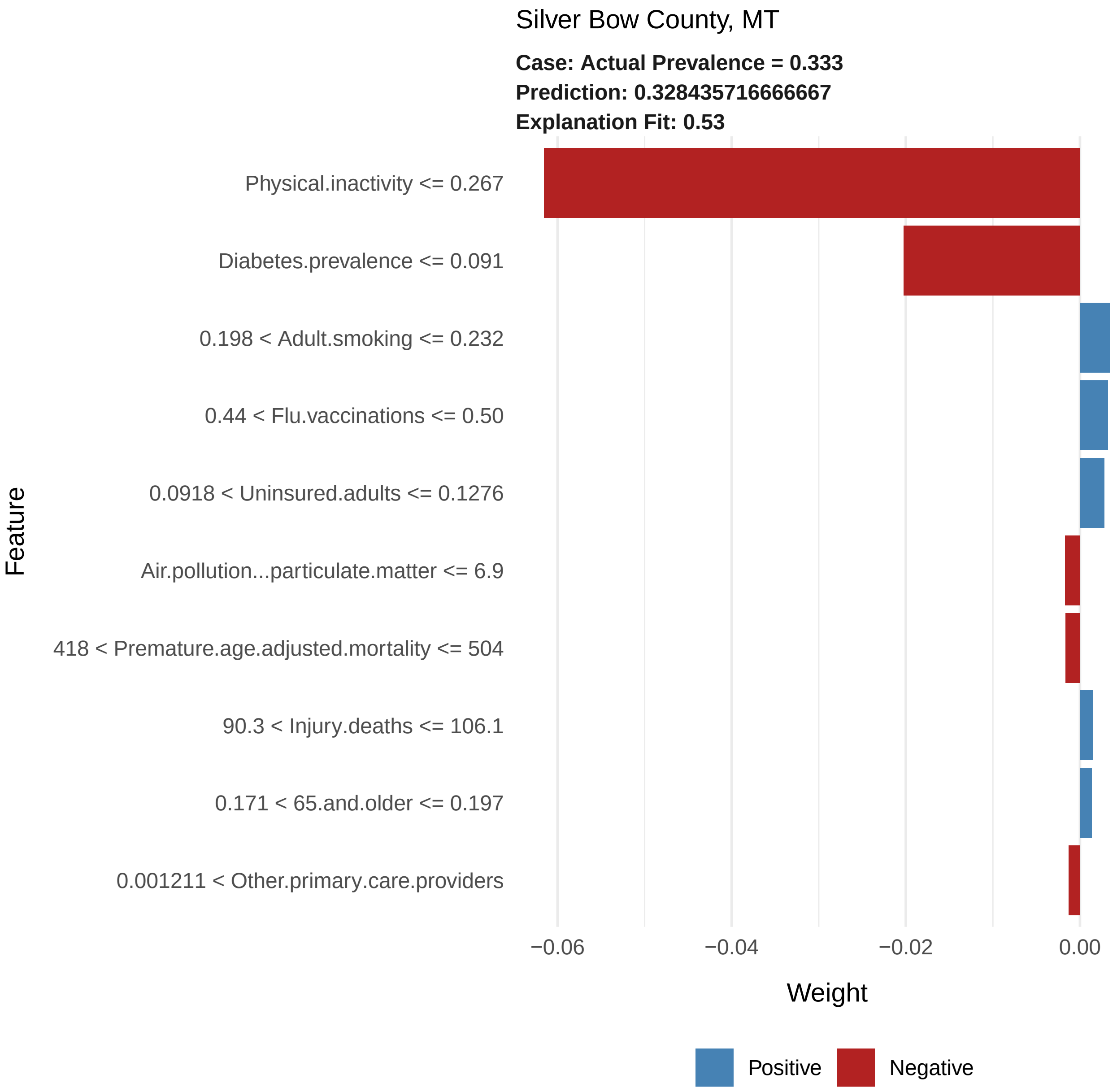

Silver Bow County, MT
Case: Actual Prevalence = 0.333
Prediction: 0.328435716666667
Explanation Fit: 0.53
Physical.inactivity <= 0.267
Diabetes.prevalence <= 0.091
0.198 < Adult.smoking <= 0.232
0.44 < Flu.vaccinations <= 0.50
0.0918 < Uninsured.adults <= 0.1276
Air.pollution...particulate.matter <= 6.9
418 < Premature.age.adjusted.mortality <= 504
90.3 < Injury.deaths <= 106.1
0.171 < 65.and.older <= 0.197
0.001211 < Other.primary.care.providers
Feature
−0.06
−0.04
−0.02
0.00
Weight
Positive
Negative

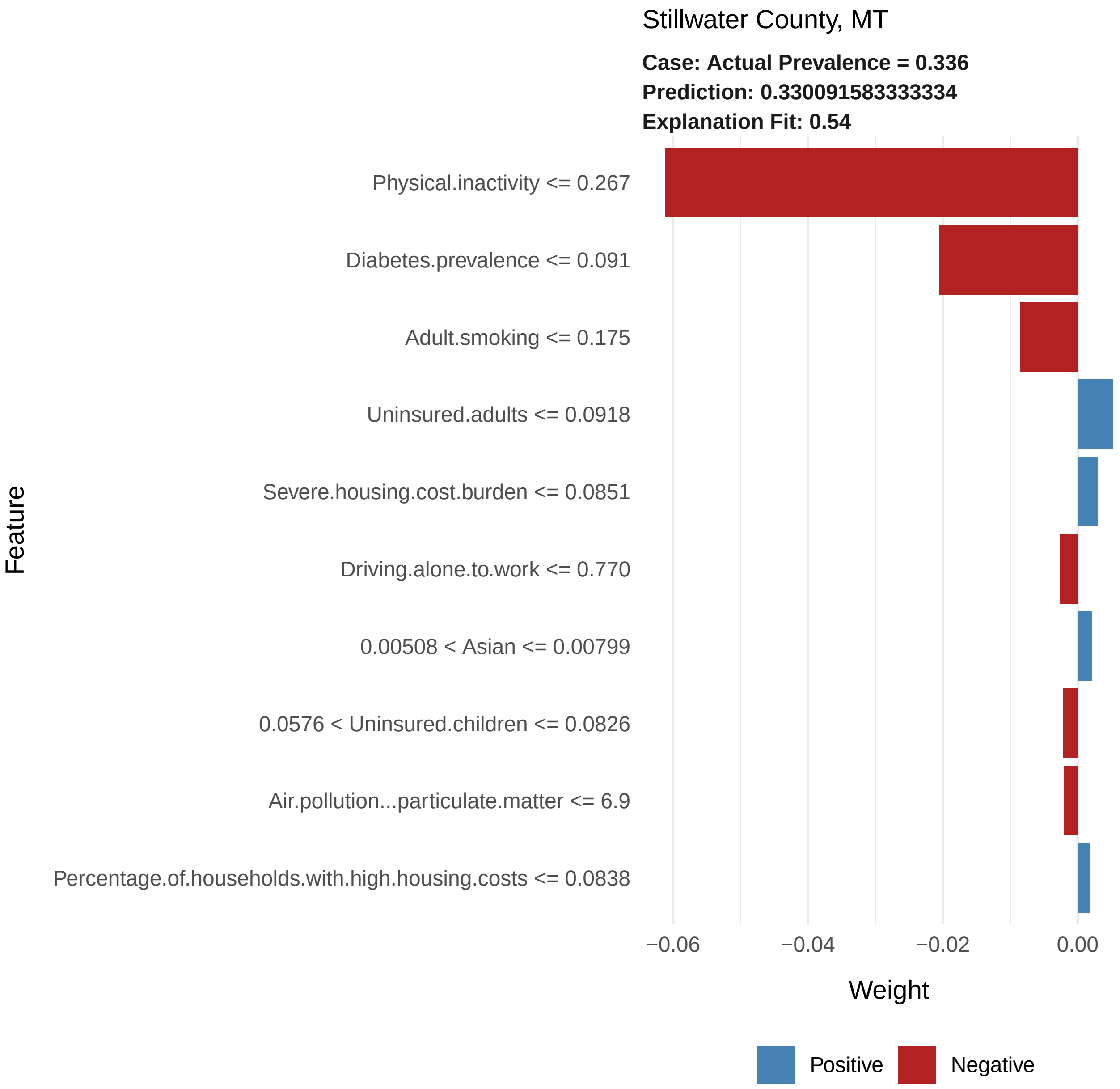

Stillwater County, MT
Case: Actual Prevalence = 0.336
Prediction: 0.330091583333334
Explanation Fit: 0.54
Physical.inactivity <= 0.267
Diabetes.prevalence <= 0.091
Adult.smoking <= 0.175
Uninsured.adults <= 0.0918
Severe.housing.cost.burden <= 0.0851
Driving.alone.to.work <= 0.770
0.00508 < Asian <= 0.00799
0.0576 < Uninsured.children <= 0.0826
Air.pollution...particulate.matter <= 6.9
Percentage.of.households.with.high.housing.costs <= 0.0838
Feature
−0.06
−0.04
−0.02
0.00
Weight
Positive
Negative

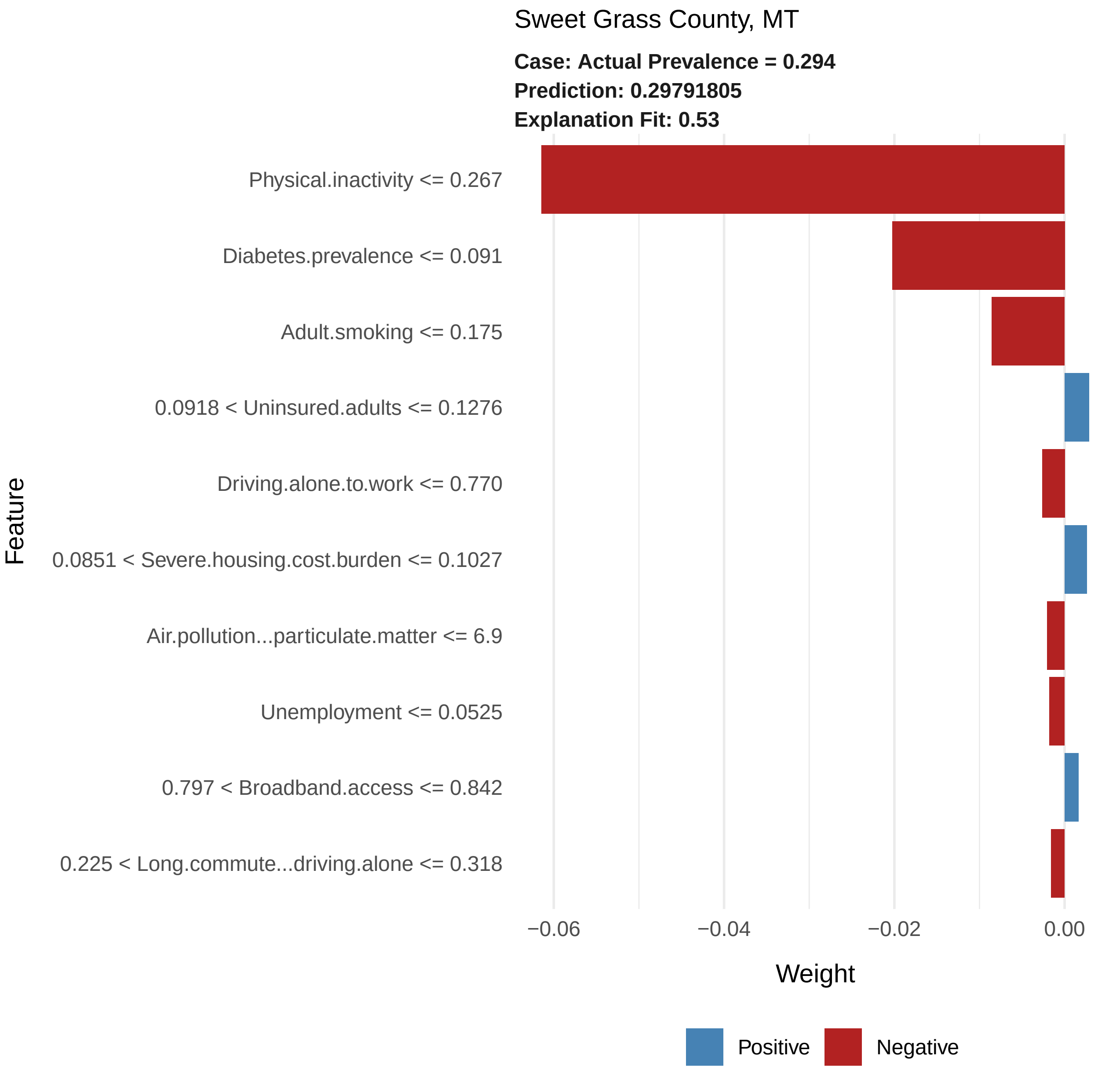

Sweet Grass County, MT
Case: Actual Prevalence = 0.294
Prediction: 0.29791805
Explanation Fit: 0.53
Physical.inactivity <= 0.267
Diabetes.prevalence <= 0.091
Adult.smoking <= 0.175
0.0918 < Uninsured.adults <= 0.1276
Driving.alone.to.work <= 0.770
0.0851 < Severe.housing.cost.burden <= 0.1027
Air.pollution...particulate.matter <= 6.9
Unemployment <= 0.0525
0.797 < Broadband.access <= 0.842
0.225 < Long.commute...driving.alone <= 0.318
Feature
−0.06
−0.04
−0.02
0.00
Weight
Positive
Negative

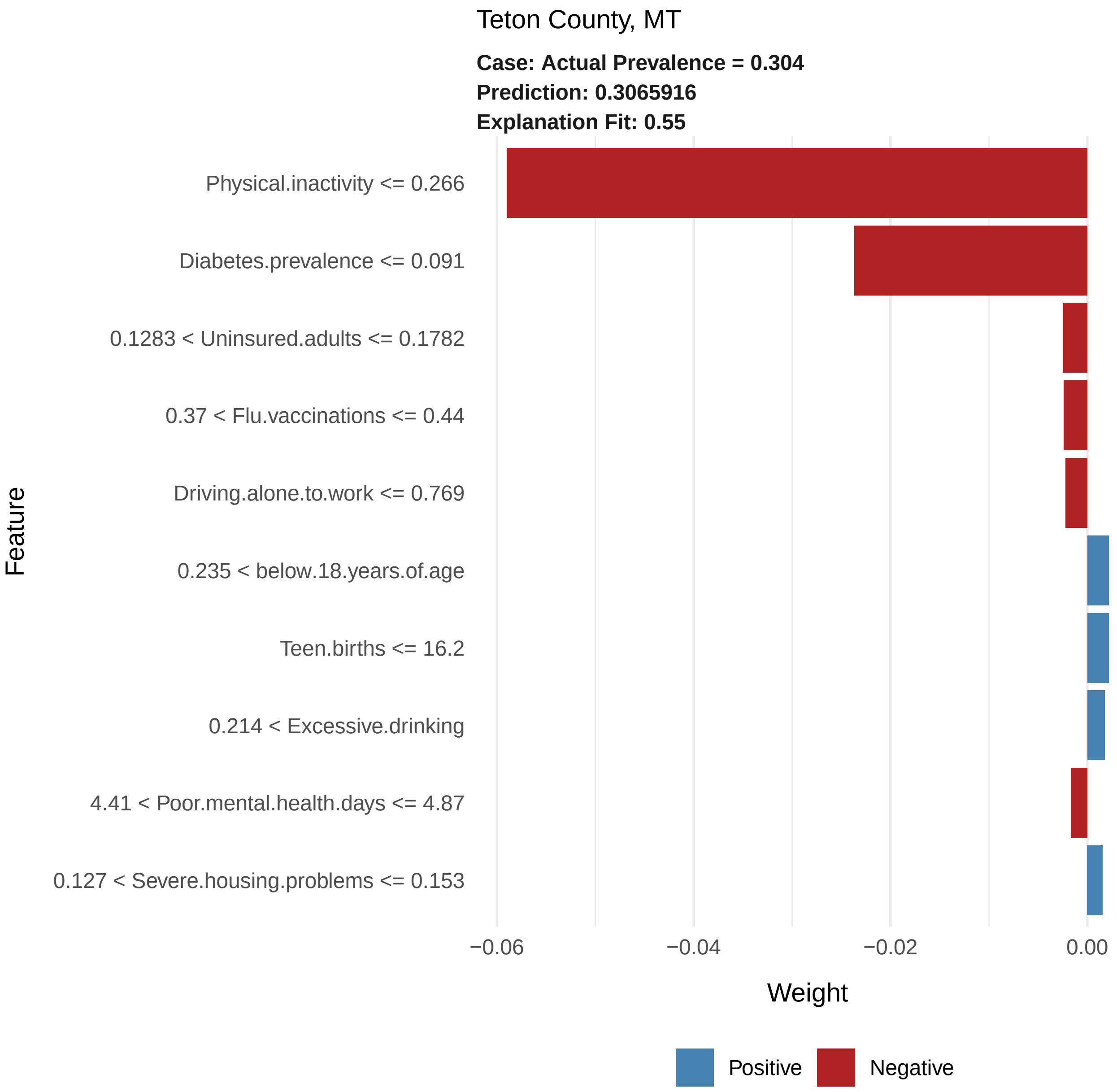

Teton County, MT
Case: Actual Prevalence = 0.304
Prediction: 0.3065916
Explanation Fit: 0.55
Physical.inactivity <= 0.266
Diabetes.prevalence <= 0.091
0.1283 < Uninsured.adults <= 0.1782
0.37 < Flu.vaccinations <= 0.44
Driving.alone.to.work <= 0.769
0.235 < below.18.years.of.age
Teen.births <= 16.2
0.214 < Excessive.drinking
4.41 < Poor.mental.health.days <= 4.87
0.127 < Severe.housing.problems <= 0.153
Feature
−0.06
−0.04
−0.02
0.00
Weight
Positive
Negative

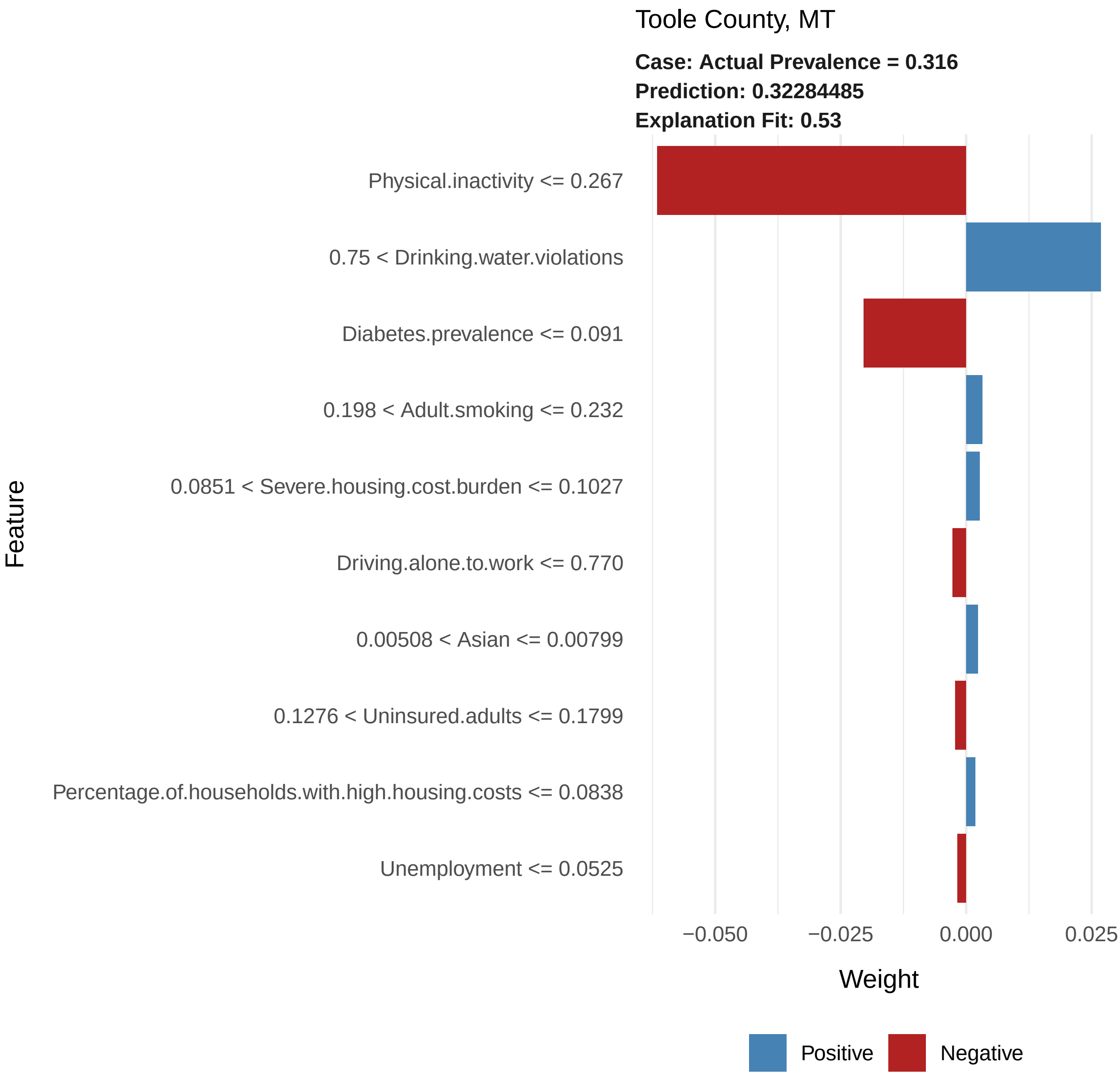

Toole County, MT
Case: Actual Prevalence = 0.316
Prediction: 0.32284485
Explanation Fit: 0.53
Physical.inactivity <= 0.267
0.75 < Drinking.water.violations
Diabetes.prevalence <= 0.091
0.198 < Adult.smoking <= 0.232
0.0851 < Severe.housing.cost.burden <= 0.1027
Driving.alone.to.work <= 0.770
0.00508 < Asian <= 0.00799
0.1276 < Uninsured.adults <= 0.1799
Percentage.of.households.with.high.housing.costs <= 0.0838
Unemployment <= 0.0525
Feature
−0.050
−0.025
0.000
0.025
Weight
Positive
Negative

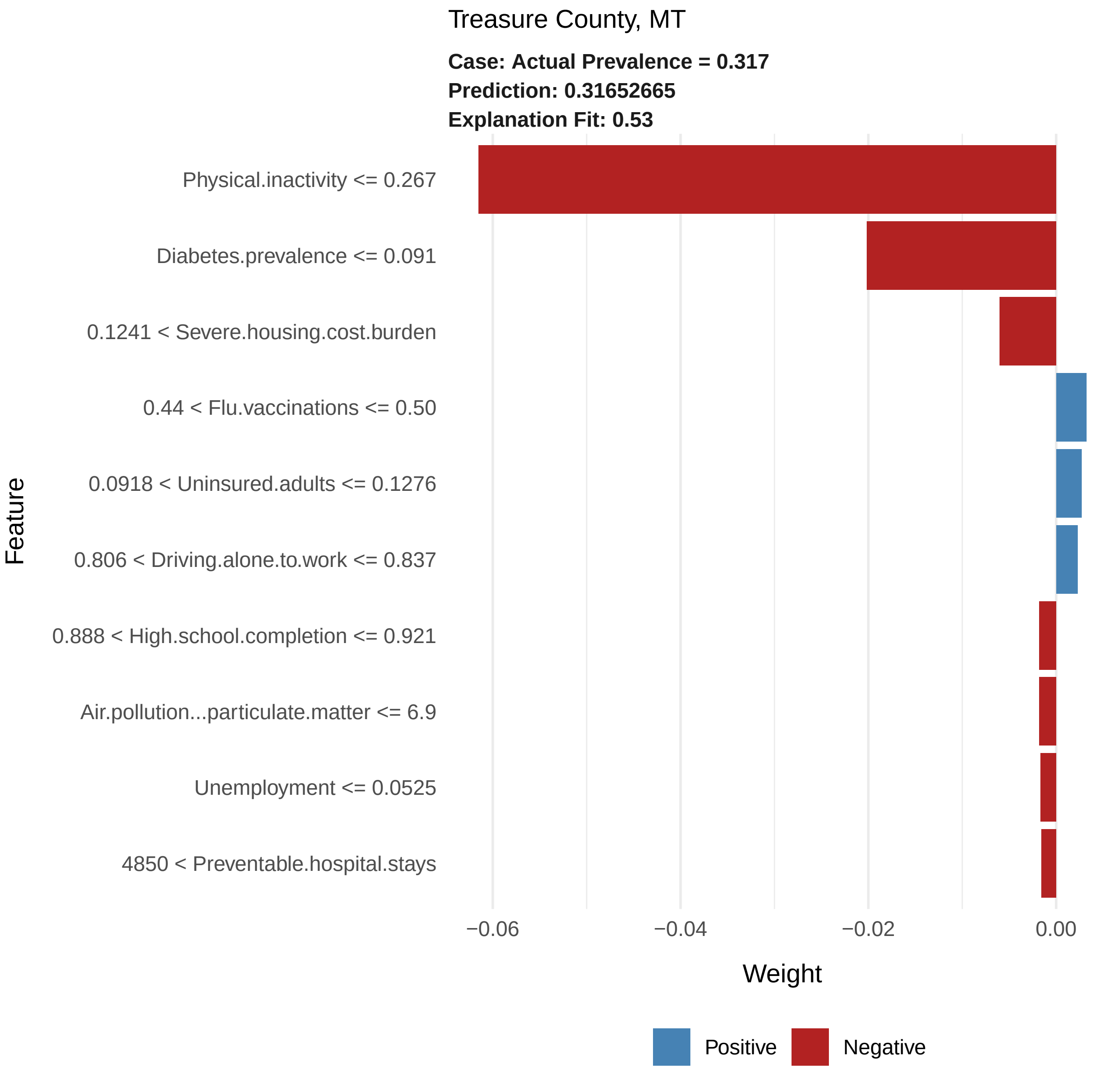

Treasure County, MT
Case: Actual Prevalence = 0.317
Prediction: 0.31652665
Explanation Fit: 0.53
Physical.inactivity <= 0.267
Diabetes.prevalence <= 0.091
0.1241 < Severe.housing.cost.burden
0.44 < Flu.vaccinations <= 0.50
0.0918 < Uninsured.adults <= 0.1276
0.806 < Driving.alone.to.work <= 0.837
0.888 < High.school.completion <= 0.921
Air.pollution...particulate.matter <= 6.9
Unemployment <= 0.0525
4850 < Preventable.hospital.stays
Feature
−0.06
−0.04
−0.02
0.00
Weight
Positive
Negative

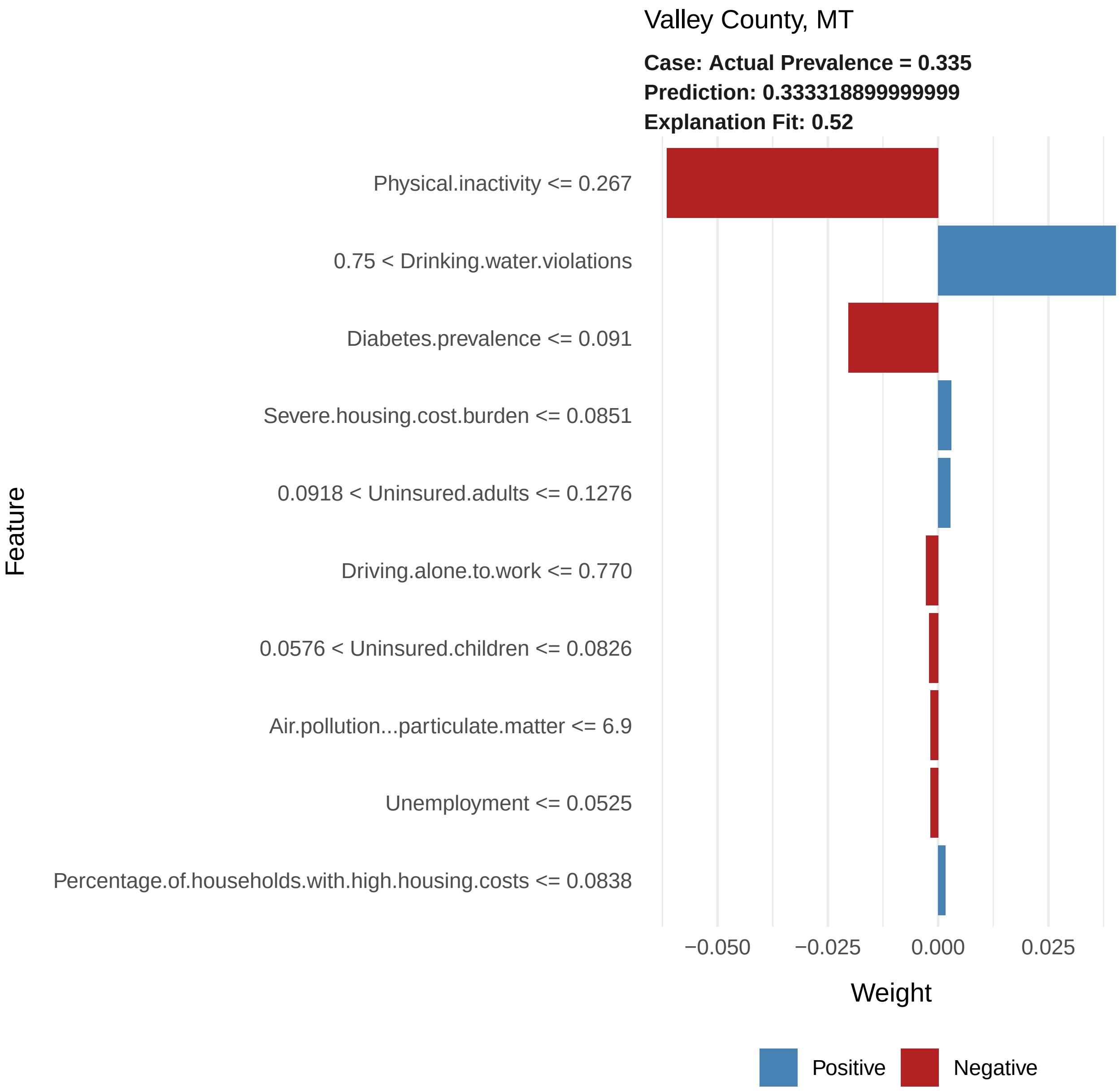

Valley County, MT
Case: Actual Prevalence = 0.335
Prediction: 0.333318899999999
Explanation Fit: 0.52
Physical.inactivity <= 0.267
0.75 < Drinking.water.violations
Diabetes.prevalence <= 0.091
Severe.housing.cost.burden <= 0.0851
0.0918 < Uninsured.adults <= 0.1276
Driving.alone.to.work <= 0.770
0.0576 < Uninsured.children <= 0.0826
Air.pollution...particulate.matter <= 6.9
Unemployment <= 0.0525
Percentage.of.households.with.high.housing.costs <= 0.0838
Feature
−0.050
−0.025
0.000
0.025
Weight
Positive
Negative

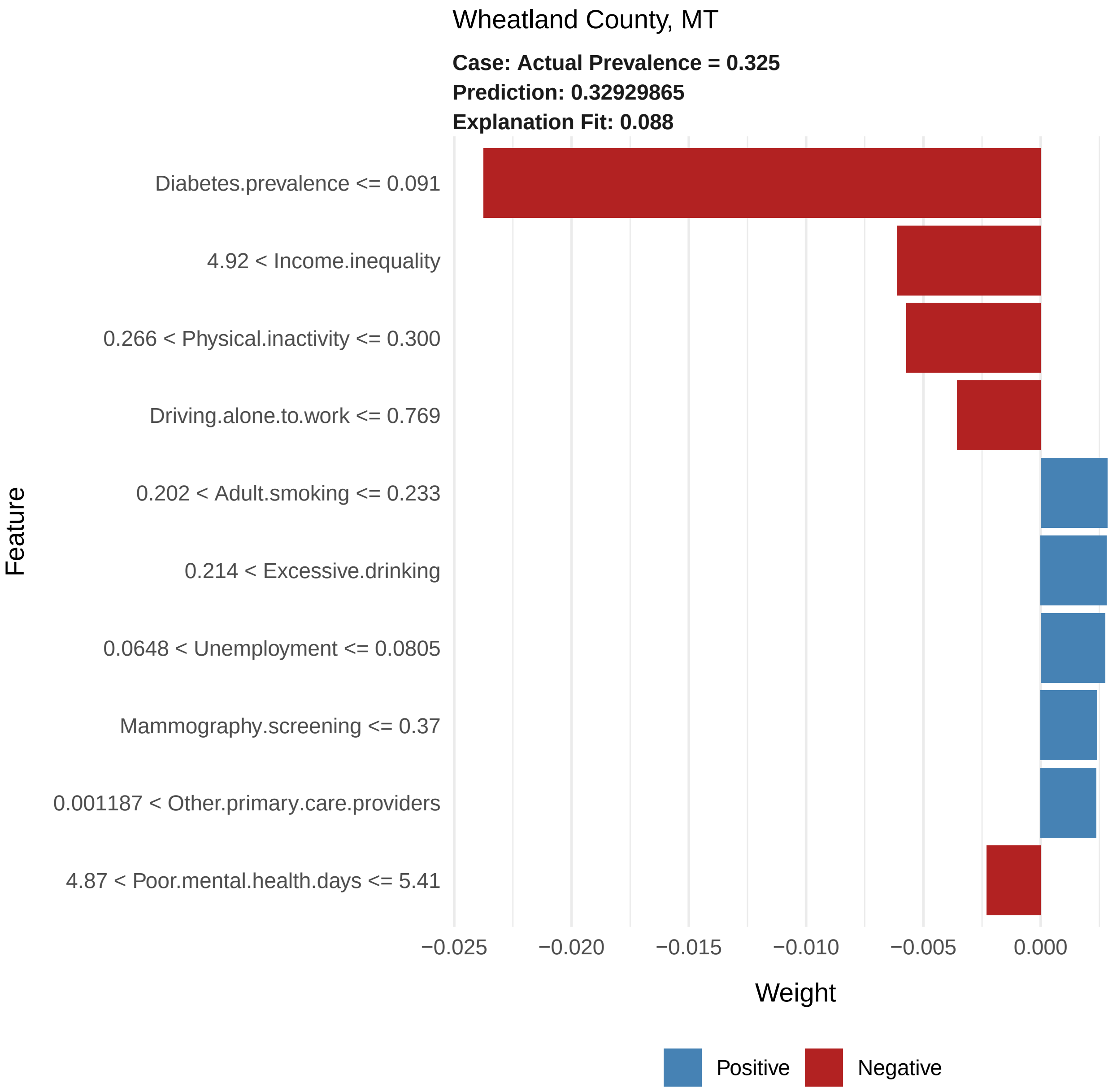
Wheatland County, MT
Case: Actual Prevalence = 0.325
Prediction: 0.32929865
Explanation Fit: 0.088
Diabetes.prevalence <= 0.091
4.92 < Income.inequality
0.266 < Physical.inactivity <= 0.300
Driving.alone.to.work <= 0.769
0.202 < Adult.smoking <= 0.233
0.214 < Excessive.drinking
0.0648 < Unemployment <= 0.0805
Mammography.screening <= 0.37
0.001187 < Other.primary.care.providers
4.87 < Poor.mental.health.days <= 5.41
Feature
−0.025
−0.020
−0.015
−0.010
−0.005
0.000
Weight
Positive
Negative

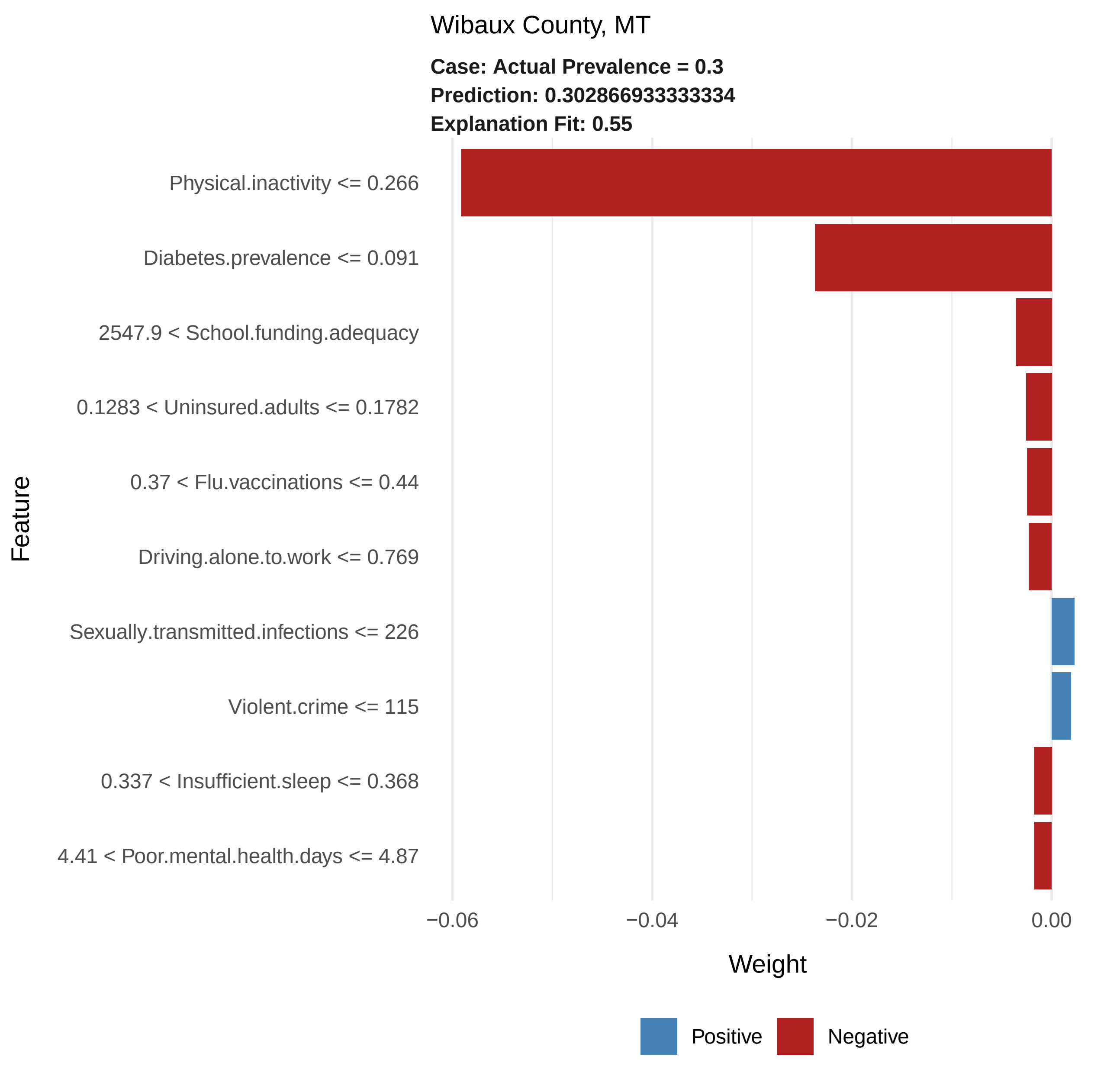

Wibaux County, MT
Case: Actual Prevalence = 0.3
Prediction: 0.302866933333334
Explanation Fit: 0.55
Physical.inactivity <= 0.266
Diabetes.prevalence <= 0.091
2547.9 < School.funding.adequacy
0.1283 < Uninsured.adults <= 0.1782
0.37 < Flu.vaccinations <= 0.44
Driving.alone.to.work <= 0.769
Sexually.transmitted.infections <= 226
Violent.crime <= 115
0.337 < Insufficient.sleep <= 0.368
4.41 < Poor.mental.health.days <= 4.87
Feature
−0.06
−0.04
−0.02
0.00
Weight
Positive
Negative

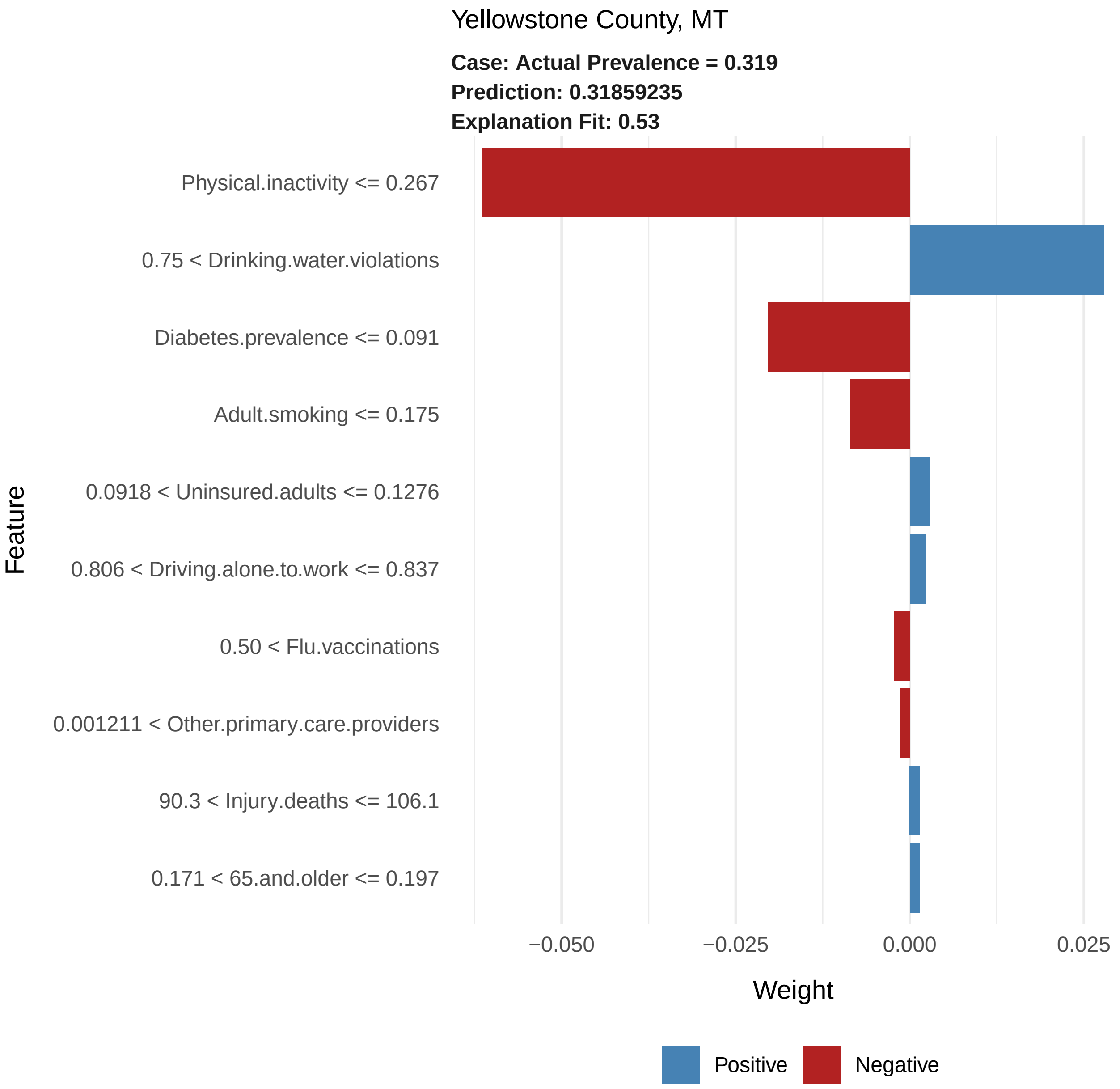

Yellowstone County, MT
Case: Actual Prevalence = 0.319
Prediction: 0.31859235
Explanation Fit: 0.53
Physical.inactivity <= 0.267
0.75 < Drinking.water.violations
Diabetes.prevalence <= 0.091
Adult.smoking <= 0.175
0.0918 < Uninsured.adults <= 0.1276
0.806 < Driving.alone.to.work <= 0.837
0.50 < Flu.vaccinations
0.001211 < Other.primary.care.providers
90.3 < Injury.deaths <= 106.1
0.171 < 65.and.older <= 0.197
Feature
−0.050
−0.025
0.000
0.025
Weight
Positive
Negative

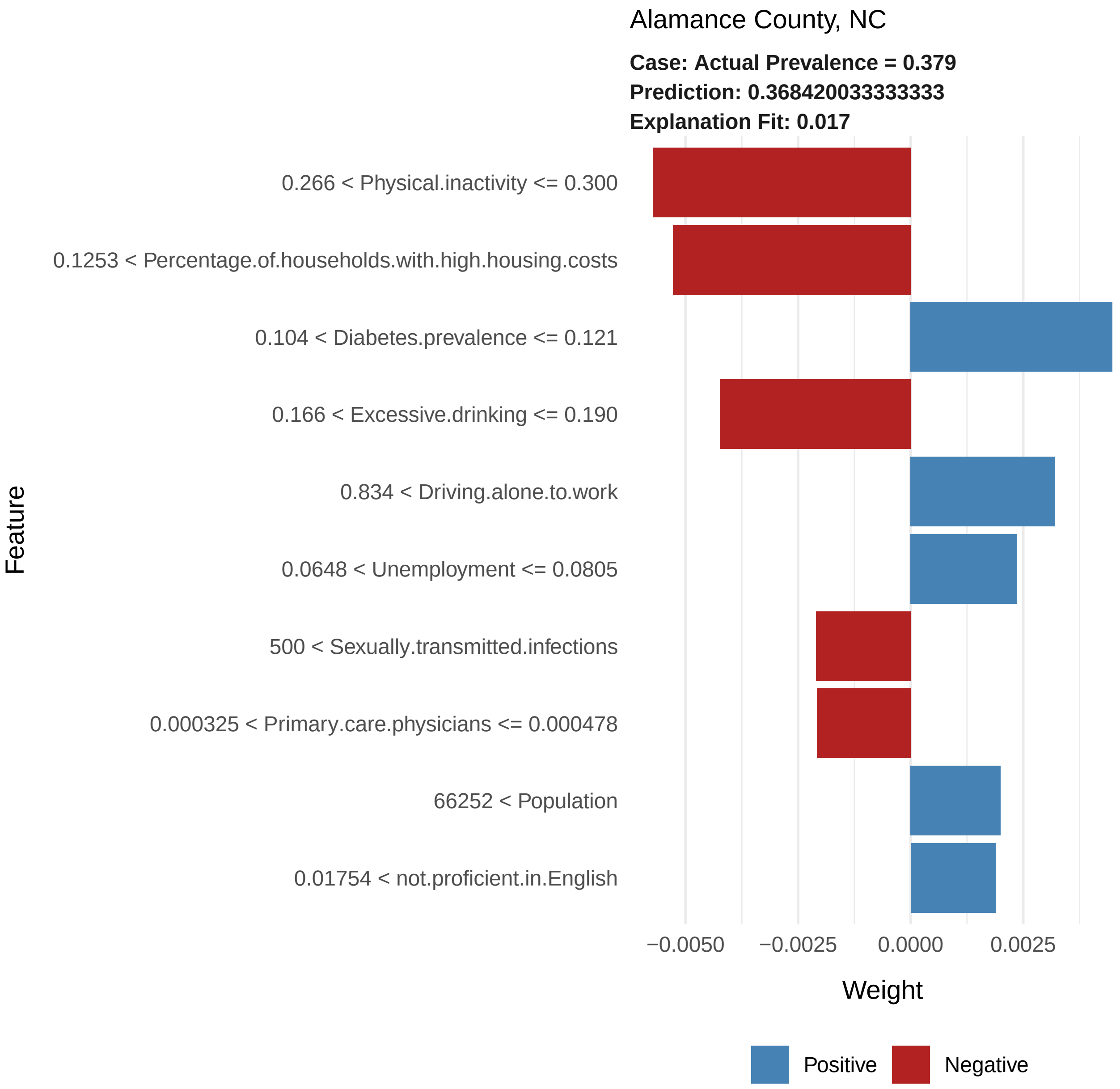

Alamance County, NC
Case: Actual Prevalence = 0.379
Prediction: 0.368420033333333
Explanation Fit: 0.017
0.266 < Physical.inactivity <= 0.300
0.1253 < Percentage.of.households.with.high.housing.costs
0.104 < Diabetes.prevalence <= 0.121
0.166 < Excessive.drinking <= 0.190
0.834 < Driving.alone.to.work
0.0648 < Unemployment <= 0.0805
500 < Sexually.transmitted.infections
0.000325 < Primary.care.physicians <= 0.000478
66252 < Population
0.01754 < not.proficient.in.English
Feature
−0.0050
−0.0025
0.0000
0.0025
Weight
Positive
Negative

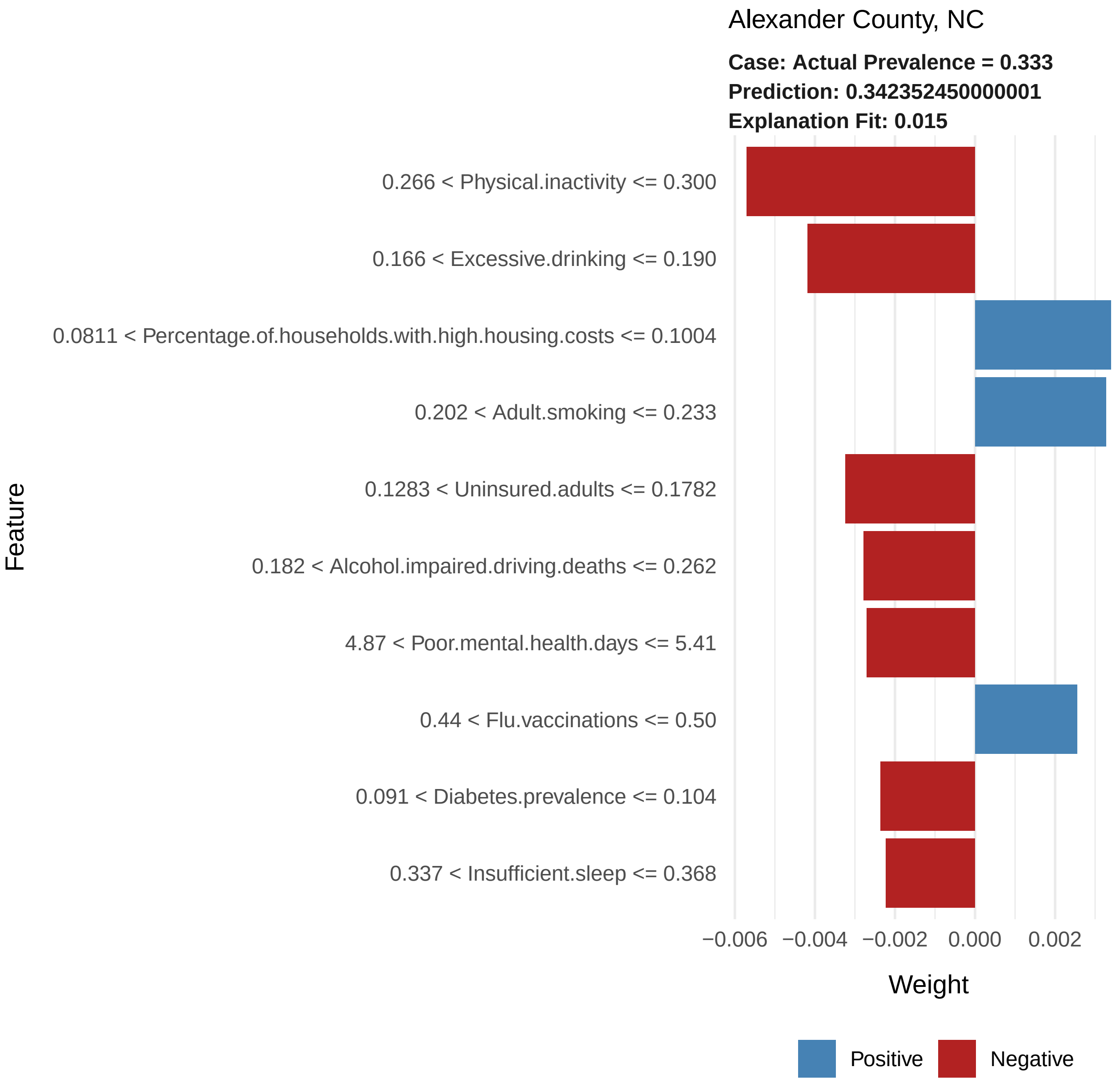
Alexander County, NC
Case: Actual Prevalence = 0.333
Prediction: 0.342352450000001
Explanation Fit: 0.015
0.266 < Physical.inactivity <= 0.300
0.166 < Excessive.drinking <= 0.190
0.0811 < Percentage.of.households.with.high.housing.costs <= 0.1004
0.202 < Adult.smoking <= 0.233
0.1283 < Uninsured.adults <= 0.1782
0.182 < Alcohol.impaired.driving.deaths <= 0.262
4.87 < Poor.mental.health.days <= 5.41
0.44 < Flu.vaccinations <= 0.50
0.091 < Diabetes.prevalence <= 0.104
0.337 < Insufficient.sleep <= 0.368
Feature
−0.006
−0.004
−0.002
0.000
0.002
Weight
Positive
Negative

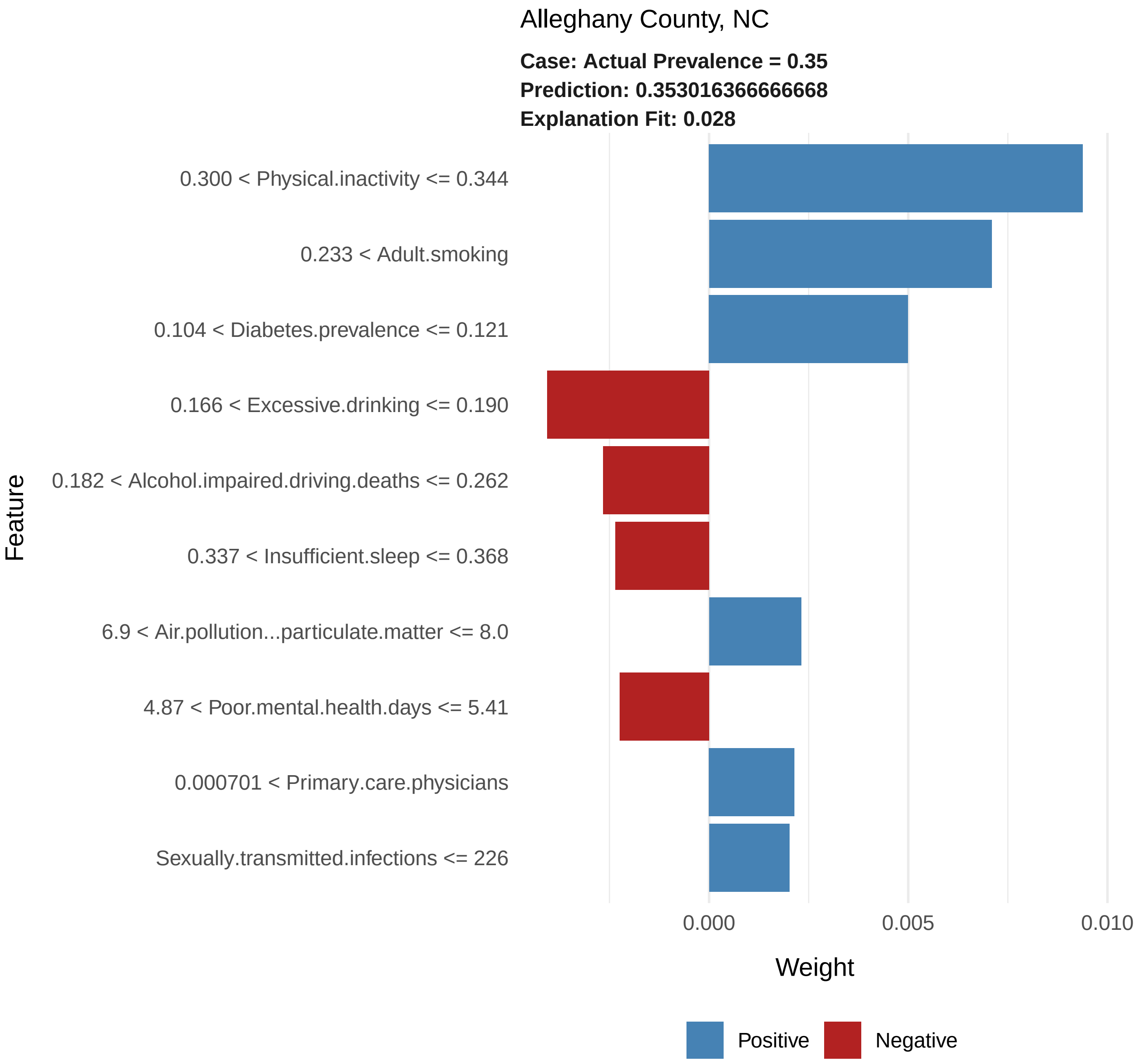

Allleghany County, NC
Case: Actual Prevalence = 0.35
Prediction: 0.353016366666668
Explanation Fit: 0.028
0.300 < Physical.inactivity <= 0.344
0.233 < Adult.smoking
0.104 < Diabetes.prevalence <= 0.121
0.166 < Excessive.drinking <= 0.190
0.182 < Alcohol.impaired.driving.deaths <= 0.262
0.337 < Insufficient.sleep <= 0.368
6.9 < Air.pollution...particulate.matter <= 8.0
4.87 < Poor.mental.health.days <= 5.41
0.000701 < Primary.care.physicians
Sexually.transmitted.infections <= 226
Feature
0.000
0.005
0.010
Weight
Positive
Negative

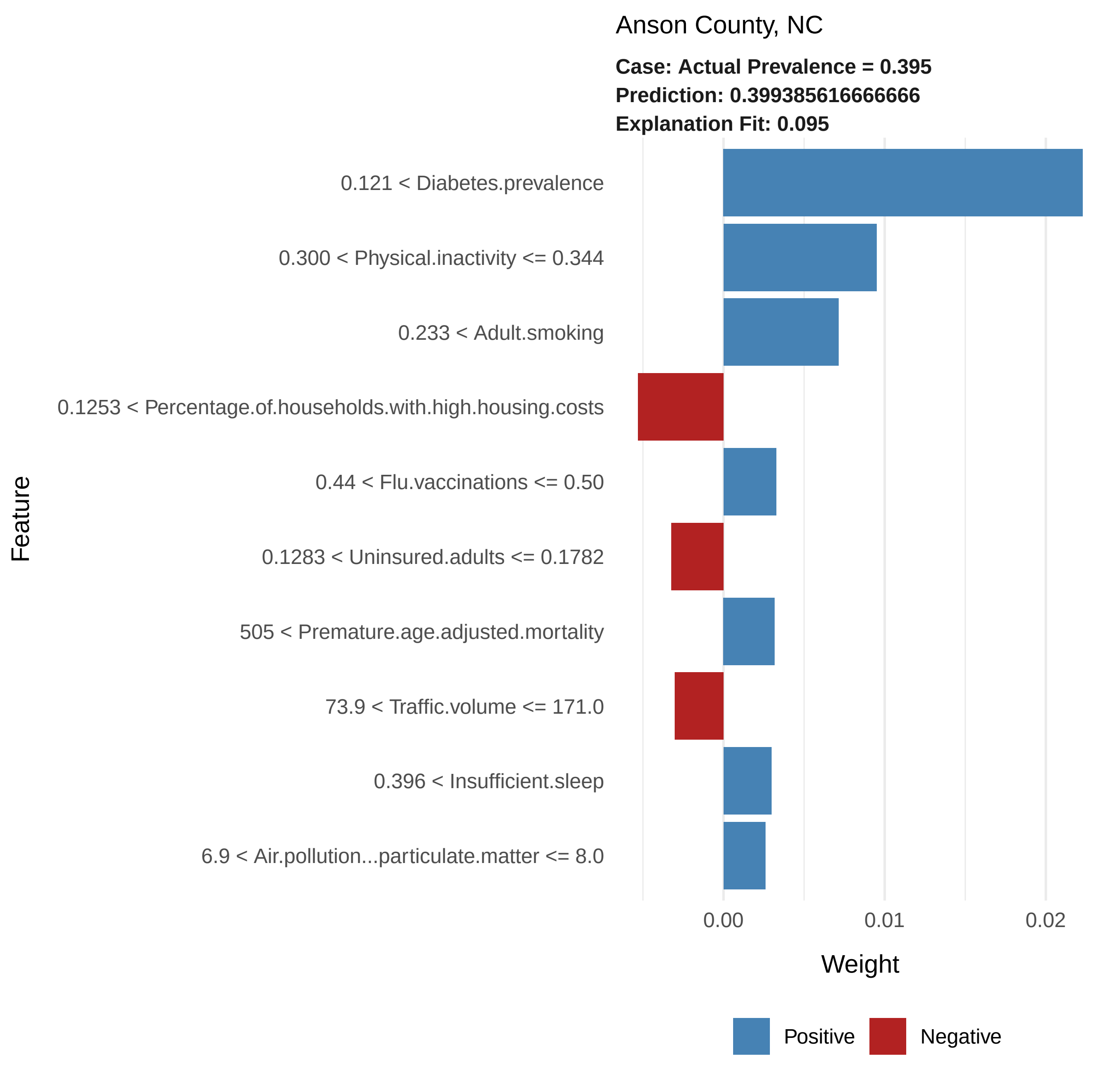

Anson County, NC
Case: Actual Prevalence = 0.395
Prediction: 0.399385616666666
Explanation Fit: 0.095
0.121 < Diabetes.prevalence
0.300 < Physical.inactivity <= 0.344
0.233 < Adult.smoking
0.1253 < Percentage.of.households.with.high.housing.costs
0.44 < Flu.vaccinations <= 0.50
0.1283 < Uninsured.adults <= 0.1782
505 < Premature.age.adjusted.mortality
73.9 < Traffic.volume <= 171.0
0.396 < Insufficient.sleep
6.9 < Air.pollution...particulate.matter <= 8.0
Feature
0.00
0.01
0.02
Weight
Positive
Negative

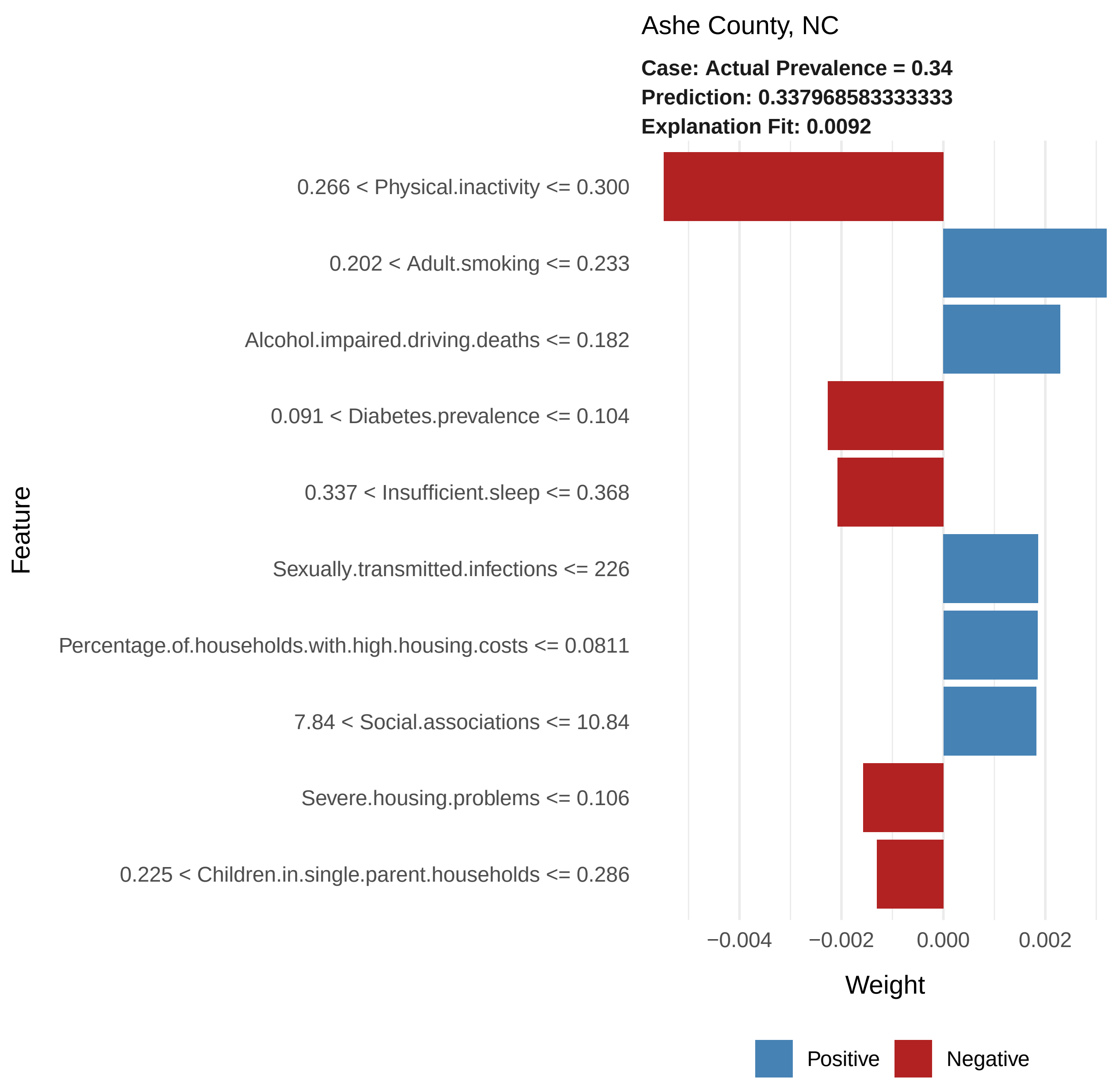

Ashe County, NC
Case: Actual Prevalence = 0.34
Prediction: 0.337968583333333
Explanation Fit: 0.0092
0.266 < Physical.inactivity <= 0.300
0.202 < Adult.smoking <= 0.233
Alcohol.impaired.driving.deaths <= 0.182
0.091 < Diabetes.prevalence <= 0.104
0.337 < Insufficient.sleep <= 0.368
Sexually.transmitted.infections <= 226
Percentage.of.households.with.high.housing.costs <= 0.0811
7.84 < Social.associations <= 10.84
Severe.housing.problems <= 0.106
0.225 < Children.in.single.parent.households <= 0.286
Feature
−0.004
−0.002
0.000
0.002
Weight
Positive
Negative

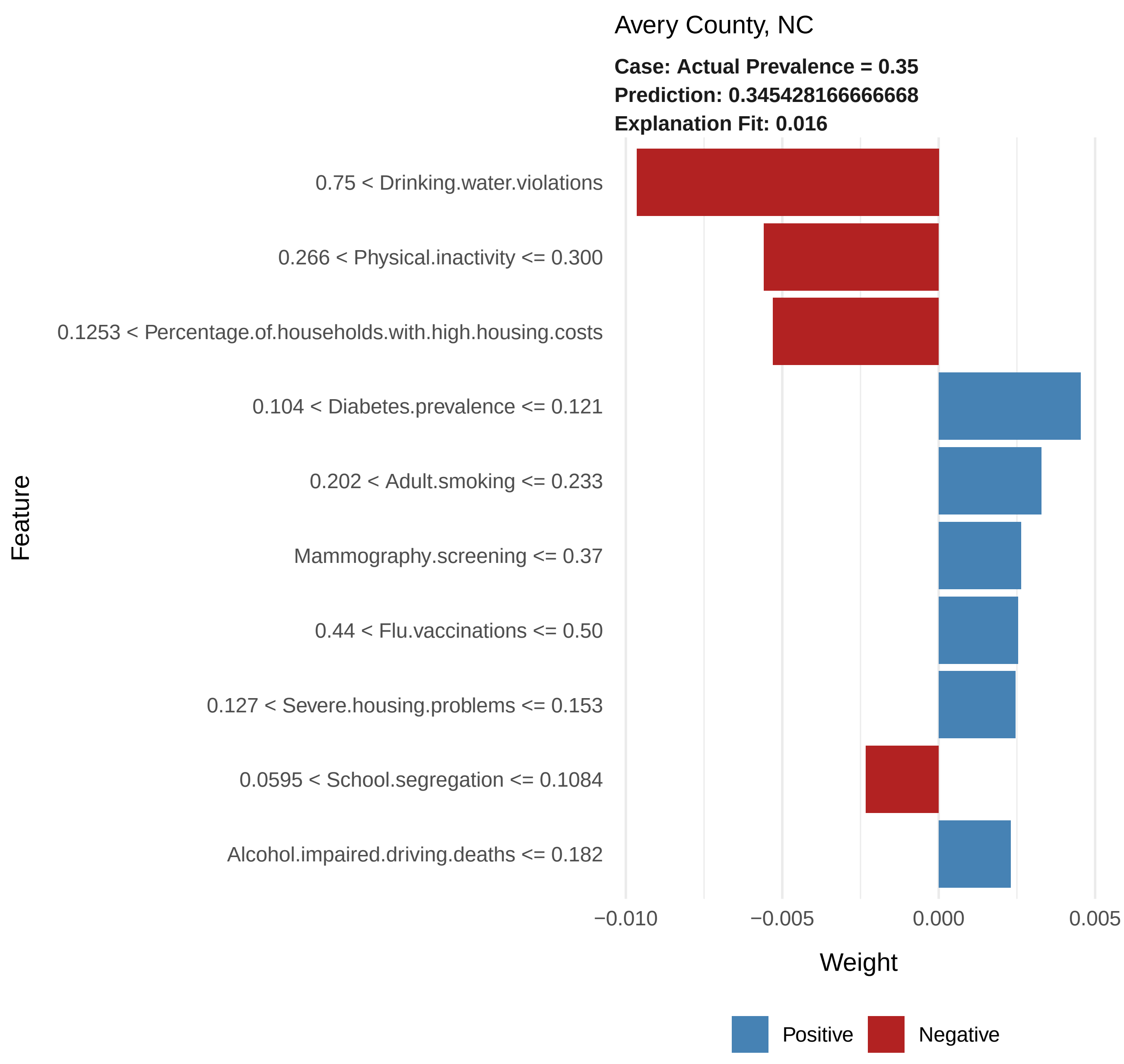
Avery County, NC
Case: Actual Prevalence = 0.35
Prediction: 0.345428166666668
Explanation Fit: 0.016
0.75 < Drinking.water.violations
0.266 < Physical.inactivity <= 0.300
0.1253 < Percentage.of.households.with.high.housing.costs
0.104 < Diabetes.prevalence <= 0.121
0.202 < Adult.smoking <= 0.233
Mammography.screening <= 0.37
0.44 < Flu.vaccinations <= 0.50
0.127 < Severe.housing.problems <= 0.153
0.0595 < School.segregation <= 0.1084
Alcohol.impaired.driving.deaths <= 0.182
Feature
−0.010
−0.005
0.000
0.005
Weight
Positive
Negative

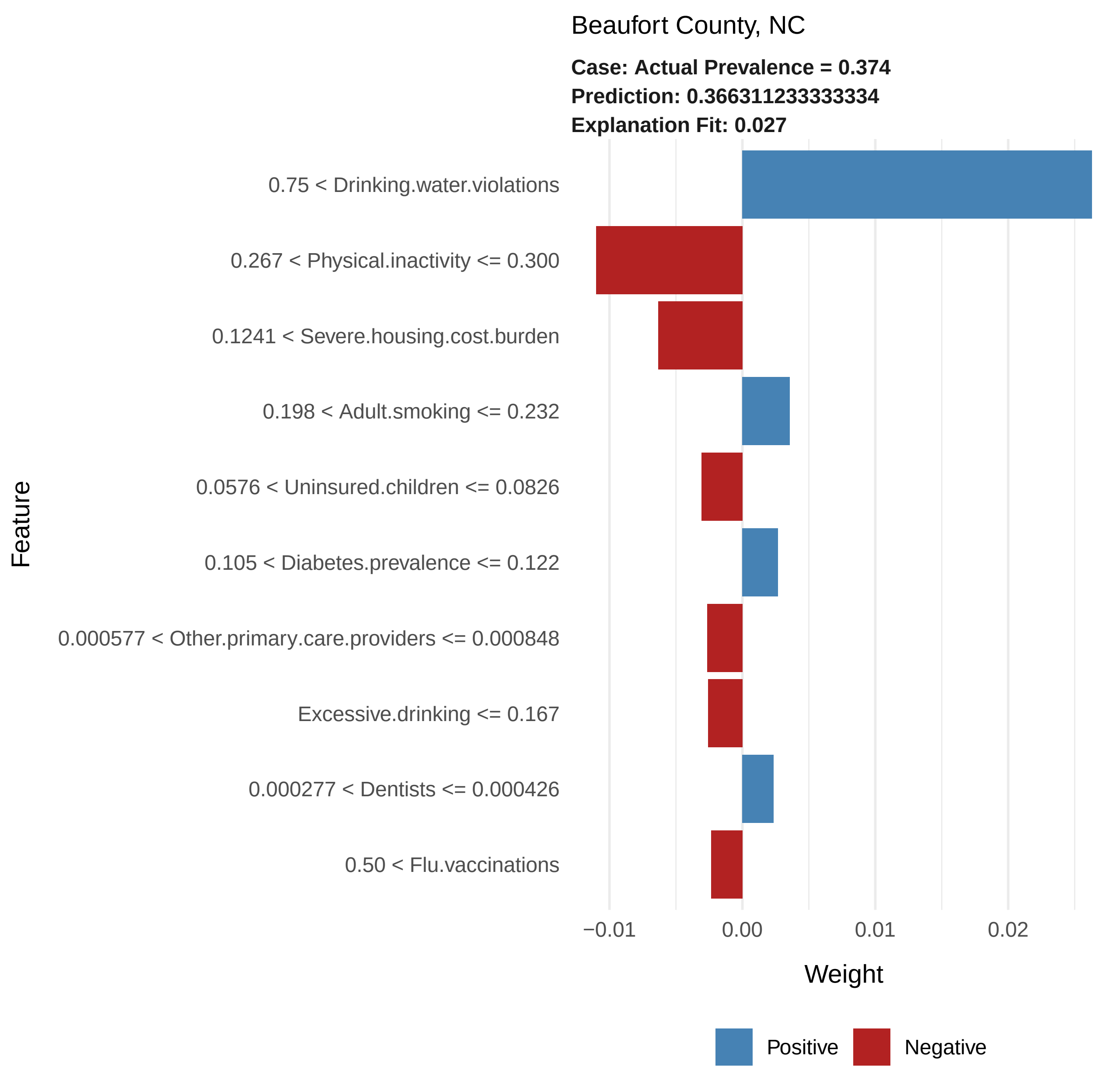

Beaufort County, NC
Case: Actual Prevalence = 0.374
Prediction: 0.366311233333334
Explanation Fit: 0.027
0.75 < Drinking.water.violations
0.267 < Physical.inactivity <= 0.300
0.1241 < Severe.housing.cost.burden
0.198 < Adult.smoking <= 0.232
0.0576 < Uninsured.children <= 0.0826
0.105 < Diabetes.prevalence <= 0.122
0.000577 < Other.primary.care.providers <= 0.000848
Excessive.drinking <= 0.167
0.000277 < Dentists <= 0.000426
0.50 < Flu.vaccinations
Feature
−0.01
0.00
0.01
0.02
Weight
Positive
Negative

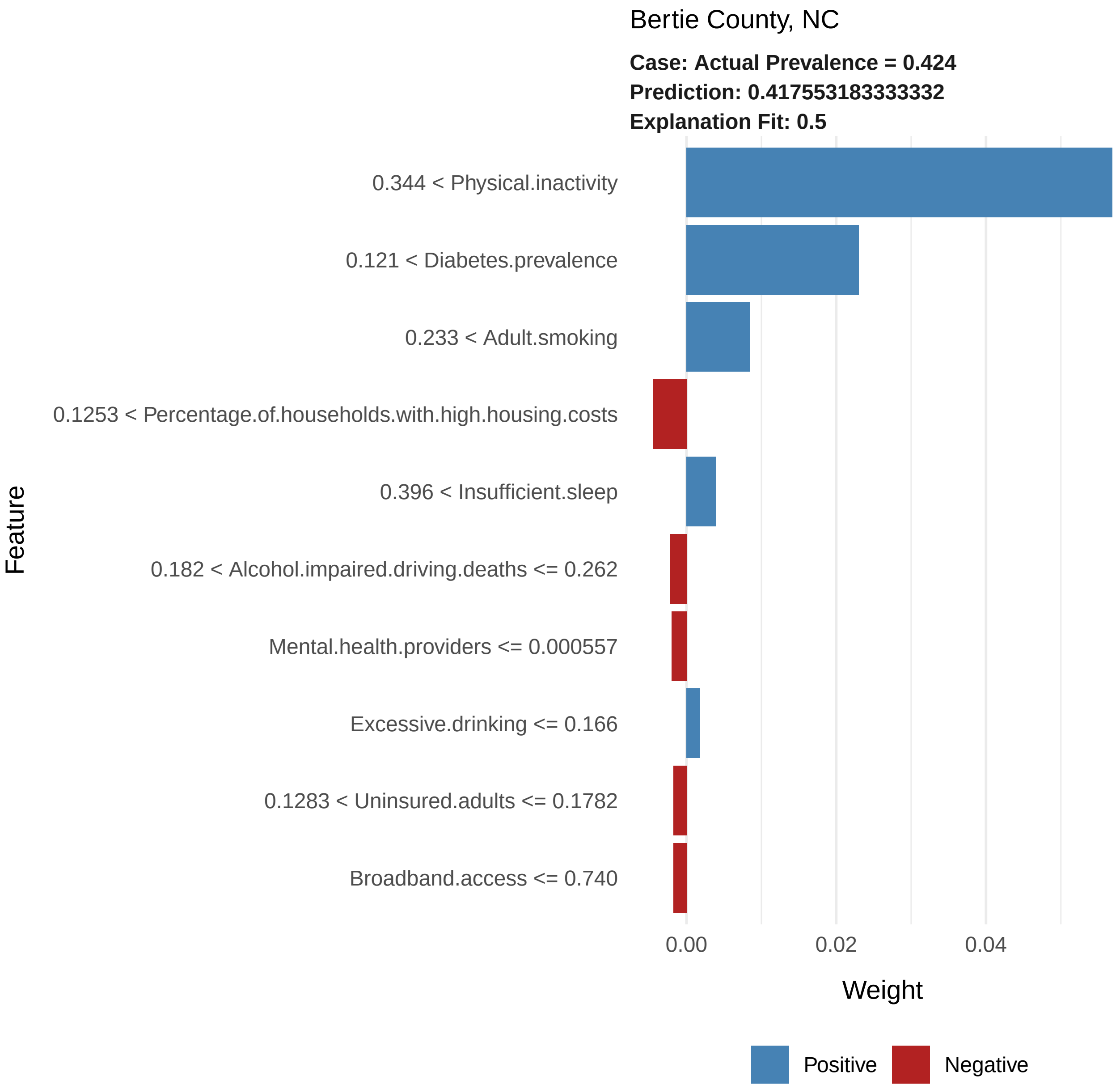

Bertie County, NC
Case: Actual Prevalence = 0.424
Prediction: 0.417553183333332
Explanation Fit: 0.5
0.344 < Physical.inactivity
0.121 < Diabetes.prevalence
0.233 < Adult.smoking
0.1253 < Percentage.of.households.with.high.housing.costs
0.396 < Insufficient.sleep
0.182 < Alcohol.impaired.driving.deaths <= 0.262
Mental.health.providers <= 0.000557
Excessive.drinking <= 0.166
0.1283 < Uninsured.adults <= 0.1782
Broadband.access <= 0.740
Feature
0.00
0.02
0.04
Weight
Positive
Negative

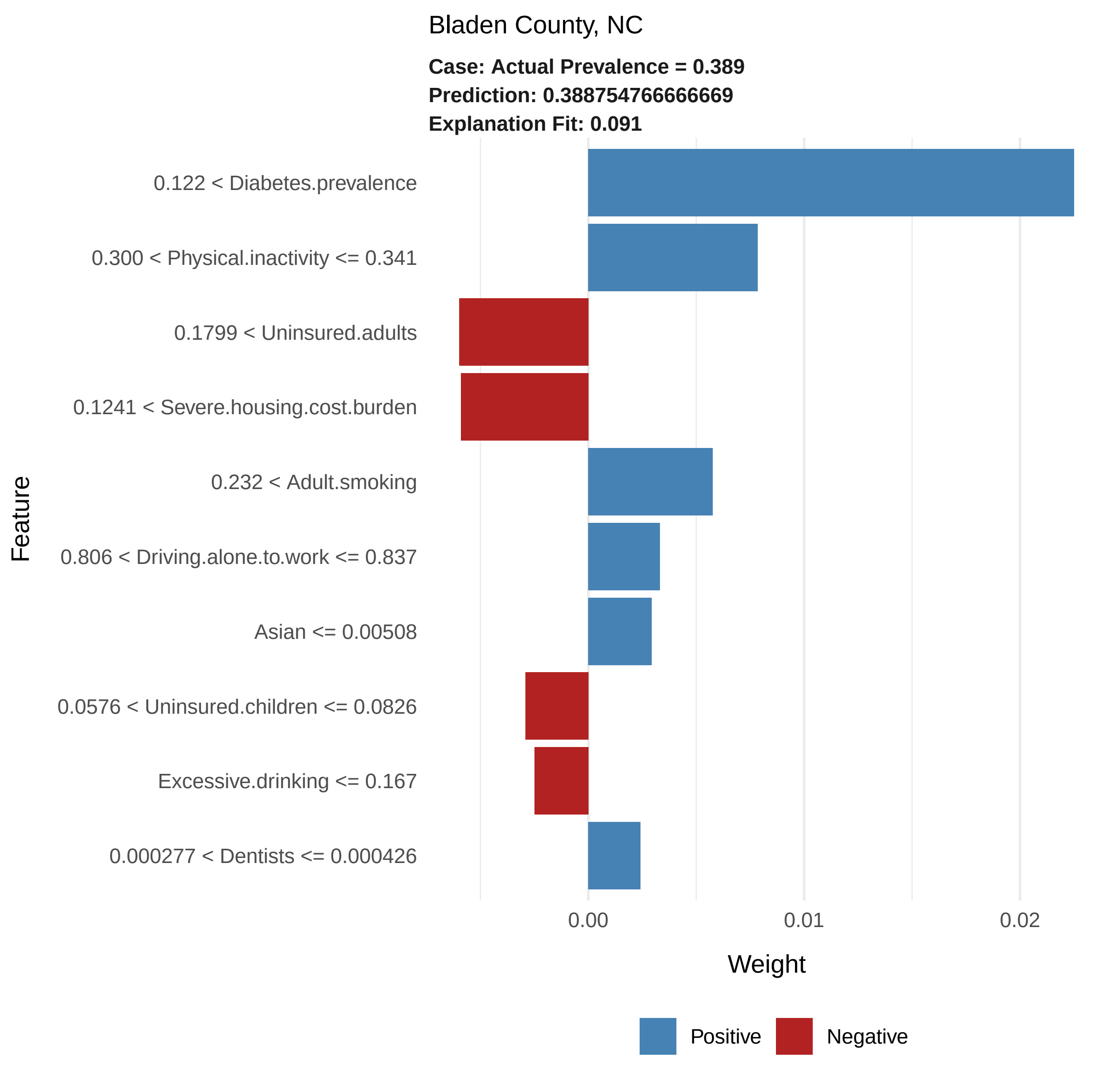
Bladen County, NC
Case: Actual Prevalence = 0.389
Prediction: 0.388754766666669
Explanation Fit: 0.091
0.122 < Diabetes.prevalence
0.300 < Physical.inactivity <= 0.341
0.1799 < Uninsured.adults
0.1241 < Severe.housing.cost.burden
0.232 < Adult.smoking
0.806 < Driving.alone.to.work <= 0.837
Asian <= 0.00508
0.0576 < Uninsured.children <= 0.0826
Excessive.drinking <= 0.167
0.000277 < Dentists <= 0.000426
Feature
0.00
0.01
0.02
Weight
Positive
Negative

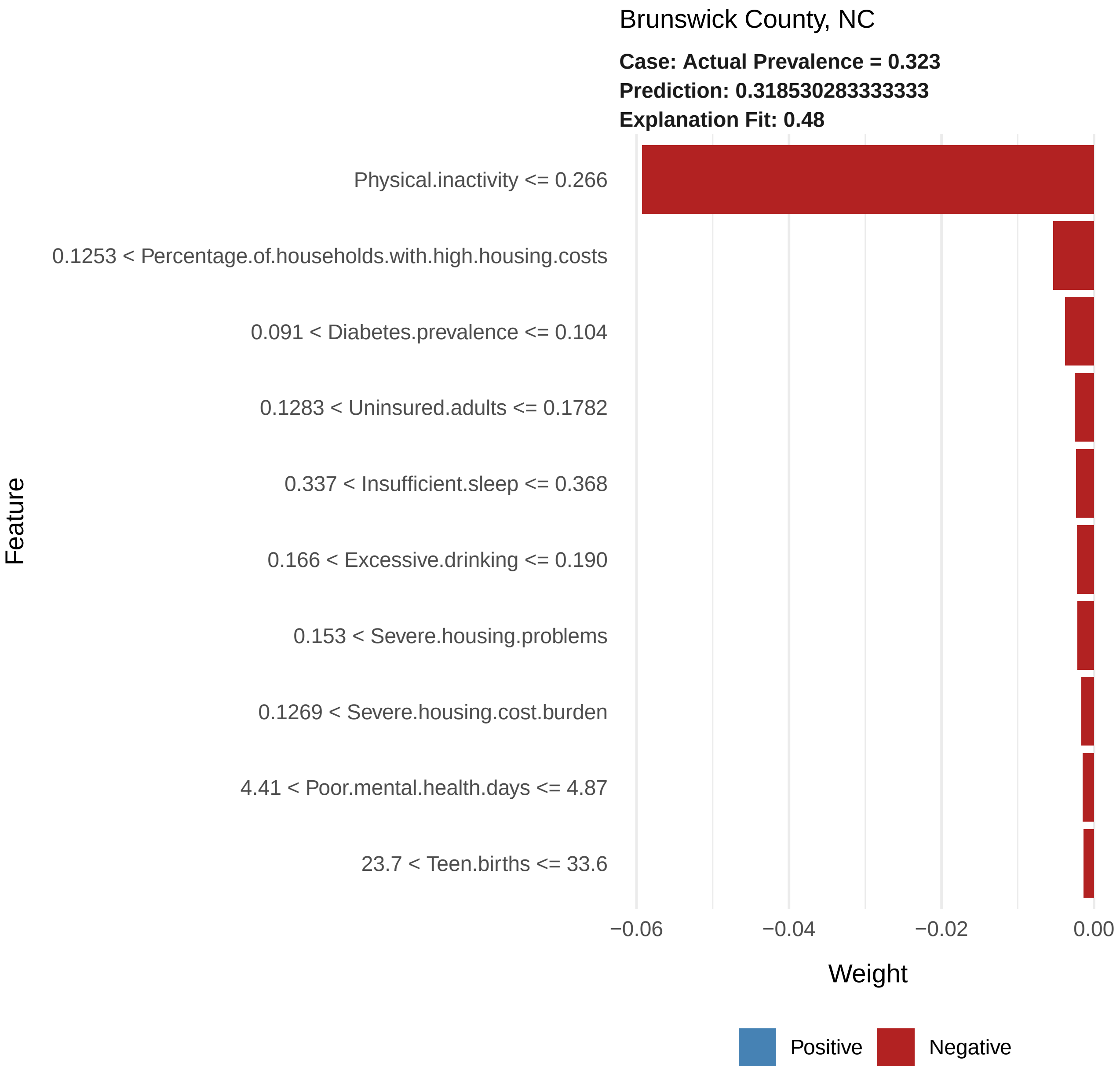
Brunswick County, NC
Case: Actual Prevalence = 0.323
Prediction: 0.318530283333333
Explanation Fit: 0.48
Physical.inactivity <= 0.266
0.1253 < Percentage.of.households.with.high.housing.costs
0.091 < Diabetes.prevalence <= 0.104
0.1283 < Uninsured.adults <= 0.1782
0.337 < Insufficient.sleep <= 0.368
0.166 < Excessive.drinking <= 0.190
0.153 < Severe.housing.problems
0.1269 < Severe.housing.cost.burden
4.41 < Poor.mental.health.days <= 4.87
23.7 < Teen.births <= 33.6
Feature
−0.06
−0.04
−0.02
0.00
Weight
Positive
Negative

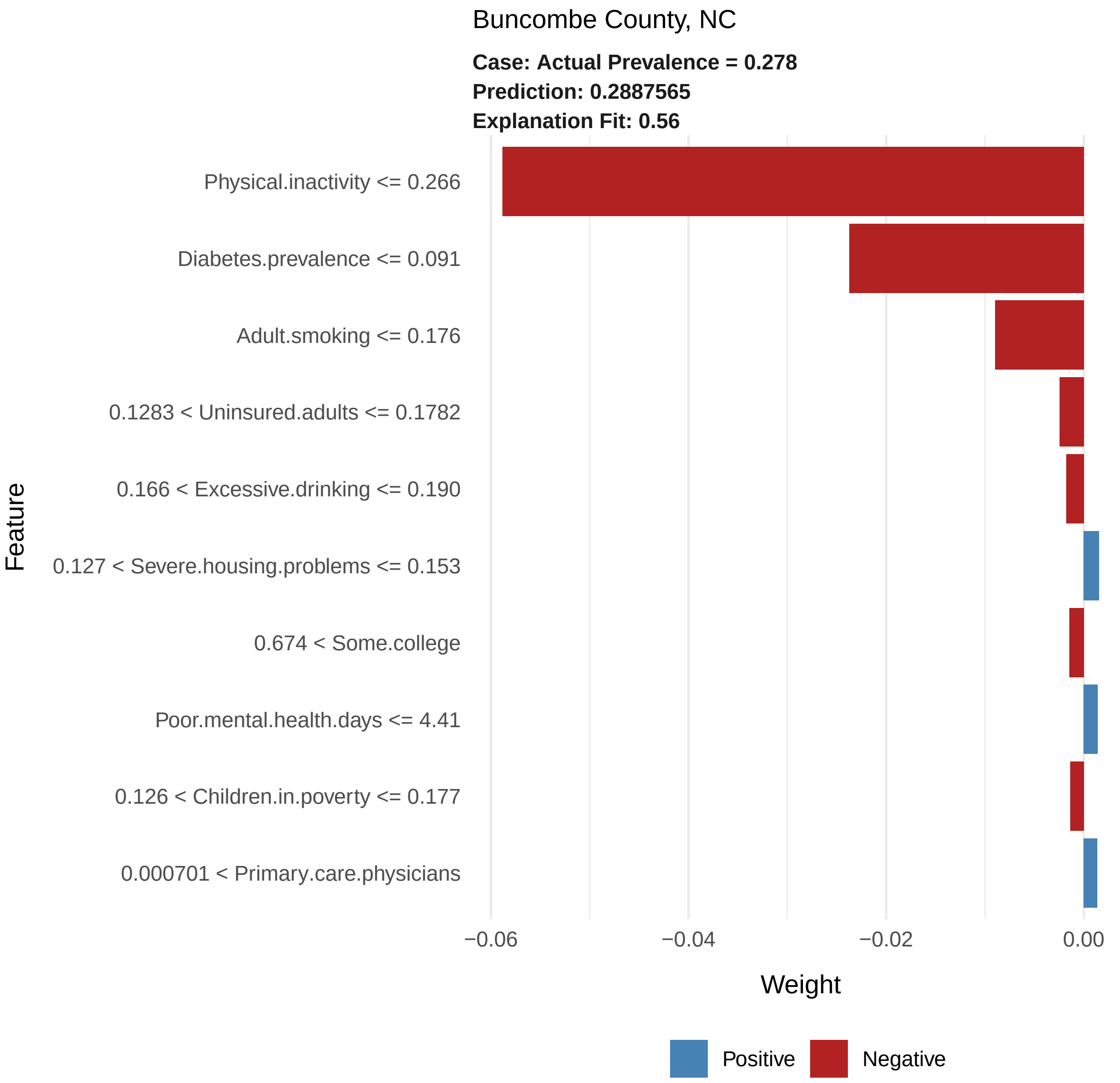

Buncombe County, NC
Case: Actual Prevalence = 0.278
Prediction: 0.2887565
Explanation Fit: 0.56
Physical.inactivity <= 0.266
Diabetes.prevalence <= 0.091
Adult.smoking <= 0.176
0.1283 < Uninsured.adults <= 0.1782
0.166 < Excessive.drinking <= 0.190
0.127 < Severe.housing.problems <= 0.153
0.674 < Some.college
Poor.mental.health.days <= 4.41
0.126 < Children.in.poverty <= 0.177
0.000701 < Primary.care.physicians
Feature
−0.06
−0.04
−0.02
0.00
Weight
Positive
Negative

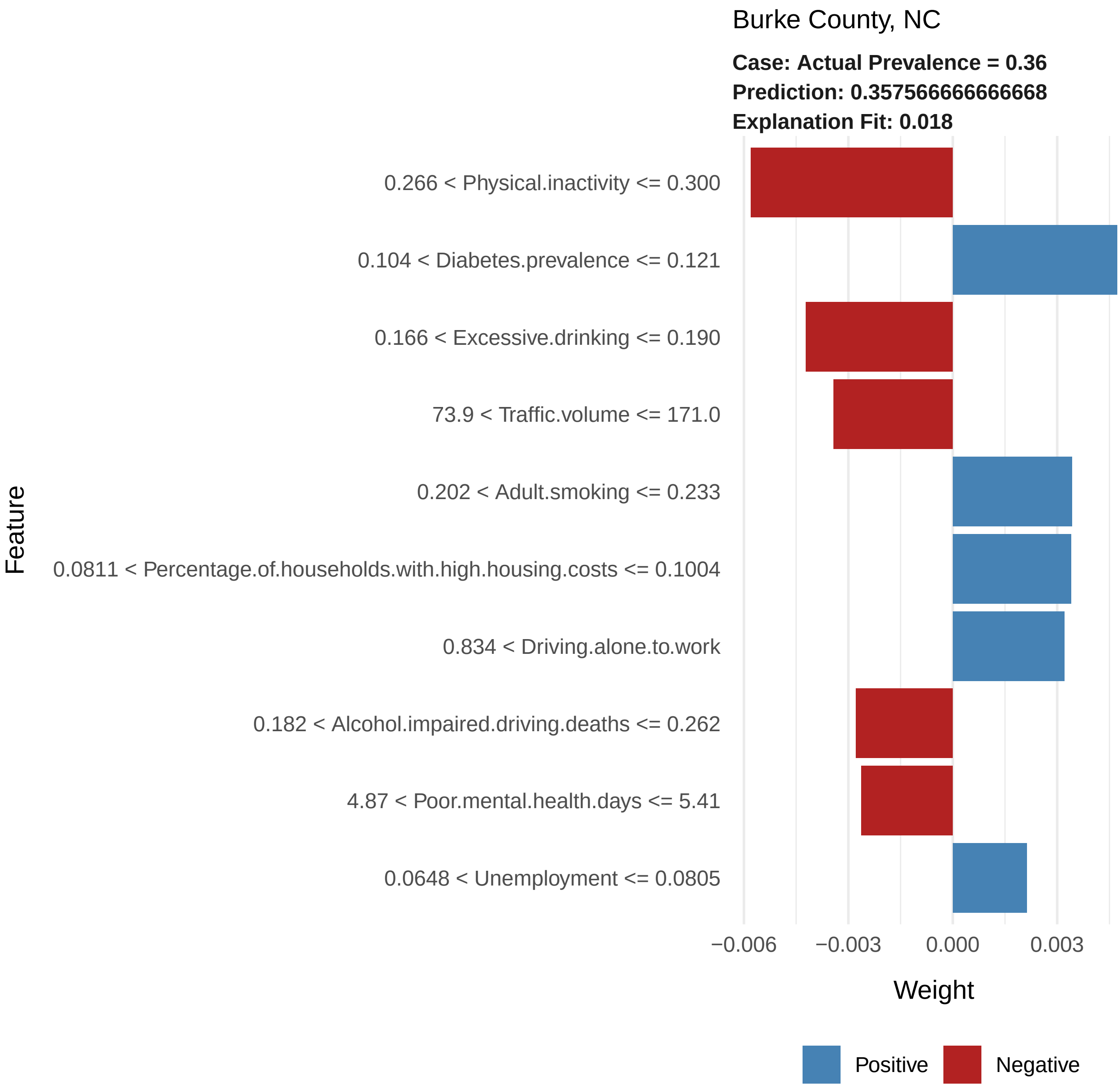
Burke County, NC
Case: Actual Prevalence = 0.36
Prediction: 0.357566666666668
Explanation Fit: 0.018
0.266 < Physical.inactivity <= 0.300
0.104 < Diabetes.prevalence <= 0.121
0.166 < Excessive.drinking <= 0.190
73.9 < Traffic.volume <= 171.0
0.202 < Adult.smoking <= 0.233
0.0811 < Percentage.of.households.with.high.housing.costs <= 0.1004
0.834 < Driving.alone.to.work
0.182 < Alcohol.impaired.driving.deaths <= 0.262
4.87 < Poor.mental.health.days <= 5.41
0.0648 < Unemployment <= 0.0805
Feature
−0.006
−0.003
0.000
0.003
Weight
Positive
Negative

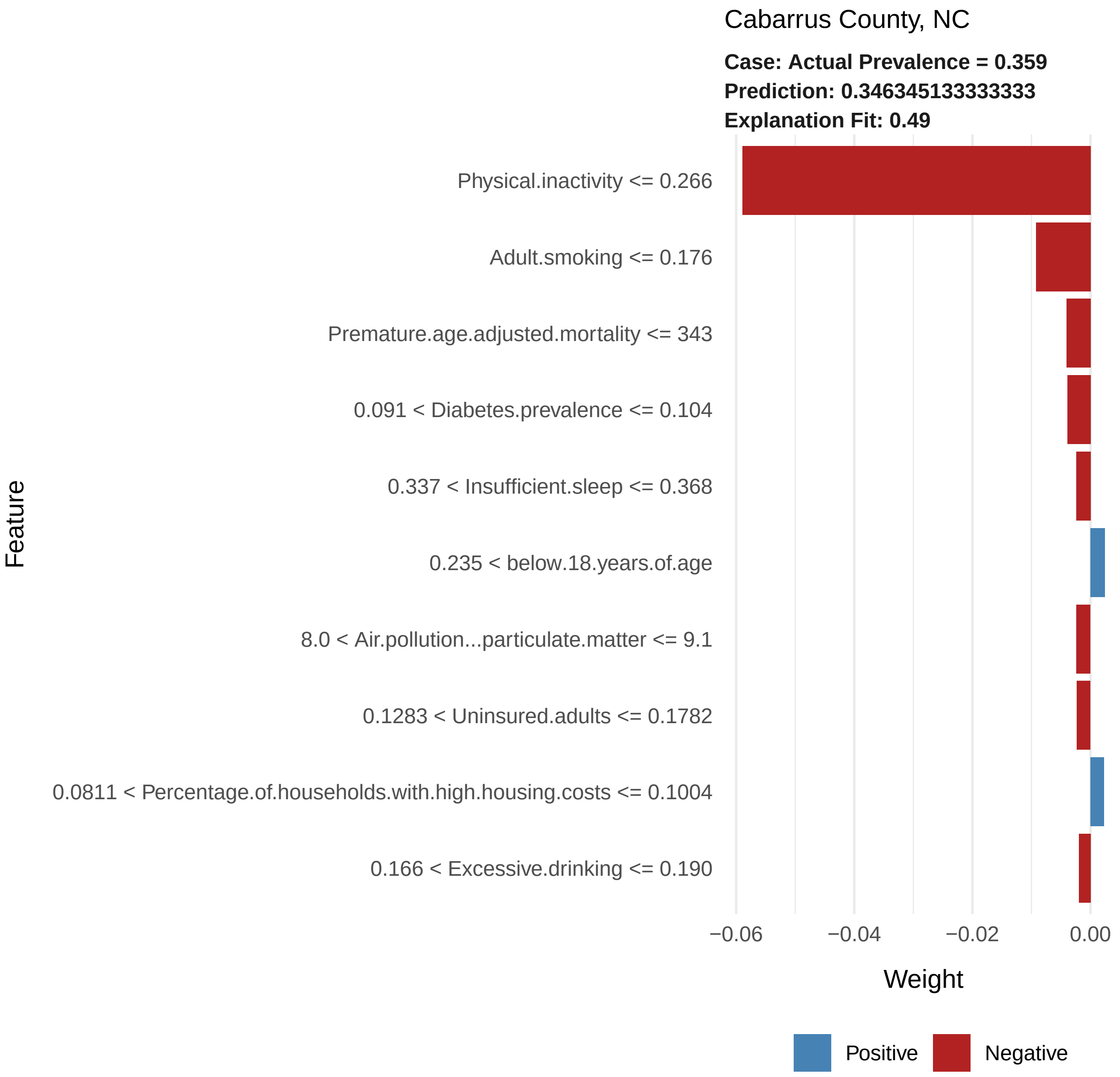
Cabarrus County, NC
Case: Actual Prevalence = 0.359
Prediction: 0.346345133333333
Explanation Fit: 0.49
Physical.inactivity <= 0.266
Adult.smoking <= 0.176
Premature.age.adjusted.mortality <= 343
0.091 < Diabetes.prevalence <= 0.104
0.337 < Insufficient.sleep <= 0.368
0.235 < below.18.years.of.age
8.0 < Air.pollution...particulate.matter <= 9.1
0.1283 < Uninsured.adults <= 0.1782
0.0811 < Percentage.of.households.with.high.housing.costs <= 0.1004
0.166 < Excessive.drinking <= 0.190
Feature
−0.06
−0.04
−0.02
0.00
Weight
Positive
Negative

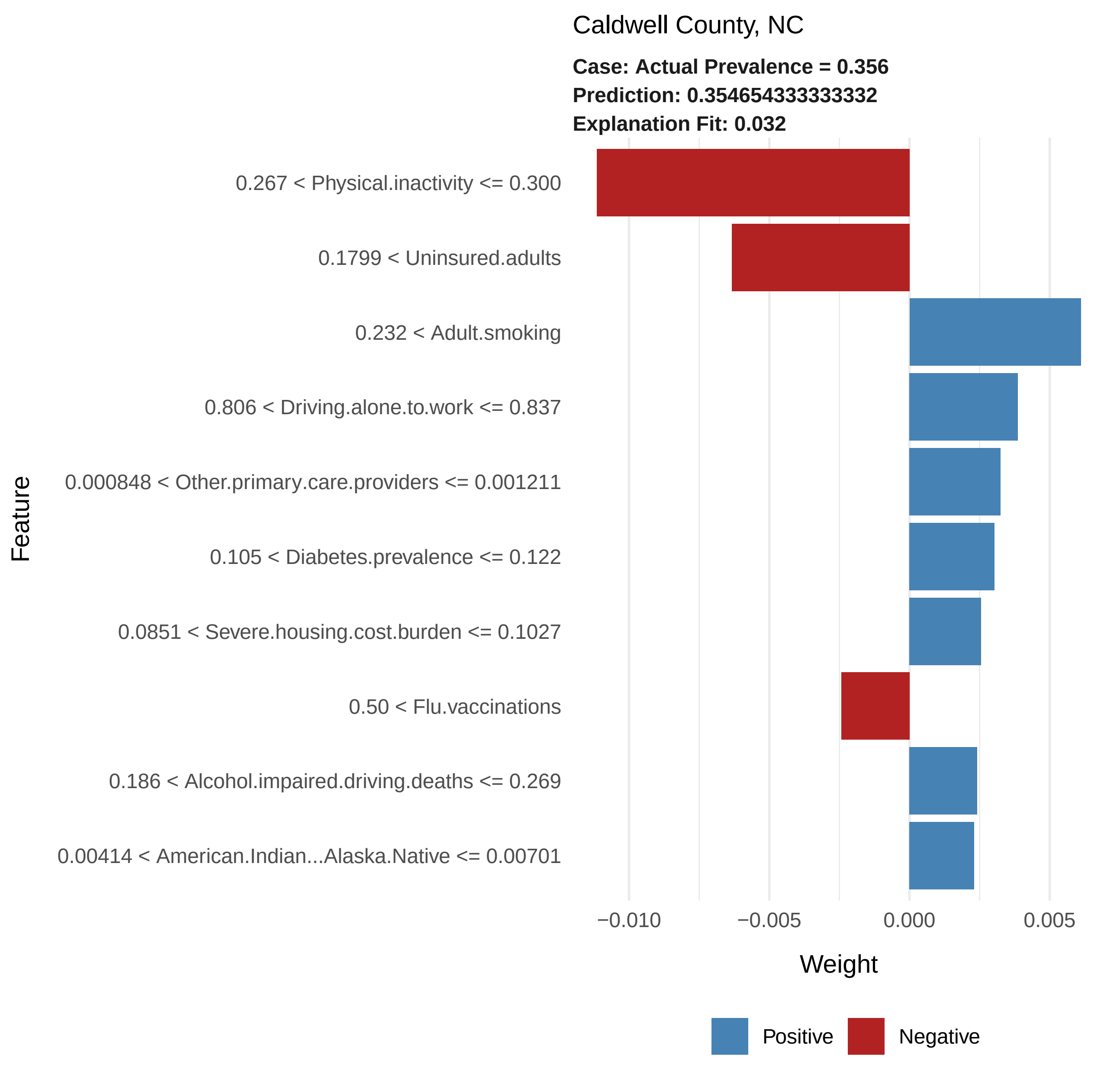

Caldwell County, NC
Case: Actual Prevalence = 0.356
Prediction: 0.354654333333332
Explanation Fit: 0.032
0.267 < Physical.inactivity <= 0.300
0.1799 < Uninsured.adults
0.232 < Adult.smoking
0.806 < Driving.alone.to.work <= 0.837
0.000848 < Other.primary.care.providers <= 0.001211
0.105 < Diabetes.prevalence <= 0.122
0.0851 < Severe.housing.cost.burden <= 0.1027
0.50 < Flu.vaccinations
0.186 < Alcohol.impaired.driving.deaths <= 0.269
0.00414 < American.Indian...Alaska.Native <= 0.00701
Feature
−0.010
−0.005
0.000
0.005
Weight
Positive
Negative

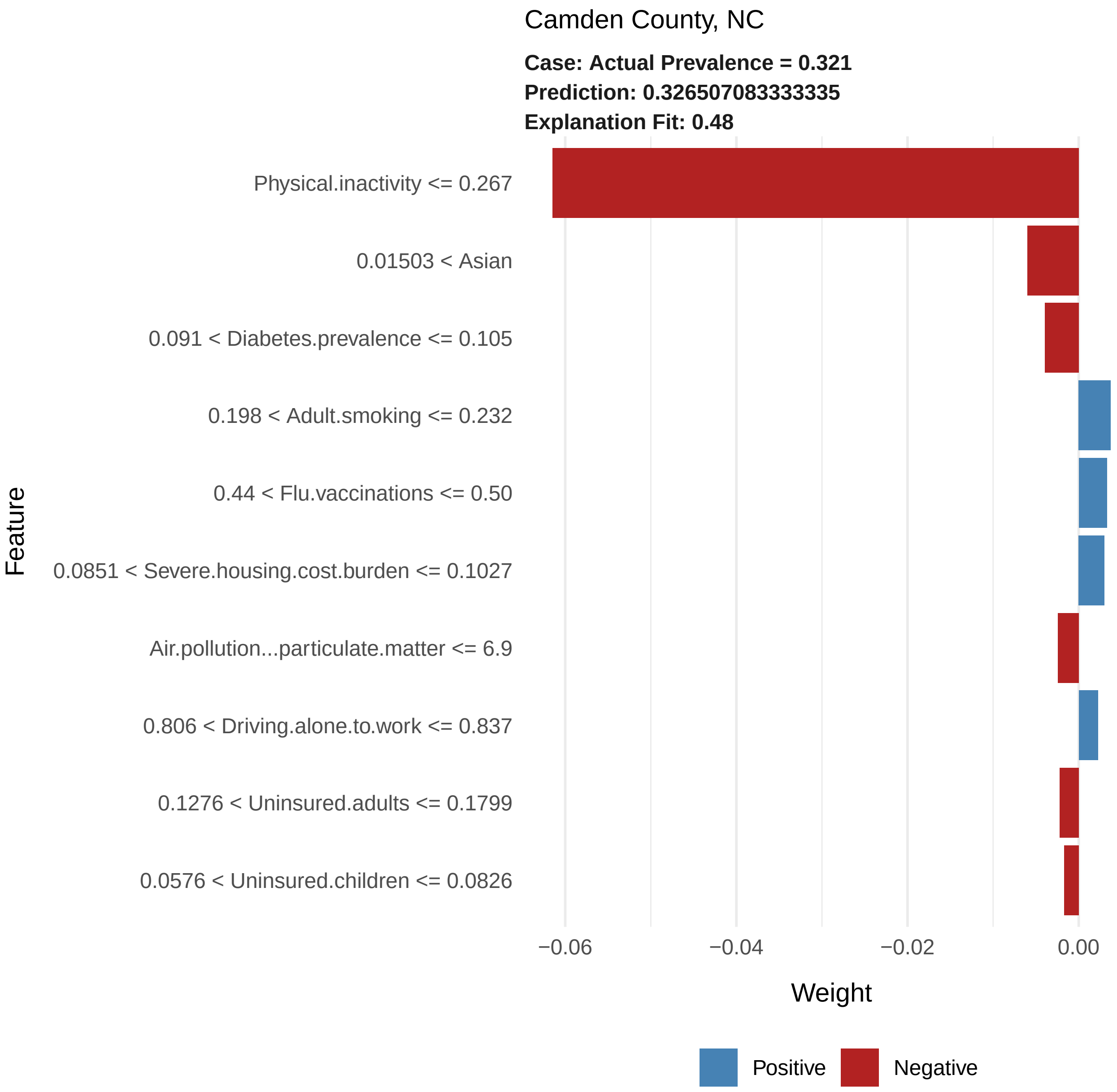

Camden County, NC
Case: Actual Prevalence = 0.321
Prediction: 0.326507083333335
Explanation Fit: 0.48
Physical.inactivity <= 0.267
0.01503 < Asian
0.091 < Diabetes.prevalence <= 0.105
0.198 < Adult.smoking <= 0.232
0.44 < Flu.vaccinations <= 0.50
0.0851 < Severe.housing.cost.burden <= 0.1027
Air.pollution...particulate.matter <= 6.9
0.806 < Driving.alone.to.work <= 0.837
0.1276 < Uninsured.adults <= 0.1799
0.0576 < Uninsured.children <= 0.0826
Feature
−0.06
−0.04
−0.02
0.00
Weight
Positive
Negative

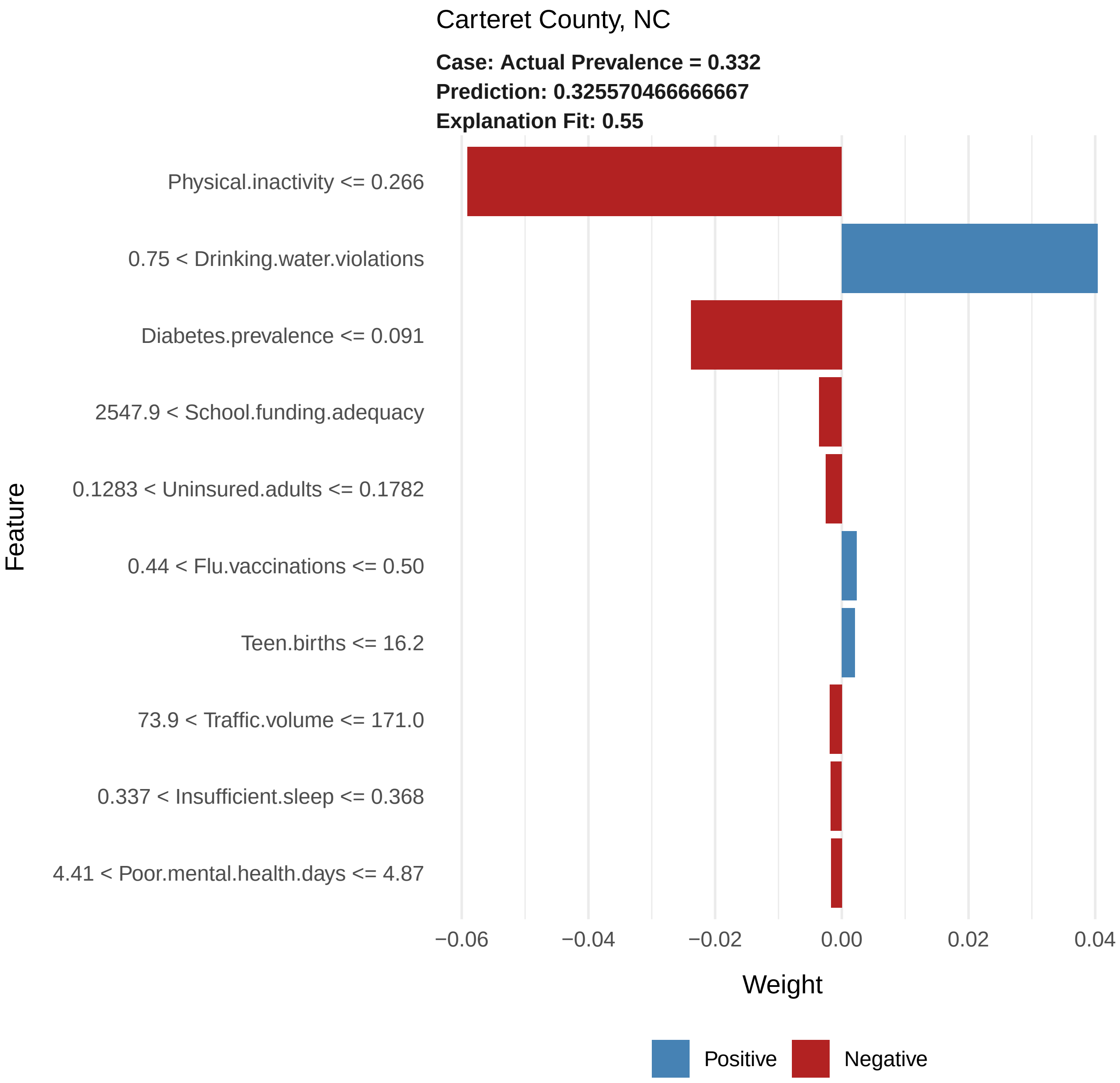
Carteret County, NC
Case: Actual Prevalence = 0.332
Prediction: 0.325570466666667
Explanation Fit: 0.55
Physical.inactivity <= 0.266
0.75 < Drinking.water.violations
Diabetes.prevalence <= 0.091
2547.9 < School.funding.adequacy
0.1283 < Uninsured.adults <= 0.1782
0.44 < Flu.vaccinations <= 0.50
Teen.births <= 16.2
73.9 < Traffic.volume <= 171.0
0.337 < Insufficient.sleep <= 0.368
4.41 < Poor.mental.health.days <= 4.87
Feature
−0.06
−0.04
−0.02
0.00
0.02
0.04
Weight
Positive
Negative

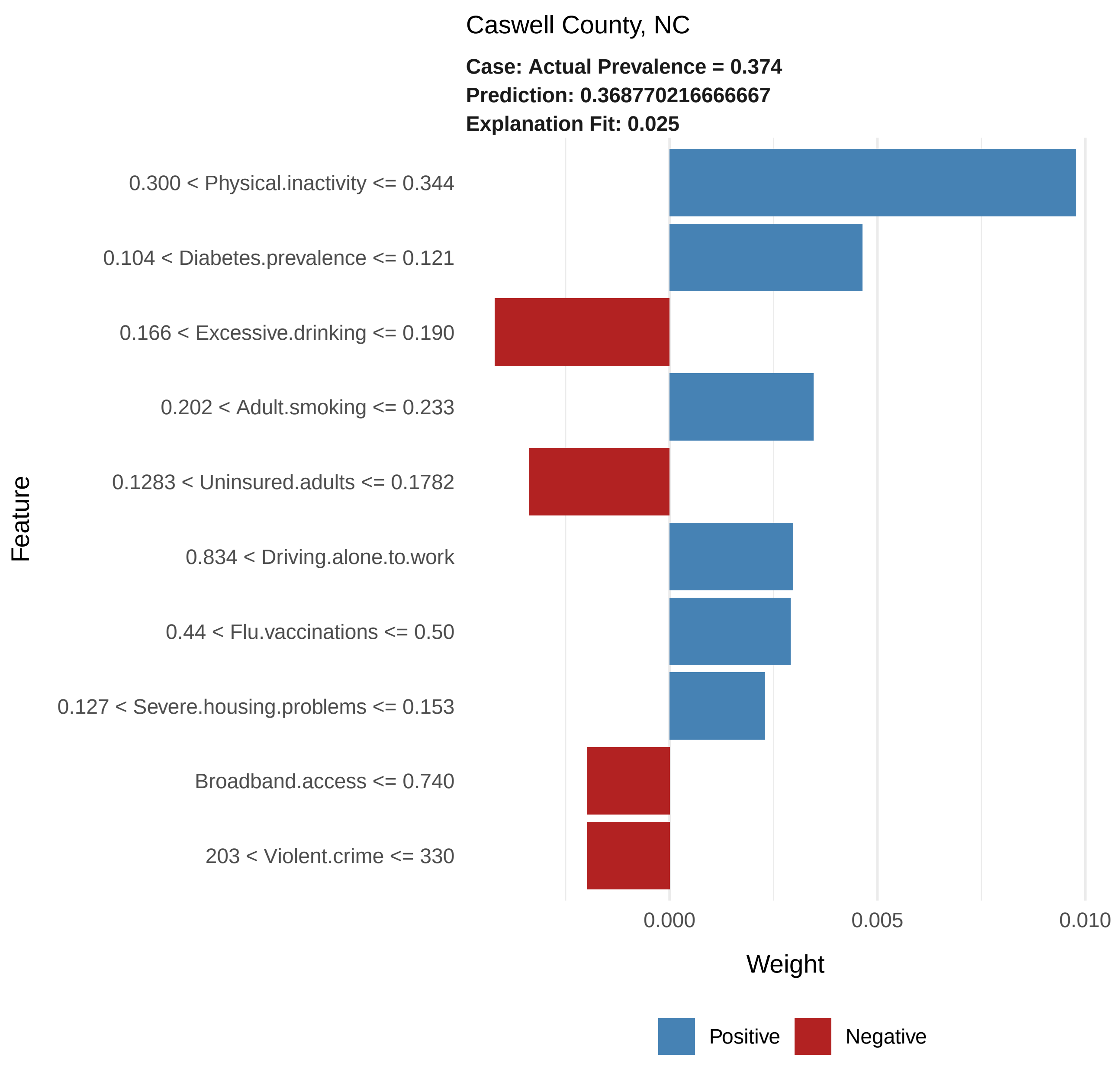
Caswell County, NC
Case: Actual Prevalence = 0.374
Prediction: 0.368770216666667
Explanation Fit: 0.025
0.300 < Physical.inactivity <= 0.344
0.104 < Diabetes.prevalence <= 0.121
0.166 < Excessive.drinking <= 0.190
0.202 < Adult.smoking <= 0.233
0.1283 < Uninsured.adults <= 0.1782
0.834 < Driving.alone.to.work
0.44 < Flu.vaccinations <= 0.50
0.127 < Severe.housing.problems <= 0.153
Broadband.access <= 0.740
203 < Violent.crime <= 330
Feature
0.000
0.005
0.010
Weight
Positive
Negative

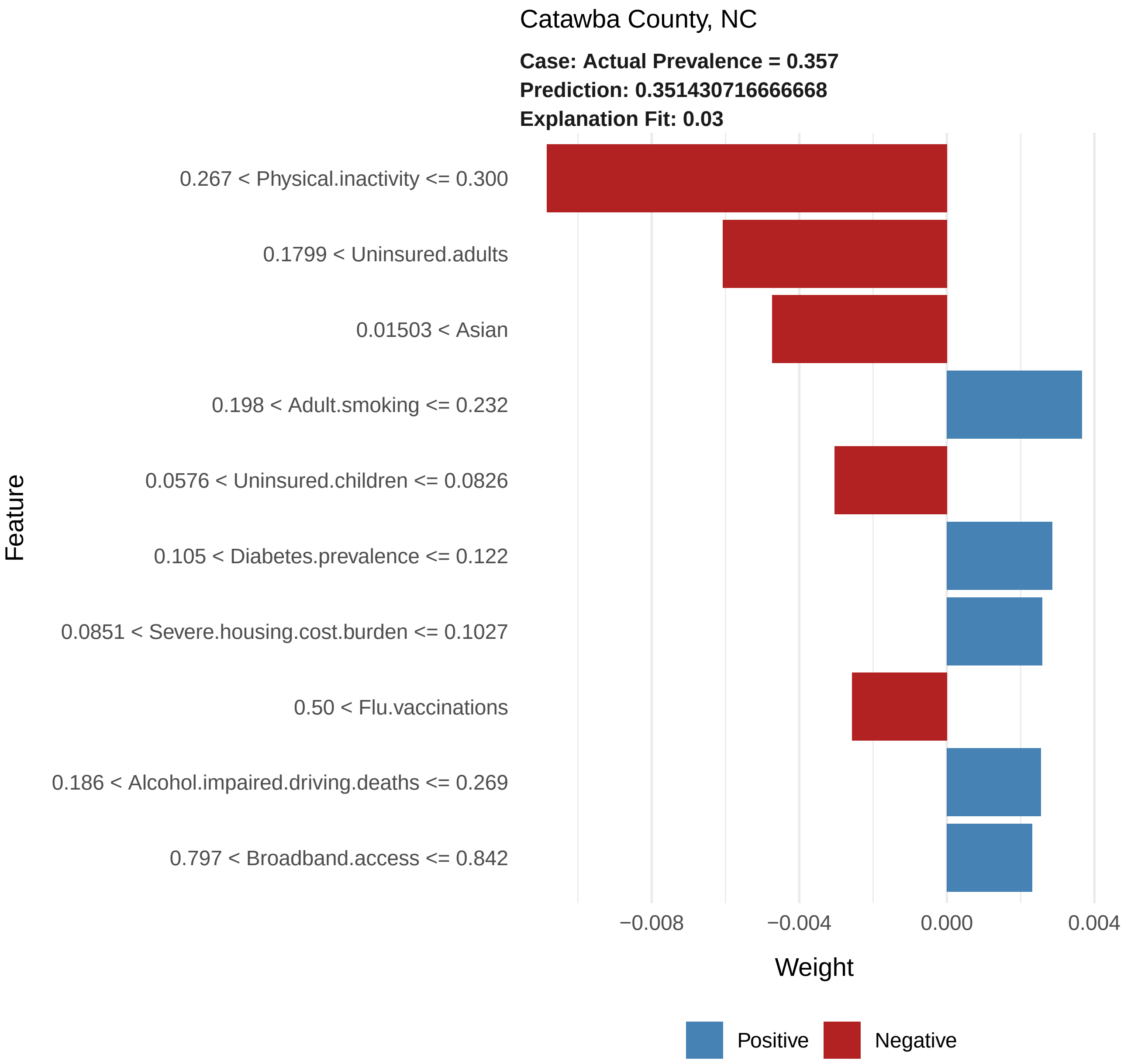

Catawba County, NC
Case: Actual Prevalence = 0.357
Prediction: 0.351430716666668
Explanation Fit: 0.03
0.267 < Physical.inactivity <= 0.300
0.1799 < Uninsured.adults
0.01503 < Asian
0.198 < Adult.smoking <= 0.232
0.0576 < Uninsured.children <= 0.0826
0.105 < Diabetes.prevalence <= 0.122
0.0851 < Severe.housing.cost.burden <= 0.1027
0.50 < Flu.vaccinations
0.186 < Alcohol.impaired.driving.deaths <= 0.269
0.797 < Broadband.access <= 0.842
Feature
−0.008
−0.004
0.000
0.004
Weight
Positive
Negative

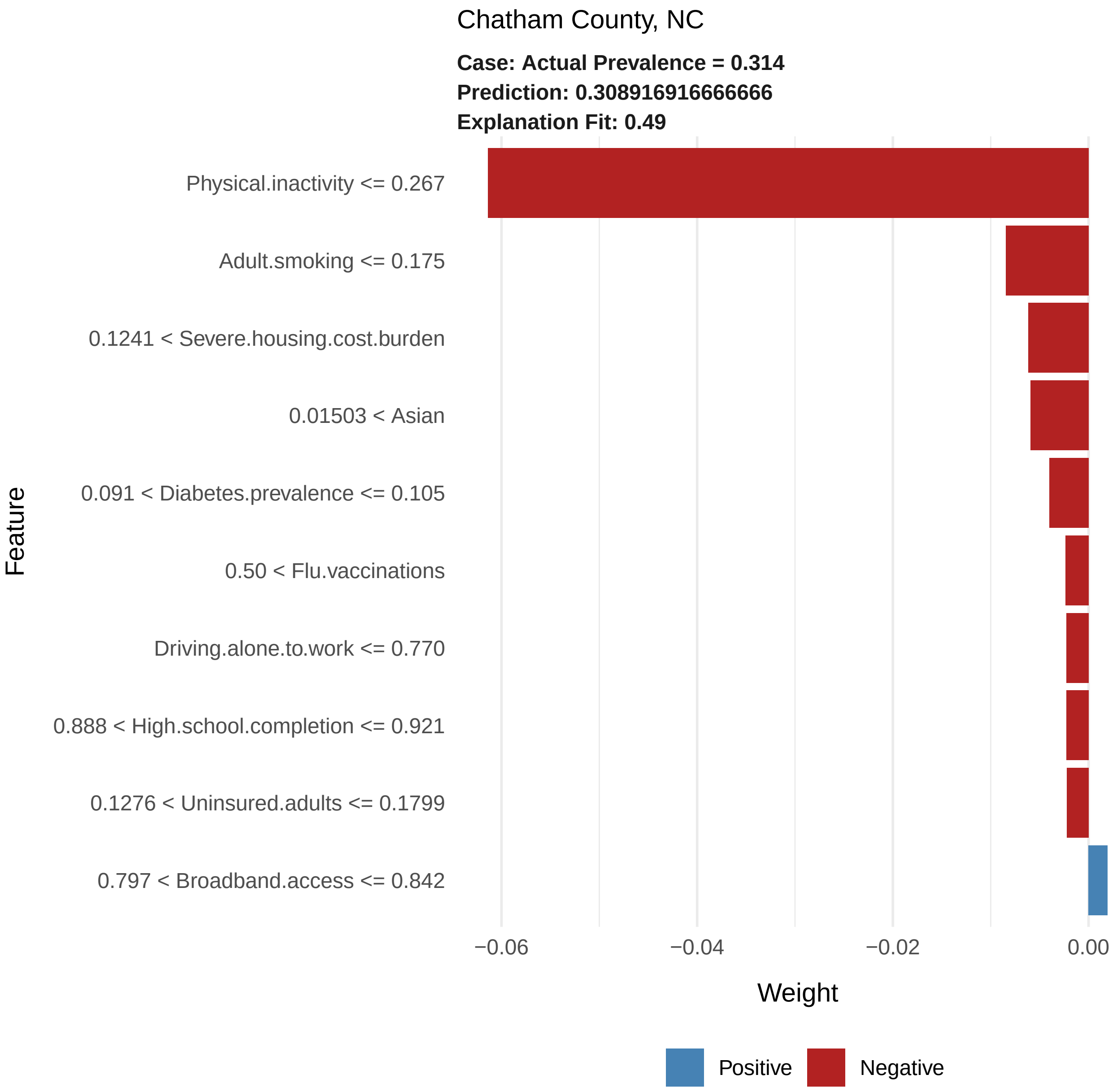
Chatham County, NC
Case: Actual Prevalence = 0.314
Prediction: 0.308916916666666
Explanation Fit: 0.49
Physical.inactivity <= 0.267
Adult.smoking <= 0.175
0.1241 < Severe.housing.cost.burden
0.01503 < Asian
0.091 < Diabetes.prevalence <= 0.105
0.50 < Flu.vaccinations
Driving.alone.to.work <= 0.770
0.888 < High.school.completion <= 0.921
0.1276 < Uninsured.adults <= 0.1799
0.797 < Broadband.access <= 0.842
Feature
−0.06
−0.04
−0.02
0.00
Weight
Positive
Negative

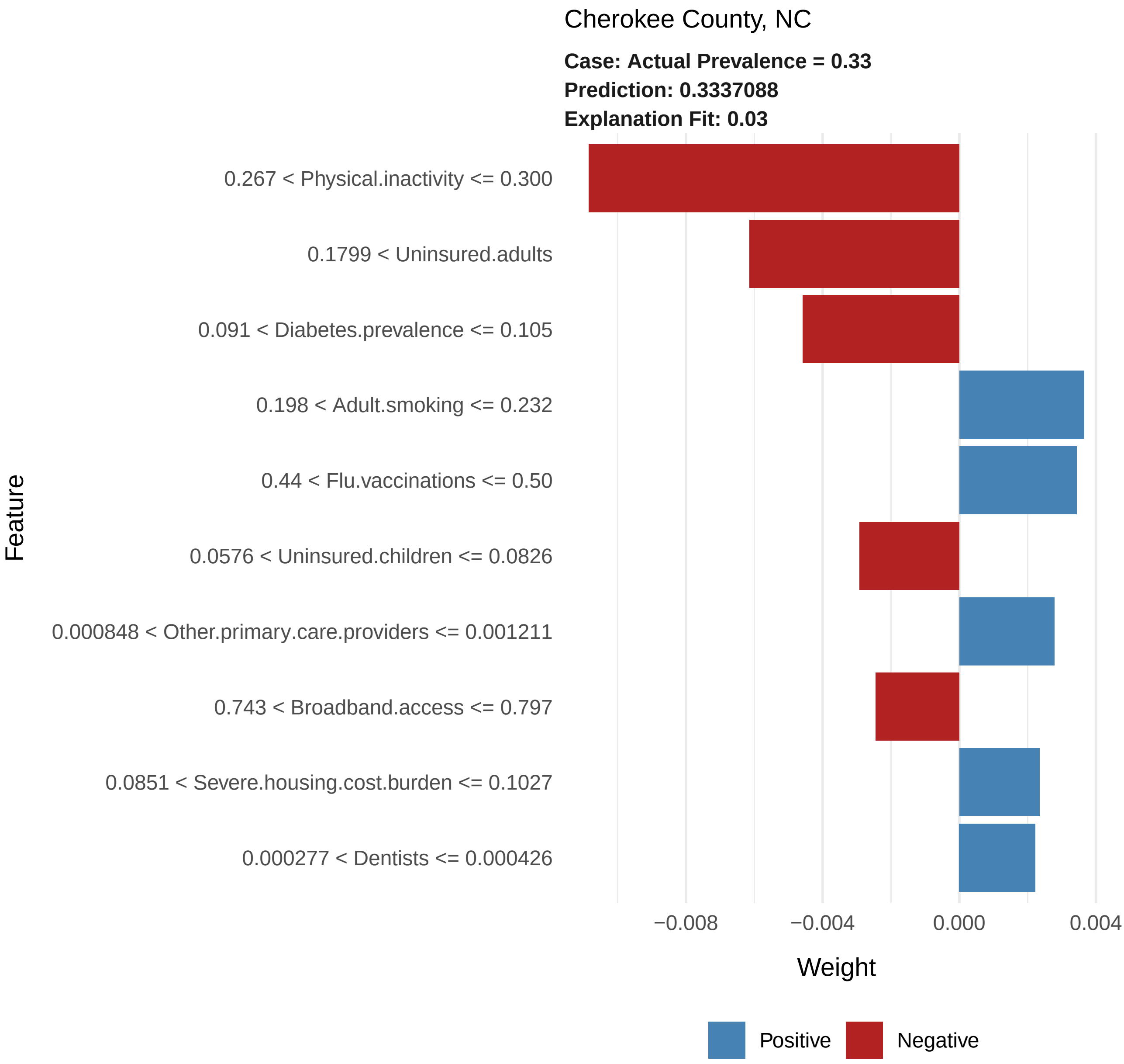

Cherokee County, NC
Case: Actual Prevalence = 0.33
Prediction: 0.3337088
Explanation Fit: 0.03
0.267 < Physical.inactivity <= 0.300
0.1799 < Uninsured.adults
0.091 < Diabetes.prevalence <= 0.105
0.198 < Adult.smoking <= 0.232
0.44 < Flu.vaccinations <= 0.50
0.0576 < Uninsured.children <= 0.0826
0.000848 < Other.primary.care.providers <= 0.001211
0.743 < Broadband.access <= 0.797
0.0851 < Severe.housing.cost.burden <= 0.1027
0.000277 < Dentists <= 0.000426
Feature
−0.008
−0.004
0.000
0.004
Weight
Positive
Negative

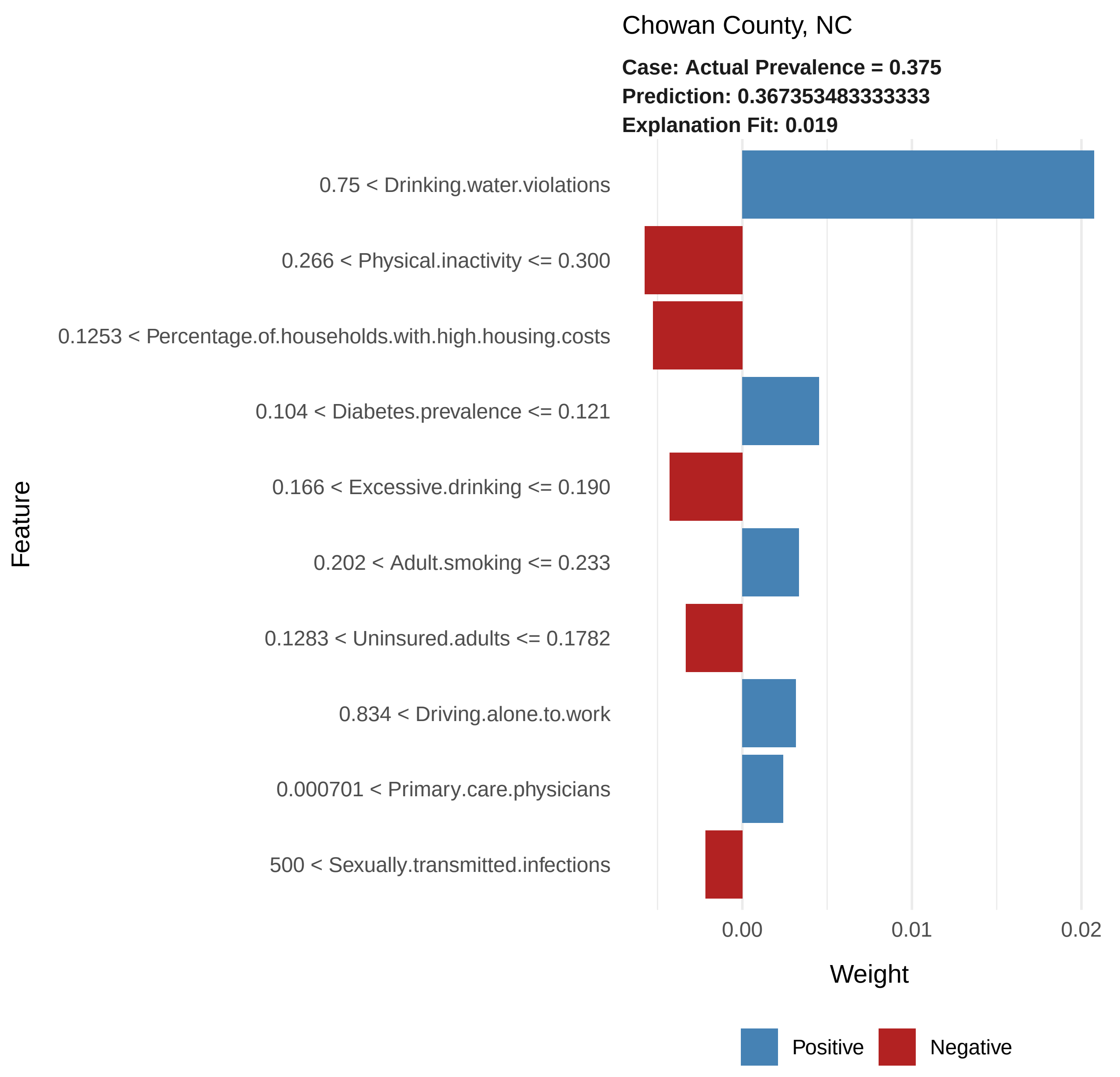

Chowan County, NC
Case: Actual Prevalence = 0.375
Prediction: 0.367353483333333
Explanation Fit: 0.019
0.75 < Drinking.water.violations
0.266 < Physical.inactivity <= 0.300
0.1253 < Percentage.of.households.with.high.housing.costs
0.104 < Diabetes.prevalence <= 0.121
0.166 < Excessive.drinking <= 0.190
0.202 < Adult.smoking <= 0.233
0.1283 < Uninsured.adults <= 0.1782
0.834 < Driving.alone.to.work
0.000701 < Primary.care.physicians
500 < Sexually.transmitted.infections
Feature
0.00
0.01
0.02
Weight
Positive
Negative

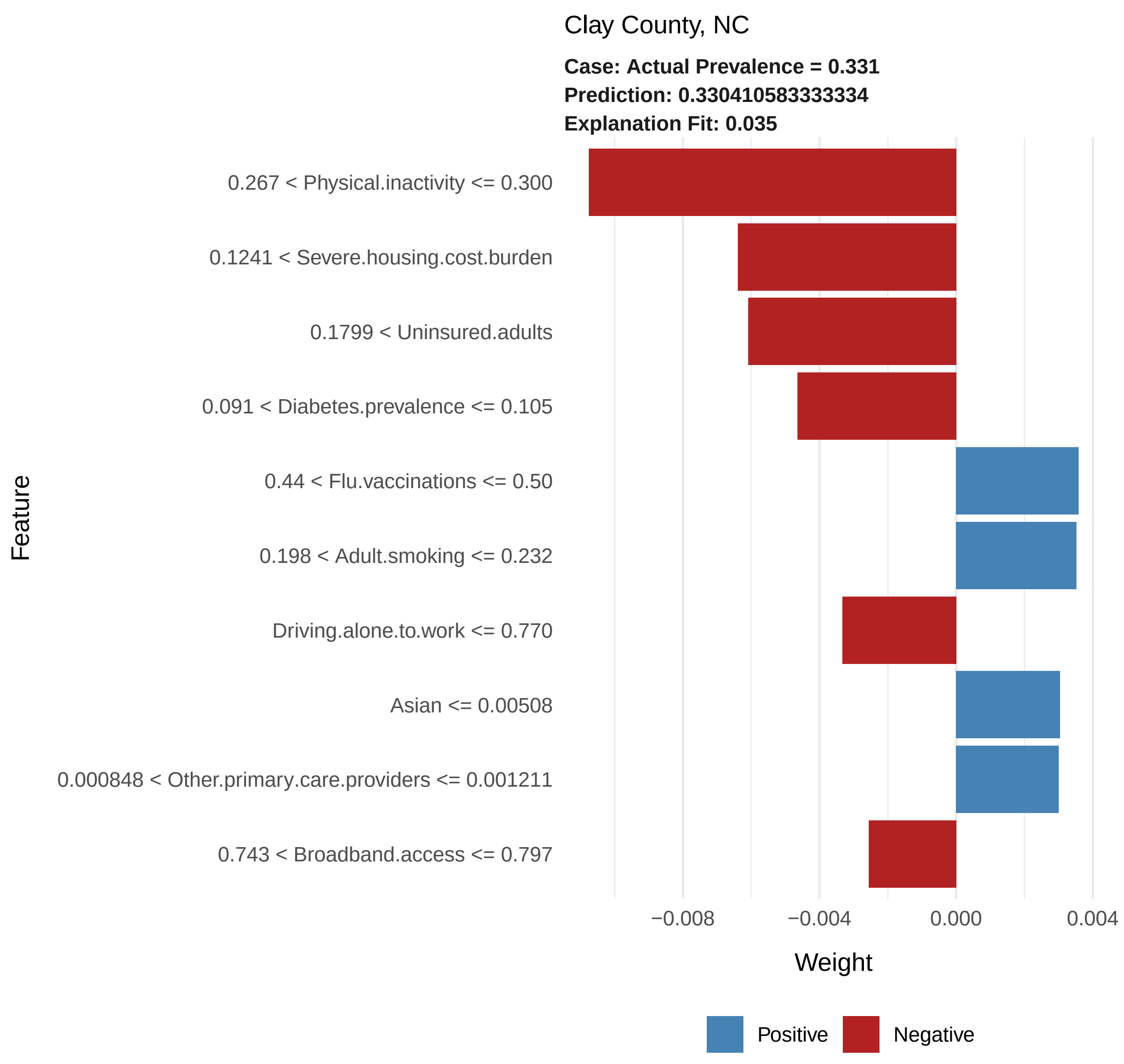

Clay County, NC
Case: Actual Prevalence = 0.331
Prediction: 0.330410583333334
Explanation Fit: 0.035
0.267 < Physical.inactivity <= 0.300
0.1241 < Severe.housing.cost.burden
0.1799 < Uninsured.adults
0.091 < Diabetes.prevalence <= 0.105
0.44 < Flu.vaccinations <= 0.50
0.198 < Adult.smoking <= 0.232
Driving.alone.to.work <= 0.770
Asian <= 0.00508
0.000848 < Other.primary.care.providers <= 0.001211
0.743 < Broadband.access <= 0.797
Feature
−0.008
−0.004
0.000
0.004
Weight
Positive
Negative

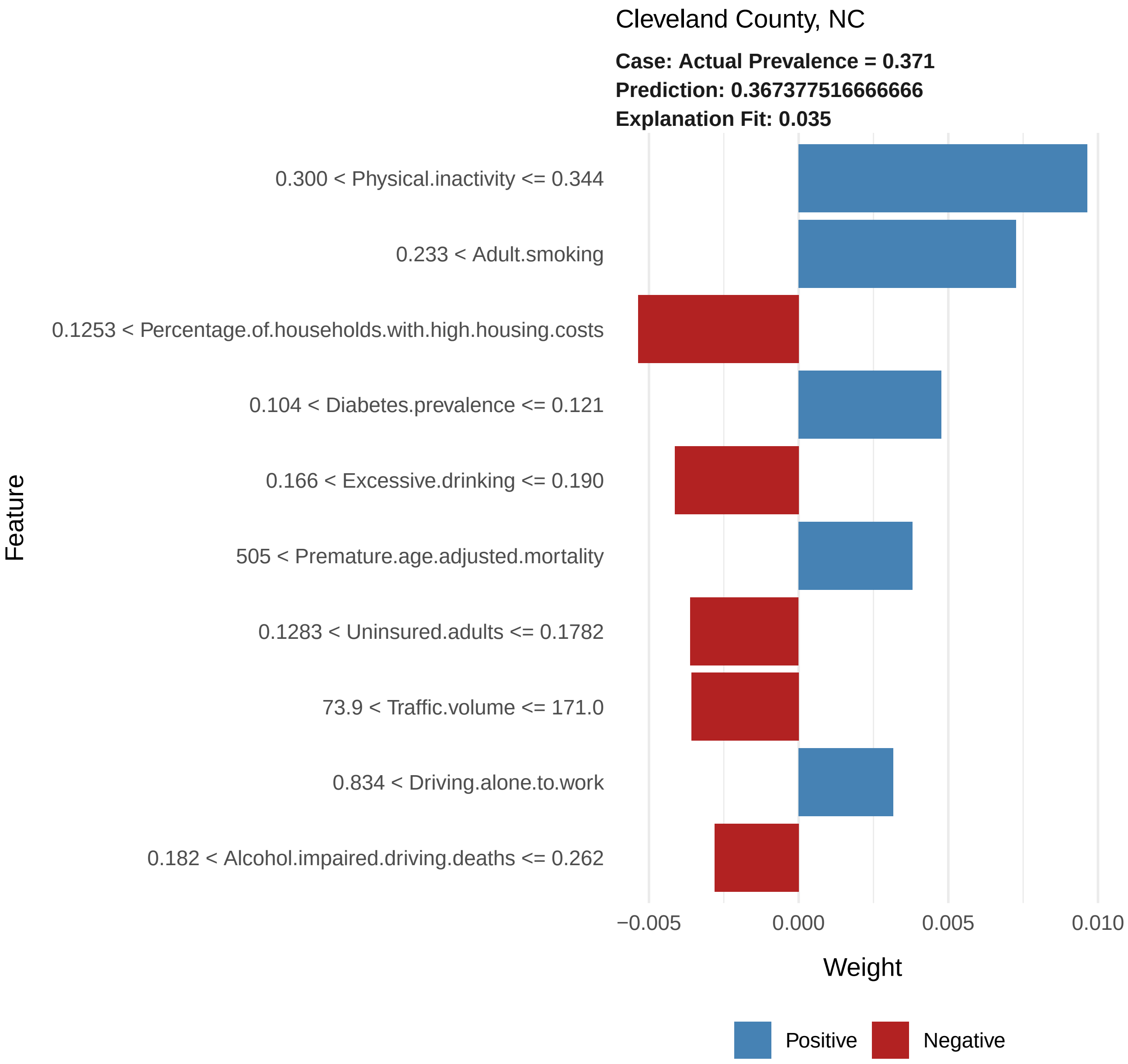
Cleveland County, NC
Case: Actual Prevalence = 0.371
Prediction: 0.367377516666666
Explanation Fit: 0.035
0.300 < Physical.inactivity <= 0.344
0.233 < Adult.smoking
0.1253 < Percentage.of.households.with.high.housing.costs
0.104 < Diabetes.prevalence <= 0.121
0.166 < Excessive.drinking <= 0.190
505 < Premature.age.adjusted.mortality
0.1283 < Uninsured.adults <= 0.1782
73.9 < Traffic.volume <= 171.0
0.834 < Driving.alone.to.work
0.182 < Alcohol.impaired.driving.deaths <= 0.262
Feature
−0.005
0.000
0.005
0.010
Weight
Positive
Negative

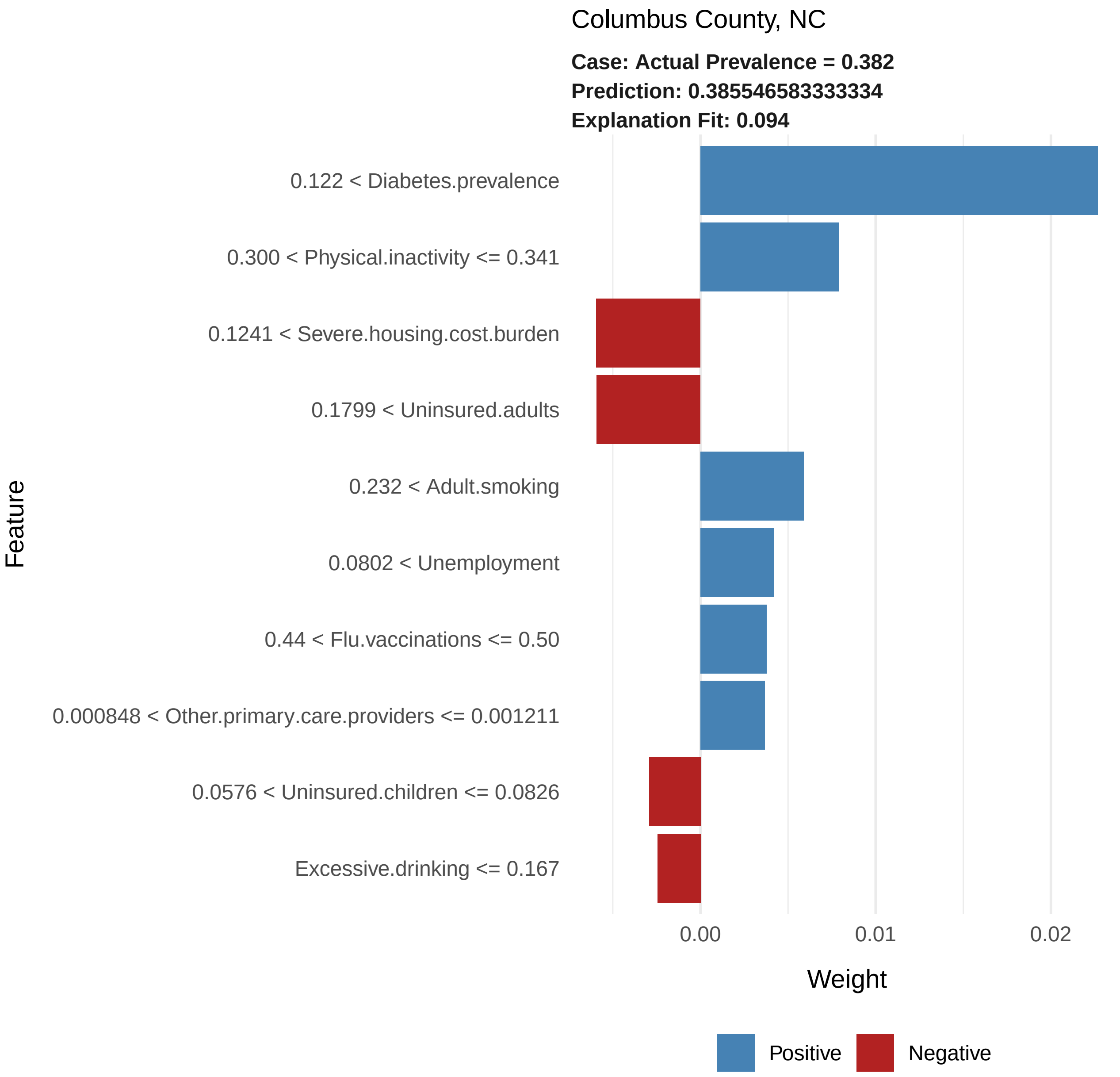
Columbus County, NC
Case: Actual Prevalence = 0.382
Prediction: 0.385546583333334
Explanation Fit: 0.094
0.122 < Diabetes.prevalence
0.300 < Physical.inactivity <= 0.341
0.1241 < Severe.housing.cost.burden
0.1799 < Uninsured.adults
0.232 < Adult.smoking
0.0802 < Unemployment
0.44 < Flu.vaccinations <= 0.50
0.000848 < Other.primary.care.providers <= 0.001211
0.0576 < Uninsured.children <= 0.0826
Excessive.drinking <= 0.167
Feature
0.00
0.01
0.02
Weight
Positive
Negative

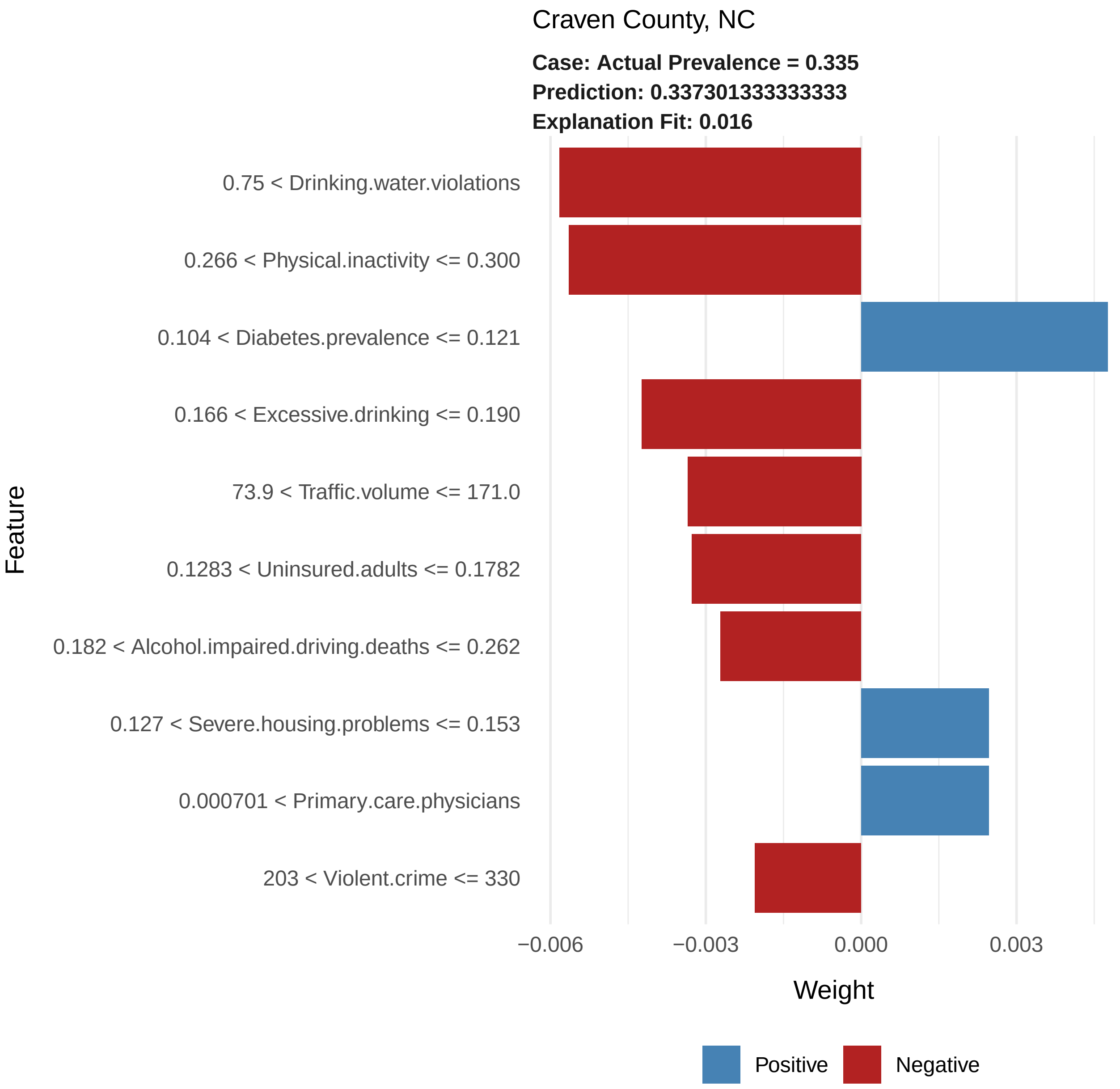

Craven County, NC
Case: Actual Prevalence = 0.335
Prediction: 0.337301333333333
Explanation Fit: 0.016
0.75 < Drinking.water.violations
0.266 < Physical.inactivity <= 0.300
0.104 < Diabetes.prevalence <= 0.121
0.166 < Excessive.drinking <= 0.190
73.9 < Traffic.volume <= 171.0
0.1283 < Uninsured.adults <= 0.1782
0.182 < Alcohol.impaired.driving.deaths <= 0.262
0.127 < Severe.housing.problems <= 0.153
0.000701 < Primary.care.physicians
203 < Violent.crime <= 330
Feature
−0.006
−0.003
0.000
0.003
Weight
Positive
Negative

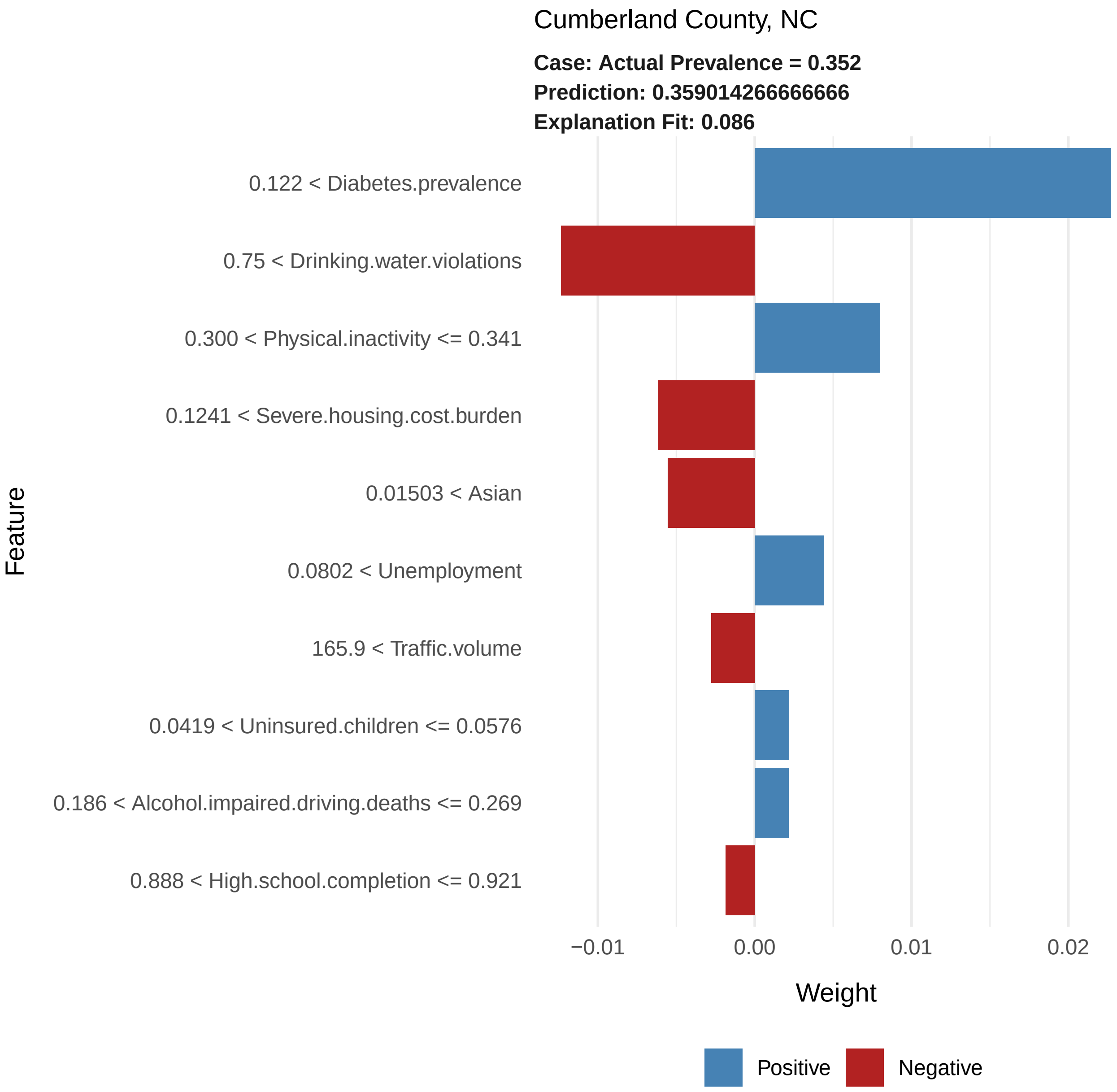

Cumberland County, NC
Case: Actual Prevalence = 0.352
Prediction: 0.359014266666666
Explanation Fit: 0.086
0.122 < Diabetes.prevalence
0.75 < Drinking.water.violations
0.300 < Physical.inactivity <= 0.341
0.1241 < Severe.housing.cost.burden
0.01503 < Asian
0.0802 < Unemployment
165.9 < Traffic.volume
0.0419 < Uninsured.children <= 0.0576
0.186 < Alcohol.impaired.driving.deaths <= 0.269
0.888 < High.school.completion <= 0.921
Feature
−0.01
0.00
0.01
0.02
Weight
Positive
Negative

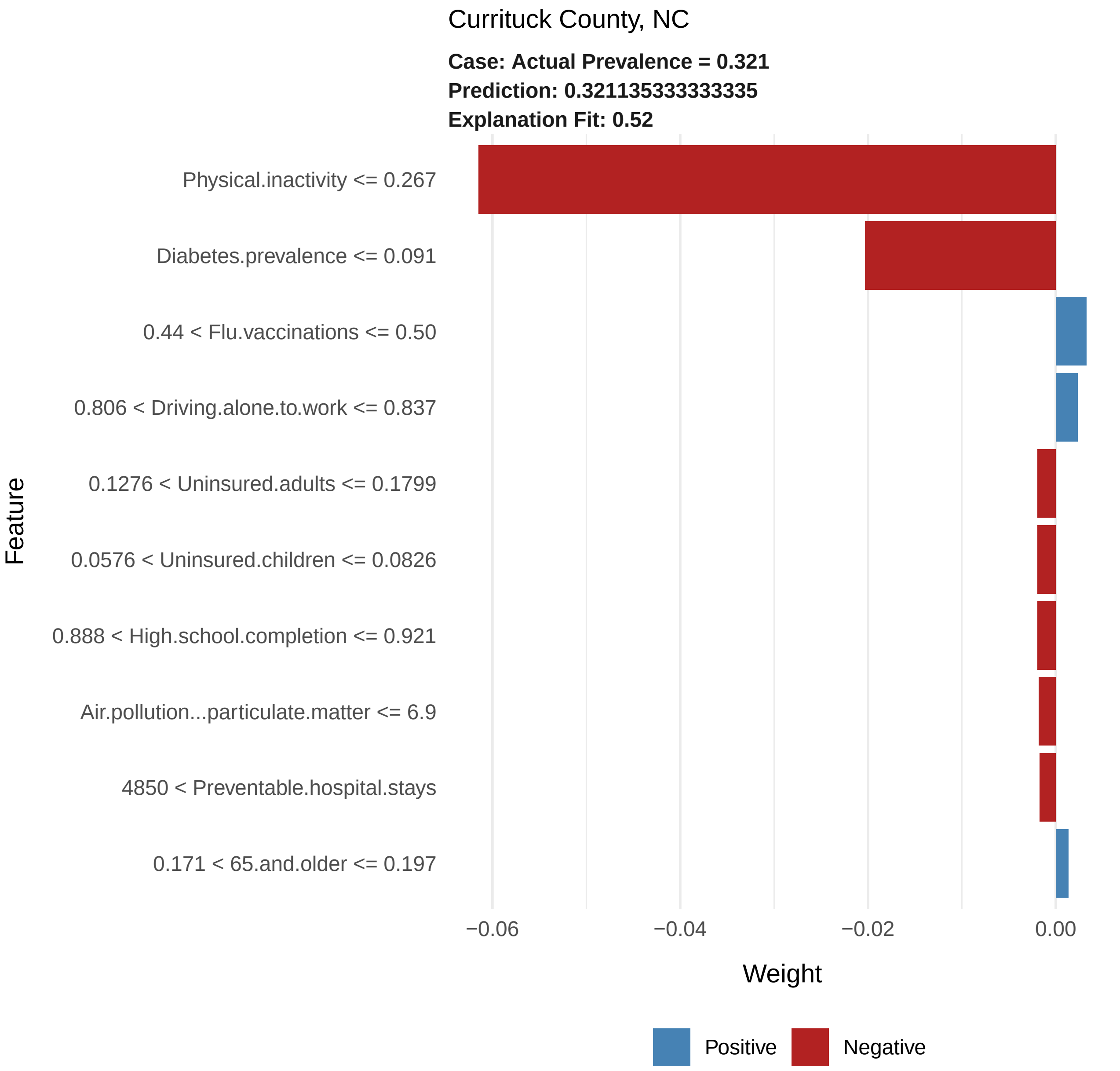
Currituck County, NC
Case: Actual Prevalence = 0.321
Prediction: 0.321135333333335
Explanation Fit: 0.52
Physical.inactivity <= 0.267
Diabetes.prevalence <= 0.091
0.44 < Flu.vaccinations <= 0.50
0.806 < Driving.alone.to.work <= 0.837
0.1276 < Uninsured.adults <= 0.1799
0.0576 < Uninsured.children <= 0.0826
0.888 < High.school.completion <= 0.921
Air.pollution...particulate.matter <= 6.9
4850 < Preventable.hospital.stays
0.171 < 65.and.older <= 0.197
Feature
−0.06
−0.04
−0.02
0.00
Weight
Positive
Negative

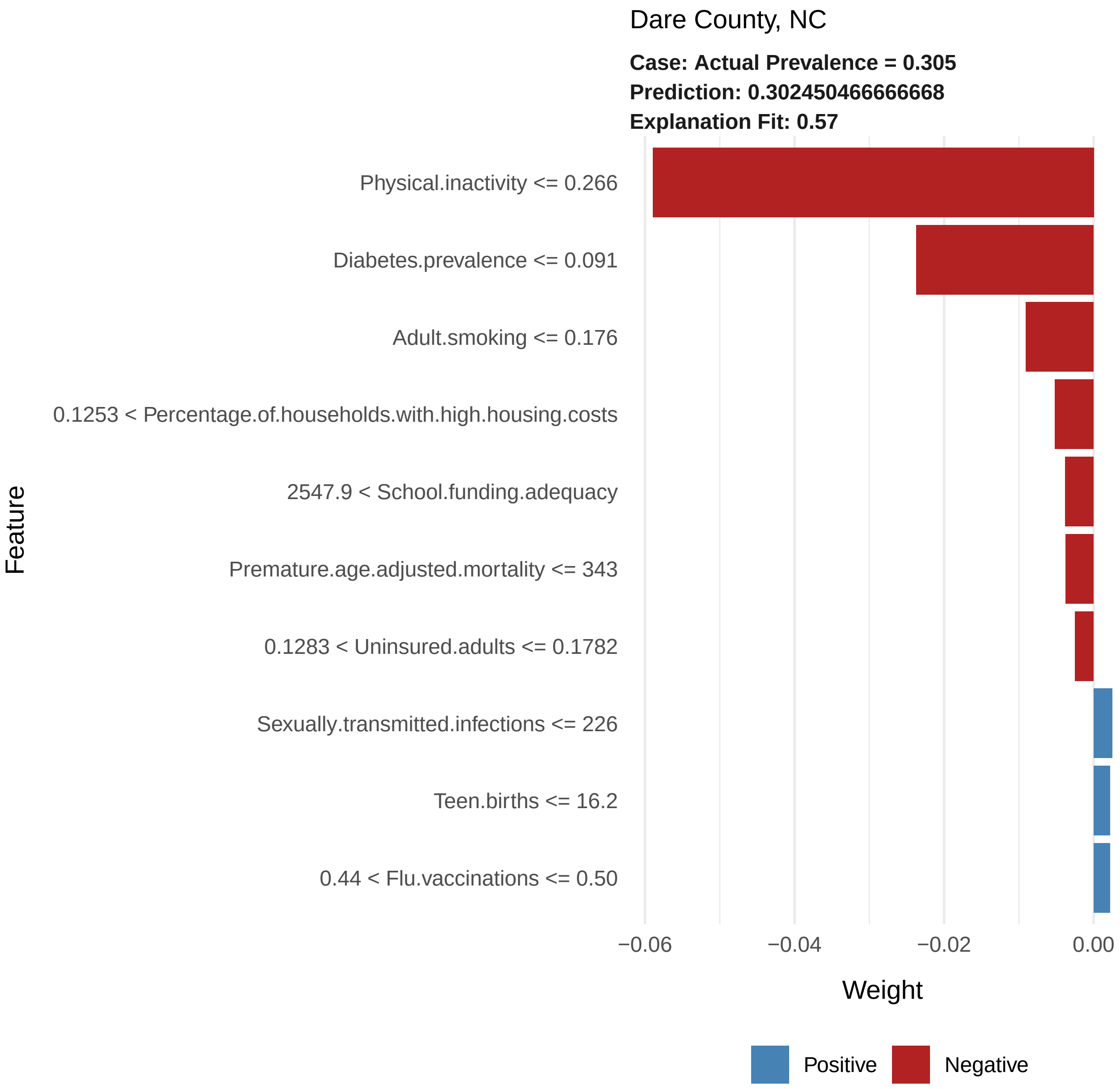
Dare County, NC
Case: Actual Prevalence = 0.305
Prediction: 0.302450466666668
Explanation Fit: 0.57
Physical.inactivity <= 0.266
Diabetes.prevalence <= 0.091
Adult.smoking <= 0.176
0.1253 < Percentage.of.households.with.high.housing.costs
2547.9 < School.funding.adequacy
Premature.age.adjusted.mortality <= 343
0.1283 < Uninsured.adults <= 0.1782
Sexually.transmitted.infections <= 226
Teen.births <= 16.2
0.44 < Flu.vaccinations <= 0.50
Feature
−0.06
−0.04
−0.02
0.00
Weight
Positive
Negative

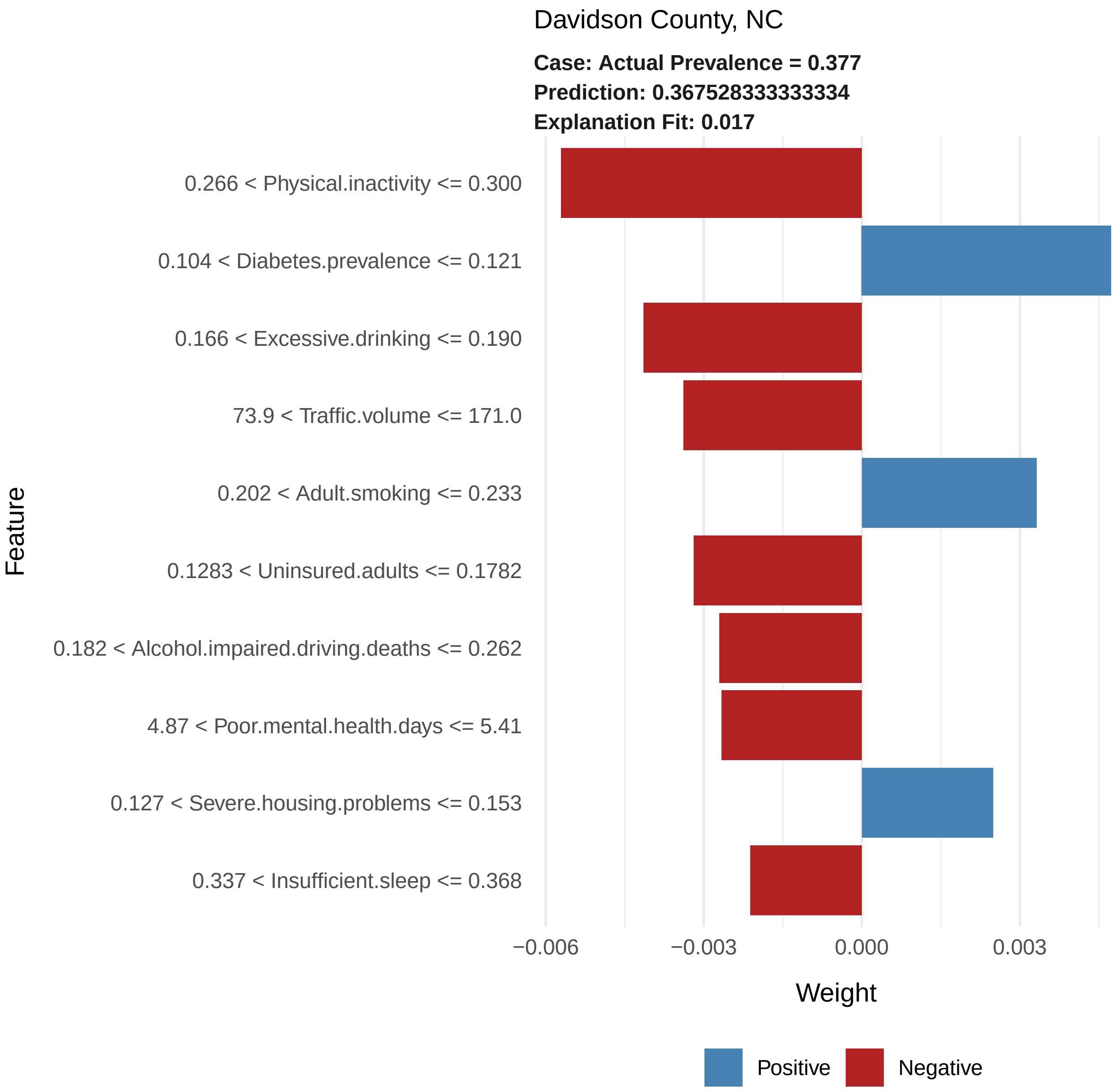

Davidson County, NC
Case: Actual Prevalence = 0.377
Prediction: 0.367528333333334
Explanation Fit: 0.017
0.266 < Physical.inactivity <= 0.300
0.104 < Diabetes.prevalence <= 0.121
0.166 < Excessive.drinking <= 0.190
73.9 < Traffic.volume <= 171.0
0.202 < Adult.smoking <= 0.233
0.1283 < Uninsured.adults <= 0.1782
0.182 < Alcohol.impaired.driving.deaths <= 0.262
4.87 < Poor.mental.health.days <= 5.41
0.127 < Severe.housing.problems <= 0.153
0.337 < Insufficient.sleep <= 0.368
Feature
−0.006
−0.003
0.000
0.003
Weight
Positive
Negative

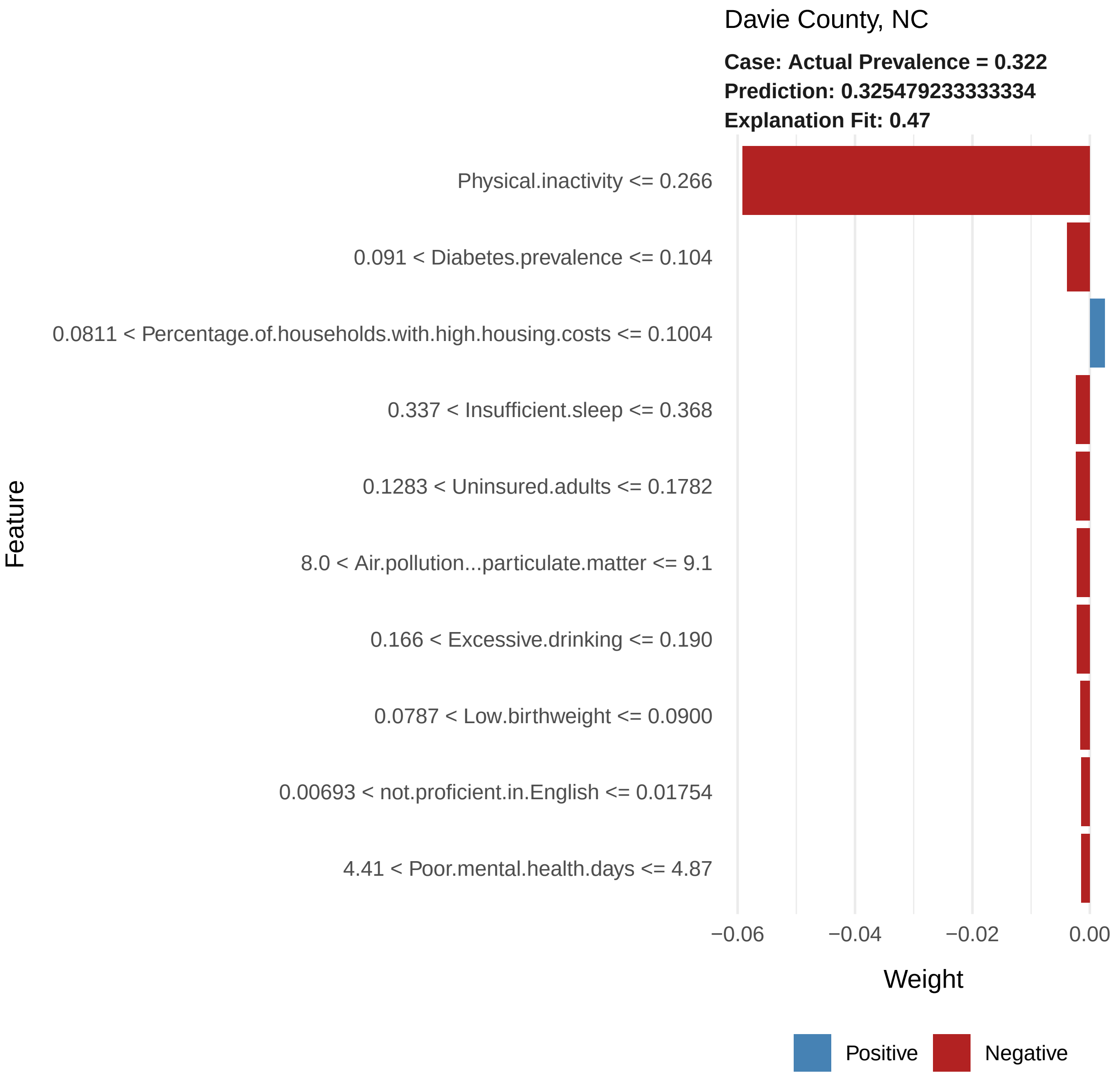
Davie County, NC
Case: Actual Prevalence = 0.322
Prediction: 0.325479233333334
Explanation Fit: 0.47
Physical.inactivity <= 0.266
0.091 < Diabetes.prevalence <= 0.104
0.0811 < Percentage.of.households.with.high.housing.costs <= 0.1004
0.337 < Insufficient.sleep <= 0.368
0.1283 < Uninsured.adults <= 0.1782
8.0 < Air.pollution...particulate.matter <= 9.1
0.166 < Excessive.drinking <= 0.190
0.0787 < Low.birthweight <= 0.0900
0.00693 < not.proficient.in.English <= 0.01754
4.41 < Poor.mental.health.days <= 4.87
Feature
−0.06
−0.04
−0.02
0.00
Weight
Positive
Negative

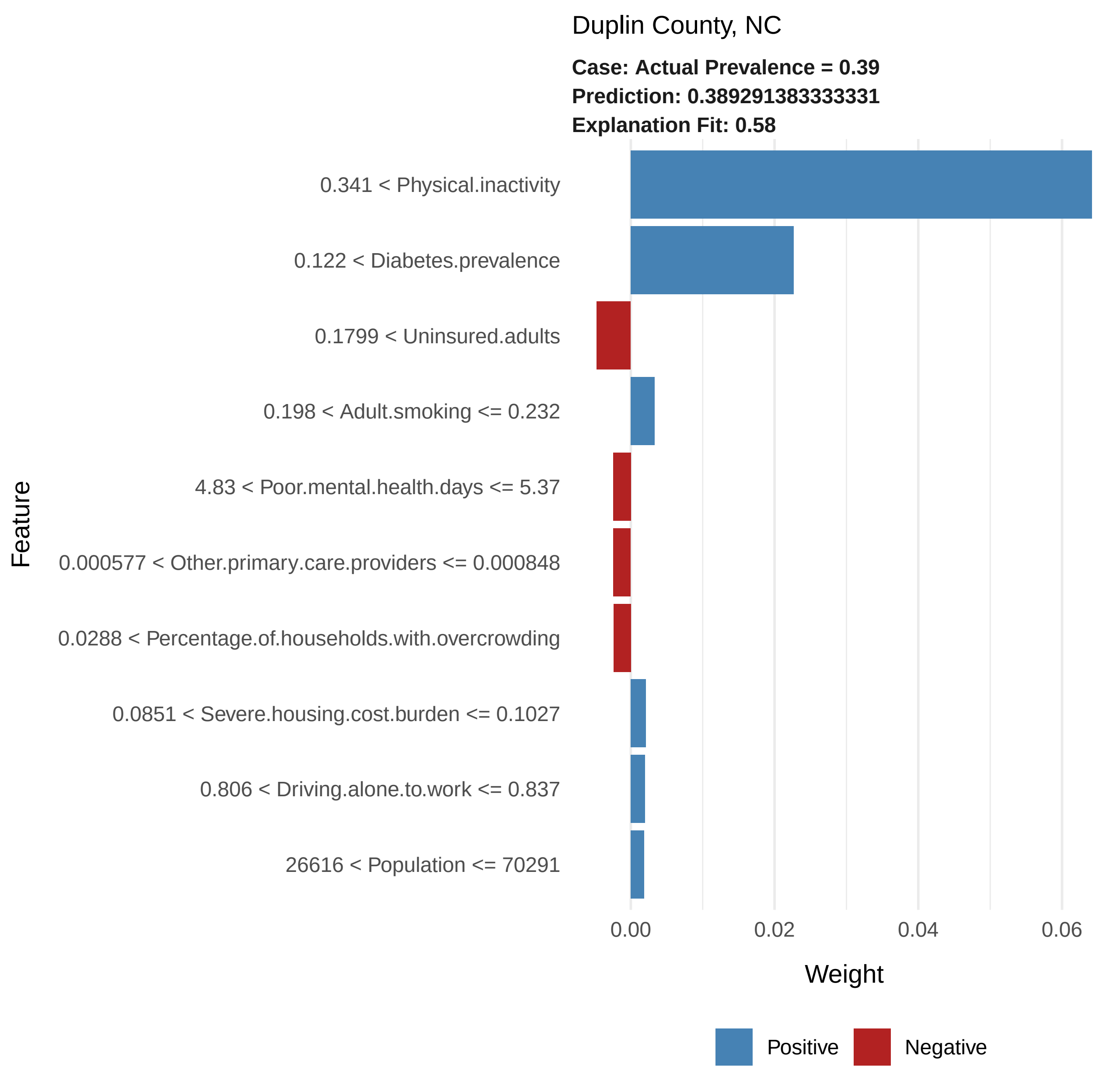

Duplin County, NC
Case: Actual Prevalence = 0.39
Prediction: 0.389291383333331
Explanation Fit: 0.58
0.341 < Physical.inactivity
0.122 < Diabetes.prevalence
0.1799 < Uninsured.adults
0.198 < Adult.smoking <= 0.232
4.83 < Poor.mental.health.days <= 5.37
0.000577 < Other.primary.care.providers <= 0.000848
0.0288 < Percentage.of.households.with.overcrowding
0.0851 < Severe.housing.cost.burden <= 0.1027
0.806 < Driving.alone.to.work <= 0.837
26616 < Population <= 70291
Feature
0.00
0.02
0.04
0.06
Weight
Positive
Negative

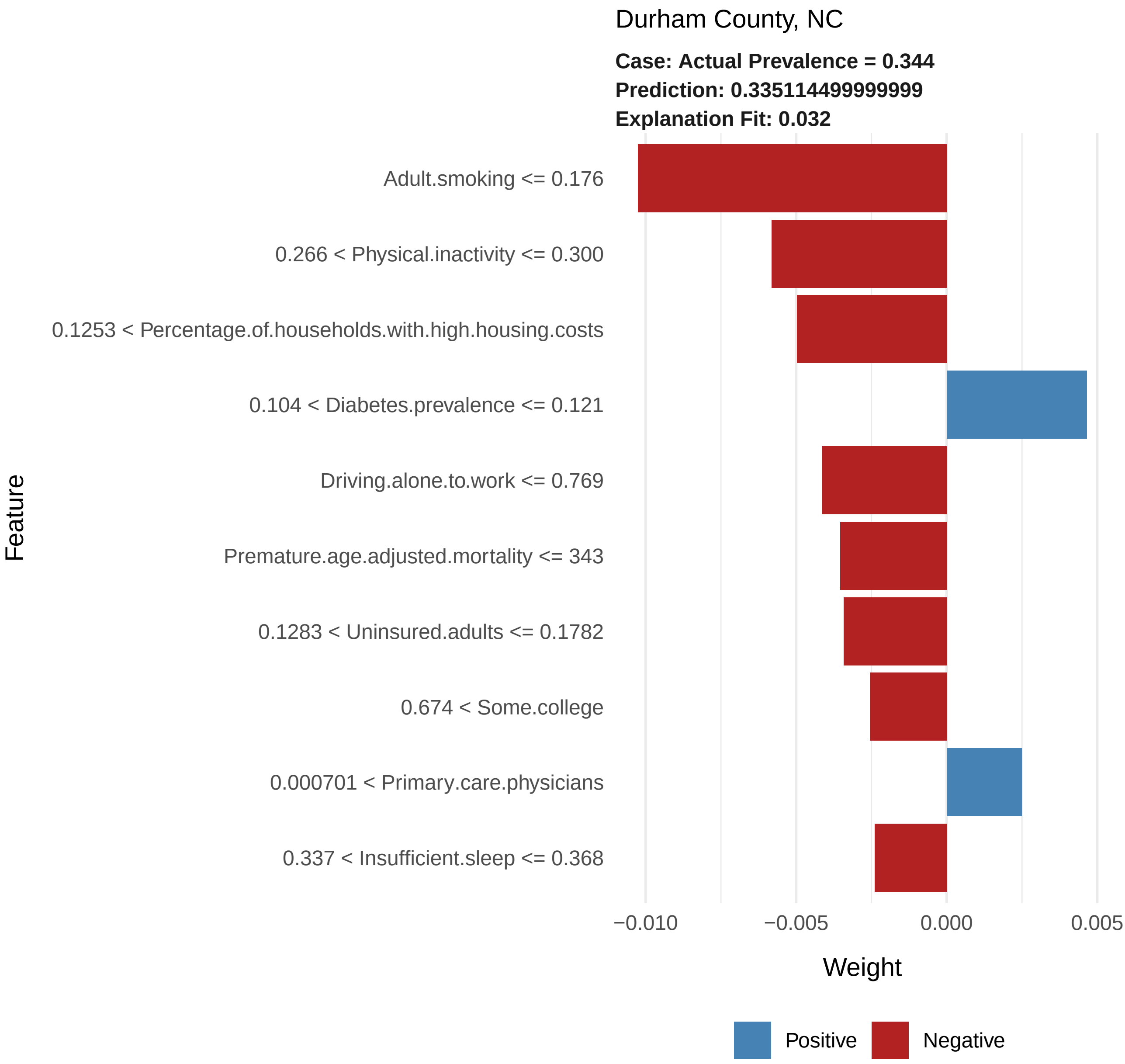
Durham County, NC
Case: Actual Prevalence = 0.344
Prediction: 0.335114499999999
Explanation Fit: 0.032
Adult.smoking <= 0.176
0.266 < Physical.inactivity <= 0.300
0.1253 < Percentage.of.households.with.high.housing.costs
0.104 < Diabetes.prevalence <= 0.121
Driving.alone.to.work <= 0.769
Premature.age.adjusted.mortality <= 343
0.1283 < Uninsured.adults <= 0.1782
0.674 < Some.college
0.000701 < Primary.care.physicians
0.337 < Insufficient.sleep <= 0.368
Feature
−0.010
−0.005
0.000
0.005
Weight
Positive
Negative

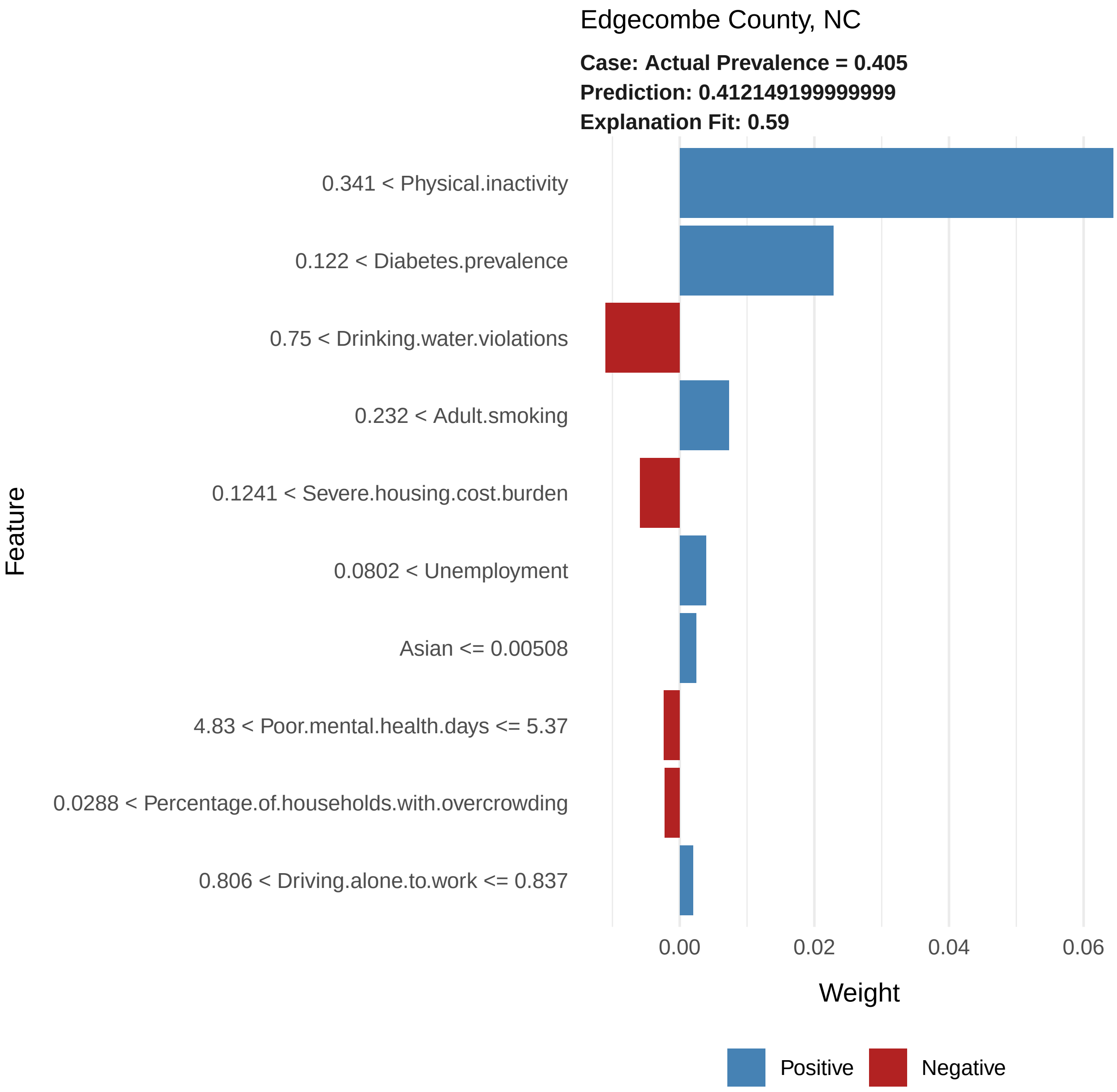

Edgecombe County, NC
Case: Actual Prevalence = 0.405
Prediction: 0.412149199999999
Explanation Fit: 0.59
0.341 < Physical.inactivity
0.122 < Diabetes.prevalence
0.75 < Drinking.water.violations
0.232 < Adult.smoking
0.1241 < Severe.housing.cost.burden
0.0802 < Unemployment
Asian <= 0.00508
4.83 < Poor.mental.health.days <= 5.37
0.0288 < Percentage.of.households.with.overcrowding
0.806 < Driving.alone.to.work <= 0.837
Feature
0.00
0.02
0.04
0.06
Weight
Positive
Negative

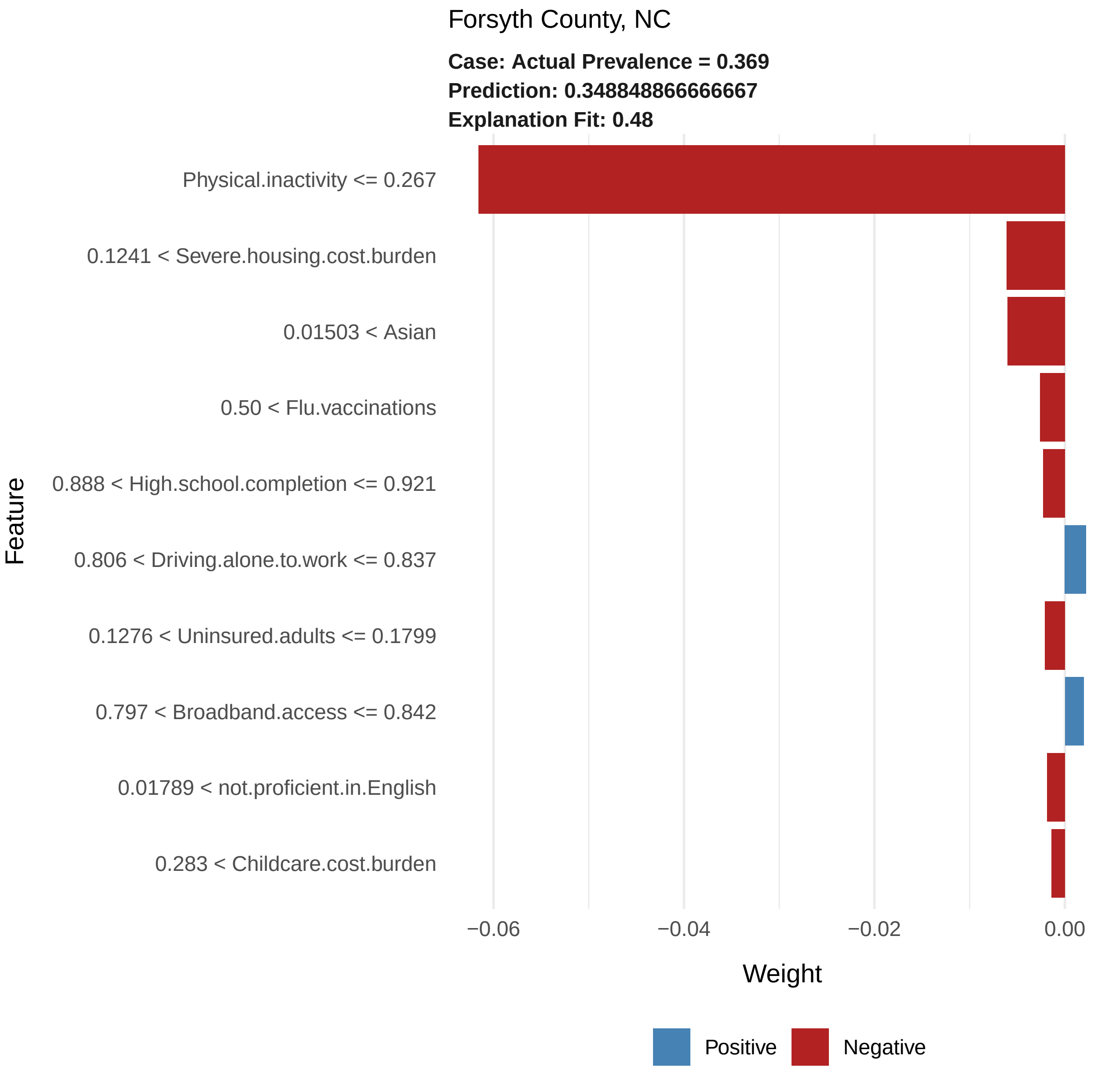
Forsyth County, NC
Case: Actual Prevalence = 0.369
Prediction: 0.348848866666667
Explanation Fit: 0.48
Physical.inactivity <= 0.267
0.1241 < Severe.housing.cost.burden
0.01503 < Asian
0.50 < Flu.vaccinations
0.888 < High.school.completion <= 0.921
0.806 < Driving.alone.to.work <= 0.837
0.1276 < Uninsured.adults <= 0.1799
0.797 < Broadband.access <= 0.842
0.01789 < not.proficient.in.English
0.283 < Childcare.cost.burden
Feature
−0.06
−0.04
−0.02
0.00
Weight
Positive
Negative

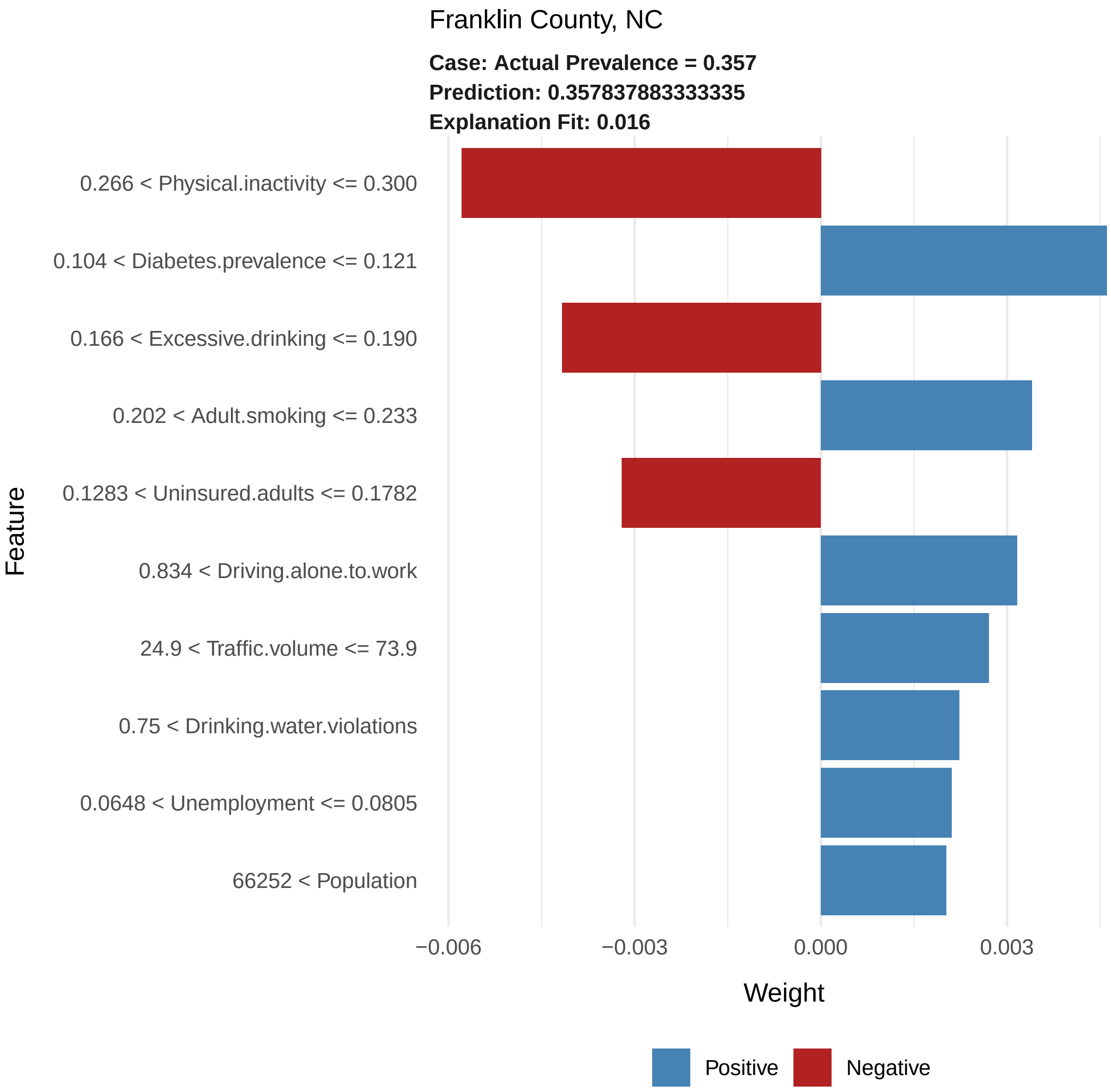

Franklin County, NC
Case: Actual Prevalence = 0.357
Prediction: 0.357837883333335
Explanation Fit: 0.016
0.266 < Physical.inactivity <= 0.300
0.104 < Diabetes.prevalence <= 0.121
0.166 < Excessive.drinking <= 0.190
0.202 < Adult.smoking <= 0.233
0.1283 < Uninsured.adults <= 0.1782
0.834 < Driving.alone.to.work
24.9 < Traffic.volume <= 73.9
0.75 < Drinking.water.violations
0.0648 < Unemployment <= 0.0805
66252 < Population
Feature
−0.006
−0.003
0.000
0.003
Weight
Positive
Negative

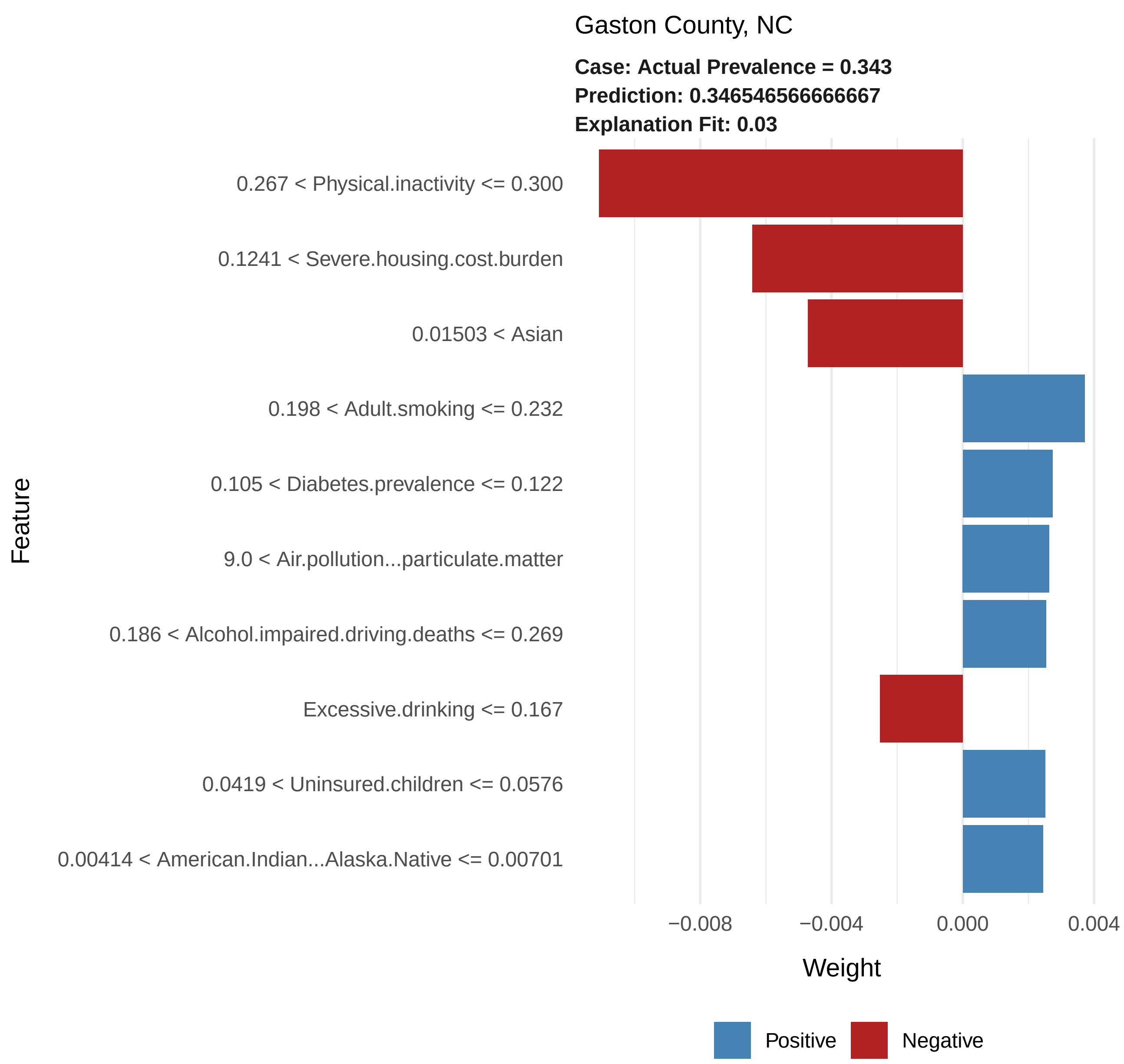
Gaston County, NC
Case: Actual Prevalence = 0.343
Prediction: 0.346546566666667
Explanation Fit: 0.03
0.267 < Physical.inactivity <= 0.300
0.1241 < Severe.housing.cost.burden
0.01503 < Asian
0.198 < Adult.smoking <= 0.232
0.105 < Diabetes.prevalence <= 0.122
9.0 < Air.pollution...particulate.matter
0.186 < Alcohol.impaired.driving.deaths <= 0.269
Excessive.drinking <= 0.167
0.0419 < Uninsured.children <= 0.0576
0.00414 < American.Indian...Alaska.Native <= 0.00701
Feature
−0.008
−0.004
0.000
0.004
Weight
Positive
Negative

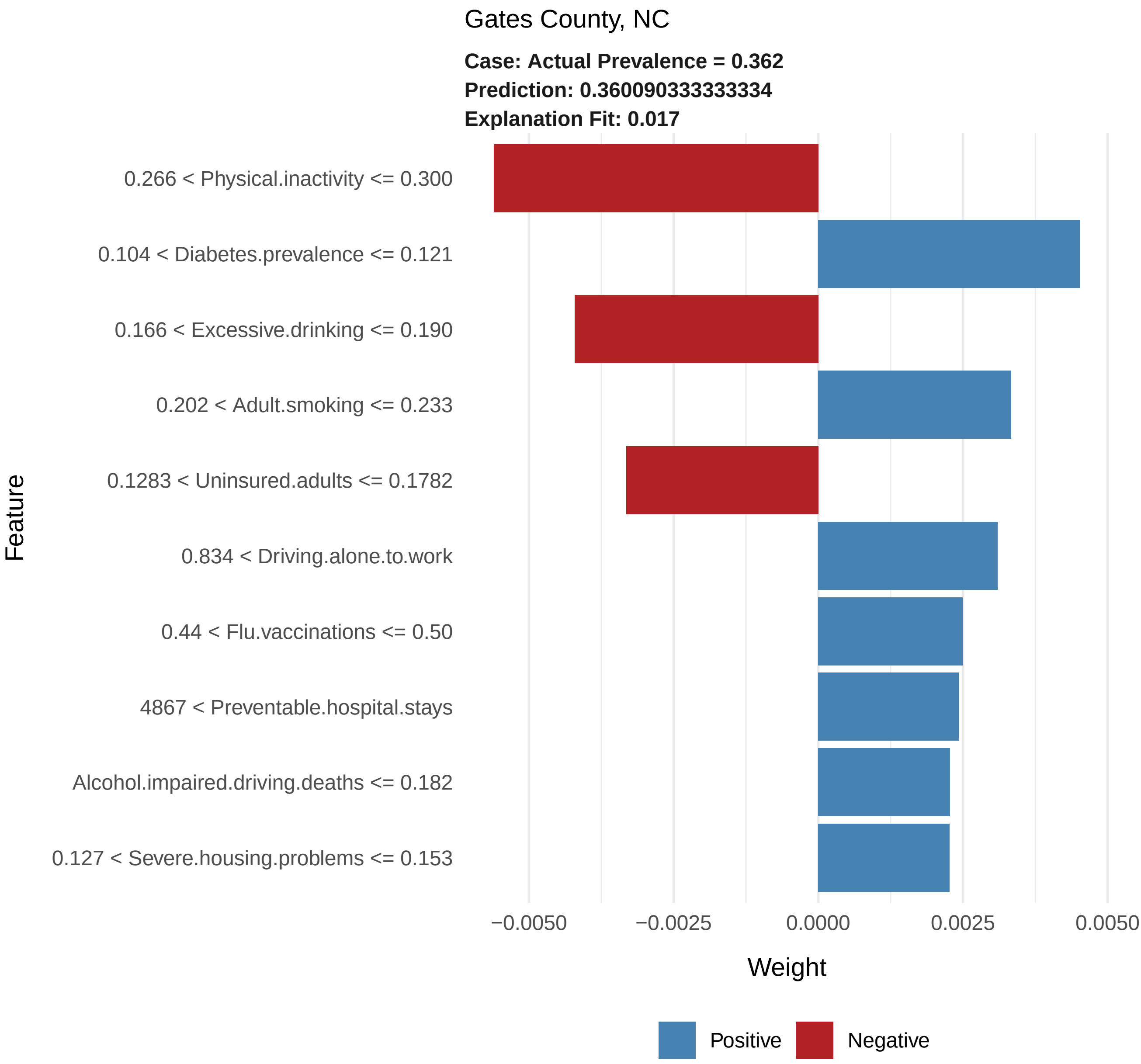
Gates County, NC
Case: Actual Prevalence = 0.362
Prediction: 0.360090333333334
Explanation Fit: 0.017
0.266 < Physical.inactivity <= 0.300
0.104 < Diabetes.prevalence <= 0.121
0.166 < Excessive.drinking <= 0.190
0.202 < Adult.smoking <= 0.233
0.1283 < Uninsured.adults <= 0.1782
0.834 < Driving.alone.to.work
0.44 < Flu.vaccinations <= 0.50
4867 < Preventable.hospital.stays
Alcohol.impaired.driving.deaths <= 0.182
0.127 < Severe.housing.problems <= 0.153
Feature
−0.0050
−0.0025
0.0000
0.0025
0.0050
Weight
Positive
Negative

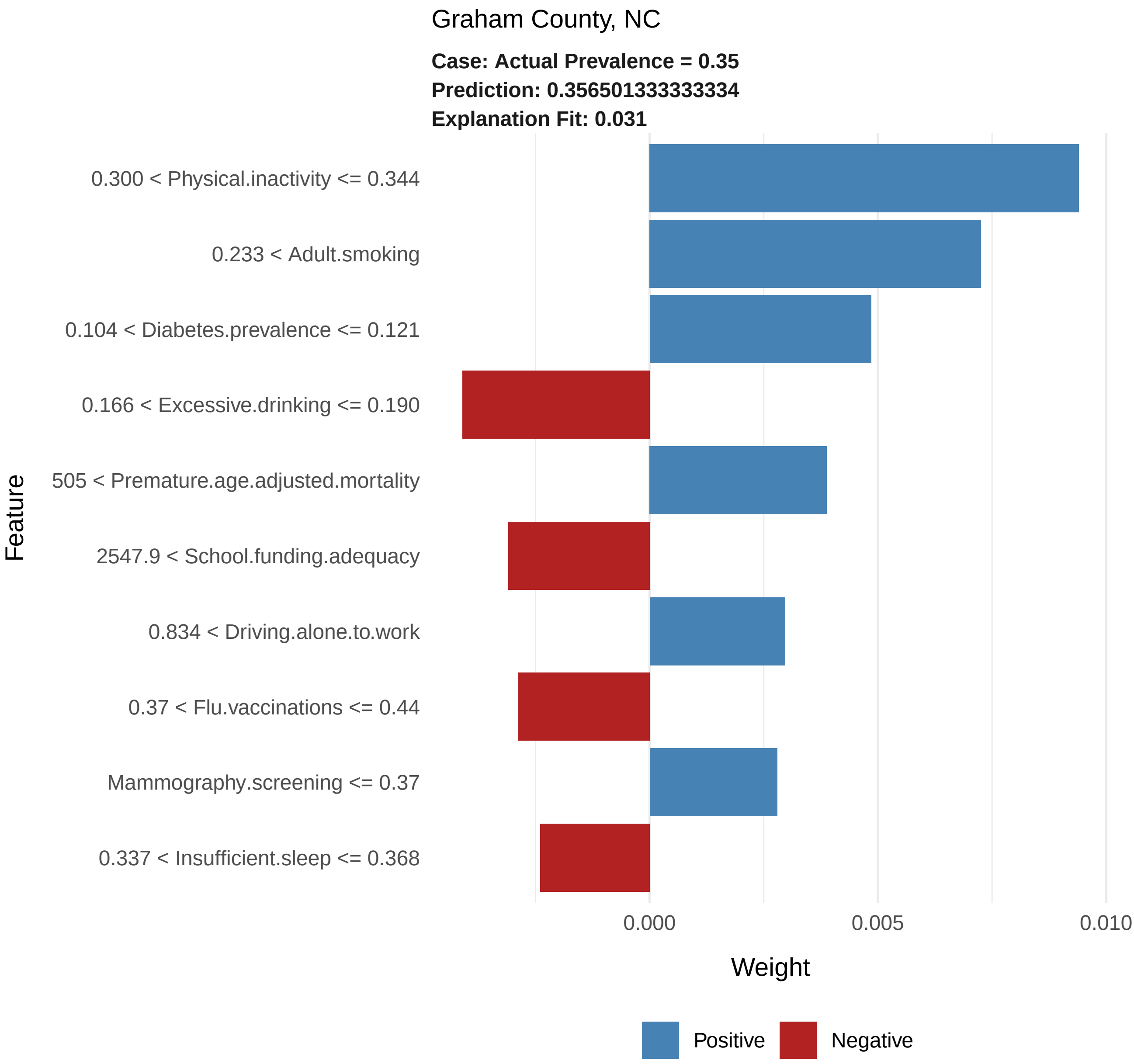

Graham County, NC
Case: Actual Prevalence = 0.35
Prediction: 0.356501333333334
Explanation Fit: 0.031
0.300 < Physical.inactivity <= 0.344
0.233 < Adult.smoking
0.104 < Diabetes.prevalence <= 0.121
0.166 < Excessive.drinking <= 0.190
505 < Premature.age.adjusted.mortality
2547.9 < School.funding.adequacy
0.834 < Driving.alone.to.work
0.37 < Flu.vaccinations <= 0.44
Mammography.screening <= 0.37
0.337 < Insufficient.sleep <= 0.368
Feature
0.000
0.005
0.010
Weight
Positive
Negative

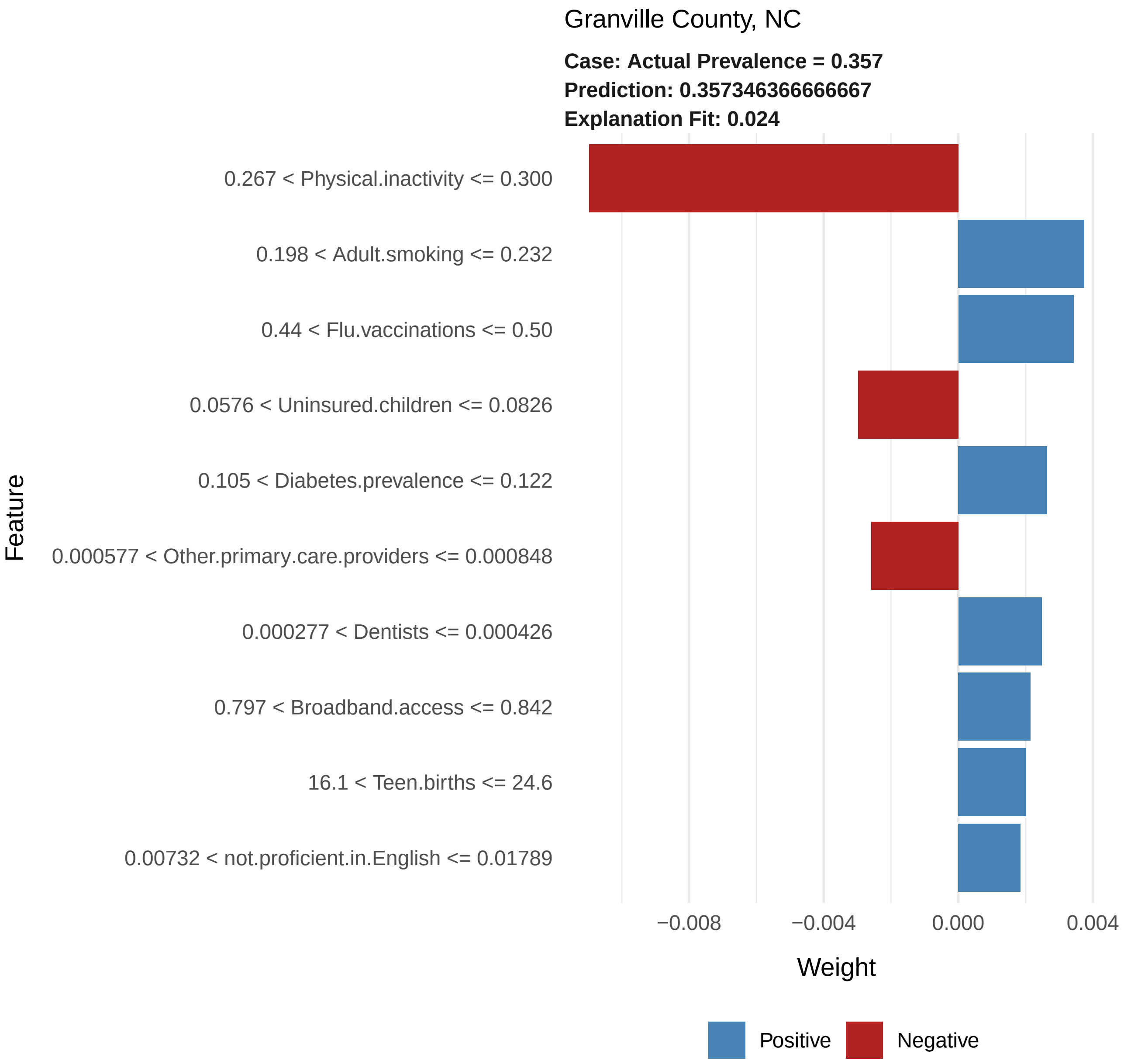

Granville County, NC
Case: Actual Prevalence = 0.357
Prediction: 0.357346366666667
Explanation Fit: 0.024
0.267 < Physical.inactivity <= 0.300
0.198 < Adult.smoking <= 0.232
0.44 < Flu.vaccinations <= 0.50
0.0576 < Uninsured.children <= 0.0826
0.105 < Diabetes.prevalence <= 0.122
0.000577 < Other.primary.care.providers <= 0.000848
0.000277 < Dentists <= 0.000426
0.797 < Broadband.access <= 0.842
16.1 < Teen.births <= 24.6
0.00732 < not.proficient.in.English <= 0.01789
Feature
−0.008
−0.004
0.000
0.004
Weight
Positive
Negative

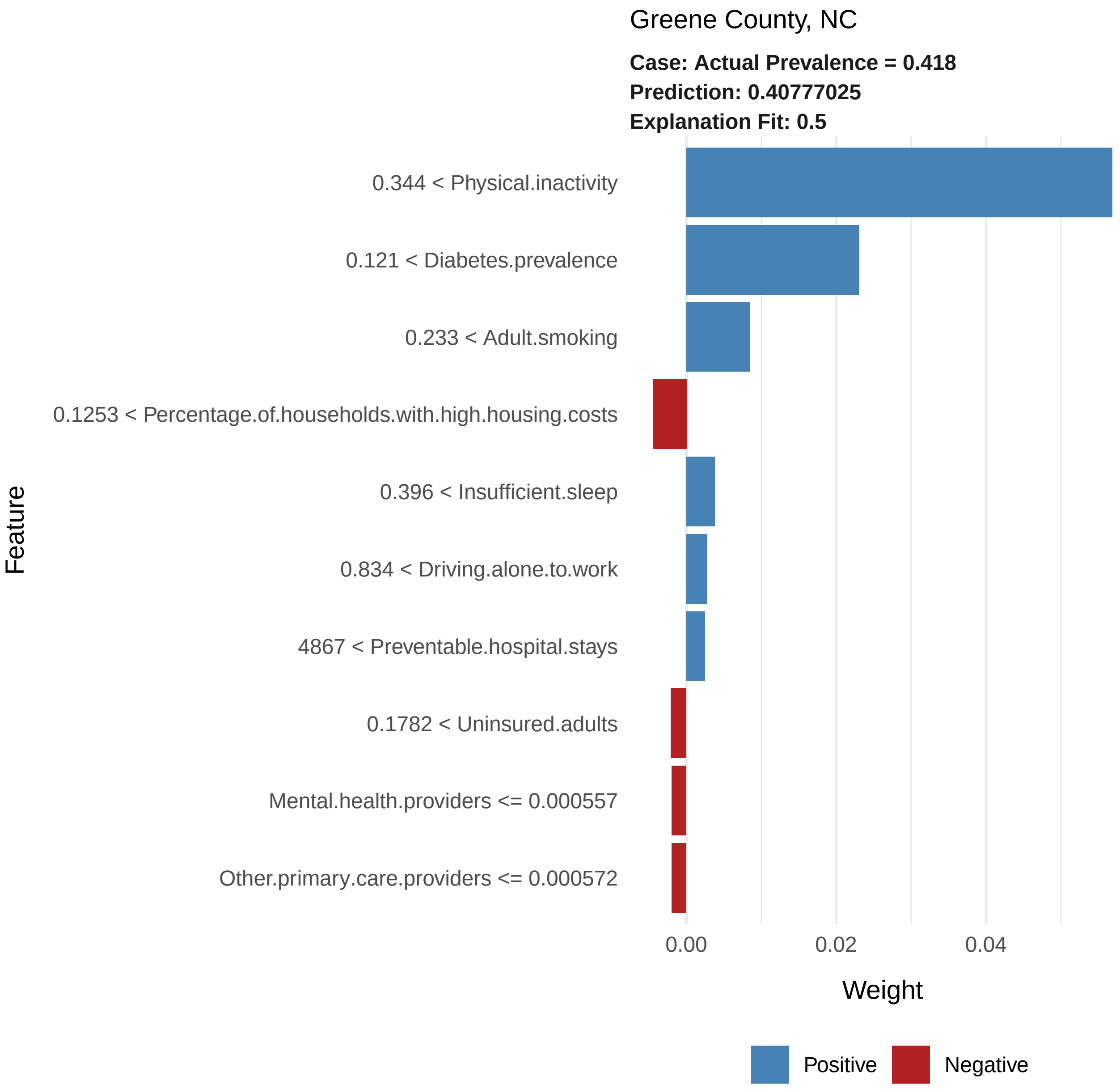

Greene County, NC
Case: Actual Prevalence = 0.418
Prediction: 0.40777025
Explanation Fit: 0.5
0.344 < Physical.inactivity
0.121 < Diabetes.prevalence
0.233 < Adult.smoking
0.1253 < Percentage.of.households.with.high.housing.costs
0.396 < Insufficient.sleep
0.834 < Driving.alone.to.work
4867 < Preventable.hospital.stays
0.1782 < Uninsured.adults
Mental.health.providers <= 0.000557
Other.primary.care.providers <= 0.000572
Feature
0.00
0.02
0.04
Weight
Positive
Negative

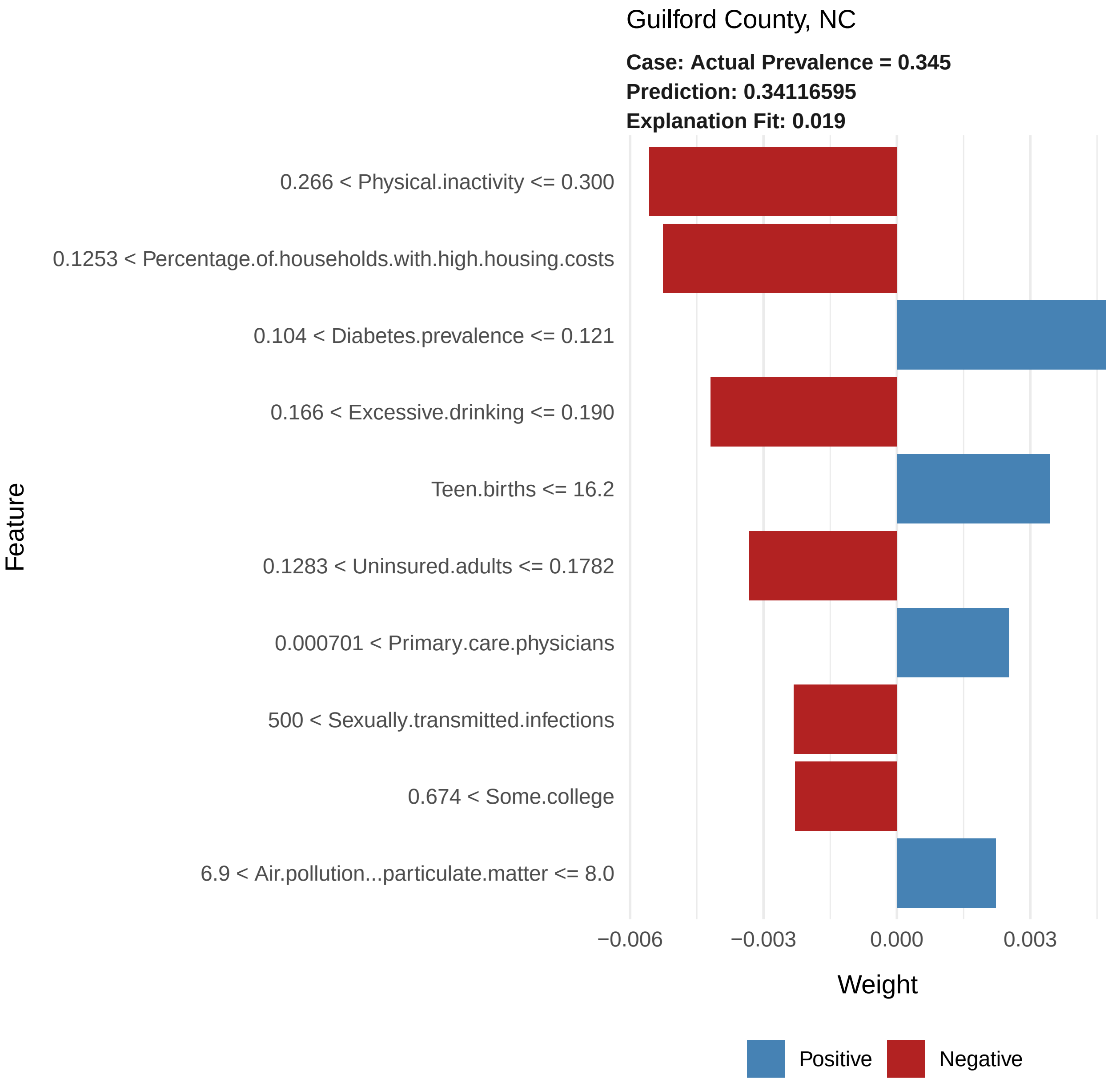
Guilford County, NC
Case: Actual Prevalence = 0.345
Prediction: 0.34116595
Explanation Fit: 0.019
0.266 < Physical.inactivity <= 0.300
0.1253 < Percentage.of.households.with.high.housing.costs
0.104 < Diabetes.prevalence <= 0.121
0.166 < Excessive.drinking <= 0.190
Teen.births <= 16.2
0.1283 < Uninsured.adults <= 0.1782
0.000701 < Primary.care.physicians
500 < Sexually.transmitted.infections
0.674 < Some.college
6.9 < Air.pollution...particulate.matter <= 8.0
Feature
−0.006
−0.003
0.000
0.003
Weight
Positive
Negative

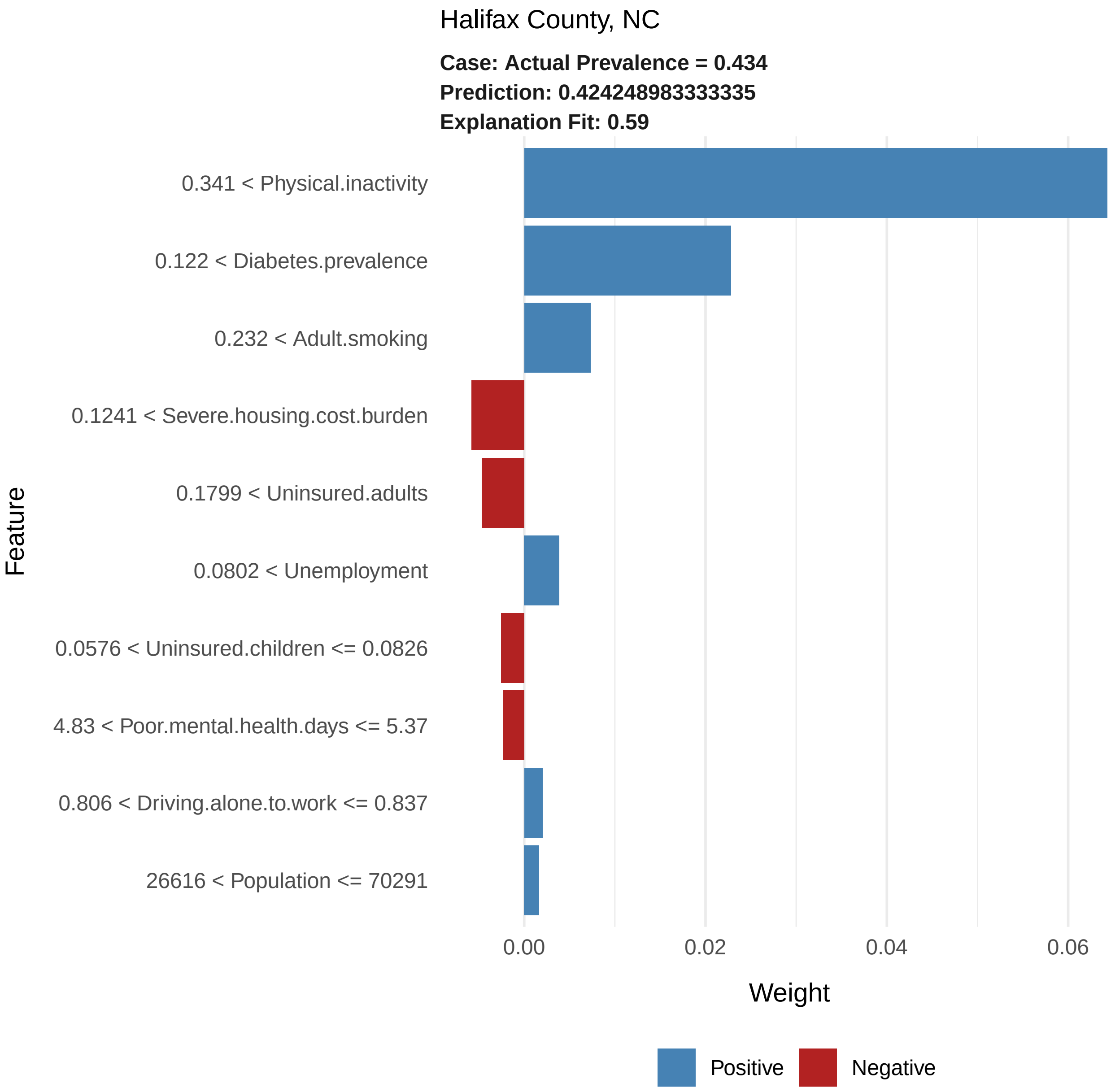

Halifax County, NC
Case: Actual Prevalence = 0.434
Prediction: 0.424248983333335
Explanation Fit: 0.59
Feature
0.341 < Physical.inactivity
0.122 < Diabetes.prevalence
0.232 < Adult.smoking
0.1241 < Severe.housing.cost.burden
0.1799 < Uninsured.adults
0.0802 < Unemployment
0.0576 < Uninsured.children <= 0.0826
4.83 < Poor.mental.health.days <= 5.37
0.806 < Driving.alone.to.work <= 0.837
26616 < Population <= 70291
0.00
0.02
0.04
0.06
Weight
Positive
Negative

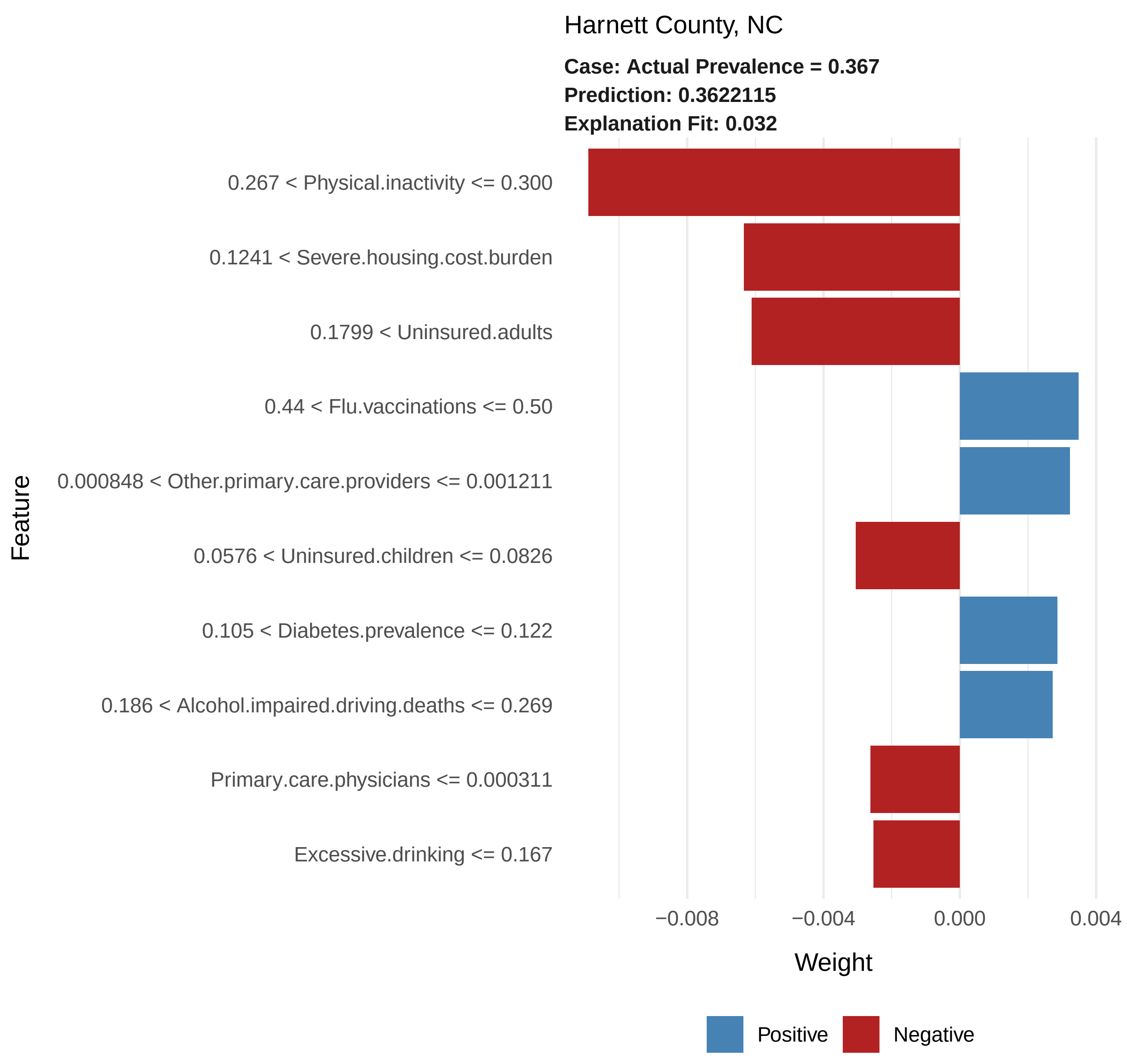
Harnett County, NC
Case: Actual Prevalence = 0.367
Prediction: 0.3622115
Explanation Fit: 0.032
0.267 < Physical.inactivity <= 0.300
0.1241 < Severe.housing.cost.burden
0.1799 < Uninsured.adults
0.44 < Flu.vaccinations <= 0.50
0.000848 < Other.primary.care.providers <= 0.001211
0.0576 < Uninsured.children <= 0.0826
0.105 < Diabetes.prevalence <= 0.122
0.186 < Alcohol.impaired.driving.deaths <= 0.269
Primary.care.physicians <= 0.000311
Excessive.drinking <= 0.167
Feature
−0.008
−0.004
0.000
0.004
Weight
Positive
Negative

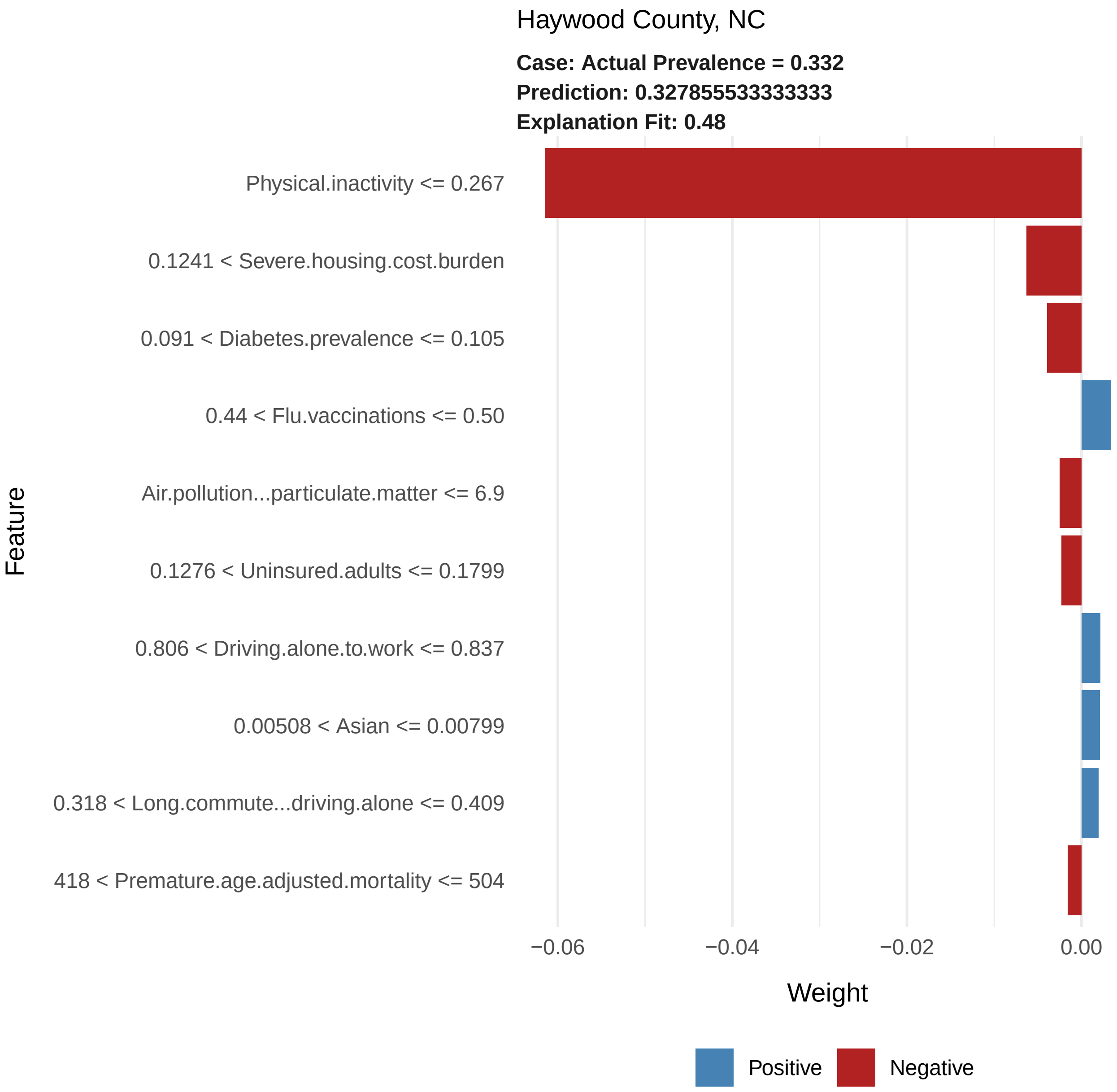

Haywood County, NC
Case: Actual Prevalence = 0.332
Prediction: 0.327855533333333
Explanation Fit: 0.48
Physical.inactivity <= 0.267
0.1241 < Severe.housing.cost.burden
0.091 < Diabetes.prevalence <= 0.105
0.44 < Flu.vaccinations <= 0.50
Air.pollution...particulate.matter <= 6.9
0.1276 < Uninsured.adults <= 0.1799
0.806 < Driving.alone.to.work <= 0.837
0.00508 < Asian <= 0.00799
0.318 < Long.commute...driving.alone <= 0.409
418 < Premature.age.adjusted.mortality <= 504
Feature
−0.06
−0.04
−0.02
0.00
Weight
Positive
Negative

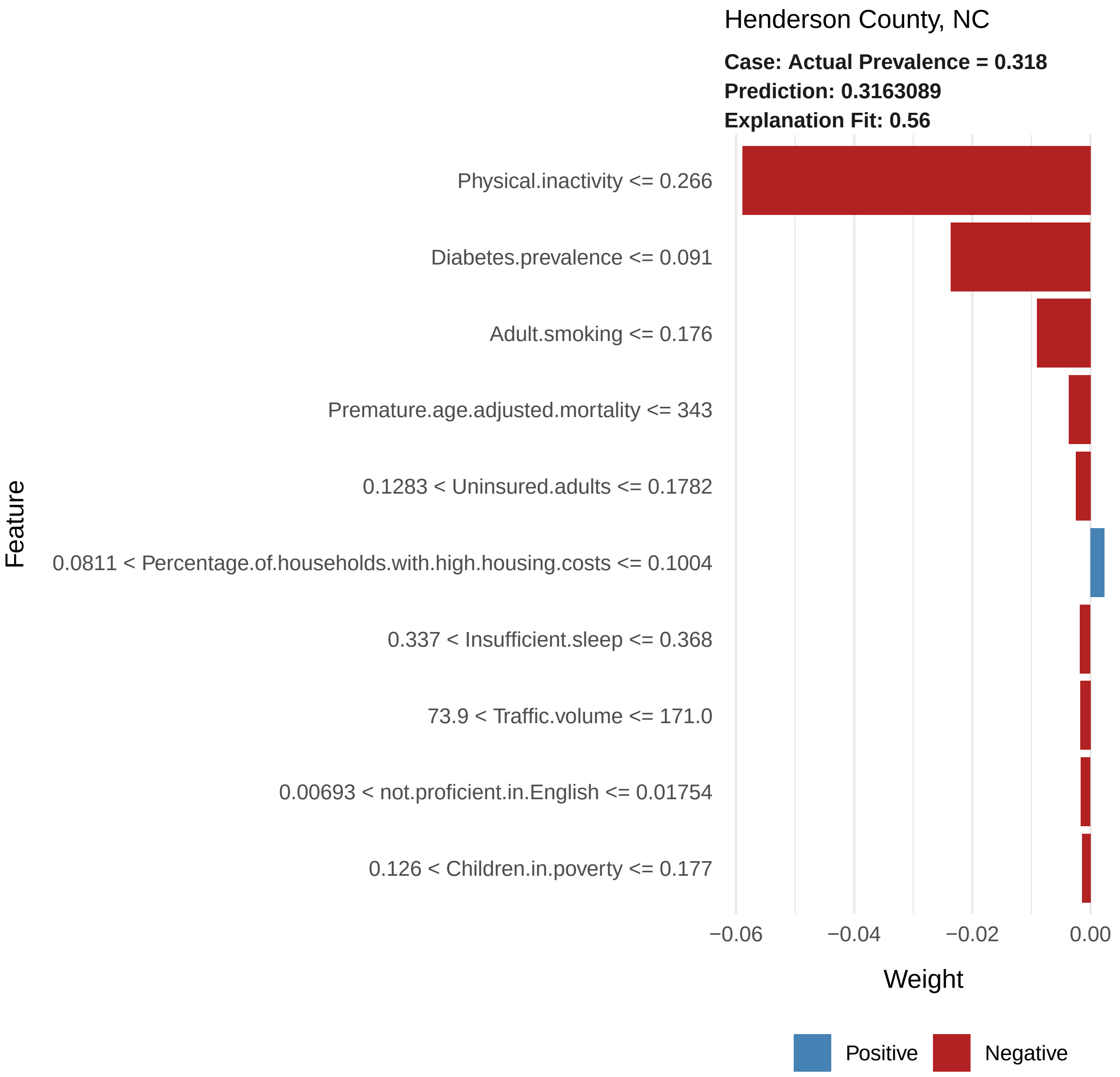
Henderson County, NC
Case: Actual Prevalence = 0.318
Prediction: 0.3163089
Explanation Fit: 0.56
Physical.inactivity <= 0.266
Diabetes.prevalence <= 0.091
Adult.smoking <= 0.176
Premature.age.adjusted.mortality <= 343
0.1283 < Uninsured.adults <= 0.1782
0.0811 < Percentage.of.households.with.high.housing.costs <= 0.1004
0.337 < Insufficient.sleep <= 0.368
73.9 < Traffic.volume <= 171.0
0.00693 < not.proficient.in.English <= 0.01754
0.126 < Children.in.poverty <= 0.177
Feature
−0.06
−0.04
−0.02
0.00
Weight
Positive
Negative

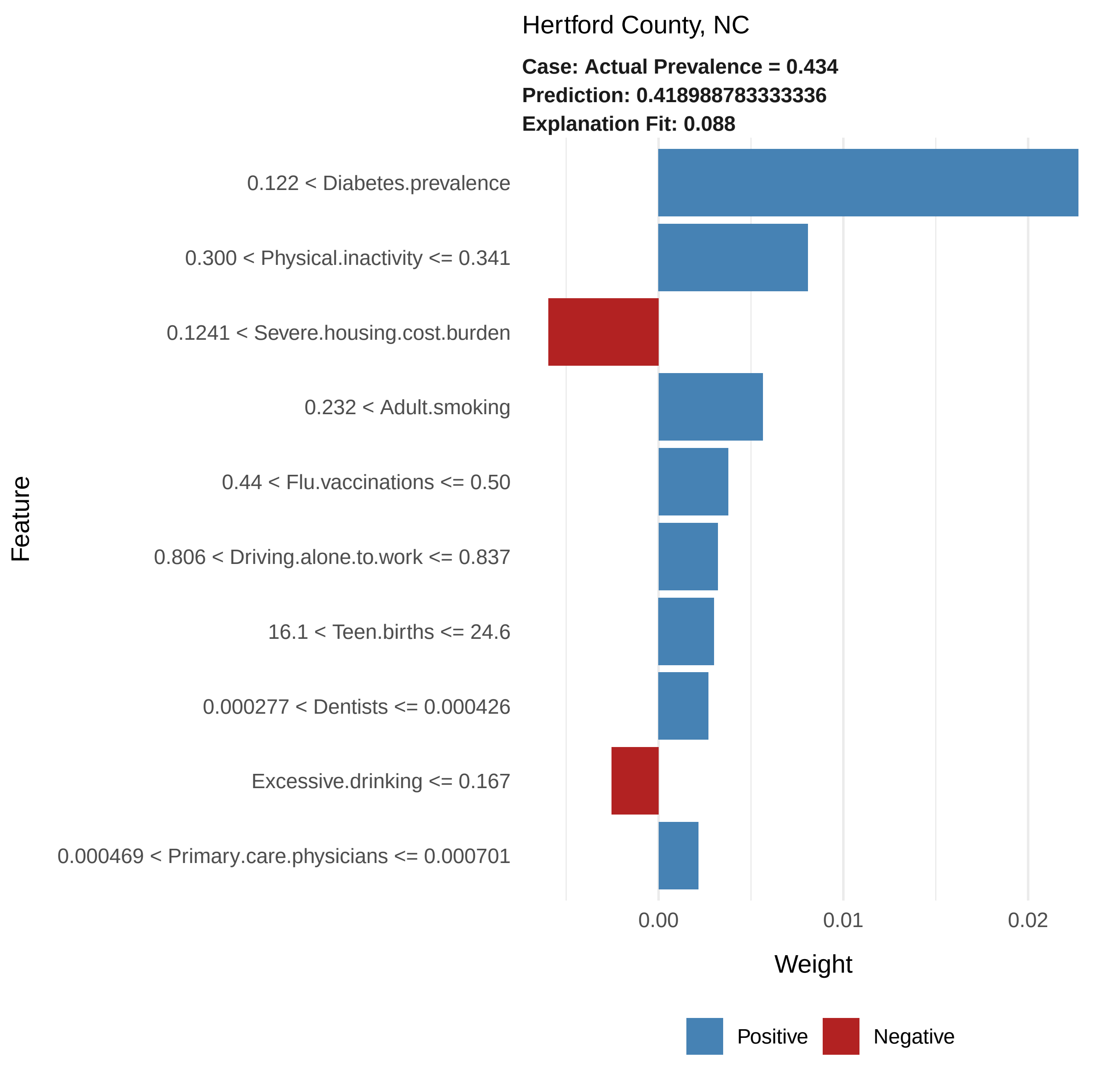

Hertford County, NC
Case: Actual Prevalence = 0.434
Prediction: 0.418988783333336
Explanation Fit: 0.088
0.122 < Diabetes.prevalence
0.300 < Physical.inactivity <= 0.341
0.1241 < Severe.housing.cost.burden
0.232 < Adult.smoking
0.44 < Flu.vaccinations <= 0.50
0.806 < Driving.alone.to.work <= 0.837
16.1 < Teen.births <= 24.6
0.000277 < Dentists <= 0.000426
Excessive.drinking <= 0.167
0.000469 < Primary.care.physicians <= 0.000701
Feature
0.00
0.01
0.02
Weight
Positive
Negative

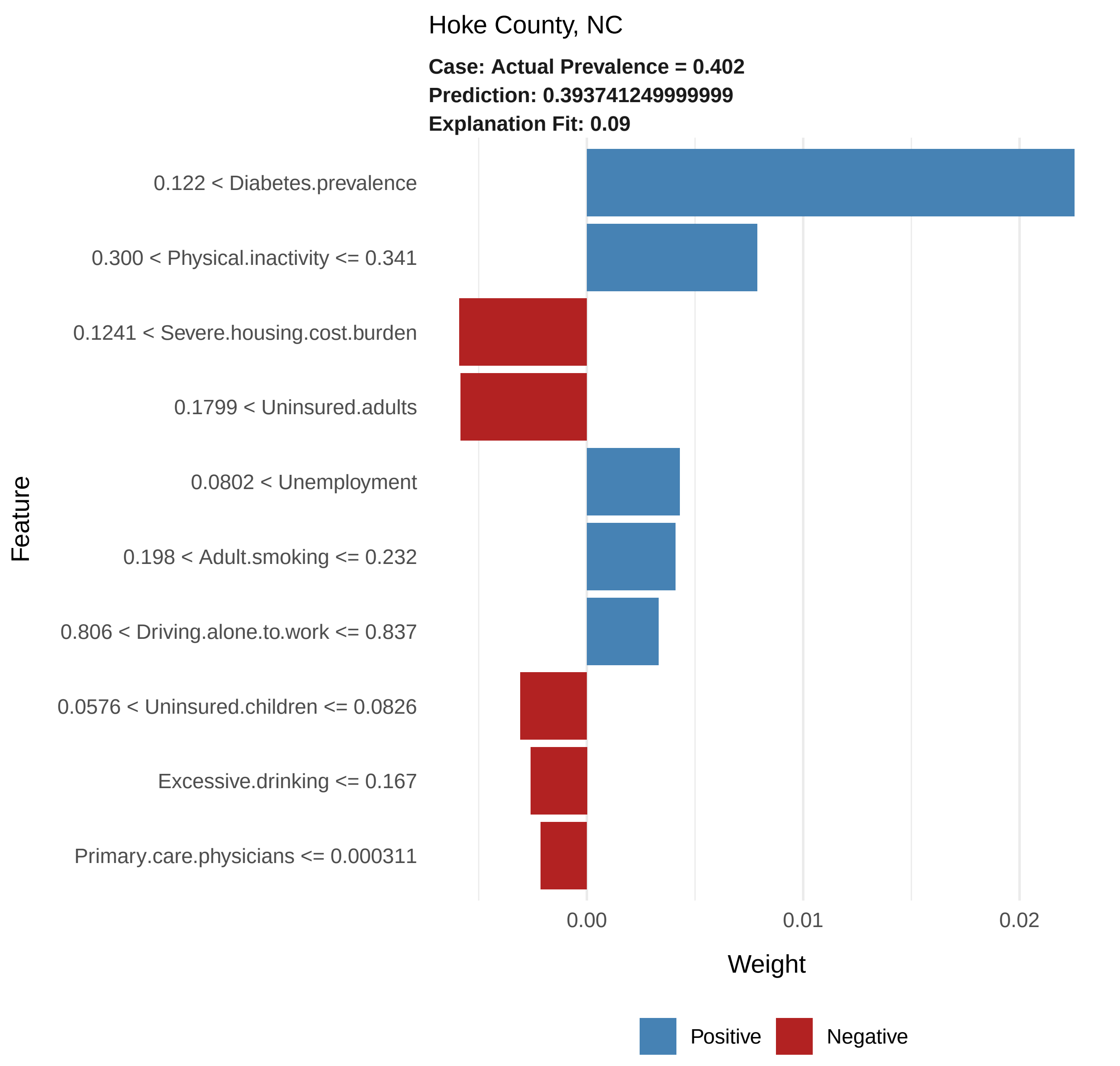
Hoke County, NC
Case: Actual Prevalence = 0.402
Prediction: 0.393741249999999
Explanation Fit: 0.09
0.122 < Diabetes.prevalence
0.300 < Physical.inactivity <= 0.341
0.1241 < Severe.housing.cost.burden
0.1799 < Uninsured.adults
0.0802 < Unemployment
0.198 < Adult.smoking <= 0.232
0.806 < Driving.alone.to.work <= 0.837
0.0576 < Uninsured.children <= 0.0826
Excessive.drinking <= 0.167
Primary.care.physicians <= 0.000311
Feature
0.00
0.01
0.02
Weight
Positive
Negative

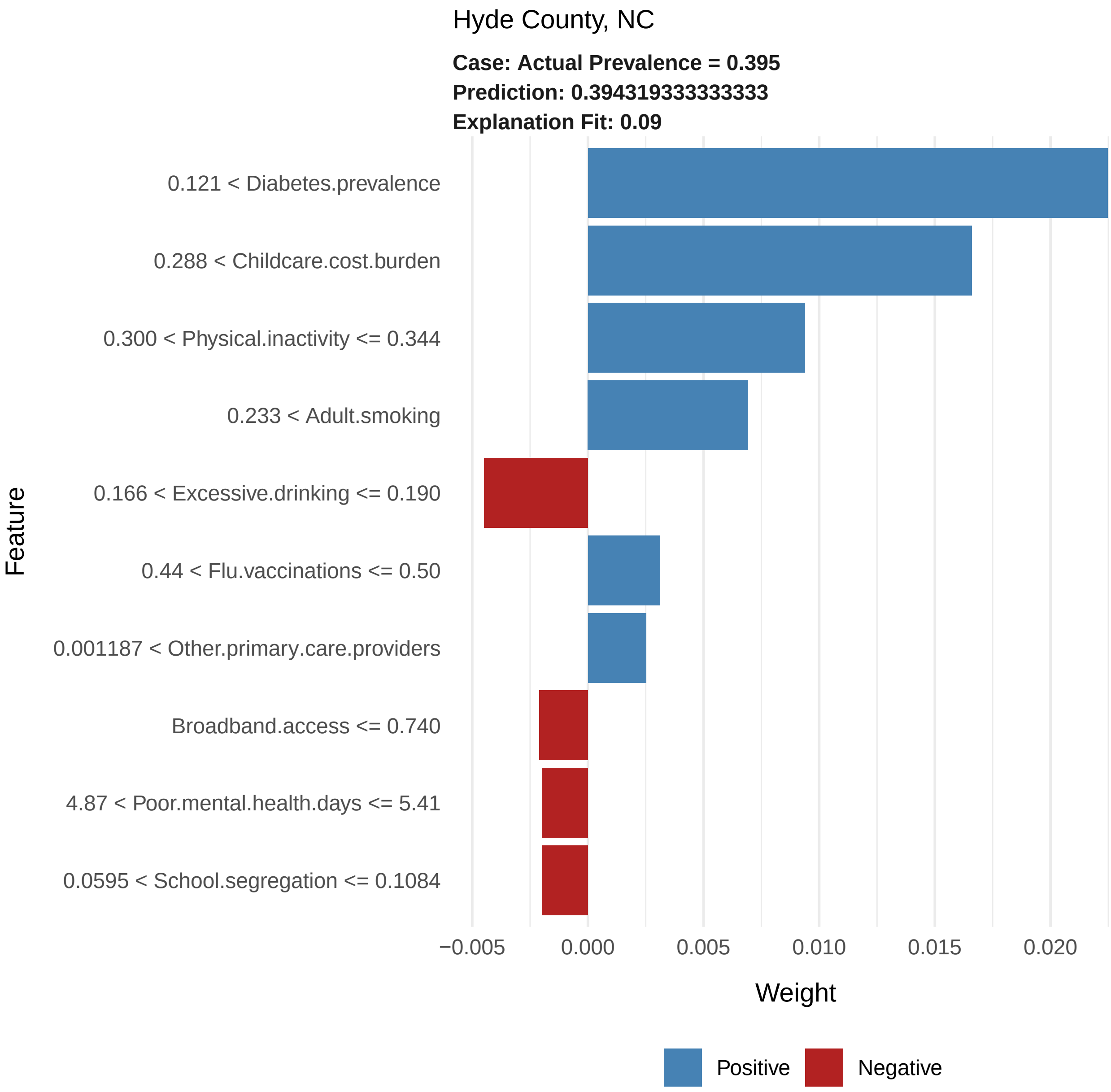
Hyde County, NC
Case: Actual Prevalence = 0.395
Prediction: 0.394319333333333
Explanation Fit: 0.09
0.121 < Diabetes.prevalence
0.288 < Childcare.cost.burden
0.300 < Physical.inactivity <= 0.344
0.233 < Adult.smoking
0.166 < Excessive.drinking <= 0.190
0.44 < Flu.vaccinations <= 0.50
0.001187 < Other.primary.care.providers
Broadband.access <= 0.740
4.87 < Poor.mental.health.days <= 5.41
0.0595 < School.segregation <= 0.1084
Feature
−0.005
0.000
0.005
0.010
0.015
0.020
Weight
Positive
Negative

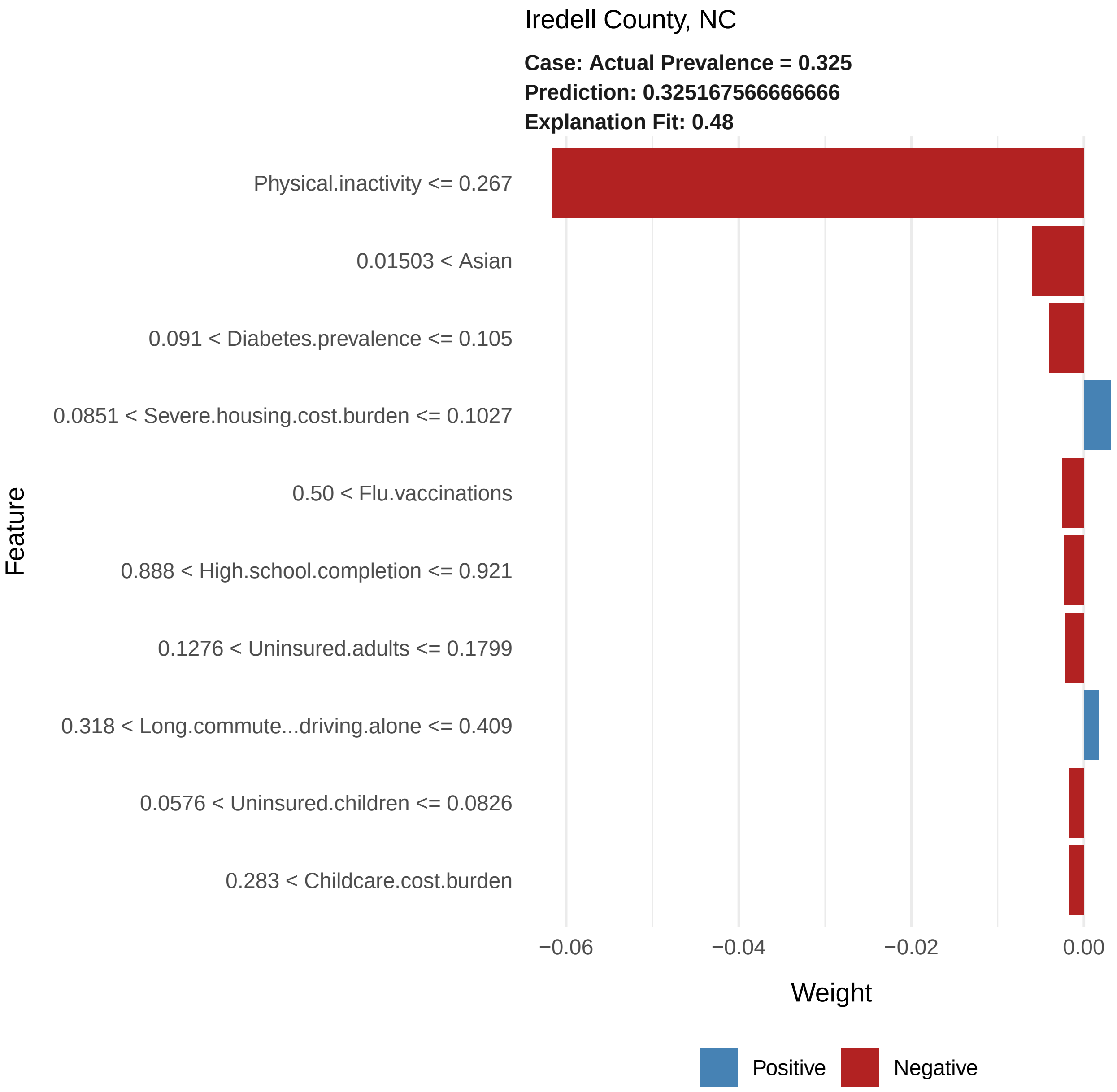
Iredell County, NC
Case: Actual Prevalence = 0.325
Prediction: 0.325167566666666
Explanation Fit: 0.48
Physical.inactivity <= 0.267
0.01503 < Asian
0.091 < Diabetes.prevalence <= 0.105
0.0851 < Severe.housing.cost.burden <= 0.1027
0.50 < Flu.vaccinations
0.888 < High.school.completion <= 0.921
0.1276 < Uninsured.adults <= 0.1799
0.318 < Long.commute...driving.alone <= 0.409
0.0576 < Uninsured.children <= 0.0826
0.283 < Childcare.cost.burden
Feature
−0.06
−0.04
−0.02
0.00
Weight
Positive
Negative

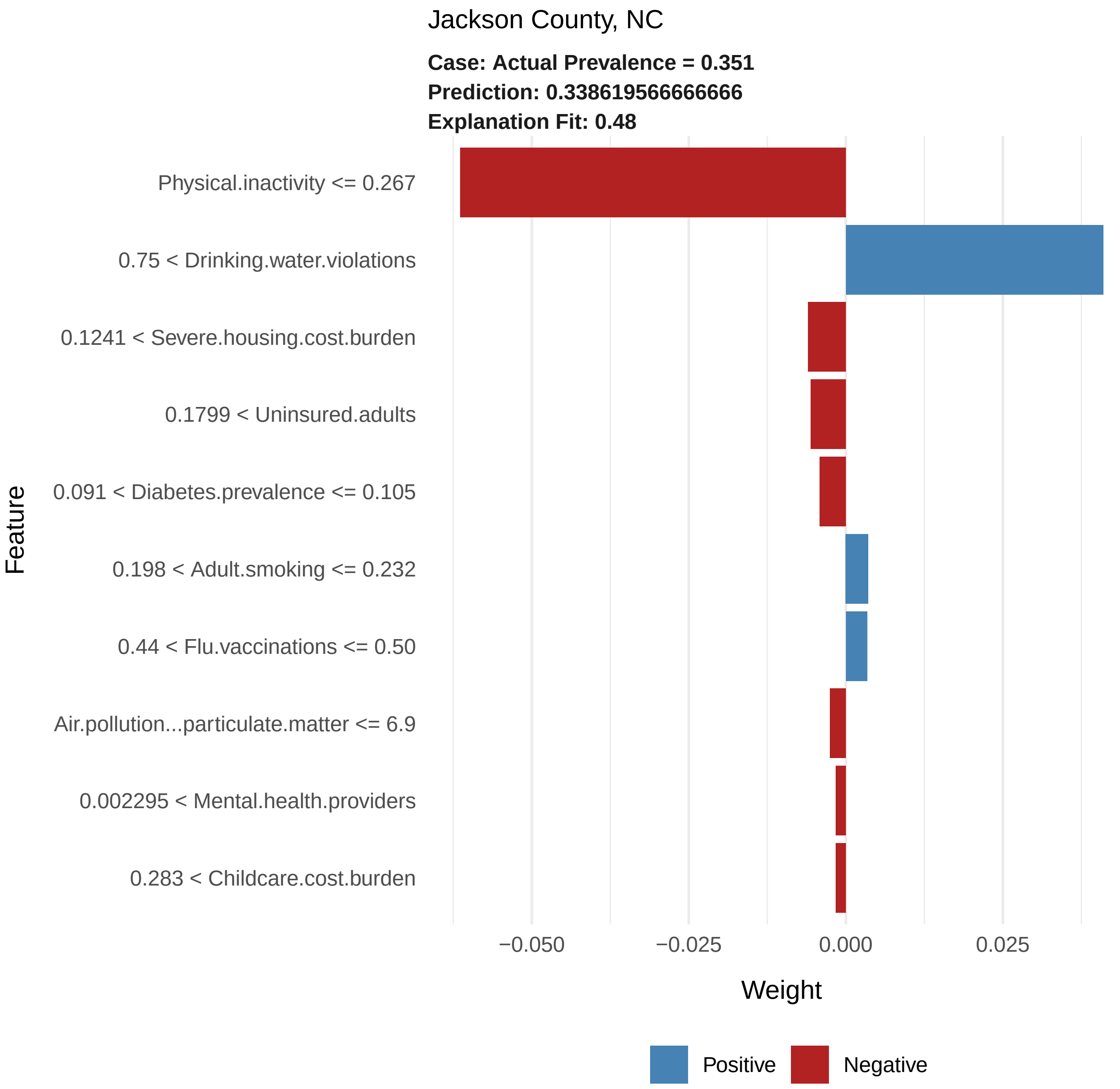

Jackson County, NC
Case: Actual Prevalence = 0.351
Prediction: 0.338619566666666
Explanation Fit: 0.48
Physical.inactivity <= 0.267
0.75 < Drinking.water.violations
0.1241 < Severe.housing.cost.burden
0.1799 < Uninsured.adults
0.091 < Diabetes.prevalence <= 0.105
0.198 < Adult.smoking <= 0.232
0.44 < Flu.vaccinations <= 0.50
Air.pollution...particulate.matter <= 6.9
0.002295 < Mental.health.providers
0.283 < Childcare.cost.burden
Feature
−0.050
−0.025
0.000
0.025
Weight
Positive
Negative

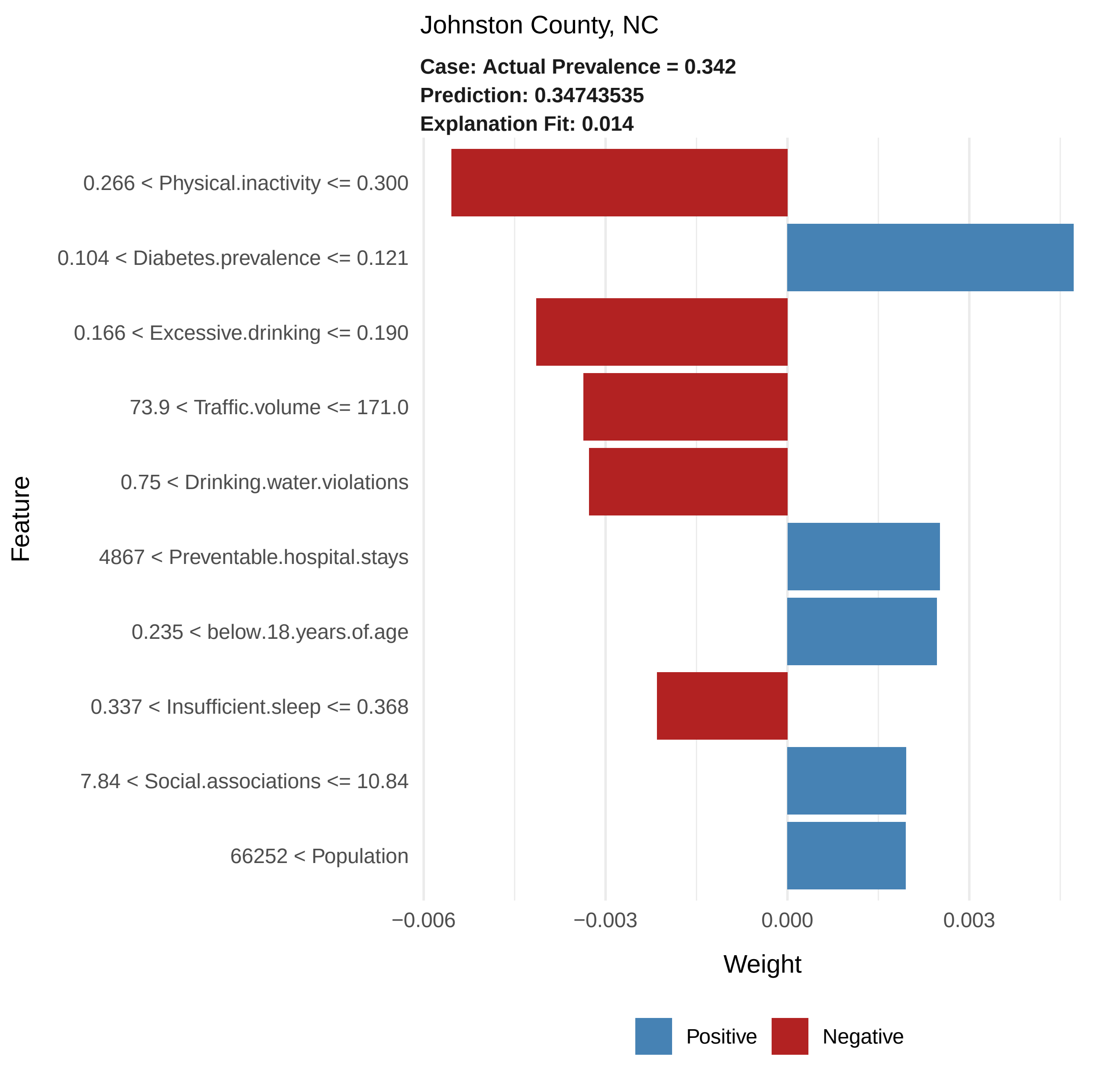

Johnston County, NC
Case: Actual Prevalence = 0.342
Prediction: 0.34743535
Explanation Fit: 0.014
0.266 < Physical.inactivity <= 0.300
0.104 < Diabetes.prevalence <= 0.121
0.166 < Excessive.drinking <= 0.190
73.9 < Traffic.volume <= 171.0
0.75 < Drinking.water.violations
4867 < Preventable.hospital.stays
0.235 < below.18.years.of.age
0.337 < Insufficient.sleep <= 0.368
7.84 < Social.associations <= 10.84
66252 < Population
Feature
−0.006
−0.003
0.000
0.003
Weight
Positive
Negative

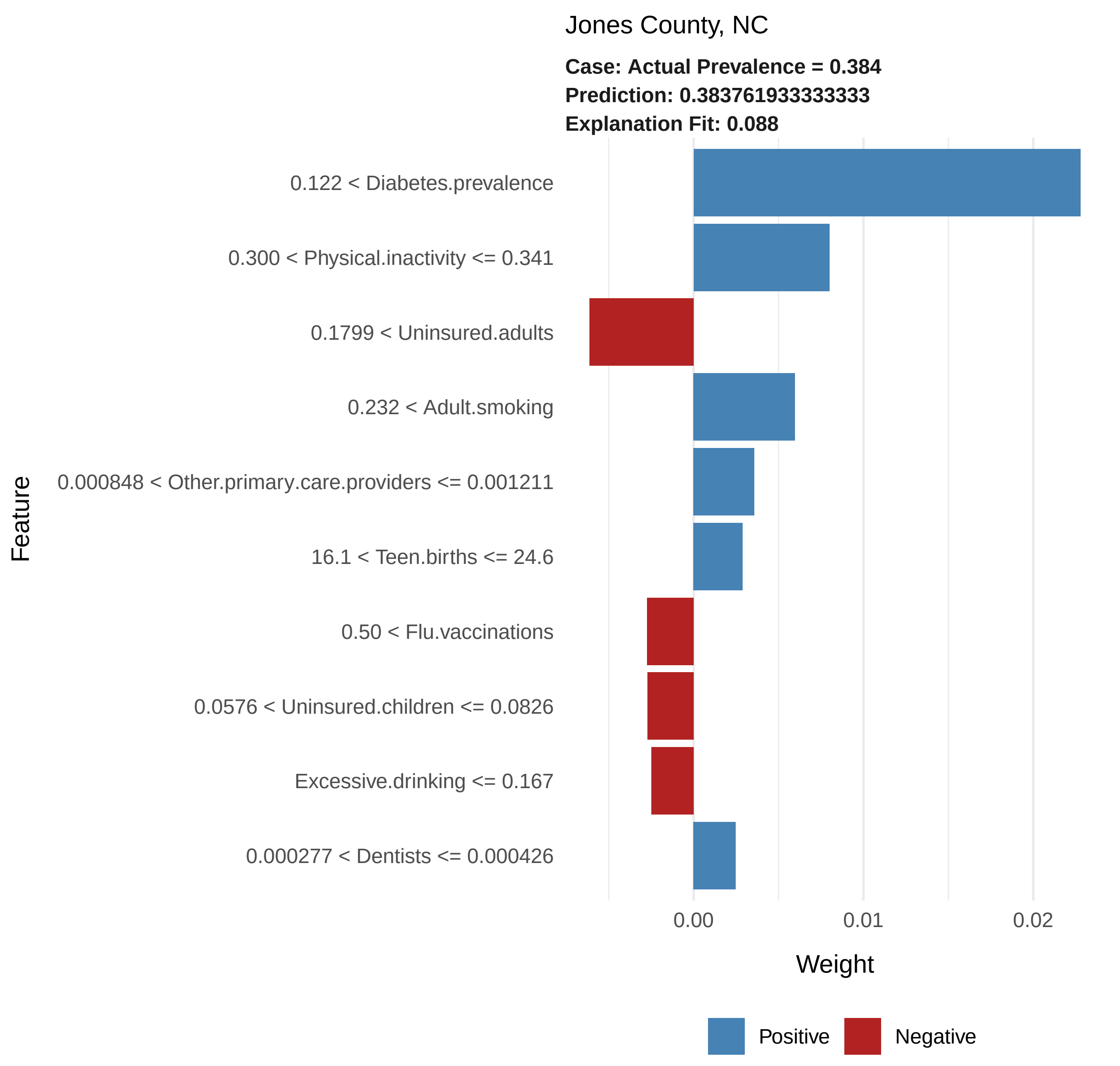

Jones County, NC
Case: Actual Prevalence = 0.384
Prediction: 0.383761933333333
Explanation Fit: 0.088
0.122 < Diabetes.prevalence
0.300 < Physical.inactivity <= 0.341
0.1799 < Uninsured.adults
0.232 < Adult.smoking
0.000848 < Other.primary.care.providers <= 0.001211
16.1 < Teen.births <= 24.6
0.50 < Flu.vaccinations
0.0576 < Uninsured.children <= 0.0826
Excessive.drinking <= 0.167
0.000277 < Dentists <= 0.000426
Feature
0.00
0.01
0.02
Weight
Positive
Negative

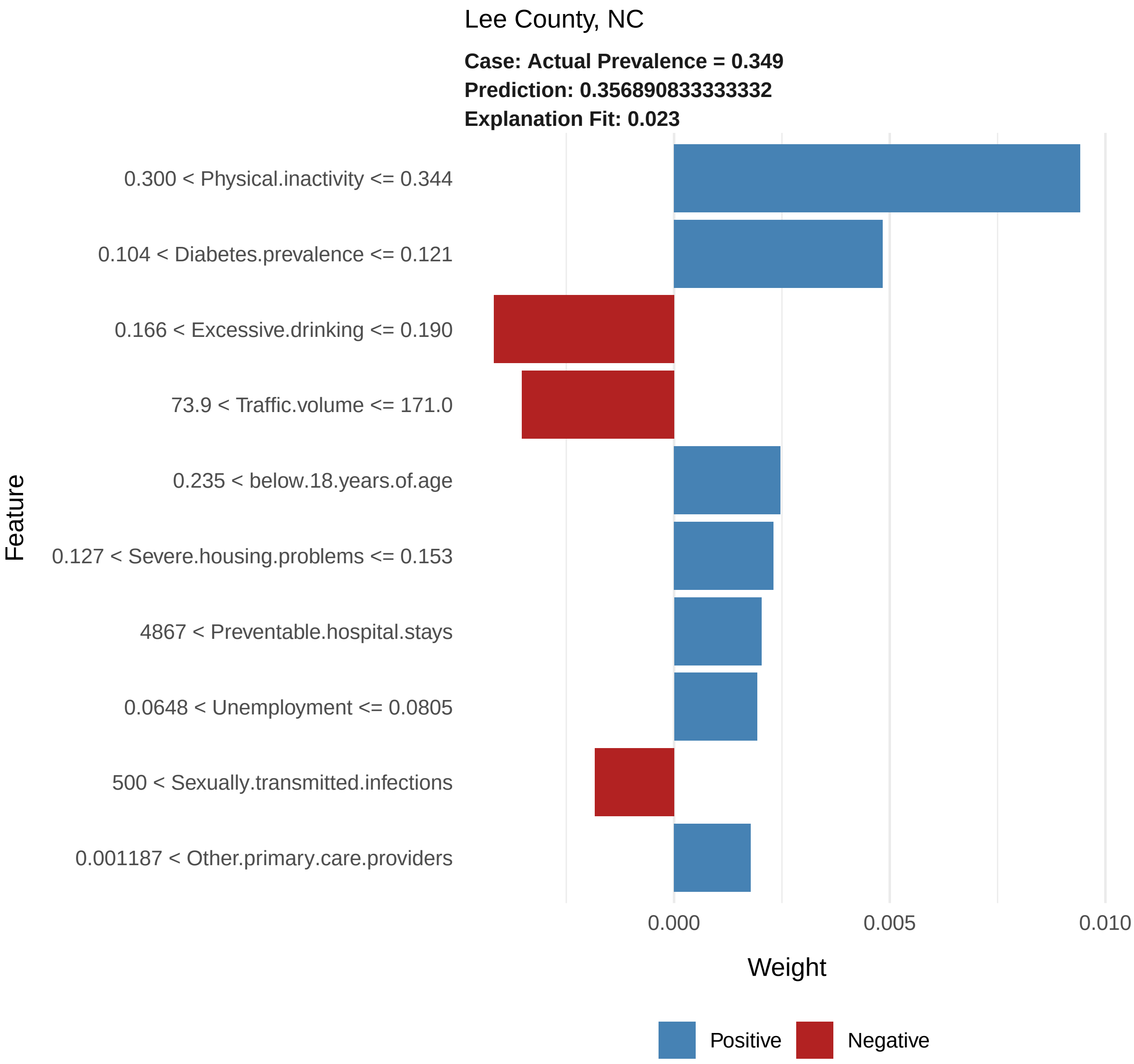

Lee County, NC
Case: Actual Prevalence = 0.349
Prediction: 0.356890833333332
Explanation Fit: 0.023
0.300 < Physical.inactivity <= 0.344
0.104 < Diabetes.prevalence <= 0.121
0.166 < Excessive.drinking <= 0.190
73.9 < Traffic.volume <= 171.0
0.235 < below.18.years.of.age
0.127 < Severe.housing.problems <= 0.153
4867 < Preventable.hospital.stays
0.0648 < Unemployment <= 0.0805
500 < Sexually.transmitted.infections
0.001187 < Other.primary.care.providers
Feature
0.000
0.005
0.010
Weight
Positive
Negative

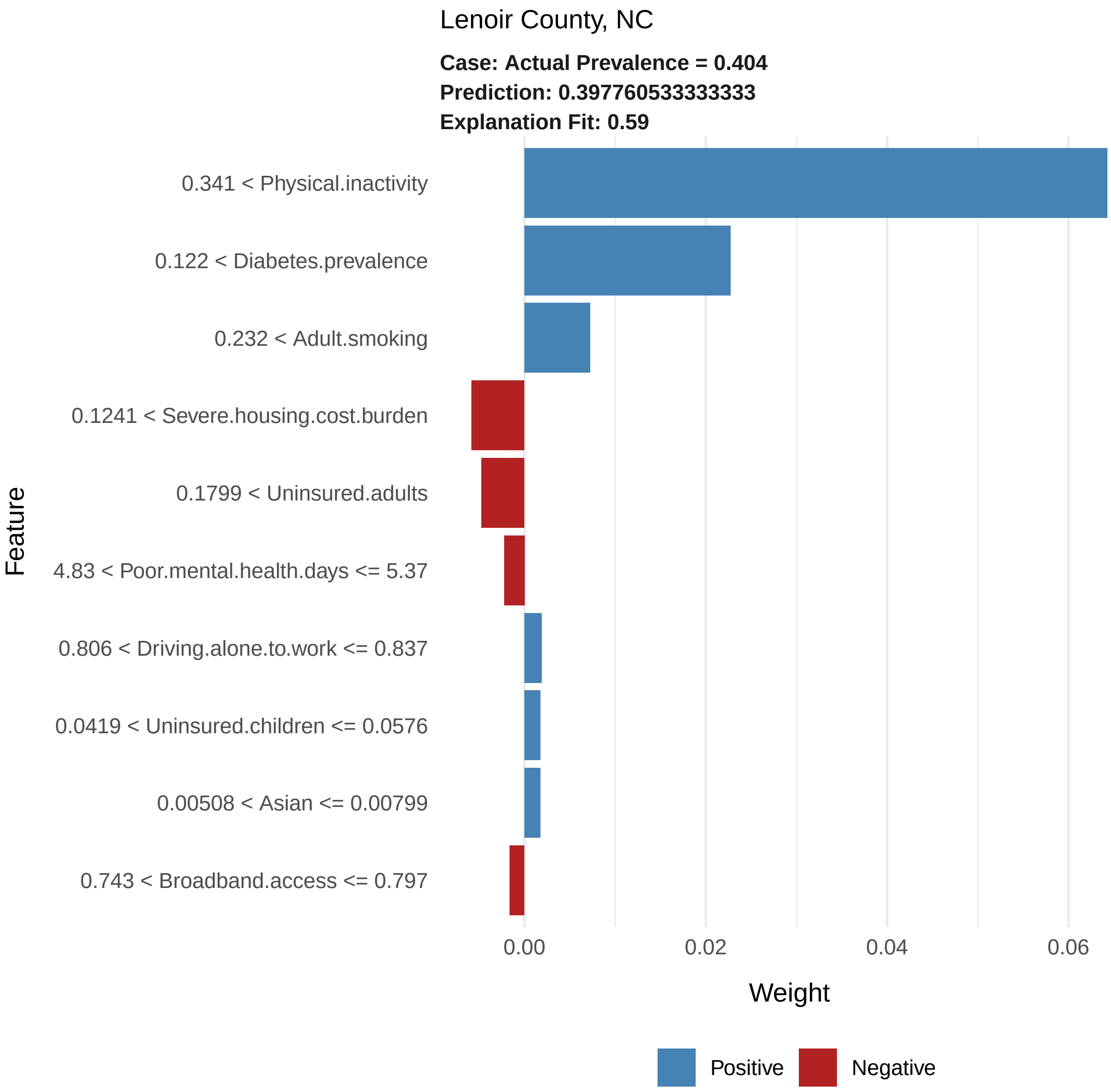
Lenoir County, NC
Case: Actual Prevalence = 0.404
Prediction: 0.397760533333333
Explanation Fit: 0.59
0.341 < Physical.inactivity
0.122 < Diabetes.prevalence
0.232 < Adult.smoking
0.1241 < Severe.housing.cost.burden
0.1799 < Uninsured.adults
4.83 < Poor.mental.health.days <= 5.37
0.806 < Driving.alone.to.work <= 0.837
0.0419 < Uninsured.children <= 0.0576
0.00508 < Asian <= 0.00799
0.743 < Broadband.access <= 0.797
Feature
0.00
0.02
0.04
0.06
Weight
Positive
Negative

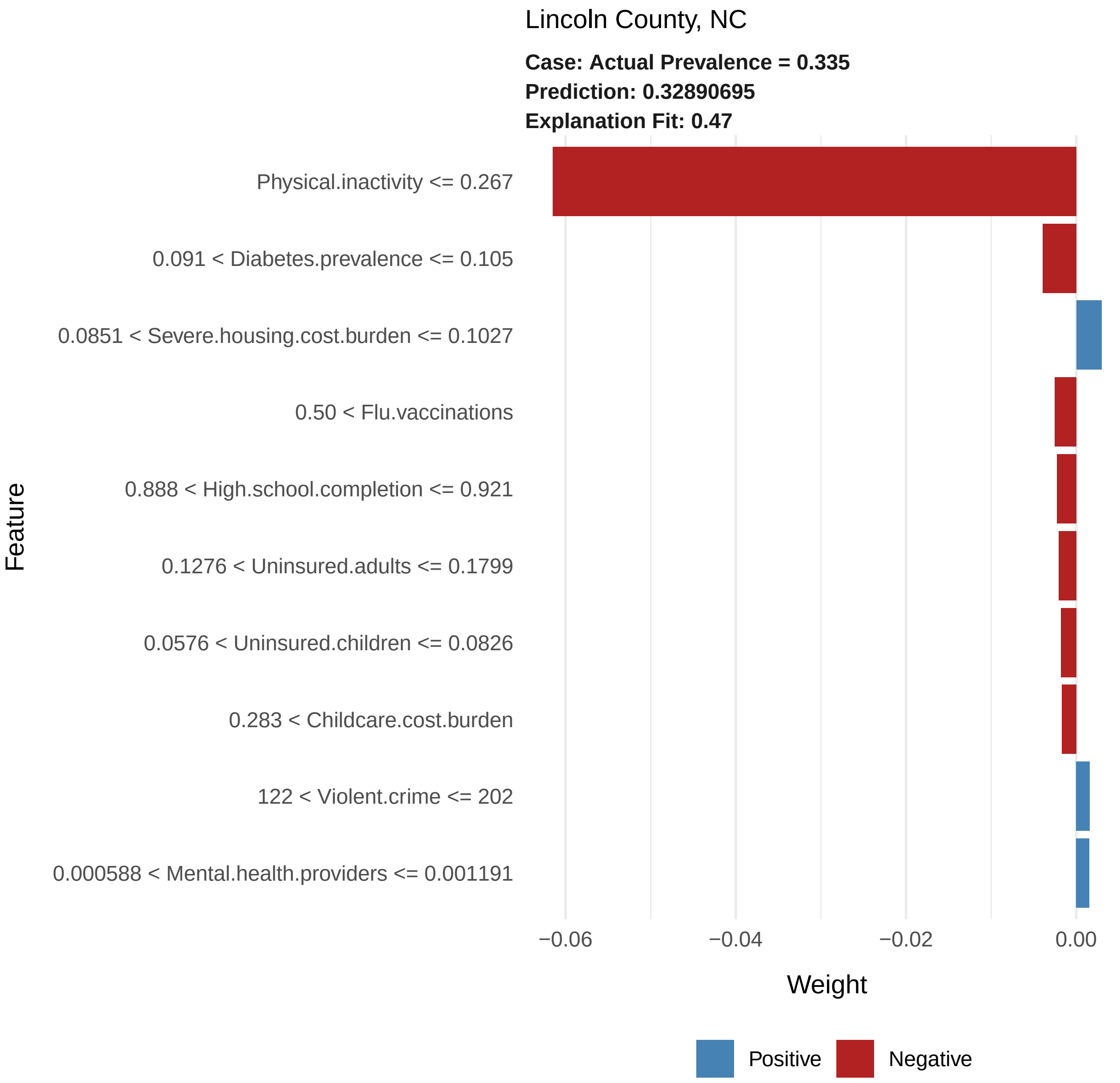
Lincoln County, NC
Case: Actual Prevalence = 0.335
Prediction: 0.32890695
Explanation Fit: 0.47
Physical.inactivity <= 0.267
0.091 < Diabetes.prevalence <= 0.105
0.0851 < Severe.housing.cost.burden <= 0.1027
0.50 < Flu.vaccinations
0.888 < High.school.completion <= 0.921
0.1276 < Uninsured.adults <= 0.1799
0.0576 < Uninsured.children <= 0.0826
0.283 < Childcare.cost.burden
122 < Violent.crime <= 202
0.000588 < Mental.health.providers <= 0.001191
Feature
−0.06
−0.04
−0.02
0.00
Weight
Positive
Negative

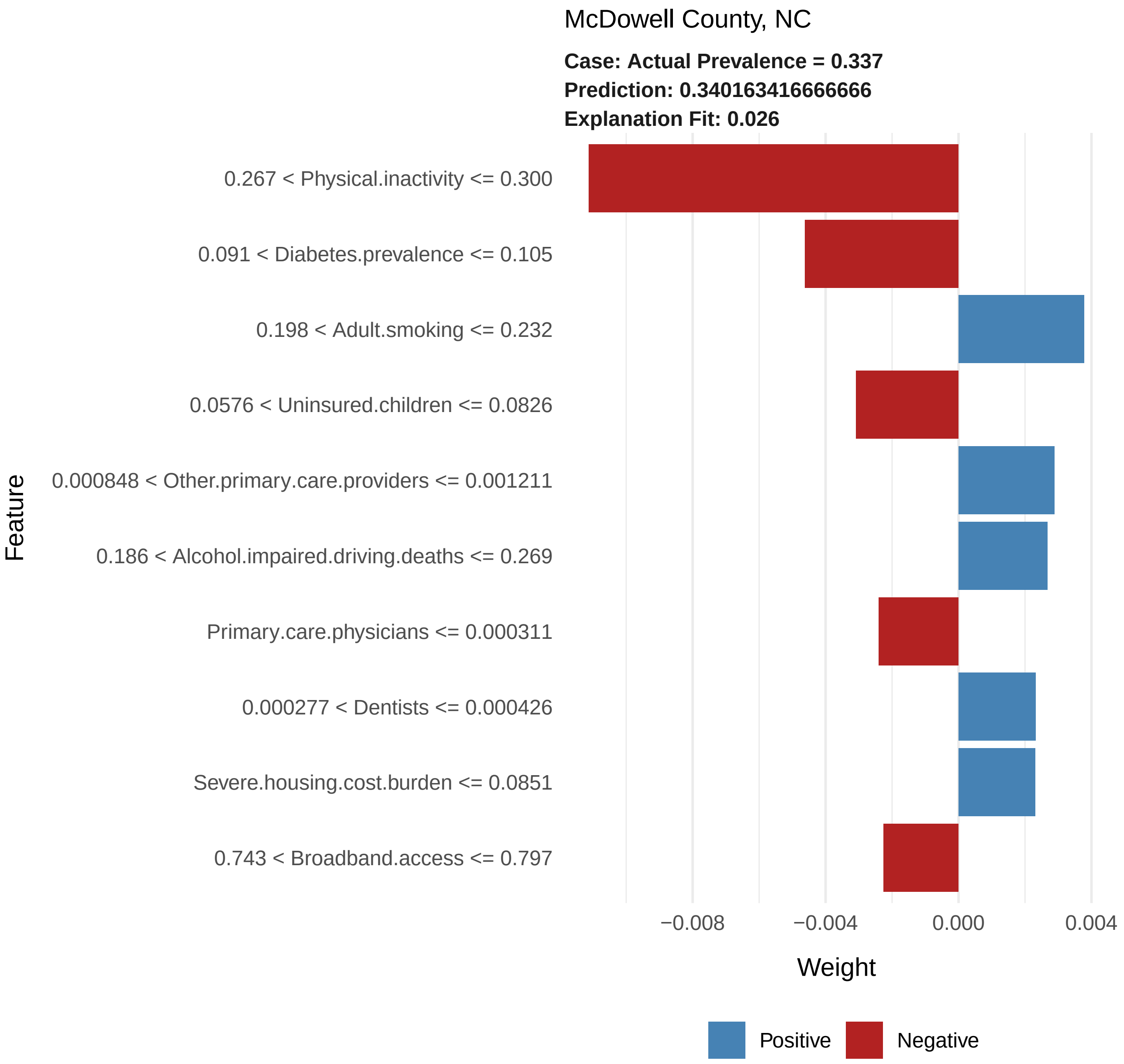
McDowell County, NC
Case: Actual Prevalence = 0.337
Prediction: 0.340163416666666
Explanation Fit: 0.026
0.267 < Physical.inactivity <= 0.300
0.091 < Diabetes.prevalence <= 0.105
0.198 < Adult.smoking <= 0.232
0.0576 < Uninsured.children <= 0.0826
0.000848 < Other.primary.care.providers <= 0.001211
0.186 < Alcohol.impaired.driving.deaths <= 0.269
Primary.care.physicians <= 0.000311
0.000277 < Dentists <= 0.000426
Severe.housing.cost.burden <= 0.0851
0.743 < Broadband.access <= 0.797
Feature
−0.008
−0.004
0.000
0.004
Weight
Positive
Negative

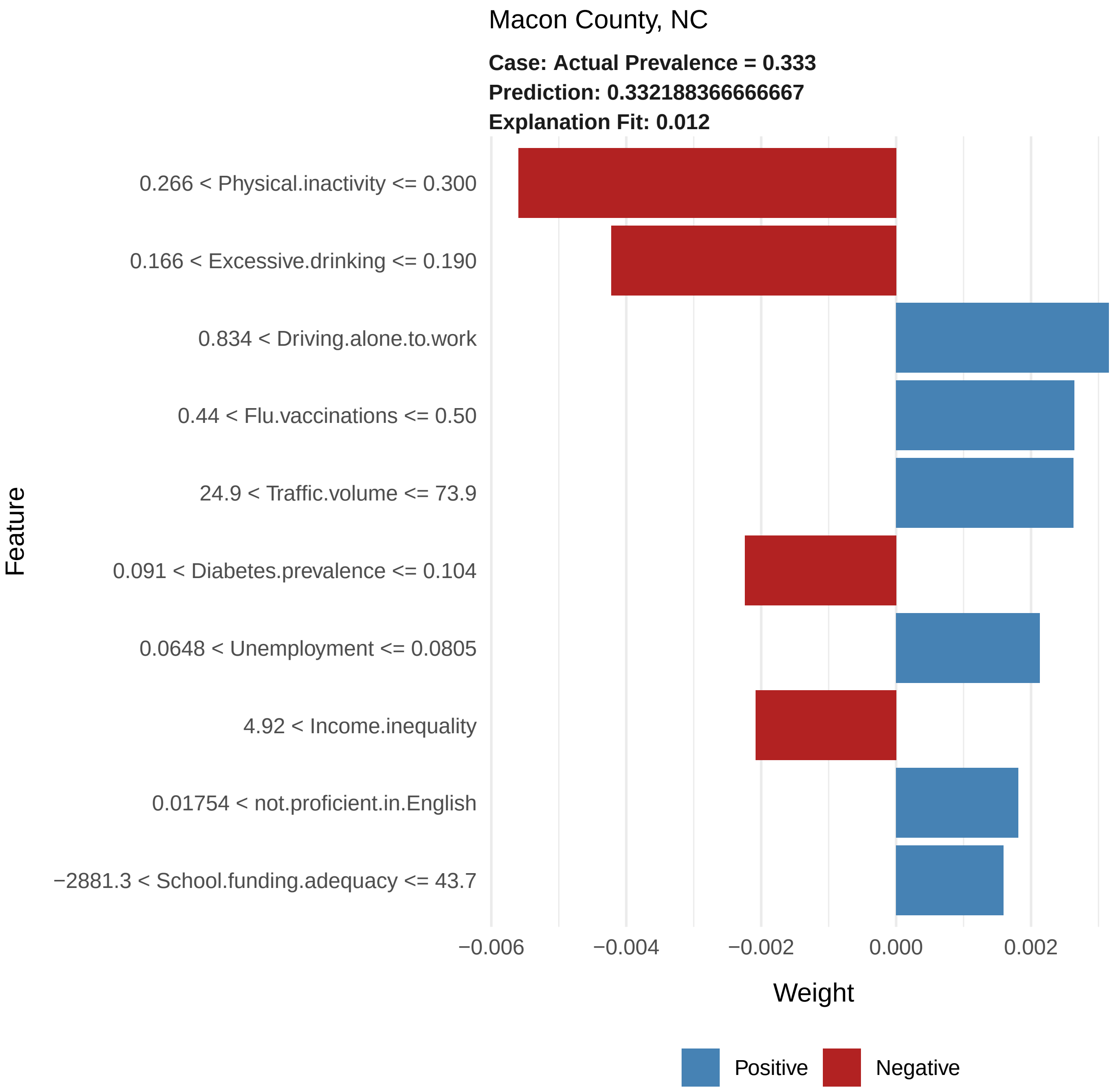

Macon County, NC
Case: Actual Prevalence = 0.333
Prediction: 0.332188366666667
Explanation Fit: 0.012
0.266 < Physical.inactivity <= 0.300
0.166 < Excessive.drinking <= 0.190
0.834 < Driving.alone.to.work
0.44 < Flu.vaccinations <= 0.50
24.9 < Traffic.volume <= 73.9
0.091 < Diabetes.prevalence <= 0.104
0.0648 < Unemployment <= 0.0805
4.92 < Income.inequality
0.01754 < not.proficient.in.English
−2881.3 < School.funding.adequacy <= 43.7
Feature
−0.006
−0.004
−0.002
0.000
0.002
Weight
Positive
Negative

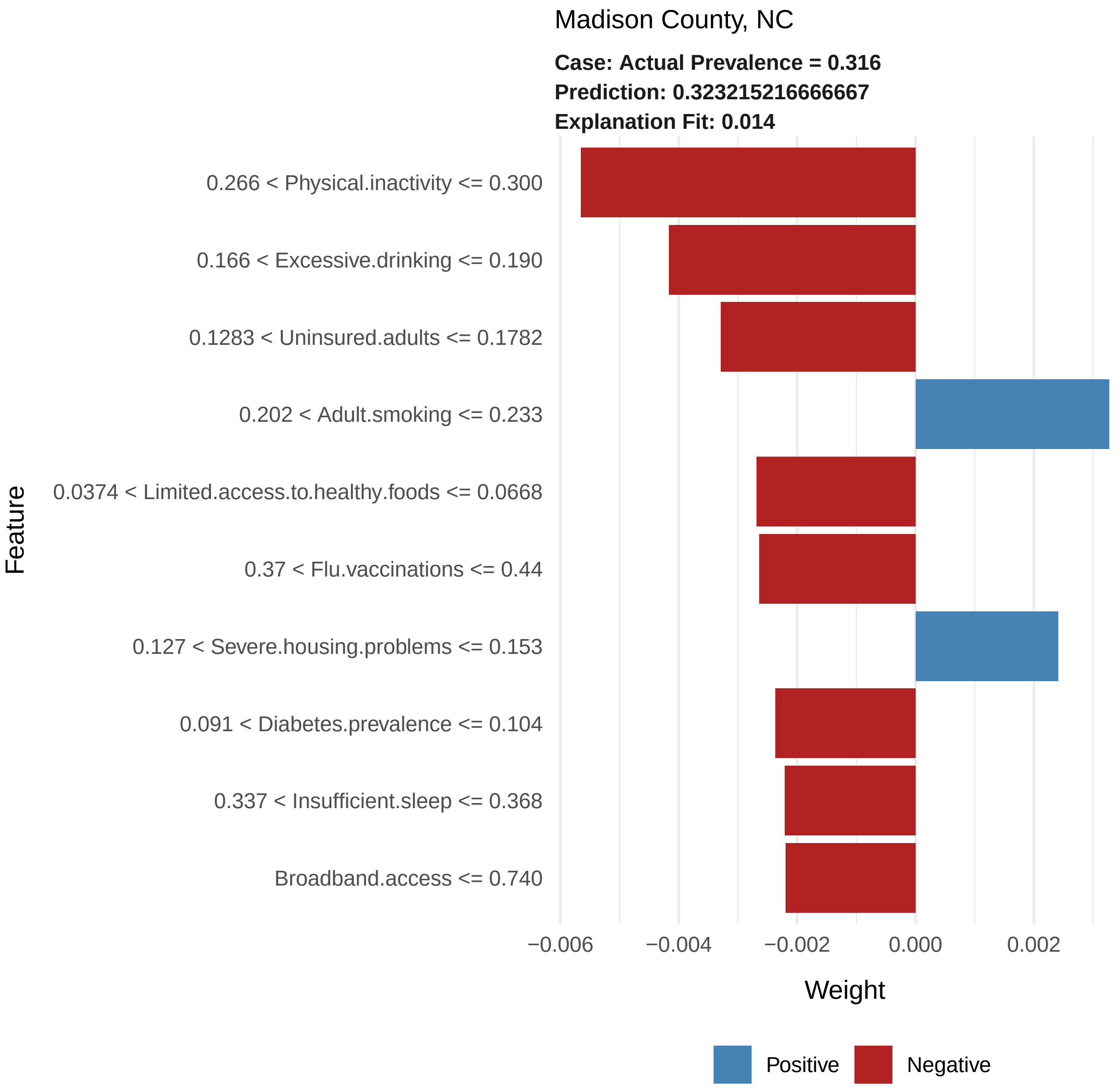

Madison County, NC
Case: Actual Prevalence = 0.316
Prediction: 0.323215216666667
Explanation Fit: 0.014
0.266 < Physical.inactivity <= 0.300
0.166 < Excessive.drinking <= 0.190
0.1283 < Uninsured.adults <= 0.1782
0.202 < Adult.smoking <= 0.233
0.0374 < Limited.access.to.healthy.foods <= 0.0668
0.37 < Flu.vaccinations <= 0.44
0.127 < Severe.housing.problems <= 0.153
0.091 < Diabetes.prevalence <= 0.104
0.337 < Insufficient.sleep <= 0.368
Broadband.access <= 0.740
Feature
−0.006
−0.004
−0.002
0.000
0.002
Weight
Positive
Negative

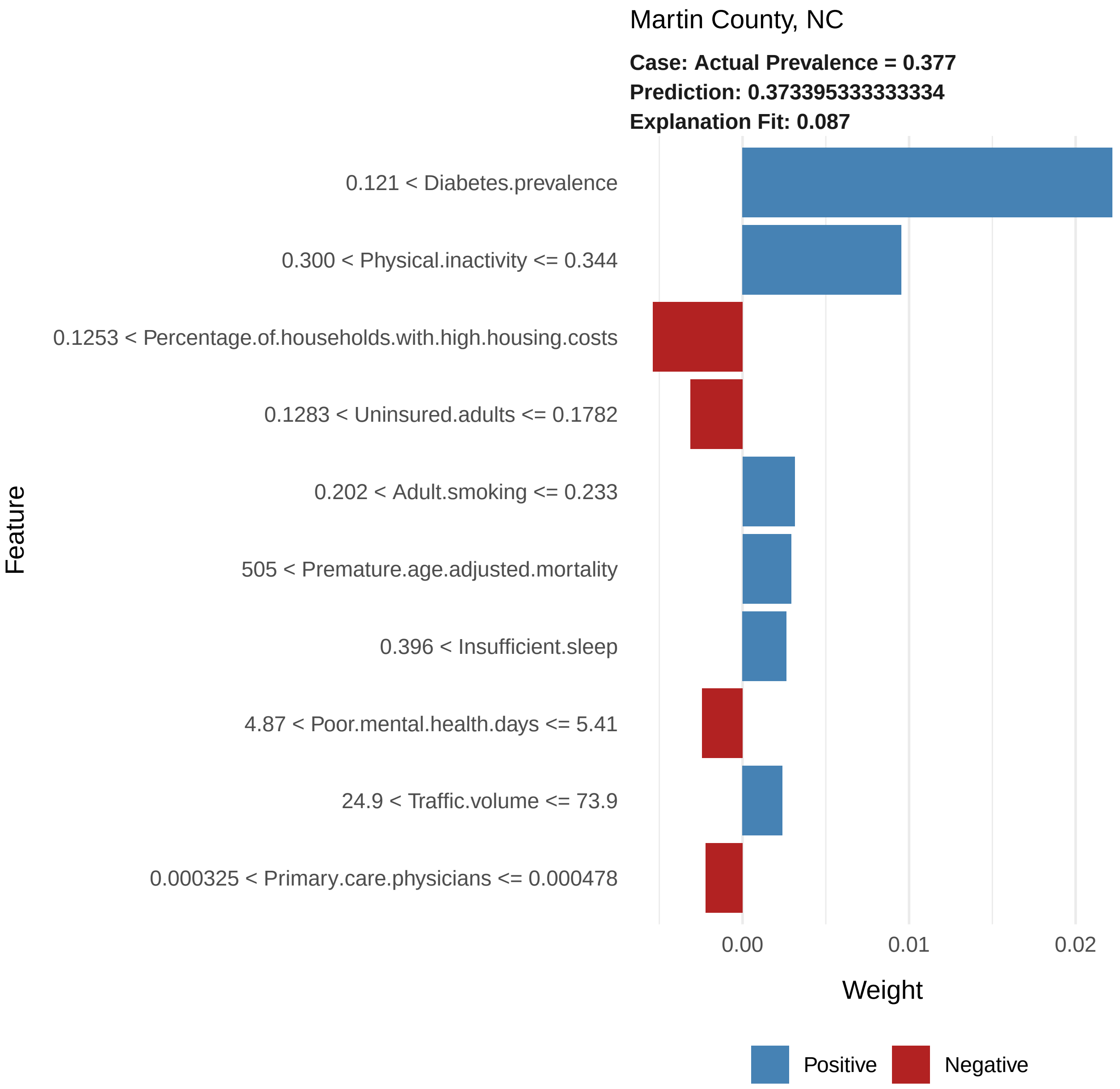
Martin County, NC
Case: Actual Prevalence = 0.377
Prediction: 0.373395333333334
Explanation Fit: 0.087
Feature
0.121 < Diabetes.prevalence
0.300 < Physical.inactivity <= 0.344
0.1253 < Percentage.of.households.with.high.housing.costs
0.1283 < Uninsured.adults <= 0.1782
0.202 < Adult.smoking <= 0.233
505 < Premature.age.adjusted.mortality
0.396 < Insufficient.sleep
4.87 < Poor.mental.health.days <= 5.41
24.9 < Traffic.volume <= 73.9
0.000325 < Primary.care.physicians <= 0.000478
0.00
0.01
0.02
Weight
Positive
Negative

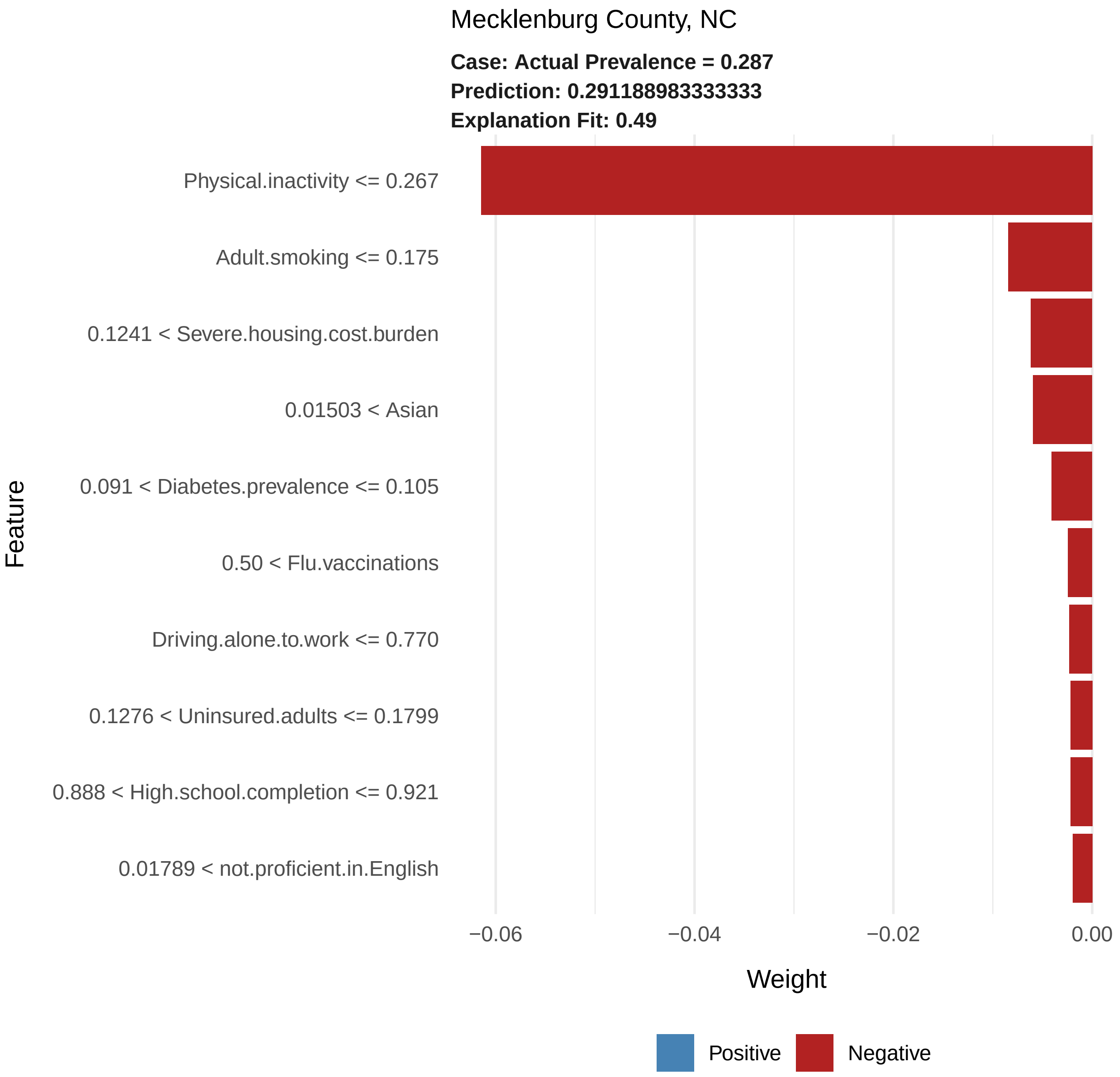

Mecklenburg County, NC
Case: Actual Prevalence = 0.287
Prediction: 0.291188983333333
Explanation Fit: 0.49
Physical.inactivity <= 0.267
Adult.smoking <= 0.175
0.1241 < Severe.housing.cost.burden
0.01503 < Asian
0.091 < Diabetes.prevalence <= 0.105
0.50 < Flu.vaccinations
Driving.alone.to.work <= 0.770
0.1276 < Uninsured.adults <= 0.1799
0.888 < High.school.completion <= 0.921
0.01789 < not.proficient.in.English
Feature
−0.06
−0.04
−0.02
0.00
Weight
Positive
Negative

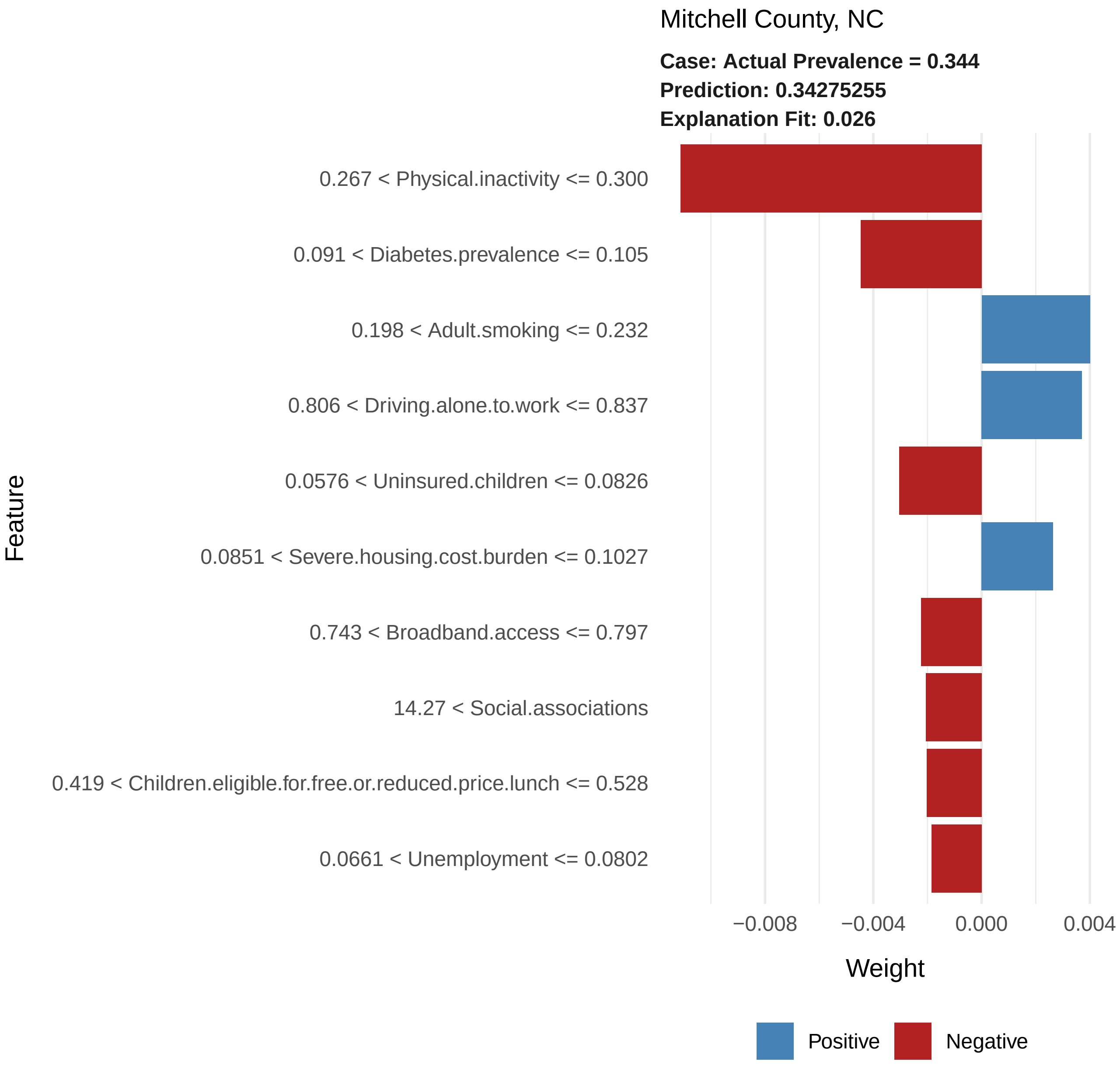

Mitchell County, NC
Case: Actual Prevalence = 0.344
Prediction: 0.34275255
Explanation Fit: 0.026
0.267 < Physical.inactivity <= 0.300
0.091 < Diabetes.prevalence <= 0.105
0.198 < Adult.smoking <= 0.232
0.806 < Driving.alone.to.work <= 0.837
0.0576 < Uninsured.children <= 0.0826
0.0851 < Severe.housing.cost.burden <= 0.1027
0.743 < Broadband.access <= 0.797
14.27 < Social.associations
0.419 < Children.eligible.for.free.or.reduced.price.lunch <= 0.528
0.0661 < Unemployment <= 0.0802
Feature
−0.008
−0.004
0.000
0.004
Weight
Positive
Negative

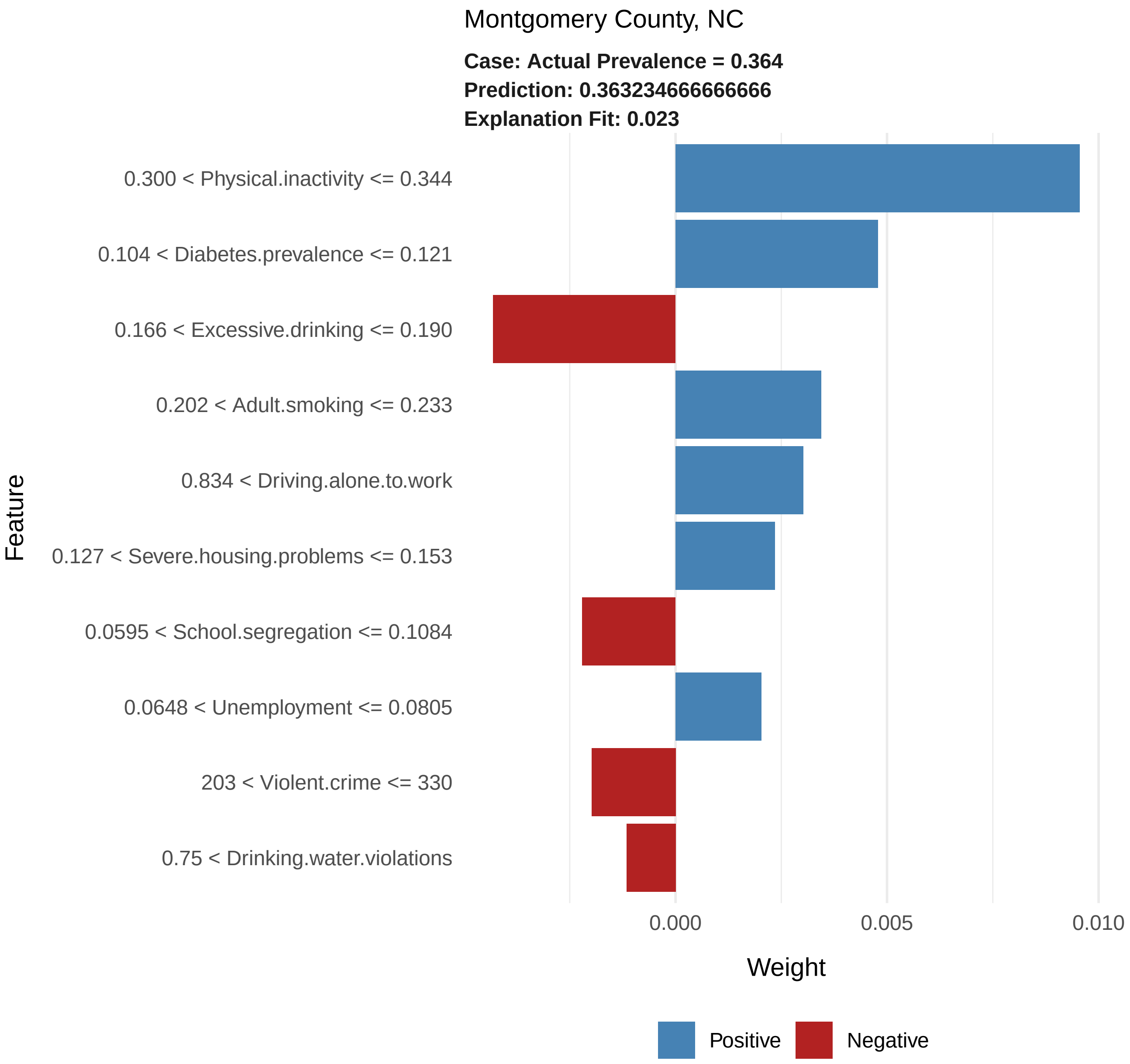

Montgomery County, NC
Case: Actual Prevalence = 0.364
Prediction: 0.363234666666666
Explanation Fit: 0.023
0.300 < Physical.inactivity <= 0.344
0.104 < Diabetes.prevalence <= 0.121
0.166 < Excessive.drinking <= 0.190
0.202 < Adult.smoking <= 0.233
0.834 < Driving.alone.to.work
0.127 < Severe.housing.problems <= 0.153
0.0595 < School.segregation <= 0.1084
0.0648 < Unemployment <= 0.0805
203 < Violent.crime <= 330
0.75 < Drinking.water.violations
Feature
0.000
0.005
0.010
Weight
Positive
Negative

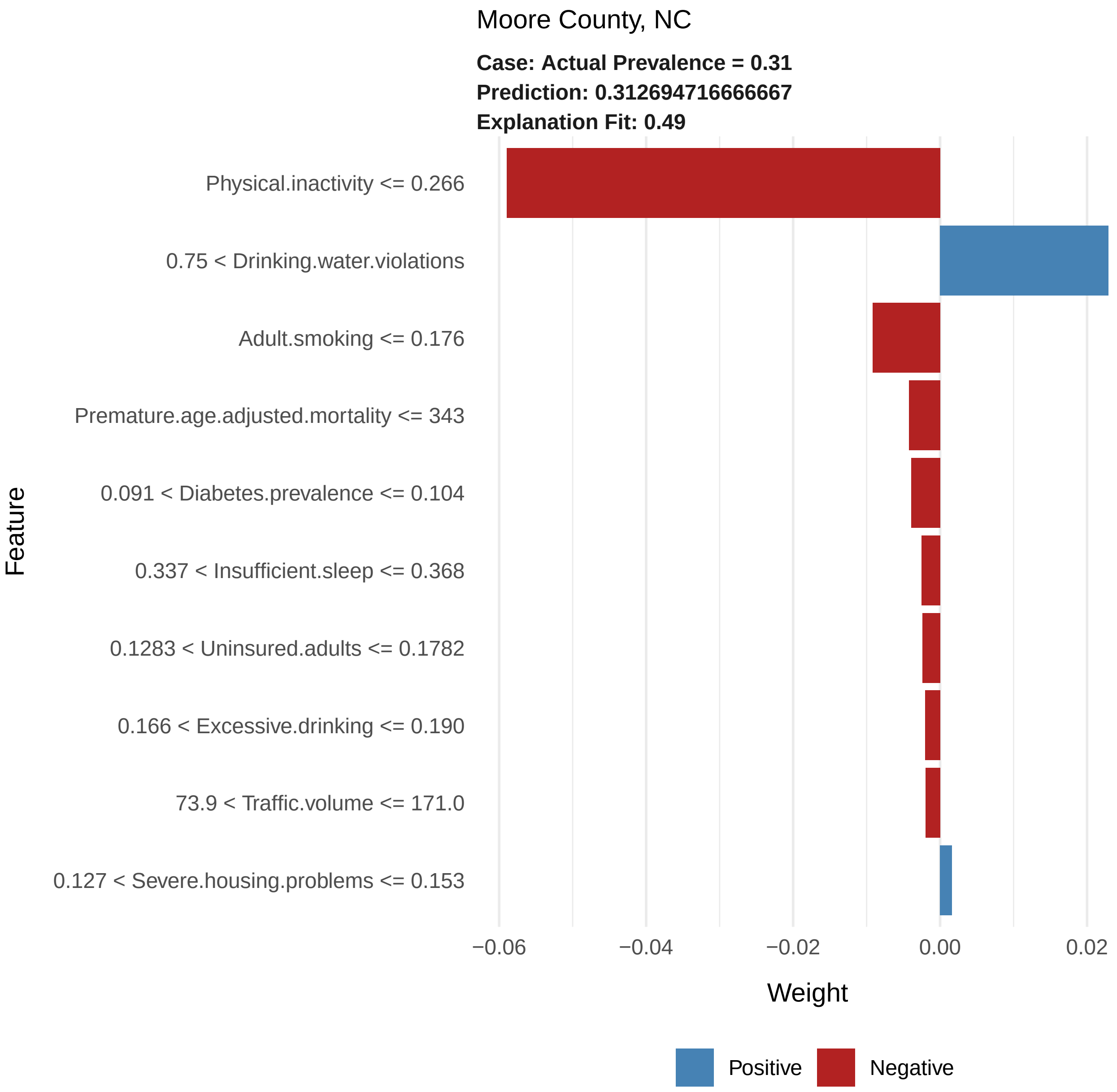

Moore County, NC
Case: Actual Prevalence = 0.31
Prediction: 0.312694716666667
Explanation Fit: 0.49
Physical.inactivity <= 0.266
0.75 < Drinking.water.violations
Adult.smoking <= 0.176
Premature.age.adjusted.mortality <= 343
0.091 < Diabetes.prevalence <= 0.104
0.337 < Insufficient.sleep <= 0.368
0.1283 < Uninsured.adults <= 0.1782
0.166 < Excessive.drinking <= 0.190
73.9 < Traffic.volume <= 171.0
0.127 < Severe.housing.problems <= 0.153
Feature
−0.06
−0.04
−0.02
0.00
0.02
Weight
Positive
Negative

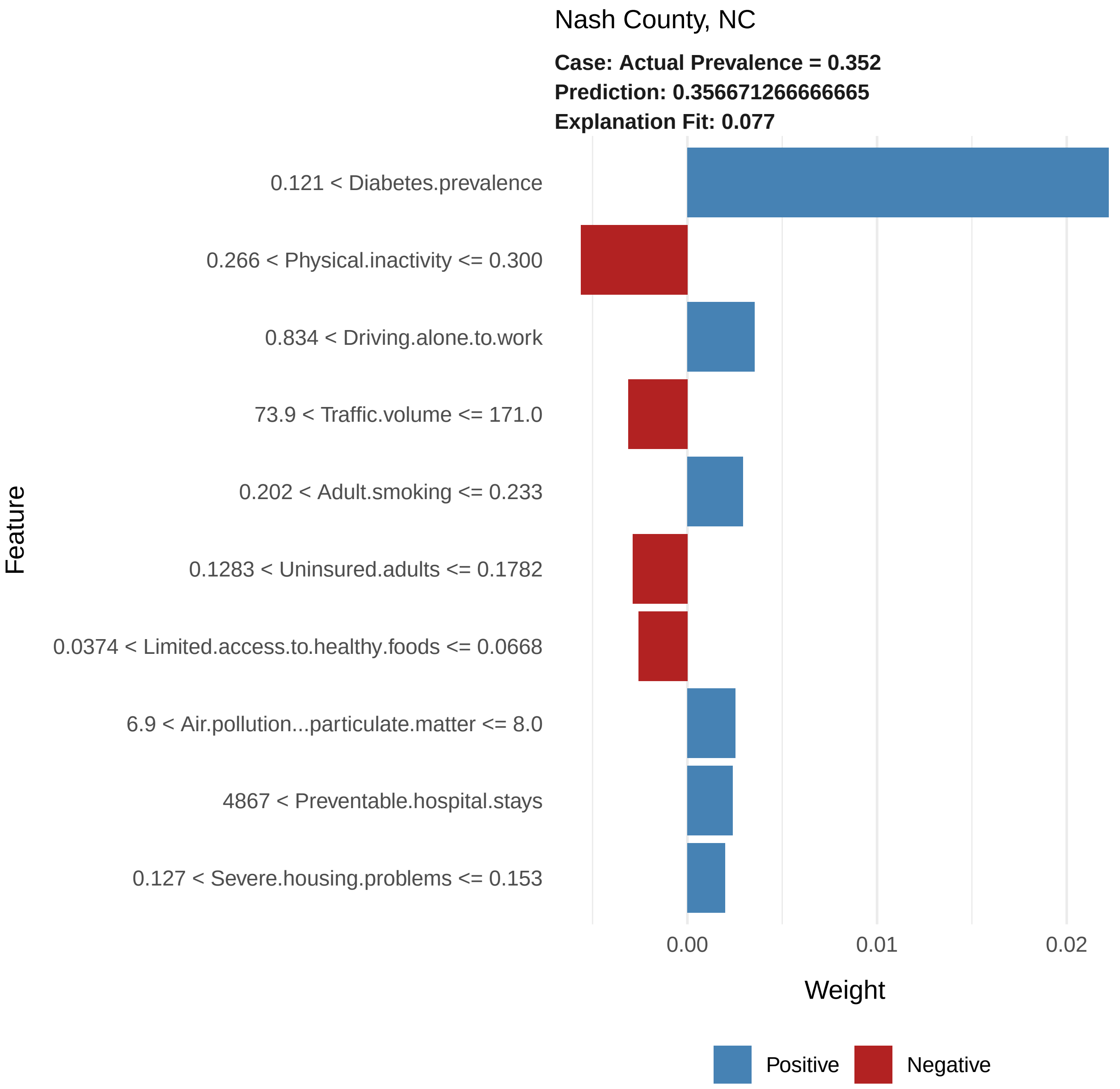
Nash County, NC
Case: Actual Prevalence = 0.352
Prediction: 0.356671266666665
Explanation Fit: 0.077
0.121 < Diabetes.prevalence
0.266 < Physical.inactivity <= 0.300
0.834 < Driving.alone.to.work
73.9 < Traffic.volume <= 171.0
0.202 < Adult.smoking <= 0.233
0.1283 < Uninsured.adults <= 0.1782
0.0374 < Limited.access.to.healthy.foods <= 0.0668
6.9 < Air.pollution...particulate.matter <= 8.0
4867 < Preventable.hospital.stays
0.127 < Severe.housing.problems <= 0.153
Feature
0.00
0.01
0.02
Weight
Positive
Negative

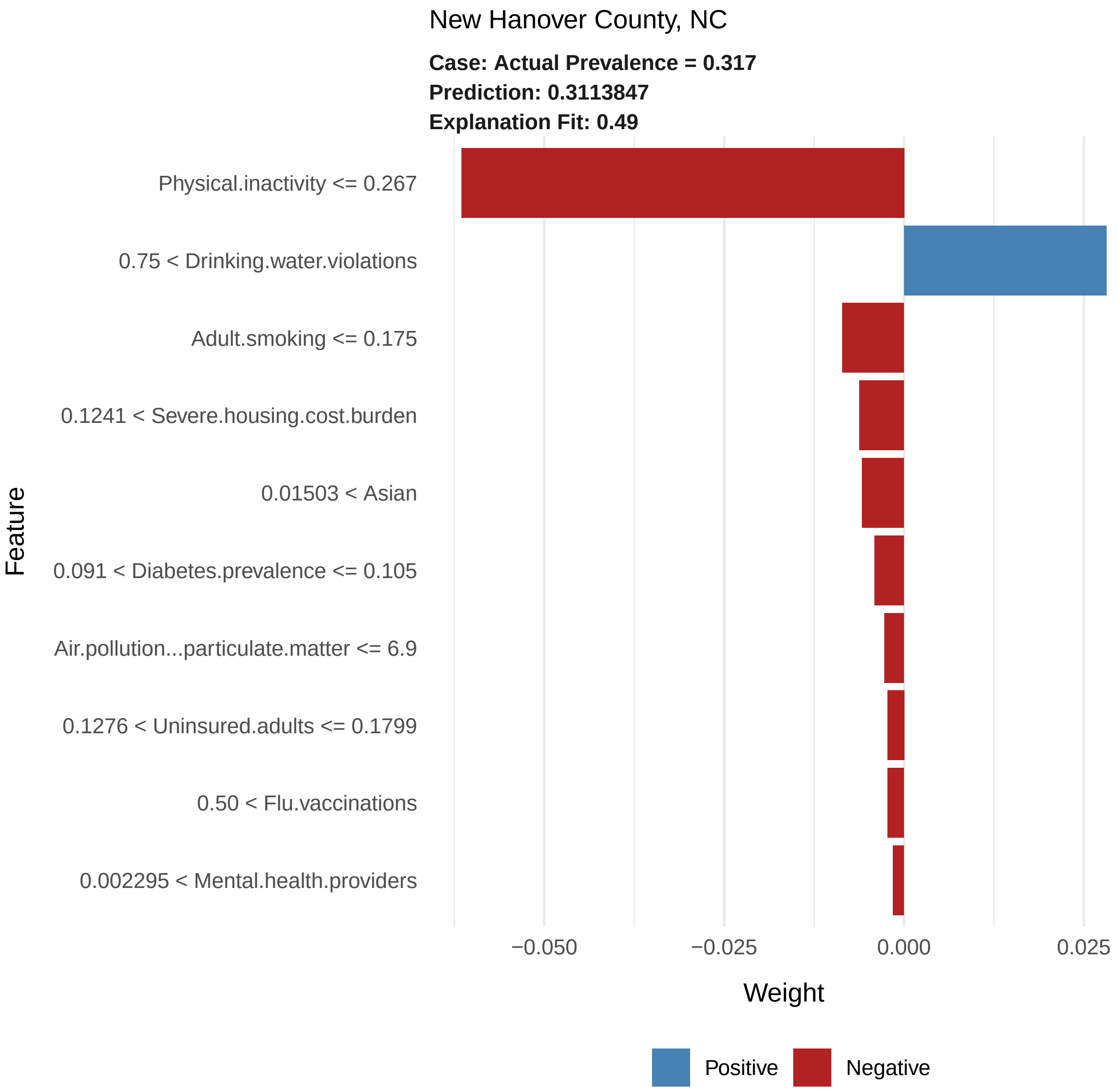

New Hanover County, NC
Case: Actual Prevalence = 0.317
Prediction: 0.3113847
Explanation Fit: 0.49
Physical.inactivity <= 0.267
0.75 < Drinking.water.violations
Adult.smoking <= 0.175
0.1241 < Severe.housing.cost.burden
0.01503 < Asian
0.091 < Diabetes.prevalence <= 0.105
Air.pollution...particulate.matter <= 6.9
0.1276 < Uninsured.adults <= 0.1799
0.50 < Flu.vaccinations
0.002295 < Mental.health.providers
Feature
−0.050
−0.025
0.000
0.025
Weight
Positive
Negative

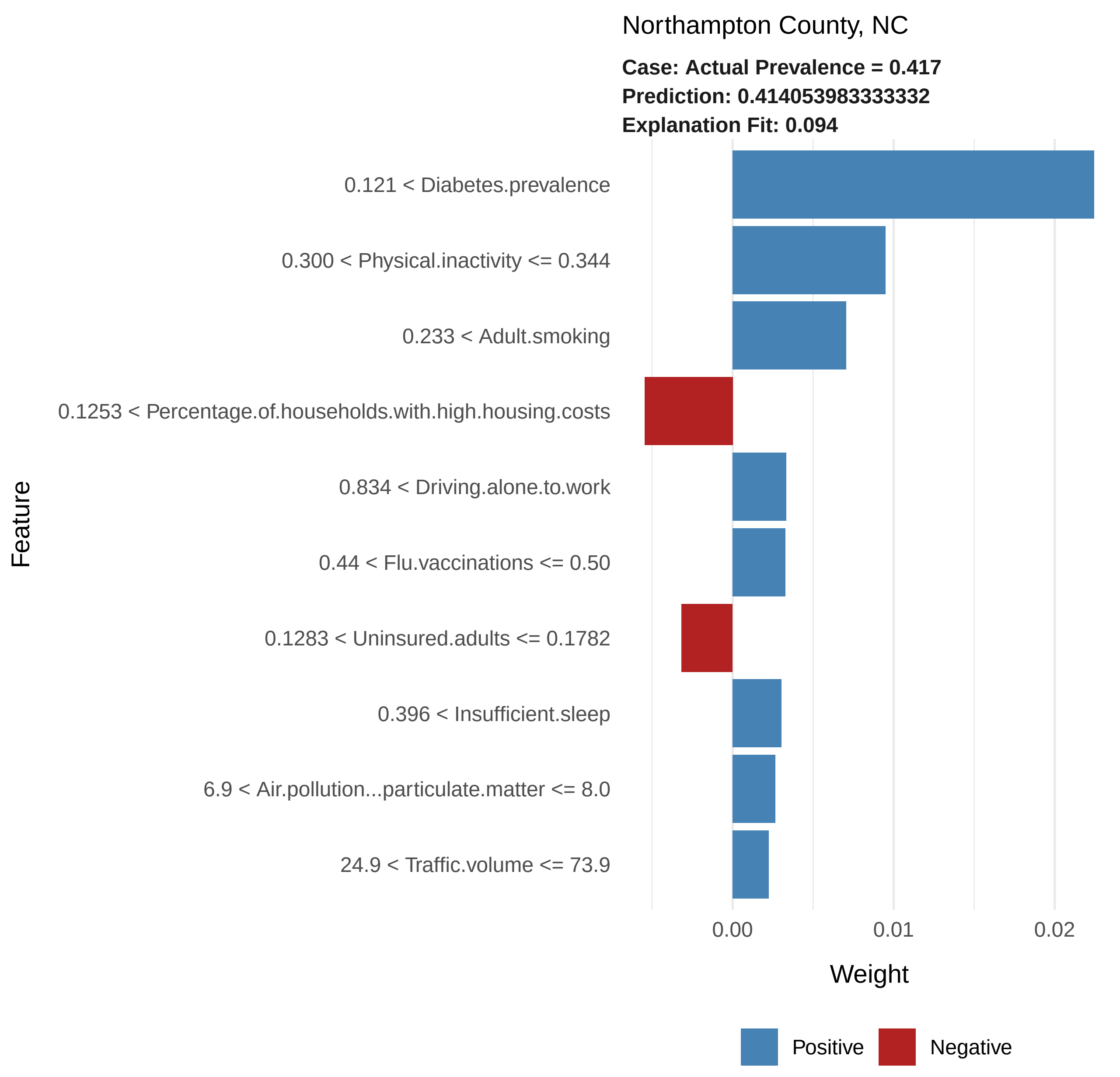

Northampton County, NC
Case: Actual Prevalence = 0.417
Prediction: 0.414053983333332
Explanation Fit: 0.094
0.121 < Diabetes.prevalence
0.300 < Physical.inactivity <= 0.344
0.233 < Adult.smoking
0.1253 < Percentage.of.households.with.high.housing.costs
0.834 < Driving.alone.to.work
0.44 < Flu.vaccinations <= 0.50
0.1283 < Uninsured.adults <= 0.1782
0.396 < Insufficient.sleep
6.9 < Air.pollution...particulate.matter <= 8.0
24.9 < Traffic.volume <= 73.9
Feature
0.00
0.01
0.02
Weight
Positive
Negative

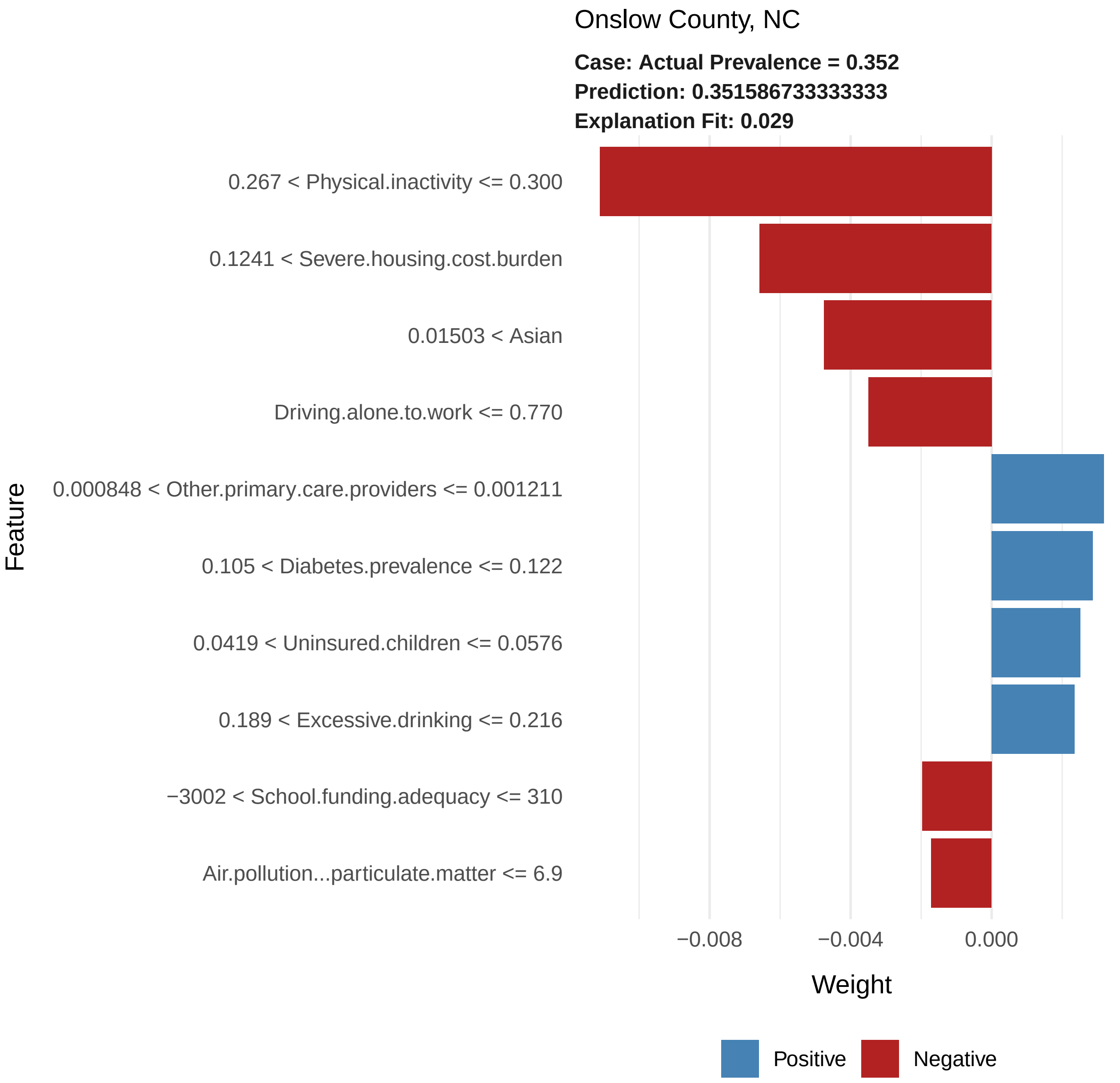

Onslow County, NC
Case: Actual Prevalence = 0.352
Prediction: 0.351586733333333
Explanation Fit: 0.029
0.267 < Physical.inactivity <= 0.300
0.1241 < Severe.housing.cost.burden
0.01503 < Asian
Driving.alone.to.work <= 0.770
0.000848 < Other.primary.care.providers <= 0.001211
0.105 < Diabetes.prevalence <= 0.122
0.0419 < Uninsured.children <= 0.0576
0.189 < Excessive.drinking <= 0.216
−3002 < School.funding.adequacy <= 310
Air.pollution...particulate.matter <= 6.9
Feature
−0.008
−0.004
0.000
Weight
Positive
Negative

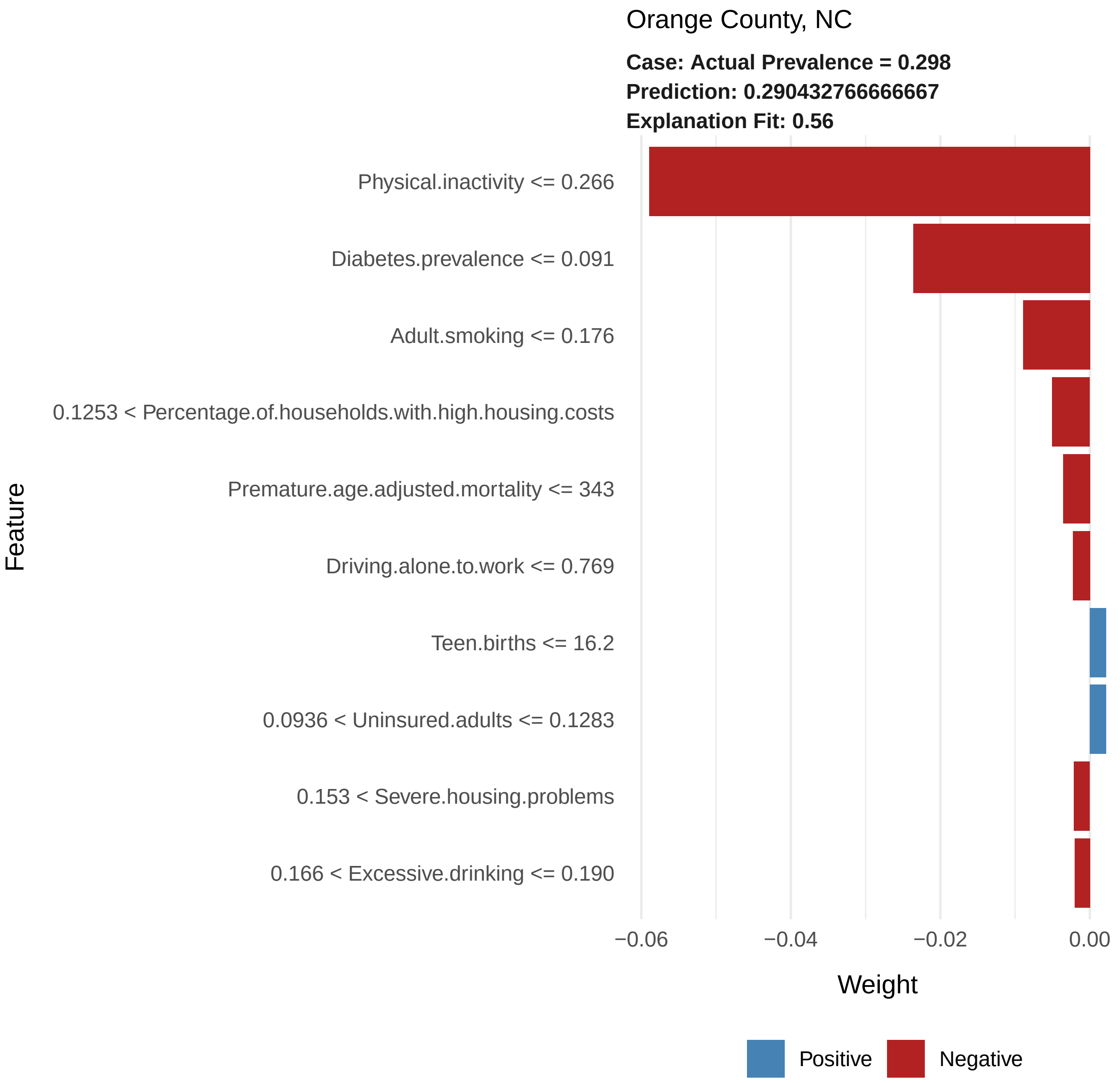

Orange County, NC
Case: Actual Prevalence = 0.298
Prediction: 0.290432766666667
Explanation Fit: 0.56
Physical.inactivity <= 0.266
Diabetes.prevalence <= 0.091
Adult.smoking <= 0.176
0.1253 < Percentage.of.households.with.high.housing.costs
Premature.age.adjusted.mortality <= 343
Driving.alone.to.work <= 0.769
Teen.births <= 16.2
0.0936 < Uninsured.adults <= 0.1283
0.153 < Severe.housing.problems
0.166 < Excessive.drinking <= 0.190
Feature
−0.06
−0.04
−0.02
0.00
Weight
Positive
Negative

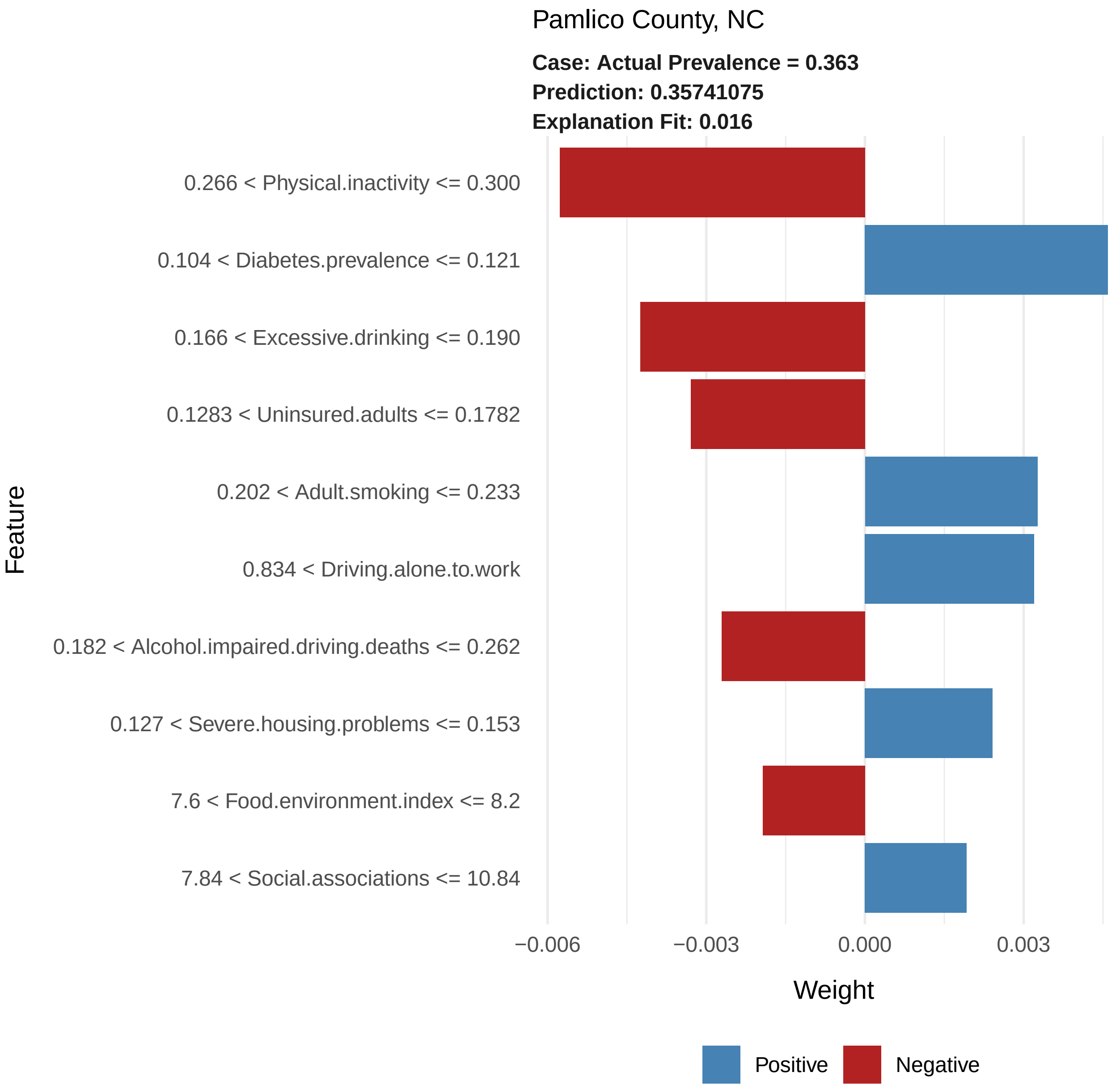
Pamlico County, NC
Case: Actual Prevalence = 0.363
Prediction: 0.35741075
Explanation Fit: 0.016
0.266 < Physical.inactivity <= 0.300
0.104 < Diabetes.prevalence <= 0.121
0.166 < Excessive.drinking <= 0.190
0.1283 < Uninsured.adults <= 0.1782
0.202 < Adult.smoking <= 0.233
0.834 < Driving.alone.to.work
0.182 < Alcohol.impaired.driving.deaths <= 0.262
0.127 < Severe.housing.problems <= 0.153
7.6 < Food.environment.index <= 8.2
7.84 < Social.associations <= 10.84
Feature
−0.006
−0.003
0.000
0.003
Weight
Positive
Negative

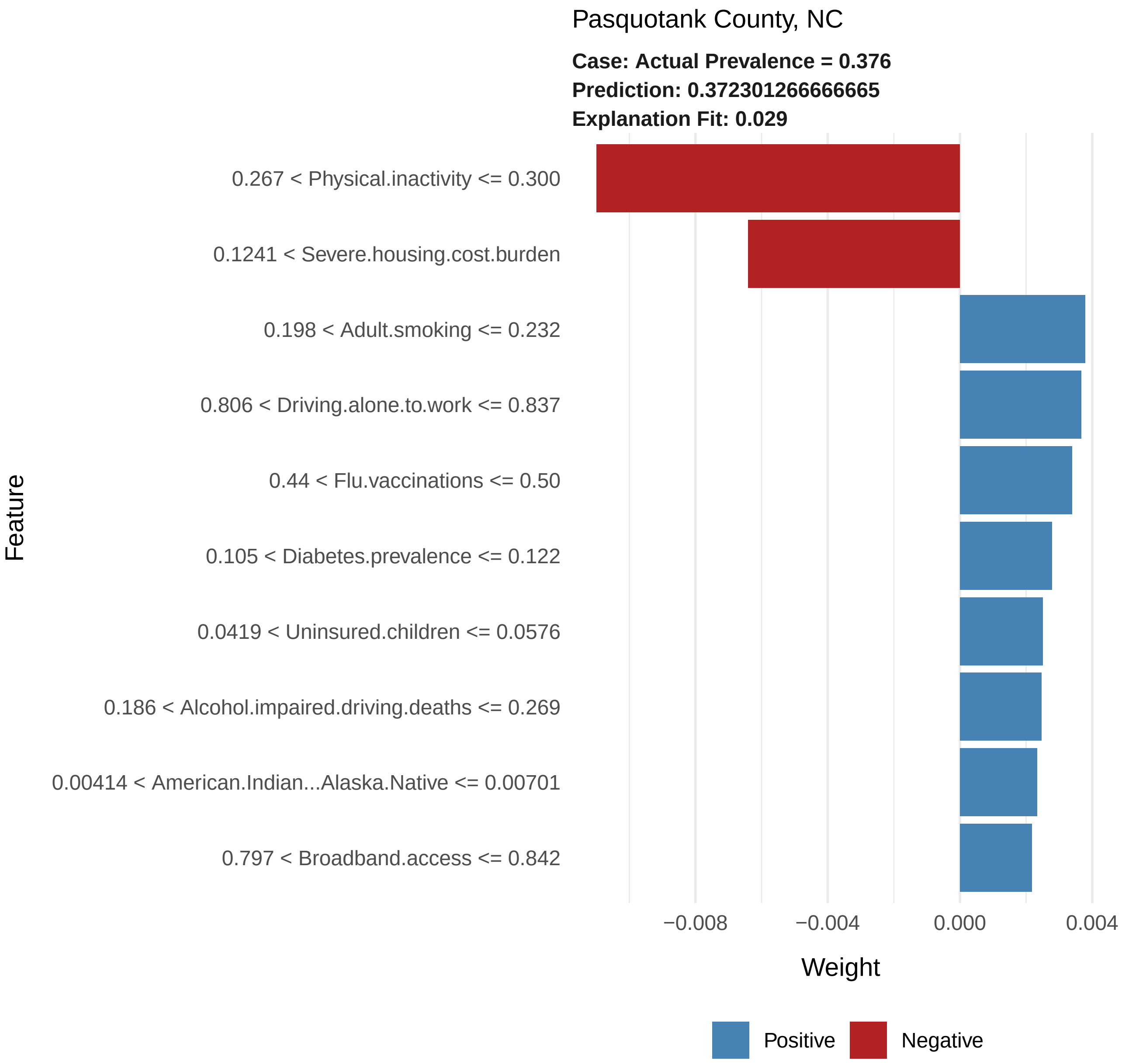
Pasquotank County, NC
Case: Actual Prevalence = 0.376
Prediction: 0.372301266666665
Explanation Fit: 0.029
0.267 < Physical.inactivity <= 0.300
0.1241 < Severe.housing.cost.burden
0.198 < Adult.smoking <= 0.232
0.806 < Driving.alone.to.work <= 0.837
0.44 < Flu.vaccinations <= 0.50
0.105 < Diabetes.prevalence <= 0.122
0.0419 < Uninsured.children <= 0.0576
0.186 < Alcohol.impaired.driving.deaths <= 0.269
0.00414 < American.Indian...Alaska.Native <= 0.00701
0.797 < Broadband.access <= 0.842
Feature
−0.008
−0.004
0.000
0.004
Weight
Positive
Negative

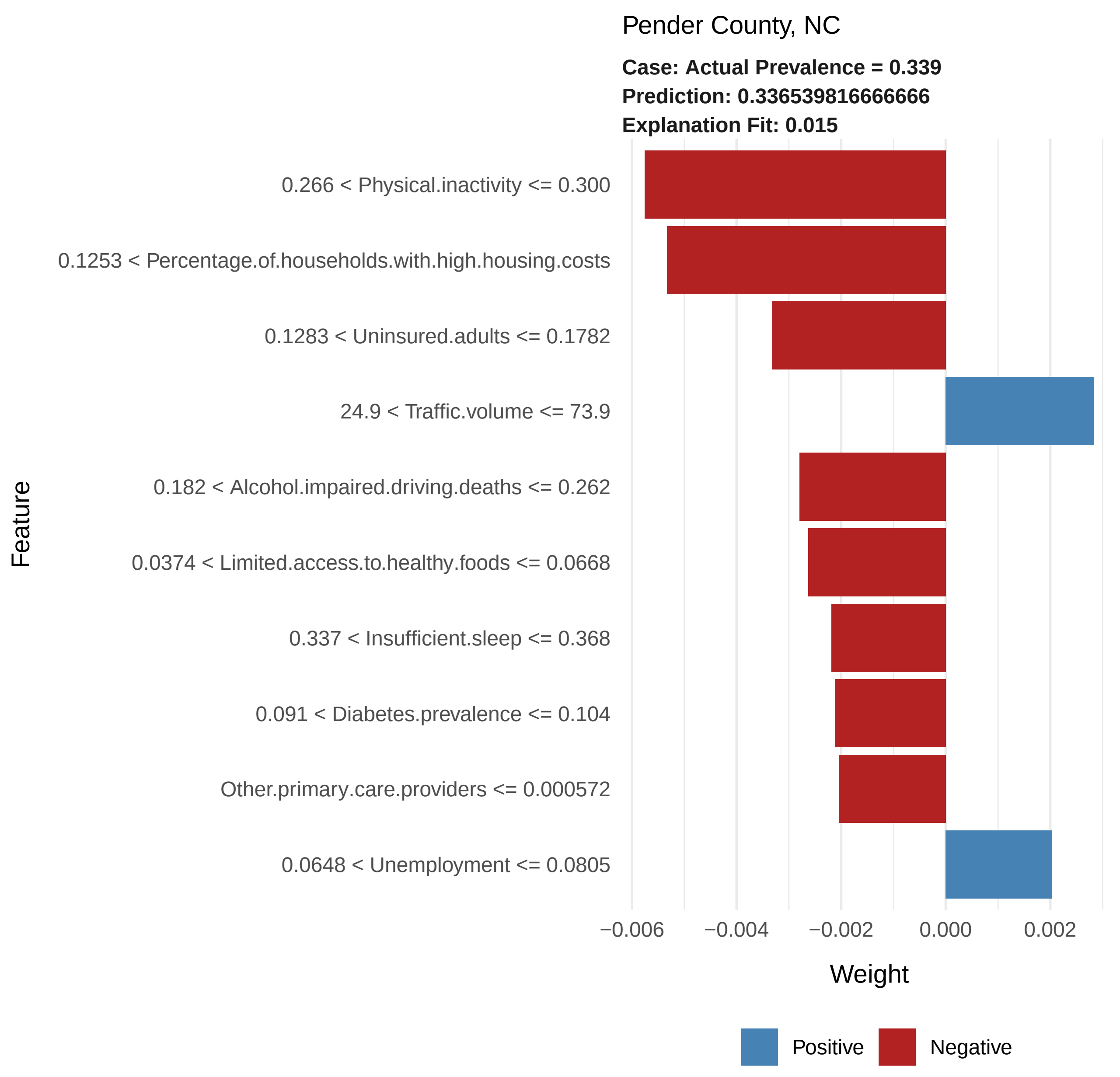

Pender County, NC
Case: Actual Prevalence = 0.339
Prediction: 0.336539816666666
Explanation Fit: 0.015
0.266 < Physical.inactivity <= 0.300
0.1253 < Percentage.of.households.with.high.housing.costs
0.1283 < Uninsured.adults <= 0.1782
24.9 < Traffic.volume <= 73.9
0.182 < Alcohol.impaired.driving.deaths <= 0.262
0.0374 < Limited.access.to.healthy.foods <= 0.0668
0.337 < Insufficient.sleep <= 0.368
0.091 < Diabetes.prevalence <= 0.104
Other.primary.care.providers <= 0.000572
0.0648 < Unemployment <= 0.0805
Feature
−0.006
−0.004
−0.002
0.000
0.002
Weight
Positive
Negative

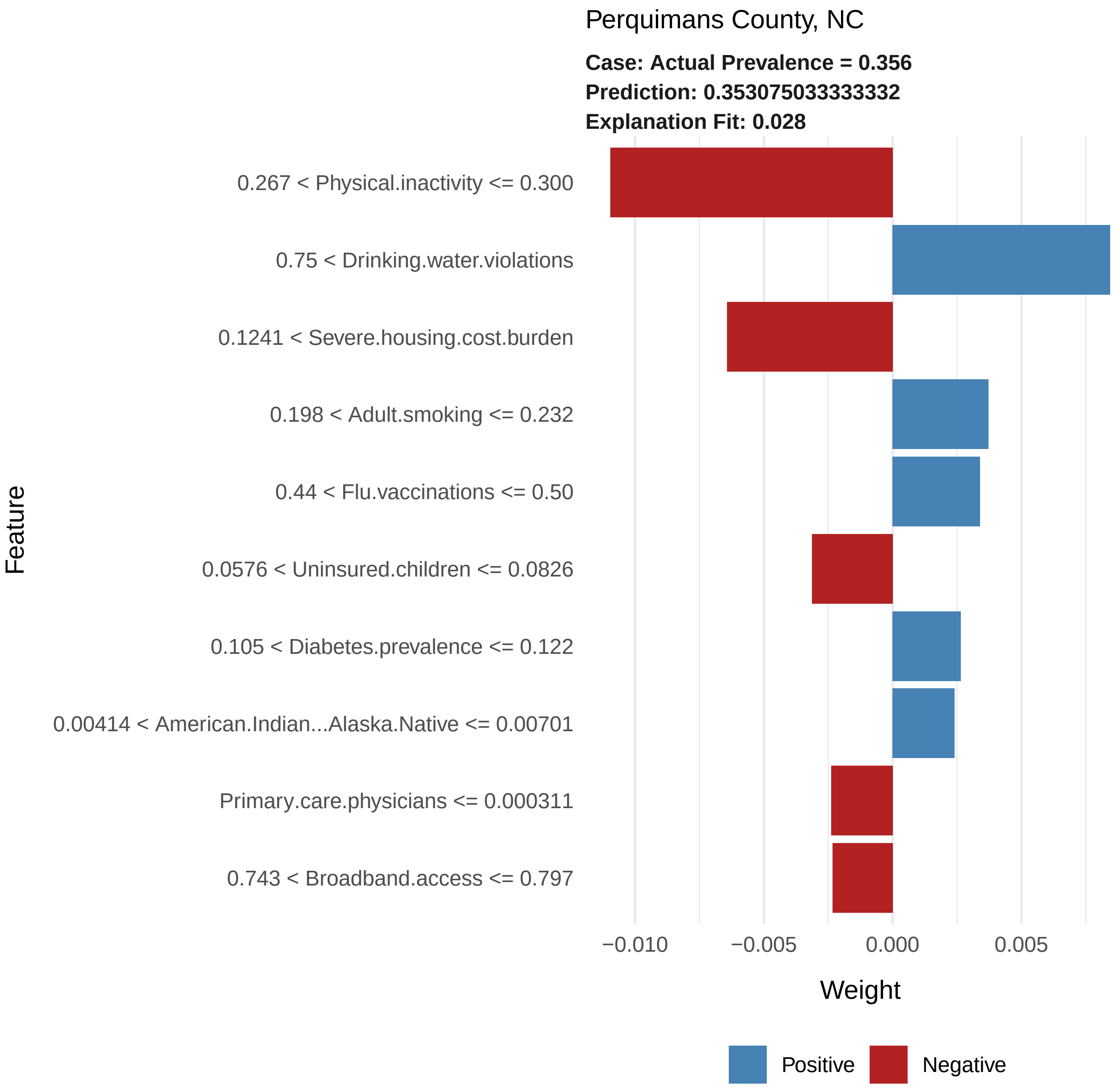
Perquimans County, NC
Case: Actual Prevalence = 0.356
Prediction: 0.353075033333332
Explanation Fit: 0.028
0.267 < Physical.inactivity <= 0.300
0.75 < Drinking.water.violations
0.1241 < Severe.housing.cost.burden
0.198 < Adult.smoking <= 0.232
0.44 < Flu.vaccinations <= 0.50
0.0576 < Uninsured.children <= 0.0826
0.105 < Diabetes.prevalence <= 0.122
0.00414 < American.Indian...Alaska.Native <= 0.00701
Primary.care.physicians <= 0.000311
0.743 < Broadband.access <= 0.797
Feature
−0.010
−0.005
0.000
0.005
Weight
Positive
Negative

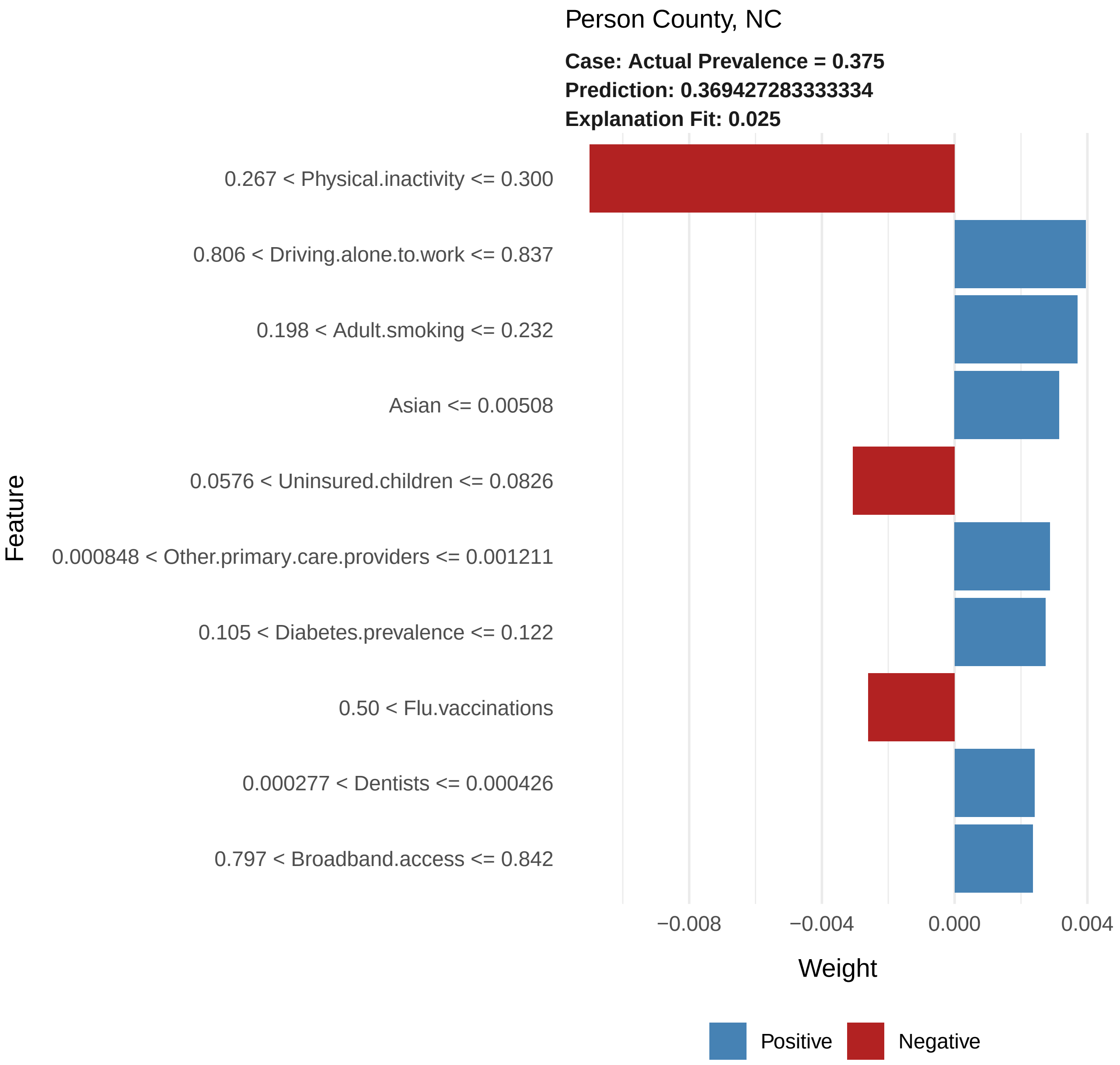

Person County, NC
Case: Actual Prevalence = 0.375
Prediction: 0.369427283333334
Explanation Fit: 0.025
0.267 < Physical.inactivity <= 0.300
0.806 < Driving.alone.to.work <= 0.837
0.198 < Adult.smoking <= 0.232
Asian <= 0.00508
0.0576 < Uninsured.children <= 0.0826
0.000848 < Other.primary.care.providers <= 0.001211
0.105 < Diabetes.prevalence <= 0.122
0.50 < Flu.vaccinations
0.000277 < Dentists <= 0.000426
0.797 < Broadband.access <= 0.842
Feature
−0.008
−0.004
0.000
0.004
Weight
Positive
Negative

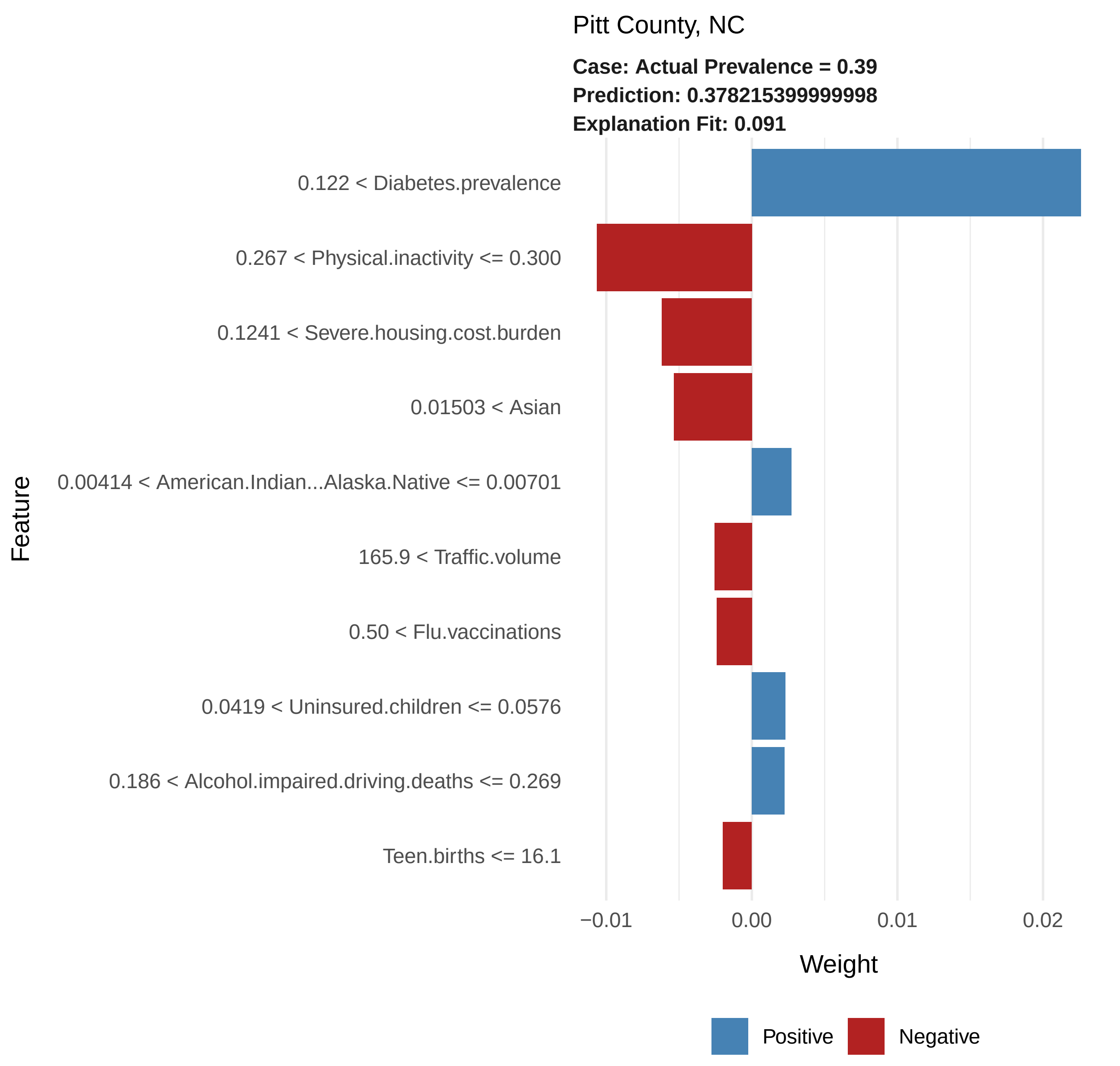

Pitt County, NC
Case: Actual Prevalence = 0.39
Prediction: 0.378215399999998
Explanation Fit: 0.091
0.122 < Diabetes.prevalence
0.267 < Physical.inactivity <= 0.300
0.1241 < Severe.housing.cost.burden
0.01503 < Asian
0.00414 < American.Indian...Alaska.Native <= 0.00701
165.9 < Traffic.volume
0.50 < Flu.vaccinations
0.0419 < Uninsured.children <= 0.0576
0.186 < Alcohol.impaired.driving.deaths <= 0.269
Teen.births <= 16.1
Feature
−0.01
0.00
0.01
0.02
Weight
Positive
Negative

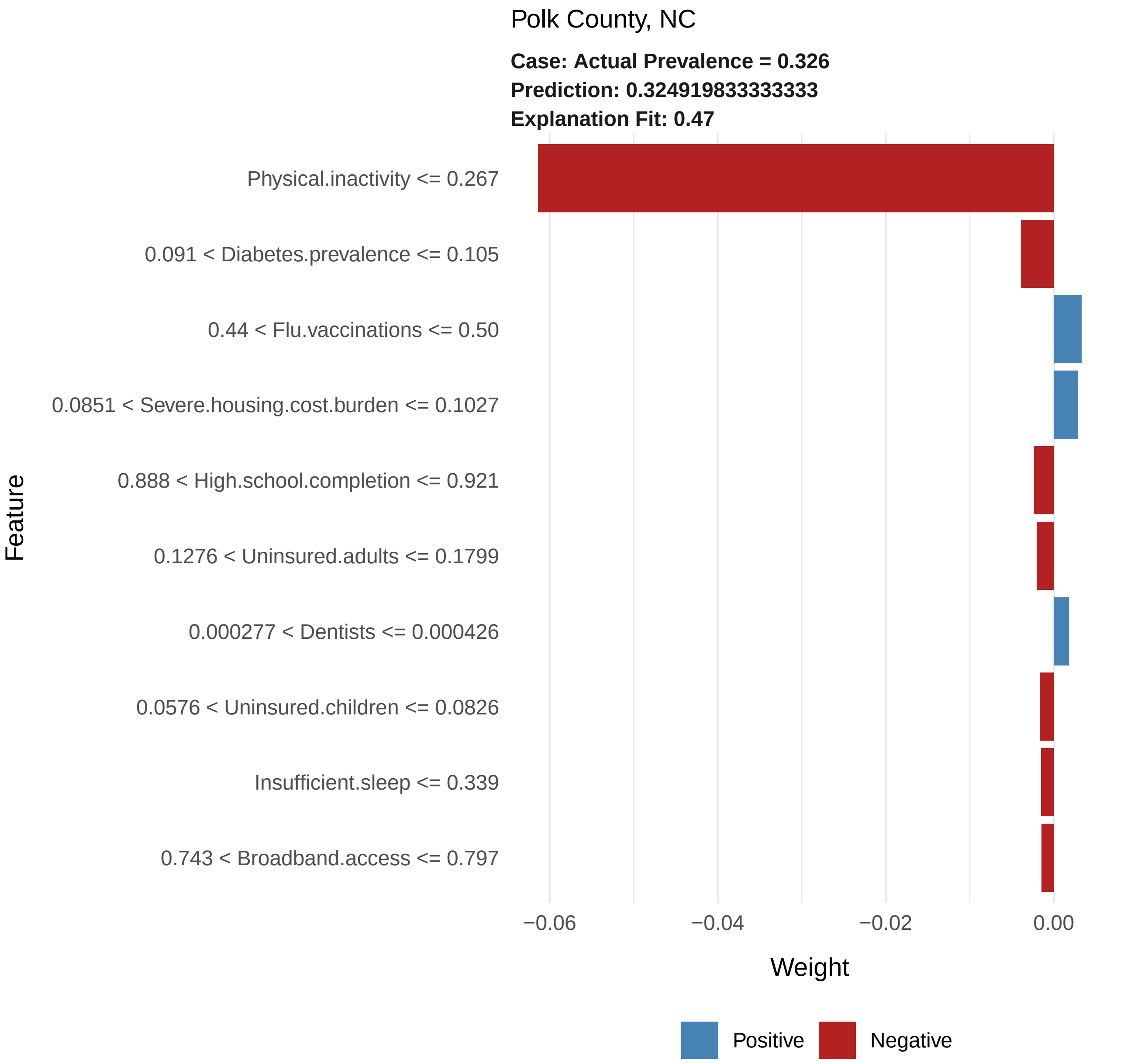

Polk County, NC
Case: Actual Prevalence = 0.326
Prediction: 0.324919833333333
Explanation Fit: 0.47
Physical.inactivity <= 0.267
0.091 < Diabetes.prevalence <= 0.105
0.44 < Flu.vaccinations <= 0.50
0.0851 < Severe.housing.cost.burden <= 0.1027
0.888 < High.school.completion <= 0.921
0.1276 < Uninsured.adults <= 0.1799
0.000277 < Dentists <= 0.000426
0.0576 < Uninsured.children <= 0.0826
Insufficient.sleep <= 0.339
0.743 < Broadband.access <= 0.797
Feature
−0.06
−0.04
−0.02
0.00
Weight
Positive
Negative

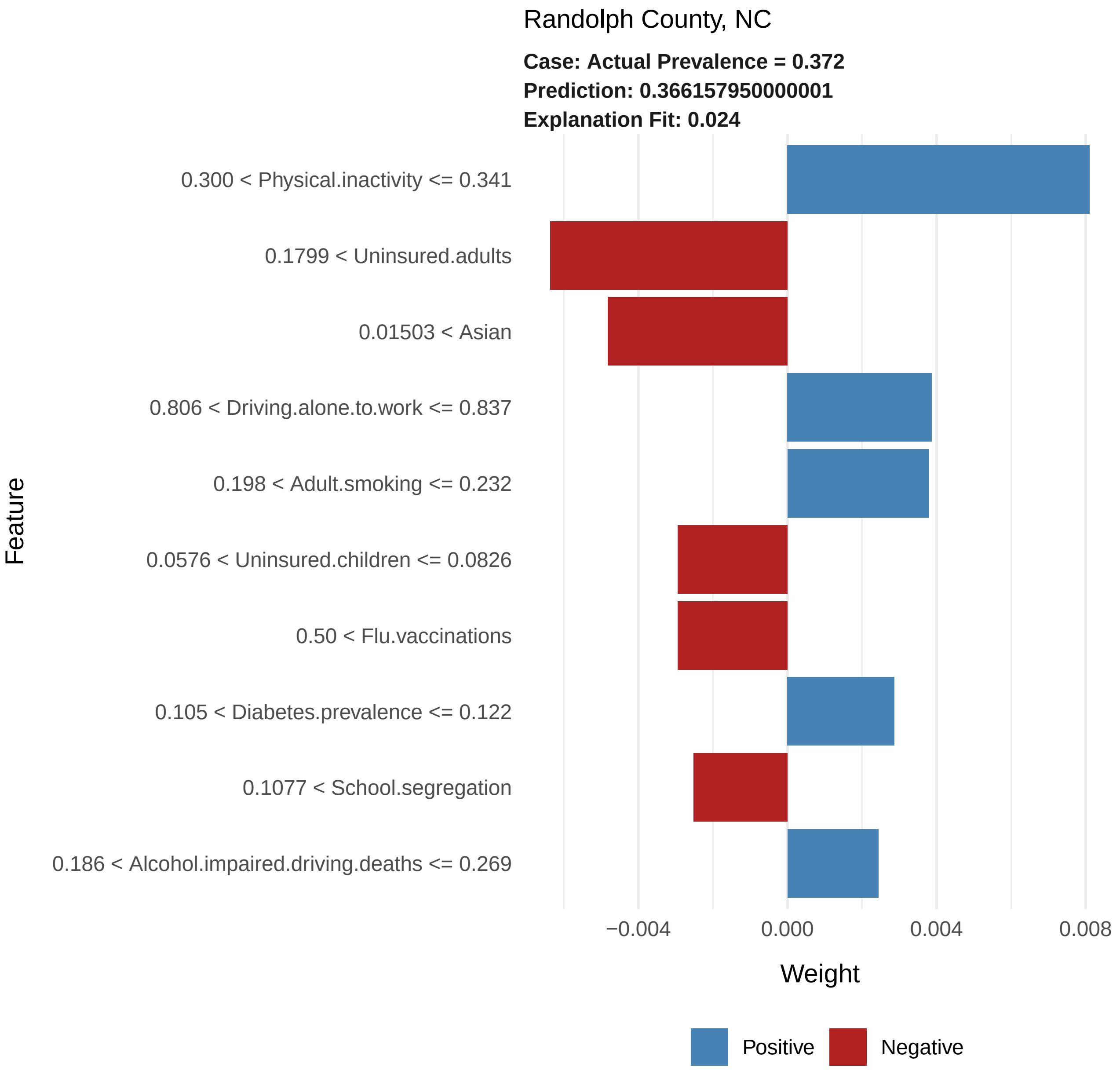
Randolph County, NC
Case: Actual Prevalence = 0.372
Prediction: 0.366157950000001
Explanation Fit: 0.024
0.300 < Physical.inactivity <= 0.341
0.1799 < Uninsured.adults
0.01503 < Asian
0.806 < Driving.alone.to.work <= 0.837
0.198 < Adult.smoking <= 0.232
0.0576 < Uninsured.children <= 0.0826
0.50 < Flu.vaccinations
0.105 < Diabetes.prevalence <= 0.122
0.1077 < School.segregation
0.186 < Alcohol.impaired.driving.deaths <= 0.269
Feature
−0.004
0.000
0.004
0.008
Weight
Positive
Negative

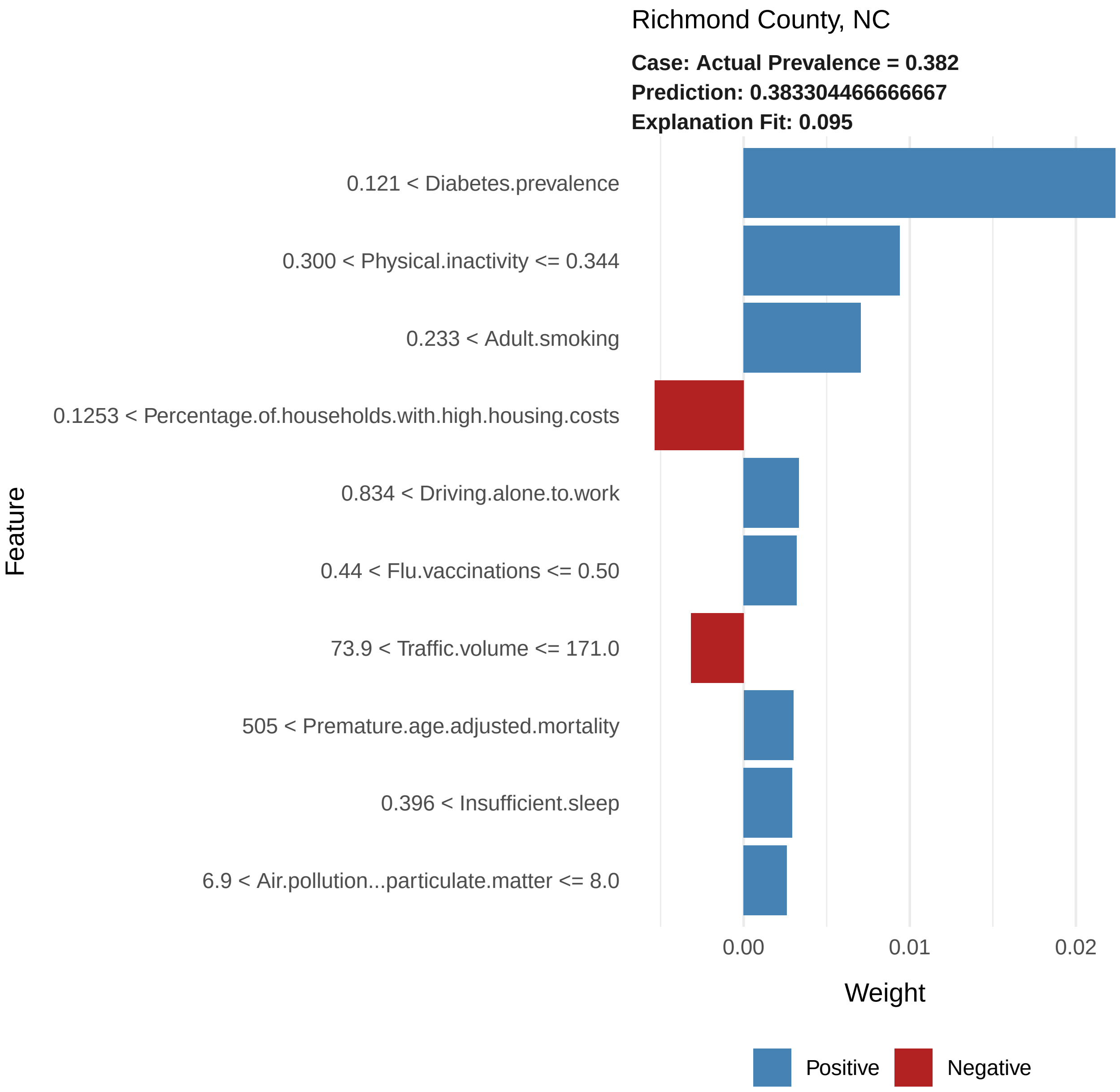

Richmond County, NC
Case: Actual Prevalence = 0.382
Prediction: 0.383304466666667
Explanation Fit: 0.095
0.121 < Diabetes.prevalence
0.300 < Physical.inactivity <= 0.344
0.233 < Adult.smoking
0.1253 < Percentage.of.households.with.high.housing.costs
0.834 < Driving.alone.to.work
0.44 < Flu.vaccinations <= 0.50
73.9 < Traffic.volume <= 171.0
505 < Premature.age.adjusted.mortality
0.396 < Insufficient.sleep
6.9 < Air.pollution...particulate.matter <= 8.0
Feature
0.00
0.01
0.02
Weight
Positive
Negative

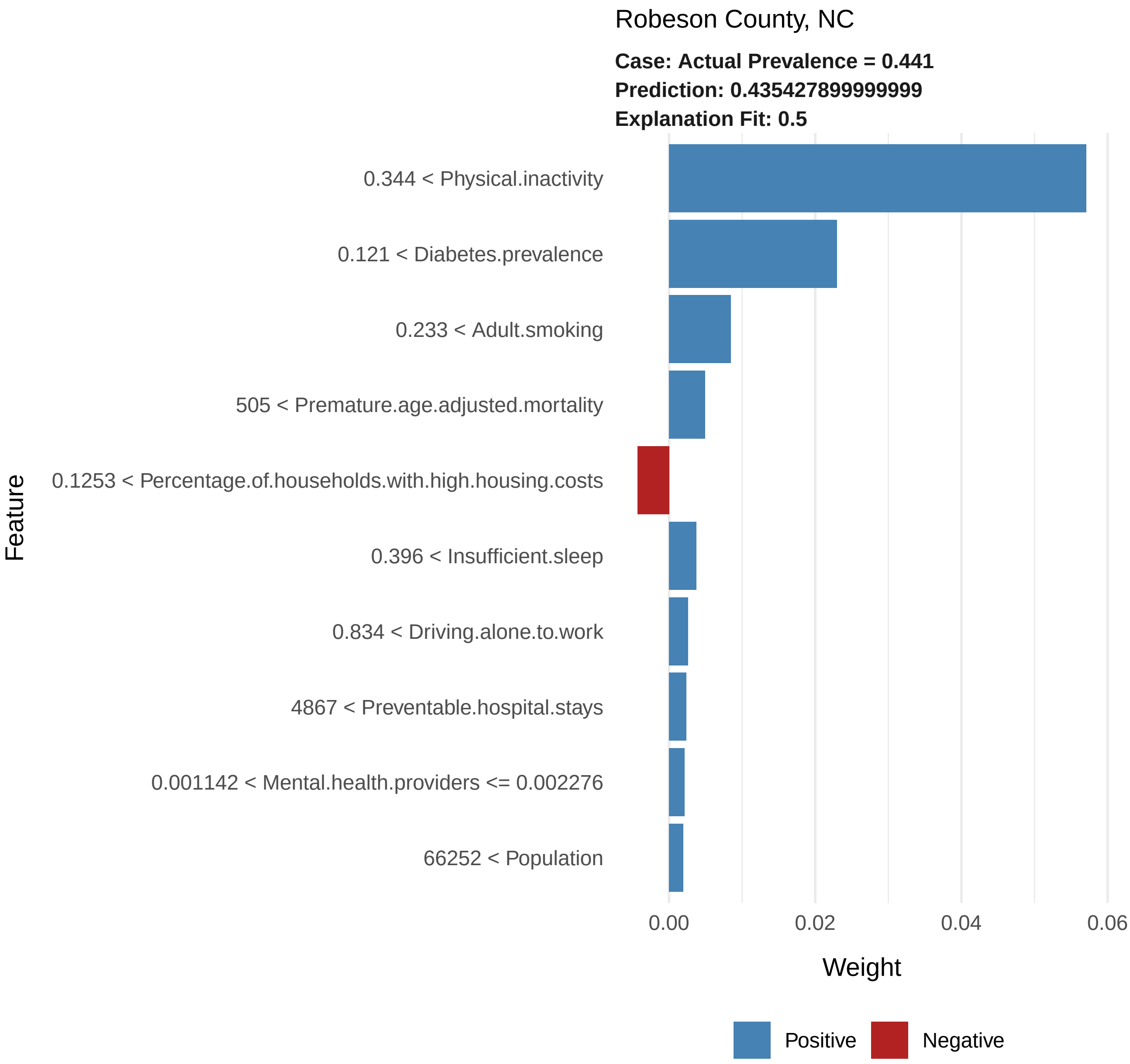
Robeson County, NC
Case: Actual Prevalence = 0.441
Prediction: 0.435427899999999
Explanation Fit: 0.5
0.344 < Physical.inactivity
0.121 < Diabetes.prevalence
0.233 < Adult.smoking
505 < Premature.age.adjusted.mortality
0.1253 < Percentage.of.households.with.high.housing.costs
0.396 < Insufficient.sleep
0.834 < Driving.alone.to.work
4867 < Preventable.hospital.stays
0.001142 < Mental.health.providers <= 0.002276
66252 < Population
Feature
0.00
0.02
0.04
0.06
Weight
Positive
Negative

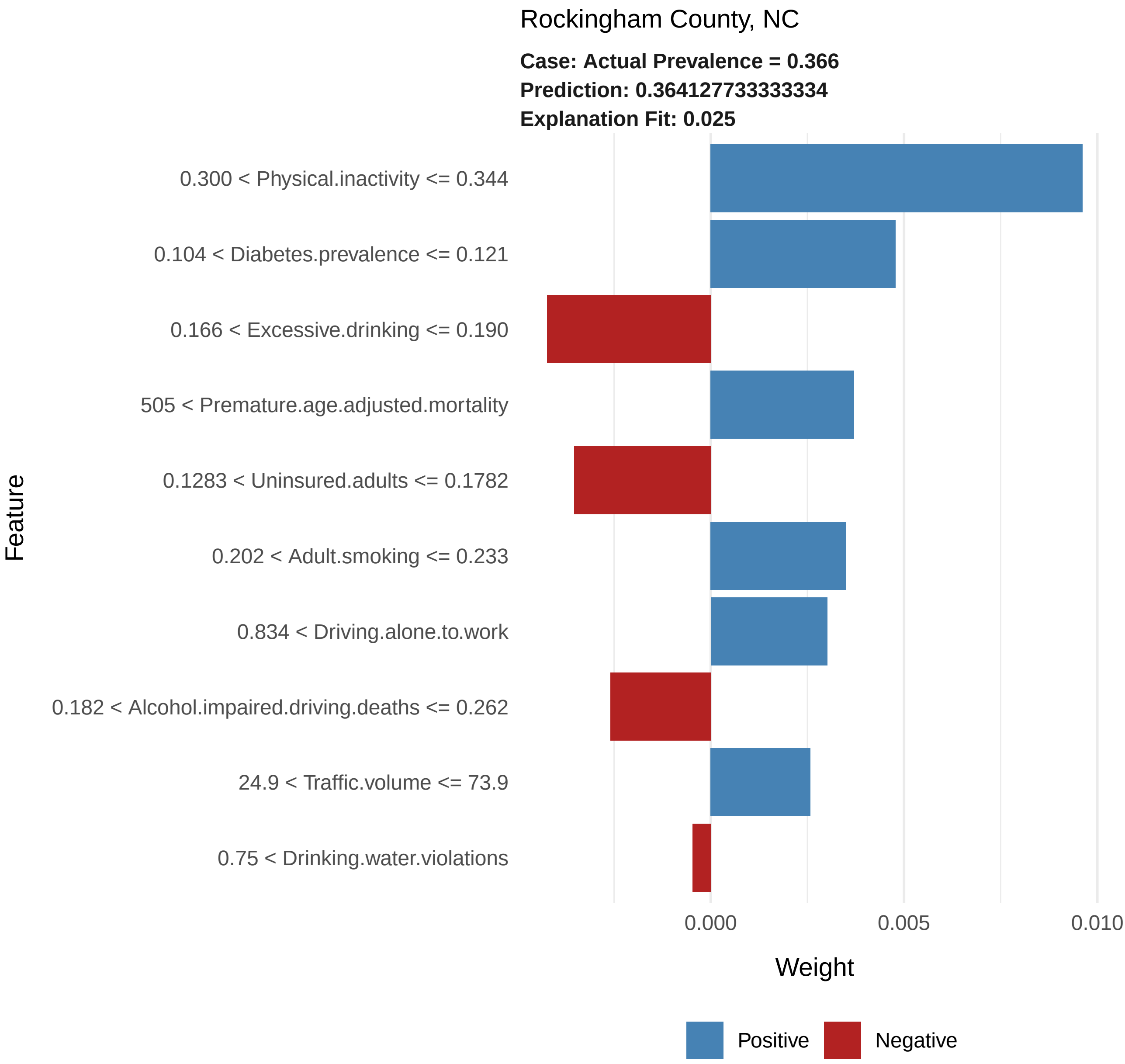

Rockingham County, NC
Case: Actual Prevalence = 0.366
Prediction: 0.364127733333334
Explanation Fit: 0.025
0.300 < Physical.inactivity <= 0.344
0.104 < Diabetes.prevalence <= 0.121
0.166 < Excessive.drinking <= 0.190
505 < Premature.age.adjusted.mortality
0.1283 < Uninsured.adults <= 0.1782
0.202 < Adult.smoking <= 0.233
0.834 < Driving.alone.to.work
0.182 < Alcohol.impaired.driving.deaths <= 0.262
24.9 < Traffic.volume <= 73.9
0.75 < Drinking.water.violations
Feature
0.000
0.005
0.010
Weight
Positive
Negative

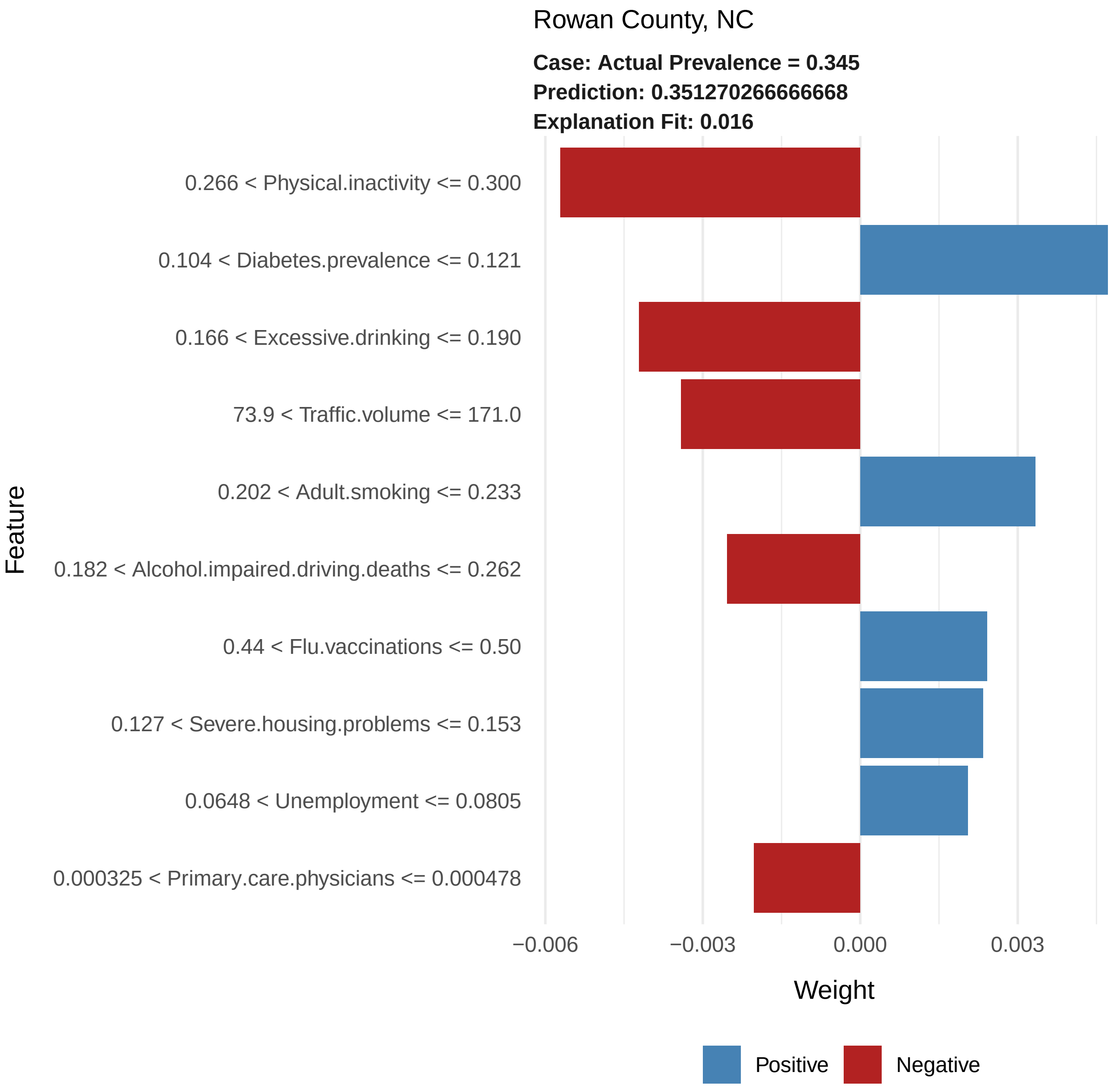
Rowan County, NC
Case: Actual Prevalence = 0.345
Prediction: 0.351270266666668
Explanation Fit: 0.016
0.266 < Physical.inactivity <= 0.300
0.104 < Diabetes.prevalence <= 0.121
0.166 < Excessive.drinking <= 0.190
73.9 < Traffic.volume <= 171.0
0.202 < Adult.smoking <= 0.233
0.182 < Alcohol.impaired.driving.deaths <= 0.262
0.44 < Flu.vaccinations <= 0.50
0.127 < Severe.housing.problems <= 0.153
0.0648 < Unemployment <= 0.0805
0.000325 < Primary.care.physicians <= 0.000478
Feature
−0.006
−0.003
0.000
0.003
Weight
Positive
Negative

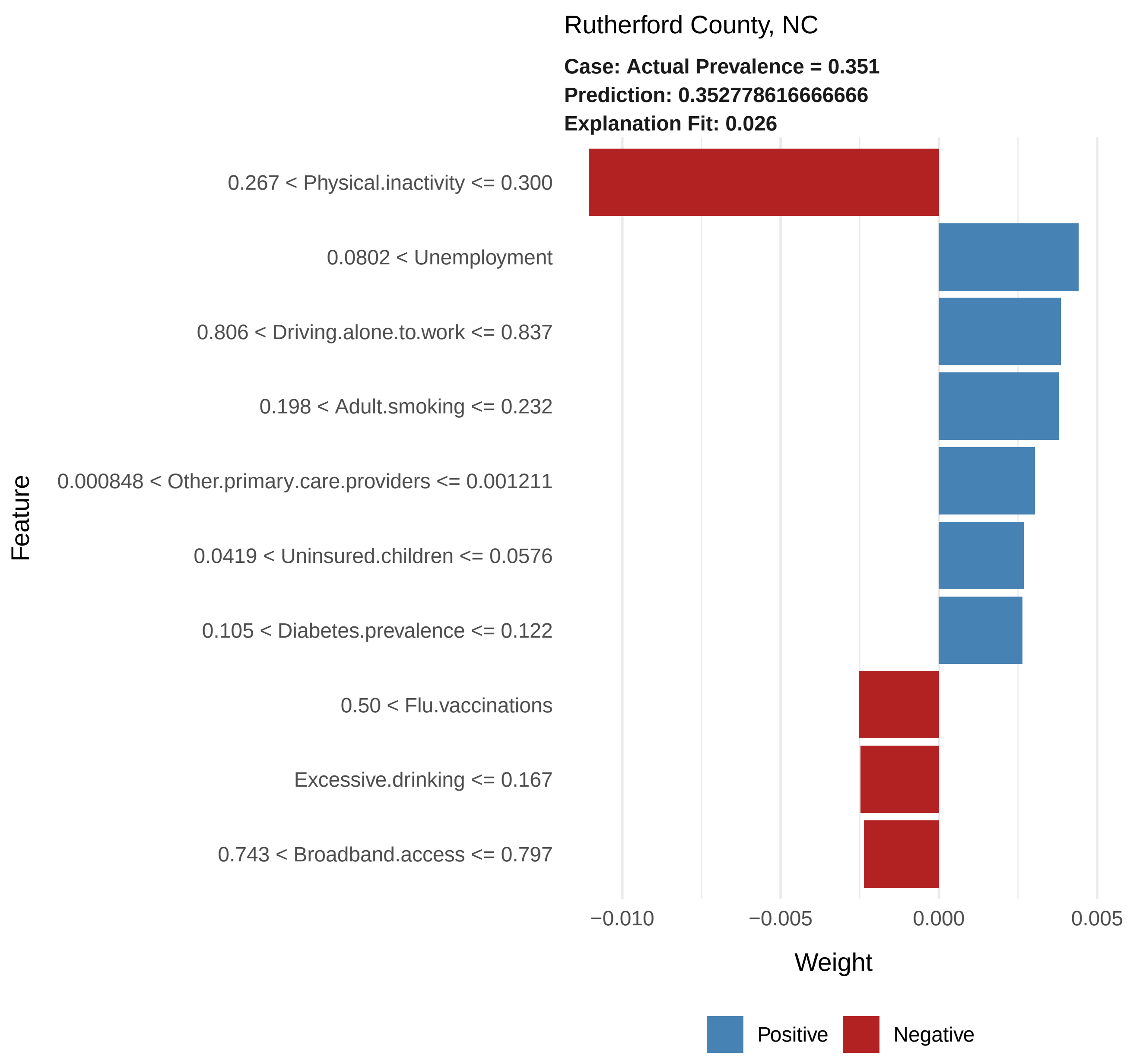
Rutherford County, NC
Case: Actual Prevalence = 0.351
Prediction: 0.352778616666666
Explanation Fit: 0.026
0.267 < Physical.inactivity <= 0.300
0.0802 < Unemployment
0.806 < Driving.alone.to.work <= 0.837
0.198 < Adult.smoking <= 0.232
0.000848 < Other.primary.care.providers <= 0.001211
0.0419 < Uninsured.children <= 0.0576
0.105 < Diabetes.prevalence <= 0.122
0.50 < Flu.vaccinations
Excessive.drinking <= 0.167
0.743 < Broadband.access <= 0.797
Feature
−0.010
−0.005
0.000
0.005
Weight
Positive
Negative

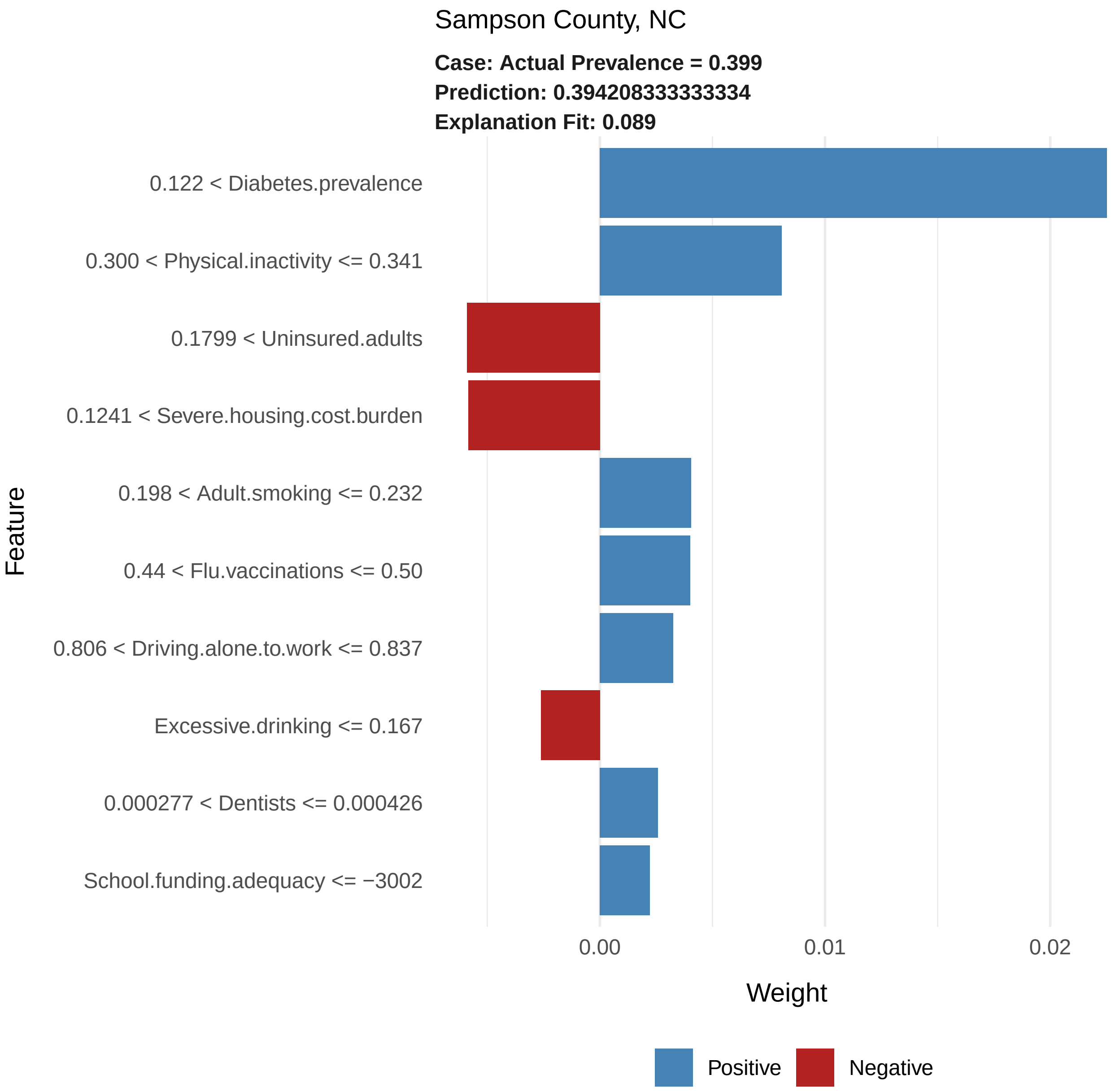

Sampson County, NC
Case: Actual Prevalence = 0.399
Prediction: 0.394208333333334
Explanation Fit: 0.089
0.122 < Diabetes.prevalence
0.300 < Physical.inactivity <= 0.341
0.1799 < Uninsured.adults
0.1241 < Severe.housing.cost.burden
0.198 < Adult.smoking <= 0.232
0.44 < Flu.vaccinations <= 0.50
0.806 < Driving.alone.to.work <= 0.837
Excessive.drinking <= 0.167
0.000277 < Dentists <= 0.000426
School.funding.adequacy <= −3002
Feature
0.00
0.01
0.02
Weight
Positive
Negative

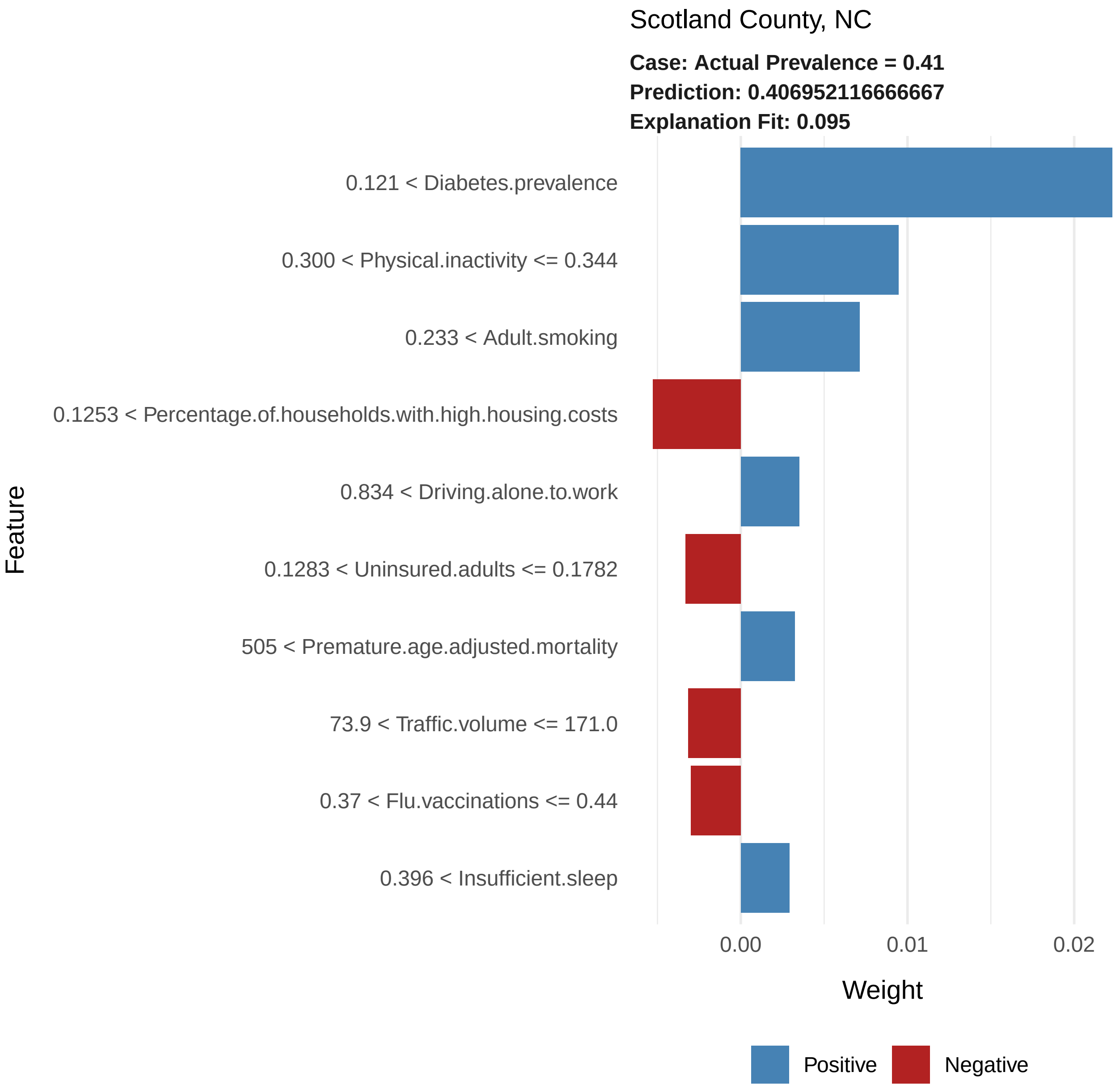

Scotland County, NC
Case: Actual Prevalence = 0.41
Prediction: 0.406952116666667
Explanation Fit: 0.095
0.121 < Diabetes.prevalence
0.300 < Physical.inactivity <= 0.344
0.233 < Adult.smoking
0.1253 < Percentage.of.households.with.high.housing.costs
0.834 < Driving.alone.to.work
0.1283 < Uninsured.adults <= 0.1782
505 < Premature.age.adjusted.mortality
73.9 < Traffic.volume <= 171.0
0.37 < Flu.vaccinations <= 0.44
0.396 < Insufficient.sleep
Feature
0.00
0.01
0.02
Weight
Positive
Negative

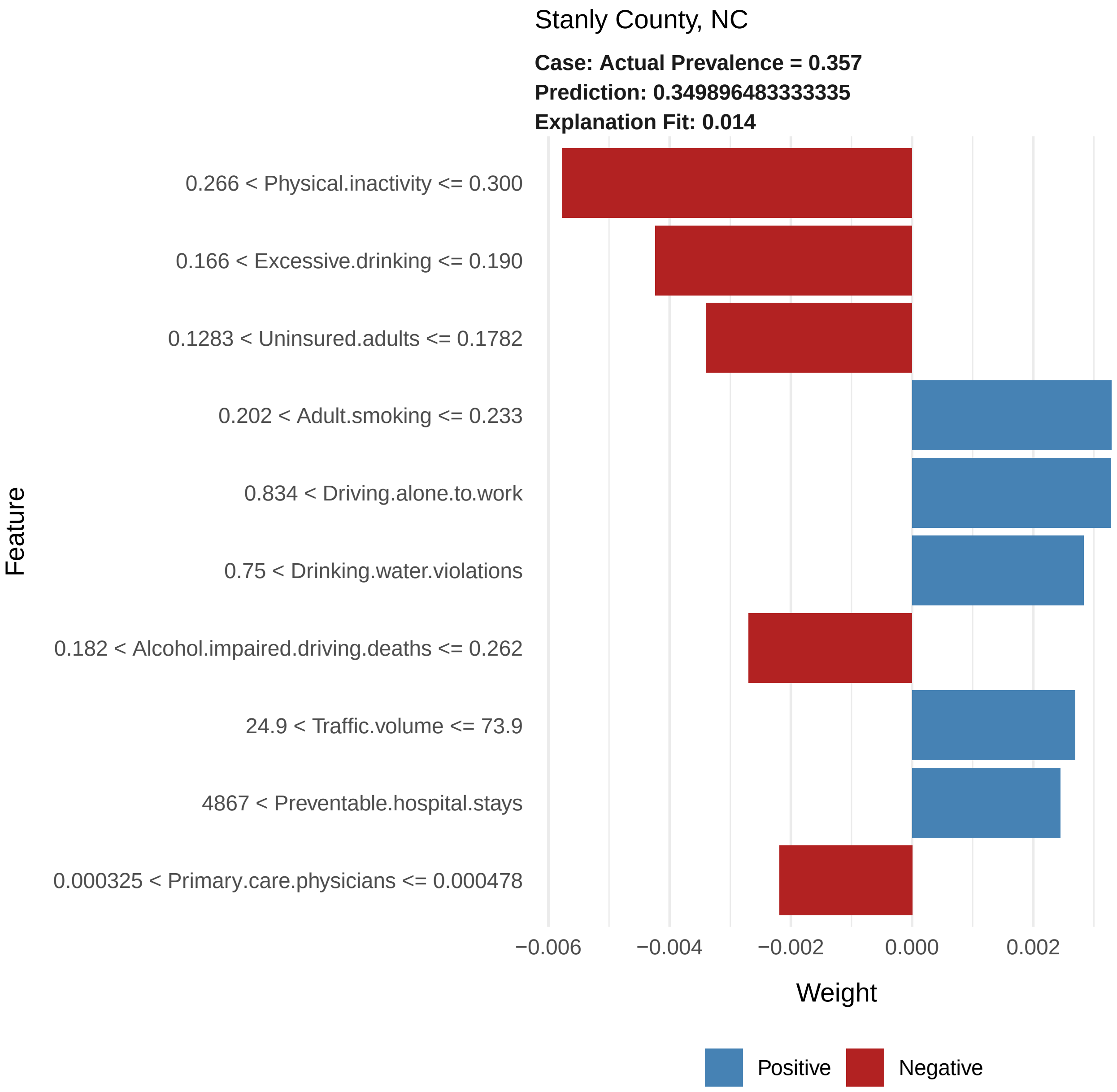
Stanly County, NC
Case: Actual Prevalence = 0.357
Prediction: 0.349896483333335
Explanation Fit: 0.014
0.266 < Physical.inactivity <= 0.300
0.166 < Excessive.drinking <= 0.190
0.1283 < Uninsured.adults <= 0.1782
0.202 < Adult.smoking <= 0.233
0.834 < Driving.alone.to.work
0.75 < Drinking.water.violations
0.182 < Alcohol.impaired.driving.deaths <= 0.262
24.9 < Traffic.volume <= 73.9
4867 < Preventable.hospital.stays
0.000325 < Primary.care.physicians <= 0.000478
Feature
−0.006
−0.004
−0.002
0.000
0.002
Weight
Positive
Negative

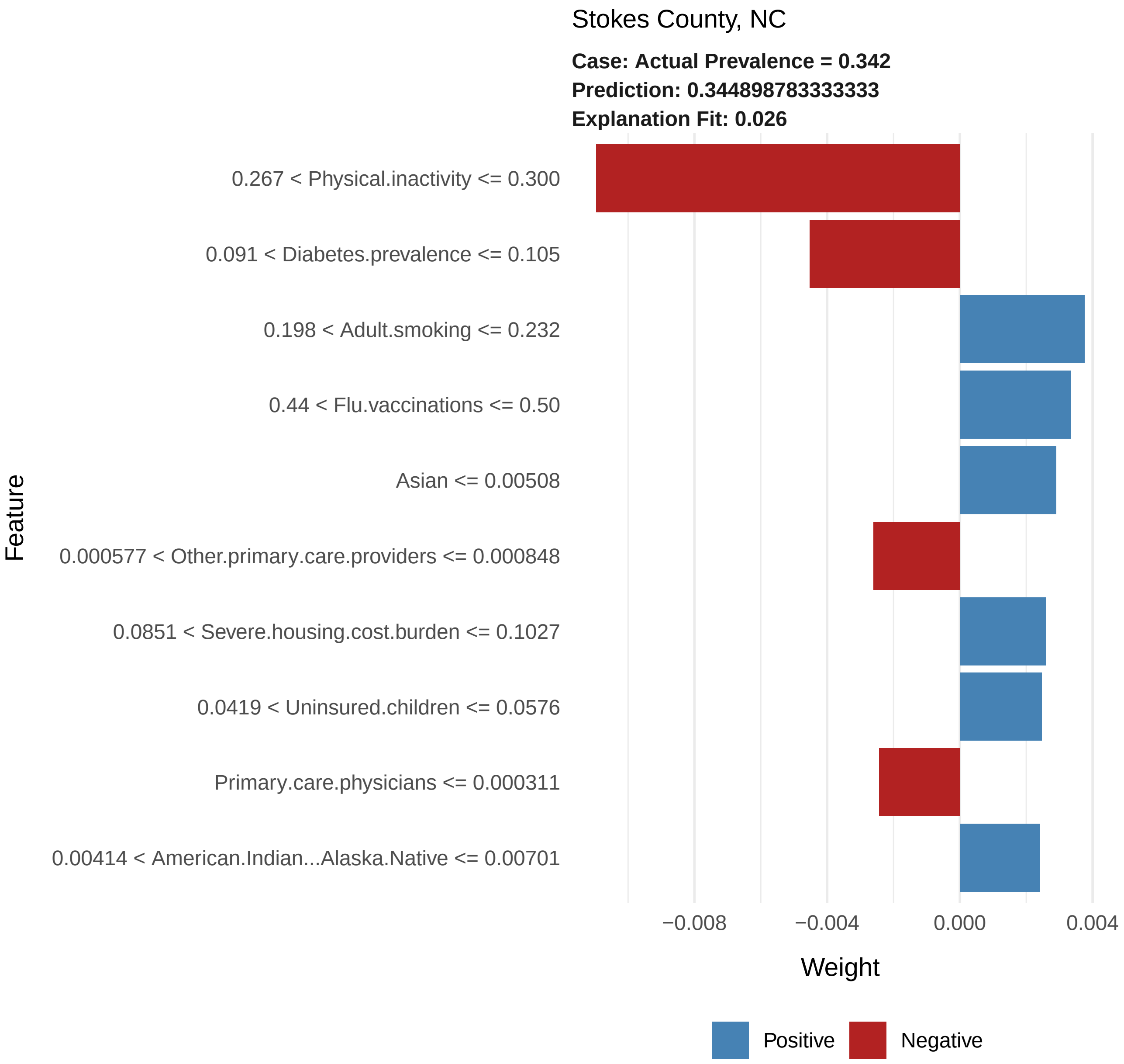

Stokes County, NC
Case: Actual Prevalence = 0.342
Prediction: 0.344898783333333
Explanation Fit: 0.026
0.267 < Physical.inactivity <= 0.300
0.091 < Diabetes.prevalence <= 0.105
0.198 < Adult.smoking <= 0.232
0.44 < Flu.vaccinations <= 0.50
Asian <= 0.00508
0.000577 < Other.primary.care.providers <= 0.000848
0.0851 < Severe.housing.cost.burden <= 0.1027
0.0419 < Uninsured.children <= 0.0576
Primary.care.physicians <= 0.000311
0.00414 < American.Indian...Alaska.Native <= 0.00701
Feature
−0.008
−0.004
0.000
0.004
Weight
Positive
Negative

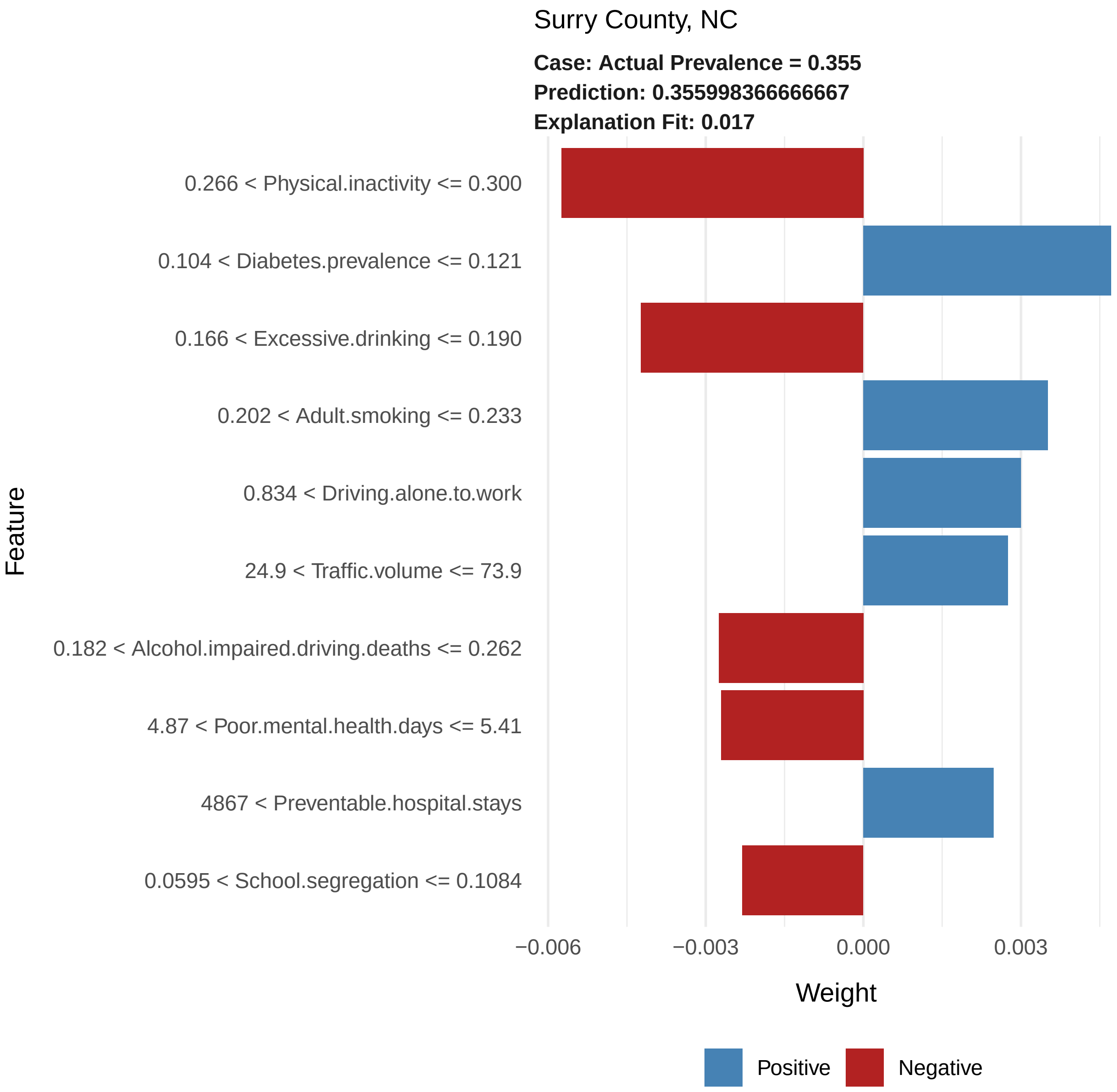

Surry County, NC
Case: Actual Prevalence = 0.355
Prediction: 0.355998366666667
Explanation Fit: 0.017
0.266 < Physical.inactivity <= 0.300
0.104 < Diabetes.prevalence <= 0.121
0.166 < Excessive.drinking <= 0.190
0.202 < Adult.smoking <= 0.233
0.834 < Driving.alone.to.work
24.9 < Traffic.volume <= 73.9
0.182 < Alcohol.impaired.driving.deaths <= 0.262
4.87 < Poor.mental.health.days <= 5.41
4867 < Preventable.hospital.stays
0.0595 < School.segregation <= 0.1084
Feature
−0.006
−0.003
0.000
0.003
Weight
Positive
Negative

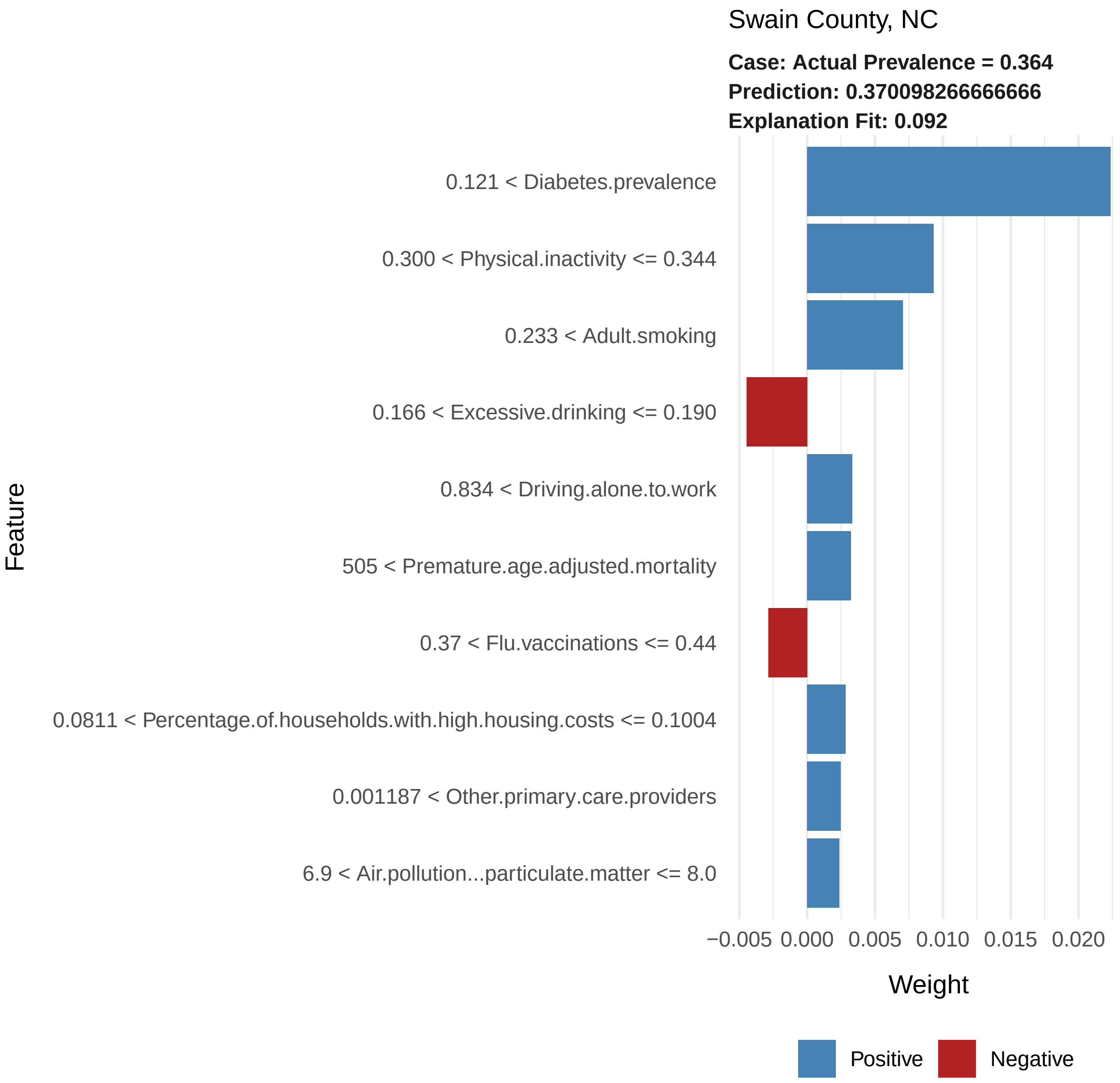

Swain County, NC
Case: Actual Prevalence = 0.364
Prediction: 0.370098266666666
Explanation Fit: 0.092
0.121 < Diabetes.prevalence
0.300 < Physical.inactivity <= 0.344
0.233 < Adult.smoking
0.166 < Excessive.drinking <= 0.190
0.834 < Driving.alone.to.work
505 < Premature.age.adjusted.mortality
0.37 < Flu.vaccinations <= 0.44
0.0811 < Percentage.of.households.with.high.housing.costs <= 0.1004
0.001187 < Other.primary.care.providers
6.9 < Air.pollution...particulate.matter <= 8.0
Feature
−0.005
0.000
0.005
0.010
0.015
0.020
Weight
Positive
Negative

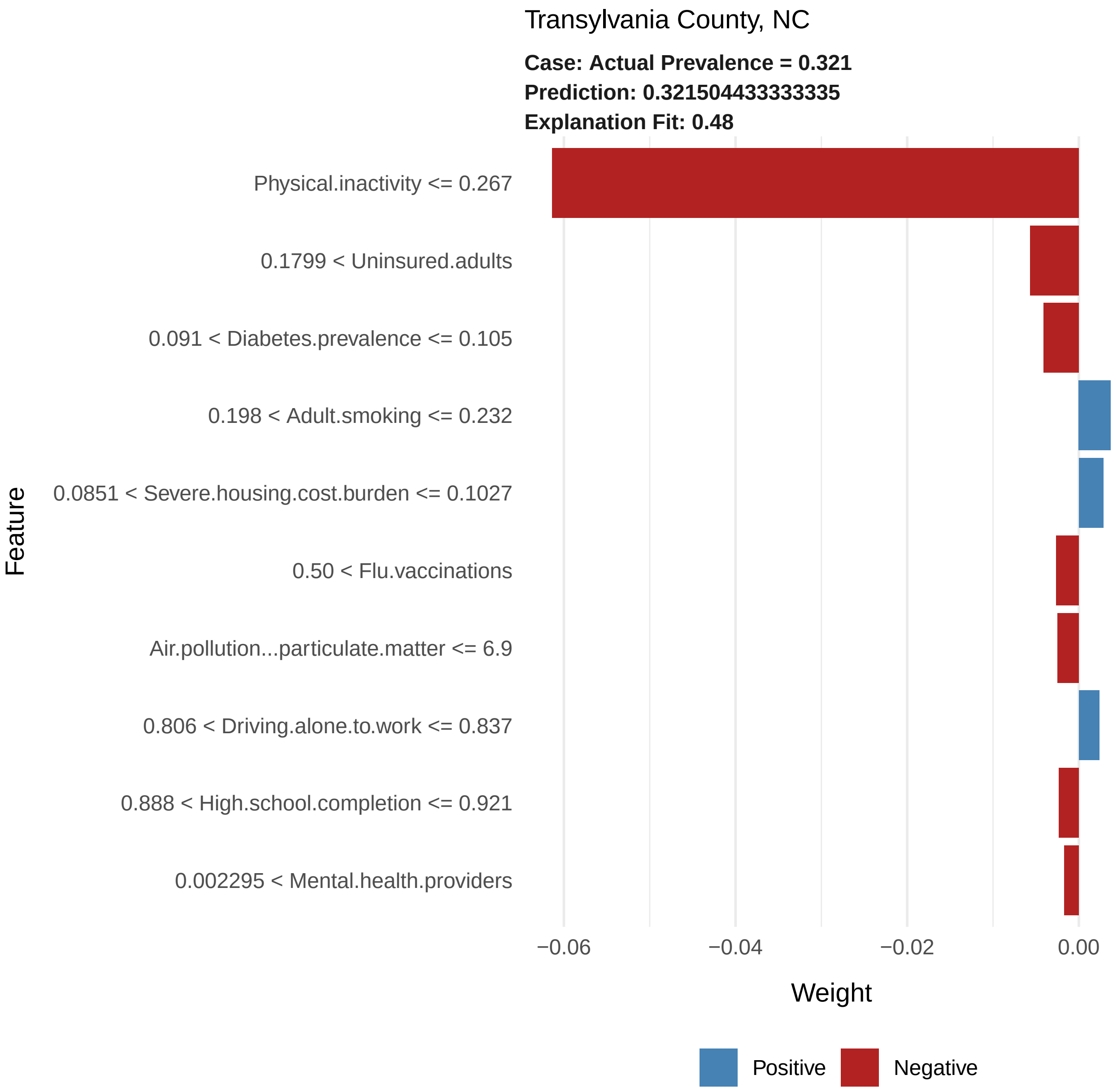

Transylvania County, NC
Case: Actual Prevalence = 0.321
Prediction: 0.321504433333335
Explanation Fit: 0.48
Physical.inactivity <= 0.267
0.1799 < Uninsured.adults
0.091 < Diabetes.prevalence <= 0.105
0.198 < Adult.smoking <= 0.232
0.0851 < Severe.housing.cost.burden <= 0.1027
0.50 < Flu.vaccinations
Air.pollution...particulate.matter <= 6.9
0.806 < Driving.alone.to.work <= 0.837
0.888 < High.school.completion <= 0.921
0.002295 < Mental.health.providers
Feature
−0.06
−0.04
−0.02
0.00
Weight
Positive
Negative

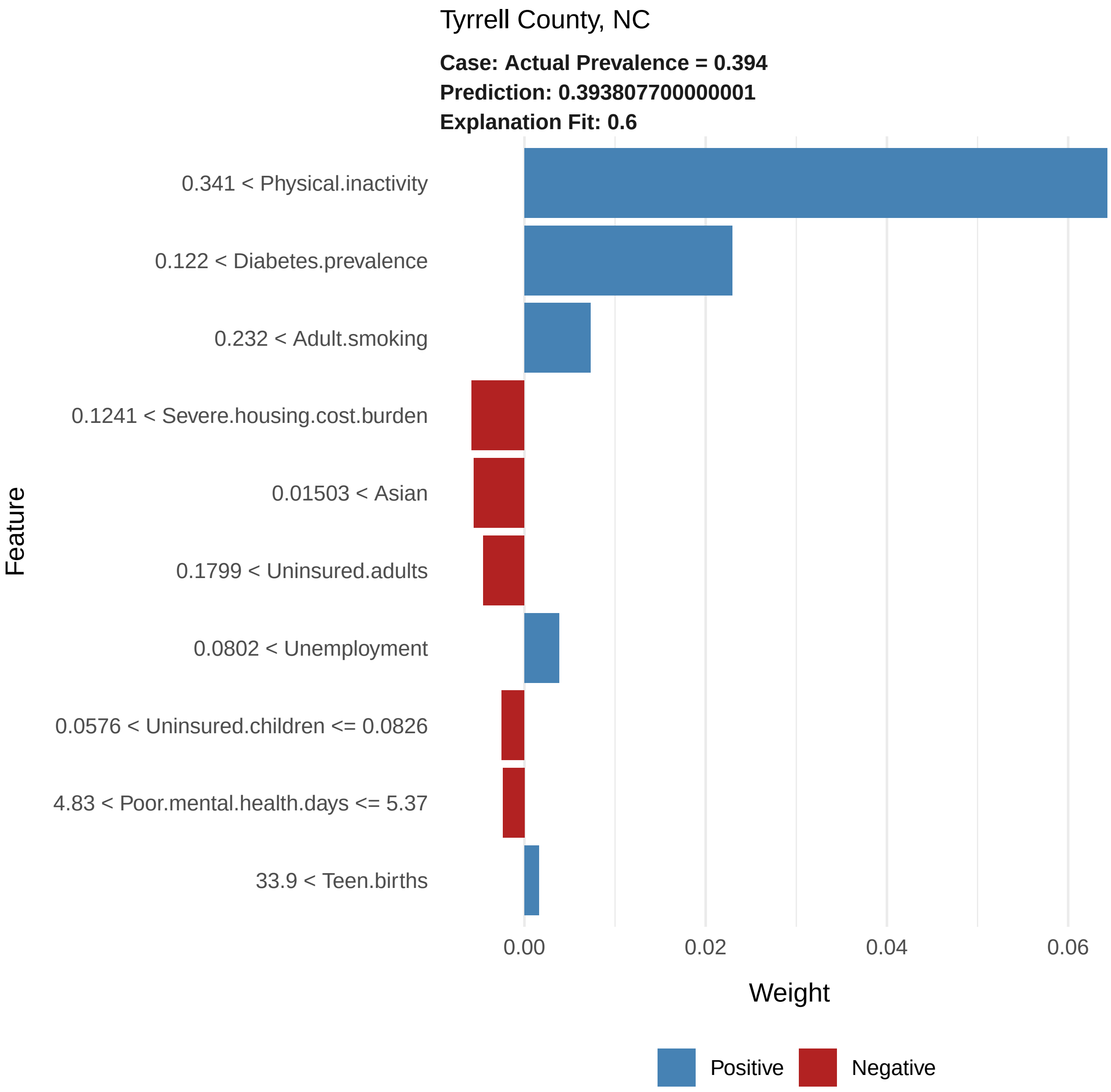

Tyrrell County, NC
Case: Actual Prevalence = 0.394
Prediction: 0.393807700000001
Explanation Fit: 0.6
0.341 < Physical.inactivity
0.122 < Diabetes.prevalence
0.232 < Adult.smoking
0.1241 < Severe.housing.cost.burden
0.01503 < Asian
0.1799 < Uninsured.adults
0.0802 < Unemployment
0.0576 < Uninsured.children <= 0.0826
4.83 < Poor.mental.health.days <= 5.37
33.9 < Teen.births
Feature
0.00
0.02
0.04
0.06
Weight
Positive
Negative

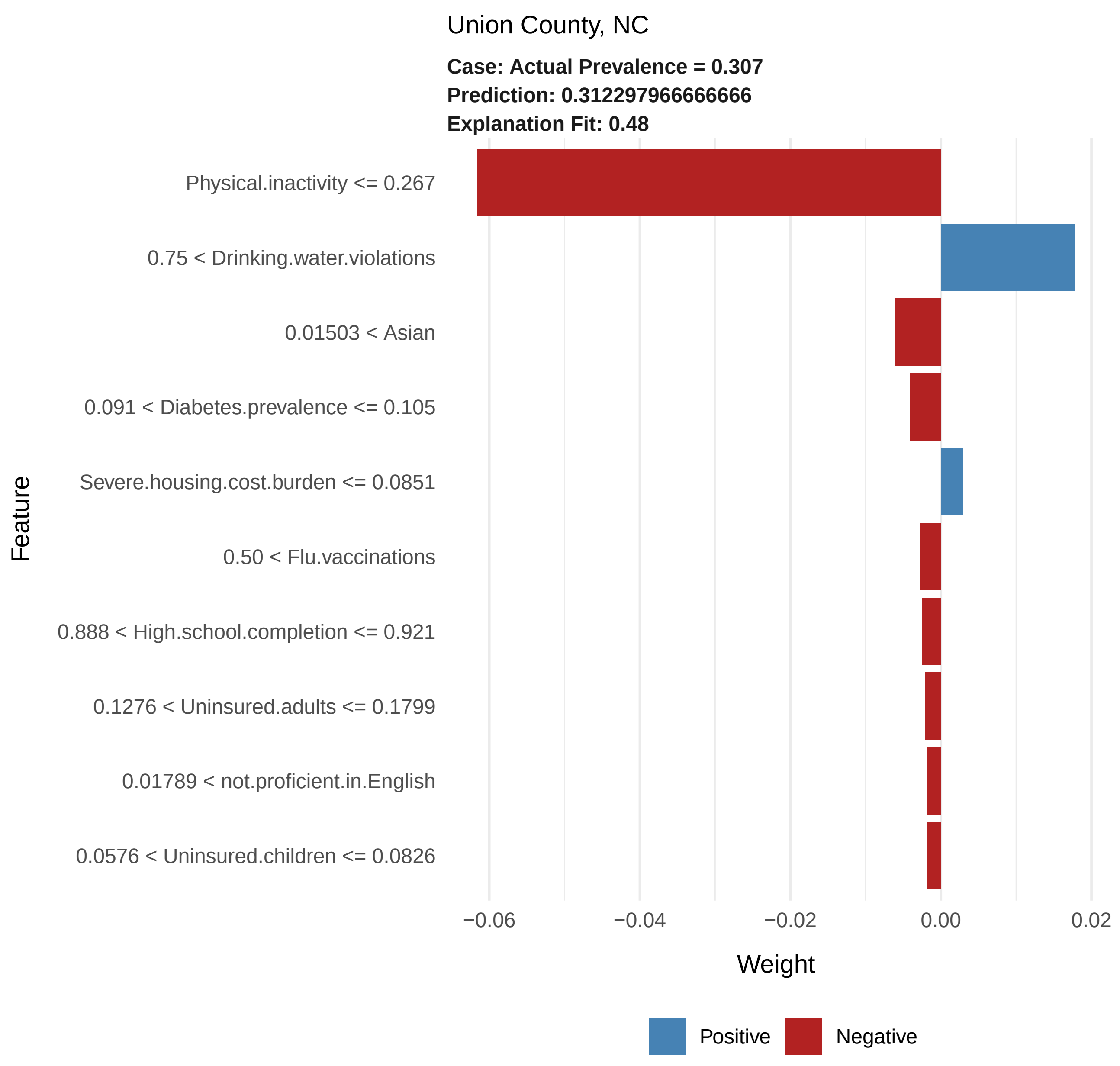

Union County, NC
Case: Actual Prevalence = 0.307
Prediction: 0.312297966666666
Explanation Fit: 0.48
Physical.inactivity <= 0.267
0.75 < Drinking.water.violations
0.01503 < Asian
0.091 < Diabetes.prevalence <= 0.105
Severe.housing.cost.burden <= 0.0851
0.50 < Flu.vaccinations
0.888 < High.school.completion <= 0.921
0.1276 < Uninsured.adults <= 0.1799
0.01789 < not.proficient.in.English
0.0576 < Uninsured.children <= 0.0826
Feature
−0.06
−0.04
−0.02
0.00
0.02
Weight
Positive
Negative

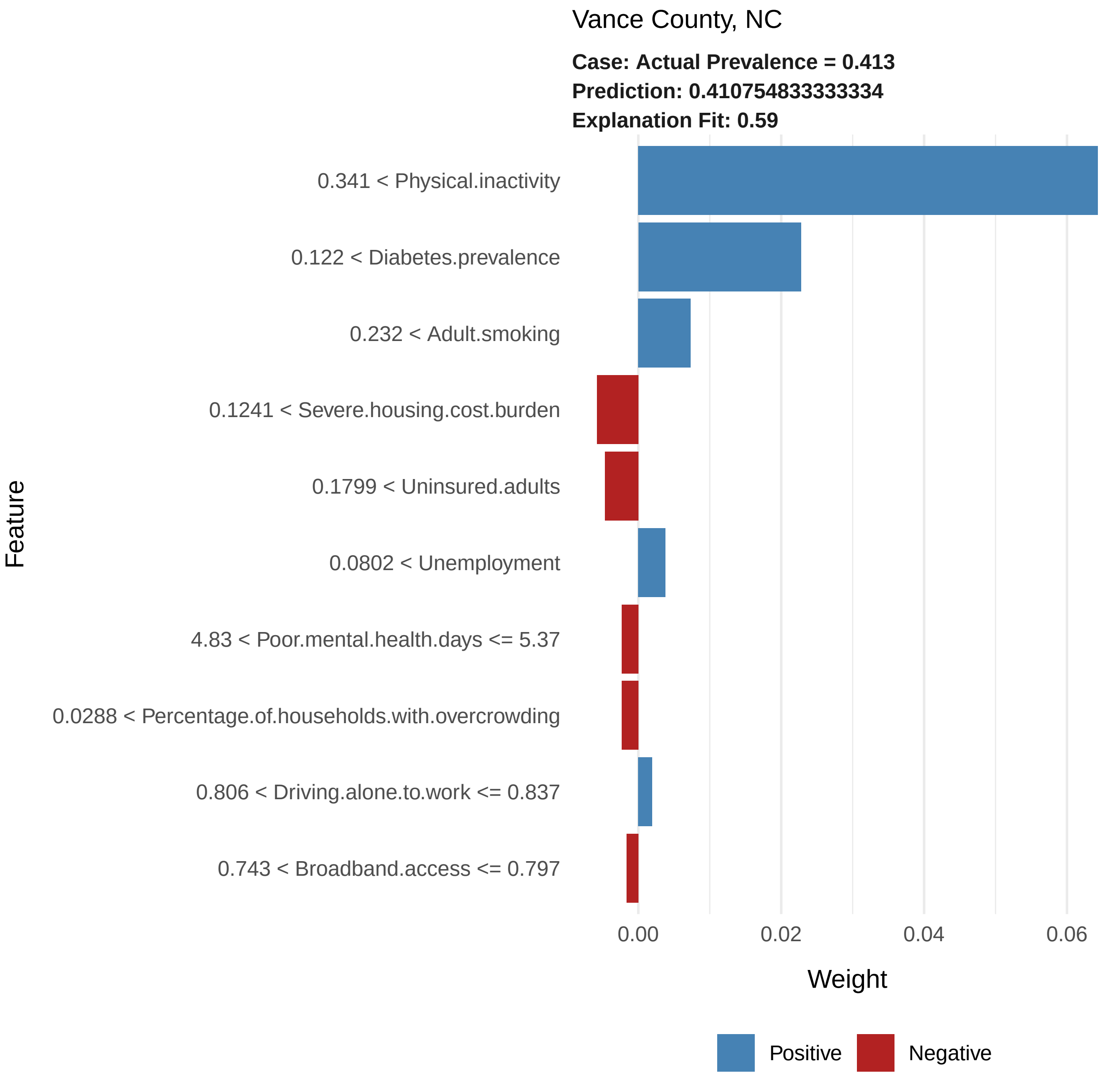

Vance County, NC
Case: Actual Prevalence = 0.413
Prediction: 0.410754833333334
Explanation Fit: 0.59
0.341 < Physical.inactivity
0.122 < Diabetes.prevalence
0.232 < Adult.smoking
0.1241 < Severe.housing.cost.burden
0.1799 < Uninsured.adults
0.0802 < Unemployment
4.83 < Poor.mental.health.days <= 5.37
0.0288 < Percentage.of.households.with.overcrowding
0.806 < Driving.alone.to.work <= 0.837
0.743 < Broadband.access <= 0.797
Feature
0.00
0.02
0.04
0.06
Weight
Positive
Negative

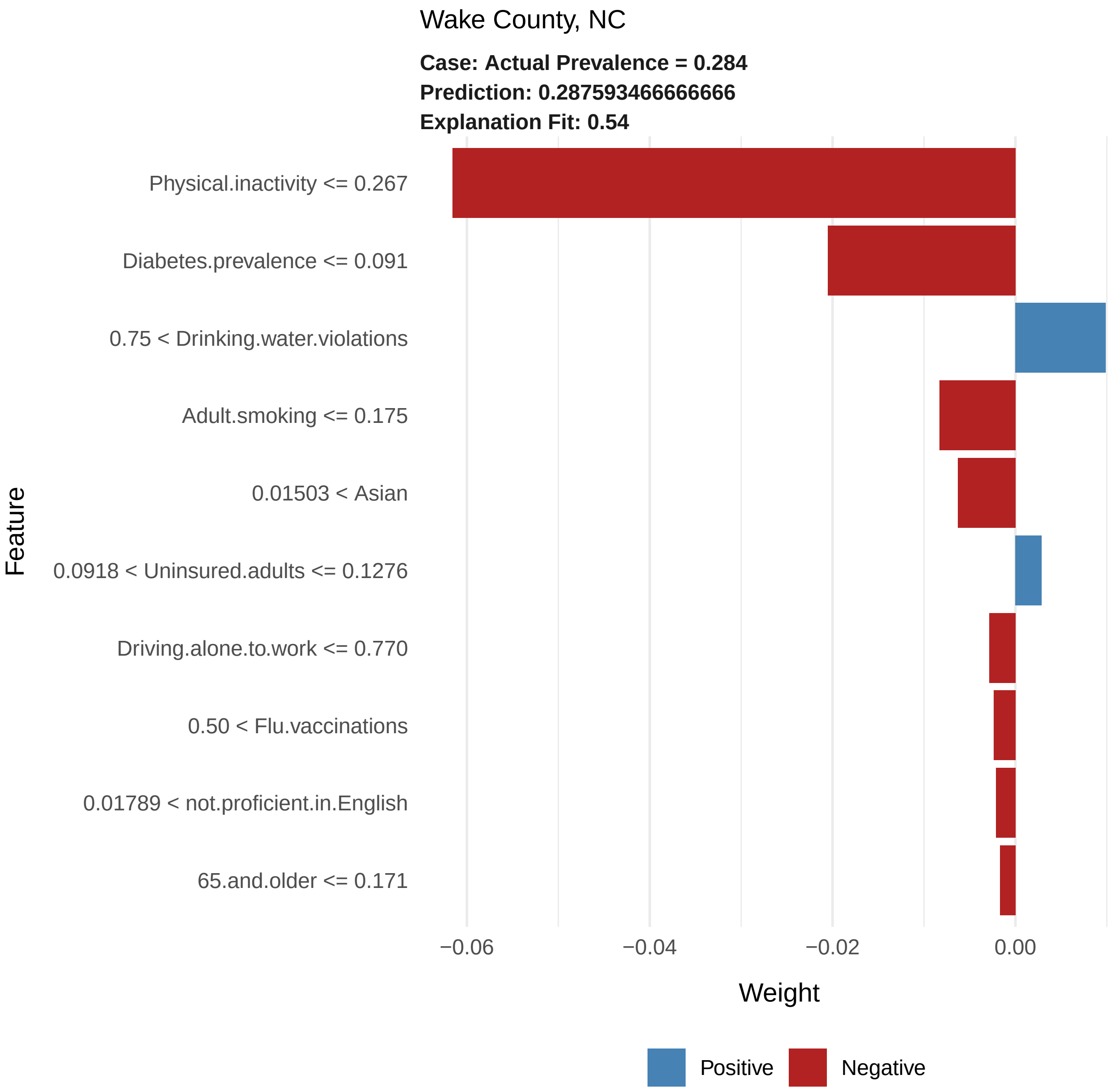

Wake County, NC
Case: Actual Prevalence = 0.284
Prediction: 0.287593466666666
Explanation Fit: 0.54
Feature
Physical.inactivity <= 0.267
Diabetes.prevalence <= 0.091
0.75 < Drinking.water.violations
Adult.smoking <= 0.175
0.01503 < Asian
0.0918 < Uninsured.adults <= 0.1276
Driving.alone.to.work <= 0.770
0.50 < Flu.vaccinations
0.01789 < not.proficient.in.English
65.and.older <= 0.171
−0.06
−0.04
−0.02
0.00
Weight
Positive
Negative

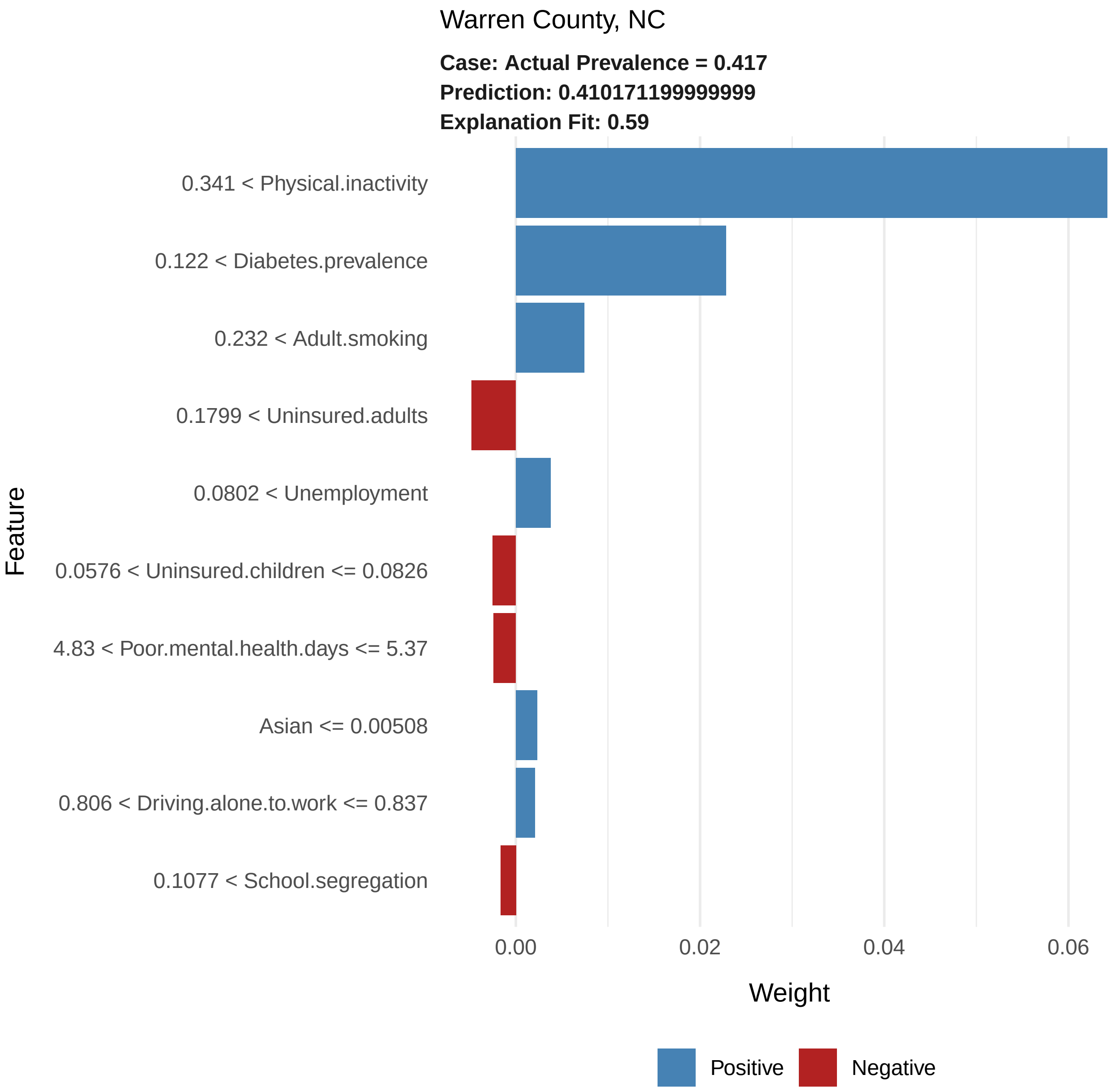

Warren County, NC
Case: Actual Prevalence = 0.417
Prediction: 0.410171199999999
Explanation Fit: 0.59
0.341 < Physical.inactivity
0.122 < Diabetes.prevalence
0.232 < Adult.smoking
0.1799 < Uninsured.adults
0.0802 < Unemployment
0.0576 < Uninsured.children <= 0.0826
4.83 < Poor.mental.health.days <= 5.37
Asian <= 0.00508
0.806 < Driving.alone.to.work <= 0.837
0.1077 < School.segregation
Feature
0.00
0.02
0.04
0.06
Weight
Positive
Negative

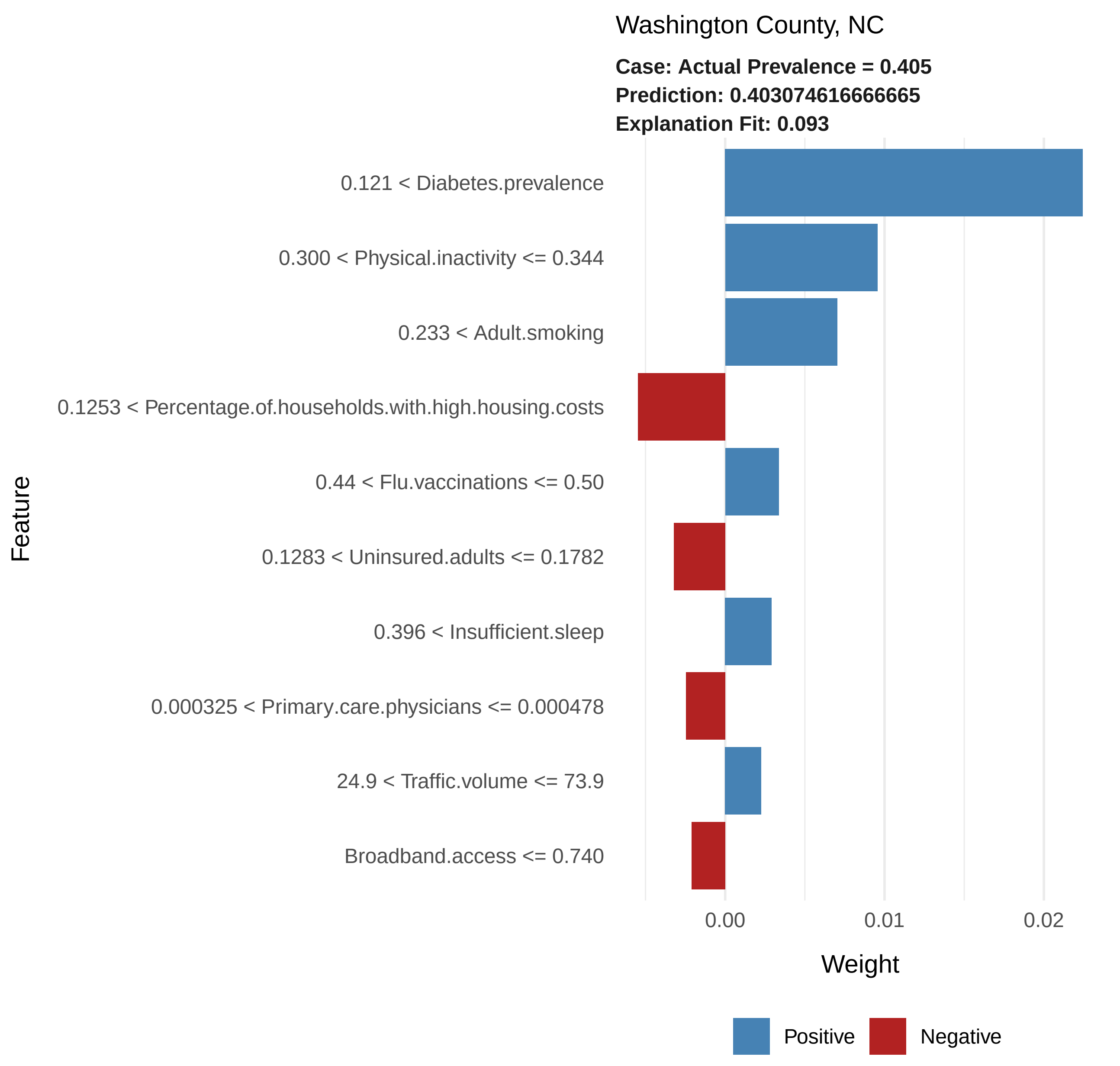
Washington County, NC
Case: Actual Prevalence = 0.405
Prediction: 0.403074616666665
Explanation Fit: 0.093
0.121 < Diabetes.prevalence
0.300 < Physical.inactivity <= 0.344
0.233 < Adult.smoking
0.1253 < Percentage.of.households.with.high.housing.costs
0.44 < Flu.vaccinations <= 0.50
0.1283 < Uninsured.adults <= 0.1782
0.396 < Insufficient.sleep
0.000325 < Primary.care.physicians <= 0.000478
24.9 < Traffic.volume <= 73.9
Broadband.access <= 0.740
Feature
0.00
0.01
0.02
Weight
Positive
Negative

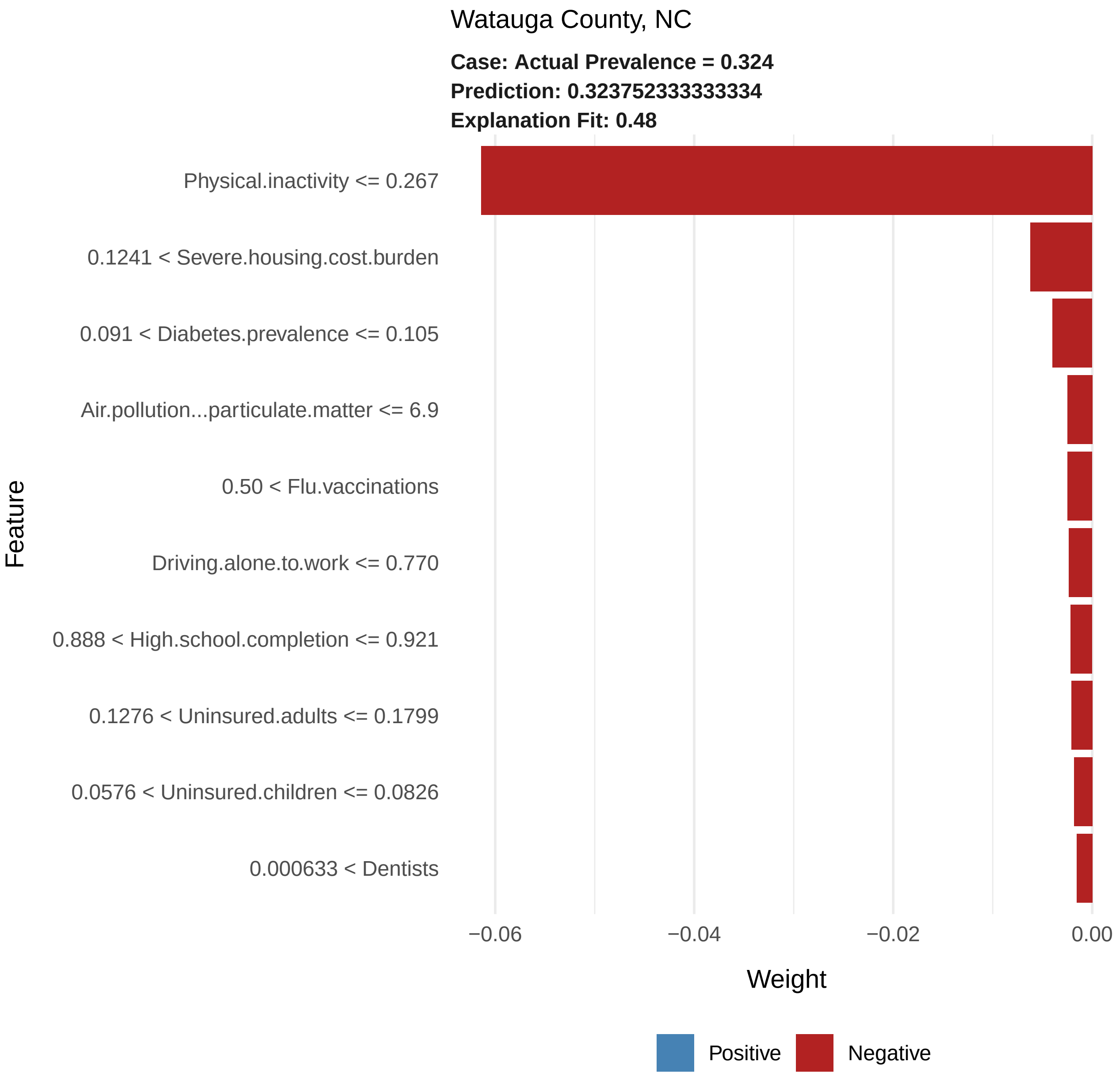
Watauga County, NC
Case: Actual Prevalence = 0.324
Prediction: 0.323752333333334
Explanation Fit: 0.48
Physical.inactivity <= 0.267
0.1241 < Severe.housing.cost.burden
0.091 < Diabetes.prevalence <= 0.105
Air.pollution...particulate.matter <= 6.9
0.50 < Flu.vaccinations
Driving.alone.to.work <= 0.770
0.888 < High.school.completion <= 0.921
0.1276 < Uninsured.adults <= 0.1799
0.0576 < Uninsured.children <= 0.0826
0.000633 < Dentists
Feature
−0.06
−0.04
−0.02
0.00
Weight
Positive
Negative

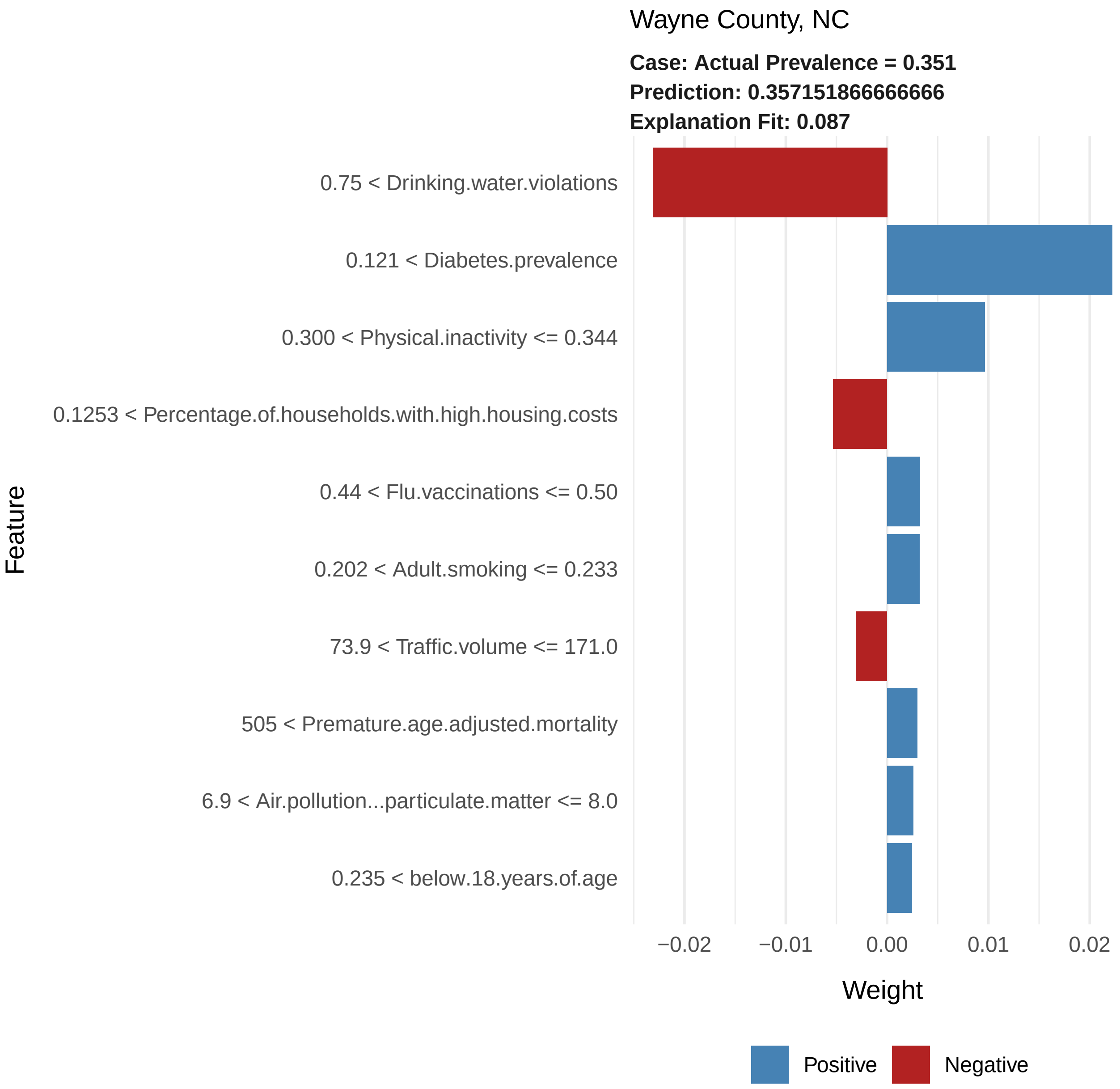
Wayne County, NC
Case: Actual Prevalence = 0.351
Prediction: 0.357151866666666
Explanation Fit: 0.087
0.75 < Drinking.water.violations
0.121 < Diabetes.prevalence
0.300 < Physical.inactivity <= 0.344
0.1253 < Percentage.of.households.with.high.housing.costs
0.44 < Flu.vaccinations <= 0.50
0.202 < Adult.smoking <= 0.233
73.9 < Traffic.volume <= 171.0
505 < Premature.age.adjusted.mortality
6.9 < Air.pollution...particulate.matter <= 8.0
0.235 < below.18.years.of.age
Feature
−0.02
−0.01
0.00
0.01
0.02
Weight
Positive
Negative

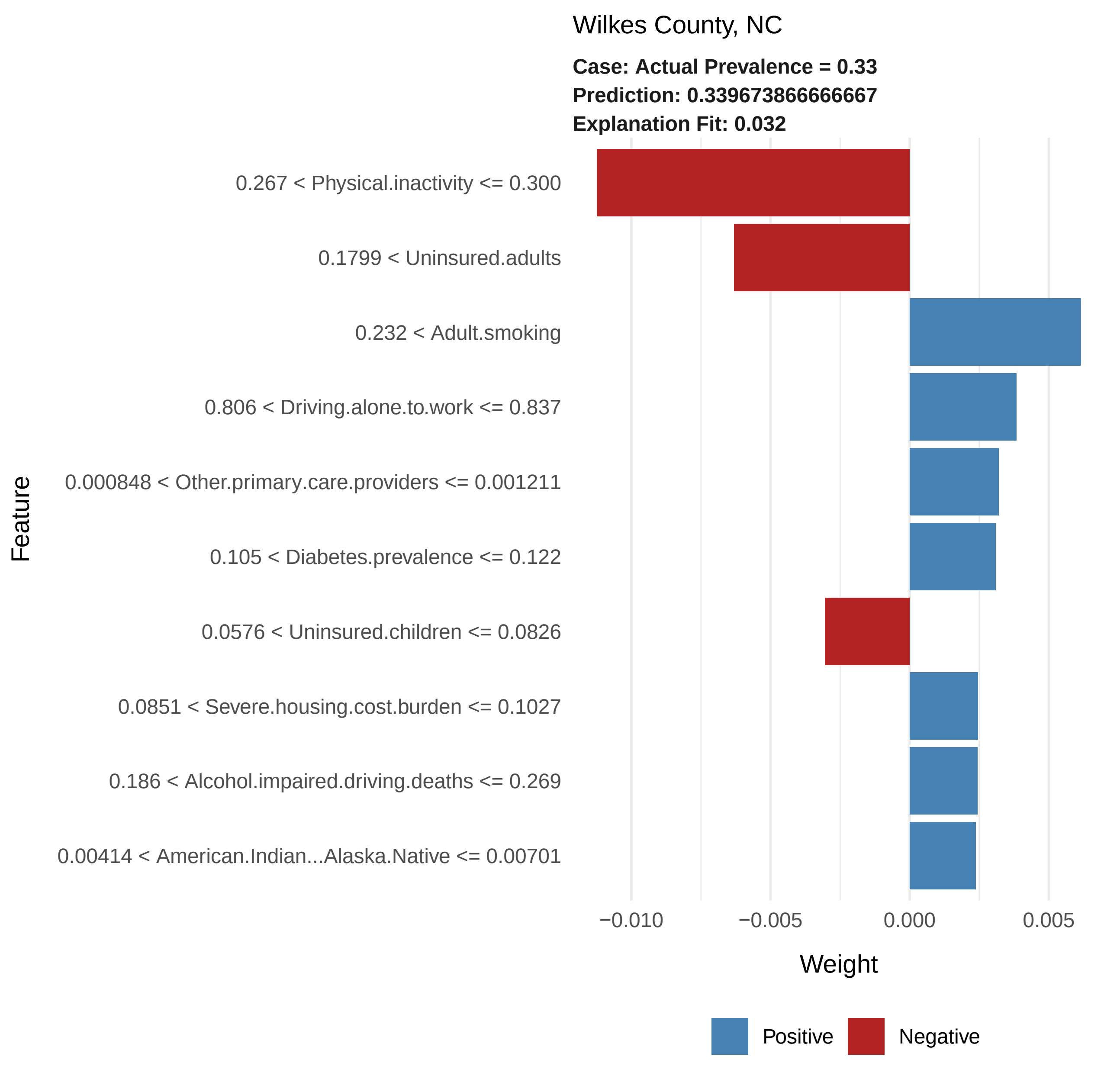
Wilkes County, NC
Case: Actual Prevalence = 0.33
Prediction: 0.339673866666667
Explanation Fit: 0.032
0.267 < Physical.inactivity <= 0.300
0.1799 < Uninsured.adults
0.232 < Adult.smoking
0.806 < Driving.alone.to.work <= 0.837
0.000848 < Other.primary.care.providers <= 0.001211
0.105 < Diabetes.prevalence <= 0.122
0.0576 < Uninsured.children <= 0.0826
0.0851 < Severe.housing.cost.burden <= 0.1027
0.186 < Alcohol.impaired.driving.deaths <= 0.269
0.00414 < American.Indian...Alaska.Native <= 0.00701
Feature
−0.010
−0.005
0.000
0.005
Weight
Positive
Negative

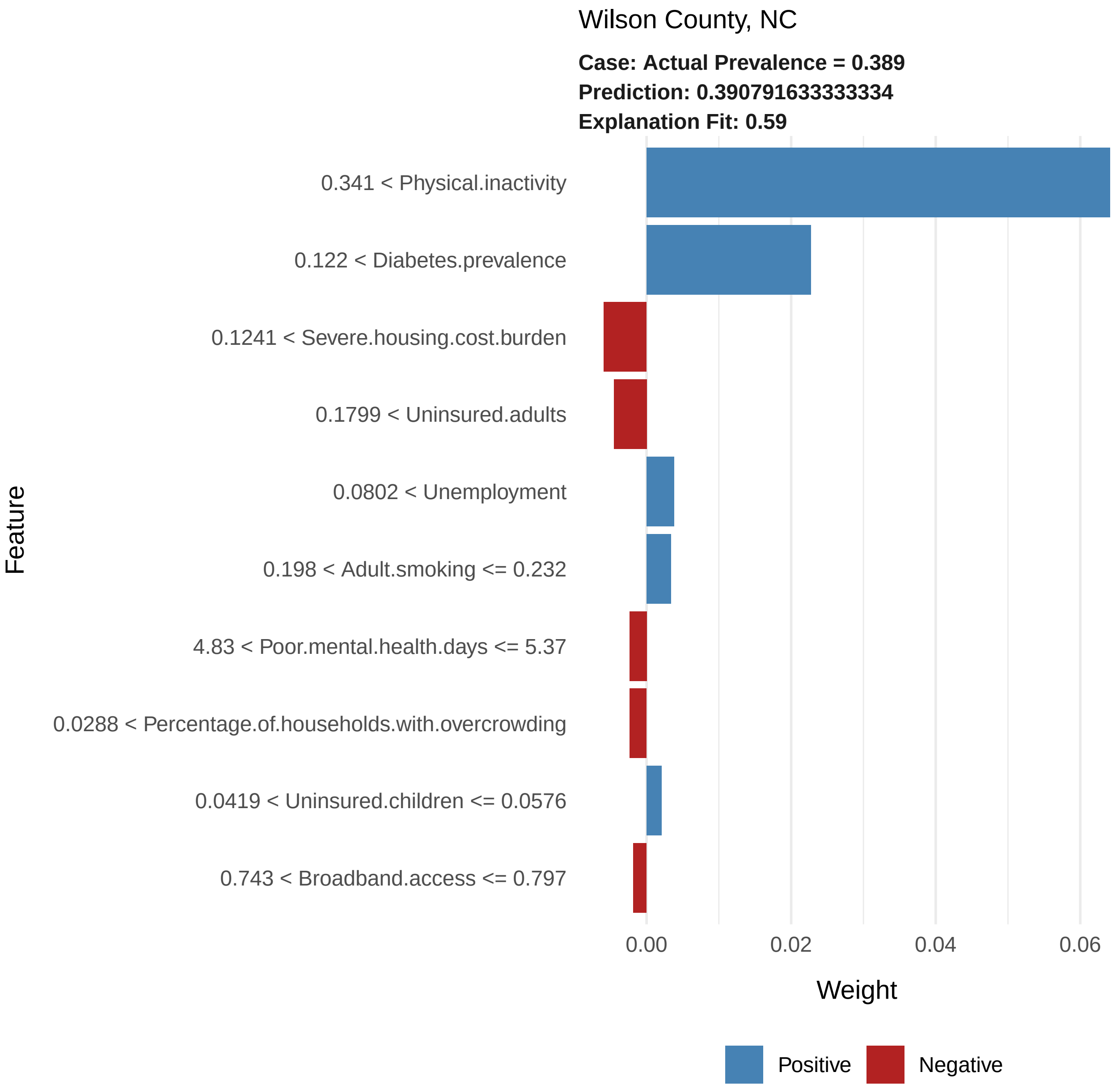
Wilson County, NC
Case: Actual Prevalence = 0.389
Prediction: 0.390791633333334
Explanation Fit: 0.59
0.341 < Physical.inactivity
0.122 < Diabetes.prevalence
0.1241 < Severe.housing.cost.burden
0.1799 < Uninsured.adults
0.0802 < Unemployment
0.198 < Adult.smoking <= 0.232
4.83 < Poor.mental.health.days <= 5.37
0.0288 < Percentage.of.households.with.overcrowding
0.0419 < Uninsured.children <= 0.0576
0.743 < Broadband.access <= 0.797
Feature
0.00
0.02
0.04
0.06
Weight
Positive
Negative

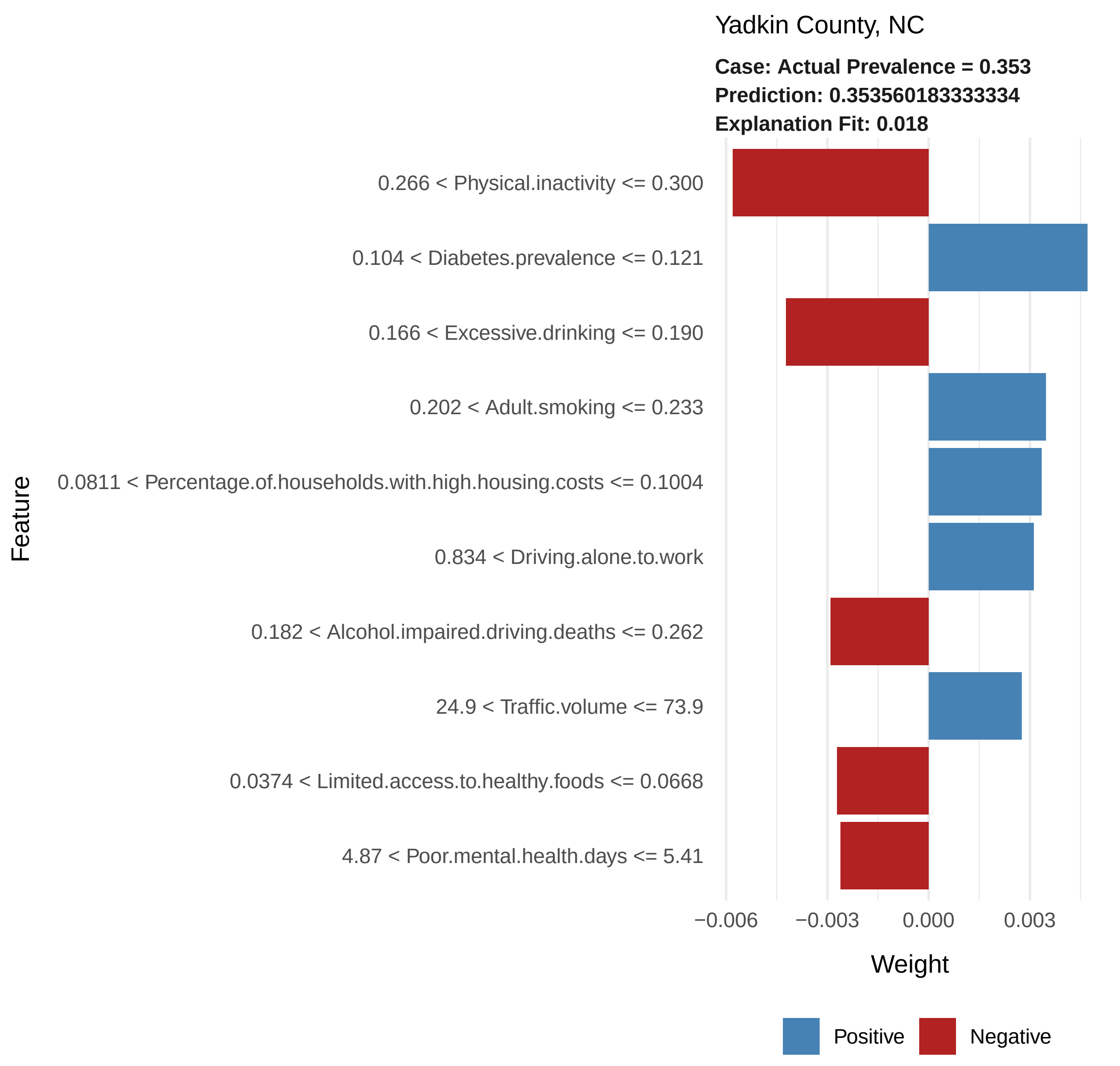

Yadkin County, NC
Case: Actual Prevalence = 0.353
Prediction: 0.353560183333334
Explanation Fit: 0.018
0.266 < Physical.inactivity <= 0.300
0.104 < Diabetes.prevalence <= 0.121
0.166 < Excessive.drinking <= 0.190
0.202 < Adult.smoking <= 0.233
0.0811 < Percentage.of.households.with.high.housing.costs <= 0.1004
0.834 < Driving.alone.to.work
0.182 < Alcohol.impaired.driving.deaths <= 0.262
24.9 < Traffic.volume <= 73.9
0.0374 < Limited.access.to.healthy.foods <= 0.0668
4.87 < Poor.mental.health.days <= 5.41
Feature
−0.006
−0.003
0.000
0.003
Weight
Positive
Negative

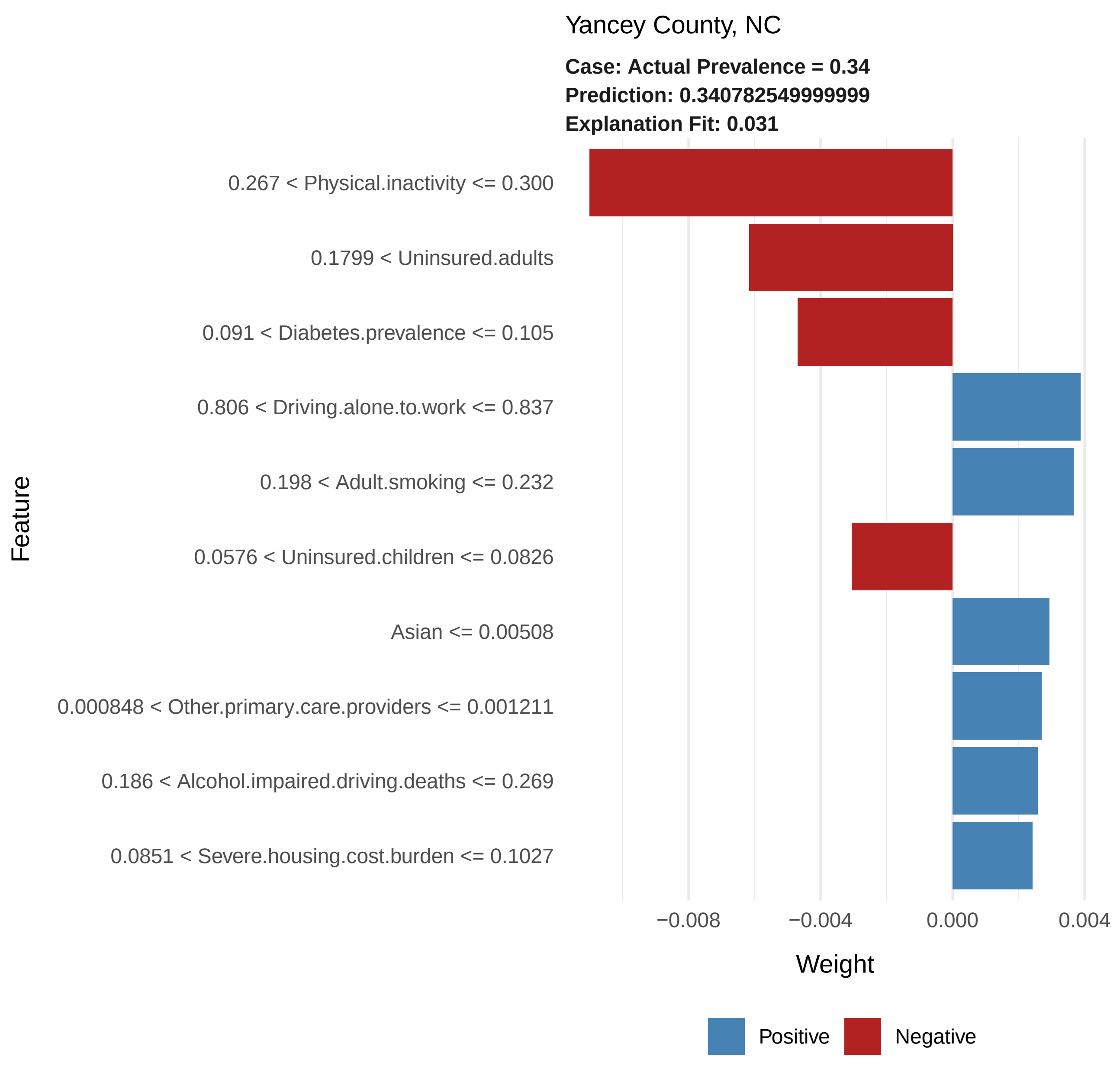
Yancey County, NC
Case: Actual Prevalence = 0.34
Prediction: 0.340782549999999
Explanation Fit: 0.031
0.267 < Physical.inactivity <= 0.300
0.1799 < Uninsured.adults
0.091 < Diabetes.prevalence <= 0.105
0.806 < Driving.alone.to.work <= 0.837
0.198 < Adult.smoking <= 0.232
0.0576 < Uninsured.children <= 0.0826
Asian <= 0.00508
0.000848 < Other.primary.care.providers <= 0.001211
0.186 < Alcohol.impaired.driving.deaths <= 0.269
0.0851 < Severe.housing.cost.burden <= 0.1027
Feature
−0.008
−0.004
0.000
0.004
Weight
Positive
Negative

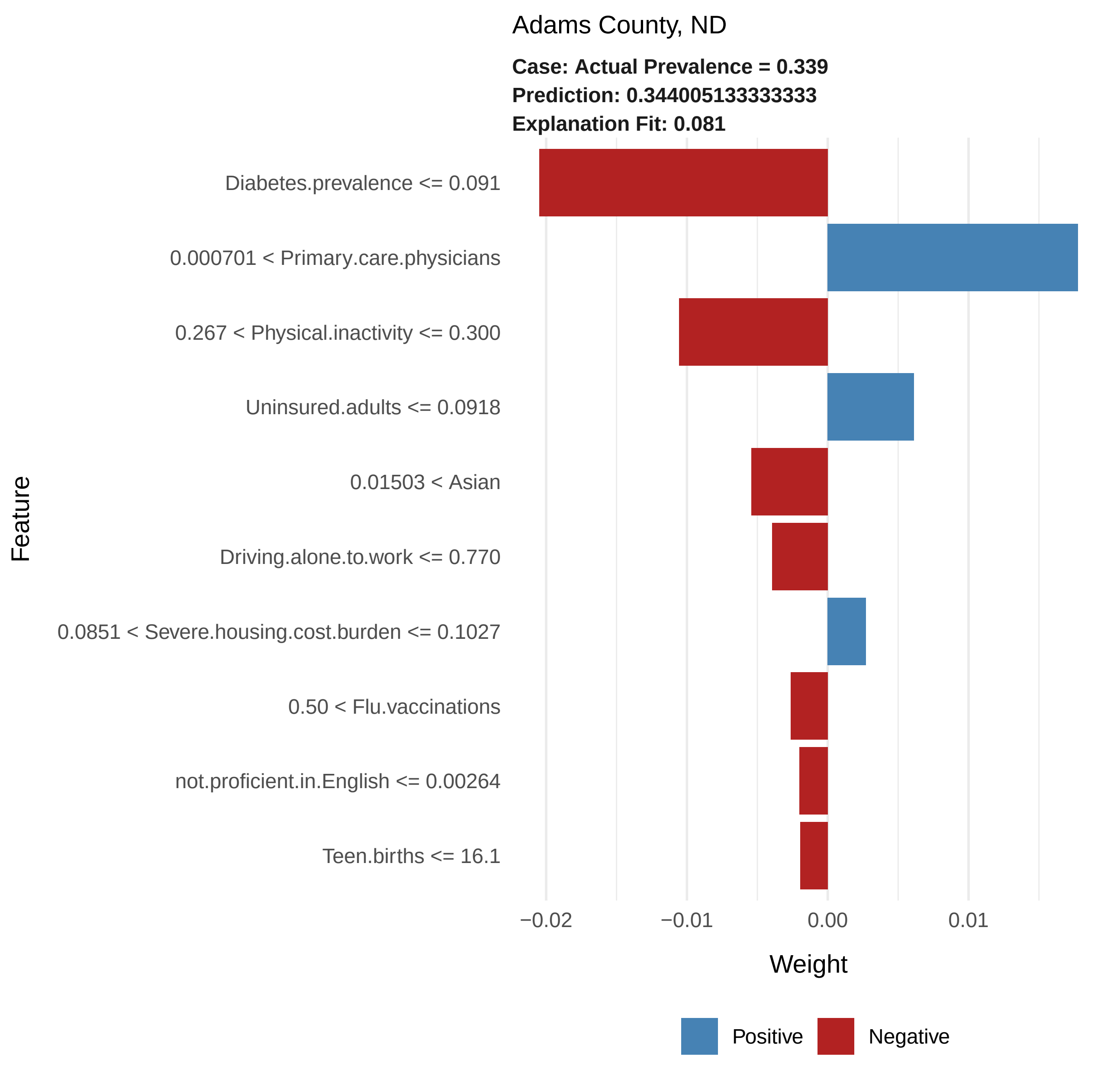

Adams County, ND
Case: Actual Prevalence = 0.339
Prediction: 0.344005133333333
Explanation Fit: 0.081
Diabetes.prevalence <= 0.091
0.000701 < Primary.care.physicians
0.267 < Physical.inactivity <= 0.300
Uninsured.adults <= 0.0918
0.01503 < Asian
Driving.alone.to.work <= 0.770
0.0851 < Severe.housing.cost.burden <= 0.1027
0.50 < Flu.vaccinations
not.proficient.in.English <= 0.00264
Teen.births <= 16.1
Feature
−0.02
−0.01
0.00
0.01
Weight
Positive
Negative

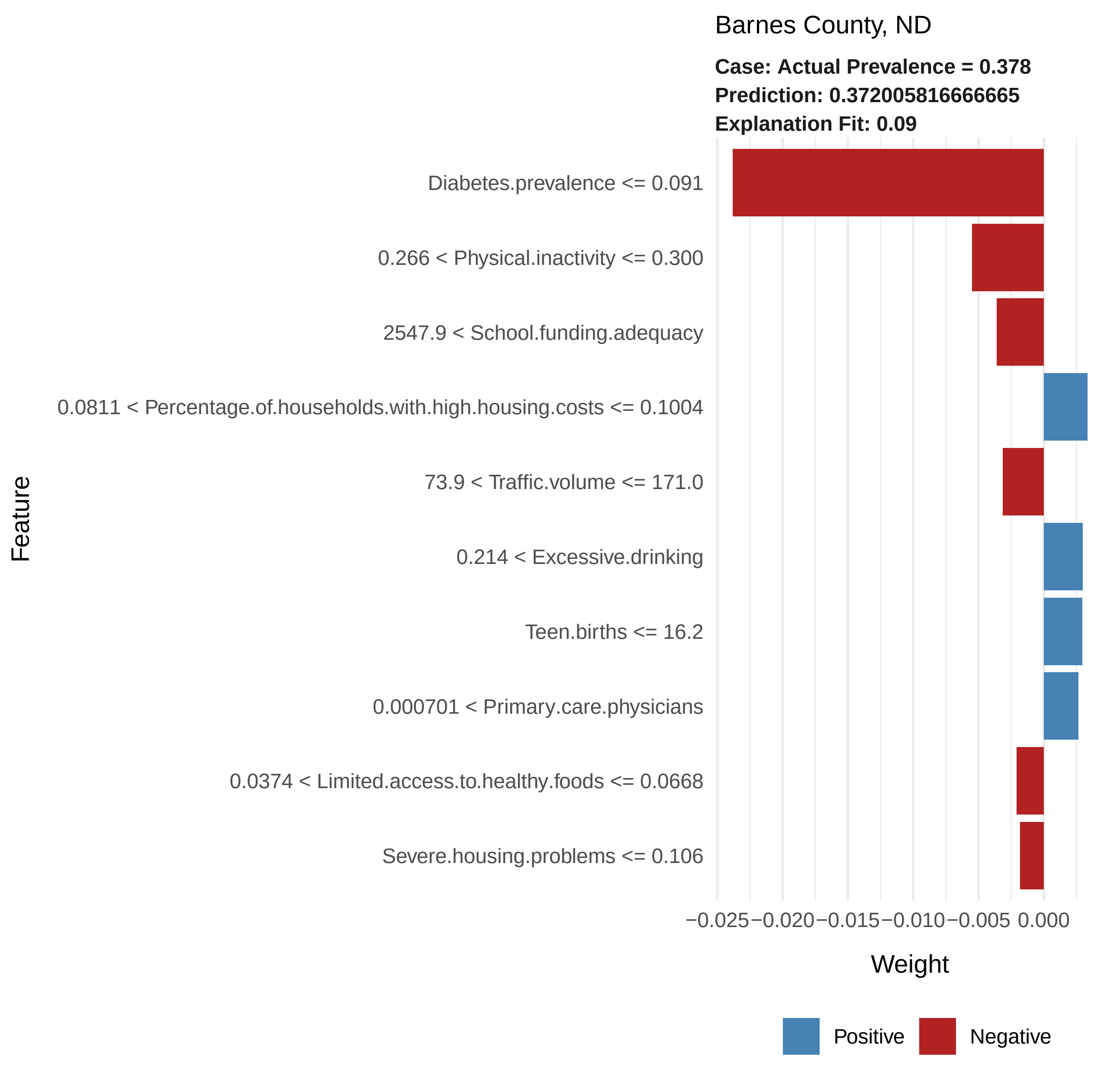
Barnes County, ND
Case: Actual Prevalence = 0.378
Prediction: 0.372005816666665
Explanation Fit: 0.09
Diabetes.prevalence <= 0.091
0.266 < Physical.inactivity <= 0.300
2547.9 < School.funding.adequacy
0.0811 < Percentage.of.households.with.high.housing.costs <= 0.1004
73.9 < Traffic.volume <= 171.0
0.214 < Excessive.drinking
Teen.births <= 16.2
0.000701 < Primary.care.physicians
0.0374 < Limited.access.to.healthy.foods <= 0.0668
Severe.housing.problems <= 0.106
Feature
−0.025
−0.020
−0.015
−0.010
−0.005
0.000
Weight
Positive
Negative

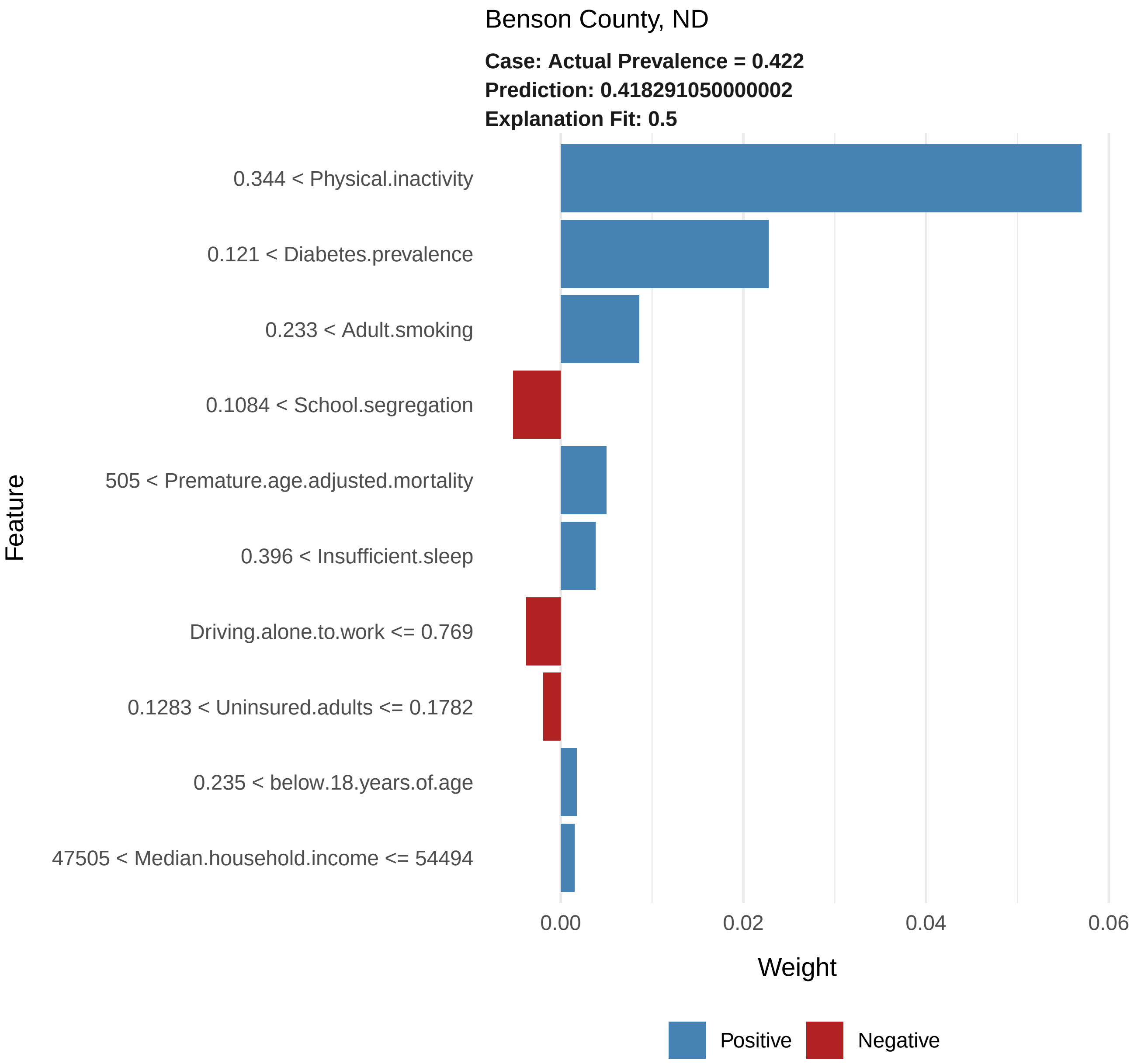
Benson County, ND
Case: Actual Prevalence = 0.422
Prediction: 0.418291050000002
Explanation Fit: 0.5
0.344 < Physical.inactivity
0.121 < Diabetes.prevalence
0.233 < Adult.smoking
0.1084 < School.segregation
505 < Premature.age.adjusted.mortality
0.396 < Insufficient.sleep
Driving.alone.to.work <= 0.769
0.1283 < Uninsured.adults <= 0.1782
0.235 < below.18.years.of.age
47505 < Median.household.income <= 54494
Feature
0.00
0.02
0.04
0.06
Weight
Positive
Negative

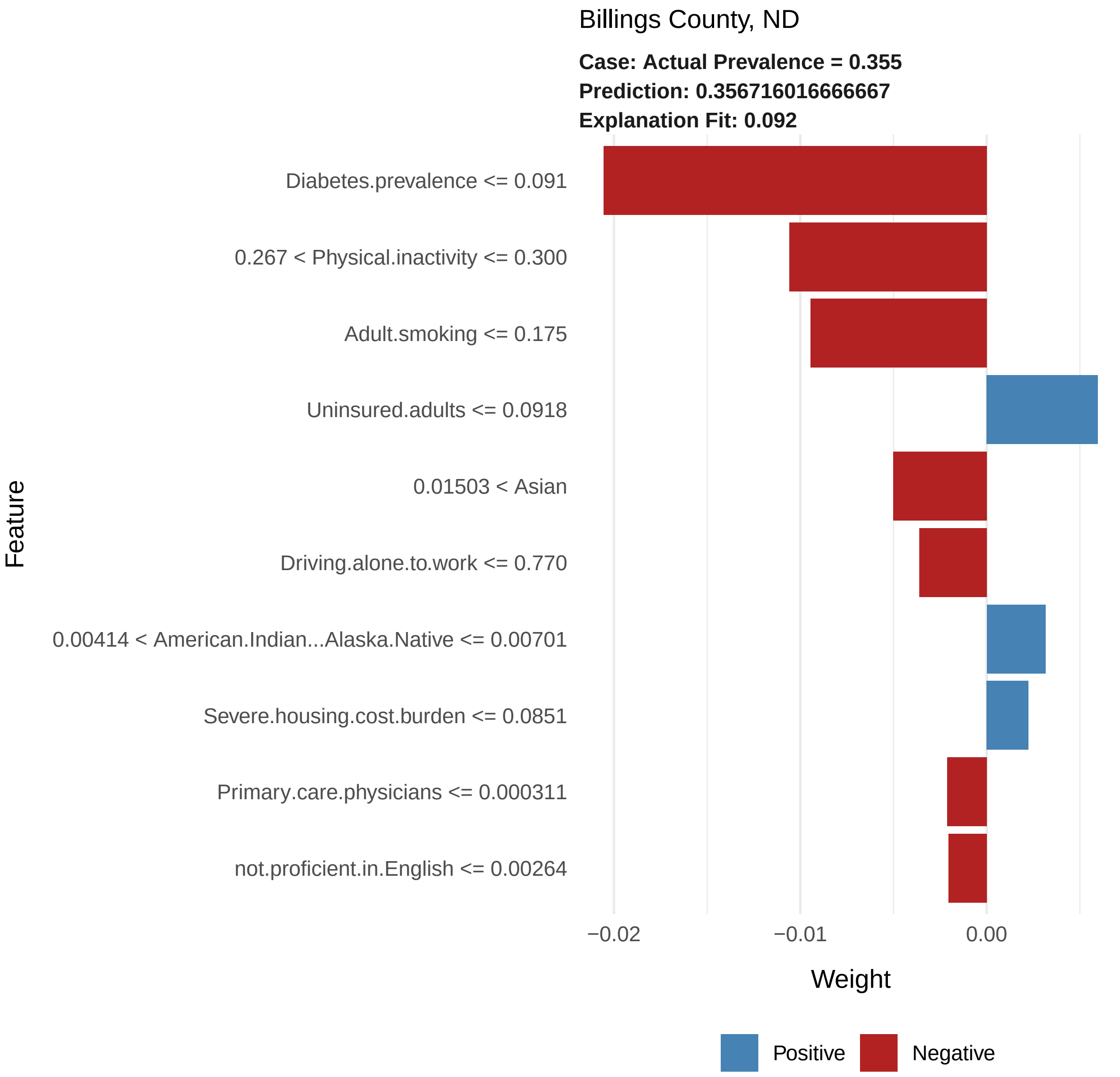
Billings County, ND
Case: Actual Prevalence = 0.355
Prediction: 0.356716016666667
Explanation Fit: 0.092
Diabetes.prevalence <= 0.091
0.267 < Physical.inactivity <= 0.300
Adult.smoking <= 0.175
Uninsured.adults <= 0.0918
0.01503 < Asian
Driving.alone.to.work <= 0.770
0.00414 < American.Indian...Alaska.Native <= 0.00701
Severe.housing.cost.burden <= 0.0851
Primary.care.physicians <= 0.000311
not.proficient.in.English <= 0.00264
Feature
−0.02
−0.01
0.00
Weight
Positive
Negative

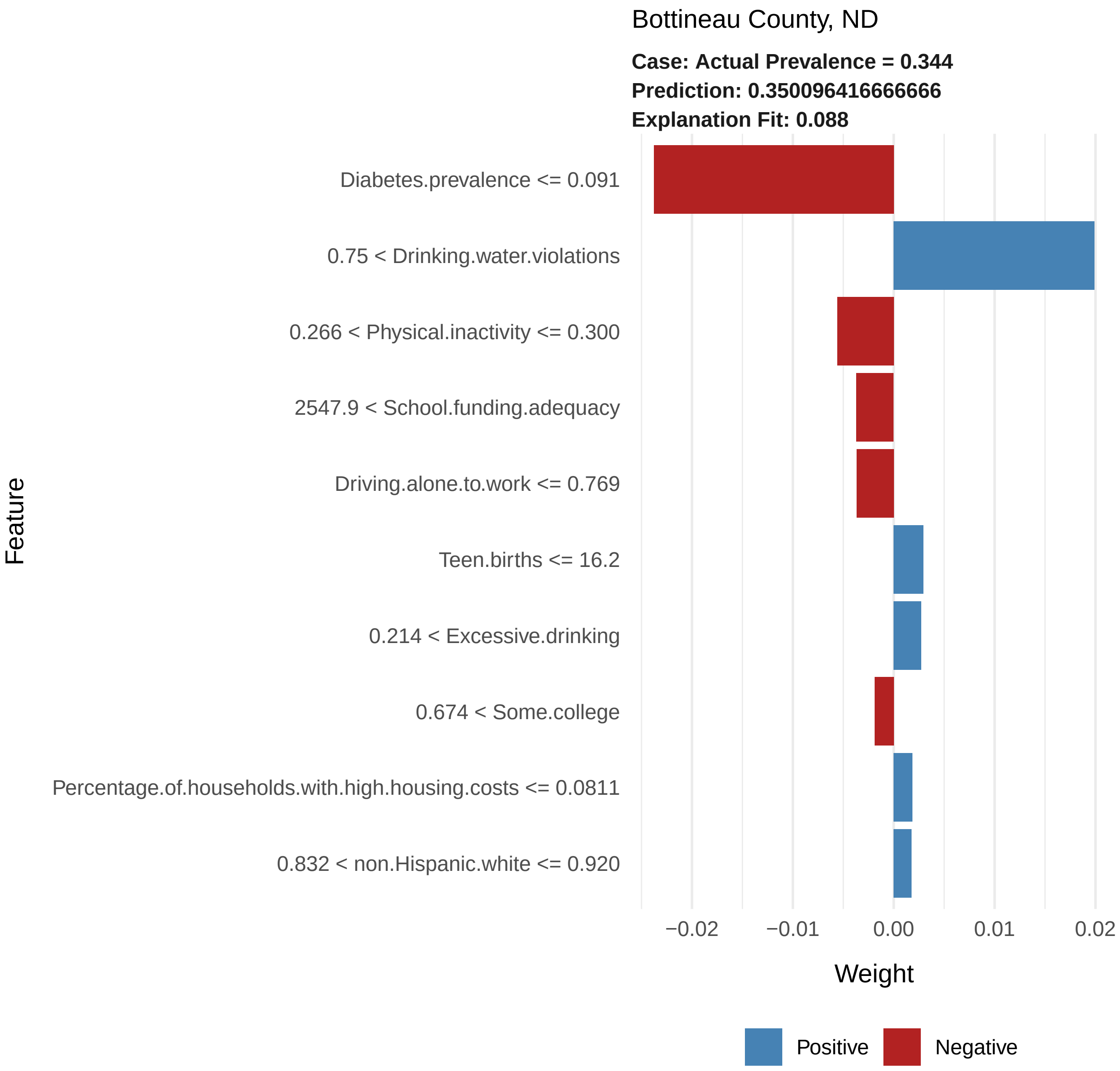

Bottineau County, ND
Case: Actual Prevalence = 0.344
Prediction: 0.350096416666666
Explanation Fit: 0.088
Diabetes.prevalence <= 0.091
0.75 < Drinking.water.violations
0.266 < Physical.inactivity <= 0.300
2547.9 < School.funding.adequacy
Driving.alone.to.work <= 0.769
Teen.births <= 16.2
0.214 < Excessive.drinking
0.674 < Some.college
Percentage.of.households.with.high.housing.costs <= 0.0811
0.832 < non.Hispanic.white <= 0.920
Feature
−0.02
−0.01
0.00
0.01
0.02
Weight
Positive
Negative

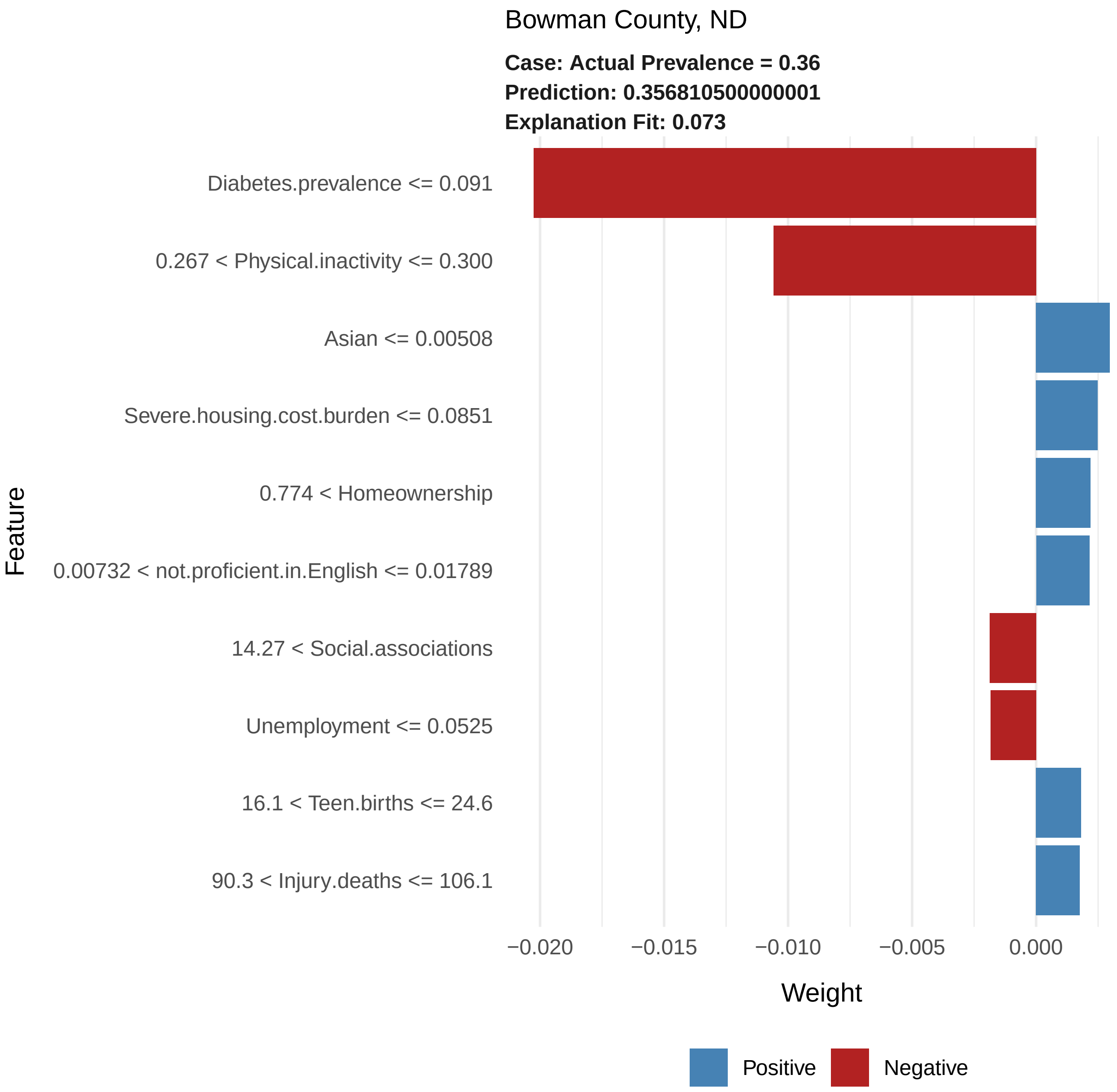

Bowman County, ND
Case: Actual Prevalence = 0.36
Prediction: 0.356810500000001
Explanation Fit: 0.073
Diabetes.prevalence <= 0.091
0.267 < Physical.inactivity <= 0.300
Asian <= 0.00508
Severe.housing.cost.burden <= 0.0851
0.774 < Homeownership
0.00732 < not.proficient.in.English <= 0.01789
14.27 < Social.associations
Unemployment <= 0.0525
16.1 < Teen.births <= 24.6
90.3 < Injury.deaths <= 106.1
Feature
−0.020
−0.015
−0.010
−0.005
0.000
Weight
Positive
Negative

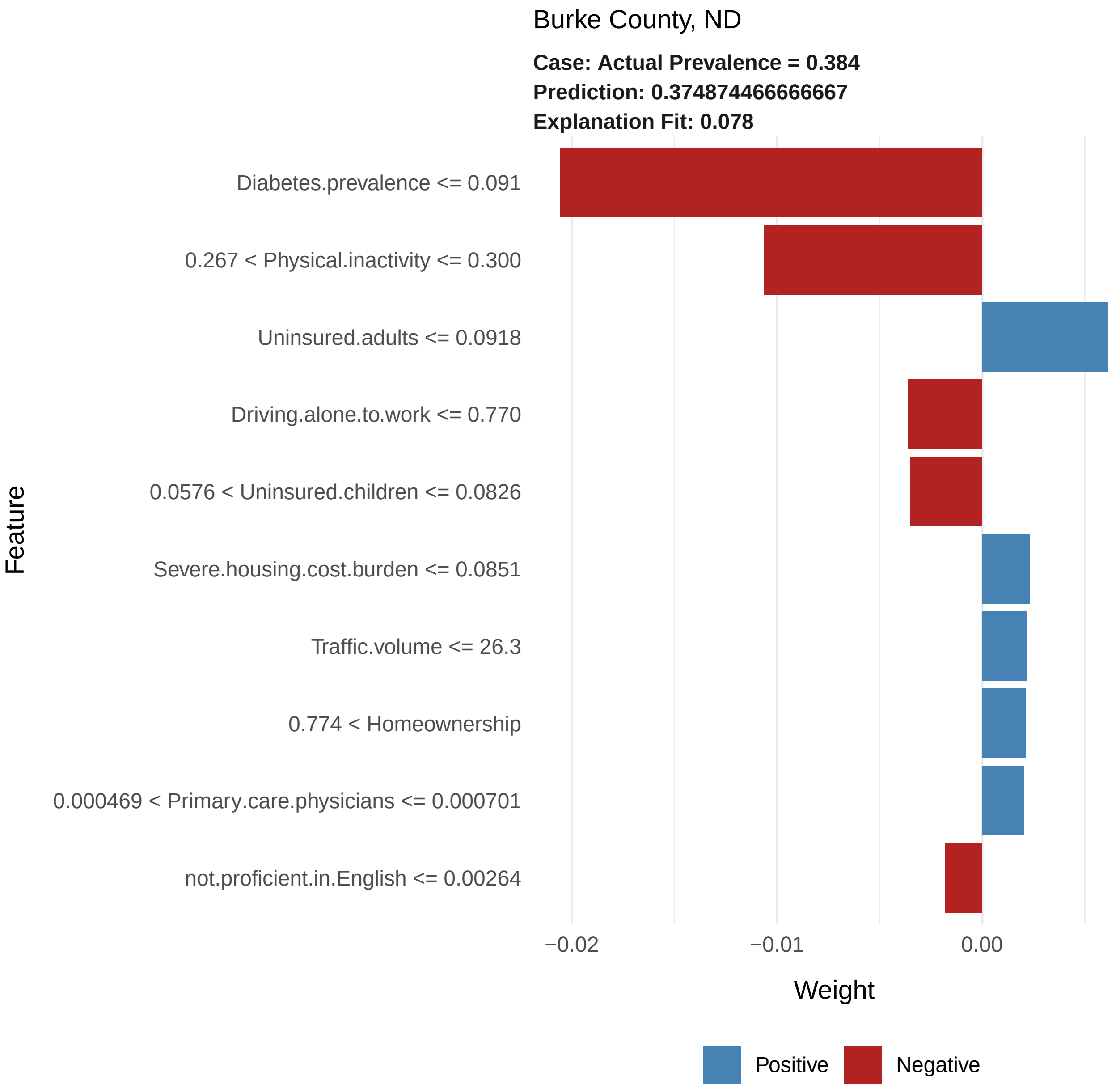

Burke County, ND
Case: Actual Prevalence = 0.384
Prediction: 0.374874466666667
Explanation Fit: 0.078
Diabetes.prevalence <= 0.091
0.267 < Physical.inactivity <= 0.300
Uninsured.adults <= 0.0918
Driving.alone.to.work <= 0.770
0.0576 < Uninsured.children <= 0.0826
Severe.housing.cost.burden <= 0.0851
Traffic.volume <= 26.3
0.774 < Homeownership
0.000469 < Primary.care.physicians <= 0.000701
not.proficient.in.English <= 0.00264
Feature
−0.02
−0.01
0.00
Weight
Positive
Negative

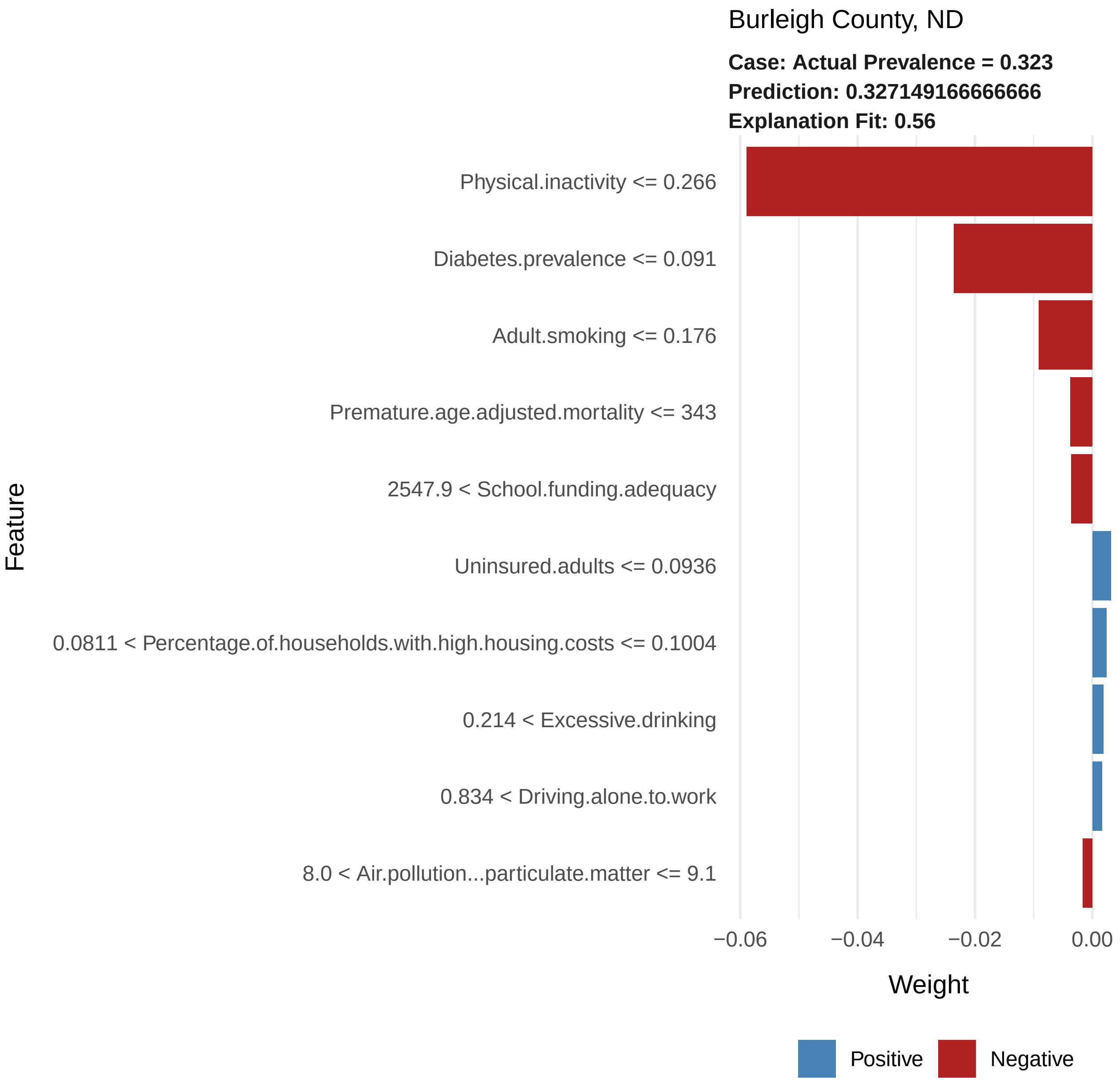
Burleigh County, ND
Case: Actual Prevalence = 0.323
Prediction: 0.327149166666666
Explanation Fit: 0.56
Physical.inactivity <= 0.266
Diabetes.prevalence <= 0.091
Adult.smoking <= 0.176
Premature.age.adjusted.mortality <= 343
2547.9 < School.funding.adequacy
Uninsured.adults <= 0.0936
0.0811 < Percentage.of.households.with.high.housing.costs <= 0.1004
0.214 < Excessive.drinking
0.834 < Driving.alone.to.work
8.0 < Air.pollution...particulate.matter <= 9.1
Feature
−0.06
−0.04
−0.02
0.00
Weight
Positive
Negative

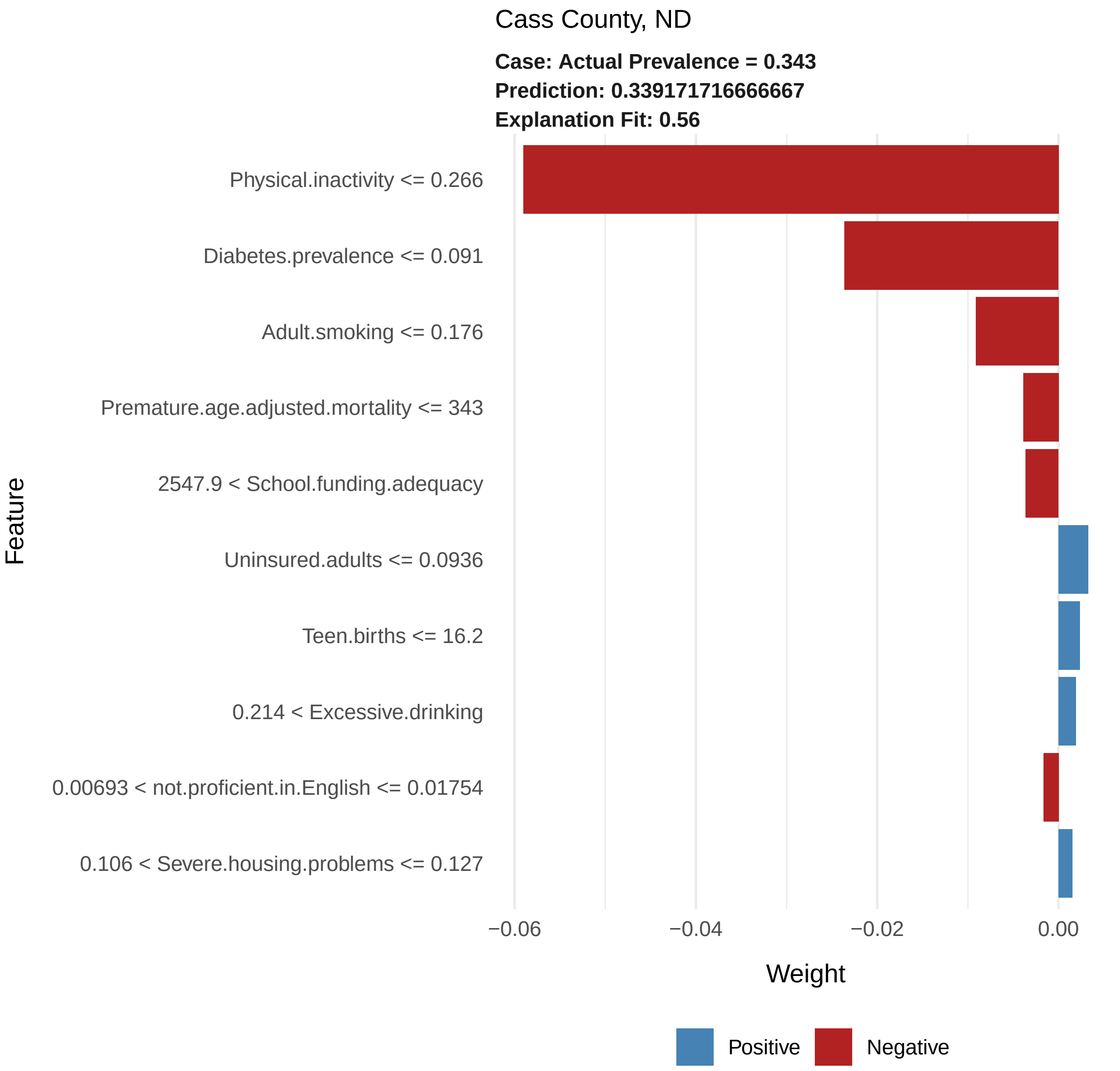
Cass County, ND
Case: Actual Prevalence = 0.343
Prediction: 0.339171716666667
Explanation Fit: 0.56
Physical.inactivity <= 0.266
Diabetes.prevalence <= 0.091
Adult.smoking <= 0.176
Premature.age.adjusted.mortality <= 343
2547.9 < School.funding.adequacy
Uninsured.adults <= 0.0936
Teen.births <= 16.2
0.214 < Excessive.drinking
0.00693 < not.proficient.in.English <= 0.01754
0.106 < Severe.housing.problems <= 0.127
Feature
−0.06
−0.04
−0.02
0.00
Weight
Positive
Negative

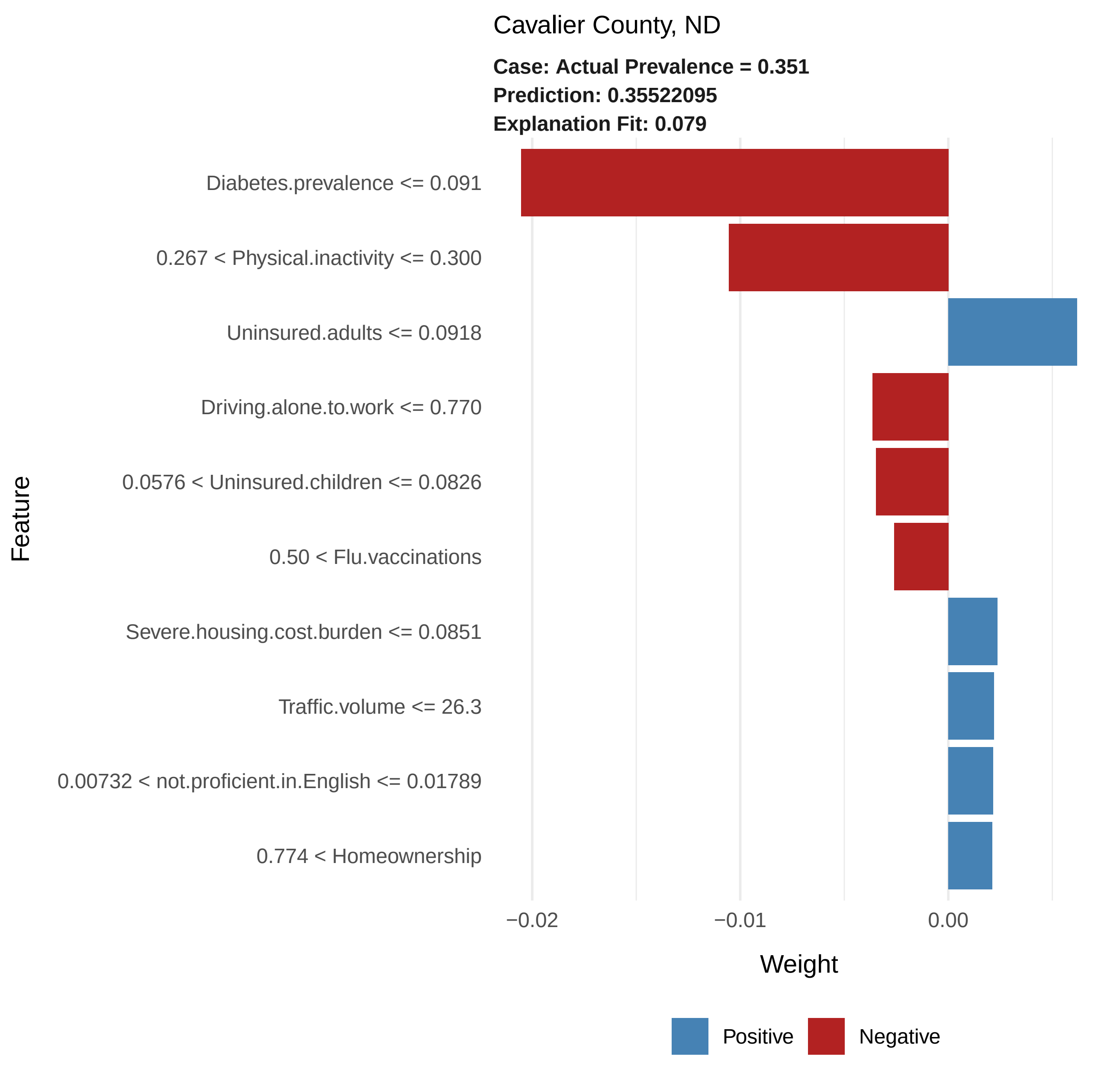
Cavalier County, ND
Case: Actual Prevalence = 0.351
Prediction: 0.35522095
Explanation Fit: 0.079
Diabetes.prevalence <= 0.091
0.267 < Physical.inactivity <= 0.300
Uninsured.adults <= 0.0918
Driving.alone.to.work <= 0.770
0.0576 < Uninsured.children <= 0.0826
0.50 < Flu.vaccinations
Severe.housing.cost.burden <= 0.0851
Traffic.volume <= 26.3
0.00732 < not.proficient.in.English <= 0.01789
0.774 < Homeownership
Feature
−0.02
−0.01
0.00
Weight
Positive
Negative

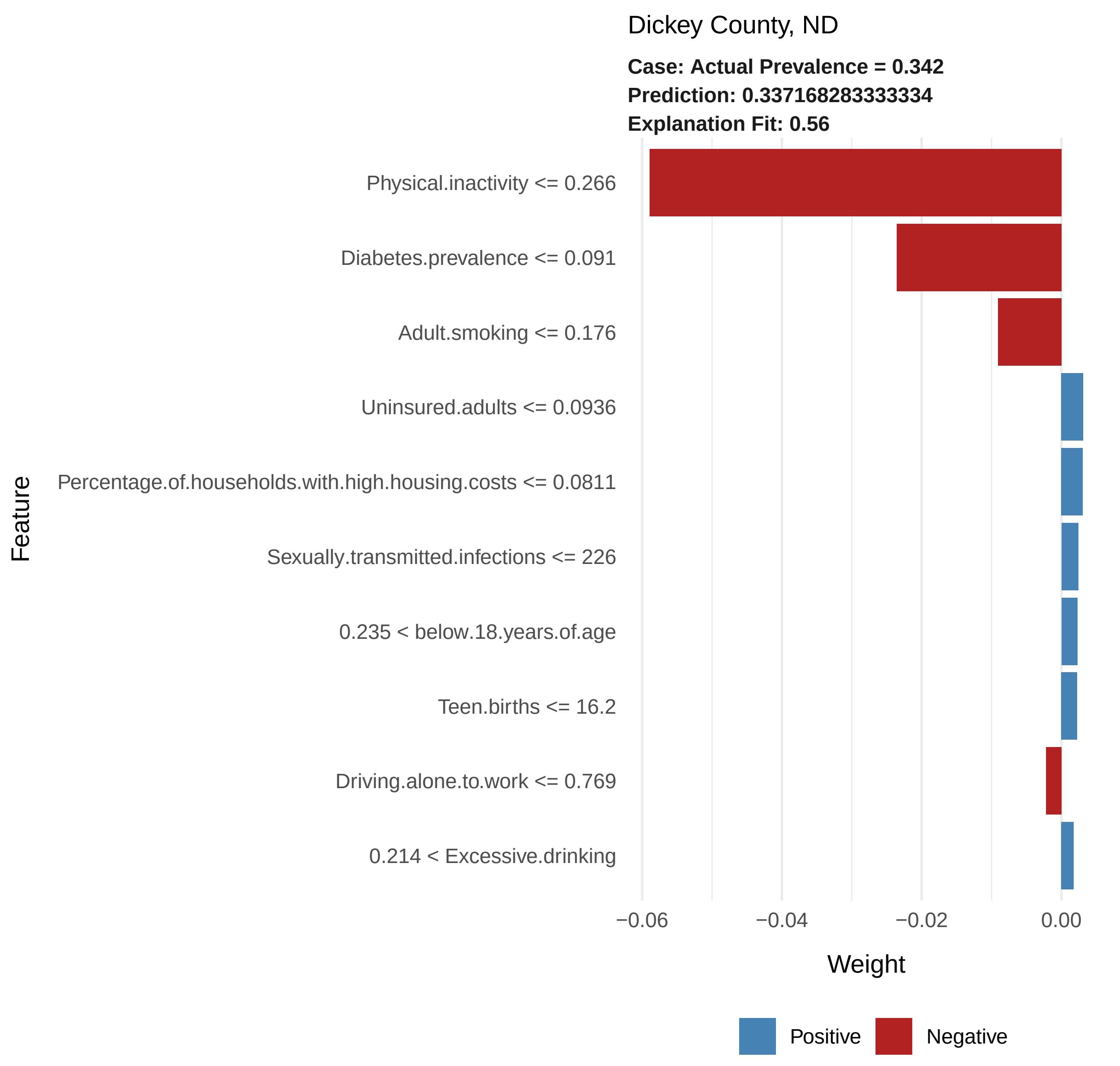

Dickey County, ND
Case: Actual Prevalence = 0.342
Prediction: 0.337168283333334
Explanation Fit: 0.56
Physical.inactivity <= 0.266
Diabetes.prevalence <= 0.091
Adult.smoking <= 0.176
Uninsured.adults <= 0.0936
Percentage.of.households.with.high.housing.costs <= 0.0811
Sexually.transmitted.infections <= 226
0.235 < below.18.years.of.age
Teen.births <= 16.2
Driving.alone.to.work <= 0.769
0.214 < Excessive.drinking
Feature
−0.06
−0.04
−0.02
0.00
Weight
Positive
Negative

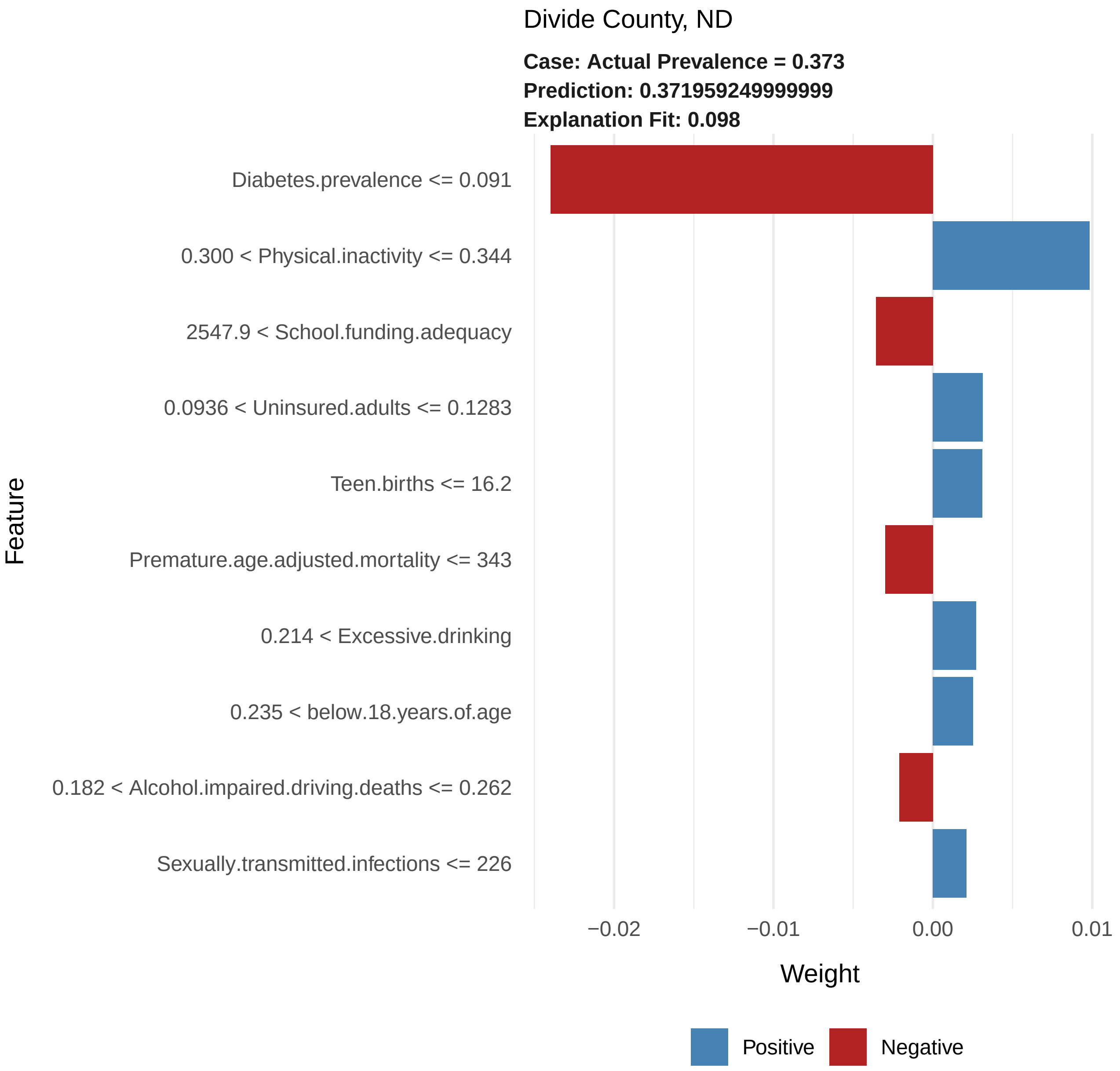
Divide County, ND
Case: Actual Prevalence = 0.373
Prediction: 0.371959249999999
Explanation Fit: 0.098
Diabetes.prevalence <= 0.091
0.300 < Physical.inactivity <= 0.344
2547.9 < School.funding.adequacy
0.0936 < Uninsured.adults <= 0.1283
Teen.births <= 16.2
Premature.age.adjusted.mortality <= 343
0.214 < Excessive.drinking
0.235 < below.18.years.of.age
0.182 < Alcohol.impaired.driving.deaths <= 0.262
Sexually.transmitted.infections <= 226
Feature
−0.02
−0.01
0.00
0.01
Weight
Positive
Negative

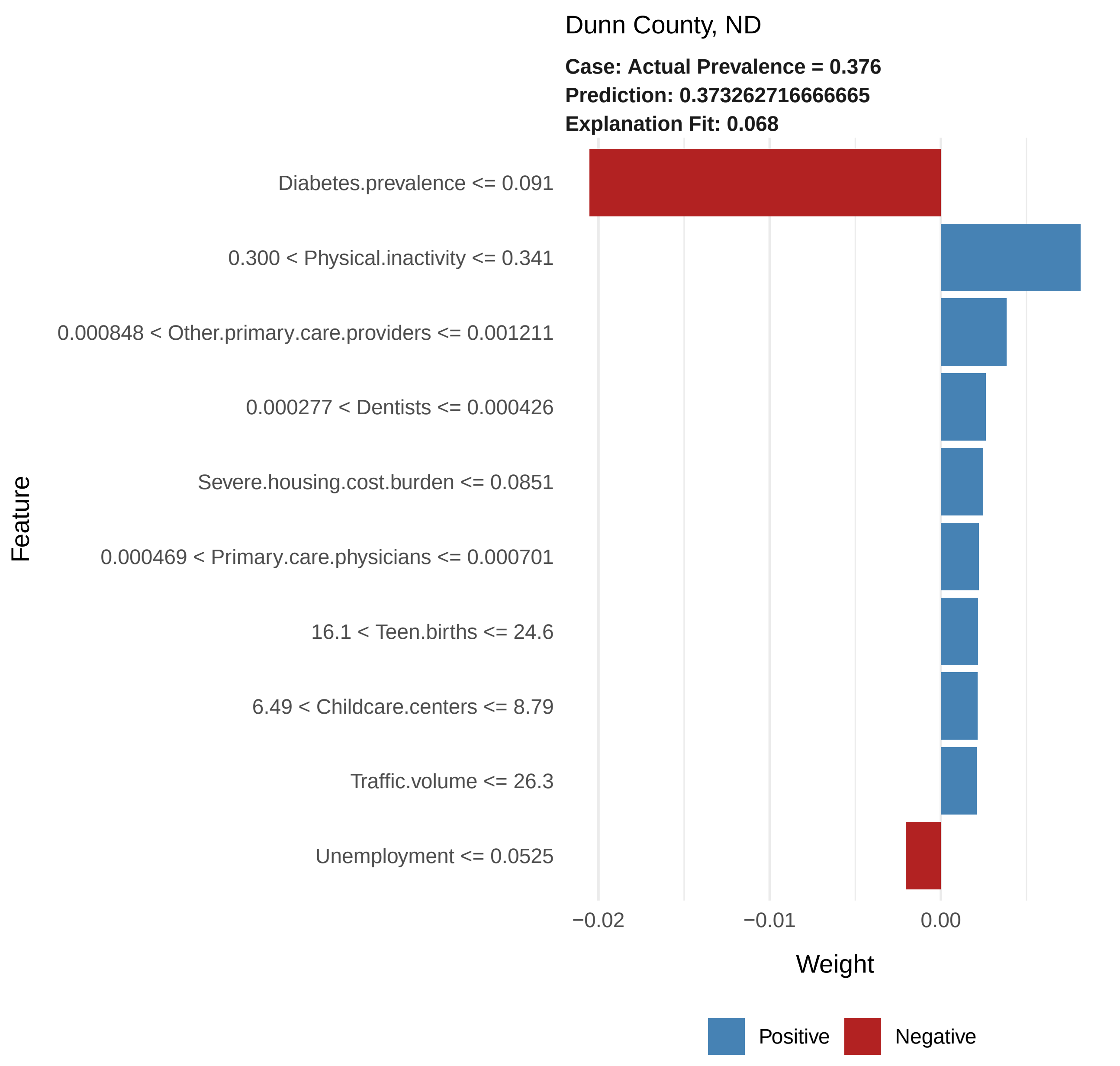
Dunn County, ND
Case: Actual Prevalence = 0.376
Prediction: 0.373262716666665
Explanation Fit: 0.068
Diabetes.prevalence <= 0.091
0.300 < Physical.inactivity <= 0.341
0.000848 < Other.primary.care.providers <= 0.001211
0.000277 < Dentists <= 0.000426
Severe.housing.cost.burden <= 0.0851
0.000469 < Primary.care.physicians <= 0.000701
16.1 < Teen.births <= 24.6
6.49 < Childcare.centers <= 8.79
Traffic.volume <= 26.3
Unemployment <= 0.0525
Feature
−0.02
−0.01
0.00
Weight
Positive
Negative

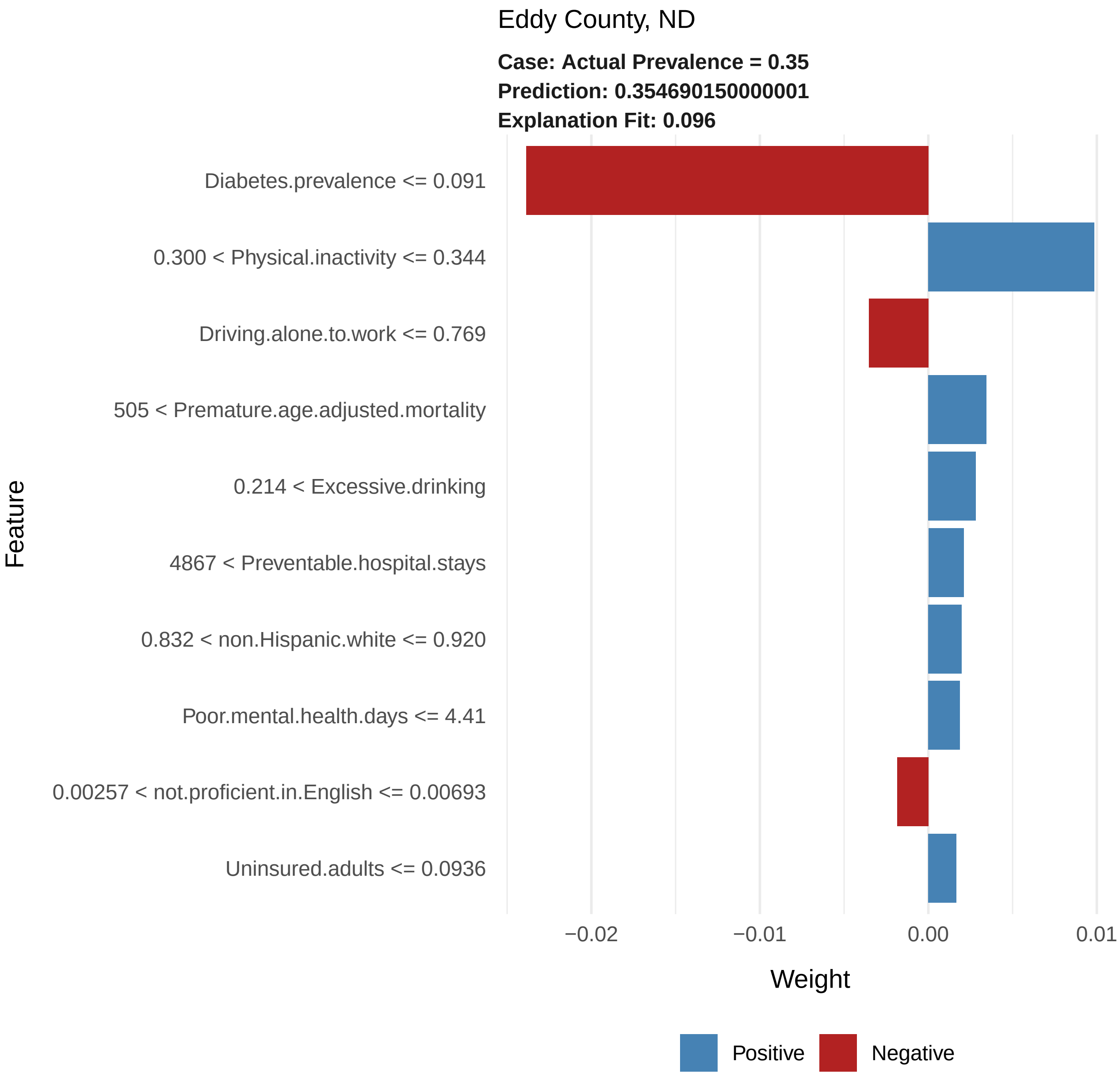

Eddy County, ND
Case: Actual Prevalence = 0.35
Prediction: 0.354690150000001
Explanation Fit: 0.096
Diabetes.prevalence <= 0.091
0.300 < Physical.inactivity <= 0.344
Driving.alone.to.work <= 0.769
505 < Premature.age.adjusted.mortality
0.214 < Excessive.drinking
4867 < Preventable.hospital.stays
0.832 < non.Hispanic.white <= 0.920
Poor.mental.health.days <= 4.41
0.00257 < not.proficient.in.English <= 0.00693
Uninsured.adults <= 0.0936
Feature
−0.02
−0.01
0.00
0.01
Weight
Positive
Negative

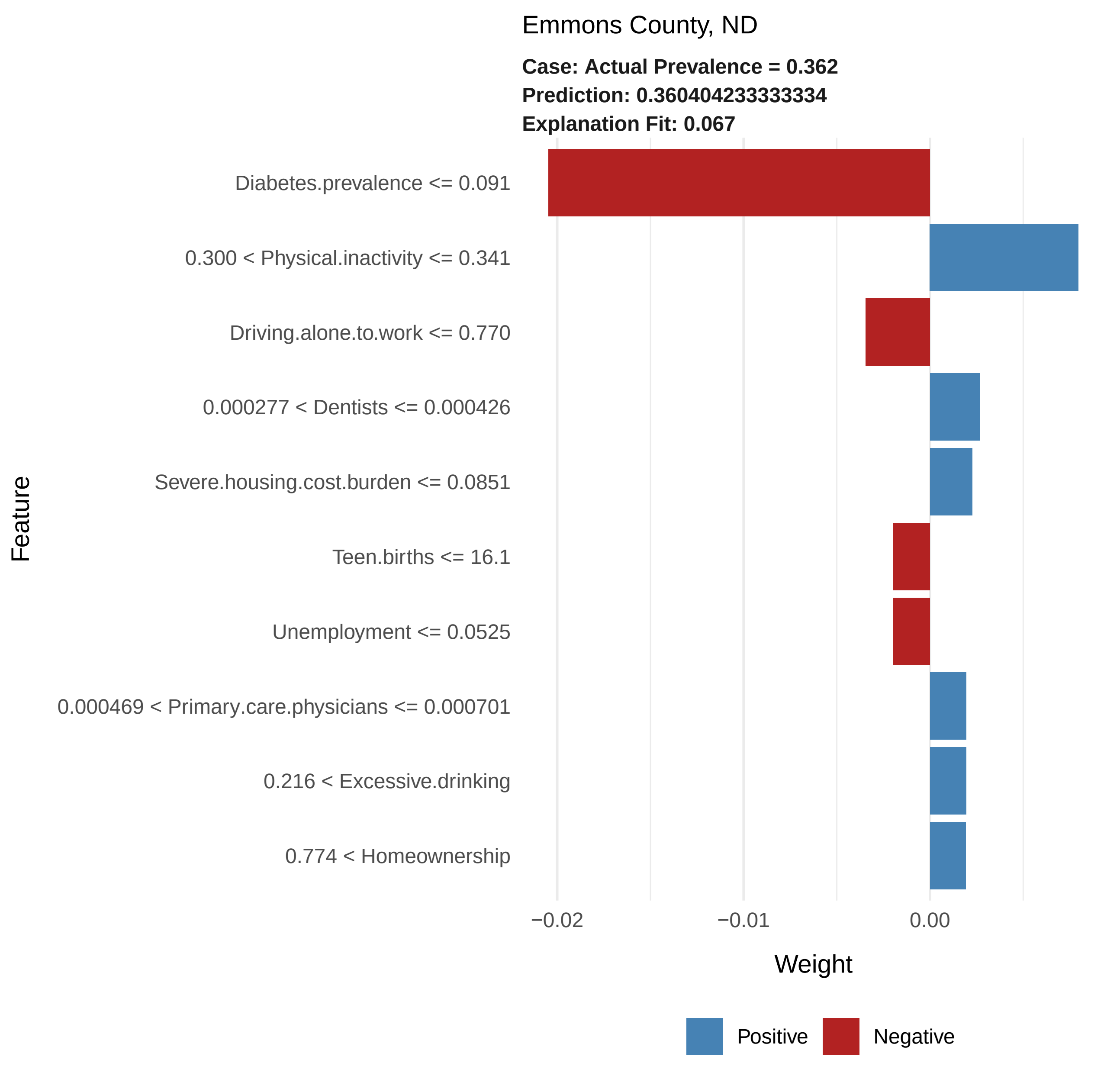
Emmons County, ND
Case: Actual Prevalence = 0.362
Prediction: 0.360404233333334
Explanation Fit: 0.067
Diabetes.prevalence <= 0.091
0.300 < Physical.inactivity <= 0.341
Driving.alone.to.work <= 0.770
0.000277 < Dentists <= 0.000426
Severe.housing.cost.burden <= 0.0851
Teen.births <= 16.1
Unemployment <= 0.0525
0.000469 < Primary.care.physicians <= 0.000701
0.216 < Excessive.drinking
0.774 < Homeownership
Feature
−0.02
−0.01
0.00
Weight
Positive
Negative

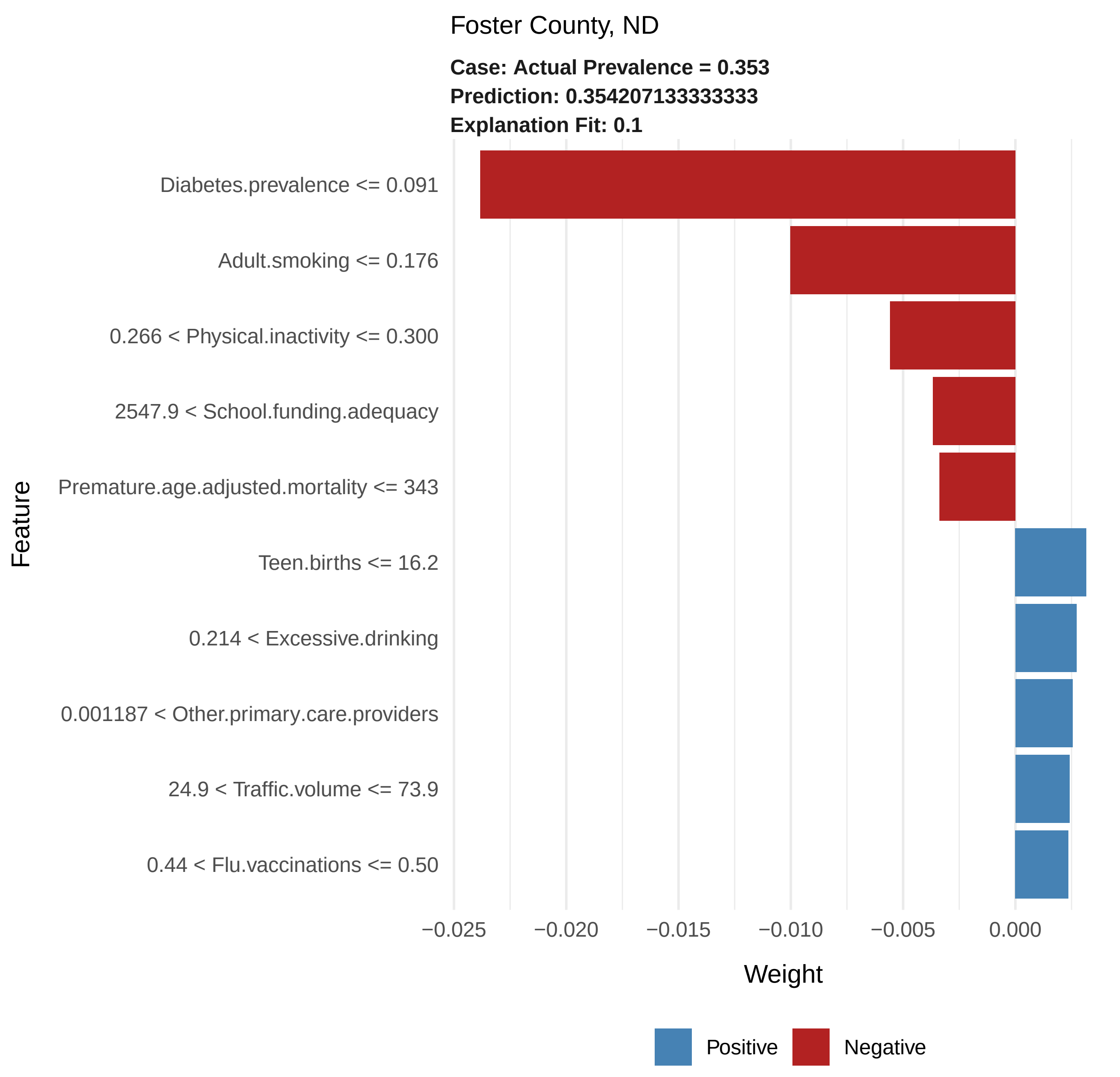

Foster County, ND
Case: Actual Prevalence = 0.353
Prediction: 0.354207133333333
Explanation Fit: 0.1
Diabetes.prevalence <= 0.091
Adult.smoking <= 0.176
0.266 < Physical.inactivity <= 0.300
2547.9 < School.funding.adequacy
Premature.age.adjusted.mortality <= 343
Teen.births <= 16.2
0.214 < Excessive.drinking
0.001187 < Other.primary.care.providers
24.9 < Traffic.volume <= 73.9
0.44 < Flu.vaccinations <= 0.50
Feature
−0.025
−0.020
−0.015
−0.010
−0.005
0.000
Weight
Positive
Negative

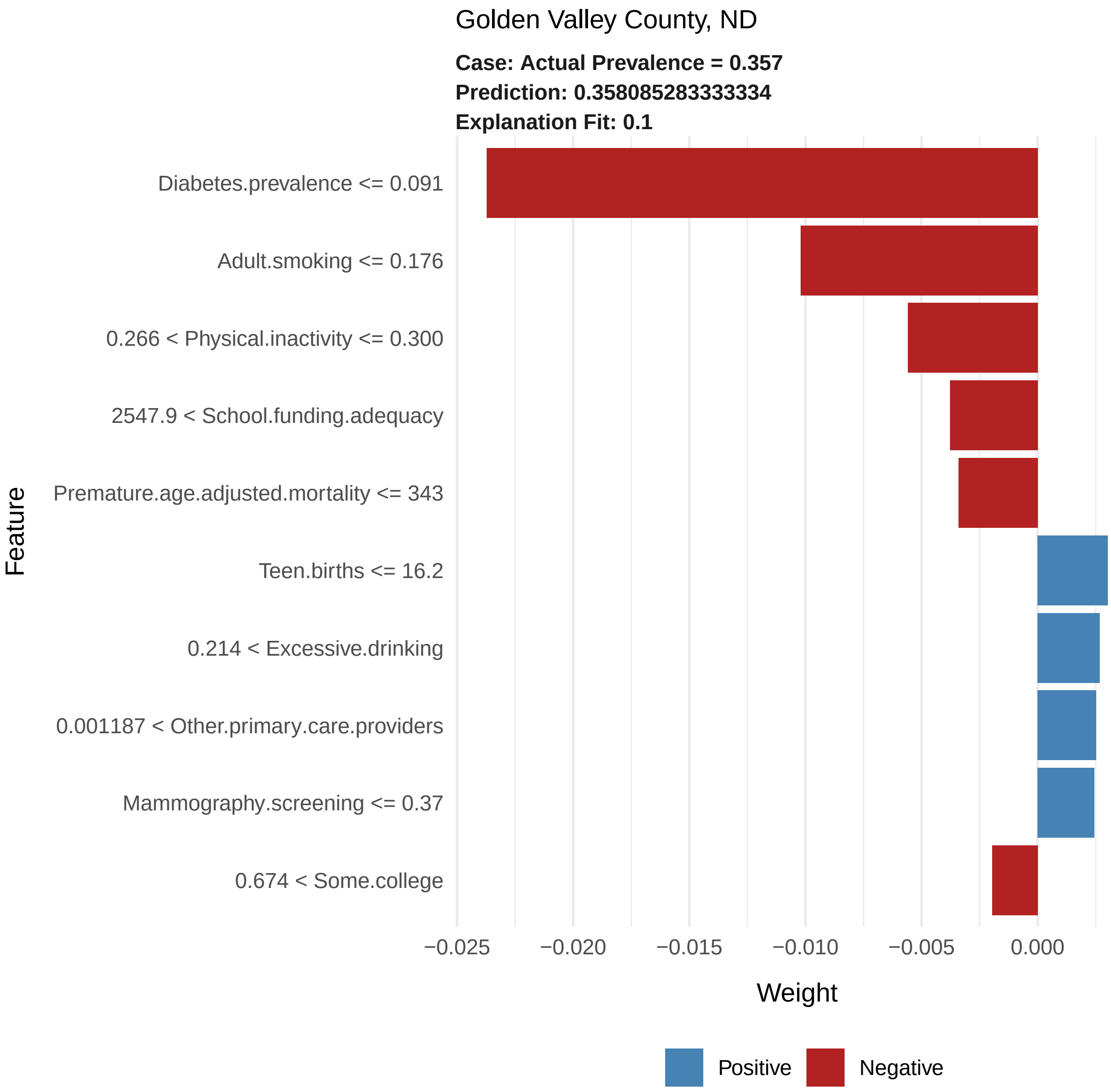

Golden Valley County, ND
Case: Actual Prevalence = 0.357
Prediction: 0.358085283333334
Explanation Fit: 0.1
Diabetes.prevalence <= 0.091
Adult.smoking <= 0.176
0.266 < Physical.inactivity <= 0.300
2547.9 < School.funding.adequacy
Premature.age.adjusted.mortality <= 343
Teen.births <= 16.2
0.214 < Excessive.drinking
0.001187 < Other.primary.care.providers
Mammography.screening <= 0.37
0.674 < Some.college
Feature
−0.025
−0.020
−0.015
−0.010
−0.005
0.000
Weight
Positive
Negative

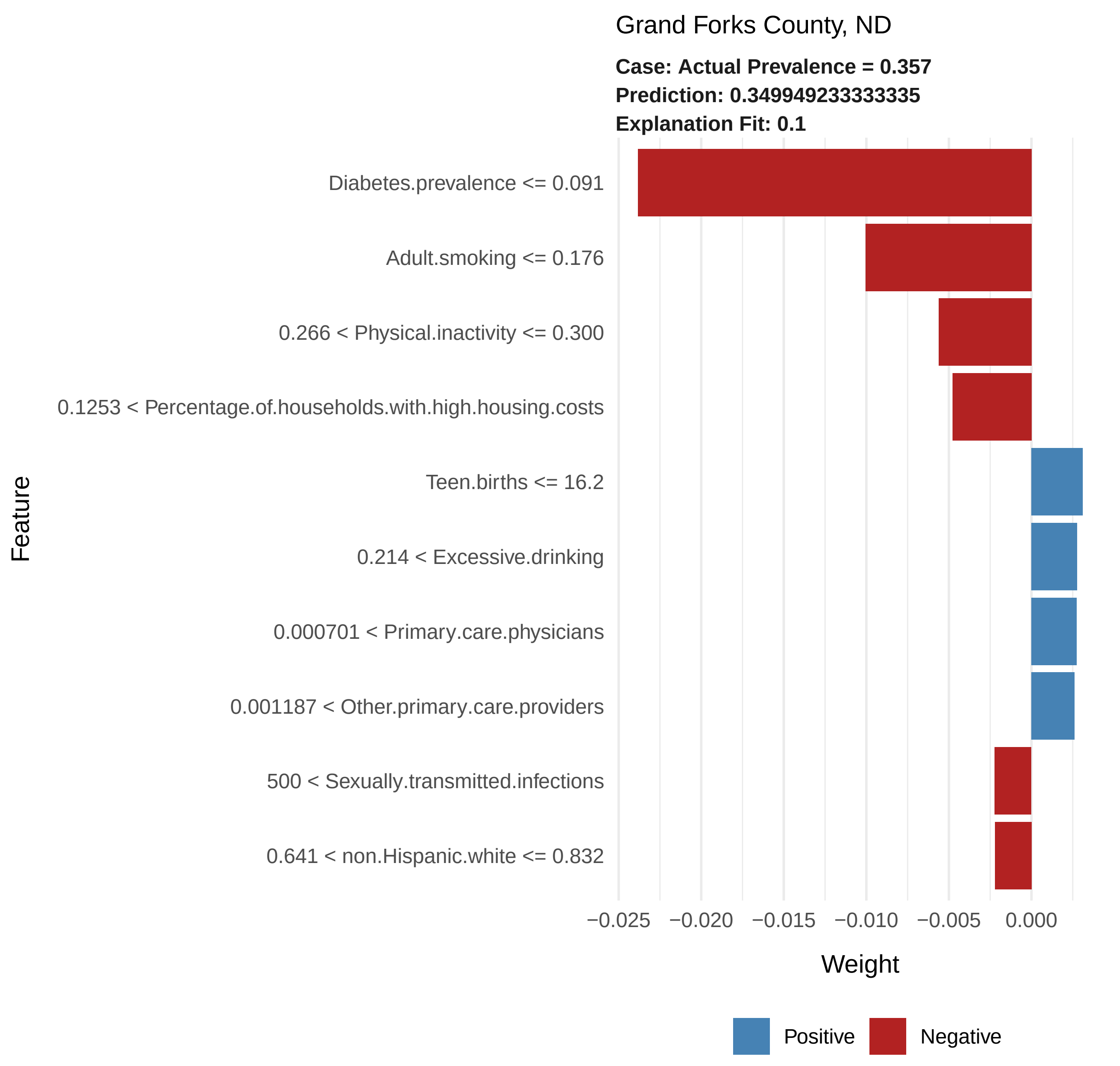

Grand Forks County, ND
Case: Actual Prevalence = 0.357
Prediction: 0.349949233333335
Explanation Fit: 0.1
Diabetes.prevalence <= 0.091
Adult.smoking <= 0.176
0.266 < Physical.inactivity <= 0.300
0.1253 < Percentage.of.households.with.high.housing.costs
Teen.births <= 16.2
0.214 < Excessive.drinking
0.000701 < Primary.care.physicians
0.001187 < Other.primary.care.providers
500 < Sexually.transmitted.infections
0.641 < non.Hispanic.white <= 0.832
Feature
−0.025
−0.020
−0.015
−0.010
−0.005
0.000
Weight
Positive
Negative

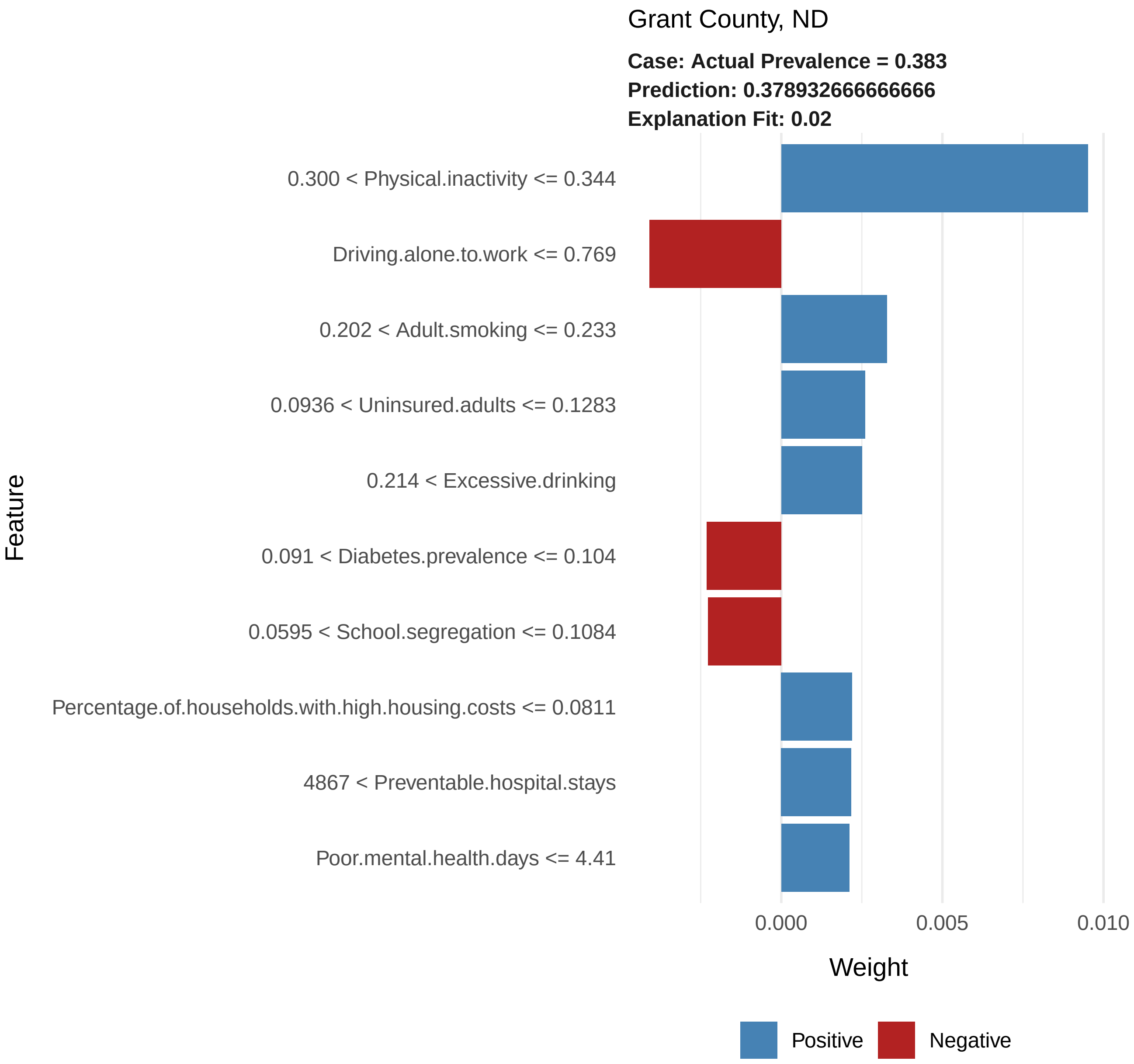

Grant County, ND
Case: Actual Prevalence = 0.383
Prediction: 0.378932666666666
Explanation Fit: 0.02
0.300 < Physical.inactivity <= 0.344
Driving.alone.to.work <= 0.769
0.202 < Adult.smoking <= 0.233
0.0936 < Uninsured.adults <= 0.1283
0.214 < Excessive.drinking
0.091 < Diabetes.prevalence <= 0.104
0.0595 < School.segregation <= 0.1084
Percentage.of.households.with.high.housing.costs <= 0.0811
4867 < Preventable.hospital.stays
Poor.mental.health.days <= 4.41
Feature
0.000
0.005
0.010
Weight
Positive
Negative

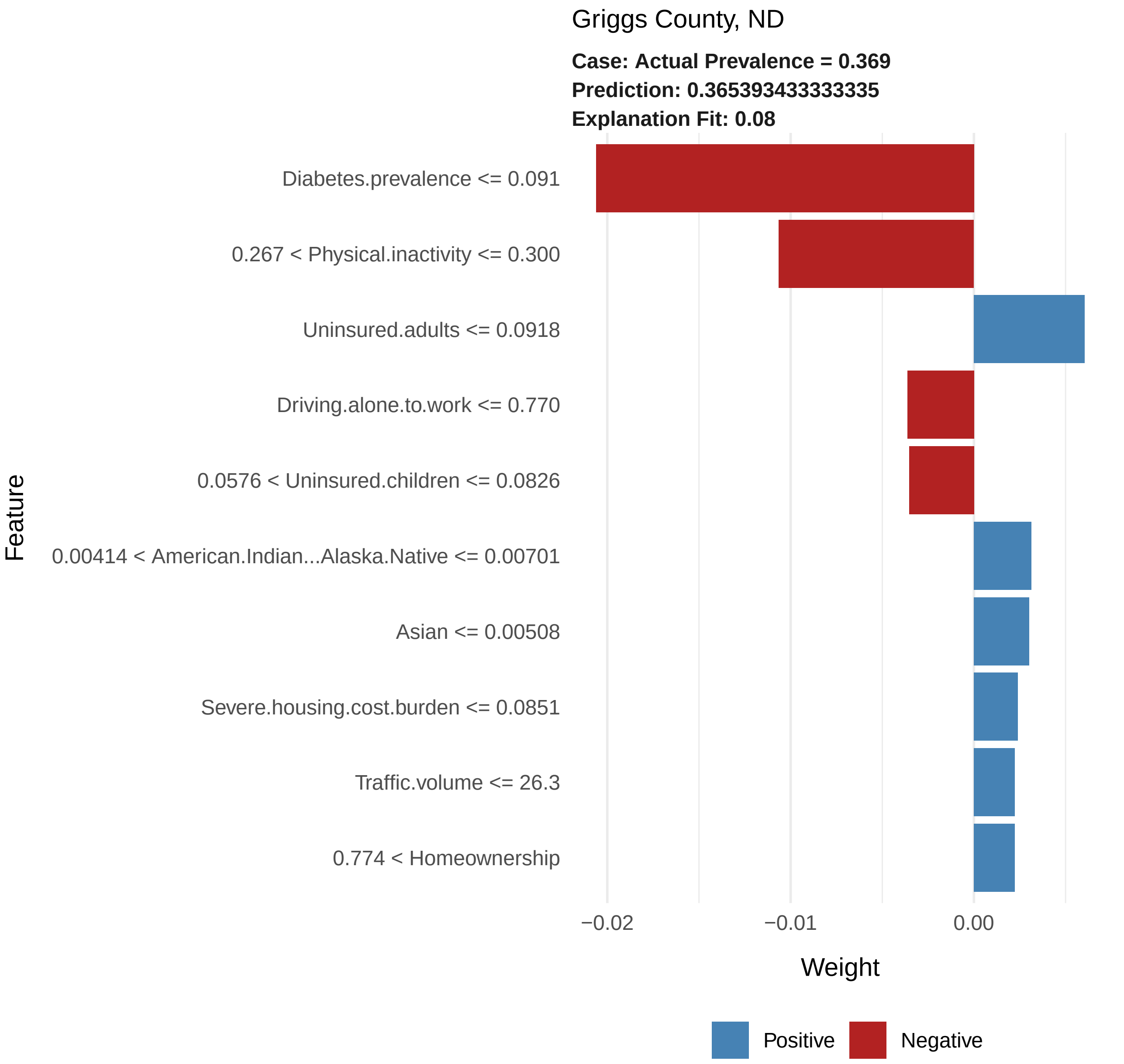

Griggs County, ND
Case: Actual Prevalence = 0.369
Prediction: 0.365393433333335
Explanation Fit: 0.08
Diabetes.prevalence <= 0.091
0.267 < Physical.inactivity <= 0.300
Uninsured.adults <= 0.0918
Driving.alone.to.work <= 0.770
0.0576 < Uninsured.children <= 0.0826
0.00414 < American.Indian...Alaska.Native <= 0.00701
Asian <= 0.00508
Severe.housing.cost.burden <= 0.0851
Traffic.volume <= 26.3
0.774 < Homeownership
Feature
−0.02
−0.01
0.00
Weight
Positive
Negative

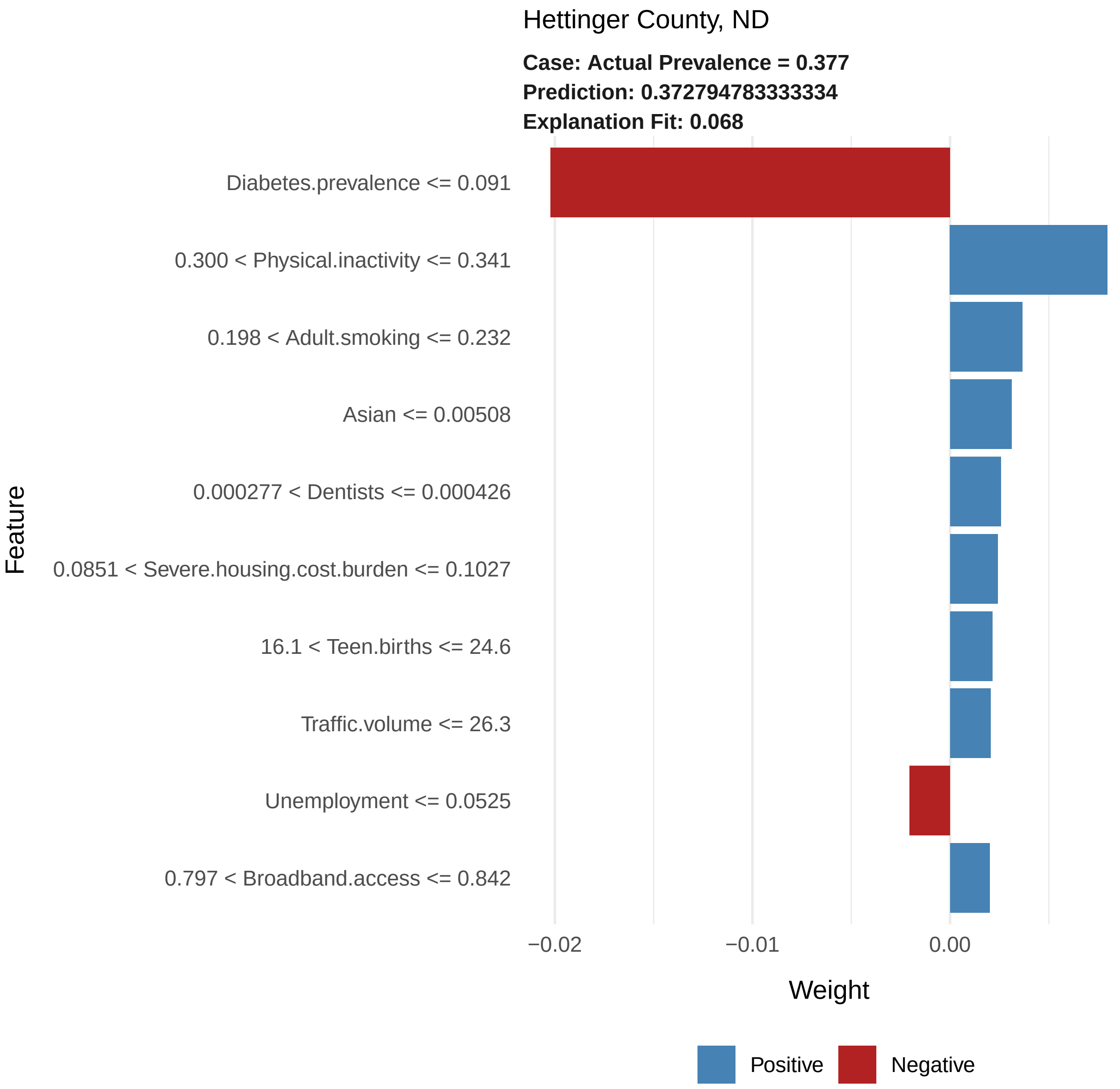

Hettinger County, ND
Case: Actual Prevalence = 0.377
Prediction: 0.372794783333334
Explanation Fit: 0.068
Diabetes.prevalence <= 0.091
0.300 < Physical.inactivity <= 0.341
0.198 < Adult.smoking <= 0.232
Asian <= 0.00508
0.000277 < Dentists <= 0.000426
0.0851 < Severe.housing.cost.burden <= 0.1027
16.1 < Teen.births <= 24.6
Traffic.volume <= 26.3
Unemployment <= 0.0525
0.797 < Broadband.access <= 0.842
Feature
−0.02
−0.01
0.00
Weight
Positive
Negative

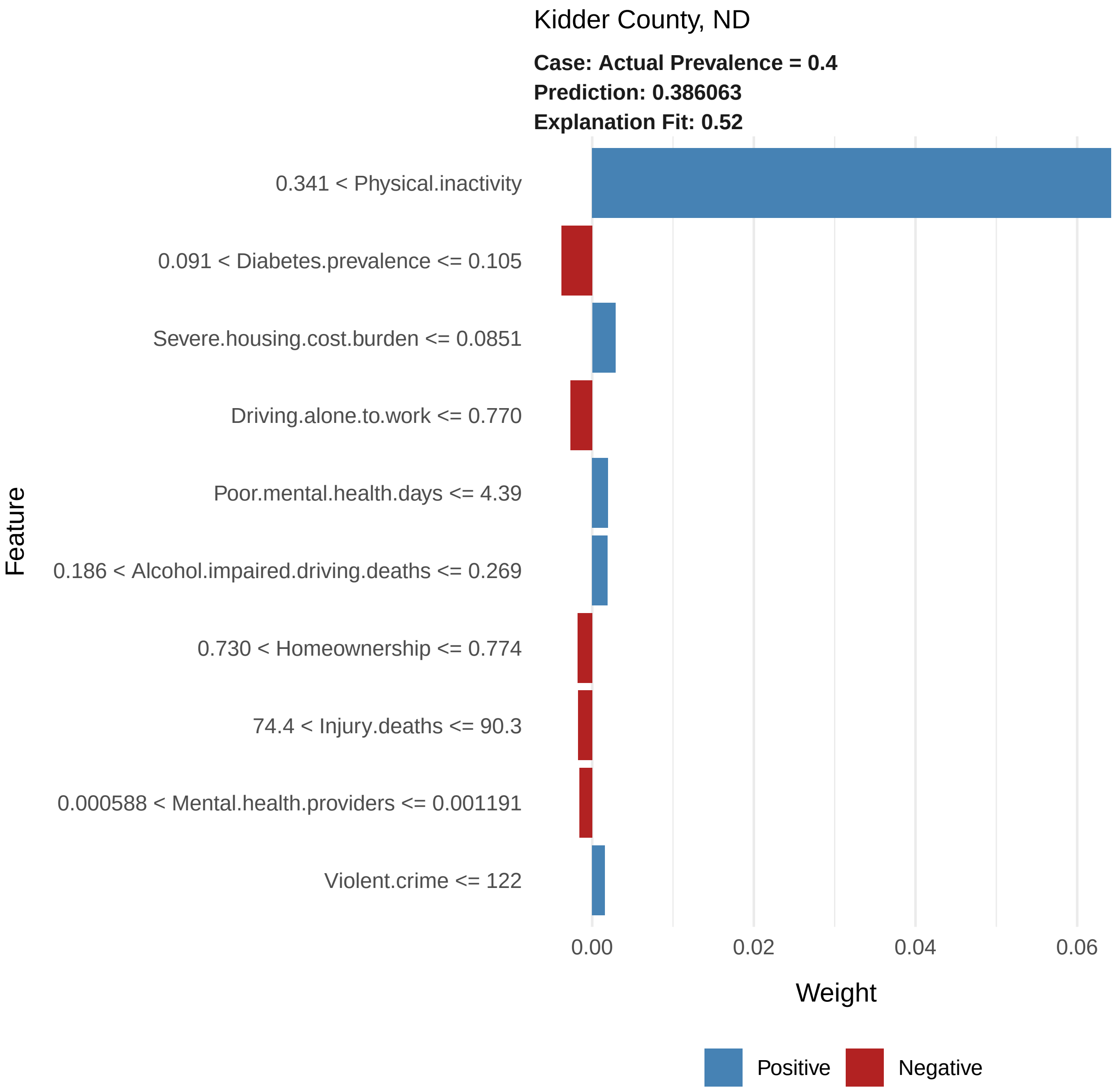

Kidder County, ND
Case: Actual Prevalence = 0.4
Prediction: 0.386063
Explanation Fit: 0.52
0.341 < Physical.inactivity
0.091 < Diabetes.prevalence <= 0.105
Severe.housing.cost.burden <= 0.0851
Driving.alone.to.work <= 0.770
Poor.mental.health.days <= 4.39
0.186 < Alcohol.impaired.driving.deaths <= 0.269
0.730 < Homeownership <= 0.774
74.4 < Injury.deaths <= 90.3
0.000588 < Mental.health.providers <= 0.001191
Violent.crime <= 122
Feature
0.00
0.02
0.04
0.06
Weight
Positive
Negative

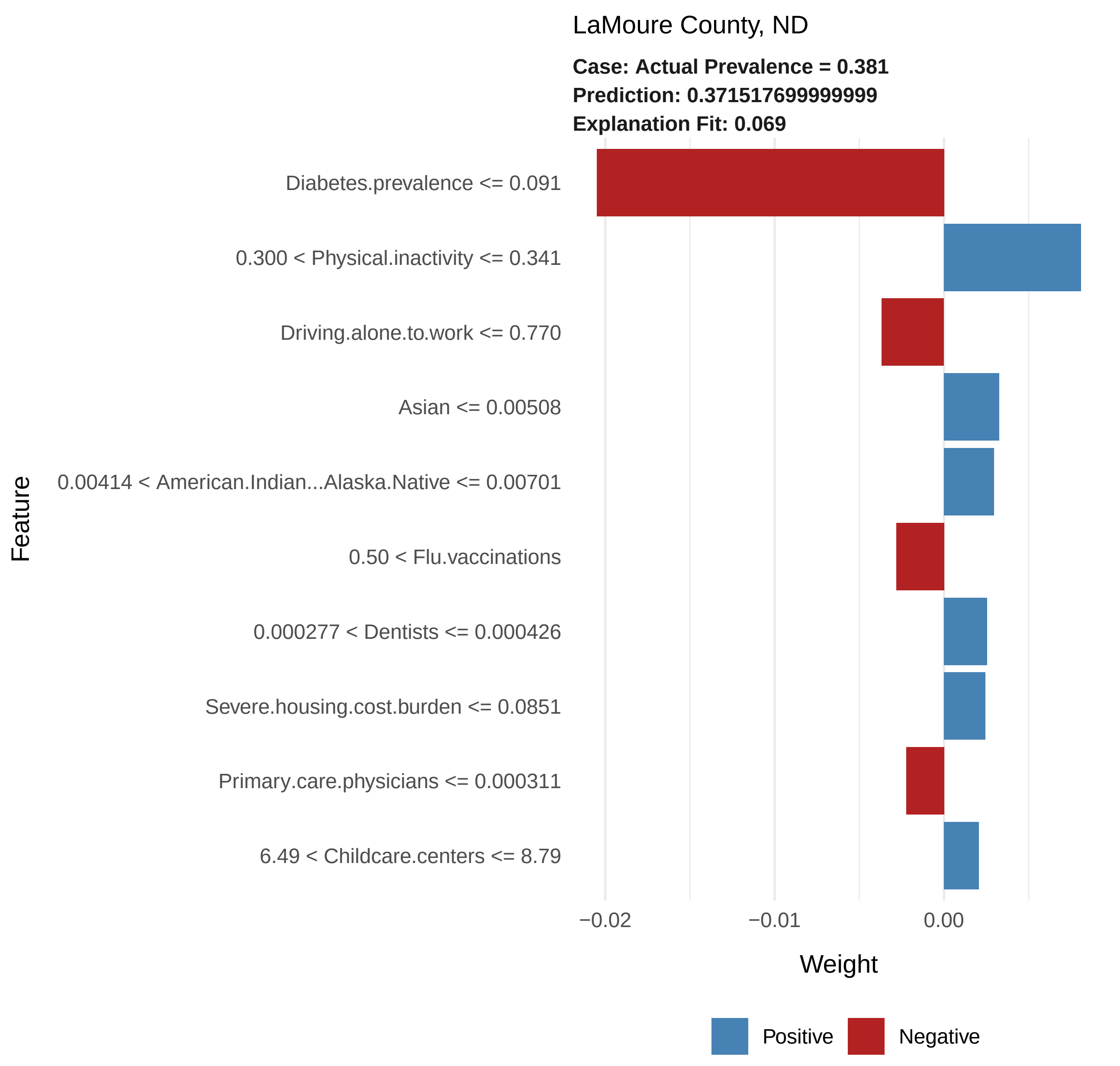

LaMoure County, ND
Case: Actual Prevalence = 0.381
Prediction: 0.371517699999999
Explanation Fit: 0.069
Diabetes.prevalence <= 0.091
0.300 < Physical.inactivity <= 0.341
Driving.alone.to.work <= 0.770
Asian <= 0.00508
0.00414 < American.Indian...Alaska.Native <= 0.00701
0.50 < Flu.vaccinations
0.000277 < Dentists <= 0.000426
Severe.housing.cost.burden <= 0.0851
Primary.care.physicians <= 0.000311
6.49 < Childcare.centers <= 8.79
Feature
−0.02
−0.01
0.00
Weight
Positive
Negative

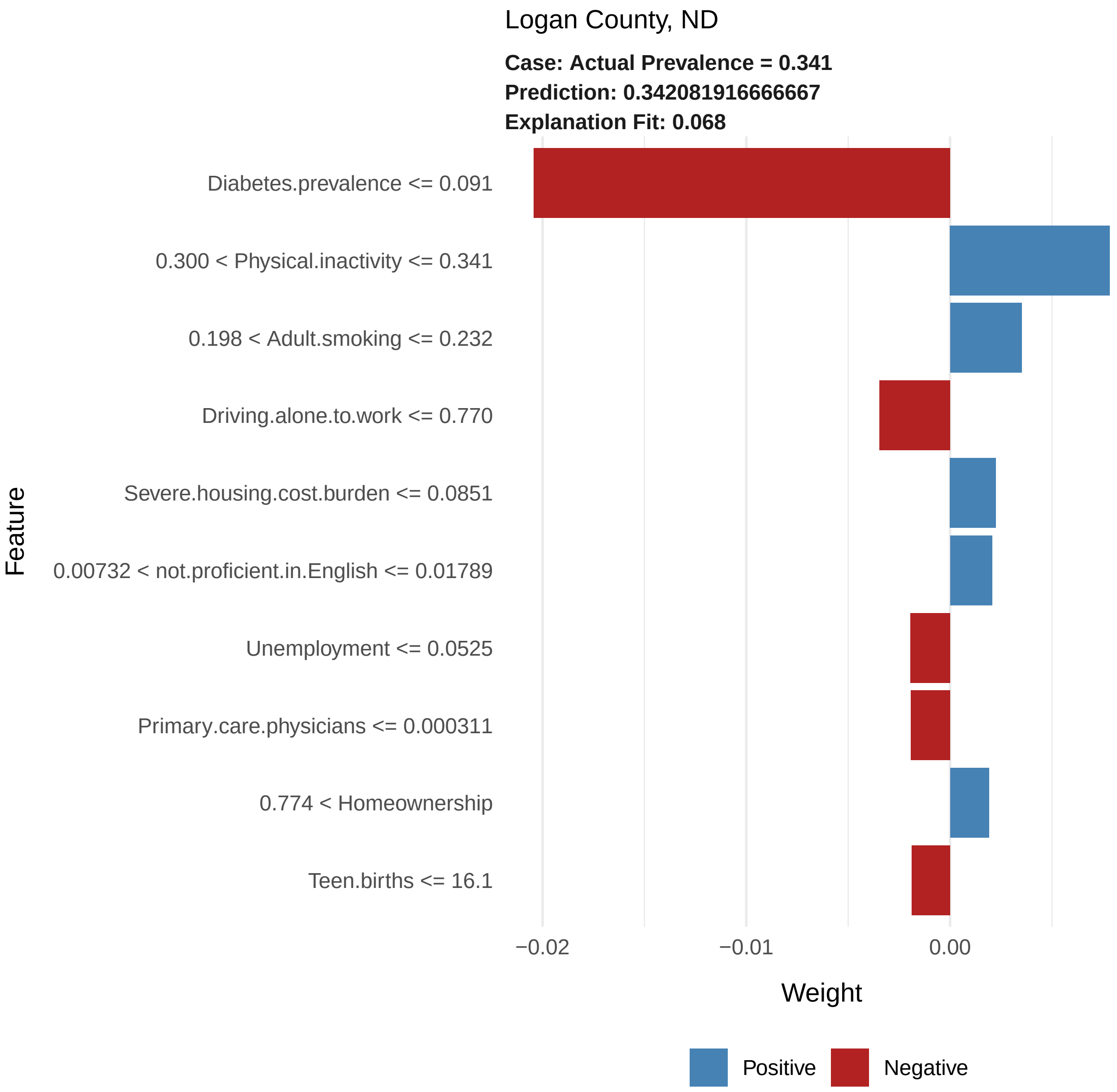

Logan County, ND
Case: Actual Prevalence = 0.341
Prediction: 0.342081916666667
Explanation Fit: 0.068
Diabetes.prevalence <= 0.091
0.300 < Physical.inactivity <= 0.341
0.198 < Adult.smoking <= 0.232
Driving.alone.to.work <= 0.770
Severe.housing.cost.burden <= 0.0851
0.00732 < not.proficient.in.English <= 0.01789
Unemployment <= 0.0525
Primary.care.physicians <= 0.000311
0.774 < Homeownership
Teen.births <= 16.1
Feature
−0.02
−0.01
0.00
Weight
Positive
Negative

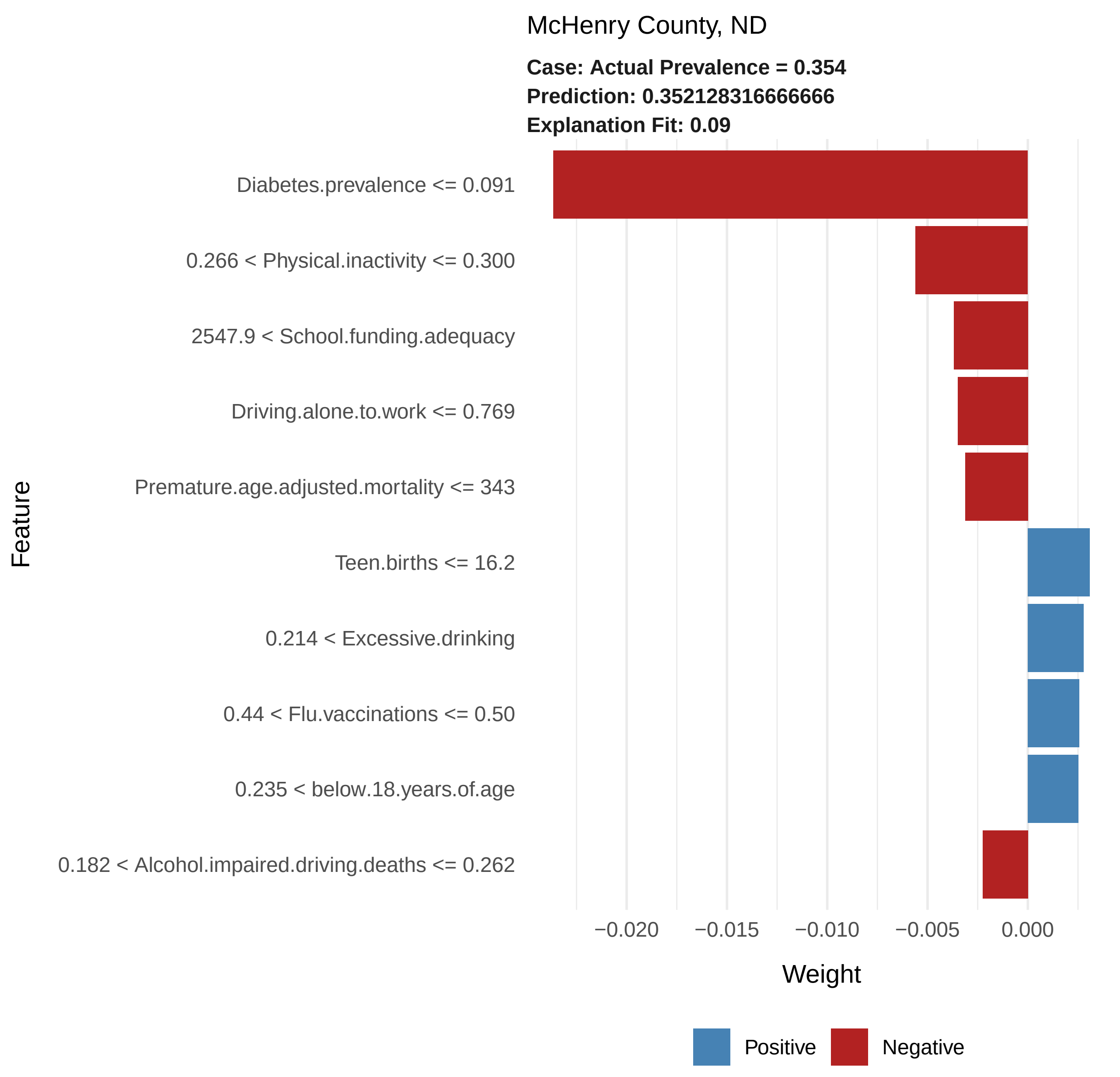
McHenry County, ND
Case: Actual Prevalence = 0.354
Prediction: 0.352128316666666
Explanation Fit: 0.09
Diabetes.prevalence <= 0.091
0.266 < Physical.inactivity <= 0.300
2547.9 < School.funding.adequacy
Driving.alone.to.work <= 0.769
Premature.age.adjusted.mortality <= 343
Teen.births <= 16.2
0.214 < Excessive.drinking
0.44 < Flu.vaccinations <= 0.50
0.235 < below.18.years.of.age
0.182 < Alcohol.impaired.driving.deaths <= 0.262
Feature
−0.020
−0.015
−0.010
−0.005
0.000
Weight
Positive
Negative

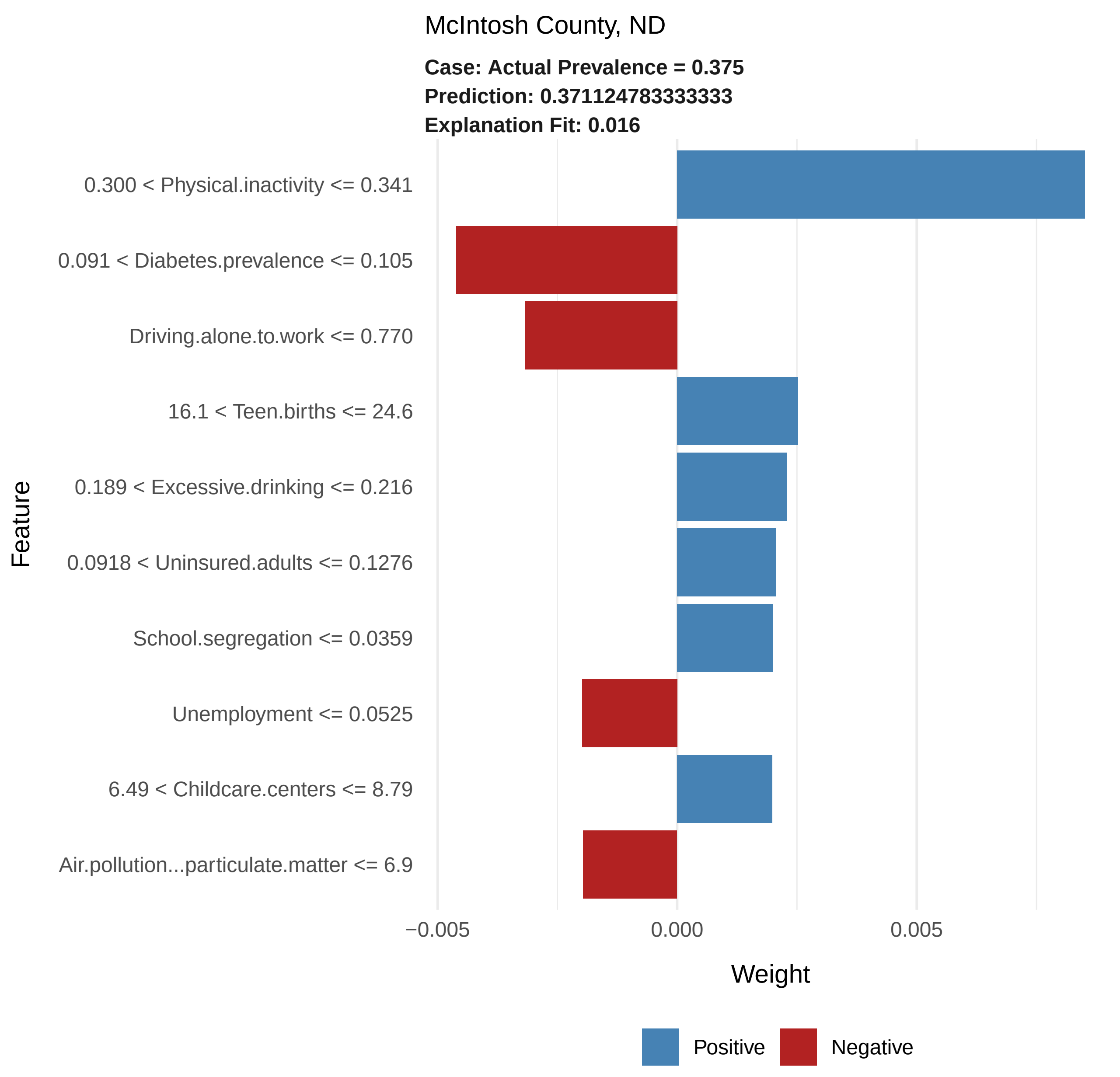

McIntosh County, ND
Case: Actual Prevalence = 0.375
Prediction: 0.371124783333333
Explanation Fit: 0.016
0.300 < Physical.inactivity <= 0.341
0.091 < Diabetes.prevalence <= 0.105
Driving.alone.to.work <= 0.770
16.1 < Teen.births <= 24.6
0.189 < Excessive.drinking <= 0.216
0.0918 < Uninsured.adults <= 0.1276
School.segregation <= 0.0359
Unemployment <= 0.0525
6.49 < Childcare.centers <= 8.79
Air.pollution...particulate.matter <= 6.9
Feature
−0.005
0.000
0.005
Weight
Positive
Negative

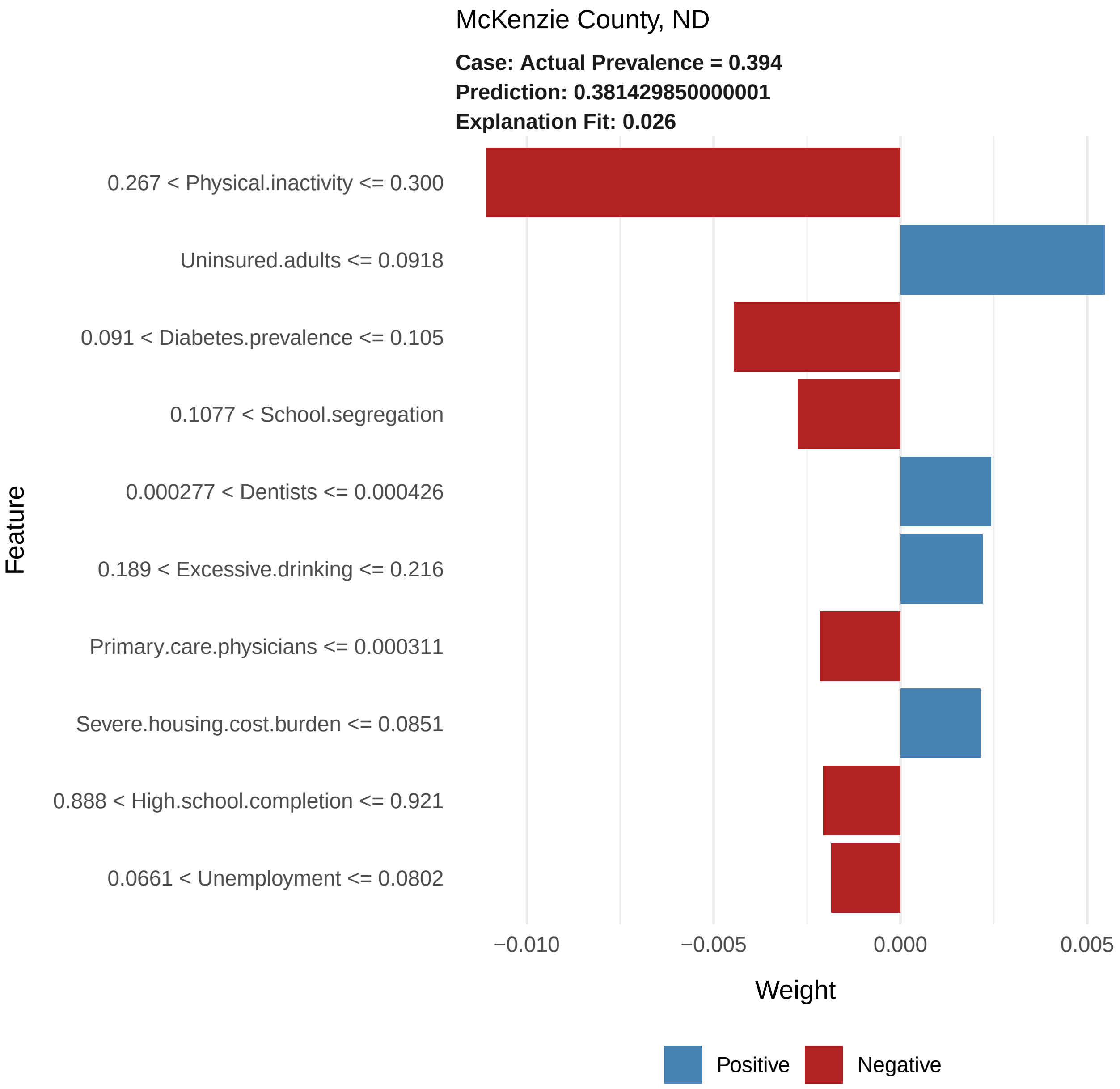
McKenzie County, ND
Case: Actual Prevalence = 0.394
Prediction: 0.381429850000001
Explanation Fit: 0.026
0.267 < Physical.inactivity <= 0.300
Uninsured.adults <= 0.0918
0.091 < Diabetes.prevalence <= 0.105
0.1077 < School.segregation
0.000277 < Dentists <= 0.000426
0.189 < Excessive.drinking <= 0.216
Primary.care.physicians <= 0.000311
Severe.housing.cost.burden <= 0.0851
0.888 < High.school.completion <= 0.921
0.0661 < Unemployment <= 0.0802
Feature
−0.010
−0.005
0.000
0.005
Weight
Positive
Negative

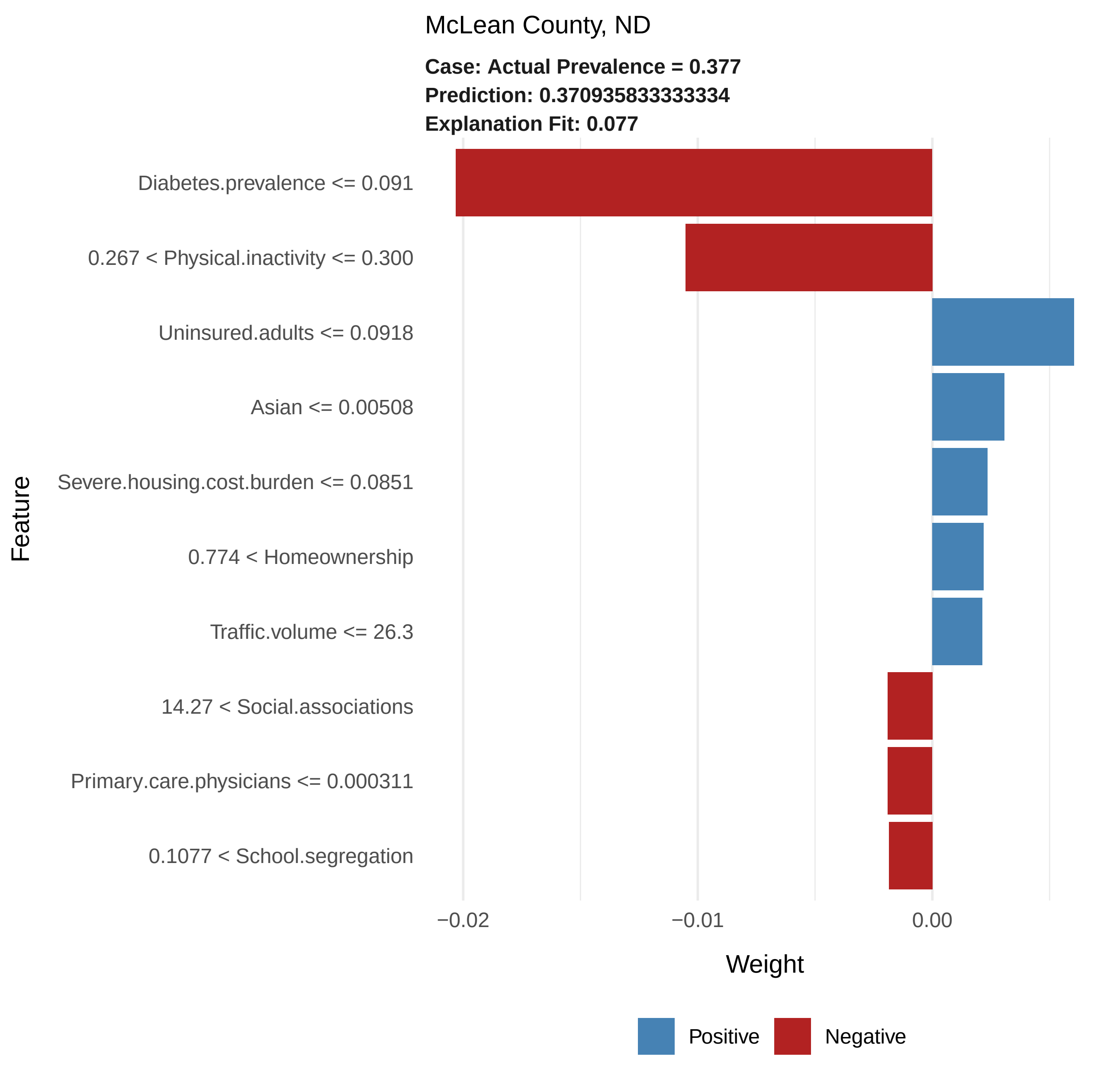

McLean County, ND
Case: Actual Prevalence = 0.377
Prediction: 0.370935833333334
Explanation Fit: 0.077
Diabetes.prevalence <= 0.091
0.267 < Physical.inactivity <= 0.300
Uninsured.adults <= 0.0918
Asian <= 0.00508
Severe.housing.cost.burden <= 0.0851
0.774 < Homeownership
Traffic.volume <= 26.3
14.27 < Social.associations
Primary.care.physicians <= 0.000311
0.1077 < School.segregation
Feature
−0.02
−0.01
0.00
Weight
Positive
Negative

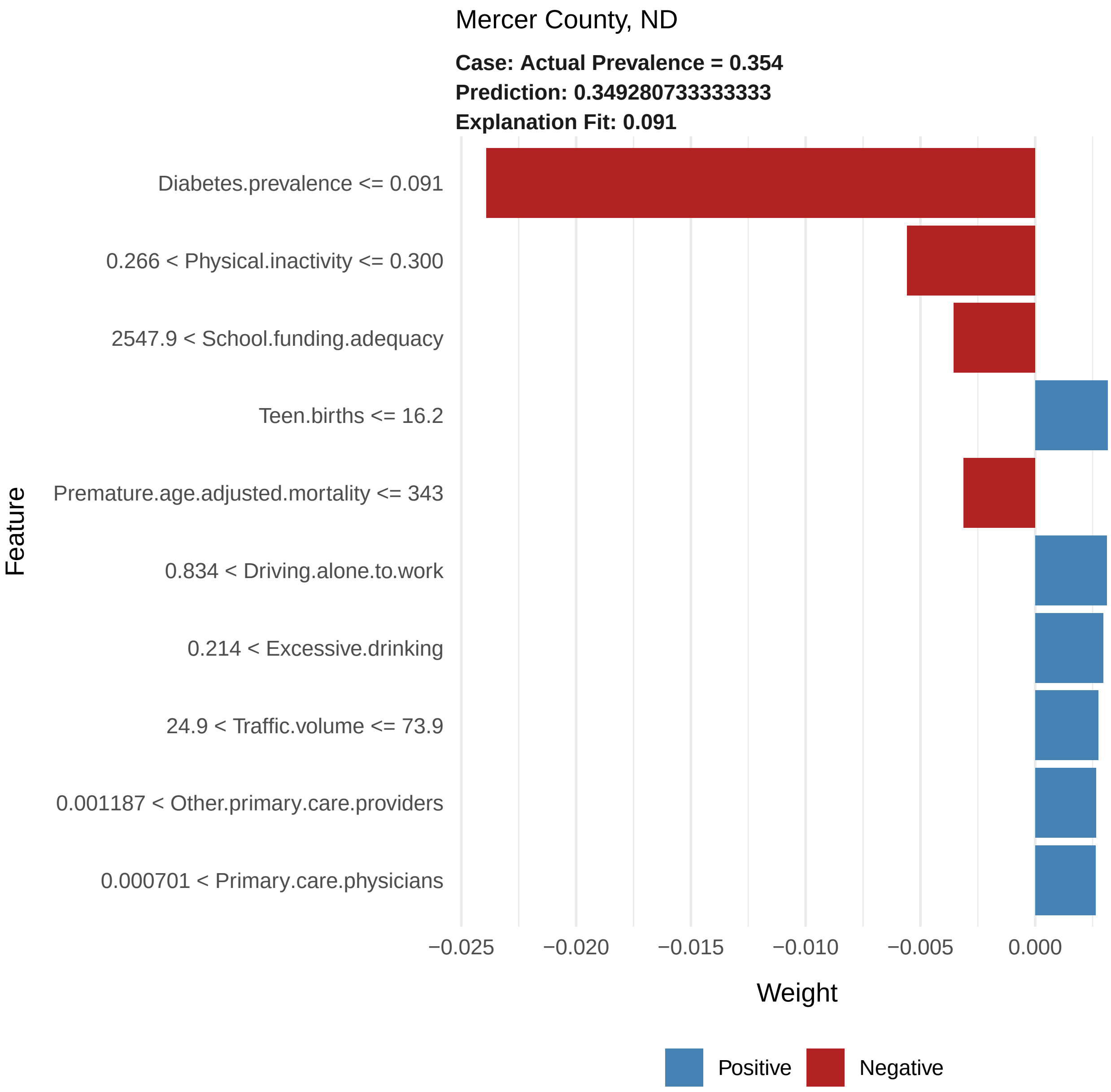

Mercer County, ND
Case: Actual Prevalence = 0.354
Prediction: 0.349280733333333
Explanation Fit: 0.091
Diabetes.prevalence <= 0.091
0.266 < Physical.inactivity <= 0.300
2547.9 < School.funding.adequacy
Teen.births <= 16.2
Premature.age.adjusted.mortality <= 343
0.834 < Driving.alone.to.work
0.214 < Excessive.drinking
24.9 < Traffic.volume <= 73.9
0.001187 < Other.primary.care.providers
0.000701 < Primary.care.physicians
Feature
−0.025
−0.020
−0.015
−0.010
−0.005
0.000
Weight
Positive
Negative

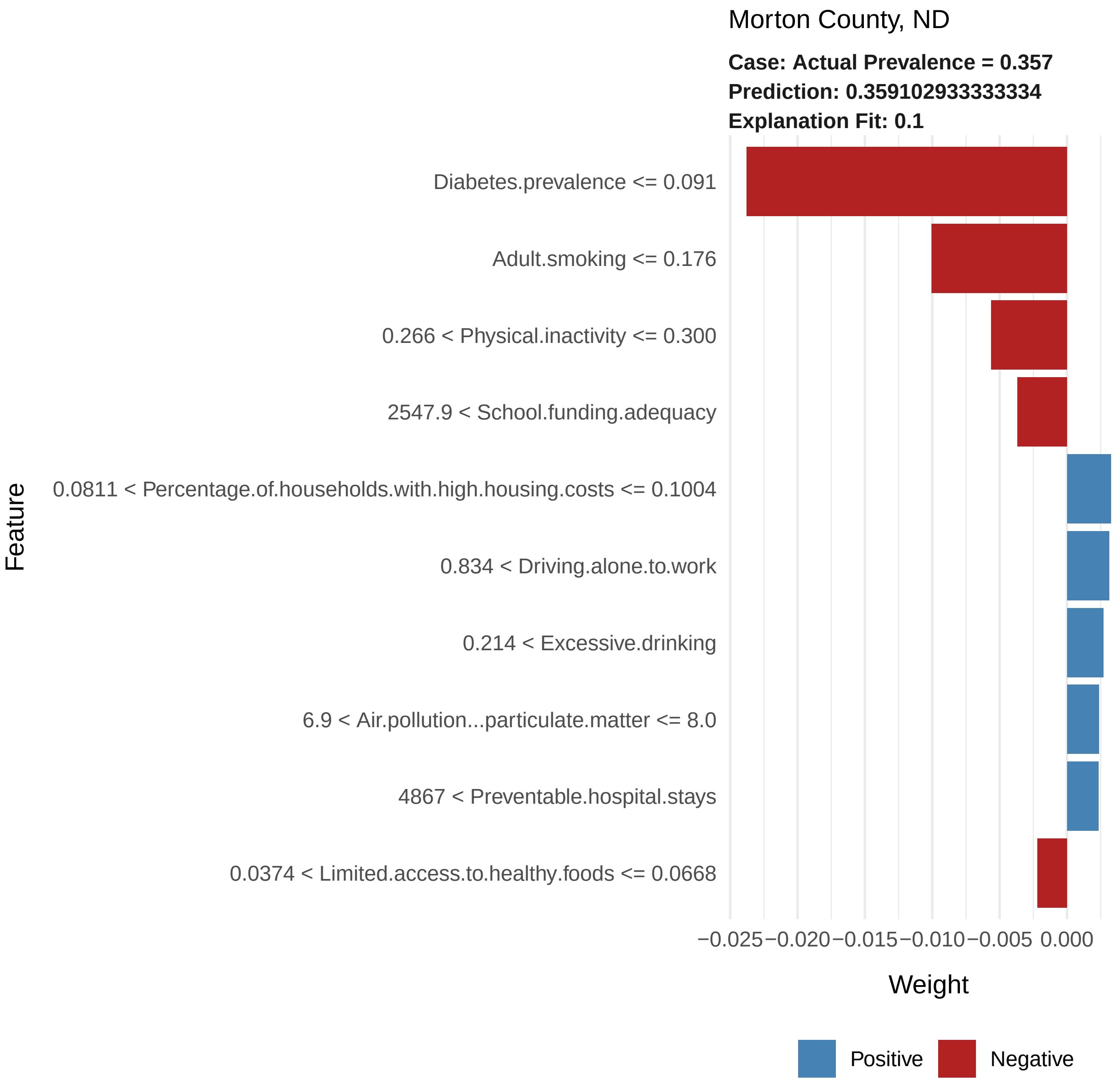
Morton County, ND
Case: Actual Prevalence = 0.357
Prediction: 0.359102933333334
Explanation Fit: 0.1
Diabetes.prevalence <= 0.091
Adult.smoking <= 0.176
0.266 < Physical.inactivity <= 0.300
2547.9 < School.funding.adequacy
0.0811 < Percentage.of.households.with.high.housing.costs <= 0.1004
0.834 < Driving.alone.to.work
0.214 < Excessive.drinking
6.9 < Air.pollution...particulate.matter <= 8.0
4867 < Preventable.hospital.stays
0.0374 < Limited.access.to.healthy.foods <= 0.0668
Feature
−0.025 −0.020 −0.015 −0.010 −0.005 0.000
Weight
Positive
Negative

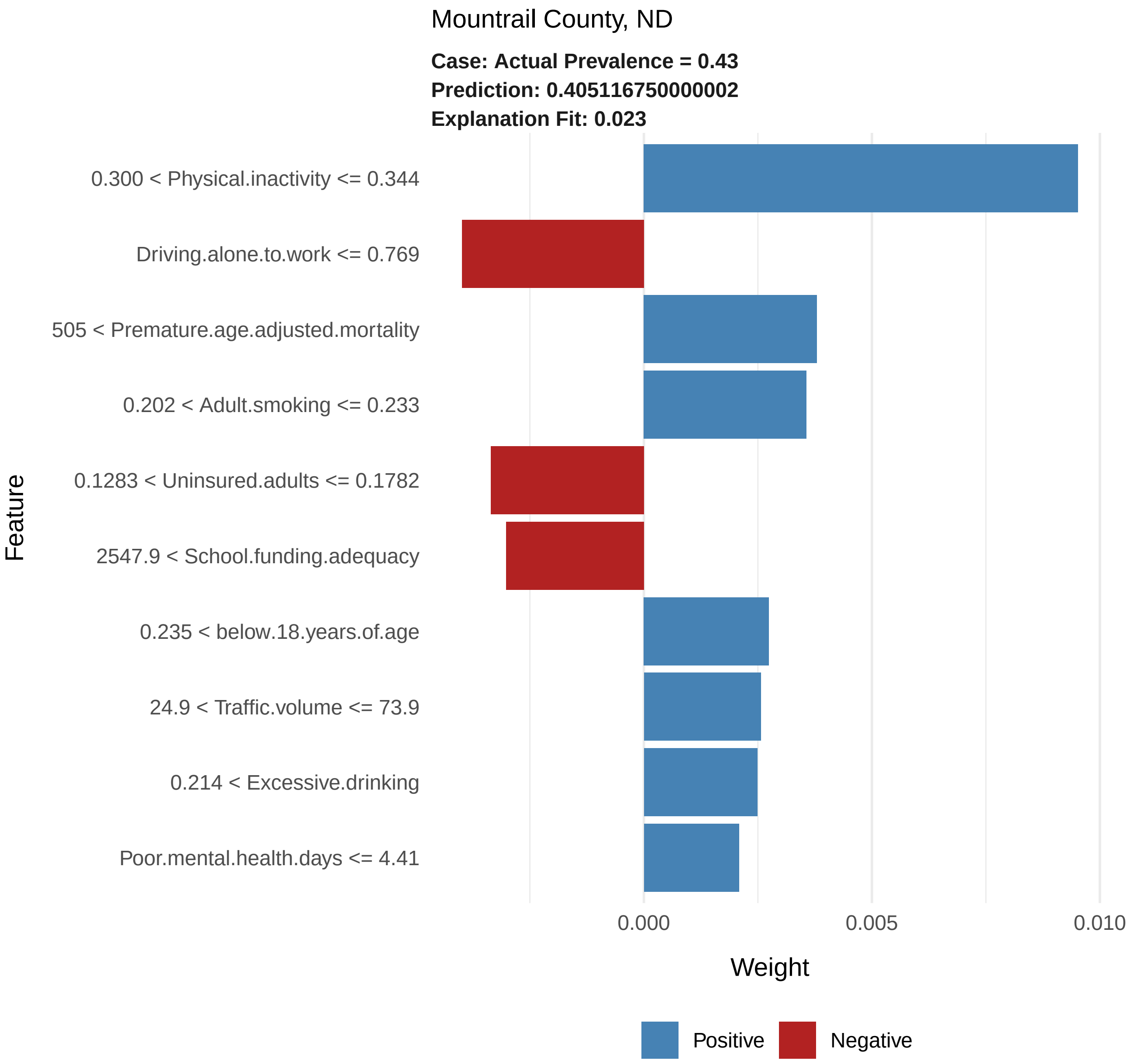

Mountrail County, ND
Case: Actual Prevalence = 0.43
Prediction: 0.405116750000002
Explanation Fit: 0.023
0.300 < Physical.inactivity <= 0.344
Driving.alone.to.work <= 0.769
505 < Premature.age.adjusted.mortality
0.202 < Adult.smoking <= 0.233
0.1283 < Uninsured.adults <= 0.1782
2547.9 < School.funding.adequacy
0.235 < below.18.years.of.age
24.9 < Traffic.volume <= 73.9
0.214 < Excessive.drinking
Poor.mental.health.days <= 4.41
Feature
0.000
0.005
0.010
Weight
Positive
Negative

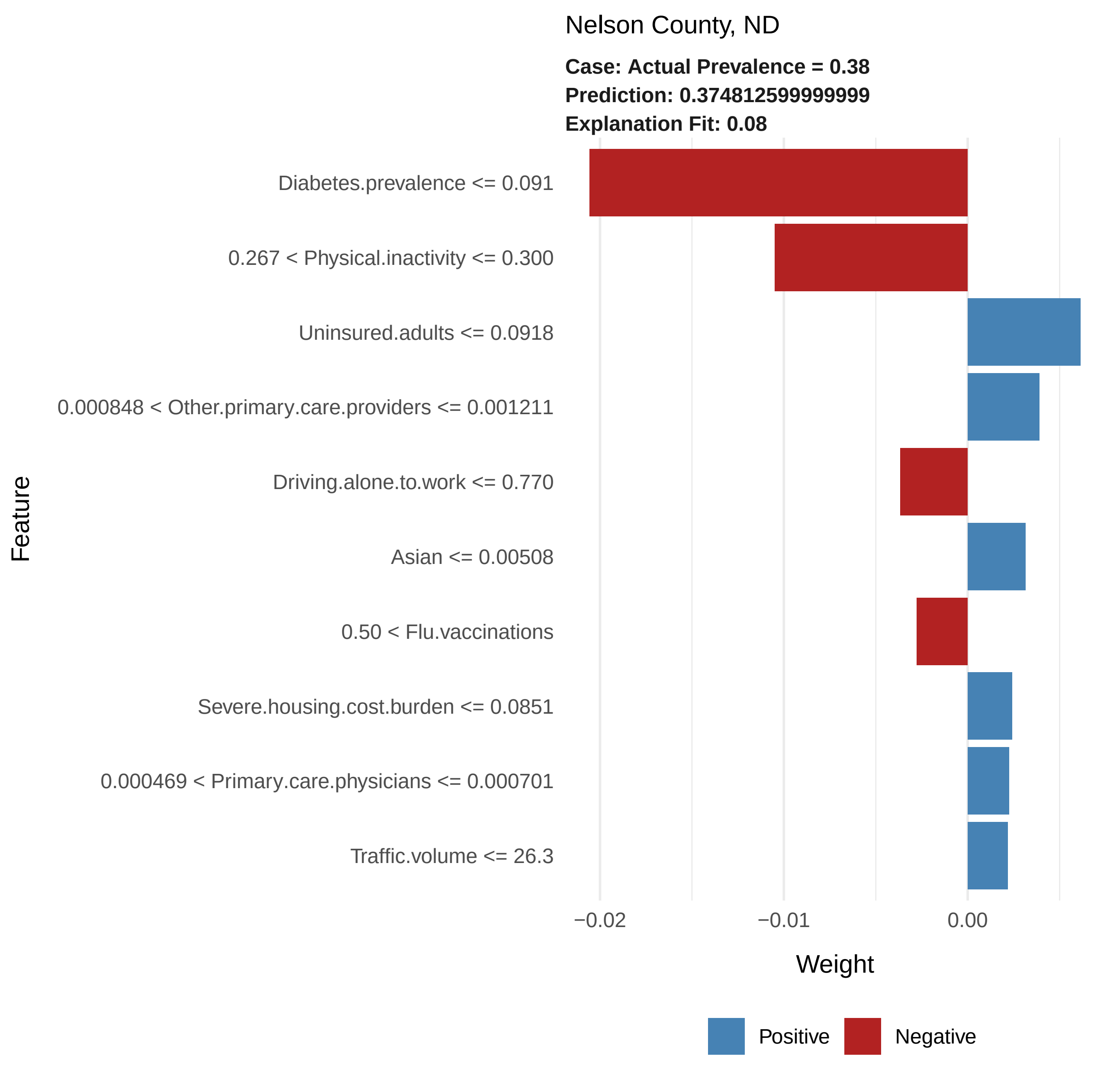
Nelson County, ND
Case: Actual Prevalence = 0.38
Prediction: 0.374812599999999
Explanation Fit: 0.08
Diabetes.prevalence <= 0.091
0.267 < Physical.inactivity <= 0.300
Uninsured.adults <= 0.0918
0.000848 < Other.primary.care.providers <= 0.001211
Driving.alone.to.work <= 0.770
Asian <= 0.00508
0.50 < Flu.vaccinations
Severe.housing.cost.burden <= 0.0851
0.000469 < Primary.care.physicians <= 0.000701
Traffic.volume <= 26.3
Feature
−0.02
−0.01
0.00
Weight
Positive
Negative

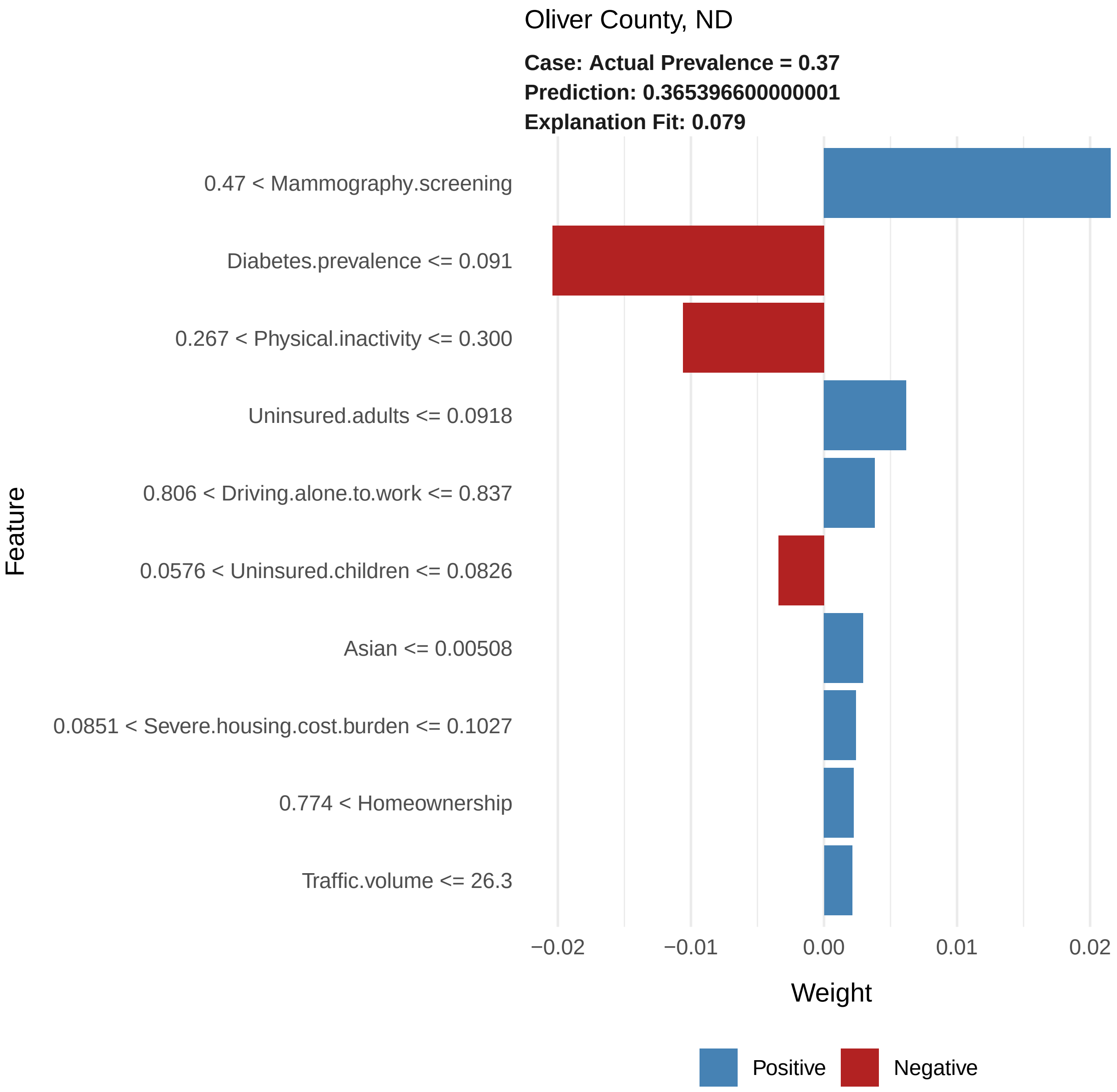

Oliver County, ND
Case: Actual Prevalence = 0.37
Prediction: 0.365396600000001
Explanation Fit: 0.079
0.47 < Mammography.screening
Diabetes.prevalence <= 0.091
0.267 < Physical.inactivity <= 0.300
Uninsured.adults <= 0.0918
0.806 < Driving.alone.to.work <= 0.837
0.0576 < Uninsured.children <= 0.0826
Asian <= 0.00508
0.0851 < Severe.housing.cost.burden <= 0.1027
0.774 < Homeownership
Traffic.volume <= 26.3
Feature
−0.02
−0.01
0.00
0.01
0.02
Weight
Positive
Negative

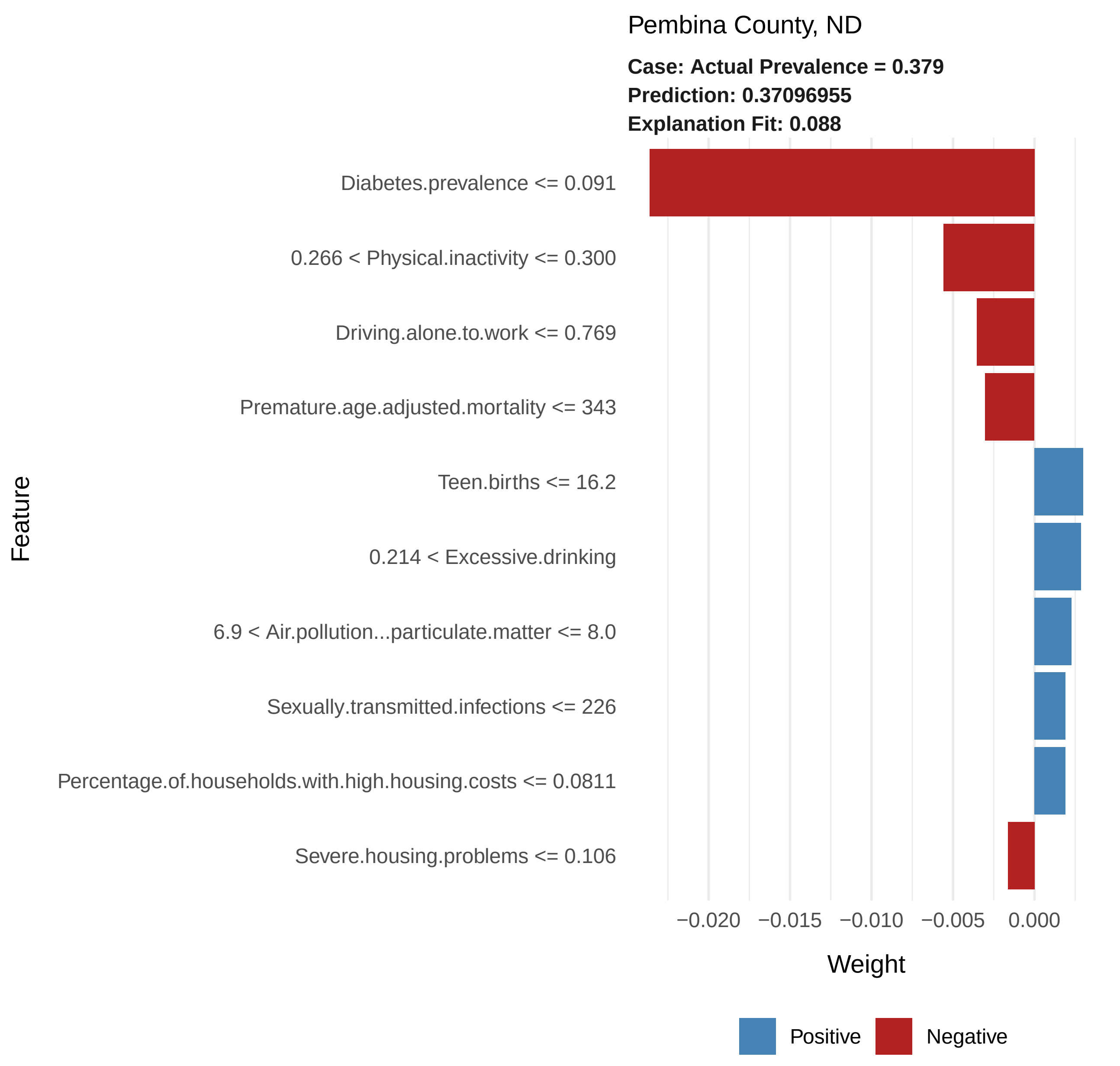

Pembina County, ND
Case: Actual Prevalence = 0.379
Prediction: 0.37096955
Explanation Fit: 0.088
Diabetes.prevalence <= 0.091
0.266 < Physical.inactivity <= 0.300
Driving.alone.to.work <= 0.769
Premature.age.adjusted.mortality <= 343
Teen.births <= 16.2
0.214 < Excessive.drinking
6.9 < Air.pollution...particulate.matter <= 8.0
Sexually.transmitted.infections <= 226
Percentage.of.households.with.high.housing.costs <= 0.0811
Severe.housing.problems <= 0.106
Feature
−0.020
−0.015
−0.010
−0.005
0.000
Weight
Positive
Negative

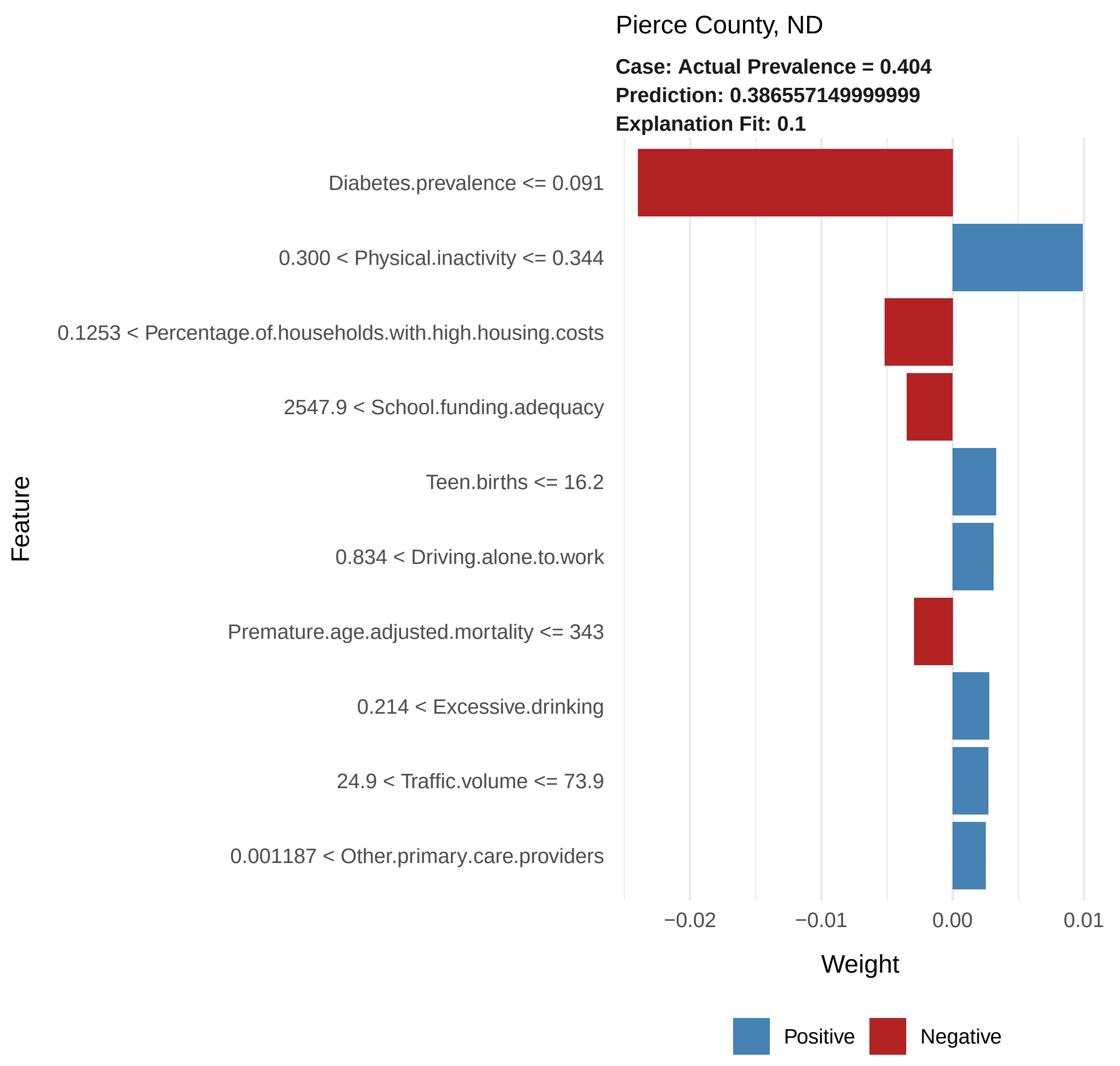

Pierce County, ND
Case: Actual Prevalence = 0.404
Prediction: 0.386557149999999
Explanation Fit: 0.1
Diabetes.prevalence <= 0.091
0.300 < Physical.inactivity <= 0.344
0.1253 < Percentage.of.households.with.high.housing.costs
2547.9 < School.funding.adequacy
Teen.births <= 16.2
0.834 < Driving.alone.to.work
Premature.age.adjusted.mortality <= 343
0.214 < Excessive.drinking
24.9 < Traffic.volume <= 73.9
0.001187 < Other.primary.care.providers
Feature
−0.02
−0.01
0.00
0.01
Weight
Positive
Negative

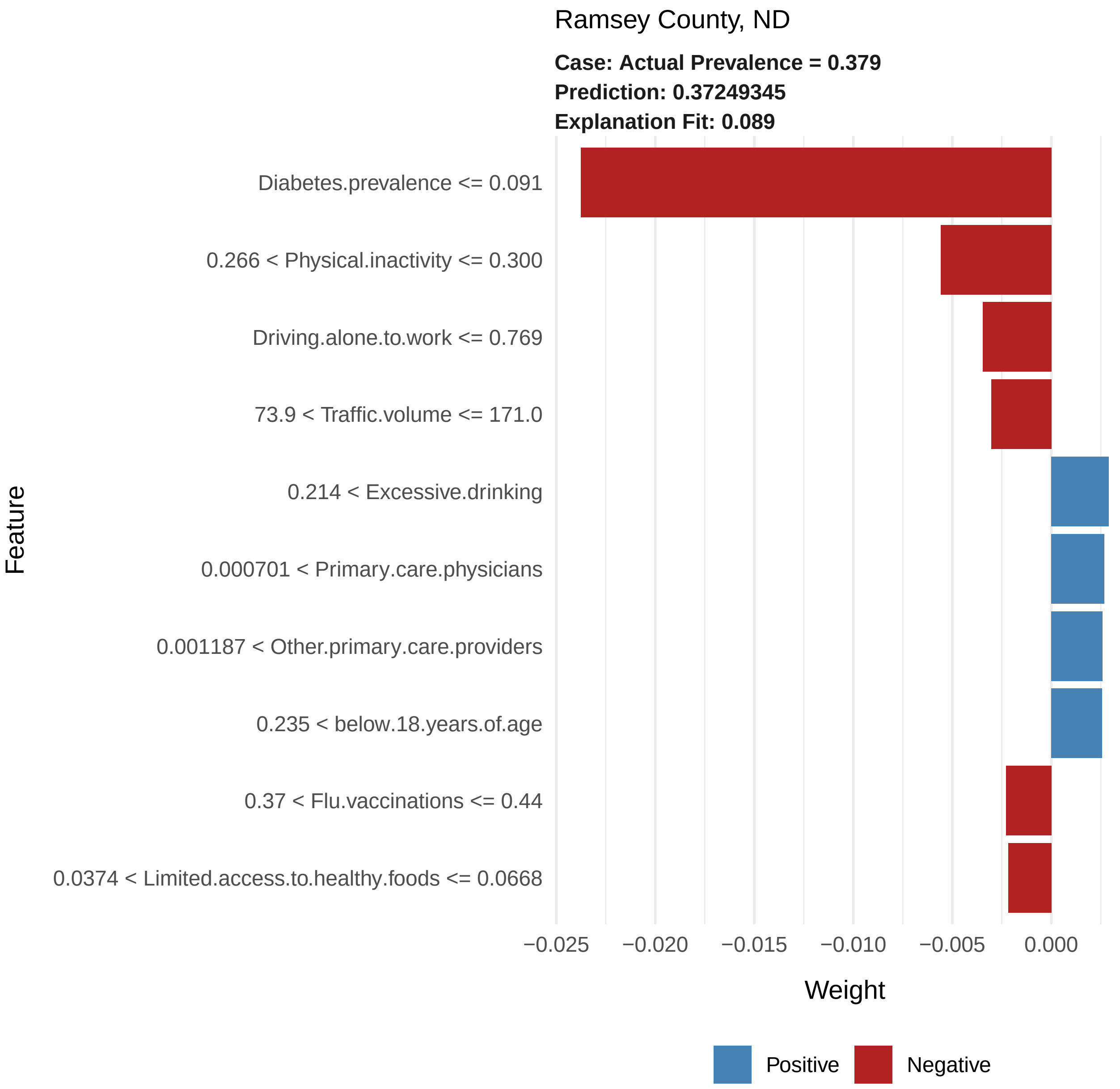
Ramsey County, ND
Case: Actual Prevalence = 0.379
Prediction: 0.37249345
Explanation Fit: 0.089
Diabetes.prevalence <= 0.091
0.266 < Physical.inactivity <= 0.300
Driving.alone.to.work <= 0.769
73.9 < Traffic.volume <= 171.0
0.214 < Excessive.drinking
0.000701 < Primary.care.physicians
0.001187 < Other.primary.care.providers
0.235 < below.18.years.of.age
0.37 < Flu.vaccinations <= 0.44
0.0374 < Limited.access.to.healthy.foods <= 0.0668
Feature
−0.025
−0.020
−0.015
−0.010
−0.005
0.000
Weight
Positive
Negative

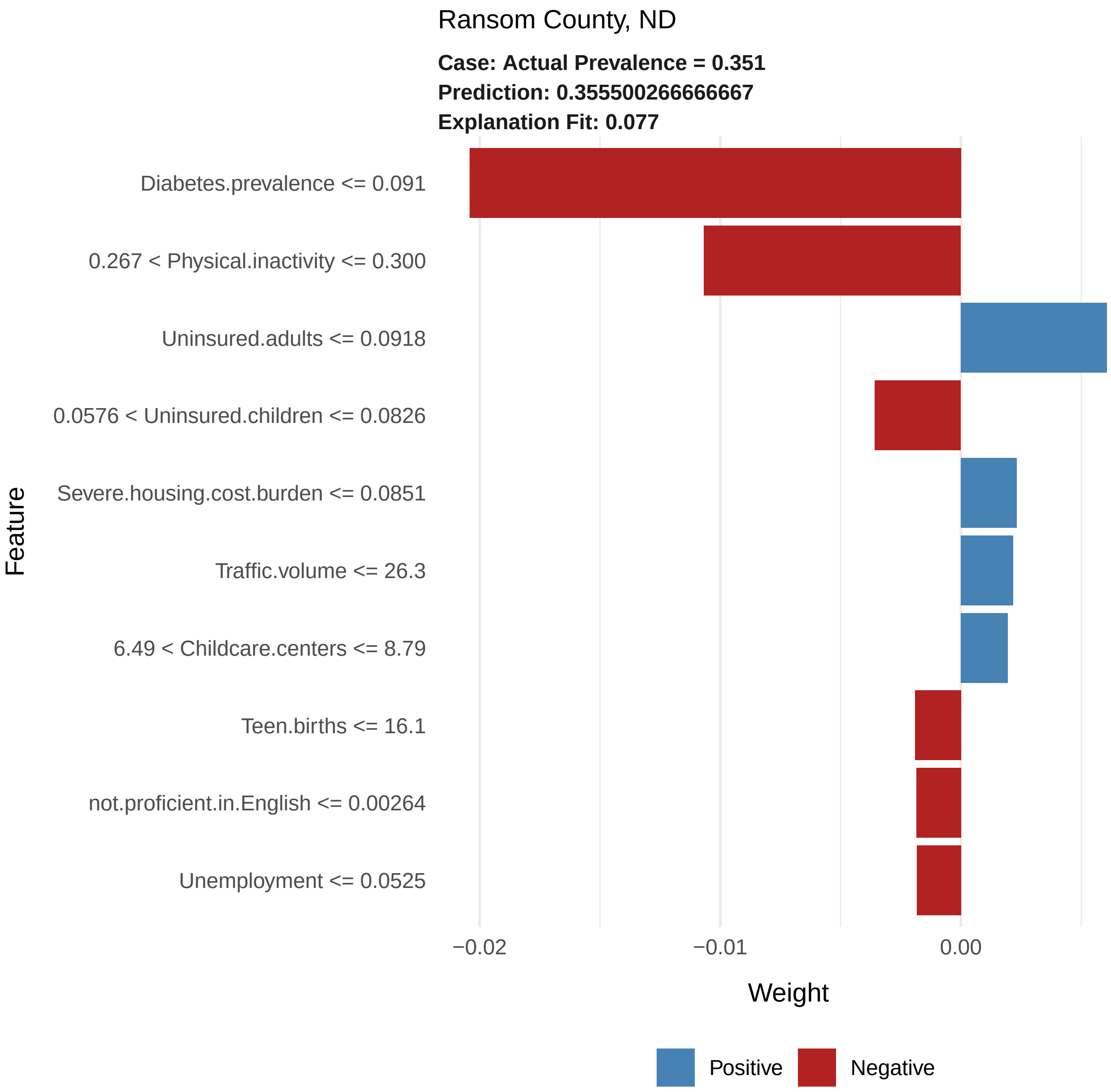

Ransom County, ND
Case: Actual Prevalence = 0.351
Prediction: 0.355500266666667
Explanation Fit: 0.077
Diabetes.prevalence <= 0.091
0.267 < Physical.inactivity <= 0.300
Uninsured.adults <= 0.0918
0.0576 < Uninsured.children <= 0.0826
Severe.housing.cost.burden <= 0.0851
Traffic.volume <= 26.3
6.49 < Childcare.centers <= 8.79
Teen.births <= 16.1
not.proficient.in.English <= 0.00264
Unemployment <= 0.0525
Feature
−0.02
−0.01
0.00
Weight
Positive
Negative

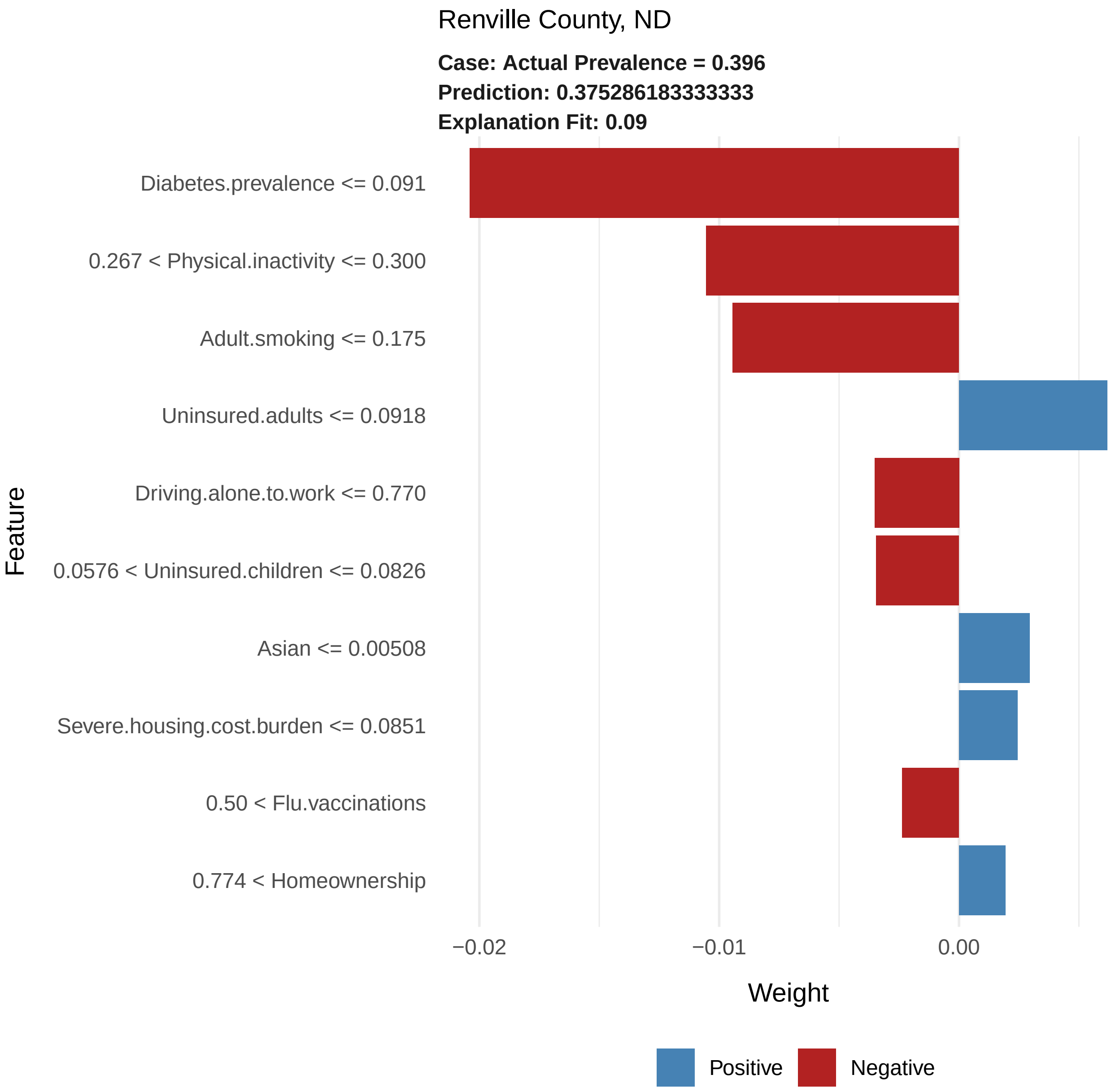

Renville County, ND
Case: Actual Prevalence = 0.396
Prediction: 0.375286183333333
Explanation Fit: 0.09
Diabetes.prevalence <= 0.091
0.267 < Physical.inactivity <= 0.300
Adult.smoking <= 0.175
Uninsured.adults <= 0.0918
Driving.alone.to.work <= 0.770
0.0576 < Uninsured.children <= 0.0826
Asian <= 0.00508
Severe.housing.cost.burden <= 0.0851
0.50 < Flu.vaccinations
0.774 < Homeownership
Feature
−0.02
−0.01
0.00
Weight
Positive
Negative

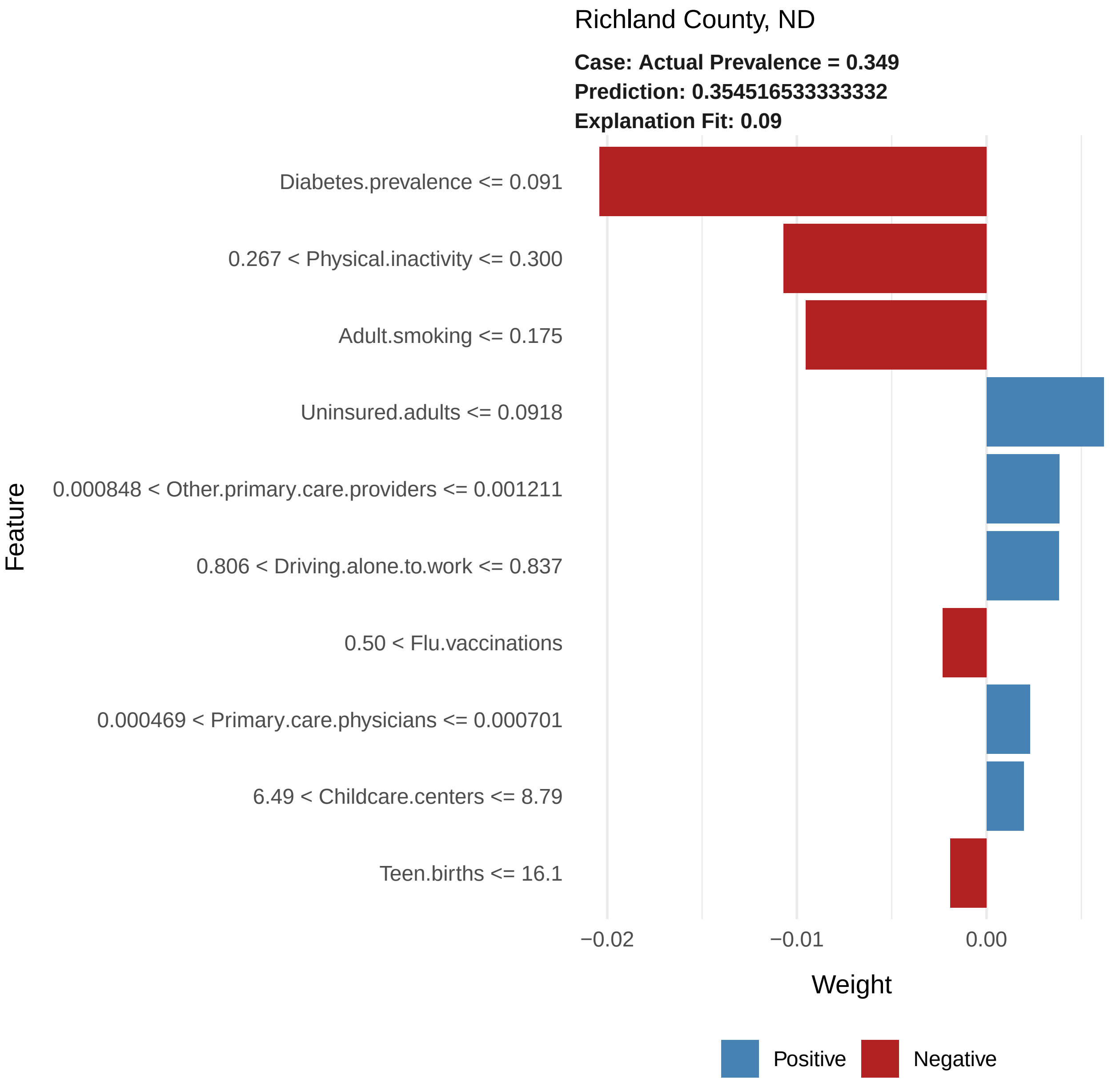
Richland County, ND
Case: Actual Prevalence = 0.349
Prediction: 0.354516533333332
Explanation Fit: 0.09
Diabetes.prevalence <= 0.091
0.267 < Physical.inactivity <= 0.300
Adult.smoking <= 0.175
Uninsured.adults <= 0.0918
0.000848 < Other.primary.care.providers <= 0.001211
0.806 < Driving.alone.to.work <= 0.837
0.50 < Flu.vaccinations
0.000469 < Primary.care.physicians <= 0.000701
6.49 < Childcare.centers <= 8.79
Teen.births <= 16.1
Feature
−0.02
−0.01
0.00
Weight
Positive
Negative

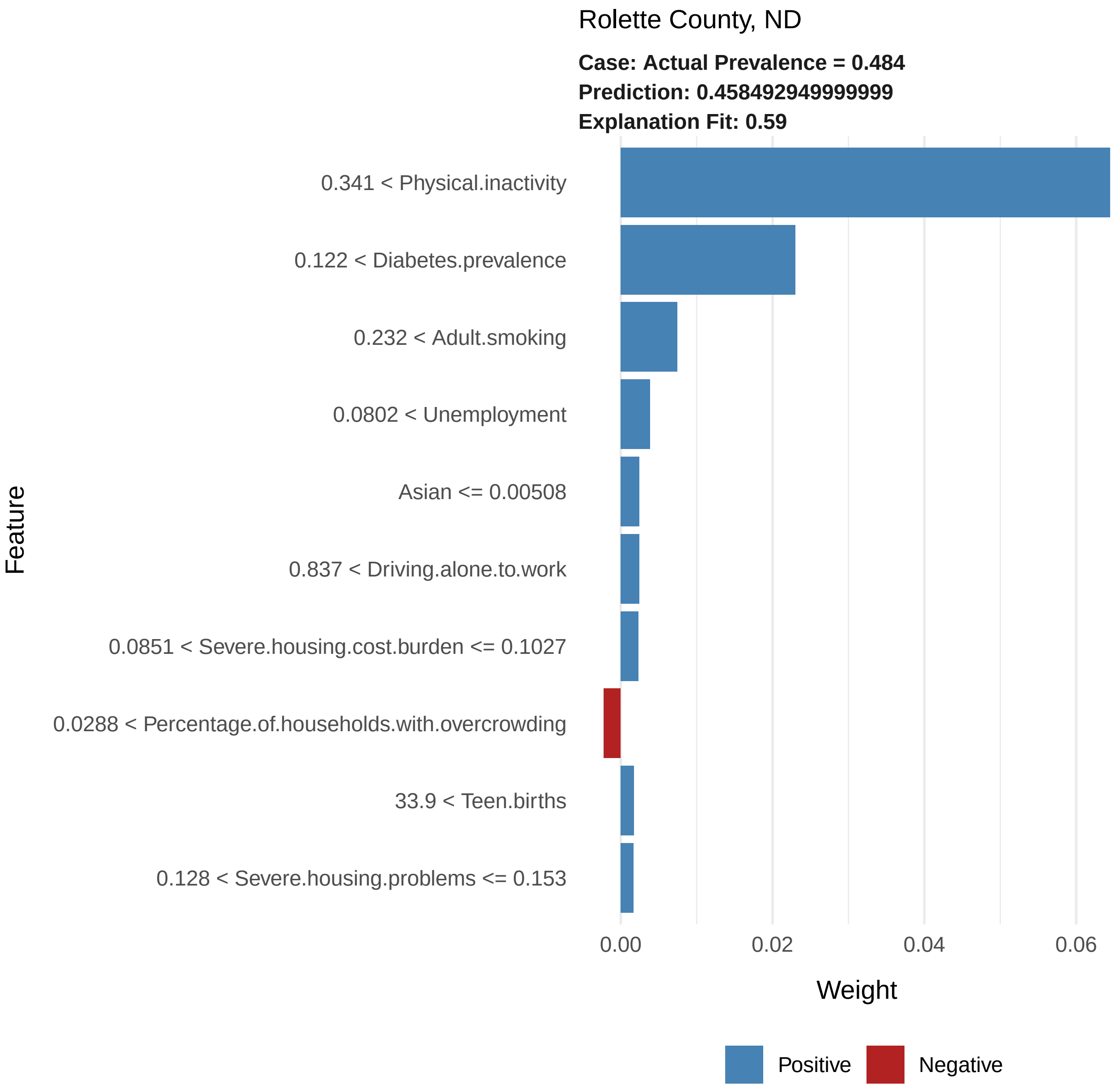

Rolette County, ND
Case: Actual Prevalence = 0.484
Prediction: 0.458492949999999
Explanation Fit: 0.59
0.341 < Physical.inactivity
0.122 < Diabetes.prevalence
0.232 < Adult.smoking
0.0802 < Unemployment
Asian <= 0.00508
0.837 < Driving.alone.to.work
0.0851 < Severe.housing.cost.burden <= 0.1027
0.0288 < Percentage.of.households.with.overcrowding
33.9 < Teen.births
0.128 < Severe.housing.problems <= 0.153
Feature
0.00
0.02
0.04
0.06
Weight
Positive
Negative

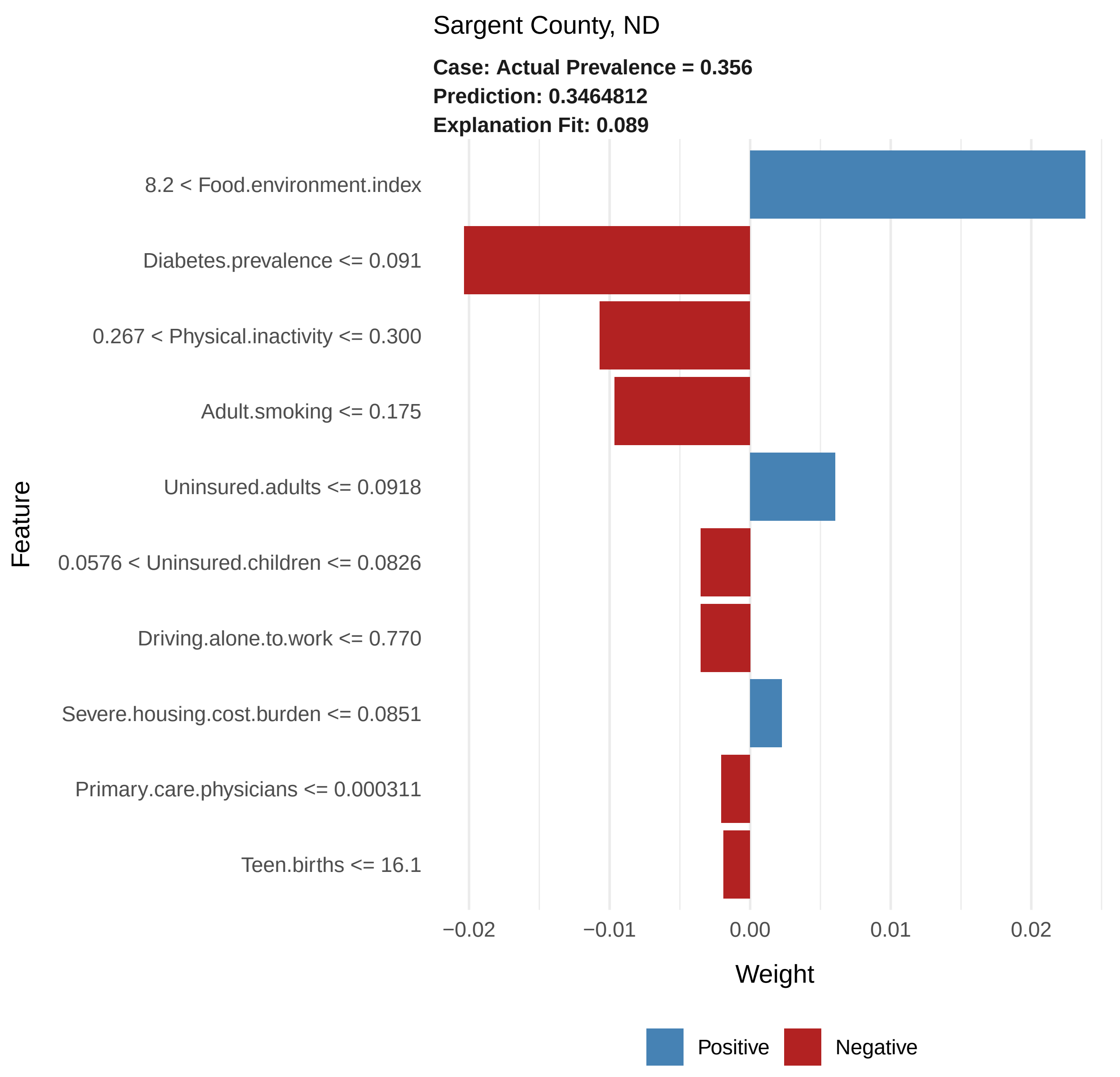

Sargent County, ND
Case: Actual Prevalence = 0.356
Prediction: 0.3464812
Explanation Fit: 0.089
8.2 < Food.environment.index
Diabetes.prevalence <= 0.091
0.267 < Physical.inactivity <= 0.300
Adult.smoking <= 0.175
Uninsured.adults <= 0.0918
0.0576 < Uninsured.children <= 0.0826
Driving.alone.to.work <= 0.770
Severe.housing.cost.burden <= 0.0851
Primary.care.physicians <= 0.000311
Teen.births <= 16.1
Feature
−0.02
−0.01
0.00
0.01
0.02
Weight
Positive
Negative

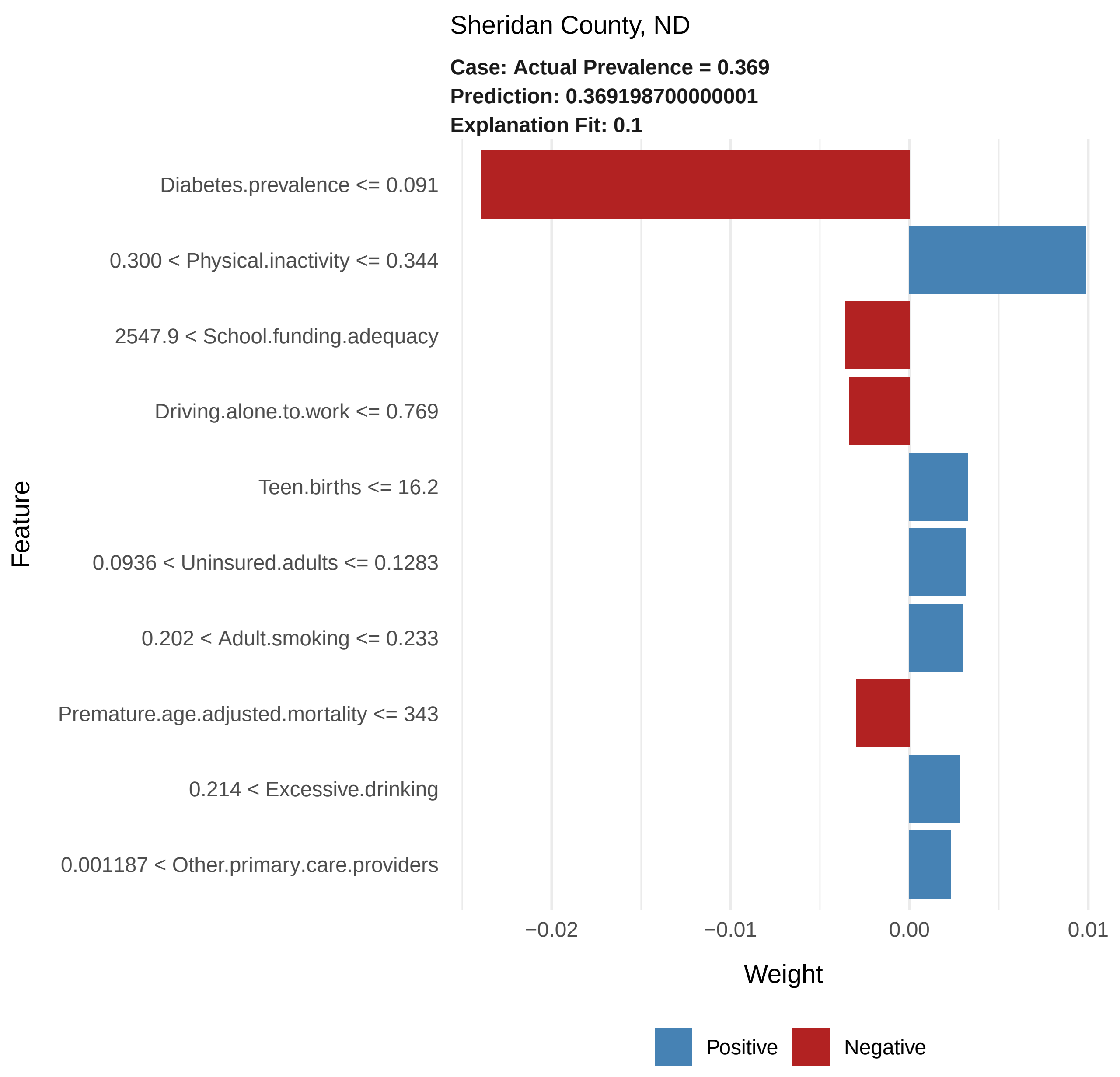

Sheridan County, ND
Case: Actual Prevalence = 0.369
Prediction: 0.369198700000001
Explanation Fit: 0.1
Diabetes.prevalence <= 0.091
0.300 < Physical.inactivity <= 0.344
2547.9 < School.funding.adequacy
Driving.alone.to.work <= 0.769
Teen.births <= 16.2
0.0936 < Uninsured.adults <= 0.1283
0.202 < Adult.smoking <= 0.233
Premature.age.adjusted.mortality <= 343
0.214 < Excessive.drinking
0.001187 < Other.primary.care.providers
Feature
−0.02
−0.01
0.00
0.01
Weight
Positive
Negative

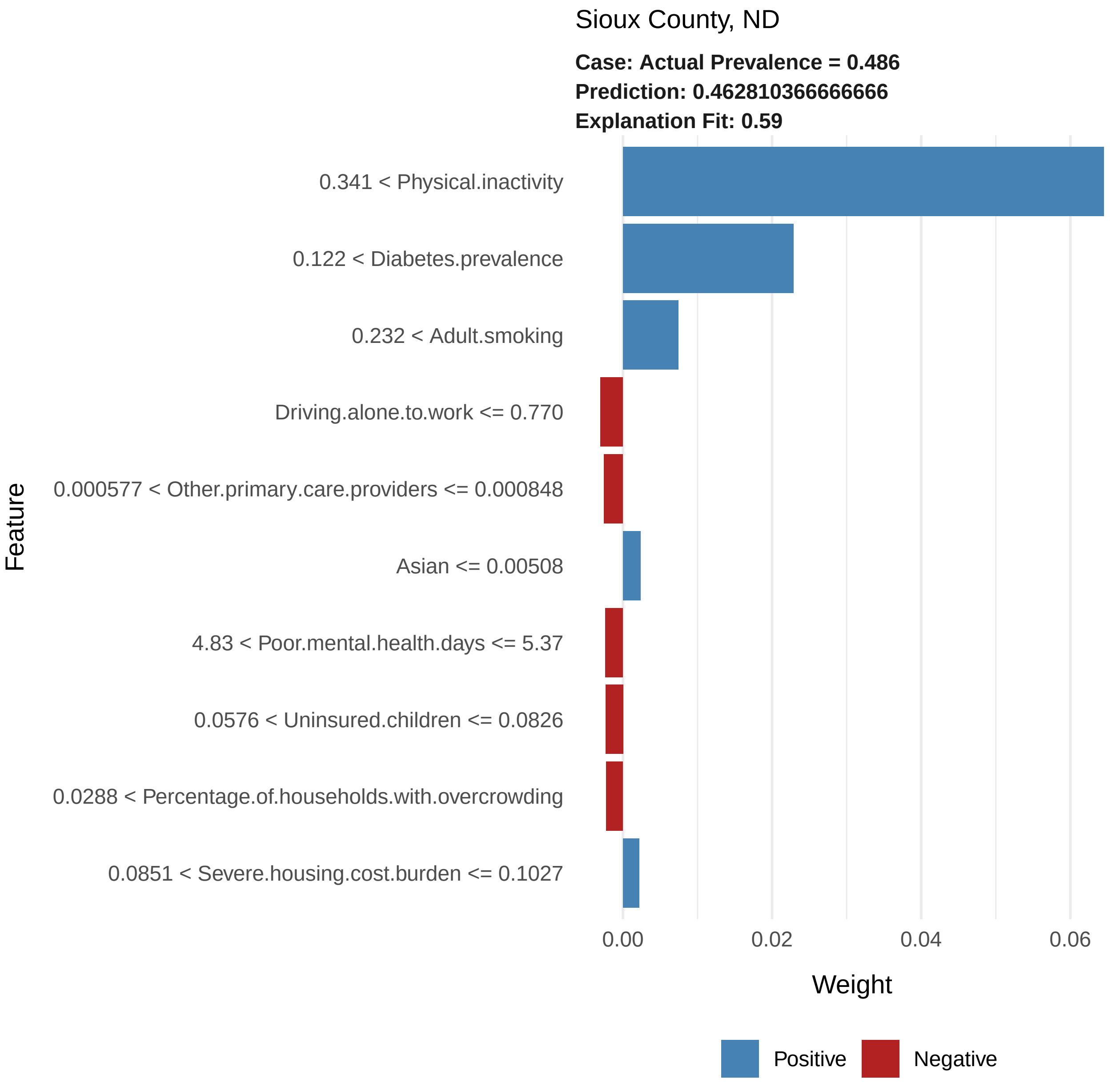
Sioux County, ND
Case: Actual Prevalence = 0.486
Prediction: 0.462810366666666
Explanation Fit: 0.59
0.341 < Physical.inactivity
0.122 < Diabetes.prevalence
0.232 < Adult.smoking
Driving.alone.to.work <= 0.770
0.000577 < Other.primary.care.providers <= 0.000848
Asian <= 0.00508
4.83 < Poor.mental.health.days <= 5.37
0.0576 < Uninsured.children <= 0.0826
0.0288 < Percentage.of.households.with.overcrowding
0.0851 < Severe.housing.cost.burden <= 0.1027
Feature
0.00
0.02
0.04
0.06
Weight
Positive
Negative

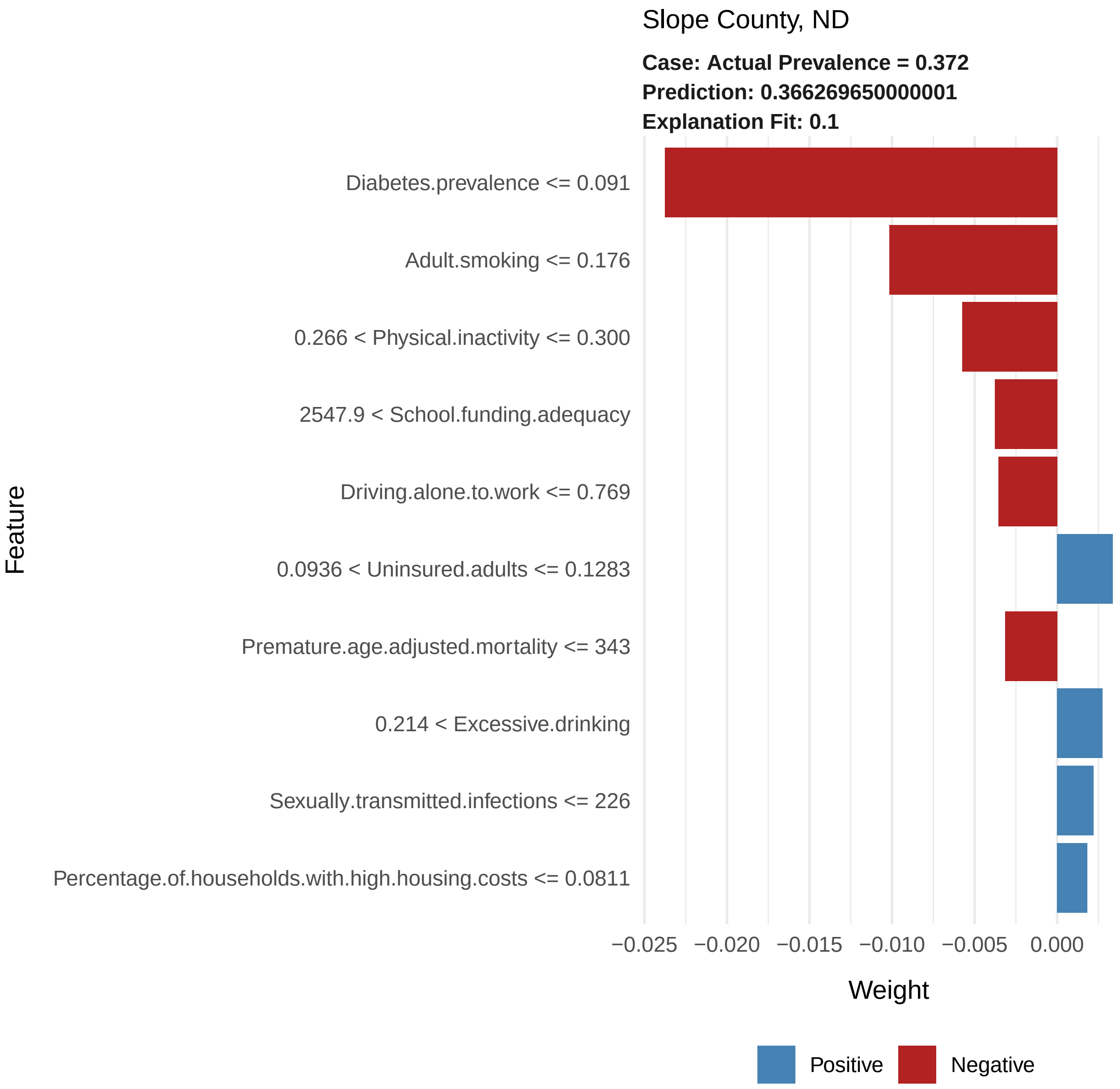
Slope County, ND
Case: Actual Prevalence = 0.372
Prediction: 0.366269650000001
Explanation Fit: 0.1
Diabetes.prevalence <= 0.091
Adult.smoking <= 0.176
0.266 < Physical.inactivity <= 0.300
2547.9 < School.funding.adequacy
Driving.alone.to.work <= 0.769
0.0936 < Uninsured.adults <= 0.1283
Premature.age.adjusted.mortality <= 343
0.214 < Excessive.drinking
Sexually.transmitted.infections <= 226
Percentage.of.households.with.high.housing.costs <= 0.0811
Feature
−0.025
−0.020
−0.015
−0.010
−0.005
0.000
Weight
Positive
Negative

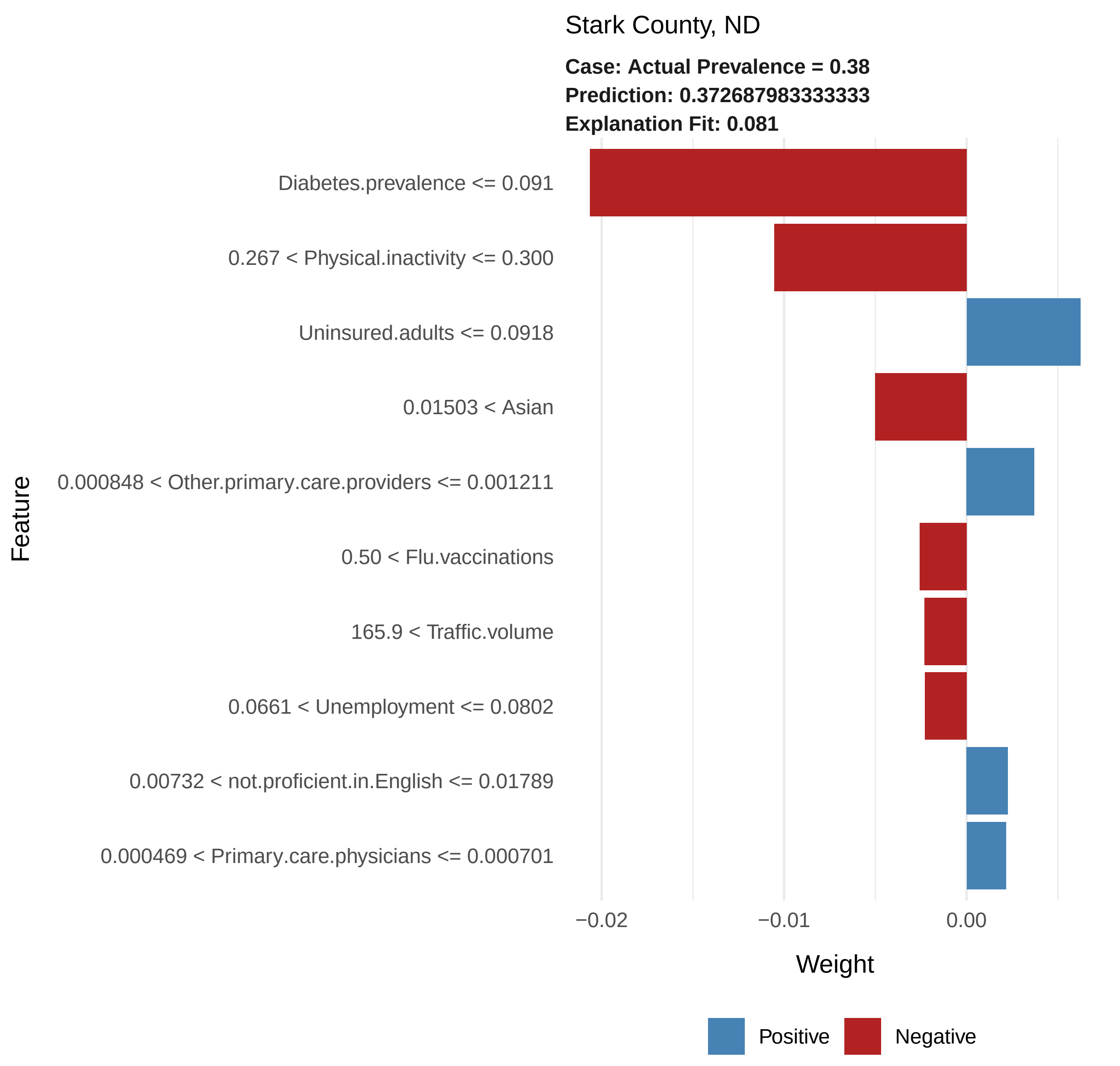
Stark County, ND
Case: Actual Prevalence = 0.38
Prediction: 0.372687983333333
Explanation Fit: 0.081
Diabetes.prevalence <= 0.091
0.267 < Physical.inactivity <= 0.300
Uninsured.adults <= 0.0918
0.01503 < Asian
0.000848 < Other.primary.care.providers <= 0.001211
0.50 < Flu.vaccinations
165.9 < Traffic.volume
0.0661 < Unemployment <= 0.0802
0.00732 < not.proficient.in.English <= 0.01789
0.000469 < Primary.care.physicians <= 0.000701
Feature
−0.02
−0.01
0.00
Weight
Positive
Negative

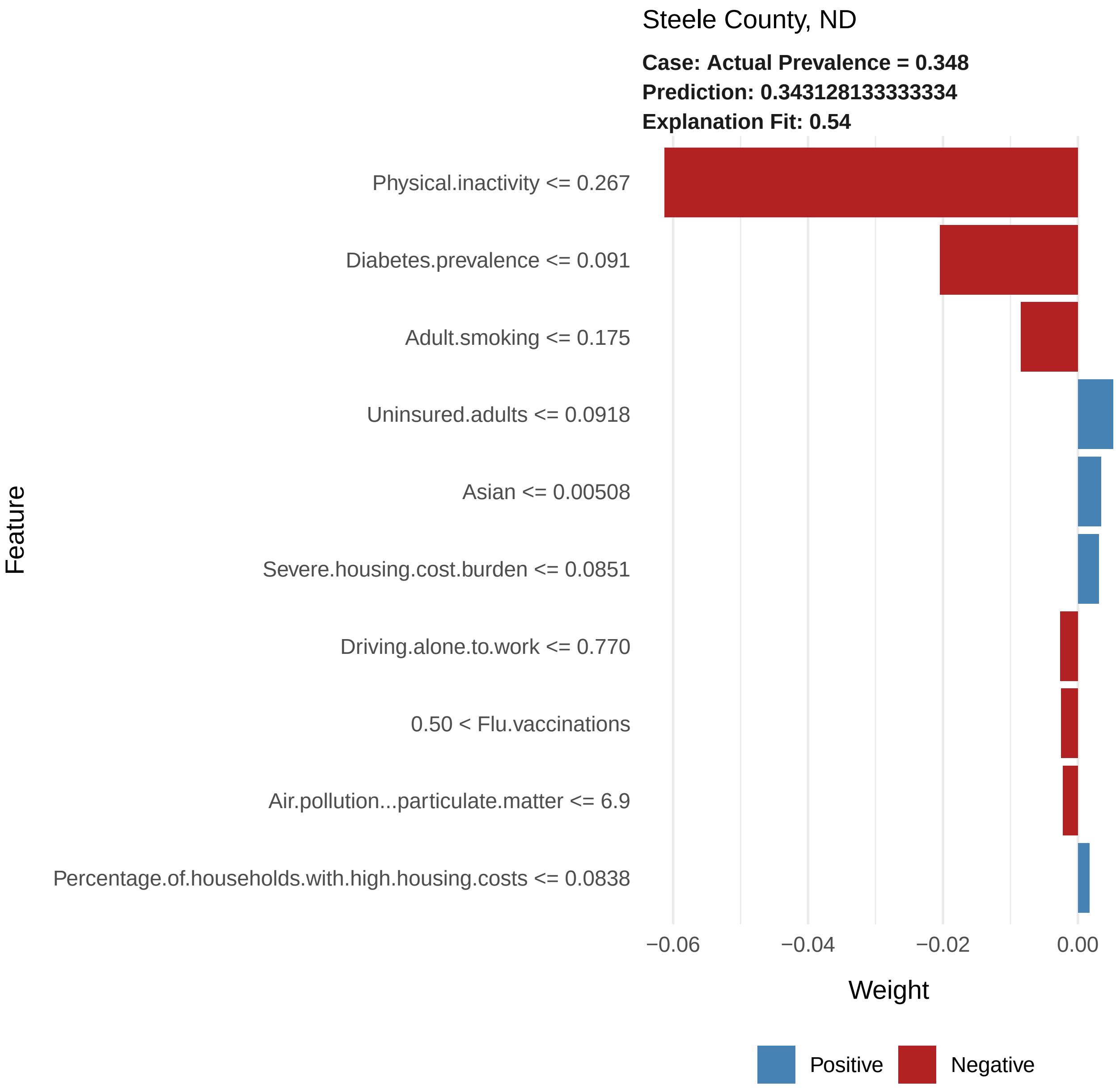
Steele County, ND
Case: Actual Prevalence = 0.348
Prediction: 0.343128133333334
Explanation Fit: 0.54
Physical.inactivity <= 0.267
Diabetes.prevalence <= 0.091
Adult.smoking <= 0.175
Uninsured.adults <= 0.0918
Asian <= 0.00508
Severe.housing.cost.burden <= 0.0851
Driving.alone.to.work <= 0.770
0.50 < Flu.vaccinations
Air.pollution...particulate.matter <= 6.9
Percentage.of.households.with.high.housing.costs <= 0.0838
Feature
−0.06
−0.04
−0.02
0.00
Weight
Positive
Negative

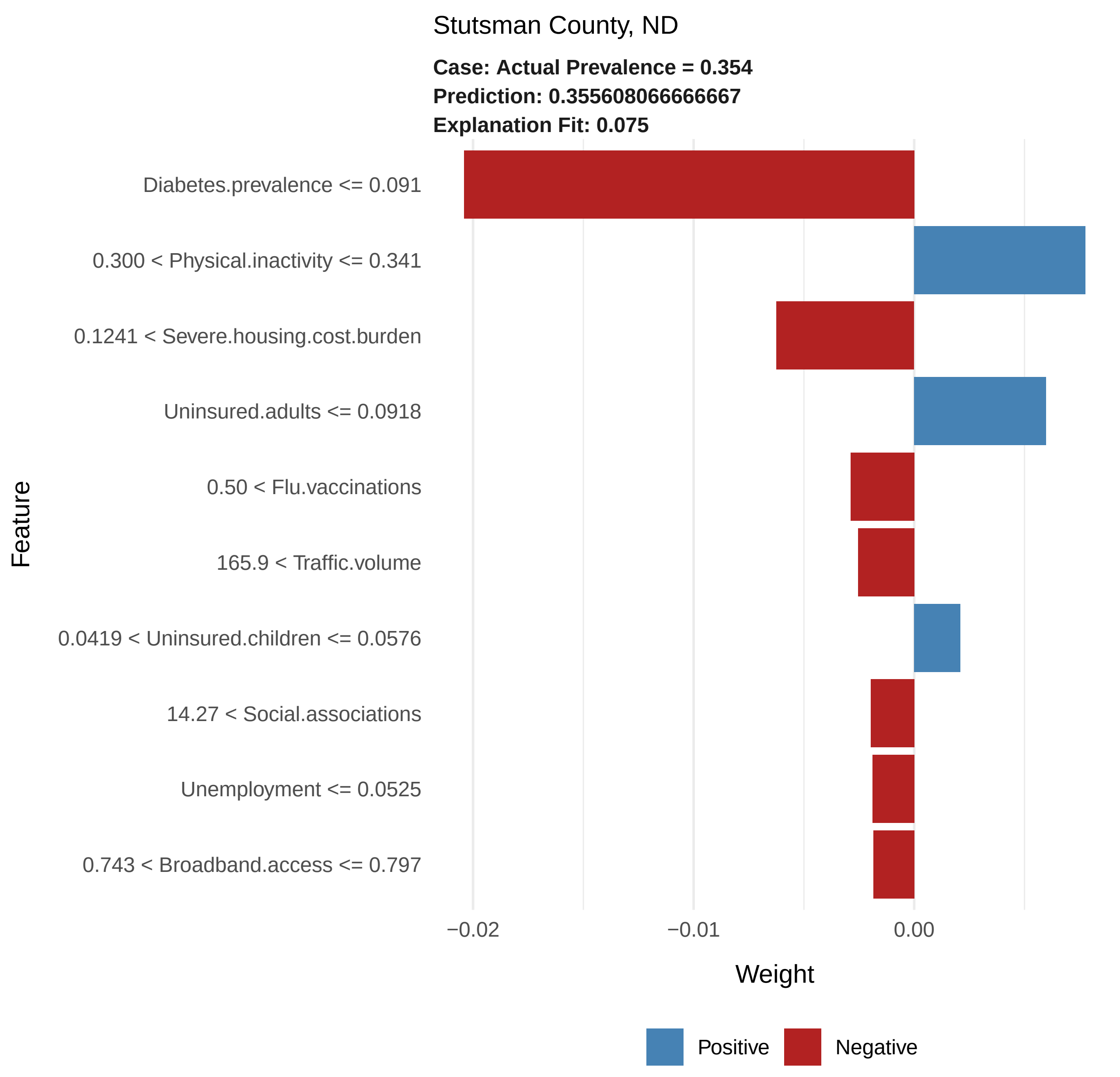

Stutsman County, ND
Case: Actual Prevalence = 0.354
Prediction: 0.355608066666667
Explanation Fit: 0.075
Diabetes.prevalence <= 0.091
0.300 < Physical.inactivity <= 0.341
0.1241 < Severe.housing.cost.burden
Uninsured.adults <= 0.0918
0.50 < Flu.vaccinations
165.9 < Traffic.volume
0.0419 < Uninsured.children <= 0.0576
14.27 < Social.associations
Unemployment <= 0.0525
0.743 < Broadband.access <= 0.797
Feature
−0.02
−0.01
0.00
Weight
Positive
Negative

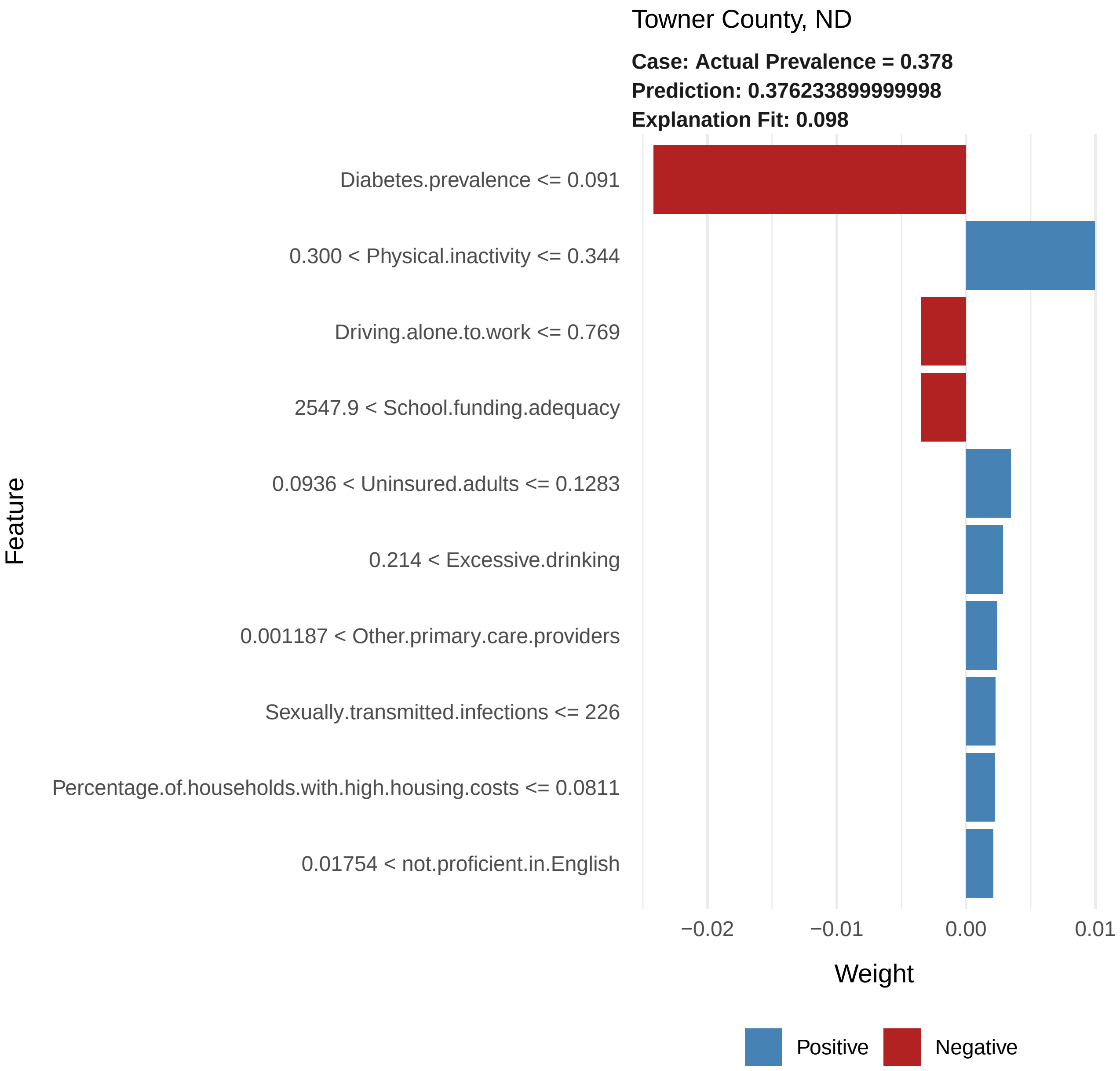

Towner County, ND
Case: Actual Prevalence = 0.378
Prediction: 0.376233899999998
Explanation Fit: 0.098
Diabetes.prevalence <= 0.091
0.300 < Physical.inactivity <= 0.344
Driving.alone.to.work <= 0.769
2547.9 < School.funding.adequacy
0.0936 < Uninsured.adults <= 0.1283
0.214 < Excessive.drinking
0.001187 < Other.primary.care.providers
Sexually.transmitted.infections <= 226
Percentage.of.households.with.high.housing.costs <= 0.0811
0.01754 < not.proficient.in.English
Feature
−0.02
−0.01
0.00
0.01
Weight
Positive
Negative

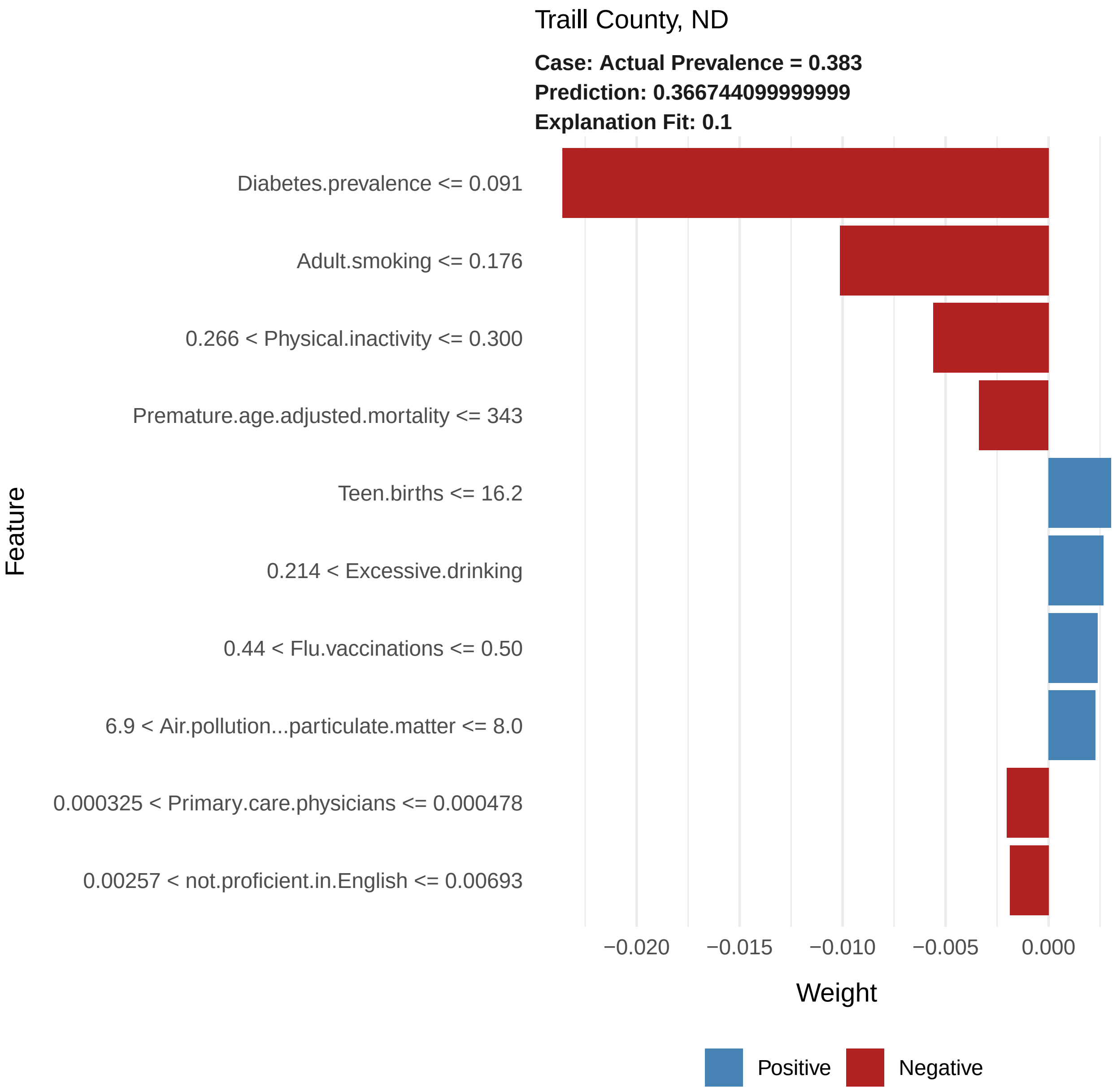

Traill County, ND
Case: Actual Prevalence = 0.383
Prediction: 0.366744099999999
Explanation Fit: 0.1
Feature
Diabetes.prevalence <= 0.091
Adult.smoking <= 0.176
0.266 < Physical.inactivity <= 0.300
Premature.age.adjusted.mortality <= 343
Teen.births <= 16.2
0.214 < Excessive.drinking
0.44 < Flu.vaccinations <= 0.50
6.9 < Air.pollution...particulate.matter <= 8.0
0.000325 < Primary.care.physicians <= 0.000478
0.00257 < not.proficient.in.English <= 0.00693
−0.020
−0.015
−0.010
−0.005
0.000
Weight
Positive
Negative

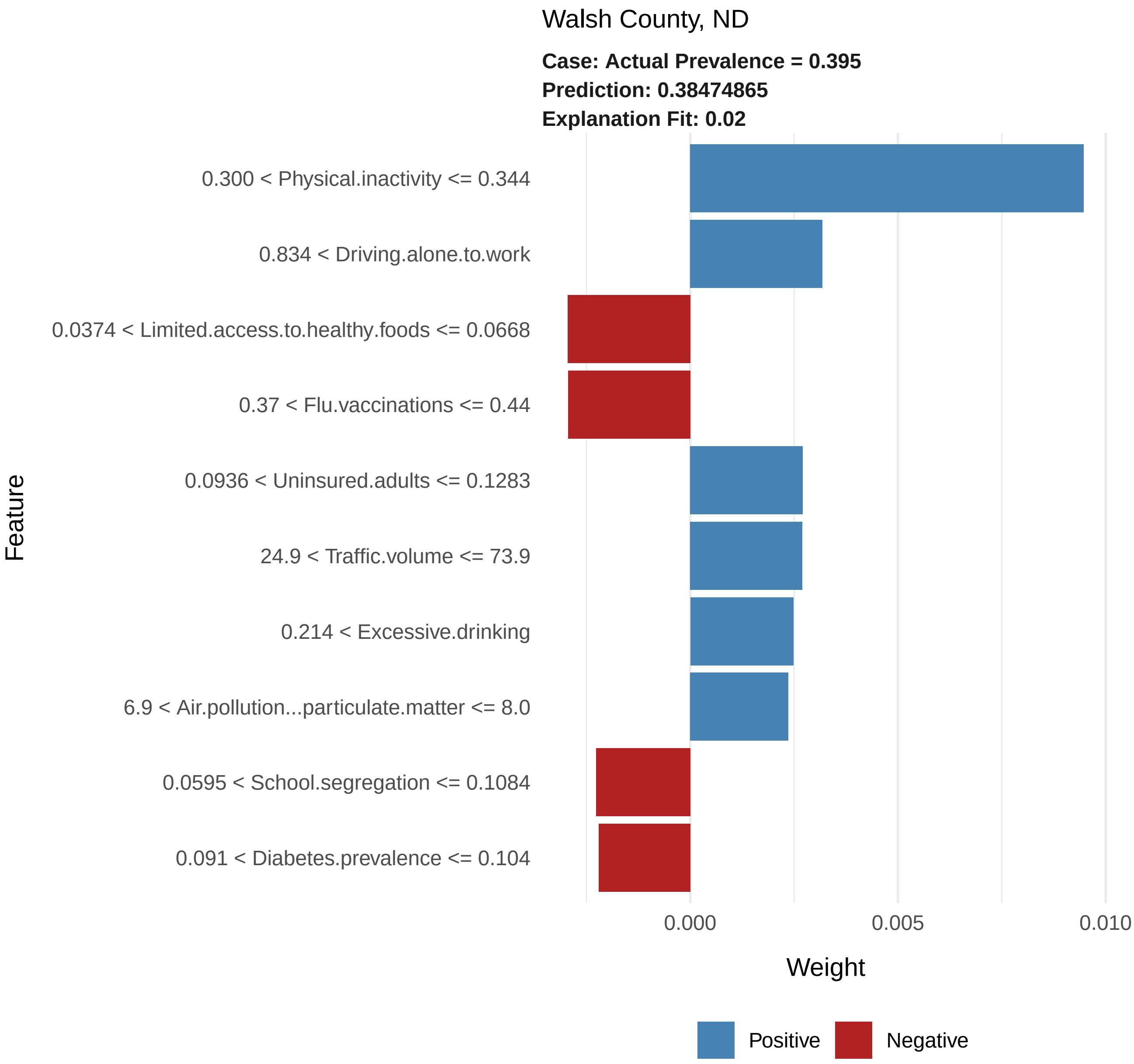

Walsh County, ND
Case: Actual Prevalence = 0.395
Prediction: 0.38474865
Explanation Fit: 0.02
0.300 < Physical.inactivity <= 0.344
0.834 < Driving.alone.to.work
0.0374 < Limited.access.to.healthy.foods <= 0.0668
0.37 < Flu.vaccinations <= 0.44
0.0936 < Uninsured.adults <= 0.1283
24.9 < Traffic.volume <= 73.9
0.214 < Excessive.drinking
6.9 < Air.pollution...particulate.matter <= 8.0
0.0595 < School.segregation <= 0.1084
0.091 < Diabetes.prevalence <= 0.104
Feature
0.000
0.005
0.010
Weight
Positive
Negative

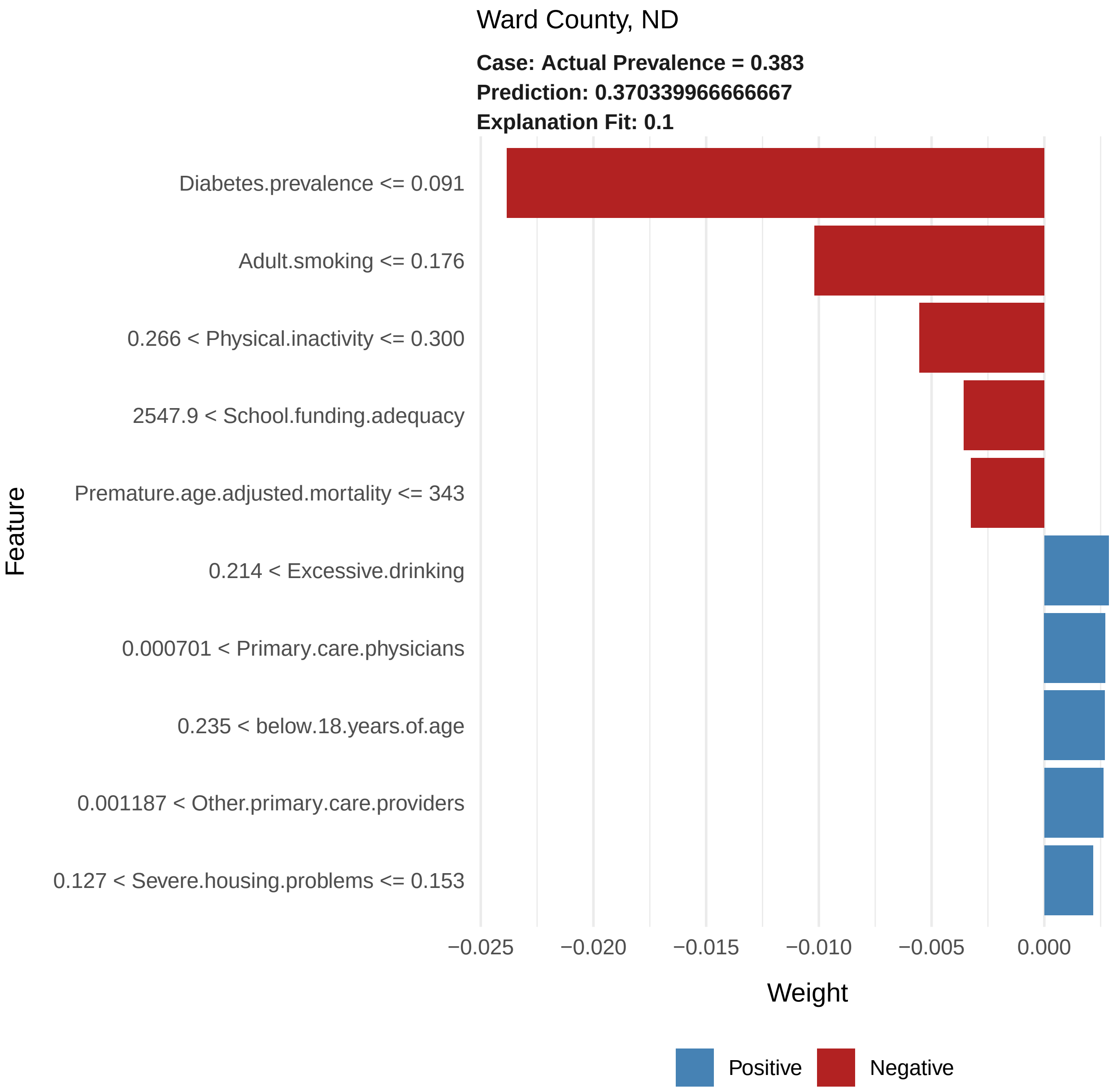

Ward County, ND
Case: Actual Prevalence = 0.383
Prediction: 0.370339966666667
Explanation Fit: 0.1
Diabetes.prevalence <= 0.091
Adult.smoking <= 0.176
0.266 < Physical.inactivity <= 0.300
2547.9 < School.funding.adequacy
Premature.age.adjusted.mortality <= 343
0.214 < Excessive.drinking
0.000701 < Primary.care.physicians
0.235 < below.18.years.of.age
0.001187 < Other.primary.care.providers
0.127 < Severe.housing.problems <= 0.153
Feature
−0.025
−0.020
−0.015
−0.010
−0.005
0.000
Weight
Positive
Negative

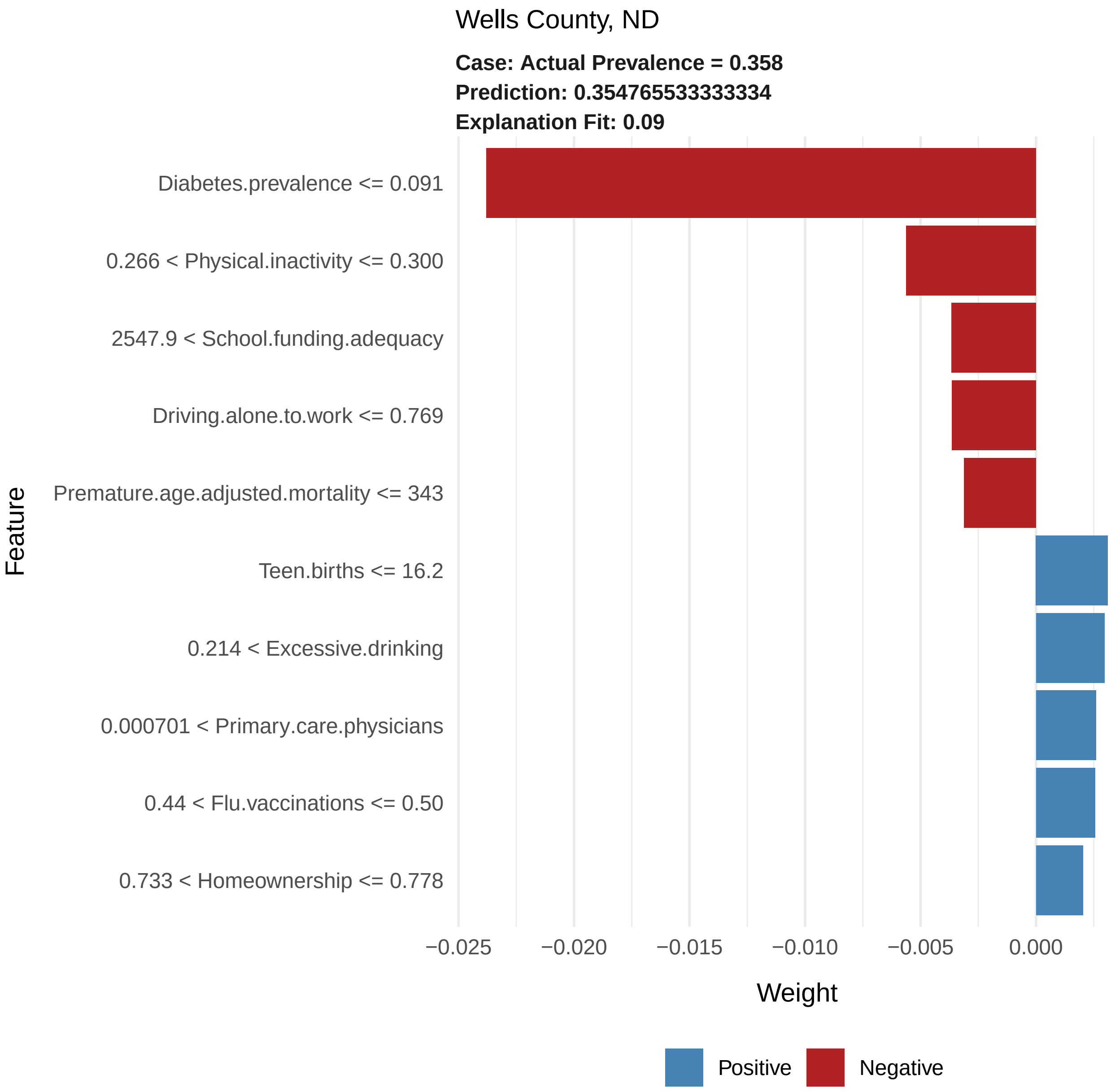
Wells County, ND
Case: Actual Prevalence = 0.358
Prediction: 0.354765533333334
Explanation Fit: 0.09
Diabetes.prevalence <= 0.091
0.266 < Physical.inactivity <= 0.300
2547.9 < School.funding.adequacy
Driving.alone.to.work <= 0.769
Premature.age.adjusted.mortality <= 343
Teen.births <= 16.2
0.214 < Excessive.drinking
0.000701 < Primary.care.physicians
0.44 < Flu.vaccinations <= 0.50
0.733 < Homeownership <= 0.778
Feature
−0.025
−0.020
−0.015
−0.010
−0.005
0.000
Weight
Positive
Negative

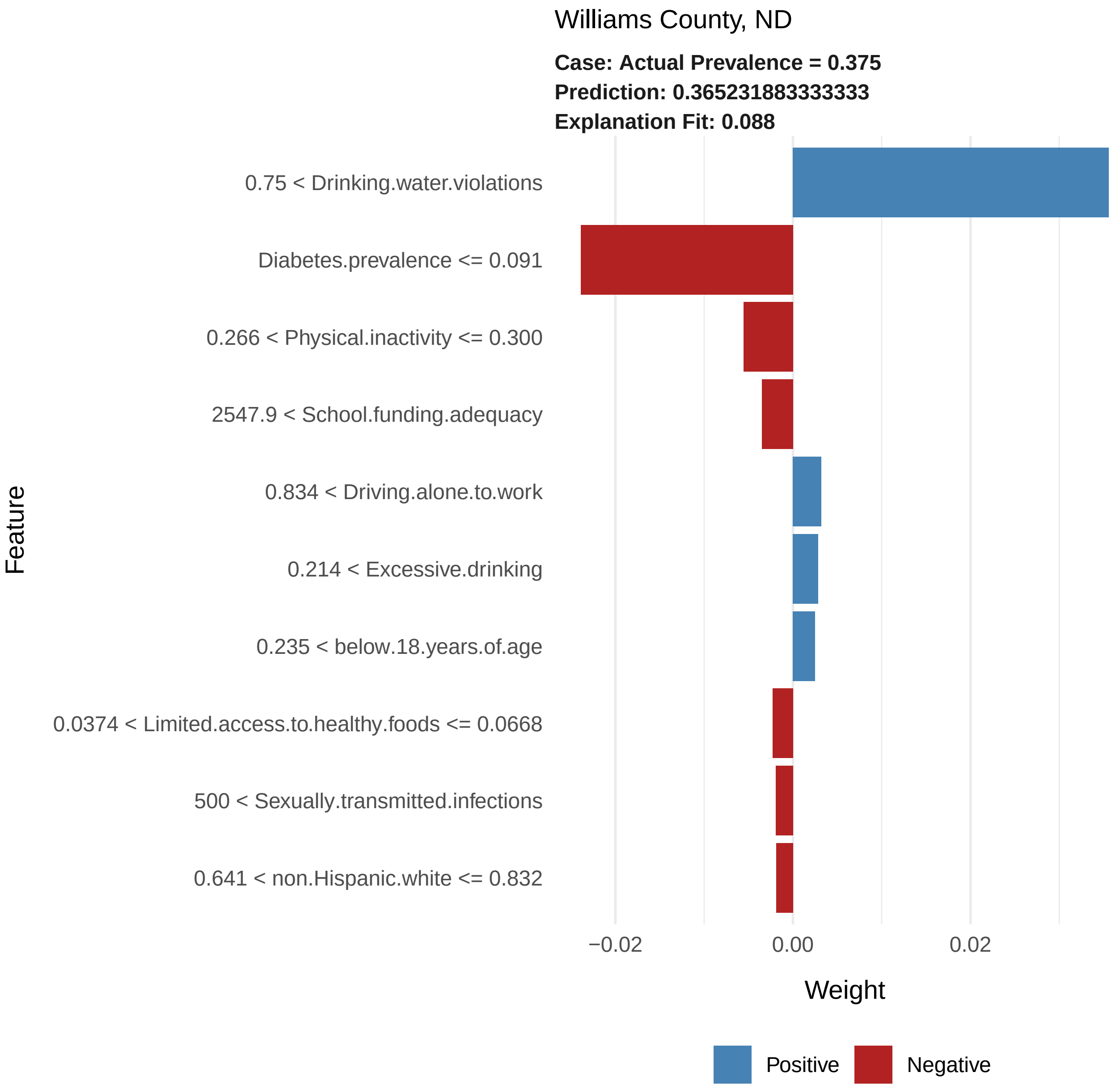
Williams County, ND
Case: Actual Prevalence = 0.375
Prediction: 0.365231883333333
Explanation Fit: 0.088
0.75 < Drinking.water.violations
Diabetes.prevalence <= 0.091
0.266 < Physical.inactivity <= 0.300
2547.9 < School.funding.adequacy
0.834 < Driving.alone.to.work
0.214 < Excessive.drinking
0.235 < below.18.years.of.age
0.0374 < Limited.access.to.healthy.foods <= 0.0668
500 < Sexually.transmitted.infections
0.641 < non.Hispanic.white <= 0.832
Feature
−0.02
0.00
0.02
Weight
Positive
Negative

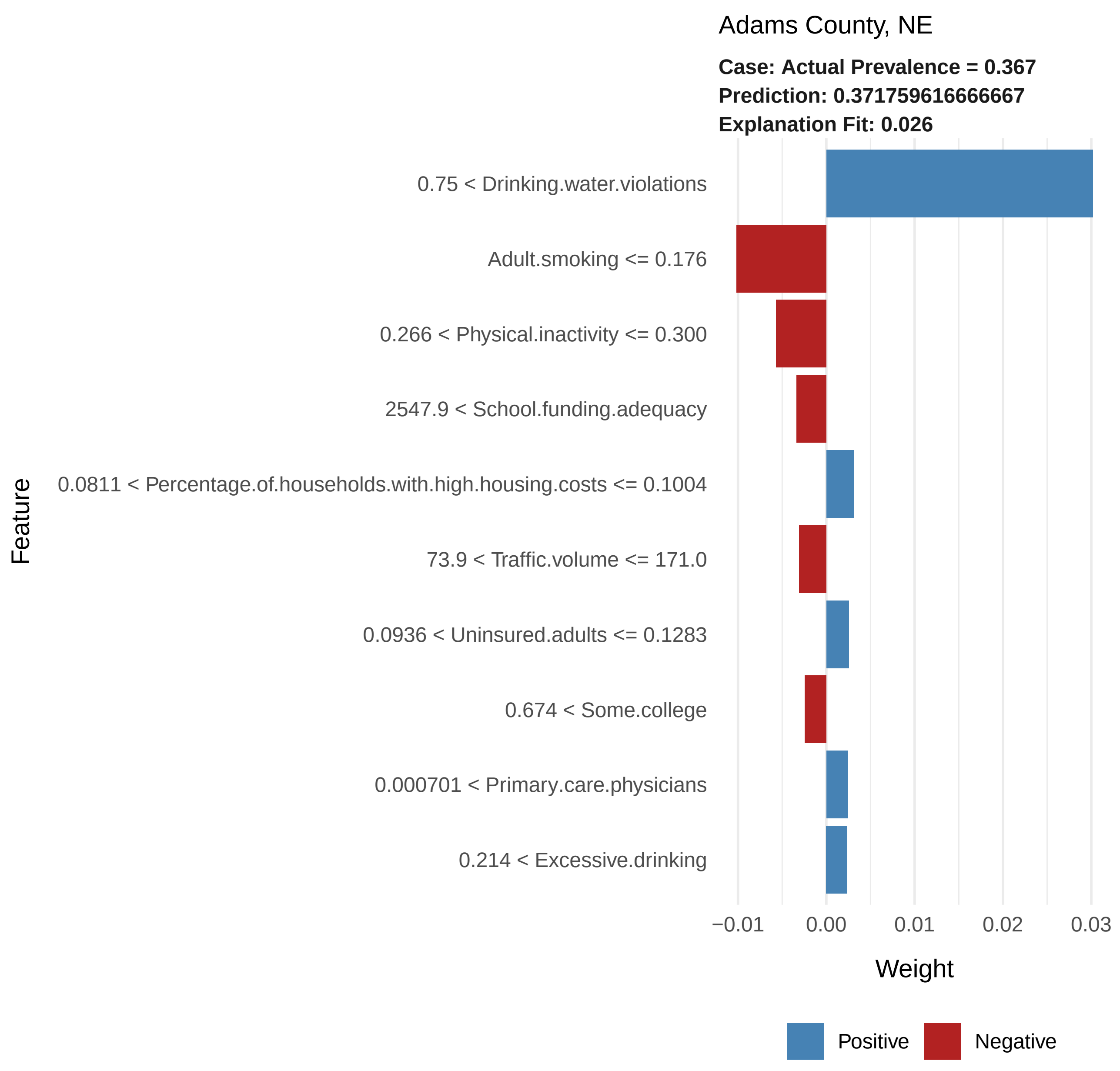

Adams County, NE
Case: Actual Prevalence = 0.367
Prediction: 0.371759616666667
Explanation Fit: 0.026
0.75 < Drinking.water.violations
Adult.smoking <= 0.176
0.266 < Physical.inactivity <= 0.300
2547.9 < School.funding.adequacy
0.0811 < Percentage.of.households.with.high.housing.costs <= 0.1004
73.9 < Traffic.volume <= 171.0
0.0936 < Uninsured.adults <= 0.1283
0.674 < Some.college
0.000701 < Primary.care.physicians
0.214 < Excessive.drinking
Feature
−0.01
0.00
0.01
0.02
0.03
Weight
Positive
Negative

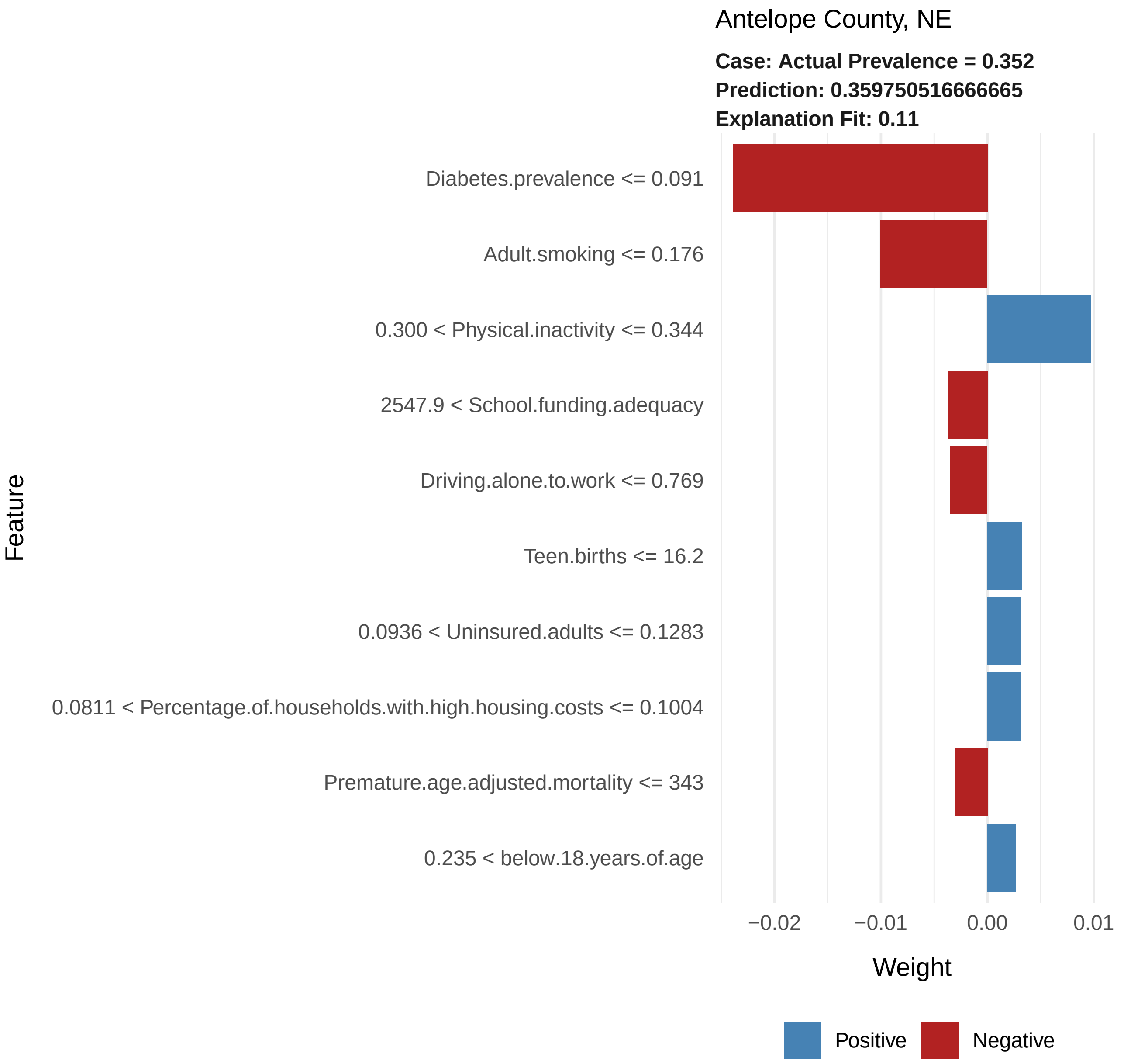

Antelope County, NE
Case: Actual Prevalence = 0.352
Prediction: 0.359750516666665
Explanation Fit: 0.11
Diabetes.prevalence <= 0.091
Adult.smoking <= 0.176
0.300 < Physical.inactivity <= 0.344
2547.9 < School.funding.adequacy
Driving.alone.to.work <= 0.769
Teen.births <= 16.2
0.0936 < Uninsured.adults <= 0.1283
0.0811 < Percentage.of.households.with.high.housing.costs <= 0.1004
Premature.age.adjusted.mortality <= 343
0.235 < below.18.years.of.age
Feature
−0.02
−0.01
0.00
0.01
Weight
Positive
Negative

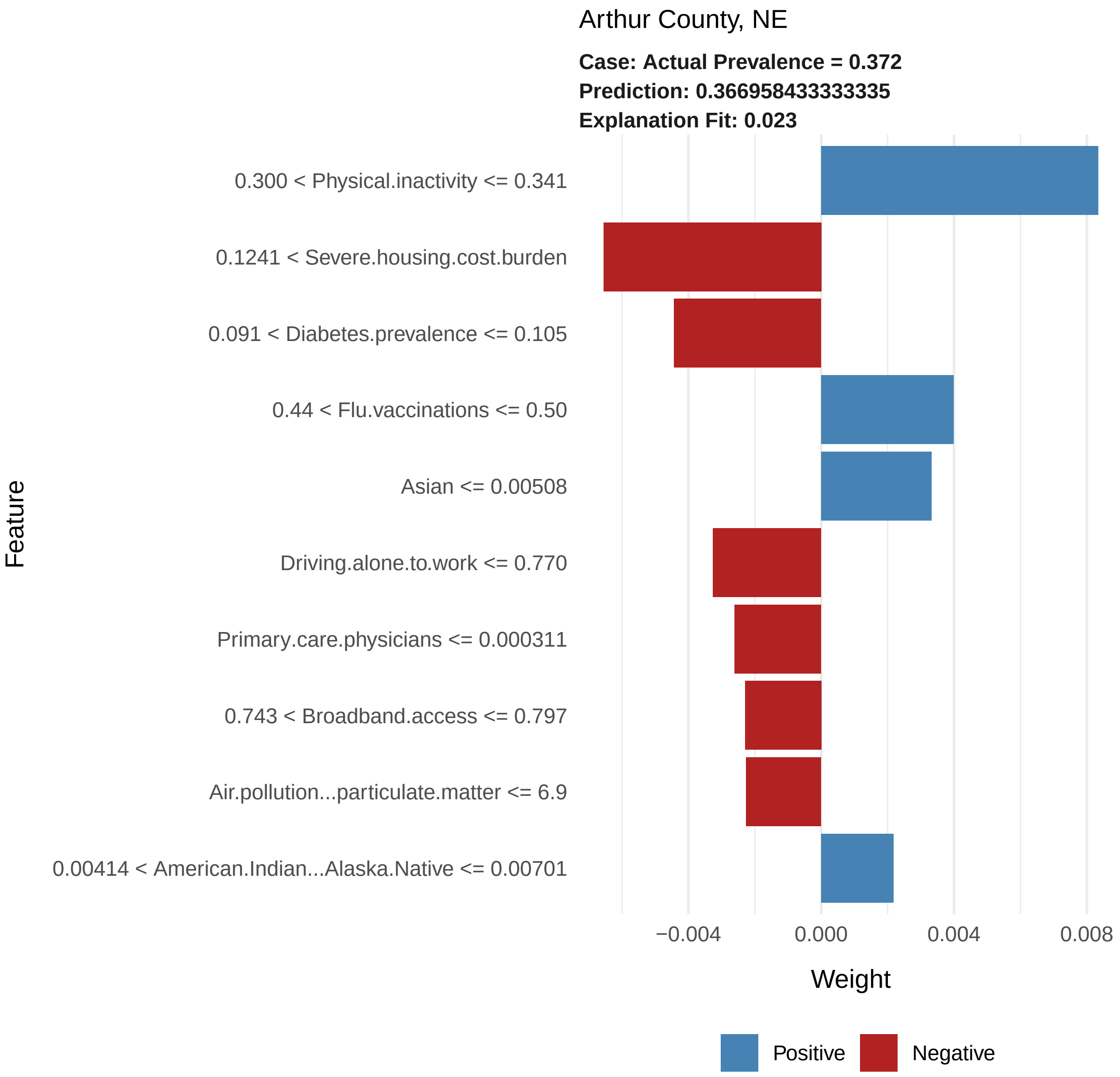

Arthur County, NE
Case: Actual Prevalence = 0.372
Prediction: 0.366958433333335
Explanation Fit: 0.023
0.300 < Physical.inactivity <= 0.341
0.1241 < Severe.housing.cost.burden
0.091 < Diabetes.prevalence <= 0.105
0.44 < Flu.vaccinations <= 0.50
Asian <= 0.00508
Driving.alone.to.work <= 0.770
Primary.care.physicians <= 0.000311
0.743 < Broadband.access <= 0.797
Air.pollution...particulate.matter <= 6.9
0.00414 < American.Indian...Alaska.Native <= 0.00701
Feature
−0.004
0.000
0.004
0.008
Weight
Positive
Negative

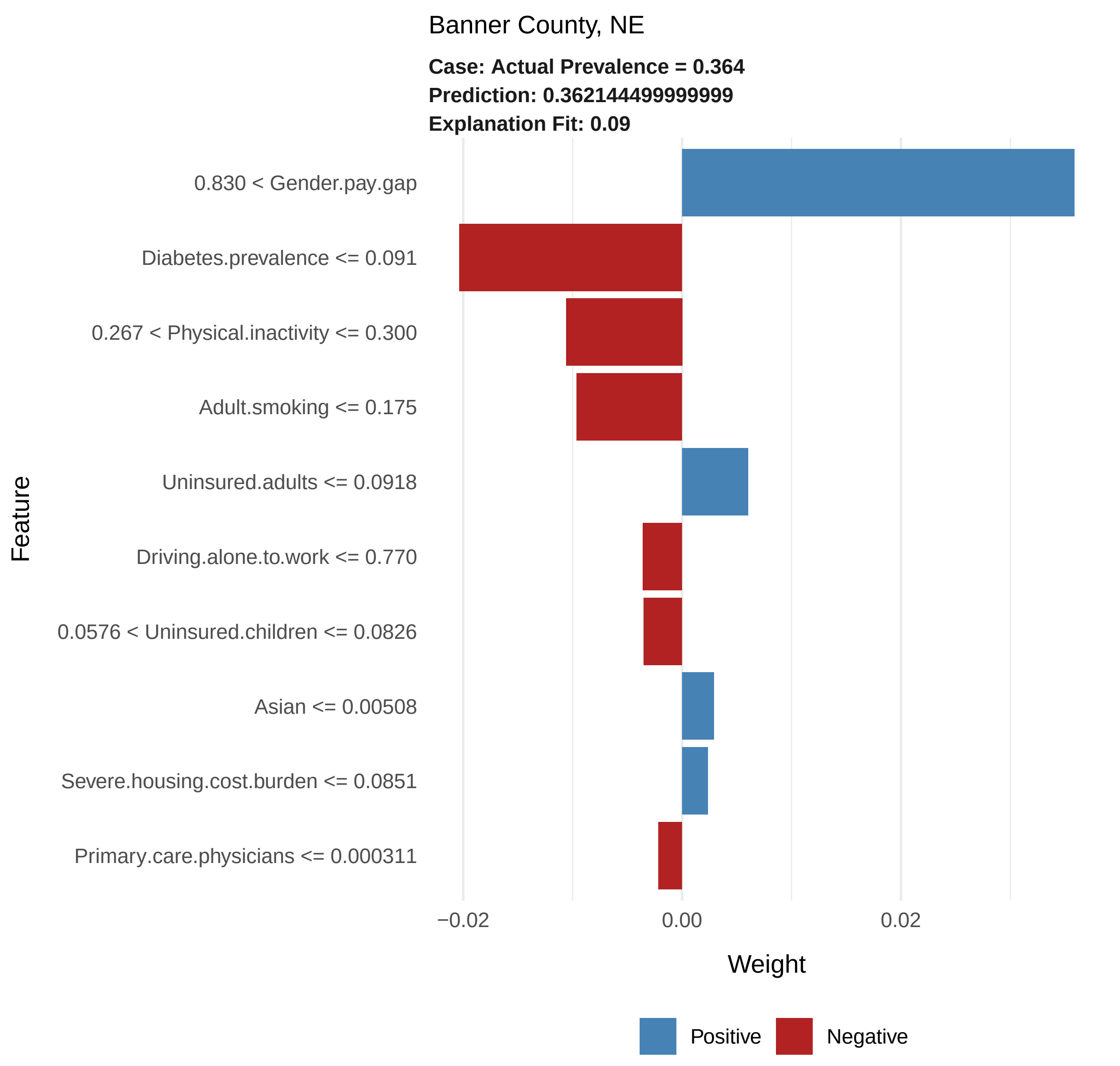
Banner County, NE
Case: Actual Prevalence = 0.364
Prediction: 0.362144499999999
Explanation Fit: 0.09
0.830 < Gender.pay.gap
Diabetes.prevalence <= 0.091
0.267 < Physical.inactivity <= 0.300
Adult.smoking <= 0.175
Uninsured.adults <= 0.0918
Driving.alone.to.work <= 0.770
0.0576 < Uninsured.children <= 0.0826
Asian <= 0.00508
Severe.housing.cost.burden <= 0.0851
Primary.care.physicians <= 0.000311
Feature
−0.02
0.00
0.02
Weight
Positive
Negative

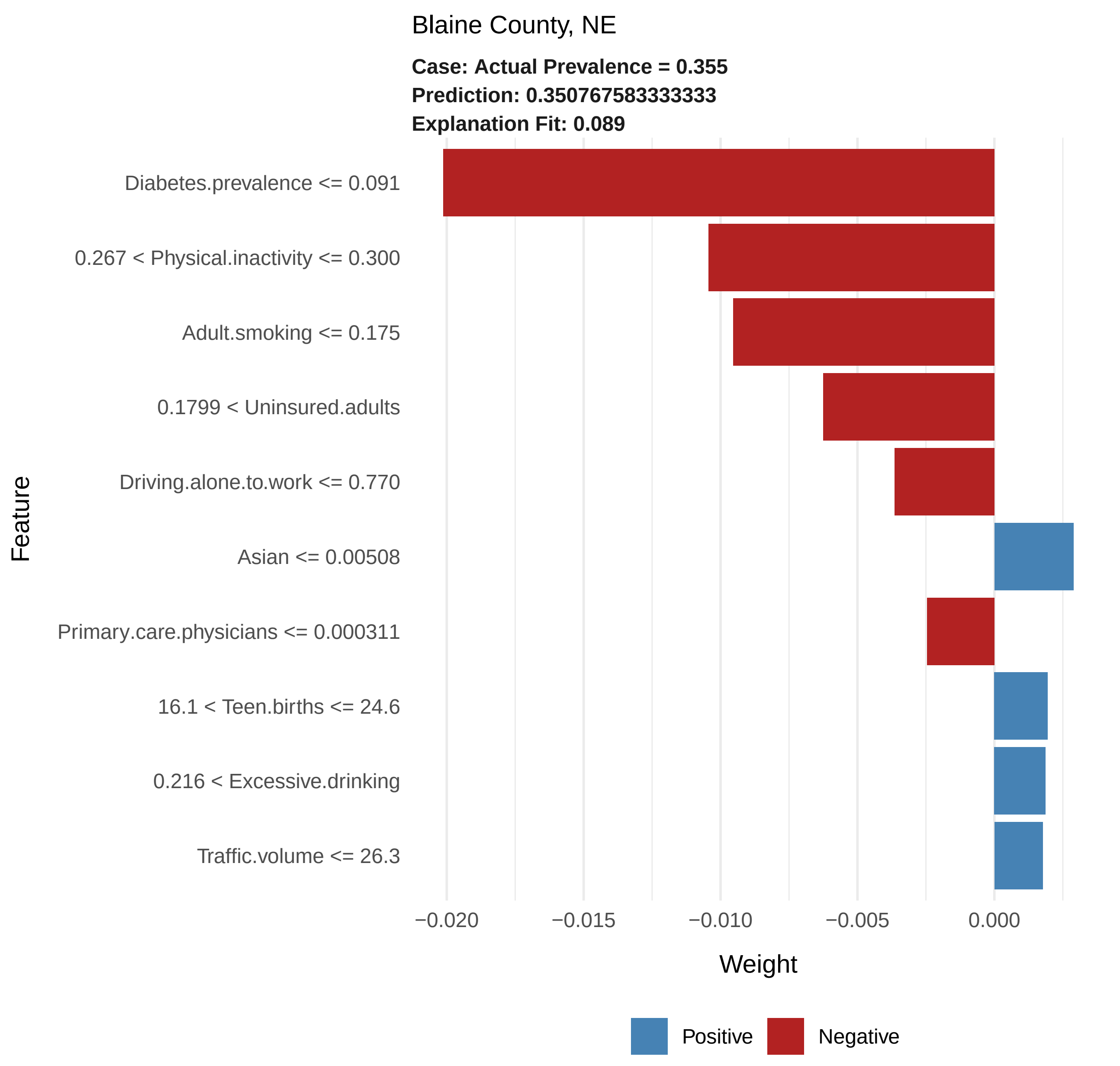
Blaine County, NE
Case: Actual Prevalence = 0.355
Prediction: 0.350767583333333
Explanation Fit: 0.089
Diabetes.prevalence <= 0.091
0.267 < Physical.inactivity <= 0.300
Adult.smoking <= 0.175
0.1799 < Uninsured.adults
Driving.alone.to.work <= 0.770
Asian <= 0.00508
Primary.care.physicians <= 0.000311
16.1 < Teen.births <= 24.6
0.216 < Excessive.drinking
Traffic.volume <= 26.3
Feature
−0.020
−0.015
−0.010
−0.005
0.000
Weight
Positive
Negative

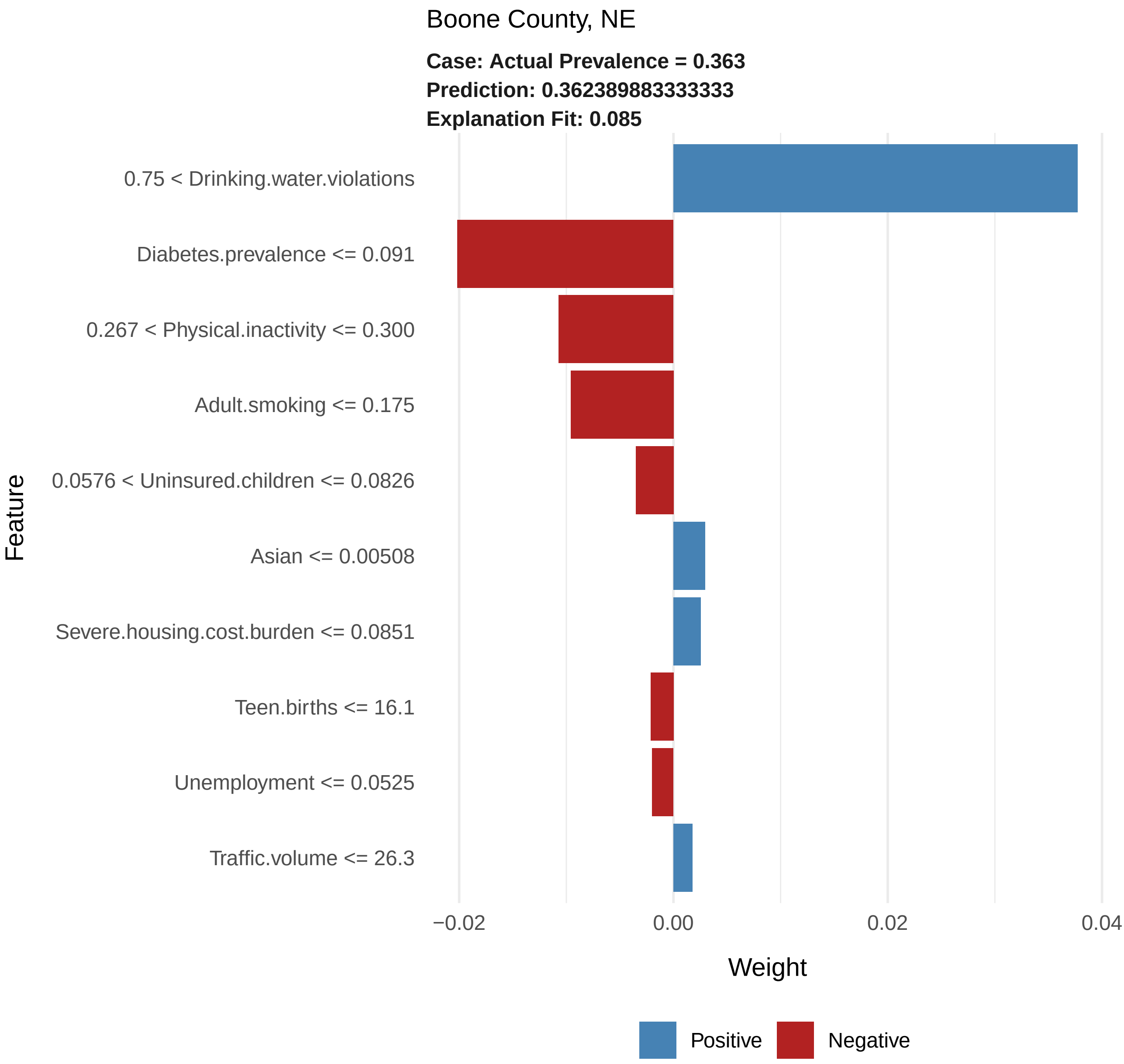

Boone County, NE
Case: Actual Prevalence = 0.363
Prediction: 0.362389883333333
Explanation Fit: 0.085
0.75 < Drinking.water.violations
Diabetes.prevalence <= 0.091
0.267 < Physical.inactivity <= 0.300
Adult.smoking <= 0.175
0.0576 < Uninsured.children <= 0.0826
Asian <= 0.00508
Severe.housing.cost.burden <= 0.0851
Teen.births <= 16.1
Unemployment <= 0.0525
Traffic.volume <= 26.3
Feature
−0.02
0.00
0.02
0.04
Weight
Positive
Negative

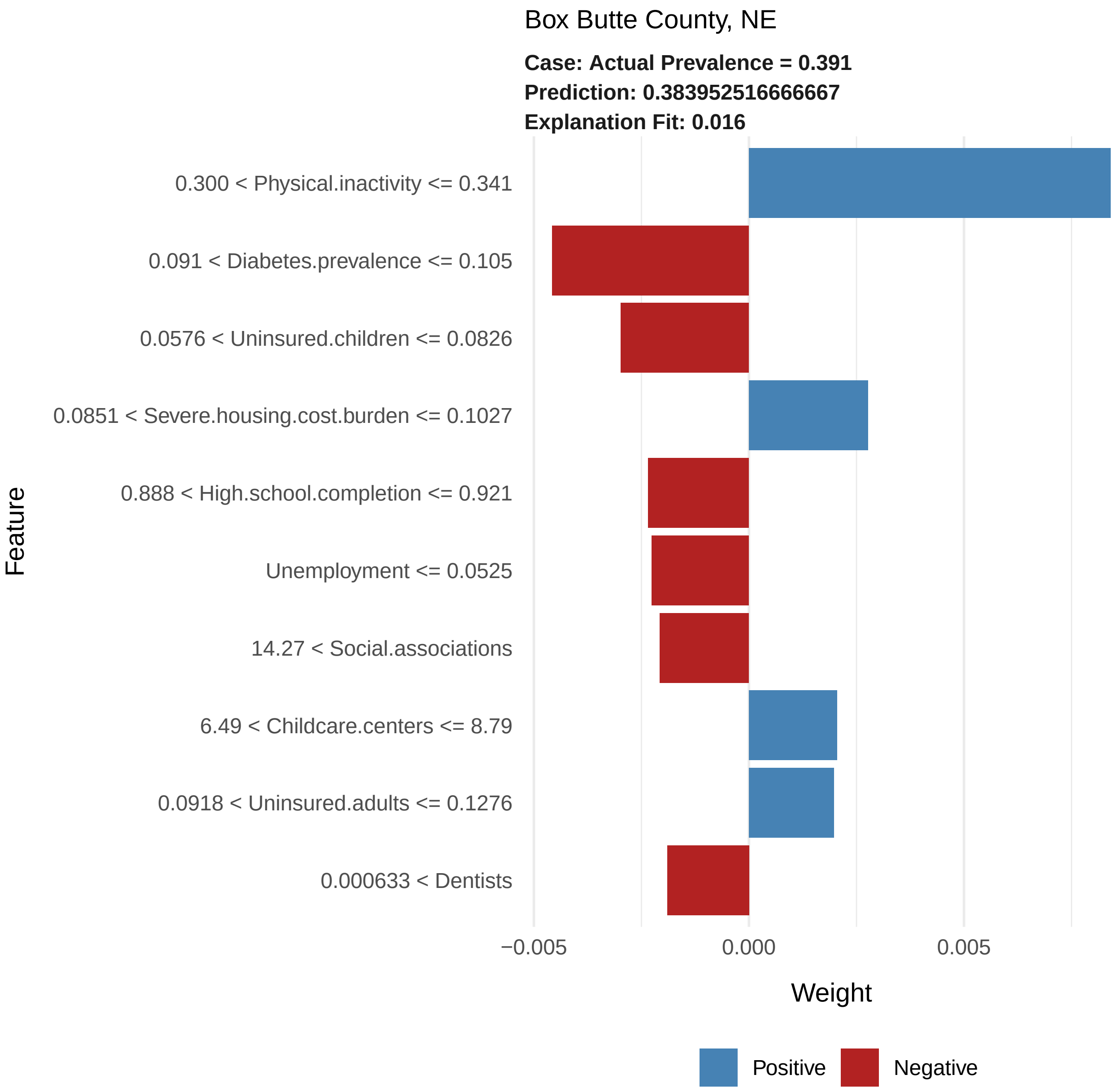

Box Butte County, NE
Case: Actual Prevalence = 0.391
Prediction: 0.383952516666667
Explanation Fit: 0.016
0.300 < Physical.inactivity <= 0.341
0.091 < Diabetes.prevalence <= 0.105
0.0576 < Uninsured.children <= 0.0826
0.0851 < Severe.housing.cost.burden <= 0.1027
0.888 < High.school.completion <= 0.921
Unemployment <= 0.0525
14.27 < Social.associations
6.49 < Childcare.centers <= 8.79
0.0918 < Uninsured.adults <= 0.1276
0.000633 < Dentists
Feature
−0.005
0.000
0.005
Weight
Positive
Negative

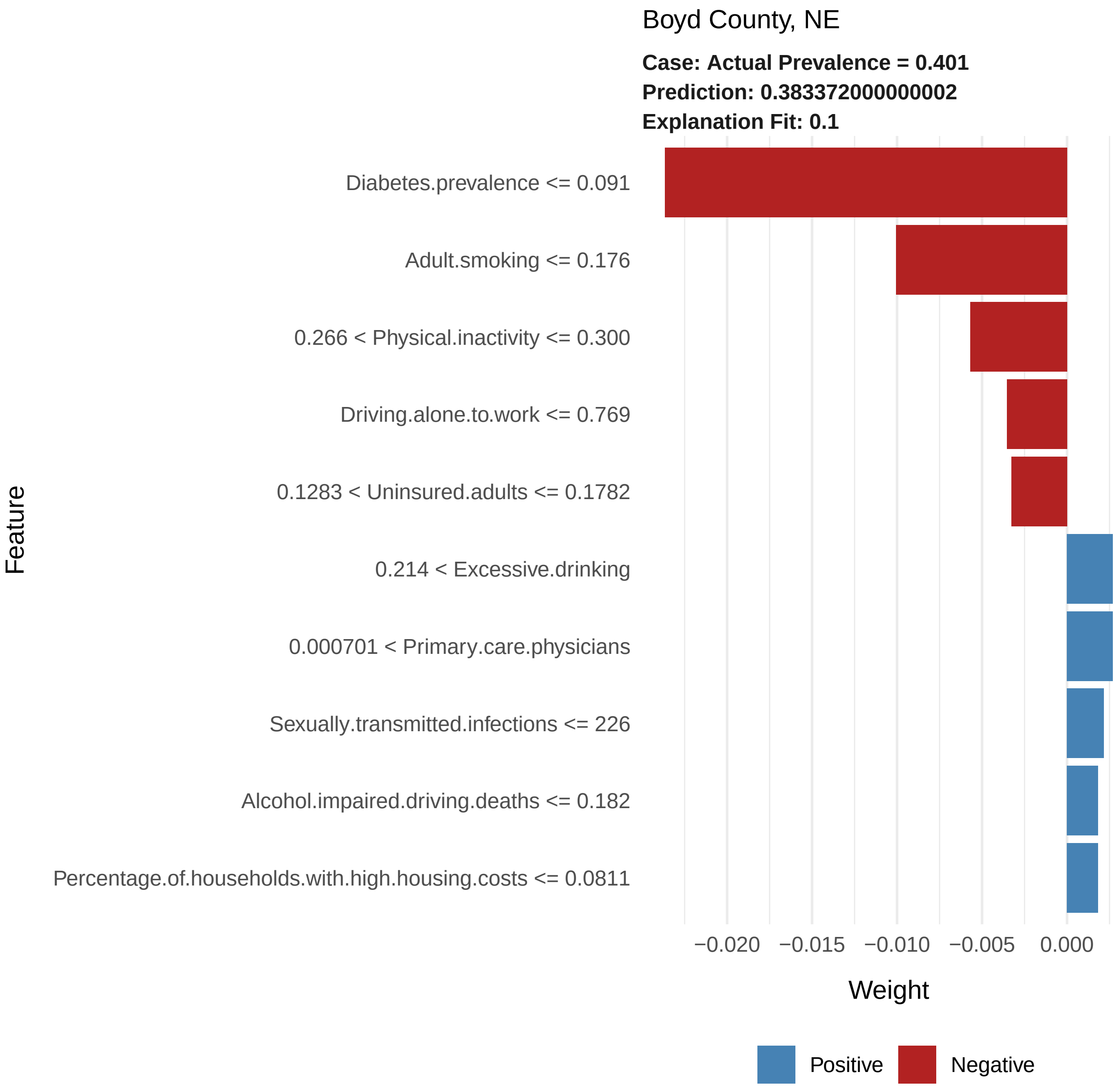
Boyd County, NE
Case: Actual Prevalence = 0.401
Prediction: 0.383372000000002
Explanation Fit: 0.1
Diabetes.prevalence <= 0.091
Adult.smoking <= 0.176
0.266 < Physical.inactivity <= 0.300
Driving.alone.to.work <= 0.769
0.1283 < Uninsured.adults <= 0.1782
0.214 < Excessive.drinking
0.000701 < Primary.care.physicians
Sexually.transmitted.infections <= 226
Alcohol.impaired.driving.deaths <= 0.182
Percentage.of.households.with.high.housing.costs <= 0.0811
Feature
−0.020
−0.015
−0.010
−0.005
0.000
Weight
Positive
Negative

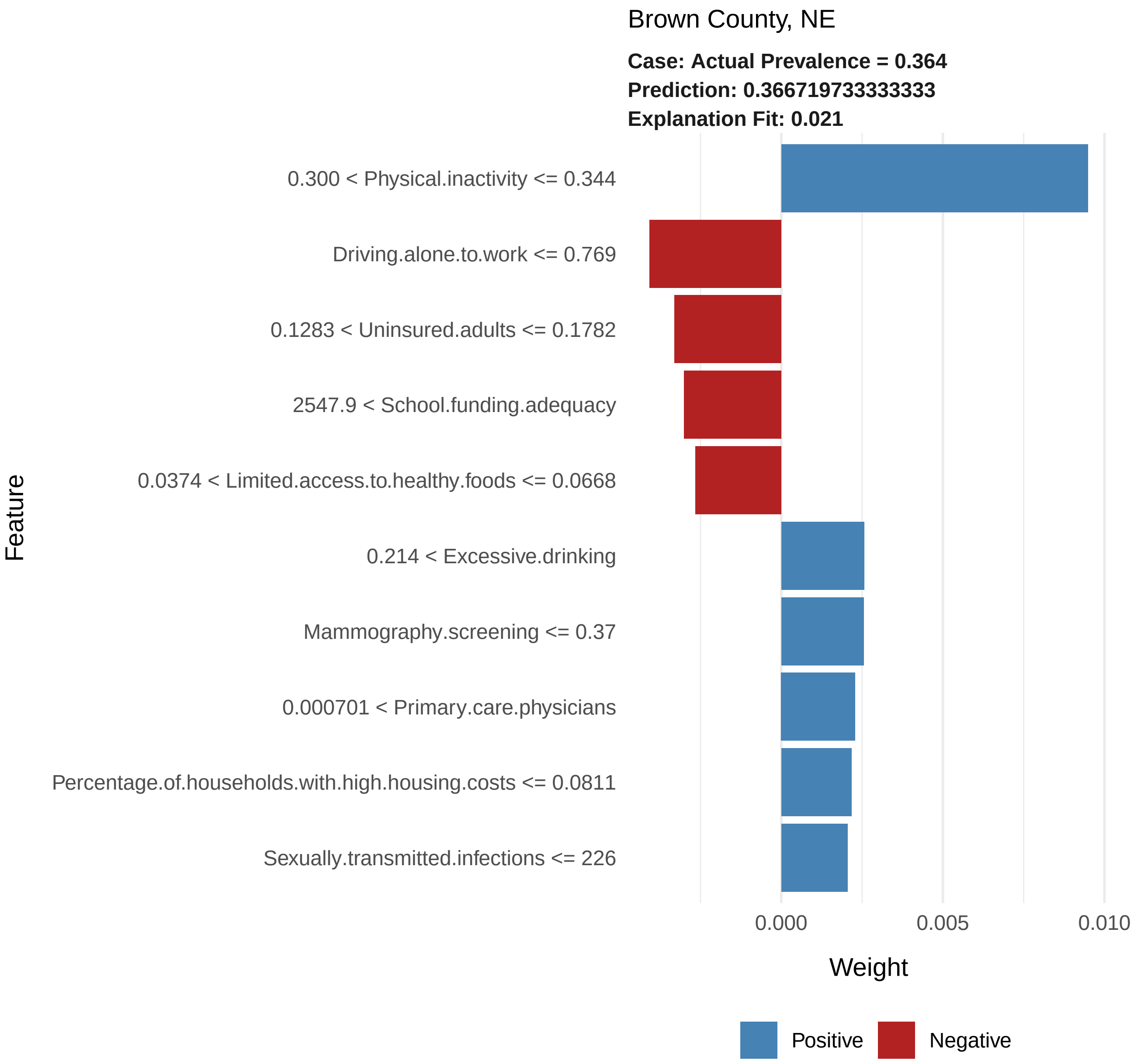
Brown County, NE
Case: Actual Prevalence = 0.364
Prediction: 0.366719733333333
Explanation Fit: 0.021
Feature
0.300 < Physical.inactivity <= 0.344
Driving.alone.to.work <= 0.769
0.1283 < Uninsured.adults <= 0.1782
2547.9 < School.funding.adequacy
0.0374 < Limited.access.to.healthy.foods <= 0.0668
0.214 < Excessive.drinking
Mammography.screening <= 0.37
0.000701 < Primary.care.physicians
Percentage.of.households.with.high.housing.costs <= 0.0811
Sexually.transmitted.infections <= 226
0.000
0.005
0.010
Weight
Positive
Negative

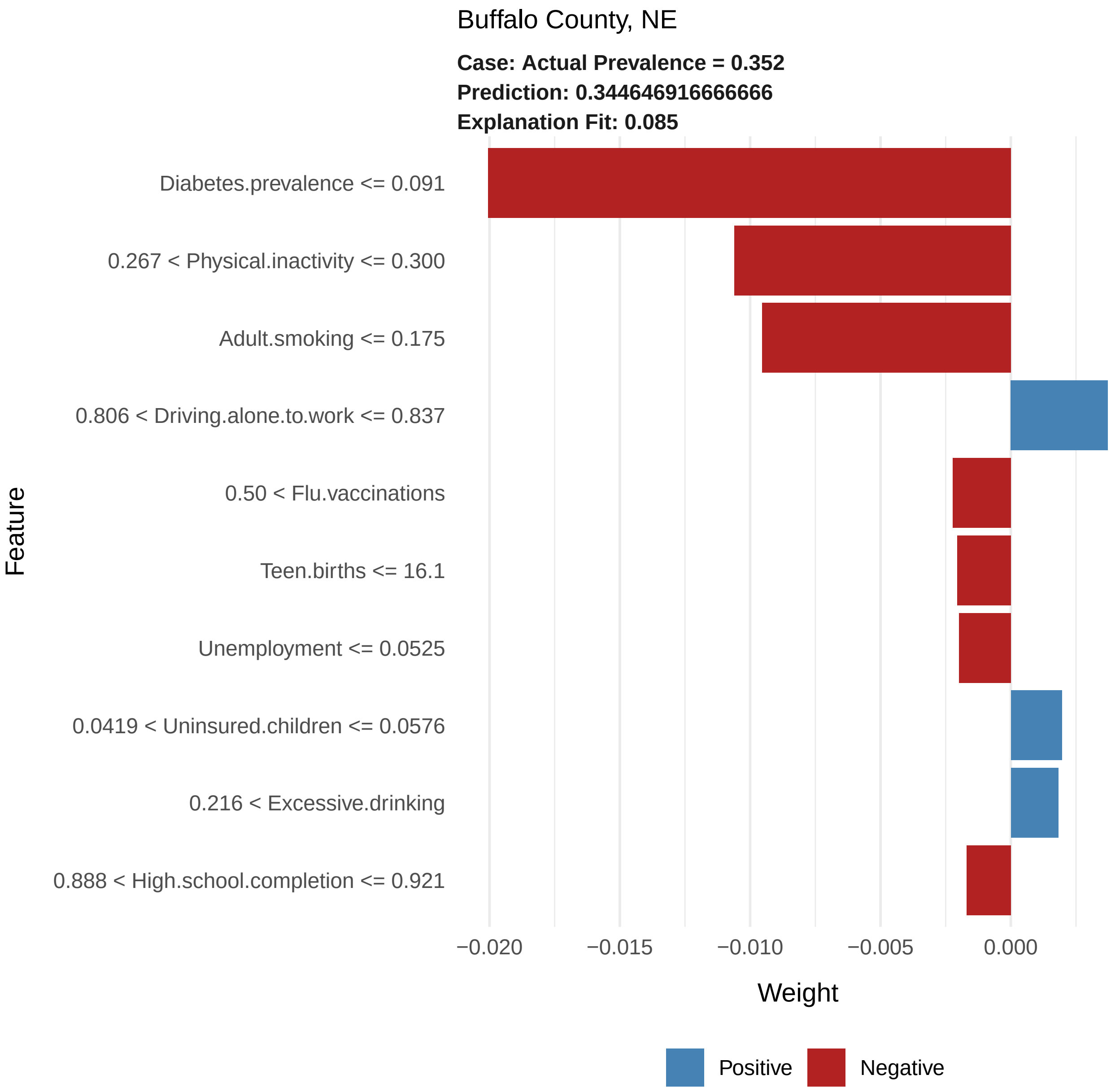

Buffalo County, NE
Case: Actual Prevalence = 0.352
Prediction: 0.344646916666666
Explanation Fit: 0.085
Diabetes.prevalence <= 0.091
0.267 < Physical.inactivity <= 0.300
Adult.smoking <= 0.175
0.806 < Driving.alone.to.work <= 0.837
0.50 < Flu.vaccinations
Teen.births <= 16.1
Unemployment <= 0.0525
0.0419 < Uninsured.children <= 0.0576
0.216 < Excessive.drinking
0.888 < High.school.completion <= 0.921
Feature
−0.020
−0.015
−0.010
−0.005
0.000
Weight
Positive
Negative

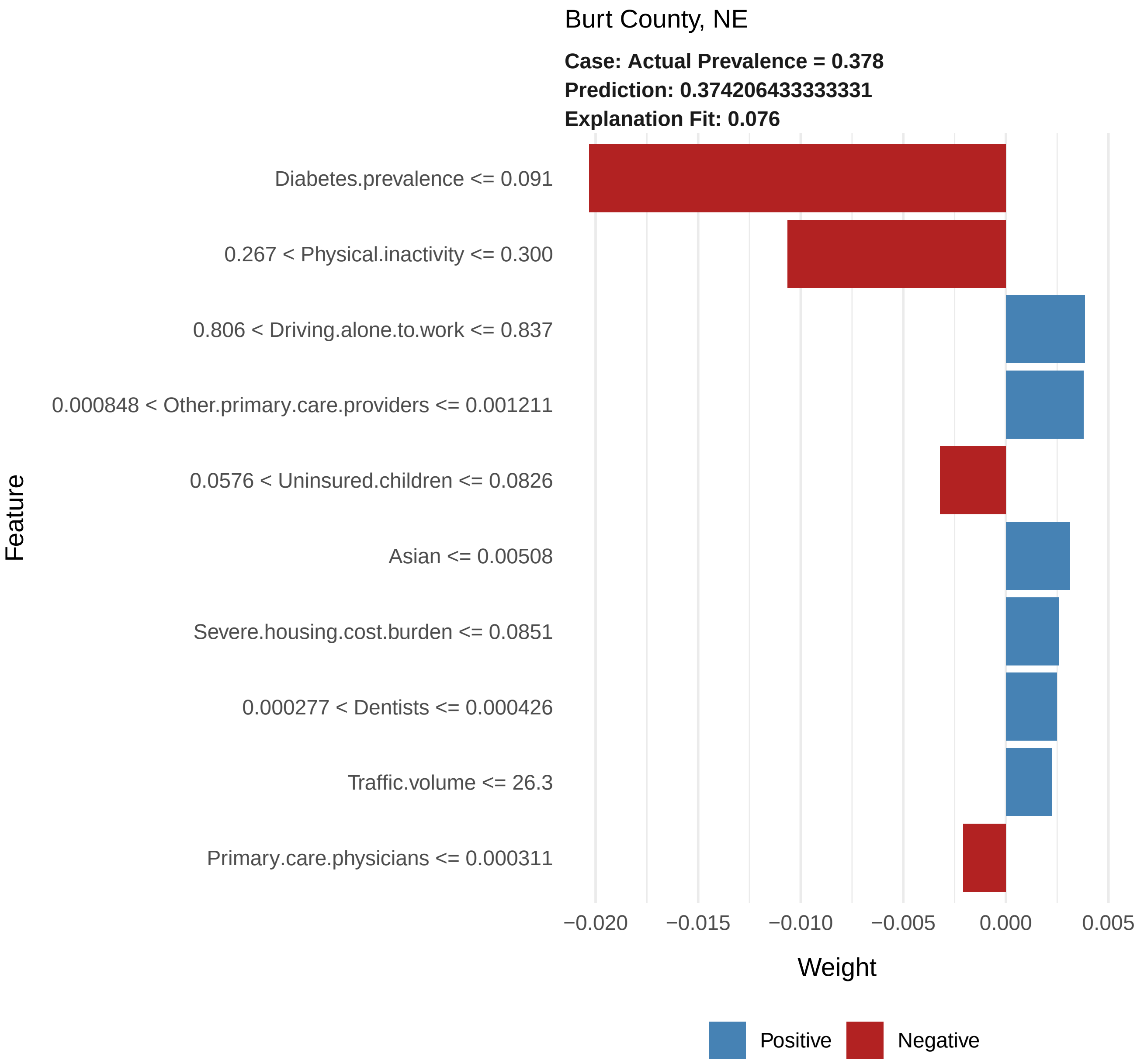

Burt County, NE
Case: Actual Prevalence = 0.378
Prediction: 0.374206433333331
Explanation Fit: 0.076
Diabetes.prevalence <= 0.091
0.267 < Physical.inactivity <= 0.300
0.806 < Driving.alone.to.work <= 0.837
0.000848 < Other.primary.care.providers <= 0.001211
0.0576 < Uninsured.children <= 0.0826
Asian <= 0.00508
Severe.housing.cost.burden <= 0.0851
0.000277 < Dentists <= 0.000426
Traffic.volume <= 26.3
Primary.care.physicians <= 0.000311
Feature
−0.020
−0.015
−0.010
−0.005
0.000
0.005
Weight
Positive
Negative

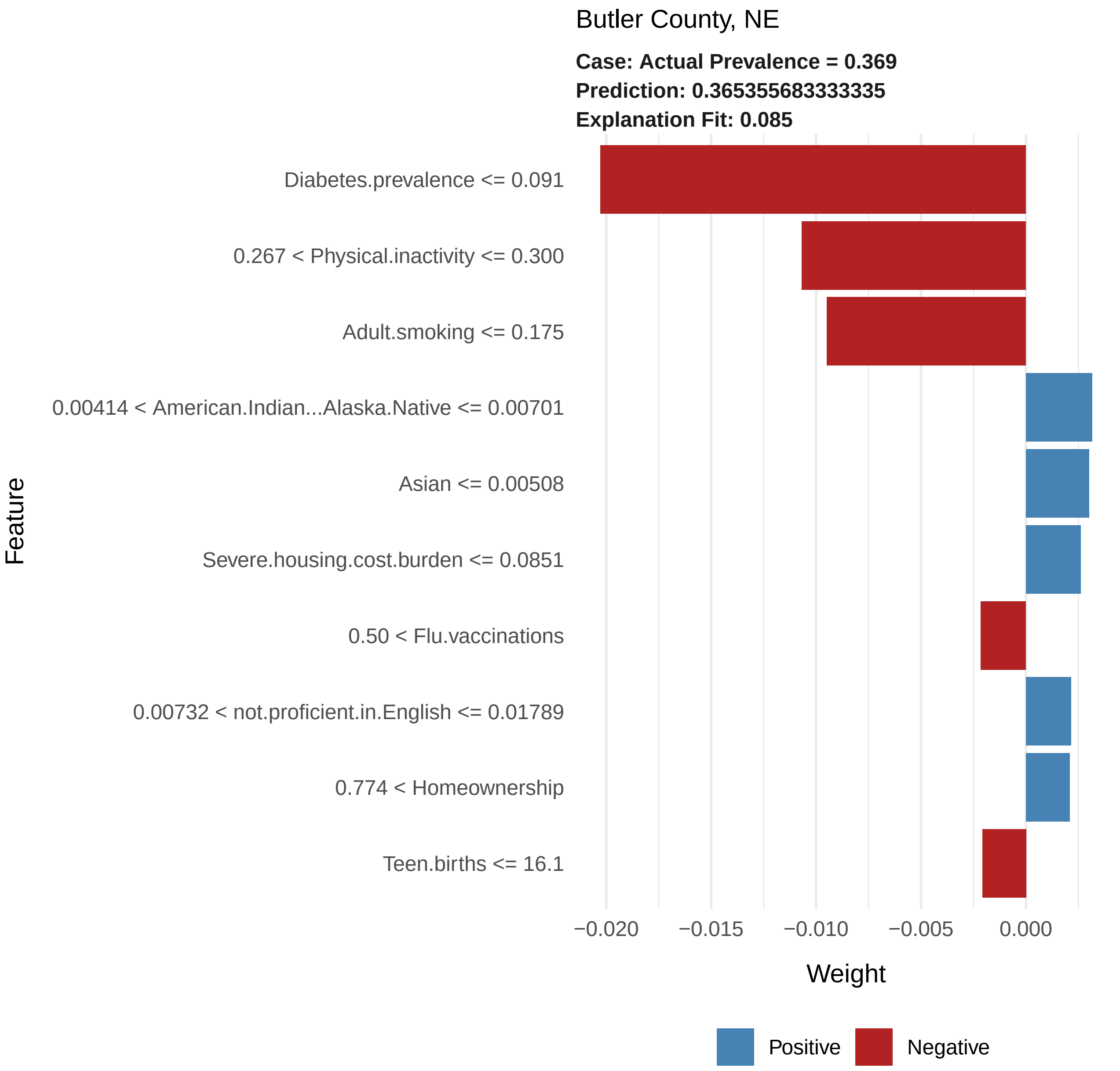
Butler County, NE
Case: Actual Prevalence = 0.369
Prediction: 0.365355683333335
Explanation Fit: 0.085
Diabetes.prevalence <= 0.091
0.267 < Physical.inactivity <= 0.300
Adult.smoking <= 0.175
0.00414 < American.Indian...Alaska.Native <= 0.00701
Asian <= 0.00508
Severe.housing.cost.burden <= 0.0851
0.50 < Flu.vaccinations
0.00732 < not.proficient.in.English <= 0.01789
0.774 < Homeownership
Teen.births <= 16.1
Feature
−0.020
−0.015
−0.010
−0.005
0.000
Weight
Positive
Negative

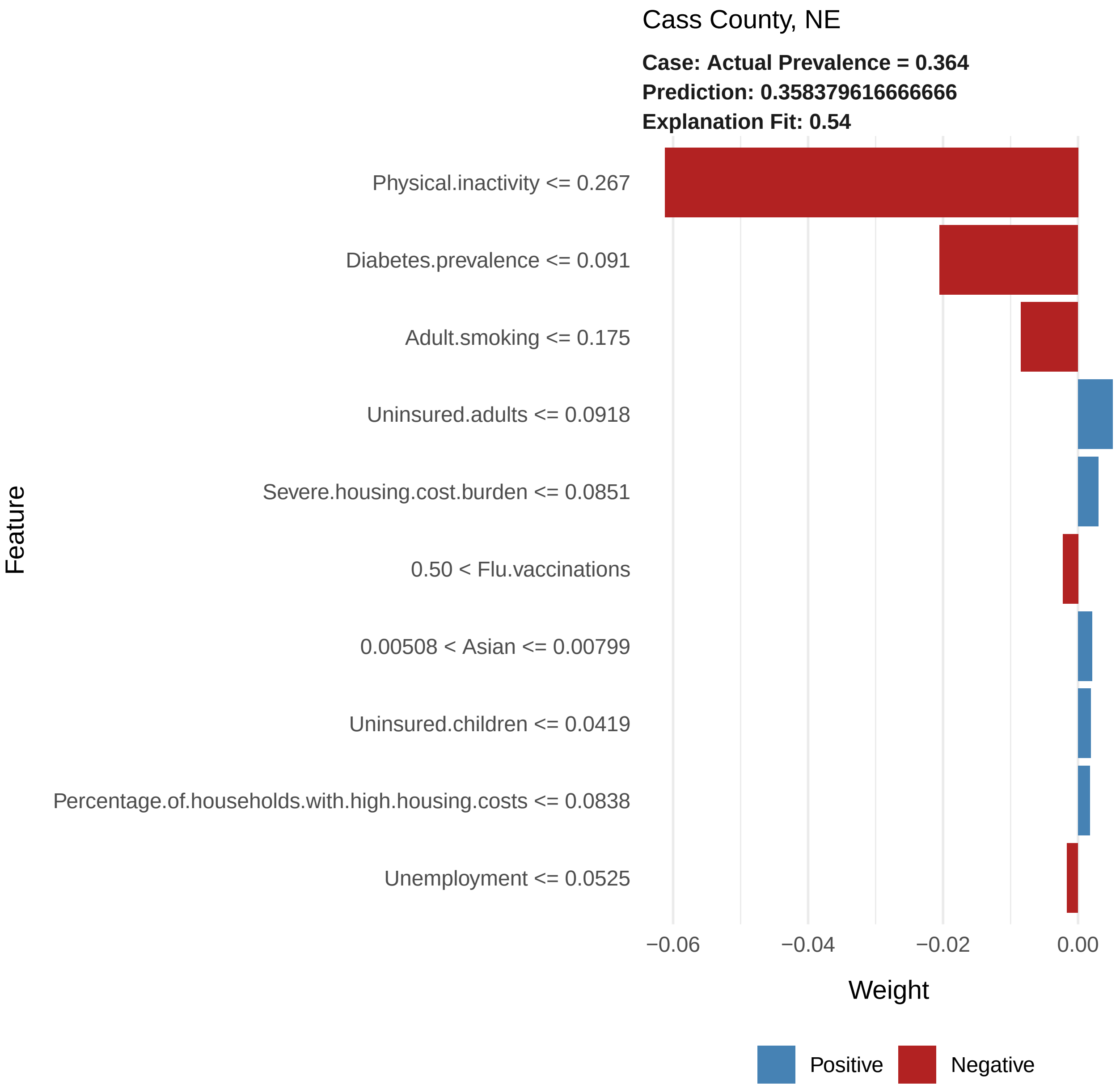
Cass County, NE
Case: Actual Prevalence = 0.364
Prediction: 0.358379616666666
Explanation Fit: 0.54
Physical.inactivity <= 0.267
Diabetes.prevalence <= 0.091
Adult.smoking <= 0.175
Uninsured.adults <= 0.0918
Severe.housing.cost.burden <= 0.0851
0.50 < Flu.vaccinations
0.00508 < Asian <= 0.00799
Uninsured.children <= 0.0419
Percentage.of.households.with.high.housing.costs <= 0.0838
Unemployment <= 0.0525
Feature
−0.06
−0.04
−0.02
0.00
Weight
Positive
Negative

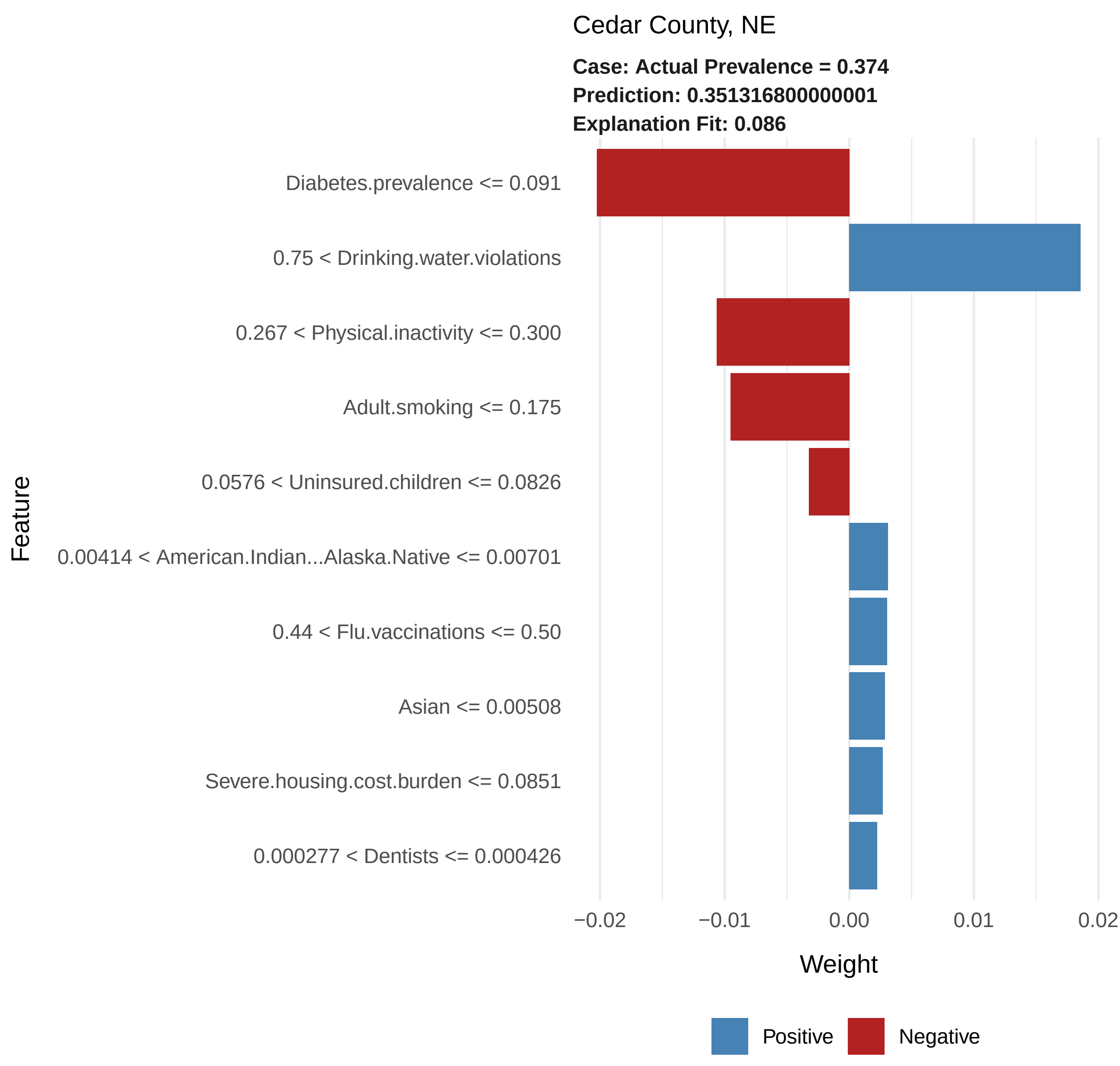
Cedar County, NE
Case: Actual Prevalence = 0.374
Prediction: 0.351316800000001
Explanation Fit: 0.086
Diabetes.prevalence <= 0.091
0.75 < Drinking.water.violations
0.267 < Physical.inactivity <= 0.300
Adult.smoking <= 0.175
0.0576 < Uninsured.children <= 0.0826
0.00414 < American.Indian...Alaska.Native <= 0.00701
0.44 < Flu.vaccinations <= 0.50
Asian <= 0.00508
Severe.housing.cost.burden <= 0.0851
0.000277 < Dentists <= 0.000426
Feature
−0.02
−0.01
0.00
0.01
0.02
Weight
Positive
Negative

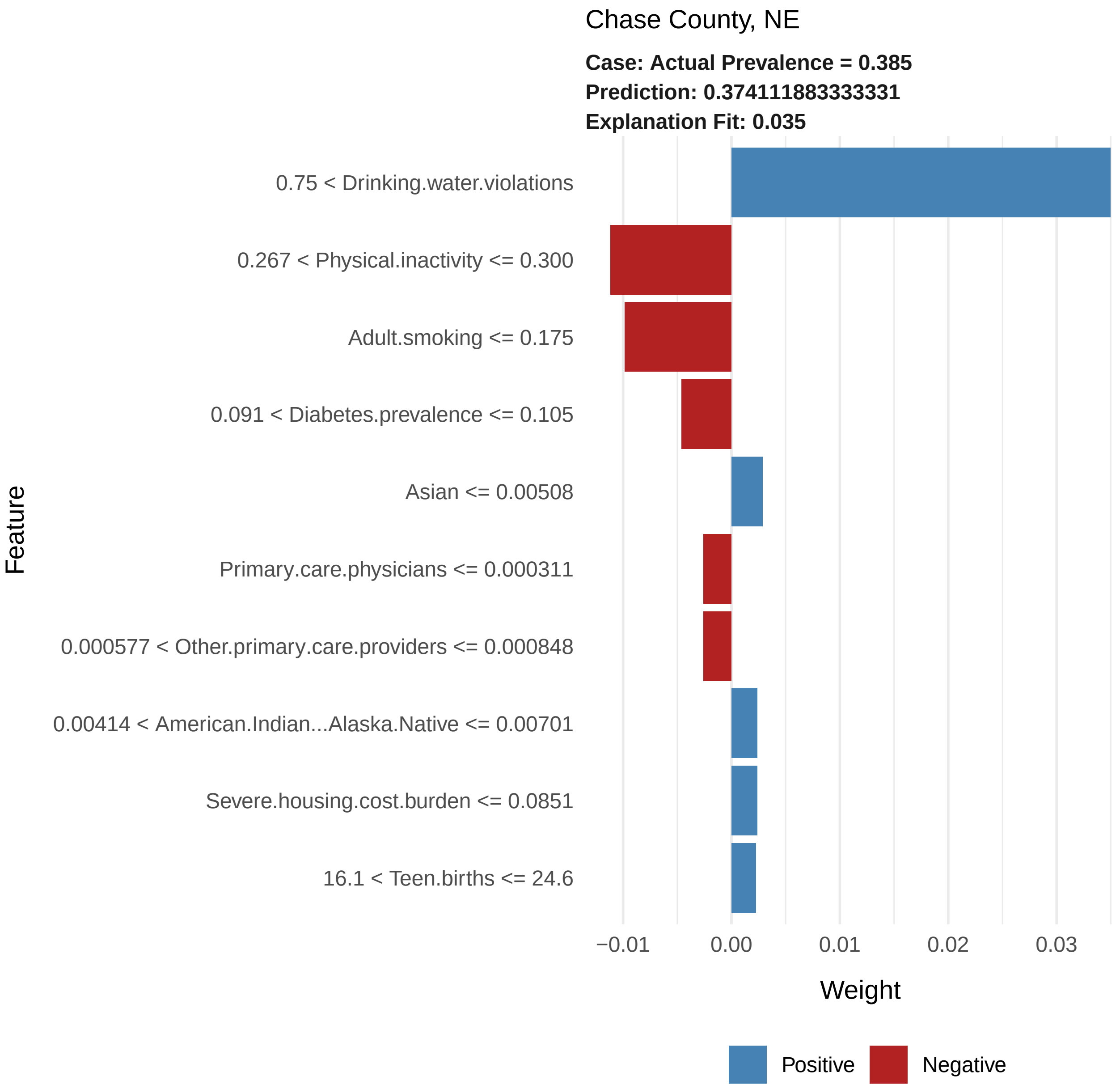

Chase County, NE
Case: Actual Prevalence = 0.385
Prediction: 0.374111883333331
Explanation Fit: 0.035
0.75 < Drinking.water.violations
0.267 < Physical.inactivity <= 0.300
Adult.smoking <= 0.175
0.091 < Diabetes.prevalence <= 0.105
Asian <= 0.00508
Primary.care.physicians <= 0.000311
0.000577 < Other.primary.care.providers <= 0.000848
0.00414 < American.Indian...Alaska.Native <= 0.00701
Severe.housing.cost.burden <= 0.0851
16.1 < Teen.births <= 24.6
Feature
−0.01
0.00
0.01
0.02
0.03
Weight
Positive
Negative

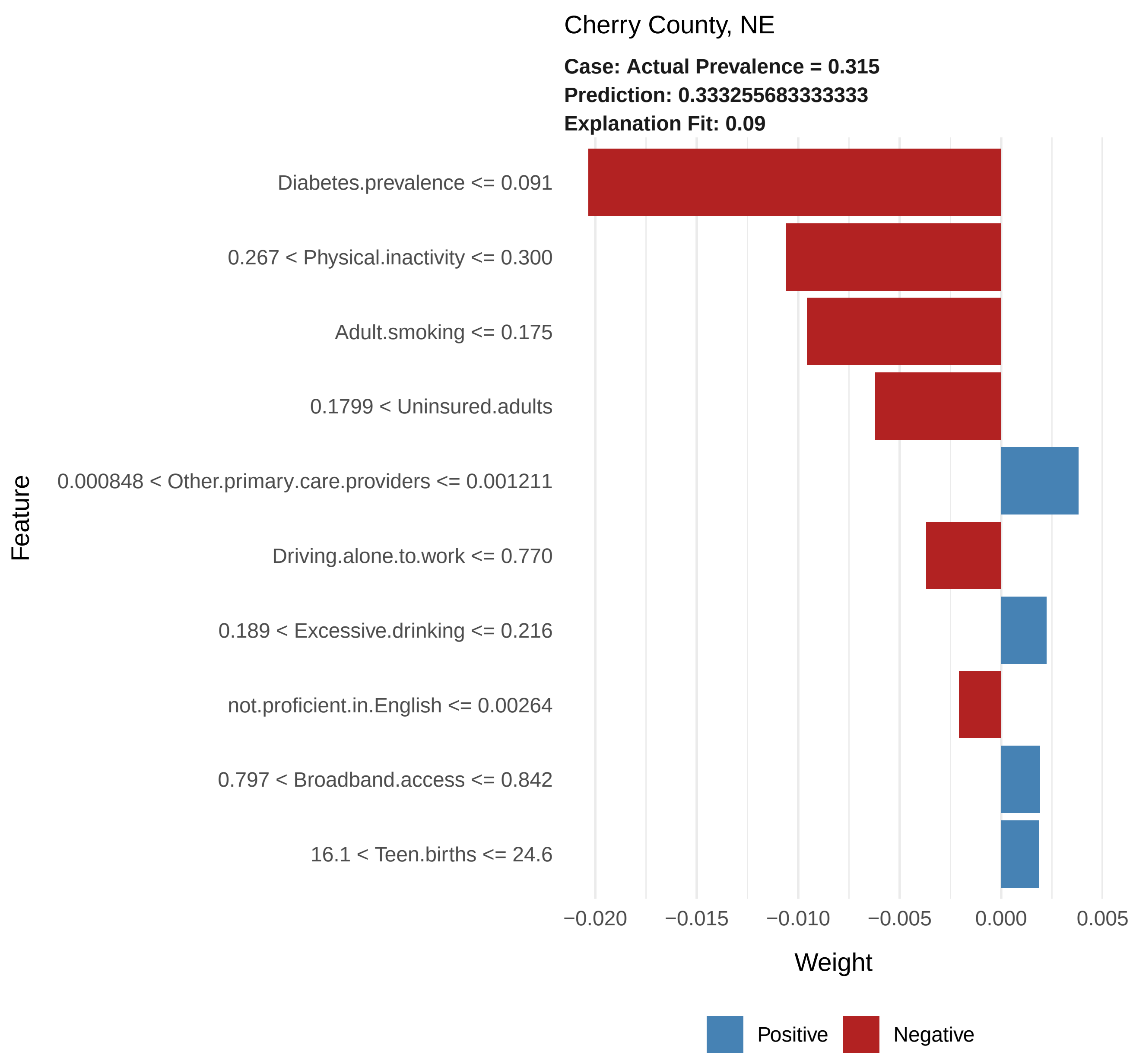

Cherry County, NE
Case: Actual Prevalence = 0.315
Prediction: 0.333255683333333
Explanation Fit: 0.09
Diabetes.prevalence <= 0.091
0.267 < Physical.inactivity <= 0.300
Adult.smoking <= 0.175
0.1799 < Uninsured.adults
0.000848 < Other.primary.care.providers <= 0.001211
Driving.alone.to.work <= 0.770
0.189 < Excessive.drinking <= 0.216
not.proficient.in.English <= 0.00264
0.797 < Broadband.access <= 0.842
16.1 < Teen.births <= 24.6
Feature
−0.020
−0.015
−0.010
−0.005
0.000
0.005
Weight
Positive
Negative

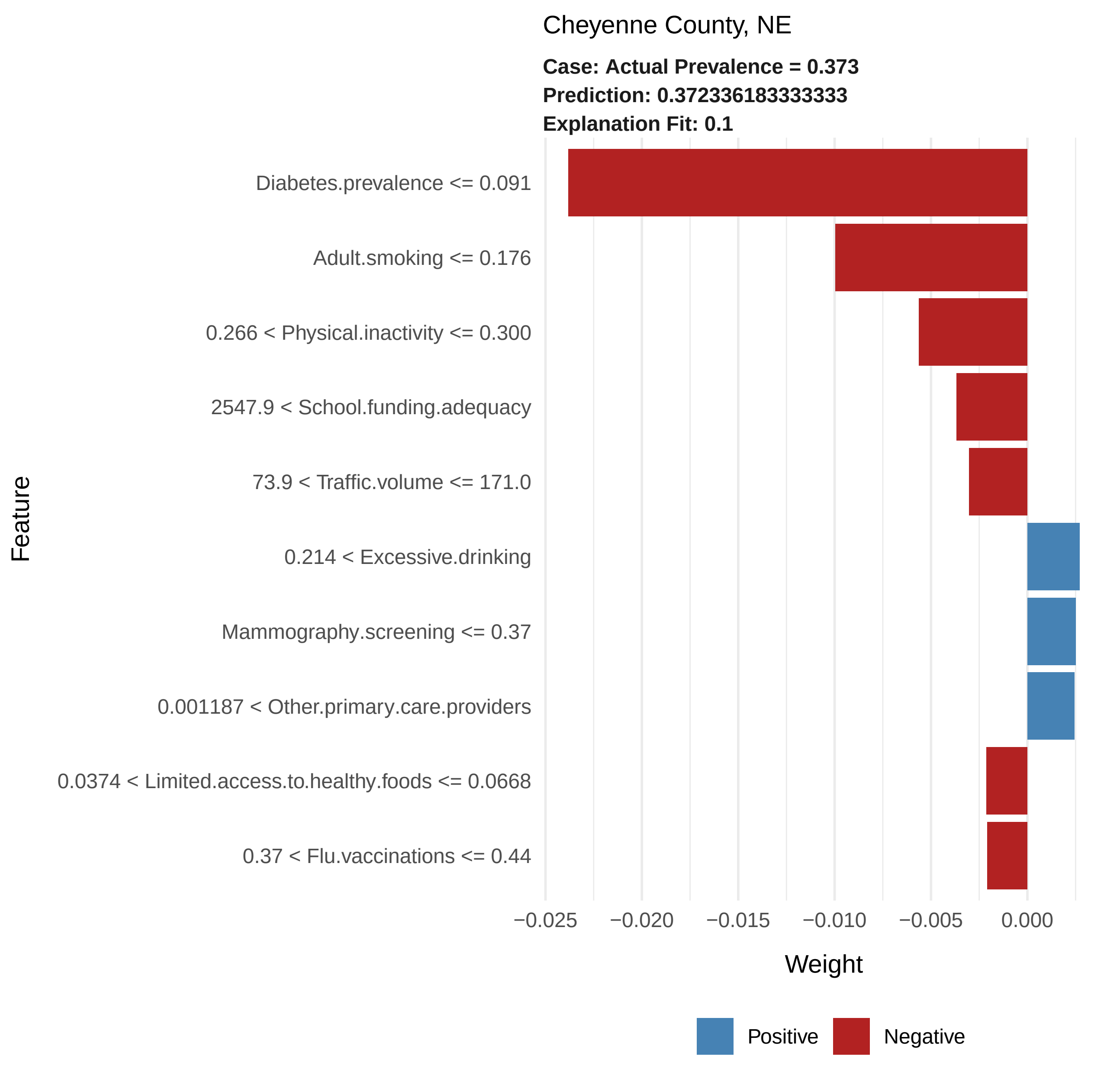
Cheyenne County, NE
Case: Actual Prevalence = 0.373
Prediction: 0.372336183333333
Explanation Fit: 0.1
Diabetes.prevalence <= 0.091
Adult.smoking <= 0.176
0.266 < Physical.inactivity <= 0.300
2547.9 < School.funding.adequacy
73.9 < Traffic.volume <= 171.0
0.214 < Excessive.drinking
Mammography.screening <= 0.37
0.001187 < Other.primary.care.providers
0.0374 < Limited.access.to.healthy.foods <= 0.0668
0.37 < Flu.vaccinations <= 0.44
Feature
−0.025
−0.020
−0.015
−0.010
−0.005
0.000
Weight
Positive
Negative

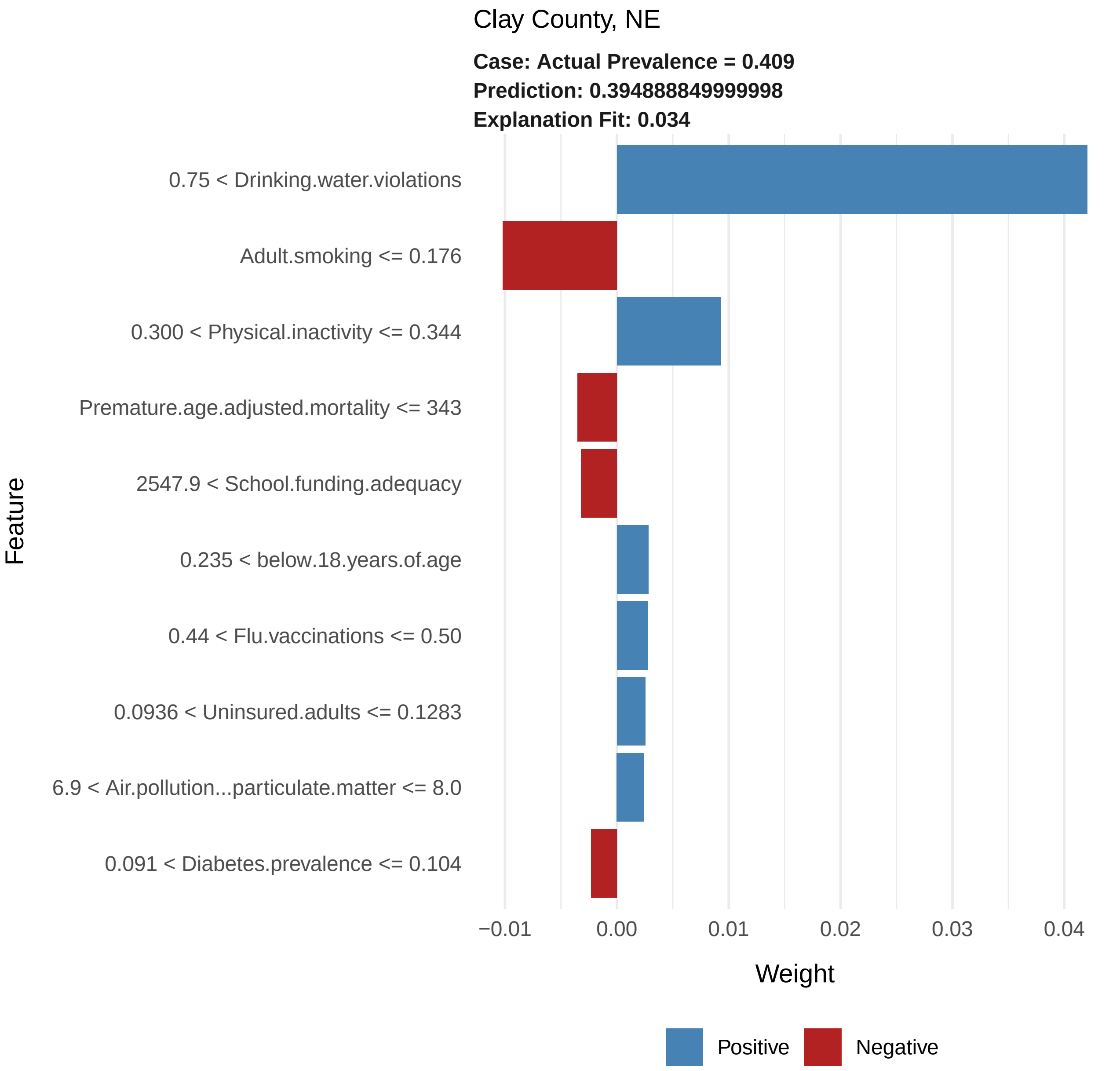
Clay County, NE
Case: Actual Prevalence = 0.409
Prediction: 0.394888849999998
Explanation Fit: 0.034
0.75 < Drinking.water.violations
Adult.smoking <= 0.176
0.300 < Physical.inactivity <= 0.344
Premature.age.adjusted.mortality <= 343
2547.9 < School.funding.adequacy
0.235 < below.18.years.of.age
0.44 < Flu.vaccinations <= 0.50
0.0936 < Uninsured.adults <= 0.1283
6.9 < Air.pollution...particulate.matter <= 8.0
0.091 < Diabetes.prevalence <= 0.104
Feature
−0.01
0.00
0.01
0.02
0.03
0.04
Weight
Positive
Negative

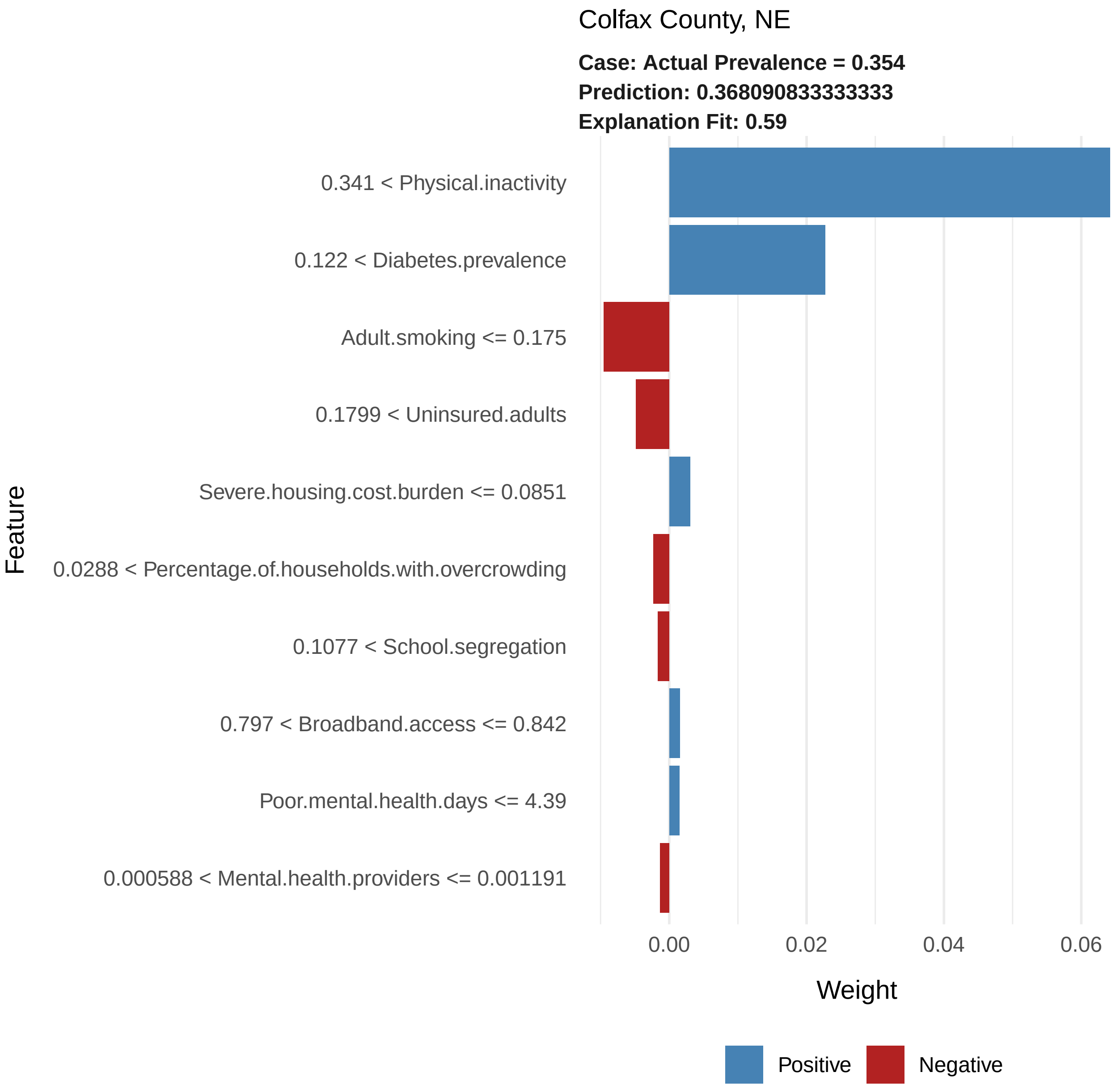

Colfax County, NE
Case: Actual Prevalence = 0.354
Prediction: 0.368090833333333
Explanation Fit: 0.59
0.341 < Physical.inactivity
0.122 < Diabetes.prevalence
Adult.smoking <= 0.175
0.1799 < Uninsured.adults
Severe.housing.cost.burden <= 0.0851
0.0288 < Percentage.of.households.with.overcrowding
0.1077 < School.segregation
0.797 < Broadband.access <= 0.842
Poor.mental.health.days <= 4.39
0.000588 < Mental.health.providers <= 0.001191
Feature
0.00
0.02
0.04
0.06
Weight
Positive
Negative

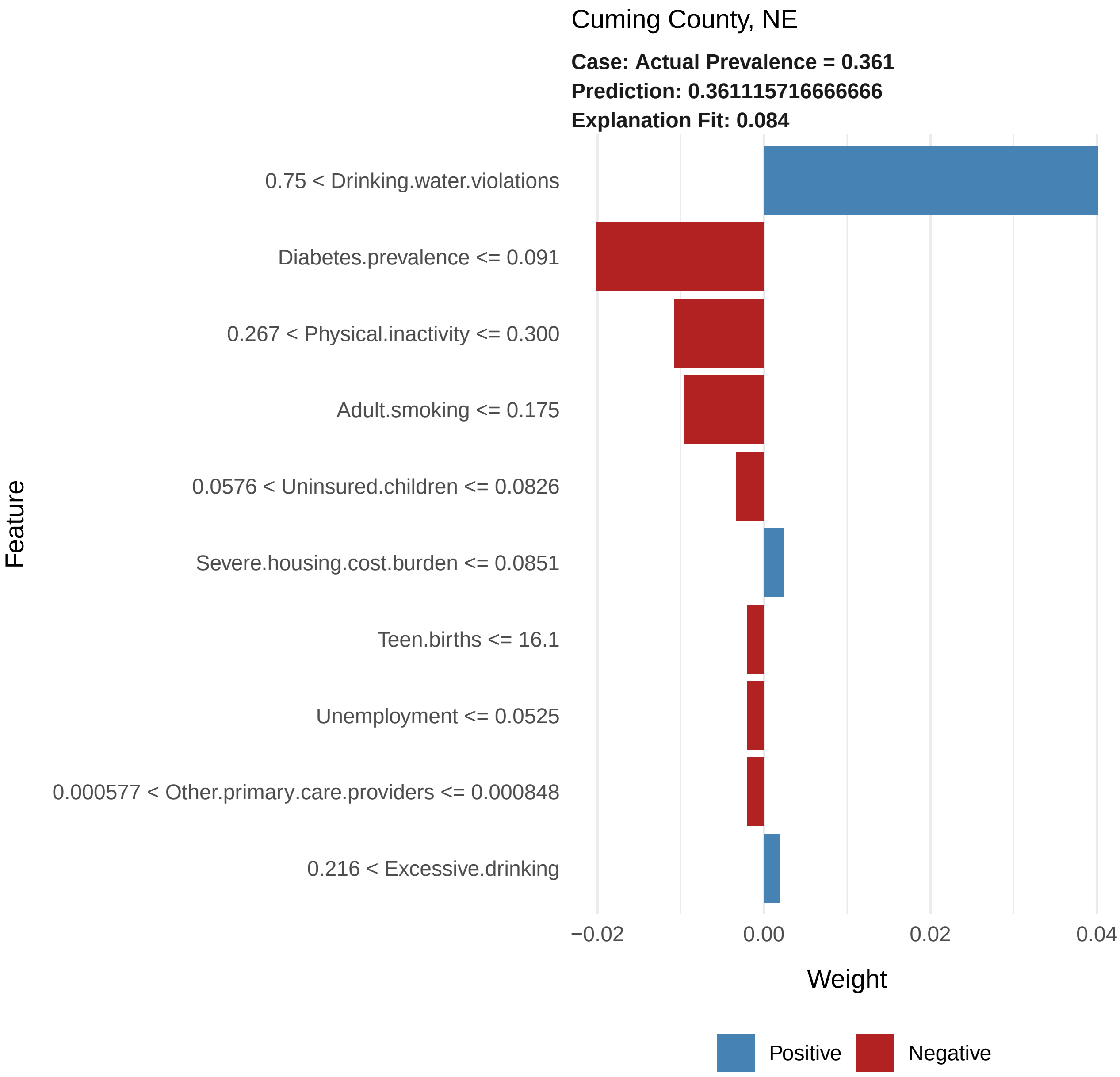

Cuming County, NE
Case: Actual Prevalence = 0.361
Prediction: 0.361115716666666
Explanation Fit: 0.084
0.75 < Drinking.water.violations
Diabetes.prevalence <= 0.091
0.267 < Physical.inactivity <= 0.300
Adult.smoking <= 0.175
0.0576 < Uninsured.children <= 0.0826
Severe.housing.cost.burden <= 0.0851
Teen.births <= 16.1
Unemployment <= 0.0525
0.000577 < Other.primary.care.providers <= 0.000848
0.216 < Excessive.drinking
Feature
−0.02
0.00
0.02
0.04
Weight
Positive
Negative

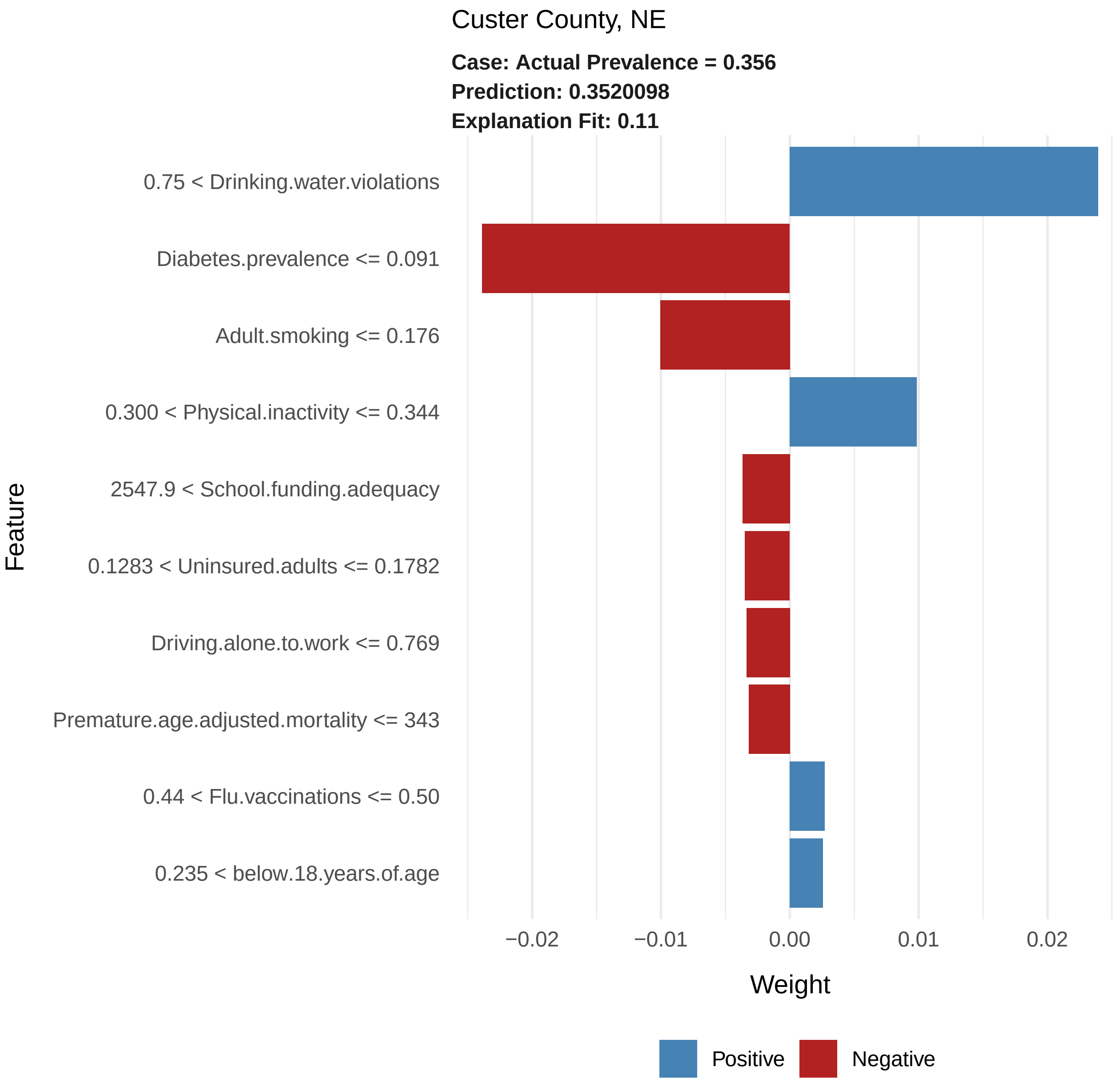

Custer County, NE
Case: Actual Prevalence = 0.356
Prediction: 0.3520098
Explanation Fit: 0.11
0.75 < Drinking.water.violations
Diabetes.prevalence <= 0.091
Adult.smoking <= 0.176
0.300 < Physical.inactivity <= 0.344
2547.9 < School.funding.adequacy
0.1283 < Uninsured.adults <= 0.1782
Driving.alone.to.work <= 0.769
Premature.age.adjusted.mortality <= 343
0.44 < Flu.vaccinations <= 0.50
0.235 < below.18.years.of.age
Feature
−0.02
−0.01
0.00
0.01
0.02
Weight
Positive
Negative

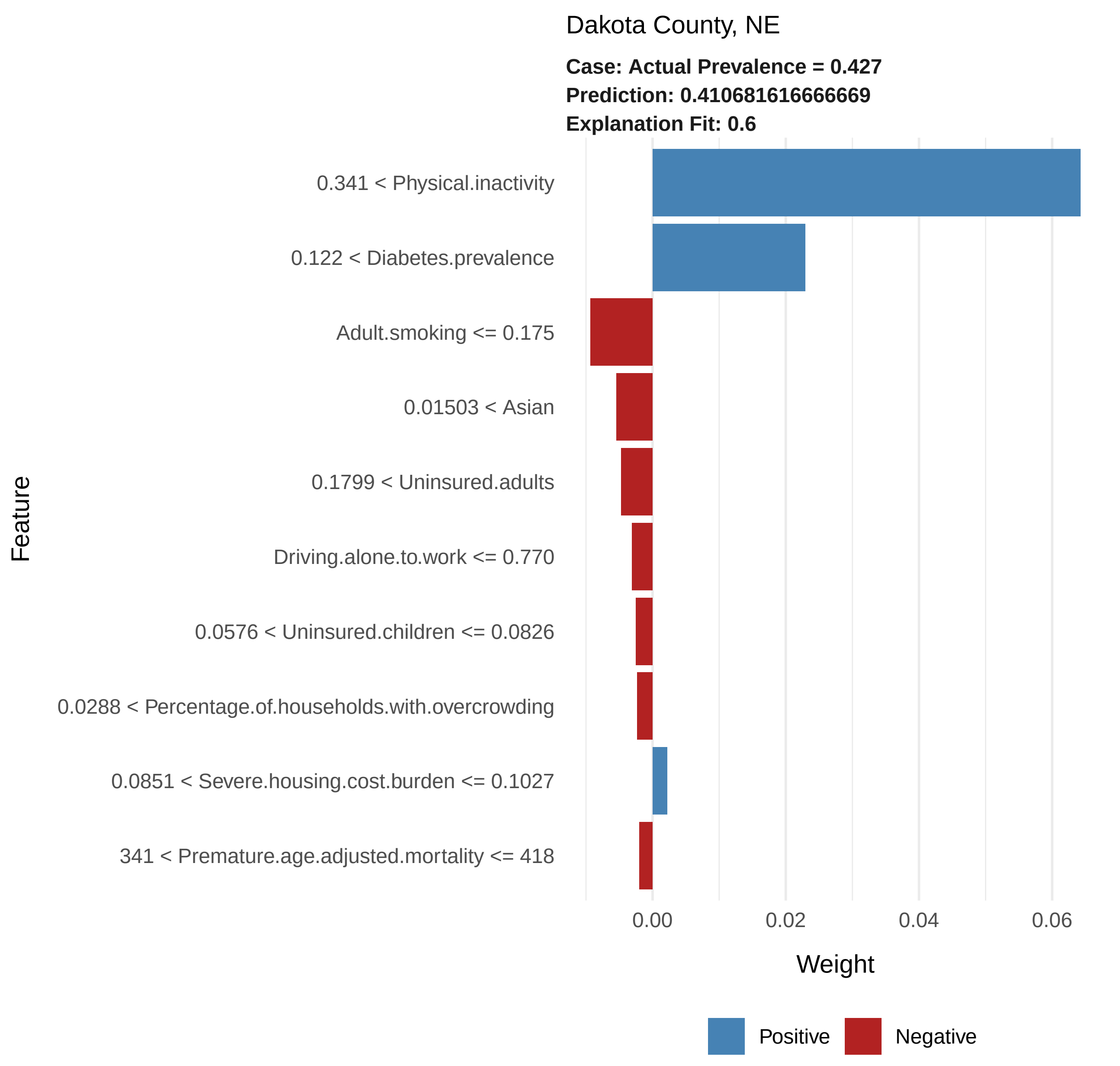

Dakota County, NE
Case: Actual Prevalence = 0.427
Prediction: 0.410681616666669
Explanation Fit: 0.6
0.341 < Physical.inactivity
0.122 < Diabetes.prevalence
Adult.smoking <= 0.175
0.01503 < Asian
0.1799 < Uninsured.adults
Driving.alone.to.work <= 0.770
0.0576 < Uninsured.children <= 0.0826
0.0288 < Percentage.of.households.with.overcrowding
0.0851 < Severe.housing.cost.burden <= 0.1027
341 < Premature.age.adjusted.mortality <= 418
Feature
0.00
0.02
0.04
0.06
Weight
Positive
Negative

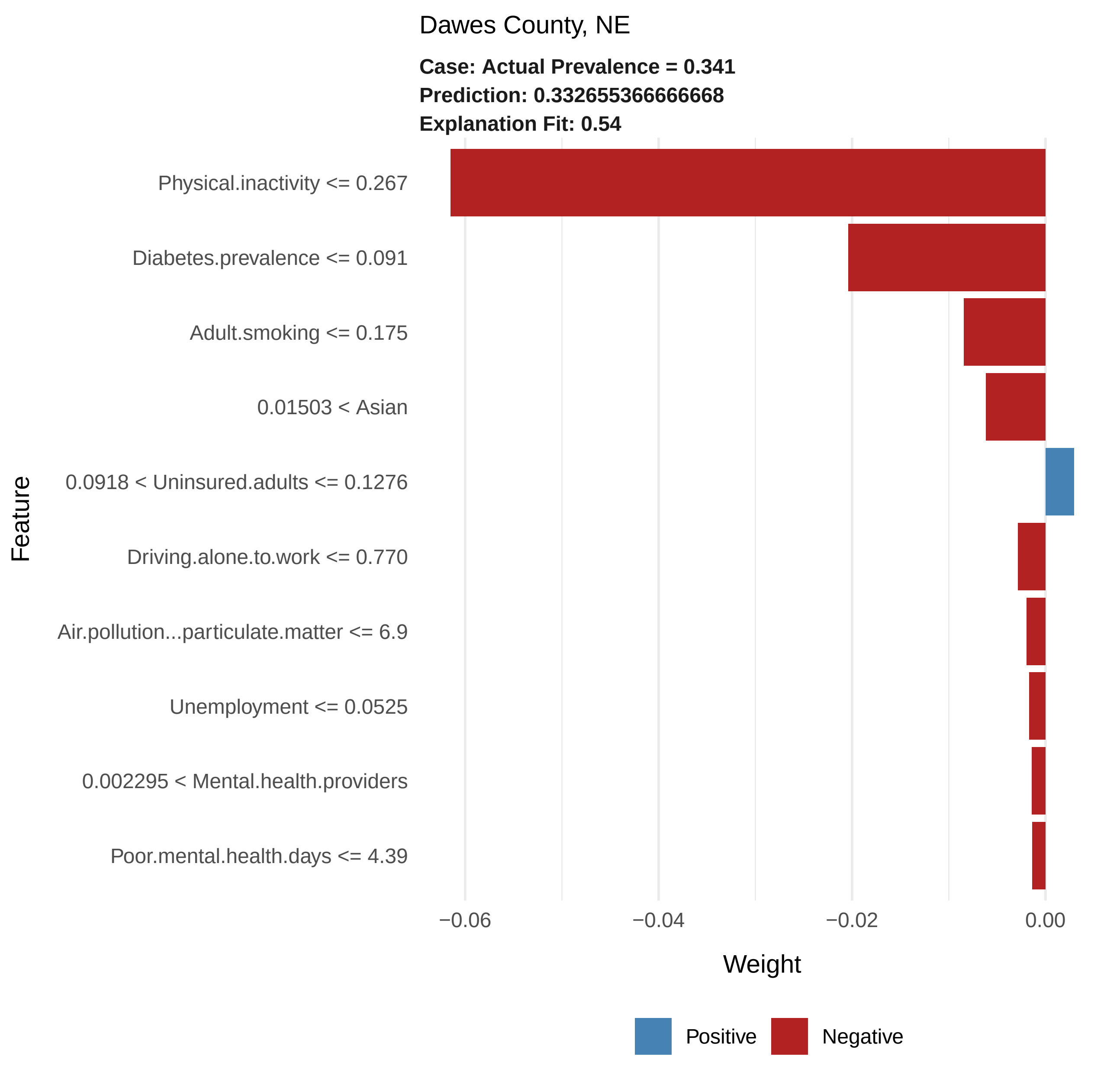

Dawes County, NE
Case: Actual Prevalence = 0.341
Prediction: 0.332655366666668
Explanation Fit: 0.54
Physical.inactivity <= 0.267
Diabetes.prevalence <= 0.091
Adult.smoking <= 0.175
0.01503 < Asian
0.0918 < Uninsured.adults <= 0.1276
Driving.alone.to.work <= 0.770
Air.pollution...particulate.matter <= 6.9
Unemployment <= 0.0525
0.002295 < Mental.health.providers
Poor.mental.health.days <= 4.39
Feature
−0.06
−0.04
−0.02
0.00
Weight
Positive
Negative

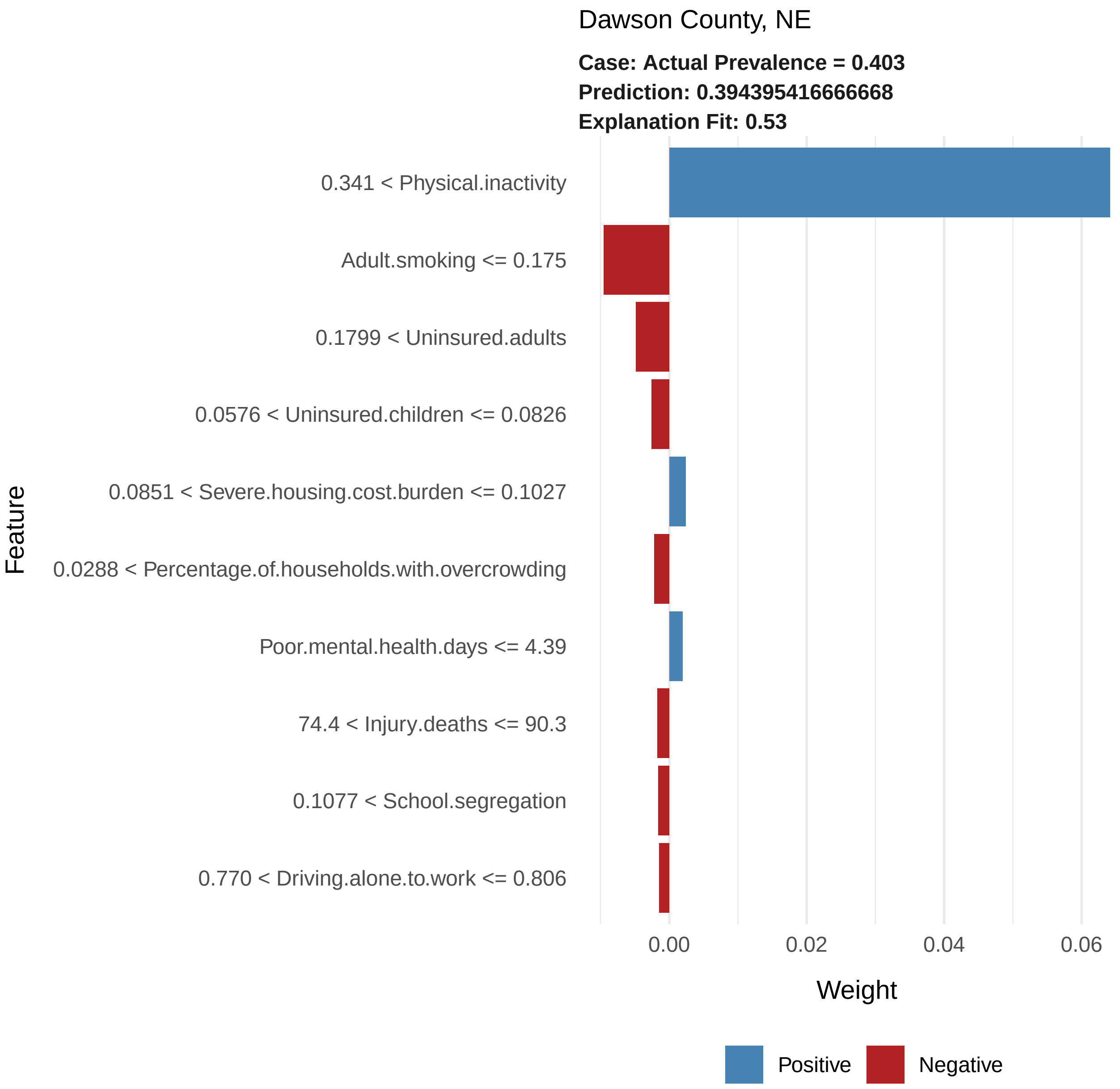
Dawson County, NE
Case: Actual Prevalence = 0.403
Prediction: 0.394395416666668
Explanation Fit: 0.53
0.341 < Physical.inactivity
Adult.smoking <= 0.175
0.1799 < Uninsured.adults
0.0576 < Uninsured.children <= 0.0826
0.0851 < Severe.housing.cost.burden <= 0.1027
0.0288 < Percentage.of.households.with.overcrowding
Poor.mental.health.days <= 4.39
74.4 < Injury.deaths <= 90.3
0.1077 < School.segregation
0.770 < Driving.alone.to.work <= 0.806
Feature
0.00
0.02
0.04
0.06
Weight
Positive
Negative

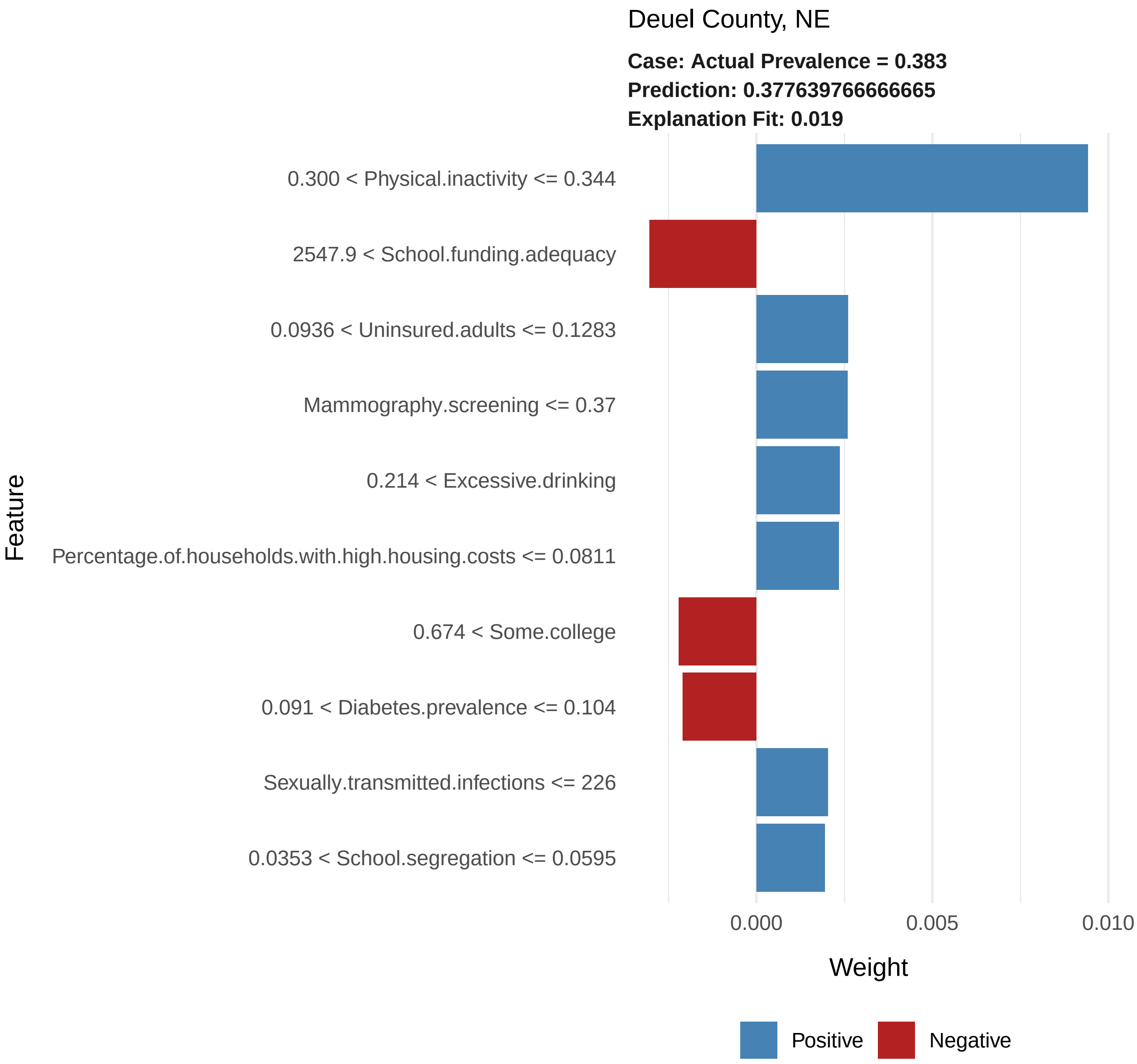

Deuel County, NE
Case: Actual Prevalence = 0.383
Prediction: 0.377639766666665
Explanation Fit: 0.019
0.300 < Physical.inactivity <= 0.344
2547.9 < School.funding.adequacy
0.0936 < Uninsured.adults <= 0.1283
Mammography.screening <= 0.37
0.214 < Excessive.drinking
Percentage.of.households.with.high.housing.costs <= 0.0811
0.674 < Some.college
0.091 < Diabetes.prevalence <= 0.104
Sexually.transmitted.infections <= 226
0.0353 < School.segregation <= 0.0595
Feature
0.000
0.005
0.010
Weight
Positive
Negative

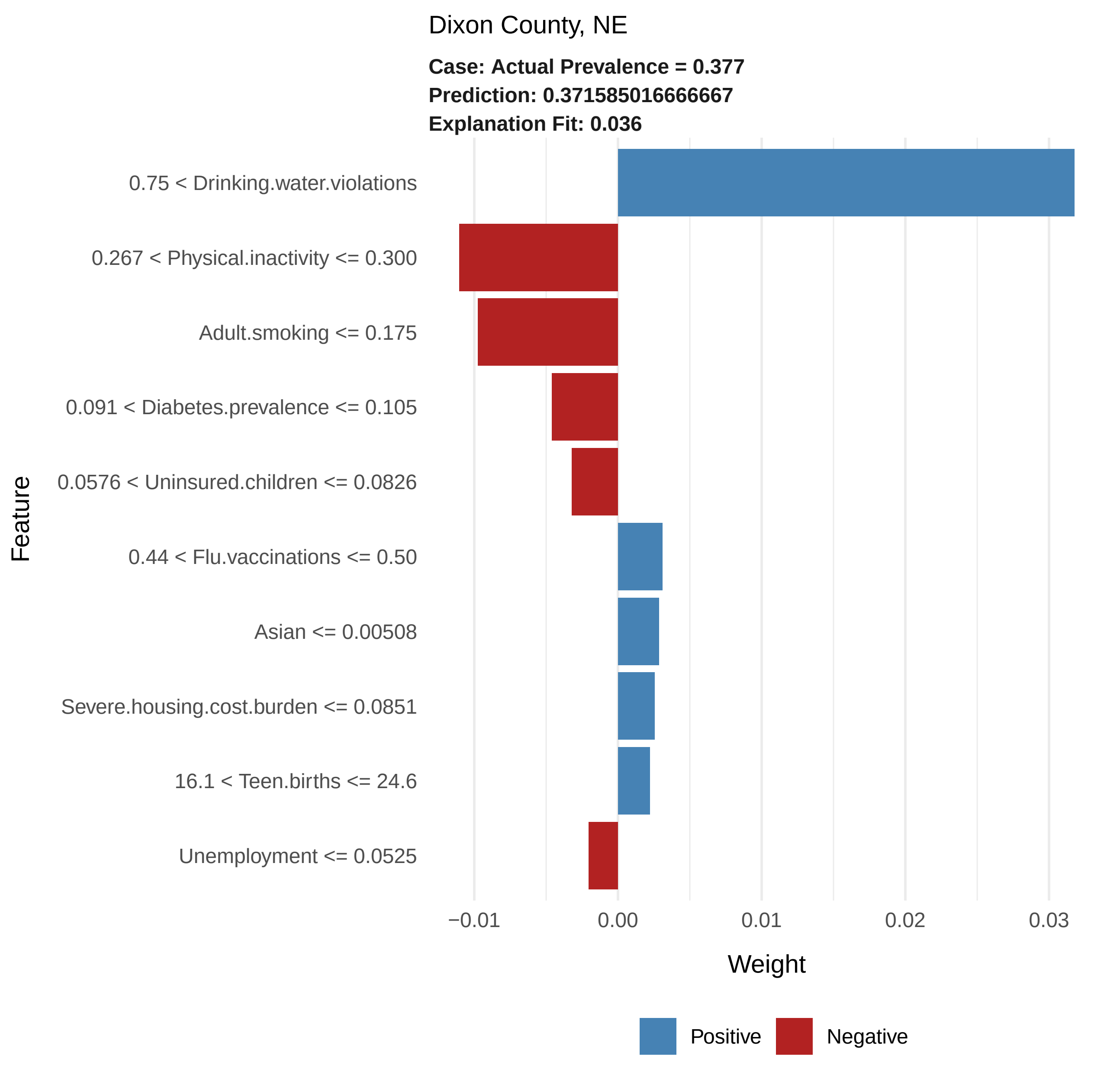

Dixon County, NE
Case: Actual Prevalence = 0.377
Prediction: 0.371585016666667
Explanation Fit: 0.036
0.75 < Drinking.water.violations
0.267 < Physical.inactivity <= 0.300
Adult.smoking <= 0.175
0.091 < Diabetes.prevalence <= 0.105
0.0576 < Uninsured.children <= 0.0826
0.44 < Flu.vaccinations <= 0.50
Asian <= 0.00508
Severe.housing.cost.burden <= 0.0851
16.1 < Teen.births <= 24.6
Unemployment <= 0.0525
Feature
−0.01
0.00
0.01
0.02
0.03
Weight
Positive
Negative

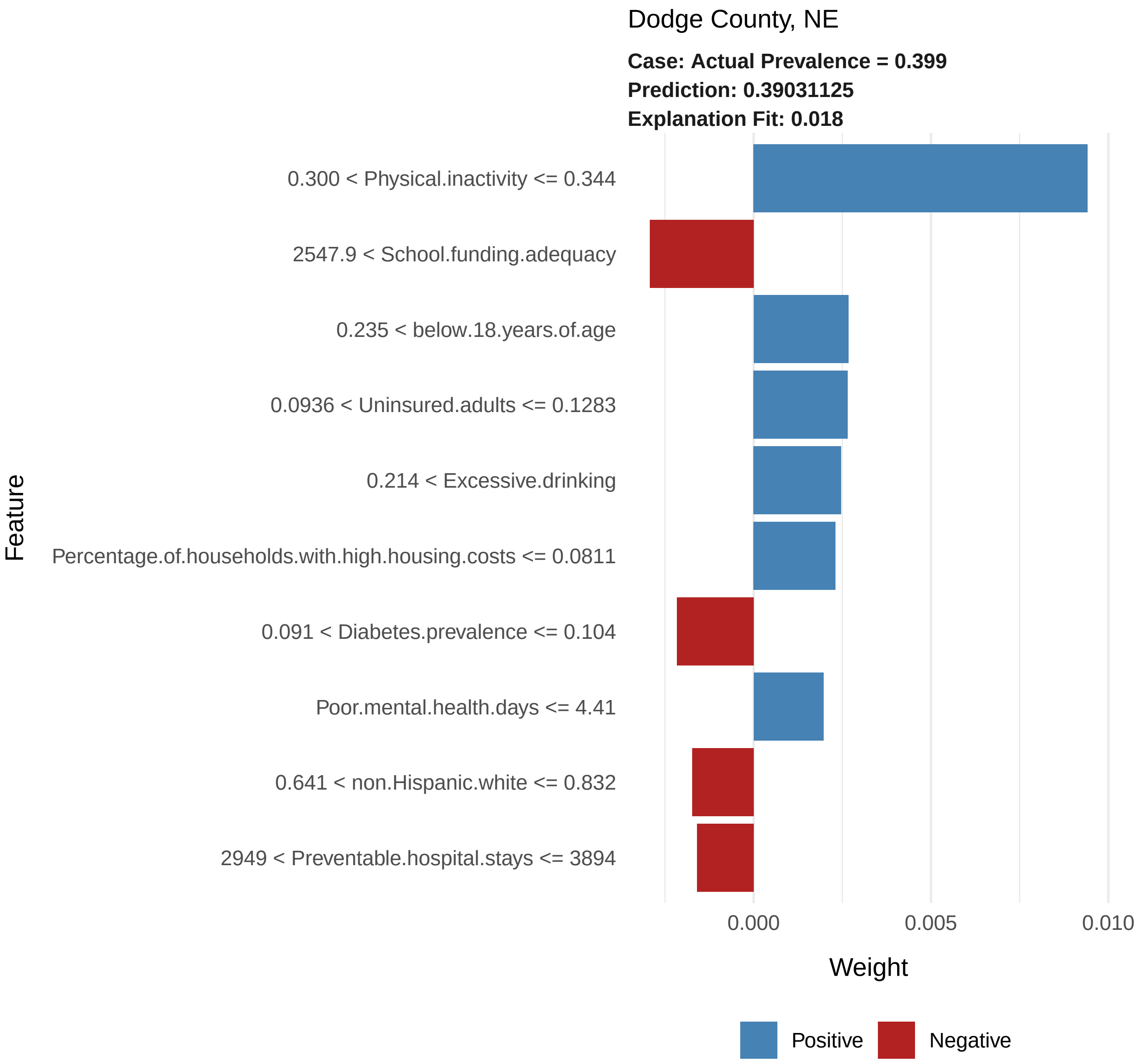

Dodge County, NE
Case: Actual Prevalence = 0.399
Prediction: 0.39031125
Explanation Fit: 0.018
0.300 < Physical.inactivity <= 0.344
2547.9 < School.funding.adequacy
0.235 < below.18.years.of.age
0.0936 < Uninsured.adults <= 0.1283
0.214 < Excessive.drinking
Percentage.of.households.with.high.housing.costs <= 0.0811
0.091 < Diabetes.prevalence <= 0.104
Poor.mental.health.days <= 4.41
0.641 < non.Hispanic.white <= 0.832
2949 < Preventable.hospital.stays <= 3894
Feature
0.000
0.005
0.010
Weight
Positive
Negative

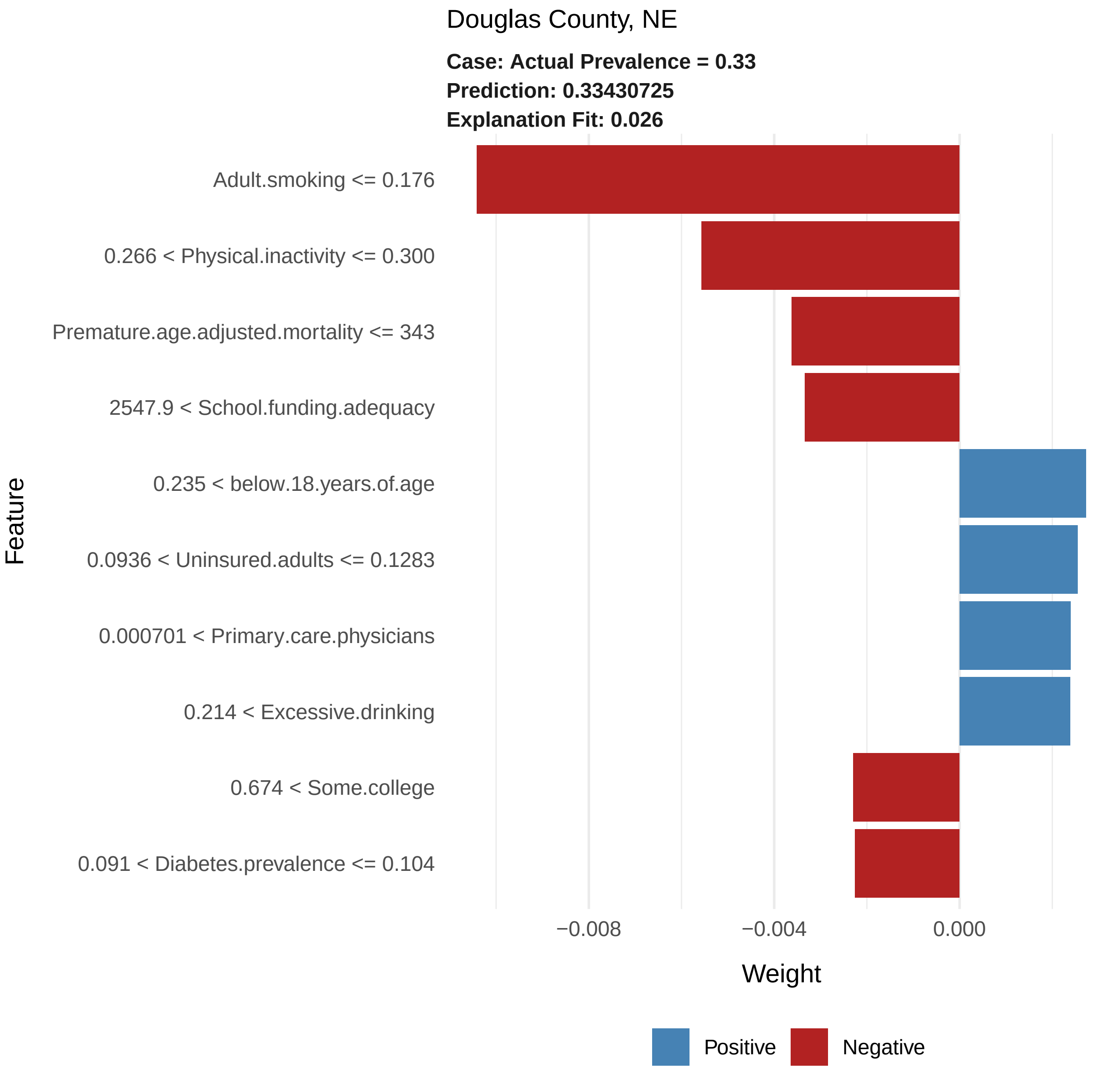

Douglas County, NE
Case: Actual Prevalence = 0.33
Prediction: 0.33430725
Explanation Fit: 0.026
Adult.smoking <= 0.176
0.266 < Physical.inactivity <= 0.300
Premature.age.adjusted.mortality <= 343
2547.9 < School.funding.adequacy
0.235 < below.18.years.of.age
0.0936 < Uninsured.adults <= 0.1283
0.000701 < Primary.care.physicians
0.214 < Excessive.drinking
0.674 < Some.college
0.091 < Diabetes.prevalence <= 0.104
Feature
−0.008
−0.004
0.000
Weight
Positive
Negative

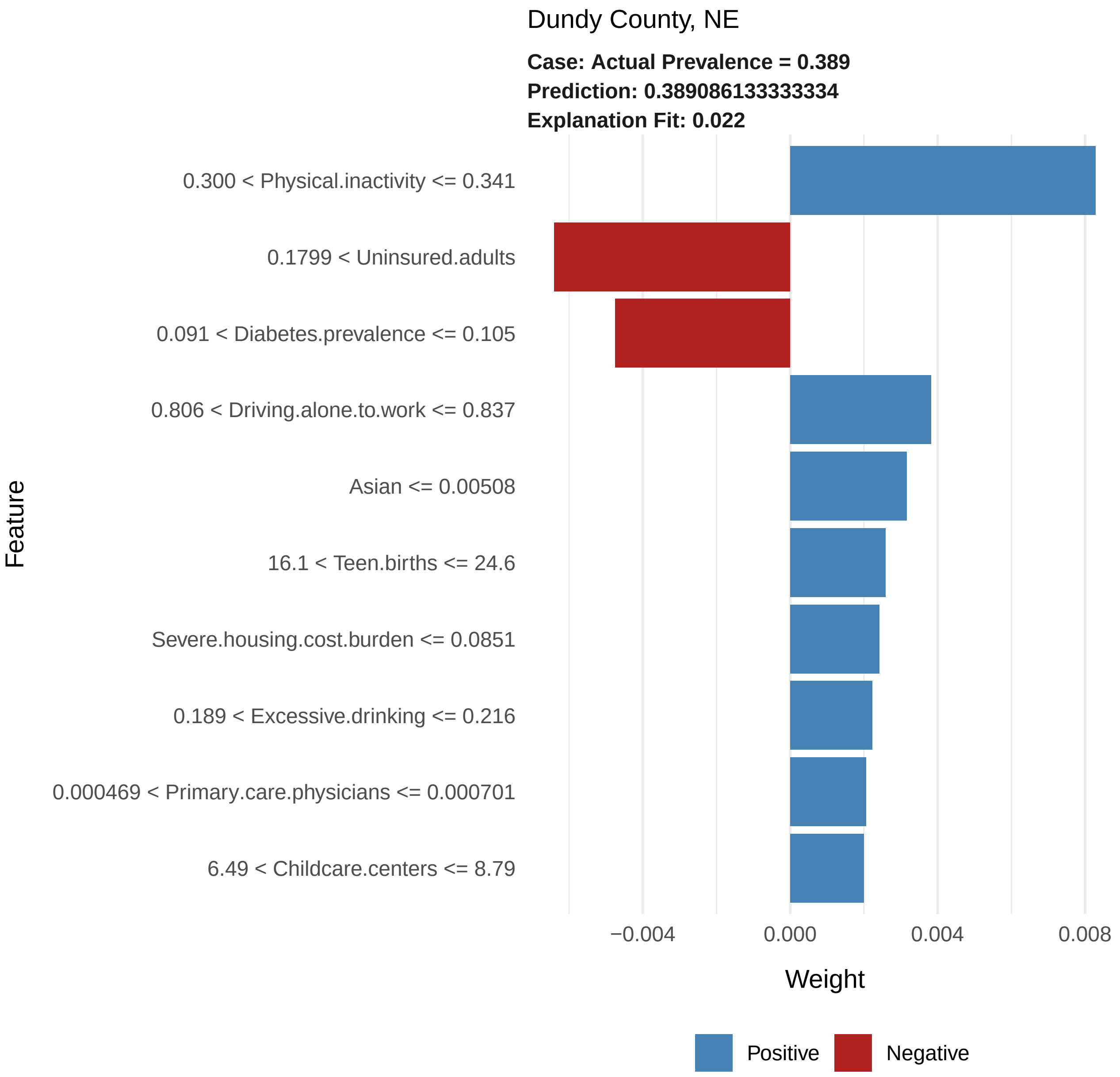

Dundy County, NE
Case: Actual Prevalence = 0.389
Prediction: 0.389086133333334
Explanation Fit: 0.022
0.300 < Physical.inactivity <= 0.341
0.1799 < Uninsured.adults
0.091 < Diabetes.prevalence <= 0.105
0.806 < Driving.alone.to.work <= 0.837
Asian <= 0.00508
16.1 < Teen.births <= 24.6
Severe.housing.cost.burden <= 0.0851
0.189 < Excessive.drinking <= 0.216
0.000469 < Primary.care.physicians <= 0.000701
6.49 < Childcare.centers <= 8.79
Feature
−0.004
0.000
0.004
0.008
Weight
Positive
Negative

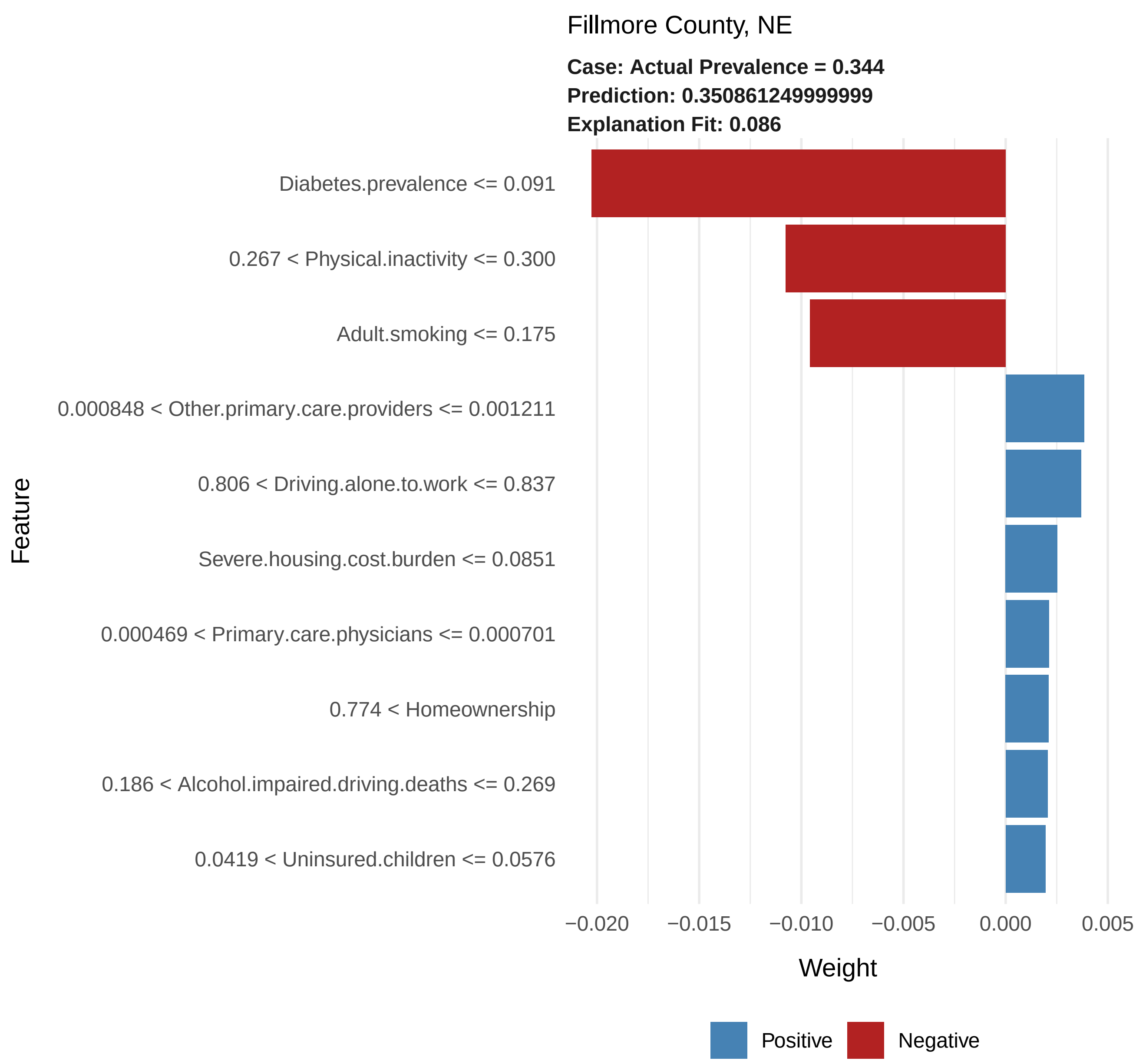

Fillmore County, NE
Case: Actual Prevalence = 0.344
Prediction: 0.350861249999999
Explanation Fit: 0.086
Diabetes.prevalence <= 0.091
0.267 < Physical.inactivity <= 0.300
Adult.smoking <= 0.175
0.000848 < Other.primary.care.providers <= 0.001211
0.806 < Driving.alone.to.work <= 0.837
Severe.housing.cost.burden <= 0.0851
0.000469 < Primary.care.physicians <= 0.000701
0.774 < Homeownership
0.186 < Alcohol.impaired.driving.deaths <= 0.269
0.0419 < Uninsured.children <= 0.0576
Feature
−0.020
−0.015
−0.010
−0.005
0.000
0.005
Weight
Positive
Negative

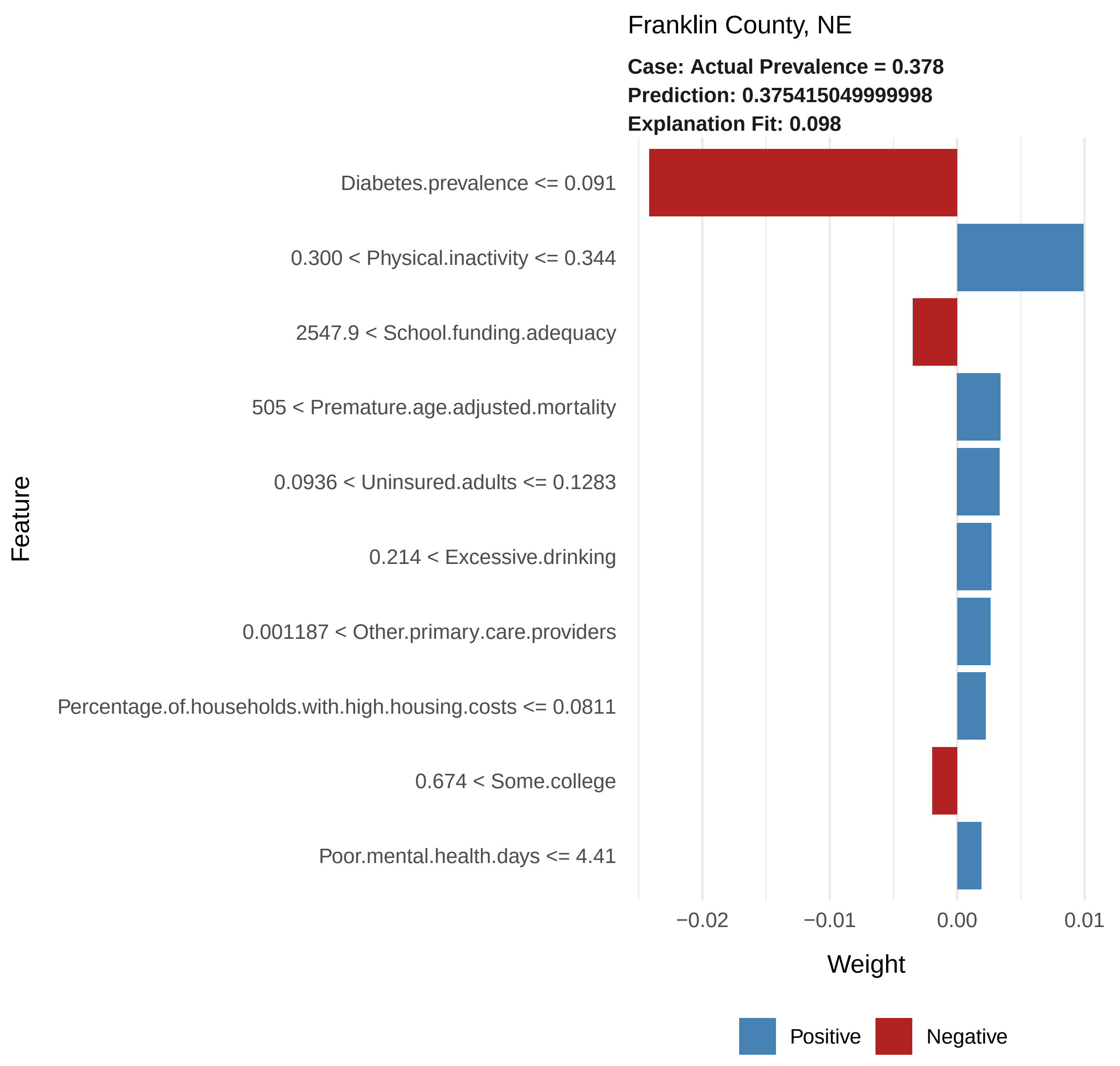

Franklin County, NE
Case: Actual Prevalence = 0.378
Prediction: 0.375415049999998
Explanation Fit: 0.098
Diabetes.prevalence <= 0.091
0.300 < Physical.inactivity <= 0.344
2547.9 < School.funding.adequacy
505 < Premature.age.adjusted.mortality
0.0936 < Uninsured.adults <= 0.1283
0.214 < Excessive.drinking
0.001187 < Other.primary.care.providers
Percentage.of.households.with.high.housing.costs <= 0.0811
0.674 < Some.college
Poor.mental.health.days <= 4.41
Feature
−0.02
−0.01
0.00
0.01
Weight
Positive
Negative

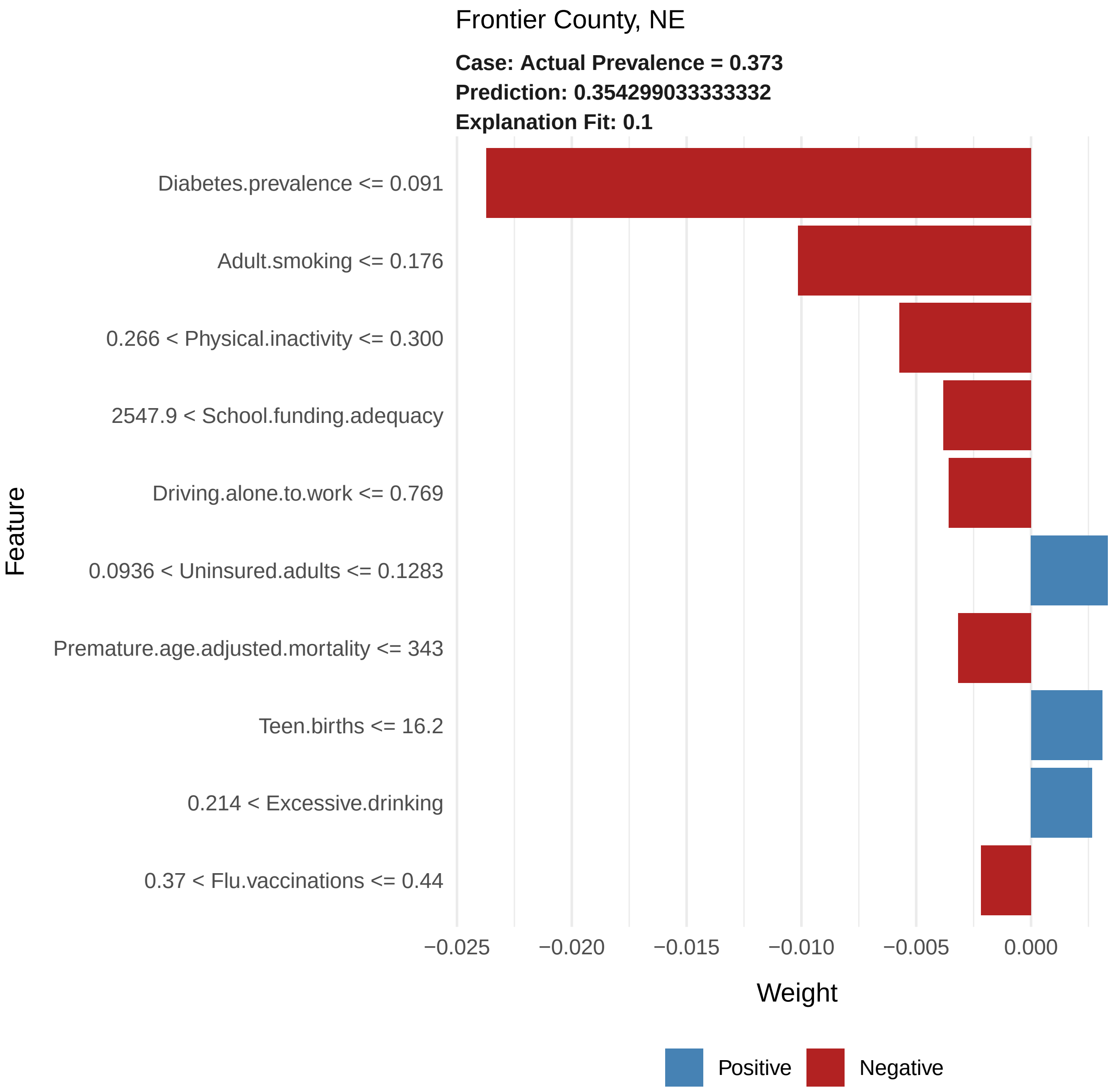
Frontier County, NE
Case: Actual Prevalence = 0.373
Prediction: 0.354299033333332
Explanation Fit: 0.1
Diabetes.prevalence <= 0.091
Adult.smoking <= 0.176
0.266 < Physical.inactivity <= 0.300
2547.9 < School.funding.adequacy
Driving.alone.to.work <= 0.769
0.0936 < Uninsured.adults <= 0.1283
Premature.age.adjusted.mortality <= 343
Teen.births <= 16.2
0.214 < Excessive.drinking
0.37 < Flu.vaccinations <= 0.44
Feature
−0.025
−0.020
−0.015
−0.010
−0.005
0.000
Weight
Positive
Negative

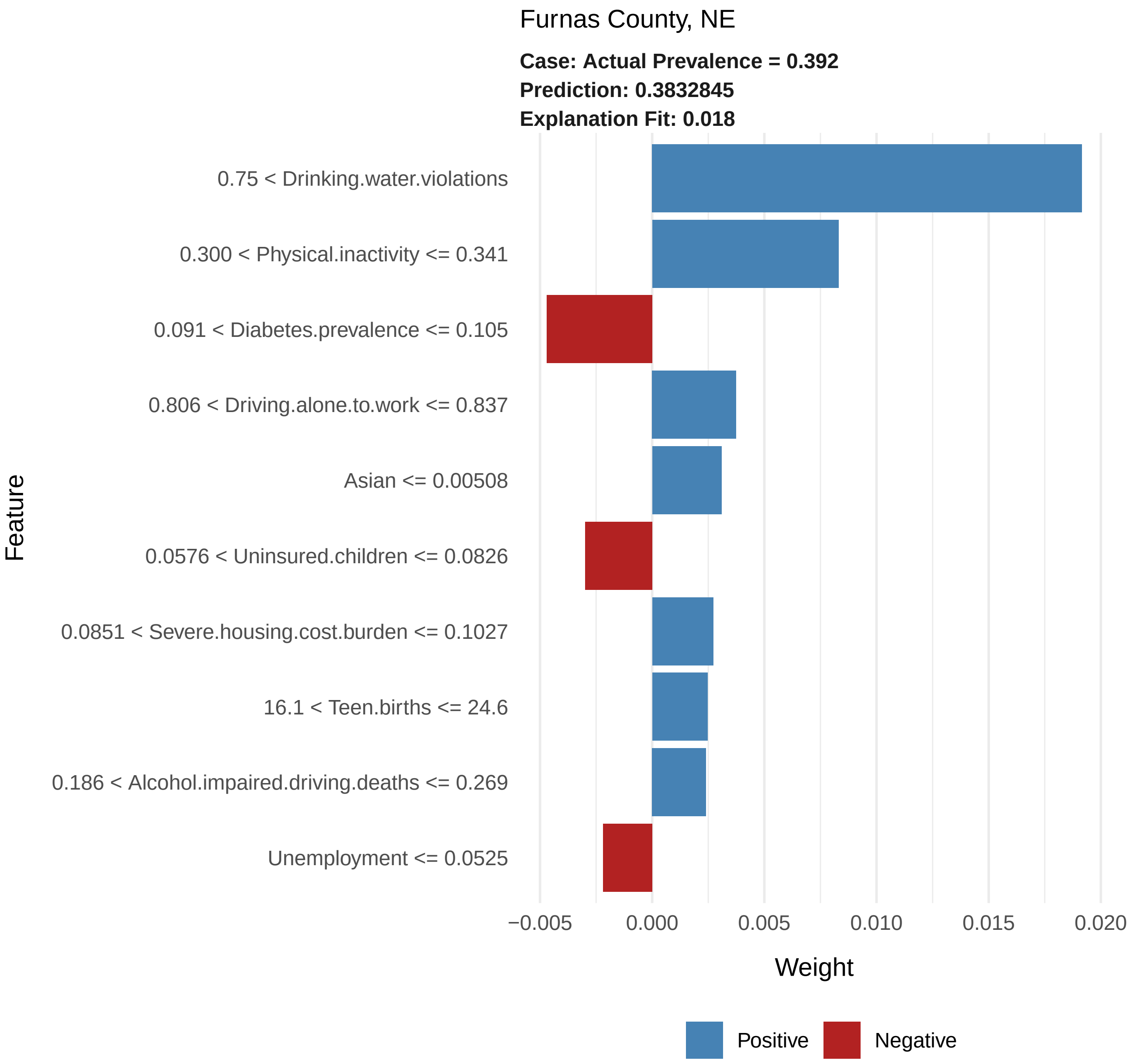
Furnas County, NE
Case: Actual Prevalence = 0.392
Prediction: 0.3832845
Explanation Fit: 0.018
0.75 < Drinking.water.violations
0.300 < Physical.inactivity <= 0.341
0.091 < Diabetes.prevalence <= 0.105
0.806 < Driving.alone.to.work <= 0.837
Asian <= 0.00508
0.0576 < Uninsured.children <= 0.0826
0.0851 < Severe.housing.cost.burden <= 0.1027
16.1 < Teen.births <= 24.6
0.186 < Alcohol.impaired.driving.deaths <= 0.269
Unemployment <= 0.0525
Feature
−0.005
0.000
0.005
0.010
0.015
0.020
Weight
Positive
Negative

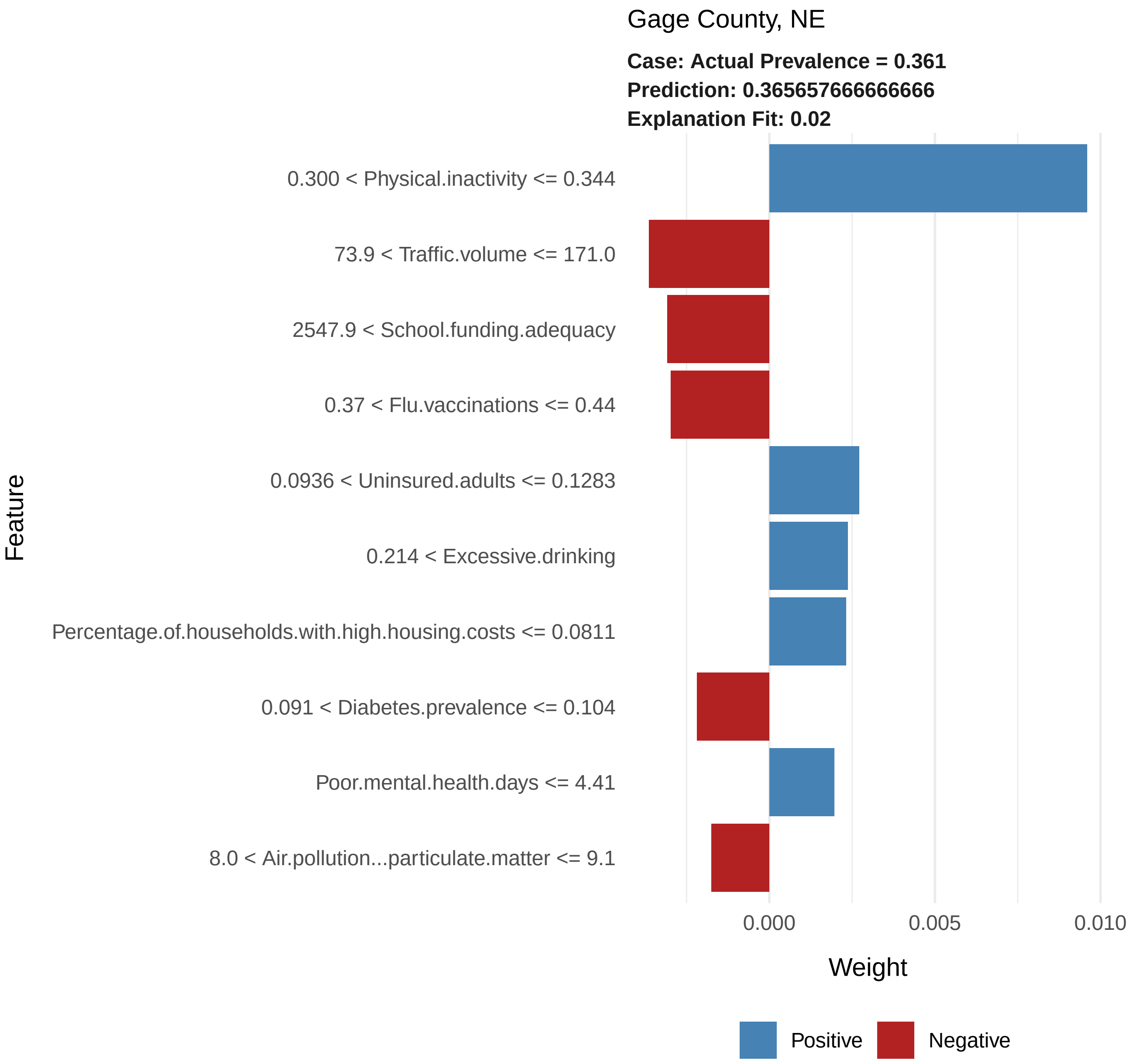
Gage County, NE
Case: Actual Prevalence = 0.361
Prediction: 0.365657666666666
Explanation Fit: 0.02
0.300 < Physical.inactivity <= 0.344
73.9 < Traffic.volume <= 171.0
2547.9 < School.funding.adequacy
0.37 < Flu.vaccinations <= 0.44
0.0936 < Uninsured.adults <= 0.1283
0.214 < Excessive.drinking
Percentage.of.households.with.high.housing.costs <= 0.0811
0.091 < Diabetes.prevalence <= 0.104
Poor.mental.health.days <= 4.41
8.0 < Air.pollution...particulate.matter <= 9.1
Feature
0.000
0.005
0.010
Weight
Positive
Negative

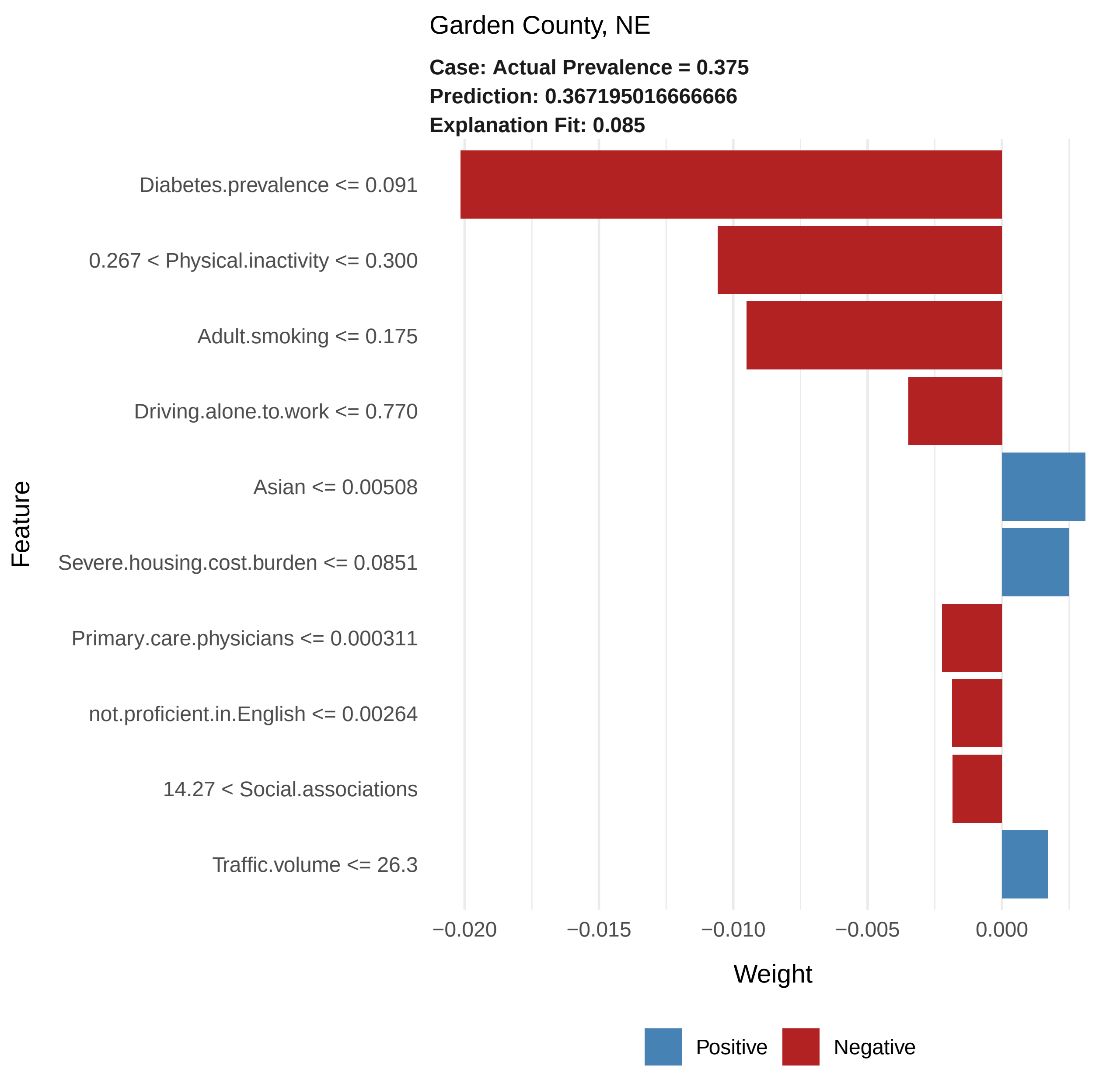

Garden County, NE
Case: Actual Prevalence = 0.375
Prediction: 0.367195016666666
Explanation Fit: 0.085
Diabetes.prevalence <= 0.091
0.267 < Physical.inactivity <= 0.300
Adult.smoking <= 0.175
Driving.alone.to.work <= 0.770
Asian <= 0.00508
Severe.housing.cost.burden <= 0.0851
Primary.care.physicians <= 0.000311
not.proficient.in.English <= 0.00264
14.27 < Social.associations
Traffic.volume <= 26.3
Feature
−0.020
−0.015
−0.010
−0.005
0.000
Weight
Positive
Negative

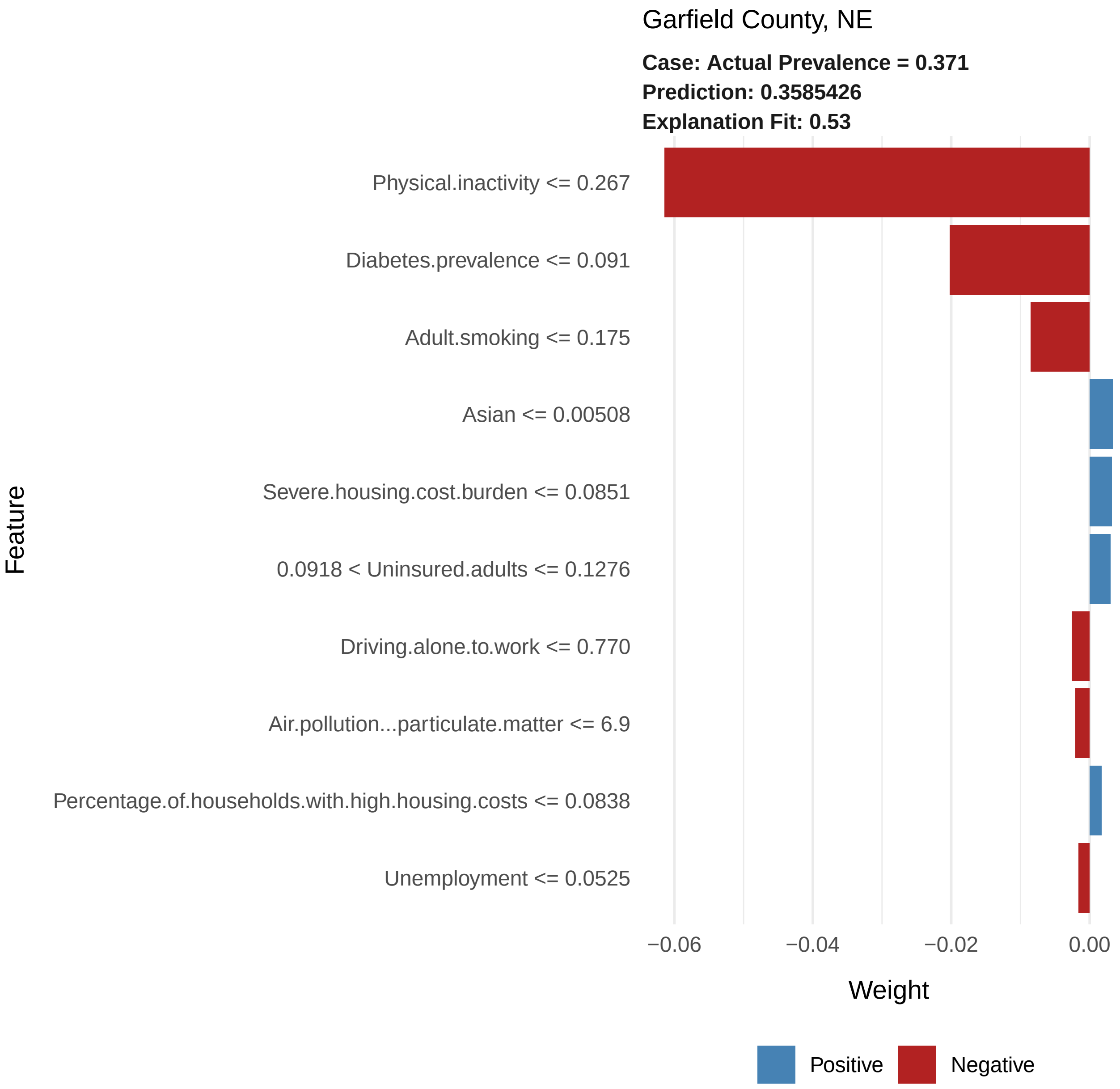
Garfield County, NE
Case: Actual Prevalence = 0.371
Prediction: 0.3585426
Explanation Fit: 0.53
Feature
Physical.inactivity <= 0.267
Diabetes.prevalence <= 0.091
Adult.smoking <= 0.175
Asian <= 0.00508
Severe.housing.cost.burden <= 0.0851
0.0918 < Uninsured.adults <= 0.1276
Driving.alone.to.work <= 0.770
Air.pollution...particulate.matter <= 6.9
Percentage.of.households.with.high.housing.costs <= 0.0838
Unemployment <= 0.0525
−0.06
−0.04
−0.02
0.00
Weight
Positive
Negative

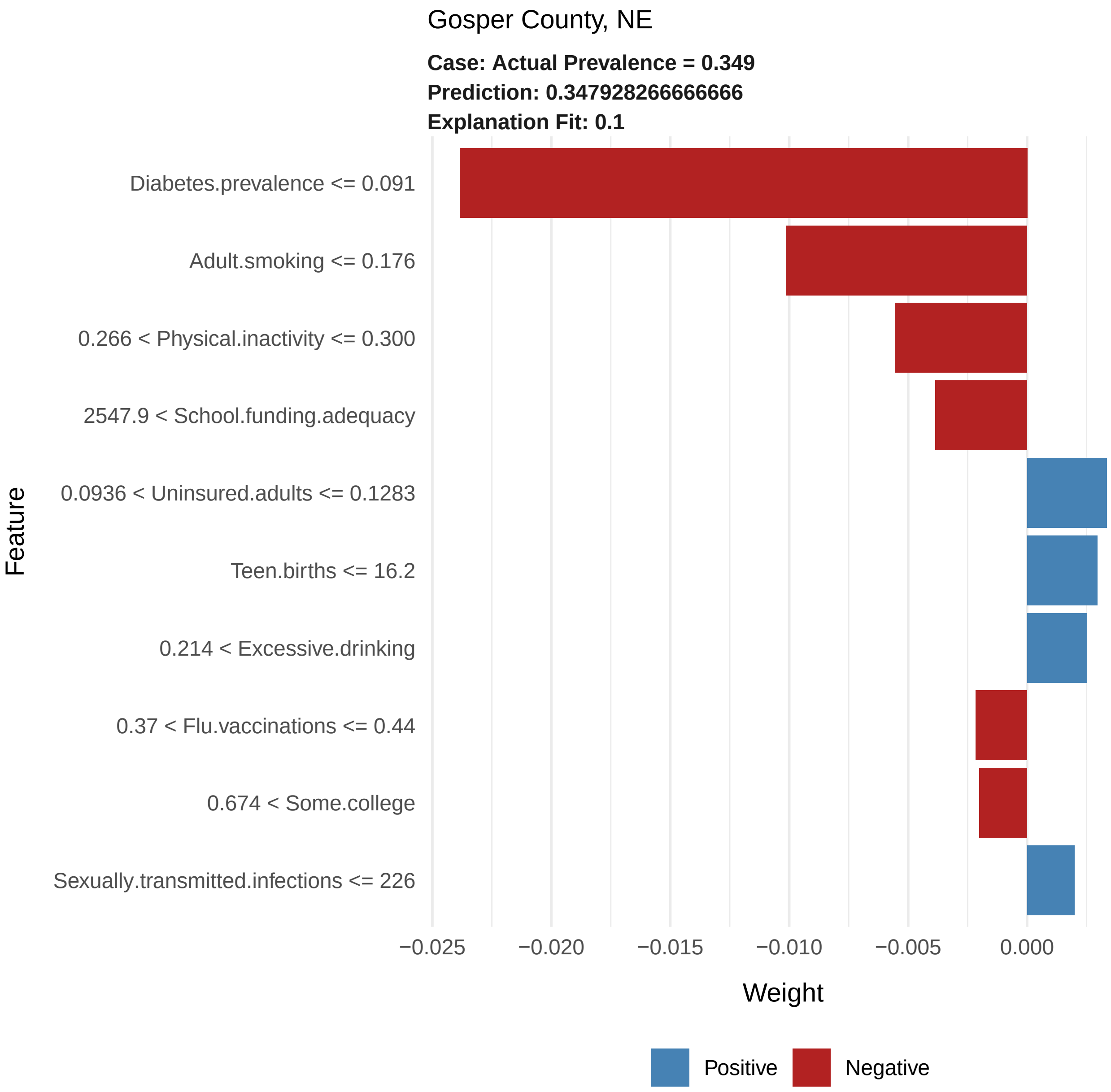

Gosper County, NE
Case: Actual Prevalence = 0.349
Prediction: 0.347928266666666
Explanation Fit: 0.1
Diabetes.prevalence <= 0.091
Adult.smoking <= 0.176
0.266 < Physical.inactivity <= 0.300
2547.9 < School.funding.adequacy
0.0936 < Uninsured.adults <= 0.1283
Teen.births <= 16.2
0.214 < Excessive.drinking
0.37 < Flu.vaccinations <= 0.44
0.674 < Some.college
Sexually.transmitted.infections <= 226
Feature
−0.025
−0.020
−0.015
−0.010
−0.005
0.000
Weight
Positive
Negative

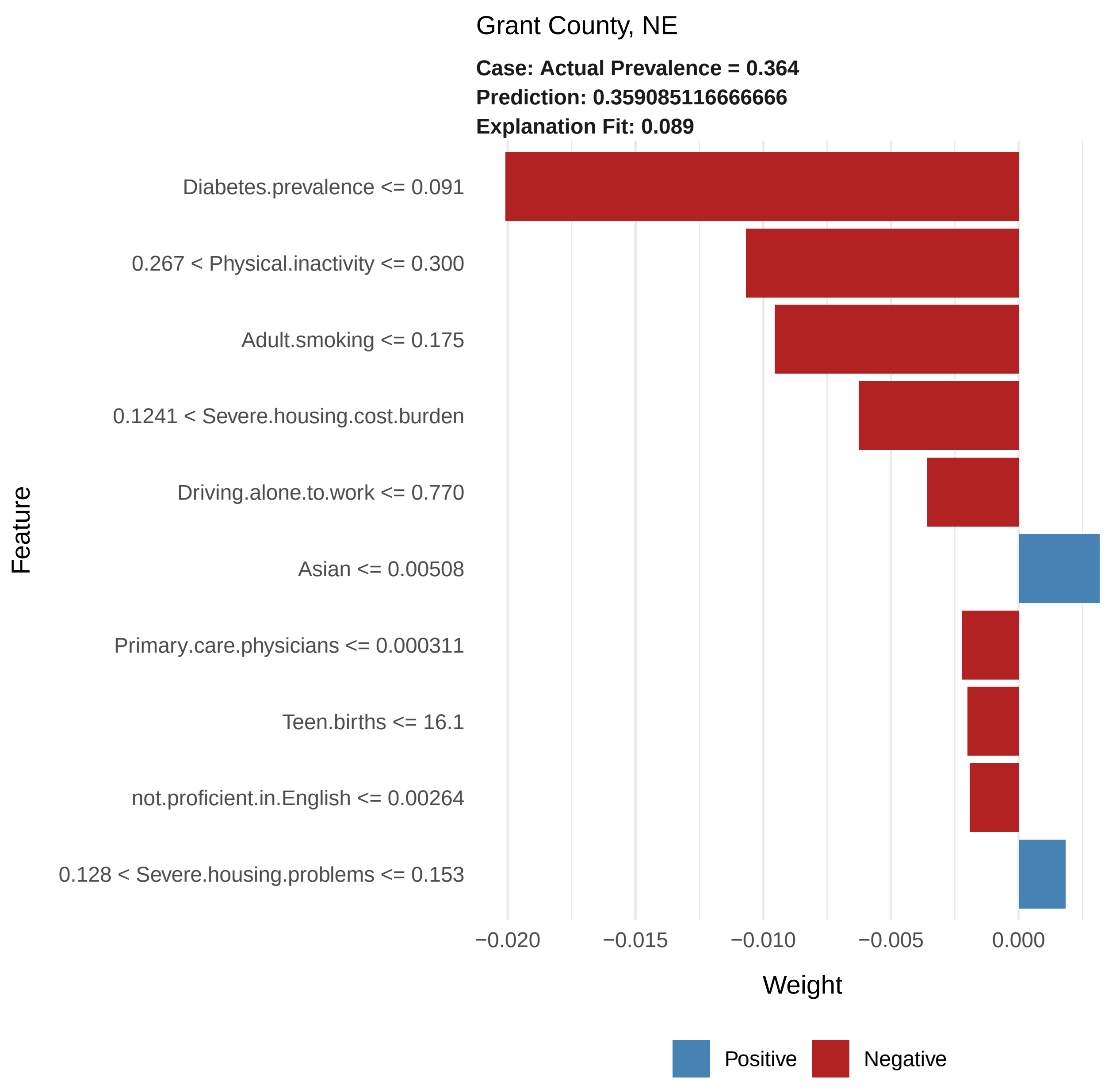

Grant County, NE
Case: Actual Prevalence = 0.364
Prediction: 0.359085116666666
Explanation Fit: 0.089
Feature
Diabetes.prevalence <= 0.091
0.267 < Physical.inactivity <= 0.300
Adult.smoking <= 0.175
0.1241 < Severe.housing.cost.burden
Driving.alone.to.work <= 0.770
Asian <= 0.00508
Primary.care.physicians <= 0.000311
Teen.births <= 16.1
not.proficient.in.English <= 0.00264
0.128 < Severe.housing.problems <= 0.153
−0.020
−0.015
−0.010
−0.005
0.000
Weight
Positive
Negative

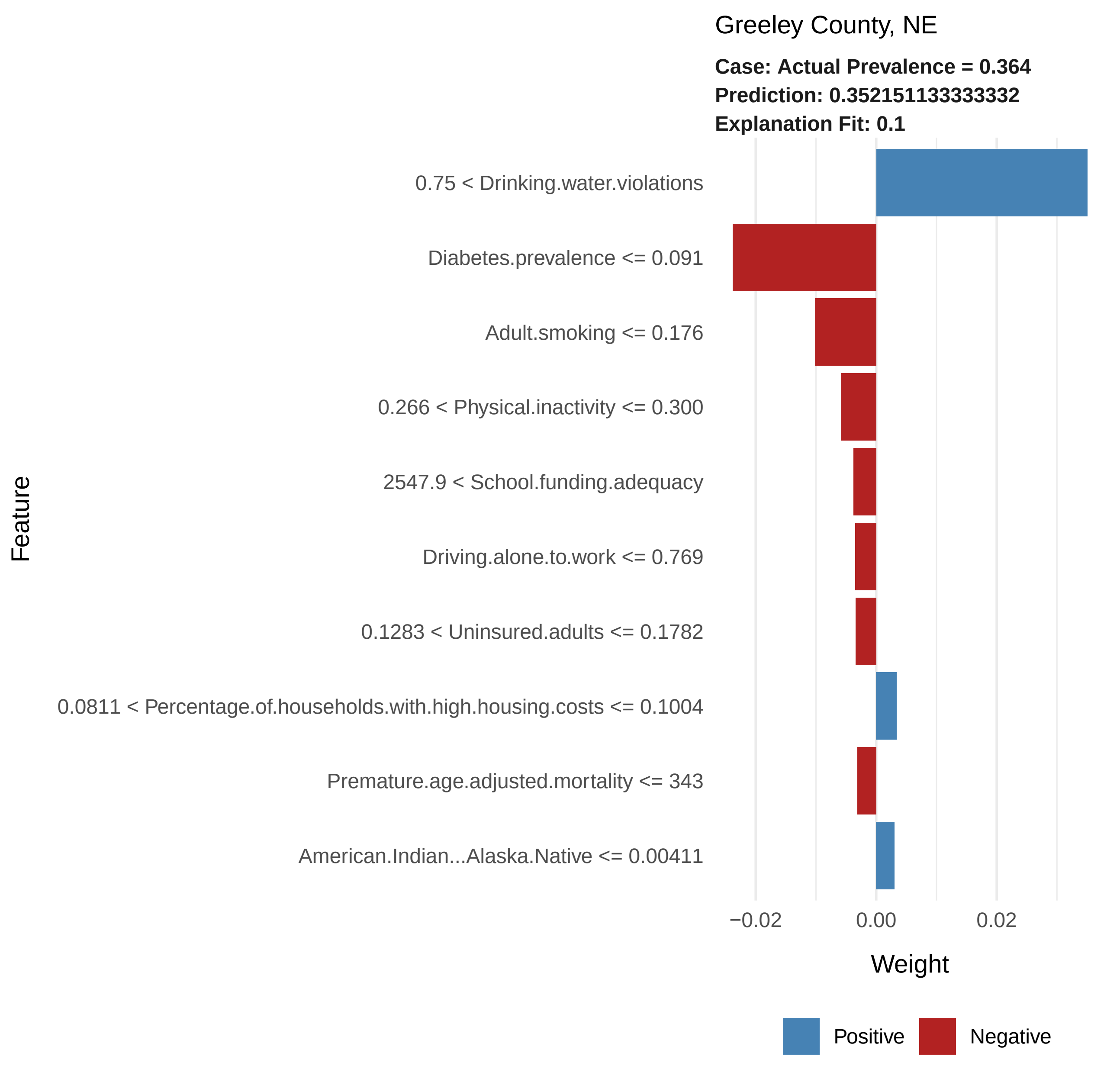

Greeley County, NE
Case: Actual Prevalence = 0.364
Prediction: 0.352151133333332
Explanation Fit: 0.1
0.75 < Drinking.water.violations
Diabetes.prevalence <= 0.091
Adult.smoking <= 0.176
0.266 < Physical.inactivity <= 0.300
2547.9 < School.funding.adequacy
Driving.alone.to.work <= 0.769
0.1283 < Uninsured.adults <= 0.1782
0.0811 < Percentage.of.households.with.high.housing.costs <= 0.1004
Premature.age.adjusted.mortality <= 343
American.Indian...Alaska.Native <= 0.00411
Feature
−0.02
0.00
0.02
Weight
Positive
Negative

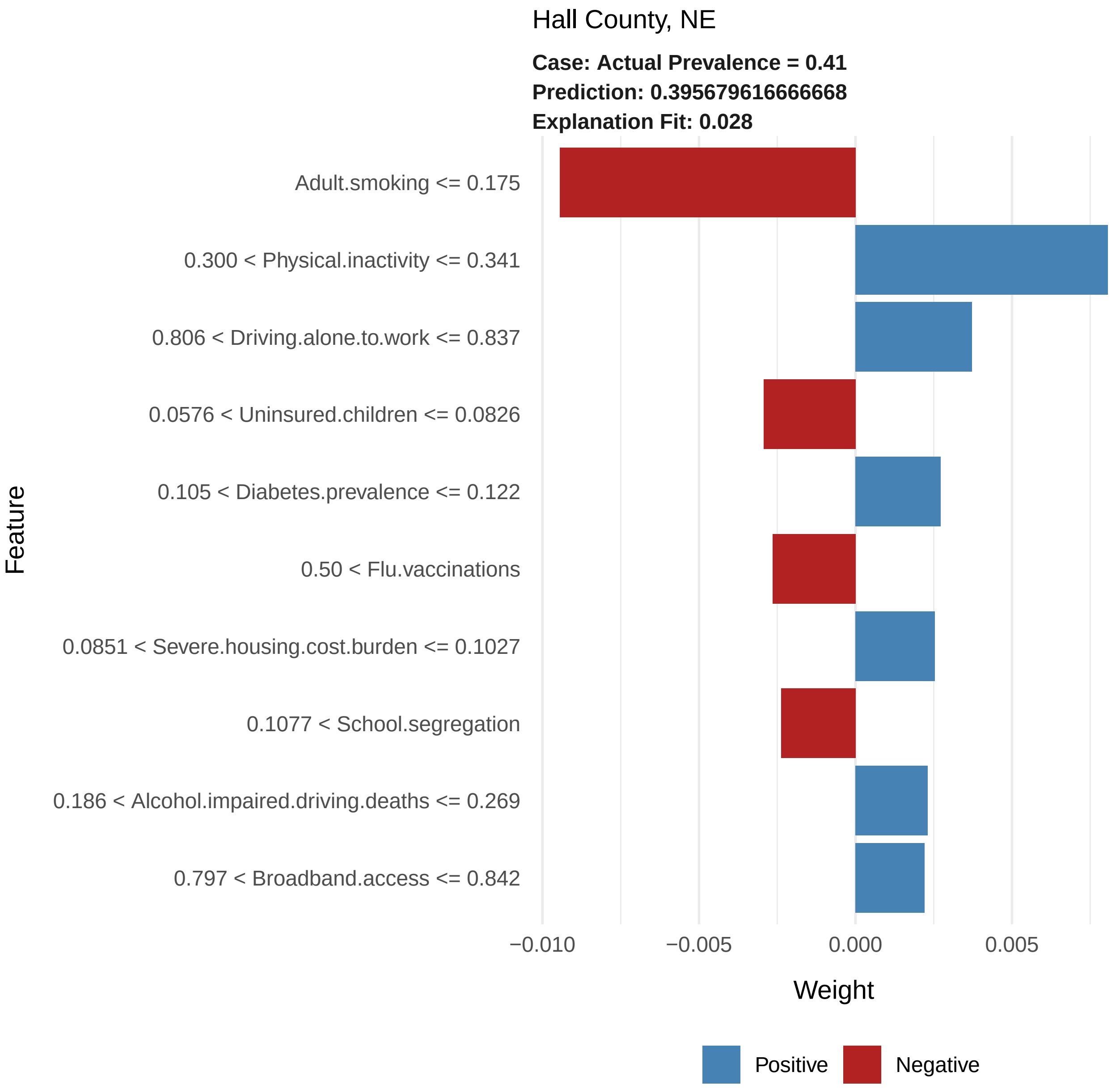

Hall County, NE
Case: Actual Prevalence = 0.41
Prediction: 0.395679616666668
Explanation Fit: 0.028
Adult.smoking <= 0.175
0.300 < Physical.inactivity <= 0.341
0.806 < Driving.alone.to.work <= 0.837
0.0576 < Uninsured.children <= 0.0826
0.105 < Diabetes.prevalence <= 0.122
0.50 < Flu.vaccinations
0.0851 < Severe.housing.cost.burden <= 0.1027
0.1077 < School.segregation
0.186 < Alcohol.impaired.driving.deaths <= 0.269
0.797 < Broadband.access <= 0.842
Feature
−0.010
−0.005
0.000
0.005
Weight
Positive
Negative

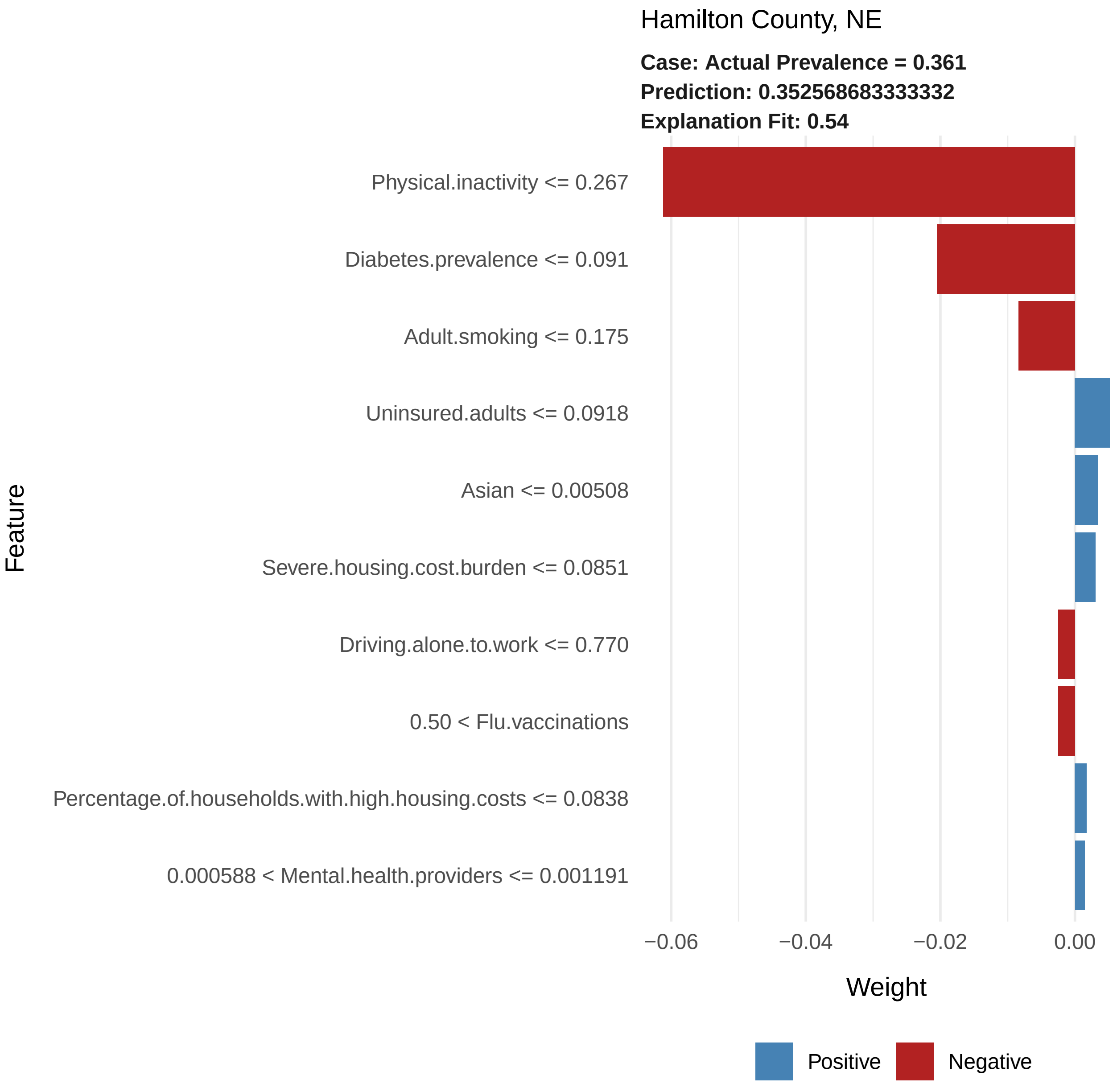

Hamilton County, NE
Case: Actual Prevalence = 0.361
Prediction: 0.352568683333332
Explanation Fit: 0.54
Physical.inactivity <= 0.267
Diabetes.prevalence <= 0.091
Adult.smoking <= 0.175
Uninsured.adults <= 0.0918
Asian <= 0.00508
Severe.housing.cost.burden <= 0.0851
Driving.alone.to.work <= 0.770
0.50 < Flu.vaccinations
Percentage.of.households.with.high.housing.costs <= 0.0838
0.000588 < Mental.health.providers <= 0.001191
Feature
−0.06
−0.04
−0.02
0.00
Weight
Positive
Negative

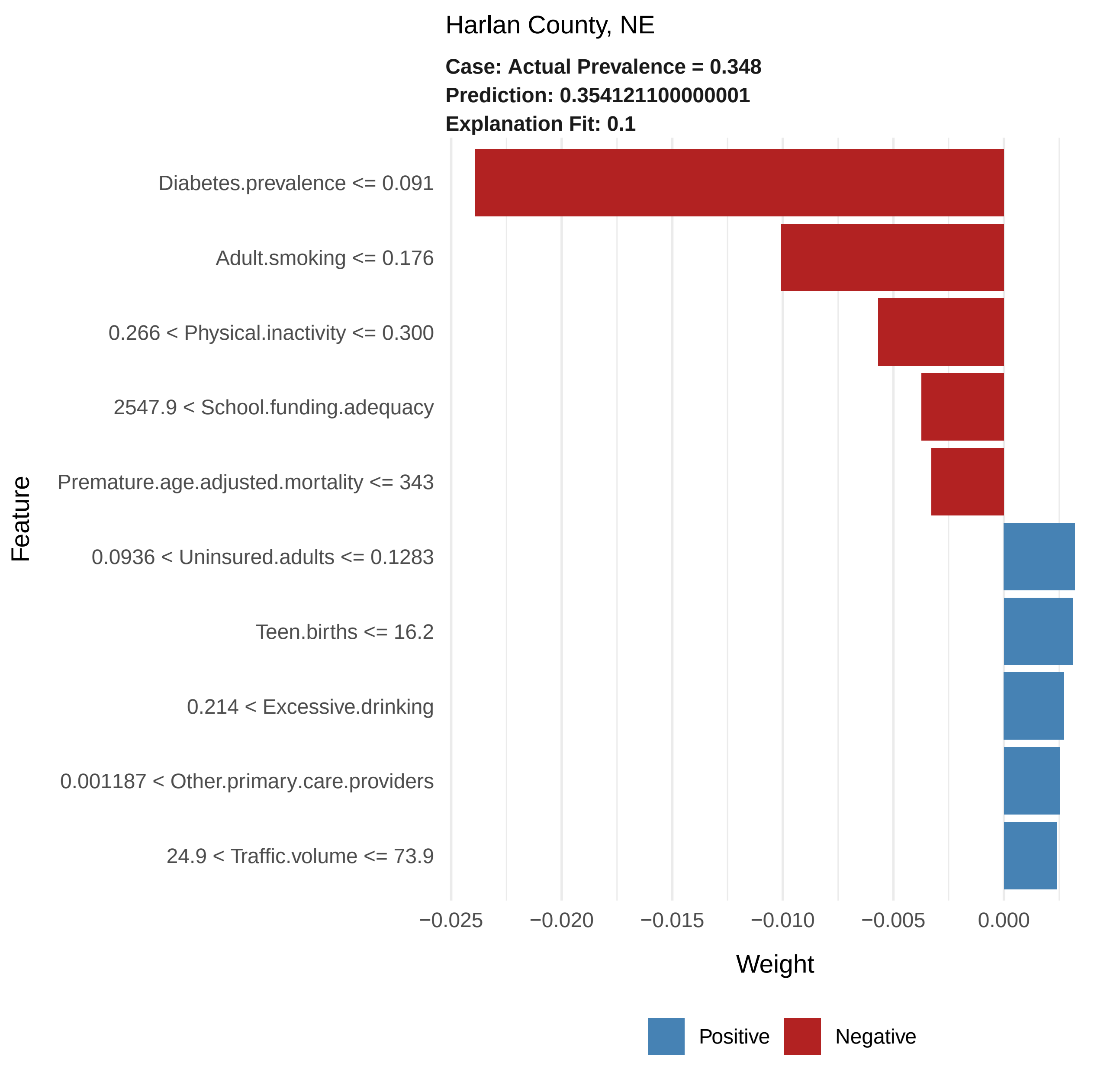
Harlan County, NE
Case: Actual Prevalence = 0.348
Prediction: 0.354121100000001
Explanation Fit: 0.1
Diabetes.prevalence <= 0.091
Adult.smoking <= 0.176
0.266 < Physical.inactivity <= 0.300
2547.9 < School.funding.adequacy
Premature.age.adjusted.mortality <= 343
0.0936 < Uninsured.adults <= 0.1283
Teen.births <= 16.2
0.214 < Excessive.drinking
0.001187 < Other.primary.care.providers
24.9 < Traffic.volume <= 73.9
Feature
−0.025
−0.020
−0.015
−0.010
−0.005
0.000
Weight
Positive
Negative

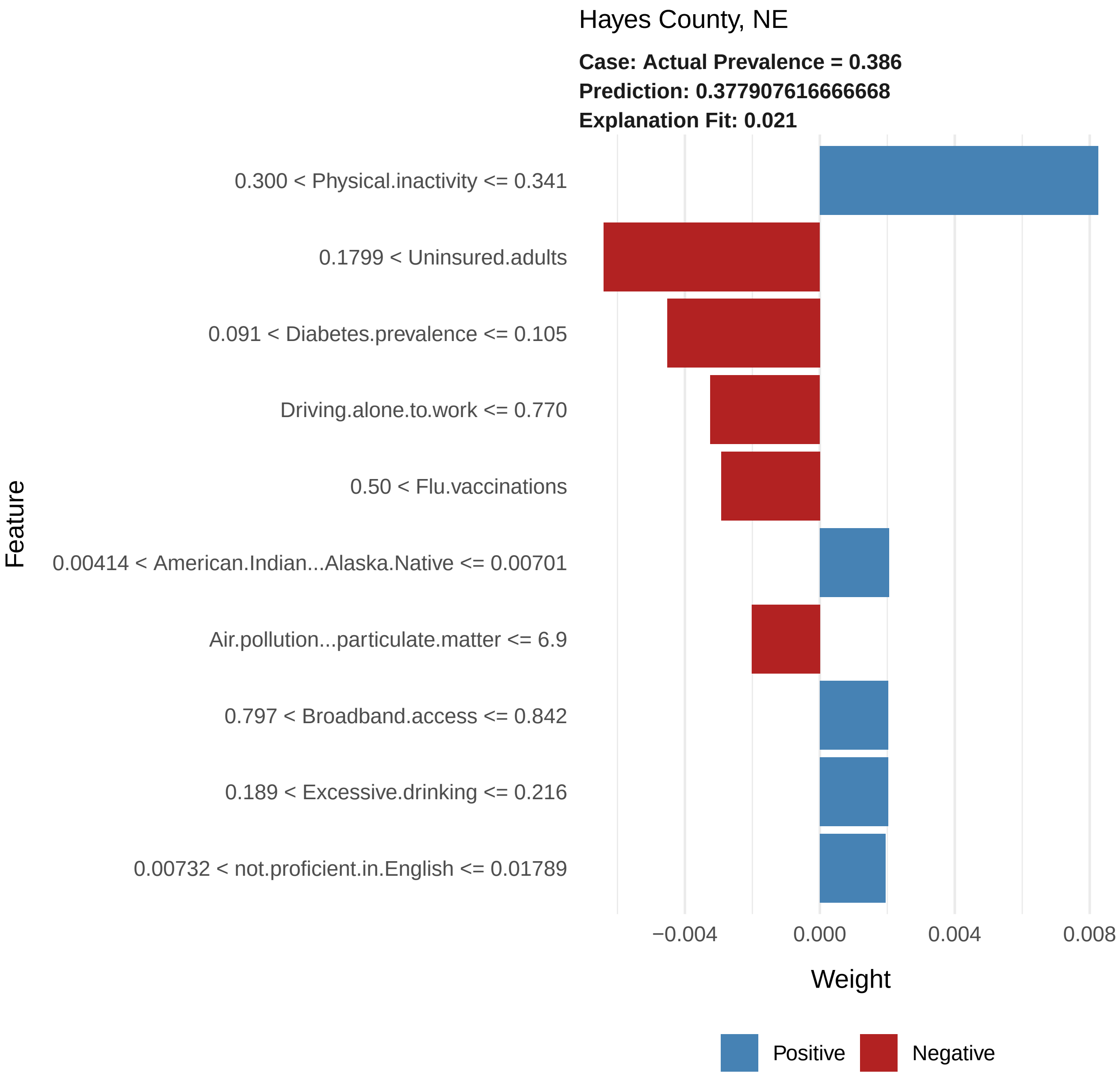

Hayes County, NE
Case: Actual Prevalence = 0.386
Prediction: 0.377907616666668
Explanation Fit: 0.021
0.300 < Physical.inactivity <= 0.341
0.1799 < Uninsured.adults
0.091 < Diabetes.prevalence <= 0.105
Driving.alone.to.work <= 0.770
0.50 < Flu.vaccinations
0.00414 < American.Indian...Alaska.Native <= 0.00701
Air.pollution...particulate.matter <= 6.9
0.797 < Broadband.access <= 0.842
0.189 < Excessive.drinking <= 0.216
0.00732 < not.proficient.in.English <= 0.01789
Feature
−0.004
0.000
0.004
0.008
Weight
Positive
Negative

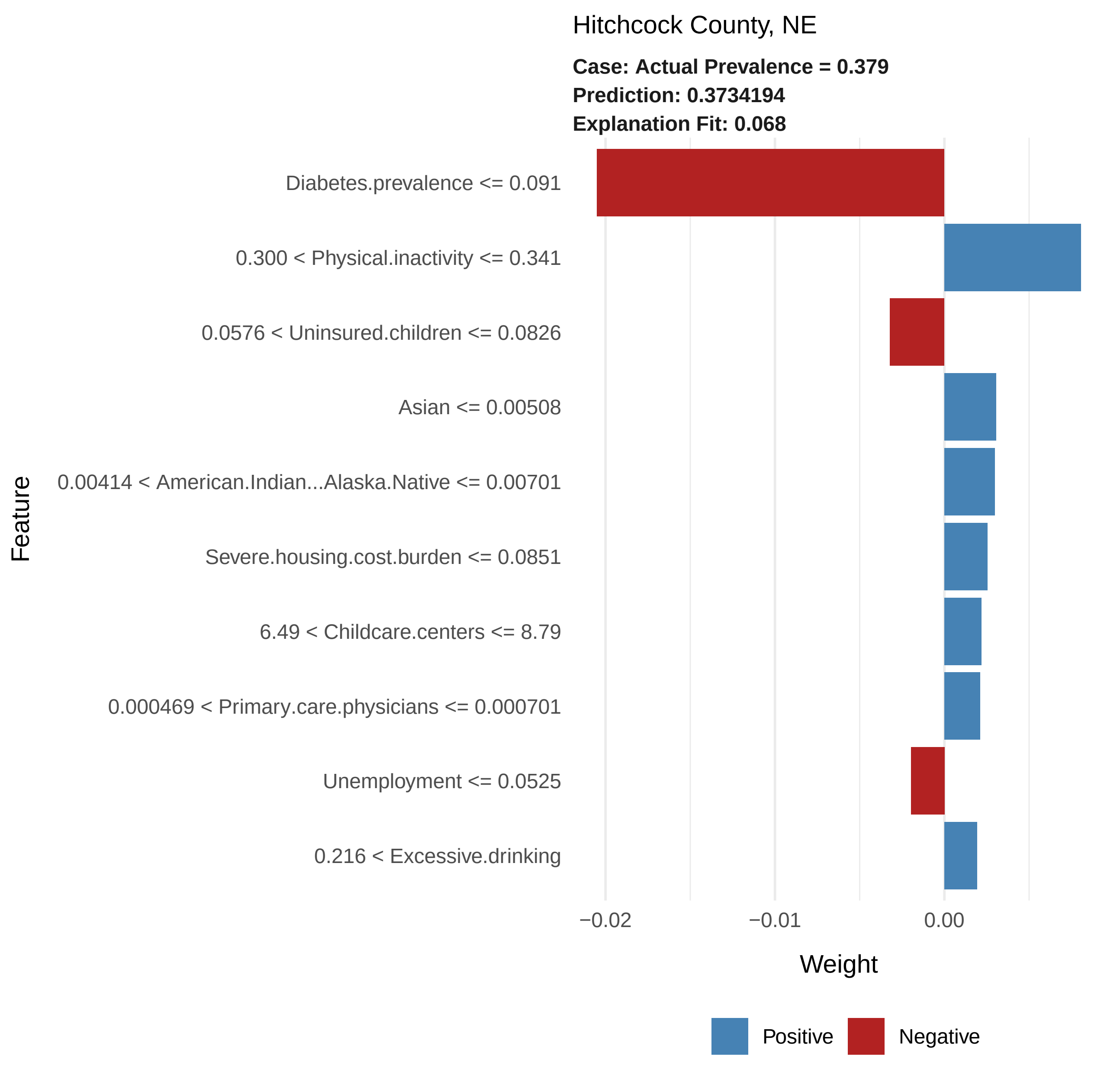
Hitchcock County, NE
Case: Actual Prevalence = 0.379
Prediction: 0.3734194
Explanation Fit: 0.068
Diabetes.prevalence <= 0.091
0.300 < Physical.inactivity <= 0.341
0.0576 < Uninsured.children <= 0.0826
Asian <= 0.00508
0.00414 < American.Indian...Alaska.Native <= 0.00701
Severe.housing.cost.burden <= 0.0851
6.49 < Childcare.centers <= 8.79
0.000469 < Primary.care.physicians <= 0.000701
Unemployment <= 0.0525
0.216 < Excessive.drinking
Feature
−0.02
−0.01
0.00
Weight
Positive
Negative

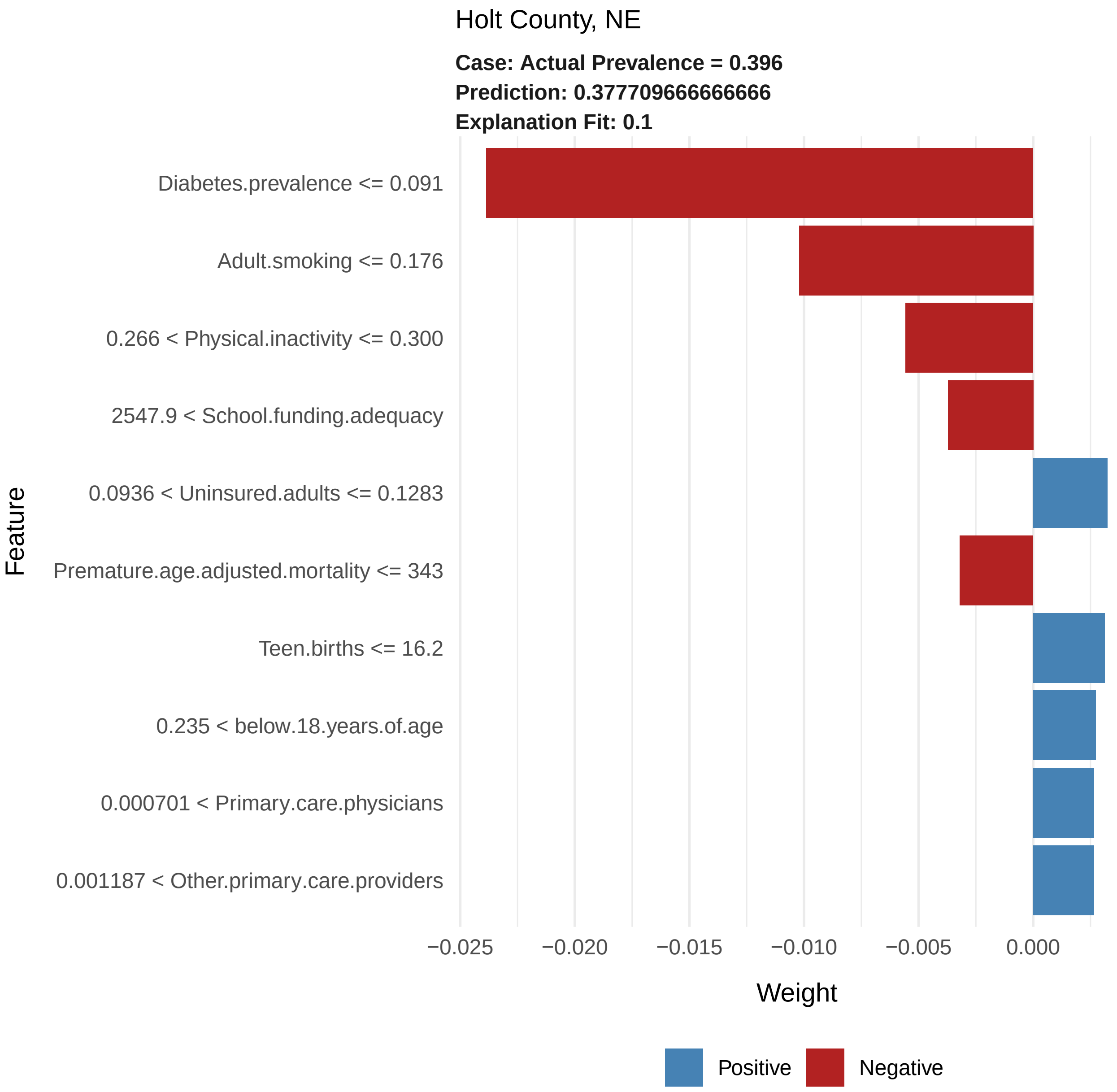

Holt County, NE
Case: Actual Prevalence = 0.396
Prediction: 0.377709666666666
Explanation Fit: 0.1
Diabetes.prevalence <= 0.091
Adult.smoking <= 0.176
0.266 < Physical.inactivity <= 0.300
2547.9 < School.funding.adequacy
0.0936 < Uninsured.adults <= 0.1283
Premature.age.adjusted.mortality <= 343
Teen.births <= 16.2
0.235 < below.18.years.of.age
0.000701 < Primary.care.physicians
0.001187 < Other.primary.care.providers
Feature
−0.025
−0.020
−0.015
−0.010
−0.005
0.000
Weight
Positive
Negative

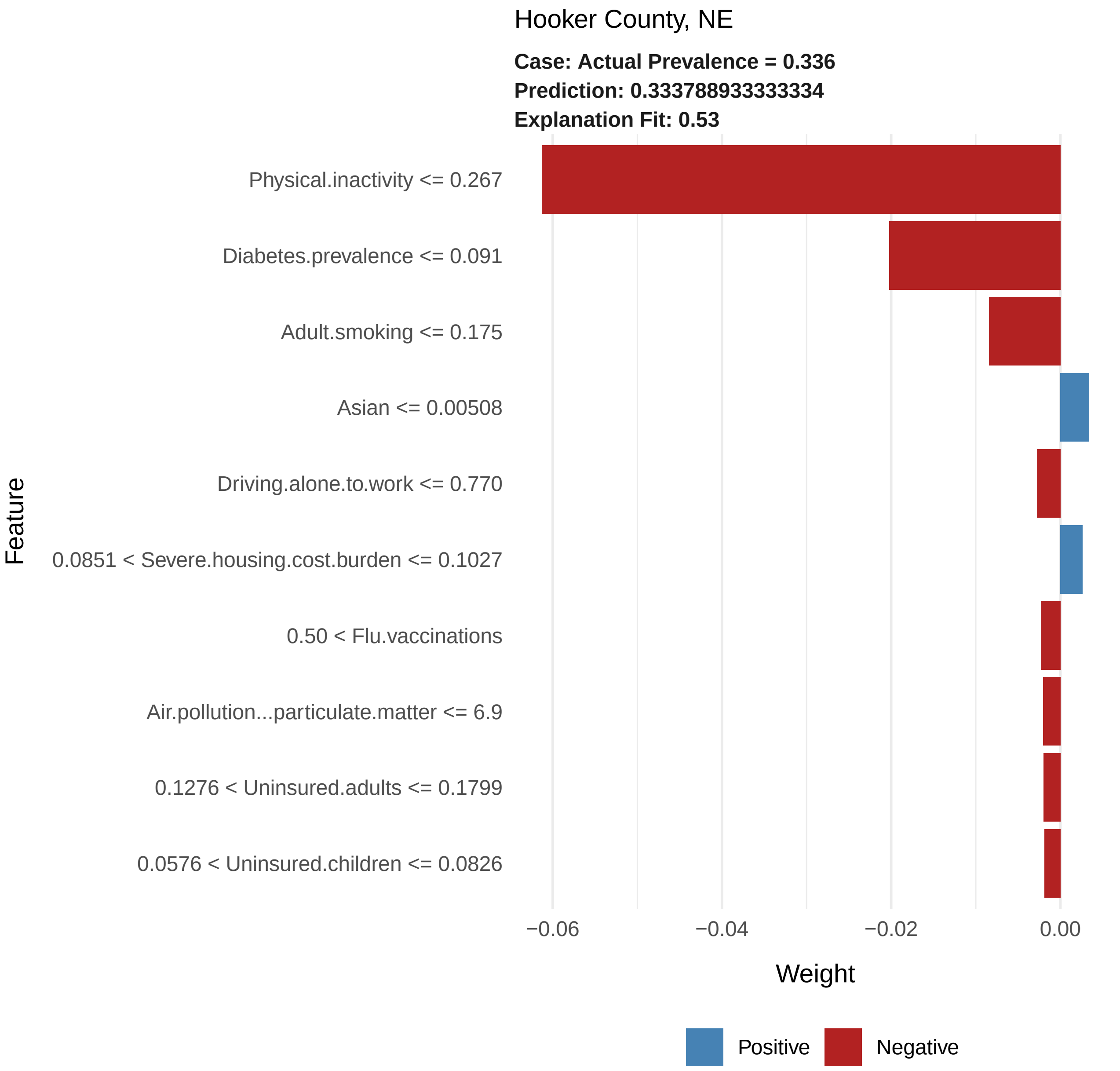

Hooker County, NE
Case: Actual Prevalence = 0.336
Prediction: 0.333788933333334
Explanation Fit: 0.53
Physical.inactivity <= 0.267
Diabetes.prevalence <= 0.091
Adult.smoking <= 0.175
Asian <= 0.00508
Driving.alone.to.work <= 0.770
0.0851 < Severe.housing.cost.burden <= 0.1027
0.50 < Flu.vaccinations
Air.pollution...particulate.matter <= 6.9
0.1276 < Uninsured.adults <= 0.1799
0.0576 < Uninsured.children <= 0.0826
Feature
−0.06
−0.04
−0.02
0.00
Weight
Positive
Negative

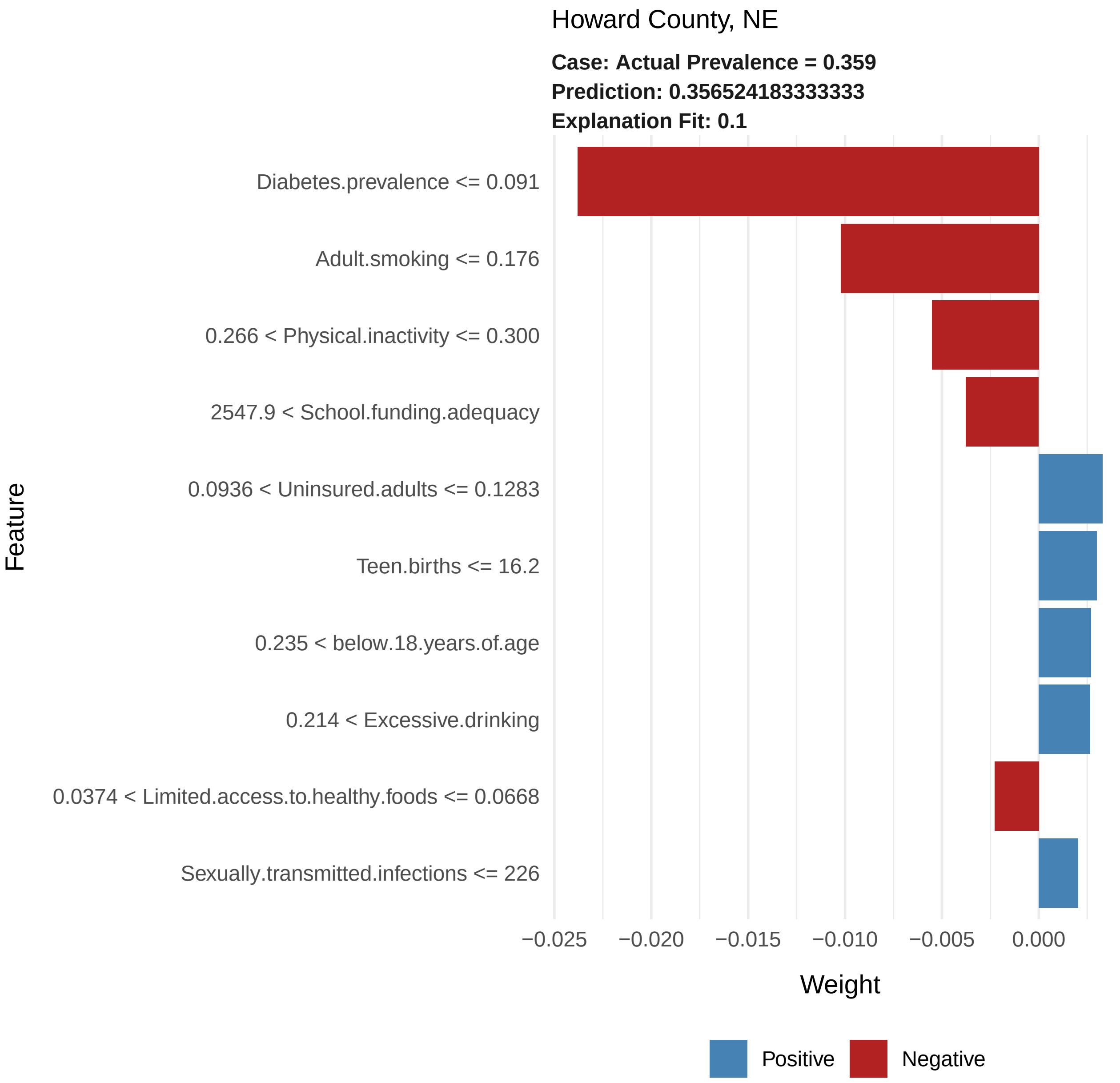

Howard County, NE
Case: Actual Prevalence = 0.359
Prediction: 0.356524183333333
Explanation Fit: 0.1
Diabetes.prevalence <= 0.091
Adult.smoking <= 0.176
0.266 < Physical.inactivity <= 0.300
2547.9 < School.funding.adequacy
0.0936 < Uninsured.adults <= 0.1283
Teen.births <= 16.2
0.235 < below.18.years.of.age
0.214 < Excessive.drinking
0.0374 < Limited.access.to.healthy.foods <= 0.0668
Sexually.transmitted.infections <= 226
Feature
−0.025
−0.020
−0.015
−0.010
−0.005
0.000
Weight
Positive
Negative

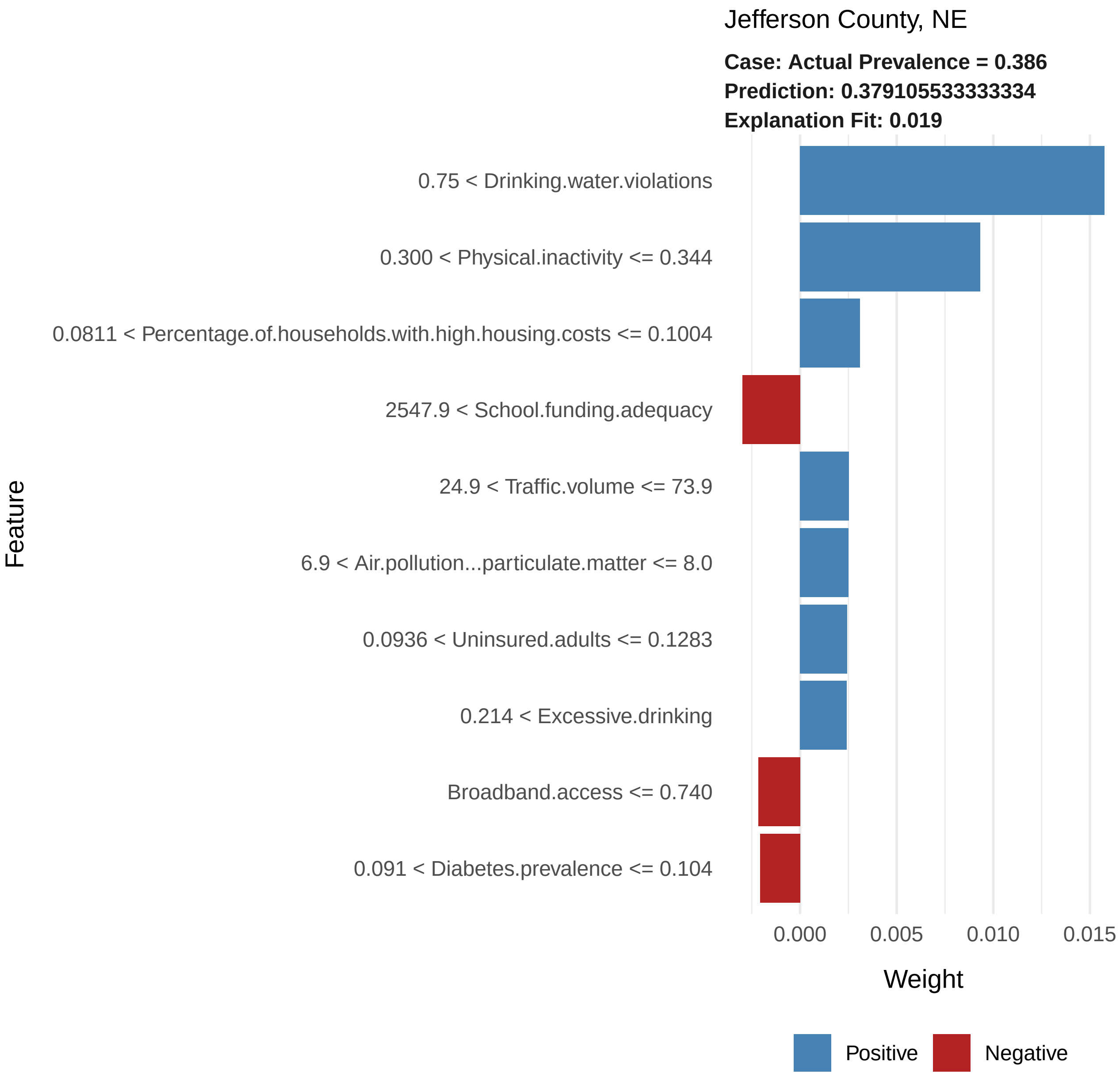

Jefferson County, NE
Case: Actual Prevalence = 0.386
Prediction: 0.379105533333334
Explanation Fit: 0.019
0.75 < Drinking.water.violations
0.300 < Physical.inactivity <= 0.344
0.0811 < Percentage.of.households.with.high.housing.costs <= 0.1004
2547.9 < School.funding.adequacy
24.9 < Traffic.volume <= 73.9
6.9 < Air.pollution...particulate.matter <= 8.0
0.0936 < Uninsured.adults <= 0.1283
0.214 < Excessive.drinking
Broadband.access <= 0.740
0.091 < Diabetes.prevalence <= 0.104
Feature
0.000
0.005
0.010
0.015
Weight
Positive
Negative

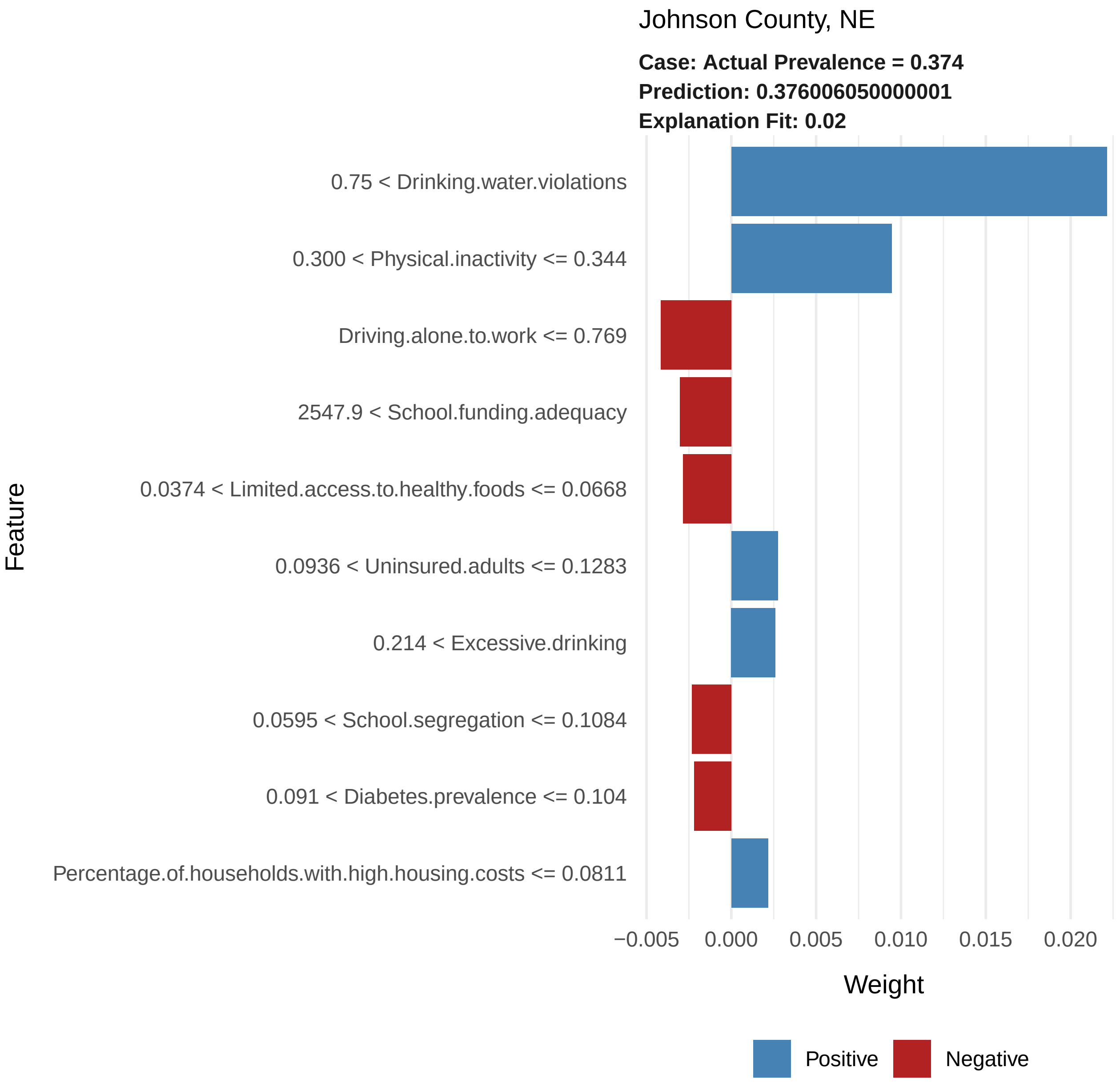
Johnson County, NE
Case: Actual Prevalence = 0.374
Prediction: 0.376006050000001
Explanation Fit: 0.02
0.75 < Drinking.water.violations
0.300 < Physical.inactivity <= 0.344
Driving.alone.to.work <= 0.769
2547.9 < School.funding.adequacy
0.0374 < Limited.access.to.healthy.foods <= 0.0668
0.0936 < Uninsured.adults <= 0.1283
0.214 < Excessive.drinking
0.0595 < School.segregation <= 0.1084
0.091 < Diabetes.prevalence <= 0.104
Percentage.of.households.with.high.housing.costs <= 0.0811
Feature
−0.005
0.000
0.005
0.010
0.015
0.020
Weight
Positive
Negative

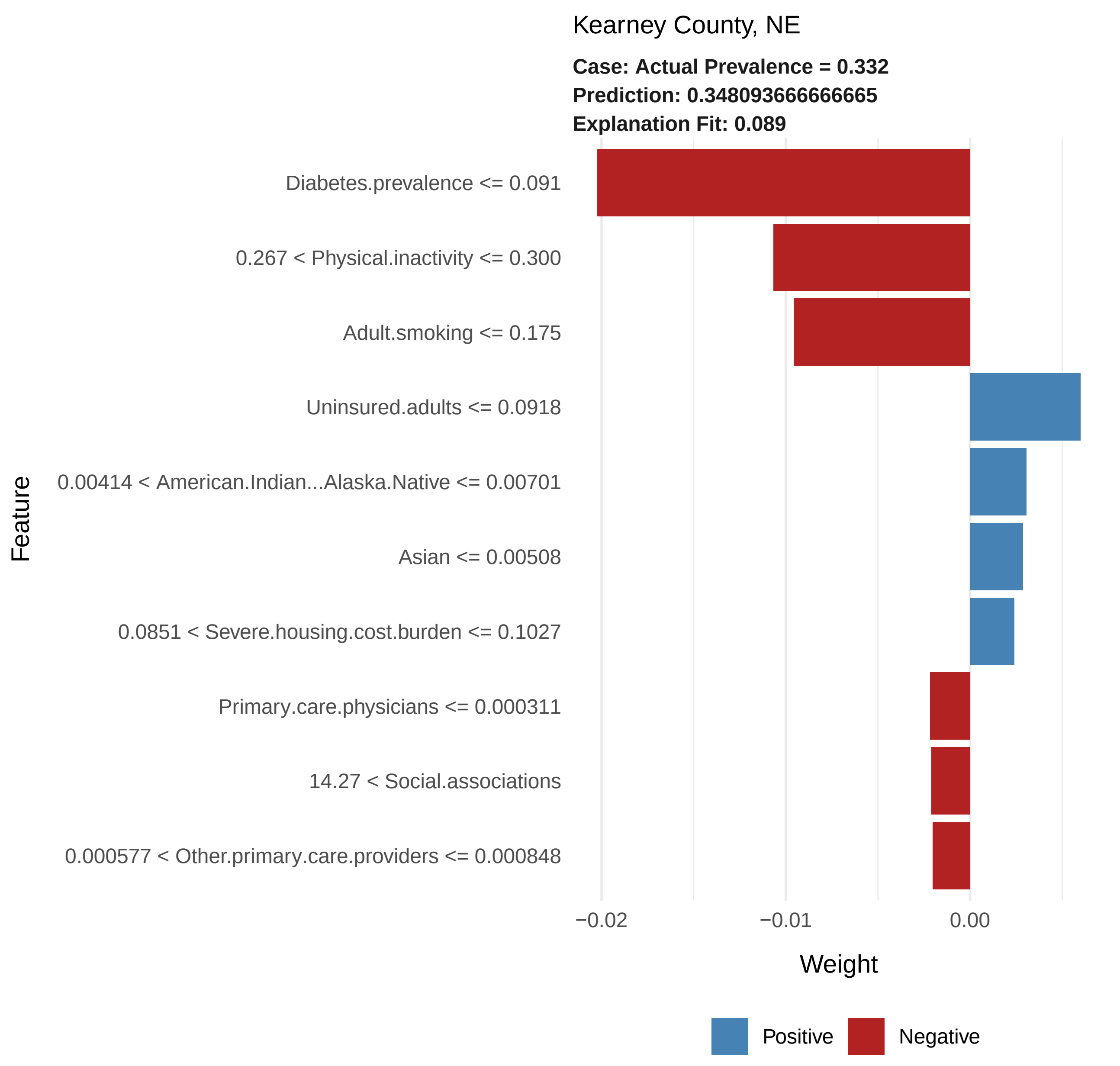

Kearney County, NE
Case: Actual Prevalence = 0.332
Prediction: 0.348093666666665
Explanation Fit: 0.089
Diabetes.prevalence <= 0.091
0.267 < Physical.inactivity <= 0.300
Adult.smoking <= 0.175
Uninsured.adults <= 0.0918
0.00414 < American.Indian...Alaska.Native <= 0.00701
Asian <= 0.00508
0.0851 < Severe.housing.cost.burden <= 0.1027
Primary.care.physicians <= 0.000311
14.27 < Social.associations
0.000577 < Other.primary.care.providers <= 0.000848
Feature
−0.02
−0.01
0.00
Weight
Positive
Negative

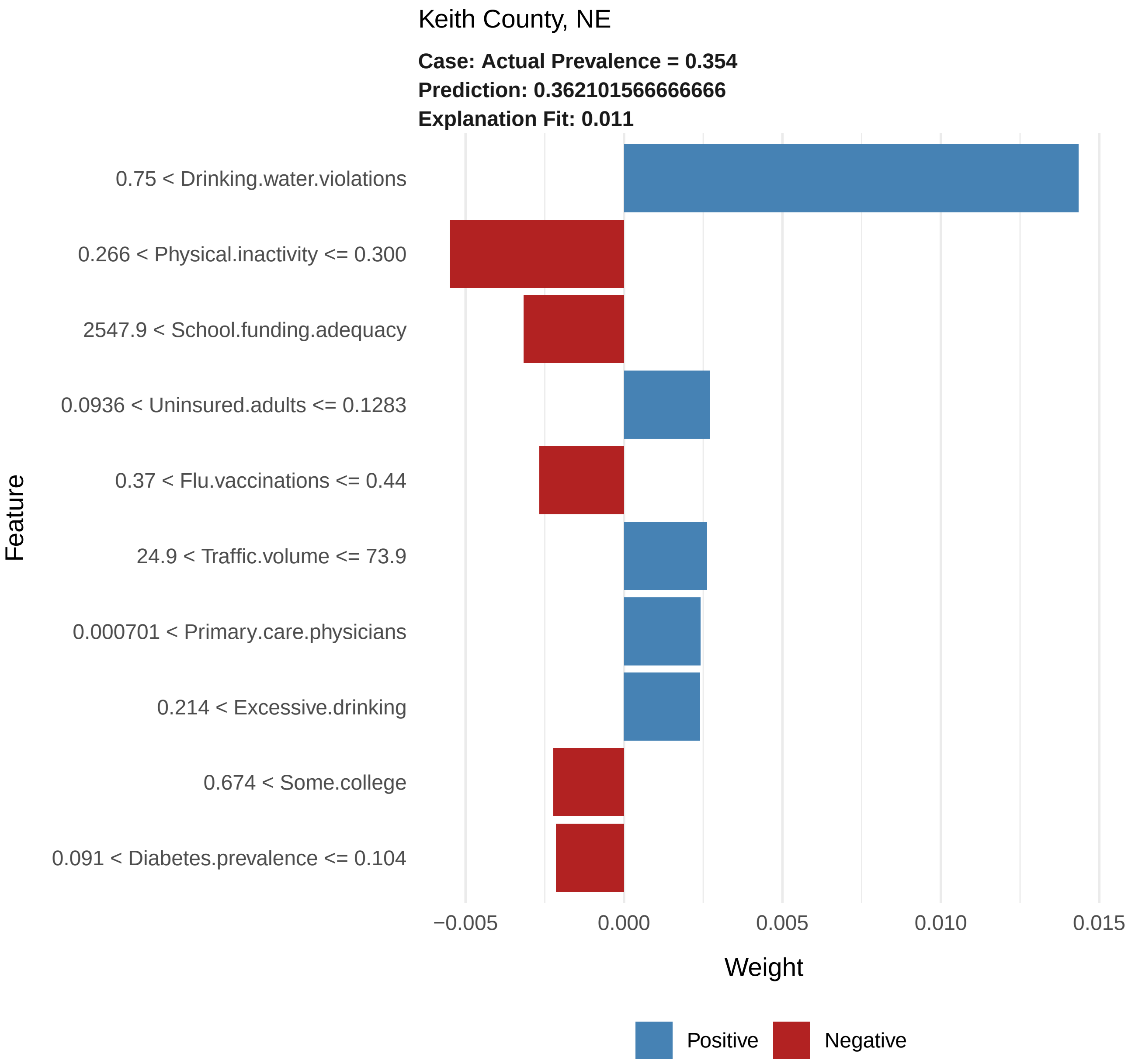
Keith County, NE
Case: Actual Prevalence = 0.354
Prediction: 0.362101566666666
Explanation Fit: 0.011
0.75 < Drinking.water.violations
0.266 < Physical.inactivity <= 0.300
2547.9 < School.funding.adequacy
0.0936 < Uninsured.adults <= 0.1283
0.37 < Flu.vaccinations <= 0.44
24.9 < Traffic.volume <= 73.9
0.000701 < Primary.care.physicians
0.214 < Excessive.drinking
0.674 < Some.college
0.091 < Diabetes.prevalence <= 0.104
Feature
−0.005
0.000
0.005
0.010
0.015
Weight
Positive
Negative

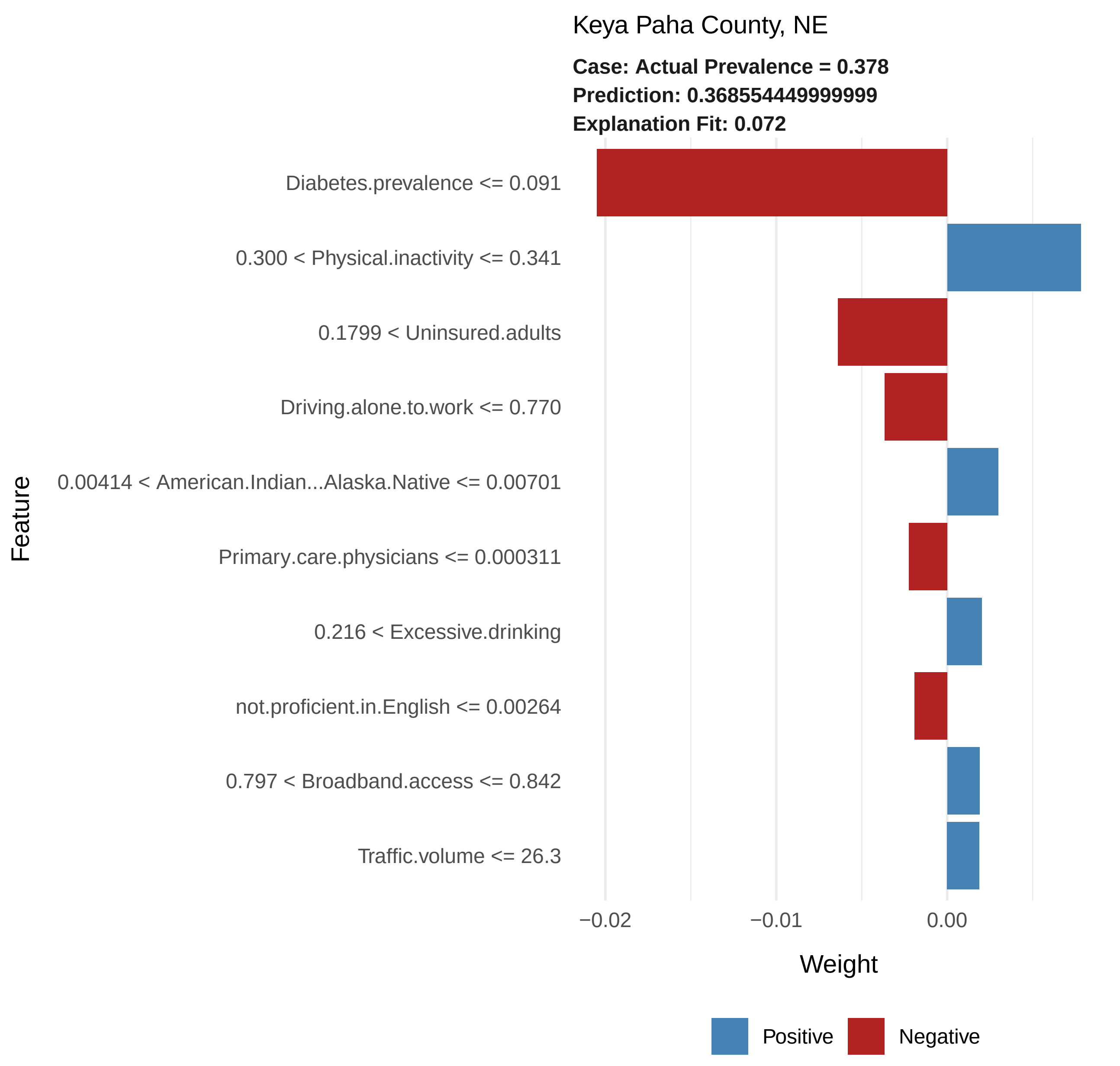

Keya Paha County, NE
Case: Actual Prevalence = 0.378
Prediction: 0.368554449999999
Explanation Fit: 0.072
Diabetes.prevalence <= 0.091
0.300 < Physical.inactivity <= 0.341
0.1799 < Uninsured.adults
Driving.alone.to.work <= 0.770
0.00414 < American.Indian...Alaska.Native <= 0.00701
Primary.care.physicians <= 0.000311
0.216 < Excessive.drinking
not.proficient.in.English <= 0.00264
0.797 < Broadband.access <= 0.842
Traffic.volume <= 26.3
Feature
−0.02
−0.01
0.00
Weight
Positive
Negative

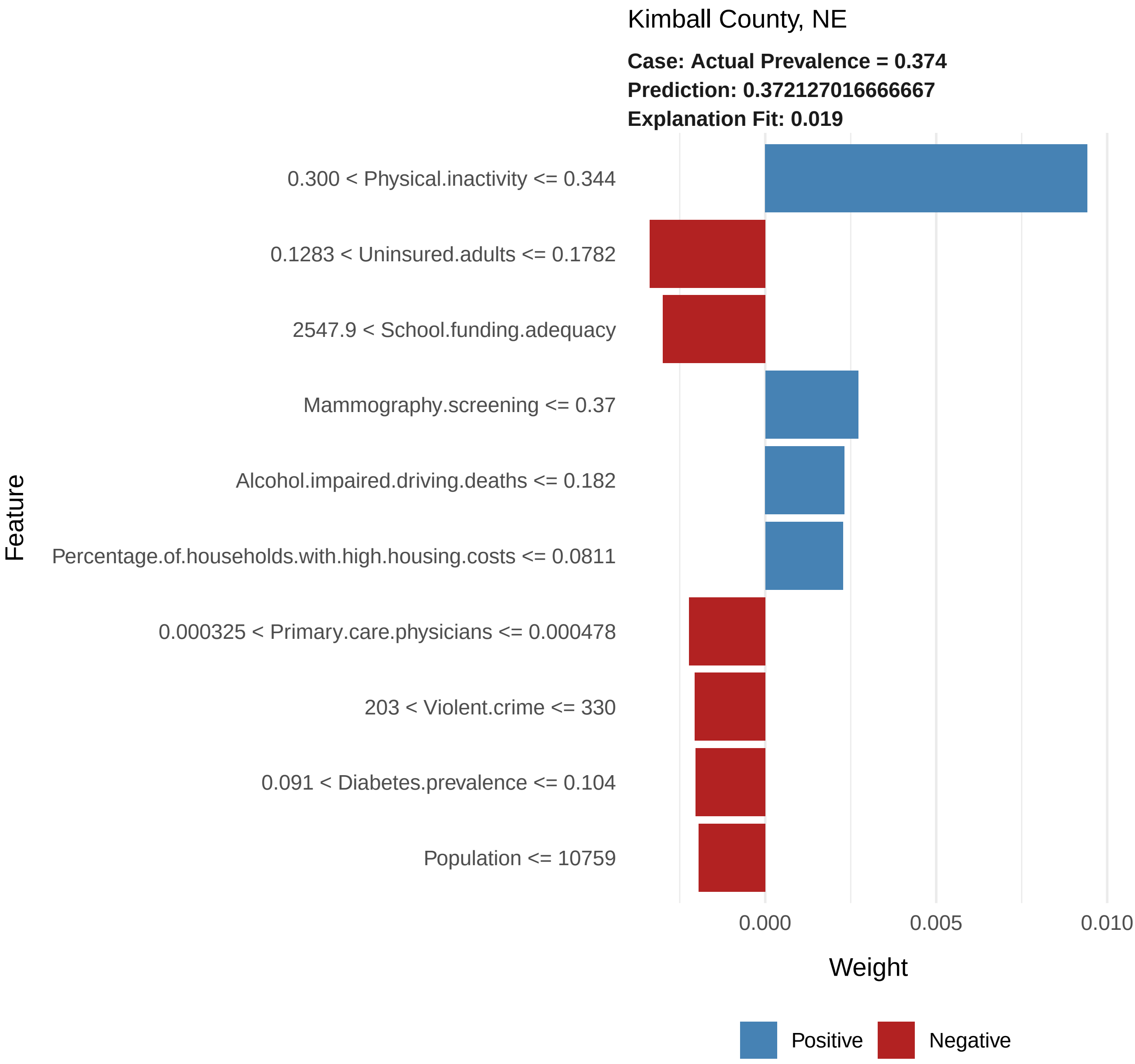
Kimball County, NE
Case: Actual Prevalence = 0.374
Prediction: 0.372127016666667
Explanation Fit: 0.019
0.300 < Physical.inactivity <= 0.344
0.1283 < Uninsured.adults <= 0.1782
2547.9 < School.funding.adequacy
Mammography.screening <= 0.37
Alcohol.impaired.driving.deaths <= 0.182
Percentage.of.households.with.high.housing.costs <= 0.0811
0.000325 < Primary.care.physicians <= 0.000478
203 < Violent.crime <= 330
0.091 < Diabetes.prevalence <= 0.104
Population <= 10759
Feature
0.000
0.005
0.010
Weight
Positive
Negative

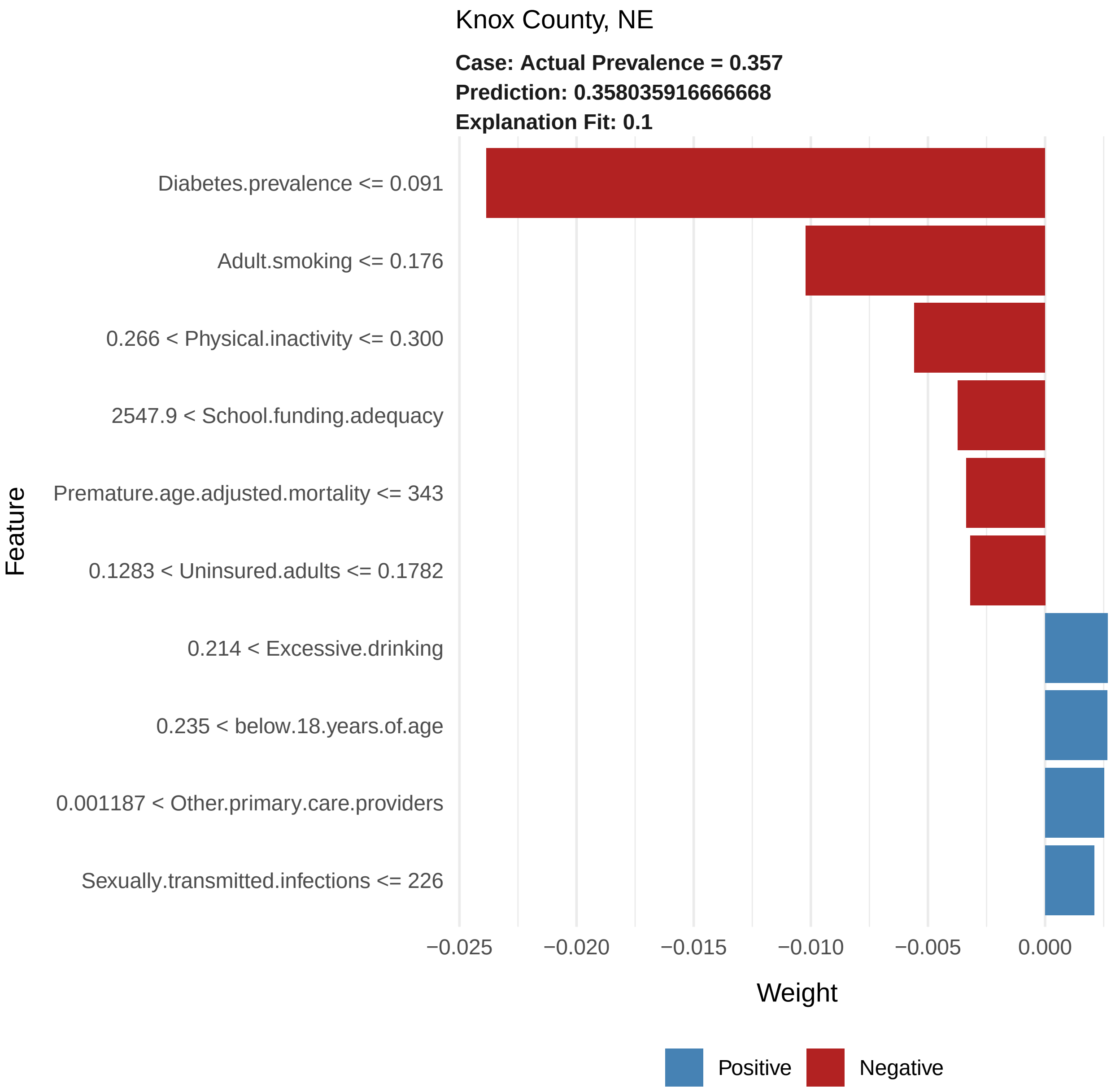

Knox County, NE
Case: Actual Prevalence = 0.357
Prediction: 0.358035916666668
Explanation Fit: 0.1
Diabetes.prevalence <= 0.091
Adult.smoking <= 0.176
0.266 < Physical.inactivity <= 0.300
2547.9 < School.funding.adequacy
Premature.age.adjusted.mortality <= 343
0.1283 < Uninsured.adults <= 0.1782
0.214 < Excessive.drinking
0.235 < below.18.years.of.age
0.001187 < Other.primary.care.providers
Sexually.transmitted.infections <= 226
Feature
−0.025
−0.020
−0.015
−0.010
−0.005
0.000
Weight
Positive
Negative

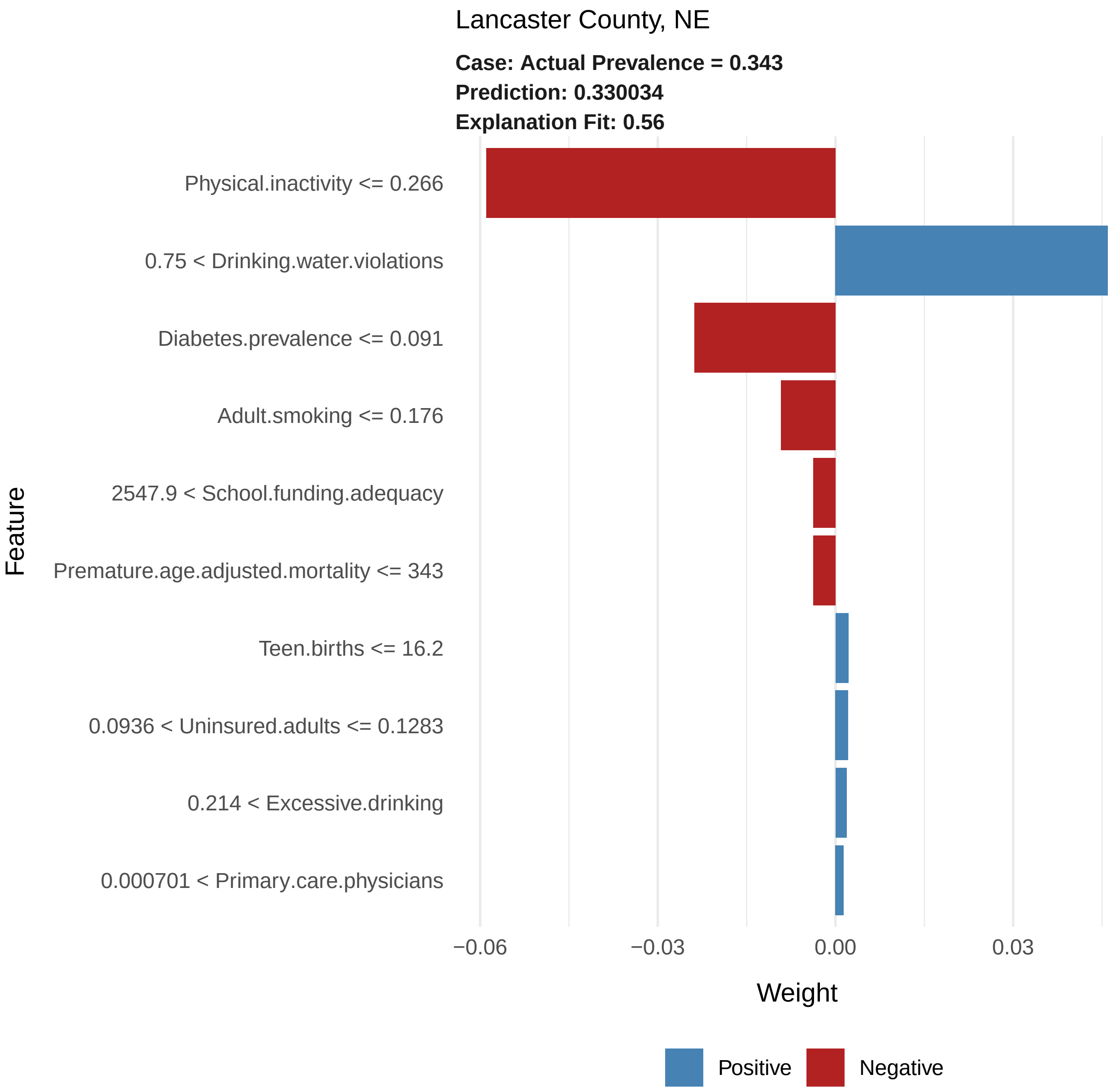

Lancaster County, NE
Case: Actual Prevalence = 0.343
Prediction: 0.330034
Explanation Fit: 0.56
Physical.inactivity <= 0.266
0.75 < Drinking.water.violations
Diabetes.prevalence <= 0.091
Adult.smoking <= 0.176
2547.9 < School.funding.adequacy
Premature.age.adjusted.mortality <= 343
Teen.births <= 16.2
0.0936 < Uninsured.adults <= 0.1283
0.214 < Excessive.drinking
0.000701 < Primary.care.physicians
Feature
−0.06
−0.03
0.00
0.03
Weight
Positive
Negative

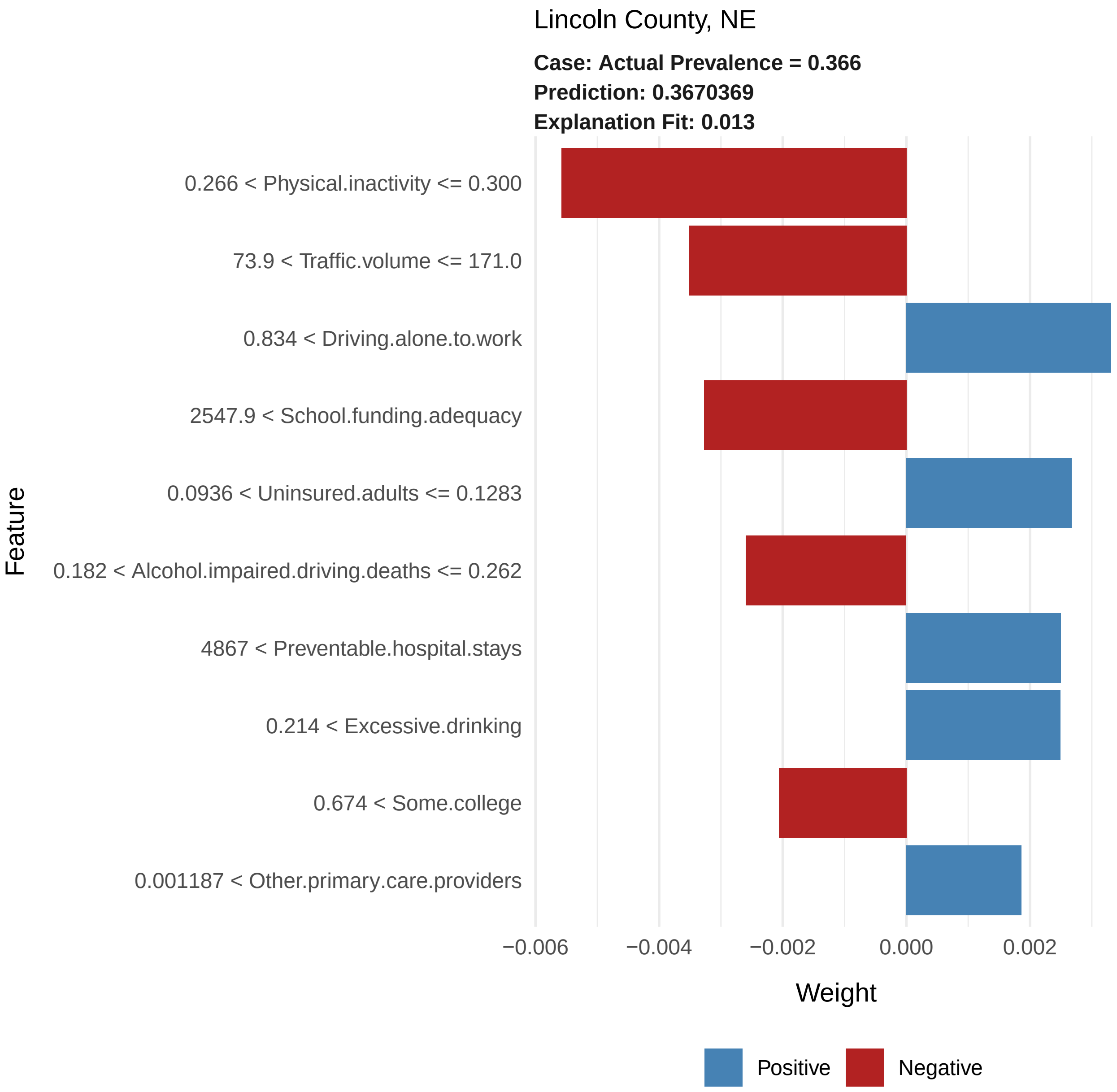
Lincoln County, NE
Case: Actual Prevalence = 0.366
Prediction: 0.3670369
Explanation Fit: 0.013
0.266 < Physical.inactivity <= 0.300
73.9 < Traffic.volume <= 171.0
0.834 < Driving.alone.to.work
2547.9 < School.funding.adequacy
0.0936 < Uninsured.adults <= 0.1283
0.182 < Alcohol.impaired.driving.deaths <= 0.262
4867 < Preventable.hospital.stays
0.214 < Excessive.drinking
0.674 < Some.college
0.001187 < Other.primary.care.providers
Feature
−0.006
−0.004
−0.002
0.000
0.002
Weight
Positive
Negative

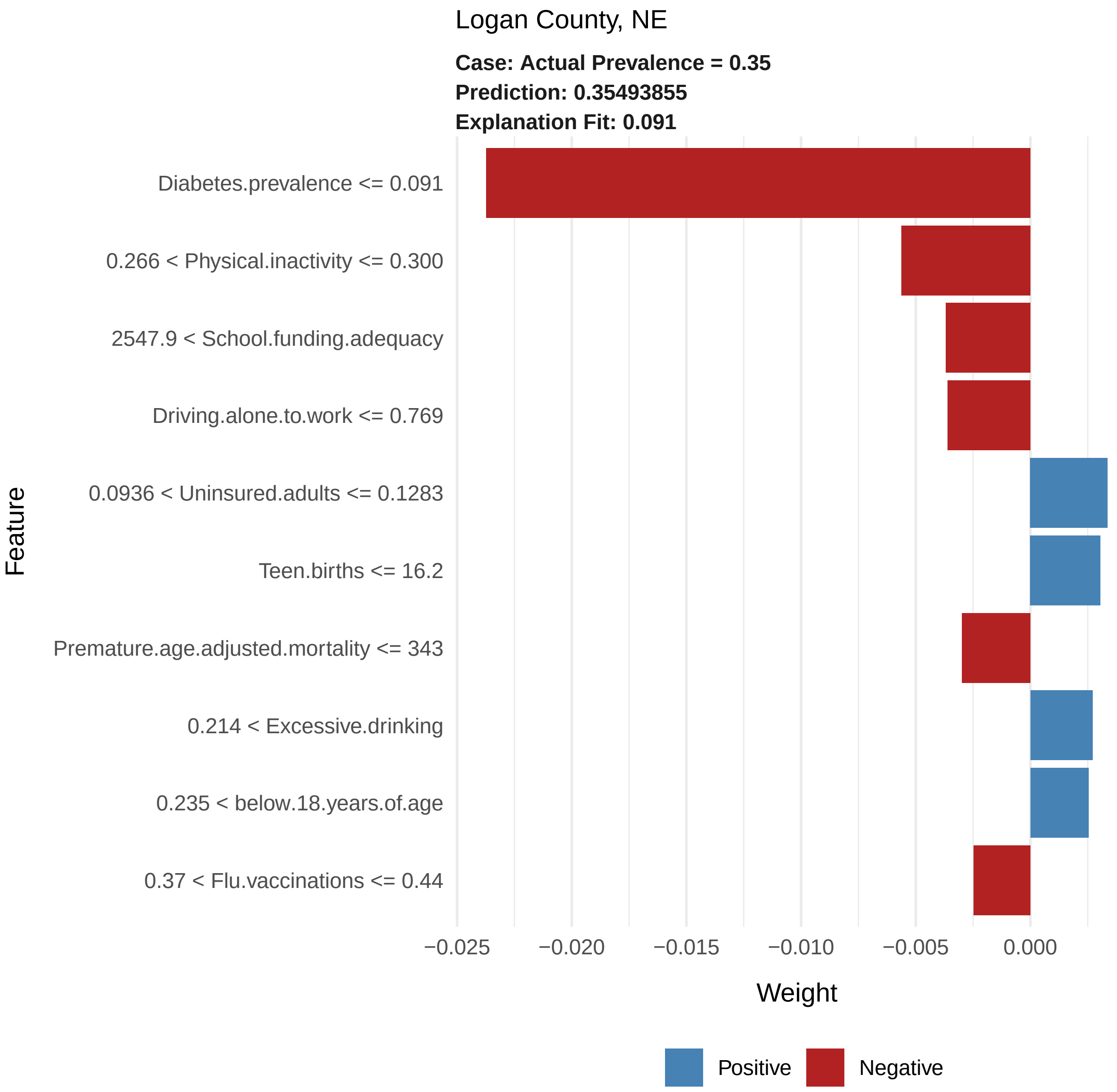

Logan County, NE
Case: Actual Prevalence = 0.35
Prediction: 0.35493855
Explanation Fit: 0.091
Diabetes.prevalence <= 0.091
0.266 < Physical.inactivity <= 0.300
2547.9 < School.funding.adequacy
Driving.alone.to.work <= 0.769
0.0936 < Uninsured.adults <= 0.1283
Teen.births <= 16.2
Premature.age.adjusted.mortality <= 343
0.214 < Excessive.drinking
0.235 < below.18.years.of.age
0.37 < Flu.vaccinations <= 0.44
Feature
−0.025
−0.020
−0.015
−0.010
−0.005
0.000
Weight
Positive
Negative

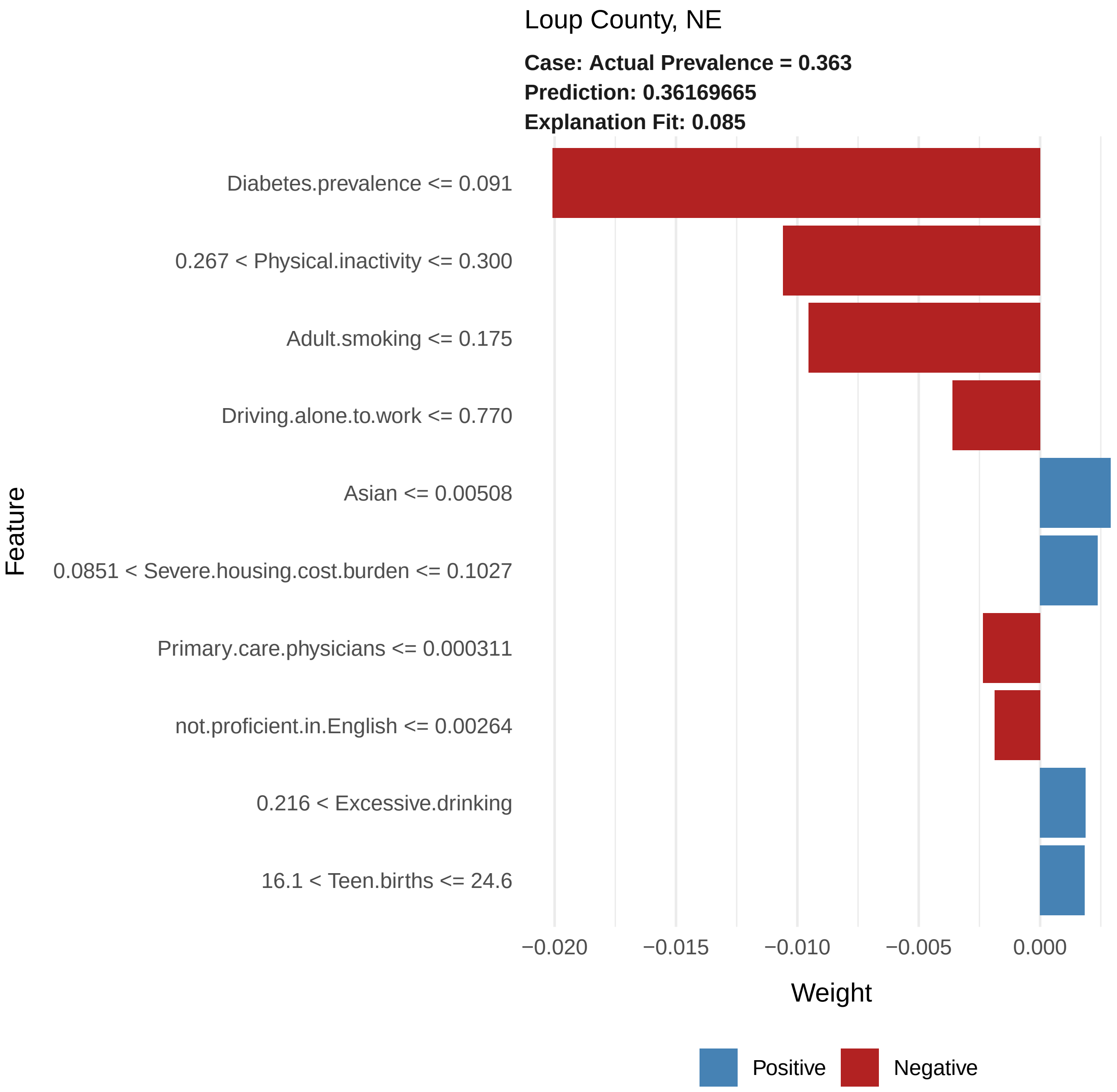

Loup County, NE
Case: Actual Prevalence = 0.363
Prediction: 0.36169665
Explanation Fit: 0.085
Feature
Diabetes.prevalence <= 0.091
0.267 < Physical.inactivity <= 0.300
Adult.smoking <= 0.175
Driving.alone.to.work <= 0.770
Asian <= 0.00508
0.0851 < Severe.housing.cost.burden <= 0.1027
Primary.care.physicians <= 0.000311
not.proficient.in.English <= 0.00264
0.216 < Excessive.drinking
16.1 < Teen.births <= 24.6
−0.020
−0.015
−0.010
−0.005
0.000
Weight
Positive
Negative

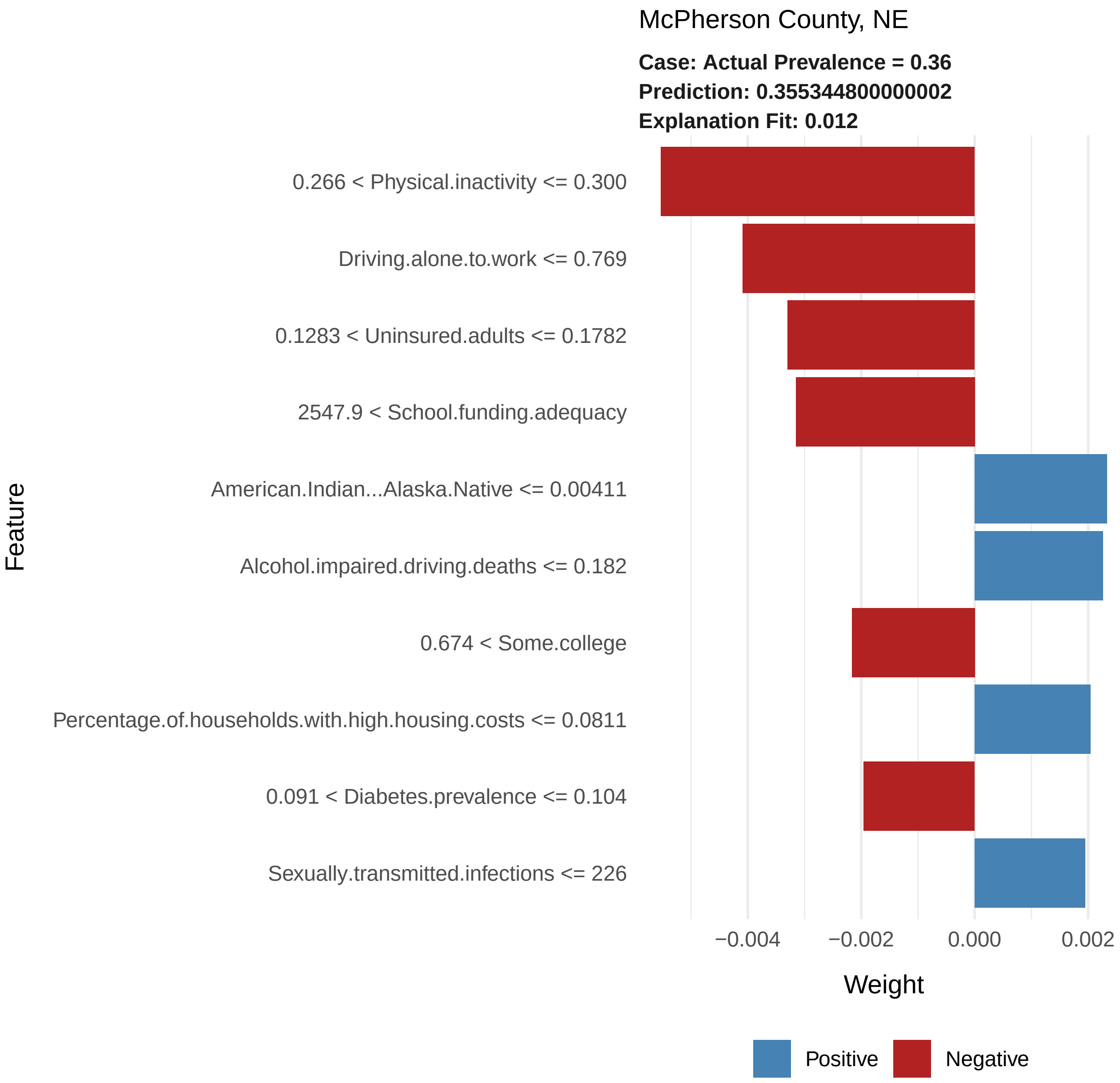
McPherson County, NE
Case: Actual Prevalence = 0.36
Prediction: 0.355344800000002
Explanation Fit: 0.012
0.266 < Physical.inactivity <= 0.300
Driving.alone.to.work <= 0.769
0.1283 < Uninsured.adults <= 0.1782
2547.9 < School.funding.adequacy
American.Indian...Alaska.Native <= 0.00411
Alcohol.impaired.driving.deaths <= 0.182
0.674 < Some.college
Percentage.of.households.with.high.housing.costs <= 0.0811
0.091 < Diabetes.prevalence <= 0.104
Sexually.transmitted.infections <= 226
Feature
−0.004
−0.002
0.000
0.002
Weight
Positive
Negative

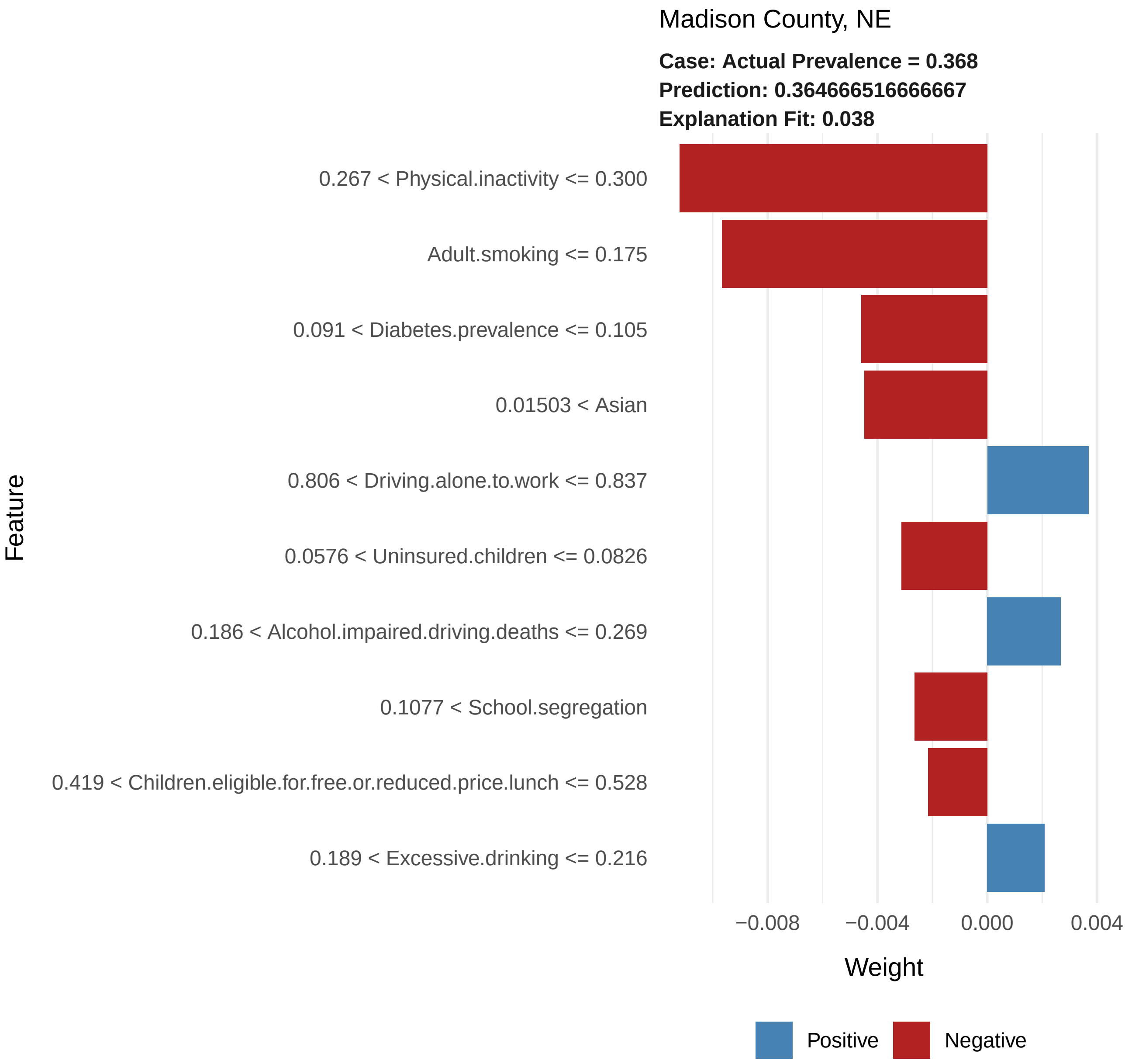

Madison County, NE
Case: Actual Prevalence = 0.368
Prediction: 0.364666516666667
Explanation Fit: 0.038
0.267 < Physical.inactivity <= 0.300
Adult.smoking <= 0.175
0.091 < Diabetes.prevalence <= 0.105
0.01503 < Asian
0.806 < Driving.alone.to.work <= 0.837
0.0576 < Uninsured.children <= 0.0826
0.186 < Alcohol.impaired.driving.deaths <= 0.269
0.1077 < School.segregation
0.419 < Children.eligible.for.free.or.reduced.price.lunch <= 0.528
0.189 < Excessive.drinking <= 0.216
Feature
−0.008
−0.004
0.000
0.004
Weight
Positive
Negative

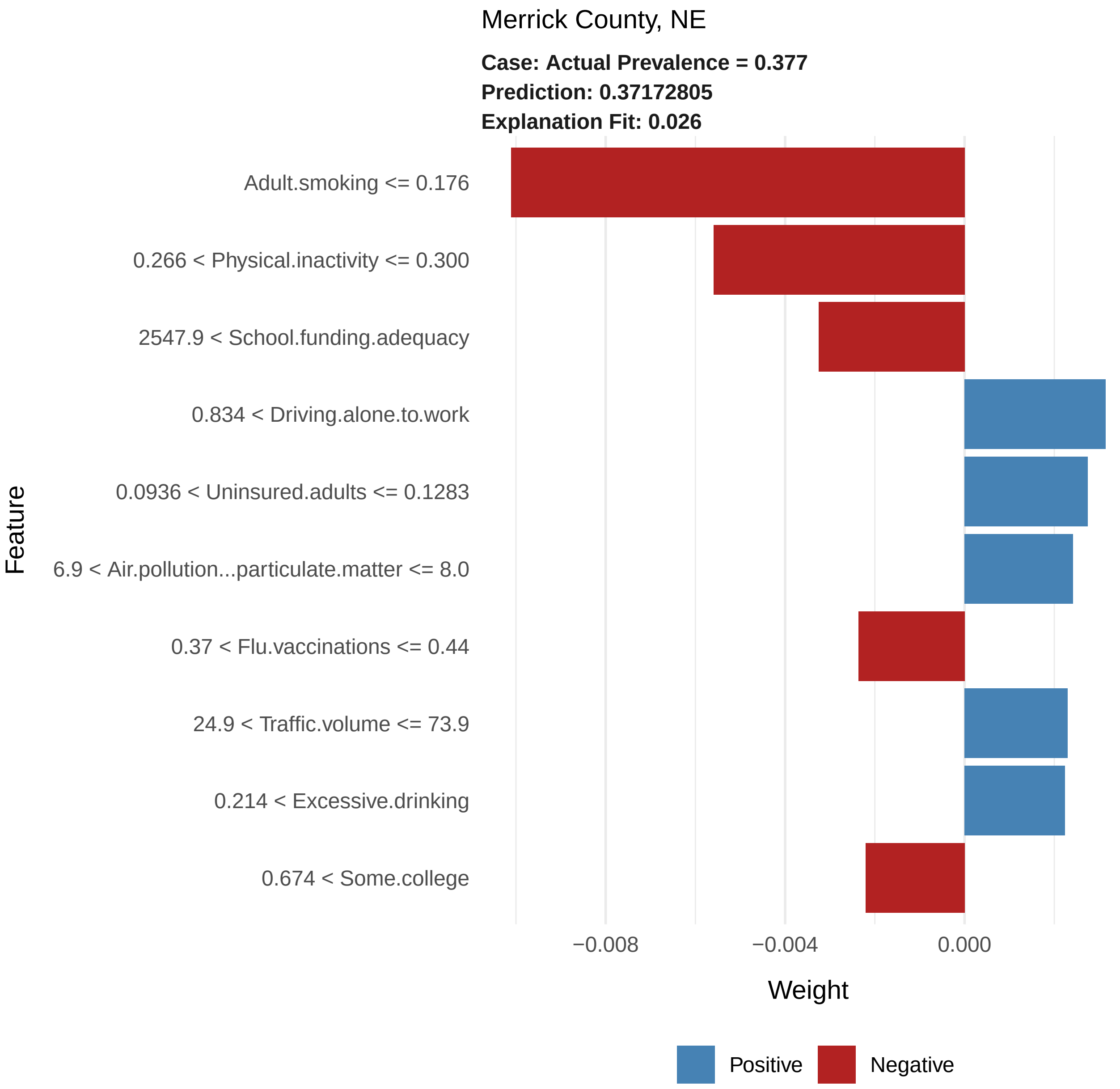
Merrick County, NE
Case: Actual Prevalence = 0.377
Prediction: 0.37172805
Explanation Fit: 0.026
Adult.smoking <= 0.176
0.266 < Physical.inactivity <= 0.300
2547.9 < School.funding.adequacy
0.834 < Driving.alone.to.work
0.0936 < Uninsured.adults <= 0.1283
6.9 < Air.pollution...particulate.matter <= 8.0
0.37 < Flu.vaccinations <= 0.44
24.9 < Traffic.volume <= 73.9
0.214 < Excessive.drinking
0.674 < Some.college
Feature
−0.008
−0.004
0.000
Weight
Positive
Negative

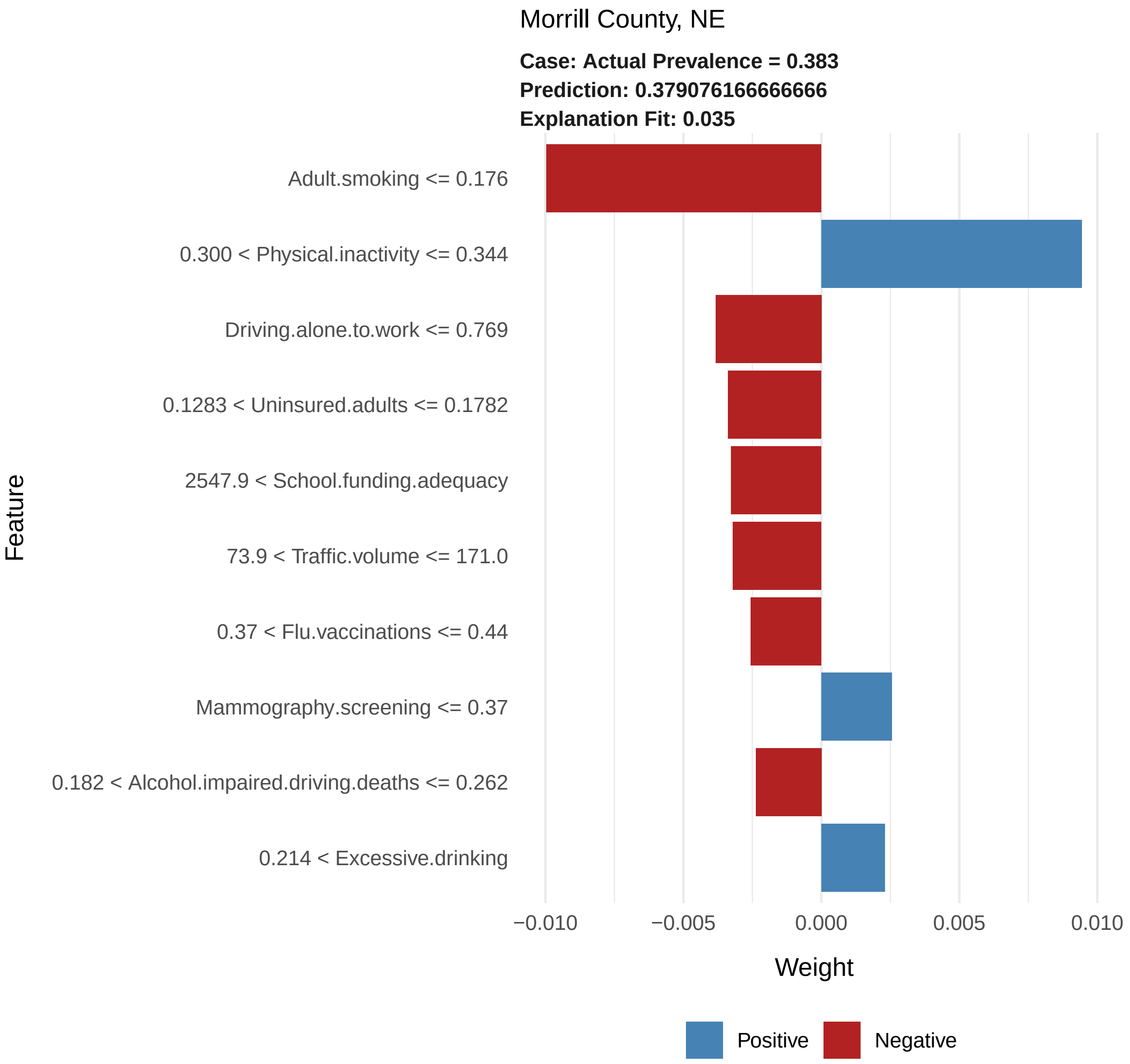

Morrill County, NE
Case: Actual Prevalence = 0.383
Prediction: 0.379076166666666
Explanation Fit: 0.035
Adult.smoking <= 0.176
0.300 < Physical.inactivity <= 0.344
Driving.alone.to.work <= 0.769
0.1283 < Uninsured.adults <= 0.1782
2547.9 < School.funding.adequacy
73.9 < Traffic.volume <= 171.0
0.37 < Flu.vaccinations <= 0.44
Mammography.screening <= 0.37
0.182 < Alcohol.impaired.driving.deaths <= 0.262
0.214 < Excessive.drinking
Feature
−0.010
−0.005
0.000
0.005
0.010
Weight
Positive
Negative

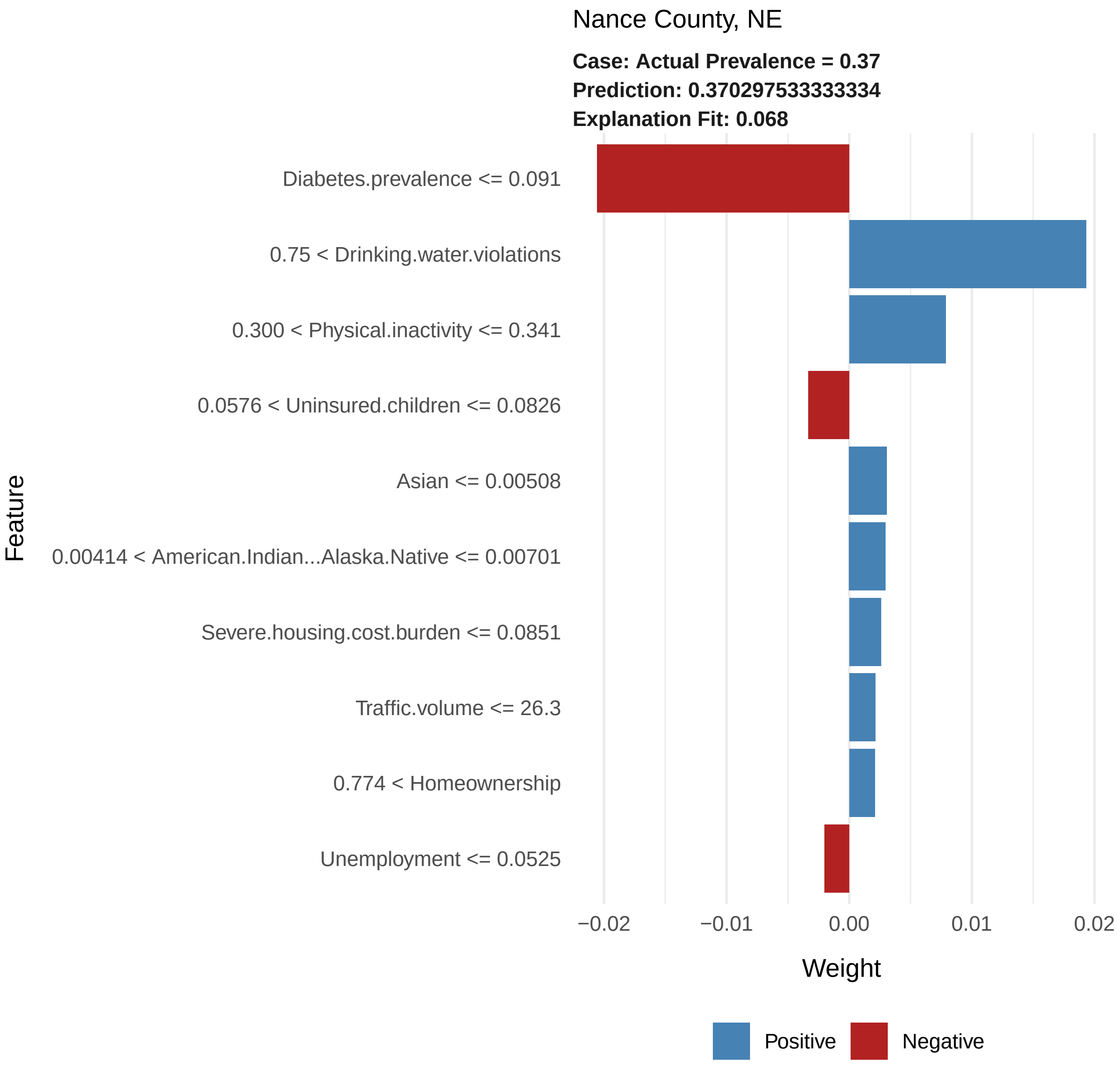

Nance County, NE
Case: Actual Prevalence = 0.37
Prediction: 0.370297533333334
Explanation Fit: 0.068
Diabetes.prevalence <= 0.091
0.75 < Drinking.water.violations
0.300 < Physical.inactivity <= 0.341
0.0576 < Uninsured.children <= 0.0826
Asian <= 0.00508
0.00414 < American.Indian...Alaska.Native <= 0.00701
Severe.housing.cost.burden <= 0.0851
Traffic.volume <= 26.3
0.774 < Homeownership
Unemployment <= 0.0525
Feature
−0.02
−0.01
0.00
0.01
0.02
Weight
Positive
Negative

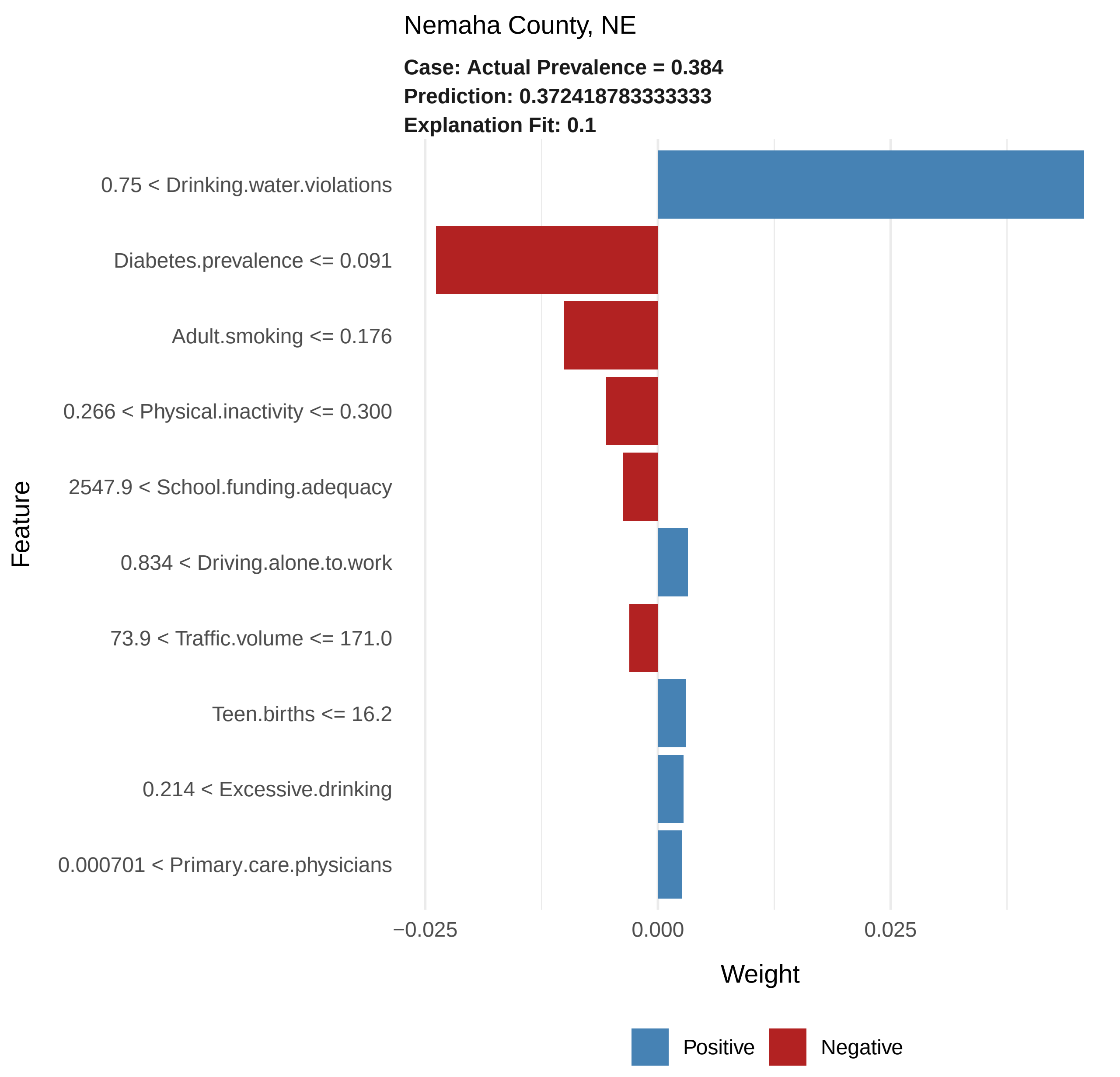

Nemaha County, NE
Case: Actual Prevalence = 0.384
Prediction: 0.372418783333333
Explanation Fit: 0.1
0.75 < Drinking.water.violations
Diabetes.prevalence <= 0.091
Adult.smoking <= 0.176
0.266 < Physical.inactivity <= 0.300
2547.9 < School.funding.adequacy
0.834 < Driving.alone.to.work
73.9 < Traffic.volume <= 171.0
Teen.births <= 16.2
0.214 < Excessive.drinking
0.000701 < Primary.care.physicians
Feature
−0.025
0.000
0.025
Weight
Positive
Negative

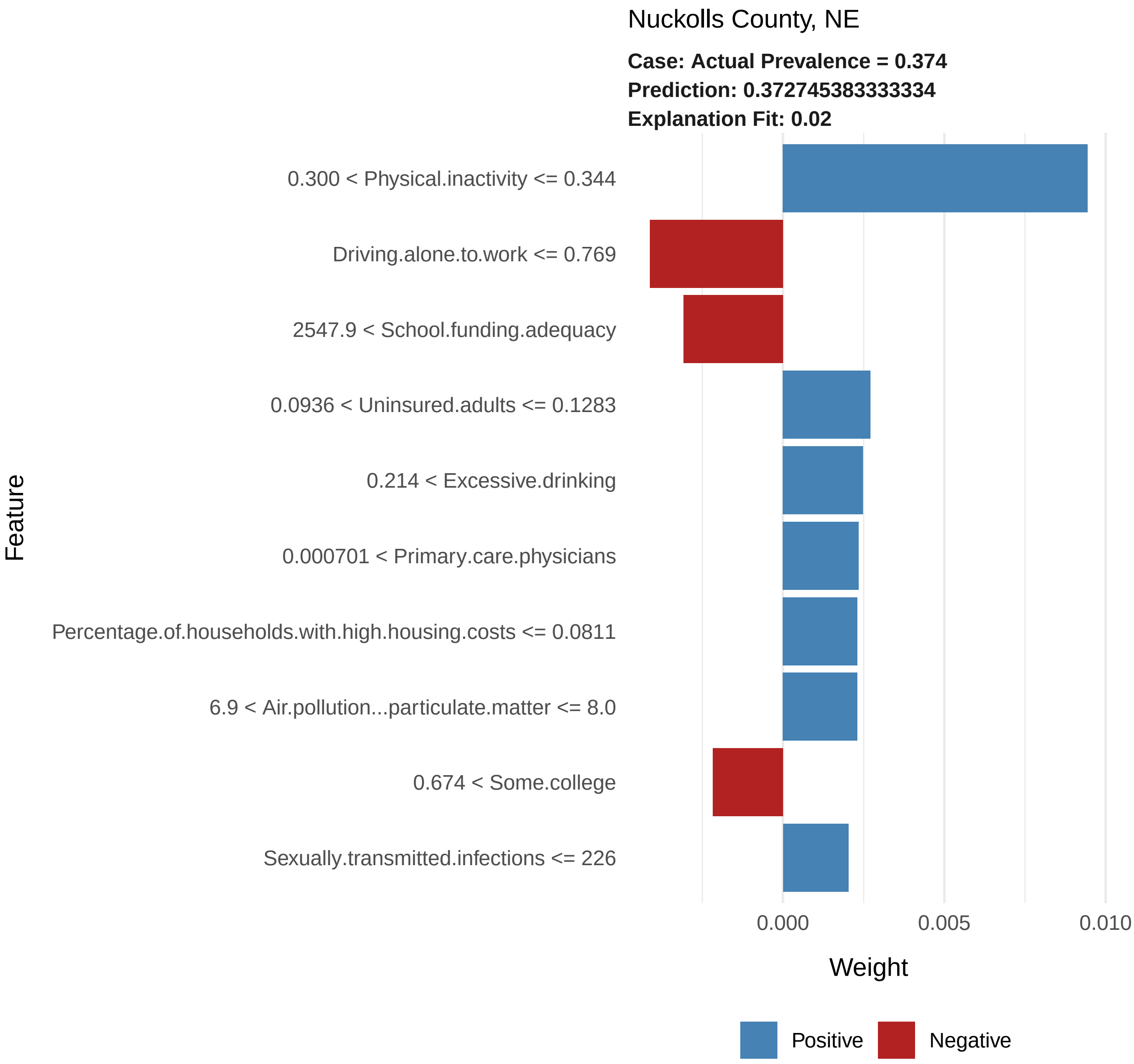
Nuckolls County, NE
Case: Actual Prevalence = 0.374
Prediction: 0.372745383333334
Explanation Fit: 0.02
0.300 < Physical.inactivity <= 0.344
Driving.alone.to.work <= 0.769
2547.9 < School.funding.adequacy
0.0936 < Uninsured.adults <= 0.1283
0.214 < Excessive.drinking
0.000701 < Primary.care.physicians
Percentage.of.households.with.high.housing.costs <= 0.0811
6.9 < Air.pollution...particulate.matter <= 8.0
0.674 < Some.college
Sexually.transmitted.infections <= 226
Feature
0.000
0.005
0.010
Weight
Positive
Negative

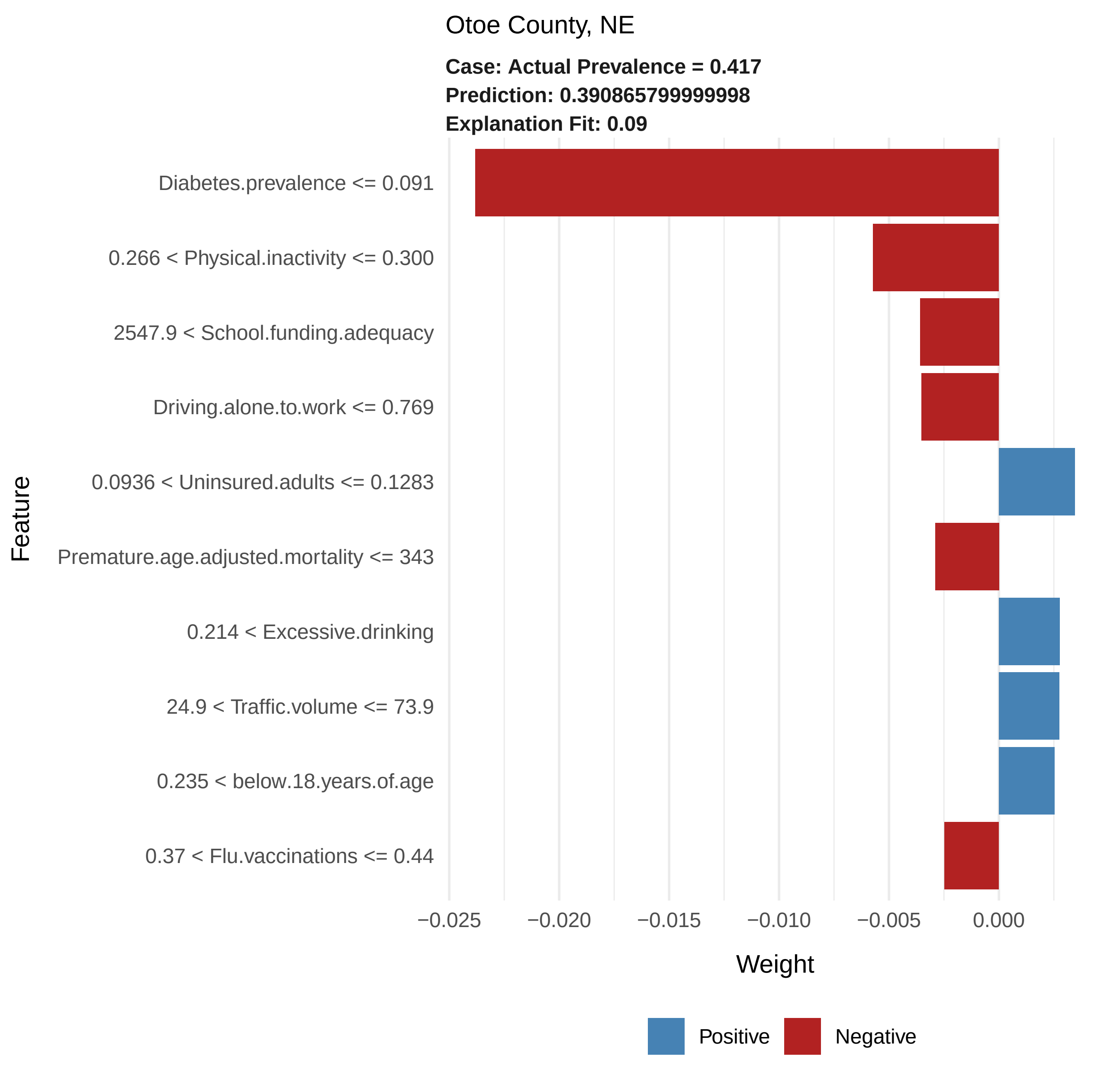

Otoe County, NE
Case: Actual Prevalence = 0.417
Prediction: 0.390865799999998
Explanation Fit: 0.09
Diabetes.prevalence <= 0.091
0.266 < Physical.inactivity <= 0.300
2547.9 < School.funding.adequacy
Driving.alone.to.work <= 0.769
0.0936 < Uninsured.adults <= 0.1283
Premature.age.adjusted.mortality <= 343
0.214 < Excessive.drinking
24.9 < Traffic.volume <= 73.9
0.235 < below.18.years.of.age
0.37 < Flu.vaccinations <= 0.44
Feature
−0.025
−0.020
−0.015
−0.010
−0.005
0.000
Weight
Positive
Negative

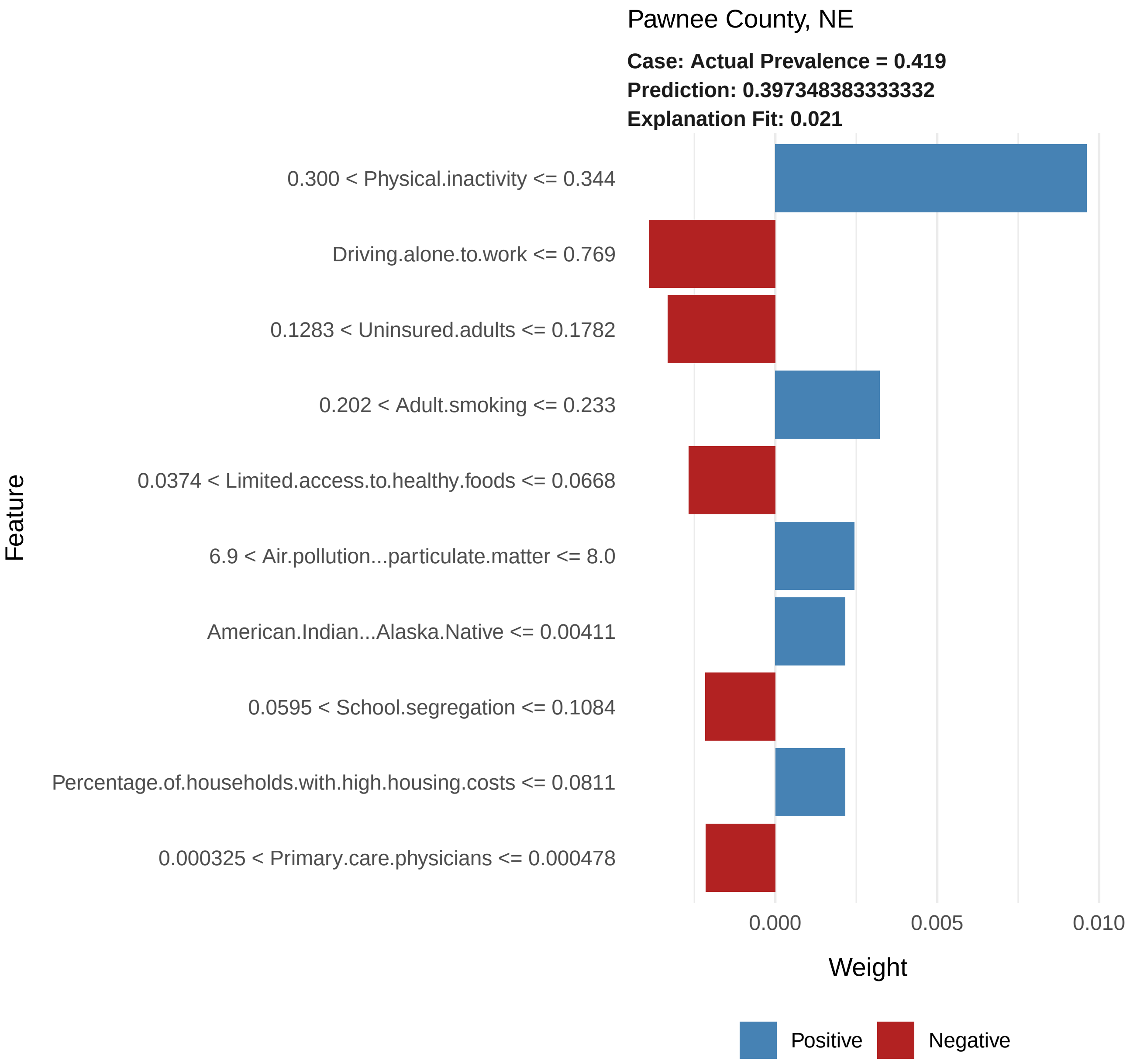
Pawnee County, NE
Case: Actual Prevalence = 0.419
Prediction: 0.397348383333332
Explanation Fit: 0.021
0.300 < Physical.inactivity <= 0.344
Driving.alone.to.work <= 0.769
0.1283 < Uninsured.adults <= 0.1782
0.202 < Adult.smoking <= 0.233
0.0374 < Limited.access.to.healthy.foods <= 0.0668
6.9 < Air.pollution...particulate.matter <= 8.0
American.Indian...Alaska.Native <= 0.00411
0.0595 < School.segregation <= 0.1084
Percentage.of.households.with.high.housing.costs <= 0.0811
0.000325 < Primary.care.physicians <= 0.000478
Feature
0.000
0.005
0.010
Weight
Positive
Negative

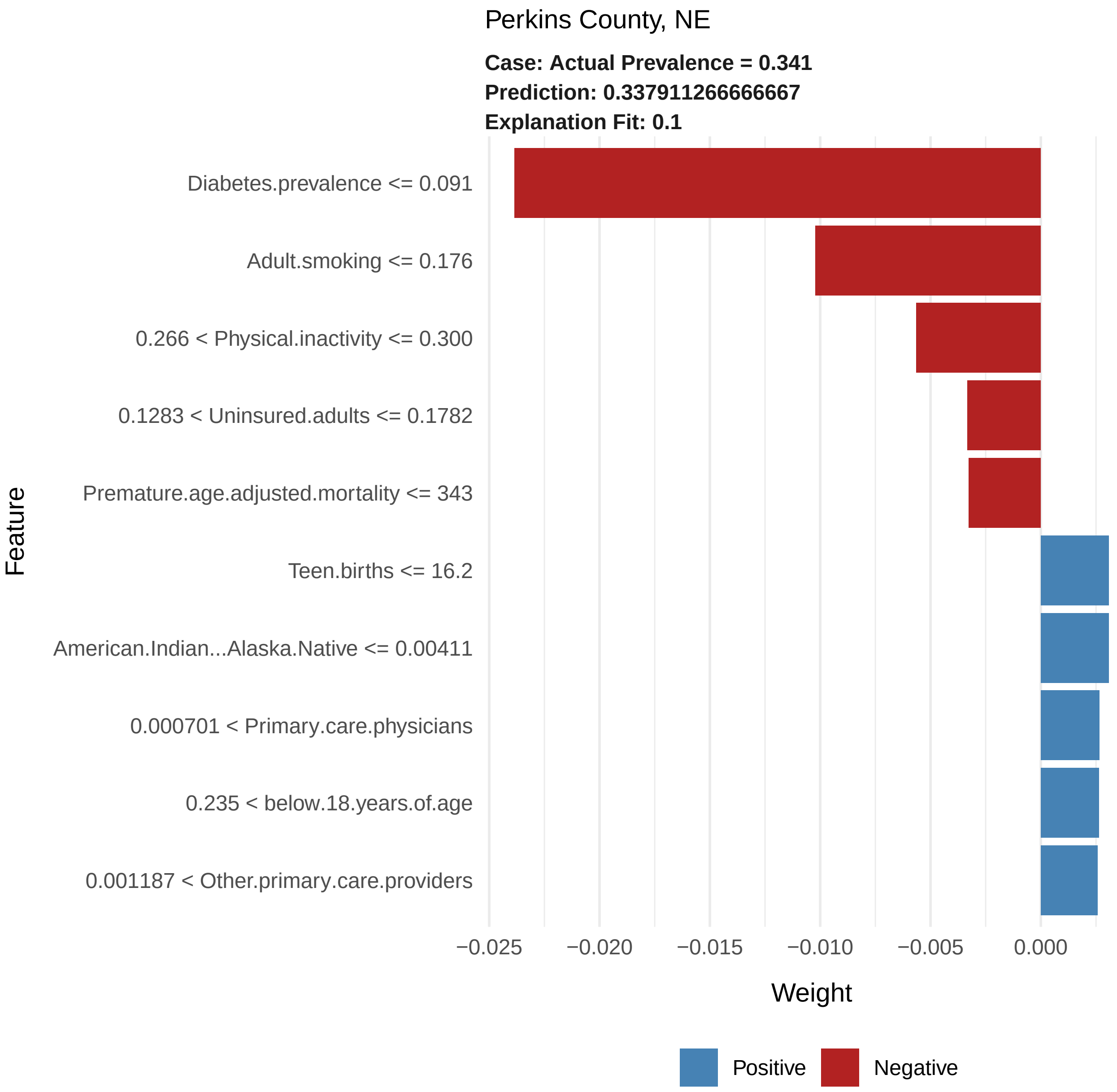
Perkins County, NE
Case: Actual Prevalence = 0.341
Prediction: 0.337911266666667
Explanation Fit: 0.1
Diabetes.prevalence <= 0.091
Adult.smoking <= 0.176
0.266 < Physical.inactivity <= 0.300
0.1283 < Uninsured.adults <= 0.1782
Premature.age.adjusted.mortality <= 343
Teen.births <= 16.2
American.Indian...Alaska.Native <= 0.00411
0.000701 < Primary.care.physicians
0.235 < below.18.years.of.age
0.001187 < Other.primary.care.providers
Feature
−0.025
−0.020
−0.015
−0.010
−0.005
0.000
Weight
Positive
Negative

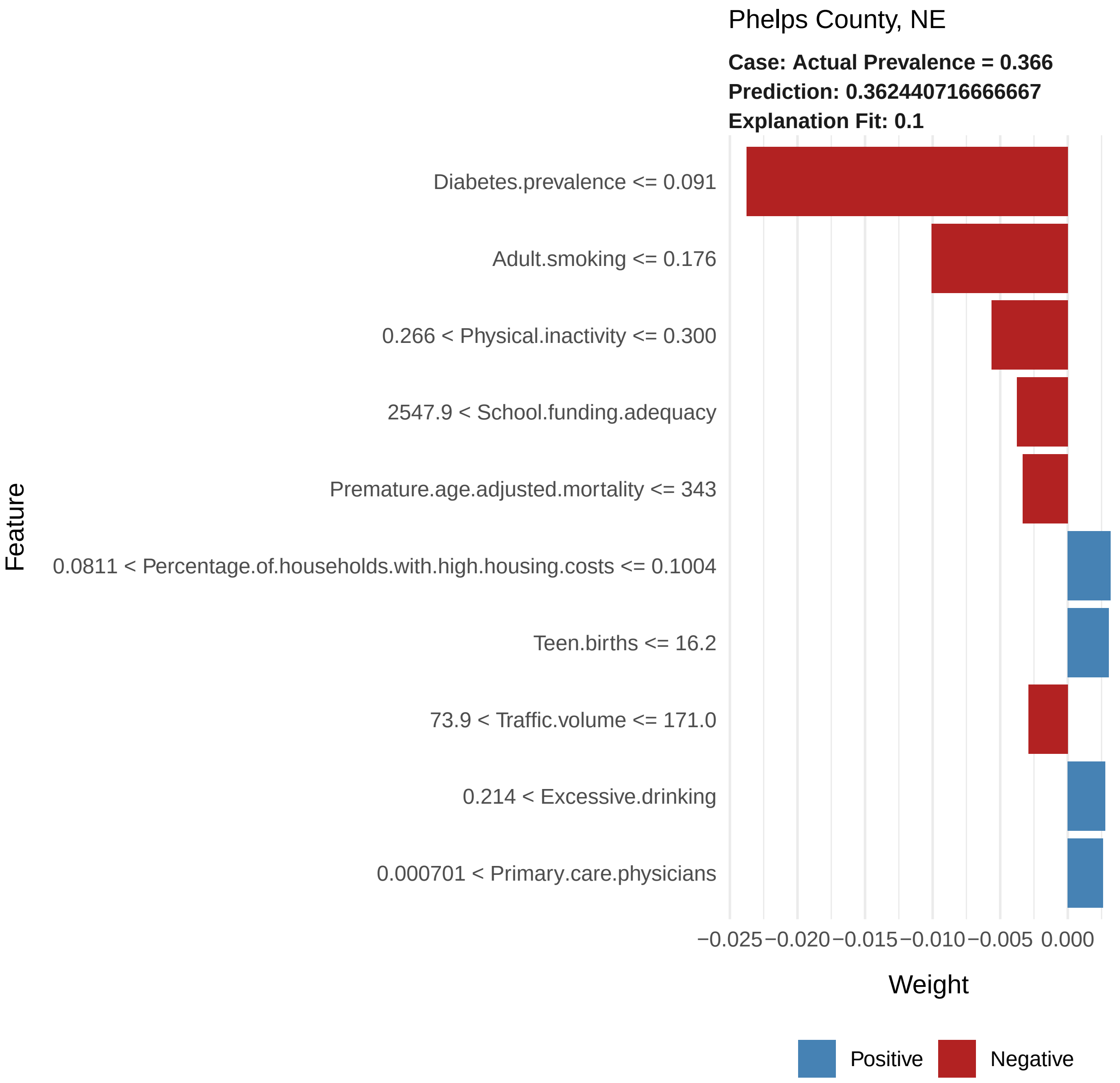
Phelps County, NE
Case: Actual Prevalence = 0.366
Prediction: 0.362440716666667
Explanation Fit: 0.1
Diabetes.prevalence <= 0.091
Adult.smoking <= 0.176
0.266 < Physical.inactivity <= 0.300
2547.9 < School.funding.adequacy
Premature.age.adjusted.mortality <= 343
0.0811 < Percentage.of.households.with.high.housing.costs <= 0.1004
Teen.births <= 16.2
73.9 < Traffic.volume <= 171.0
0.214 < Excessive.drinking
0.000701 < Primary.care.physicians
Feature
−0.025
−0.020
−0.015
−0.010
−0.005
0.000
Weight
Positive
Negative

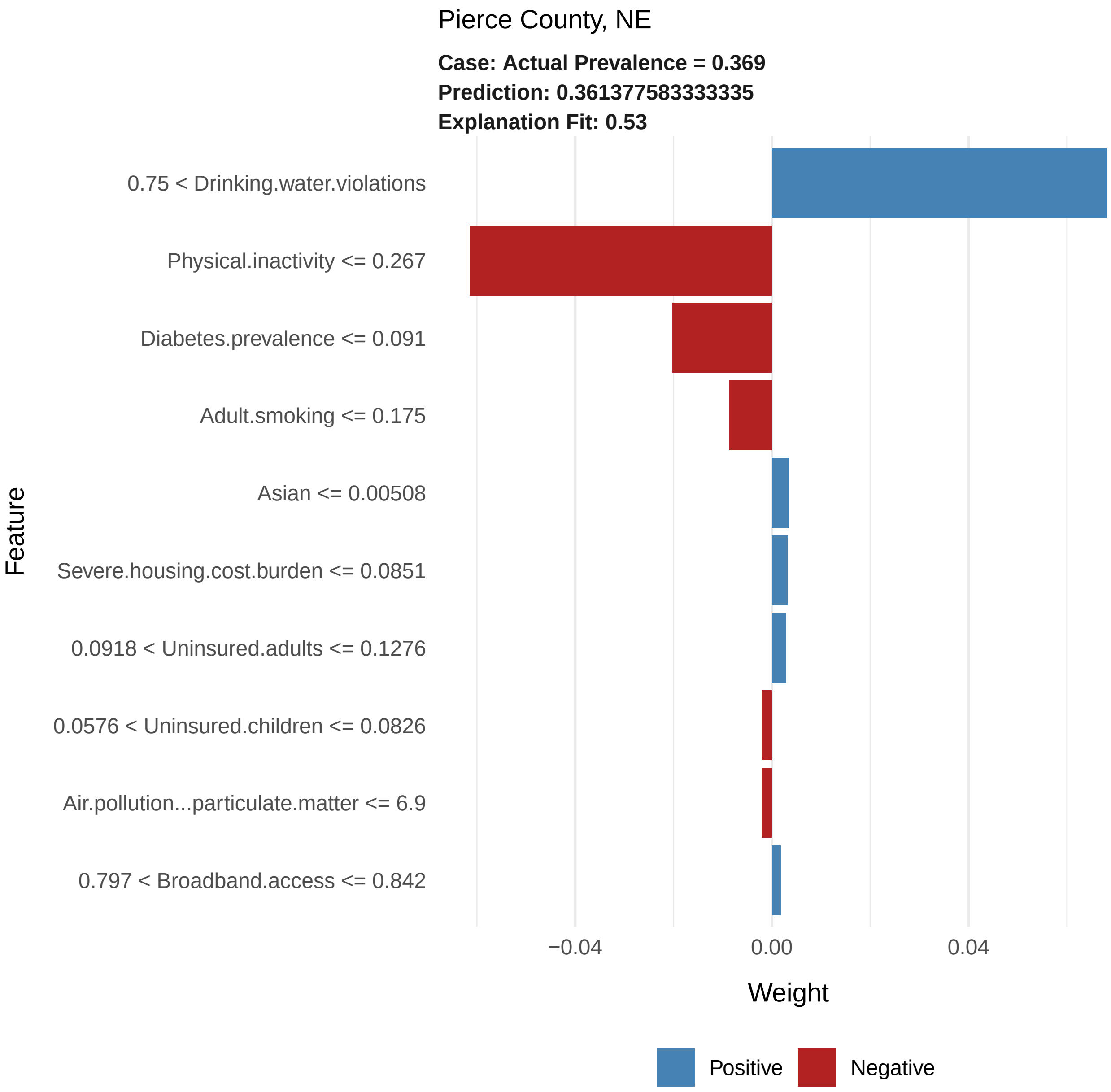

Pierce County, NE
Case: Actual Prevalence = 0.369
Prediction: 0.361377583333335
Explanation Fit: 0.53
0.75 < Drinking.water.violations
Physical.inactivity <= 0.267
Diabetes.prevalence <= 0.091
Adult.smoking <= 0.175
Asian <= 0.00508
Severe.housing.cost.burden <= 0.0851
0.0918 < Uninsured.adults <= 0.1276
0.0576 < Uninsured.children <= 0.0826
Air.pollution...particulate.matter <= 6.9
0.797 < Broadband.access <= 0.842
Feature
−0.04
0.00
0.04
Weight
Positive
Negative

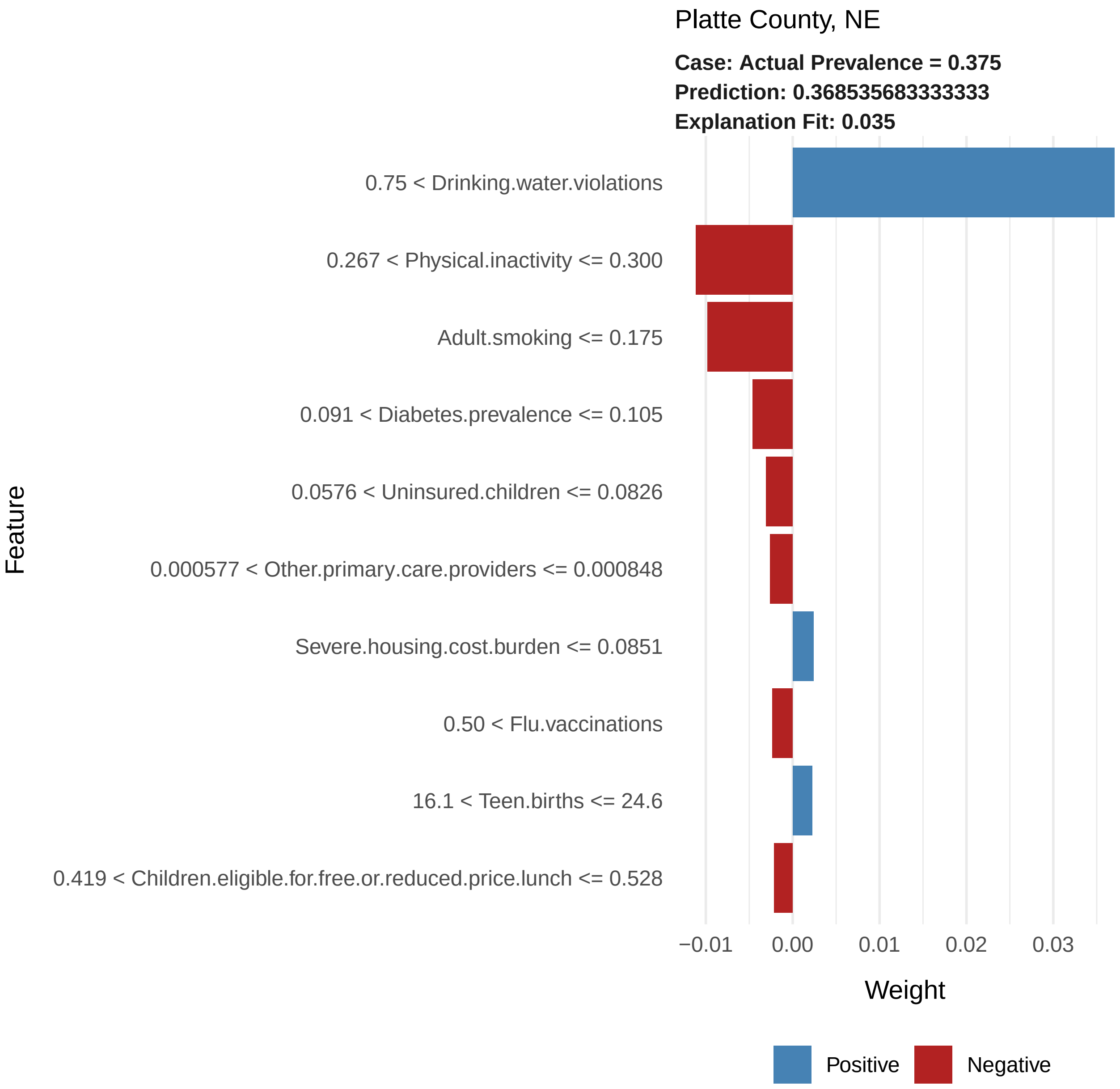
Platte County, NE
Case: Actual Prevalence = 0.375
Prediction: 0.368535683333333
Explanation Fit: 0.035
0.75 < Drinking.water.violations
0.267 < Physical.inactivity <= 0.300
Adult.smoking <= 0.175
0.091 < Diabetes.prevalence <= 0.105
0.0576 < Uninsured.children <= 0.0826
0.000577 < Other.primary.care.providers <= 0.000848
Severe.housing.cost.burden <= 0.0851
0.50 < Flu.vaccinations
16.1 < Teen.births <= 24.6
0.419 < Children.eligible.for.free.or.reduced.price.lunch <= 0.528
Feature
−0.01
0.00
0.01
0.02
0.03
Weight
Positive
Negative

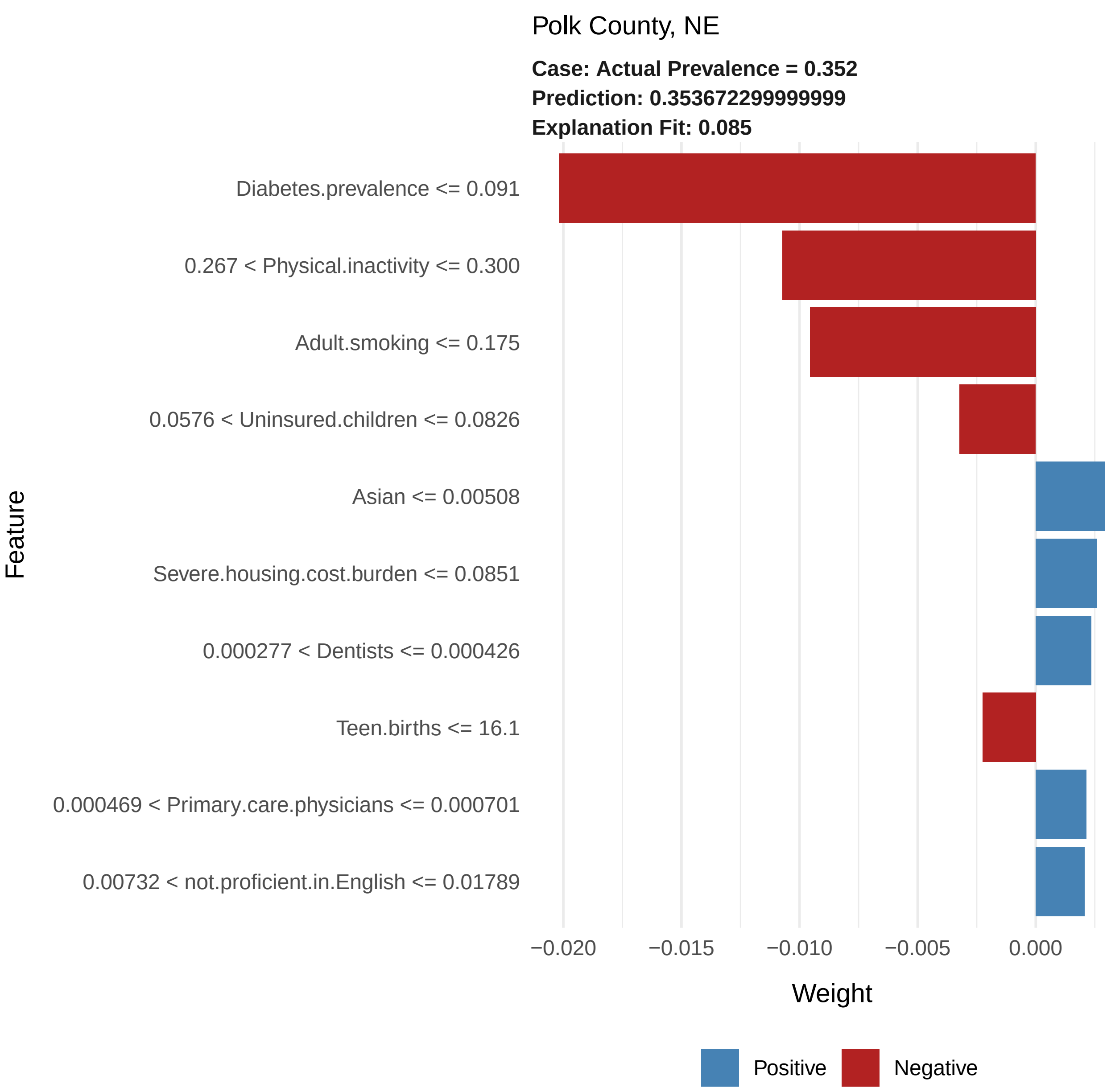

Polk County, NE
Case: Actual Prevalence = 0.352
Prediction: 0.353672299999999
Explanation Fit: 0.085
Diabetes.prevalence <= 0.091
0.267 < Physical.inactivity <= 0.300
Adult.smoking <= 0.175
0.0576 < Uninsured.children <= 0.0826
Asian <= 0.00508
Severe.housing.cost.burden <= 0.0851
0.000277 < Dentists <= 0.000426
Teen.births <= 16.1
0.000469 < Primary.care.physicians <= 0.000701
0.00732 < not.proficient.in.English <= 0.01789
Feature
−0.020
−0.015
−0.010
−0.005
0.000
Weight
Positive
Negative

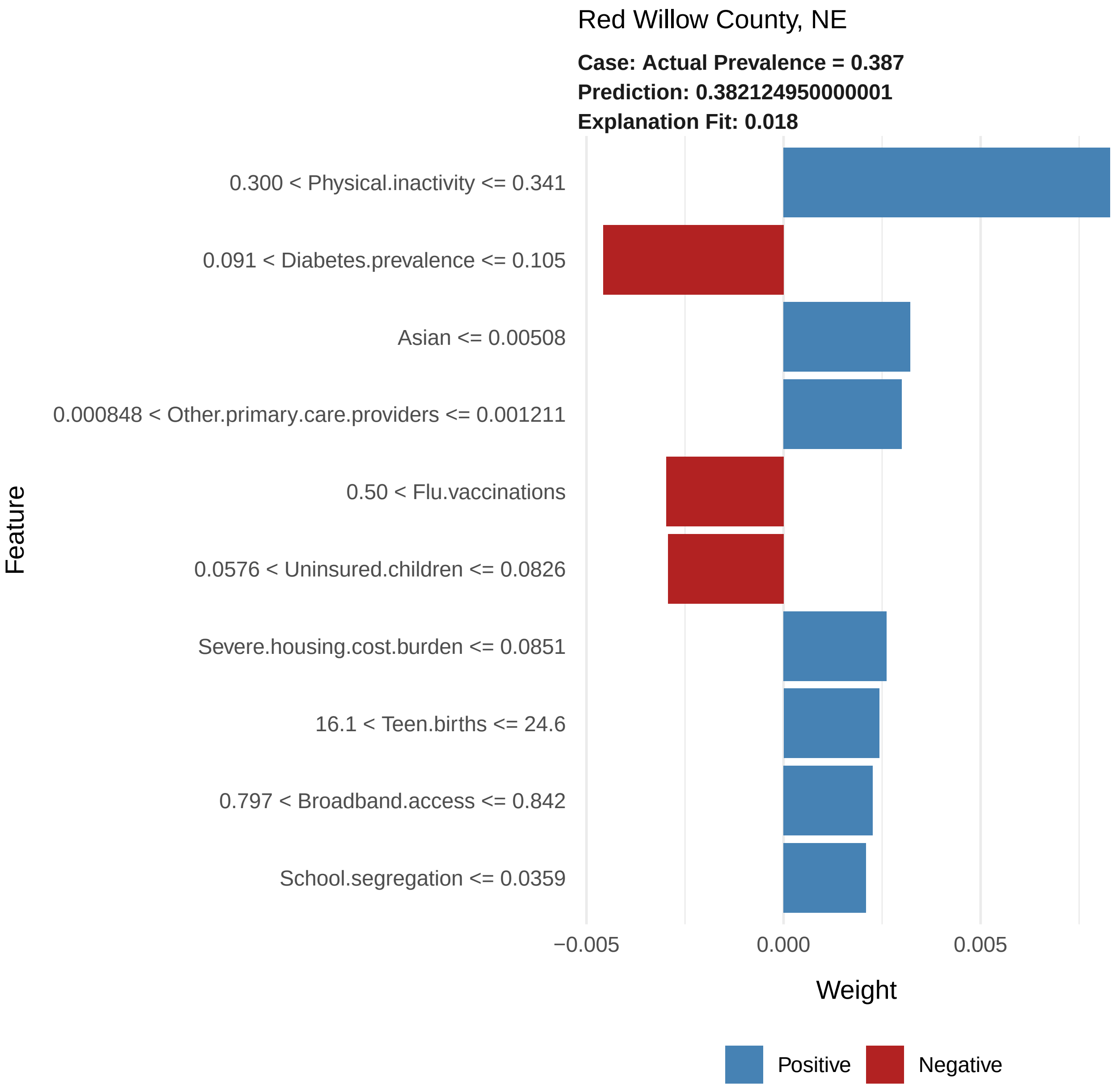
Red Willow County, NE
Case: Actual Prevalence = 0.387
Prediction: 0.382124950000001
Explanation Fit: 0.018
0.300 < Physical.inactivity <= 0.341
0.091 < Diabetes.prevalence <= 0.105
Asian <= 0.00508
0.000848 < Other.primary.care.providers <= 0.001211
0.50 < Flu.vaccinations
0.0576 < Uninsured.children <= 0.0826
Severe.housing.cost.burden <= 0.0851
16.1 < Teen.births <= 24.6
0.797 < Broadband.access <= 0.842
School.segregation <= 0.0359
Feature
−0.005
0.000
0.005
Weight
Positive
Negative

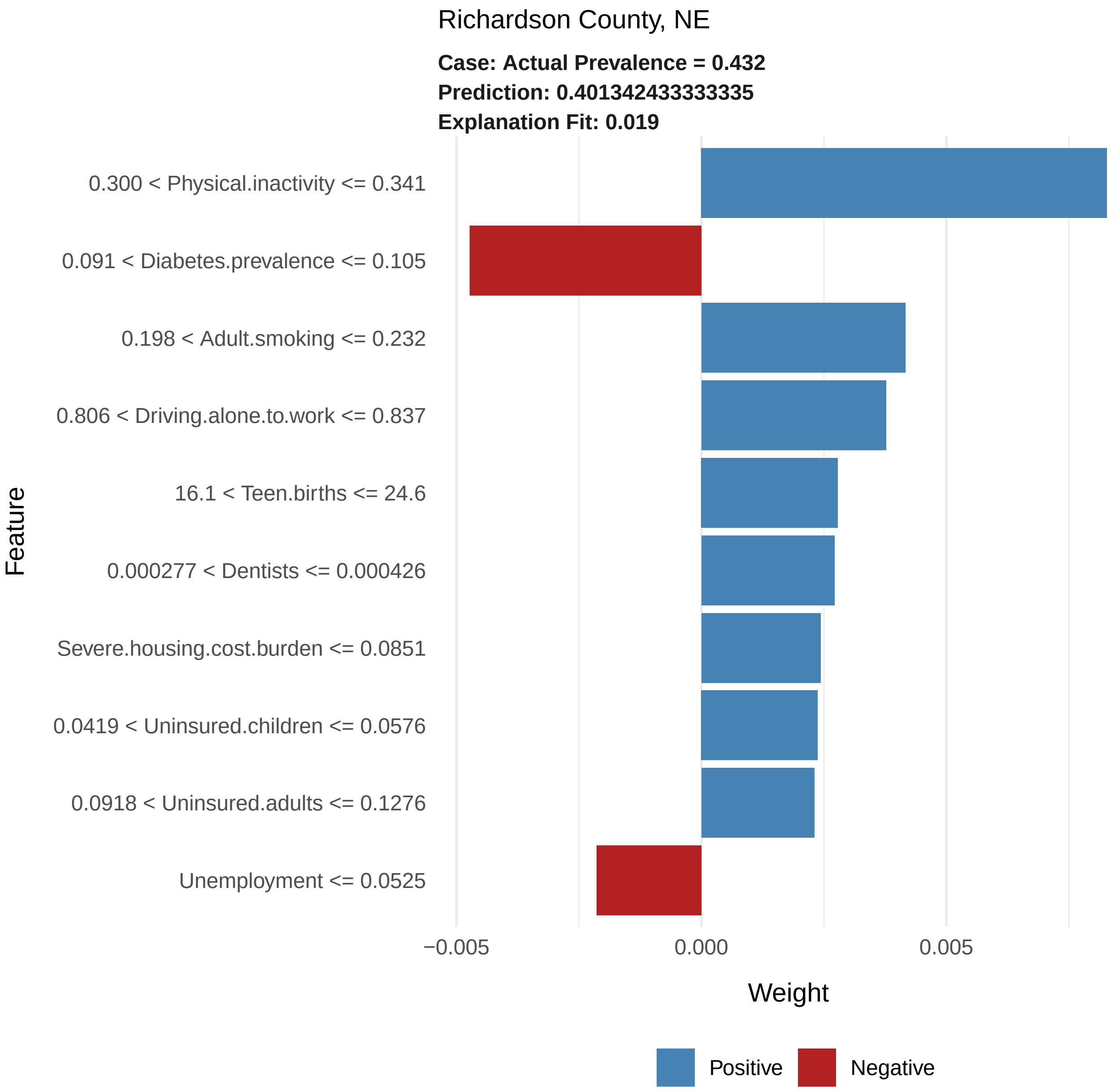

Richardson County, NE
Case: Actual Prevalence = 0.432
Prediction: 0.401342433333335
Explanation Fit: 0.019
Feature
0.300 < Physical.inactivity <= 0.341
0.091 < Diabetes.prevalence <= 0.105
0.198 < Adult.smoking <= 0.232
0.806 < Driving.alone.to.work <= 0.837
16.1 < Teen.births <= 24.6
0.000277 < Dentists <= 0.000426
Severe.housing.cost.burden <= 0.0851
0.0419 < Uninsured.children <= 0.0576
0.0918 < Uninsured.adults <= 0.1276
Unemployment <= 0.0525
−0.005
0.000
0.005
Weight
Positive
Negative

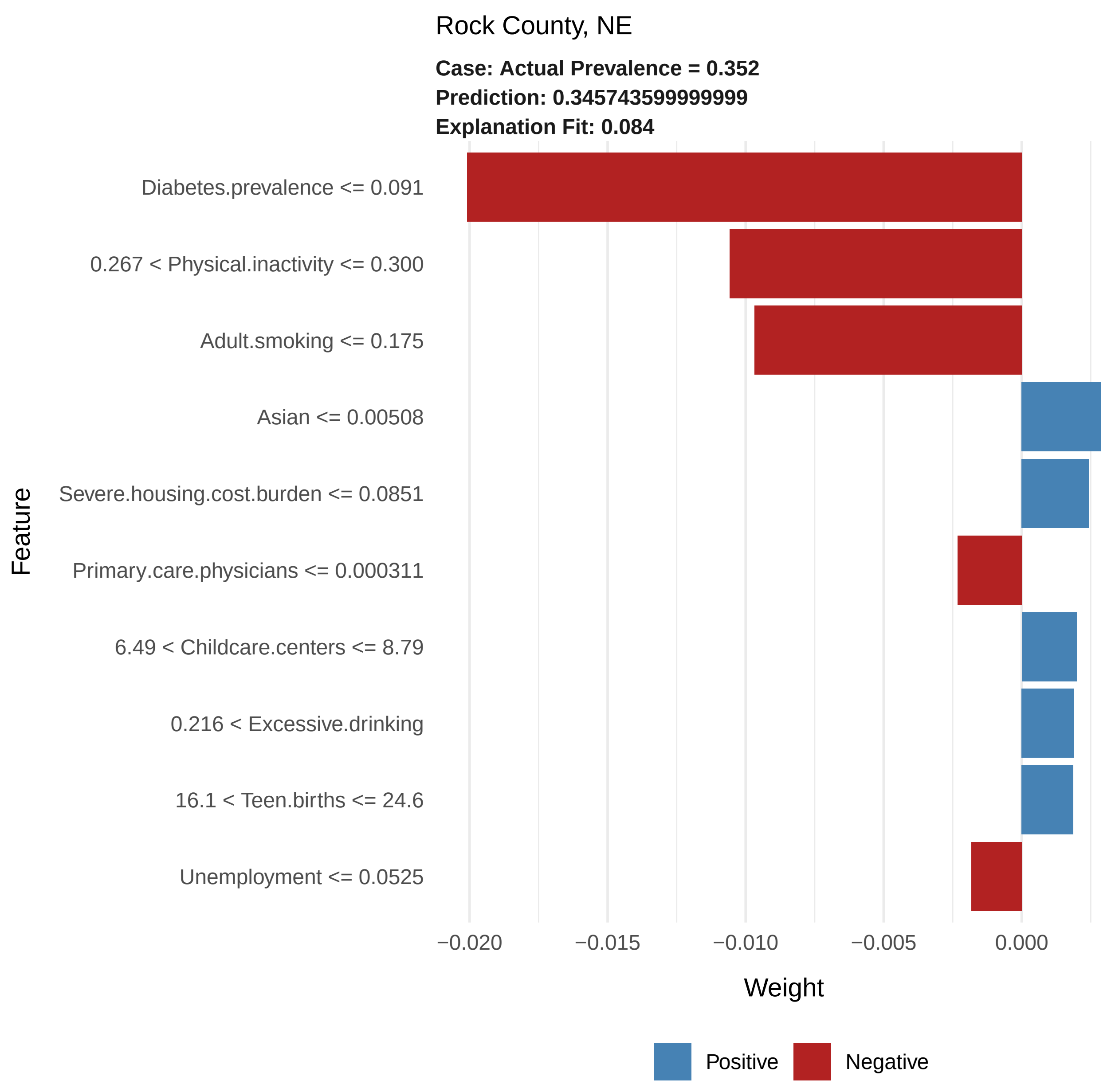

Rock County, NE
Case: Actual Prevalence = 0.352
Prediction: 0.345743599999999
Explanation Fit: 0.084
Diabetes.prevalence <= 0.091
0.267 < Physical.inactivity <= 0.300
Adult.smoking <= 0.175
Asian <= 0.00508
Severe.housing.cost.burden <= 0.0851
Primary.care.physicians <= 0.000311
6.49 < Childcare.centers <= 8.79
0.216 < Excessive.drinking
16.1 < Teen.births <= 24.6
Unemployment <= 0.0525
Feature
−0.020
−0.015
−0.010
−0.005
0.000
Weight
Positive
Negative

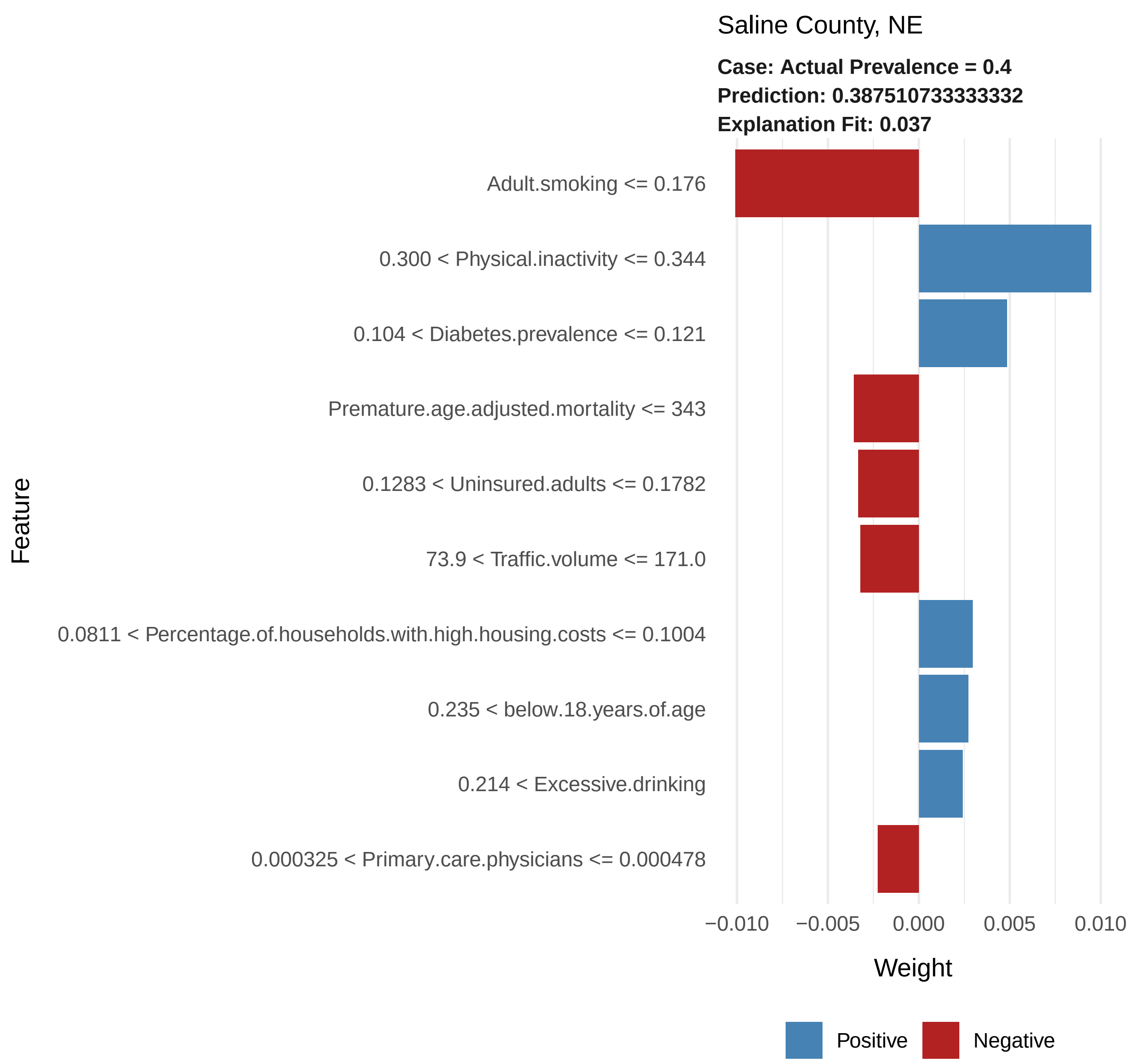

Saline County, NE
Case: Actual Prevalence = 0.4
Prediction: 0.387510733333332
Explanation Fit: 0.037
Adult.smoking <= 0.176
0.300 < Physical.inactivity <= 0.344
0.104 < Diabetes.prevalence <= 0.121
Premature.age.adjusted.mortality <= 343
0.1283 < Uninsured.adults <= 0.1782
73.9 < Traffic.volume <= 171.0
0.0811 < Percentage.of.households.with.high.housing.costs <= 0.1004
0.235 < below.18.years.of.age
0.214 < Excessive.drinking
0.000325 < Primary.care.physicians <= 0.000478
Feature
−0.010
−0.005
0.000
0.005
0.010
Weight
Positive
Negative

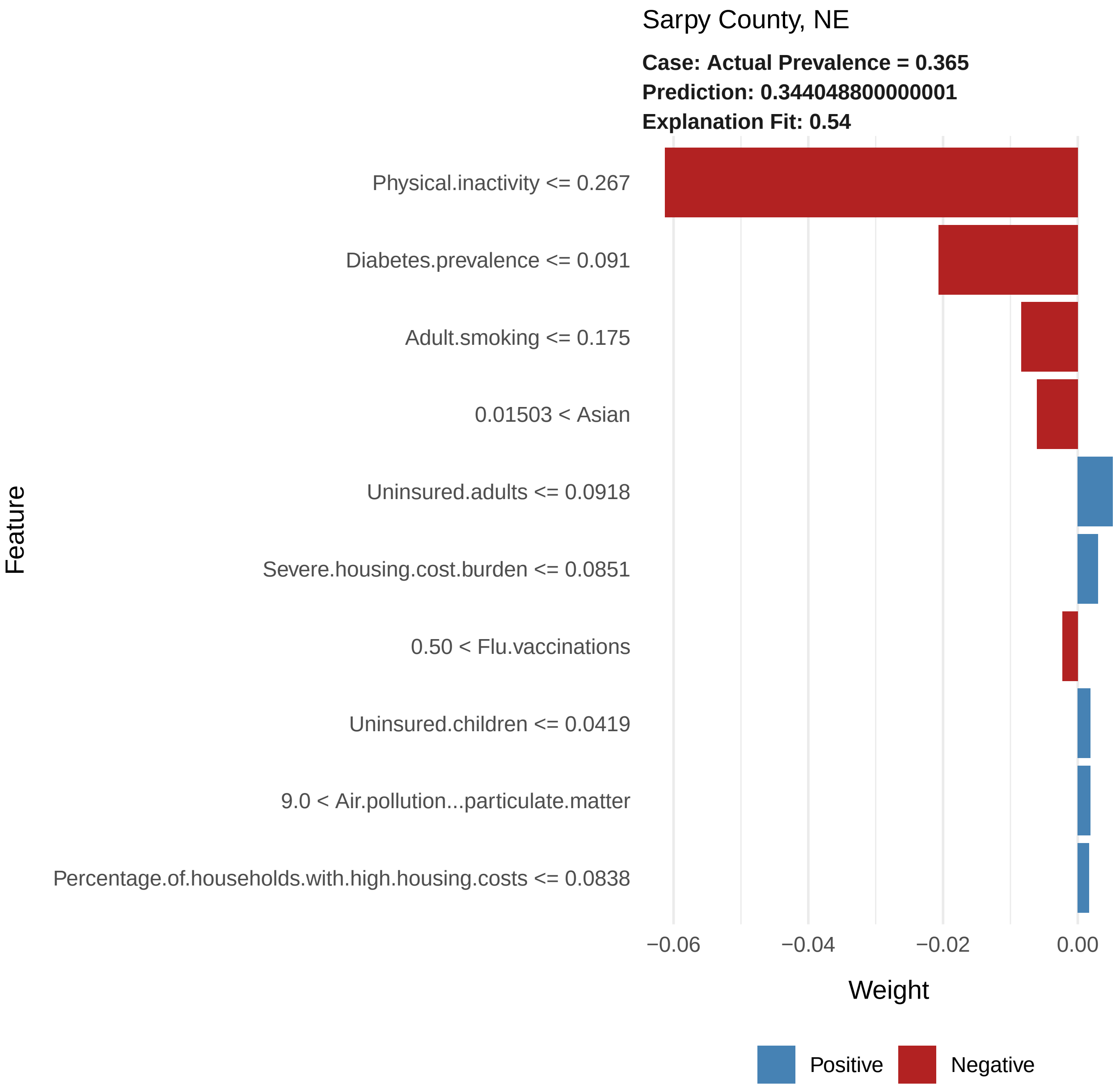
Sarpy County, NE
Case: Actual Prevalence = 0.365
Prediction: 0.344048800000001
Explanation Fit: 0.54
Feature
Physical.inactivity <= 0.267
Diabetes.prevalence <= 0.091
Adult.smoking <= 0.175
0.01503 < Asian
Uninsured.adults <= 0.0918
Severe.housing.cost.burden <= 0.0851
0.50 < Flu.vaccinations
Uninsured.children <= 0.0419
9.0 < Air.pollution...particulate.matter
Percentage.of.households.with.high.housing.costs <= 0.0838
−0.06
−0.04
−0.02
0.00
Weight
Positive
Negative

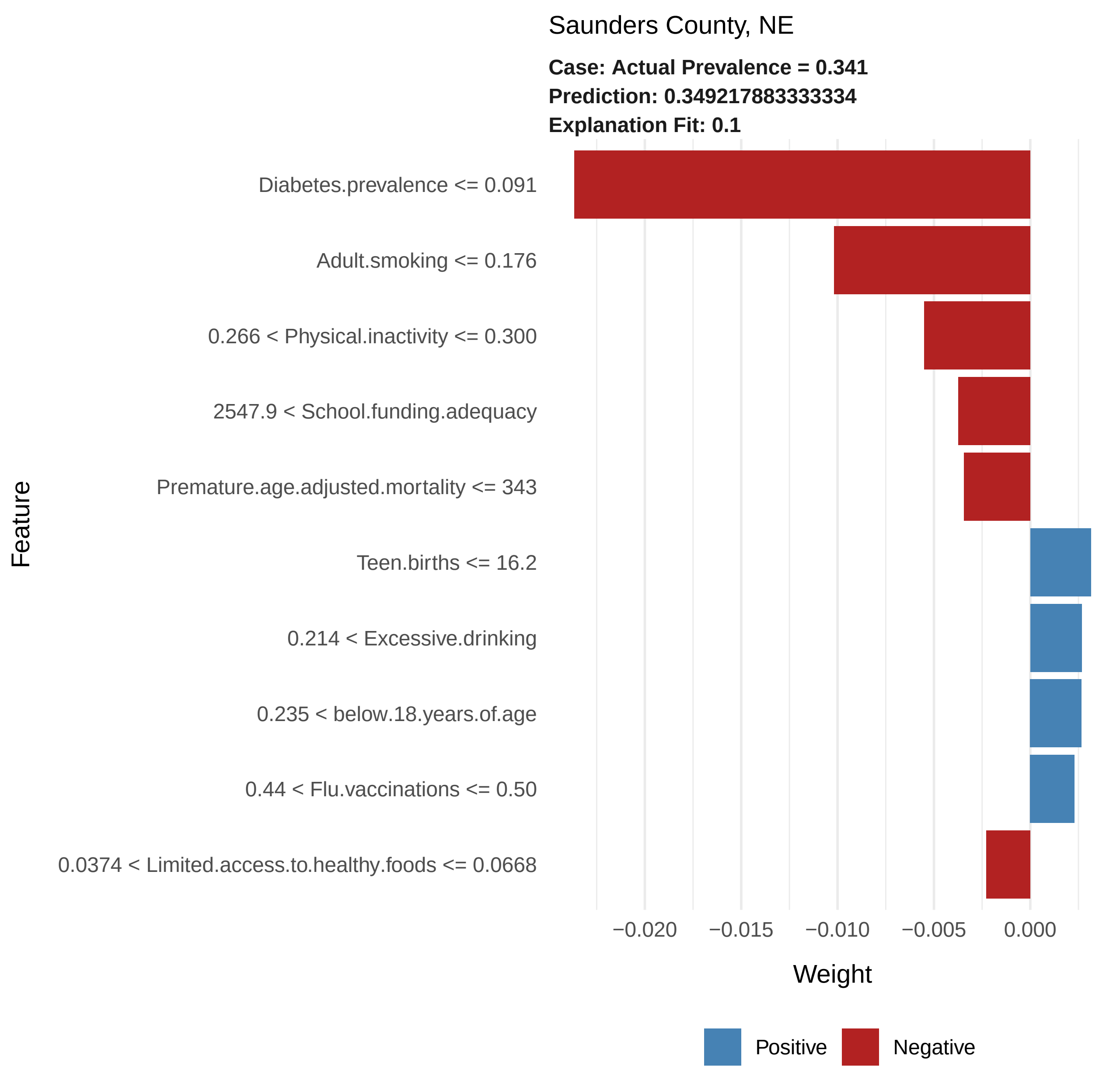
Saunders County, NE
Case: Actual Prevalence = 0.341
Prediction: 0.349217883333334
Explanation Fit: 0.1
Diabetes.prevalence <= 0.091
Adult.smoking <= 0.176
0.266 < Physical.inactivity <= 0.300
2547.9 < School.funding.adequacy
Premature.age.adjusted.mortality <= 343
Teen.births <= 16.2
0.214 < Excessive.drinking
0.235 < below.18.years.of.age
0.44 < Flu.vaccinations <= 0.50
0.0374 < Limited.access.to.healthy.foods <= 0.0668
Feature
−0.020
−0.015
−0.010
−0.005
0.000
Weight
Positive
Negative

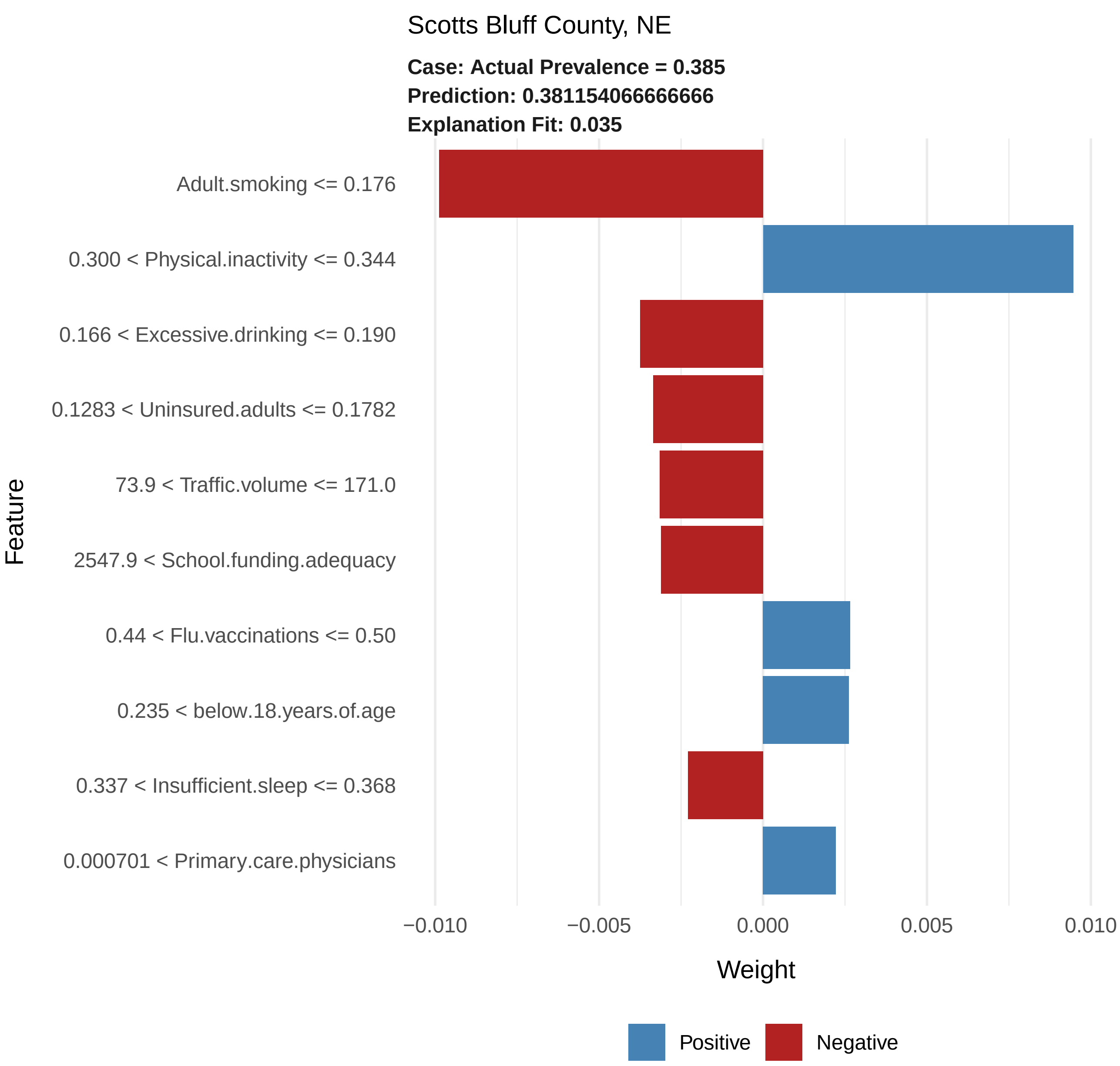

Scotts Bluff County, NE
Case: Actual Prevalence = 0.385
Prediction: 0.381154066666666
Explanation Fit: 0.035
Feature
Adult.smoking <= 0.176
0.300 < Physical.inactivity <= 0.344
0.166 < Excessive.drinking <= 0.190
0.1283 < Uninsured.adults <= 0.1782
73.9 < Traffic.volume <= 171.0
2547.9 < School.funding.adequacy
0.44 < Flu.vaccinations <= 0.50
0.235 < below.18.years.of.age
0.337 < Insufficient.sleep <= 0.368
0.000701 < Primary.care.physicians
−0.010
−0.005
0.000
0.005
0.010
Weight
Positive
Negative

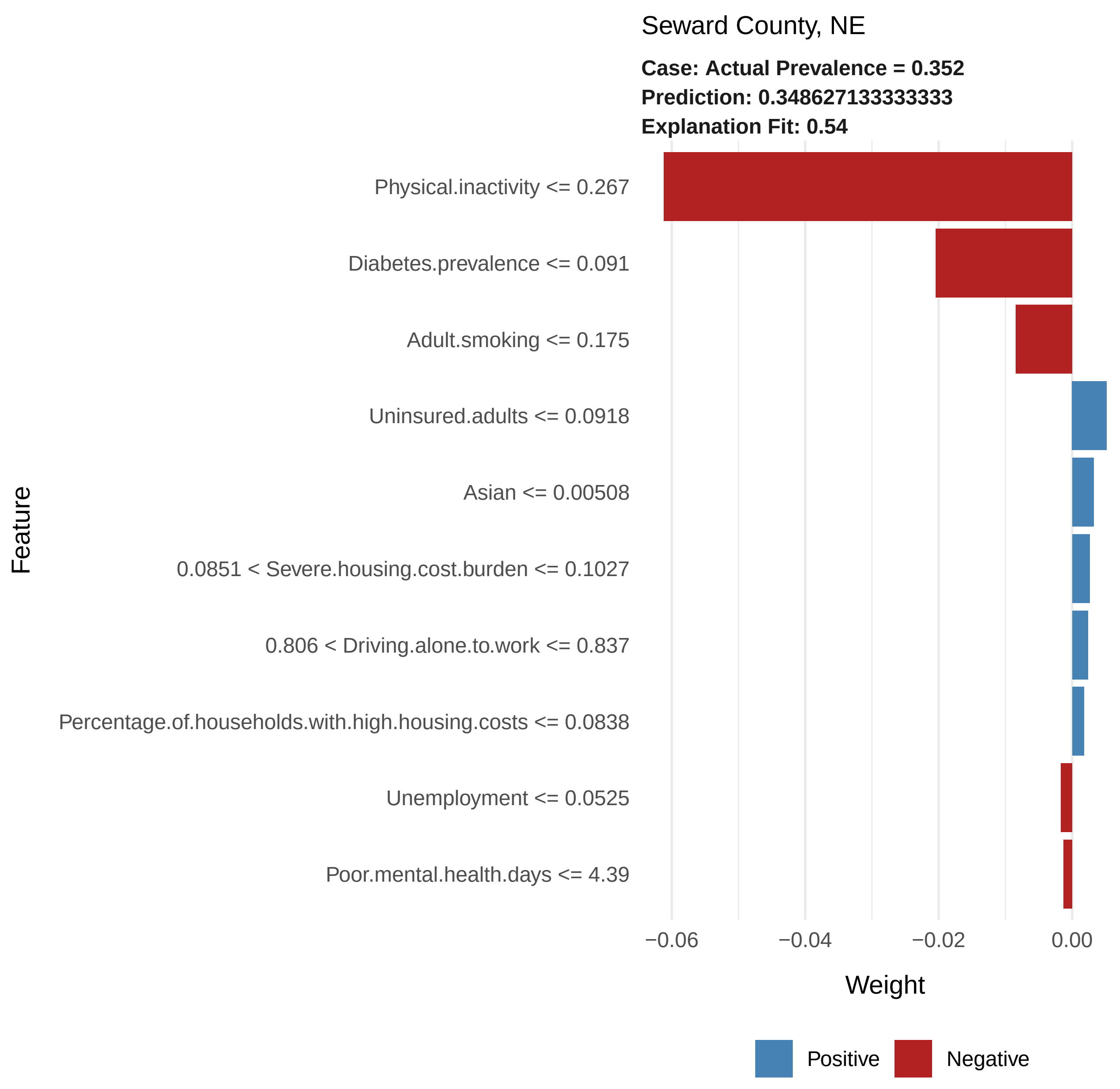

Seward County, NE
Case: Actual Prevalence = 0.352
Prediction: 0.348627133333333
Explanation Fit: 0.54
Physical.inactivity <= 0.267
Diabetes.prevalence <= 0.091
Adult.smoking <= 0.175
Uninsured.adults <= 0.0918
Asian <= 0.00508
0.0851 < Severe.housing.cost.burden <= 0.1027
0.806 < Driving.alone.to.work <= 0.837
Percentage.of.households.with.high.housing.costs <= 0.0838
Unemployment <= 0.0525
Poor.mental.health.days <= 4.39
Feature
−0.06
−0.04
−0.02
0.00
Weight
Positive
Negative

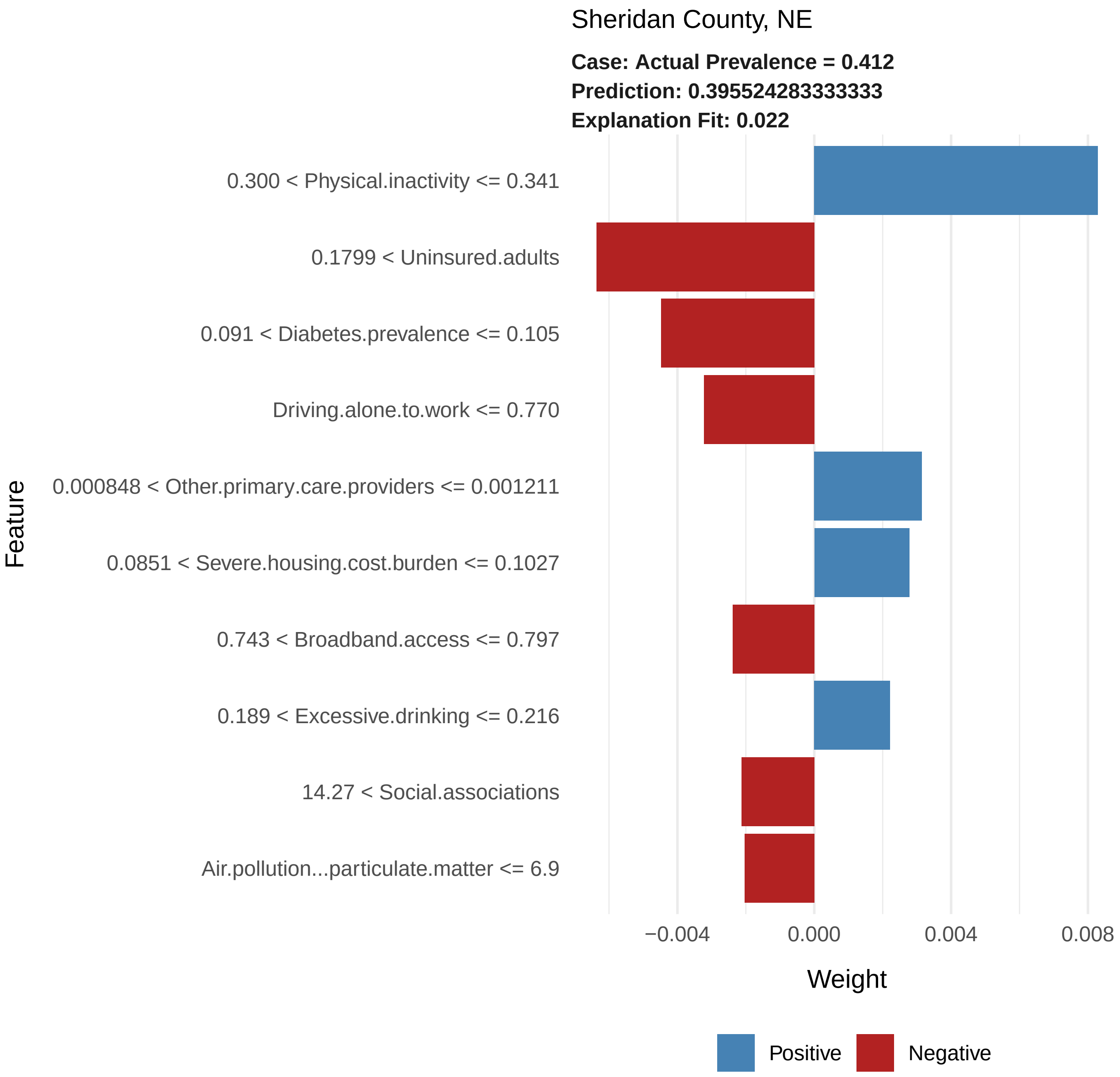

Sheridan County, NE
Case: Actual Prevalence = 0.412
Prediction: 0.395524283333333
Explanation Fit: 0.022
0.300 < Physical.inactivity <= 0.341
0.1799 < Uninsured.adults
0.091 < Diabetes.prevalence <= 0.105
Driving.alone.to.work <= 0.770
0.000848 < Other.primary.care.providers <= 0.001211
0.0851 < Severe.housing.cost.burden <= 0.1027
0.743 < Broadband.access <= 0.797
0.189 < Excessive.drinking <= 0.216
14.27 < Social.associations
Air.pollution...particulate.matter <= 6.9
Feature
−0.004
0.000
0.004
0.008
Weight
Positive
Negative

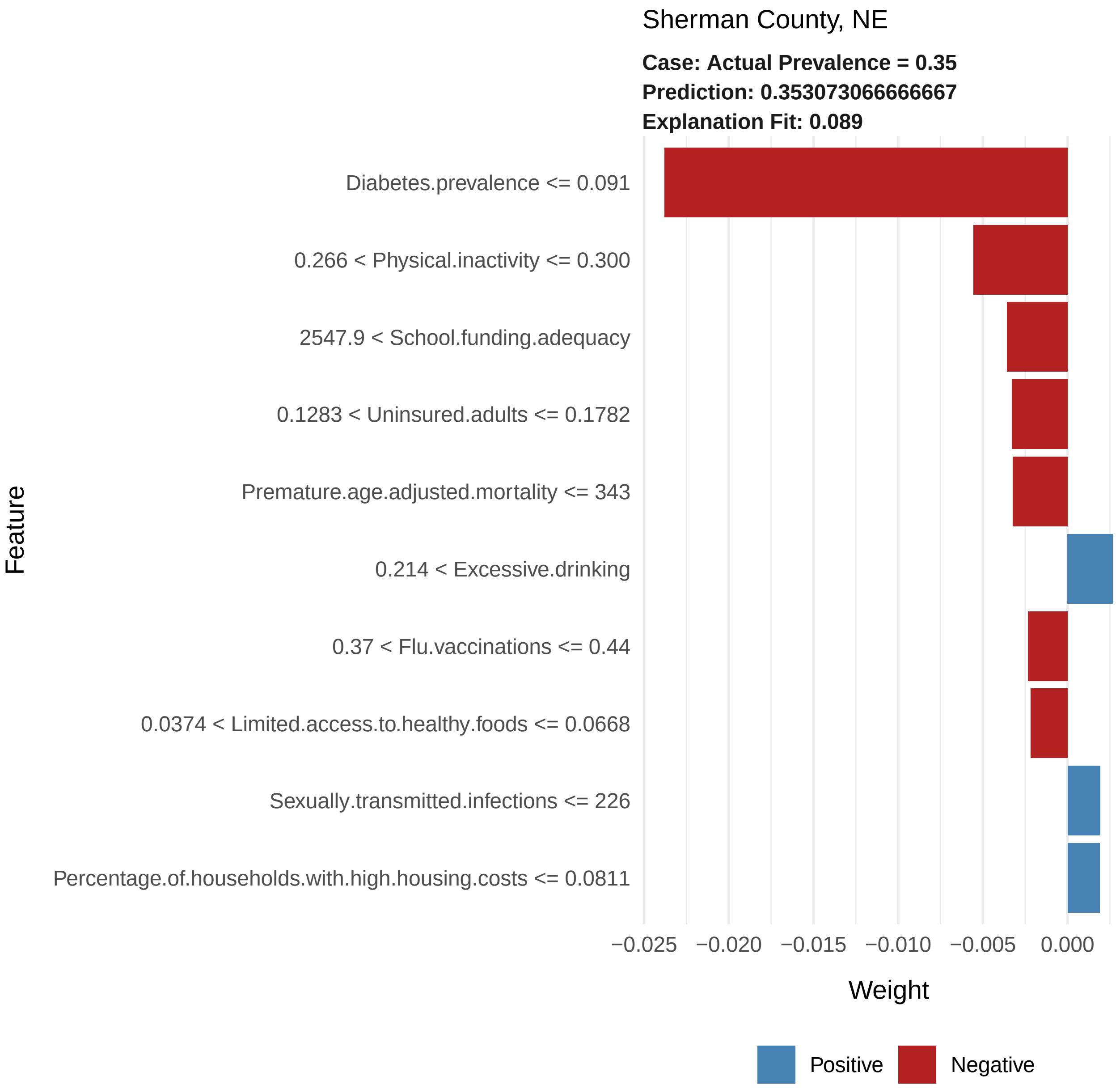

Sherman County, NE
Case: Actual Prevalence = 0.35
Prediction: 0.353073066666667
Explanation Fit: 0.089
Feature
Diabetes.prevalence <= 0.091
0.266 < Physical.inactivity <= 0.300
2547.9 < School.funding.adequacy
0.1283 < Uninsured.adults <= 0.1782
Premature.age.adjusted.mortality <= 343
0.214 < Excessive.drinking
0.37 < Flu.vaccinations <= 0.44
0.0374 < Limited.access.to.healthy.foods <= 0.0668
Sexually.transmitted.infections <= 226
Percentage.of.households.with.high.housing.costs <= 0.0811
−0.025
−0.020
−0.015
−0.010
−0.005
0.000
Weight
Positive
Negative

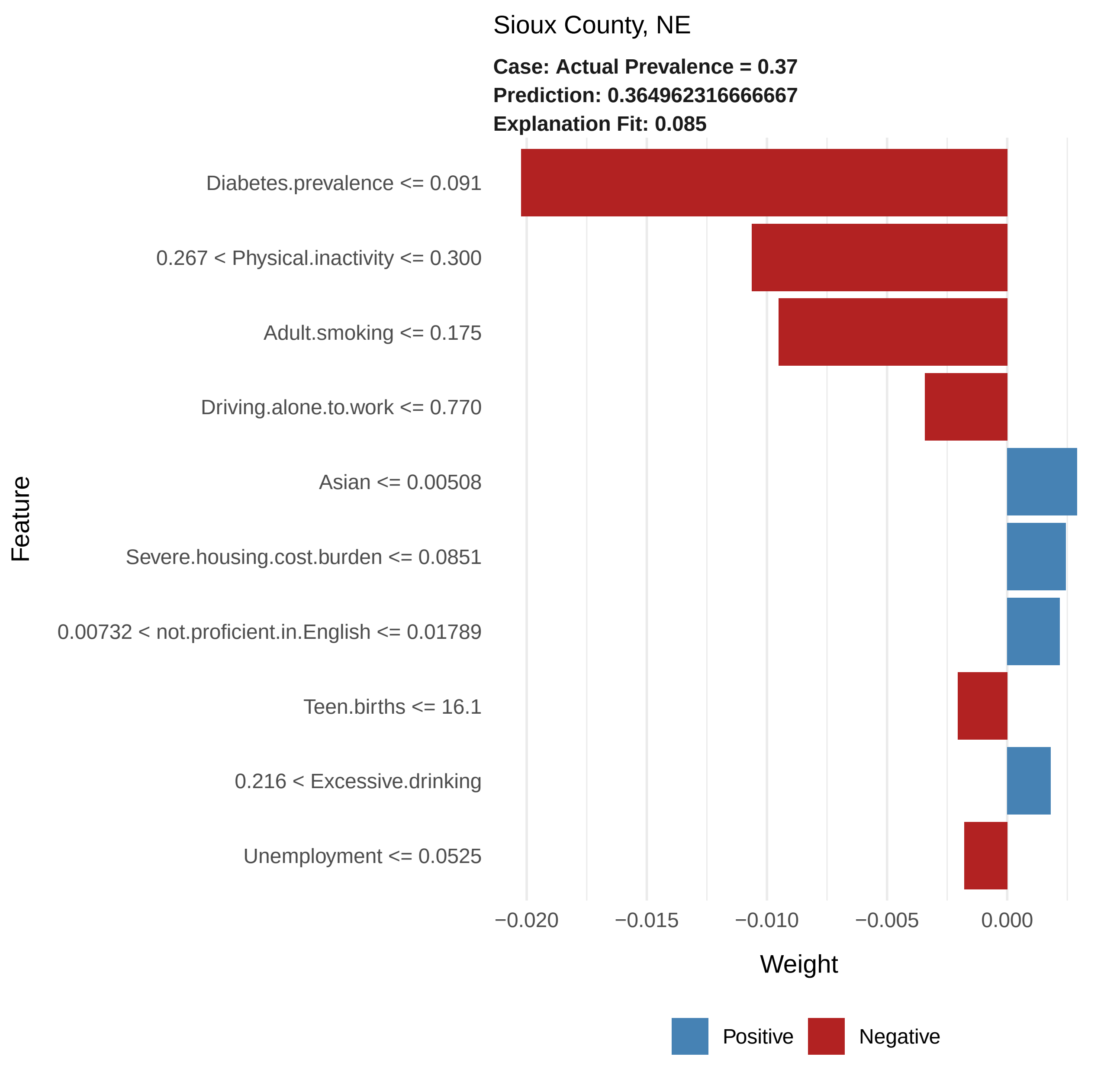
Sioux County, NE
Case: Actual Prevalence = 0.37
Prediction: 0.364962316666667
Explanation Fit: 0.085
Diabetes.prevalence <= 0.091
0.267 < Physical.inactivity <= 0.300
Adult.smoking <= 0.175
Driving.alone.to.work <= 0.770
Asian <= 0.00508
Severe.housing.cost.burden <= 0.0851
0.00732 < not.proficient.in.English <= 0.01789
Teen.births <= 16.1
0.216 < Excessive.drinking
Unemployment <= 0.0525
Feature
−0.020
−0.015
−0.010
−0.005
0.000
Weight
Positive
Negative

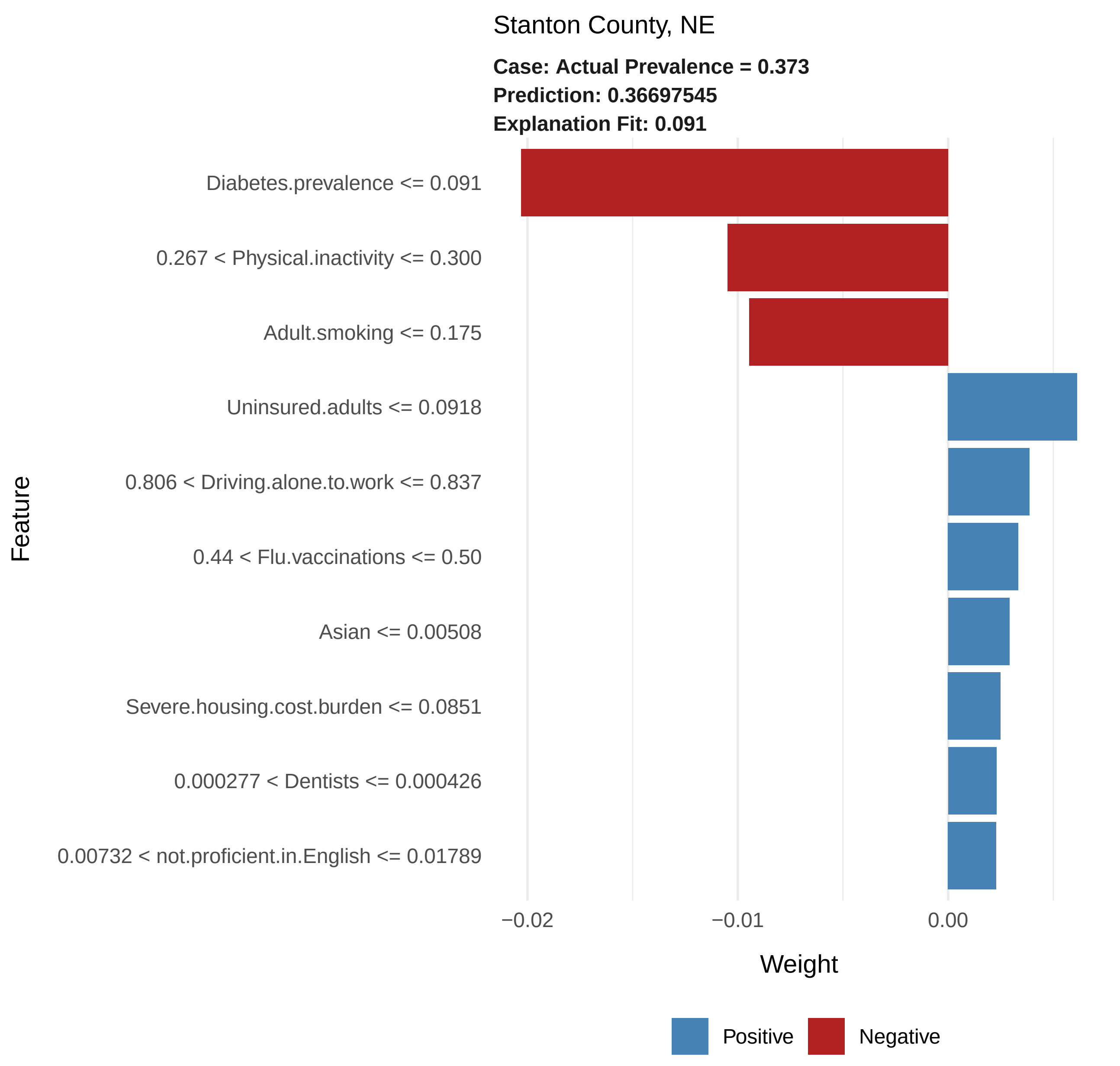

Stanton County, NE
Case: Actual Prevalence = 0.373
Prediction: 0.36697545
Explanation Fit: 0.091
Diabetes.prevalence <= 0.091
0.267 < Physical.inactivity <= 0.300
Adult.smoking <= 0.175
Uninsured.adults <= 0.0918
0.806 < Driving.alone.to.work <= 0.837
0.44 < Flu.vaccinations <= 0.50
Asian <= 0.00508
Severe.housing.cost.burden <= 0.0851
0.000277 < Dentists <= 0.000426
0.00732 < not.proficient.in.English <= 0.01789
Feature
−0.02
−0.01
0.00
Weight
Positive
Negative

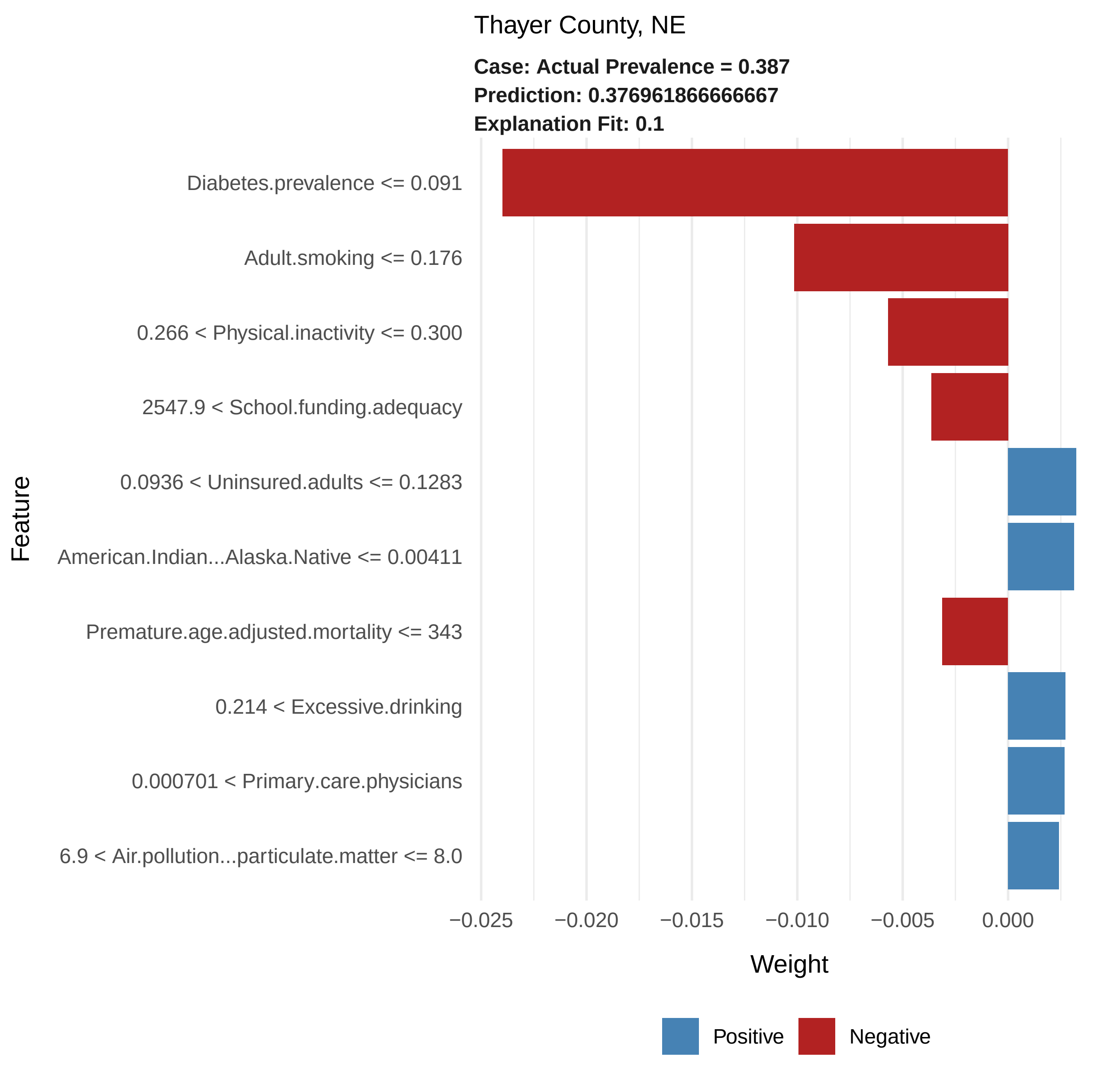

Thayer County, NE
Case: Actual Prevalence = 0.387
Prediction: 0.376961866666667
Explanation Fit: 0.1
Diabetes.prevalence <= 0.091
Adult.smoking <= 0.176
0.266 < Physical.inactivity <= 0.300
2547.9 < School.funding.adequacy
0.0936 < Uninsured.adults <= 0.1283
American.Indian...Alaska.Native <= 0.00411
Premature.age.adjusted.mortality <= 343
0.214 < Excessive.drinking
0.000701 < Primary.care.physicians
6.9 < Air.pollution...particulate.matter <= 8.0
Feature
−0.025
−0.020
−0.015
−0.010
−0.005
0.000
Weight
Positive
Negative

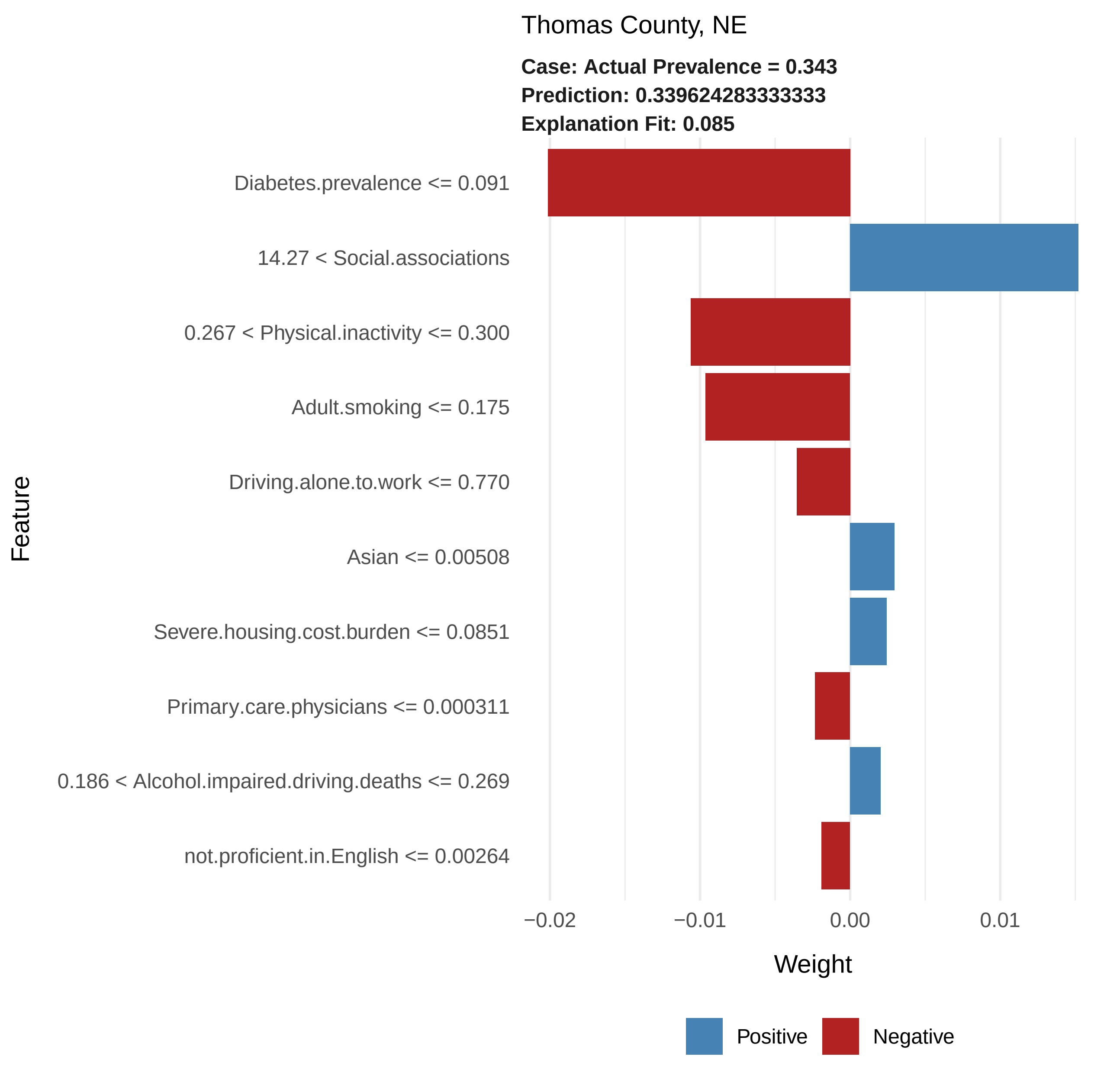

Thomas County, NE
Case: Actual Prevalence = 0.343
Prediction: 0.339624283333333
Explanation Fit: 0.085
Feature
Diabetes.prevalence <= 0.091
14.27 < Social.associations
0.267 < Physical.inactivity <= 0.300
Adult.smoking <= 0.175
Driving.alone.to.work <= 0.770
Asian <= 0.00508
Severe.housing.cost.burden <= 0.0851
Primary.care.physicians <= 0.000311
0.186 < Alcohol.impaired.driving.deaths <= 0.269
not.proficient.in.English <= 0.00264
−0.02
−0.01
0.00
0.01
Weight
Positive
Negative

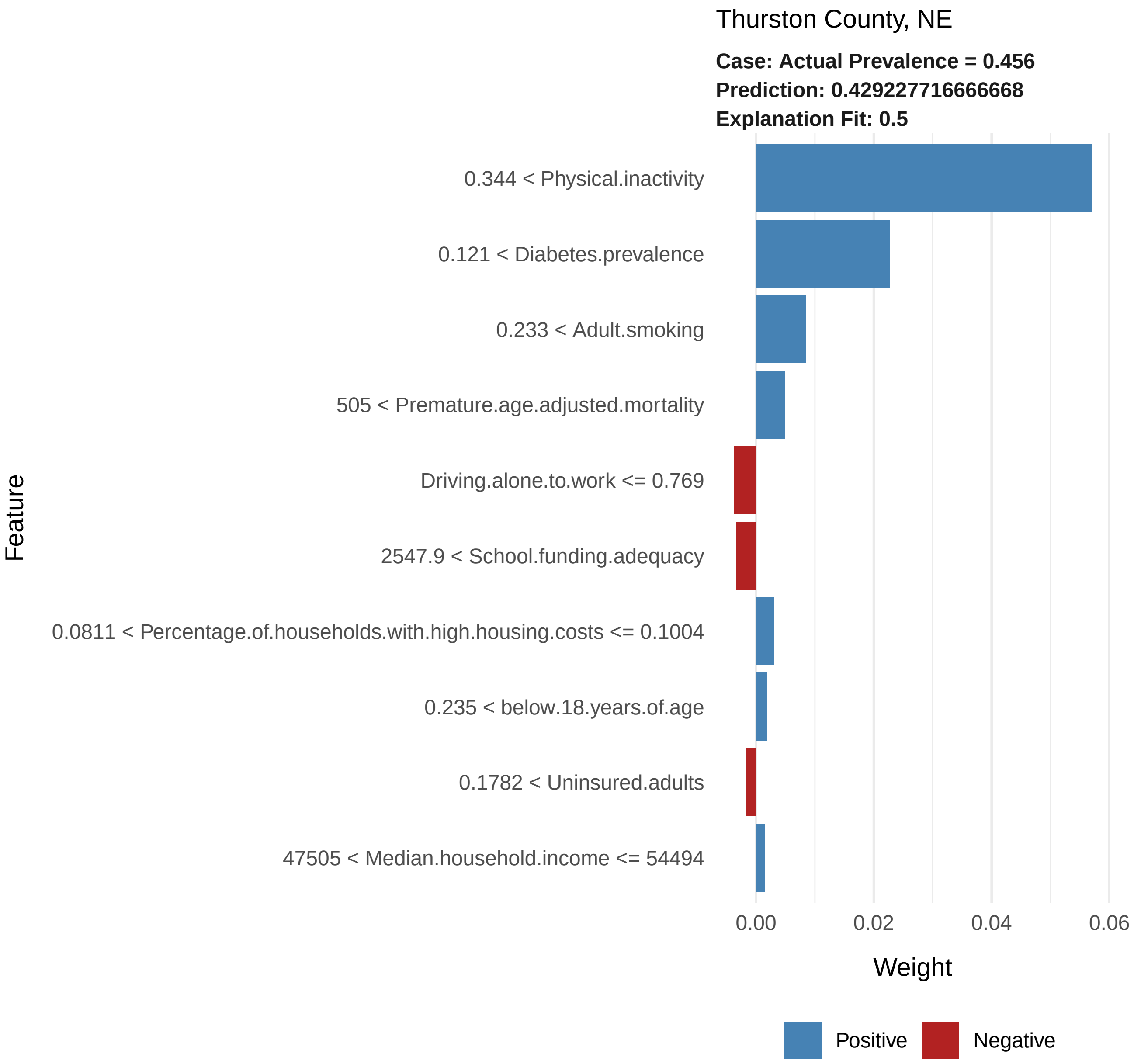

Thurston County, NE
Case: Actual Prevalence = 0.456
Prediction: 0.429227716666668
Explanation Fit: 0.5
0.344 < Physical.inactivity
0.121 < Diabetes.prevalence
0.233 < Adult.smoking
505 < Premature.age.adjusted.mortality
Driving.alone.to.work <= 0.769
2547.9 < School.funding.adequacy
0.0811 < Percentage.of.households.with.high.housing.costs <= 0.1004
0.235 < below.18.years.of.age
0.1782 < Uninsured.adults
47505 < Median.household.income <= 54494
Feature
0.00
0.02
0.04
0.06
Weight
Positive
Negative

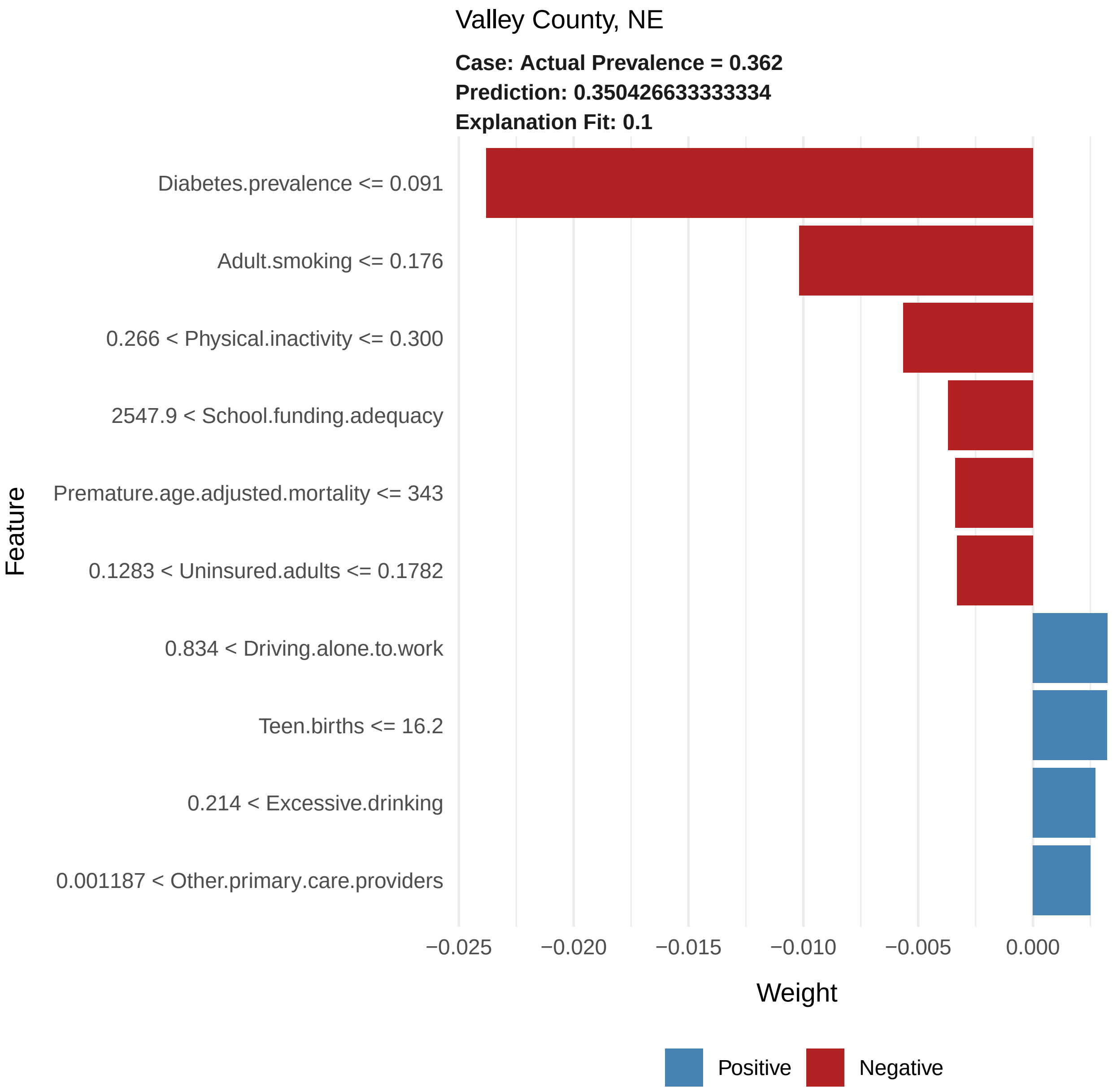

Valley County, NE
Case: Actual Prevalence = 0.362
Prediction: 0.350426633333334
Explanation Fit: 0.1
Diabetes.prevalence <= 0.091
Adult.smoking <= 0.176
0.266 < Physical.inactivity <= 0.300
2547.9 < School.funding.adequacy
Premature.age.adjusted.mortality <= 343
0.1283 < Uninsured.adults <= 0.1782
0.834 < Driving.alone.to.work
Teen.births <= 16.2
0.214 < Excessive.drinking
0.001187 < Other.primary.care.providers
Feature
−0.025
−0.020
−0.015
−0.010
−0.005
0.000
Weight
Positive
Negative

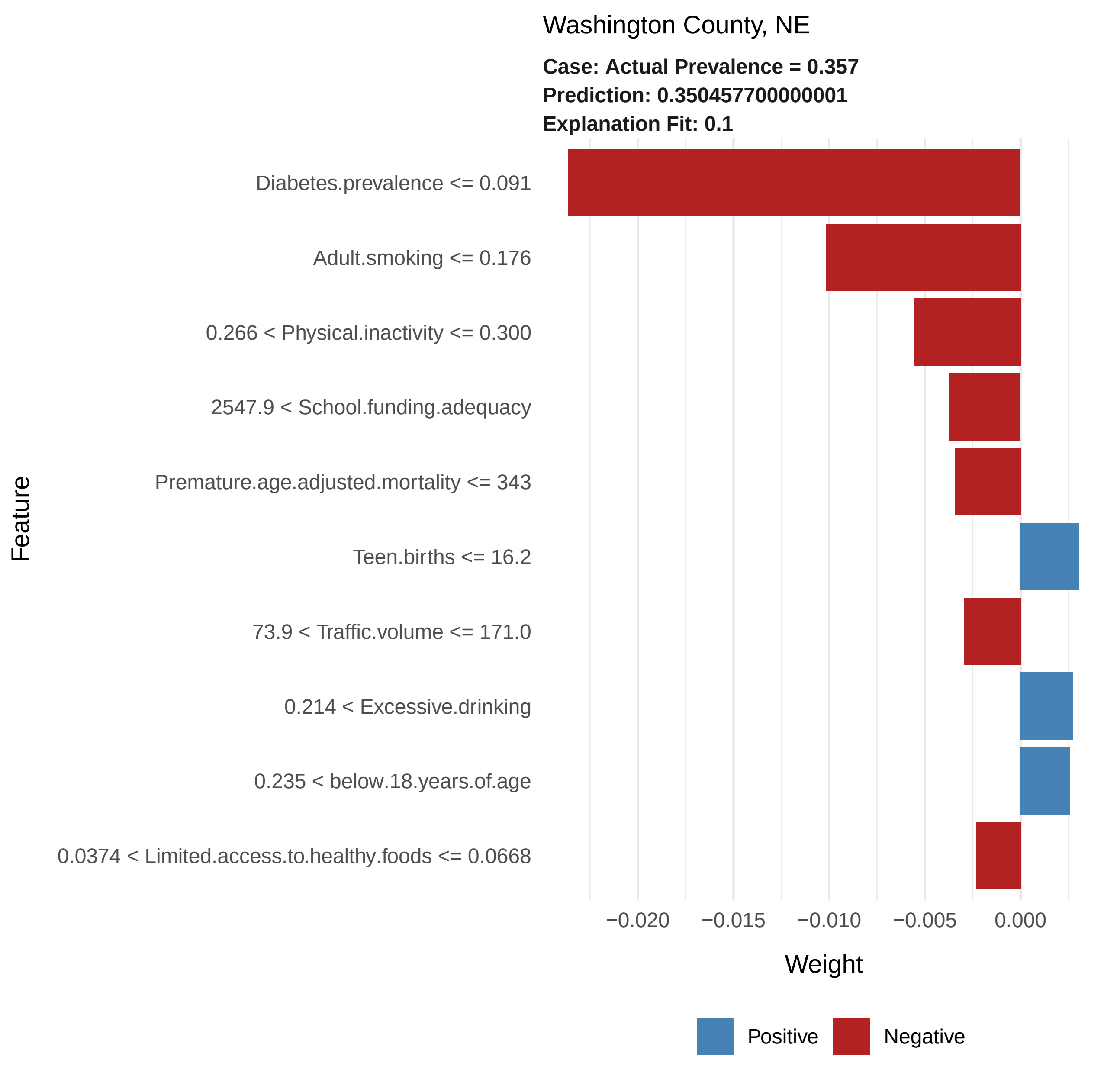

Washington County, NE
Case: Actual Prevalence = 0.357
Prediction: 0.350457700000001
Explanation Fit: 0.1
Feature
Diabetes.prevalence <= 0.091
Adult.smoking <= 0.176
0.266 < Physical.inactivity <= 0.300
2547.9 < School.funding.adequacy
Premature.age.adjusted.mortality <= 343
Teen.births <= 16.2
73.9 < Traffic.volume <= 171.0
0.214 < Excessive.drinking
0.235 < below.18.years.of.age
0.0374 < Limited.access.to.healthy.foods <= 0.0668
−0.020
−0.015
−0.010
−0.005
0.000
Weight
Positive
Negative

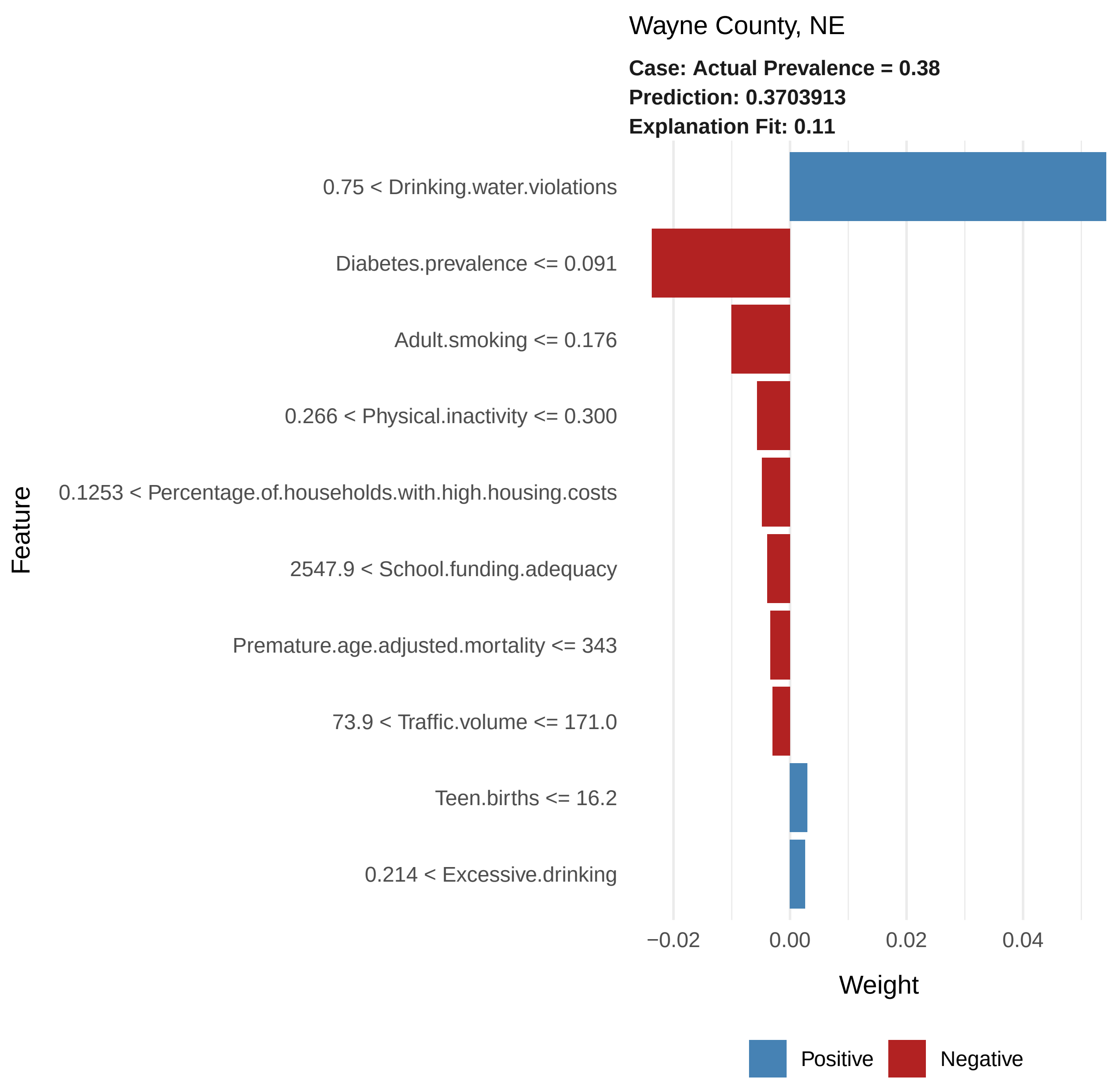
Wayne County, NE
Case: Actual Prevalence = 0.38
Prediction: 0.3703913
Explanation Fit: 0.11
0.75 < Drinking.water.violations
Diabetes.prevalence <= 0.091
Adult.smoking <= 0.176
0.266 < Physical.inactivity <= 0.300
0.1253 < Percentage.of.households.with.high.housing.costs
2547.9 < School.funding.adequacy
Premature.age.adjusted.mortality <= 343
73.9 < Traffic.volume <= 171.0
Teen.births <= 16.2
0.214 < Excessive.drinking
Feature
−0.02
0.00
0.02
0.04
Weight
Positive
Negative

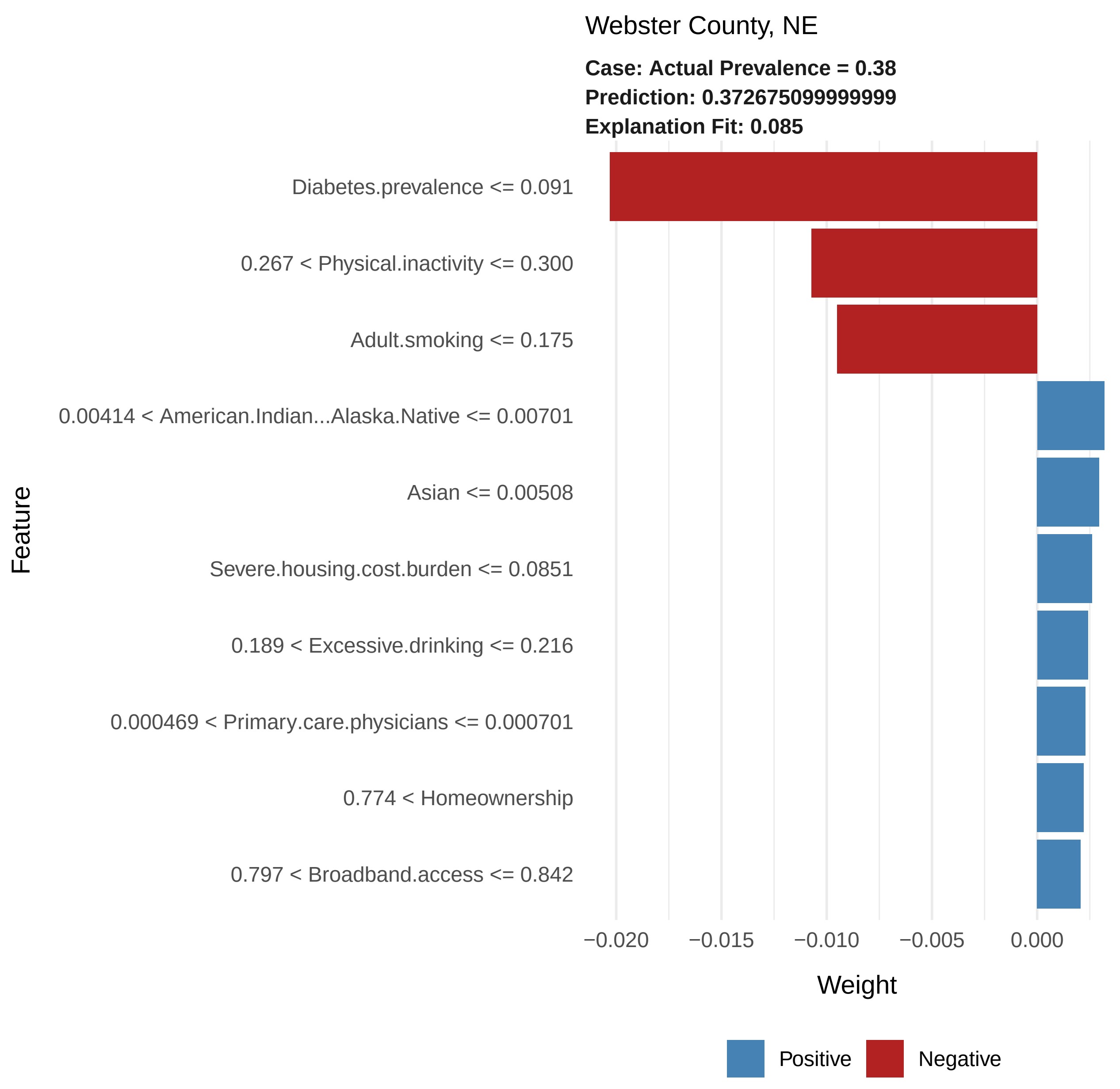
Webster County, NE
Case: Actual Prevalence = 0.38
Prediction: 0.372675099999999
Explanation Fit: 0.085
Diabetes.prevalence <= 0.091
0.267 < Physical.inactivity <= 0.300
Adult.smoking <= 0.175
0.00414 < American.Indian...Alaska.Native <= 0.00701
Asian <= 0.00508
Severe.housing.cost.burden <= 0.0851
0.189 < Excessive.drinking <= 0.216
0.000469 < Primary.care.physicians <= 0.000701
0.774 < Homeownership
0.797 < Broadband.access <= 0.842
Feature
−0.020
−0.015
−0.010
−0.005
0.000
Weight
Positive
Negative

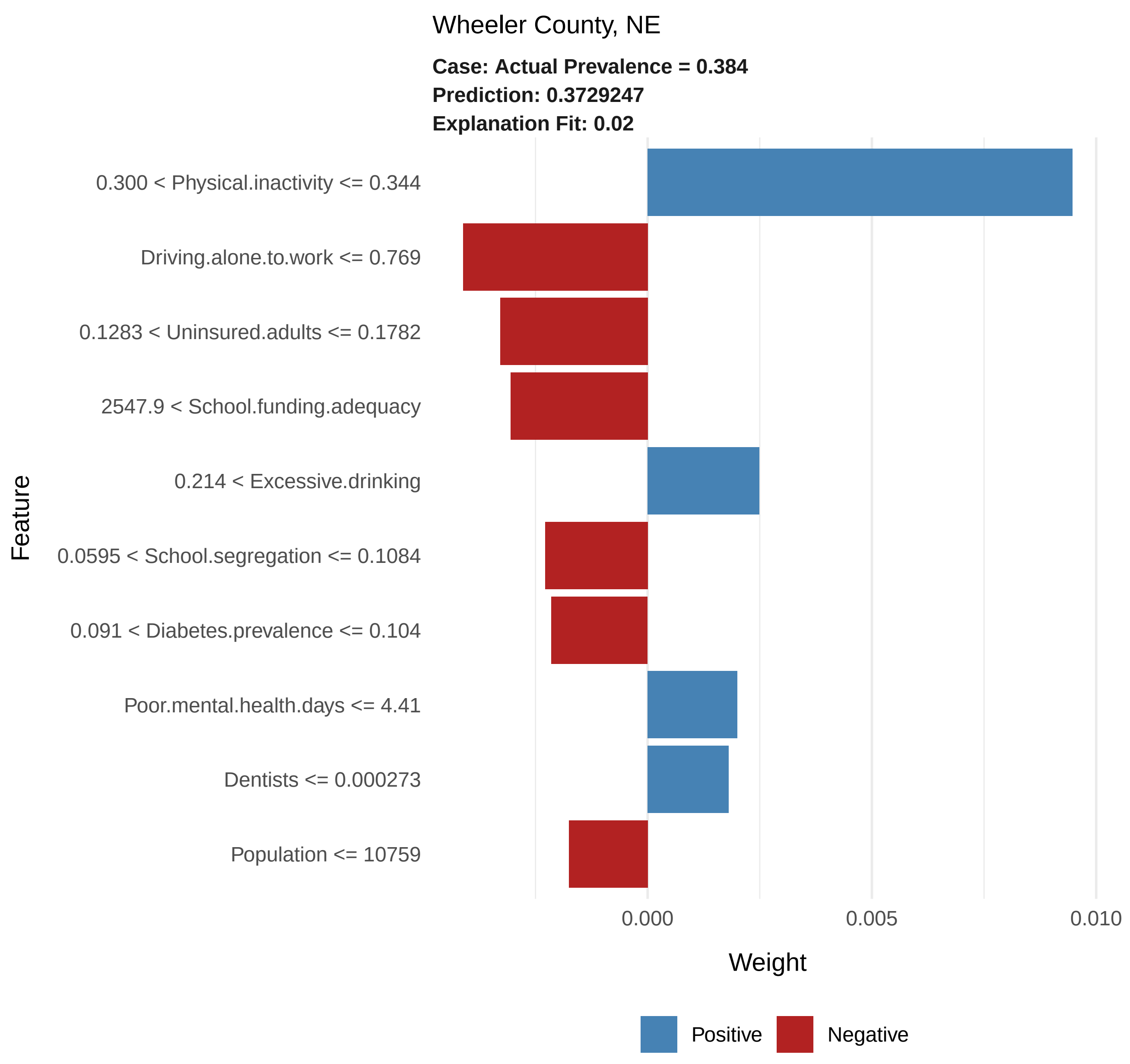

Wheeler County, NE
Case: Actual Prevalence = 0.384
Prediction: 0.3729247
Explanation Fit: 0.02
0.300 < Physical.inactivity <= 0.344
Driving.alone.to.work <= 0.769
0.1283 < Uninsured.adults <= 0.1782
2547.9 < School.funding.adequacy
0.214 < Excessive.drinking
0.0595 < School.segregation <= 0.1084
0.091 < Diabetes.prevalence <= 0.104
Poor.mental.health.days <= 4.41
Dentists <= 0.000273
Population <= 10759
Feature
0.000
0.005
0.010
Weight
Positive
Negative

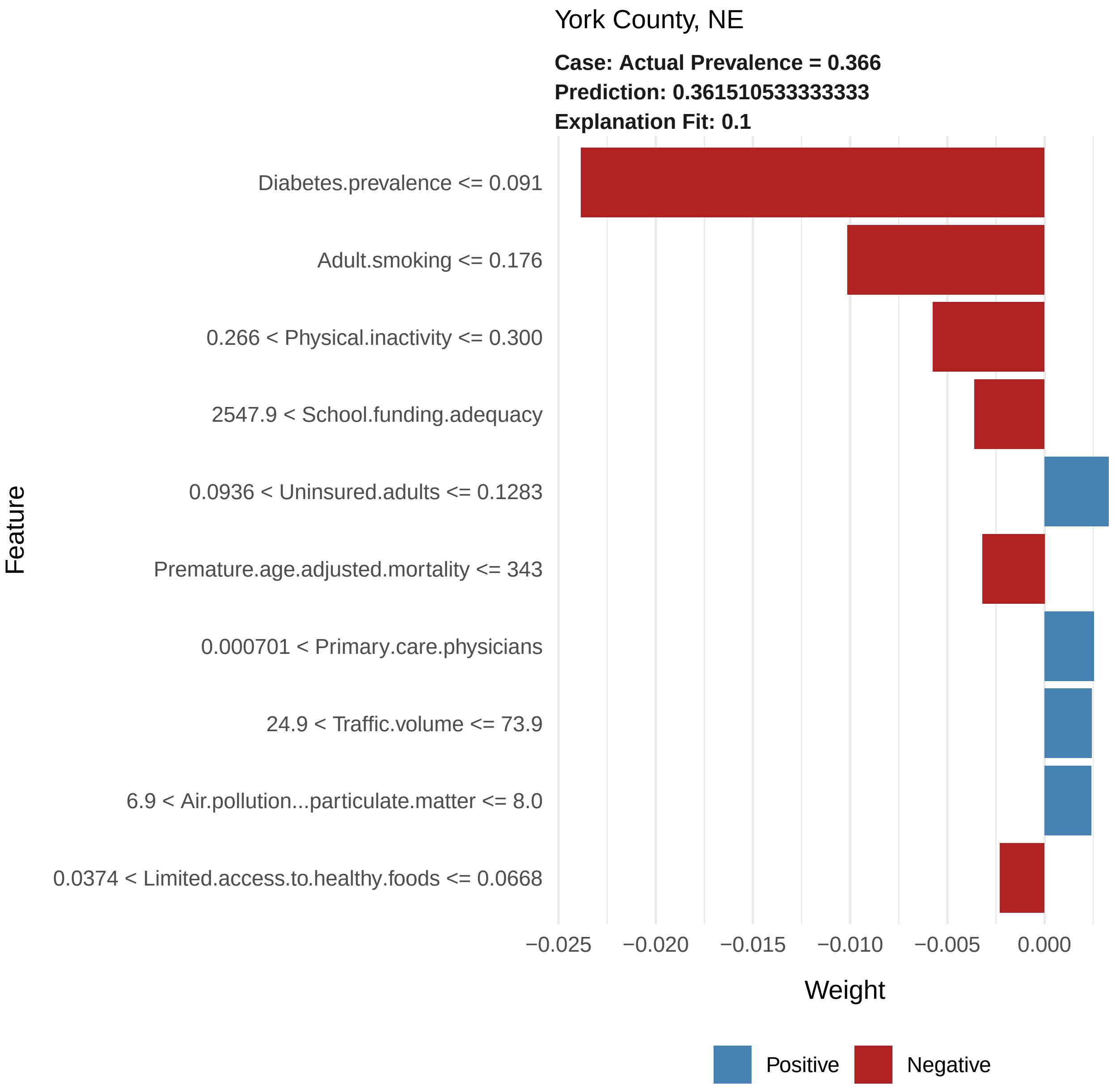

York County, NE
Case: Actual Prevalence = 0.366
Prediction: 0.361510533333333
Explanation Fit: 0.1
Diabetes.prevalence <= 0.091
Adult.smoking <= 0.176
0.266 < Physical.inactivity <= 0.300
2547.9 < School.funding.adequacy
0.0936 < Uninsured.adults <= 0.1283
Premature.age.adjusted.mortality <= 343
0.000701 < Primary.care.physicians
24.9 < Traffic.volume <= 73.9
6.9 < Air.pollution...particulate.matter <= 8.0
0.0374 < Limited.access.to.healthy.foods <= 0.0668
Feature
−0.025
−0.020
−0.015
−0.010
−0.005
0.000
Weight
Positive
Negative

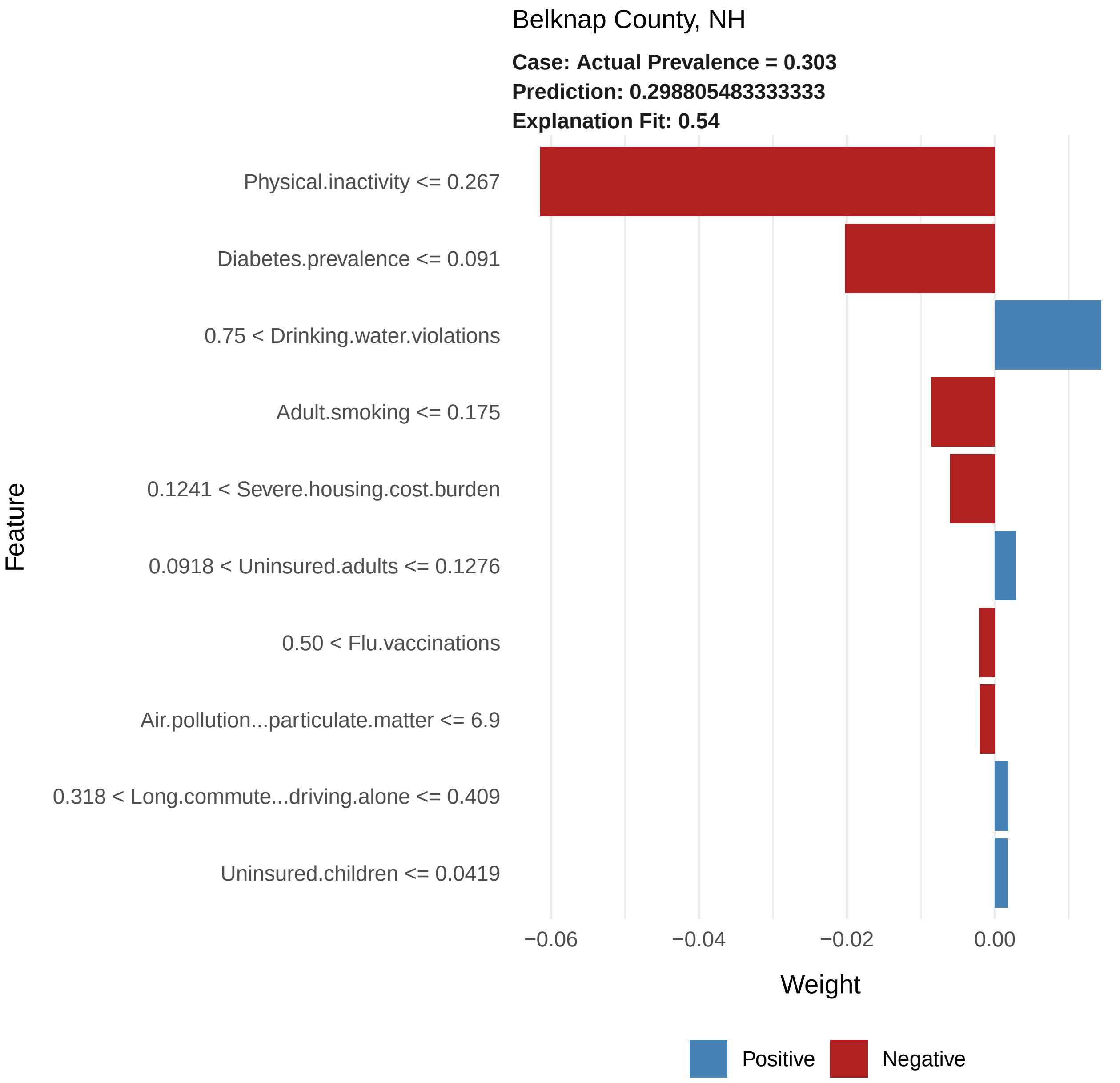
Belknap County, NH
Case: Actual Prevalence = 0.303
Prediction: 0.298805483333333
Explanation Fit: 0.54
Physical.inactivity <= 0.267
Diabetes.prevalence <= 0.091
0.75 < Drinking.water.violations
Adult.smoking <= 0.175
0.1241 < Severe.housing.cost.burden
0.0918 < Uninsured.adults <= 0.1276
0.50 < Flu.vaccinations
Air.pollution...particulate.matter <= 6.9
0.318 < Long.commute...driving.alone <= 0.409
Uninsured.children <= 0.0419
Feature
−0.06
−0.04
−0.02
0.00
Weight
Positive
Negative

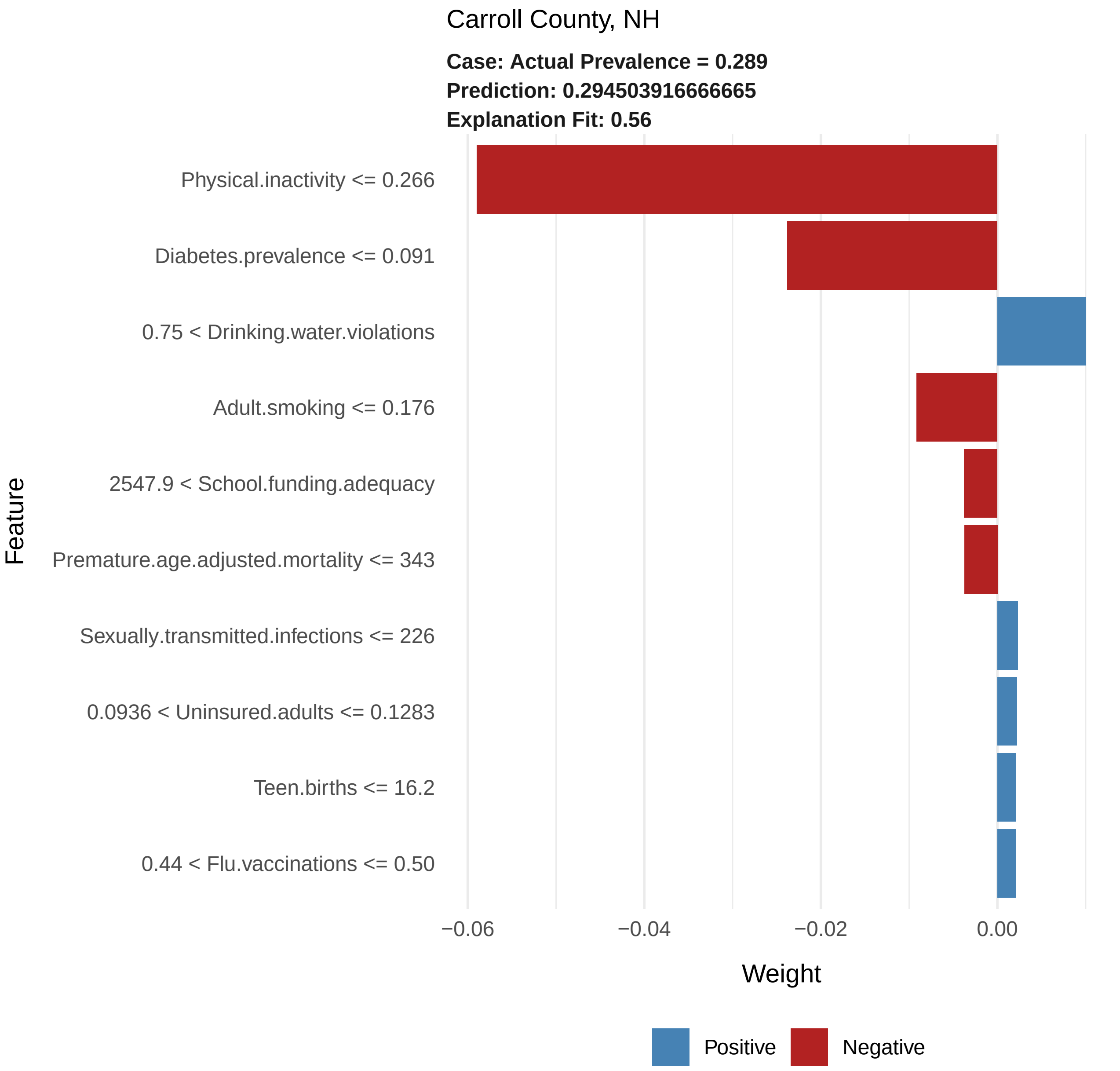

Carroll County, NH
Case: Actual Prevalence = 0.289
Prediction: 0.294503916666665
Explanation Fit: 0.56
Physical.inactivity <= 0.266
Diabetes.prevalence <= 0.091
0.75 < Drinking.water.violations
Adult.smoking <= 0.176
2547.9 < School.funding.adequacy
Premature.age.adjusted.mortality <= 343
Sexually.transmitted.infections <= 226
0.0936 < Uninsured.adults <= 0.1283
Teen.births <= 16.2
0.44 < Flu.vaccinations <= 0.50
Feature
−0.06
−0.04
−0.02
0.00
Weight
Positive
Negative

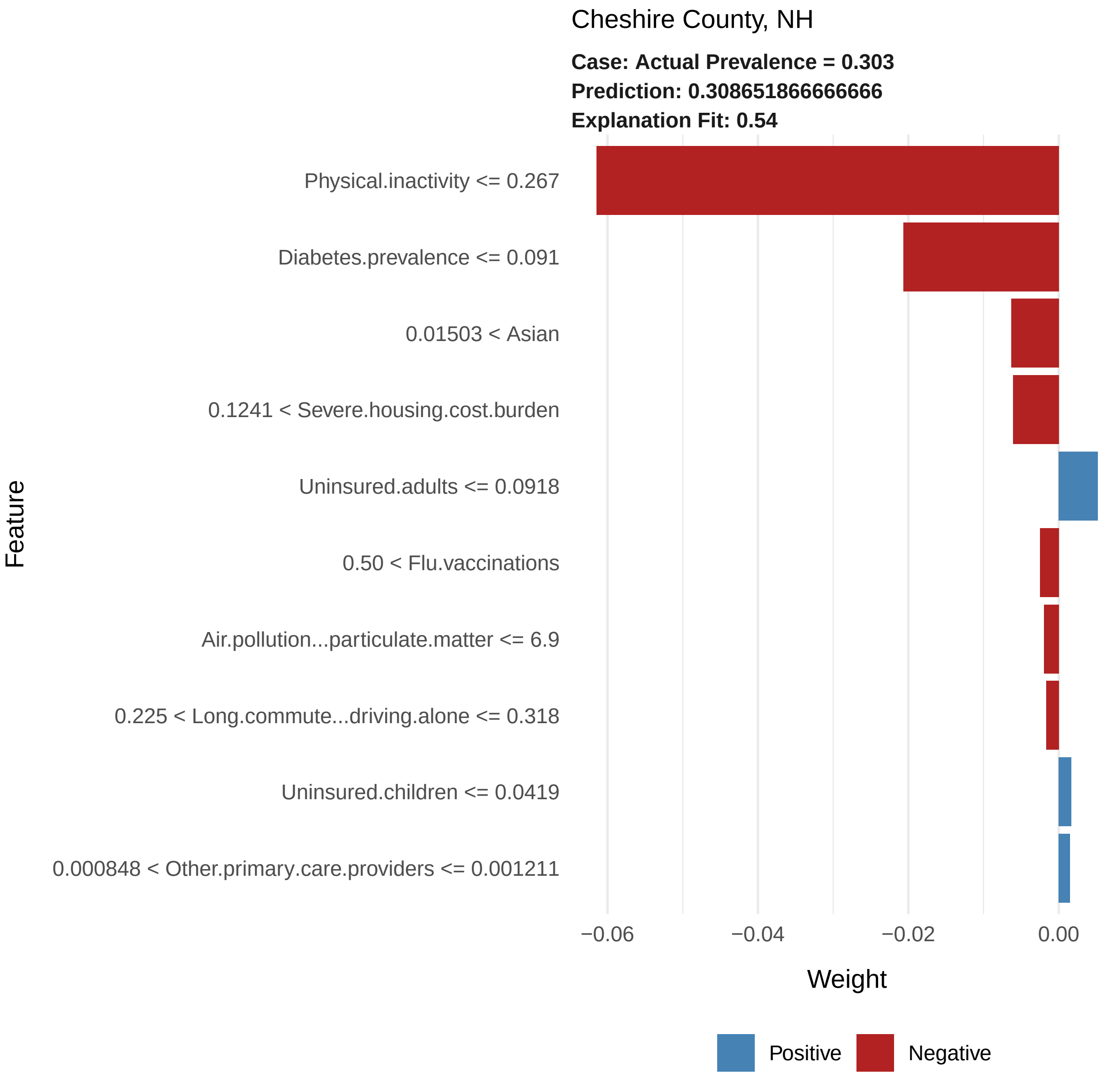
Cheshire County, NH
Case: Actual Prevalence = 0.303
Prediction: 0.308651866666666
Explanation Fit: 0.54
Physical.inactivity <= 0.267
Diabetes.prevalence <= 0.091
0.01503 < Asian
0.1241 < Severe.housing.cost.burden
Uninsured.adults <= 0.0918
0.50 < Flu.vaccinations
Air.pollution...particulate.matter <= 6.9
0.225 < Long.commute...driving.alone <= 0.318
Uninsured.children <= 0.0419
0.000848 < Other.primary.care.providers <= 0.001211
Feature
−0.06
−0.04
−0.02
0.00
Weight
Positive
Negative

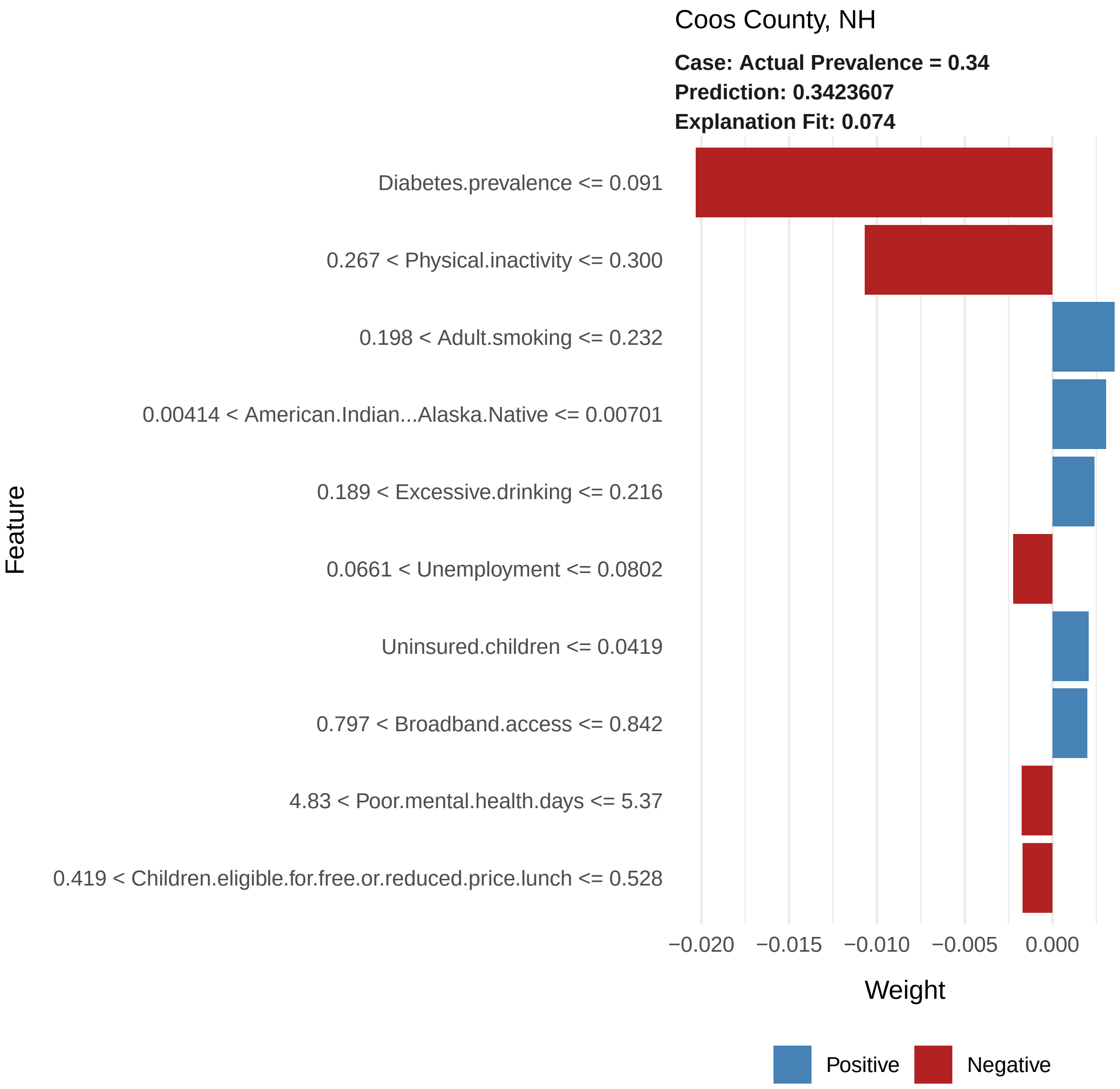
Coos County, NH
Case: Actual Prevalence = 0.34
Prediction: 0.3423607
Explanation Fit: 0.074
Diabetes.prevalence <= 0.091
0.267 < Physical.inactivity <= 0.300
0.198 < Adult.smoking <= 0.232
0.00414 < American.Indian...Alaska.Native <= 0.00701
0.189 < Excessive.drinking <= 0.216
0.0661 < Unemployment <= 0.0802
Uninsured.children <= 0.0419
0.797 < Broadband.access <= 0.842
4.83 < Poor.mental.health.days <= 5.37
0.419 < Children.eligible.for.free.or.reduced.price.lunch <= 0.528
Feature
−0.020
−0.015
−0.010
−0.005
0.000
Weight
Positive
Negative

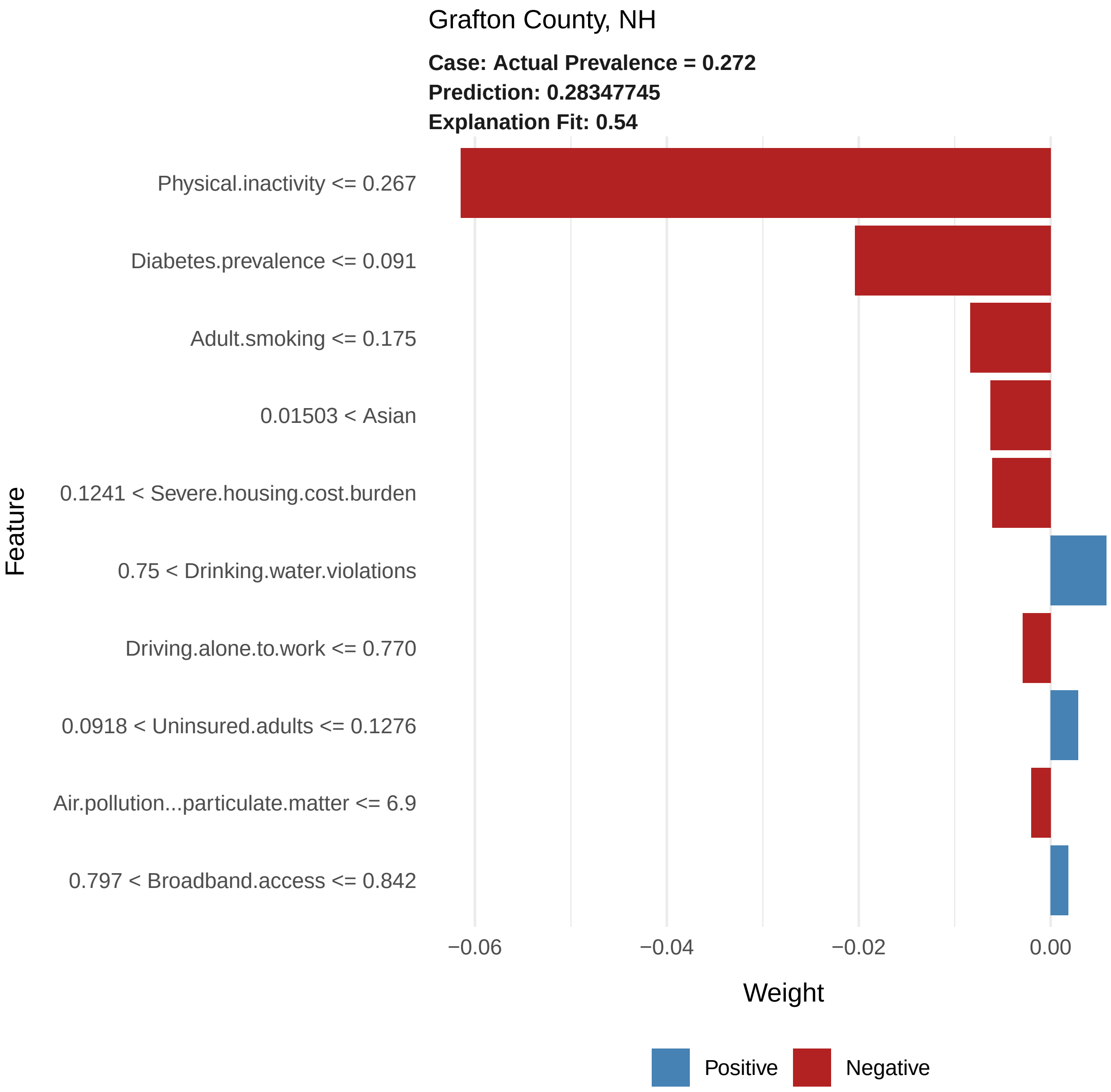

Grafton County, NH
Case: Actual Prevalence = 0.272
Prediction: 0.28347745
Explanation Fit: 0.54
Physical.inactivity <= 0.267
Diabetes.prevalence <= 0.091
Adult.smoking <= 0.175
0.01503 < Asian
0.1241 < Severe.housing.cost.burden
0.75 < Drinking.water.violations
Driving.alone.to.work <= 0.770
0.0918 < Uninsured.adults <= 0.1276
Air.pollution...particulate.matter <= 6.9
0.797 < Broadband.access <= 0.842
Feature
−0.06
−0.04
−0.02
0.00
Weight
Positive
Negative

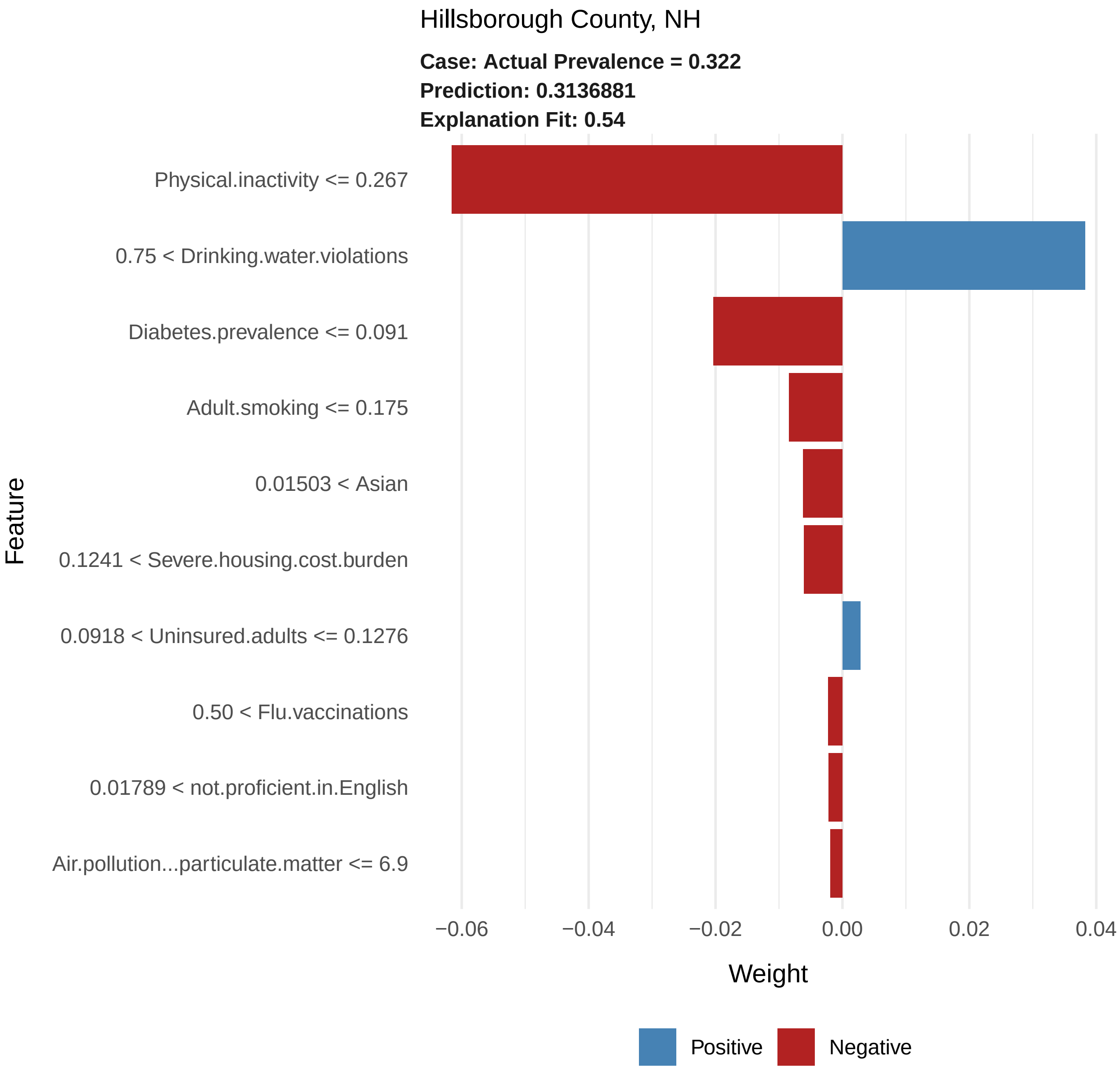
Hillsborough County, NH
Case: Actual Prevalence = 0.322
Prediction: 0.3136881
Explanation Fit: 0.54
Physical.inactivity <= 0.267
0.75 < Drinking.water.violations
Diabetes.prevalence <= 0.091
Adult.smoking <= 0.175
0.01503 < Asian
0.1241 < Severe.housing.cost.burden
0.0918 < Uninsured.adults <= 0.1276
0.50 < Flu.vaccinations
0.01789 < not.proficient.in.English
Air.pollution...particulate.matter <= 6.9
Feature
−0.06
−0.04
−0.02
0.00
0.02
0.04
Weight
Positive
Negative

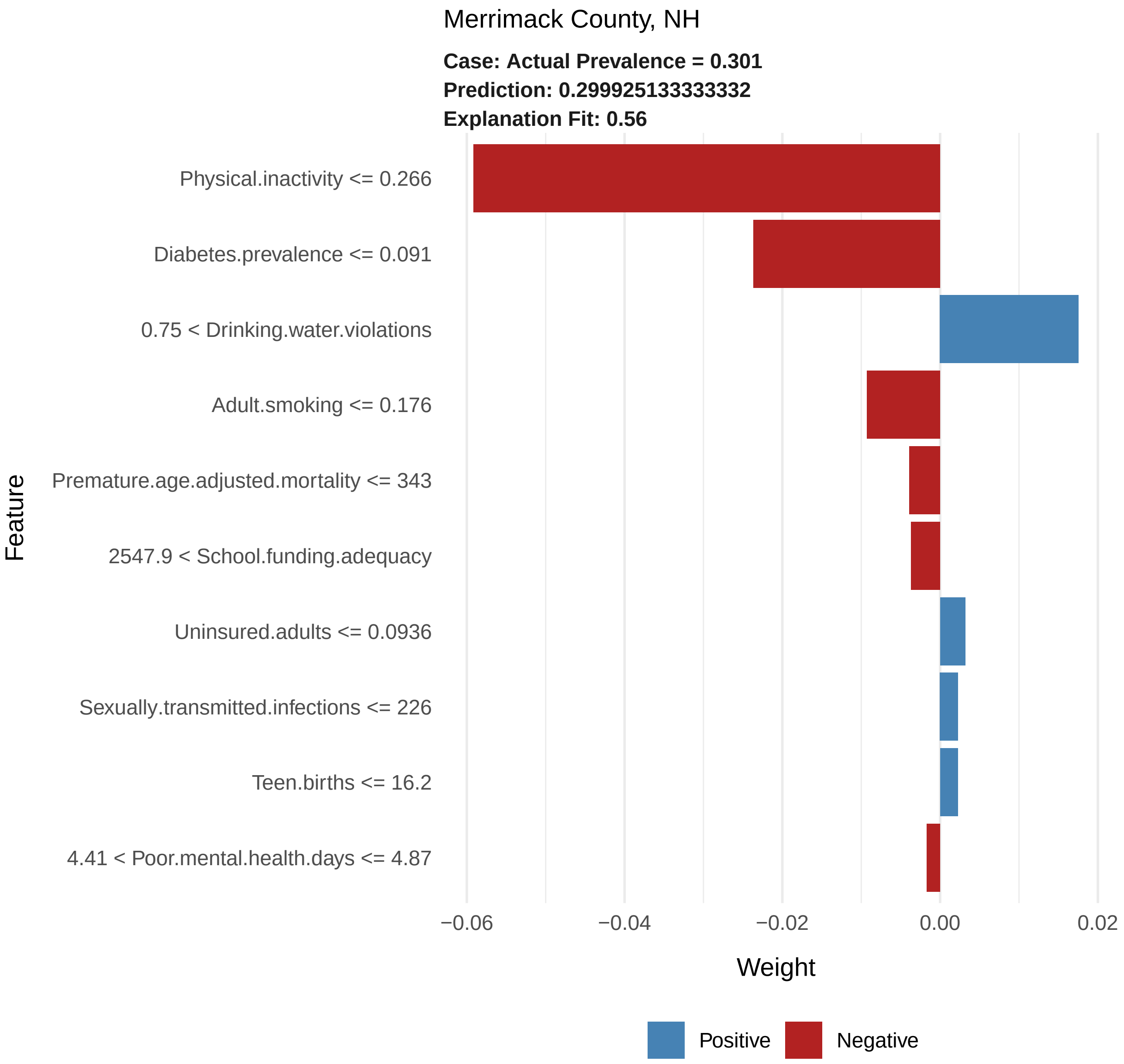
Merrimack County, NH
Case: Actual Prevalence = 0.301
Prediction: 0.299925133333332
Explanation Fit: 0.56
Physical.inactivity <= 0.266
Diabetes.prevalence <= 0.091
0.75 < Drinking.water.violations
Adult.smoking <= 0.176
Premature.age.adjusted.mortality <= 343
2547.9 < School.funding.adequacy
Uninsured.adults <= 0.0936
Sexually.transmitted.infections <= 226
Teen.births <= 16.2
4.41 < Poor.mental.health.days <= 4.87
Feature
−0.06
−0.04
−0.02
0.00
0.02
Weight
Positive
Negative

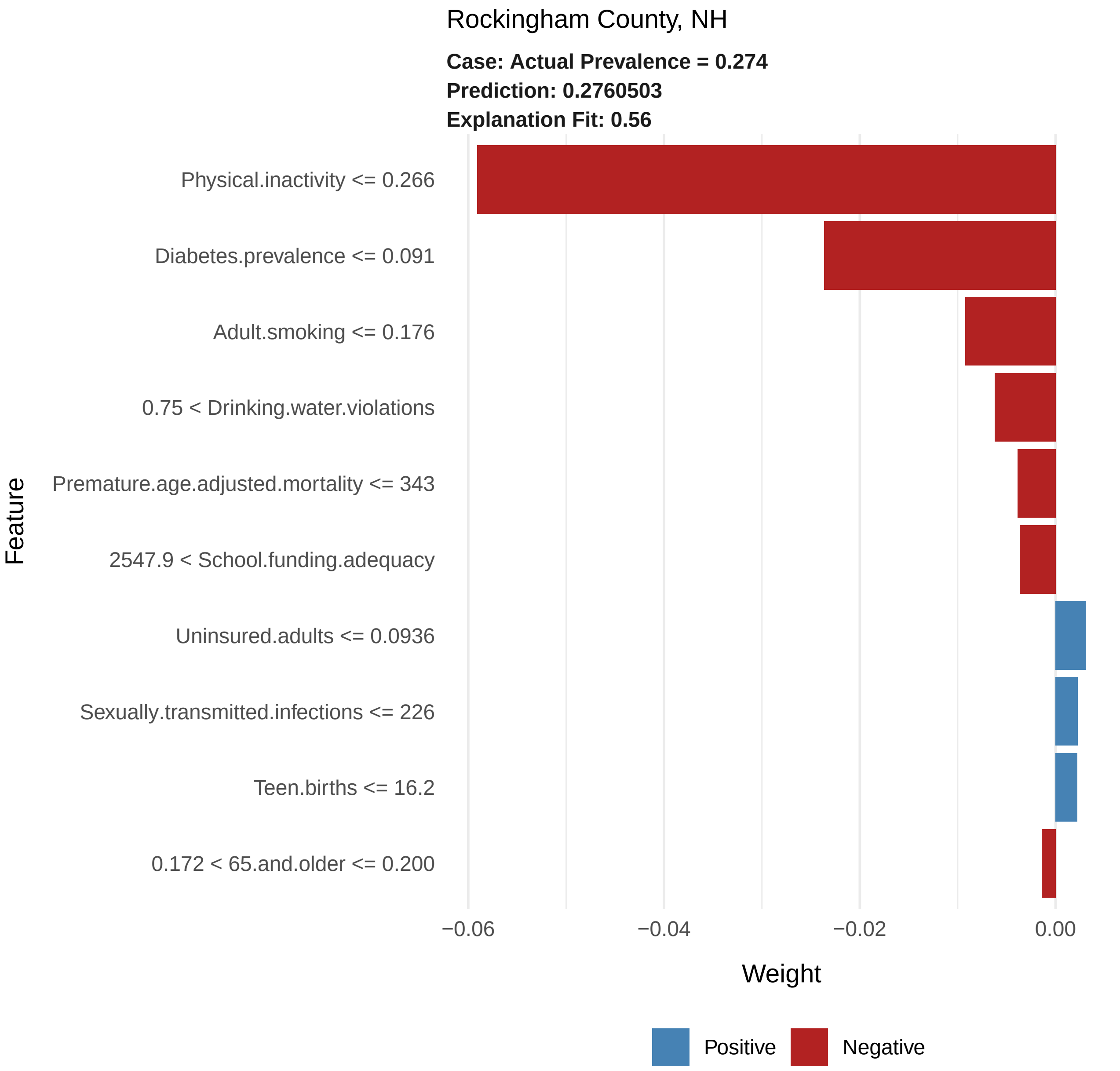

Rockingham County, NH
Case: Actual Prevalence = 0.274
Prediction: 0.2760503
Explanation Fit: 0.56
Physical.inactivity <= 0.266
Diabetes.prevalence <= 0.091
Adult.smoking <= 0.176
0.75 < Drinking.water.violations
Premature.age.adjusted.mortality <= 343
2547.9 < School.funding.adequacy
Uninsured.adults <= 0.0936
Sexually.transmitted.infections <= 226
Teen.births <= 16.2
0.172 < 65.and.older <= 0.200
Feature
−0.06
−0.04
−0.02
0.00
Weight
Positive
Negative

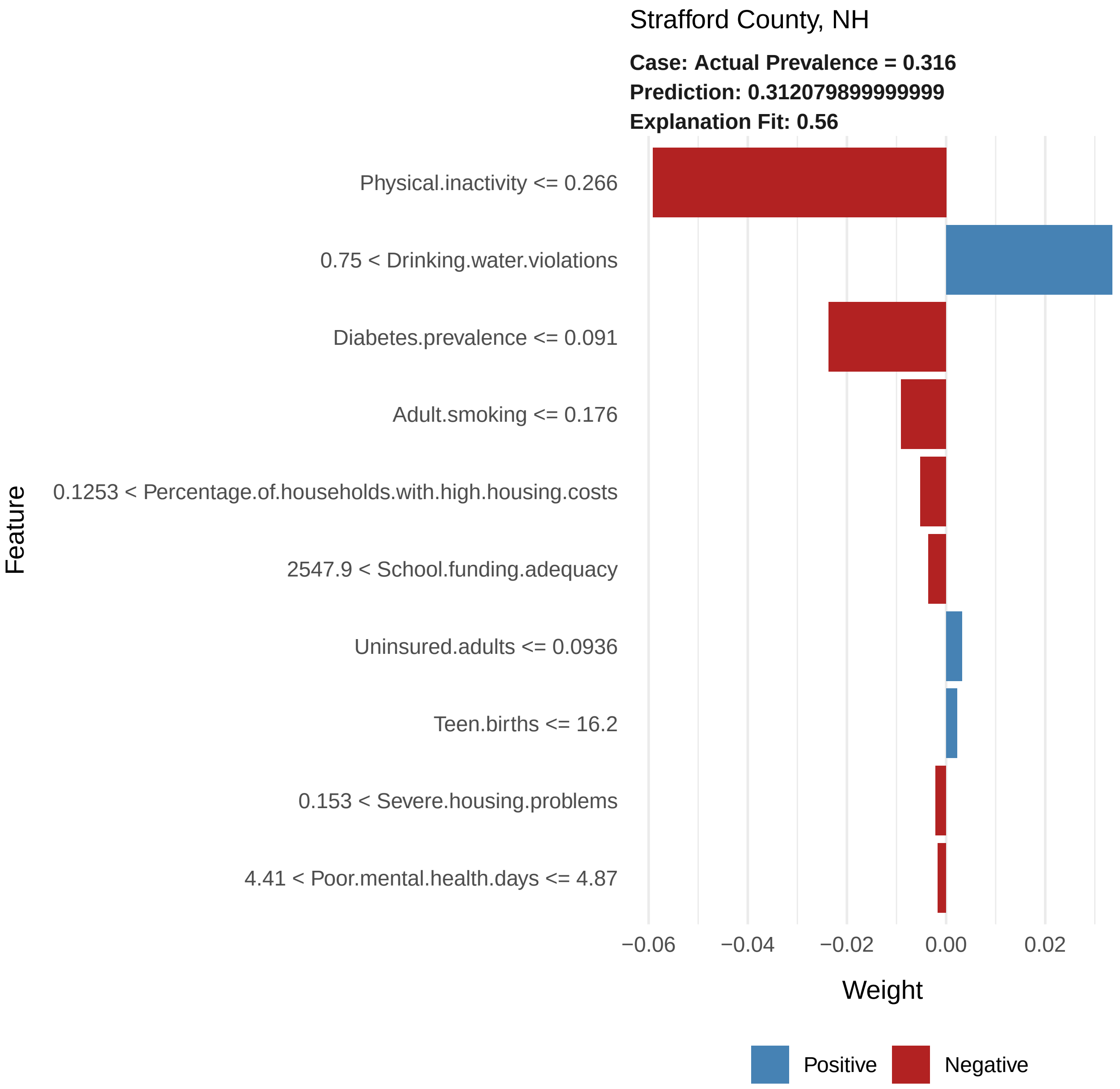

Strafford County, NH
Case: Actual Prevalence = 0.316
Prediction: 0.312079899999999
Explanation Fit: 0.56
Physical.inactivity <= 0.266
0.75 < Drinking.water.violations
Diabetes.prevalence <= 0.091
Adult.smoking <= 0.176
0.1253 < Percentage.of.households.with.high.housing.costs
2547.9 < School.funding.adequacy
Uninsured.adults <= 0.0936
Teen.births <= 16.2
0.153 < Severe.housing.problems
4.41 < Poor.mental.health.days <= 4.87
Feature
−0.06
−0.04
−0.02
0.00
0.02
Weight
Positive
Negative

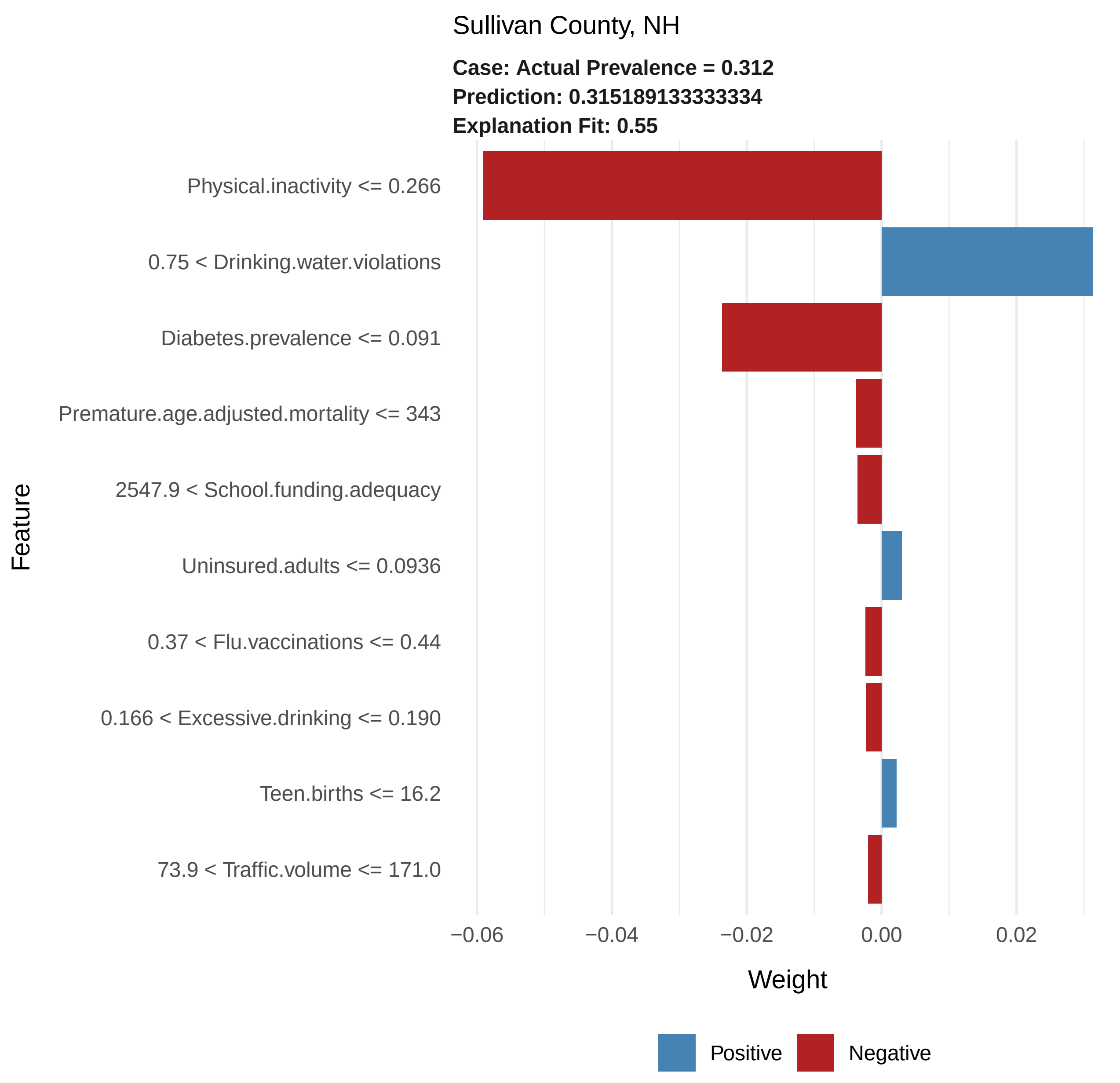
Sullivan County, NH
Case: Actual Prevalence = 0.312
Prediction: 0.315189133333334
Explanation Fit: 0.55
Physical.inactivity <= 0.266
0.75 < Drinking.water.violations
Diabetes.prevalence <= 0.091
Premature.age.adjusted.mortality <= 343
2547.9 < School.funding.adequacy
Uninsured.adults <= 0.0936
0.37 < Flu.vaccinations <= 0.44
0.166 < Excessive.drinking <= 0.190
Teen.births <= 16.2
73.9 < Traffic.volume <= 171.0
Feature
−0.06
−0.04
−0.02
0.00
0.02
Weight
Positive
Negative

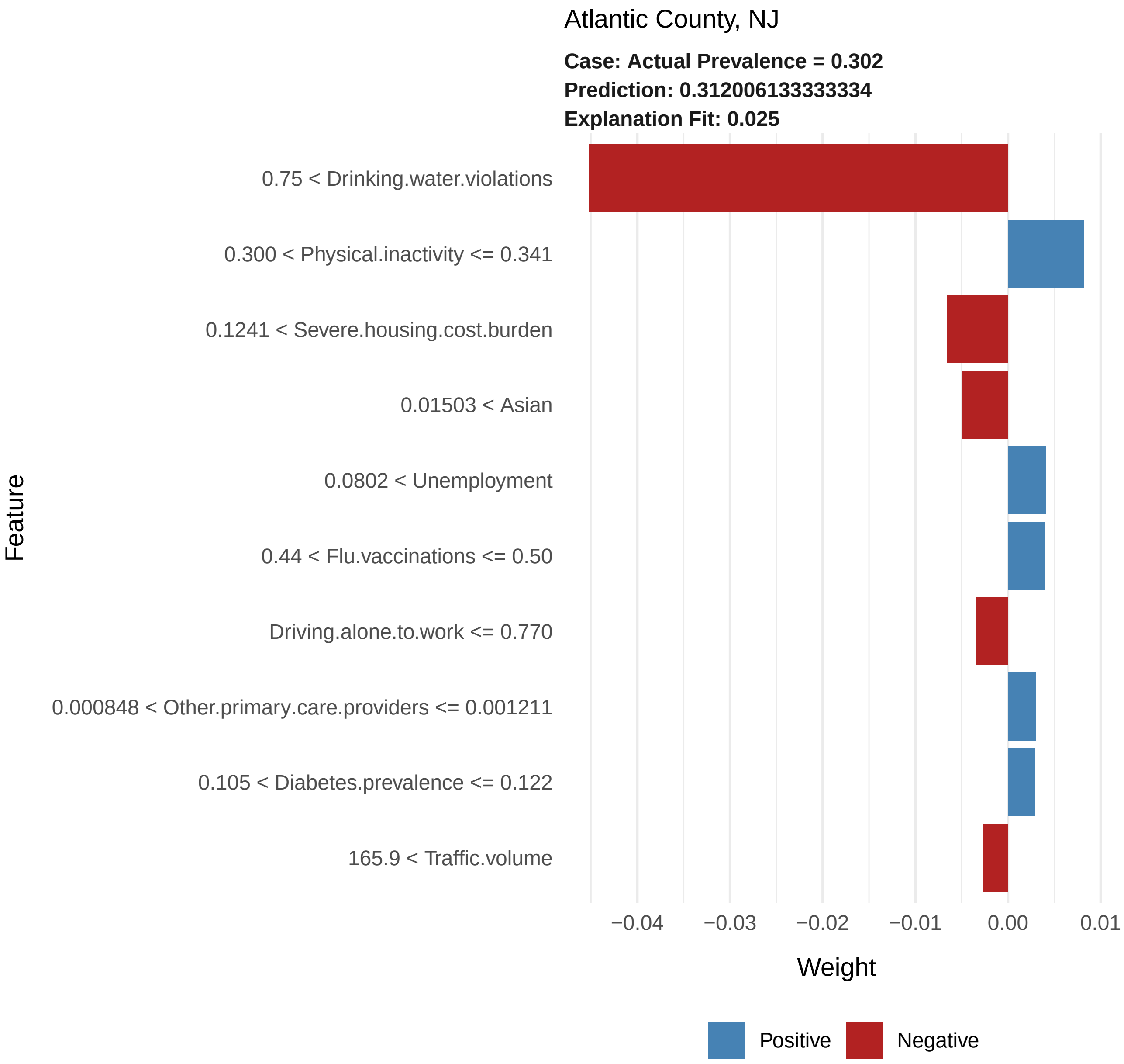

Atlantic County, NJ
Case: Actual Prevalence = 0.302
Prediction: 0.312006133333334
Explanation Fit: 0.025
0.75 < Drinking.water.violations
0.300 < Physical.inactivity <= 0.341
0.1241 < Severe.housing.cost.burden
0.01503 < Asian
0.0802 < Unemployment
0.44 < Flu.vaccinations <= 0.50
Driving.alone.to.work <= 0.770
0.000848 < Other.primary.care.providers <= 0.001211
0.105 < Diabetes.prevalence <= 0.122
165.9 < Traffic.volume
Feature
−0.04
−0.03
−0.02
−0.01
0.00
0.01
Weight
Positive
Negative

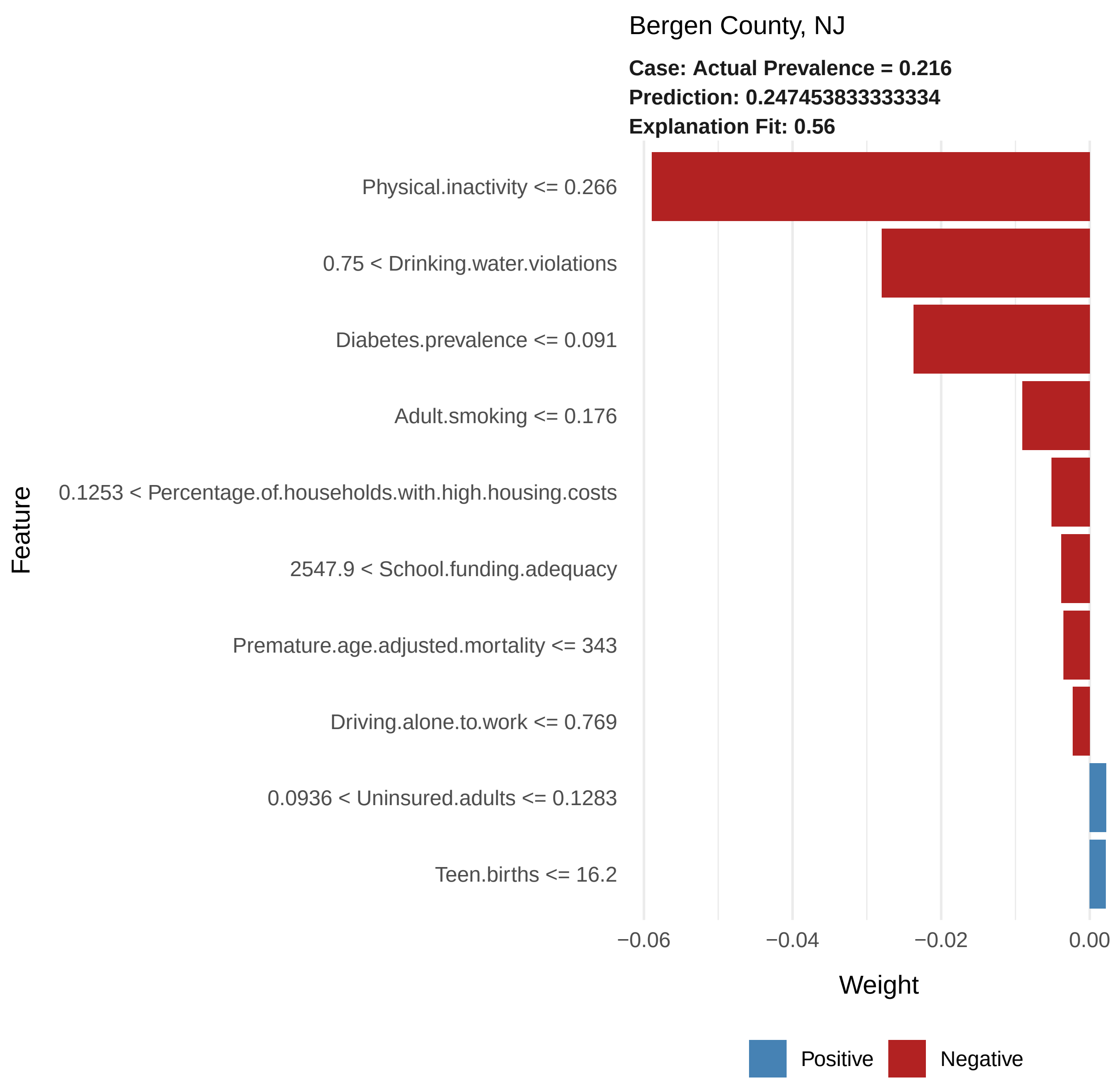

Bergen County, NJ
Case: Actual Prevalence = 0.216
Prediction: 0.247453833333334
Explanation Fit: 0.56
Physical.inactivity <= 0.266
0.75 < Drinking.water.violations
Diabetes.prevalence <= 0.091
Adult.smoking <= 0.176
0.1253 < Percentage.of.households.with.high.housing.costs
2547.9 < School.funding.adequacy
Premature.age.adjusted.mortality <= 343
Driving.alone.to.work <= 0.769
0.0936 < Uninsured.adults <= 0.1283
Teen.births <= 16.2
Feature
−0.06
−0.04
−0.02
0.00
Weight
Positive
Negative

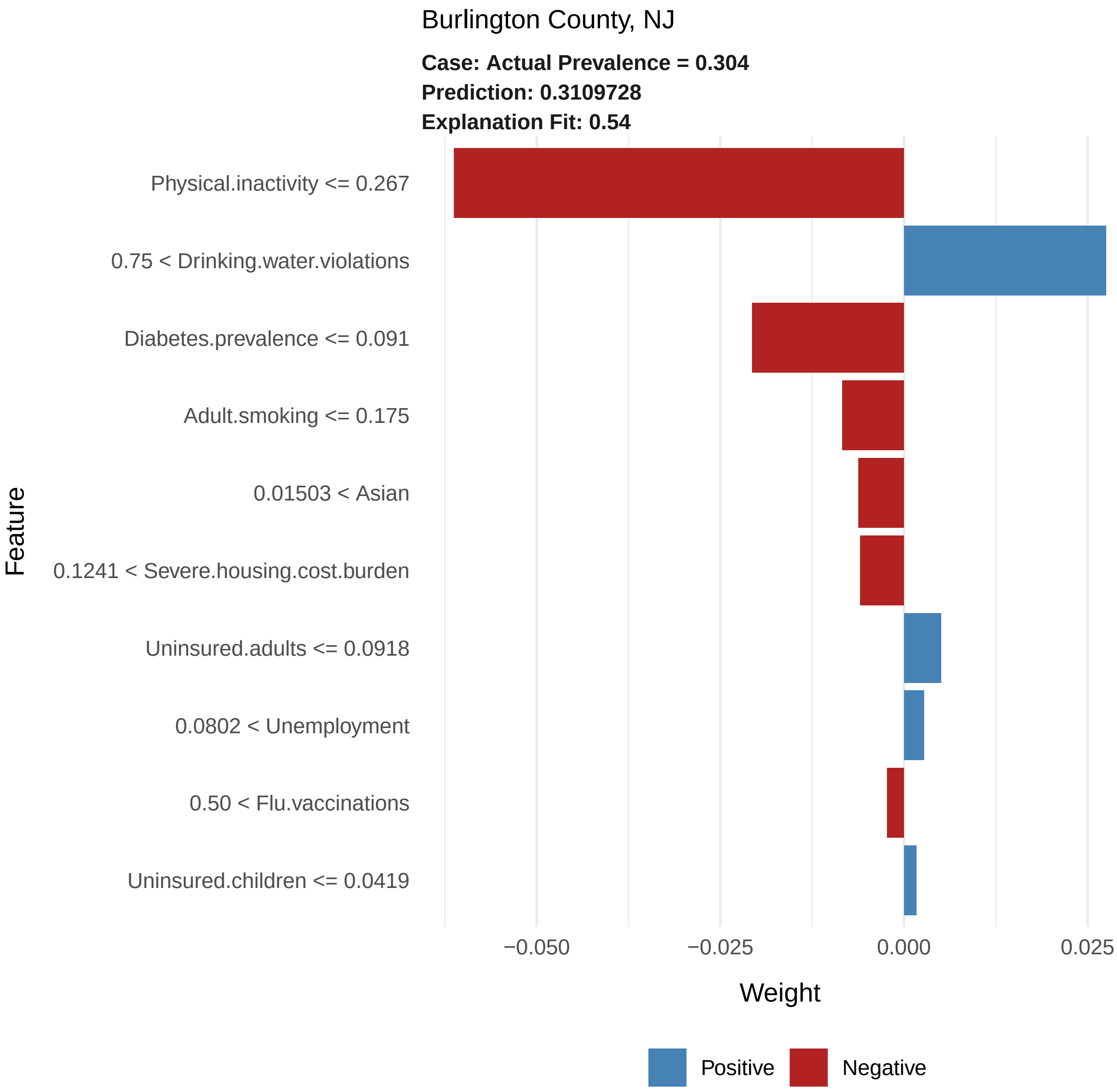
Burlington County, NJ
Case: Actual Prevalence = 0.304
Prediction: 0.3109728
Explanation Fit: 0.54
Physical.inactivity <= 0.267
0.75 < Drinking.water.violations
Diabetes.prevalence <= 0.091
Adult.smoking <= 0.175
0.01503 < Asian
0.1241 < Severe.housing.cost.burden
Uninsured.adults <= 0.0918
0.0802 < Unemployment
0.50 < Flu.vaccinations
Uninsured.children <= 0.0419
Feature
−0.050
−0.025
0.000
0.025
Weight
Positive
Negative

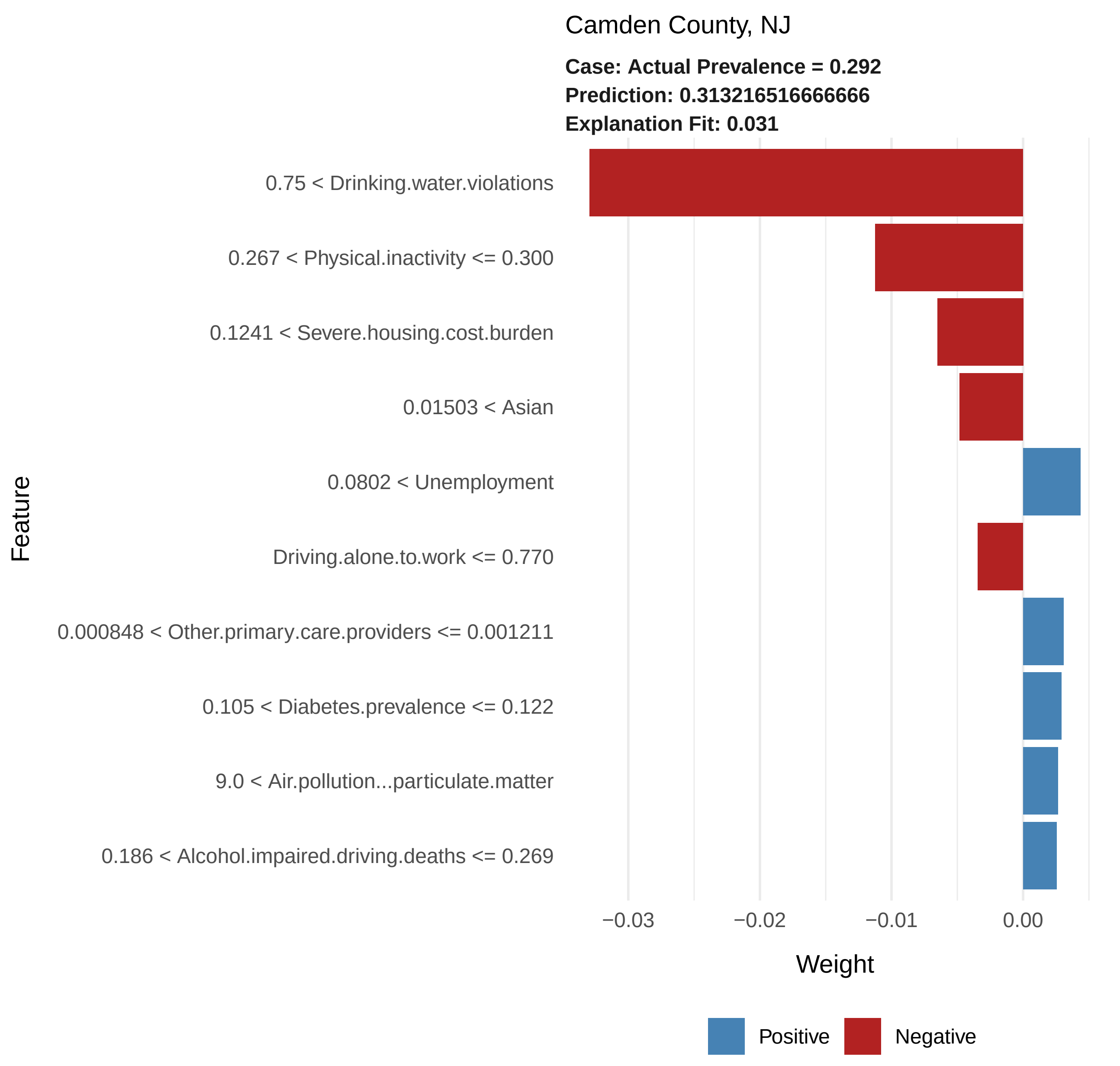
Camden County, NJ
Case: Actual Prevalence = 0.292
Prediction: 0.313216516666666
Explanation Fit: 0.031
0.75 < Drinking.water.violations
0.267 < Physical.inactivity <= 0.300
0.1241 < Severe.housing.cost.burden
0.01503 < Asian
0.0802 < Unemployment
Driving.alone.to.work <= 0.770
0.000848 < Other.primary.care.providers <= 0.001211
0.105 < Diabetes.prevalence <= 0.122
9.0 < Air.pollution...particulate.matter
0.186 < Alcohol.impaired.driving.deaths <= 0.269
Feature
−0.03
−0.02
−0.01
0.00
Weight
Positive
Negative

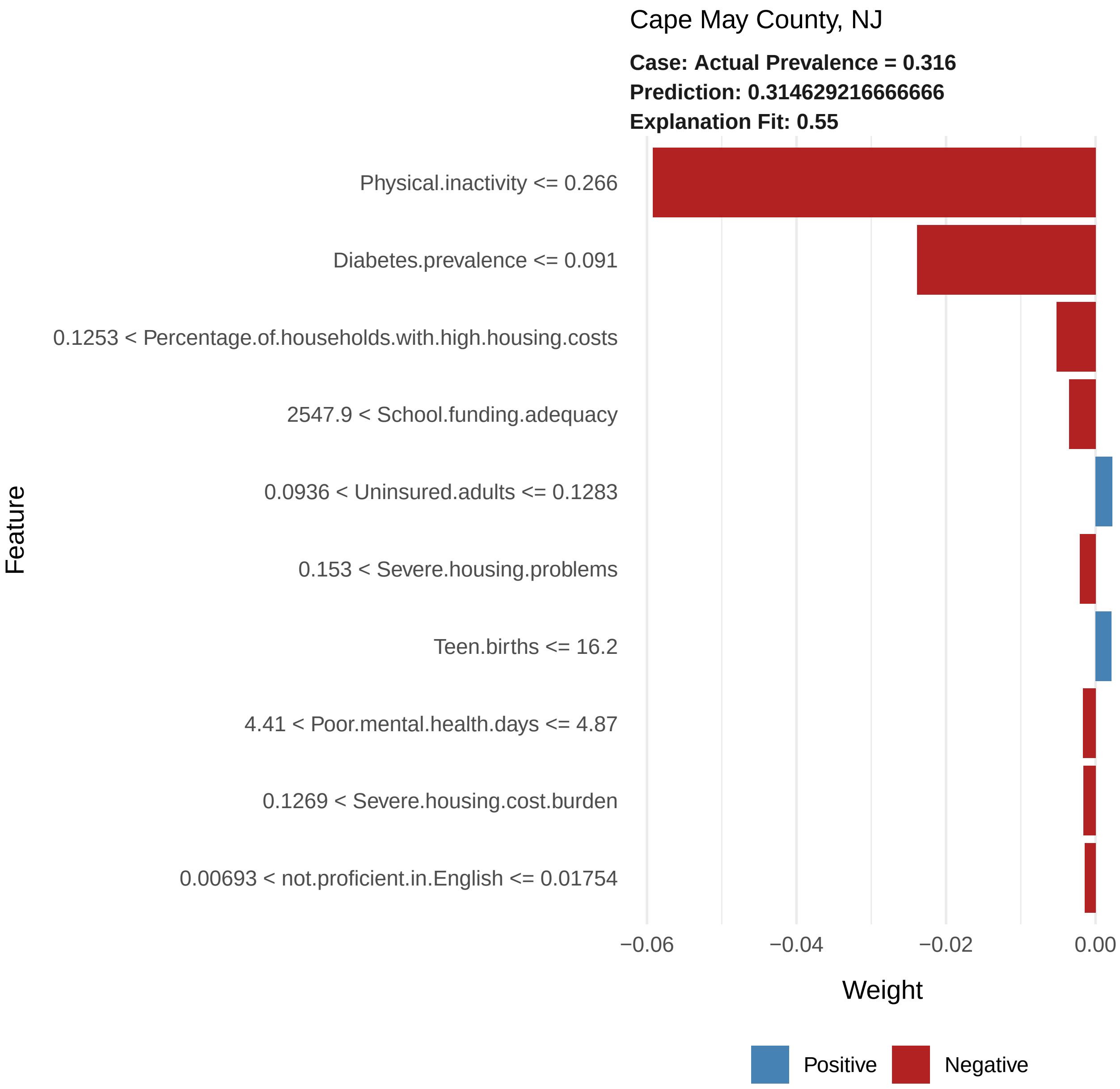

Cape May County, NJ
Case: Actual Prevalence = 0.316
Prediction: 0.314629216666666
Explanation Fit: 0.55
Physical.inactivity <= 0.266
Diabetes.prevalence <= 0.091
0.1253 < Percentage.of.households.with.high.housing.costs
2547.9 < School.funding.adequacy
0.0936 < Uninsured.adults <= 0.1283
0.153 < Severe.housing.problems
Teen.births <= 16.2
4.41 < Poor.mental.health.days <= 4.87
0.1269 < Severe.housing.cost.burden
0.00693 < not.proficient.in.English <= 0.01754
Feature
−0.06
−0.04
−0.02
0.00
Weight
Positive
Negative

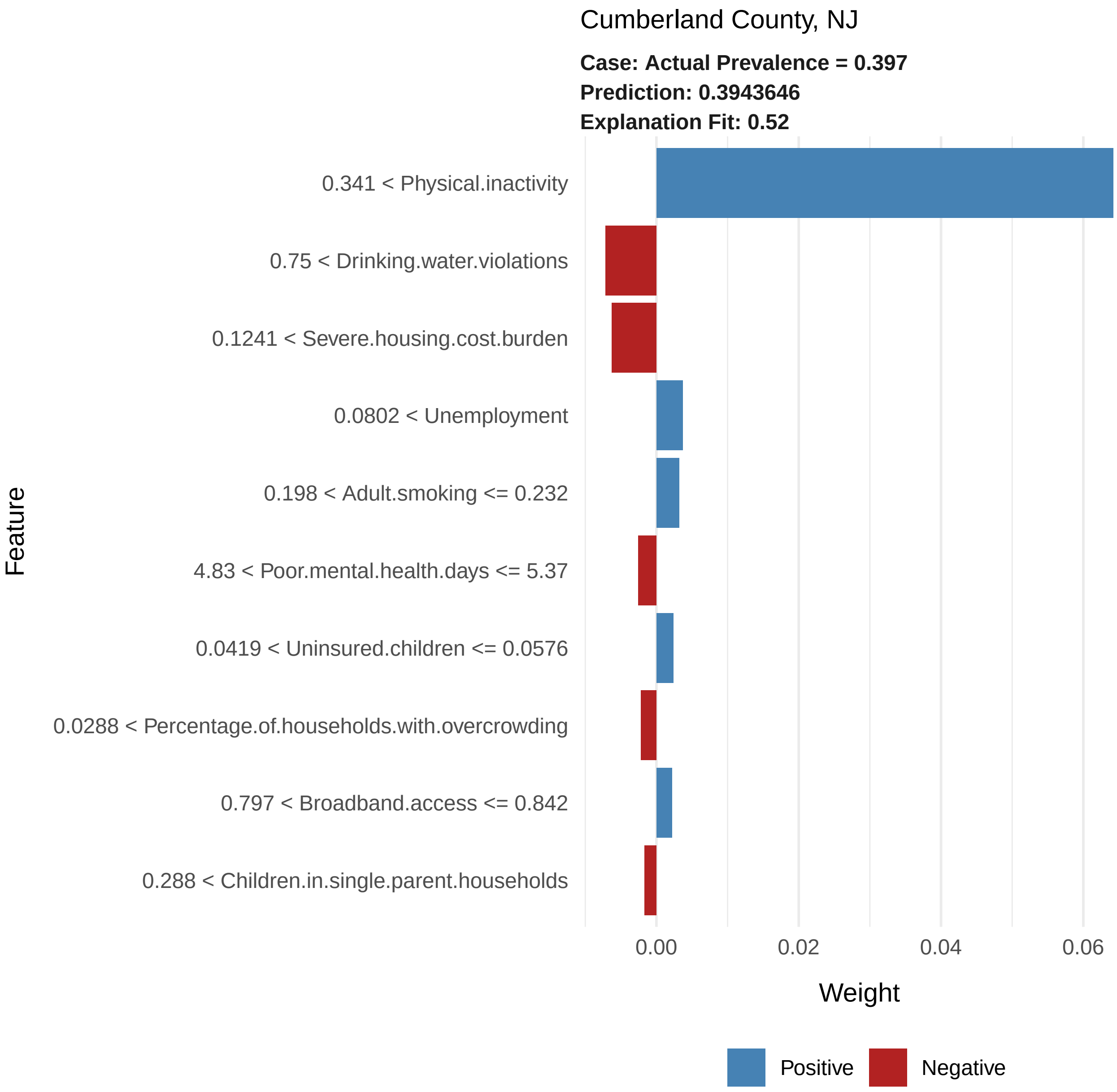
Cumberland County, NJ
Case: Actual Prevalence = 0.397
Prediction: 0.3943646
Explanation Fit: 0.52
0.341 < Physical.inactivity
0.75 < Drinking.water.violations
0.1241 < Severe.housing.cost.burden
0.0802 < Unemployment
0.198 < Adult.smoking <= 0.232
4.83 < Poor.mental.health.days <= 5.37
0.0419 < Uninsured.children <= 0.0576
0.0288 < Percentage.of.households.with.overcrowding
0.797 < Broadband.access <= 0.842
0.288 < Children.in.single.parent.households
Feature
0.00
0.02
0.04
0.06
Weight
Positive
Negative

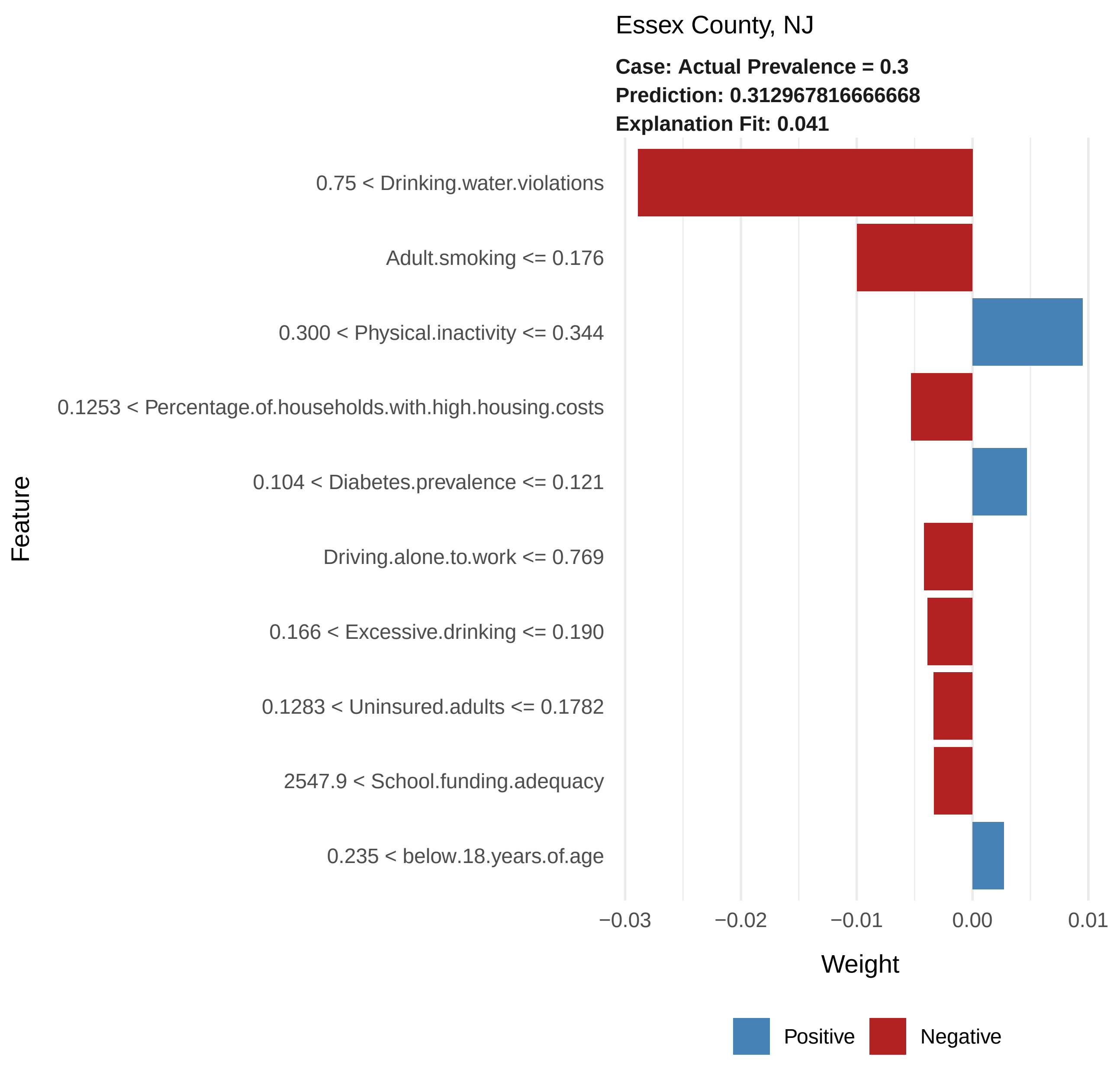
Essex County, NJ
Case: Actual Prevalence = 0.3
Prediction: 0.312967816666668
Explanation Fit: 0.041
0.75 < Drinking.water.violations
Adult.smoking <= 0.176
0.300 < Physical.inactivity <= 0.344
0.1253 < Percentage.of.households.with.high.housing.costs
0.104 < Diabetes.prevalence <= 0.121
Driving.alone.to.work <= 0.769
0.166 < Excessive.drinking <= 0.190
0.1283 < Uninsured.adults <= 0.1782
2547.9 < School.funding.adequacy
0.235 < below.18.years.of.age
Feature
−0.03
−0.02
−0.01
0.00
0.01
Weight
Positive
Negative

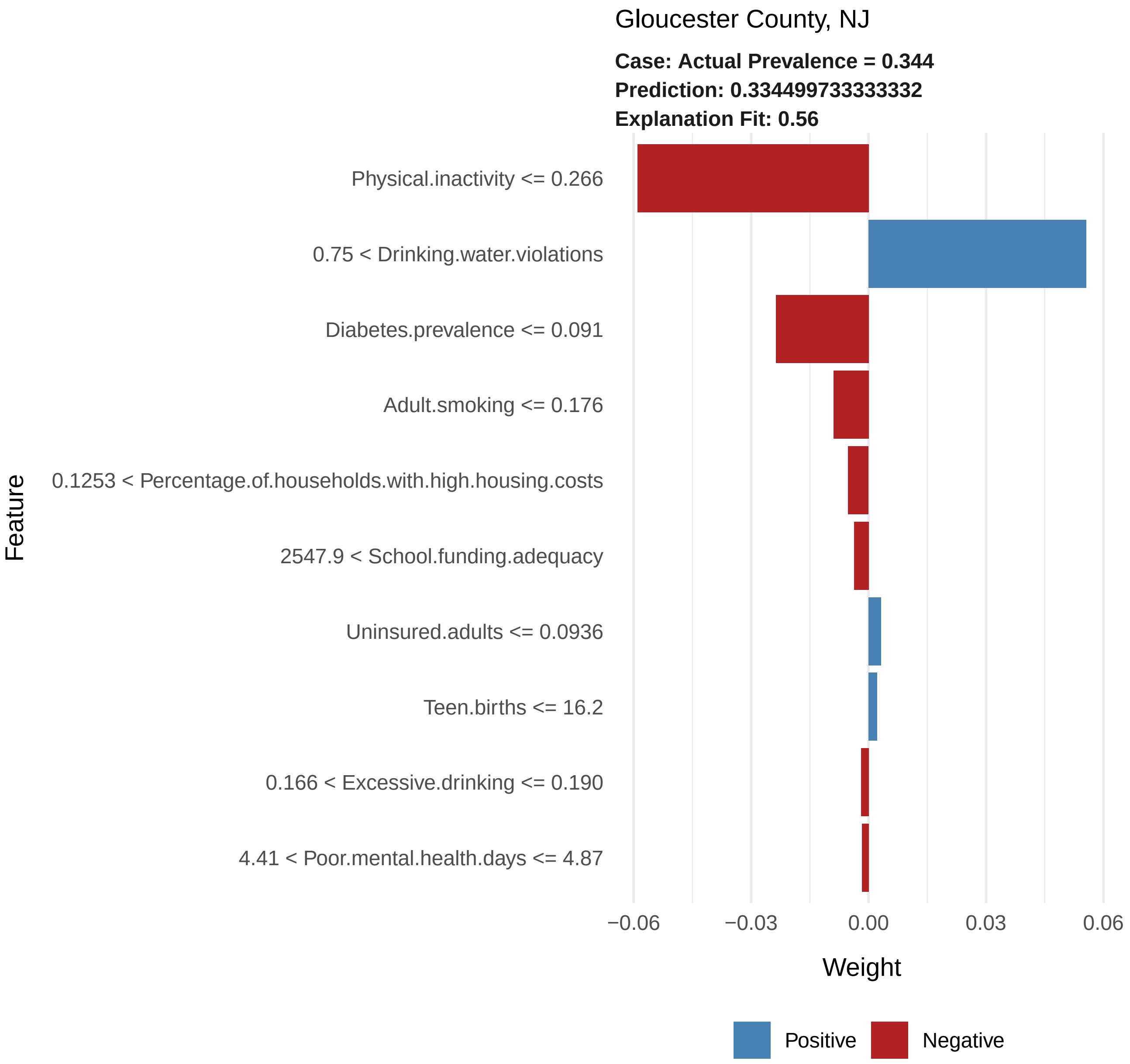
Gloucester County, NJ
Case: Actual Prevalence = 0.344
Prediction: 0.334499733333332
Explanation Fit: 0.56
Physical.inactivity <= 0.266
0.75 < Drinking.water.violations
Diabetes.prevalence <= 0.091
Adult.smoking <= 0.176
0.1253 < Percentage.of.households.with.high.housing.costs
2547.9 < School.funding.adequacy
Uninsured.adults <= 0.0936
Teen.births <= 16.2
0.166 < Excessive.drinking <= 0.190
4.41 < Poor.mental.health.days <= 4.87
Feature
−0.06
−0.03
0.00
0.03
0.06
Weight
Positive
Negative

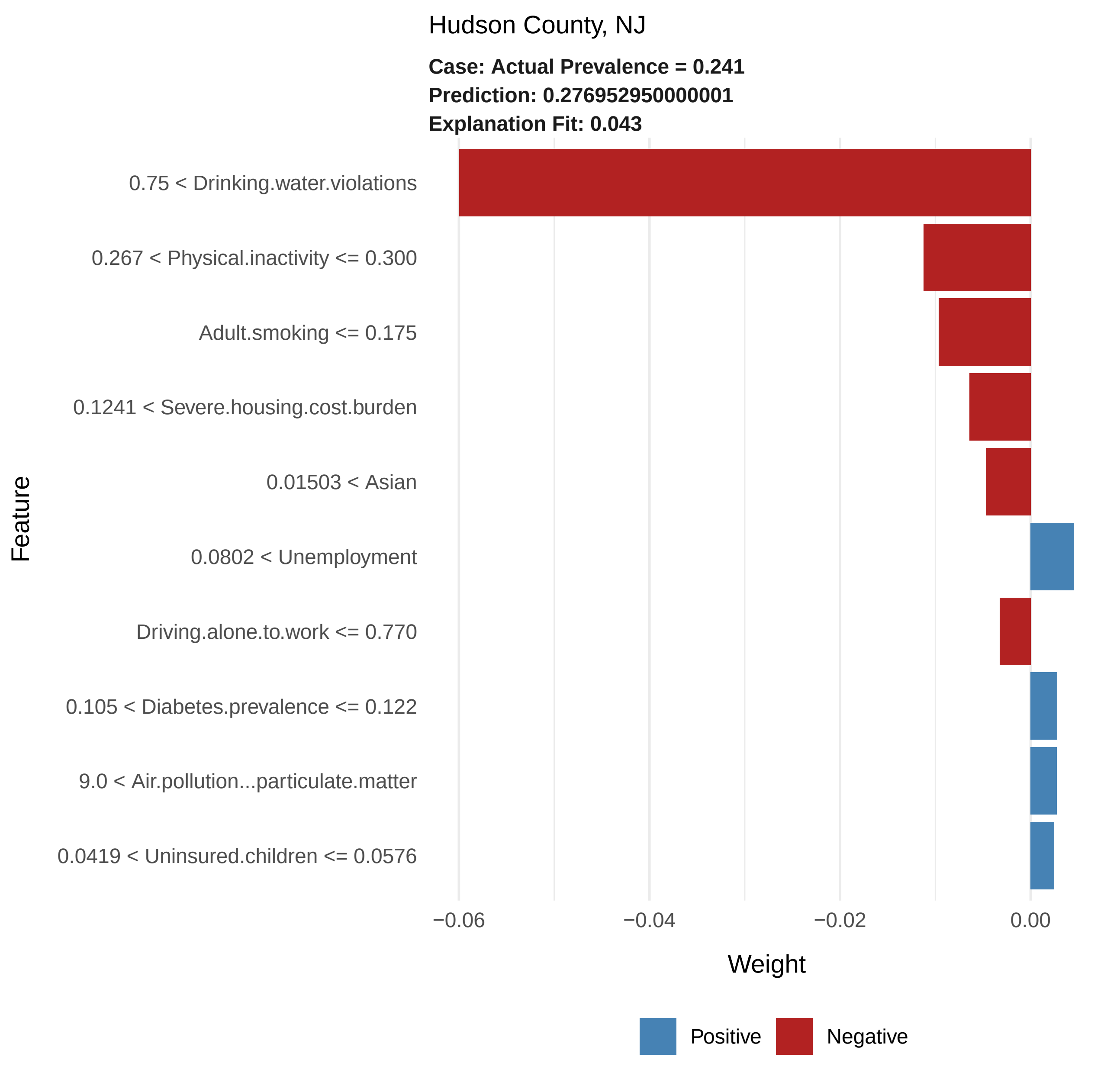

Hudson County, NJ
Case: Actual Prevalence = 0.241
Prediction: 0.276952950000001
Explanation Fit: 0.043
0.75 < Drinking.water.violations
0.267 < Physical.inactivity <= 0.300
Adult.smoking <= 0.175
0.1241 < Severe.housing.cost.burden
0.01503 < Asian
0.0802 < Unemployment
Driving.alone.to.work <= 0.770
0.105 < Diabetes.prevalence <= 0.122
9.0 < Air.pollution...particulate.matter
0.0419 < Uninsured.children <= 0.0576
Feature
−0.06
−0.04
−0.02
0.00
Weight
Positive
Negative

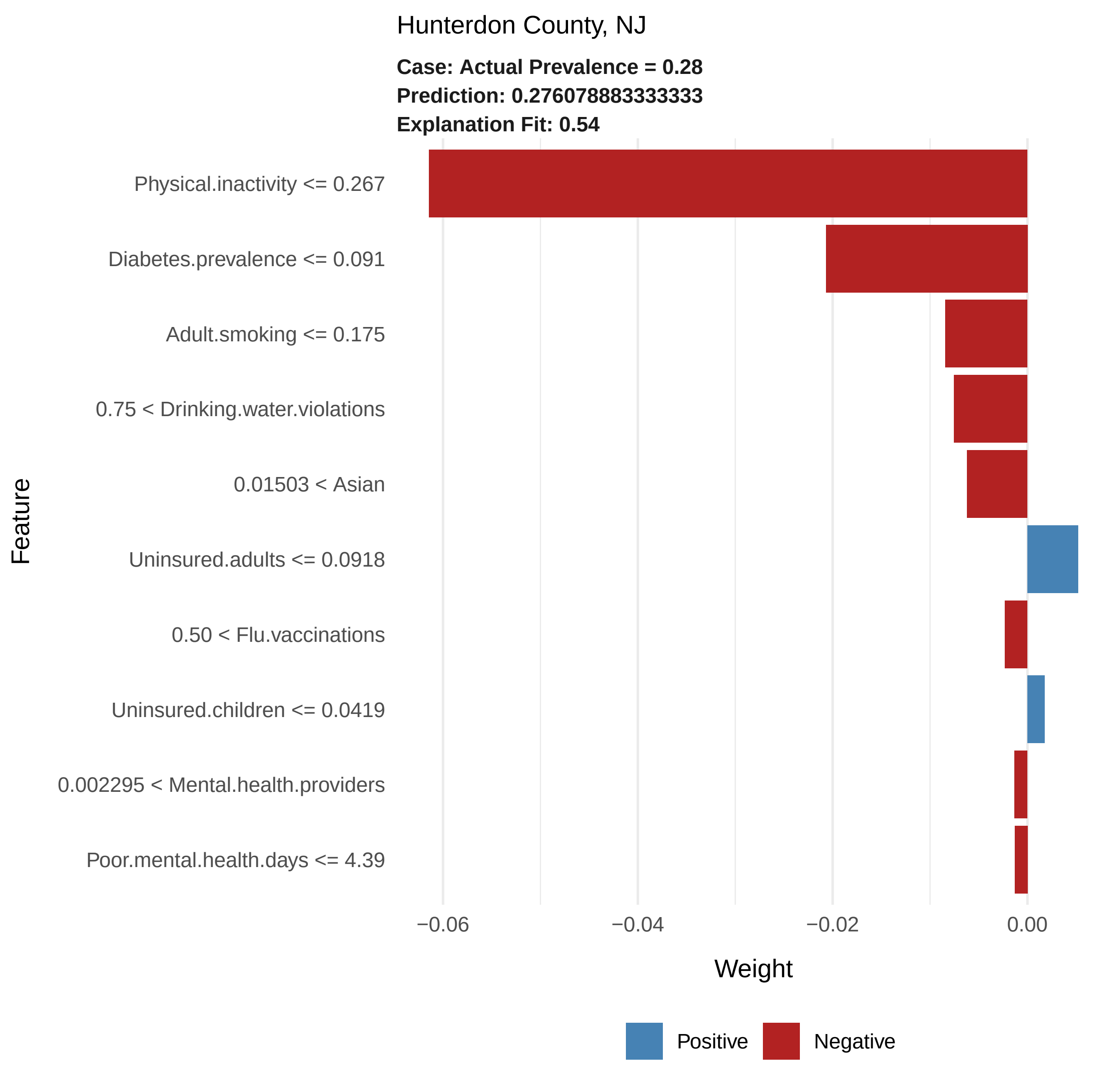

Hunterdon County, NJ
Case: Actual Prevalence = 0.28
Prediction: 0.276078883333333
Explanation Fit: 0.54
Physical.inactivity <= 0.267
Diabetes.prevalence <= 0.091
Adult.smoking <= 0.175
0.75 < Drinking.water.violations
0.01503 < Asian
Uninsured.adults <= 0.0918
0.50 < Flu.vaccinations
Uninsured.children <= 0.0419
0.002295 < Mental.health.providers
Poor.mental.health.days <= 4.39
Feature
−0.06
−0.04
−0.02
0.00
Weight
Positive
Negative

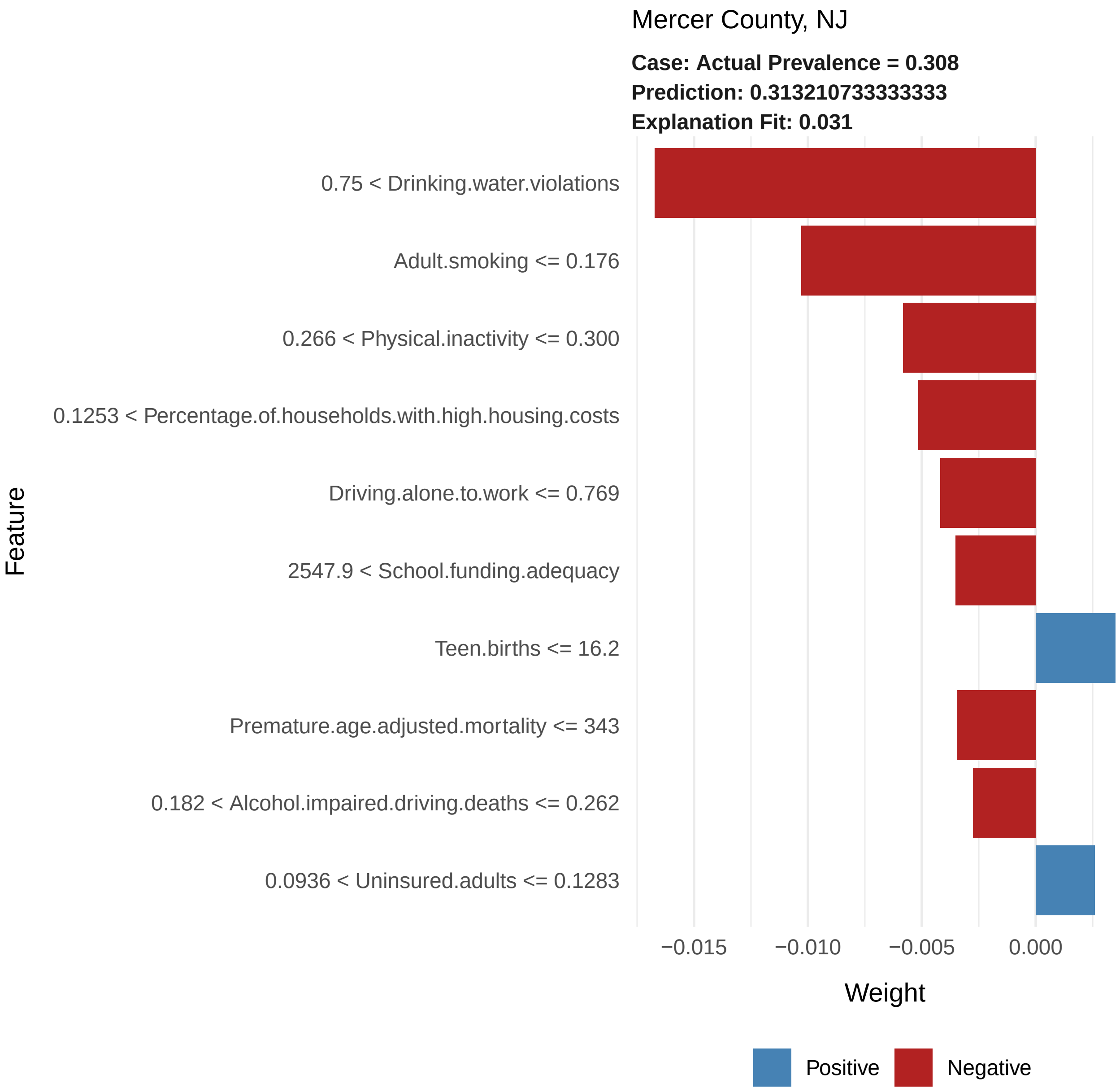
Mercer County, NJ
Case: Actual Prevalence = 0.308
Prediction: 0.313210733333333
Explanation Fit: 0.031
0.75 < Drinking.water.violations
Adult.smoking <= 0.176
0.266 < Physical.inactivity <= 0.300
0.1253 < Percentage.of.households.with.high.housing.costs
Driving.alone.to.work <= 0.769
2547.9 < School.funding.adequacy
Teen.births <= 16.2
Premature.age.adjusted.mortality <= 343
0.182 < Alcohol.impaired.driving.deaths <= 0.262
0.0936 < Uninsured.adults <= 0.1283
Feature
−0.015
−0.010
−0.005
0.000
Weight
Positive
Negative

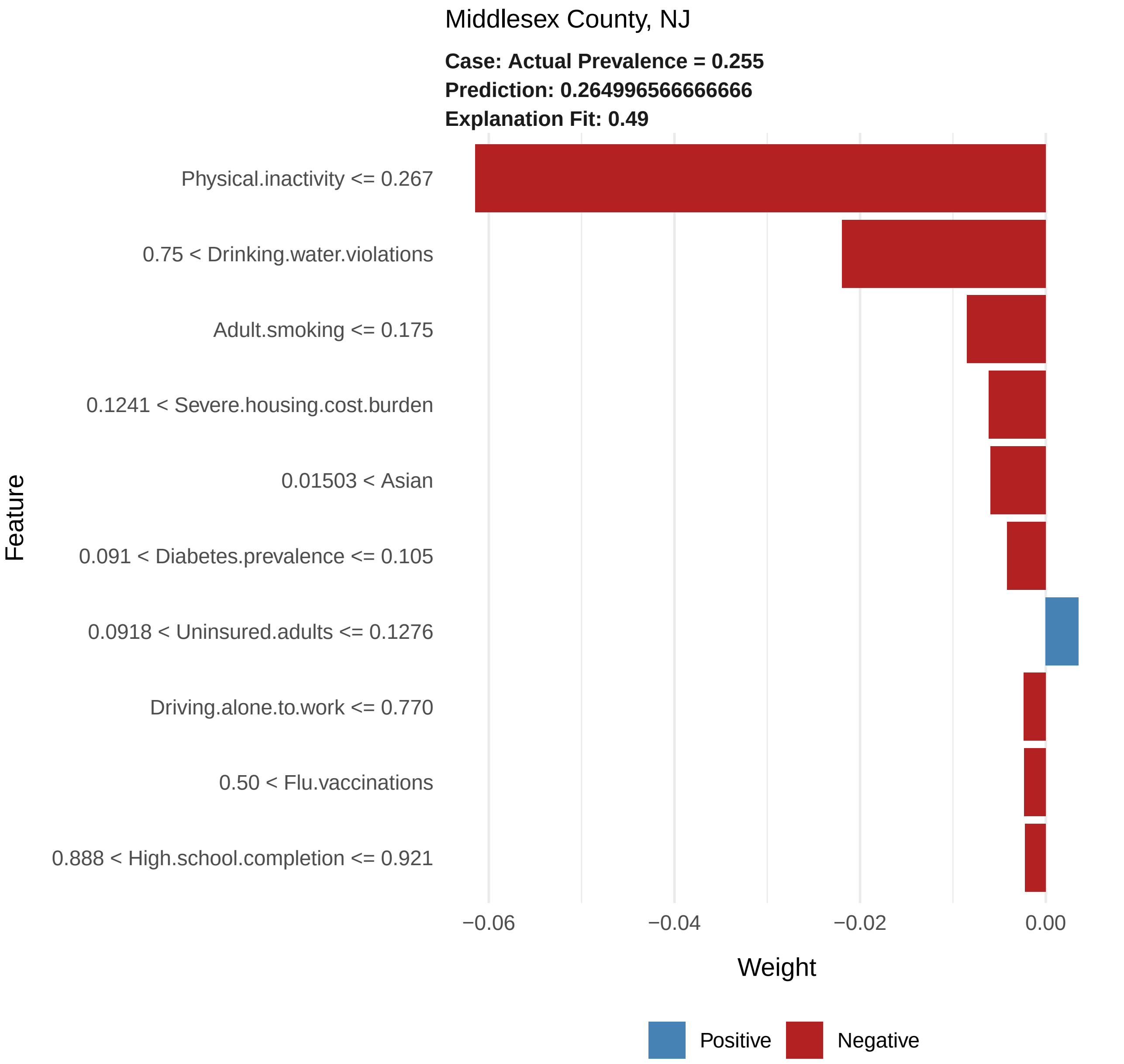

Middlesex County, NJ
Case: Actual Prevalence = 0.255
Prediction: 0.264996566666666
Explanation Fit: 0.49
Physical.inactivity <= 0.267
0.75 < Drinking.water.violations
Adult.smoking <= 0.175
0.1241 < Severe.housing.cost.burden
0.01503 < Asian
0.091 < Diabetes.prevalence <= 0.105
0.0918 < Uninsured.adults <= 0.1276
Driving.alone.to.work <= 0.770
0.50 < Flu.vaccinations
0.888 < High.school.completion <= 0.921
Feature
−0.06
−0.04
−0.02
0.00
Weight
Positive
Negative

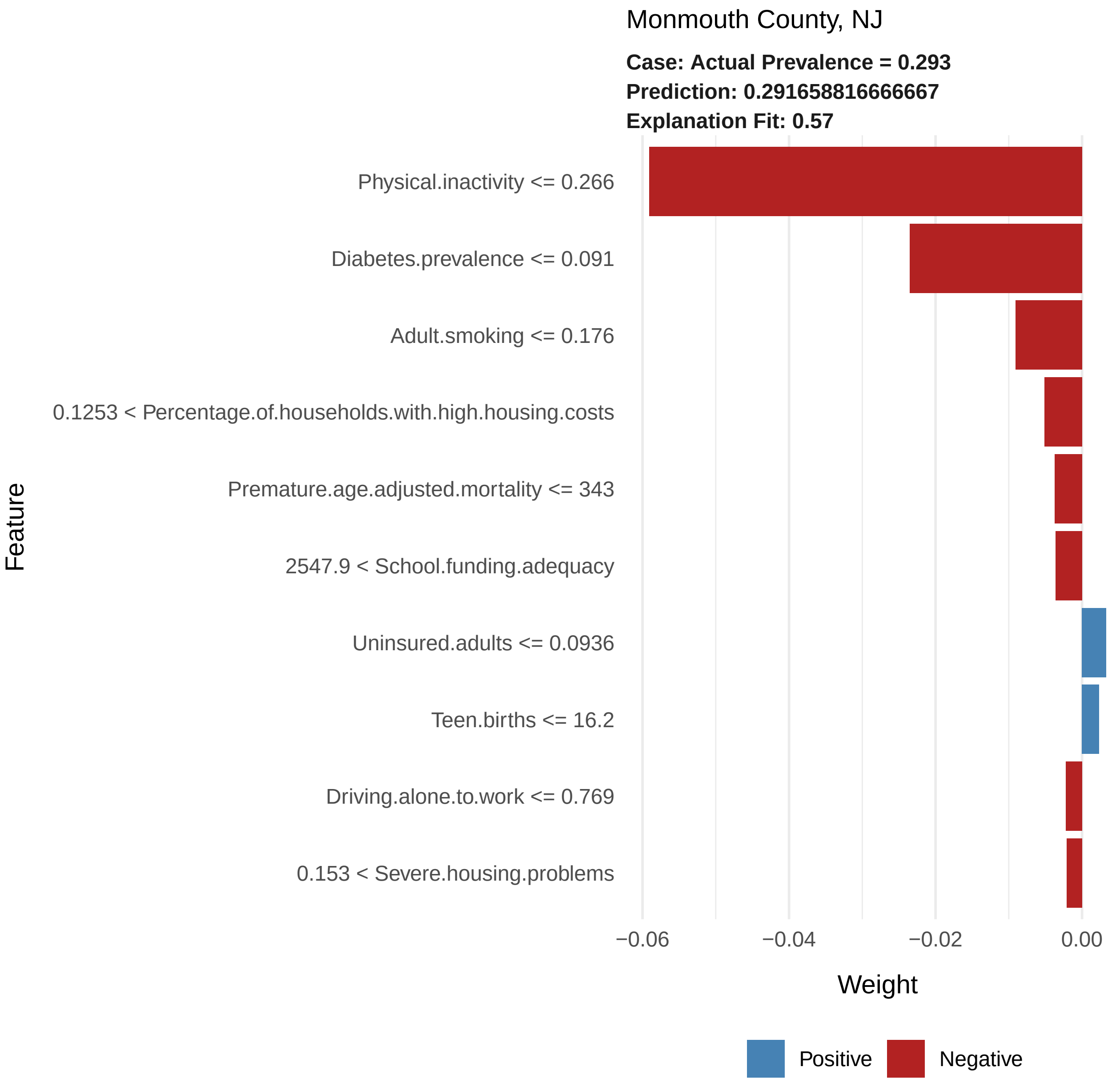
Monmouth County, NJ
Case: Actual Prevalence = 0.293
Prediction: 0.291658816666667
Explanation Fit: 0.57
Physical.inactivity <= 0.266
Diabetes.prevalence <= 0.091
Adult.smoking <= 0.176
0.1253 < Percentage.of.households.with.high.housing.costs
Premature.age.adjusted.mortality <= 343
2547.9 < School.funding.adequacy
Uninsured.adults <= 0.0936
Teen.births <= 16.2
Driving.alone.to.work <= 0.769
0.153 < Severe.housing.problems
Feature
−0.06
−0.04
−0.02
0.00
Weight
Positive
Negative

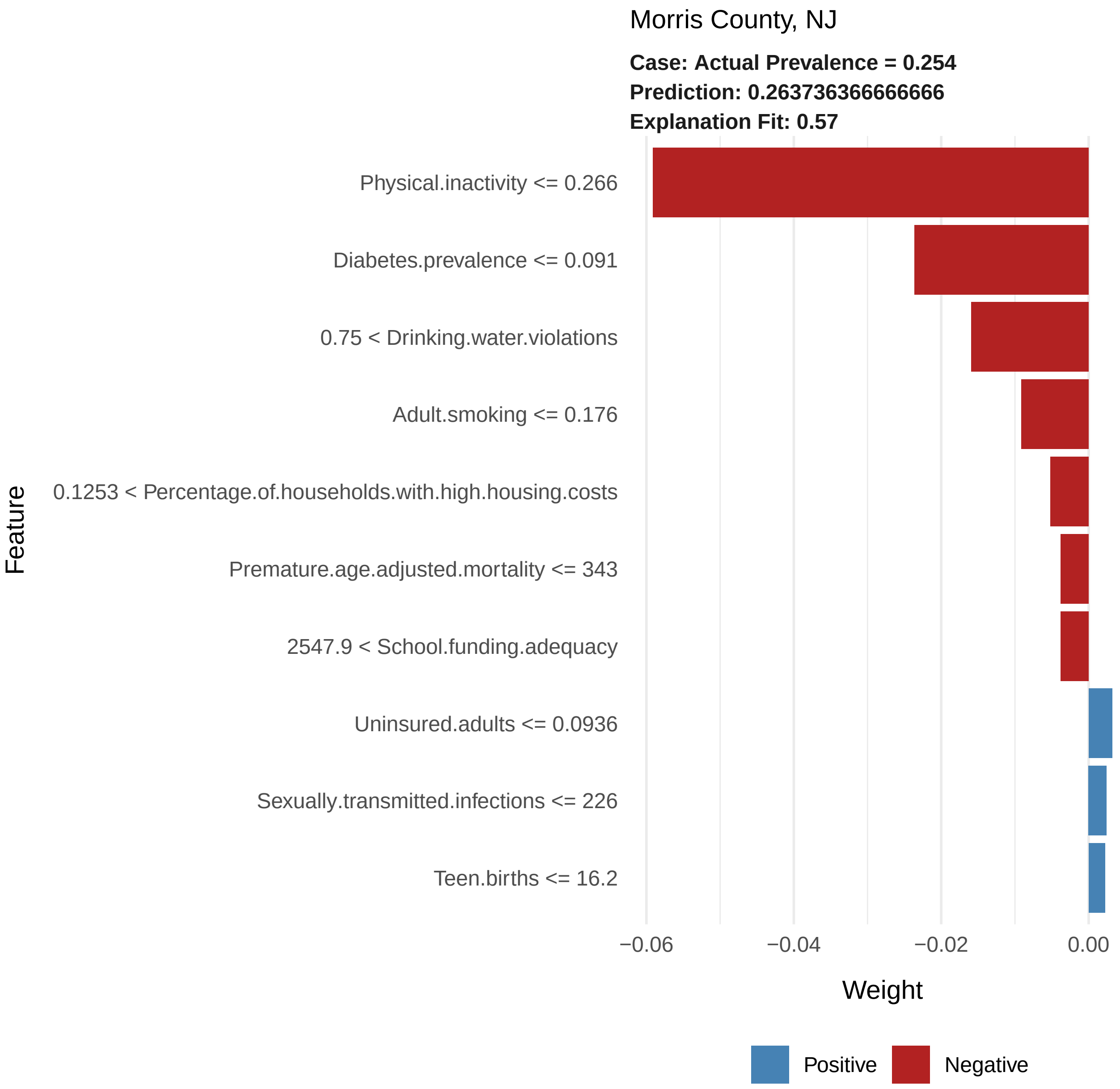

Morris County, NJ
Case: Actual Prevalence = 0.254
Prediction: 0.263736366666666
Explanation Fit: 0.57
Physical.inactivity <= 0.266
Diabetes.prevalence <= 0.091
0.75 < Drinking.water.violations
Adult.smoking <= 0.176
0.1253 < Percentage.of.households.with.high.housing.costs
Premature.age.adjusted.mortality <= 343
2547.9 < School.funding.adequacy
Uninsured.adults <= 0.0936
Sexually.transmitted.infections <= 226
Teen.births <= 16.2
Feature
−0.06
−0.04
−0.02
0.00
Weight
Positive
Negative

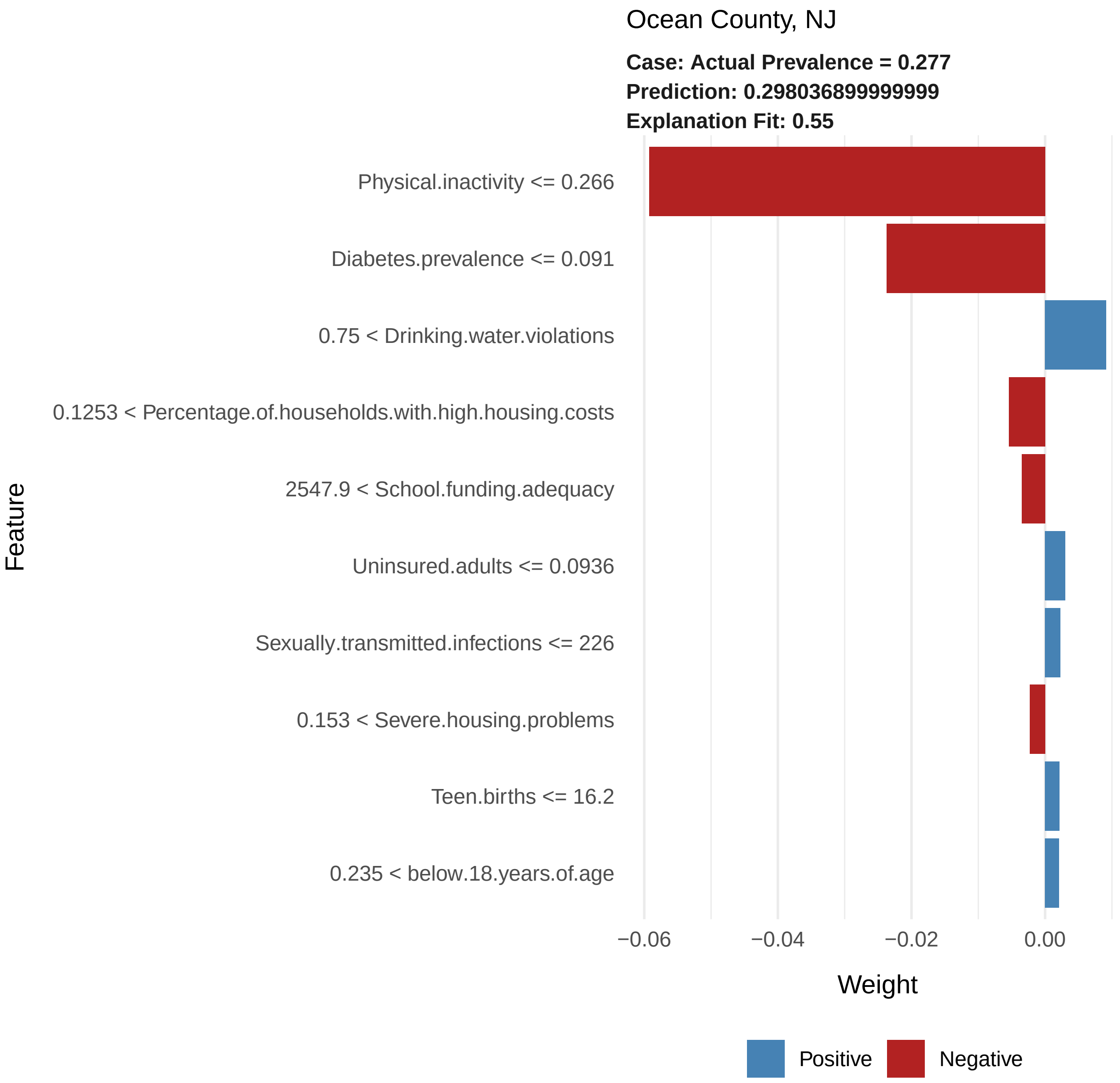
Ocean County, NJ
Case: Actual Prevalence = 0.277
Prediction: 0.298036899999999
Explanation Fit: 0.55
Physical.inactivity <= 0.266
Diabetes.prevalence <= 0.091
0.75 < Drinking.water.violations
0.1253 < Percentage.of.households.with.high.housing.costs
2547.9 < School.funding.adequacy
Uninsured.adults <= 0.0936
Sexually.transmitted.infections <= 226
0.153 < Severe.housing.problems
Teen.births <= 16.2
0.235 < below.18.years.of.age
Feature
−0.06
−0.04
−0.02
0.00
Weight
Positive
Negative

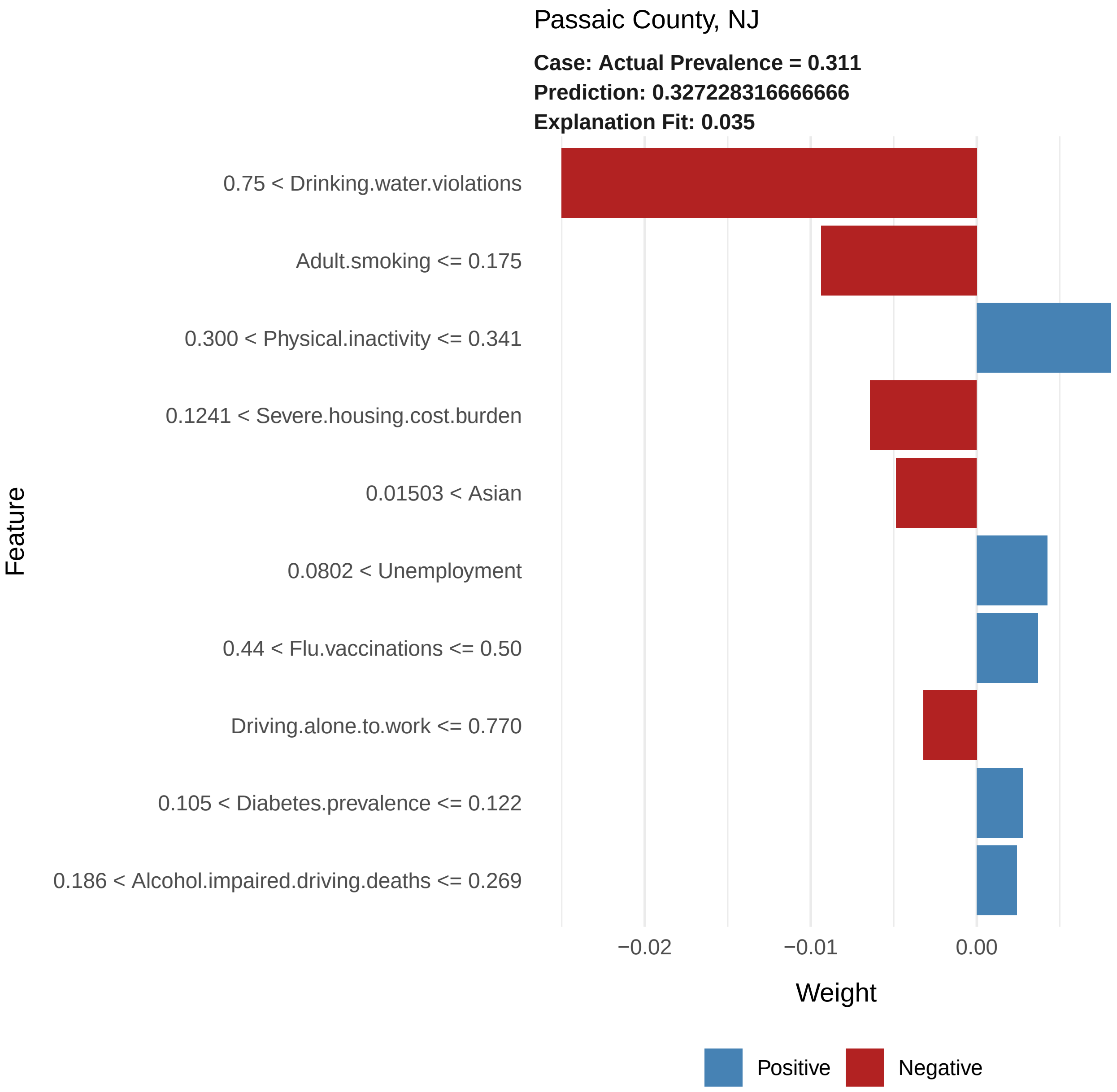

Passaic County, NJ
Case: Actual Prevalence = 0.311
Prediction: 0.327228316666666
Explanation Fit: 0.035
0.75 < Drinking.water.violations
Adult.smoking <= 0.175
0.300 < Physical.inactivity <= 0.341
0.1241 < Severe.housing.cost.burden
0.01503 < Asian
0.0802 < Unemployment
0.44 < Flu.vaccinations <= 0.50
Driving.alone.to.work <= 0.770
0.105 < Diabetes.prevalence <= 0.122
0.186 < Alcohol.impaired.driving.deaths <= 0.269
Feature
−0.02
−0.01
0.00
Weight
Positive
Negative

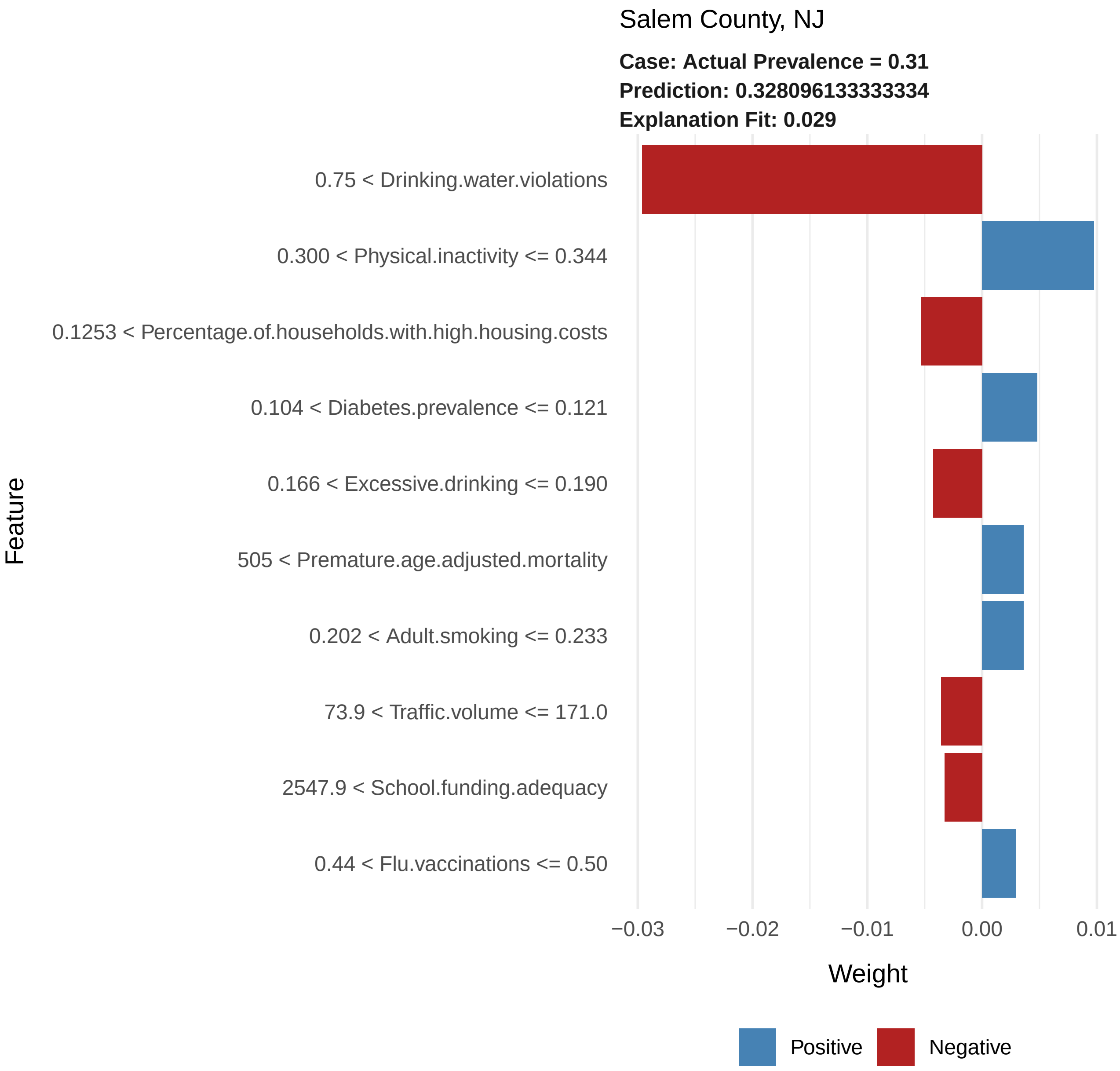

Salem County, NJ
Case: Actual Prevalence = 0.31
Prediction: 0.328096133333334
Explanation Fit: 0.029
0.75 < Drinking.water.violations
0.300 < Physical.inactivity <= 0.344
0.1253 < Percentage.of.households.with.high.housing.costs
0.104 < Diabetes.prevalence <= 0.121
0.166 < Excessive.drinking <= 0.190
505 < Premature.age.adjusted.mortality
0.202 < Adult.smoking <= 0.233
73.9 < Traffic.volume <= 171.0
2547.9 < School.funding.adequacy
0.44 < Flu.vaccinations <= 0.50
Feature
−0.03
−0.02
−0.01
0.00
0.01
Weight
Positive
Negative

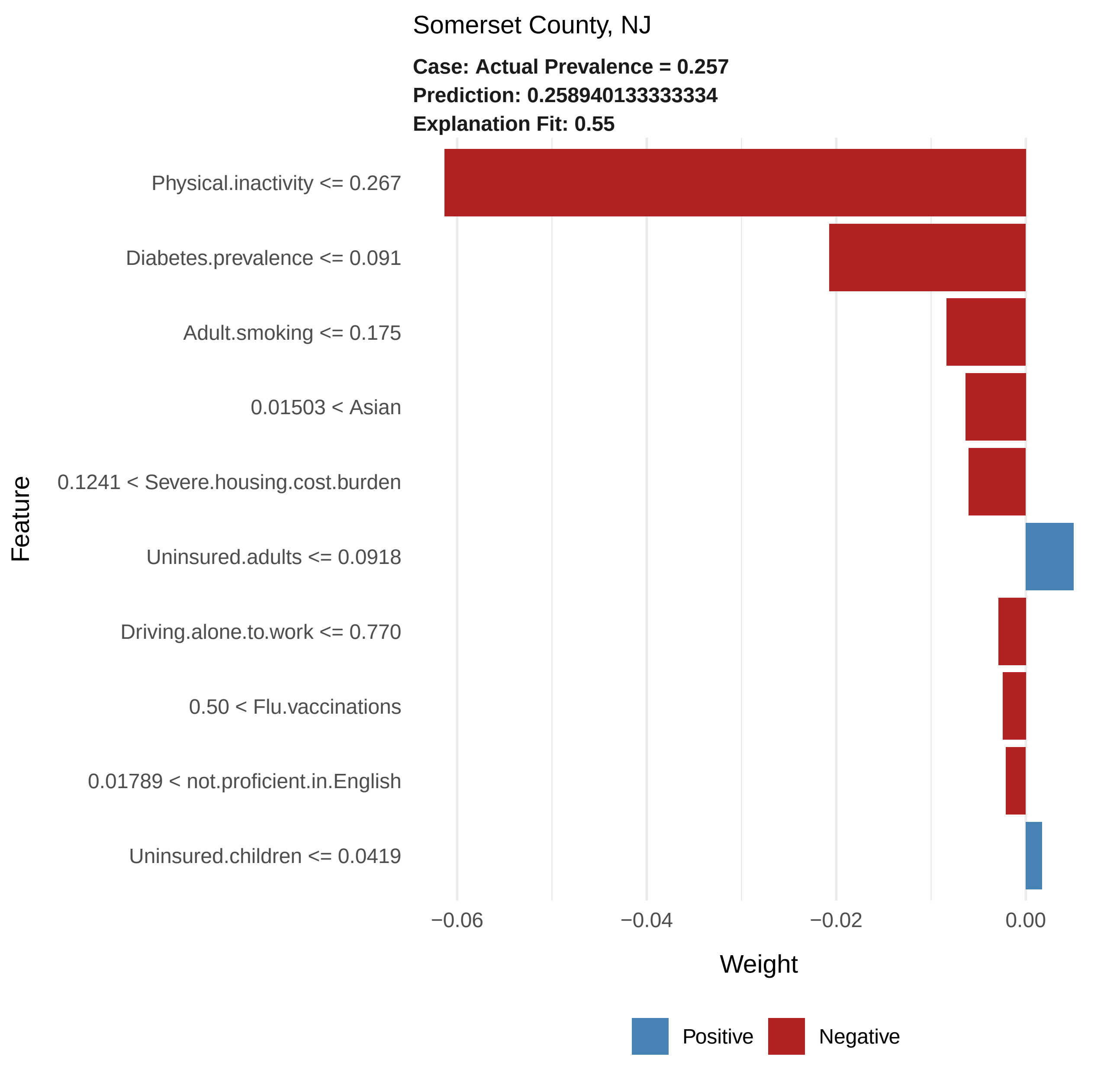
Somerset County, NJ
Case: Actual Prevalence = 0.257
Prediction: 0.258940133333334
Explanation Fit: 0.55
Physical.inactivity <= 0.267
Diabetes.prevalence <= 0.091
Adult.smoking <= 0.175
0.01503 < Asian
0.1241 < Severe.housing.cost.burden
Uninsured.adults <= 0.0918
Driving.alone.to.work <= 0.770
0.50 < Flu.vaccinations
0.01789 < not.proficient.in.English
Uninsured.children <= 0.0419
Feature
−0.06
−0.04
−0.02
0.00
Weight
Positive
Negative

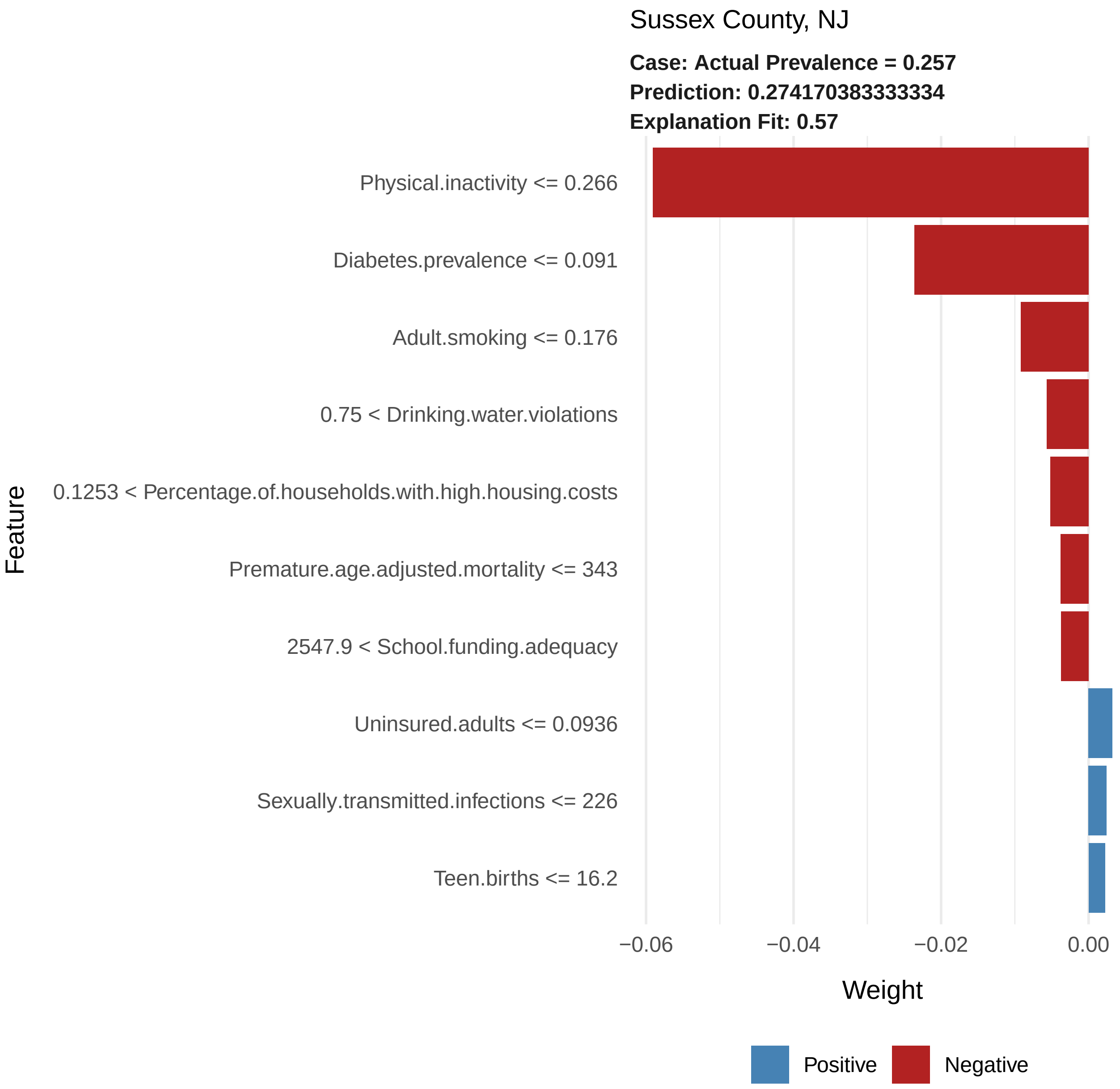
Sussex County, NJ
Case: Actual Prevalence = 0.257
Prediction: 0.274170383333334
Explanation Fit: 0.57
Physical.inactivity <= 0.266
Diabetes.prevalence <= 0.091
Adult.smoking <= 0.176
0.75 < Drinking.water.violations
0.1253 < Percentage.of.households.with.high.housing.costs
Premature.age.adjusted.mortality <= 343
2547.9 < School.funding.adequacy
Uninsured.adults <= 0.0936
Sexually.transmitted.infections <= 226
Teen.births <= 16.2
Feature
−0.06
−0.04
−0.02
0.00
Weight
Positive
Negative

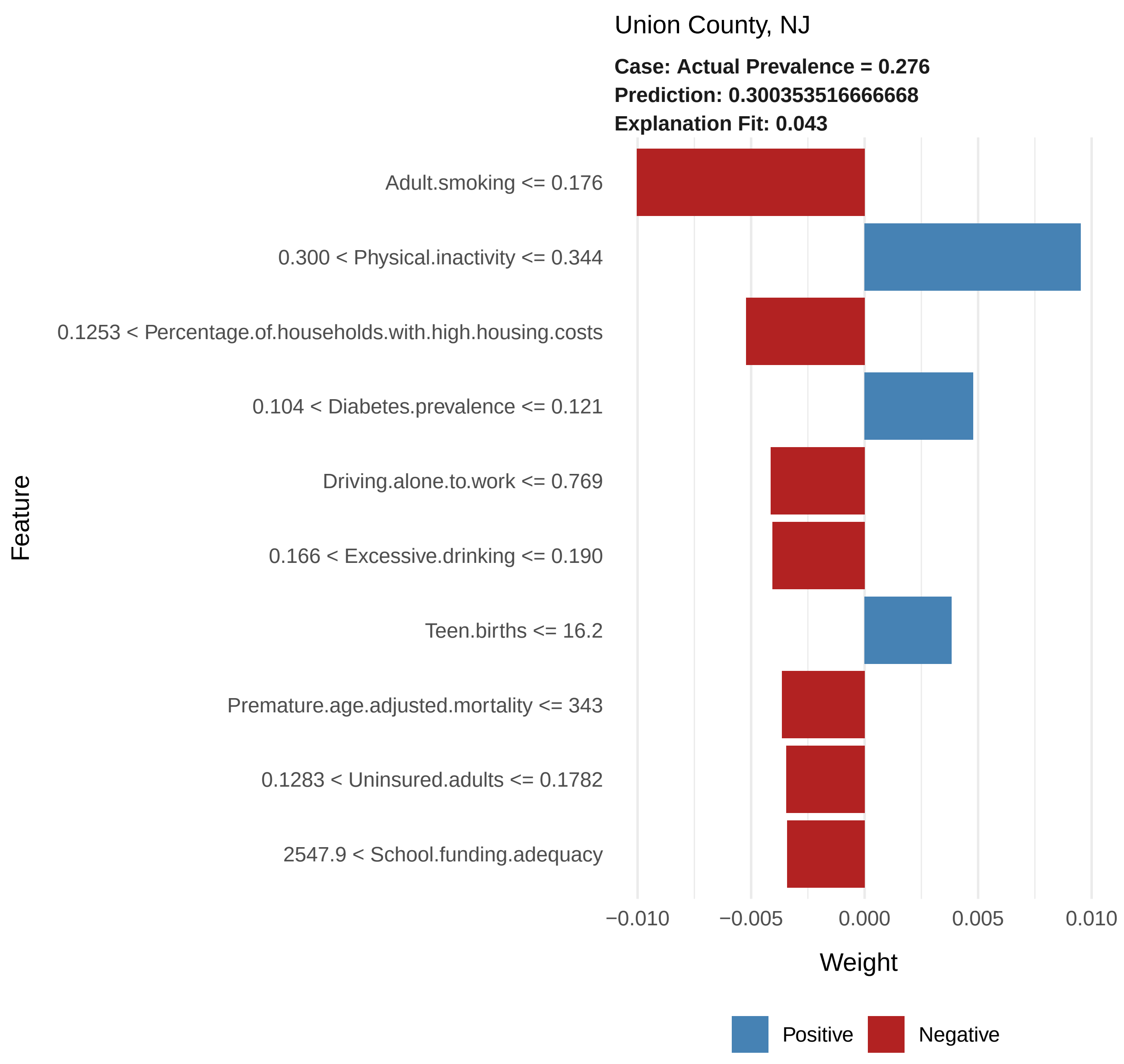
Union County, NJ
Case: Actual Prevalence = 0.276
Prediction: 0.300353516666668
Explanation Fit: 0.043
Adult.smoking <= 0.176
0.300 < Physical.inactivity <= 0.344
0.1253 < Percentage.of.households.with.high.housing.costs
0.104 < Diabetes.prevalence <= 0.121
Driving.alone.to.work <= 0.769
0.166 < Excessive.drinking <= 0.190
Teen.births <= 16.2
Premature.age.adjusted.mortality <= 343
0.1283 < Uninsured.adults <= 0.1782
2547.9 < School.funding.adequacy
Feature
−0.010
−0.005
0.000
0.005
0.010
Weight
Positive
Negative

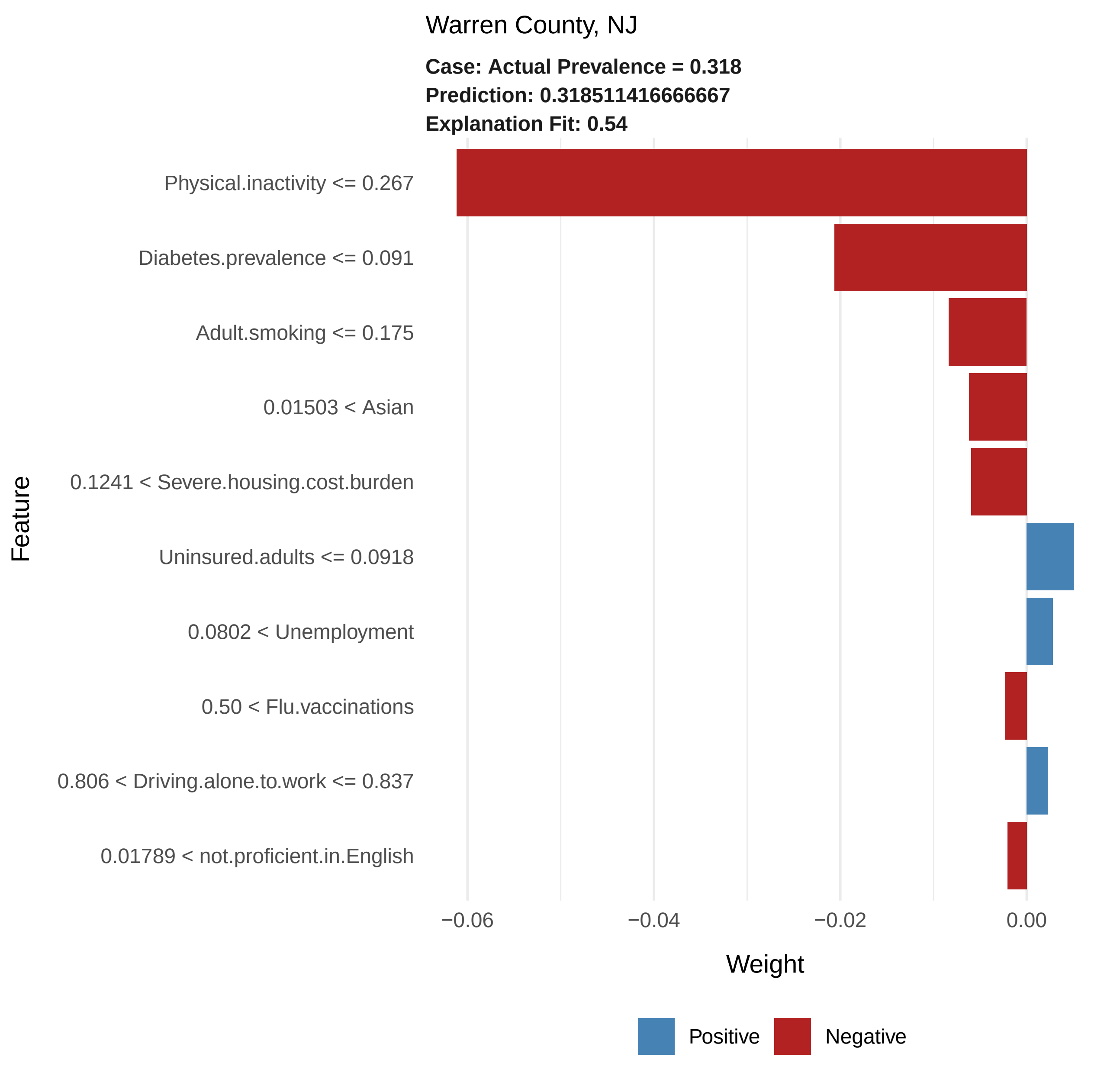
Warren County, NJ
Case: Actual Prevalence = 0.318
Prediction: 0.318511416666667
Explanation Fit: 0.54
Physical.inactivity <= 0.267
Diabetes.prevalence <= 0.091
Adult.smoking <= 0.175
0.01503 < Asian
0.1241 < Severe.housing.cost.burden
Uninsured.adults <= 0.0918
0.0802 < Unemployment
0.50 < Flu.vaccinations
0.806 < Driving.alone.to.work <= 0.837
0.01789 < not.proficient.in.English
Feature
−0.06
−0.04
−0.02
0.00
Weight
Positive
Negative

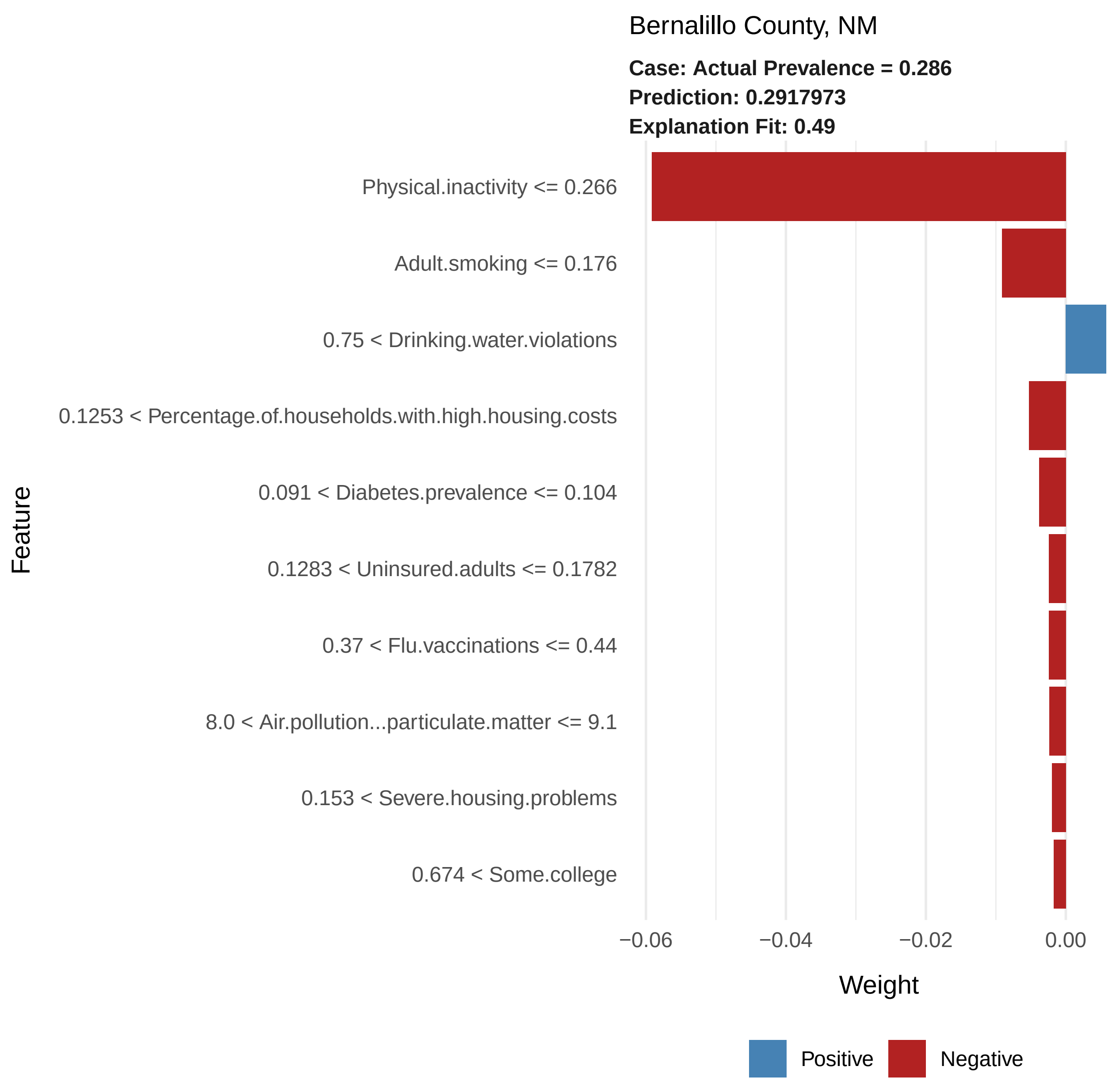

Bernalillo County, NM
Case: Actual Prevalence = 0.286
Prediction: 0.2917973
Explanation Fit: 0.49
Physical.inactivity <= 0.266
Adult.smoking <= 0.176
0.75 < Drinking.water.violations
0.1253 < Percentage.of.households.with.high.housing.costs
0.091 < Diabetes.prevalence <= 0.104
0.1283 < Uninsured.adults <= 0.1782
0.37 < Flu.vaccinations <= 0.44
8.0 < Air.pollution...particulate.matter <= 9.1
0.153 < Severe.housing.problems
0.674 < Some.college
Feature
−0.06
−0.04
−0.02
0.00
Weight
Positive
Negative

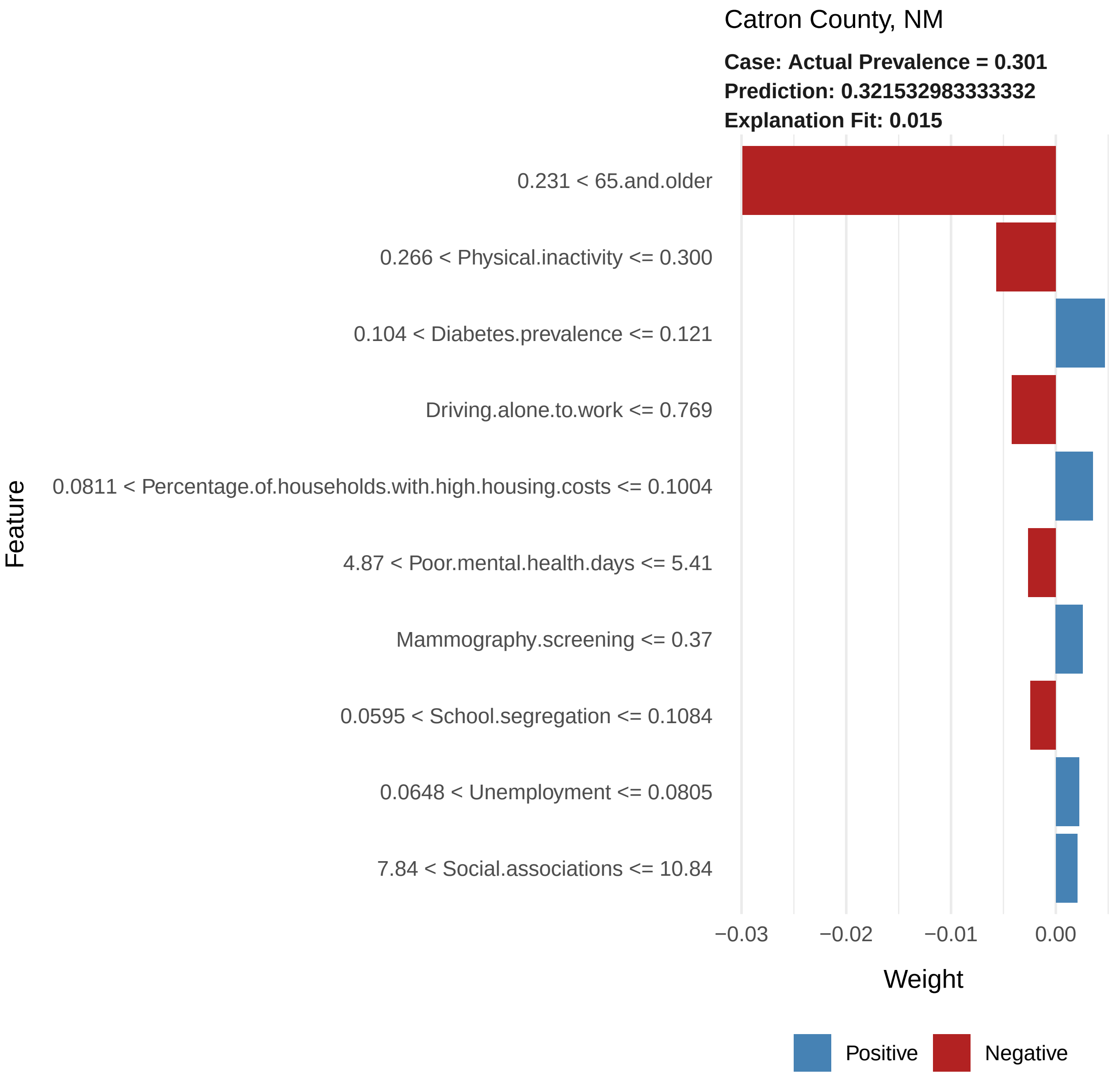

Catron County, NM
Case: Actual Prevalence = 0.301
Prediction: 0.321532983333332
Explanation Fit: 0.015
0.231 < 65.and.older
0.266 < Physical.inactivity <= 0.300
0.104 < Diabetes.prevalence <= 0.121
Driving.alone.to.work <= 0.769
0.0811 < Percentage.of.households.with.high.housing.costs <= 0.1004
4.87 < Poor.mental.health.days <= 5.41
Mammography.screening <= 0.37
0.0595 < School.segregation <= 0.1084
0.0648 < Unemployment <= 0.0805
7.84 < Social.associations <= 10.84
Feature
−0.03
−0.02
−0.01
0.00
Weight
Positive
Negative

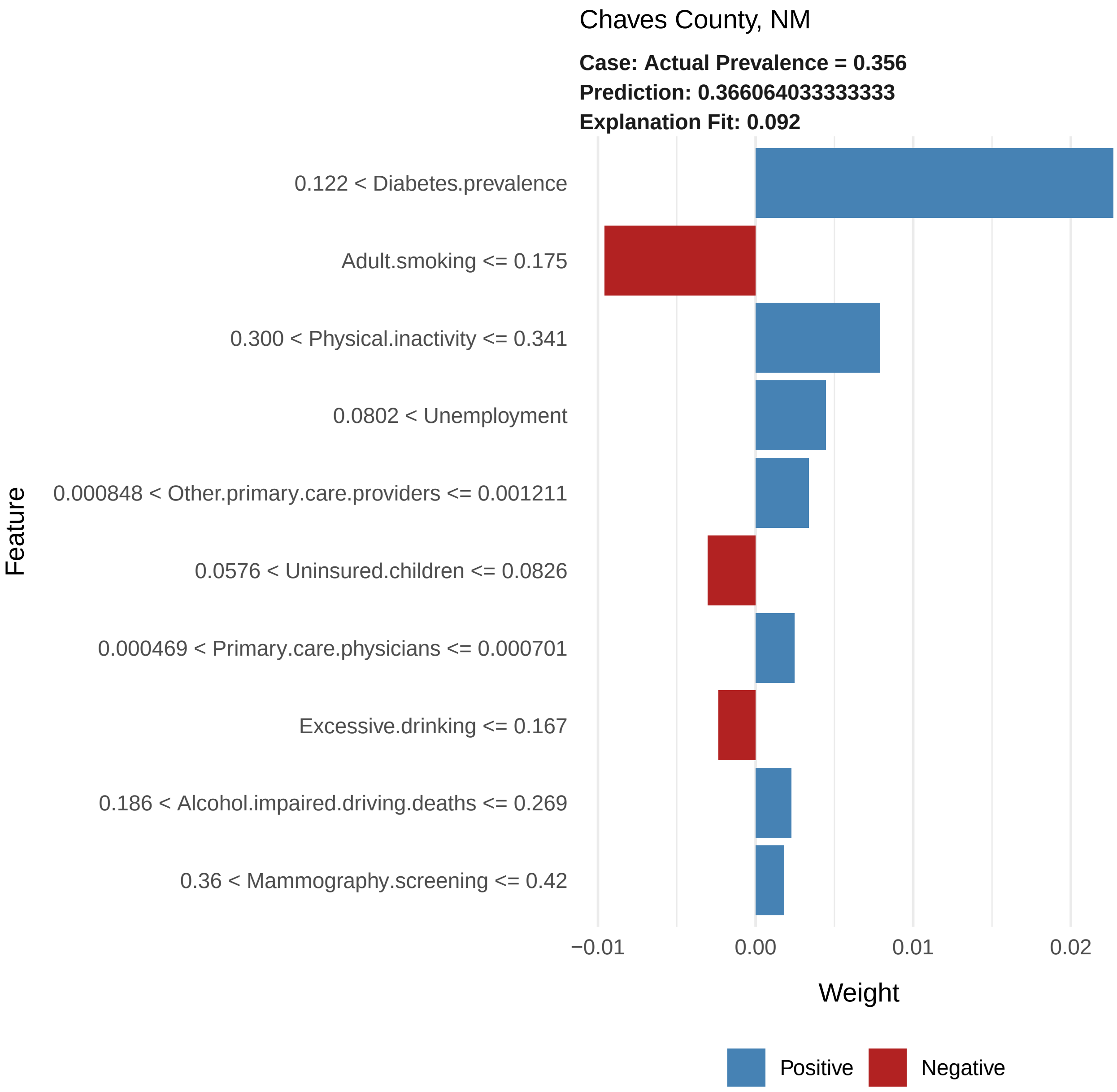

Chaves County, NM
Case: Actual Prevalence = 0.356
Prediction: 0.366064033333333
Explanation Fit: 0.092
0.122 < Diabetes.prevalence
Adult.smoking <= 0.175
0.300 < Physical.inactivity <= 0.341
0.0802 < Unemployment
0.000848 < Other.primary.care.providers <= 0.001211
0.0576 < Uninsured.children <= 0.0826
0.000469 < Primary.care.physicians <= 0.000701
Excessive.drinking <= 0.167
0.186 < Alcohol.impaired.driving.deaths <= 0.269
0.36 < Mammography.screening <= 0.42
Feature
−0.01
0.00
0.01
0.02
Weight
Positive
Negative

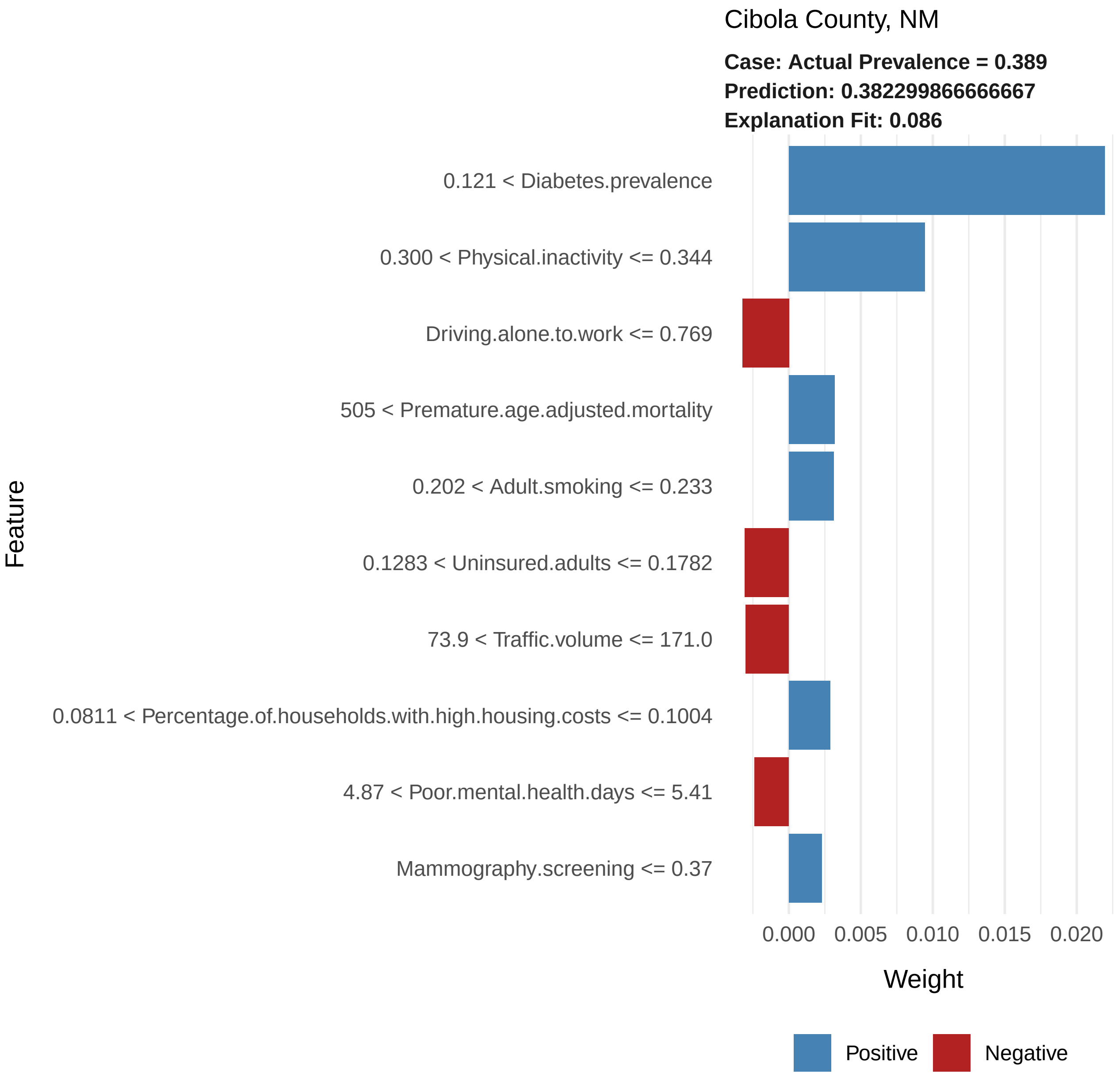

Cibola County, NM
Case: Actual Prevalence = 0.389
Prediction: 0.382299866666667
Explanation Fit: 0.086
0.121 < Diabetes.prevalence
0.300 < Physical.inactivity <= 0.344
Driving.alone.to.work <= 0.769
505 < Premature.age.adjusted.mortality
0.202 < Adult.smoking <= 0.233
0.1283 < Uninsured.adults <= 0.1782
73.9 < Traffic.volume <= 171.0
0.0811 < Percentage.of.households.with.high.housing.costs <= 0.1004
4.87 < Poor.mental.health.days <= 5.41
Mammography.screening <= 0.37
Feature
0.000
0.005
0.010
0.015
0.020
Weight
Positive
Negative

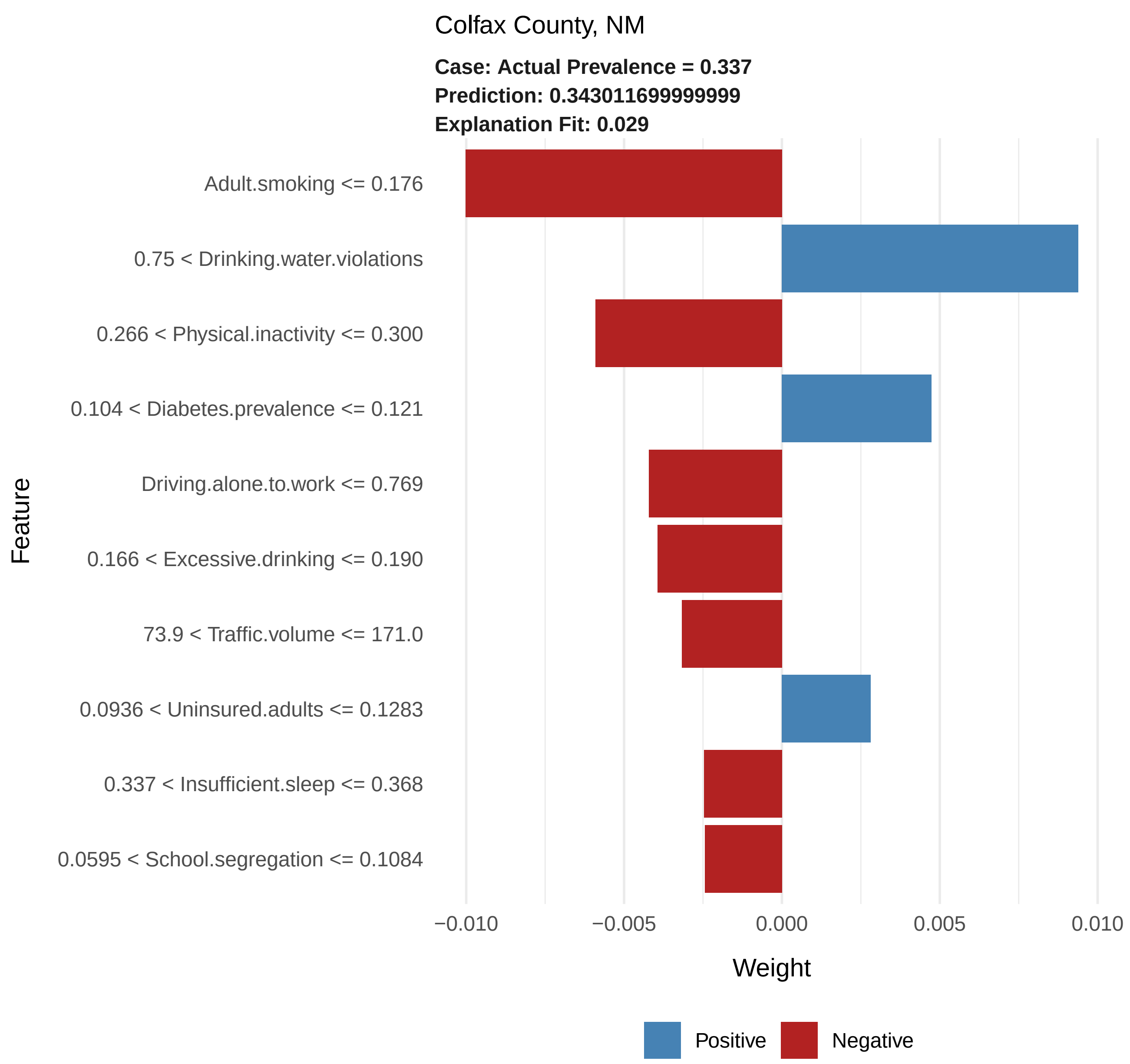
Colfax County, NM
Case: Actual Prevalence = 0.337
Prediction: 0.343011699999999
Explanation Fit: 0.029
Adult.smoking <= 0.176
0.75 < Drinking.water.violations
0.266 < Physical.inactivity <= 0.300
0.104 < Diabetes.prevalence <= 0.121
Driving.alone.to.work <= 0.769
0.166 < Excessive.drinking <= 0.190
73.9 < Traffic.volume <= 171.0
0.0936 < Uninsured.adults <= 0.1283
0.337 < Insufficient.sleep <= 0.368
0.0595 < School.segregation <= 0.1084
Feature
−0.010
−0.005
0.000
0.005
0.010
Weight
Positive
Negative

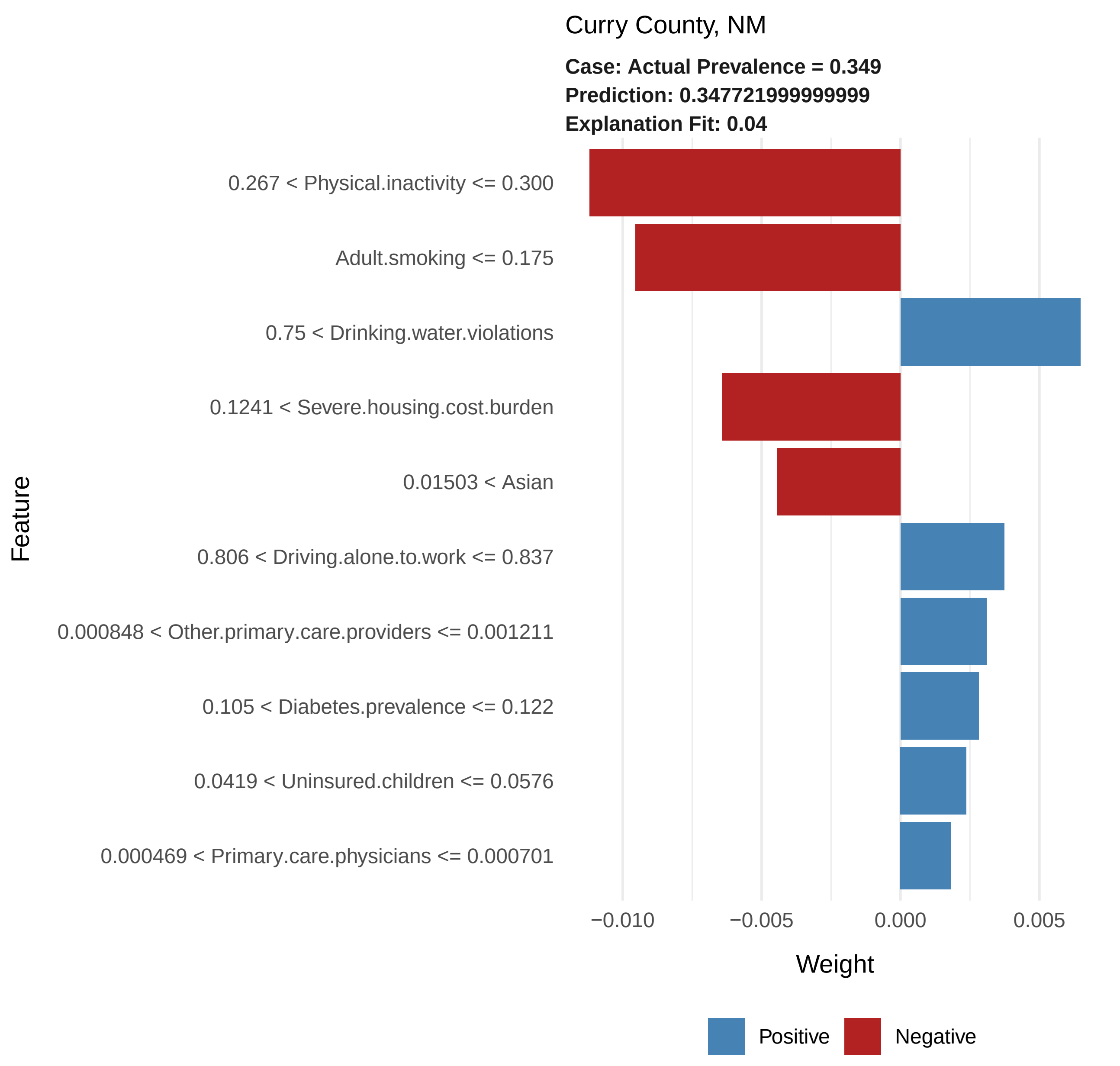

Curry County, NM
Case: Actual Prevalence = 0.349
Prediction: 0.347721999999999
Explanation Fit: 0.04
0.267 < Physical.inactivity <= 0.300
Adult.smoking <= 0.175
0.75 < Drinking.water.violations
0.1241 < Severe.housing.cost.burden
0.01503 < Asian
0.806 < Driving.alone.to.work <= 0.837
0.000848 < Other.primary.care.providers <= 0.001211
0.105 < Diabetes.prevalence <= 0.122
0.0419 < Uninsured.children <= 0.0576
0.000469 < Primary.care.physicians <= 0.000701
Feature
−0.010
−0.005
0.000
0.005
Weight
Positive
Negative

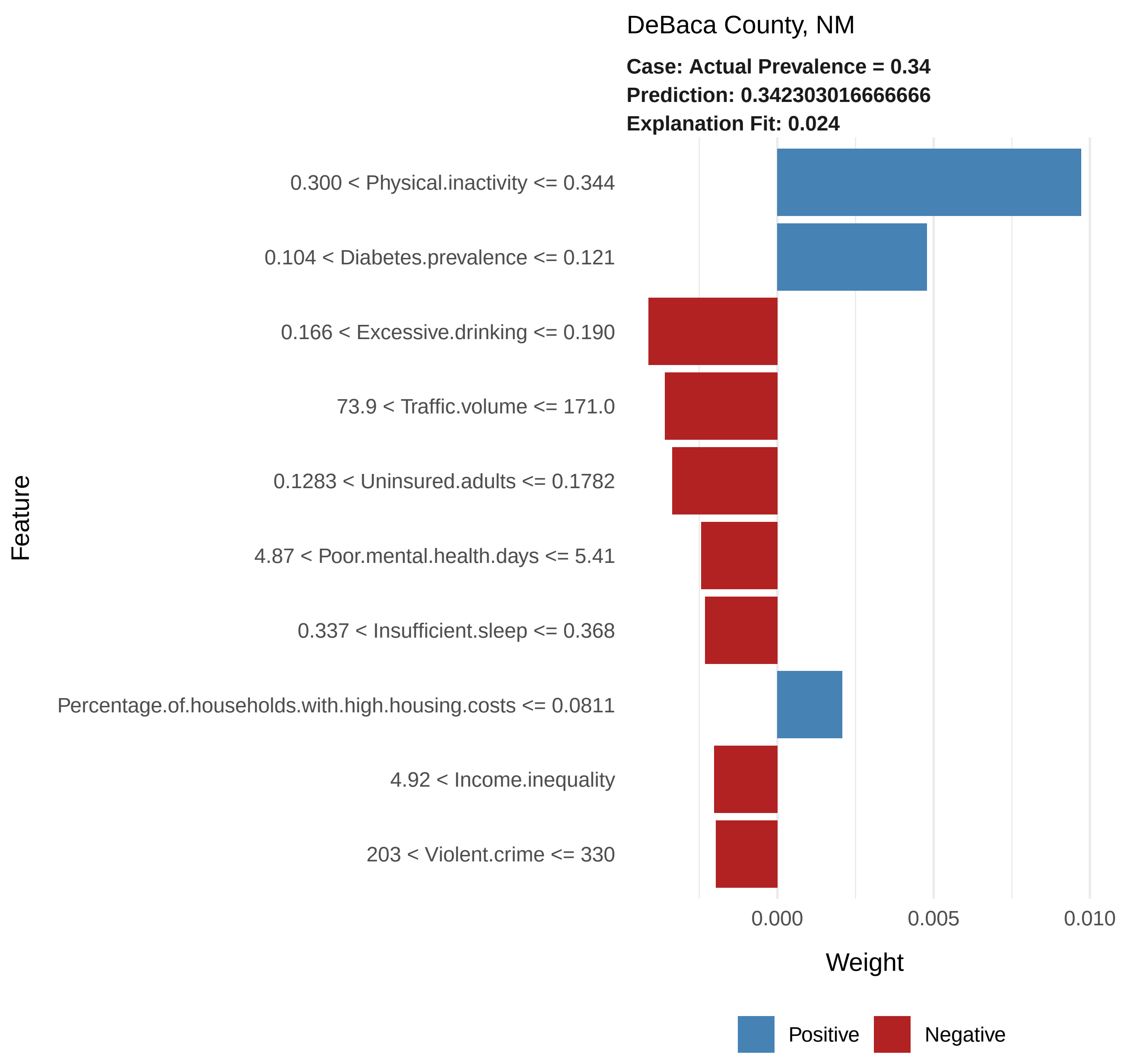
DeBaca County, NM
Case: Actual Prevalence = 0.34
Prediction: 0.342303016666666
Explanation Fit: 0.024
0.300 < Physical.inactivity <= 0.344
0.104 < Diabetes.prevalence <= 0.121
0.166 < Excessive.drinking <= 0.190
73.9 < Traffic.volume <= 171.0
0.1283 < Uninsured.adults <= 0.1782
4.87 < Poor.mental.health.days <= 5.41
0.337 < Insufficient.sleep <= 0.368
Percentage.of.households.with.high.housing.costs <= 0.0811
4.92 < Income.inequality
203 < Violent.crime <= 330
Feature
0.000
0.005
0.010
Weight
Positive
Negative

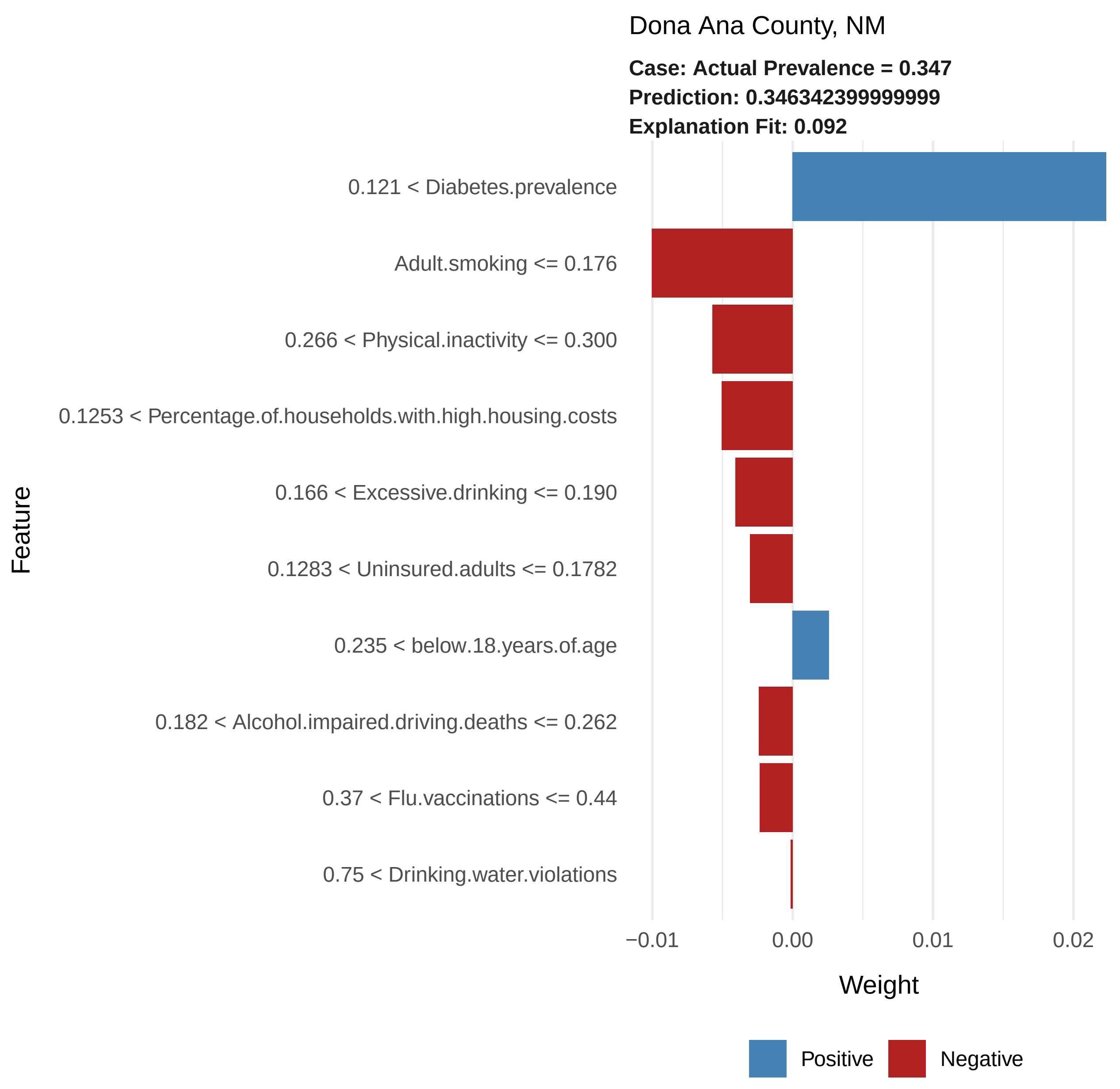

Dona Ana County, NM
Case: Actual Prevalence = 0.347
Prediction: 0.346342399999999
Explanation Fit: 0.092
0.121 < Diabetes.prevalence
Adult.smoking <= 0.176
0.266 < Physical.inactivity <= 0.300
0.1253 < Percentage.of.households.with.high.housing.costs
0.166 < Excessive.drinking <= 0.190
0.1283 < Uninsured.adults <= 0.1782
0.235 < below.18.years.of.age
0.182 < Alcohol.impaired.driving.deaths <= 0.262
0.37 < Flu.vaccinations <= 0.44
0.75 < Drinking.water.violations
Feature
−0.01
0.00
0.01
0.02
Weight
Positive
Negative

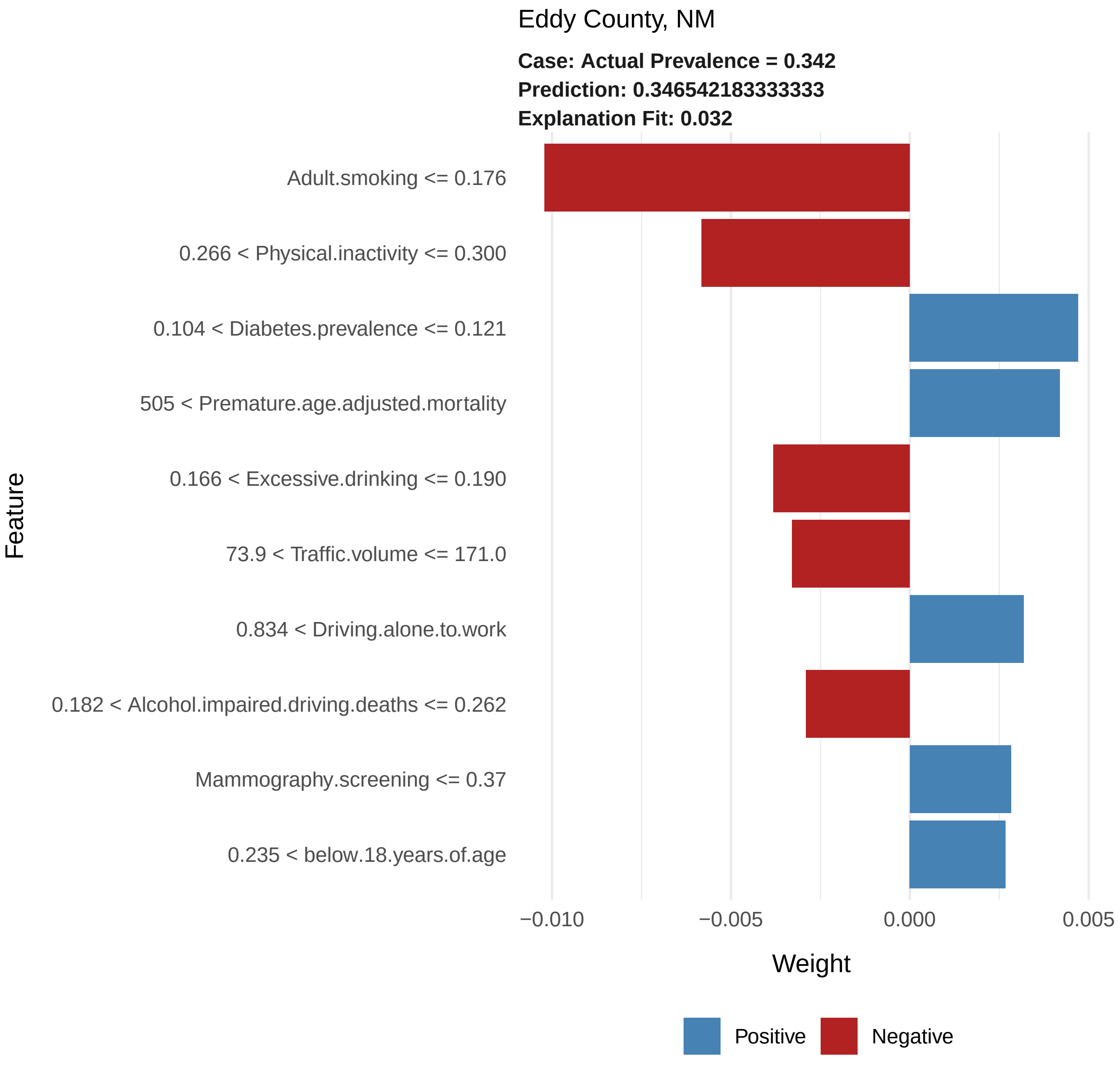
Eddy County, NM
Case: Actual Prevalence = 0.342
Prediction: 0.346542183333333
Explanation Fit: 0.032
Adult.smoking <= 0.176
0.266 < Physical.inactivity <= 0.300
0.104 < Diabetes.prevalence <= 0.121
505 < Premature.age.adjusted.mortality
0.166 < Excessive.drinking <= 0.190
73.9 < Traffic.volume <= 171.0
0.834 < Driving.alone.to.work
0.182 < Alcohol.impaired.driving.deaths <= 0.262
Mammography.screening <= 0.37
0.235 < below.18.years.of.age
Feature
−0.010
−0.005
0.000
0.005
Weight
Positive
Negative

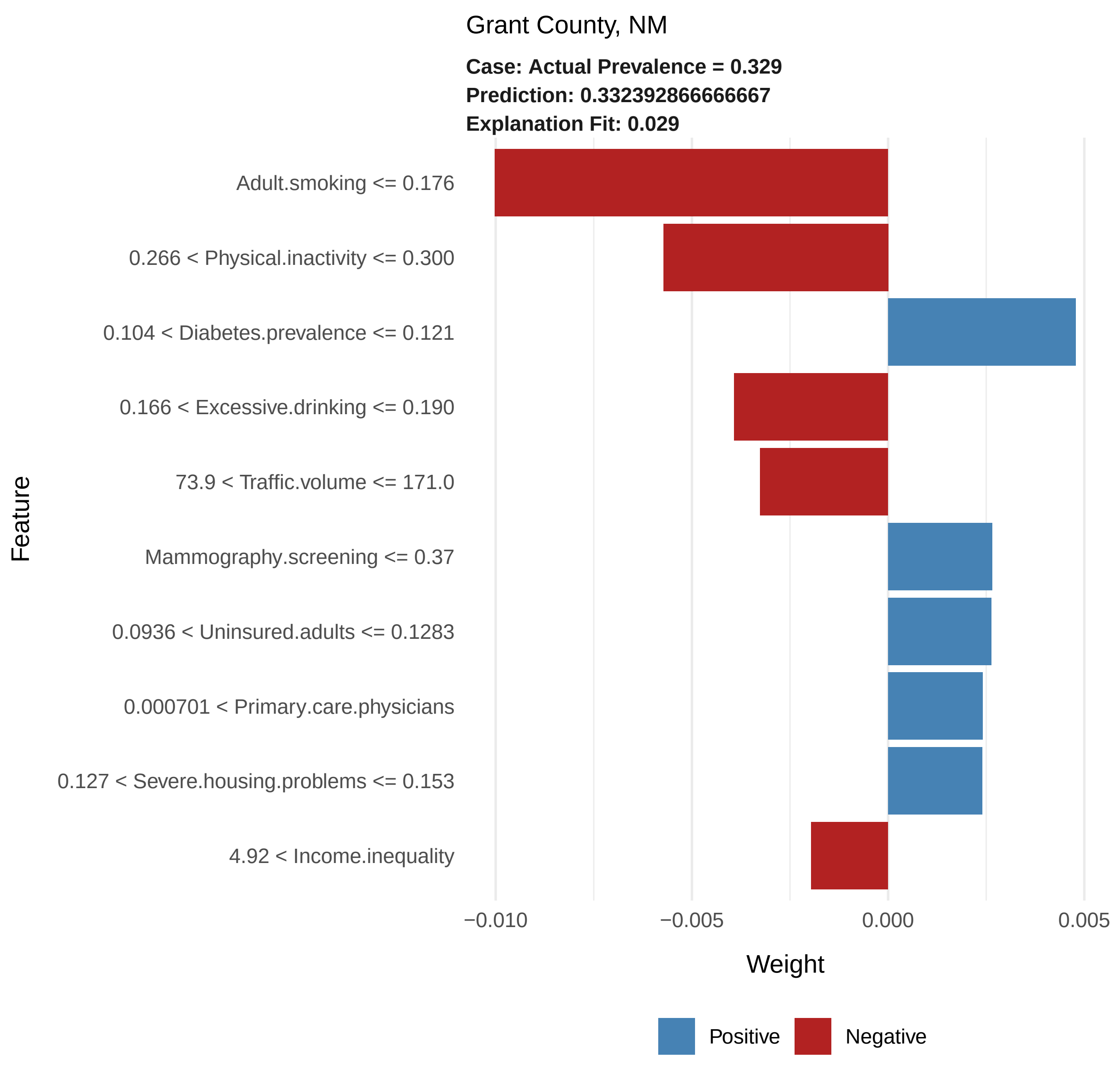

Grant County, NM
Case: Actual Prevalence = 0.329
Prediction: 0.332392866666667
Explanation Fit: 0.029
Adult.smoking <= 0.176
0.266 < Physical.inactivity <= 0.300
0.104 < Diabetes.prevalence <= 0.121
0.166 < Excessive.drinking <= 0.190
73.9 < Traffic.volume <= 171.0
Mammography.screening <= 0.37
0.0936 < Uninsured.adults <= 0.1283
0.000701 < Primary.care.physicians
0.127 < Severe.housing.problems <= 0.153
4.92 < Income.inequality
Feature
−0.010
−0.005
0.000
0.005
Weight
Positive
Negative

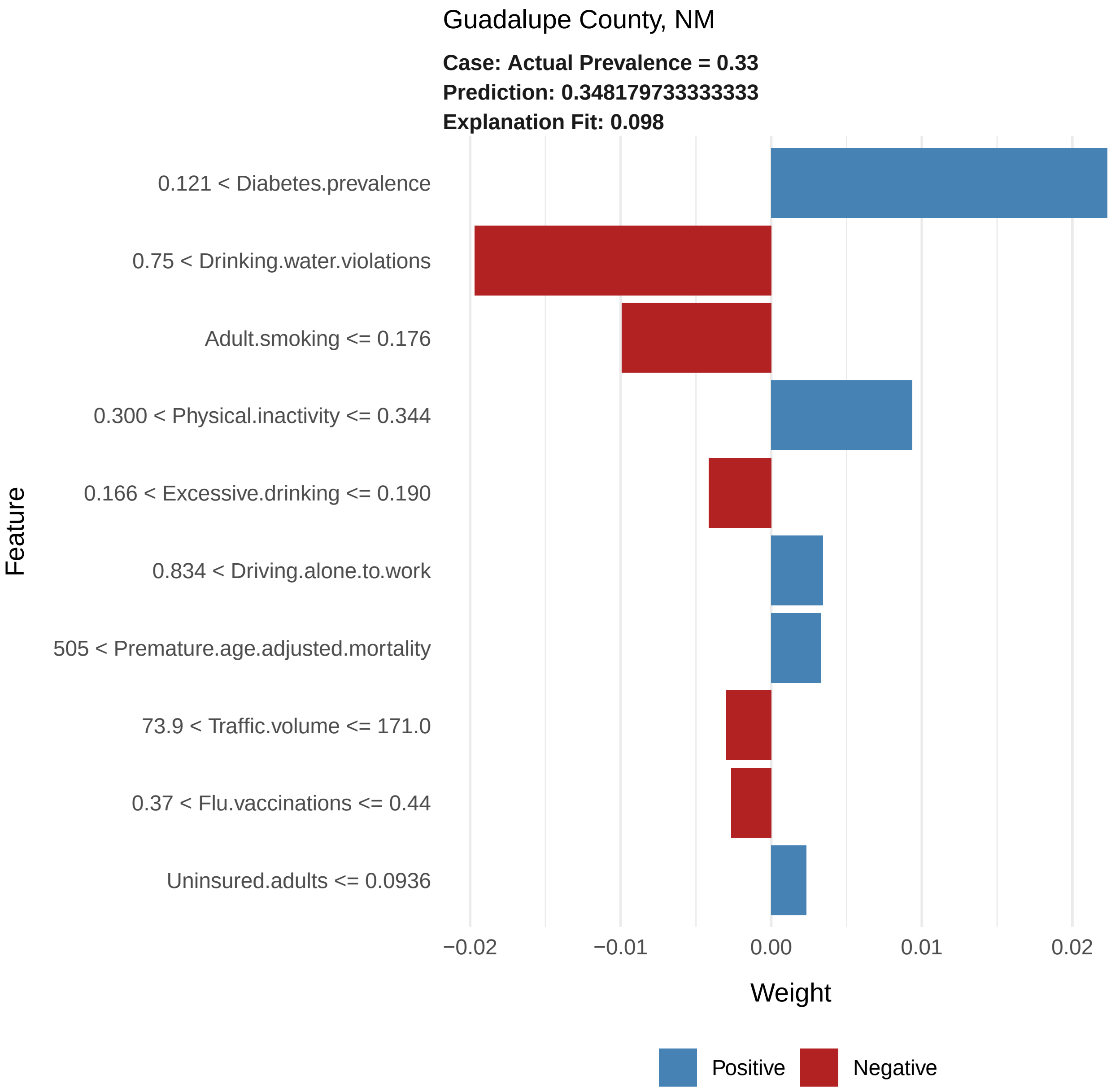
Guadalupe County, NM
Case: Actual Prevalence = 0.33
Prediction: 0.348179733333333
Explanation Fit: 0.098
0.121 < Diabetes.prevalence
0.75 < Drinking.water.violations
Adult.smoking <= 0.176
0.300 < Physical.inactivity <= 0.344
0.166 < Excessive.drinking <= 0.190
0.834 < Driving.alone.to.work
505 < Premature.age.adjusted.mortality
73.9 < Traffic.volume <= 171.0
0.37 < Flu.vaccinations <= 0.44
Uninsured.adults <= 0.0936
Feature
−0.02
−0.01
0.00
0.01
0.02
Weight
Positive
Negative

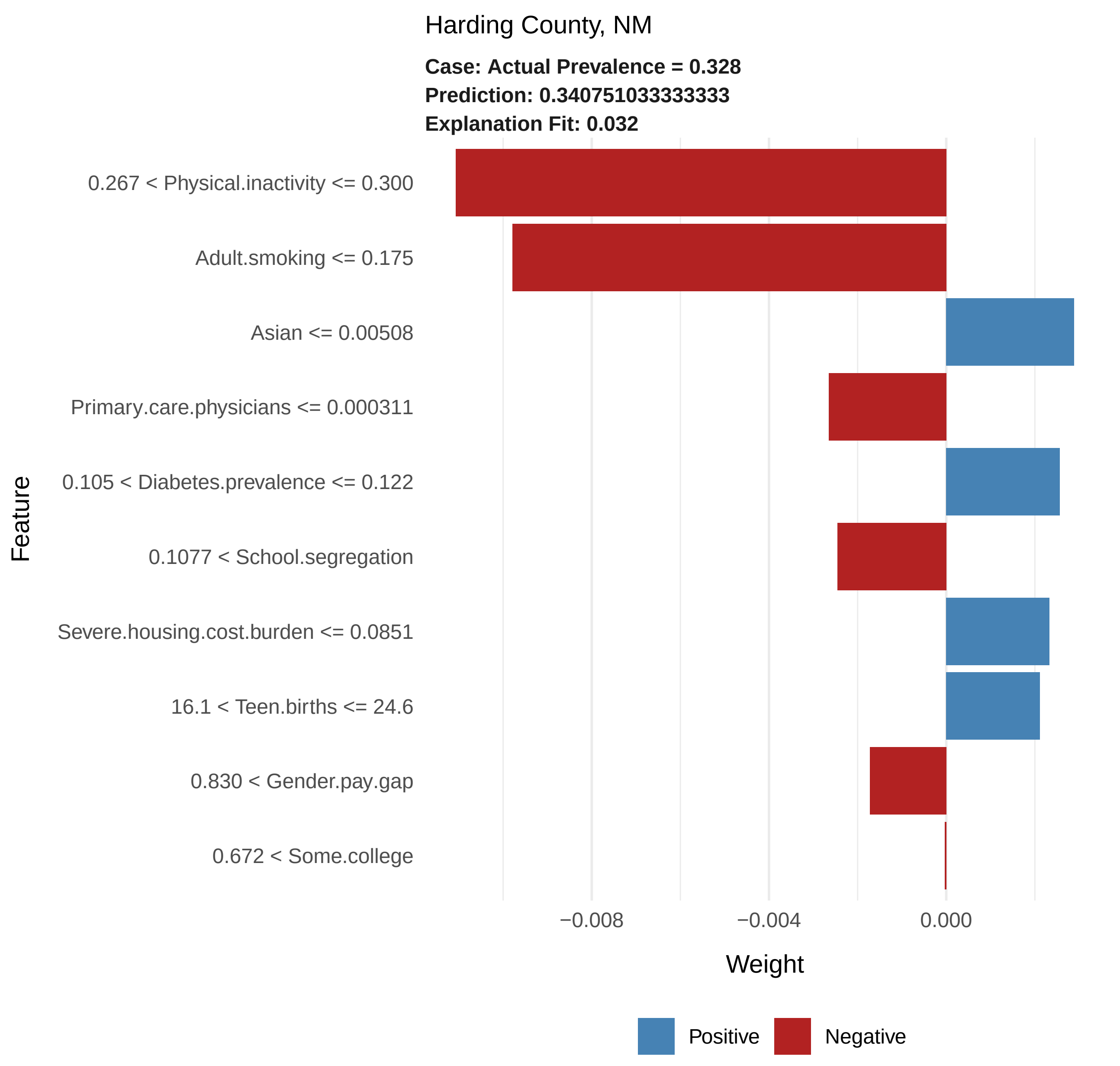

Harding County, NM
Case: Actual Prevalence = 0.328
Prediction: 0.340751033333333
Explanation Fit: 0.032
0.267 < Physical.inactivity <= 0.300
Adult.smoking <= 0.175
Asian <= 0.00508
Primary.care.physicians <= 0.000311
0.105 < Diabetes.prevalence <= 0.122
0.1077 < School.segregation
Severe.housing.cost.burden <= 0.0851
16.1 < Teen.births <= 24.6
0.830 < Gender.pay.gap
0.672 < Some.college
Feature
−0.008
−0.004
0.000
Weight
Positive
Negative

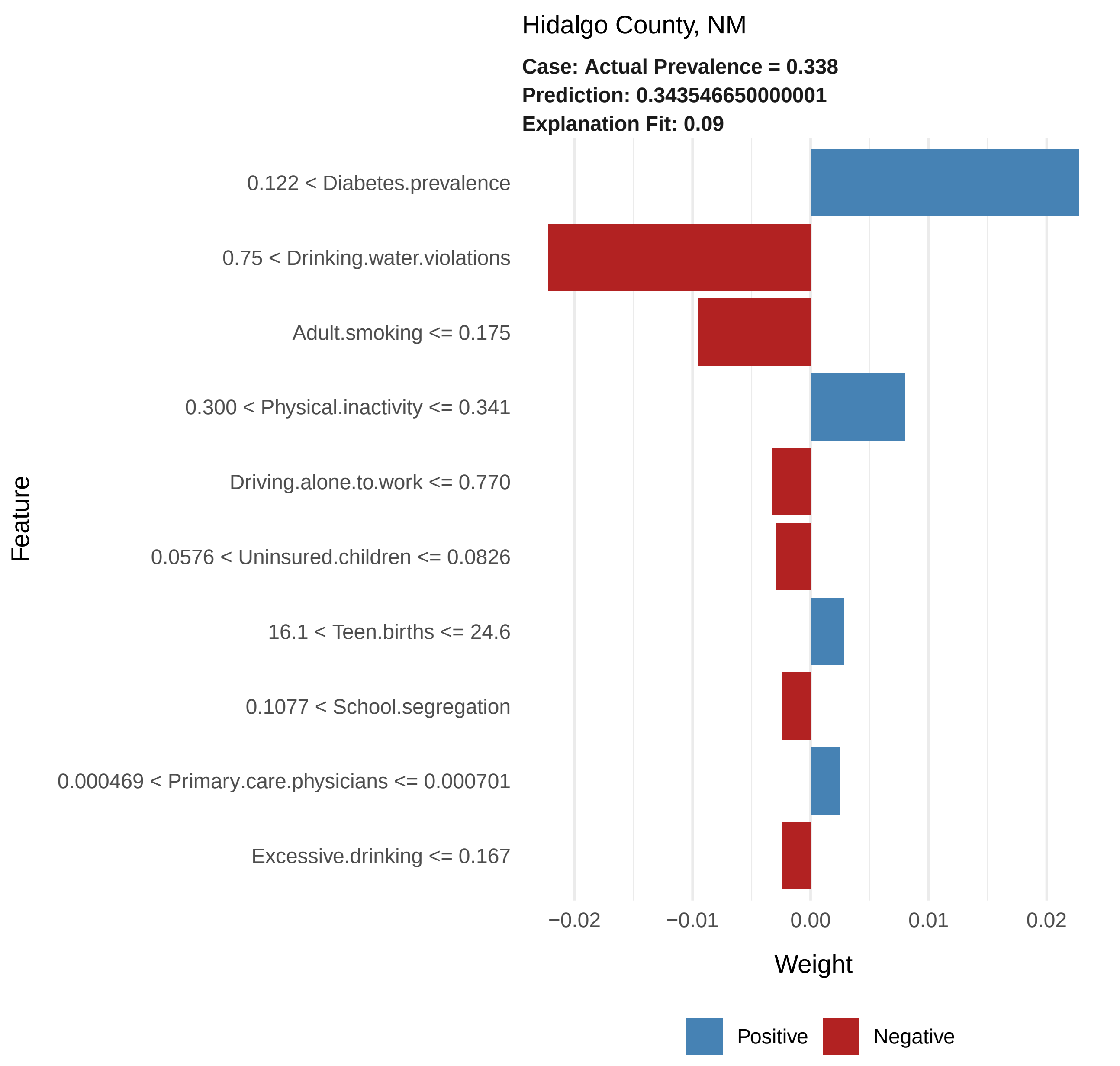
Hidalgo County, NM
Case: Actual Prevalence = 0.338
Prediction: 0.343546650000001
Explanation Fit: 0.09
0.122 < Diabetes.prevalence
0.75 < Drinking.water.violations
Adult.smoking <= 0.175
0.300 < Physical.inactivity <= 0.341
Driving.alone.to.work <= 0.770
0.0576 < Uninsured.children <= 0.0826
16.1 < Teen.births <= 24.6
0.1077 < School.segregation
0.000469 < Primary.care.physicians <= 0.000701
Excessive.drinking <= 0.167
Feature
−0.02
−0.01
0.00
0.01
0.02
Weight
Positive
Negative

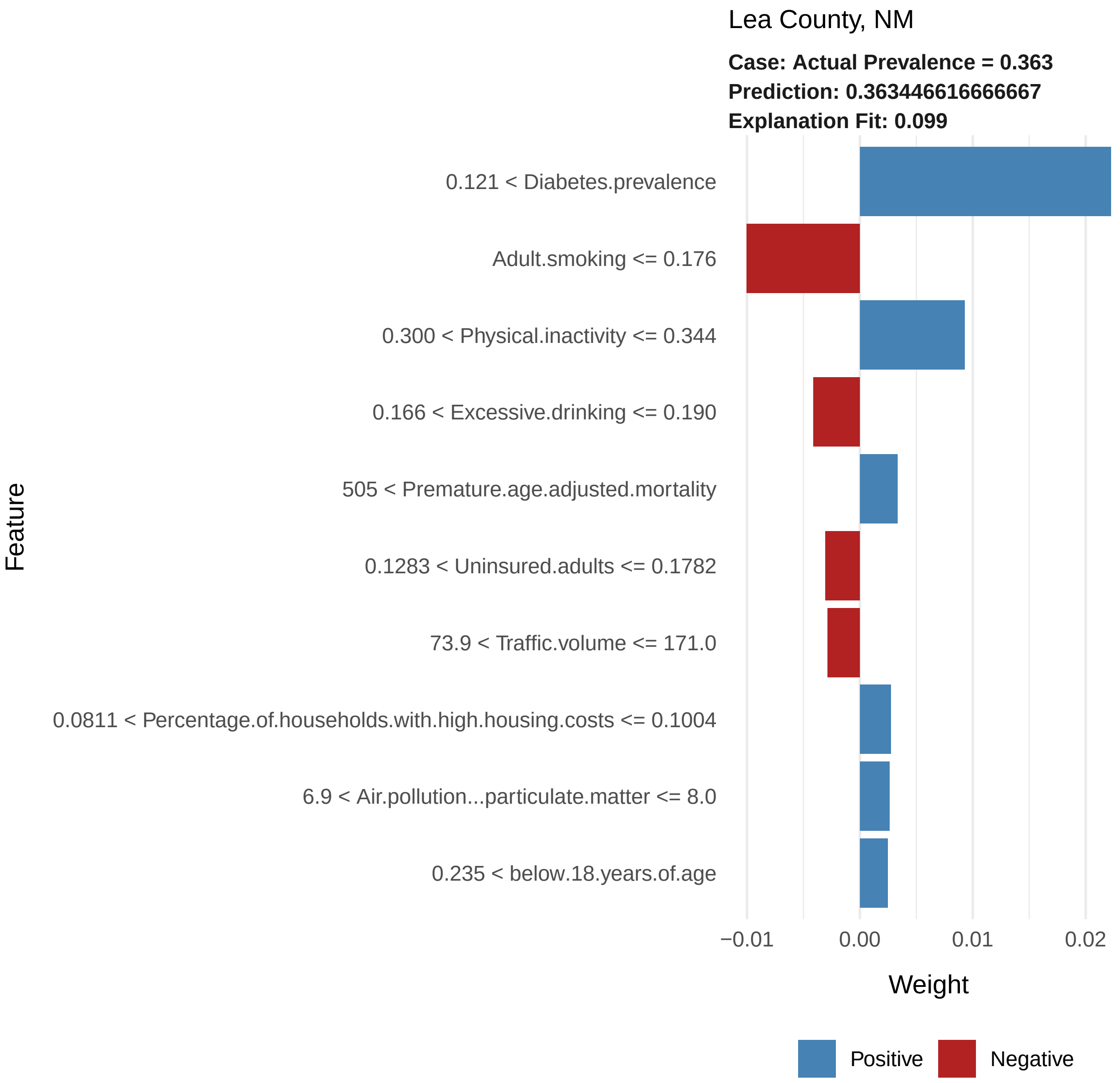

Lea County, NM
Case: Actual Prevalence = 0.363
Prediction: 0.363446616666667
Explanation Fit: 0.099
0.121 < Diabetes.prevalence
Adult.smoking <= 0.176
0.300 < Physical.inactivity <= 0.344
0.166 < Excessive.drinking <= 0.190
505 < Premature.age.adjusted.mortality
0.1283 < Uninsured.adults <= 0.1782
73.9 < Traffic.volume <= 171.0
0.0811 < Percentage.of.households.with.high.housing.costs <= 0.1004
6.9 < Air.pollution...particulate.matter <= 8.0
0.235 < below.18.years.of.age
Feature
−0.01
0.00
0.01
0.02
Weight
Positive
Negative

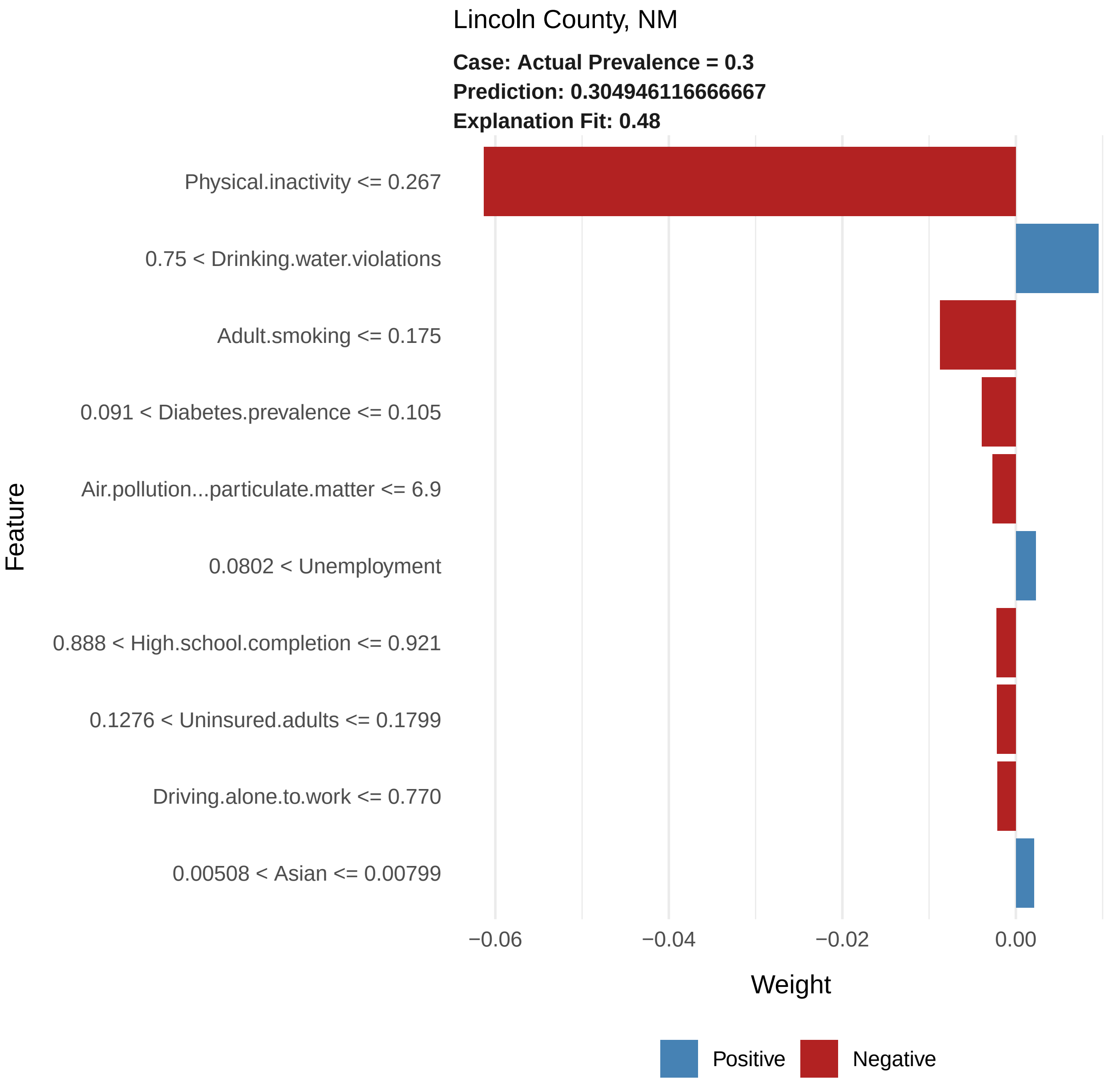
Lincoln County, NM
Case: Actual Prevalence = 0.3
Prediction: 0.304946116666667
Explanation Fit: 0.48
Physical.inactivity <= 0.267
0.75 < Drinking.water.violations
Adult.smoking <= 0.175
0.091 < Diabetes.prevalence <= 0.105
Air.pollution...particulate.matter <= 6.9
0.0802 < Unemployment
0.888 < High.school.completion <= 0.921
0.1276 < Uninsured.adults <= 0.1799
Driving.alone.to.work <= 0.770
0.00508 < Asian <= 0.00799
Feature
−0.06
−0.04
−0.02
0.00
Weight
Positive
Negative

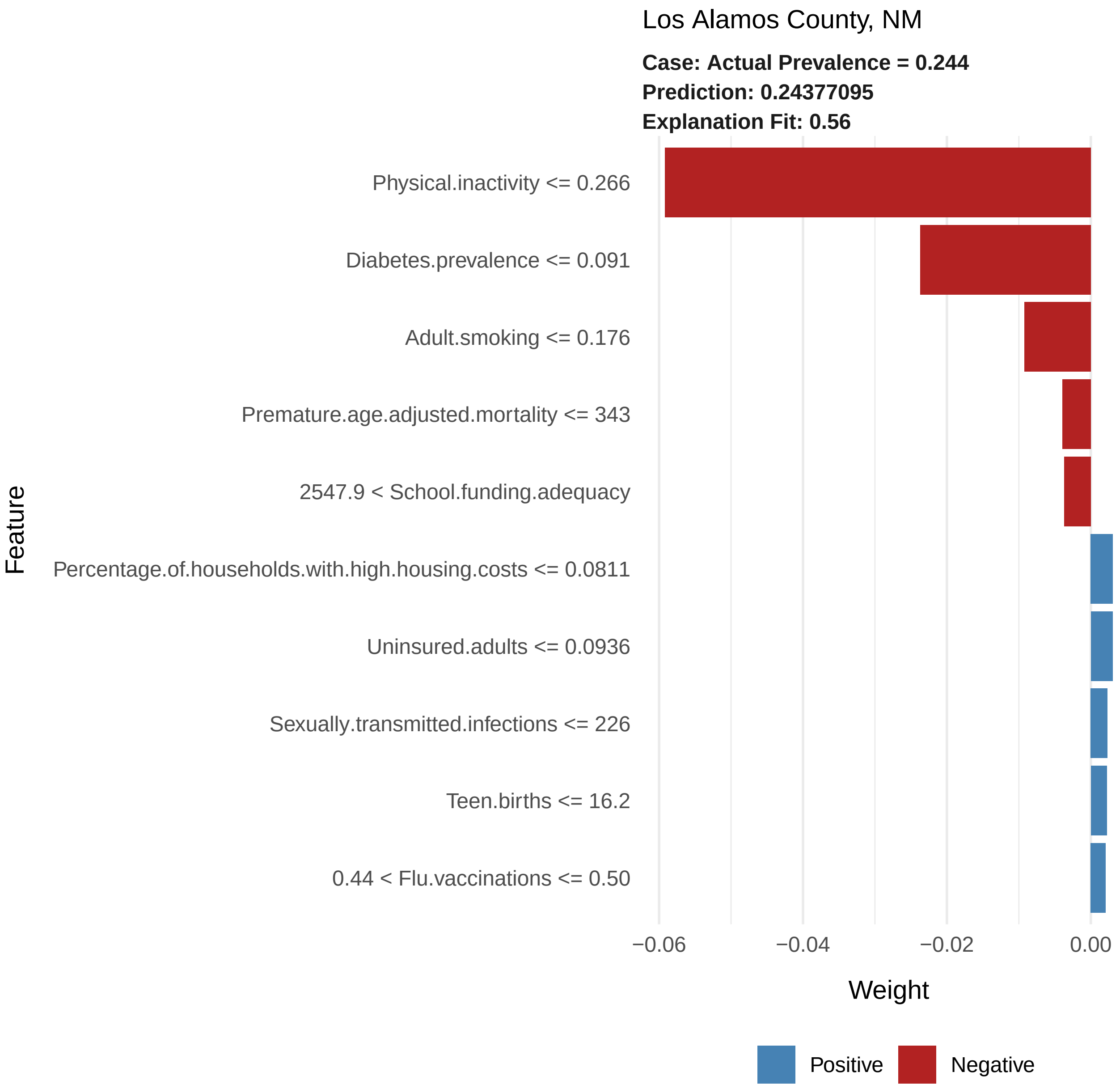

Los Alamos County, NM
Case: Actual Prevalence = 0.244
Prediction: 0.24377095
Explanation Fit: 0.56
Physical.inactivity <= 0.266
Diabetes.prevalence <= 0.091
Adult.smoking <= 0.176
Premature.age.adjusted.mortality <= 343
2547.9 < School.funding.adequacy
Percentage.of.households.with.high.housing.costs <= 0.0811
Uninsured.adults <= 0.0936
Sexually.transmitted.infections <= 226
Teen.births <= 16.2
0.44 < Flu.vaccinations <= 0.50
Feature
−0.06
−0.04
−0.02
0.00
Weight
Positive
Negative

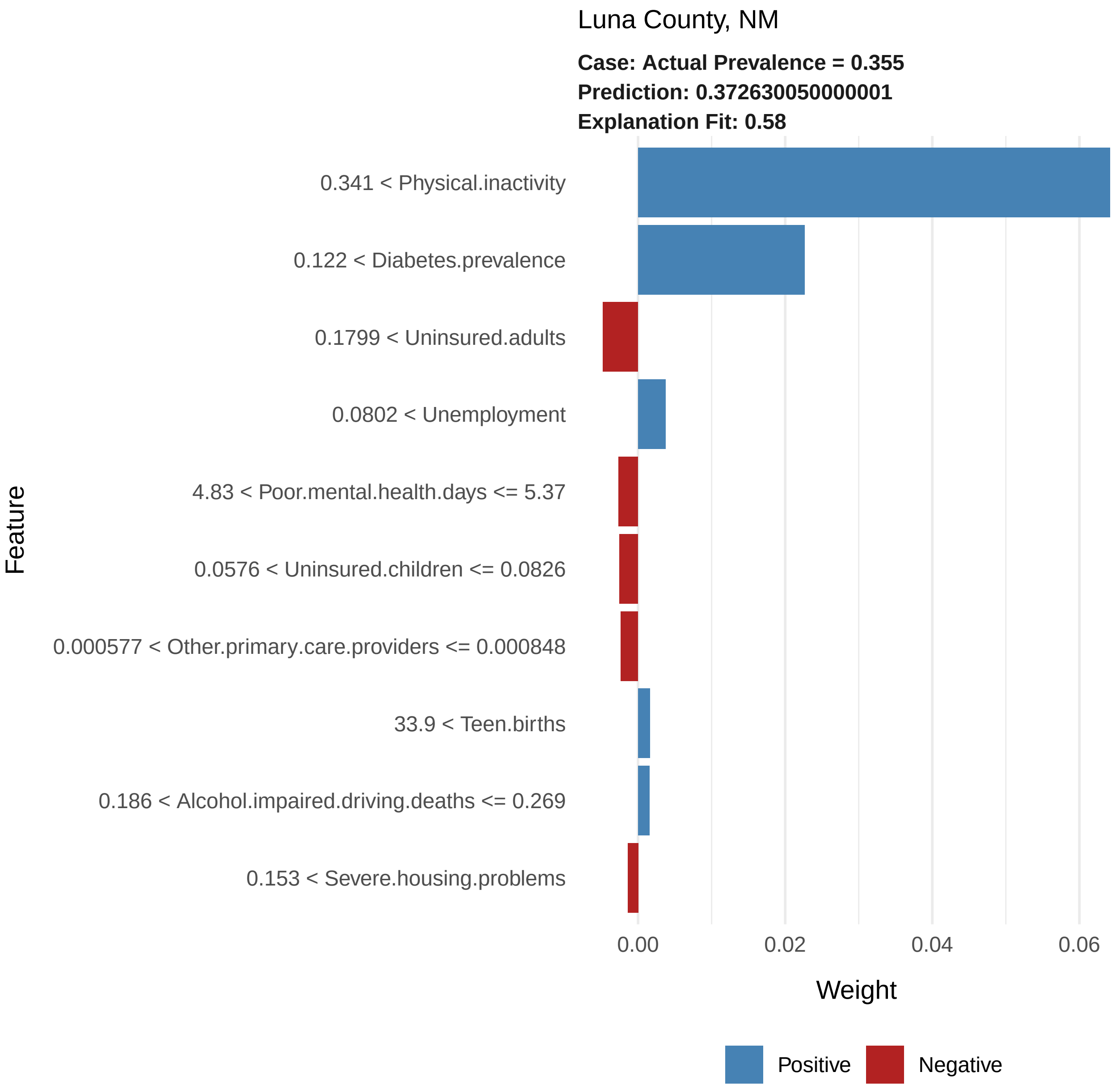

Luna County, NM
Case: Actual Prevalence = 0.355
Prediction: 0.372630050000001
Explanation Fit: 0.58
0.341 < Physical.inactivity
0.122 < Diabetes.prevalence
0.1799 < Uninsured.adults
0.0802 < Unemployment
4.83 < Poor.mental.health.days <= 5.37
0.0576 < Uninsured.children <= 0.0826
0.000577 < Other.primary.care.providers <= 0.000848
33.9 < Teen.births
0.186 < Alcohol.impaired.driving.deaths <= 0.269
0.153 < Severe.housing.problems
Feature
0.00
0.02
0.04
0.06
Weight
Positive
Negative

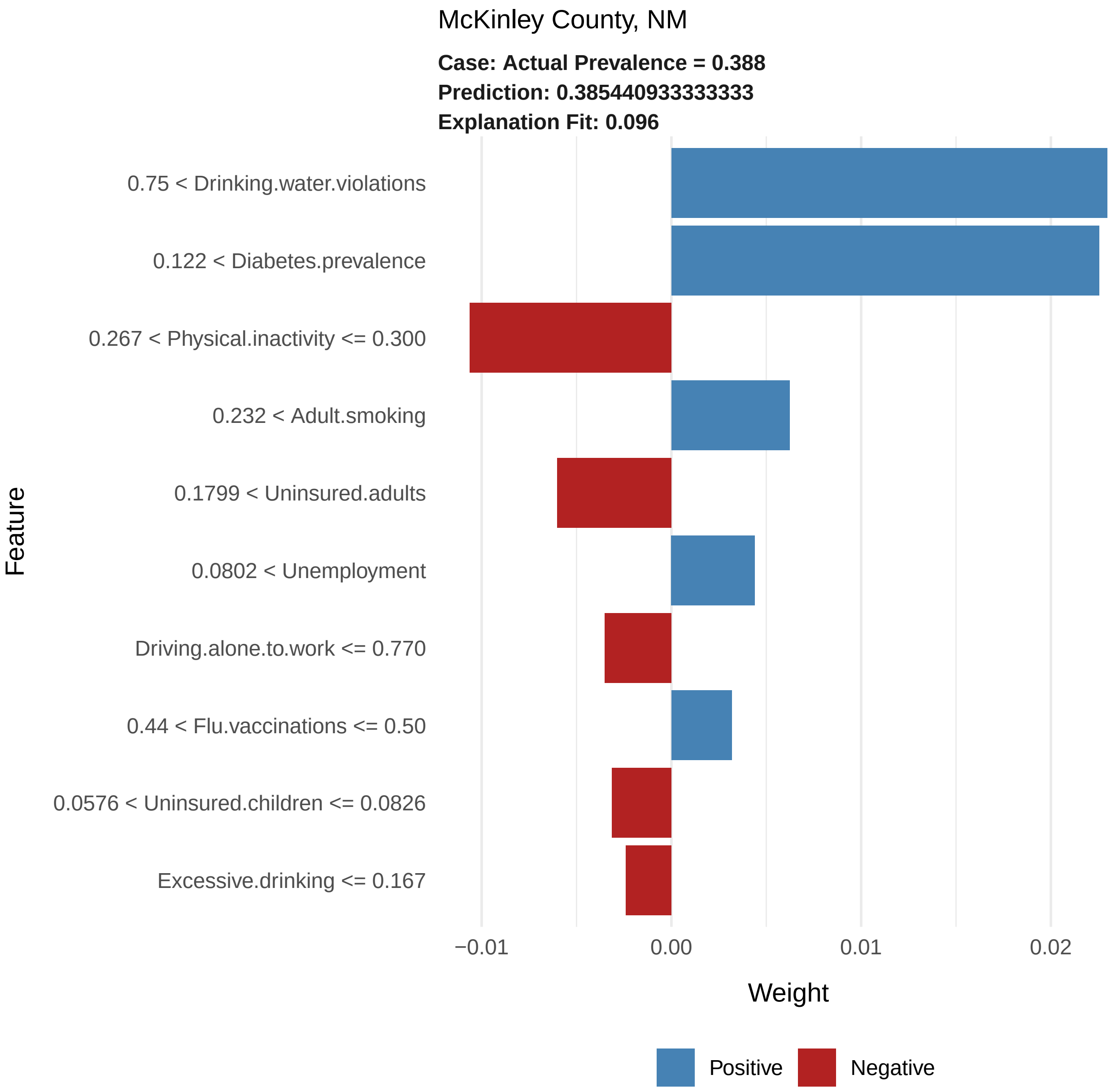
McKinley County, NM
Case: Actual Prevalence = 0.388
Prediction: 0.385440933333333
Explanation Fit: 0.096
0.75 < Drinking.water.violations
0.122 < Diabetes.prevalence
0.267 < Physical.inactivity <= 0.300
0.232 < Adult.smoking
0.1799 < Uninsured.adults
0.0802 < Unemployment
Driving.alone.to.work <= 0.770
0.44 < Flu.vaccinations <= 0.50
0.0576 < Uninsured.children <= 0.0826
Excessive.drinking <= 0.167
Feature
−0.01
0.00
0.01
0.02
Weight
Positive
Negative

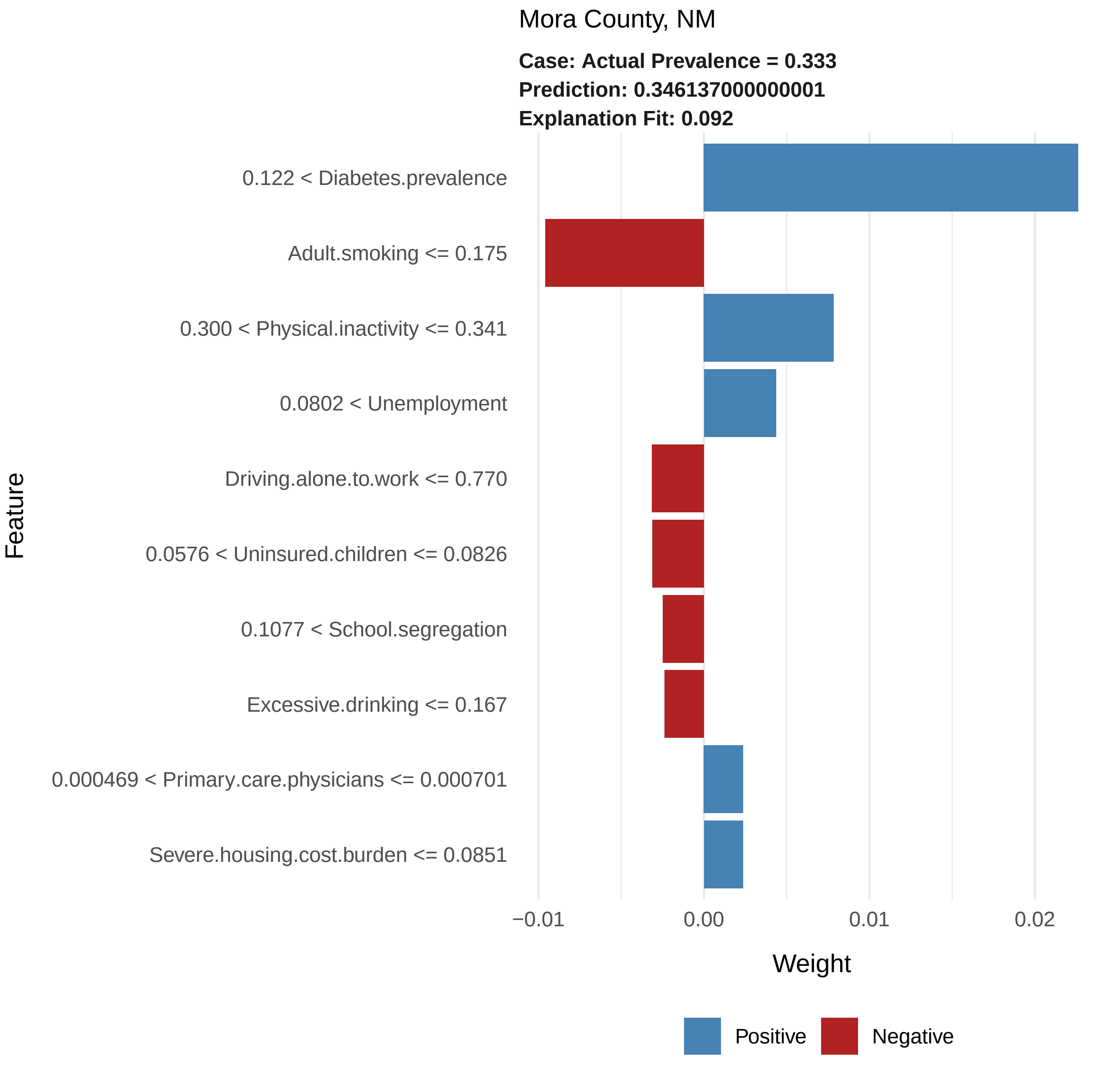

Mora County, NM
Case: Actual Prevalence = 0.333
Prediction: 0.346137000000001
Explanation Fit: 0.092
0.122 < Diabetes.prevalence
Adult.smoking <= 0.175
0.300 < Physical.inactivity <= 0.341
0.0802 < Unemployment
Driving.alone.to.work <= 0.770
0.0576 < Uninsured.children <= 0.0826
0.1077 < School.segregation
Excessive.drinking <= 0.167
0.000469 < Primary.care.physicians <= 0.000701
Severe.housing.cost.burden <= 0.0851
Feature
−0.01
0.00
0.01
0.02
Weight
Positive
Negative

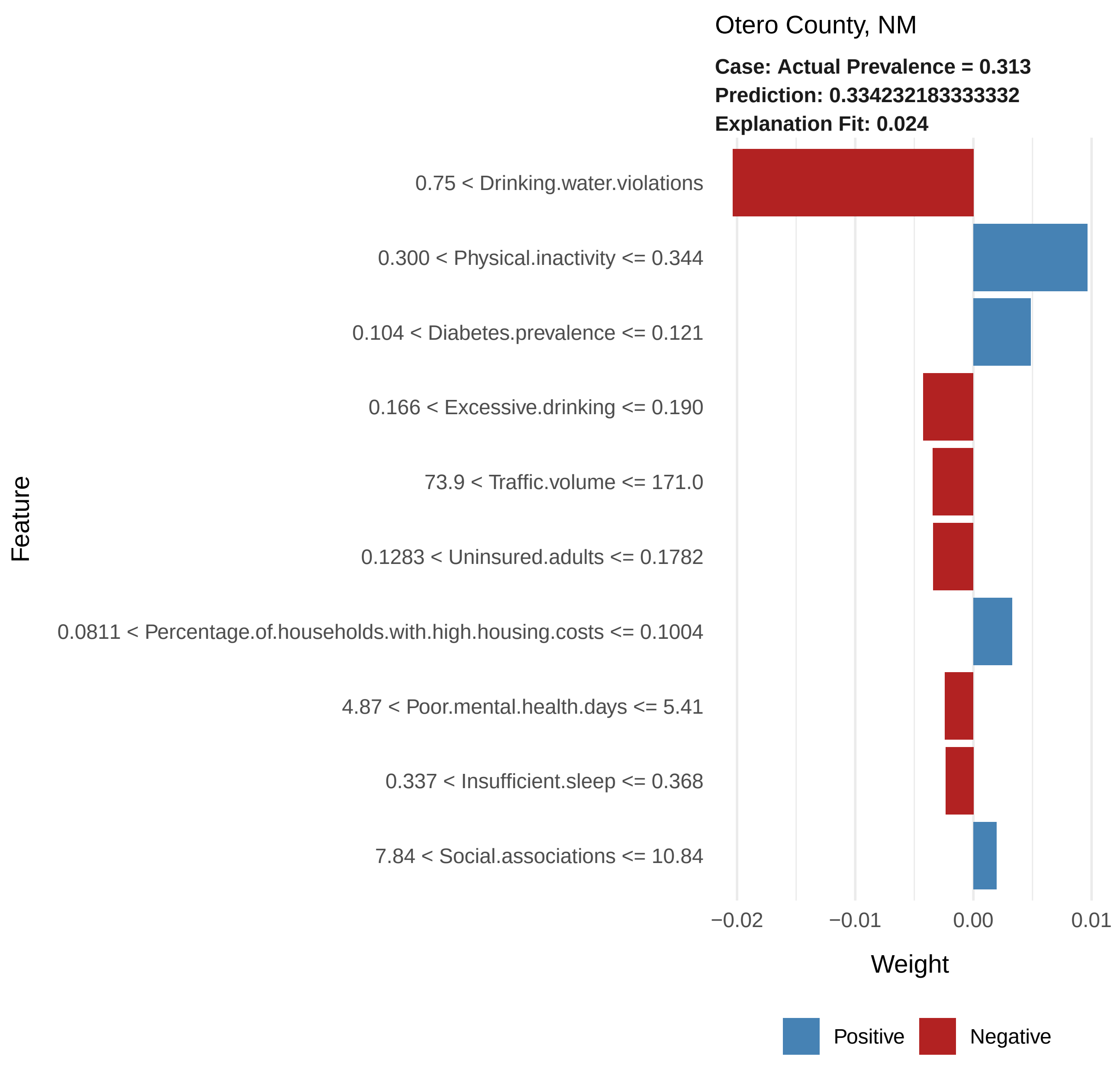

Otero County, NM
Case: Actual Prevalence = 0.313
Prediction: 0.334232183333332
Explanation Fit: 0.024
0.75 < Drinking.water.violations
0.300 < Physical.inactivity <= 0.344
0.104 < Diabetes.prevalence <= 0.121
0.166 < Excessive.drinking <= 0.190
73.9 < Traffic.volume <= 171.0
0.1283 < Uninsured.adults <= 0.1782
0.0811 < Percentage.of.households.with.high.housing.costs <= 0.1004
4.87 < Poor.mental.health.days <= 5.41
0.337 < Insufficient.sleep <= 0.368
7.84 < Social.associations <= 10.84
Feature
−0.02
−0.01
0.00
0.01
Weight
Positive
Negative

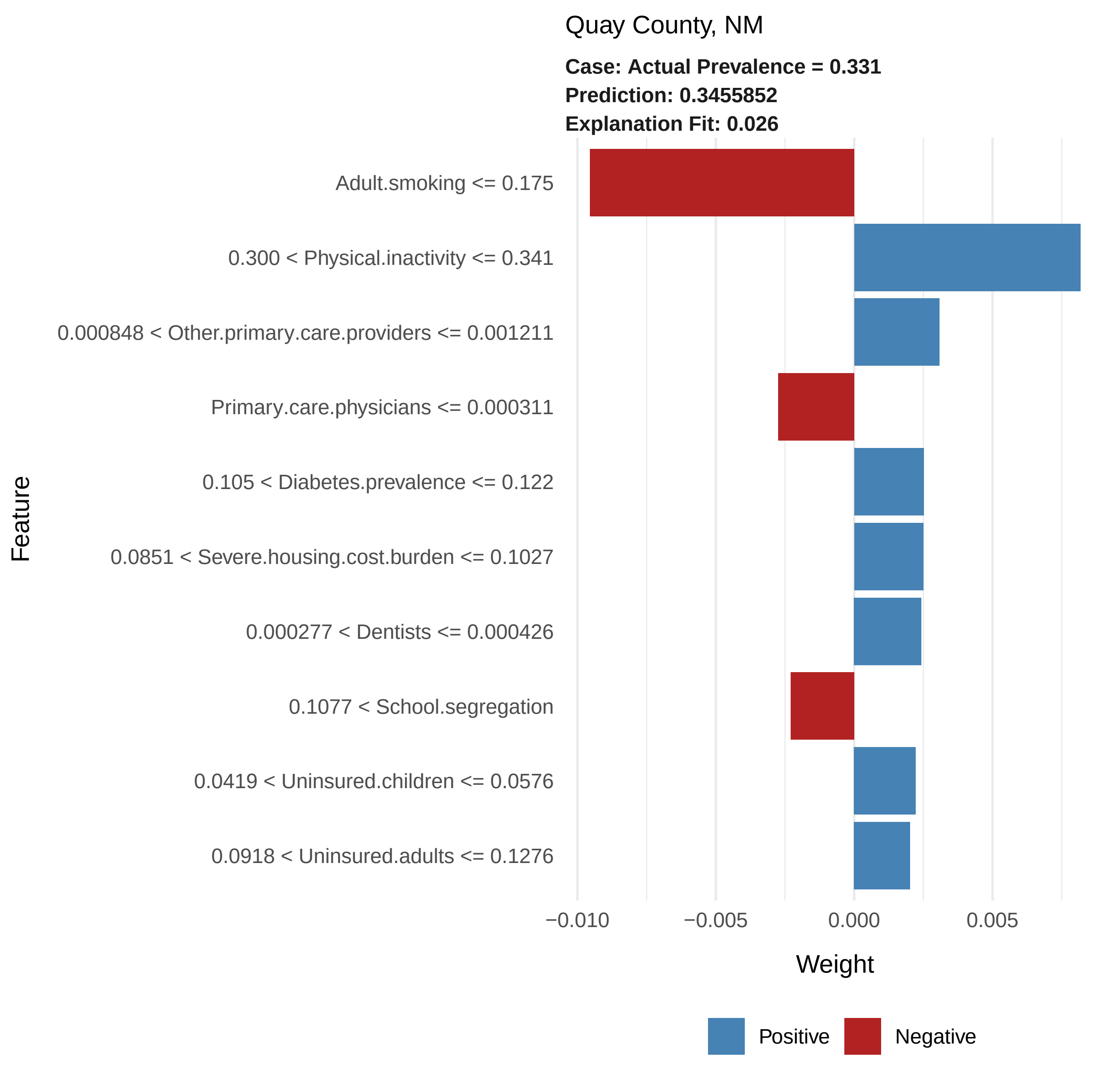
Quay County, NM
Case: Actual Prevalence = 0.331
Prediction: 0.3455852
Explanation Fit: 0.026
Adult.smoking <= 0.175
0.300 < Physical.inactivity <= 0.341
0.000848 < Other.primary.care.providers <= 0.001211
Primary.care.physicians <= 0.000311
0.105 < Diabetes.prevalence <= 0.122
0.0851 < Severe.housing.cost.burden <= 0.1027
0.000277 < Dentists <= 0.000426
0.1077 < School.segregation
0.0419 < Uninsured.children <= 0.0576
0.0918 < Uninsured.adults <= 0.1276
Feature
−0.010
−0.005
0.000
0.005
Weight
Positive
Negative

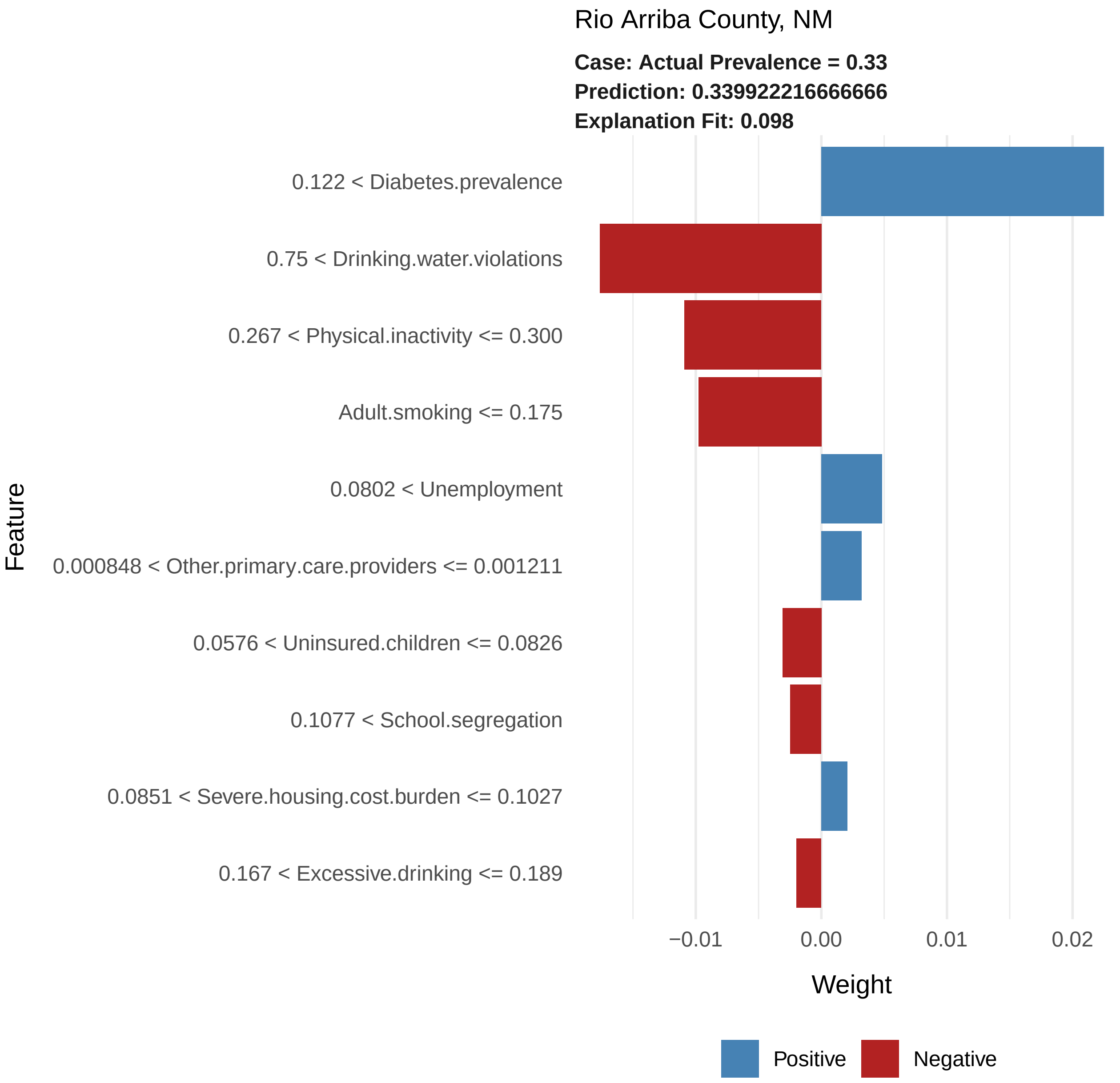
Rio Arriba County, NM
Case: Actual Prevalence = 0.33
Prediction: 0.339922216666666
Explanation Fit: 0.098
0.122 < Diabetes.prevalence
0.75 < Drinking.water.violations
0.267 < Physical.inactivity <= 0.300
Adult.smoking <= 0.175
0.0802 < Unemployment
0.000848 < Other.primary.care.providers <= 0.001211
0.0576 < Uninsured.children <= 0.0826
0.1077 < School.segregation
0.0851 < Severe.housing.cost.burden <= 0.1027
0.167 < Excessive.drinking <= 0.189
Feature
−0.01
0.00
0.01
0.02
Weight
Positive
Negative

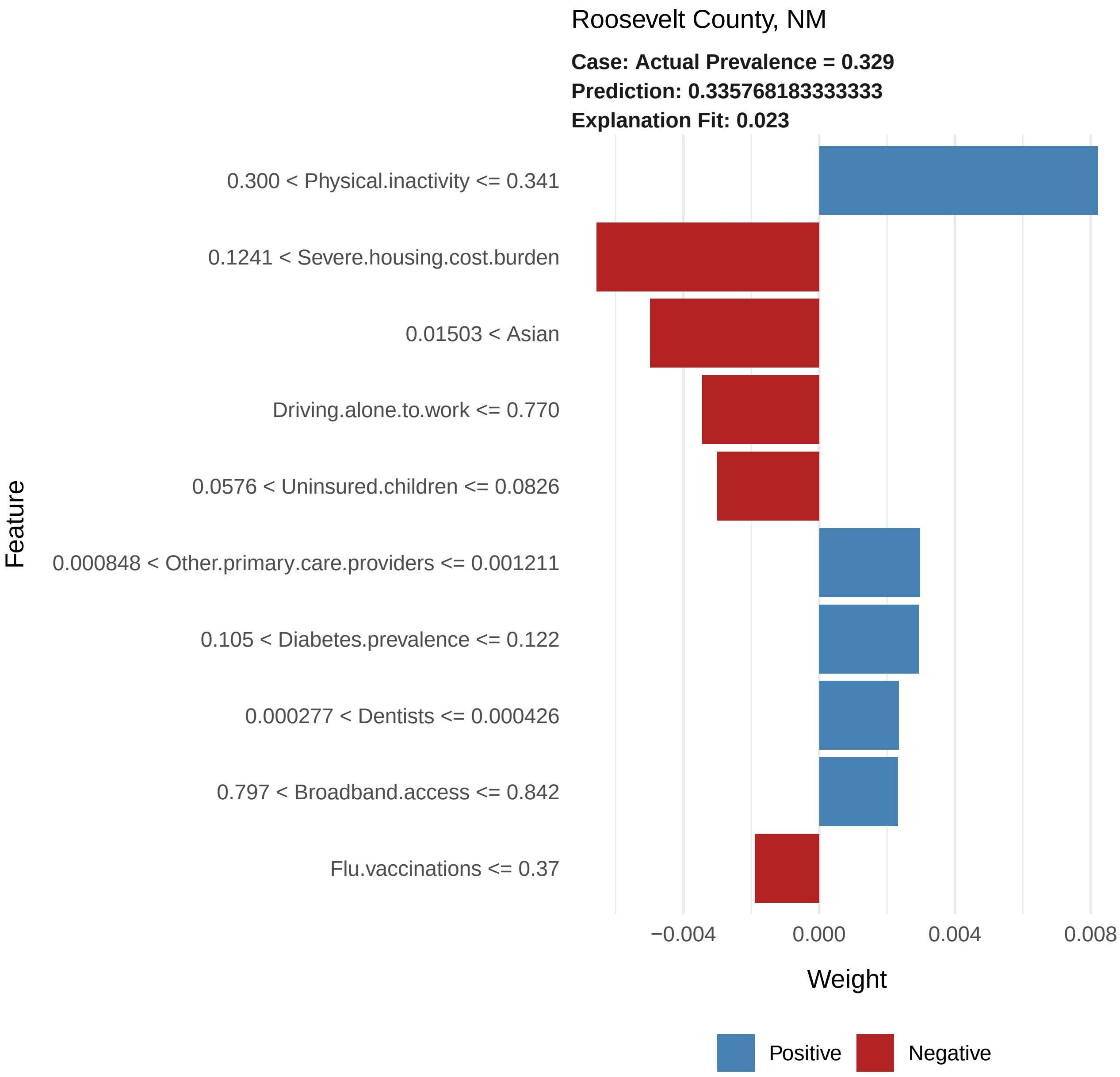
Roosevelt County, NM
Case: Actual Prevalence = 0.329
Prediction: 0.335768183333333
Explanation Fit: 0.023
0.300 < Physical.inactivity <= 0.341
0.1241 < Severe.housing.cost.burden
0.01503 < Asian
Driving.alone.to.work <= 0.770
0.0576 < Uninsured.children <= 0.0826
0.000848 < Other.primary.care.providers <= 0.001211
0.105 < Diabetes.prevalence <= 0.122
0.000277 < Dentists <= 0.000426
0.797 < Broadband.access <= 0.842
Flu.vaccinations <= 0.37
Feature
−0.004
0.000
0.004
0.008
Weight
Positive
Negative

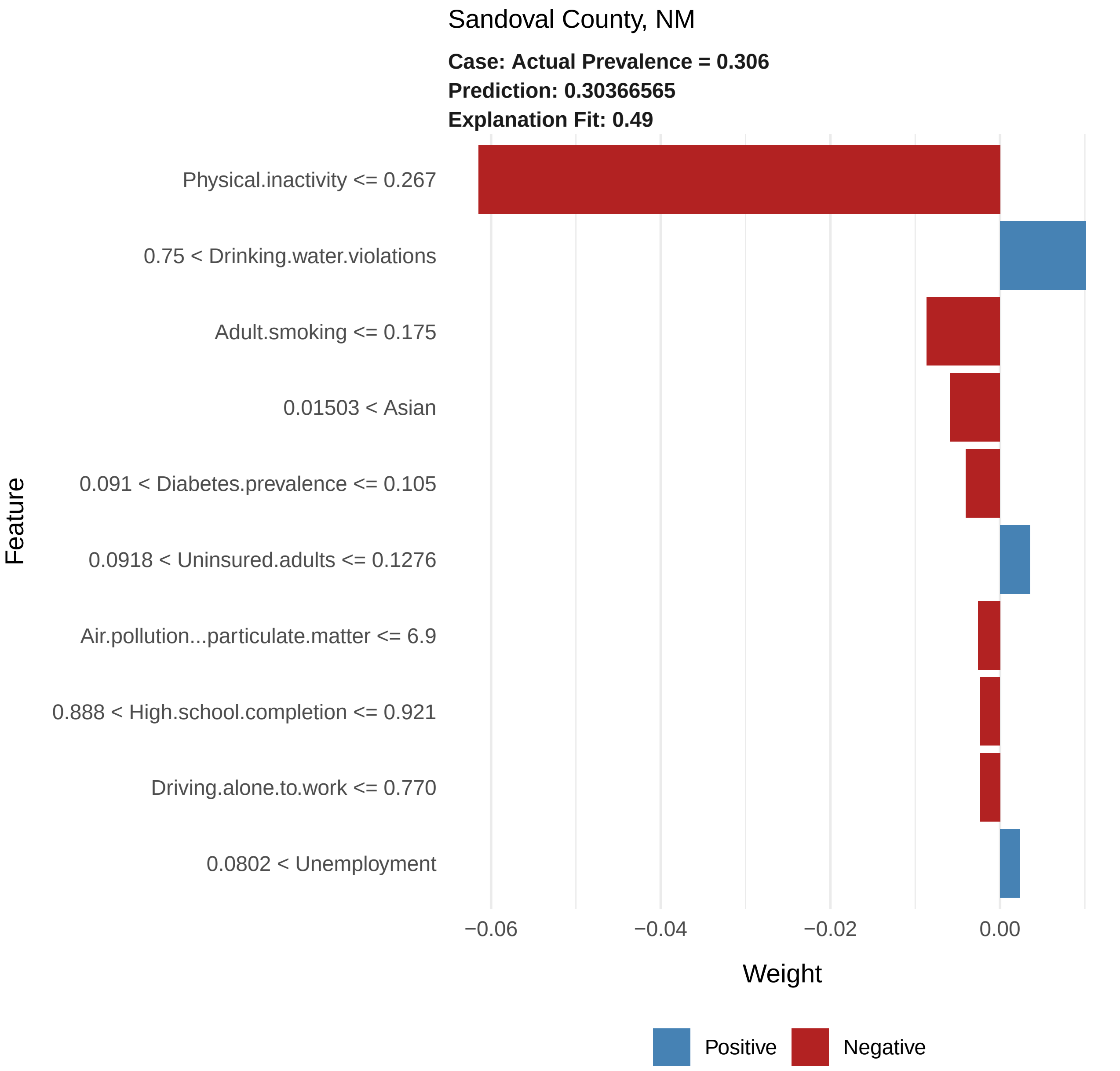

Sandoval County, NM
Case: Actual Prevalence = 0.306
Prediction: 0.30366565
Explanation Fit: 0.49
Physical.inactivity <= 0.267
0.75 < Drinking.water.violations
Adult.smoking <= 0.175
0.01503 < Asian
0.091 < Diabetes.prevalence <= 0.105
0.0918 < Uninsured.adults <= 0.1276
Air.pollution...particulate.matter <= 6.9
0.888 < High.school.completion <= 0.921
Driving.alone.to.work <= 0.770
0.0802 < Unemployment
Feature
−0.06
−0.04
−0.02
0.00
Weight
Positive
Negative

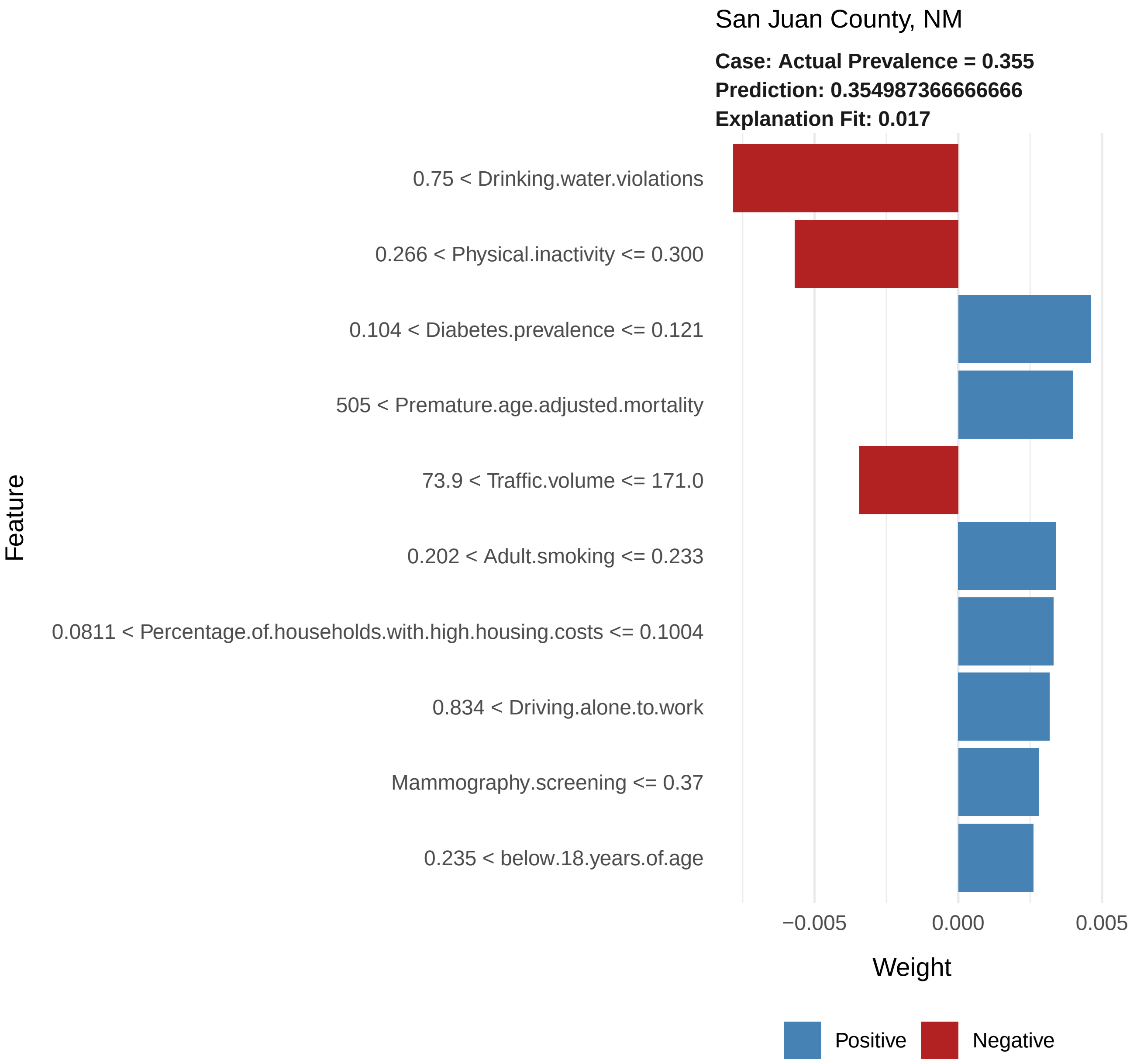
San Juan County, NM
Case: Actual Prevalence = 0.355
Prediction: 0.354987366666666
Explanation Fit: 0.017
0.75 < Drinking.water.violations
0.266 < Physical.inactivity <= 0.300
0.104 < Diabetes.prevalence <= 0.121
505 < Premature.age.adjusted.mortality
73.9 < Traffic.volume <= 171.0
0.202 < Adult.smoking <= 0.233
0.0811 < Percentage.of.households.with.high.housing.costs <= 0.1004
0.834 < Driving.alone.to.work
Mammography.screening <= 0.37
0.235 < below.18.years.of.age
Feature
−0.005
0.000
0.005
Weight
Positive
Negative

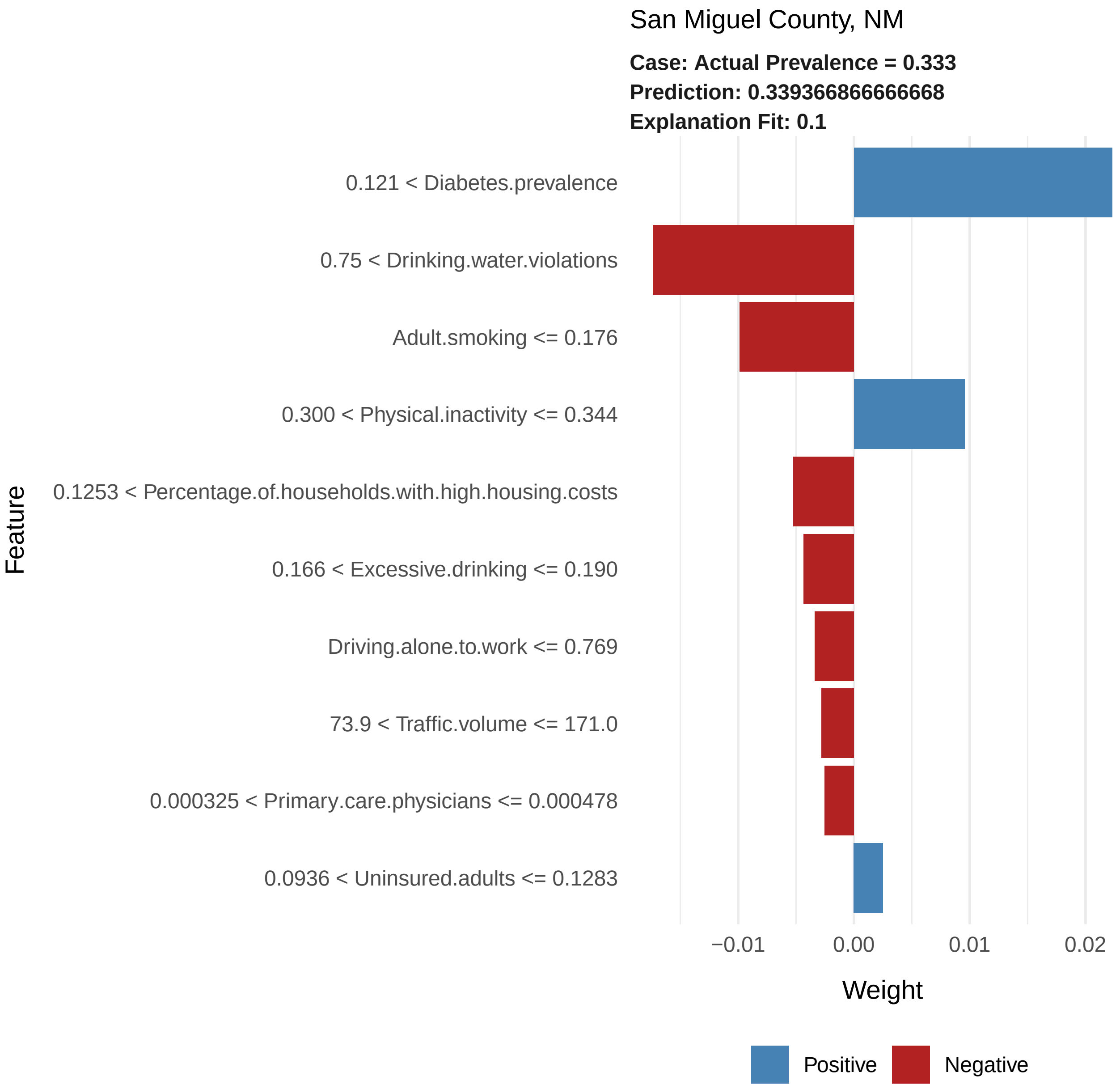

San Miguel County, NM
Case: Actual Prevalence = 0.333
Prediction: 0.339366866666668
Explanation Fit: 0.1
0.121 < Diabetes.prevalence
0.75 < Drinking.water.violations
Adult.smoking <= 0.176
0.300 < Physical.inactivity <= 0.344
0.1253 < Percentage.of.households.with.high.housing.costs
0.166 < Excessive.drinking <= 0.190
Driving.alone.to.work <= 0.769
73.9 < Traffic.volume <= 171.0
0.000325 < Primary.care.physicians <= 0.000478
0.0936 < Uninsured.adults <= 0.1283
Feature
−0.01
0.00
0.01
0.02
Weight
Positive
Negative

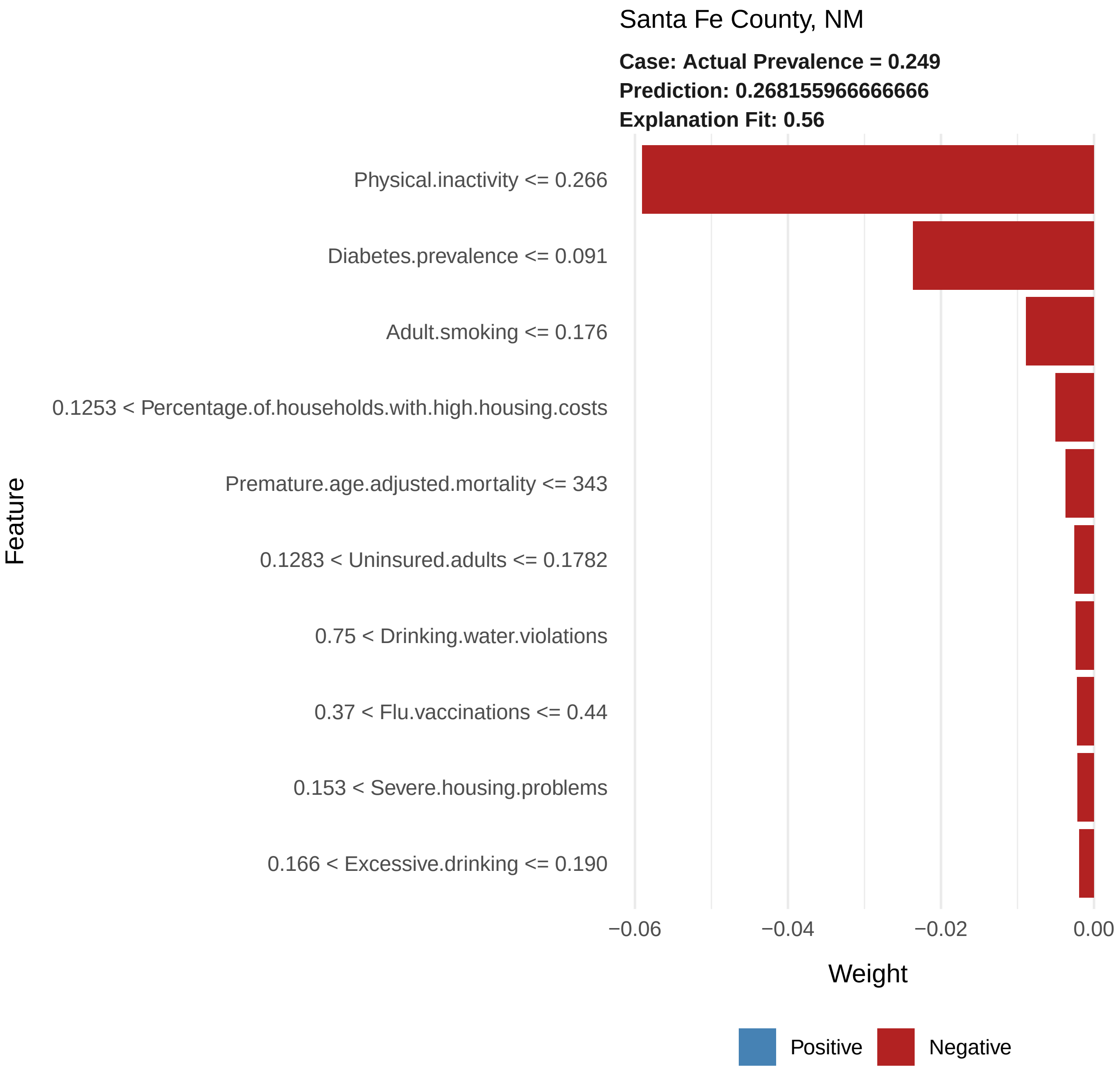

Santa Fe County, NM
Case: Actual Prevalence = 0.249
Prediction: 0.268155966666666
Explanation Fit: 0.56
Physical.inactivity <= 0.266
Diabetes.prevalence <= 0.091
Adult.smoking <= 0.176
0.1253 < Percentage.of.households.with.high.housing.costs
Premature.age.adjusted.mortality <= 343
0.1283 < Uninsured.adults <= 0.1782
0.75 < Drinking.water.violations
0.37 < Flu.vaccinations <= 0.44
0.153 < Severe.housing.problems
0.166 < Excessive.drinking <= 0.190
Feature
−0.06
−0.04
−0.02
0.00
Weight
Positive
Negative

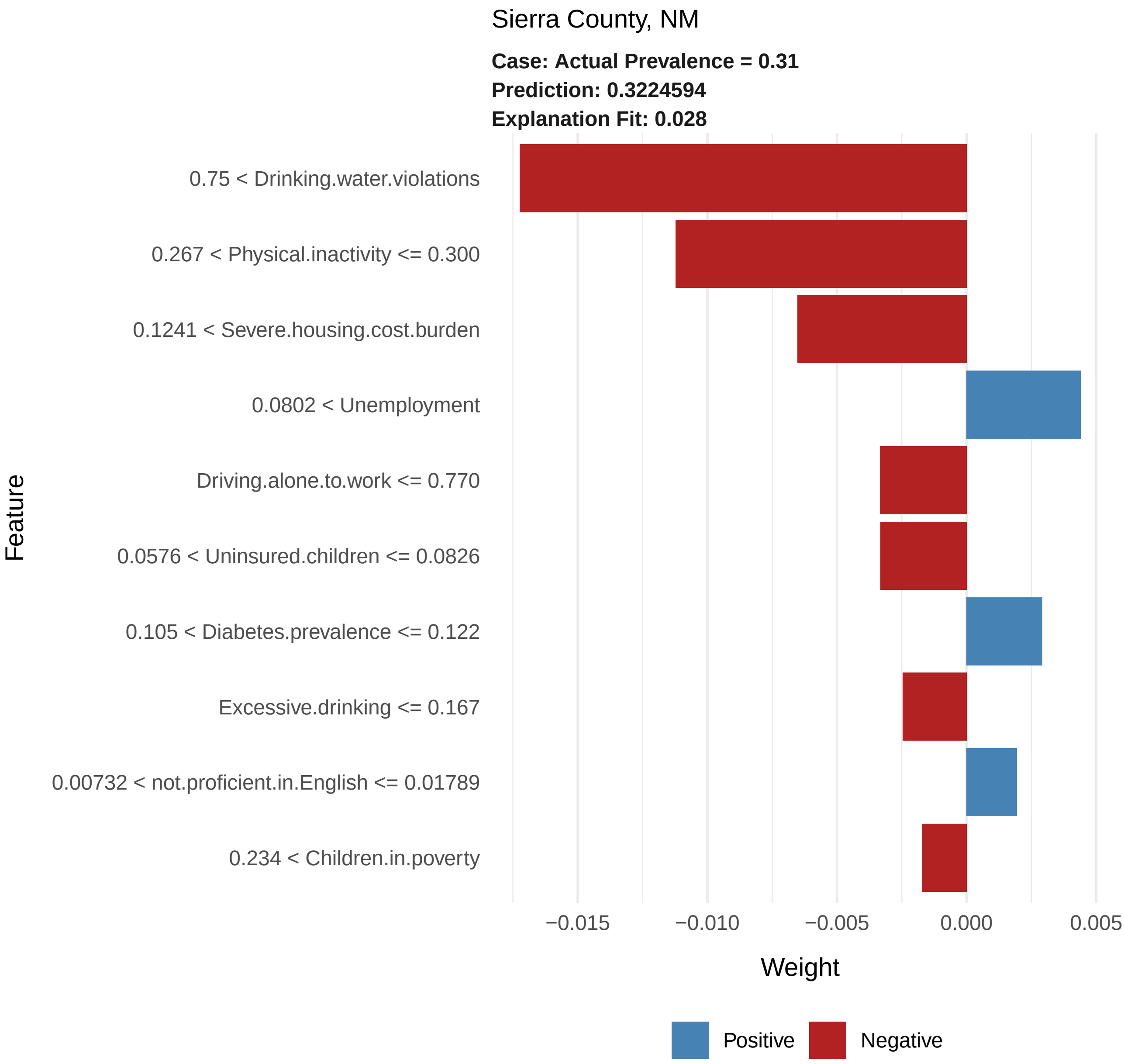
Sierra County, NM
Case: Actual Prevalence = 0.31
Prediction: 0.3224594
Explanation Fit: 0.028
0.75 < Drinking.water.violations
0.267 < Physical.inactivity <= 0.300
0.1241 < Severe.housing.cost.burden
0.0802 < Unemployment
Driving.alone.to.work <= 0.770
0.0576 < Uninsured.children <= 0.0826
0.105 < Diabetes.prevalence <= 0.122
Excessive.drinking <= 0.167
0.00732 < not.proficient.in.English <= 0.01789
0.234 < Children.in.poverty
Feature
−0.015
−0.010
−0.005
0.000
0.005
Weight
Positive
Negative

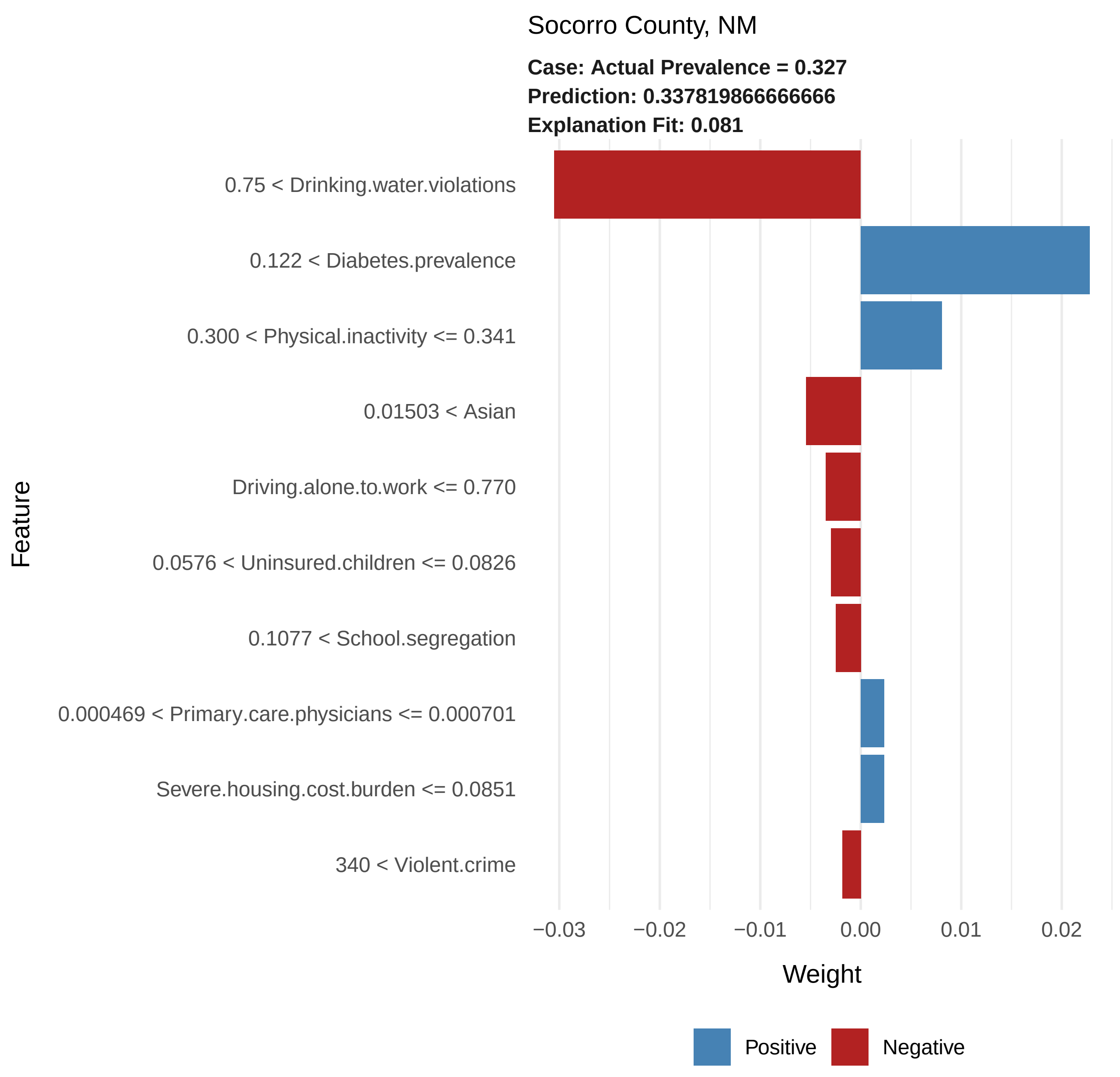

Socorro County, NM
Case: Actual Prevalence = 0.327
Prediction: 0.337819866666666
Explanation Fit: 0.081
0.75 < Drinking.water.violations
0.122 < Diabetes.prevalence
0.300 < Physical.inactivity <= 0.341
0.01503 < Asian
Driving.alone.to.work <= 0.770
0.0576 < Uninsured.children <= 0.0826
0.1077 < School.segregation
0.000469 < Primary.care.physicians <= 0.000701
Severe.housing.cost.burden <= 0.0851
340 < Violent.crime
Feature
−0.03
−0.02
−0.01
0.00
0.01
0.02
Weight
Positive
Negative

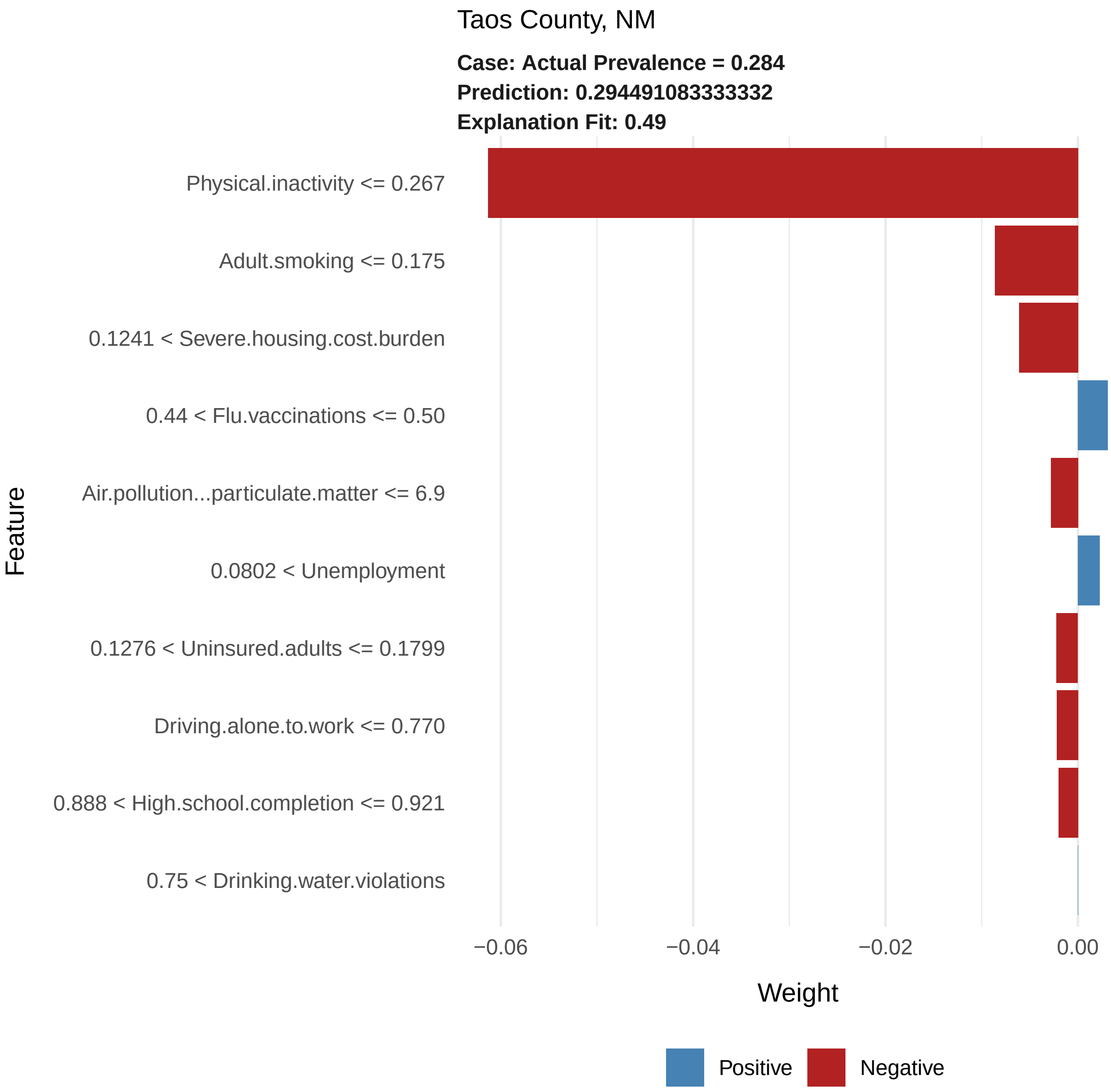
Taos County, NM
Case: Actual Prevalence = 0.284
Prediction: 0.294491083333332
Explanation Fit: 0.49
Physical.inactivity <= 0.267
Adult.smoking <= 0.175
0.1241 < Severe.housing.cost.burden
0.44 < Flu.vaccinations <= 0.50
Air.pollution...particulate.matter <= 6.9
0.0802 < Unemployment
0.1276 < Uninsured.adults <= 0.1799
Driving.alone.to.work <= 0.770
0.888 < High.school.completion <= 0.921
0.75 < Drinking.water.violations
Feature
−0.06
−0.04
−0.02
0.00
Weight
Positive
Negative

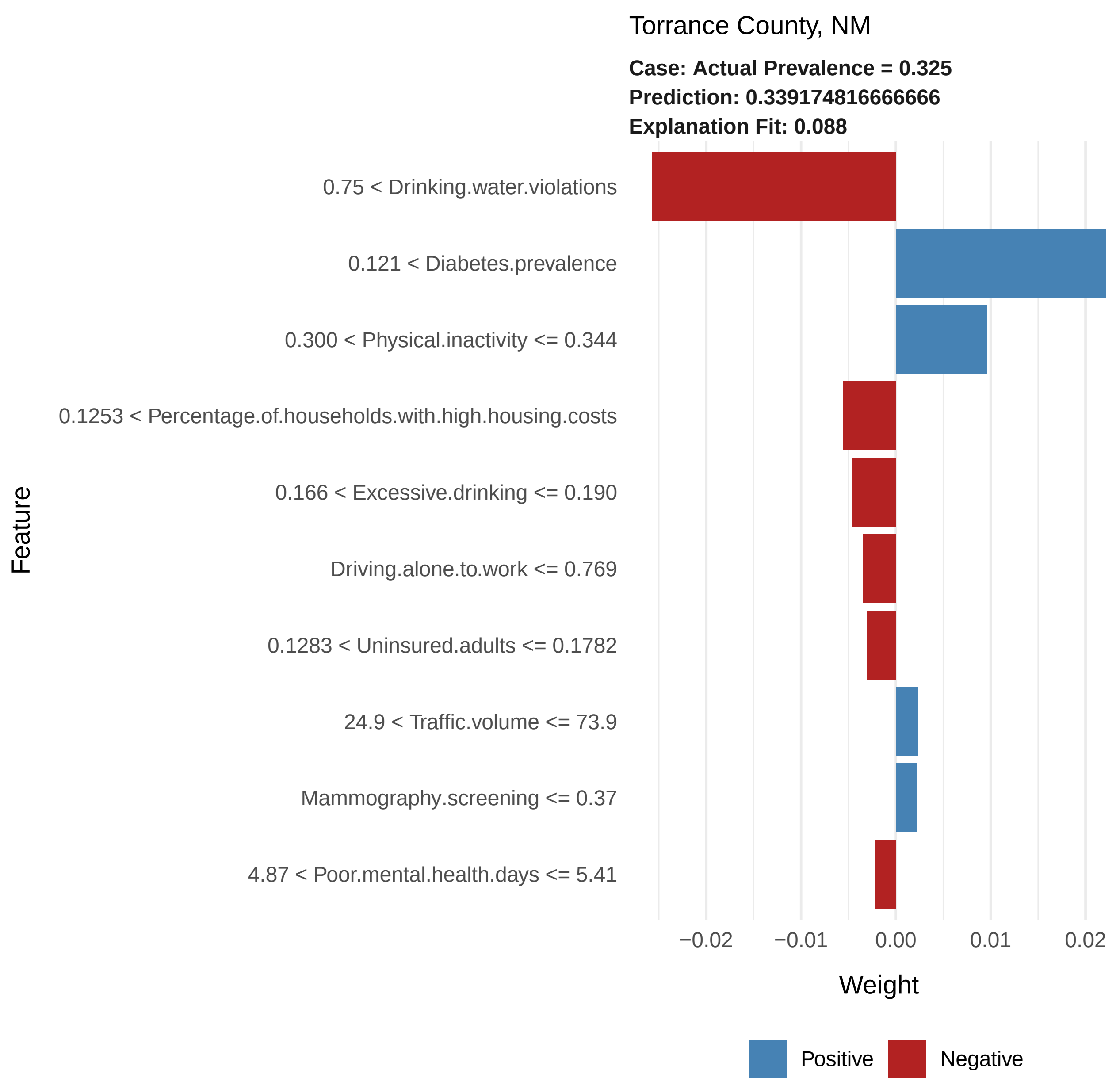
Torrance County, NM
Case: Actual Prevalence = 0.325
Prediction: 0.339174816666666
Explanation Fit: 0.088
Feature
0.75 < Drinking.water.violations
0.121 < Diabetes.prevalence
0.300 < Physical.inactivity <= 0.344
0.1253 < Percentage.of.households.with.high.housing.costs
0.166 < Excessive.drinking <= 0.190
Driving.alone.to.work <= 0.769
0.1283 < Uninsured.adults <= 0.1782
24.9 < Traffic.volume <= 73.9
Mammography.screening <= 0.37
4.87 < Poor.mental.health.days <= 5.41
−0.02
−0.01
0.00
0.01
0.02
Weight
Positive
Negative

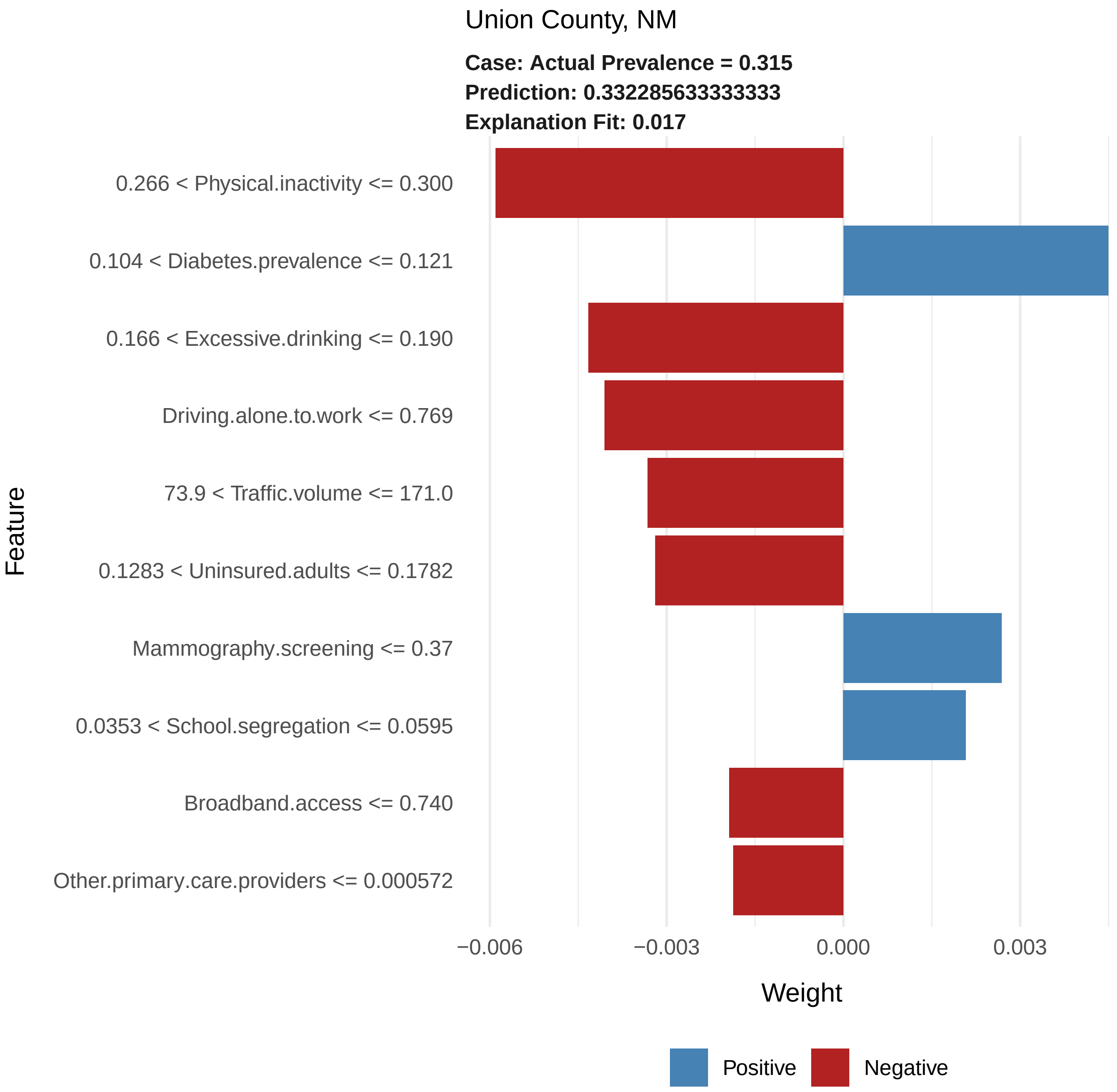
Union County, NM
Case: Actual Prevalence = 0.315
Prediction: 0.332285633333333
Explanation Fit: 0.017
0.266 < Physical.inactivity <= 0.300
0.104 < Diabetes.prevalence <= 0.121
0.166 < Excessive.drinking <= 0.190
Driving.alone.to.work <= 0.769
73.9 < Traffic.volume <= 171.0
0.1283 < Uninsured.adults <= 0.1782
Mammography.screening <= 0.37
0.0353 < School.segregation <= 0.0595
Broadband.access <= 0.740
Other.primary.care.providers <= 0.000572
Feature
−0.006
−0.003
0.000
0.003
Weight
Positive
Negative

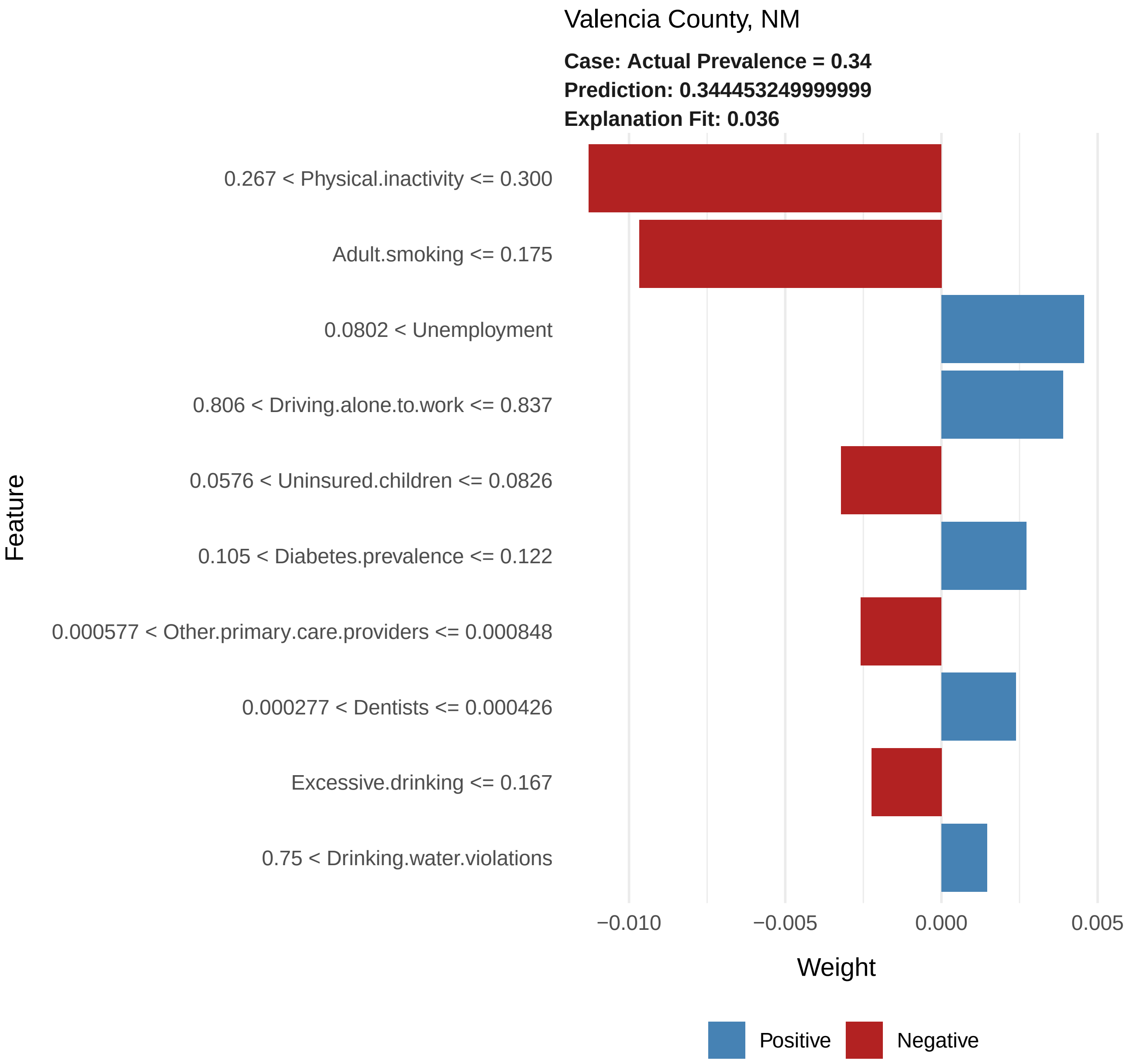
Valencia County, NM
Case: Actual Prevalence = 0.34
Prediction: 0.344453249999999
Explanation Fit: 0.036
0.267 < Physical.inactivity <= 0.300
Adult.smoking <= 0.175
0.0802 < Unemployment
0.806 < Driving.alone.to.work <= 0.837
0.0576 < Uninsured.children <= 0.0826
0.105 < Diabetes.prevalence <= 0.122
0.000577 < Other.primary.care.providers <= 0.000848
0.000277 < Dentists <= 0.000426
Excessive.drinking <= 0.167
0.75 < Drinking.water.violations
Feature
−0.010
−0.005
0.000
0.005
Weight
Positive
Negative

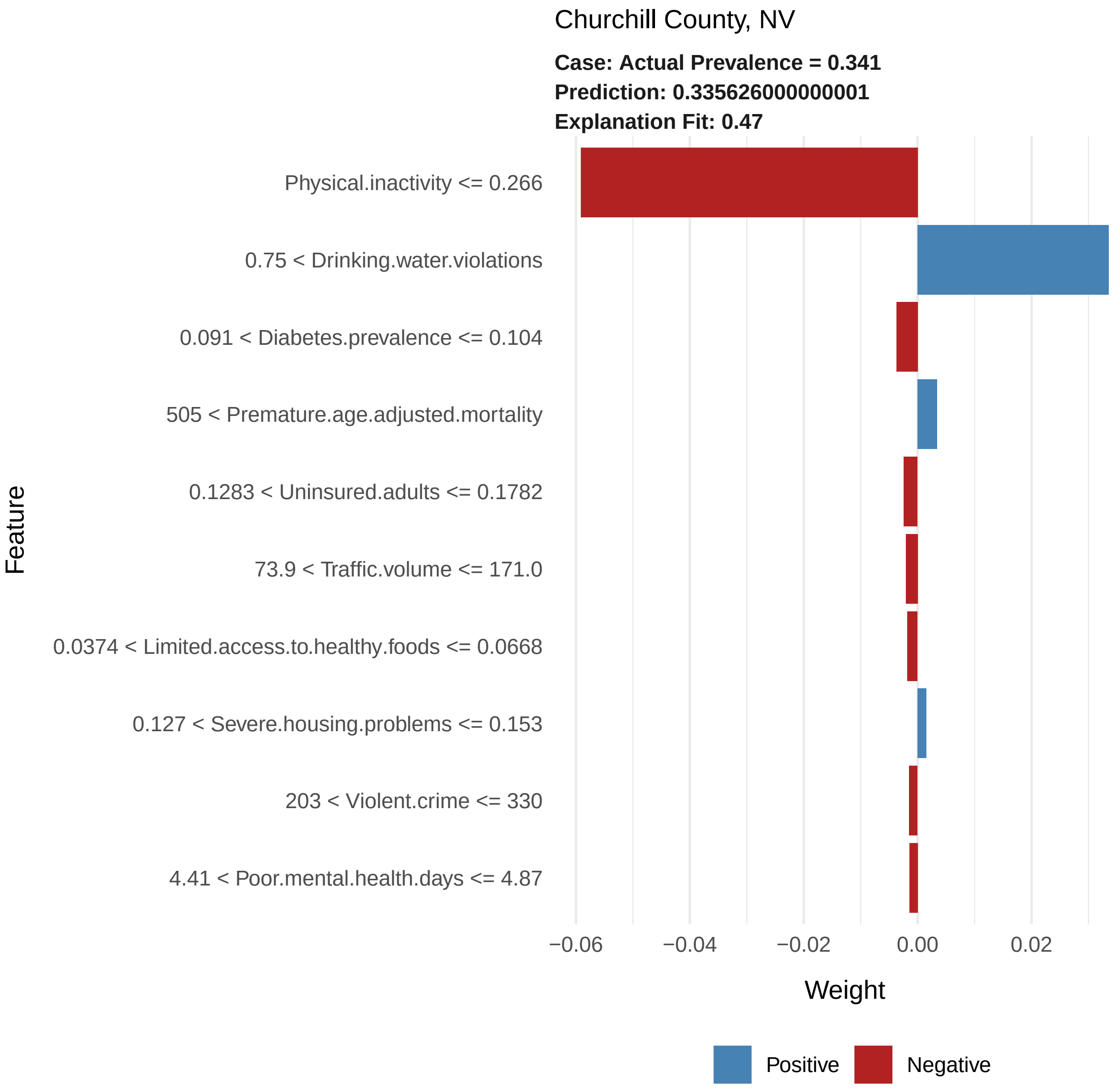

Churchill County, NV
Case: Actual Prevalence = 0.341
Prediction: 0.335626000000001
Explanation Fit: 0.47
Physical.inactivity <= 0.266
0.75 < Drinking.water.violations
0.091 < Diabetes.prevalence <= 0.104
505 < Premature.age.adjusted.mortality
0.1283 < Uninsured.adults <= 0.1782
73.9 < Traffic.volume <= 171.0
0.0374 < Limited.access.to.healthy.foods <= 0.0668
0.127 < Severe.housing.problems <= 0.153
203 < Violent.crime <= 330
4.41 < Poor.mental.health.days <= 4.87
Feature
−0.06
−0.04
−0.02
0.00
0.02
Weight
Positive
Negative

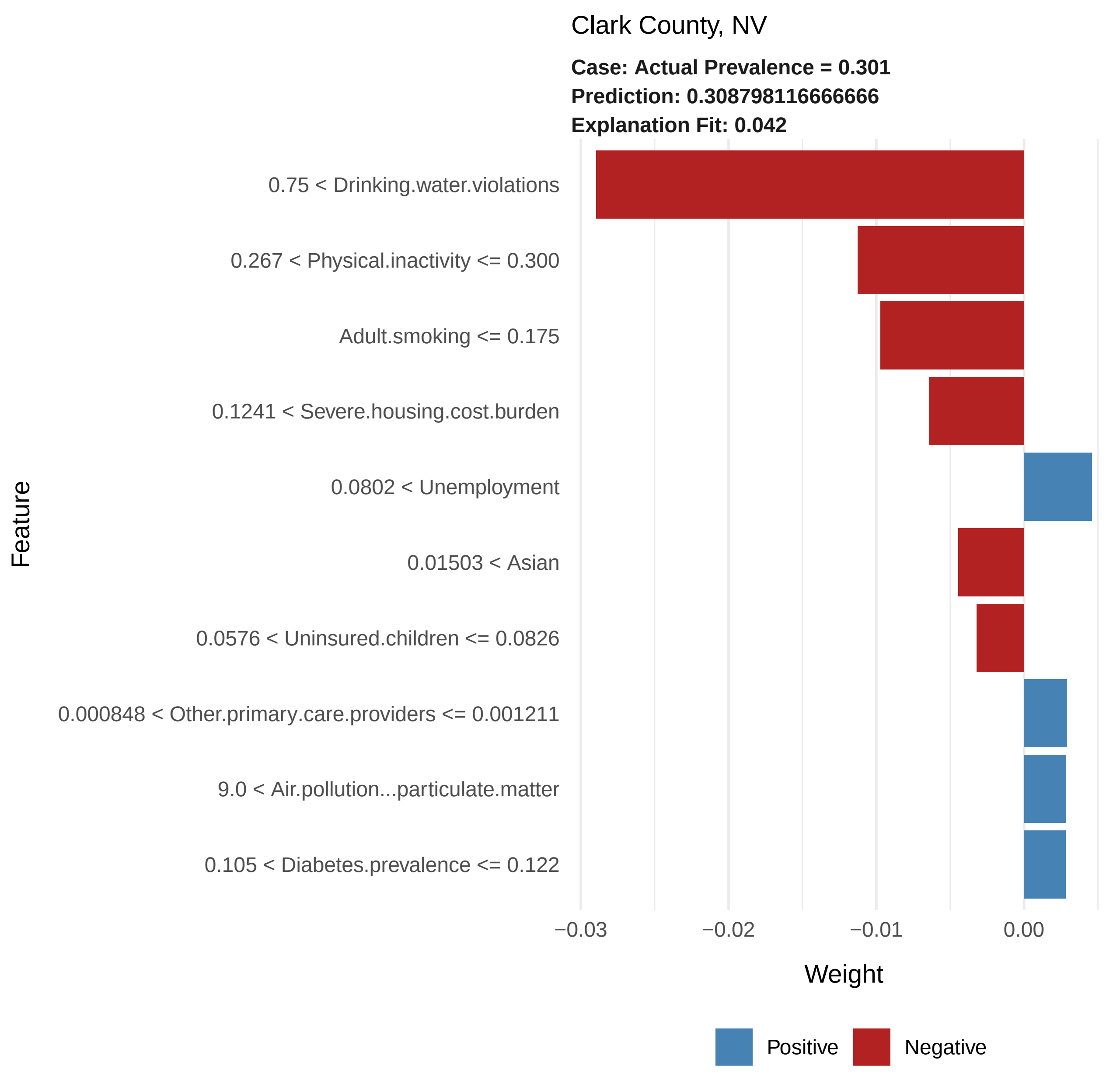
Clark County, NV
Case: Actual Prevalence = 0.301
Prediction: 0.308798116666666
Explanation Fit: 0.042
0.75 < Drinking.water.violations
0.267 < Physical.inactivity <= 0.300
Adult.smoking <= 0.175
0.1241 < Severe.housing.cost.burden
0.0802 < Unemployment
0.01503 < Asian
0.0576 < Uninsured.children <= 0.0826
0.000848 < Other.primary.care.providers <= 0.001211
9.0 < Air.pollution...particulate.matter
0.105 < Diabetes.prevalence <= 0.122
Feature
−0.03
−0.02
−0.01
0.00
Weight
Positive
Negative

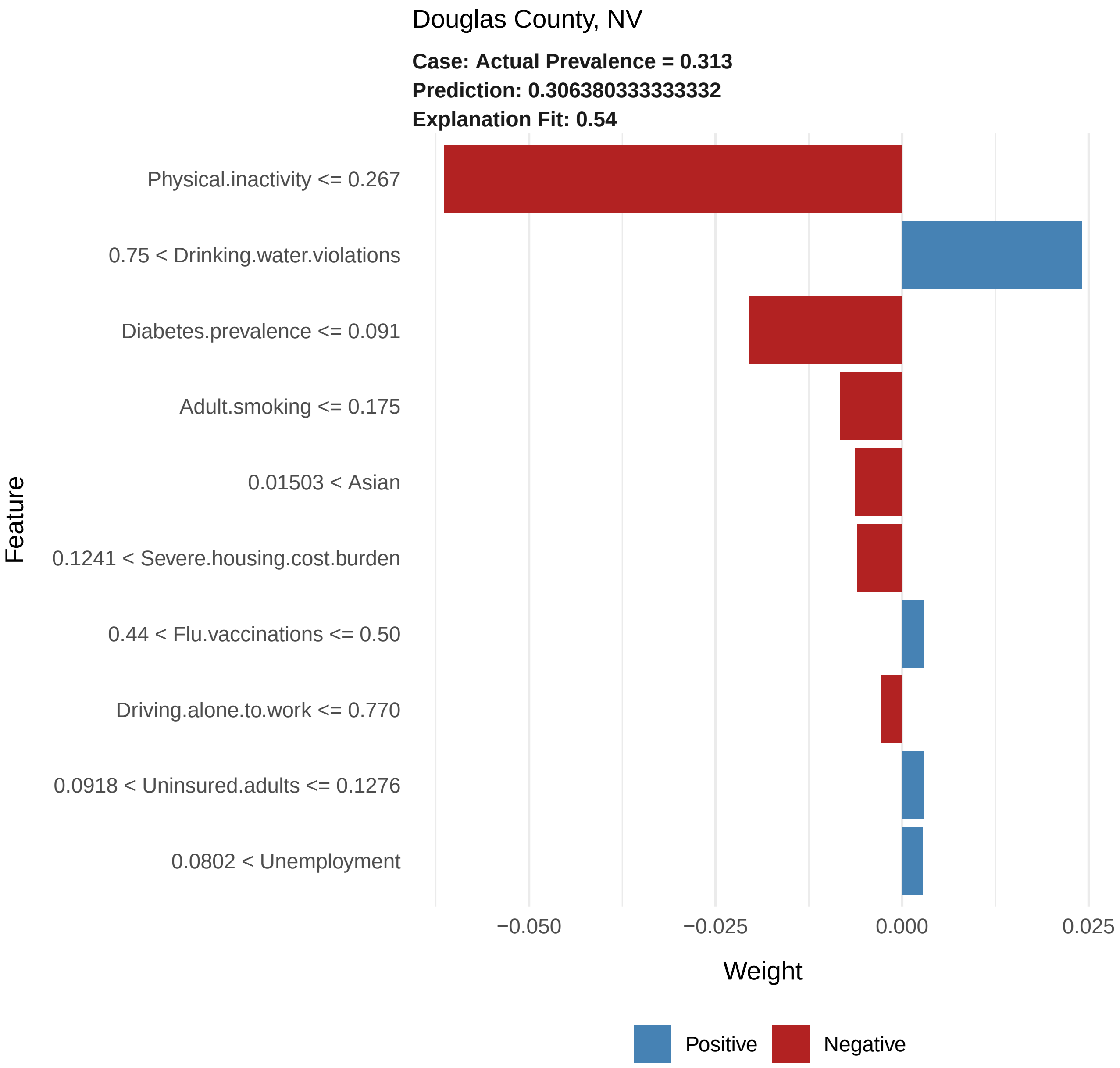

Douglas County, NV
Case: Actual Prevalence = 0.313
Prediction: 0.306380333333332
Explanation Fit: 0.54
Physical.inactivity <= 0.267
0.75 < Drinking.water.violations
Diabetes.prevalence <= 0.091
Adult.smoking <= 0.175
0.01503 < Asian
0.1241 < Severe.housing.cost.burden
0.44 < Flu.vaccinations <= 0.50
Driving.alone.to.work <= 0.770
0.0918 < Uninsured.adults <= 0.1276
0.0802 < Unemployment
Feature
−0.050
−0.025
0.000
0.025
Weight
Positive
Negative

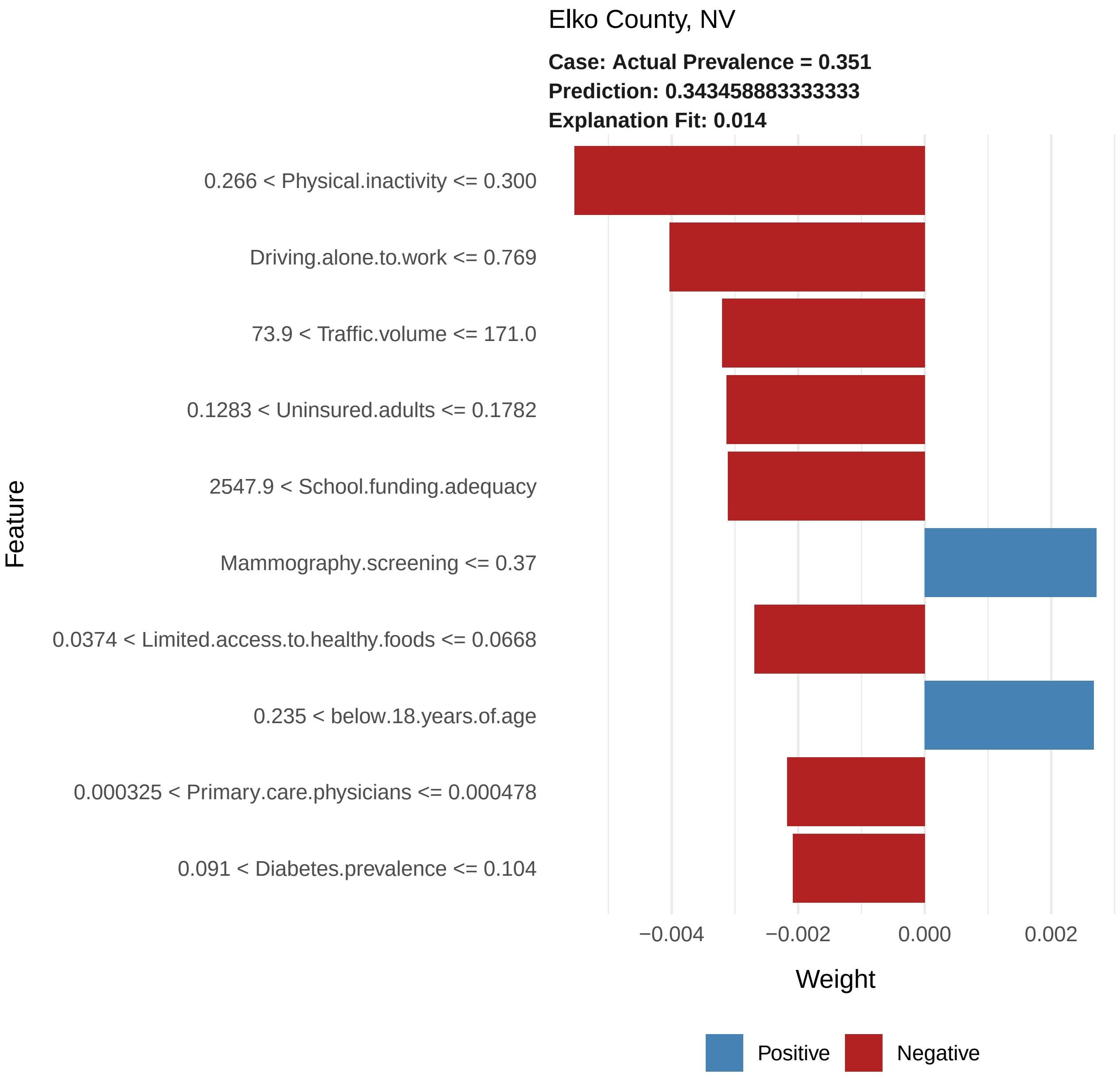

Elko County, NV
Case: Actual Prevalence = 0.351
Prediction: 0.343458883333333
Explanation Fit: 0.014
0.266 < Physical.inactivity <= 0.300
Driving.alone.to.work <= 0.769
73.9 < Traffic.volume <= 171.0
0.1283 < Uninsured.adults <= 0.1782
2547.9 < School.funding.adequacy
Mammography.screening <= 0.37
0.0374 < Limited.access.to.healthy.foods <= 0.0668
0.235 < below.18.years.of.age
0.000325 < Primary.care.physicians <= 0.000478
0.091 < Diabetes.prevalence <= 0.104
Feature
−0.004
−0.002
0.000
0.002
Weight
Positive
Negative

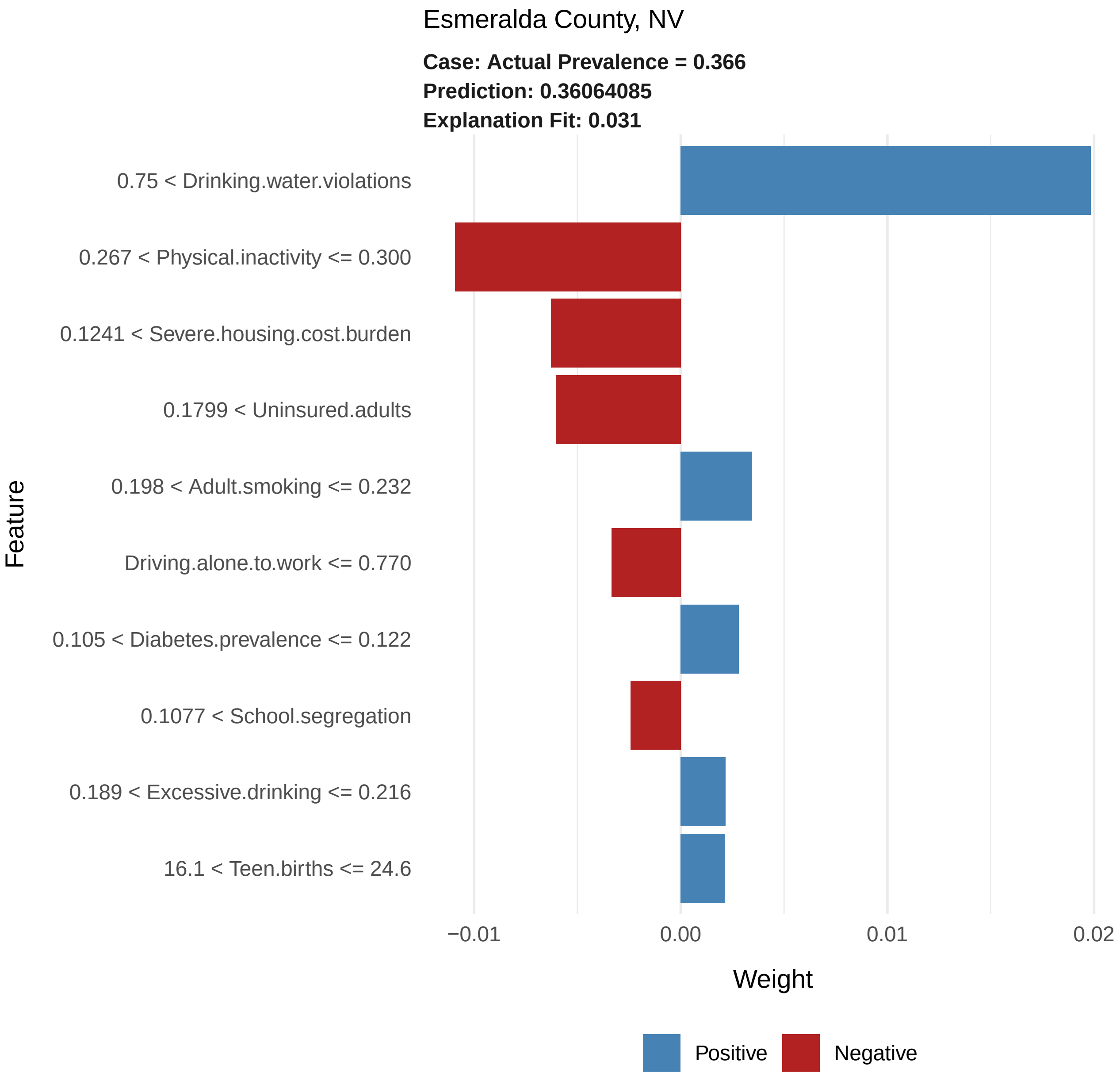

Esmeralda County, NV
Case: Actual Prevalence = 0.366
Prediction: 0.36064085
Explanation Fit: 0.031
0.75 < Drinking.water.violations
0.267 < Physical.inactivity <= 0.300
0.1241 < Severe.housing.cost.burden
0.1799 < Uninsured.adults
0.198 < Adult.smoking <= 0.232
Driving.alone.to.work <= 0.770
0.105 < Diabetes.prevalence <= 0.122
0.1077 < School.segregation
0.189 < Excessive.drinking <= 0.216
16.1 < Teen.births <= 24.6
Feature
−0.01
0.00
0.01
0.02
Weight
Positive
Negative

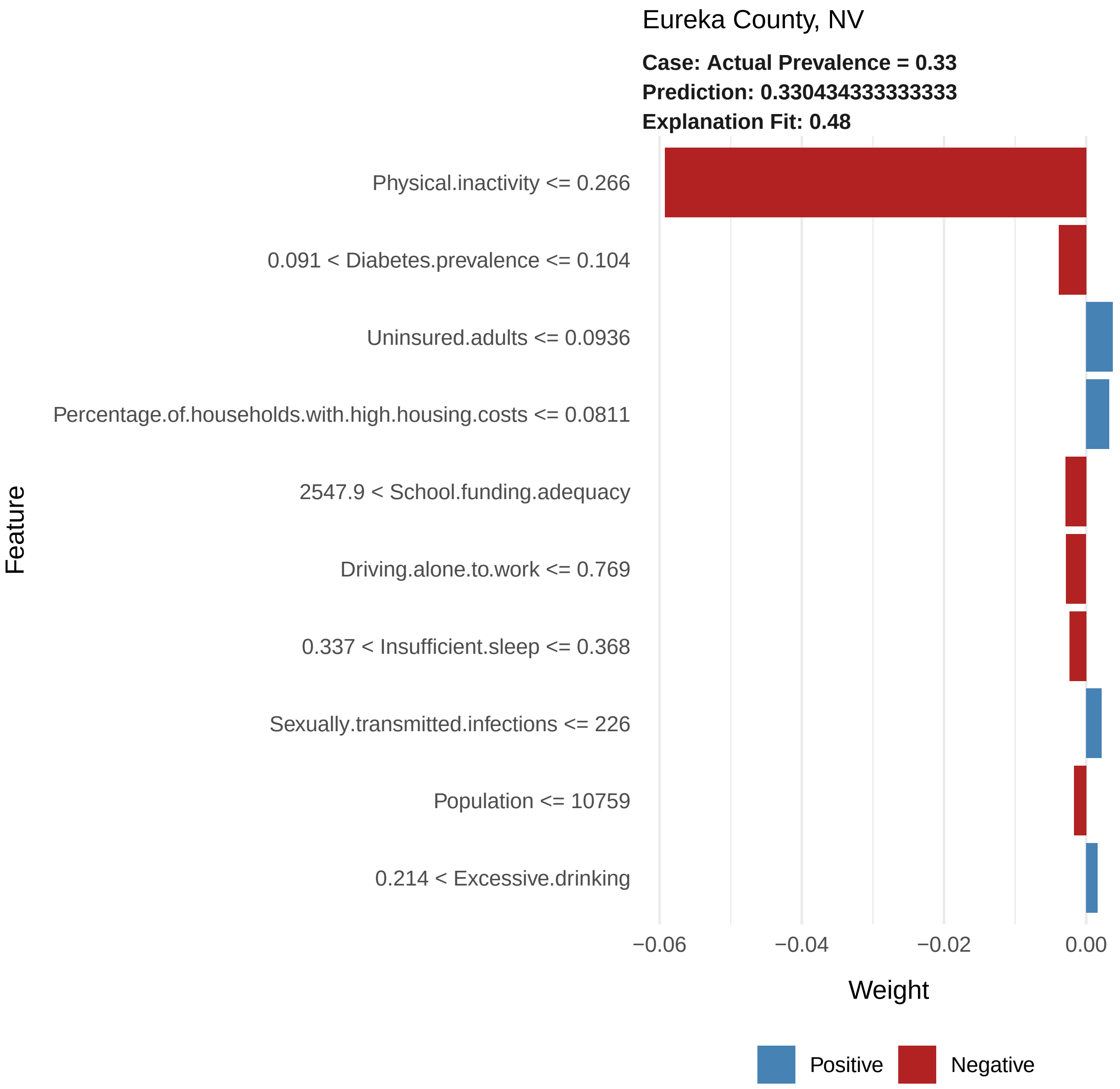

Eureka County, NV
Case: Actual Prevalence = 0.33
Prediction: 0.330434333333333
Explanation Fit: 0.48
Physical.inactivity <= 0.266
0.091 < Diabetes.prevalence <= 0.104
Uninsured.adults <= 0.0936
Percentage.of.households.with.high.housing.costs <= 0.0811
2547.9 < School.funding.adequacy
Driving.alone.to.work <= 0.769
0.337 < Insufficient.sleep <= 0.368
Sexually.transmitted.infections <= 226
Population <= 10759
0.214 < Excessive.drinking
Feature
−0.06
−0.04
−0.02
0.00
Weight
Positive
Negative

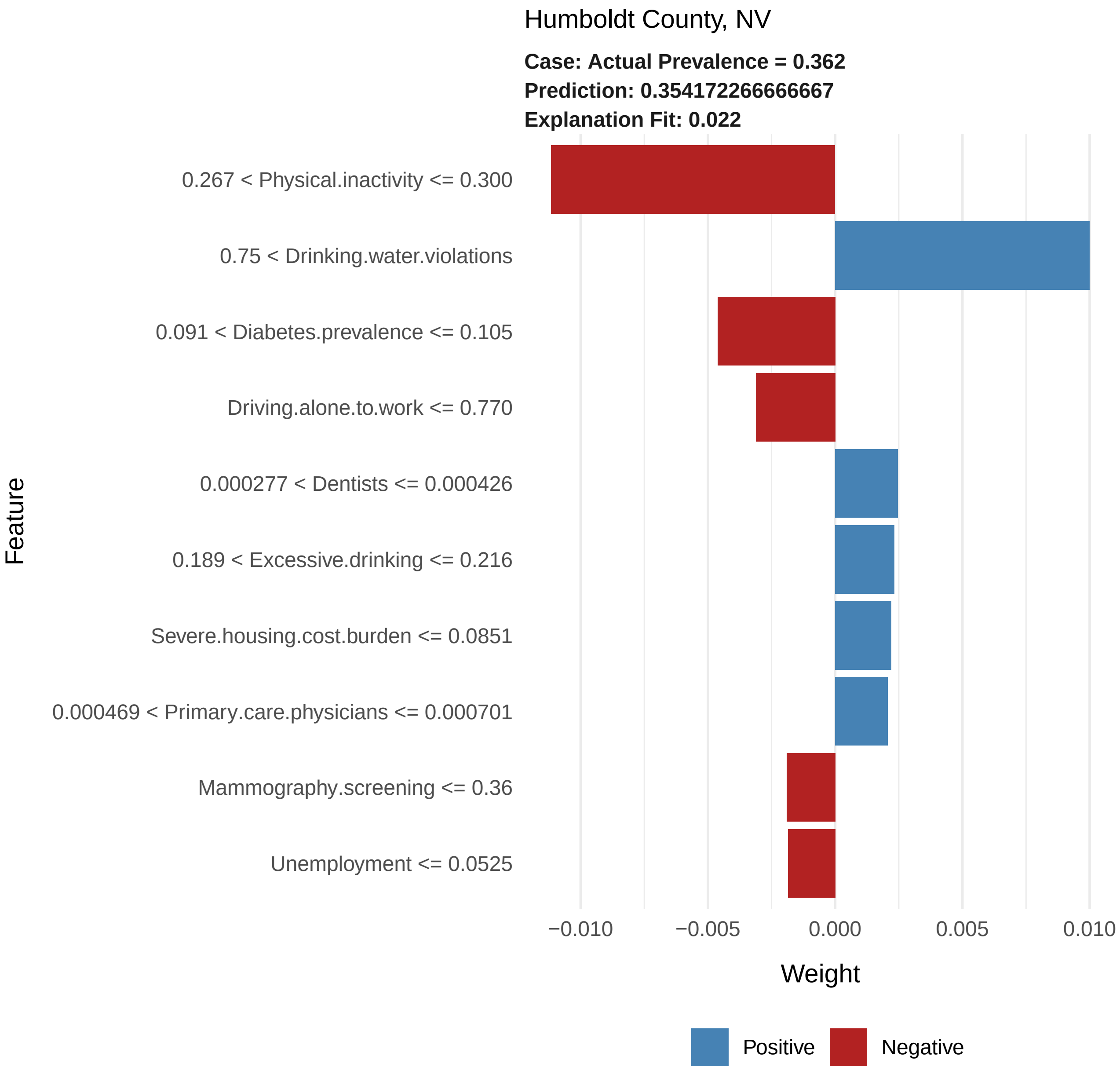

Humboldt County, NV
Case: Actual Prevalence = 0.362
Prediction: 0.354172266666667
Explanation Fit: 0.022
0.267 < Physical.inactivity <= 0.300
0.75 < Drinking.water.violations
0.091 < Diabetes.prevalence <= 0.105
Driving.alone.to.work <= 0.770
0.000277 < Dentists <= 0.000426
0.189 < Excessive.drinking <= 0.216
Severe.housing.cost.burden <= 0.0851
0.000469 < Primary.care.physicians <= 0.000701
Mammography.screening <= 0.36
Unemployment <= 0.0525
Feature
−0.010
−0.005
0.000
0.005
0.010
Weight
Positive
Negative

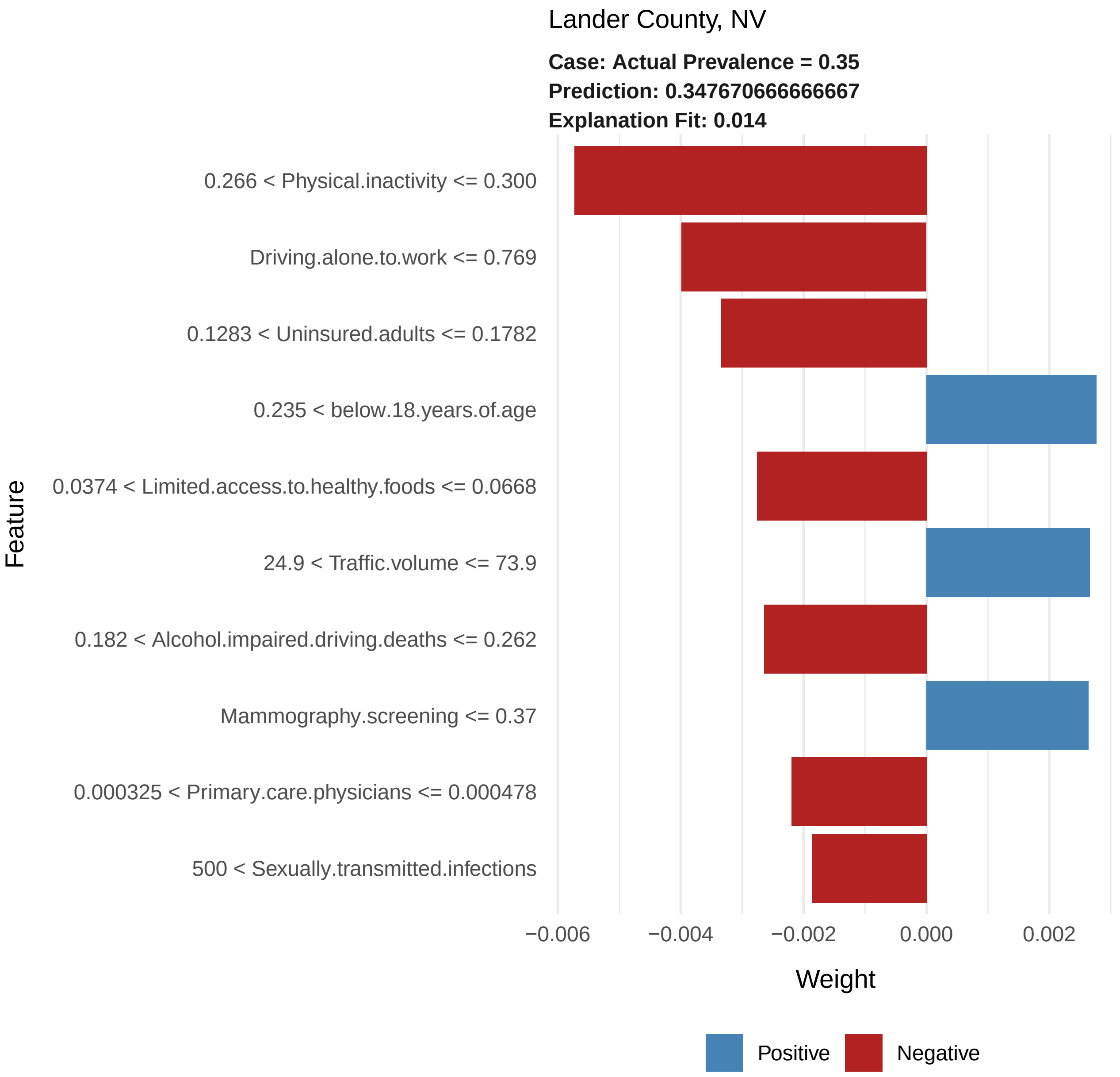

Lander County, NV
Case: Actual Prevalence = 0.35
Prediction: 0.347670666666667
Explanation Fit: 0.014
0.266 < Physical.inactivity <= 0.300
Driving.alone.to.work <= 0.769
0.1283 < Uninsured.adults <= 0.1782
0.235 < below.18.years.of.age
0.0374 < Limited.access.to.healthy.foods <= 0.0668
24.9 < Traffic.volume <= 73.9
0.182 < Alcohol.impaired.driving.deaths <= 0.262
Mammography.screening <= 0.37
0.000325 < Primary.care.physicians <= 0.000478
500 < Sexually.transmitted.infections
Feature
−0.006
−0.004
−0.002
0.000
0.002
Weight
Positive
Negative

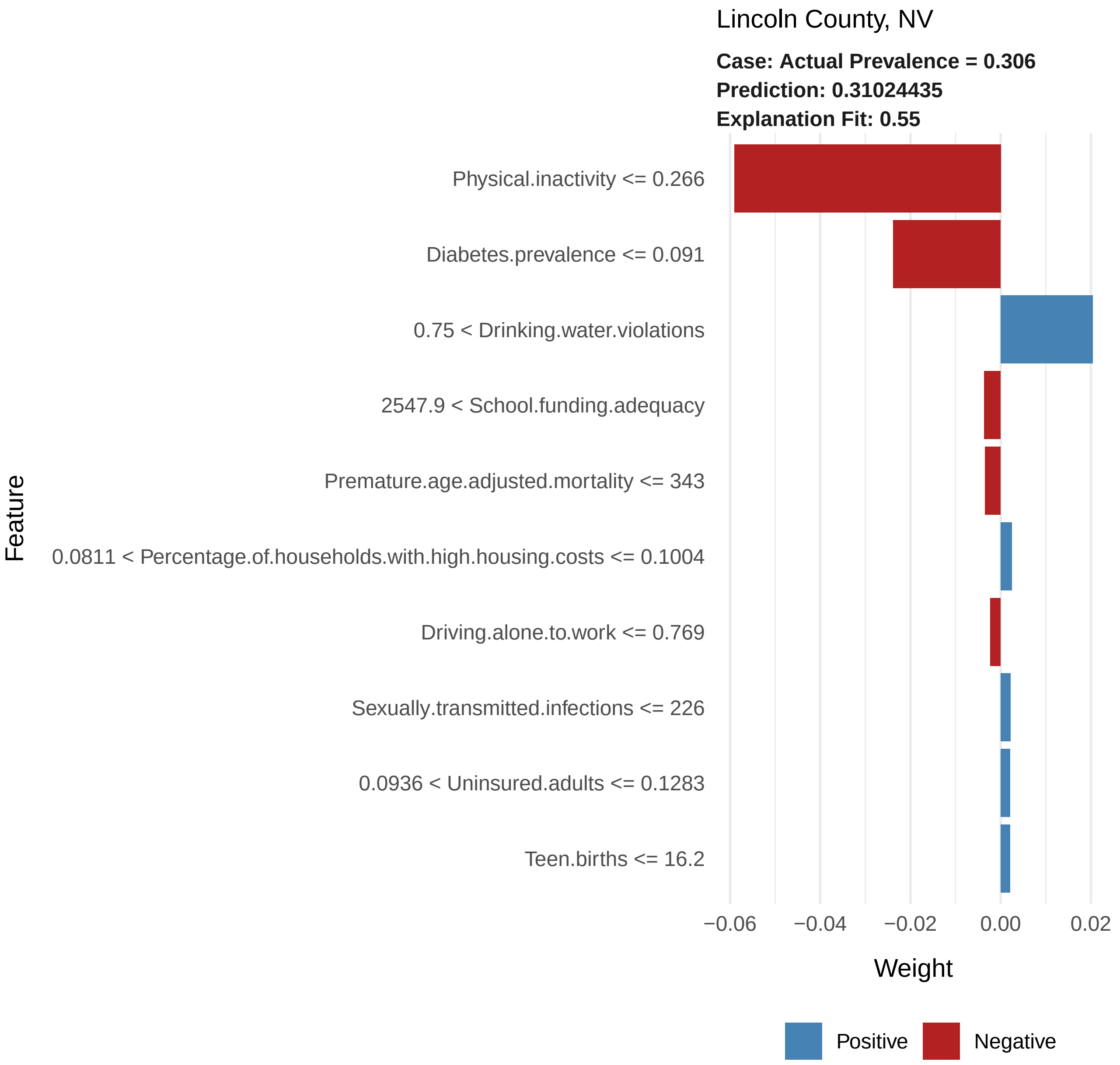
Lincoln County, NV
Case: Actual Prevalence = 0.306
Prediction: 0.31024435
Explanation Fit: 0.55
Physical.inactivity <= 0.266
Diabetes.prevalence <= 0.091
0.75 < Drinking.water.violations
2547.9 < School.funding.adequacy
Premature.age.adjusted.mortality <= 343
0.0811 < Percentage.of.households.with.high.housing.costs <= 0.1004
Driving.alone.to.work <= 0.769
Sexually.transmitted.infections <= 226
0.0936 < Uninsured.adults <= 0.1283
Teen.births <= 16.2
Feature
−0.06
−0.04
−0.02
0.00
0.02
Weight
Positive
Negative

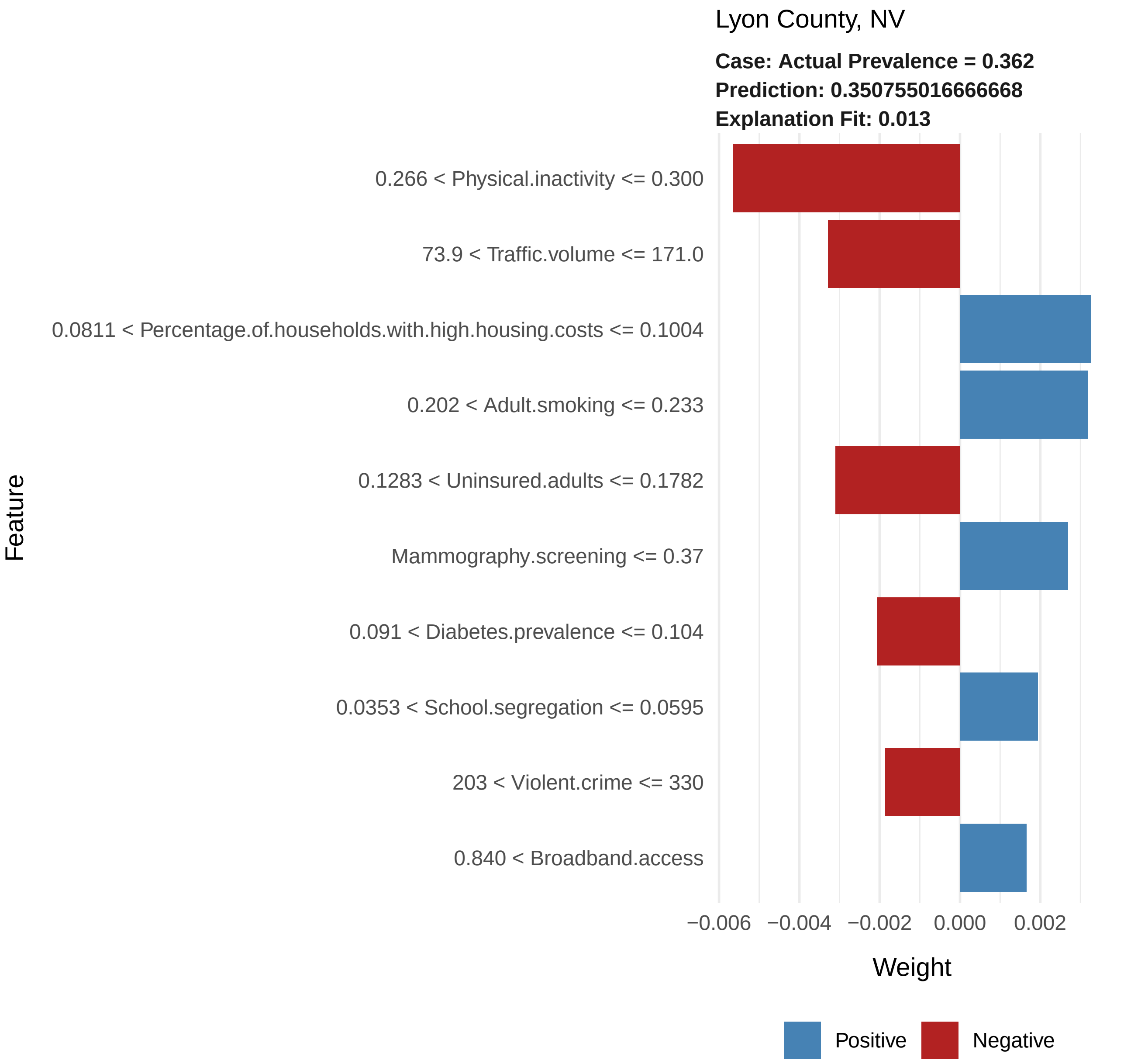

Lyon County, NV
Case: Actual Prevalence = 0.362
Prediction: 0.350755016666668
Explanation Fit: 0.013
0.266 < Physical.inactivity <= 0.300
73.9 < Traffic.volume <= 171.0
0.0811 < Percentage.of.households.with.high.housing.costs <= 0.1004
0.202 < Adult.smoking <= 0.233
0.1283 < Uninsured.adults <= 0.1782
Mammography.screening <= 0.37
0.091 < Diabetes.prevalence <= 0.104
0.0353 < School.segregation <= 0.0595
203 < Violent.crime <= 330
0.840 < Broadband.access
Feature
−0.006
−0.004
−0.002
0.000
0.002
Weight
Positive
Negative

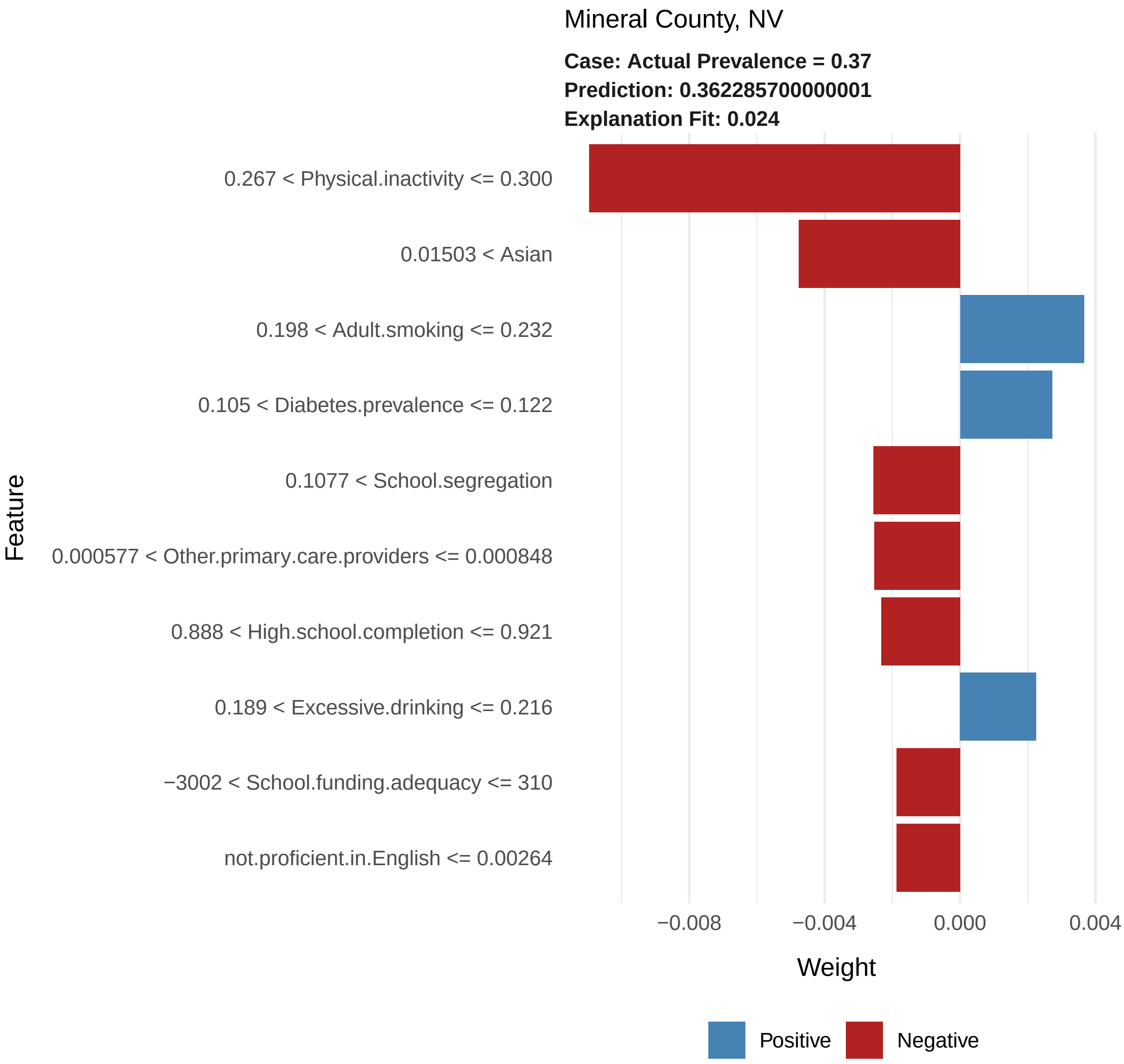
Mineral County, NV
Case: Actual Prevalence = 0.37
Prediction: 0.362285700000001
Explanation Fit: 0.024
0.267 < Physical.inactivity <= 0.300
0.01503 < Asian
0.198 < Adult.smoking <= 0.232
0.105 < Diabetes.prevalence <= 0.122
0.1077 < School.segregation
0.000577 < Other.primary.care.providers <= 0.000848
0.888 < High.school.completion <= 0.921
0.189 < Excessive.drinking <= 0.216
−3002 < School.funding.adequacy <= 310
not.proficient.in.English <= 0.00264
Feature
−0.008
−0.004
0.000
0.004
Weight
Positive
Negative

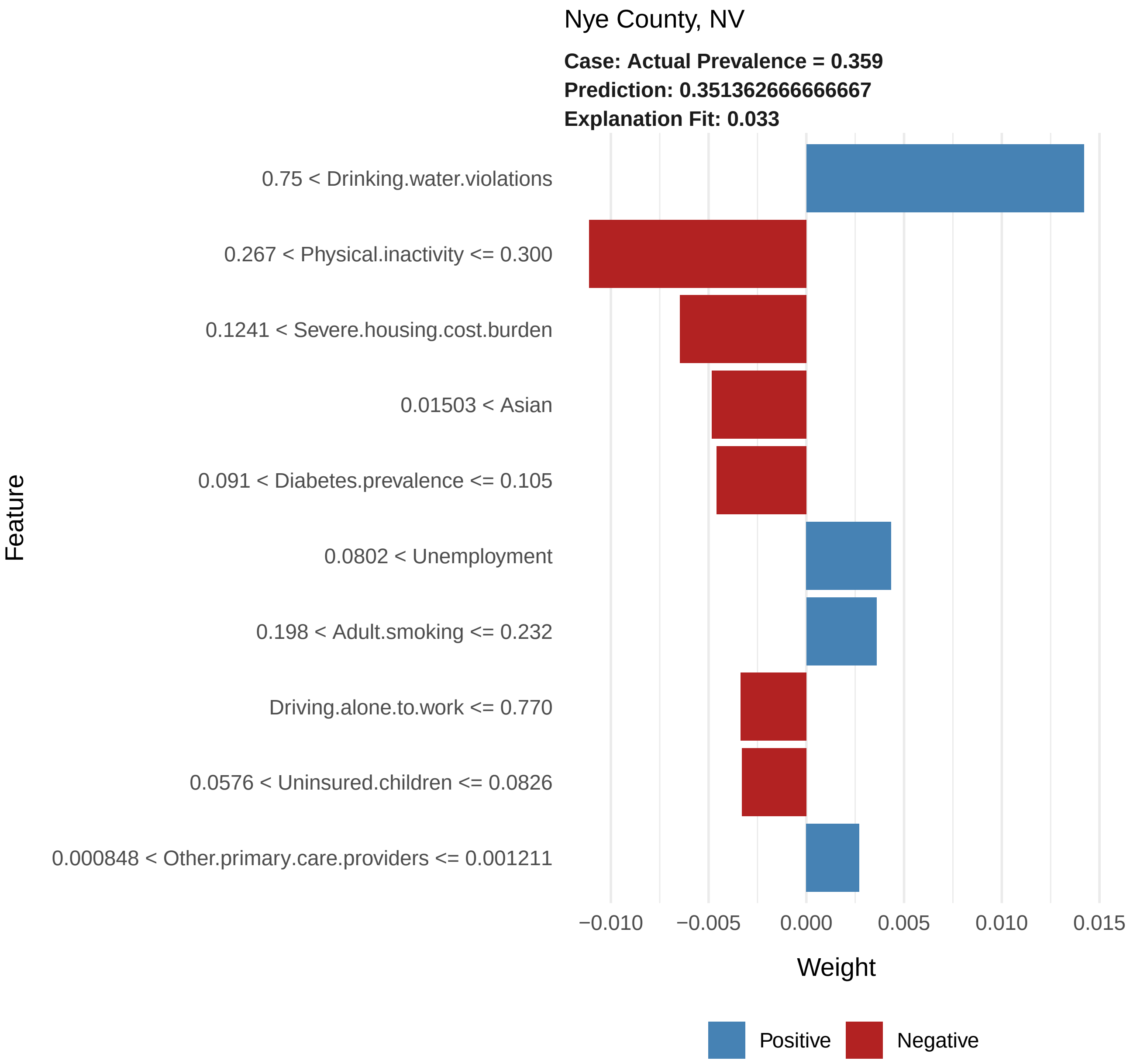

Nye County, NV
Case: Actual Prevalence = 0.359
Prediction: 0.351362666666667
Explanation Fit: 0.033
0.75 < Drinking.water.violations
0.267 < Physical.inactivity <= 0.300
0.1241 < Severe.housing.cost.burden
0.01503 < Asian
0.091 < Diabetes.prevalence <= 0.105
0.0802 < Unemployment
0.198 < Adult.smoking <= 0.232
Driving.alone.to.work <= 0.770
0.0576 < Uninsured.children <= 0.0826
0.000848 < Other.primary.care.providers <= 0.001211
Feature
−0.010
−0.005
0.000
0.005
0.010
0.015
Weight
Positive
Negative

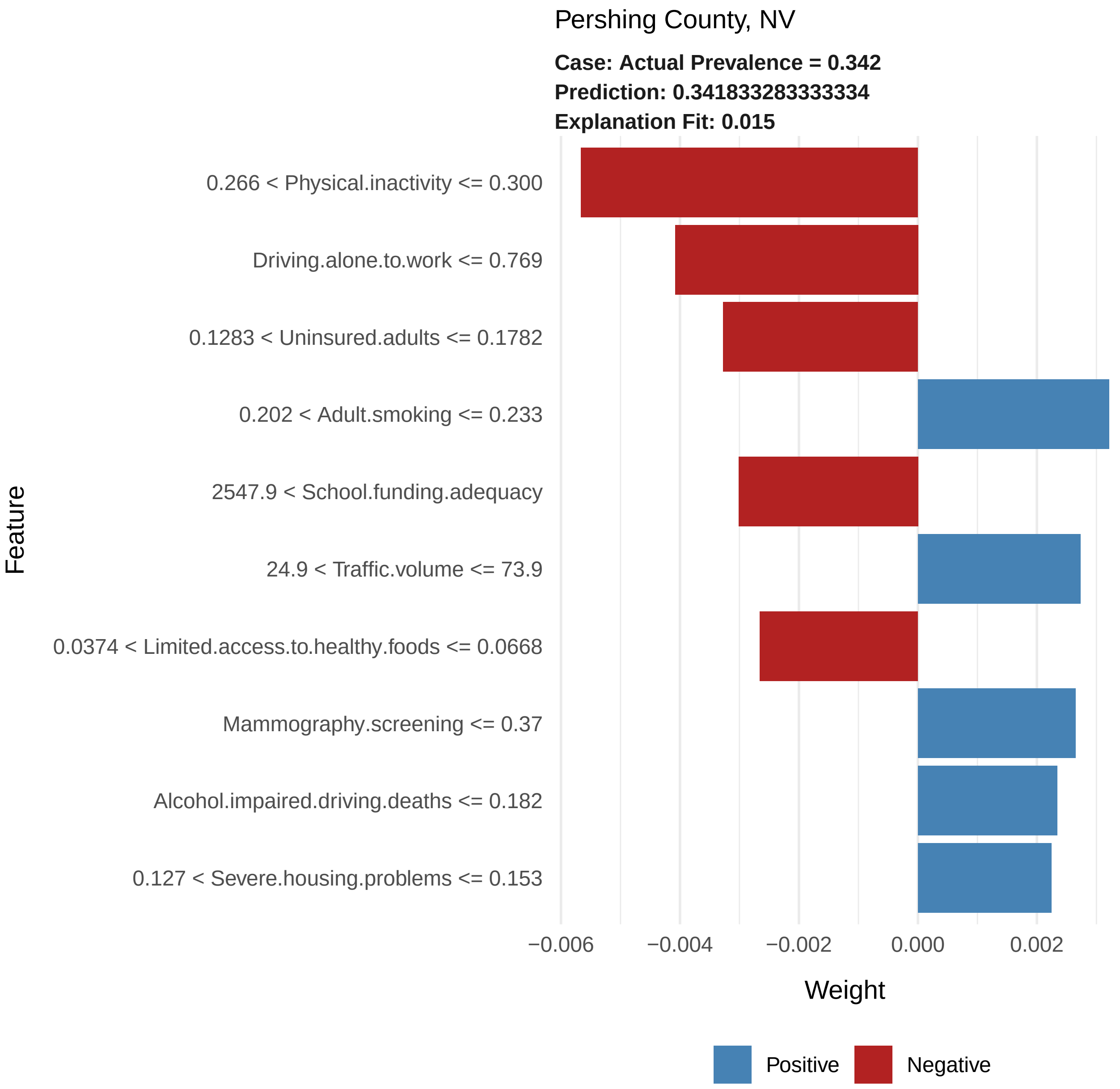

Pershing County, NV
Case: Actual Prevalence = 0.342
Prediction: 0.341833283333334
Explanation Fit: 0.015
0.266 < Physical.inactivity <= 0.300
Driving.alone.to.work <= 0.769
0.1283 < Uninsured.adults <= 0.1782
0.202 < Adult.smoking <= 0.233
2547.9 < School.funding.adequacy
24.9 < Traffic.volume <= 73.9
0.0374 < Limited.access.to.healthy.foods <= 0.0668
Mammography.screening <= 0.37
Alcohol.impaired.driving.deaths <= 0.182
0.127 < Severe.housing.problems <= 0.153
Feature
−0.006
−0.004
−0.002
0.000
0.002
Weight
Positive
Negative

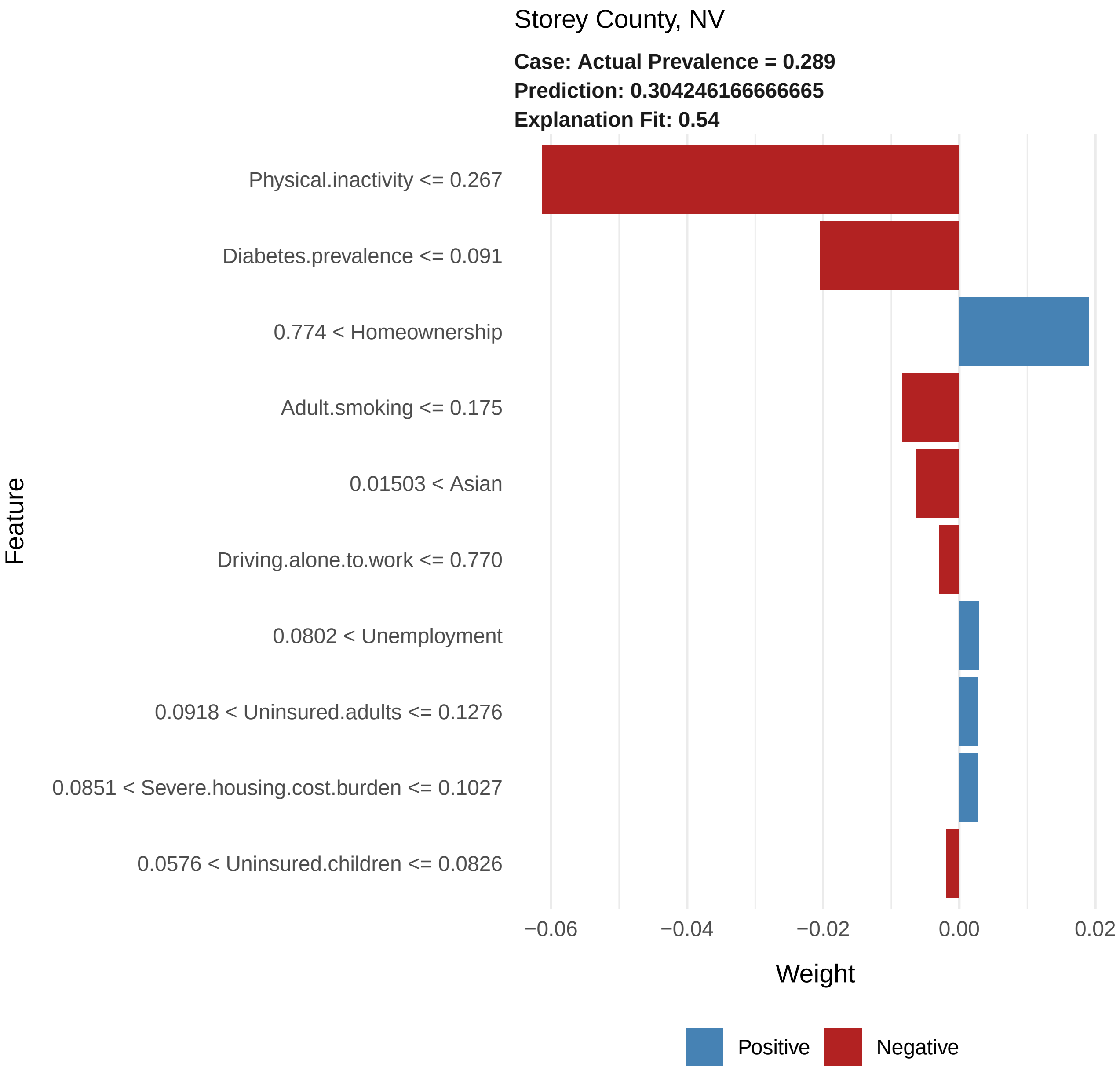
Storey County, NV
Case: Actual Prevalence = 0.289
Prediction: 0.304246166666665
Explanation Fit: 0.54
Physical.inactivity <= 0.267
Diabetes.prevalence <= 0.091
0.774 < Homeownership
Adult.smoking <= 0.175
0.01503 < Asian
Driving.alone.to.work <= 0.770
0.0802 < Unemployment
0.0918 < Uninsured.adults <= 0.1276
0.0851 < Severe.housing.cost.burden <= 0.1027
0.0576 < Uninsured.children <= 0.0826
Feature
−0.06
−0.04
−0.02
0.00
0.02
Weight
Positive
Negative

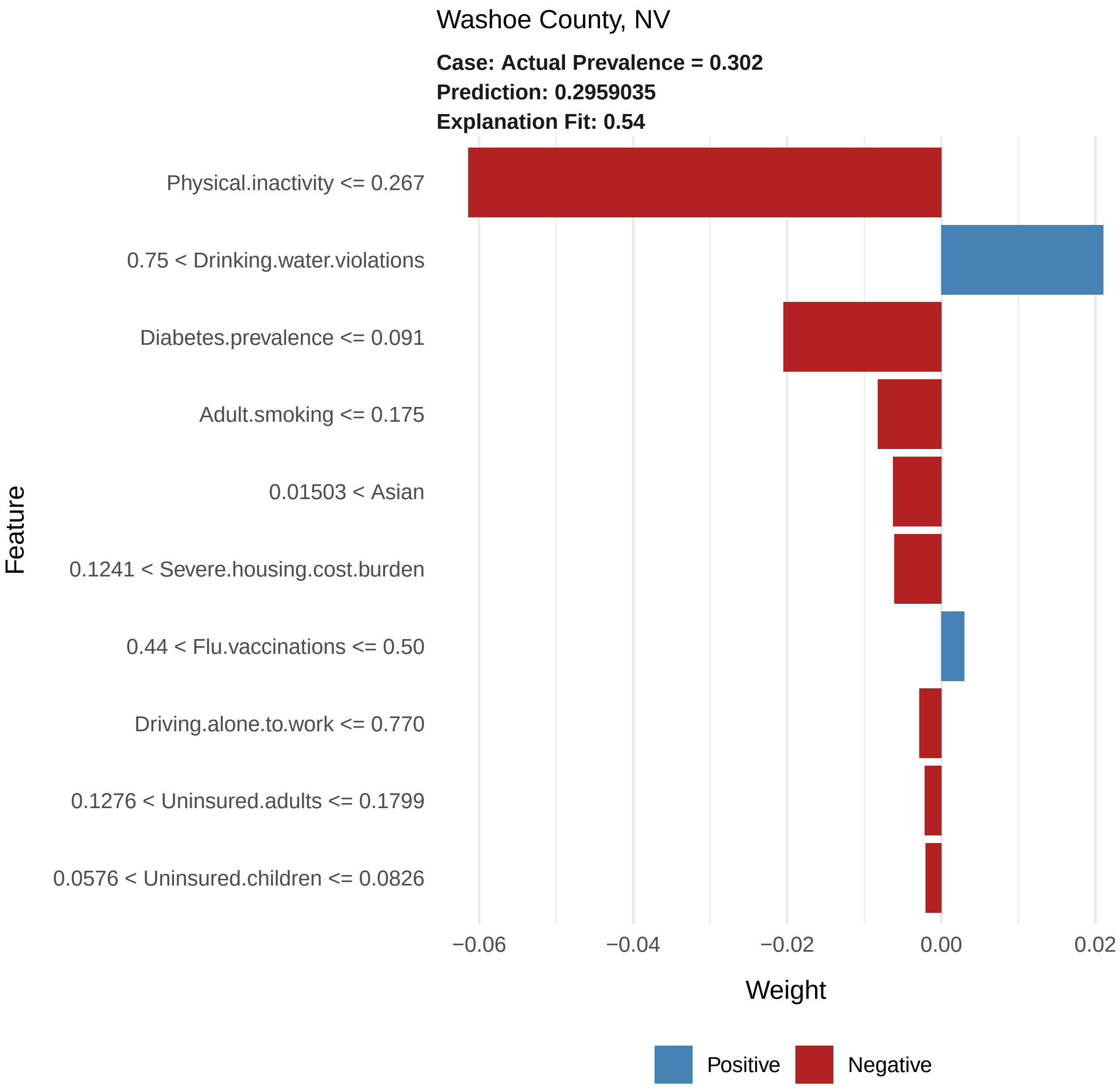
Washoe County, NV
Case: Actual Prevalence = 0.302
Prediction: 0.2959035
Explanation Fit: 0.54
Physical.inactivity <= 0.267
0.75 < Drinking.water.violations
Diabetes.prevalence <= 0.091
Adult.smoking <= 0.175
0.01503 < Asian
0.1241 < Severe.housing.cost.burden
0.44 < Flu.vaccinations <= 0.50
Driving.alone.to.work <= 0.770
0.1276 < Uninsured.adults <= 0.1799
0.0576 < Uninsured.children <= 0.0826
Feature
−0.06
−0.04
−0.02
0.00
0.02
Weight
Positive
Negative

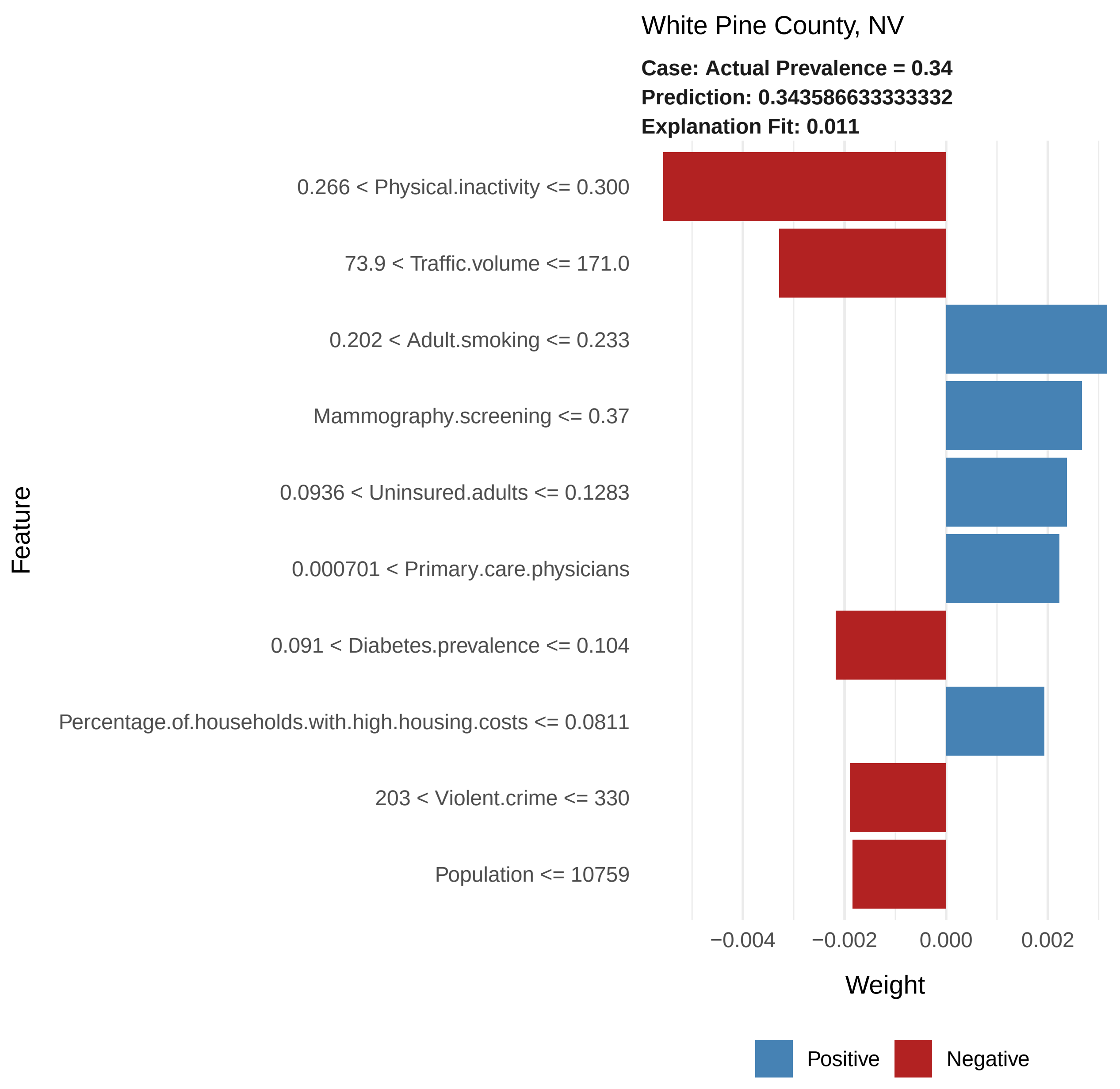
White Pine County, NV
Case: Actual Prevalence = 0.34
Prediction: 0.343586633333332
Explanation Fit: 0.011
0.266 < Physical.inactivity <= 0.300
73.9 < Traffic.volume <= 171.0
0.202 < Adult.smoking <= 0.233
Mammography.screening <= 0.37
0.0936 < Uninsured.adults <= 0.1283
0.000701 < Primary.care.physicians
0.091 < Diabetes.prevalence <= 0.104
Percentage.of.households.with.high.housing.costs <= 0.0811
203 < Violent.crime <= 330
Population <= 10759
Feature
−0.004
−0.002
0.000
0.002
Weight
Positive
Negative

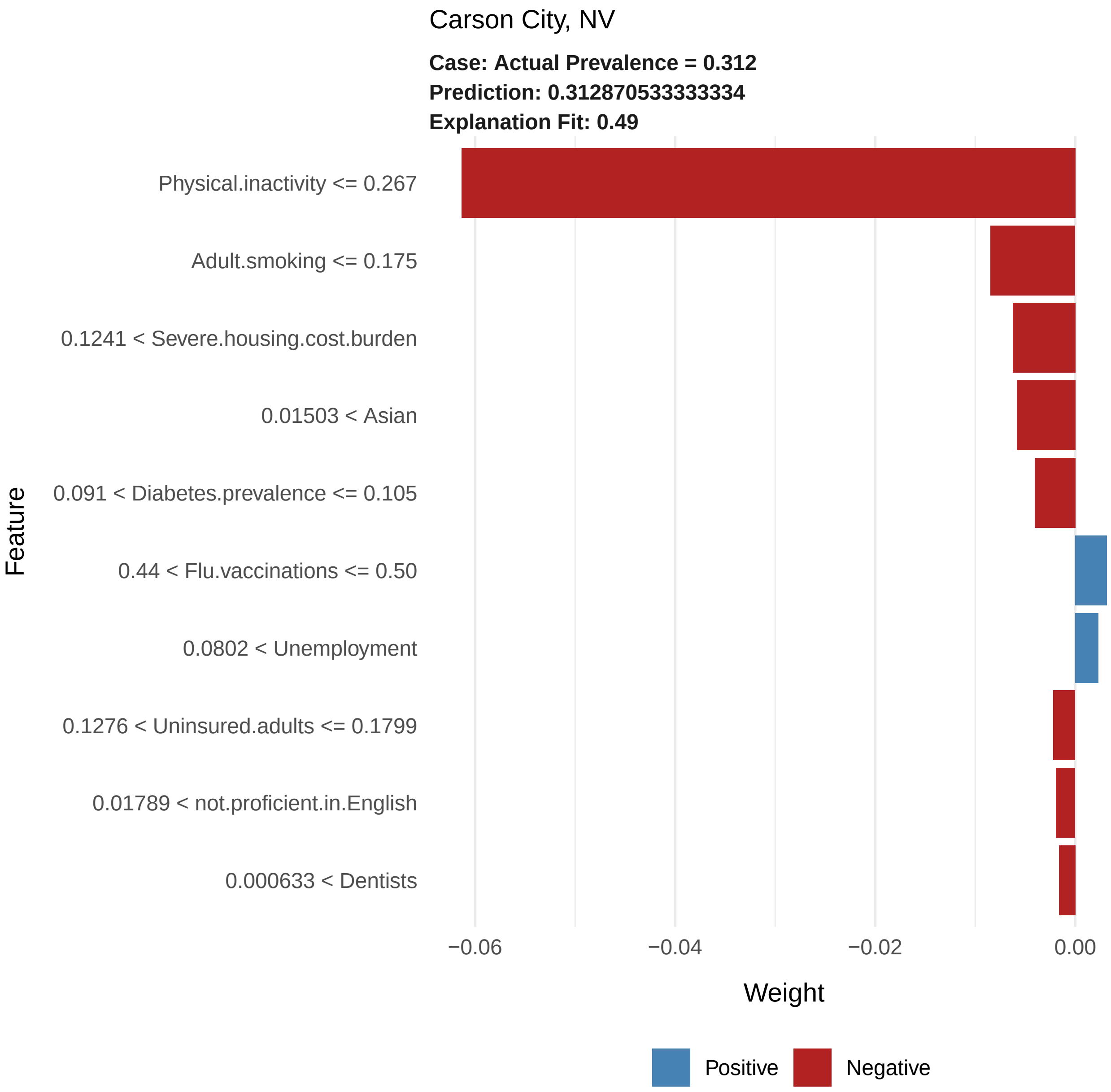

Carson City, NV
Case: Actual Prevalence = 0.312
Prediction: 0.312870533333334
Explanation Fit: 0.49
Physical.inactivity <= 0.267
Adult.smoking <= 0.175
0.1241 < Severe.housing.cost.burden
0.01503 < Asian
0.091 < Diabetes.prevalence <= 0.105
0.44 < Flu.vaccinations <= 0.50
0.0802 < Unemployment
0.1276 < Uninsured.adults <= 0.1799
0.01789 < not.proficient.in.English
0.000633 < Dentists
Feature
−0.06
−0.04
−0.02
0.00
Weight
Positive
Negative

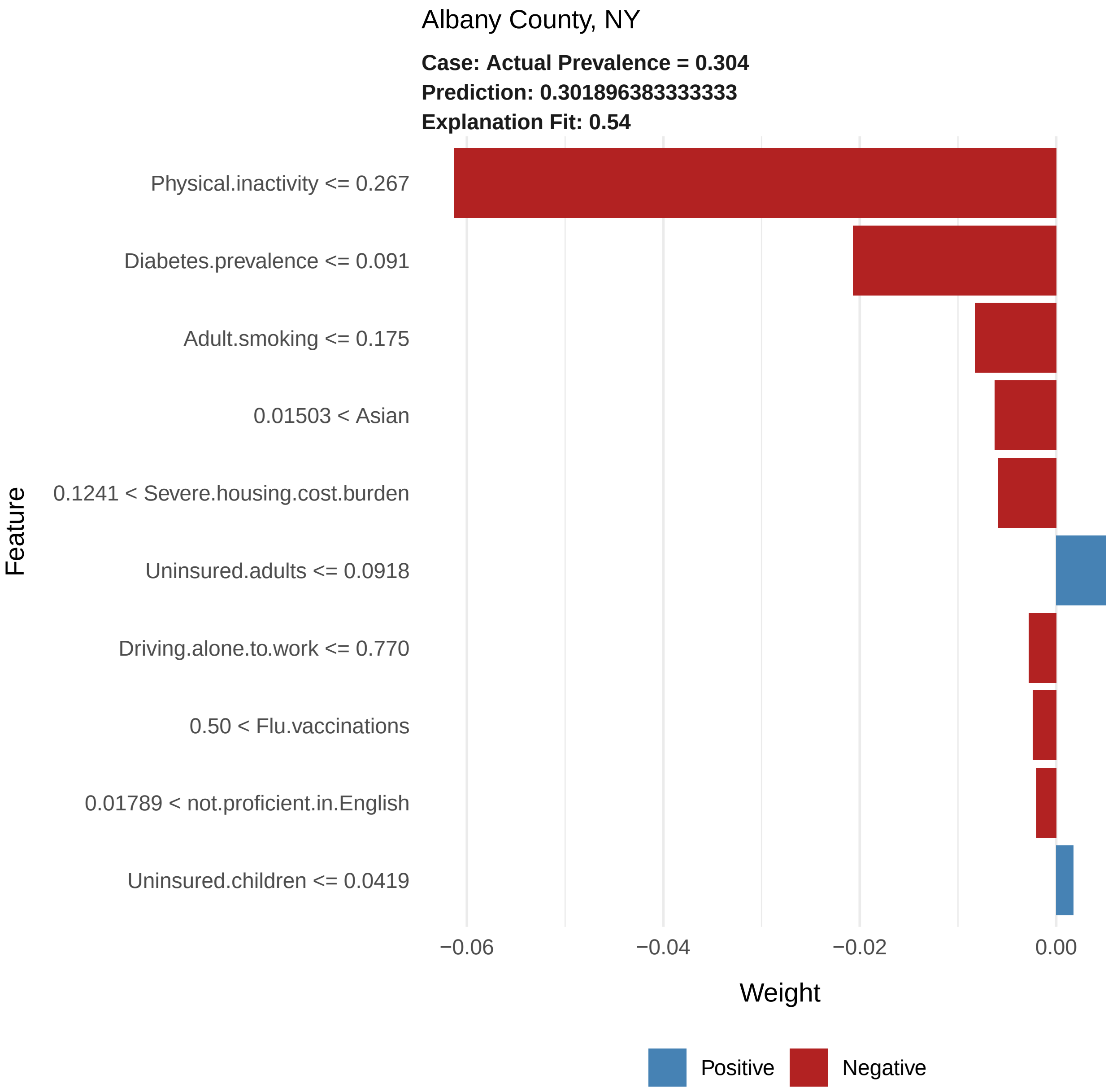
Albany County, NY
Case: Actual Prevalence = 0.304
Prediction: 0.301896383333333
Explanation Fit: 0.54
Physical.inactivity <= 0.267
Diabetes.prevalence <= 0.091
Adult.smoking <= 0.175
0.01503 < Asian
0.1241 < Severe.housing.cost.burden
Uninsured.adults <= 0.0918
Driving.alone.to.work <= 0.770
0.50 < Flu.vaccinations
0.01789 < not.proficient.in.English
Uninsured.children <= 0.0419
Feature
−0.06
−0.04
−0.02
0.00
Weight
Positive
Negative

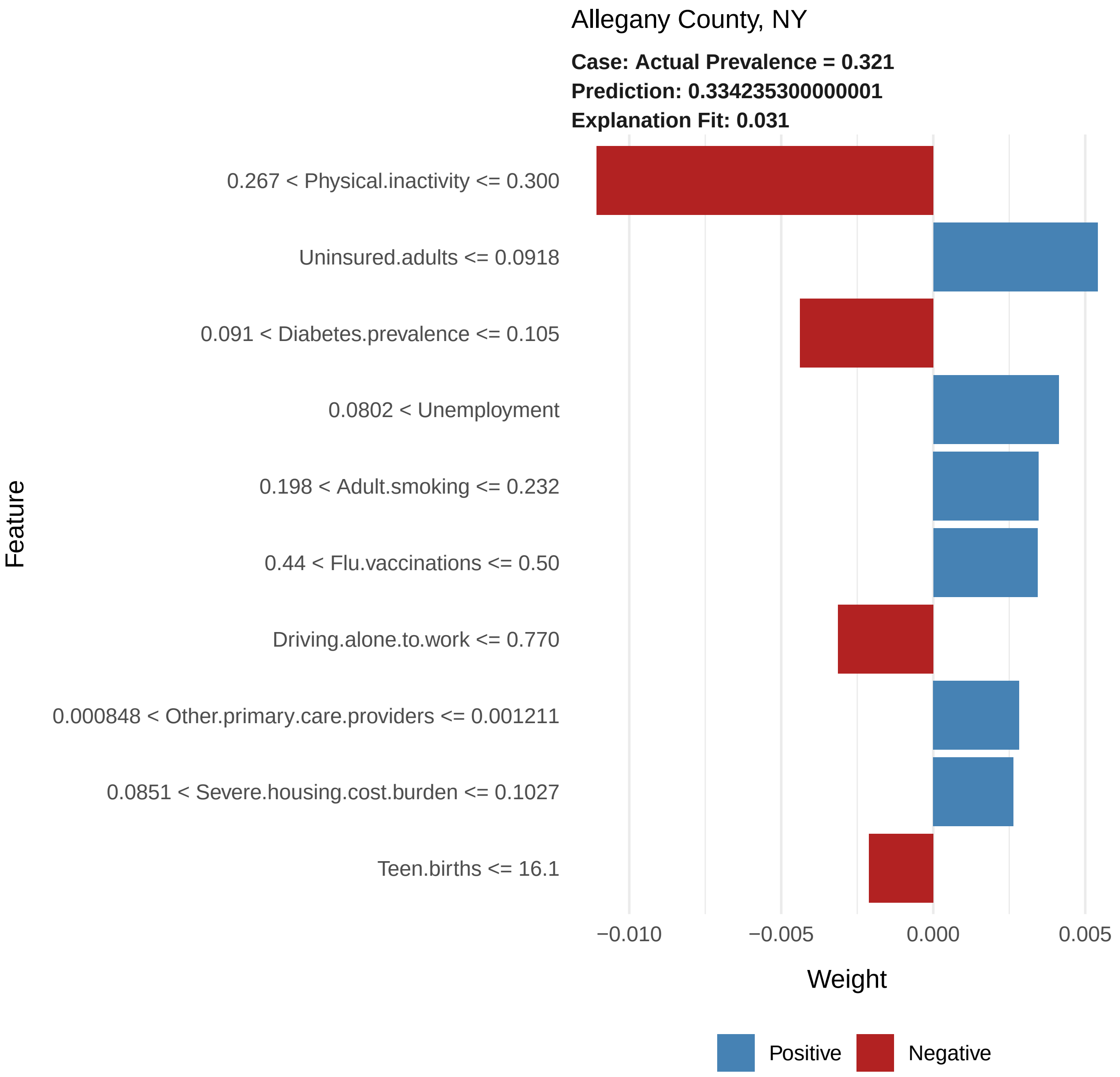
Allegany County, NY
Case: Actual Prevalence = 0.321
Prediction: 0.334235300000001
Explanation Fit: 0.031
0.267 < Physical.inactivity <= 0.300
Uninsured.adults <= 0.0918
0.091 < Diabetes.prevalence <= 0.105
0.0802 < Unemployment
0.198 < Adult.smoking <= 0.232
0.44 < Flu.vaccinations <= 0.50
Driving.alone.to.work <= 0.770
0.000848 < Other.primary.care.providers <= 0.001211
0.0851 < Severe.housing.cost.burden <= 0.1027
Teen.births <= 16.1
Feature
−0.010
−0.005
0.000
0.005
Weight
Positive
Negative

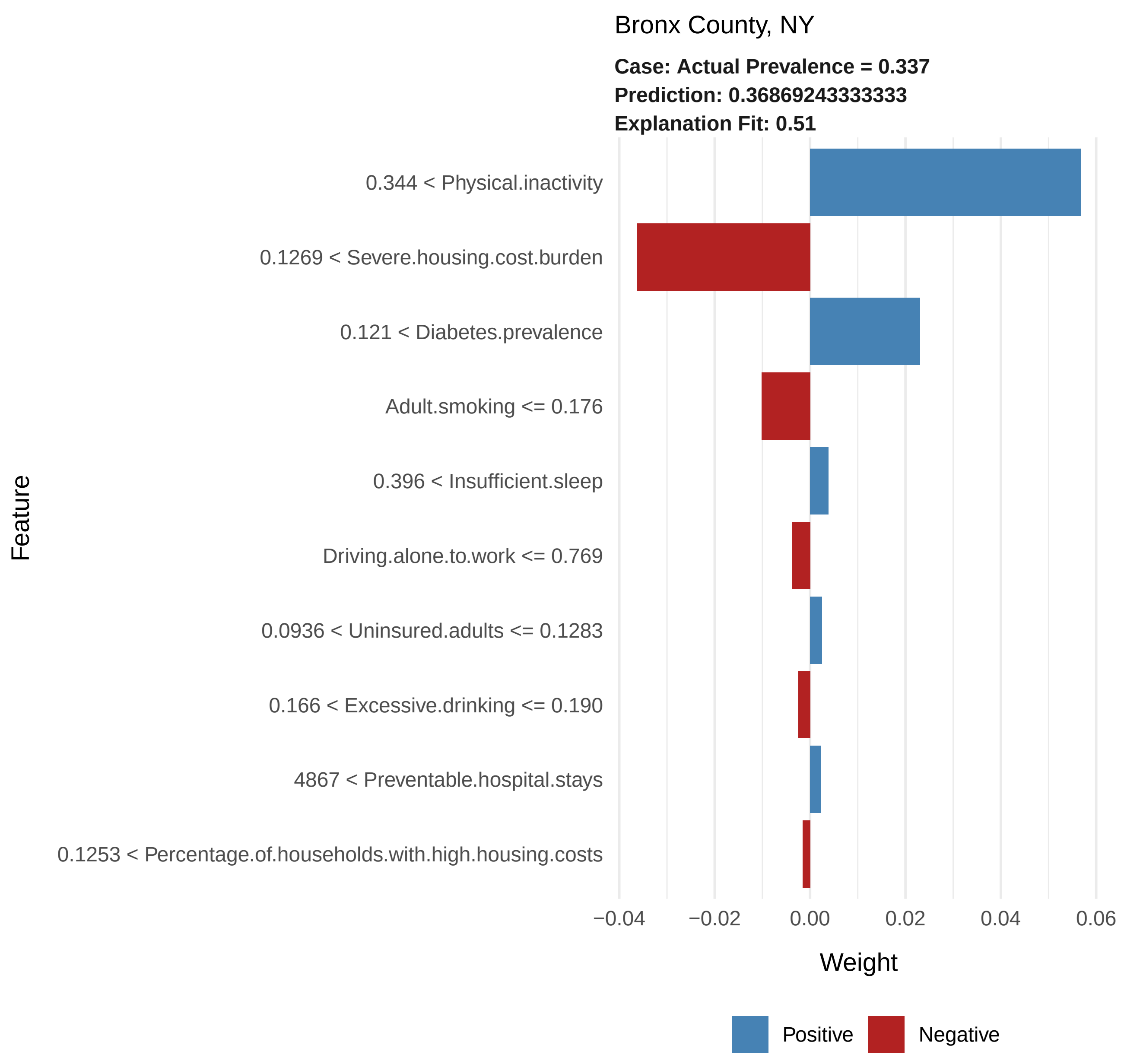
Bronx County, NY
Case: Actual Prevalence = 0.337
Prediction: 0.368692433333333
Explanation Fit: 0.51
0.344 < Physical.inactivity
0.1269 < Severe.housing.cost.burden
0.121 < Diabetes.prevalence
Adult.smoking <= 0.176
0.396 < Insufficient.sleep
Driving.alone.to.work <= 0.769
0.0936 < Uninsured.adults <= 0.1283
0.166 < Excessive.drinking <= 0.190
4867 < Preventable.hospital.stays
0.1253 < Percentage.of.households.with.high.housing.costs
Feature
−0.04
−0.02
0.00
0.02
0.04
0.06
Weight
Positive
Negative

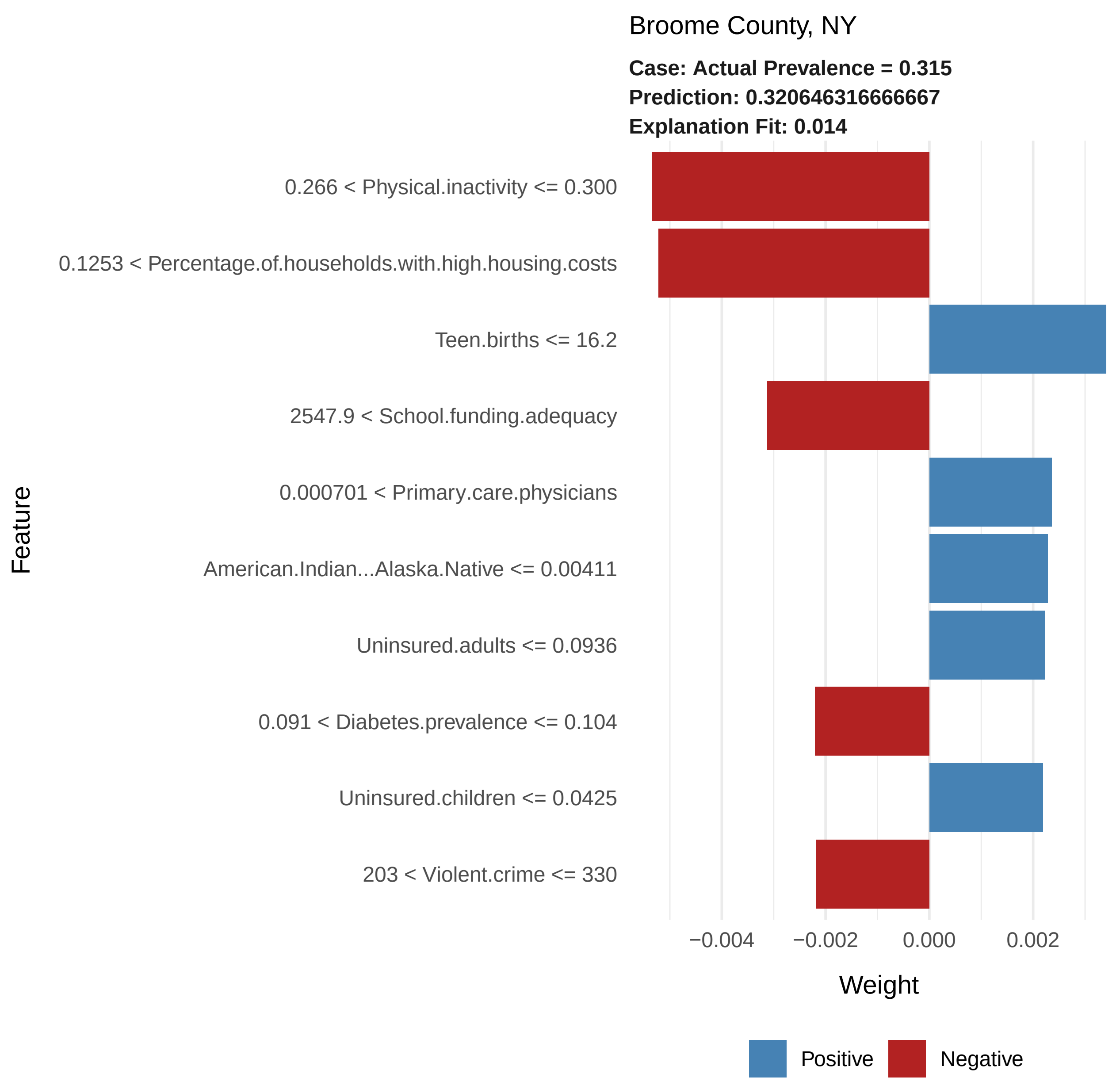

Broome County, NY
Case: Actual Prevalence = 0.315
Prediction: 0.320646316666667
Explanation Fit: 0.014
0.266 < Physical.inactivity <= 0.300
0.1253 < Percentage.of.households.with.high.housing.costs
Teen.births <= 16.2
2547.9 < School.funding.adequacy
0.000701 < Primary.care.physicians
American.Indian...Alaska.Native <= 0.00411
Uninsured.adults <= 0.0936
0.091 < Diabetes.prevalence <= 0.104
Uninsured.children <= 0.0425
203 < Violent.crime <= 330
Feature
−0.004
−0.002
0.000
0.002
Weight
Positive
Negative

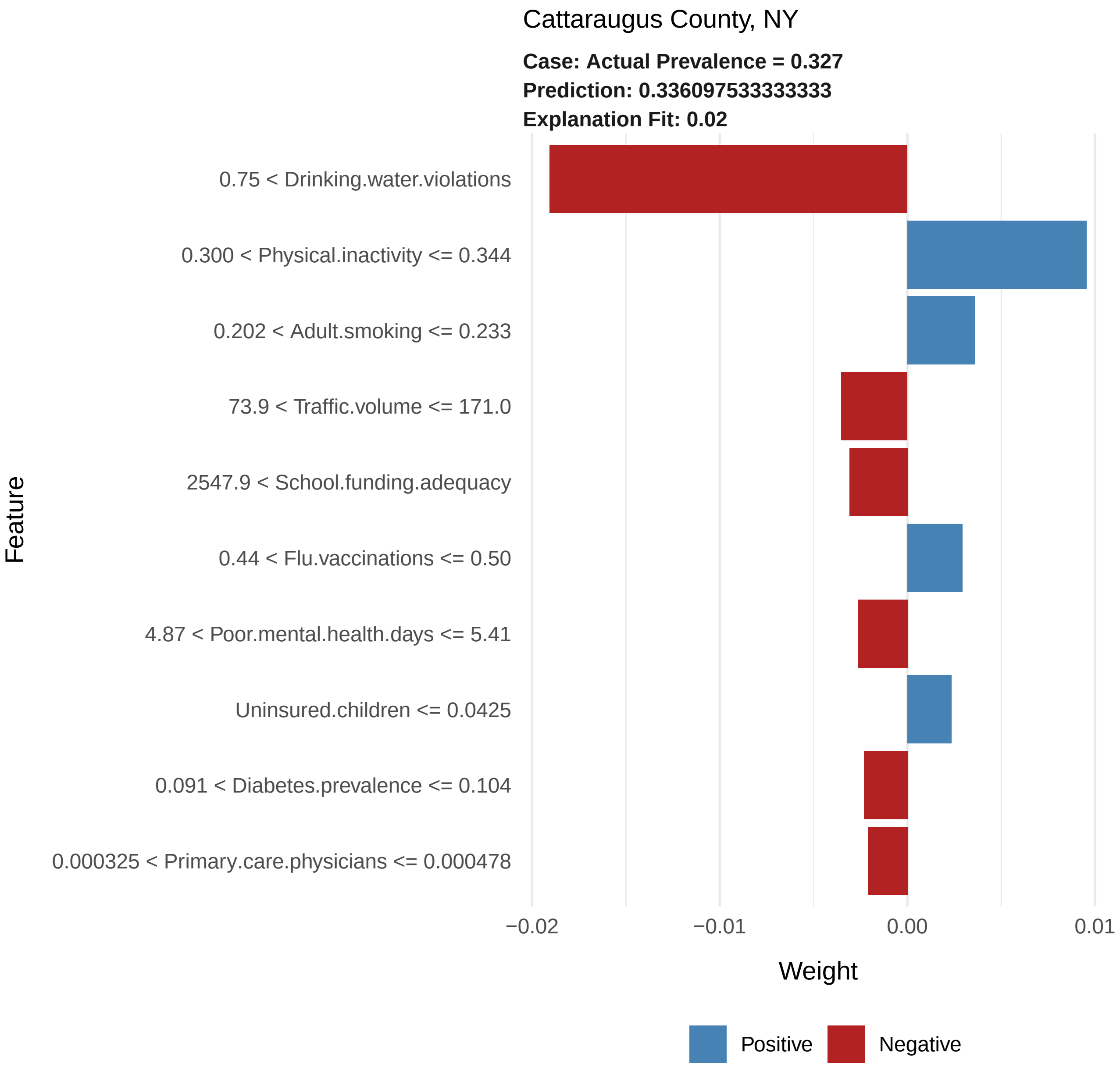

Cattaraugus County, NY
Case: Actual Prevalence = 0.327
Prediction: 0.336097533333333
Explanation Fit: 0.02
0.75 < Drinking.water.violations
0.300 < Physical.inactivity <= 0.344
0.202 < Adult.smoking <= 0.233
73.9 < Traffic.volume <= 171.0
2547.9 < School.funding.adequacy
0.44 < Flu.vaccinations <= 0.50
4.87 < Poor.mental.health.days <= 5.41
Uninsured.children <= 0.0425
0.091 < Diabetes.prevalence <= 0.104
0.000325 < Primary.care.physicians <= 0.000478
Feature
−0.02
−0.01
0.00
0.01
Weight
Positive
Negative

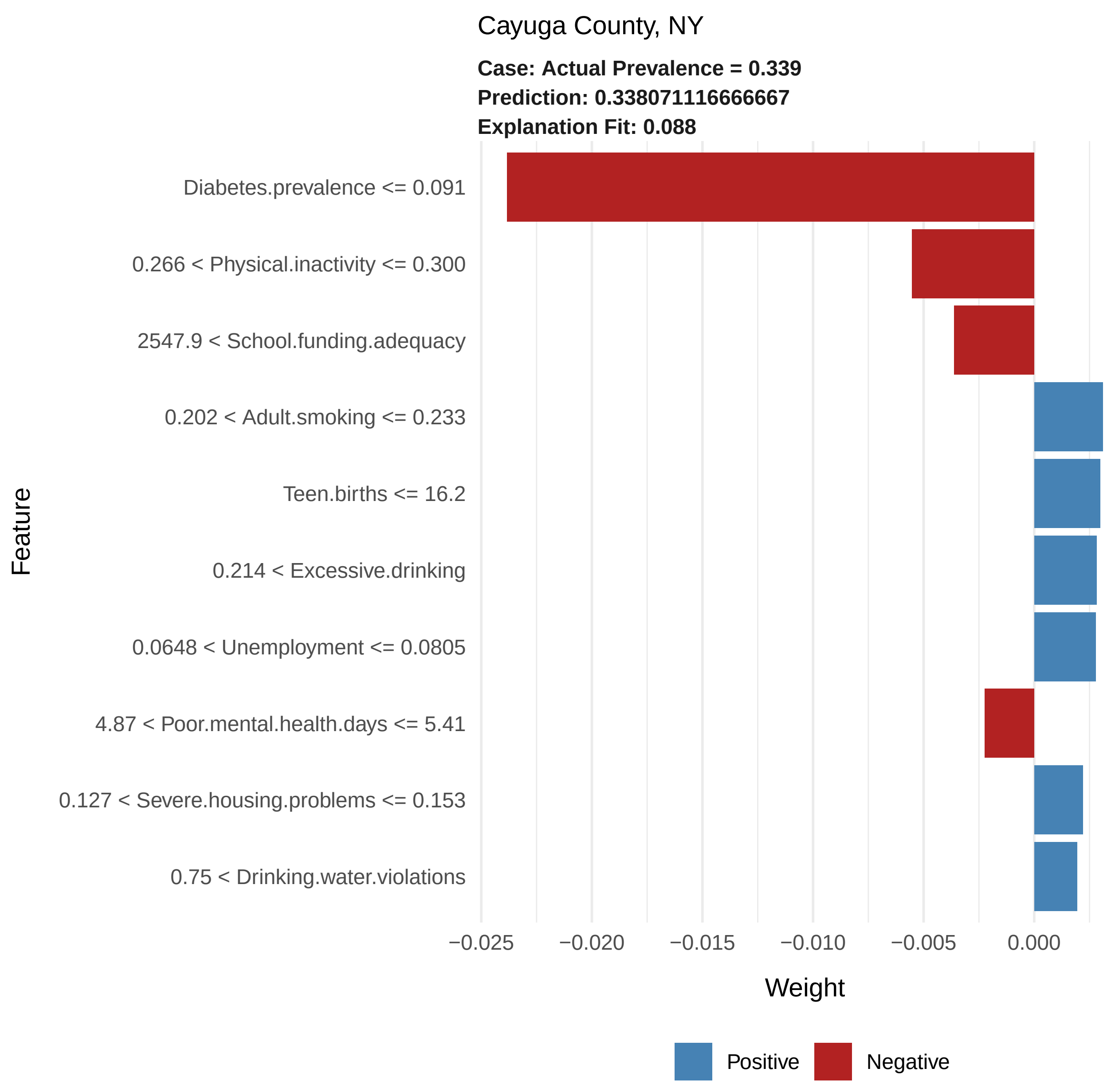

Cayuga County, NY
Case: Actual Prevalence = 0.339
Prediction: 0.338071116666667
Explanation Fit: 0.088
Diabetes.prevalence <= 0.091
0.266 < Physical.inactivity <= 0.300
2547.9 < School.funding.adequacy
0.202 < Adult.smoking <= 0.233
Teen.births <= 16.2
0.214 < Excessive.drinking
0.0648 < Unemployment <= 0.0805
4.87 < Poor.mental.health.days <= 5.41
0.127 < Severe.housing.problems <= 0.153
0.75 < Drinking.water.violations
Feature
−0.025
−0.020
−0.015
−0.010
−0.005
0.000
Weight
Positive
Negative

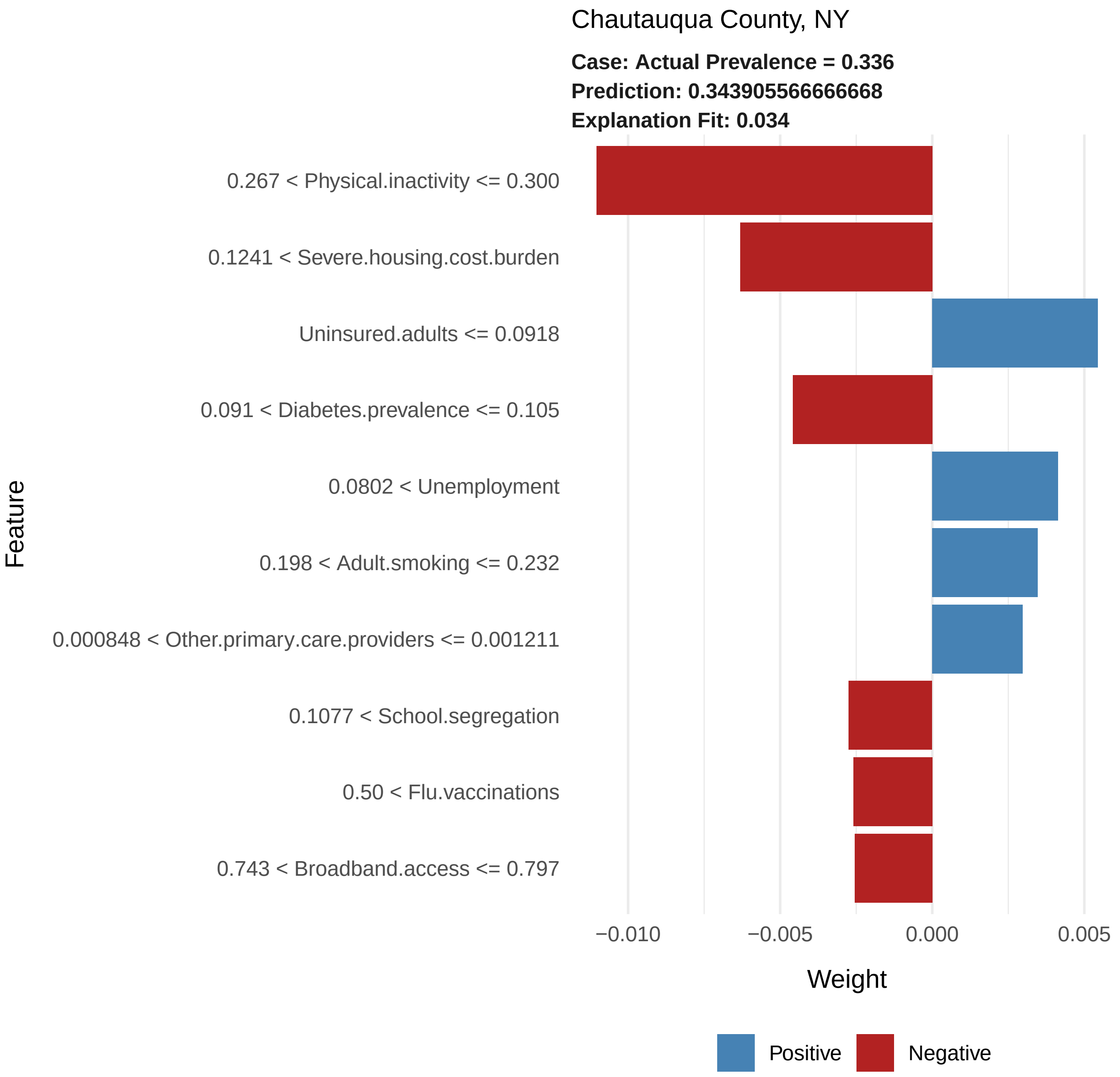
Chautauqua County, NY
Case: Actual Prevalence = 0.336
Prediction: 0.343905566666668
Explanation Fit: 0.034
0.267 < Physical.inactivity <= 0.300
0.1241 < Severe.housing.cost.burden
Uninsured.adults <= 0.0918
0.091 < Diabetes.prevalence <= 0.105
0.0802 < Unemployment
0.198 < Adult.smoking <= 0.232
0.000848 < Other.primary.care.providers <= 0.001211
0.1077 < School.segregation
0.50 < Flu.vaccinations
0.743 < Broadband.access <= 0.797
Feature
−0.010
−0.005
0.000
0.005
Weight
Positive
Negative

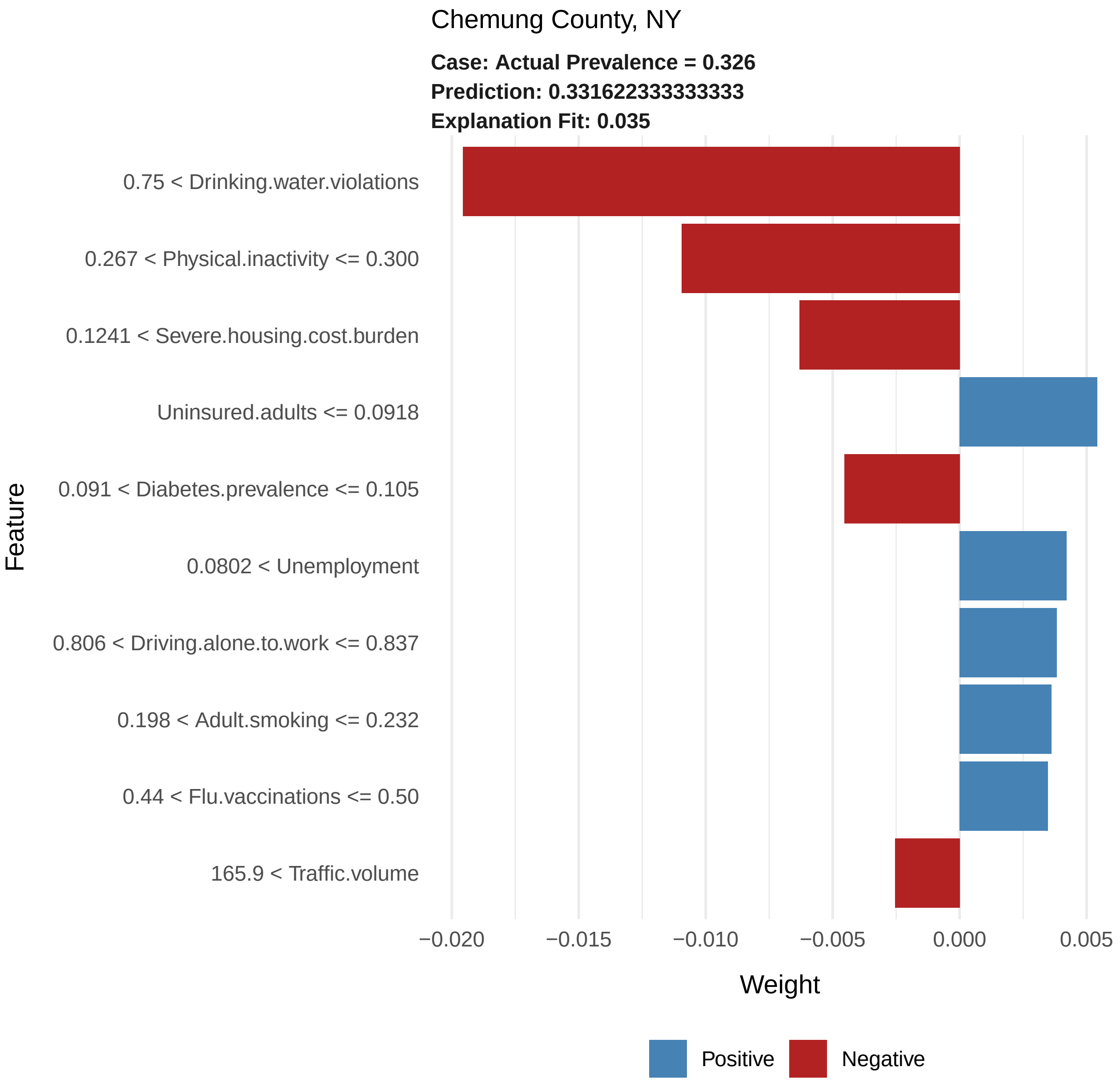

Chemung County, NY
Case: Actual Prevalence = 0.326
Prediction: 0.331622333333333
Explanation Fit: 0.035
0.75 < Drinking.water.violations
0.267 < Physical.inactivity <= 0.300
0.1241 < Severe.housing.cost.burden
Uninsured.adults <= 0.0918
0.091 < Diabetes.prevalence <= 0.105
0.0802 < Unemployment
0.806 < Driving.alone.to.work <= 0.837
0.198 < Adult.smoking <= 0.232
0.44 < Flu.vaccinations <= 0.50
165.9 < Traffic.volume
Feature
−0.020
−0.015
−0.010
−0.005
0.000
0.005
Weight
Positive
Negative

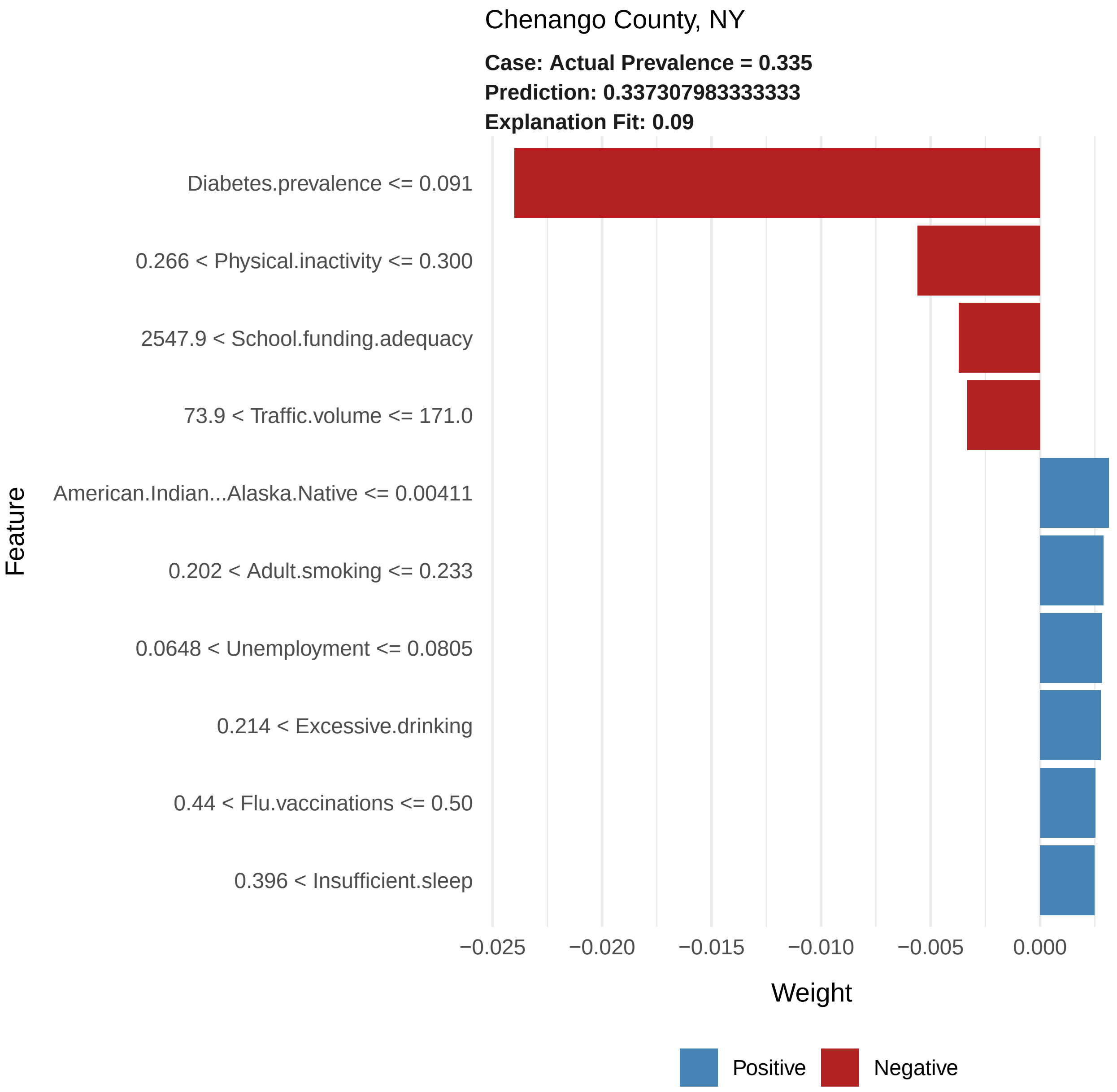

Chenango County, NY
Case: Actual Prevalence = 0.335
Prediction: 0.337307983333333
Explanation Fit: 0.09
Diabetes.prevalence <= 0.091
0.266 < Physical.inactivity <= 0.300
2547.9 < School.funding.adequacy
73.9 < Traffic.volume <= 171.0
American.Indian...Alaska.Native <= 0.00411
0.202 < Adult.smoking <= 0.233
0.0648 < Unemployment <= 0.0805
0.214 < Excessive.drinking
0.44 < Flu.vaccinations <= 0.50
0.396 < Insufficient.sleep
Feature
−0.025
−0.020
−0.015
−0.010
−0.005
0.000
Weight
Positive
Negative

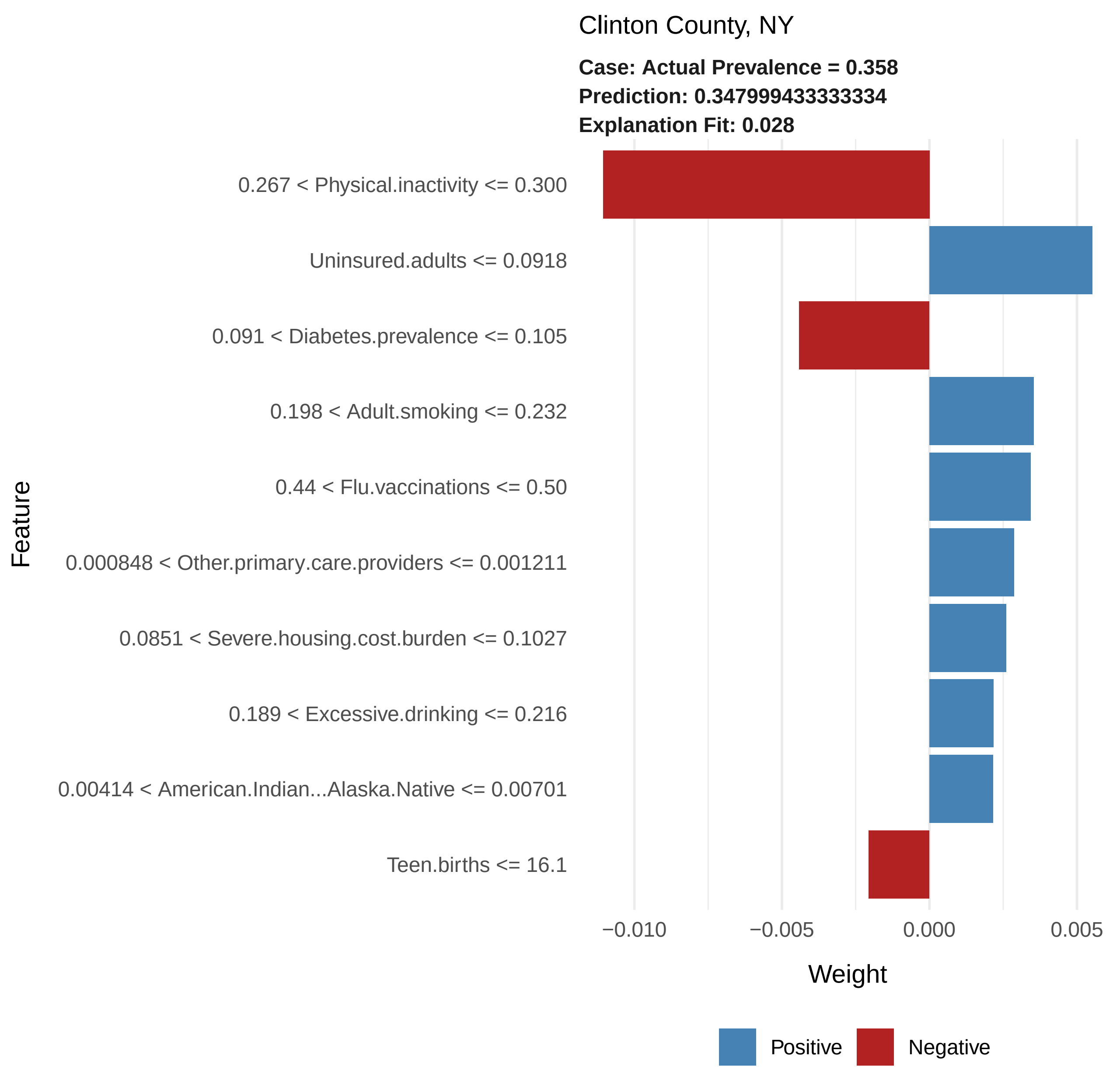
Clinton County, NY
Case: Actual Prevalence = 0.358
Prediction: 0.347999433333334
Explanation Fit: 0.028
0.267 < Physical.inactivity <= 0.300
Uninsured.adults <= 0.0918
0.091 < Diabetes.prevalence <= 0.105
0.198 < Adult.smoking <= 0.232
0.44 < Flu.vaccinations <= 0.50
0.000848 < Other.primary.care.providers <= 0.001211
0.0851 < Severe.housing.cost.burden <= 0.1027
0.189 < Excessive.drinking <= 0.216
0.00414 < American.Indian...Alaska.Native <= 0.00701
Teen.births <= 16.1
Feature
−0.010
−0.005
0.000
0.005
Weight
Positive
Negative

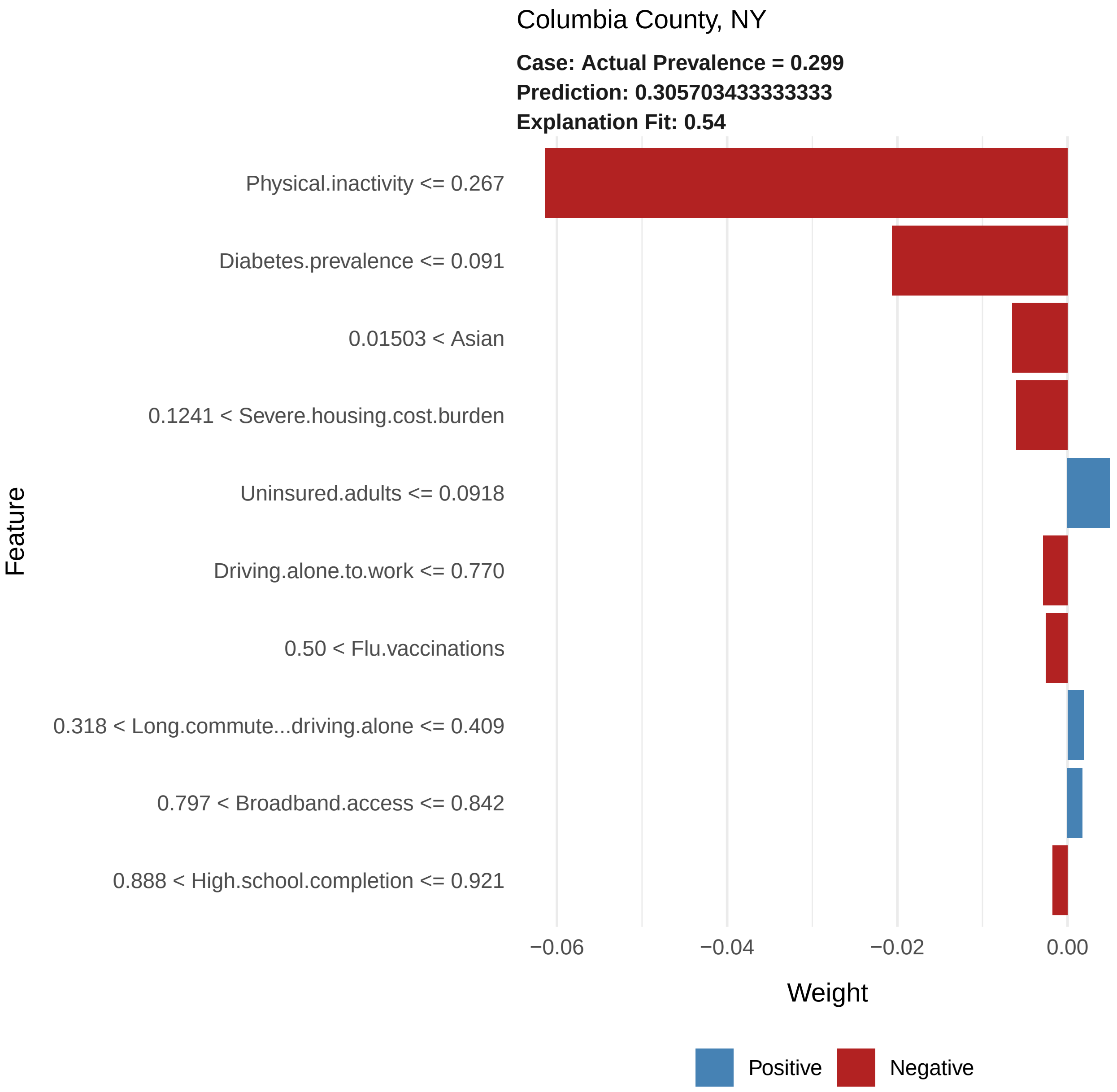

Columbia County, NY
Case: Actual Prevalence = 0.299
Prediction: 0.305703433333333
Explanation Fit: 0.54
Physical.inactivity <= 0.267
Diabetes.prevalence <= 0.091
0.01503 < Asian
0.1241 < Severe.housing.cost.burden
Uninsured.adults <= 0.0918
Driving.alone.to.work <= 0.770
0.50 < Flu.vaccinations
0.318 < Long.commute...driving.alone <= 0.409
0.797 < Broadband.access <= 0.842
0.888 < High.school.completion <= 0.921
Feature
−0.06
−0.04
−0.02
0.00
Weight
Positive
Negative

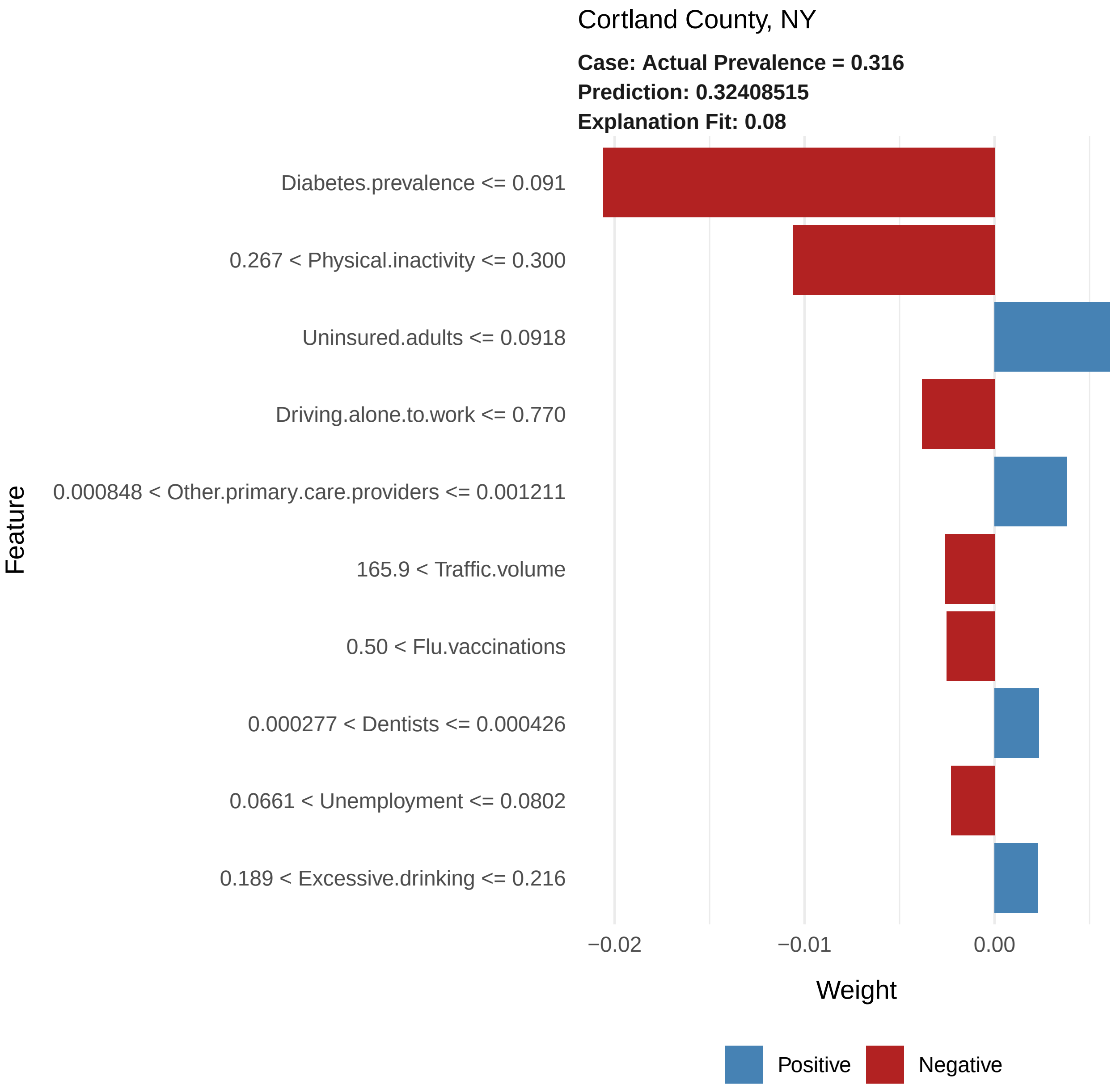
Cortland County, NY
Case: Actual Prevalence = 0.316
Prediction: 0.32408515
Explanation Fit: 0.08
Diabetes.prevalence <= 0.091
0.267 < Physical.inactivity <= 0.300
Uninsured.adults <= 0.0918
Driving.alone.to.work <= 0.770
0.000848 < Other.primary.care.providers <= 0.001211
165.9 < Traffic.volume
0.50 < Flu.vaccinations
0.000277 < Dentists <= 0.000426
0.0661 < Unemployment <= 0.0802
0.189 < Excessive.drinking <= 0.216
Feature
−0.02
−0.01
0.00
Weight
Positive
Negative

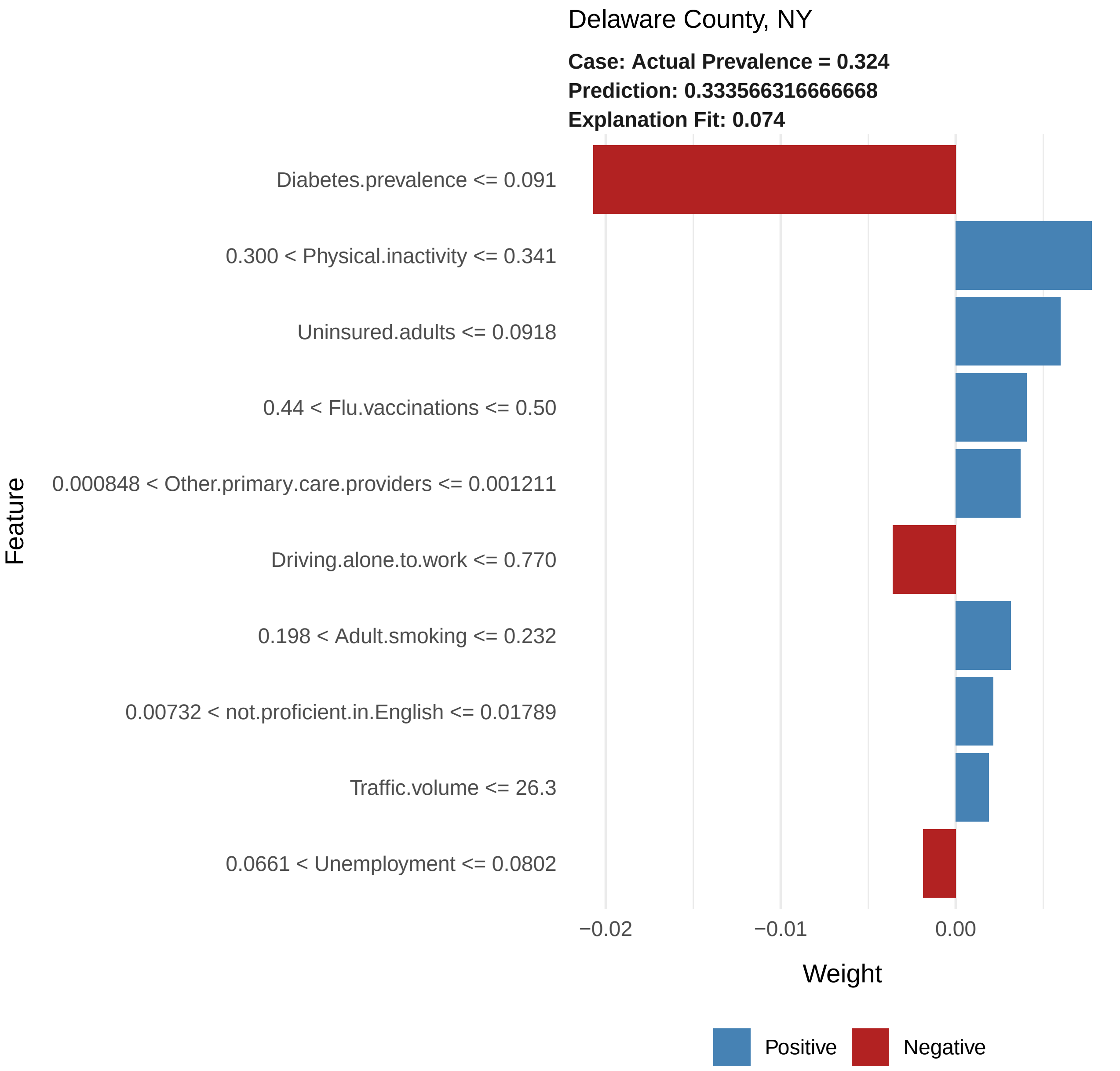

Delaware County, NY
Case: Actual Prevalence = 0.324
Prediction: 0.333566316666668
Explanation Fit: 0.074
Diabetes.prevalence <= 0.091
0.300 < Physical.inactivity <= 0.341
Uninsured.adults <= 0.0918
0.44 < Flu.vaccinations <= 0.50
0.000848 < Other.primary.care.providers <= 0.001211
Driving.alone.to.work <= 0.770
0.198 < Adult.smoking <= 0.232
0.00732 < not.proficient.in.English <= 0.01789
Traffic.volume <= 26.3
0.0661 < Unemployment <= 0.0802
Feature
−0.02
−0.01
0.00
Weight
Positive
Negative

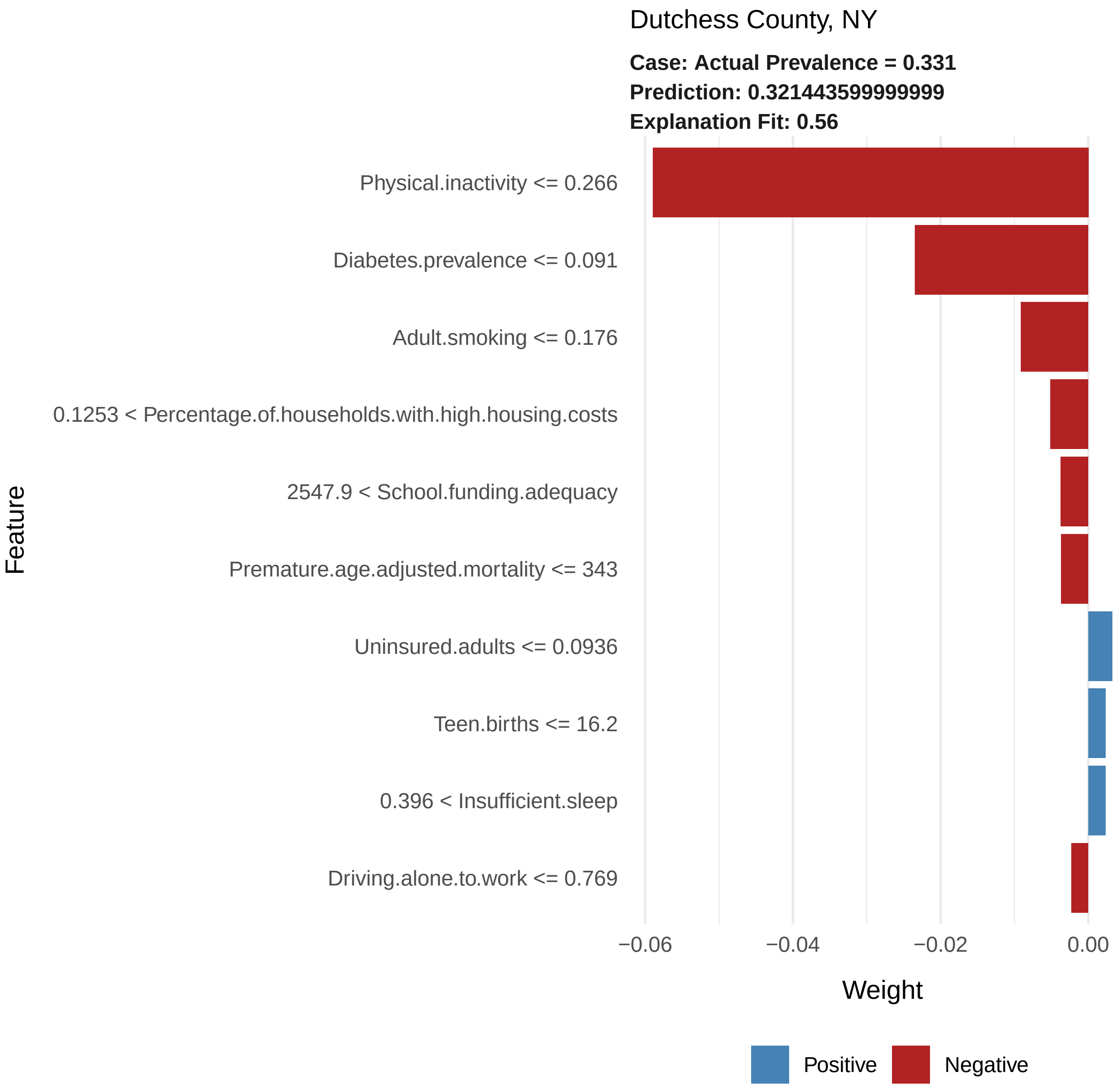
Dutchess County, NY
Case: Actual Prevalence = 0.331
Prediction: 0.321443599999999
Explanation Fit: 0.56
Physical.inactivity <= 0.266
Diabetes.prevalence <= 0.091
Adult.smoking <= 0.176
0.1253 < Percentage.of.households.with.high.housing.costs
2547.9 < School.funding.adequacy
Premature.age.adjusted.mortality <= 343
Uninsured.adults <= 0.0936
Teen.births <= 16.2
0.396 < Insufficient.sleep
Driving.alone.to.work <= 0.769
Feature
−0.06
−0.04
−0.02
0.00
Weight
Positive
Negative

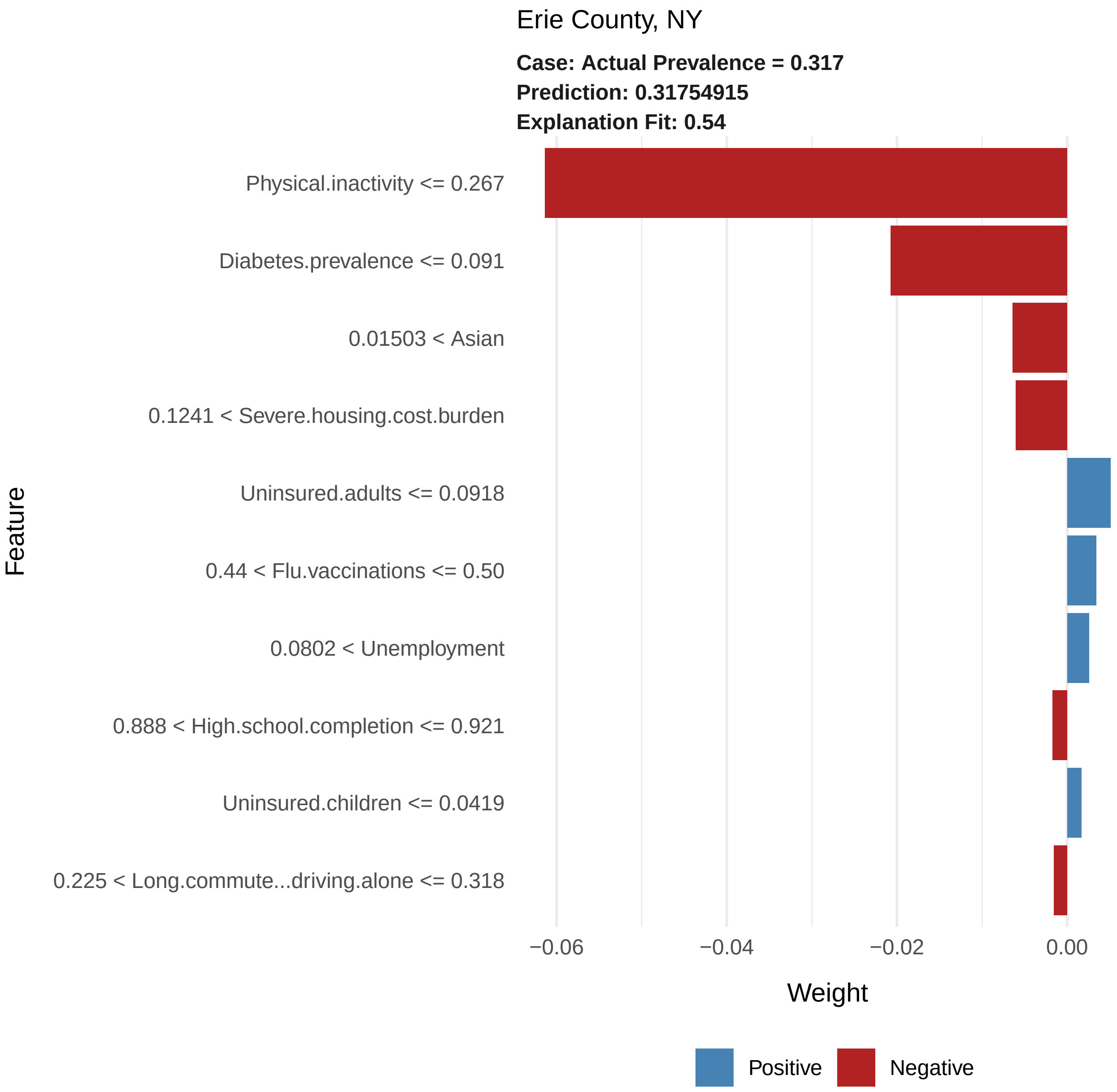

Erie County, NY
Case: Actual Prevalence = 0.317
Prediction: 0.31754915
Explanation Fit: 0.54
Physical.inactivity <= 0.267
Diabetes.prevalence <= 0.091
0.01503 < Asian
0.1241 < Severe.housing.cost.burden
Uninsured.adults <= 0.0918
0.44 < Flu.vaccinations <= 0.50
0.0802 < Unemployment
0.888 < High.school.completion <= 0.921
Uninsured.children <= 0.0419
0.225 < Long.commute...driving.alone <= 0.318
Feature
−0.06
−0.04
−0.02
0.00
Weight
Positive
Negative

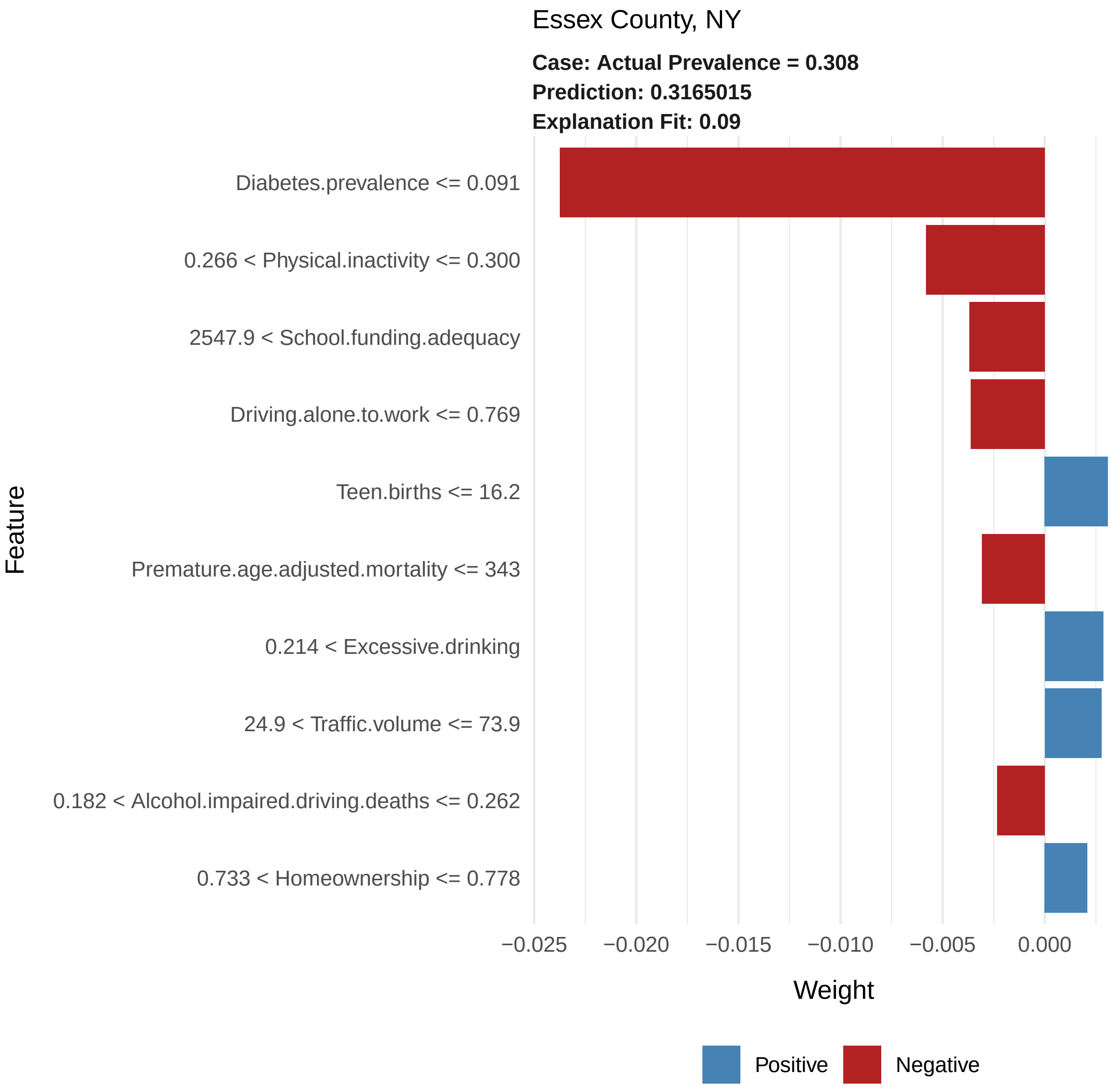

Essex County, NY
Case: Actual Prevalence = 0.308
Prediction: 0.3165015
Explanation Fit: 0.09
Diabetes.prevalence <= 0.091
0.266 < Physical.inactivity <= 0.300
2547.9 < School.funding.adequacy
Driving.alone.to.work <= 0.769
Teen.births <= 16.2
Premature.age.adjusted.mortality <= 343
0.214 < Excessive.drinking
24.9 < Traffic.volume <= 73.9
0.182 < Alcohol.impaired.driving.deaths <= 0.262
0.733 < Homeownership <= 0.778
Feature
−0.025
−0.020
−0.015
−0.010
−0.005
0.000
Weight
Positive
Negative

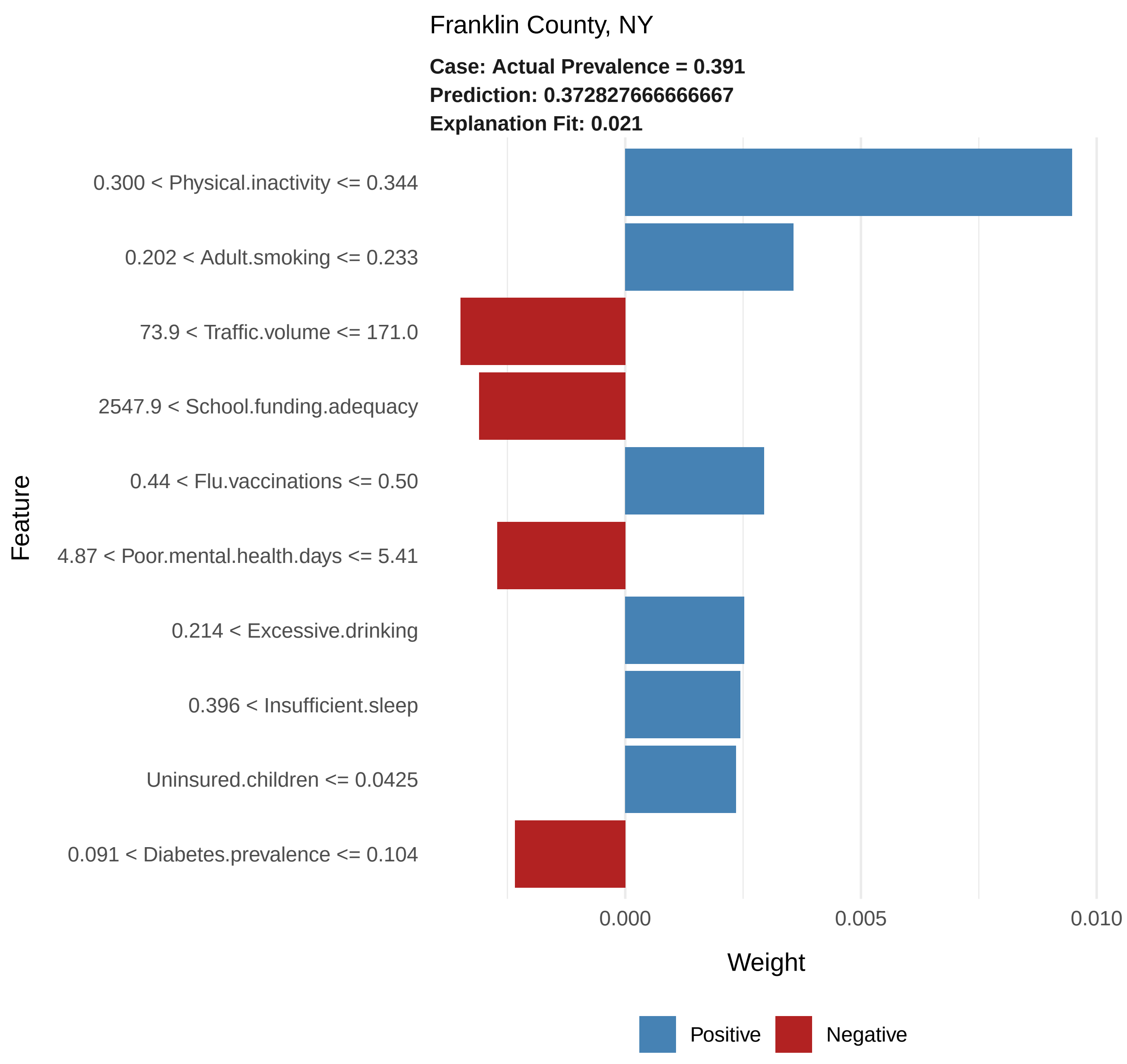

Franklin County, NY
Case: Actual Prevalence = 0.391
Prediction: 0.372827666666667
Explanation Fit: 0.021
Feature
0.300 < Physical.inactivity <= 0.344
0.202 < Adult.smoking <= 0.233
73.9 < Traffic.volume <= 171.0
2547.9 < School.funding.adequacy
0.44 < Flu.vaccinations <= 0.50
4.87 < Poor.mental.health.days <= 5.41
0.214 < Excessive.drinking
0.396 < Insufficient.sleep
Uninsured.children <= 0.0425
0.091 < Diabetes.prevalence <= 0.104
0.000
0.005
0.010
Weight
Positive
Negative

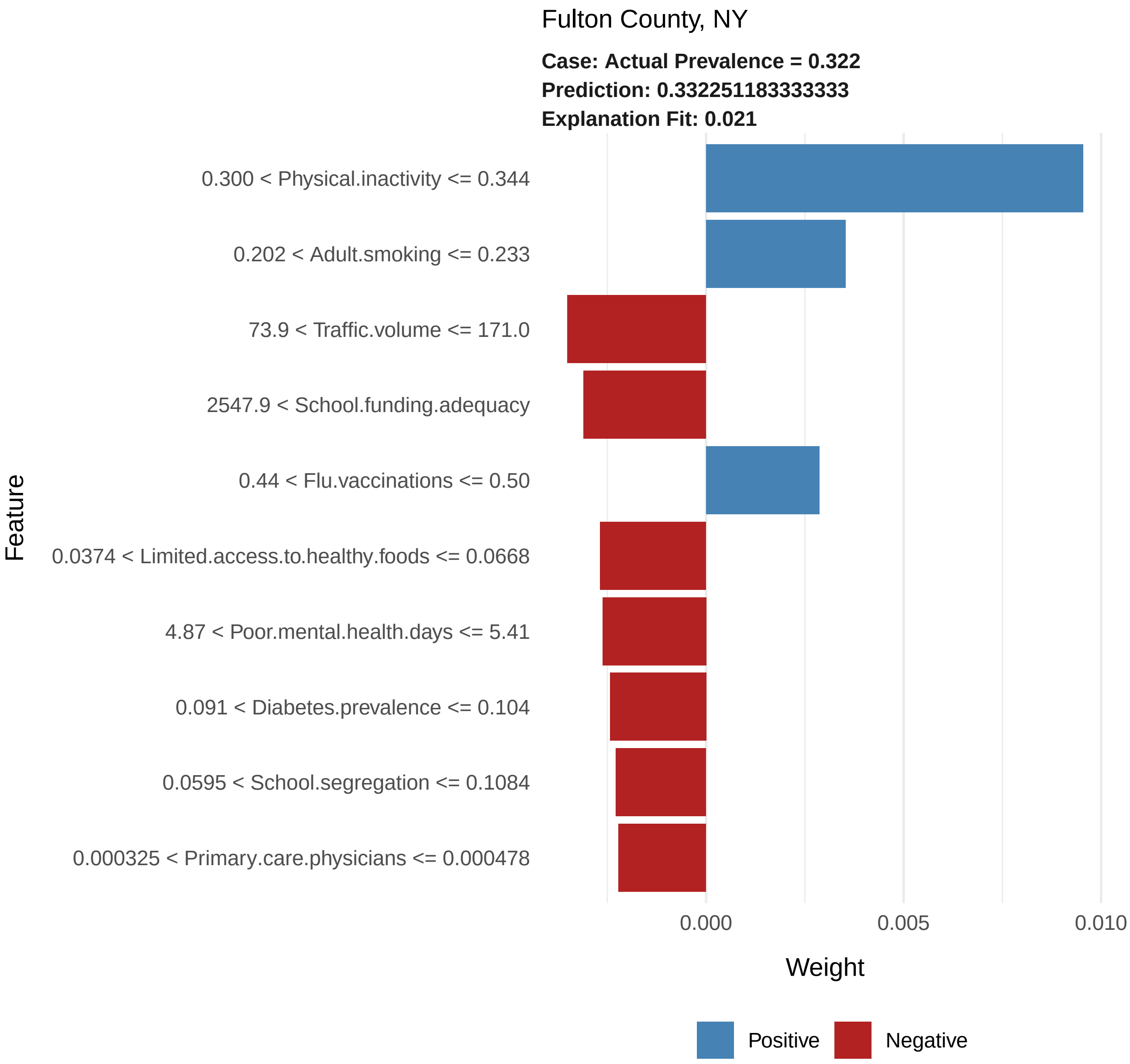

Fulton County, NY
Case: Actual Prevalence = 0.322
Prediction: 0.332251183333333
Explanation Fit: 0.021
0.300 < Physical.inactivity <= 0.344
0.202 < Adult.smoking <= 0.233
73.9 < Traffic.volume <= 171.0
2547.9 < School.funding.adequacy
0.44 < Flu.vaccinations <= 0.50
0.0374 < Limited.access.to.healthy.foods <= 0.0668
4.87 < Poor.mental.health.days <= 5.41
0.091 < Diabetes.prevalence <= 0.104
0.0595 < School.segregation <= 0.1084
0.000325 < Primary.care.physicians <= 0.000478
Feature
0.000
0.005
0.010
Weight
Positive
Negative

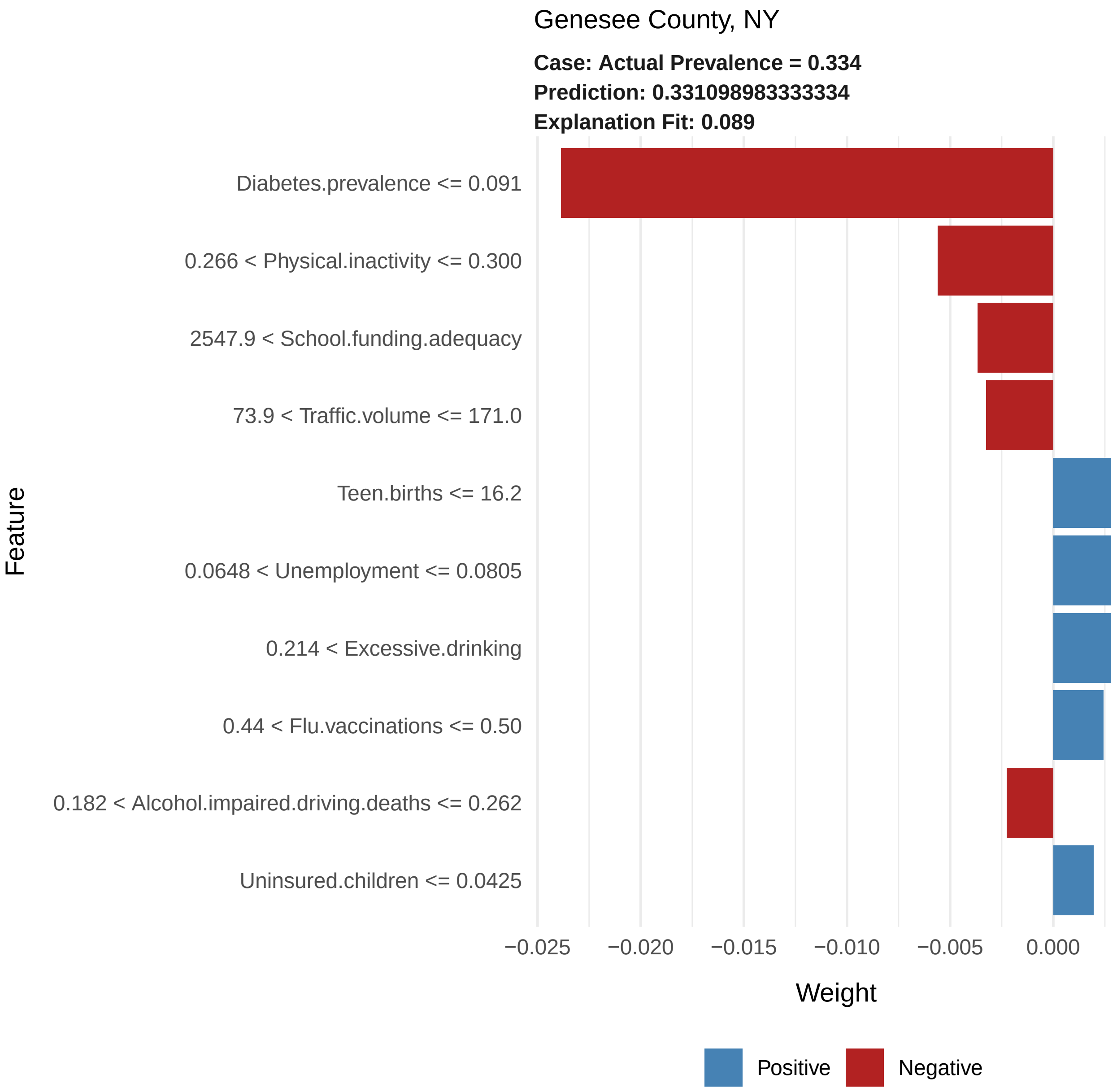
Genesee County, NY
Case: Actual Prevalence = 0.334
Prediction: 0.331098983333334
Explanation Fit: 0.089
Diabetes.prevalence <= 0.091
0.266 < Physical.inactivity <= 0.300
2547.9 < School.funding.adequacy
73.9 < Traffic.volume <= 171.0
Teen.births <= 16.2
0.0648 < Unemployment <= 0.0805
0.214 < Excessive.drinking
0.44 < Flu.vaccinations <= 0.50
0.182 < Alcohol.impaired.driving.deaths <= 0.262
Uninsured.children <= 0.0425
Feature
−0.025
−0.020
−0.015
−0.010
−0.005
0.000
Weight
Positive
Negative

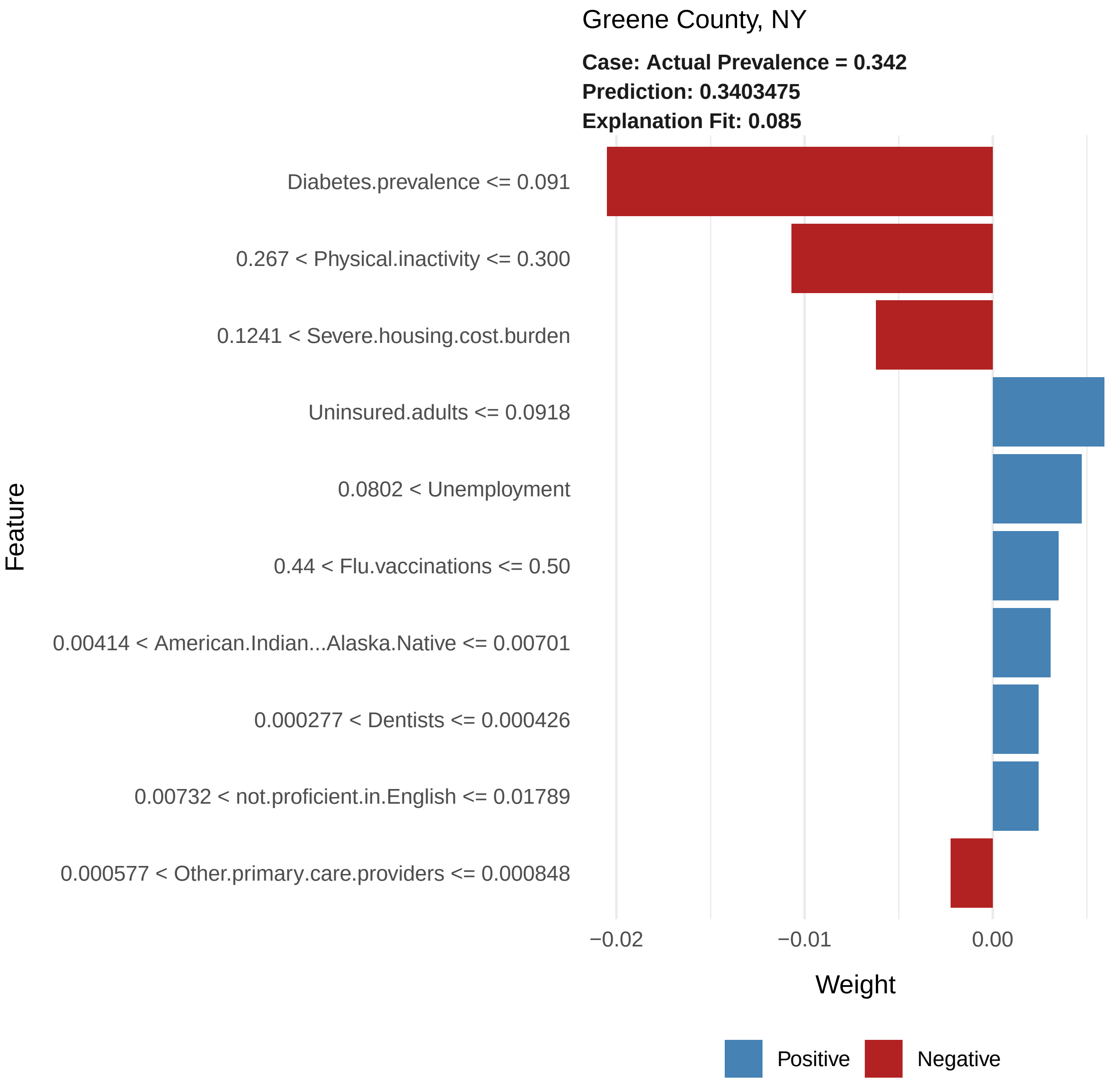
Greene County, NY
Case: Actual Prevalence = 0.342
Prediction: 0.3403475
Explanation Fit: 0.085
Diabetes.prevalence <= 0.091
0.267 < Physical.inactivity <= 0.300
0.1241 < Severe.housing.cost.burden
Uninsured.adults <= 0.0918
0.0802 < Unemployment
0.44 < Flu.vaccinations <= 0.50
0.00414 < American.Indian...Alaska.Native <= 0.00701
0.000277 < Dentists <= 0.000426
0.00732 < not.proficient.in.English <= 0.01789
0.000577 < Other.primary.care.providers <= 0.000848
Feature
−0.02
−0.01
0.00
Weight
Positive
Negative

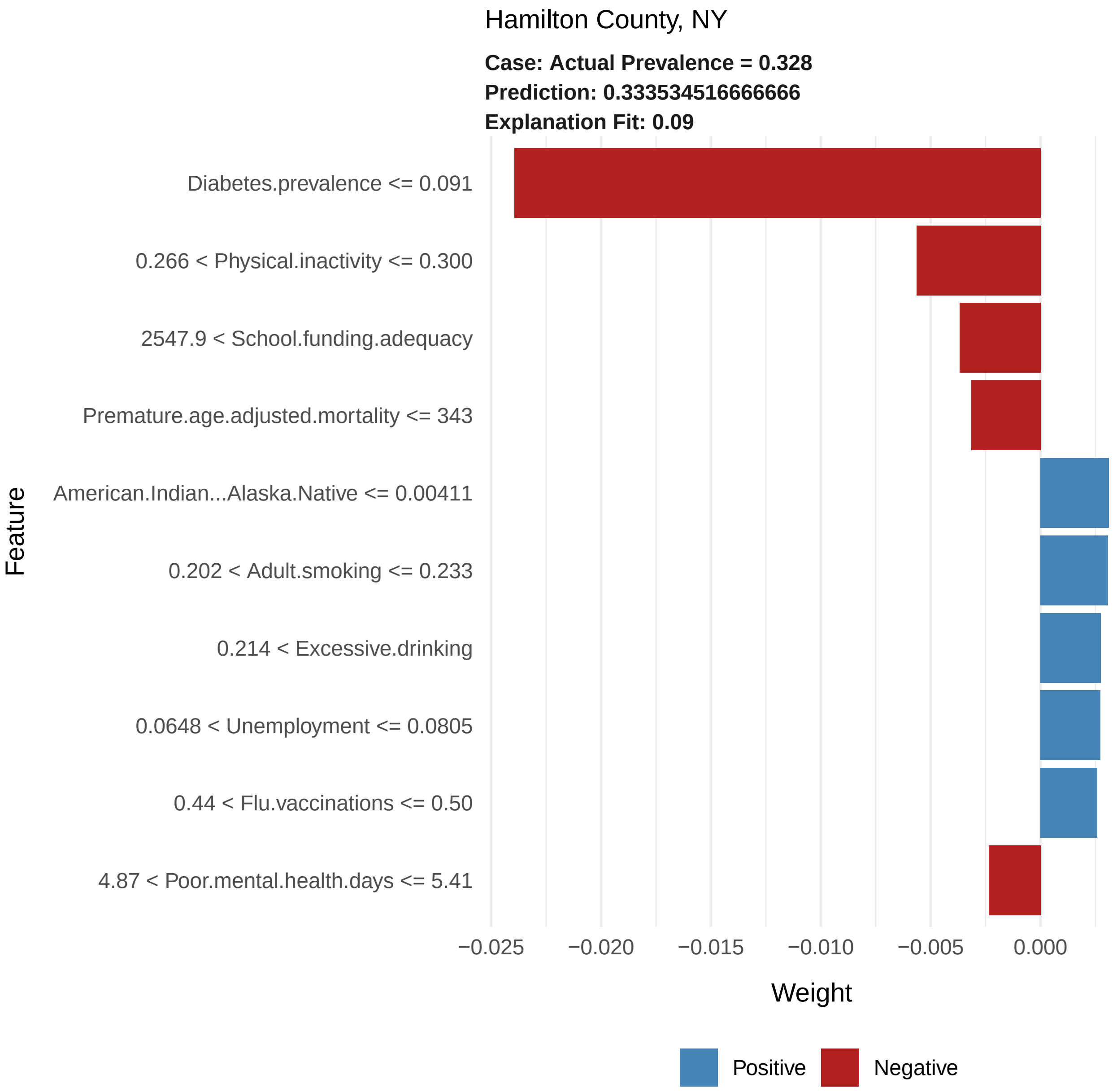

Hamilton County, NY
Case: Actual Prevalence = 0.328
Prediction: 0.333534516666666
Explanation Fit: 0.09
Diabetes.prevalence <= 0.091
0.266 < Physical.inactivity <= 0.300
2547.9 < School.funding.adequacy
Premature.age.adjusted.mortality <= 343
American.Indian...Alaska.Native <= 0.00411
0.202 < Adult.smoking <= 0.233
0.214 < Excessive.drinking
0.0648 < Unemployment <= 0.0805
0.44 < Flu.vaccinations <= 0.50
4.87 < Poor.mental.health.days <= 5.41
Feature
−0.025
−0.020
−0.015
−0.010
−0.005
0.000
Weight
Positive
Negative

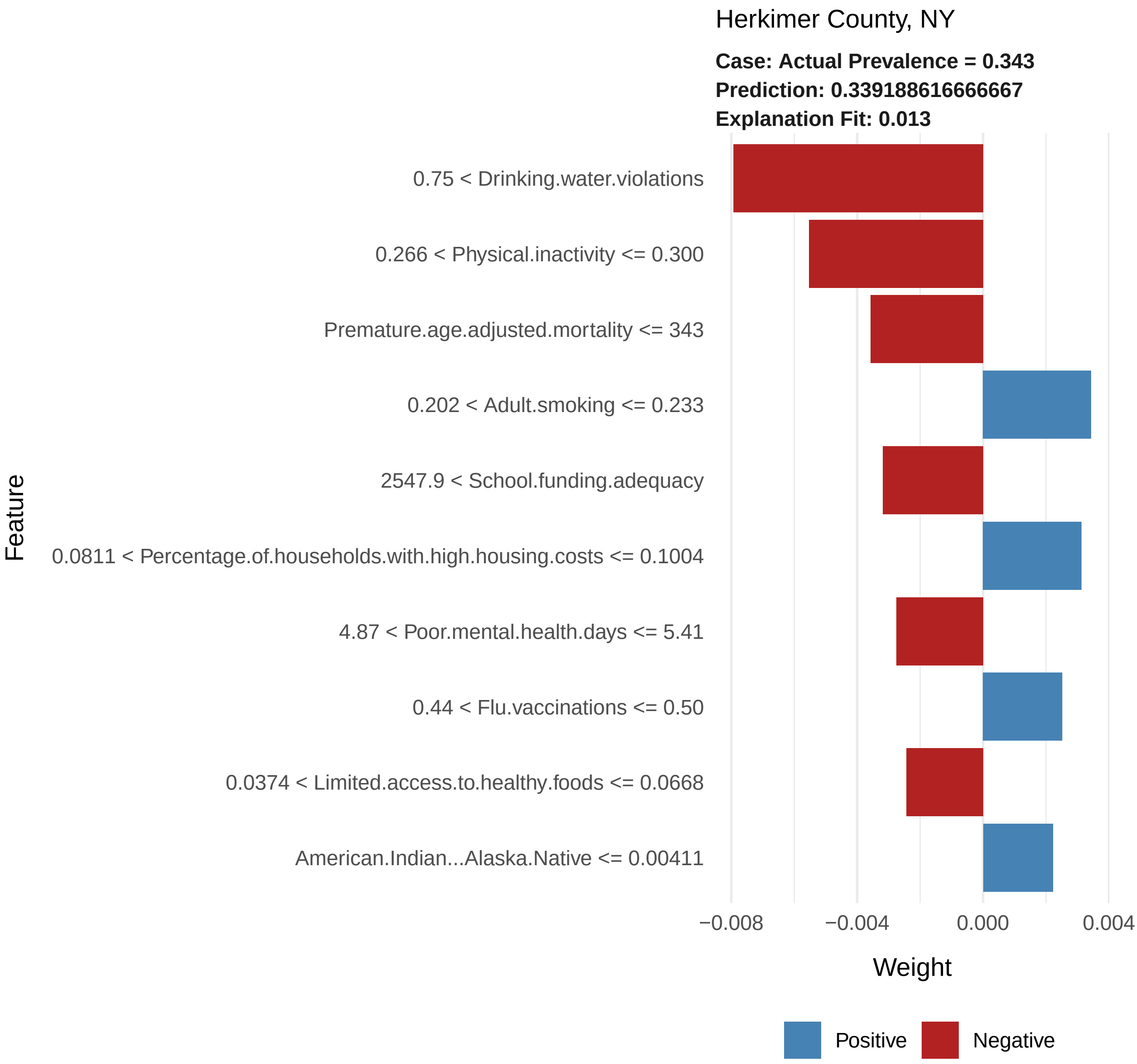
Herkimer County, NY
Case: Actual Prevalence = 0.343
Prediction: 0.339188616666667
Explanation Fit: 0.013
Feature
0.75 < Drinking.water.violations
0.266 < Physical.inactivity <= 0.300
Premature.age.adjusted.mortality <= 343
0.202 < Adult.smoking <= 0.233
2547.9 < School.funding.adequacy
0.0811 < Percentage.of.households.with.high.housing.costs <= 0.1004
4.87 < Poor.mental.health.days <= 5.41
0.44 < Flu.vaccinations <= 0.50
0.0374 < Limited.access.to.healthy.foods <= 0.0668
American.Indian...Alaska.Native <= 0.00411
−0.008
−0.004
0.000
0.004
Weight
Positive
Negative

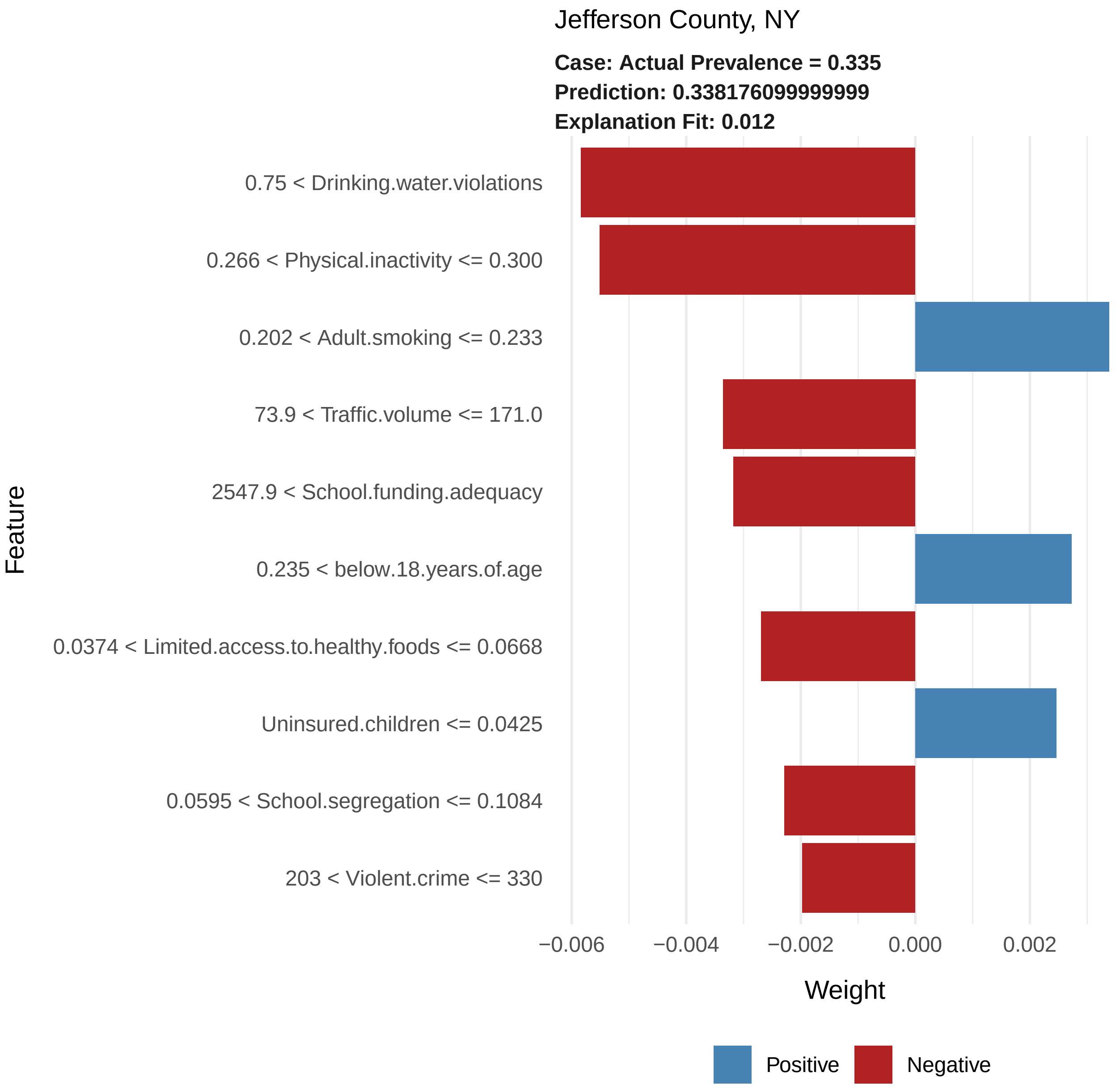
Jefferson County, NY
Case: Actual Prevalence = 0.335
Prediction: 0.338176099999999
Explanation Fit: 0.012
0.75 < Drinking.water.violations
0.266 < Physical.inactivity <= 0.300
0.202 < Adult.smoking <= 0.233
73.9 < Traffic.volume <= 171.0
2547.9 < School.funding.adequacy
0.235 < below.18.years.of.age
0.0374 < Limited.access.to.healthy.foods <= 0.0668
Uninsured.children <= 0.0425
0.0595 < School.segregation <= 0.1084
203 < Violent.crime <= 330
Feature
−0.006
−0.004
−0.002
0.000
0.002
Weight
Positive
Negative

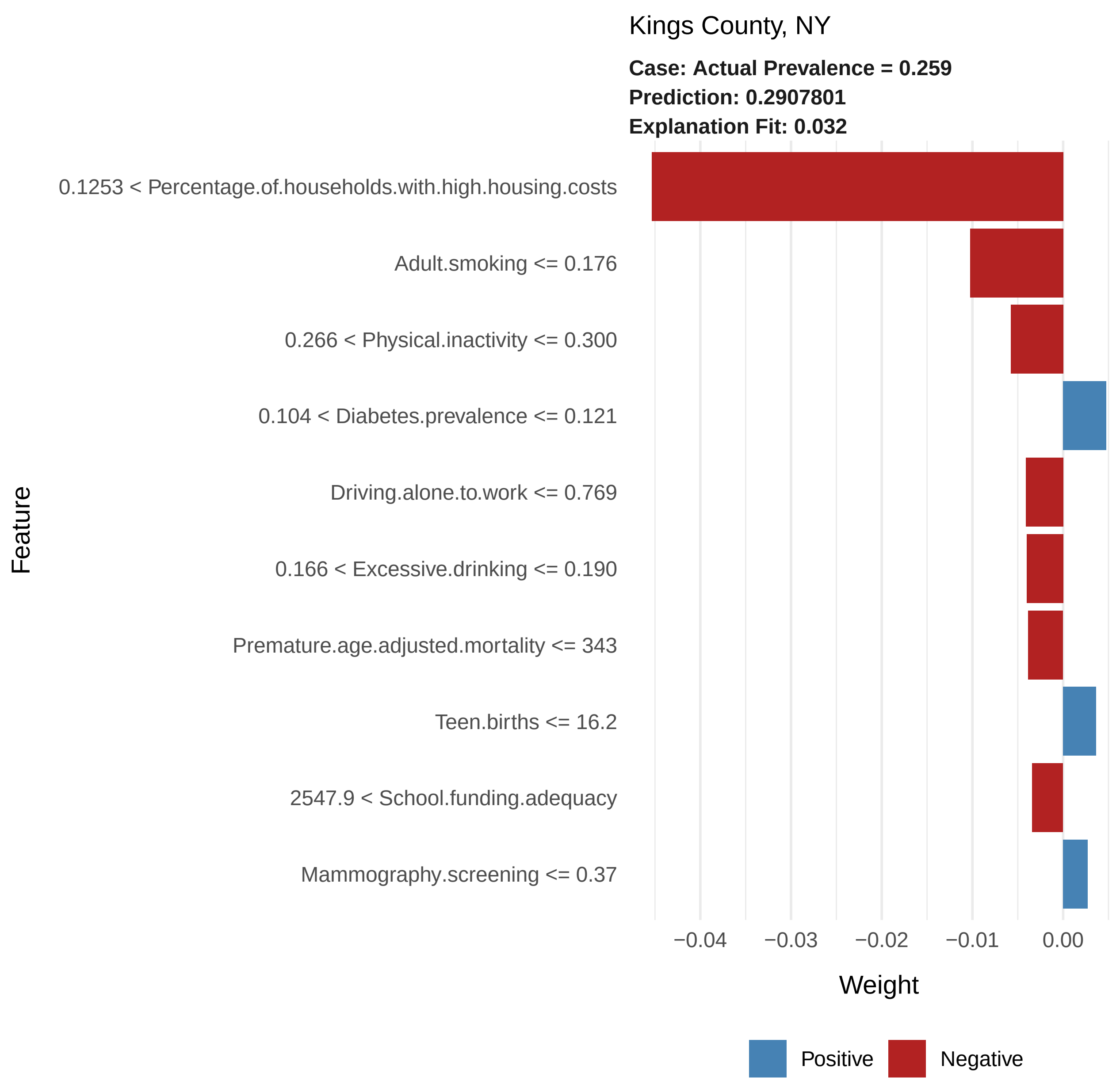
Kings County, NY
Case: Actual Prevalence = 0.259
Prediction: 0.2907801
Explanation Fit: 0.032
0.1253 < Percentage.of.households.with.high.housing.costs
Adult.smoking <= 0.176
0.266 < Physical.inactivity <= 0.300
0.104 < Diabetes.prevalence <= 0.121
Driving.alone.to.work <= 0.769
0.166 < Excessive.drinking <= 0.190
Premature.age.adjusted.mortality <= 343
Teen.births <= 16.2
2547.9 < School.funding.adequacy
Mammography.screening <= 0.37
Feature
−0.04
−0.03
−0.02
−0.01
0.00
Weight
Positive
Negative

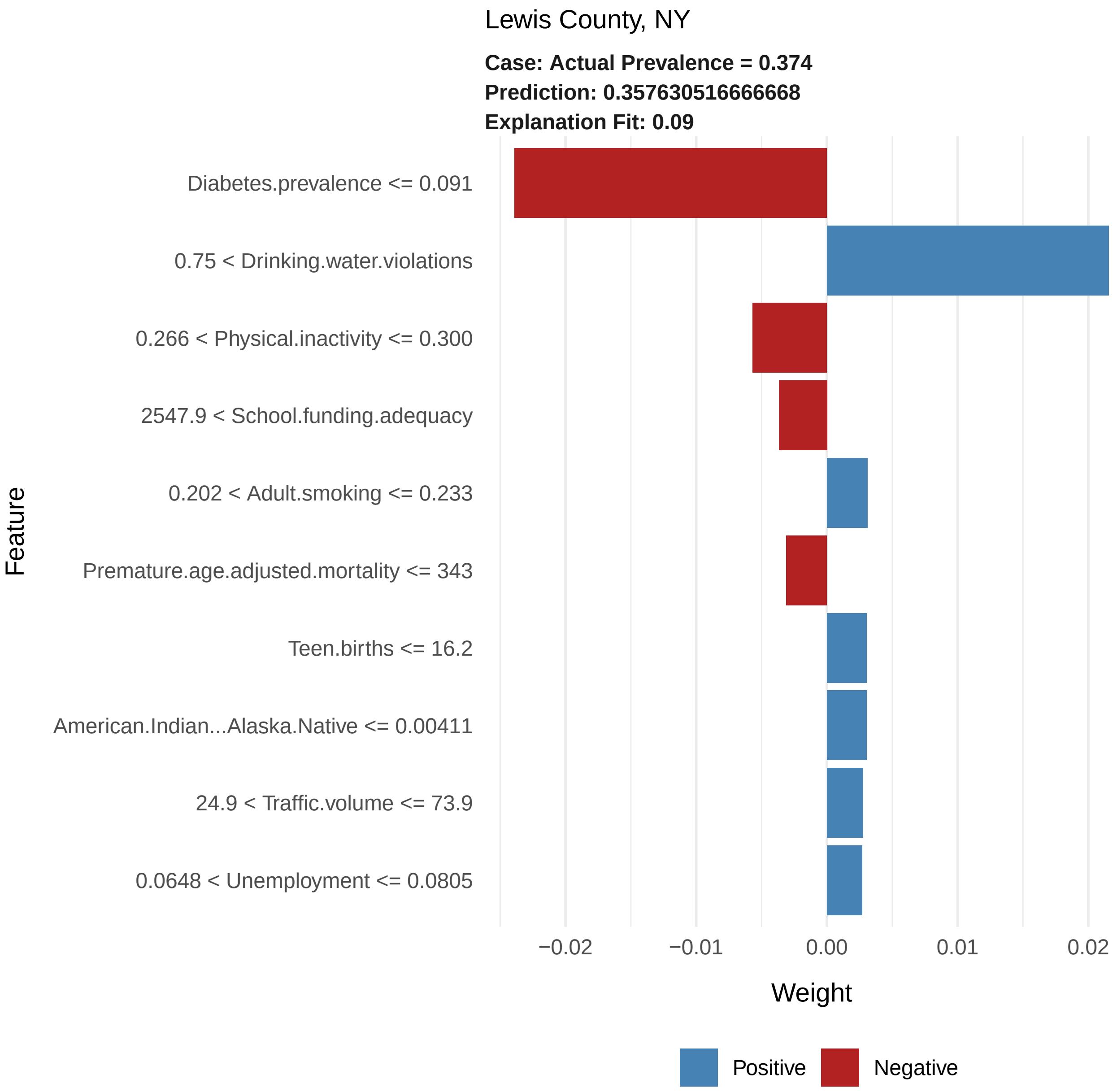

Lewis County, NY
Case: Actual Prevalence = 0.374
Prediction: 0.357630516666668
Explanation Fit: 0.09
Diabetes.prevalence <= 0.091
0.75 < Drinking.water.violations
0.266 < Physical.inactivity <= 0.300
2547.9 < School.funding.adequacy
0.202 < Adult.smoking <= 0.233
Premature.age.adjusted.mortality <= 343
Teen.births <= 16.2
American.Indian...Alaska.Native <= 0.00411
24.9 < Traffic.volume <= 73.9
0.0648 < Unemployment <= 0.0805
Feature
−0.02
−0.01
0.00
0.01
0.02
Weight
Positive
Negative

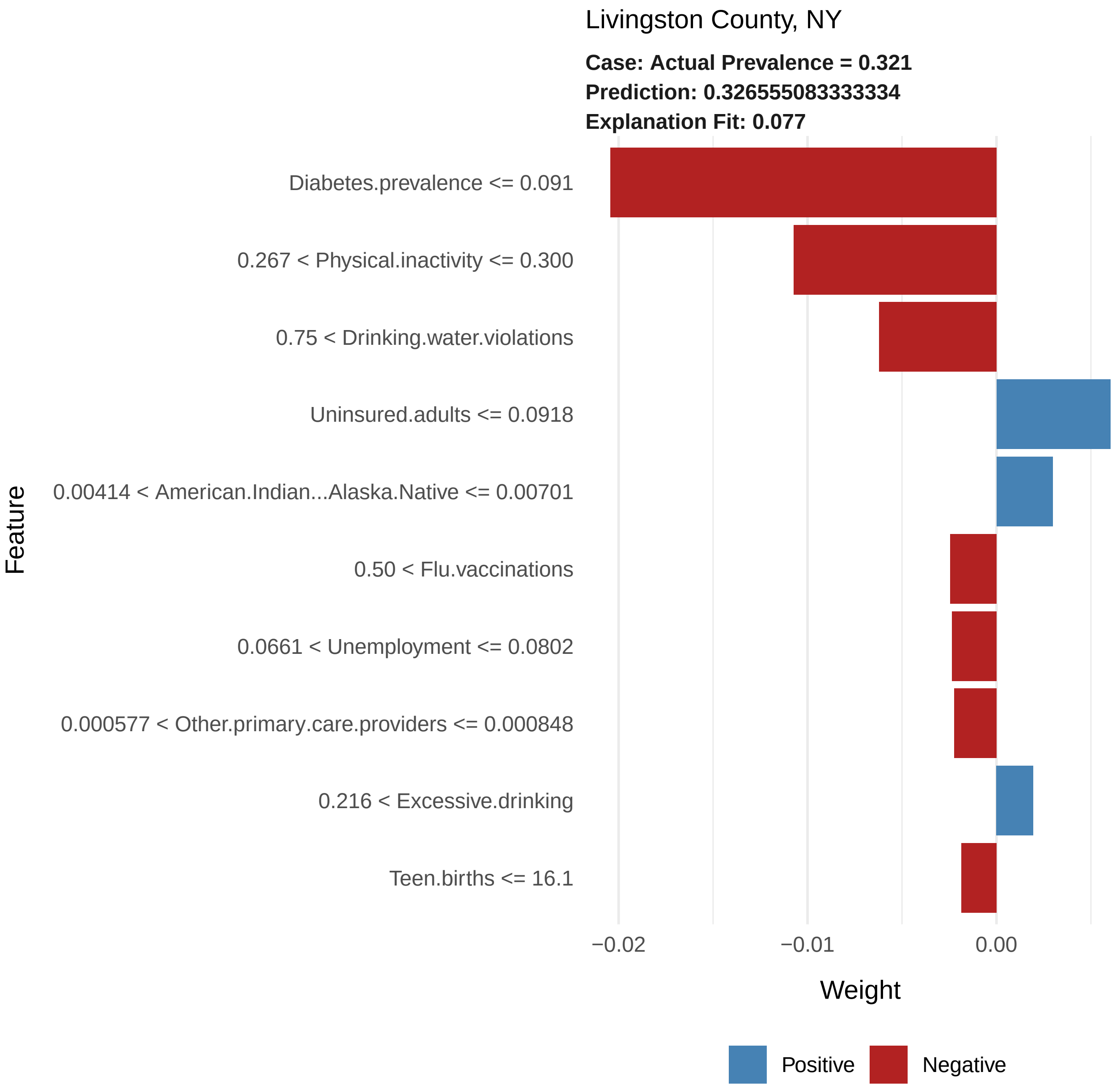
Livingston County, NY
Case: Actual Prevalence = 0.321
Prediction: 0.326555083333334
Explanation Fit: 0.077
Diabetes.prevalence <= 0.091
0.267 < Physical.inactivity <= 0.300
0.75 < Drinking.water.violations
Uninsured.adults <= 0.0918
0.00414 < American.Indian...Alaska.Native <= 0.00701
0.50 < Flu.vaccinations
0.0661 < Unemployment <= 0.0802
0.000577 < Other.primary.care.providers <= 0.000848
0.216 < Excessive.drinking
Teen.births <= 16.1
Feature
−0.02
−0.01
0.00
Weight
Positive
Negative

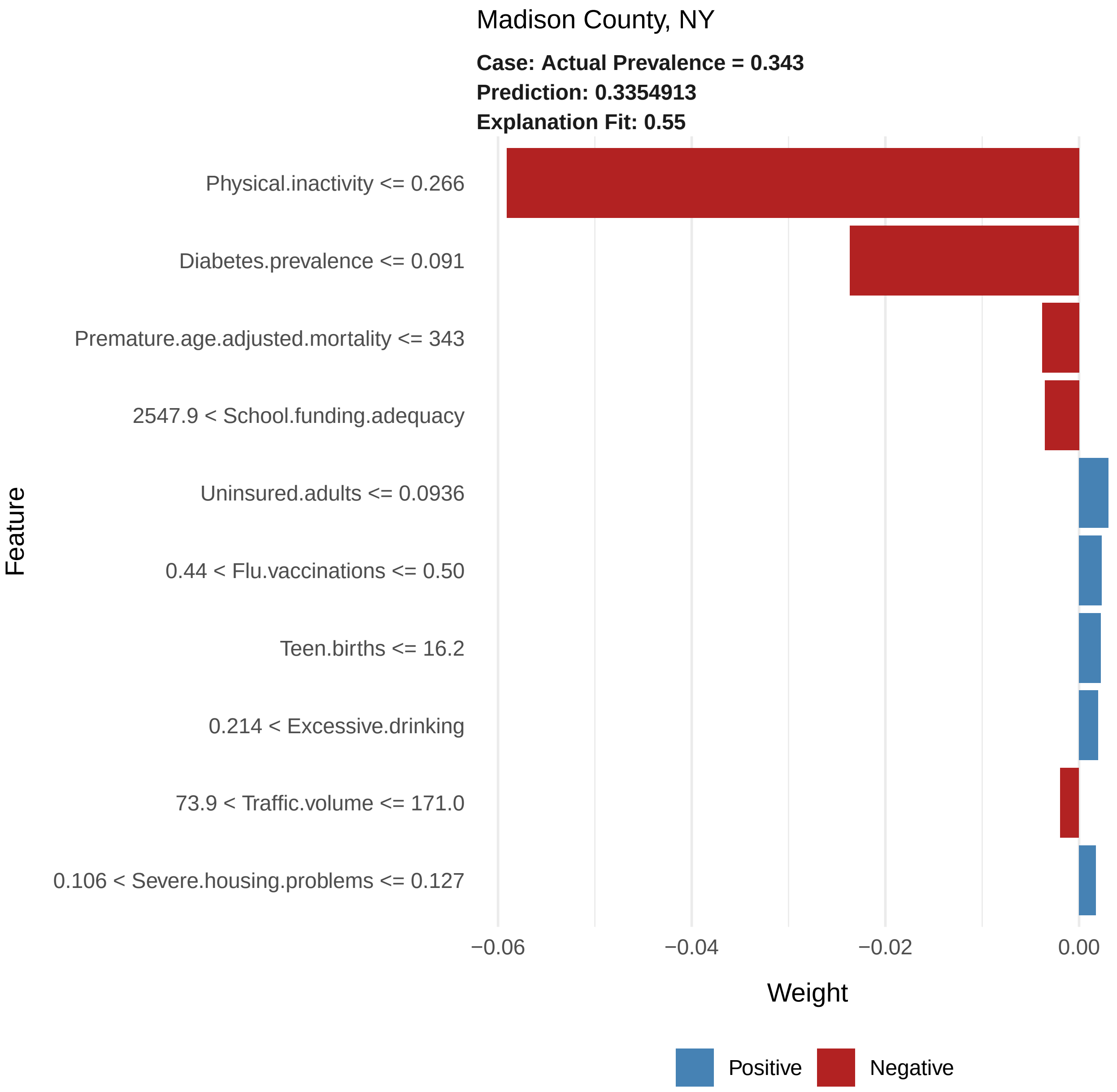

Madison County, NY
Case: Actual Prevalence = 0.343
Prediction: 0.3354913
Explanation Fit: 0.55
Physical.inactivity <= 0.266
Diabetes.prevalence <= 0.091
Premature.age.adjusted.mortality <= 343
2547.9 < School.funding.adequacy
Uninsured.adults <= 0.0936
0.44 < Flu.vaccinations <= 0.50
Teen.births <= 16.2
0.214 < Excessive.drinking
73.9 < Traffic.volume <= 171.0
0.106 < Severe.housing.problems <= 0.127
Feature
−0.06
−0.04
−0.02
0.00
Weight
Positive
Negative

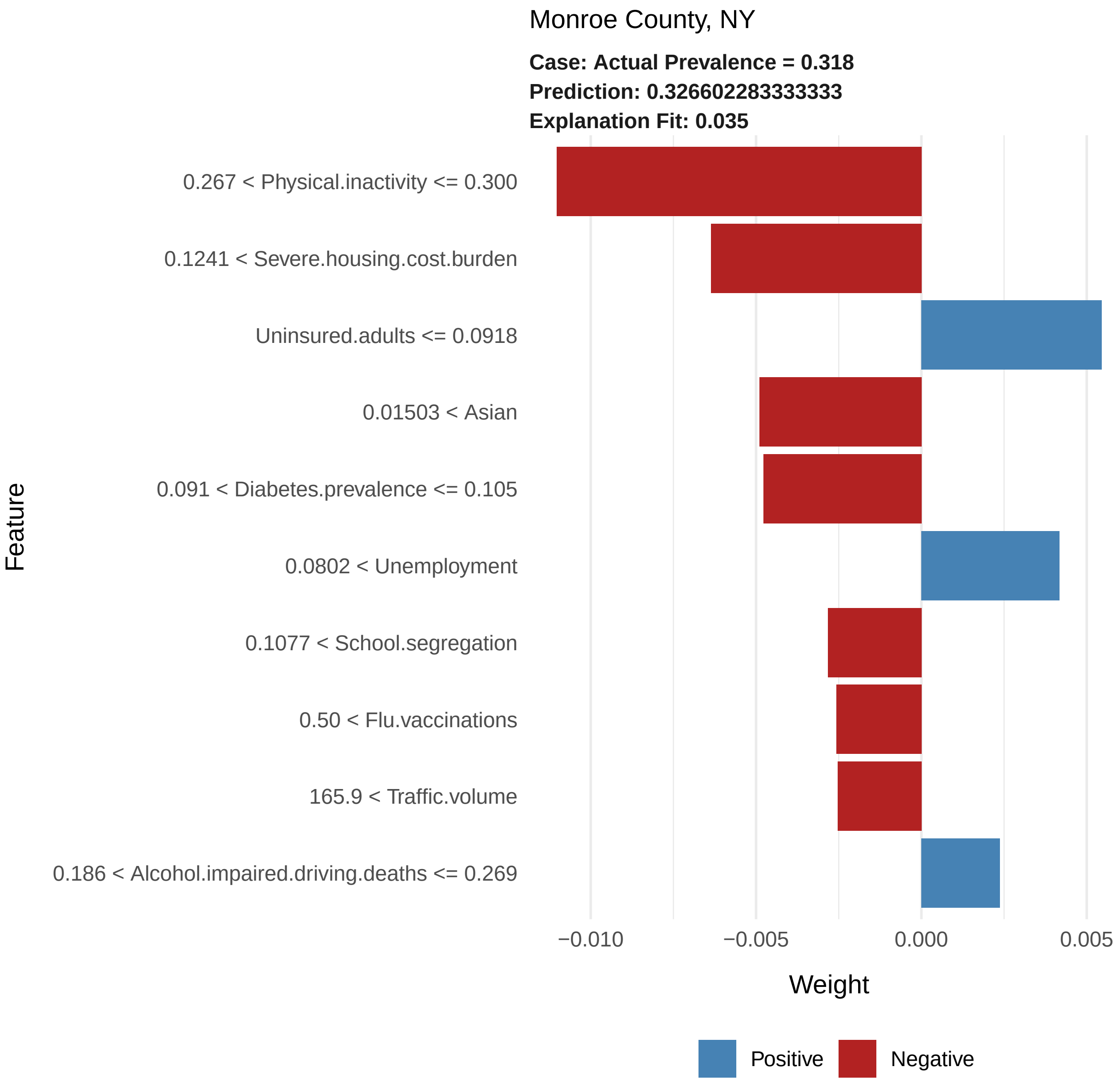

Monroe County, NY
Case: Actual Prevalence = 0.318
Prediction: 0.326602283333333
Explanation Fit: 0.035
0.267 < Physical.inactivity <= 0.300
0.1241 < Severe.housing.cost.burden
Uninsured.adults <= 0.0918
0.01503 < Asian
0.091 < Diabetes.prevalence <= 0.105
0.0802 < Unemployment
0.1077 < School.segregation
0.50 < Flu.vaccinations
165.9 < Traffic.volume
0.186 < Alcohol.impaired.driving.deaths <= 0.269
Feature
−0.010
−0.005
0.000
0.005
Weight
Positive
Negative

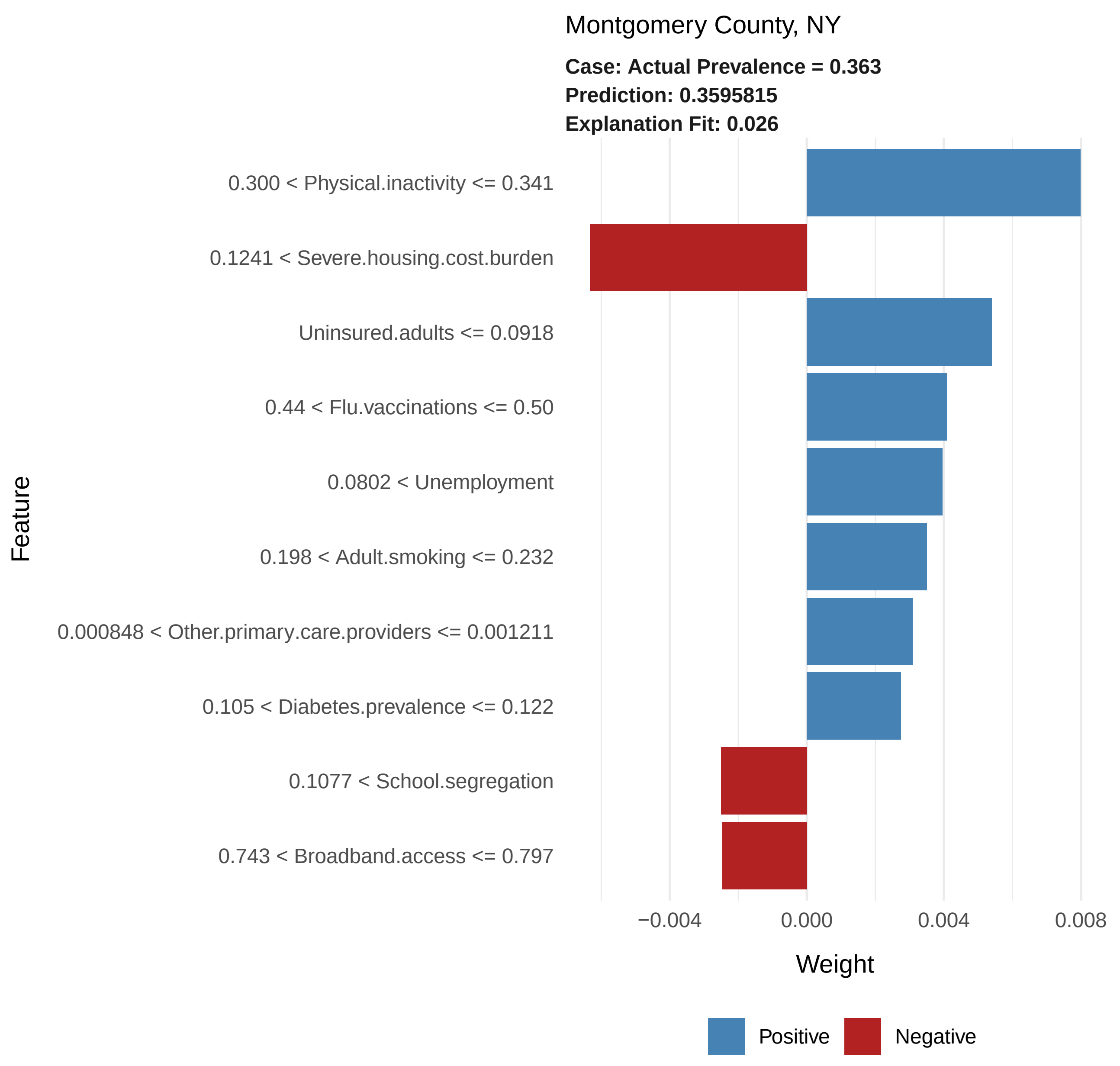
Montgomery County, NY
Case: Actual Prevalence = 0.363
Prediction: 0.3595815
Explanation Fit: 0.026
0.300 < Physical.inactivity <= 0.341
0.1241 < Severe.housing.cost.burden
Uninsured.adults <= 0.0918
0.44 < Flu.vaccinations <= 0.50
0.0802 < Unemployment
0.198 < Adult.smoking <= 0.232
0.000848 < Other.primary.care.providers <= 0.001211
0.105 < Diabetes.prevalence <= 0.122
0.1077 < School.segregation
0.743 < Broadband.access <= 0.797
Feature
−0.004
0.000
0.004
0.008
Weight
Positive
Negative

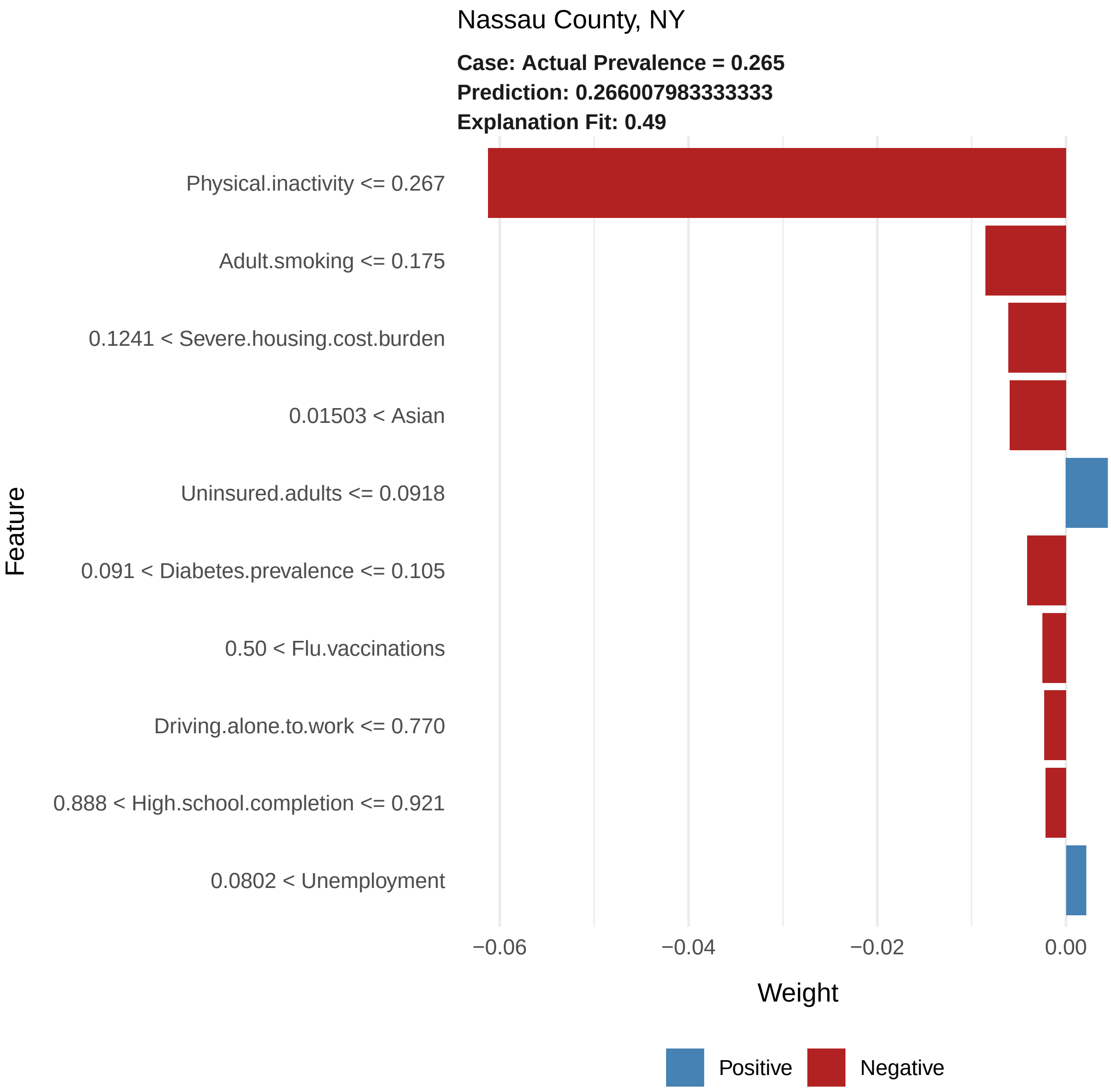

Nassau County, NY
Case: Actual Prevalence = 0.265
Prediction: 0.266007983333333
Explanation Fit: 0.49
Physical.inactivity <= 0.267
Adult.smoking <= 0.175
0.1241 < Severe.housing.cost.burden
0.01503 < Asian
Uninsured.adults <= 0.0918
0.091 < Diabetes.prevalence <= 0.105
0.50 < Flu.vaccinations
Driving.alone.to.work <= 0.770
0.888 < High.school.completion <= 0.921
0.0802 < Unemployment
Feature
−0.06
−0.04
−0.02
0.00
Weight
Positive
Negative

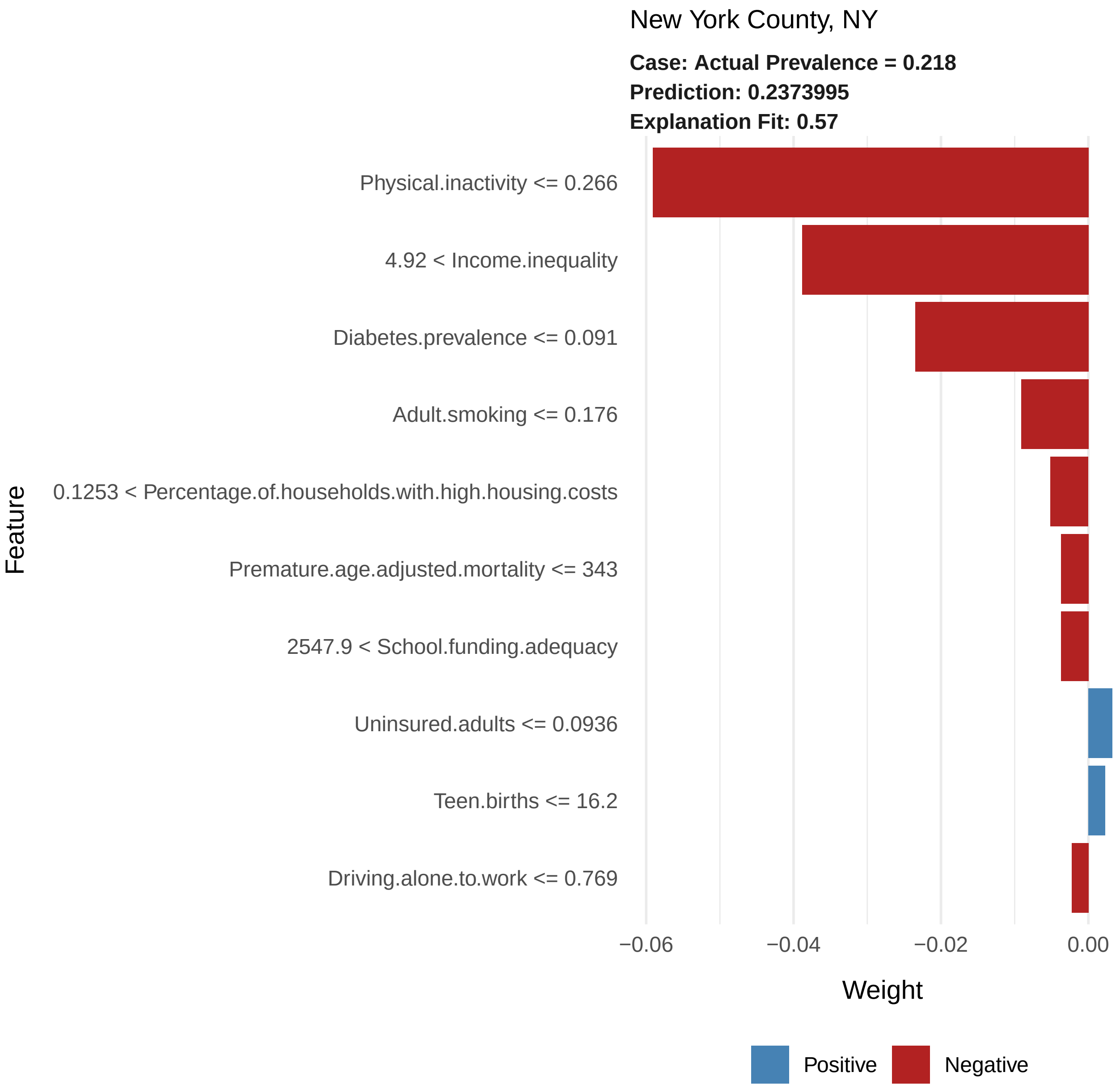
New York County, NY
Case: Actual Prevalence = 0.218
Prediction: 0.2373995
Explanation Fit: 0.57
Physical.inactivity <= 0.266
4.92 < Income.inequality
Diabetes.prevalence <= 0.091
Adult.smoking <= 0.176
0.1253 < Percentage.of.households.with.high.housing.costs
Premature.age.adjusted.mortality <= 343
2547.9 < School.funding.adequacy
Uninsured.adults <= 0.0936
Teen.births <= 16.2
Driving.alone.to.work <= 0.769
Feature
−0.06
−0.04
−0.02
0.00
Weight
Positive
Negative

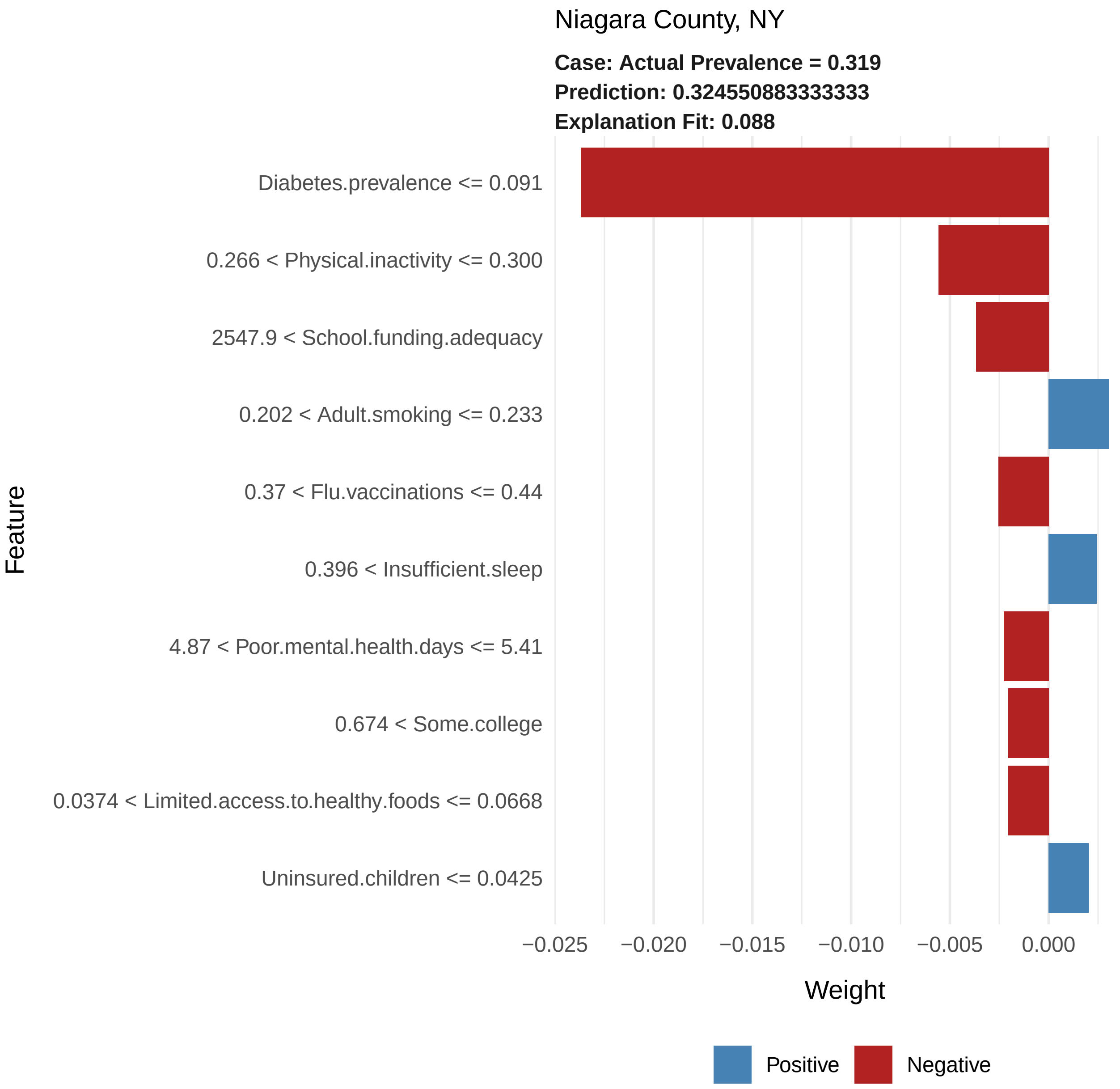
Niagara County, NY
Case: Actual Prevalence = 0.319
Prediction: 0.324550883333333
Explanation Fit: 0.088
Diabetes.prevalence <= 0.091
0.266 < Physical.inactivity <= 0.300
2547.9 < School.funding.adequacy
0.202 < Adult.smoking <= 0.233
0.37 < Flu.vaccinations <= 0.44
0.396 < Insufficient.sleep
4.87 < Poor.mental.health.days <= 5.41
0.674 < Some.college
0.0374 < Limited.access.to.healthy.foods <= 0.0668
Uninsured.children <= 0.0425
Feature
−0.025
−0.020
−0.015
−0.010
−0.005
0.000
Weight
Positive
Negative

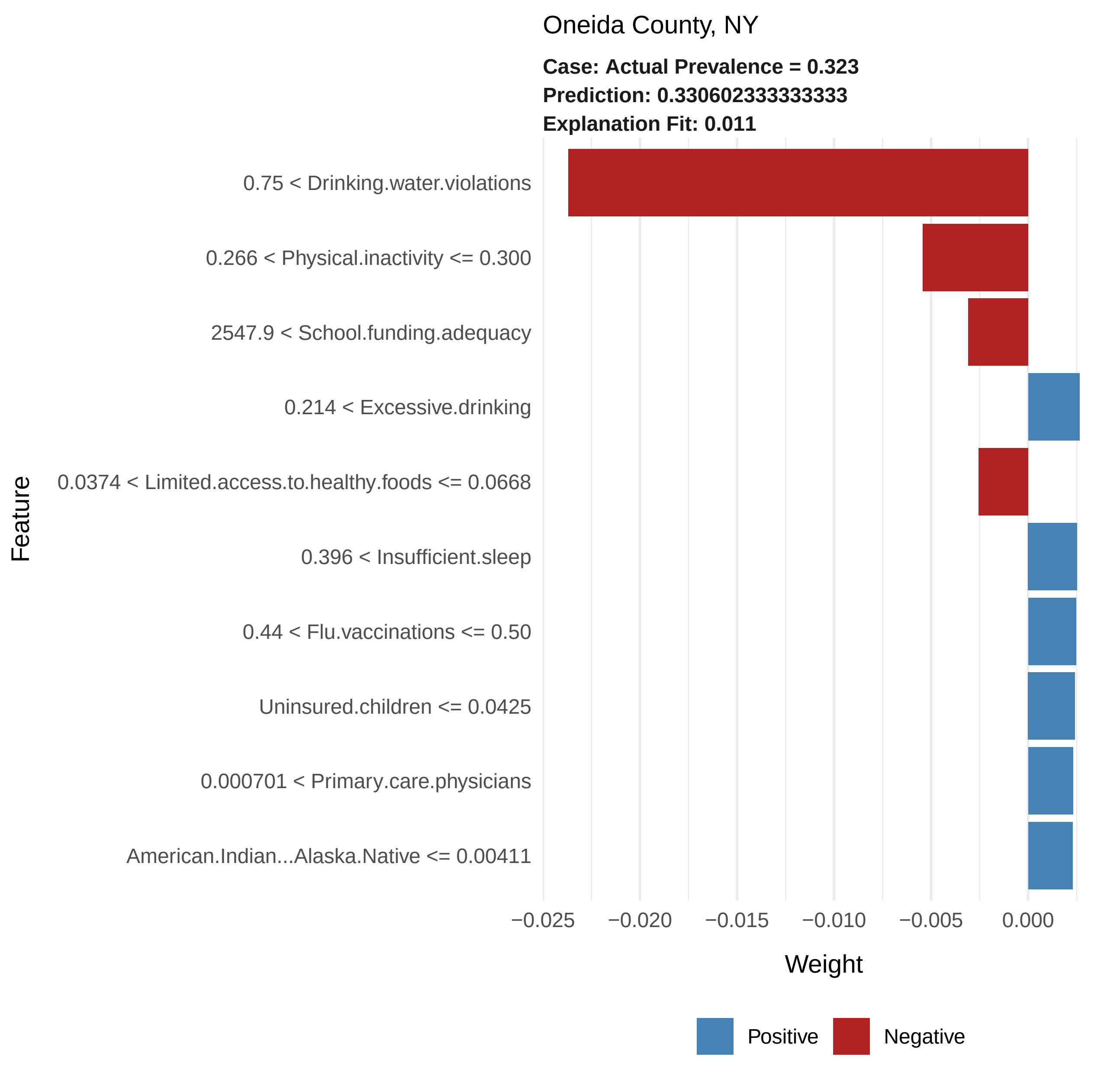

Oneida County, NY
Case: Actual Prevalence = 0.323
Prediction: 0.330602333333333
Explanation Fit: 0.011
0.75 < Drinking.water.violations
0.266 < Physical.inactivity <= 0.300
2547.9 < School.funding.adequacy
0.214 < Excessive.drinking
0.0374 < Limited.access.to.healthy.foods <= 0.0668
0.396 < Insufficient.sleep
0.44 < Flu.vaccinations <= 0.50
Uninsured.children <= 0.0425
0.000701 < Primary.care.physicians
American.Indian...Alaska.Native <= 0.00411
Feature
−0.025
−0.020
−0.015
−0.010
−0.005
0.000
Weight
Positive
Negative

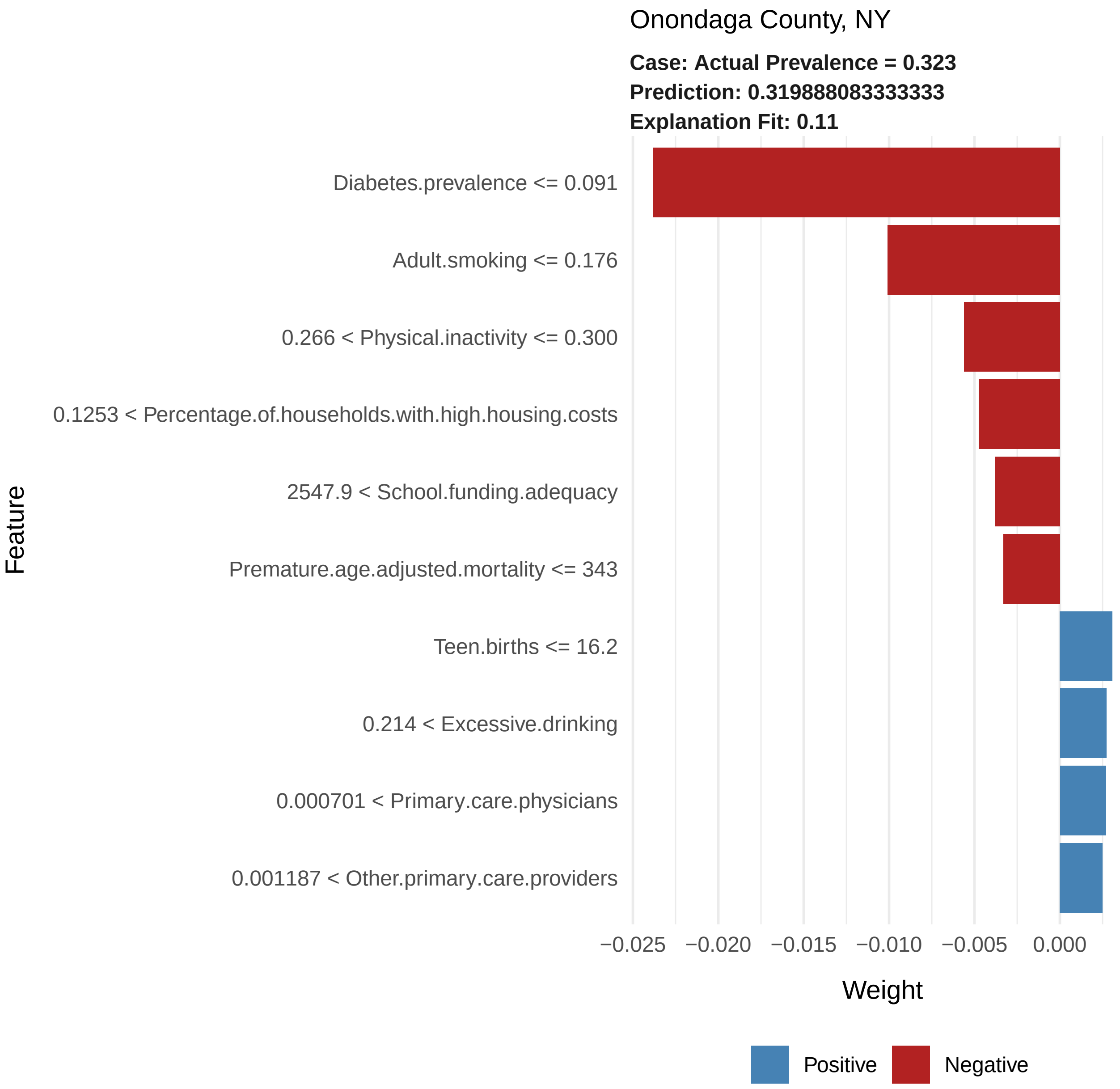

Onondaga County, NY
Case: Actual Prevalence = 0.323
Prediction: 0.319888083333333
Explanation Fit: 0.11
Diabetes.prevalence <= 0.091
Adult.smoking <= 0.176
0.266 < Physical.inactivity <= 0.300
0.1253 < Percentage.of.households.with.high.housing.costs
2547.9 < School.funding.adequacy
Premature.age.adjusted.mortality <= 343
Teen.births <= 16.2
0.214 < Excessive.drinking
0.000701 < Primary.care.physicians
0.001187 < Other.primary.care.providers
Feature
−0.025
−0.020
−0.015
−0.010
−0.005
0.000
Weight
Positive
Negative

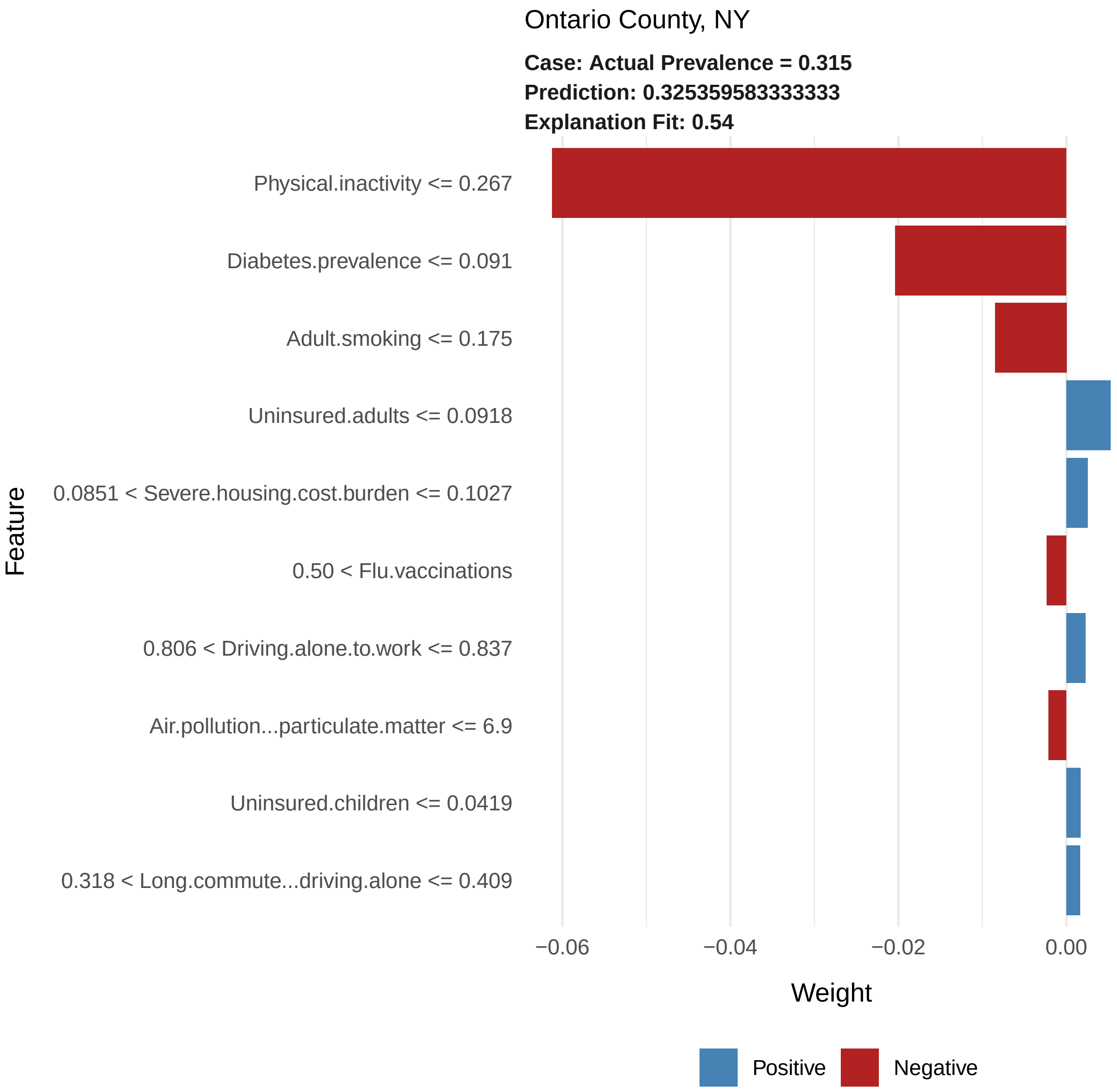

Ontario County, NY
Case: Actual Prevalence = 0.315
Prediction: 0.325359583333333
Explanation Fit: 0.54
Physical.inactivity <= 0.267
Diabetes.prevalence <= 0.091
Adult.smoking <= 0.175
Uninsured.adults <= 0.0918
0.0851 < Severe.housing.cost.burden <= 0.1027
0.50 < Flu.vaccinations
0.806 < Driving.alone.to.work <= 0.837
Air.pollution...particulate.matter <= 6.9
Uninsured.children <= 0.0419
0.318 < Long.commute...driving.alone <= 0.409
Feature
−0.06
−0.04
−0.02
0.00
Weight
Positive
Negative

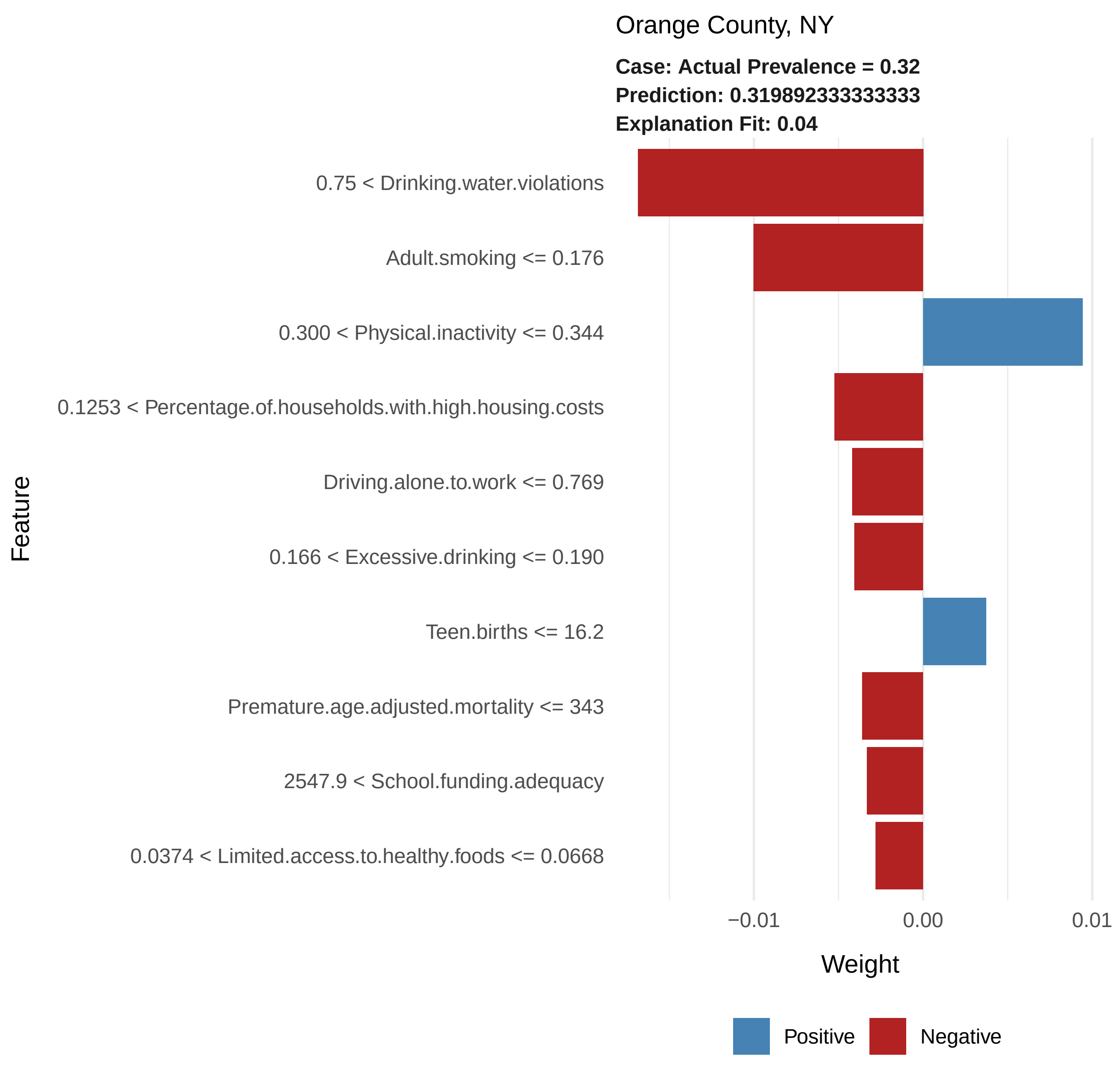
Orange County, NY
Case: Actual Prevalence = 0.32
Prediction: 0.319892333333333
Explanation Fit: 0.04
0.75 < Drinking.water.violations
Adult.smoking <= 0.176
0.300 < Physical.inactivity <= 0.344
0.1253 < Percentage.of.households.with.high.housing.costs
Driving.alone.to.work <= 0.769
0.166 < Excessive.drinking <= 0.190
Teen.births <= 16.2
Premature.age.adjusted.mortality <= 343
2547.9 < School.funding.adequacy
0.0374 < Limited.access.to.healthy.foods <= 0.0668
Feature
−0.01
0.00
0.01
Weight
Positive
Negative

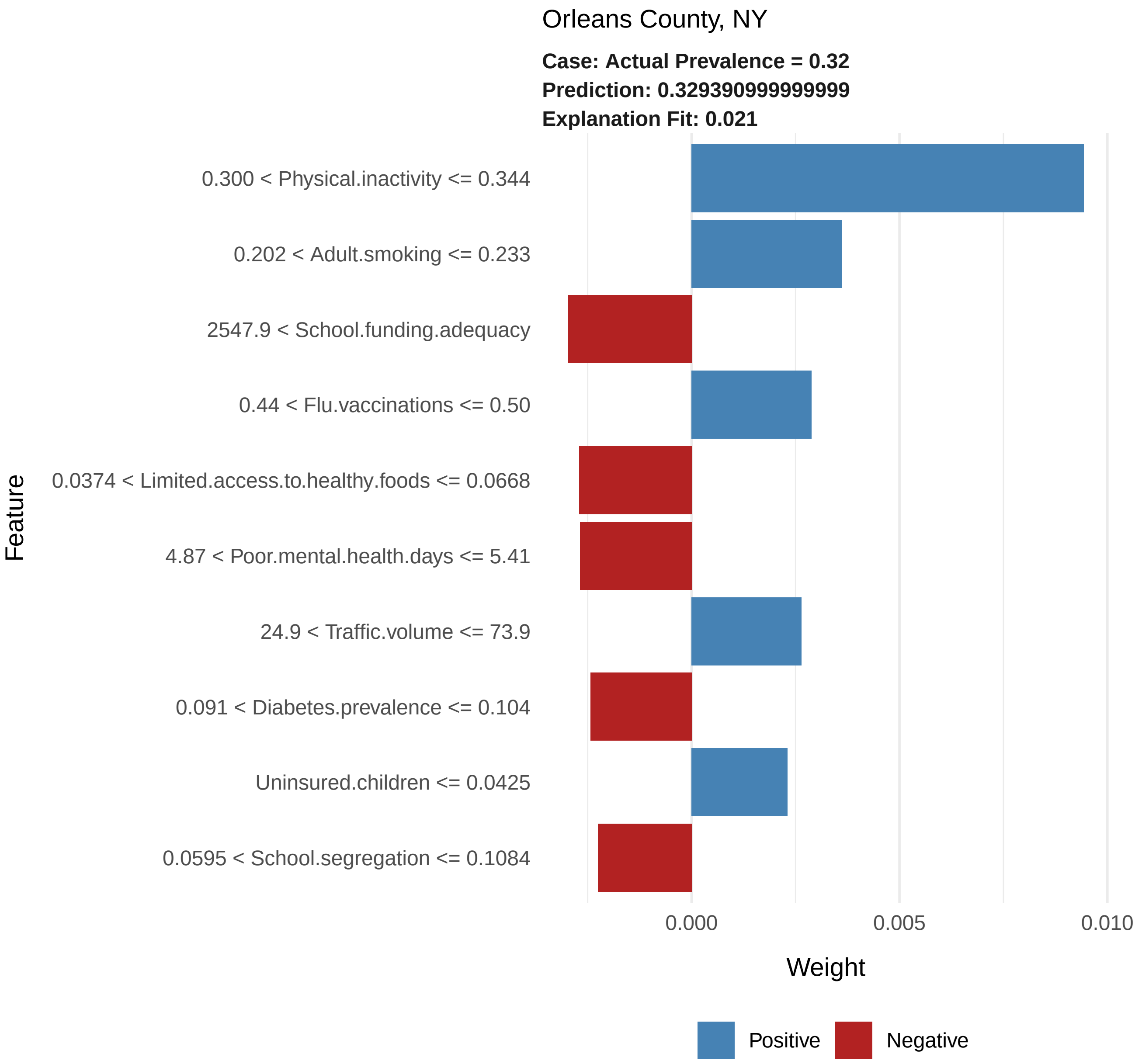

Orleans County, NY
Case: Actual Prevalence = 0.32
Prediction: 0.329390999999999
Explanation Fit: 0.021
0.300 < Physical.inactivity <= 0.344
0.202 < Adult.smoking <= 0.233
2547.9 < School.funding.adequacy
0.44 < Flu.vaccinations <= 0.50
0.0374 < Limited.access.to.healthy.foods <= 0.0668
4.87 < Poor.mental.health.days <= 5.41
24.9 < Traffic.volume <= 73.9
0.091 < Diabetes.prevalence <= 0.104
Uninsured.children <= 0.0425
0.0595 < School.segregation <= 0.1084
Feature
0.000
0.005
0.010
Weight
Positive
Negative

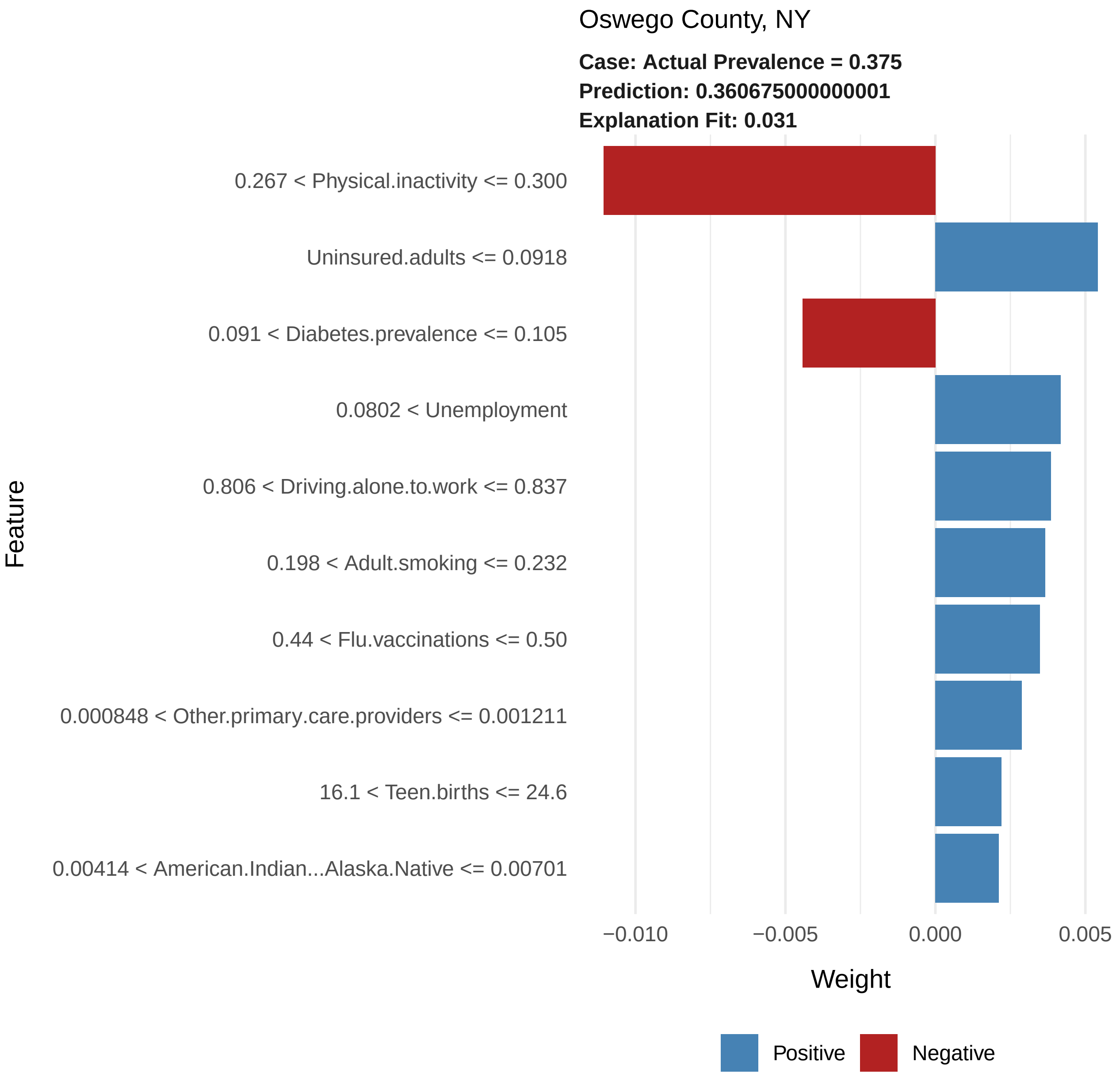

Oswego County, NY
Case: Actual Prevalence = 0.375
Prediction: 0.360675000000001
Explanation Fit: 0.031
0.267 < Physical.inactivity <= 0.300
Uninsured.adults <= 0.0918
0.091 < Diabetes.prevalence <= 0.105
0.0802 < Unemployment
0.806 < Driving.alone.to.work <= 0.837
0.198 < Adult.smoking <= 0.232
0.44 < Flu.vaccinations <= 0.50
0.000848 < Other.primary.care.providers <= 0.001211
16.1 < Teen.births <= 24.6
0.00414 < American.Indian...Alaska.Native <= 0.00701
Feature
−0.010
−0.005
0.000
0.005
Weight
Positive
Negative

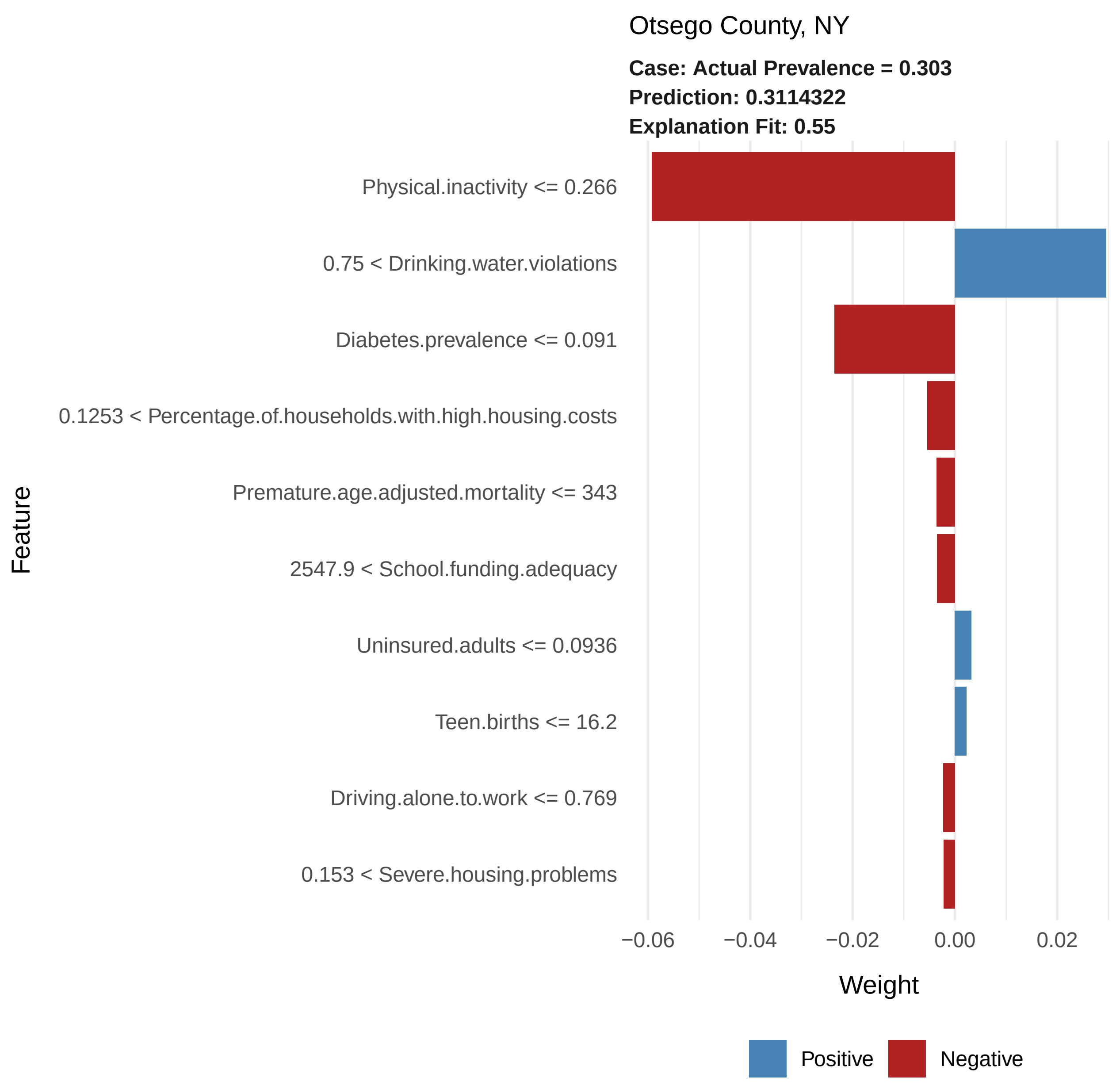

Otsego County, NY
Case: Actual Prevalence = 0.303
Prediction: 0.3114322
Explanation Fit: 0.55
Physical.inactivity <= 0.266
0.75 < Drinking.water.violations
Diabetes.prevalence <= 0.091
0.1253 < Percentage.of.households.with.high.housing.costs
Premature.age.adjusted.mortality <= 343
2547.9 < School.funding.adequacy
Uninsured.adults <= 0.0936
Teen.births <= 16.2
Driving.alone.to.work <= 0.769
0.153 < Severe.housing.problems
Feature
−0.06
−0.04
−0.02
0.00
0.02
Weight
Positive
Negative

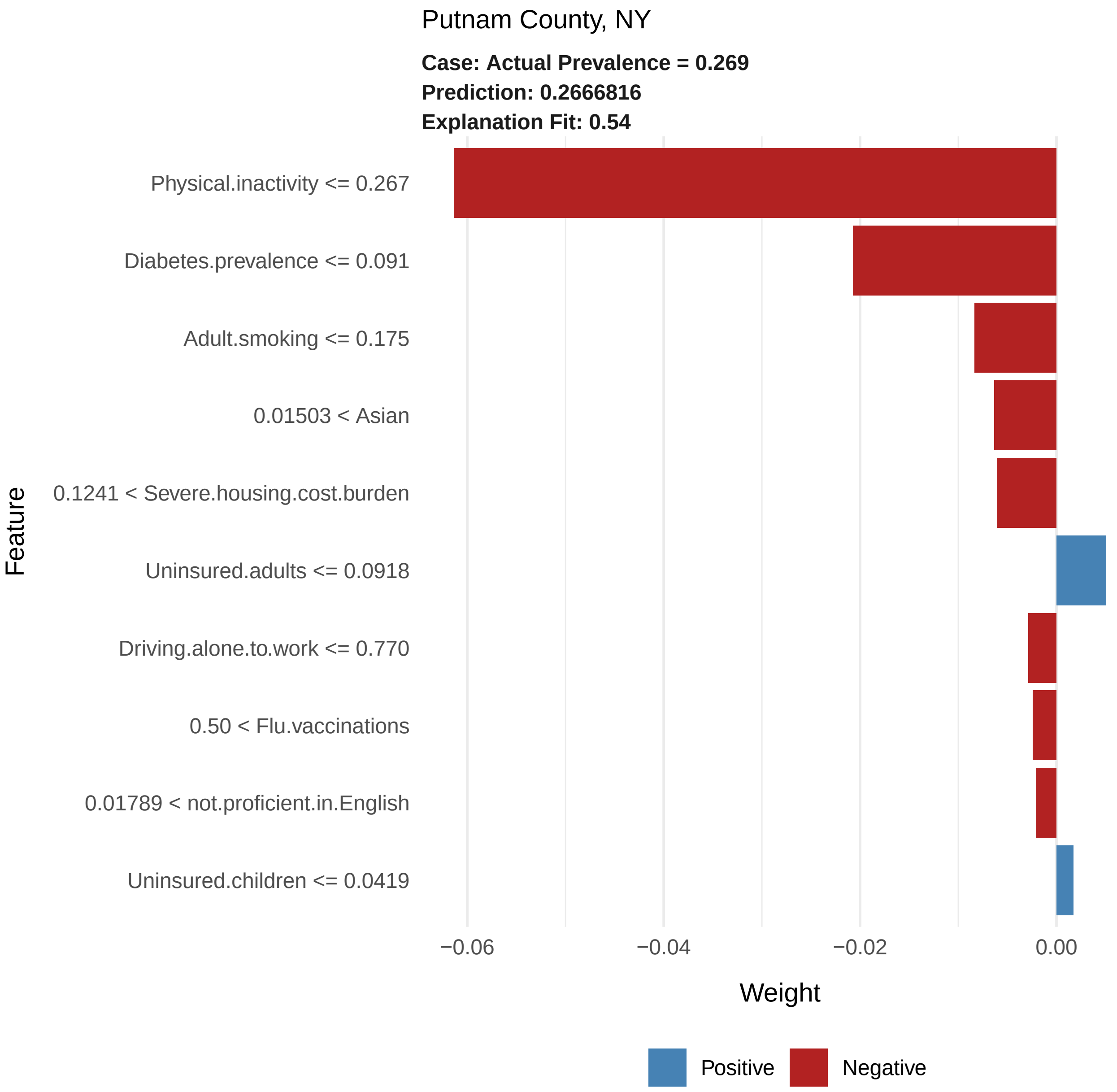
Putnam County, NY
Case: Actual Prevalence = 0.269
Prediction: 0.2666816
Explanation Fit: 0.54
Physical.inactivity <= 0.267
Diabetes.prevalence <= 0.091
Adult.smoking <= 0.175
0.01503 < Asian
0.1241 < Severe.housing.cost.burden
Uninsured.adults <= 0.0918
Driving.alone.to.work <= 0.770
0.50 < Flu.vaccinations
0.01789 < not.proficient.in.English
Uninsured.children <= 0.0419
Feature
−0.06
−0.04
−0.02
0.00
Weight
Positive
Negative

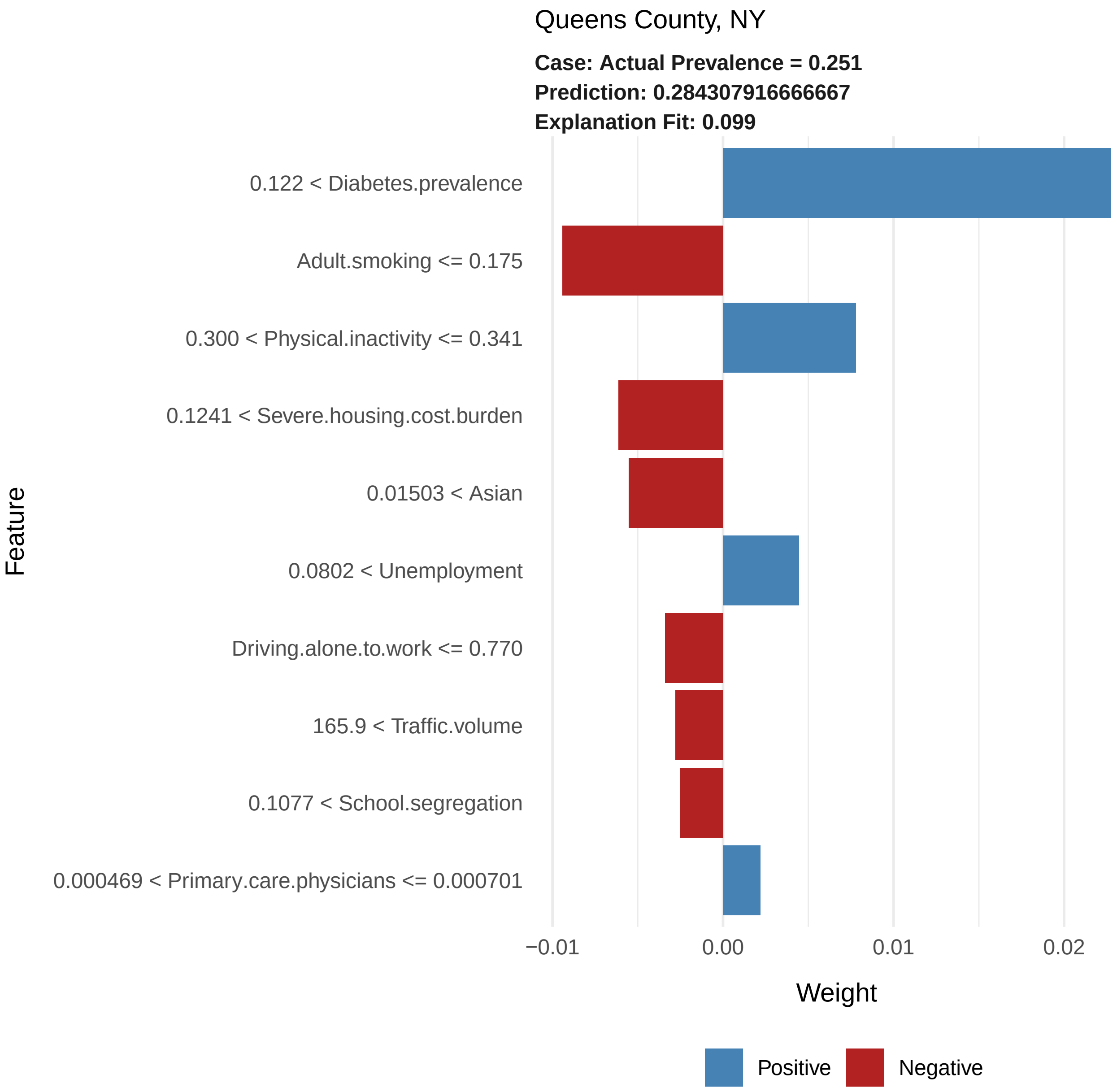

Queens County, NY
Case: Actual Prevalence = 0.251
Prediction: 0.284307916666667
Explanation Fit: 0.099
0.122 < Diabetes.prevalence
Adult.smoking <= 0.175
0.300 < Physical.inactivity <= 0.341
0.1241 < Severe.housing.cost.burden
0.01503 < Asian
0.0802 < Unemployment
Driving.alone.to.work <= 0.770
165.9 < Traffic.volume
0.1077 < School.segregation
0.000469 < Primary.care.physicians <= 0.000701
Feature
−0.01
0.00
0.01
0.02
Weight
Positive
Negative

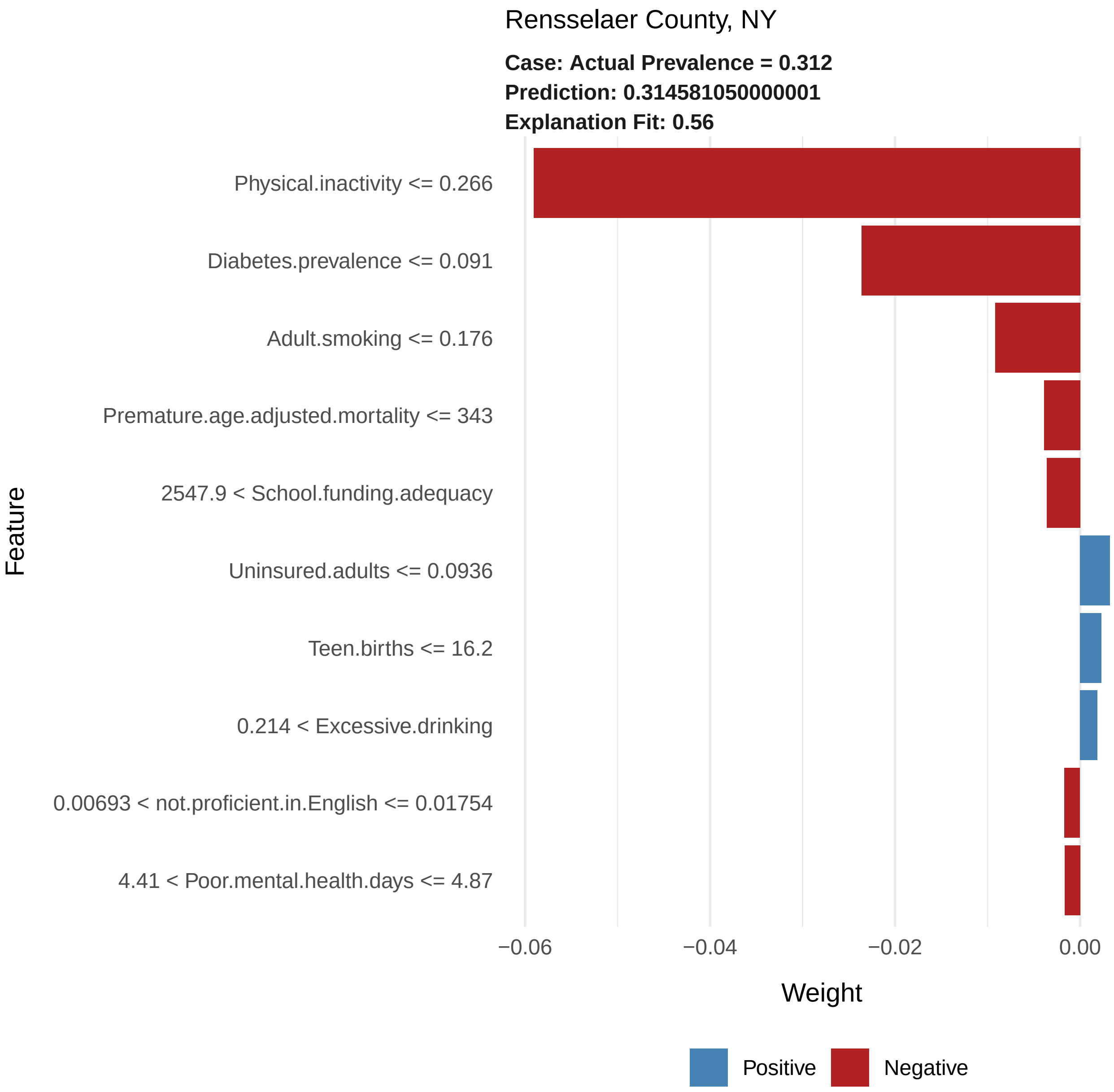
Rensselaer County, NY
Case: Actual Prevalence = 0.312
Prediction: 0.314581050000001
Explanation Fit: 0.56
Physical.inactivity <= 0.266
Diabetes.prevalence <= 0.091
Adult.smoking <= 0.176
Premature.age.adjusted.mortality <= 343
2547.9 < School.funding.adequacy
Uninsured.adults <= 0.0936
Teen.births <= 16.2
0.214 < Excessive.drinking
0.00693 < not.proficient.in.English <= 0.01754
4.41 < Poor.mental.health.days <= 4.87
Feature
−0.06
−0.04
−0.02
0.00
Weight
Positive
Negative

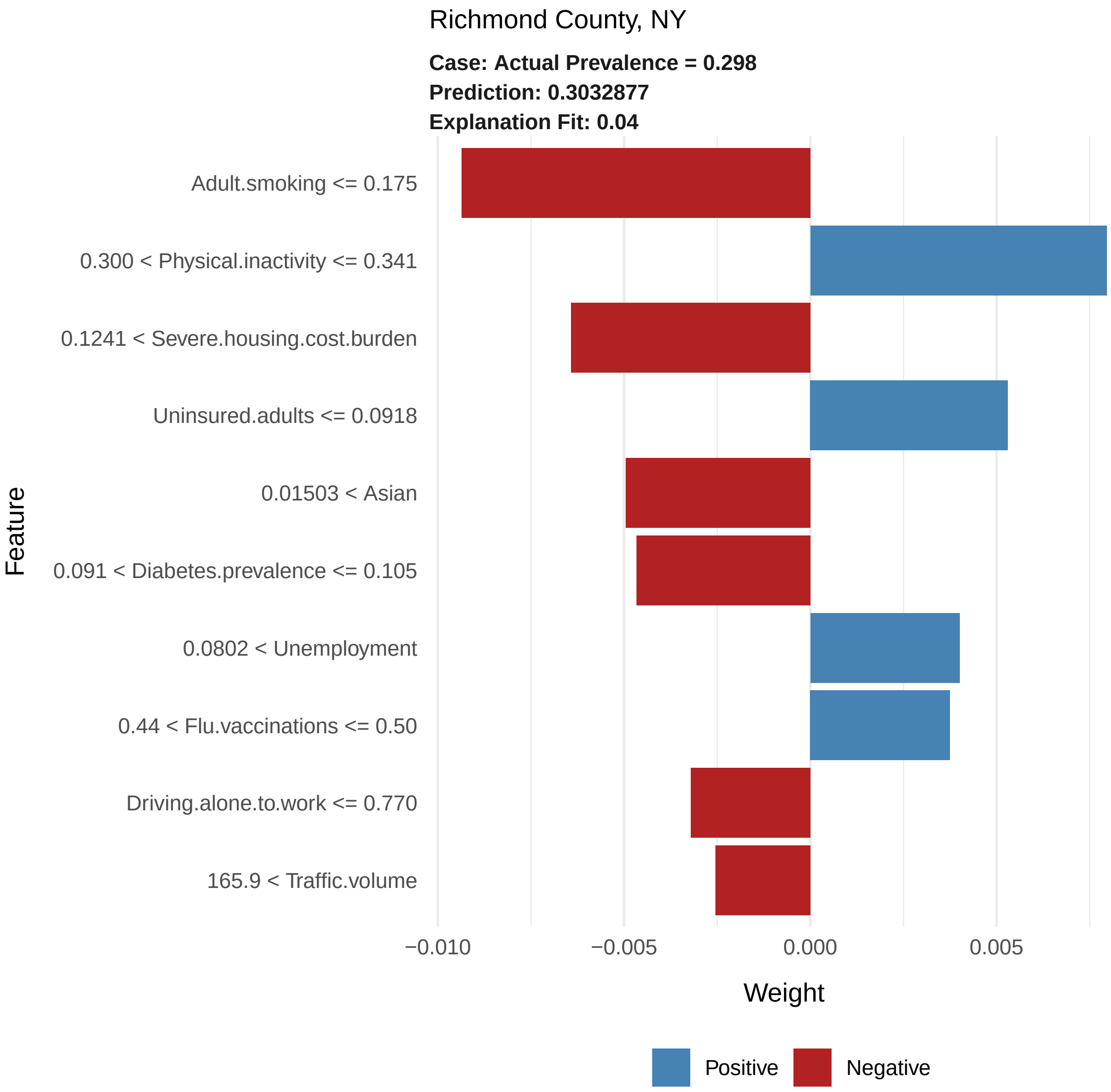
Richmond County, NY
Case: Actual Prevalence = 0.298
Prediction: 0.3032877
Explanation Fit: 0.04
Adult.smoking <= 0.175
0.300 < Physical.inactivity <= 0.341
0.1241 < Severe.housing.cost.burden
Uninsured.adults <= 0.0918
0.01503 < Asian
0.091 < Diabetes.prevalence <= 0.105
0.0802 < Unemployment
0.44 < Flu.vaccinations <= 0.50
Driving.alone.to.work <= 0.770
165.9 < Traffic.volume
Feature
−0.010
−0.005
0.000
0.005
Weight
Positive
Negative

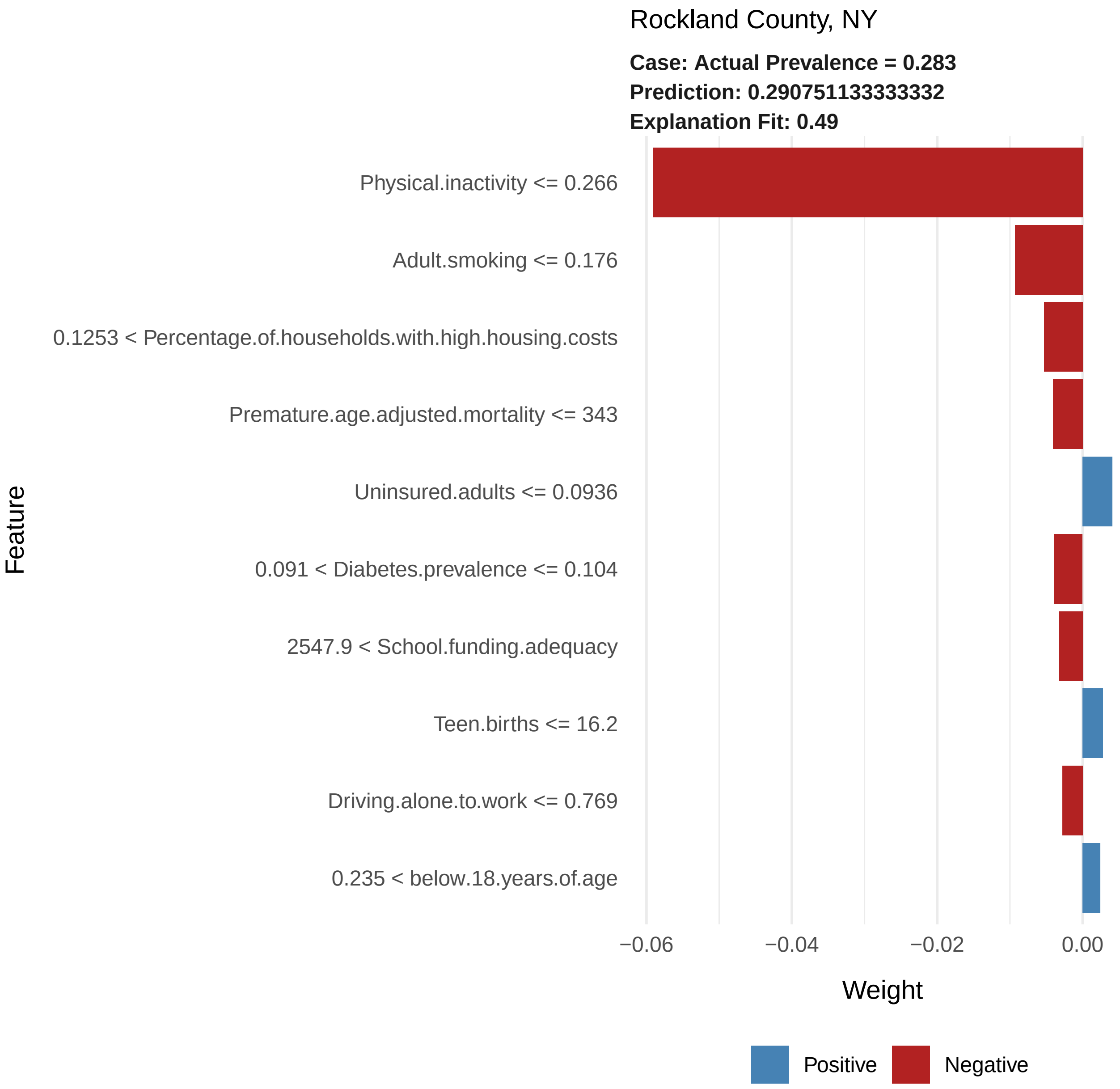
Rockland County, NY
Case: Actual Prevalence = 0.283
Prediction: 0.290751133333332
Explanation Fit: 0.49
Physical.inactivity <= 0.266
Adult.smoking <= 0.176
0.1253 < Percentage.of.households.with.high.housing.costs
Premature.age.adjusted.mortality <= 343
Uninsured.adults <= 0.0936
0.091 < Diabetes.prevalence <= 0.104
2547.9 < School.funding.adequacy
Teen.births <= 16.2
Driving.alone.to.work <= 0.769
0.235 < below.18.years.of.age
Feature
−0.06
−0.04
−0.02
0.00
Weight
Positive
Negative

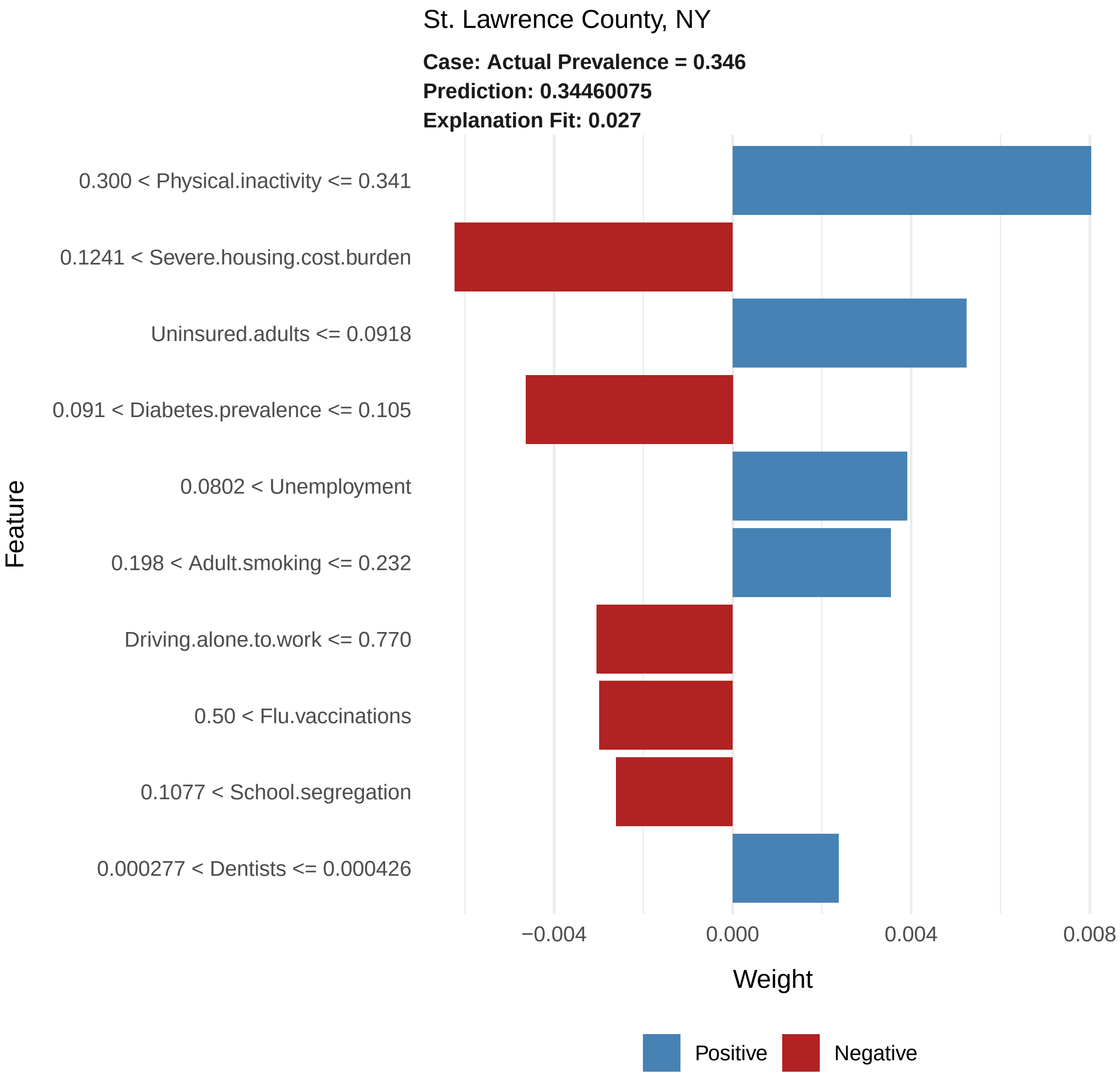

St. Lawrence County, NY
Case: Actual Prevalence = 0.346
Prediction: 0.34460075
Explanation Fit: 0.027
0.300 < Physical.inactivity <= 0.341
0.1241 < Severe.housing.cost.burden
Uninsured.adults <= 0.0918
0.091 < Diabetes.prevalence <= 0.105
0.0802 < Unemployment
0.198 < Adult.smoking <= 0.232
Driving.alone.to.work <= 0.770
0.50 < Flu.vaccinations
0.1077 < School.segregation
0.000277 < Dentists <= 0.000426
Feature
−0.004
0.000
0.004
0.008
Weight
Positive
Negative

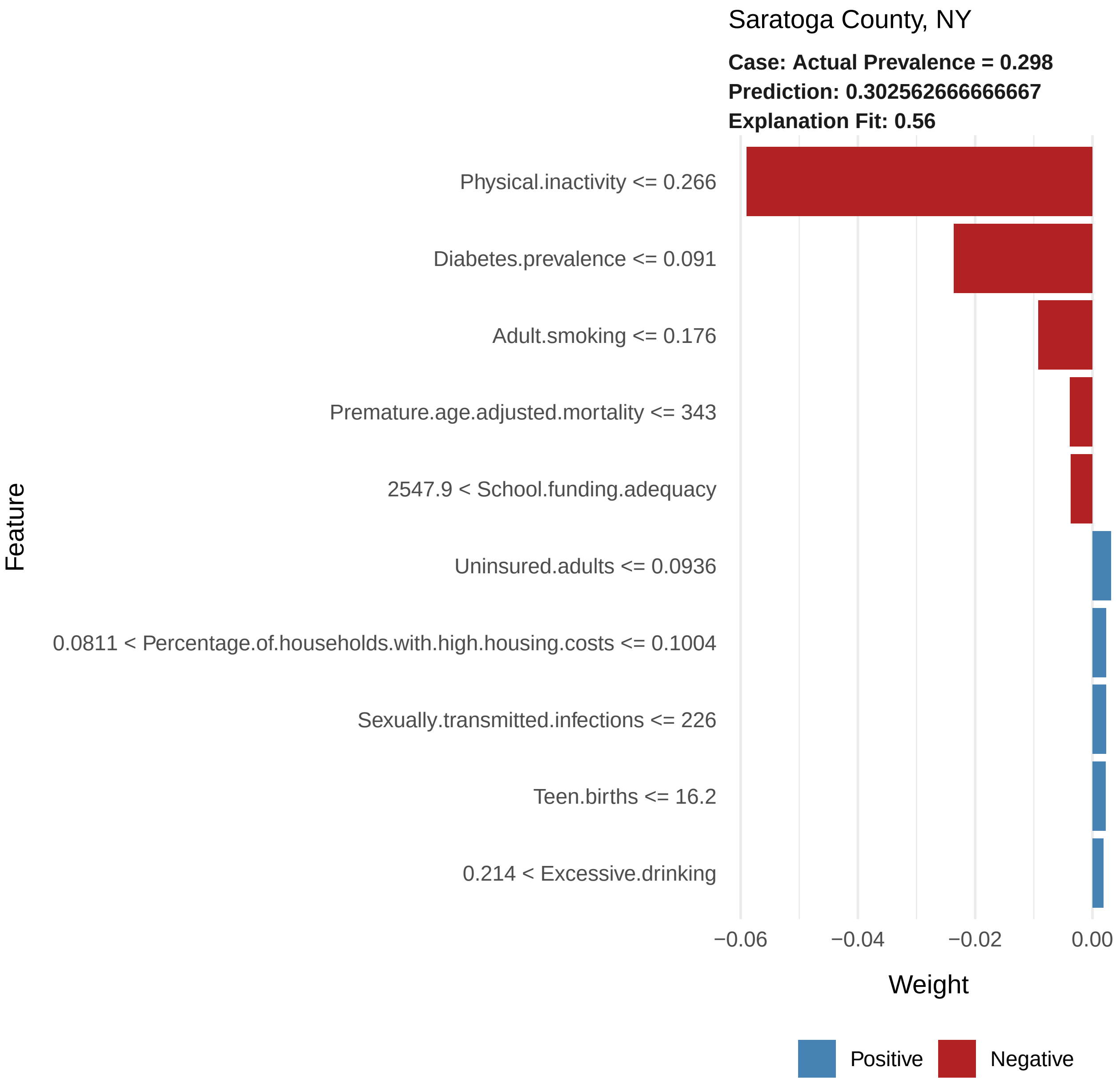
Saratoga County, NY
Case: Actual Prevalence = 0.298
Prediction: 0.302562666666667
Explanation Fit: 0.56
Physical.inactivity <= 0.266
Diabetes.prevalence <= 0.091
Adult.smoking <= 0.176
Premature.age.adjusted.mortality <= 343
2547.9 < School.funding.adequacy
Uninsured.adults <= 0.0936
0.0811 < Percentage.of.households.with.high.housing.costs <= 0.1004
Sexually.transmitted.infections <= 226
Teen.births <= 16.2
0.214 < Excessive.drinking
Feature
−0.06
−0.04
−0.02
0.00
Weight
Positive
Negative

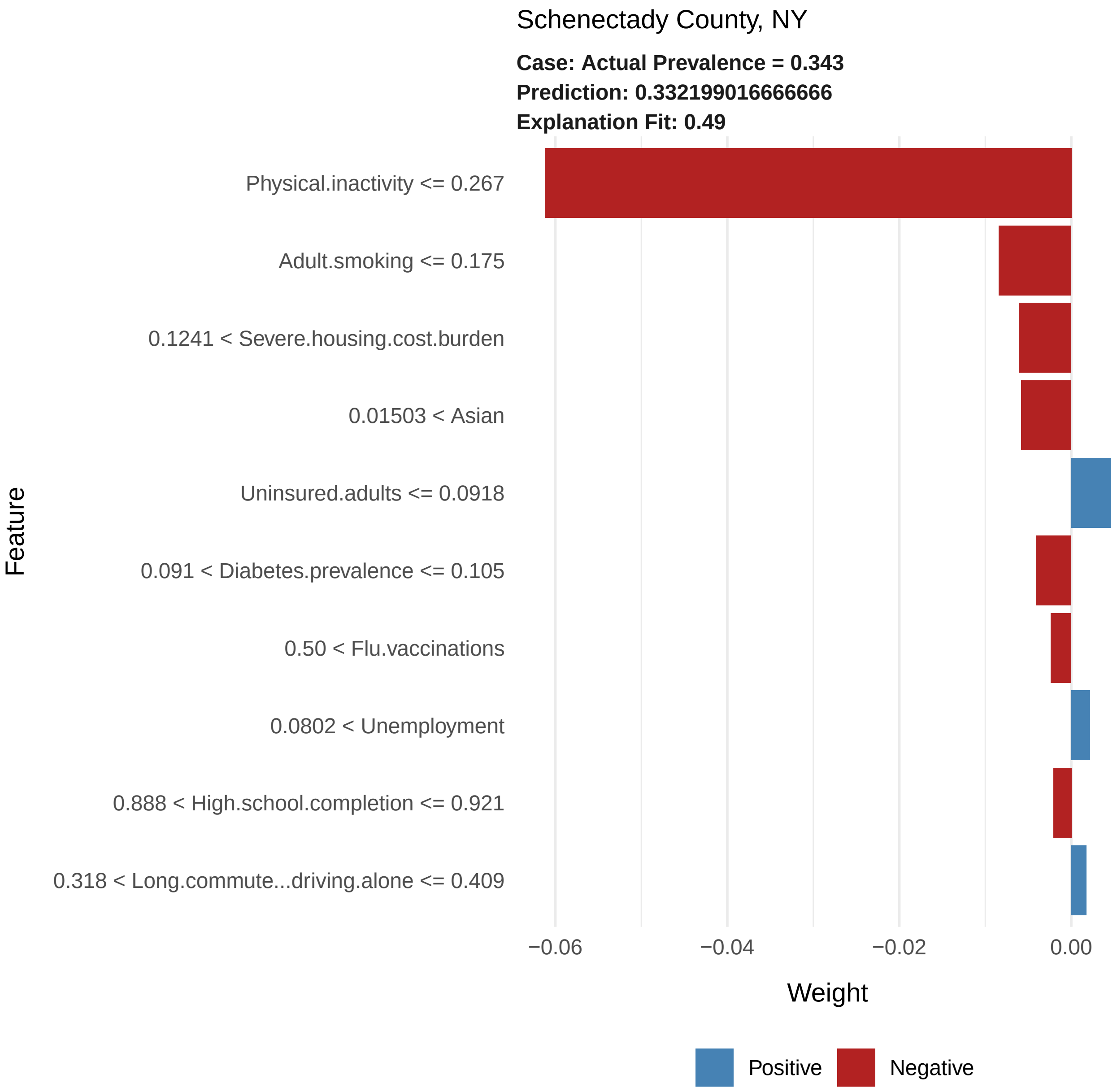
Schenectady County, NY
Case: Actual Prevalence = 0.343
Prediction: 0.332199016666666
Explanation Fit: 0.49
Physical.inactivity <= 0.267
Adult.smoking <= 0.175
0.1241 < Severe.housing.cost.burden
0.01503 < Asian
Uninsured.adults <= 0.0918
0.091 < Diabetes.prevalence <= 0.105
0.50 < Flu.vaccinations
0.0802 < Unemployment
0.888 < High.school.completion <= 0.921
0.318 < Long.commute...driving.alone <= 0.409
Feature
−0.06
−0.04
−0.02
0.00
Weight
Positive
Negative

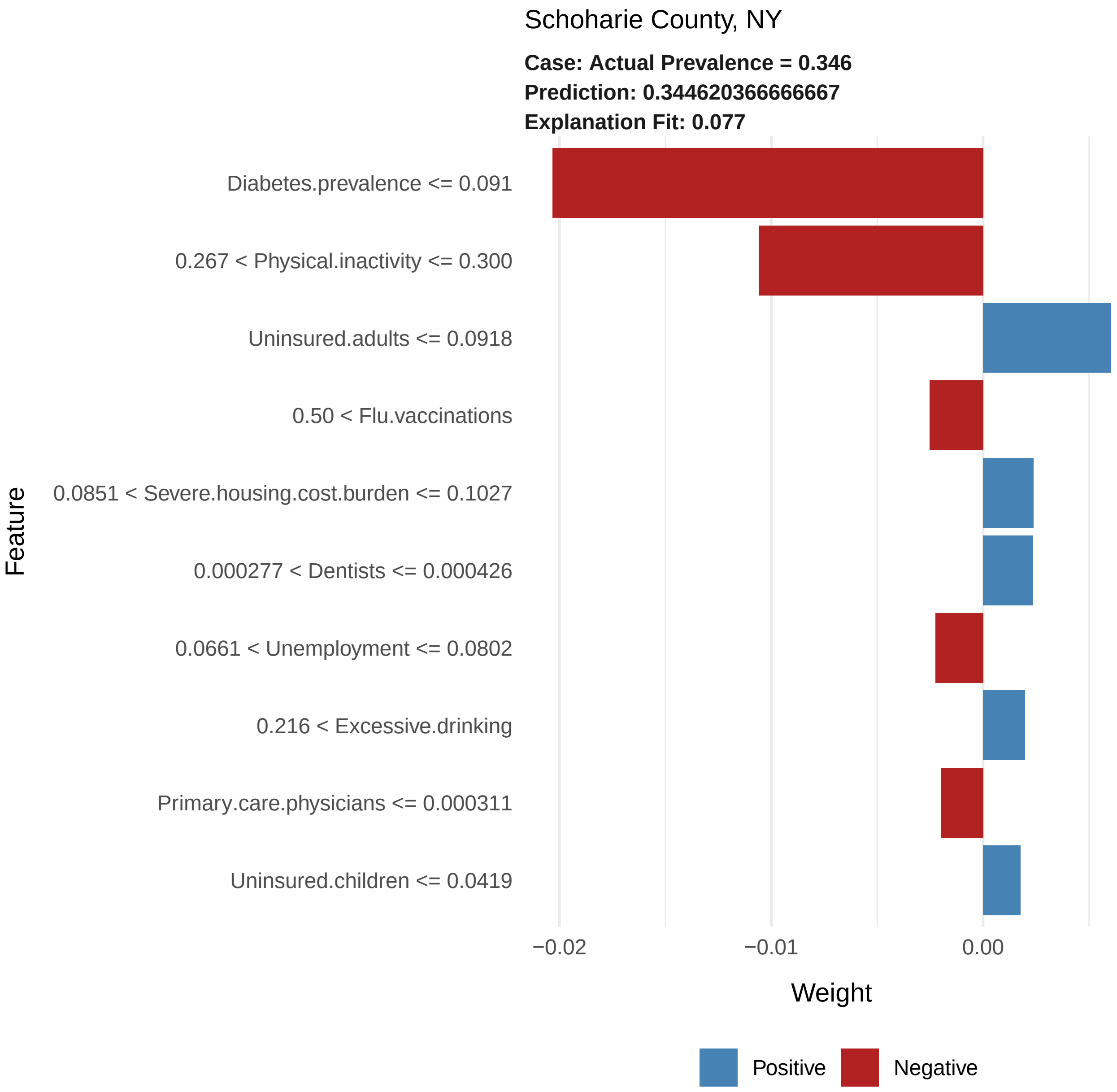
Schoharie County, NY
Case: Actual Prevalence = 0.346
Prediction: 0.344620366666667
Explanation Fit: 0.077
Diabetes.prevalence <= 0.091
0.267 < Physical.inactivity <= 0.300
Uninsured.adults <= 0.0918
0.50 < Flu.vaccinations
0.0851 < Severe.housing.cost.burden <= 0.1027
0.000277 < Dentists <= 0.000426
0.0661 < Unemployment <= 0.0802
0.216 < Excessive.drinking
Primary.care.physicians <= 0.000311
Uninsured.children <= 0.0419
Feature
−0.02
−0.01
0.00
Weight
Positive
Negative

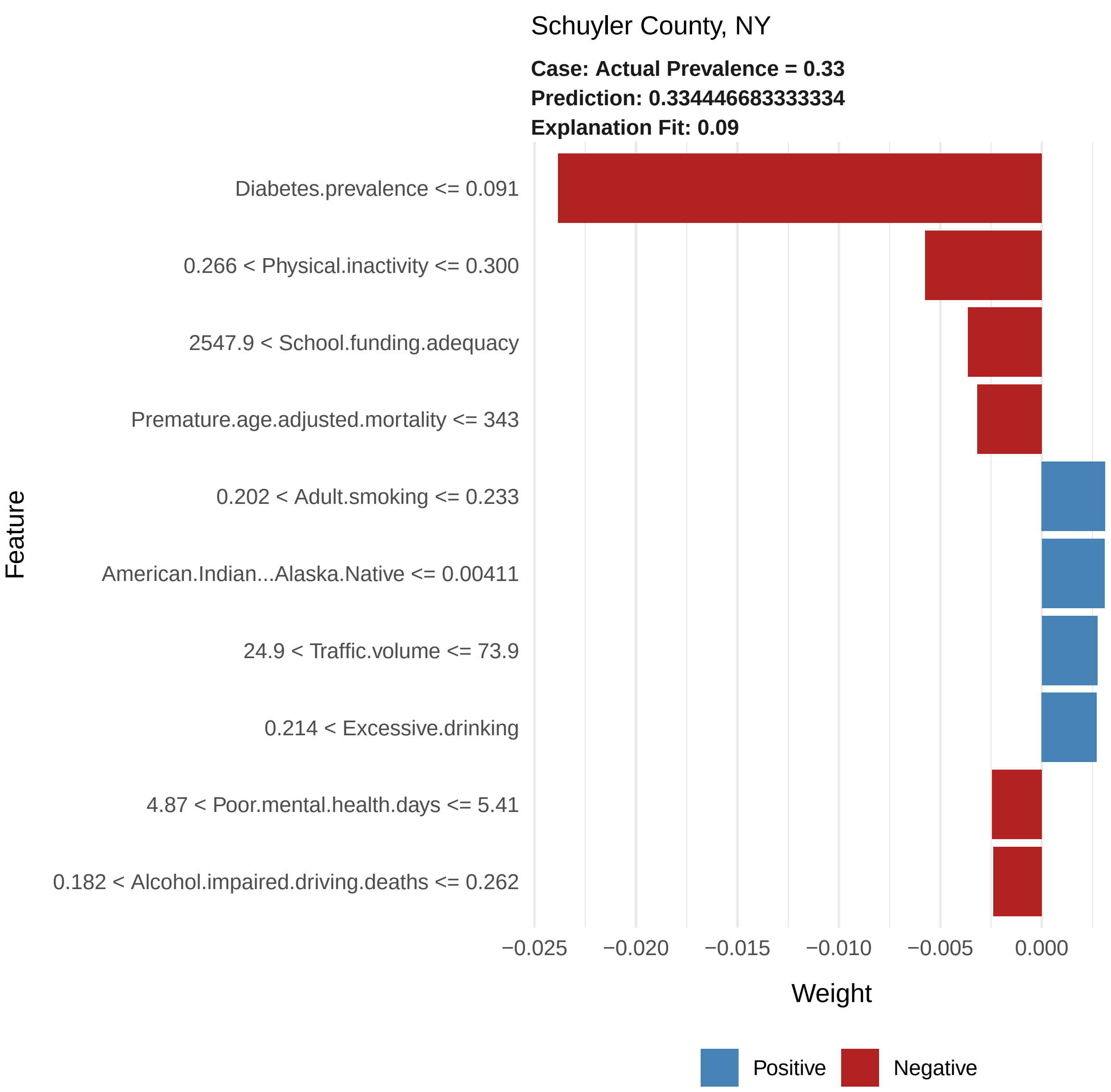

Schuyler County, NY
Case: Actual Prevalence = 0.33
Prediction: 0.334446683333334
Explanation Fit: 0.09
Diabetes.prevalence <= 0.091
0.266 < Physical.inactivity <= 0.300
2547.9 < School.funding.adequacy
Premature.age.adjusted.mortality <= 343
0.202 < Adult.smoking <= 0.233
American.Indian...Alaska.Native <= 0.00411
24.9 < Traffic.volume <= 73.9
0.214 < Excessive.drinking
4.87 < Poor.mental.health.days <= 5.41
0.182 < Alcohol.impaired.driving.deaths <= 0.262
Feature
−0.025
−0.020
−0.015
−0.010
−0.005
0.000
Weight
Positive
Negative

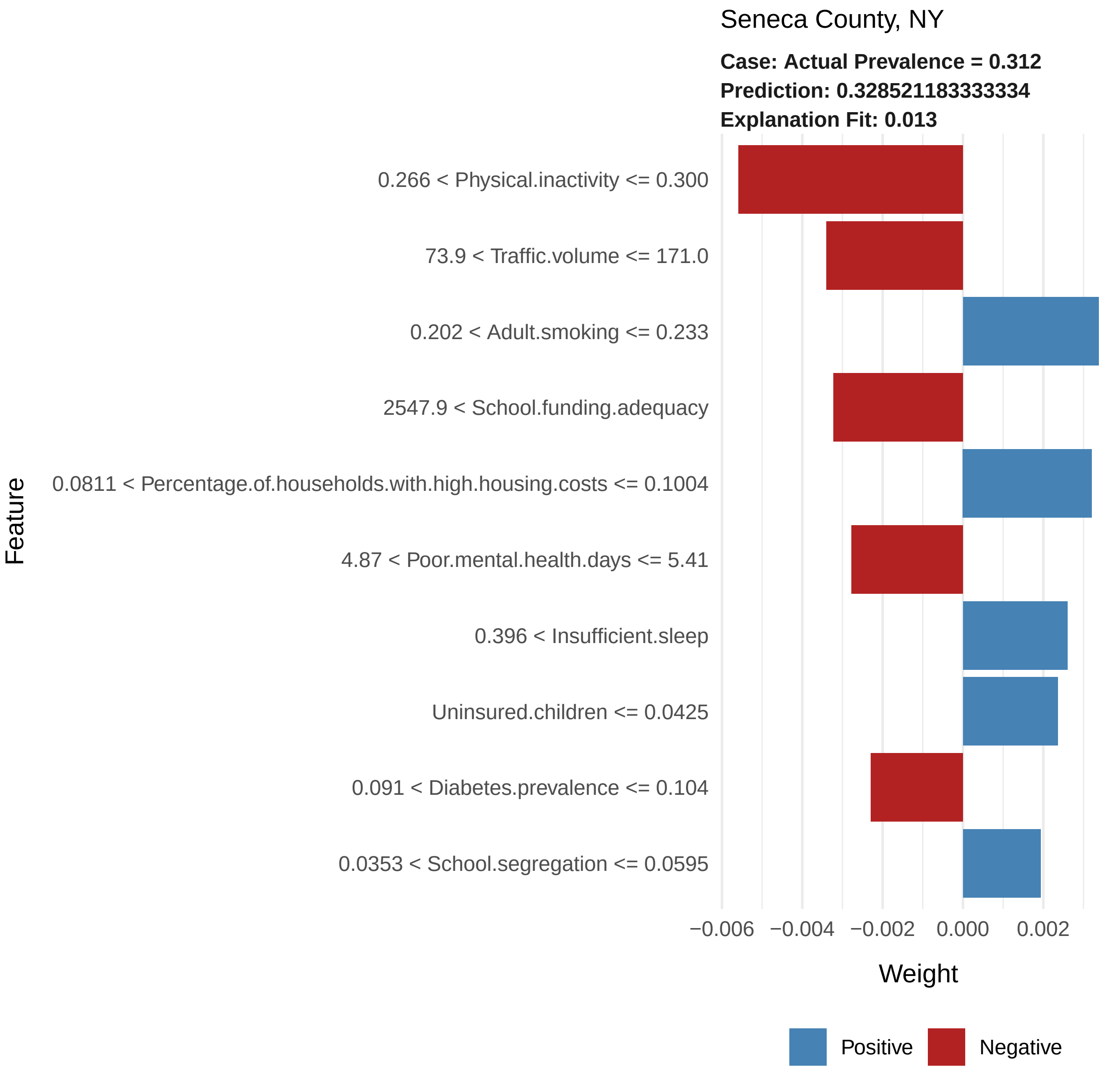

Seneca County, NY
Case: Actual Prevalence = 0.312
Prediction: 0.328521183333334
Explanation Fit: 0.013
0.266 < Physical.inactivity <= 0.300
73.9 < Traffic.volume <= 171.0
0.202 < Adult.smoking <= 0.233
2547.9 < School.funding.adequacy
0.0811 < Percentage.of.households.with.high.housing.costs <= 0.1004
4.87 < Poor.mental.health.days <= 5.41
0.396 < Insufficient.sleep
Uninsured.children <= 0.0425
0.091 < Diabetes.prevalence <= 0.104
0.0353 < School.segregation <= 0.0595
Feature
−0.006
−0.004
−0.002
0.000
0.002
Weight
Positive
Negative

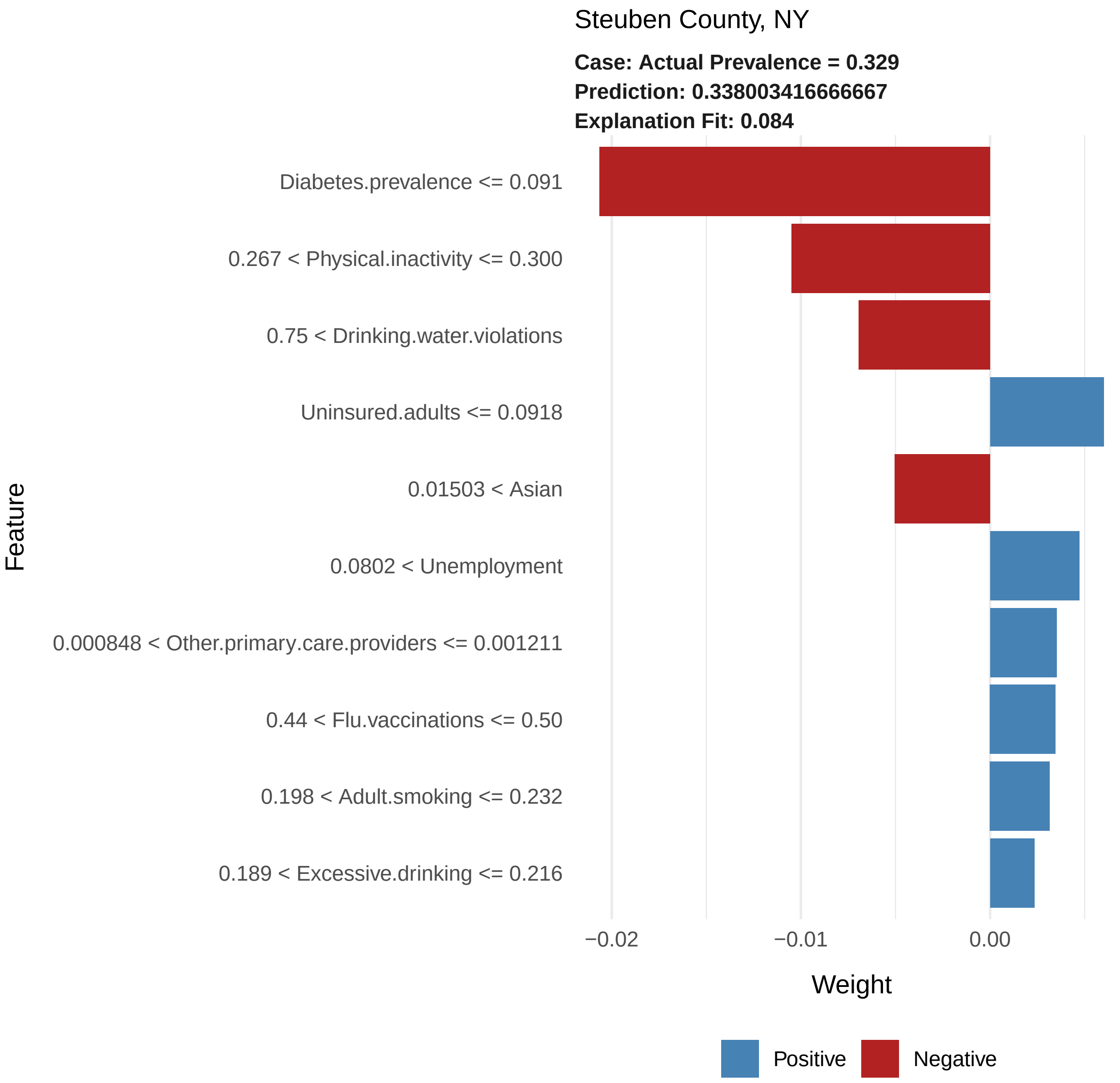

Steuben County, NY
Case: Actual Prevalence = 0.329
Prediction: 0.338003416666667
Explanation Fit: 0.084
Diabetes.prevalence <= 0.091
0.267 < Physical.inactivity <= 0.300
0.75 < Drinking.water.violations
Uninsured.adults <= 0.0918
0.01503 < Asian
0.0802 < Unemployment
0.000848 < Other.primary.care.providers <= 0.001211
0.44 < Flu.vaccinations <= 0.50
0.198 < Adult.smoking <= 0.232
0.189 < Excessive.drinking <= 0.216
Feature
−0.02
−0.01
0.00
Weight
Positive
Negative

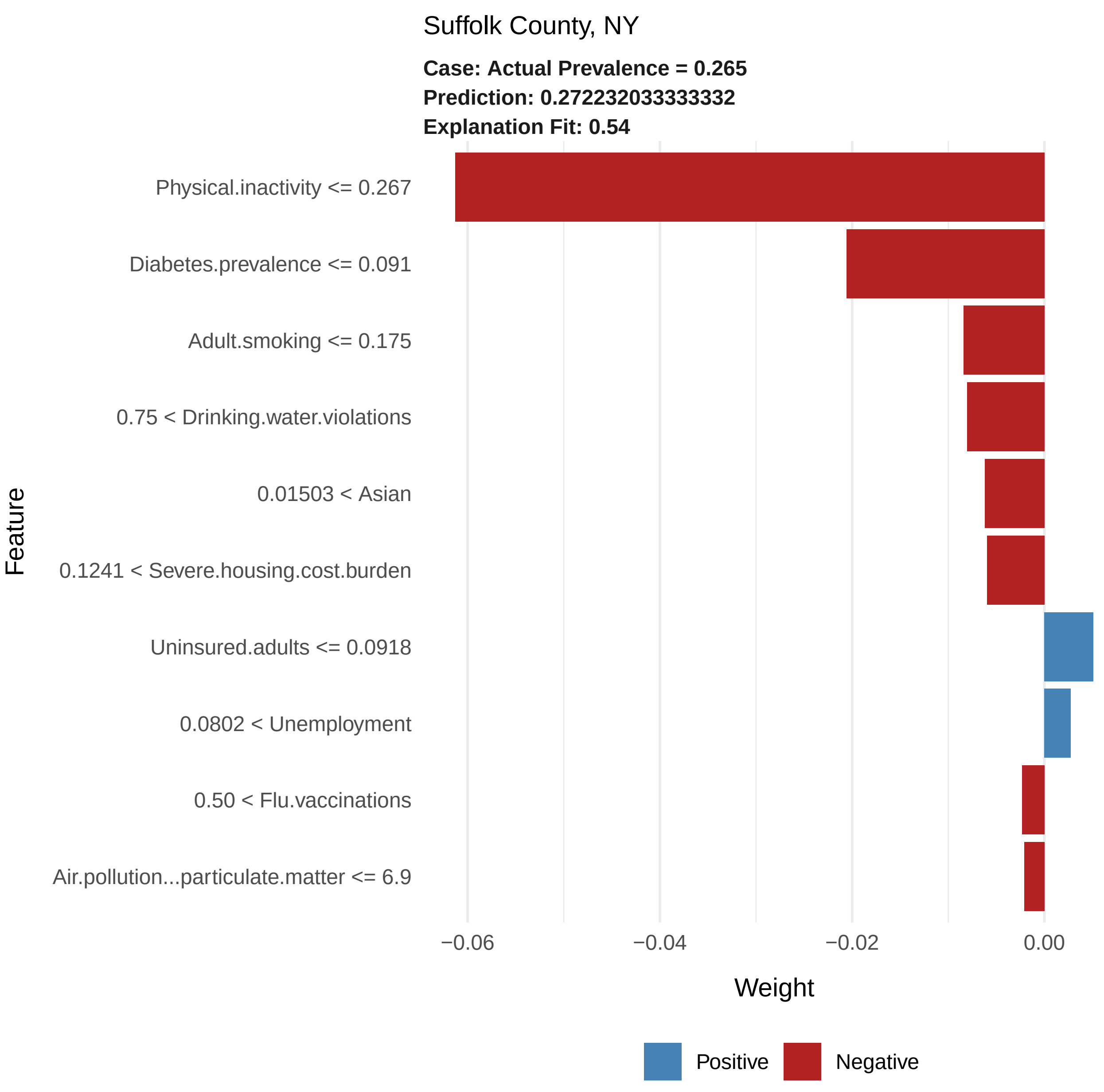
Suffolk County, NY
Case: Actual Prevalence = 0.265
Prediction: 0.272232033333332
Explanation Fit: 0.54
Physical.inactivity <= 0.267
Diabetes.prevalence <= 0.091
Adult.smoking <= 0.175
0.75 < Drinking.water.violations
0.01503 < Asian
0.1241 < Severe.housing.cost.burden
Uninsured.adults <= 0.0918
0.0802 < Unemployment
0.50 < Flu.vaccinations
Air.pollution...particulate.matter <= 6.9
Feature
−0.06
−0.04
−0.02
0.00
Weight
Positive
Negative

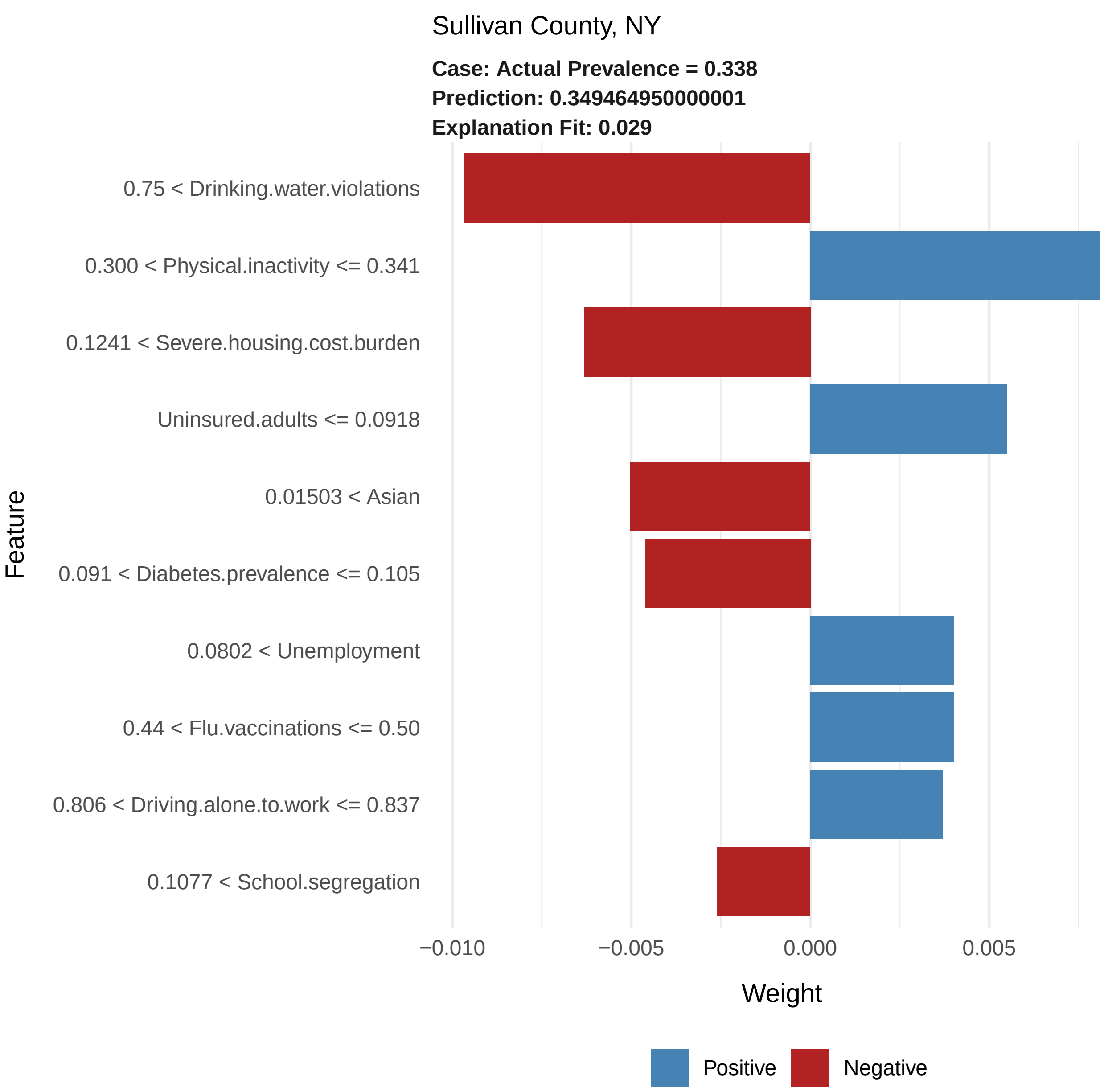
Sullivan County, NY
Case: Actual Prevalence = 0.338
Prediction: 0.349464950000001
Explanation Fit: 0.029
0.75 < Drinking.water.violations
0.300 < Physical.inactivity <= 0.341
0.1241 < Severe.housing.cost.burden
Uninsured.adults <= 0.0918
0.01503 < Asian
0.091 < Diabetes.prevalence <= 0.105
0.0802 < Unemployment
0.44 < Flu.vaccinations <= 0.50
0.806 < Driving.alone.to.work <= 0.837
0.1077 < School.segregation
Feature
−0.010
−0.005
0.000
0.005
Weight
Positive
Negative

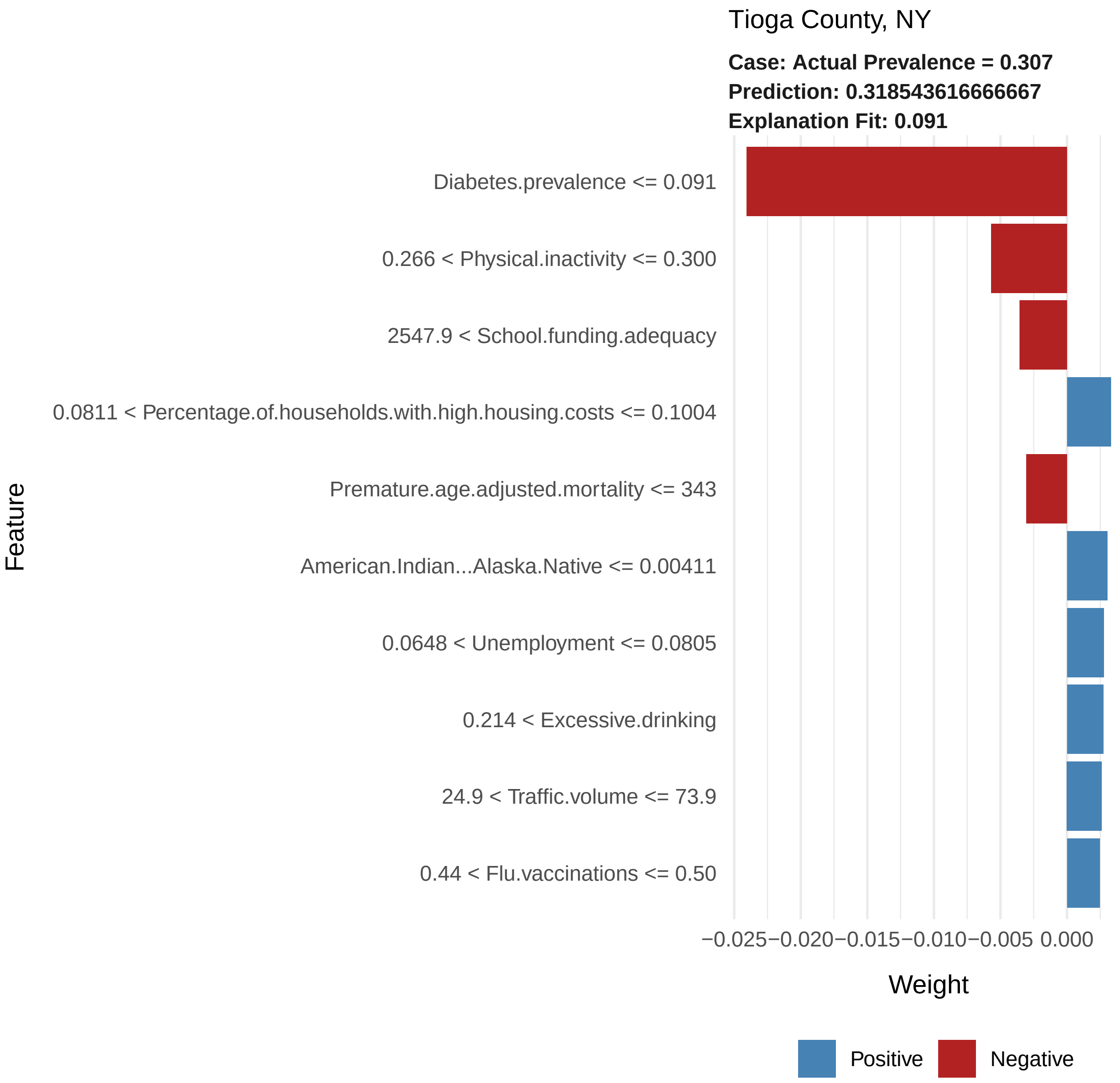
Tioga County, NY
Case: Actual Prevalence = 0.307
Prediction: 0.318543616666667
Explanation Fit: 0.091
Feature
Diabetes.prevalence <= 0.091
0.266 < Physical.inactivity <= 0.300
2547.9 < School.funding.adequacy
0.0811 < Percentage.of.households.with.high.housing.costs <= 0.1004
Premature.age.adjusted.mortality <= 343
American.Indian...Alaska.Native <= 0.00411
0.0648 < Unemployment <= 0.0805
0.214 < Excessive.drinking
24.9 < Traffic.volume <= 73.9
0.44 < Flu.vaccinations <= 0.50
−0.025
−0.020
−0.015
−0.010
−0.005
0.000
Weight
Positive
Negative

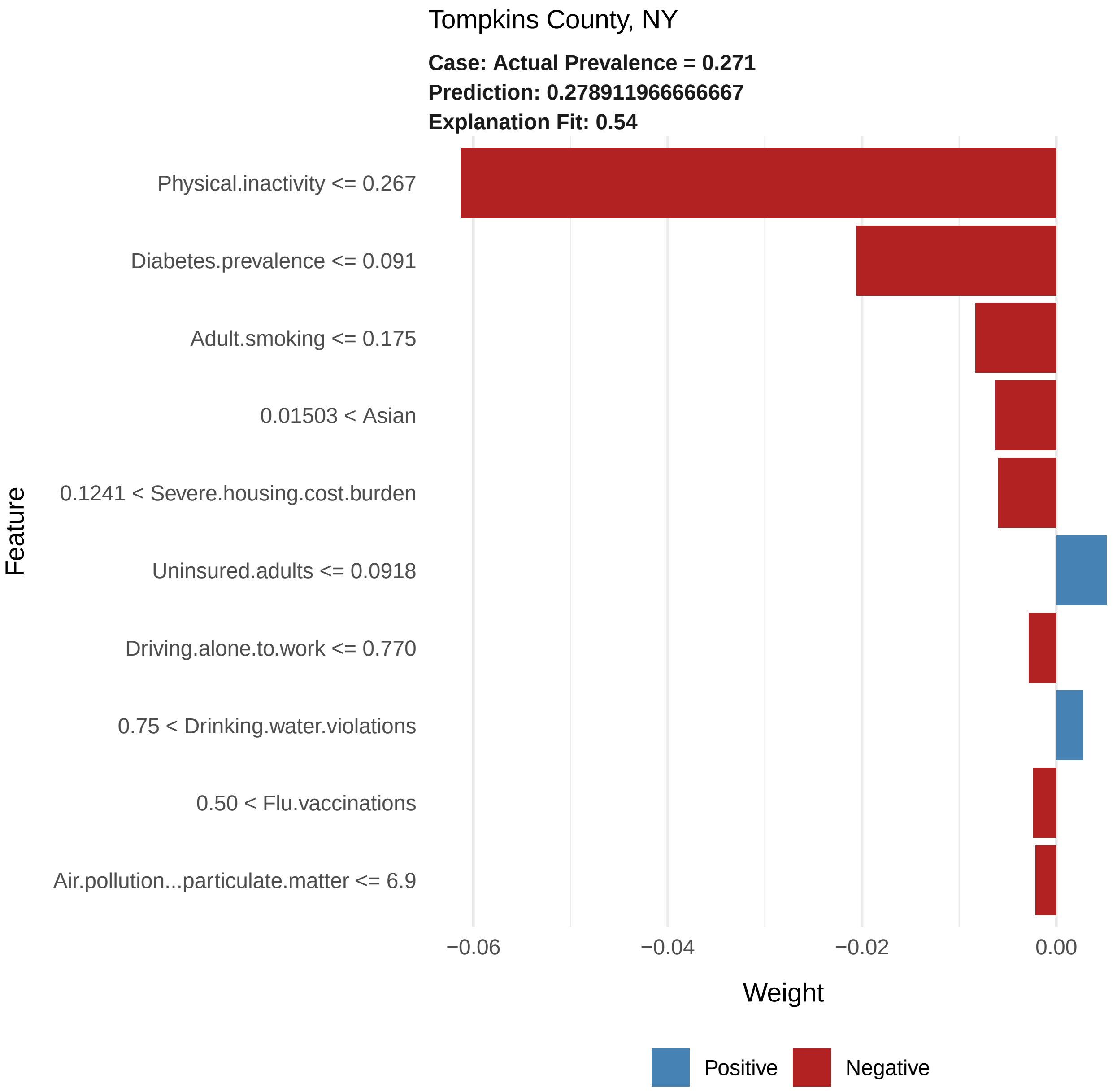
Tompkins County, NY
Case: Actual Prevalence = 0.271
Prediction: 0.278911966666667
Explanation Fit: 0.54
Physical.inactivity <= 0.267
Diabetes.prevalence <= 0.091
Adult.smoking <= 0.175
0.01503 < Asian
0.1241 < Severe.housing.cost.burden
Uninsured.adults <= 0.0918
Driving.alone.to.work <= 0.770
0.75 < Drinking.water.violations
0.50 < Flu.vaccinations
Air.pollution...particulate.matter <= 6.9
Feature
−0.06
−0.04
−0.02
0.00
Weight
Positive
Negative

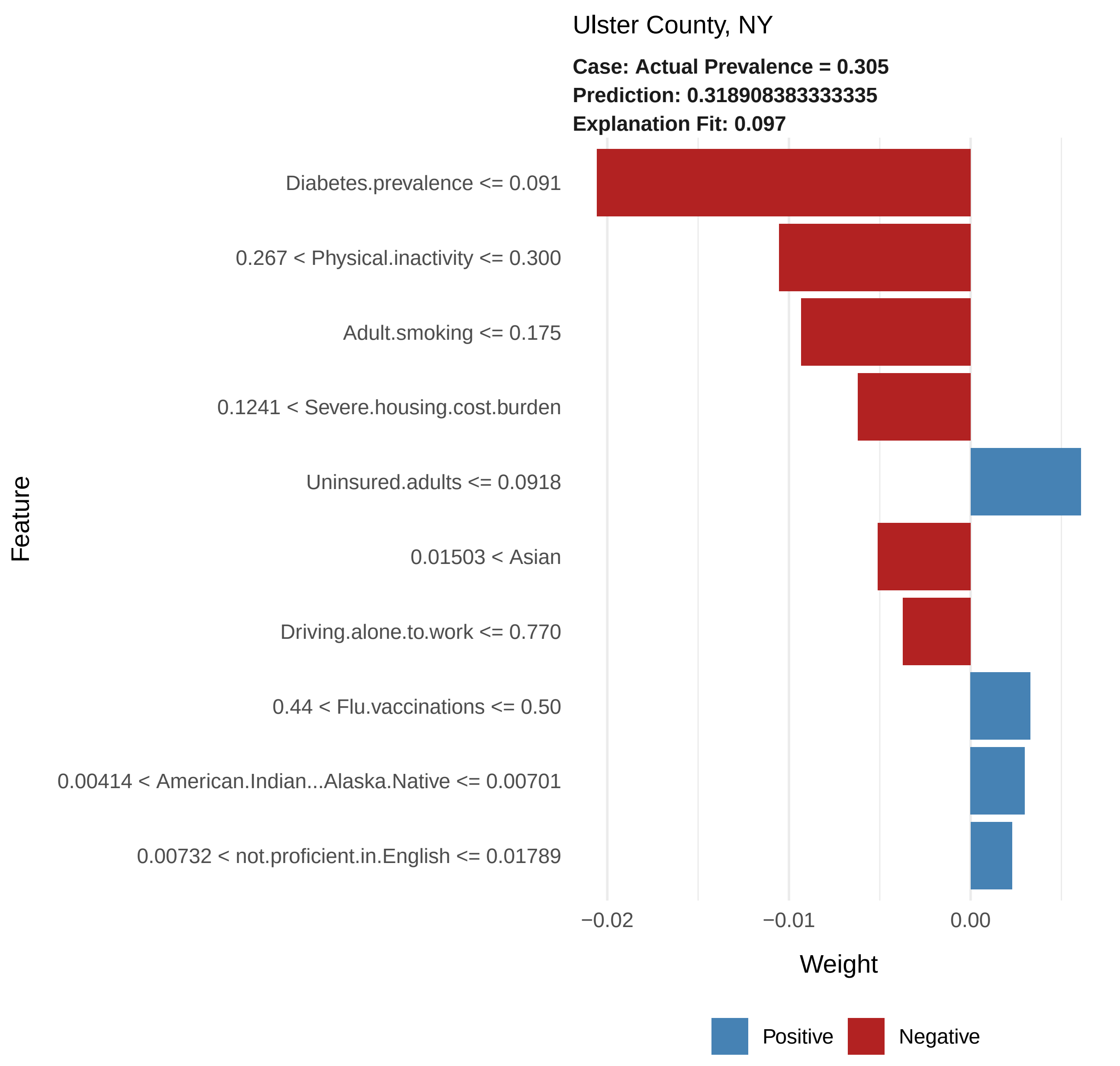
Ulster County, NY
Case: Actual Prevalence = 0.305
Prediction: 0.318908383333335
Explanation Fit: 0.097
Diabetes.prevalence <= 0.091
0.267 < Physical.inactivity <= 0.300
Adult.smoking <= 0.175
0.1241 < Severe.housing.cost.burden
Uninsured.adults <= 0.0918
0.01503 < Asian
Driving.alone.to.work <= 0.770
0.44 < Flu.vaccinations <= 0.50
0.00414 < American.Indian...Alaska.Native <= 0.00701
0.00732 < not.proficient.in.English <= 0.01789
Feature
−0.02
−0.01
0.00
Weight
Positive
Negative

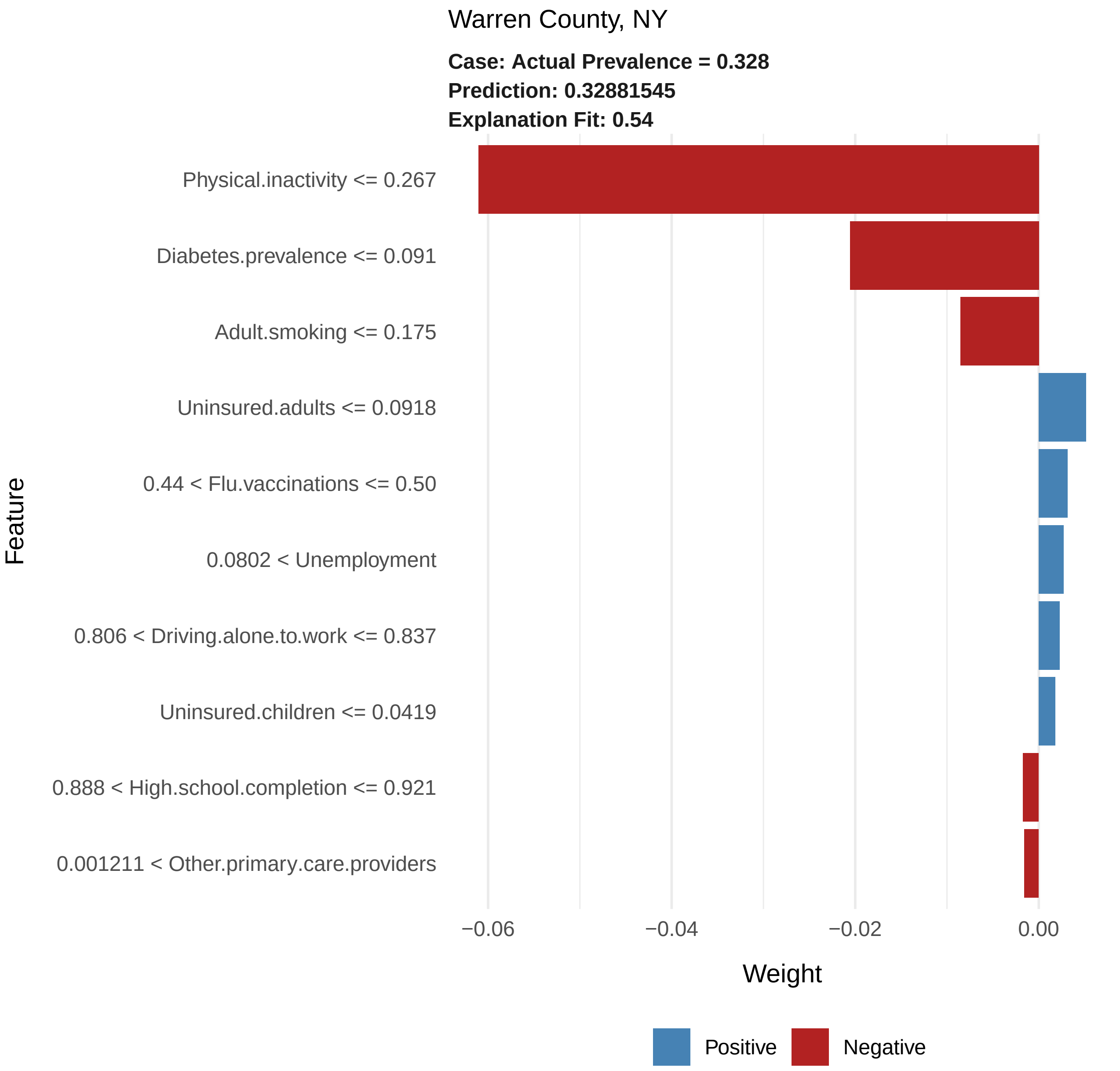
Warren County, NY
Case: Actual Prevalence = 0.328
Prediction: 0.32881545
Explanation Fit: 0.54
Physical.inactivity <= 0.267
Diabetes.prevalence <= 0.091
Adult.smoking <= 0.175
Uninsured.adults <= 0.0918
0.44 < Flu.vaccinations <= 0.50
0.0802 < Unemployment
0.806 < Driving.alone.to.work <= 0.837
Uninsured.children <= 0.0419
0.888 < High.school.completion <= 0.921
0.001211 < Other.primary.care.providers
Feature
−0.06
−0.04
−0.02
0.00
Weight
Positive
Negative

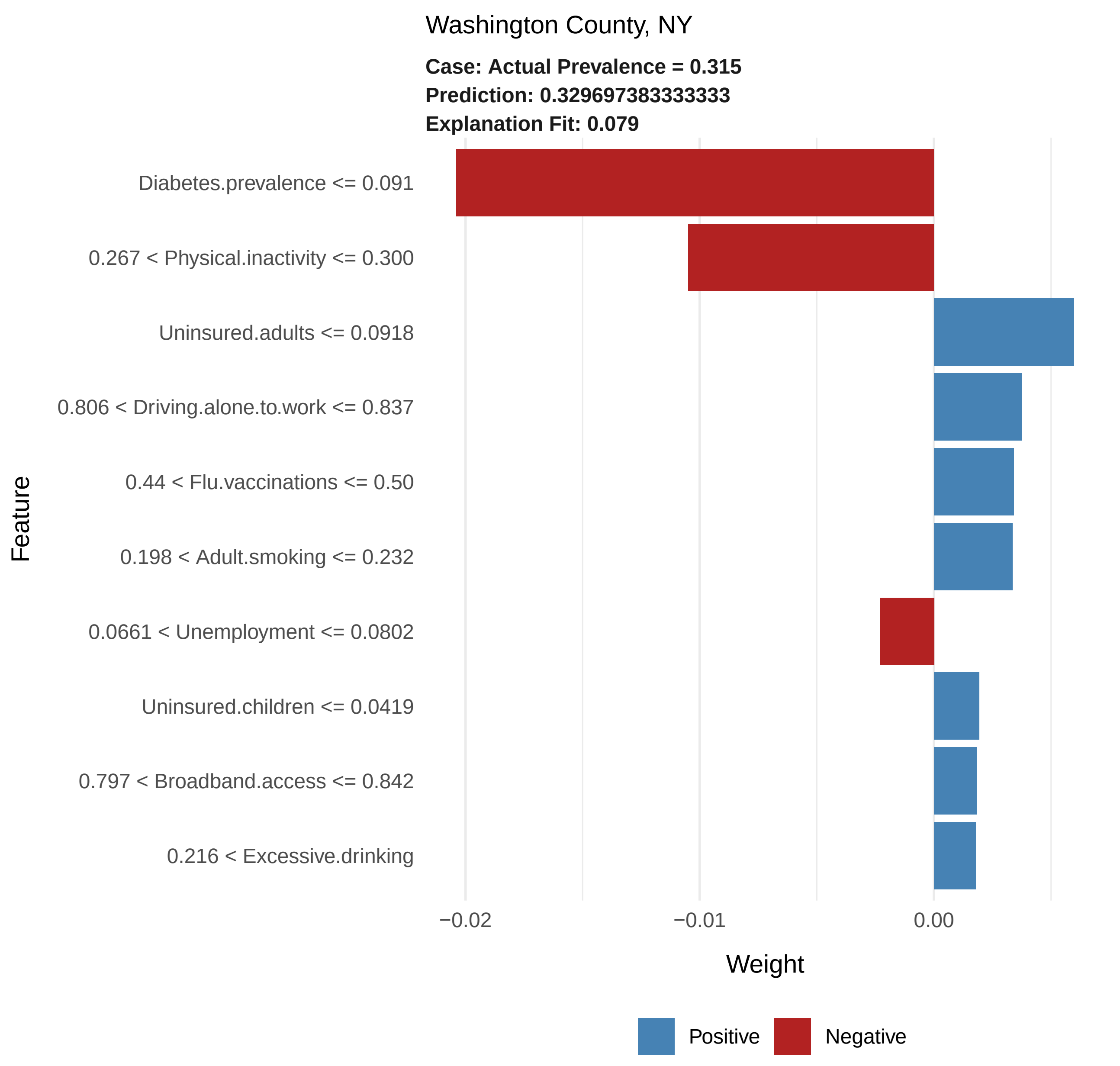
Washington County, NY
Case: Actual Prevalence = 0.315
Prediction: 0.329697383333333
Explanation Fit: 0.079
Diabetes.prevalence <= 0.091
0.267 < Physical.inactivity <= 0.300
Uninsured.adults <= 0.0918
0.806 < Driving.alone.to.work <= 0.837
0.44 < Flu.vaccinations <= 0.50
0.198 < Adult.smoking <= 0.232
0.0661 < Unemployment <= 0.0802
Uninsured.children <= 0.0419
0.797 < Broadband.access <= 0.842
0.216 < Excessive.drinking
Feature
−0.02
−0.01
0.00
Weight
Positive
Negative

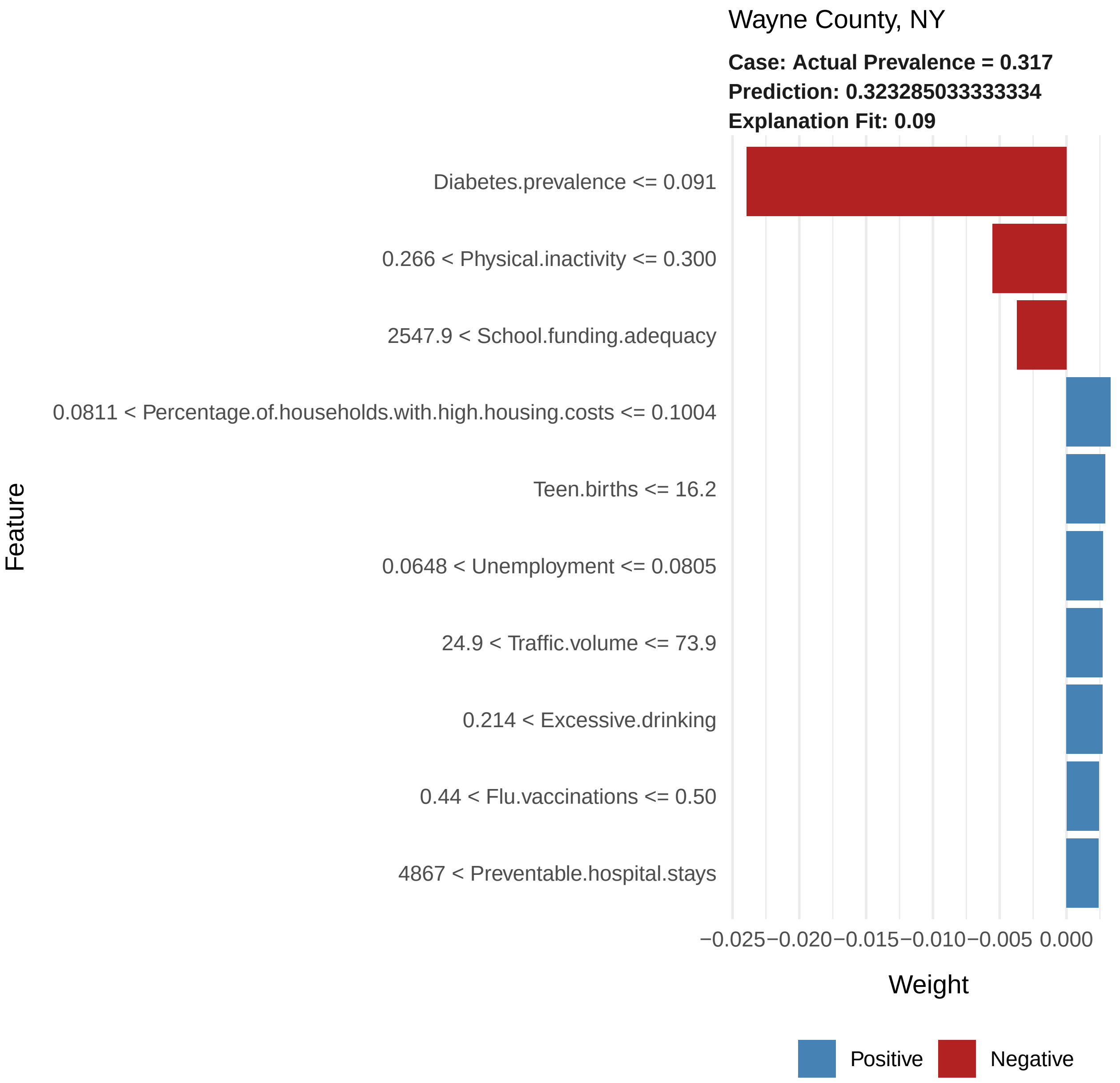

Wayne County, NY
Case: Actual Prevalence = 0.317
Prediction: 0.323285033333334
Explanation Fit: 0.09
Diabetes.prevalence <= 0.091
0.266 < Physical.inactivity <= 0.300
2547.9 < School.funding.adequacy
0.0811 < Percentage.of.households.with.high.housing.costs <= 0.1004
Teen.births <= 16.2
0.0648 < Unemployment <= 0.0805
24.9 < Traffic.volume <= 73.9
0.214 < Excessive.drinking
0.44 < Flu.vaccinations <= 0.50
4867 < Preventable.hospital.stays
Feature
−0.025
−0.020
−0.015
−0.010
−0.005
0.000
Weight
Positive
Negative

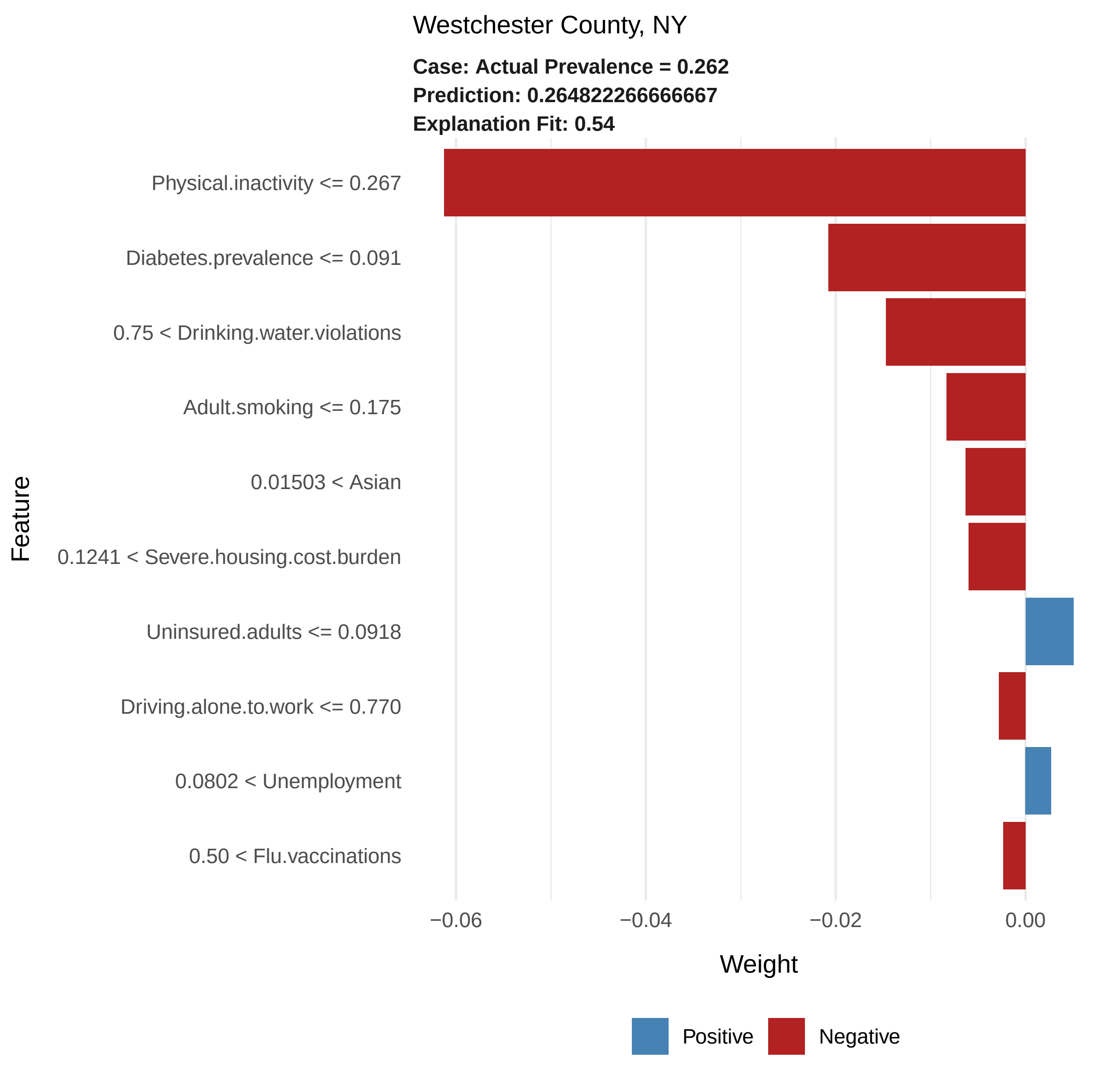
Westchester County, NY
Case: Actual Prevalence = 0.262
Prediction: 0.264822266666667
Explanation Fit: 0.54
Physical.inactivity <= 0.267
Diabetes.prevalence <= 0.091
0.75 < Drinking.water.violations
Adult.smoking <= 0.175
0.01503 < Asian
0.1241 < Severe.housing.cost.burden
Uninsured.adults <= 0.0918
Driving.alone.to.work <= 0.770
0.0802 < Unemployment
0.50 < Flu.vaccinations
Feature
−0.06
−0.04
−0.02
0.00
Weight
Positive
Negative

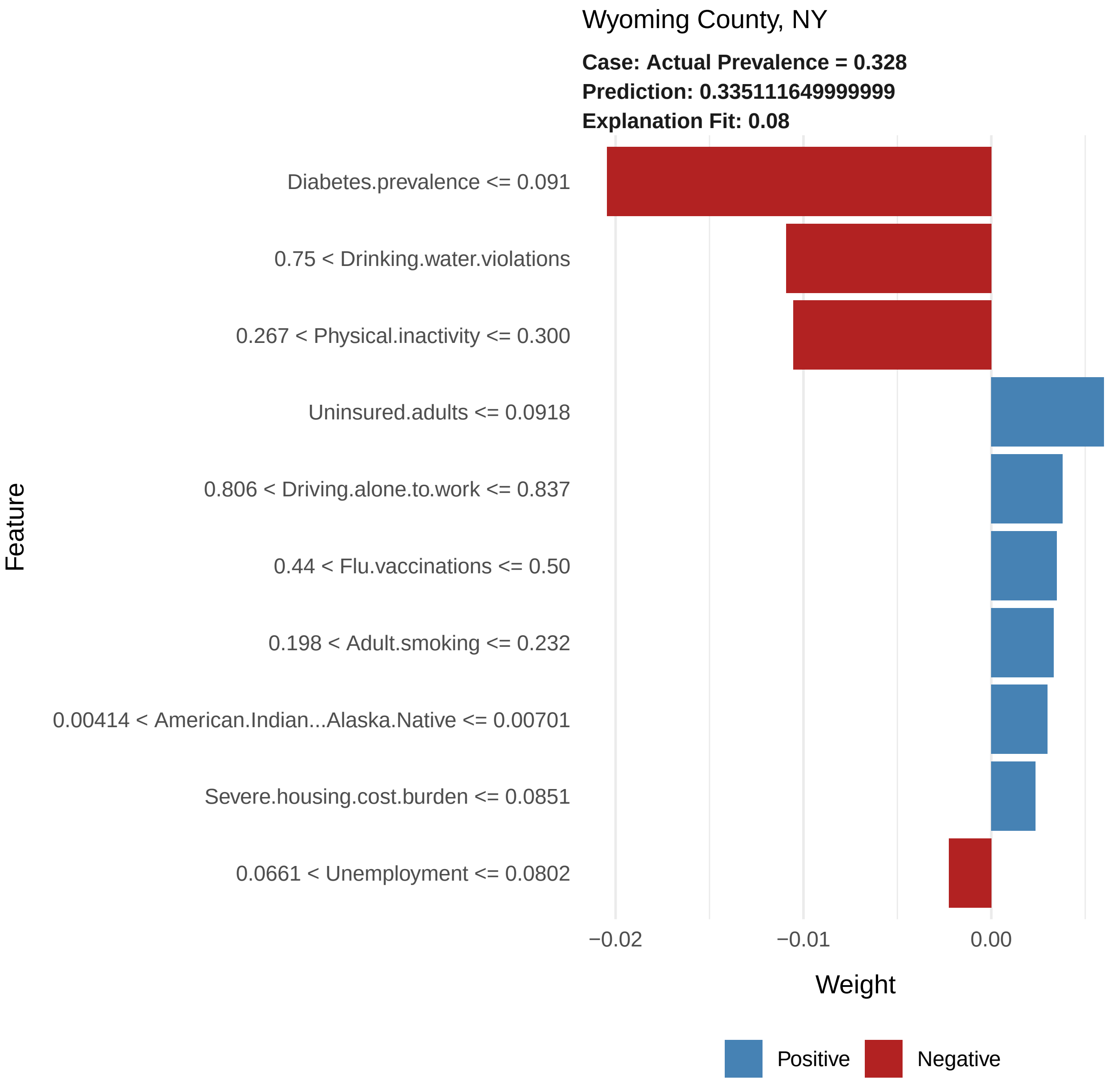

Wyoming County, NY
Case: Actual Prevalence = 0.328
Prediction: 0.335111649999999
Explanation Fit: 0.08
Diabetes.prevalence <= 0.091
0.75 < Drinking.water.violations
0.267 < Physical.inactivity <= 0.300
Uninsured.adults <= 0.0918
0.806 < Driving.alone.to.work <= 0.837
0.44 < Flu.vaccinations <= 0.50
0.198 < Adult.smoking <= 0.232
0.00414 < American.Indian...Alaska.Native <= 0.00701
Severe.housing.cost.burden <= 0.0851
0.0661 < Unemployment <= 0.0802
Feature
−0.02
−0.01
0.00
Weight
Positive
Negative

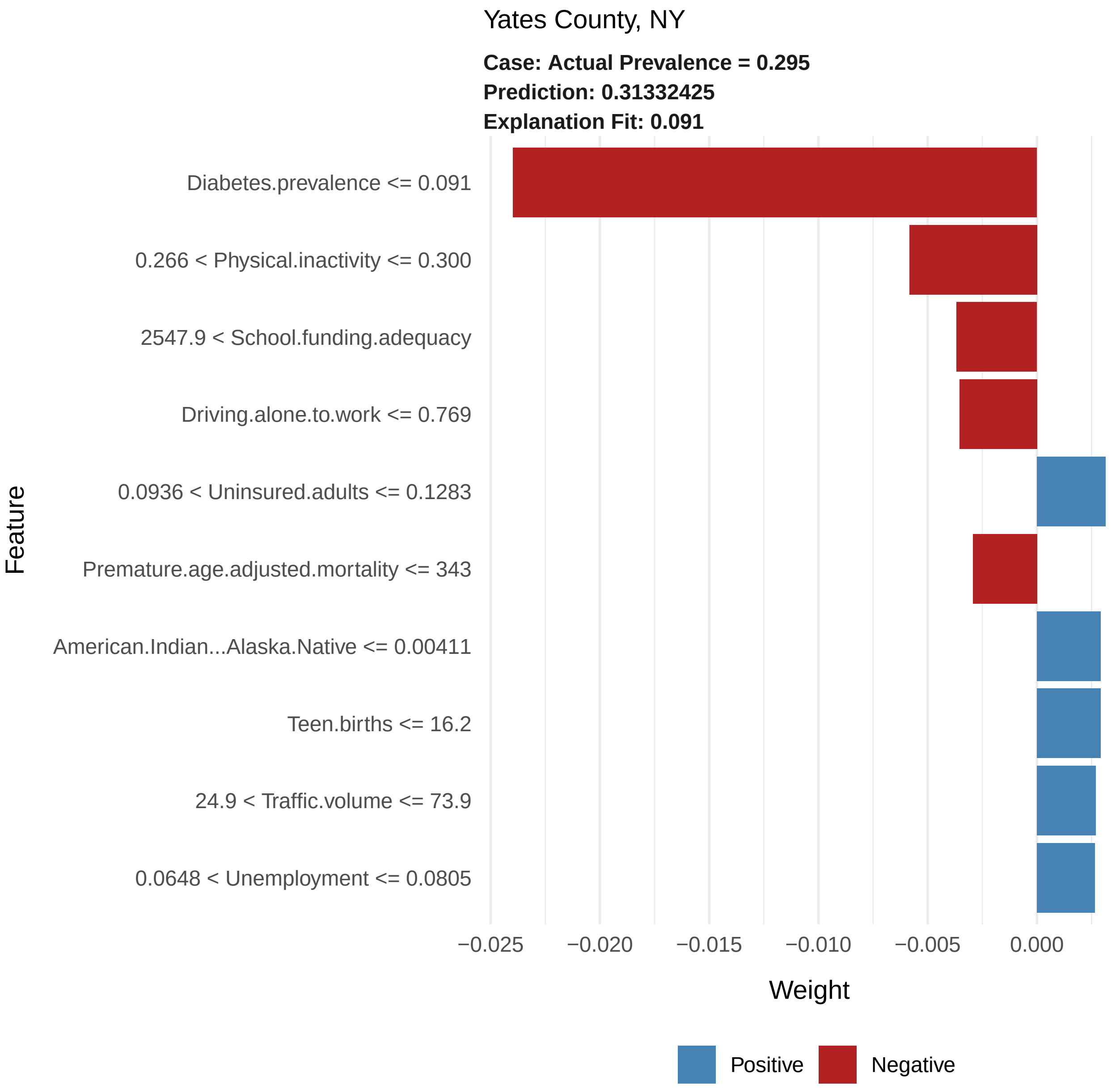

Yates County, NY
Case: Actual Prevalence = 0.295
Prediction: 0.31332425
Explanation Fit: 0.091
Diabetes.prevalence <= 0.091
0.266 < Physical.inactivity <= 0.300
2547.9 < School.funding.adequacy
Driving.alone.to.work <= 0.769
0.0936 < Uninsured.adults <= 0.1283
Premature.age.adjusted.mortality <= 343
American.Indian...Alaska.Native <= 0.00411
Teen.births <= 16.2
24.9 < Traffic.volume <= 73.9
0.0648 < Unemployment <= 0.0805
Feature
−0.025
−0.020
−0.015
−0.010
−0.005
0.000
Weight
Positive
Negative

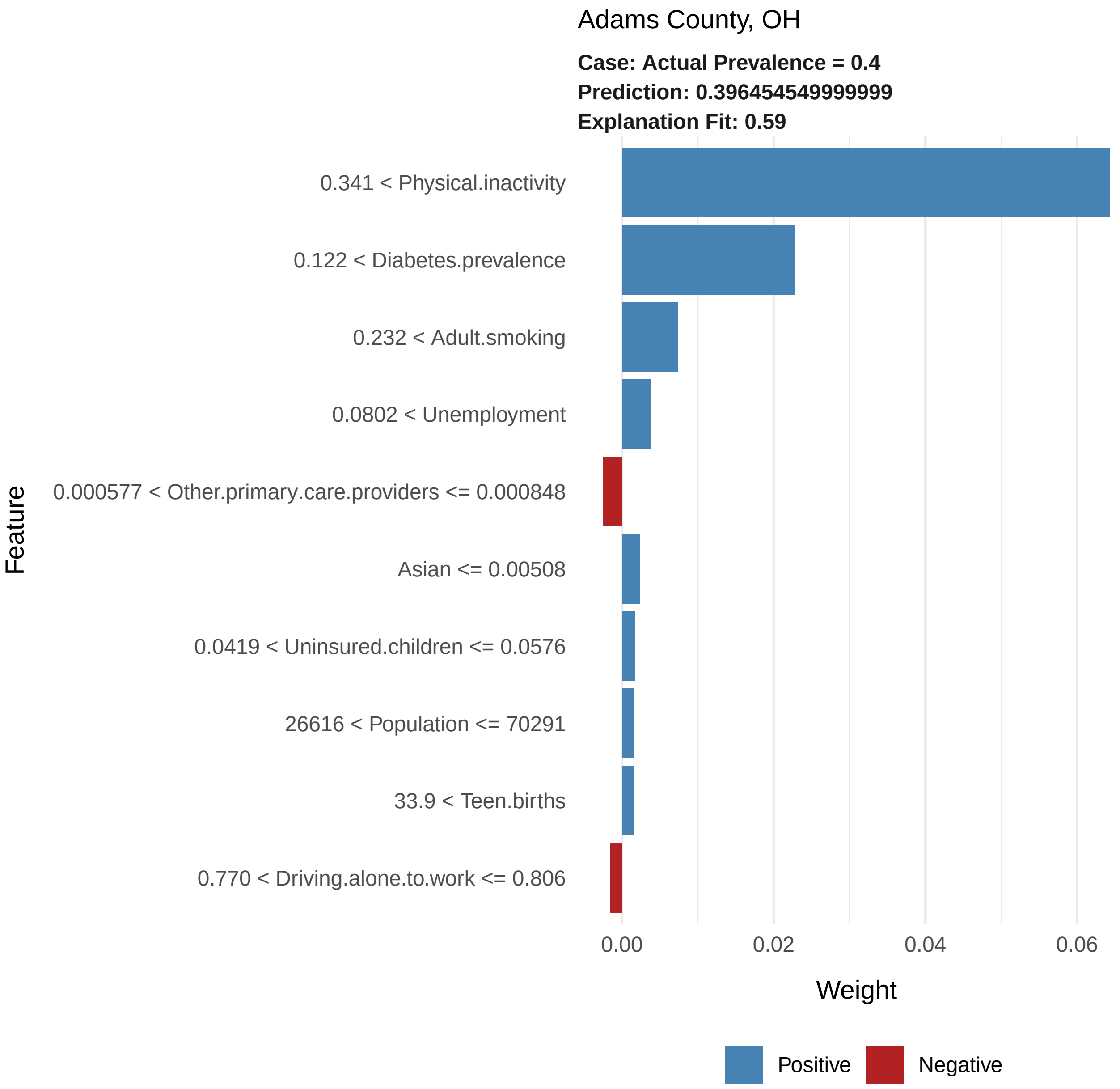
Adams County, OH
Case: Actual Prevalence = 0.4
Prediction: 0.396454549999999
Explanation Fit: 0.59
0.341 < Physical.inactivity
0.122 < Diabetes.prevalence
0.232 < Adult.smoking
0.0802 < Unemployment
0.000577 < Other.primary.care.providers <= 0.000848
Asian <= 0.00508
0.0419 < Uninsured.children <= 0.0576
26616 < Population <= 70291
33.9 < Teen.births
0.770 < Driving.alone.to.work <= 0.806
Feature
0.00
0.02
0.04
0.06
Weight
Positive
Negative

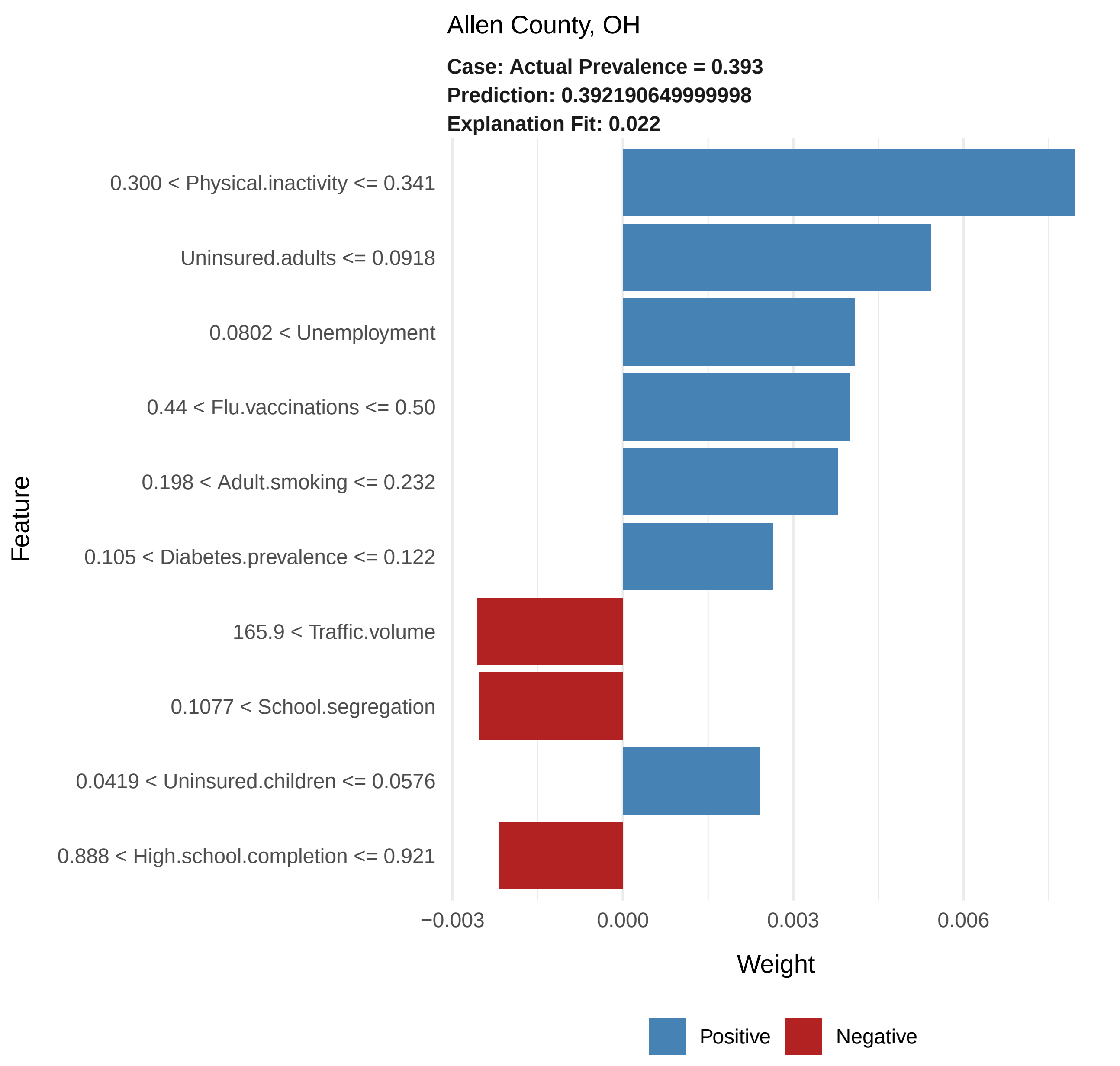

Allen County, OH
Case: Actual Prevalence = 0.393
Prediction: 0.392190649999998
Explanation Fit: 0.022
0.300 < Physical.inactivity <= 0.341
Uninsured.adults <= 0.0918
0.0802 < Unemployment
0.44 < Flu.vaccinations <= 0.50
0.198 < Adult.smoking <= 0.232
0.105 < Diabetes.prevalence <= 0.122
165.9 < Traffic.volume
0.1077 < School.segregation
0.0419 < Uninsured.children <= 0.0576
0.888 < High.school.completion <= 0.921
Feature
−0.003
0.000
0.003
0.006
Weight
Positive
Negative

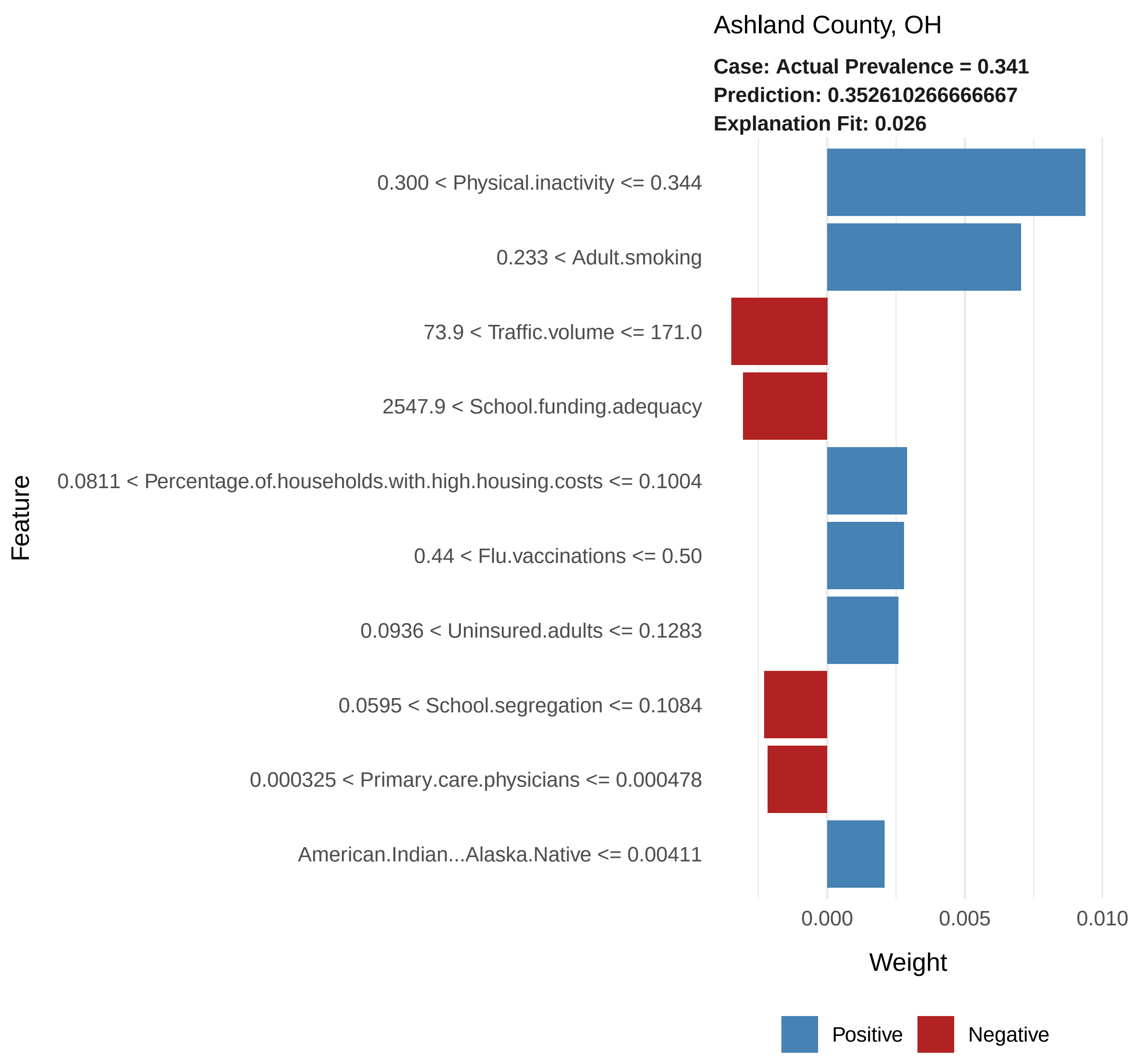
Ashland County, OH
Case: Actual Prevalence = 0.341
Prediction: 0.352610266666667
Explanation Fit: 0.026
0.300 < Physical.inactivity <= 0.344
0.233 < Adult.smoking
73.9 < Traffic.volume <= 171.0
2547.9 < School.funding.adequacy
0.0811 < Percentage.of.households.with.high.housing.costs <= 0.1004
0.44 < Flu.vaccinations <= 0.50
0.0936 < Uninsured.adults <= 0.1283
0.0595 < School.segregation <= 0.1084
0.000325 < Primary.care.physicians <= 0.000478
American.Indian...Alaska.Native <= 0.00411
Feature
0.000
0.005
0.010
Weight
Positive
Negative

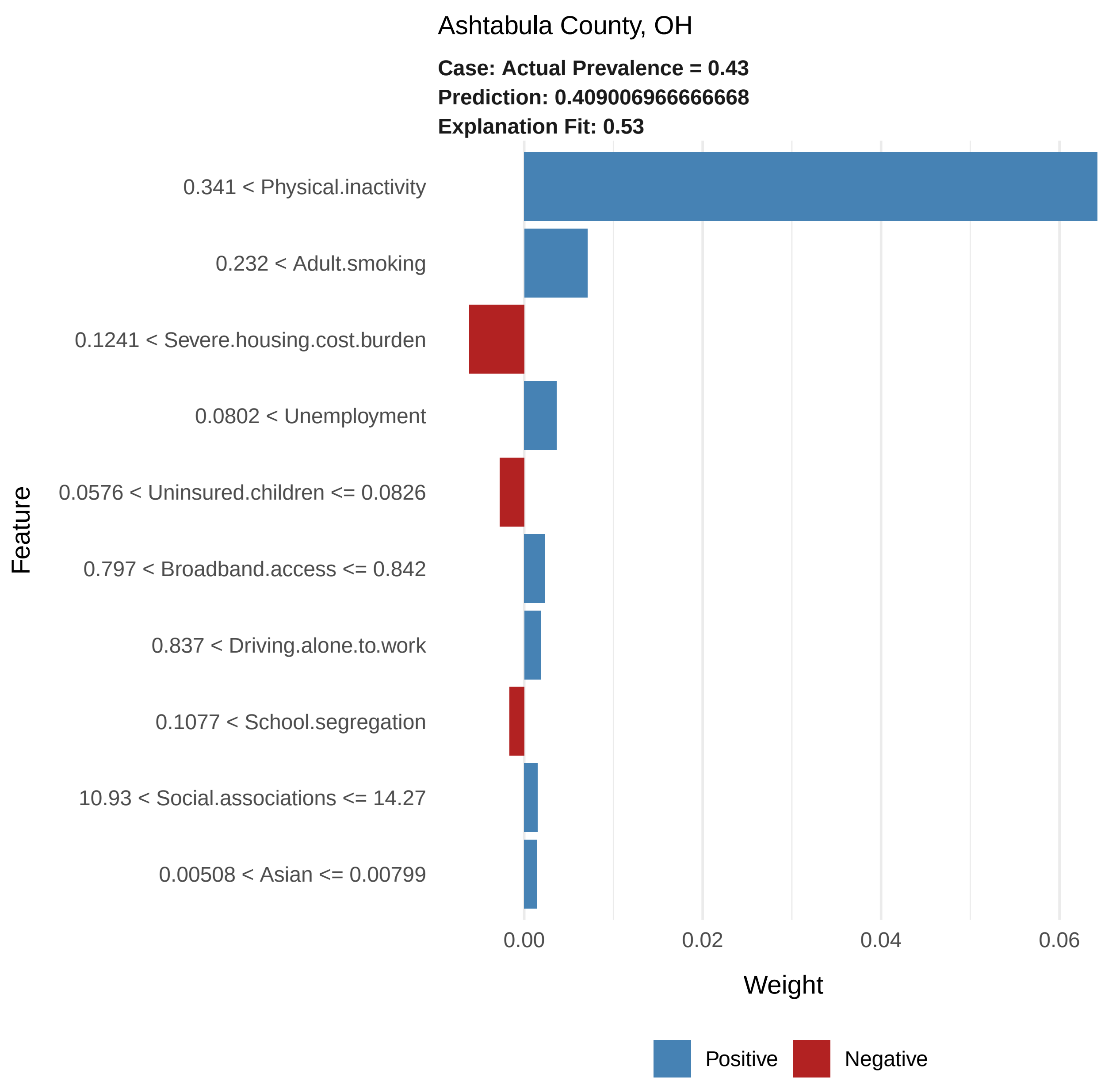
Ashtabula County, OH
Case: Actual Prevalence = 0.43
Prediction: 0.409006966666668
Explanation Fit: 0.53
0.341 < Physical.inactivity
0.232 < Adult.smoking
0.1241 < Severe.housing.cost.burden
0.0802 < Unemployment
0.0576 < Uninsured.children <= 0.0826
0.797 < Broadband.access <= 0.842
0.837 < Driving.alone.to.work
0.1077 < School.segregation
10.93 < Social.associations <= 14.27
0.00508 < Asian <= 0.00799
Feature
0.00
0.02
0.04
0.06
Weight
Positive
Negative

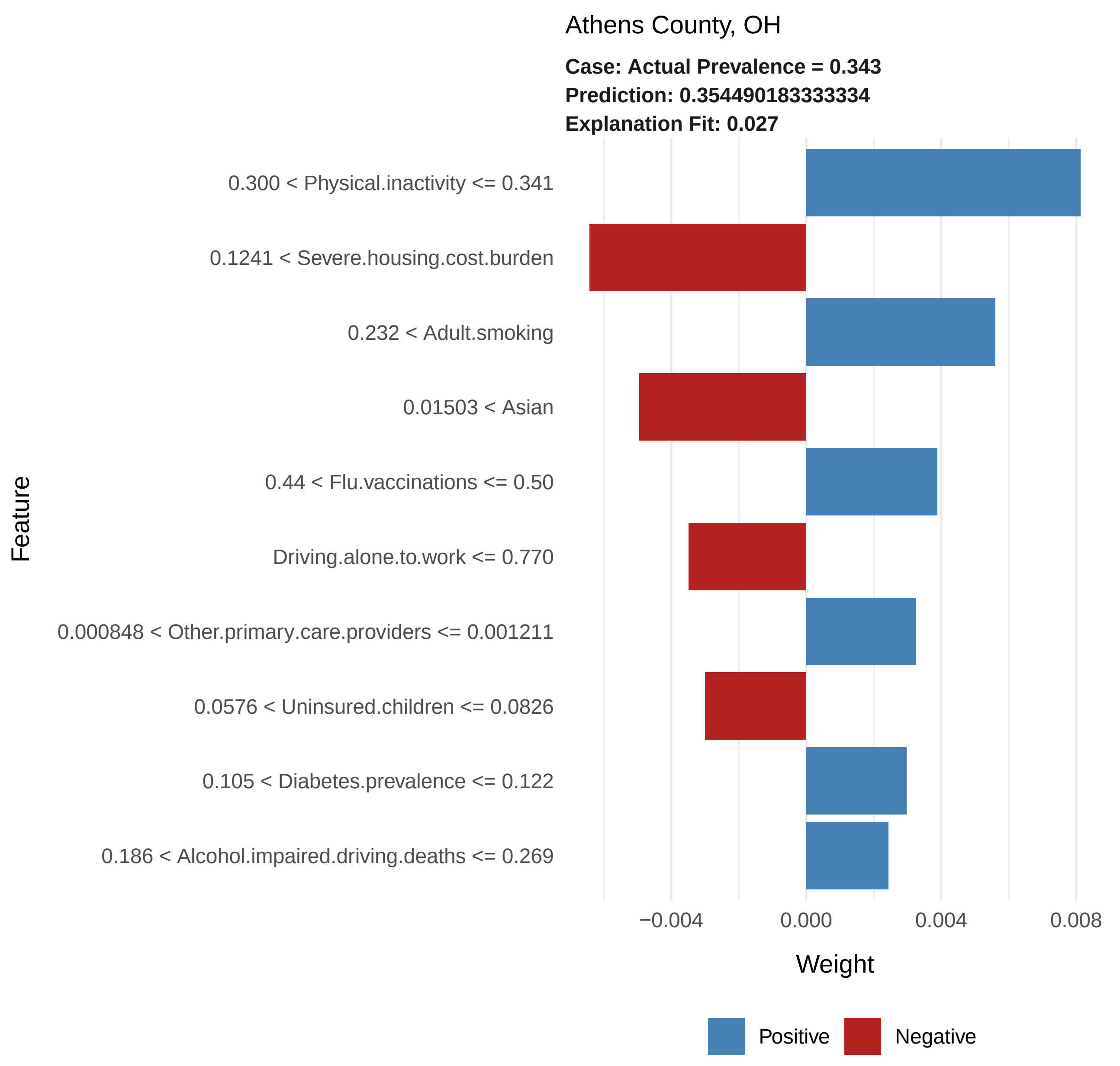

Athens County, OH
Case: Actual Prevalence = 0.343
Prediction: 0.354490183333334
Explanation Fit: 0.027
0.300 < Physical.inactivity <= 0.341
0.1241 < Severe.housing.cost.burden
0.232 < Adult.smoking
0.01503 < Asian
0.44 < Flu.vaccinations <= 0.50
Driving.alone.to.work <= 0.770
0.000848 < Other.primary.care.providers <= 0.001211
0.0576 < Uninsured.children <= 0.0826
0.105 < Diabetes.prevalence <= 0.122
0.186 < Alcohol.impaired.driving.deaths <= 0.269
Feature
−0.004
0.000
0.004
0.008
Weight
Positive
Negative

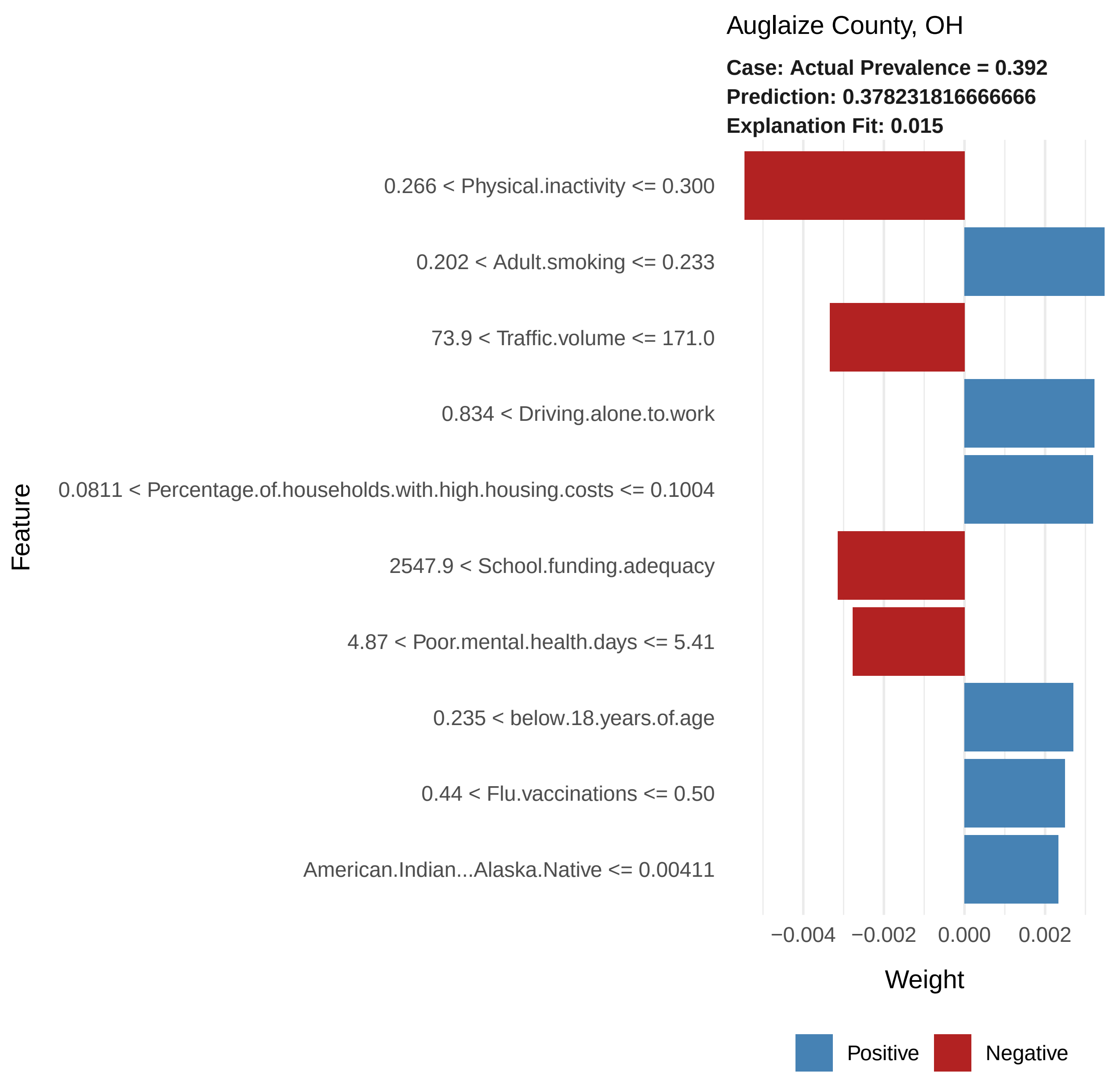

Auglaize County, OH
Case: Actual Prevalence = 0.392
Prediction: 0.378231816666666
Explanation Fit: 0.015
0.266 < Physical.inactivity <= 0.300
0.202 < Adult.smoking <= 0.233
73.9 < Traffic.volume <= 171.0
0.834 < Driving.alone.to.work
0.0811 < Percentage.of.households.with.high.housing.costs <= 0.1004
2547.9 < School.funding.adequacy
4.87 < Poor.mental.health.days <= 5.41
0.235 < below.18.years.of.age
0.44 < Flu.vaccinations <= 0.50
American.Indian...Alaska.Native <= 0.00411
Feature
−0.004
−0.002
0.000
0.002
Weight
Positive
Negative

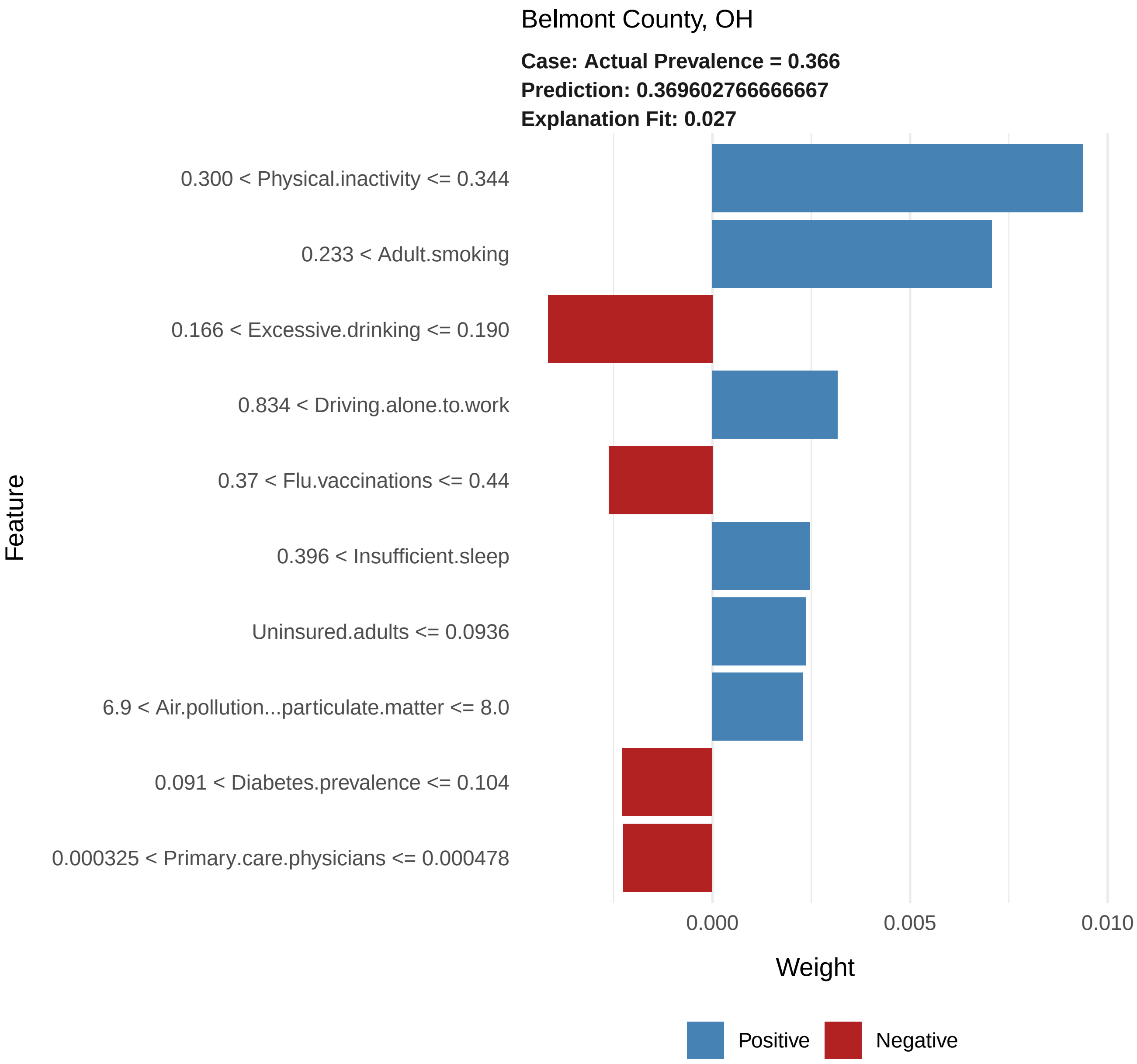

Belmont County, OH
Case: Actual Prevalence = 0.366
Prediction: 0.369602766666667
Explanation Fit: 0.027
0.300 < Physical.inactivity <= 0.344
0.233 < Adult.smoking
0.166 < Excessive.drinking <= 0.190
0.834 < Driving.alone.to.work
0.37 < Flu.vaccinations <= 0.44
0.396 < Insufficient.sleep
Uninsured.adults <= 0.0936
6.9 < Air.pollution...particulate.matter <= 8.0
0.091 < Diabetes.prevalence <= 0.104
0.000325 < Primary.care.physicians <= 0.000478
Feature
0.000
0.005
0.010
Weight
Positive
Negative

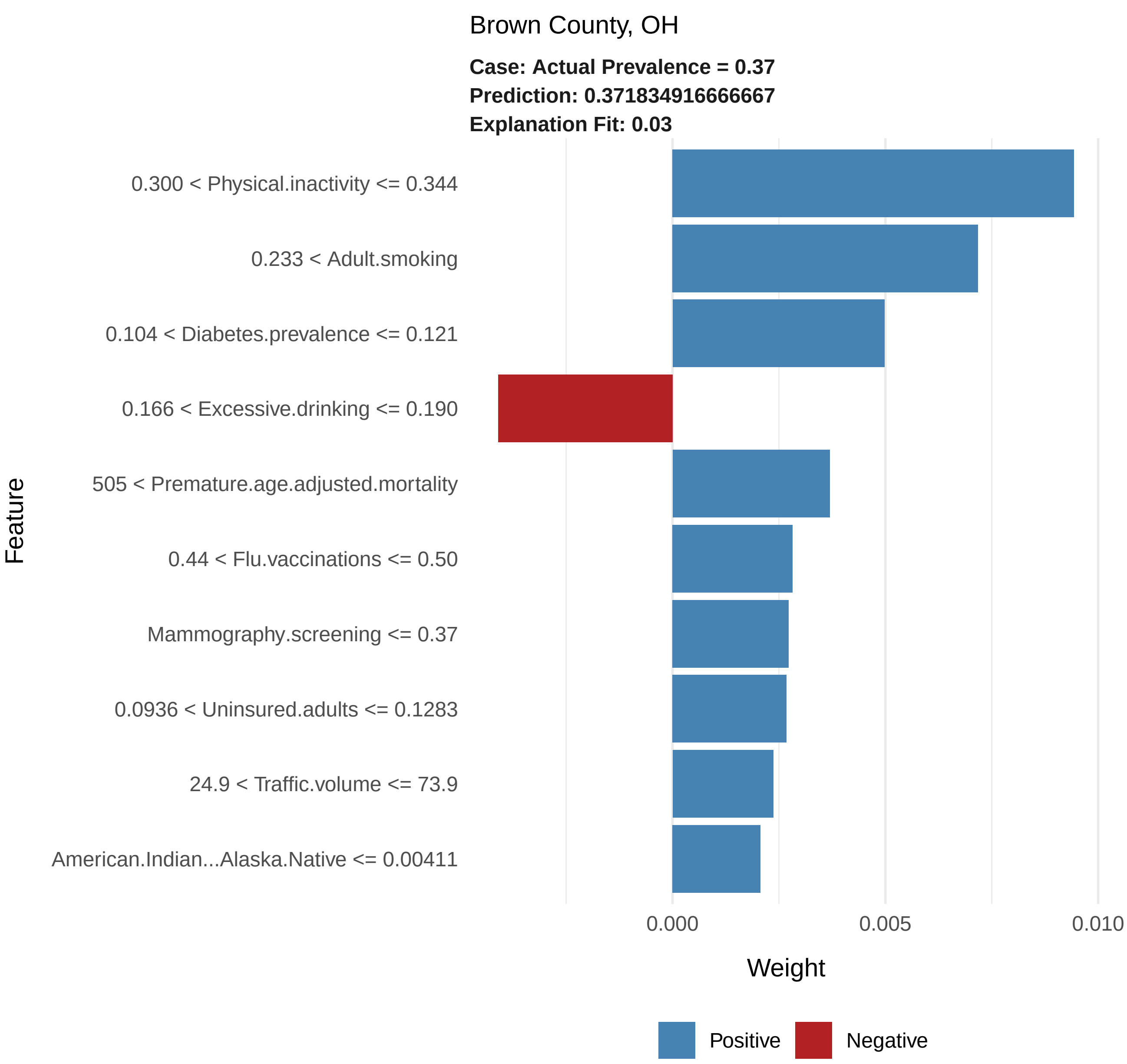

Brown County, OH
Case: Actual Prevalence = 0.37
Prediction: 0.371834916666667
Explanation Fit: 0.03
0.300 < Physical.inactivity <= 0.344
0.233 < Adult.smoking
0.104 < Diabetes.prevalence <= 0.121
0.166 < Excessive.drinking <= 0.190
505 < Premature.age.adjusted.mortality
0.44 < Flu.vaccinations <= 0.50
Mammography.screening <= 0.37
0.0936 < Uninsured.adults <= 0.1283
24.9 < Traffic.volume <= 73.9
American.Indian...Alaska.Native <= 0.00411
Feature
0.000
0.005
0.010
Weight
Positive
Negative

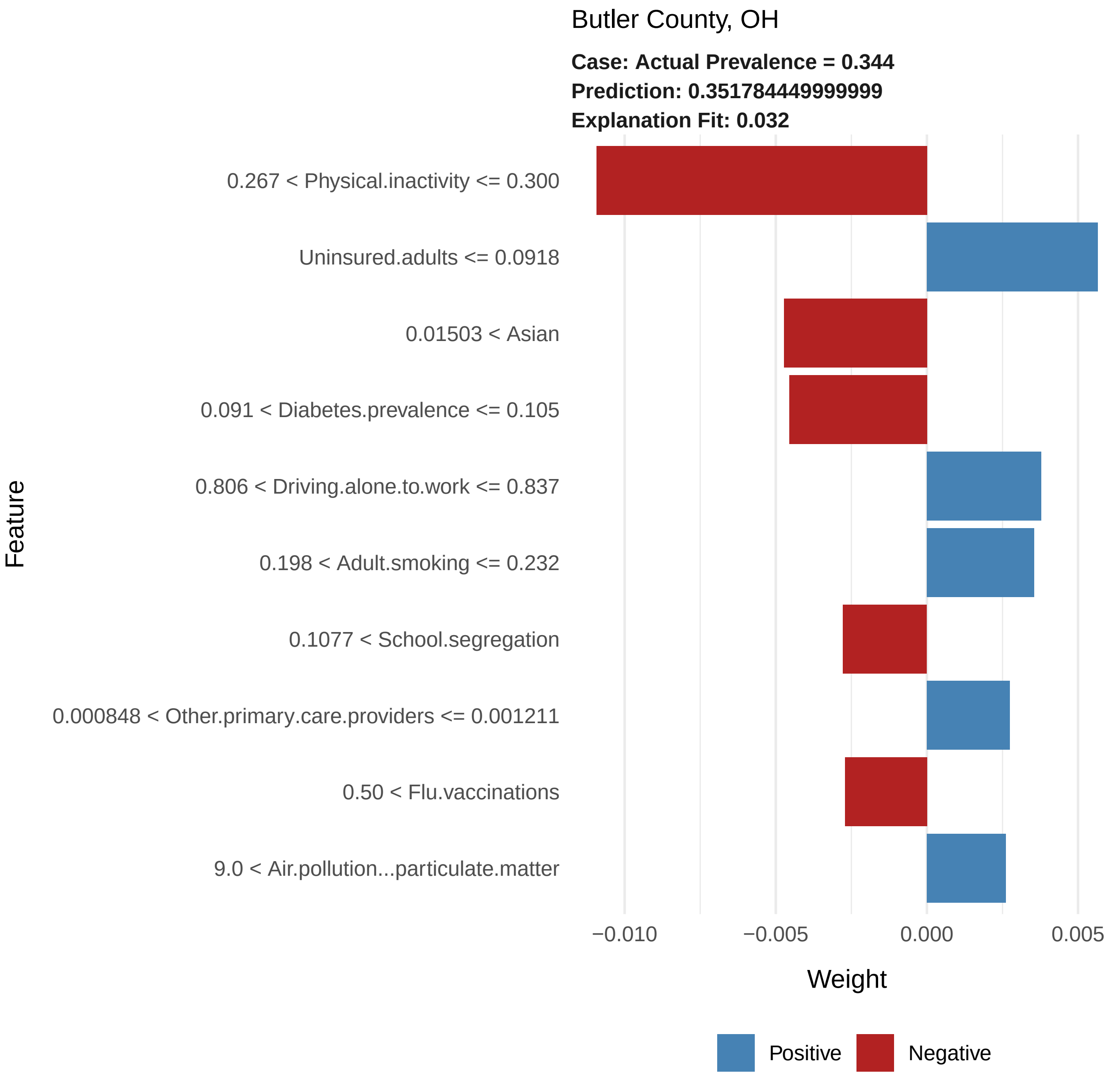
Butler County, OH
Case: Actual Prevalence = 0.344
Prediction: 0.351784449999999
Explanation Fit: 0.032
0.267 < Physical.inactivity <= 0.300
Uninsured.adults <= 0.0918
0.01503 < Asian
0.091 < Diabetes.prevalence <= 0.105
0.806 < Driving.alone.to.work <= 0.837
0.198 < Adult.smoking <= 0.232
0.1077 < School.segregation
0.000848 < Other.primary.care.providers <= 0.001211
0.50 < Flu.vaccinations
9.0 < Air.pollution...particulate.matter
Feature
−0.010
−0.005
0.000
0.005
Weight
Positive
Negative

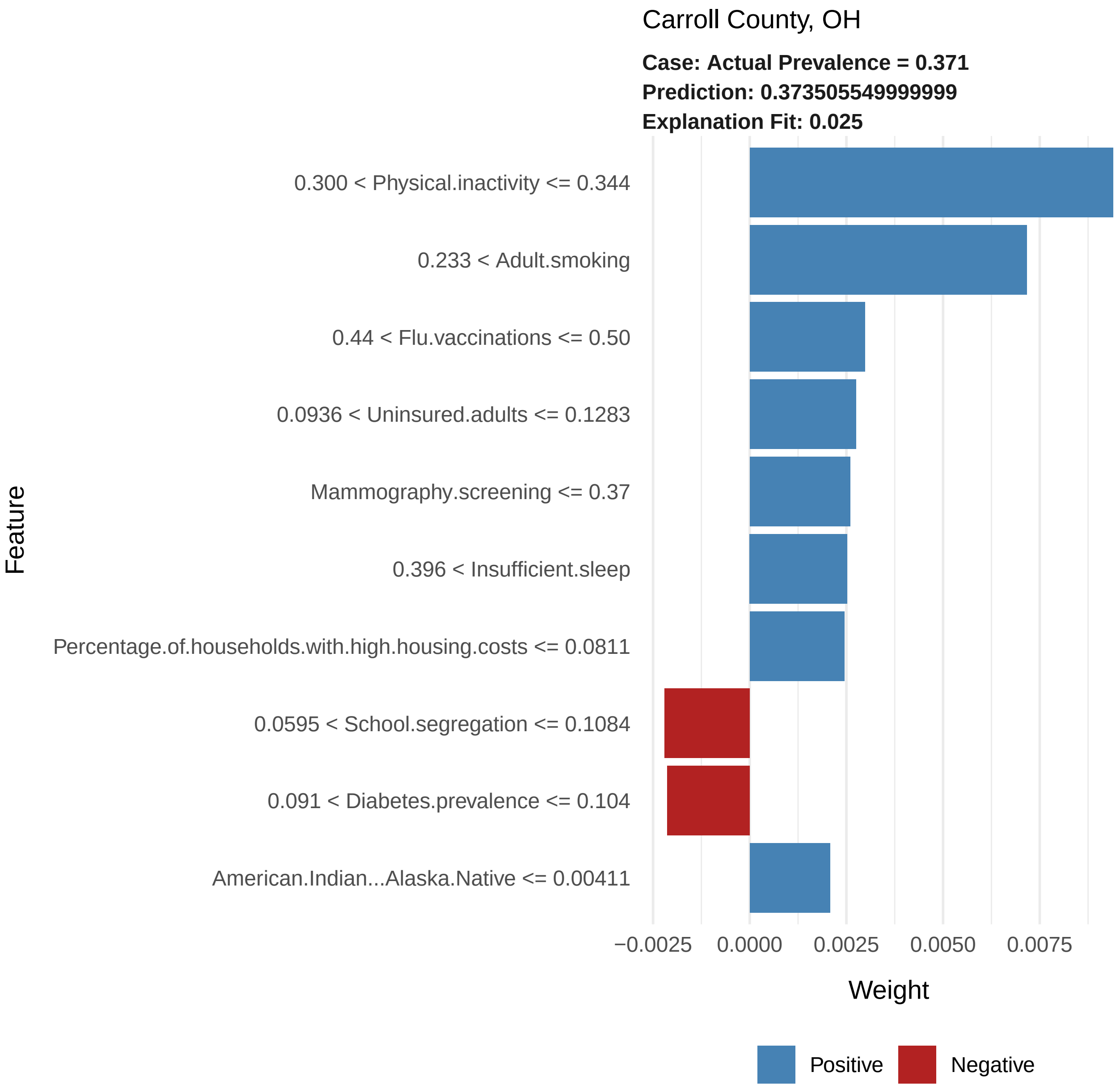

Carroll County, OH
Case: Actual Prevalence = 0.371
Prediction: 0.373505549999999
Explanation Fit: 0.025
Feature
0.300 < Physical.inactivity <= 0.344
0.233 < Adult.smoking
0.44 < Flu.vaccinations <= 0.50
0.0936 < Uninsured.adults <= 0.1283
Mammography.screening <= 0.37
0.396 < Insufficient.sleep
Percentage.of.households.with.high.housing.costs <= 0.0811
0.0595 < School.segregation <= 0.1084
0.091 < Diabetes.prevalence <= 0.104
American.Indian...Alaska.Native <= 0.00411
−0.0025
0.0000
0.0025
0.0050
0.0075
Weight
Positive
Negative

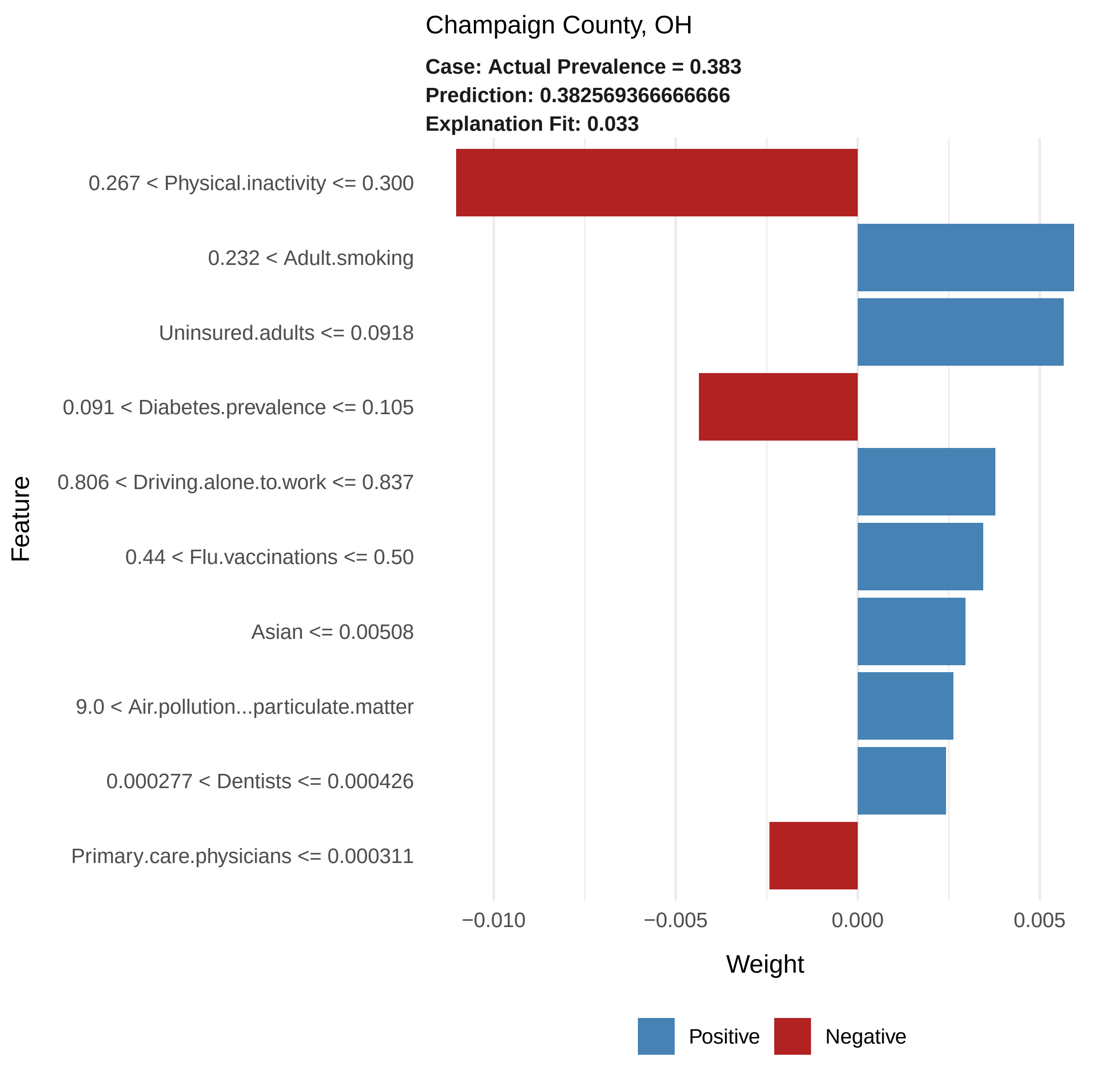

Champaign County, OH
Case: Actual Prevalence = 0.383
Prediction: 0.382569366666666
Explanation Fit: 0.033
0.267 < Physical.inactivity <= 0.300
0.232 < Adult.smoking
Uninsured.adults <= 0.0918
0.091 < Diabetes.prevalence <= 0.105
0.806 < Driving.alone.to.work <= 0.837
0.44 < Flu.vaccinations <= 0.50
Asian <= 0.00508
9.0 < Air.pollution...particulate.matter
0.000277 < Dentists <= 0.000426
Primary.care.physicians <= 0.000311
Feature
−0.010
−0.005
0.000
0.005
Weight
Positive
Negative

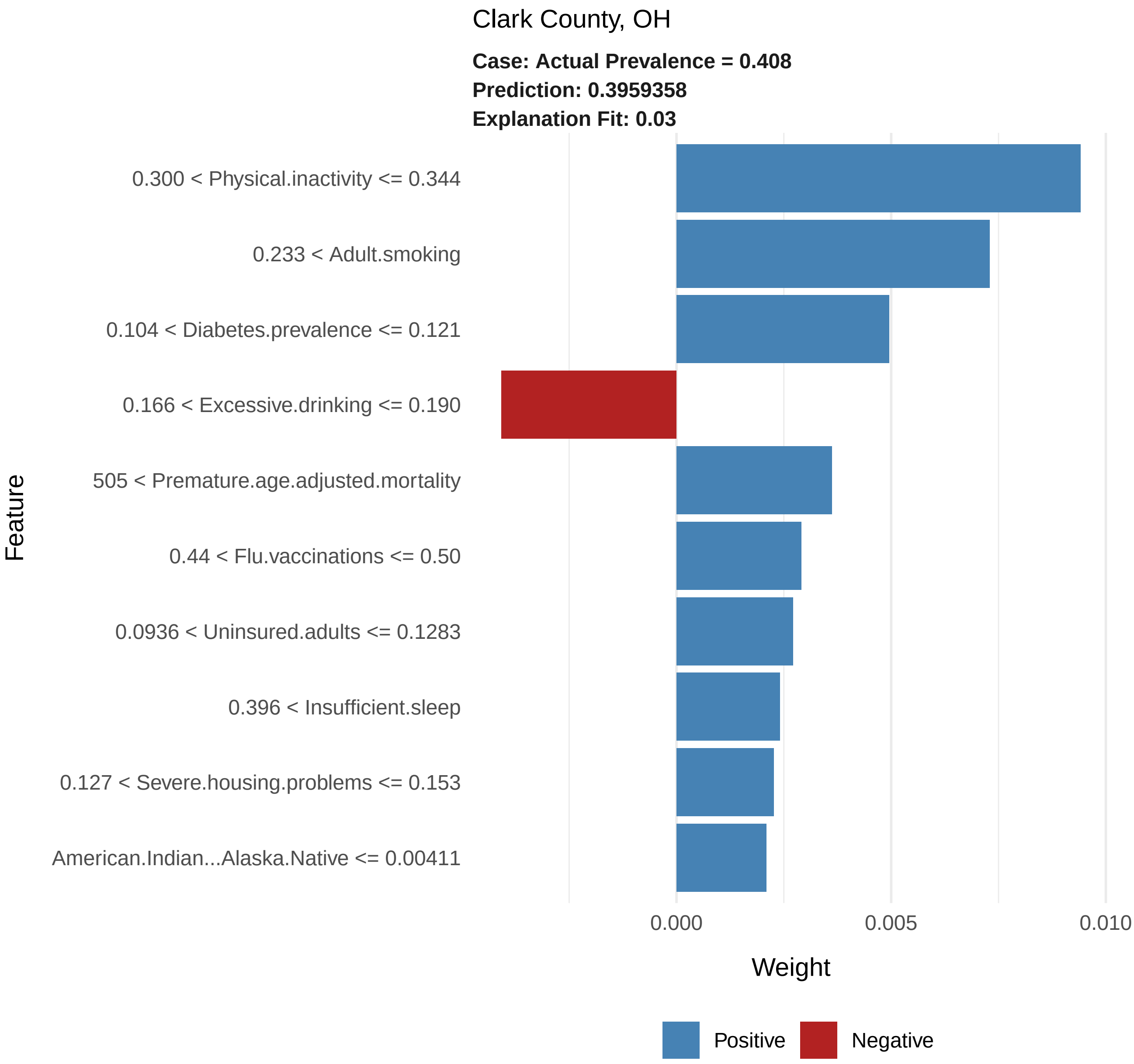
Clark County, OH
Case: Actual Prevalence = 0.408
Prediction: 0.3959358
Explanation Fit: 0.03
0.300 < Physical.inactivity <= 0.344
0.233 < Adult.smoking
0.104 < Diabetes.prevalence <= 0.121
0.166 < Excessive.drinking <= 0.190
505 < Premature.age.adjusted.mortality
0.44 < Flu.vaccinations <= 0.50
0.0936 < Uninsured.adults <= 0.1283
0.396 < Insufficient.sleep
0.127 < Severe.housing.problems <= 0.153
American.Indian...Alaska.Native <= 0.00411
Feature
0.000
0.005
0.010
Weight
Positive
Negative

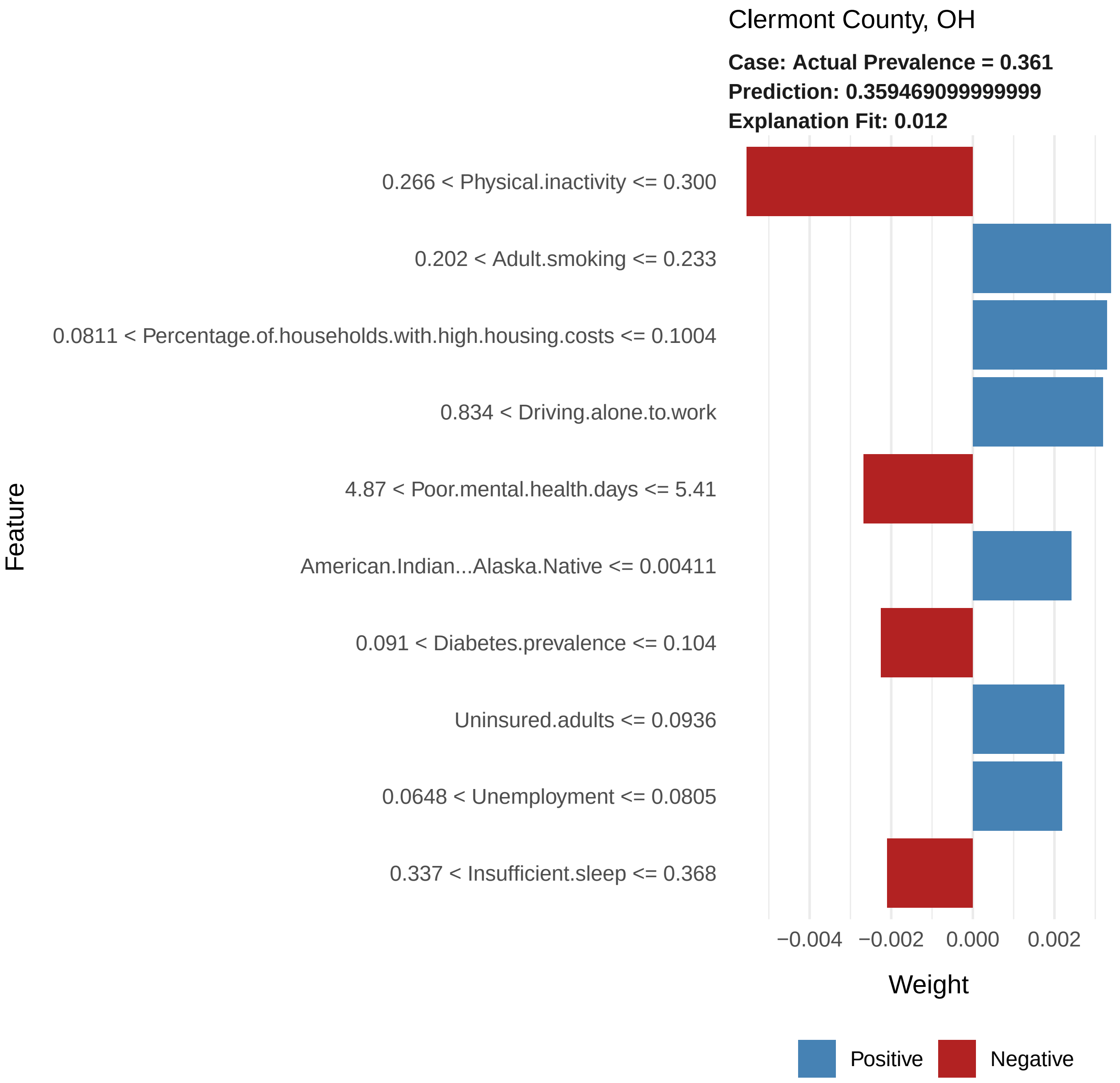

Clermont County, OH
Case: Actual Prevalence = 0.361
Prediction: 0.359469099999999
Explanation Fit: 0.012
0.266 < Physical.inactivity <= 0.300
0.202 < Adult.smoking <= 0.233
0.0811 < Percentage.of.households.with.high.housing.costs <= 0.1004
0.834 < Driving.alone.to.work
4.87 < Poor.mental.health.days <= 5.41
American.Indian...Alaska.Native <= 0.00411
0.091 < Diabetes.prevalence <= 0.104
Uninsured.adults <= 0.0936
0.0648 < Unemployment <= 0.0805
0.337 < Insufficient.sleep <= 0.368
Feature
−0.004
−0.002
0.000
0.002
Weight
Positive
Negative

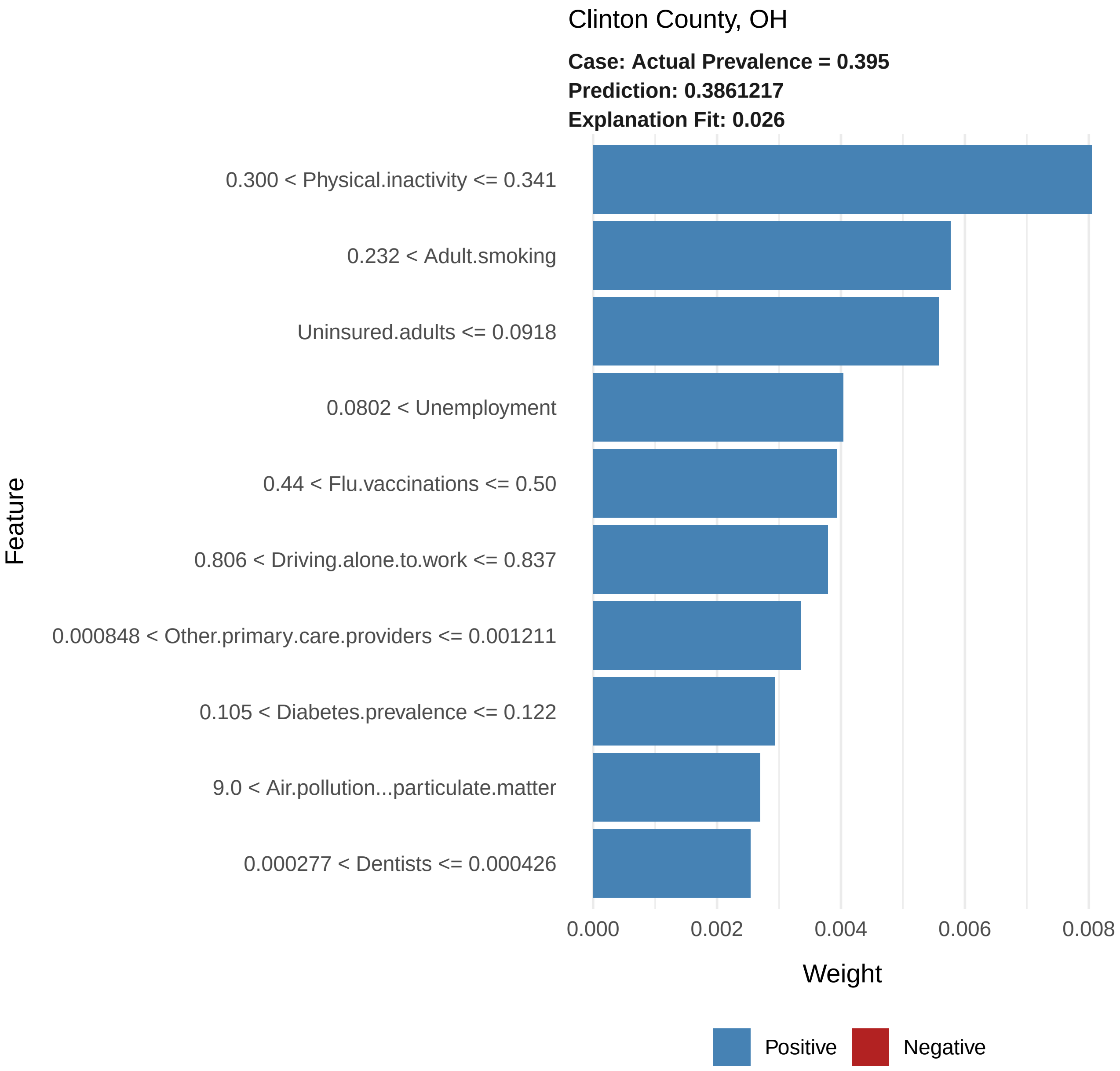
Clinton County, OH
Case: Actual Prevalence = 0.395
Prediction: 0.3861217
Explanation Fit: 0.026
0.300 < Physical.inactivity <= 0.341
0.232 < Adult.smoking
Uninsured.adults <= 0.0918
0.0802 < Unemployment
0.44 < Flu.vaccinations <= 0.50
0.806 < Driving.alone.to.work <= 0.837
0.000848 < Other.primary.care.providers <= 0.001211
0.105 < Diabetes.prevalence <= 0.122
9.0 < Air.pollution...particulate.matter
0.000277 < Dentists <= 0.000426
Feature
0.000
0.002
0.004
0.006
0.008
Weight
Positive
Negative

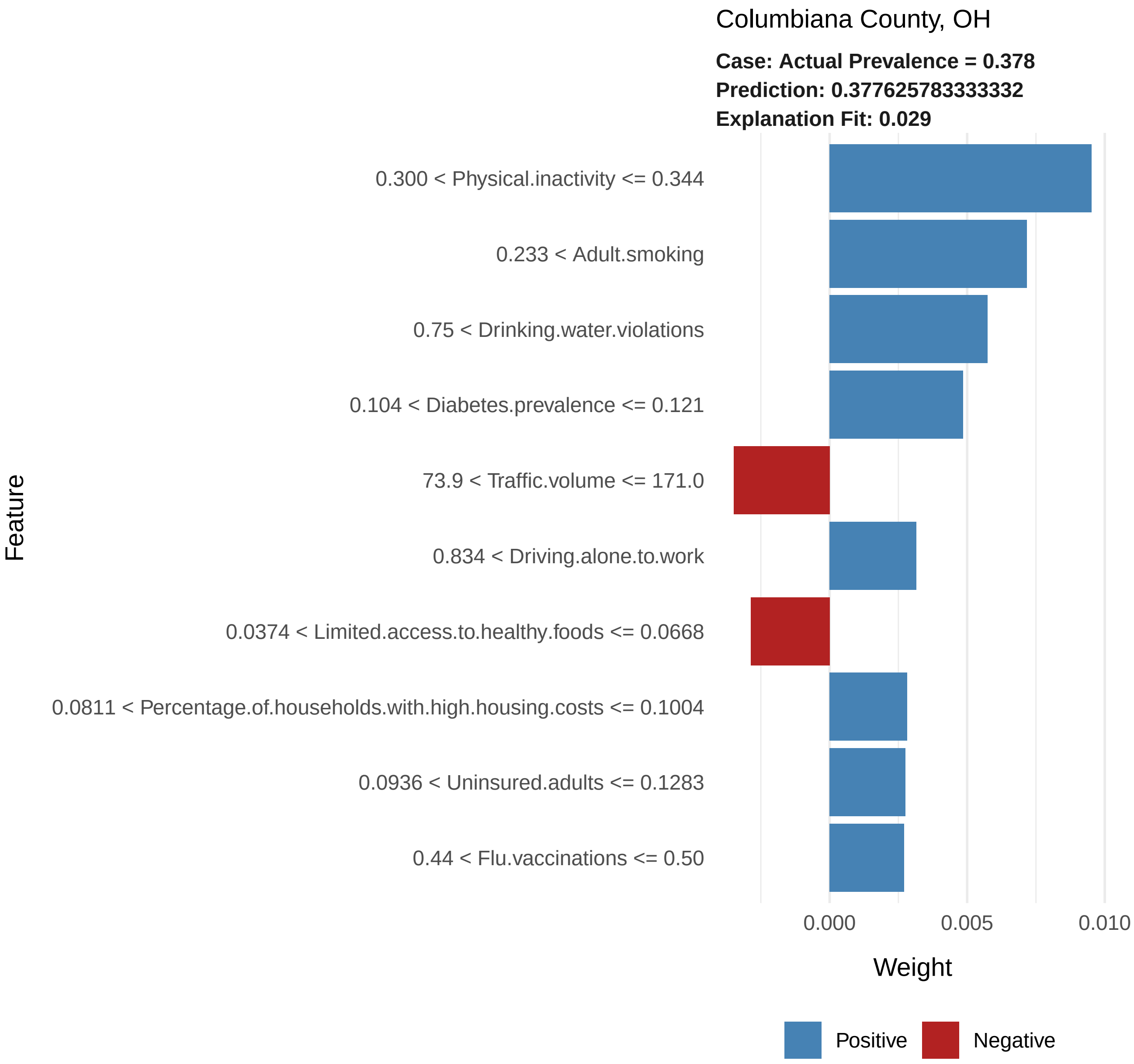
Columbiana County, OH
Case: Actual Prevalence = 0.378
Prediction: 0.377625783333332
Explanation Fit: 0.029
Feature
0.300 < Physical.inactivity <= 0.344
0.233 < Adult.smoking
0.75 < Drinking.water.violations
0.104 < Diabetes.prevalence <= 0.121
73.9 < Traffic.volume <= 171.0
0.834 < Driving.alone.to.work
0.0374 < Limited.access.to.healthy.foods <= 0.0668
0.0811 < Percentage.of.households.with.high.housing.costs <= 0.1004
0.0936 < Uninsured.adults <= 0.1283
0.44 < Flu.vaccinations <= 0.50
0.000
0.005
0.010
Weight
Positive
Negative

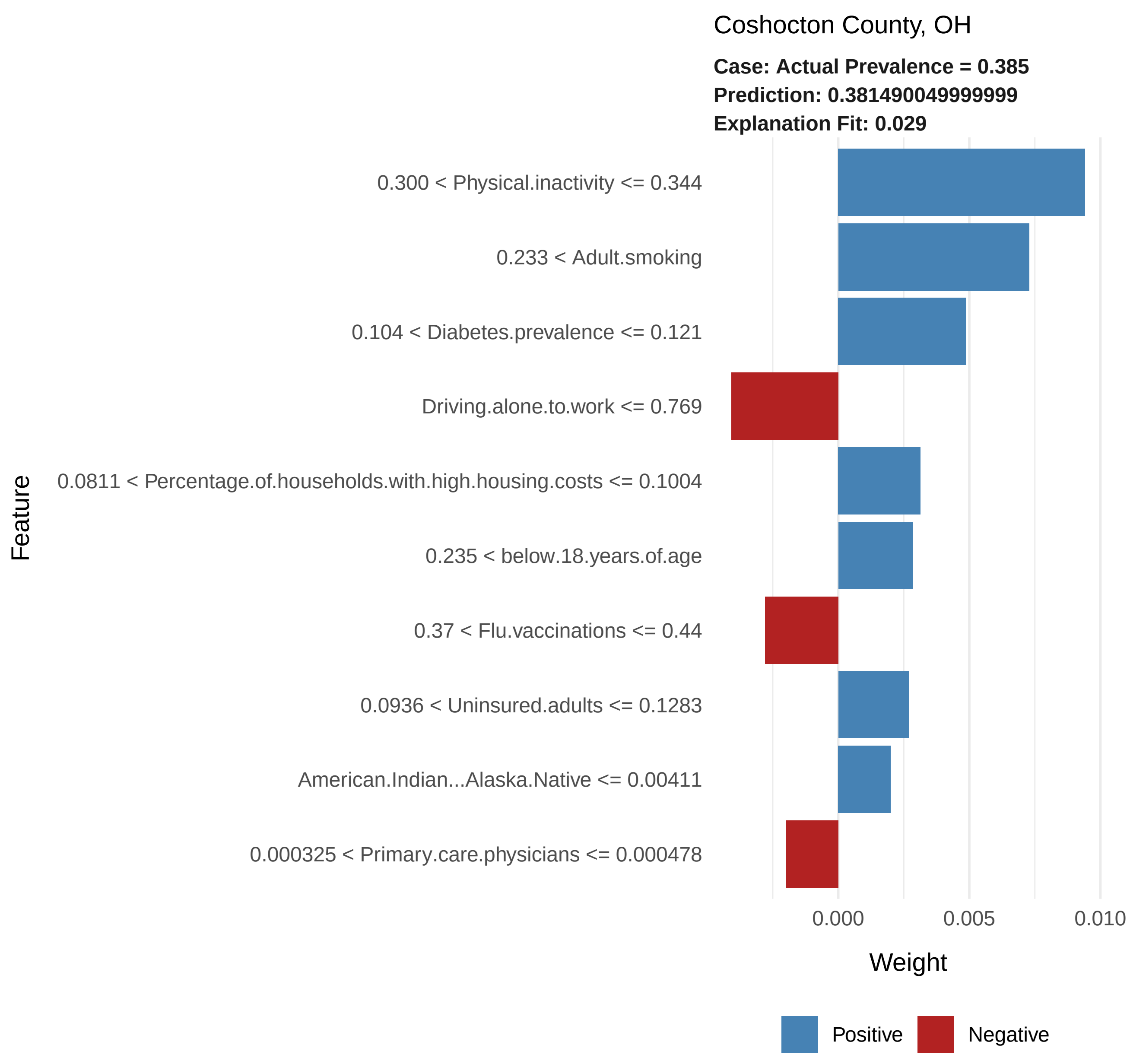
Coshocton County, OH
Case: Actual Prevalence = 0.385
Prediction: 0.381490049999999
Explanation Fit: 0.029
0.300 < Physical.inactivity <= 0.344
0.233 < Adult.smoking
0.104 < Diabetes.prevalence <= 0.121
Driving.alone.to.work <= 0.769
0.0811 < Percentage.of.households.with.high.housing.costs <= 0.1004
0.235 < below.18.years.of.age
0.37 < Flu.vaccinations <= 0.44
0.0936 < Uninsured.adults <= 0.1283
American.Indian...Alaska.Native <= 0.00411
0.000325 < Primary.care.physicians <= 0.000478
Feature
0.000
0.005
0.010
Weight
Positive
Negative

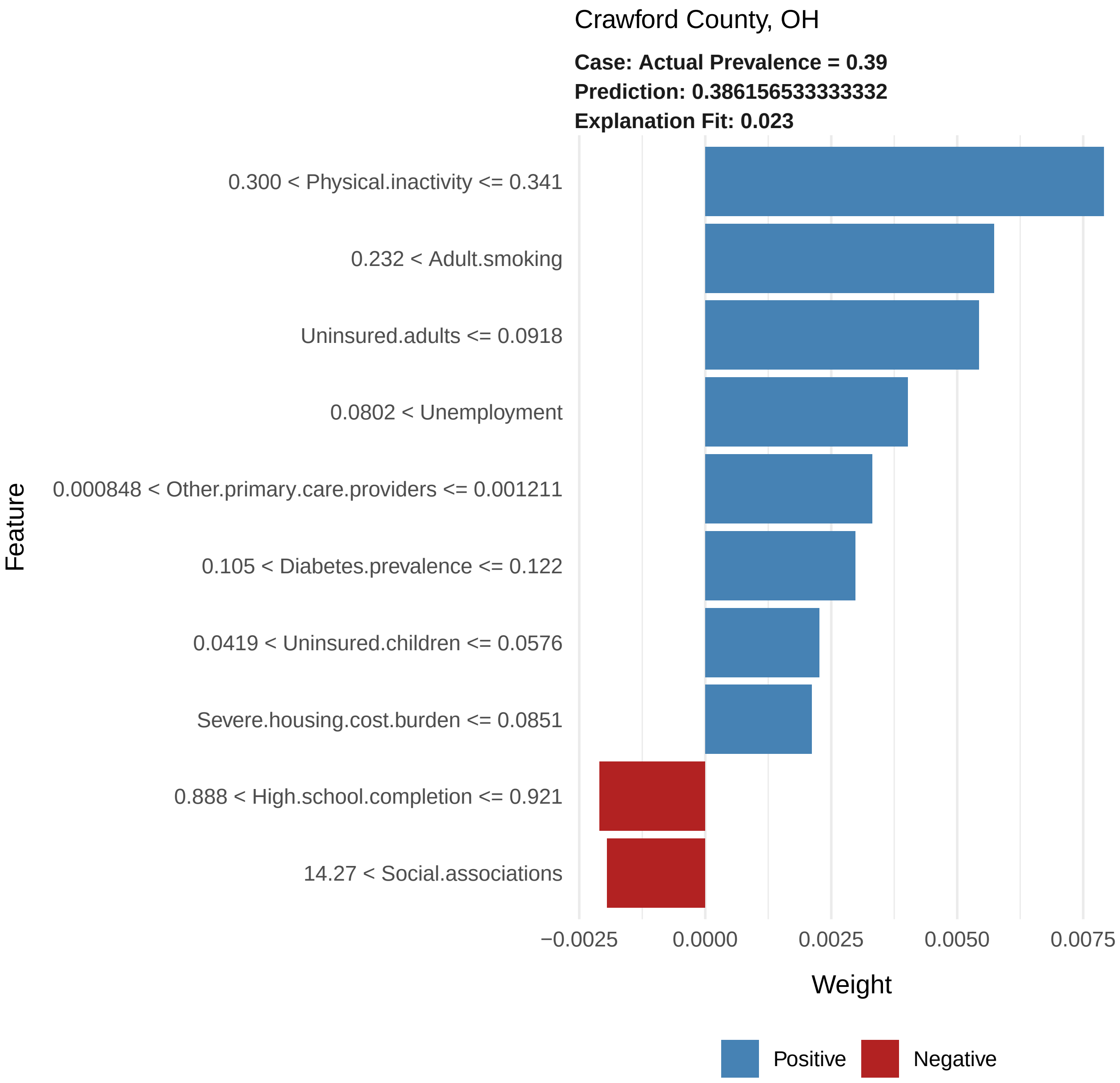
Crawford County, OH
Case: Actual Prevalence = 0.39
Prediction: 0.386156533333332
Explanation Fit: 0.023
0.300 < Physical.inactivity <= 0.341
0.232 < Adult.smoking
Uninsured.adults <= 0.0918
0.0802 < Unemployment
0.000848 < Other.primary.care.providers <= 0.001211
0.105 < Diabetes.prevalence <= 0.122
0.0419 < Uninsured.children <= 0.0576
Severe.housing.cost.burden <= 0.0851
0.888 < High.school.completion <= 0.921
14.27 < Social.associations
Feature
−0.0025
0.0000
0.0025
0.0050
0.0075
Weight
Positive
Negative

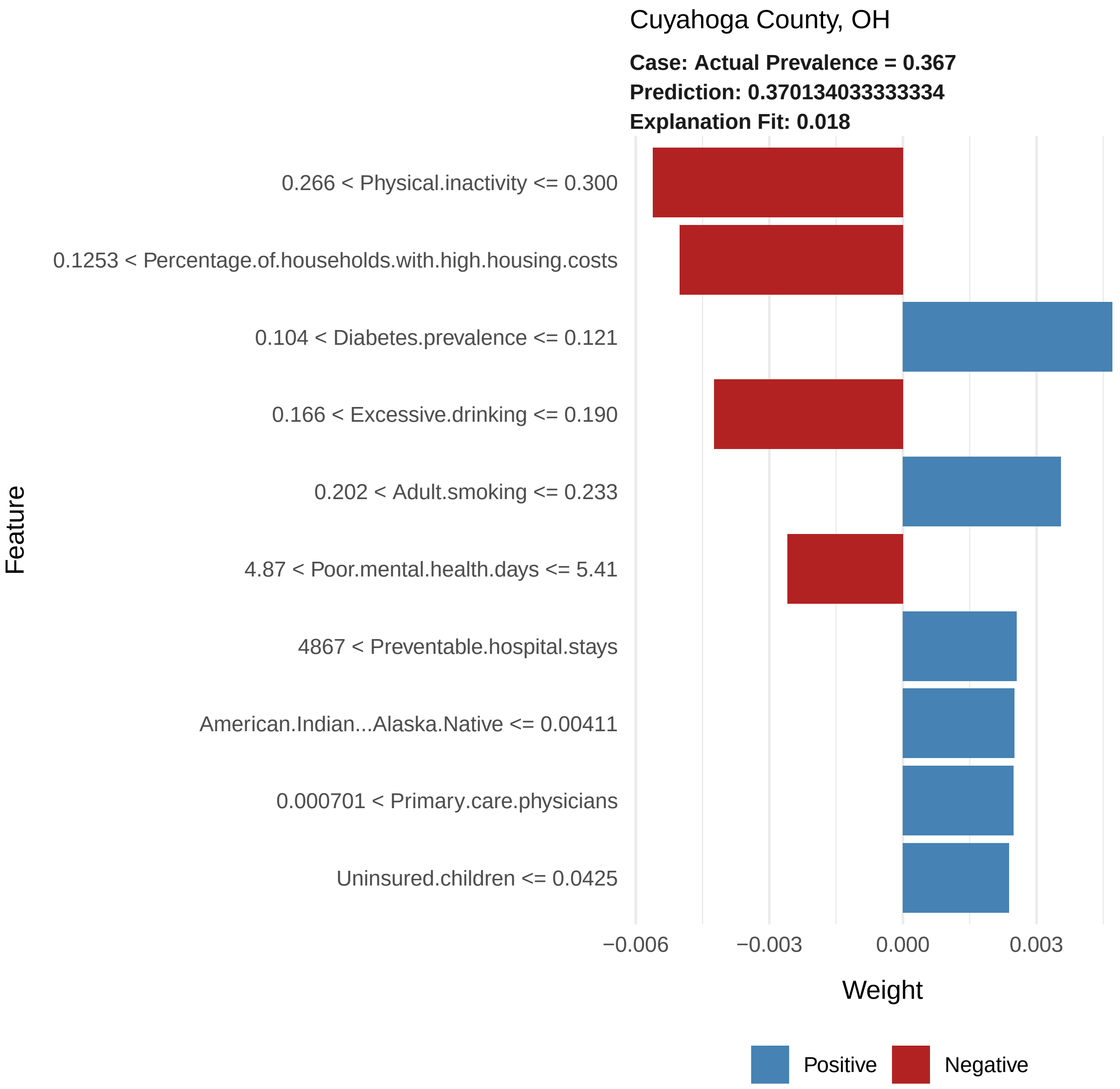

Cuyahoga County, OH
Case: Actual Prevalence = 0.367
Prediction: 0.370134033333334
Explanation Fit: 0.018
0.266 < Physical.inactivity <= 0.300
0.1253 < Percentage.of.households.with.high.housing.costs
0.104 < Diabetes.prevalence <= 0.121
0.166 < Excessive.drinking <= 0.190
0.202 < Adult.smoking <= 0.233
4.87 < Poor.mental.health.days <= 5.41
4867 < Preventable.hospital.stays
American.Indian...Alaska.Native <= 0.00411
0.000701 < Primary.care.physicians
Uninsured.children <= 0.0425
Feature
−0.006
−0.003
0.000
0.003
Weight
Positive
Negative

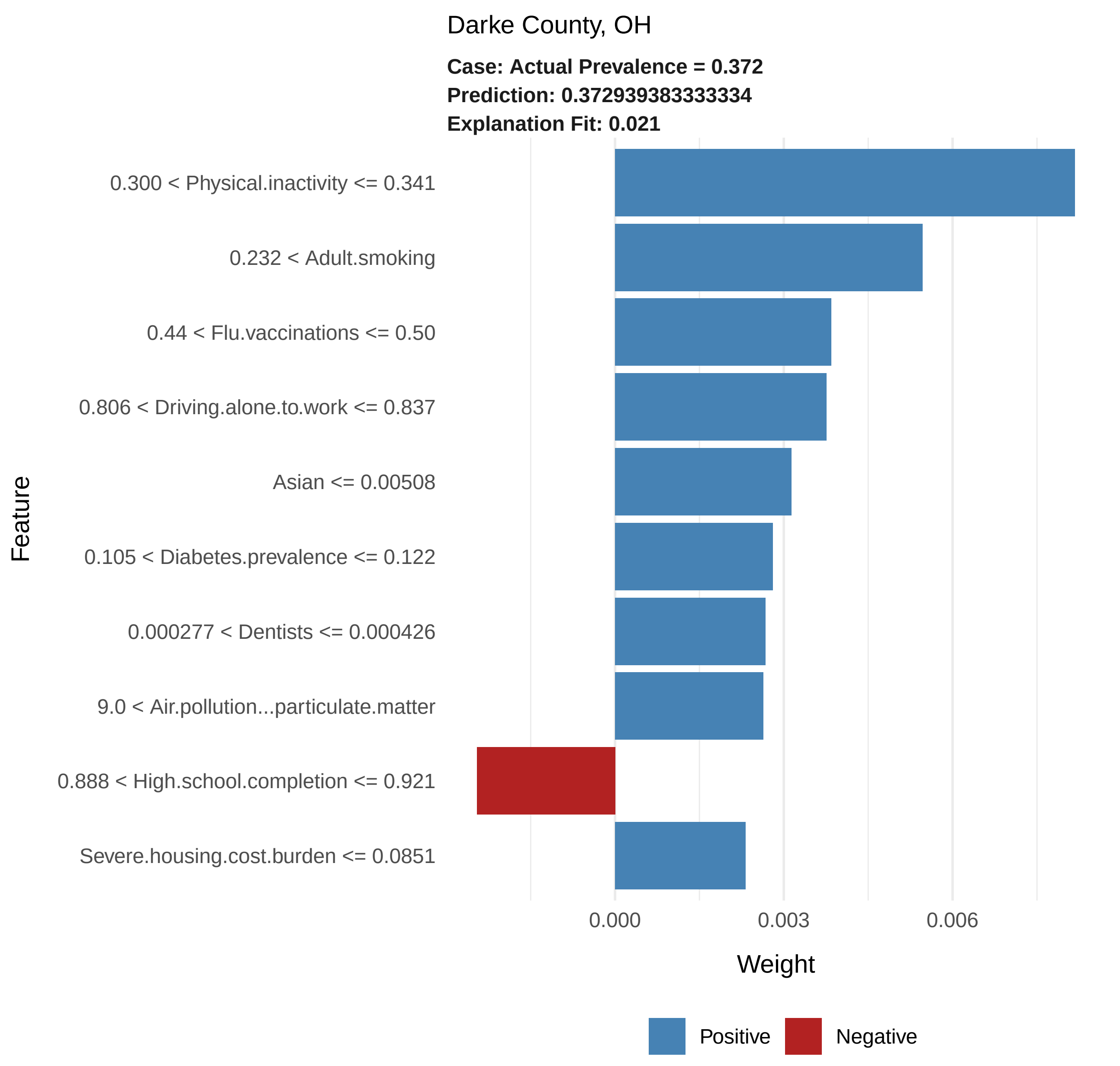
Darke County, OH
Case: Actual Prevalence = 0.372
Prediction: 0.372939383333334
Explanation Fit: 0.021
0.300 < Physical.inactivity <= 0.341
0.232 < Adult.smoking
0.44 < Flu.vaccinations <= 0.50
0.806 < Driving.alone.to.work <= 0.837
Asian <= 0.00508
0.105 < Diabetes.prevalence <= 0.122
0.000277 < Dentists <= 0.000426
9.0 < Air.pollution...particulate.matter
0.888 < High.school.completion <= 0.921
Severe.housing.cost.burden <= 0.0851
Feature
0.000
0.003
0.006
Weight
Positive
Negative

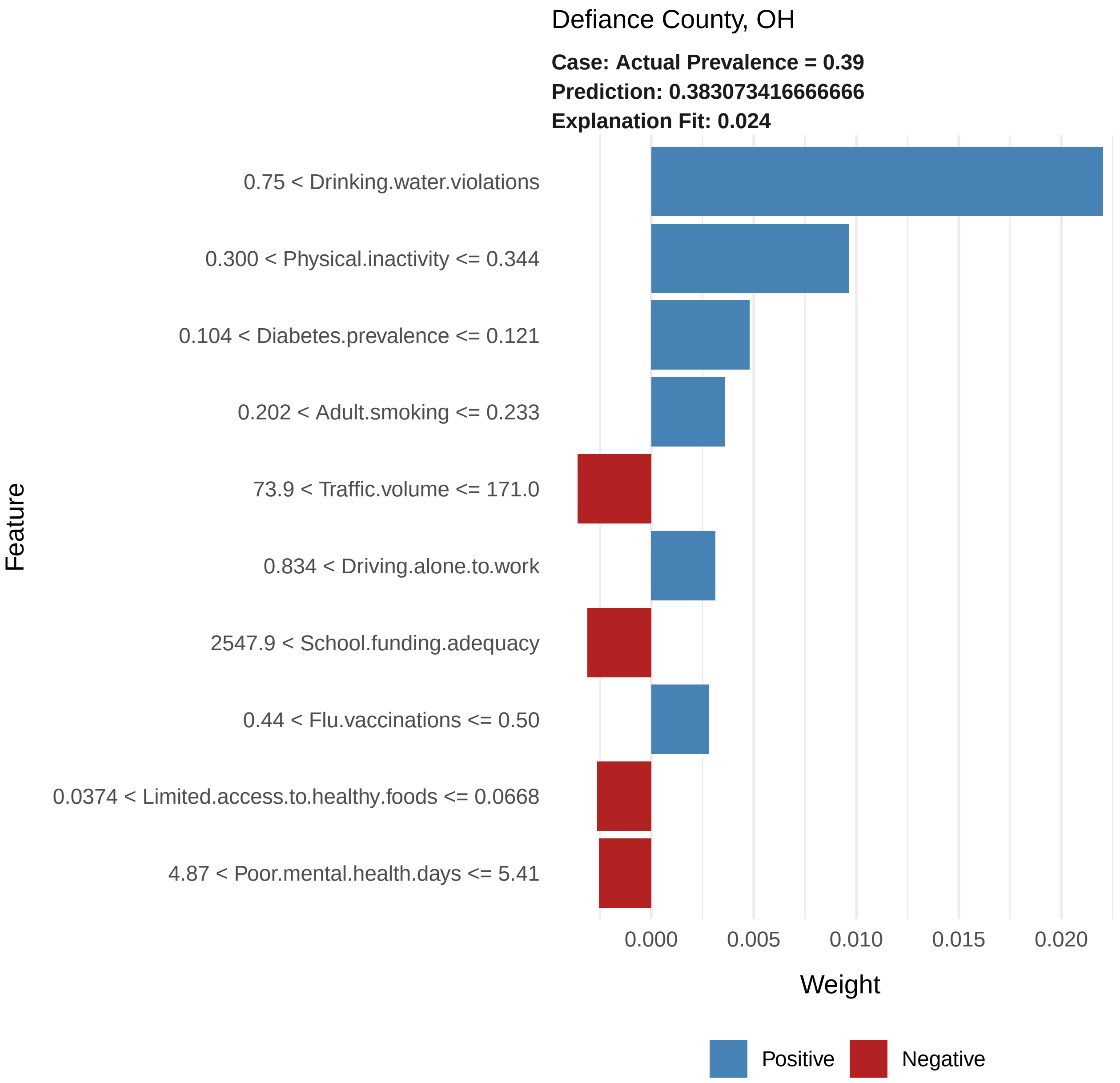
Defiance County, OH
Case: Actual Prevalence = 0.39
Prediction: 0.383073416666666
Explanation Fit: 0.024
0.75 < Drinking.water.violations
0.300 < Physical.inactivity <= 0.344
0.104 < Diabetes.prevalence <= 0.121
0.202 < Adult.smoking <= 0.233
73.9 < Traffic.volume <= 171.0
0.834 < Driving.alone.to.work
2547.9 < School.funding.adequacy
0.44 < Flu.vaccinations <= 0.50
0.0374 < Limited.access.to.healthy.foods <= 0.0668
4.87 < Poor.mental.health.days <= 5.41
Feature
0.000
0.005
0.010
0.015
0.020
Weight
Positive
Negative

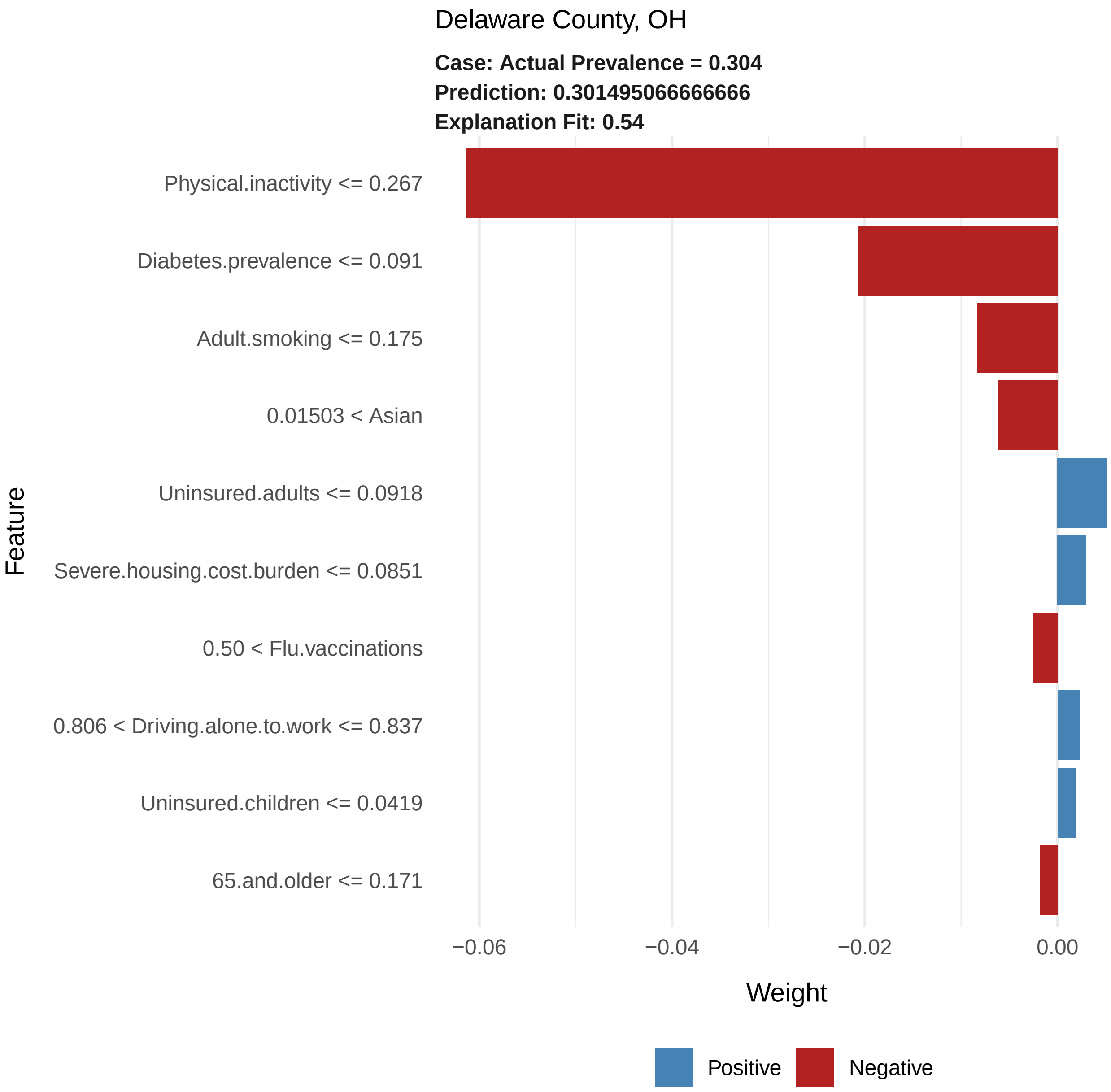

Delaware County, OH
Case: Actual Prevalence = 0.304
Prediction: 0.301495066666666
Explanation Fit: 0.54
Physical.inactivity <= 0.267
Diabetes.prevalence <= 0.091
Adult.smoking <= 0.175
0.01503 < Asian
Uninsured.adults <= 0.0918
Severe.housing.cost.burden <= 0.0851
0.50 < Flu.vaccinations
0.806 < Driving.alone.to.work <= 0.837
Uninsured.children <= 0.0419
65.and.older <= 0.171
Feature
−0.06
−0.04
−0.02
0.00
Weight
Positive
Negative

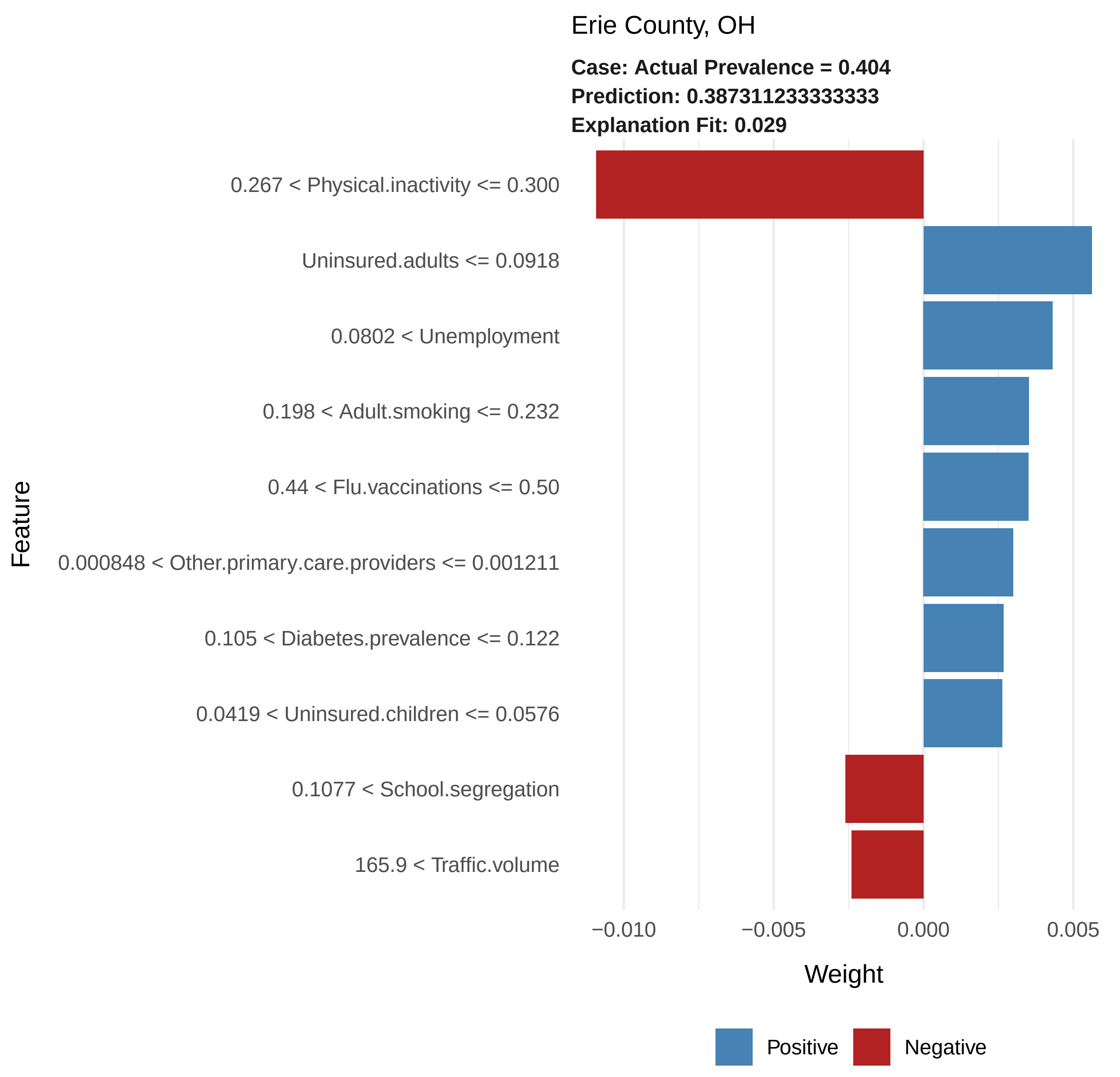
Erie County, OH
Case: Actual Prevalence = 0.404
Prediction: 0.387311233333333
Explanation Fit: 0.029
0.267 < Physical.inactivity <= 0.300
Uninsured.adults <= 0.0918
0.0802 < Unemployment
0.198 < Adult.smoking <= 0.232
0.44 < Flu.vaccinations <= 0.50
0.000848 < Other.primary.care.providers <= 0.001211
0.105 < Diabetes.prevalence <= 0.122
0.0419 < Uninsured.children <= 0.0576
0.1077 < School.segregation
165.9 < Traffic.volume
Feature
−0.010
−0.005
0.000
0.005
Weight
Positive
Negative

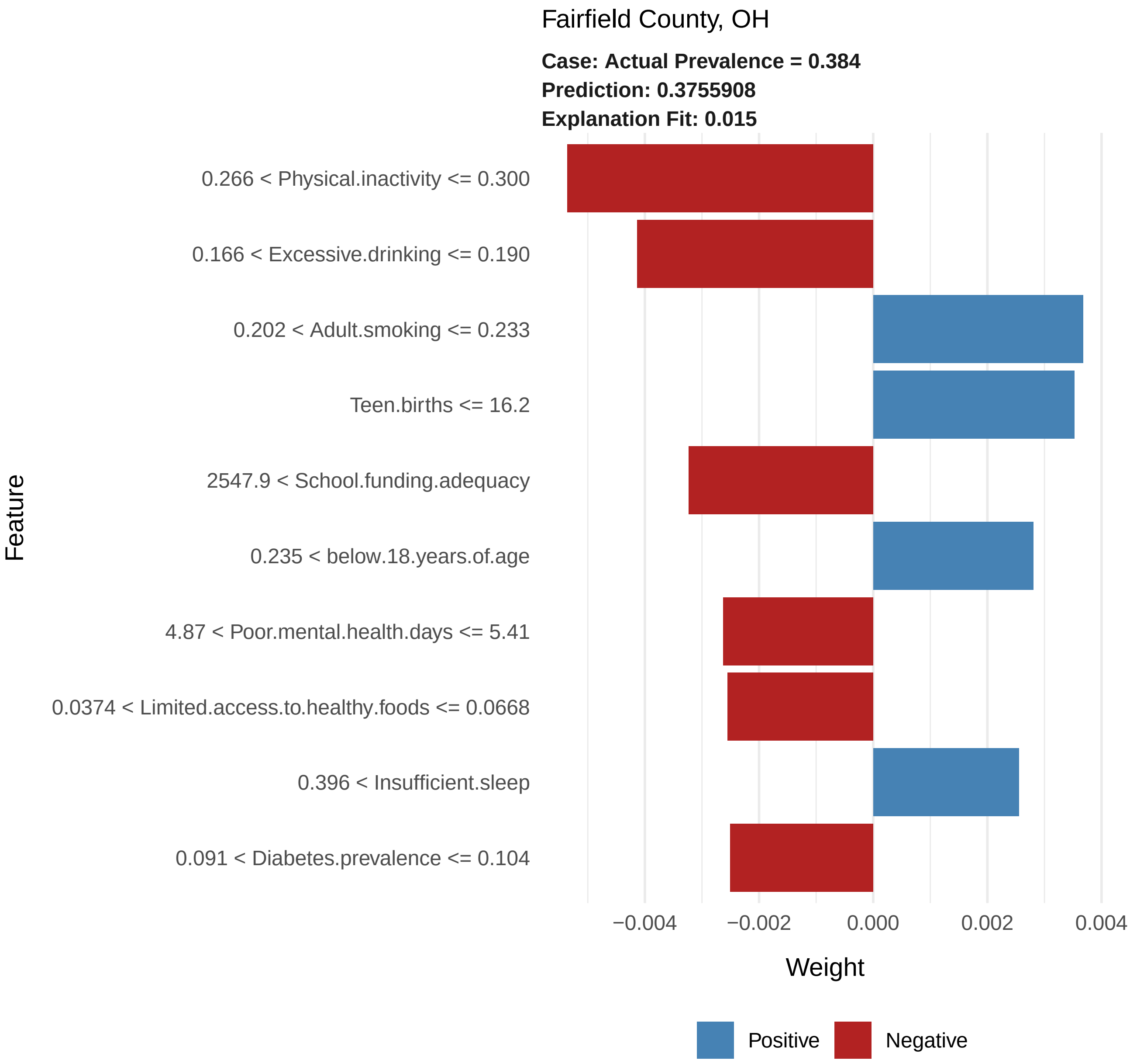

Fairfield County, OH
Case: Actual Prevalence = 0.384
Prediction: 0.3755908
Explanation Fit: 0.015
Feature
0.266 < Physical.inactivity <= 0.300
0.166 < Excessive.drinking <= 0.190
0.202 < Adult.smoking <= 0.233
Teen.births <= 16.2
2547.9 < School.funding.adequacy
0.235 < below.18.years.of.age
4.87 < Poor.mental.health.days <= 5.41
0.0374 < Limited.access.to.healthy.foods <= 0.0668
0.396 < Insufficient.sleep
0.091 < Diabetes.prevalence <= 0.104
−0.004
−0.002
0.000
0.002
0.004
Weight
Positive
Negative

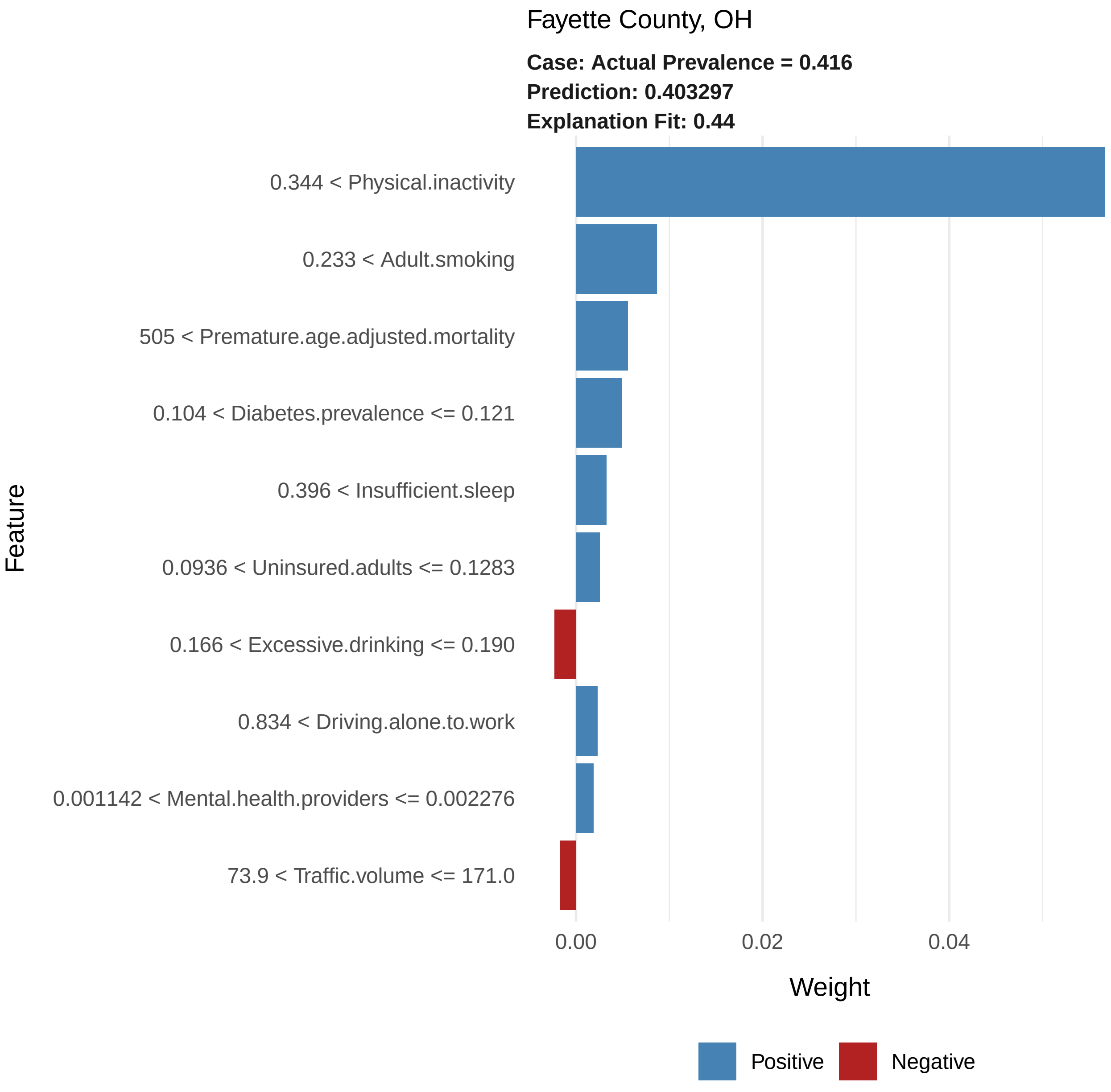
Fayette County, OH
Case: Actual Prevalence = 0.416
Prediction: 0.403297
Explanation Fit: 0.44
0.344 < Physical.inactivity
0.233 < Adult.smoking
505 < Premature.age.adjusted.mortality
0.104 < Diabetes.prevalence <= 0.121
0.396 < Insufficient.sleep
0.0936 < Uninsured.adults <= 0.1283
0.166 < Excessive.drinking <= 0.190
0.834 < Driving.alone.to.work
0.001142 < Mental.health.providers <= 0.002276
73.9 < Traffic.volume <= 171.0
Feature
0.00
0.02
0.04
Weight
Positive
Negative

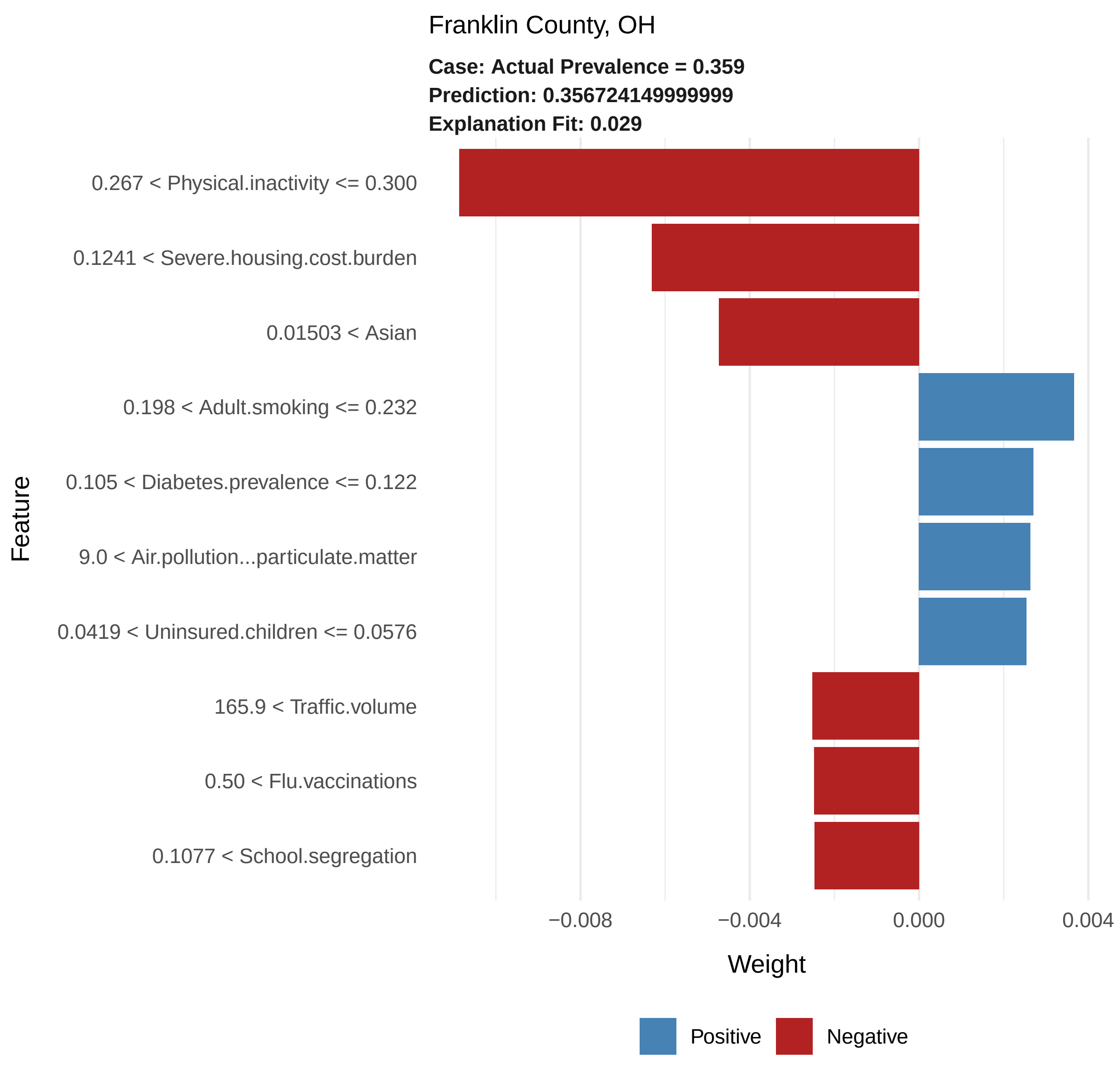
Franklin County, OH
Case: Actual Prevalence = 0.359
Prediction: 0.356724149999999
Explanation Fit: 0.029
0.267 < Physical.inactivity <= 0.300
0.1241 < Severe.housing.cost.burden
0.01503 < Asian
0.198 < Adult.smoking <= 0.232
0.105 < Diabetes.prevalence <= 0.122
9.0 < Air.pollution...particulate.matter
0.0419 < Uninsured.children <= 0.0576
165.9 < Traffic.volume
0.50 < Flu.vaccinations
0.1077 < School.segregation
Feature
−0.008
−0.004
0.000
0.004
Weight
Positive
Negative

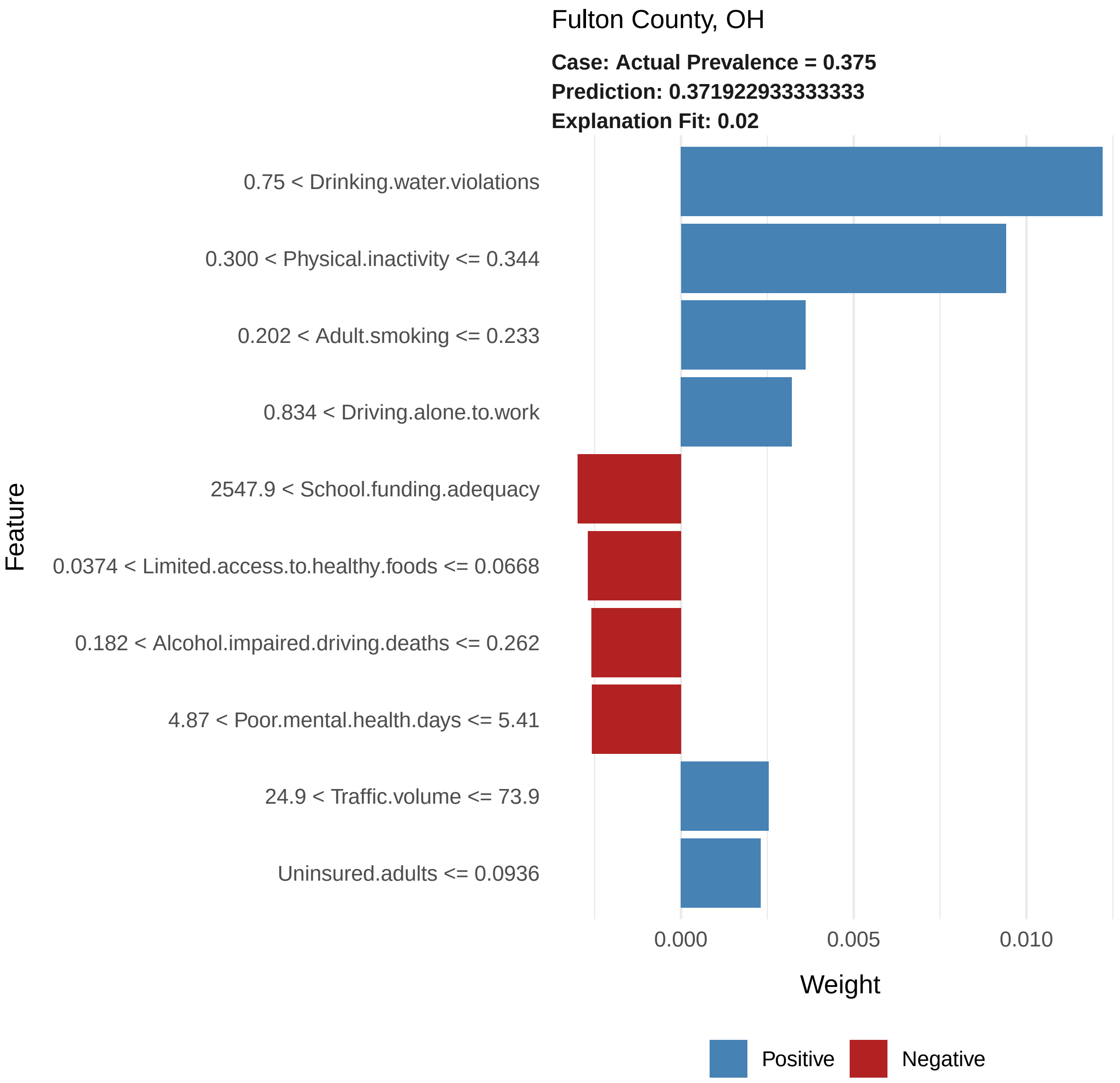

Fulton County, OH
Case: Actual Prevalence = 0.375
Prediction: 0.371922933333333
Explanation Fit: 0.02
0.75 < Drinking.water.violations
0.300 < Physical.inactivity <= 0.344
0.202 < Adult.smoking <= 0.233
0.834 < Driving.alone.to.work
2547.9 < School.funding.adequacy
0.0374 < Limited.access.to.healthy.foods <= 0.0668
0.182 < Alcohol.impaired.driving.deaths <= 0.262
4.87 < Poor.mental.health.days <= 5.41
24.9 < Traffic.volume <= 73.9
Uninsured.adults <= 0.0936
Feature
0.000
0.005
0.010
Weight
Positive
Negative

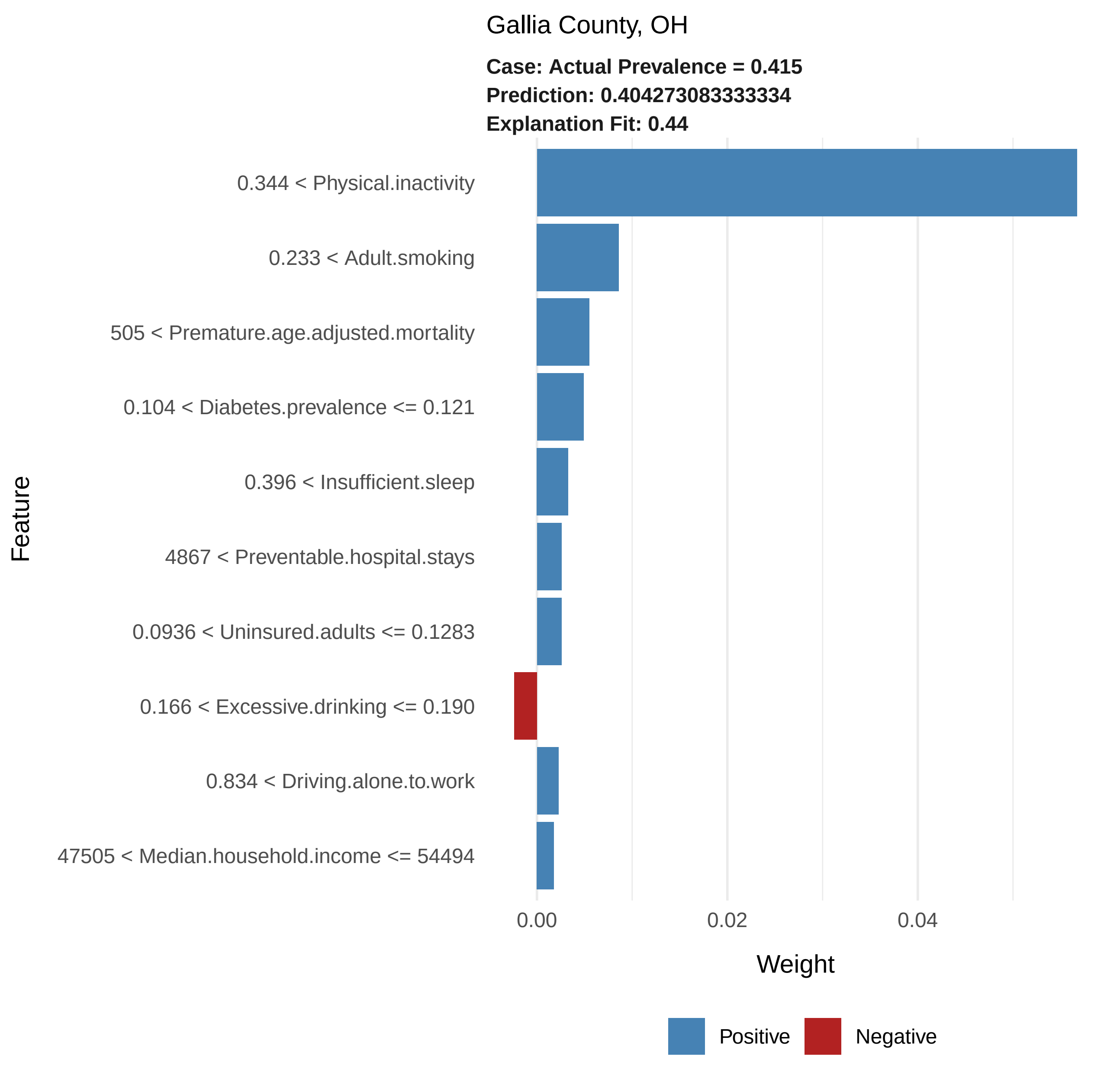
Gallia County, OH
Case: Actual Prevalence = 0.415
Prediction: 0.404273083333334
Explanation Fit: 0.44
0.344 < Physical.inactivity
0.233 < Adult.smoking
505 < Premature.age.adjusted.mortality
0.104 < Diabetes.prevalence <= 0.121
0.396 < Insufficient.sleep
4867 < Preventable.hospital.stays
0.0936 < Uninsured.adults <= 0.1283
0.166 < Excessive.drinking <= 0.190
0.834 < Driving.alone.to.work
47505 < Median.household.income <= 54494
Feature
0.00
0.02
0.04
Weight
Positive
Negative

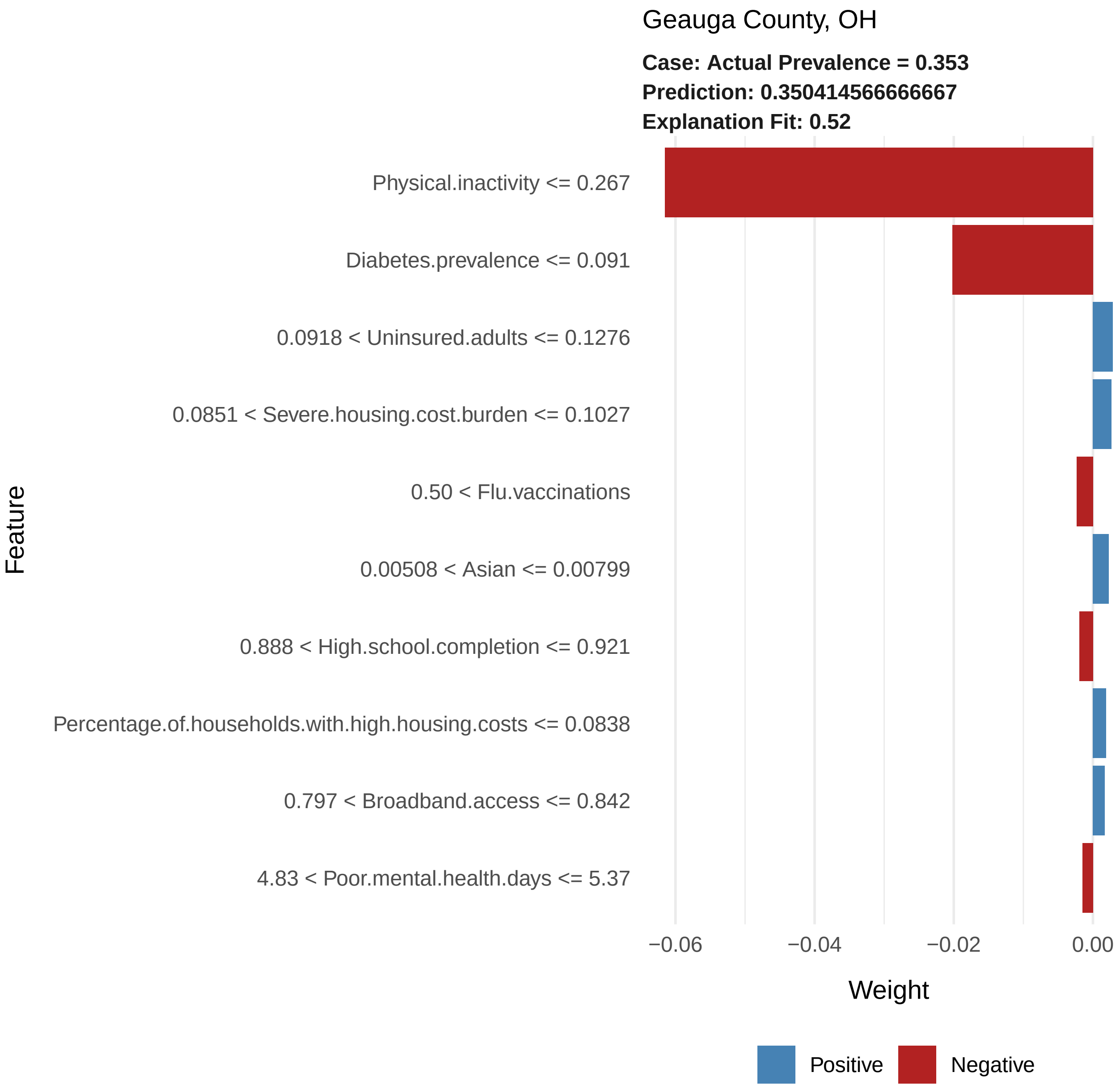
Geauga County, OH
Case: Actual Prevalence = 0.353
Prediction: 0.350414566666667
Explanation Fit: 0.52
Physical.inactivity <= 0.267
Diabetes.prevalence <= 0.091
0.0918 < Uninsured.adults <= 0.1276
0.0851 < Severe.housing.cost.burden <= 0.1027
0.50 < Flu.vaccinations
0.00508 < Asian <= 0.00799
0.888 < High.school.completion <= 0.921
Percentage.of.households.with.high.housing.costs <= 0.0838
0.797 < Broadband.access <= 0.842
4.83 < Poor.mental.health.days <= 5.37
Feature
−0.06
−0.04
−0.02
0.00
Weight
Positive
Negative

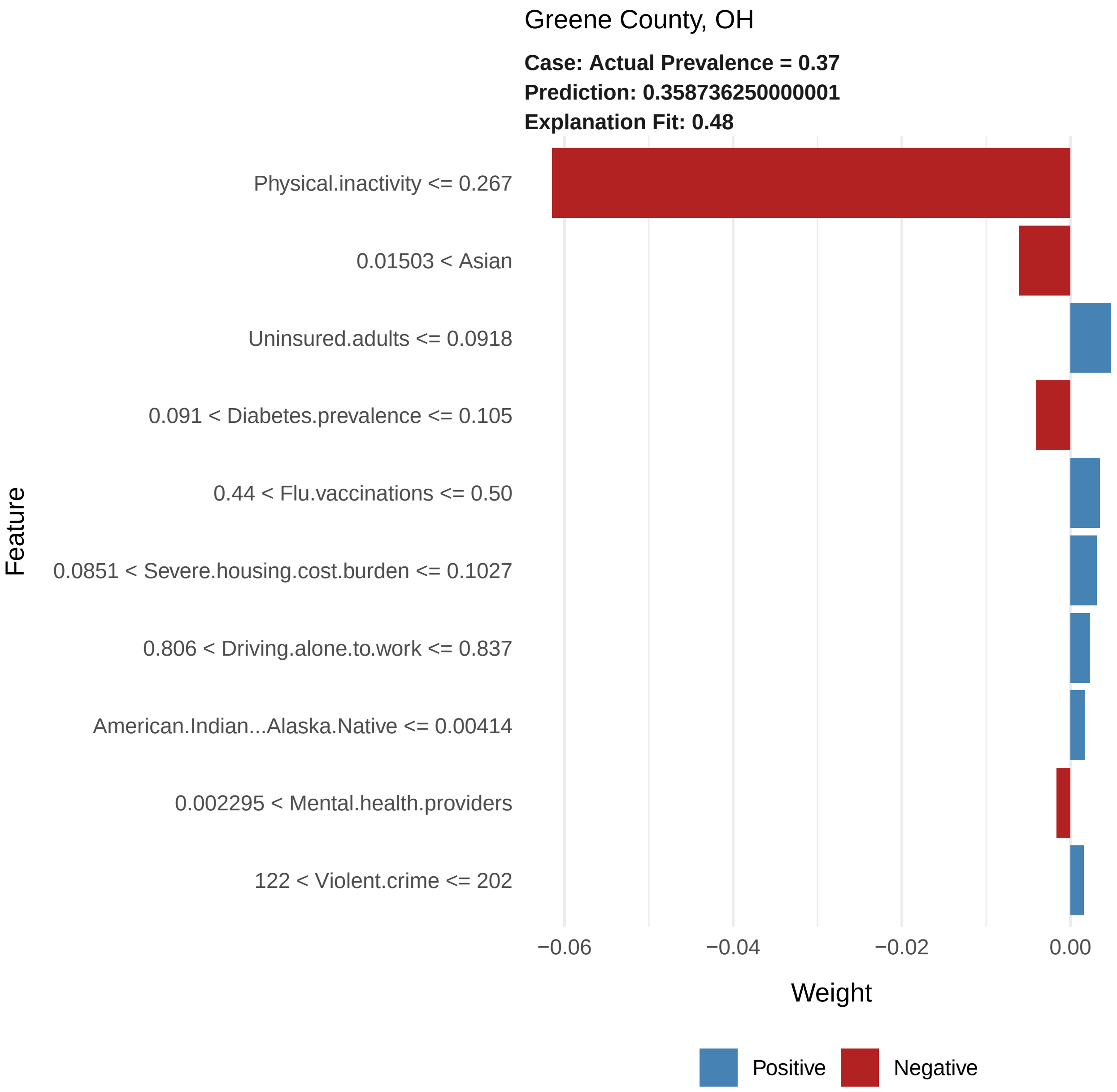

Greene County, OH
Case: Actual Prevalence = 0.37
Prediction: 0.358736250000001
Explanation Fit: 0.48
Feature
Physical.inactivity <= 0.267
0.01503 < Asian
Uninsured.adults <= 0.0918
0.091 < Diabetes.prevalence <= 0.105
0.44 < Flu.vaccinations <= 0.50
0.0851 < Severe.housing.cost.burden <= 0.1027
0.806 < Driving.alone.to.work <= 0.837
American.Indian...Alaska.Native <= 0.00414
0.002295 < Mental.health.providers
122 < Violent.crime <= 202
−0.06
−0.04
−0.02
0.00
Weight
Positive
Negative

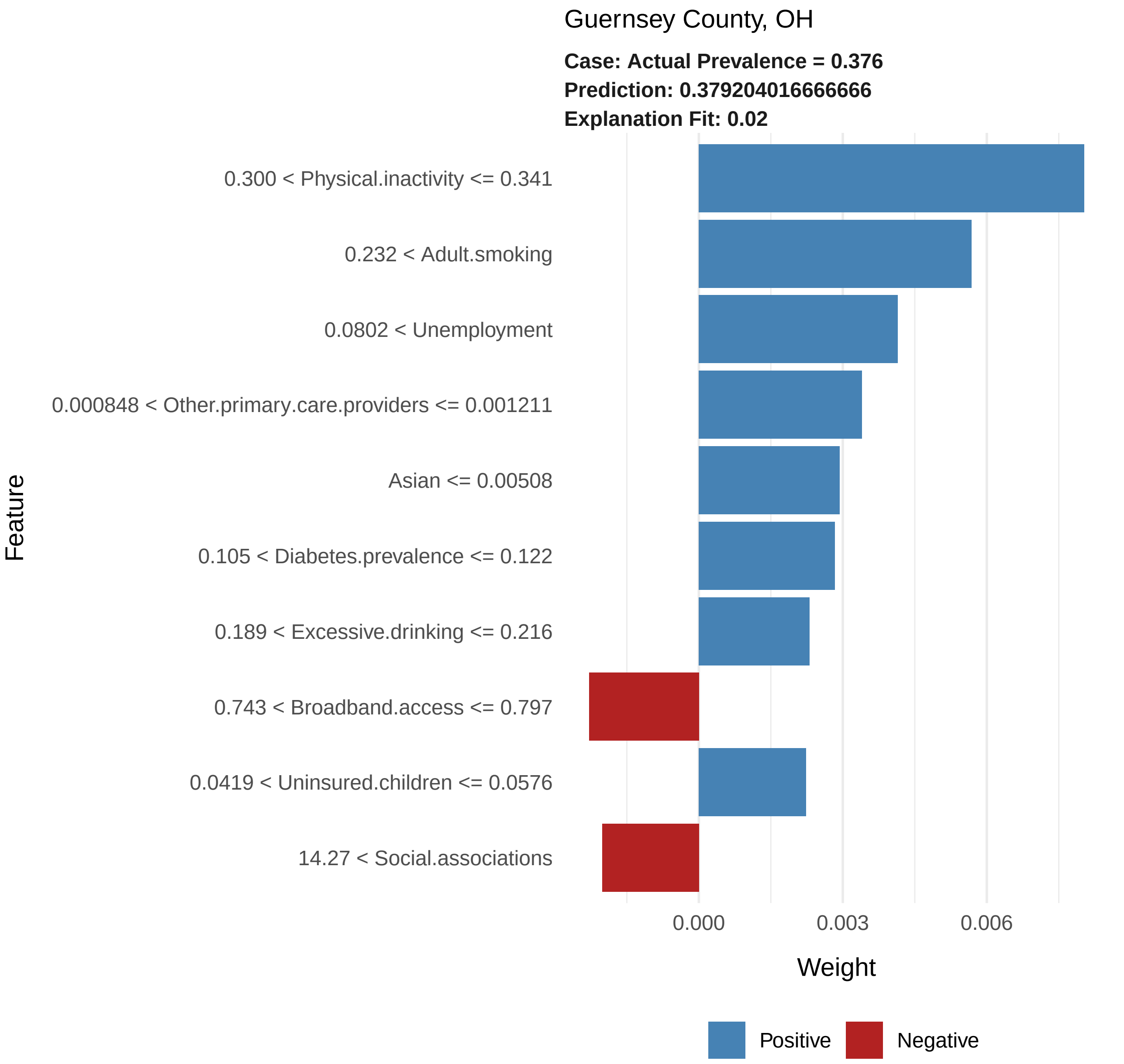

Guernsey County, OH
Case: Actual Prevalence = 0.376
Prediction: 0.379204016666666
Explanation Fit: 0.02
0.300 < Physical.inactivity <= 0.341
0.232 < Adult.smoking
0.0802 < Unemployment
0.000848 < Other.primary.care.providers <= 0.001211
Asian <= 0.00508
0.105 < Diabetes.prevalence <= 0.122
0.189 < Excessive.drinking <= 0.216
0.743 < Broadband.access <= 0.797
0.0419 < Uninsured.children <= 0.0576
14.27 < Social.associations
Feature
0.000
0.003
0.006
Weight
Positive
Negative

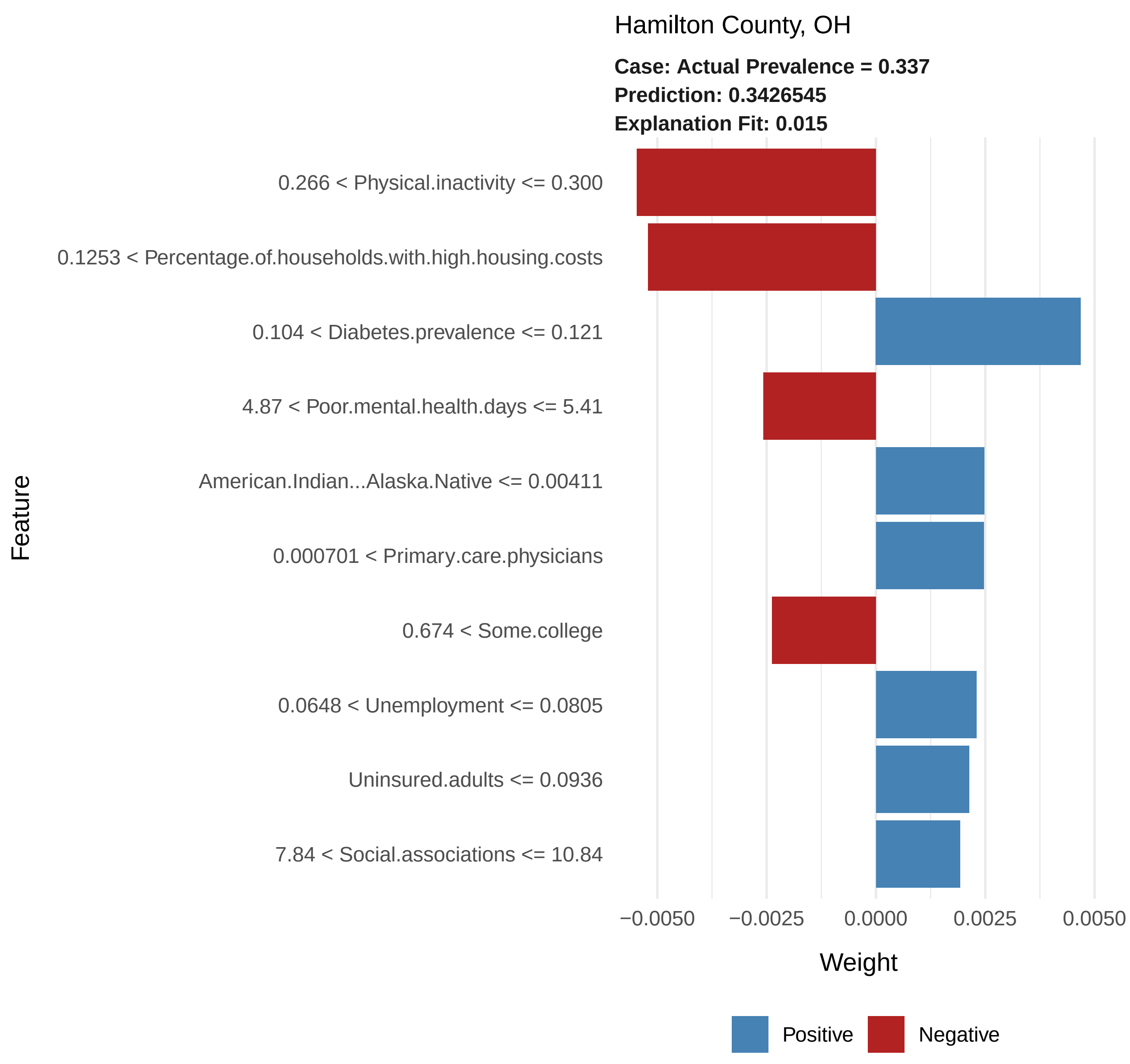
Hamilton County, OH
Case: Actual Prevalence = 0.337
Prediction: 0.3426545
Explanation Fit: 0.015
0.266 < Physical.inactivity <= 0.300
0.1253 < Percentage.of.households.with.high.housing.costs
0.104 < Diabetes.prevalence <= 0.121
4.87 < Poor.mental.health.days <= 5.41
American.Indian...Alaska.Native <= 0.00411
0.000701 < Primary.care.physicians
0.674 < Some.college
0.0648 < Unemployment <= 0.0805
Uninsured.adults <= 0.0936
7.84 < Social.associations <= 10.84
Feature
−0.0050
−0.0025
0.0000
0.0025
0.0050
Weight
Positive
Negative

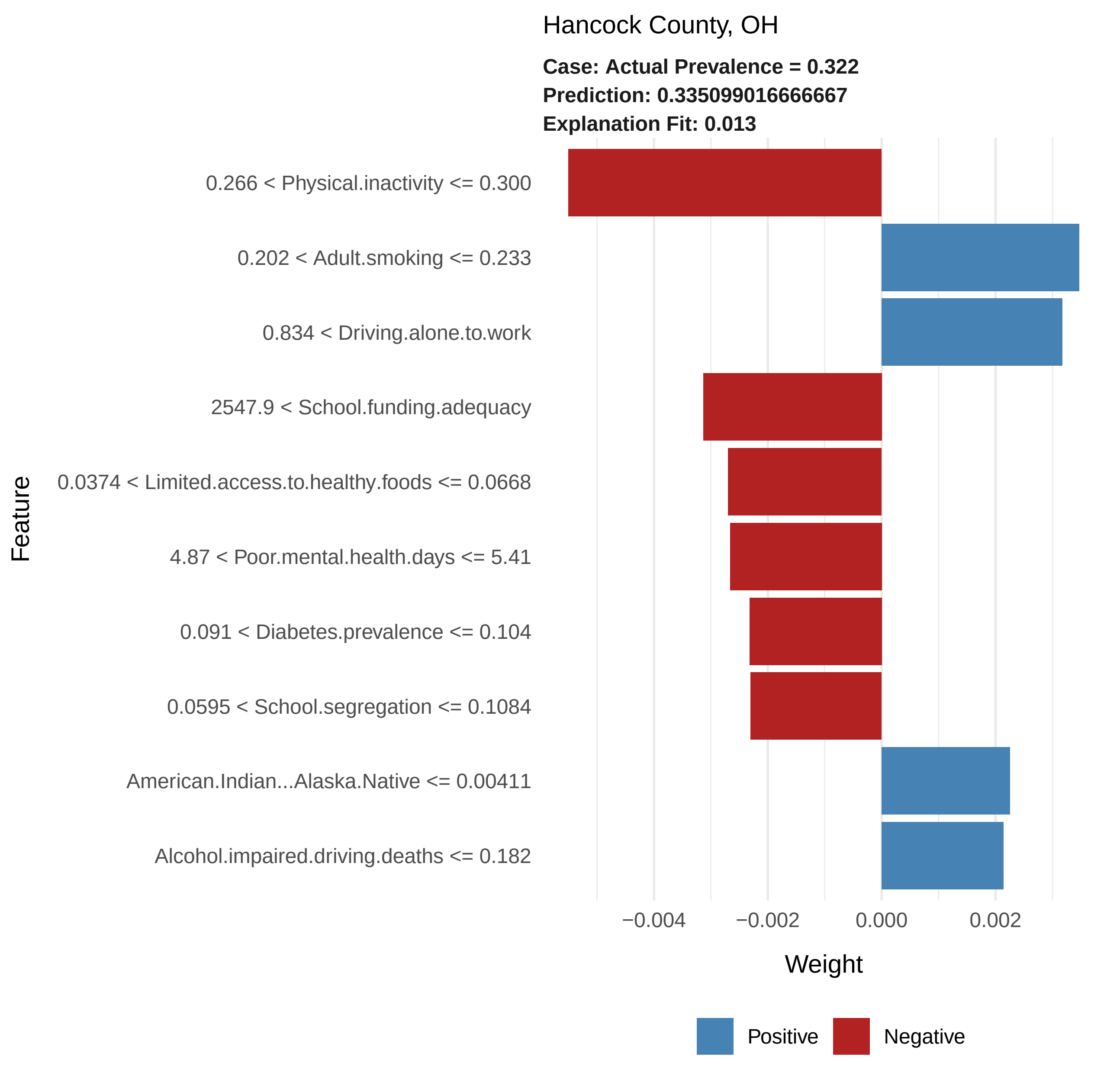

Hancock County, OH
Case: Actual Prevalence = 0.322
Prediction: 0.335099016666667
Explanation Fit: 0.013
Feature
0.266 < Physical.inactivity <= 0.300
0.202 < Adult.smoking <= 0.233
0.834 < Driving.alone.to.work
2547.9 < School.funding.adequacy
0.0374 < Limited.access.to.healthy.foods <= 0.0668
4.87 < Poor.mental.health.days <= 5.41
0.091 < Diabetes.prevalence <= 0.104
0.0595 < School.segregation <= 0.1084
American.Indian...Alaska.Native <= 0.00411
Alcohol.impaired.driving.deaths <= 0.182
−0.004
−0.002
0.000
0.002
Weight
Positive
Negative

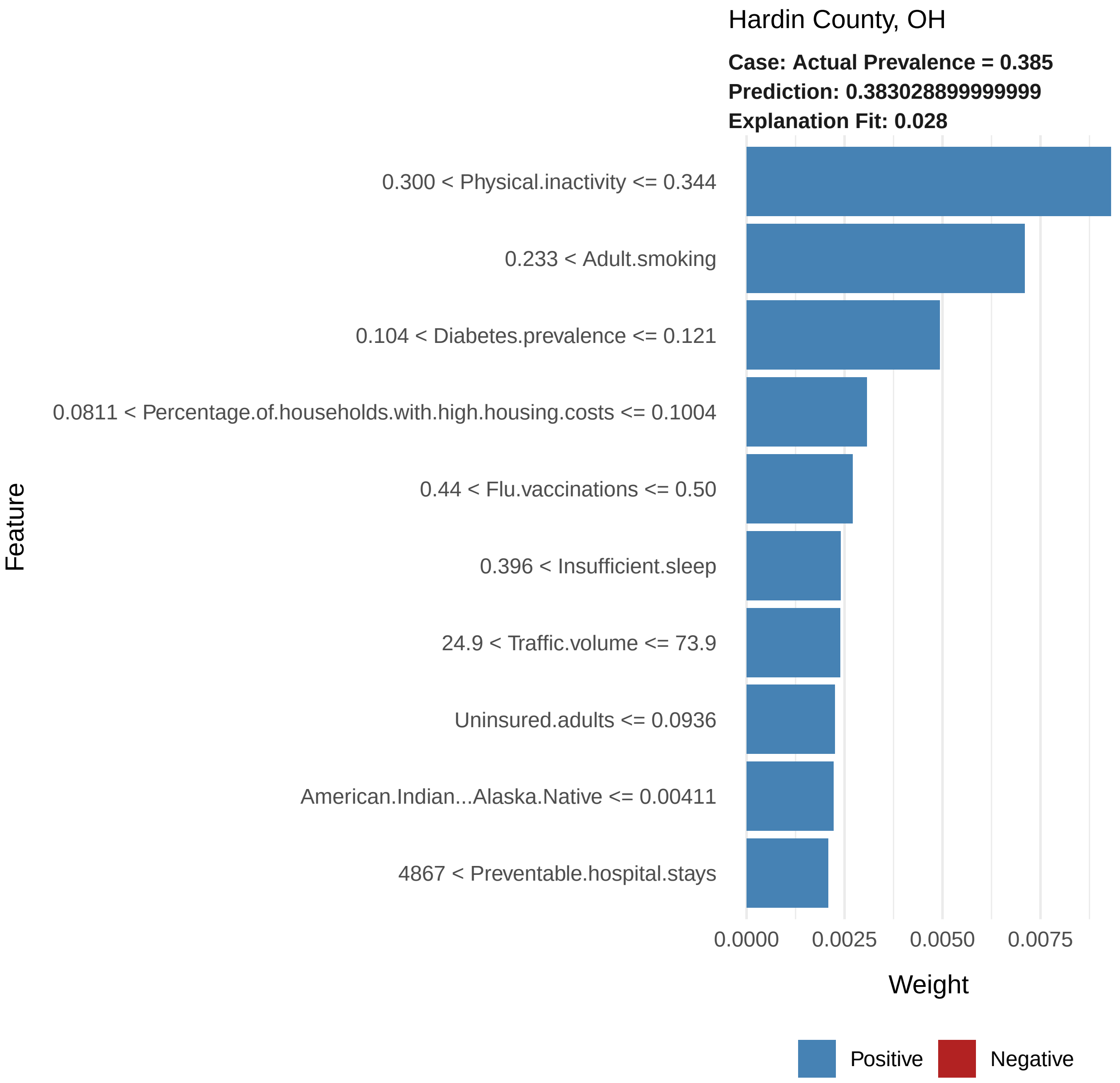

Hardin County, OH
Case: Actual Prevalence = 0.385
Prediction: 0.383028899999999
Explanation Fit: 0.028
0.300 < Physical.inactivity <= 0.344
0.233 < Adult.smoking
0.104 < Diabetes.prevalence <= 0.121
0.0811 < Percentage.of.households.with.high.housing.costs <= 0.1004
0.44 < Flu.vaccinations <= 0.50
0.396 < Insufficient.sleep
24.9 < Traffic.volume <= 73.9
Uninsured.adults <= 0.0936
American.Indian...Alaska.Native <= 0.00411
4867 < Preventable.hospital.stays
Feature
0.0000
0.0025
0.0050
0.0075
Weight
Positive
Negative

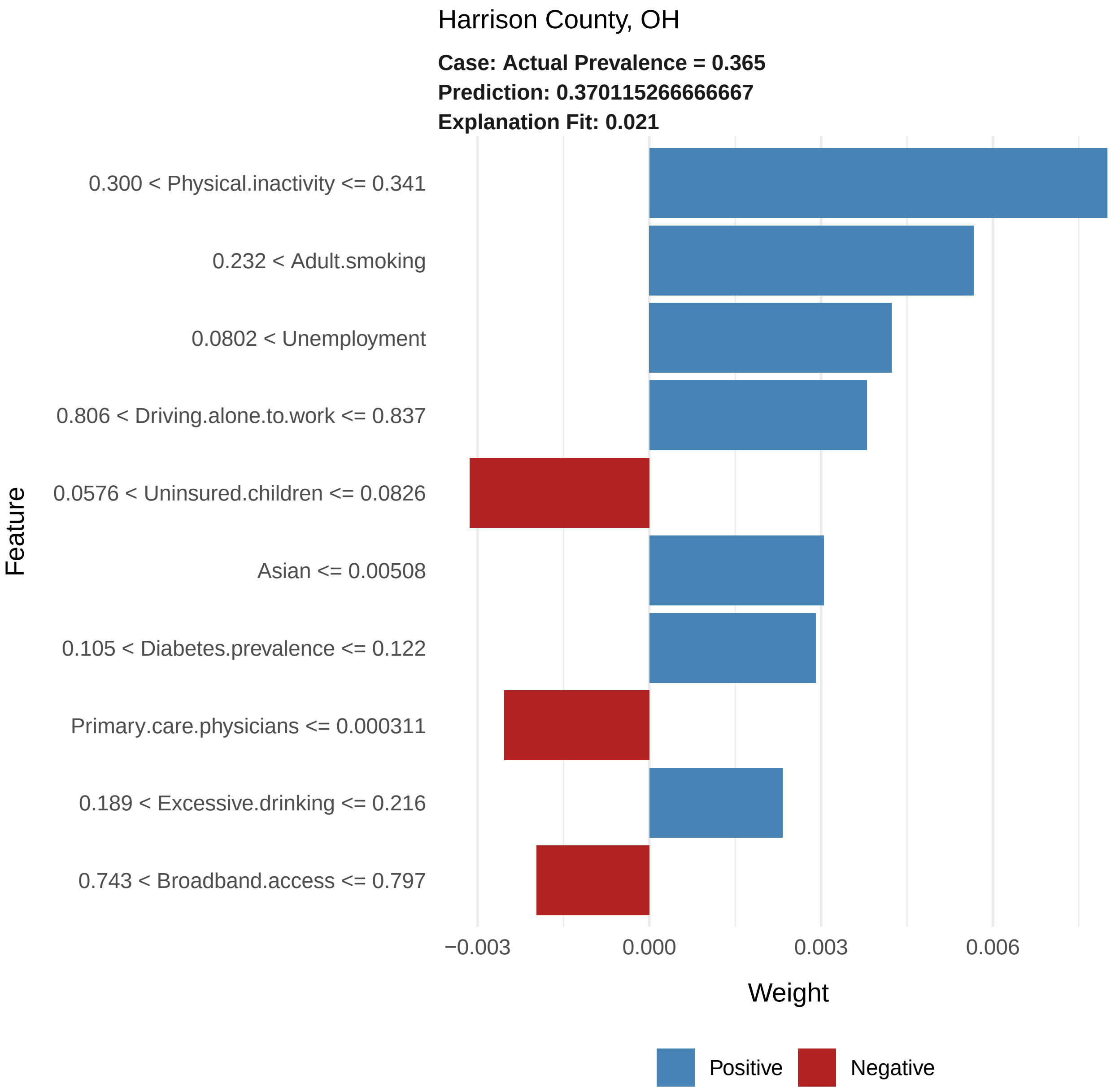
Harrison County, OH
Case: Actual Prevalence = 0.365
Prediction: 0.370115266666667
Explanation Fit: 0.021
0.300 < Physical.inactivity <= 0.341
0.232 < Adult.smoking
0.0802 < Unemployment
0.806 < Driving.alone.to.work <= 0.837
0.0576 < Uninsured.children <= 0.0826
Asian <= 0.00508
0.105 < Diabetes.prevalence <= 0.122
Primary.care.physicians <= 0.000311
0.189 < Excessive.drinking <= 0.216
0.743 < Broadband.access <= 0.797
Feature
−0.003
0.000
0.003
0.006
Weight
Positive
Negative

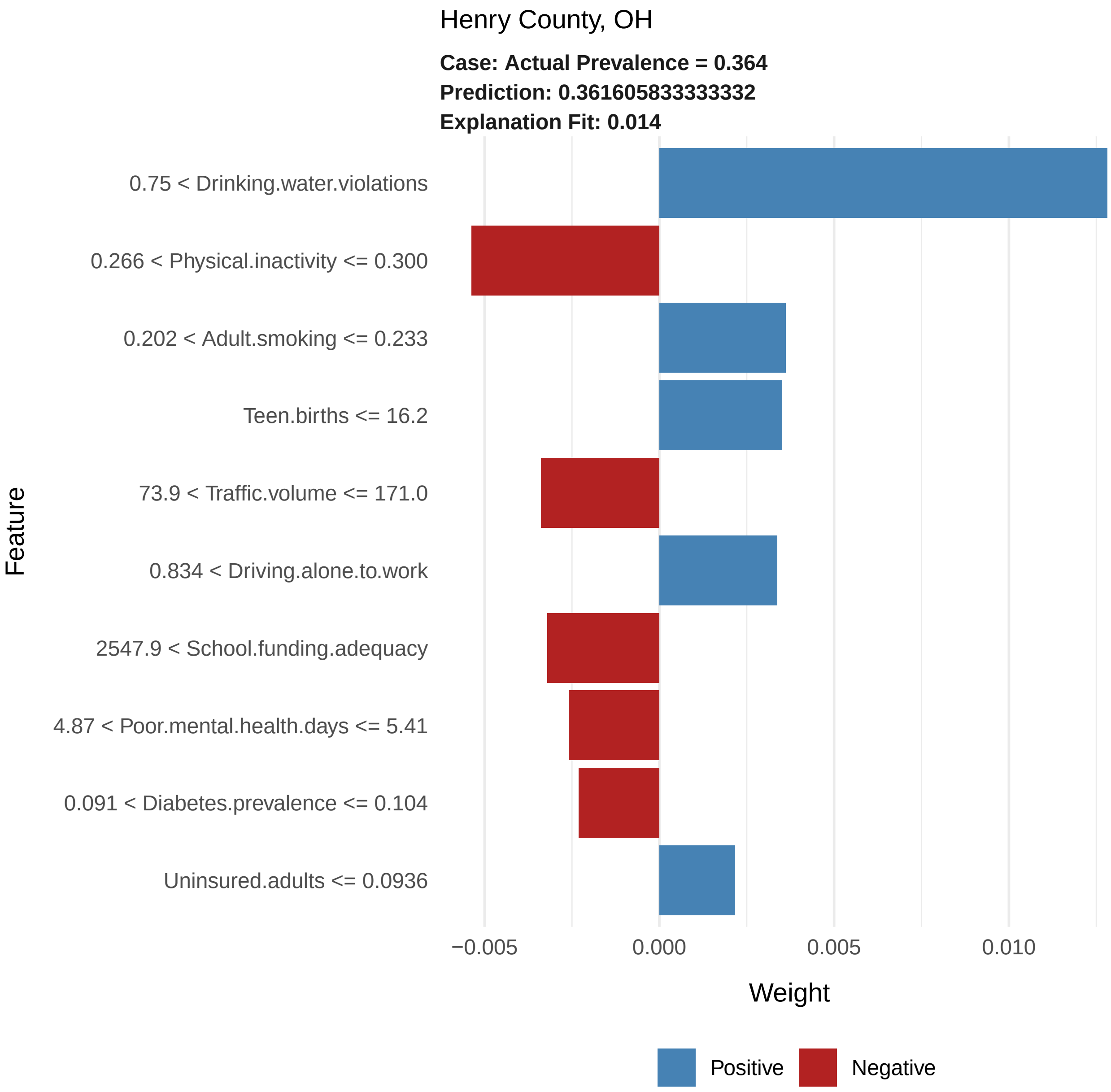
Henry County, OH
Case: Actual Prevalence = 0.364
Prediction: 0.361605833333332
Explanation Fit: 0.014
0.75 < Drinking.water.violations
0.266 < Physical.inactivity <= 0.300
0.202 < Adult.smoking <= 0.233
Teen.births <= 16.2
73.9 < Traffic.volume <= 171.0
0.834 < Driving.alone.to.work
2547.9 < School.funding.adequacy
4.87 < Poor.mental.health.days <= 5.41
0.091 < Diabetes.prevalence <= 0.104
Uninsured.adults <= 0.0936
Feature
−0.005
0.000
0.005
0.010
Weight
Positive
Negative

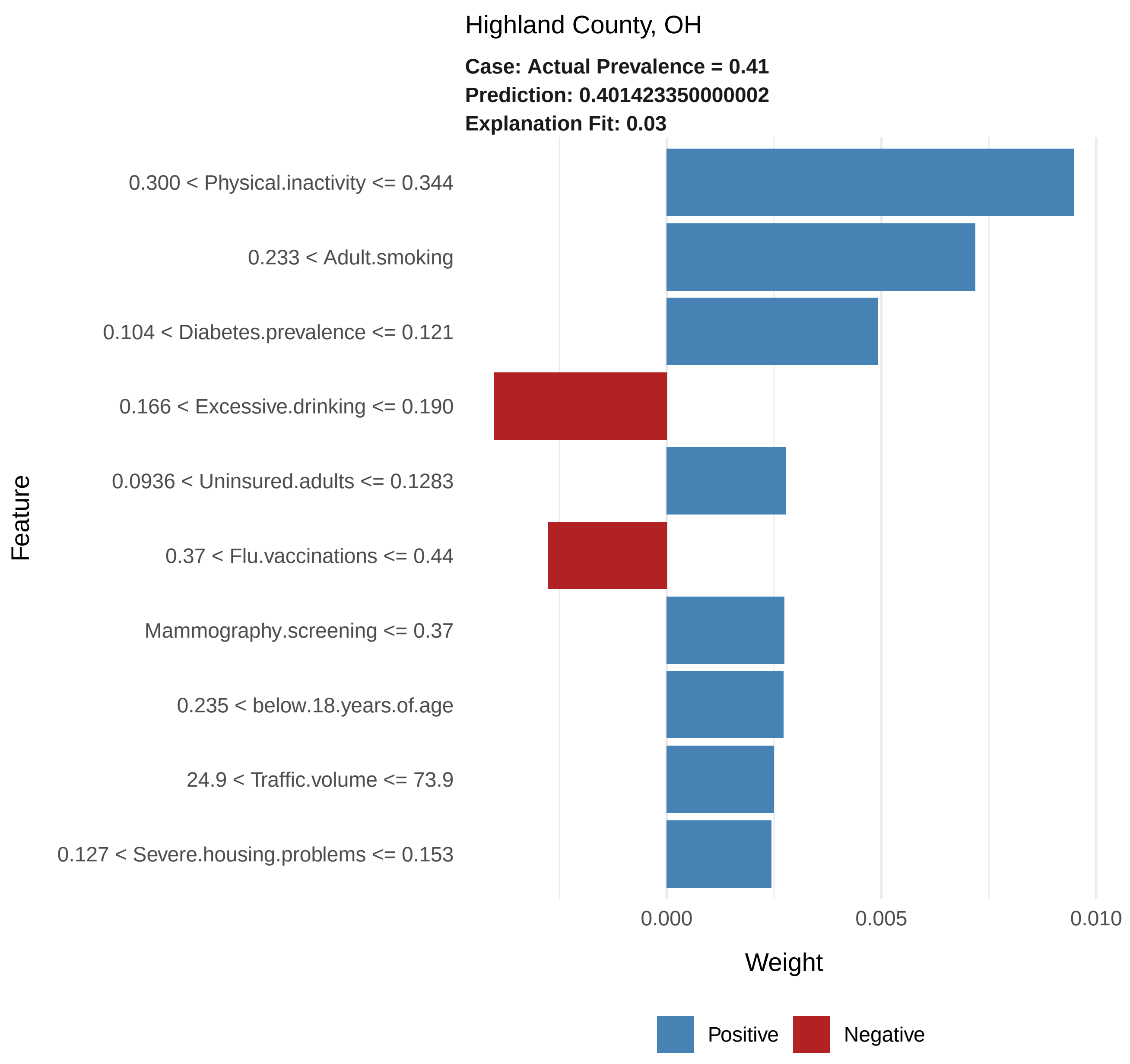
Highland County, OH
Case: Actual Prevalence = 0.41
Prediction: 0.401423350000002
Explanation Fit: 0.03
0.300 < Physical.inactivity <= 0.344
0.233 < Adult.smoking
0.104 < Diabetes.prevalence <= 0.121
0.166 < Excessive.drinking <= 0.190
0.0936 < Uninsured.adults <= 0.1283
0.37 < Flu.vaccinations <= 0.44
Mammography.screening <= 0.37
0.235 < below.18.years.of.age
24.9 < Traffic.volume <= 73.9
0.127 < Severe.housing.problems <= 0.153
Feature
0.000
0.005
0.010
Weight
Positive
Negative

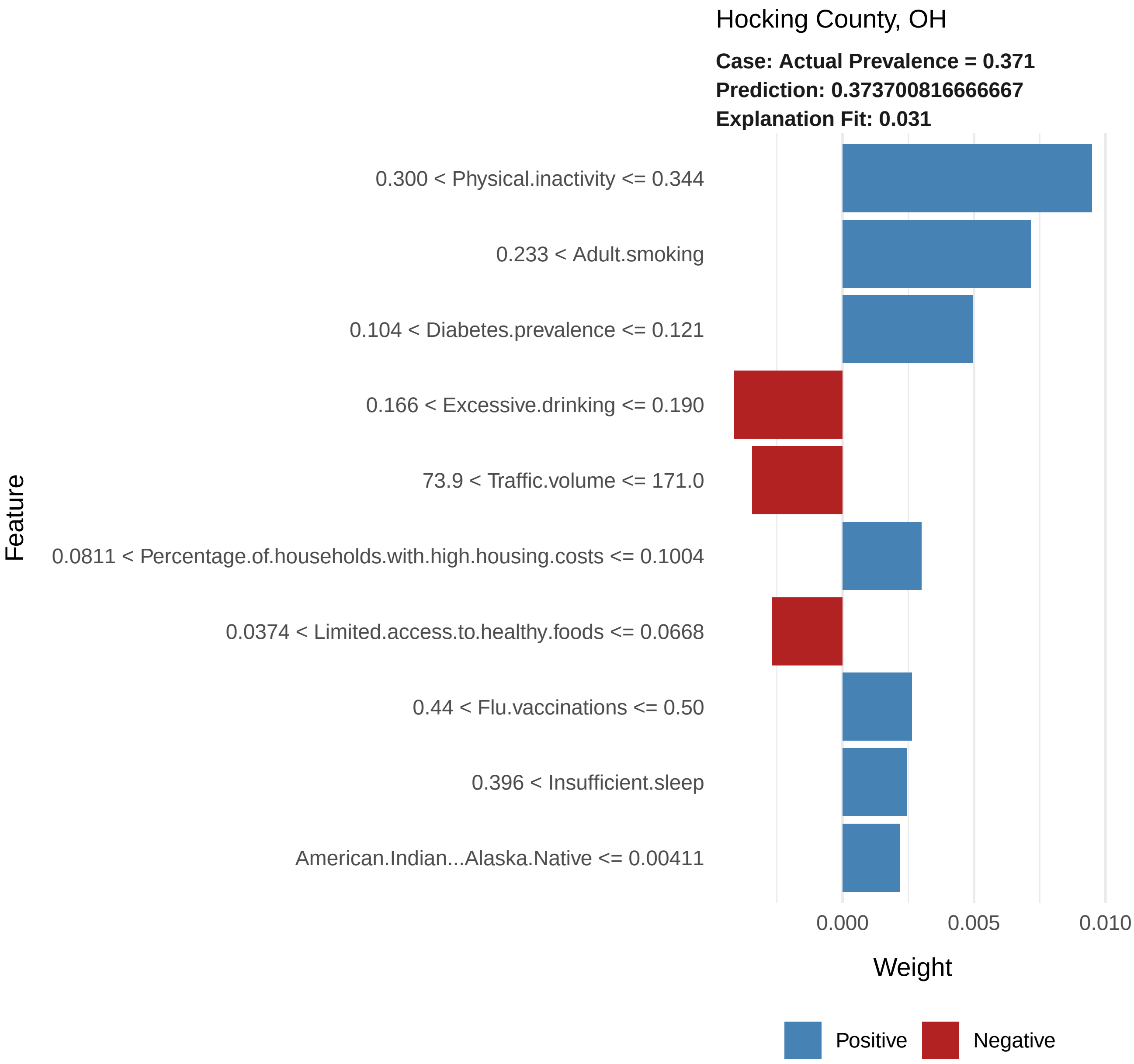
Hocking County, OH
Case: Actual Prevalence = 0.371
Prediction: 0.373700816666667
Explanation Fit: 0.031
0.300 < Physical.inactivity <= 0.344
0.233 < Adult.smoking
0.104 < Diabetes.prevalence <= 0.121
0.166 < Excessive.drinking <= 0.190
73.9 < Traffic.volume <= 171.0
0.0811 < Percentage.of.households.with.high.housing.costs <= 0.1004
0.0374 < Limited.access.to.healthy.foods <= 0.0668
0.44 < Flu.vaccinations <= 0.50
0.396 < Insufficient.sleep
American.Indian...Alaska.Native <= 0.00411
Feature
0.000
0.005
0.010
Weight
Positive
Negative

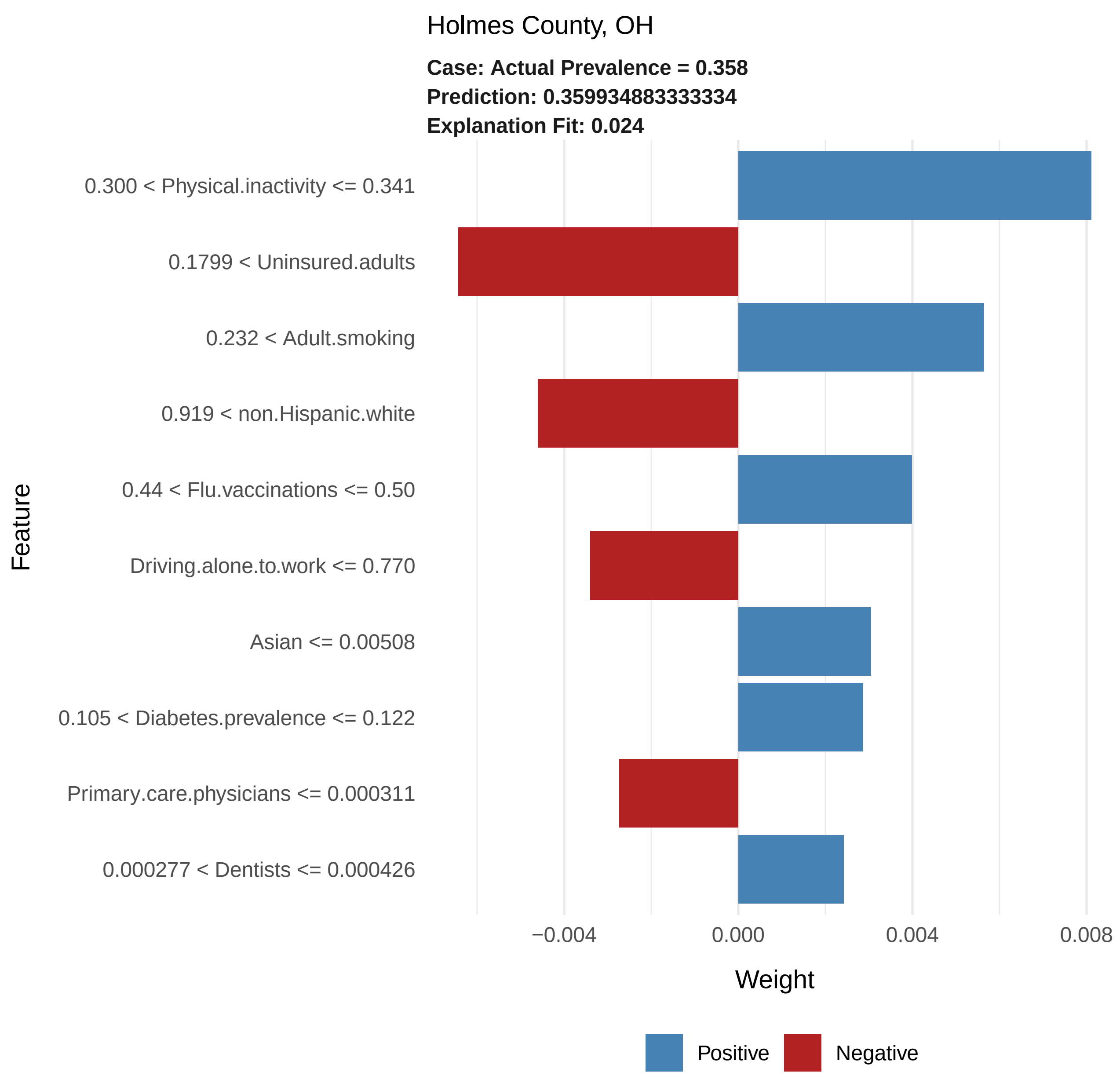

Holmes County, OH
Case: Actual Prevalence = 0.358
Prediction: 0.359934883333334
Explanation Fit: 0.024
0.300 < Physical.inactivity <= 0.341
0.1799 < Uninsured.adults
0.232 < Adult.smoking
0.919 < non.Hispanic.white
0.44 < Flu.vaccinations <= 0.50
Driving.alone.to.work <= 0.770
Asian <= 0.00508
0.105 < Diabetes.prevalence <= 0.122
Primary.care.physicians <= 0.000311
0.000277 < Dentists <= 0.000426
Feature
−0.004
0.000
0.004
0.008
Weight
Positive
Negative

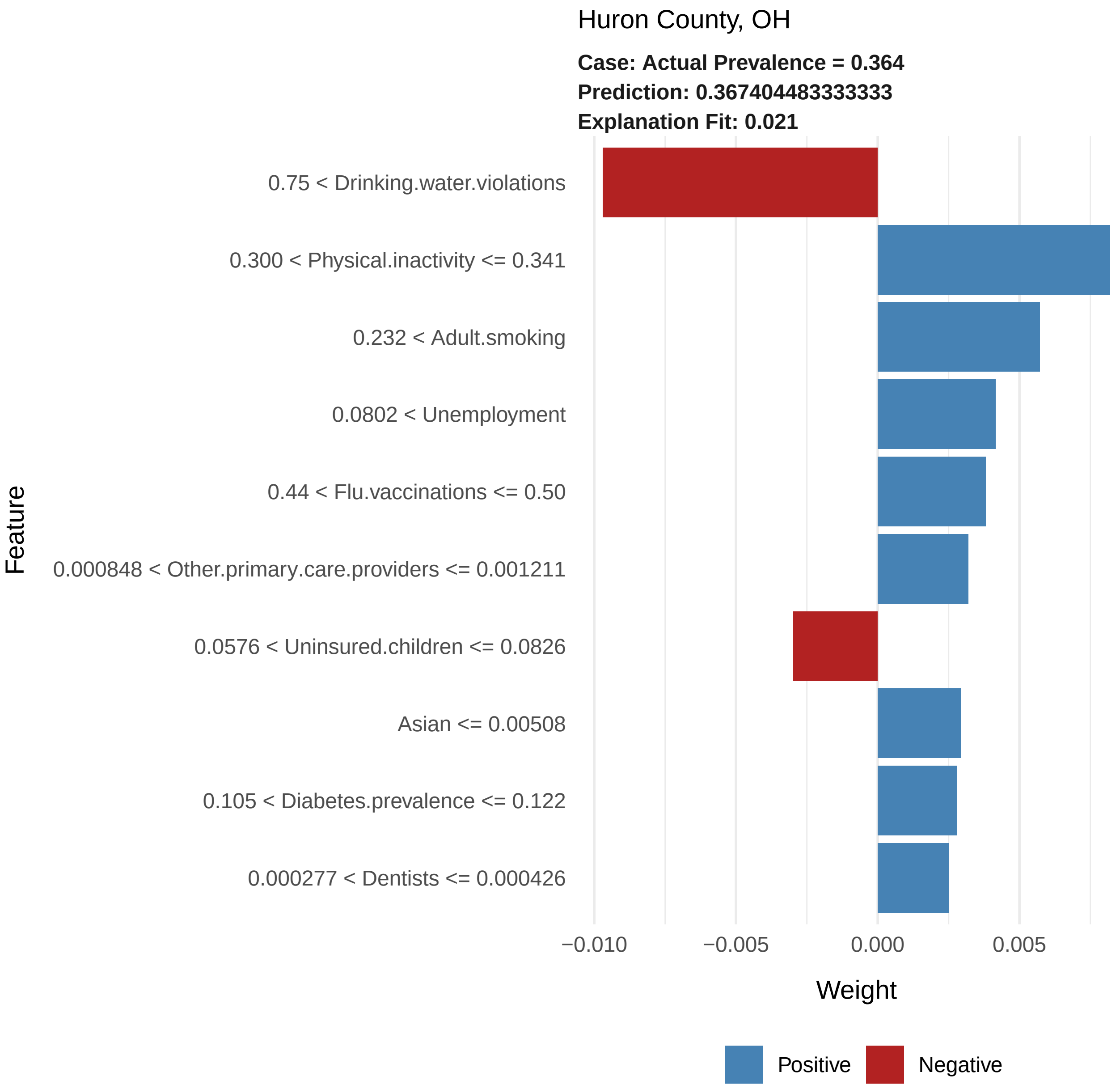

Huron County, OH
Case: Actual Prevalence = 0.364
Prediction: 0.367404483333333
Explanation Fit: 0.021
0.75 < Drinking.water.violations
0.300 < Physical.inactivity <= 0.341
0.232 < Adult.smoking
0.0802 < Unemployment
0.44 < Flu.vaccinations <= 0.50
0.000848 < Other.primary.care.providers <= 0.001211
0.0576 < Uninsured.children <= 0.0826
Asian <= 0.00508
0.105 < Diabetes.prevalence <= 0.122
0.000277 < Dentists <= 0.000426
Feature
−0.010
−0.005
0.000
0.005
Weight
Positive
Negative

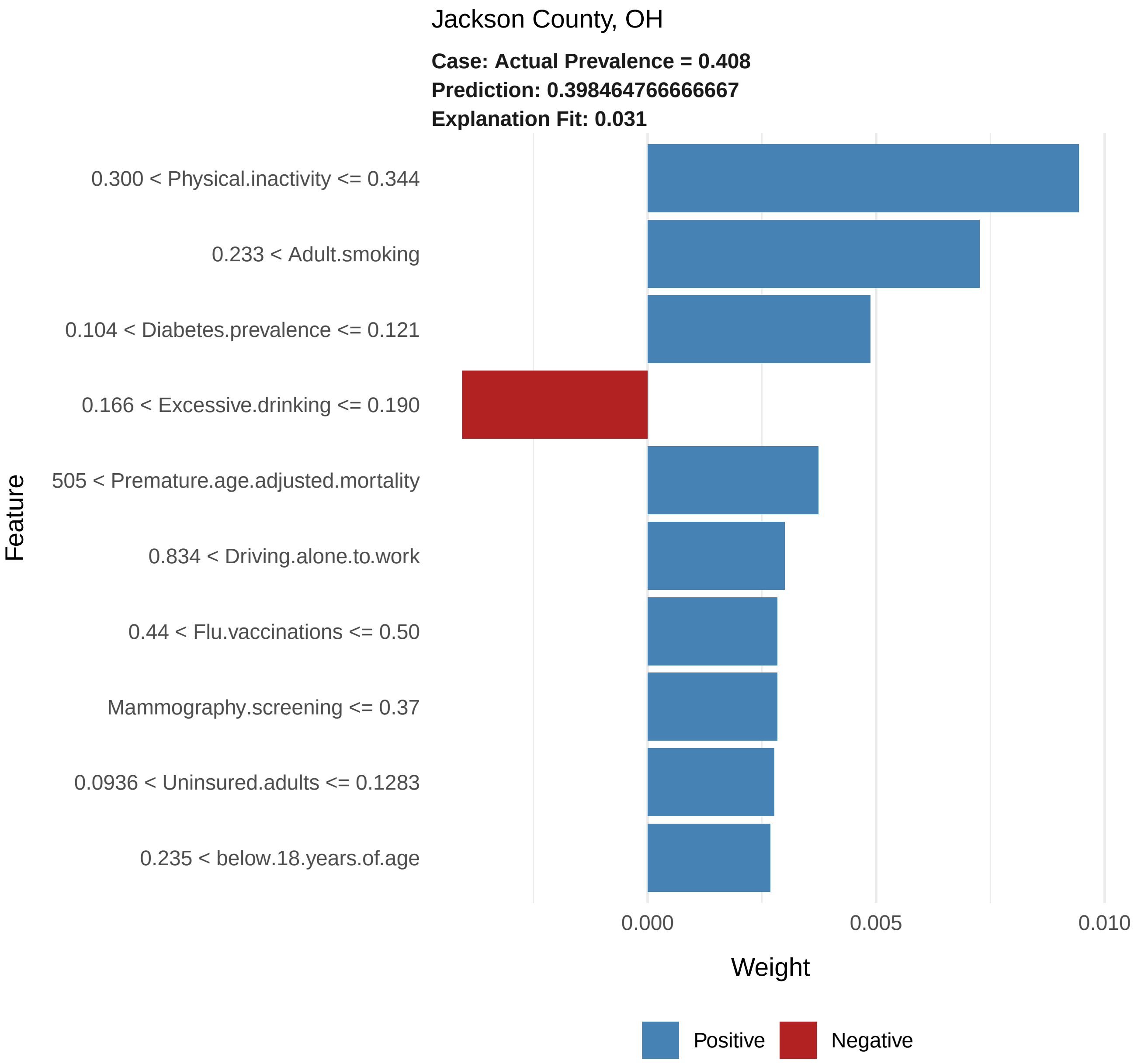

Jackson County, OH
Case: Actual Prevalence = 0.408
Prediction: 0.398464766666667
Explanation Fit: 0.031
0.300 < Physical.inactivity <= 0.344
0.233 < Adult.smoking
0.104 < Diabetes.prevalence <= 0.121
0.166 < Excessive.drinking <= 0.190
505 < Premature.age.adjusted.mortality
0.834 < Driving.alone.to.work
0.44 < Flu.vaccinations <= 0.50
Mammography.screening <= 0.37
0.0936 < Uninsured.adults <= 0.1283
0.235 < below.18.years.of.age
Feature
0.000
0.005
0.010
Weight
Positive
Negative

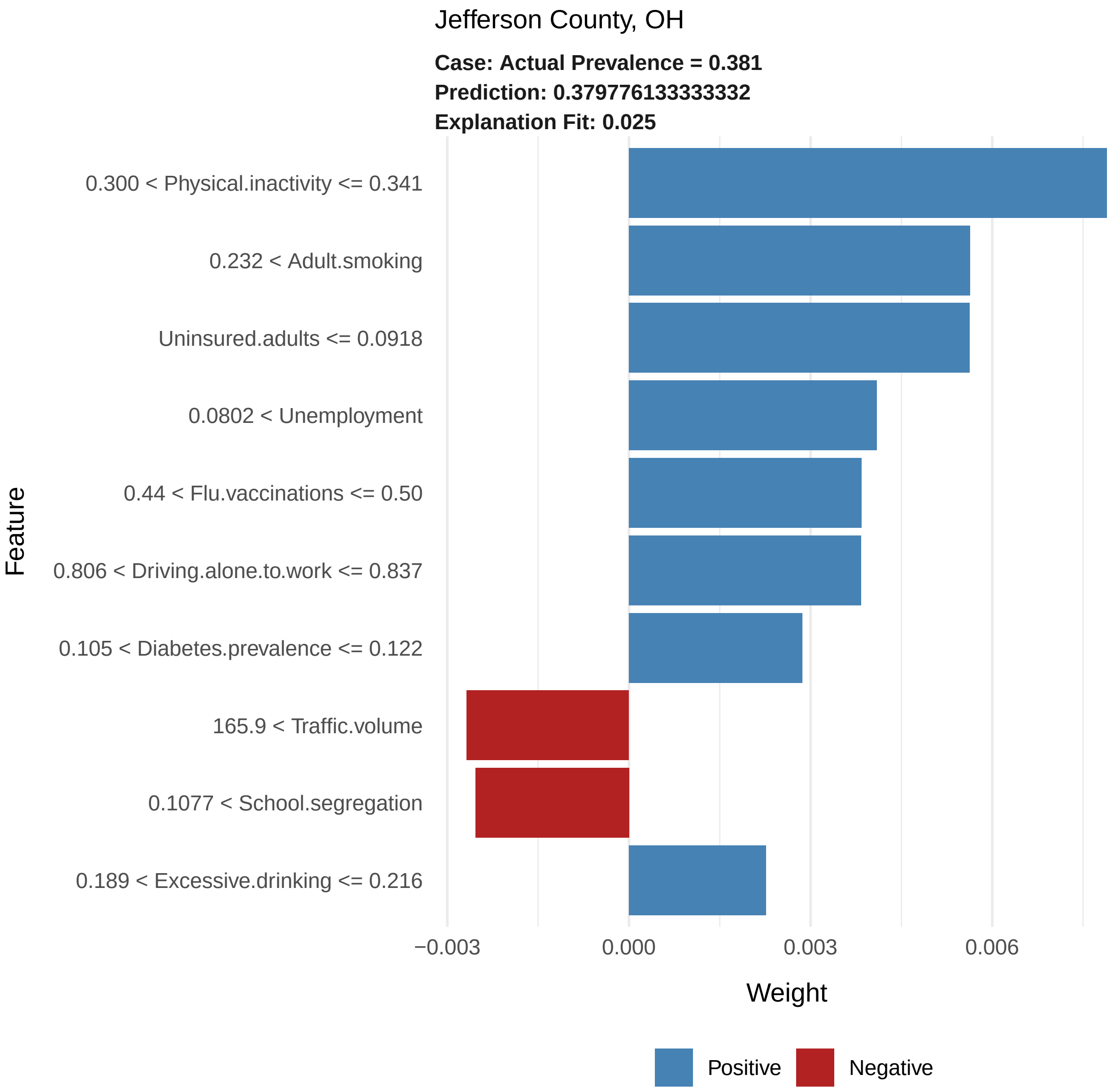

Jefferson County, OH
Case: Actual Prevalence = 0.381
Prediction: 0.379776133333332
Explanation Fit: 0.025
0.300 < Physical.inactivity <= 0.341
0.232 < Adult.smoking
Uninsured.adults <= 0.0918
0.0802 < Unemployment
0.44 < Flu.vaccinations <= 0.50
0.806 < Driving.alone.to.work <= 0.837
0.105 < Diabetes.prevalence <= 0.122
165.9 < Traffic.volume
0.1077 < School.segregation
0.189 < Excessive.drinking <= 0.216
Feature
−0.003
0.000
0.003
0.006
Weight
Positive
Negative

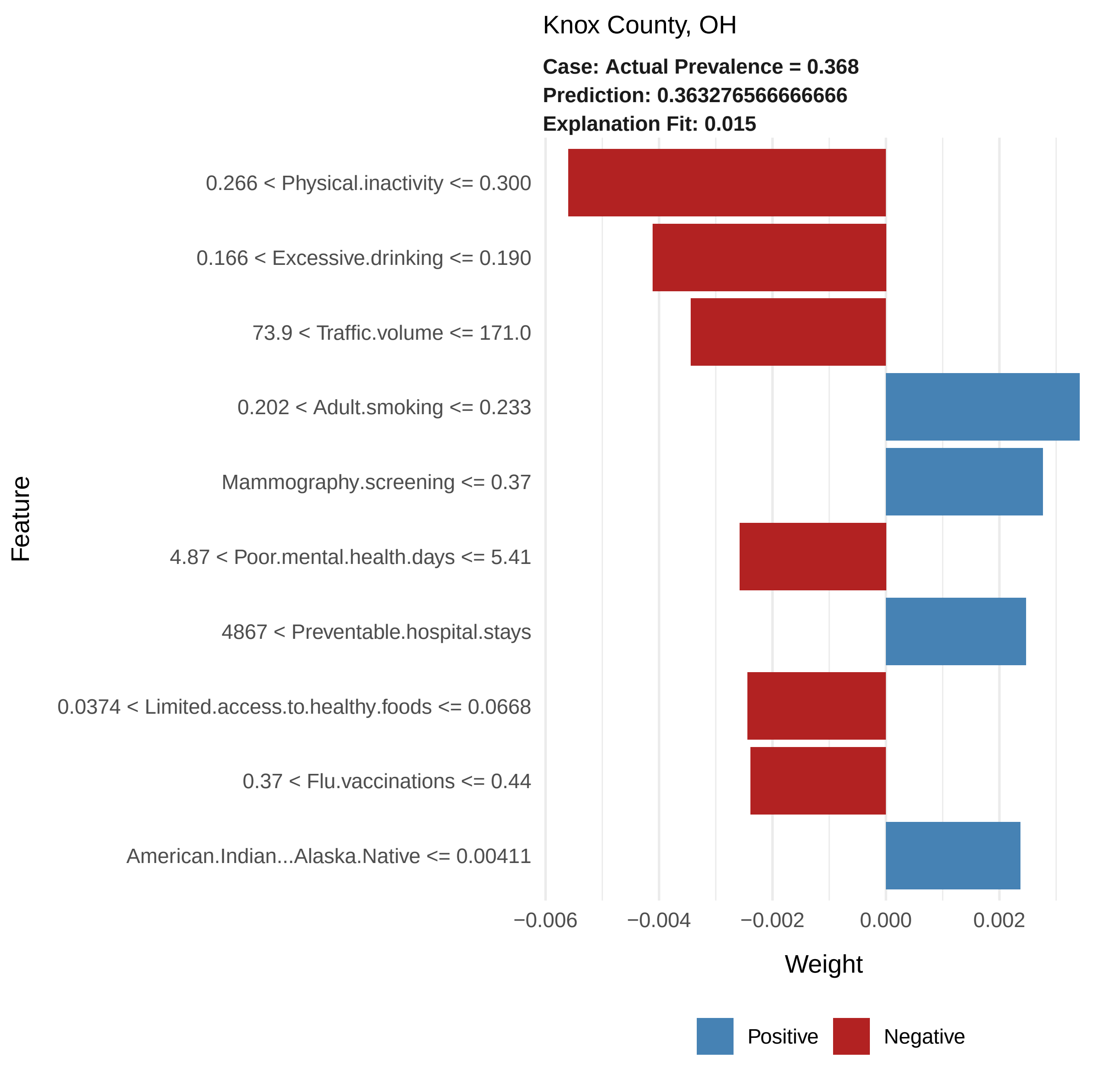
Knox County, OH
Case: Actual Prevalence = 0.368
Prediction: 0.363276566666666
Explanation Fit: 0.015
0.266 < Physical.inactivity <= 0.300
0.166 < Excessive.drinking <= 0.190
73.9 < Traffic.volume <= 171.0
0.202 < Adult.smoking <= 0.233
Mammography.screening <= 0.37
4.87 < Poor.mental.health.days <= 5.41
4867 < Preventable.hospital.stays
0.0374 < Limited.access.to.healthy.foods <= 0.0668
0.37 < Flu.vaccinations <= 0.44
American.Indian...Alaska.Native <= 0.00411
Feature
−0.006
−0.004
−0.002
0.000
0.002
Weight
Positive
Negative

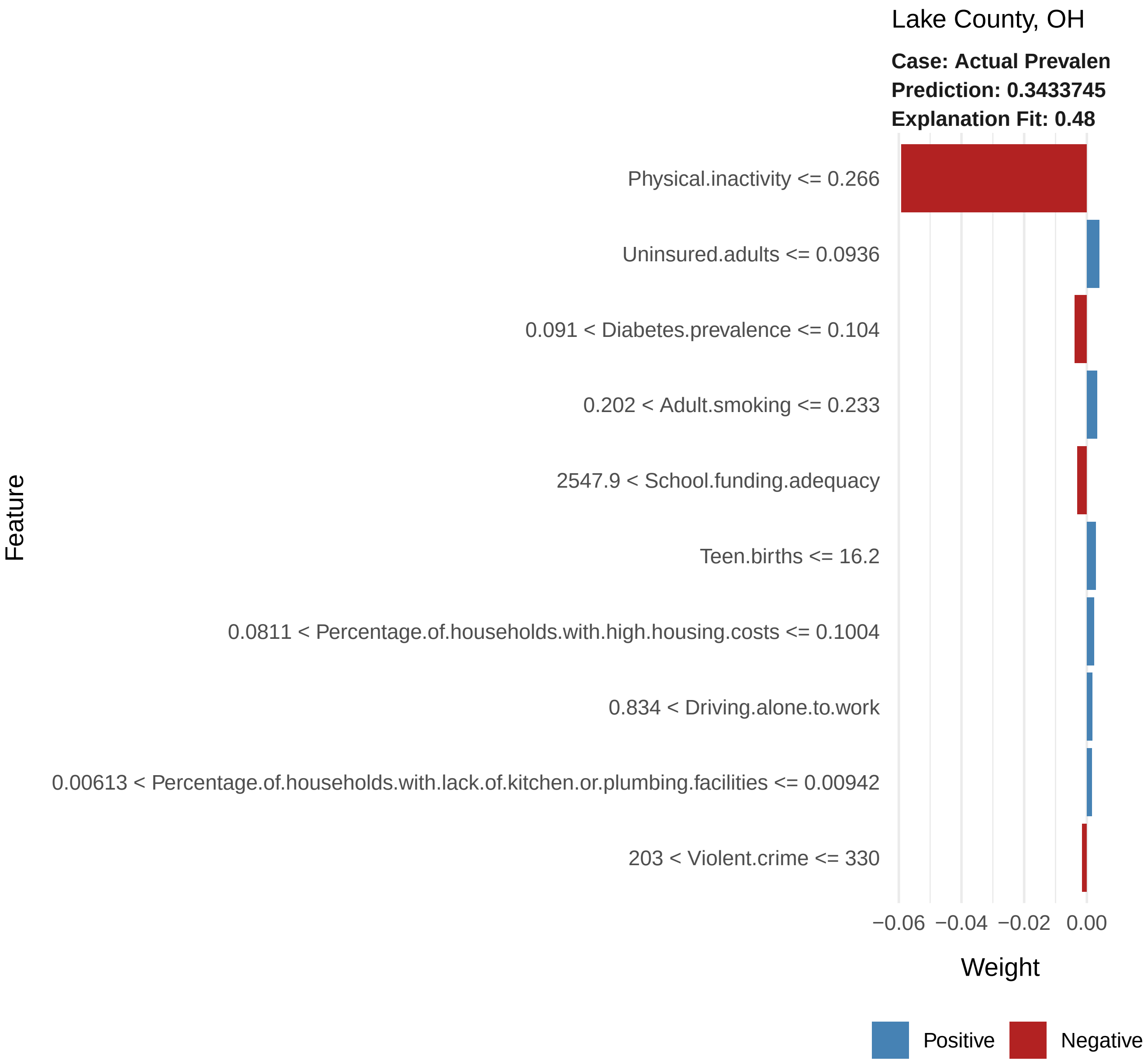

Lake County, OH
Case: Actual Prevalen
Prediction: 0.3433745
Explanation Fit: 0.48
Physical.inactivity <= 0.266
Uninsured.adults <= 0.0936
0.091 < Diabetes.prevalence <= 0.104
0.202 < Adult.smoking <= 0.233
2547.9 < School.funding.adequacy
Teen.births <= 16.2
0.0811 < Percentage.of.households.with.high.housing.costs <= 0.1004
0.834 < Driving.alone.to.work
0.00613 < Percentage.of.households.with.lack.of.kitchen.or.plumbing.facilities <= 0.00942
203 < Violent.crime <= 330
Feature
−0.06 −0.04 −0.02 0.00
Weight
Positive
Negative

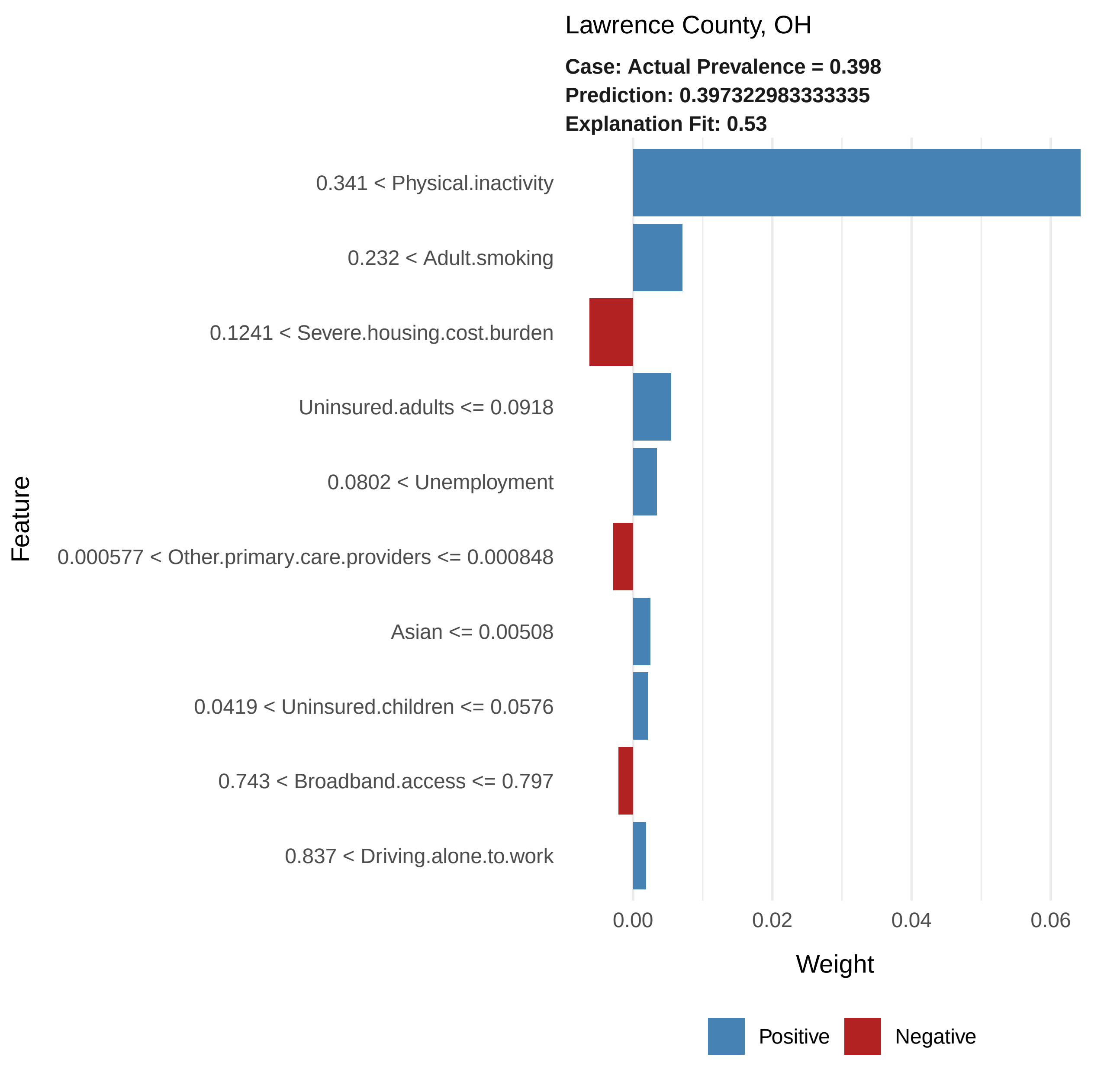

Lawrence County, OH
Case: Actual Prevalence = 0.398
Prediction: 0.397322983333335
Explanation Fit: 0.53
0.341 < Physical.inactivity
0.232 < Adult.smoking
0.1241 < Severe.housing.cost.burden
Uninsured.adults <= 0.0918
0.0802 < Unemployment
0.000577 < Other.primary.care.providers <= 0.000848
Asian <= 0.00508
0.0419 < Uninsured.children <= 0.0576
0.743 < Broadband.access <= 0.797
0.837 < Driving.alone.to.work
Feature
0.00
0.02
0.04
0.06
Weight
Positive
Negative

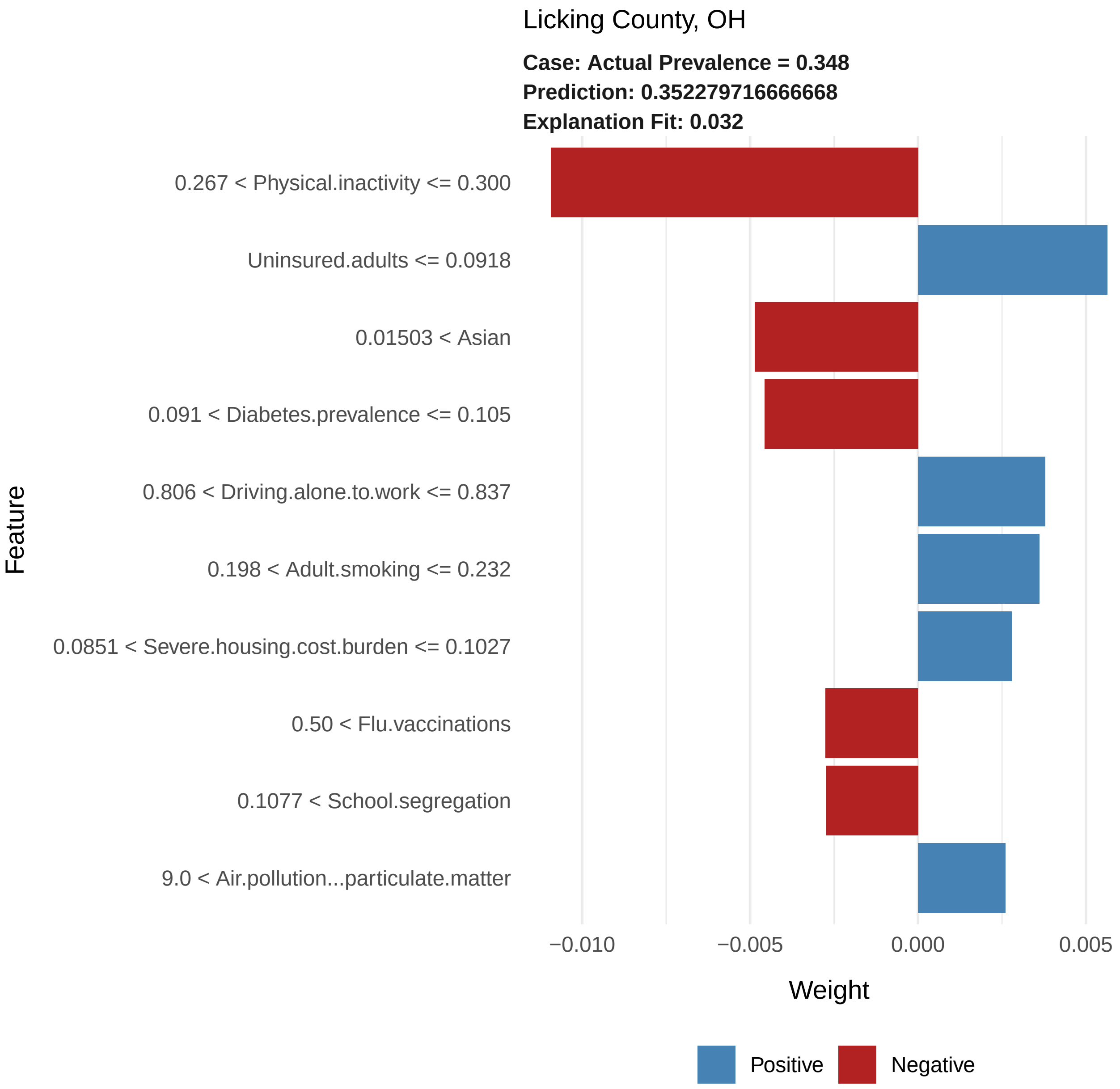
Licking County, OH
Case: Actual Prevalence = 0.348
Prediction: 0.352279716666668
Explanation Fit: 0.032
0.267 < Physical.inactivity <= 0.300
Uninsured.adults <= 0.0918
0.01503 < Asian
0.091 < Diabetes.prevalence <= 0.105
0.806 < Driving.alone.to.work <= 0.837
0.198 < Adult.smoking <= 0.232
0.0851 < Severe.housing.cost.burden <= 0.1027
0.50 < Flu.vaccinations
0.1077 < School.segregation
9.0 < Air.pollution...particulate.matter
Feature
−0.010
−0.005
0.000
0.005
Weight
Positive
Negative

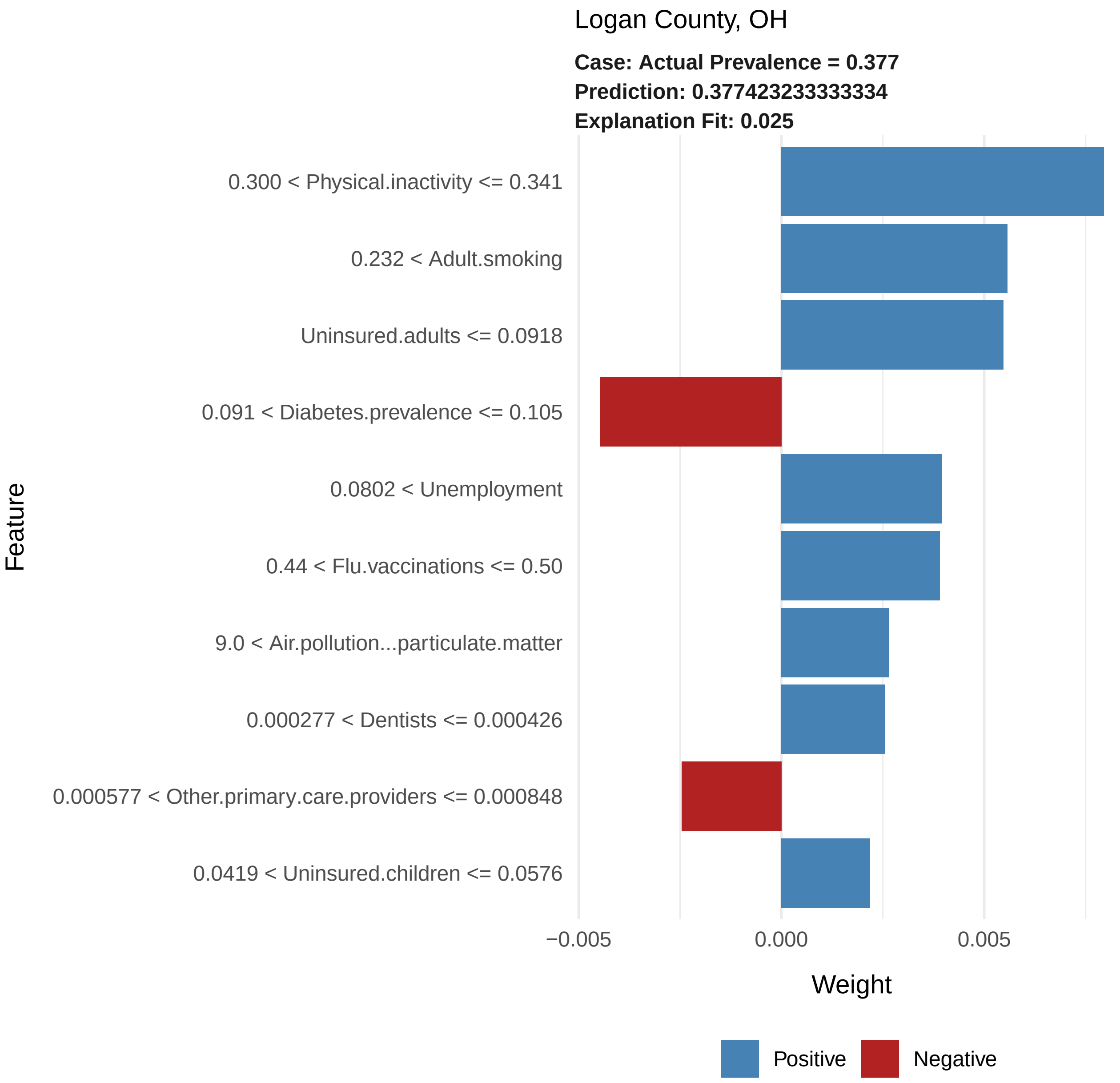

Logan County, OH
Case: Actual Prevalence = 0.377
Prediction: 0.377423233333334
Explanation Fit: 0.025
0.300 < Physical.inactivity <= 0.341
0.232 < Adult.smoking
Uninsured.adults <= 0.0918
0.091 < Diabetes.prevalence <= 0.105
0.0802 < Unemployment
0.44 < Flu.vaccinations <= 0.50
9.0 < Air.pollution...particulate.matter
0.000277 < Dentists <= 0.000426
0.000577 < Other.primary.care.providers <= 0.000848
0.0419 < Uninsured.children <= 0.0576
Feature
−0.005
0.000
0.005
Weight
Positive
Negative

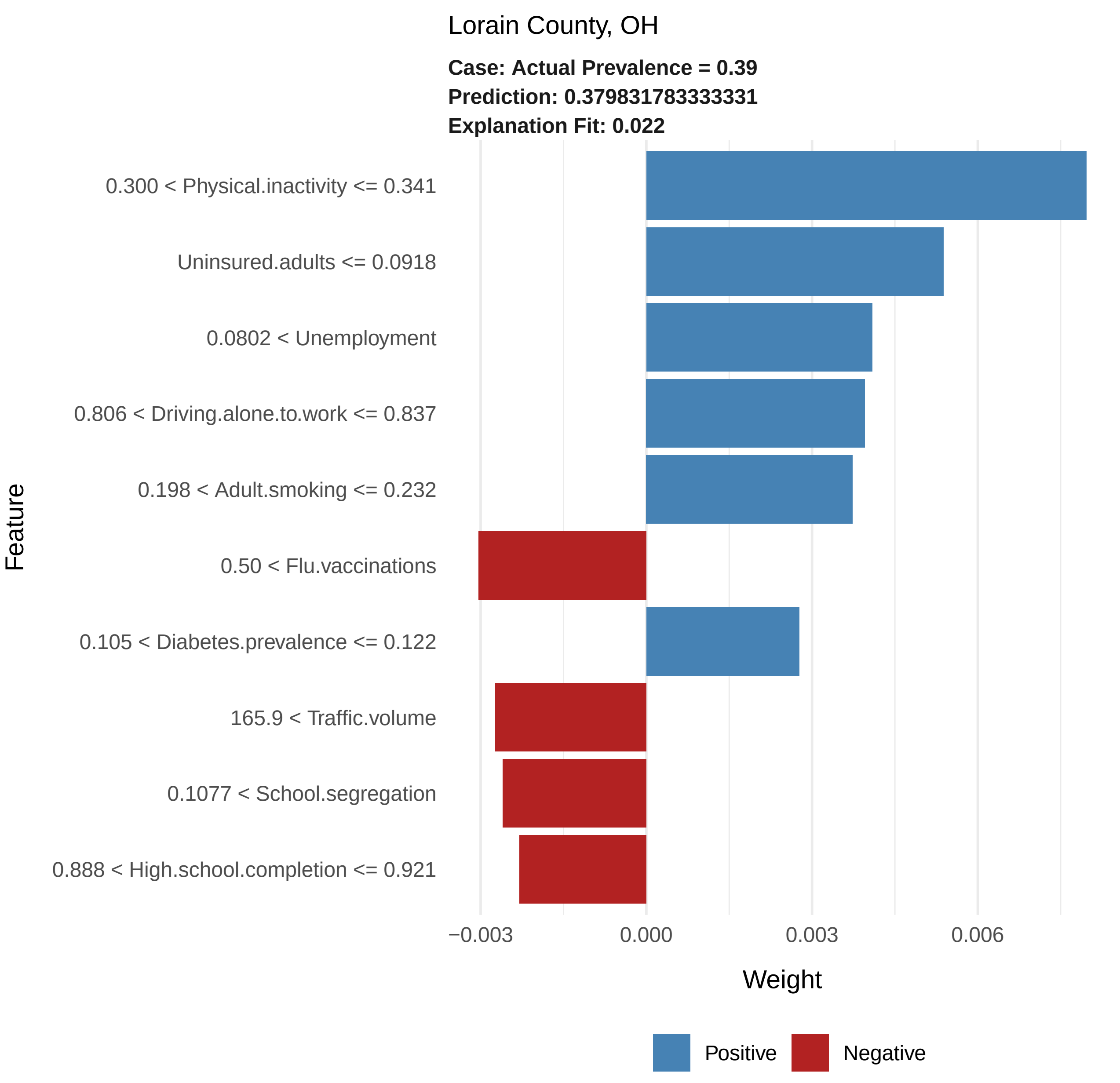
Lorain County, OH
Case: Actual Prevalence = 0.39
Prediction: 0.379831783333331
Explanation Fit: 0.022
0.300 < Physical.inactivity <= 0.341
Uninsured.adults <= 0.0918
0.0802 < Unemployment
0.806 < Driving.alone.to.work <= 0.837
0.198 < Adult.smoking <= 0.232
0.50 < Flu.vaccinations
0.105 < Diabetes.prevalence <= 0.122
165.9 < Traffic.volume
0.1077 < School.segregation
0.888 < High.school.completion <= 0.921
Feature
−0.003
0.000
0.003
0.006
Weight
Positive
Negative

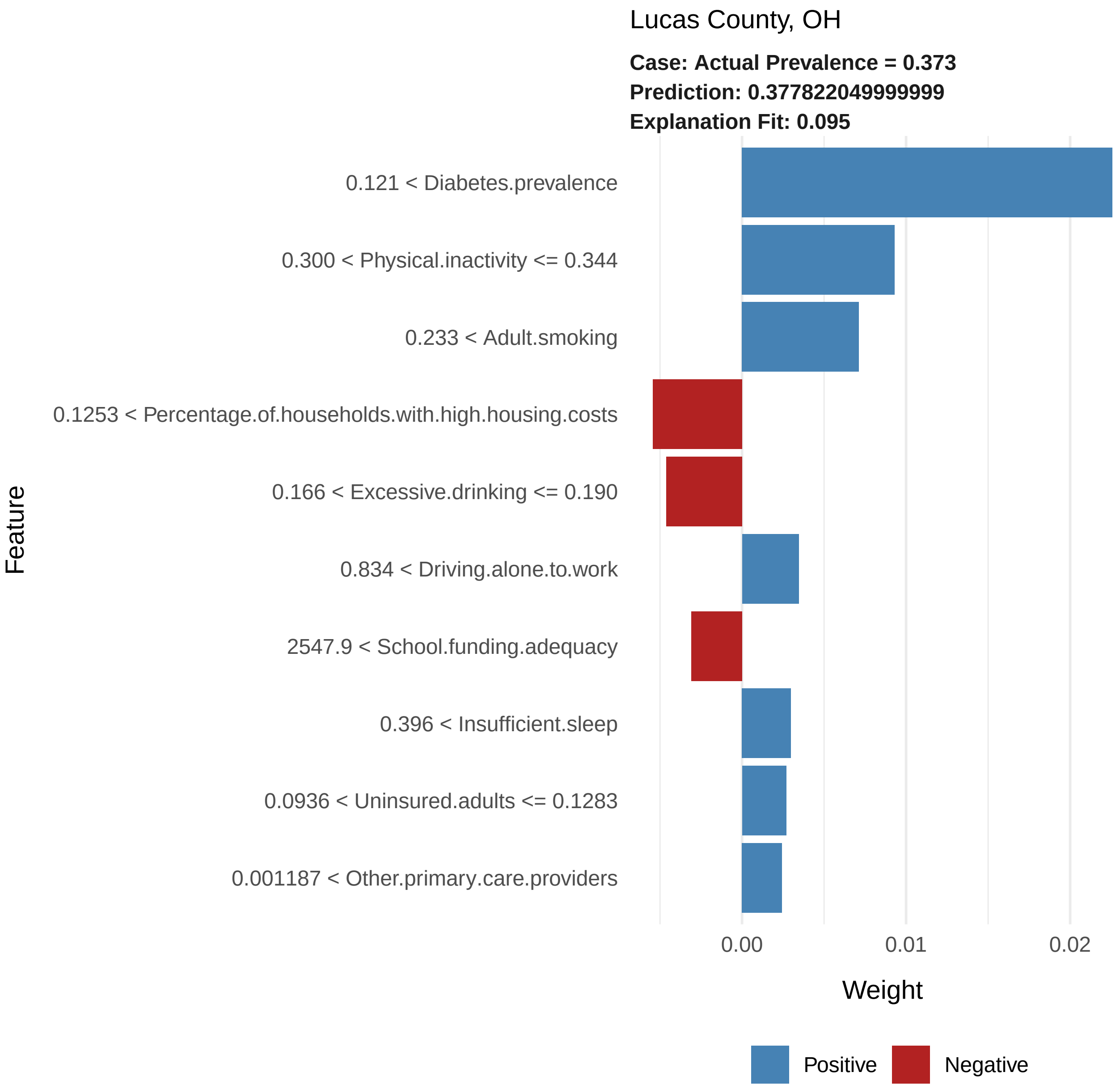

Lucas County, OH
Case: Actual Prevalence = 0.373
Prediction: 0.377822049999999
Explanation Fit: 0.095
0.121 < Diabetes.prevalence
0.300 < Physical.inactivity <= 0.344
0.233 < Adult.smoking
0.1253 < Percentage.of.households.with.high.housing.costs
0.166 < Excessive.drinking <= 0.190
0.834 < Driving.alone.to.work
2547.9 < School.funding.adequacy
0.396 < Insufficient.sleep
0.0936 < Uninsured.adults <= 0.1283
0.001187 < Other.primary.care.providers
Feature
0.00
0.01
0.02
Weight
Positive
Negative

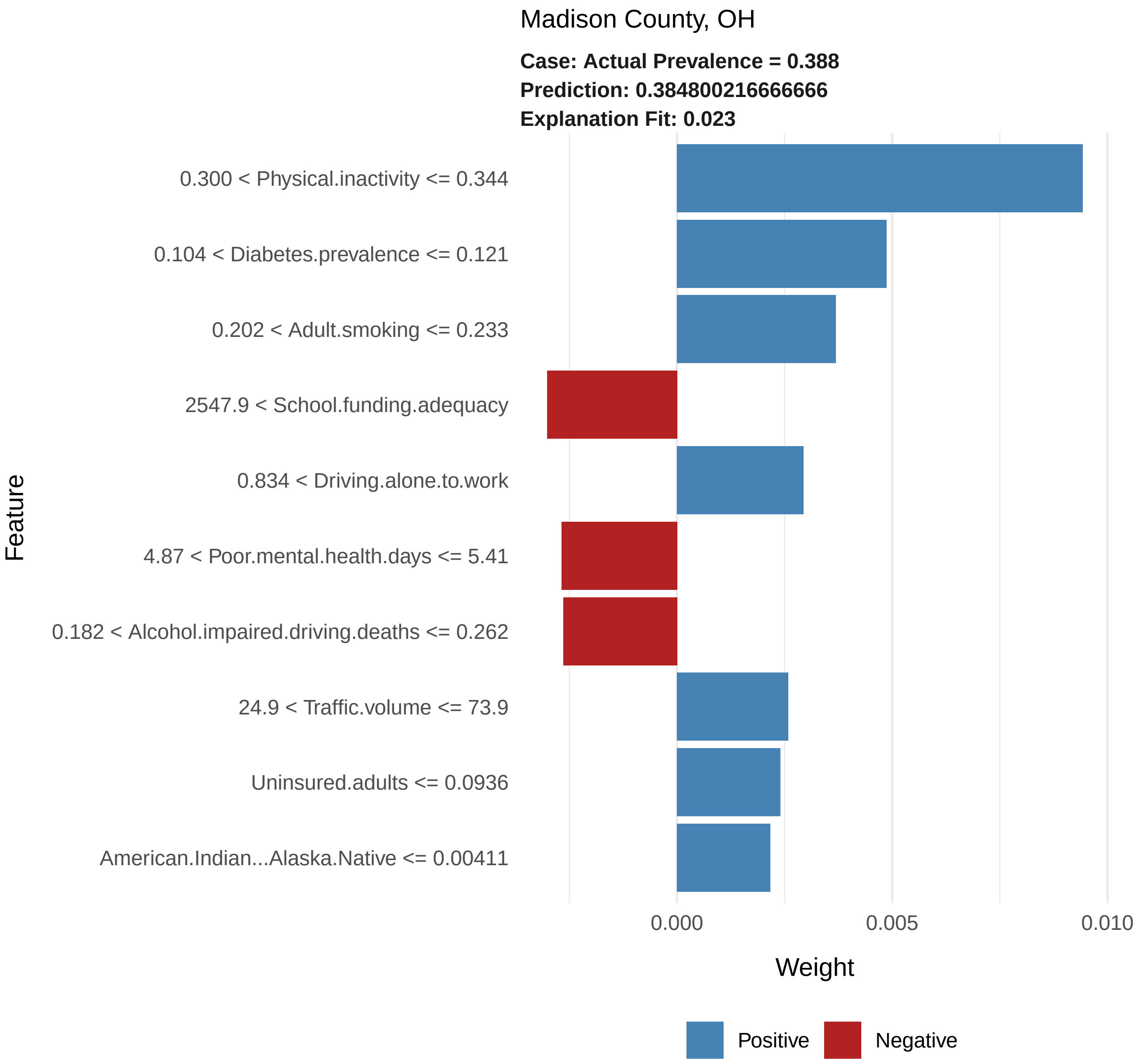
Madison County, OH
Case: Actual Prevalence = 0.388
Prediction: 0.384800216666666
Explanation Fit: 0.023
0.300 < Physical.inactivity <= 0.344
0.104 < Diabetes.prevalence <= 0.121
0.202 < Adult.smoking <= 0.233
2547.9 < School.funding.adequacy
0.834 < Driving.alone.to.work
4.87 < Poor.mental.health.days <= 5.41
0.182 < Alcohol.impaired.driving.deaths <= 0.262
24.9 < Traffic.volume <= 73.9
Uninsured.adults <= 0.0936
American.Indian...Alaska.Native <= 0.00411
Feature
0.000
0.005
0.010
Weight
Positive
Negative

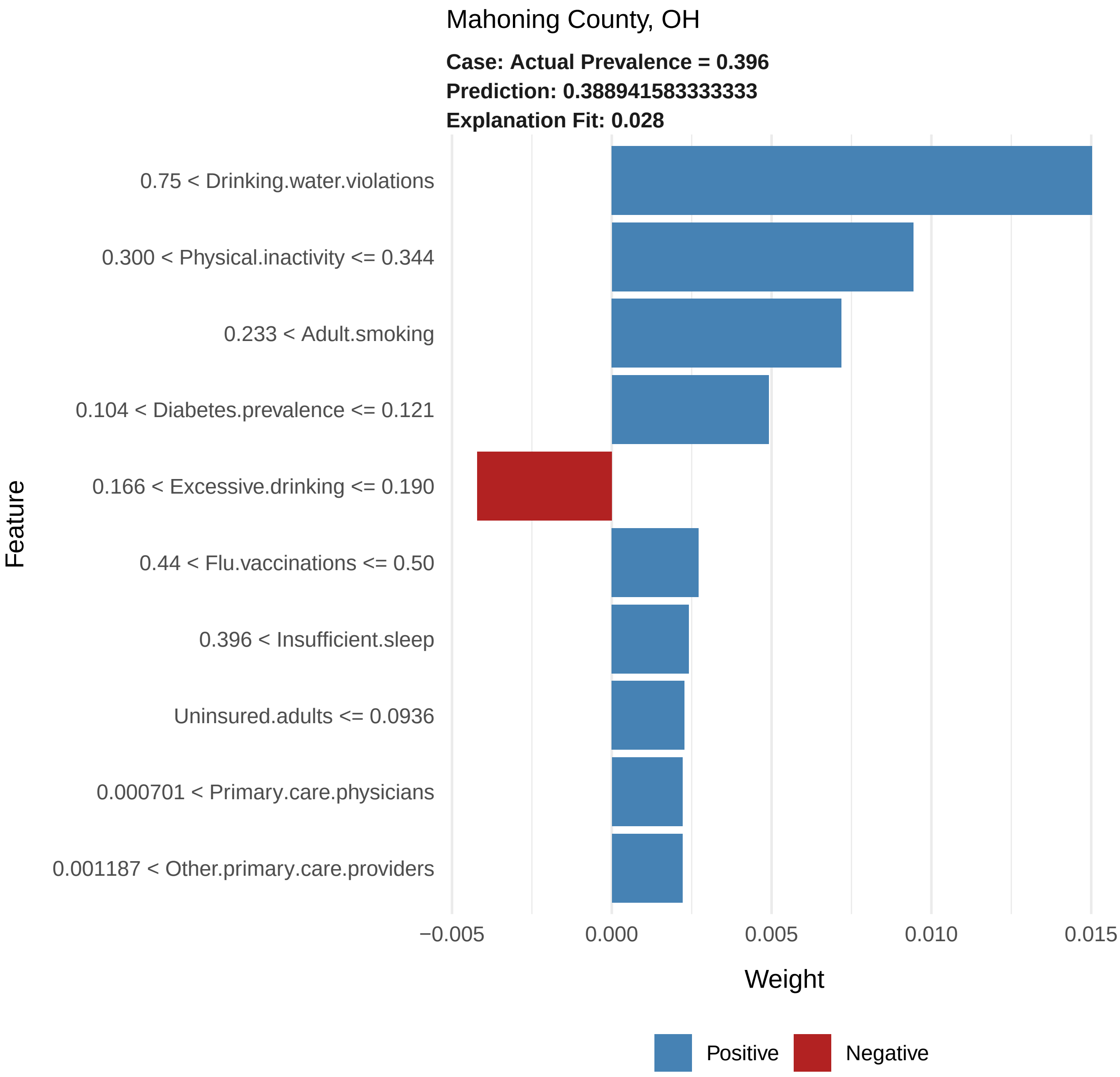

Mahoning County, OH
Case: Actual Prevalence = 0.396
Prediction: 0.388941583333333
Explanation Fit: 0.028
0.75 < Drinking.water.violations
0.300 < Physical.inactivity <= 0.344
0.233 < Adult.smoking
0.104 < Diabetes.prevalence <= 0.121
0.166 < Excessive.drinking <= 0.190
0.44 < Flu.vaccinations <= 0.50
0.396 < Insufficient.sleep
Uninsured.adults <= 0.0936
0.000701 < Primary.care.physicians
0.001187 < Other.primary.care.providers
Feature
−0.005
0.000
0.005
0.010
0.015
Weight
Positive
Negative

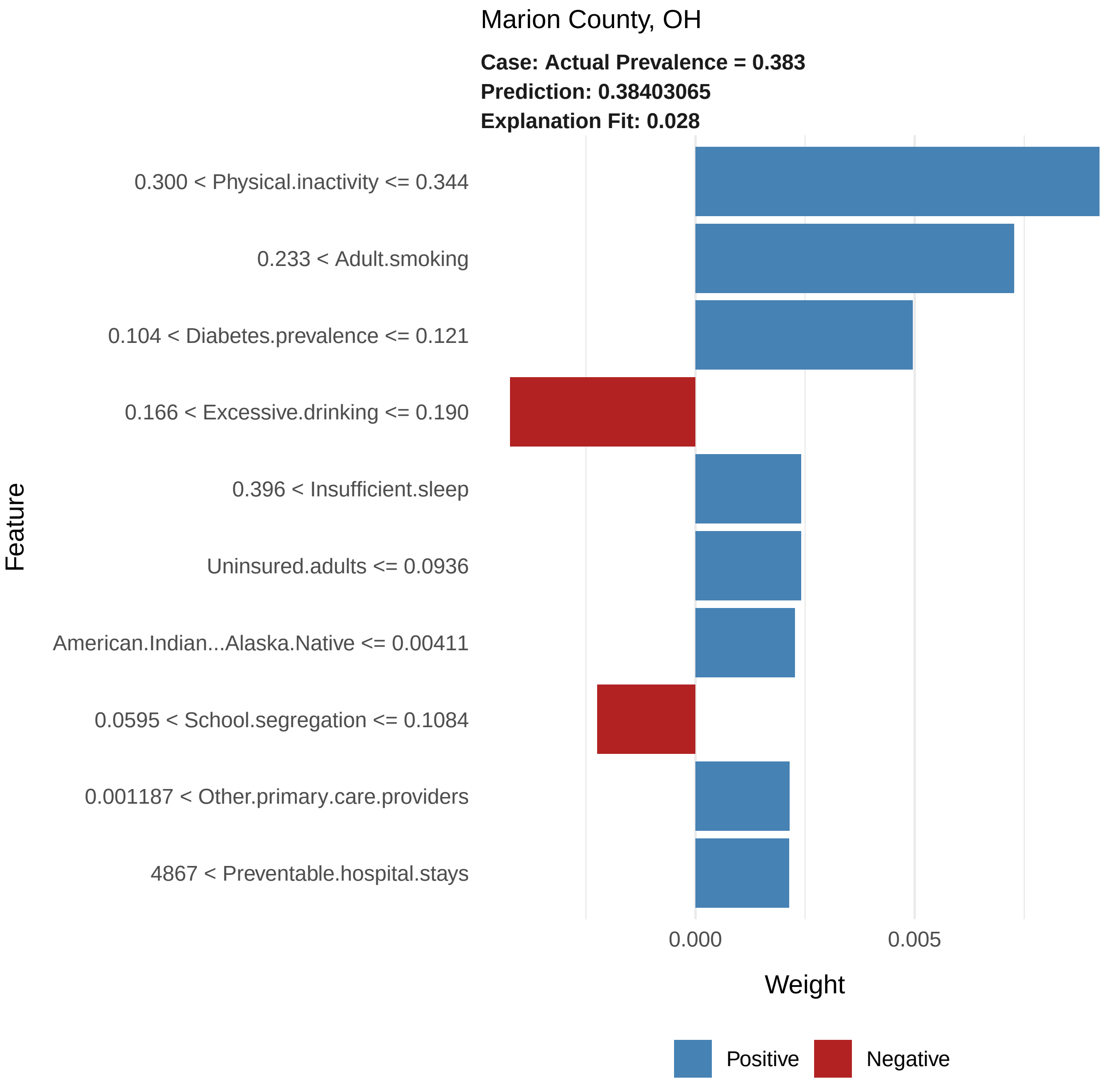
Marion County, OH
Case: Actual Prevalence = 0.383
Prediction: 0.38403065
Explanation Fit: 0.028
0.300 < Physical.inactivity <= 0.344
0.233 < Adult.smoking
0.104 < Diabetes.prevalence <= 0.121
0.166 < Excessive.drinking <= 0.190
0.396 < Insufficient.sleep
Uninsured.adults <= 0.0936
American.Indian...Alaska.Native <= 0.00411
0.0595 < School.segregation <= 0.1084
0.001187 < Other.primary.care.providers
4867 < Preventable.hospital.stays
Feature
0.000
0.005
Weight
Positive
Negative

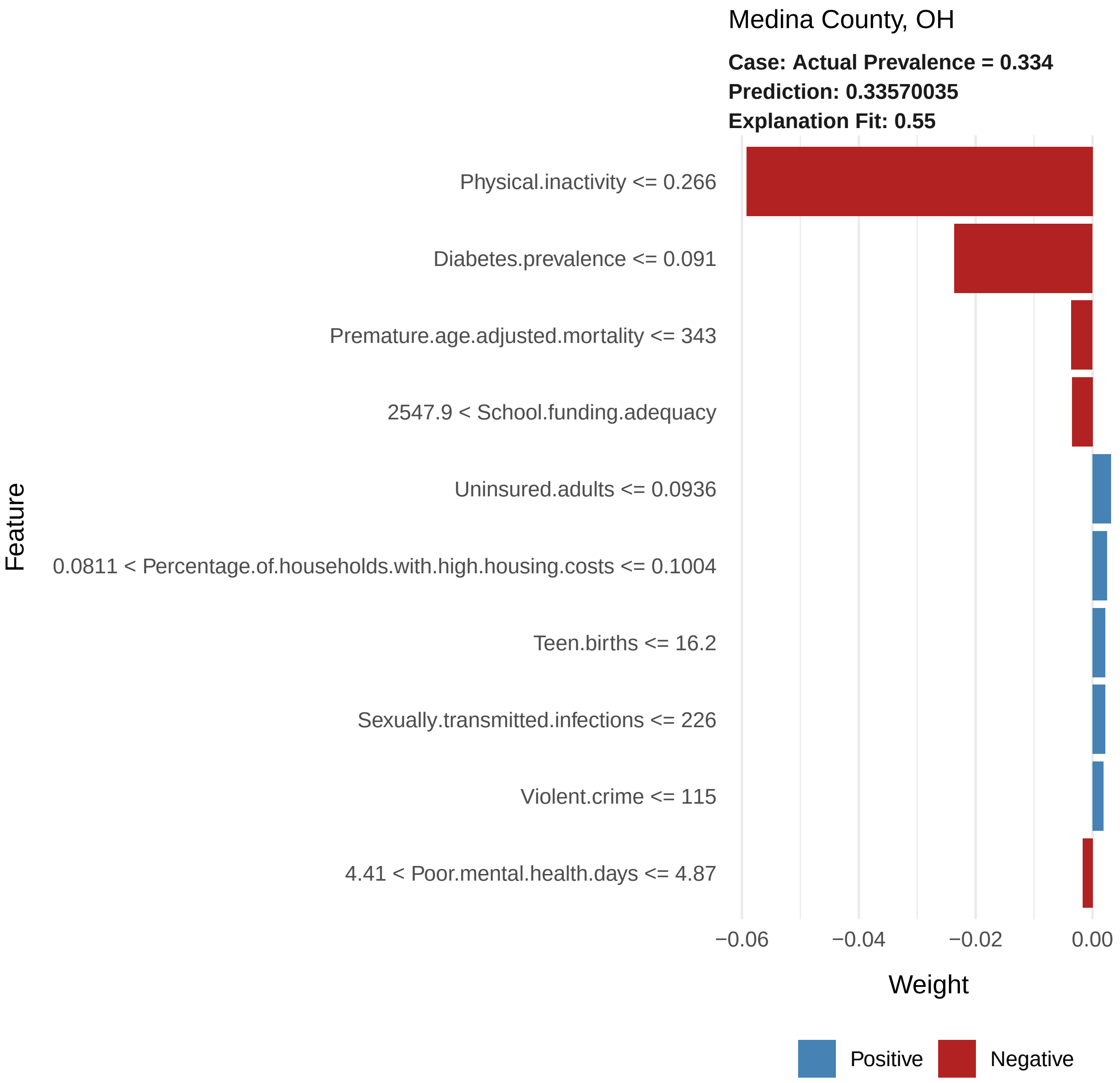

Medina County, OH
Case: Actual Prevalence = 0.334
Prediction: 0.33570035
Explanation Fit: 0.55
Physical.inactivity <= 0.266
Diabetes.prevalence <= 0.091
Premature.age.adjusted.mortality <= 343
2547.9 < School.funding.adequacy
Uninsured.adults <= 0.0936
0.0811 < Percentage.of.households.with.high.housing.costs <= 0.1004
Teen.births <= 16.2
Sexually.transmitted.infections <= 226
Violent.crime <= 115
4.41 < Poor.mental.health.days <= 4.87
Feature
−0.06
−0.04
−0.02
0.00
Weight
Positive
Negative

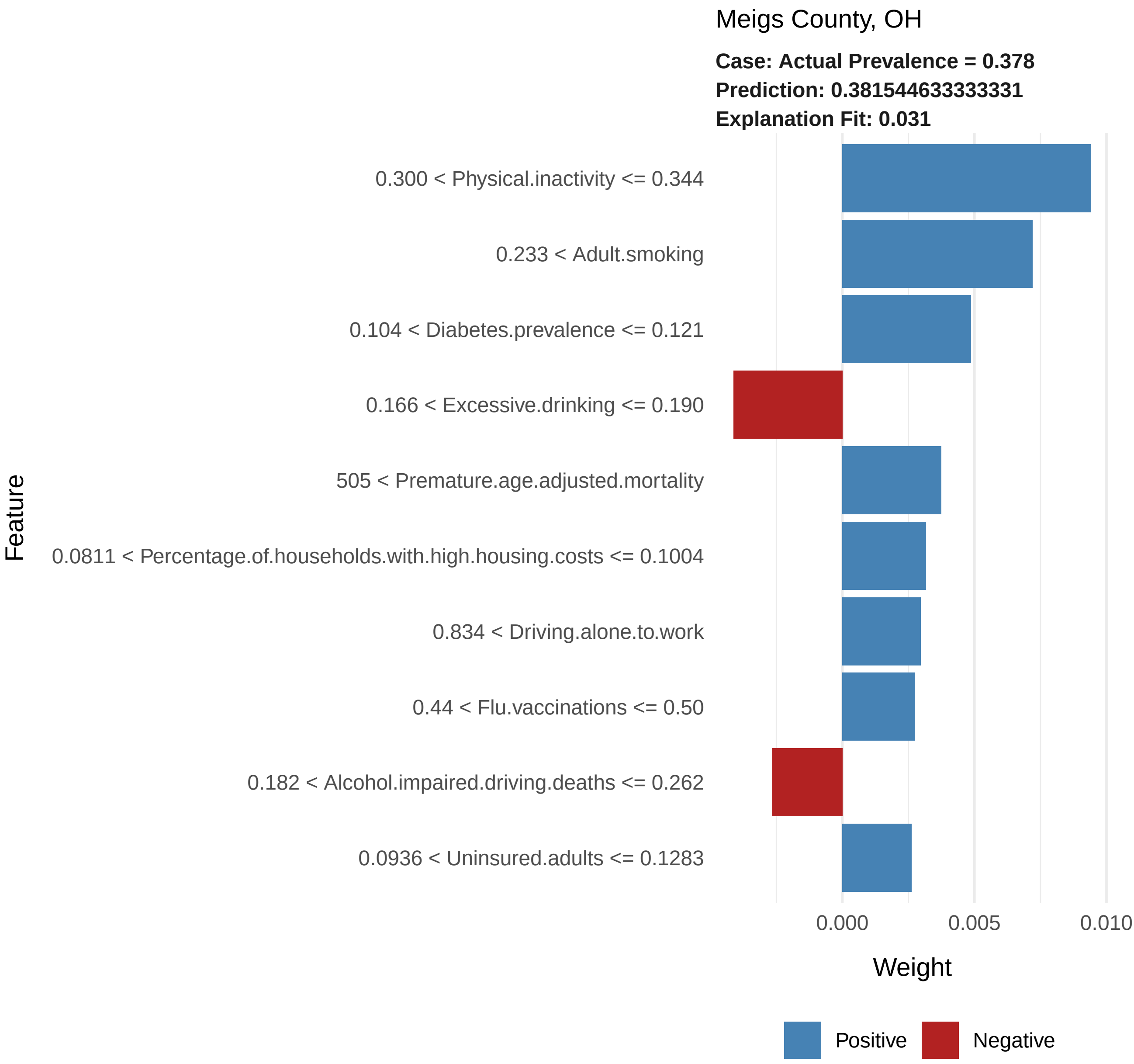
Meigs County, OH
Case: Actual Prevalence = 0.378
Prediction: 0.381544633333331
Explanation Fit: 0.031
0.300 < Physical.inactivity <= 0.344
0.233 < Adult.smoking
0.104 < Diabetes.prevalence <= 0.121
0.166 < Excessive.drinking <= 0.190
505 < Premature.age.adjusted.mortality
0.0811 < Percentage.of.households.with.high.housing.costs <= 0.1004
0.834 < Driving.alone.to.work
0.44 < Flu.vaccinations <= 0.50
0.182 < Alcohol.impaired.driving.deaths <= 0.262
0.0936 < Uninsured.adults <= 0.1283
Feature
0.000
0.005
0.010
Weight
Positive
Negative

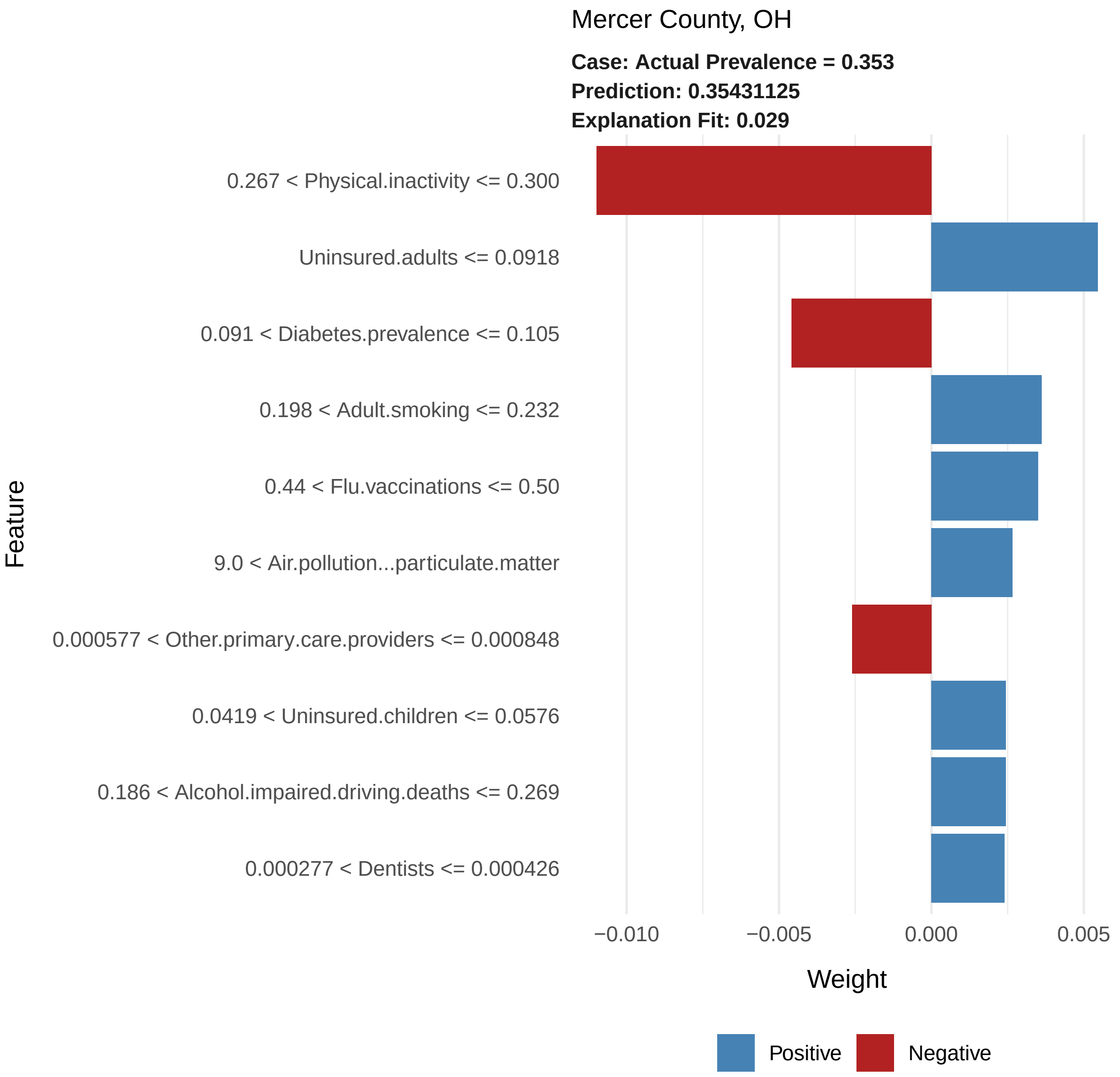

Mercer County, OH
Case: Actual Prevalence = 0.353
Prediction: 0.35431125
Explanation Fit: 0.029
0.267 < Physical.inactivity <= 0.300
Uninsured.adults <= 0.0918
0.091 < Diabetes.prevalence <= 0.105
0.198 < Adult.smoking <= 0.232
0.44 < Flu.vaccinations <= 0.50
9.0 < Air.pollution...particulate.matter
0.000577 < Other.primary.care.providers <= 0.000848
0.0419 < Uninsured.children <= 0.0576
0.186 < Alcohol.impaired.driving.deaths <= 0.269
0.000277 < Dentists <= 0.000426
Feature
−0.010
−0.005
0.000
0.005
Weight
Positive
Negative

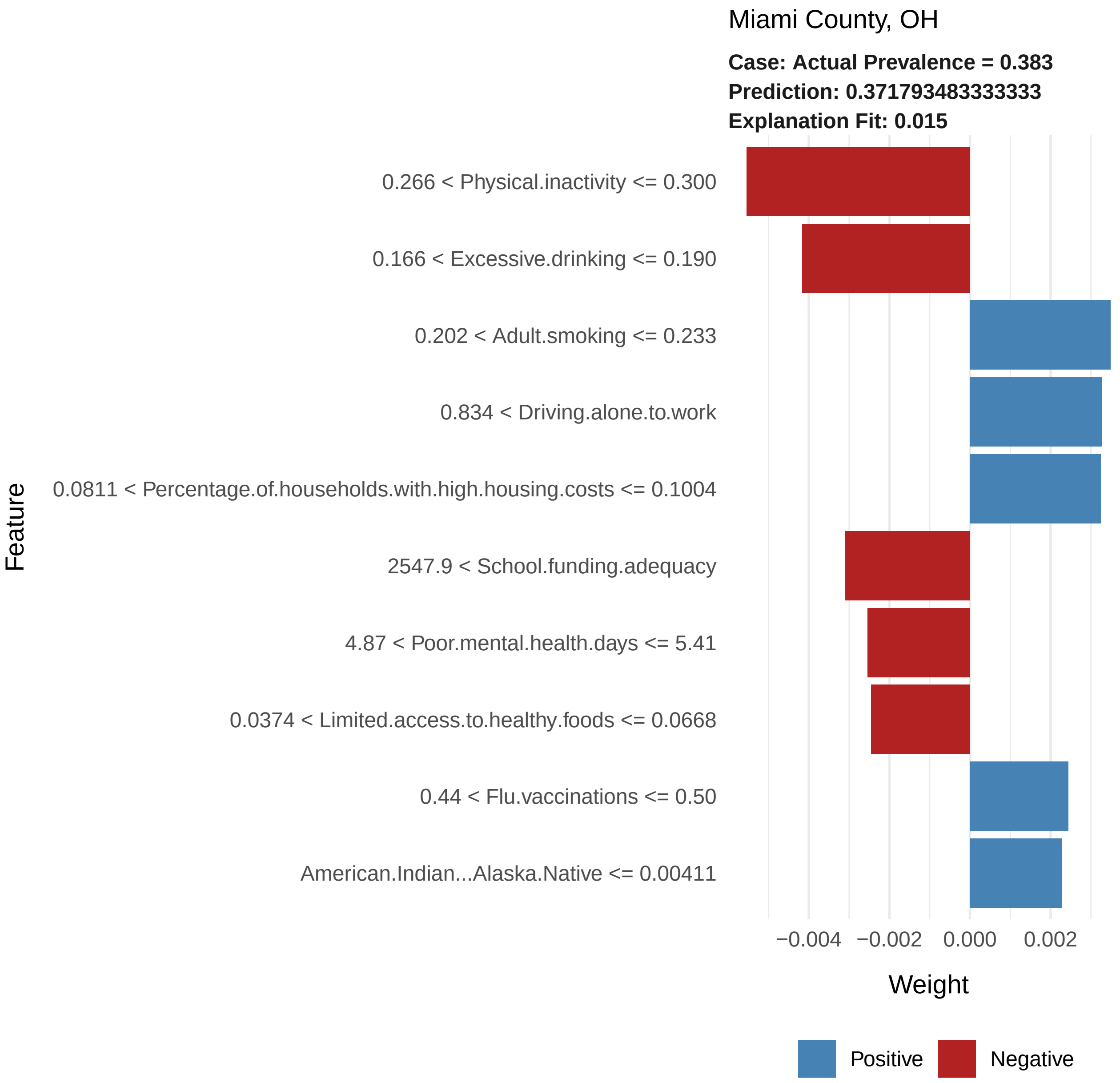

Miami County, OH
Case: Actual Prevalence = 0.383
Prediction: 0.371793483333333
Explanation Fit: 0.015
0.266 < Physical.inactivity <= 0.300
0.166 < Excessive.drinking <= 0.190
0.202 < Adult.smoking <= 0.233
0.834 < Driving.alone.to.work
0.0811 < Percentage.of.households.with.high.housing.costs <= 0.1004
2547.9 < School.funding.adequacy
4.87 < Poor.mental.health.days <= 5.41
0.0374 < Limited.access.to.healthy.foods <= 0.0668
0.44 < Flu.vaccinations <= 0.50
American.Indian...Alaska.Native <= 0.00411
Feature
−0.004
−0.002
0.000
0.002
Weight
Positive
Negative

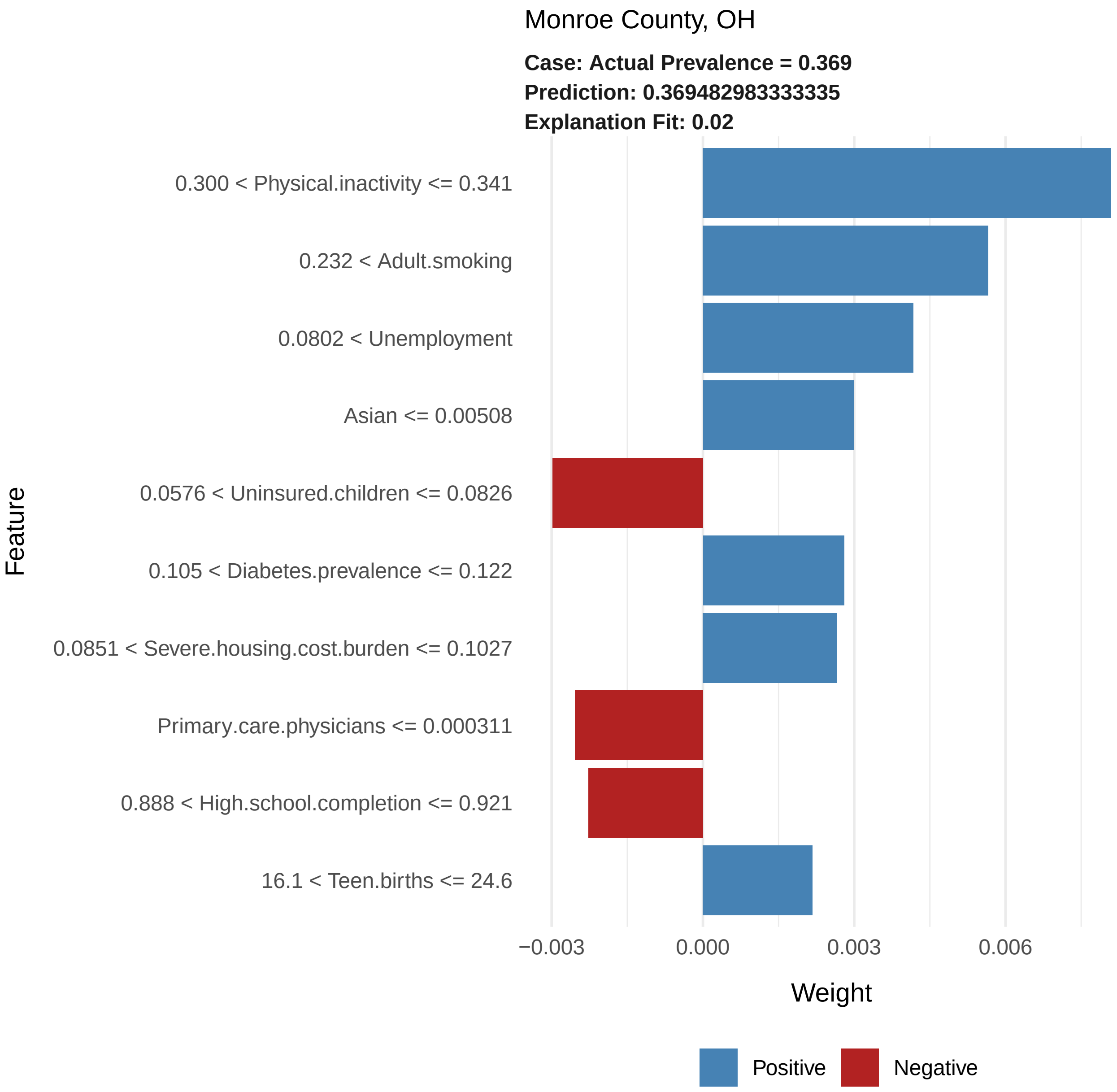

Monroe County, OH
Case: Actual Prevalence = 0.369
Prediction: 0.369482983333335
Explanation Fit: 0.02
0.300 < Physical.inactivity <= 0.341
0.232 < Adult.smoking
0.0802 < Unemployment
Asian <= 0.00508
0.0576 < Uninsured.children <= 0.0826
0.105 < Diabetes.prevalence <= 0.122
0.0851 < Severe.housing.cost.burden <= 0.1027
Primary.care.physicians <= 0.000311
0.888 < High.school.completion <= 0.921
16.1 < Teen.births <= 24.6
Feature
−0.003
0.000
0.003
0.006
Weight
Positive
Negative

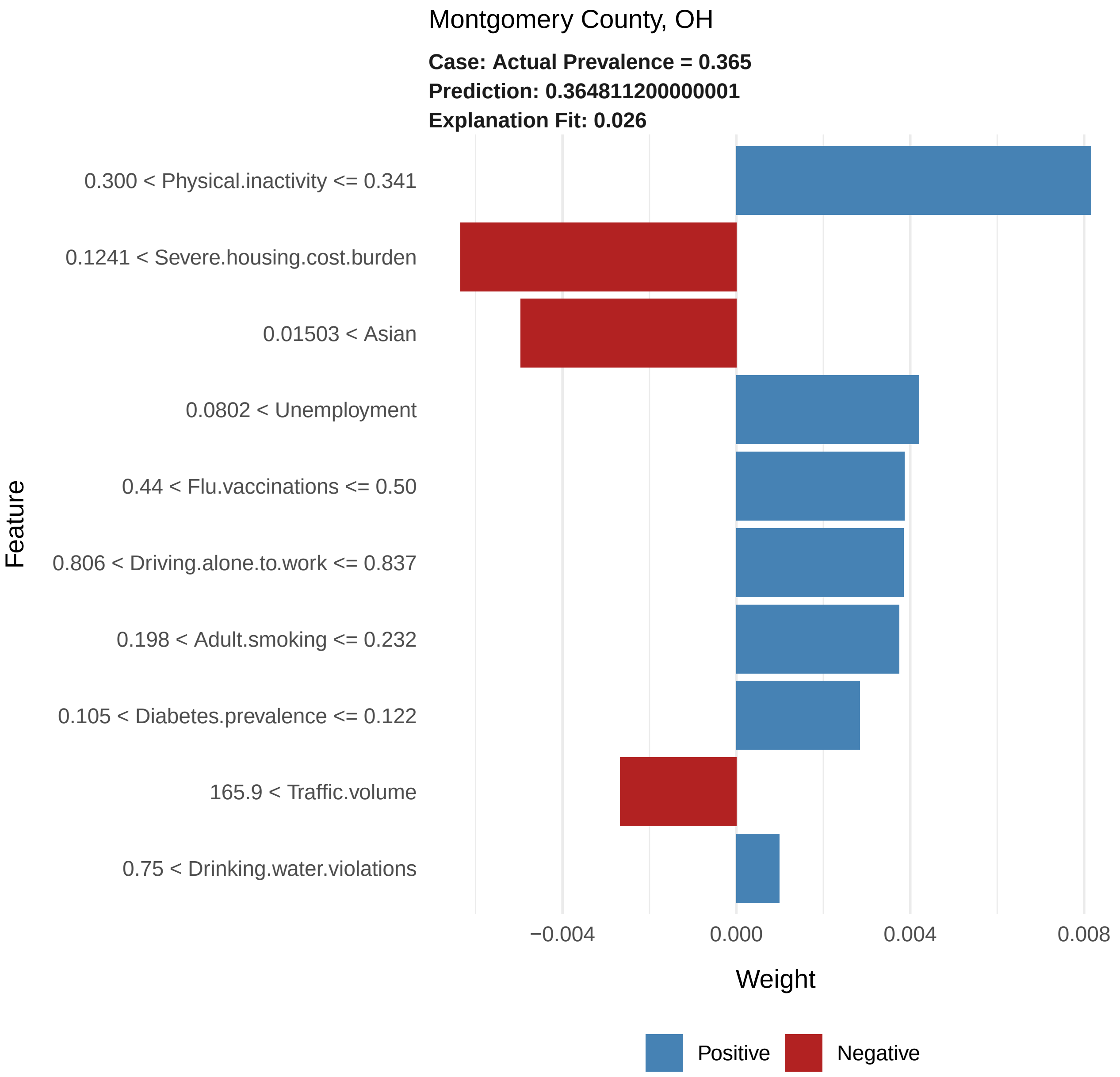
Montgomery County, OH
Case: Actual Prevalence = 0.365
Prediction: 0.364811200000001
Explanation Fit: 0.026
0.300 < Physical.inactivity <= 0.341
0.1241 < Severe.housing.cost.burden
0.01503 < Asian
0.0802 < Unemployment
0.44 < Flu.vaccinations <= 0.50
0.806 < Driving.alone.to.work <= 0.837
0.198 < Adult.smoking <= 0.232
0.105 < Diabetes.prevalence <= 0.122
165.9 < Traffic.volume
0.75 < Drinking.water.violations
Feature
−0.004
0.000
0.004
0.008
Weight
Positive
Negative

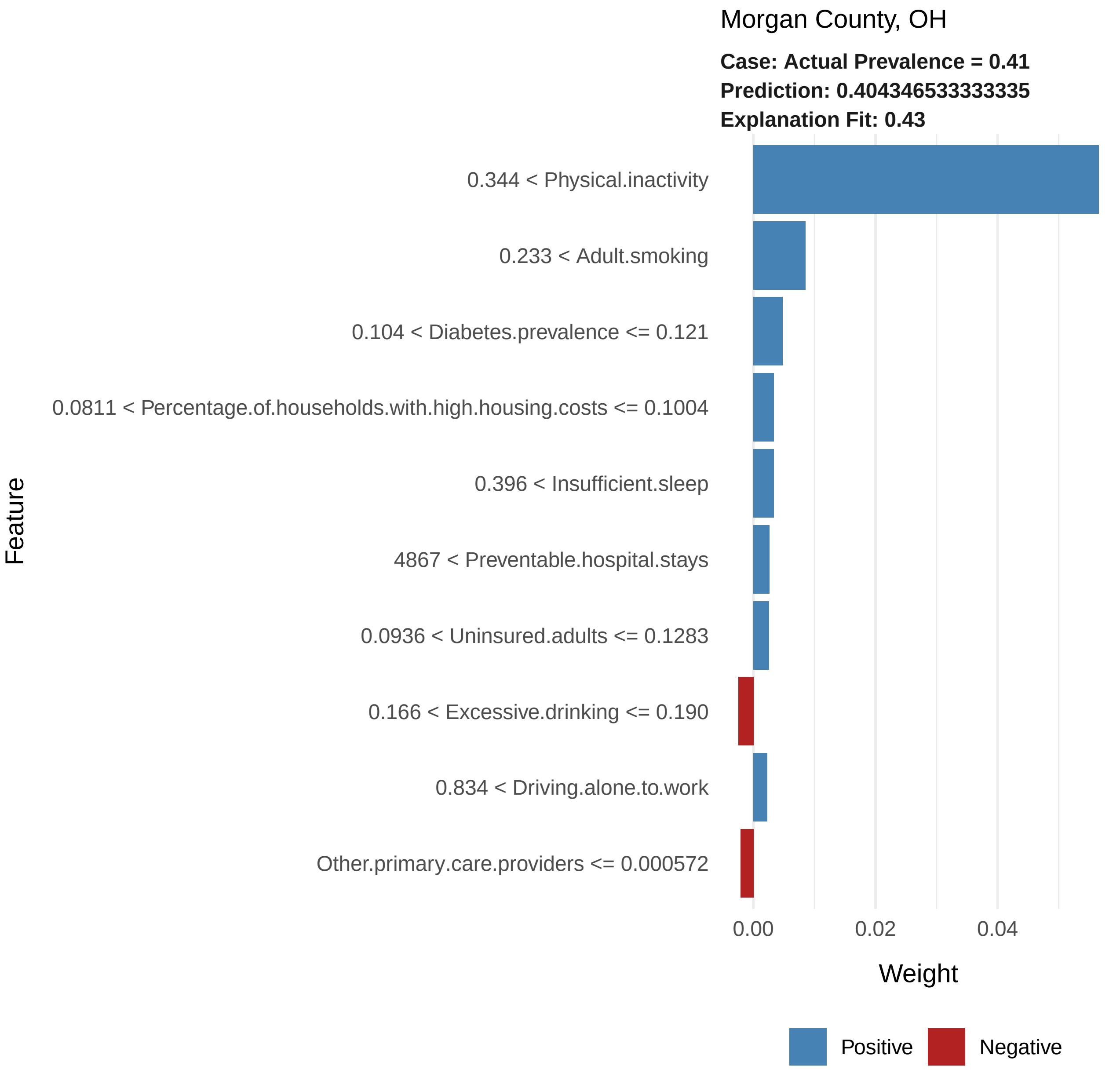

Morgan County, OH
Case: Actual Prevalence = 0.41
Prediction: 0.404346533333335
Explanation Fit: 0.43
0.344 < Physical.inactivity
0.233 < Adult.smoking
0.104 < Diabetes.prevalence <= 0.121
0.0811 < Percentage.of.households.with.high.housing.costs <= 0.1004
0.396 < Insufficient.sleep
4867 < Preventable.hospital.stays
0.0936 < Uninsured.adults <= 0.1283
0.166 < Excessive.drinking <= 0.190
0.834 < Driving.alone.to.work
Other.primary.care.providers <= 0.000572
Feature
0.00
0.02
0.04
Weight
Positive
Negative

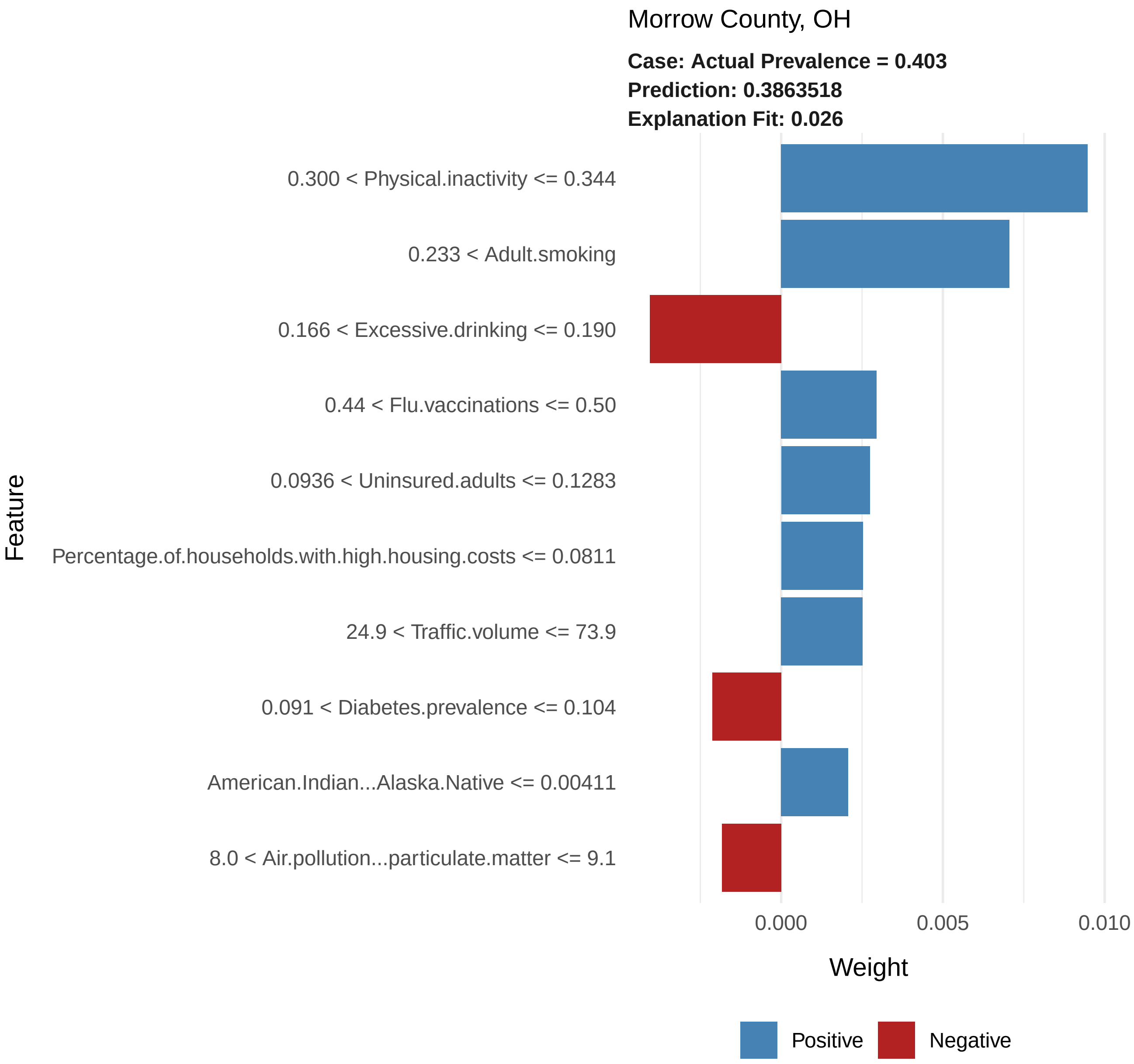

Morrow County, OH
Case: Actual Prevalence = 0.403
Prediction: 0.3863518
Explanation Fit: 0.026
0.300 < Physical.inactivity <= 0.344
0.233 < Adult.smoking
0.166 < Excessive.drinking <= 0.190
0.44 < Flu.vaccinations <= 0.50
0.0936 < Uninsured.adults <= 0.1283
Percentage.of.households.with.high.housing.costs <= 0.0811
24.9 < Traffic.volume <= 73.9
0.091 < Diabetes.prevalence <= 0.104
American.Indian...Alaska.Native <= 0.00411
8.0 < Air.pollution...particulate.matter <= 9.1
Feature
0.000
0.005
0.010
Weight
Positive
Negative

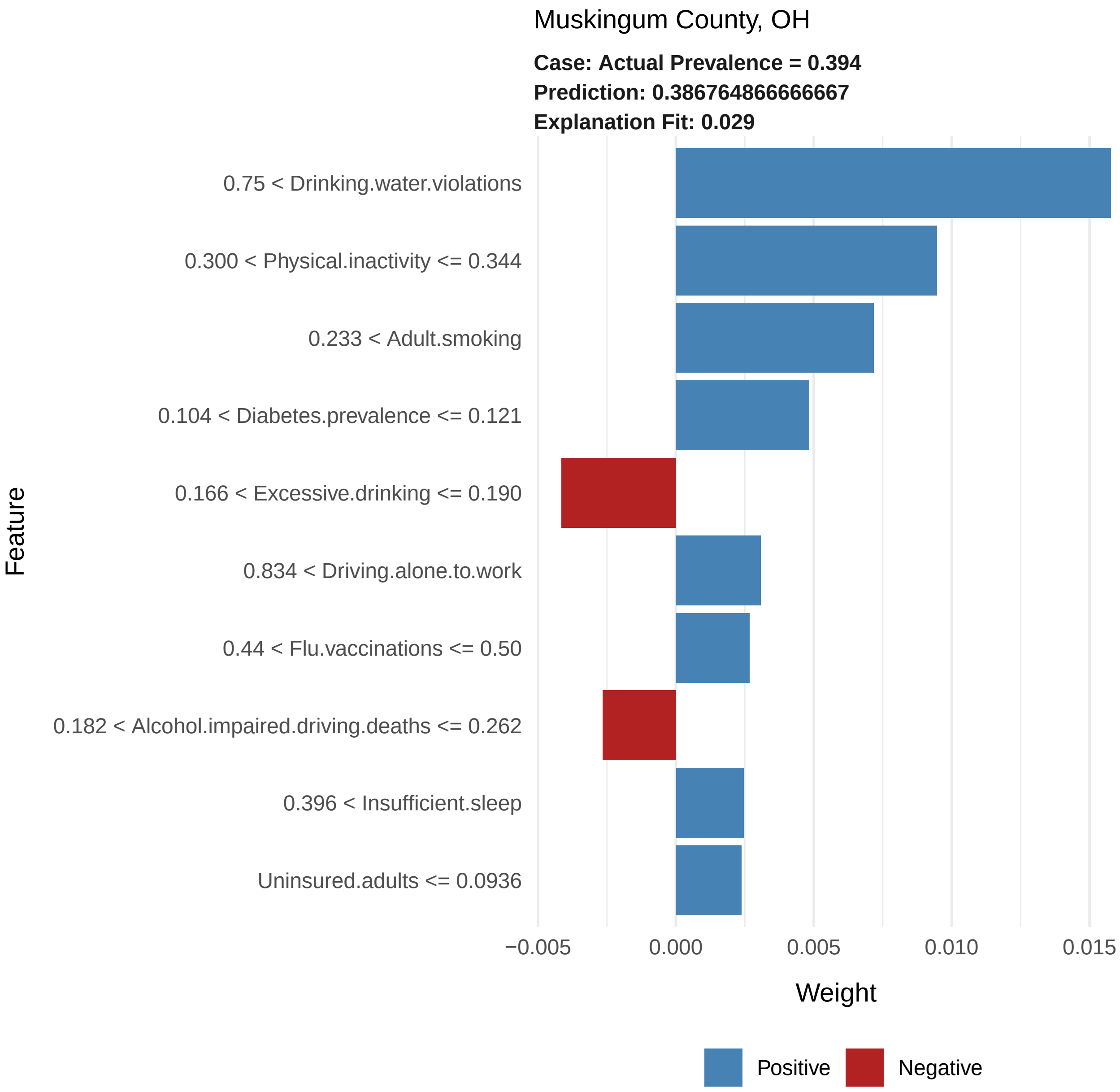
Muskingum County, OH
Case: Actual Prevalence = 0.394
Prediction: 0.386764866666667
Explanation Fit: 0.029
0.75 < Drinking.water.violations
0.300 < Physical.inactivity <= 0.344
0.233 < Adult.smoking
0.104 < Diabetes.prevalence <= 0.121
0.166 < Excessive.drinking <= 0.190
0.834 < Driving.alone.to.work
0.44 < Flu.vaccinations <= 0.50
0.182 < Alcohol.impaired.driving.deaths <= 0.262
0.396 < Insufficient.sleep
Uninsured.adults <= 0.0936
Feature
−0.005
0.000
0.005
0.010
0.015
Weight
Positive
Negative

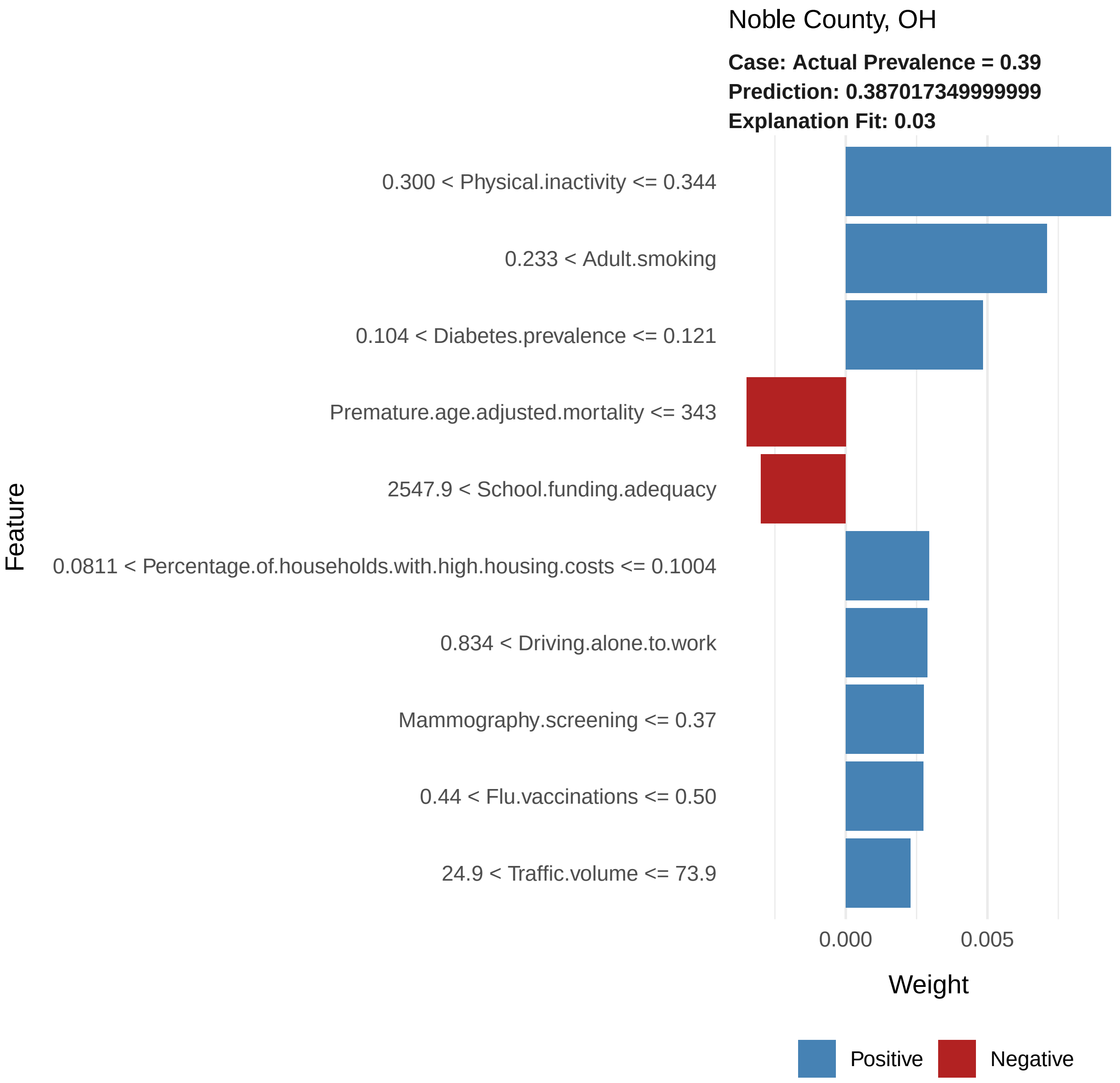
Noble County, OH
Case: Actual Prevalence = 0.39
Prediction: 0.387017349999999
Explanation Fit: 0.03
0.300 < Physical.inactivity <= 0.344
0.233 < Adult.smoking
0.104 < Diabetes.prevalence <= 0.121
Premature.age.adjusted.mortality <= 343
2547.9 < School.funding.adequacy
0.0811 < Percentage.of.households.with.high.housing.costs <= 0.1004
0.834 < Driving.alone.to.work
Mammography.screening <= 0.37
0.44 < Flu.vaccinations <= 0.50
24.9 < Traffic.volume <= 73.9
Feature
0.000
0.005
Weight
Positive
Negative

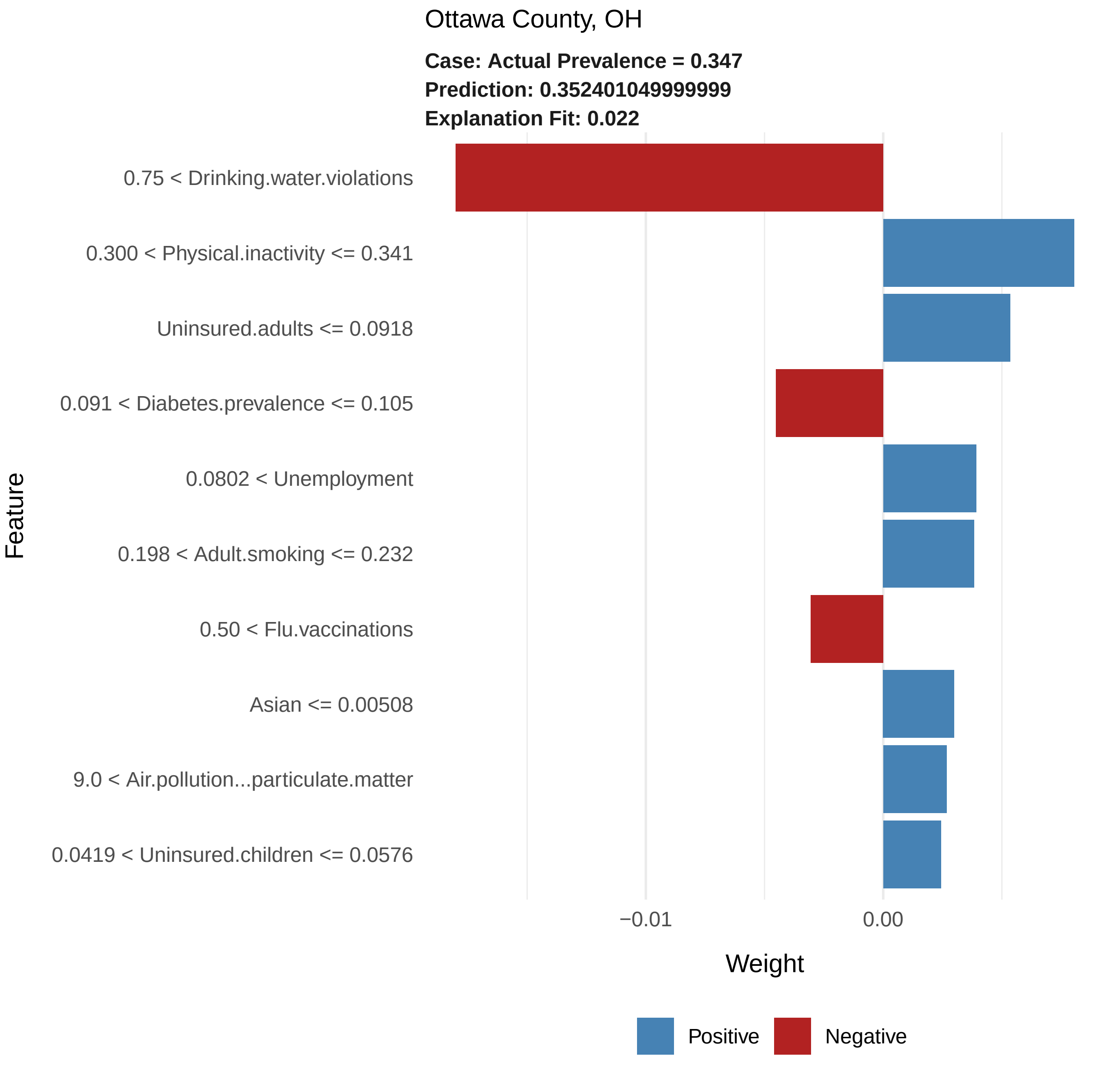

Ottawa County, OH
Case: Actual Prevalence = 0.347
Prediction: 0.352401049999999
Explanation Fit: 0.022
0.75 < Drinking.water.violations
0.300 < Physical.inactivity <= 0.341
Uninsured.adults <= 0.0918
0.091 < Diabetes.prevalence <= 0.105
0.0802 < Unemployment
0.198 < Adult.smoking <= 0.232
0.50 < Flu.vaccinations
Asian <= 0.00508
9.0 < Air.pollution...particulate.matter
0.0419 < Uninsured.children <= 0.0576
Feature
−0.01
0.00
Weight
Positive
Negative

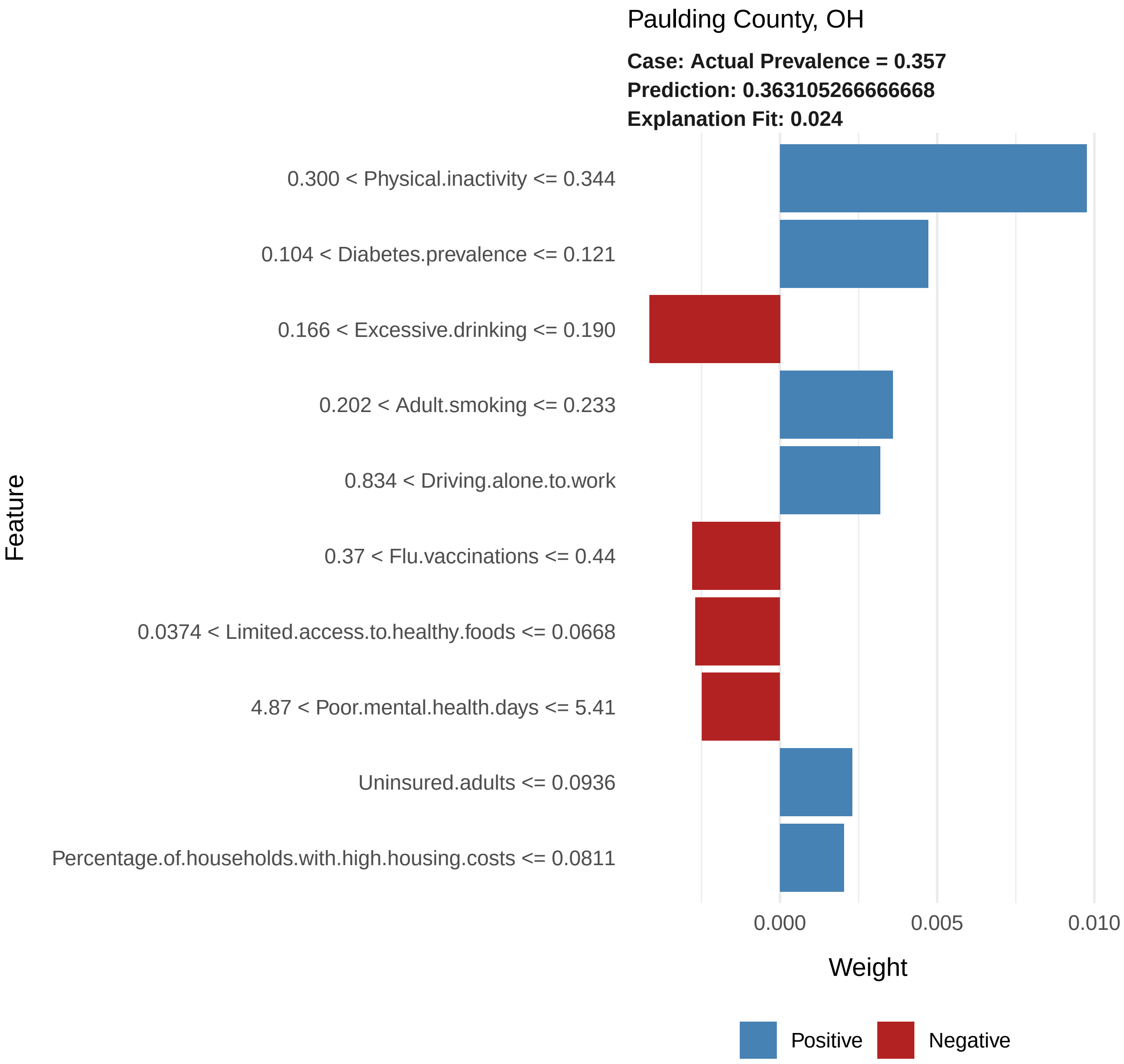

Paulding County, OH
Case: Actual Prevalence = 0.357
Prediction: 0.363105266666668
Explanation Fit: 0.024
0.300 < Physical.inactivity <= 0.344
0.104 < Diabetes.prevalence <= 0.121
0.166 < Excessive.drinking <= 0.190
0.202 < Adult.smoking <= 0.233
0.834 < Driving.alone.to.work
0.37 < Flu.vaccinations <= 0.44
0.0374 < Limited.access.to.healthy.foods <= 0.0668
4.87 < Poor.mental.health.days <= 5.41
Uninsured.adults <= 0.0936
Percentage.of.households.with.high.housing.costs <= 0.0811
Feature
0.000
0.005
0.010
Weight
Positive
Negative

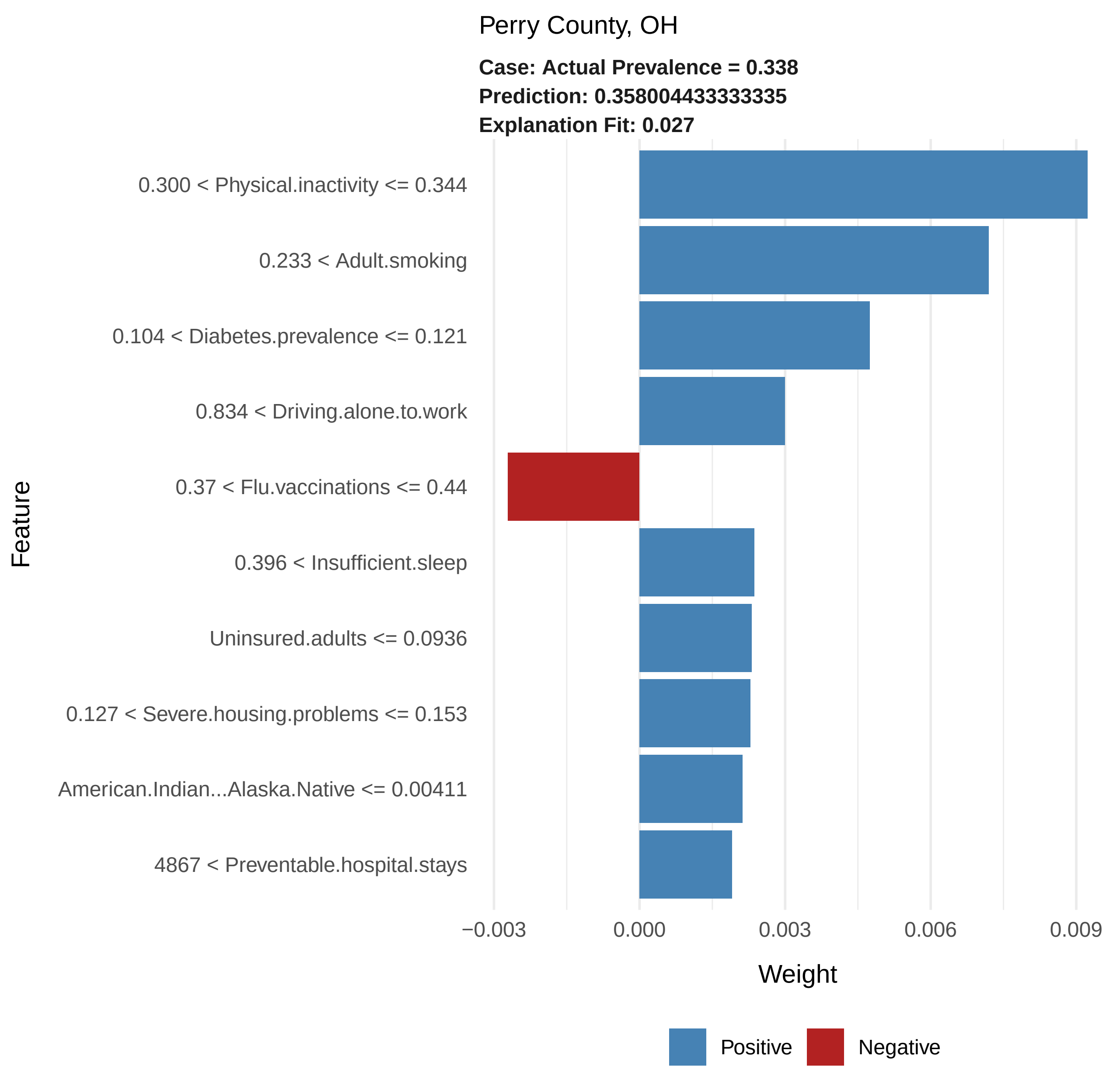

Perry County, OH
Case: Actual Prevalence = 0.338
Prediction: 0.358004433333335
Explanation Fit: 0.027
0.300 < Physical.inactivity <= 0.344
0.233 < Adult.smoking
0.104 < Diabetes.prevalence <= 0.121
0.834 < Driving.alone.to.work
0.37 < Flu.vaccinations <= 0.44
0.396 < Insufficient.sleep
Uninsured.adults <= 0.0936
0.127 < Severe.housing.problems <= 0.153
American.Indian...Alaska.Native <= 0.00411
4867 < Preventable.hospital.stays
Feature
−0.003
0.000
0.003
0.006
0.009
Weight
Positive
Negative

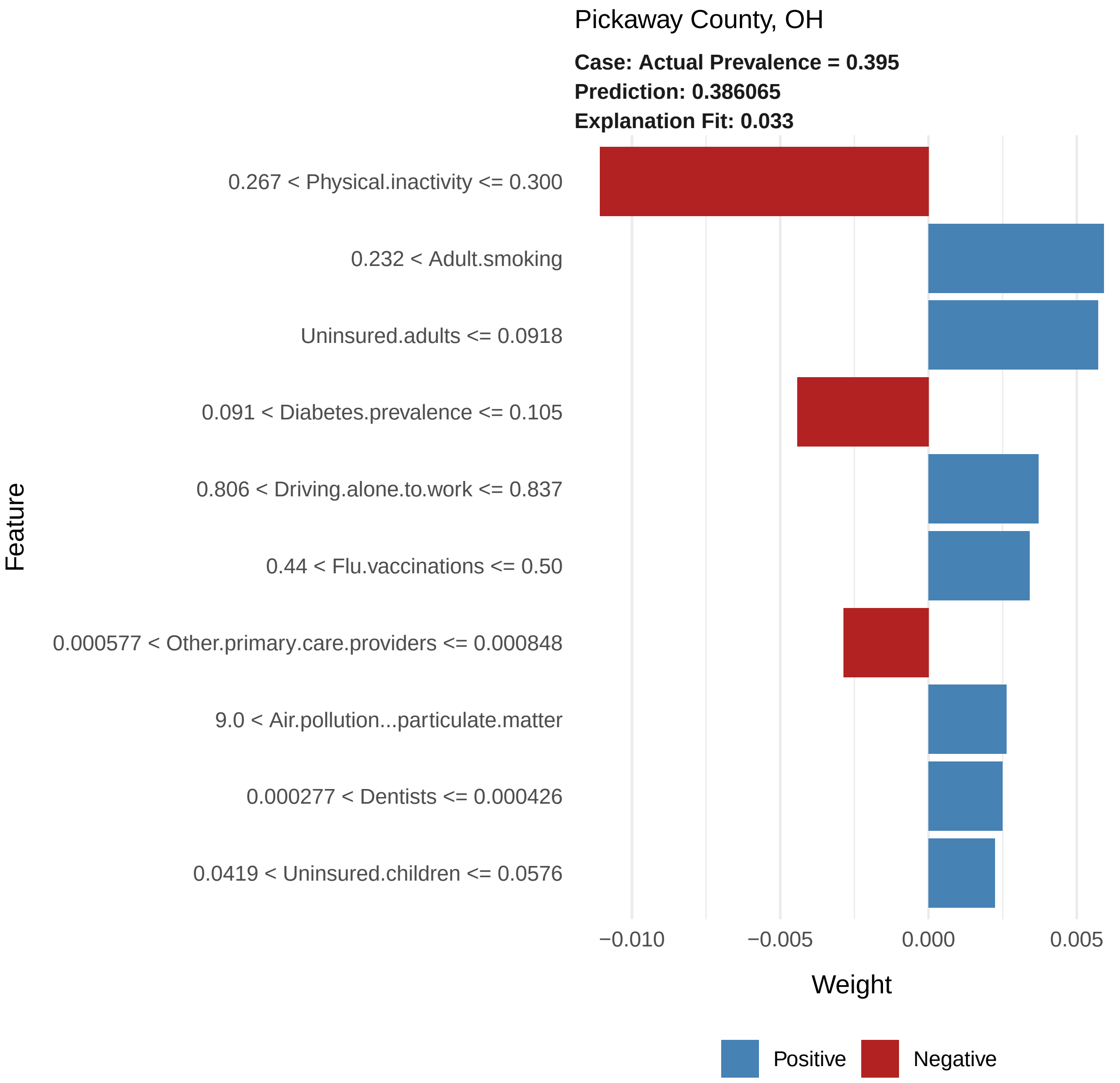

Pickaway County, OH
Case: Actual Prevalence = 0.395
Prediction: 0.386065
Explanation Fit: 0.033
Feature
0.267 < Physical.inactivity <= 0.300
0.232 < Adult.smoking
Uninsured.adults <= 0.0918
0.091 < Diabetes.prevalence <= 0.105
0.806 < Driving.alone.to.work <= 0.837
0.44 < Flu.vaccinations <= 0.50
0.000577 < Other.primary.care.providers <= 0.000848
9.0 < Air.pollution...particulate.matter
0.000277 < Dentists <= 0.000426
0.0419 < Uninsured.children <= 0.0576
−0.010
−0.005
0.000
0.005
Weight
Positive
Negative

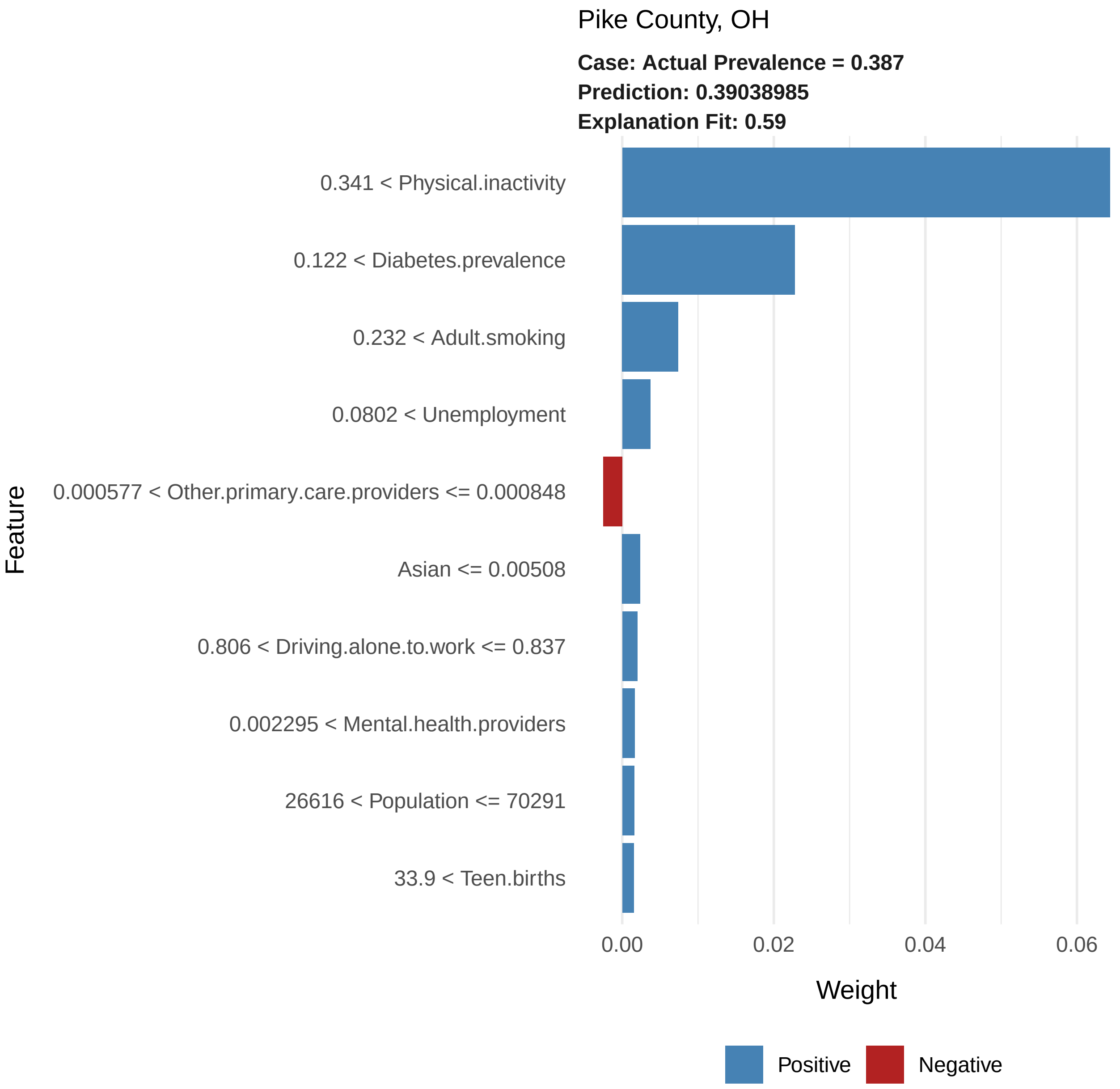

Pike County, OH
Case: Actual Prevalence = 0.387
Prediction: 0.39038985
Explanation Fit: 0.59
0.341 < Physical.inactivity
0.122 < Diabetes.prevalence
0.232 < Adult.smoking
0.0802 < Unemployment
0.000577 < Other.primary.care.providers <= 0.000848
Asian <= 0.00508
0.806 < Driving.alone.to.work <= 0.837
0.002295 < Mental.health.providers
26616 < Population <= 70291
33.9 < Teen.births
Feature
0.00
0.02
0.04
0.06
Weight
Positive
Negative

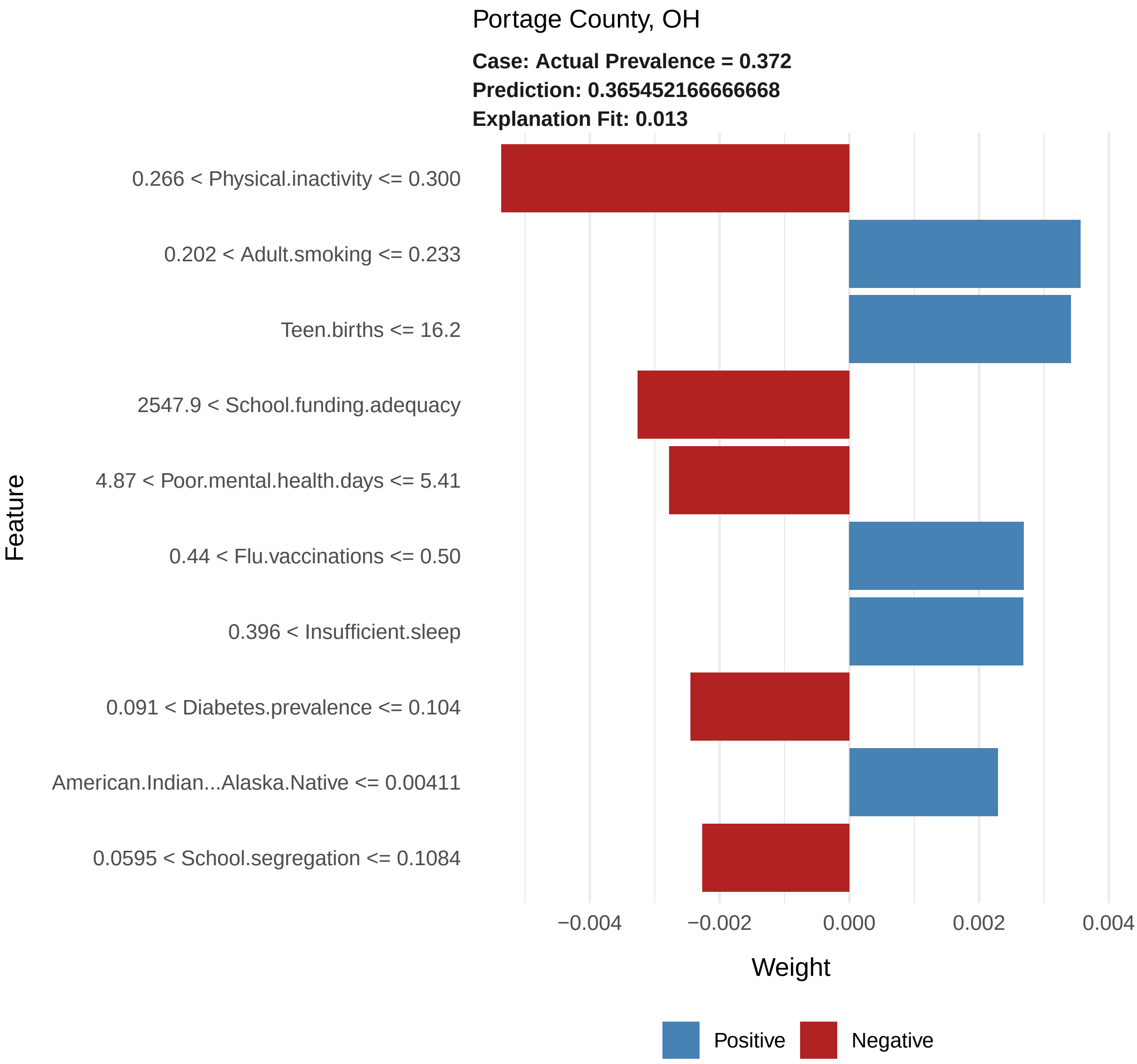
Portage County, OH
Case: Actual Prevalence = 0.372
Prediction: 0.365452166666668
Explanation Fit: 0.013
0.266 < Physical.inactivity <= 0.300
0.202 < Adult.smoking <= 0.233
Teen.births <= 16.2
2547.9 < School.funding.adequacy
4.87 < Poor.mental.health.days <= 5.41
0.44 < Flu.vaccinations <= 0.50
0.396 < Insufficient.sleep
0.091 < Diabetes.prevalence <= 0.104
American.Indian...Alaska.Native <= 0.00411
0.0595 < School.segregation <= 0.1084
Feature
−0.004
−0.002
0.000
0.002
0.004
Weight
Positive
Negative

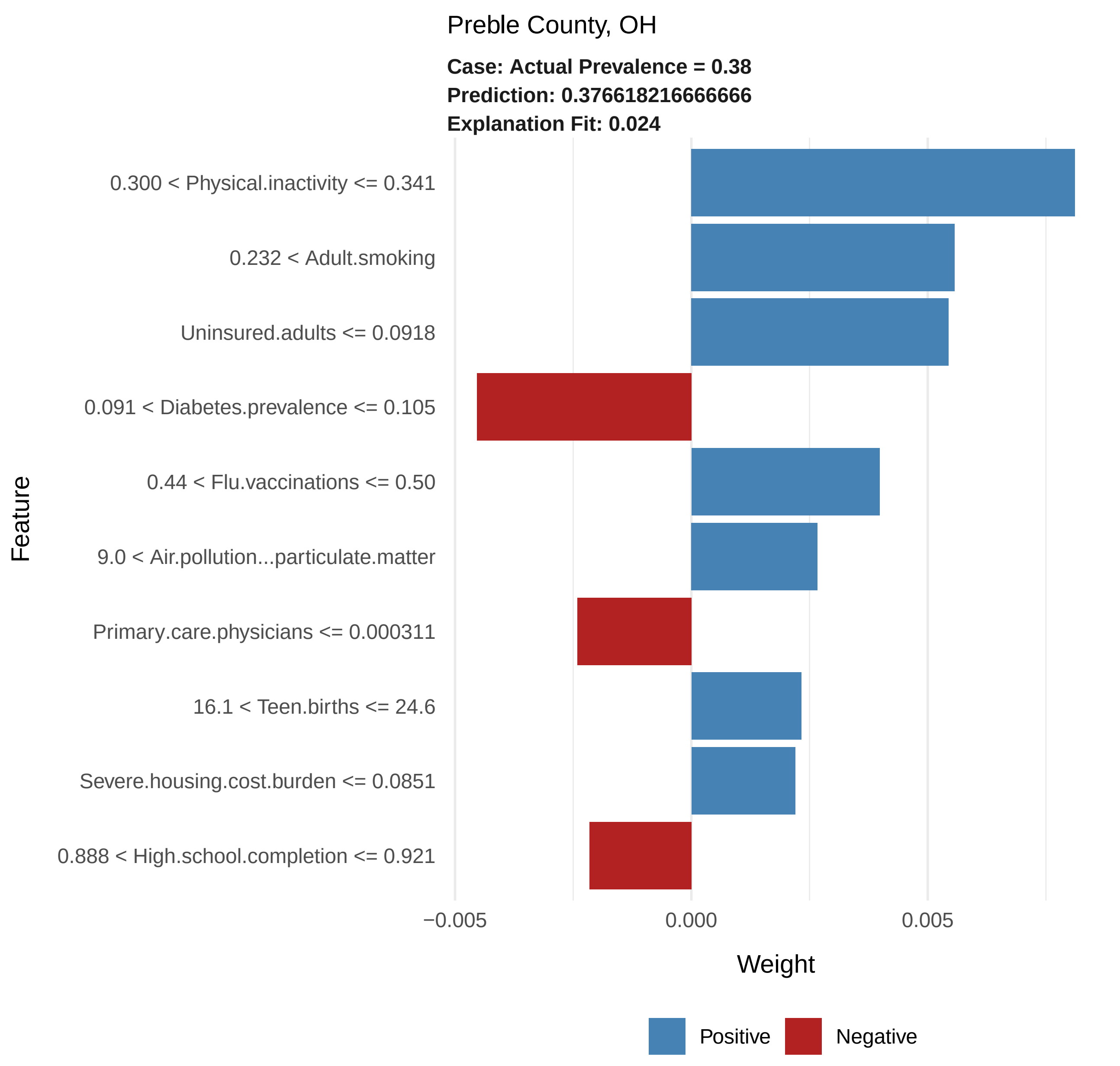
Preble County, OH
Case: Actual Prevalence = 0.38
Prediction: 0.376618216666666
Explanation Fit: 0.024
0.300 < Physical.inactivity <= 0.341
0.232 < Adult.smoking
Uninsured.adults <= 0.0918
0.091 < Diabetes.prevalence <= 0.105
0.44 < Flu.vaccinations <= 0.50
9.0 < Air.pollution...particulate.matter
Primary.care.physicians <= 0.000311
16.1 < Teen.births <= 24.6
Severe.housing.cost.burden <= 0.0851
0.888 < High.school.completion <= 0.921
Feature
−0.005
0.000
0.005
Weight
Positive
Negative

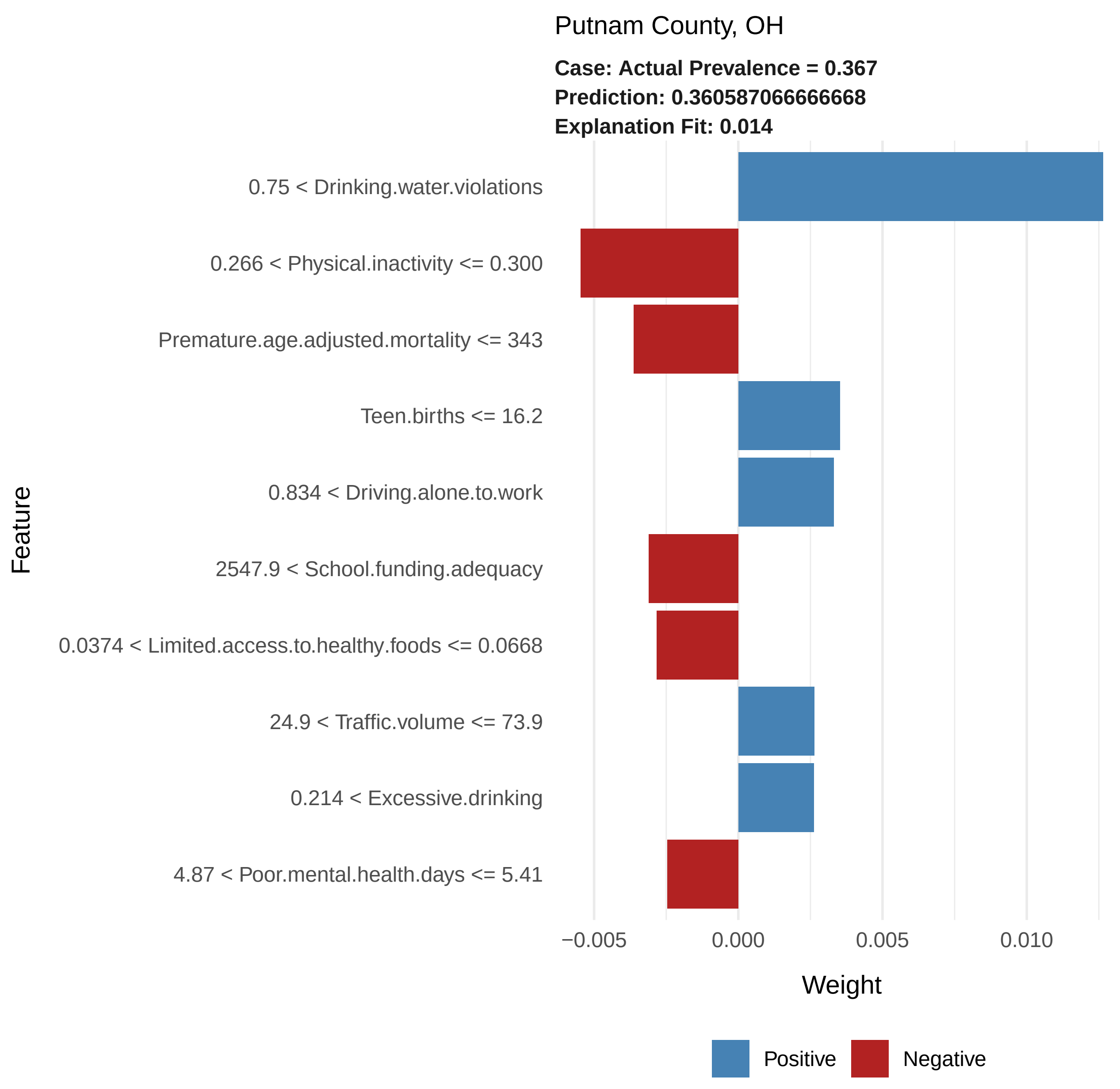
Putnam County, OH
Case: Actual Prevalence = 0.367
Prediction: 0.360587066666668
Explanation Fit: 0.014
0.75 < Drinking.water.violations
0.266 < Physical.inactivity <= 0.300
Premature.age.adjusted.mortality <= 343
Teen.births <= 16.2
0.834 < Driving.alone.to.work
2547.9 < School.funding.adequacy
0.0374 < Limited.access.to.healthy.foods <= 0.0668
24.9 < Traffic.volume <= 73.9
0.214 < Excessive.drinking
4.87 < Poor.mental.health.days <= 5.41
Feature
−0.005
0.000
0.005
0.010
Weight
Positive
Negative

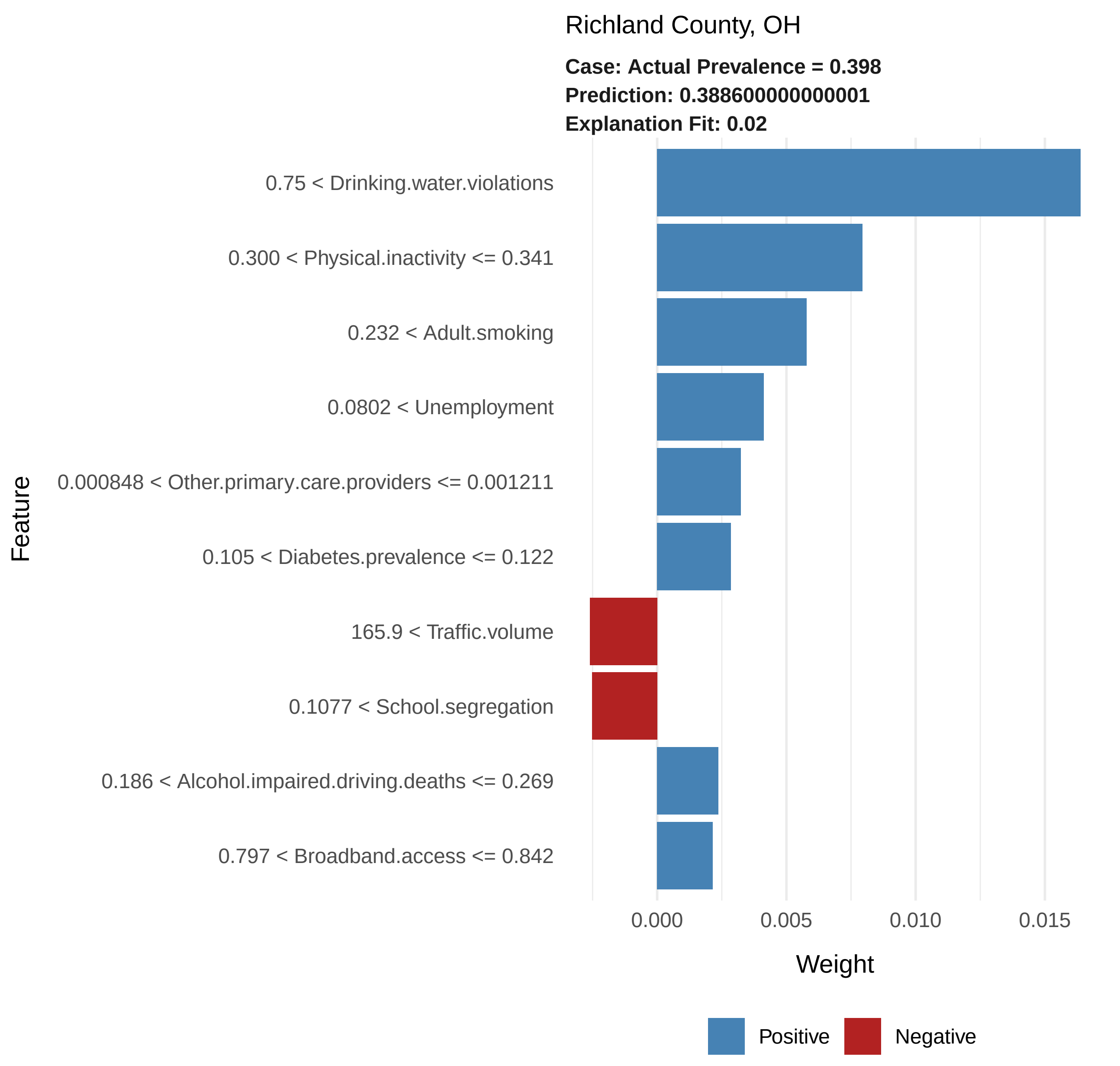

Richland County, OH
Case: Actual Prevalence = 0.398
Prediction: 0.388600000000001
Explanation Fit: 0.02
0.75 < Drinking.water.violations
0.300 < Physical.inactivity <= 0.341
0.232 < Adult.smoking
0.0802 < Unemployment
0.000848 < Other.primary.care.providers <= 0.001211
0.105 < Diabetes.prevalence <= 0.122
165.9 < Traffic.volume
0.1077 < School.segregation
0.186 < Alcohol.impaired.driving.deaths <= 0.269
0.797 < Broadband.access <= 0.842
Feature
0.000
0.005
0.010
0.015
Weight
Positive
Negative

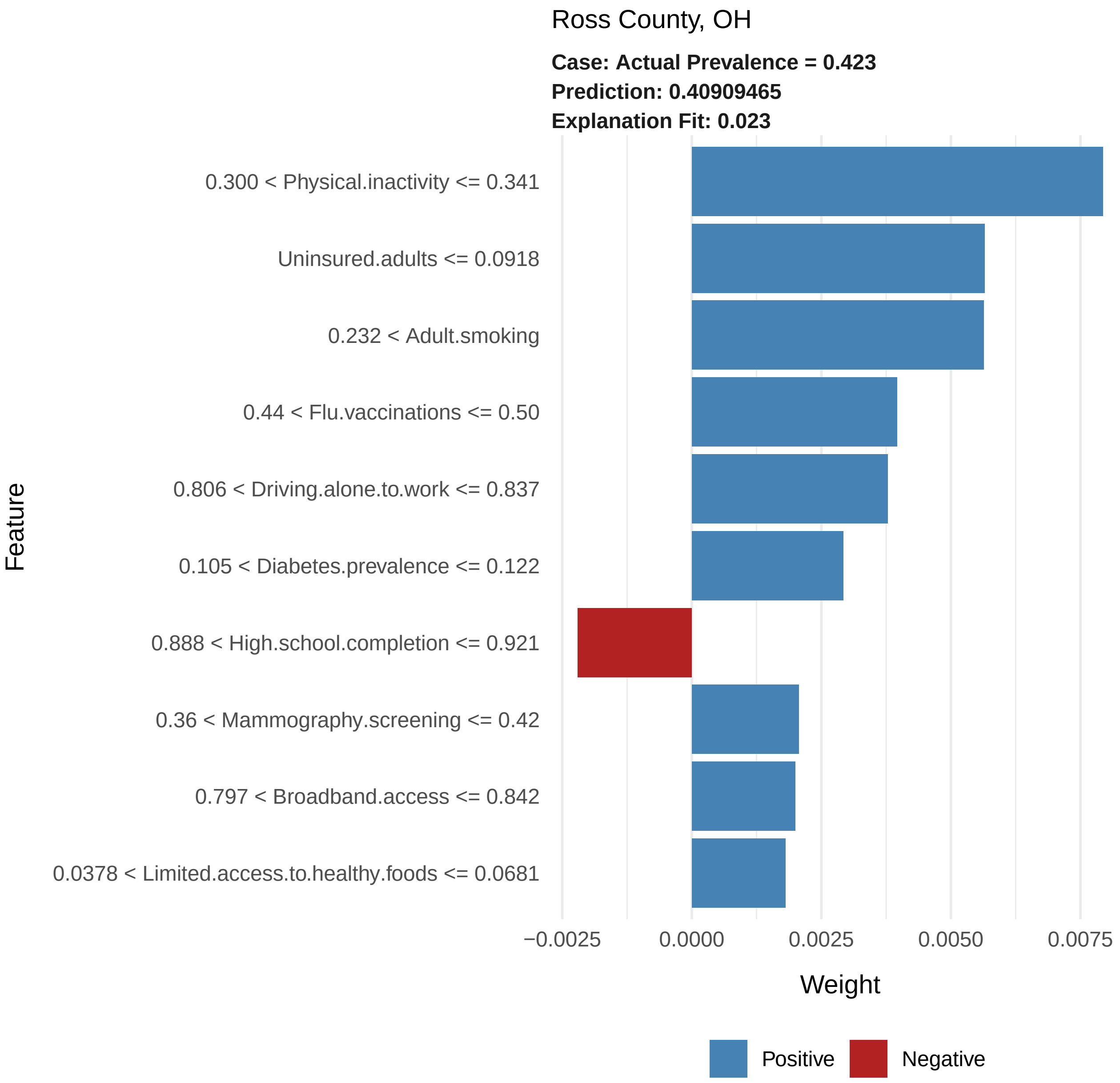

Ross County, OH
Case: Actual Prevalence = 0.423
Prediction: 0.40909465
Explanation Fit: 0.023
0.300 < Physical.inactivity <= 0.341
Uninsured.adults <= 0.0918
0.232 < Adult.smoking
0.44 < Flu.vaccinations <= 0.50
0.806 < Driving.alone.to.work <= 0.837
0.105 < Diabetes.prevalence <= 0.122
0.888 < High.school.completion <= 0.921
0.36 < Mammography.screening <= 0.42
0.797 < Broadband.access <= 0.842
0.0378 < Limited.access.to.healthy.foods <= 0.0681
Feature
−0.0025
0.0000
0.0025
0.0050
0.0075
Weight
Positive
Negative

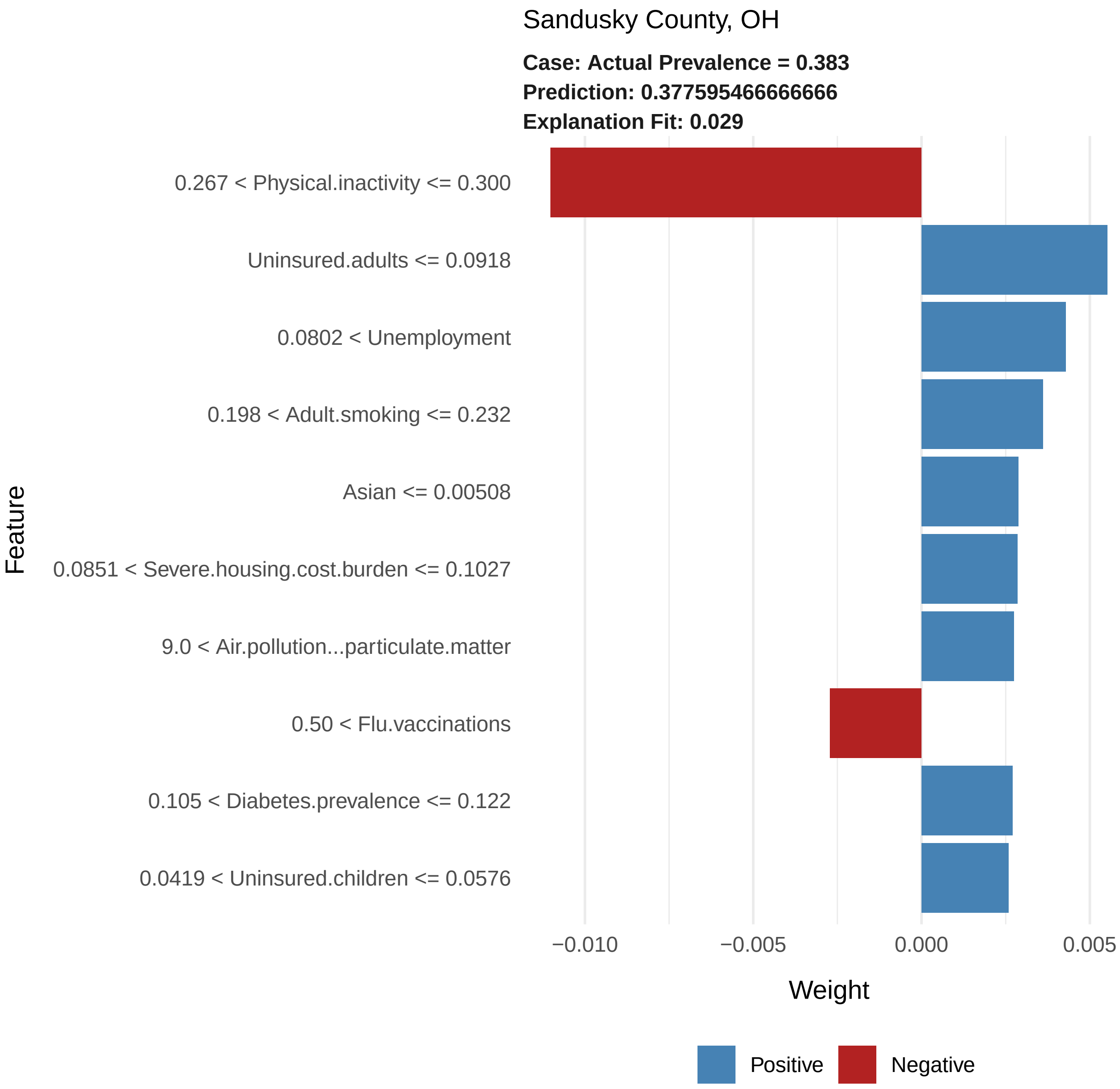

Sandusky County, OH
Case: Actual Prevalence = 0.383
Prediction: 0.377595466666666
Explanation Fit: 0.029
0.267 < Physical.inactivity <= 0.300
Uninsured.adults <= 0.0918
0.0802 < Unemployment
0.198 < Adult.smoking <= 0.232
Asian <= 0.00508
0.0851 < Severe.housing.cost.burden <= 0.1027
9.0 < Air.pollution...particulate.matter
0.50 < Flu.vaccinations
0.105 < Diabetes.prevalence <= 0.122
0.0419 < Uninsured.children <= 0.0576
Feature
−0.010
−0.005
0.000
0.005
Weight
Positive
Negative

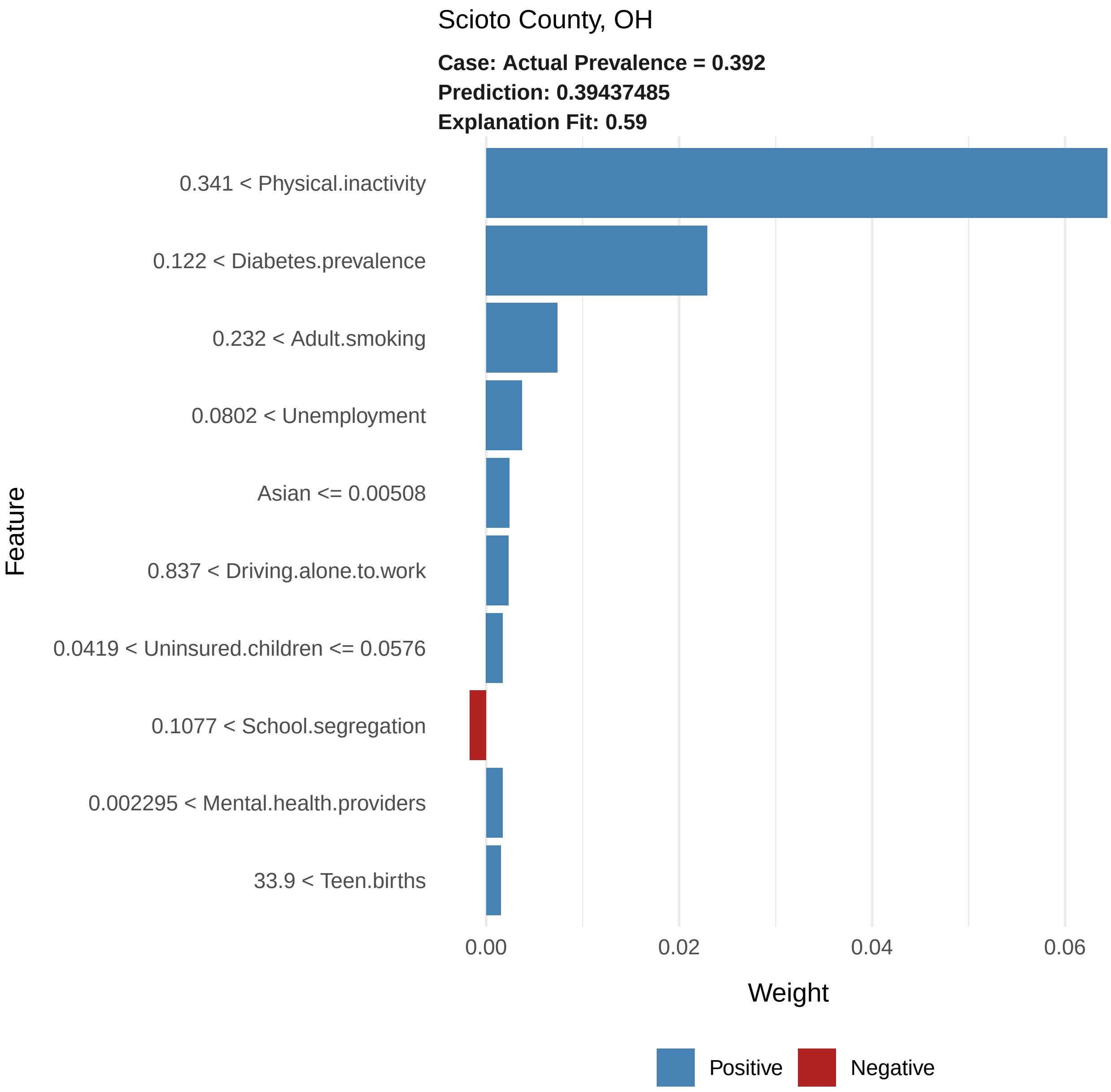
Scioto County, OH
Case: Actual Prevalence = 0.392
Prediction: 0.39437485
Explanation Fit: 0.59
0.341 < Physical.inactivity
0.122 < Diabetes.prevalence
0.232 < Adult.smoking
0.0802 < Unemployment
Asian <= 0.00508
0.837 < Driving.alone.to.work
0.0419 < Uninsured.children <= 0.0576
0.1077 < School.segregation
0.002295 < Mental.health.providers
33.9 < Teen.births
Feature
0.00
0.02
0.04
0.06
Weight
Positive
Negative

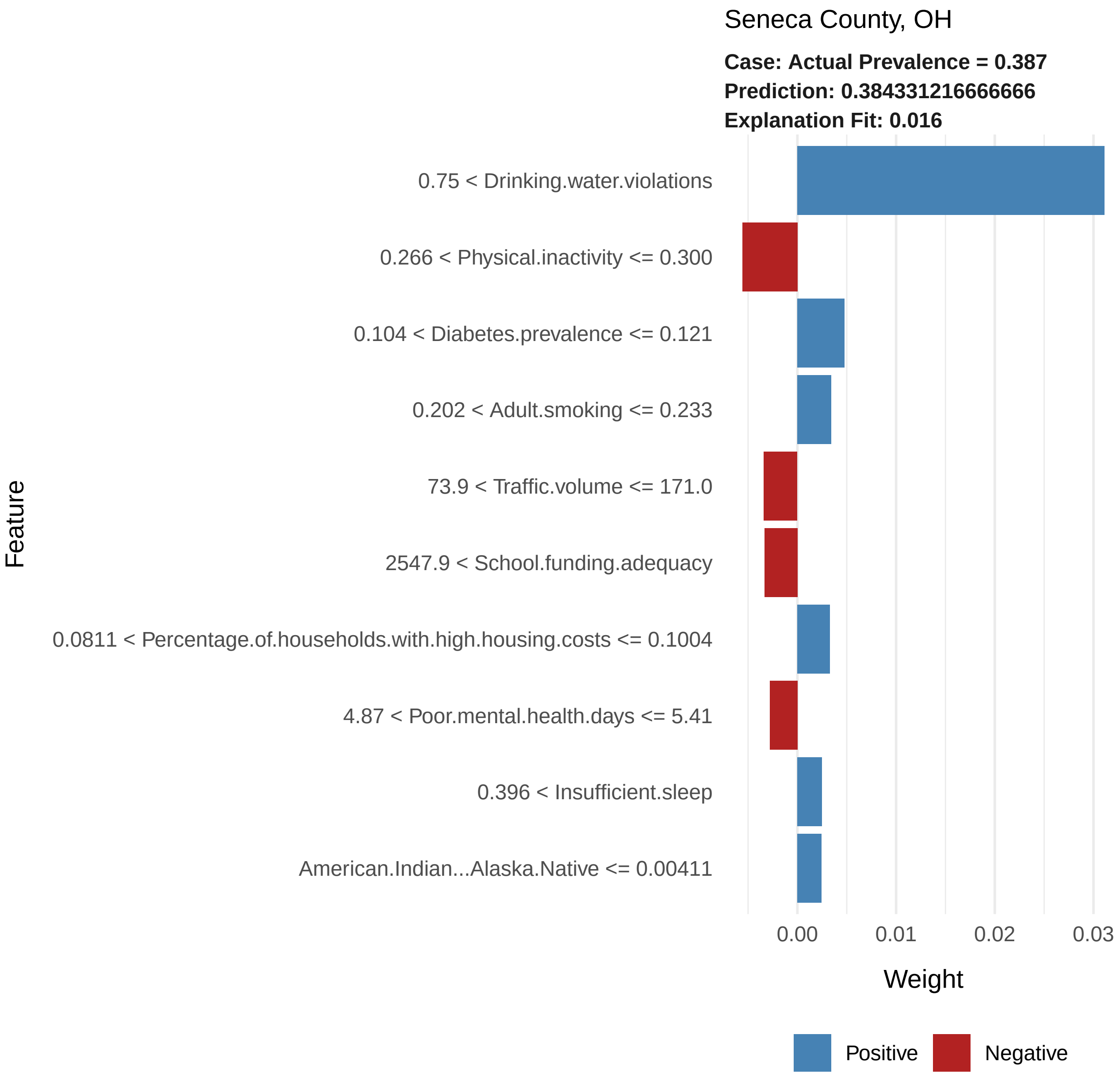

Seneca County, OH
Case: Actual Prevalence = 0.387
Prediction: 0.384331216666666
Explanation Fit: 0.016
0.75 < Drinking.water.violations
0.266 < Physical.inactivity <= 0.300
0.104 < Diabetes.prevalence <= 0.121
0.202 < Adult.smoking <= 0.233
73.9 < Traffic.volume <= 171.0
2547.9 < School.funding.adequacy
0.0811 < Percentage.of.households.with.high.housing.costs <= 0.1004
4.87 < Poor.mental.health.days <= 5.41
0.396 < Insufficient.sleep
American.Indian...Alaska.Native <= 0.00411
Feature
0.00
0.01
0.02
0.03
Weight
Positive
Negative

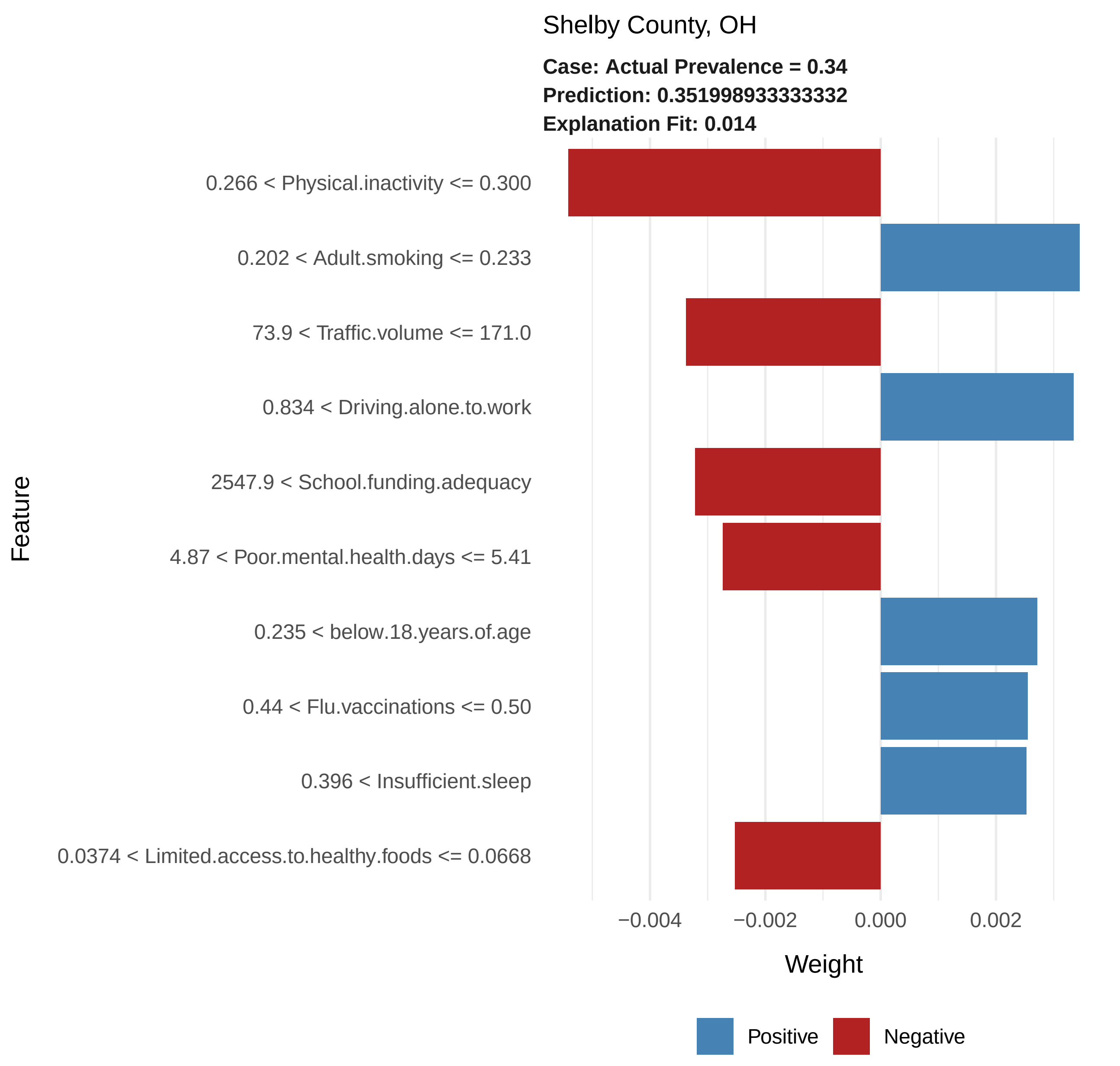
Shelby County, OH
Case: Actual Prevalence = 0.34
Prediction: 0.351998933333332
Explanation Fit: 0.014
0.266 < Physical.inactivity <= 0.300
0.202 < Adult.smoking <= 0.233
73.9 < Traffic.volume <= 171.0
0.834 < Driving.alone.to.work
2547.9 < School.funding.adequacy
4.87 < Poor.mental.health.days <= 5.41
0.235 < below.18.years.of.age
0.44 < Flu.vaccinations <= 0.50
0.396 < Insufficient.sleep
0.0374 < Limited.access.to.healthy.foods <= 0.0668
Feature
−0.004
−0.002
0.000
0.002
Weight
Positive
Negative

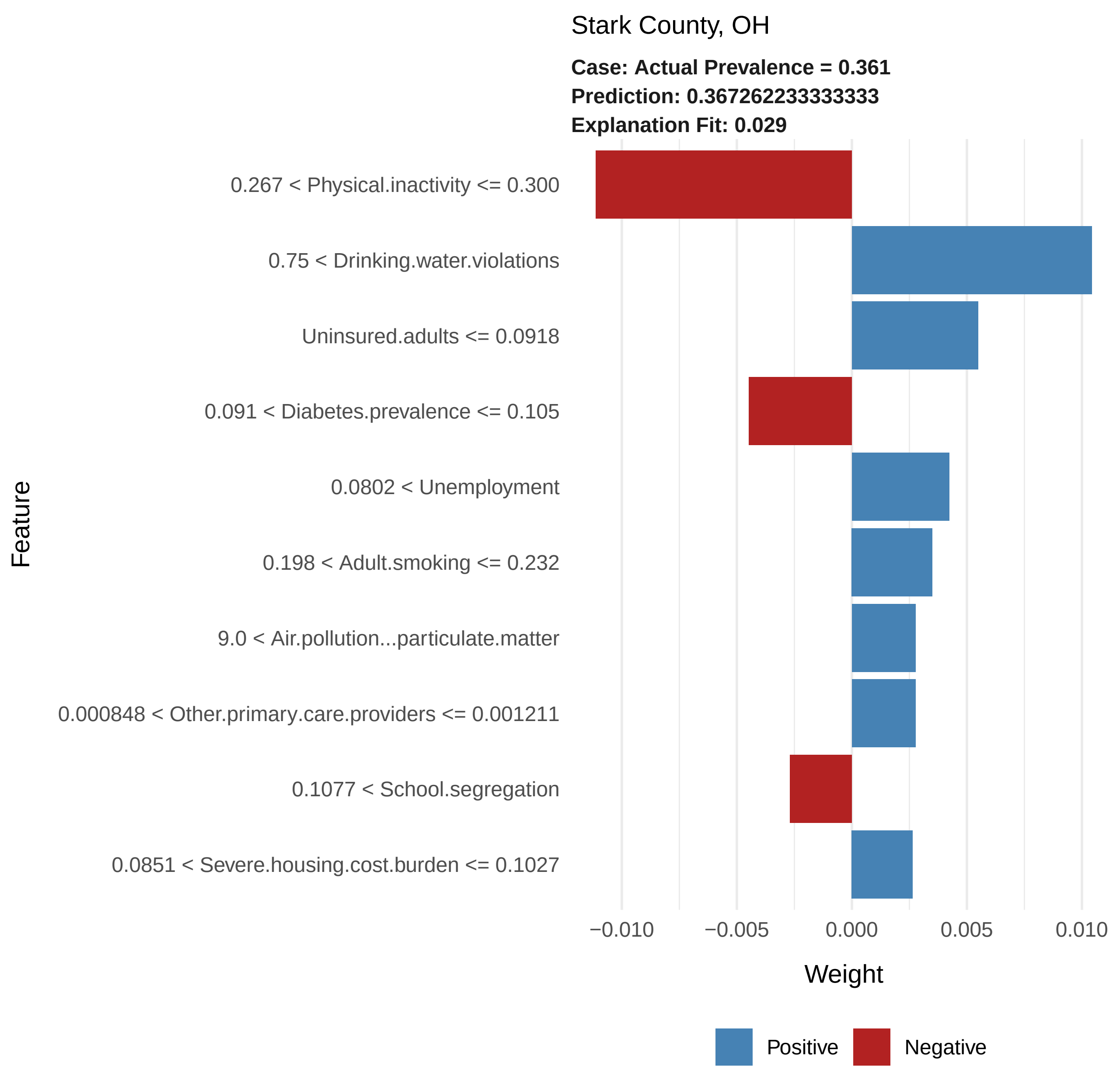
Stark County, OH
Case: Actual Prevalence = 0.361
Prediction: 0.367262233333333
Explanation Fit: 0.029
0.267 < Physical.inactivity <= 0.300
0.75 < Drinking.water.violations
Uninsured.adults <= 0.0918
0.091 < Diabetes.prevalence <= 0.105
0.0802 < Unemployment
0.198 < Adult.smoking <= 0.232
9.0 < Air.pollution...particulate.matter
0.000848 < Other.primary.care.providers <= 0.001211
0.1077 < School.segregation
0.0851 < Severe.housing.cost.burden <= 0.1027
Feature
−0.010
−0.005
0.000
0.005
0.010
Weight
Positive
Negative

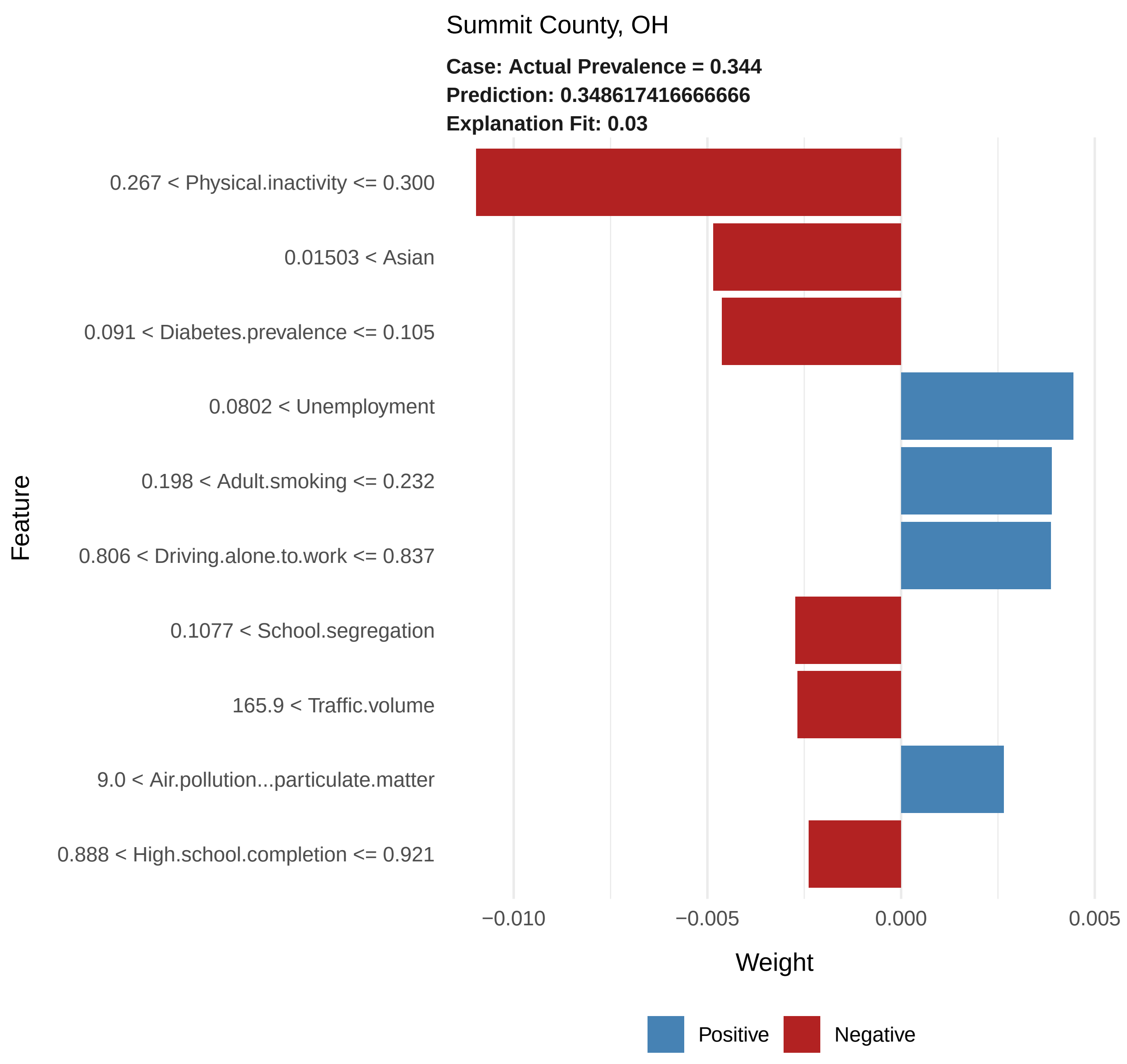
Summit County, OH
Case: Actual Prevalence = 0.344
Prediction: 0.348617416666666
Explanation Fit: 0.03
0.267 < Physical.inactivity <= 0.300
0.01503 < Asian
0.091 < Diabetes.prevalence <= 0.105
0.0802 < Unemployment
0.198 < Adult.smoking <= 0.232
0.806 < Driving.alone.to.work <= 0.837
0.1077 < School.segregation
165.9 < Traffic.volume
9.0 < Air.pollution...particulate.matter
0.888 < High.school.completion <= 0.921
Feature
−0.010
−0.005
0.000
0.005
Weight
Positive
Negative

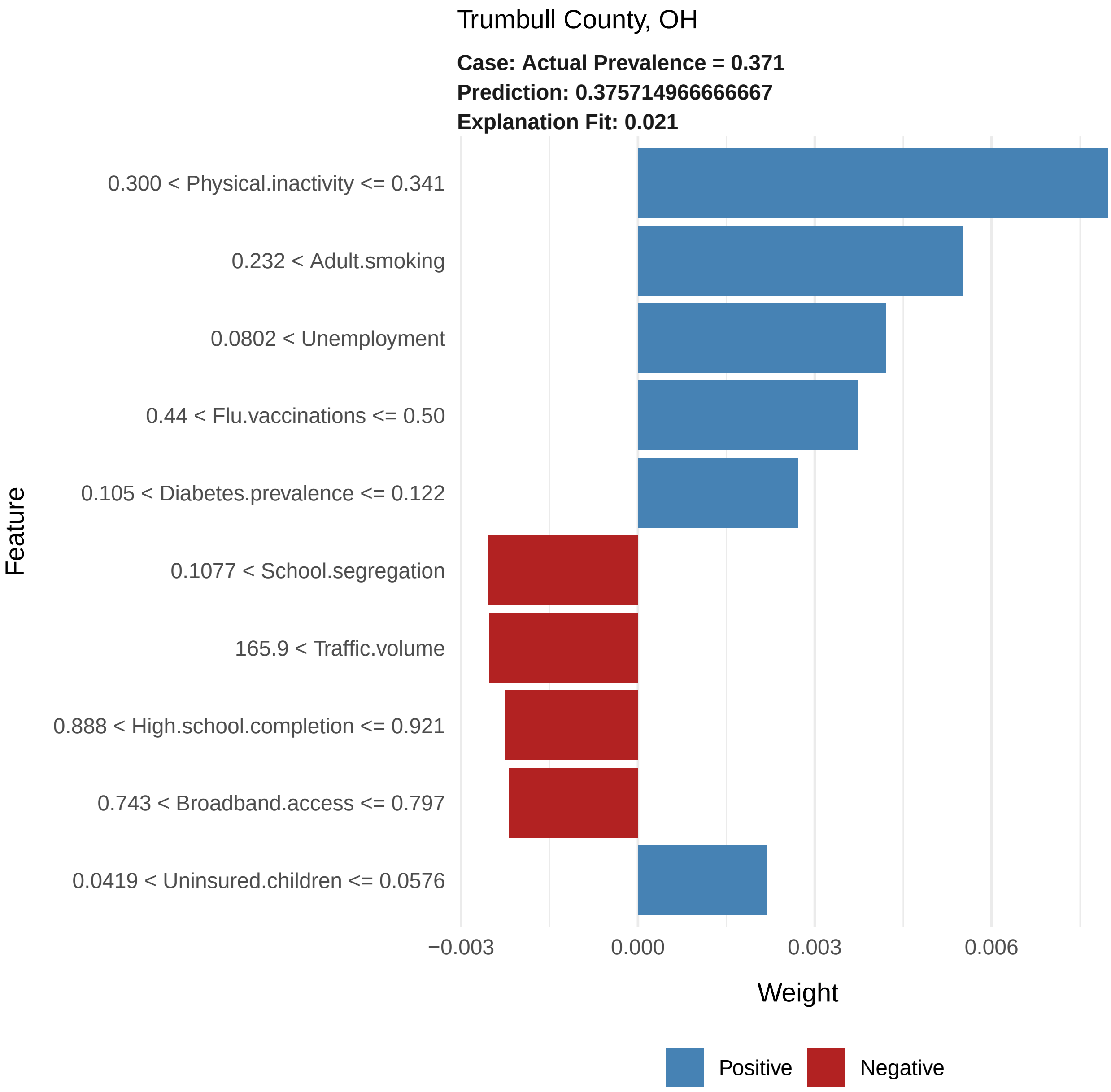

Trumbull County, OH
Case: Actual Prevalence = 0.371
Prediction: 0.375714966666667
Explanation Fit: 0.021
0.300 < Physical.inactivity <= 0.341
0.232 < Adult.smoking
0.0802 < Unemployment
0.44 < Flu.vaccinations <= 0.50
0.105 < Diabetes.prevalence <= 0.122
0.1077 < School.segregation
165.9 < Traffic.volume
0.888 < High.school.completion <= 0.921
0.743 < Broadband.access <= 0.797
0.0419 < Uninsured.children <= 0.0576
Feature
−0.003
0.000
0.003
0.006
Weight
Positive
Negative

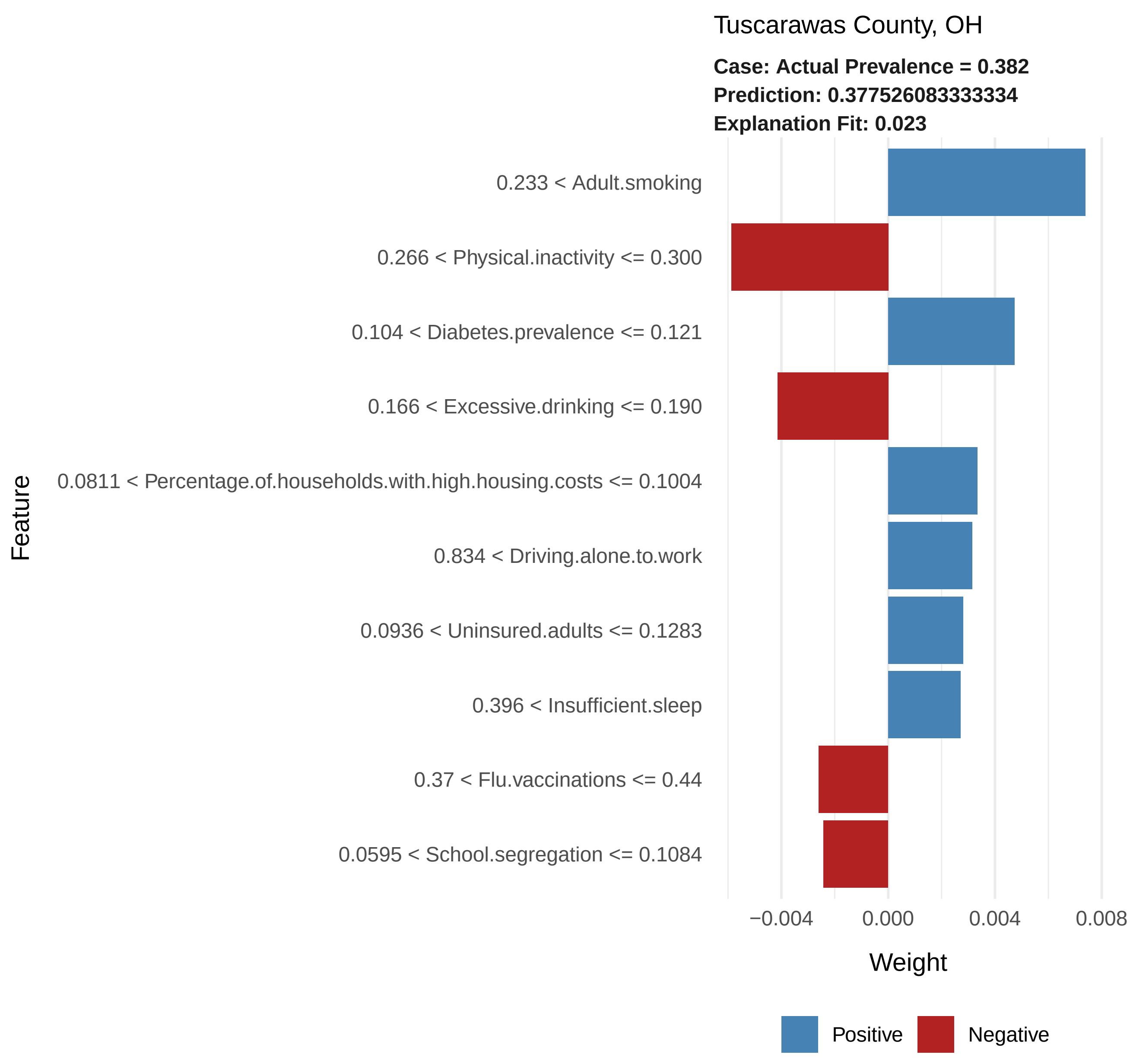

Tuscarawas County, OH
Case: Actual Prevalence = 0.382
Prediction: 0.377526083333334
Explanation Fit: 0.023
0.233 < Adult.smoking
0.266 < Physical.inactivity <= 0.300
0.104 < Diabetes.prevalence <= 0.121
0.166 < Excessive.drinking <= 0.190
0.0811 < Percentage.of.households.with.high.housing.costs <= 0.1004
0.834 < Driving.alone.to.work
0.0936 < Uninsured.adults <= 0.1283
0.396 < Insufficient.sleep
0.37 < Flu.vaccinations <= 0.44
0.0595 < School.segregation <= 0.1084
Feature
−0.004
0.000
0.004
0.008
Weight
Positive
Negative

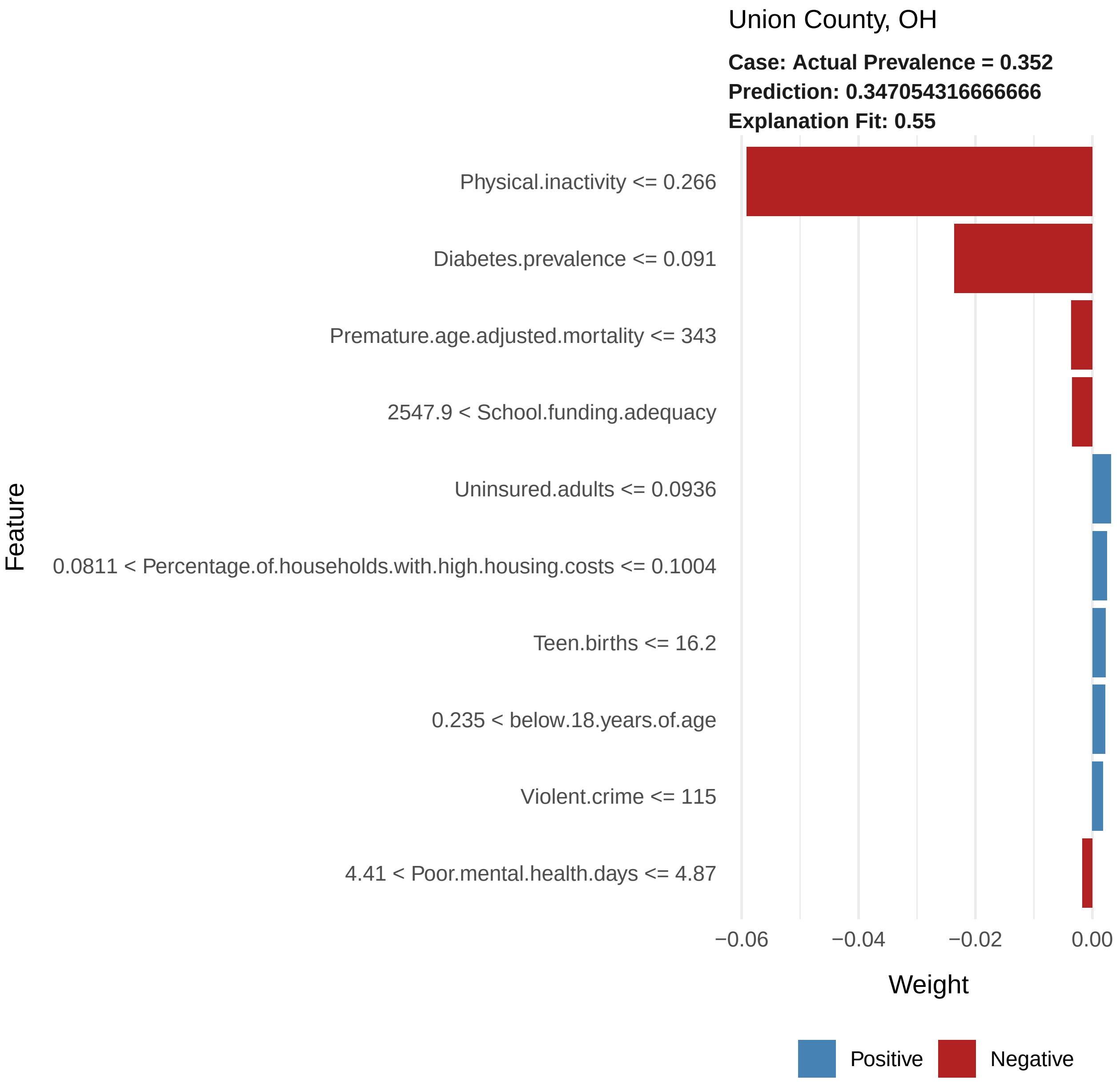
Union County, OH
Case: Actual Prevalence = 0.352
Prediction: 0.347054316666666
Explanation Fit: 0.55
Physical.inactivity <= 0.266
Diabetes.prevalence <= 0.091
Premature.age.adjusted.mortality <= 343
2547.9 < School.funding.adequacy
Uninsured.adults <= 0.0936
0.0811 < Percentage.of.households.with.high.housing.costs <= 0.1004
Teen.births <= 16.2
0.235 < below.18.years.of.age
Violent.crime <= 115
4.41 < Poor.mental.health.days <= 4.87
Feature
−0.06
−0.04
−0.02
0.00
Weight
Positive
Negative

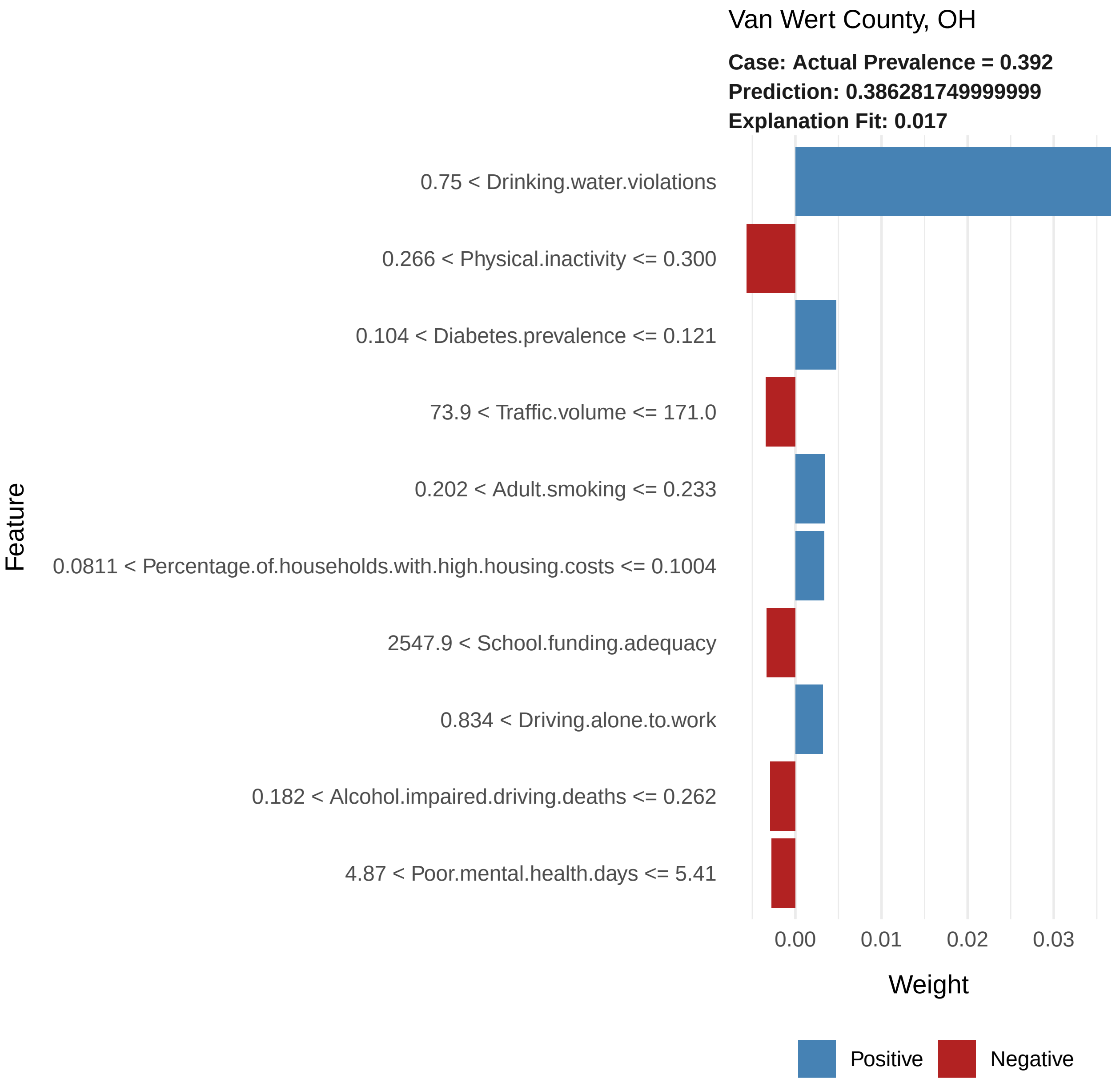
Van Wert County, OH
Case: Actual Prevalence = 0.392
Prediction: 0.386281749999999
Explanation Fit: 0.017
0.75 < Drinking.water.violations
0.266 < Physical.inactivity <= 0.300
0.104 < Diabetes.prevalence <= 0.121
73.9 < Traffic.volume <= 171.0
0.202 < Adult.smoking <= 0.233
0.0811 < Percentage.of.households.with.high.housing.costs <= 0.1004
2547.9 < School.funding.adequacy
0.834 < Driving.alone.to.work
0.182 < Alcohol.impaired.driving.deaths <= 0.262
4.87 < Poor.mental.health.days <= 5.41
Feature
0.00
0.01
0.02
0.03
Weight
Positive
Negative

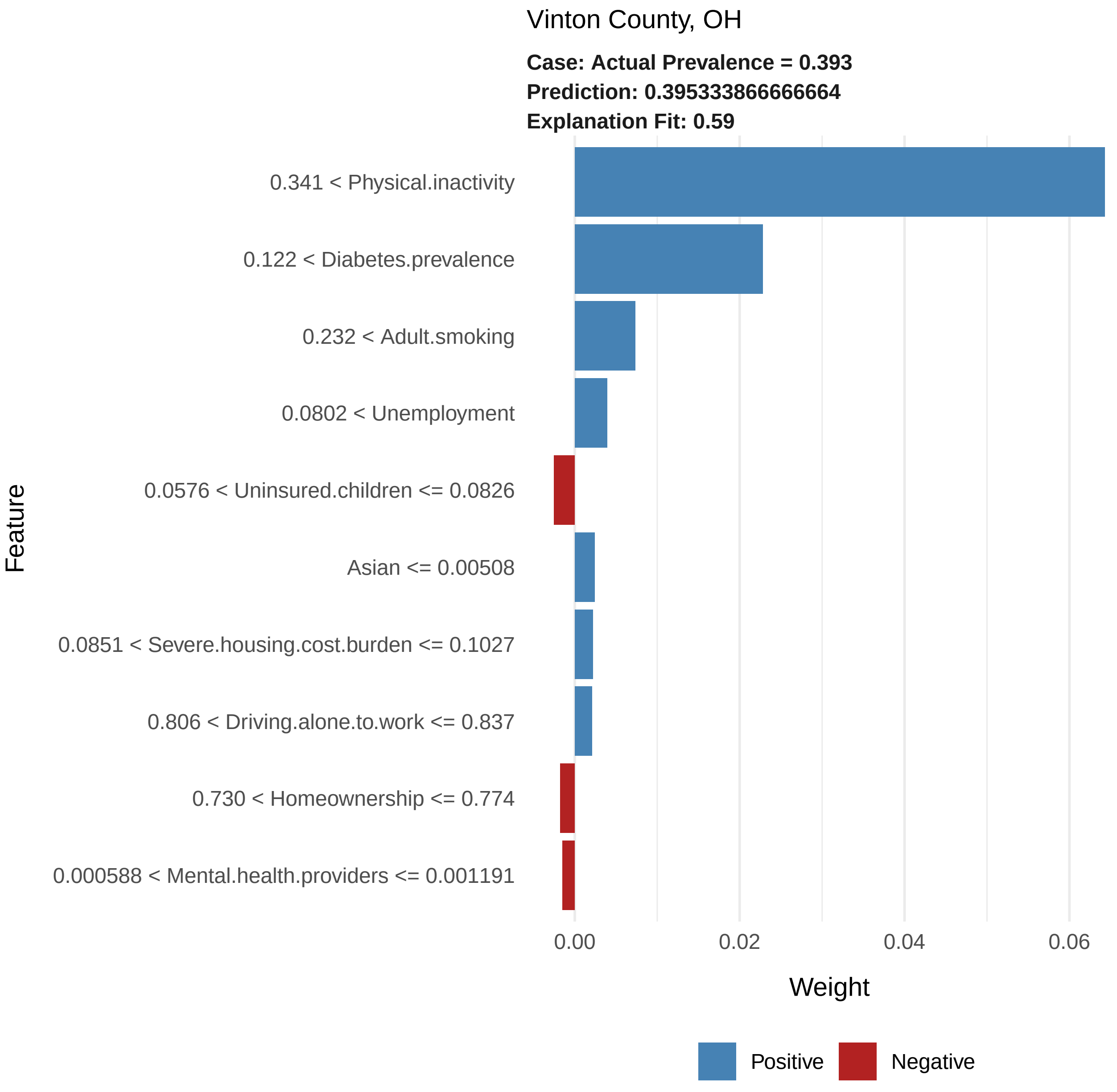

Vinton County, OH
Case: Actual Prevalence = 0.393
Prediction: 0.395333866666664
Explanation Fit: 0.59
0.341 < Physical.inactivity
0.122 < Diabetes.prevalence
0.232 < Adult.smoking
0.0802 < Unemployment
0.0576 < Uninsured.children <= 0.0826
Asian <= 0.00508
0.0851 < Severe.housing.cost.burden <= 0.1027
0.806 < Driving.alone.to.work <= 0.837
0.730 < Homeownership <= 0.774
0.000588 < Mental.health.providers <= 0.001191
Feature
0.00
0.02
0.04
0.06
Weight
Positive
Negative

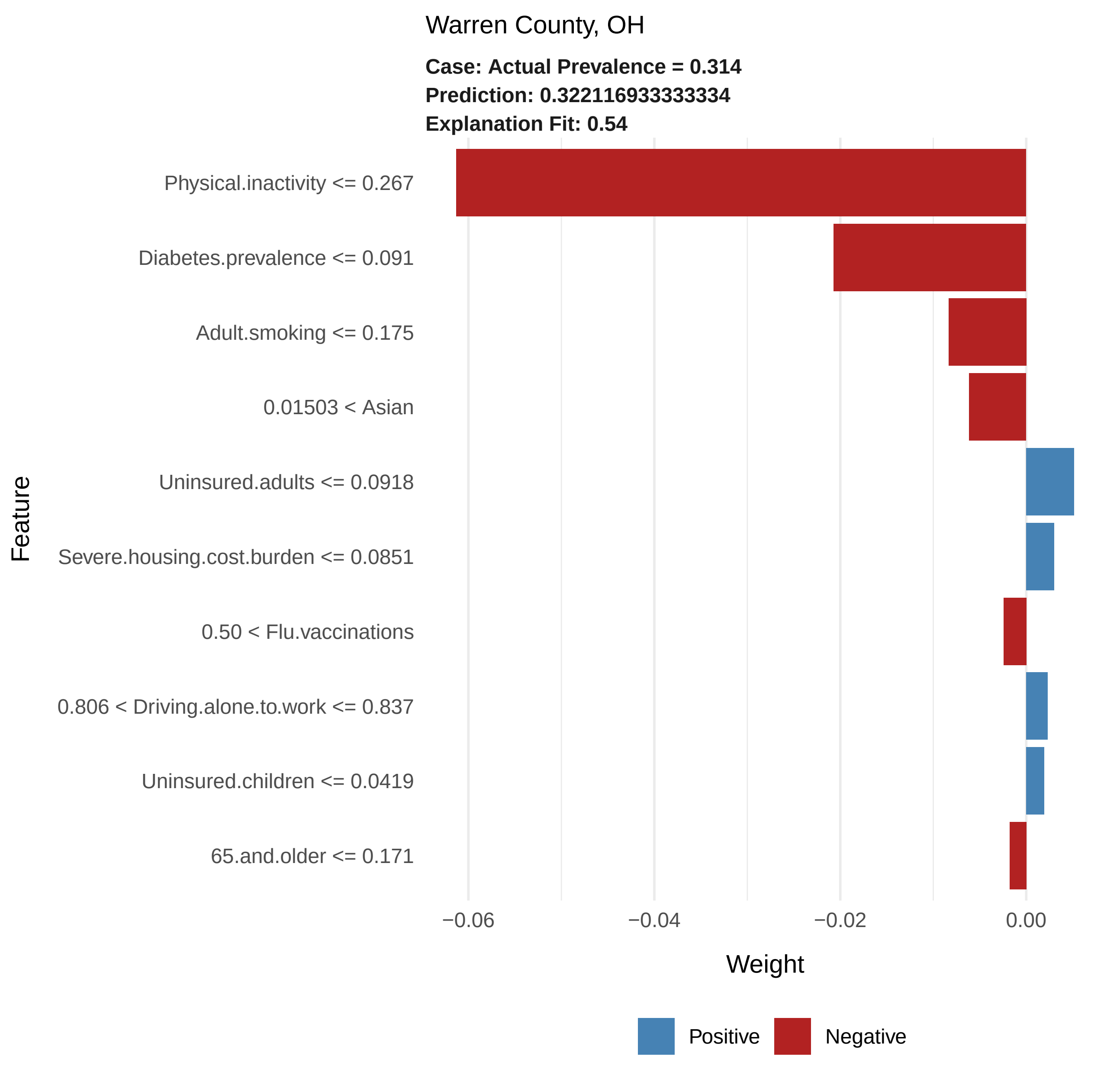

Warren County, OH
Case: Actual Prevalence = 0.314
Prediction: 0.322116933333334
Explanation Fit: 0.54
Physical.inactivity <= 0.267
Diabetes.prevalence <= 0.091
Adult.smoking <= 0.175
0.01503 < Asian
Uninsured.adults <= 0.0918
Severe.housing.cost.burden <= 0.0851
0.50 < Flu.vaccinations
0.806 < Driving.alone.to.work <= 0.837
Uninsured.children <= 0.0419
65.and.older <= 0.171
Feature
−0.06
−0.04
−0.02
0.00
Weight
Positive
Negative

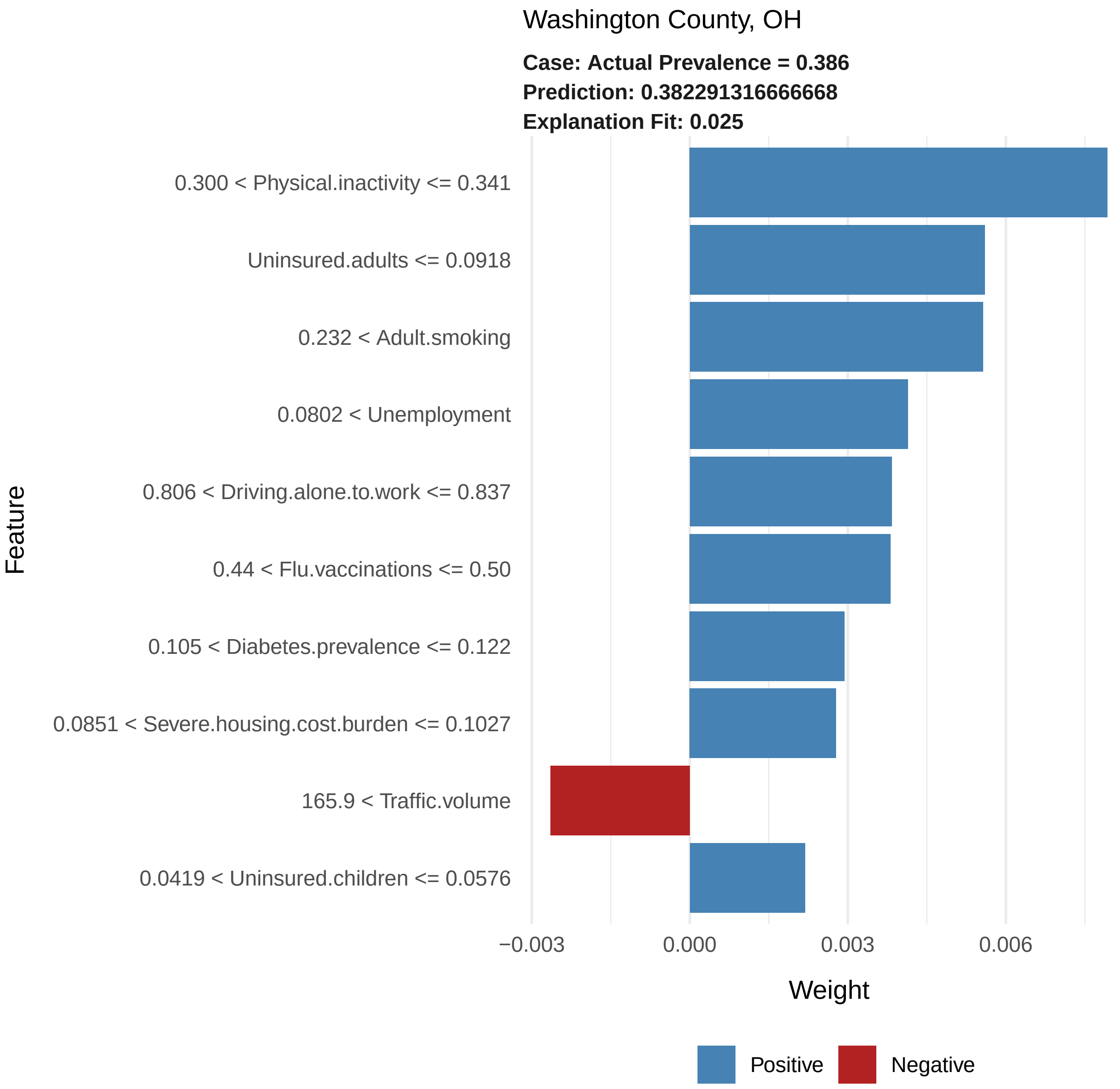
Washington County, OH
Case: Actual Prevalence = 0.386
Prediction: 0.382291316666668
Explanation Fit: 0.025
0.300 < Physical.inactivity <= 0.341
Uninsured.adults <= 0.0918
0.232 < Adult.smoking
0.0802 < Unemployment
0.806 < Driving.alone.to.work <= 0.837
0.44 < Flu.vaccinations <= 0.50
0.105 < Diabetes.prevalence <= 0.122
0.0851 < Severe.housing.cost.burden <= 0.1027
165.9 < Traffic.volume
0.0419 < Uninsured.children <= 0.0576
Feature
−0.003
0.000
0.003
0.006
Weight
Positive
Negative

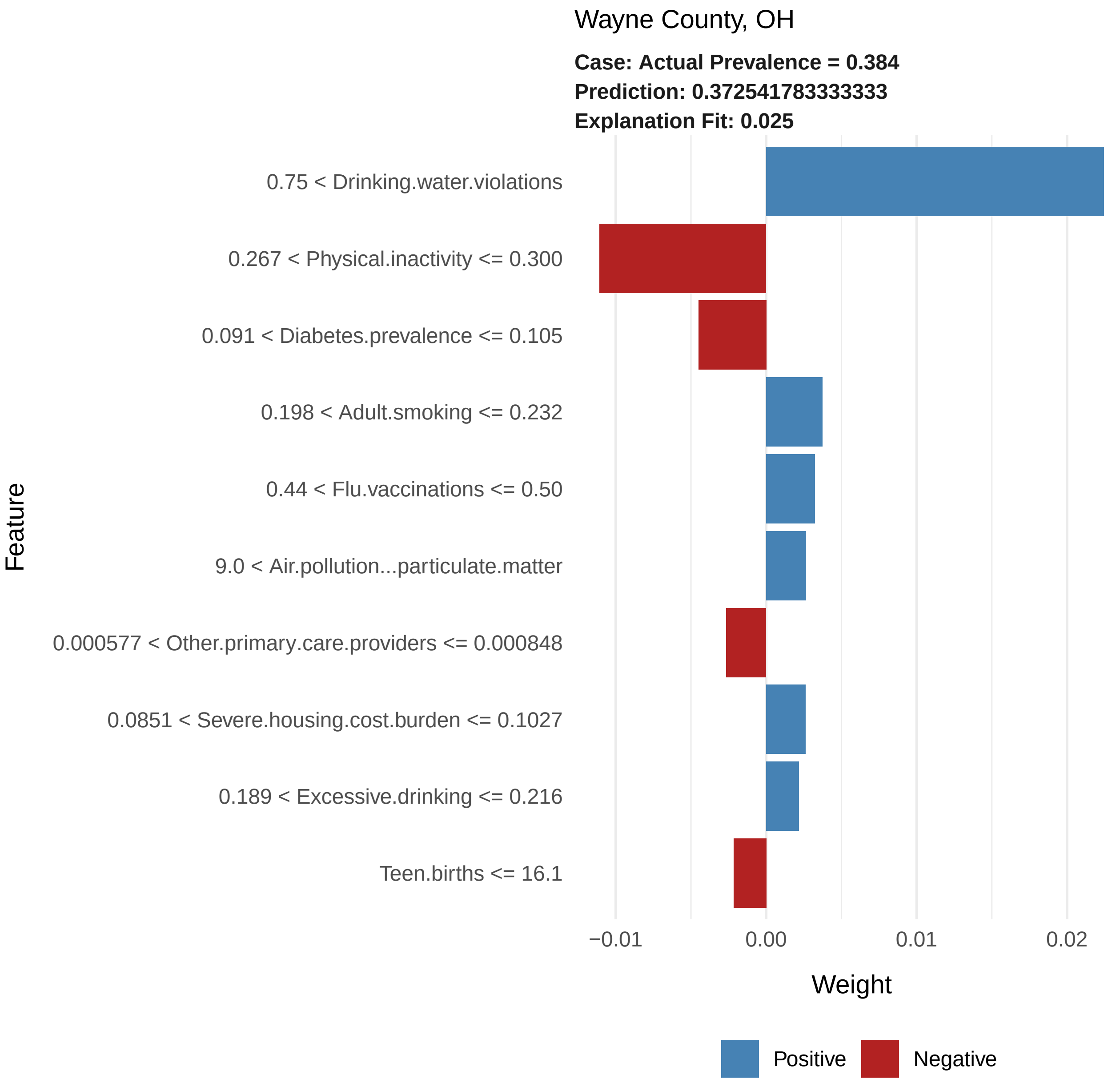

Wayne County, OH
Case: Actual Prevalence = 0.384
Prediction: 0.372541783333333
Explanation Fit: 0.025
0.75 < Drinking.water.violations
0.267 < Physical.inactivity <= 0.300
0.091 < Diabetes.prevalence <= 0.105
0.198 < Adult.smoking <= 0.232
0.44 < Flu.vaccinations <= 0.50
9.0 < Air.pollution...particulate.matter
0.000577 < Other.primary.care.providers <= 0.000848
0.0851 < Severe.housing.cost.burden <= 0.1027
0.189 < Excessive.drinking <= 0.216
Teen.births <= 16.1
Feature
−0.01
0.00
0.01
0.02
Weight
Positive
Negative

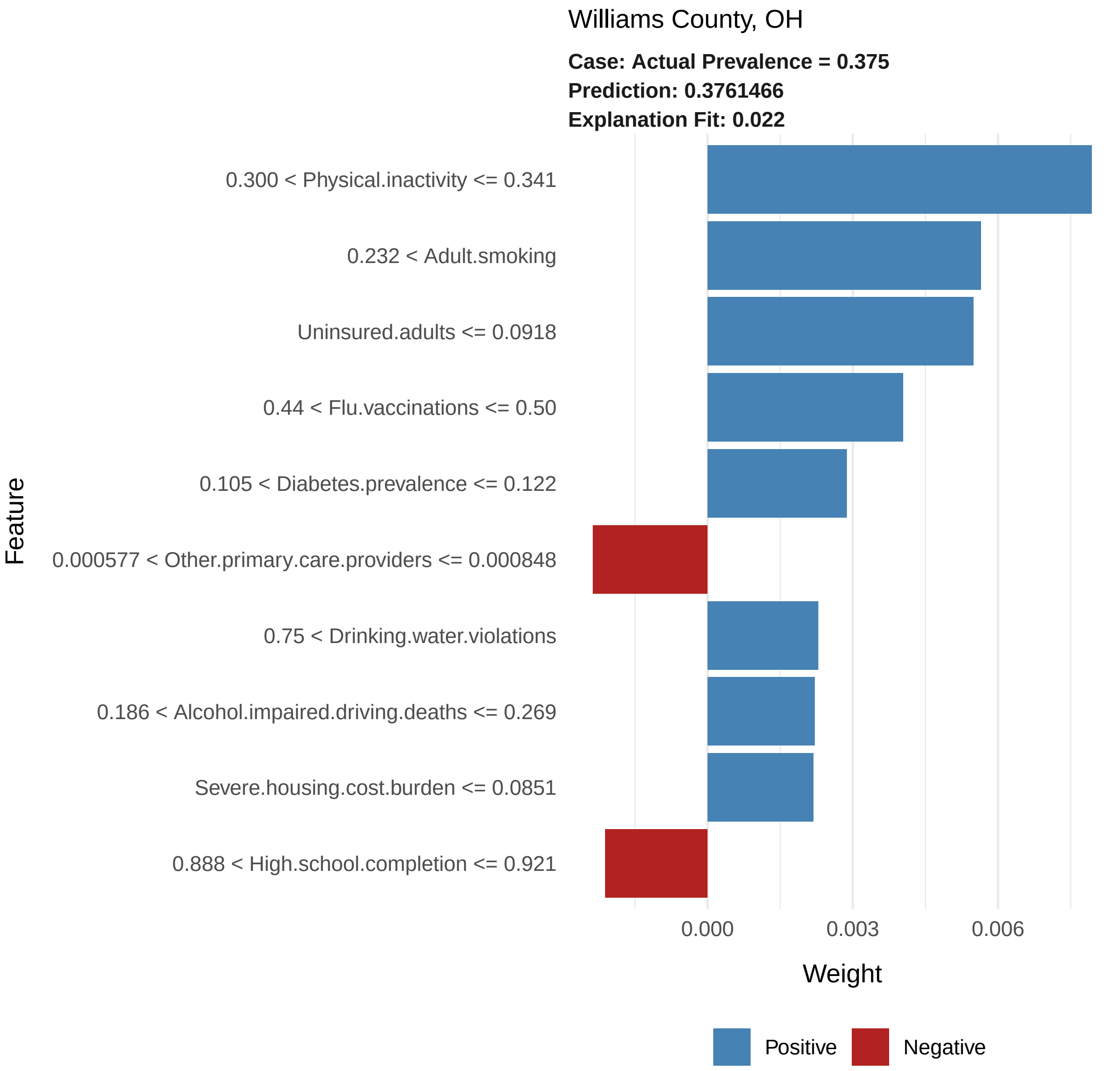

Williams County, OH
Case: Actual Prevalence = 0.375
Prediction: 0.3761466
Explanation Fit: 0.022
0.300 < Physical.inactivity <= 0.341
0.232 < Adult.smoking
Uninsured.adults <= 0.0918
0.44 < Flu.vaccinations <= 0.50
0.105 < Diabetes.prevalence <= 0.122
0.000577 < Other.primary.care.providers <= 0.000848
0.75 < Drinking.water.violations
0.186 < Alcohol.impaired.driving.deaths <= 0.269
Severe.housing.cost.burden <= 0.0851
0.888 < High.school.completion <= 0.921
Feature
0.000
0.003
0.006
Weight
Positive
Negative

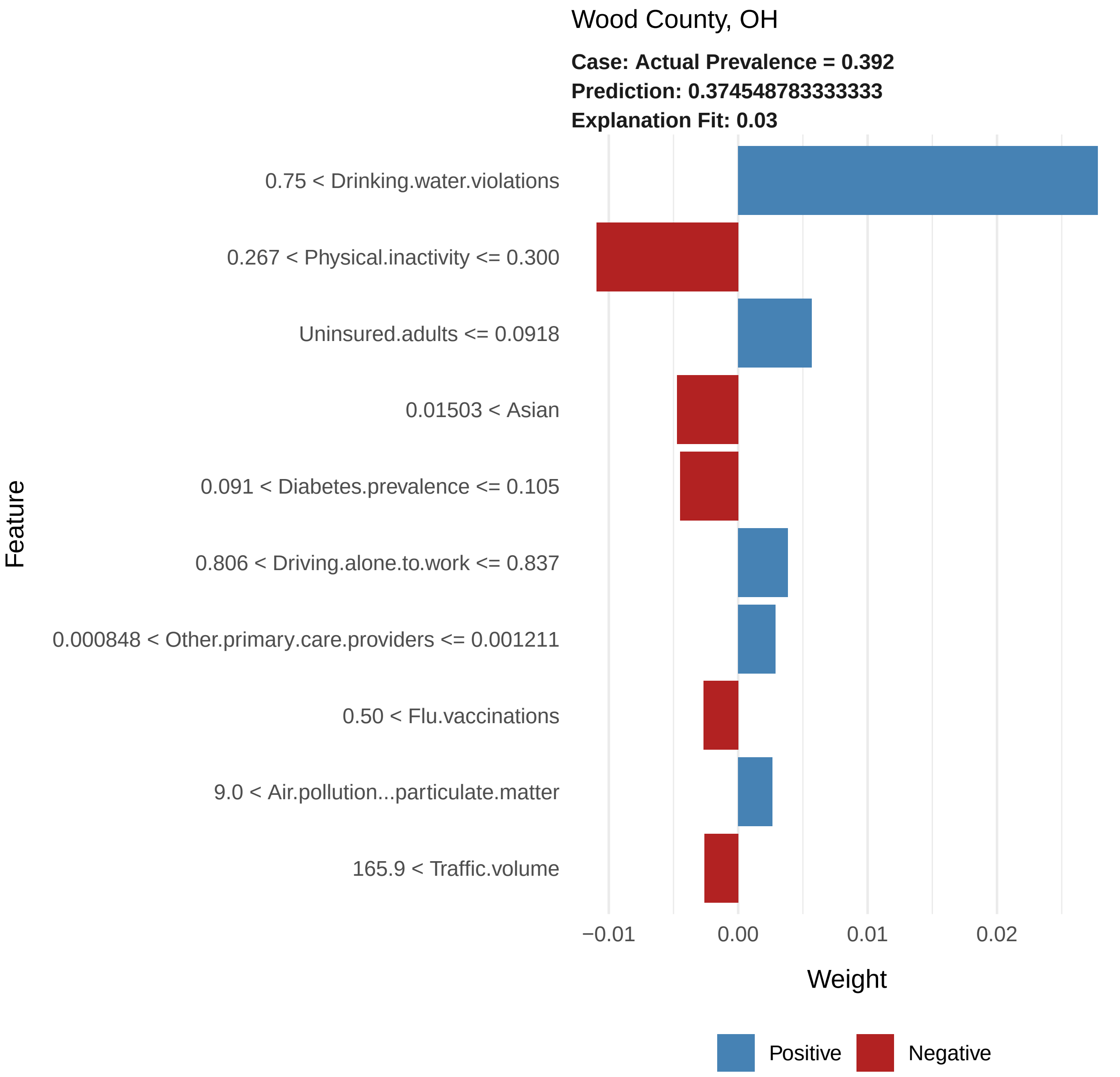
Wood County, OH
Case: Actual Prevalence = 0.392
Prediction: 0.374548783333333
Explanation Fit: 0.03
0.75 < Drinking.water.violations
0.267 < Physical.inactivity <= 0.300
Uninsured.adults <= 0.0918
0.01503 < Asian
0.091 < Diabetes.prevalence <= 0.105
0.806 < Driving.alone.to.work <= 0.837
0.000848 < Other.primary.care.providers <= 0.001211
0.50 < Flu.vaccinations
9.0 < Air.pollution...particulate.matter
165.9 < Traffic.volume
Feature
−0.01
0.00
0.01
0.02
Weight
Positive
Negative

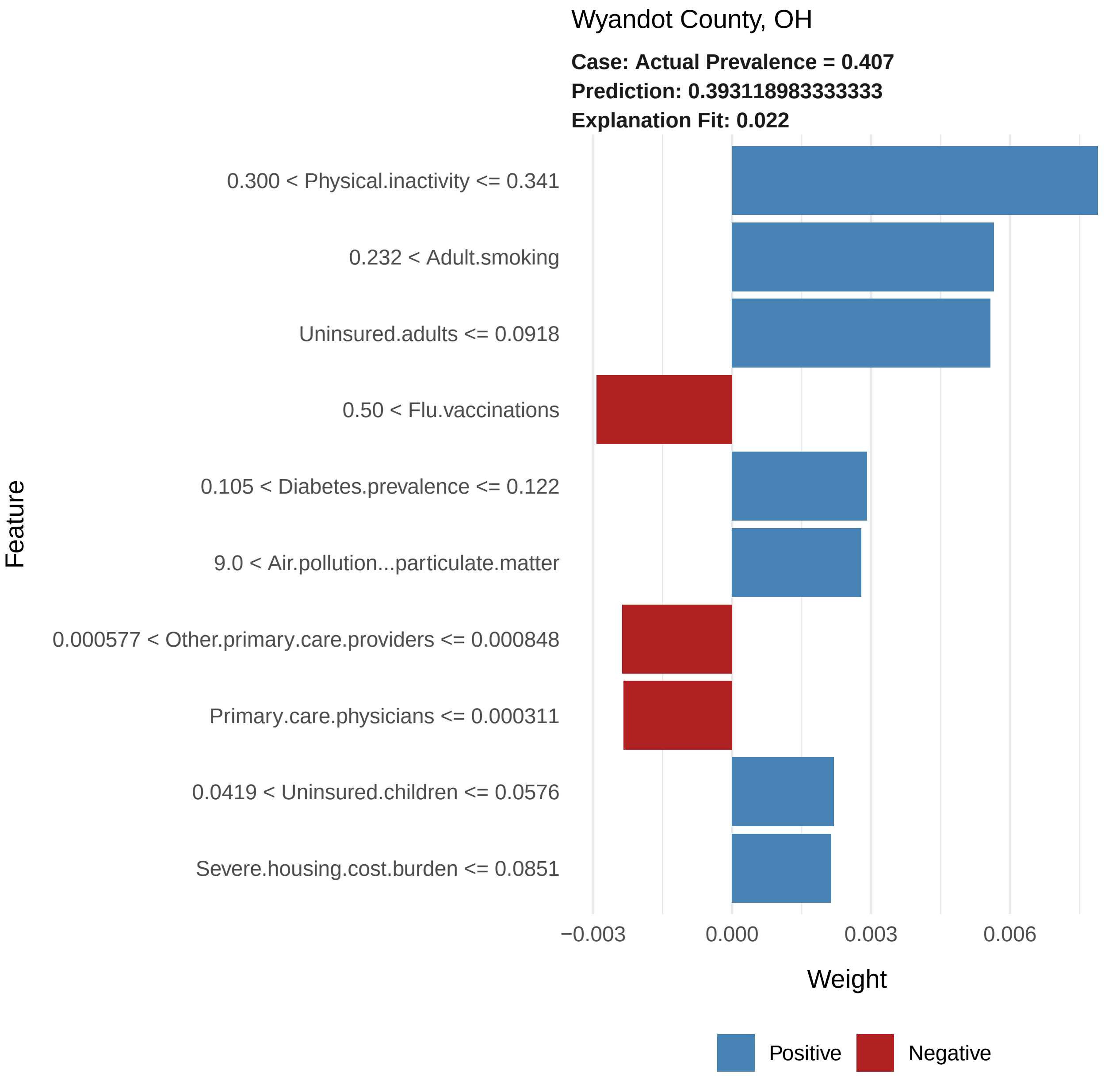
Wyandot County, OH
Case: Actual Prevalence = 0.407
Prediction: 0.393118983333333
Explanation Fit: 0.022
0.300 < Physical.inactivity <= 0.341
0.232 < Adult.smoking
Uninsured.adults <= 0.0918
0.50 < Flu.vaccinations
0.105 < Diabetes.prevalence <= 0.122
9.0 < Air.pollution...particulate.matter
0.000577 < Other.primary.care.providers <= 0.000848
Primary.care.physicians <= 0.000311
0.0419 < Uninsured.children <= 0.0576
Severe.housing.cost.burden <= 0.0851
Feature
−0.003
0.000
0.003
0.006
Weight
Positive
Negative

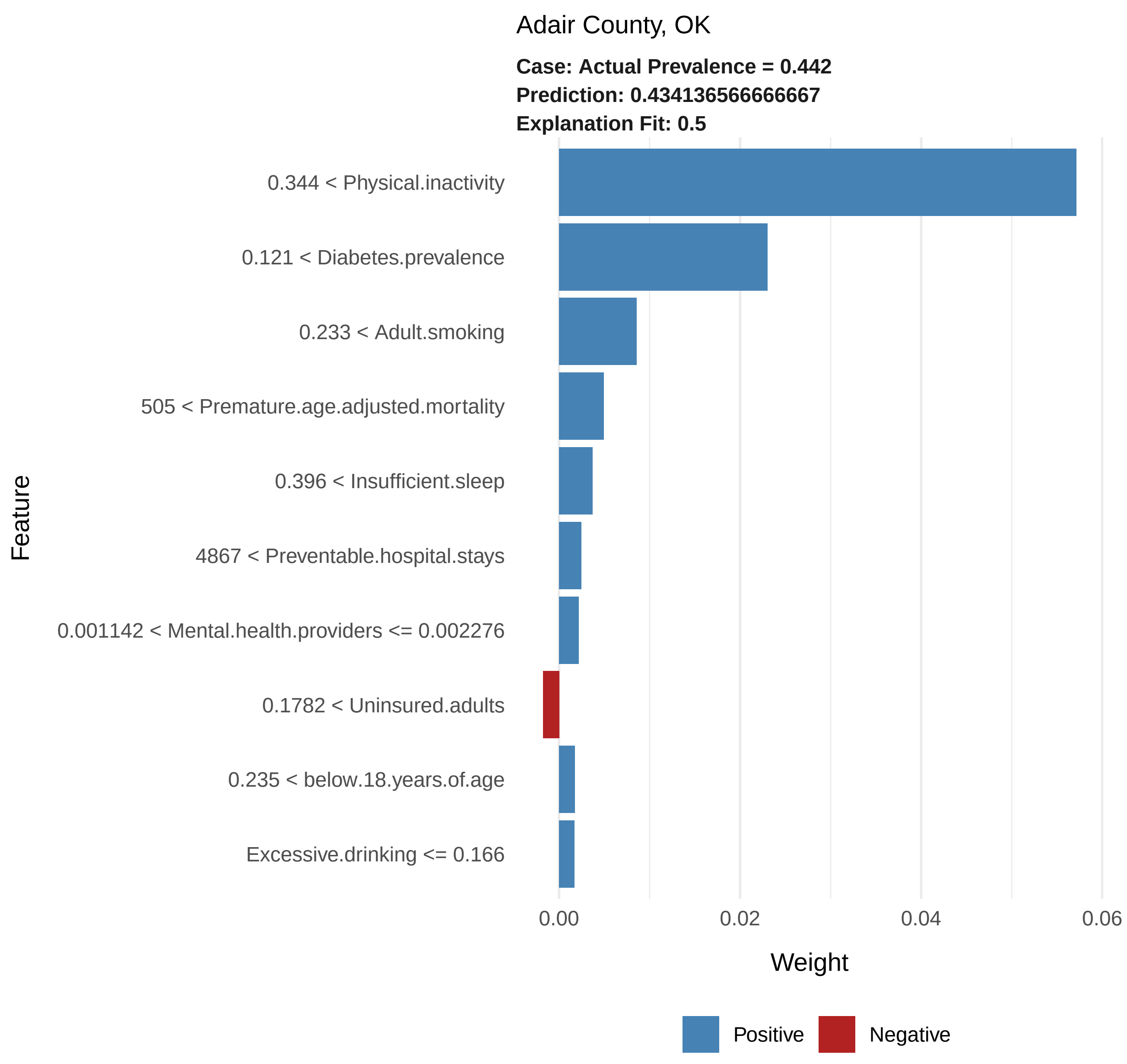

Adair County, OK
Case: Actual Prevalence = 0.442
Prediction: 0.434136566666667
Explanation Fit: 0.5
0.344 < Physical.inactivity
0.121 < Diabetes.prevalence
0.233 < Adult.smoking
505 < Premature.age.adjusted.mortality
0.396 < Insufficient.sleep
4867 < Preventable.hospital.stays
0.001142 < Mental.health.providers <= 0.002276
0.1782 < Uninsured.adults
0.235 < below.18.years.of.age
Excessive.drinking <= 0.166
Feature
0.00
0.02
0.04
0.06
Weight
Positive
Negative

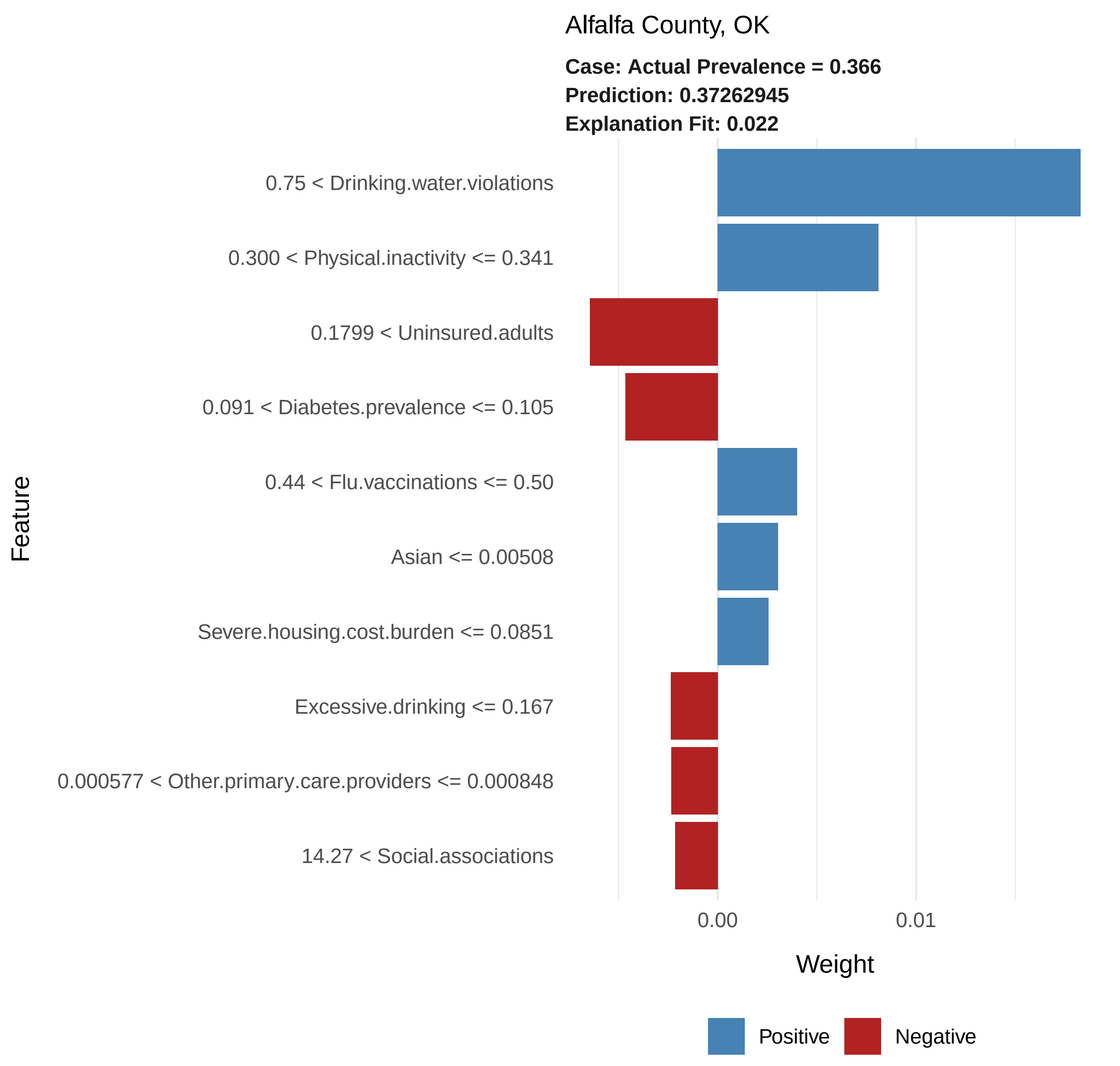

Alfalfa County, OK
Case: Actual Prevalence = 0.366
Prediction: 0.37262945
Explanation Fit: 0.022
0.75 < Drinking.water.violations
0.300 < Physical.inactivity <= 0.341
0.1799 < Uninsured.adults
0.091 < Diabetes.prevalence <= 0.105
0.44 < Flu.vaccinations <= 0.50
Asian <= 0.00508
Severe.housing.cost.burden <= 0.0851
Excessive.drinking <= 0.167
0.000577 < Other.primary.care.providers <= 0.000848
14.27 < Social.associations
Feature
0.00
0.01
Weight
Positive
Negative

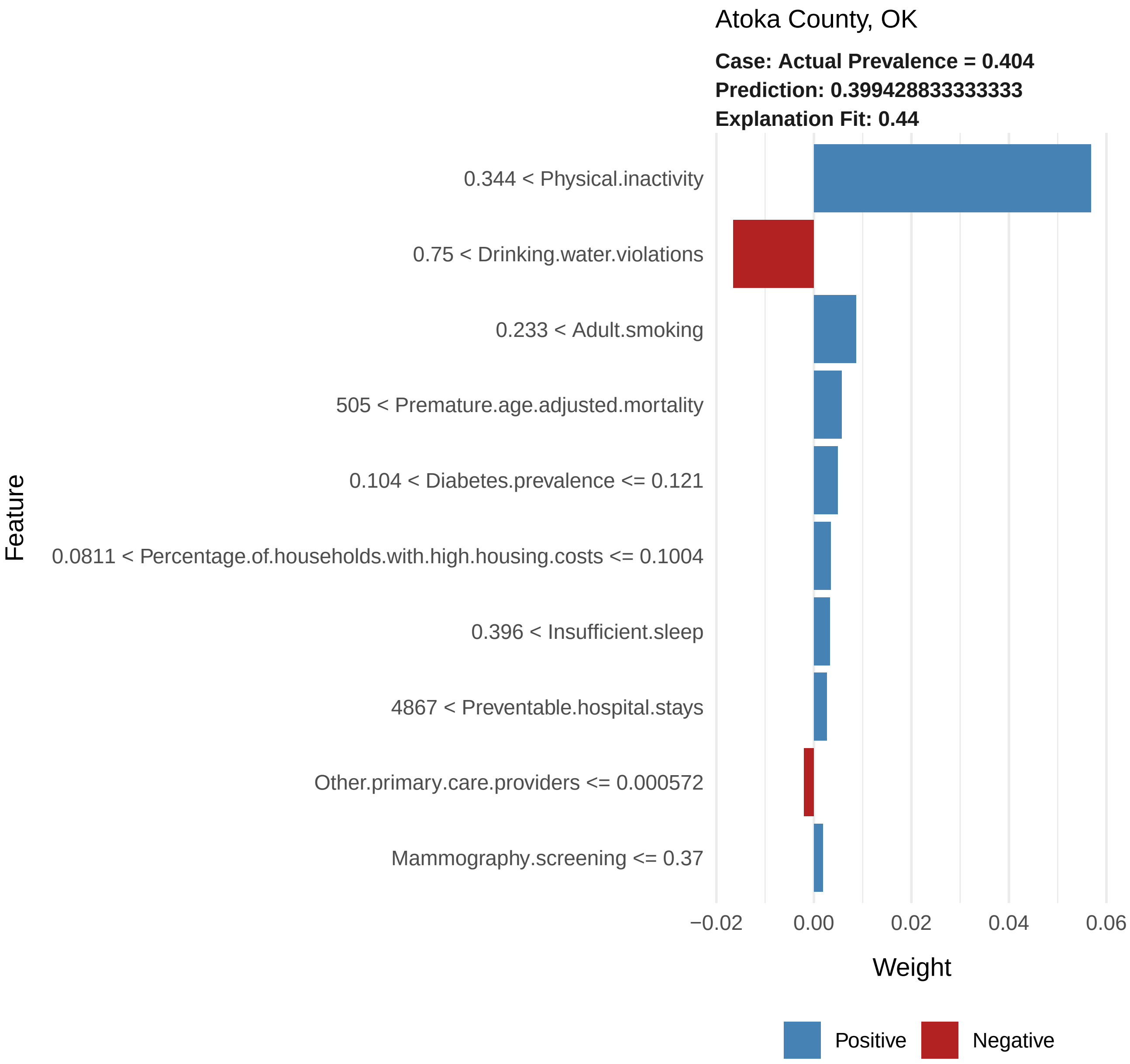
Atoka County, OK
Case: Actual Prevalence = 0.404
Prediction: 0.399428833333333
Explanation Fit: 0.44
0.344 < Physical.inactivity
0.75 < Drinking.water.violations
0.233 < Adult.smoking
505 < Premature.age.adjusted.mortality
0.104 < Diabetes.prevalence <= 0.121
0.0811 < Percentage.of.households.with.high.housing.costs <= 0.1004
0.396 < Insufficient.sleep
4867 < Preventable.hospital.stays
Other.primary.care.providers <= 0.000572
Mammography.screening <= 0.37
Feature
−0.02
0.00
0.02
0.04
0.06
Weight
Positive
Negative

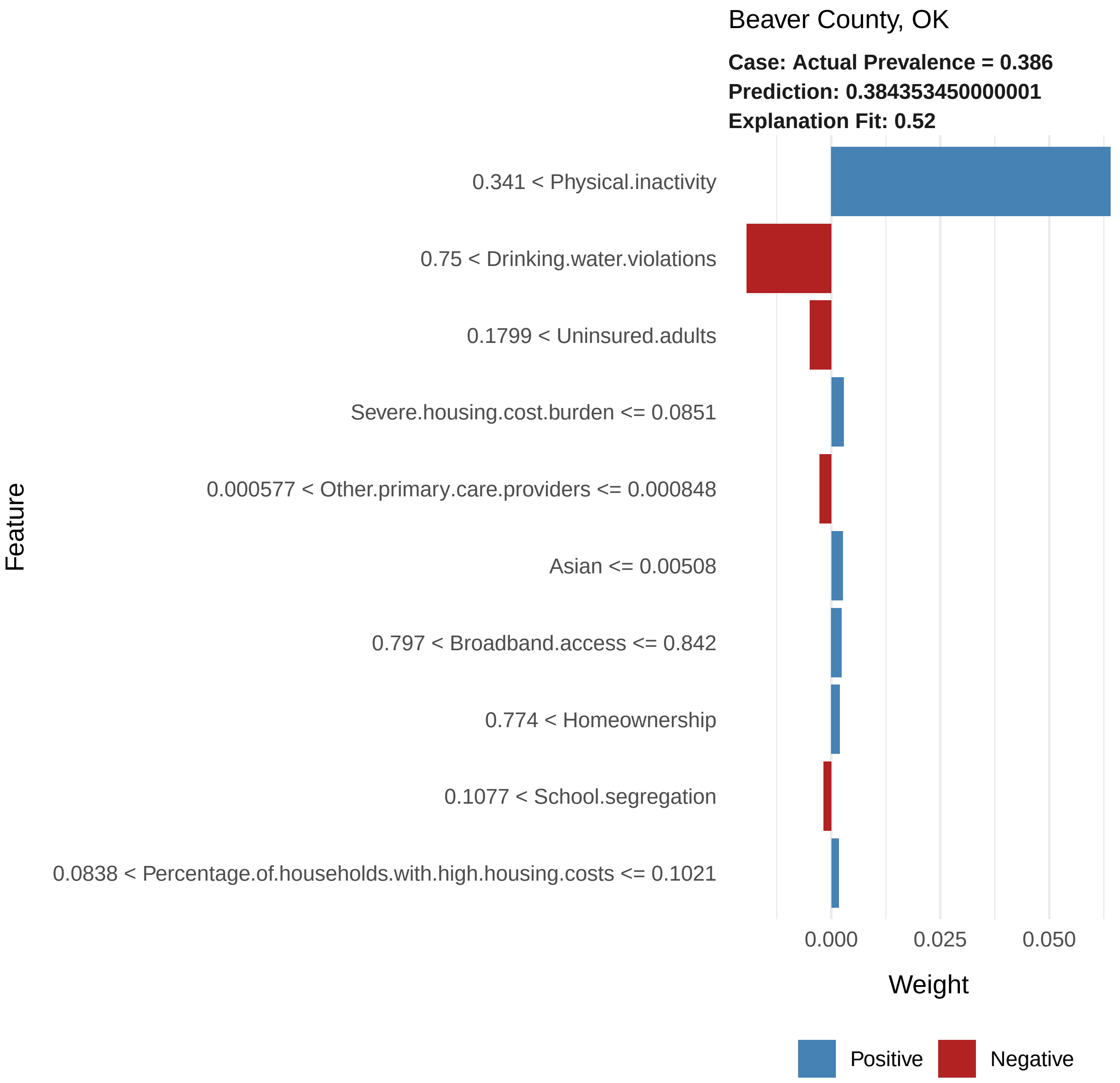

Beaver County, OK
Case: Actual Prevalence = 0.386
Prediction: 0.384353450000001
Explanation Fit: 0.52
0.341 < Physical.inactivity
0.75 < Drinking.water.violations
0.1799 < Uninsured.adults
Severe.housing.cost.burden <= 0.0851
0.000577 < Other.primary.care.providers <= 0.000848
Asian <= 0.00508
0.797 < Broadband.access <= 0.842
0.774 < Homeownership
0.1077 < School.segregation
0.0838 < Percentage.of.households.with.high.housing.costs <= 0.1021
Feature
0.000
0.025
0.050
Weight
Positive
Negative

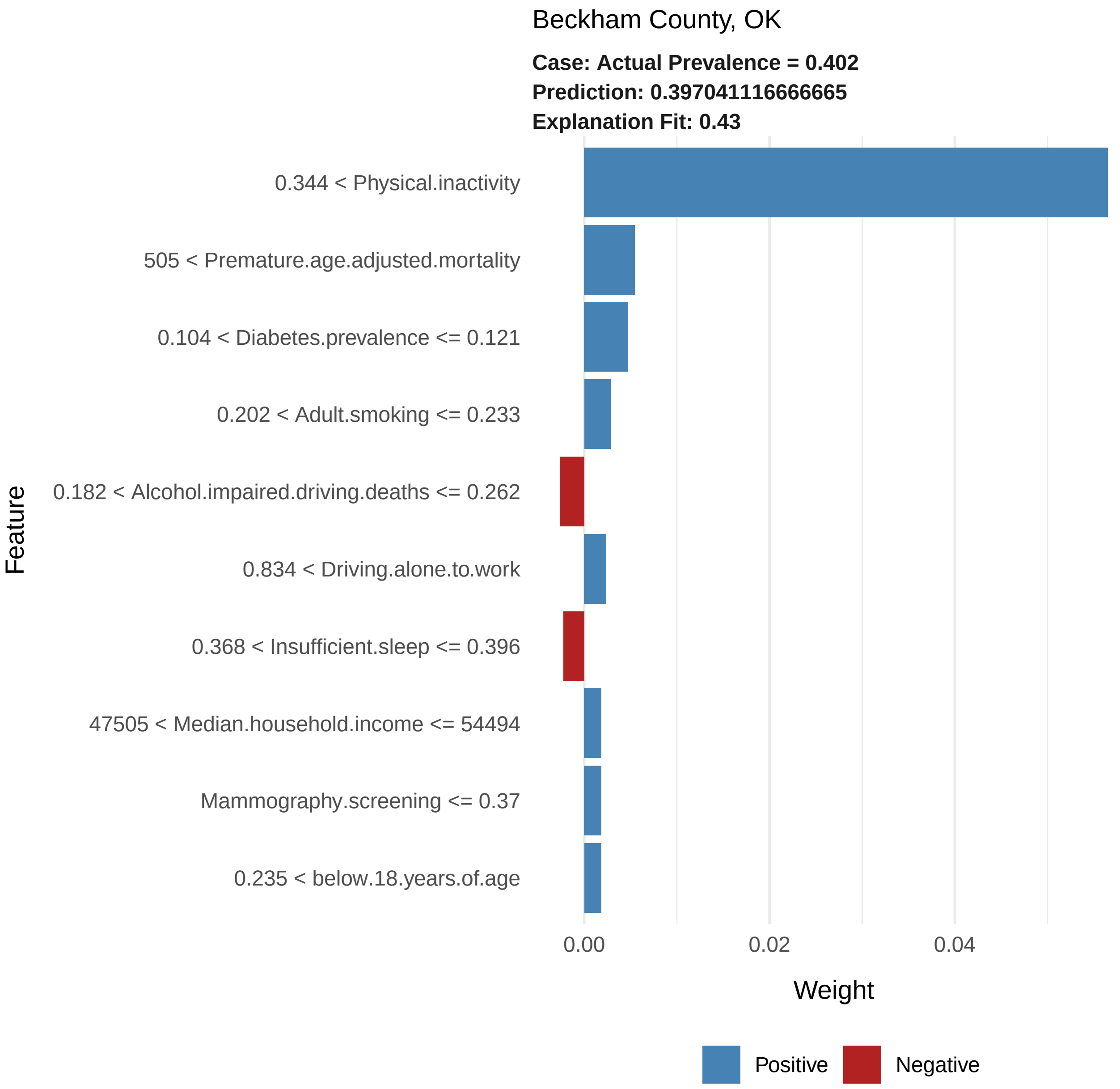

Beckham County, OK
Case: Actual Prevalence = 0.402
Prediction: 0.397041116666665
Explanation Fit: 0.43
0.344 < Physical.inactivity
505 < Premature.age.adjusted.mortality
0.104 < Diabetes.prevalence <= 0.121
0.202 < Adult.smoking <= 0.233
0.182 < Alcohol.impaired.driving.deaths <= 0.262
0.834 < Driving.alone.to.work
0.368 < Insufficient.sleep <= 0.396
47505 < Median.household.income <= 54494
Mammography.screening <= 0.37
0.235 < below.18.years.of.age
Feature
0.00
0.02
0.04
Weight
Positive
Negative

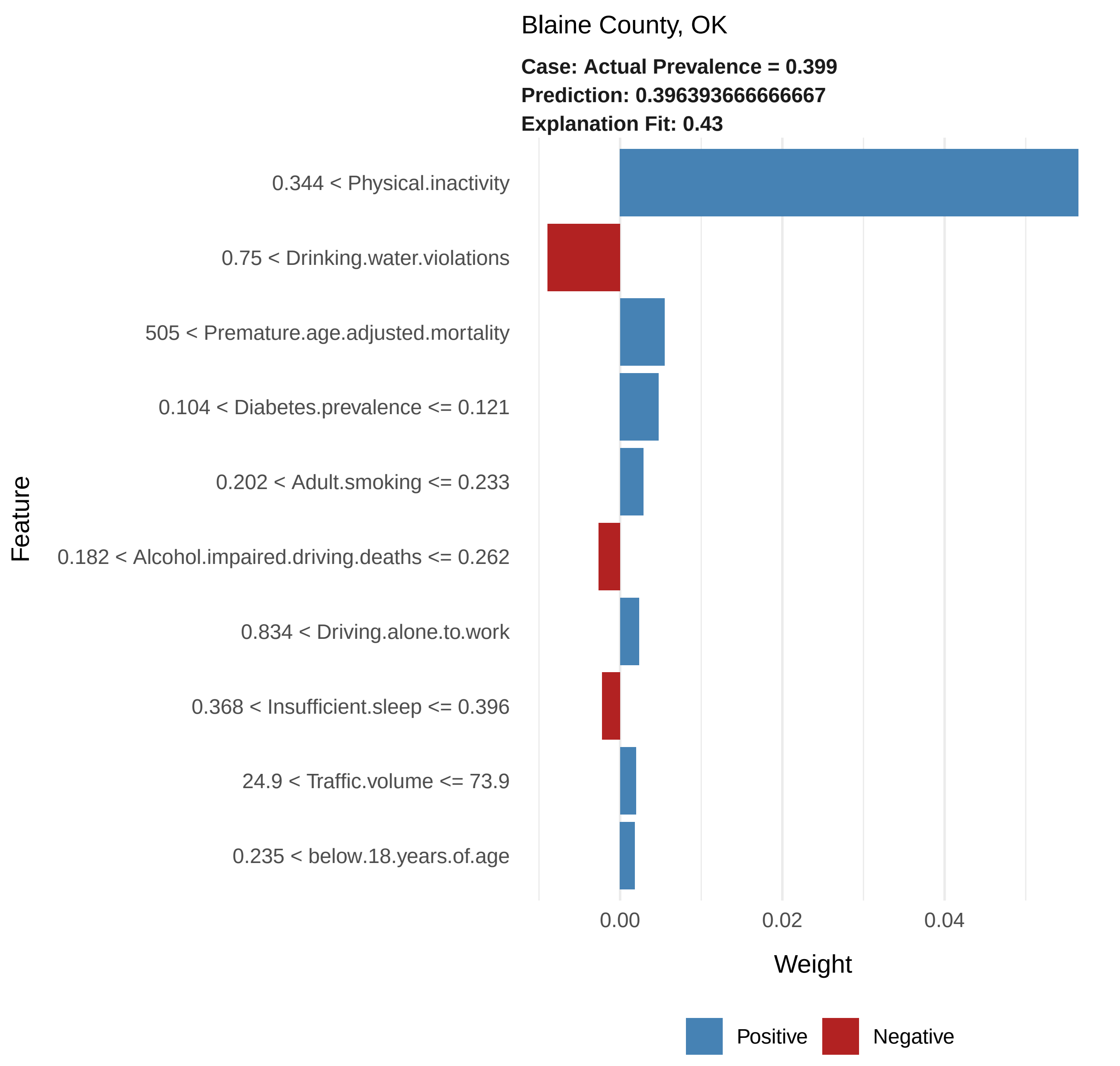

Blaine County, OK
Case: Actual Prevalence = 0.399
Prediction: 0.396393666666667
Explanation Fit: 0.43
0.344 < Physical.inactivity
0.75 < Drinking.water.violations
505 < Premature.age.adjusted.mortality
0.104 < Diabetes.prevalence <= 0.121
0.202 < Adult.smoking <= 0.233
0.182 < Alcohol.impaired.driving.deaths <= 0.262
0.834 < Driving.alone.to.work
0.368 < Insufficient.sleep <= 0.396
24.9 < Traffic.volume <= 73.9
0.235 < below.18.years.of.age
Feature
0.00
0.02
0.04
Weight
Positive
Negative

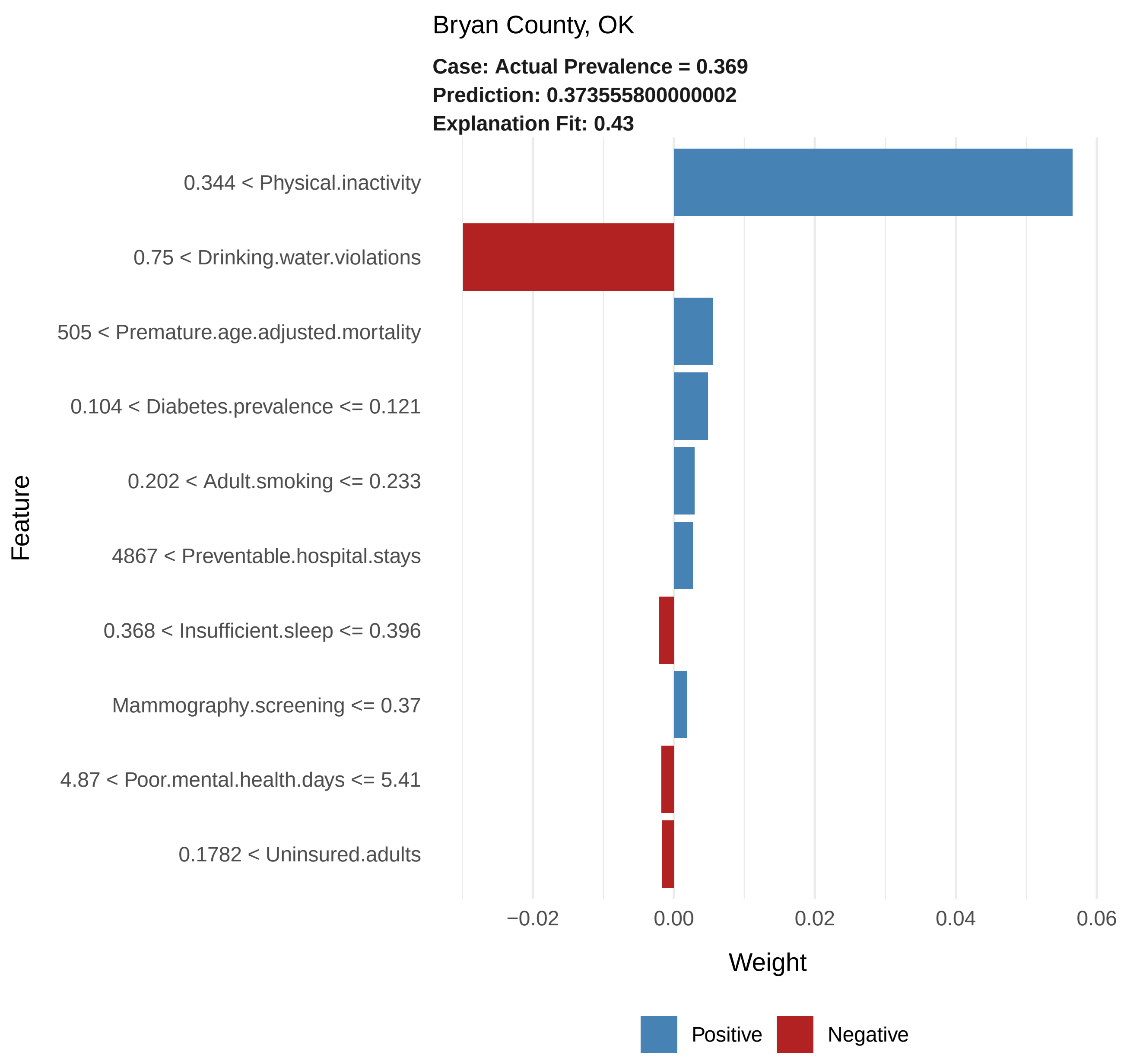

Bryan County, OK
Case: Actual Prevalence = 0.369
Prediction: 0.373555800000002
Explanation Fit: 0.43
Feature
0.344 < Physical.inactivity
0.75 < Drinking.water.violations
505 < Premature.age.adjusted.mortality
0.104 < Diabetes.prevalence <= 0.121
0.202 < Adult.smoking <= 0.233
4867 < Preventable.hospital.stays
0.368 < Insufficient.sleep <= 0.396
Mammography.screening <= 0.37
4.87 < Poor.mental.health.days <= 5.41
0.1782 < Uninsured.adults
−0.02
0.00
0.02
0.04
0.06
Weight
Positive
Negative

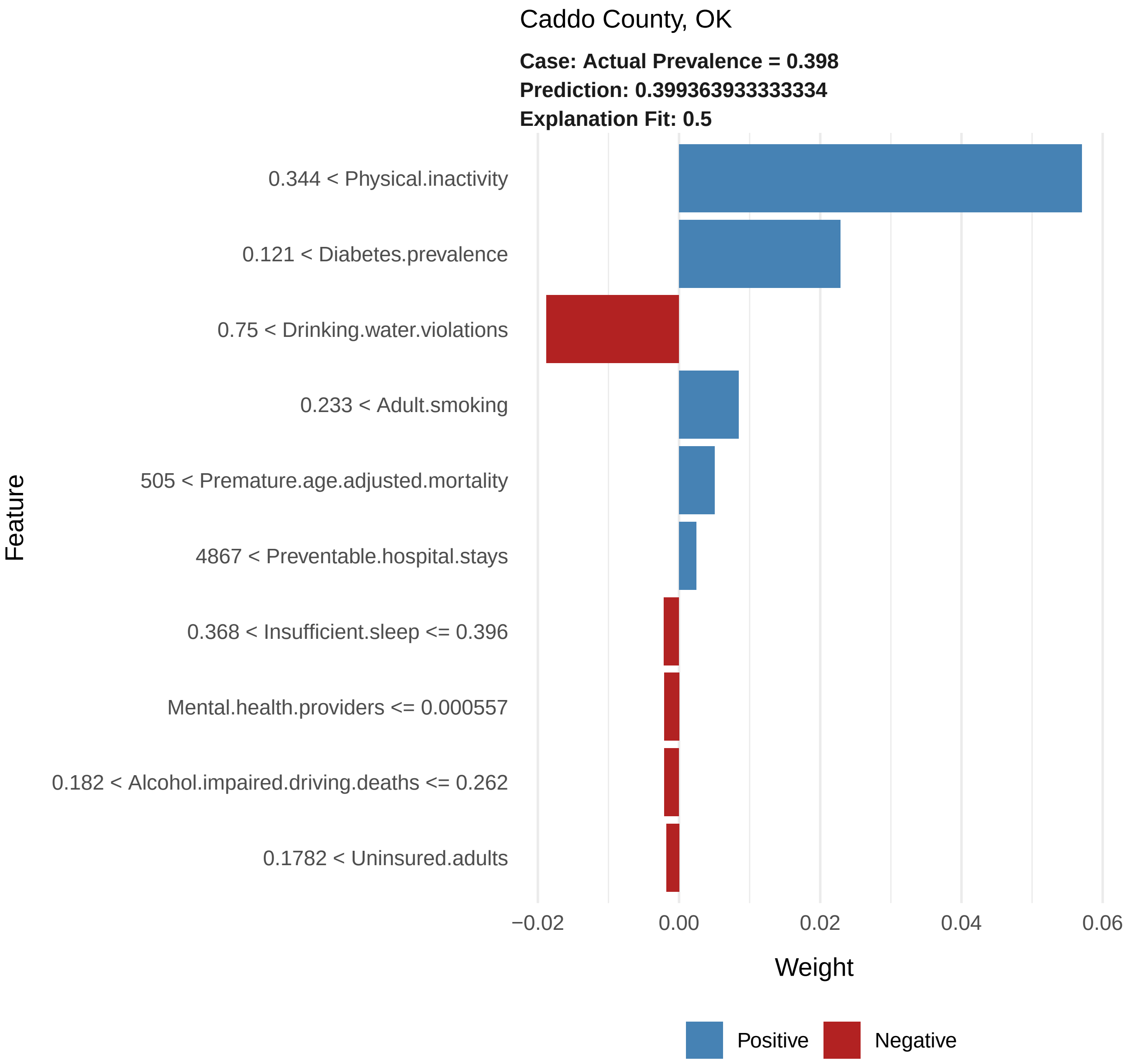

Caddo County, OK
Case: Actual Prevalence = 0.398
Prediction: 0.399363933333334
Explanation Fit: 0.5
0.344 < Physical.inactivity
0.121 < Diabetes.prevalence
0.75 < Drinking.water.violations
0.233 < Adult.smoking
505 < Premature.age.adjusted.mortality
4867 < Preventable.hospital.stays
0.368 < Insufficient.sleep <= 0.396
Mental.health.providers <= 0.000557
0.182 < Alcohol.impaired.driving.deaths <= 0.262
0.1782 < Uninsured.adults
Feature
−0.02
0.00
0.02
0.04
0.06
Weight
Positive
Negative

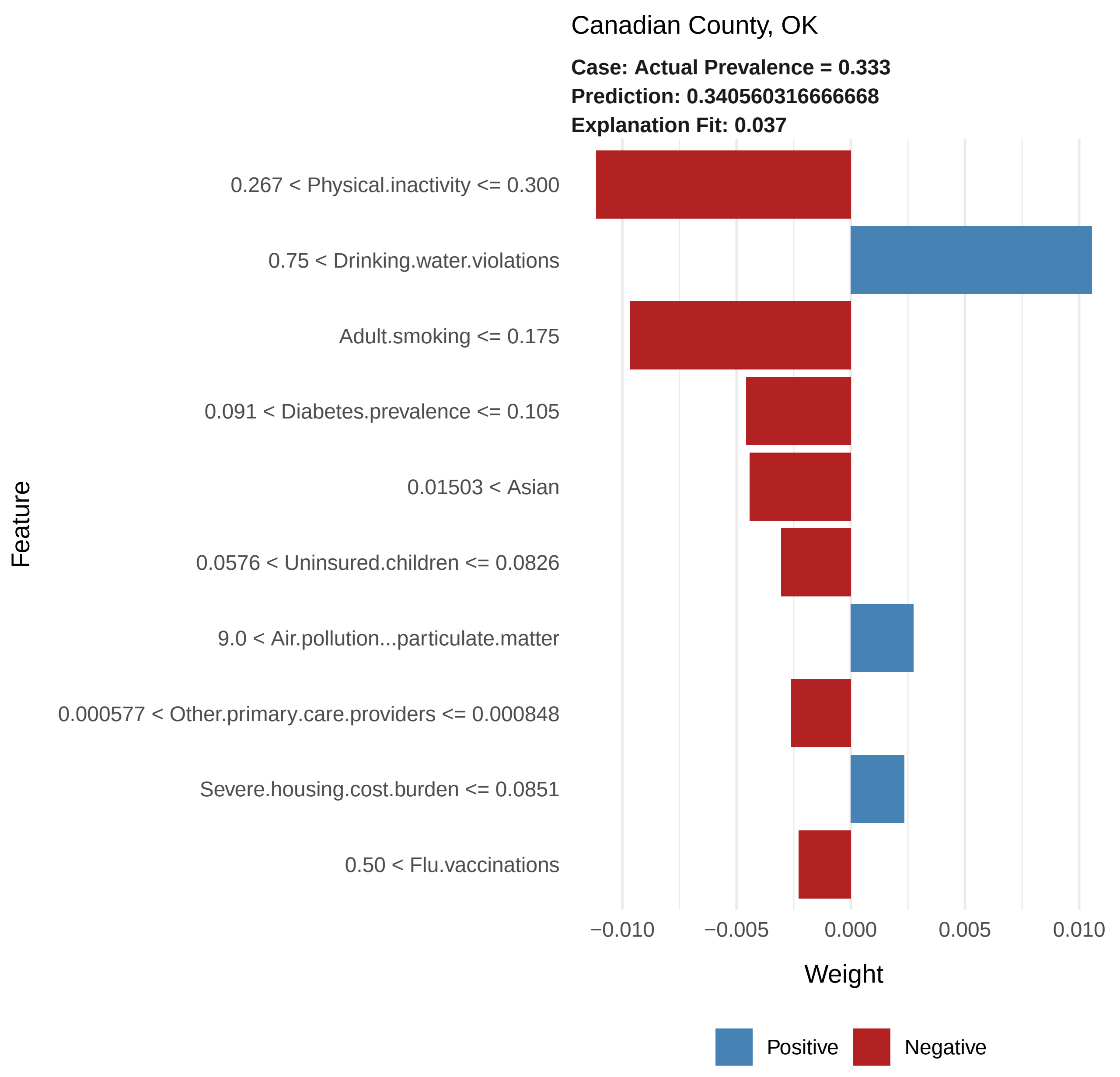
Canadian County, OK
Case: Actual Prevalence = 0.333
Prediction: 0.340560316666668
Explanation Fit: 0.037
0.267 < Physical.inactivity <= 0.300
0.75 < Drinking.water.violations
Adult.smoking <= 0.175
0.091 < Diabetes.prevalence <= 0.105
0.01503 < Asian
0.0576 < Uninsured.children <= 0.0826
9.0 < Air.pollution...particulate.matter
0.000577 < Other.primary.care.providers <= 0.000848
Severe.housing.cost.burden <= 0.0851
0.50 < Flu.vaccinations
Feature
−0.010
−0.005
0.000
0.005
0.010
Weight
Positive
Negative

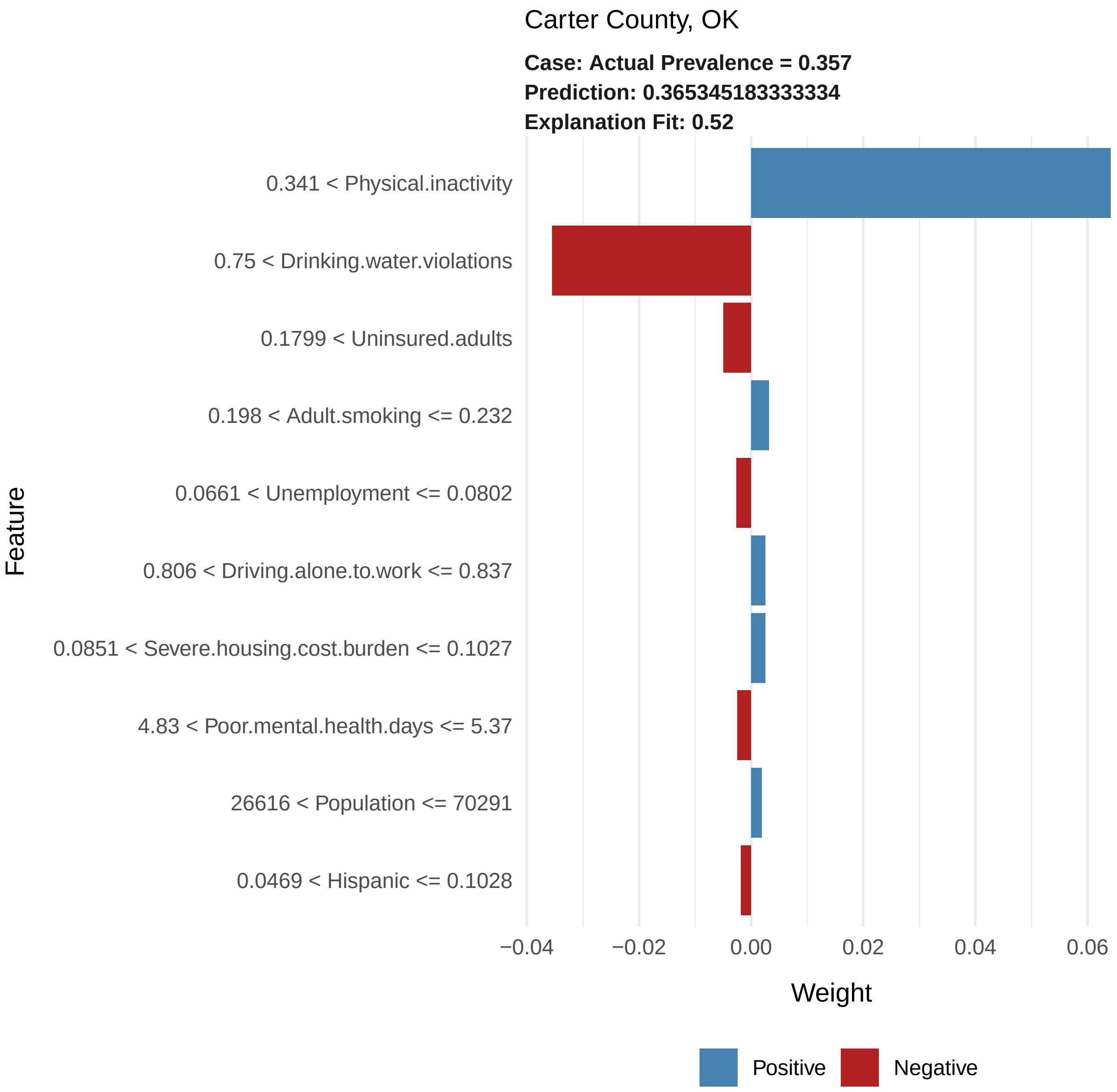

Carter County, OK
Case: Actual Prevalence = 0.357
Prediction: 0.365345183333334
Explanation Fit: 0.52
0.341 < Physical.inactivity
0.75 < Drinking.water.violations
0.1799 < Uninsured.adults
0.198 < Adult.smoking <= 0.232
0.0661 < Unemployment <= 0.0802
0.806 < Driving.alone.to.work <= 0.837
0.0851 < Severe.housing.cost.burden <= 0.1027
4.83 < Poor.mental.health.days <= 5.37
26616 < Population <= 70291
0.0469 < Hispanic <= 0.1028
Feature
−0.04
−0.02
0.00
0.02
0.04
0.06
Weight
Positive
Negative

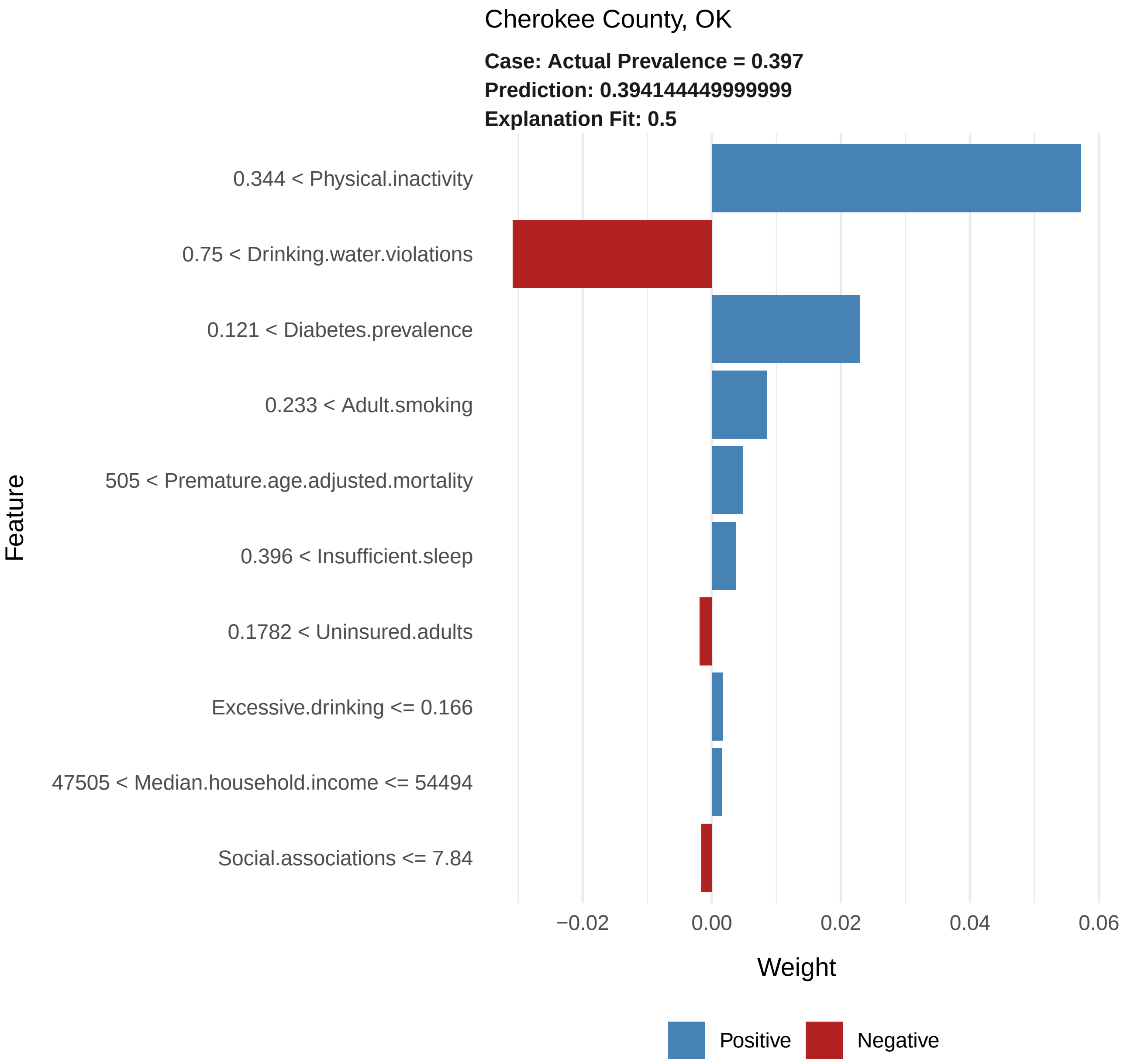
Cherokee County, OK
Case: Actual Prevalence = 0.397
Prediction: 0.394144449999999
Explanation Fit: 0.5
0.344 < Physical.inactivity
0.75 < Drinking.water.violations
0.121 < Diabetes.prevalence
0.233 < Adult.smoking
505 < Premature.age.adjusted.mortality
0.396 < Insufficient.sleep
0.1782 < Uninsured.adults
Excessive.drinking <= 0.166
47505 < Median.household.income <= 54494
Social.associations <= 7.84
Feature
−0.02
0.00
0.02
0.04
0.06
Weight
Positive
Negative

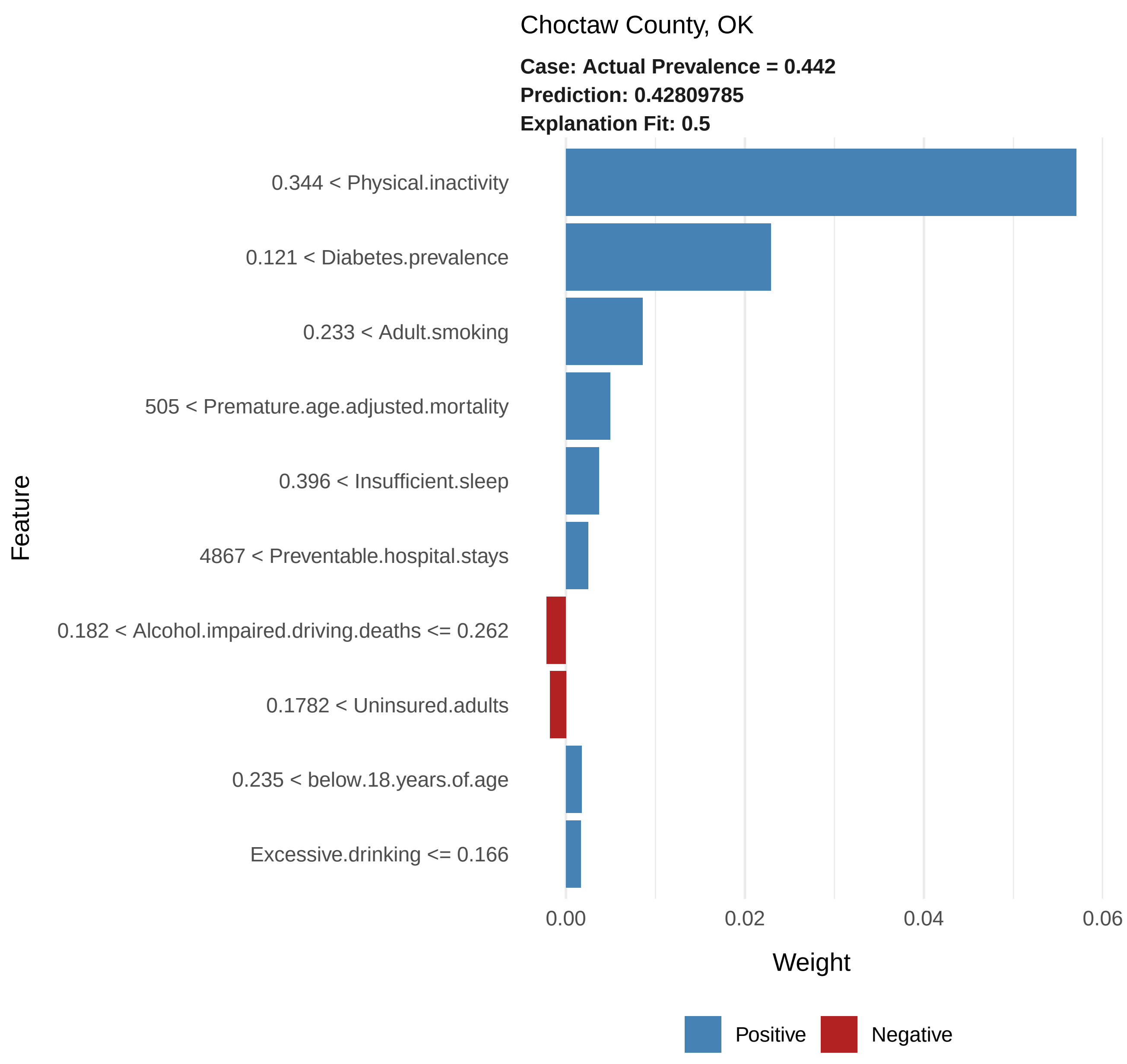
Choctaw County, OK
Case: Actual Prevalence = 0.442
Prediction: 0.42809785
Explanation Fit: 0.5
0.344 < Physical.inactivity
0.121 < Diabetes.prevalence
0.233 < Adult.smoking
505 < Premature.age.adjusted.mortality
0.396 < Insufficient.sleep
4867 < Preventable.hospital.stays
0.182 < Alcohol.impaired.driving.deaths <= 0.262
0.1782 < Uninsured.adults
0.235 < below.18.years.of.age
Excessive.drinking <= 0.166
Feature
0.00
0.02
0.04
0.06
Weight
Positive
Negative

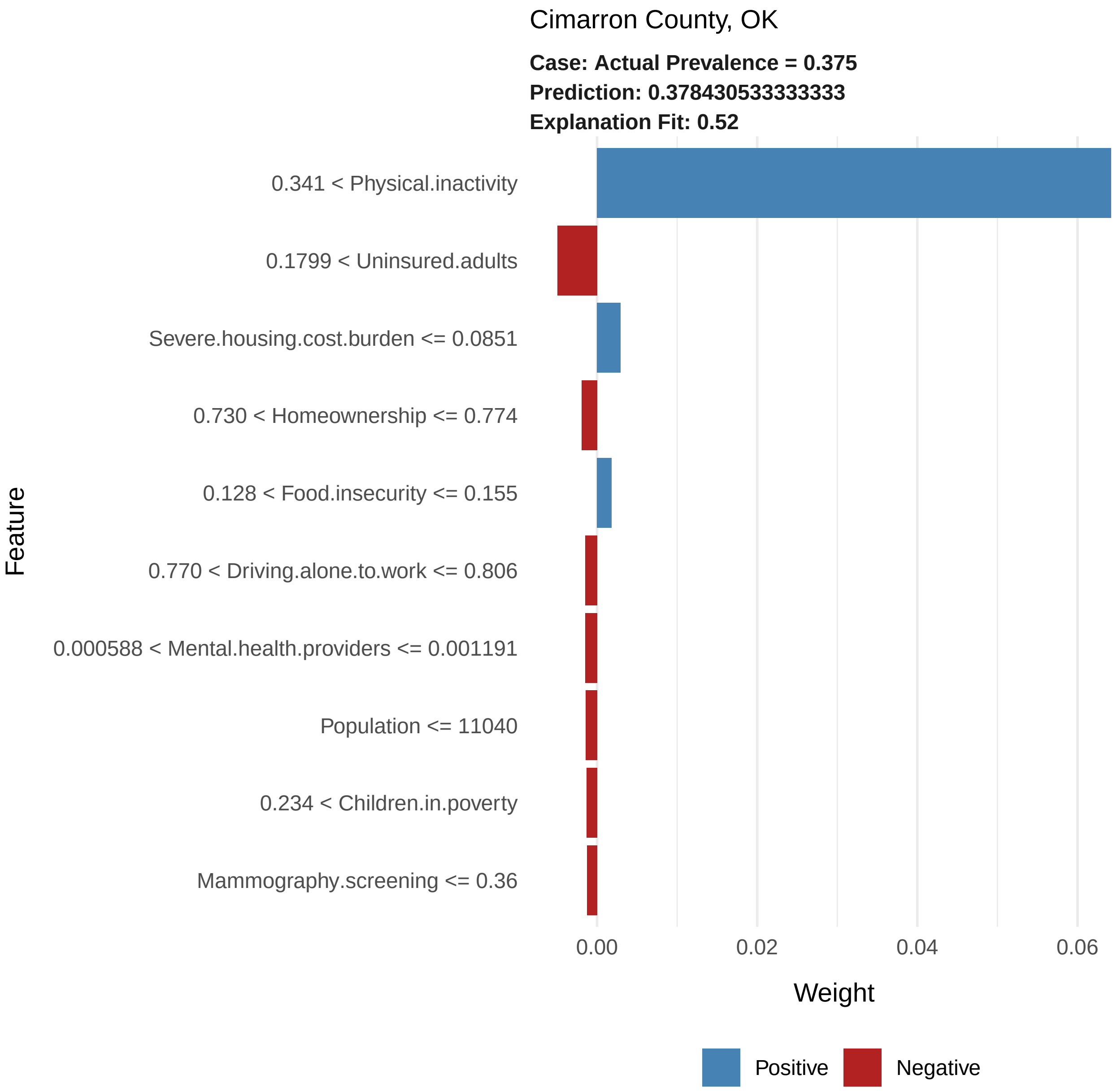

Cimarron County, OK
Case: Actual Prevalence = 0.375
Prediction: 0.378430533333333
Explanation Fit: 0.52
0.341 < Physical.inactivity
0.1799 < Uninsured.adults
Severe.housing.cost.burden <= 0.0851
0.730 < Homeownership <= 0.774
0.128 < Food.insecurity <= 0.155
0.770 < Driving.alone.to.work <= 0.806
0.000588 < Mental.health.providers <= 0.001191
Population <= 11040
0.234 < Children.in.poverty
Mammography.screening <= 0.36
Feature
0.00
0.02
0.04
0.06
Weight
Positive
Negative

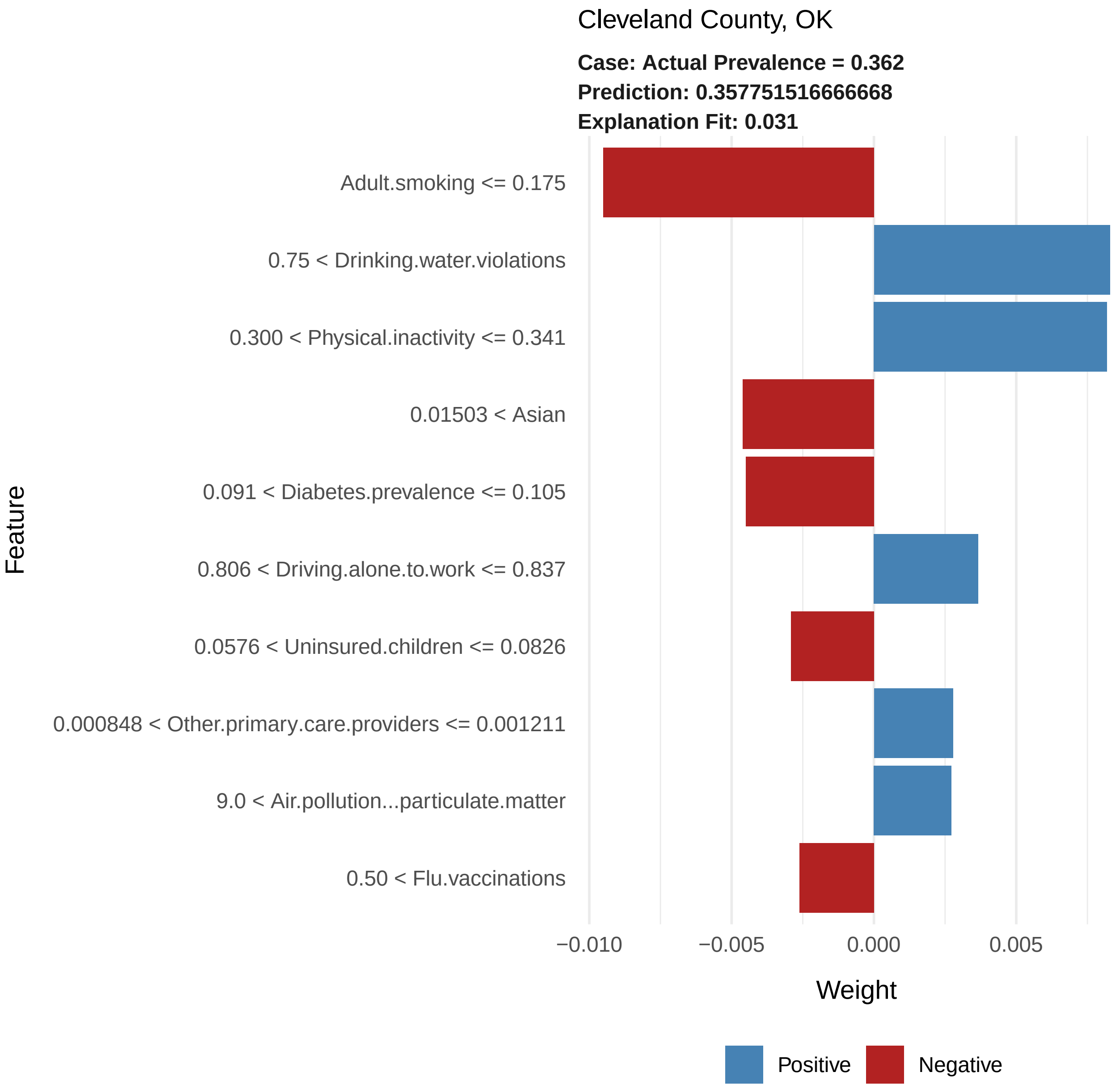
Cleveland County, OK
Case: Actual Prevalence = 0.362
Prediction: 0.357751516666668
Explanation Fit: 0.031
Adult.smoking <= 0.175
0.75 < Drinking.water.violations
0.300 < Physical.inactivity <= 0.341
0.01503 < Asian
0.091 < Diabetes.prevalence <= 0.105
0.806 < Driving.alone.to.work <= 0.837
0.0576 < Uninsured.children <= 0.0826
0.000848 < Other.primary.care.providers <= 0.001211
9.0 < Air.pollution...particulate.matter
0.50 < Flu.vaccinations
Feature
−0.010
−0.005
0.000
0.005
Weight
Positive
Negative

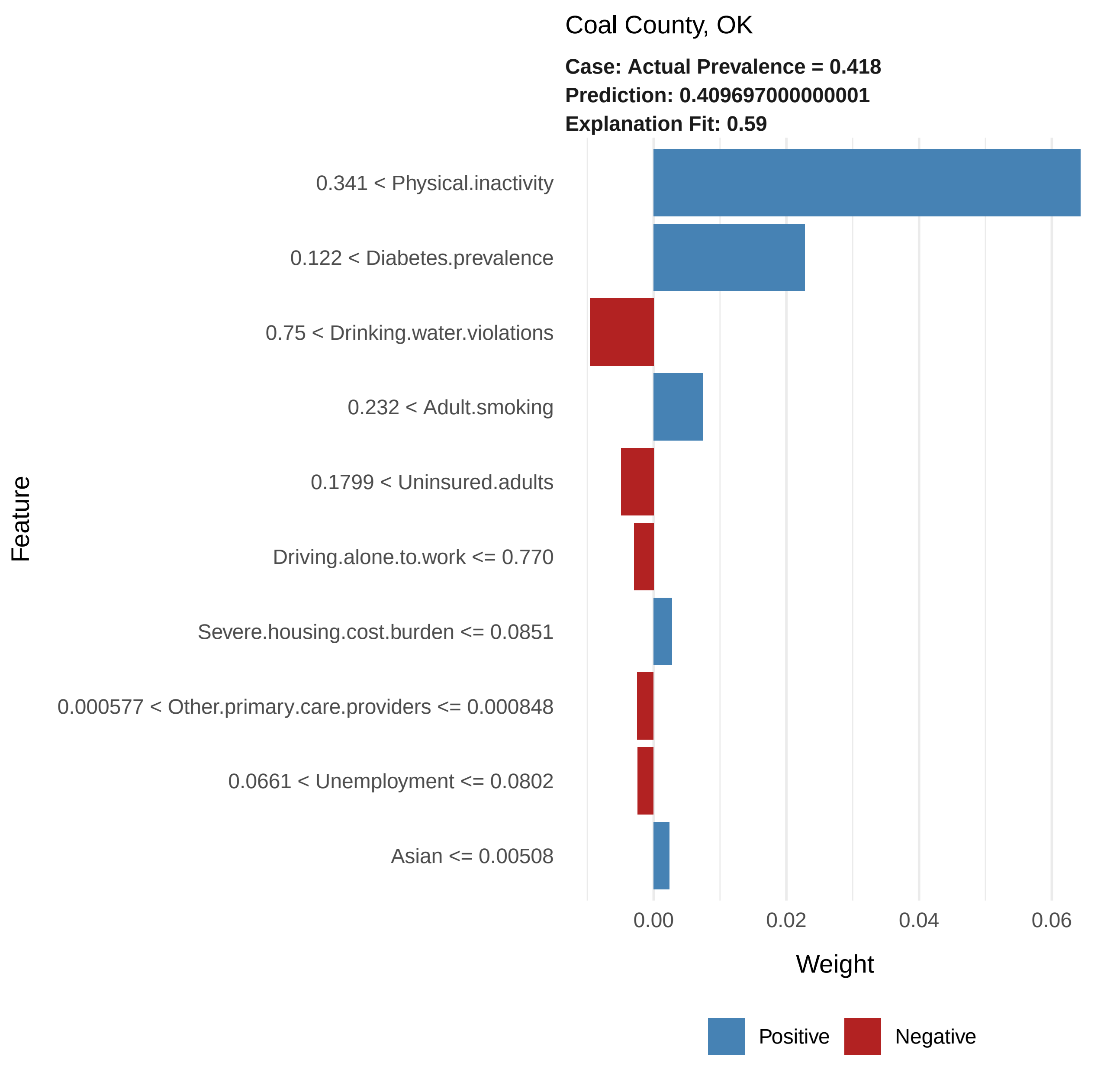
Coal County, OK
Case: Actual Prevalence = 0.418
Prediction: 0.409697000000001
Explanation Fit: 0.59
0.341 < Physical.inactivity
0.122 < Diabetes.prevalence
0.75 < Drinking.water.violations
0.232 < Adult.smoking
0.1799 < Uninsured.adults
Driving.alone.to.work <= 0.770
Severe.housing.cost.burden <= 0.0851
0.000577 < Other.primary.care.providers <= 0.000848
0.0661 < Unemployment <= 0.0802
Asian <= 0.00508
Feature
0.00
0.02
0.04
0.06
Weight
Positive
Negative

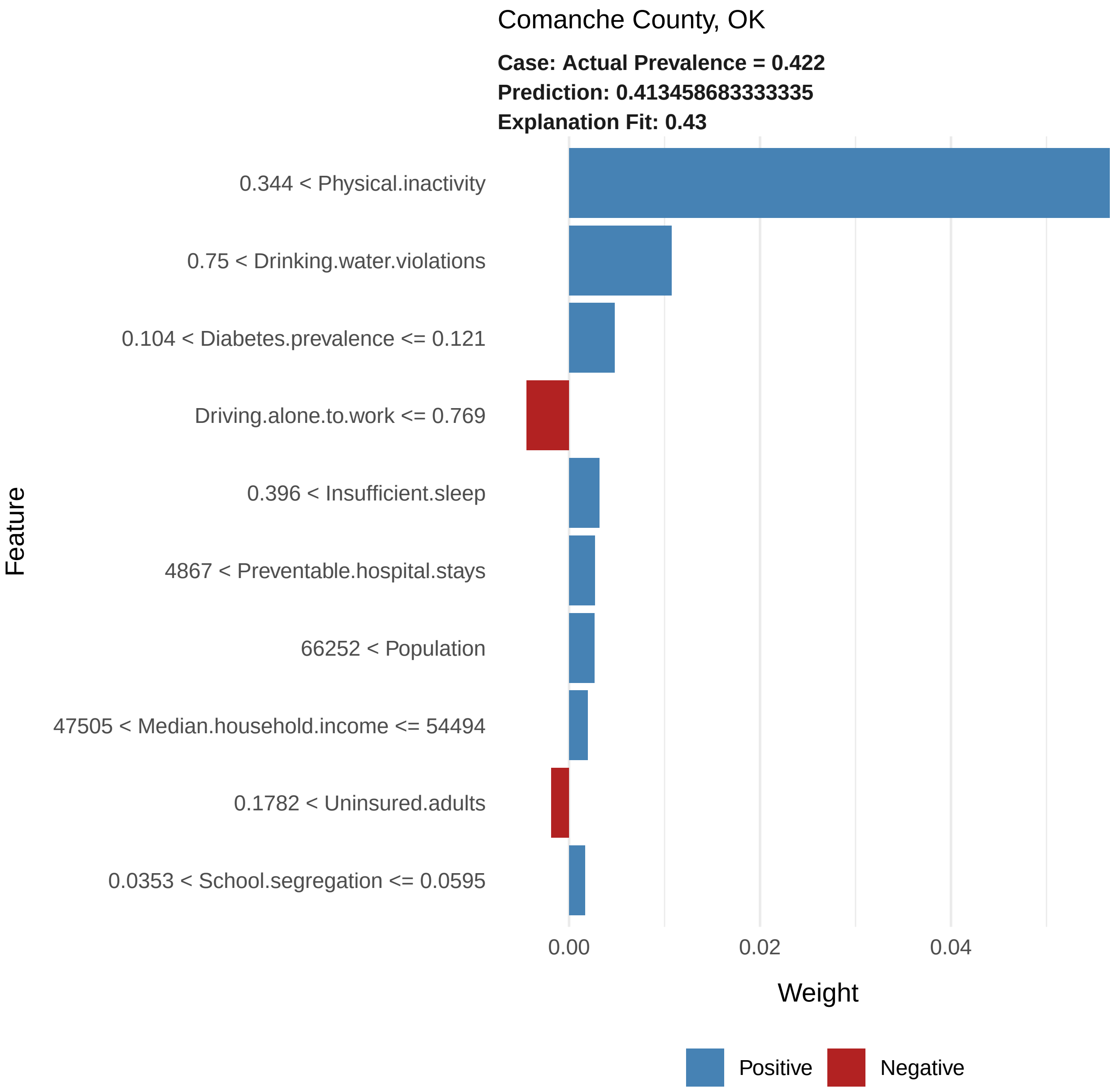

Comanche County, OK
Case: Actual Prevalence = 0.422
Prediction: 0.413458683333335
Explanation Fit: 0.43
0.344 < Physical.inactivity
0.75 < Drinking.water.violations
0.104 < Diabetes.prevalence <= 0.121
Driving.alone.to.work <= 0.769
0.396 < Insufficient.sleep
4867 < Preventable.hospital.stays
66252 < Population
47505 < Median.household.income <= 54494
0.1782 < Uninsured.adults
0.0353 < School.segregation <= 0.0595
Feature
0.00
0.02
0.04
Weight
Positive
Negative

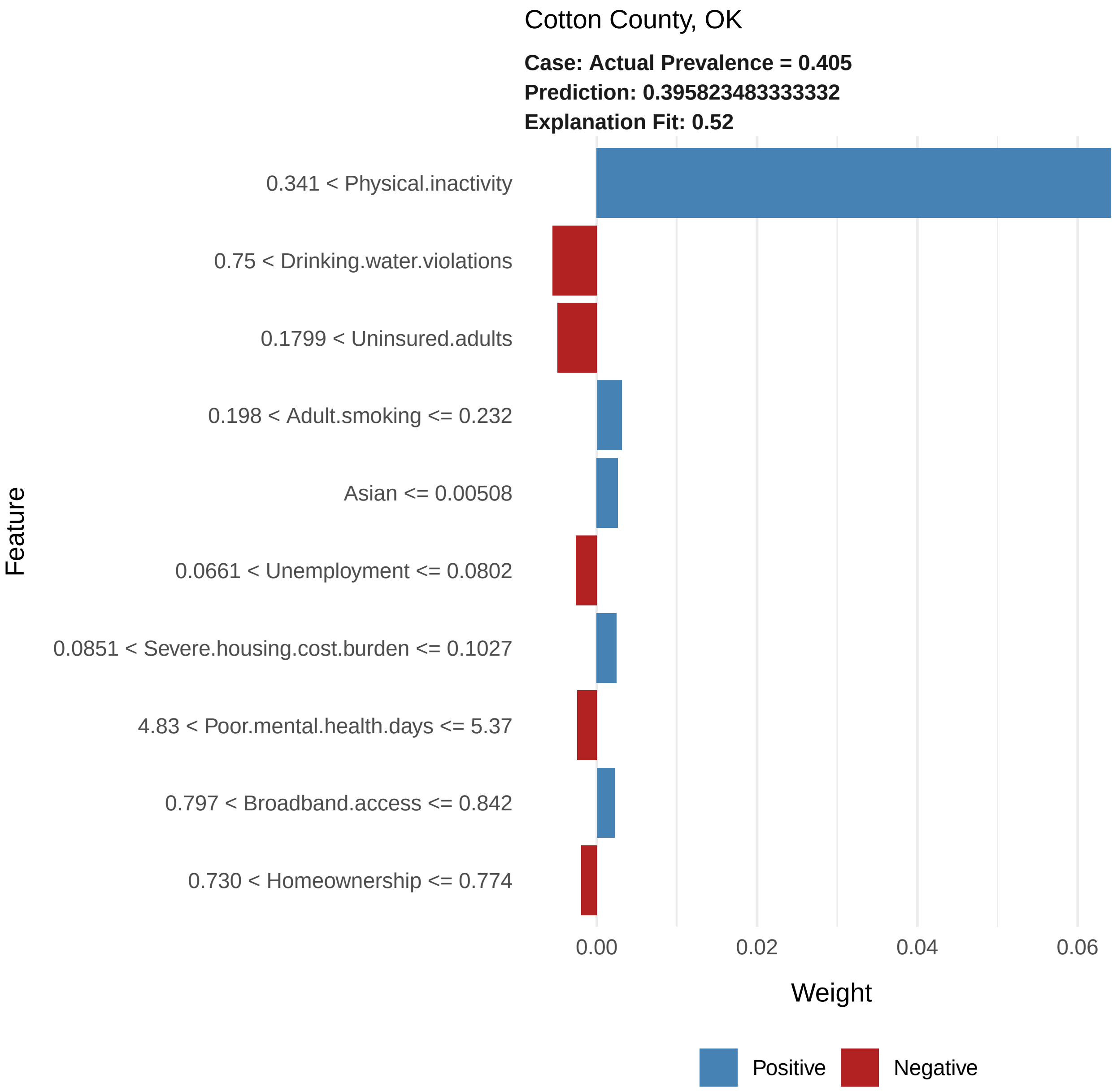
Cotton County, OK
Case: Actual Prevalence = 0.405
Prediction: 0.395823483333332
Explanation Fit: 0.52
0.341 < Physical.inactivity
0.75 < Drinking.water.violations
0.1799 < Uninsured.adults
0.198 < Adult.smoking <= 0.232
Asian <= 0.00508
0.0661 < Unemployment <= 0.0802
0.0851 < Severe.housing.cost.burden <= 0.1027
4.83 < Poor.mental.health.days <= 5.37
0.797 < Broadband.access <= 0.842
0.730 < Homeownership <= 0.774
Feature
0.00
0.02
0.04
0.06
Weight
Positive
Negative

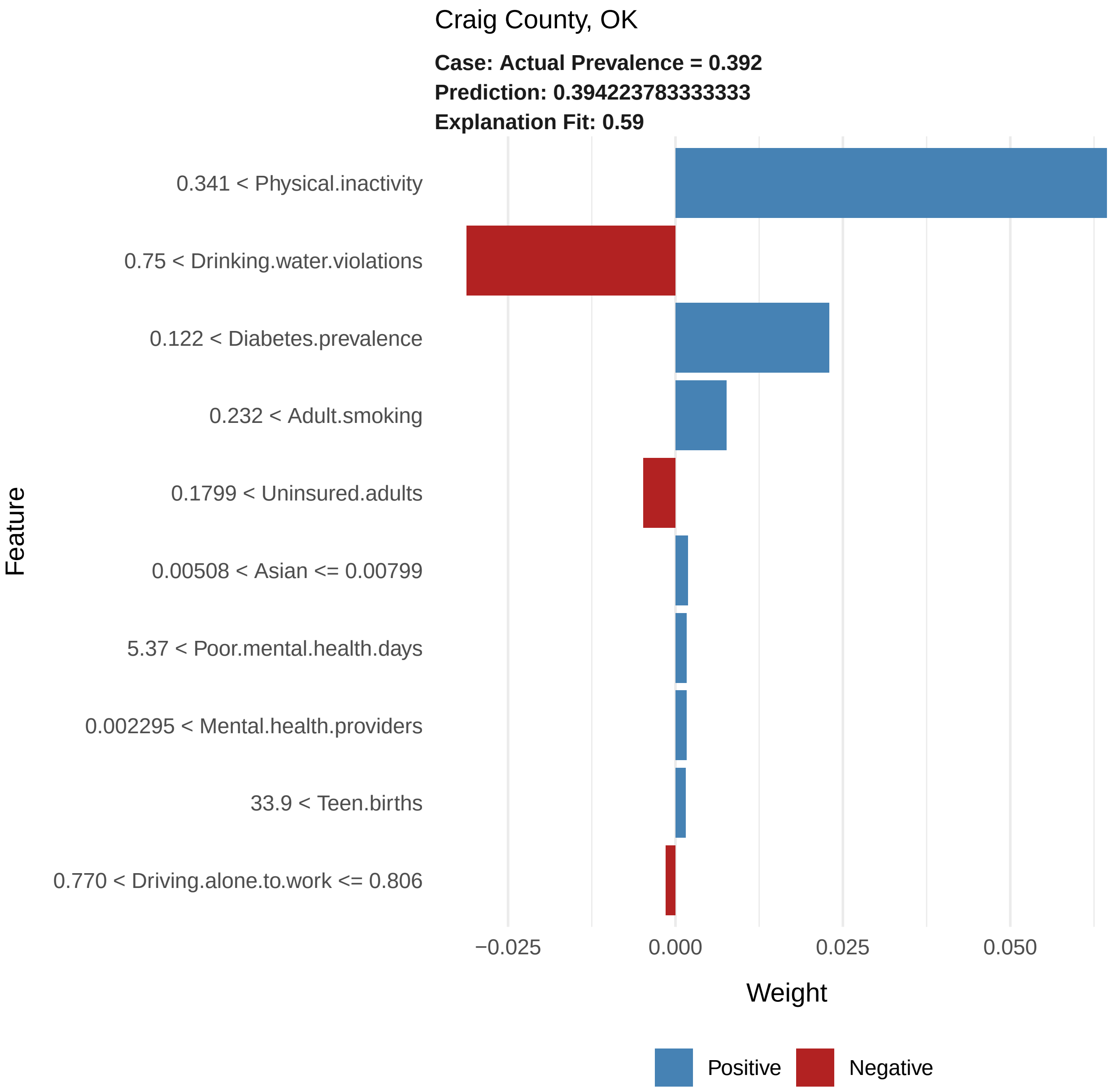

Craig County, OK
Case: Actual Prevalence = 0.392
Prediction: 0.394223783333333
Explanation Fit: 0.59
0.341 < Physical.inactivity
0.75 < Drinking.water.violations
0.122 < Diabetes.prevalence
0.232 < Adult.smoking
0.1799 < Uninsured.adults
0.00508 < Asian <= 0.00799
5.37 < Poor.mental.health.days
0.002295 < Mental.health.providers
33.9 < Teen.births
0.770 < Driving.alone.to.work <= 0.806
Feature
−0.025
0.000
0.025
0.050
Weight
Positive
Negative

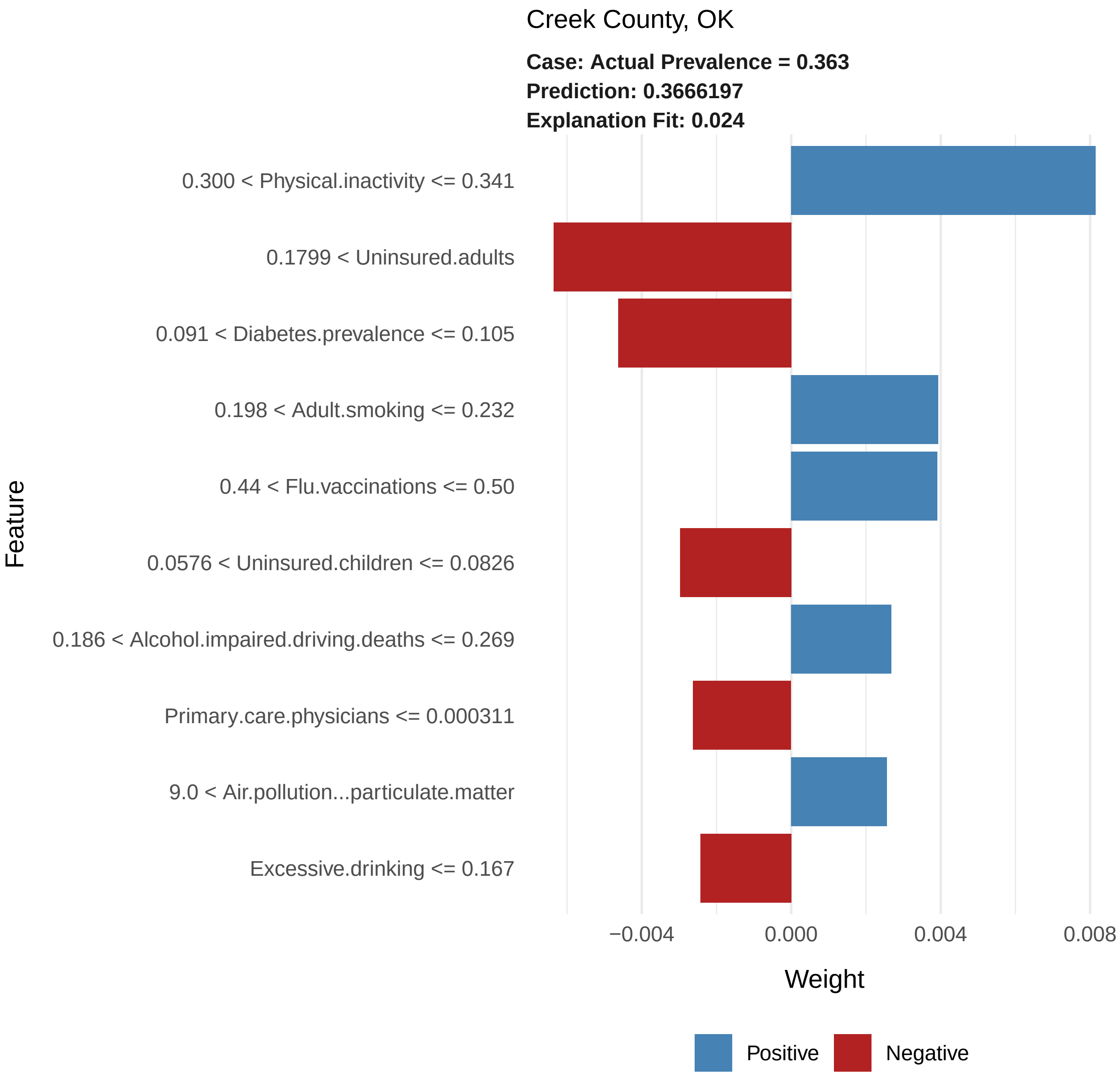

Creek County, OK
Case: Actual Prevalence = 0.363
Prediction: 0.3666197
Explanation Fit: 0.024
0.300 < Physical.inactivity <= 0.341
0.1799 < Uninsured.adults
0.091 < Diabetes.prevalence <= 0.105
0.198 < Adult.smoking <= 0.232
0.44 < Flu.vaccinations <= 0.50
0.0576 < Uninsured.children <= 0.0826
0.186 < Alcohol.impaired.driving.deaths <= 0.269
Primary.care.physicians <= 0.000311
9.0 < Air.pollution...particulate.matter
Excessive.drinking <= 0.167
Feature
−0.004
0.000
0.004
0.008
Weight
Positive
Negative

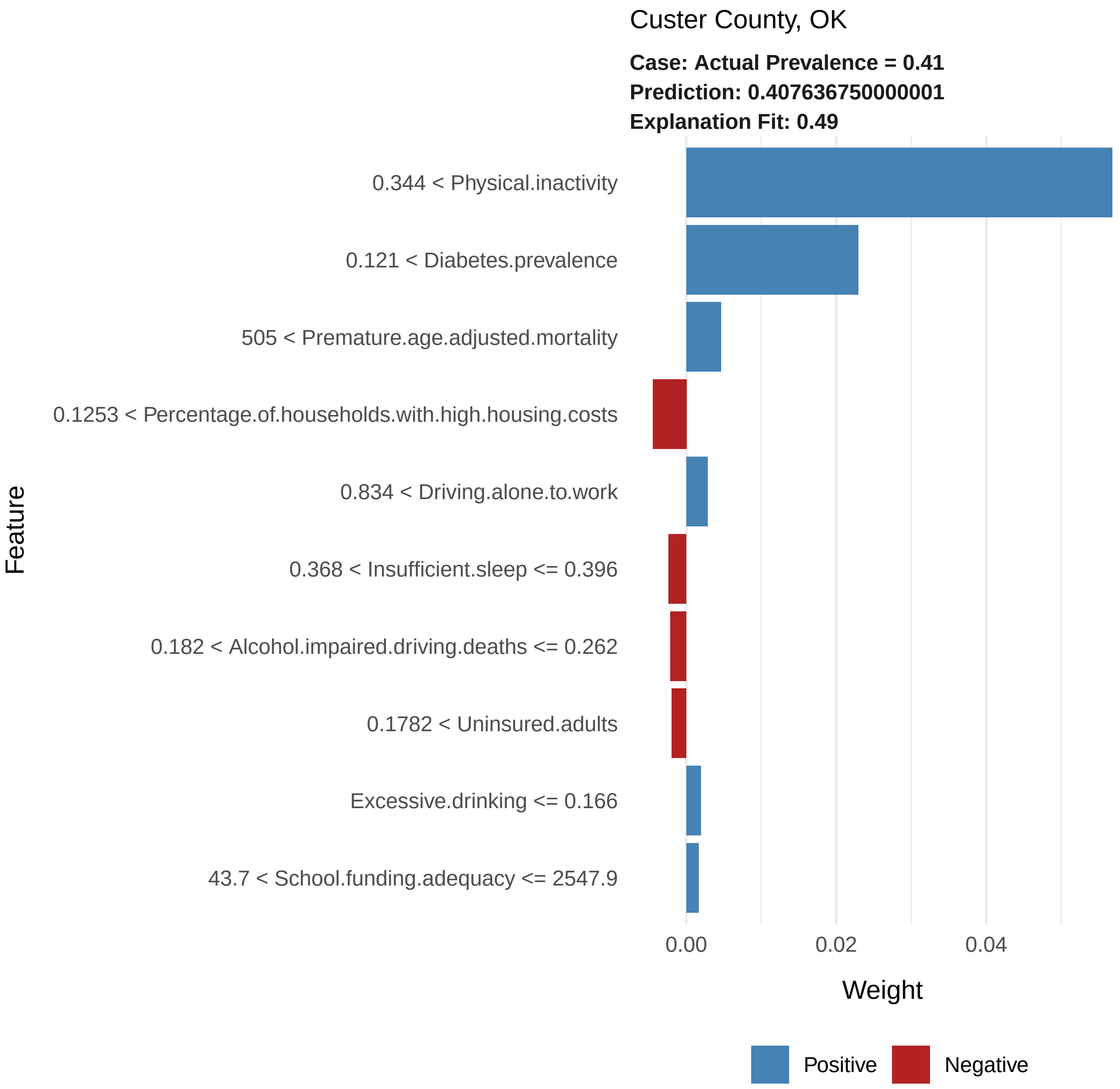

Custer County, OK
Case: Actual Prevalence = 0.41
Prediction: 0.407636750000001
Explanation Fit: 0.49
0.344 < Physical.inactivity
0.121 < Diabetes.prevalence
505 < Premature.age.adjusted.mortality
0.1253 < Percentage.of.households.with.high.housing.costs
0.834 < Driving.alone.to.work
0.368 < Insufficient.sleep <= 0.396
0.182 < Alcohol.impaired.driving.deaths <= 0.262
0.1782 < Uninsured.adults
Excessive.drinking <= 0.166
43.7 < School.funding.adequacy <= 2547.9
Feature
0.00
0.02
0.04
Weight
Positive
Negative

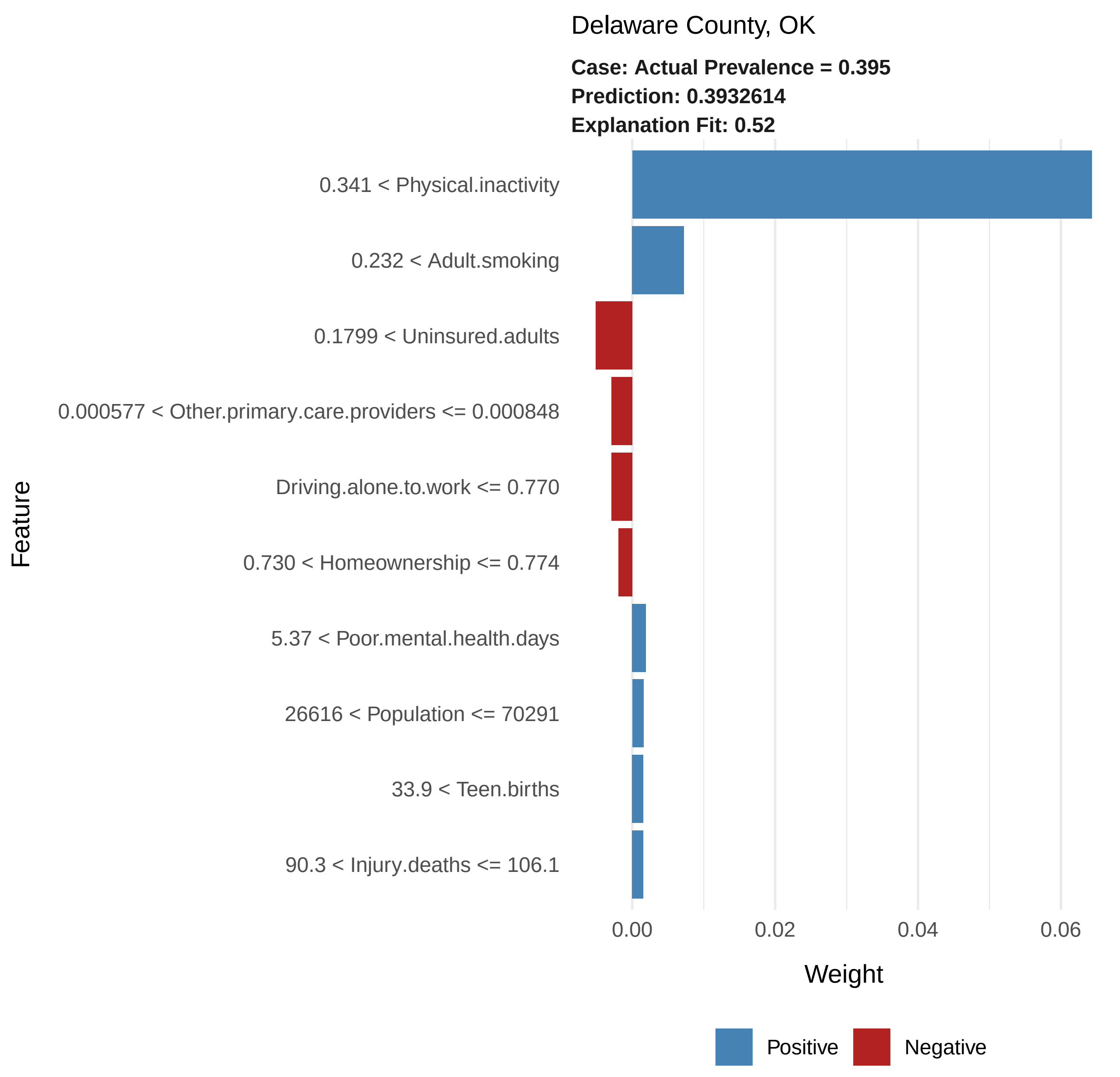
Delaware County, OK
Case: Actual Prevalence = 0.395
Prediction: 0.3932614
Explanation Fit: 0.52
0.341 < Physical.inactivity
0.232 < Adult.smoking
0.1799 < Uninsured.adults
0.000577 < Other.primary.care.providers <= 0.000848
Driving.alone.to.work <= 0.770
0.730 < Homeownership <= 0.774
5.37 < Poor.mental.health.days
26616 < Population <= 70291
33.9 < Teen.births
90.3 < Injury.deaths <= 106.1
Feature
0.00
0.02
0.04
0.06
Weight
Positive
Negative

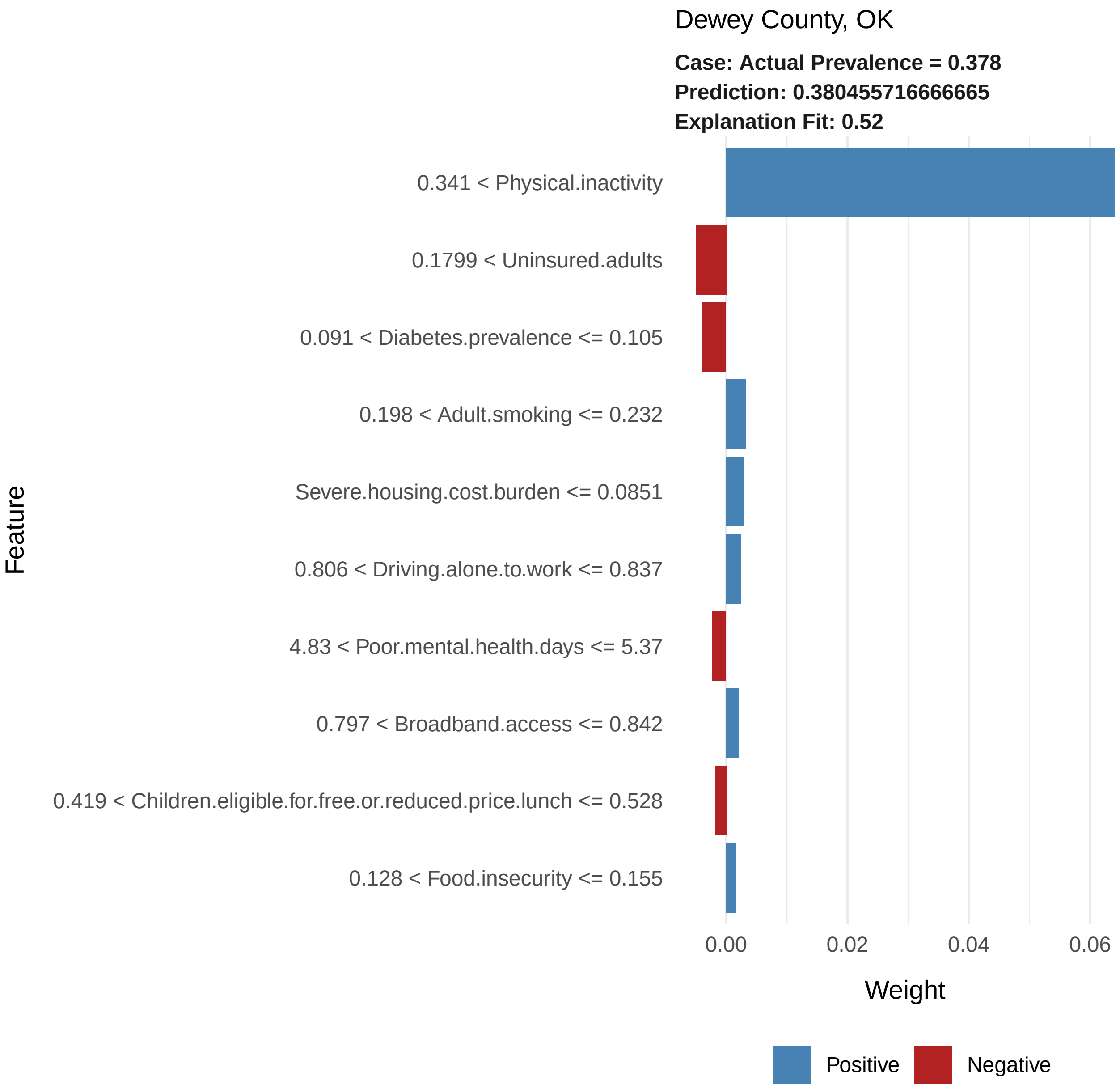

Dewey County, OK
Case: Actual Prevalence = 0.378
Prediction: 0.380455716666665
Explanation Fit: 0.52
0.341 < Physical.inactivity
0.1799 < Uninsured.adults
0.091 < Diabetes.prevalence <= 0.105
0.198 < Adult.smoking <= 0.232
Severe.housing.cost.burden <= 0.0851
0.806 < Driving.alone.to.work <= 0.837
4.83 < Poor.mental.health.days <= 5.37
0.797 < Broadband.access <= 0.842
0.419 < Children.eligible.for.free.or.reduced.price.lunch <= 0.528
0.128 < Food.insecurity <= 0.155
Feature
0.00
0.02
0.04
0.06
Weight
Positive
Negative

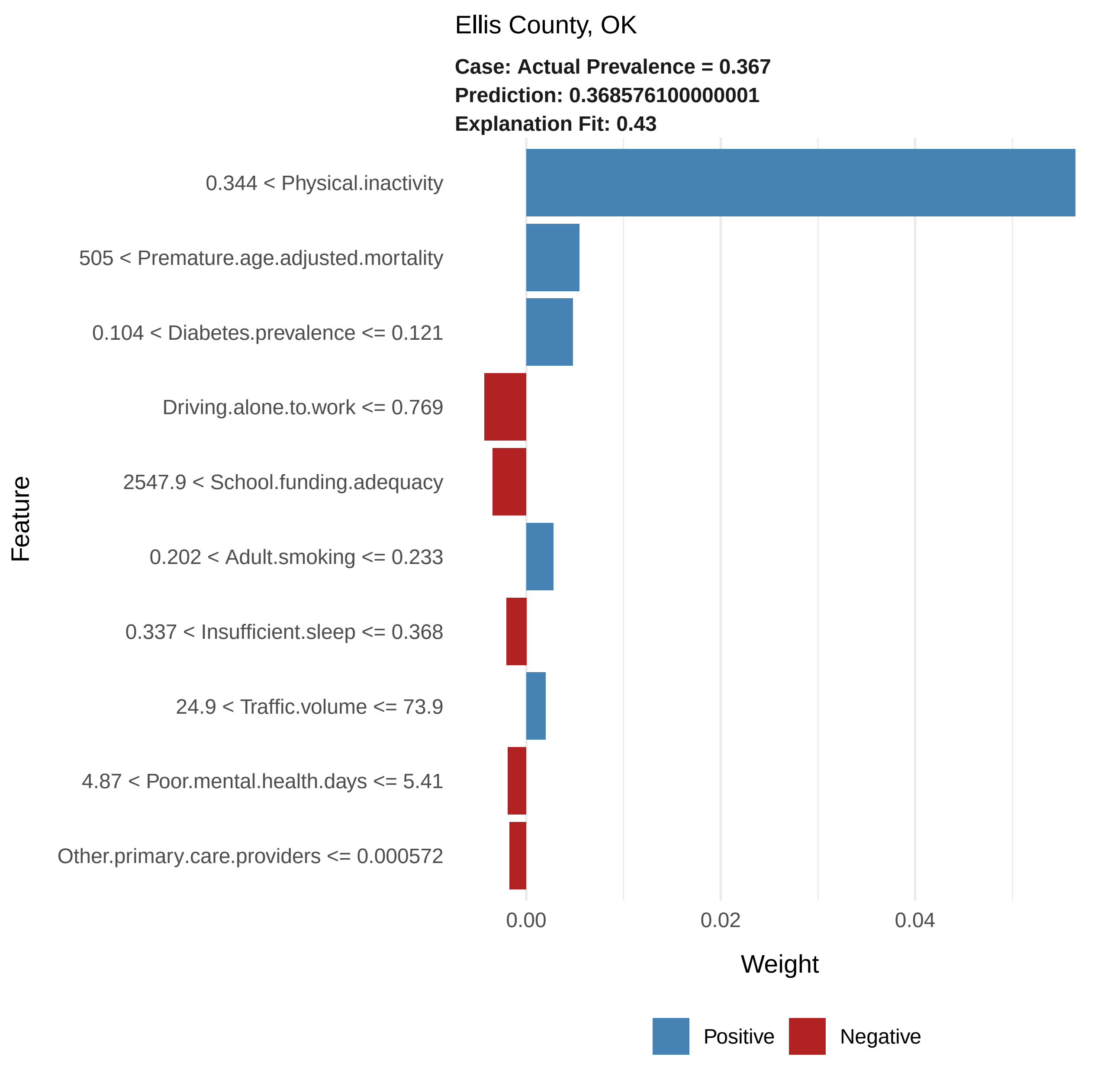

Ellis County, OK
Case: Actual Prevalence = 0.367
Prediction: 0.368576100000001
Explanation Fit: 0.43
0.344 < Physical.inactivity
505 < Premature.age.adjusted.mortality
0.104 < Diabetes.prevalence <= 0.121
Driving.alone.to.work <= 0.769
2547.9 < School.funding.adequacy
0.202 < Adult.smoking <= 0.233
0.337 < Insufficient.sleep <= 0.368
24.9 < Traffic.volume <= 73.9
4.87 < Poor.mental.health.days <= 5.41
Other.primary.care.providers <= 0.000572
Feature
0.00
0.02
0.04
Weight
Positive
Negative

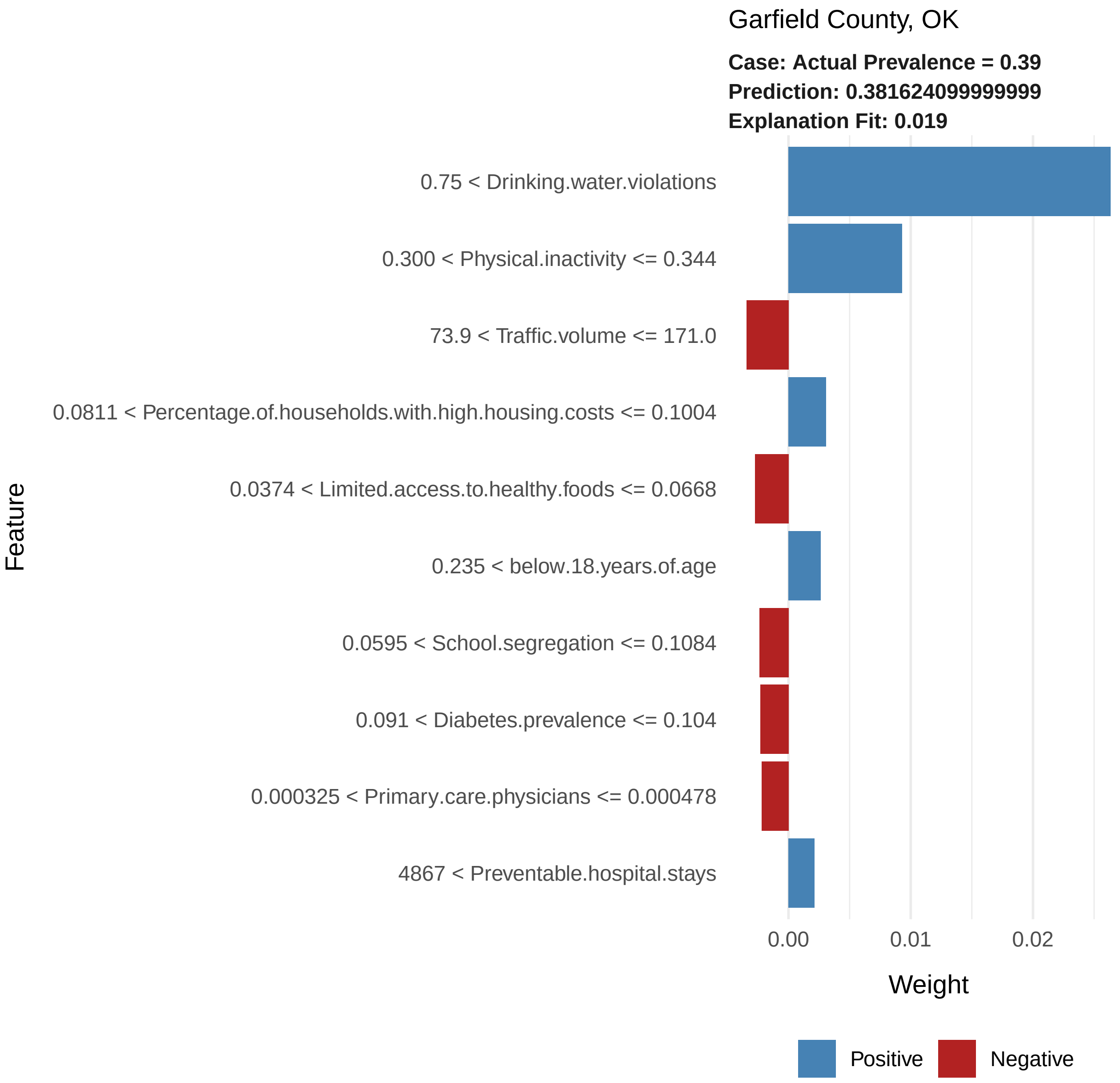

Garfield County, OK
Case: Actual Prevalence = 0.39
Prediction: 0.381624099999999
Explanation Fit: 0.019
0.75 < Drinking.water.violations
0.300 < Physical.inactivity <= 0.344
73.9 < Traffic.volume <= 171.0
0.0811 < Percentage.of.households.with.high.housing.costs <= 0.1004
0.0374 < Limited.access.to.healthy.foods <= 0.0668
0.235 < below.18.years.of.age
0.0595 < School.segregation <= 0.1084
0.091 < Diabetes.prevalence <= 0.104
0.000325 < Primary.care.physicians <= 0.000478
4867 < Preventable.hospital.stays
Feature
0.00
0.01
0.02
Weight
Positive
Negative

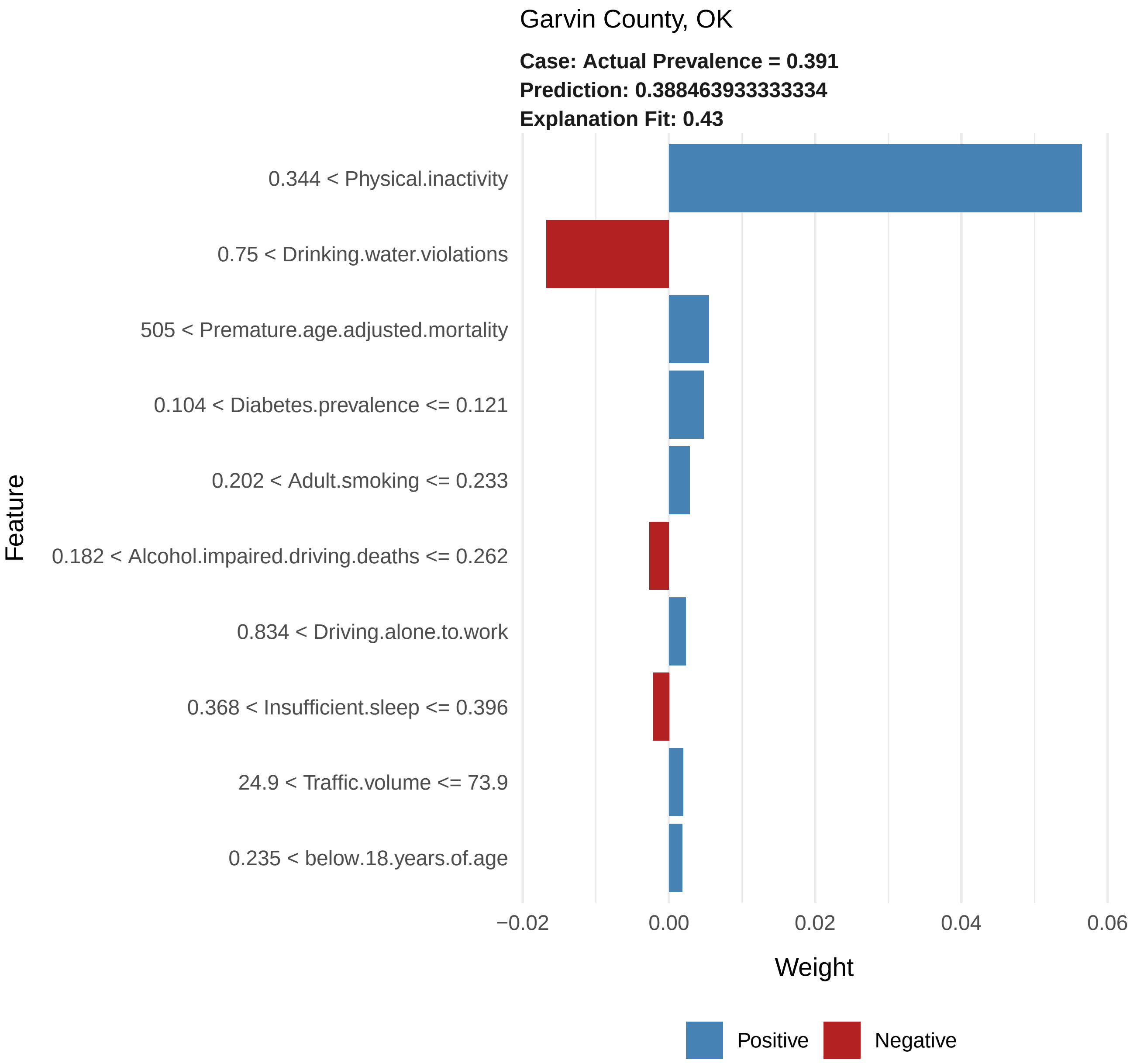
Garvin County, OK
Case: Actual Prevalence = 0.391
Prediction: 0.388463933333334
Explanation Fit: 0.43
0.344 < Physical.inactivity
0.75 < Drinking.water.violations
505 < Premature.age.adjusted.mortality
0.104 < Diabetes.prevalence <= 0.121
0.202 < Adult.smoking <= 0.233
0.182 < Alcohol.impaired.driving.deaths <= 0.262
0.834 < Driving.alone.to.work
0.368 < Insufficient.sleep <= 0.396
24.9 < Traffic.volume <= 73.9
0.235 < below.18.years.of.age
Feature
−0.02
0.00
0.02
0.04
0.06
Weight
Positive
Negative

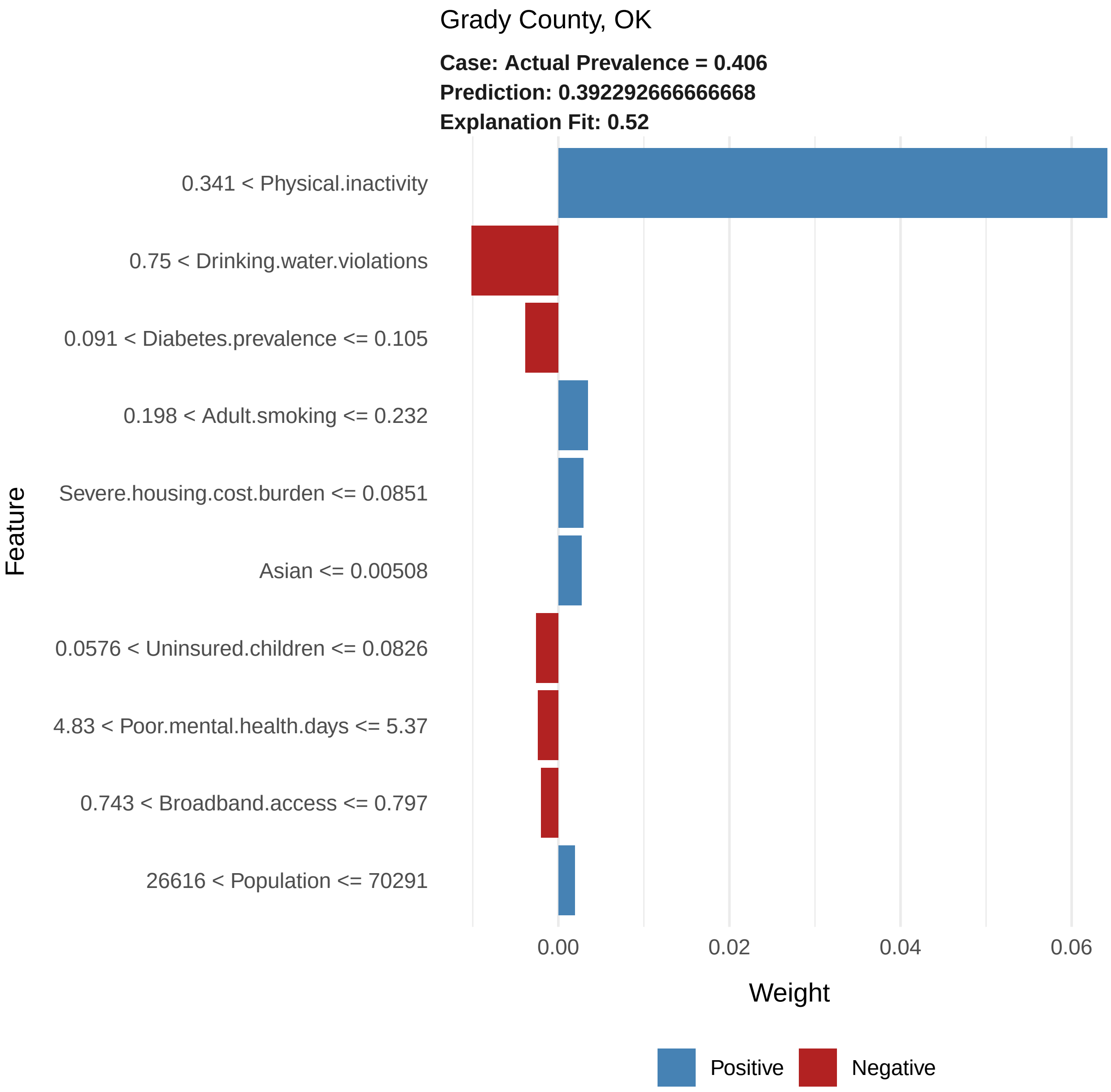

Grady County, OK
Case: Actual Prevalence = 0.406
Prediction: 0.392292666666668
Explanation Fit: 0.52
0.341 < Physical.inactivity
0.75 < Drinking.water.violations
0.091 < Diabetes.prevalence <= 0.105
0.198 < Adult.smoking <= 0.232
Severe.housing.cost.burden <= 0.0851
Asian <= 0.00508
0.0576 < Uninsured.children <= 0.0826
4.83 < Poor.mental.health.days <= 5.37
0.743 < Broadband.access <= 0.797
26616 < Population <= 70291
Feature
0.00
0.02
0.04
0.06
Weight
Positive
Negative

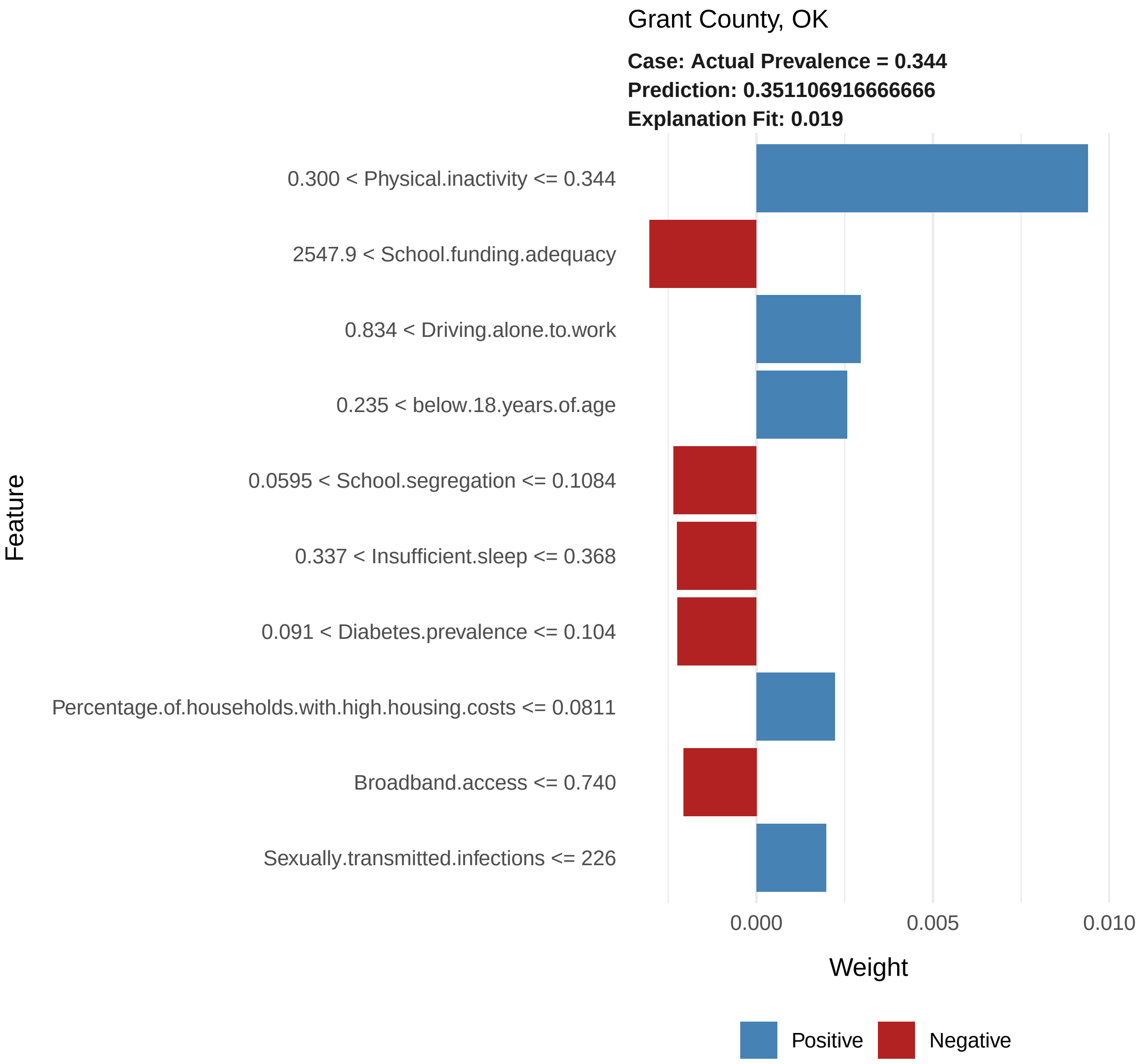

Grant County, OK
Case: Actual Prevalence = 0.344
Prediction: 0.351106916666666
Explanation Fit: 0.019
0.300 < Physical.inactivity <= 0.344
2547.9 < School.funding.adequacy
0.834 < Driving.alone.to.work
0.235 < below.18.years.of.age
0.0595 < School.segregation <= 0.1084
0.337 < Insufficient.sleep <= 0.368
0.091 < Diabetes.prevalence <= 0.104
Percentage.of.households.with.high.housing.costs <= 0.0811
Broadband.access <= 0.740
Sexually.transmitted.infections <= 226
Feature
0.000
0.005
0.010
Weight
Positive
Negative

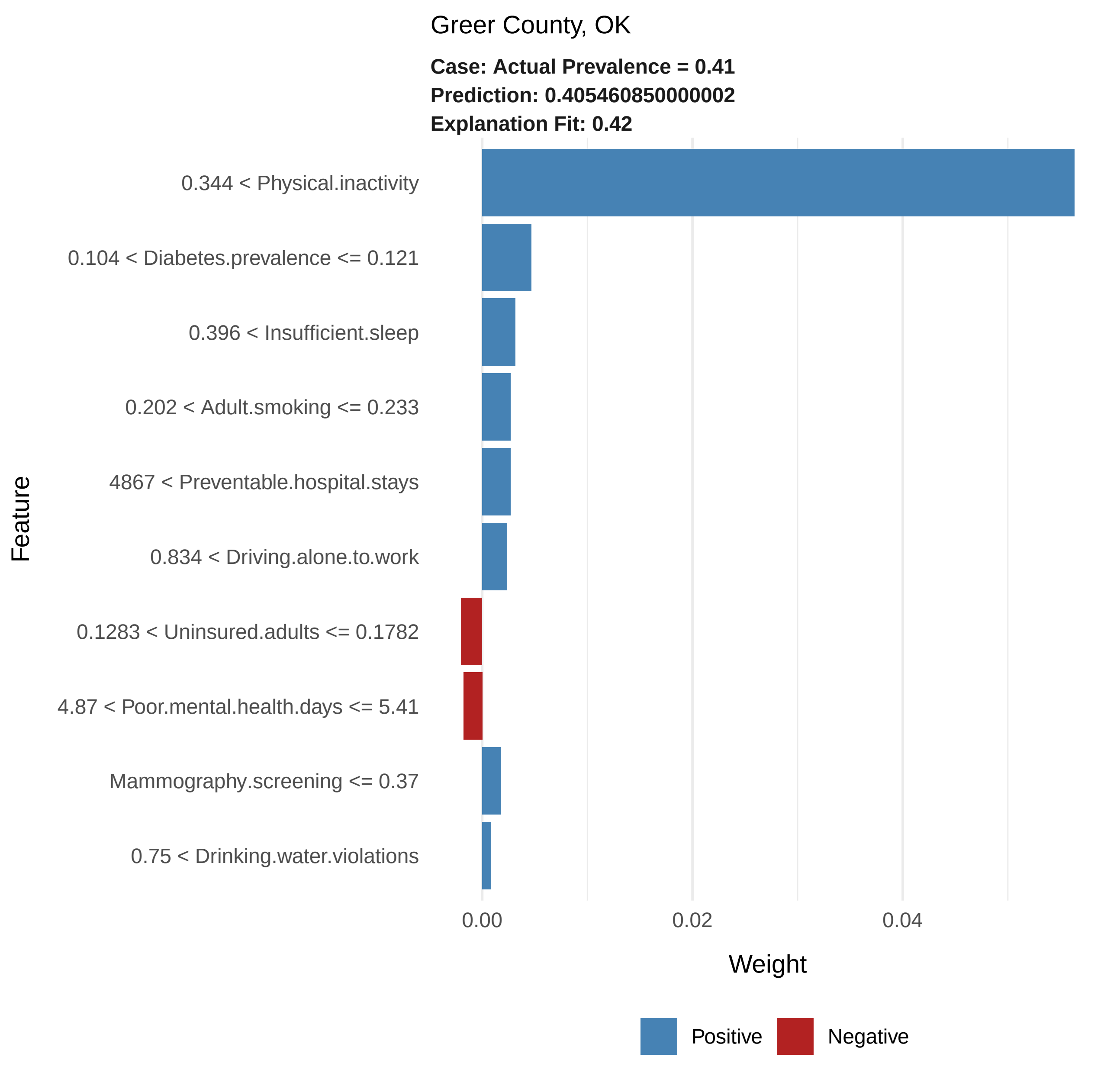
Greer County, OK
Case: Actual Prevalence = 0.41
Prediction: 0.405460850000002
Explanation Fit: 0.42
0.344 < Physical.inactivity
0.104 < Diabetes.prevalence <= 0.121
0.396 < Insufficient.sleep
0.202 < Adult.smoking <= 0.233
4867 < Preventable.hospital.stays
0.834 < Driving.alone.to.work
0.1283 < Uninsured.adults <= 0.1782
4.87 < Poor.mental.health.days <= 5.41
Mammography.screening <= 0.37
0.75 < Drinking.water.violations
Feature
0.00
0.02
0.04
Weight
Positive
Negative

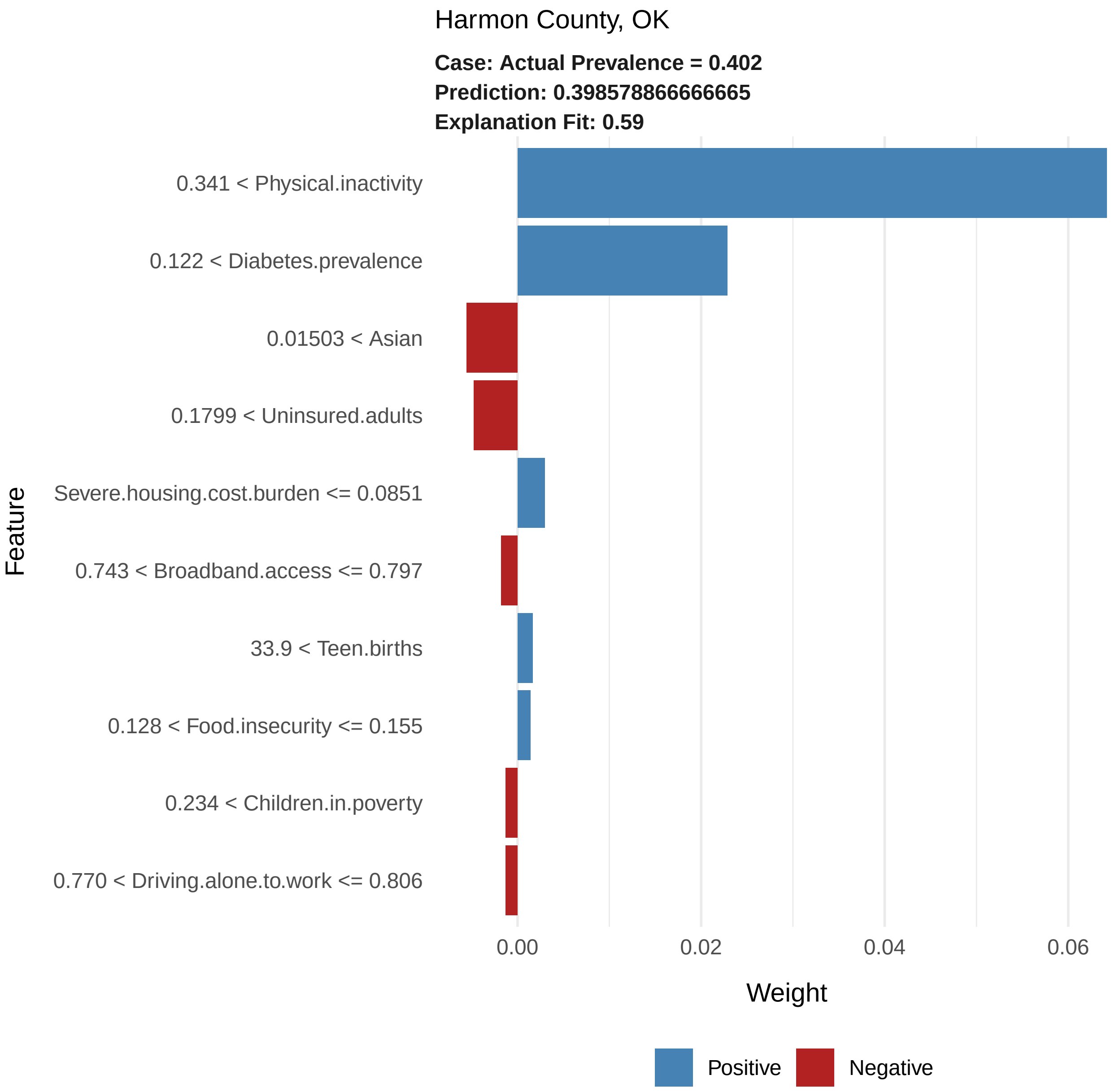

Harmon County, OK
Case: Actual Prevalence = 0.402
Prediction: 0.398578866666665
Explanation Fit: 0.59
0.341 < Physical.inactivity
0.122 < Diabetes.prevalence
0.01503 < Asian
0.1799 < Uninsured.adults
Severe.housing.cost.burden <= 0.0851
0.743 < Broadband.access <= 0.797
33.9 < Teen.births
0.128 < Food.insecurity <= 0.155
0.234 < Children.in.poverty
0.770 < Driving.alone.to.work <= 0.806
Feature
0.00
0.02
0.04
0.06
Weight
Positive
Negative

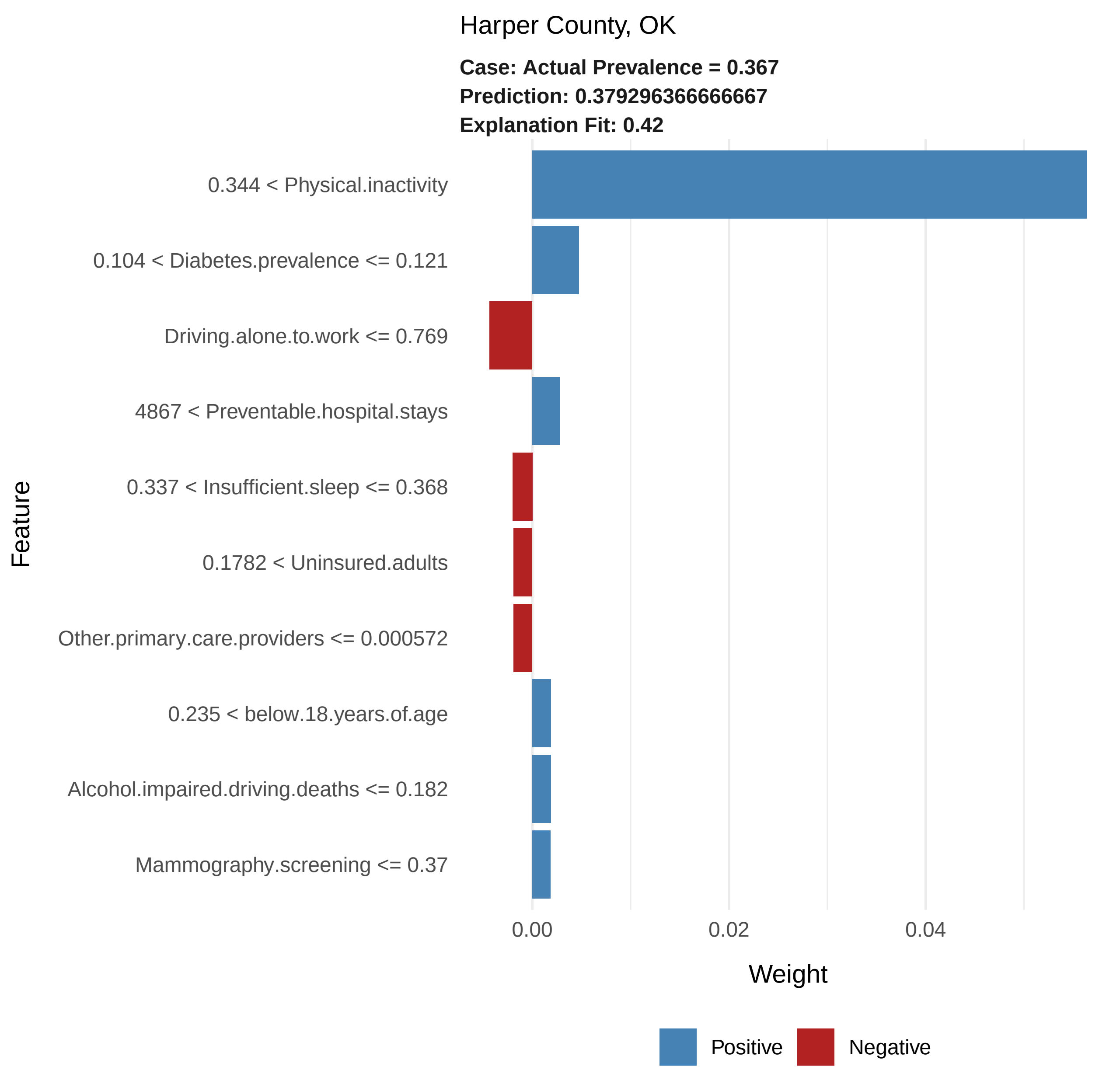

Harper County, OK
Case: Actual Prevalence = 0.367
Prediction: 0.379296366666667
Explanation Fit: 0.42
0.344 < Physical.inactivity
0.104 < Diabetes.prevalence <= 0.121
Driving.alone.to.work <= 0.769
4867 < Preventable.hospital.stays
0.337 < Insufficient.sleep <= 0.368
0.1782 < Uninsured.adults
Other.primary.care.providers <= 0.000572
0.235 < below.18.years.of.age
Alcohol.impaired.driving.deaths <= 0.182
Mammography.screening <= 0.37
Feature
0.00
0.02
0.04
Weight
Positive
Negative

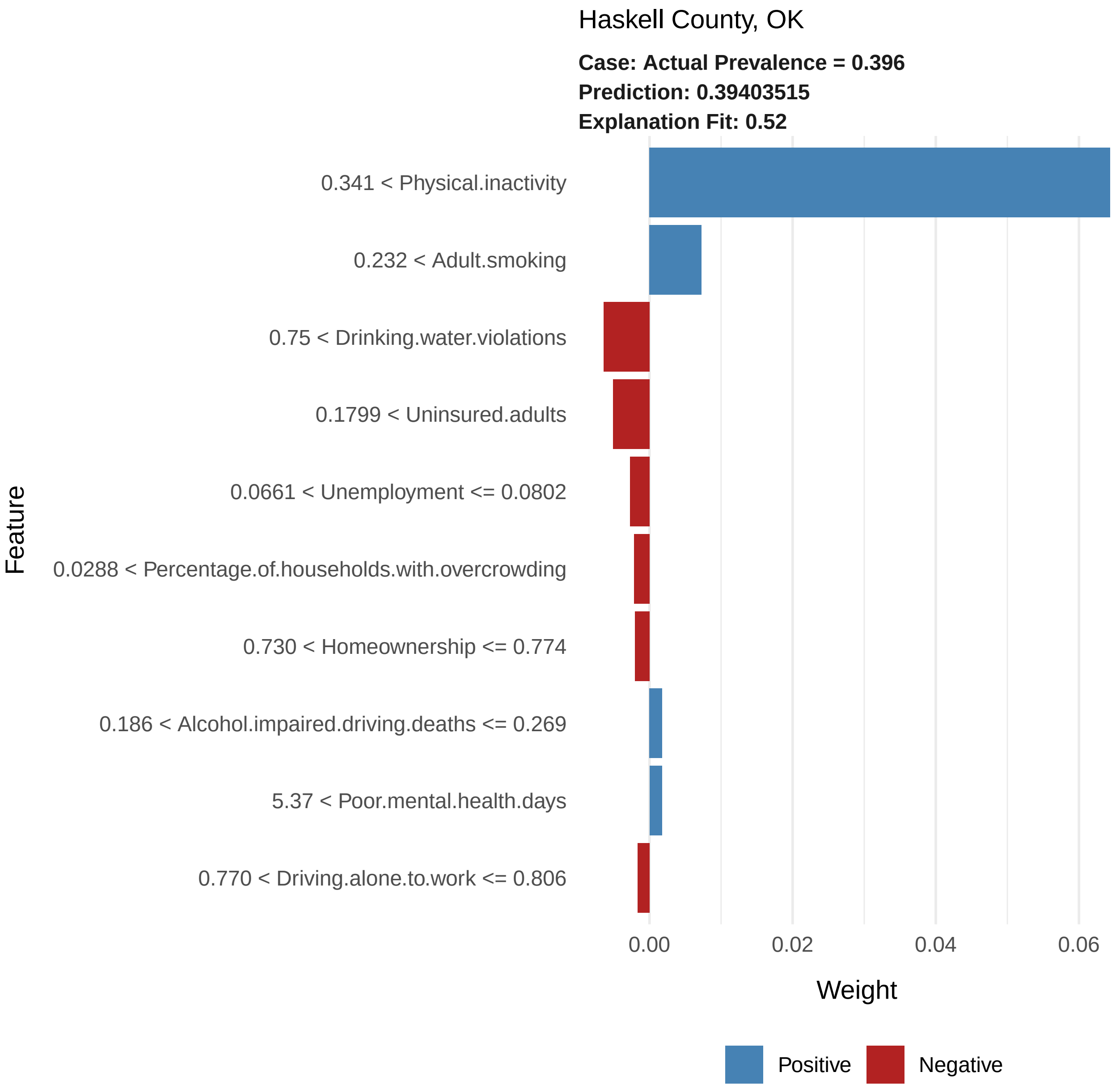

Haskell County, OK
Case: Actual Prevalence = 0.396
Prediction: 0.39403515
Explanation Fit: 0.52
0.341 < Physical.inactivity
0.232 < Adult.smoking
0.75 < Drinking.water.violations
0.1799 < Uninsured.adults
0.0661 < Unemployment <= 0.0802
0.0288 < Percentage.of.households.with.overcrowding
0.730 < Homeownership <= 0.774
0.186 < Alcohol.impaired.driving.deaths <= 0.269
5.37 < Poor.mental.health.days
0.770 < Driving.alone.to.work <= 0.806
Feature
0.00
0.02
0.04
0.06
Weight
Positive
Negative

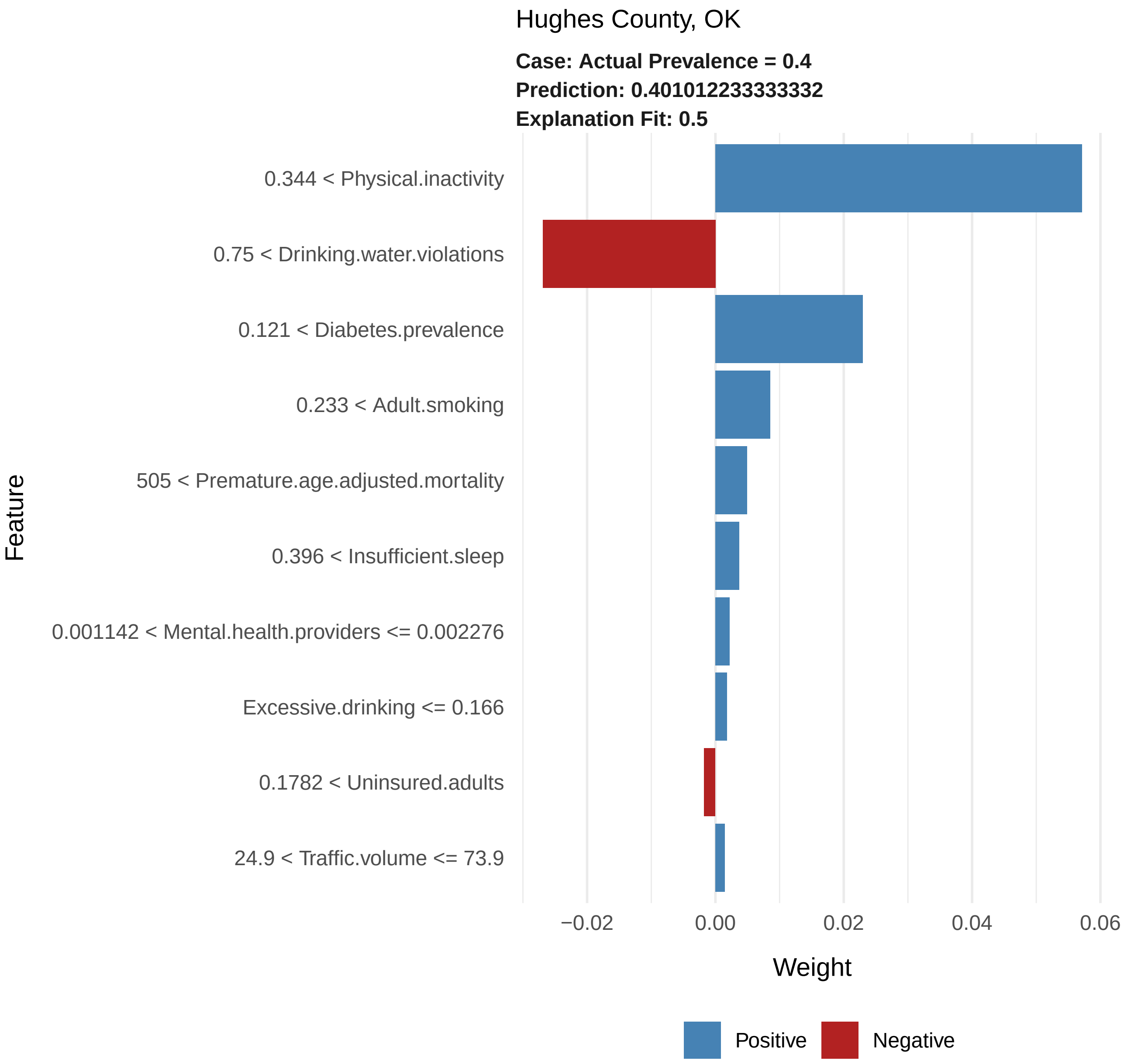
Hughes County, OK
Case: Actual Prevalence = 0.4
Prediction: 0.401012233333332
Explanation Fit: 0.5
0.344 < Physical.inactivity
0.75 < Drinking.water.violations
0.121 < Diabetes.prevalence
0.233 < Adult.smoking
505 < Premature.age.adjusted.mortality
0.396 < Insufficient.sleep
0.001142 < Mental.health.providers <= 0.002276
Excessive.drinking <= 0.166
0.1782 < Uninsured.adults
24.9 < Traffic.volume <= 73.9
Feature
−0.02
0.00
0.02
0.04
0.06
Weight
Positive
Negative

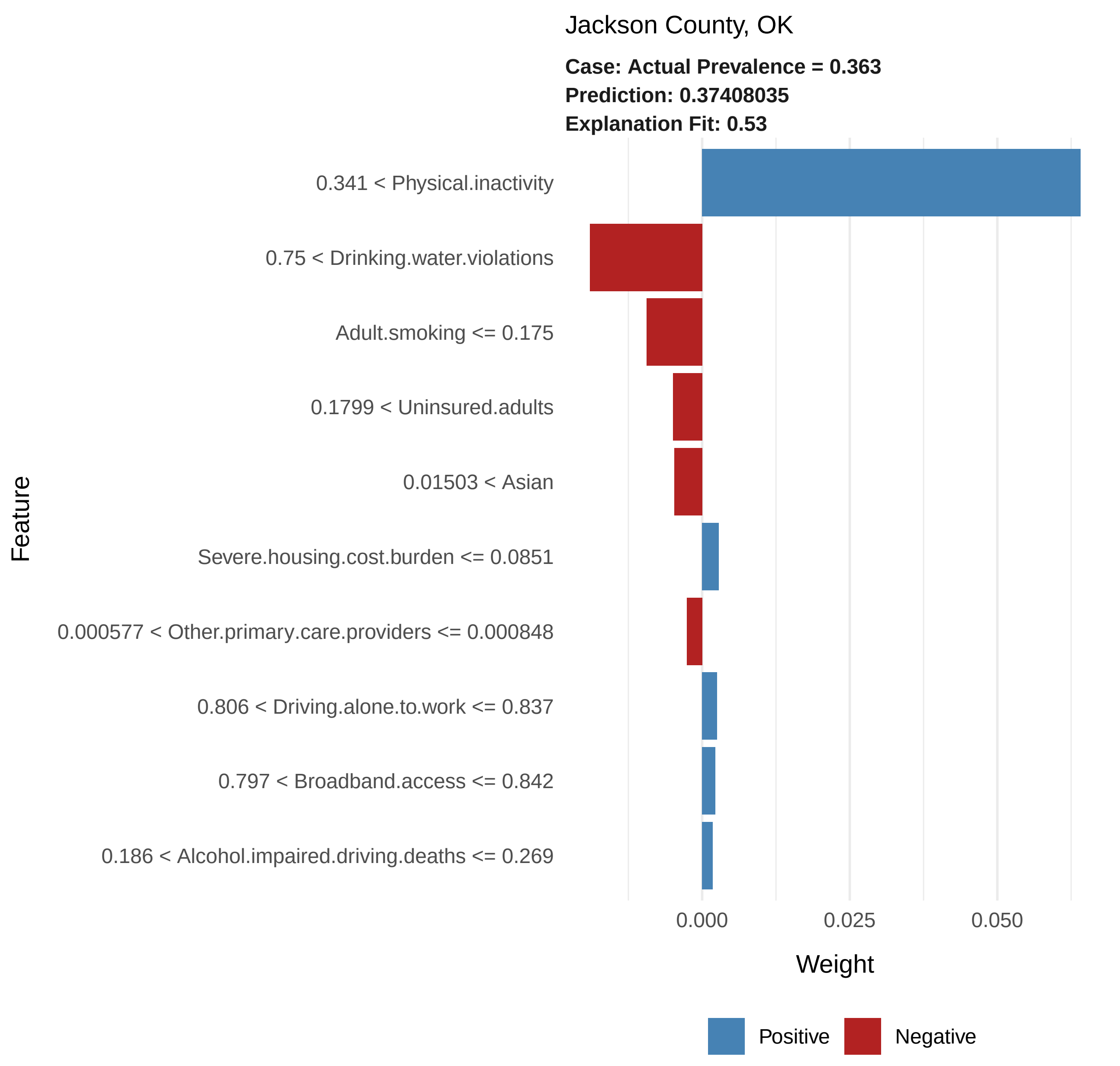

Jackson County, OK
Case: Actual Prevalence = 0.363
Prediction: 0.37408035
Explanation Fit: 0.53
Feature
0.341 < Physical.inactivity
0.75 < Drinking.water.violations
Adult.smoking <= 0.175
0.1799 < Uninsured.adults
0.01503 < Asian
Severe.housing.cost.burden <= 0.0851
0.000577 < Other.primary.care.providers <= 0.000848
0.806 < Driving.alone.to.work <= 0.837
0.797 < Broadband.access <= 0.842
0.186 < Alcohol.impaired.driving.deaths <= 0.269
0.000
0.025
0.050
Weight
Positive
Negative

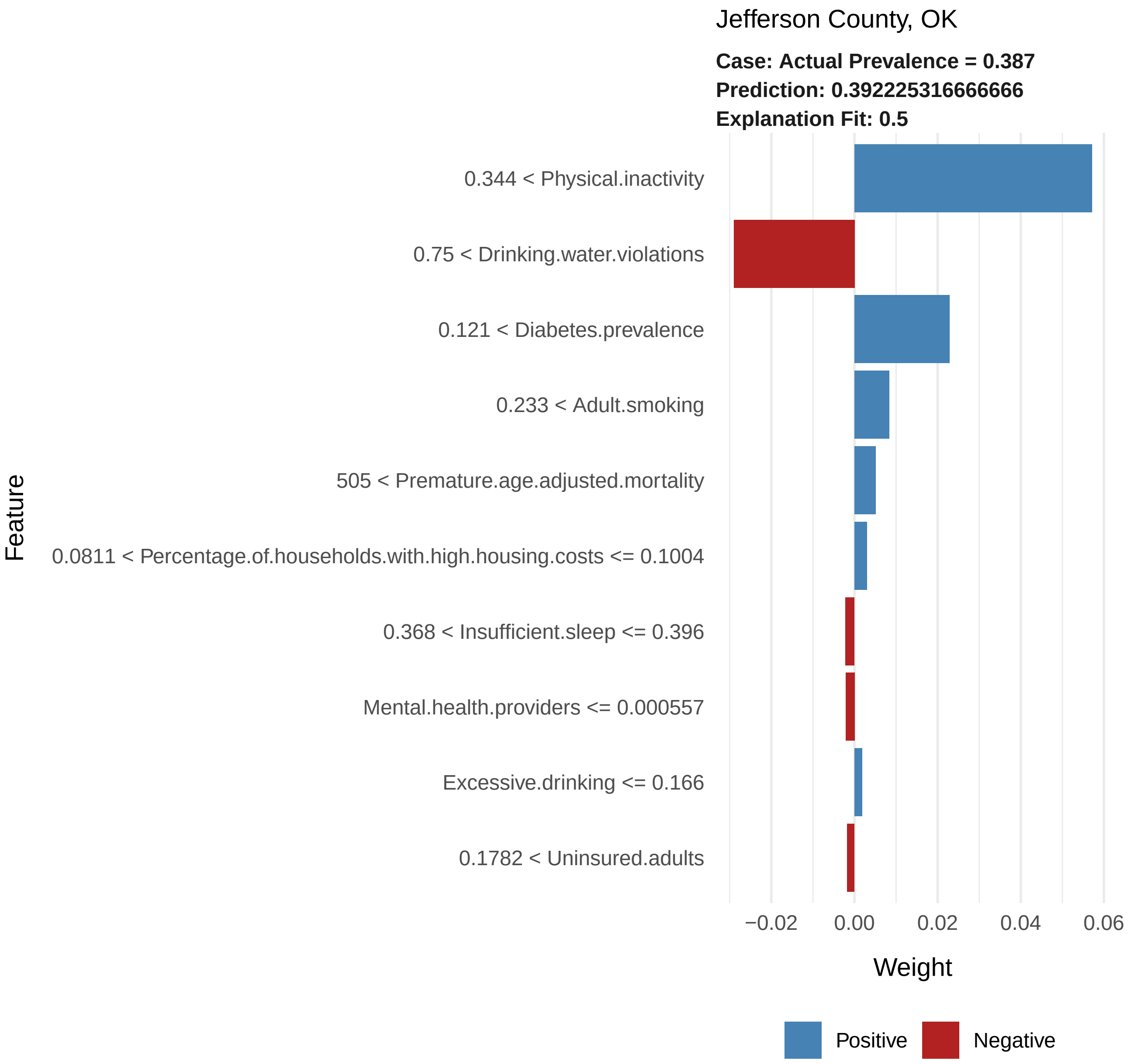

Jefferson County, OK
Case: Actual Prevalence = 0.387
Prediction: 0.392225316666666
Explanation Fit: 0.5
0.344 < Physical.inactivity
0.75 < Drinking.water.violations
0.121 < Diabetes.prevalence
0.233 < Adult.smoking
505 < Premature.age.adjusted.mortality
0.0811 < Percentage.of.households.with.high.housing.costs <= 0.1004
0.368 < Insufficient.sleep <= 0.396
Mental.health.providers <= 0.000557
Excessive.drinking <= 0.166
0.1782 < Uninsured.adults
Feature
−0.02
0.00
0.02
0.04
0.06
Weight
Positive
Negative

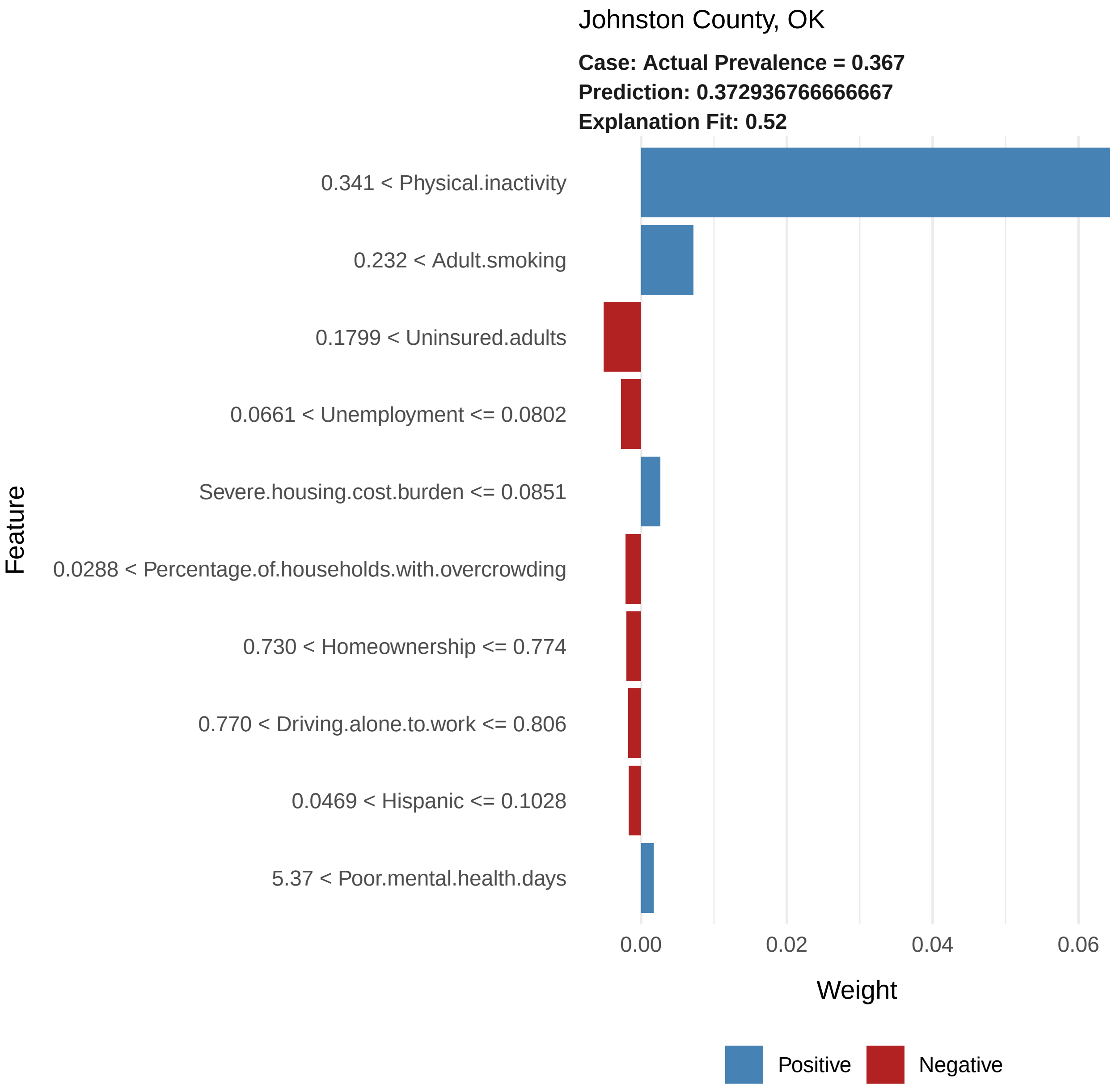

Johnston County, OK
Case: Actual Prevalence = 0.367
Prediction: 0.372936766666667
Explanation Fit: 0.52
0.341 < Physical.inactivity
0.232 < Adult.smoking
0.1799 < Uninsured.adults
0.0661 < Unemployment <= 0.0802
Severe.housing.cost.burden <= 0.0851
0.0288 < Percentage.of.households.with.overcrowding
0.730 < Homeownership <= 0.774
0.770 < Driving.alone.to.work <= 0.806
0.0469 < Hispanic <= 0.1028
5.37 < Poor.mental.health.days
Feature
0.00
0.02
0.04
0.06
Weight
Positive
Negative

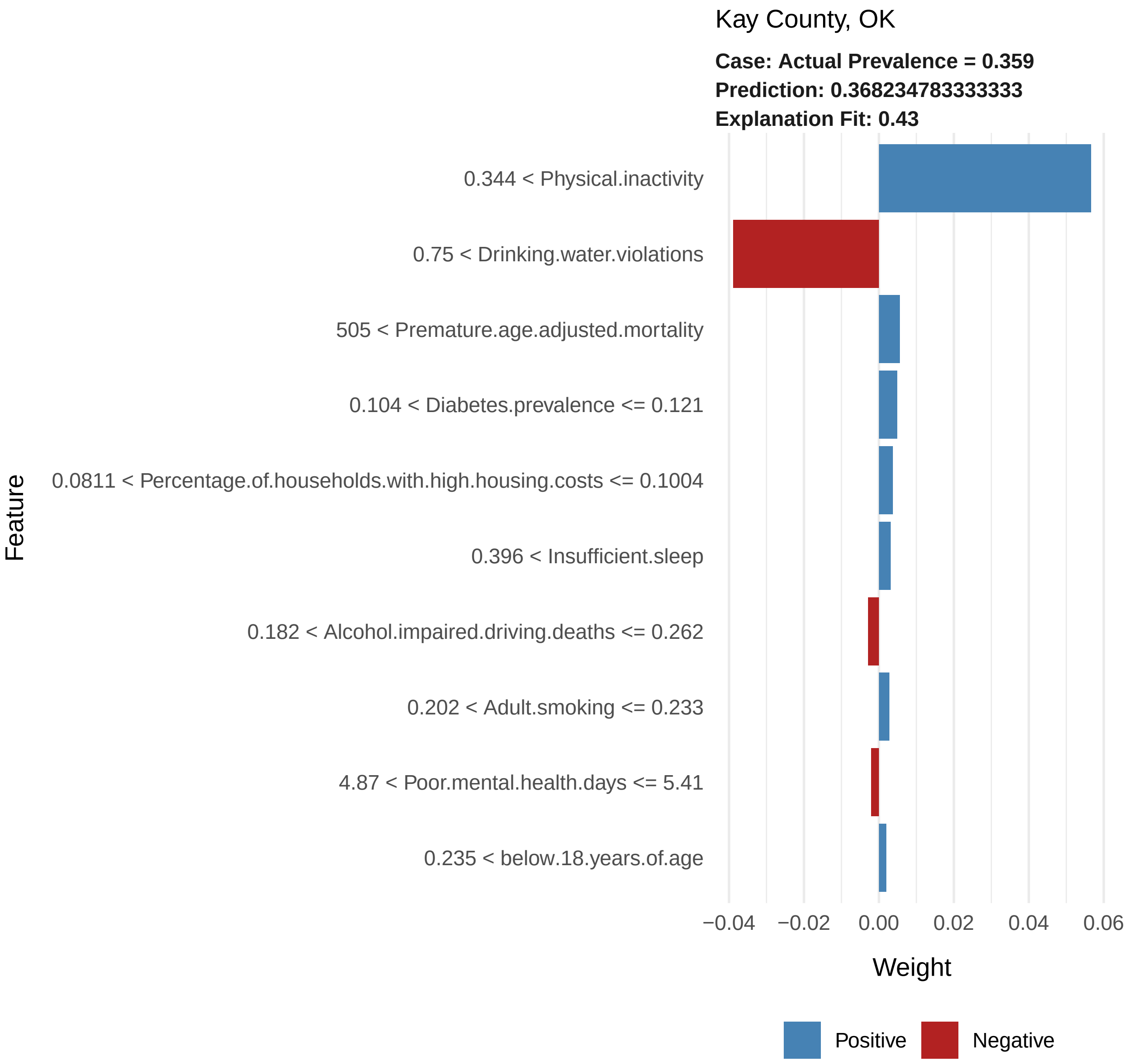
Kay County, OK
Case: Actual Prevalence = 0.359
Prediction: 0.368234783333333
Explanation Fit: 0.43
0.344 < Physical.inactivity
0.75 < Drinking.water.violations
505 < Premature.age.adjusted.mortality
0.104 < Diabetes.prevalence <= 0.121
0.0811 < Percentage.of.households.with.high.housing.costs <= 0.1004
0.396 < Insufficient.sleep
0.182 < Alcohol.impaired.driving.deaths <= 0.262
0.202 < Adult.smoking <= 0.233
4.87 < Poor.mental.health.days <= 5.41
0.235 < below.18.years.of.age
Feature
−0.04
−0.02
0.00
0.02
0.04
0.06
Weight
Positive
Negative

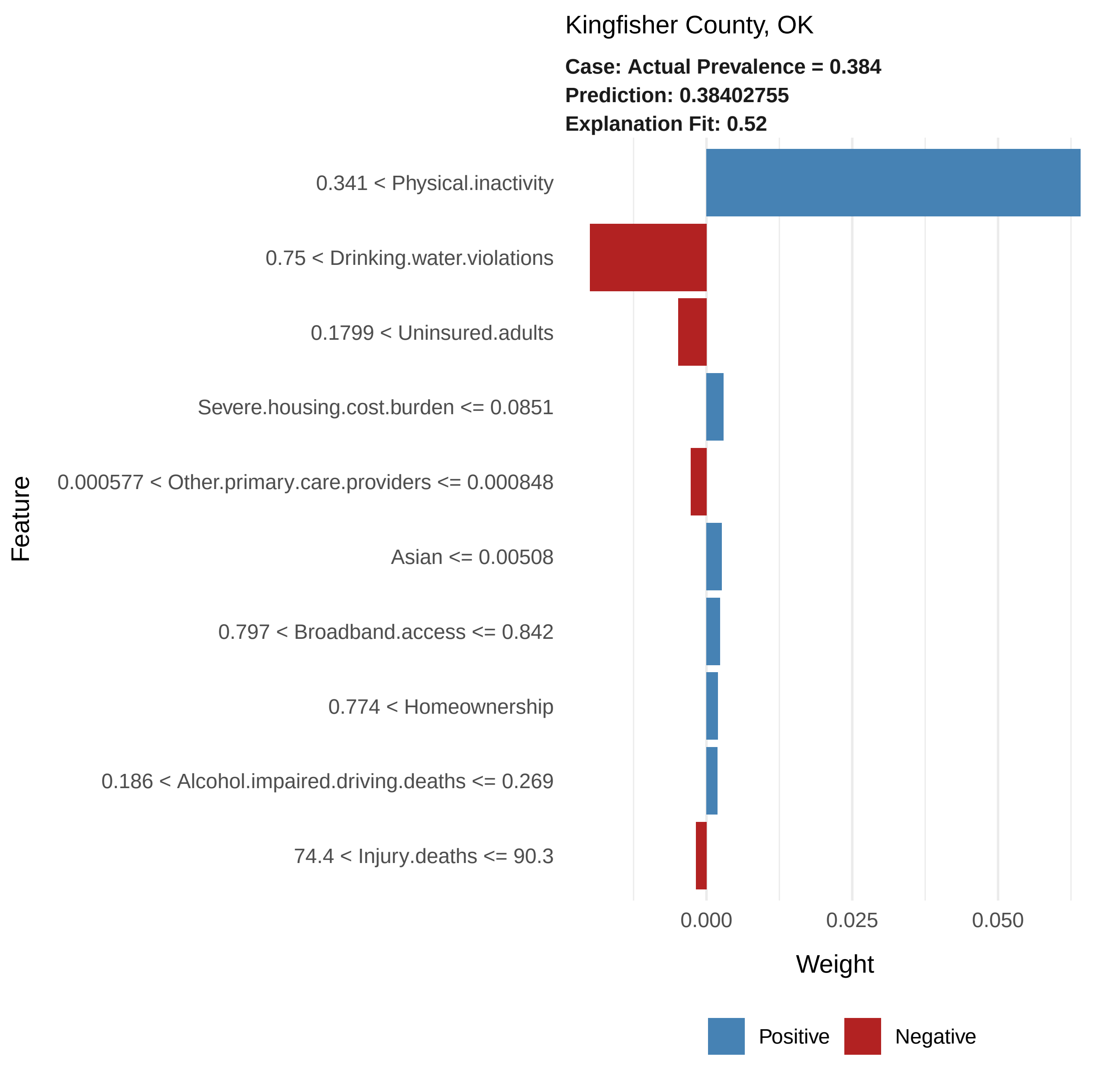

Kingfisher County, OK
Case: Actual Prevalence = 0.384
Prediction: 0.38402755
Explanation Fit: 0.52
0.341 < Physical.inactivity
0.75 < Drinking.water.violations
0.1799 < Uninsured.adults
Severe.housing.cost.burden <= 0.0851
0.000577 < Other.primary.care.providers <= 0.000848
Asian <= 0.00508
0.797 < Broadband.access <= 0.842
0.774 < Homeownership
0.186 < Alcohol.impaired.driving.deaths <= 0.269
74.4 < Injury.deaths <= 90.3
Feature
0.000
0.025
0.050
Weight
Positive
Negative

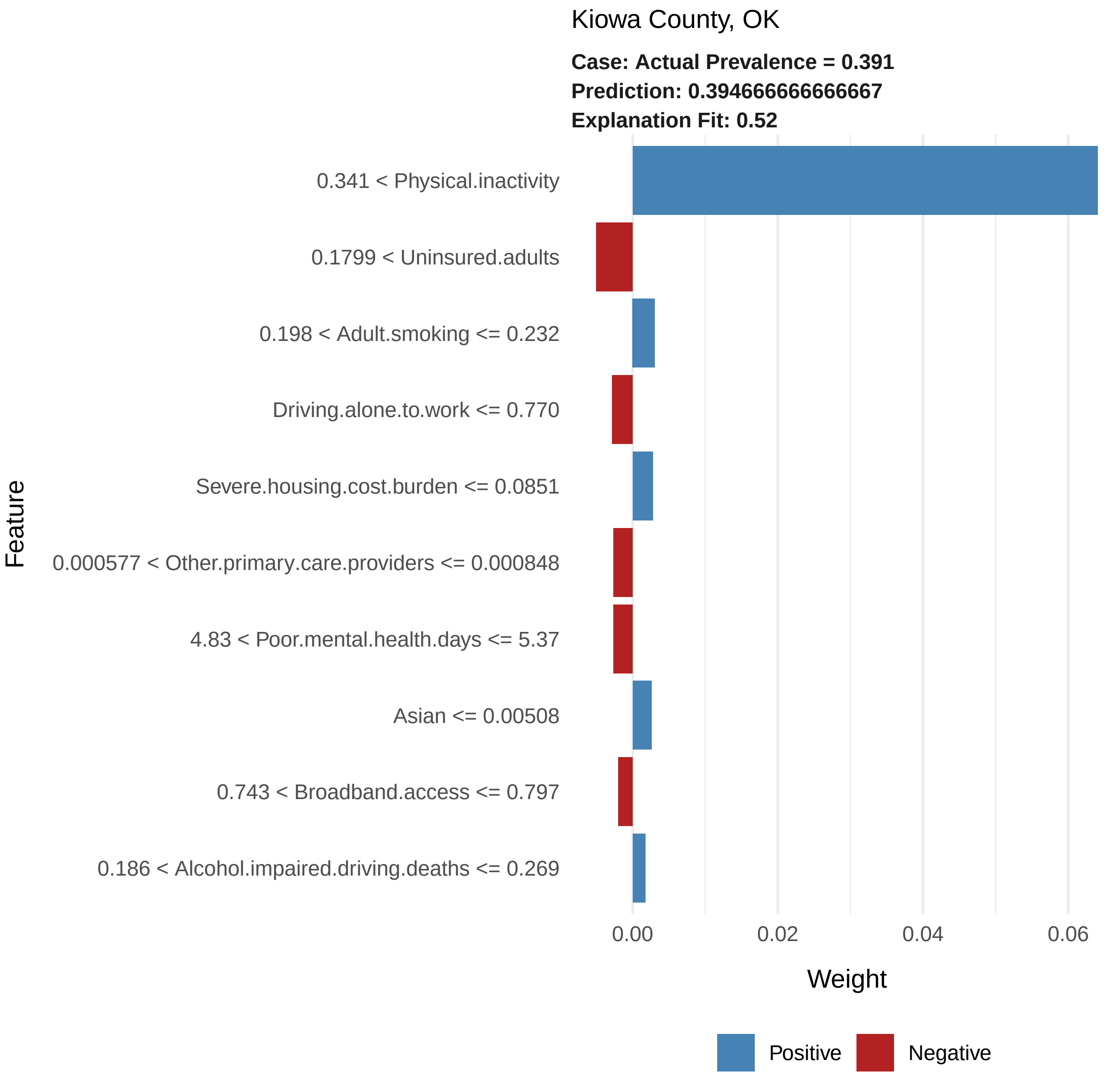

Kiowa County, OK
Case: Actual Prevalence = 0.391
Prediction: 0.394666666666667
Explanation Fit: 0.52
0.341 < Physical.inactivity
0.1799 < Uninsured.adults
0.198 < Adult.smoking <= 0.232
Driving.alone.to.work <= 0.770
Severe.housing.cost.burden <= 0.0851
0.000577 < Other.primary.care.providers <= 0.000848
4.83 < Poor.mental.health.days <= 5.37
Asian <= 0.00508
0.743 < Broadband.access <= 0.797
0.186 < Alcohol.impaired.driving.deaths <= 0.269
Feature
0.00
0.02
0.04
0.06
Weight
Positive
Negative

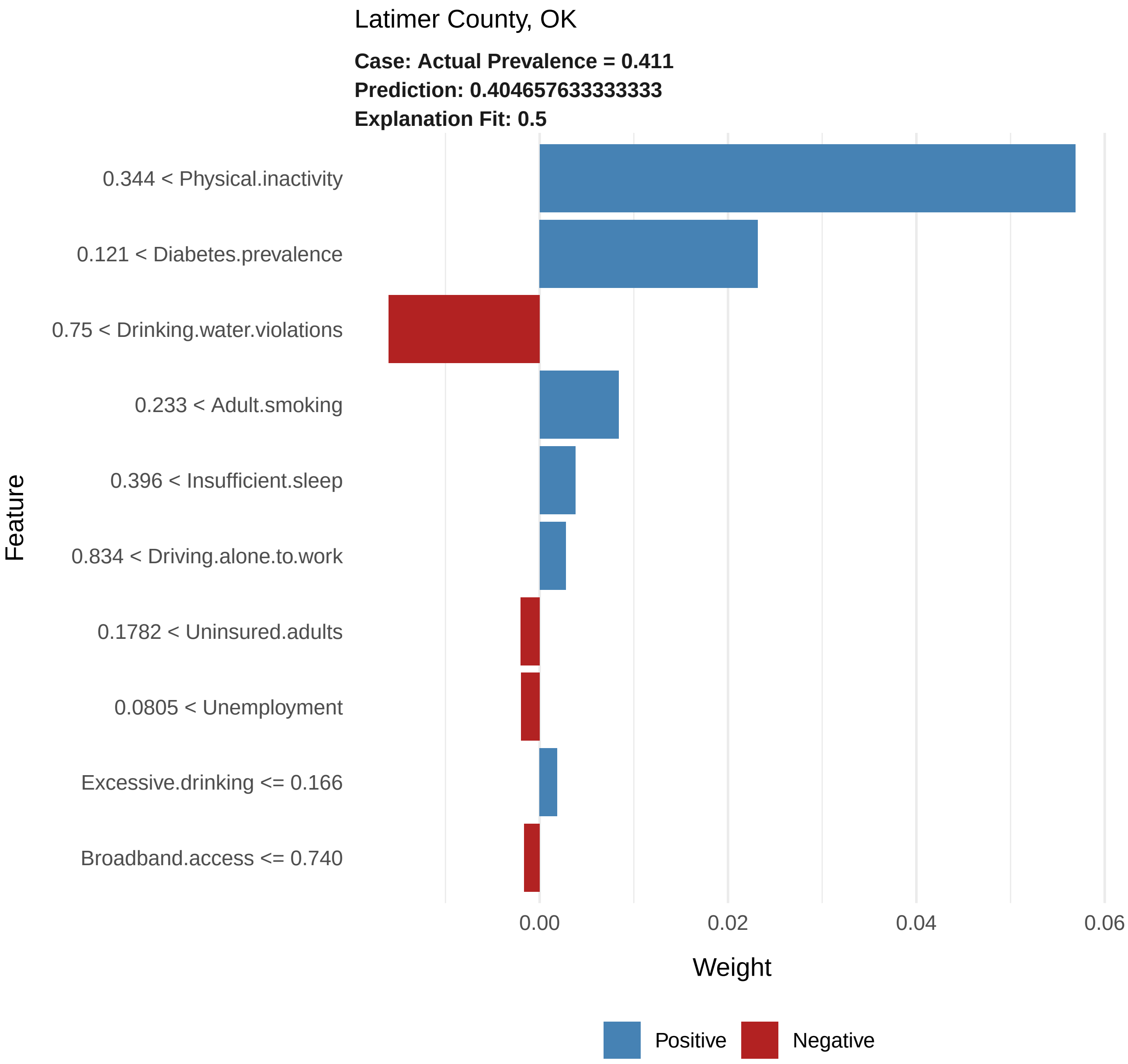

Latimer County, OK
Case: Actual Prevalence = 0.411
Prediction: 0.404657633333333
Explanation Fit: 0.5
0.344 < Physical.inactivity
0.121 < Diabetes.prevalence
0.75 < Drinking.water.violations
0.233 < Adult.smoking
0.396 < Insufficient.sleep
0.834 < Driving.alone.to.work
0.1782 < Uninsured.adults
0.0805 < Unemployment
Excessive.drinking <= 0.166
Broadband.access <= 0.740
Feature
0.00
0.02
0.04
0.06
Weight
Positive
Negative

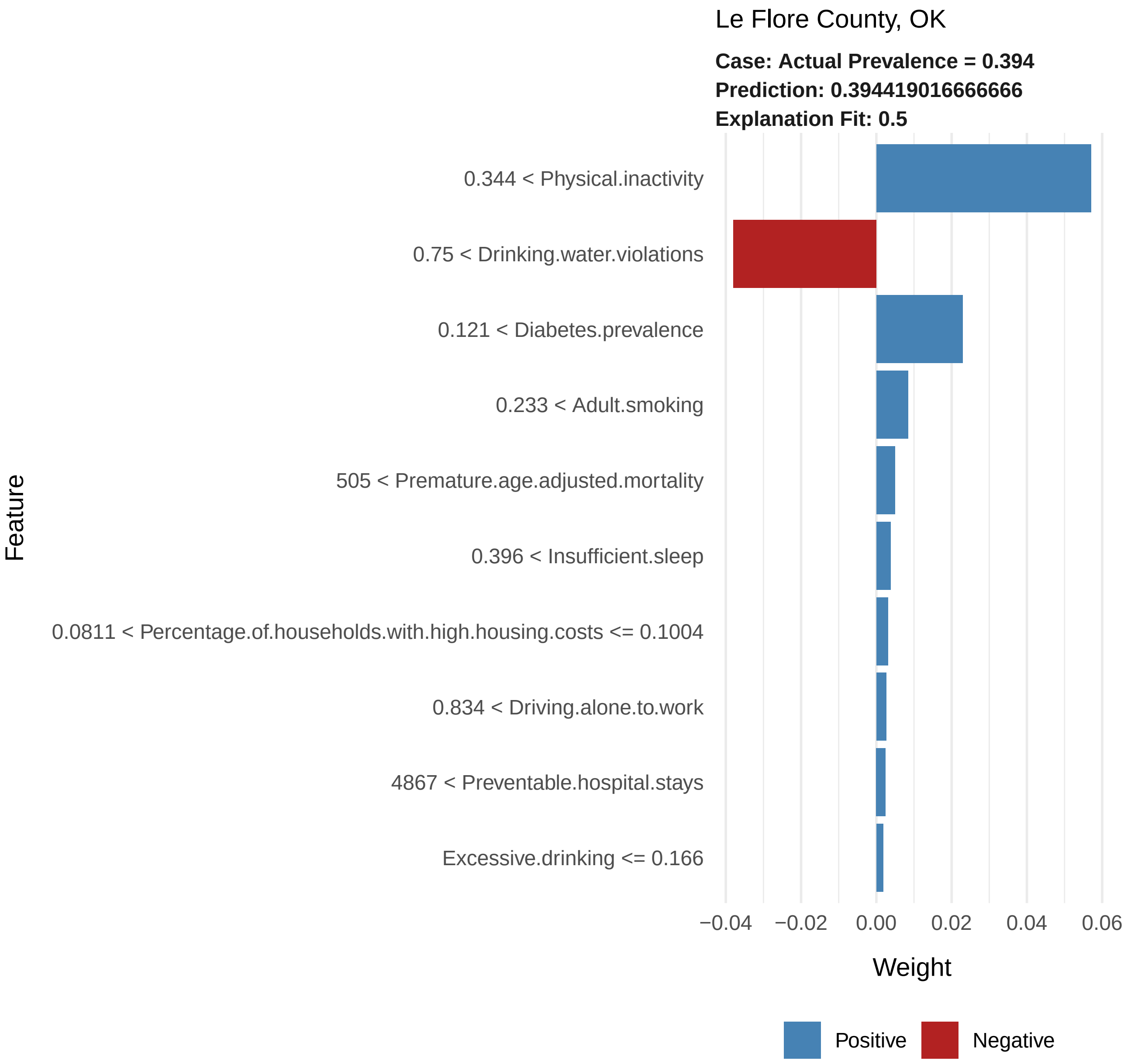

Le Flore County, OK
Case: Actual Prevalence = 0.394
Prediction: 0.394419016666666
Explanation Fit: 0.5
0.344 < Physical.inactivity
0.75 < Drinking.water.violations
0.121 < Diabetes.prevalence
0.233 < Adult.smoking
505 < Premature.age.adjusted.mortality
0.396 < Insufficient.sleep
0.0811 < Percentage.of.households.with.high.housing.costs <= 0.1004
0.834 < Driving.alone.to.work
4867 < Preventable.hospital.stays
Excessive.drinking <= 0.166
Feature
−0.04
−0.02
0.00
0.02
0.04
0.06
Weight
Positive
Negative

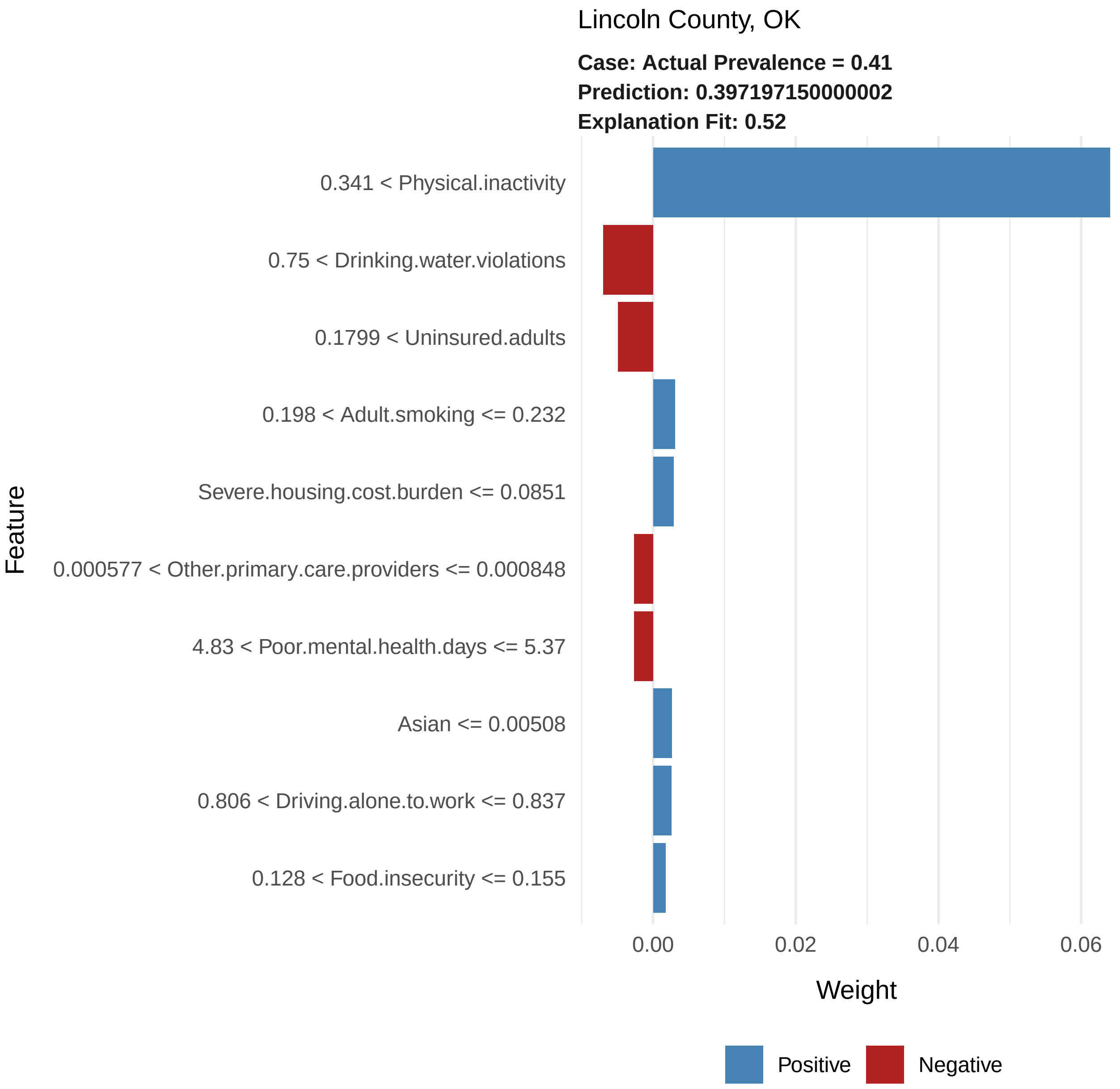
Lincoln County, OK
Case: Actual Prevalence = 0.41
Prediction: 0.397197150000002
Explanation Fit: 0.52
0.341 < Physical.inactivity
0.75 < Drinking.water.violations
0.1799 < Uninsured.adults
0.198 < Adult.smoking <= 0.232
Severe.housing.cost.burden <= 0.0851
0.000577 < Other.primary.care.providers <= 0.000848
4.83 < Poor.mental.health.days <= 5.37
Asian <= 0.00508
0.806 < Driving.alone.to.work <= 0.837
0.128 < Food.insecurity <= 0.155
Feature
0.00
0.02
0.04
0.06
Weight
Positive
Negative

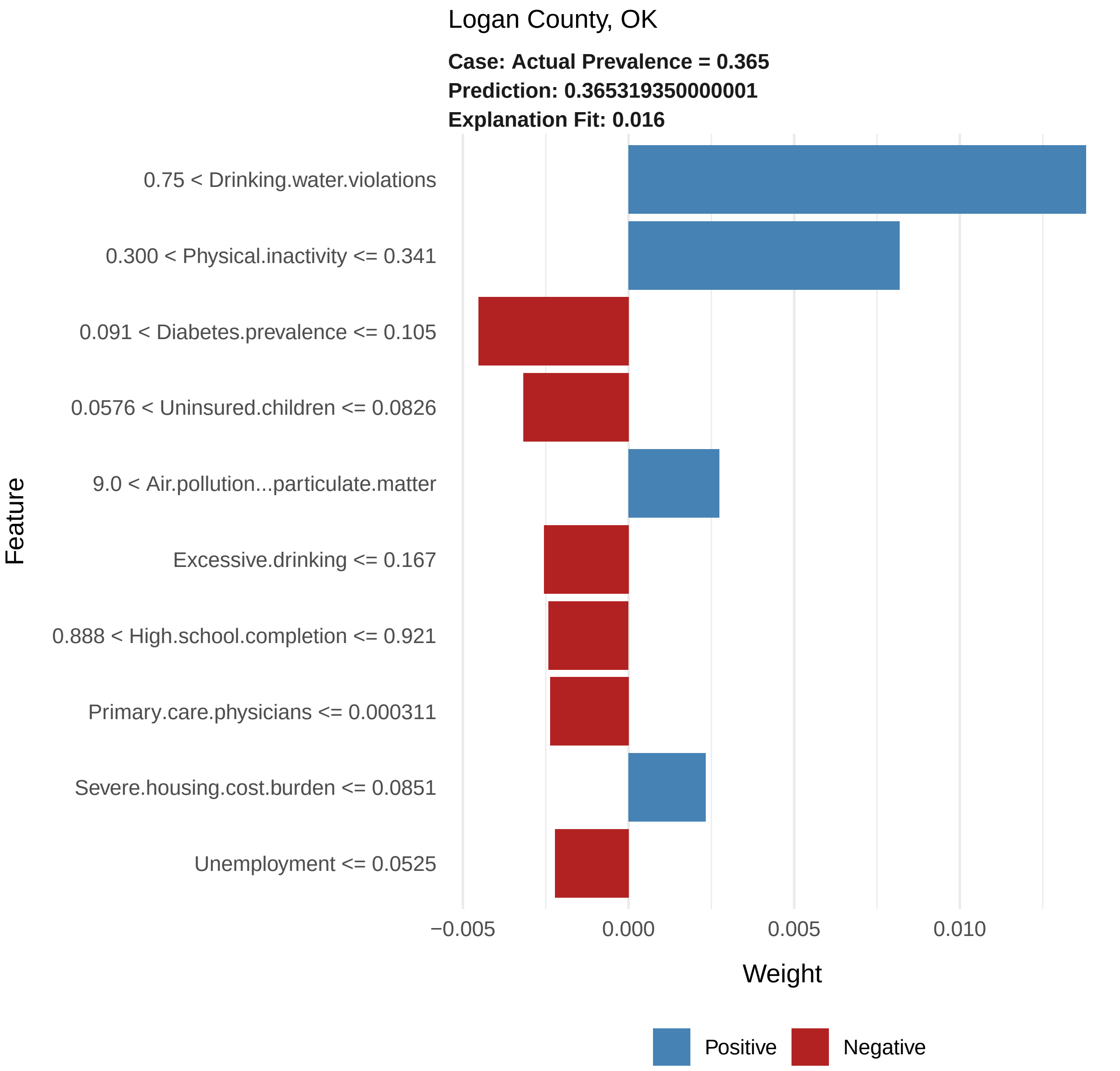

Logan County, OK
Case: Actual Prevalence = 0.365
Prediction: 0.365319350000001
Explanation Fit: 0.016
0.75 < Drinking.water.violations
0.300 < Physical.inactivity <= 0.341
0.091 < Diabetes.prevalence <= 0.105
0.0576 < Uninsured.children <= 0.0826
9.0 < Air.pollution...particulate.matter
Excessive.drinking <= 0.167
0.888 < High.school.completion <= 0.921
Primary.care.physicians <= 0.000311
Severe.housing.cost.burden <= 0.0851
Unemployment <= 0.0525
Feature
−0.005
0.000
0.005
0.010
Weight
Positive
Negative

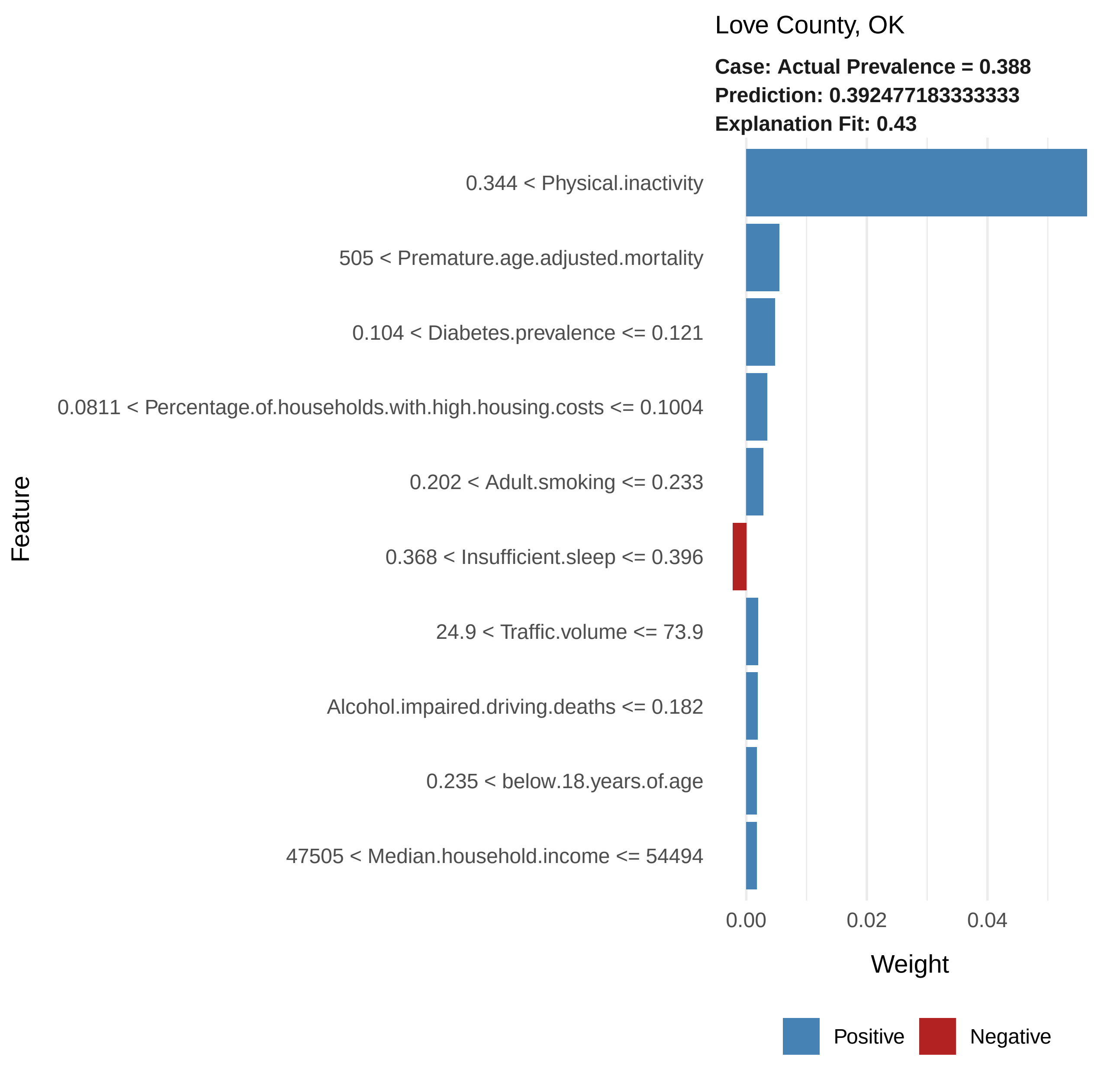

Love County, OK
Case: Actual Prevalence = 0.388
Prediction: 0.392477183333333
Explanation Fit: 0.43
0.344 < Physical.inactivity
505 < Premature.age.adjusted.mortality
0.104 < Diabetes.prevalence <= 0.121
0.0811 < Percentage.of.households.with.high.housing.costs <= 0.1004
0.202 < Adult.smoking <= 0.233
0.368 < Insufficient.sleep <= 0.396
24.9 < Traffic.volume <= 73.9
Alcohol.impaired.driving.deaths <= 0.182
0.235 < below.18.years.of.age
47505 < Median.household.income <= 54494
Feature
0.00
0.02
0.04
Weight
Positive
Negative

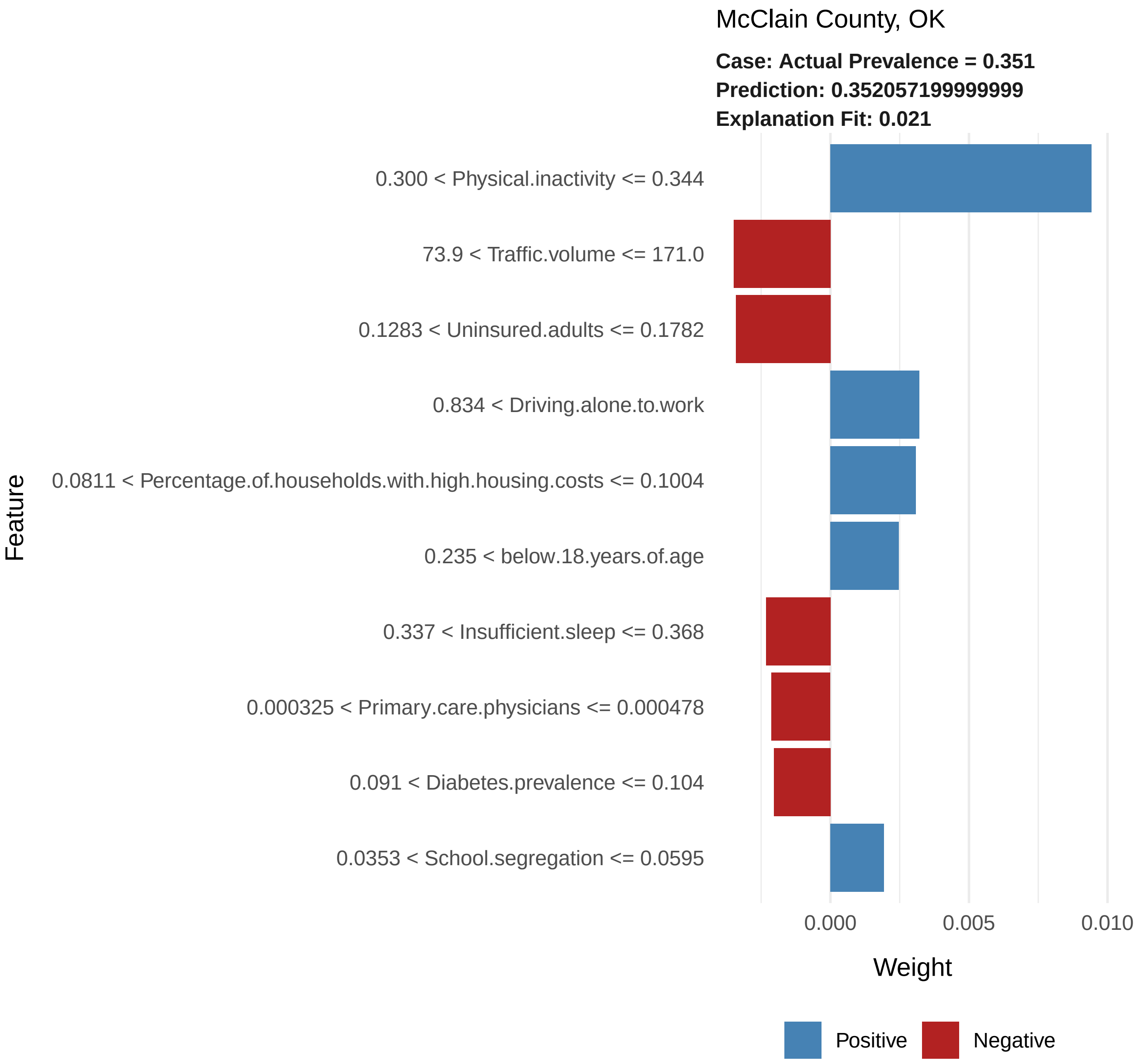

McClain County, OK
Case: Actual Prevalence = 0.351
Prediction: 0.352057199999999
Explanation Fit: 0.021
0.300 < Physical.inactivity <= 0.344
73.9 < Traffic.volume <= 171.0
0.1283 < Uninsured.adults <= 0.1782
0.834 < Driving.alone.to.work
0.0811 < Percentage.of.households.with.high.housing.costs <= 0.1004
0.235 < below.18.years.of.age
0.337 < Insufficient.sleep <= 0.368
0.000325 < Primary.care.physicians <= 0.000478
0.091 < Diabetes.prevalence <= 0.104
0.0353 < School.segregation <= 0.0595
Feature
0.000
0.005
0.010
Weight
Positive
Negative

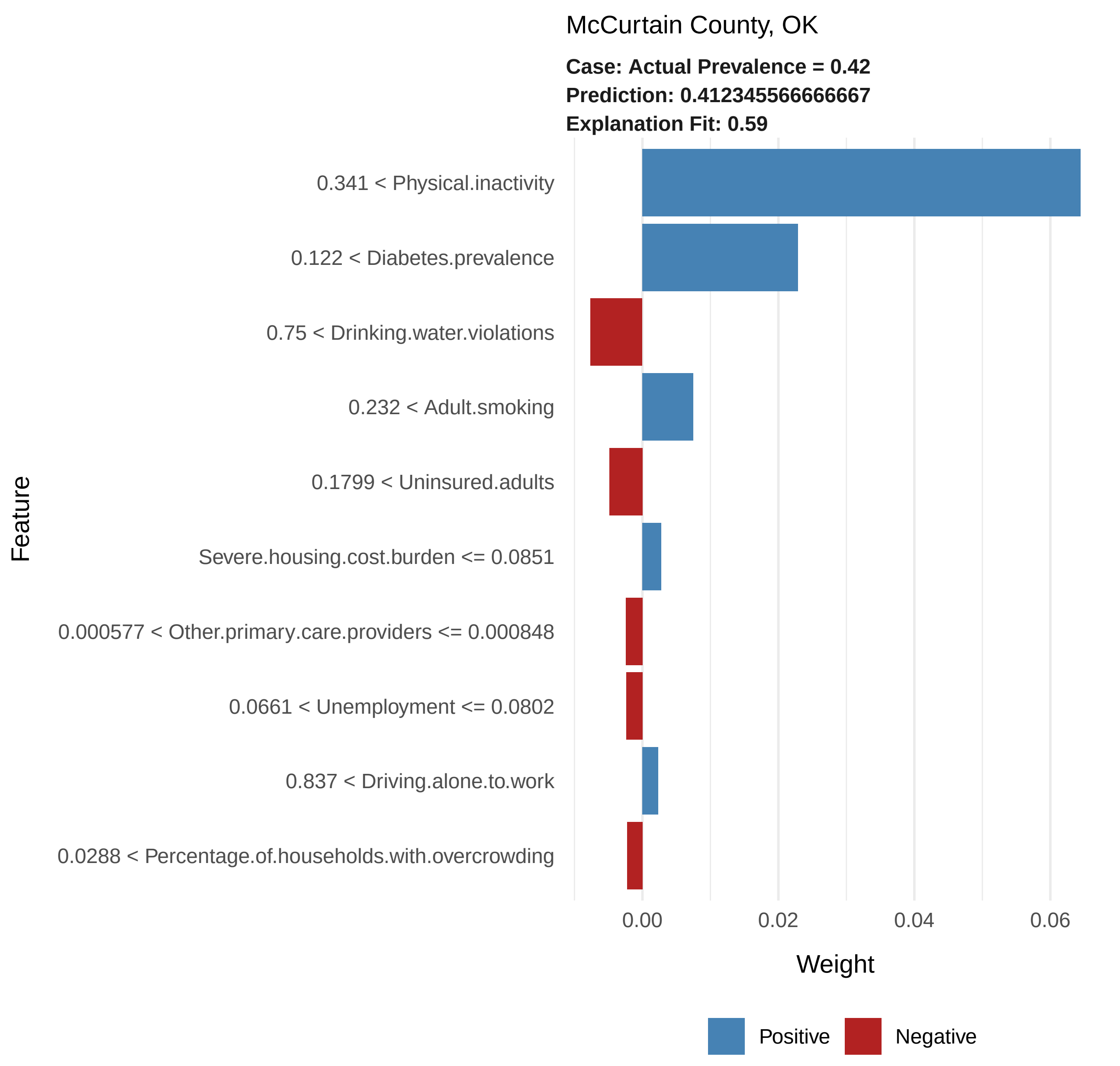
McCurtain County, OK
Case: Actual Prevalence = 0.42
Prediction: 0.412345566666667
Explanation Fit: 0.59
0.341 < Physical.inactivity
0.122 < Diabetes.prevalence
0.75 < Drinking.water.violations
0.232 < Adult.smoking
0.1799 < Uninsured.adults
Severe.housing.cost.burden <= 0.0851
0.000577 < Other.primary.care.providers <= 0.000848
0.0661 < Unemployment <= 0.0802
0.837 < Driving.alone.to.work
0.0288 < Percentage.of.households.with.overcrowding
Feature
0.00
0.02
0.04
0.06
Weight
Positive
Negative

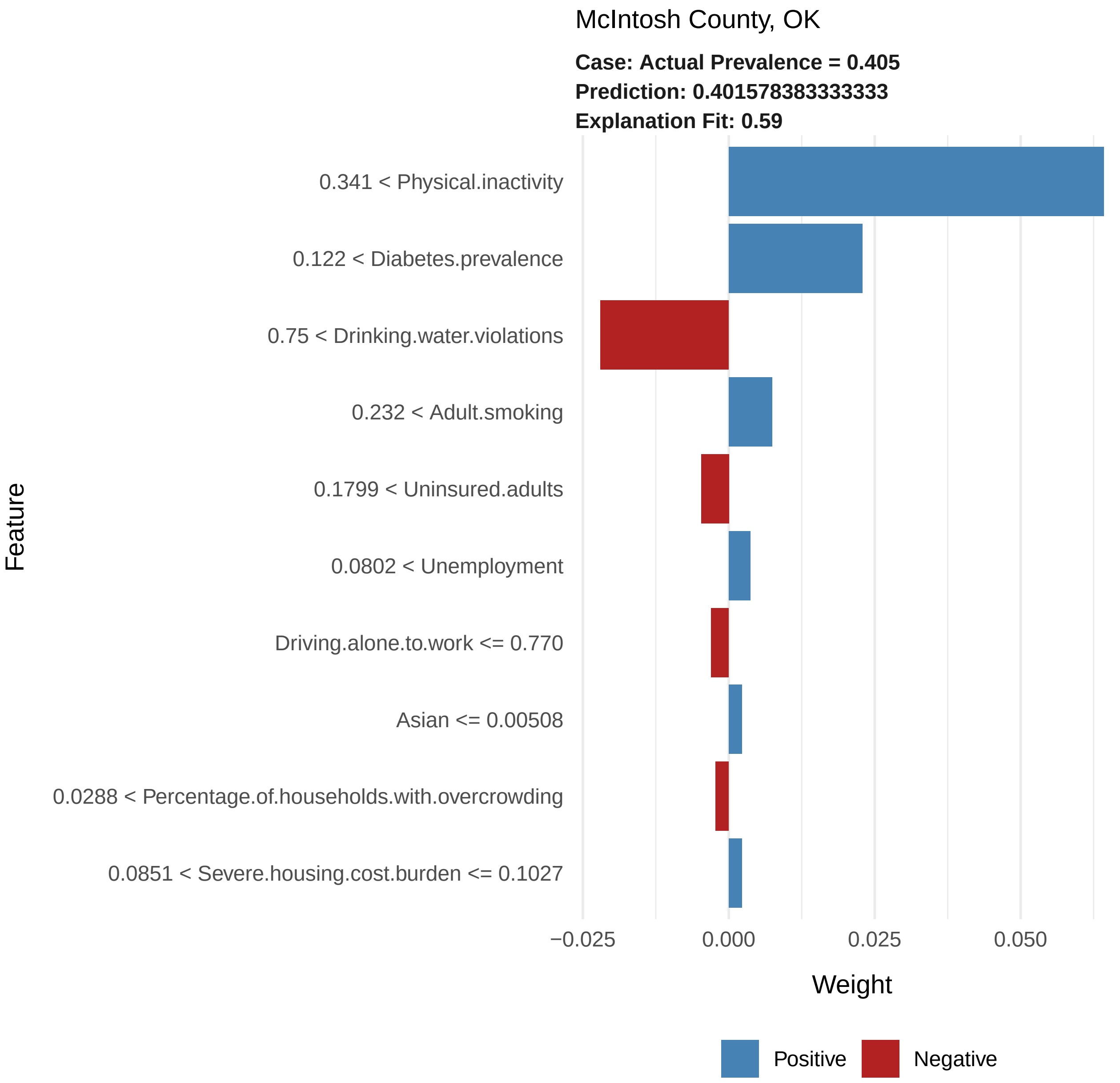

McIntosh County, OK
Case: Actual Prevalence = 0.405
Prediction: 0.401578383333333
Explanation Fit: 0.59
0.341 < Physical.inactivity
0.122 < Diabetes.prevalence
0.75 < Drinking.water.violations
0.232 < Adult.smoking
0.1799 < Uninsured.adults
0.0802 < Unemployment
Driving.alone.to.work <= 0.770
Asian <= 0.00508
0.0288 < Percentage.of.households.with.overcrowding
0.0851 < Severe.housing.cost.burden <= 0.1027
Feature
−0.025
0.000
0.025
0.050
Weight
Positive
Negative

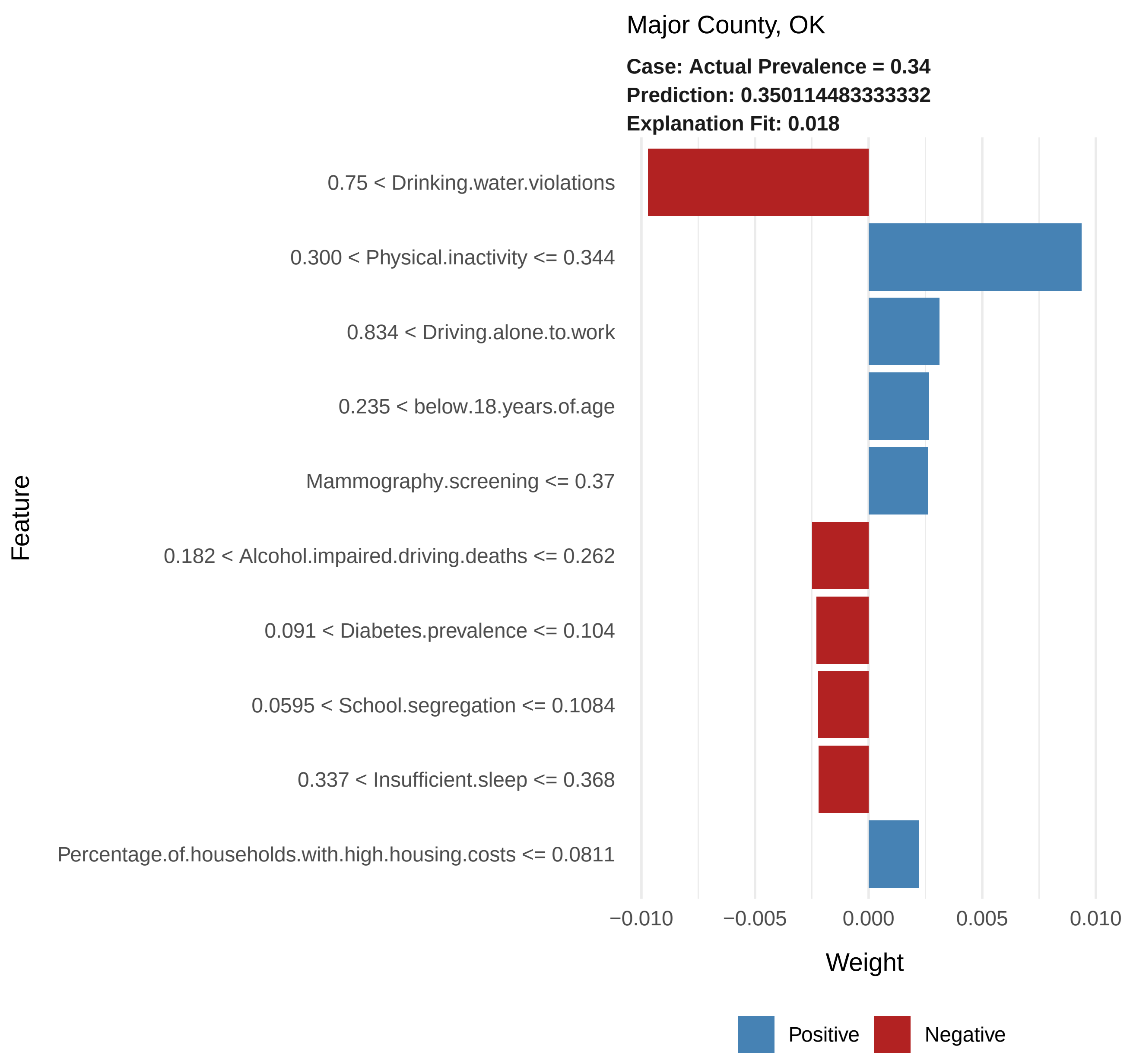
Major County, OK
Case: Actual Prevalence = 0.34
Prediction: 0.350114483333332
Explanation Fit: 0.018
0.75 < Drinking.water.violations
0.300 < Physical.inactivity <= 0.344
0.834 < Driving.alone.to.work
0.235 < below.18.years.of.age
Mammography.screening <= 0.37
0.182 < Alcohol.impaired.driving.deaths <= 0.262
0.091 < Diabetes.prevalence <= 0.104
0.0595 < School.segregation <= 0.1084
0.337 < Insufficient.sleep <= 0.368
Percentage.of.households.with.high.housing.costs <= 0.0811
Feature
−0.010
−0.005
0.000
0.005
0.010
Weight
Positive
Negative

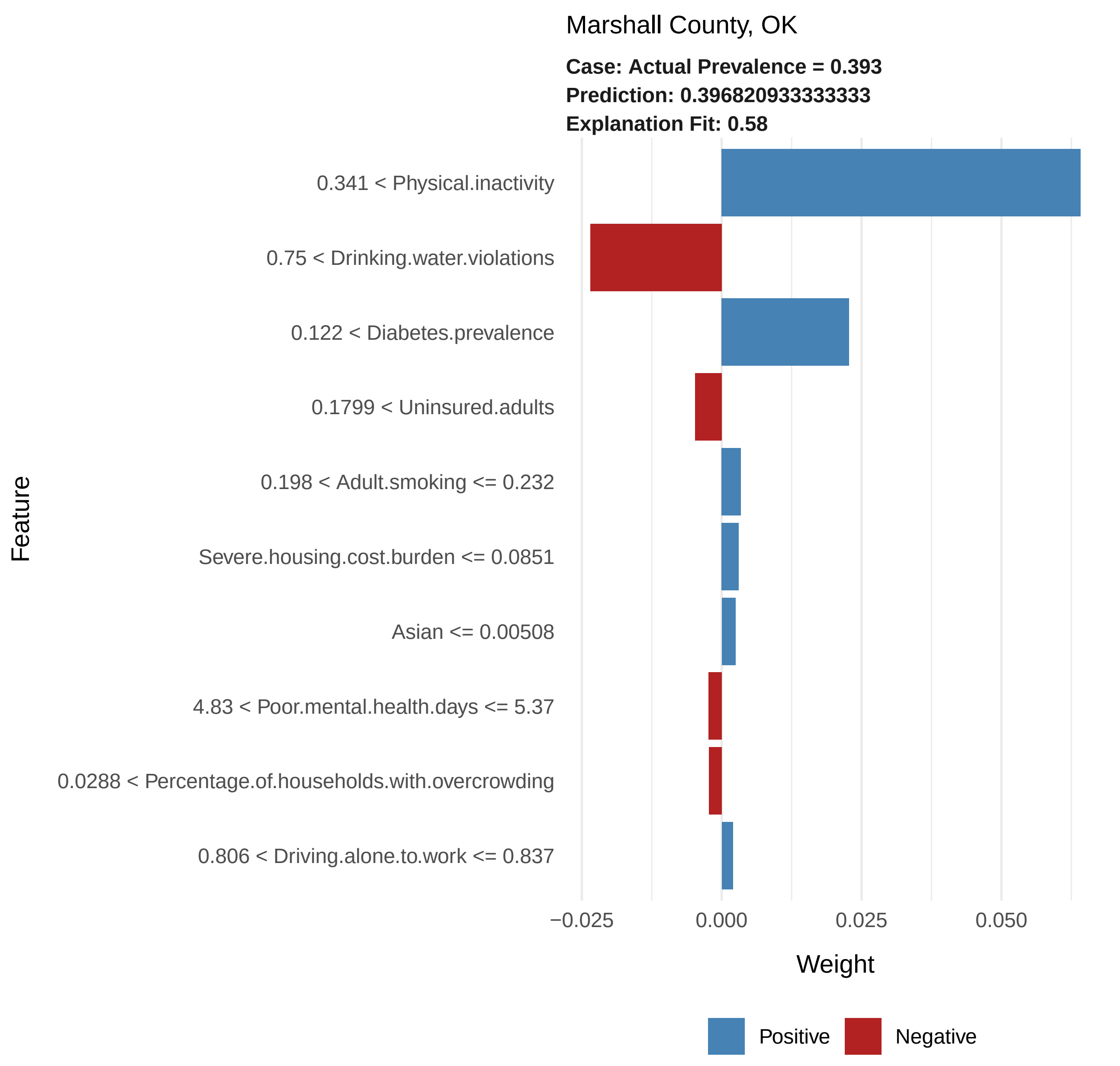

Marshall County, OK
Case: Actual Prevalence = 0.393
Prediction: 0.396820933333333
Explanation Fit: 0.58
0.341 < Physical.inactivity
0.75 < Drinking.water.violations
0.122 < Diabetes.prevalence
0.1799 < Uninsured.adults
0.198 < Adult.smoking <= 0.232
Severe.housing.cost.burden <= 0.0851
Asian <= 0.00508
4.83 < Poor.mental.health.days <= 5.37
0.0288 < Percentage.of.households.with.overcrowding
0.806 < Driving.alone.to.work <= 0.837
Feature
−0.025
0.000
0.025
0.050
Weight
Positive
Negative

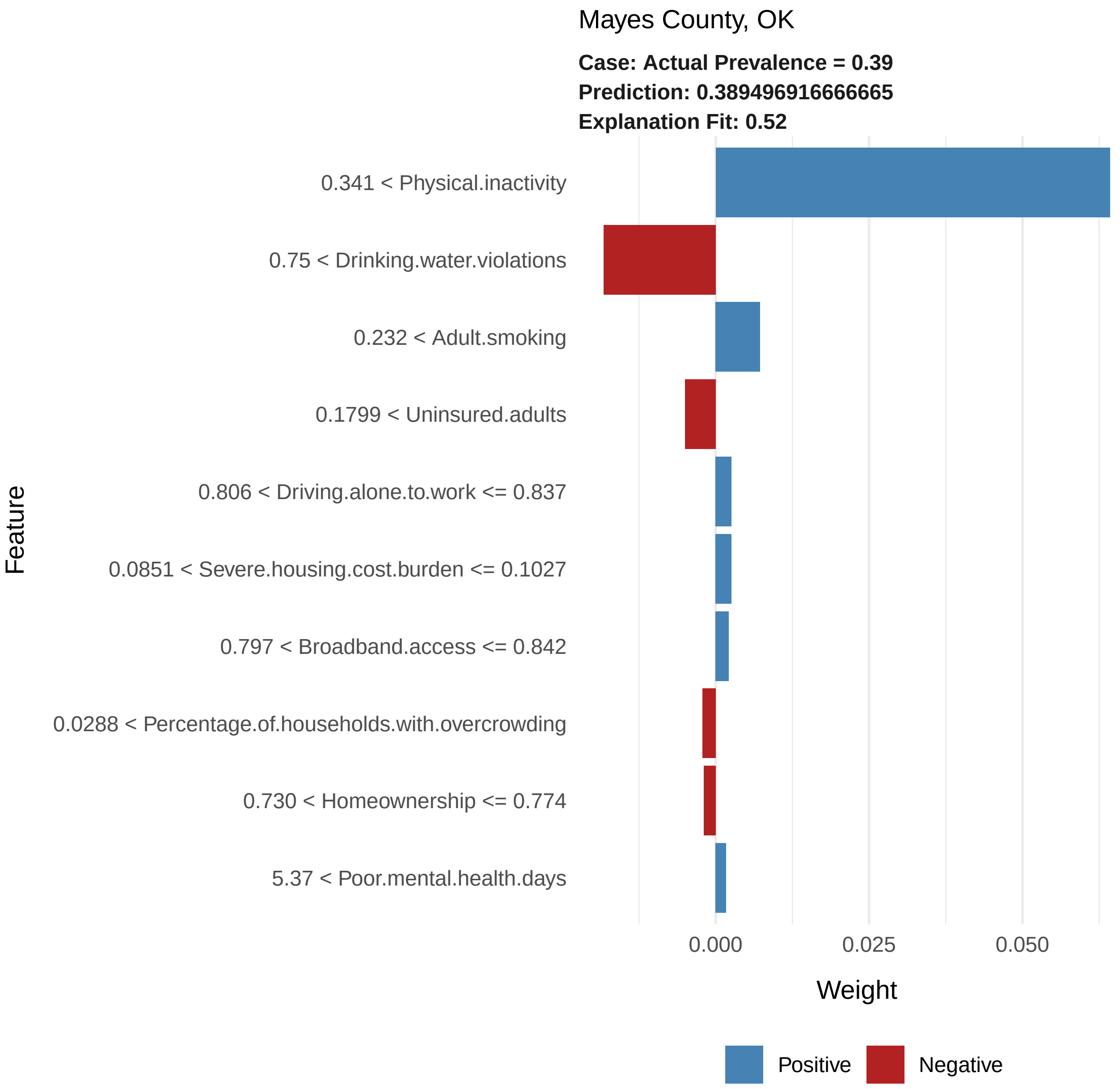
Mayes County, OK
Case: Actual Prevalence = 0.39
Prediction: 0.389496916666665
Explanation Fit: 0.52
0.341 < Physical.inactivity
0.75 < Drinking.water.violations
0.232 < Adult.smoking
0.1799 < Uninsured.adults
0.806 < Driving.alone.to.work <= 0.837
0.0851 < Severe.housing.cost.burden <= 0.1027
0.797 < Broadband.access <= 0.842
0.0288 < Percentage.of.households.with.overcrowding
0.730 < Homeownership <= 0.774
5.37 < Poor.mental.health.days
Feature
0.000
0.025
0.050
Weight
Positive
Negative

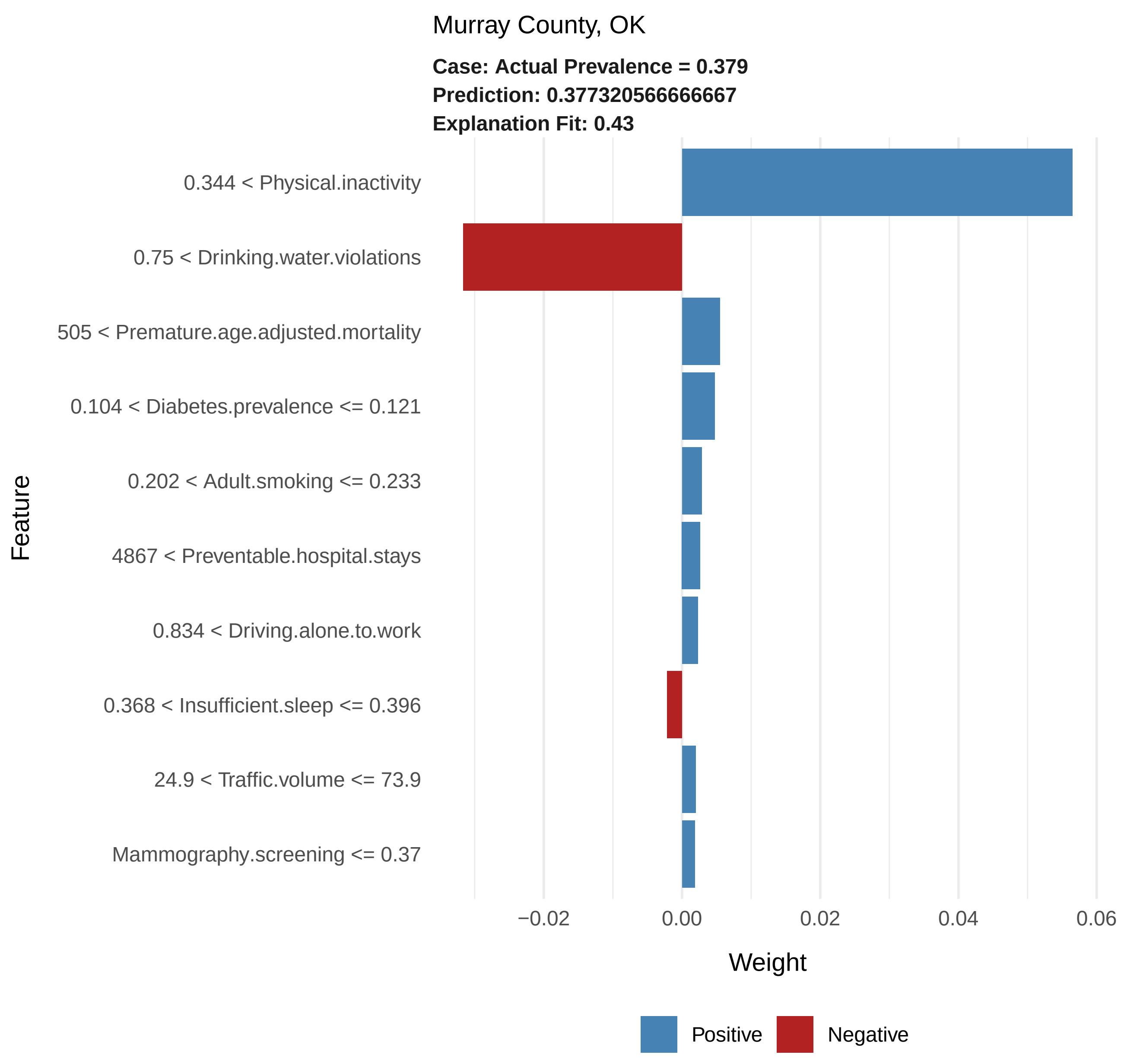

Murray County, OK
Case: Actual Prevalence = 0.379
Prediction: 0.377320566666667
Explanation Fit: 0.43
0.344 < Physical.inactivity
0.75 < Drinking.water.violations
505 < Premature.age.adjusted.mortality
0.104 < Diabetes.prevalence <= 0.121
0.202 < Adult.smoking <= 0.233
4867 < Preventable.hospital.stays
0.834 < Driving.alone.to.work
0.368 < Insufficient.sleep <= 0.396
24.9 < Traffic.volume <= 73.9
Mammography.screening <= 0.37
Feature
−0.02
0.00
0.02
0.04
0.06
Weight
Positive
Negative

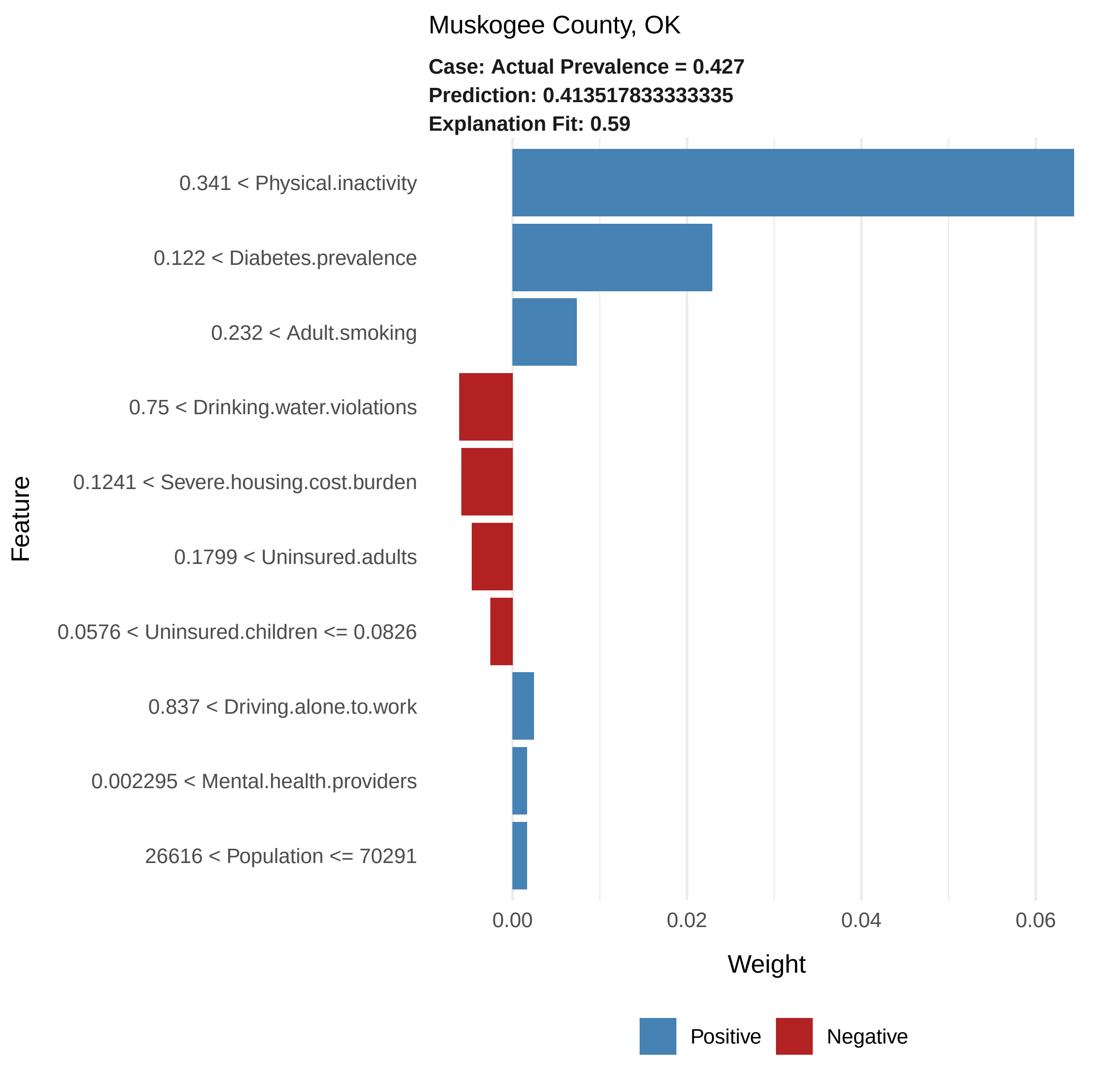

Muskogee County, OK
Case: Actual Prevalence = 0.427
Prediction: 0.413517833333335
Explanation Fit: 0.59
0.341 < Physical.inactivity
0.122 < Diabetes.prevalence
0.232 < Adult.smoking
0.75 < Drinking.water.violations
0.1241 < Severe.housing.cost.burden
0.1799 < Uninsured.adults
0.0576 < Uninsured.children <= 0.0826
0.837 < Driving.alone.to.work
0.002295 < Mental.health.providers
26616 < Population <= 70291
Feature
0.00
0.02
0.04
0.06
Weight
Positive
Negative

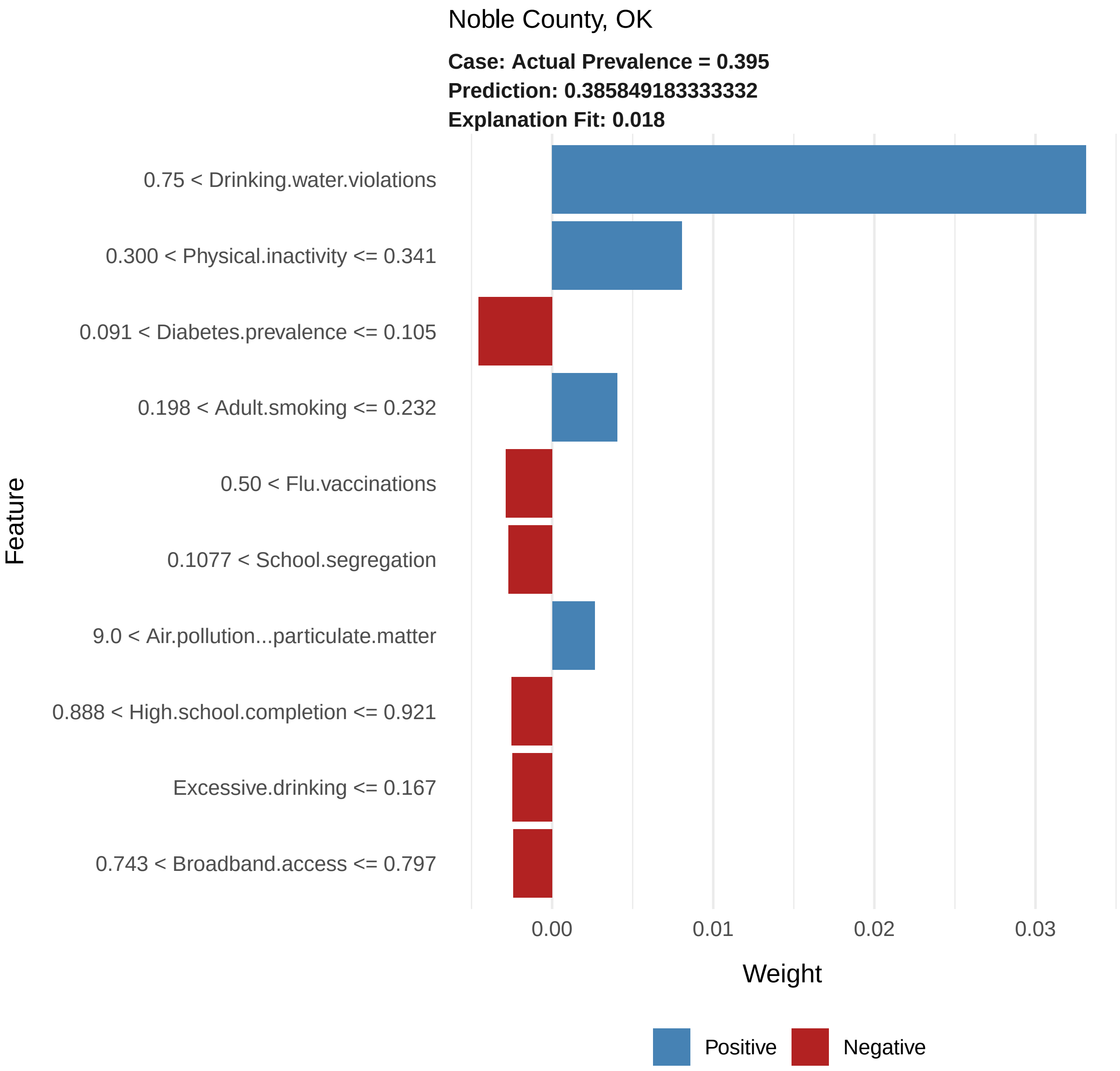
Noble County, OK
Case: Actual Prevalence = 0.395
Prediction: 0.385849183333332
Explanation Fit: 0.018
0.75 < Drinking.water.violations
0.300 < Physical.inactivity <= 0.341
0.091 < Diabetes.prevalence <= 0.105
0.198 < Adult.smoking <= 0.232
0.50 < Flu.vaccinations
0.1077 < School.segregation
9.0 < Air.pollution...particulate.matter
0.888 < High.school.completion <= 0.921
Excessive.drinking <= 0.167
0.743 < Broadband.access <= 0.797
Feature
0.00
0.01
0.02
0.03
Weight
Positive
Negative

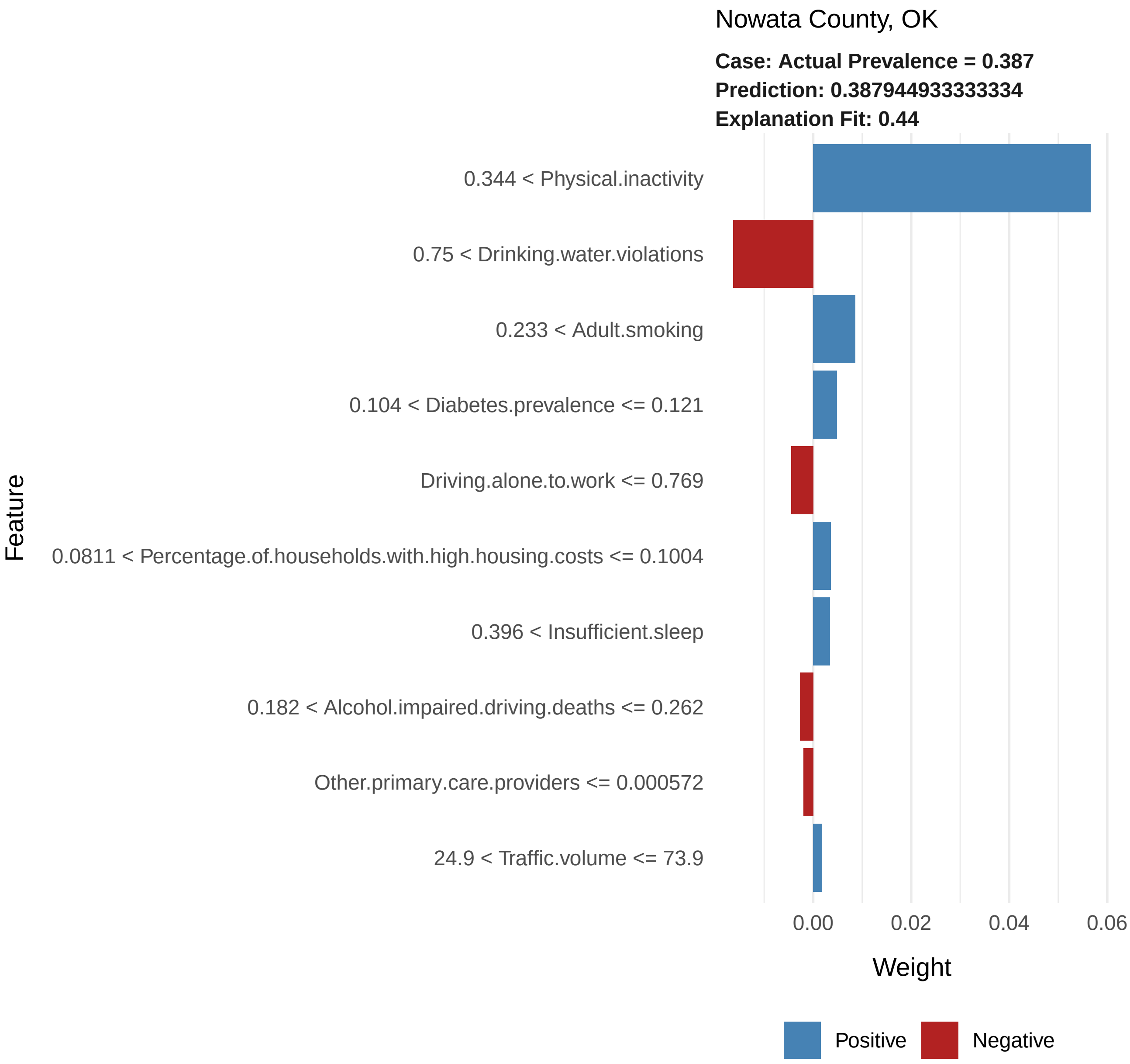
Nowata County, OK
Case: Actual Prevalence = 0.387
Prediction: 0.387944933333334
Explanation Fit: 0.44
0.344 < Physical.inactivity
0.75 < Drinking.water.violations
0.233 < Adult.smoking
0.104 < Diabetes.prevalence <= 0.121
Driving.alone.to.work <= 0.769
0.0811 < Percentage.of.households.with.high.housing.costs <= 0.1004
0.396 < Insufficient.sleep
0.182 < Alcohol.impaired.driving.deaths <= 0.262
Other.primary.care.providers <= 0.000572
24.9 < Traffic.volume <= 73.9
Feature
0.00
0.02
0.04
0.06
Weight
Positive
Negative

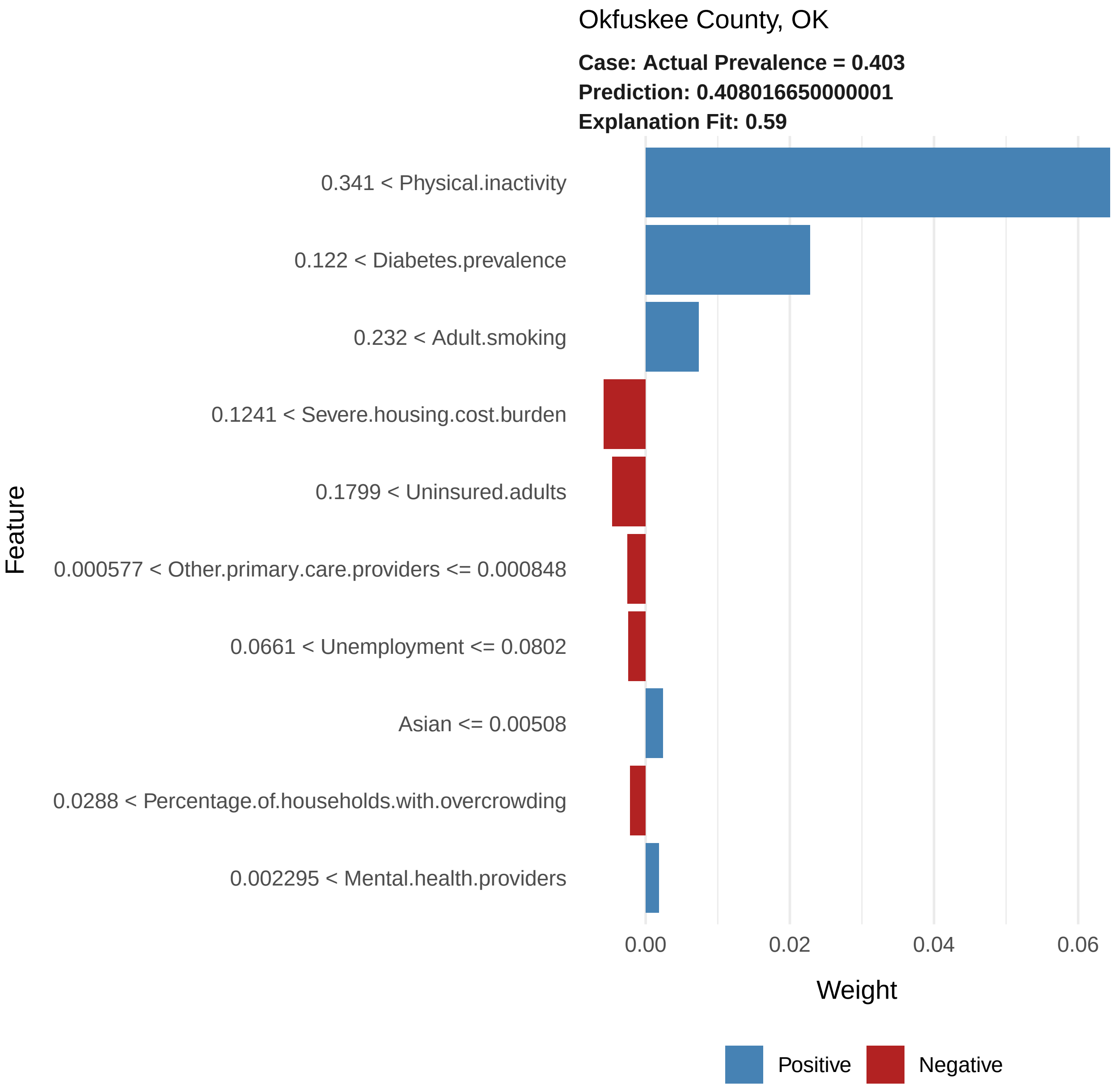
Okfuskee County, OK
Case: Actual Prevalence = 0.403
Prediction: 0.408016650000001
Explanation Fit: 0.59
0.341 < Physical.inactivity
0.122 < Diabetes.prevalence
0.232 < Adult.smoking
0.1241 < Severe.housing.cost.burden
0.1799 < Uninsured.adults
0.000577 < Other.primary.care.providers <= 0.000848
0.0661 < Unemployment <= 0.0802
Asian <= 0.00508
0.0288 < Percentage.of.households.with.overcrowding
0.002295 < Mental.health.providers
Feature
0.00
0.02
0.04
0.06
Weight
Positive
Negative

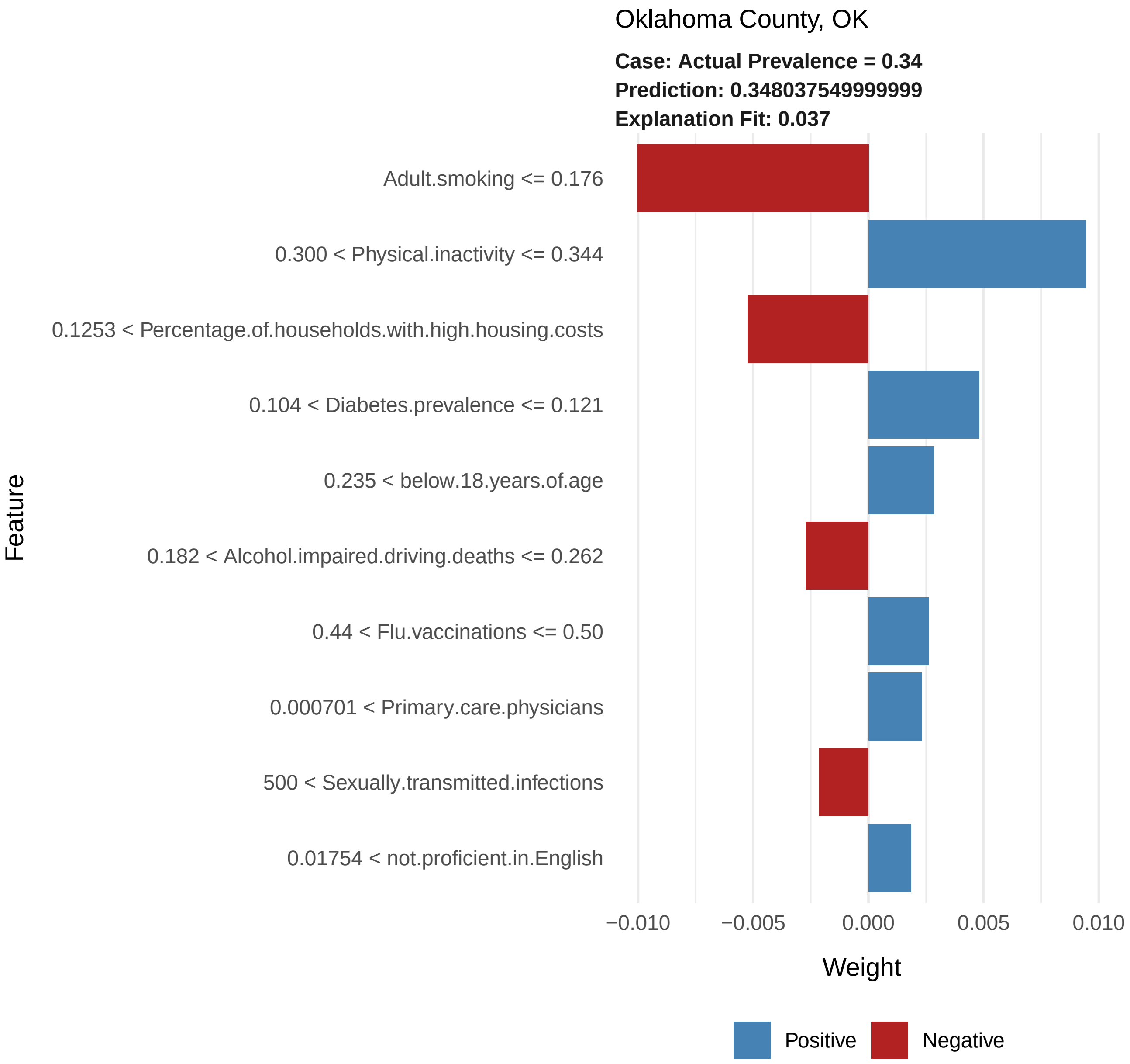
Oklahoma County, OK
Case: Actual Prevalence = 0.34
Prediction: 0.348037549999999
Explanation Fit: 0.037
Adult.smoking <= 0.176
0.300 < Physical.inactivity <= 0.344
0.1253 < Percentage.of.households.with.high.housing.costs
0.104 < Diabetes.prevalence <= 0.121
0.235 < below.18.years.of.age
0.182 < Alcohol.impaired.driving.deaths <= 0.262
0.44 < Flu.vaccinations <= 0.50
0.000701 < Primary.care.physicians
500 < Sexually.transmitted.infections
0.01754 < not.proficient.in.English
Feature
−0.010
−0.005
0.000
0.005
0.010
Weight
Positive
Negative

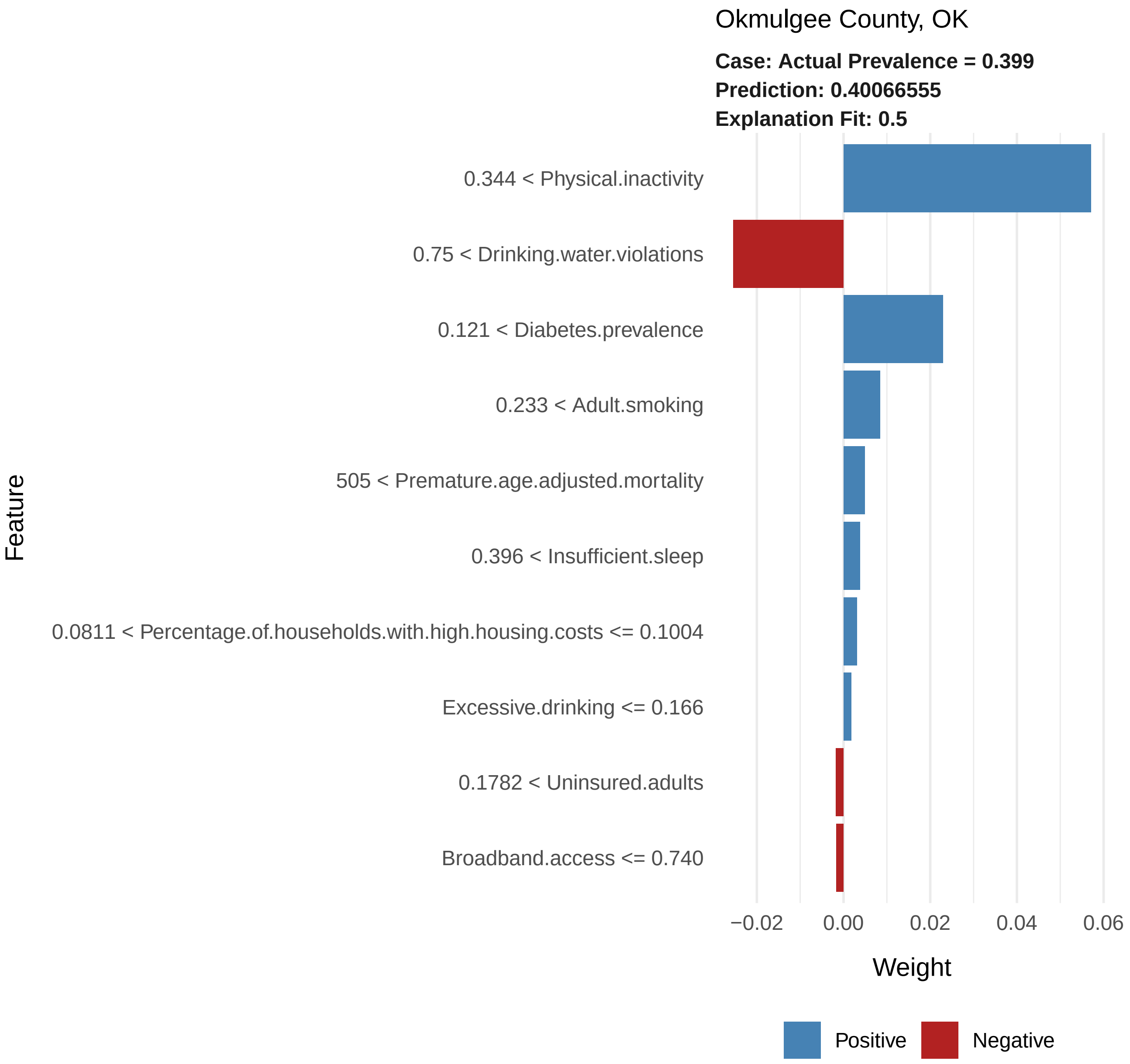

Okmulgee County, OK
Case: Actual Prevalence = 0.399
Prediction: 0.40066555
Explanation Fit: 0.5
0.344 < Physical.inactivity
0.75 < Drinking.water.violations
0.121 < Diabetes.prevalence
0.233 < Adult.smoking
505 < Premature.age.adjusted.mortality
0.396 < Insufficient.sleep
0.0811 < Percentage.of.households.with.high.housing.costs <= 0.1004
Excessive.drinking <= 0.166
0.1782 < Uninsured.adults
Broadband.access <= 0.740
Feature
−0.02
0.00
0.02
0.04
0.06
Weight
Positive
Negative

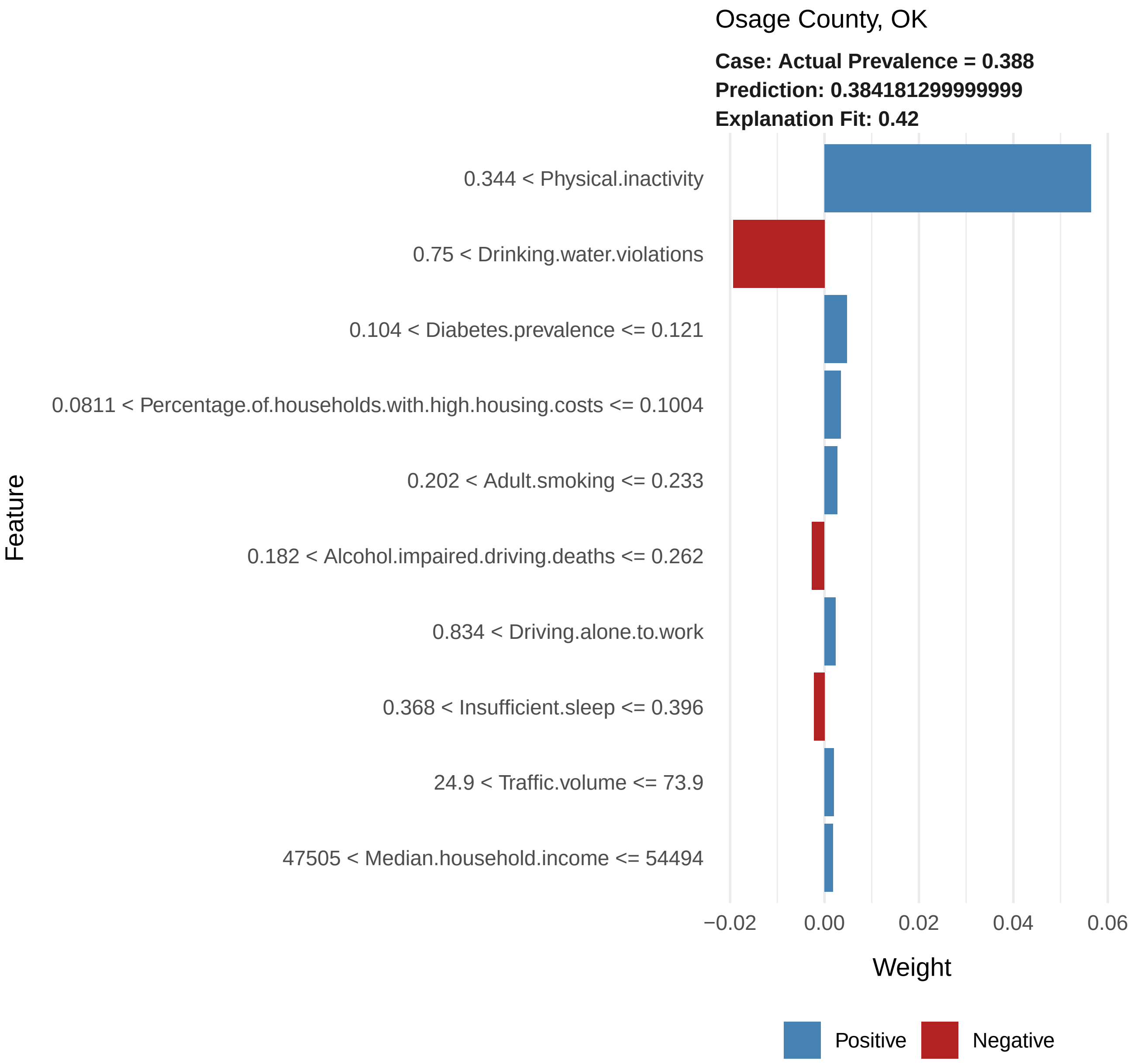

Osage County, OK
Case: Actual Prevalence = 0.388
Prediction: 0.384181299999999
Explanation Fit: 0.42
0.344 < Physical.inactivity
0.75 < Drinking.water.violations
0.104 < Diabetes.prevalence <= 0.121
0.0811 < Percentage.of.households.with.high.housing.costs <= 0.1004
0.202 < Adult.smoking <= 0.233
0.182 < Alcohol.impaired.driving.deaths <= 0.262
0.834 < Driving.alone.to.work
0.368 < Insufficient.sleep <= 0.396
24.9 < Traffic.volume <= 73.9
47505 < Median.household.income <= 54494
Feature
−0.02
0.00
0.02
0.04
0.06
Weight
Positive
Negative

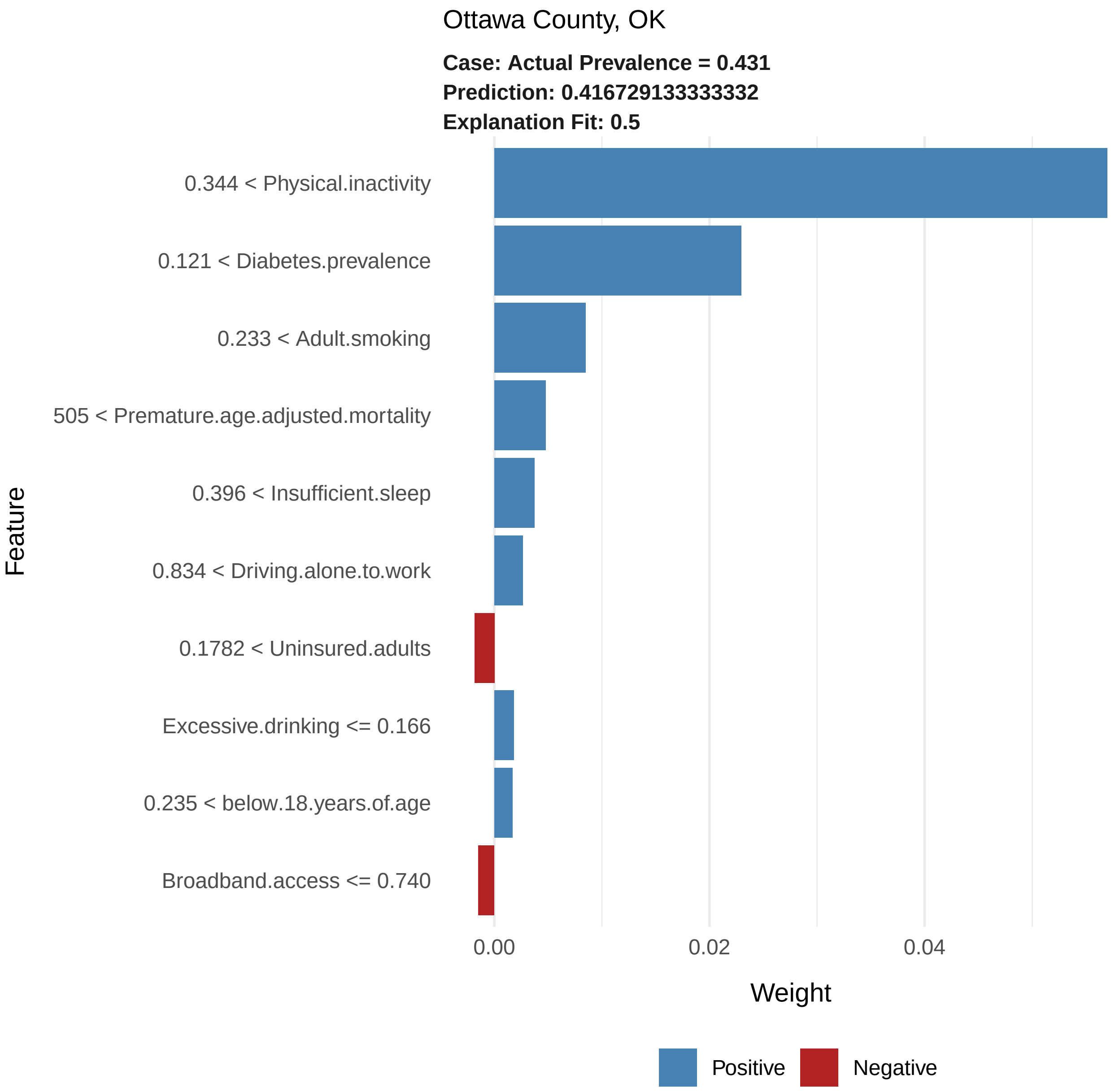

Ottawa County, OK
Case: Actual Prevalence = 0.431
Prediction: 0.416729133333332
Explanation Fit: 0.5
0.344 < Physical.inactivity
0.121 < Diabetes.prevalence
0.233 < Adult.smoking
505 < Premature.age.adjusted.mortality
0.396 < Insufficient.sleep
0.834 < Driving.alone.to.work
0.1782 < Uninsured.adults
Excessive.drinking <= 0.166
0.235 < below.18.years.of.age
Broadband.access <= 0.740
Feature
0.00
0.02
0.04
Weight
Positive
Negative

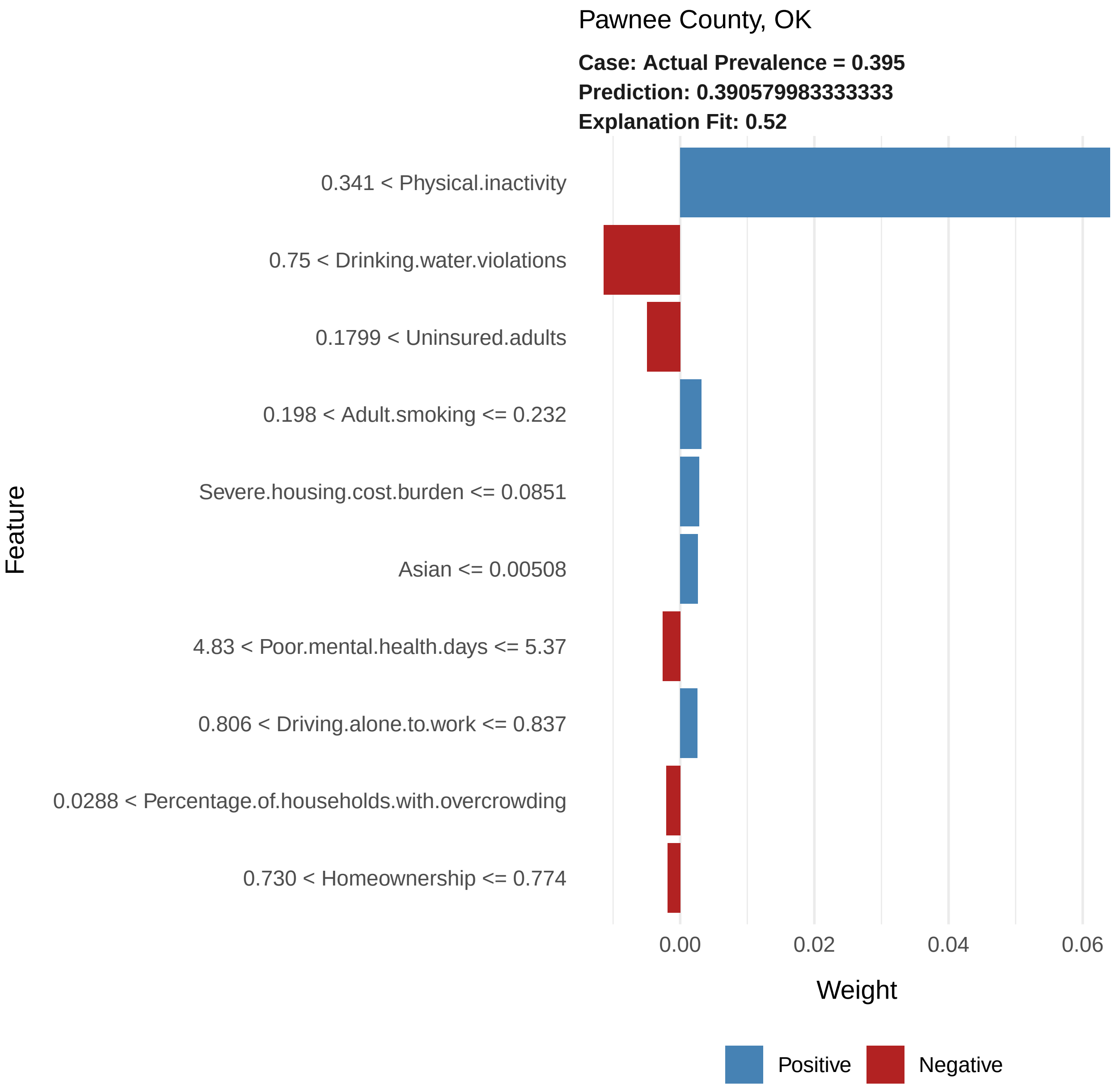

Pawnee County, OK
Case: Actual Prevalence = 0.395
Prediction: 0.390579983333333
Explanation Fit: 0.52
0.341 < Physical.inactivity
0.75 < Drinking.water.violations
0.1799 < Uninsured.adults
0.198 < Adult.smoking <= 0.232
Severe.housing.cost.burden <= 0.0851
Asian <= 0.00508
4.83 < Poor.mental.health.days <= 5.37
0.806 < Driving.alone.to.work <= 0.837
0.0288 < Percentage.of.households.with.overcrowding
0.730 < Homeownership <= 0.774
Feature
0.00
0.02
0.04
0.06
Weight
Positive
Negative

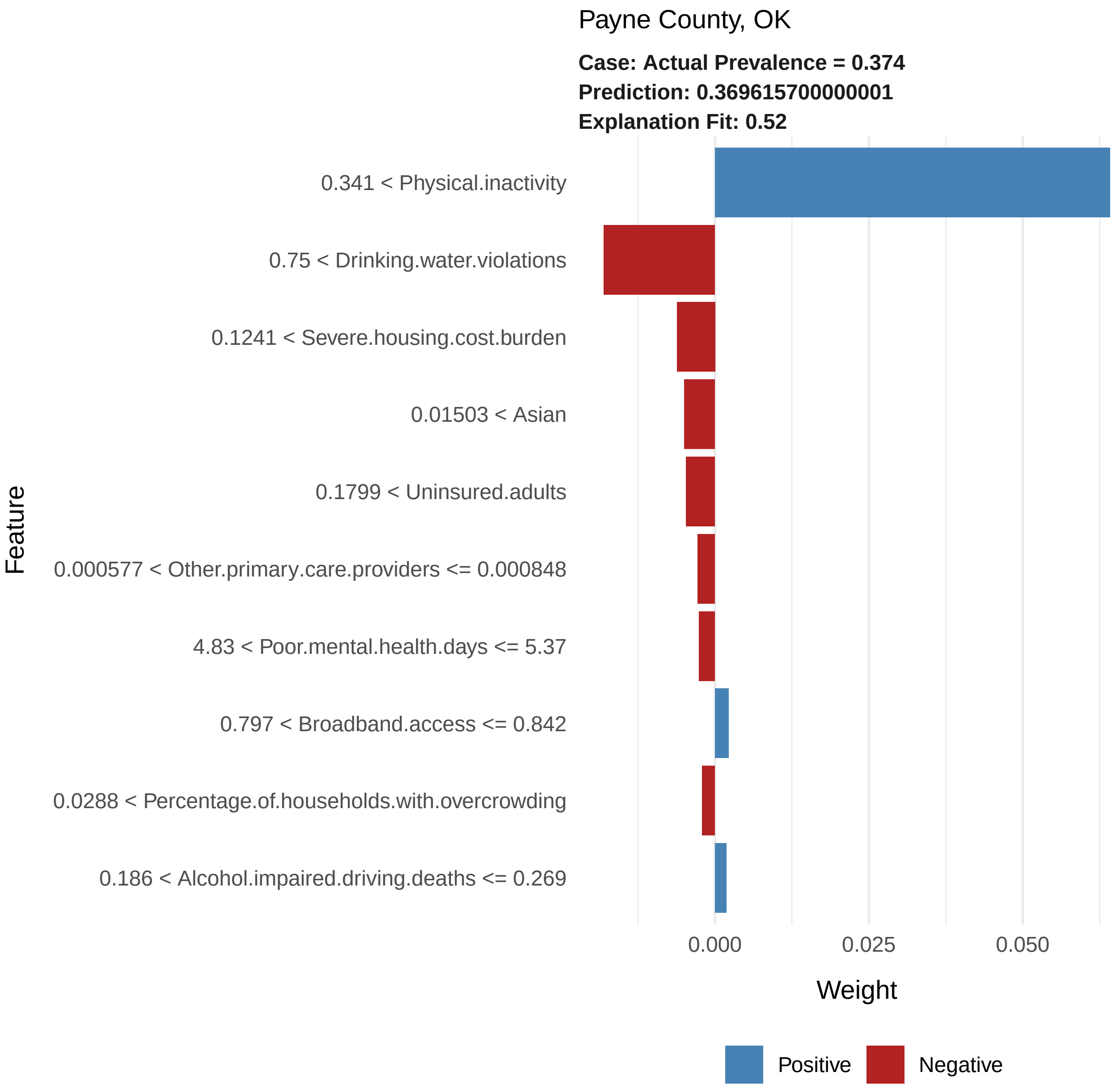
Payne County, OK
Case: Actual Prevalence = 0.374
Prediction: 0.369615700000001
Explanation Fit: 0.52
0.341 < Physical.inactivity
0.75 < Drinking.water.violations
0.1241 < Severe.housing.cost.burden
0.01503 < Asian
0.1799 < Uninsured.adults
0.000577 < Other.primary.care.providers <= 0.000848
4.83 < Poor.mental.health.days <= 5.37
0.797 < Broadband.access <= 0.842
0.0288 < Percentage.of.households.with.overcrowding
0.186 < Alcohol.impaired.driving.deaths <= 0.269
Feature
0.000
0.025
0.050
Weight
Positive
Negative

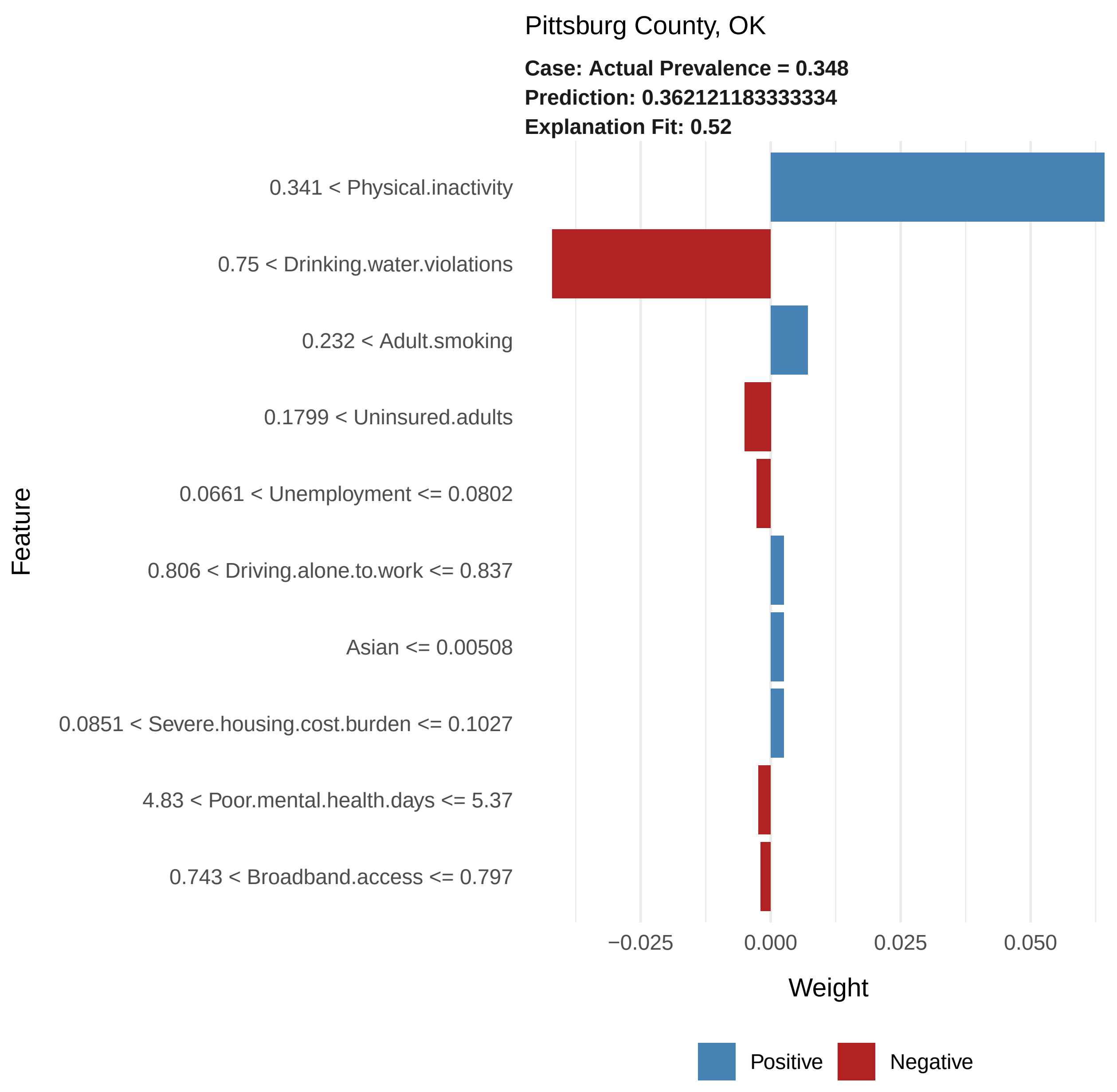
Pittsburg County, OK
Case: Actual Prevalence = 0.348
Prediction: 0.362121183333334
Explanation Fit: 0.52
0.341 < Physical.inactivity
0.75 < Drinking.water.violations
0.232 < Adult.smoking
0.1799 < Uninsured.adults
0.0661 < Unemployment <= 0.0802
0.806 < Driving.alone.to.work <= 0.837
Asian <= 0.00508
0.0851 < Severe.housing.cost.burden <= 0.1027
4.83 < Poor.mental.health.days <= 5.37
0.743 < Broadband.access <= 0.797
Feature
−0.025
0.000
0.025
0.050
Weight
Positive
Negative

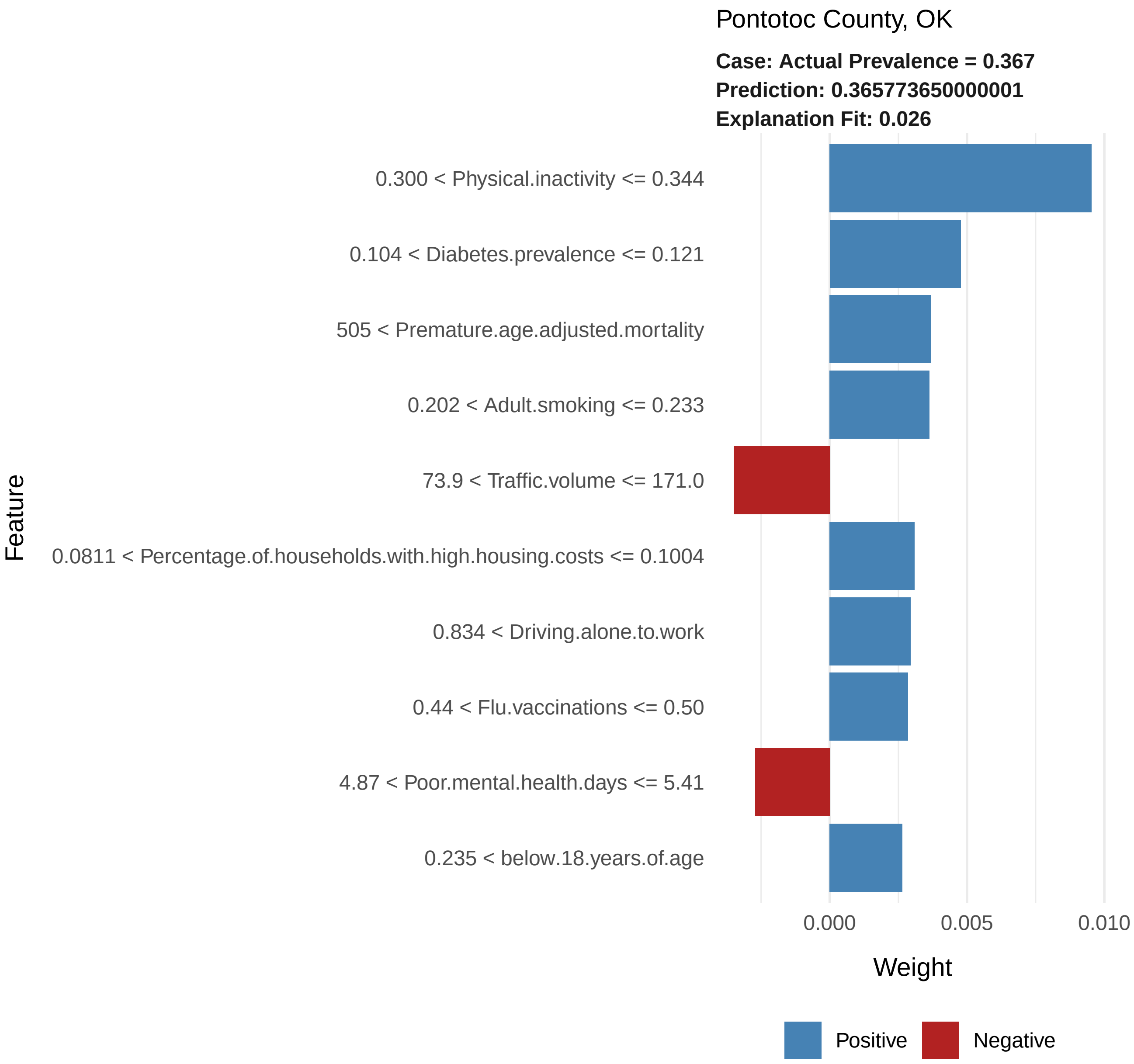

Pontotoc County, OK
Case: Actual Prevalence = 0.367
Prediction: 0.365773650000001
Explanation Fit: 0.026
0.300 < Physical.inactivity <= 0.344
0.104 < Diabetes.prevalence <= 0.121
505 < Premature.age.adjusted.mortality
0.202 < Adult.smoking <= 0.233
73.9 < Traffic.volume <= 171.0
0.0811 < Percentage.of.households.with.high.housing.costs <= 0.1004
0.834 < Driving.alone.to.work
0.44 < Flu.vaccinations <= 0.50
4.87 < Poor.mental.health.days <= 5.41
0.235 < below.18.years.of.age
Feature
0.000
0.005
0.010
Weight
Positive
Negative

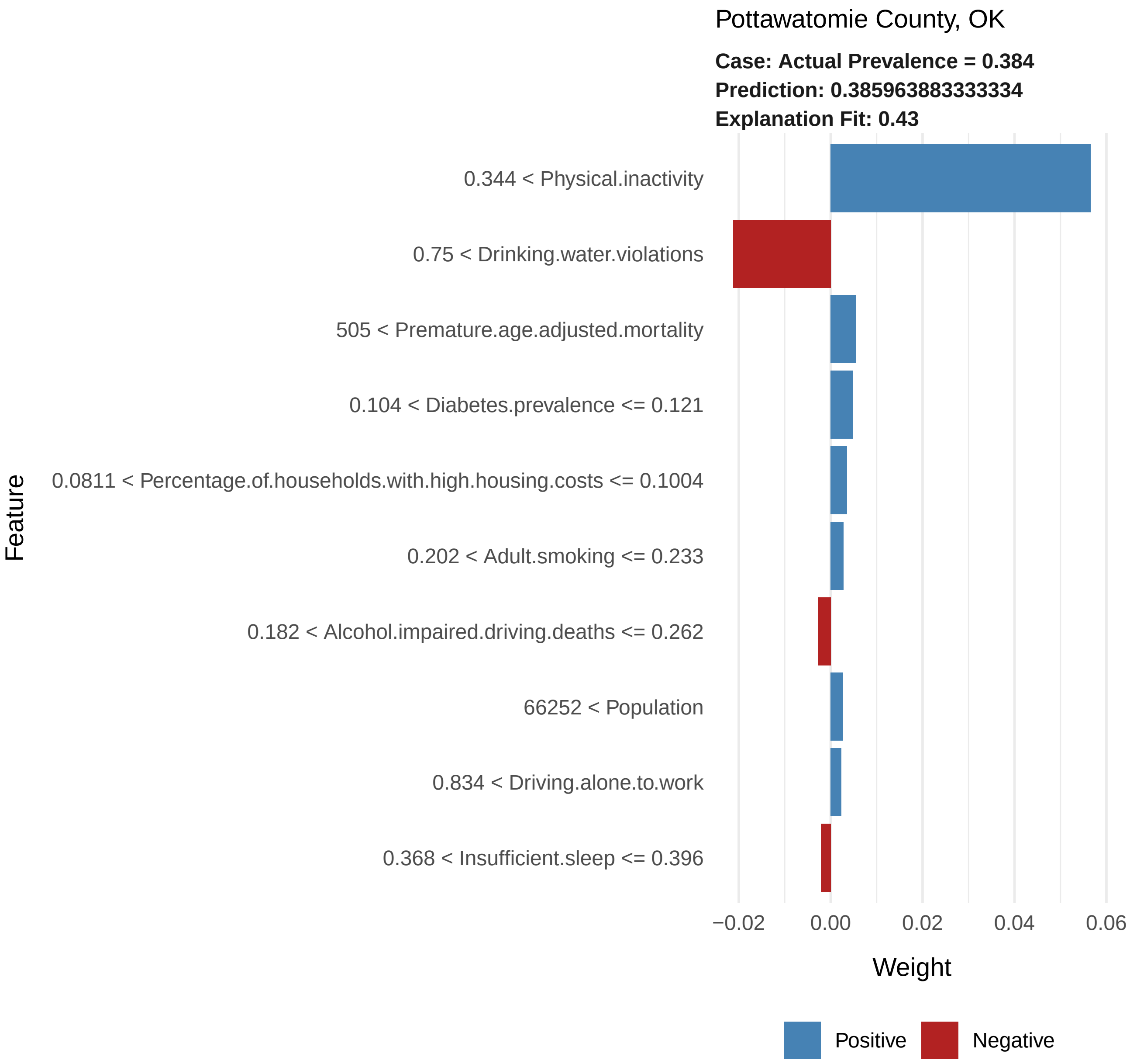

Pottawatomie County, OK
Case: Actual Prevalence = 0.384
Prediction: 0.385963883333334
Explanation Fit: 0.43
0.344 < Physical.inactivity
0.75 < Drinking.water.violations
505 < Premature.age.adjusted.mortality
0.104 < Diabetes.prevalence <= 0.121
0.0811 < Percentage.of.households.with.high.housing.costs <= 0.1004
0.202 < Adult.smoking <= 0.233
0.182 < Alcohol.impaired.driving.deaths <= 0.262
66252 < Population
0.834 < Driving.alone.to.work
0.368 < Insufficient.sleep <= 0.396
Feature
−0.02
0.00
0.02
0.04
0.06
Weight
Positive
Negative

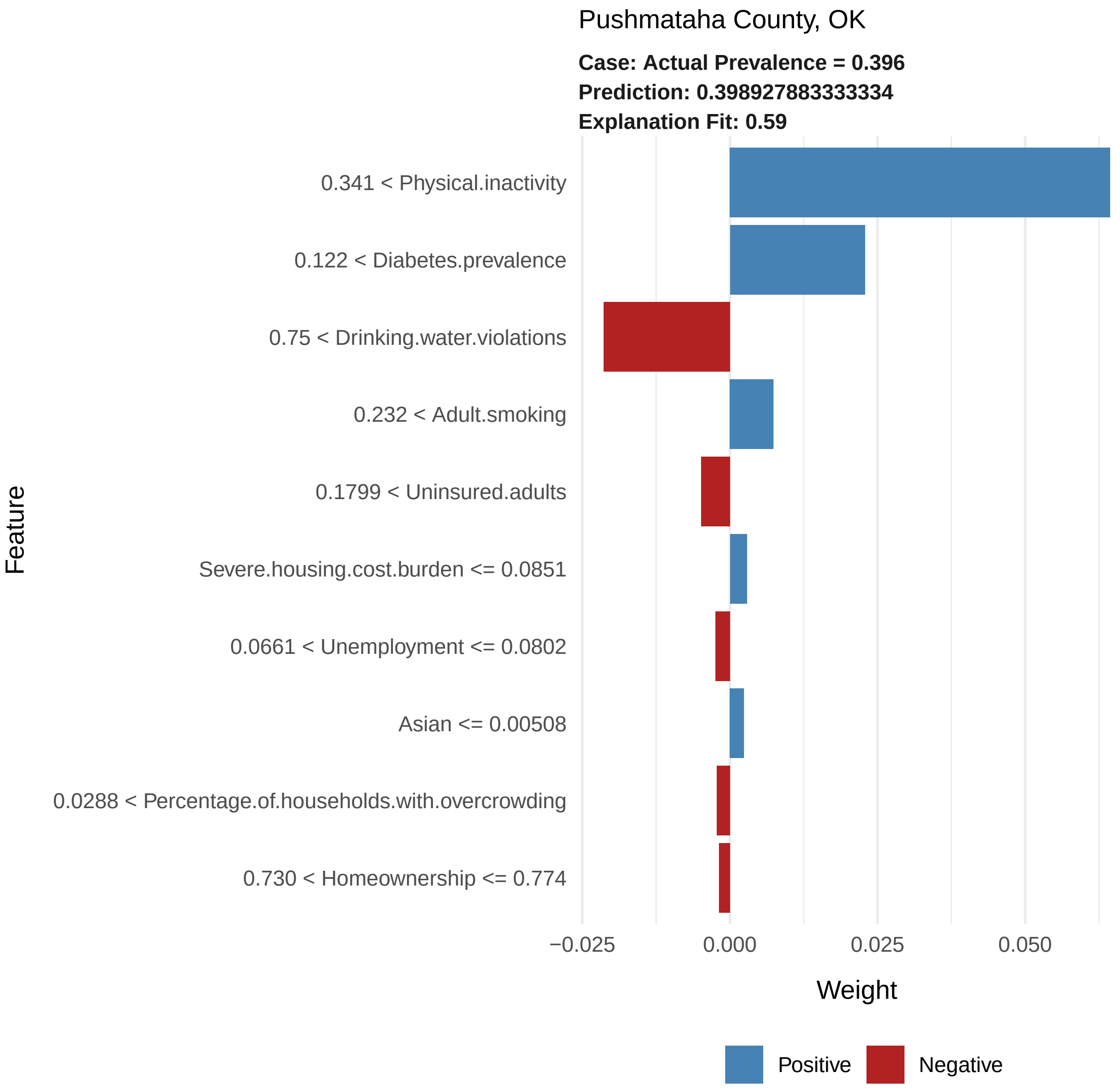
Pushmataha County, OK
Case: Actual Prevalence = 0.396
Prediction: 0.398927883333334
Explanation Fit: 0.59
0.341 < Physical.inactivity
0.122 < Diabetes.prevalence
0.75 < Drinking.water.violations
0.232 < Adult.smoking
0.1799 < Uninsured.adults
Severe.housing.cost.burden <= 0.0851
0.0661 < Unemployment <= 0.0802
Asian <= 0.00508
0.0288 < Percentage.of.households.with.overcrowding
0.730 < Homeownership <= 0.774
Feature
−0.025
0.000
0.025
0.050
Weight
Positive
Negative

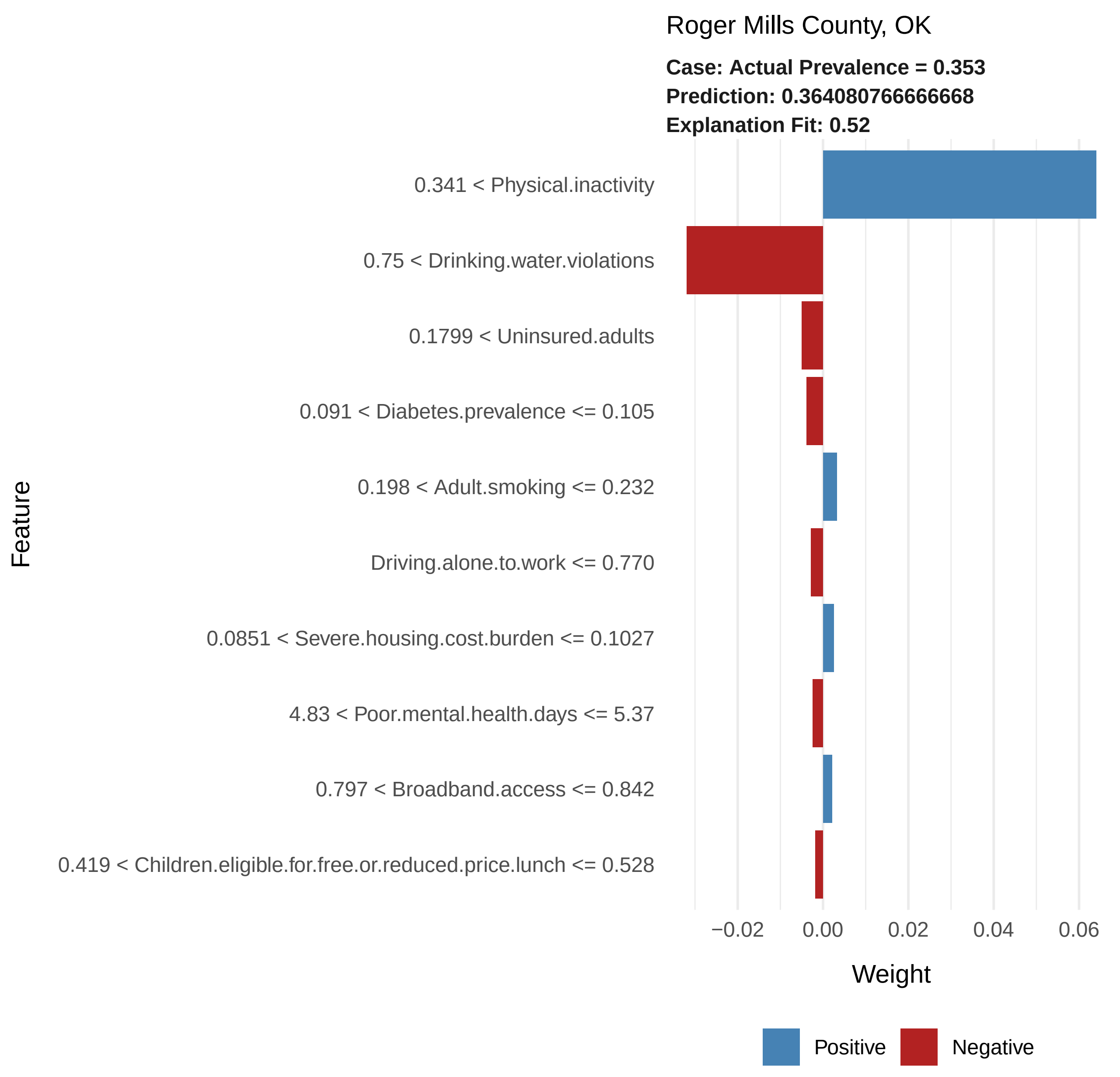
Roger Mills County, OK
Case: Actual Prevalence = 0.353
Prediction: 0.364080766666668
Explanation Fit: 0.52
0.341 < Physical.inactivity
0.75 < Drinking.water.violations
0.1799 < Uninsured.adults
0.091 < Diabetes.prevalence <= 0.105
0.198 < Adult.smoking <= 0.232
Driving.alone.to.work <= 0.770
0.0851 < Severe.housing.cost.burden <= 0.1027
4.83 < Poor.mental.health.days <= 5.37
0.797 < Broadband.access <= 0.842
0.419 < Children.eligible.for.free.or.reduced.price.lunch <= 0.528
Feature
−0.02
0.00
0.02
0.04
0.06
Weight
Positive
Negative

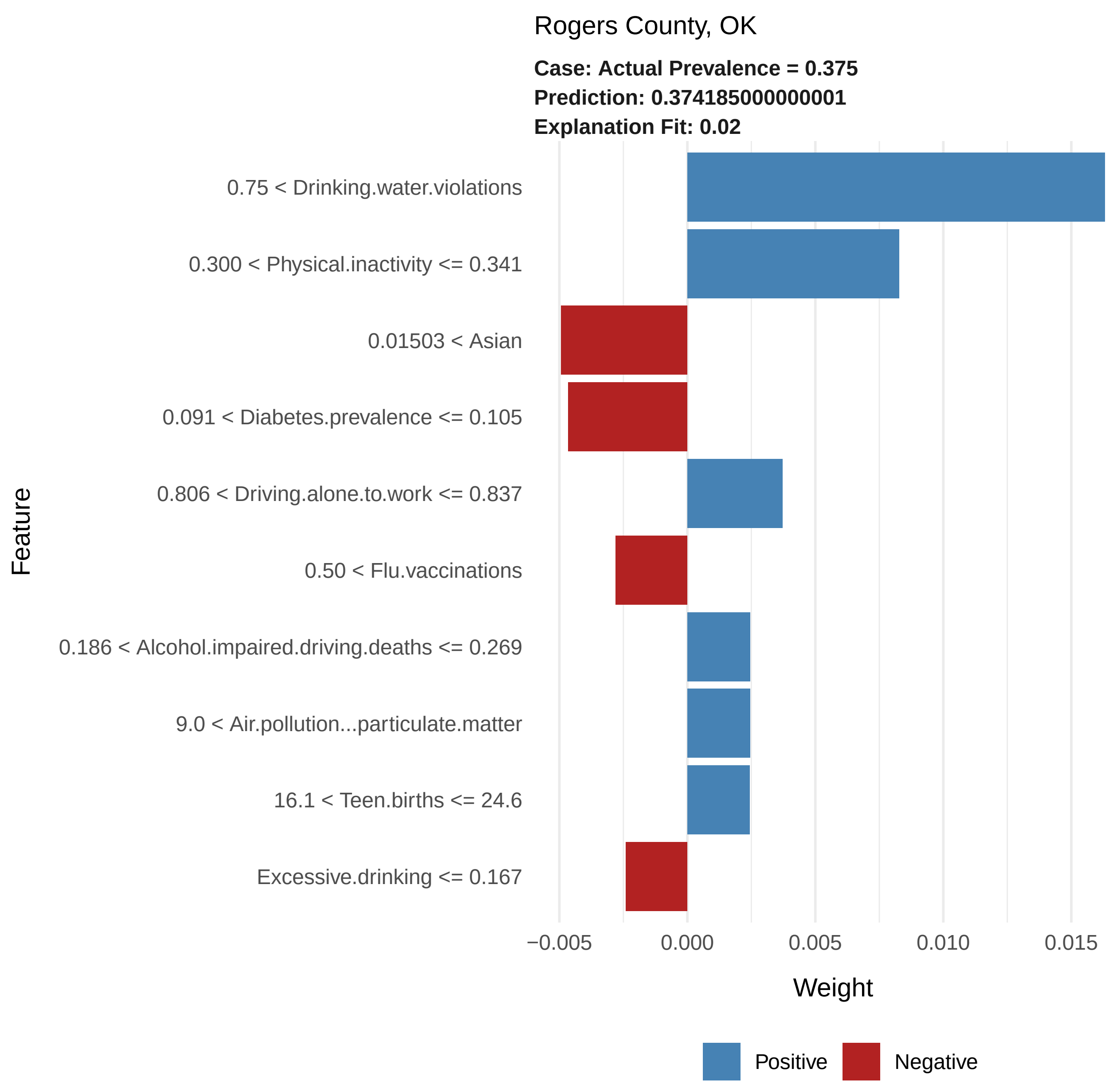

Rogers County, OK
Case: Actual Prevalence = 0.375
Prediction: 0.374185000000001
Explanation Fit: 0.02
0.75 < Drinking.water.violations
0.300 < Physical.inactivity <= 0.341
0.01503 < Asian
0.091 < Diabetes.prevalence <= 0.105
0.806 < Driving.alone.to.work <= 0.837
0.50 < Flu.vaccinations
0.186 < Alcohol.impaired.driving.deaths <= 0.269
9.0 < Air.pollution...particulate.matter
16.1 < Teen.births <= 24.6
Excessive.drinking <= 0.167
Feature
−0.005
0.000
0.005
0.010
0.015
Weight
Positive
Negative

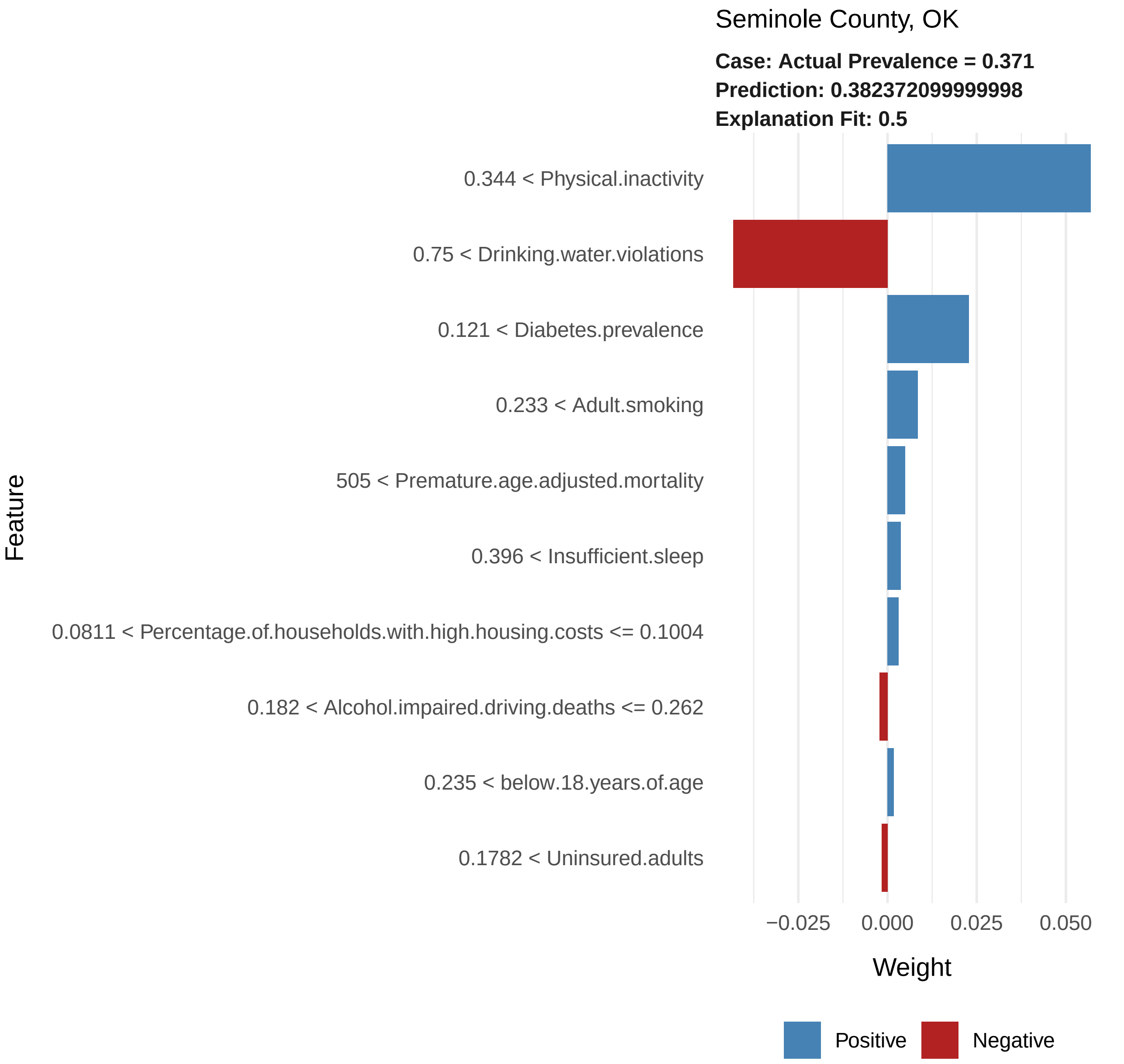

Seminole County, OK
Case: Actual Prevalence = 0.371
Prediction: 0.382372099999998
Explanation Fit: 0.5
0.344 < Physical.inactivity
0.75 < Drinking.water.violations
0.121 < Diabetes.prevalence
0.233 < Adult.smoking
505 < Premature.age.adjusted.mortality
0.396 < Insufficient.sleep
0.0811 < Percentage.of.households.with.high.housing.costs <= 0.1004
0.182 < Alcohol.impaired.driving.deaths <= 0.262
0.235 < below.18.years.of.age
0.1782 < Uninsured.adults
Feature
−0.025
0.000
0.025
0.050
Weight
Positive
Negative

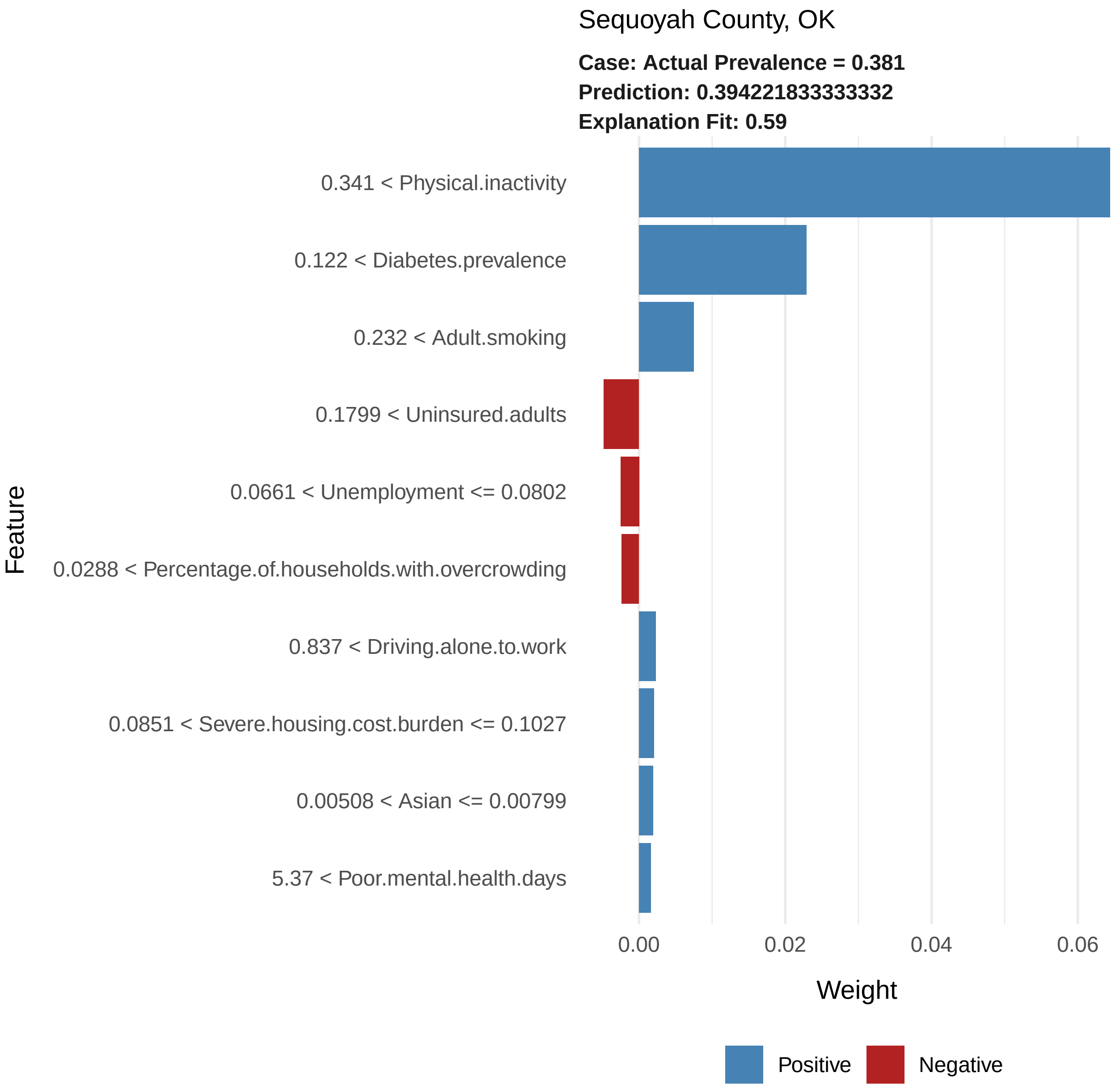

Sequoyah County, OK
Case: Actual Prevalence = 0.381
Prediction: 0.394221833333332
Explanation Fit: 0.59
0.341 < Physical.inactivity
0.122 < Diabetes.prevalence
0.232 < Adult.smoking
0.1799 < Uninsured.adults
0.0661 < Unemployment <= 0.0802
0.0288 < Percentage.of.households.with.overcrowding
0.837 < Driving.alone.to.work
0.0851 < Severe.housing.cost.burden <= 0.1027
0.00508 < Asian <= 0.00799
5.37 < Poor.mental.health.days
Feature
0.00
0.02
0.04
0.06
Weight
Positive
Negative

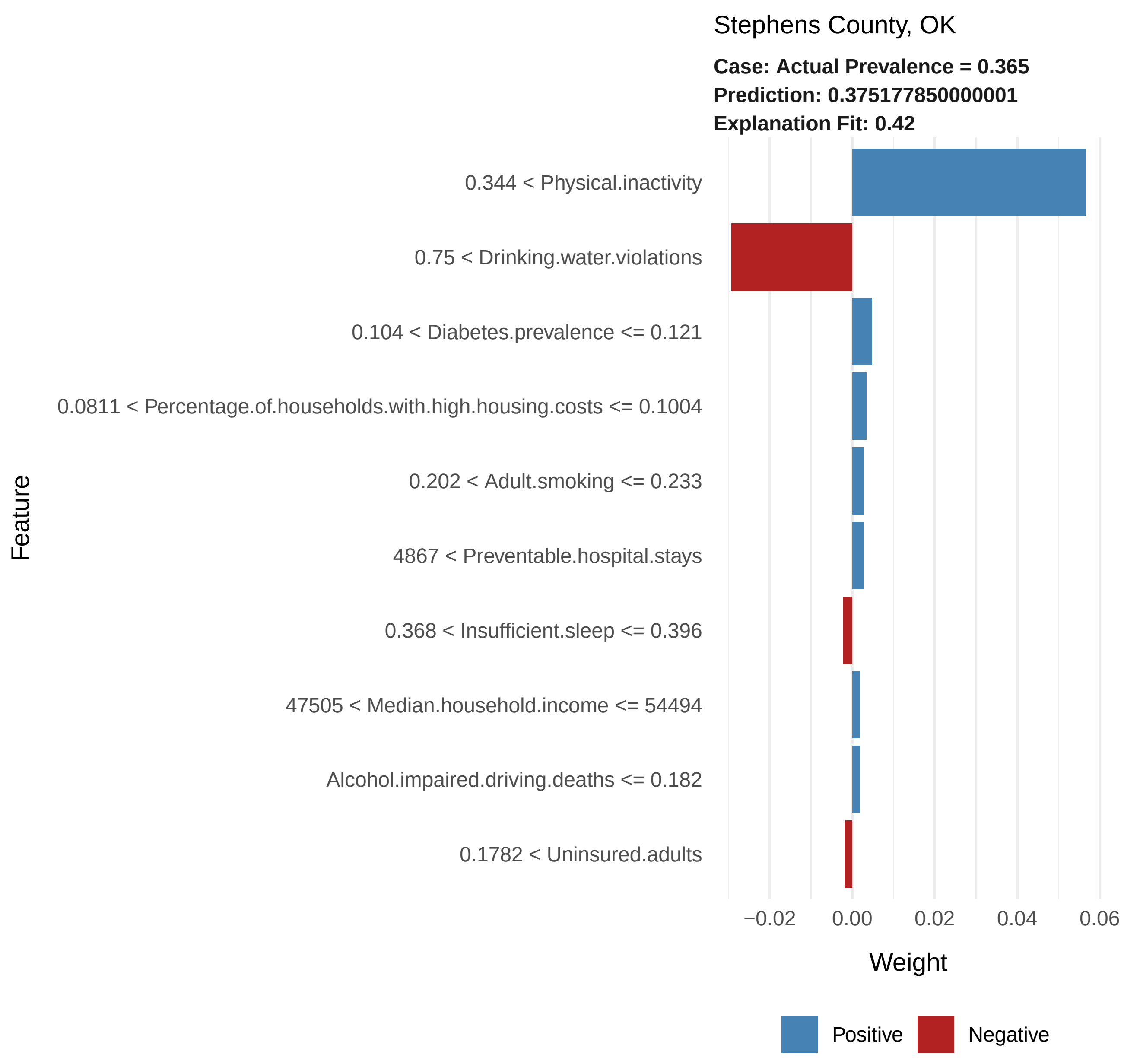

Stephens County, OK
Case: Actual Prevalence = 0.365
Prediction: 0.375177850000001
Explanation Fit: 0.42
0.344 < Physical.inactivity
0.75 < Drinking.water.violations
0.104 < Diabetes.prevalence <= 0.121
0.0811 < Percentage.of.households.with.high.housing.costs <= 0.1004
0.202 < Adult.smoking <= 0.233
4867 < Preventable.hospital.stays
0.368 < Insufficient.sleep <= 0.396
47505 < Median.household.income <= 54494
Alcohol.impaired.driving.deaths <= 0.182
0.1782 < Uninsured.adults
Feature
−0.02
0.00
0.02
0.04
0.06
Weight
Positive
Negative

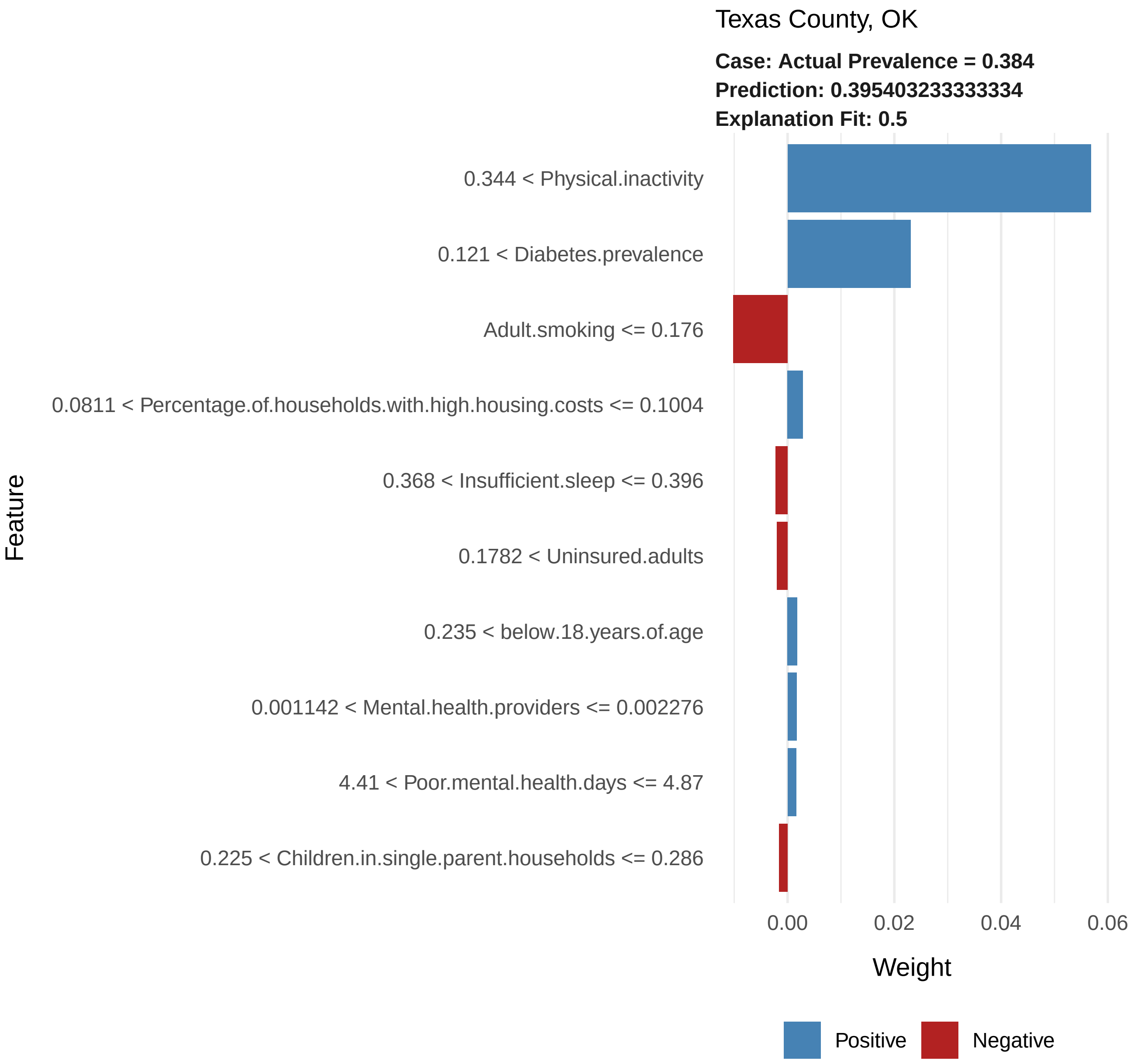
Texas County, OK
Case: Actual Prevalence = 0.384
Prediction: 0.395403233333334
Explanation Fit: 0.5
0.344 < Physical.inactivity
0.121 < Diabetes.prevalence
Adult.smoking <= 0.176
0.0811 < Percentage.of.households.with.high.housing.costs <= 0.1004
0.368 < Insufficient.sleep <= 0.396
0.1782 < Uninsured.adults
0.235 < below.18.years.of.age
0.001142 < Mental.health.providers <= 0.002276
4.41 < Poor.mental.health.days <= 4.87
0.225 < Children.in.single.parent.households <= 0.286
Feature
0.00
0.02
0.04
0.06
Weight
Positive
Negative

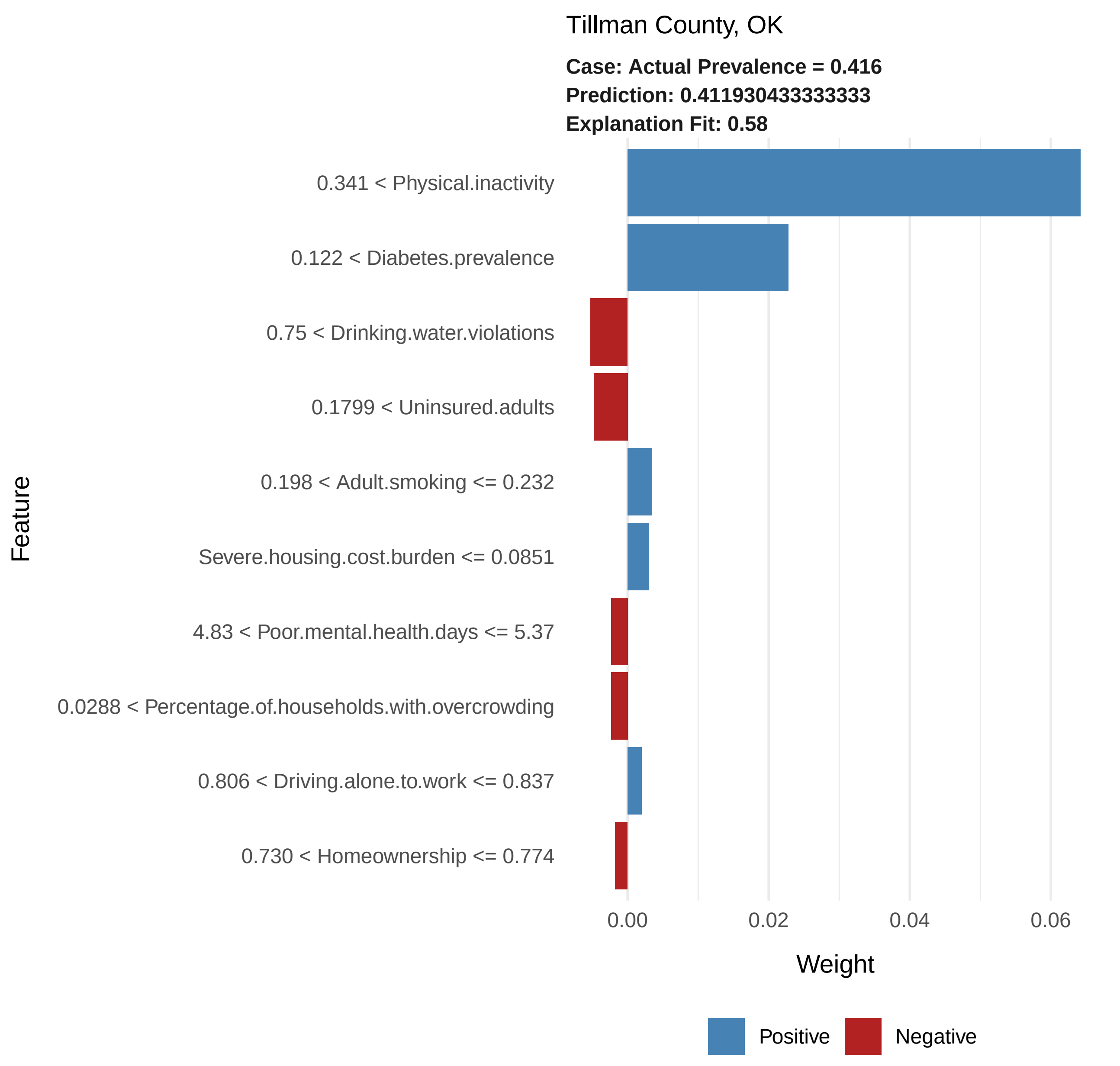

Tillman County, OK
Case: Actual Prevalence = 0.416
Prediction: 0.411930433333333
Explanation Fit: 0.58
0.341 < Physical.inactivity
0.122 < Diabetes.prevalence
0.75 < Drinking.water.violations
0.1799 < Uninsured.adults
0.198 < Adult.smoking <= 0.232
Severe.housing.cost.burden <= 0.0851
4.83 < Poor.mental.health.days <= 5.37
0.0288 < Percentage.of.households.with.overcrowding
0.806 < Driving.alone.to.work <= 0.837
0.730 < Homeownership <= 0.774
Feature
0.00
0.02
0.04
0.06
Weight
Positive
Negative

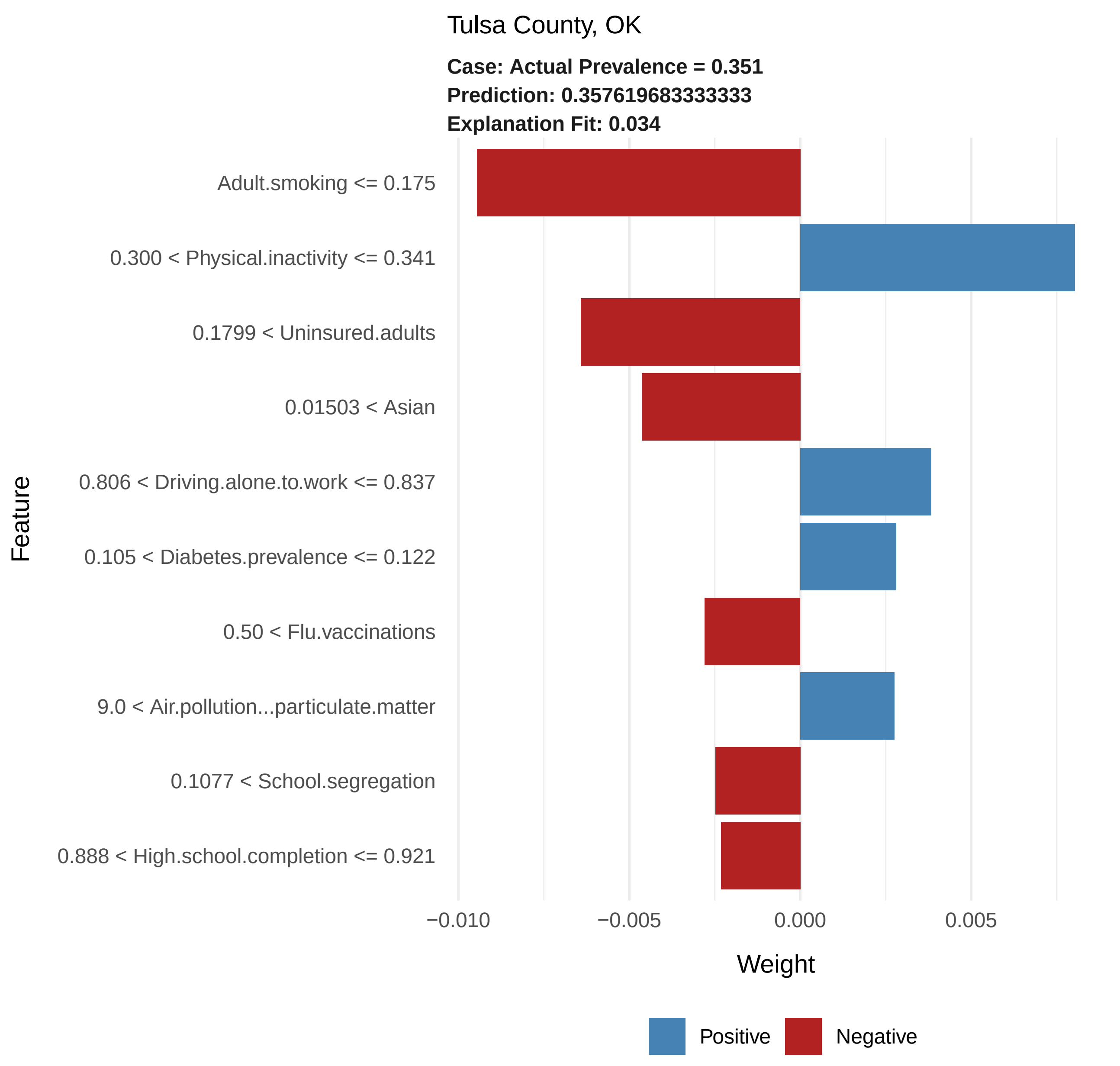
Tulsa County, OK
Case: Actual Prevalence = 0.351
Prediction: 0.357619683333333
Explanation Fit: 0.034
Adult.smoking <= 0.175
0.300 < Physical.inactivity <= 0.341
0.1799 < Uninsured.adults
0.01503 < Asian
0.806 < Driving.alone.to.work <= 0.837
0.105 < Diabetes.prevalence <= 0.122
0.50 < Flu.vaccinations
9.0 < Air.pollution...particulate.matter
0.1077 < School.segregation
0.888 < High.school.completion <= 0.921
Feature
−0.010
−0.005
0.000
0.005
Weight
Positive
Negative

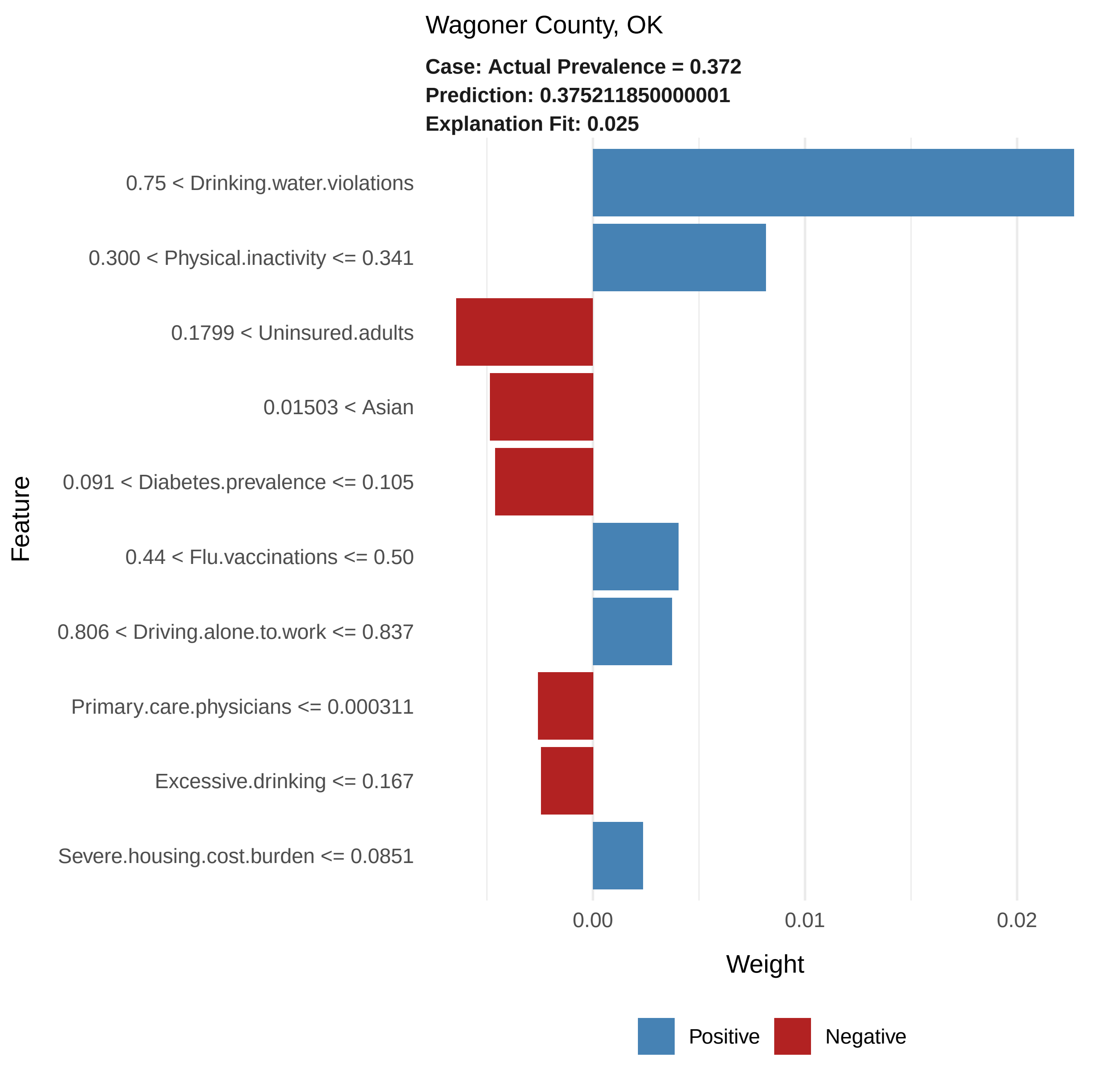
Wagoner County, OK
Case: Actual Prevalence = 0.372
Prediction: 0.375211850000001
Explanation Fit: 0.025
0.75 < Drinking.water.violations
0.300 < Physical.inactivity <= 0.341
0.1799 < Uninsured.adults
0.01503 < Asian
0.091 < Diabetes.prevalence <= 0.105
0.44 < Flu.vaccinations <= 0.50
0.806 < Driving.alone.to.work <= 0.837
Primary.care.physicians <= 0.000311
Excessive.drinking <= 0.167
Severe.housing.cost.burden <= 0.0851
Feature
0.00
0.01
0.02
Weight
Positive
Negative

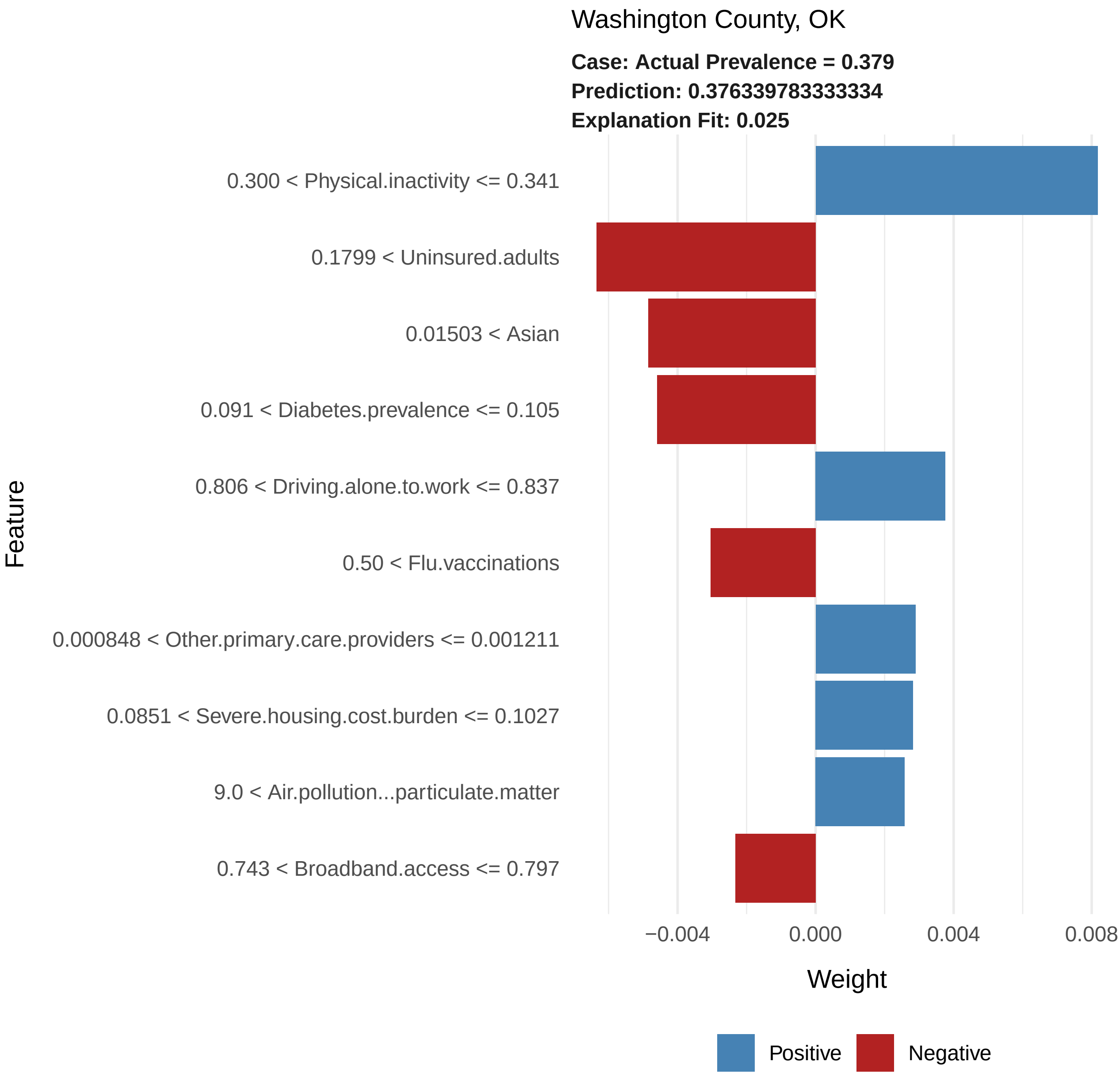

Washington County, OK
Case: Actual Prevalence = 0.379
Prediction: 0.376339783333334
Explanation Fit: 0.025
0.300 < Physical.inactivity <= 0.341
0.1799 < Uninsured.adults
0.01503 < Asian
0.091 < Diabetes.prevalence <= 0.105
0.806 < Driving.alone.to.work <= 0.837
0.50 < Flu.vaccinations
0.000848 < Other.primary.care.providers <= 0.001211
0.0851 < Severe.housing.cost.burden <= 0.1027
9.0 < Air.pollution...particulate.matter
0.743 < Broadband.access <= 0.797
Feature
−0.004
0.000
0.004
0.008
Weight
Positive
Negative

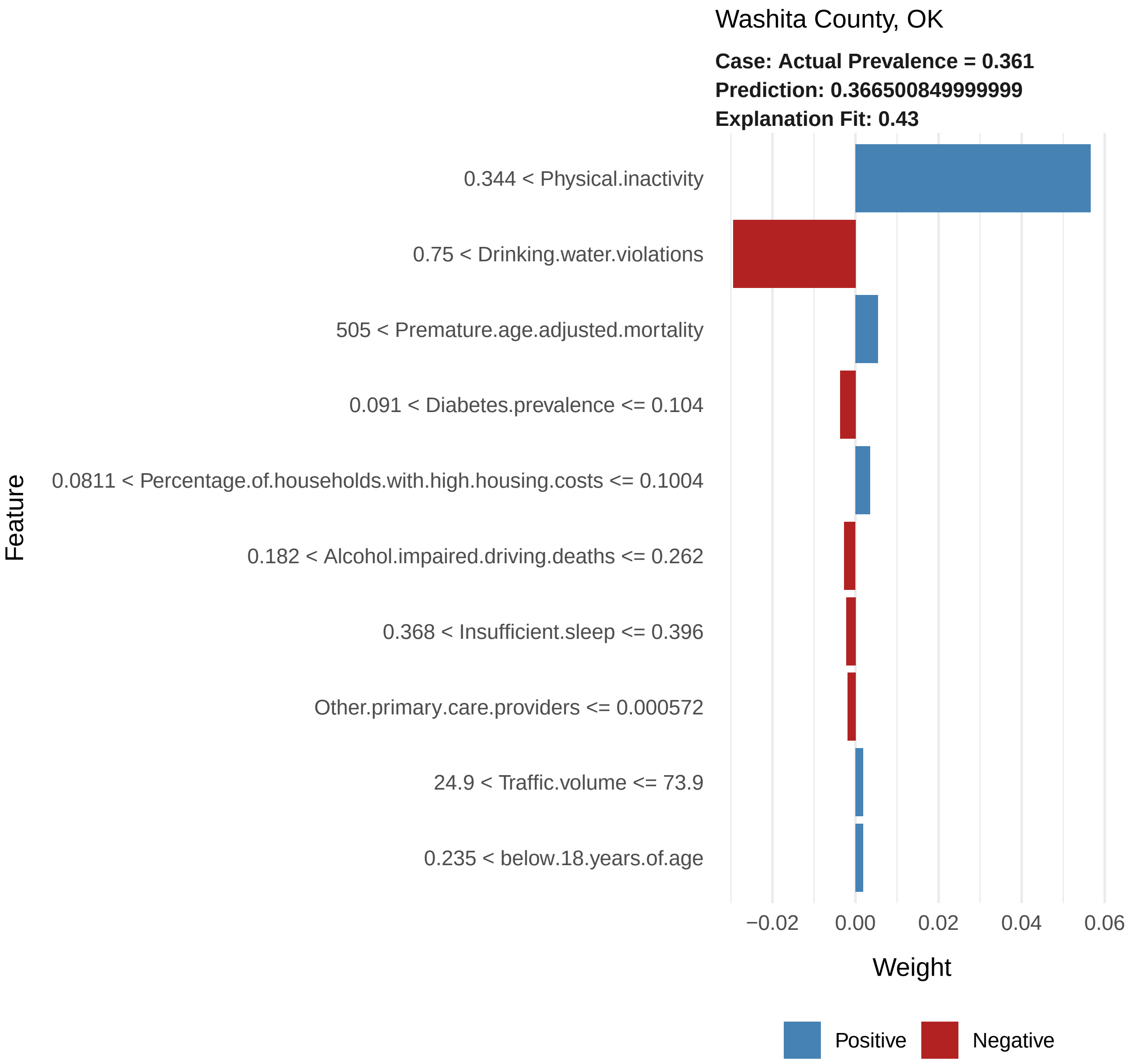

Washita County, OK
Case: Actual Prevalence = 0.361
Prediction: 0.366500849999999
Explanation Fit: 0.43
0.344 < Physical.inactivity
0.75 < Drinking.water.violations
505 < Premature.age.adjusted.mortality
0.091 < Diabetes.prevalence <= 0.104
0.0811 < Percentage.of.households.with.high.housing.costs <= 0.1004
0.182 < Alcohol.impaired.driving.deaths <= 0.262
0.368 < Insufficient.sleep <= 0.396
Other.primary.care.providers <= 0.000572
24.9 < Traffic.volume <= 73.9
0.235 < below.18.years.of.age
Feature
−0.02
0.00
0.02
0.04
0.06
Weight
Positive
Negative

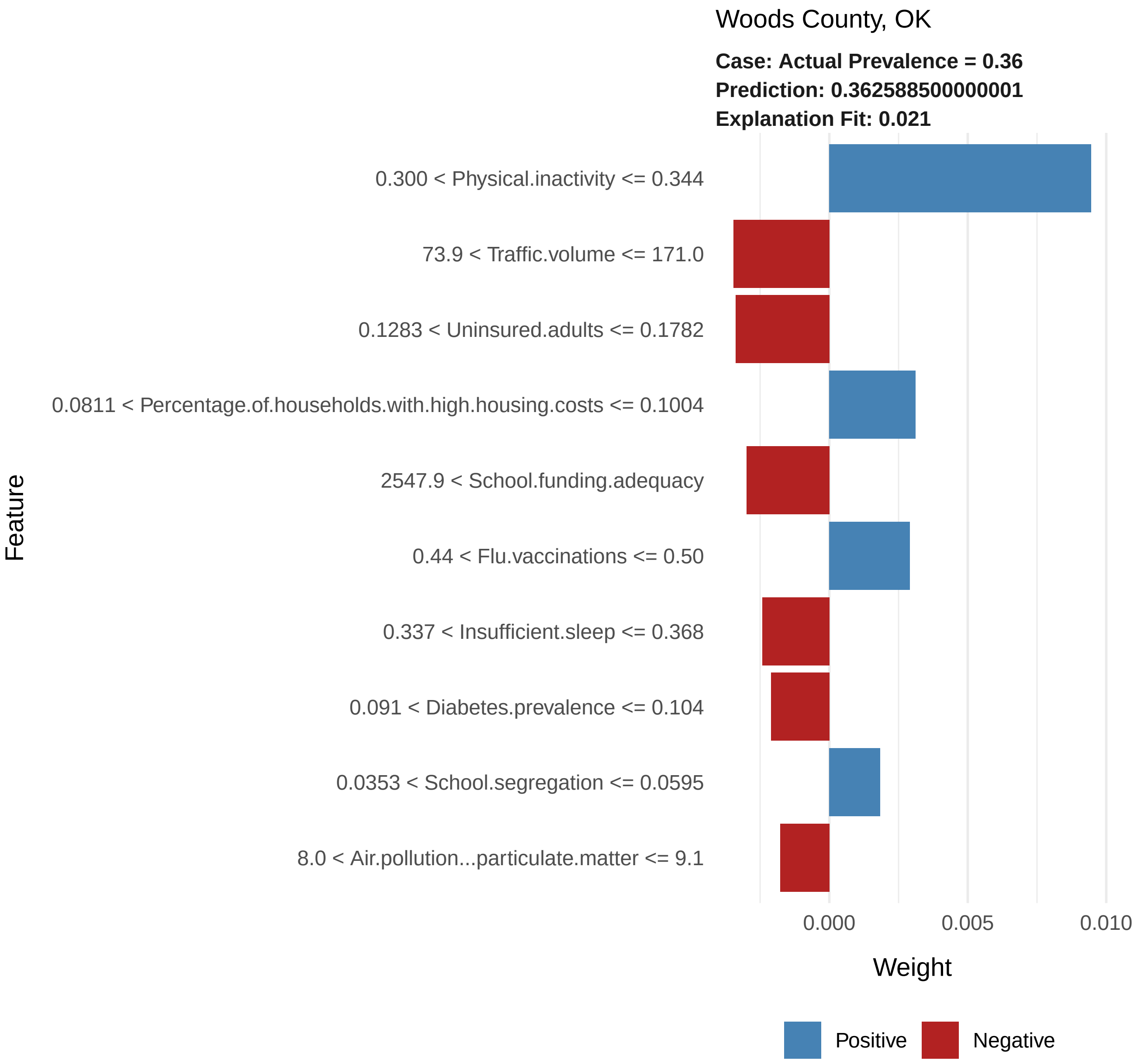

Woods County, OK
Case: Actual Prevalence = 0.36
Prediction: 0.362588500000001
Explanation Fit: 0.021
0.300 < Physical.inactivity <= 0.344
73.9 < Traffic.volume <= 171.0
0.1283 < Uninsured.adults <= 0.1782
0.0811 < Percentage.of.households.with.high.housing.costs <= 0.1004
2547.9 < School.funding.adequacy
0.44 < Flu.vaccinations <= 0.50
0.337 < Insufficient.sleep <= 0.368
0.091 < Diabetes.prevalence <= 0.104
0.0353 < School.segregation <= 0.0595
8.0 < Air.pollution...particulate.matter <= 9.1
Feature
0.000
0.005
0.010
Weight
Positive
Negative

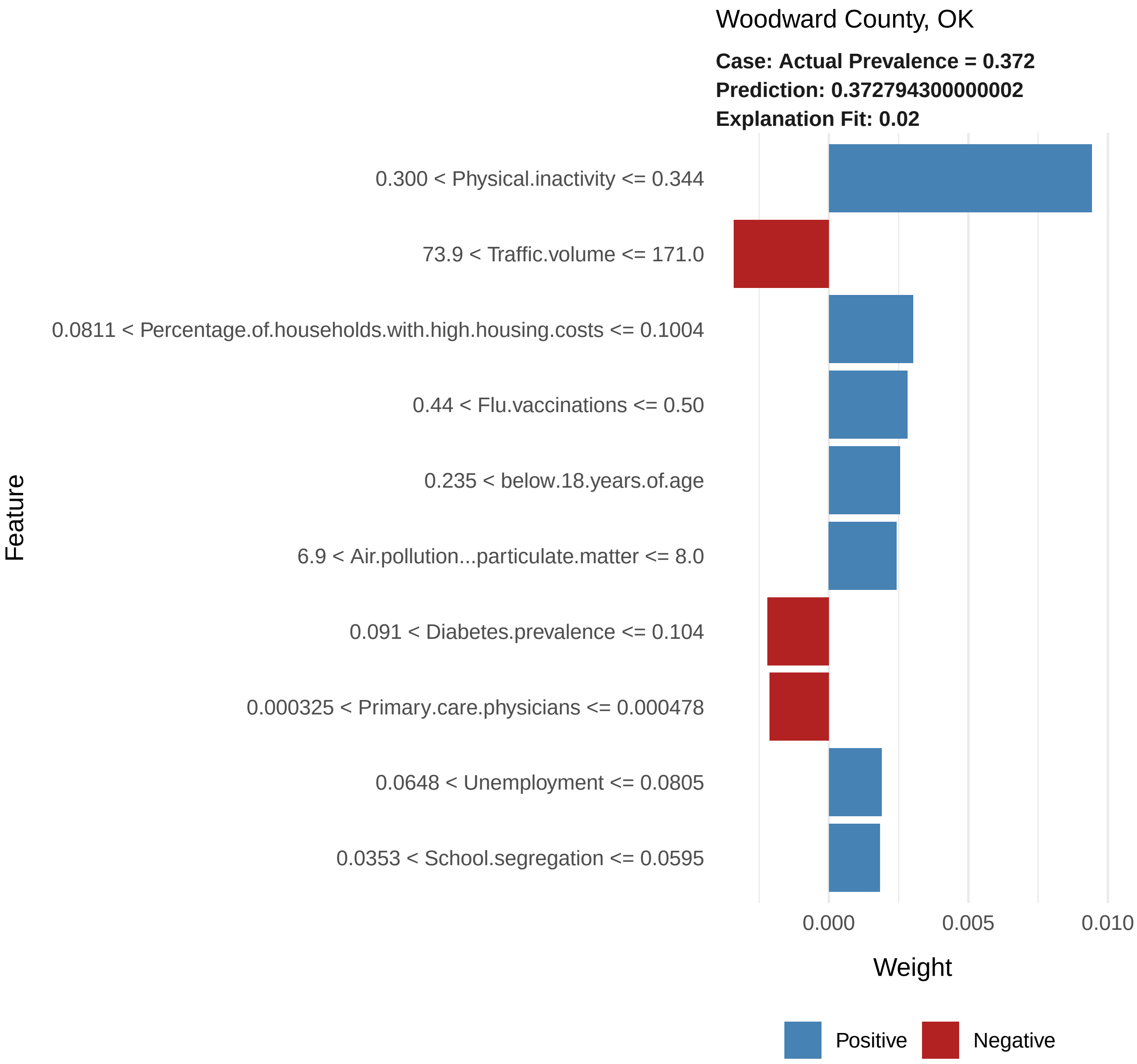
Woodward County, OK
Case: Actual Prevalence = 0.372
Prediction: 0.372794300000002
Explanation Fit: 0.02
0.300 < Physical.inactivity <= 0.344
73.9 < Traffic.volume <= 171.0
0.0811 < Percentage.of.households.with.high.housing.costs <= 0.1004
0.44 < Flu.vaccinations <= 0.50
0.235 < below.18.years.of.age
6.9 < Air.pollution...particulate.matter <= 8.0
0.091 < Diabetes.prevalence <= 0.104
0.000325 < Primary.care.physicians <= 0.000478
0.0648 < Unemployment <= 0.0805
0.0353 < School.segregation <= 0.0595
Feature
0.000
0.005
0.010
Weight
Positive
Negative

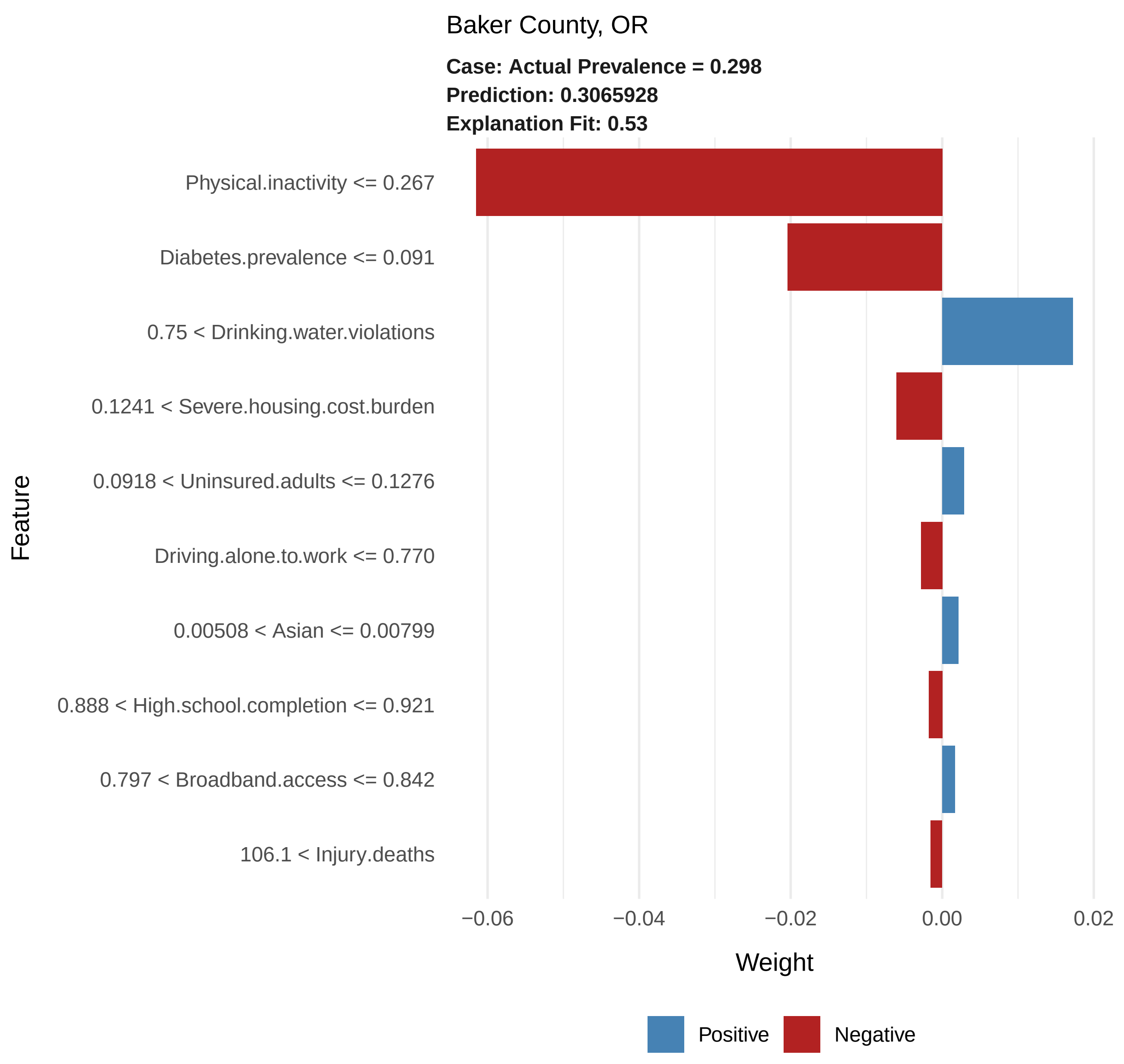

Baker County, OR
Case: Actual Prevalence = 0.298
Prediction: 0.3065928
Explanation Fit: 0.53
Physical.inactivity <= 0.267
Diabetes.prevalence <= 0.091
0.75 < Drinking.water.violations
0.1241 < Severe.housing.cost.burden
0.0918 < Uninsured.adults <= 0.1276
Driving.alone.to.work <= 0.770
0.00508 < Asian <= 0.00799
0.888 < High.school.completion <= 0.921
0.797 < Broadband.access <= 0.842
106.1 < Injury.deaths
Feature
−0.06
−0.04
−0.02
0.00
0.02
Weight
Positive
Negative

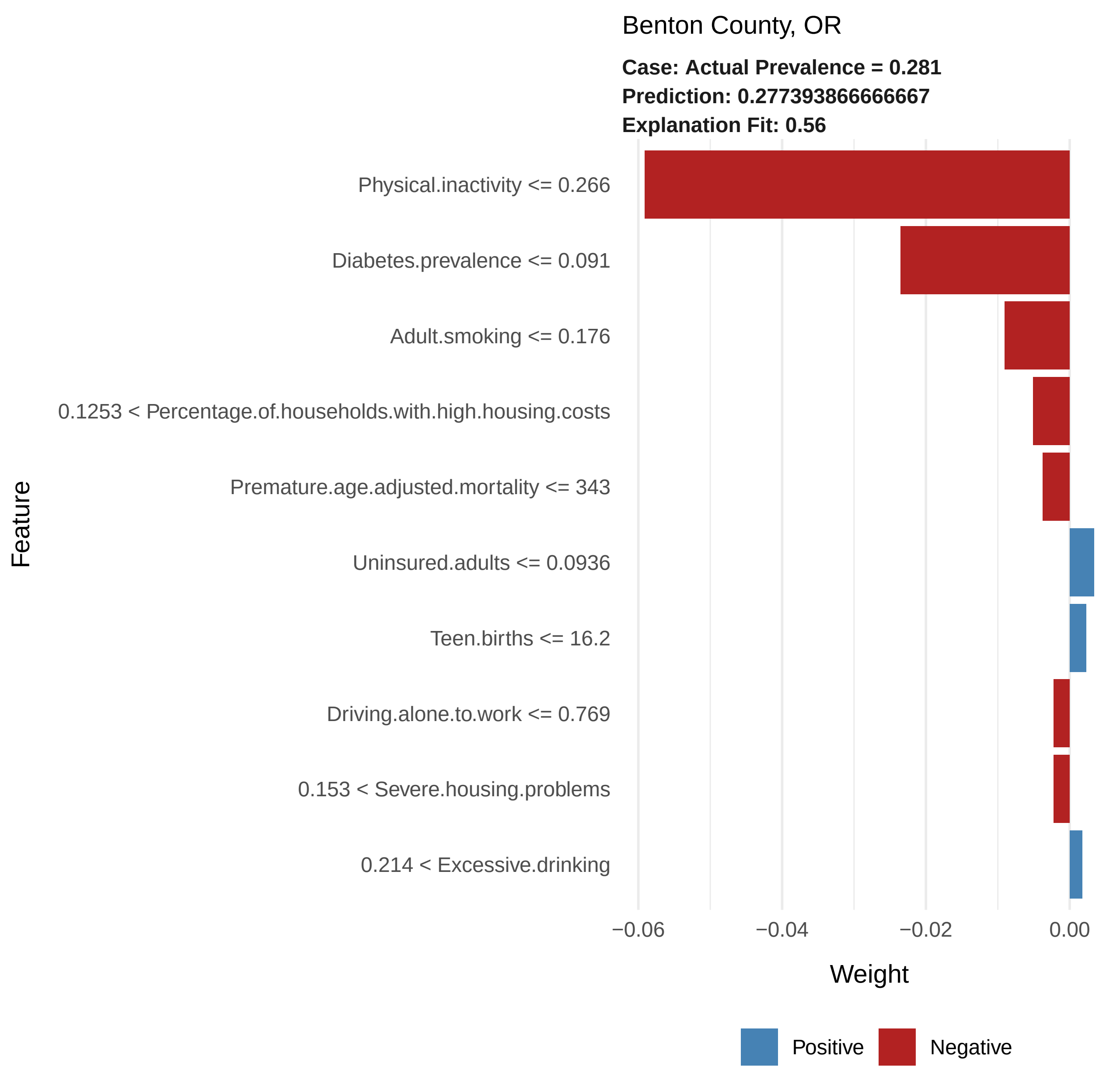
Benton County, OR
Case: Actual Prevalence = 0.281
Prediction: 0.277393866666667
Explanation Fit: 0.56
Physical.inactivity <= 0.266
Diabetes.prevalence <= 0.091
Adult.smoking <= 0.176
0.1253 < Percentage.of.households.with.high.housing.costs
Premature.age.adjusted.mortality <= 343
Uninsured.adults <= 0.0936
Teen.births <= 16.2
Driving.alone.to.work <= 0.769
0.153 < Severe.housing.problems
0.214 < Excessive.drinking
Feature
−0.06
−0.04
−0.02
0.00
Weight
Positive
Negative

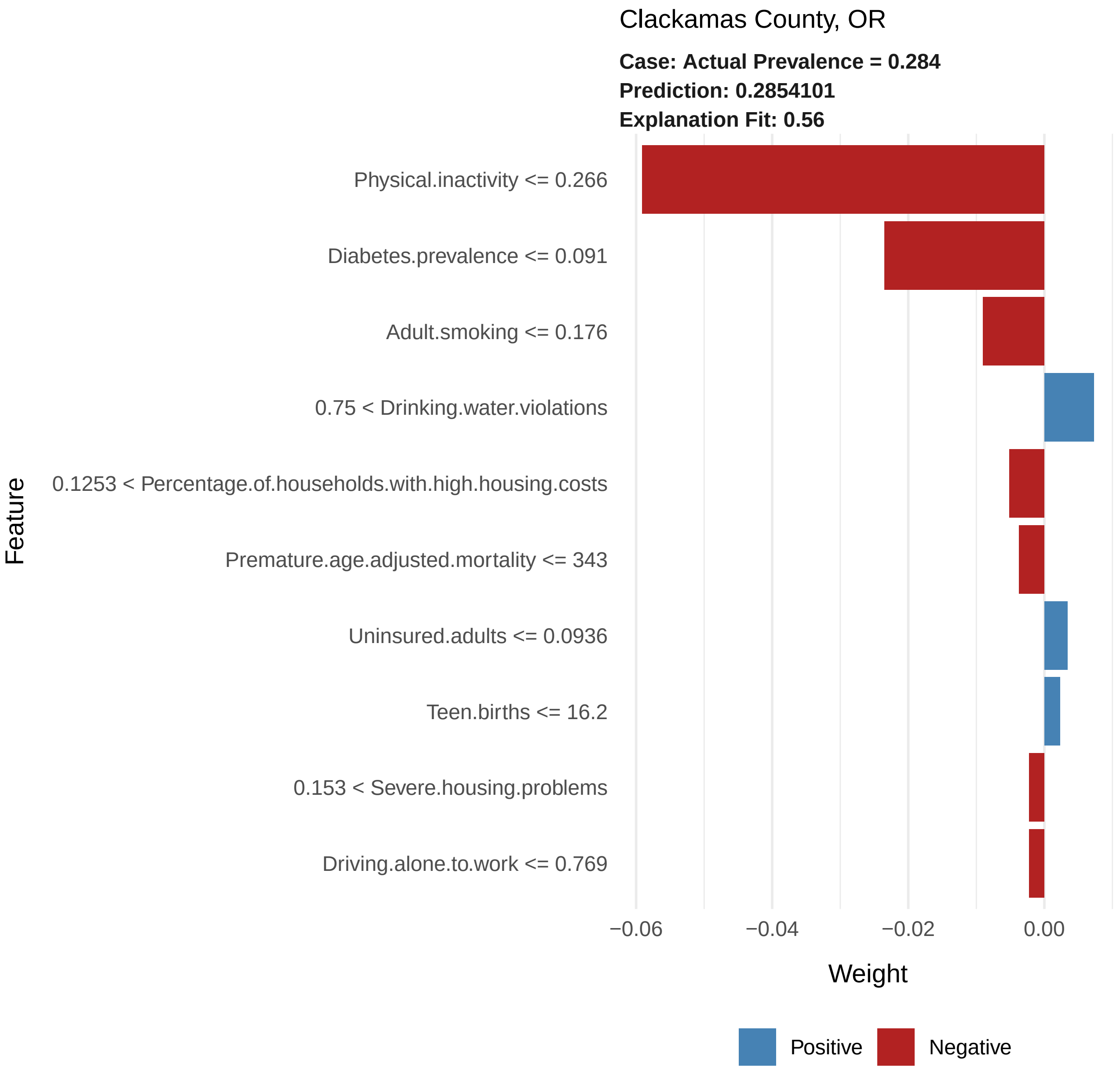
Clackamas County, OR
Case: Actual Prevalence = 0.284
Prediction: 0.2854101
Explanation Fit: 0.56
Physical.inactivity <= 0.266
Diabetes.prevalence <= 0.091
Adult.smoking <= 0.176
0.75 < Drinking.water.violations
0.1253 < Percentage.of.households.with.high.housing.costs
Premature.age.adjusted.mortality <= 343
Uninsured.adults <= 0.0936
Teen.births <= 16.2
0.153 < Severe.housing.problems
Driving.alone.to.work <= 0.769
Feature
−0.06
−0.04
−0.02
0.00
Weight
Positive
Negative

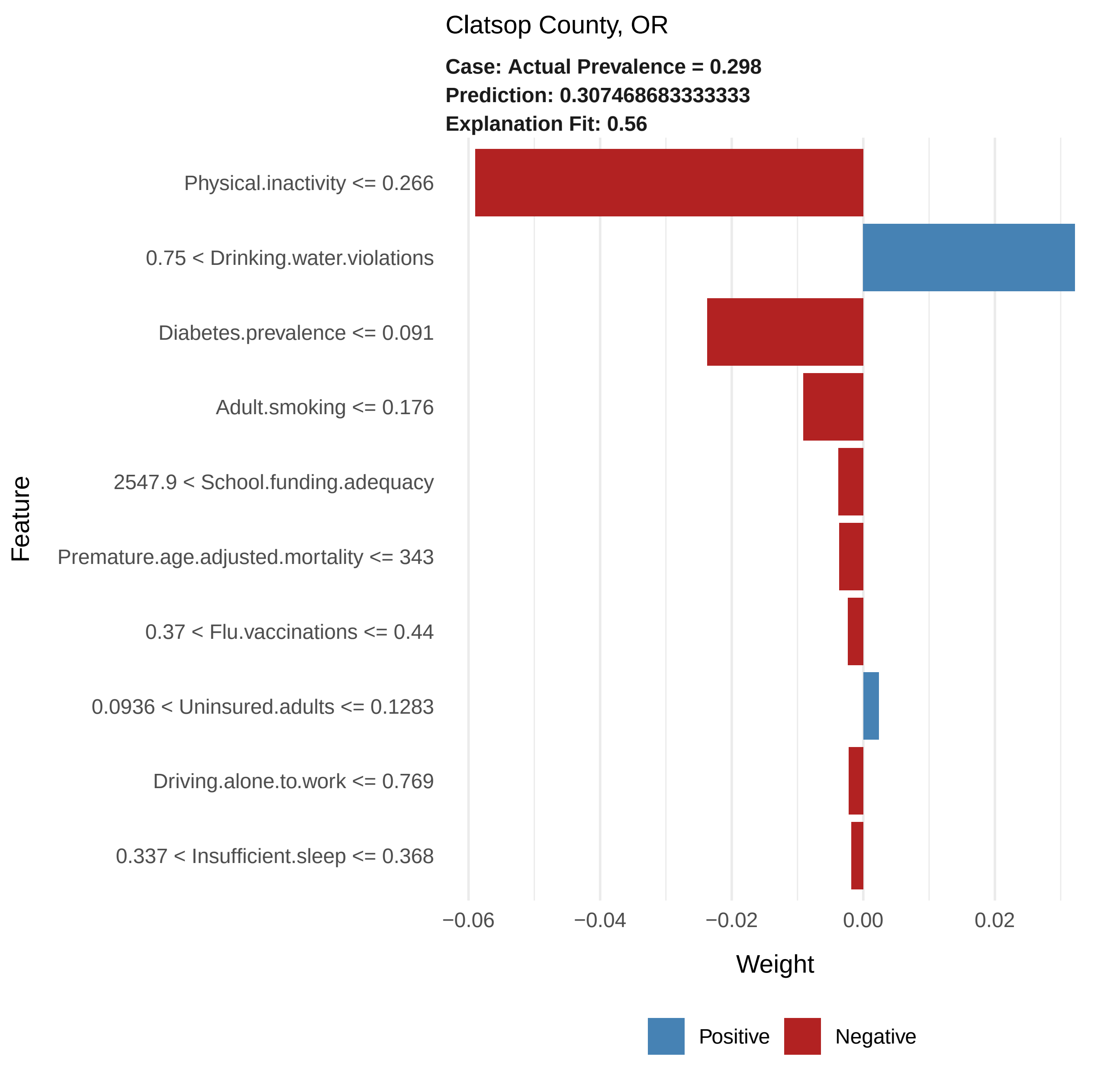
Clatsop County, OR
Case: Actual Prevalence = 0.298
Prediction: 0.307468683333333
Explanation Fit: 0.56
Physical.inactivity <= 0.266
0.75 < Drinking.water.violations
Diabetes.prevalence <= 0.091
Adult.smoking <= 0.176
2547.9 < School.funding.adequacy
Premature.age.adjusted.mortality <= 343
0.37 < Flu.vaccinations <= 0.44
0.0936 < Uninsured.adults <= 0.1283
Driving.alone.to.work <= 0.769
0.337 < Insufficient.sleep <= 0.368
Feature
−0.06
−0.04
−0.02
0.00
0.02
Weight
Positive
Negative

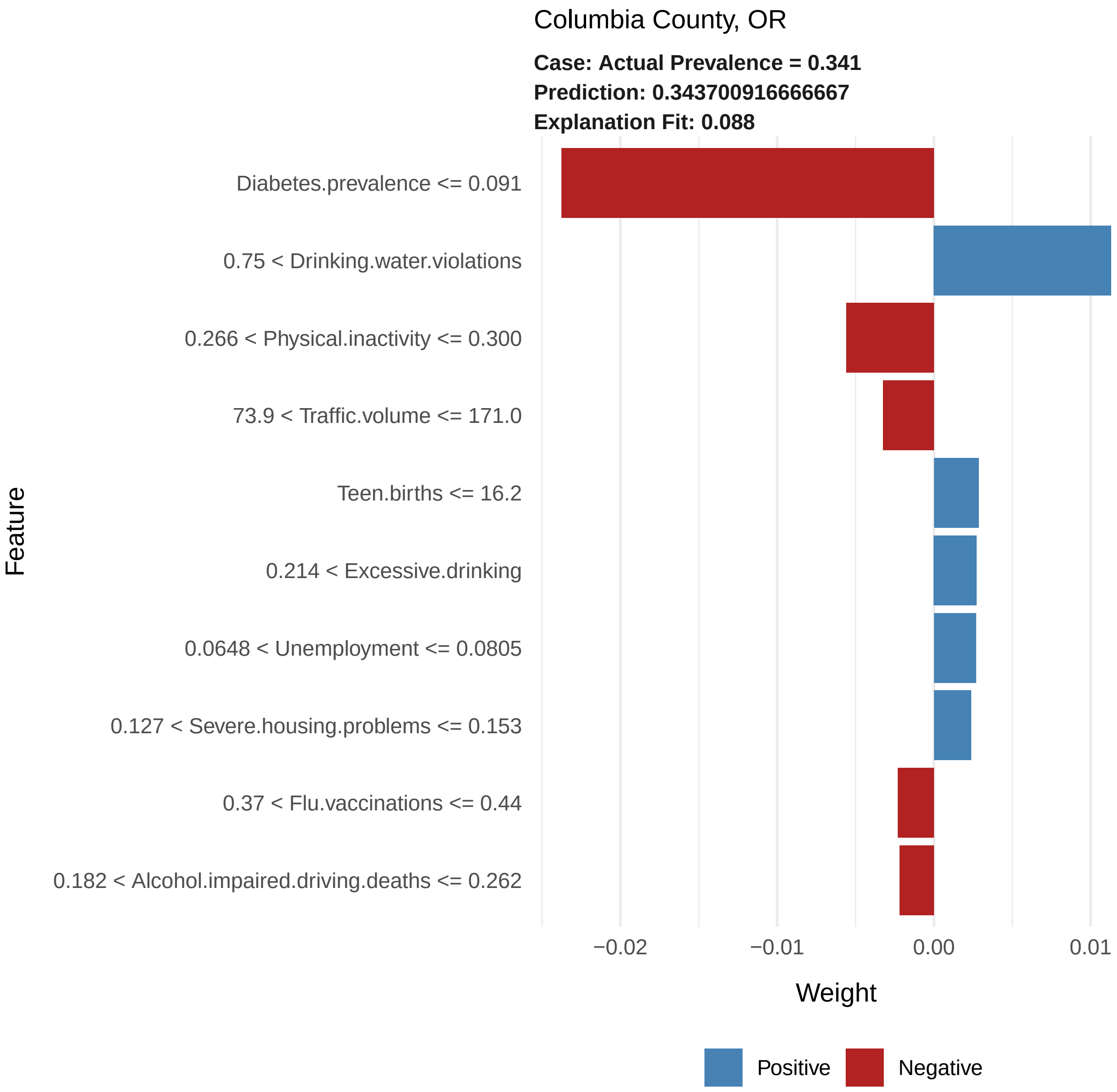
Columbia County, OR
Case: Actual Prevalence = 0.341
Prediction: 0.343700916666667
Explanation Fit: 0.088
Diabetes.prevalence <= 0.091
0.75 < Drinking.water.violations
0.266 < Physical.inactivity <= 0.300
73.9 < Traffic.volume <= 171.0
Teen.births <= 16.2
0.214 < Excessive.drinking
0.0648 < Unemployment <= 0.0805
0.127 < Severe.housing.problems <= 0.153
0.37 < Flu.vaccinations <= 0.44
0.182 < Alcohol.impaired.driving.deaths <= 0.262
Feature
−0.02
−0.01
0.00
0.01
Weight
Positive
Negative

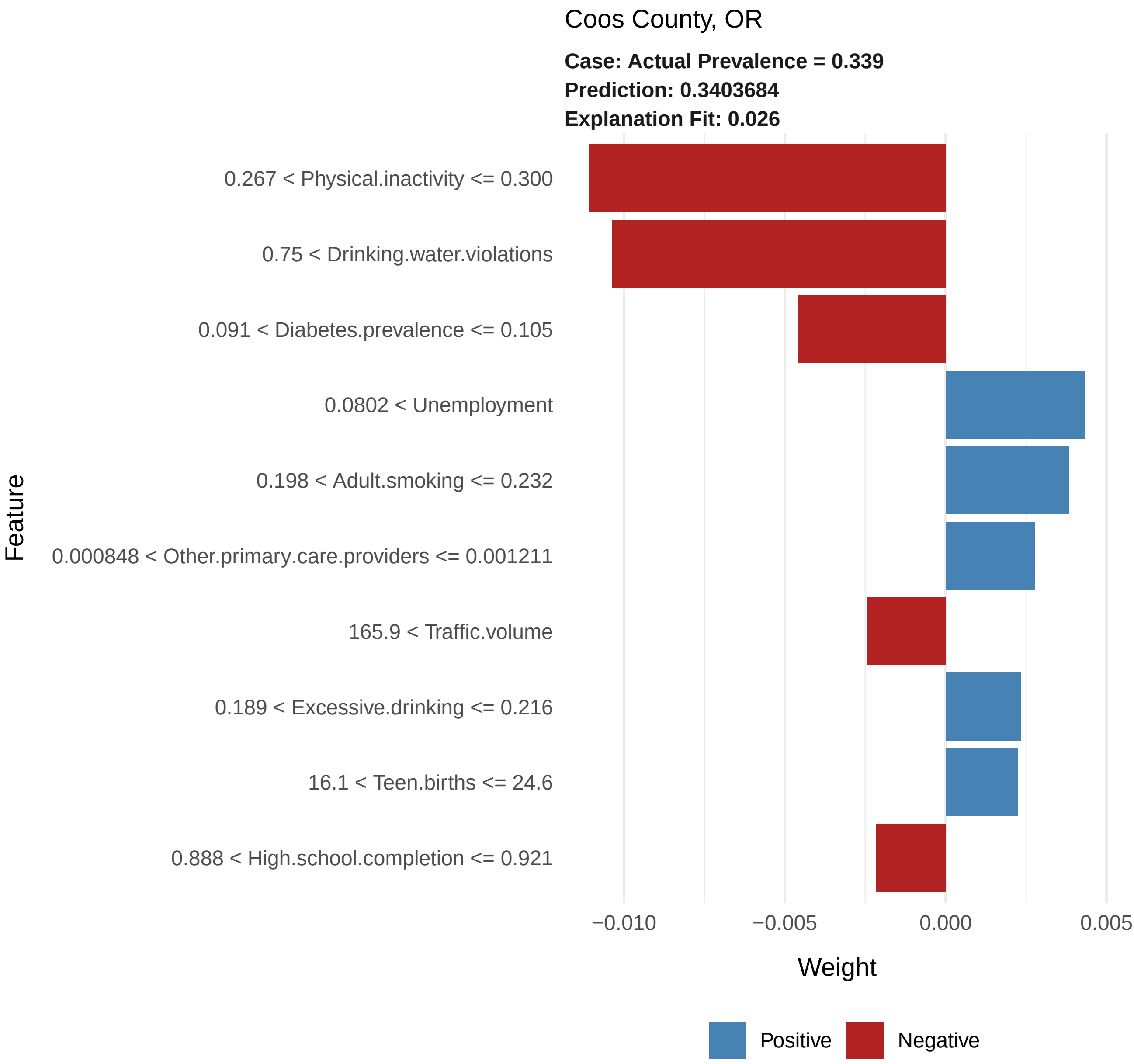
Coos County, OR
Case: Actual Prevalence = 0.339
Prediction: 0.3403684
Explanation Fit: 0.026
0.267 < Physical.inactivity <= 0.300
0.75 < Drinking.water.violations
0.091 < Diabetes.prevalence <= 0.105
0.0802 < Unemployment
0.198 < Adult.smoking <= 0.232
0.000848 < Other.primary.care.providers <= 0.001211
165.9 < Traffic.volume
0.189 < Excessive.drinking <= 0.216
16.1 < Teen.births <= 24.6
0.888 < High.school.completion <= 0.921
Feature
−0.010
−0.005
0.000
0.005
Weight
Positive
Negative

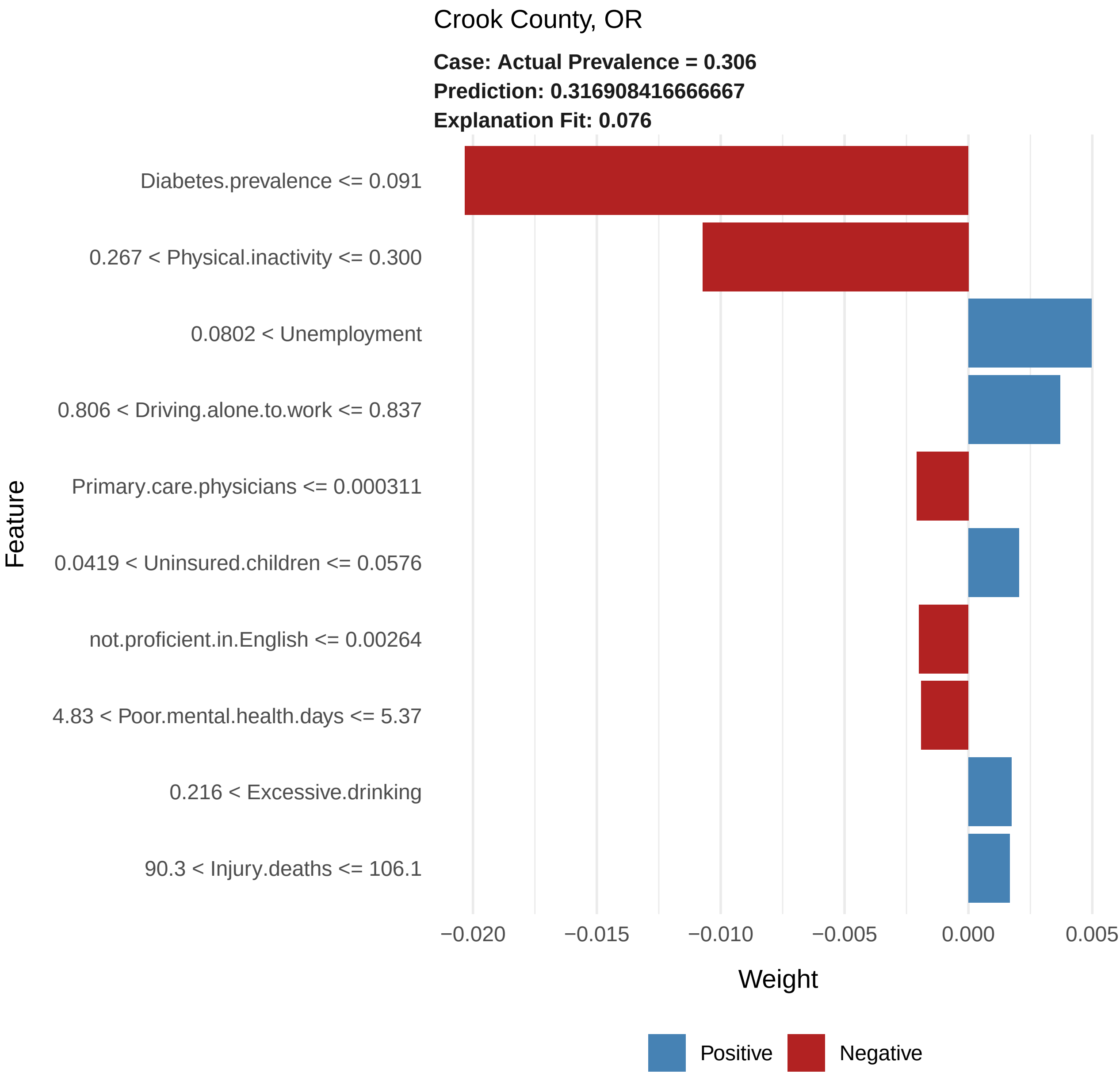

Crook County, OR
Case: Actual Prevalence = 0.306
Prediction: 0.316908416666667
Explanation Fit: 0.076
Diabetes.prevalence <= 0.091
0.267 < Physical.inactivity <= 0.300
0.0802 < Unemployment
0.806 < Driving.alone.to.work <= 0.837
Primary.care.physicians <= 0.000311
0.0419 < Uninsured.children <= 0.0576
not.proficient.in.English <= 0.00264
4.83 < Poor.mental.health.days <= 5.37
0.216 < Excessive.drinking
90.3 < Injury.deaths <= 106.1
Feature
−0.020
−0.015
−0.010
−0.005
0.000
0.005
Weight
Positive
Negative

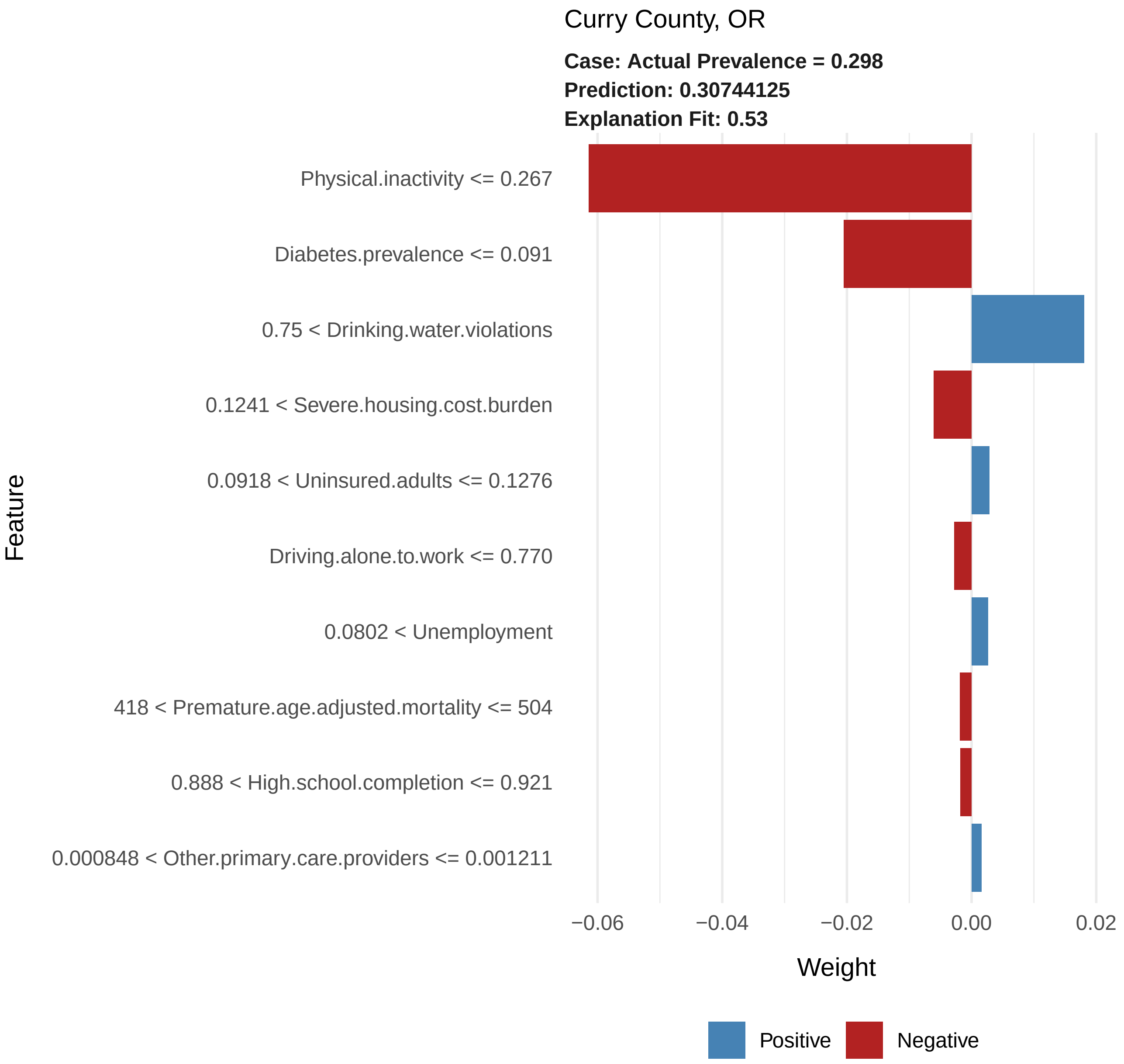
Curry County, OR
Case: Actual Prevalence = 0.298
Prediction: 0.30744125
Explanation Fit: 0.53
Physical.inactivity <= 0.267
Diabetes.prevalence <= 0.091
0.75 < Drinking.water.violations
0.1241 < Severe.housing.cost.burden
0.0918 < Uninsured.adults <= 0.1276
Driving.alone.to.work <= 0.770
0.0802 < Unemployment
418 < Premature.age.adjusted.mortality <= 504
0.888 < High.school.completion <= 0.921
0.000848 < Other.primary.care.providers <= 0.001211
Feature
−0.06
−0.04
−0.02
0.00
0.02
Weight
Positive
Negative

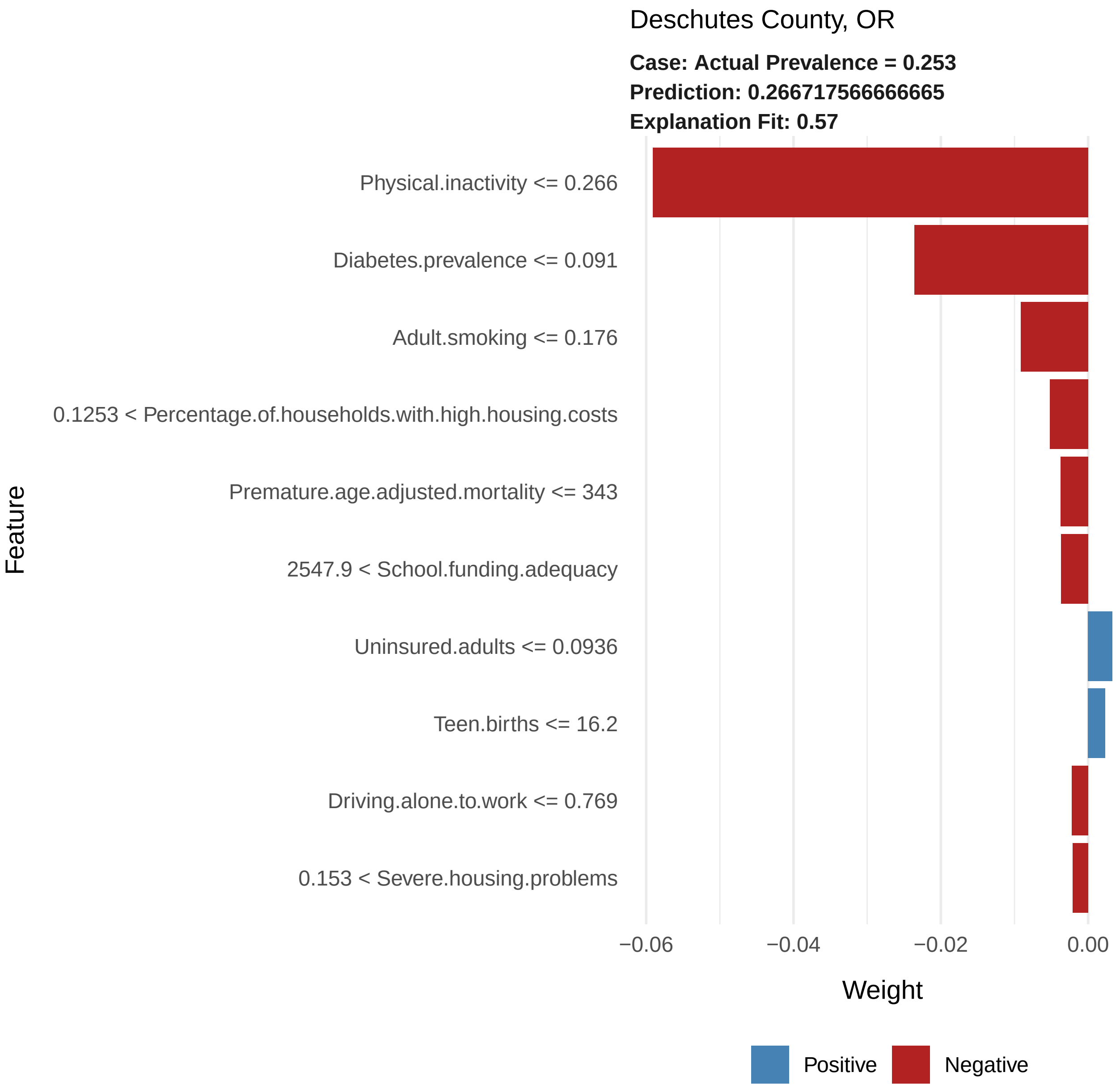
Deschutes County, OR
Case: Actual Prevalence = 0.253
Prediction: 0.266717566666665
Explanation Fit: 0.57
Physical.inactivity <= 0.266
Diabetes.prevalence <= 0.091
Adult.smoking <= 0.176
0.1253 < Percentage.of.households.with.high.housing.costs
Premature.age.adjusted.mortality <= 343
2547.9 < School.funding.adequacy
Uninsured.adults <= 0.0936
Teen.births <= 16.2
Driving.alone.to.work <= 0.769
0.153 < Severe.housing.problems
Feature
−0.06
−0.04
−0.02
0.00
Weight
Positive
Negative

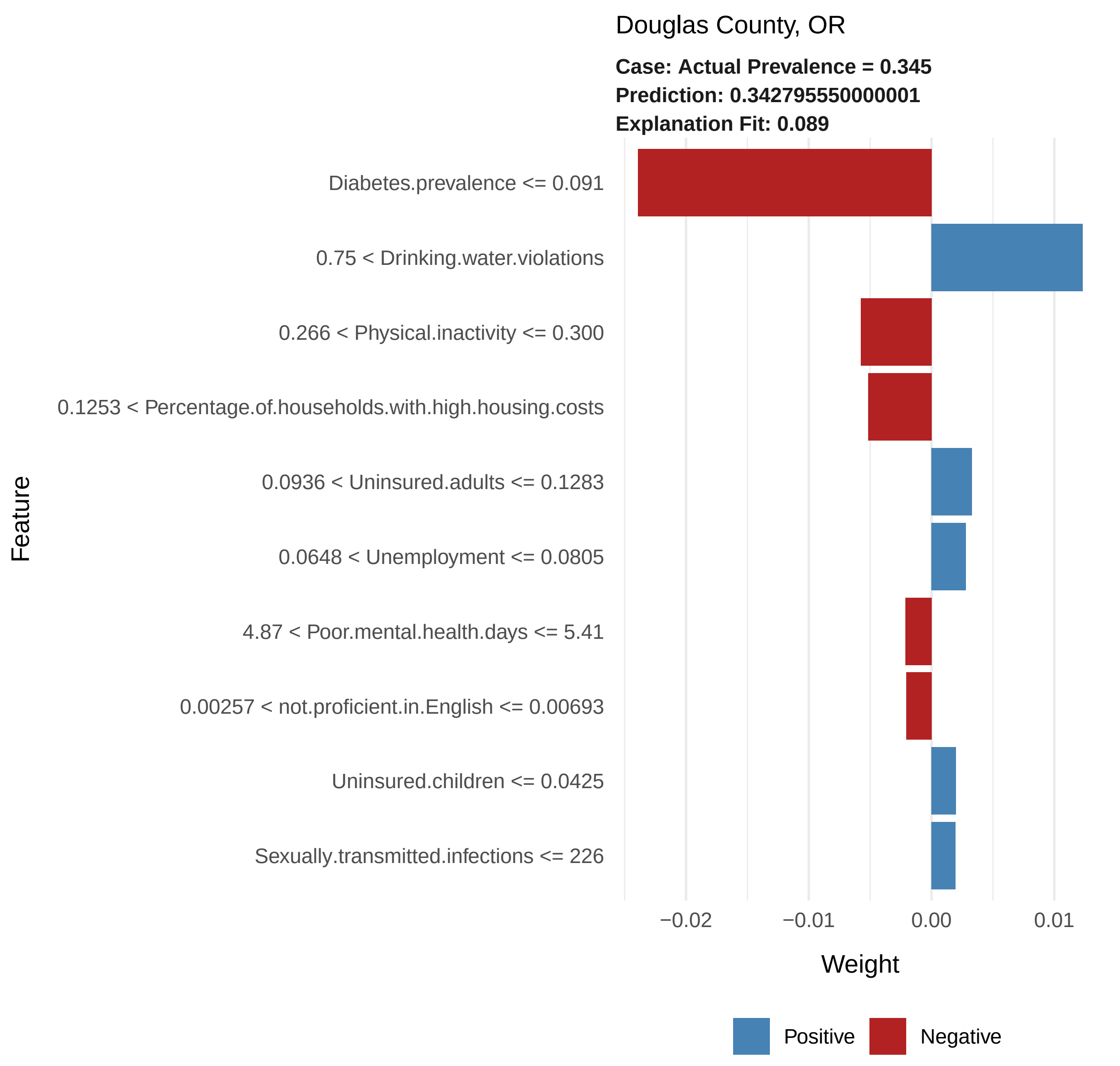
Douglas County, OR
Case: Actual Prevalence = 0.345
Prediction: 0.342795550000001
Explanation Fit: 0.089
Diabetes.prevalence <= 0.091
0.75 < Drinking.water.violations
0.266 < Physical.inactivity <= 0.300
0.1253 < Percentage.of.households.with.high.housing.costs
0.0936 < Uninsured.adults <= 0.1283
0.0648 < Unemployment <= 0.0805
4.87 < Poor.mental.health.days <= 5.41
0.00257 < not.proficient.in.English <= 0.00693
Uninsured.children <= 0.0425
Sexually.transmitted.infections <= 226
Feature
−0.02
−0.01
0.00
0.01
Weight
Positive
Negative

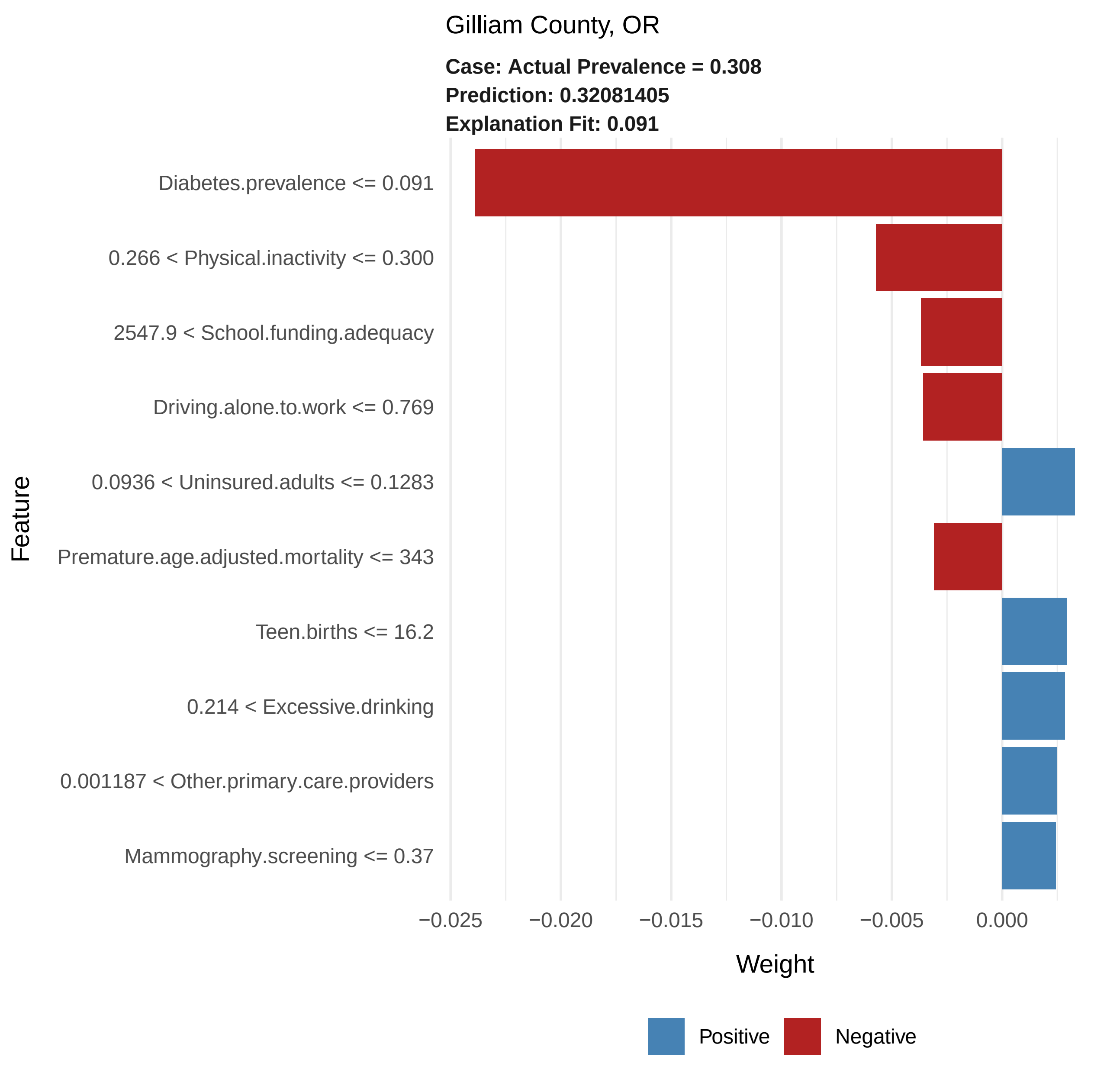
Gilliam County, OR
Case: Actual Prevalence = 0.308
Prediction: 0.32081405
Explanation Fit: 0.091
Diabetes.prevalence <= 0.091
0.266 < Physical.inactivity <= 0.300
2547.9 < School.funding.adequacy
Driving.alone.to.work <= 0.769
0.0936 < Uninsured.adults <= 0.1283
Premature.age.adjusted.mortality <= 343
Teen.births <= 16.2
0.214 < Excessive.drinking
0.001187 < Other.primary.care.providers
Mammography.screening <= 0.37
Feature
−0.025
−0.020
−0.015
−0.010
−0.005
0.000
Weight
Positive
Negative

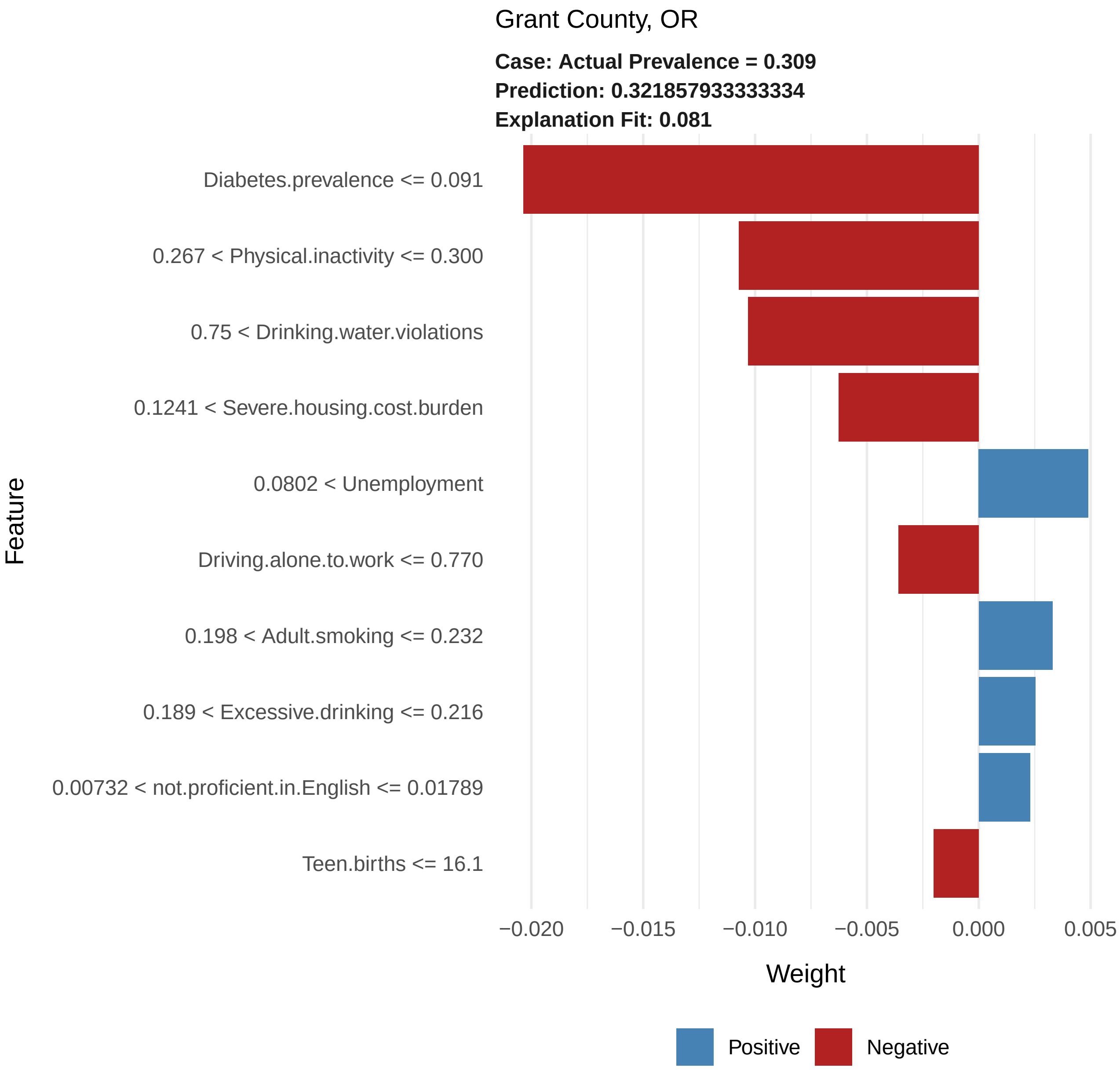
Grant County, OR
Case: Actual Prevalence = 0.309
Prediction: 0.321857933333334
Explanation Fit: 0.081
Feature
Diabetes.prevalence <= 0.091
0.267 < Physical.inactivity <= 0.300
0.75 < Drinking.water.violations
0.1241 < Severe.housing.cost.burden
0.0802 < Unemployment
Driving.alone.to.work <= 0.770
0.198 < Adult.smoking <= 0.232
0.189 < Excessive.drinking <= 0.216
0.00732 < not.proficient.in.English <= 0.01789
Teen.births <= 16.1
−0.020
−0.015
−0.010
−0.005
0.000
0.005
Weight
Positive
Negative

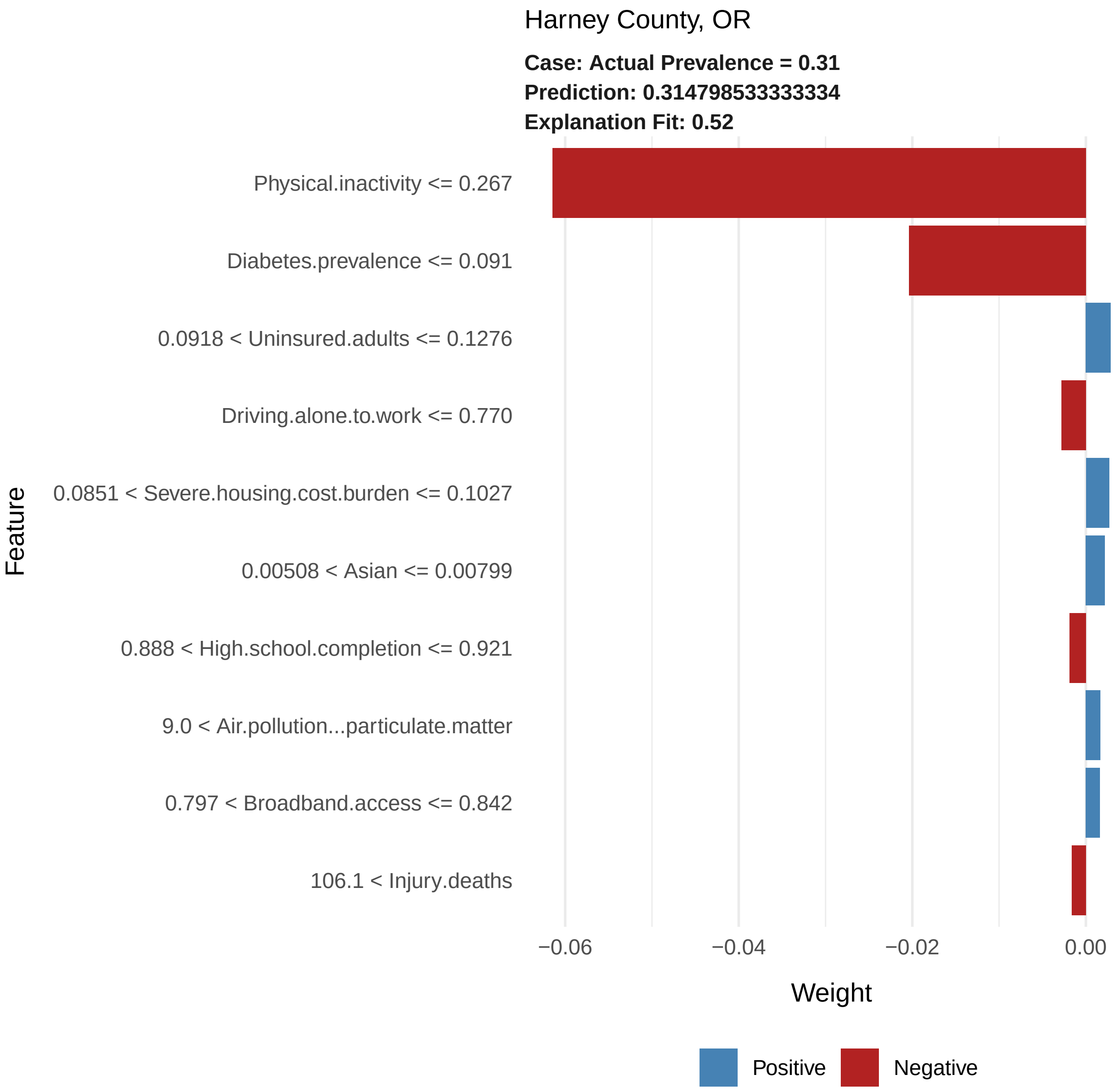

Harney County, OR
Case: Actual Prevalence = 0.31
Prediction: 0.314798533333334
Explanation Fit: 0.52
Physical.inactivity <= 0.267
Diabetes.prevalence <= 0.091
0.0918 < Uninsured.adults <= 0.1276
Driving.alone.to.work <= 0.770
0.0851 < Severe.housing.cost.burden <= 0.1027
0.00508 < Asian <= 0.00799
0.888 < High.school.completion <= 0.921
9.0 < Air.pollution...particulate.matter
0.797 < Broadband.access <= 0.842
106.1 < Injury.deaths
Feature
−0.06
−0.04
−0.02
0.00
Weight
Positive
Negative

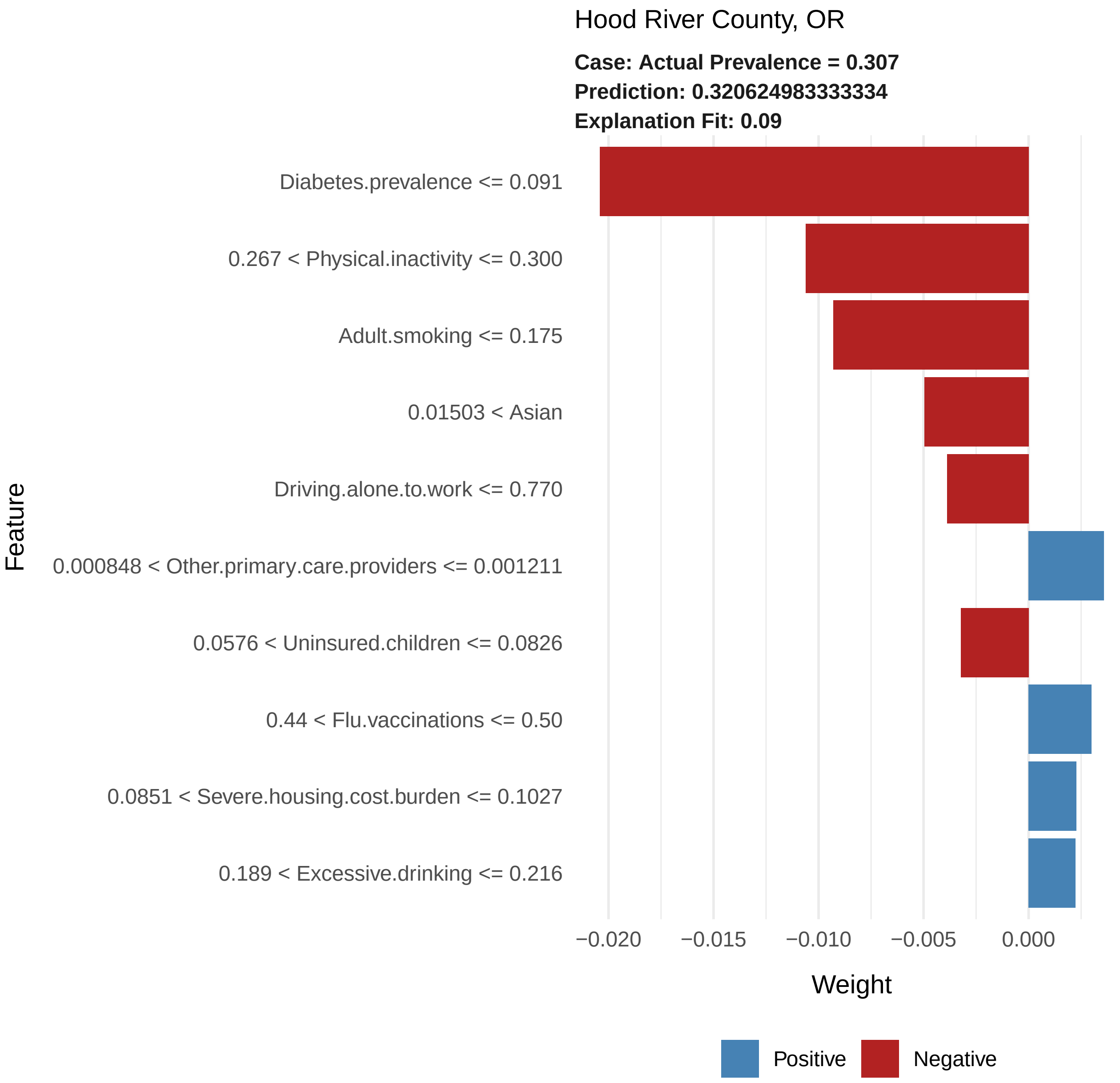
Hood River County, OR
Case: Actual Prevalence = 0.307
Prediction: 0.320624983333334
Explanation Fit: 0.09
Diabetes.prevalence <= 0.091
0.267 < Physical.inactivity <= 0.300
Adult.smoking <= 0.175
0.01503 < Asian
Driving.alone.to.work <= 0.770
0.000848 < Other.primary.care.providers <= 0.001211
0.0576 < Uninsured.children <= 0.0826
0.44 < Flu.vaccinations <= 0.50
0.0851 < Severe.housing.cost.burden <= 0.1027
0.189 < Excessive.drinking <= 0.216
Feature
−0.020
−0.015
−0.010
−0.005
0.000
Weight
Positive
Negative

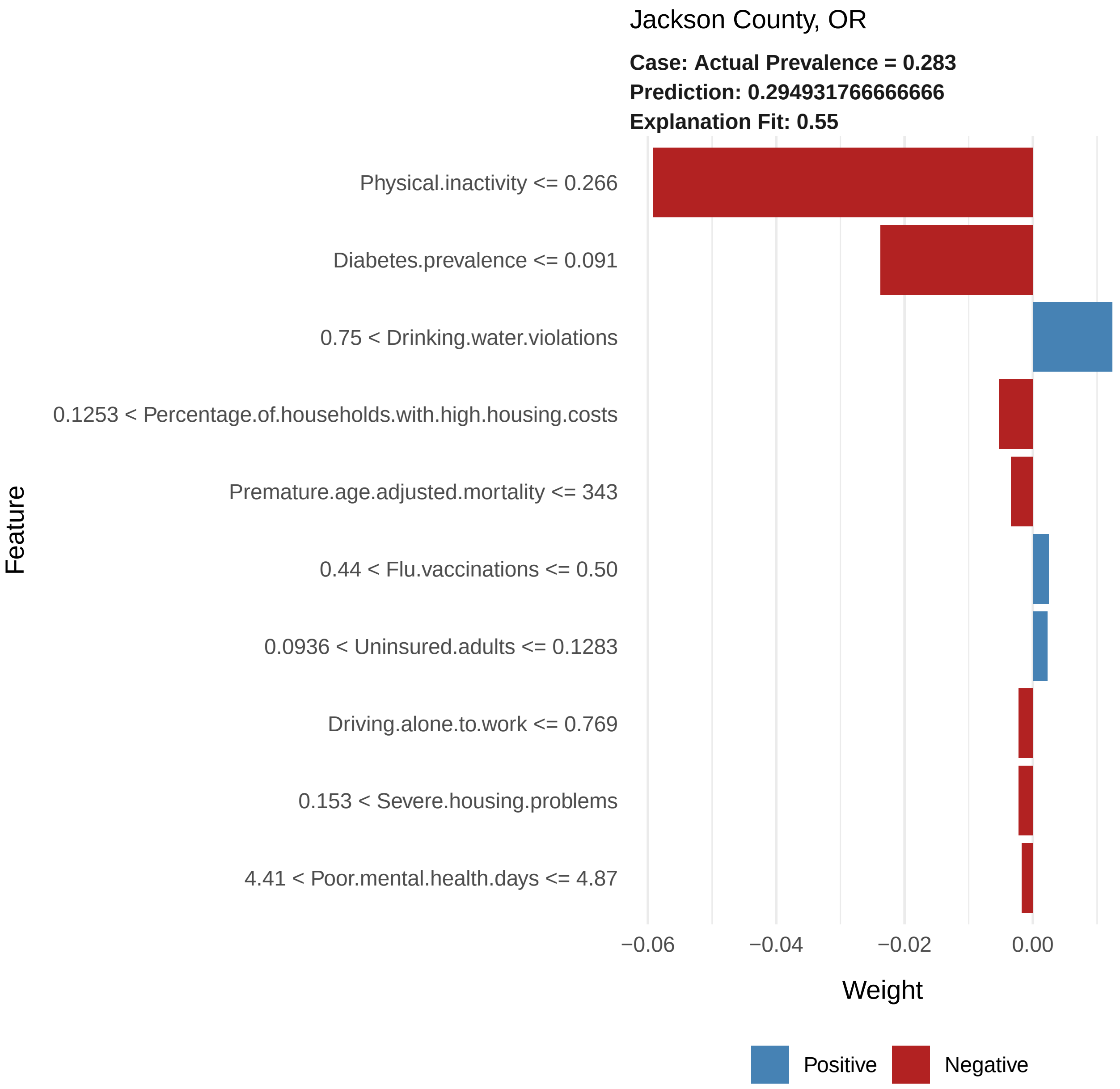

Jackson County, OR
Case: Actual Prevalence = 0.283
Prediction: 0.294931766666666
Explanation Fit: 0.55
Physical.inactivity <= 0.266
Diabetes.prevalence <= 0.091
0.75 < Drinking.water.violations
0.1253 < Percentage.of.households.with.high.housing.costs
Premature.age.adjusted.mortality <= 343
0.44 < Flu.vaccinations <= 0.50
0.0936 < Uninsured.adults <= 0.1283
Driving.alone.to.work <= 0.769
0.153 < Severe.housing.problems
4.41 < Poor.mental.health.days <= 4.87
Feature
−0.06
−0.04
−0.02
0.00
Weight
Positive
Negative

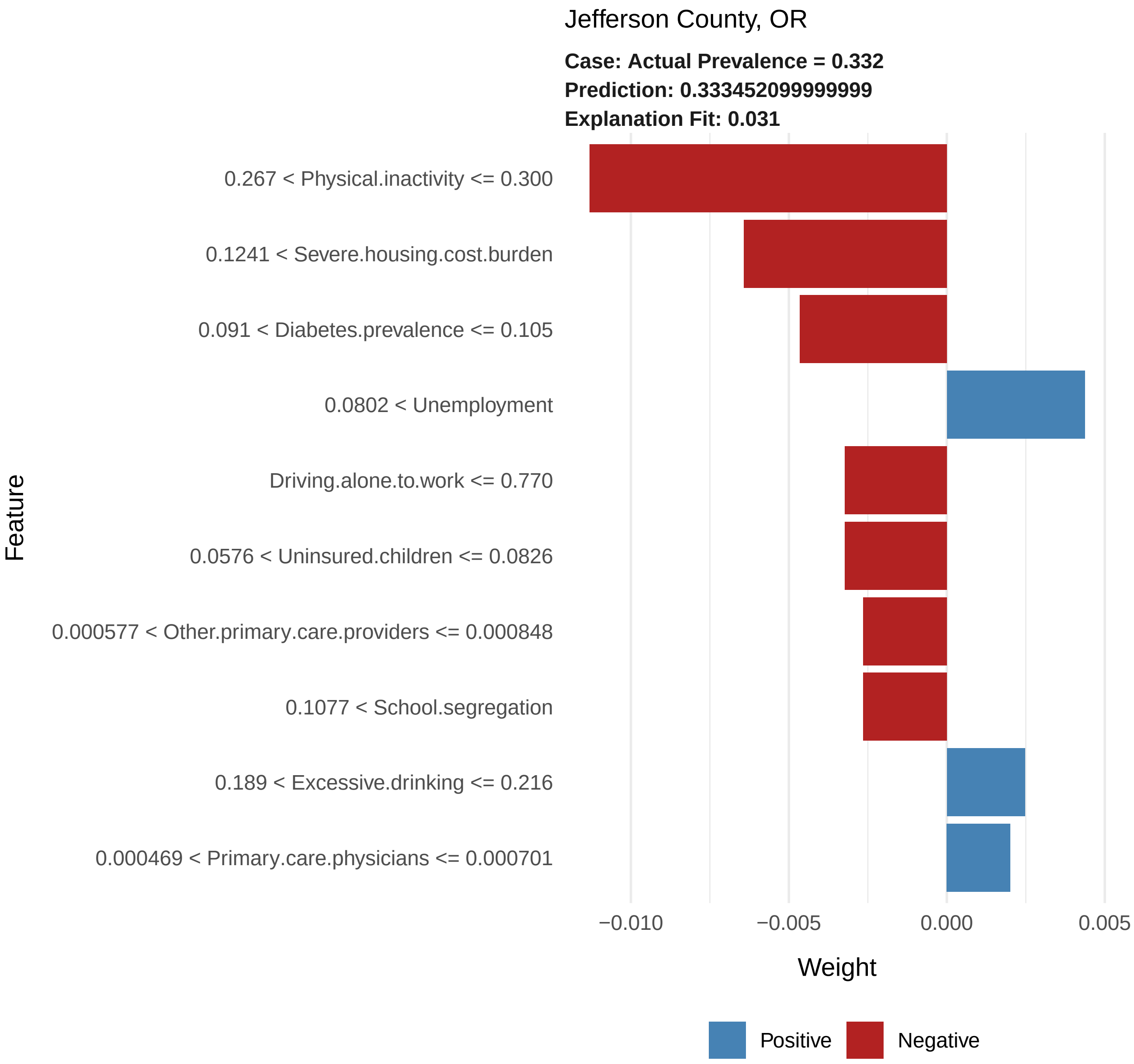

Jefferson County, OR
Case: Actual Prevalence = 0.332
Prediction: 0.333452099999999
Explanation Fit: 0.031
0.267 < Physical.inactivity <= 0.300
0.1241 < Severe.housing.cost.burden
0.091 < Diabetes.prevalence <= 0.105
0.0802 < Unemployment
Driving.alone.to.work <= 0.770
0.0576 < Uninsured.children <= 0.0826
0.000577 < Other.primary.care.providers <= 0.000848
0.1077 < School.segregation
0.189 < Excessive.drinking <= 0.216
0.000469 < Primary.care.physicians <= 0.000701
Feature
−0.010
−0.005
0.000
0.005
Weight
Positive
Negative

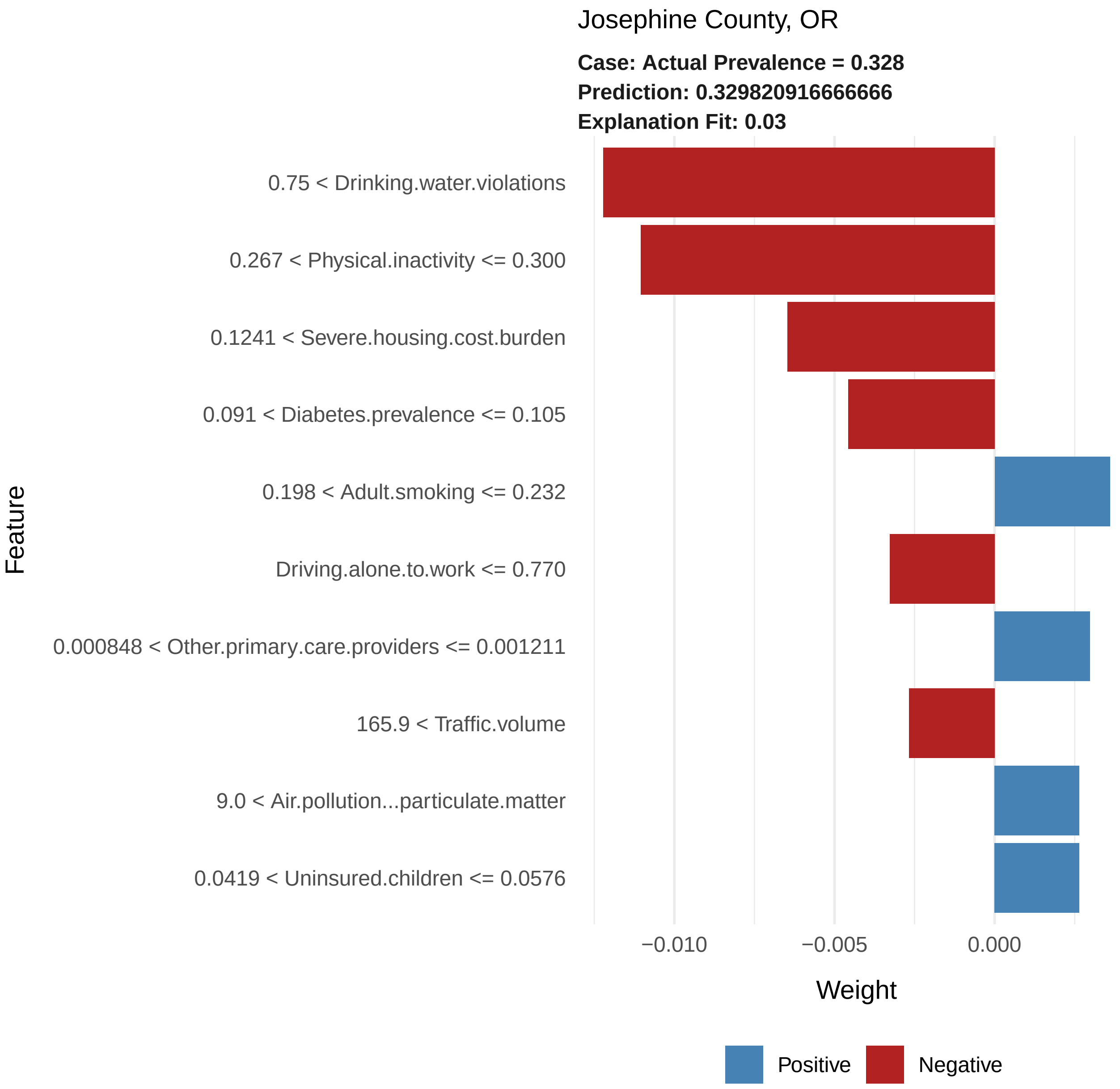
Josephine County, OR
Case: Actual Prevalence = 0.328
Prediction: 0.329820916666666
Explanation Fit: 0.03
0.75 < Drinking.water.violations
0.267 < Physical.inactivity <= 0.300
0.1241 < Severe.housing.cost.burden
0.091 < Diabetes.prevalence <= 0.105
0.198 < Adult.smoking <= 0.232
Driving.alone.to.work <= 0.770
0.000848 < Other.primary.care.providers <= 0.001211
165.9 < Traffic.volume
9.0 < Air.pollution...particulate.matter
0.0419 < Uninsured.children <= 0.0576
Feature
−0.010
−0.005
0.000
Weight
Positive
Negative

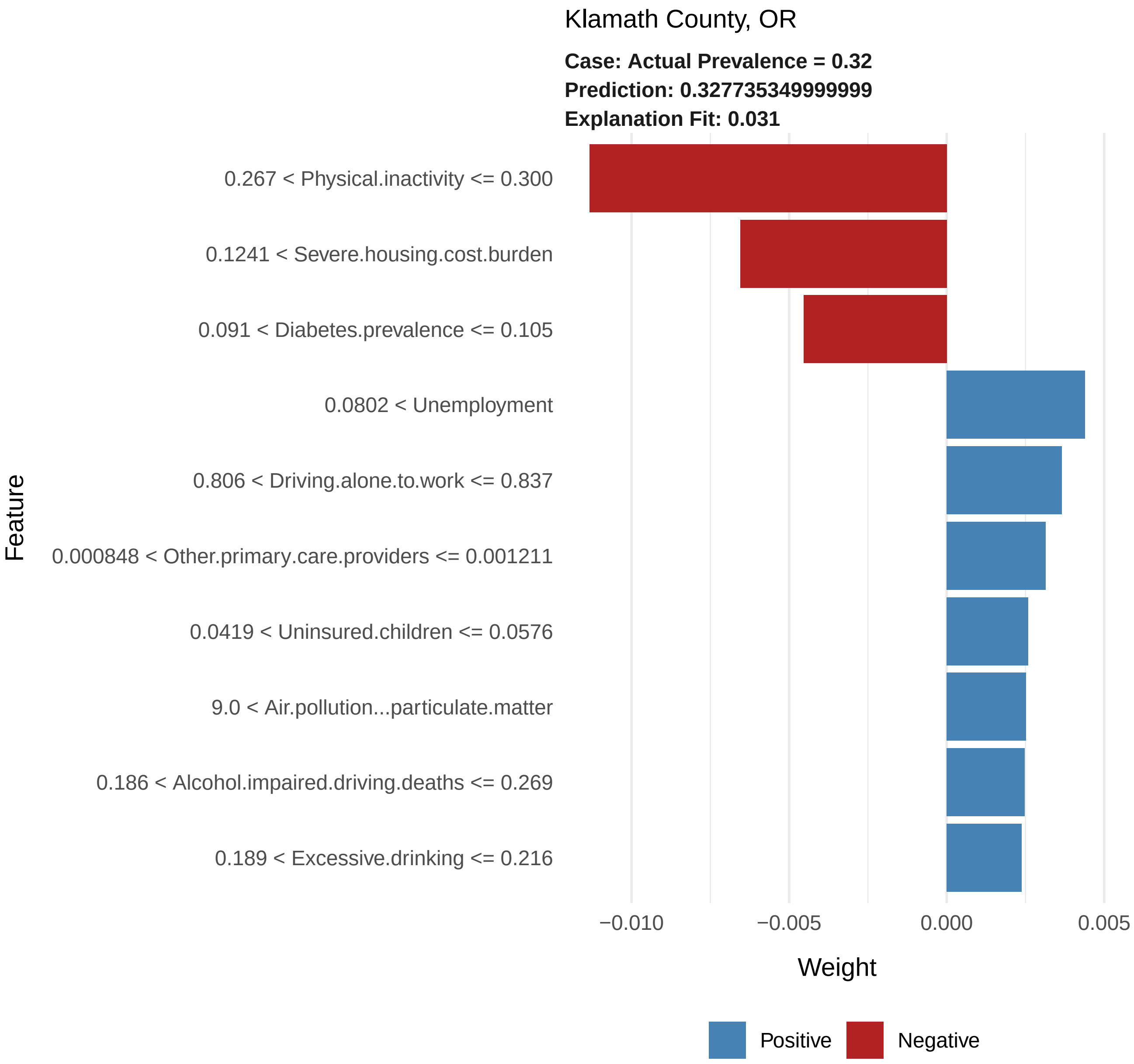

Klamath County, OR
Case: Actual Prevalence = 0.32
Prediction: 0.327735349999999
Explanation Fit: 0.031
0.267 < Physical.inactivity <= 0.300
0.1241 < Severe.housing.cost.burden
0.091 < Diabetes.prevalence <= 0.105
0.0802 < Unemployment
0.806 < Driving.alone.to.work <= 0.837
0.000848 < Other.primary.care.providers <= 0.001211
0.0419 < Uninsured.children <= 0.0576
9.0 < Air.pollution...particulate.matter
0.186 < Alcohol.impaired.driving.deaths <= 0.269
0.189 < Excessive.drinking <= 0.216
Feature
−0.010
−0.005
0.000
0.005
Weight
Positive
Negative

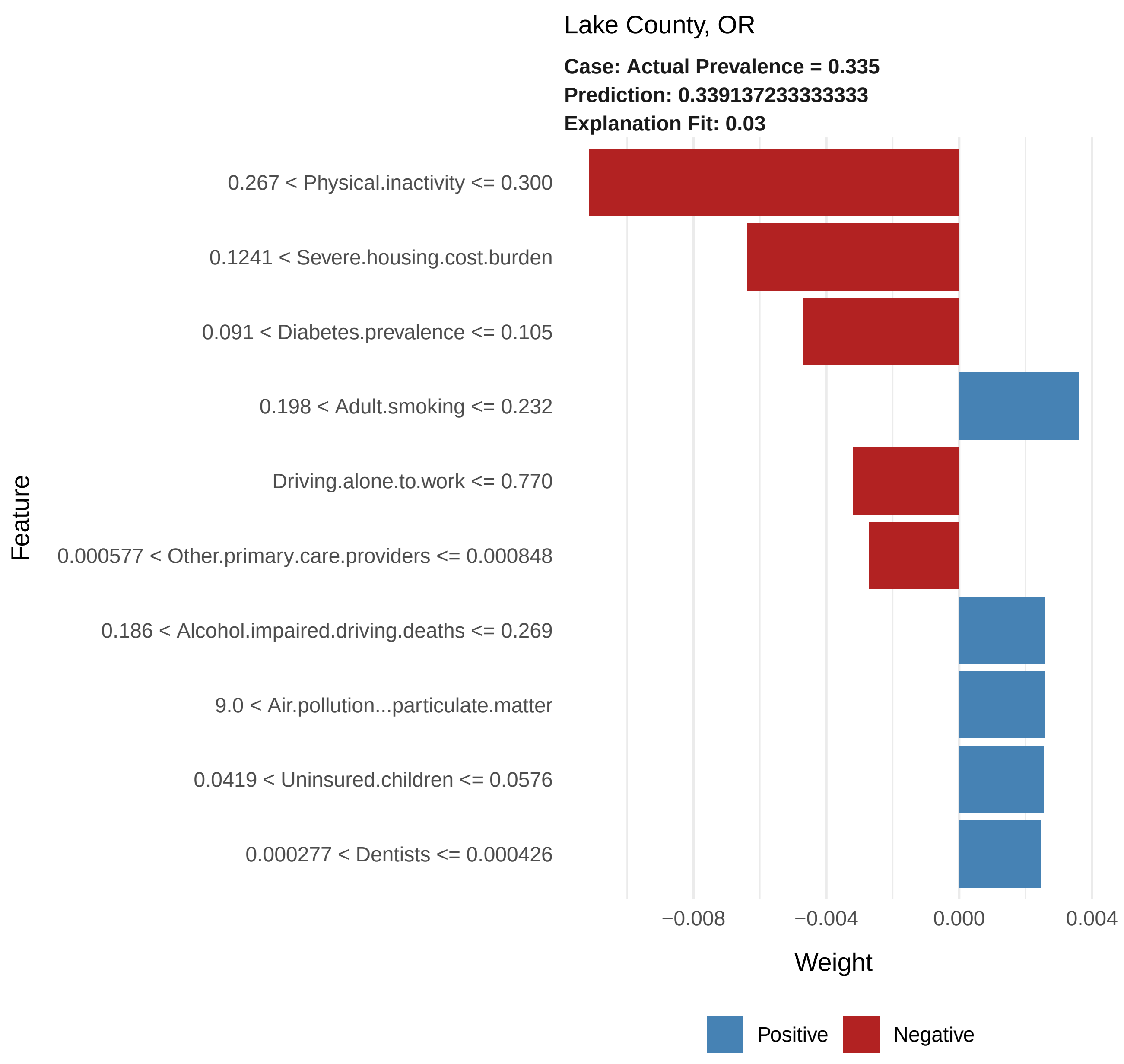
Lake County, OR
Case: Actual Prevalence = 0.335
Prediction: 0.339137233333333
Explanation Fit: 0.03
0.267 < Physical.inactivity <= 0.300
0.1241 < Severe.housing.cost.burden
0.091 < Diabetes.prevalence <= 0.105
0.198 < Adult.smoking <= 0.232
Driving.alone.to.work <= 0.770
0.000577 < Other.primary.care.providers <= 0.000848
0.186 < Alcohol.impaired.driving.deaths <= 0.269
9.0 < Air.pollution...particulate.matter
0.0419 < Uninsured.children <= 0.0576
0.000277 < Dentists <= 0.000426
Feature
−0.008
−0.004
0.000
0.004
Weight
Positive
Negative

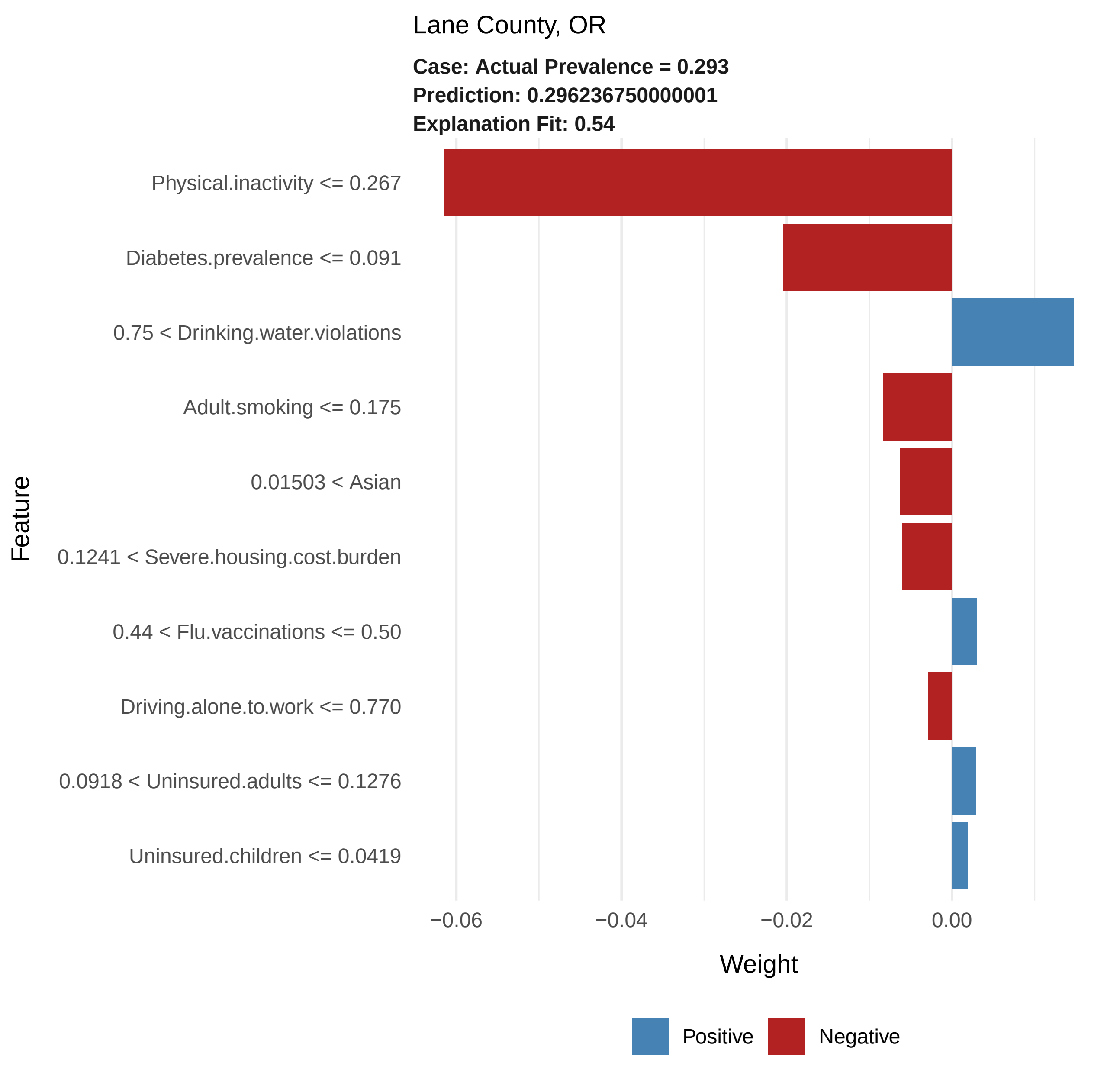

Lane County, OR
Case: Actual Prevalence = 0.293
Prediction: 0.296236750000001
Explanation Fit: 0.54
Physical.inactivity <= 0.267
Diabetes.prevalence <= 0.091
0.75 < Drinking.water.violations
Adult.smoking <= 0.175
0.01503 < Asian
0.1241 < Severe.housing.cost.burden
0.44 < Flu.vaccinations <= 0.50
Driving.alone.to.work <= 0.770
0.0918 < Uninsured.adults <= 0.1276
Uninsured.children <= 0.0419
Feature
−0.06
−0.04
−0.02
0.00
Weight
Positive
Negative

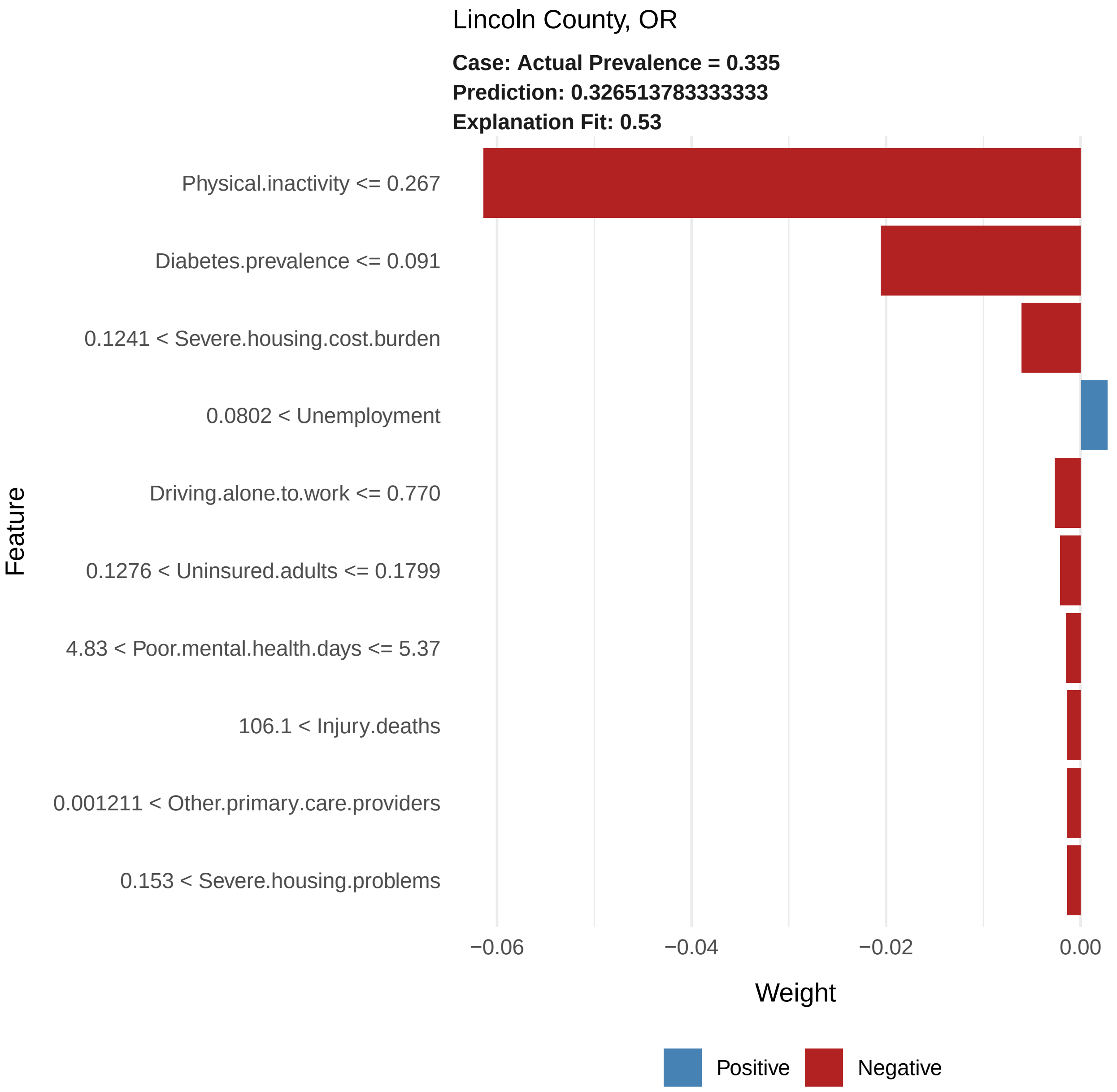
Lincoln County, OR
Case: Actual Prevalence = 0.335
Prediction: 0.326513783333333
Explanation Fit: 0.53
Physical.inactivity <= 0.267
Diabetes.prevalence <= 0.091
0.1241 < Severe.housing.cost.burden
0.0802 < Unemployment
Driving.alone.to.work <= 0.770
0.1276 < Uninsured.adults <= 0.1799
4.83 < Poor.mental.health.days <= 5.37
106.1 < Injury.deaths
0.001211 < Other.primary.care.providers
0.153 < Severe.housing.problems
Feature
−0.06
−0.04
−0.02
0.00
Weight
Positive
Negative

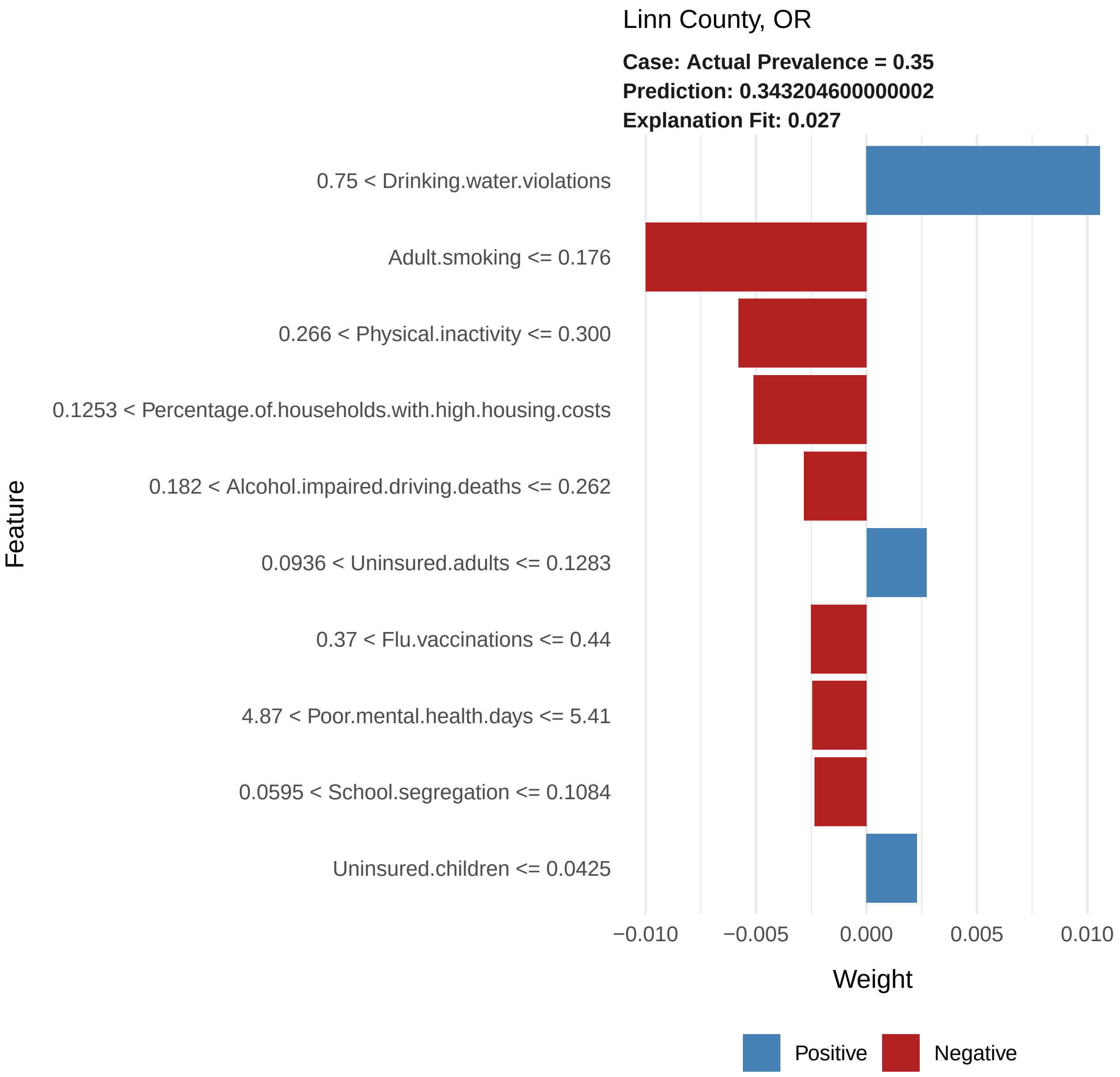

Linn County, OR
Case: Actual Prevalence = 0.35
Prediction: 0.343204600000002
Explanation Fit: 0.027
0.75 < Drinking.water.violations
Adult.smoking <= 0.176
0.266 < Physical.inactivity <= 0.300
0.1253 < Percentage.of.households.with.high.housing.costs
0.182 < Alcohol.impaired.driving.deaths <= 0.262
0.0936 < Uninsured.adults <= 0.1283
0.37 < Flu.vaccinations <= 0.44
4.87 < Poor.mental.health.days <= 5.41
0.0595 < School.segregation <= 0.1084
Uninsured.children <= 0.0425
Feature
−0.010
−0.005
0.000
0.005
0.010
Weight
Positive
Negative

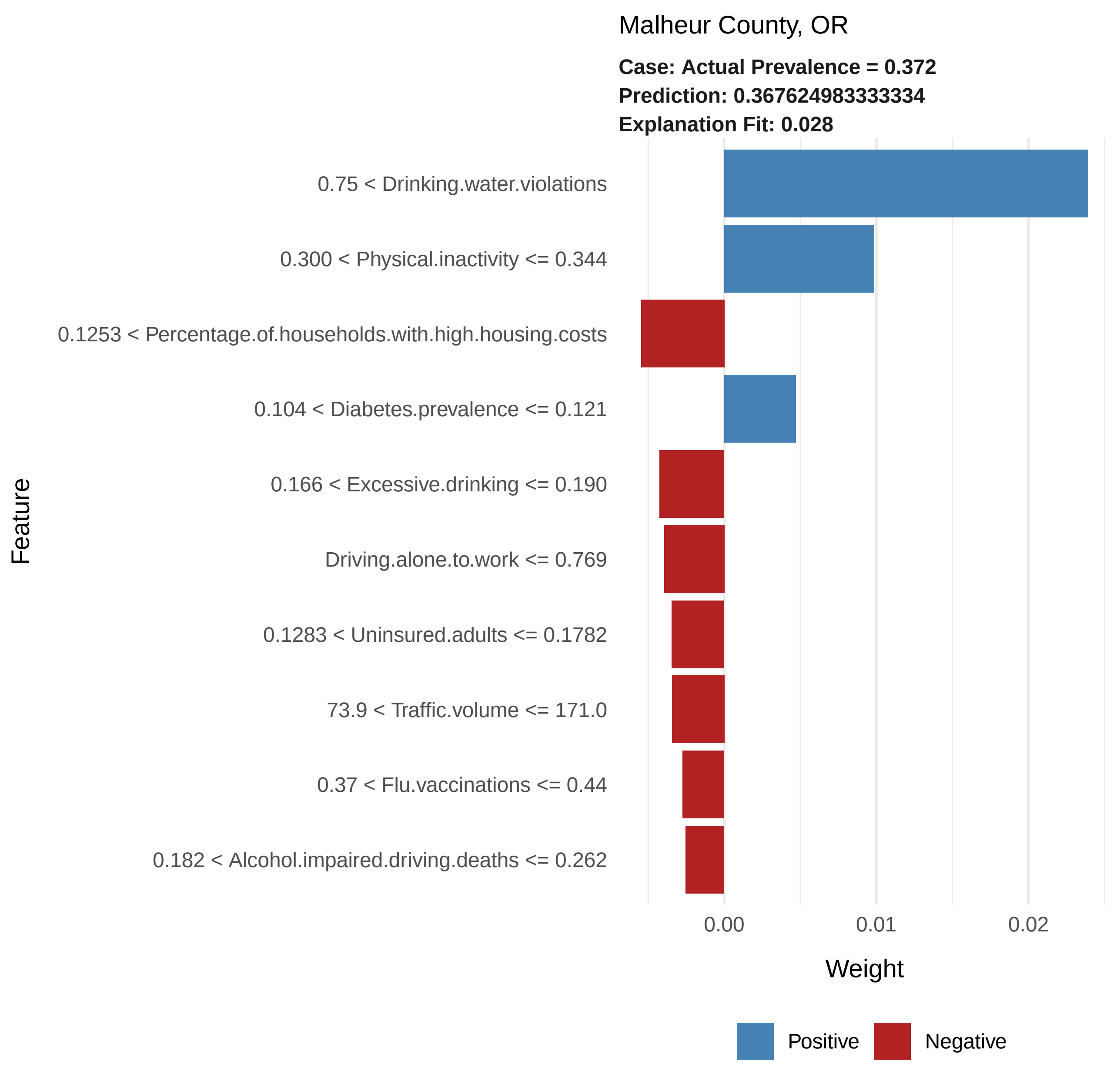

Malheur County, OR
Case: Actual Prevalence = 0.372
Prediction: 0.367624983333334
Explanation Fit: 0.028
0.75 < Drinking.water.violations
0.300 < Physical.inactivity <= 0.344
0.1253 < Percentage.of.households.with.high.housing.costs
0.104 < Diabetes.prevalence <= 0.121
0.166 < Excessive.drinking <= 0.190
Driving.alone.to.work <= 0.769
0.1283 < Uninsured.adults <= 0.1782
73.9 < Traffic.volume <= 171.0
0.37 < Flu.vaccinations <= 0.44
0.182 < Alcohol.impaired.driving.deaths <= 0.262
Feature
0.00
0.01
0.02
Weight
Positive
Negative

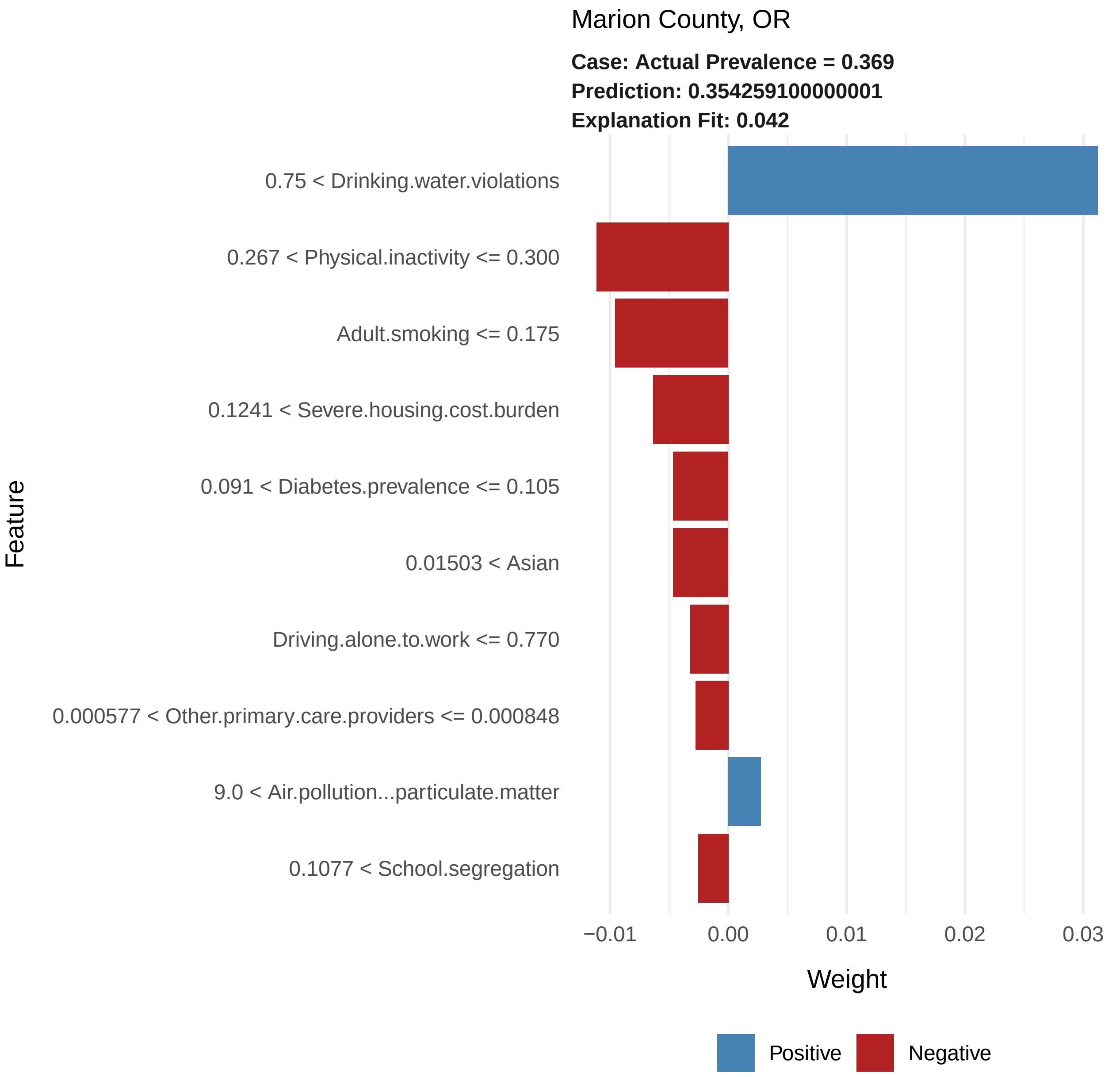

Marion County, OR
Case: Actual Prevalence = 0.369
Prediction: 0.354259100000001
Explanation Fit: 0.042
0.75 < Drinking.water.violations
0.267 < Physical.inactivity <= 0.300
Adult.smoking <= 0.175
0.1241 < Severe.housing.cost.burden
0.091 < Diabetes.prevalence <= 0.105
0.01503 < Asian
Driving.alone.to.work <= 0.770
0.000577 < Other.primary.care.providers <= 0.000848
9.0 < Air.pollution...particulate.matter
0.1077 < School.segregation
Feature
−0.01
0.00
0.01
0.02
0.03
Weight
Positive
Negative

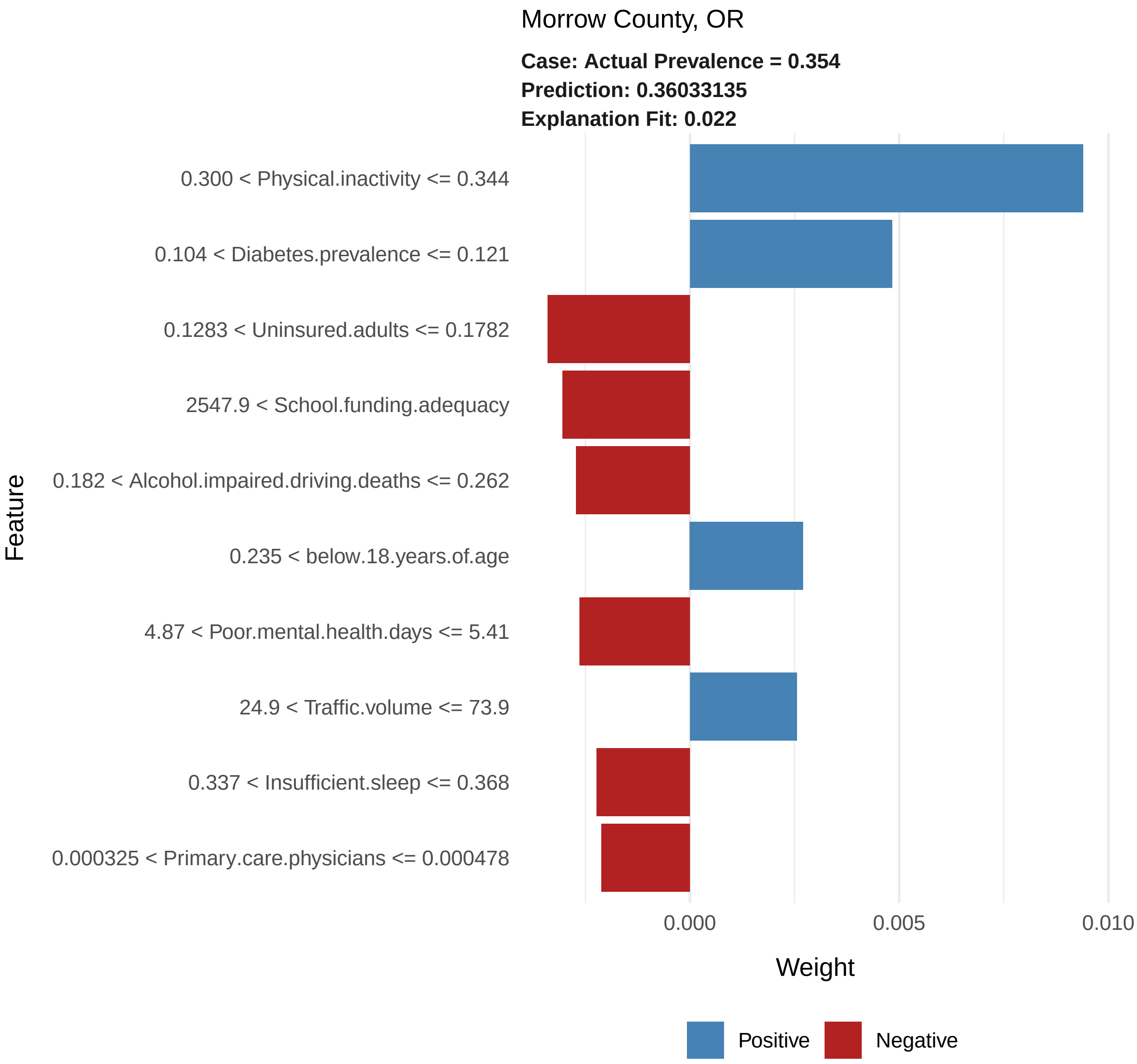

Morrow County, OR
Case: Actual Prevalence = 0.354
Prediction: 0.36033135
Explanation Fit: 0.022
0.300 < Physical.inactivity <= 0.344
0.104 < Diabetes.prevalence <= 0.121
0.1283 < Uninsured.adults <= 0.1782
2547.9 < School.funding.adequacy
0.182 < Alcohol.impaired.driving.deaths <= 0.262
0.235 < below.18.years.of.age
4.87 < Poor.mental.health.days <= 5.41
24.9 < Traffic.volume <= 73.9
0.337 < Insufficient.sleep <= 0.368
0.000325 < Primary.care.physicians <= 0.000478
Feature
0.000
0.005
0.010
Weight
Positive
Negative

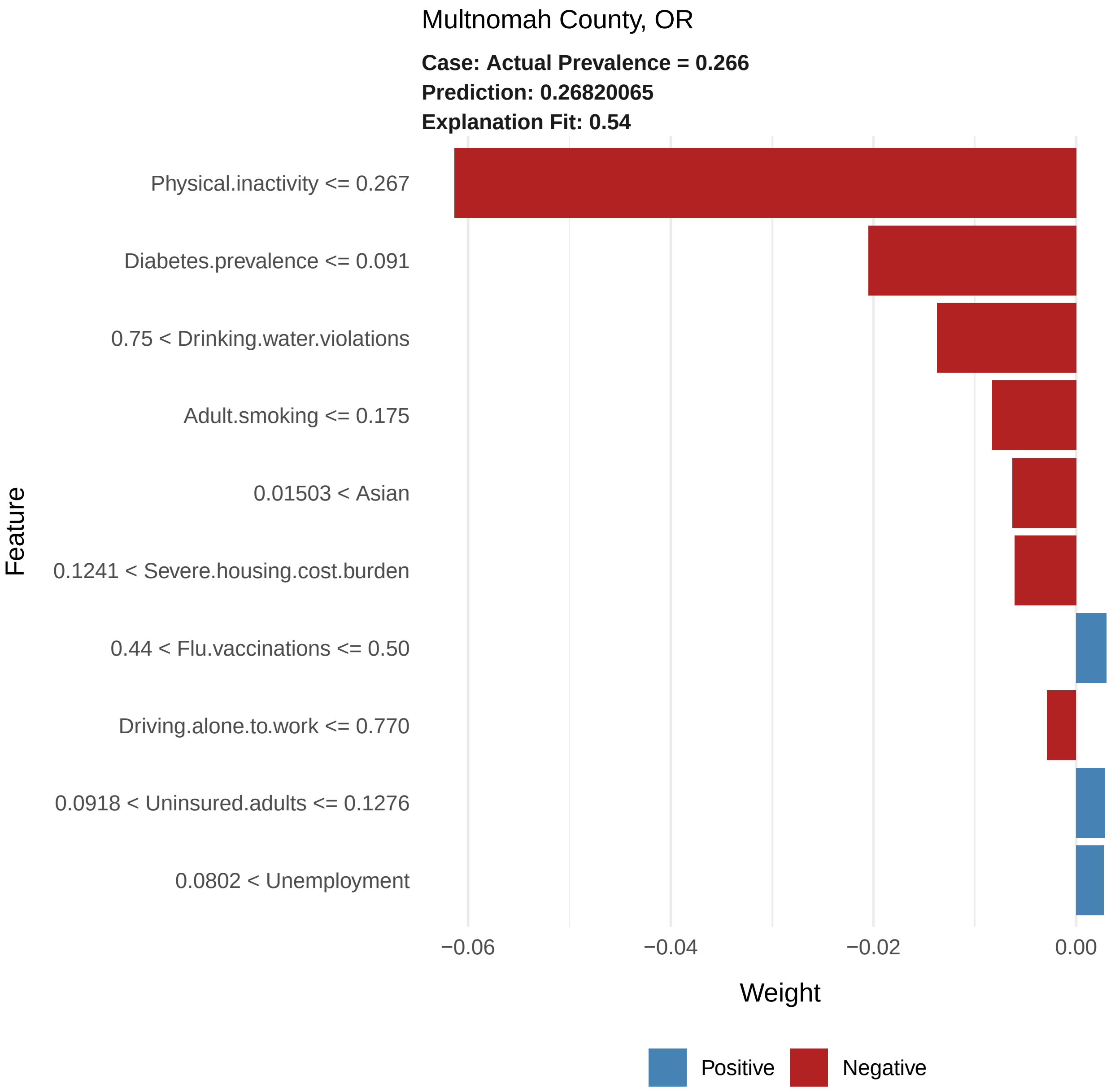

Multnomah County, OR
Case: Actual Prevalence = 0.266
Prediction: 0.26820065
Explanation Fit: 0.54
Physical.inactivity <= 0.267
Diabetes.prevalence <= 0.091
0.75 < Drinking.water.violations
Adult.smoking <= 0.175
0.01503 < Asian
0.1241 < Severe.housing.cost.burden
0.44 < Flu.vaccinations <= 0.50
Driving.alone.to.work <= 0.770
0.0918 < Uninsured.adults <= 0.1276
0.0802 < Unemployment
Feature
−0.06
−0.04
−0.02
0.00
Weight
Positive
Negative

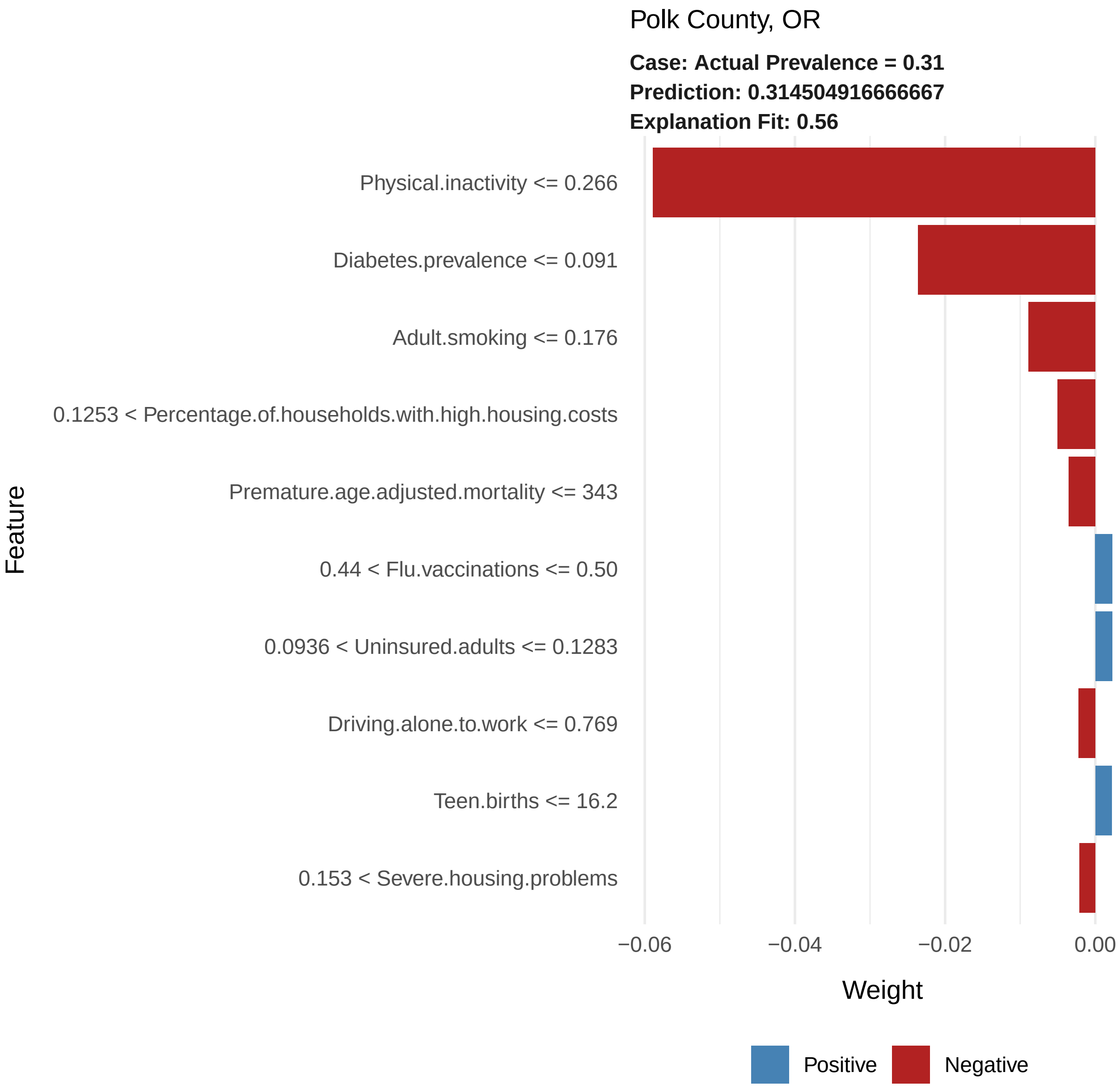
Polk County, OR
Case: Actual Prevalence = 0.31
Prediction: 0.314504916666667
Explanation Fit: 0.56
Physical.inactivity <= 0.266
Diabetes.prevalence <= 0.091
Adult.smoking <= 0.176
0.1253 < Percentage.of.households.with.high.housing.costs
Premature.age.adjusted.mortality <= 343
0.44 < Flu.vaccinations <= 0.50
0.0936 < Uninsured.adults <= 0.1283
Driving.alone.to.work <= 0.769
Teen.births <= 16.2
0.153 < Severe.housing.problems
Feature
−0.06
−0.04
−0.02
0.00
Weight
Positive
Negative

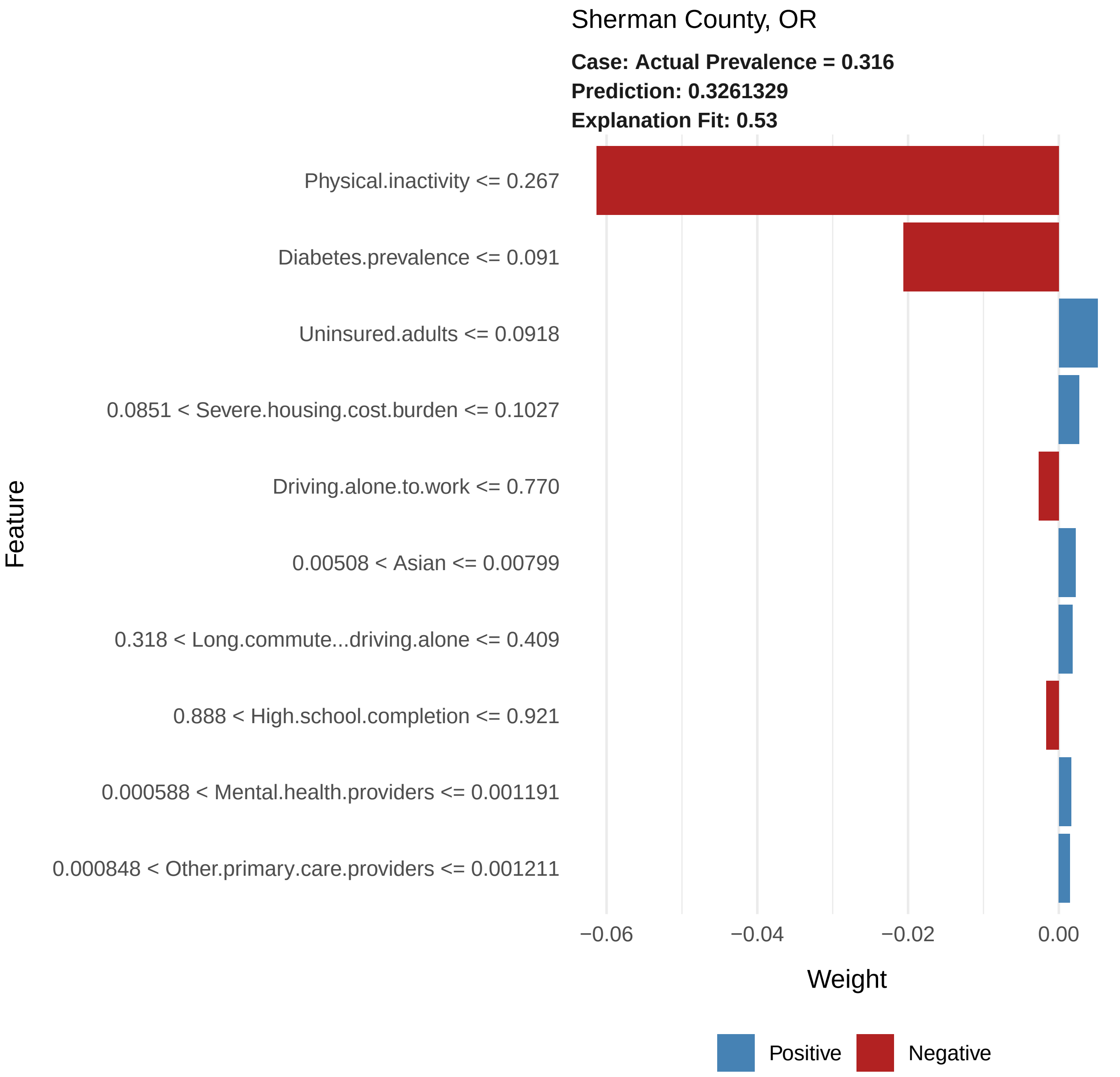
Sherman County, OR
Case: Actual Prevalence = 0.316
Prediction: 0.3261329
Explanation Fit: 0.53
Physical.inactivity <= 0.267
Diabetes.prevalence <= 0.091
Uninsured.adults <= 0.0918
0.0851 < Severe.housing.cost.burden <= 0.1027
Driving.alone.to.work <= 0.770
0.00508 < Asian <= 0.00799
0.318 < Long.commute...driving.alone <= 0.409
0.888 < High.school.completion <= 0.921
0.000588 < Mental.health.providers <= 0.001191
0.000848 < Other.primary.care.providers <= 0.001211
Feature
−0.06
−0.04
−0.02
0.00
Weight
Positive
Negative

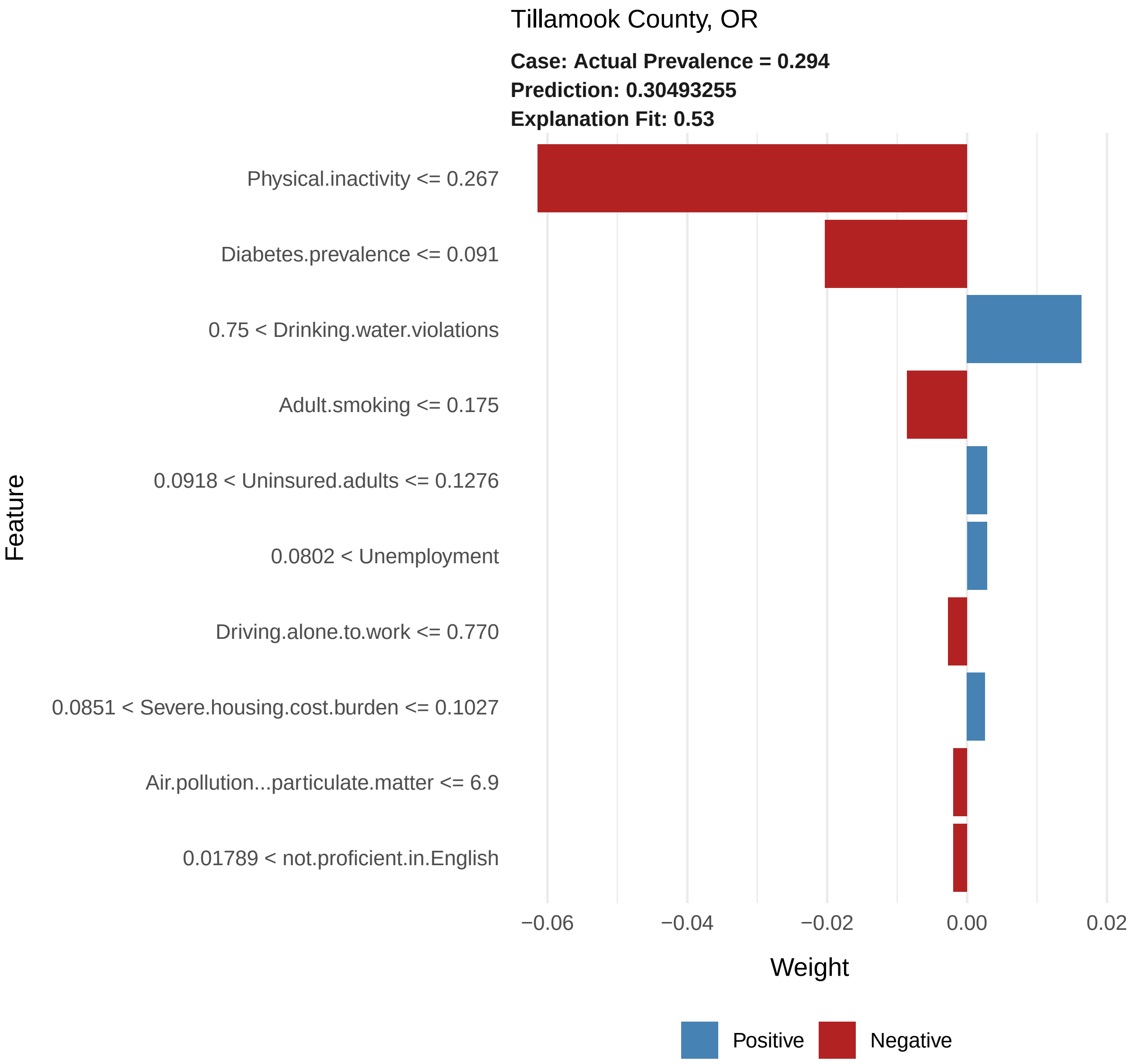

Tillamook County, OR
Case: Actual Prevalence = 0.294
Prediction: 0.30493255
Explanation Fit: 0.53
Physical.inactivity <= 0.267
Diabetes.prevalence <= 0.091
0.75 < Drinking.water.violations
Adult.smoking <= 0.175
0.0918 < Uninsured.adults <= 0.1276
0.0802 < Unemployment
Driving.alone.to.work <= 0.770
0.0851 < Severe.housing.cost.burden <= 0.1027
Air.pollution...particulate.matter <= 6.9
0.01789 < not.proficient.in.English
Feature
−0.06
−0.04
−0.02
0.00
0.02
Weight
Positive
Negative

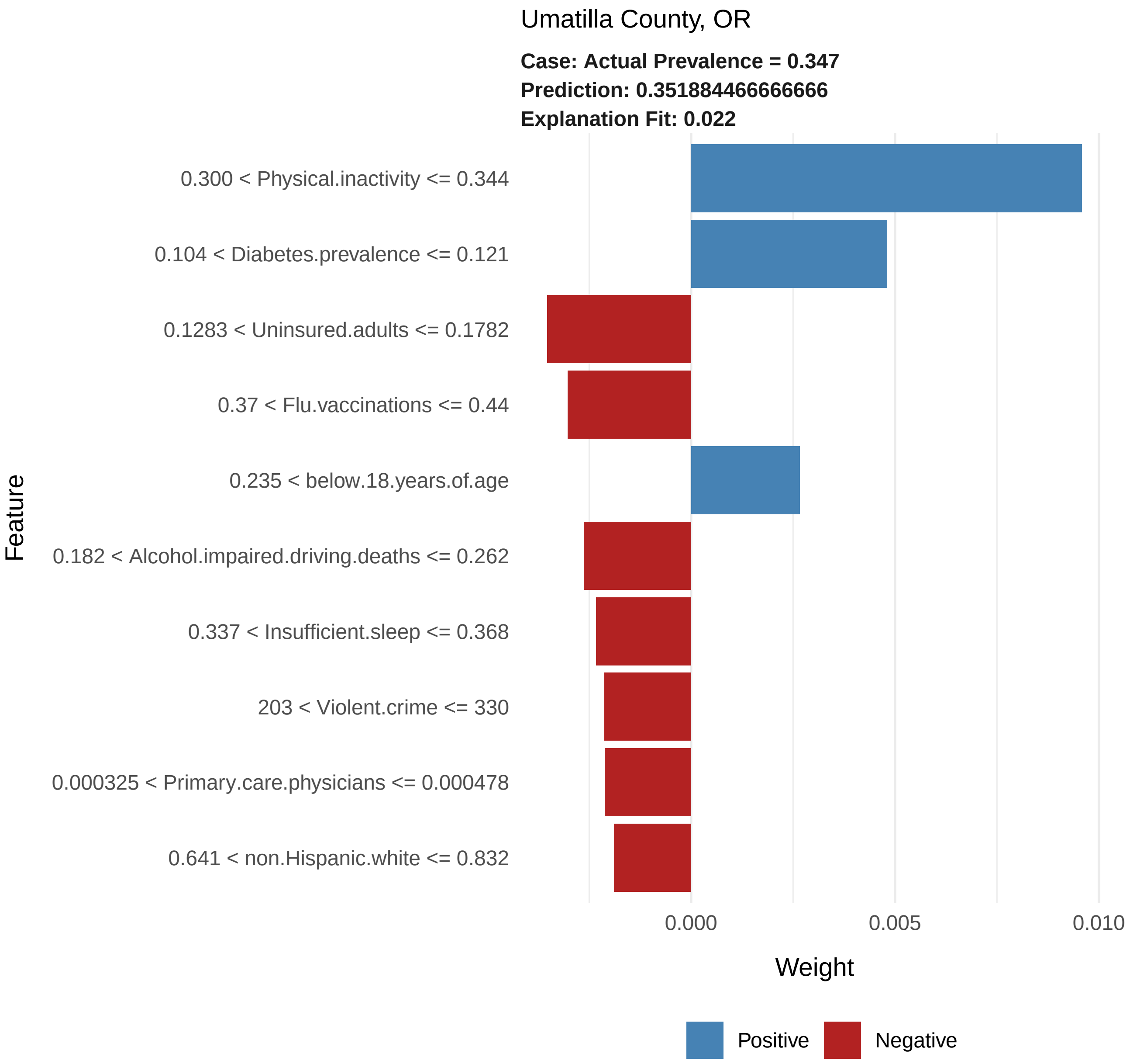
Umatilla County, OR
Case: Actual Prevalence = 0.347
Prediction: 0.351884466666666
Explanation Fit: 0.022
0.300 < Physical.inactivity <= 0.344
0.104 < Diabetes.prevalence <= 0.121
0.1283 < Uninsured.adults <= 0.1782
0.37 < Flu.vaccinations <= 0.44
0.235 < below.18.years.of.age
0.182 < Alcohol.impaired.driving.deaths <= 0.262
0.337 < Insufficient.sleep <= 0.368
203 < Violent.crime <= 330
0.000325 < Primary.care.physicians <= 0.000478
0.641 < non.Hispanic.white <= 0.832
Feature
0.000
0.005
0.010
Weight
Positive
Negative

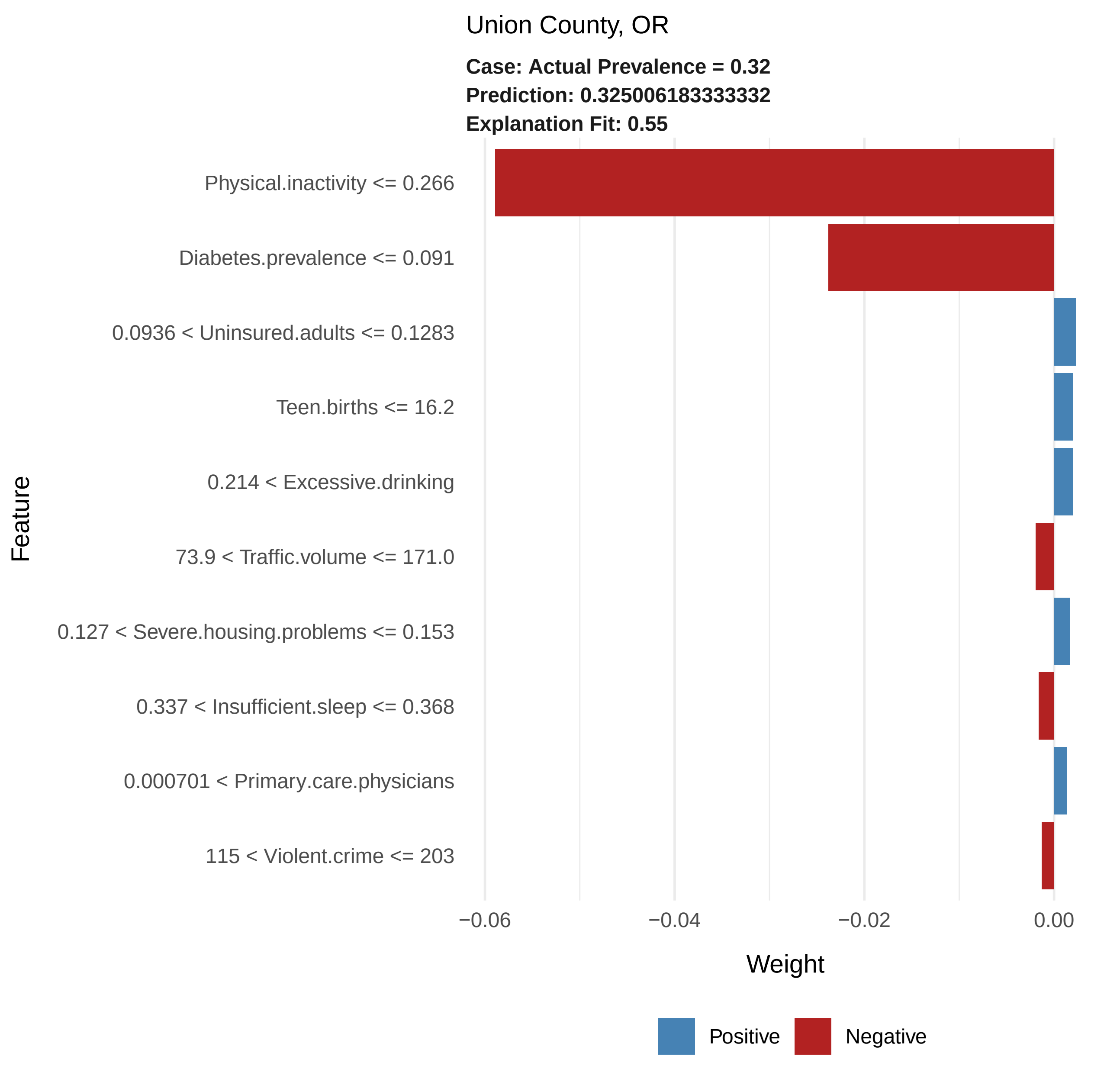

Union County, OR
Case: Actual Prevalence = 0.32
Prediction: 0.325006183333332
Explanation Fit: 0.55
Physical.inactivity <= 0.266
Diabetes.prevalence <= 0.091
0.0936 < Uninsured.adults <= 0.1283
Teen.births <= 16.2
0.214 < Excessive.drinking
73.9 < Traffic.volume <= 171.0
0.127 < Severe.housing.problems <= 0.153
0.337 < Insufficient.sleep <= 0.368
0.000701 < Primary.care.physicians
115 < Violent.crime <= 203
Feature
−0.06
−0.04
−0.02
0.00
Weight
Positive
Negative

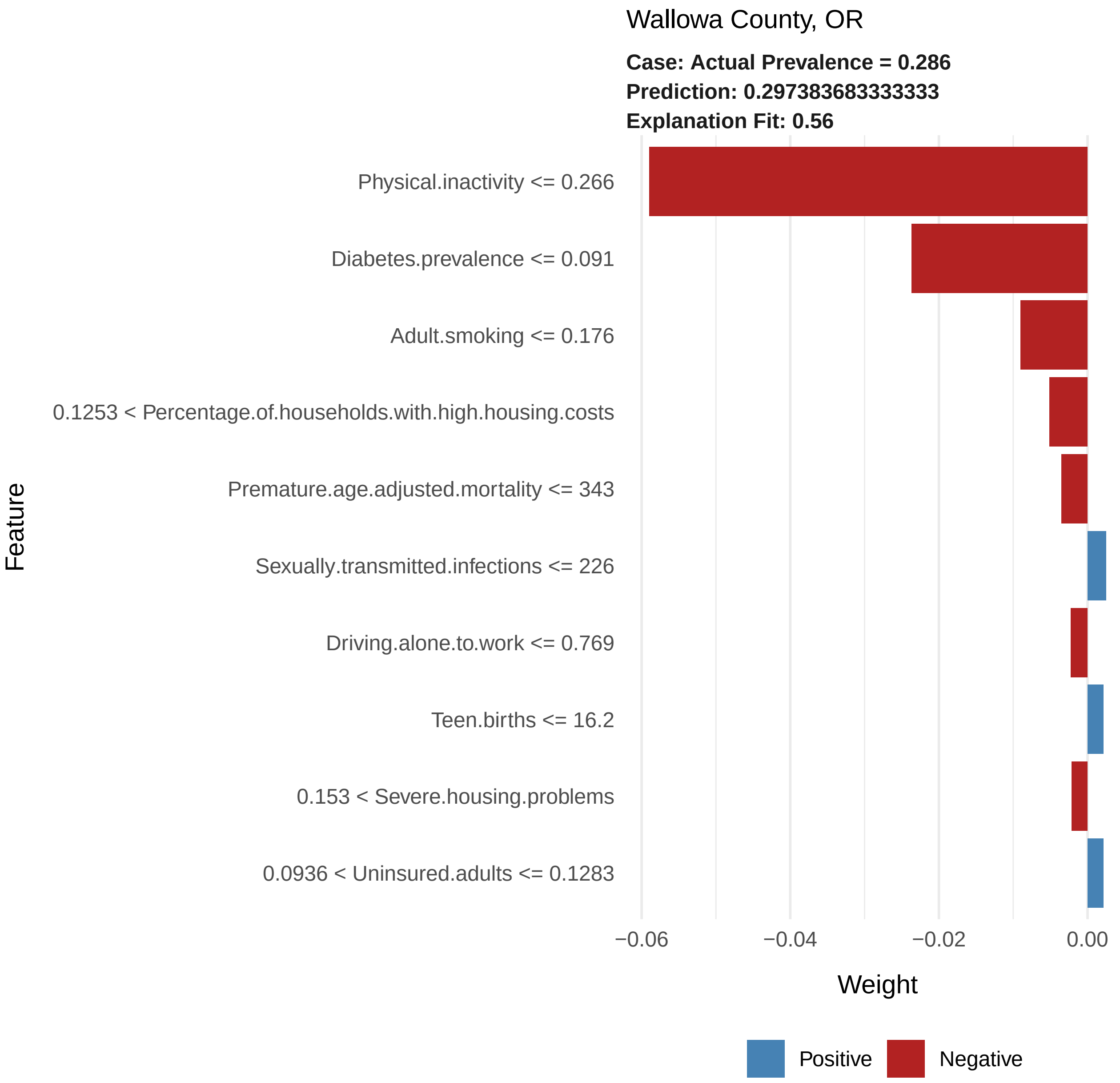

Wallowa County, OR
Case: Actual Prevalence = 0.286
Prediction: 0.297383683333333
Explanation Fit: 0.56
Physical.inactivity <= 0.266
Diabetes.prevalence <= 0.091
Adult.smoking <= 0.176
0.1253 < Percentage.of.households.with.high.housing.costs
Premature.age.adjusted.mortality <= 343
Sexually.transmitted.infections <= 226
Driving.alone.to.work <= 0.769
Teen.births <= 16.2
0.153 < Severe.housing.problems
0.0936 < Uninsured.adults <= 0.1283
Feature
−0.06
−0.04
−0.02
0.00
Weight
Positive
Negative

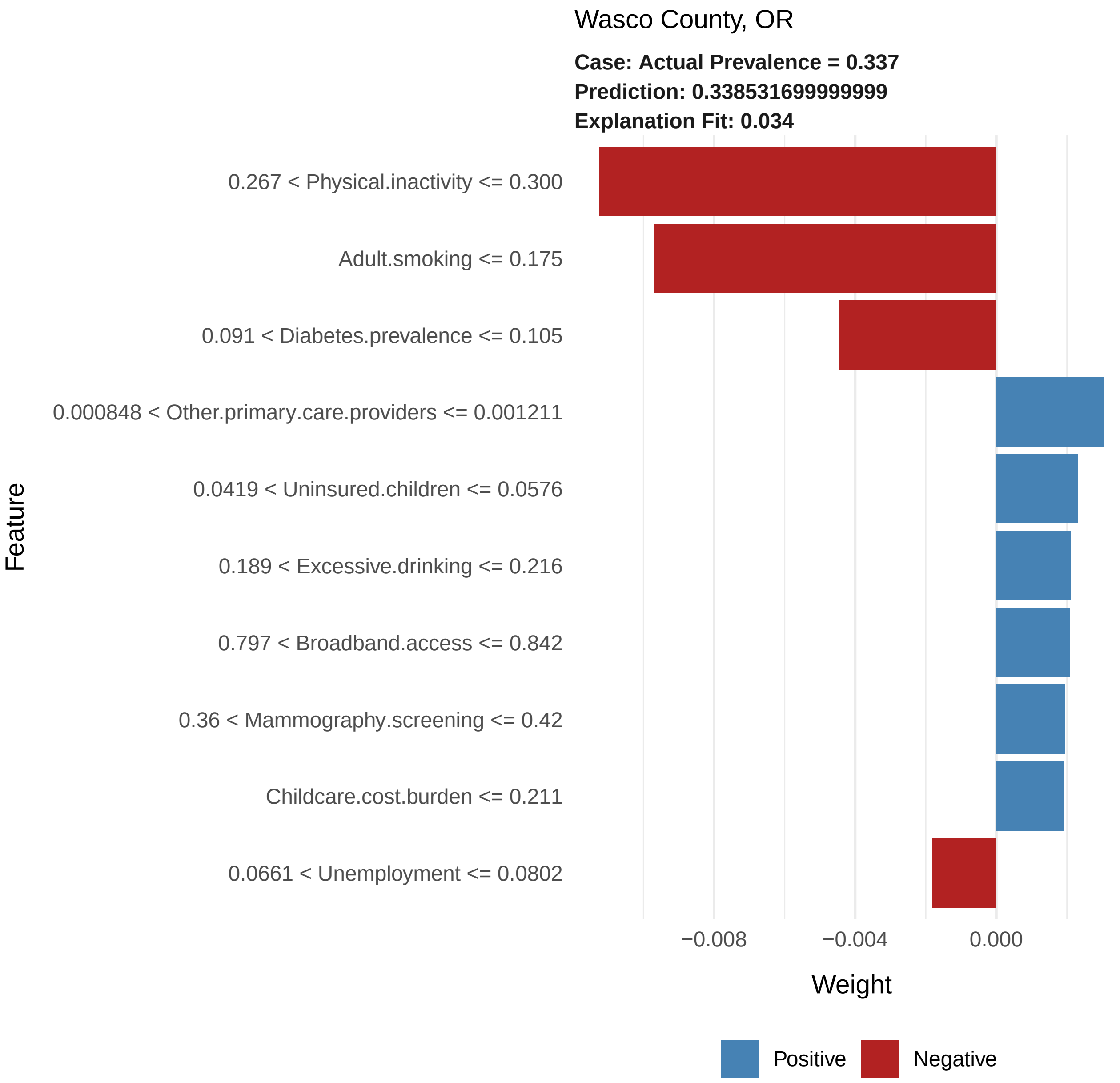

Wasco County, OR
Case: Actual Prevalence = 0.337
Prediction: 0.338531699999999
Explanation Fit: 0.034
0.267 < Physical.inactivity <= 0.300
Adult.smoking <= 0.175
0.091 < Diabetes.prevalence <= 0.105
0.000848 < Other.primary.care.providers <= 0.001211
0.0419 < Uninsured.children <= 0.0576
0.189 < Excessive.drinking <= 0.216
0.797 < Broadband.access <= 0.842
0.36 < Mammography.screening <= 0.42
Childcare.cost.burden <= 0.211
0.0661 < Unemployment <= 0.0802
Feature
−0.008
−0.004
0.000
Weight
Positive
Negative

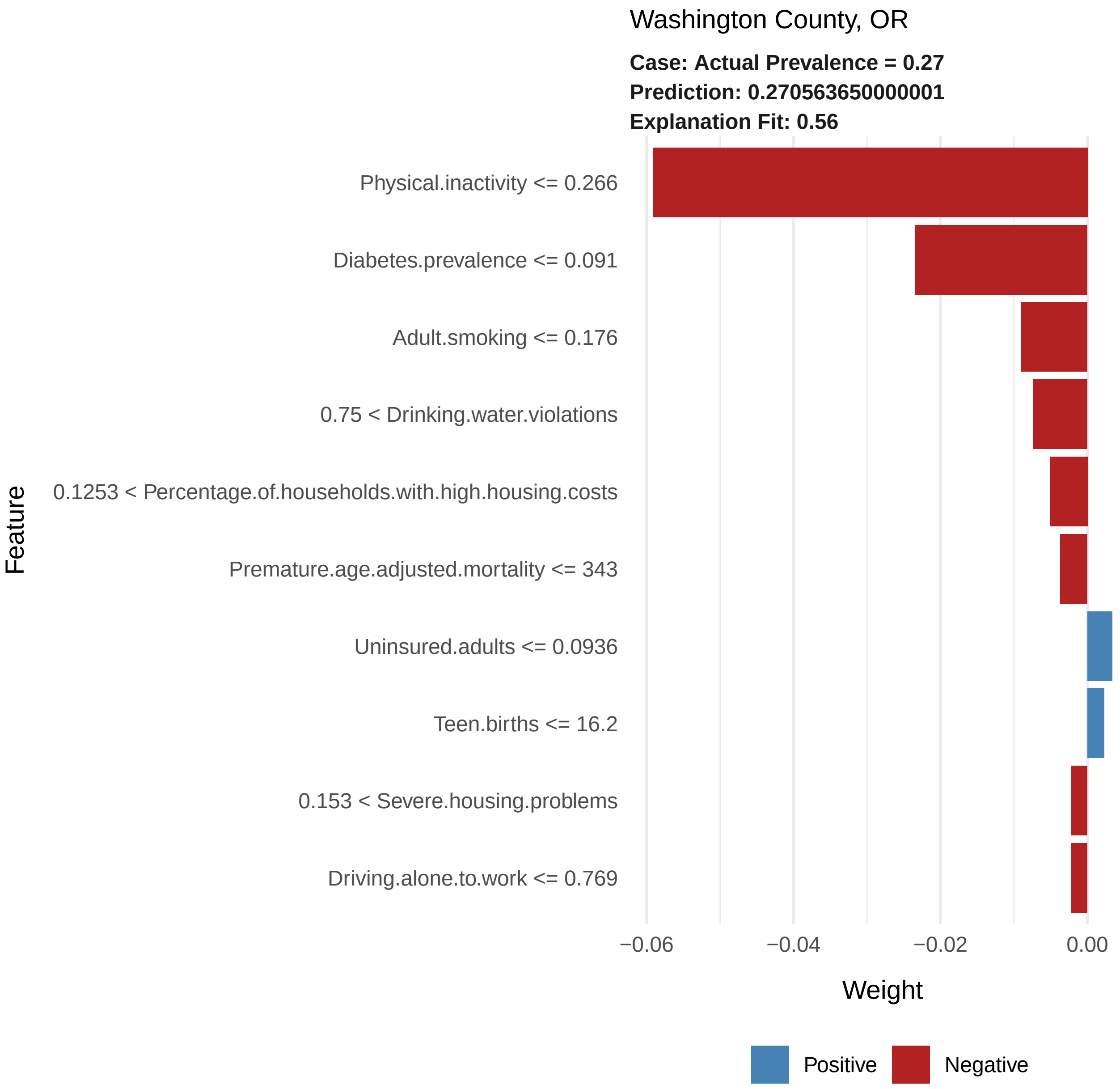

Washington County, OR
Case: Actual Prevalence = 0.27
Prediction: 0.270563650000001
Explanation Fit: 0.56
Physical.inactivity <= 0.266
Diabetes.prevalence <= 0.091
Adult.smoking <= 0.176
0.75 < Drinking.water.violations
0.1253 < Percentage.of.households.with.high.housing.costs
Premature.age.adjusted.mortality <= 343
Uninsured.adults <= 0.0936
Teen.births <= 16.2
0.153 < Severe.housing.problems
Driving.alone.to.work <= 0.769
Feature
−0.06
−0.04
−0.02
0.00
Weight
Positive
Negative

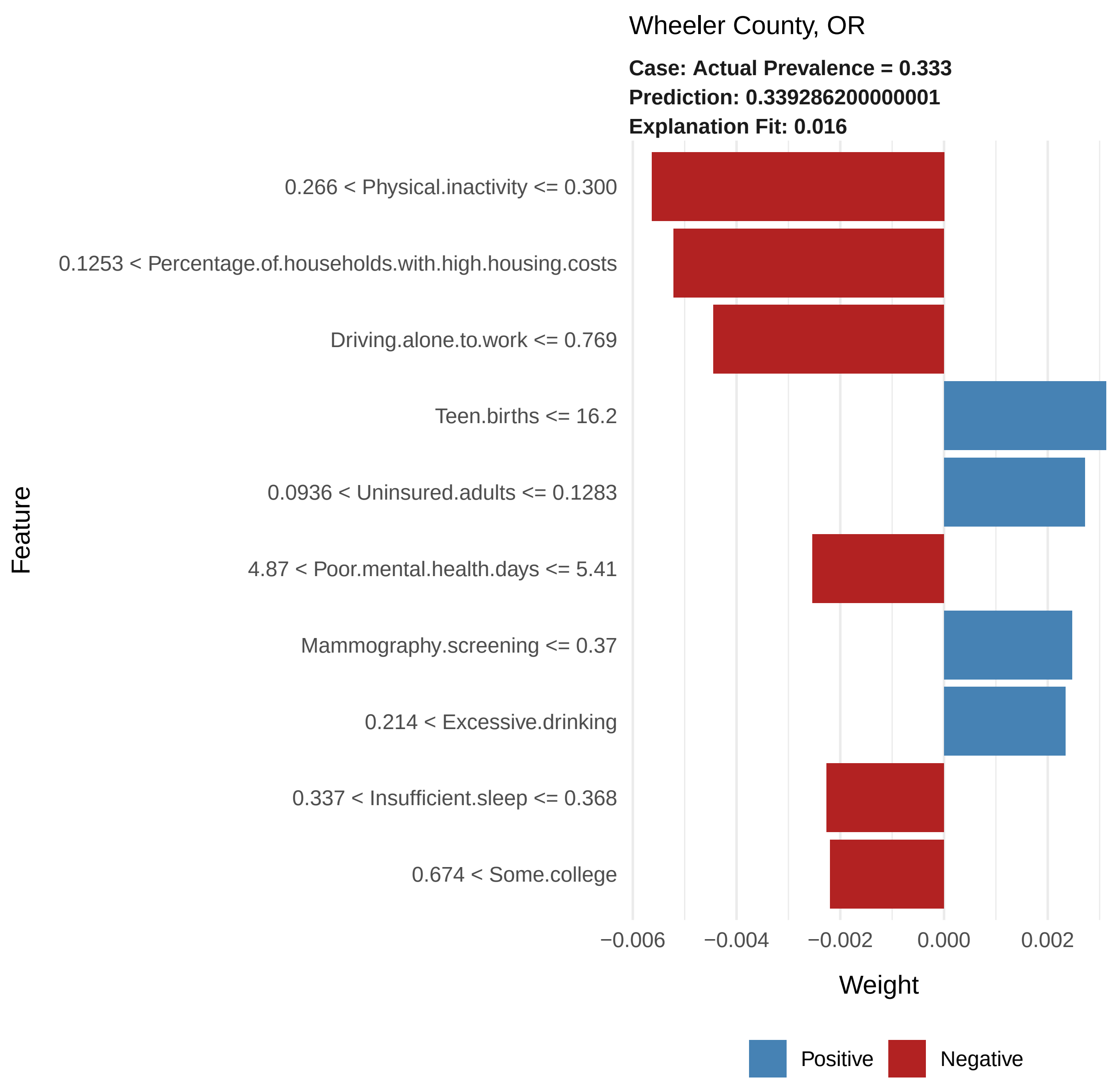

Wheeler County, OR
Case: Actual Prevalence = 0.333
Prediction: 0.339286200000001
Explanation Fit: 0.016
0.266 < Physical.inactivity <= 0.300
0.1253 < Percentage.of.households.with.high.housing.costs
Driving.alone.to.work <= 0.769
Teen.births <= 16.2
0.0936 < Uninsured.adults <= 0.1283
4.87 < Poor.mental.health.days <= 5.41
Mammography.screening <= 0.37
0.214 < Excessive.drinking
0.337 < Insufficient.sleep <= 0.368
0.674 < Some.college
Feature
−0.006
−0.004
−0.002
0.000
0.002
Weight
Positive
Negative

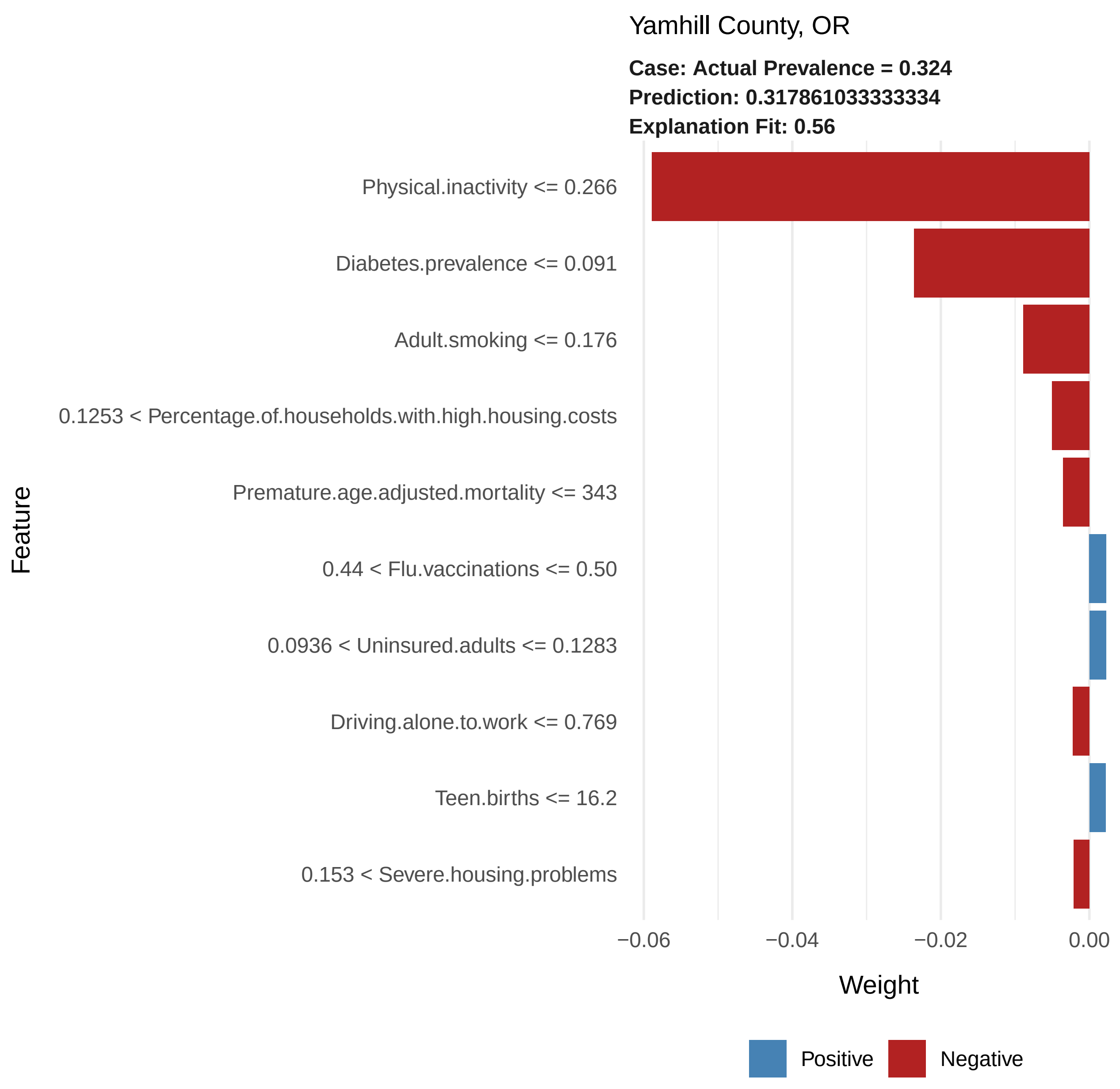

Yamhill County, OR
Case: Actual Prevalence = 0.324
Prediction: 0.317861033333334
Explanation Fit: 0.56
Physical.inactivity <= 0.266
Diabetes.prevalence <= 0.091
Adult.smoking <= 0.176
0.1253 < Percentage.of.households.with.high.housing.costs
Premature.age.adjusted.mortality <= 343
0.44 < Flu.vaccinations <= 0.50
0.0936 < Uninsured.adults <= 0.1283
Driving.alone.to.work <= 0.769
Teen.births <= 16.2
0.153 < Severe.housing.problems
Feature
−0.06
−0.04
−0.02
0.00
Weight
Positive
Negative

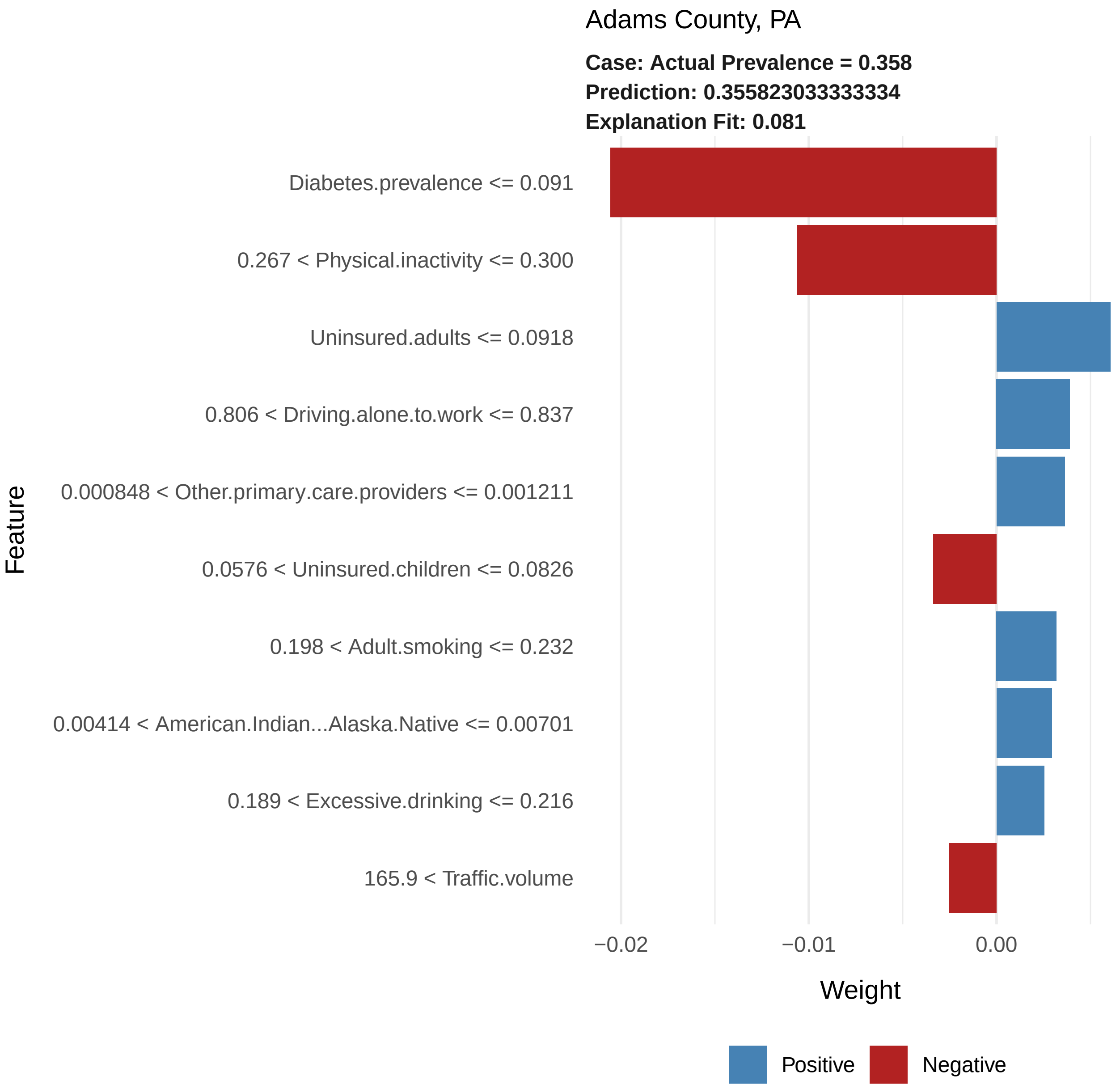
Adams County, PA
Case: Actual Prevalence = 0.358
Prediction: 0.355823033333334
Explanation Fit: 0.081
Diabetes.prevalence <= 0.091
0.267 < Physical.inactivity <= 0.300
Uninsured.adults <= 0.0918
0.806 < Driving.alone.to.work <= 0.837
0.000848 < Other.primary.care.providers <= 0.001211
0.0576 < Uninsured.children <= 0.0826
0.198 < Adult.smoking <= 0.232
0.00414 < American.Indian...Alaska.Native <= 0.00701
0.189 < Excessive.drinking <= 0.216
165.9 < Traffic.volume
Feature
−0.02
−0.01
0.00
Weight
Positive
Negative

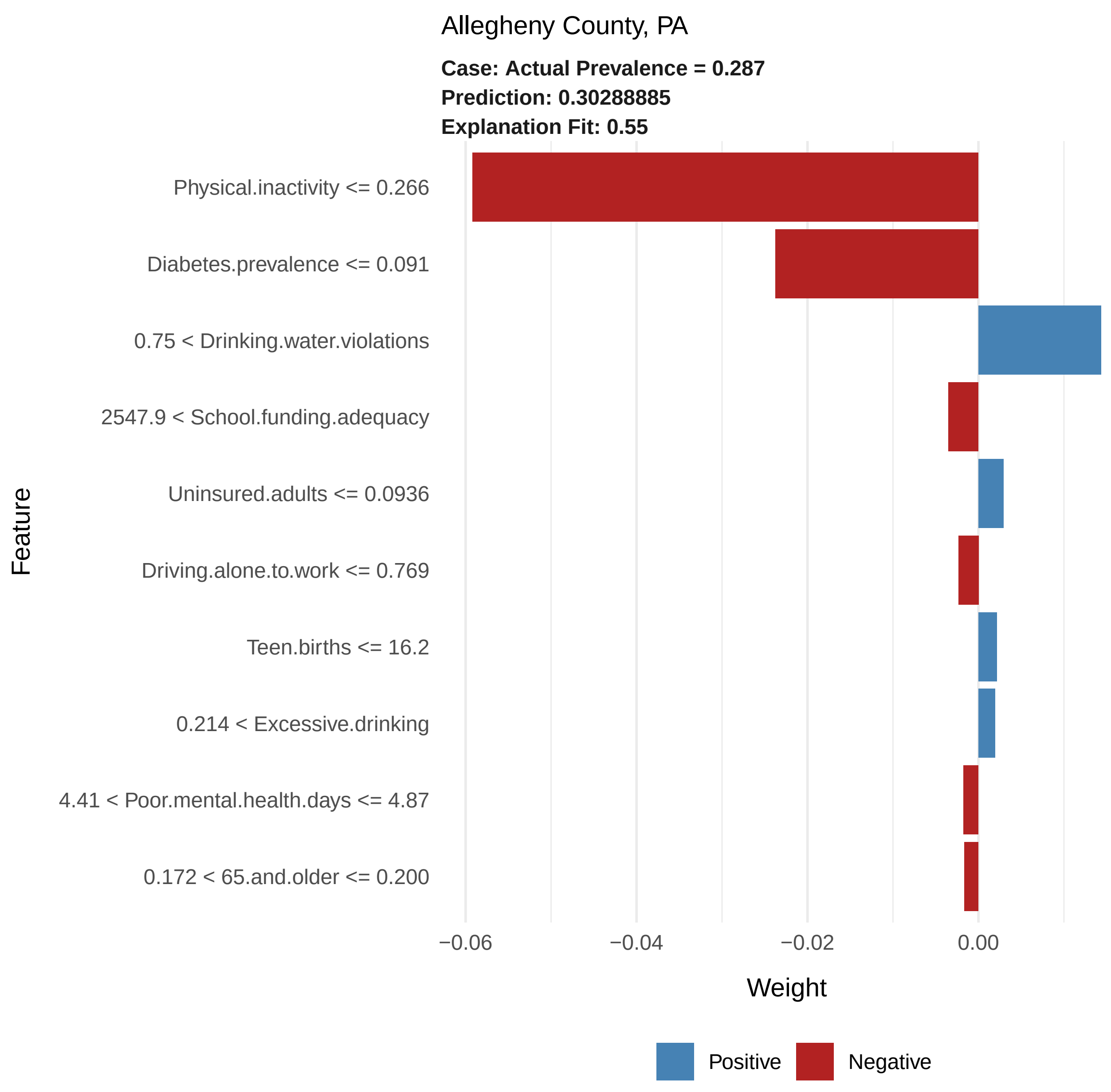

Allegheny County, PA
Case: Actual Prevalence = 0.287
Prediction: 0.30288885
Explanation Fit: 0.55
Physical.inactivity <= 0.266
Diabetes.prevalence <= 0.091
0.75 < Drinking.water.violations
2547.9 < School.funding.adequacy
Uninsured.adults <= 0.0936
Driving.alone.to.work <= 0.769
Teen.births <= 16.2
0.214 < Excessive.drinking
4.41 < Poor.mental.health.days <= 4.87
0.172 < 65.and.older <= 0.200
Feature
−0.06
−0.04
−0.02
0.00
Weight
Positive
Negative

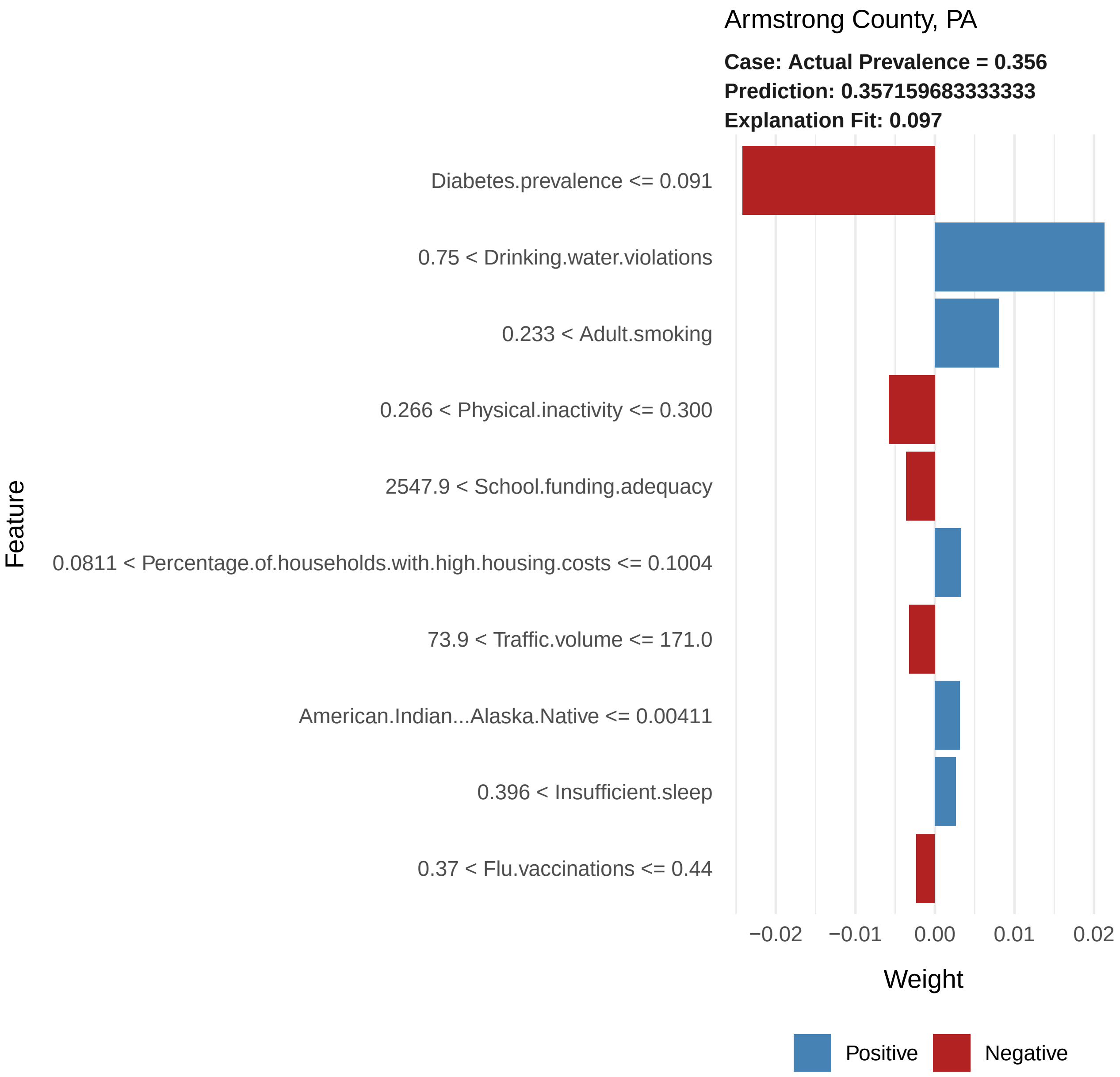
Armstrong County, PA
Case: Actual Prevalence = 0.356
Prediction: 0.357159683333333
Explanation Fit: 0.097
Diabetes.prevalence <= 0.091
0.75 < Drinking.water.violations
0.233 < Adult.smoking
0.266 < Physical.inactivity <= 0.300
2547.9 < School.funding.adequacy
0.0811 < Percentage.of.households.with.high.housing.costs <= 0.1004
73.9 < Traffic.volume <= 171.0
American.Indian...Alaska.Native <= 0.00411
0.396 < Insufficient.sleep
0.37 < Flu.vaccinations <= 0.44
Feature
−0.02
−0.01
0.00
0.01
0.02
Weight
Positive
Negative

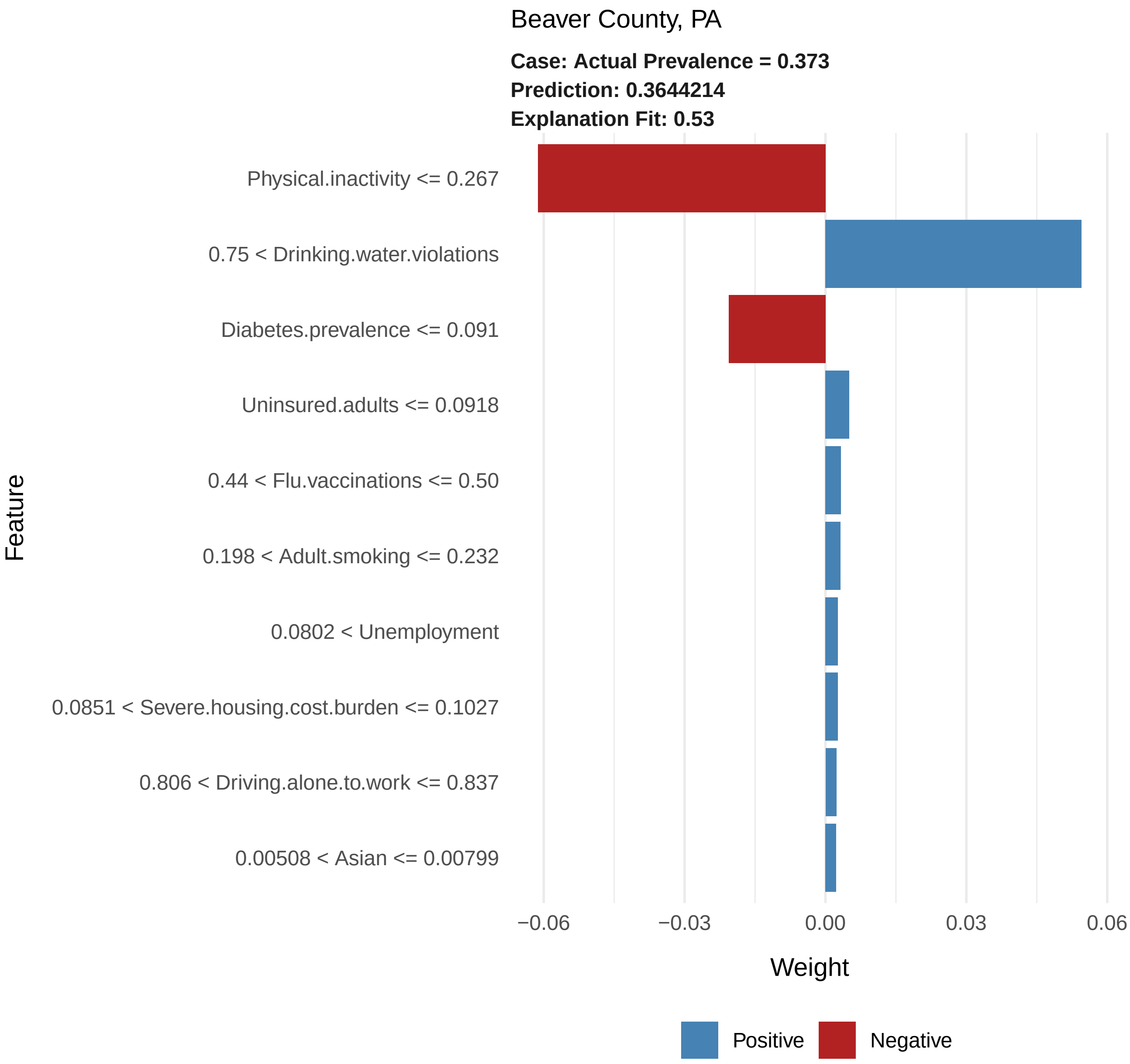

Beaver County, PA
Case: Actual Prevalence = 0.373
Prediction: 0.3644214
Explanation Fit: 0.53
Physical.inactivity <= 0.267
0.75 < Drinking.water.violations
Diabetes.prevalence <= 0.091
Uninsured.adults <= 0.0918
0.44 < Flu.vaccinations <= 0.50
0.198 < Adult.smoking <= 0.232
0.0802 < Unemployment
0.0851 < Severe.housing.cost.burden <= 0.1027
0.806 < Driving.alone.to.work <= 0.837
0.00508 < Asian <= 0.00799
Feature
−0.06
−0.03
0.00
0.03
0.06
Weight
Positive
Negative

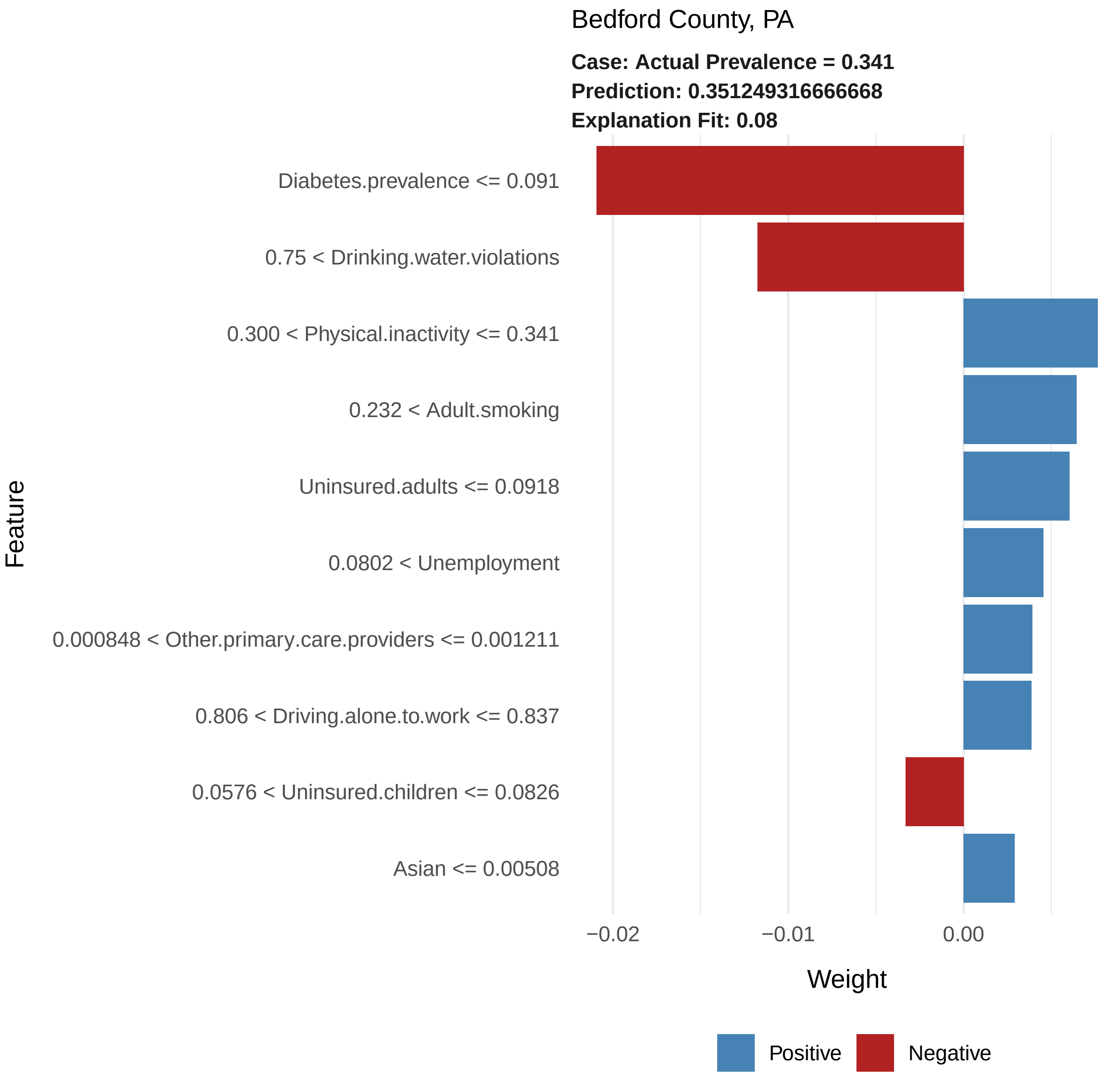

Bedford County, PA
Case: Actual Prevalence = 0.341
Prediction: 0.351249316666668
Explanation Fit: 0.08
Diabetes.prevalence <= 0.091
0.75 < Drinking.water.violations
0.300 < Physical.inactivity <= 0.341
0.232 < Adult.smoking
Uninsured.adults <= 0.0918
0.0802 < Unemployment
0.000848 < Other.primary.care.providers <= 0.001211
0.806 < Driving.alone.to.work <= 0.837
0.0576 < Uninsured.children <= 0.0826
Asian <= 0.00508
Feature
−0.02
−0.01
0.00
Weight
Positive
Negative

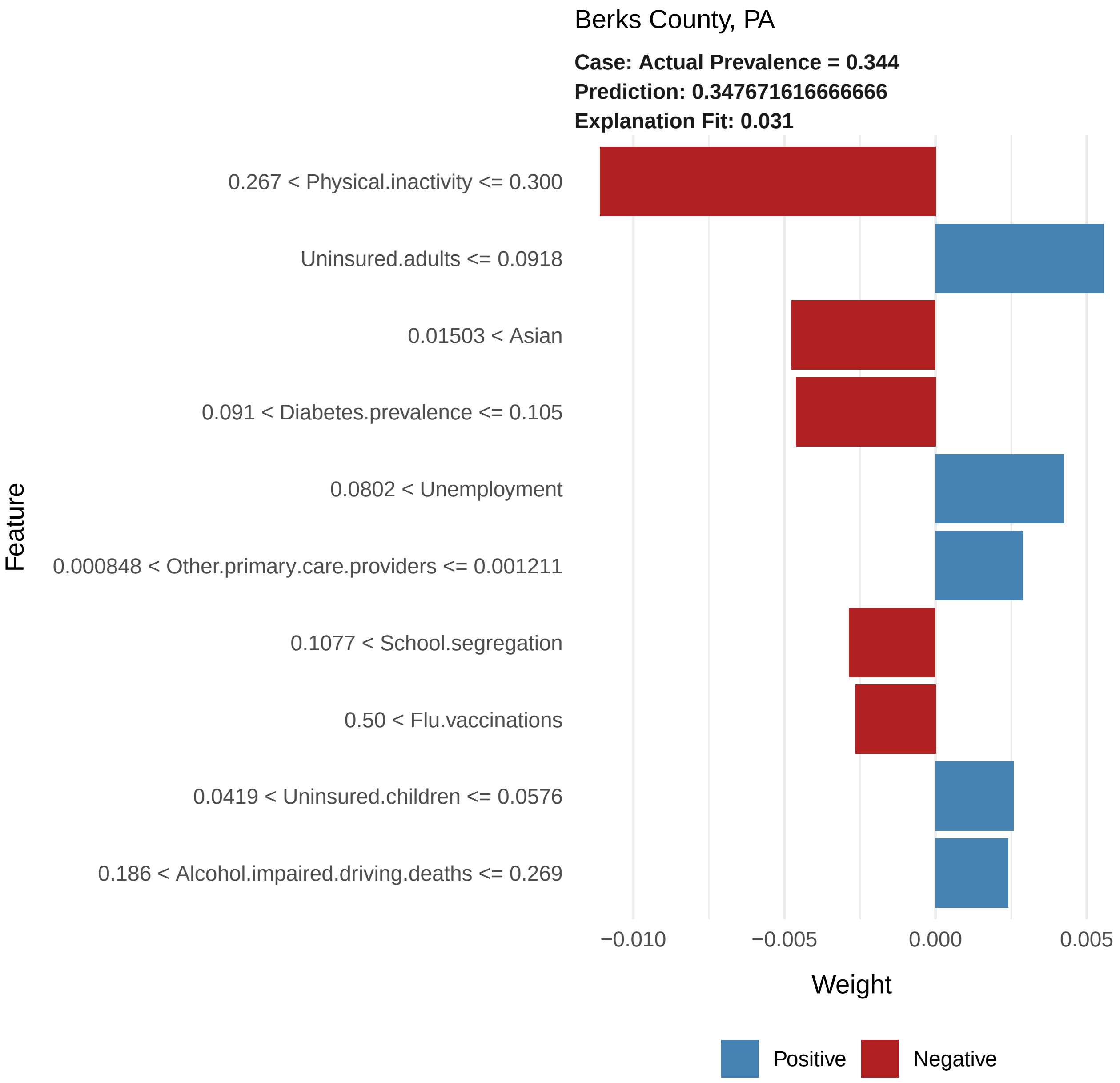
Berks County, PA
Case: Actual Prevalence = 0.344
Prediction: 0.347671616666666
Explanation Fit: 0.031
0.267 < Physical.inactivity <= 0.300
Uninsured.adults <= 0.0918
0.01503 < Asian
0.091 < Diabetes.prevalence <= 0.105
0.0802 < Unemployment
0.000848 < Other.primary.care.providers <= 0.001211
0.1077 < School.segregation
0.50 < Flu.vaccinations
0.0419 < Uninsured.children <= 0.0576
0.186 < Alcohol.impaired.driving.deaths <= 0.269
Feature
−0.010
−0.005
0.000
0.005
Weight
Positive
Negative

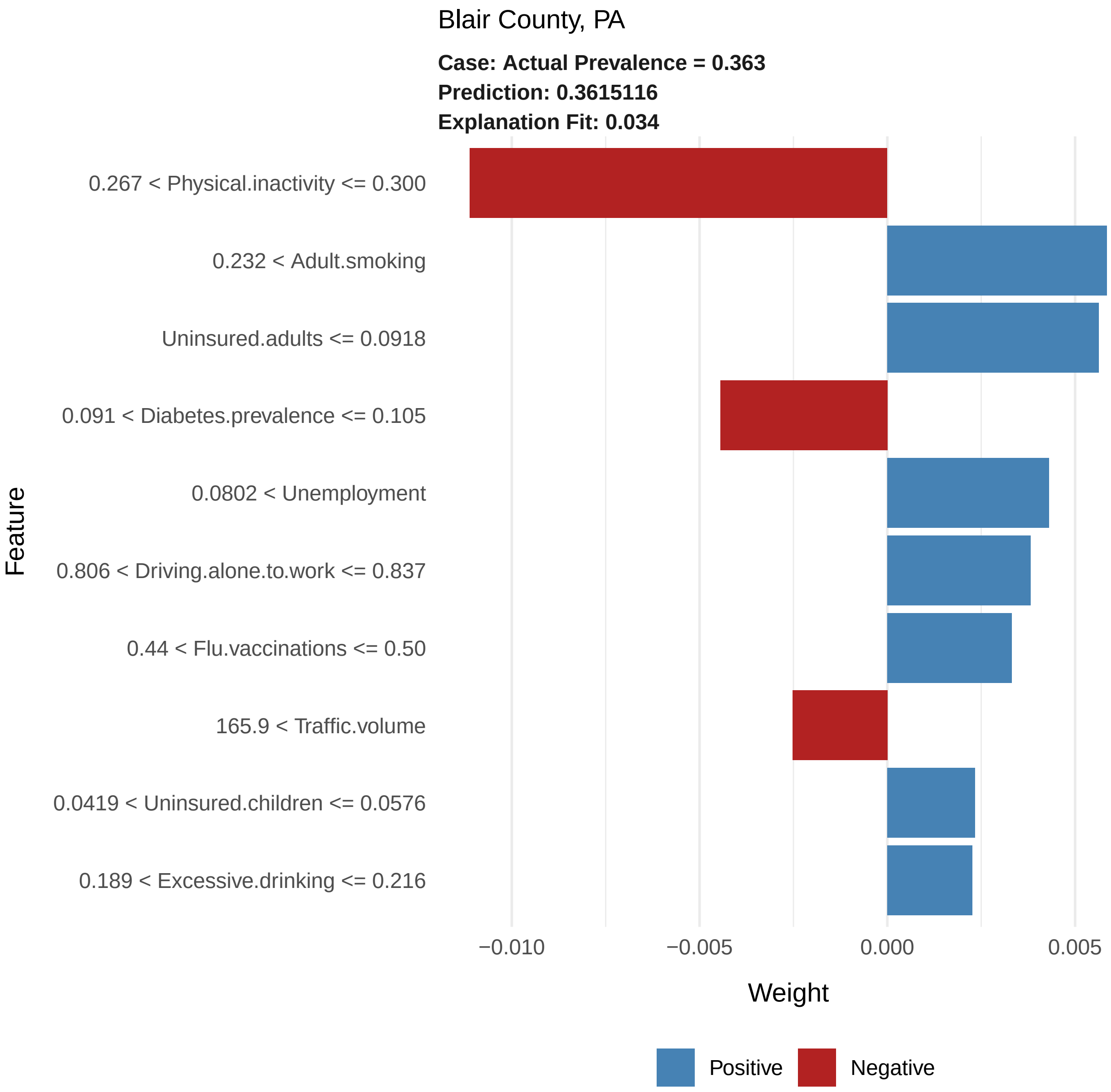

Blair County, PA
Case: Actual Prevalence = 0.363
Prediction: 0.3615116
Explanation Fit: 0.034
0.267 < Physical.inactivity <= 0.300
0.232 < Adult.smoking
Uninsured.adults <= 0.0918
0.091 < Diabetes.prevalence <= 0.105
0.0802 < Unemployment
0.806 < Driving.alone.to.work <= 0.837
0.44 < Flu.vaccinations <= 0.50
165.9 < Traffic.volume
0.0419 < Uninsured.children <= 0.0576
0.189 < Excessive.drinking <= 0.216
Feature
−0.010
−0.005
0.000
0.005
Weight
Positive
Negative

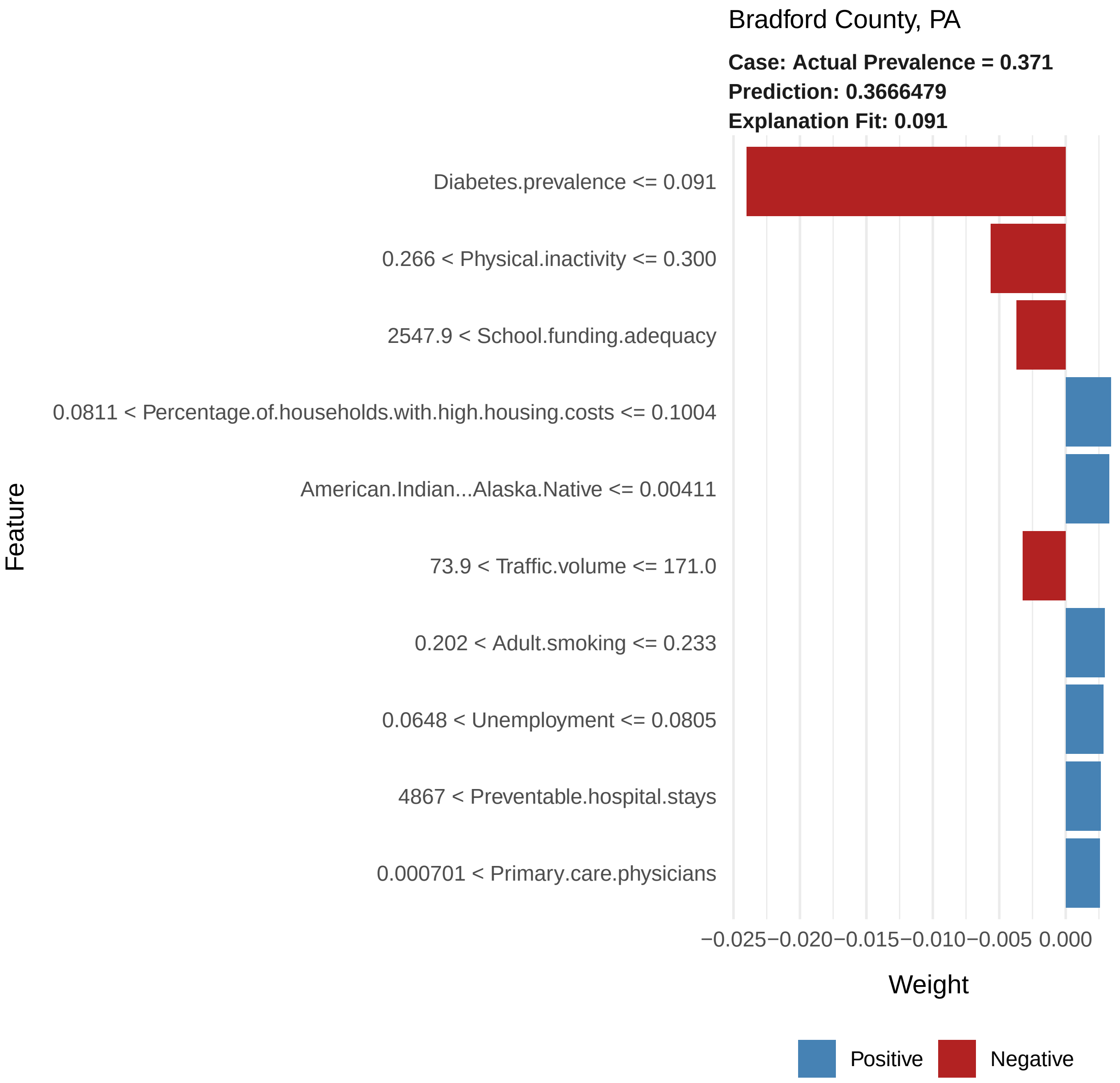
Bradford County, PA
Case: Actual Prevalence = 0.371
Prediction: 0.3666479
Explanation Fit: 0.091
Diabetes.prevalence <= 0.091
0.266 < Physical.inactivity <= 0.300
2547.9 < School.funding.adequacy
0.0811 < Percentage.of.households.with.high.housing.costs <= 0.1004
American.Indian...Alaska.Native <= 0.00411
73.9 < Traffic.volume <= 171.0
0.202 < Adult.smoking <= 0.233
0.0648 < Unemployment <= 0.0805
4867 < Preventable.hospital.stays
0.000701 < Primary.care.physicians
Feature
−0.025
−0.020
−0.015
−0.010
−0.005
0.000
Weight
Positive
Negative

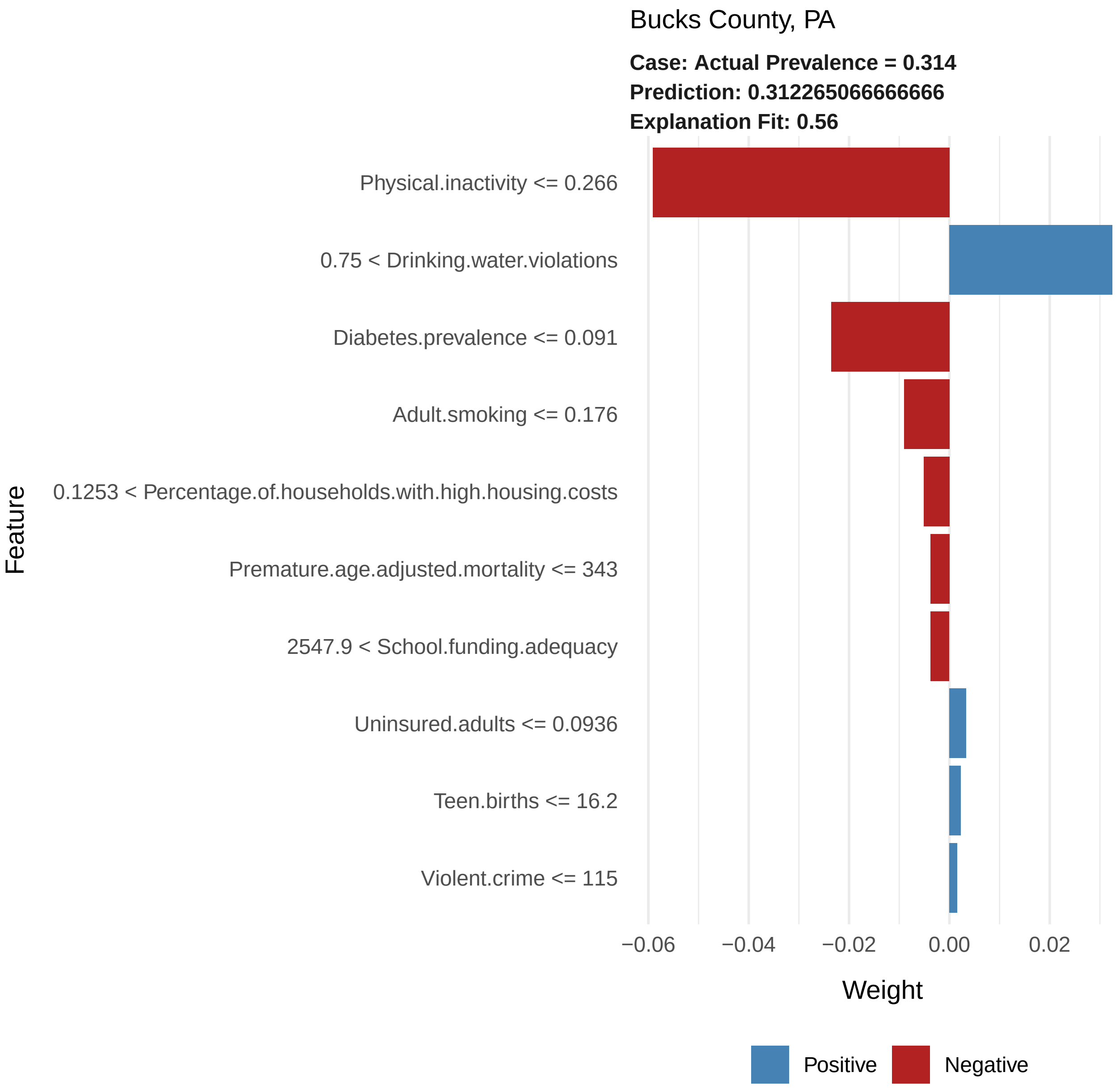

Bucks County, PA
Case: Actual Prevalence = 0.314
Prediction: 0.312265066666666
Explanation Fit: 0.56
Physical.inactivity <= 0.266
0.75 < Drinking.water.violations
Diabetes.prevalence <= 0.091
Adult.smoking <= 0.176
0.1253 < Percentage.of.households.with.high.housing.costs
Premature.age.adjusted.mortality <= 343
2547.9 < School.funding.adequacy
Uninsured.adults <= 0.0936
Teen.births <= 16.2
Violent.crime <= 115
Feature
−0.06
−0.04
−0.02
0.00
0.02
Weight
Positive
Negative

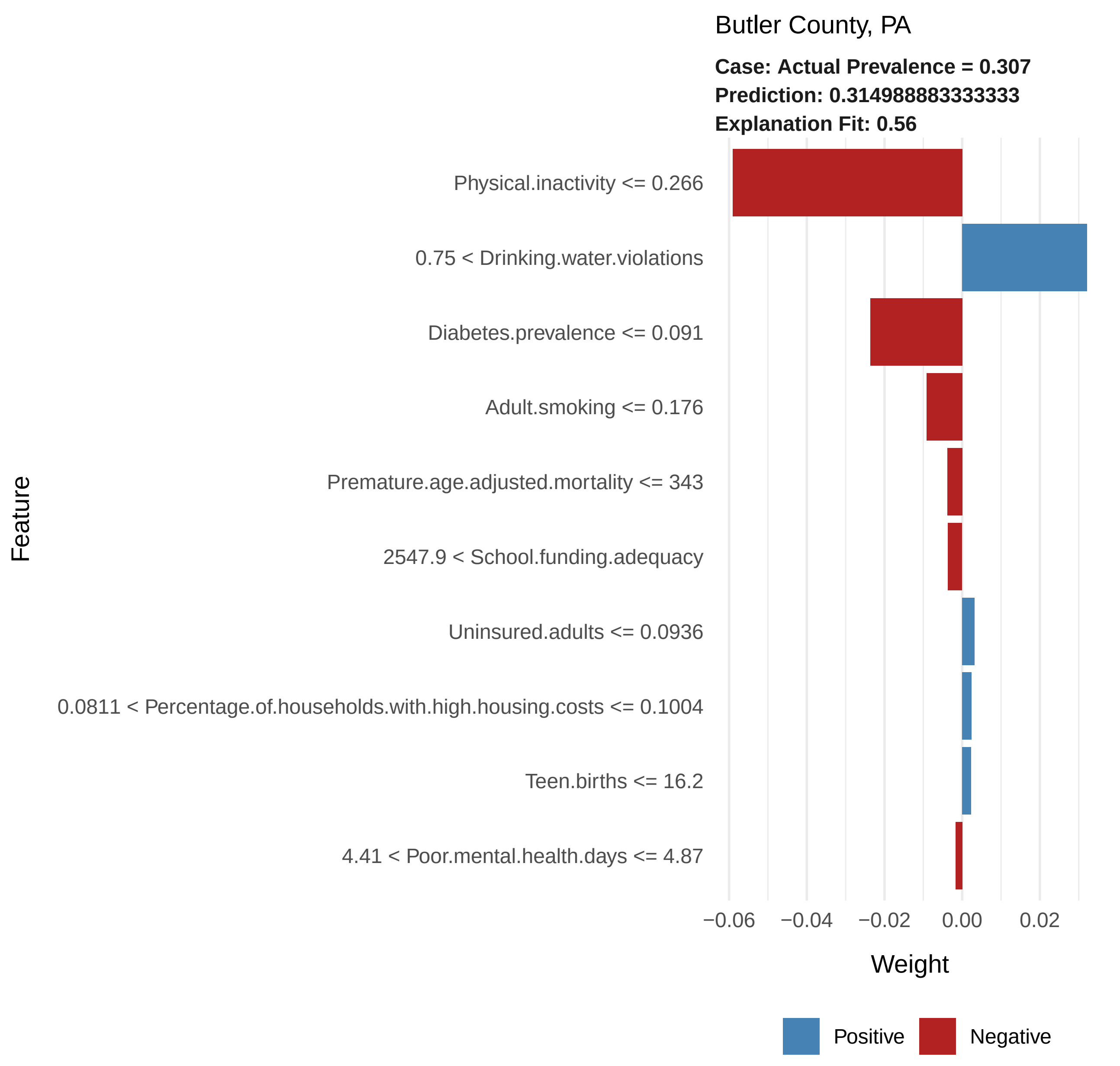
Butler County, PA
Case: Actual Prevalence = 0.307
Prediction: 0.314988883333333
Explanation Fit: 0.56
Physical.inactivity <= 0.266
0.75 < Drinking.water.violations
Diabetes.prevalence <= 0.091
Adult.smoking <= 0.176
Premature.age.adjusted.mortality <= 343
2547.9 < School.funding.adequacy
Uninsured.adults <= 0.0936
0.0811 < Percentage.of.households.with.high.housing.costs <= 0.1004
Teen.births <= 16.2
4.41 < Poor.mental.health.days <= 4.87
Feature
−0.06
−0.04
−0.02
0.00
0.02
Weight
Positive
Negative

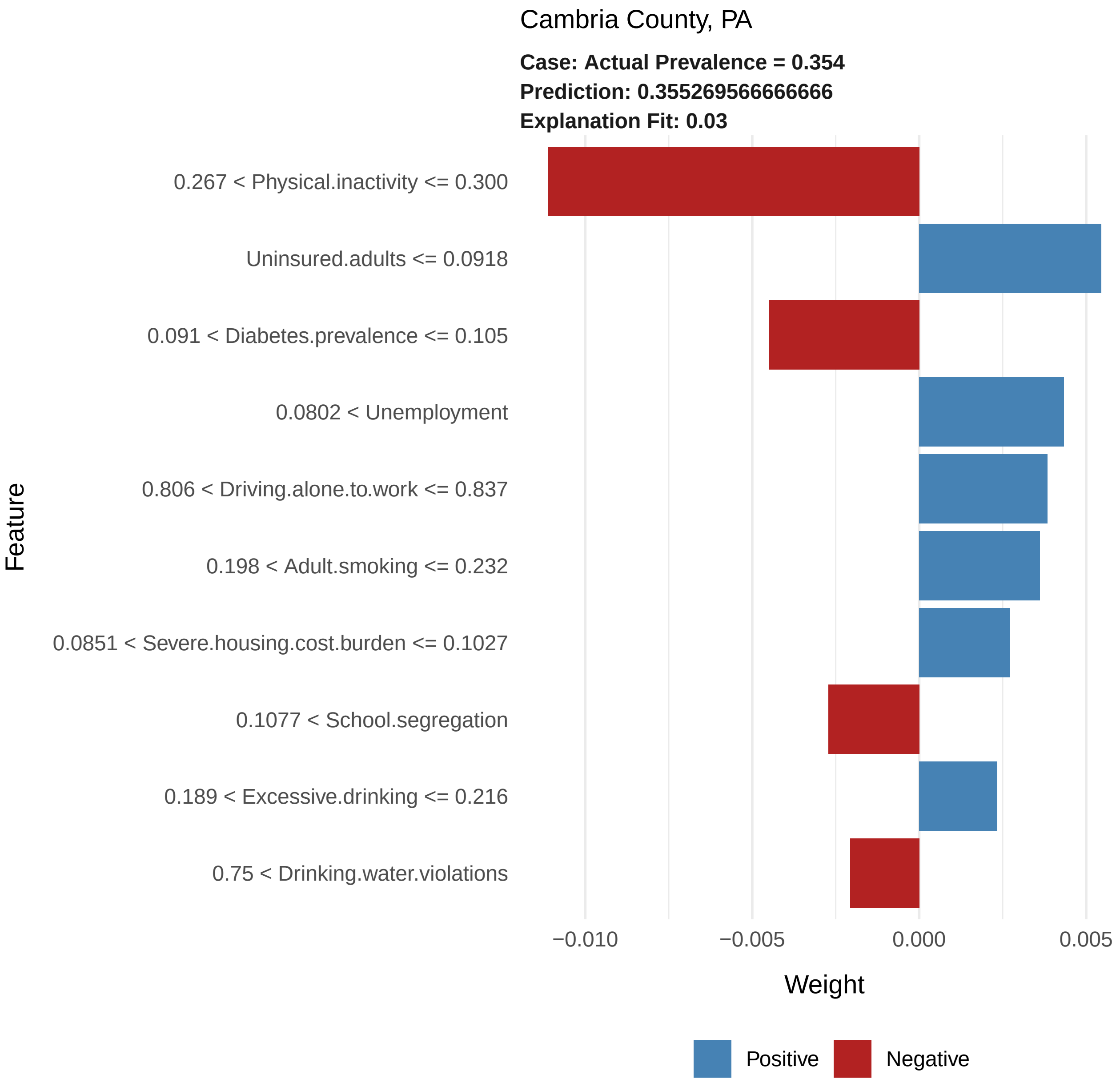

Cambria County, PA
Case: Actual Prevalence = 0.354
Prediction: 0.355269566666666
Explanation Fit: 0.03
0.267 < Physical.inactivity <= 0.300
Uninsured.adults <= 0.0918
0.091 < Diabetes.prevalence <= 0.105
0.0802 < Unemployment
0.806 < Driving.alone.to.work <= 0.837
0.198 < Adult.smoking <= 0.232
0.0851 < Severe.housing.cost.burden <= 0.1027
0.1077 < School.segregation
0.189 < Excessive.drinking <= 0.216
0.75 < Drinking.water.violations
Feature
−0.010
−0.005
0.000
0.005
Weight
Positive
Negative

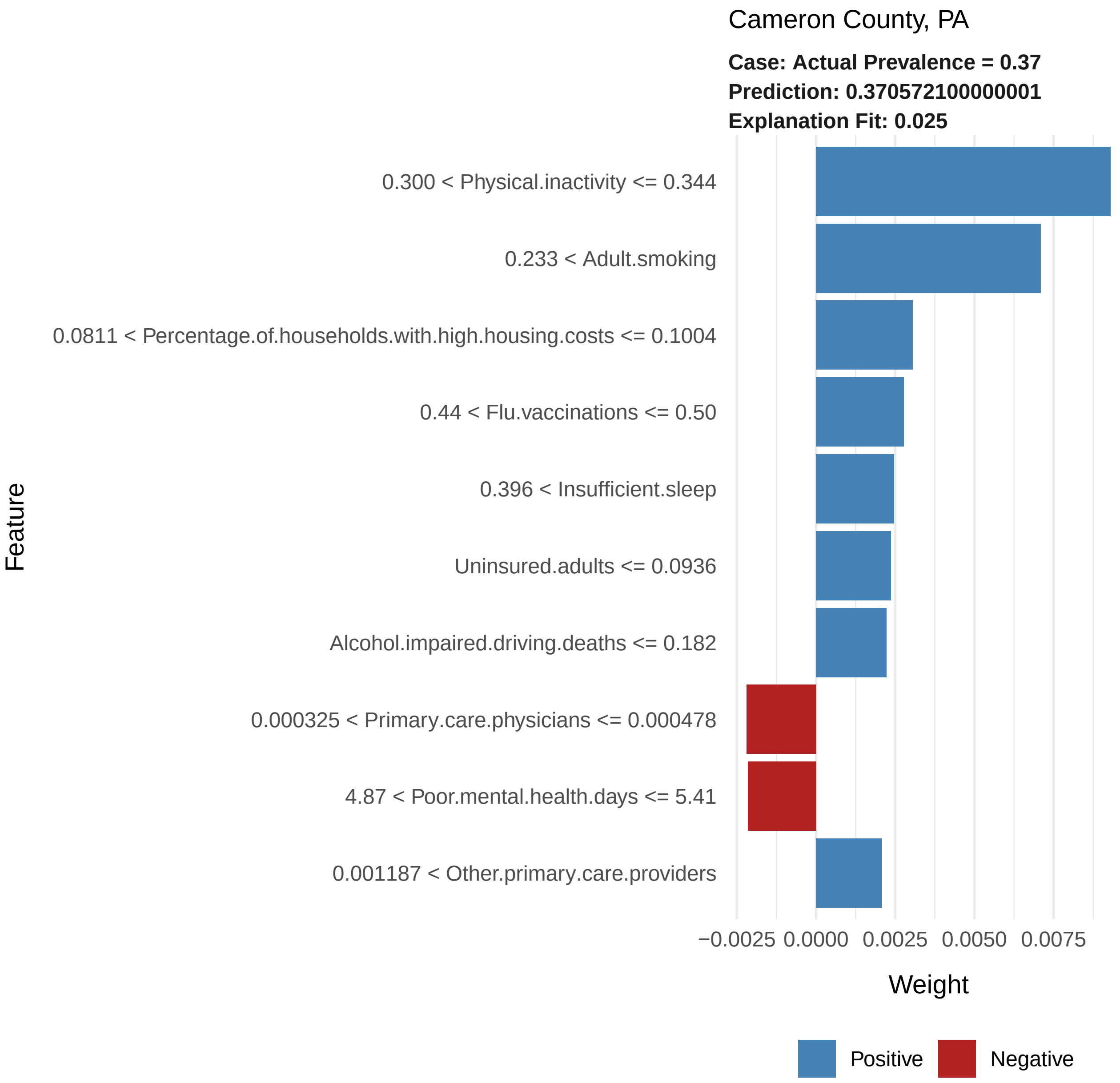
Cameron County, PA
Case: Actual Prevalence = 0.37
Prediction: 0.370572100000001
Explanation Fit: 0.025
0.300 < Physical.inactivity <= 0.344
0.233 < Adult.smoking
0.0811 < Percentage.of.households.with.high.housing.costs <= 0.1004
0.44 < Flu.vaccinations <= 0.50
0.396 < Insufficient.sleep
Uninsured.adults <= 0.0936
Alcohol.impaired.driving.deaths <= 0.182
0.000325 < Primary.care.physicians <= 0.000478
4.87 < Poor.mental.health.days <= 5.41
0.001187 < Other.primary.care.providers
Feature
−0.0025
0.0000
0.0025
0.0050
0.0075
Weight
Positive
Negative

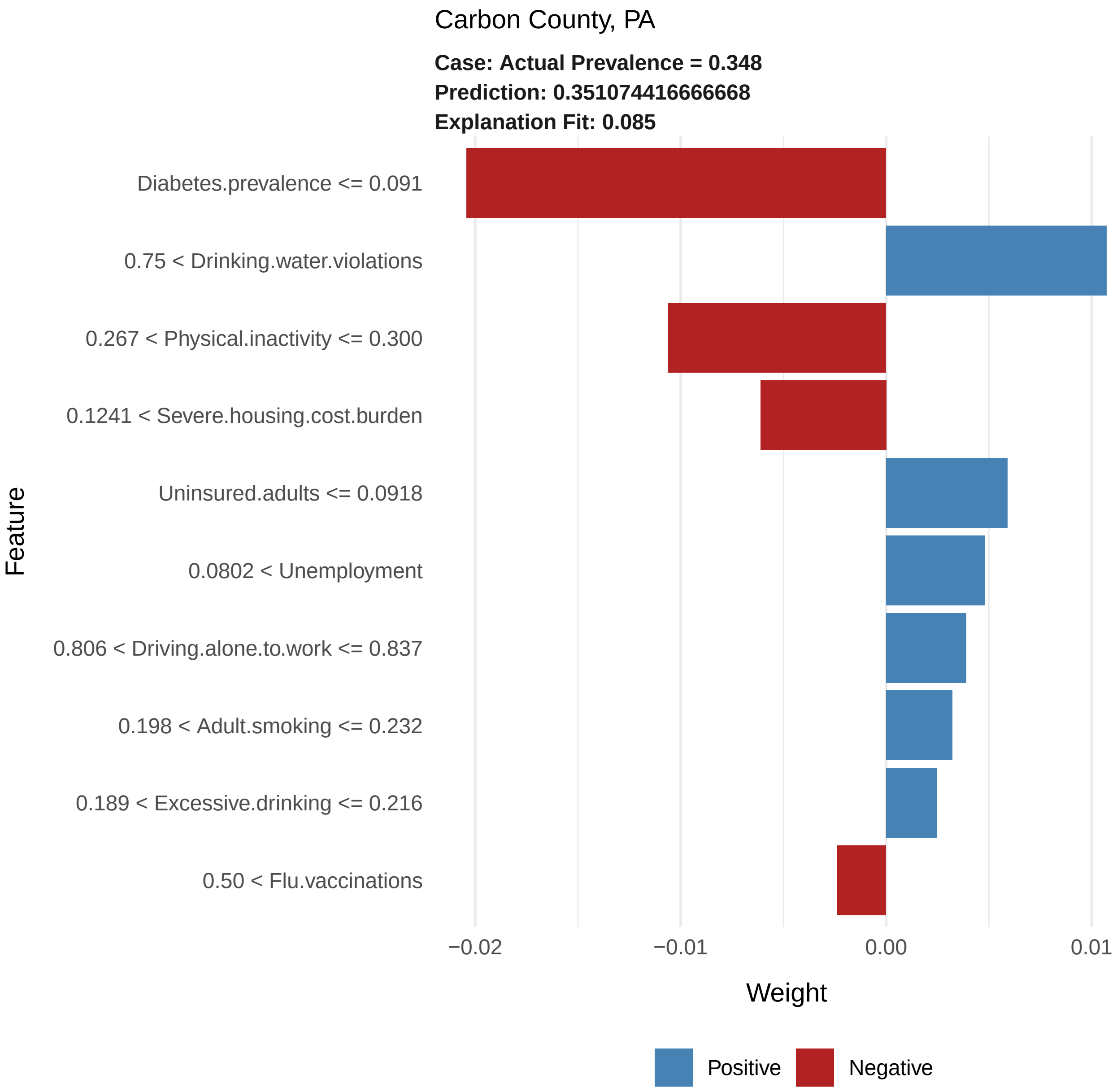

Carbon County, PA
Case: Actual Prevalence = 0.348
Prediction: 0.351074416666668
Explanation Fit: 0.085
Diabetes.prevalence <= 0.091
0.75 < Drinking.water.violations
0.267 < Physical.inactivity <= 0.300
0.1241 < Severe.housing.cost.burden
Uninsured.adults <= 0.0918
0.0802 < Unemployment
0.806 < Driving.alone.to.work <= 0.837
0.198 < Adult.smoking <= 0.232
0.189 < Excessive.drinking <= 0.216
0.50 < Flu.vaccinations
Feature
−0.02
−0.01
0.00
0.01
Weight
Positive
Negative

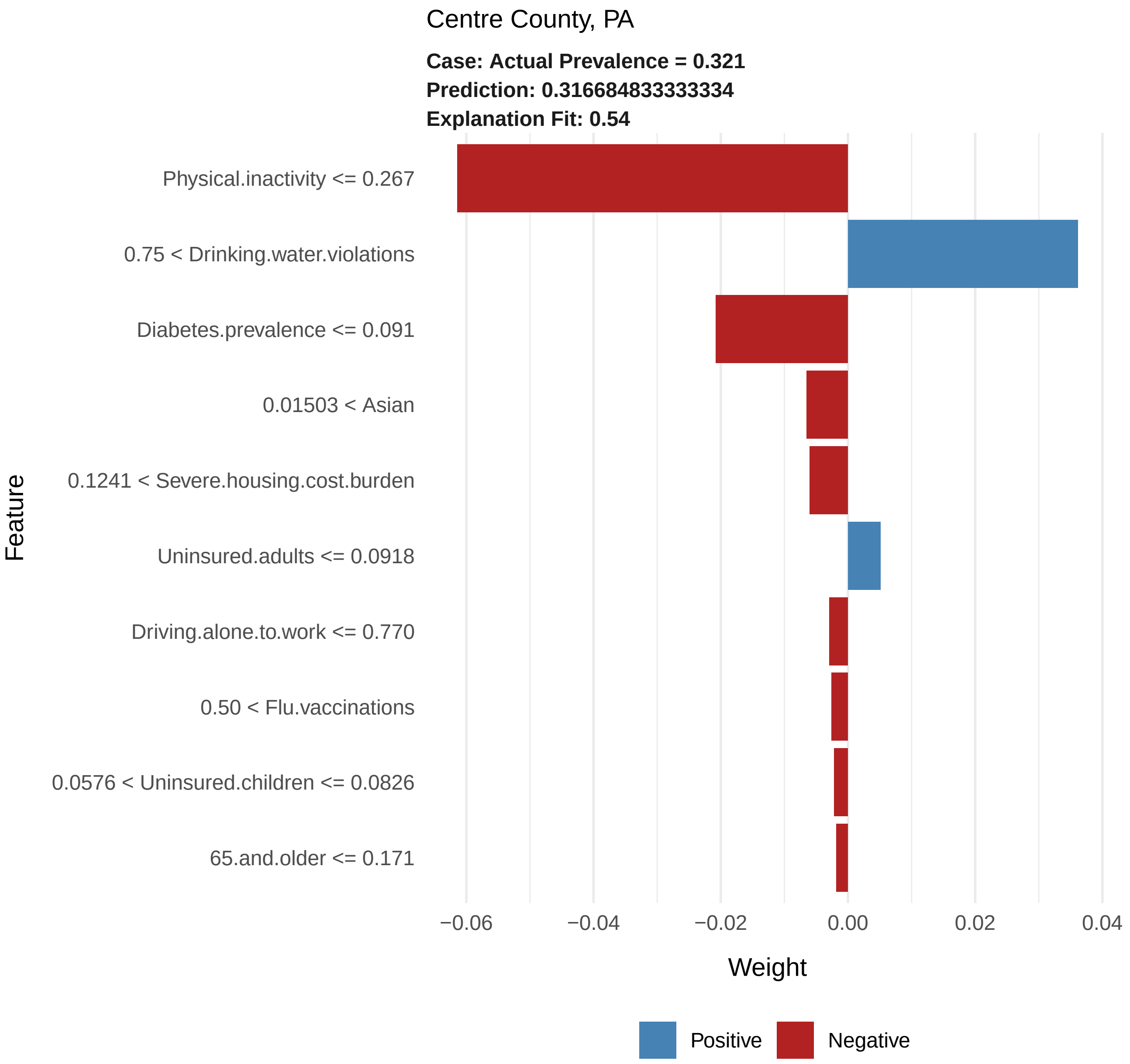

Centre County, PA
Case: Actual Prevalence = 0.321
Prediction: 0.316684833333334
Explanation Fit: 0.54
Physical.inactivity <= 0.267
0.75 < Drinking.water.violations
Diabetes.prevalence <= 0.091
0.01503 < Asian
0.1241 < Severe.housing.cost.burden
Uninsured.adults <= 0.0918
Driving.alone.to.work <= 0.770
0.50 < Flu.vaccinations
0.0576 < Uninsured.children <= 0.0826
65.and.older <= 0.171
Feature
−0.06
−0.04
−0.02
0.00
0.02
0.04
Weight
Positive
Negative

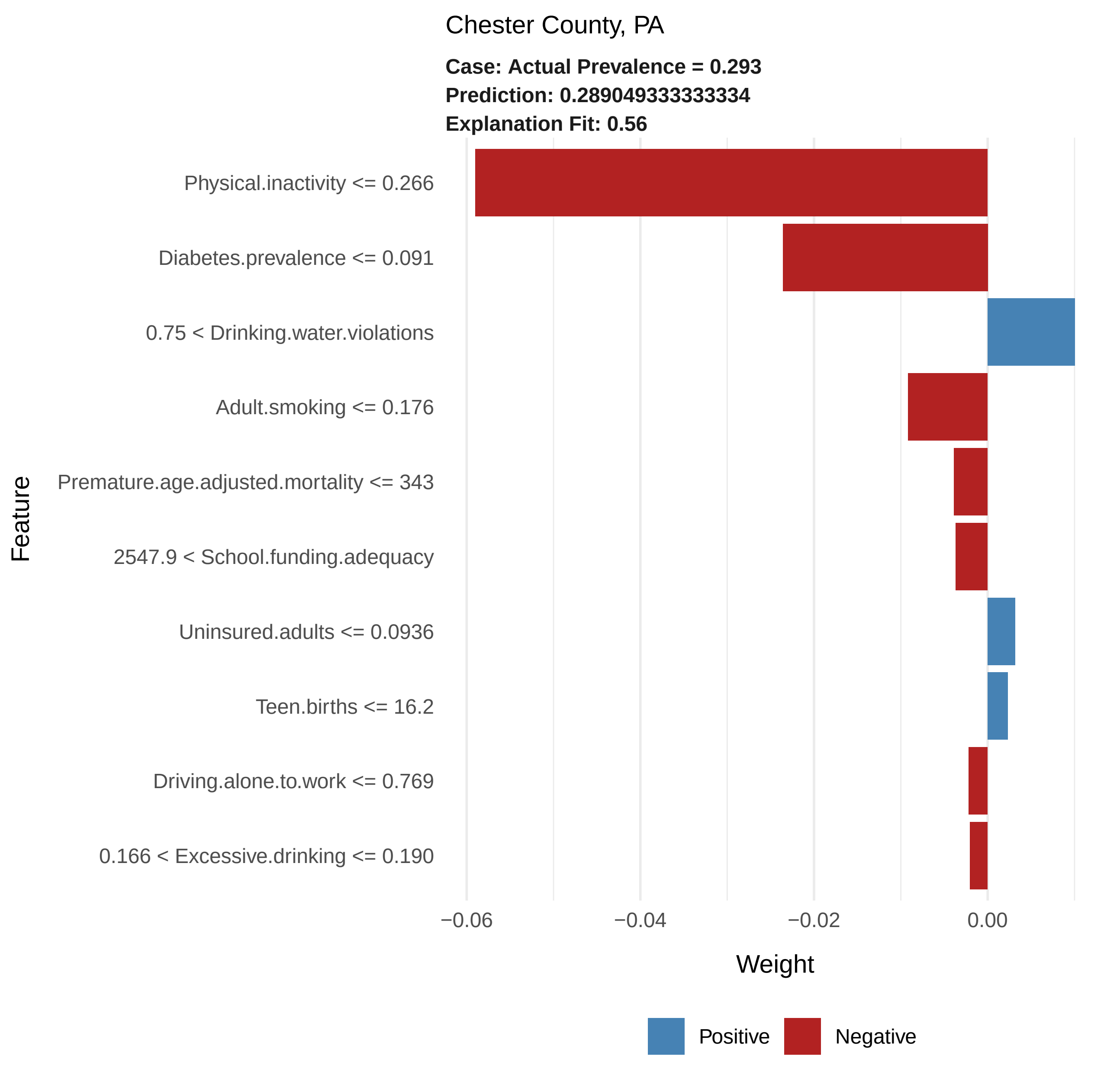
Chester County, PA
Case: Actual Prevalence = 0.293
Prediction: 0.289049333333334
Explanation Fit: 0.56
Physical.inactivity <= 0.266
Diabetes.prevalence <= 0.091
0.75 < Drinking.water.violations
Adult.smoking <= 0.176
Premature.age.adjusted.mortality <= 343
2547.9 < School.funding.adequacy
Uninsured.adults <= 0.0936
Teen.births <= 16.2
Driving.alone.to.work <= 0.769
0.166 < Excessive.drinking <= 0.190
Feature
−0.06
−0.04
−0.02
0.00
Weight
Positive
Negative

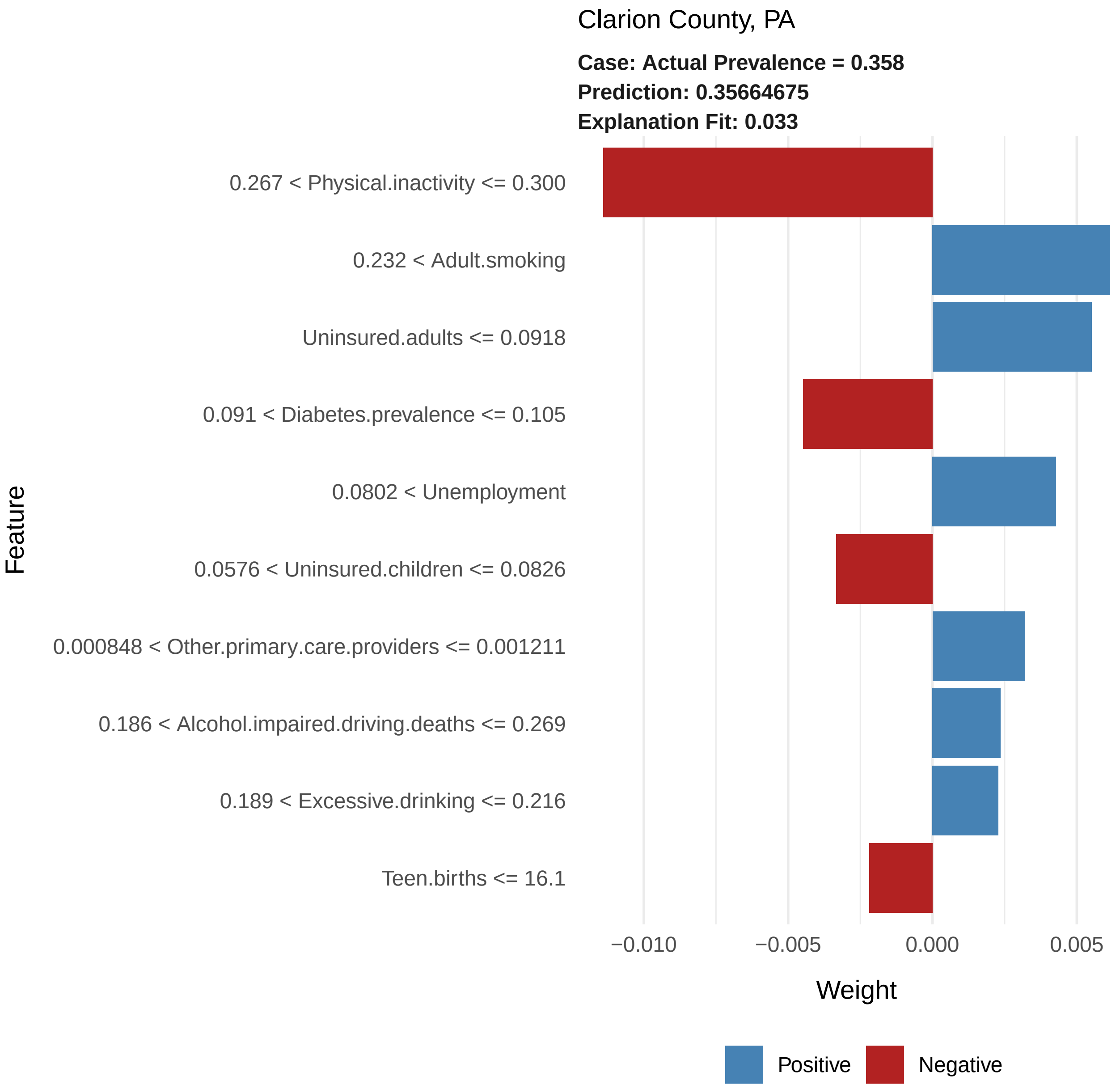
Clarion County, PA
Case: Actual Prevalence = 0.358
Prediction: 0.35664675
Explanation Fit: 0.033
0.267 < Physical.inactivity <= 0.300
0.232 < Adult.smoking
Uninsured.adults <= 0.0918
0.091 < Diabetes.prevalence <= 0.105
0.0802 < Unemployment
0.0576 < Uninsured.children <= 0.0826
0.000848 < Other.primary.care.providers <= 0.001211
0.186 < Alcohol.impaired.driving.deaths <= 0.269
0.189 < Excessive.drinking <= 0.216
Teen.births <= 16.1
Feature
−0.010
−0.005
0.000
0.005
Weight
Positive
Negative

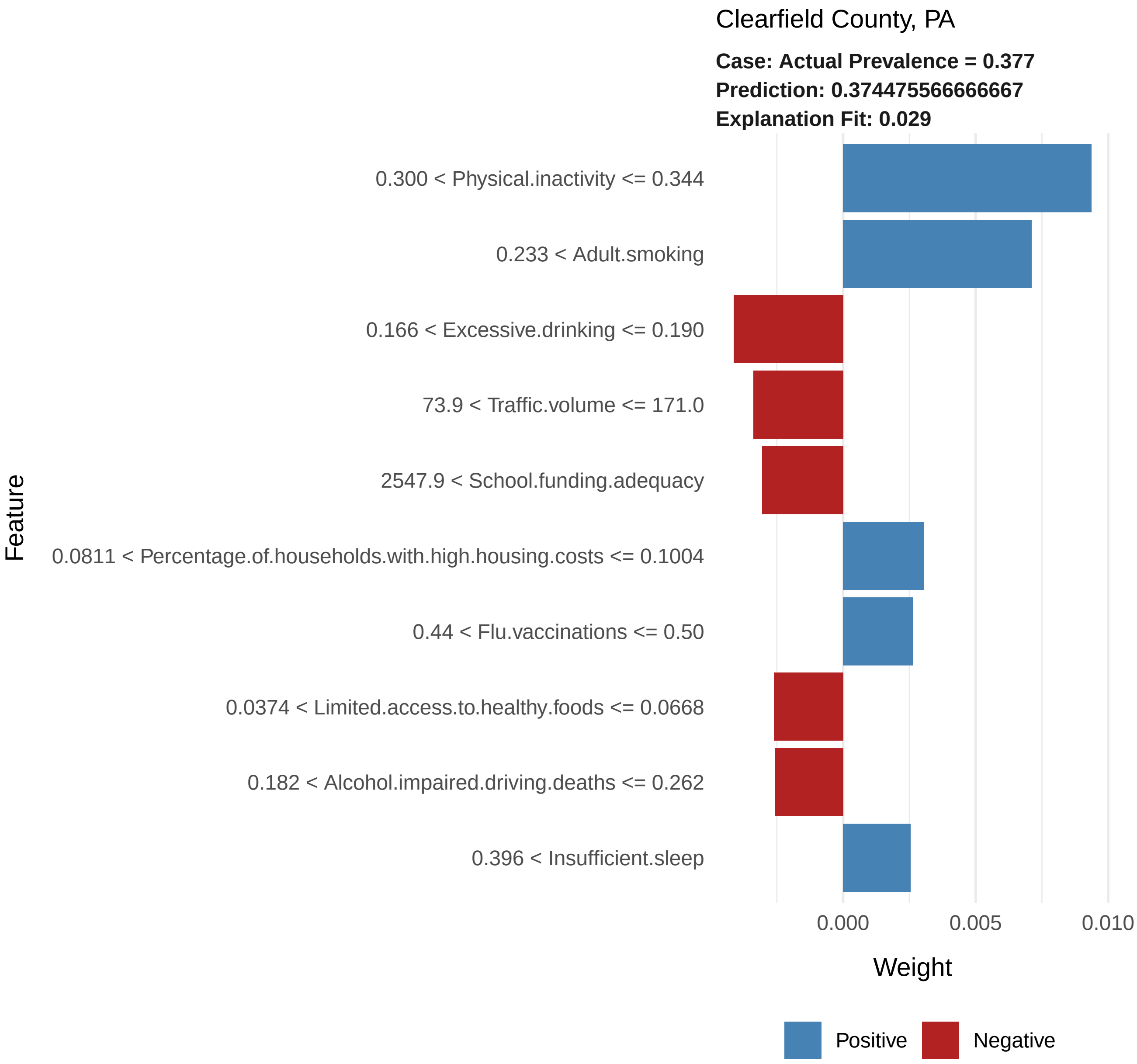

Clearfield County, PA
Case: Actual Prevalence = 0.377
Prediction: 0.374475566666667
Explanation Fit: 0.029
0.300 < Physical.inactivity <= 0.344
0.233 < Adult.smoking
0.166 < Excessive.drinking <= 0.190
73.9 < Traffic.volume <= 171.0
2547.9 < School.funding.adequacy
0.0811 < Percentage.of.households.with.high.housing.costs <= 0.1004
0.44 < Flu.vaccinations <= 0.50
0.0374 < Limited.access.to.healthy.foods <= 0.0668
0.182 < Alcohol.impaired.driving.deaths <= 0.262
0.396 < Insufficient.sleep
Feature
0.000
0.005
0.010
Weight
Positive
Negative

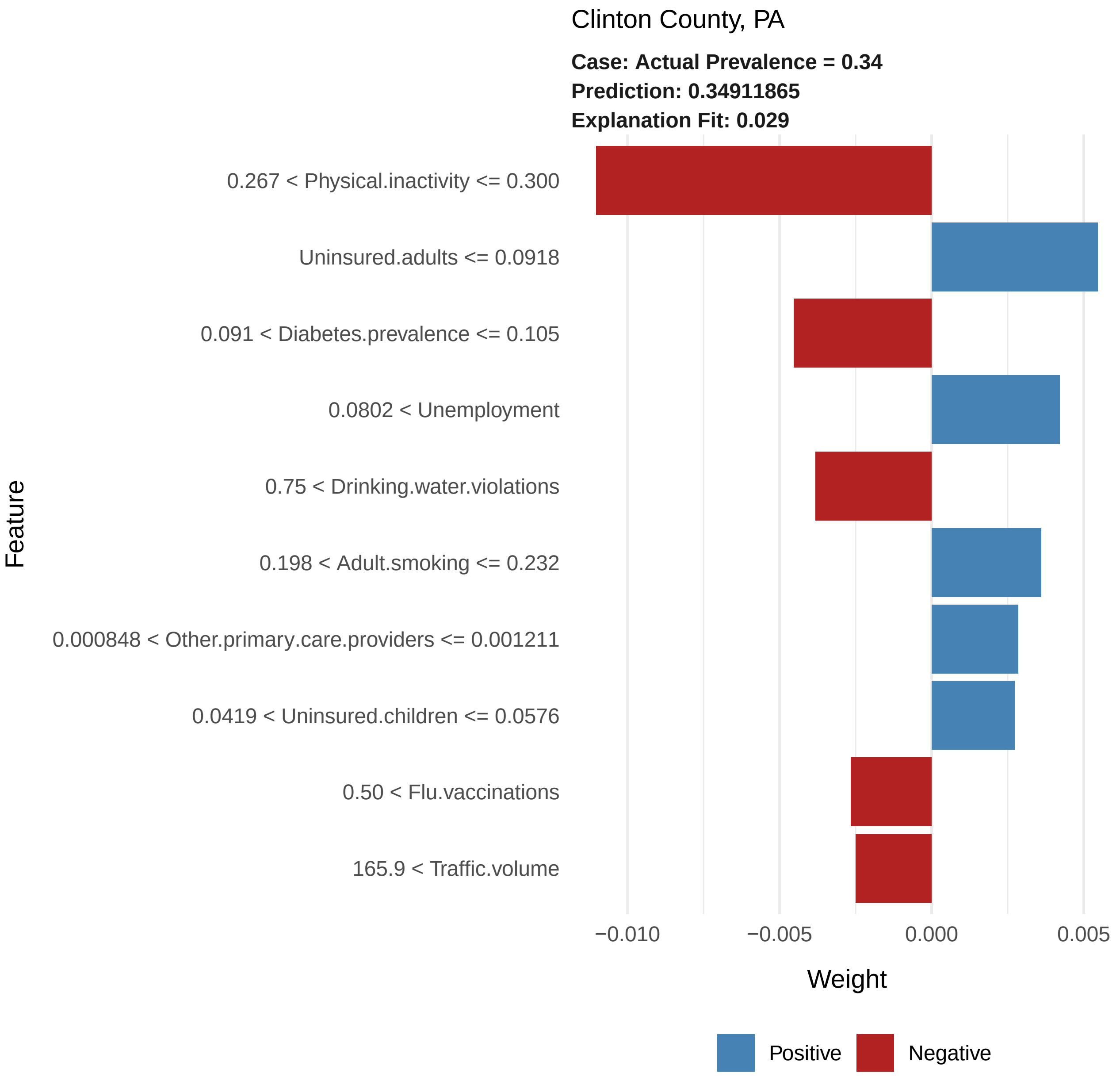

Clinton County, PA
Case: Actual Prevalence = 0.34
Prediction: 0.34911865
Explanation Fit: 0.029
0.267 < Physical.inactivity <= 0.300
Uninsured.adults <= 0.0918
0.091 < Diabetes.prevalence <= 0.105
0.0802 < Unemployment
0.75 < Drinking.water.violations
0.198 < Adult.smoking <= 0.232
0.000848 < Other.primary.care.providers <= 0.001211
0.0419 < Uninsured.children <= 0.0576
0.50 < Flu.vaccinations
165.9 < Traffic.volume
Feature
−0.010
−0.005
0.000
0.005
Weight
Positive
Negative

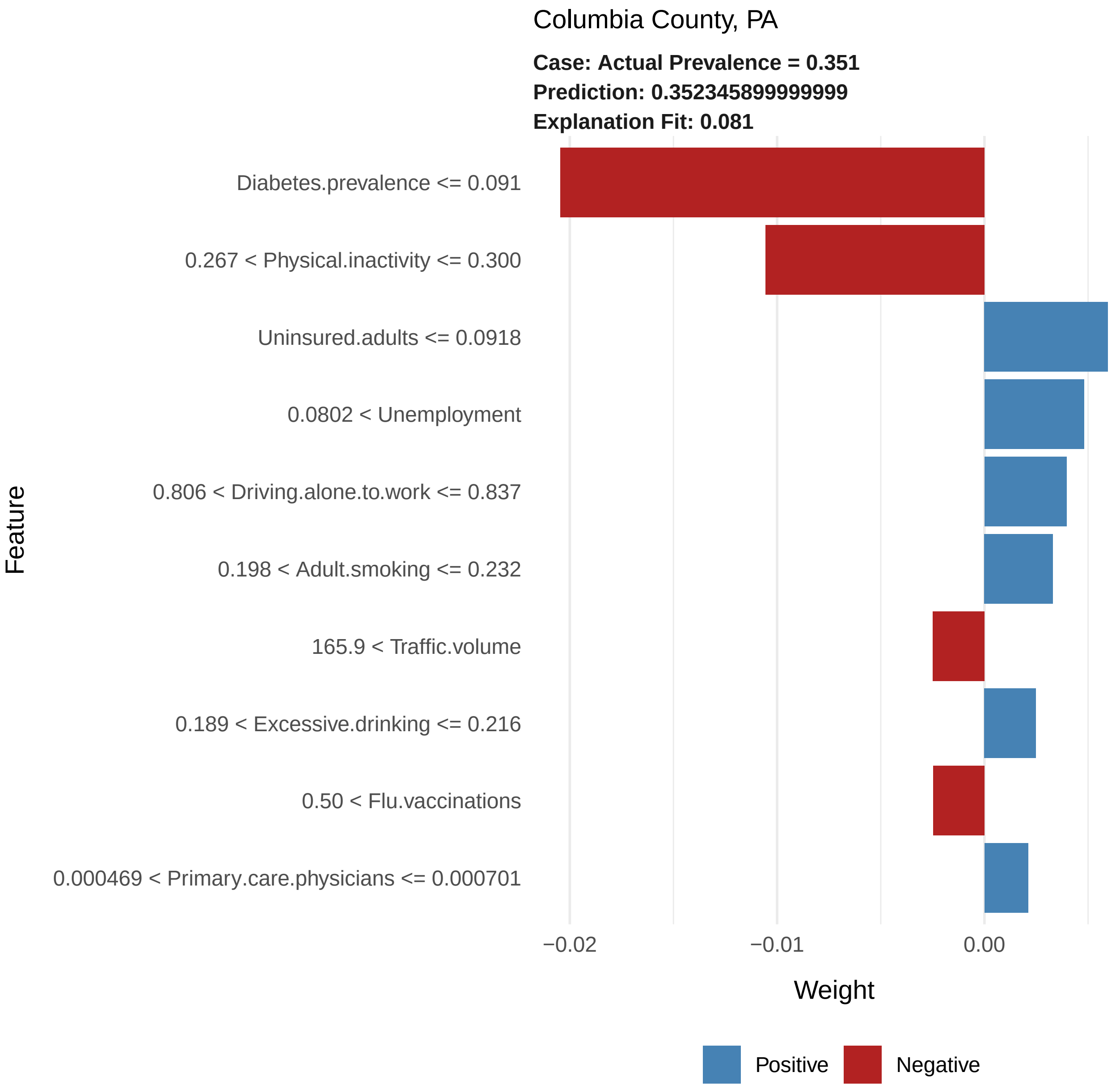

Columbia County, PA
Case: Actual Prevalence = 0.351
Prediction: 0.352345899999999
Explanation Fit: 0.081
Diabetes.prevalence <= 0.091
0.267 < Physical.inactivity <= 0.300
Uninsured.adults <= 0.0918
0.0802 < Unemployment
0.806 < Driving.alone.to.work <= 0.837
0.198 < Adult.smoking <= 0.232
165.9 < Traffic.volume
0.189 < Excessive.drinking <= 0.216
0.50 < Flu.vaccinations
0.000469 < Primary.care.physicians <= 0.000701
Feature
−0.02
−0.01
0.00
Weight
Positive
Negative

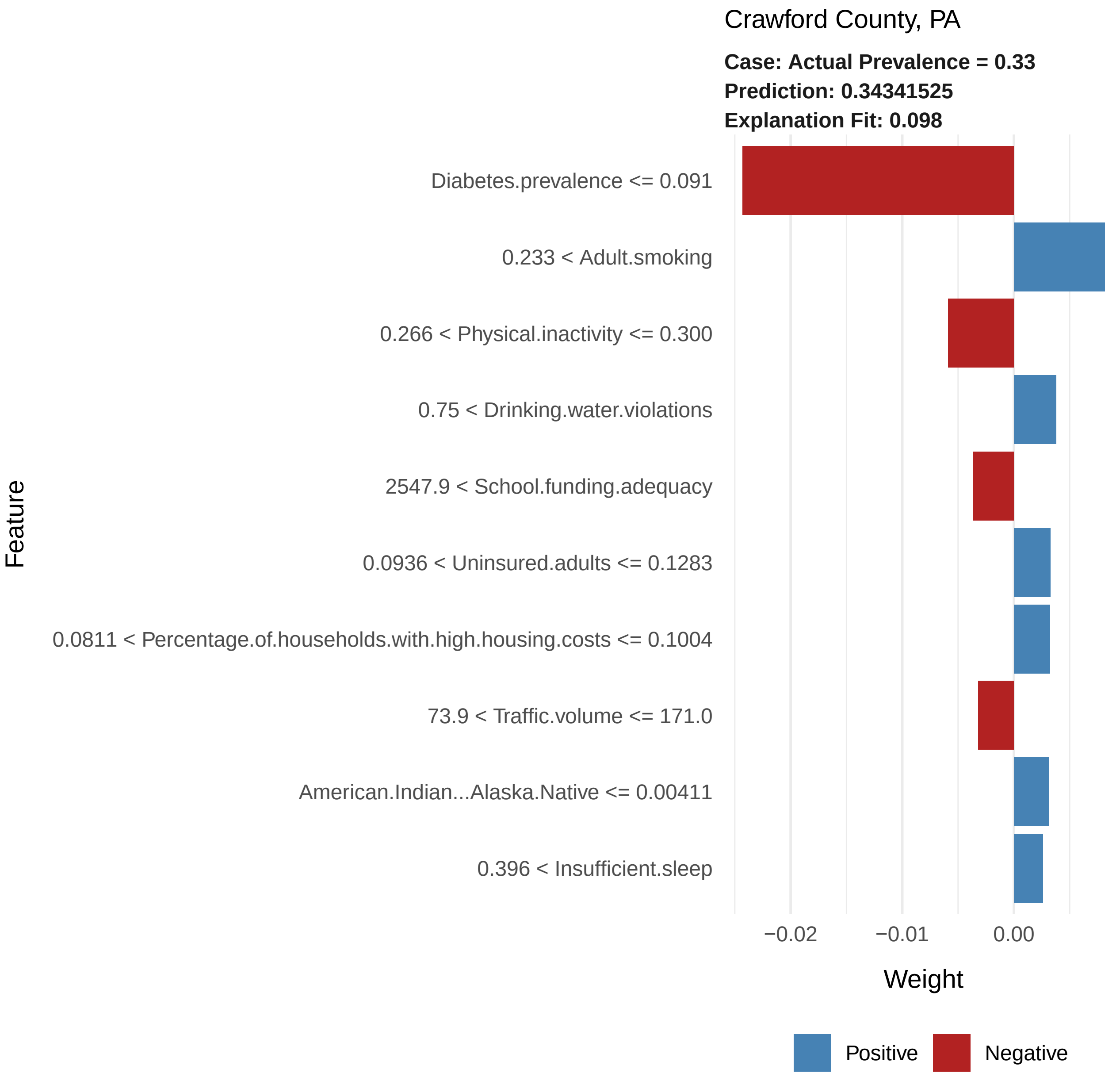
Crawford County, PA
Case: Actual Prevalence = 0.33
Prediction: 0.34341525
Explanation Fit: 0.098
Feature
Diabetes.prevalence <= 0.091
0.233 < Adult.smoking
0.266 < Physical.inactivity <= 0.300
0.75 < Drinking.water.violations
2547.9 < School.funding.adequacy
0.0936 < Uninsured.adults <= 0.1283
0.0811 < Percentage.of.households.with.high.housing.costs <= 0.1004
73.9 < Traffic.volume <= 171.0
American.Indian...Alaska.Native <= 0.00411
0.396 < Insufficient.sleep
−0.02
−0.01
0.00
Weight
Positive
Negative

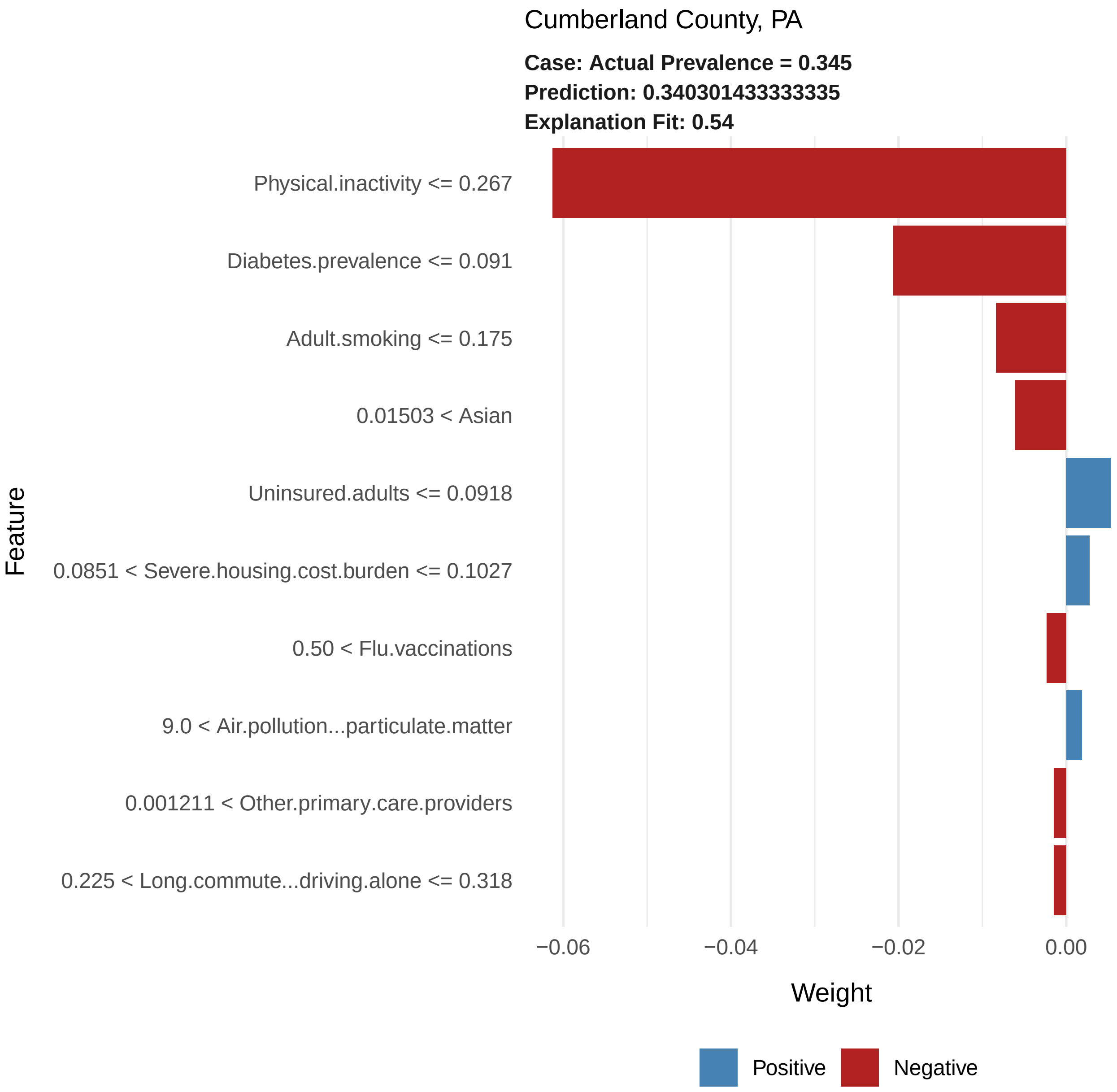

Cumberland County, PA
Case: Actual Prevalence = 0.345
Prediction: 0.340301433333335
Explanation Fit: 0.54
Physical.inactivity <= 0.267
Diabetes.prevalence <= 0.091
Adult.smoking <= 0.175
0.01503 < Asian
Uninsured.adults <= 0.0918
0.0851 < Severe.housing.cost.burden <= 0.1027
0.50 < Flu.vaccinations
9.0 < Air.pollution...particulate.matter
0.001211 < Other.primary.care.providers
0.225 < Long.commute...driving.alone <= 0.318
Feature
−0.06
−0.04
−0.02
0.00
Weight
Positive
Negative

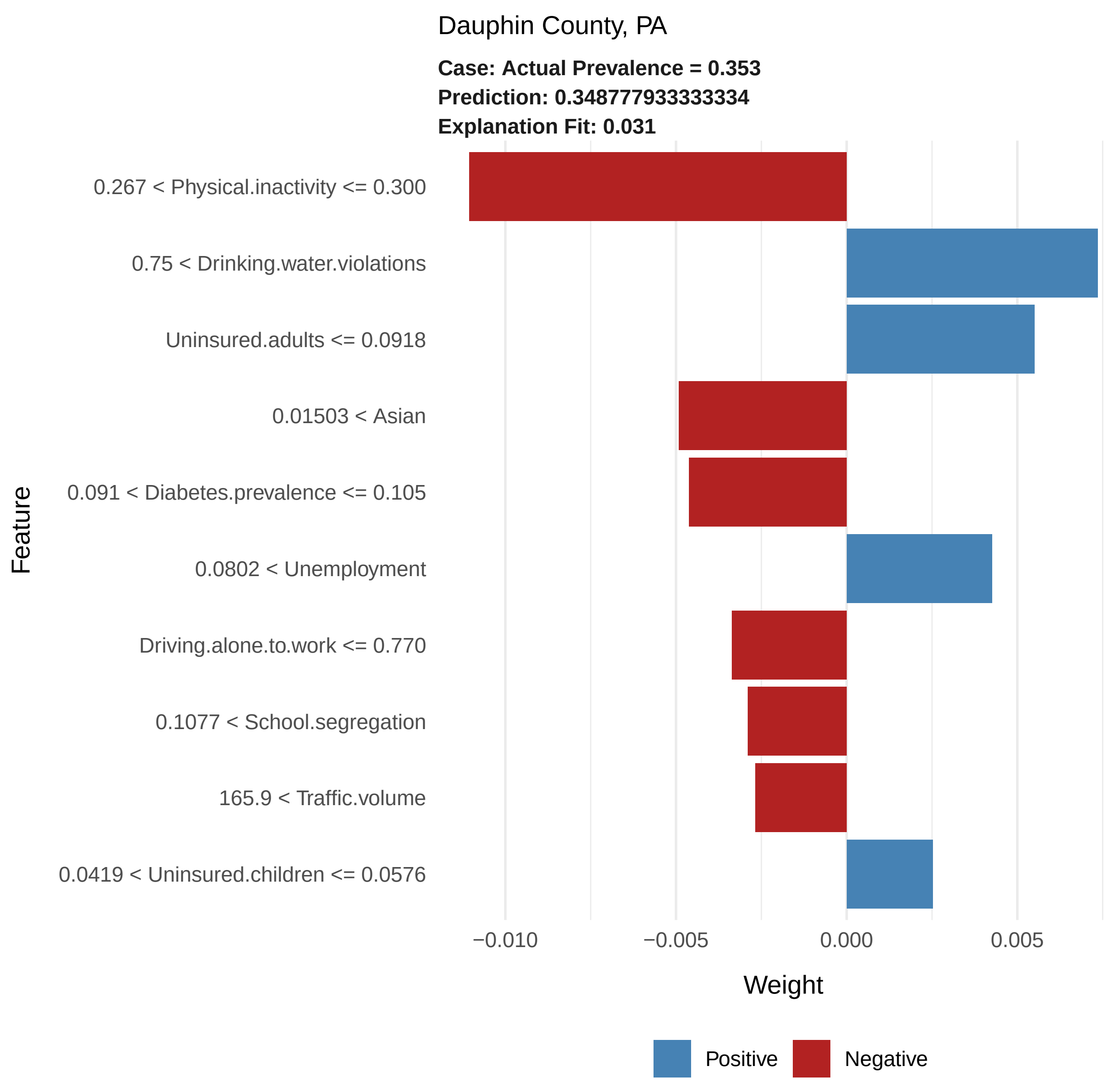

Dauphin County, PA
Case: Actual Prevalence = 0.353
Prediction: 0.348777933333334
Explanation Fit: 0.031
0.267 < Physical.inactivity <= 0.300
0.75 < Drinking.water.violations
Uninsured.adults <= 0.0918
0.01503 < Asian
0.091 < Diabetes.prevalence <= 0.105
0.0802 < Unemployment
Driving.alone.to.work <= 0.770
0.1077 < School.segregation
165.9 < Traffic.volume
0.0419 < Uninsured.children <= 0.0576
Feature
−0.010
−0.005
0.000
0.005
Weight
Positive
Negative

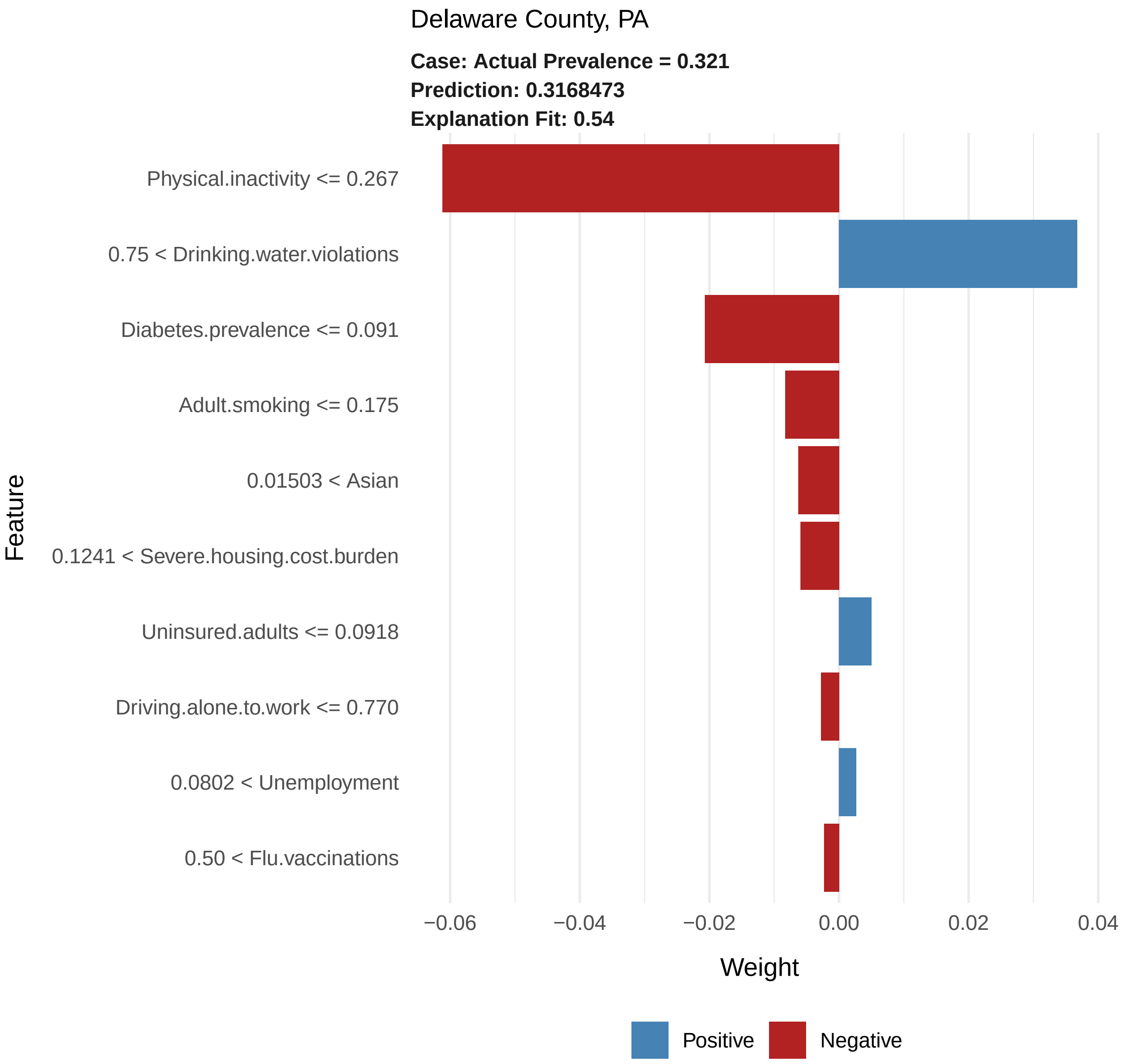

Delaware County, PA
Case: Actual Prevalence = 0.321
Prediction: 0.3168473
Explanation Fit: 0.54
Physical.inactivity <= 0.267
0.75 < Drinking.water.violations
Diabetes.prevalence <= 0.091
Adult.smoking <= 0.175
0.01503 < Asian
0.1241 < Severe.housing.cost.burden
Uninsured.adults <= 0.0918
Driving.alone.to.work <= 0.770
0.0802 < Unemployment
0.50 < Flu.vaccinations
Feature
−0.06
−0.04
−0.02
0.00
0.02
0.04
Weight
Positive
Negative

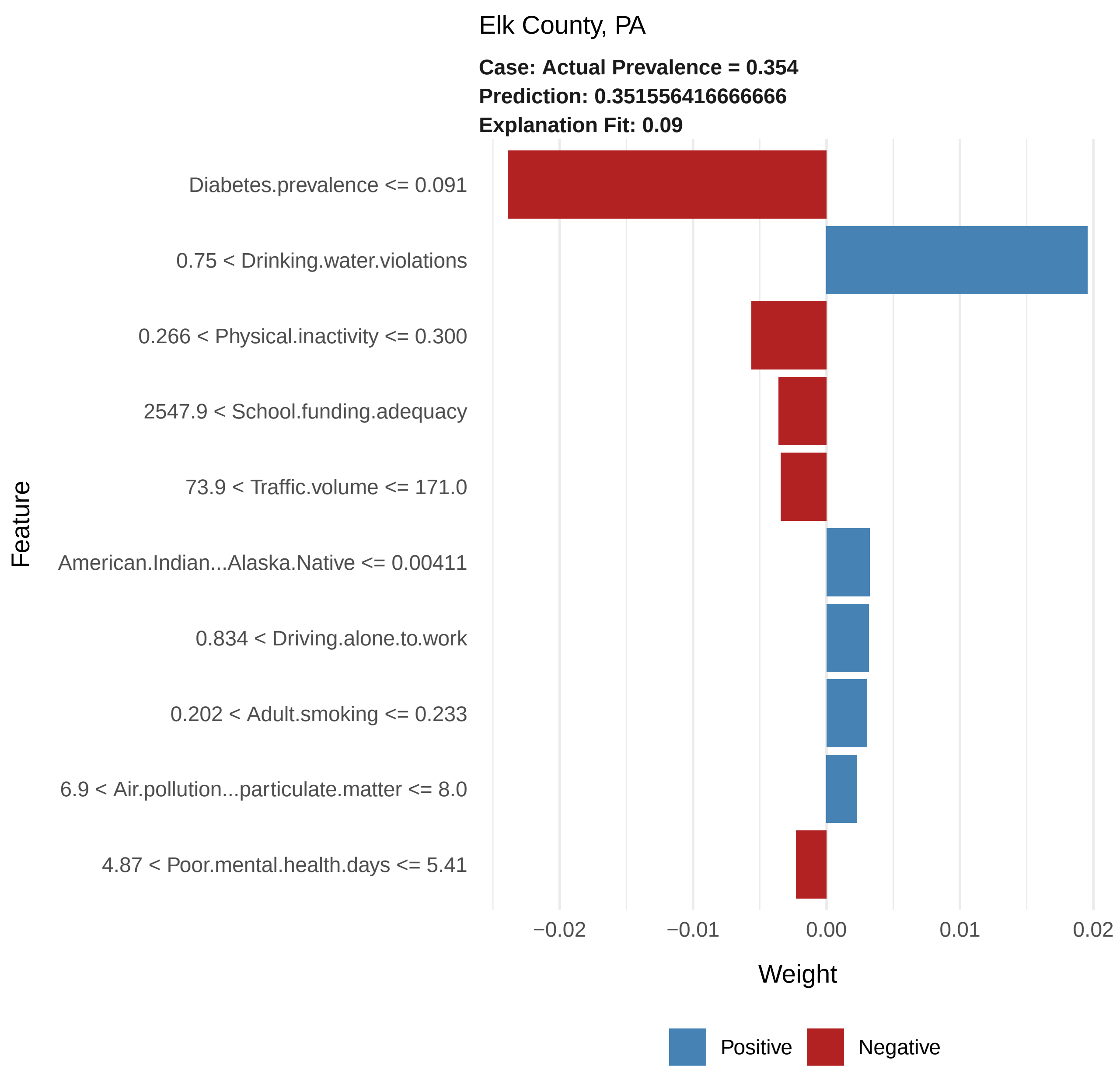
Elk County, PA
Case: Actual Prevalence = 0.354
Prediction: 0.351556416666666
Explanation Fit: 0.09
Feature
Diabetes.prevalence <= 0.091
0.75 < Drinking.water.violations
0.266 < Physical.inactivity <= 0.300
2547.9 < School.funding.adequacy
73.9 < Traffic.volume <= 171.0
American.Indian...Alaska.Native <= 0.00411
0.834 < Driving.alone.to.work
0.202 < Adult.smoking <= 0.233
6.9 < Air.pollution...particulate.matter <= 8.0
4.87 < Poor.mental.health.days <= 5.41
−0.02
−0.01
0.00
0.01
0.02
Weight
Positive
Negative

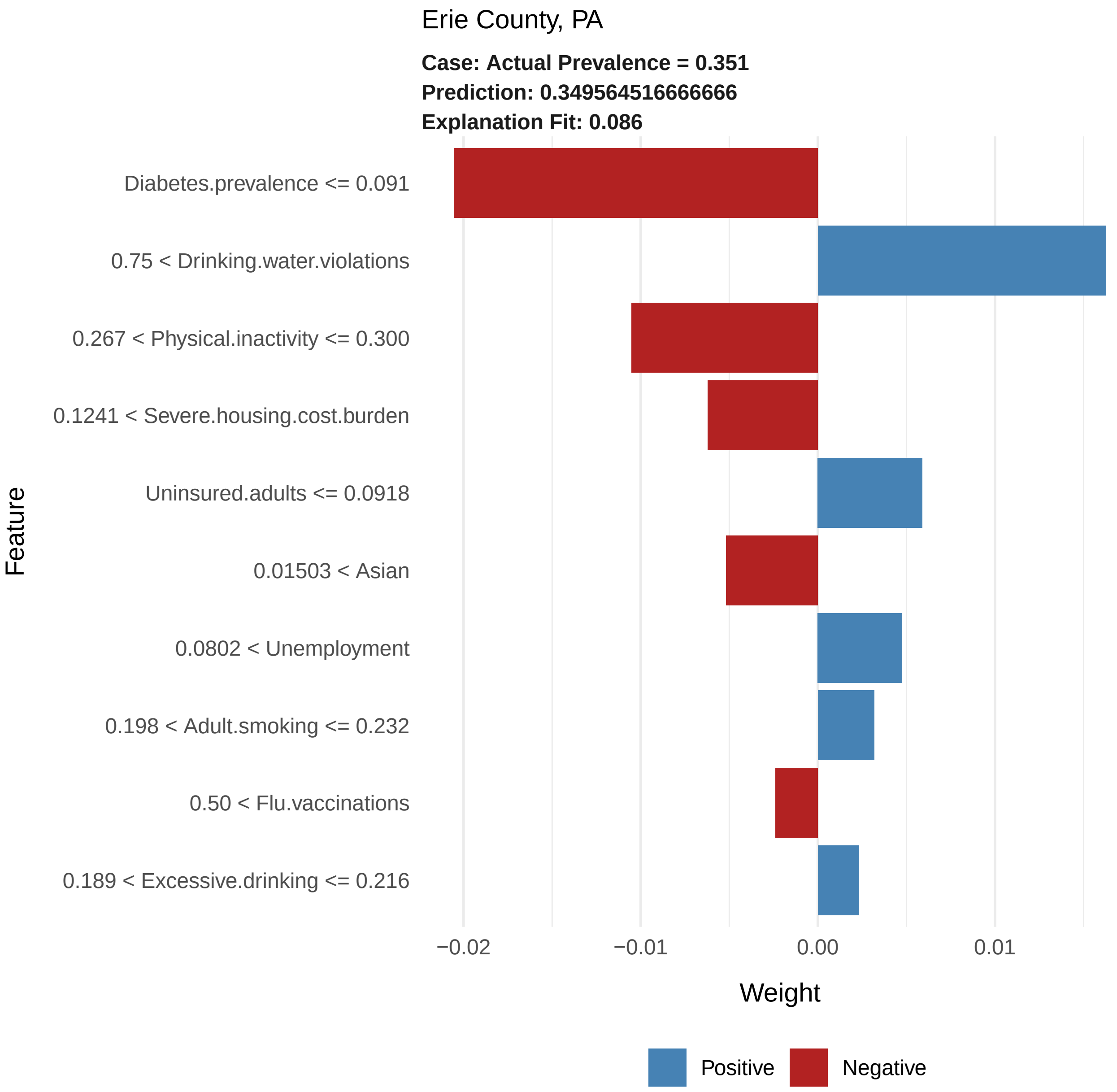

Erie County, PA
Case: Actual Prevalence = 0.351
Prediction: 0.349564516666666
Explanation Fit: 0.086
Diabetes.prevalence <= 0.091
0.75 < Drinking.water.violations
0.267 < Physical.inactivity <= 0.300
0.1241 < Severe.housing.cost.burden
Uninsured.adults <= 0.0918
0.01503 < Asian
0.0802 < Unemployment
0.198 < Adult.smoking <= 0.232
0.50 < Flu.vaccinations
0.189 < Excessive.drinking <= 0.216
Feature
−0.02
−0.01
0.00
0.01
Weight
Positive
Negative

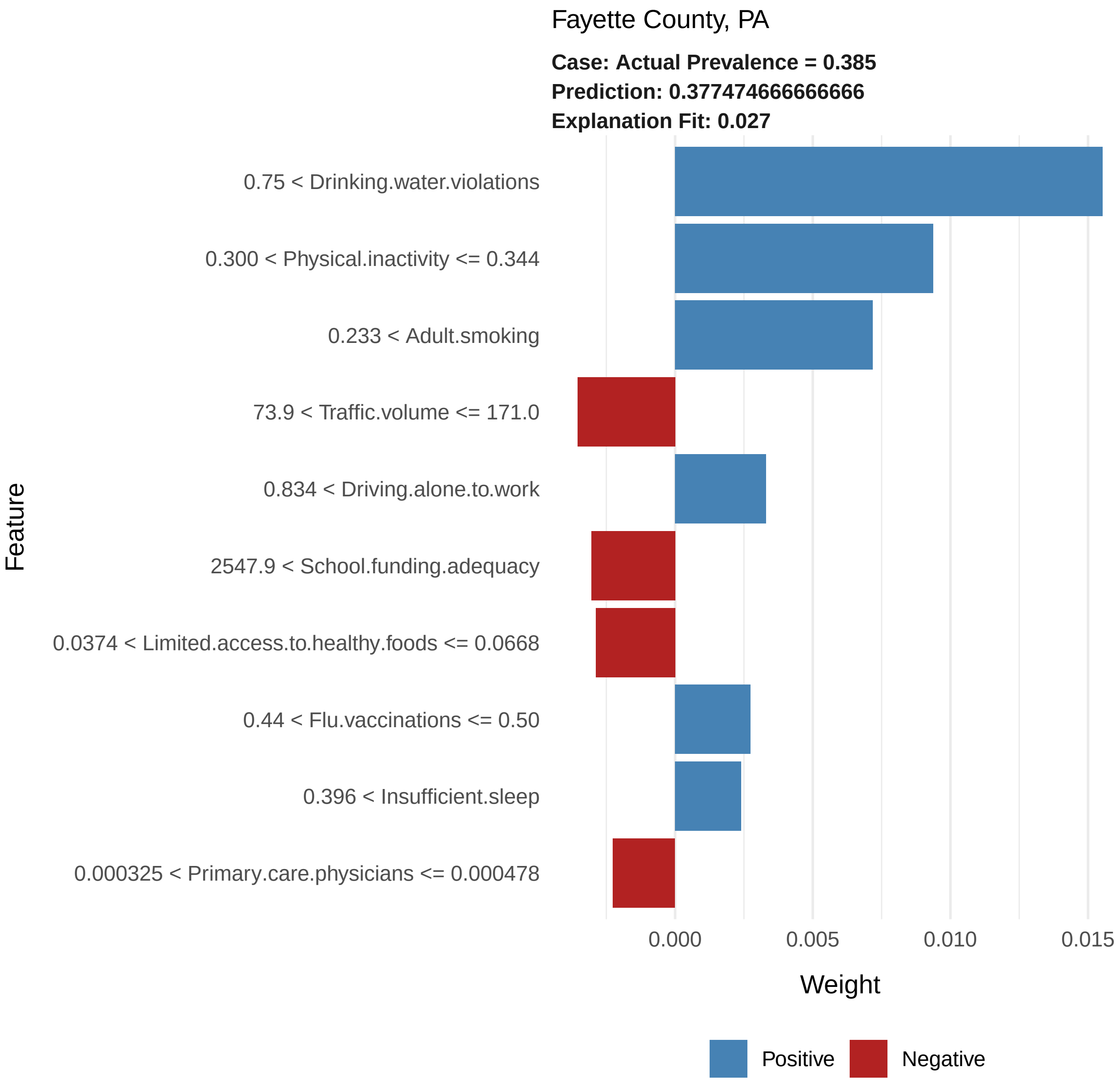
Fayette County, PA
Case: Actual Prevalence = 0.385
Prediction: 0.377474666666666
Explanation Fit: 0.027
0.75 < Drinking.water.violations
0.300 < Physical.inactivity <= 0.344
0.233 < Adult.smoking
73.9 < Traffic.volume <= 171.0
0.834 < Driving.alone.to.work
2547.9 < School.funding.adequacy
0.0374 < Limited.access.to.healthy.foods <= 0.0668
0.44 < Flu.vaccinations <= 0.50
0.396 < Insufficient.sleep
0.000325 < Primary.care.physicians <= 0.000478
Feature
0.000
0.005
0.010
0.015
Weight
Positive
Negative

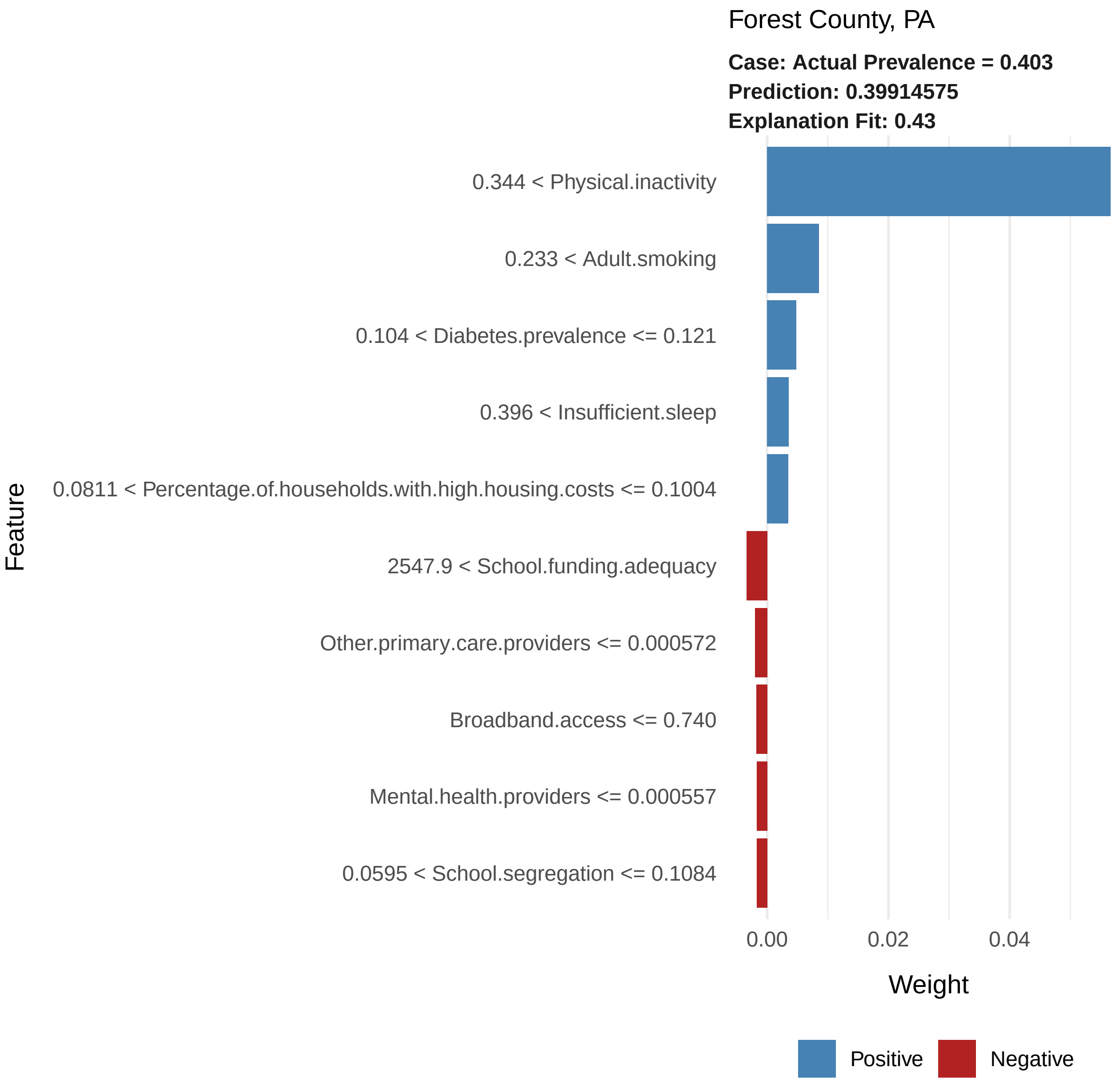
Forest County, PA
Case: Actual Prevalence = 0.403
Prediction: 0.39914575
Explanation Fit: 0.43
0.344 < Physical.inactivity
0.233 < Adult.smoking
0.104 < Diabetes.prevalence <= 0.121
0.396 < Insufficient.sleep
0.0811 < Percentage.of.households.with.high.housing.costs <= 0.1004
2547.9 < School.funding.adequacy
Other.primary.care.providers <= 0.000572
Broadband.access <= 0.740
Mental.health.providers <= 0.000557
0.0595 < School.segregation <= 0.1084
Feature
0.00
0.02
0.04
Weight
Positive
Negative

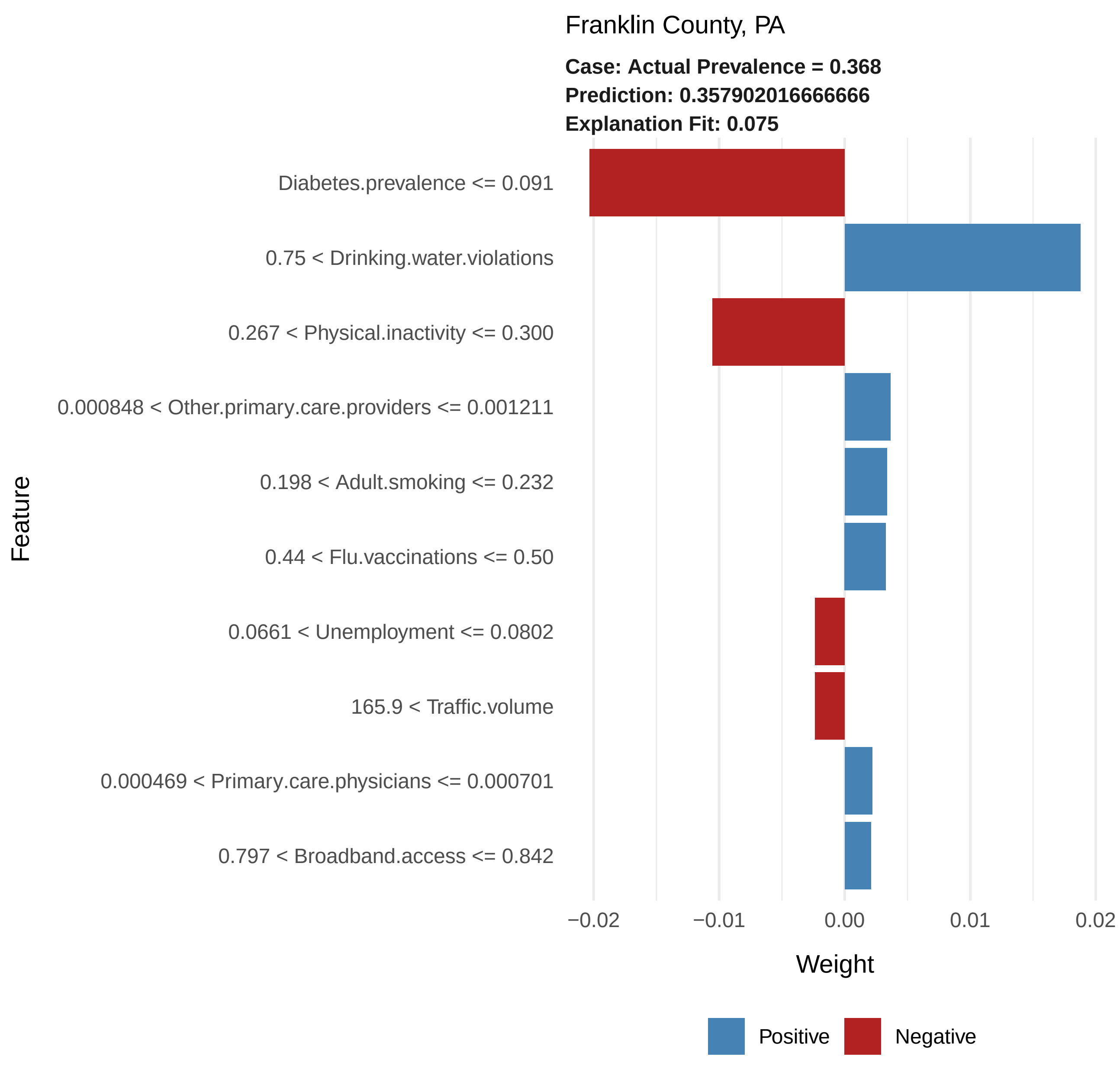

Franklin County, PA
Case: Actual Prevalence = 0.368
Prediction: 0.357902016666666
Explanation Fit: 0.075
Diabetes.prevalence <= 0.091
0.75 < Drinking.water.violations
0.267 < Physical.inactivity <= 0.300
0.000848 < Other.primary.care.providers <= 0.001211
0.198 < Adult.smoking <= 0.232
0.44 < Flu.vaccinations <= 0.50
0.0661 < Unemployment <= 0.0802
165.9 < Traffic.volume
0.000469 < Primary.care.physicians <= 0.000701
0.797 < Broadband.access <= 0.842
Feature
−0.02
−0.01
0.00
0.01
0.02
Weight
Positive
Negative

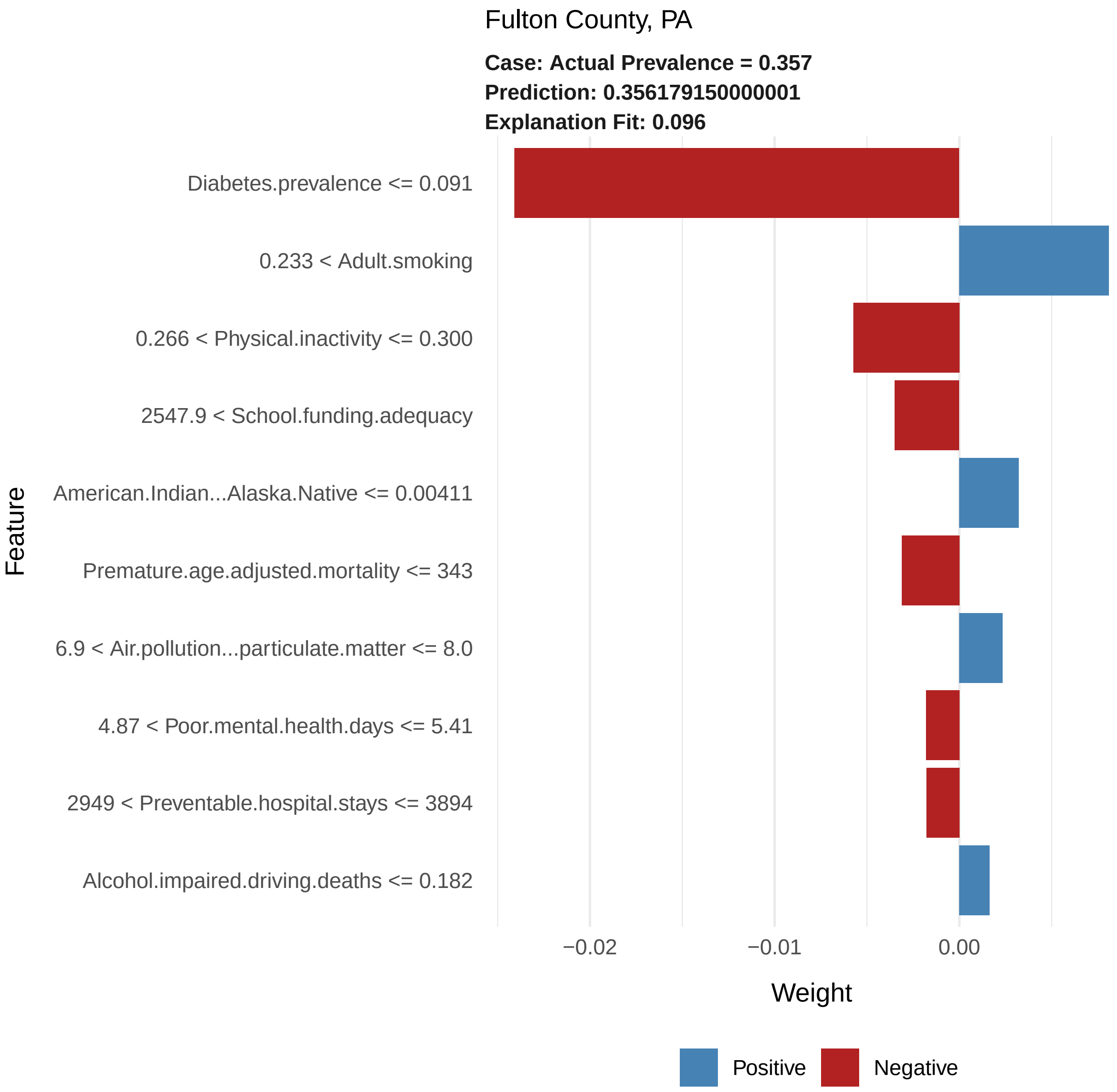

Fulton County, PA
Case: Actual Prevalence = 0.357
Prediction: 0.356179150000001
Explanation Fit: 0.096
Diabetes.prevalence <= 0.091
0.233 < Adult.smoking
0.266 < Physical.inactivity <= 0.300
2547.9 < School.funding.adequacy
American.Indian...Alaska.Native <= 0.00411
Premature.age.adjusted.mortality <= 343
6.9 < Air.pollution...particulate.matter <= 8.0
4.87 < Poor.mental.health.days <= 5.41
2949 < Preventable.hospital.stays <= 3894
Alcohol.impaired.driving.deaths <= 0.182
Feature
−0.02
−0.01
0.00
Weight
Positive
Negative

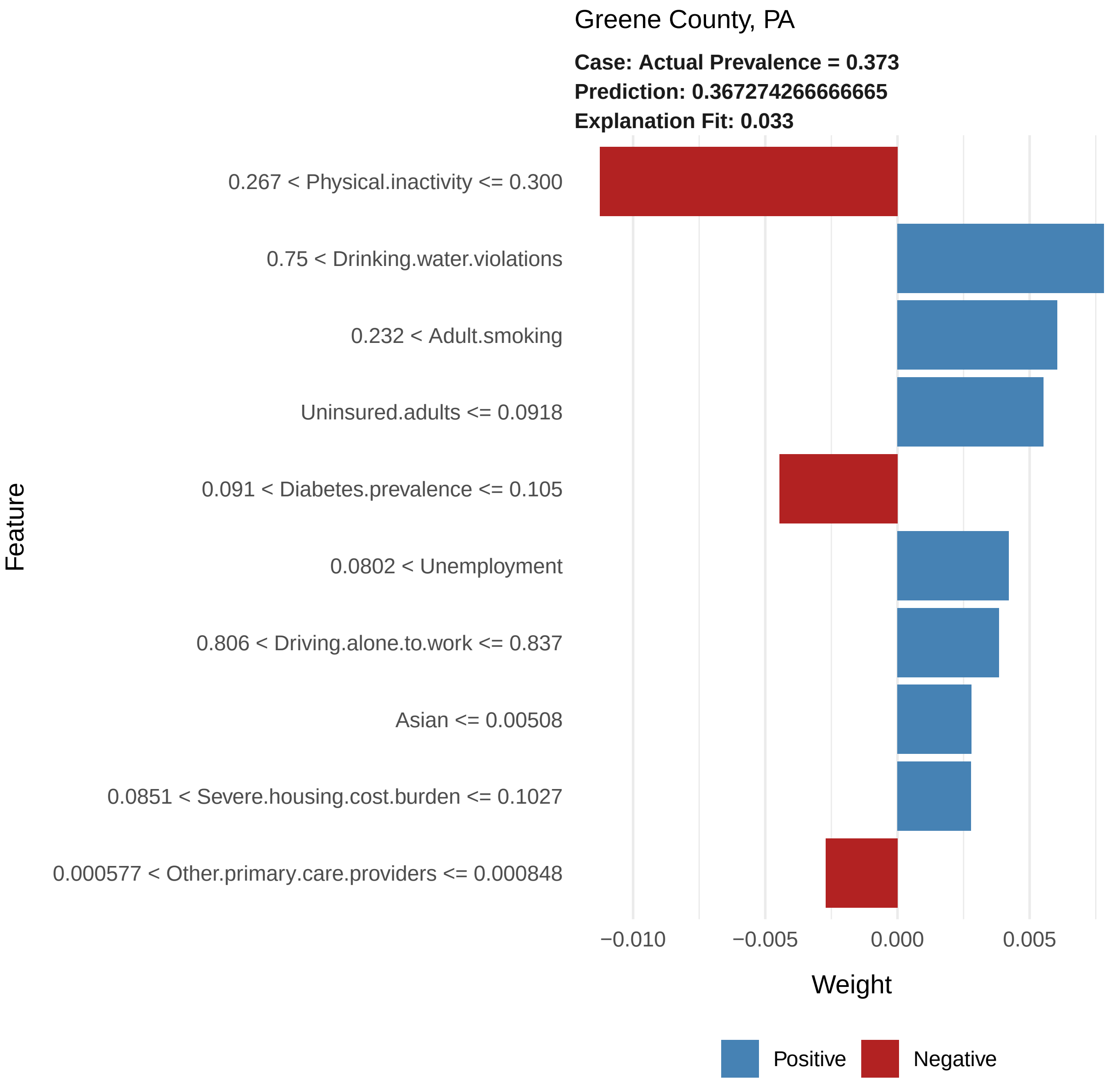

Greene County, PA
Case: Actual Prevalence = 0.373
Prediction: 0.367274266666665
Explanation Fit: 0.033
0.267 < Physical.inactivity <= 0.300
0.75 < Drinking.water.violations
0.232 < Adult.smoking
Uninsured.adults <= 0.0918
0.091 < Diabetes.prevalence <= 0.105
0.0802 < Unemployment
0.806 < Driving.alone.to.work <= 0.837
Asian <= 0.00508
0.0851 < Severe.housing.cost.burden <= 0.1027
0.000577 < Other.primary.care.providers <= 0.000848
Feature
−0.010
−0.005
0.000
0.005
Weight
Positive
Negative

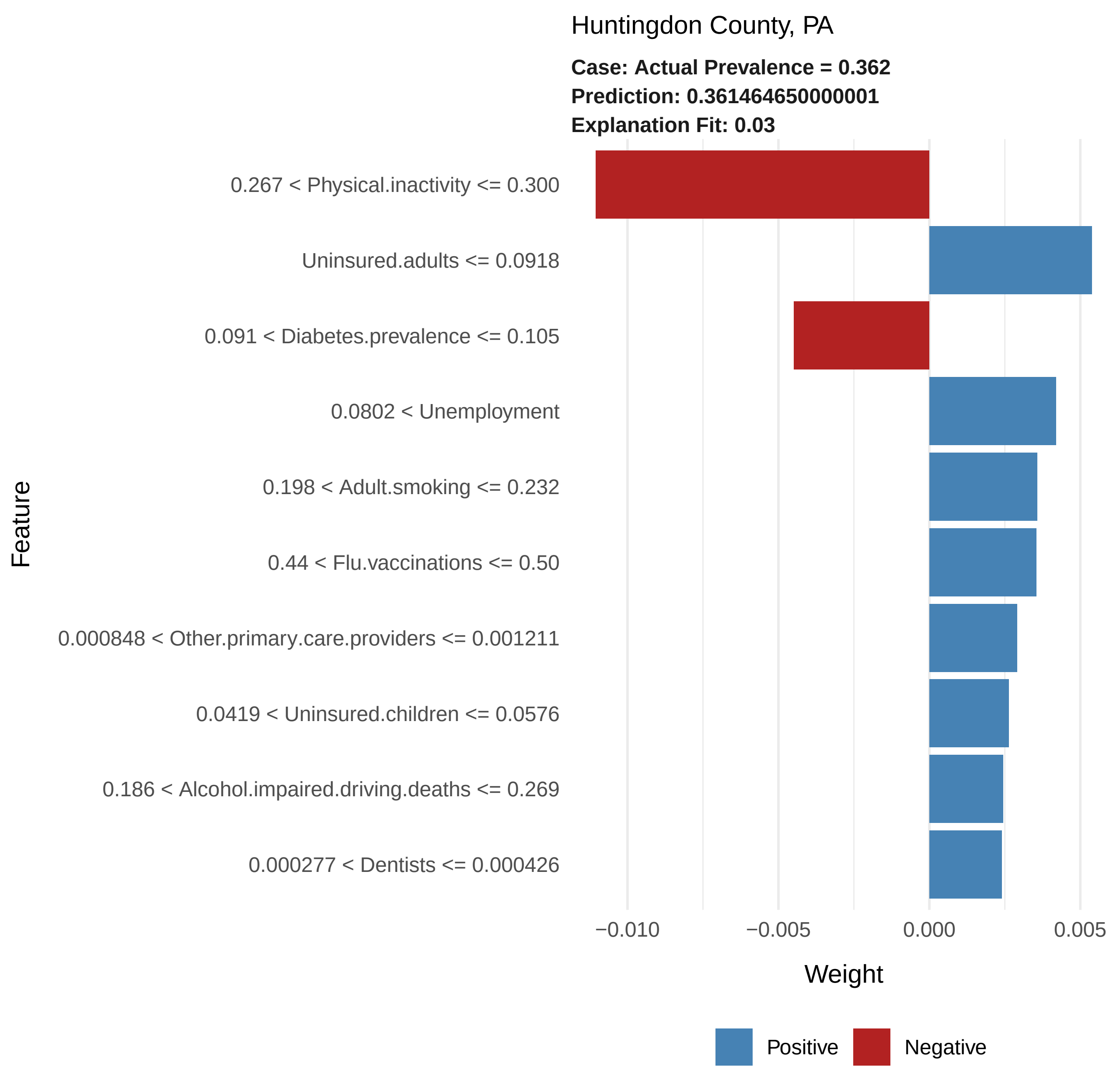
Huntingdon County, PA
Case: Actual Prevalence = 0.362
Prediction: 0.361464650000001
Explanation Fit: 0.03
0.267 < Physical.inactivity <= 0.300
Uninsured.adults <= 0.0918
0.091 < Diabetes.prevalence <= 0.105
0.0802 < Unemployment
0.198 < Adult.smoking <= 0.232
0.44 < Flu.vaccinations <= 0.50
0.000848 < Other.primary.care.providers <= 0.001211
0.0419 < Uninsured.children <= 0.0576
0.186 < Alcohol.impaired.driving.deaths <= 0.269
0.000277 < Dentists <= 0.000426
Feature
−0.010
−0.005
0.000
0.005
Weight
Positive
Negative

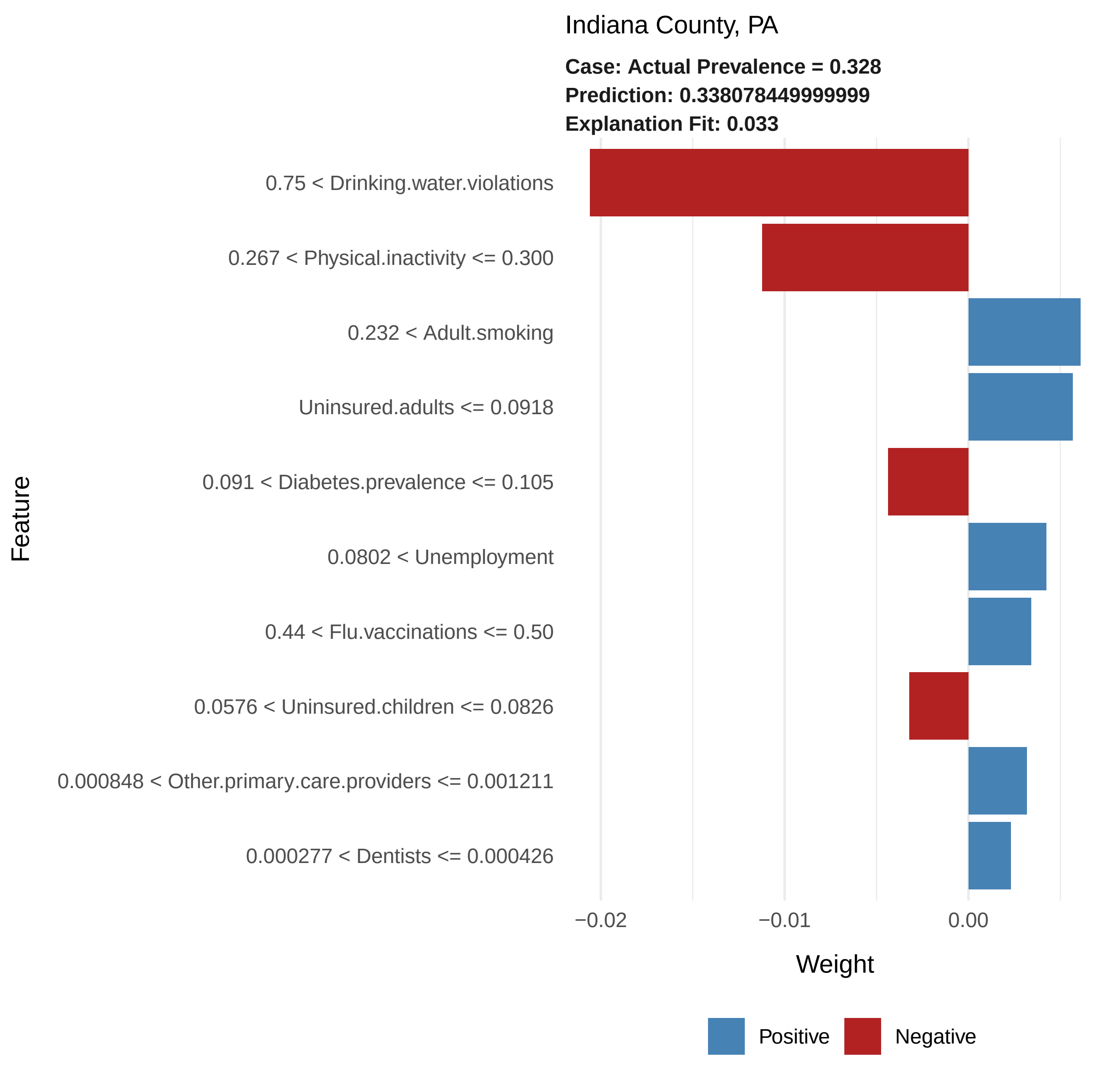

Indiana County, PA
Case: Actual Prevalence = 0.328
Prediction: 0.338078449999999
Explanation Fit: 0.033
0.75 < Drinking.water.violations
0.267 < Physical.inactivity <= 0.300
0.232 < Adult.smoking
Uninsured.adults <= 0.0918
0.091 < Diabetes.prevalence <= 0.105
0.0802 < Unemployment
0.44 < Flu.vaccinations <= 0.50
0.0576 < Uninsured.children <= 0.0826
0.000848 < Other.primary.care.providers <= 0.001211
0.000277 < Dentists <= 0.000426
Feature
−0.02
−0.01
0.00
Weight
Positive
Negative

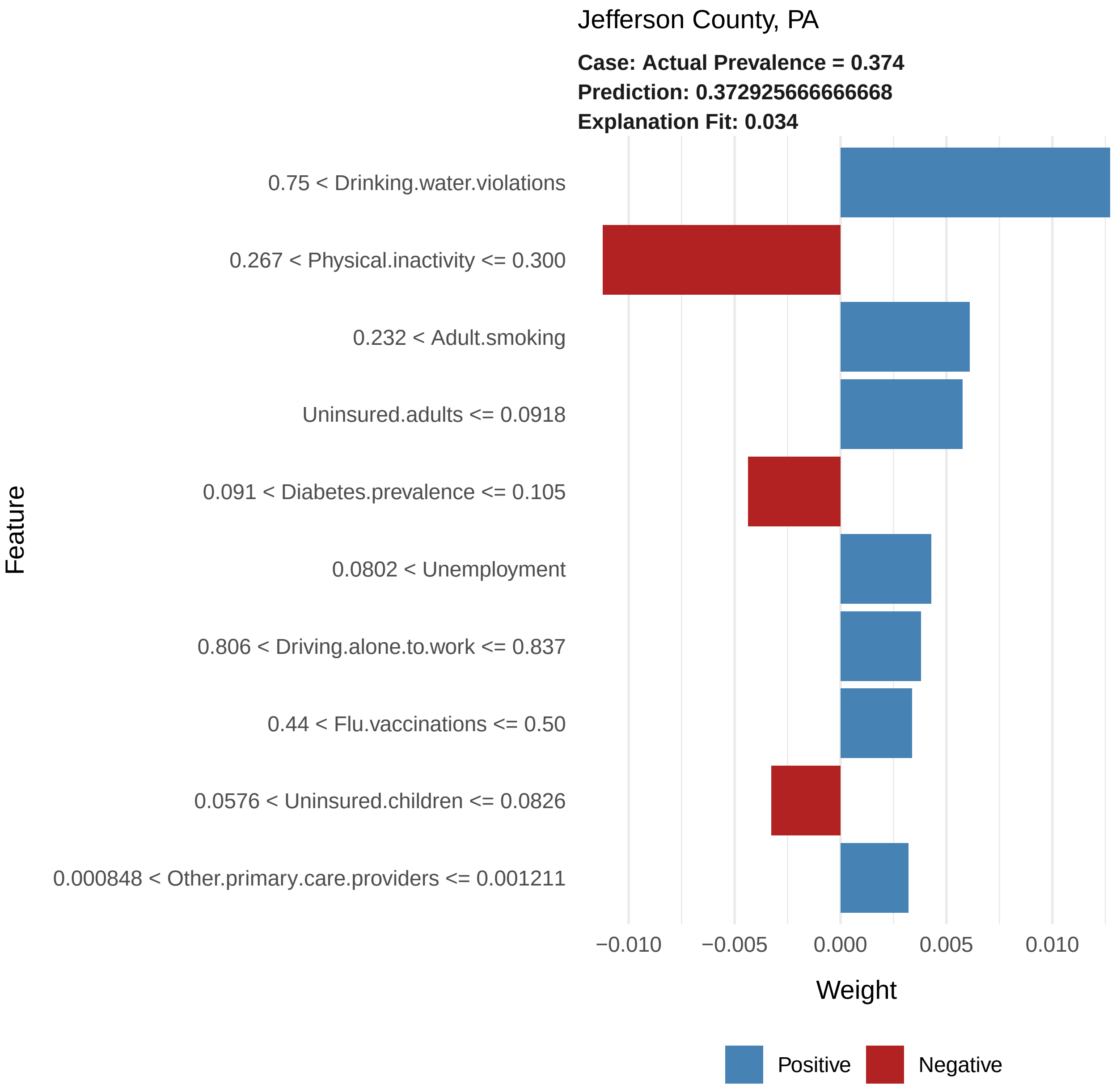

Jefferson County, PA
Case: Actual Prevalence = 0.374
Prediction: 0.372925666666668
Explanation Fit: 0.034
0.75 < Drinking.water.violations
0.267 < Physical.inactivity <= 0.300
0.232 < Adult.smoking
Uninsured.adults <= 0.0918
0.091 < Diabetes.prevalence <= 0.105
0.0802 < Unemployment
0.806 < Driving.alone.to.work <= 0.837
0.44 < Flu.vaccinations <= 0.50
0.0576 < Uninsured.children <= 0.0826
0.000848 < Other.primary.care.providers <= 0.001211
Feature
−0.010
−0.005
0.000
0.005
0.010
Weight
Positive
Negative

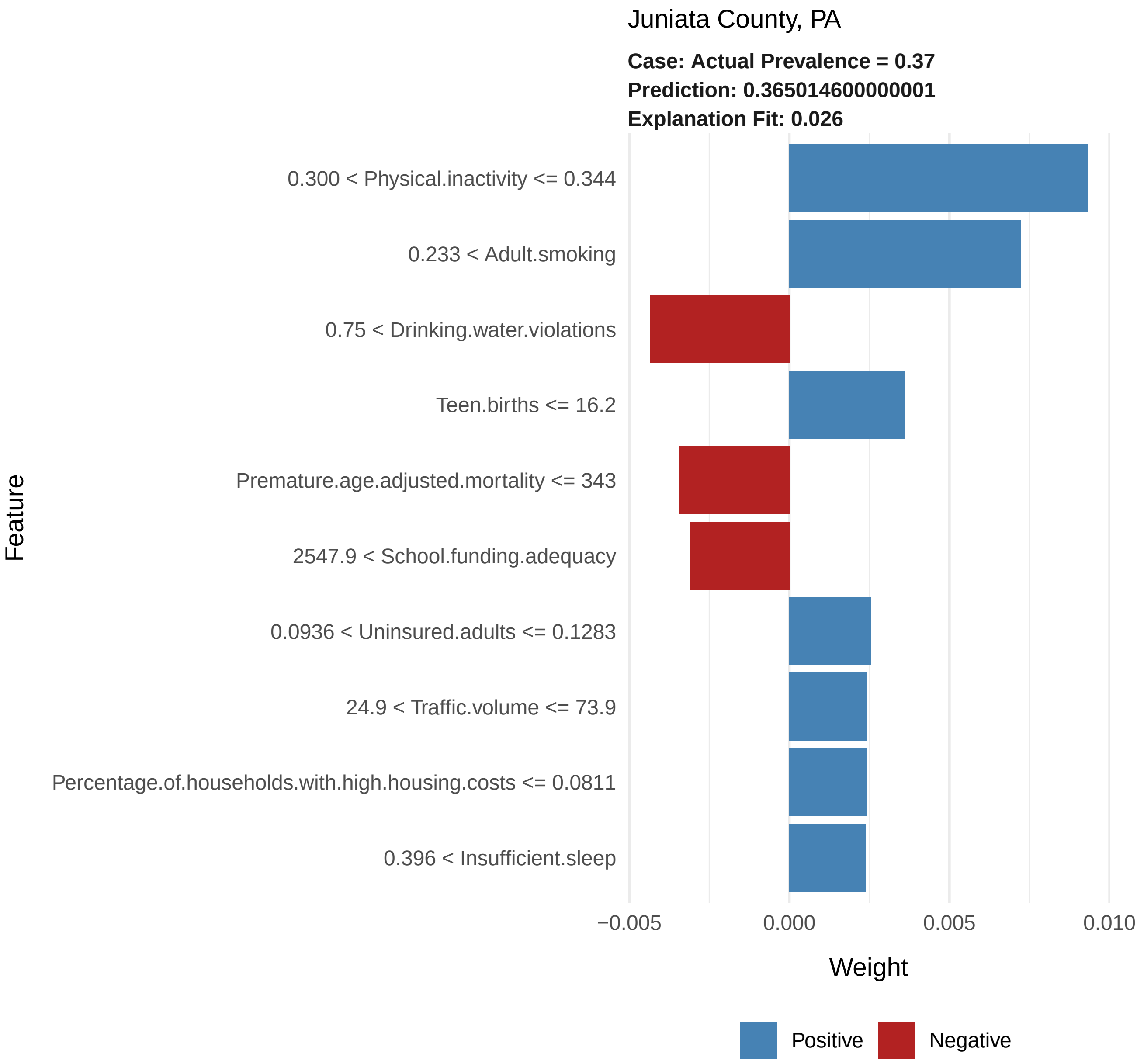

Juniata County, PA
Case: Actual Prevalence = 0.37
Prediction: 0.365014600000001
Explanation Fit: 0.026
0.300 < Physical.inactivity <= 0.344
0.233 < Adult.smoking
0.75 < Drinking.water.violations
Teen.births <= 16.2
Premature.age.adjusted.mortality <= 343
2547.9 < School.funding.adequacy
0.0936 < Uninsured.adults <= 0.1283
24.9 < Traffic.volume <= 73.9
Percentage.of.households.with.high.housing.costs <= 0.0811
0.396 < Insufficient.sleep
Feature
−0.005
0.000
0.005
0.010
Weight
Positive
Negative

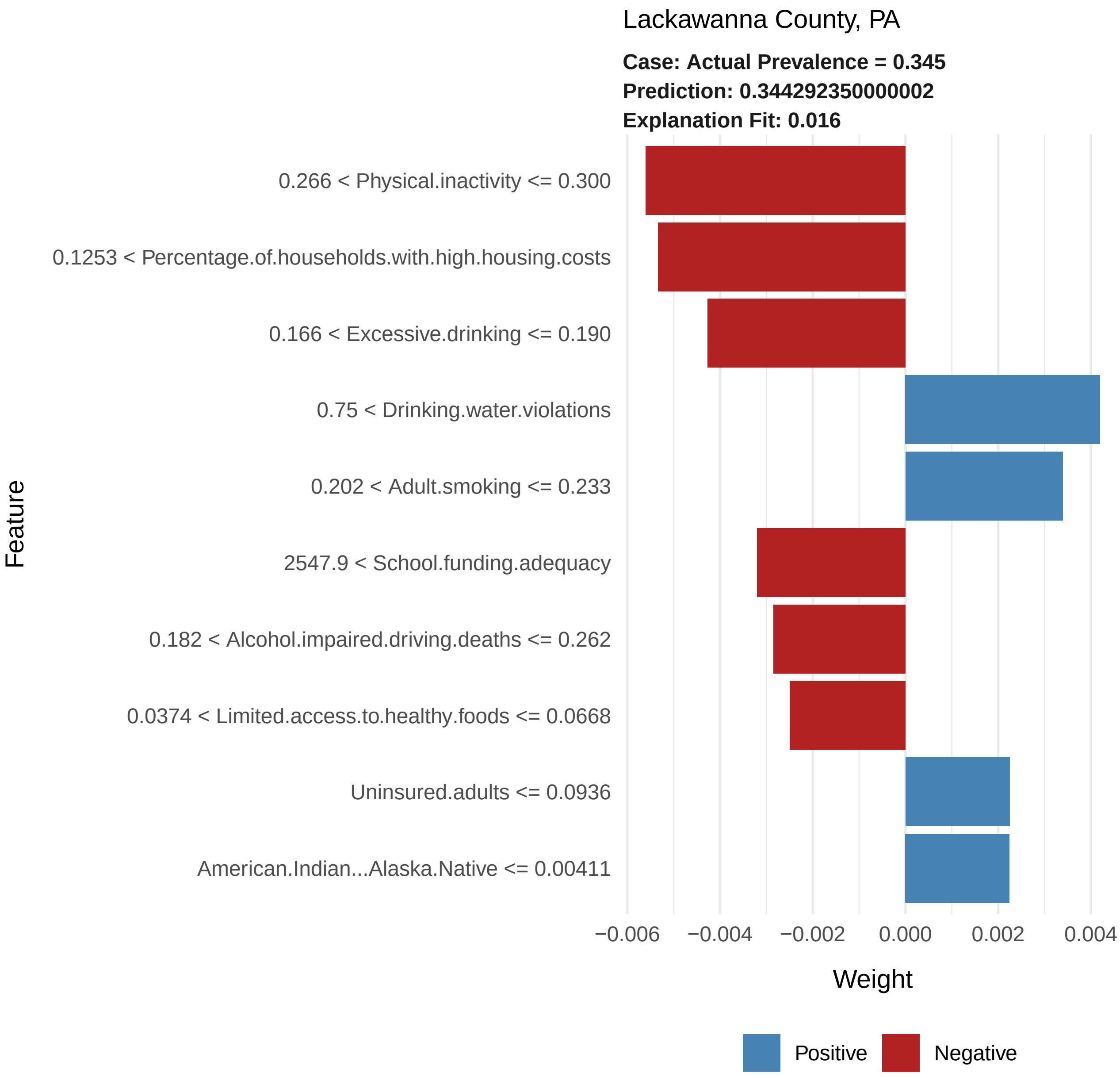
Lackawanna County, PA
Case: Actual Prevalence = 0.345
Prediction: 0.344292350000002
Explanation Fit: 0.016
0.266 < Physical.inactivity <= 0.300
0.1253 < Percentage.of.households.with.high.housing.costs
0.166 < Excessive.drinking <= 0.190
0.75 < Drinking.water.violations
0.202 < Adult.smoking <= 0.233
2547.9 < School.funding.adequacy
0.182 < Alcohol.impaired.driving.deaths <= 0.262
0.0374 < Limited.access.to.healthy.foods <= 0.0668
Uninsured.adults <= 0.0936
American.Indian...Alaska.Native <= 0.00411
Feature
−0.006
−0.004
−0.002
0.000
0.002
0.004
Weight
Positive
Negative

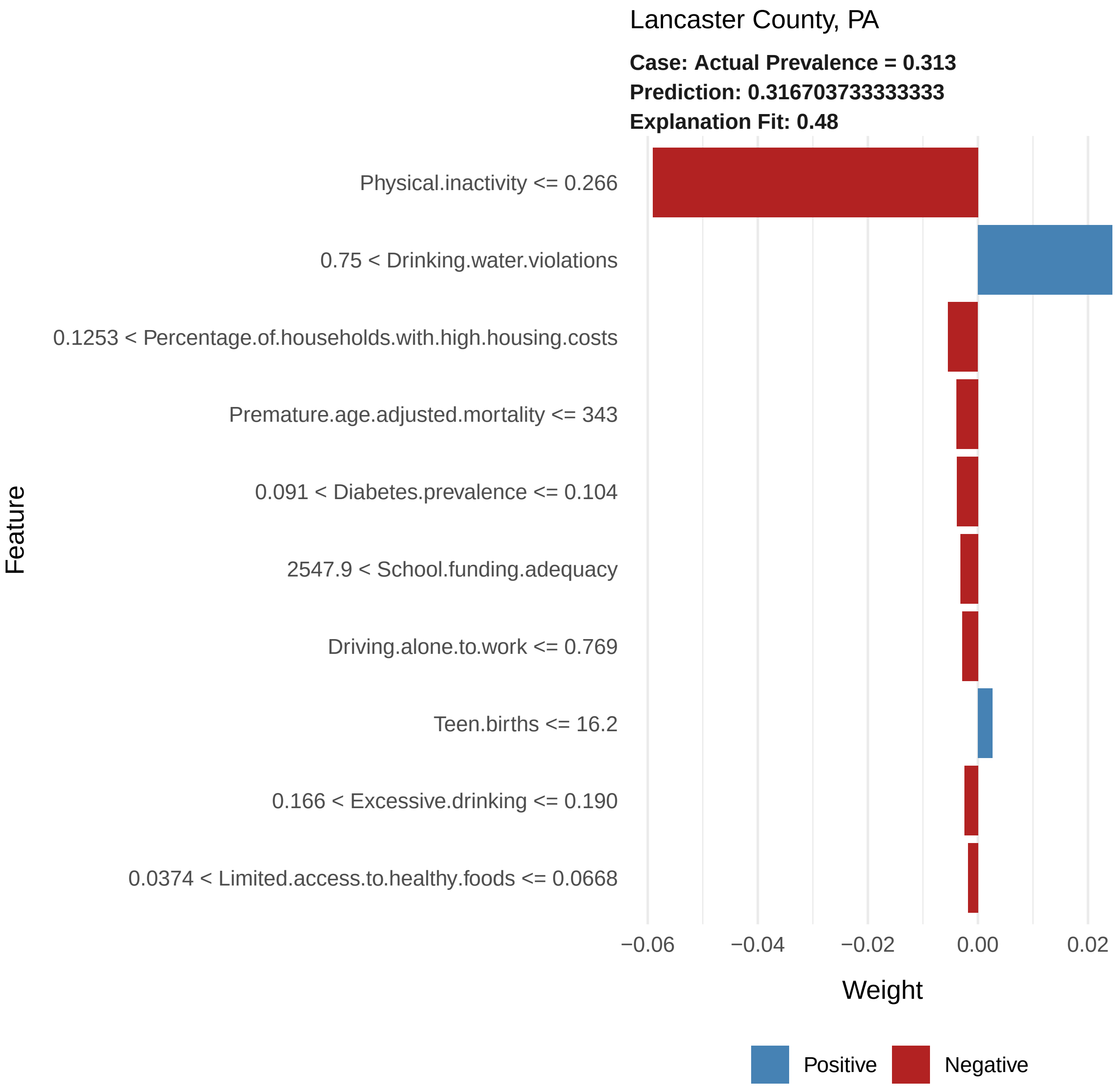
Lancaster County, PA
Case: Actual Prevalence = 0.313
Prediction: 0.316703733333333
Explanation Fit: 0.48
Physical.inactivity <= 0.266
0.75 < Drinking.water.violations
0.1253 < Percentage.of.households.with.high.housing.costs
Premature.age.adjusted.mortality <= 343
0.091 < Diabetes.prevalence <= 0.104
2547.9 < School.funding.adequacy
Driving.alone.to.work <= 0.769
Teen.births <= 16.2
0.166 < Excessive.drinking <= 0.190
0.0374 < Limited.access.to.healthy.foods <= 0.0668
Feature
−0.06
−0.04
−0.02
0.00
0.02
Weight
Positive
Negative

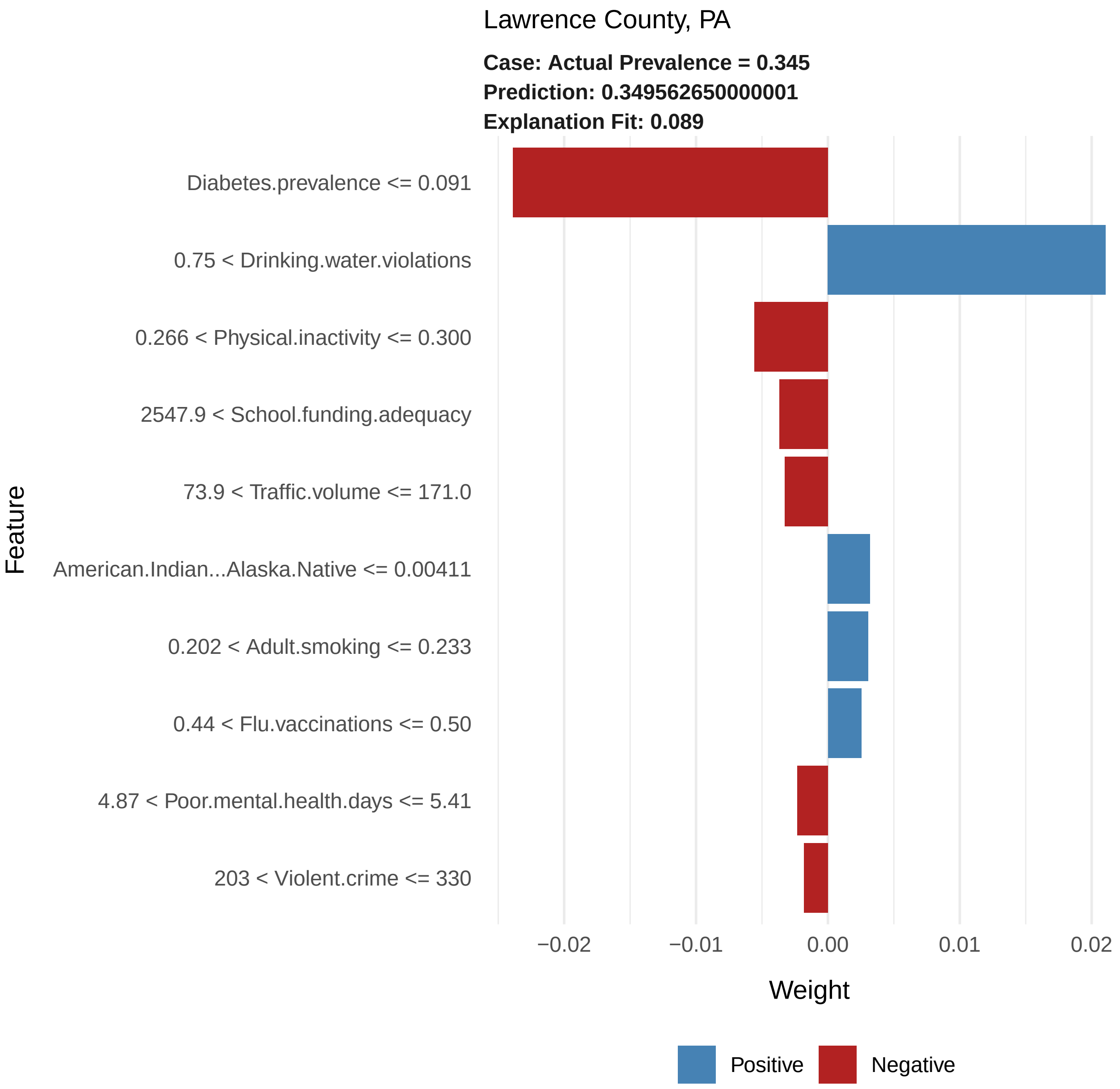

Lawrence County, PA
Case: Actual Prevalence = 0.345
Prediction: 0.349562650000001
Explanation Fit: 0.089
Diabetes.prevalence <= 0.091
0.75 < Drinking.water.violations
0.266 < Physical.inactivity <= 0.300
2547.9 < School.funding.adequacy
73.9 < Traffic.volume <= 171.0
American.Indian...Alaska.Native <= 0.00411
0.202 < Adult.smoking <= 0.233
0.44 < Flu.vaccinations <= 0.50
4.87 < Poor.mental.health.days <= 5.41
203 < Violent.crime <= 330
Feature
−0.02
−0.01
0.00
0.01
0.02
Weight
Positive
Negative

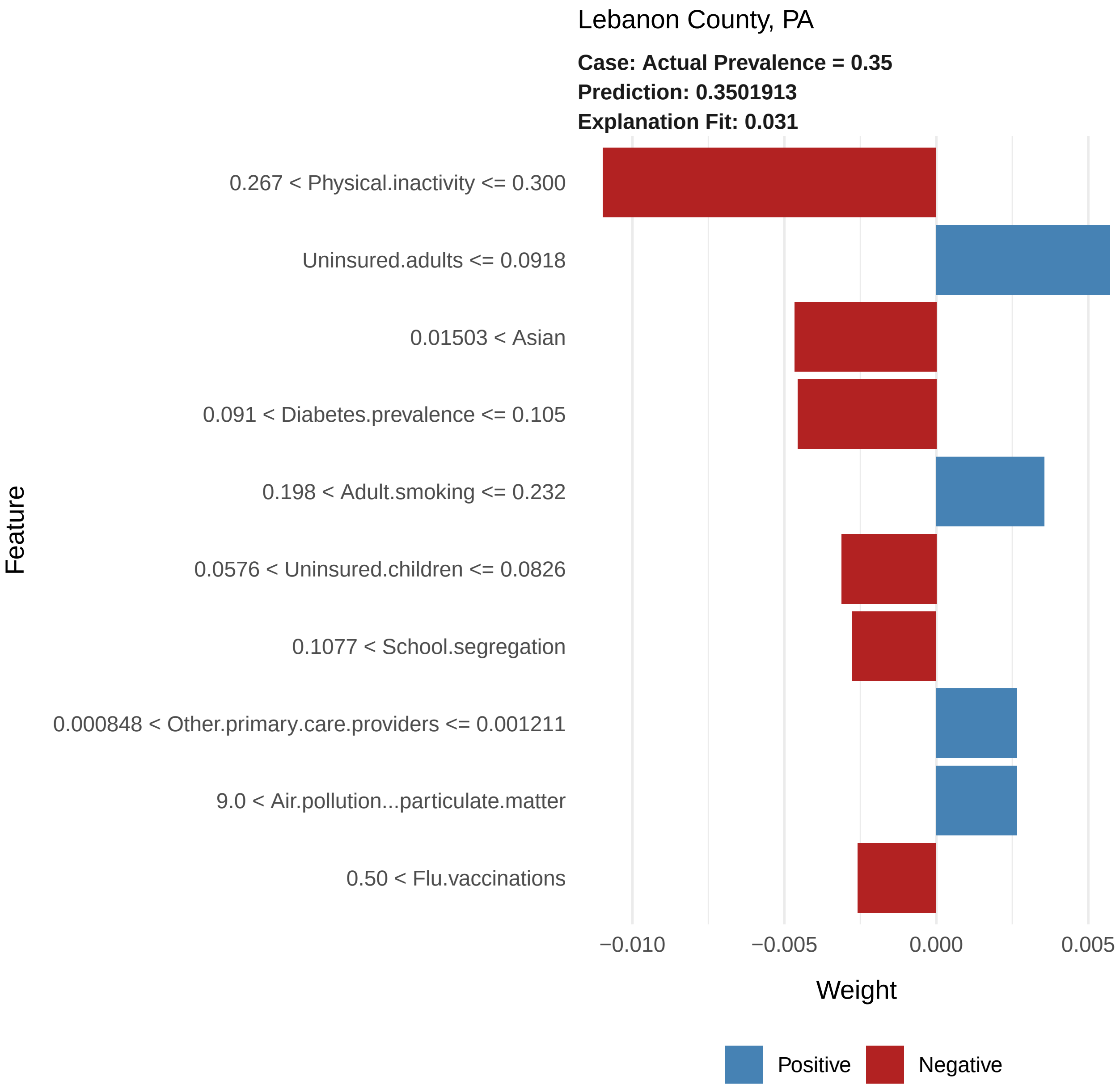

Lebanon County, PA
Case: Actual Prevalence = 0.35
Prediction: 0.3501913
Explanation Fit: 0.031
0.267 < Physical.inactivity <= 0.300
Uninsured.adults <= 0.0918
0.01503 < Asian
0.091 < Diabetes.prevalence <= 0.105
0.198 < Adult.smoking <= 0.232
0.0576 < Uninsured.children <= 0.0826
0.1077 < School.segregation
0.000848 < Other.primary.care.providers <= 0.001211
9.0 < Air.pollution...particulate.matter
0.50 < Flu.vaccinations
Feature
−0.010
−0.005
0.000
0.005
Weight
Positive
Negative

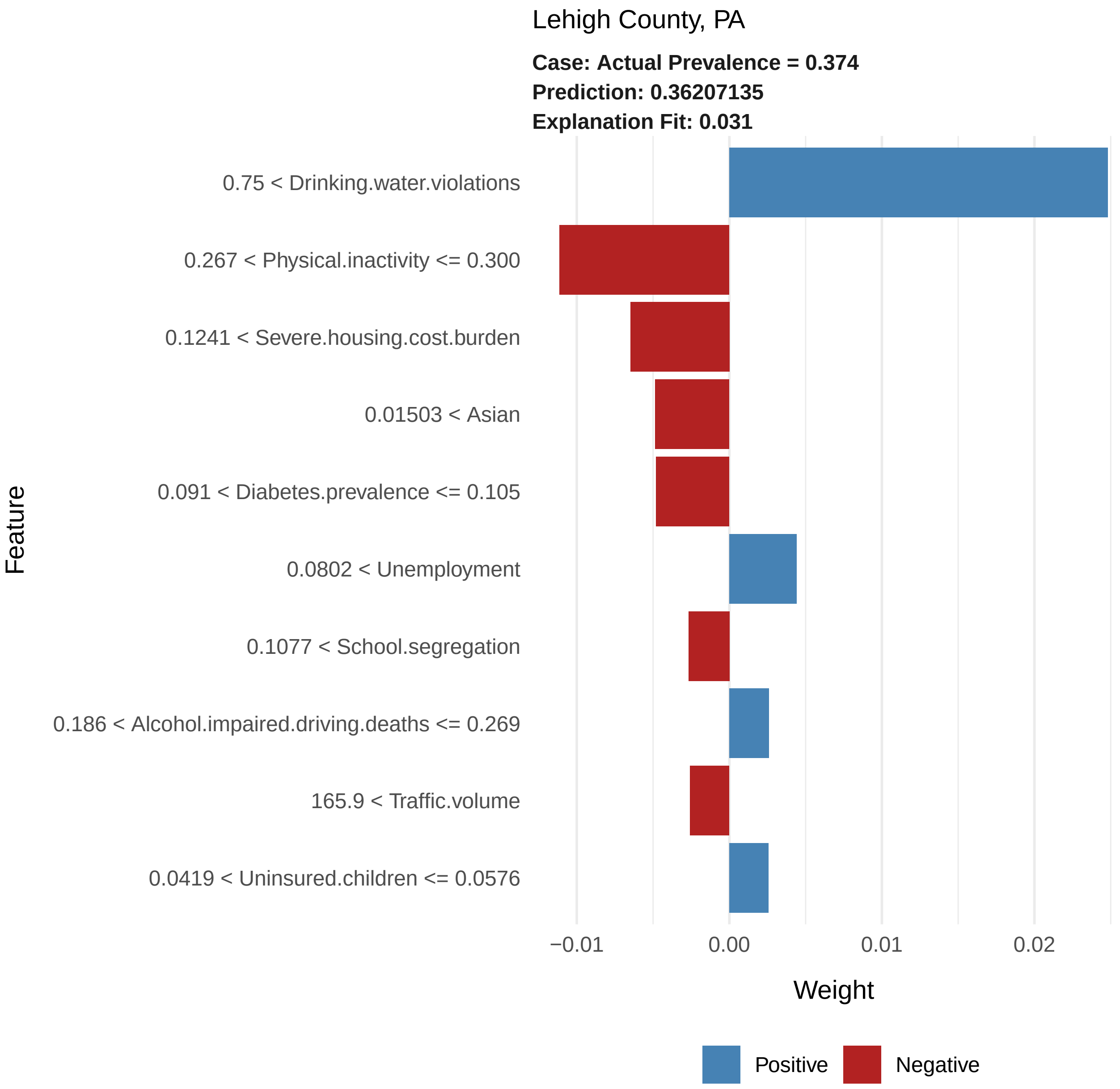

Lehigh County, PA
Case: Actual Prevalence = 0.374
Prediction: 0.36207135
Explanation Fit: 0.031
0.75 < Drinking.water.violations
0.267 < Physical.inactivity <= 0.300
0.1241 < Severe.housing.cost.burden
0.01503 < Asian
0.091 < Diabetes.prevalence <= 0.105
0.0802 < Unemployment
0.1077 < School.segregation
0.186 < Alcohol.impaired.driving.deaths <= 0.269
165.9 < Traffic.volume
0.0419 < Uninsured.children <= 0.0576
Feature
−0.01
0.00
0.01
0.02
Weight
Positive
Negative

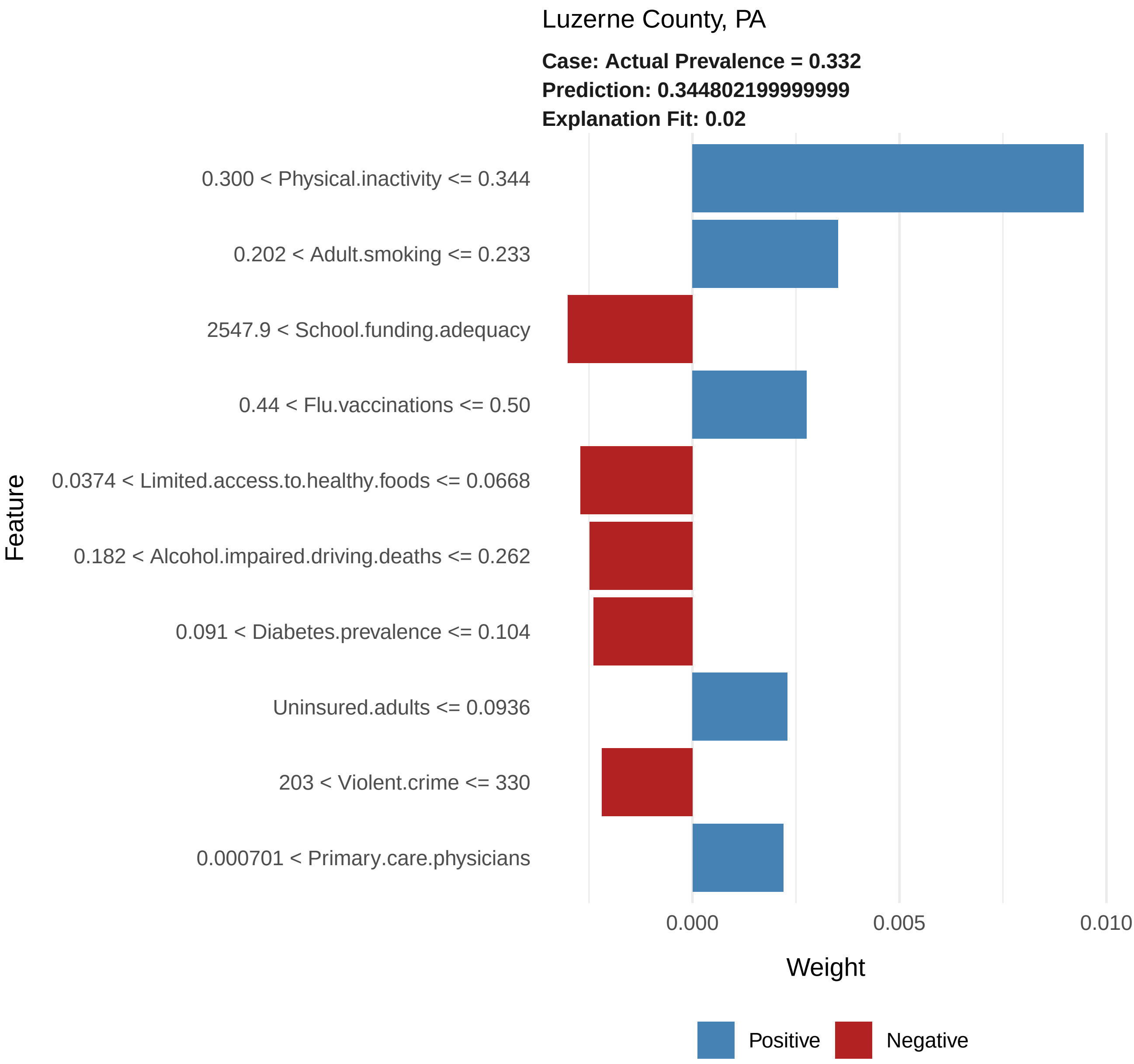
Luzerne County, PA
Case: Actual Prevalence = 0.332
Prediction: 0.344802199999999
Explanation Fit: 0.02
0.300 < Physical.inactivity <= 0.344
0.202 < Adult.smoking <= 0.233
2547.9 < School.funding.adequacy
0.44 < Flu.vaccinations <= 0.50
0.0374 < Limited.access.to.healthy.foods <= 0.0668
0.182 < Alcohol.impaired.driving.deaths <= 0.262
0.091 < Diabetes.prevalence <= 0.104
Uninsured.adults <= 0.0936
203 < Violent.crime <= 330
0.000701 < Primary.care.physicians
Feature
0.000
0.005
0.010
Weight
Positive
Negative

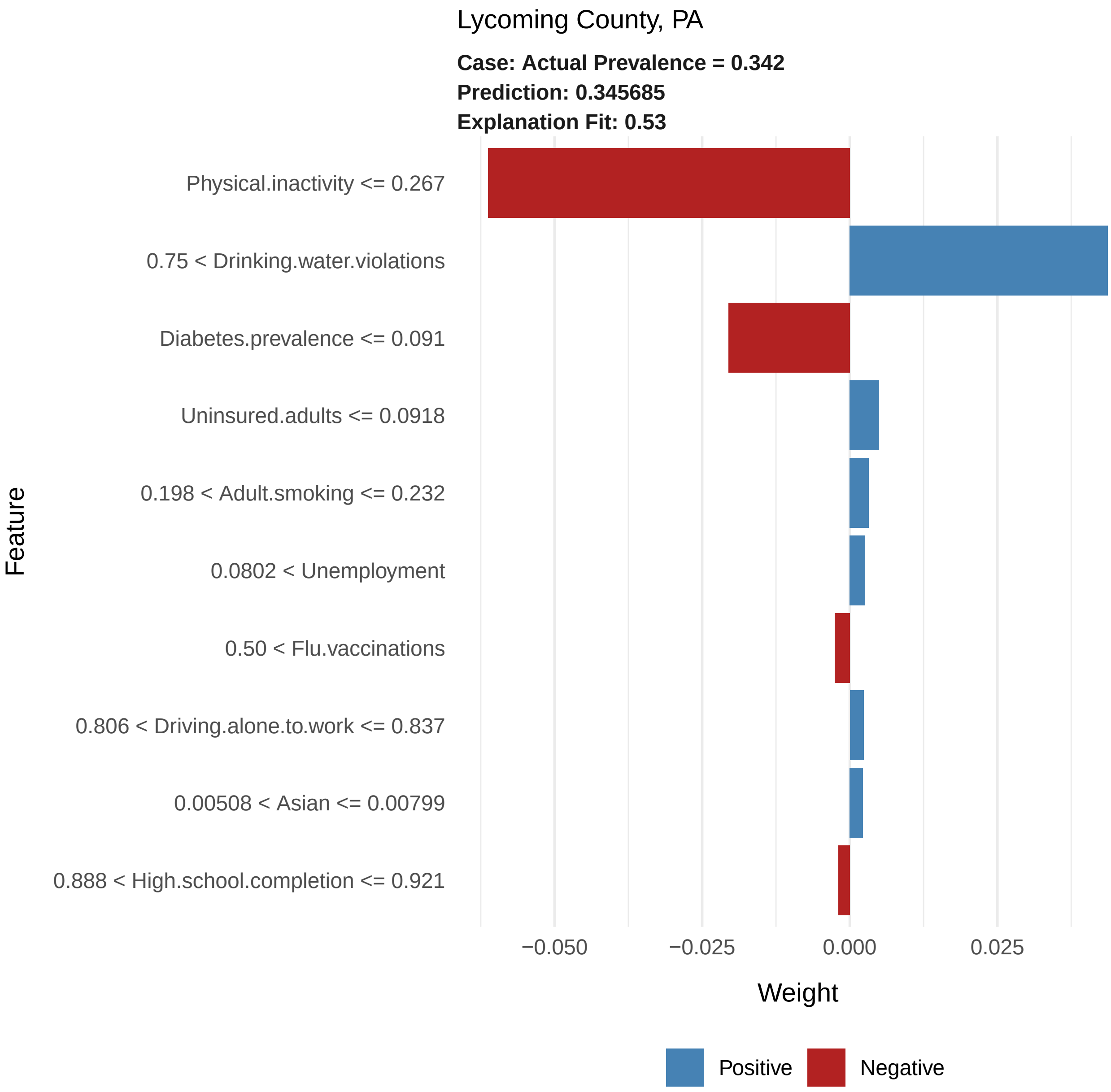
Lycoming County, PA
Case: Actual Prevalence = 0.342
Prediction: 0.345685
Explanation Fit: 0.53
Physical.inactivity <= 0.267
0.75 < Drinking.water.violations
Diabetes.prevalence <= 0.091
Uninsured.adults <= 0.0918
0.198 < Adult.smoking <= 0.232
0.0802 < Unemployment
0.50 < Flu.vaccinations
0.806 < Driving.alone.to.work <= 0.837
0.00508 < Asian <= 0.00799
0.888 < High.school.completion <= 0.921
Feature
−0.050
−0.025
0.000
0.025
Weight
Positive
Negative

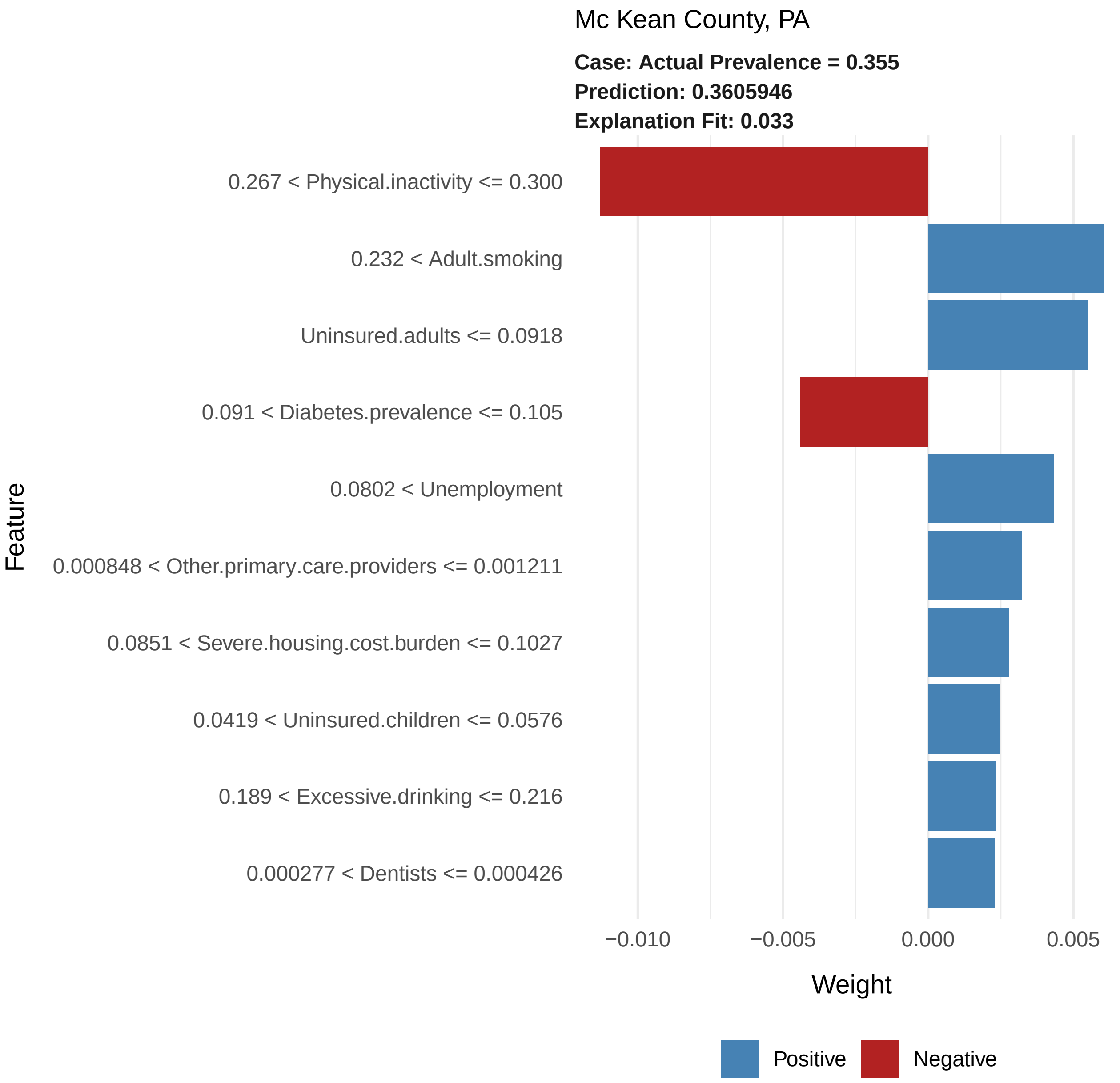
Mc Kean County, PA
Case: Actual Prevalence = 0.355
Prediction: 0.3605946
Explanation Fit: 0.033
0.267 < Physical.inactivity <= 0.300
0.232 < Adult.smoking
Uninsured.adults <= 0.0918
0.091 < Diabetes.prevalence <= 0.105
0.0802 < Unemployment
0.000848 < Other.primary.care.providers <= 0.001211
0.0851 < Severe.housing.cost.burden <= 0.1027
0.0419 < Uninsured.children <= 0.0576
0.189 < Excessive.drinking <= 0.216
0.000277 < Dentists <= 0.000426
Feature
−0.010
−0.005
0.000
0.005
Weight
Positive
Negative

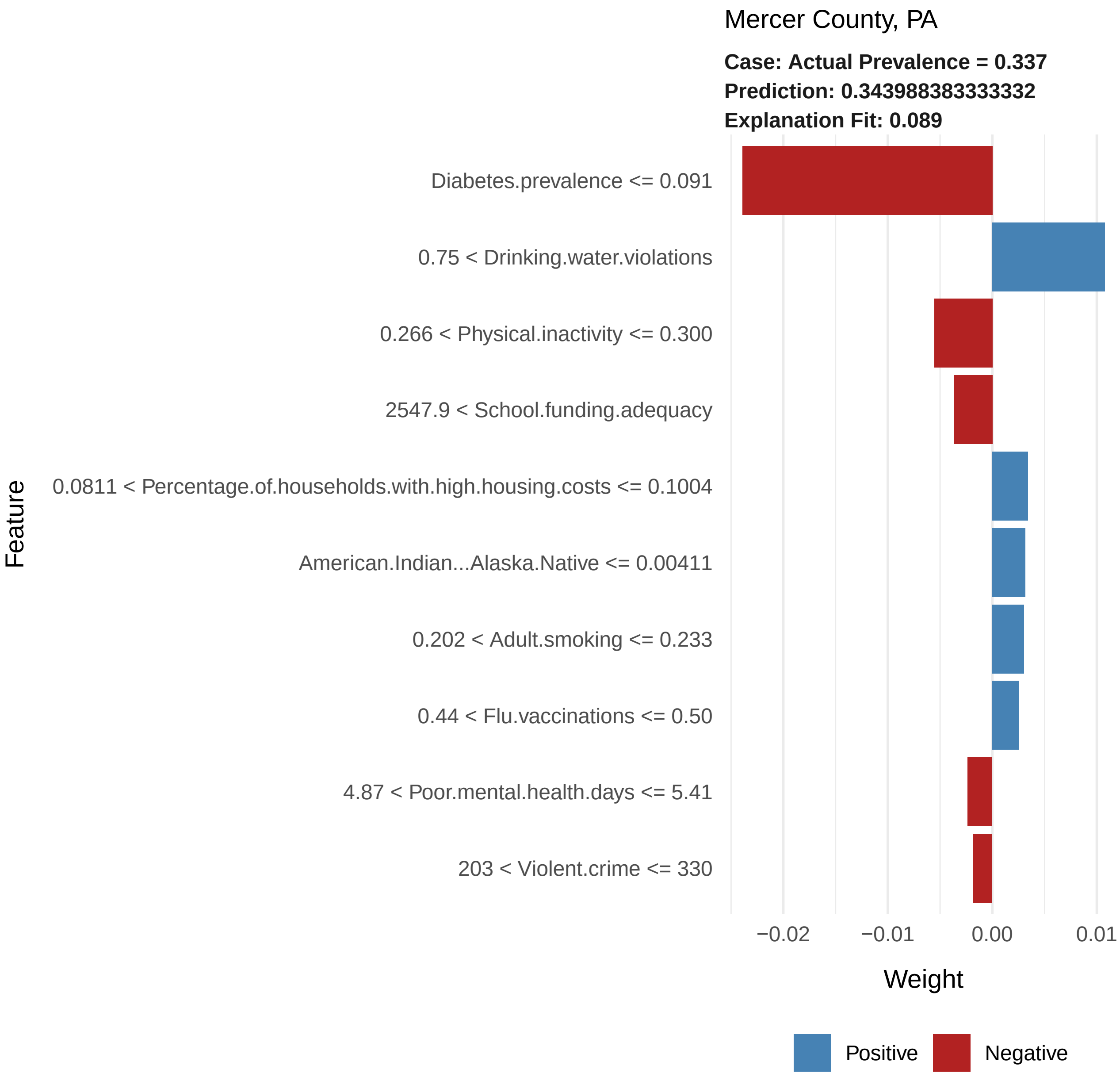
Mercer County, PA
Case: Actual Prevalence = 0.337
Prediction: 0.343988383333332
Explanation Fit: 0.089
Diabetes.prevalence <= 0.091
0.75 < Drinking.water.violations
0.266 < Physical.inactivity <= 0.300
2547.9 < School.funding.adequacy
0.0811 < Percentage.of.households.with.high.housing.costs <= 0.1004
American.Indian...Alaska.Native <= 0.00411
0.202 < Adult.smoking <= 0.233
0.44 < Flu.vaccinations <= 0.50
4.87 < Poor.mental.health.days <= 5.41
203 < Violent.crime <= 330
Feature
−0.02
−0.01
0.00
0.01
Weight
Positive
Negative

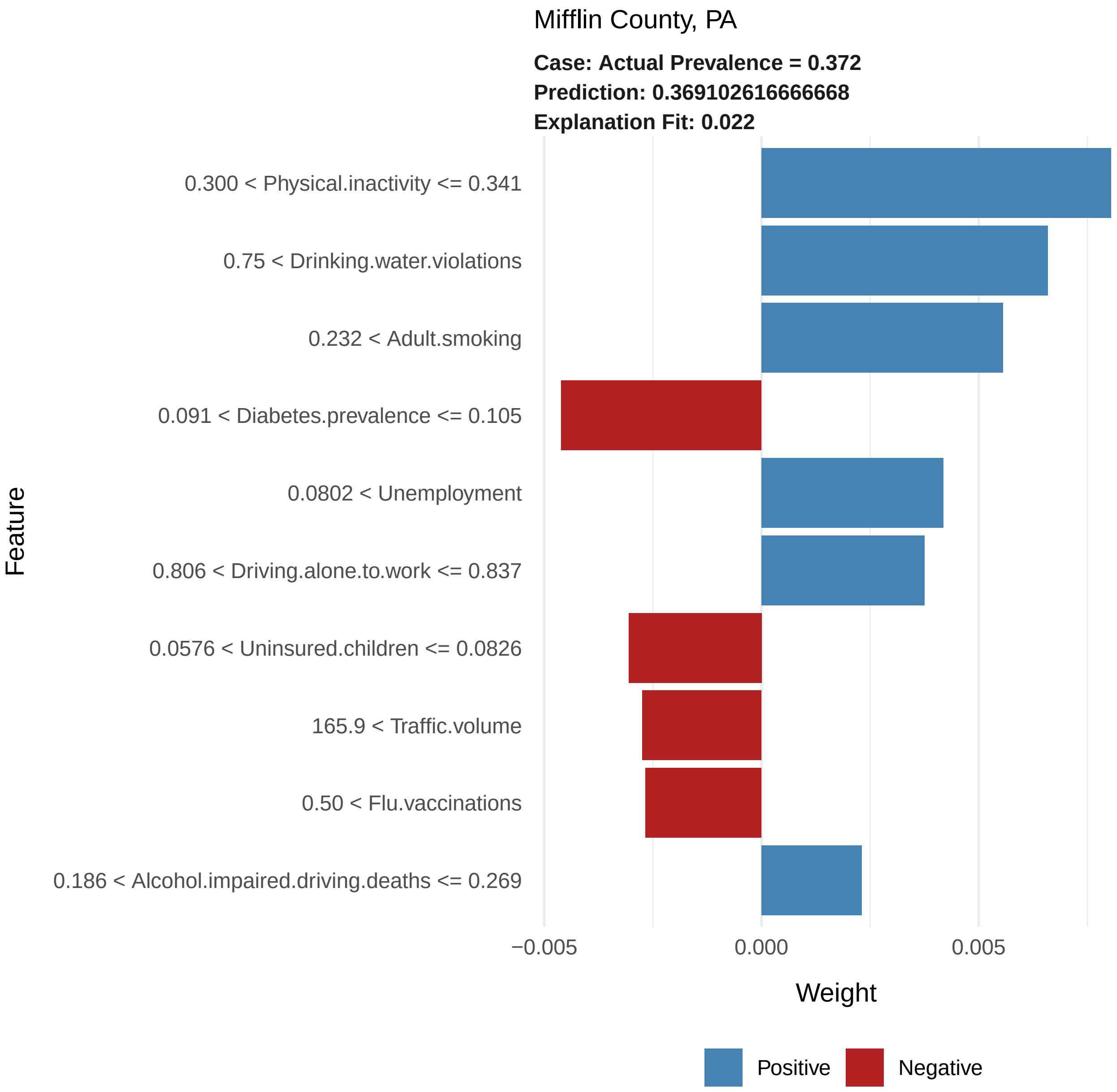
Mifflin County, PA
Case: Actual Prevalence = 0.372
Prediction: 0.369102616666668
Explanation Fit: 0.022
0.300 < Physical.inactivity <= 0.341
0.75 < Drinking.water.violations
0.232 < Adult.smoking
0.091 < Diabetes.prevalence <= 0.105
0.0802 < Unemployment
0.806 < Driving.alone.to.work <= 0.837
0.0576 < Uninsured.children <= 0.0826
165.9 < Traffic.volume
0.50 < Flu.vaccinations
0.186 < Alcohol.impaired.driving.deaths <= 0.269
Feature
−0.005
0.000
0.005
Weight
Positive
Negative

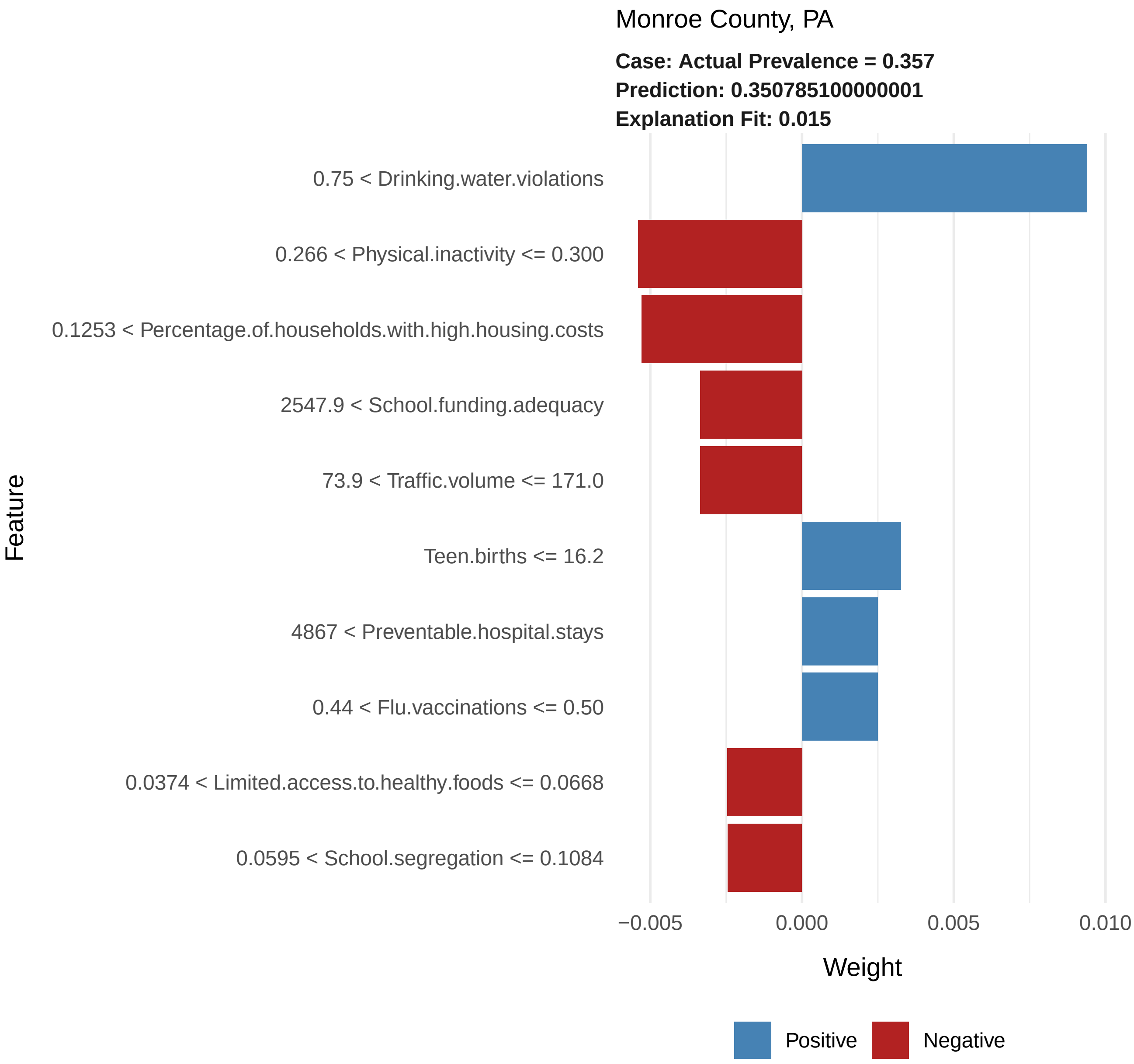
Monroe County, PA
Case: Actual Prevalence = 0.357
Prediction: 0.350785100000001
Explanation Fit: 0.015
0.75 < Drinking.water.violations
0.266 < Physical.inactivity <= 0.300
0.1253 < Percentage.of.households.with.high.housing.costs
2547.9 < School.funding.adequacy
73.9 < Traffic.volume <= 171.0
Teen.births <= 16.2
4867 < Preventable.hospital.stays
0.44 < Flu.vaccinations <= 0.50
0.0374 < Limited.access.to.healthy.foods <= 0.0668
0.0595 < School.segregation <= 0.1084
Feature
−0.005
0.000
0.005
0.010
Weight
Positive
Negative

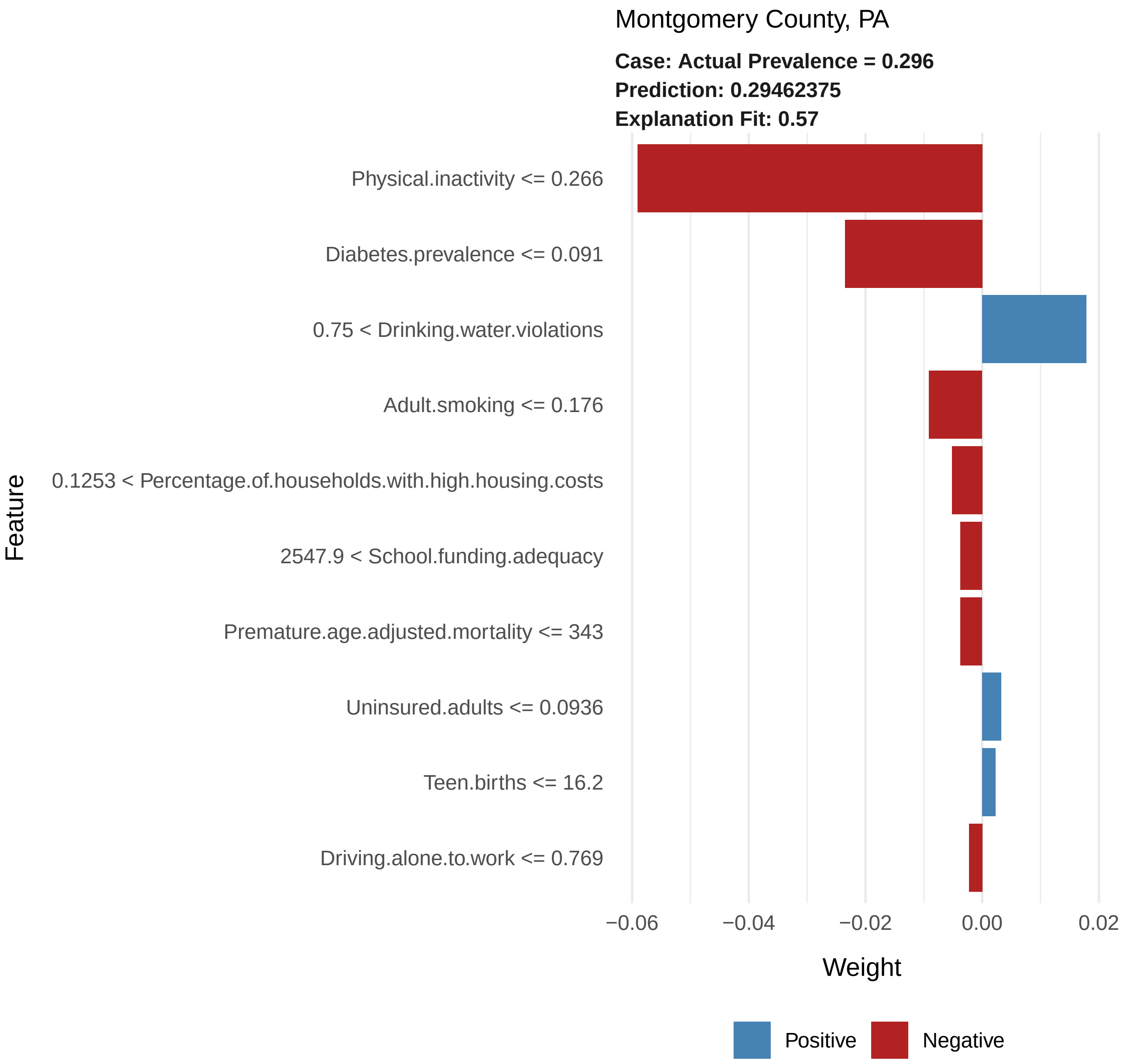

Montgomery County, PA
Case: Actual Prevalence = 0.296
Prediction: 0.29462375
Explanation Fit: 0.57
Physical.inactivity <= 0.266
Diabetes.prevalence <= 0.091
0.75 < Drinking.water.violations
Adult.smoking <= 0.176
0.1253 < Percentage.of.households.with.high.housing.costs
2547.9 < School.funding.adequacy
Premature.age.adjusted.mortality <= 343
Uninsured.adults <= 0.0936
Teen.births <= 16.2
Driving.alone.to.work <= 0.769
Feature
−0.06
−0.04
−0.02
0.00
0.02
Weight
Positive
Negative

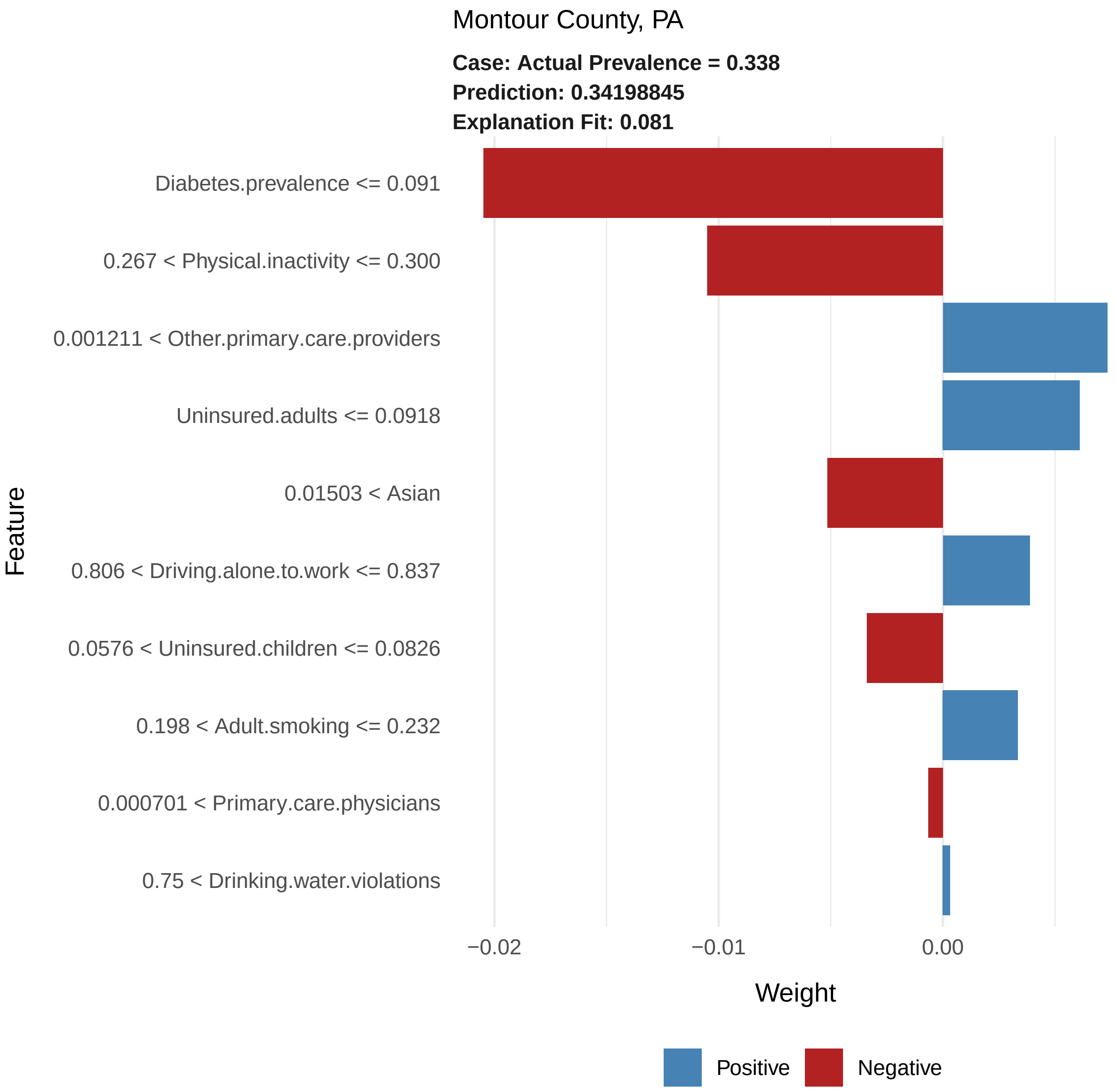

Montour County, PA
Case: Actual Prevalence = 0.338
Prediction: 0.34198845
Explanation Fit: 0.081
Diabetes.prevalence <= 0.091
0.267 < Physical.inactivity <= 0.300
0.001211 < Other.primary.care.providers
Uninsured.adults <= 0.0918
0.01503 < Asian
0.806 < Driving.alone.to.work <= 0.837
0.0576 < Uninsured.children <= 0.0826
0.198 < Adult.smoking <= 0.232
0.000701 < Primary.care.physicians
0.75 < Drinking.water.violations
Feature
−0.02
−0.01
0.00
Weight
Positive
Negative

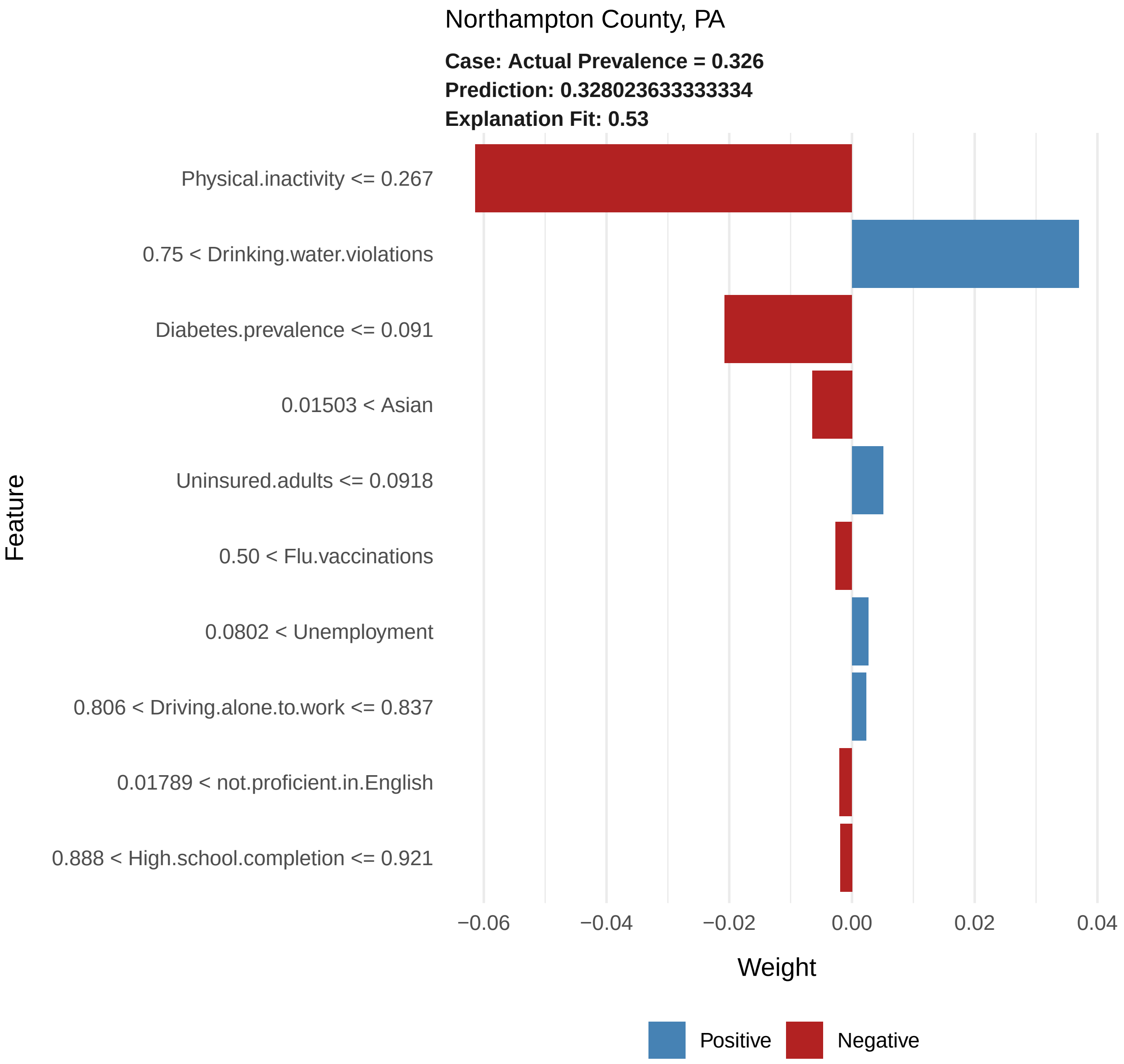
Northampton County, PA
Case: Actual Prevalence = 0.326
Prediction: 0.328023633333334
Explanation Fit: 0.53
Physical.inactivity <= 0.267
0.75 < Drinking.water.violations
Diabetes.prevalence <= 0.091
0.01503 < Asian
Uninsured.adults <= 0.0918
0.50 < Flu.vaccinations
0.0802 < Unemployment
0.806 < Driving.alone.to.work <= 0.837
0.01789 < not.proficient.in.English
0.888 < High.school.completion <= 0.921
Feature
−0.06
−0.04
−0.02
0.00
0.02
0.04
Weight
Positive
Negative

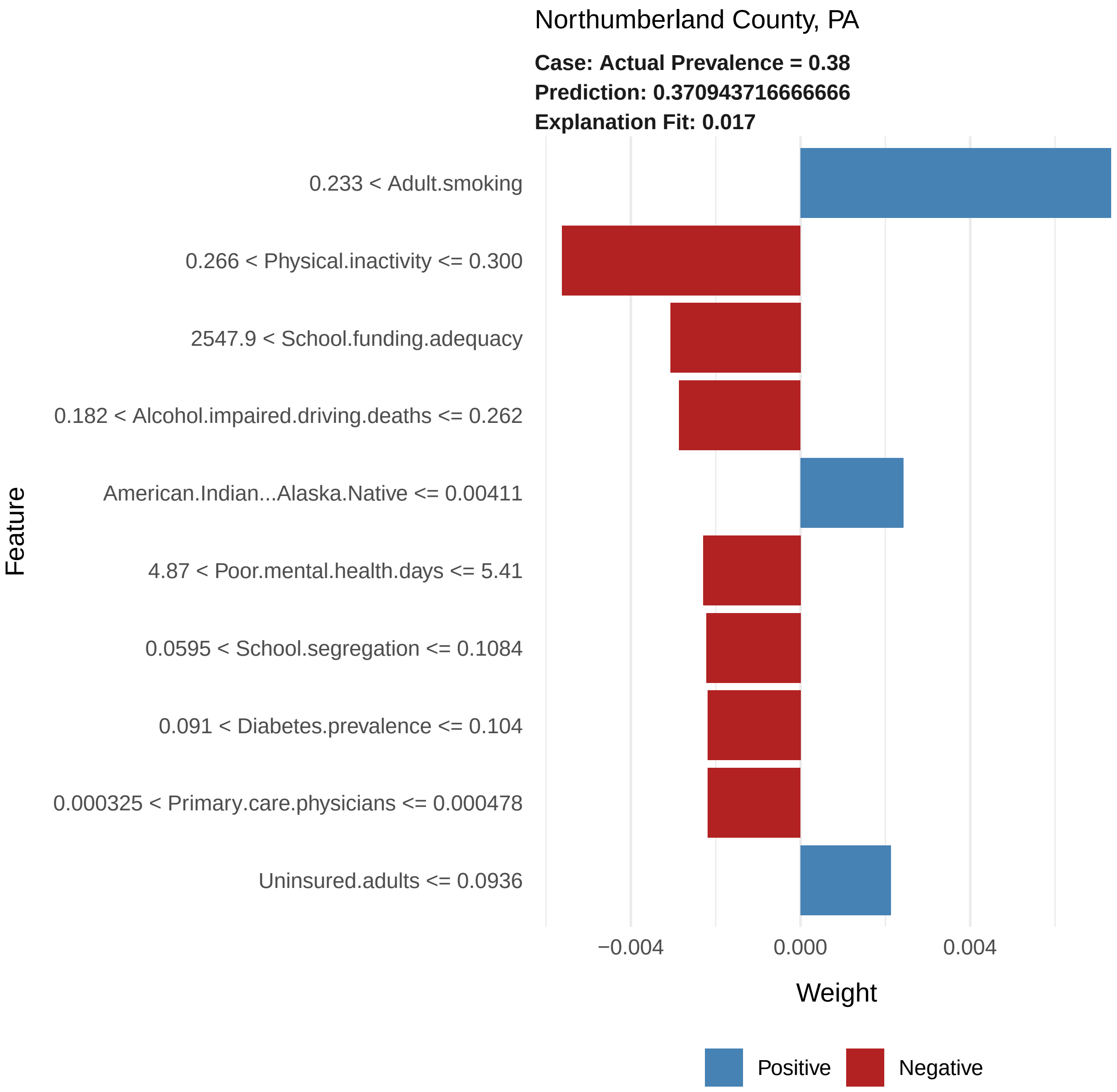

Northumberland County, PA
Case: Actual Prevalence = 0.38
Prediction: 0.370943716666666
Explanation Fit: 0.017
0.233 < Adult.smoking
0.266 < Physical.inactivity <= 0.300
2547.9 < School.funding.adequacy
0.182 < Alcohol.impaired.driving.deaths <= 0.262
American.Indian...Alaska.Native <= 0.00411
4.87 < Poor.mental.health.days <= 5.41
0.0595 < School.segregation <= 0.1084
0.091 < Diabetes.prevalence <= 0.104
0.000325 < Primary.care.physicians <= 0.000478
Uninsured.adults <= 0.0936
Feature
−0.004
0.000
0.004
Weight
Positive
Negative

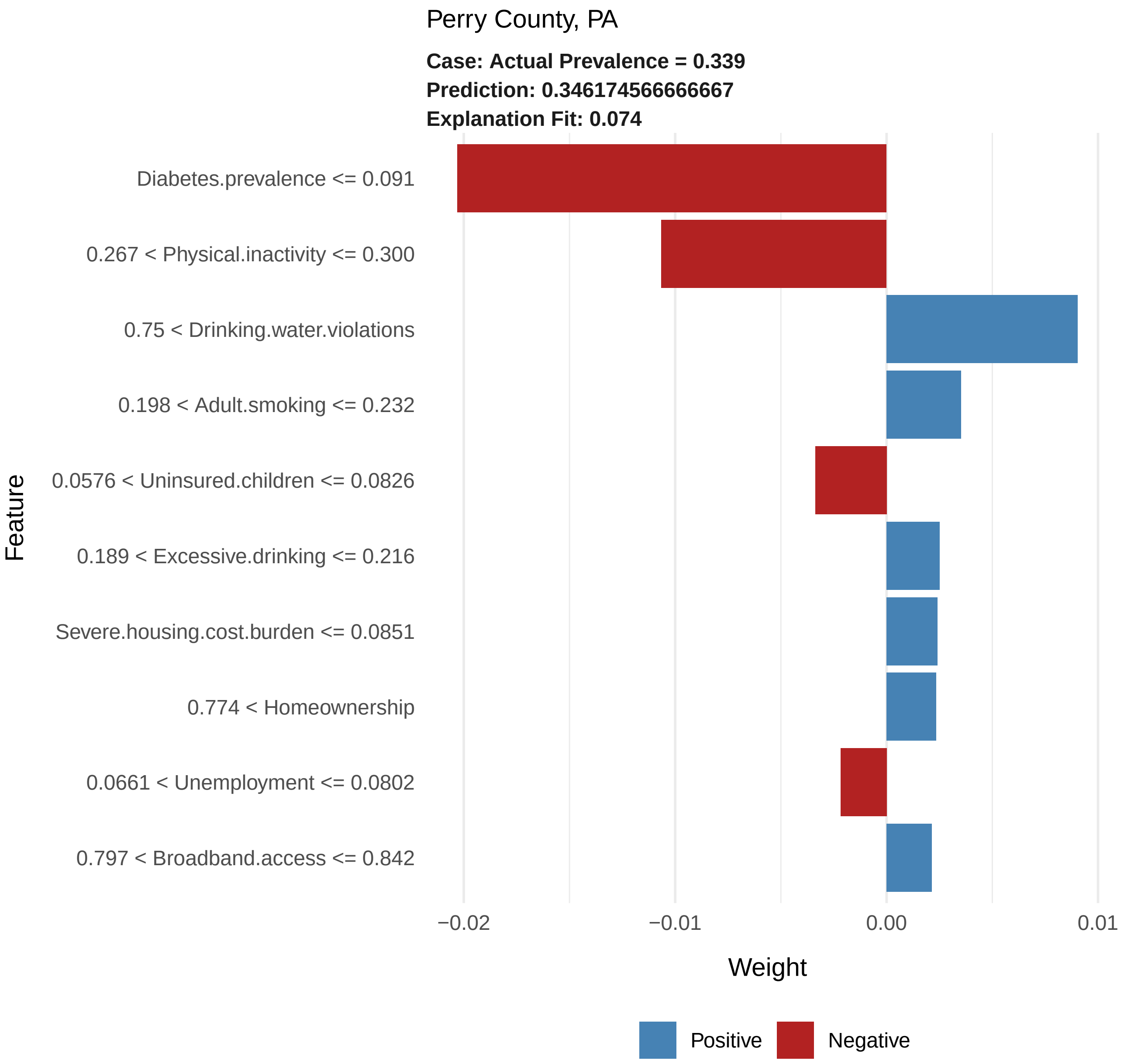

Perry County, PA
Case: Actual Prevalence = 0.339
Prediction: 0.346174566666667
Explanation Fit: 0.074
Diabetes.prevalence <= 0.091
0.267 < Physical.inactivity <= 0.300
0.75 < Drinking.water.violations
0.198 < Adult.smoking <= 0.232
0.0576 < Uninsured.children <= 0.0826
0.189 < Excessive.drinking <= 0.216
Severe.housing.cost.burden <= 0.0851
0.774 < Homeownership
0.0661 < Unemployment <= 0.0802
0.797 < Broadband.access <= 0.842
Feature
−0.02
−0.01
0.00
0.01
Weight
Positive
Negative

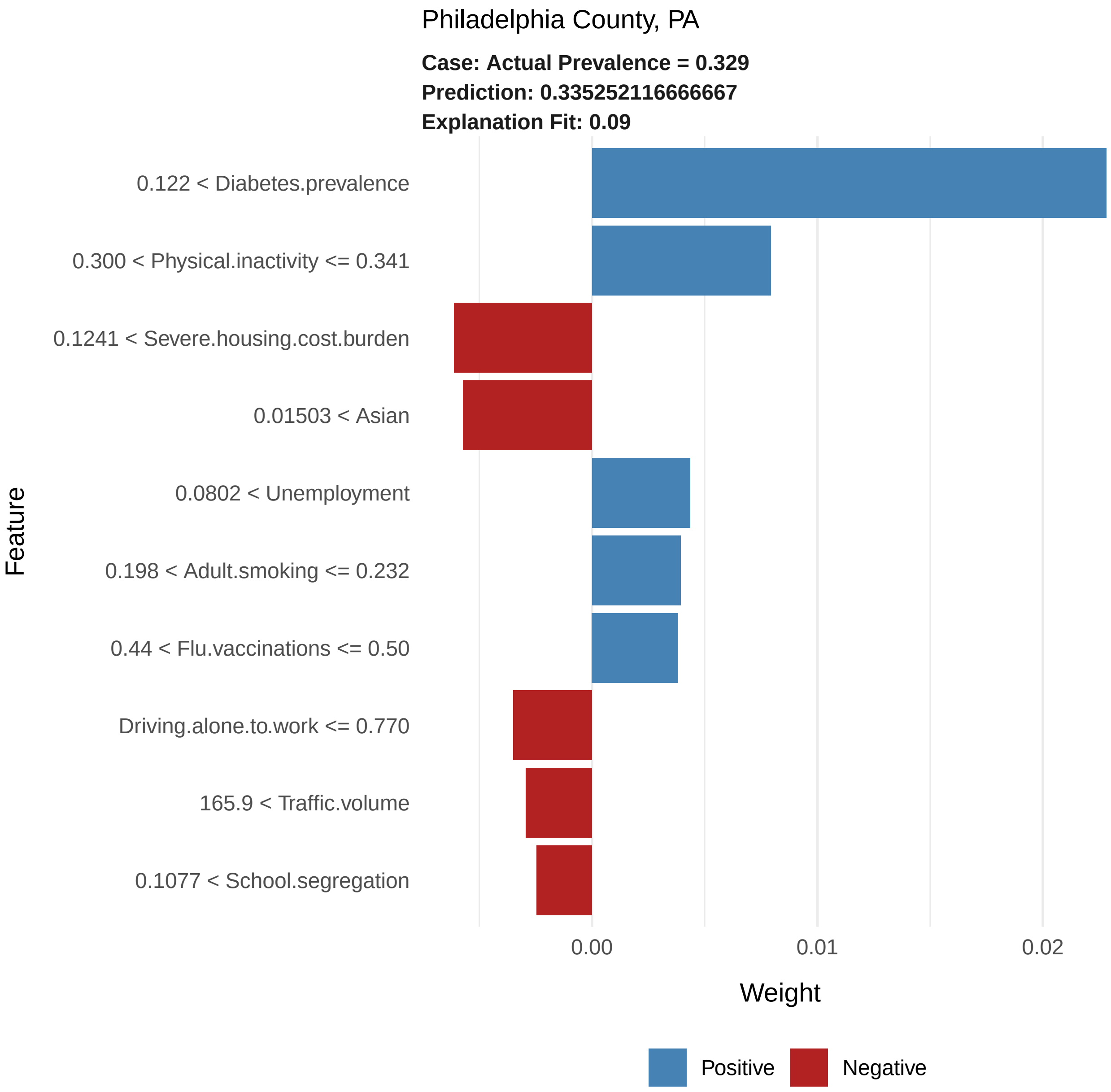
Philadelphia County, PA
Case: Actual Prevalence = 0.329
Prediction: 0.335252116666667
Explanation Fit: 0.09
0.122 < Diabetes.prevalence
0.300 < Physical.inactivity <= 0.341
0.1241 < Severe.housing.cost.burden
0.01503 < Asian
0.0802 < Unemployment
0.198 < Adult.smoking <= 0.232
0.44 < Flu.vaccinations <= 0.50
Driving.alone.to.work <= 0.770
165.9 < Traffic.volume
0.1077 < School.segregation
Feature
0.00
0.01
0.02
Weight
Positive
Negative

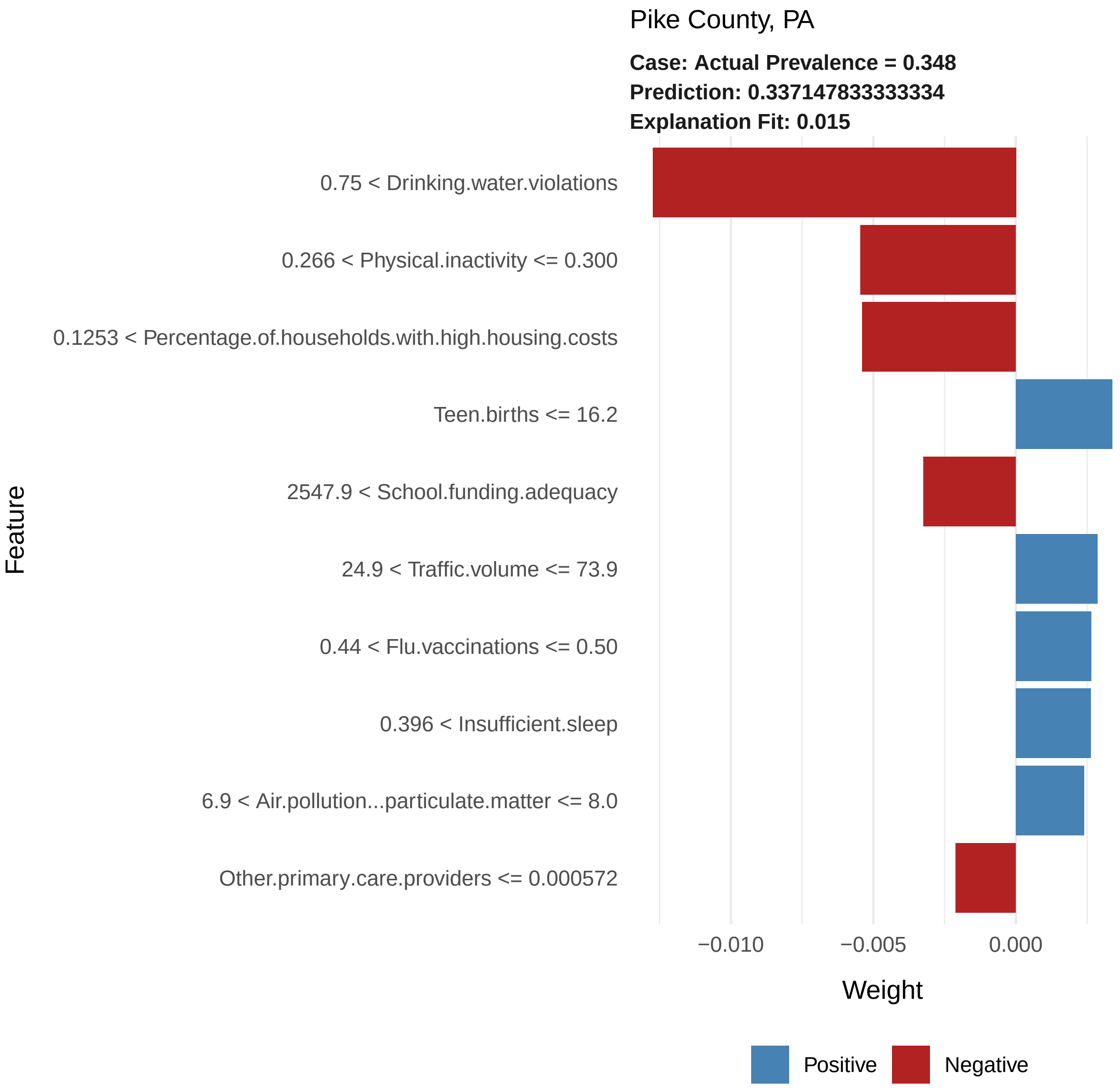

Pike County, PA
Case: Actual Prevalence = 0.348
Prediction: 0.337147833333334
Explanation Fit: 0.015
0.75 < Drinking.water.violations
0.266 < Physical.inactivity <= 0.300
0.1253 < Percentage.of.households.with.high.housing.costs
Teen.births <= 16.2
2547.9 < School.funding.adequacy
24.9 < Traffic.volume <= 73.9
0.44 < Flu.vaccinations <= 0.50
0.396 < Insufficient.sleep
6.9 < Air.pollution...particulate.matter <= 8.0
Other.primary.care.providers <= 0.000572
Feature
−0.010
−0.005
0.000
Weight
Positive
Negative

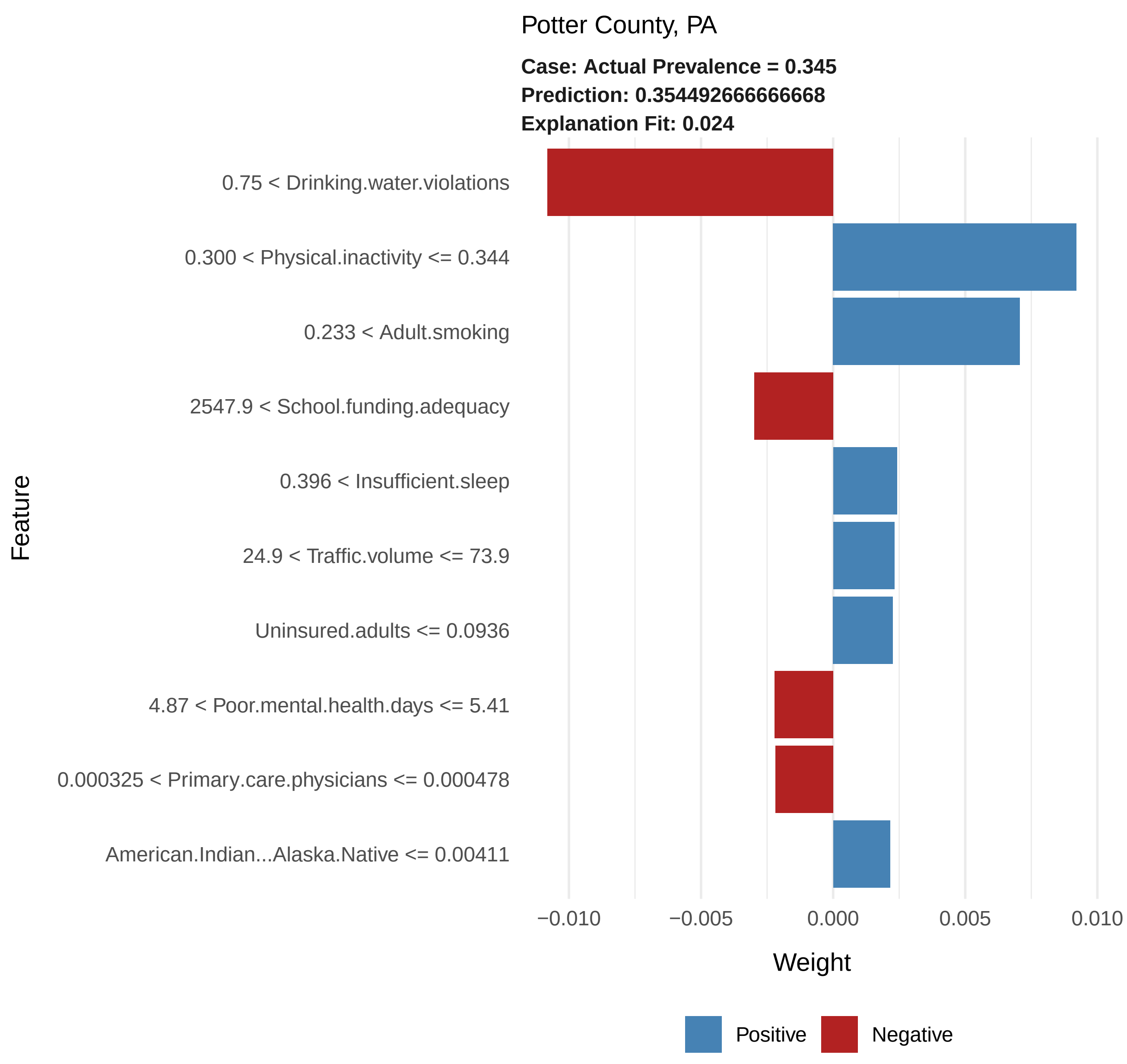
Potter County, PA
Case: Actual Prevalence = 0.345
Prediction: 0.354492666666668
Explanation Fit: 0.024
0.75 < Drinking.water.violations
0.300 < Physical.inactivity <= 0.344
0.233 < Adult.smoking
2547.9 < School.funding.adequacy
0.396 < Insufficient.sleep
24.9 < Traffic.volume <= 73.9
Uninsured.adults <= 0.0936
4.87 < Poor.mental.health.days <= 5.41
0.000325 < Primary.care.physicians <= 0.000478
American.Indian...Alaska.Native <= 0.00411
Feature
−0.010
−0.005
0.000
0.005
0.010
Weight
Positive
Negative

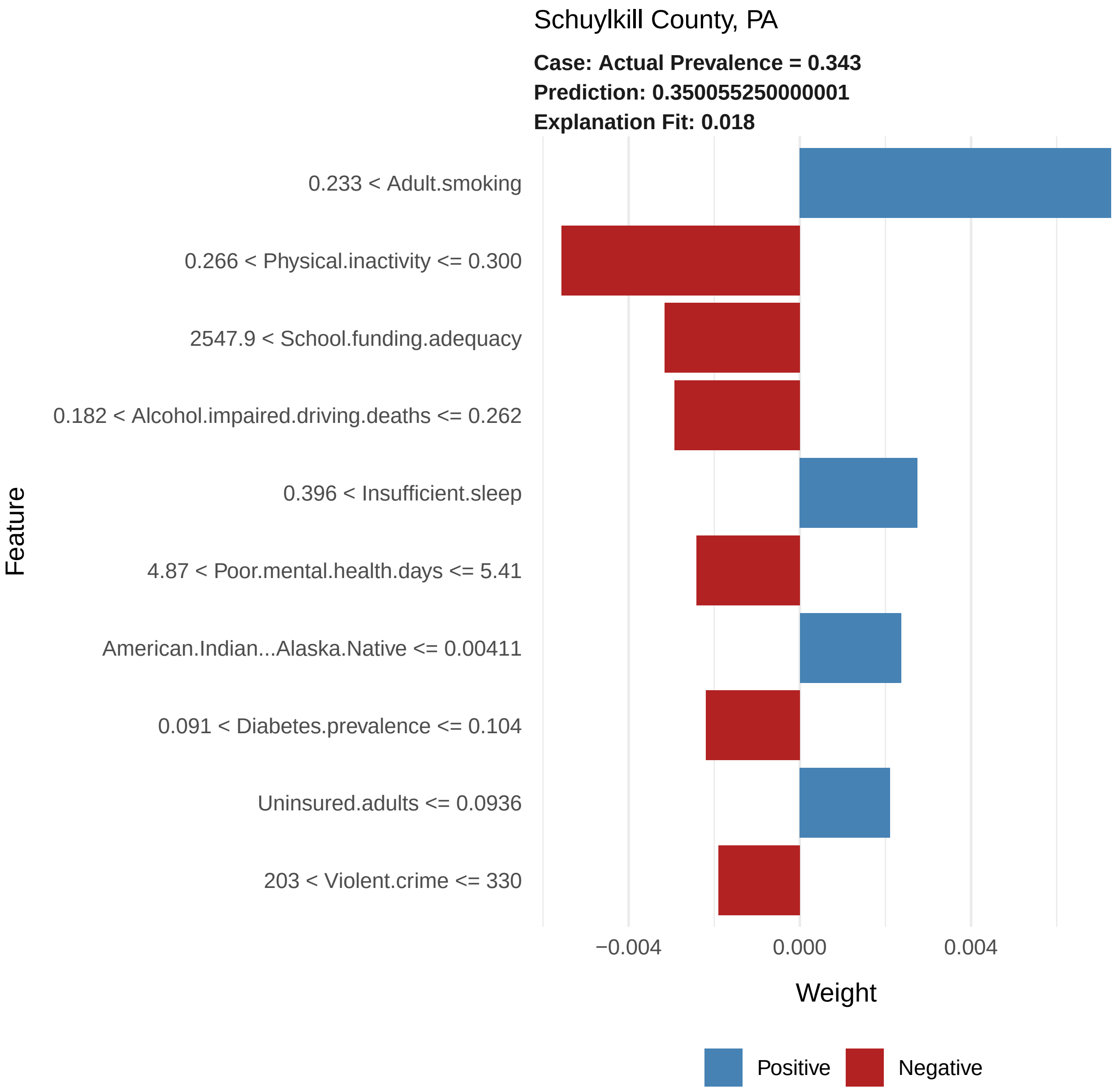
Schuylkill County, PA
Case: Actual Prevalence = 0.343
Prediction: 0.350055250000001
Explanation Fit: 0.018
0.233 < Adult.smoking
0.266 < Physical.inactivity <= 0.300
2547.9 < School.funding.adequacy
0.182 < Alcohol.impaired.driving.deaths <= 0.262
0.396 < Insufficient.sleep
4.87 < Poor.mental.health.days <= 5.41
American.Indian...Alaska.Native <= 0.00411
0.091 < Diabetes.prevalence <= 0.104
Uninsured.adults <= 0.0936
203 < Violent.crime <= 330
Feature
−0.004
0.000
0.004
Weight
Positive
Negative

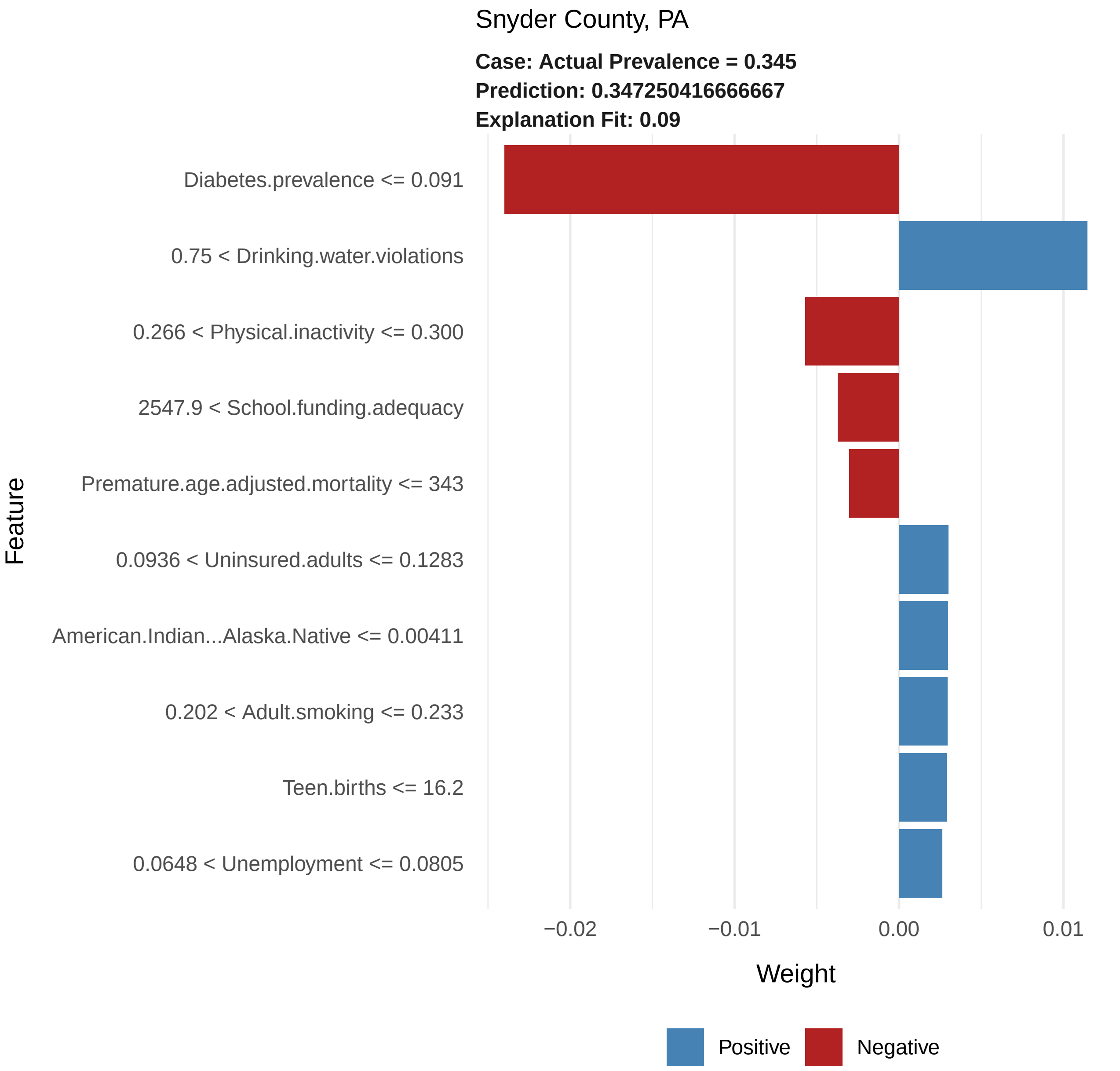

Snyder County, PA
Case: Actual Prevalence = 0.345
Prediction: 0.347250416666667
Explanation Fit: 0.09
Diabetes.prevalence <= 0.091
0.75 < Drinking.water.violations
0.266 < Physical.inactivity <= 0.300
2547.9 < School.funding.adequacy
Premature.age.adjusted.mortality <= 343
0.0936 < Uninsured.adults <= 0.1283
American.Indian...Alaska.Native <= 0.00411
0.202 < Adult.smoking <= 0.233
Teen.births <= 16.2
0.0648 < Unemployment <= 0.0805
Feature
−0.02
−0.01
0.00
0.01
Weight
Positive
Negative

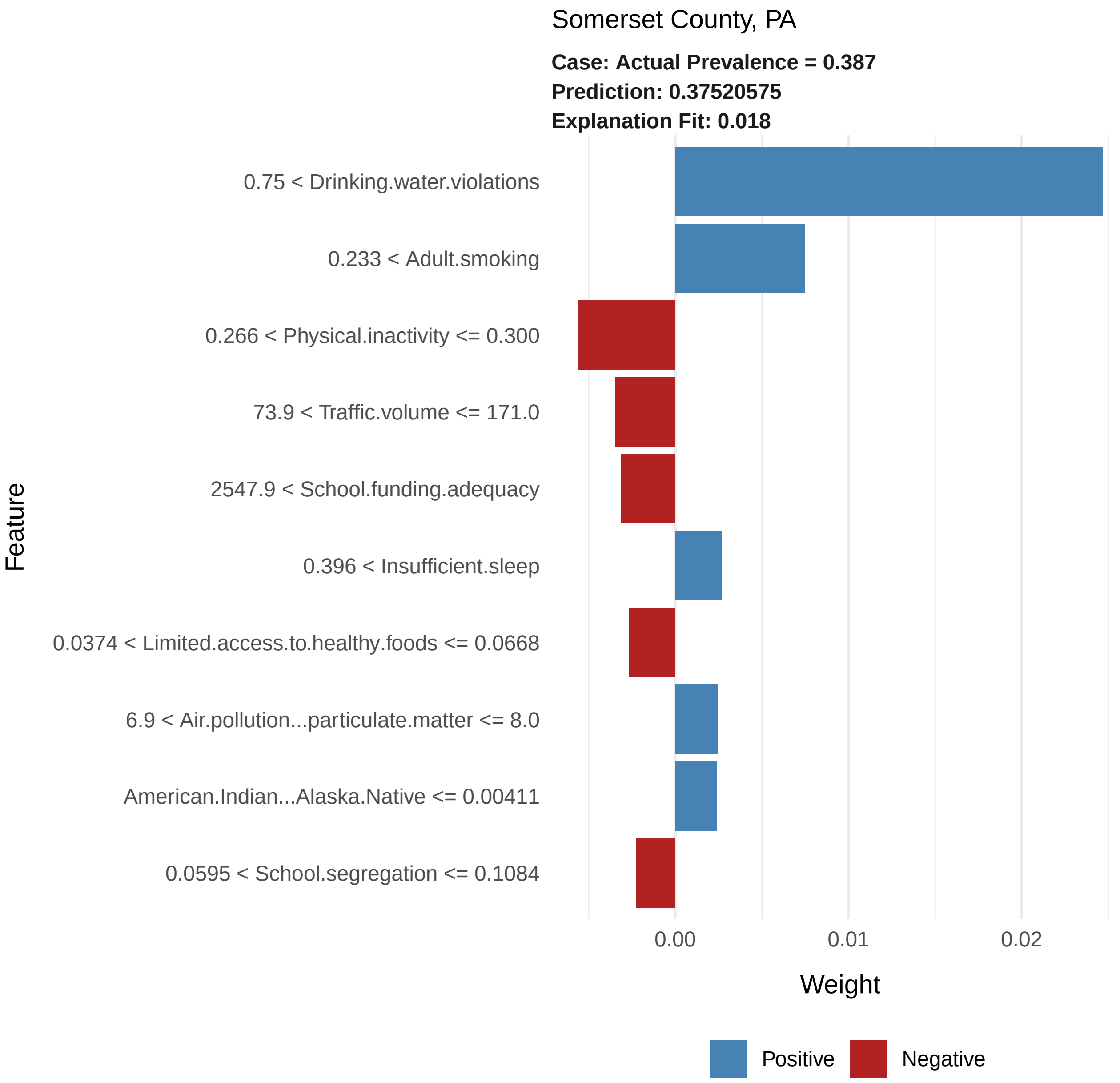
Somerset County, PA
Case: Actual Prevalence = 0.387
Prediction: 0.37520575
Explanation Fit: 0.018
0.75 < Drinking.water.violations
0.233 < Adult.smoking
0.266 < Physical.inactivity <= 0.300
73.9 < Traffic.volume <= 171.0
2547.9 < School.funding.adequacy
0.396 < Insufficient.sleep
0.0374 < Limited.access.to.healthy.foods <= 0.0668
6.9 < Air.pollution...particulate.matter <= 8.0
American.Indian...Alaska.Native <= 0.00411
0.0595 < School.segregation <= 0.1084
Feature
0.00
0.01
0.02
Weight
Positive
Negative

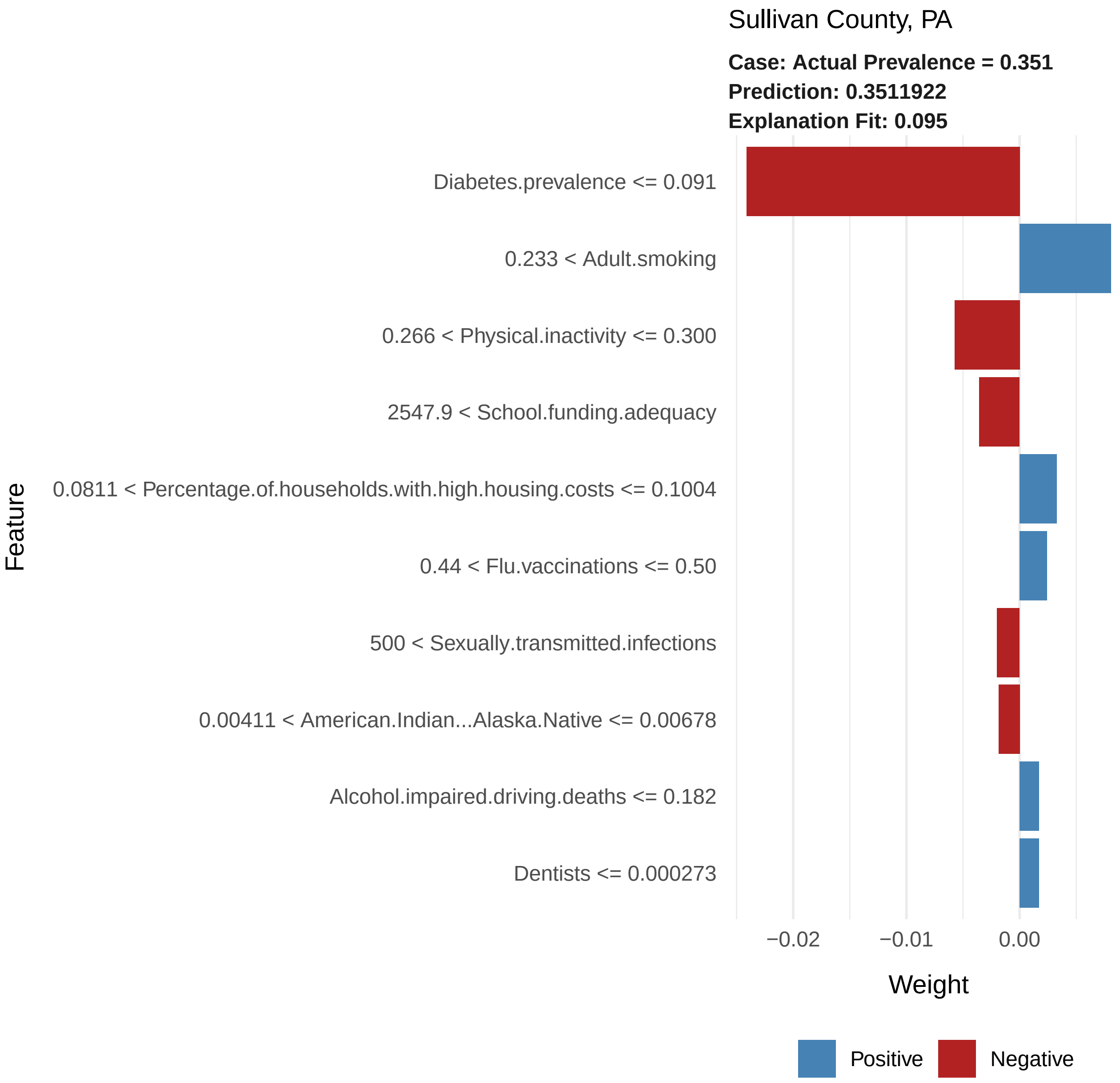

Sullivan County, PA
Case: Actual Prevalence = 0.351
Prediction: 0.3511922
Explanation Fit: 0.095
Diabetes.prevalence <= 0.091
0.233 < Adult.smoking
0.266 < Physical.inactivity <= 0.300
2547.9 < School.funding.adequacy
0.0811 < Percentage.of.households.with.high.housing.costs <= 0.1004
0.44 < Flu.vaccinations <= 0.50
500 < Sexually.transmitted.infections
0.00411 < American.Indian...Alaska.Native <= 0.00678
Alcohol.impaired.driving.deaths <= 0.182
Dentists <= 0.000273
Feature
−0.02
−0.01
0.00
Weight
Positive
Negative

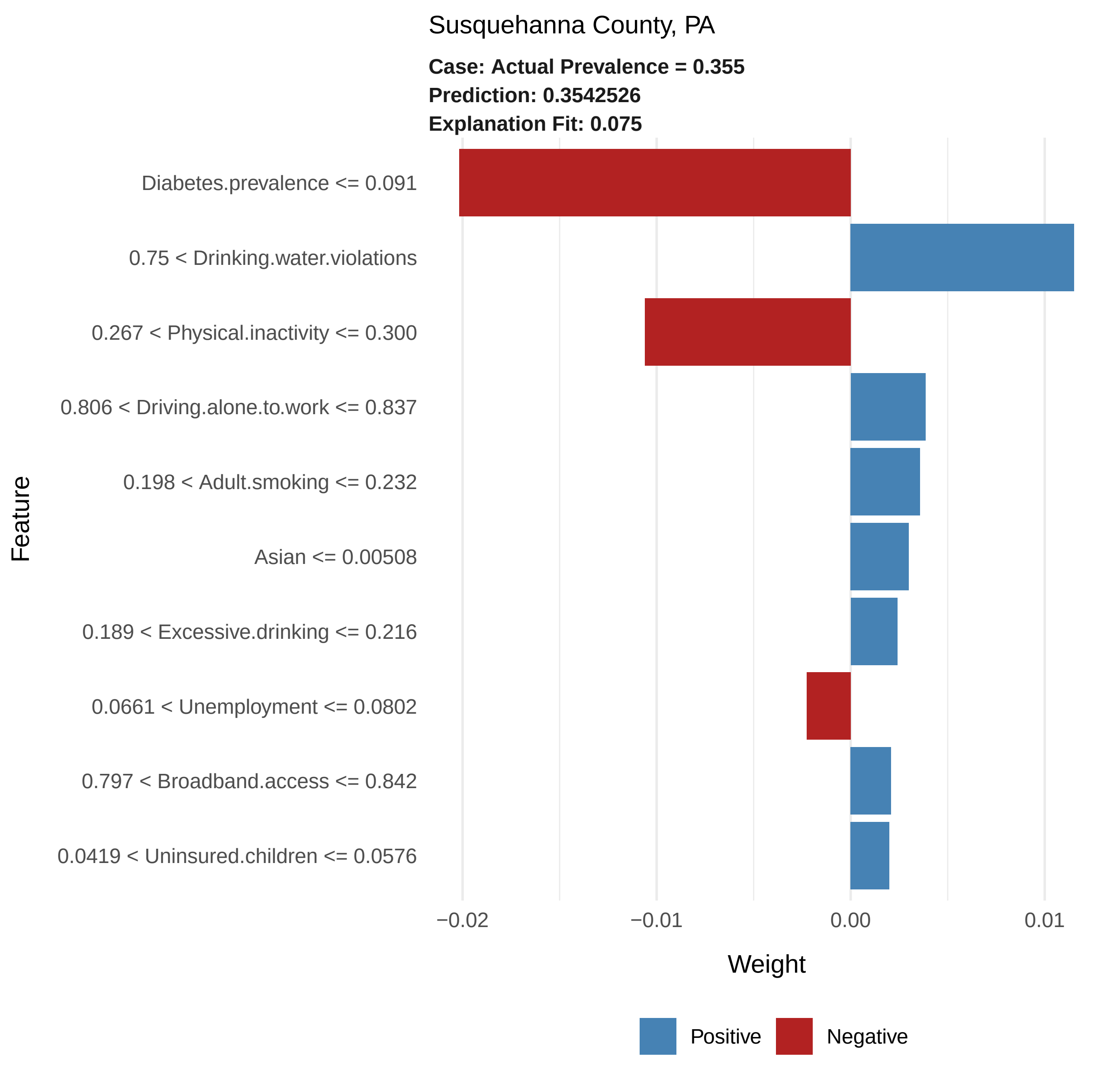

Susquehanna County, PA
Case: Actual Prevalence = 0.355
Prediction: 0.3542526
Explanation Fit: 0.075
Diabetes.prevalence <= 0.091
0.75 < Drinking.water.violations
0.267 < Physical.inactivity <= 0.300
0.806 < Driving.alone.to.work <= 0.837
0.198 < Adult.smoking <= 0.232
Asian <= 0.00508
0.189 < Excessive.drinking <= 0.216
0.0661 < Unemployment <= 0.0802
0.797 < Broadband.access <= 0.842
0.0419 < Uninsured.children <= 0.0576
Feature
−0.02
−0.01
0.00
0.01
Weight
Positive
Negative

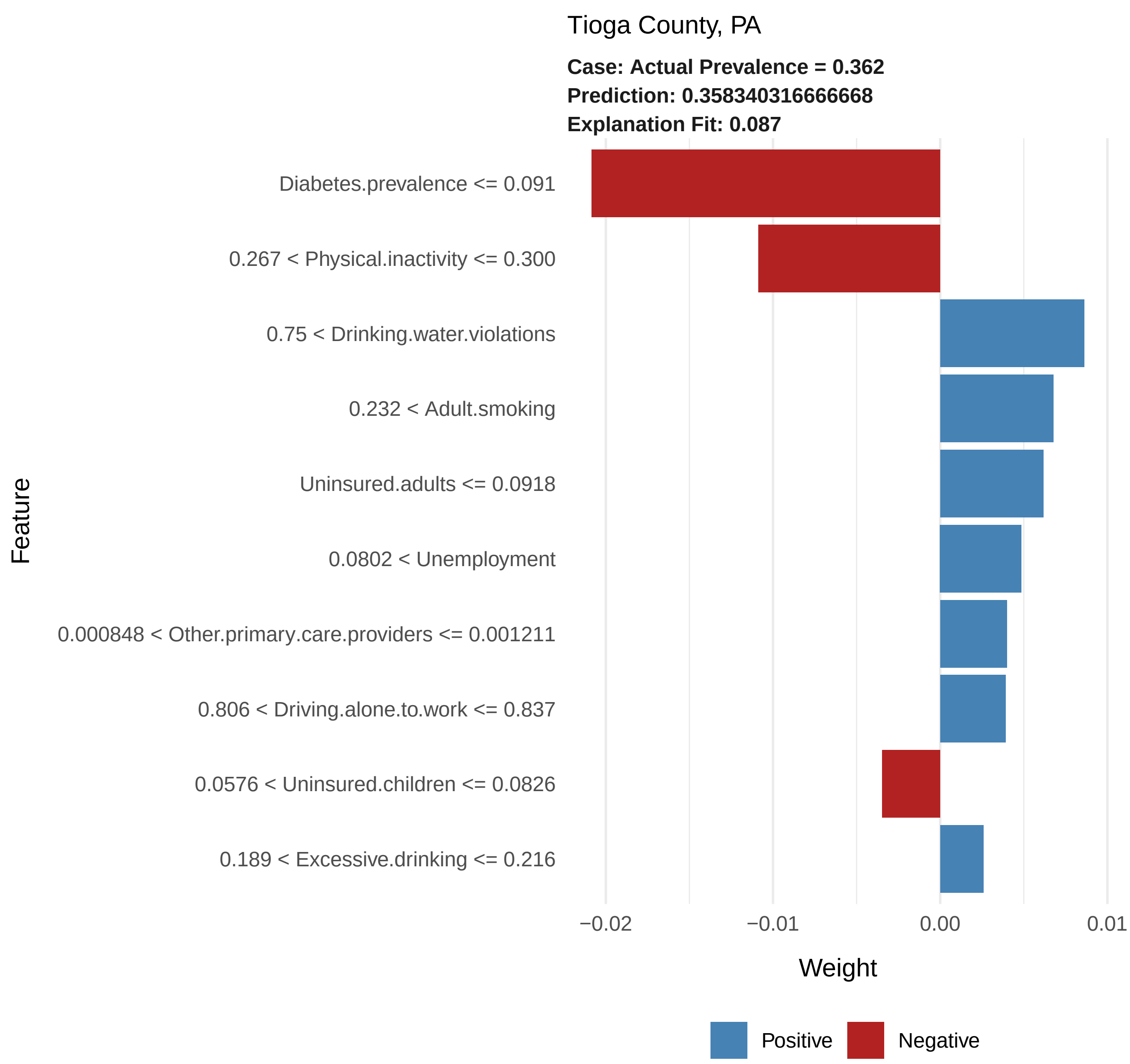

Tioga County, PA
Case: Actual Prevalence = 0.362
Prediction: 0.358340316666668
Explanation Fit: 0.087
Diabetes.prevalence <= 0.091
0.267 < Physical.inactivity <= 0.300
0.75 < Drinking.water.violations
0.232 < Adult.smoking
Uninsured.adults <= 0.0918
0.0802 < Unemployment
0.000848 < Other.primary.care.providers <= 0.001211
0.806 < Driving.alone.to.work <= 0.837
0.0576 < Uninsured.children <= 0.0826
0.189 < Excessive.drinking <= 0.216
Feature
−0.02
−0.01
0.00
0.01
Weight
Positive
Negative

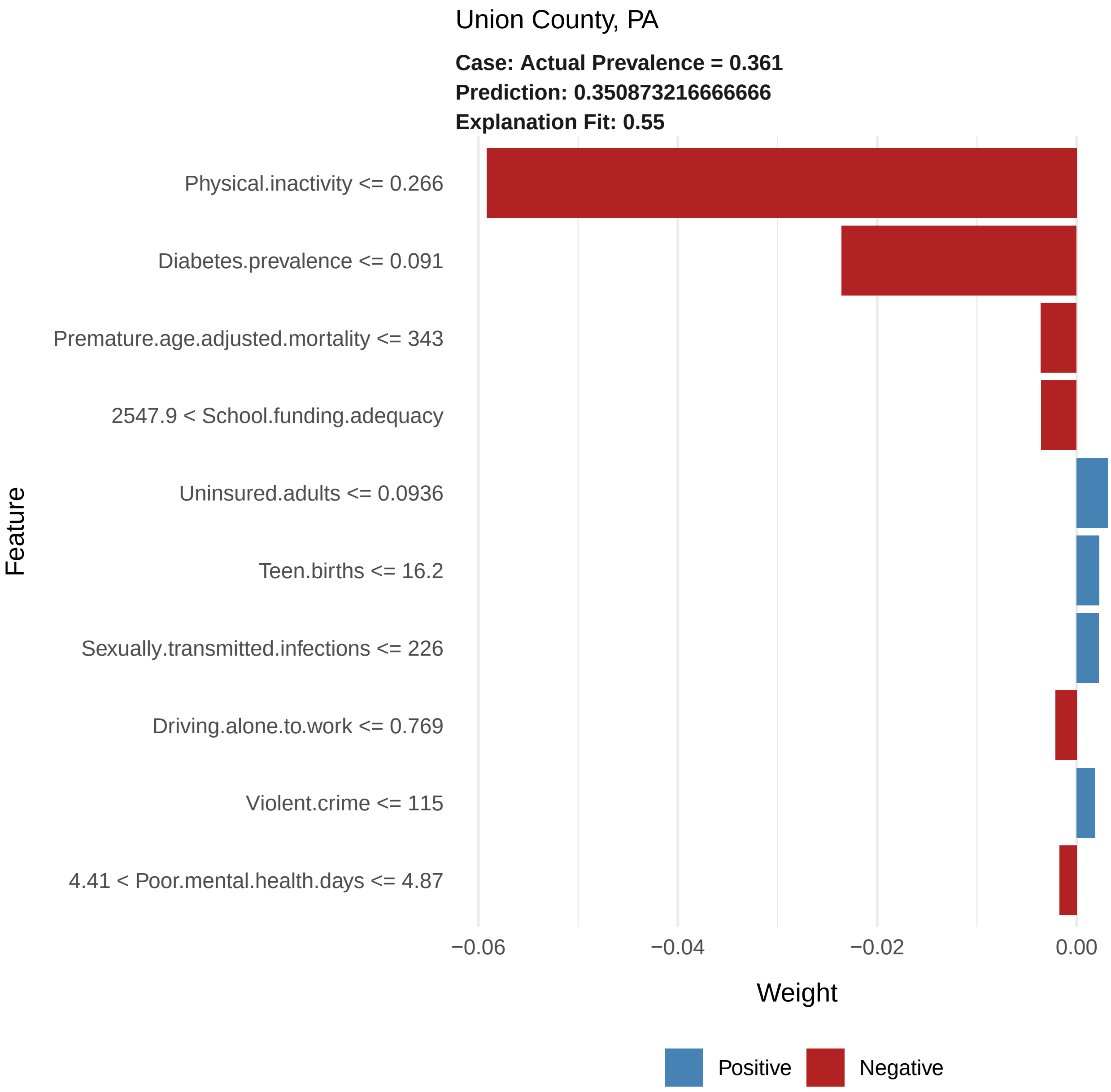
Union County, PA
Case: Actual Prevalence = 0.361
Prediction: 0.350873216666666
Explanation Fit: 0.55
Physical.inactivity <= 0.266
Diabetes.prevalence <= 0.091
Premature.age.adjusted.mortality <= 343
2547.9 < School.funding.adequacy
Uninsured.adults <= 0.0936
Teen.births <= 16.2
Sexually.transmitted.infections <= 226
Driving.alone.to.work <= 0.769
Violent.crime <= 115
4.41 < Poor.mental.health.days <= 4.87
Feature
−0.06
−0.04
−0.02
0.00
Weight
Positive
Negative

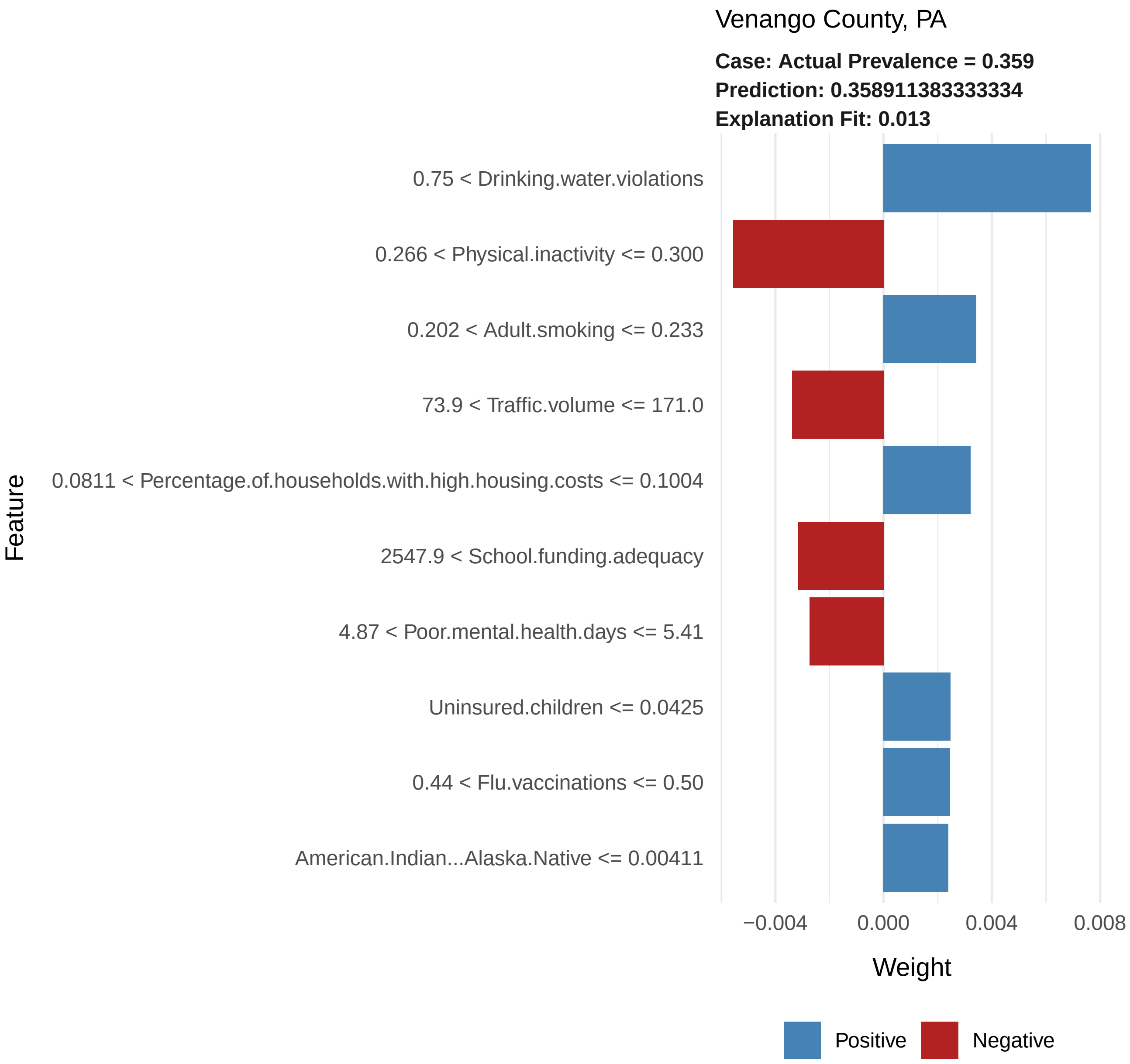
Venango County, PA
Case: Actual Prevalence = 0.359
Prediction: 0.358911383333334
Explanation Fit: 0.013
0.75 < Drinking.water.violations
0.266 < Physical.inactivity <= 0.300
0.202 < Adult.smoking <= 0.233
73.9 < Traffic.volume <= 171.0
0.0811 < Percentage.of.households.with.high.housing.costs <= 0.1004
2547.9 < School.funding.adequacy
4.87 < Poor.mental.health.days <= 5.41
Uninsured.children <= 0.0425
0.44 < Flu.vaccinations <= 0.50
American.Indian...Alaska.Native <= 0.00411
Feature
−0.004
0.000
0.004
0.008
Weight
Positive
Negative

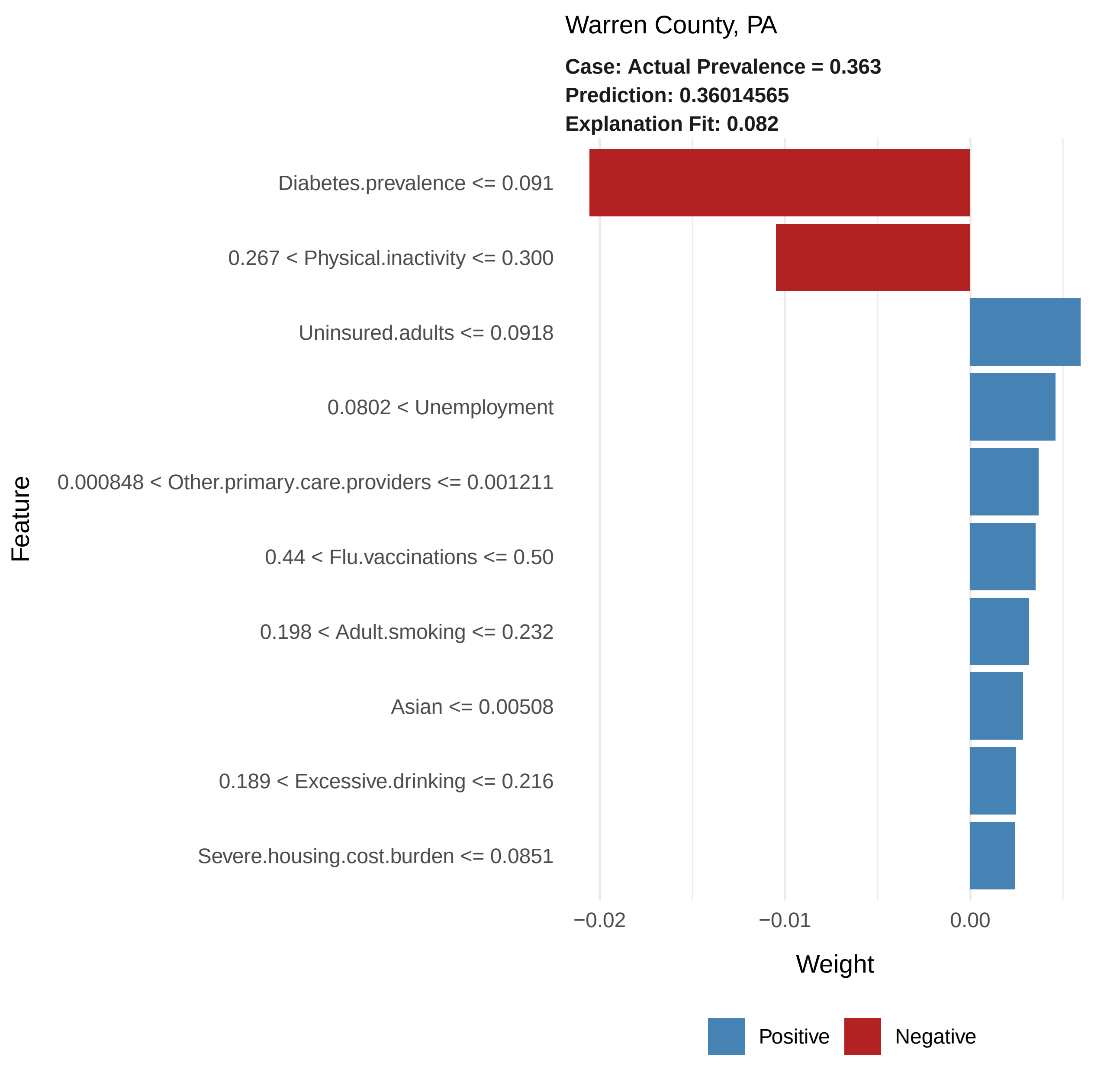

Warren County, PA
Case: Actual Prevalence = 0.363
Prediction: 0.36014565
Explanation Fit: 0.082
Diabetes.prevalence <= 0.091
0.267 < Physical.inactivity <= 0.300
Uninsured.adults <= 0.0918
0.0802 < Unemployment
0.000848 < Other.primary.care.providers <= 0.001211
0.44 < Flu.vaccinations <= 0.50
0.198 < Adult.smoking <= 0.232
Asian <= 0.00508
0.189 < Excessive.drinking <= 0.216
Severe.housing.cost.burden <= 0.0851
Feature
−0.02
−0.01
0.00
Weight
Positive
Negative

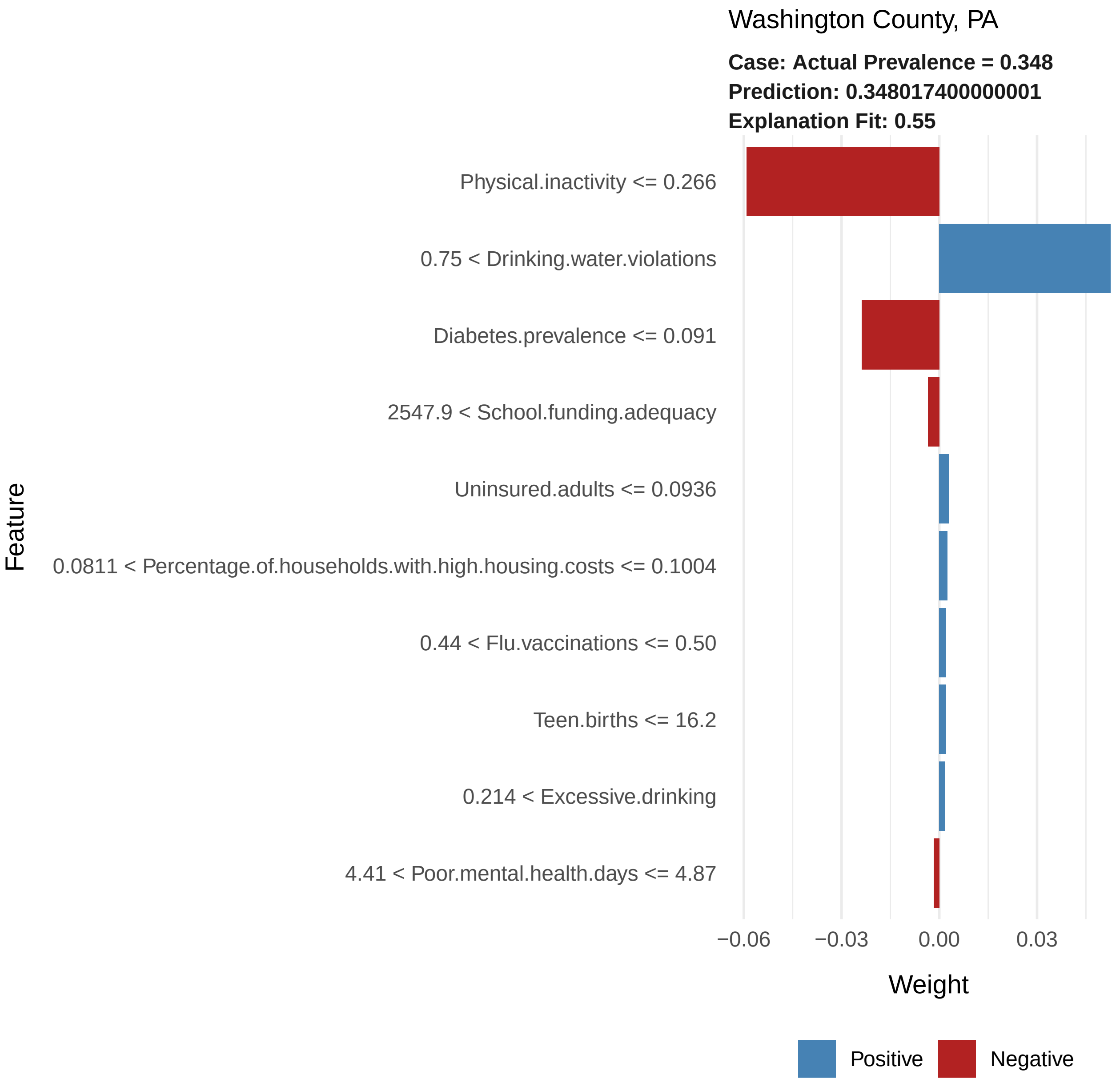

Washington County, PA
Case: Actual Prevalence = 0.348
Prediction: 0.348017400000001
Explanation Fit: 0.55
Physical.inactivity <= 0.266
0.75 < Drinking.water.violations
Diabetes.prevalence <= 0.091
2547.9 < School.funding.adequacy
Uninsured.adults <= 0.0936
0.0811 < Percentage.of.households.with.high.housing.costs <= 0.1004
0.44 < Flu.vaccinations <= 0.50
Teen.births <= 16.2
0.214 < Excessive.drinking
4.41 < Poor.mental.health.days <= 4.87
Feature
−0.06
−0.03
0.00
0.03
Weight
Positive
Negative

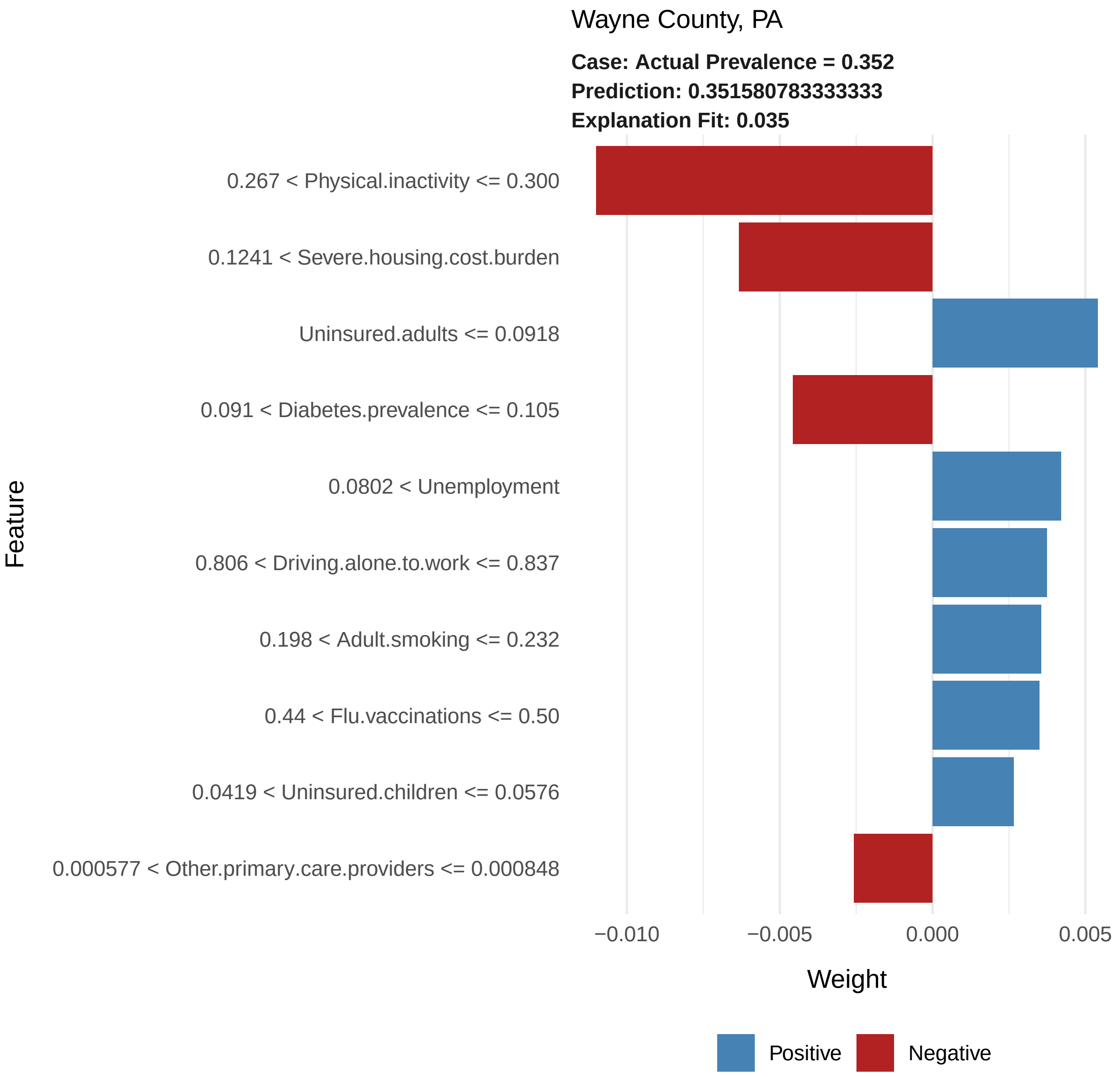
Wayne County, PA
Case: Actual Prevalence = 0.352
Prediction: 0.351580783333333
Explanation Fit: 0.035
0.267 < Physical.inactivity <= 0.300
0.1241 < Severe.housing.cost.burden
Uninsured.adults <= 0.0918
0.091 < Diabetes.prevalence <= 0.105
0.0802 < Unemployment
0.806 < Driving.alone.to.work <= 0.837
0.198 < Adult.smoking <= 0.232
0.44 < Flu.vaccinations <= 0.50
0.0419 < Uninsured.children <= 0.0576
0.000577 < Other.primary.care.providers <= 0.000848
Feature
−0.010
−0.005
0.000
0.005
Weight
Positive
Negative

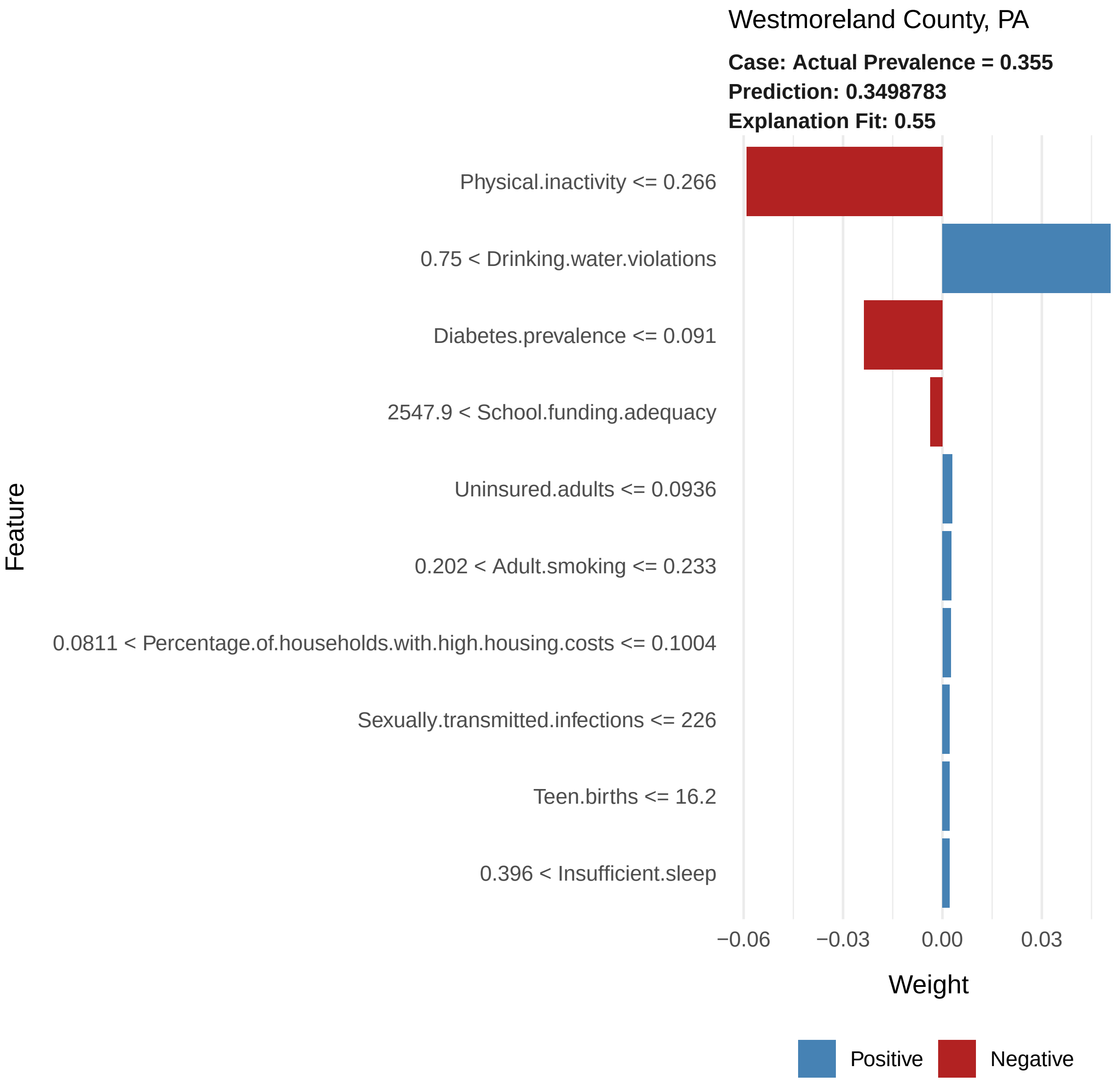

Westmoreland County, PA
Case: Actual Prevalence = 0.355
Prediction: 0.3498783
Explanation Fit: 0.55
Physical.inactivity <= 0.266
0.75 < Drinking.water.violations
Diabetes.prevalence <= 0.091
2547.9 < School.funding.adequacy
Uninsured.adults <= 0.0936
0.202 < Adult.smoking <= 0.233
0.0811 < Percentage.of.households.with.high.housing.costs <= 0.1004
Sexually.transmitted.infections <= 226
Teen.births <= 16.2
0.396 < Insufficient.sleep
Feature
−0.06
−0.03
0.00
0.03
Weight
Positive
Negative

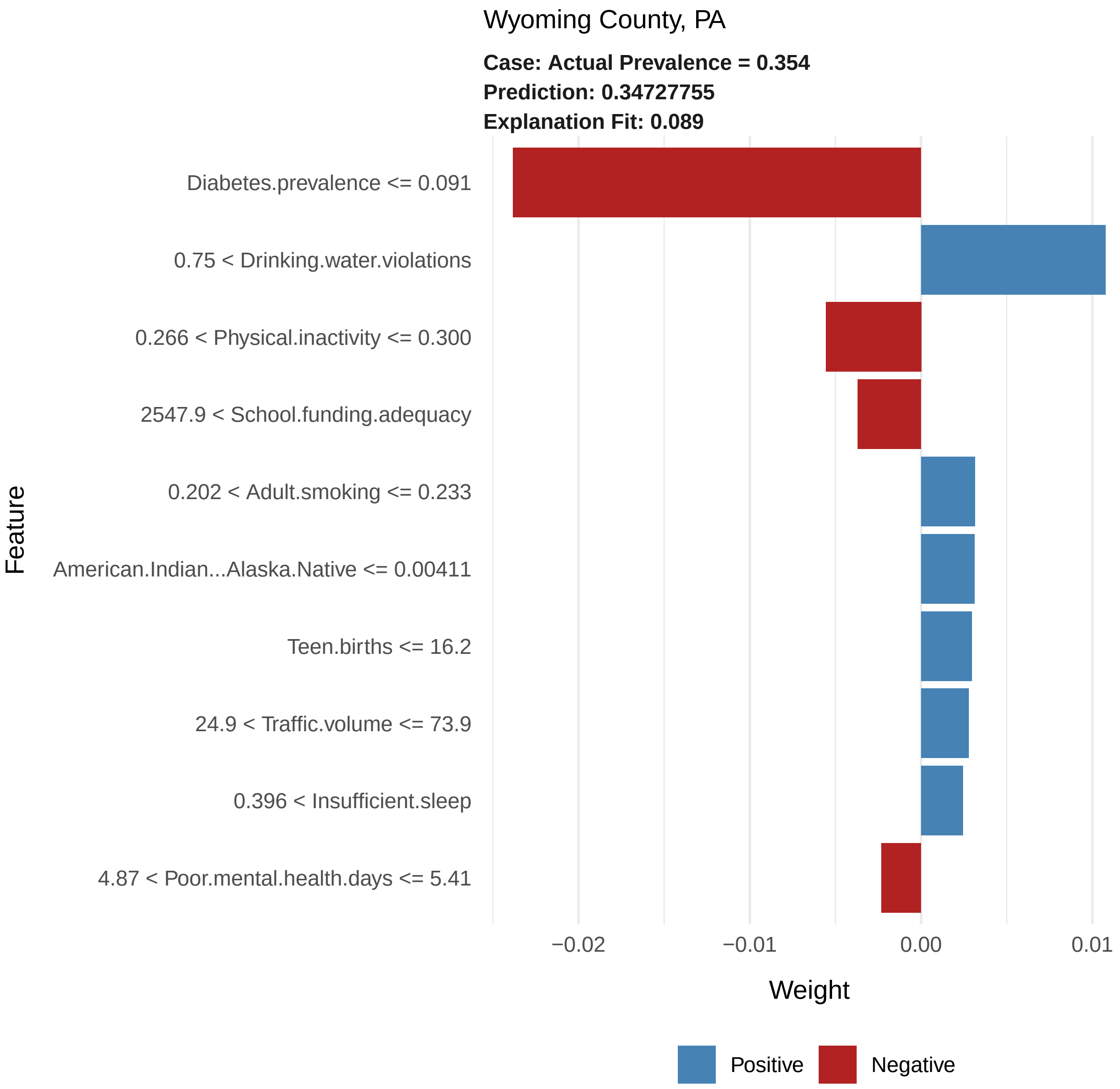

Wyoming County, PA
Case: Actual Prevalence = 0.354
Prediction: 0.34727755
Explanation Fit: 0.089
Diabetes.prevalence <= 0.091
0.75 < Drinking.water.violations
0.266 < Physical.inactivity <= 0.300
2547.9 < School.funding.adequacy
0.202 < Adult.smoking <= 0.233
American.Indian...Alaska.Native <= 0.00411
Teen.births <= 16.2
24.9 < Traffic.volume <= 73.9
0.396 < Insufficient.sleep
4.87 < Poor.mental.health.days <= 5.41
Feature
−0.02
−0.01
0.00
0.01
Weight
Positive
Negative

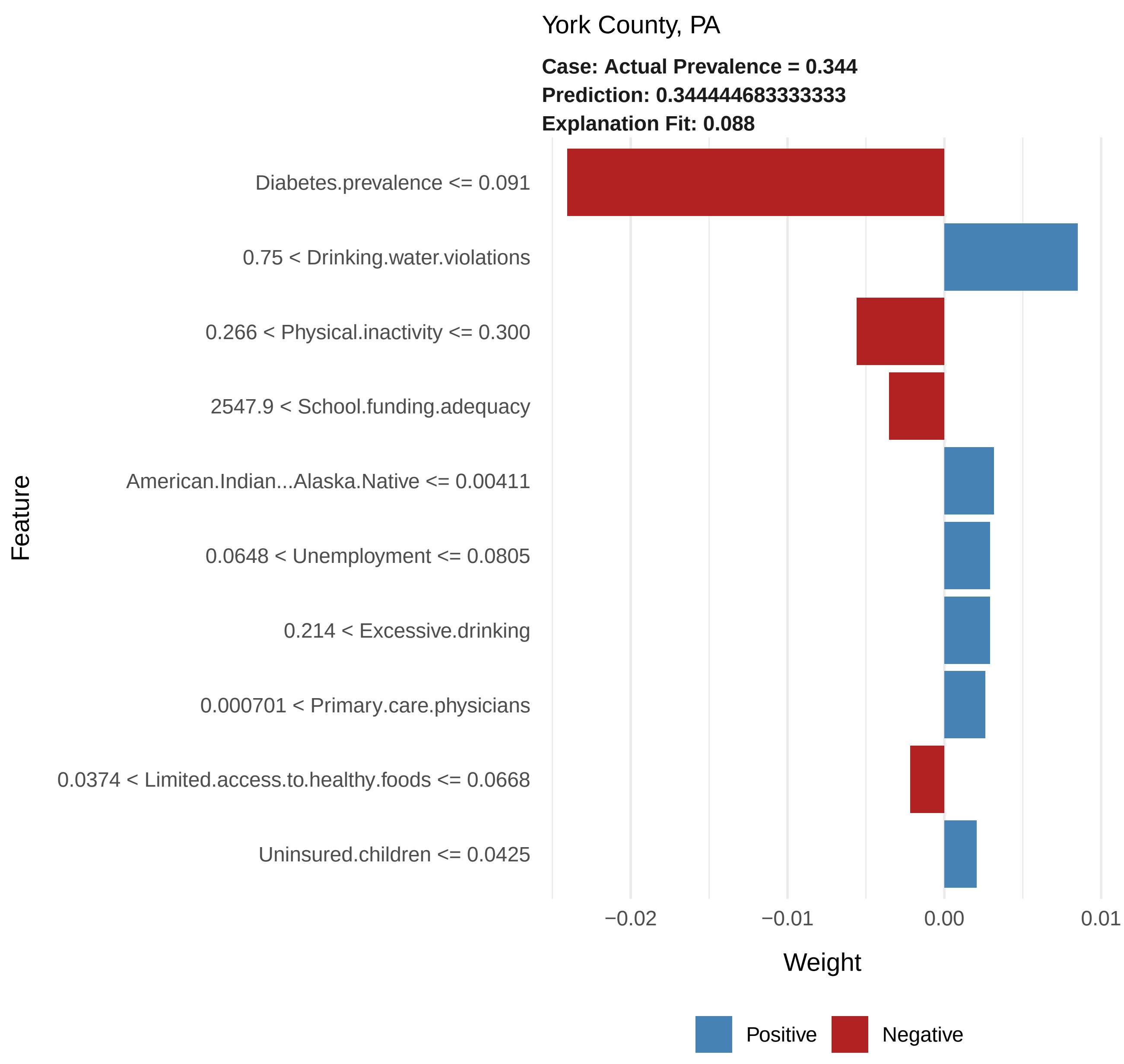
York County, PA
Case: Actual Prevalence = 0.344
Prediction: 0.344444683333333
Explanation Fit: 0.088
Diabetes.prevalence <= 0.091
0.75 < Drinking.water.violations
0.266 < Physical.inactivity <= 0.300
2547.9 < School.funding.adequacy
American.Indian...Alaska.Native <= 0.00411
0.0648 < Unemployment <= 0.0805
0.214 < Excessive.drinking
0.000701 < Primary.care.physicians
0.0374 < Limited.access.to.healthy.foods <= 0.0668
Uninsured.children <= 0.0425
Feature
−0.02
−0.01
0.00
0.01
Weight
Positive
Negative

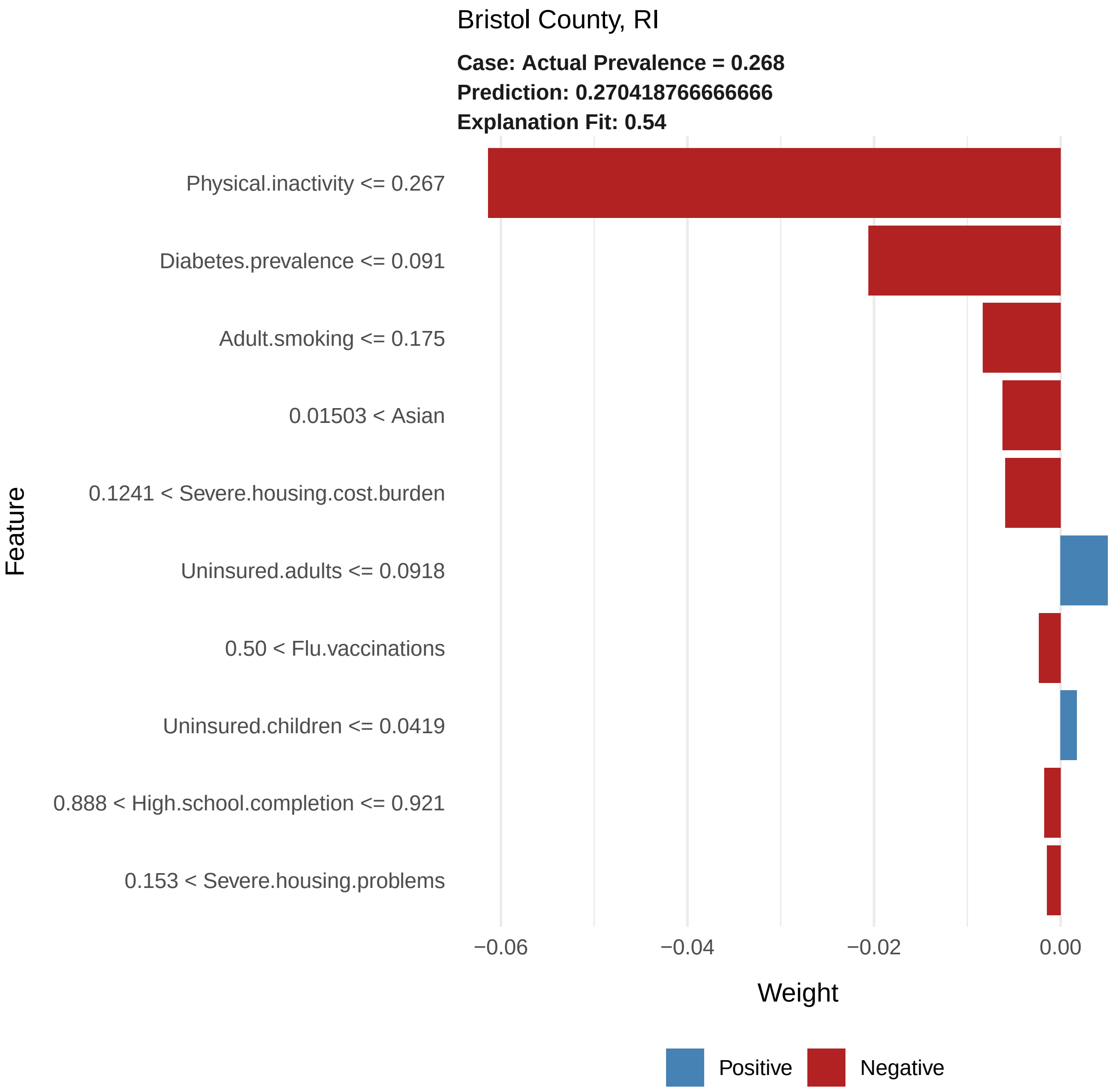

Bristol County, RI
Case: Actual Prevalence = 0.268
Prediction: 0.270418766666666
Explanation Fit: 0.54
Physical.inactivity <= 0.267
Diabetes.prevalence <= 0.091
Adult.smoking <= 0.175
0.01503 < Asian
0.1241 < Severe.housing.cost.burden
Uninsured.adults <= 0.0918
0.50 < Flu.vaccinations
Uninsured.children <= 0.0419
0.888 < High.school.completion <= 0.921
0.153 < Severe.housing.problems
Feature
−0.06
−0.04
−0.02
0.00
Weight
Positive
Negative

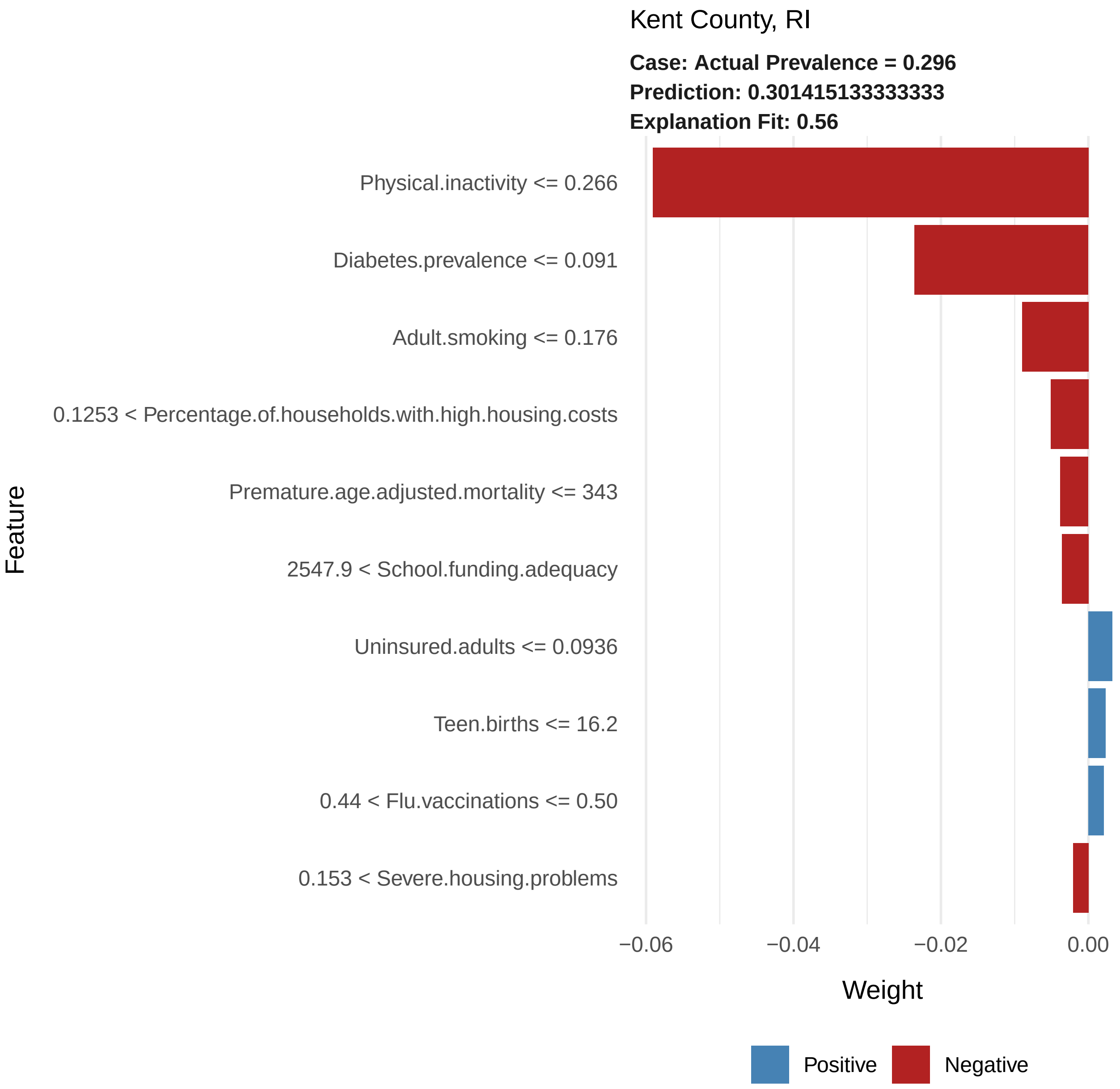

Kent County, RI
Case: Actual Prevalence = 0.296
Prediction: 0.301415133333333
Explanation Fit: 0.56
Physical.inactivity <= 0.266
Diabetes.prevalence <= 0.091
Adult.smoking <= 0.176
0.1253 < Percentage.of.households.with.high.housing.costs
Premature.age.adjusted.mortality <= 343
2547.9 < School.funding.adequacy
Uninsured.adults <= 0.0936
Teen.births <= 16.2
0.44 < Flu.vaccinations <= 0.50
0.153 < Severe.housing.problems
Feature
−0.06
−0.04
−0.02
0.00
Weight
Positive
Negative

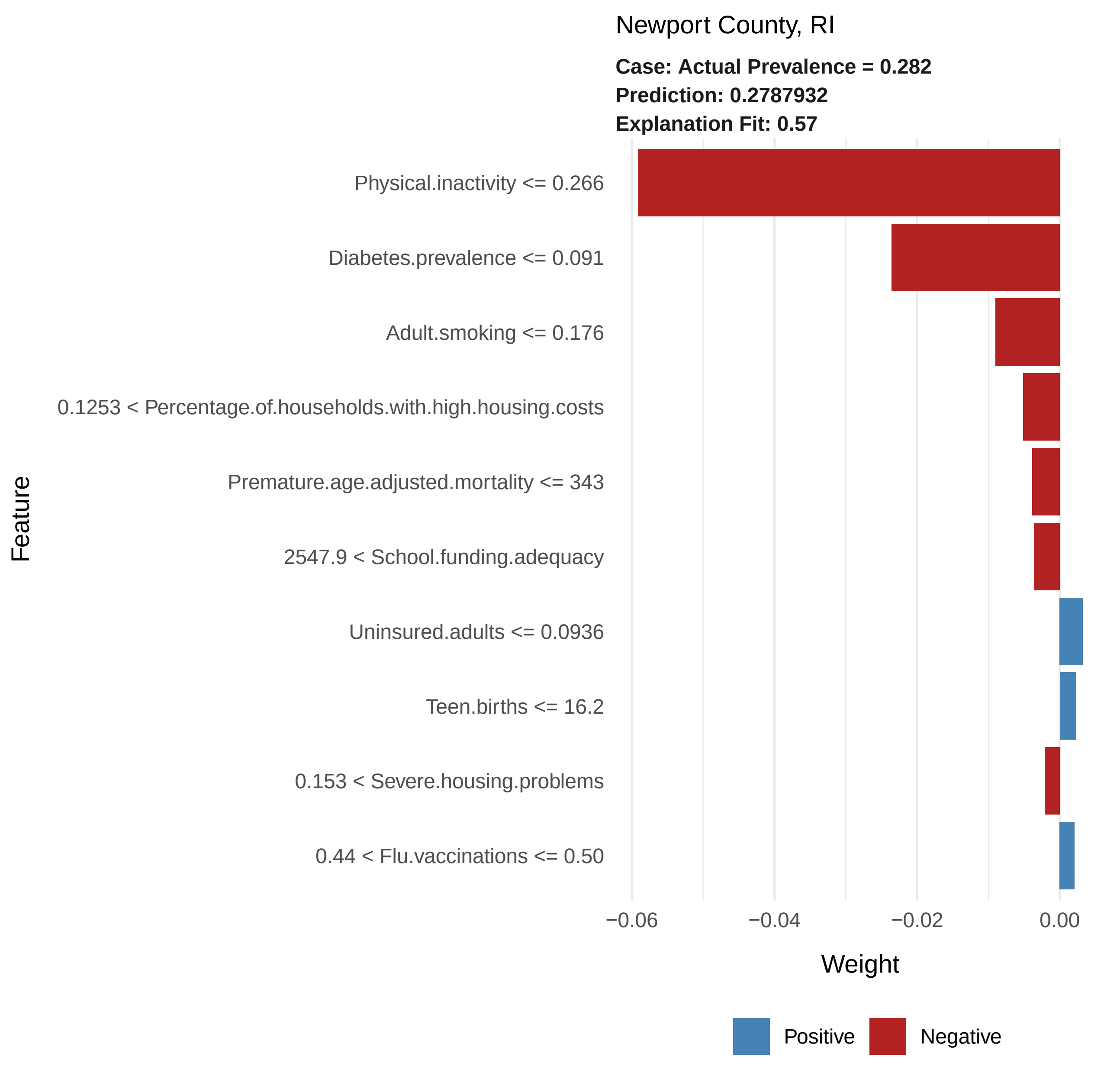
Newport County, RI
Case: Actual Prevalence = 0.282
Prediction: 0.2787932
Explanation Fit: 0.57
Physical.inactivity <= 0.266
Diabetes.prevalence <= 0.091
Adult.smoking <= 0.176
0.1253 < Percentage.of.households.with.high.housing.costs
Premature.age.adjusted.mortality <= 343
2547.9 < School.funding.adequacy
Uninsured.adults <= 0.0936
Teen.births <= 16.2
0.153 < Severe.housing.problems
0.44 < Flu.vaccinations <= 0.50
Feature
−0.06
−0.04
−0.02
0.00
Weight
Positive
Negative

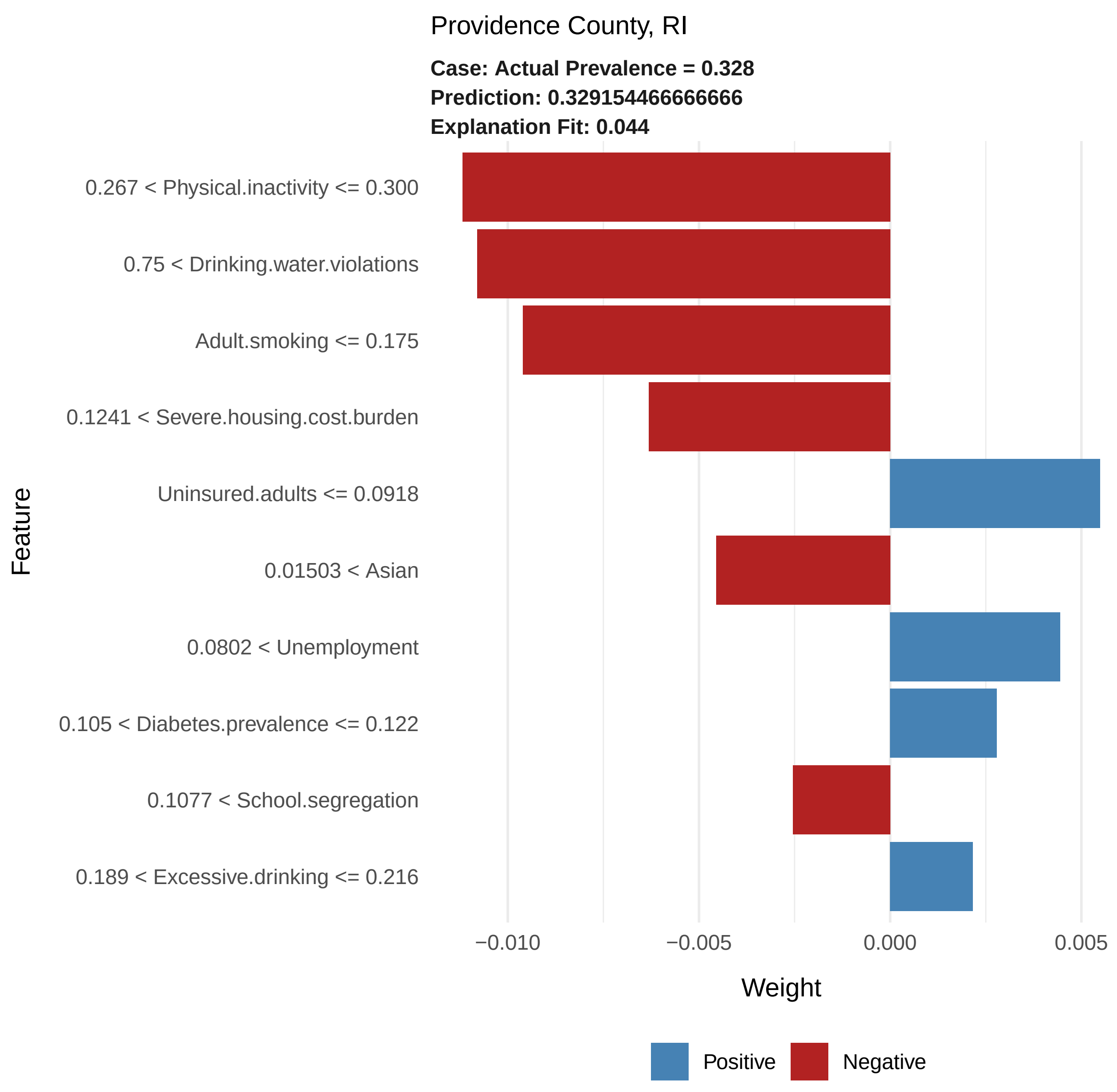
Providence County, RI
Case: Actual Prevalence = 0.328
Prediction: 0.329154466666666
Explanation Fit: 0.044
0.267 < Physical.inactivity <= 0.300
0.75 < Drinking.water.violations
Adult.smoking <= 0.175
0.1241 < Severe.housing.cost.burden
Uninsured.adults <= 0.0918
0.01503 < Asian
0.0802 < Unemployment
0.105 < Diabetes.prevalence <= 0.122
0.1077 < School.segregation
0.189 < Excessive.drinking <= 0.216
Feature
−0.010
−0.005
0.000
0.005
Weight
Positive
Negative

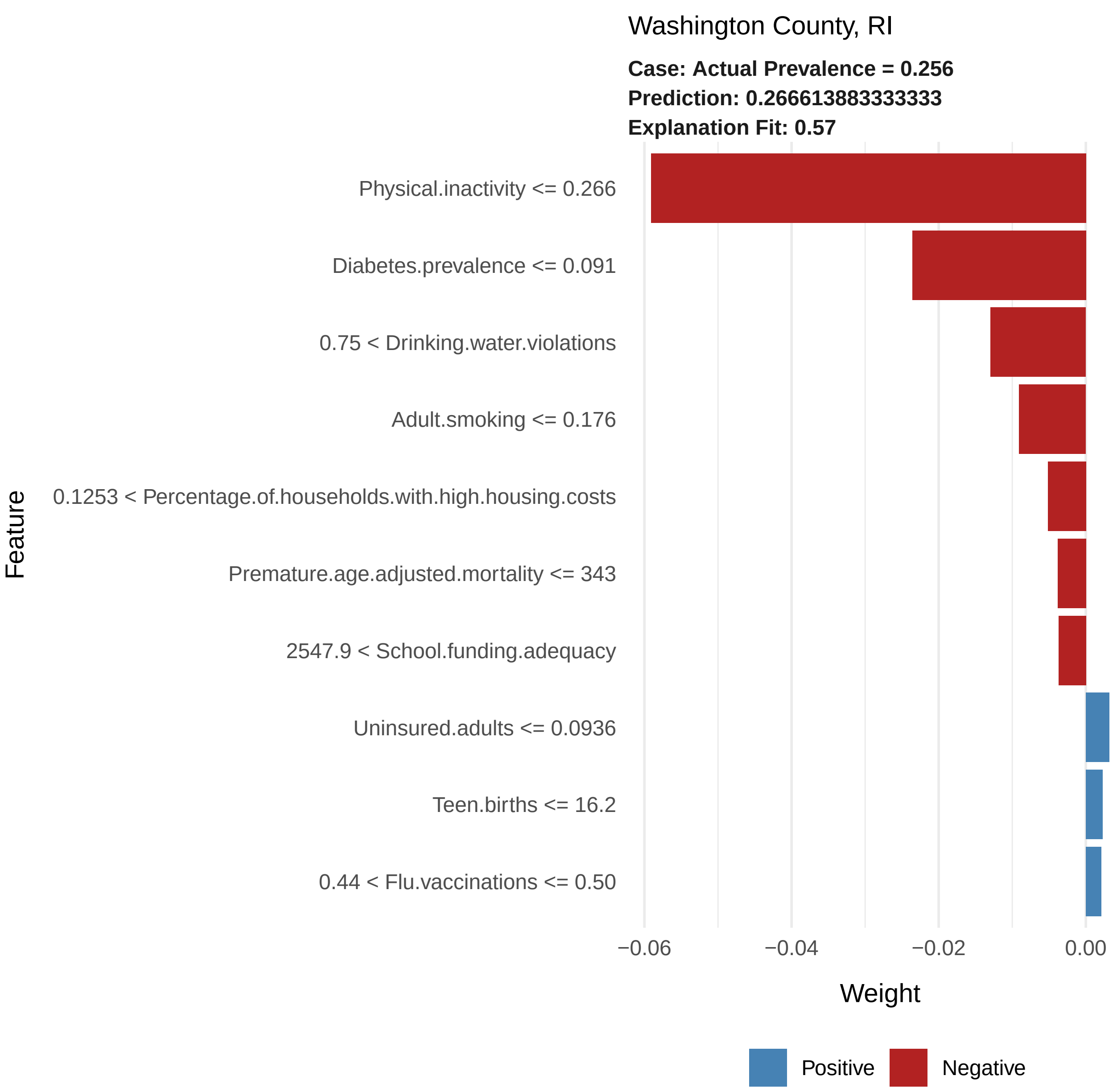
Washington County, RI
Case: Actual Prevalence = 0.256
Prediction: 0.266613883333333
Explanation Fit: 0.57
Physical.inactivity <= 0.266
Diabetes.prevalence <= 0.091
0.75 < Drinking.water.violations
Adult.smoking <= 0.176
0.1253 < Percentage.of.households.with.high.housing.costs
Premature.age.adjusted.mortality <= 343
2547.9 < School.funding.adequacy
Uninsured.adults <= 0.0936
Teen.births <= 16.2
0.44 < Flu.vaccinations <= 0.50
Feature
−0.06
−0.04
−0.02
0.00
Weight
Positive
Negative

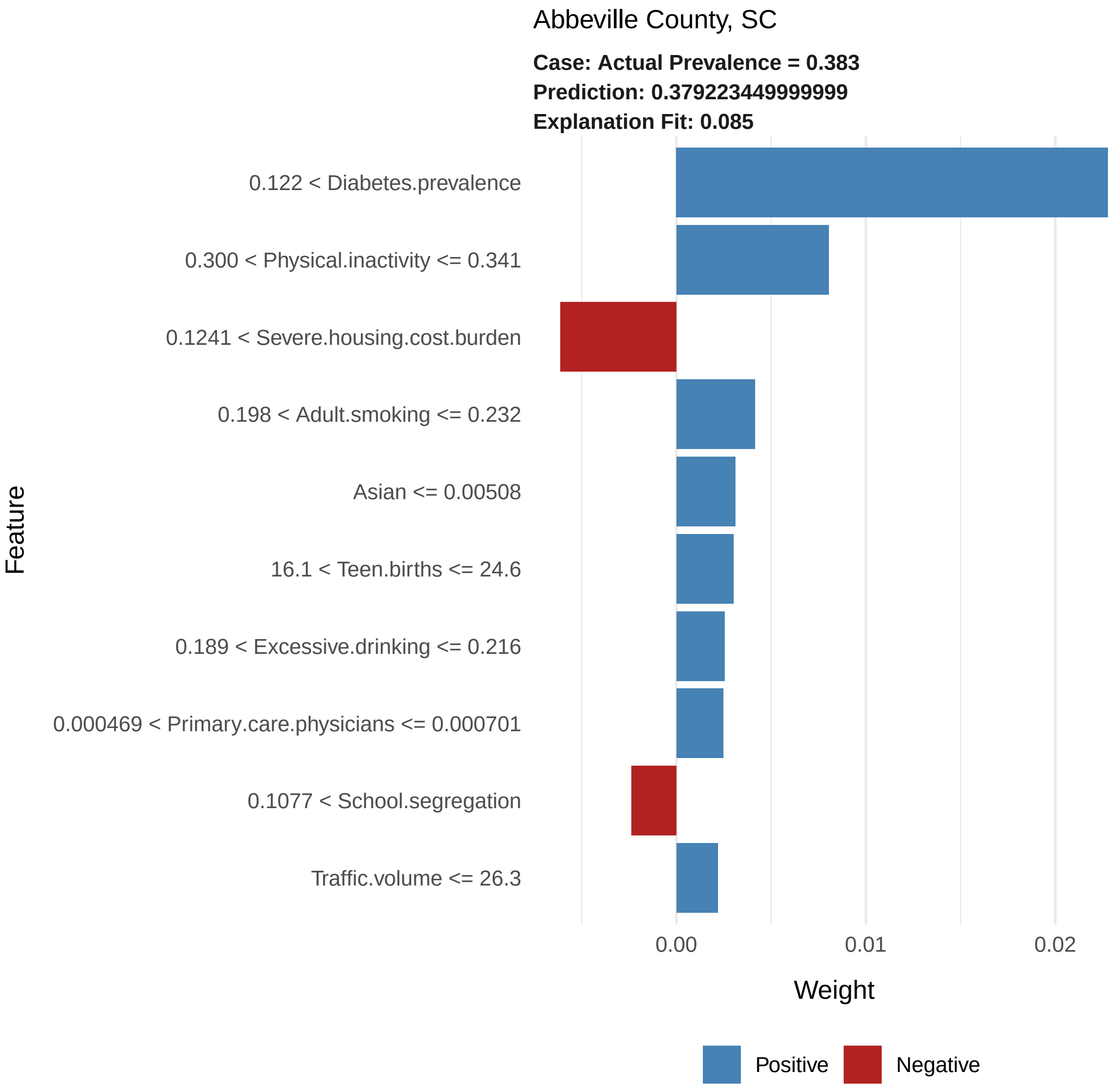
Abbeville County, SC
Case: Actual Prevalence = 0.383
Prediction: 0.379223449999999
Explanation Fit: 0.085
0.122 < Diabetes.prevalence
0.300 < Physical.inactivity <= 0.341
0.1241 < Severe.housing.cost.burden
0.198 < Adult.smoking <= 0.232
Asian <= 0.00508
16.1 < Teen.births <= 24.6
0.189 < Excessive.drinking <= 0.216
0.000469 < Primary.care.physicians <= 0.000701
0.1077 < School.segregation
Traffic.volume <= 26.3
Feature
0.00
0.01
0.02
Weight
Positive
Negative

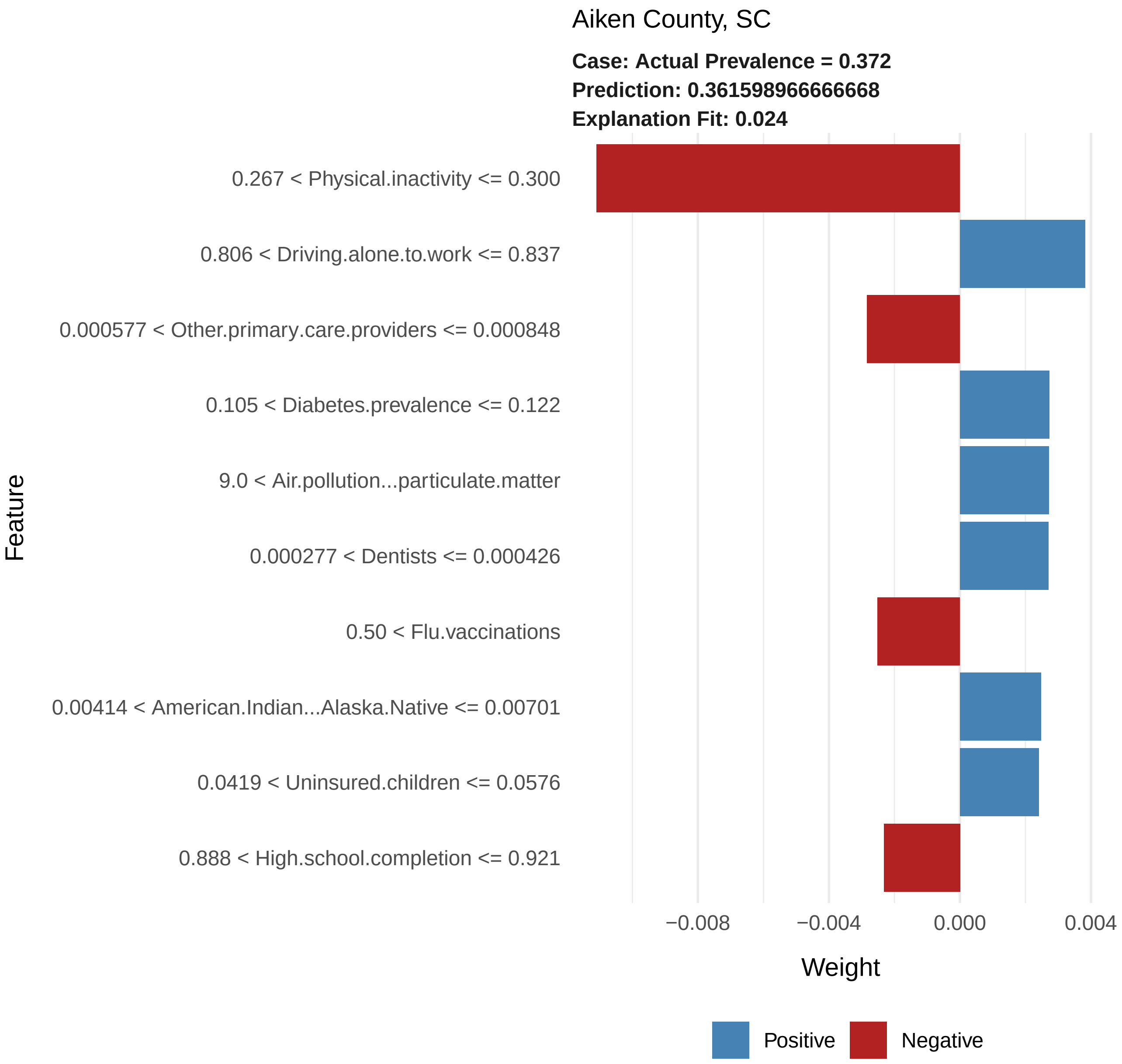
Aiken County, SC
Case: Actual Prevalence = 0.372
Prediction: 0.361598966666668
Explanation Fit: 0.024
0.267 < Physical.inactivity <= 0.300
0.806 < Driving.alone.to.work <= 0.837
0.000577 < Other.primary.care.providers <= 0.000848
0.105 < Diabetes.prevalence <= 0.122
9.0 < Air.pollution...particulate.matter
0.000277 < Dentists <= 0.000426
0.50 < Flu.vaccinations
0.00414 < American.Indian...Alaska.Native <= 0.00701
0.0419 < Uninsured.children <= 0.0576
0.888 < High.school.completion <= 0.921
Feature
−0.008
−0.004
0.000
0.004
Weight
Positive
Negative

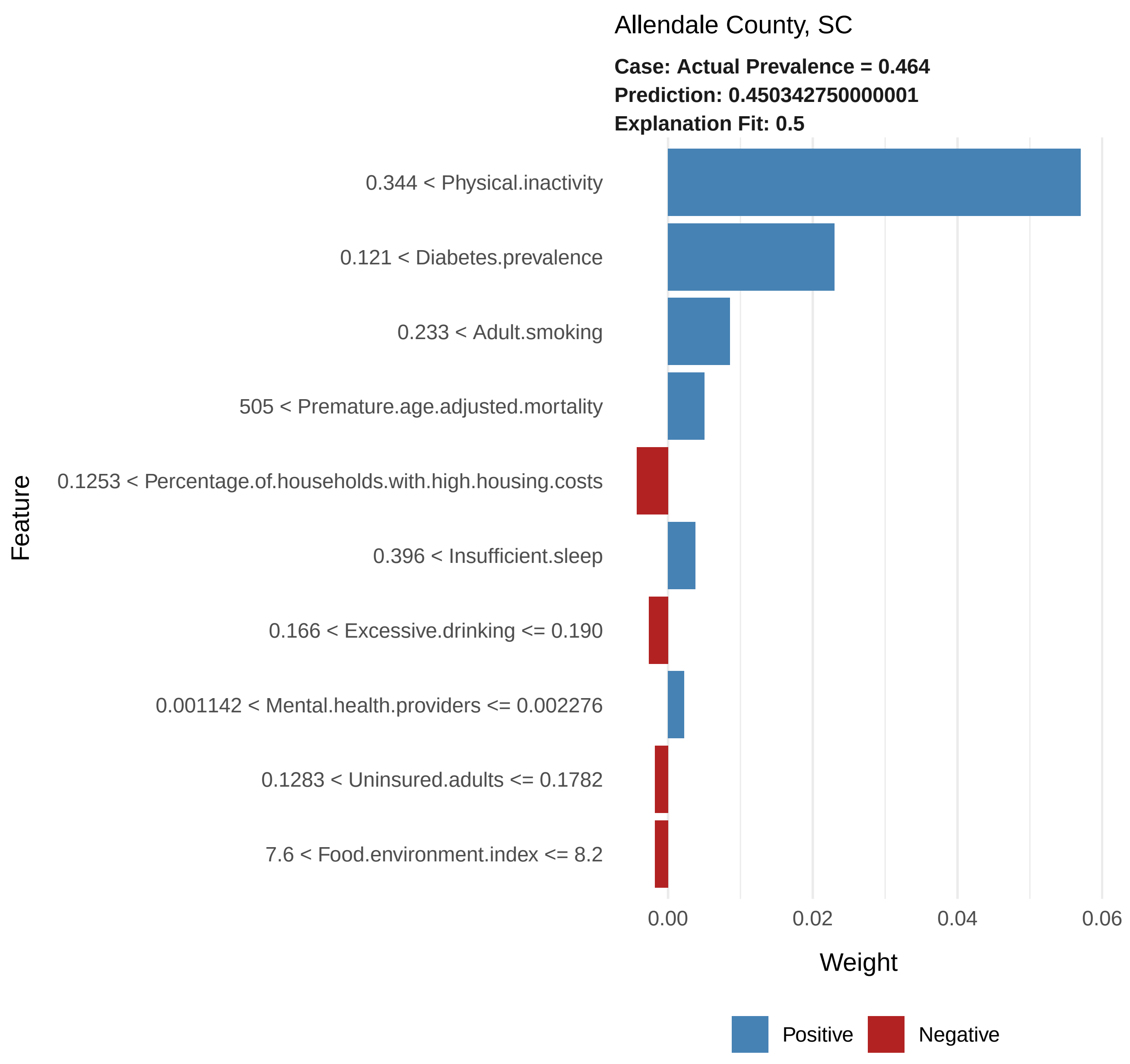

Allendale County, SC
Case: Actual Prevalence = 0.464
Prediction: 0.450342750000001
Explanation Fit: 0.5
0.344 < Physical.inactivity
0.121 < Diabetes.prevalence
0.233 < Adult.smoking
505 < Premature.age.adjusted.mortality
0.1253 < Percentage.of.households.with.high.housing.costs
0.396 < Insufficient.sleep
0.166 < Excessive.drinking <= 0.190
0.001142 < Mental.health.providers <= 0.002276
0.1283 < Uninsured.adults <= 0.1782
7.6 < Food.environment.index <= 8.2
Feature
0.00
0.02
0.04
0.06
Weight
Positive
Negative

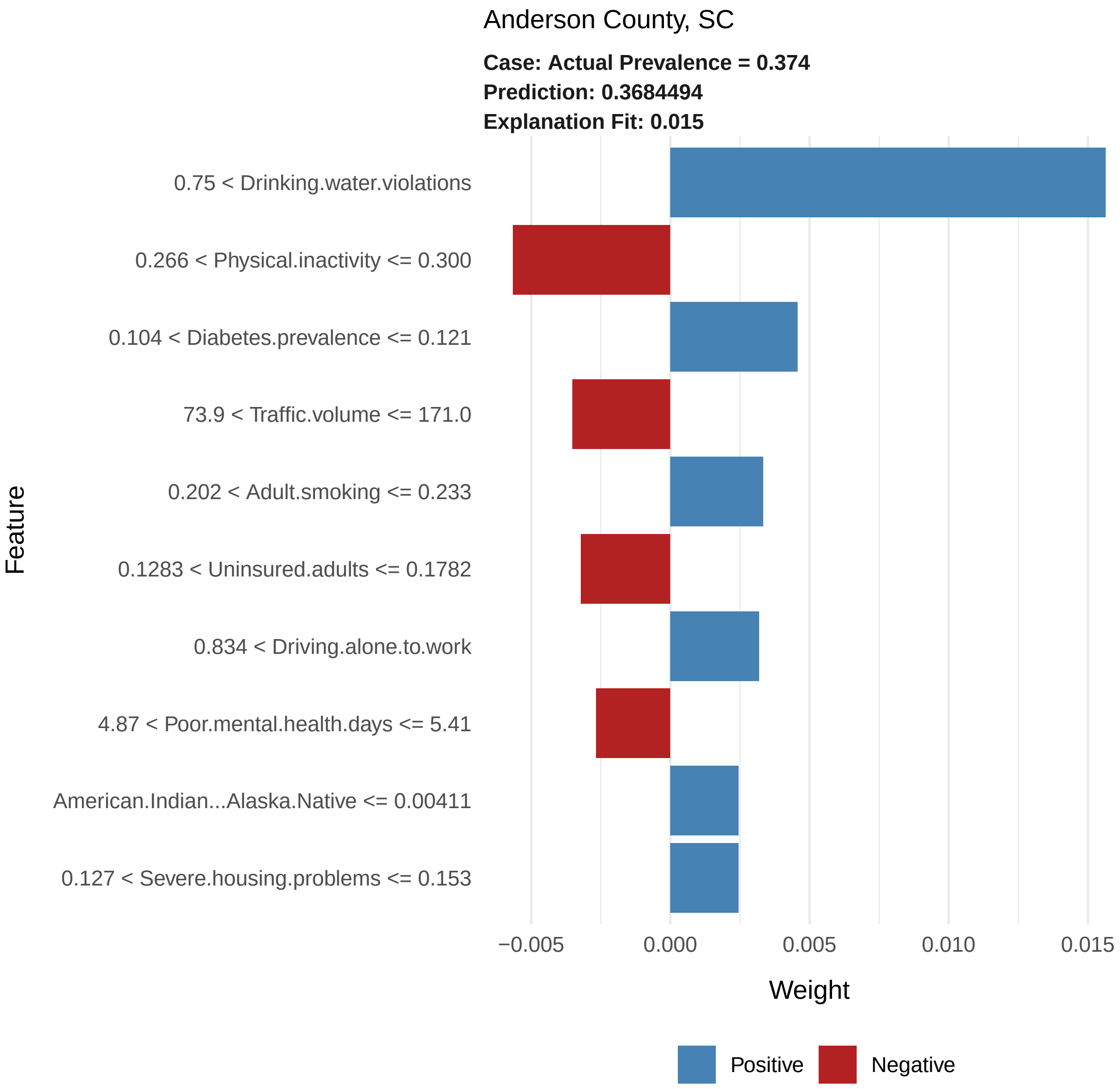
Anderson County, SC
Case: Actual Prevalence = 0.374
Prediction: 0.3684494
Explanation Fit: 0.015
0.75 < Drinking.water.violations
0.266 < Physical.inactivity <= 0.300
0.104 < Diabetes.prevalence <= 0.121
73.9 < Traffic.volume <= 171.0
0.202 < Adult.smoking <= 0.233
0.1283 < Uninsured.adults <= 0.1782
0.834 < Driving.alone.to.work
4.87 < Poor.mental.health.days <= 5.41
American.Indian...Alaska.Native <= 0.00411
0.127 < Severe.housing.problems <= 0.153
Feature
−0.005
0.000
0.005
0.010
0.015
Weight
Positive
Negative

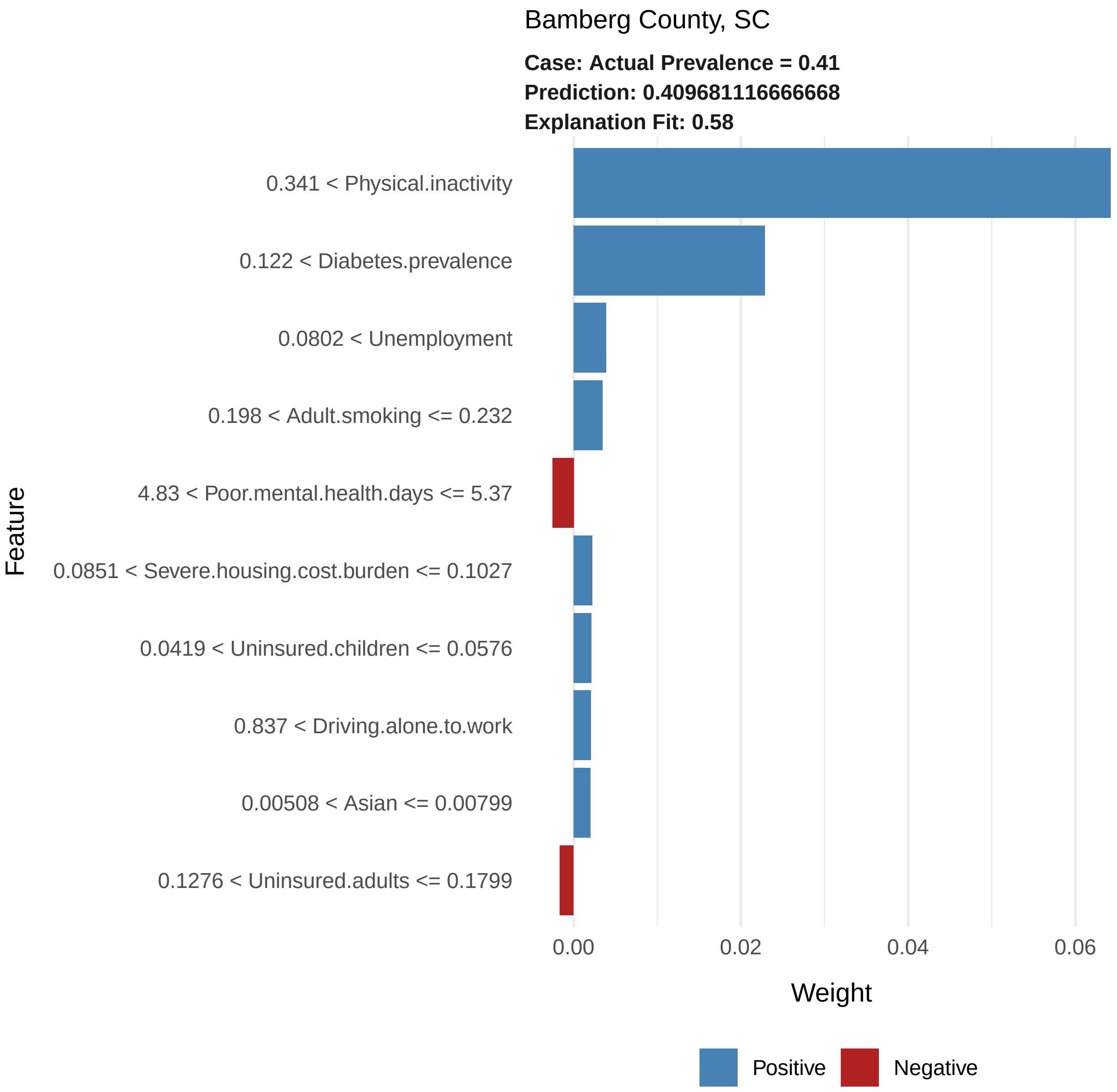

Bamberg County, SC
Case: Actual Prevalence = 0.41
Prediction: 0.409681116666668
Explanation Fit: 0.58
0.341 < Physical.inactivity
0.122 < Diabetes.prevalence
0.0802 < Unemployment
0.198 < Adult.smoking <= 0.232
4.83 < Poor.mental.health.days <= 5.37
0.0851 < Severe.housing.cost.burden <= 0.1027
0.0419 < Uninsured.children <= 0.0576
0.837 < Driving.alone.to.work
0.00508 < Asian <= 0.00799
0.1276 < Uninsured.adults <= 0.1799
Feature
0.00
0.02
0.04
0.06
Weight
Positive
Negative

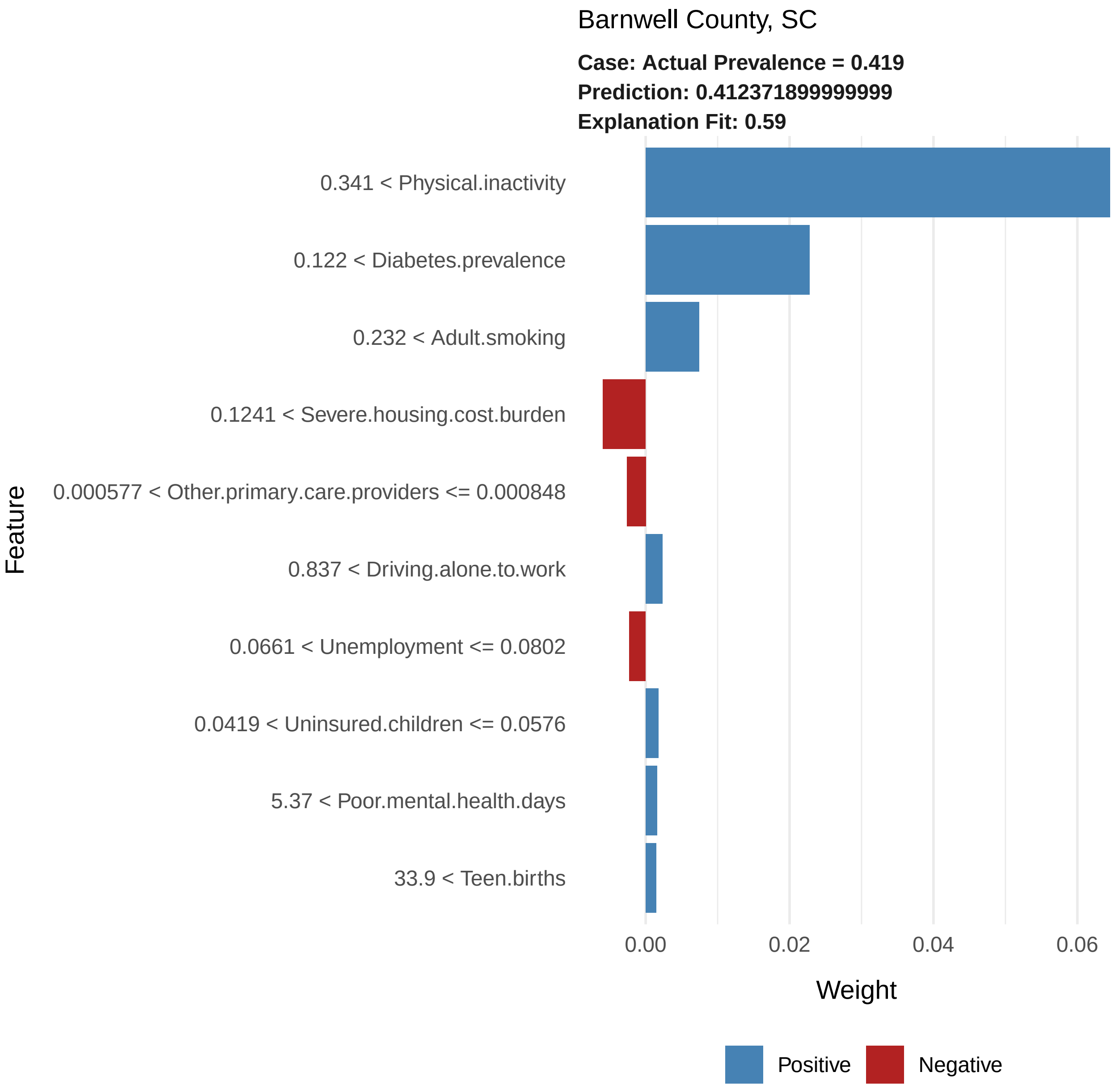
Barnwell County, SC
Case: Actual Prevalence = 0.419
Prediction: 0.412371899999999
Explanation Fit: 0.59
0.341 < Physical.inactivity
0.122 < Diabetes.prevalence
0.232 < Adult.smoking
0.1241 < Severe.housing.cost.burden
0.000577 < Other.primary.care.providers <= 0.000848
0.837 < Driving.alone.to.work
0.0661 < Unemployment <= 0.0802
0.0419 < Uninsured.children <= 0.0576
5.37 < Poor.mental.health.days
33.9 < Teen.births
Feature
0.00
0.02
0.04
0.06
Weight
Positive
Negative

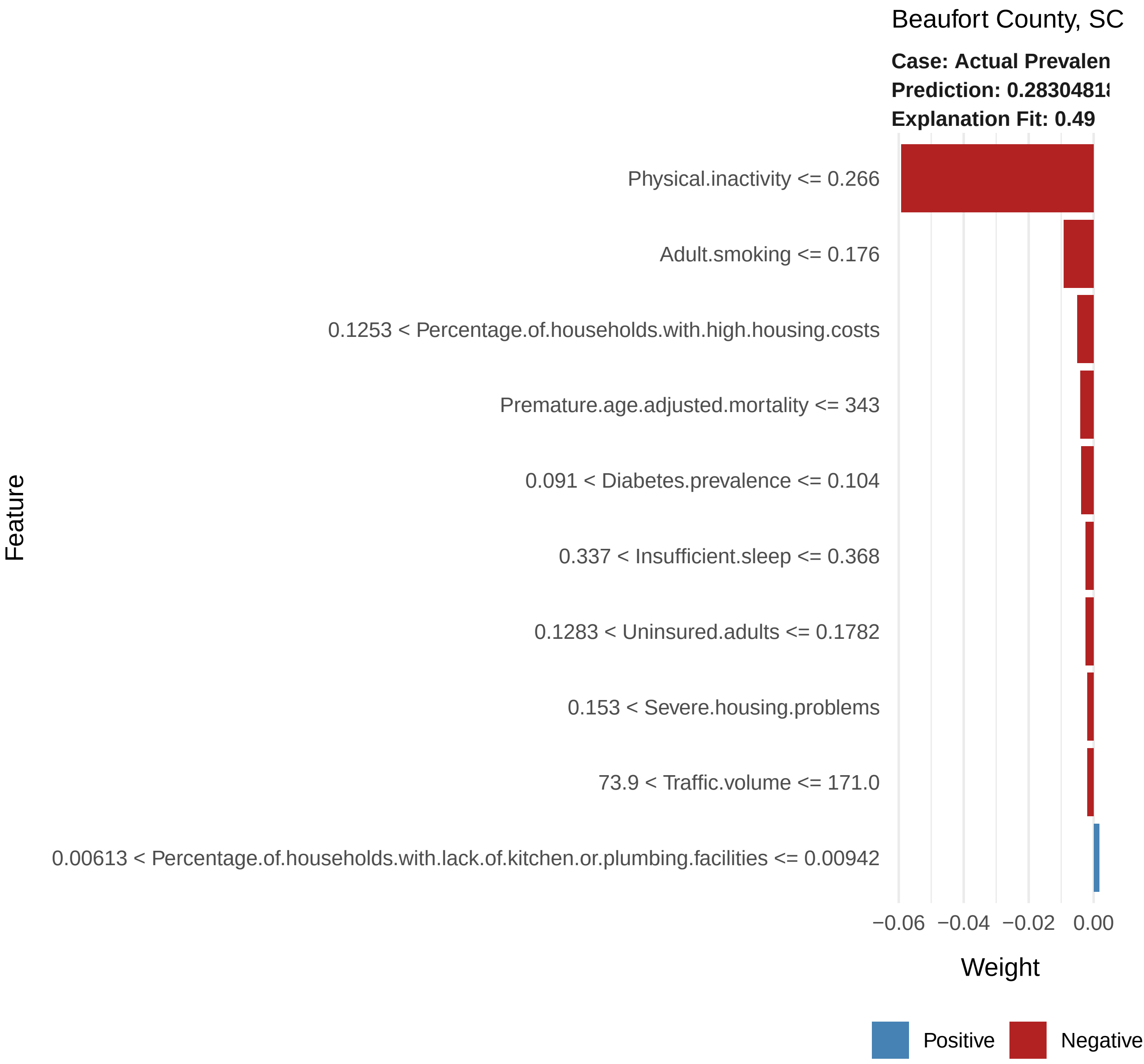

Beaufort County, SC
Case: Actual Prevalen
Prediction: 0.28304818
Explanation Fit: 0.49
Physical.inactivity <= 0.266
Adult.smoking <= 0.176
0.1253 < Percentage.of.households.with.high.housing.costs
Premature.age.adjusted.mortality <= 343
0.091 < Diabetes.prevalence <= 0.104
0.337 < Insufficient.sleep <= 0.368
0.1283 < Uninsured.adults <= 0.1782
0.153 < Severe.housing.problems
73.9 < Traffic.volume <= 171.0
0.00613 < Percentage.of.households.with.lack.of.kitchen.or.plumbing.facilities <= 0.00942
Feature
−0.06
−0.04
−0.02
0.00
Weight
Positive
Negative

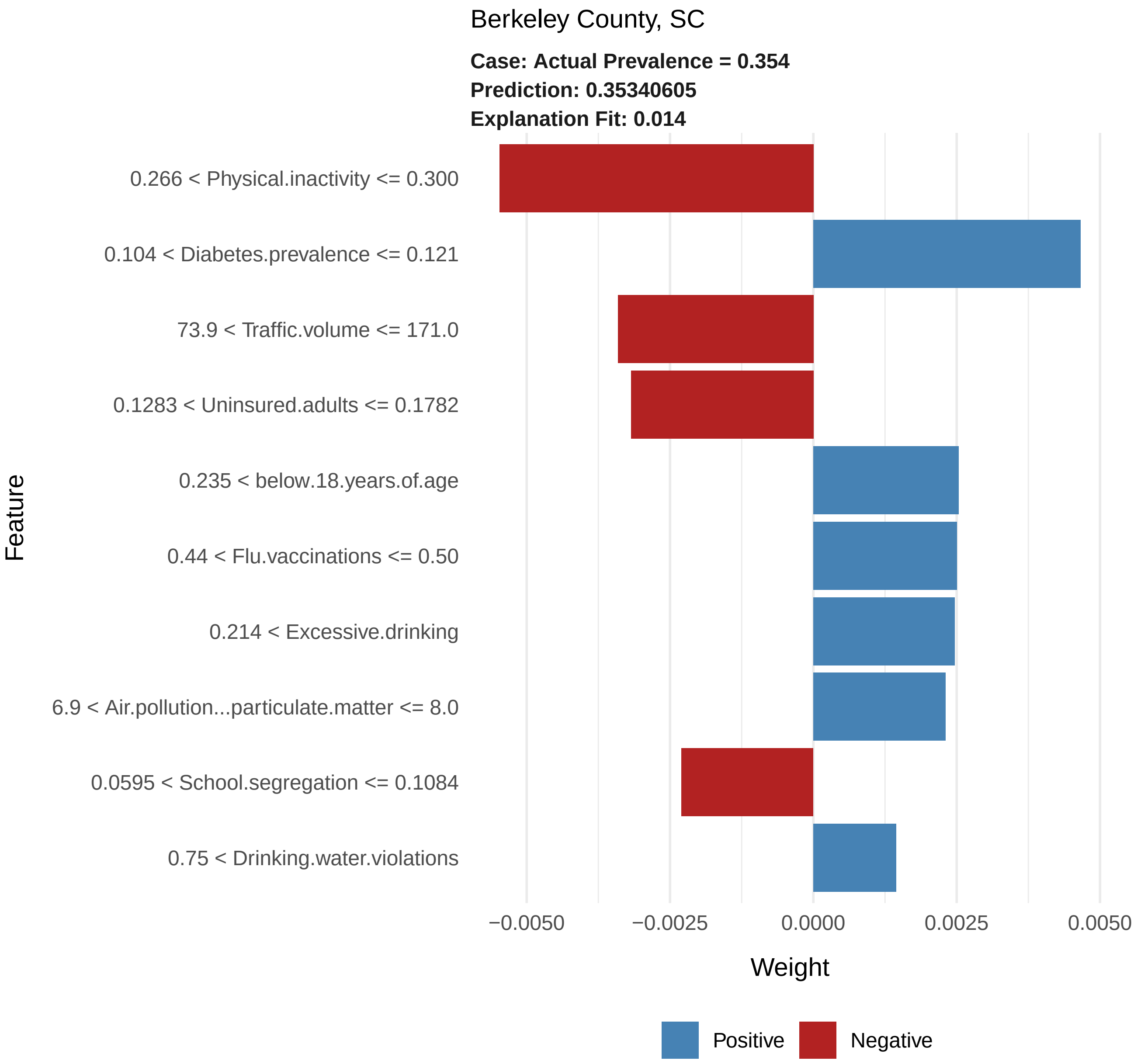

Berkeley County, SC
Case: Actual Prevalence = 0.354
Prediction: 0.35340605
Explanation Fit: 0.014
0.266 < Physical.inactivity <= 0.300
0.104 < Diabetes.prevalence <= 0.121
73.9 < Traffic.volume <= 171.0
0.1283 < Uninsured.adults <= 0.1782
0.235 < below.18.years.of.age
0.44 < Flu.vaccinations <= 0.50
0.214 < Excessive.drinking
6.9 < Air.pollution...particulate.matter <= 8.0
0.0595 < School.segregation <= 0.1084
0.75 < Drinking.water.violations
Feature
−0.0050
−0.0025
0.0000
0.0025
0.0050
Weight
Positive
Negative

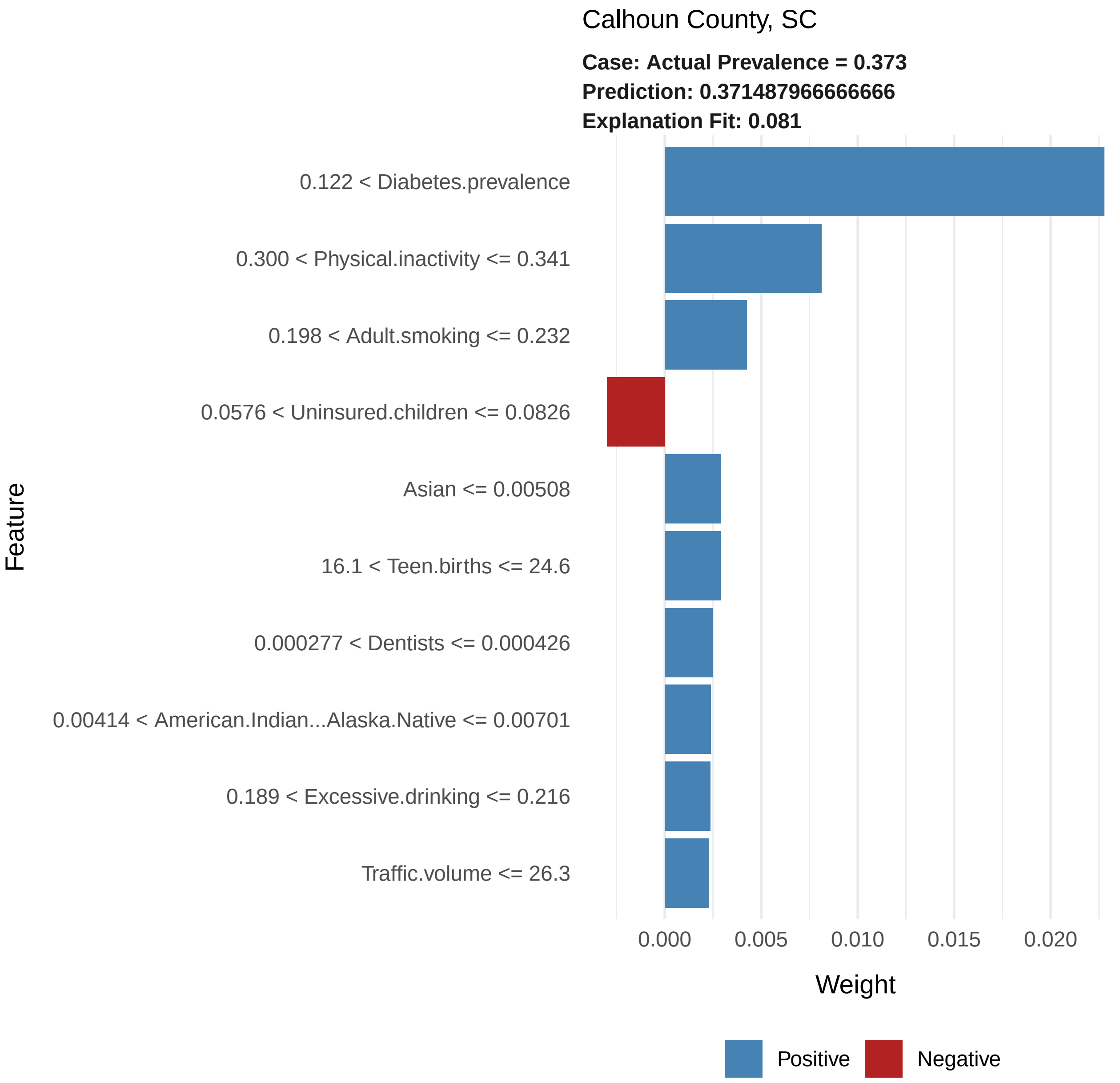

Calhoun County, SC
Case: Actual Prevalence = 0.373
Prediction: 0.371487966666666
Explanation Fit: 0.081
0.122 < Diabetes.prevalence
0.300 < Physical.inactivity <= 0.341
0.198 < Adult.smoking <= 0.232
0.0576 < Uninsured.children <= 0.0826
Asian <= 0.00508
16.1 < Teen.births <= 24.6
0.000277 < Dentists <= 0.000426
0.00414 < American.Indian...Alaska.Native <= 0.00701
0.189 < Excessive.drinking <= 0.216
Traffic.volume <= 26.3
Feature
0.000
0.005
0.010
0.015
0.020
Weight
Positive
Negative

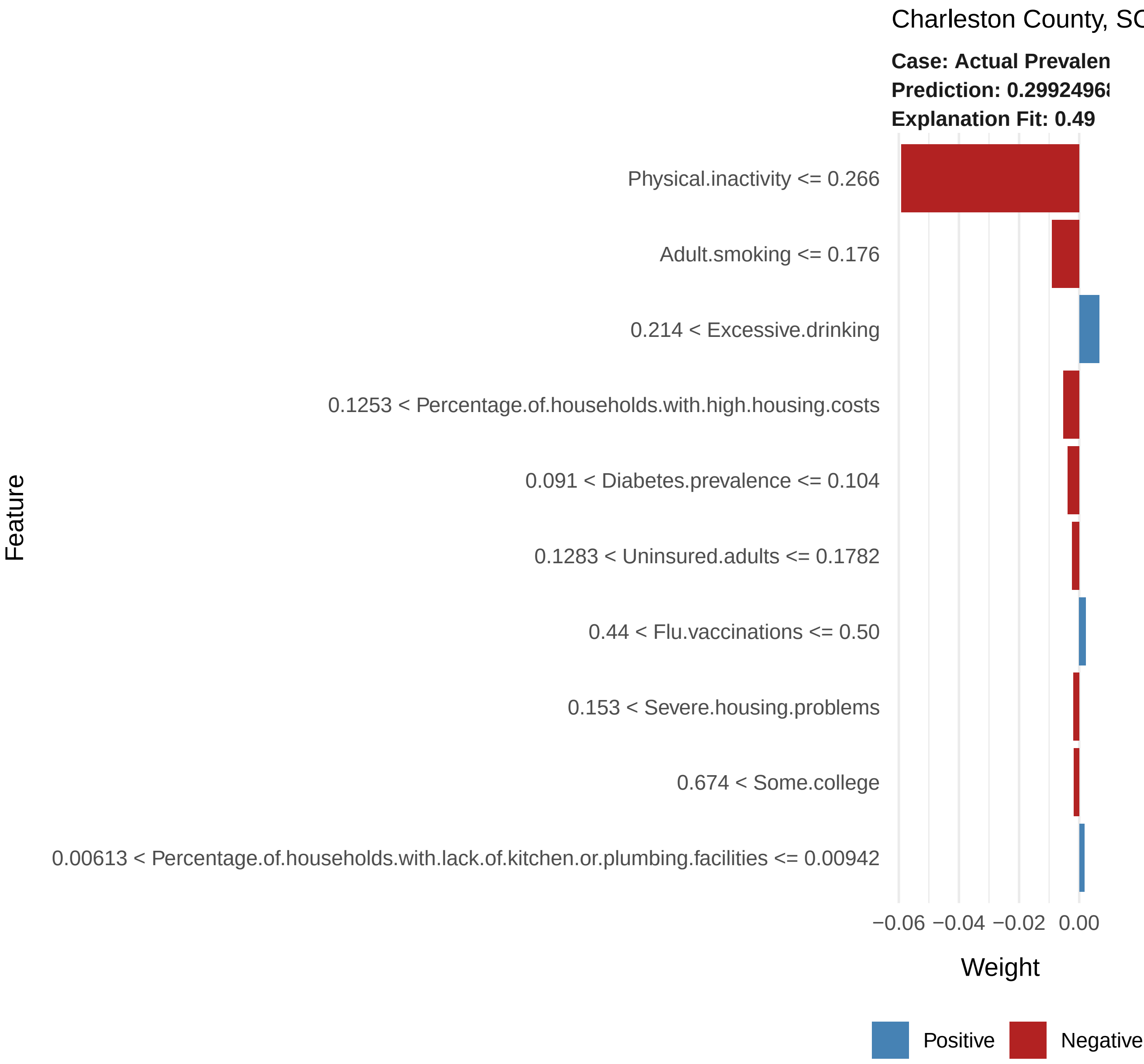

Charleston County, SC
Case: Actual Prevalen
Prediction: 0.2992496
Explanation Fit: 0.49
Physical.inactivity <= 0.266
Adult.smoking <= 0.176
0.214 < Excessive.drinking
0.1253 < Percentage.of.households.with.high.housing.costs
0.091 < Diabetes.prevalence <= 0.104
0.1283 < Uninsured.adults <= 0.1782
0.44 < Flu.vaccinations <= 0.50
0.153 < Severe.housing.problems
0.674 < Some.college
0.00613 < Percentage.of.households.with.lack.of.kitchen.or.plumbing.facilities <= 0.00942
Feature
−0.06
−0.04
−0.02
0.00
Weight
Positive
Negative

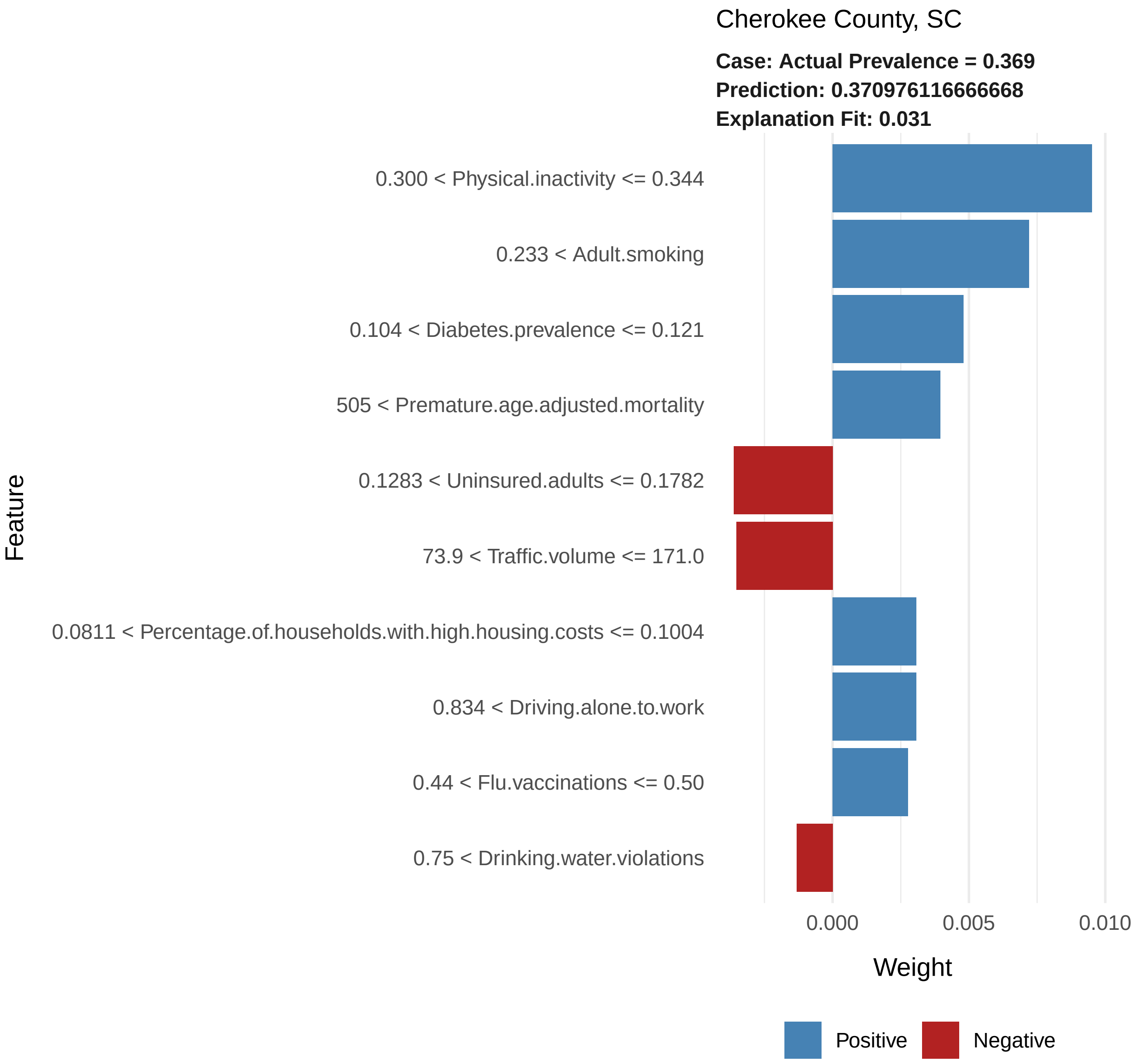
Cherokee County, SC
Case: Actual Prevalence = 0.369
Prediction: 0.370976116666668
Explanation Fit: 0.031
0.300 < Physical.inactivity <= 0.344
0.233 < Adult.smoking
0.104 < Diabetes.prevalence <= 0.121
505 < Premature.age.adjusted.mortality
0.1283 < Uninsured.adults <= 0.1782
73.9 < Traffic.volume <= 171.0
0.0811 < Percentage.of.households.with.high.housing.costs <= 0.1004
0.834 < Driving.alone.to.work
0.44 < Flu.vaccinations <= 0.50
0.75 < Drinking.water.violations
Feature
0.000
0.005
0.010
Weight
Positive
Negative

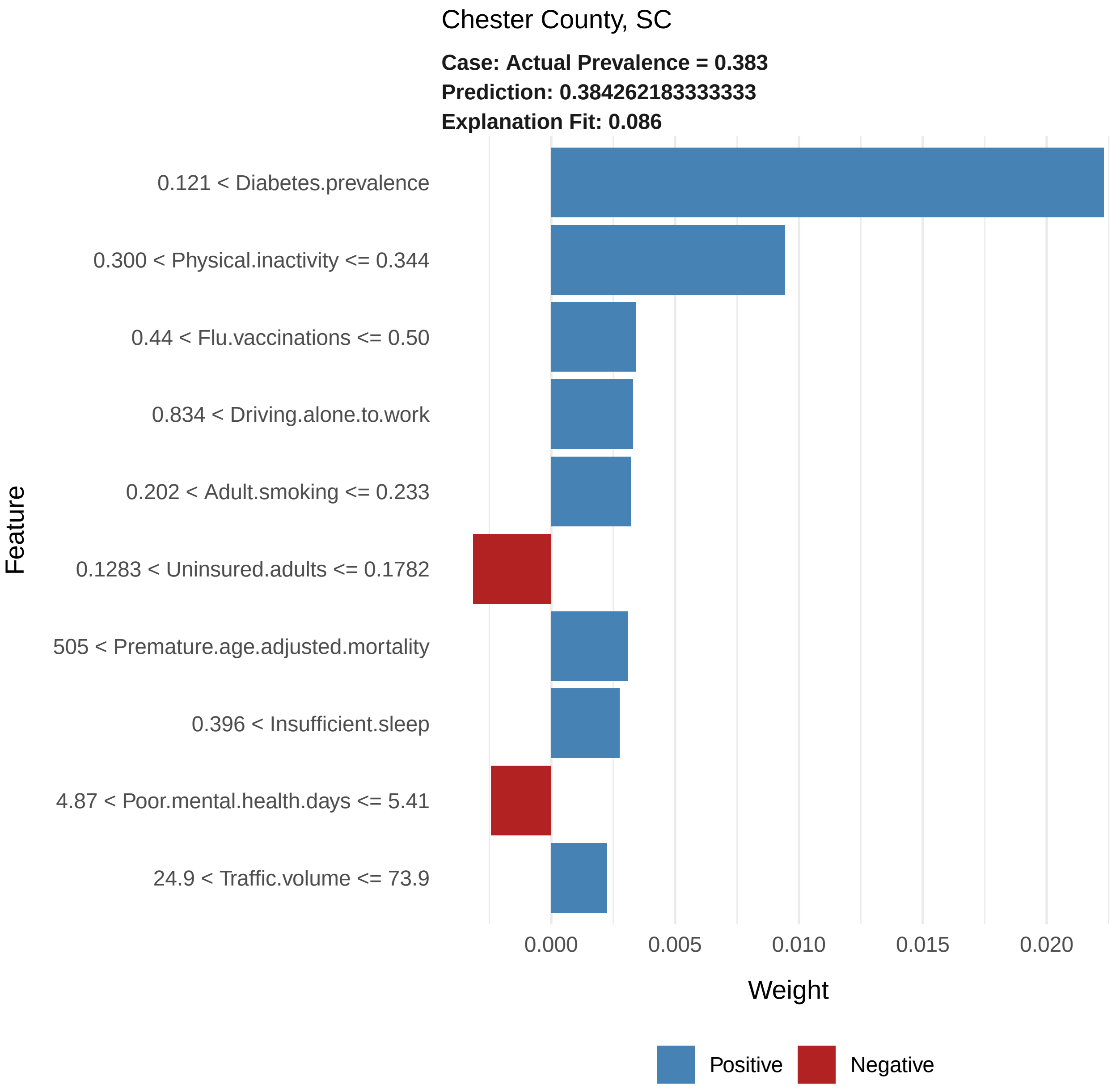
Chester County, SC
Case: Actual Prevalence = 0.383
Prediction: 0.384262183333333
Explanation Fit: 0.086
0.121 < Diabetes.prevalence
0.300 < Physical.inactivity <= 0.344
0.44 < Flu.vaccinations <= 0.50
0.834 < Driving.alone.to.work
0.202 < Adult.smoking <= 0.233
0.1283 < Uninsured.adults <= 0.1782
505 < Premature.age.adjusted.mortality
0.396 < Insufficient.sleep
4.87 < Poor.mental.health.days <= 5.41
24.9 < Traffic.volume <= 73.9
Feature
0.000
0.005
0.010
0.015
0.020
Weight
Positive
Negative

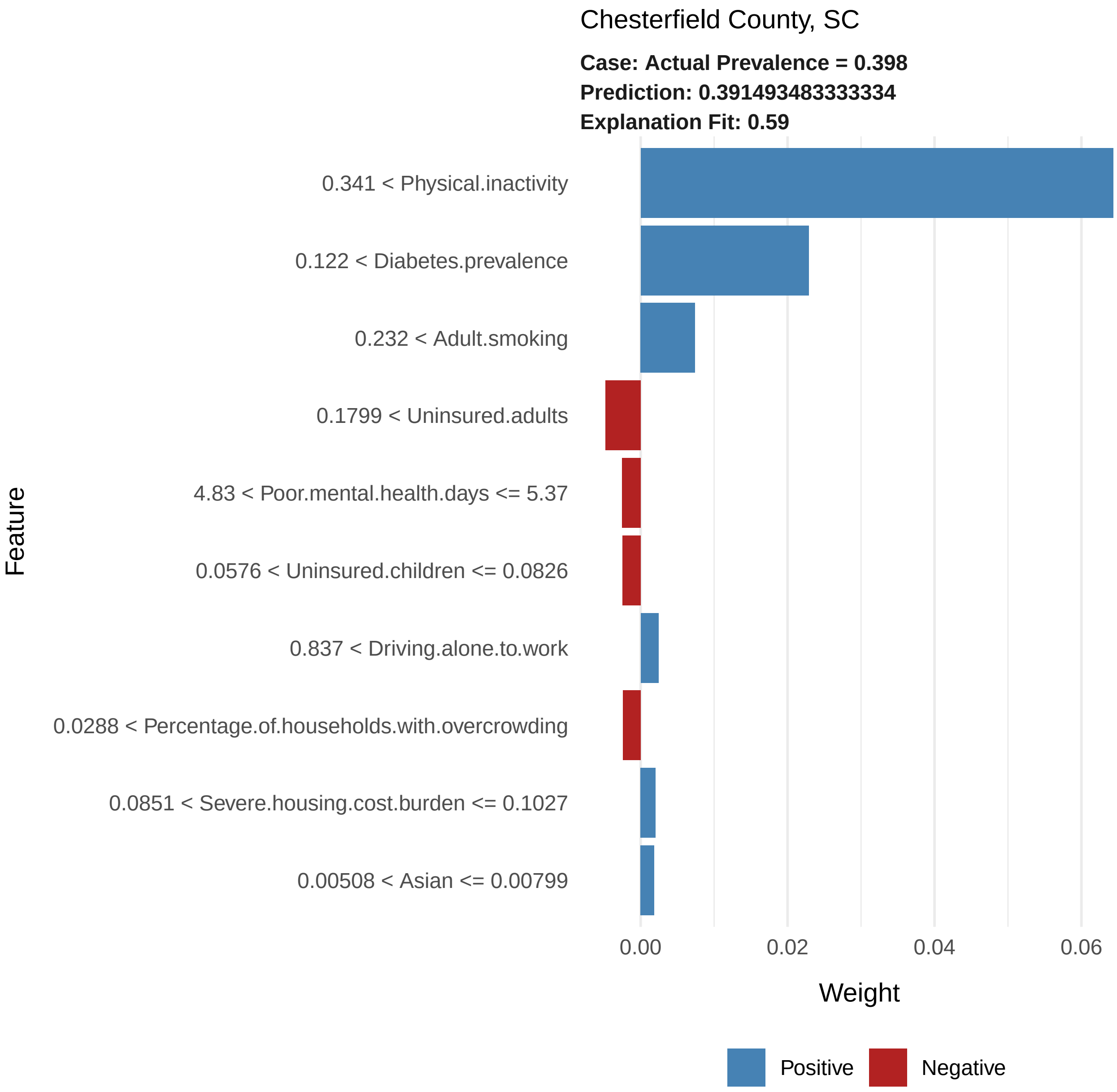
Chesterfield County, SC
Case: Actual Prevalence = 0.398
Prediction: 0.391493483333334
Explanation Fit: 0.59
0.341 < Physical.inactivity
0.122 < Diabetes.prevalence
0.232 < Adult.smoking
0.1799 < Uninsured.adults
4.83 < Poor.mental.health.days <= 5.37
0.0576 < Uninsured.children <= 0.0826
0.837 < Driving.alone.to.work
0.0288 < Percentage.of.households.with.overcrowding
0.0851 < Severe.housing.cost.burden <= 0.1027
0.00508 < Asian <= 0.00799
Feature
0.00
0.02
0.04
0.06
Weight
Positive
Negative

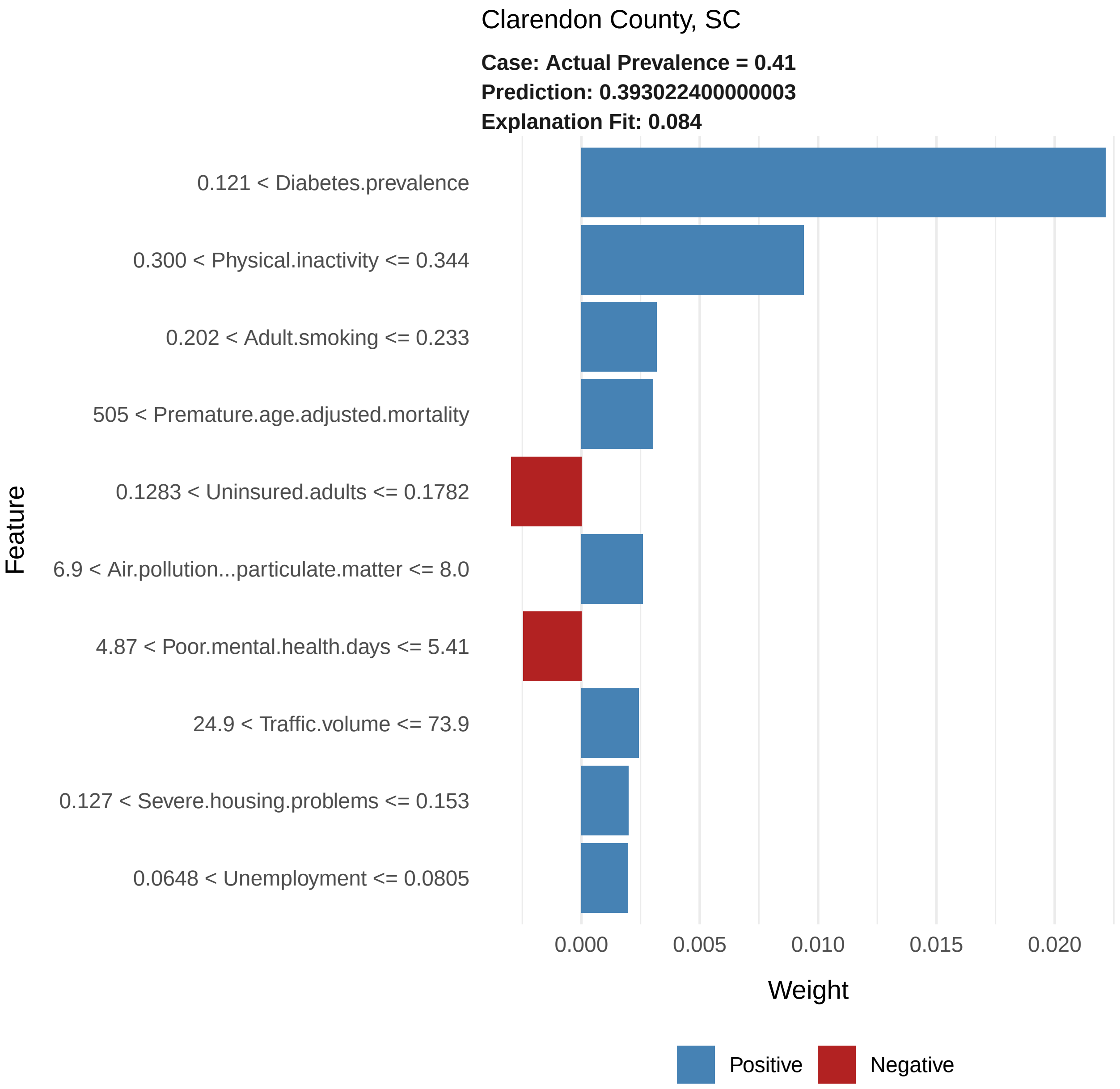

Clarendon County, SC
Case: Actual Prevalence = 0.41
Prediction: 0.393022400000003
Explanation Fit: 0.084
0.121 < Diabetes.prevalence
0.300 < Physical.inactivity <= 0.344
0.202 < Adult.smoking <= 0.233
505 < Premature.age.adjusted.mortality
0.1283 < Uninsured.adults <= 0.1782
6.9 < Air.pollution...particulate.matter <= 8.0
4.87 < Poor.mental.health.days <= 5.41
24.9 < Traffic.volume <= 73.9
0.127 < Severe.housing.problems <= 0.153
0.0648 < Unemployment <= 0.0805
Feature
0.000
0.005
0.010
0.015
0.020
Weight
Positive
Negative

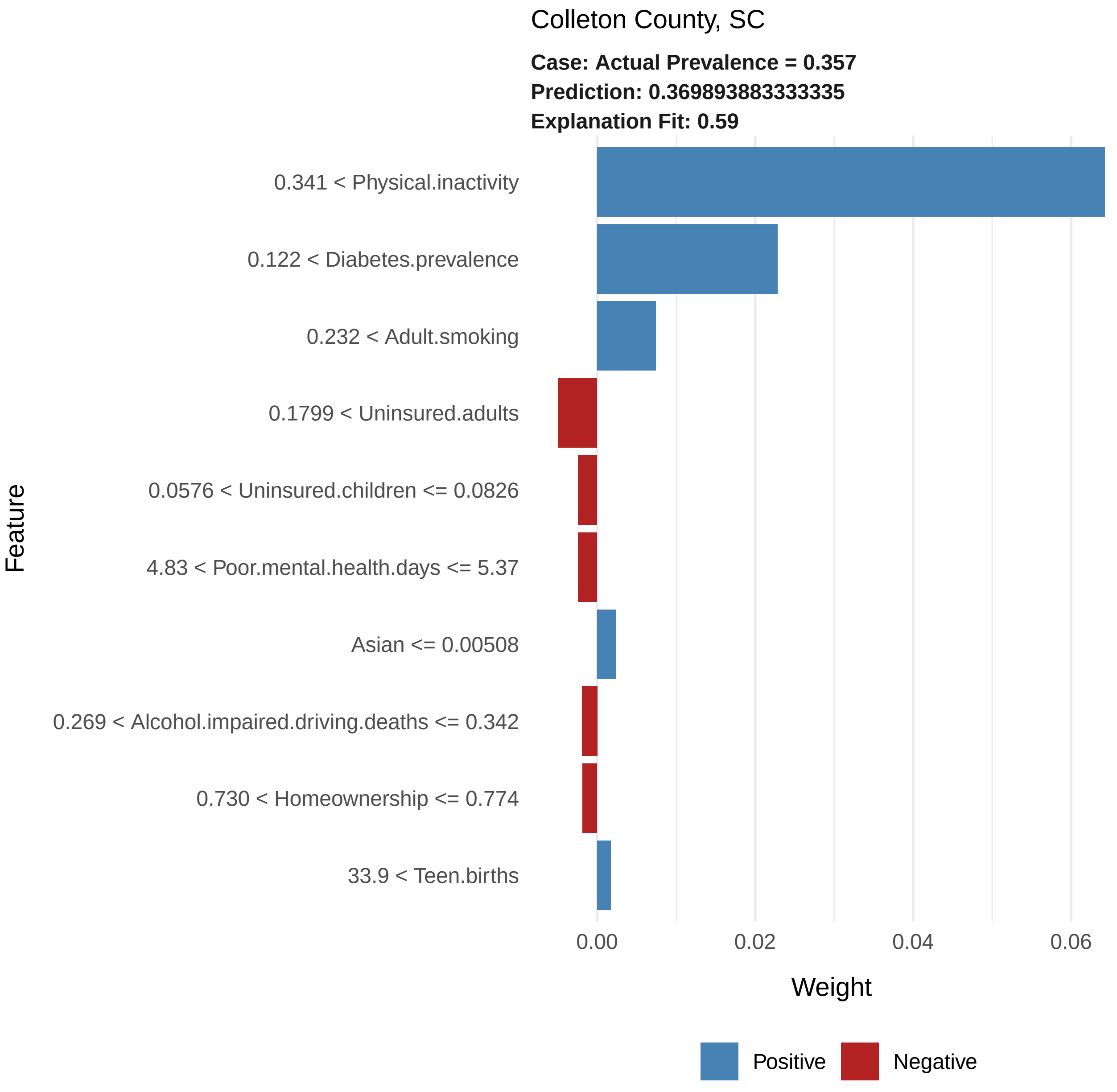
Colleton County, SC
Case: Actual Prevalence = 0.357
Prediction: 0.369893883333335
Explanation Fit: 0.59
0.341 < Physical.inactivity
0.122 < Diabetes.prevalence
0.232 < Adult.smoking
0.1799 < Uninsured.adults
0.0576 < Uninsured.children <= 0.0826
4.83 < Poor.mental.health.days <= 5.37
Asian <= 0.00508
0.269 < Alcohol.impaired.driving.deaths <= 0.342
0.730 < Homeownership <= 0.774
33.9 < Teen.births
Feature
0.00
0.02
0.04
0.06
Weight
Positive
Negative

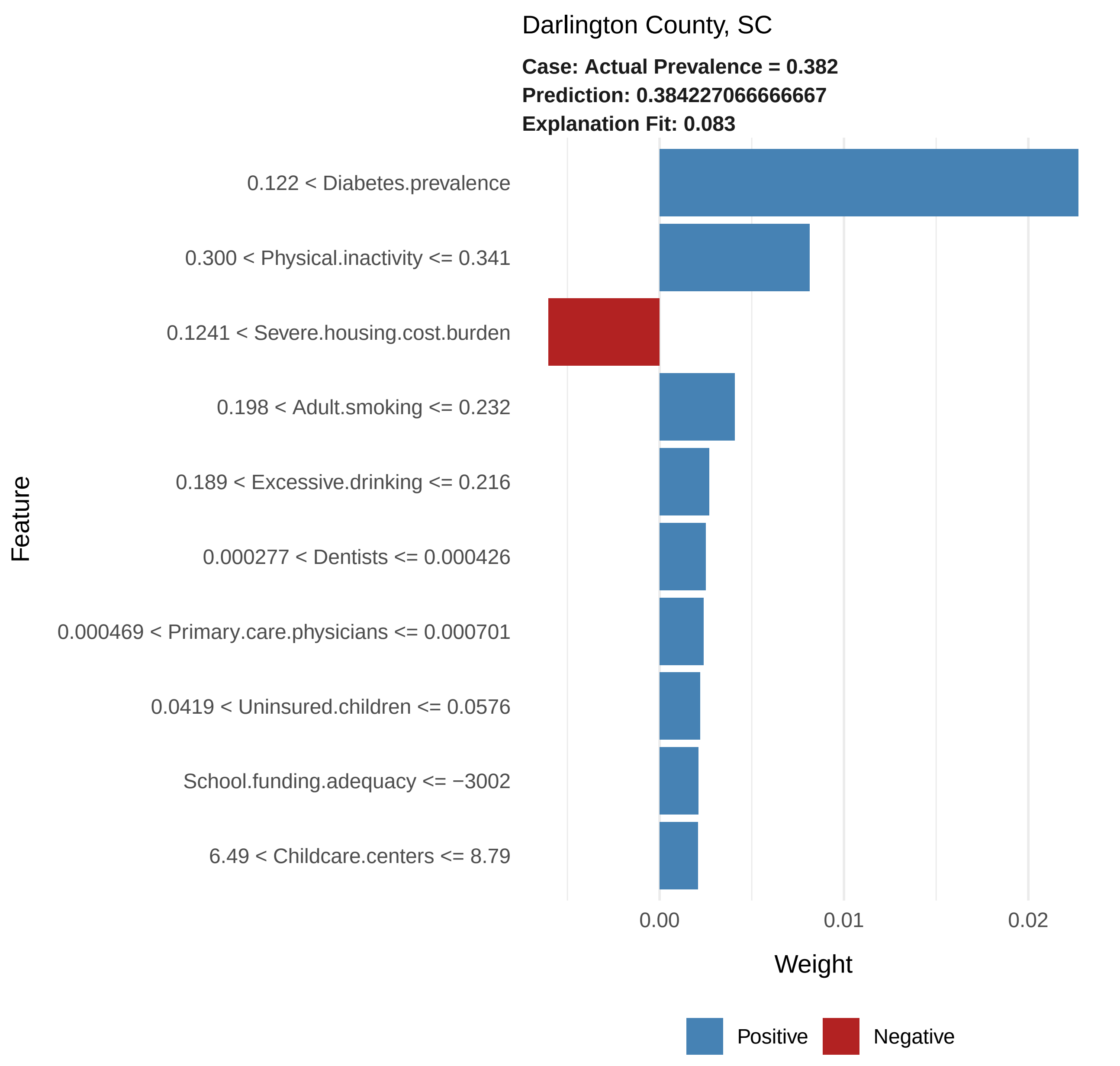
Darlington County, SC
Case: Actual Prevalence = 0.382
Prediction: 0.384227066666667
Explanation Fit: 0.083
0.122 < Diabetes.prevalence
0.300 < Physical.inactivity <= 0.341
0.1241 < Severe.housing.cost.burden
0.198 < Adult.smoking <= 0.232
0.189 < Excessive.drinking <= 0.216
0.000277 < Dentists <= 0.000426
0.000469 < Primary.care.physicians <= 0.000701
0.0419 < Uninsured.children <= 0.0576
School.funding.adequacy <= −3002
6.49 < Childcare.centers <= 8.79
Feature
0.00
0.01
0.02
Weight
Positive
Negative

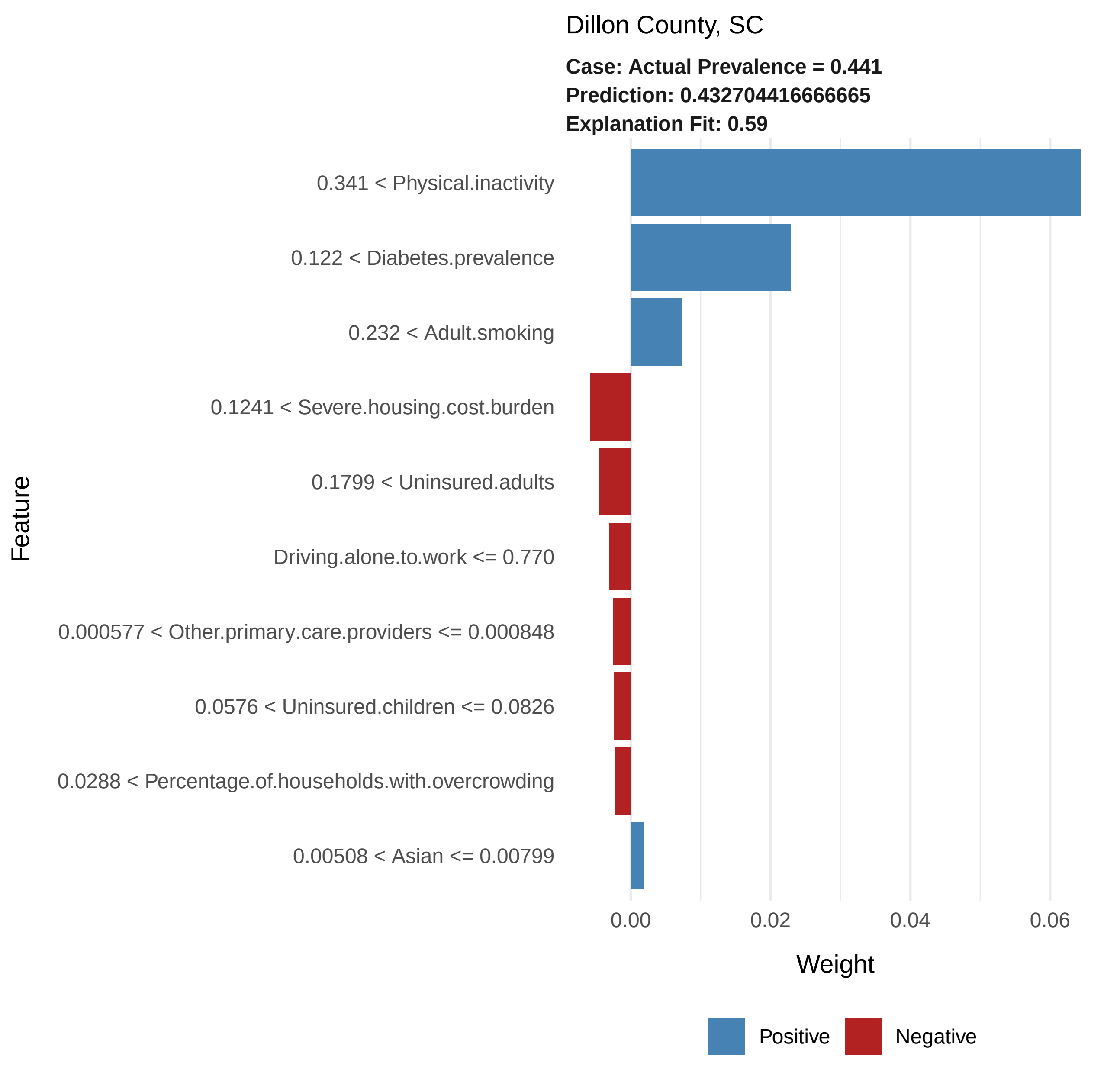
Dillon County, SC
Case: Actual Prevalence = 0.441
Prediction: 0.432704416666665
Explanation Fit: 0.59
0.341 < Physical.inactivity
0.122 < Diabetes.prevalence
0.232 < Adult.smoking
0.1241 < Severe.housing.cost.burden
0.1799 < Uninsured.adults
Driving.alone.to.work <= 0.770
0.000577 < Other.primary.care.providers <= 0.000848
0.0576 < Uninsured.children <= 0.0826
0.0288 < Percentage.of.households.with.overcrowding
0.00508 < Asian <= 0.00799
Feature
0.00
0.02
0.04
0.06
Weight
Positive
Negative

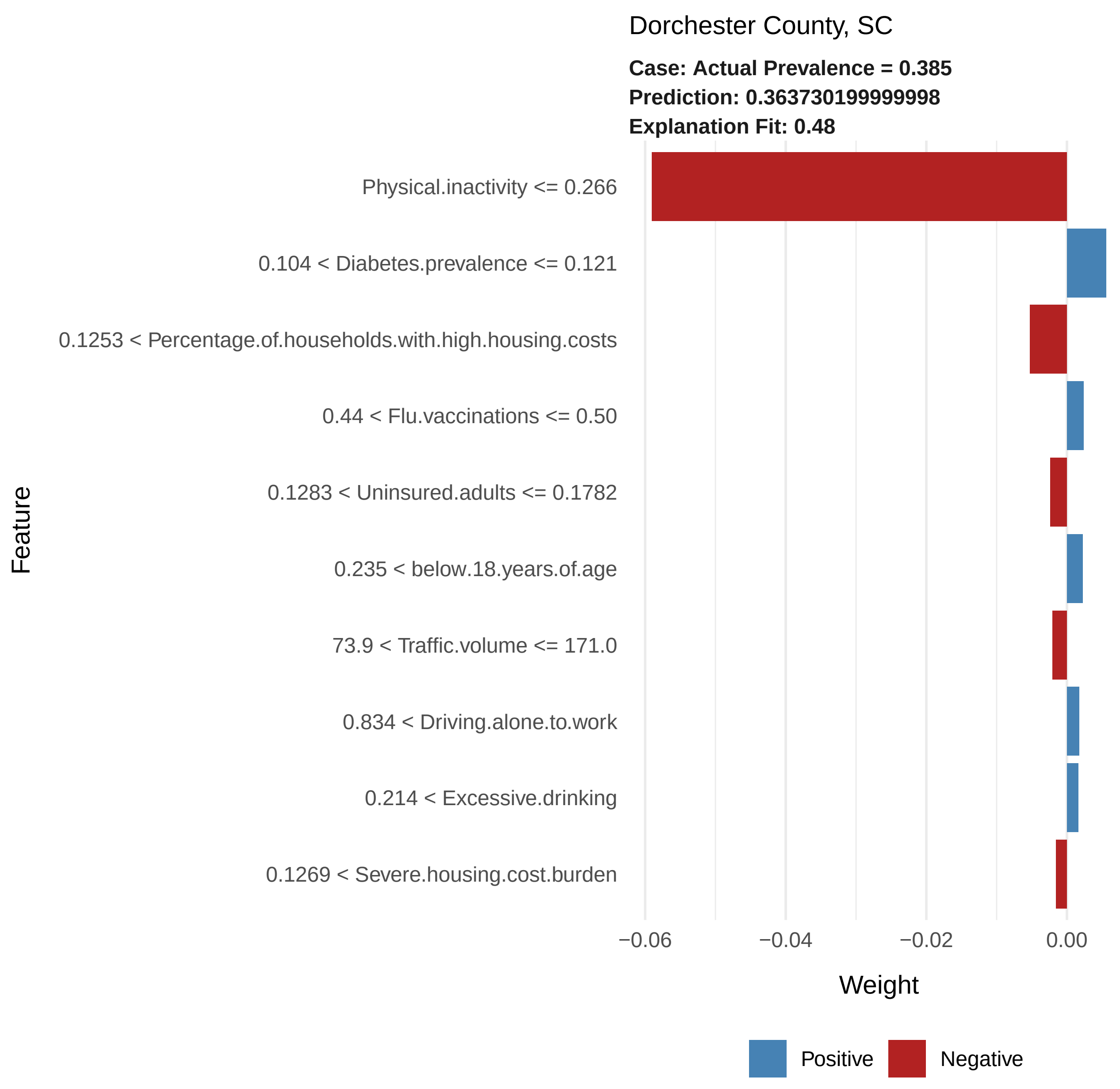

Dorchester County, SC
Case: Actual Prevalence = 0.385
Prediction: 0.363730199999998
Explanation Fit: 0.48
Physical.inactivity <= 0.266
0.104 < Diabetes.prevalence <= 0.121
0.1253 < Percentage.of.households.with.high.housing.costs
0.44 < Flu.vaccinations <= 0.50
0.1283 < Uninsured.adults <= 0.1782
0.235 < below.18.years.of.age
73.9 < Traffic.volume <= 171.0
0.834 < Driving.alone.to.work
0.214 < Excessive.drinking
0.1269 < Severe.housing.cost.burden
Feature
−0.06
−0.04
−0.02
0.00
Weight
Positive
Negative

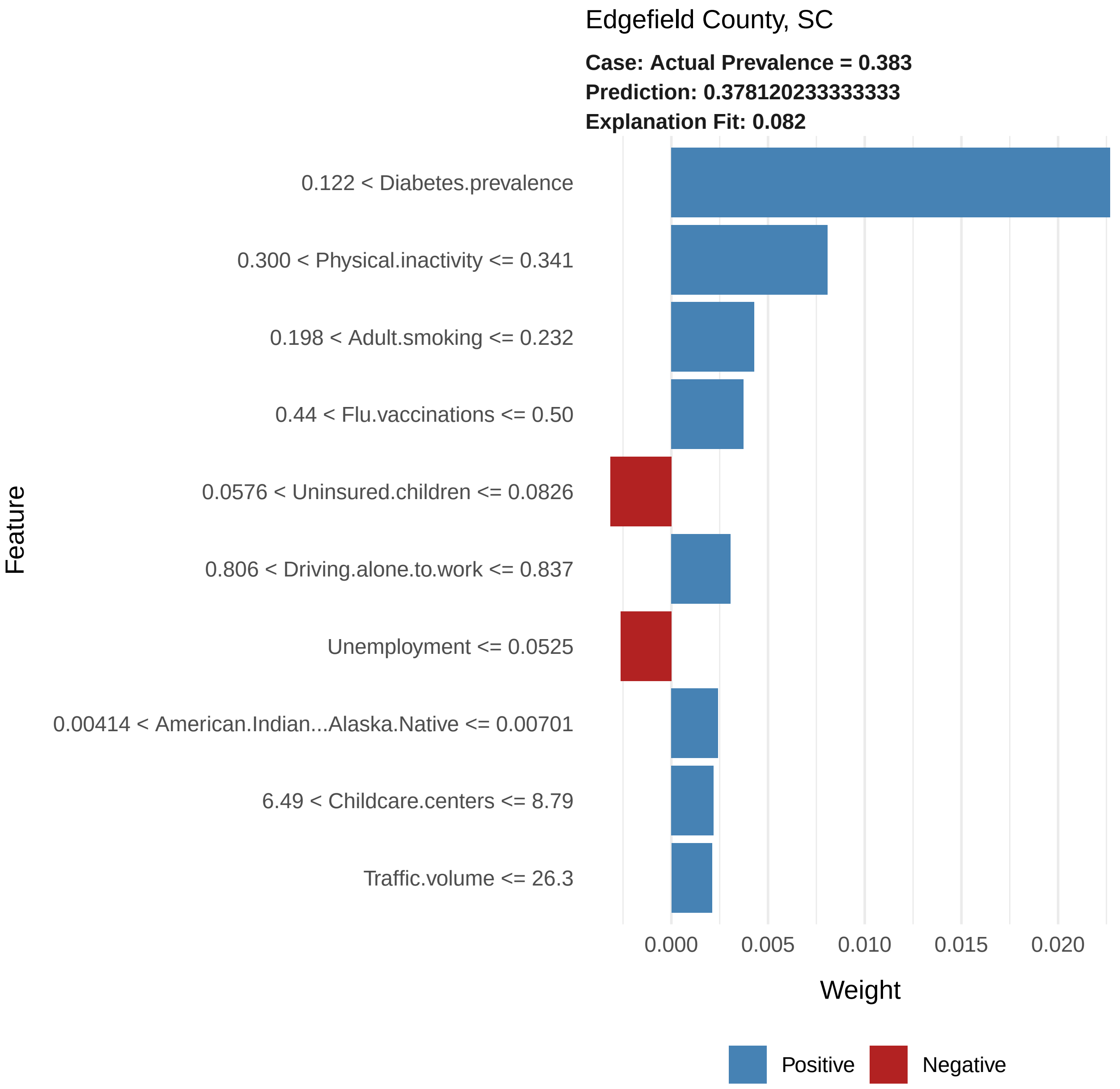
Edgefield County, SC
Case: Actual Prevalence = 0.383
Prediction: 0.378120233333333
Explanation Fit: 0.082
0.122 < Diabetes.prevalence
0.300 < Physical.inactivity <= 0.341
0.198 < Adult.smoking <= 0.232
0.44 < Flu.vaccinations <= 0.50
0.0576 < Uninsured.children <= 0.0826
0.806 < Driving.alone.to.work <= 0.837
Unemployment <= 0.0525
0.00414 < American.Indian...Alaska.Native <= 0.00701
6.49 < Childcare.centers <= 8.79
Traffic.volume <= 26.3
Feature
0.000
0.005
0.010
0.015
0.020
Weight
Positive
Negative

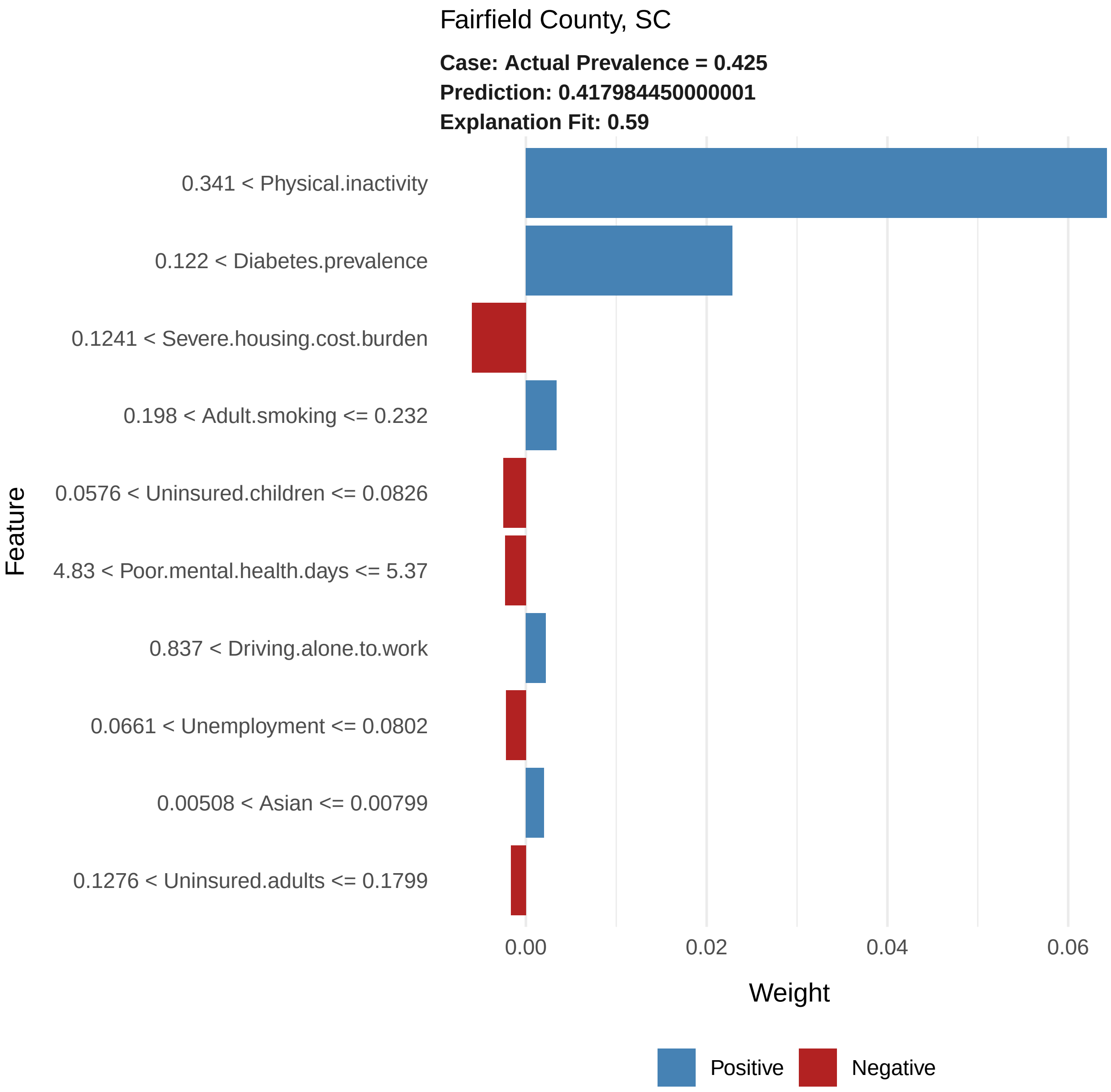
Fairfield County, SC
Case: Actual Prevalence = 0.425
Prediction: 0.417984450000001
Explanation Fit: 0.59
0.341 < Physical.inactivity
0.122 < Diabetes.prevalence
0.1241 < Severe.housing.cost.burden
0.198 < Adult.smoking <= 0.232
0.0576 < Uninsured.children <= 0.0826
4.83 < Poor.mental.health.days <= 5.37
0.837 < Driving.alone.to.work
0.0661 < Unemployment <= 0.0802
0.00508 < Asian <= 0.00799
0.1276 < Uninsured.adults <= 0.1799
Feature
0.00
0.02
0.04
0.06
Weight
Positive
Negative

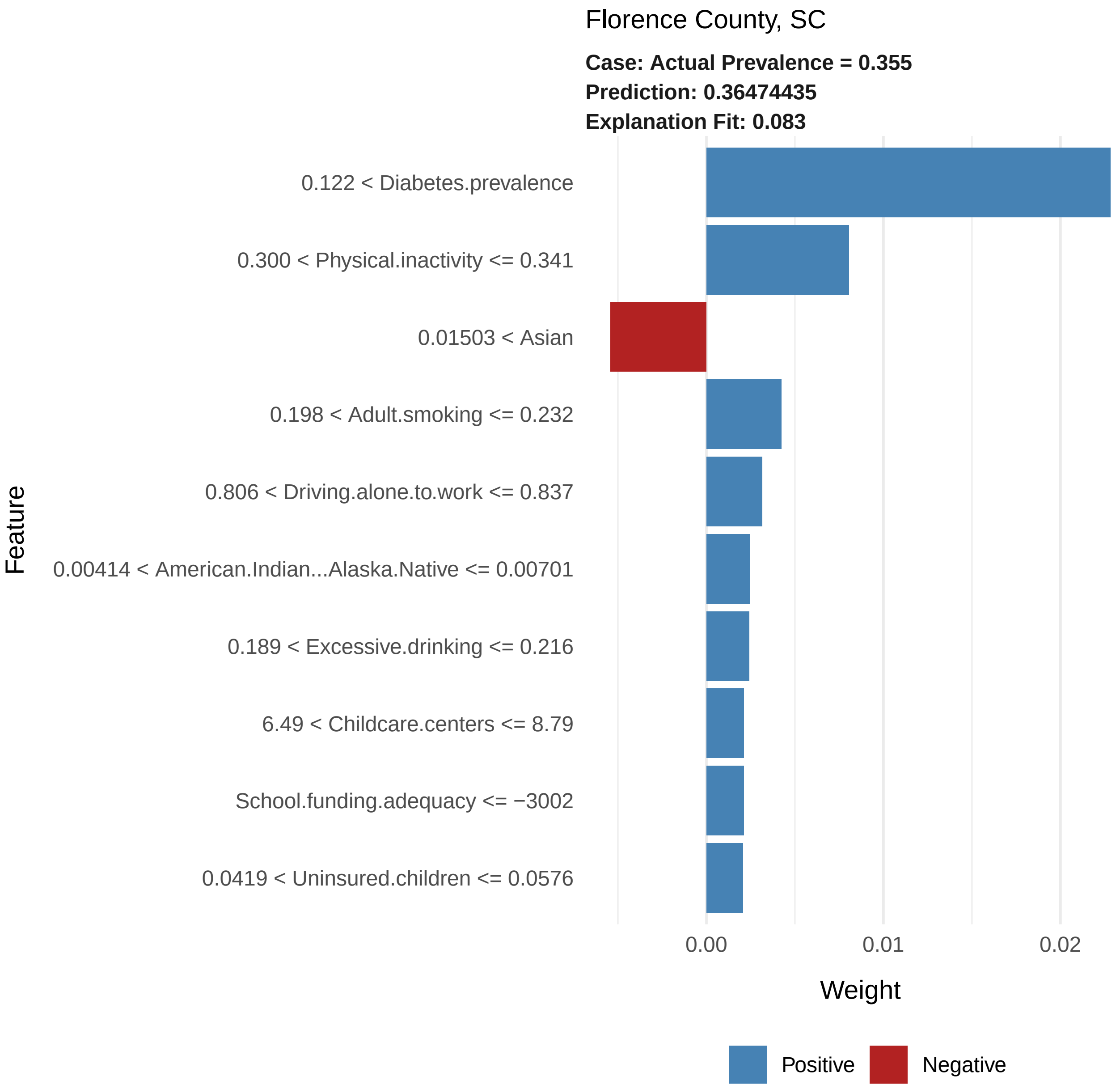
Florence County, SC
Case: Actual Prevalence = 0.355
Prediction: 0.36474435
Explanation Fit: 0.083
0.122 < Diabetes.prevalence
0.300 < Physical.inactivity <= 0.341
0.01503 < Asian
0.198 < Adult.smoking <= 0.232
0.806 < Driving.alone.to.work <= 0.837
0.00414 < American.Indian...Alaska.Native <= 0.00701
0.189 < Excessive.drinking <= 0.216
6.49 < Childcare.centers <= 8.79
School.funding.adequacy <= −3002
0.0419 < Uninsured.children <= 0.0576
Feature
0.00
0.01
0.02
Weight
Positive
Negative

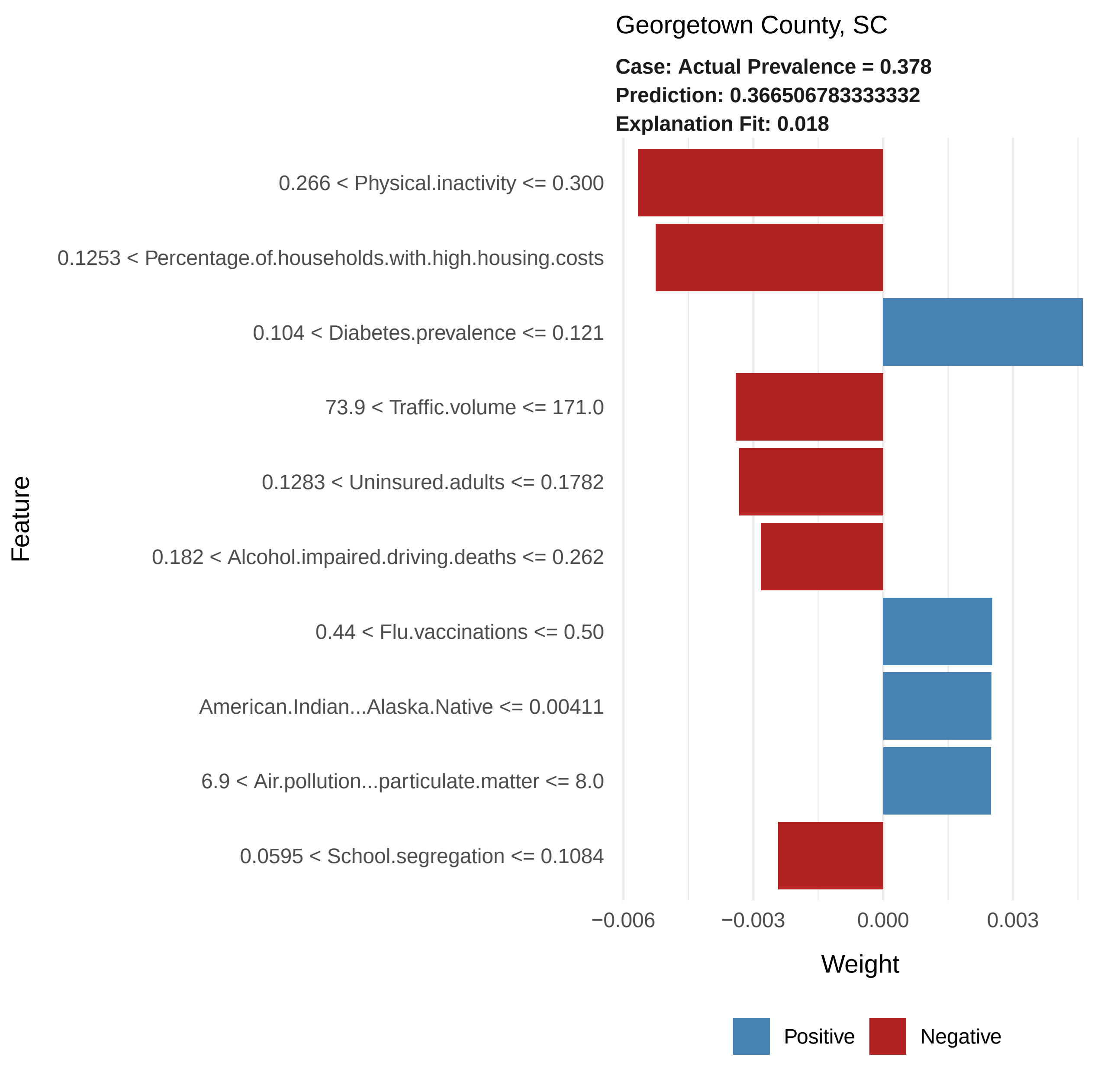
Georgetown County, SC
Case: Actual Prevalence = 0.378
Prediction: 0.366506783333332
Explanation Fit: 0.018
0.266 < Physical.inactivity <= 0.300
0.1253 < Percentage.of.households.with.high.housing.costs
0.104 < Diabetes.prevalence <= 0.121
73.9 < Traffic.volume <= 171.0
0.1283 < Uninsured.adults <= 0.1782
0.182 < Alcohol.impaired.driving.deaths <= 0.262
0.44 < Flu.vaccinations <= 0.50
American.Indian...Alaska.Native <= 0.00411
6.9 < Air.pollution...particulate.matter <= 8.0
0.0595 < School.segregation <= 0.1084
Feature
−0.006
−0.003
0.000
0.003
Weight
Positive
Negative

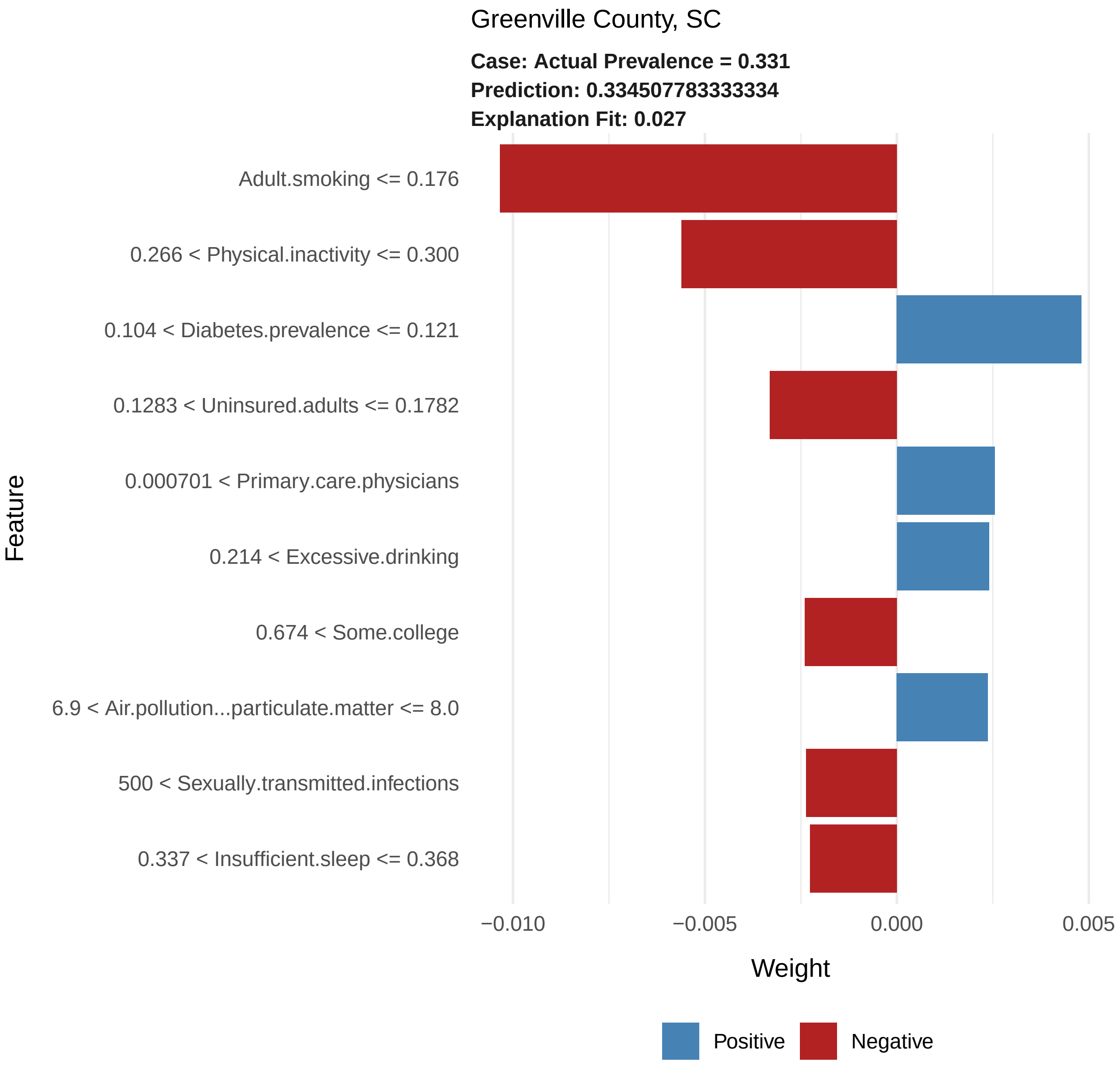
Greenville County, SC
Case: Actual Prevalence = 0.331
Prediction: 0.334507783333334
Explanation Fit: 0.027
Adult.smoking <= 0.176
0.266 < Physical.inactivity <= 0.300
0.104 < Diabetes.prevalence <= 0.121
0.1283 < Uninsured.adults <= 0.1782
0.000701 < Primary.care.physicians
0.214 < Excessive.drinking
0.674 < Some.college
6.9 < Air.pollution...particulate.matter <= 8.0
500 < Sexually.transmitted.infections
0.337 < Insufficient.sleep <= 0.368
Feature
−0.010
−0.005
0.000
0.005
Weight
Positive
Negative

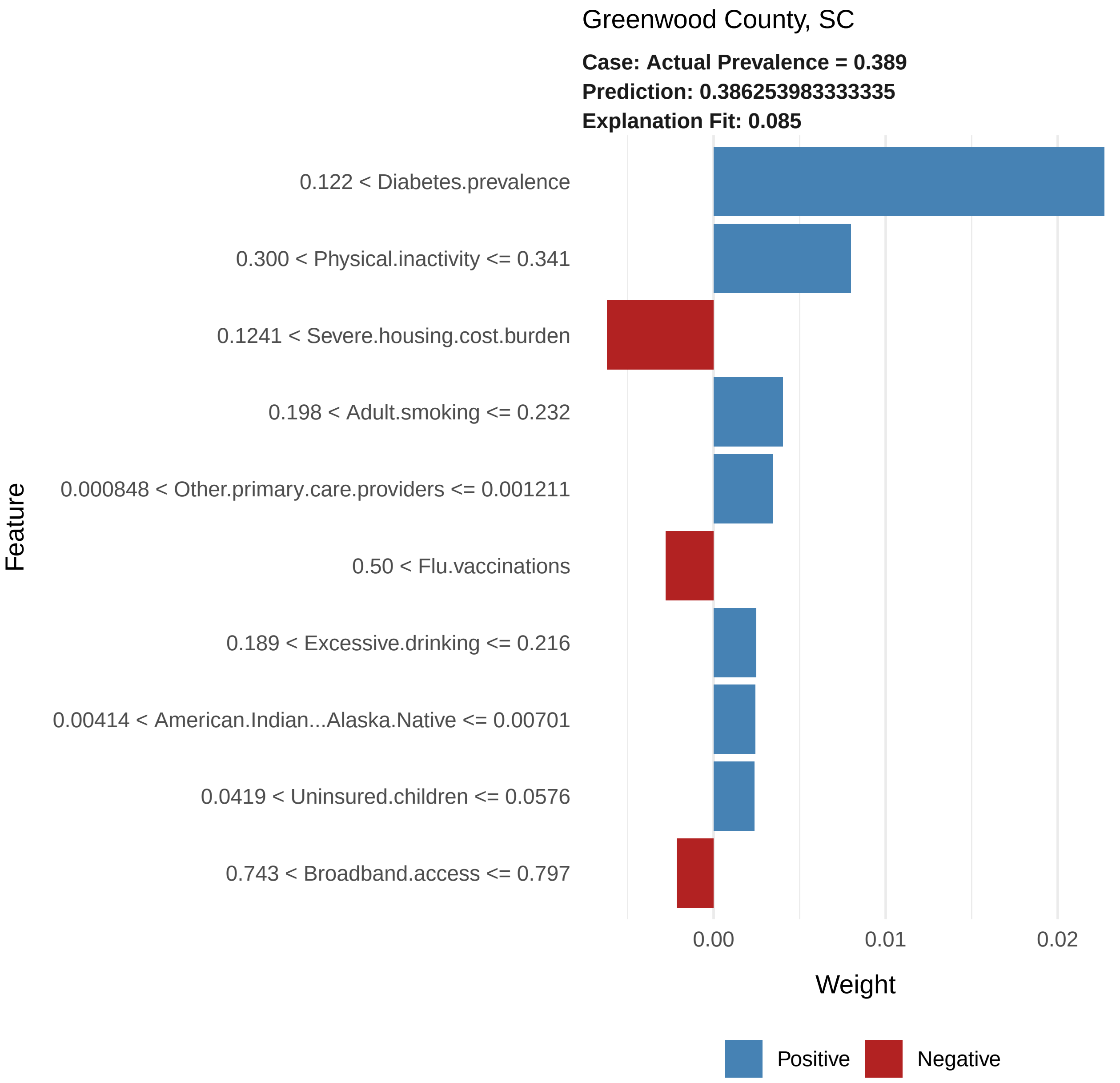

Greenwood County, SC
Case: Actual Prevalence = 0.389
Prediction: 0.386253983333335
Explanation Fit: 0.085
0.122 < Diabetes.prevalence
0.300 < Physical.inactivity <= 0.341
0.1241 < Severe.housing.cost.burden
0.198 < Adult.smoking <= 0.232
0.000848 < Other.primary.care.providers <= 0.001211
0.50 < Flu.vaccinations
0.189 < Excessive.drinking <= 0.216
0.00414 < American.Indian...Alaska.Native <= 0.00701
0.0419 < Uninsured.children <= 0.0576
0.743 < Broadband.access <= 0.797
Feature
0.00
0.01
0.02
Weight
Positive
Negative

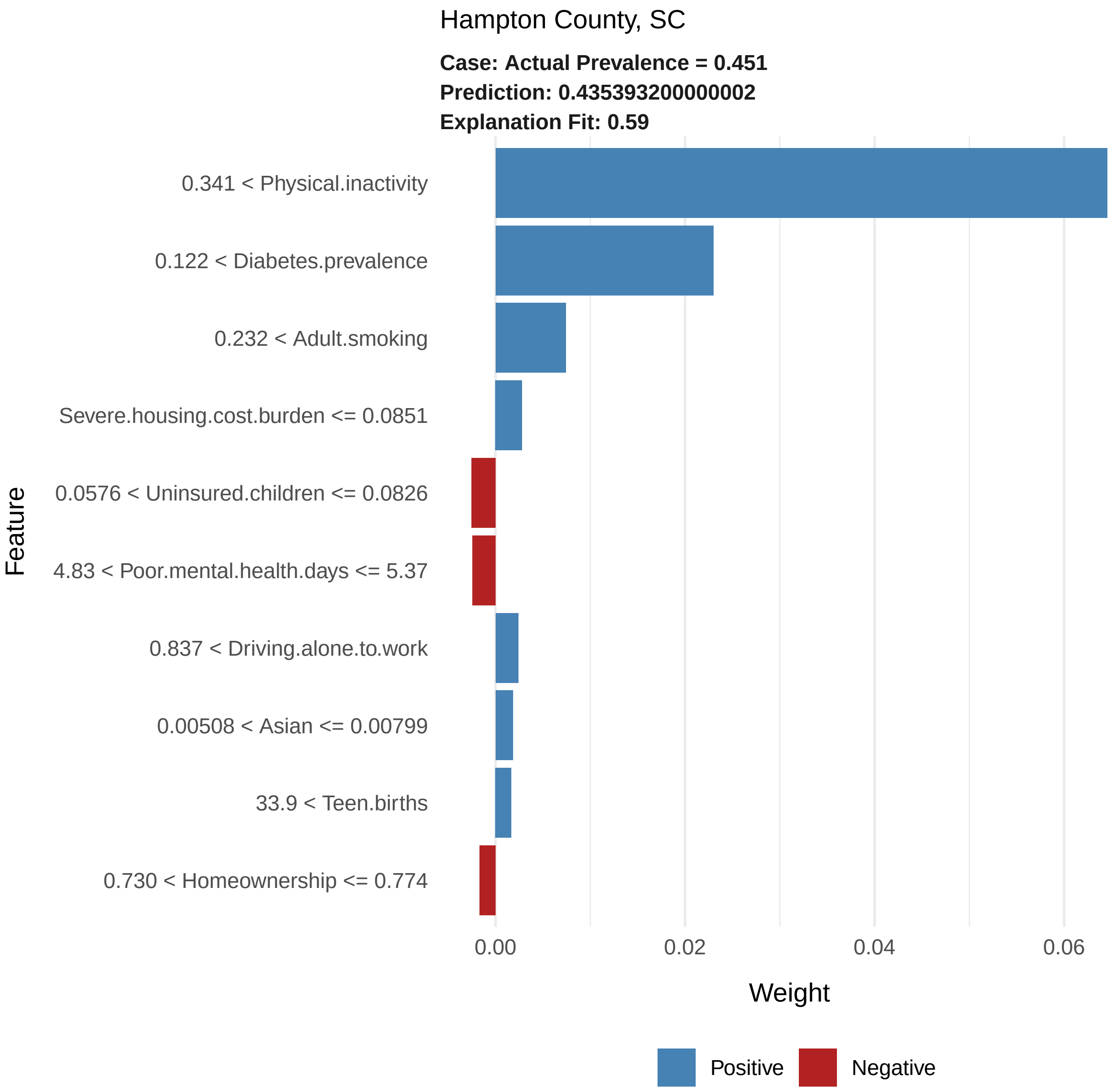
Hampton County, SC
Case: Actual Prevalence = 0.451
Prediction: 0.435393200000002
Explanation Fit: 0.59
0.341 < Physical.inactivity
0.122 < Diabetes.prevalence
0.232 < Adult.smoking
Severe.housing.cost.burden <= 0.0851
0.0576 < Uninsured.children <= 0.0826
4.83 < Poor.mental.health.days <= 5.37
0.837 < Driving.alone.to.work
0.00508 < Asian <= 0.00799
33.9 < Teen.births
0.730 < Homeownership <= 0.774
Feature
0.00
0.02
0.04
0.06
Weight
Positive
Negative

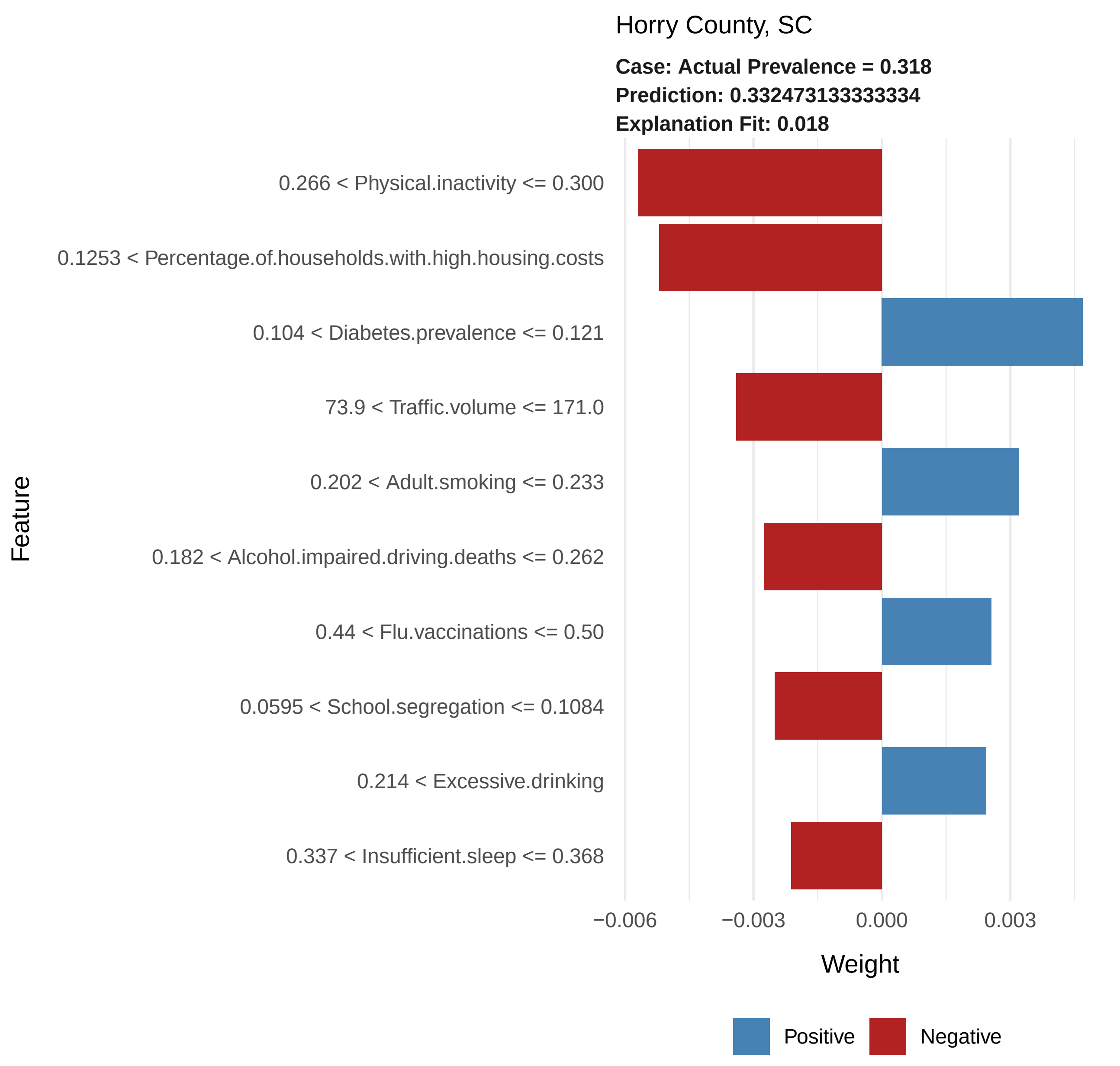

Horry County, SC
Case: Actual Prevalence = 0.318
Prediction: 0.332473133333334
Explanation Fit: 0.018
0.266 < Physical.inactivity <= 0.300
0.1253 < Percentage.of.households.with.high.housing.costs
0.104 < Diabetes.prevalence <= 0.121
73.9 < Traffic.volume <= 171.0
0.202 < Adult.smoking <= 0.233
0.182 < Alcohol.impaired.driving.deaths <= 0.262
0.44 < Flu.vaccinations <= 0.50
0.0595 < School.segregation <= 0.1084
0.214 < Excessive.drinking
0.337 < Insufficient.sleep <= 0.368
Feature
−0.006
−0.003
0.000
0.003
Weight
Positive
Negative

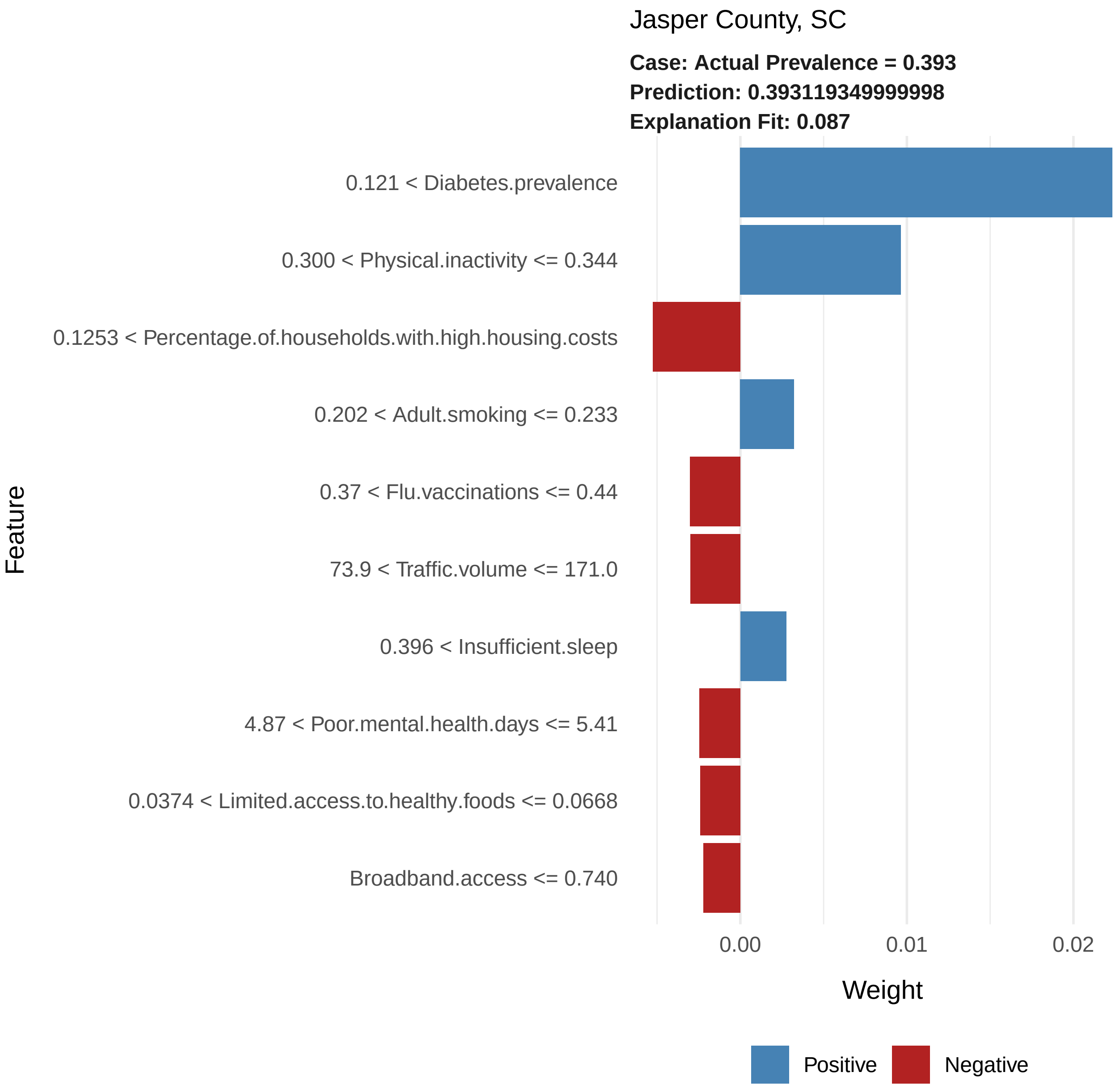

Jasper County, SC
Case: Actual Prevalence = 0.393
Prediction: 0.393119349999998
Explanation Fit: 0.087
0.121 < Diabetes.prevalence
0.300 < Physical.inactivity <= 0.344
0.1253 < Percentage.of.households.with.high.housing.costs
0.202 < Adult.smoking <= 0.233
0.37 < Flu.vaccinations <= 0.44
73.9 < Traffic.volume <= 171.0
0.396 < Insufficient.sleep
4.87 < Poor.mental.health.days <= 5.41
0.0374 < Limited.access.to.healthy.foods <= 0.0668
Broadband.access <= 0.740
Feature
0.00
0.01
0.02
Weight
Positive
Negative

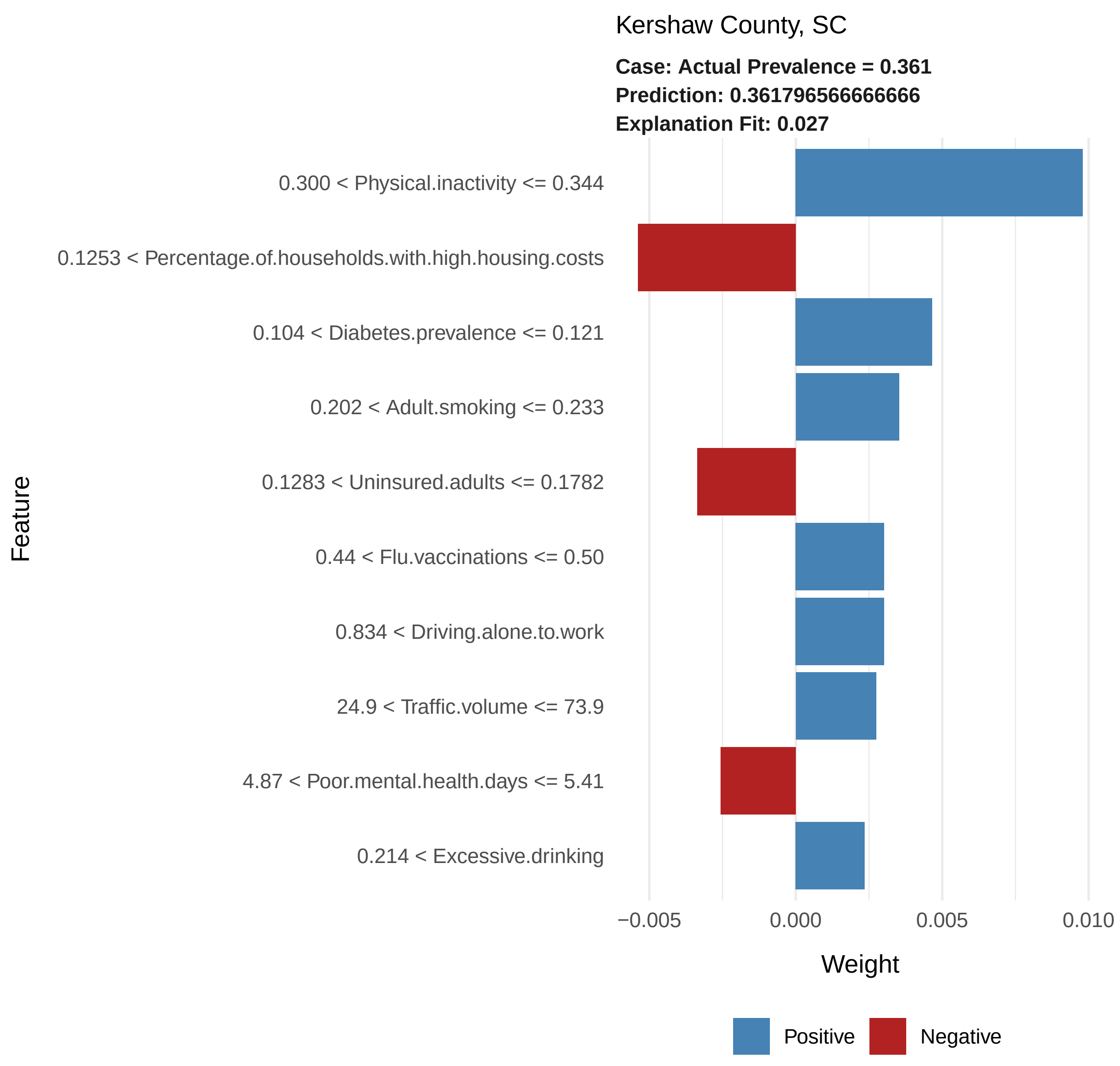

Kershaw County, SC
Case: Actual Prevalence = 0.361
Prediction: 0.361796566666666
Explanation Fit: 0.027
0.300 < Physical.inactivity <= 0.344
0.1253 < Percentage.of.households.with.high.housing.costs
0.104 < Diabetes.prevalence <= 0.121
0.202 < Adult.smoking <= 0.233
0.1283 < Uninsured.adults <= 0.1782
0.44 < Flu.vaccinations <= 0.50
0.834 < Driving.alone.to.work
24.9 < Traffic.volume <= 73.9
4.87 < Poor.mental.health.days <= 5.41
0.214 < Excessive.drinking
Feature
−0.005
0.000
0.005
0.010
Weight
Positive
Negative

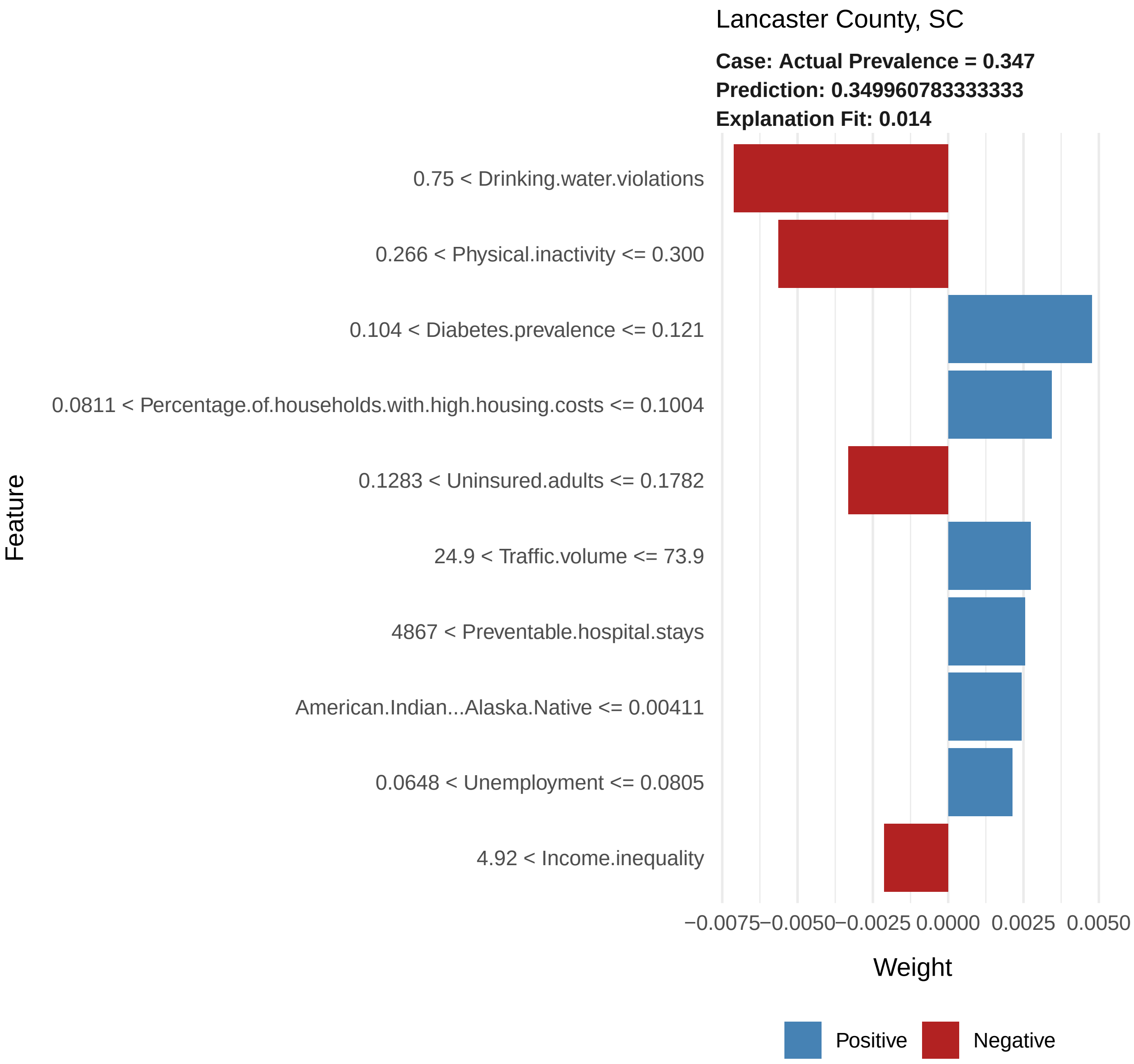
Lancaster County, SC
Case: Actual Prevalence = 0.347
Prediction: 0.349960783333333
Explanation Fit: 0.014
0.75 < Drinking.water.violations
0.266 < Physical.inactivity <= 0.300
0.104 < Diabetes.prevalence <= 0.121
0.0811 < Percentage.of.households.with.high.housing.costs <= 0.1004
0.1283 < Uninsured.adults <= 0.1782
24.9 < Traffic.volume <= 73.9
4867 < Preventable.hospital.stays
American.Indian...Alaska.Native <= 0.00411
0.0648 < Unemployment <= 0.0805
4.92 < Income.inequality
Feature
−0.0075
−0.0050
−0.0025
0.0000
0.0025
0.0050
Weight
Positive
Negative

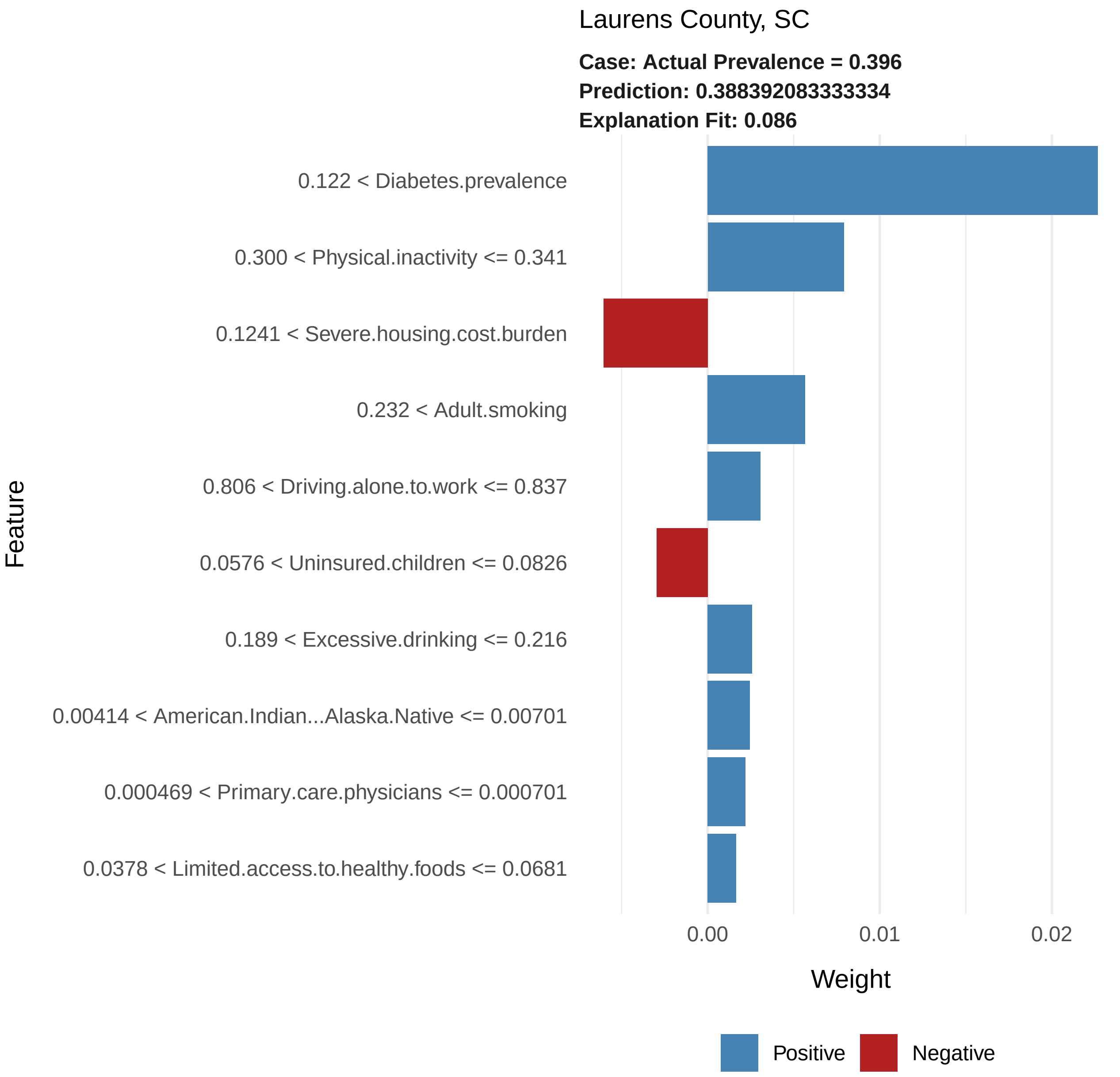

Laurens County, SC
Case: Actual Prevalence = 0.396
Prediction: 0.388392083333334
Explanation Fit: 0.086
0.122 < Diabetes.prevalence
0.300 < Physical.inactivity <= 0.341
0.1241 < Severe.housing.cost.burden
0.232 < Adult.smoking
0.806 < Driving.alone.to.work <= 0.837
0.0576 < Uninsured.children <= 0.0826
0.189 < Excessive.drinking <= 0.216
0.00414 < American.Indian...Alaska.Native <= 0.00701
0.000469 < Primary.care.physicians <= 0.000701
0.0378 < Limited.access.to.healthy.foods <= 0.0681
Feature
0.00
0.01
0.02
Weight
Positive
Negative

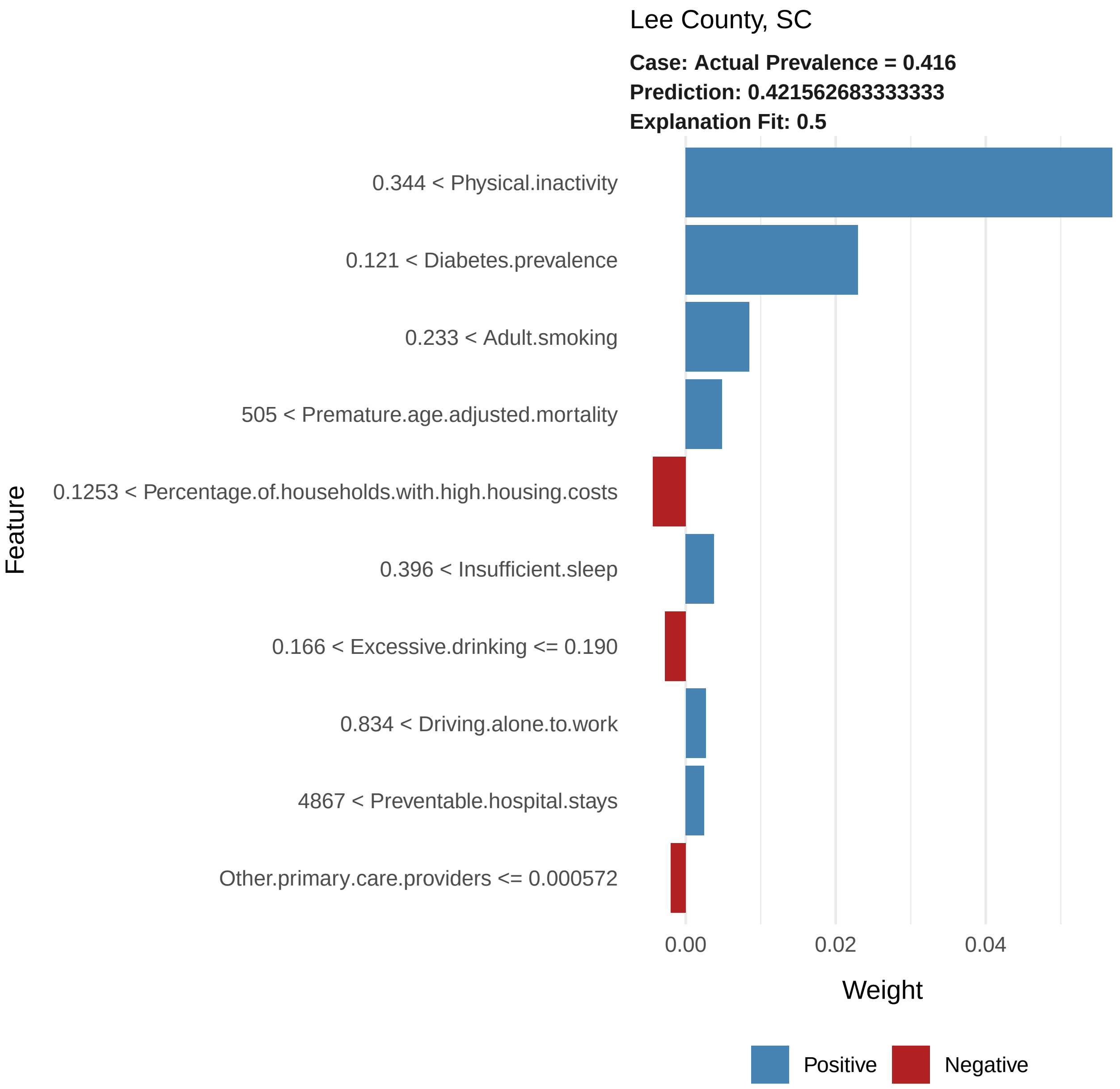
Lee County, SC
Case: Actual Prevalence = 0.416
Prediction: 0.421562683333333
Explanation Fit: 0.5
0.344 < Physical.inactivity
0.121 < Diabetes.prevalence
0.233 < Adult.smoking
505 < Premature.age.adjusted.mortality
0.1253 < Percentage.of.households.with.high.housing.costs
0.396 < Insufficient.sleep
0.166 < Excessive.drinking <= 0.190
0.834 < Driving.alone.to.work
4867 < Preventable.hospital.stays
Other.primary.care.providers <= 0.000572
Feature
0.00
0.02
0.04
Weight
Positive
Negative

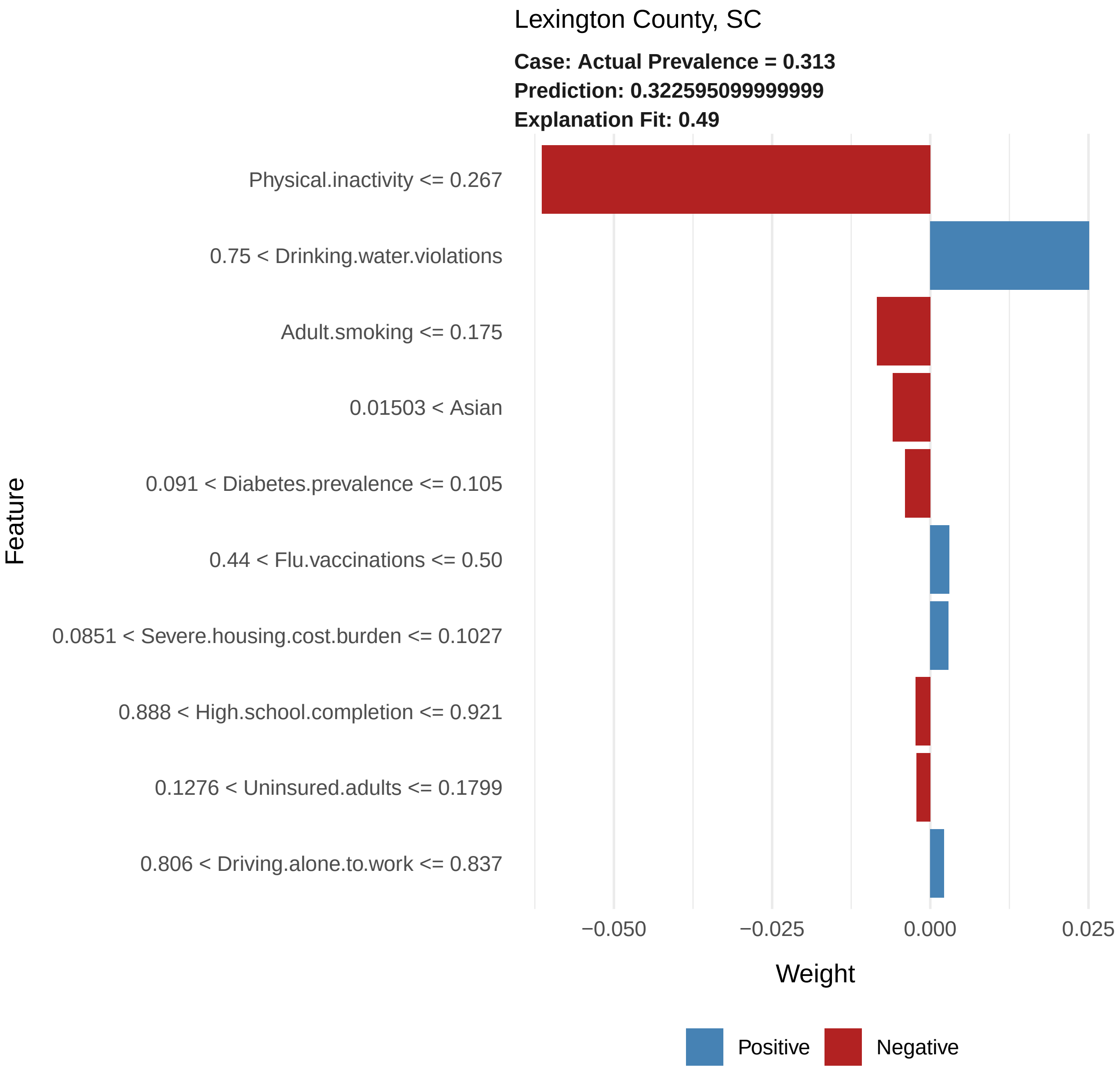

Lexington County, SC
Case: Actual Prevalence = 0.313
Prediction: 0.322595099999999
Explanation Fit: 0.49
Physical.inactivity <= 0.267
0.75 < Drinking.water.violations
Adult.smoking <= 0.175
0.01503 < Asian
0.091 < Diabetes.prevalence <= 0.105
0.44 < Flu.vaccinations <= 0.50
0.0851 < Severe.housing.cost.burden <= 0.1027
0.888 < High.school.completion <= 0.921
0.1276 < Uninsured.adults <= 0.1799
0.806 < Driving.alone.to.work <= 0.837
Feature
−0.050
−0.025
0.000
0.025
Weight
Positive
Negative

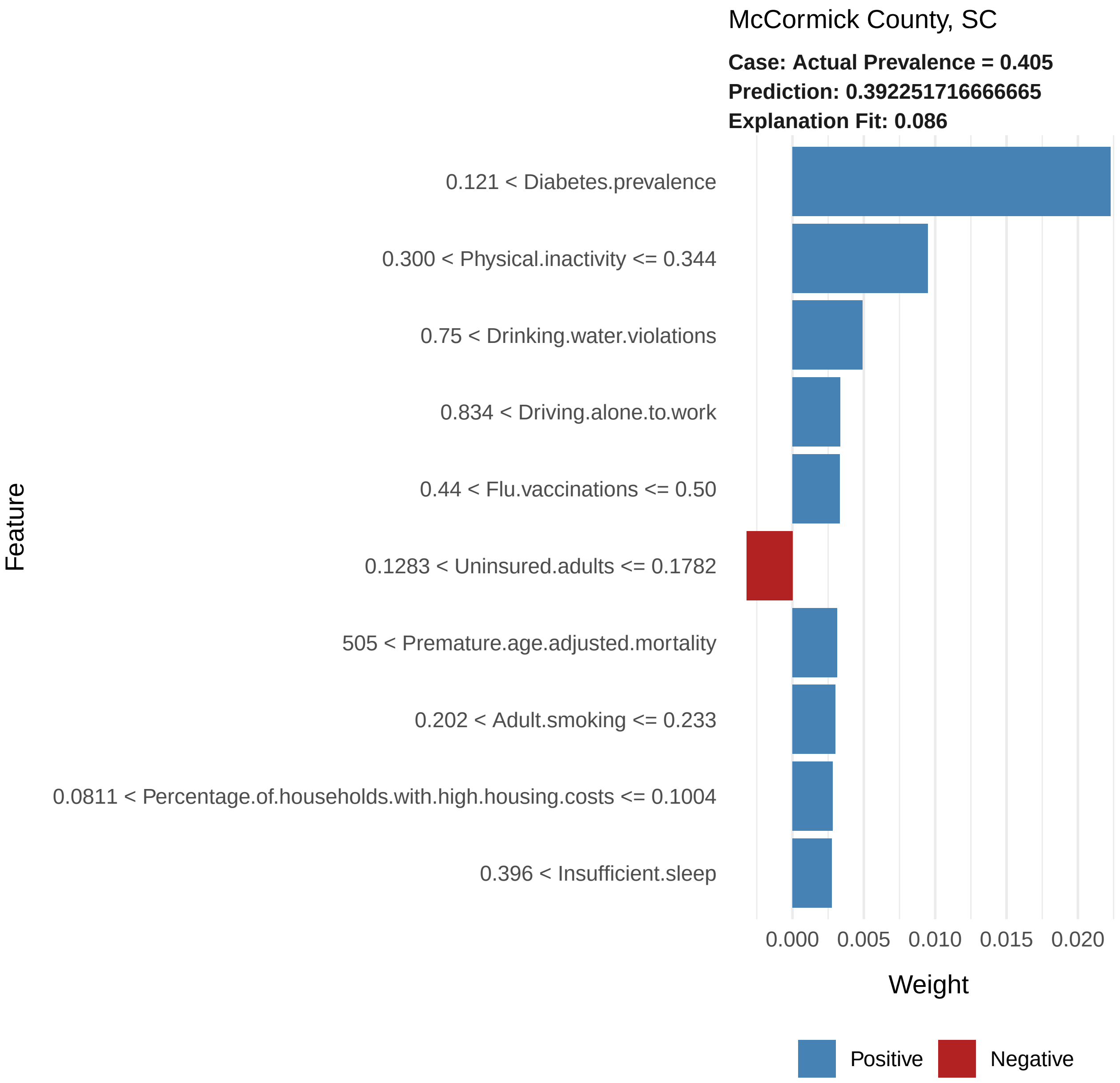
McCormick County, SC
Case: Actual Prevalence = 0.405
Prediction: 0.392251716666665
Explanation Fit: 0.086
0.121 < Diabetes.prevalence
0.300 < Physical.inactivity <= 0.344
0.75 < Drinking.water.violations
0.834 < Driving.alone.to.work
0.44 < Flu.vaccinations <= 0.50
0.1283 < Uninsured.adults <= 0.1782
505 < Premature.age.adjusted.mortality
0.202 < Adult.smoking <= 0.233
0.0811 < Percentage.of.households.with.high.housing.costs <= 0.1004
0.396 < Insufficient.sleep
Feature
0.000
0.005
0.010
0.015
0.020
Weight
Positive
Negative

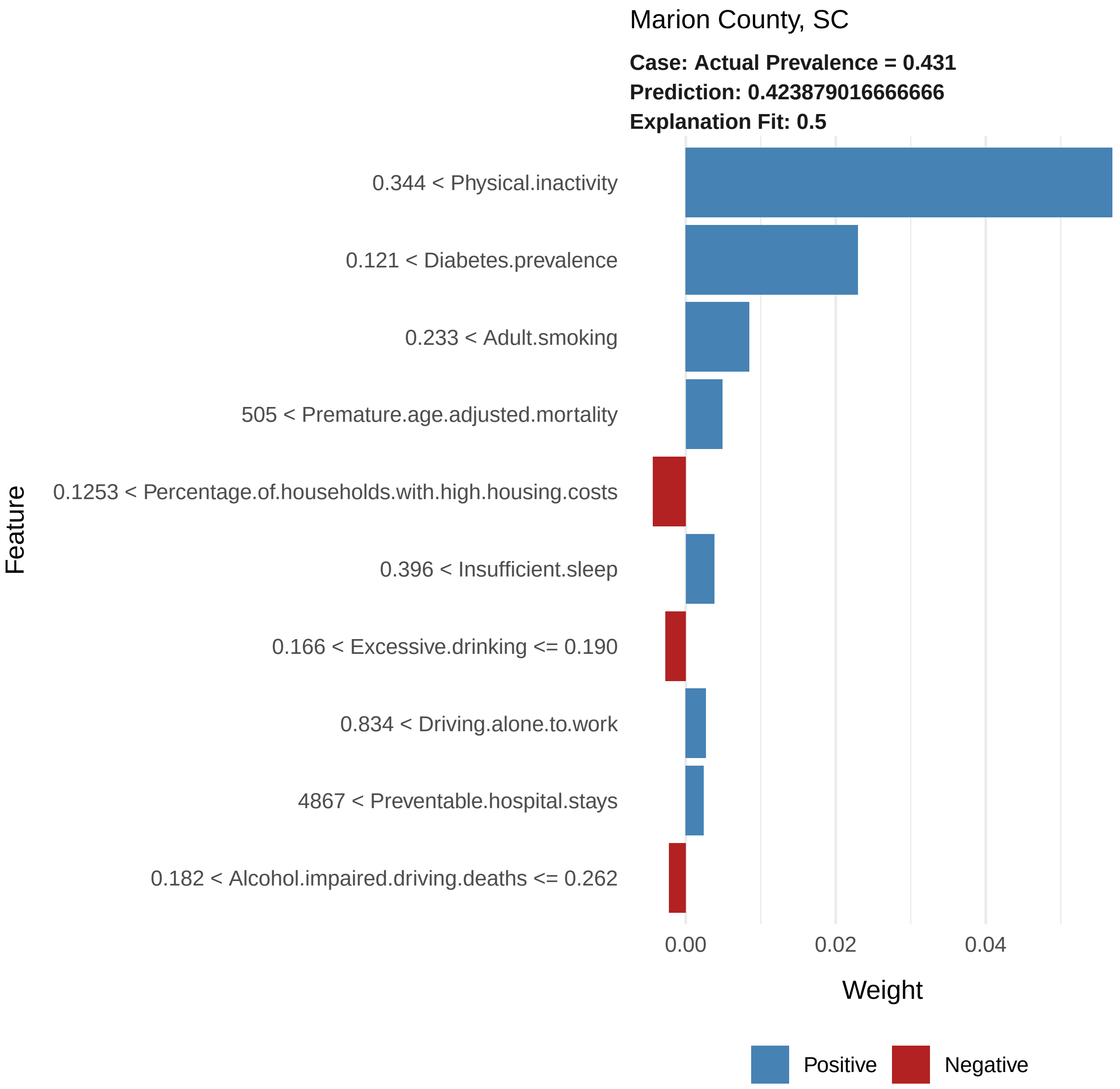
Marion County, SC
Case: Actual Prevalence = 0.431
Prediction: 0.423879016666666
Explanation Fit: 0.5
0.344 < Physical.inactivity
0.121 < Diabetes.prevalence
0.233 < Adult.smoking
505 < Premature.age.adjusted.mortality
0.1253 < Percentage.of.households.with.high.housing.costs
0.396 < Insufficient.sleep
0.166 < Excessive.drinking <= 0.190
0.834 < Driving.alone.to.work
4867 < Preventable.hospital.stays
0.182 < Alcohol.impaired.driving.deaths <= 0.262
Feature
0.00
0.02
0.04
Weight
Positive
Negative

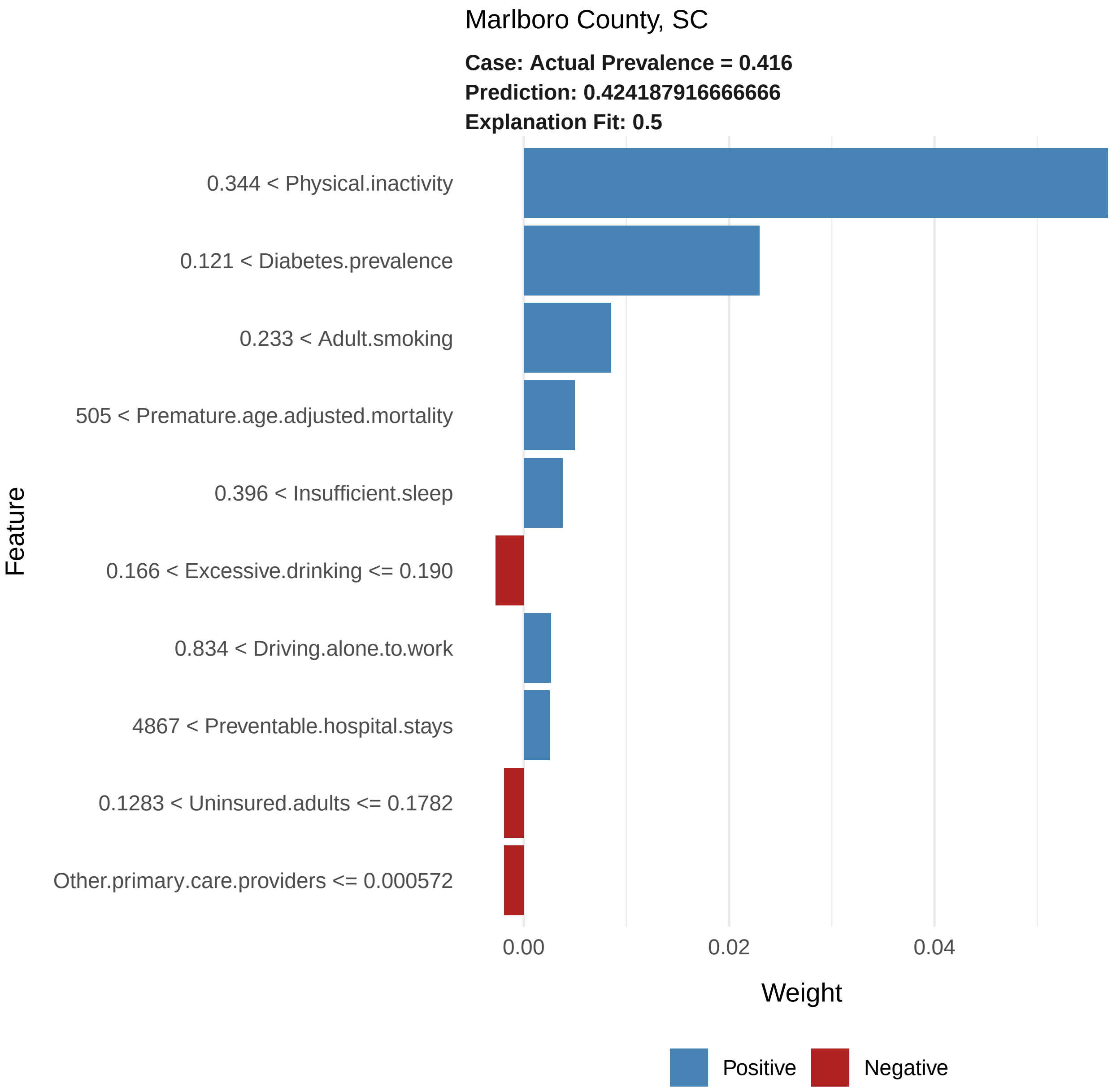

Marlboro County, SC
Case: Actual Prevalence = 0.416
Prediction: 0.424187916666666
Explanation Fit: 0.5
0.344 < Physical.inactivity
0.121 < Diabetes.prevalence
0.233 < Adult.smoking
505 < Premature.age.adjusted.mortality
0.396 < Insufficient.sleep
0.166 < Excessive.drinking <= 0.190
0.834 < Driving.alone.to.work
4867 < Preventable.hospital.stays
0.1283 < Uninsured.adults <= 0.1782
Other.primary.care.providers <= 0.000572
Feature
0.00
0.02
0.04
Weight
Positive
Negative

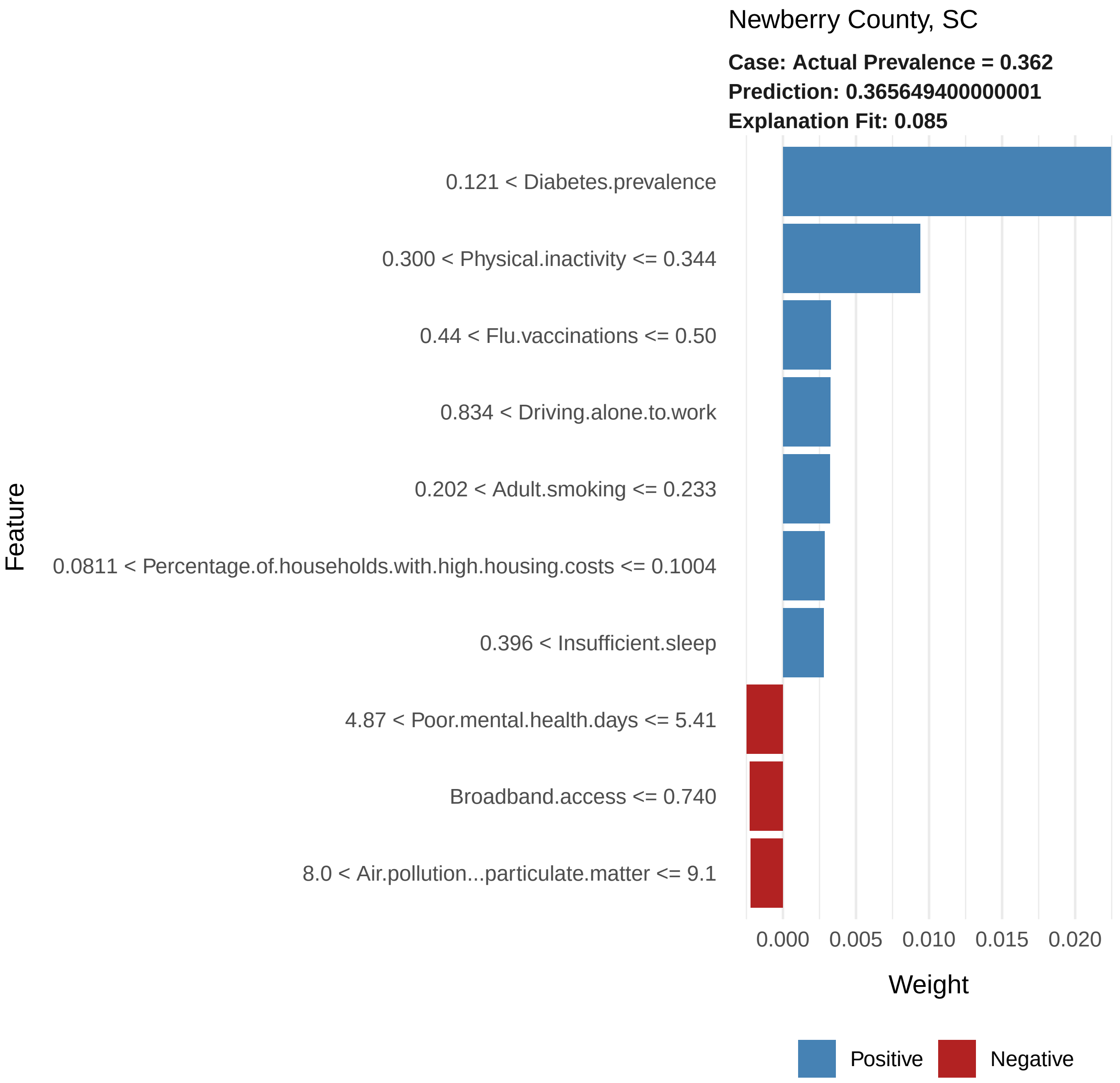

Newberry County, SC
Case: Actual Prevalence = 0.362
Prediction: 0.365649400000001
Explanation Fit: 0.085
0.121 < Diabetes.prevalence
0.300 < Physical.inactivity <= 0.344
0.44 < Flu.vaccinations <= 0.50
0.834 < Driving.alone.to.work
0.202 < Adult.smoking <= 0.233
0.0811 < Percentage.of.households.with.high.housing.costs <= 0.1004
0.396 < Insufficient.sleep
4.87 < Poor.mental.health.days <= 5.41
Broadband.access <= 0.740
8.0 < Air.pollution...particulate.matter <= 9.1
Feature
0.000
0.005
0.010
0.015
0.020
Weight
Positive
Negative

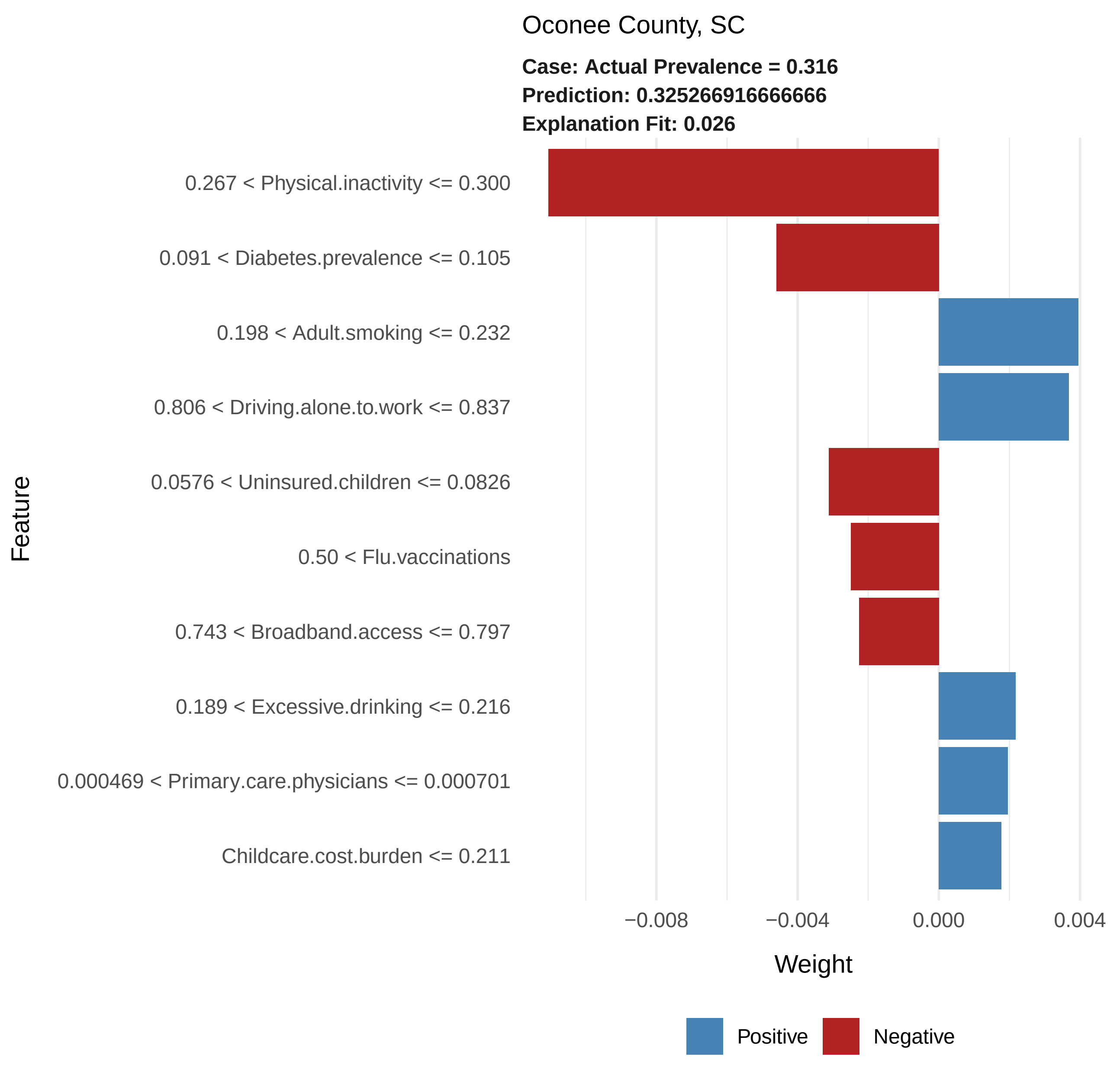

Oconee County, SC
Case: Actual Prevalence = 0.316
Prediction: 0.325266916666666
Explanation Fit: 0.026
0.267 < Physical.inactivity <= 0.300
0.091 < Diabetes.prevalence <= 0.105
0.198 < Adult.smoking <= 0.232
0.806 < Driving.alone.to.work <= 0.837
0.0576 < Uninsured.children <= 0.0826
0.50 < Flu.vaccinations
0.743 < Broadband.access <= 0.797
0.189 < Excessive.drinking <= 0.216
0.000469 < Primary.care.physicians <= 0.000701
Childcare.cost.burden <= 0.211
Feature
−0.008
−0.004
0.000
0.004
Weight
Positive
Negative

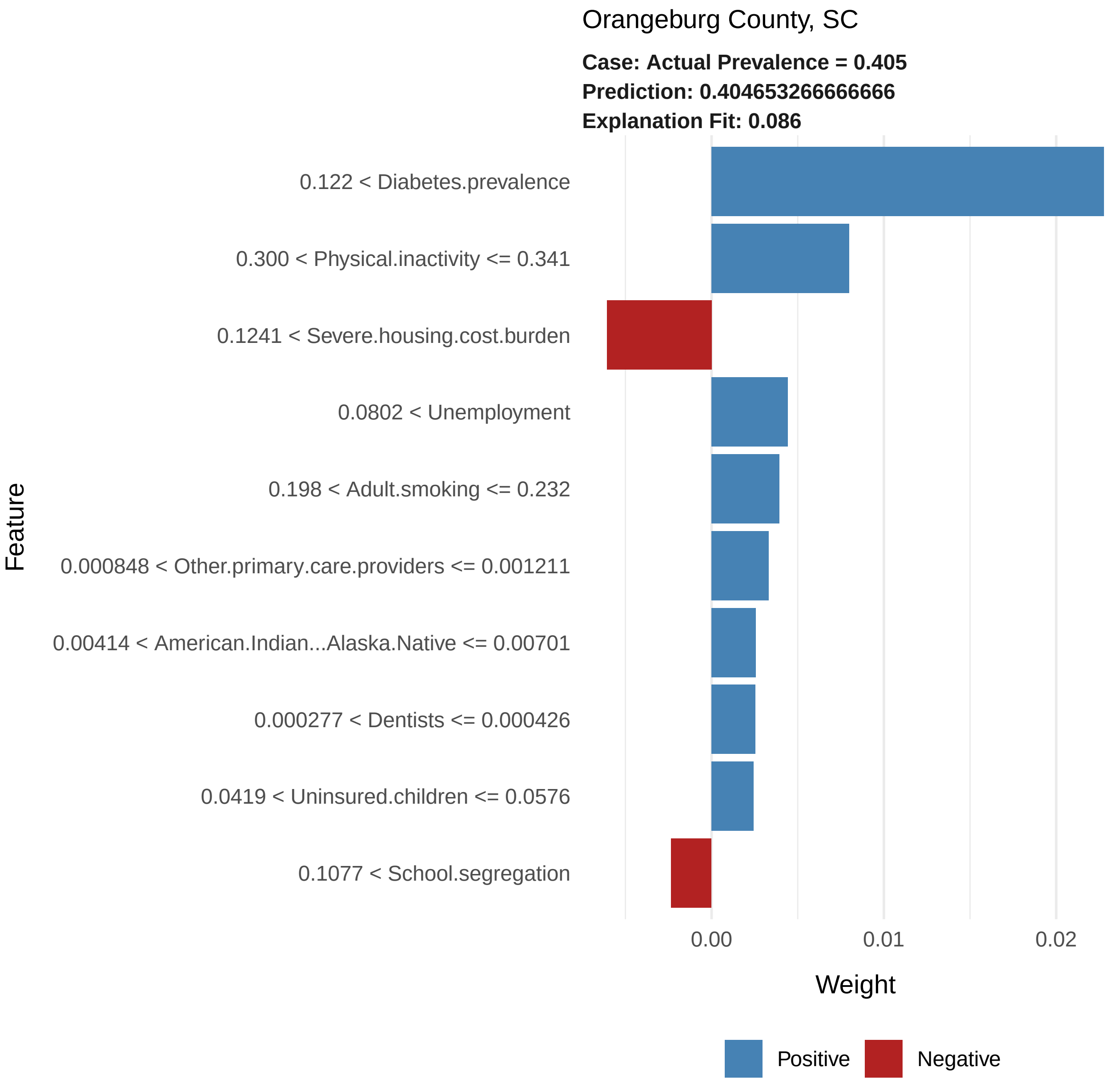
Orangeburg County, SC
Case: Actual Prevalence = 0.405
Prediction: 0.404653266666666
Explanation Fit: 0.086
0.122 < Diabetes.prevalence
0.300 < Physical.inactivity <= 0.341
0.1241 < Severe.housing.cost.burden
0.0802 < Unemployment
0.198 < Adult.smoking <= 0.232
0.000848 < Other.primary.care.providers <= 0.001211
0.00414 < American.Indian...Alaska.Native <= 0.00701
0.000277 < Dentists <= 0.000426
0.0419 < Uninsured.children <= 0.0576
0.1077 < School.segregation
Feature
0.00
0.01
0.02
Weight
Positive
Negative

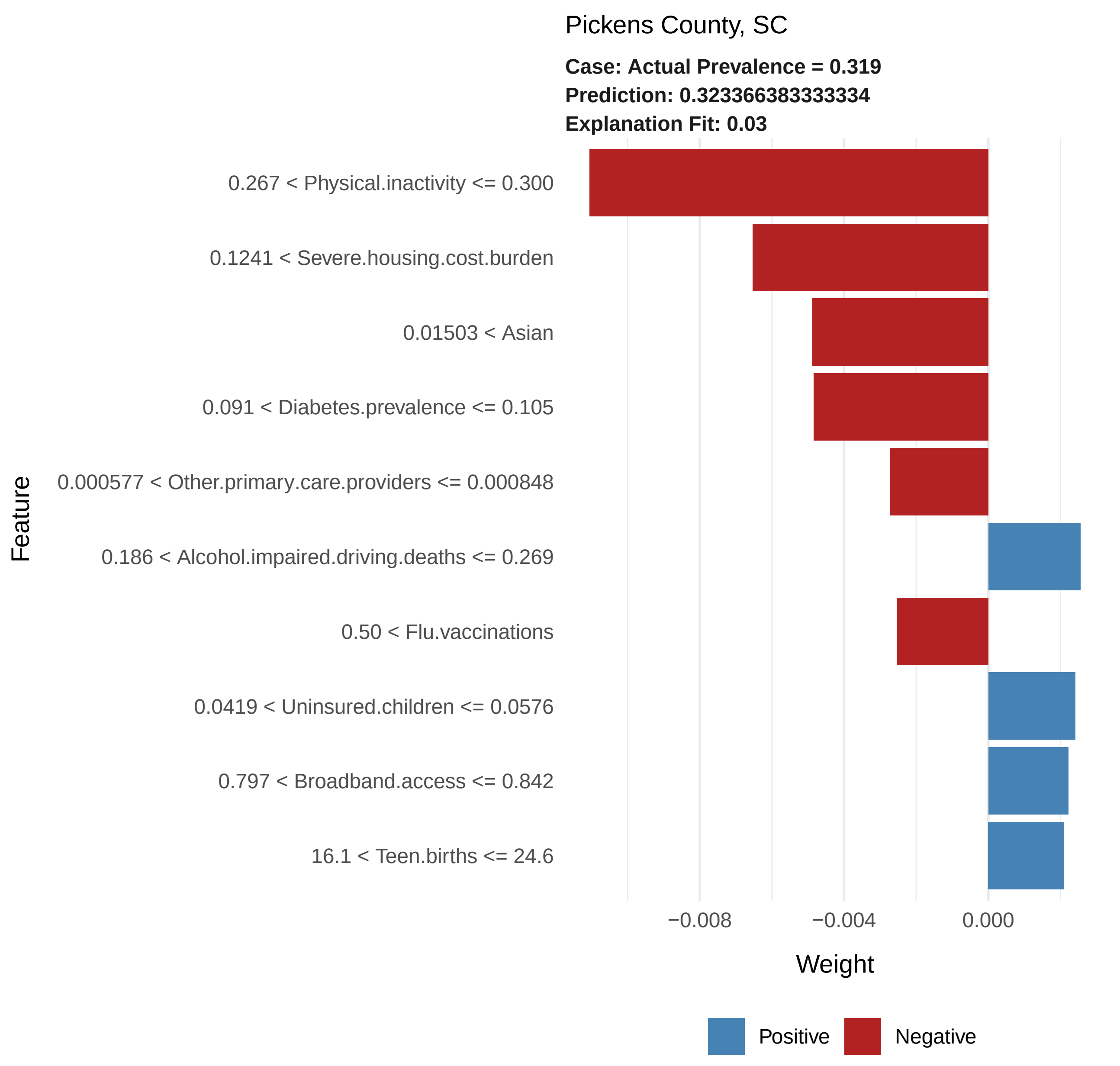

Pickens County, SC
Case: Actual Prevalence = 0.319
Prediction: 0.323366383333334
Explanation Fit: 0.03
0.267 < Physical.inactivity <= 0.300
0.1241 < Severe.housing.cost.burden
0.01503 < Asian
0.091 < Diabetes.prevalence <= 0.105
0.000577 < Other.primary.care.providers <= 0.000848
0.186 < Alcohol.impaired.driving.deaths <= 0.269
0.50 < Flu.vaccinations
0.0419 < Uninsured.children <= 0.0576
0.797 < Broadband.access <= 0.842
16.1 < Teen.births <= 24.6
Feature
−0.008
−0.004
0.000
Weight
Positive
Negative

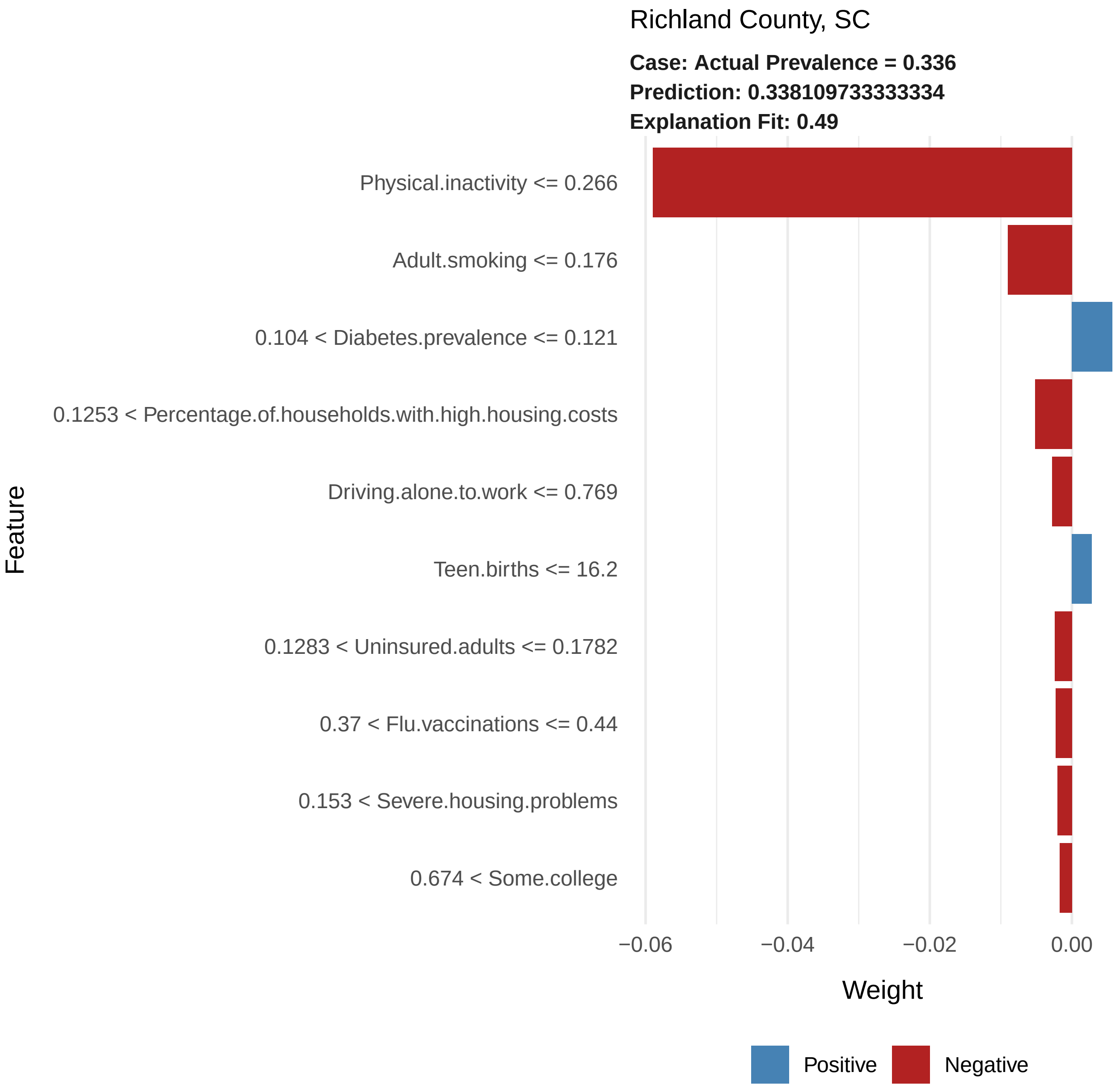

Richland County, SC
Case: Actual Prevalence = 0.336
Prediction: 0.338109733333334
Explanation Fit: 0.49
Physical.inactivity <= 0.266
Adult.smoking <= 0.176
0.104 < Diabetes.prevalence <= 0.121
0.1253 < Percentage.of.households.with.high.housing.costs
Driving.alone.to.work <= 0.769
Teen.births <= 16.2
0.1283 < Uninsured.adults <= 0.1782
0.37 < Flu.vaccinations <= 0.44
0.153 < Severe.housing.problems
0.674 < Some.college
Feature
−0.06
−0.04
−0.02
0.00
Weight
Positive
Negative

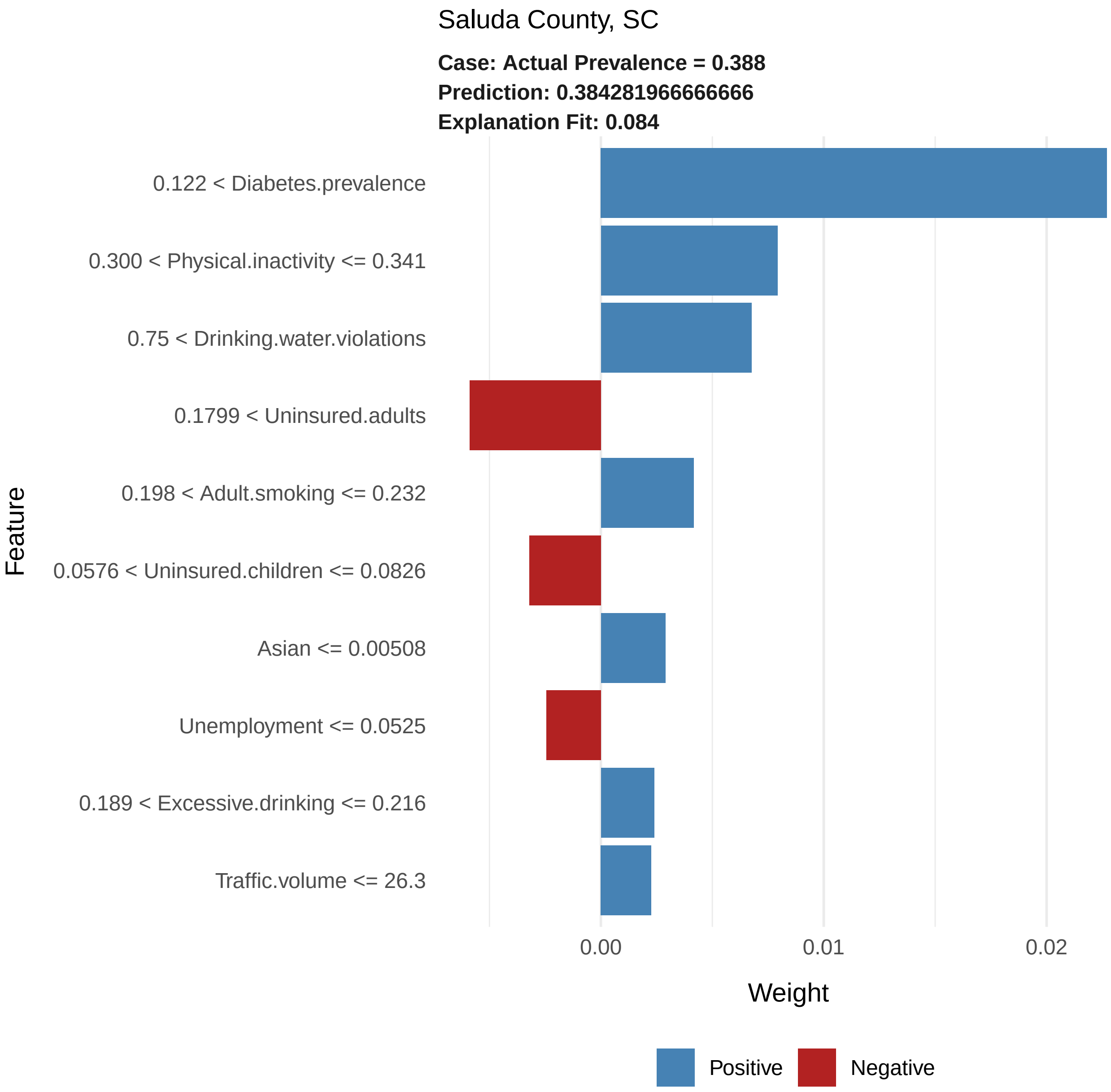

Saluda County, SC
Case: Actual Prevalence = 0.388
Prediction: 0.384281966666666
Explanation Fit: 0.084
0.122 < Diabetes.prevalence
0.300 < Physical.inactivity <= 0.341
0.75 < Drinking.water.violations
0.1799 < Uninsured.adults
0.198 < Adult.smoking <= 0.232
0.0576 < Uninsured.children <= 0.0826
Asian <= 0.00508
Unemployment <= 0.0525
0.189 < Excessive.drinking <= 0.216
Traffic.volume <= 26.3
Feature
0.00
0.01
0.02
Weight
Positive
Negative

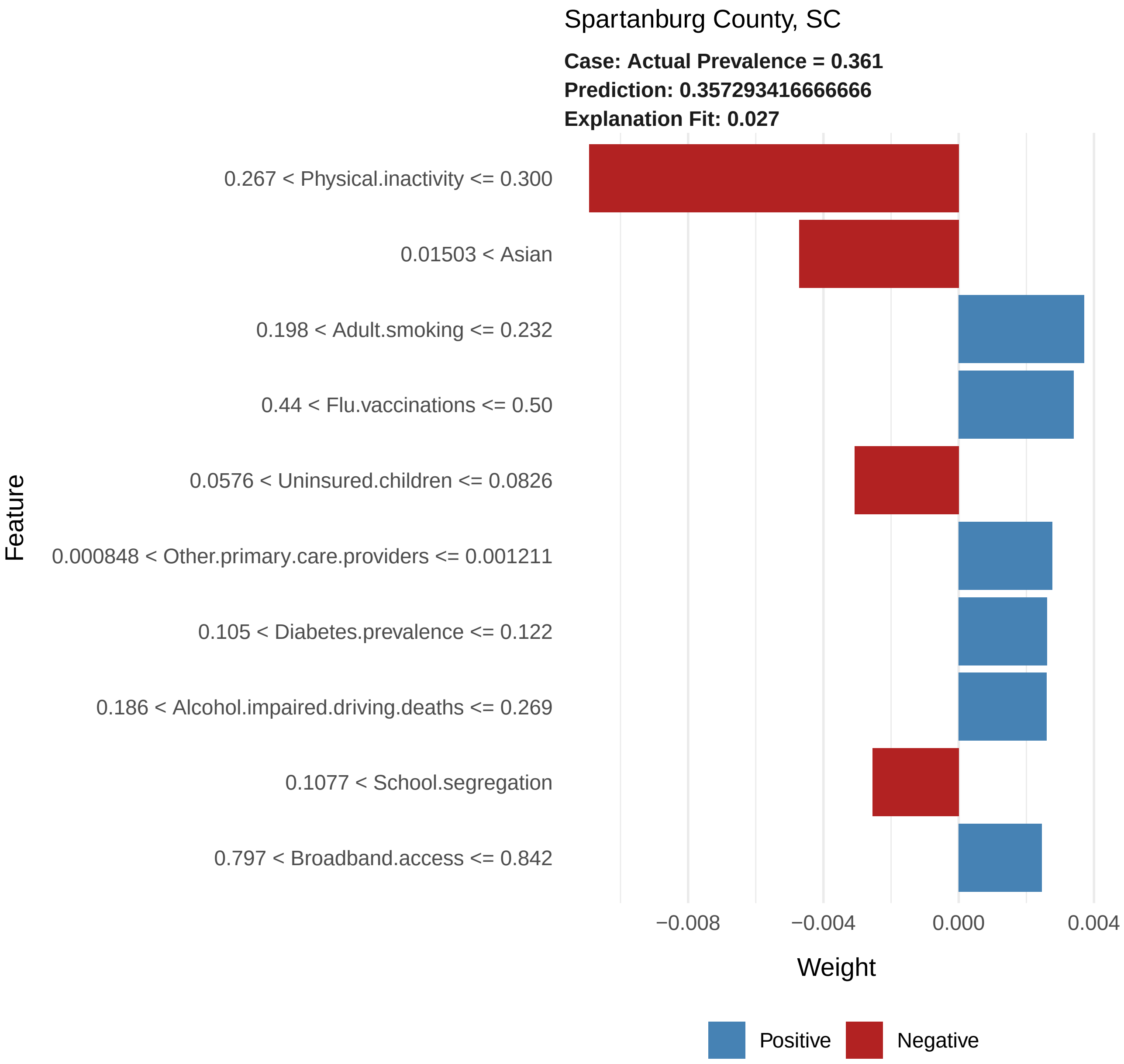

Spartanburg County, SC
Case: Actual Prevalence = 0.361
Prediction: 0.357293416666666
Explanation Fit: 0.027
0.267 < Physical.inactivity <= 0.300
0.01503 < Asian
0.198 < Adult.smoking <= 0.232
0.44 < Flu.vaccinations <= 0.50
0.0576 < Uninsured.children <= 0.0826
0.000848 < Other.primary.care.providers <= 0.001211
0.105 < Diabetes.prevalence <= 0.122
0.186 < Alcohol.impaired.driving.deaths <= 0.269
0.1077 < School.segregation
0.797 < Broadband.access <= 0.842
Feature
−0.008
−0.004
0.000
0.004
Weight
Positive
Negative

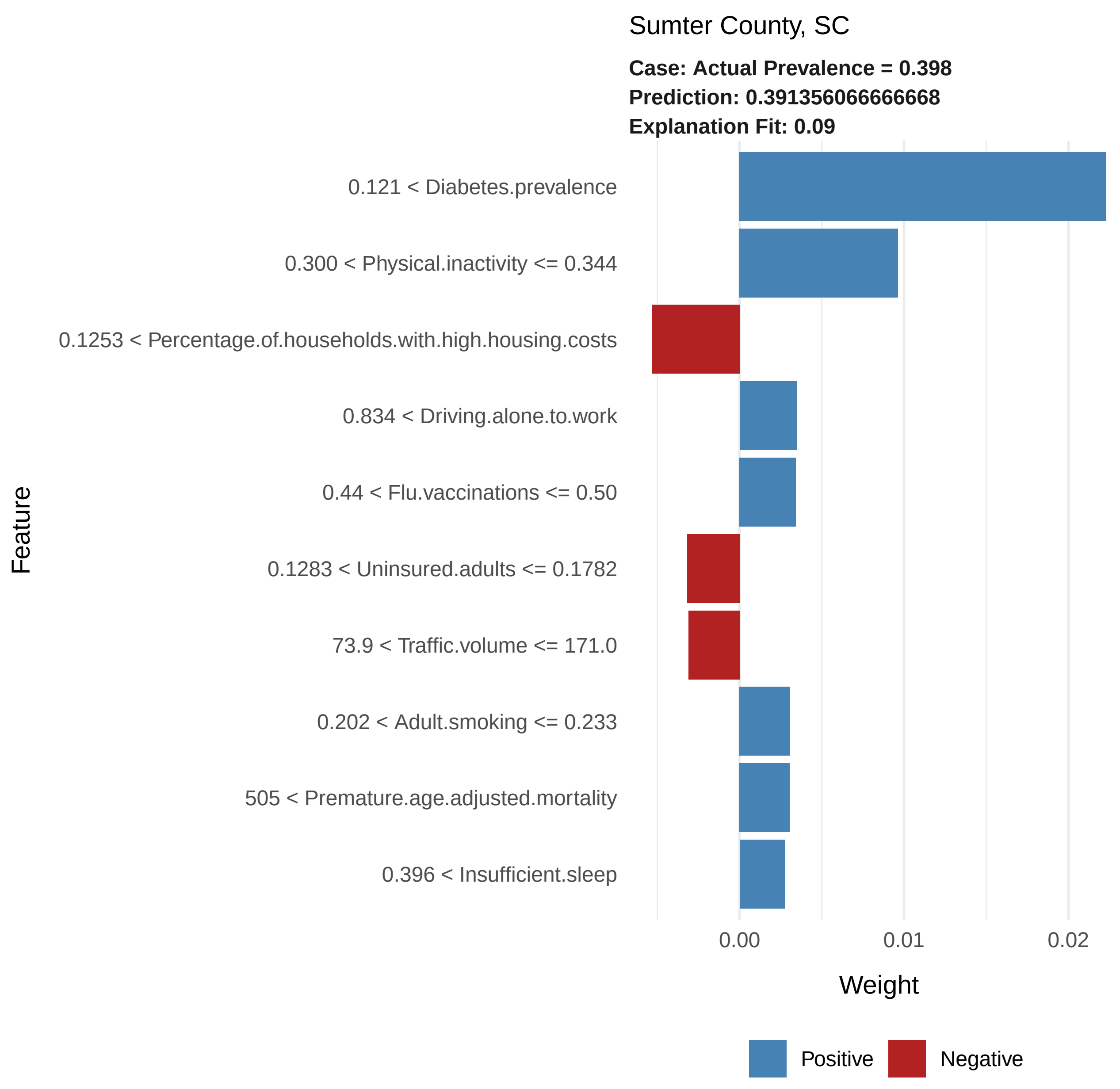
Sumter County, SC
Case: Actual Prevalence = 0.398
Prediction: 0.391356066666668
Explanation Fit: 0.09
0.121 < Diabetes.prevalence
0.300 < Physical.inactivity <= 0.344
0.1253 < Percentage.of.households.with.high.housing.costs
0.834 < Driving.alone.to.work
0.44 < Flu.vaccinations <= 0.50
0.1283 < Uninsured.adults <= 0.1782
73.9 < Traffic.volume <= 171.0
0.202 < Adult.smoking <= 0.233
505 < Premature.age.adjusted.mortality
0.396 < Insufficient.sleep
Feature
0.00
0.01
0.02
Weight
Positive
Negative

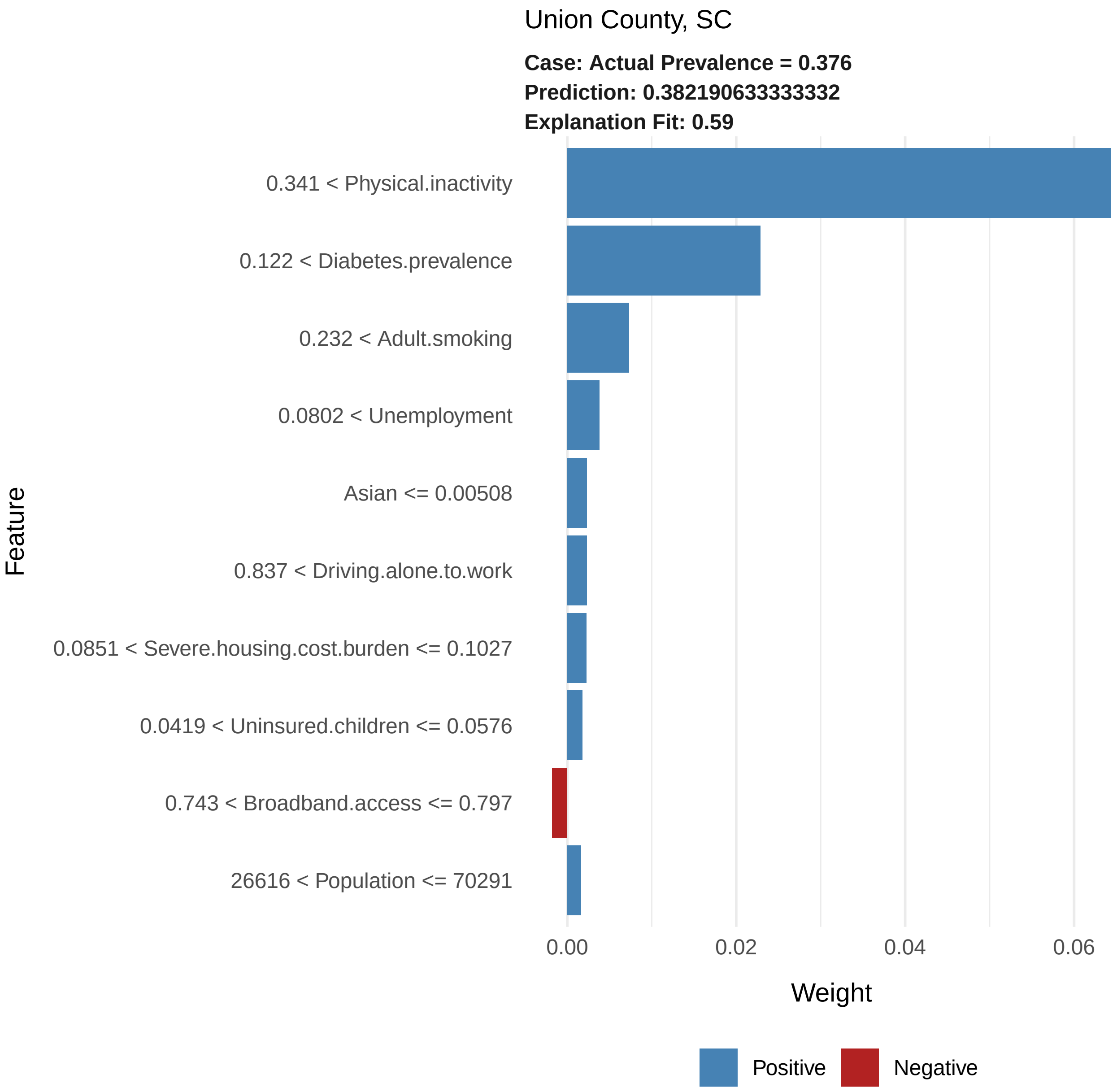

Union County, SC
Case: Actual Prevalence = 0.376
Prediction: 0.382190633333332
Explanation Fit: 0.59
0.341 < Physical.inactivity
0.122 < Diabetes.prevalence
0.232 < Adult.smoking
0.0802 < Unemployment
Asian <= 0.00508
0.837 < Driving.alone.to.work
0.0851 < Severe.housing.cost.burden <= 0.1027
0.0419 < Uninsured.children <= 0.0576
0.743 < Broadband.access <= 0.797
26616 < Population <= 70291
Feature
0.00
0.02
0.04
0.06
Weight
Positive
Negative

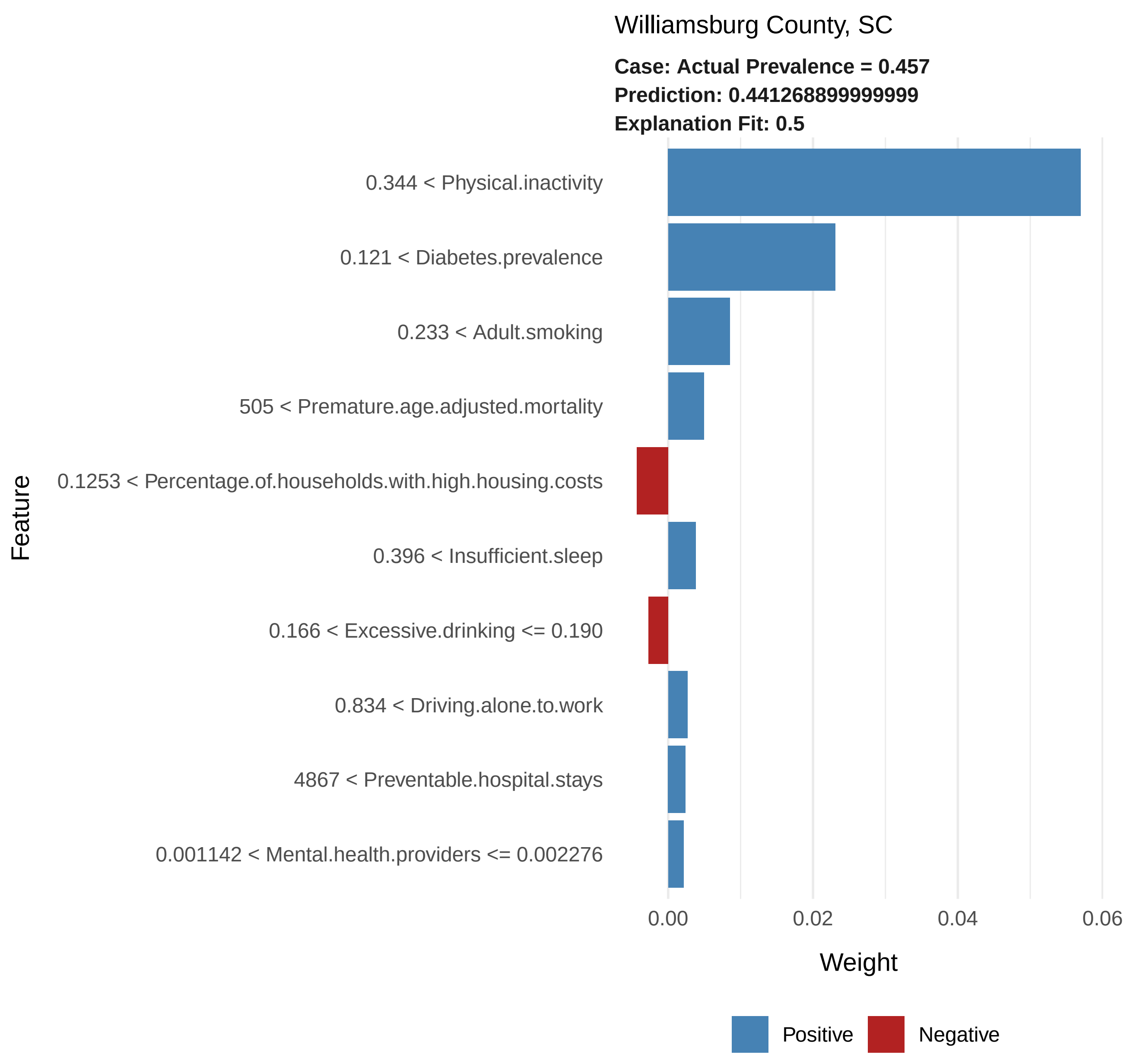

Williamsburg County, SC
Case: Actual Prevalence = 0.457
Prediction: 0.441268899999999
Explanation Fit: 0.5
0.344 < Physical.inactivity
0.121 < Diabetes.prevalence
0.233 < Adult.smoking
505 < Premature.age.adjusted.mortality
0.1253 < Percentage.of.households.with.high.housing.costs
0.396 < Insufficient.sleep
0.166 < Excessive.drinking <= 0.190
0.834 < Driving.alone.to.work
4867 < Preventable.hospital.stays
0.001142 < Mental.health.providers <= 0.002276
Feature
0.00
0.02
0.04
0.06
Weight
Positive
Negative

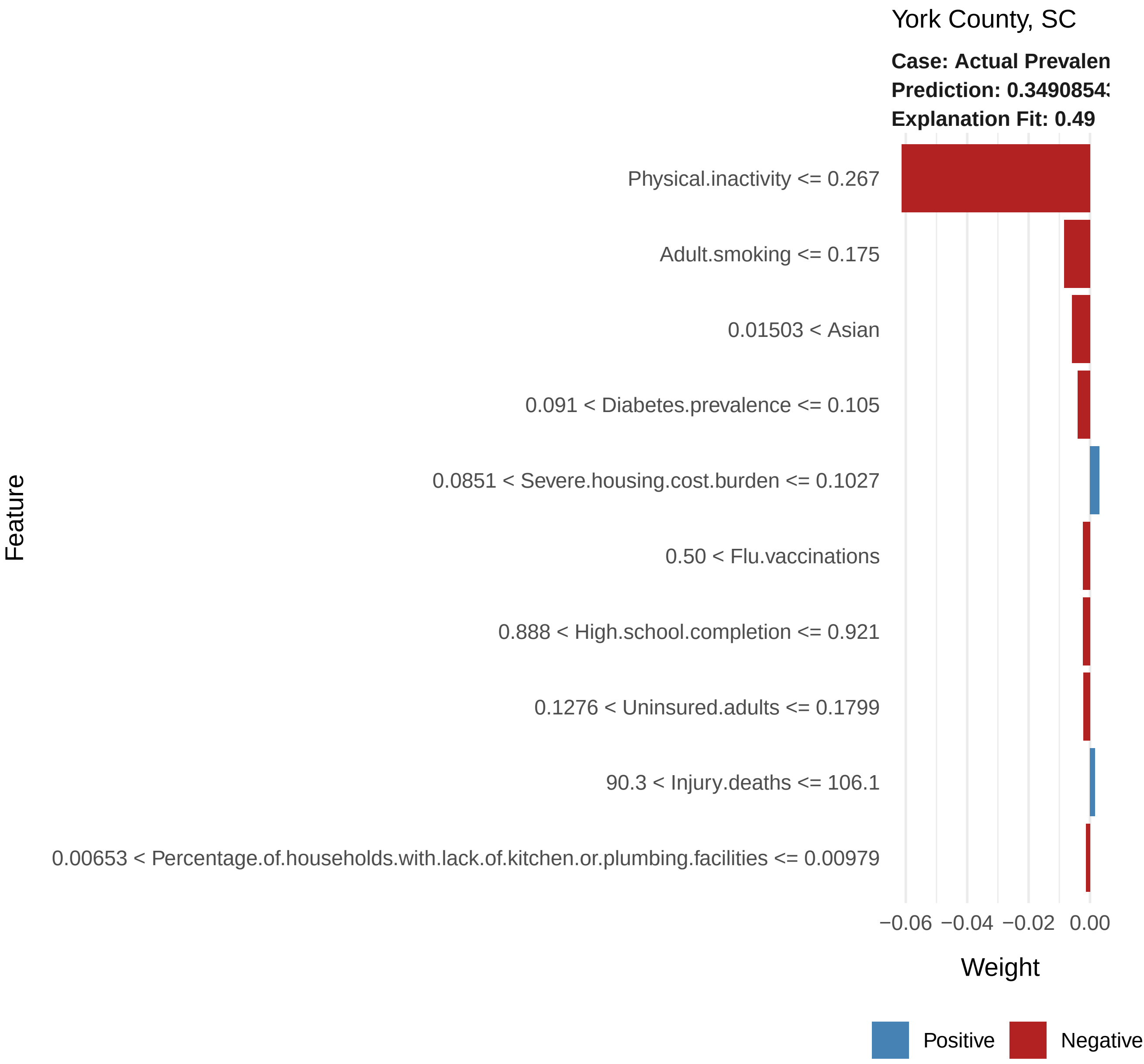
York County, SC
Case: Actual Prevalen
Prediction: 0.3490854
Explanation Fit: 0.49
Physical.inactivity <= 0.267
Adult.smoking <= 0.175
0.01503 < Asian
0.091 < Diabetes.prevalence <= 0.105
0.0851 < Severe.housing.cost.burden <= 0.1027
0.50 < Flu.vaccinations
0.888 < High.school.completion <= 0.921
0.1276 < Uninsured.adults <= 0.1799
90.3 < Injury.deaths <= 106.1
0.00653 < Percentage.of.households.with.lack.of.kitchen.or.plumbing.facilities <= 0.00979
Feature
−0.06
−0.04
−0.02
0.00
Weight
Positive
Negative

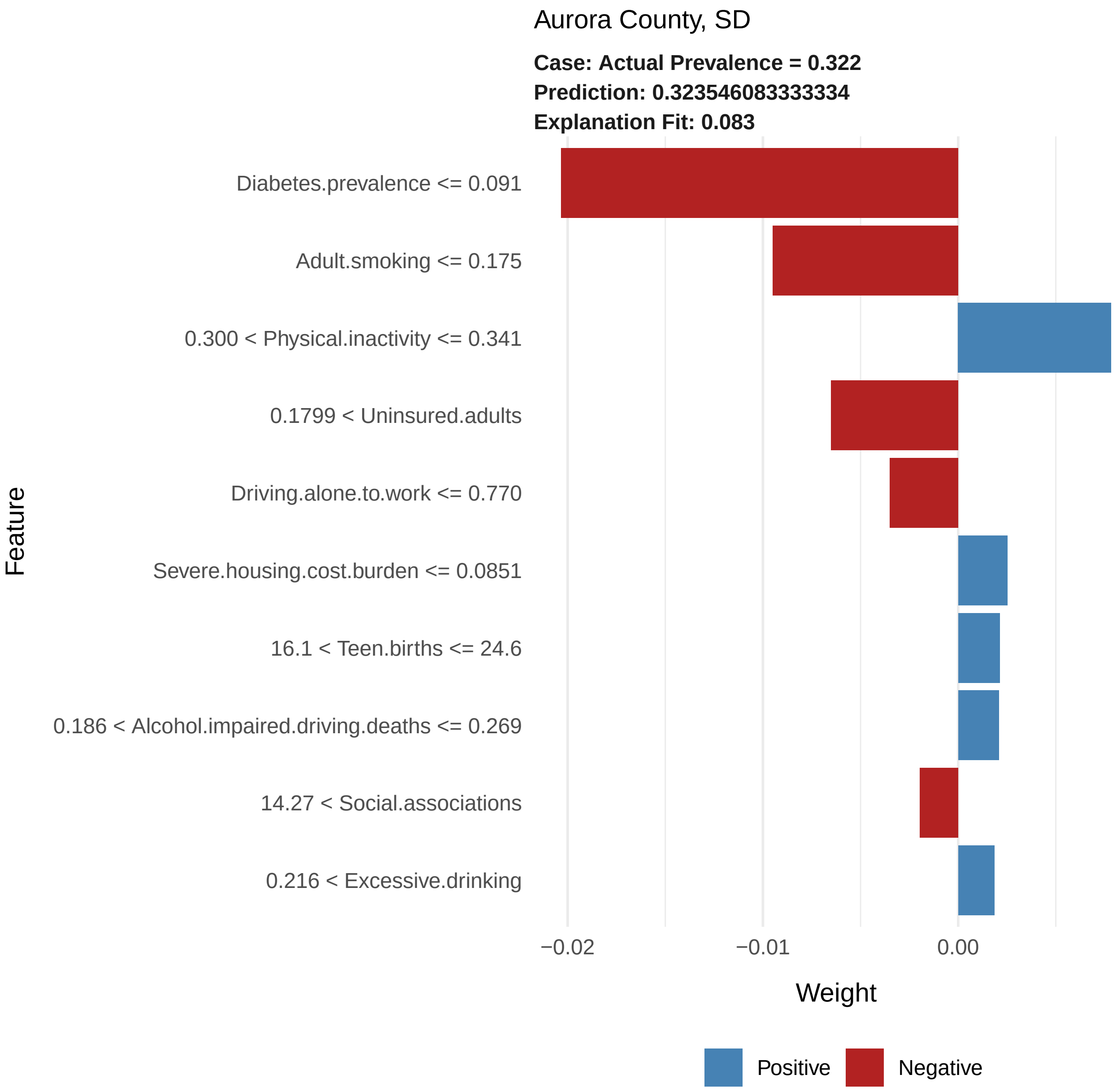
Aurora County, SD
Case: Actual Prevalence = 0.322
Prediction: 0.323546083333334
Explanation Fit: 0.083
Diabetes.prevalence <= 0.091
Adult.smoking <= 0.175
0.300 < Physical.inactivity <= 0.341
0.1799 < Uninsured.adults
Driving.alone.to.work <= 0.770
Severe.housing.cost.burden <= 0.0851
16.1 < Teen.births <= 24.6
0.186 < Alcohol.impaired.driving.deaths <= 0.269
14.27 < Social.associations
0.216 < Excessive.drinking
Feature
−0.02
−0.01
0.00
Weight
Positive
Negative

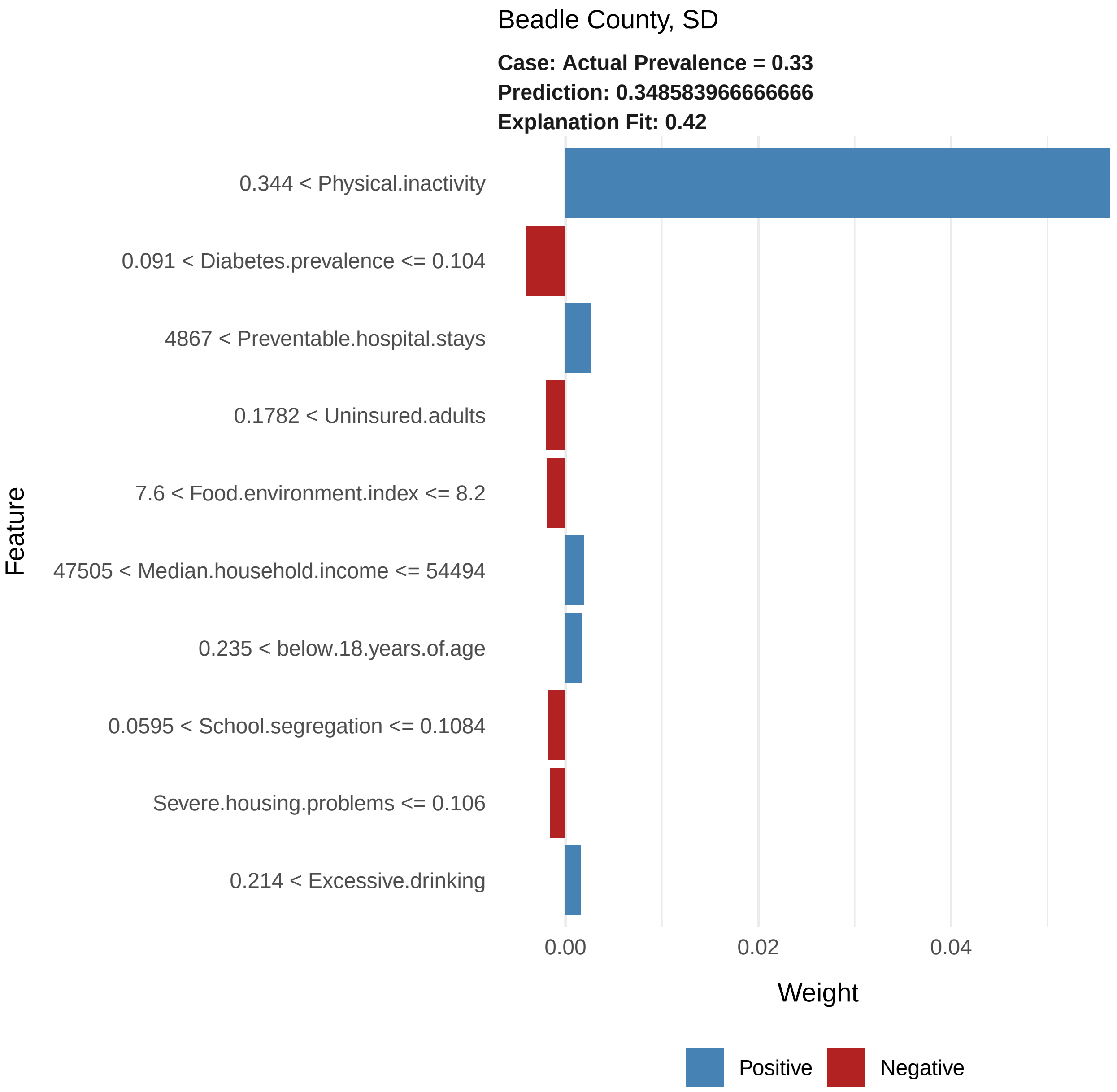

Beadle County, SD
Case: Actual Prevalence = 0.33
Prediction: 0.348583966666666
Explanation Fit: 0.42
0.344 < Physical.inactivity
0.091 < Diabetes.prevalence <= 0.104
4867 < Preventable.hospital.stays
0.1782 < Uninsured.adults
7.6 < Food.environment.index <= 8.2
47505 < Median.household.income <= 54494
0.235 < below.18.years.of.age
0.0595 < School.segregation <= 0.1084
Severe.housing.problems <= 0.106
0.214 < Excessive.drinking
Feature
0.00
0.02
0.04
Weight
Positive
Negative

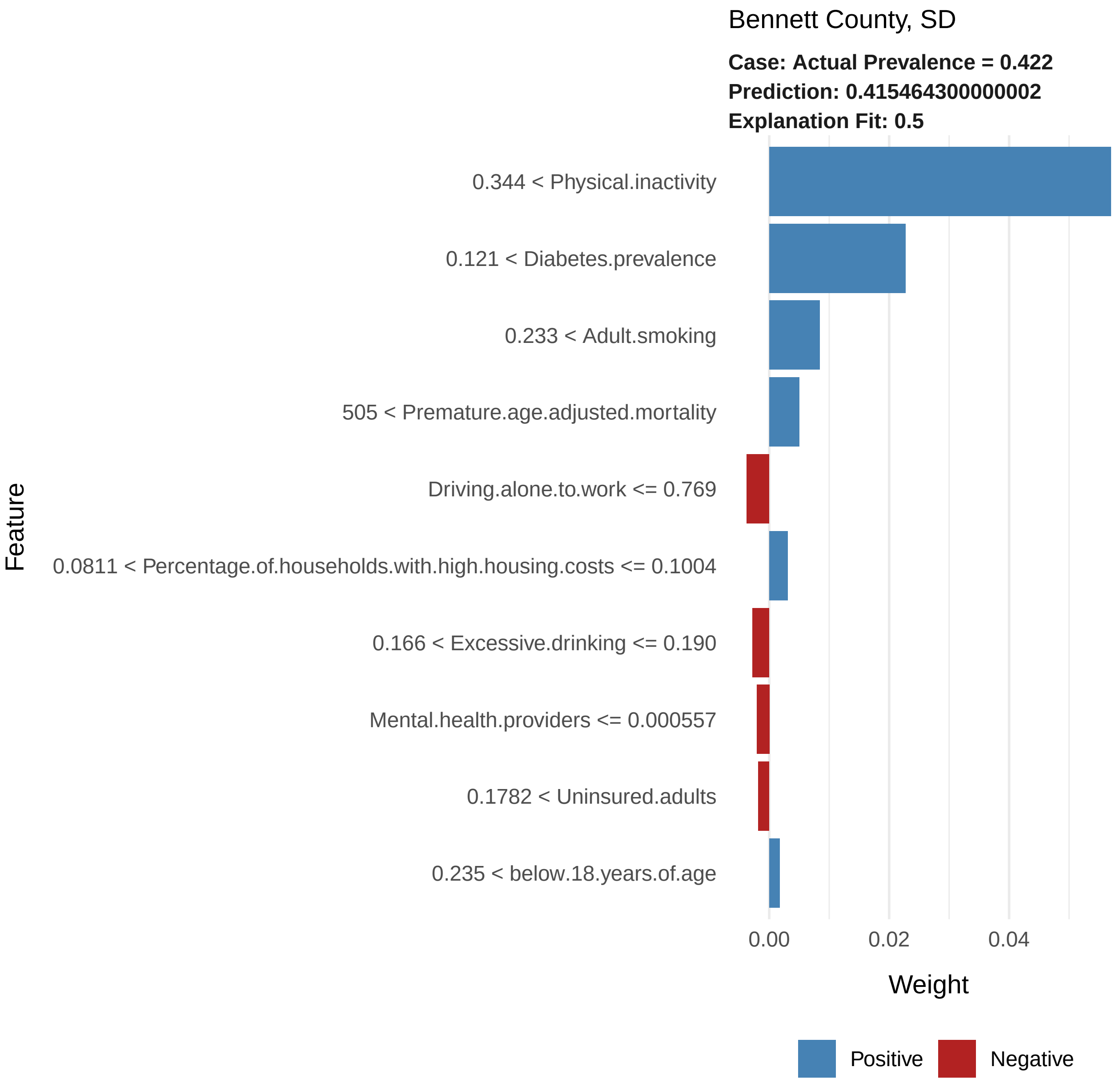

Bennett County, SD
Case: Actual Prevalence = 0.422
Prediction: 0.415464300000002
Explanation Fit: 0.5
0.344 < Physical.inactivity
0.121 < Diabetes.prevalence
0.233 < Adult.smoking
505 < Premature.age.adjusted.mortality
Driving.alone.to.work <= 0.769
0.0811 < Percentage.of.households.with.high.housing.costs <= 0.1004
0.166 < Excessive.drinking <= 0.190
Mental.health.providers <= 0.000557
0.1782 < Uninsured.adults
0.235 < below.18.years.of.age
Feature
0.00
0.02
0.04
Weight
Positive
Negative

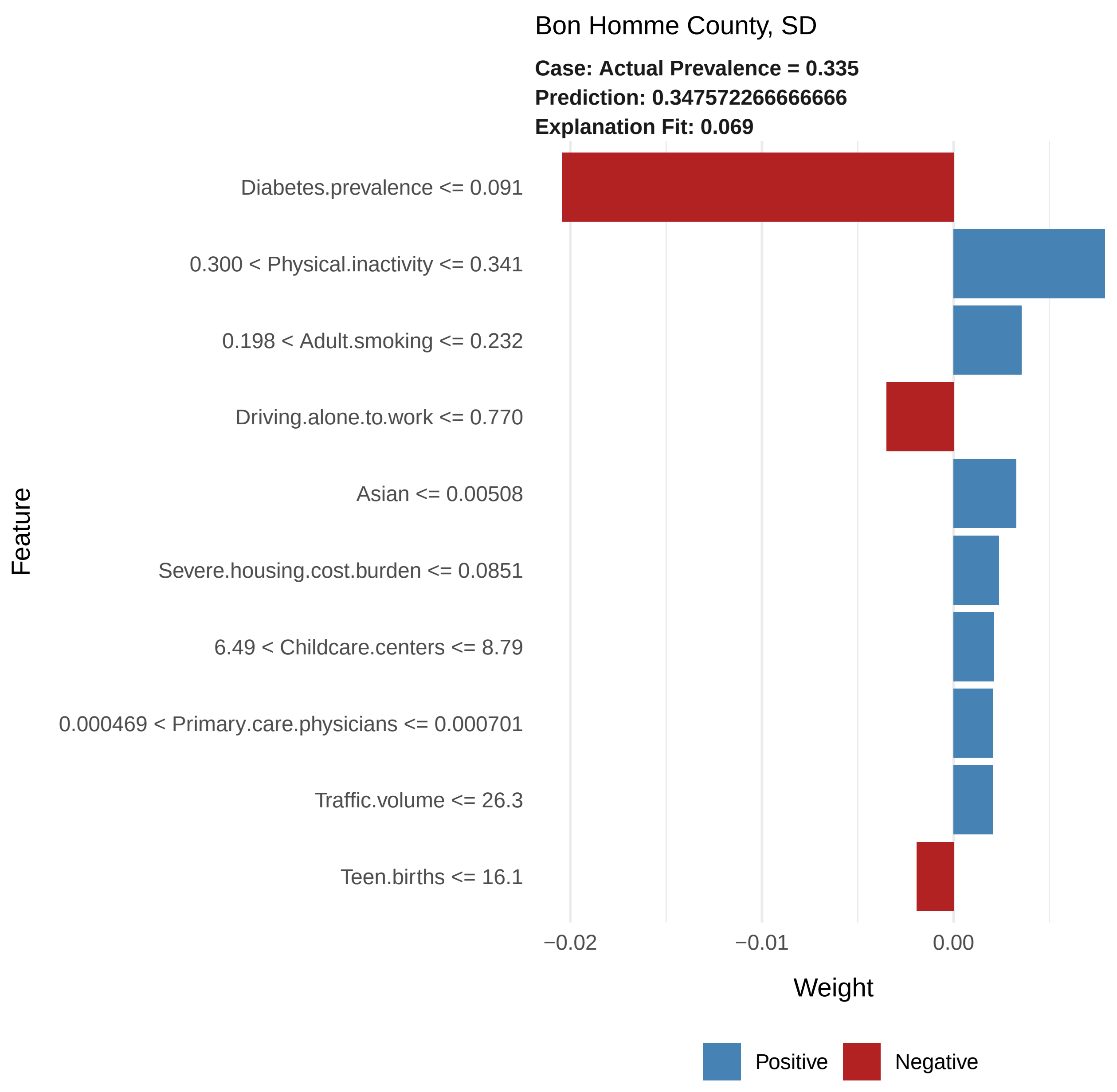
Bon Homme County, SD
Case: Actual Prevalence = 0.335
Prediction: 0.347572266666666
Explanation Fit: 0.069
Diabetes.prevalence <= 0.091
0.300 < Physical.inactivity <= 0.341
0.198 < Adult.smoking <= 0.232
Driving.alone.to.work <= 0.770
Asian <= 0.00508
Severe.housing.cost.burden <= 0.0851
6.49 < Childcare.centers <= 8.79
0.000469 < Primary.care.physicians <= 0.000701
Traffic.volume <= 26.3
Teen.births <= 16.1
Feature
−0.02
−0.01
0.00
Weight
Positive
Negative

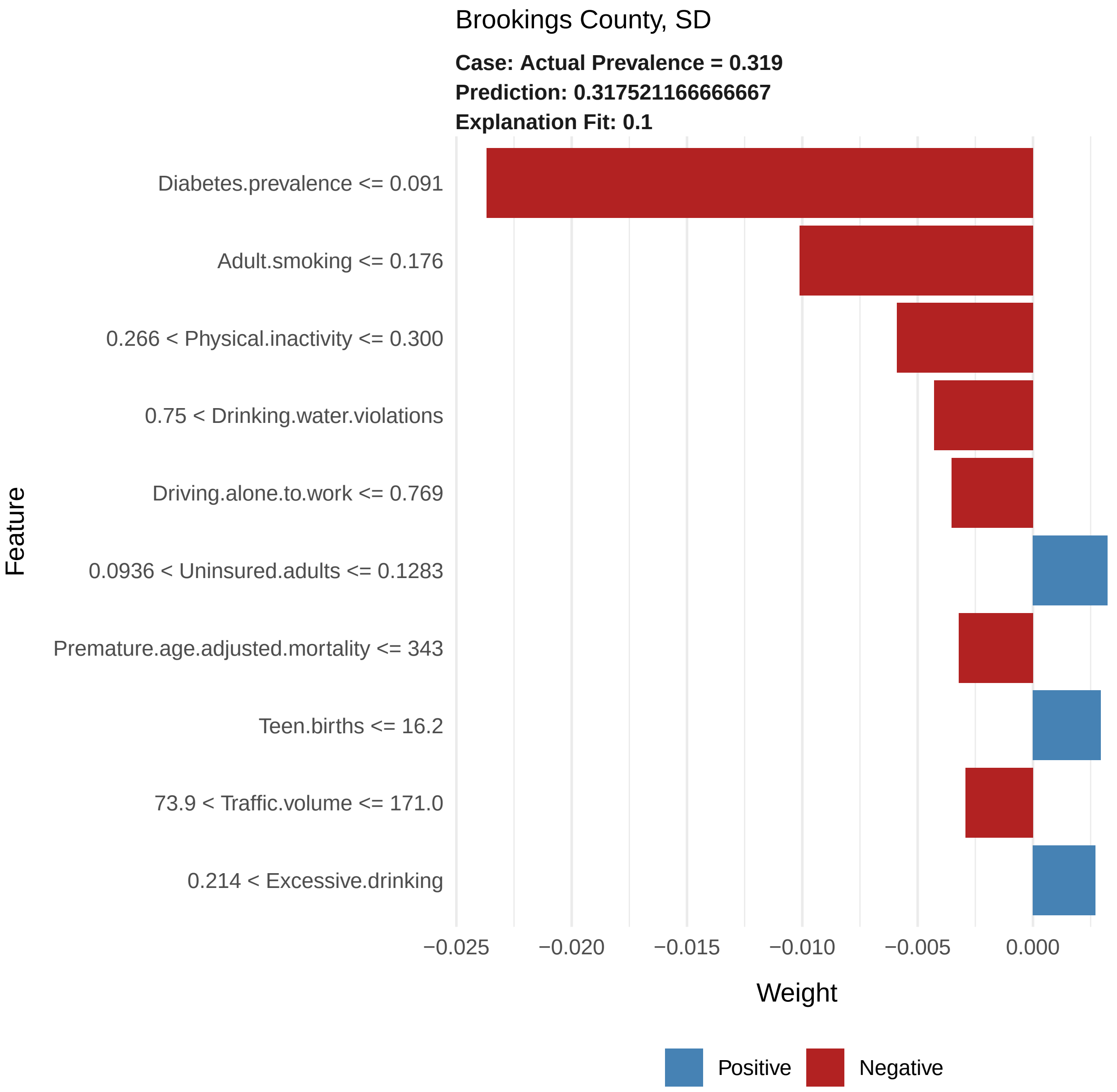

Brookings County, SD
Case: Actual Prevalence = 0.319
Prediction: 0.317521166666667
Explanation Fit: 0.1
Feature
Diabetes.prevalence <= 0.091
Adult.smoking <= 0.176
0.266 < Physical.inactivity <= 0.300
0.75 < Drinking.water.violations
Driving.alone.to.work <= 0.769
0.0936 < Uninsured.adults <= 0.1283
Premature.age.adjusted.mortality <= 343
Teen.births <= 16.2
73.9 < Traffic.volume <= 171.0
0.214 < Excessive.drinking
−0.025
−0.020
−0.015
−0.010
−0.005
0.000
Weight
Positive
Negative

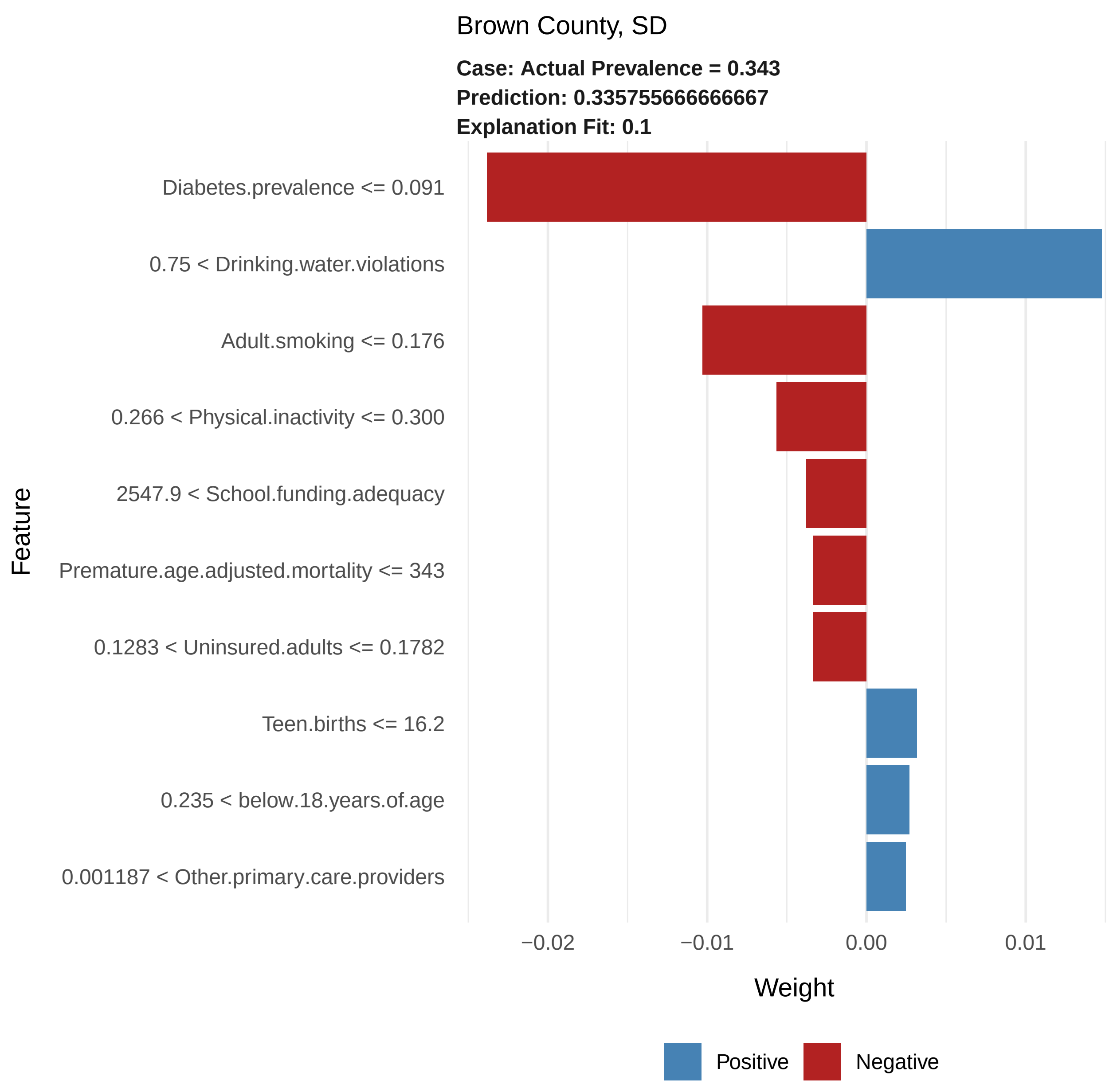
Brown County, SD
Case: Actual Prevalence = 0.343
Prediction: 0.335755666666667
Explanation Fit: 0.1
Diabetes.prevalence <= 0.091
0.75 < Drinking.water.violations
Adult.smoking <= 0.176
0.266 < Physical.inactivity <= 0.300
2547.9 < School.funding.adequacy
Premature.age.adjusted.mortality <= 343
0.1283 < Uninsured.adults <= 0.1782
Teen.births <= 16.2
0.235 < below.18.years.of.age
0.001187 < Other.primary.care.providers
Feature
−0.02
−0.01
0.00
0.01
Weight
Positive
Negative

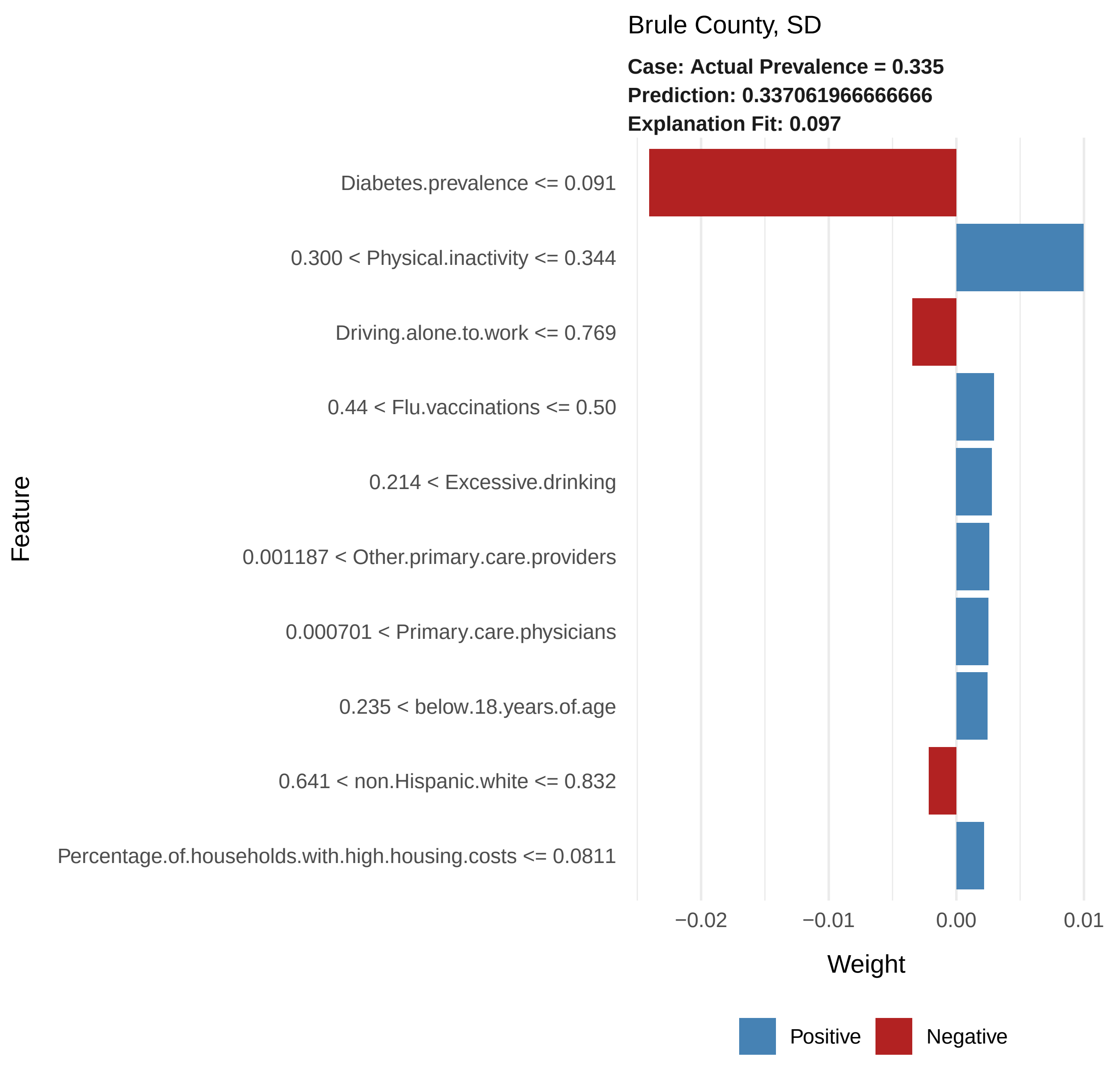

Brule County, SD
Case: Actual Prevalence = 0.335
Prediction: 0.337061966666666
Explanation Fit: 0.097
Diabetes.prevalence <= 0.091
0.300 < Physical.inactivity <= 0.344
Driving.alone.to.work <= 0.769
0.44 < Flu.vaccinations <= 0.50
0.214 < Excessive.drinking
0.001187 < Other.primary.care.providers
0.000701 < Primary.care.physicians
0.235 < below.18.years.of.age
0.641 < non.Hispanic.white <= 0.832
Percentage.of.households.with.high.housing.costs <= 0.0811
Feature
−0.02
−0.01
0.00
0.01
Weight
Positive
Negative

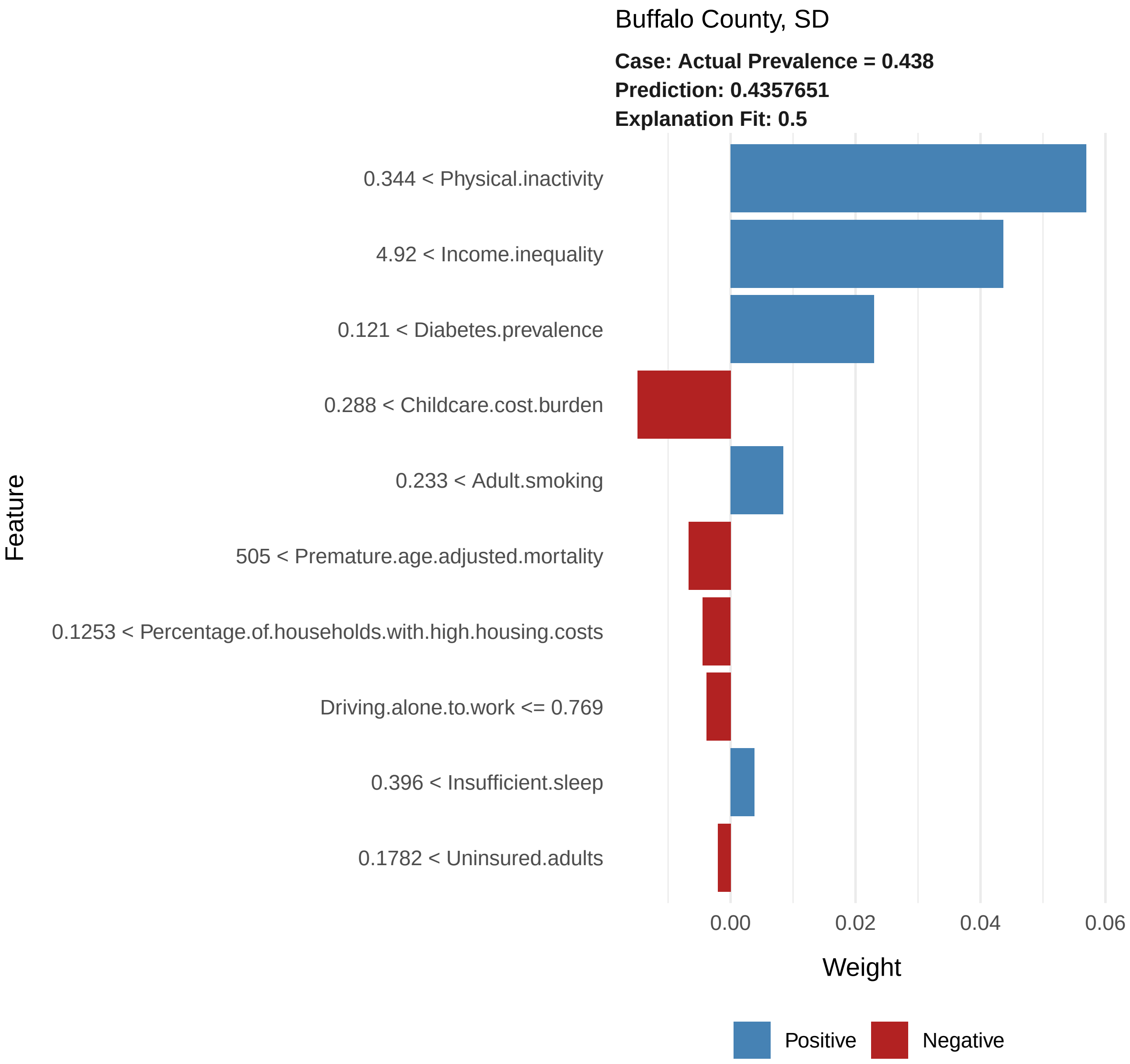
Buffalo County, SD
Case: Actual Prevalence = 0.438
Prediction: 0.4357651
Explanation Fit: 0.5
0.344 < Physical.inactivity
4.92 < Income.inequality
0.121 < Diabetes.prevalence
0.288 < Childcare.cost.burden
0.233 < Adult.smoking
505 < Premature.age.adjusted.mortality
0.1253 < Percentage.of.households.with.high.housing.costs
Driving.alone.to.work <= 0.769
0.396 < Insufficient.sleep
0.1782 < Uninsured.adults
Feature
0.00
0.02
0.04
0.06
Weight
Positive
Negative

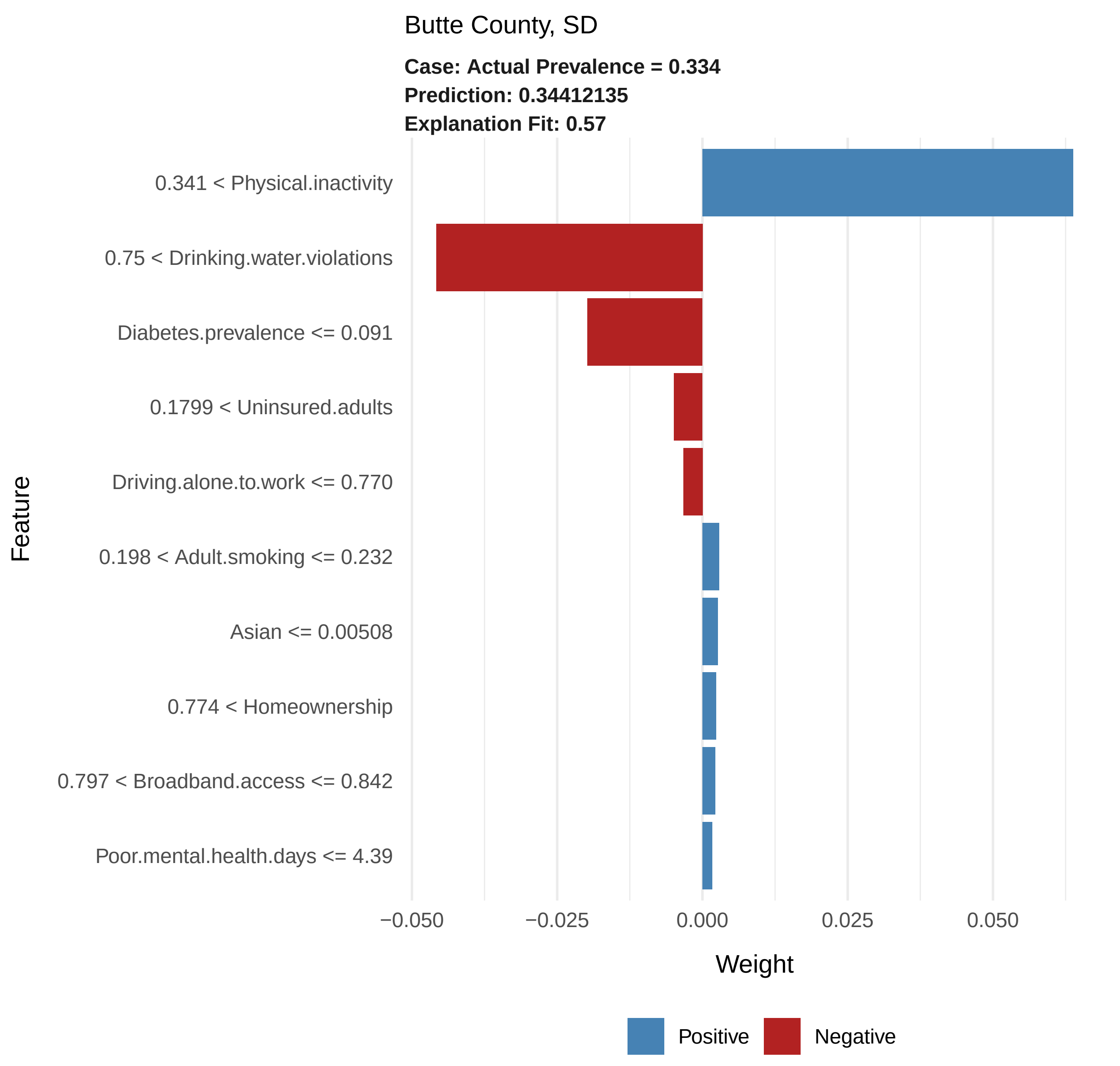
Butte County, SD
Case: Actual Prevalence = 0.334
Prediction: 0.34412135
Explanation Fit: 0.57
0.341 < Physical.inactivity
0.75 < Drinking.water.violations
Diabetes.prevalence <= 0.091
0.1799 < Uninsured.adults
Driving.alone.to.work <= 0.770
0.198 < Adult.smoking <= 0.232
Asian <= 0.00508
0.774 < Homeownership
0.797 < Broadband.access <= 0.842
Poor.mental.health.days <= 4.39
Feature
−0.050
−0.025
0.000
0.025
0.050
Weight
Positive
Negative

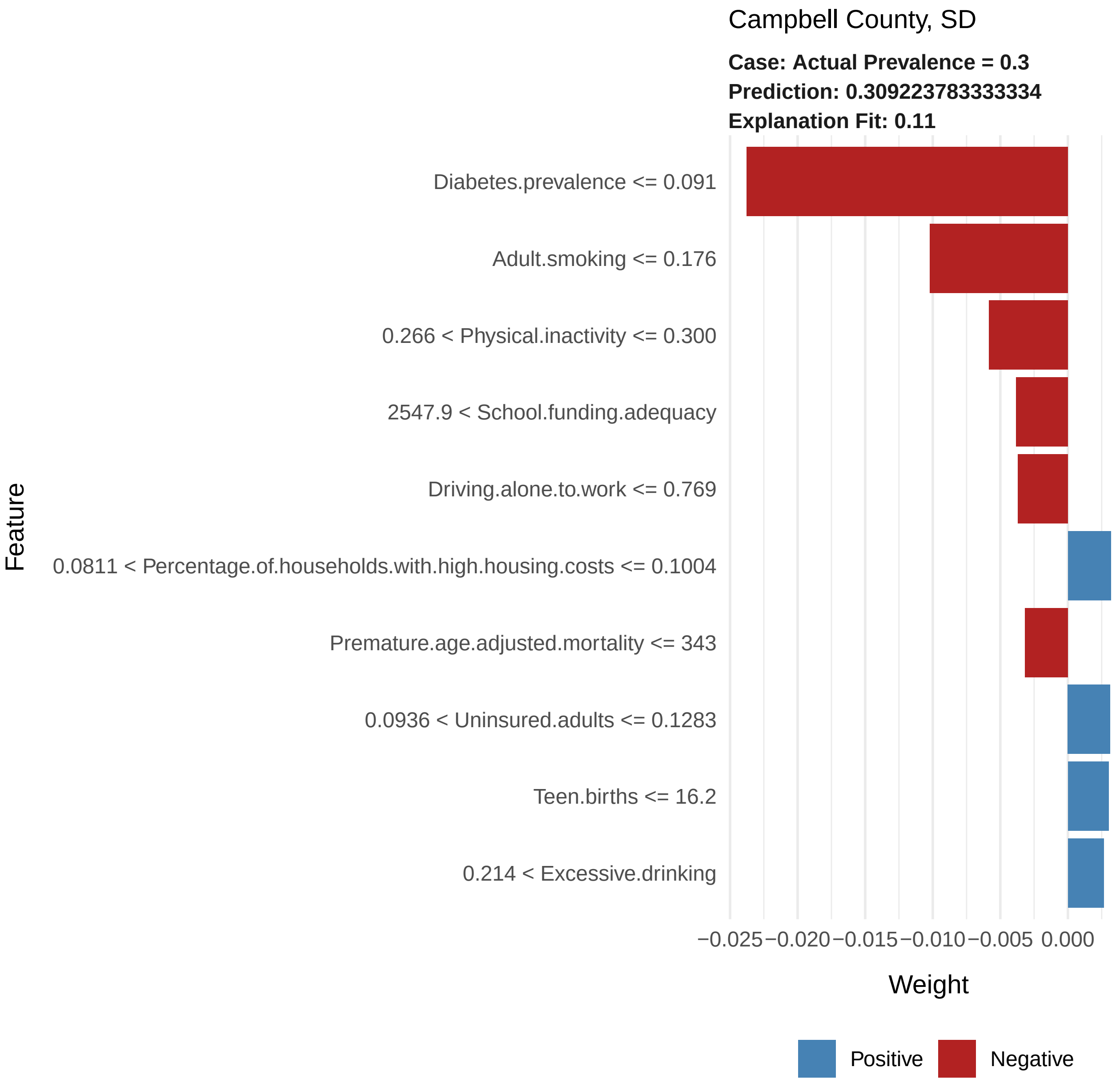

Campbell County, SD
Case: Actual Prevalence = 0.3
Prediction: 0.309223783333334
Explanation Fit: 0.11
Diabetes.prevalence <= 0.091
Adult.smoking <= 0.176
0.266 < Physical.inactivity <= 0.300
2547.9 < School.funding.adequacy
Driving.alone.to.work <= 0.769
0.0811 < Percentage.of.households.with.high.housing.costs <= 0.1004
Premature.age.adjusted.mortality <= 343
0.0936 < Uninsured.adults <= 0.1283
Teen.births <= 16.2
0.214 < Excessive.drinking
Feature
−0.025
−0.020
−0.015
−0.010
−0.005
0.000
Weight
Positive
Negative

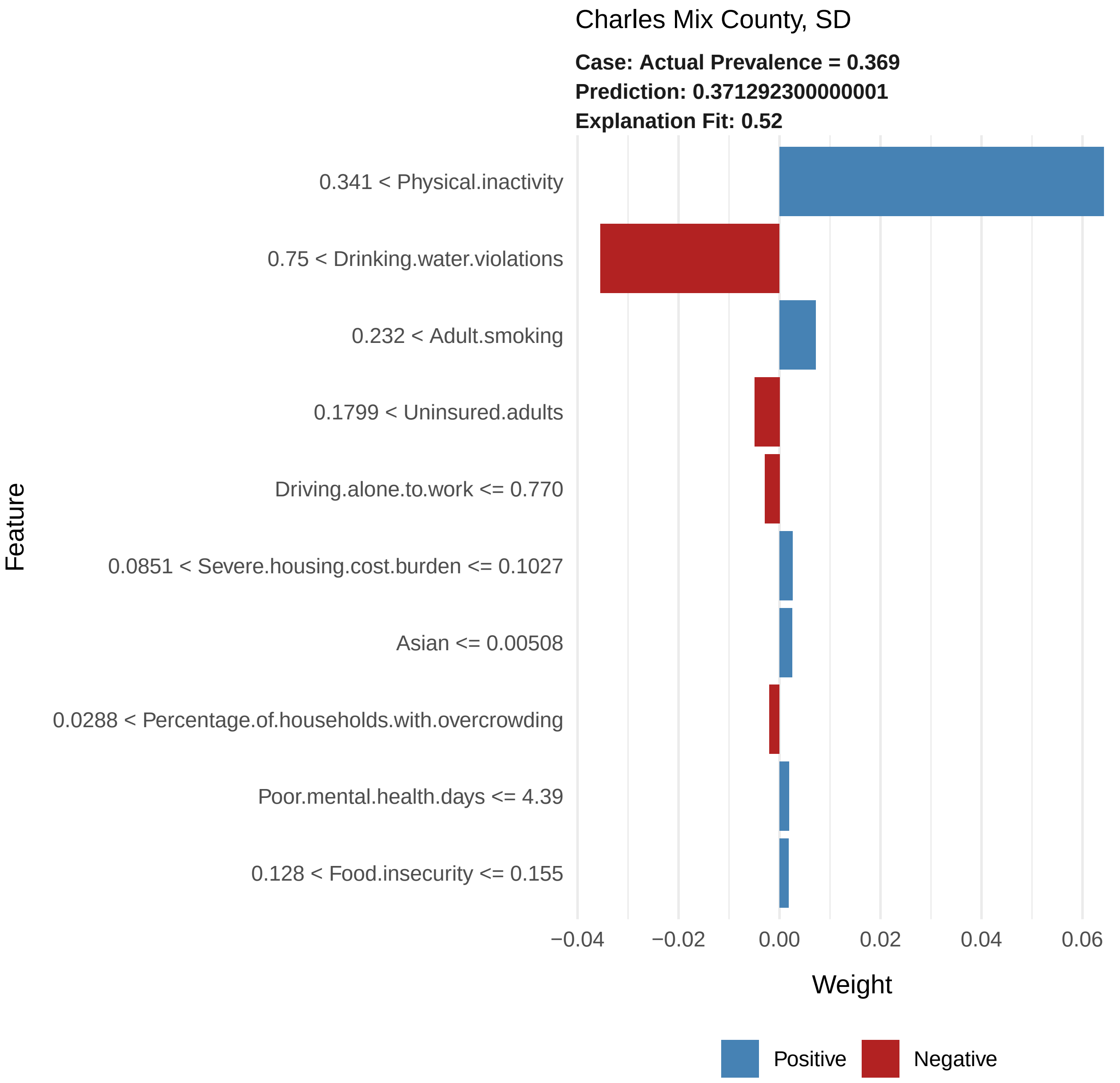
Charles Mix County, SD
Case: Actual Prevalence = 0.369
Prediction: 0.371292300000001
Explanation Fit: 0.52
0.341 < Physical.inactivity
0.75 < Drinking.water.violations
0.232 < Adult.smoking
0.1799 < Uninsured.adults
Driving.alone.to.work <= 0.770
0.0851 < Severe.housing.cost.burden <= 0.1027
Asian <= 0.00508
0.0288 < Percentage.of.households.with.overcrowding
Poor.mental.health.days <= 4.39
0.128 < Food.insecurity <= 0.155
Feature
−0.04
−0.02
0.00
0.02
0.04
0.06
Weight
Positive
Negative

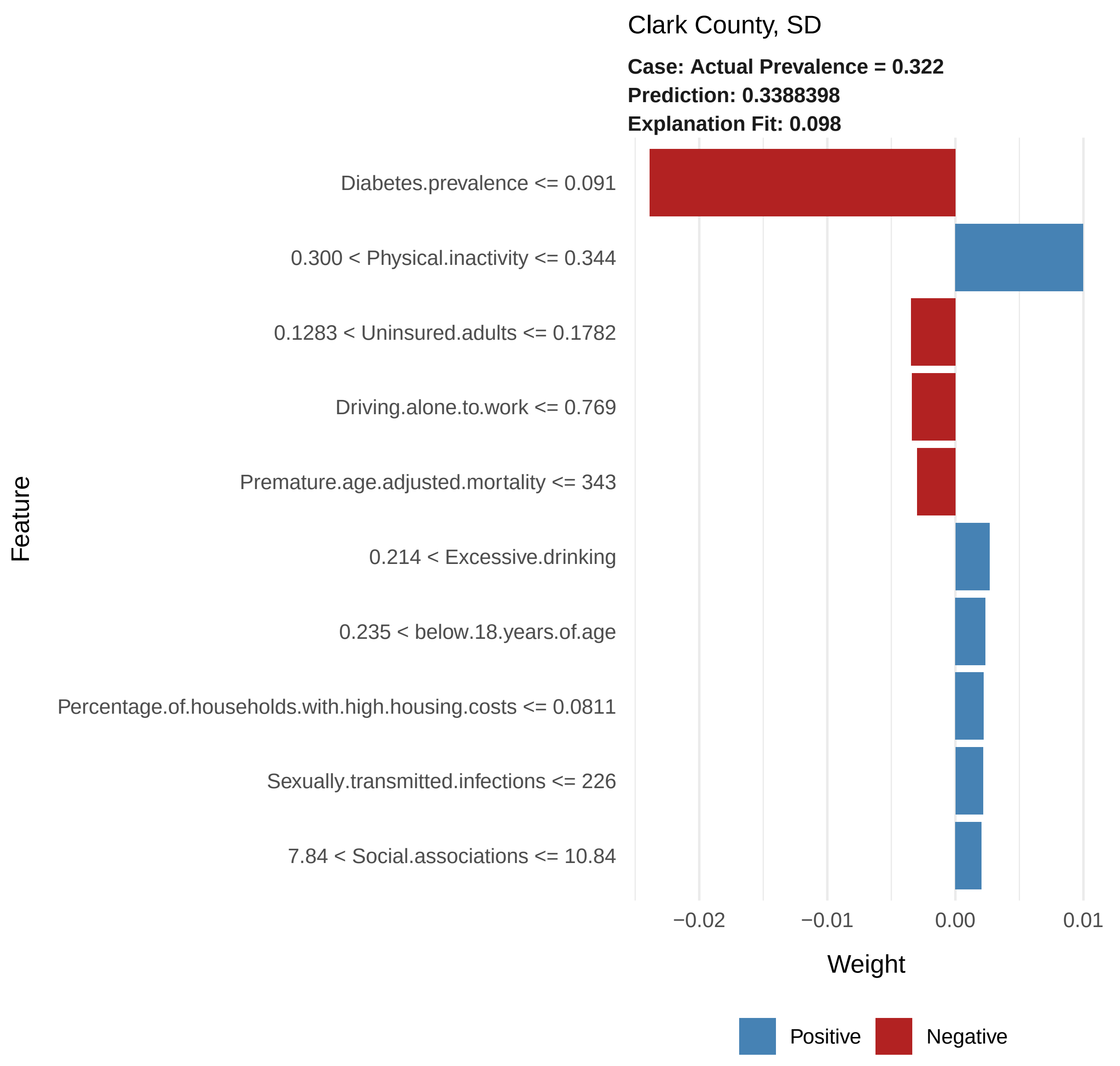

Clark County, SD
Case: Actual Prevalence = 0.322
Prediction: 0.3388398
Explanation Fit: 0.098
Diabetes.prevalence <= 0.091
0.300 < Physical.inactivity <= 0.344
0.1283 < Uninsured.adults <= 0.1782
Driving.alone.to.work <= 0.769
Premature.age.adjusted.mortality <= 343
0.214 < Excessive.drinking
0.235 < below.18.years.of.age
Percentage.of.households.with.high.housing.costs <= 0.0811
Sexually.transmitted.infections <= 226
7.84 < Social.associations <= 10.84
Feature
−0.02
−0.01
0.00
0.01
Weight
Positive
Negative

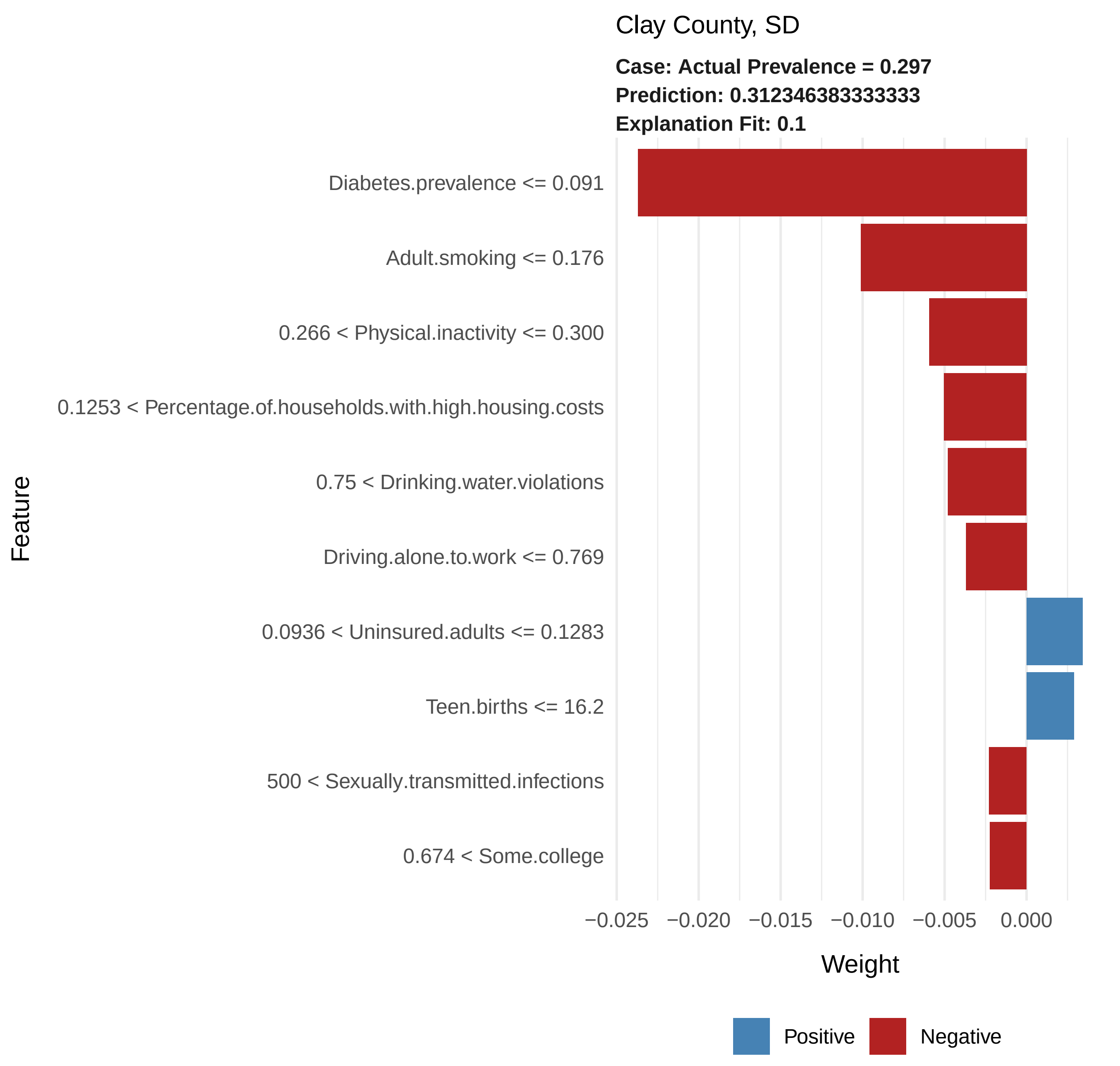
Clay County, SD
Case: Actual Prevalence = 0.297
Prediction: 0.312346383333333
Explanation Fit: 0.1
Diabetes.prevalence <= 0.091
Adult.smoking <= 0.176
0.266 < Physical.inactivity <= 0.300
0.1253 < Percentage.of.households.with.high.housing.costs
0.75 < Drinking.water.violations
Driving.alone.to.work <= 0.769
0.0936 < Uninsured.adults <= 0.1283
Teen.births <= 16.2
500 < Sexually.transmitted.infections
0.674 < Some.college
Feature
−0.025
−0.020
−0.015
−0.010
−0.005
0.000
Weight
Positive
Negative

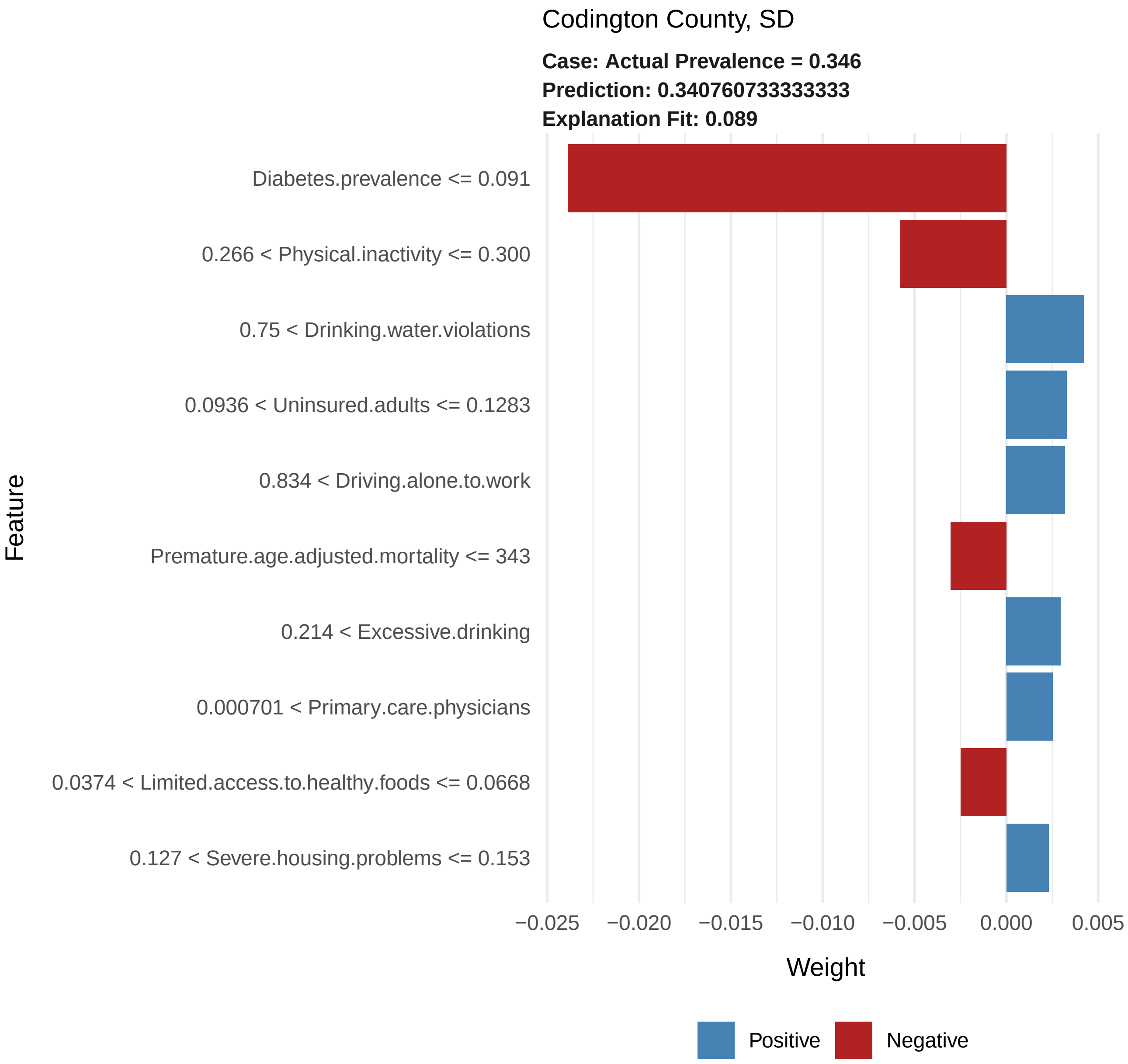

Codington County, SD
Case: Actual Prevalence = 0.346
Prediction: 0.340760733333333
Explanation Fit: 0.089
Diabetes.prevalence <= 0.091
0.266 < Physical.inactivity <= 0.300
0.75 < Drinking.water.violations
0.0936 < Uninsured.adults <= 0.1283
0.834 < Driving.alone.to.work
Premature.age.adjusted.mortality <= 343
0.214 < Excessive.drinking
0.000701 < Primary.care.physicians
0.0374 < Limited.access.to.healthy.foods <= 0.0668
0.127 < Severe.housing.problems <= 0.153
Feature
−0.025
−0.020
−0.015
−0.010
−0.005
0.000
0.005
Weight
Positive
Negative

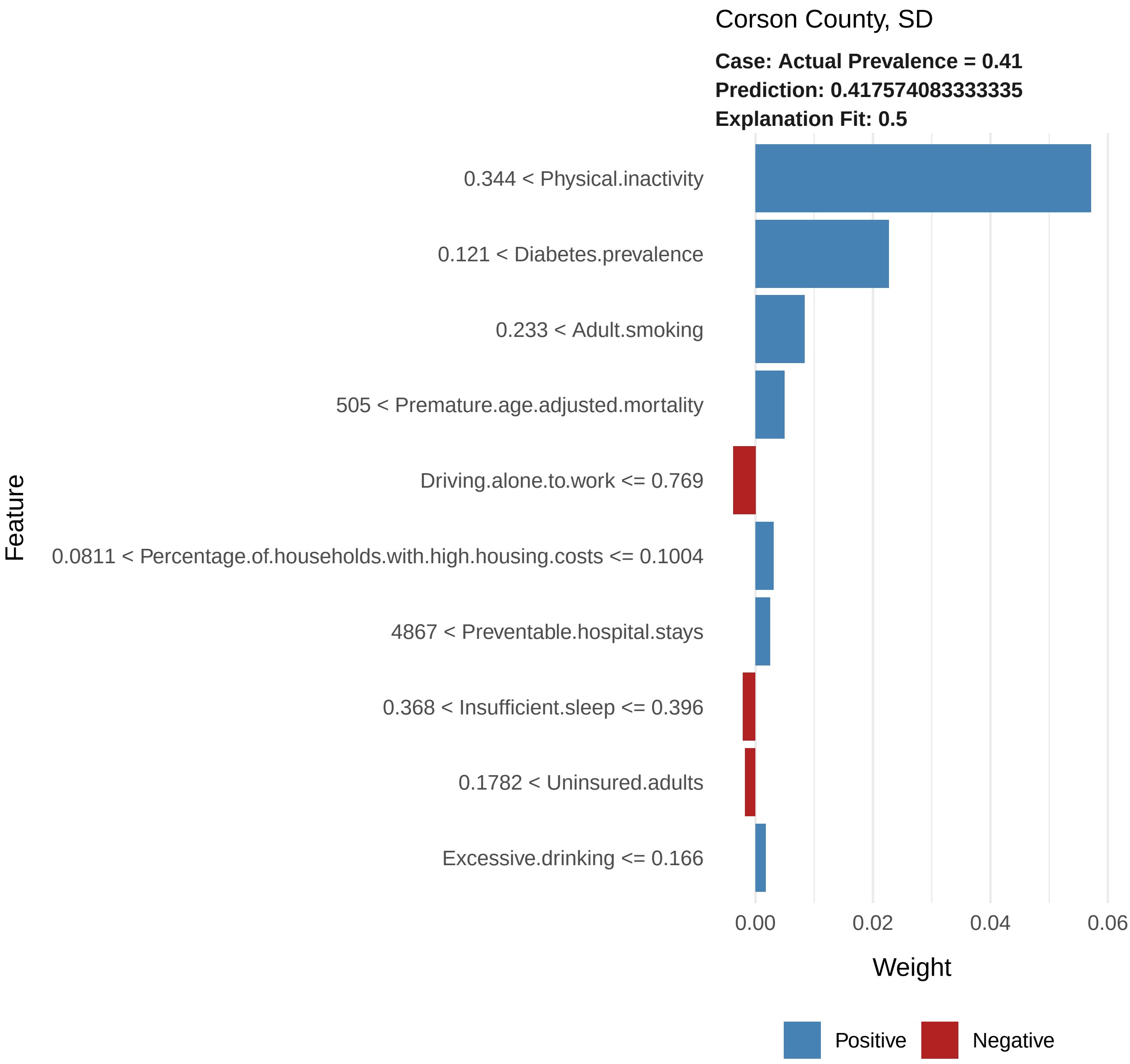
Corson County, SD
Case: Actual Prevalence = 0.41
Prediction: 0.417574083333335
Explanation Fit: 0.5
0.344 < Physical.inactivity
0.121 < Diabetes.prevalence
0.233 < Adult.smoking
505 < Premature.age.adjusted.mortality
Driving.alone.to.work <= 0.769
0.0811 < Percentage.of.households.with.high.housing.costs <= 0.1004
4867 < Preventable.hospital.stays
0.368 < Insufficient.sleep <= 0.396
0.1782 < Uninsured.adults
Excessive.drinking <= 0.166
Feature
0.00
0.02
0.04
0.06
Weight
Positive
Negative

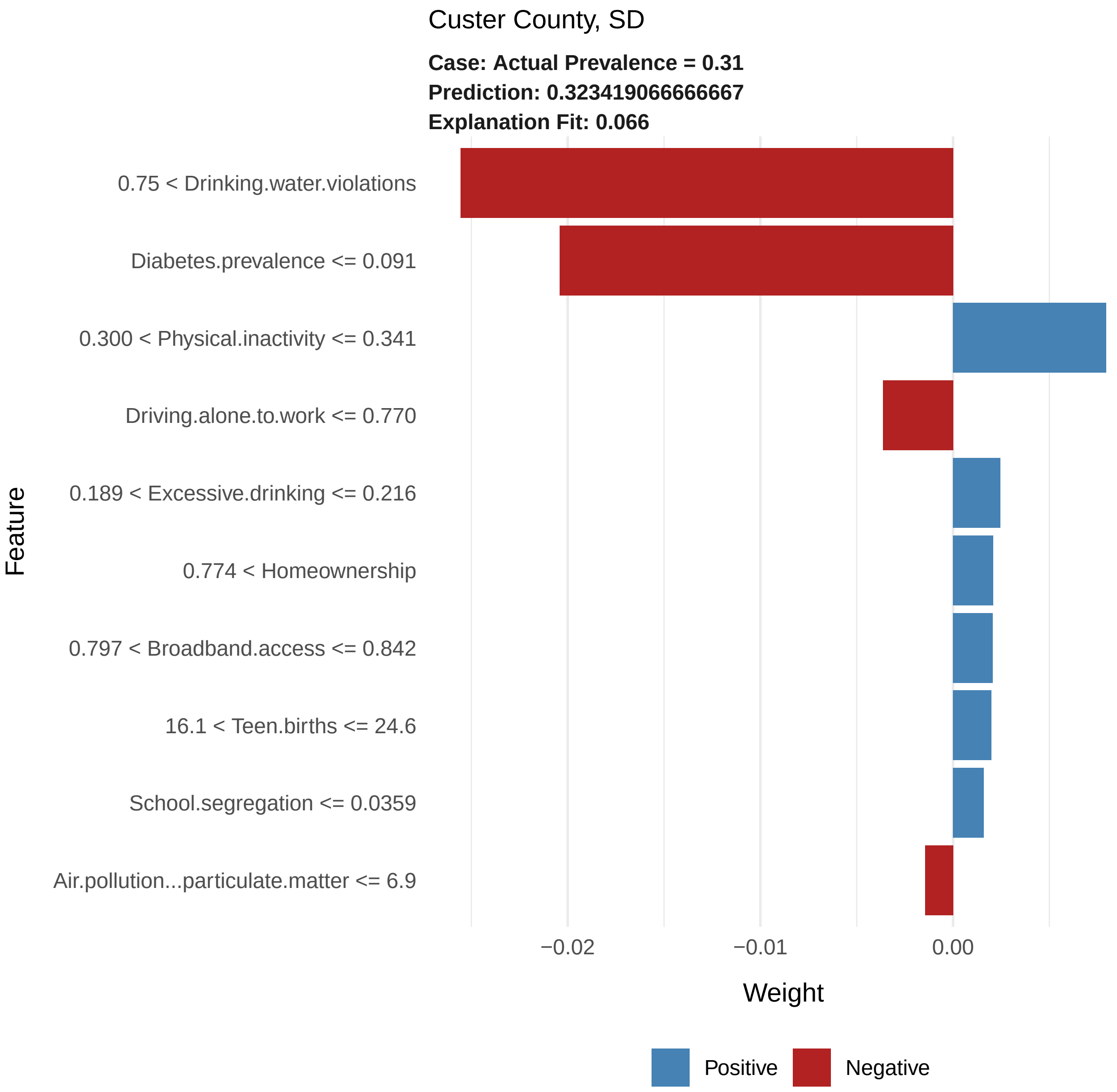

Custer County, SD
Case: Actual Prevalence = 0.31
Prediction: 0.323419066666667
Explanation Fit: 0.066
0.75 < Drinking.water.violations
Diabetes.prevalence <= 0.091
0.300 < Physical.inactivity <= 0.341
Driving.alone.to.work <= 0.770
0.189 < Excessive.drinking <= 0.216
0.774 < Homeownership
0.797 < Broadband.access <= 0.842
16.1 < Teen.births <= 24.6
School.segregation <= 0.0359
Air.pollution...particulate.matter <= 6.9
Feature
−0.02
−0.01
0.00
Weight
Positive
Negative

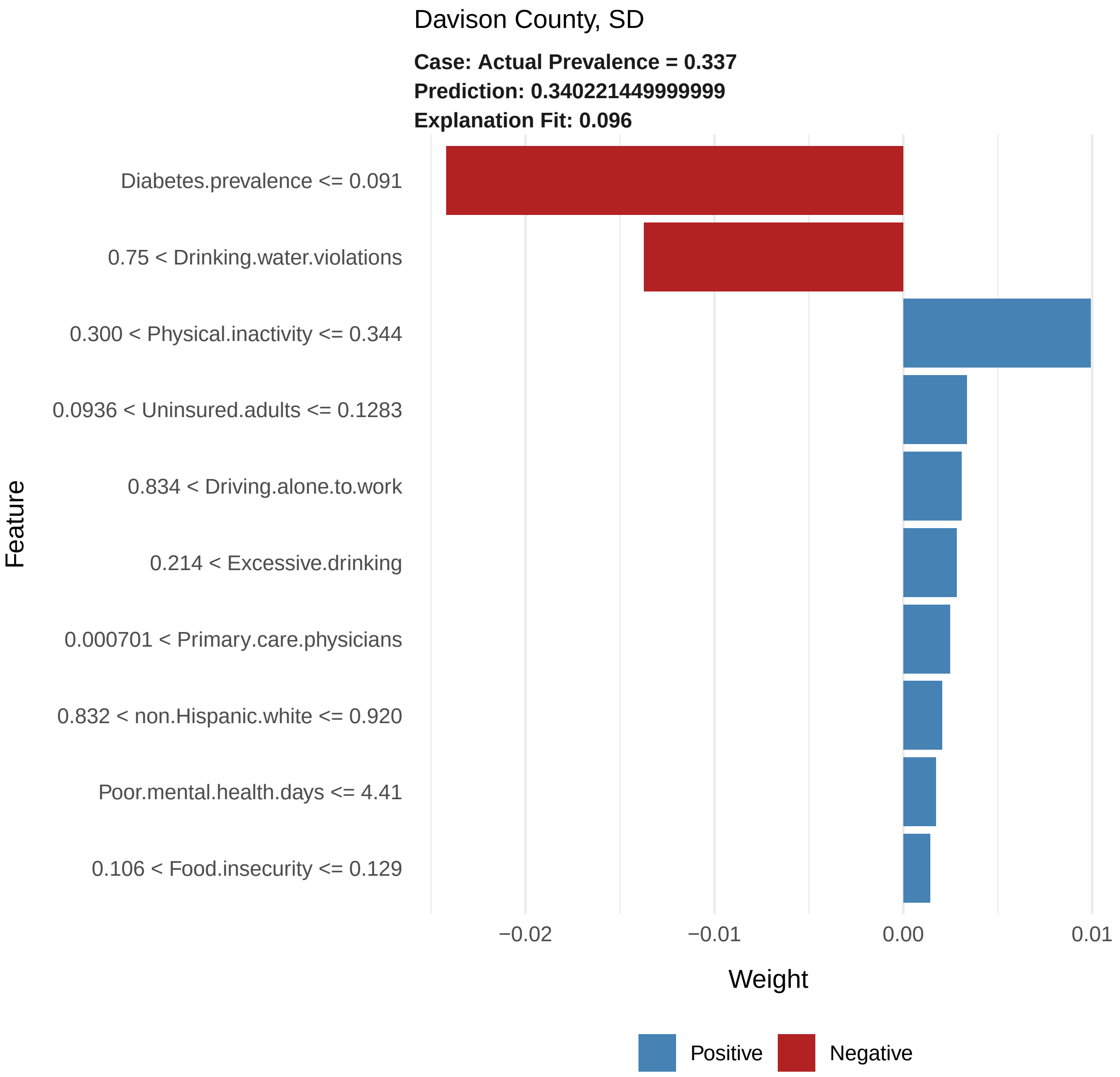

Davison County, SD
Case: Actual Prevalence = 0.337
Prediction: 0.340221449999999
Explanation Fit: 0.096
Diabetes.prevalence <= 0.091
0.75 < Drinking.water.violations
0.300 < Physical.inactivity <= 0.344
0.0936 < Uninsured.adults <= 0.1283
0.834 < Driving.alone.to.work
0.214 < Excessive.drinking
0.000701 < Primary.care.physicians
0.832 < non.Hispanic.white <= 0.920
Poor.mental.health.days <= 4.41
0.106 < Food.insecurity <= 0.129
Feature
−0.02
−0.01
0.00
0.01
Weight
Positive
Negative

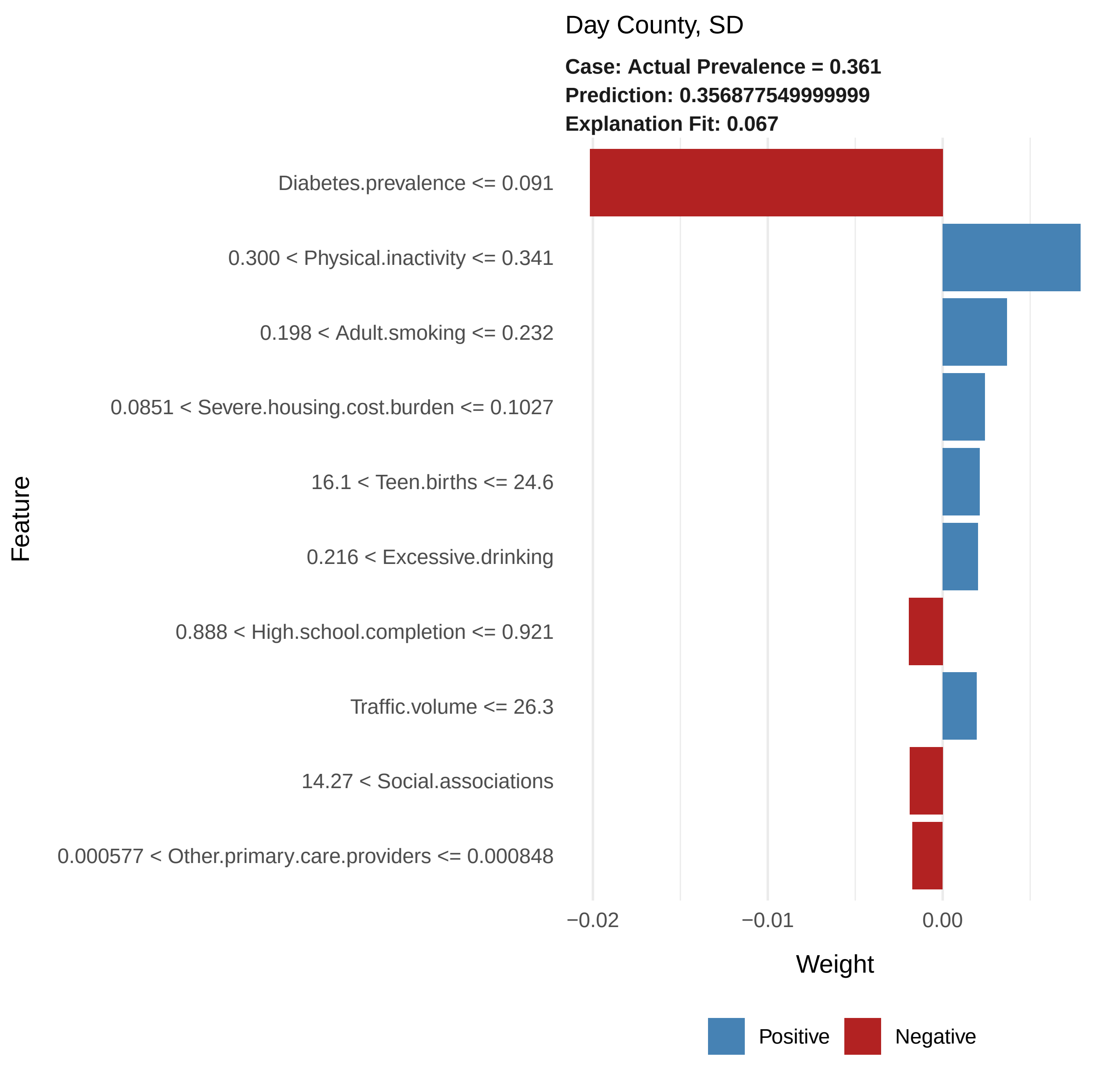

Day County, SD
Case: Actual Prevalence = 0.361
Prediction: 0.356877549999999
Explanation Fit: 0.067
Diabetes.prevalence <= 0.091
0.300 < Physical.inactivity <= 0.341
0.198 < Adult.smoking <= 0.232
0.0851 < Severe.housing.cost.burden <= 0.1027
16.1 < Teen.births <= 24.6
0.216 < Excessive.drinking
0.888 < High.school.completion <= 0.921
Traffic.volume <= 26.3
14.27 < Social.associations
0.000577 < Other.primary.care.providers <= 0.000848
Feature
−0.02
−0.01
0.00
Weight
Positive
Negative

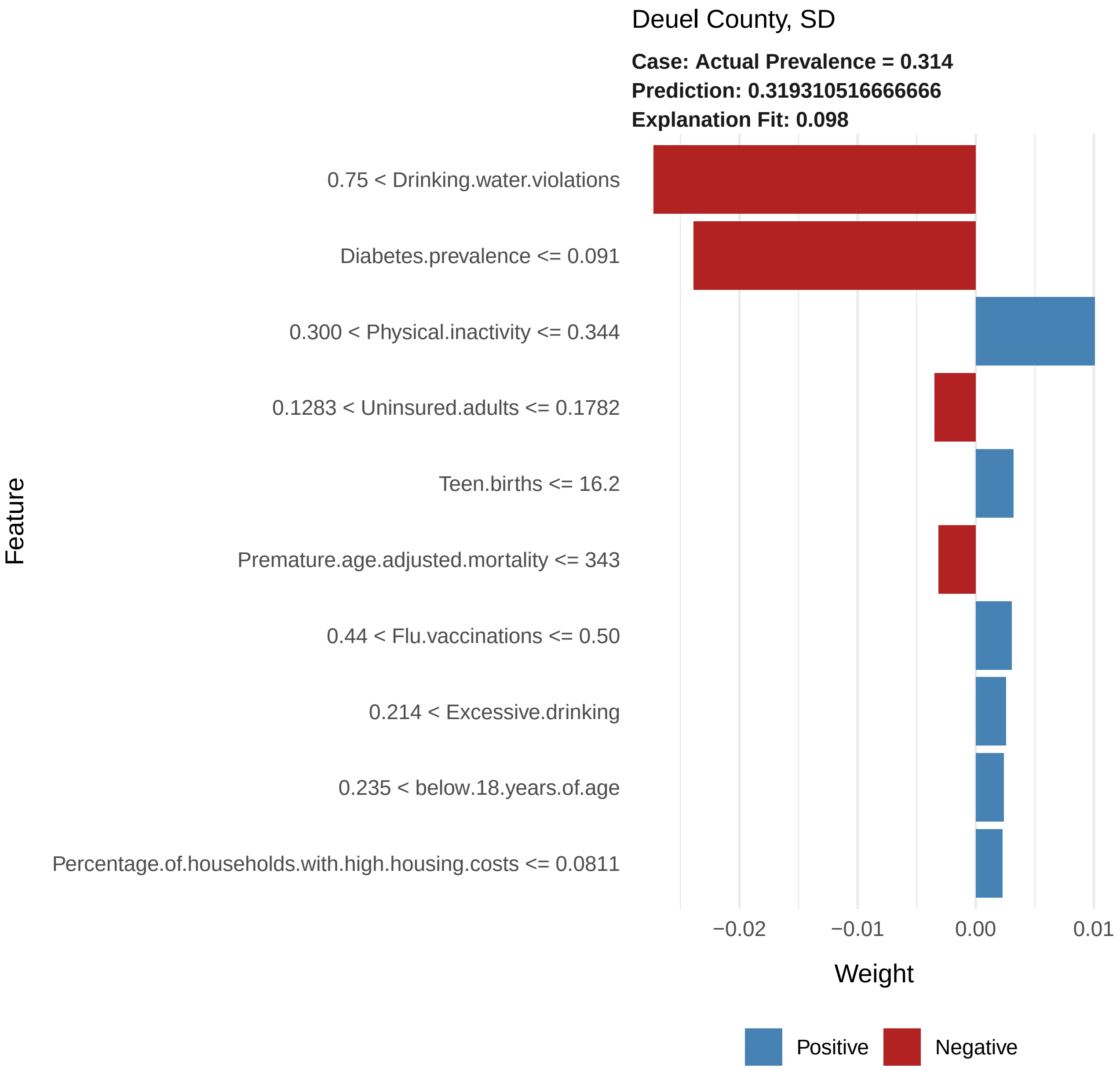

Deuel County, SD
Case: Actual Prevalence = 0.314
Prediction: 0.319310516666666
Explanation Fit: 0.098
0.75 < Drinking.water.violations
Diabetes.prevalence <= 0.091
0.300 < Physical.inactivity <= 0.344
0.1283 < Uninsured.adults <= 0.1782
Teen.births <= 16.2
Premature.age.adjusted.mortality <= 343
0.44 < Flu.vaccinations <= 0.50
0.214 < Excessive.drinking
0.235 < below.18.years.of.age
Percentage.of.households.with.high.housing.costs <= 0.0811
Feature
−0.02
−0.01
0.00
0.01
Weight
Positive
Negative

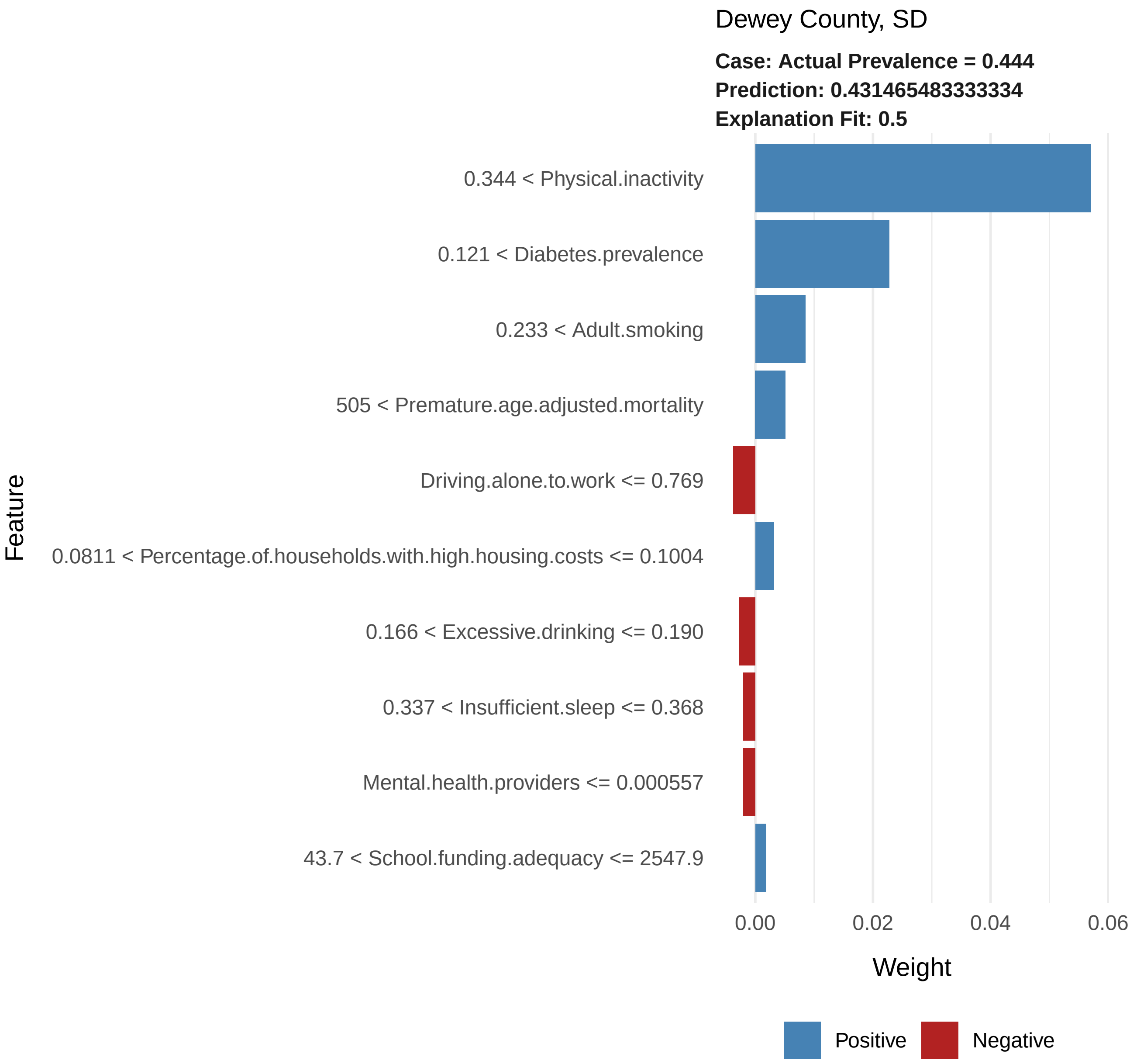
Dewey County, SD
Case: Actual Prevalence = 0.444
Prediction: 0.431465483333334
Explanation Fit: 0.5
0.344 < Physical.inactivity
0.121 < Diabetes.prevalence
0.233 < Adult.smoking
505 < Premature.age.adjusted.mortality
Driving.alone.to.work <= 0.769
0.0811 < Percentage.of.households.with.high.housing.costs <= 0.1004
0.166 < Excessive.drinking <= 0.190
0.337 < Insufficient.sleep <= 0.368
Mental.health.providers <= 0.000557
43.7 < School.funding.adequacy <= 2547.9
Feature
0.00
0.02
0.04
0.06
Weight
Positive
Negative

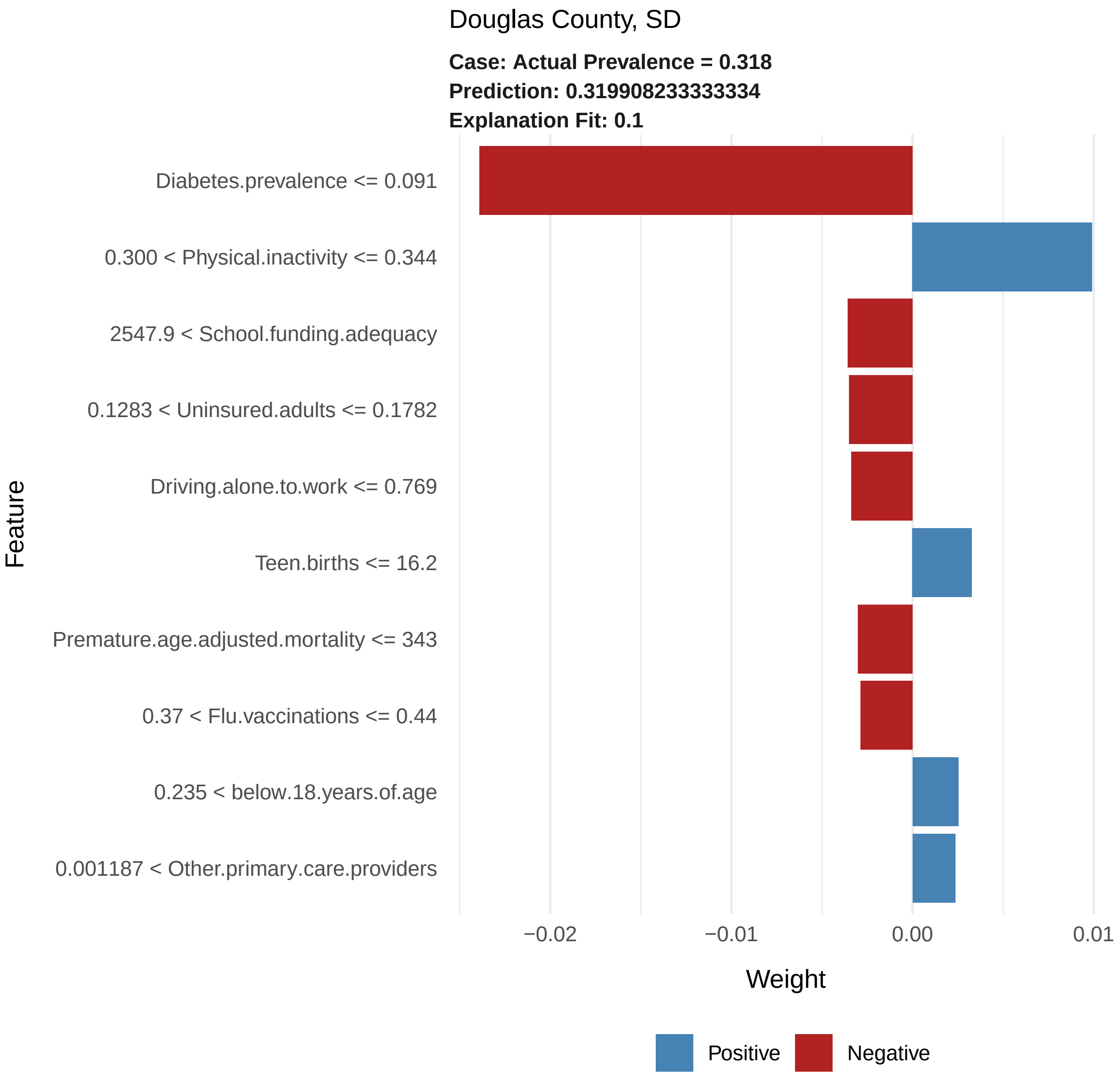
Douglas County, SD
Case: Actual Prevalence = 0.318
Prediction: 0.319908233333334
Explanation Fit: 0.1
Diabetes.prevalence <= 0.091
0.300 < Physical.inactivity <= 0.344
2547.9 < School.funding.adequacy
0.1283 < Uninsured.adults <= 0.1782
Driving.alone.to.work <= 0.769
Teen.births <= 16.2
Premature.age.adjusted.mortality <= 343
0.37 < Flu.vaccinations <= 0.44
0.235 < below.18.years.of.age
0.001187 < Other.primary.care.providers
Feature
−0.02
−0.01
0.00
0.01
Weight
Positive
Negative

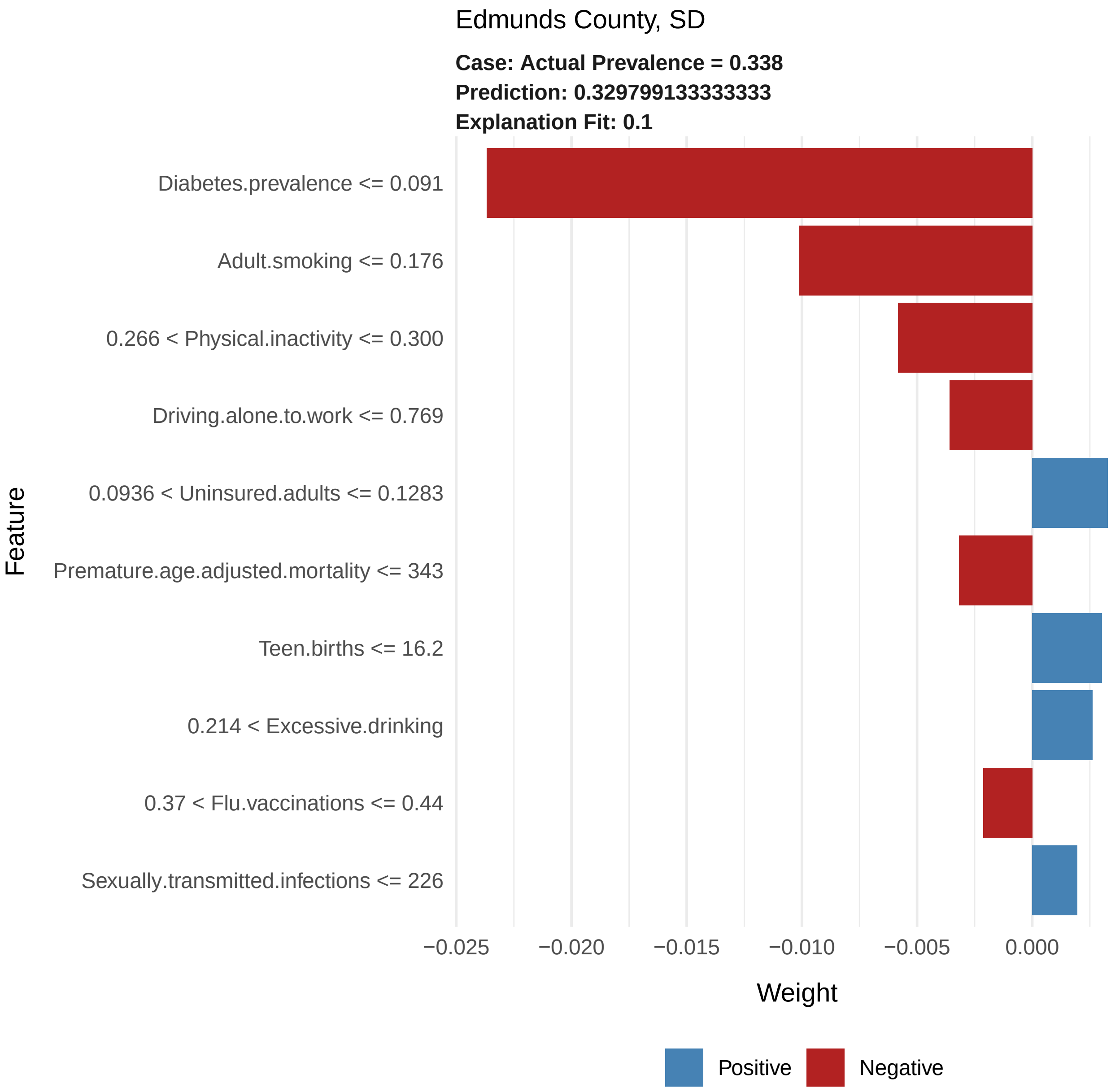

Edmunds County, SD
Case: Actual Prevalence = 0.338
Prediction: 0.329799133333333
Explanation Fit: 0.1
Diabetes.prevalence <= 0.091
Adult.smoking <= 0.176
0.266 < Physical.inactivity <= 0.300
Driving.alone.to.work <= 0.769
0.0936 < Uninsured.adults <= 0.1283
Premature.age.adjusted.mortality <= 343
Teen.births <= 16.2
0.214 < Excessive.drinking
0.37 < Flu.vaccinations <= 0.44
Sexually.transmitted.infections <= 226
Feature
−0.025
−0.020
−0.015
−0.010
−0.005
0.000
Weight
Positive
Negative

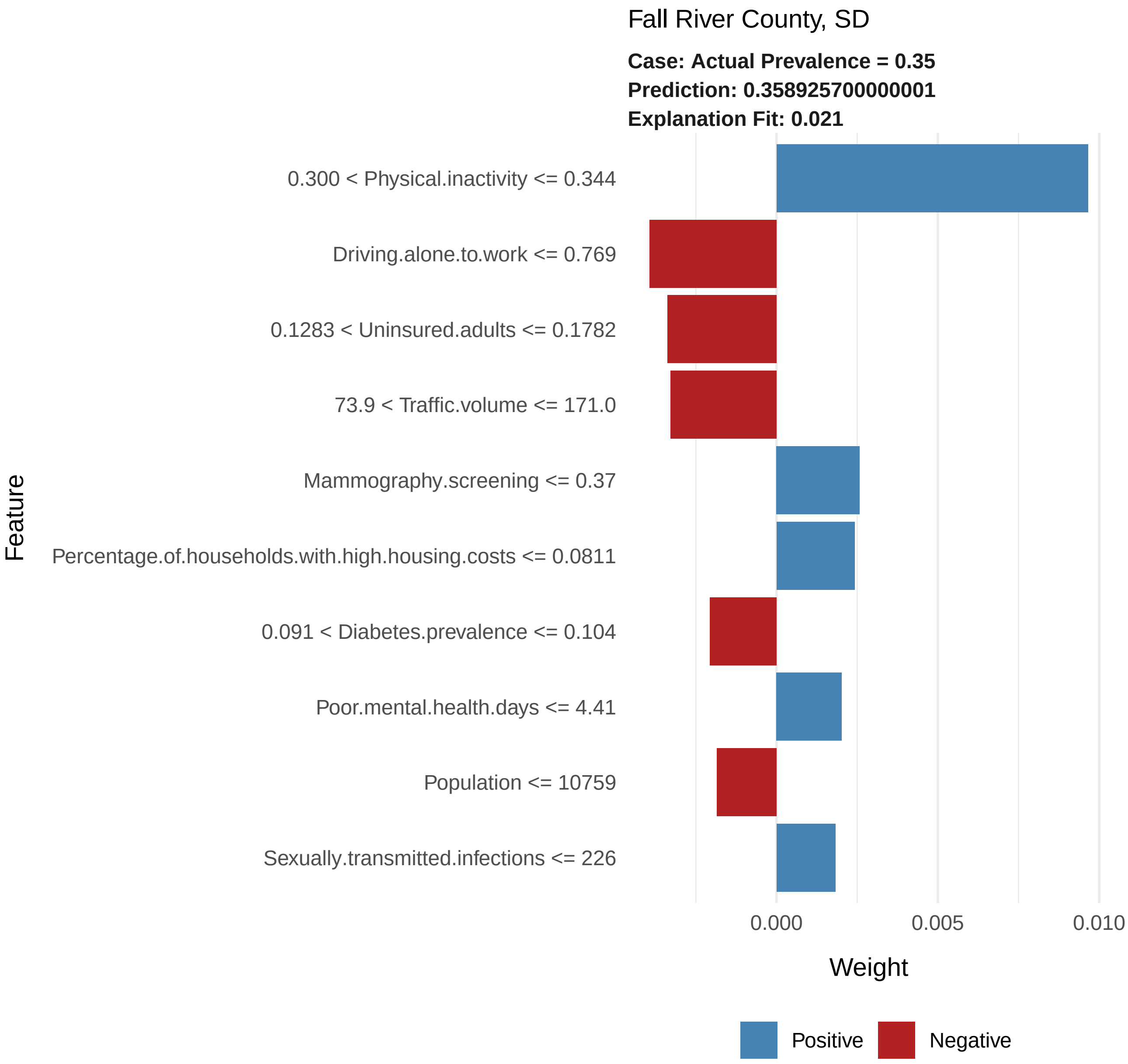

Fall River County, SD
Case: Actual Prevalence = 0.35
Prediction: 0.358925700000001
Explanation Fit: 0.021
0.300 < Physical.inactivity <= 0.344
Driving.alone.to.work <= 0.769
0.1283 < Uninsured.adults <= 0.1782
73.9 < Traffic.volume <= 171.0
Mammography.screening <= 0.37
Percentage.of.households.with.high.housing.costs <= 0.0811
0.091 < Diabetes.prevalence <= 0.104
Poor.mental.health.days <= 4.41
Population <= 10759
Sexually.transmitted.infections <= 226
Feature
0.000
0.005
0.010
Weight
Positive
Negative

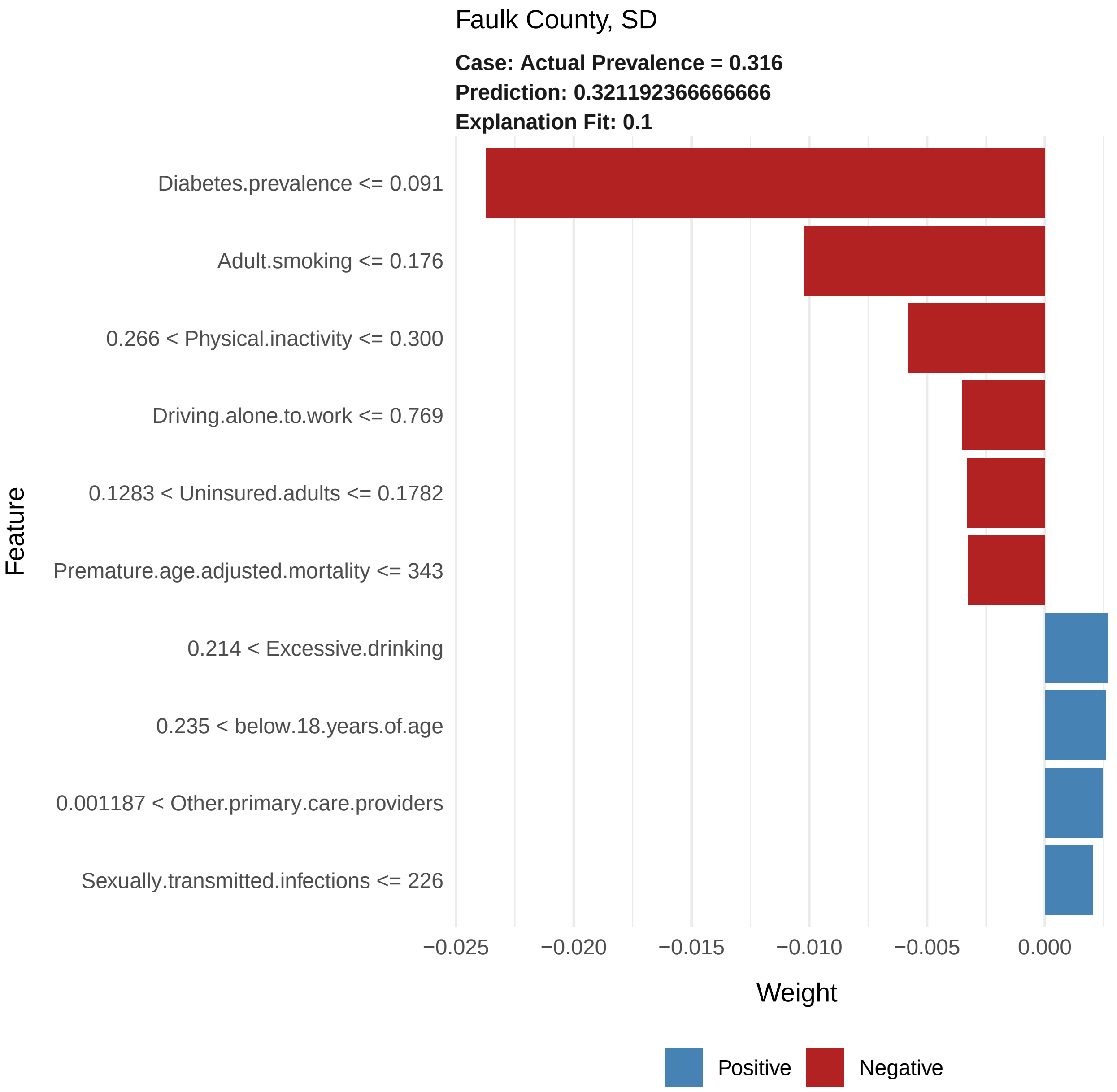

Faulk County, SD
Case: Actual Prevalence = 0.316
Prediction: 0.321192366666666
Explanation Fit: 0.1
Diabetes.prevalence <= 0.091
Adult.smoking <= 0.176
0.266 < Physical.inactivity <= 0.300
Driving.alone.to.work <= 0.769
0.1283 < Uninsured.adults <= 0.1782
Premature.age.adjusted.mortality <= 343
0.214 < Excessive.drinking
0.235 < below.18.years.of.age
0.001187 < Other.primary.care.providers
Sexually.transmitted.infections <= 226
Feature
−0.025
−0.020
−0.015
−0.010
−0.005
0.000
Weight
Positive
Negative

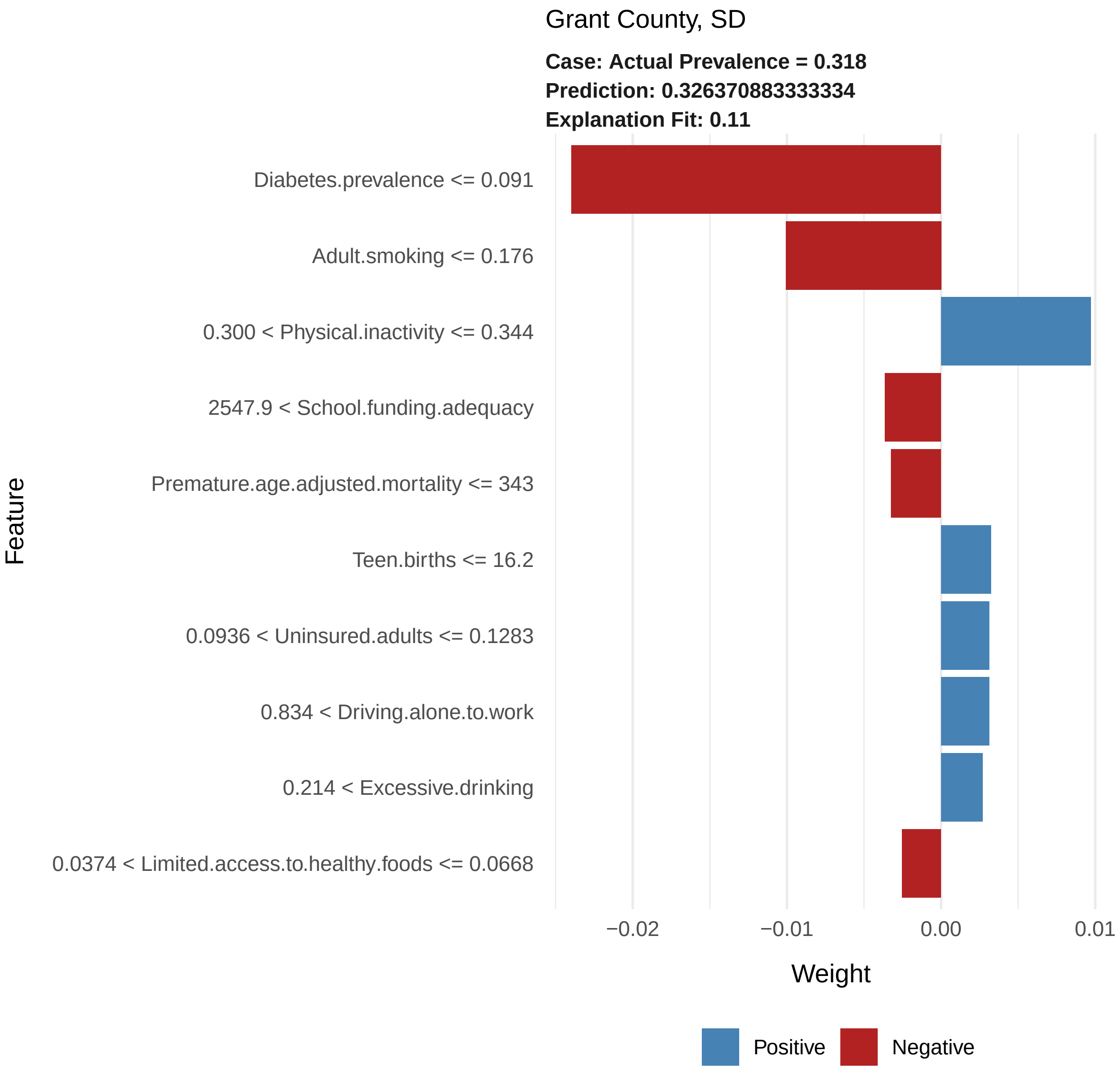
Grant County, SD
Case: Actual Prevalence = 0.318
Prediction: 0.326370883333334
Explanation Fit: 0.11
Diabetes.prevalence <= 0.091
Adult.smoking <= 0.176
0.300 < Physical.inactivity <= 0.344
2547.9 < School.funding.adequacy
Premature.age.adjusted.mortality <= 343
Teen.births <= 16.2
0.0936 < Uninsured.adults <= 0.1283
0.834 < Driving.alone.to.work
0.214 < Excessive.drinking
0.0374 < Limited.access.to.healthy.foods <= 0.0668
Feature
−0.02
−0.01
0.00
0.01
Weight
Positive
Negative

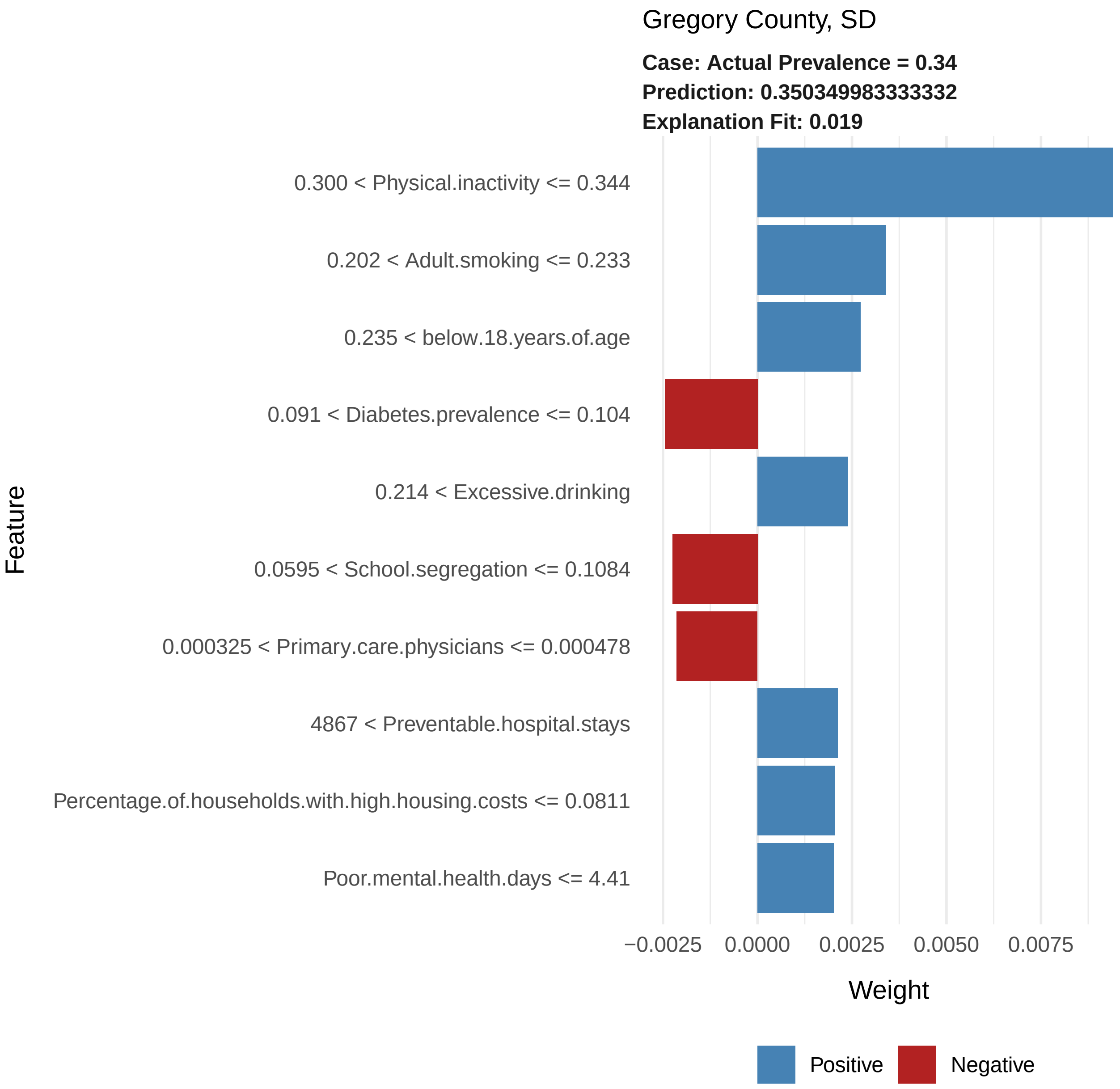
Gregory County, SD
Case: Actual Prevalence = 0.34
Prediction: 0.350349983333332
Explanation Fit: 0.019
Feature
0.300 < Physical.inactivity <= 0.344
0.202 < Adult.smoking <= 0.233
0.235 < below.18.years.of.age
0.091 < Diabetes.prevalence <= 0.104
0.214 < Excessive.drinking
0.0595 < School.segregation <= 0.1084
0.000325 < Primary.care.physicians <= 0.000478
4867 < Preventable.hospital.stays
Percentage.of.households.with.high.housing.costs <= 0.0811
Poor.mental.health.days <= 4.41
−0.0025
0.0000
0.0025
0.0050
0.0075
Weight
Positive
Negative

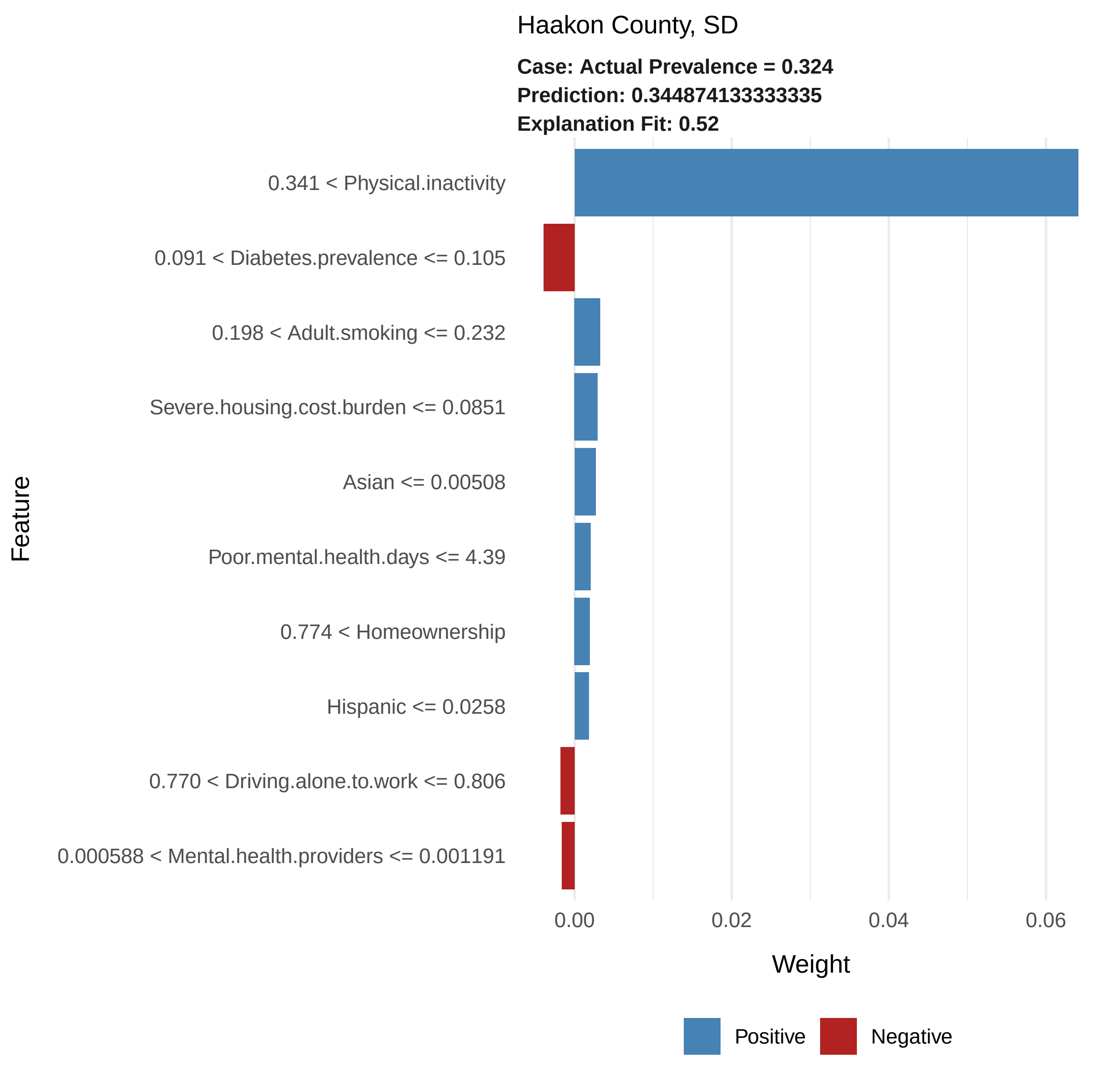
Haakon County, SD
Case: Actual Prevalence = 0.324
Prediction: 0.344874133333335
Explanation Fit: 0.52
0.341 < Physical.inactivity
0.091 < Diabetes.prevalence <= 0.105
0.198 < Adult.smoking <= 0.232
Severe.housing.cost.burden <= 0.0851
Asian <= 0.00508
Poor.mental.health.days <= 4.39
0.774 < Homeownership
Hispanic <= 0.0258
0.770 < Driving.alone.to.work <= 0.806
0.000588 < Mental.health.providers <= 0.001191
Feature
0.00
0.02
0.04
0.06
Weight
Positive
Negative

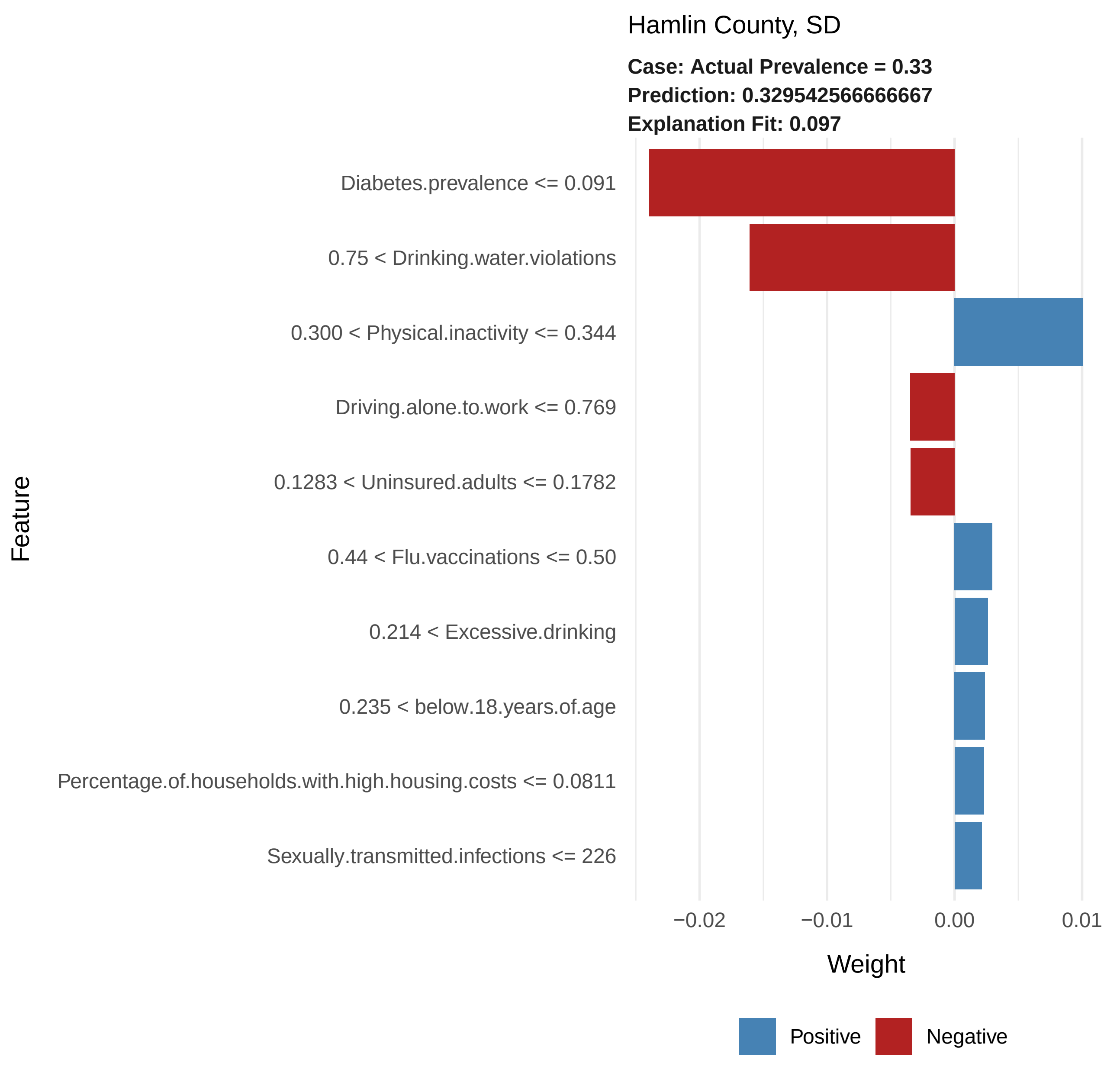

Hamlin County, SD
Case: Actual Prevalence = 0.33
Prediction: 0.329542566666667
Explanation Fit: 0.097
Diabetes.prevalence <= 0.091
0.75 < Drinking.water.violations
0.300 < Physical.inactivity <= 0.344
Driving.alone.to.work <= 0.769
0.1283 < Uninsured.adults <= 0.1782
0.44 < Flu.vaccinations <= 0.50
0.214 < Excessive.drinking
0.235 < below.18.years.of.age
Percentage.of.households.with.high.housing.costs <= 0.0811
Sexually.transmitted.infections <= 226
Feature
−0.02
−0.01
0.00
0.01
Weight
Positive
Negative

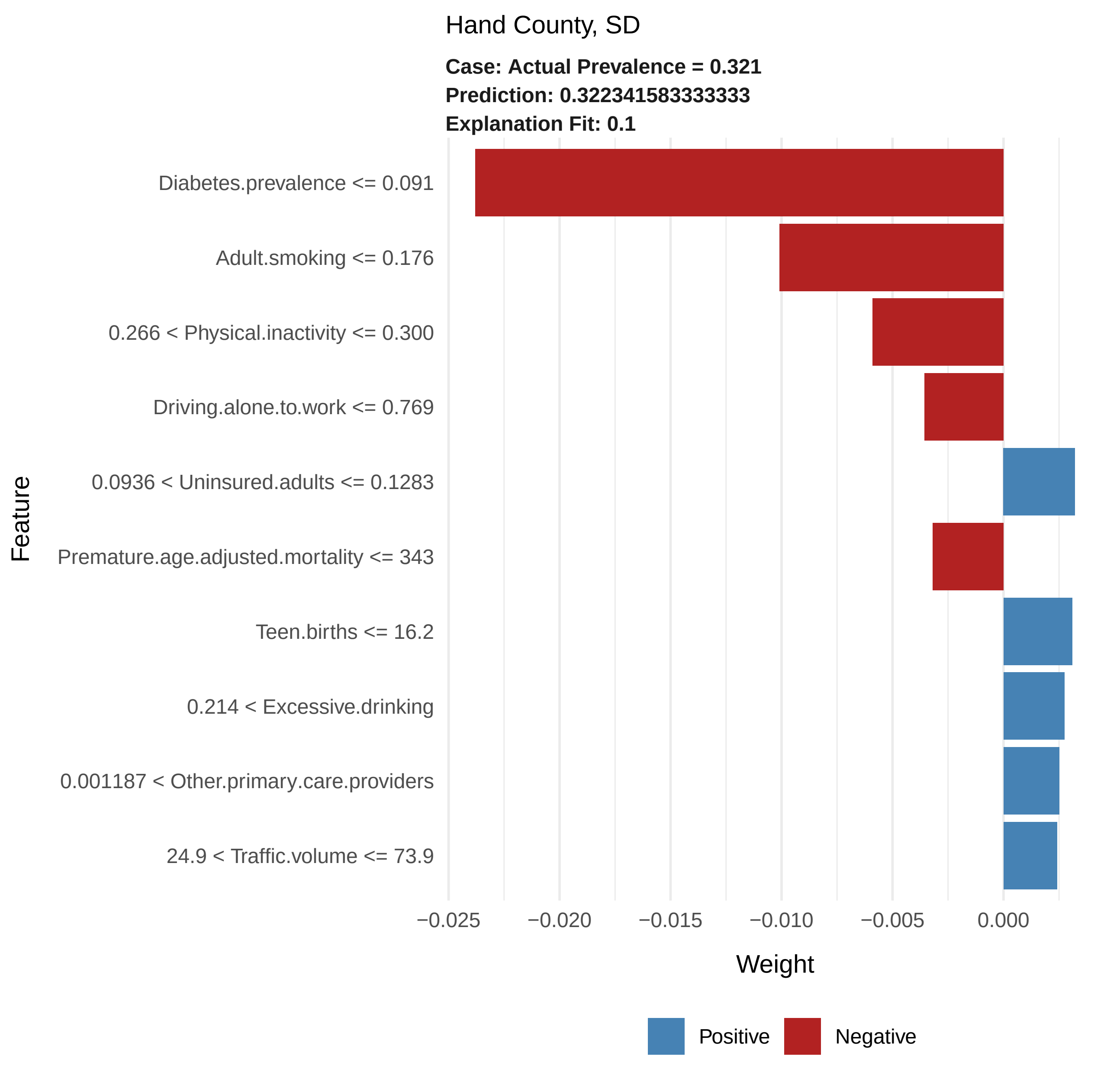

Hand County, SD
Case: Actual Prevalence = 0.321
Prediction: 0.322341583333333
Explanation Fit: 0.1
Diabetes.prevalence <= 0.091
Adult.smoking <= 0.176
0.266 < Physical.inactivity <= 0.300
Driving.alone.to.work <= 0.769
0.0936 < Uninsured.adults <= 0.1283
Premature.age.adjusted.mortality <= 343
Teen.births <= 16.2
0.214 < Excessive.drinking
0.001187 < Other.primary.care.providers
24.9 < Traffic.volume <= 73.9
Feature
−0.025
−0.020
−0.015
−0.010
−0.005
0.000
Weight
Positive
Negative

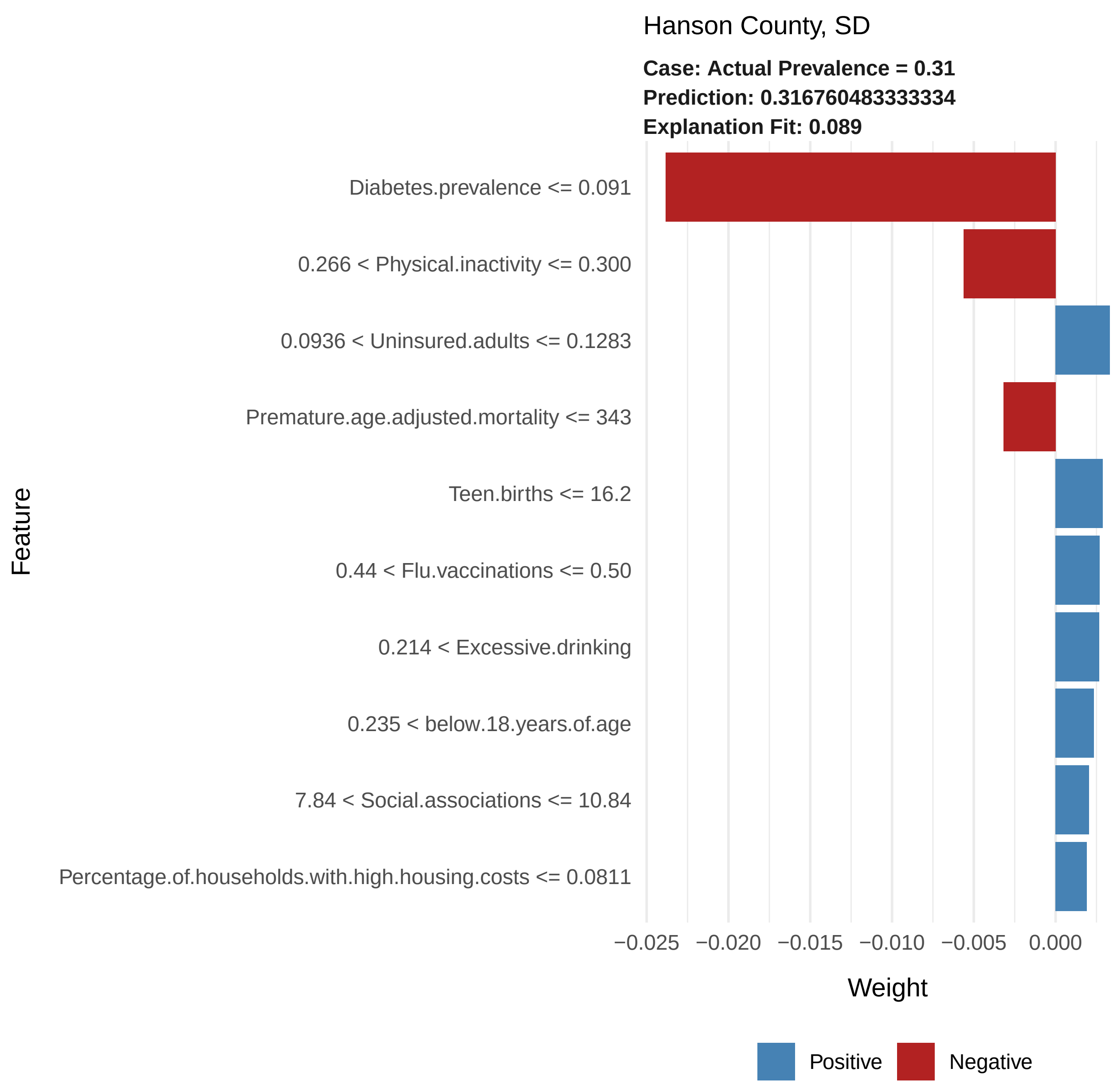
Hanson County, SD
Case: Actual Prevalence = 0.31
Prediction: 0.316760483333334
Explanation Fit: 0.089
Diabetes.prevalence <= 0.091
0.266 < Physical.inactivity <= 0.300
0.0936 < Uninsured.adults <= 0.1283
Premature.age.adjusted.mortality <= 343
Teen.births <= 16.2
0.44 < Flu.vaccinations <= 0.50
0.214 < Excessive.drinking
0.235 < below.18.years.of.age
7.84 < Social.associations <= 10.84
Percentage.of.households.with.high.housing.costs <= 0.0811
Feature
−0.025
−0.020
−0.015
−0.010
−0.005
0.000
Weight
Positive
Negative

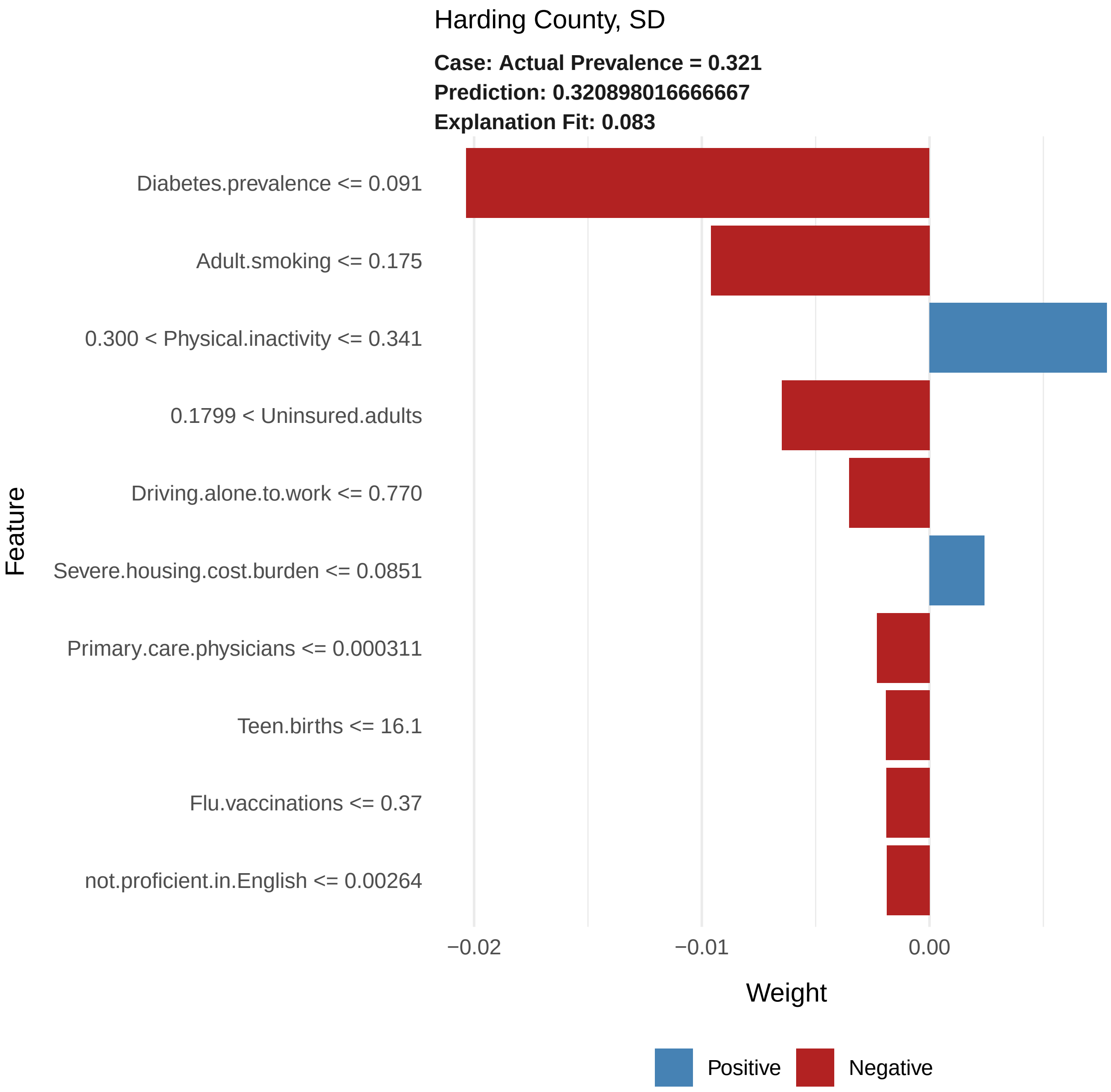

Harding County, SD
Case: Actual Prevalence = 0.321
Prediction: 0.320898016666667
Explanation Fit: 0.083
Diabetes.prevalence <= 0.091
Adult.smoking <= 0.175
0.300 < Physical.inactivity <= 0.341
0.1799 < Uninsured.adults
Driving.alone.to.work <= 0.770
Severe.housing.cost.burden <= 0.0851
Primary.care.physicians <= 0.000311
Teen.births <= 16.1
Flu.vaccinations <= 0.37
not.proficient.in.English <= 0.00264
Feature
−0.02
−0.01
0.00
Weight
Positive
Negative

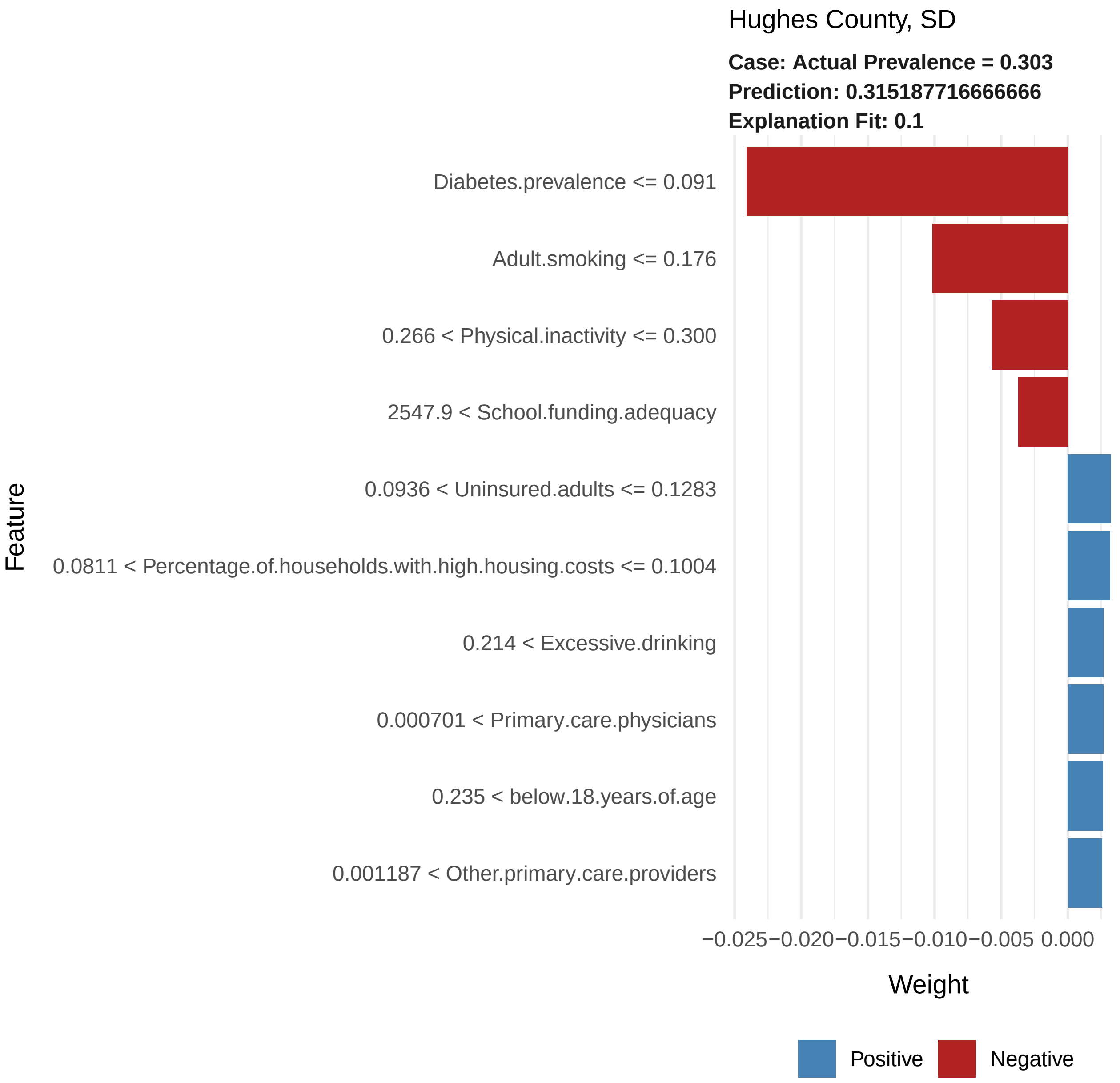
Hughes County, SD
Case: Actual Prevalence = 0.303
Prediction: 0.315187716666666
Explanation Fit: 0.1
Diabetes.prevalence <= 0.091
Adult.smoking <= 0.176
0.266 < Physical.inactivity <= 0.300
2547.9 < School.funding.adequacy
0.0936 < Uninsured.adults <= 0.1283
0.0811 < Percentage.of.households.with.high.housing.costs <= 0.1004
0.214 < Excessive.drinking
0.000701 < Primary.care.physicians
0.235 < below.18.years.of.age
0.001187 < Other.primary.care.providers
Feature
−0.025
−0.020
−0.015
−0.010
−0.005
0.000
Weight
Positive
Negative

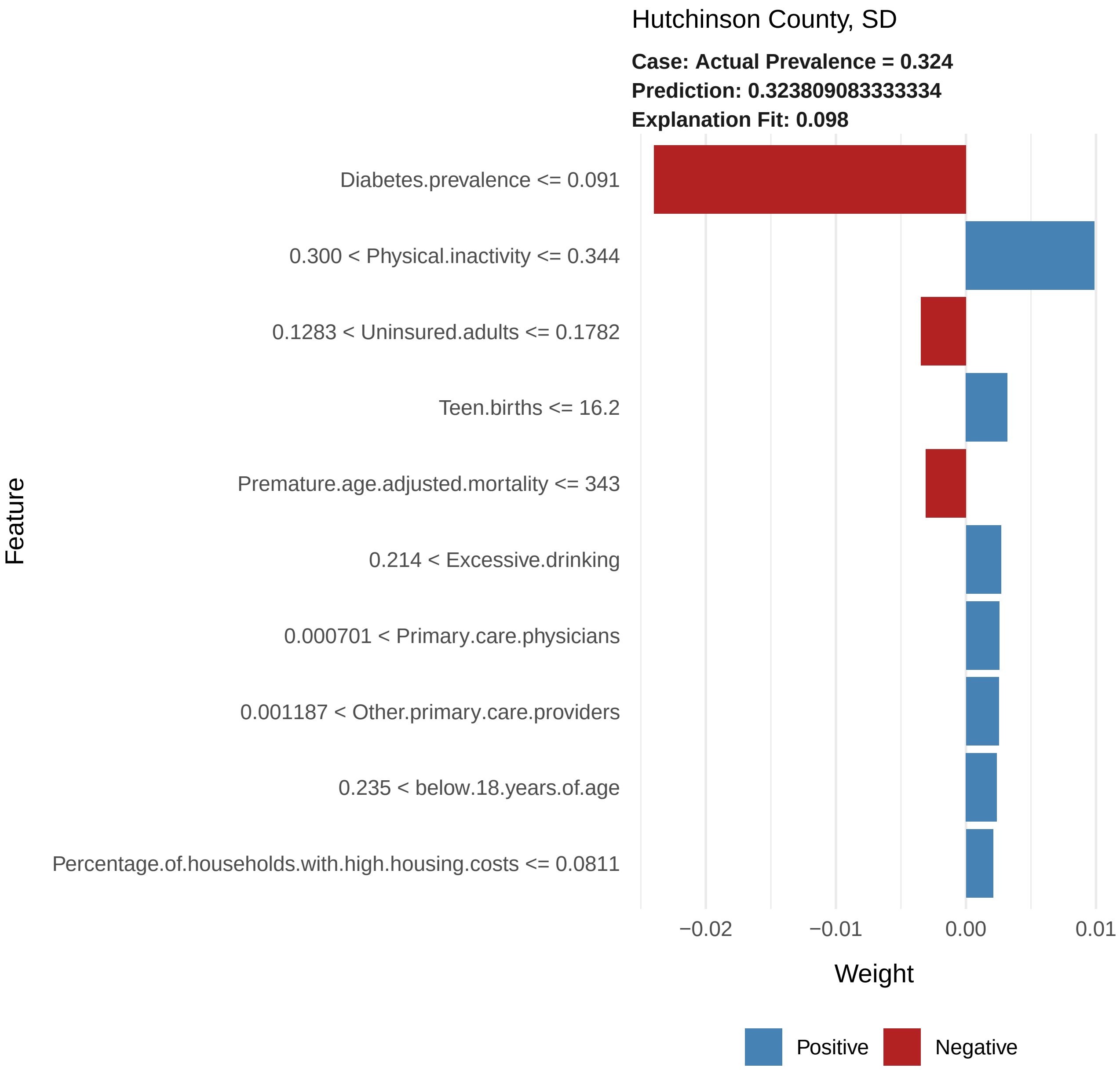

Hutchinson County, SD
Case: Actual Prevalence = 0.324
Prediction: 0.323809083333334
Explanation Fit: 0.098
Diabetes.prevalence <= 0.091
0.300 < Physical.inactivity <= 0.344
0.1283 < Uninsured.adults <= 0.1782
Teen.births <= 16.2
Premature.age.adjusted.mortality <= 343
0.214 < Excessive.drinking
0.000701 < Primary.care.physicians
0.001187 < Other.primary.care.providers
0.235 < below.18.years.of.age
Percentage.of.households.with.high.housing.costs <= 0.0811
Feature
−0.02
−0.01
0.00
0.01
Weight
Positive
Negative

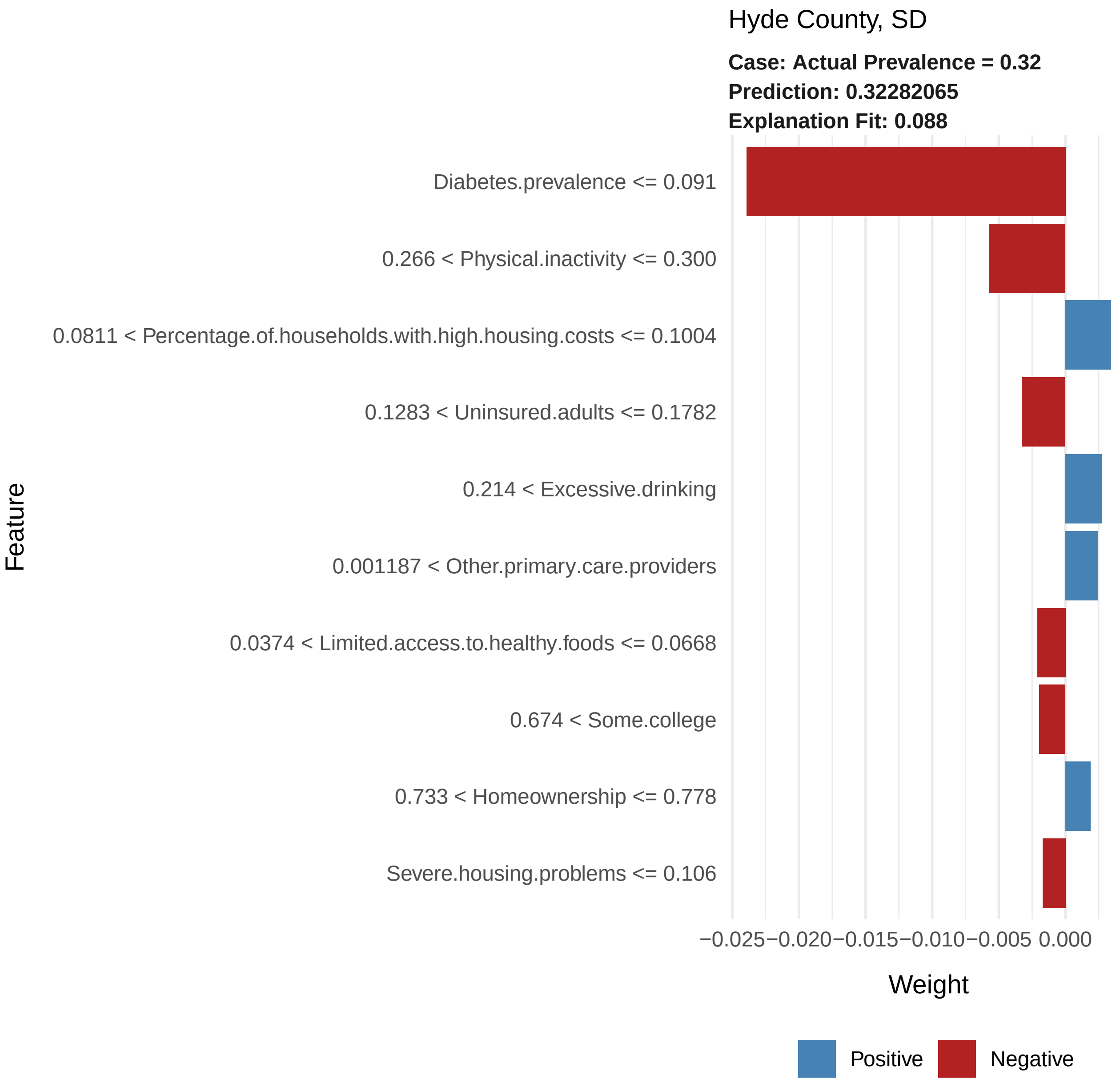

Hyde County, SD
Case: Actual Prevalence = 0.32
Prediction: 0.32282065
Explanation Fit: 0.088
Diabetes.prevalence <= 0.091
0.266 < Physical.inactivity <= 0.300
0.0811 < Percentage.of.households.with.high.housing.costs <= 0.1004
0.1283 < Uninsured.adults <= 0.1782
0.214 < Excessive.drinking
0.001187 < Other.primary.care.providers
0.0374 < Limited.access.to.healthy.foods <= 0.0668
0.674 < Some.college
0.733 < Homeownership <= 0.778
Severe.housing.problems <= 0.106
Feature
−0.025
−0.020
−0.015
−0.010
−0.005
0.000
Weight
Positive
Negative

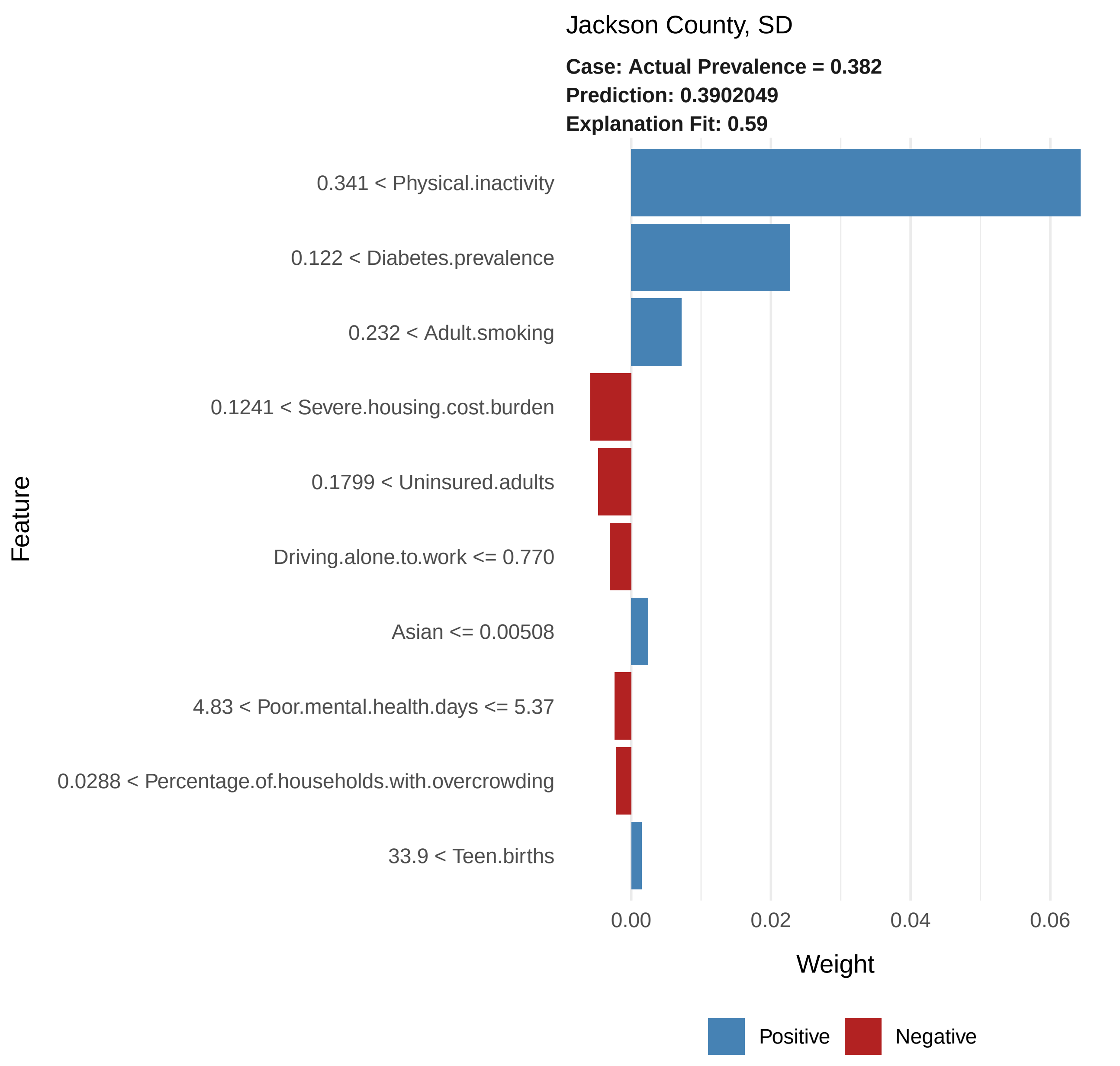

Jackson County, SD
Case: Actual Prevalence = 0.382
Prediction: 0.3902049
Explanation Fit: 0.59
0.341 < Physical.inactivity
0.122 < Diabetes.prevalence
0.232 < Adult.smoking
0.1241 < Severe.housing.cost.burden
0.1799 < Uninsured.adults
Driving.alone.to.work <= 0.770
Asian <= 0.00508
4.83 < Poor.mental.health.days <= 5.37
0.0288 < Percentage.of.households.with.overcrowding
33.9 < Teen.births
Feature
0.00
0.02
0.04
0.06
Weight
Positive
Negative

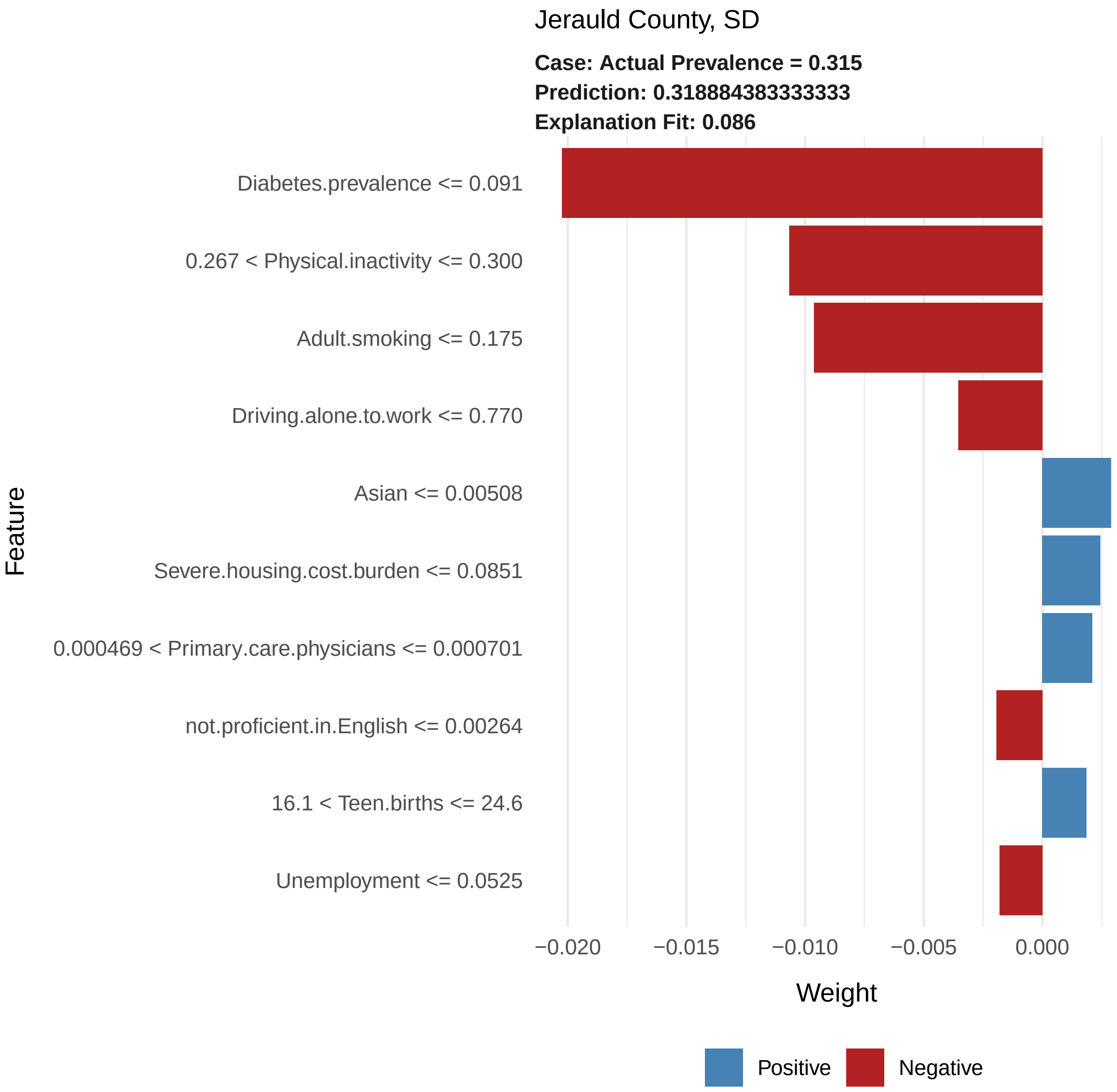
Jerauld County, SD
Case: Actual Prevalence = 0.315
Prediction: 0.318884383333333
Explanation Fit: 0.086
Diabetes.prevalence <= 0.091
0.267 < Physical.inactivity <= 0.300
Adult.smoking <= 0.175
Driving.alone.to.work <= 0.770
Asian <= 0.00508
Severe.housing.cost.burden <= 0.0851
0.000469 < Primary.care.physicians <= 0.000701
not.proficient.in.English <= 0.00264
16.1 < Teen.births <= 24.6
Unemployment <= 0.0525
Feature
−0.020
−0.015
−0.010
−0.005
0.000
Weight
Positive
Negative

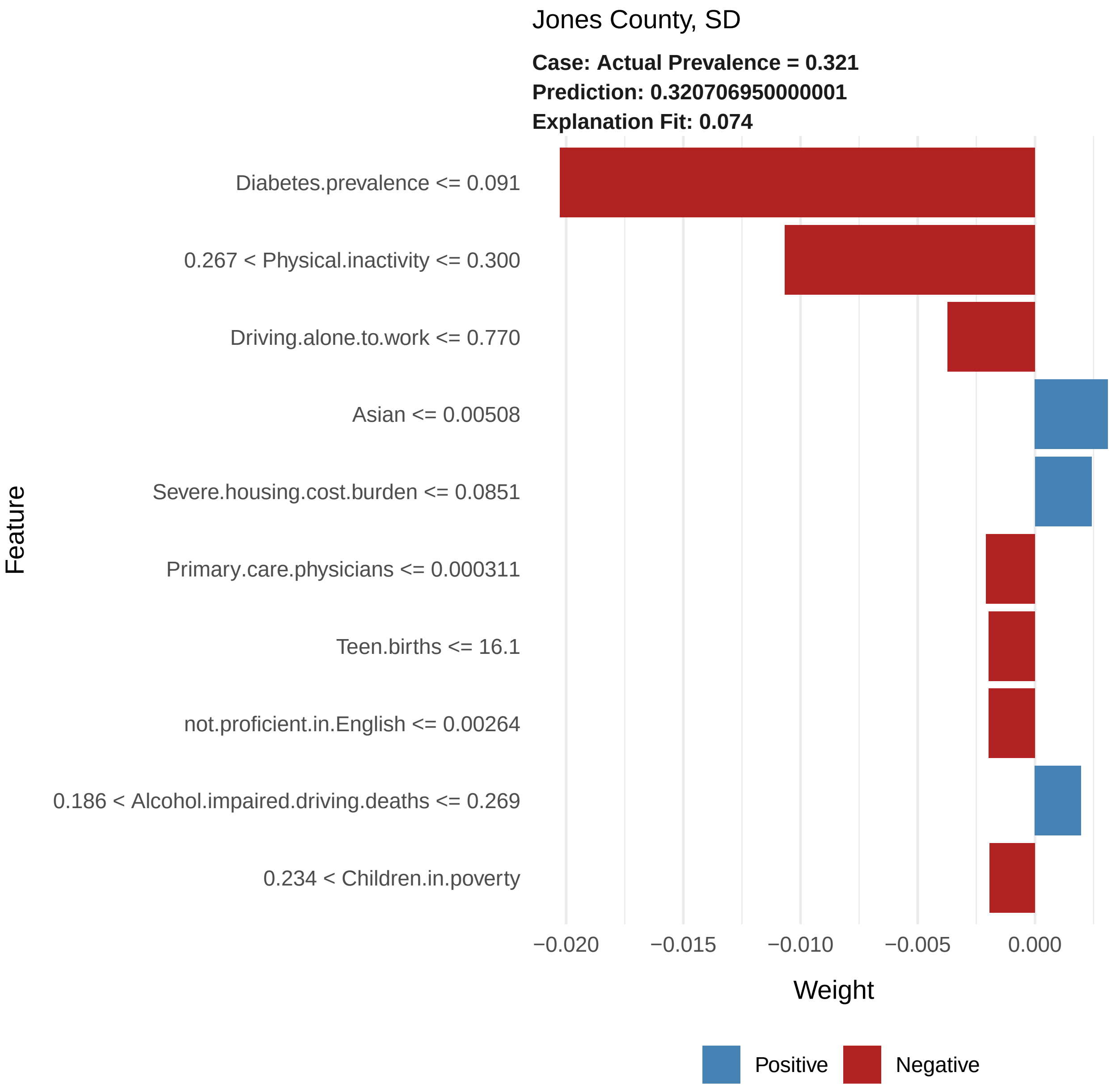

Jones County, SD
Case: Actual Prevalence = 0.321
Prediction: 0.320706950000001
Explanation Fit: 0.074
Diabetes.prevalence <= 0.091
0.267 < Physical.inactivity <= 0.300
Driving.alone.to.work <= 0.770
Asian <= 0.00508
Severe.housing.cost.burden <= 0.0851
Primary.care.physicians <= 0.000311
Teen.births <= 16.1
not.proficient.in.English <= 0.00264
0.186 < Alcohol.impaired.driving.deaths <= 0.269
0.234 < Children.in.poverty
Feature
−0.020
−0.015
−0.010
−0.005
0.000
Weight
Positive
Negative

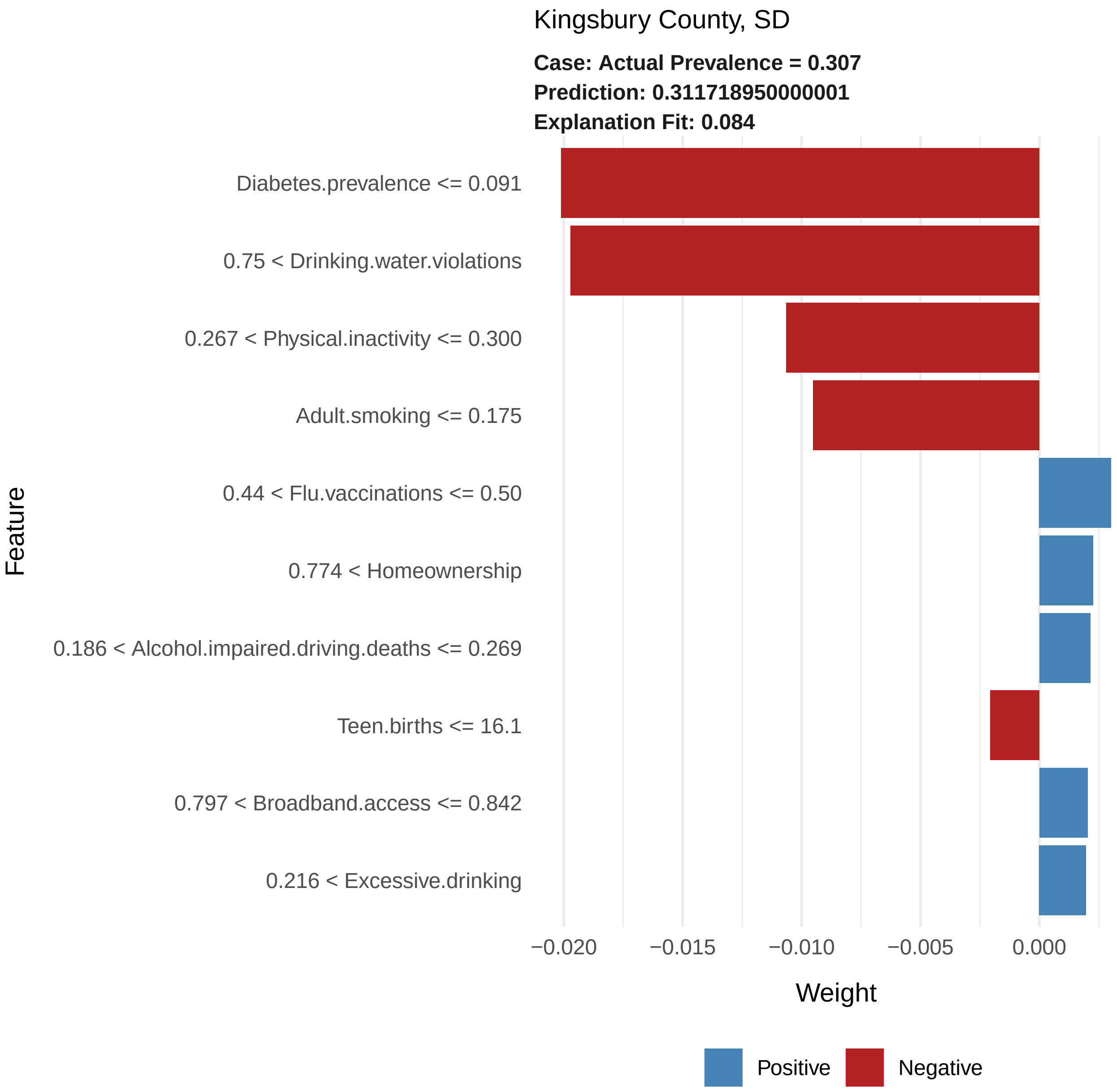

Kingsbury County, SD
Case: Actual Prevalence = 0.307
Prediction: 0.311718950000001
Explanation Fit: 0.084
Diabetes.prevalence <= 0.091
0.75 < Drinking.water.violations
0.267 < Physical.inactivity <= 0.300
Adult.smoking <= 0.175
0.44 < Flu.vaccinations <= 0.50
0.774 < Homeownership
0.186 < Alcohol.impaired.driving.deaths <= 0.269
Teen.births <= 16.1
0.797 < Broadband.access <= 0.842
0.216 < Excessive.drinking
Feature
−0.020
−0.015
−0.010
−0.005
0.000
Weight
Positive
Negative

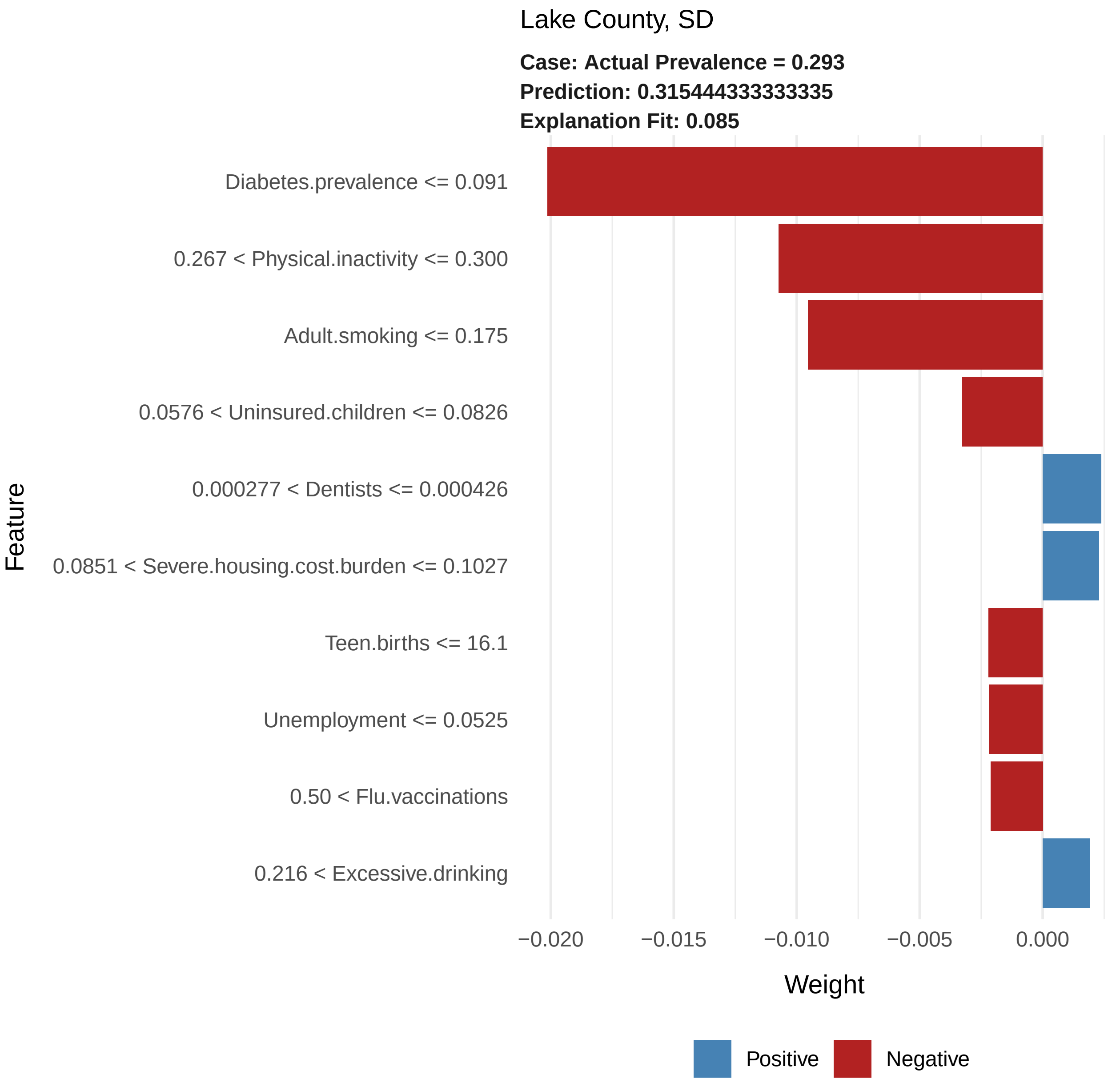

Lake County, SD
Case: Actual Prevalence = 0.293
Prediction: 0.315444333333335
Explanation Fit: 0.085
Diabetes.prevalence <= 0.091
0.267 < Physical.inactivity <= 0.300
Adult.smoking <= 0.175
0.0576 < Uninsured.children <= 0.0826
0.000277 < Dentists <= 0.000426
0.0851 < Severe.housing.cost.burden <= 0.1027
Teen.births <= 16.1
Unemployment <= 0.0525
0.50 < Flu.vaccinations
0.216 < Excessive.drinking
Feature
−0.020
−0.015
−0.010
−0.005
0.000
Weight
Positive
Negative

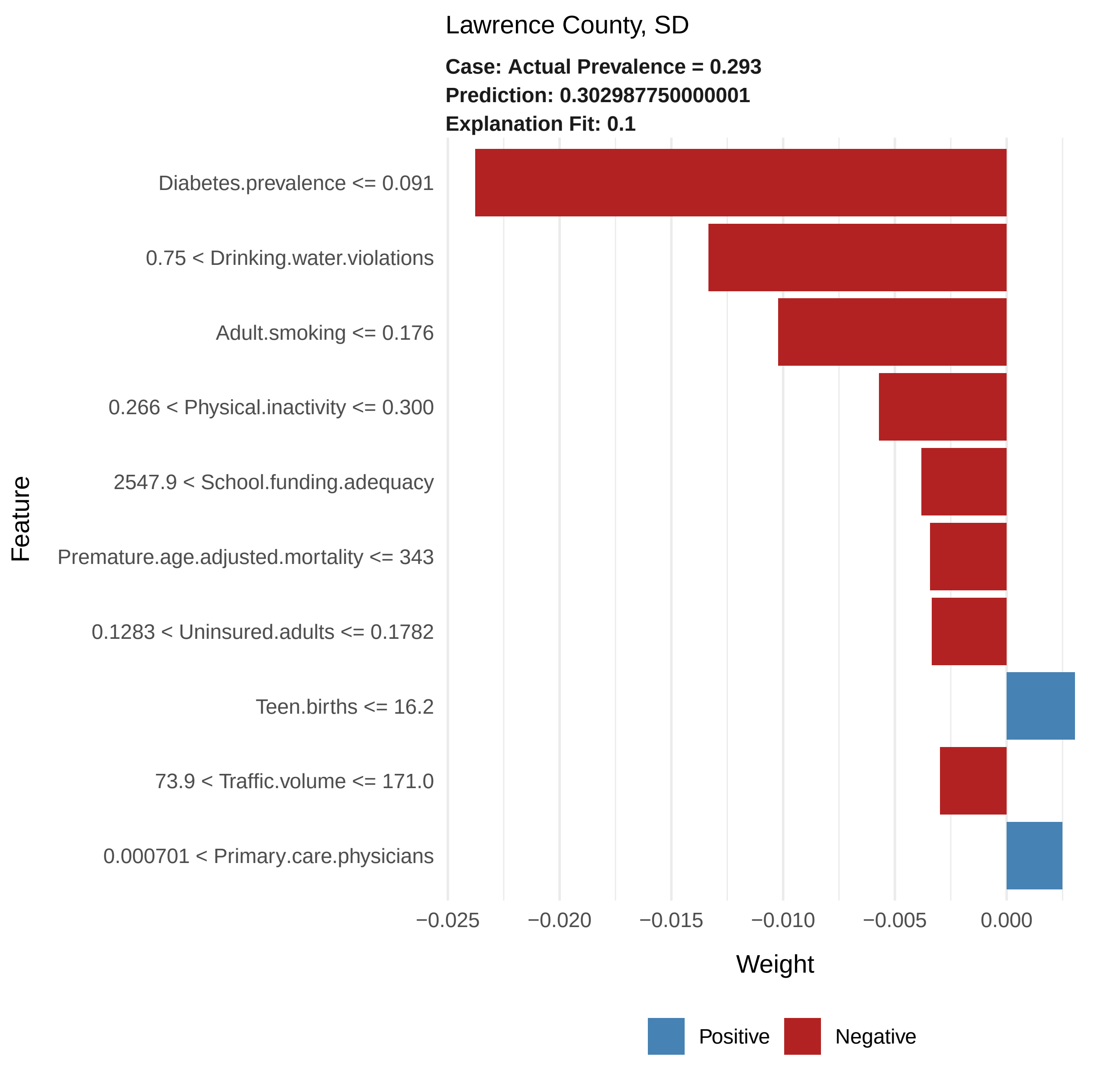

Lawrence County, SD
Case: Actual Prevalence = 0.293
Prediction: 0.302987750000001
Explanation Fit: 0.1
Feature
Diabetes.prevalence <= 0.091
0.75 < Drinking.water.violations
Adult.smoking <= 0.176
0.266 < Physical.inactivity <= 0.300
2547.9 < School.funding.adequacy
Premature.age.adjusted.mortality <= 343
0.1283 < Uninsured.adults <= 0.1782
Teen.births <= 16.2
73.9 < Traffic.volume <= 171.0
0.000701 < Primary.care.physicians
−0.025
−0.020
−0.015
−0.010
−0.005
0.000
Weight
Positive
Negative

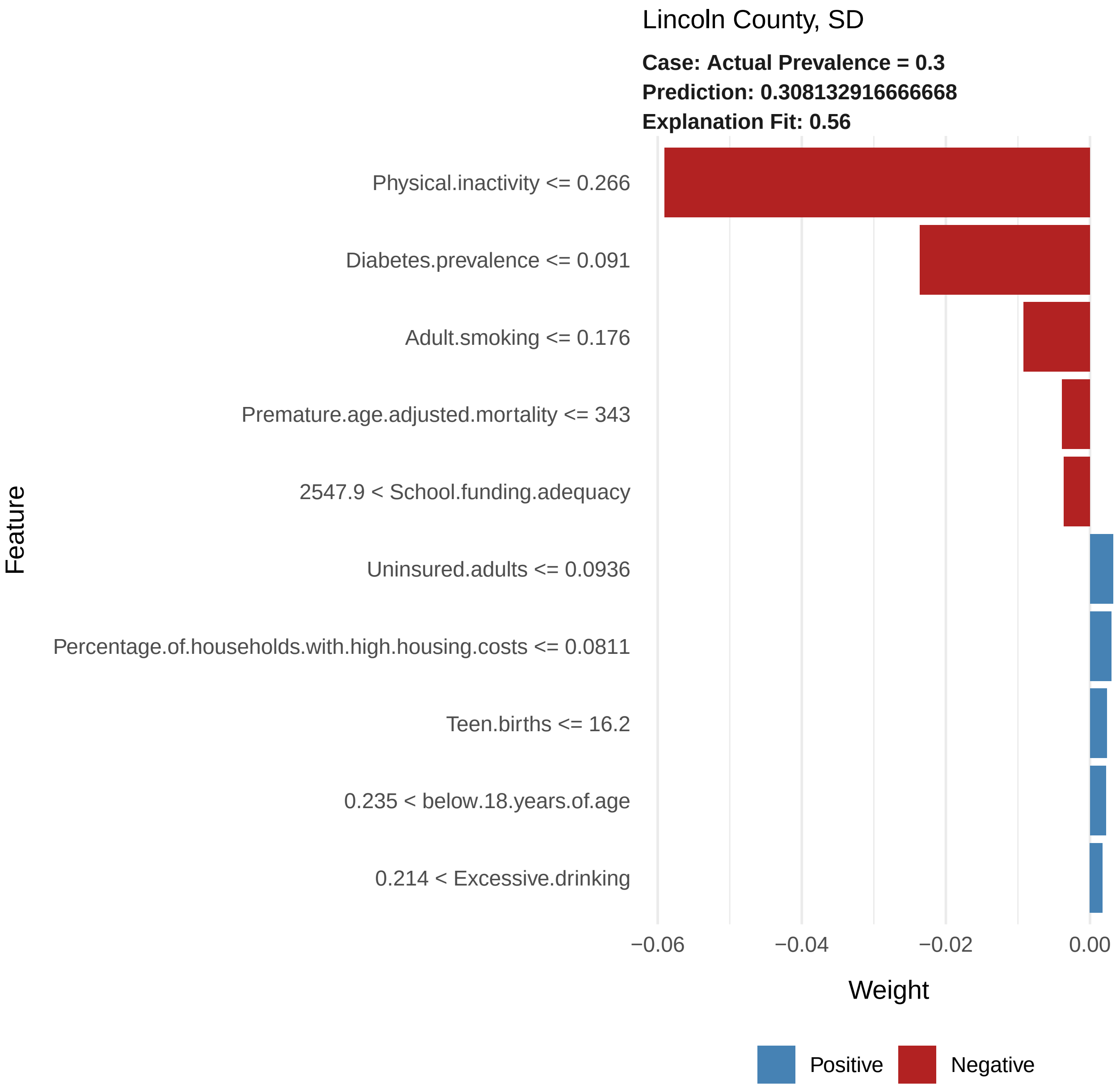

Lincoln County, SD
Case: Actual Prevalence = 0.3
Prediction: 0.308132916666668
Explanation Fit: 0.56
Physical.inactivity <= 0.266
Diabetes.prevalence <= 0.091
Adult.smoking <= 0.176
Premature.age.adjusted.mortality <= 343
2547.9 < School.funding.adequacy
Uninsured.adults <= 0.0936
Percentage.of.households.with.high.housing.costs <= 0.0811
Teen.births <= 16.2
0.235 < below.18.years.of.age
0.214 < Excessive.drinking
Feature
−0.06
−0.04
−0.02
0.00
Weight
Positive
Negative

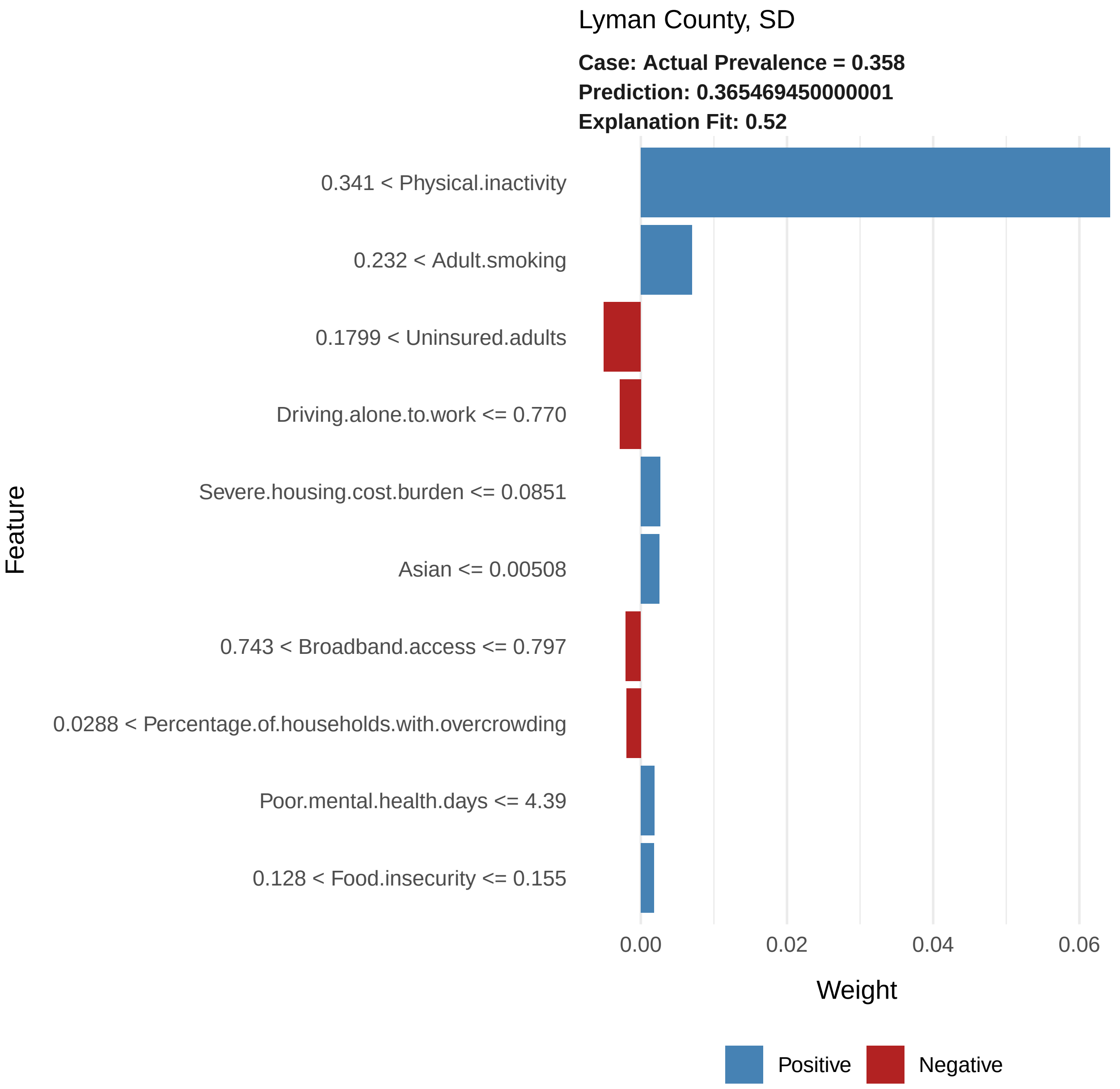
Lyman County, SD
Case: Actual Prevalence = 0.358
Prediction: 0.365469450000001
Explanation Fit: 0.52
0.341 < Physical.inactivity
0.232 < Adult.smoking
0.1799 < Uninsured.adults
Driving.alone.to.work <= 0.770
Severe.housing.cost.burden <= 0.0851
Asian <= 0.00508
0.743 < Broadband.access <= 0.797
0.0288 < Percentage.of.households.with.overcrowding
Poor.mental.health.days <= 4.39
0.128 < Food.insecurity <= 0.155
Feature
0.00
0.02
0.04
0.06
Weight
Positive
Negative

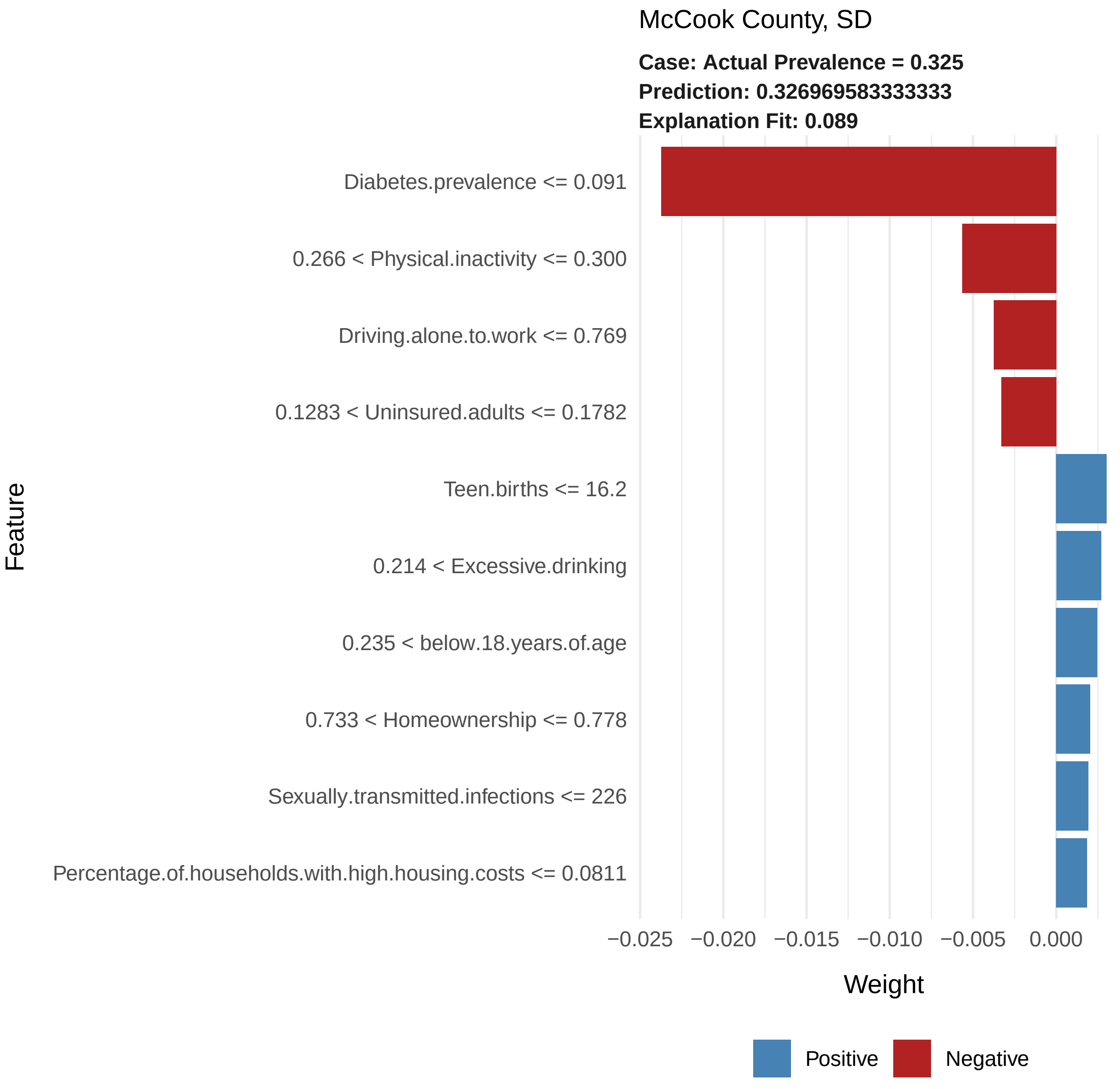
McCook County, SD
Case: Actual Prevalence = 0.325
Prediction: 0.326969583333333
Explanation Fit: 0.089
Diabetes.prevalence <= 0.091
0.266 < Physical.inactivity <= 0.300
Driving.alone.to.work <= 0.769
0.1283 < Uninsured.adults <= 0.1782
Teen.births <= 16.2
0.214 < Excessive.drinking
0.235 < below.18.years.of.age
0.733 < Homeownership <= 0.778
Sexually.transmitted.infections <= 226
Percentage.of.households.with.high.housing.costs <= 0.0811
Feature
−0.025
−0.020
−0.015
−0.010
−0.005
0.000
Weight
Positive
Negative

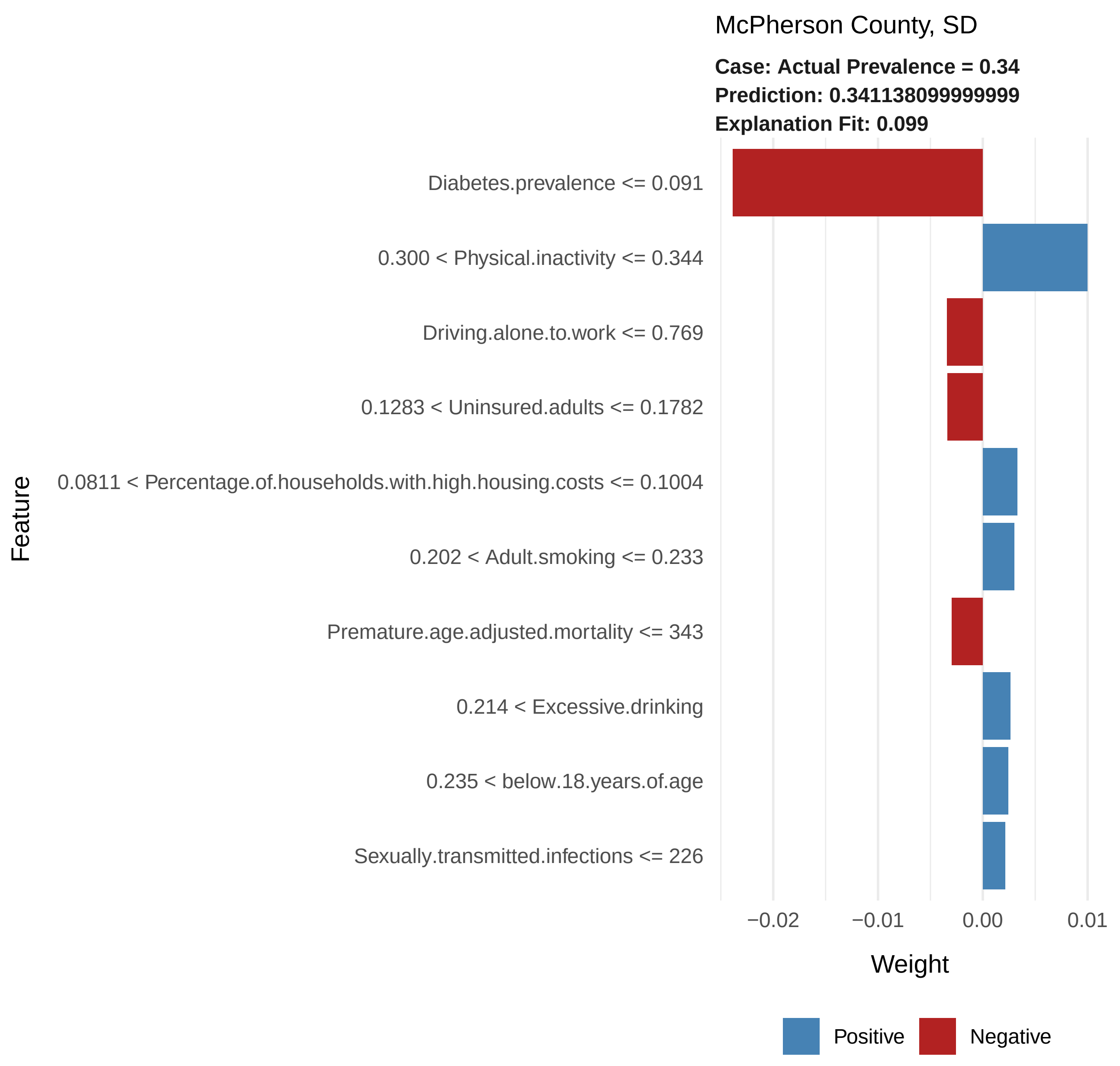
McPherson County, SD
Case: Actual Prevalence = 0.34
Prediction: 0.341138099999999
Explanation Fit: 0.099
Diabetes.prevalence <= 0.091
0.300 < Physical.inactivity <= 0.344
Driving.alone.to.work <= 0.769
0.1283 < Uninsured.adults <= 0.1782
0.0811 < Percentage.of.households.with.high.housing.costs <= 0.1004
0.202 < Adult.smoking <= 0.233
Premature.age.adjusted.mortality <= 343
0.214 < Excessive.drinking
0.235 < below.18.years.of.age
Sexually.transmitted.infections <= 226
Feature
−0.02
−0.01
0.00
0.01
Weight
Positive
Negative

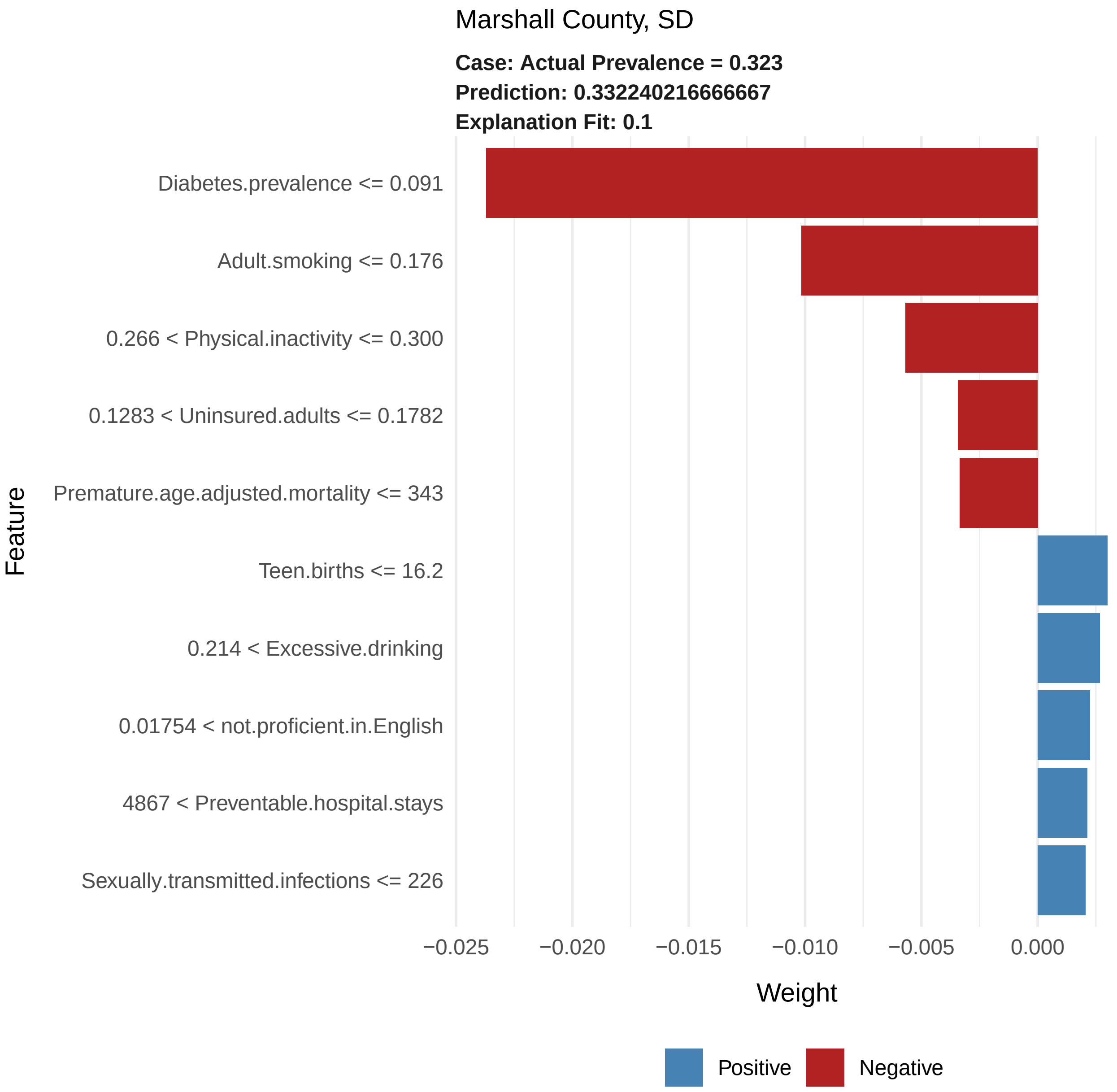
Marshall County, SD
Case: Actual Prevalence = 0.323
Prediction: 0.332240216666667
Explanation Fit: 0.1
Diabetes.prevalence <= 0.091
Adult.smoking <= 0.176
0.266 < Physical.inactivity <= 0.300
0.1283 < Uninsured.adults <= 0.1782
Premature.age.adjusted.mortality <= 343
Teen.births <= 16.2
0.214 < Excessive.drinking
0.01754 < not.proficient.in.English
4867 < Preventable.hospital.stays
Sexually.transmitted.infections <= 226
Feature
−0.025
−0.020
−0.015
−0.010
−0.005
0.000
Weight
Positive
Negative

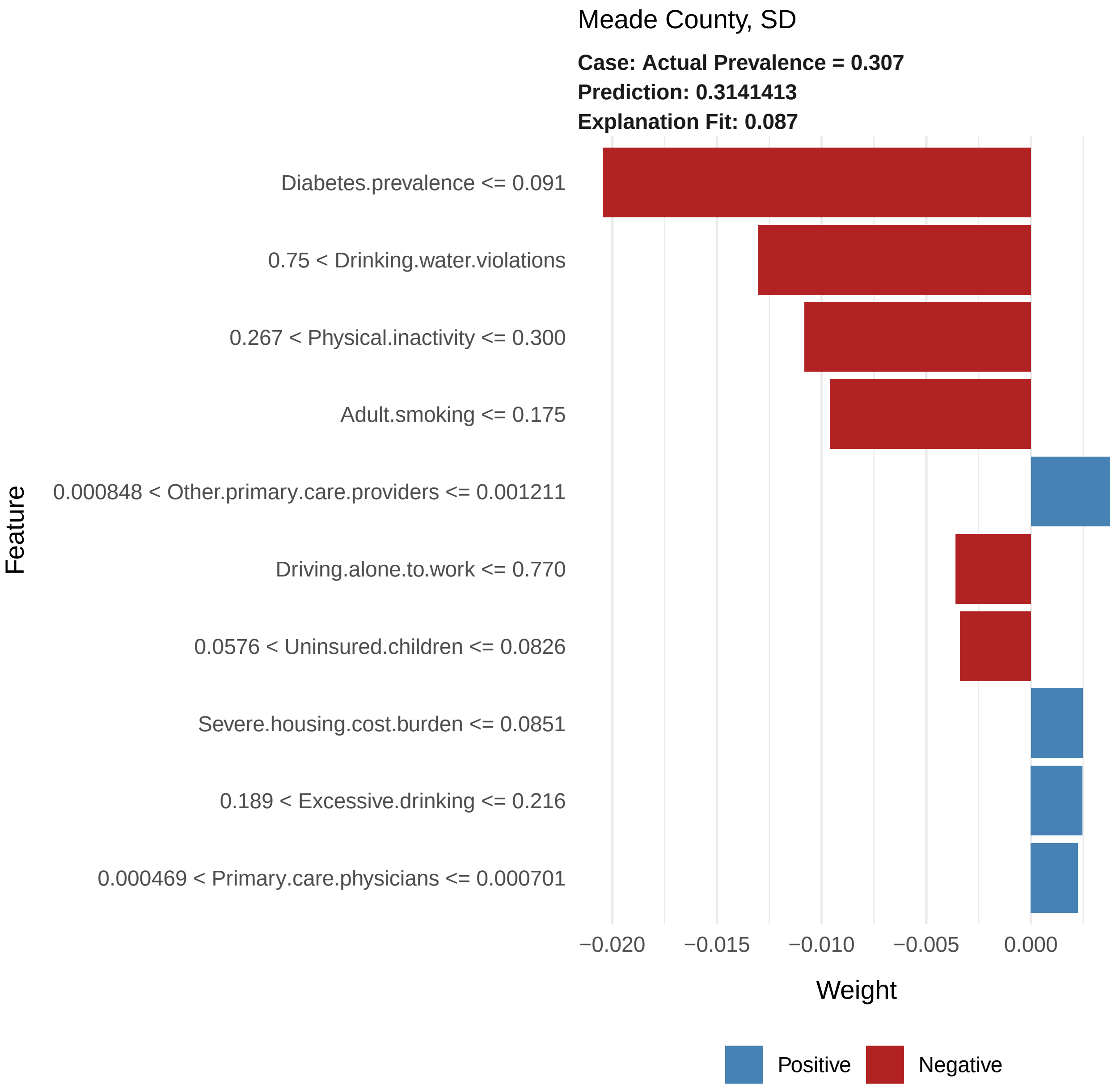
Meade County, SD
Case: Actual Prevalence = 0.307
Prediction: 0.3141413
Explanation Fit: 0.087
Diabetes.prevalence <= 0.091
0.75 < Drinking.water.violations
0.267 < Physical.inactivity <= 0.300
Adult.smoking <= 0.175
0.000848 < Other.primary.care.providers <= 0.001211
Driving.alone.to.work <= 0.770
0.0576 < Uninsured.children <= 0.0826
Severe.housing.cost.burden <= 0.0851
0.189 < Excessive.drinking <= 0.216
0.000469 < Primary.care.physicians <= 0.000701
Feature
−0.020
−0.015
−0.010
−0.005
0.000
Weight
Positive
Negative

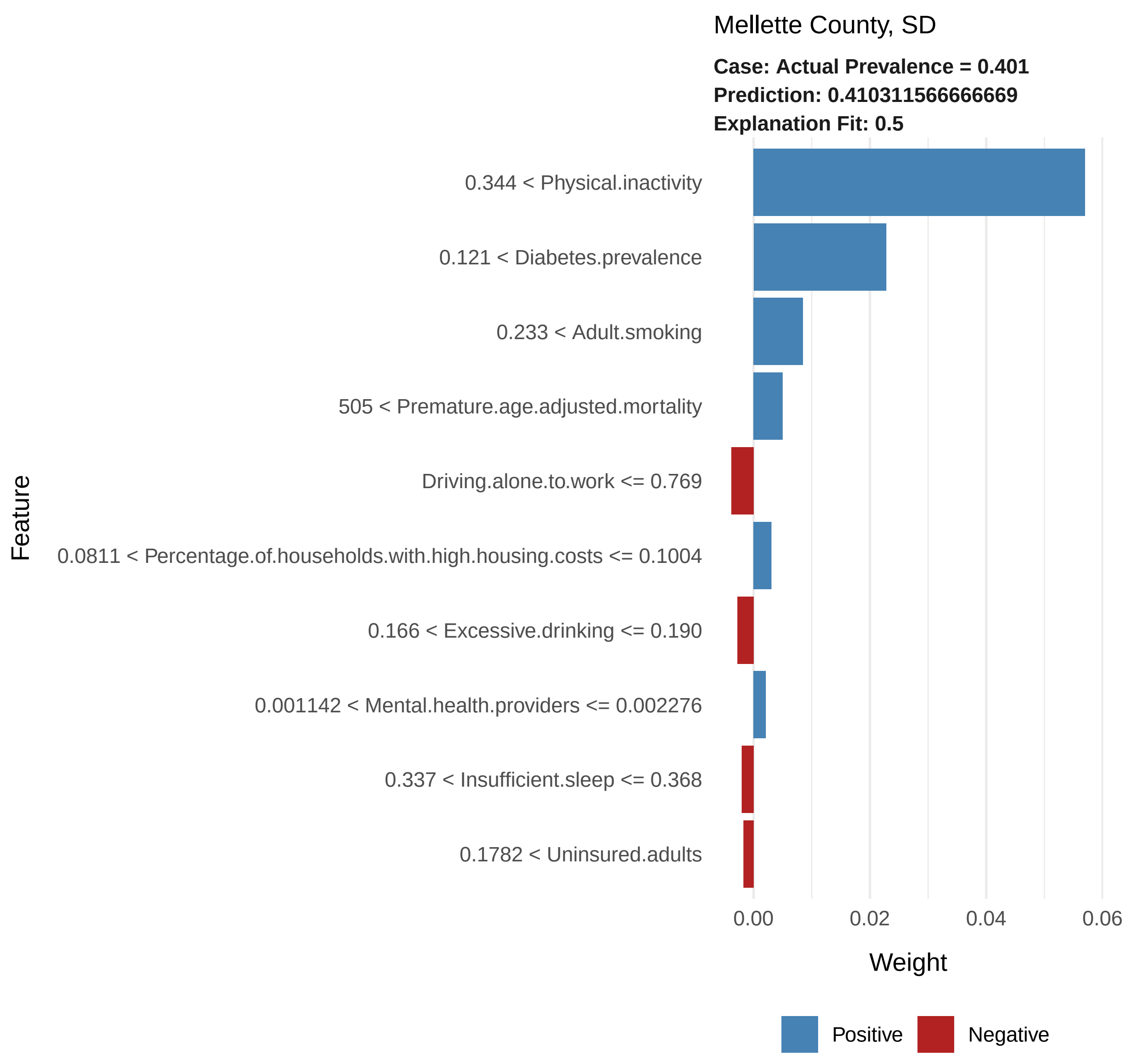

Mellette County, SD
Case: Actual Prevalence = 0.401
Prediction: 0.410311566666669
Explanation Fit: 0.5
0.344 < Physical.inactivity
0.121 < Diabetes.prevalence
0.233 < Adult.smoking
505 < Premature.age.adjusted.mortality
Driving.alone.to.work <= 0.769
0.0811 < Percentage.of.households.with.high.housing.costs <= 0.1004
0.166 < Excessive.drinking <= 0.190
0.001142 < Mental.health.providers <= 0.002276
0.337 < Insufficient.sleep <= 0.368
0.1782 < Uninsured.adults
Feature
0.00
0.02
0.04
0.06
Weight
Positive
Negative

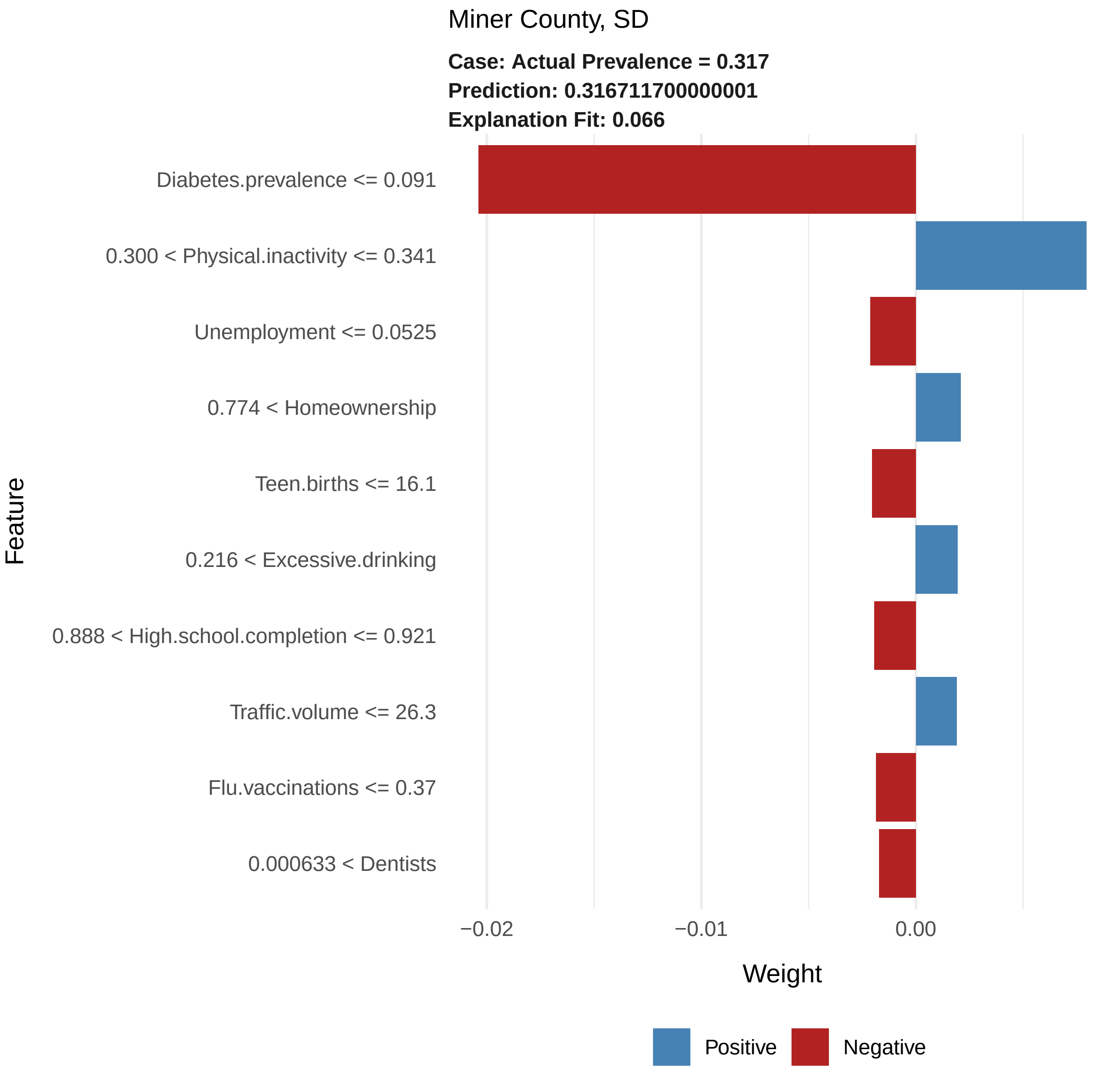

Miner County, SD
Case: Actual Prevalence = 0.317
Prediction: 0.316711700000001
Explanation Fit: 0.066
Diabetes.prevalence <= 0.091
0.300 < Physical.inactivity <= 0.341
Unemployment <= 0.0525
0.774 < Homeownership
Teen.births <= 16.1
0.216 < Excessive.drinking
0.888 < High.school.completion <= 0.921
Traffic.volume <= 26.3
Flu.vaccinations <= 0.37
0.000633 < Dentists
Feature
−0.02
−0.01
0.00
Weight
Positive
Negative

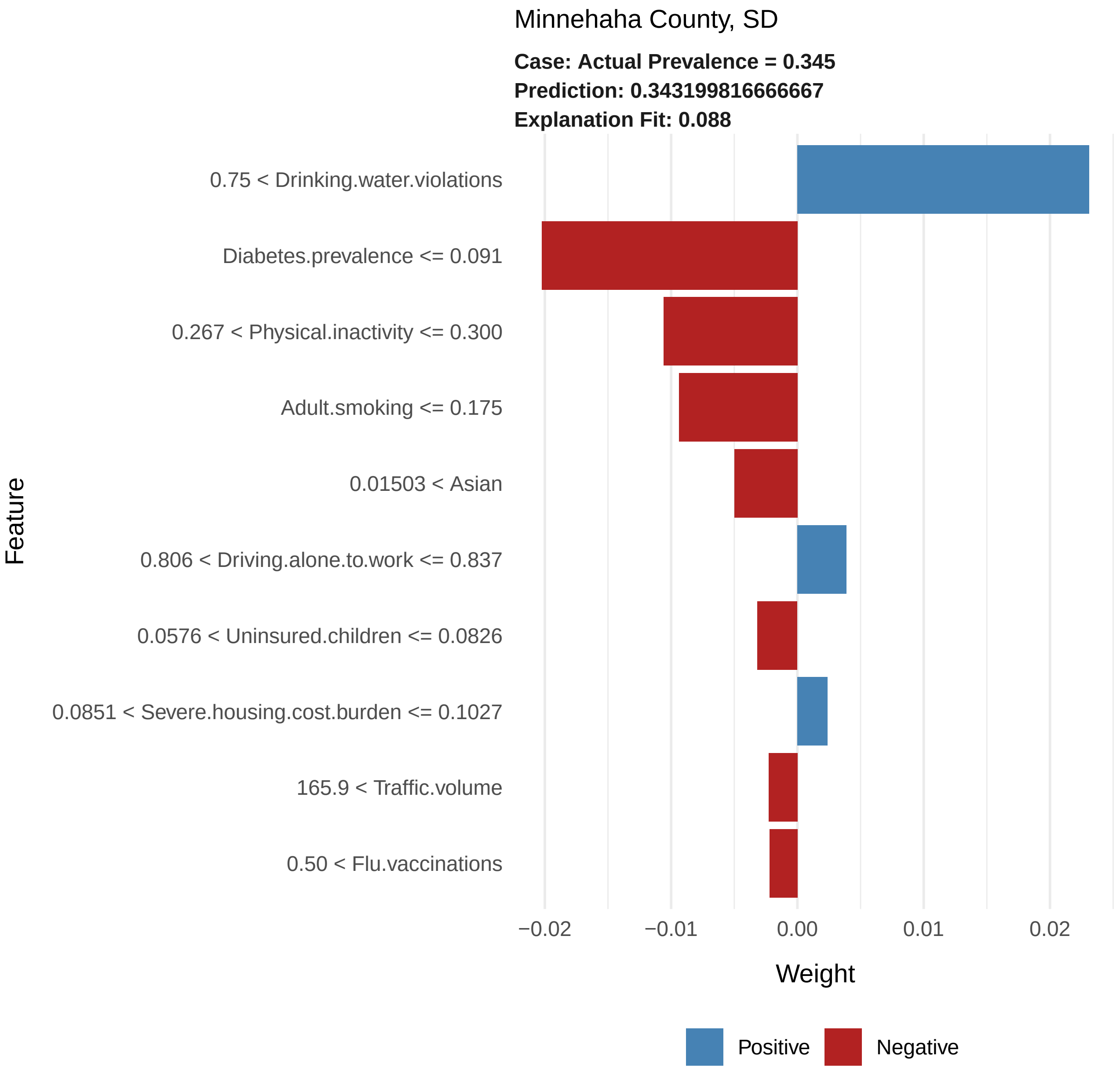

Minnehaha County, SD
Case: Actual Prevalence = 0.345
Prediction: 0.343199816666667
Explanation Fit: 0.088
0.75 < Drinking.water.violations
Diabetes.prevalence <= 0.091
0.267 < Physical.inactivity <= 0.300
Adult.smoking <= 0.175
0.01503 < Asian
0.806 < Driving.alone.to.work <= 0.837
0.0576 < Uninsured.children <= 0.0826
0.0851 < Severe.housing.cost.burden <= 0.1027
165.9 < Traffic.volume
0.50 < Flu.vaccinations
Feature
−0.02
−0.01
0.00
0.01
0.02
Weight
Positive
Negative

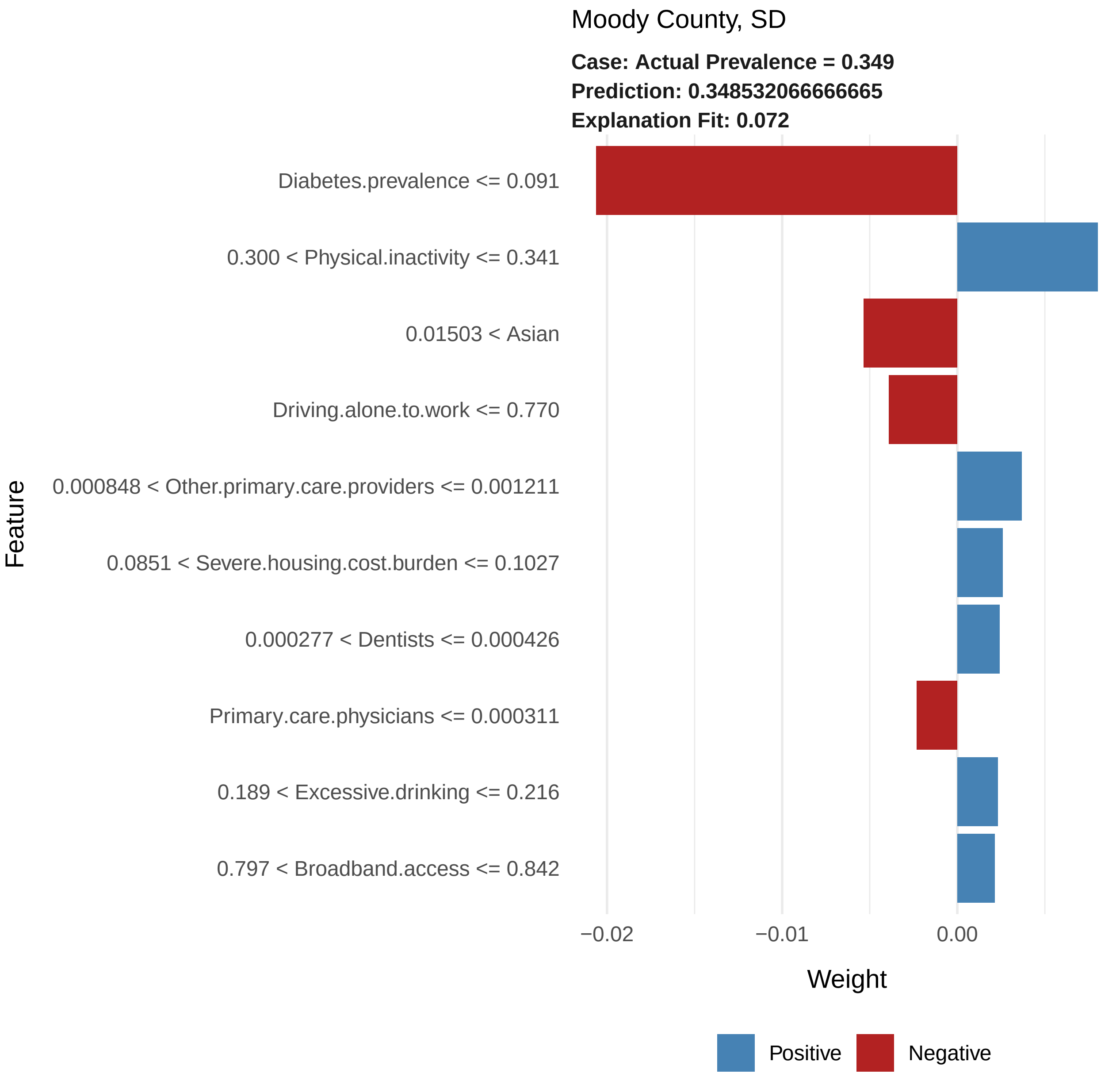

Moody County, SD
Case: Actual Prevalence = 0.349
Prediction: 0.348532066666665
Explanation Fit: 0.072
Diabetes.prevalence <= 0.091
0.300 < Physical.inactivity <= 0.341
0.01503 < Asian
Driving.alone.to.work <= 0.770
0.000848 < Other.primary.care.providers <= 0.001211
0.0851 < Severe.housing.cost.burden <= 0.1027
0.000277 < Dentists <= 0.000426
Primary.care.physicians <= 0.000311
0.189 < Excessive.drinking <= 0.216
0.797 < Broadband.access <= 0.842
Feature
−0.02
−0.01
0.00
Weight
Positive
Negative

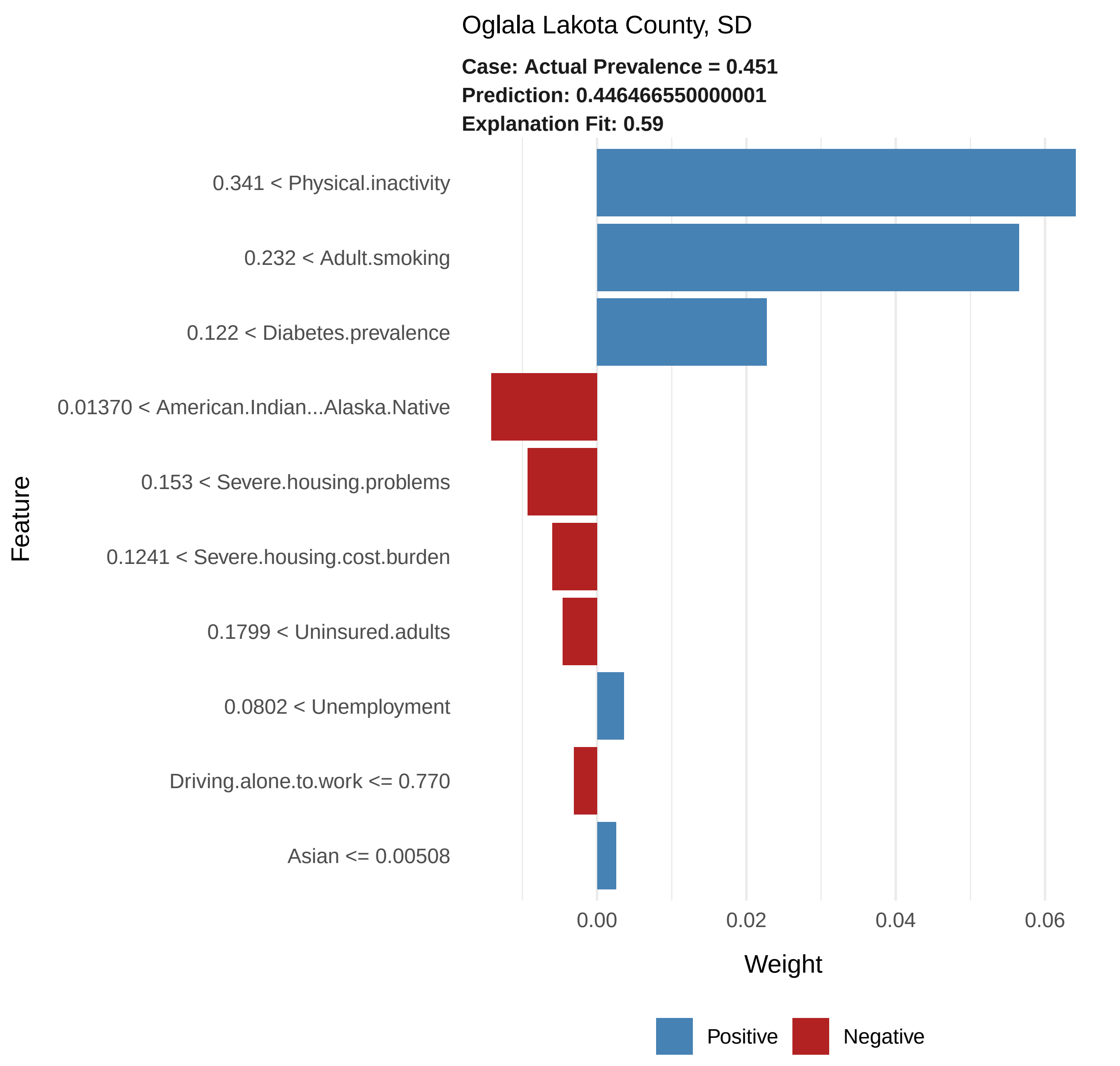
Oglala Lakota County, SD
Case: Actual Prevalence = 0.451
Prediction: 0.446466550000001
Explanation Fit: 0.59
0.341 < Physical.inactivity
0.232 < Adult.smoking
0.122 < Diabetes.prevalence
0.01370 < American.Indian...Alaska.Native
0.153 < Severe.housing.problems
0.1241 < Severe.housing.cost.burden
0.1799 < Uninsured.adults
0.0802 < Unemployment
Driving.alone.to.work <= 0.770
Asian <= 0.00508
Feature
0.00
0.02
0.04
0.06
Weight
Positive
Negative

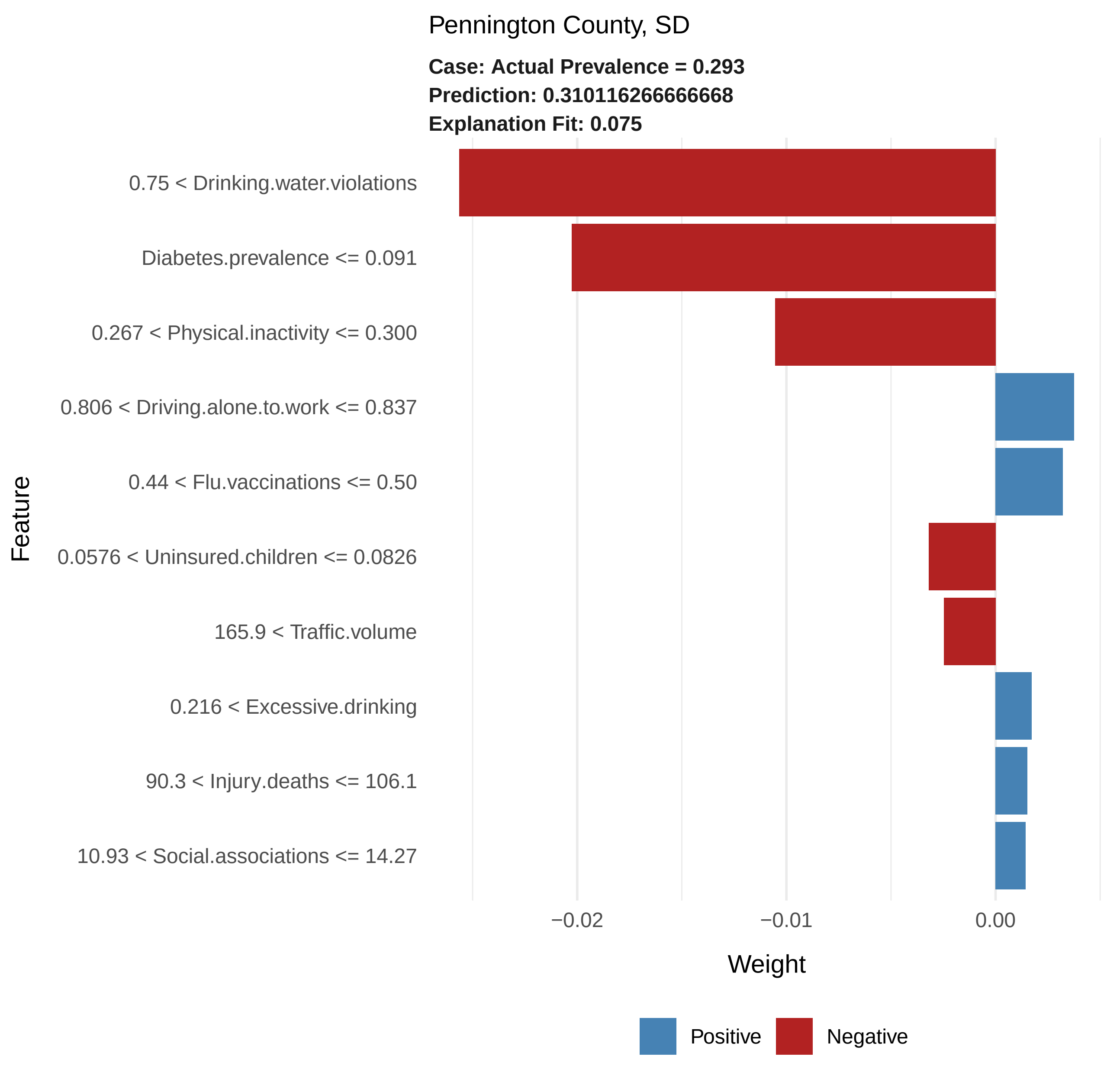
Pennington County, SD
Case: Actual Prevalence = 0.293
Prediction: 0.310116266666668
Explanation Fit: 0.075
0.75 < Drinking.water.violations
Diabetes.prevalence <= 0.091
0.267 < Physical.inactivity <= 0.300
0.806 < Driving.alone.to.work <= 0.837
0.44 < Flu.vaccinations <= 0.50
0.0576 < Uninsured.children <= 0.0826
165.9 < Traffic.volume
0.216 < Excessive.drinking
90.3 < Injury.deaths <= 106.1
10.93 < Social.associations <= 14.27
Feature
−0.02
−0.01
0.00
Weight
Positive
Negative

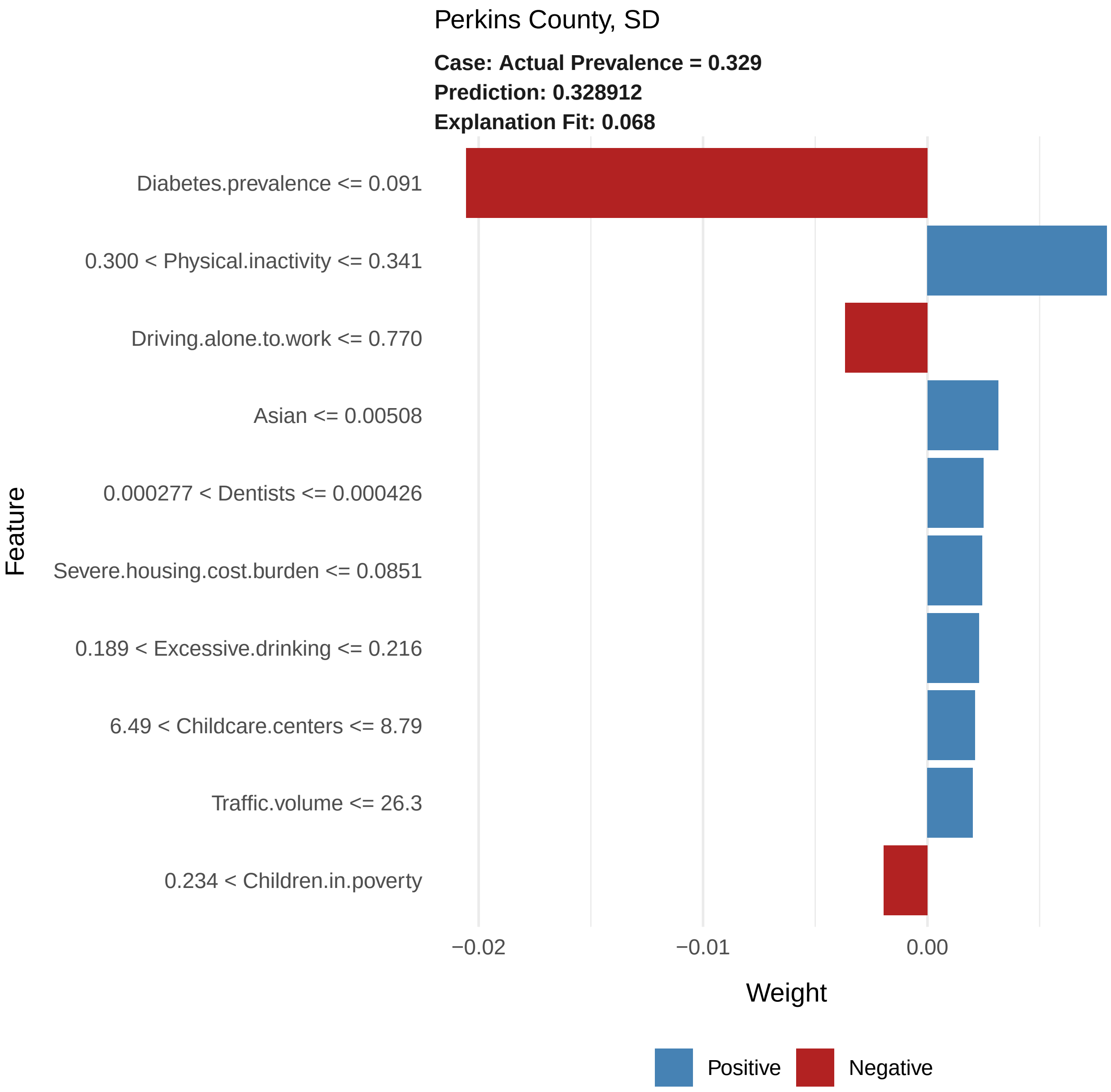
Perkins County, SD
Case: Actual Prevalence = 0.329
Prediction: 0.328912
Explanation Fit: 0.068
Diabetes.prevalence <= 0.091
0.300 < Physical.inactivity <= 0.341
Driving.alone.to.work <= 0.770
Asian <= 0.00508
0.000277 < Dentists <= 0.000426
Severe.housing.cost.burden <= 0.0851
0.189 < Excessive.drinking <= 0.216
6.49 < Childcare.centers <= 8.79
Traffic.volume <= 26.3
0.234 < Children.in.poverty
Feature
−0.02
−0.01
0.00
Weight
Positive
Negative

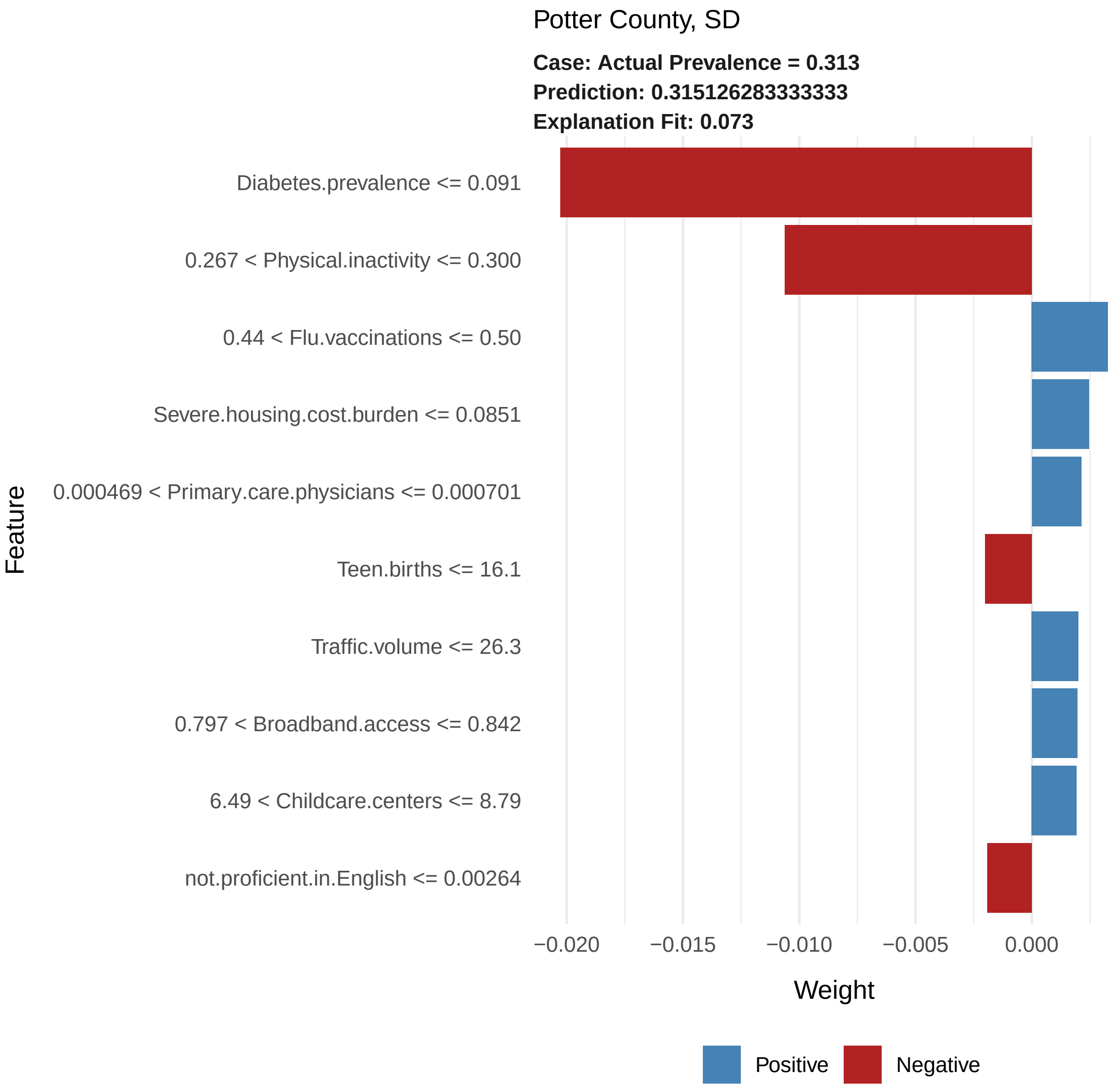

Potter County, SD
Case: Actual Prevalence = 0.313
Prediction: 0.315126283333333
Explanation Fit: 0.073
Feature
Diabetes.prevalence <= 0.091
0.267 < Physical.inactivity <= 0.300
0.44 < Flu.vaccinations <= 0.50
Severe.housing.cost.burden <= 0.0851
0.000469 < Primary.care.physicians <= 0.000701
Teen.births <= 16.1
Traffic.volume <= 26.3
0.797 < Broadband.access <= 0.842
6.49 < Childcare.centers <= 8.79
not.proficient.in.English <= 0.00264
−0.020
−0.015
−0.010
−0.005
0.000
Weight
Positive
Negative

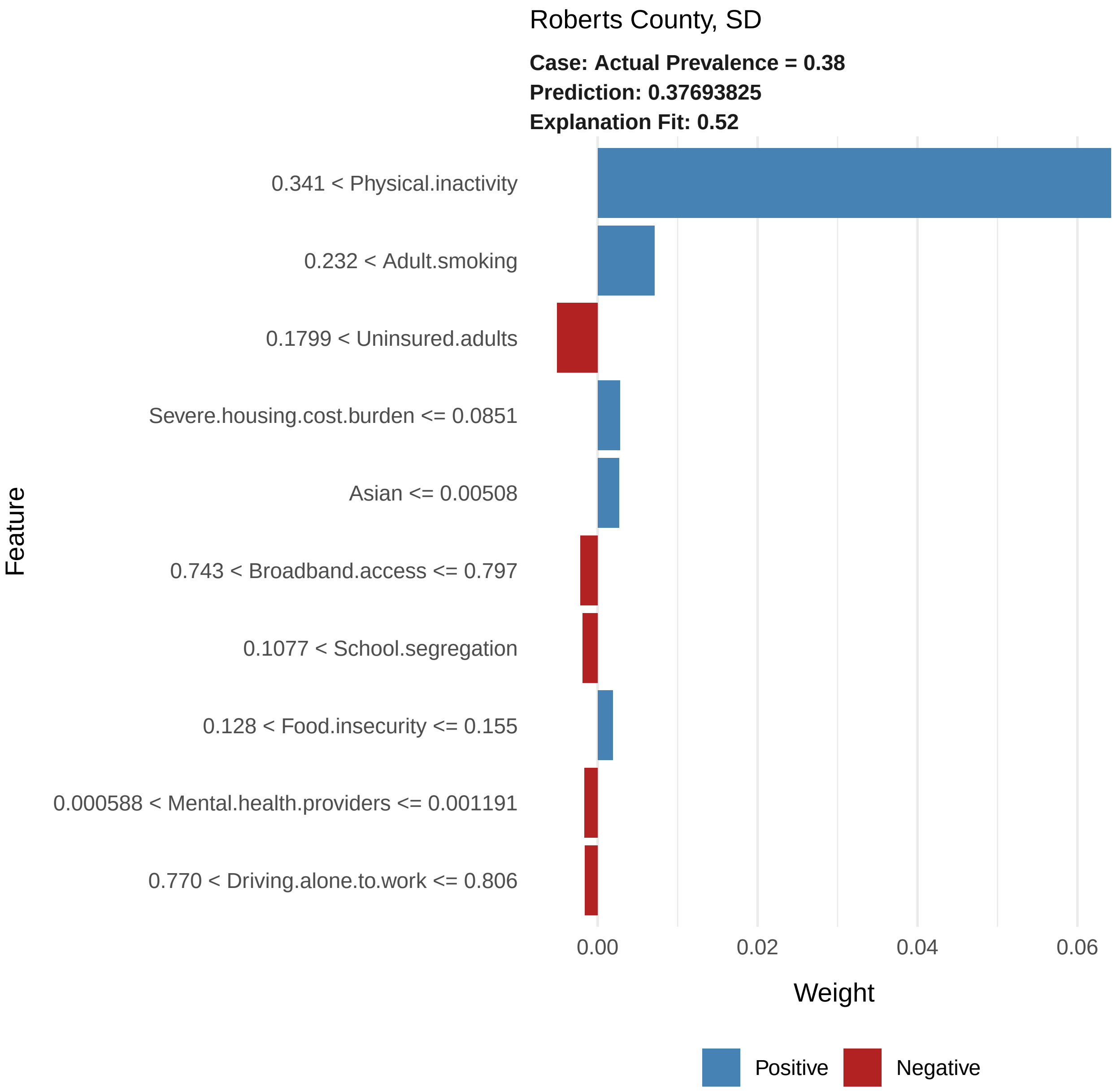
Roberts County, SD
Case: Actual Prevalence = 0.38
Prediction: 0.37693825
Explanation Fit: 0.52
0.341 < Physical.inactivity
0.232 < Adult.smoking
0.1799 < Uninsured.adults
Severe.housing.cost.burden <= 0.0851
Asian <= 0.00508
0.743 < Broadband.access <= 0.797
0.1077 < School.segregation
0.128 < Food.insecurity <= 0.155
0.000588 < Mental.health.providers <= 0.001191
0.770 < Driving.alone.to.work <= 0.806
Feature
0.00
0.02
0.04
0.06
Weight
Positive
Negative

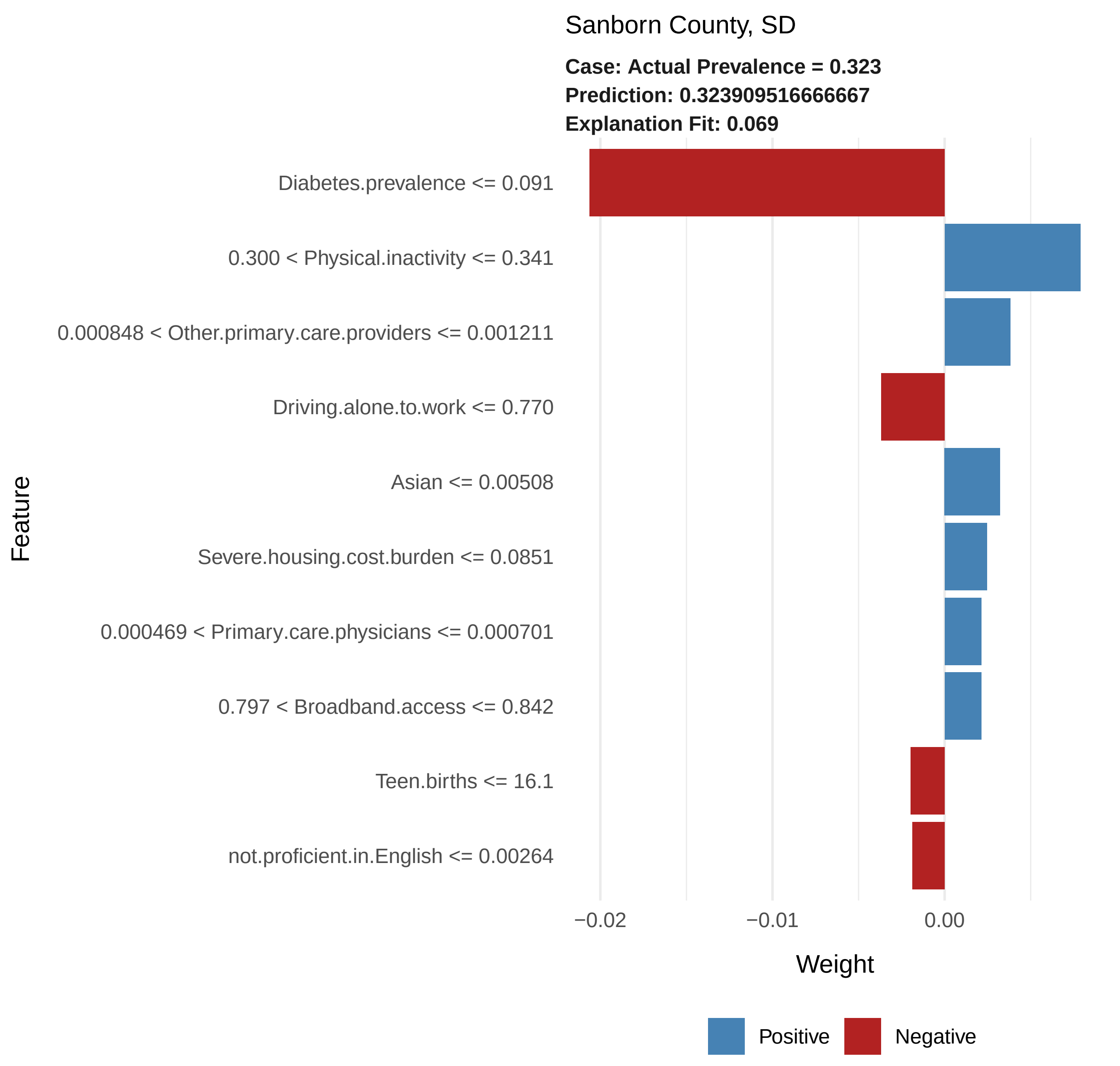

Sanborn County, SD
Case: Actual Prevalence = 0.323
Prediction: 0.323909516666667
Explanation Fit: 0.069
Diabetes.prevalence <= 0.091
0.300 < Physical.inactivity <= 0.341
0.000848 < Other.primary.care.providers <= 0.001211
Driving.alone.to.work <= 0.770
Asian <= 0.00508
Severe.housing.cost.burden <= 0.0851
0.000469 < Primary.care.physicians <= 0.000701
0.797 < Broadband.access <= 0.842
Teen.births <= 16.1
not.proficient.in.English <= 0.00264
Feature
−0.02
−0.01
0.00
Weight
Positive
Negative

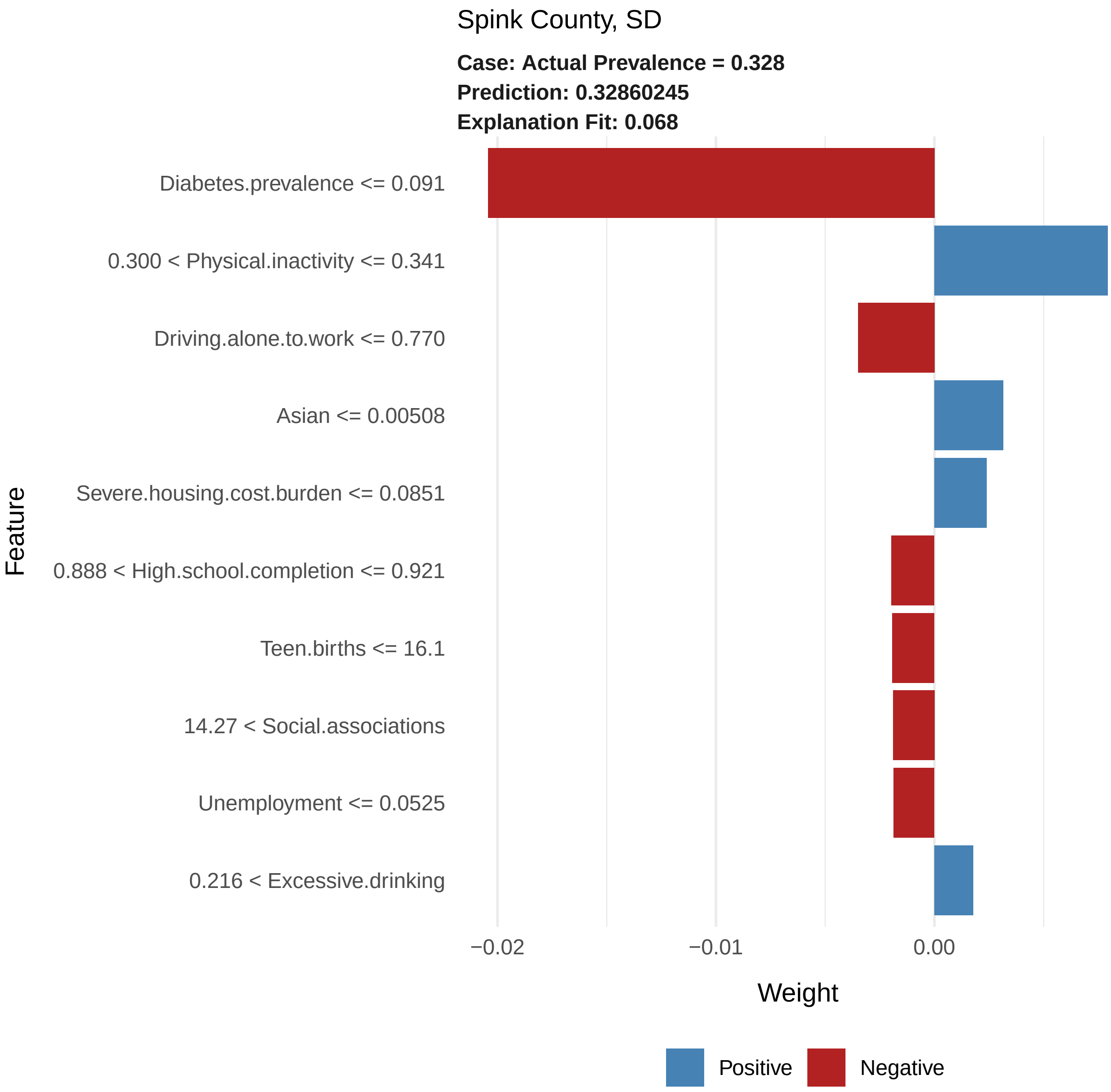
Spink County, SD
Case: Actual Prevalence = 0.328
Prediction: 0.32860245
Explanation Fit: 0.068
Feature
Diabetes.prevalence <= 0.091
0.300 < Physical.inactivity <= 0.341
Driving.alone.to.work <= 0.770
Asian <= 0.00508
Severe.housing.cost.burden <= 0.0851
0.888 < High.school.completion <= 0.921
Teen.births <= 16.1
14.27 < Social.associations
Unemployment <= 0.0525
0.216 < Excessive.drinking
−0.02
−0.01
0.00
Weight
Positive
Negative

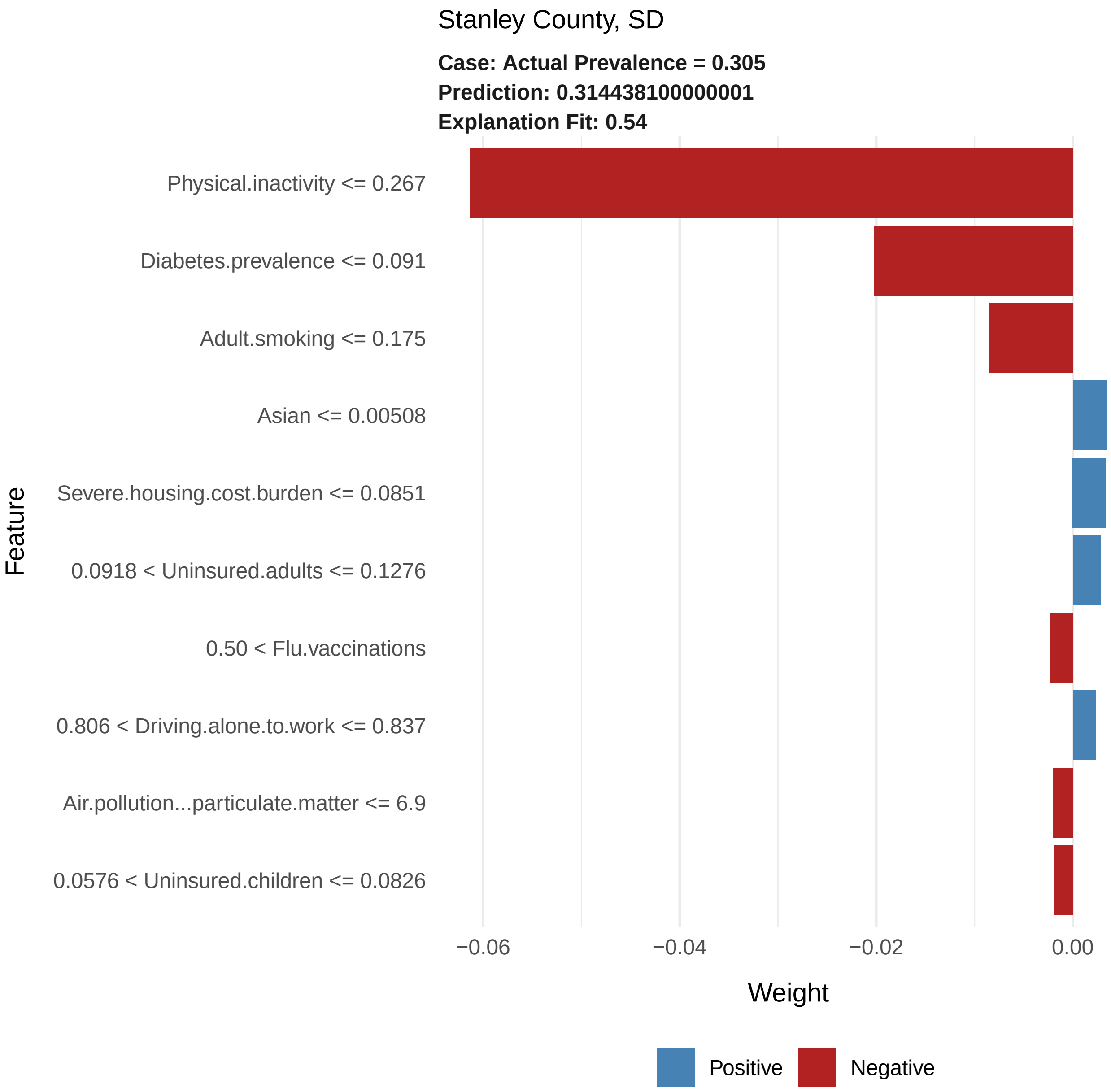

Stanley County, SD
Case: Actual Prevalence = 0.305
Prediction: 0.314438100000001
Explanation Fit: 0.54
Physical.inactivity <= 0.267
Diabetes.prevalence <= 0.091
Adult.smoking <= 0.175
Asian <= 0.00508
Severe.housing.cost.burden <= 0.0851
0.0918 < Uninsured.adults <= 0.1276
0.50 < Flu.vaccinations
0.806 < Driving.alone.to.work <= 0.837
Air.pollution...particulate.matter <= 6.9
0.0576 < Uninsured.children <= 0.0826
Feature
−0.06
−0.04
−0.02
0.00
Weight
Positive
Negative

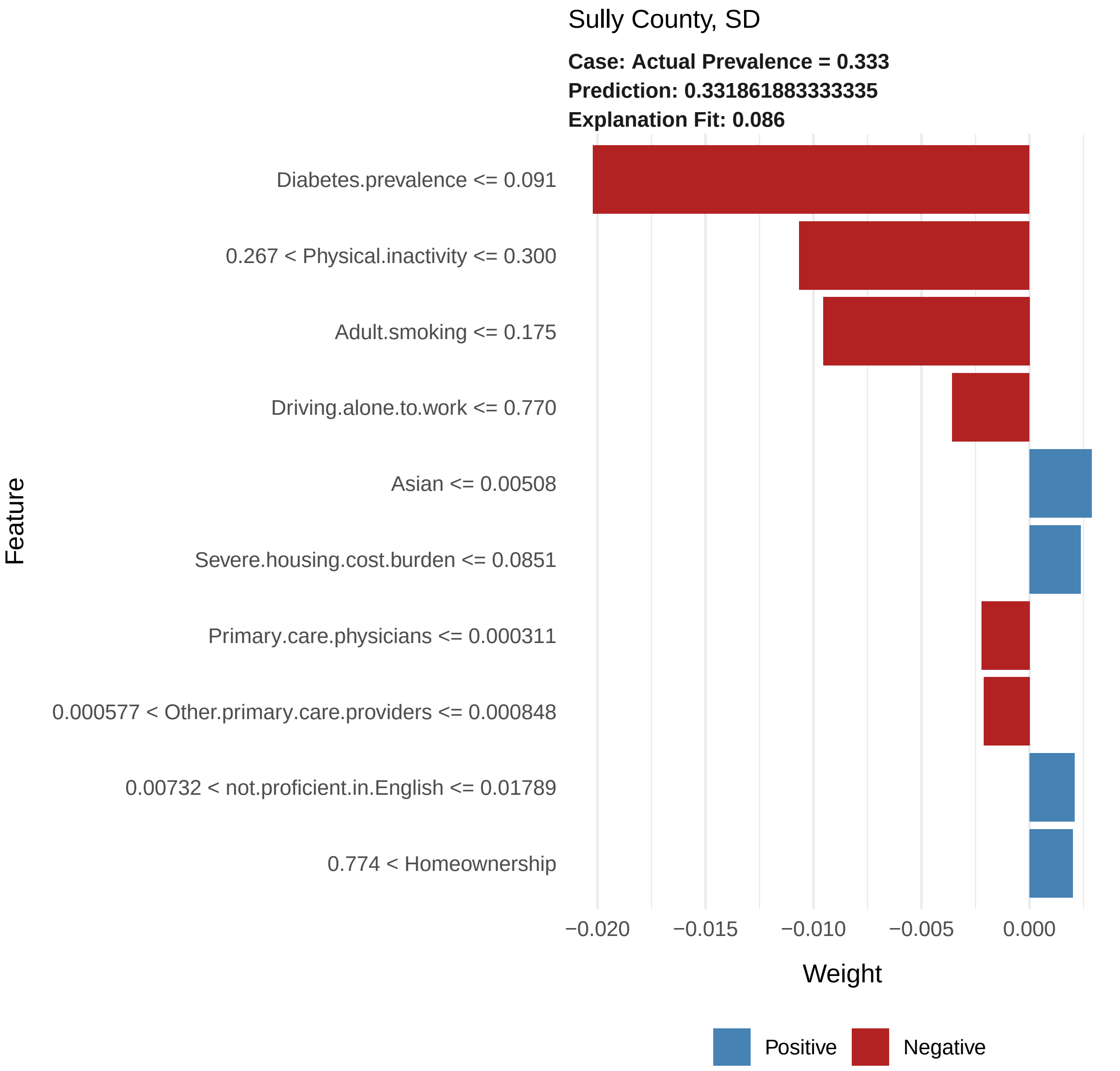
Sully County, SD
Case: Actual Prevalence = 0.333
Prediction: 0.331861883333335
Explanation Fit: 0.086
Diabetes.prevalence <= 0.091
0.267 < Physical.inactivity <= 0.300
Adult.smoking <= 0.175
Driving.alone.to.work <= 0.770
Asian <= 0.00508
Severe.housing.cost.burden <= 0.0851
Primary.care.physicians <= 0.000311
0.000577 < Other.primary.care.providers <= 0.000848
0.00732 < not.proficient.in.English <= 0.01789
0.774 < Homeownership
Feature
−0.020
−0.015
−0.010
−0.005
0.000
Weight
Positive
Negative

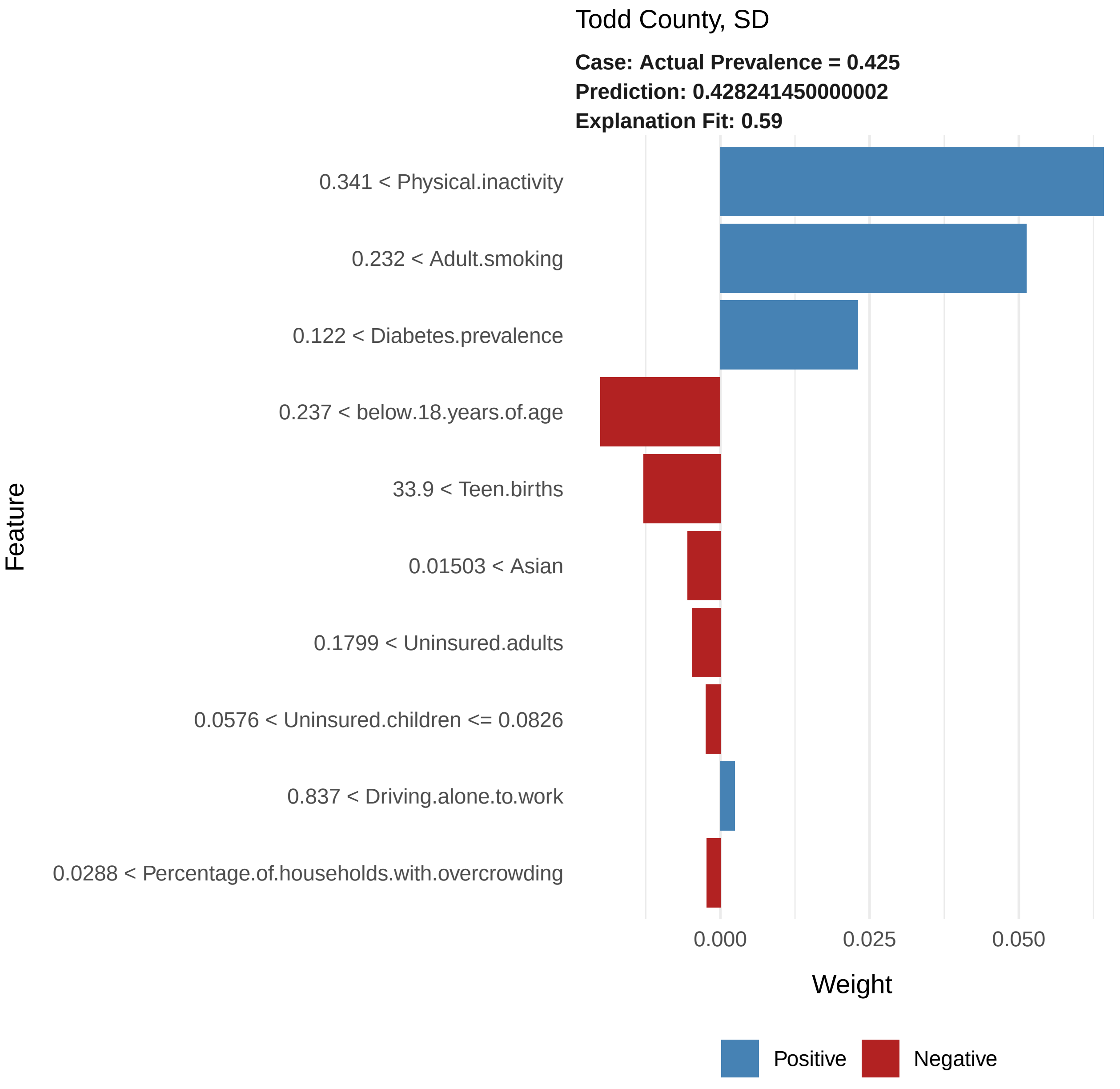
Todd County, SD
Case: Actual Prevalence = 0.425
Prediction: 0.428241450000002
Explanation Fit: 0.59
0.341 < Physical.inactivity
0.232 < Adult.smoking
0.122 < Diabetes.prevalence
0.237 < below.18.years.of.age
33.9 < Teen.births
0.01503 < Asian
0.1799 < Uninsured.adults
0.0576 < Uninsured.children <= 0.0826
0.837 < Driving.alone.to.work
0.0288 < Percentage.of.households.with.overcrowding
Feature
0.000
0.025
0.050
Weight
Positive
Negative

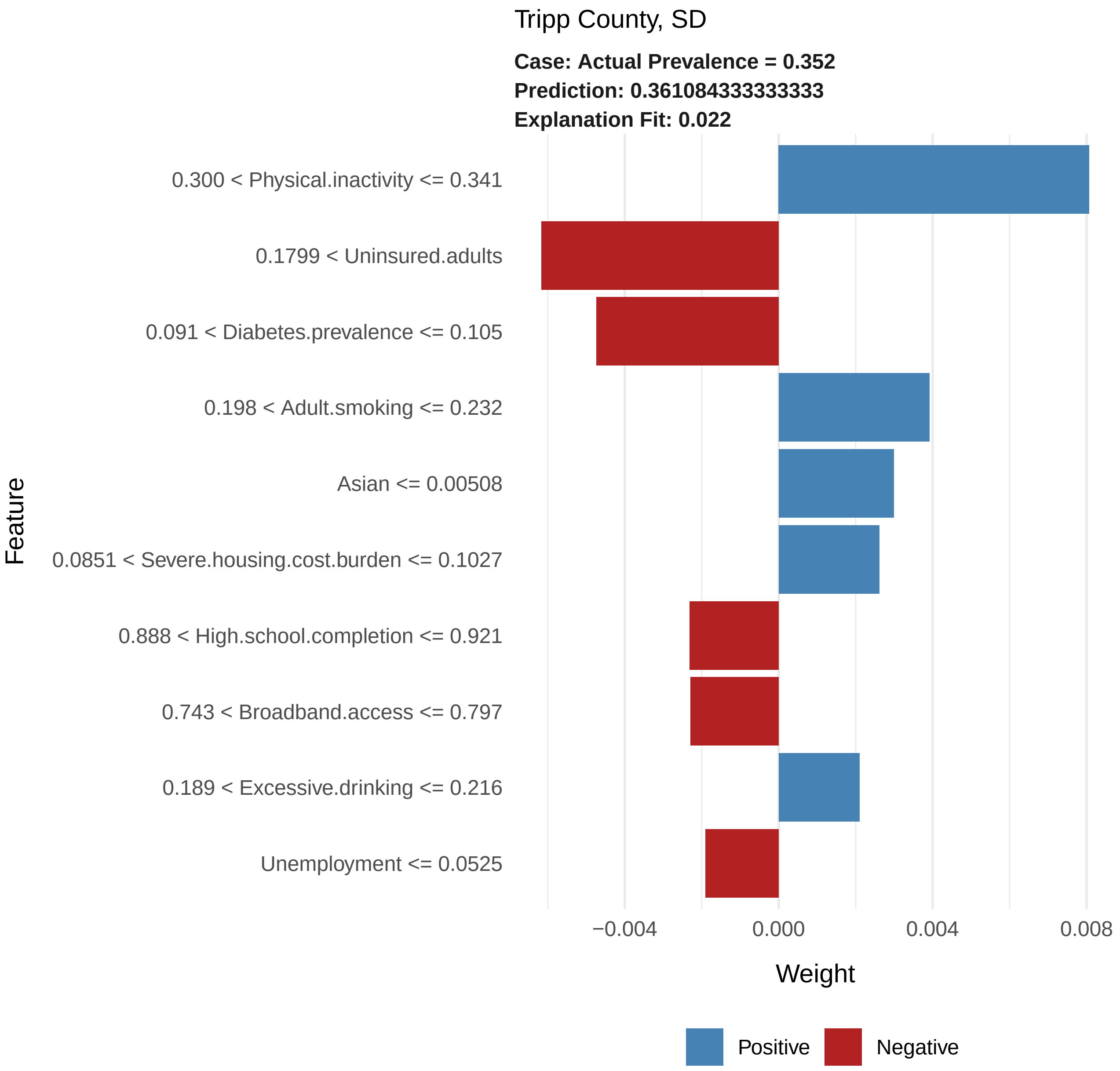

Tripp County, SD
Case: Actual Prevalence = 0.352
Prediction: 0.361084333333333
Explanation Fit: 0.022
0.300 < Physical.inactivity <= 0.341
0.1799 < Uninsured.adults
0.091 < Diabetes.prevalence <= 0.105
0.198 < Adult.smoking <= 0.232
Asian <= 0.00508
0.0851 < Severe.housing.cost.burden <= 0.1027
0.888 < High.school.completion <= 0.921
0.743 < Broadband.access <= 0.797
0.189 < Excessive.drinking <= 0.216
Unemployment <= 0.0525
Feature
−0.004
0.000
0.004
0.008
Weight
Positive
Negative

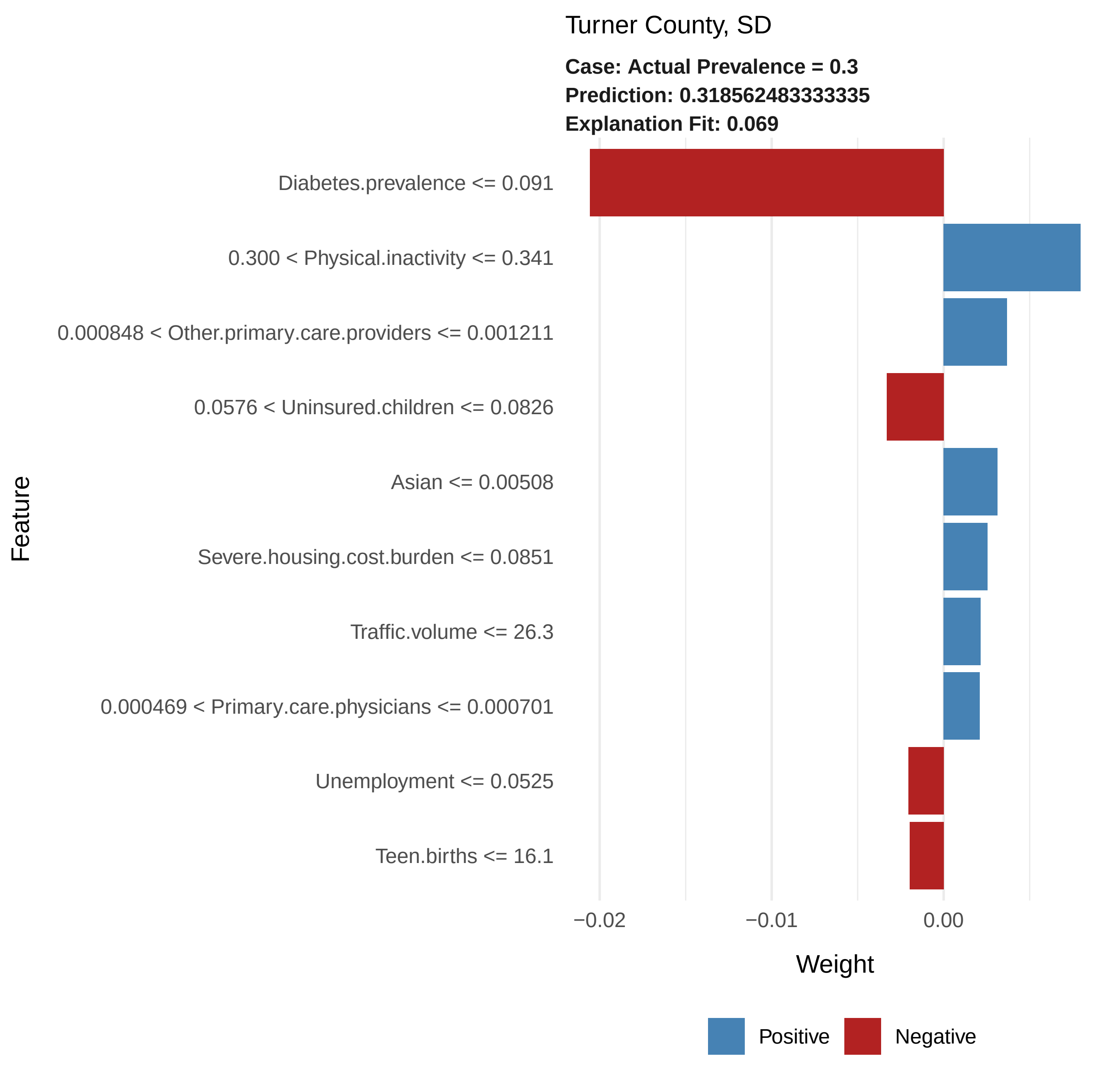

Turner County, SD
Case: Actual Prevalence = 0.3
Prediction: 0.318562483333335
Explanation Fit: 0.069
Diabetes.prevalence <= 0.091
0.300 < Physical.inactivity <= 0.341
0.000848 < Other.primary.care.providers <= 0.001211
0.0576 < Uninsured.children <= 0.0826
Asian <= 0.00508
Severe.housing.cost.burden <= 0.0851
Traffic.volume <= 26.3
0.000469 < Primary.care.physicians <= 0.000701
Unemployment <= 0.0525
Teen.births <= 16.1
Feature
−0.02
−0.01
0.00
Weight
Positive
Negative

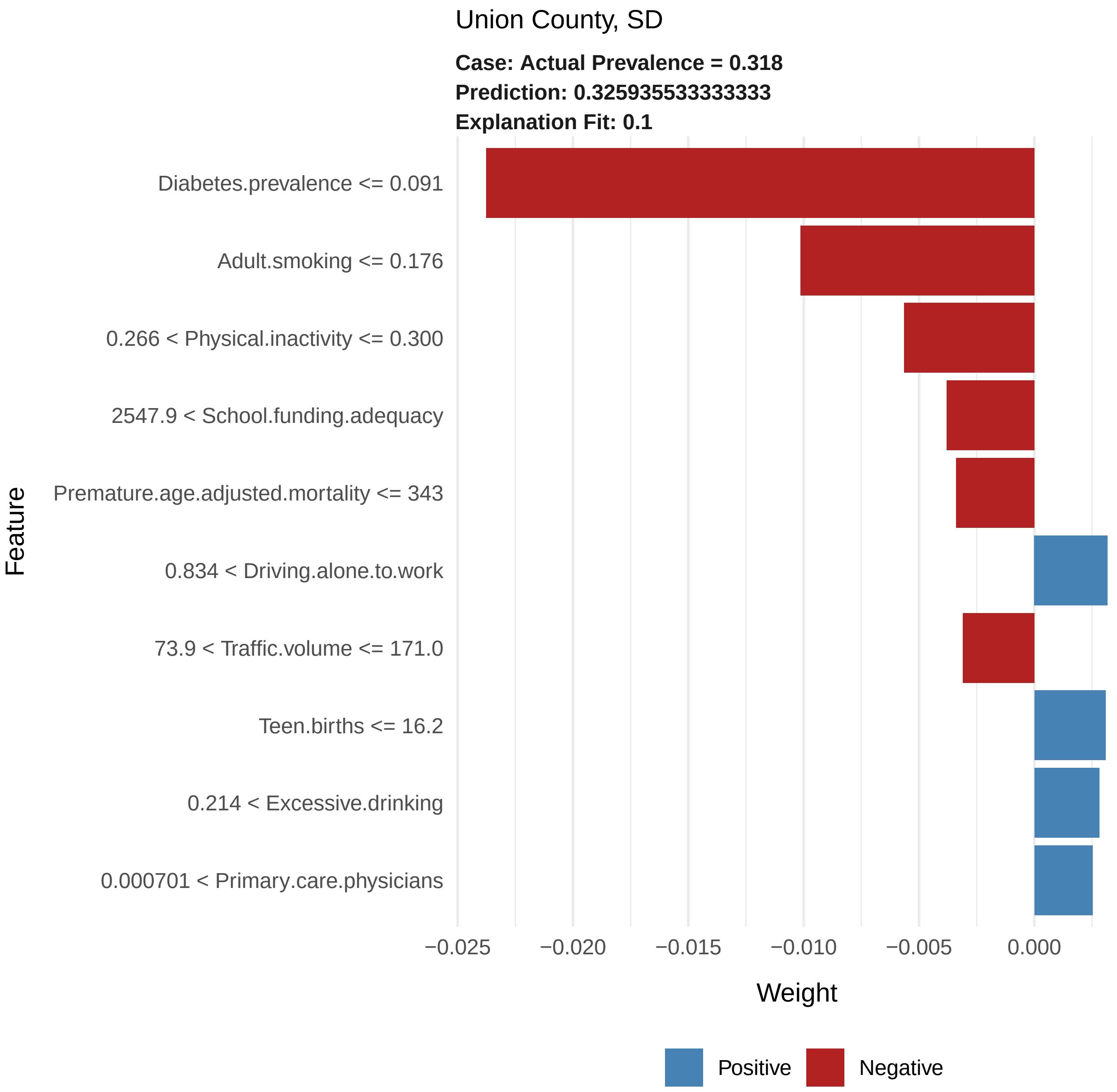

Union County, SD
Case: Actual Prevalence = 0.318
Prediction: 0.325935533333333
Explanation Fit: 0.1
Diabetes.prevalence <= 0.091
Adult.smoking <= 0.176
0.266 < Physical.inactivity <= 0.300
2547.9 < School.funding.adequacy
Premature.age.adjusted.mortality <= 343
0.834 < Driving.alone.to.work
73.9 < Traffic.volume <= 171.0
Teen.births <= 16.2
0.214 < Excessive.drinking
0.000701 < Primary.care.physicians
Feature
−0.025
−0.020
−0.015
−0.010
−0.005
0.000
Weight
Positive
Negative

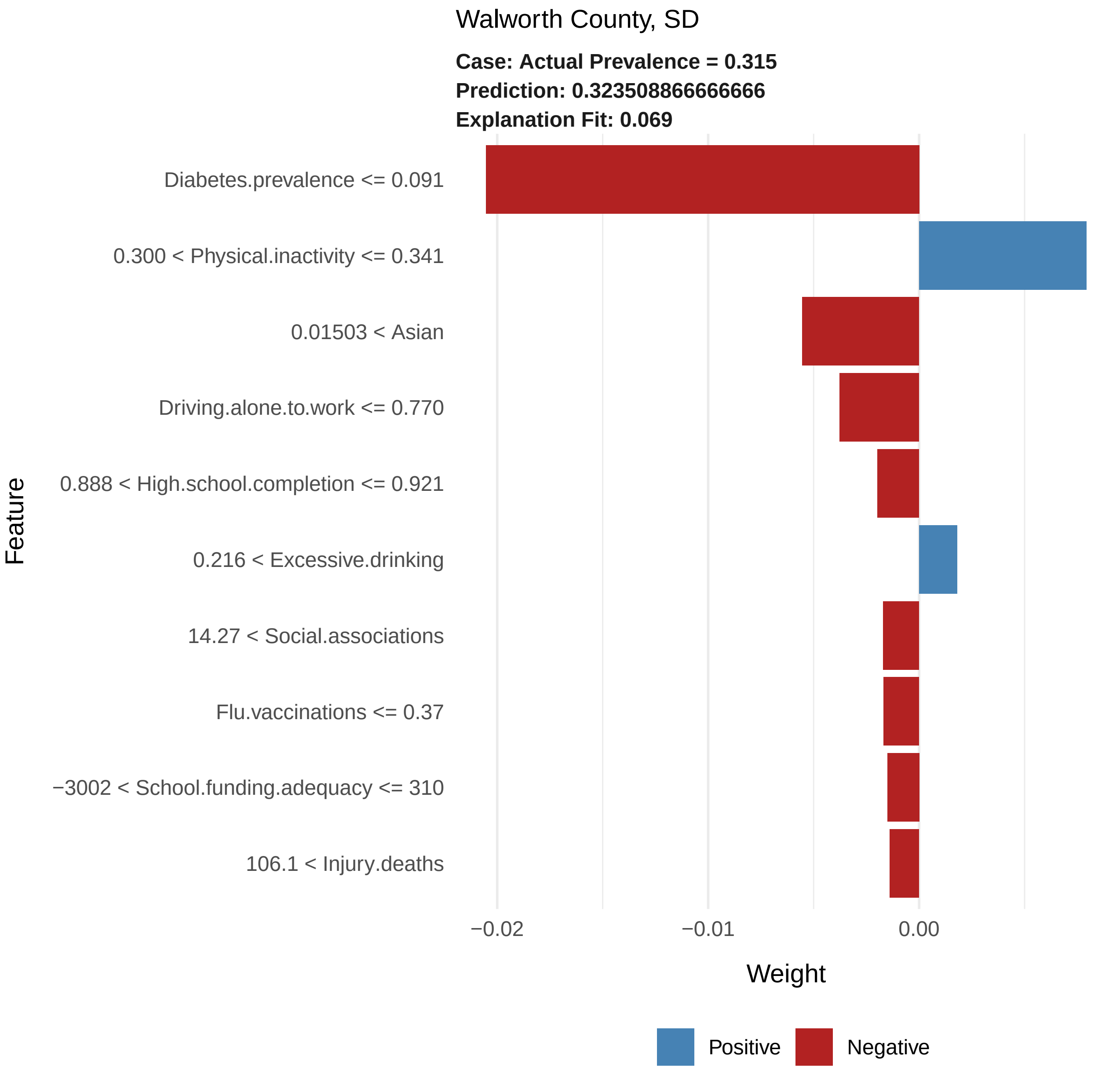
Walworth County, SD
Case: Actual Prevalence = 0.315
Prediction: 0.323508866666666
Explanation Fit: 0.069
Diabetes.prevalence <= 0.091
0.300 < Physical.inactivity <= 0.341
0.01503 < Asian
Driving.alone.to.work <= 0.770
0.888 < High.school.completion <= 0.921
0.216 < Excessive.drinking
14.27 < Social.associations
Flu.vaccinations <= 0.37
−3002 < School.funding.adequacy <= 310
106.1 < Injury.deaths
Feature
−0.02
−0.01
0.00
Weight
Positive
Negative

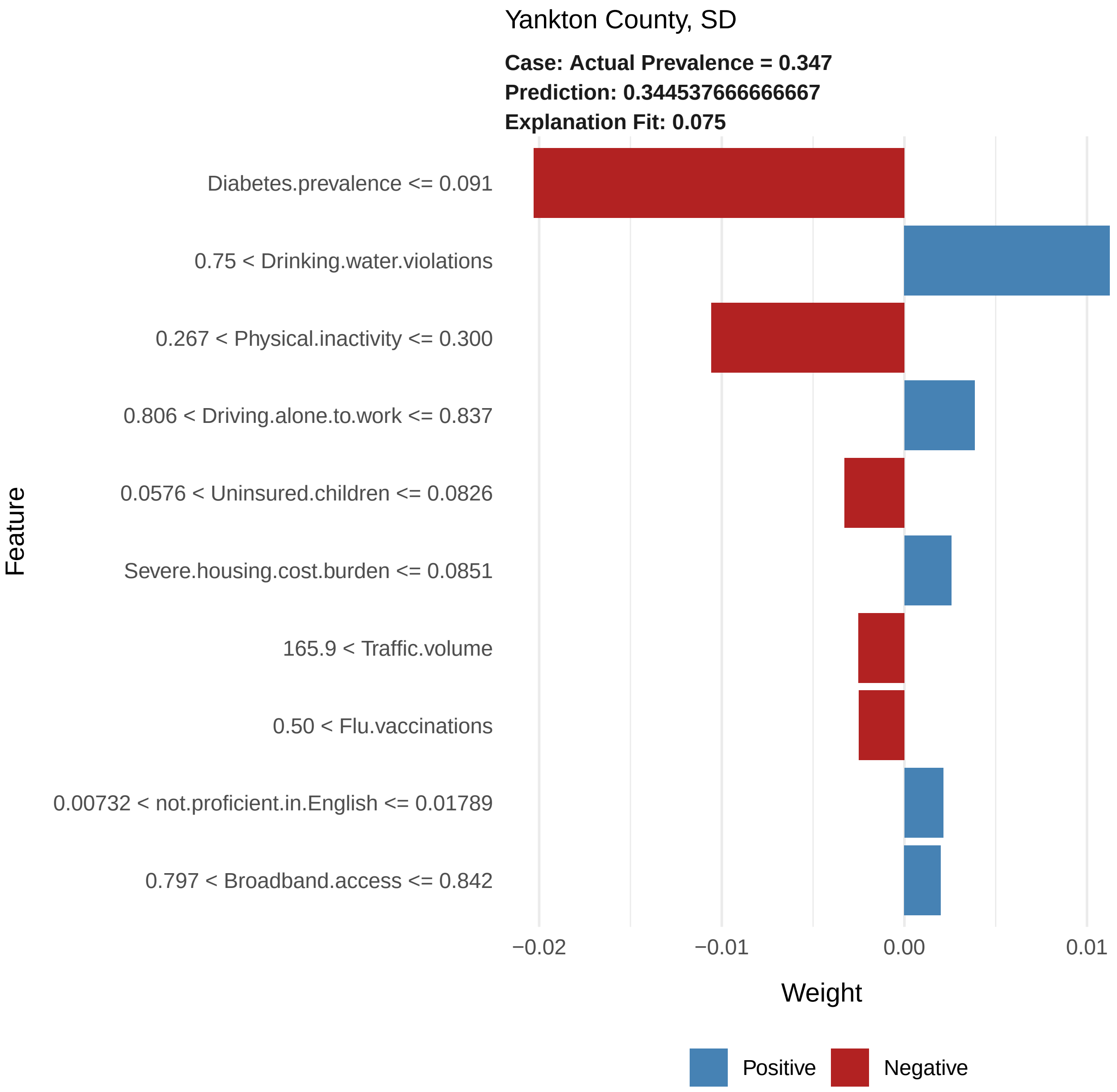
Yankton County, SD
Case: Actual Prevalence = 0.347
Prediction: 0.344537666666667
Explanation Fit: 0.075
Diabetes.prevalence <= 0.091
0.75 < Drinking.water.violations
0.267 < Physical.inactivity <= 0.300
0.806 < Driving.alone.to.work <= 0.837
0.0576 < Uninsured.children <= 0.0826
Severe.housing.cost.burden <= 0.0851
165.9 < Traffic.volume
0.50 < Flu.vaccinations
0.00732 < not.proficient.in.English <= 0.01789
0.797 < Broadband.access <= 0.842
Feature
−0.02
−0.01
0.00
0.01
Weight
Positive
Negative

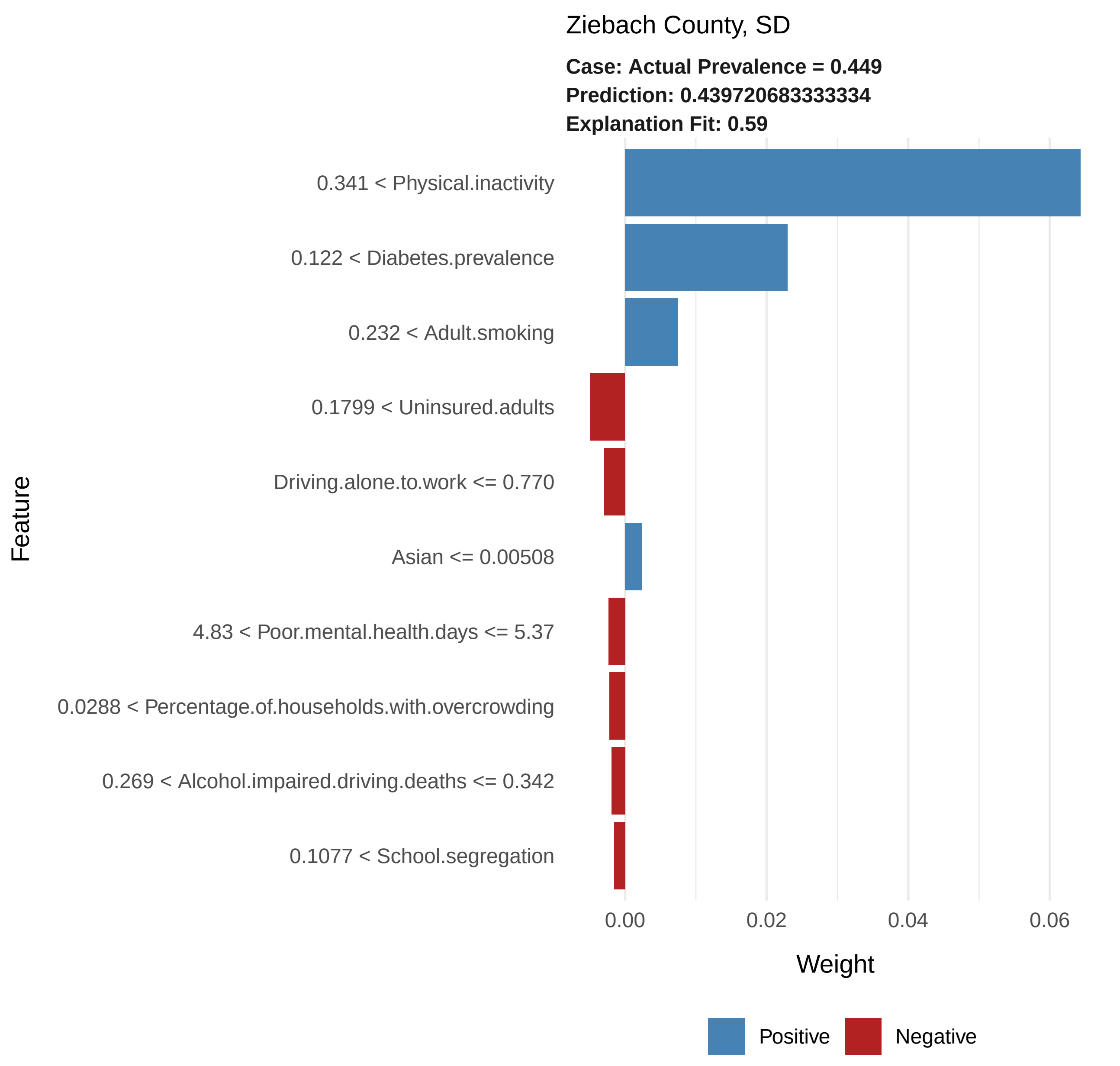

Ziebach County, SD
Case: Actual Prevalence = 0.449
Prediction: 0.439720683333334
Explanation Fit: 0.59
0.341 < Physical.inactivity
0.122 < Diabetes.prevalence
0.232 < Adult.smoking
0.1799 < Uninsured.adults
Driving.alone.to.work <= 0.770
Asian <= 0.00508
4.83 < Poor.mental.health.days <= 5.37
0.0288 < Percentage.of.households.with.overcrowding
0.269 < Alcohol.impaired.driving.deaths <= 0.342
0.1077 < School.segregation
Feature
0.00
0.02
0.04
0.06
Weight
Positive
Negative

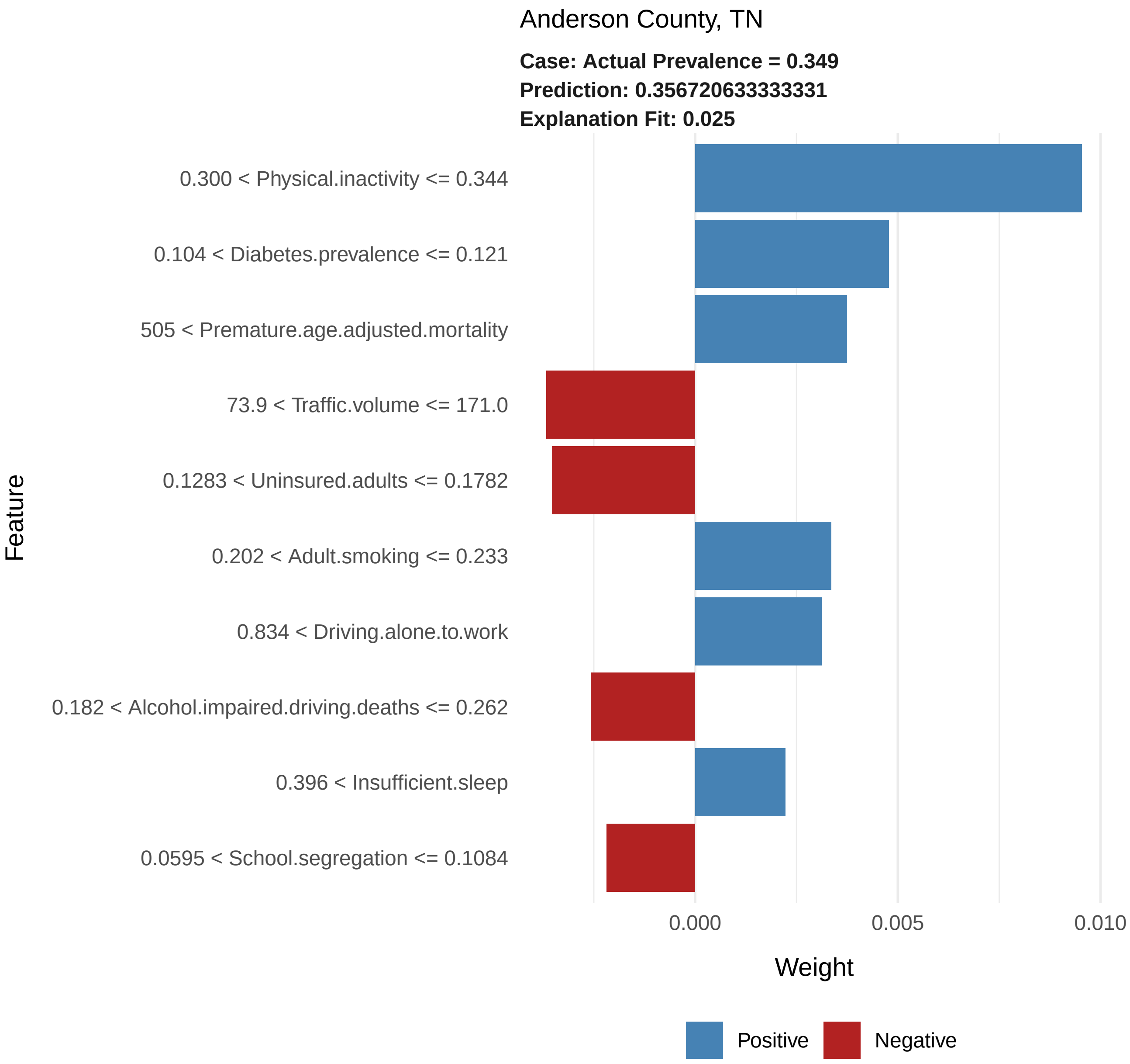
Anderson County, TN
Case: Actual Prevalence = 0.349
Prediction: 0.356720633333331
Explanation Fit: 0.025
0.300 < Physical.inactivity <= 0.344
0.104 < Diabetes.prevalence <= 0.121
505 < Premature.age.adjusted.mortality
73.9 < Traffic.volume <= 171.0
0.1283 < Uninsured.adults <= 0.1782
0.202 < Adult.smoking <= 0.233
0.834 < Driving.alone.to.work
0.182 < Alcohol.impaired.driving.deaths <= 0.262
0.396 < Insufficient.sleep
0.0595 < School.segregation <= 0.1084
Feature
0.000
0.005
0.010
Weight
Positive
Negative

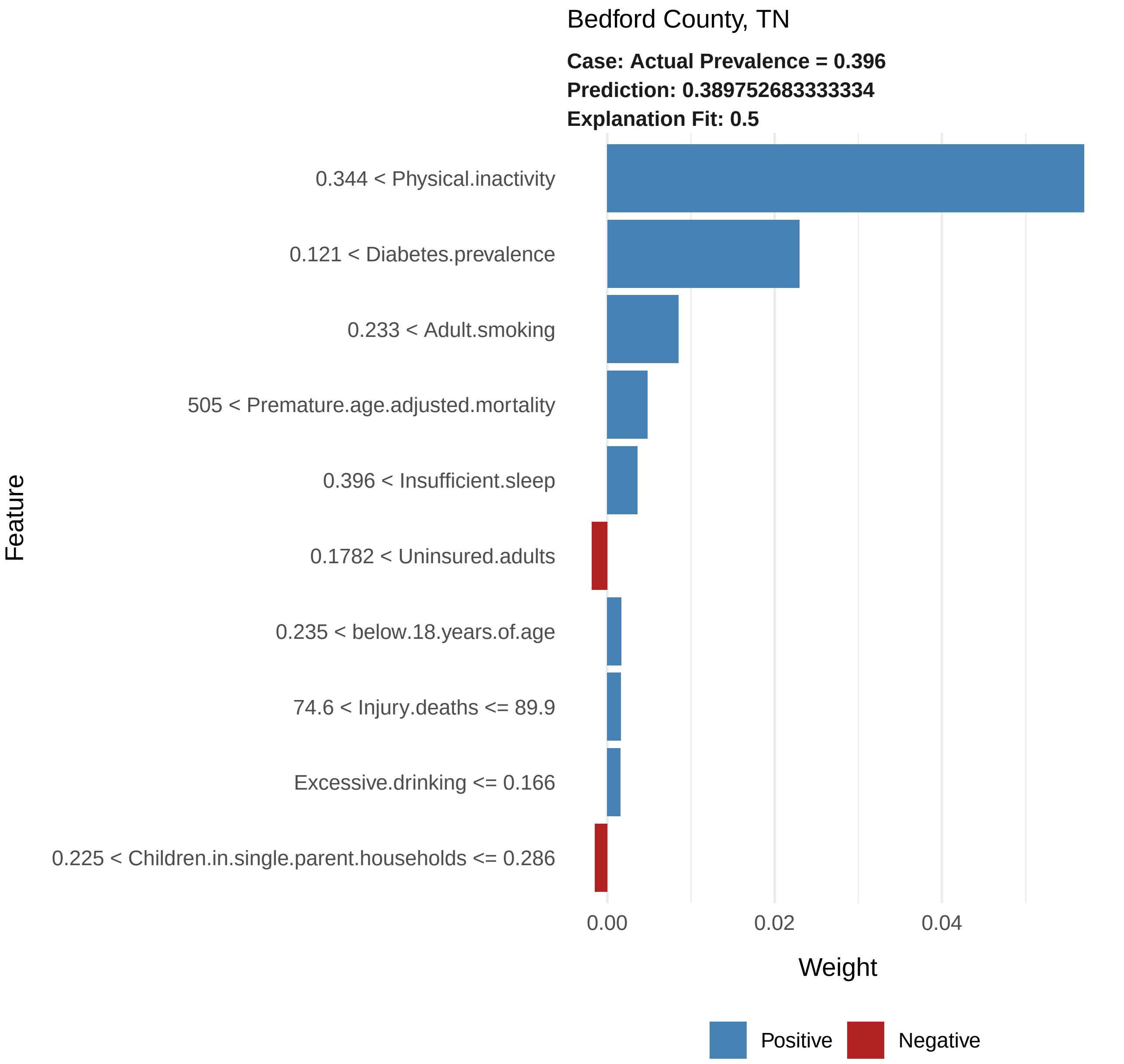
Bedford County, TN
Case: Actual Prevalence = 0.396
Prediction: 0.389752683333334
Explanation Fit: 0.5
0.344 < Physical.inactivity
0.121 < Diabetes.prevalence
0.233 < Adult.smoking
505 < Premature.age.adjusted.mortality
0.396 < Insufficient.sleep
0.1782 < Uninsured.adults
0.235 < below.18.years.of.age
74.6 < Injury.deaths <= 89.9
Excessive.drinking <= 0.166
0.225 < Children.in.single.parent.households <= 0.286
Feature
0.00
0.02
0.04
Weight
Positive
Negative

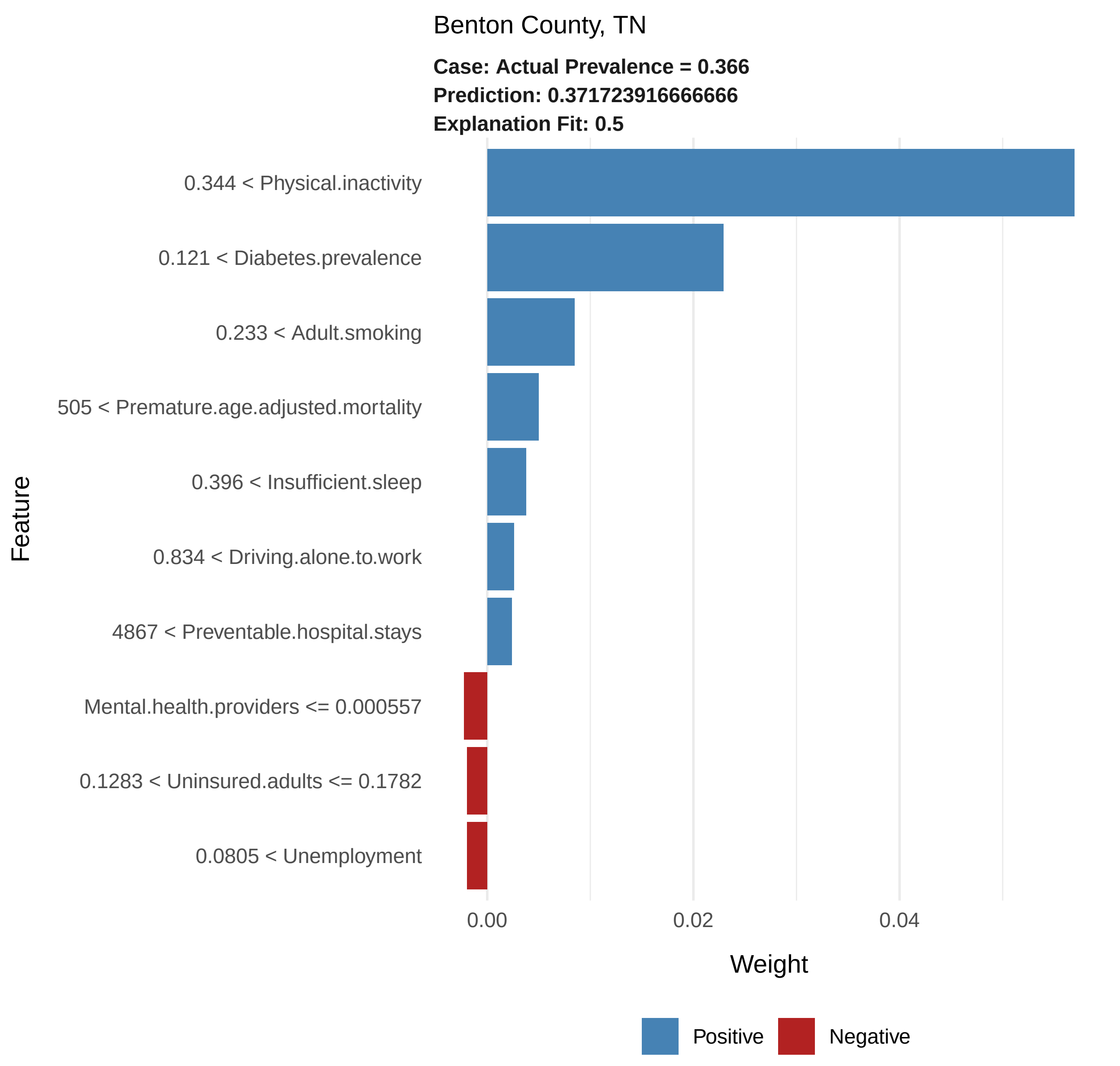
Benton County, TN
Case: Actual Prevalence = 0.366
Prediction: 0.371723916666666
Explanation Fit: 0.5
0.344 < Physical.inactivity
0.121 < Diabetes.prevalence
0.233 < Adult.smoking
505 < Premature.age.adjusted.mortality
0.396 < Insufficient.sleep
0.834 < Driving.alone.to.work
4867 < Preventable.hospital.stays
Mental.health.providers <= 0.000557
0.1283 < Uninsured.adults <= 0.1782
0.0805 < Unemployment
Feature
0.00
0.02
0.04
Weight
Positive
Negative

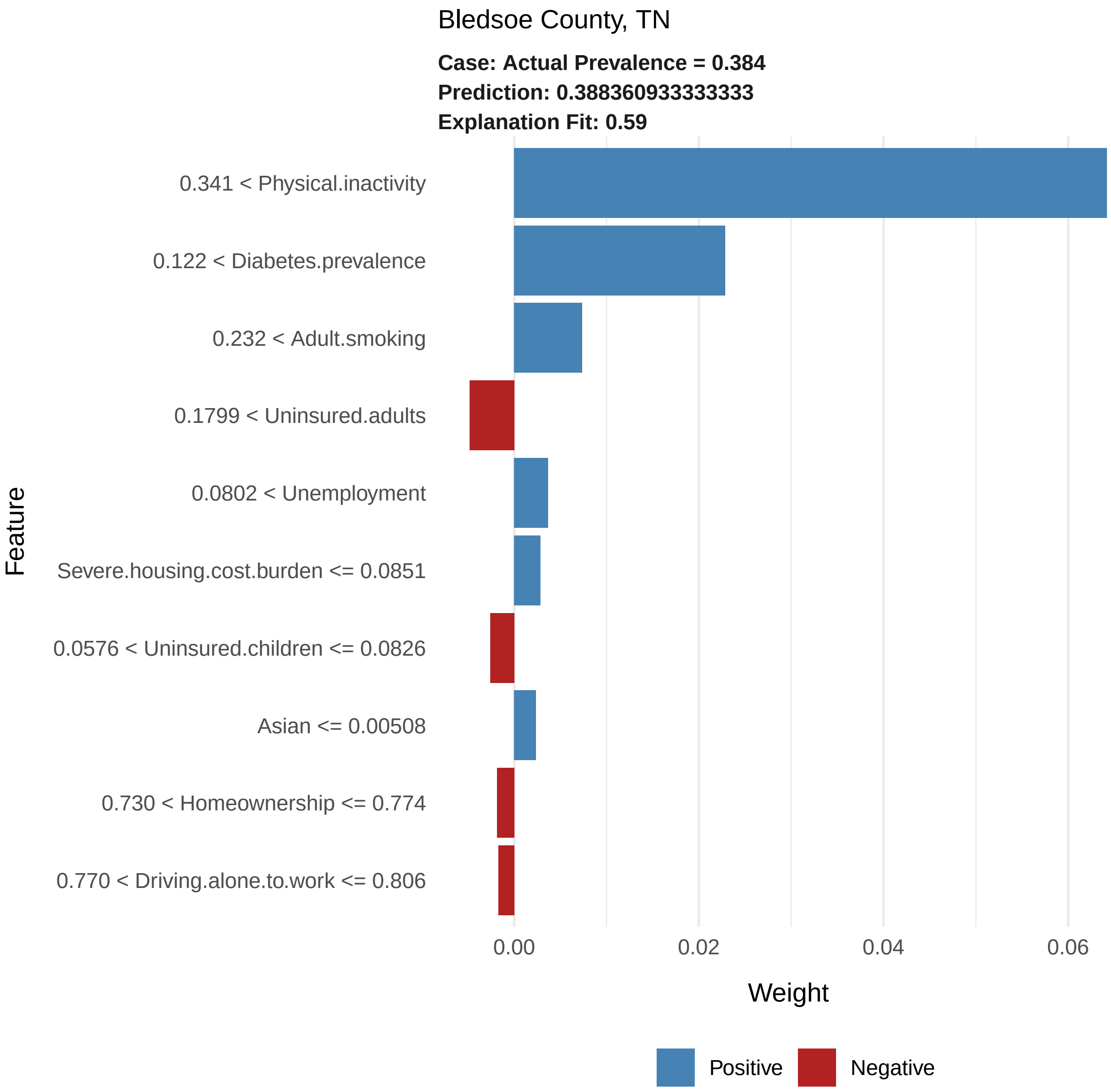
Bledsoe County, TN
Case: Actual Prevalence = 0.384
Prediction: 0.388360933333333
Explanation Fit: 0.59
0.341 < Physical.inactivity
0.122 < Diabetes.prevalence
0.232 < Adult.smoking
0.1799 < Uninsured.adults
0.0802 < Unemployment
Severe.housing.cost.burden <= 0.0851
0.0576 < Uninsured.children <= 0.0826
Asian <= 0.00508
0.730 < Homeownership <= 0.774
0.770 < Driving.alone.to.work <= 0.806
Feature
0.00
0.02
0.04
0.06
Weight
Positive
Negative

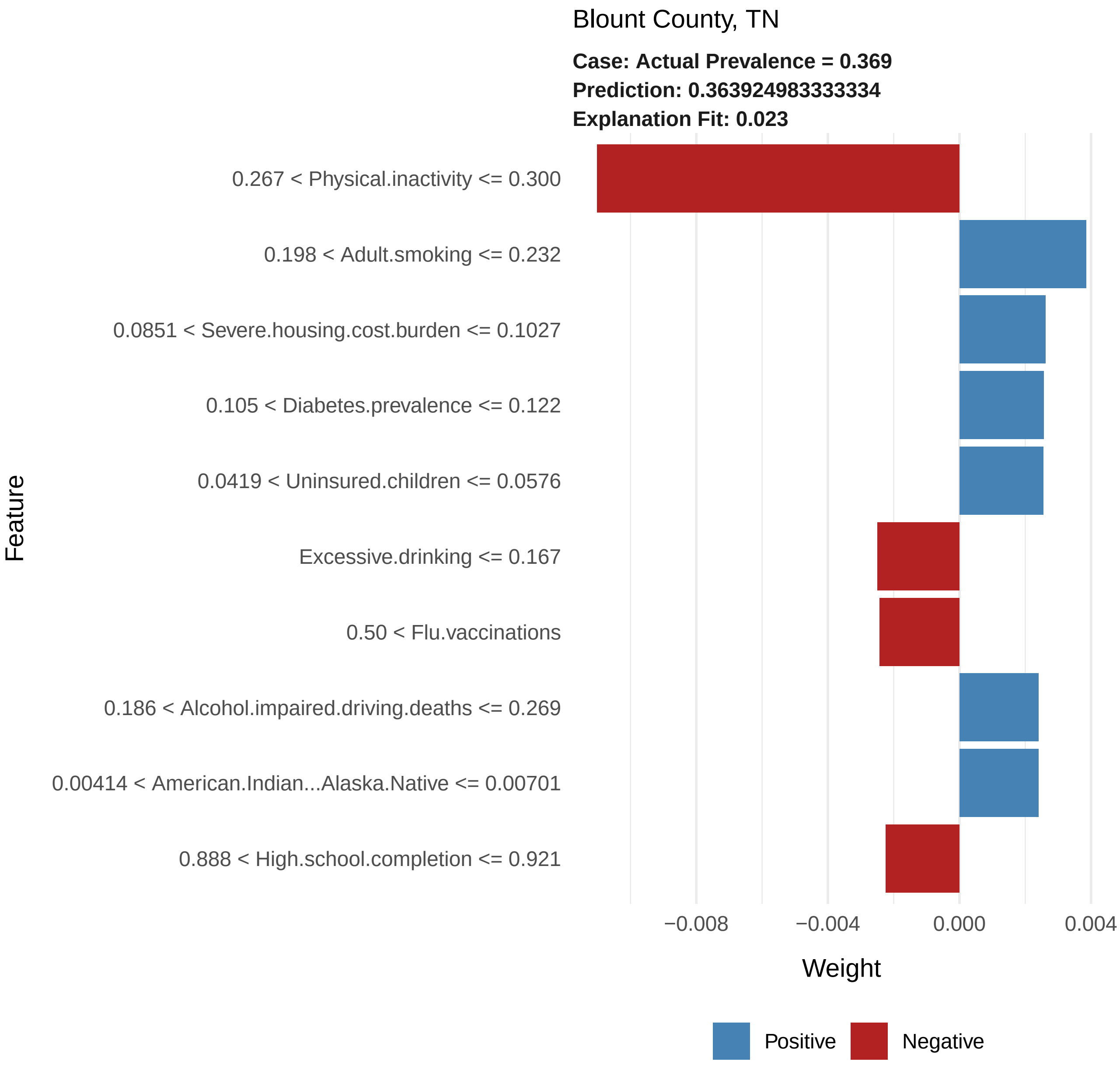

Blount County, TN
Case: Actual Prevalence = 0.369
Prediction: 0.363924983333334
Explanation Fit: 0.023
0.267 < Physical.inactivity <= 0.300
0.198 < Adult.smoking <= 0.232
0.0851 < Severe.housing.cost.burden <= 0.1027
0.105 < Diabetes.prevalence <= 0.122
0.0419 < Uninsured.children <= 0.0576
Excessive.drinking <= 0.167
0.50 < Flu.vaccinations
0.186 < Alcohol.impaired.driving.deaths <= 0.269
0.00414 < American.Indian...Alaska.Native <= 0.00701
0.888 < High.school.completion <= 0.921
Feature
−0.008
−0.004
0.000
0.004
Weight
Positive
Negative

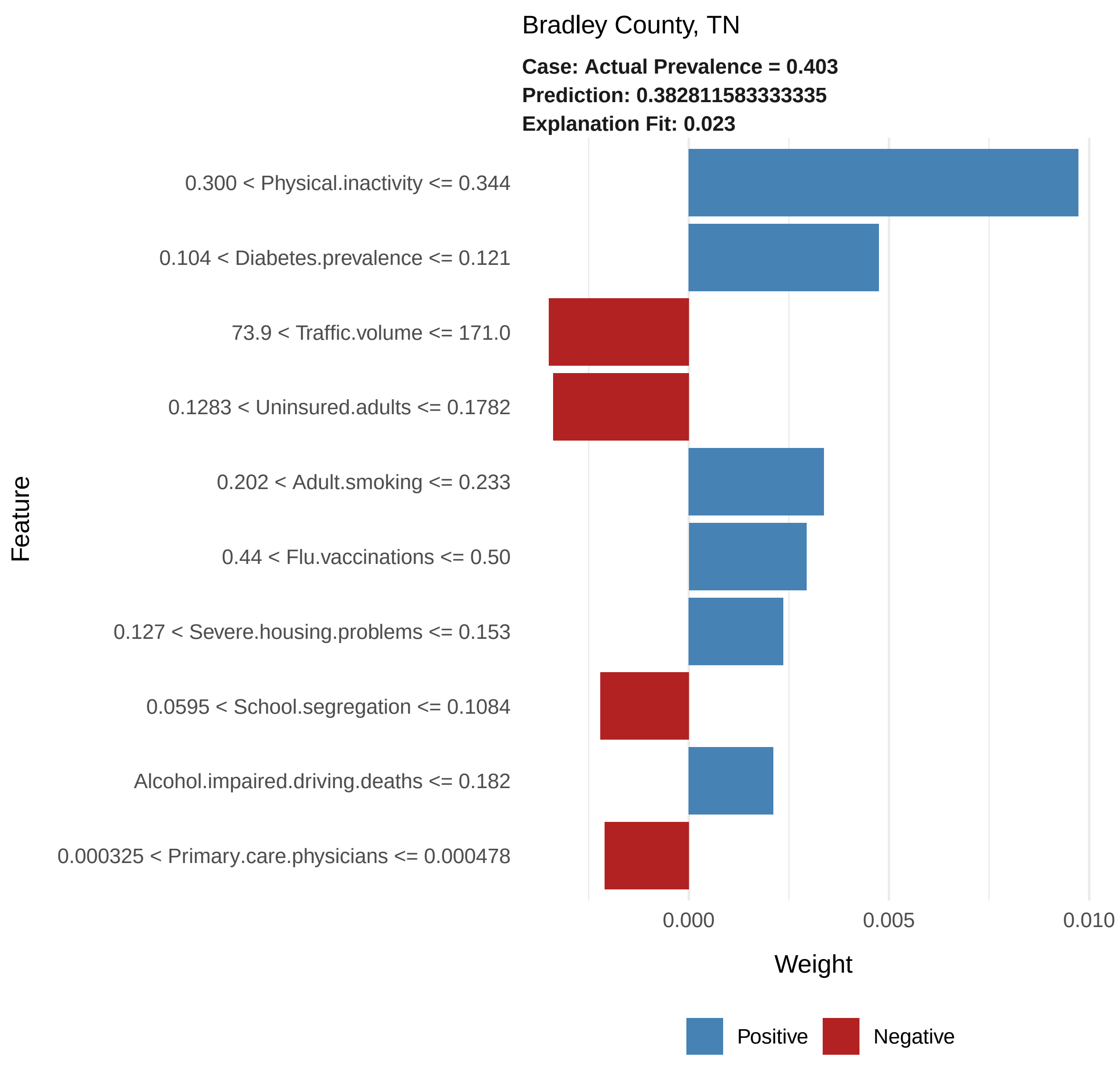

Bradley County, TN
Case: Actual Prevalence = 0.403
Prediction: 0.382811583333335
Explanation Fit: 0.023
0.300 < Physical.inactivity <= 0.344
0.104 < Diabetes.prevalence <= 0.121
73.9 < Traffic.volume <= 171.0
0.1283 < Uninsured.adults <= 0.1782
0.202 < Adult.smoking <= 0.233
0.44 < Flu.vaccinations <= 0.50
0.127 < Severe.housing.problems <= 0.153
0.0595 < School.segregation <= 0.1084
Alcohol.impaired.driving.deaths <= 0.182
0.000325 < Primary.care.physicians <= 0.000478
Feature
0.000
0.005
0.010
Weight
Positive
Negative

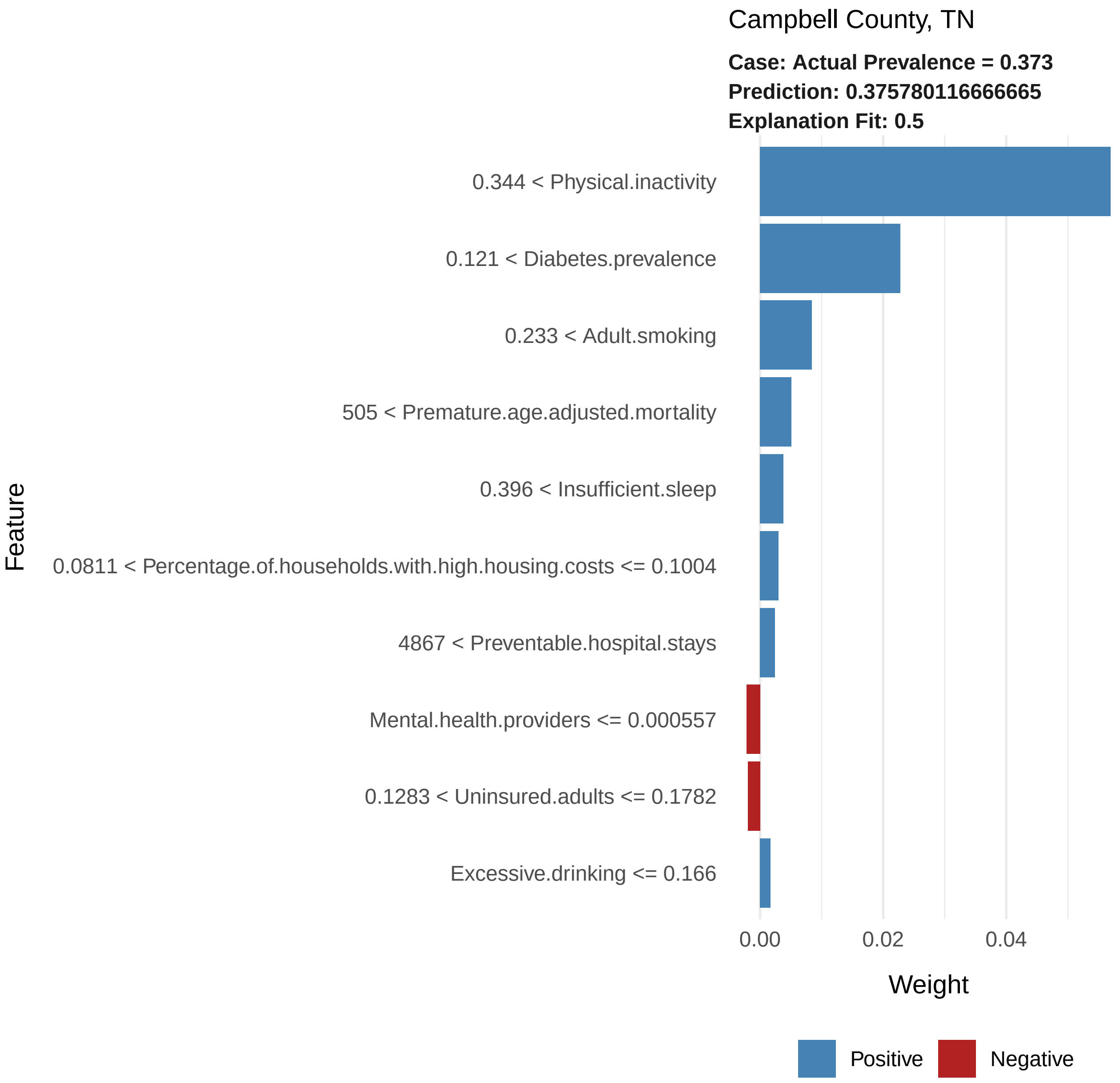
Campbell County, TN
Case: Actual Prevalence = 0.373
Prediction: 0.375780116666665
Explanation Fit: 0.5
0.344 < Physical.inactivity
0.121 < Diabetes.prevalence
0.233 < Adult.smoking
505 < Premature.age.adjusted.mortality
0.396 < Insufficient.sleep
0.0811 < Percentage.of.households.with.high.housing.costs <= 0.1004
4867 < Preventable.hospital.stays
Mental.health.providers <= 0.000557
0.1283 < Uninsured.adults <= 0.1782
Excessive.drinking <= 0.166
Feature
0.00
0.02
0.04
Weight
Positive
Negative

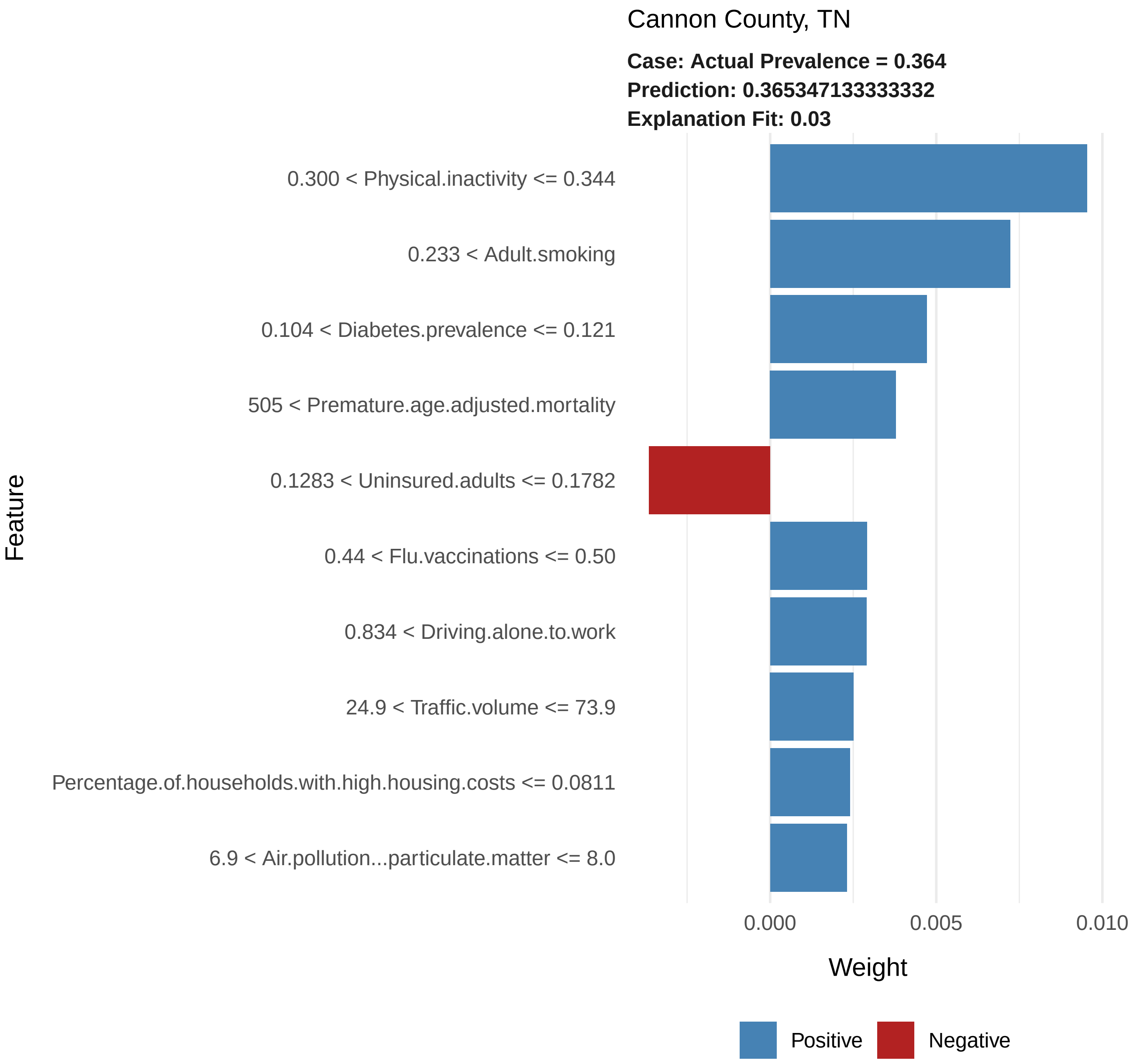

Cannon County, TN
Case: Actual Prevalence = 0.364
Prediction: 0.365347133333332
Explanation Fit: 0.03
0.300 < Physical.inactivity <= 0.344
0.233 < Adult.smoking
0.104 < Diabetes.prevalence <= 0.121
505 < Premature.age.adjusted.mortality
0.1283 < Uninsured.adults <= 0.1782
0.44 < Flu.vaccinations <= 0.50
0.834 < Driving.alone.to.work
24.9 < Traffic.volume <= 73.9
Percentage.of.households.with.high.housing.costs <= 0.0811
6.9 < Air.pollution...particulate.matter <= 8.0
Feature
0.000
0.005
0.010
Weight
Positive
Negative

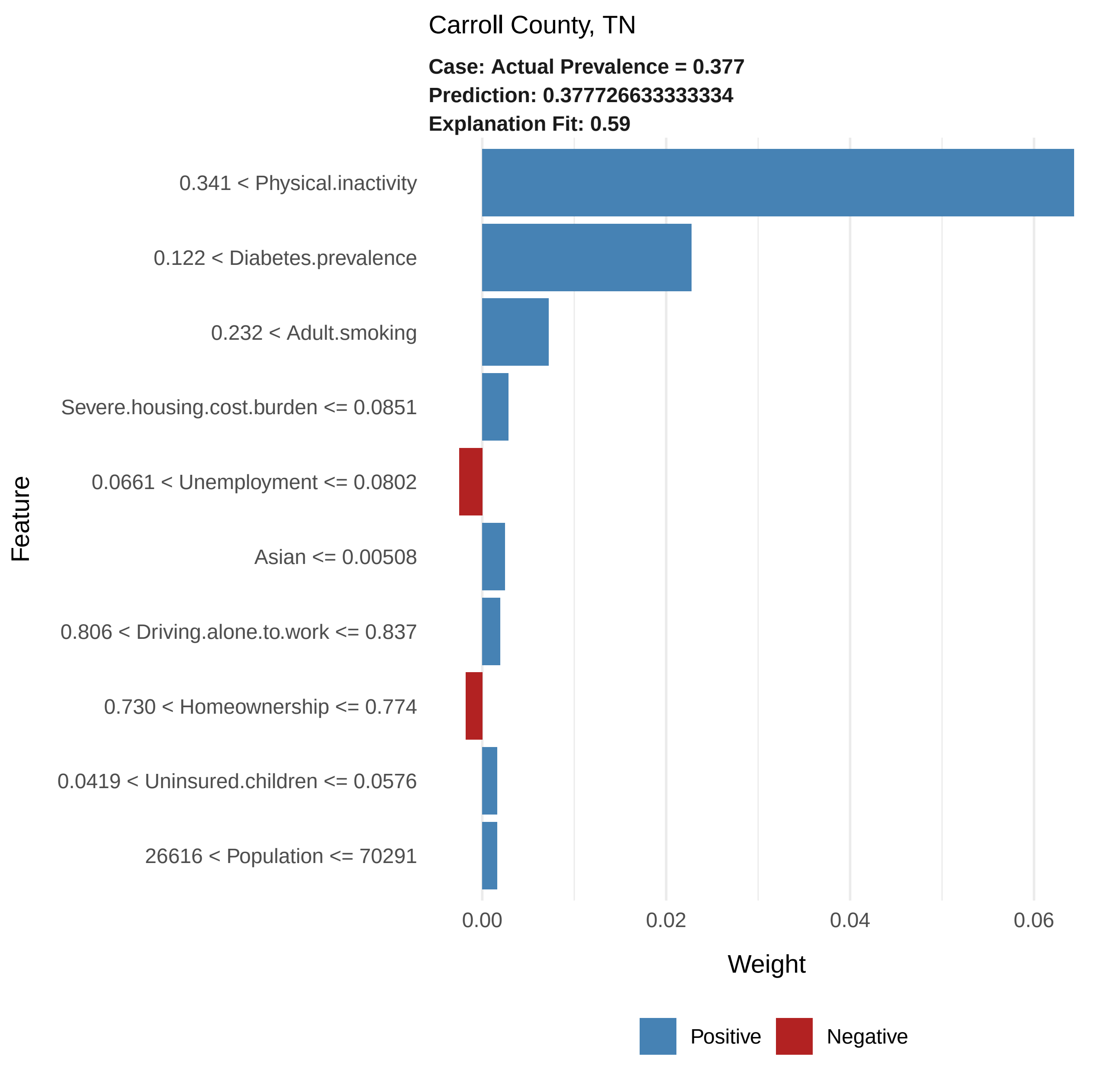

Carroll County, TN
Case: Actual Prevalence = 0.377
Prediction: 0.377726633333334
Explanation Fit: 0.59
0.341 < Physical.inactivity
0.122 < Diabetes.prevalence
0.232 < Adult.smoking
Severe.housing.cost.burden <= 0.0851
0.0661 < Unemployment <= 0.0802
Asian <= 0.00508
0.806 < Driving.alone.to.work <= 0.837
0.730 < Homeownership <= 0.774
0.0419 < Uninsured.children <= 0.0576
26616 < Population <= 70291
Feature
0.00
0.02
0.04
0.06
Weight
Positive
Negative

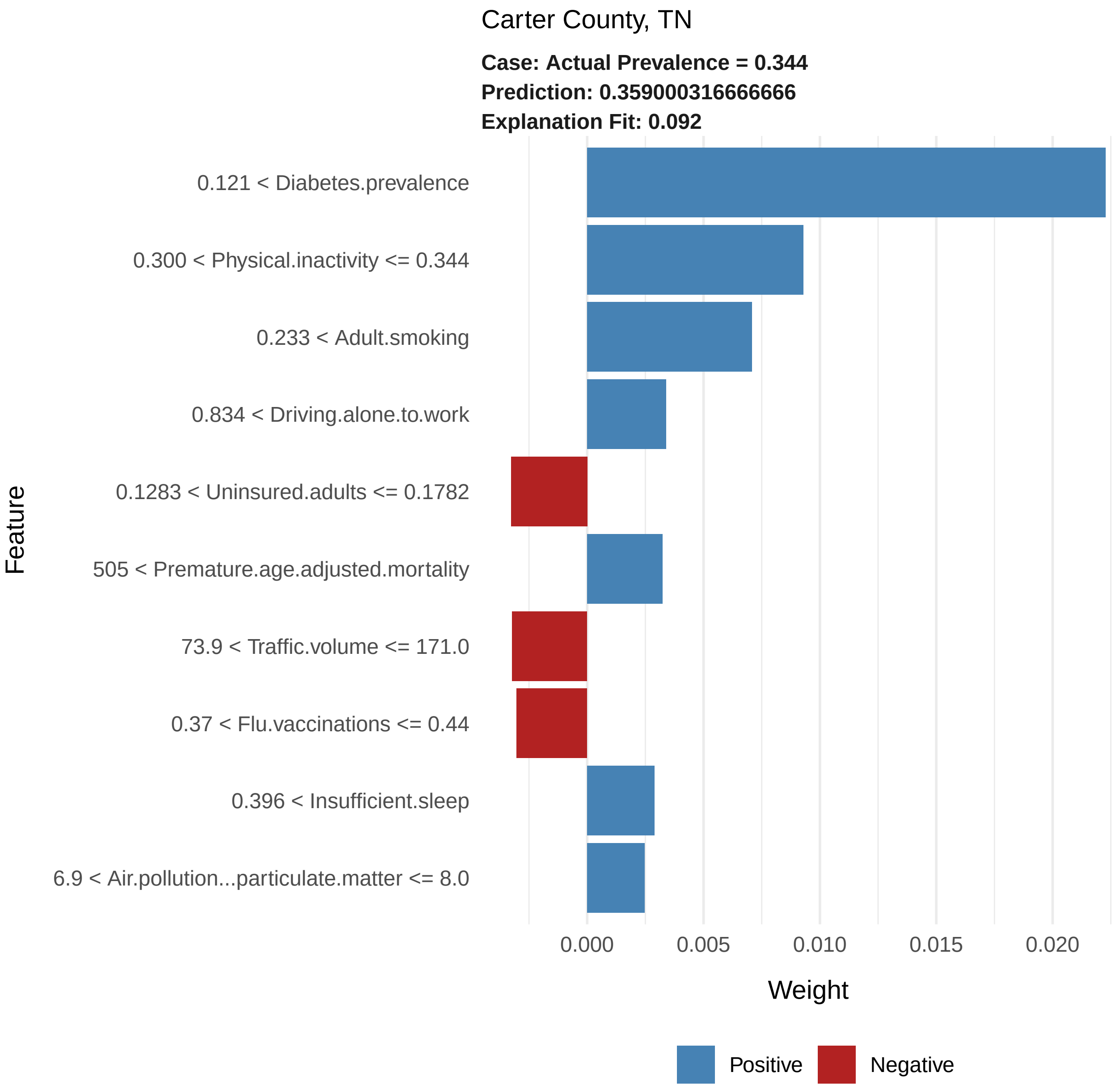

Carter County, TN
Case: Actual Prevalence = 0.344
Prediction: 0.359000316666666
Explanation Fit: 0.092
0.121 < Diabetes.prevalence
0.300 < Physical.inactivity <= 0.344
0.233 < Adult.smoking
0.834 < Driving.alone.to.work
0.1283 < Uninsured.adults <= 0.1782
505 < Premature.age.adjusted.mortality
73.9 < Traffic.volume <= 171.0
0.37 < Flu.vaccinations <= 0.44
0.396 < Insufficient.sleep
6.9 < Air.pollution...particulate.matter <= 8.0
Feature
0.000
0.005
0.010
0.015
0.020
Weight
Positive
Negative

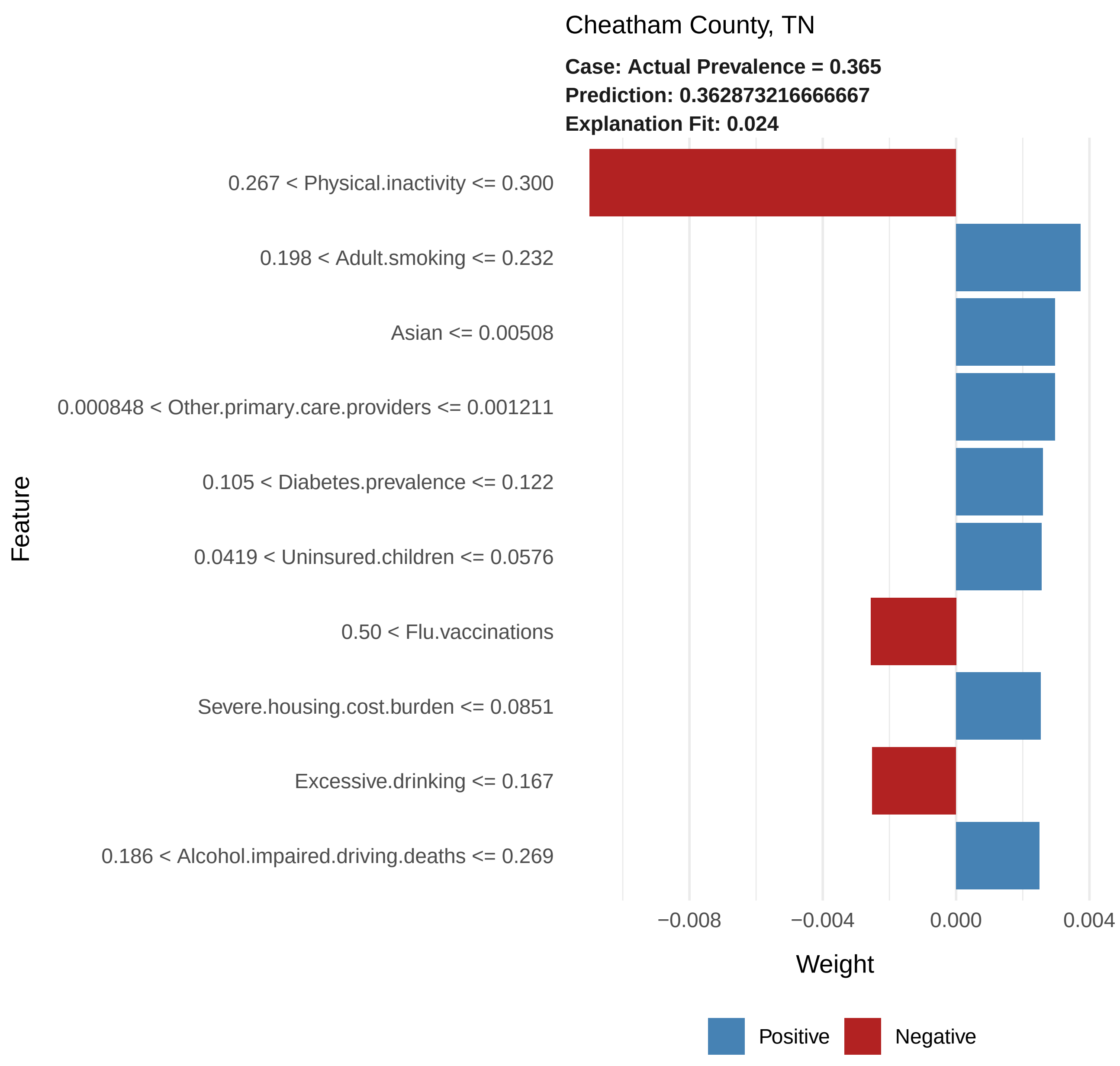
Cheatham County, TN
Case: Actual Prevalence = 0.365
Prediction: 0.362873216666667
Explanation Fit: 0.024
0.267 < Physical.inactivity <= 0.300
0.198 < Adult.smoking <= 0.232
Asian <= 0.00508
0.000848 < Other.primary.care.providers <= 0.001211
0.105 < Diabetes.prevalence <= 0.122
0.0419 < Uninsured.children <= 0.0576
0.50 < Flu.vaccinations
Severe.housing.cost.burden <= 0.0851
Excessive.drinking <= 0.167
0.186 < Alcohol.impaired.driving.deaths <= 0.269
Feature
−0.008
−0.004
0.000
0.004
Weight
Positive
Negative

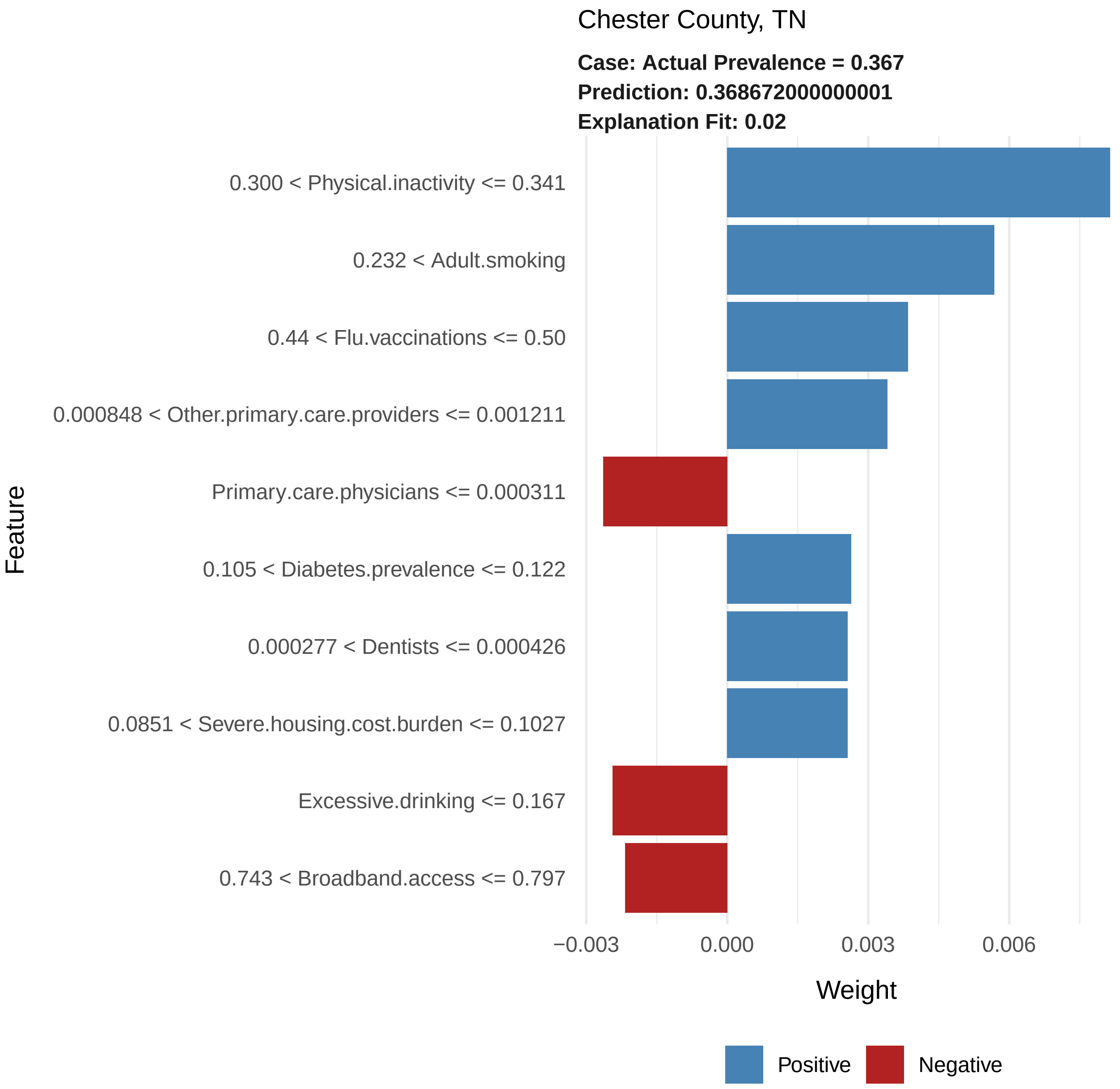

Chester County, TN
Case: Actual Prevalence = 0.367
Prediction: 0.368672000000001
Explanation Fit: 0.02
0.300 < Physical.inactivity <= 0.341
0.232 < Adult.smoking
0.44 < Flu.vaccinations <= 0.50
0.000848 < Other.primary.care.providers <= 0.001211
Primary.care.physicians <= 0.000311
0.105 < Diabetes.prevalence <= 0.122
0.000277 < Dentists <= 0.000426
0.0851 < Severe.housing.cost.burden <= 0.1027
Excessive.drinking <= 0.167
0.743 < Broadband.access <= 0.797
Feature
−0.003
0.000
0.003
0.006
Weight
Positive
Negative

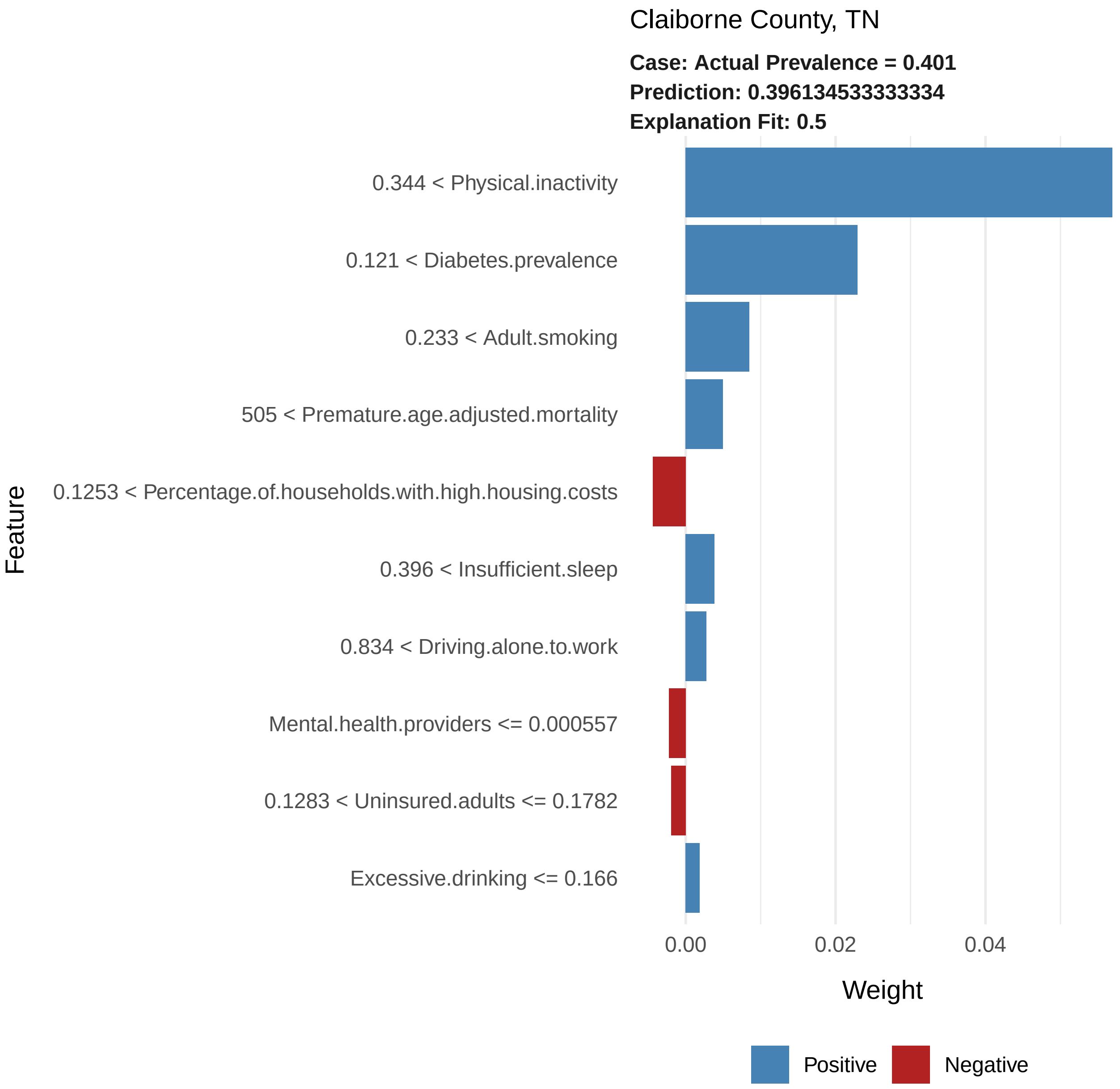
Claiborne County, TN
Case: Actual Prevalence = 0.401
Prediction: 0.396134533333334
Explanation Fit: 0.5
0.344 < Physical.inactivity
0.121 < Diabetes.prevalence
0.233 < Adult.smoking
505 < Premature.age.adjusted.mortality
0.1253 < Percentage.of.households.with.high.housing.costs
0.396 < Insufficient.sleep
0.834 < Driving.alone.to.work
Mental.health.providers <= 0.000557
0.1283 < Uninsured.adults <= 0.1782
Excessive.drinking <= 0.166
Feature
0.00
0.02
0.04
Weight
Positive
Negative

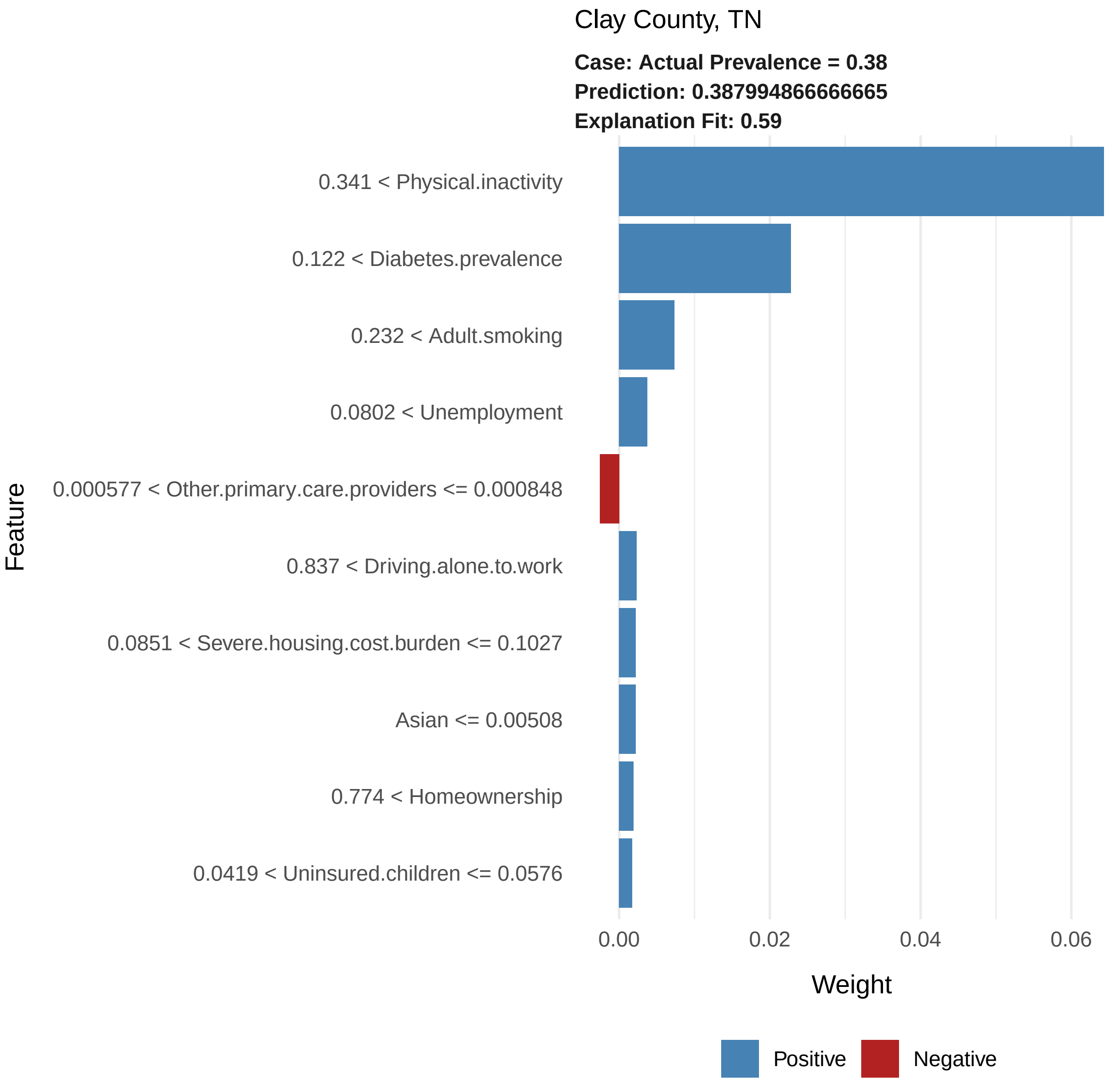
Clay County, TN
Case: Actual Prevalence = 0.38
Prediction: 0.387994866666665
Explanation Fit: 0.59
0.341 < Physical.inactivity
0.122 < Diabetes.prevalence
0.232 < Adult.smoking
0.0802 < Unemployment
0.000577 < Other.primary.care.providers <= 0.000848
0.837 < Driving.alone.to.work
0.0851 < Severe.housing.cost.burden <= 0.1027
Asian <= 0.00508
0.774 < Homeownership
0.0419 < Uninsured.children <= 0.0576
Feature
0.00
0.02
0.04
0.06
Weight
Positive
Negative

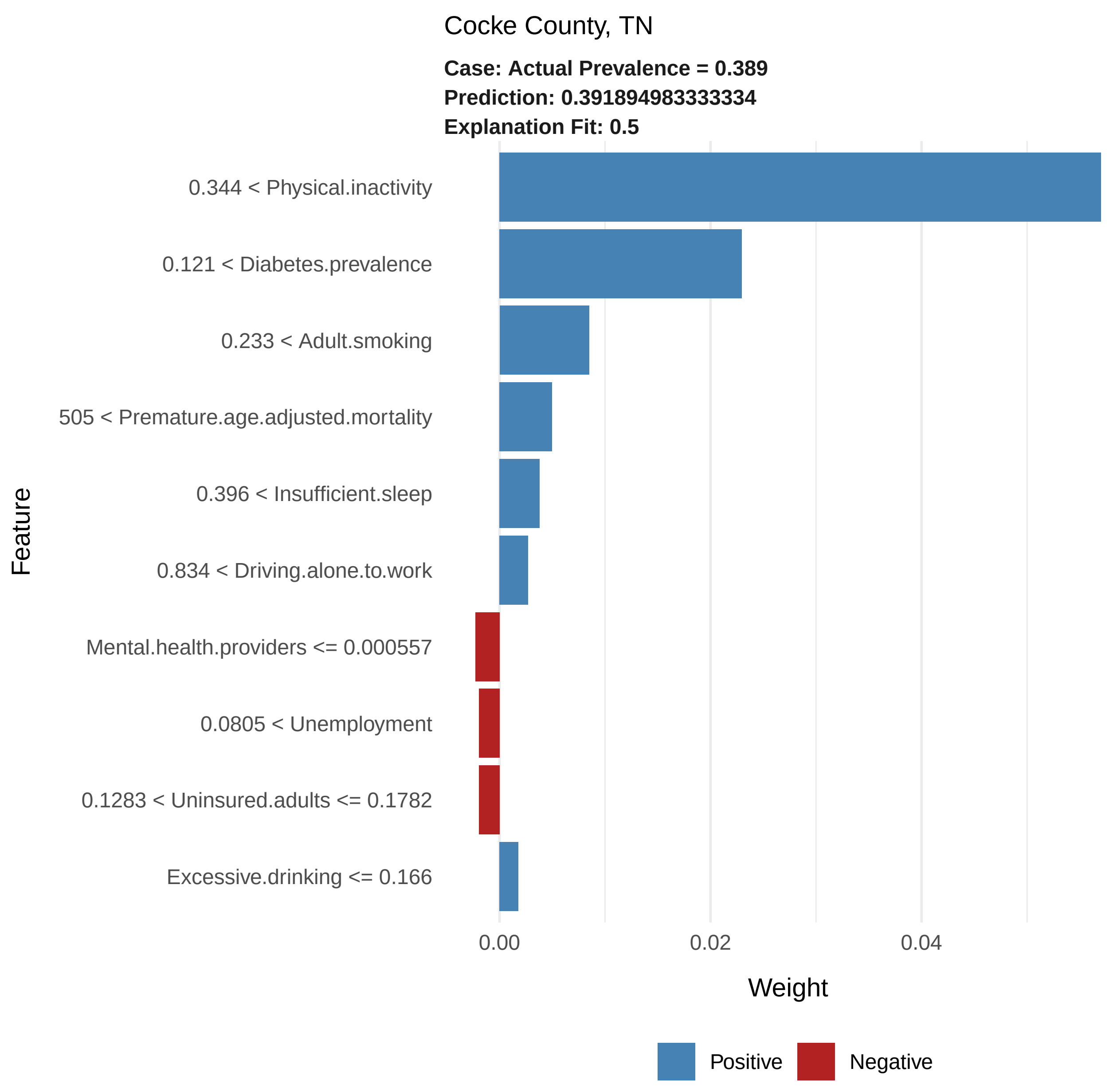
Cocke County, TN
Case: Actual Prevalence = 0.389
Prediction: 0.391894983333334
Explanation Fit: 0.5
0.344 < Physical.inactivity
0.121 < Diabetes.prevalence
0.233 < Adult.smoking
505 < Premature.age.adjusted.mortality
0.396 < Insufficient.sleep
0.834 < Driving.alone.to.work
Mental.health.providers <= 0.000557
0.0805 < Unemployment
0.1283 < Uninsured.adults <= 0.1782
Excessive.drinking <= 0.166
Feature
0.00
0.02
0.04
Weight
Positive
Negative

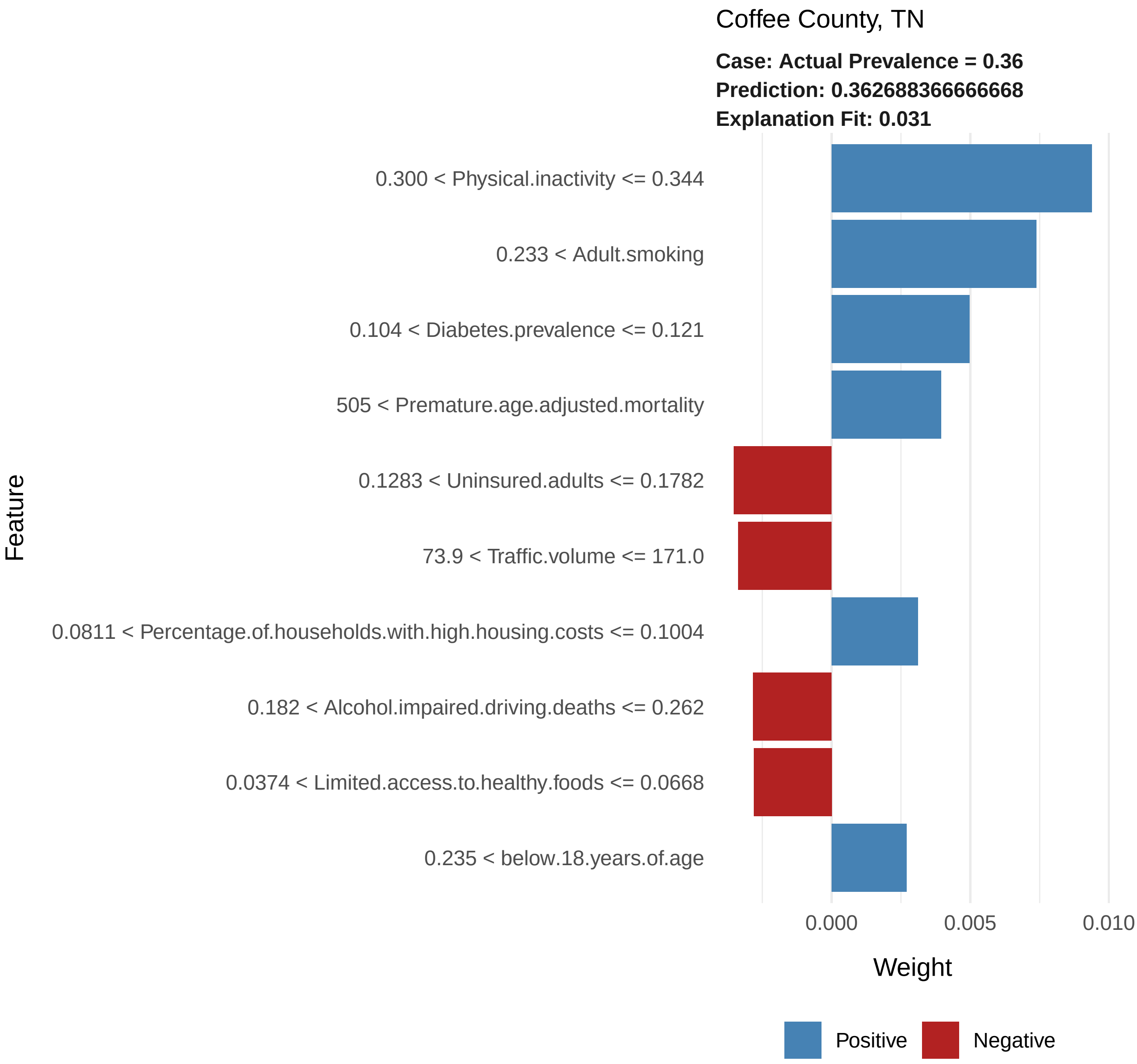

Coffee County, TN
Case: Actual Prevalence = 0.36
Prediction: 0.362688366666668
Explanation Fit: 0.031
0.300 < Physical.inactivity <= 0.344
0.233 < Adult.smoking
0.104 < Diabetes.prevalence <= 0.121
505 < Premature.age.adjusted.mortality
0.1283 < Uninsured.adults <= 0.1782
73.9 < Traffic.volume <= 171.0
0.0811 < Percentage.of.households.with.high.housing.costs <= 0.1004
0.182 < Alcohol.impaired.driving.deaths <= 0.262
0.0374 < Limited.access.to.healthy.foods <= 0.0668
0.235 < below.18.years.of.age
Feature
0.000
0.005
0.010
Weight
Positive
Negative

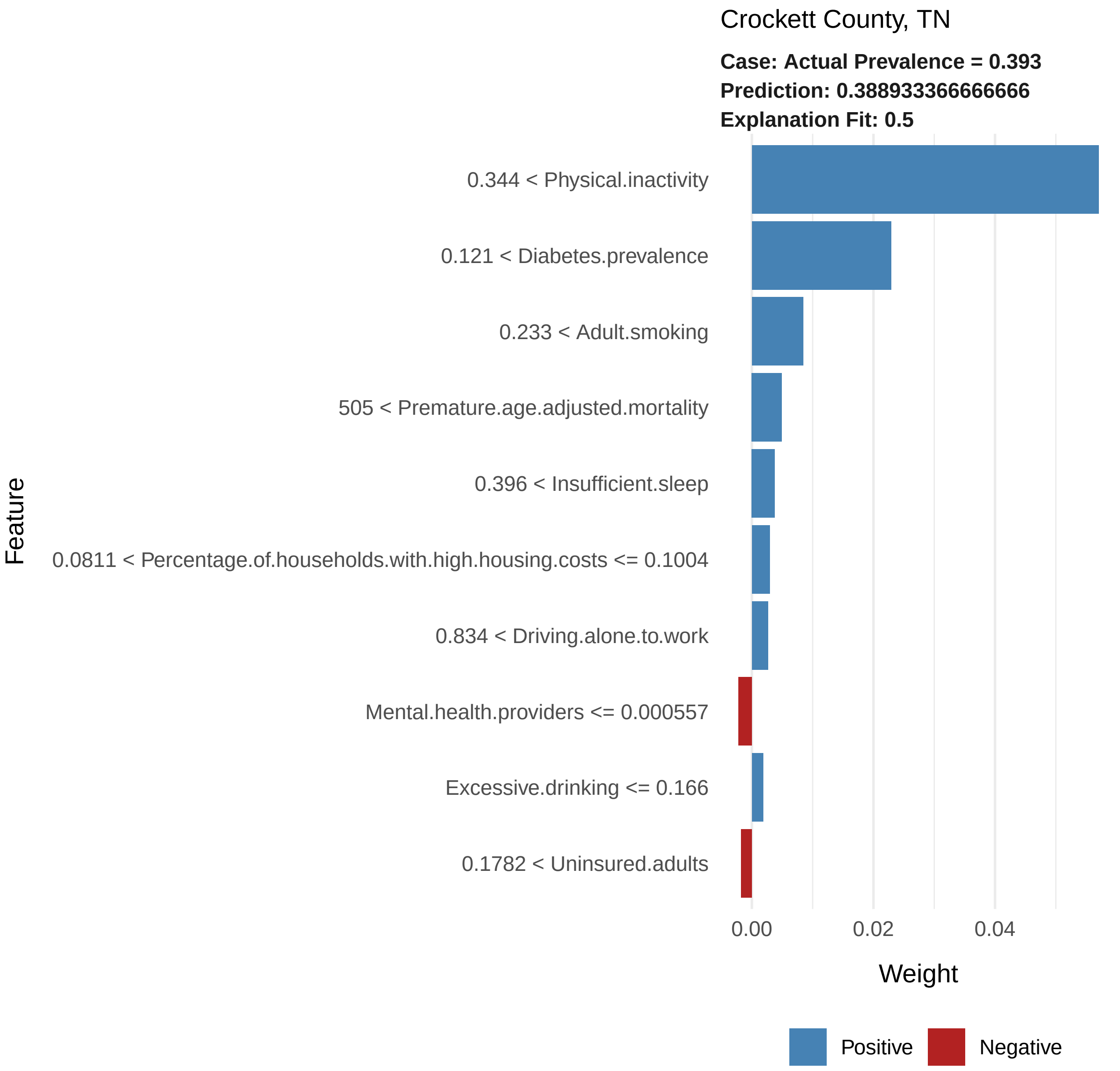

Crockett County, TN
Case: Actual Prevalence = 0.393
Prediction: 0.388933366666666
Explanation Fit: 0.5
0.344 < Physical.inactivity
0.121 < Diabetes.prevalence
0.233 < Adult.smoking
505 < Premature.age.adjusted.mortality
0.396 < Insufficient.sleep
0.0811 < Percentage.of.households.with.high.housing.costs <= 0.1004
0.834 < Driving.alone.to.work
Mental.health.providers <= 0.000557
Excessive.drinking <= 0.166
0.1782 < Uninsured.adults
Feature
0.00
0.02
0.04
Weight
Positive
Negative

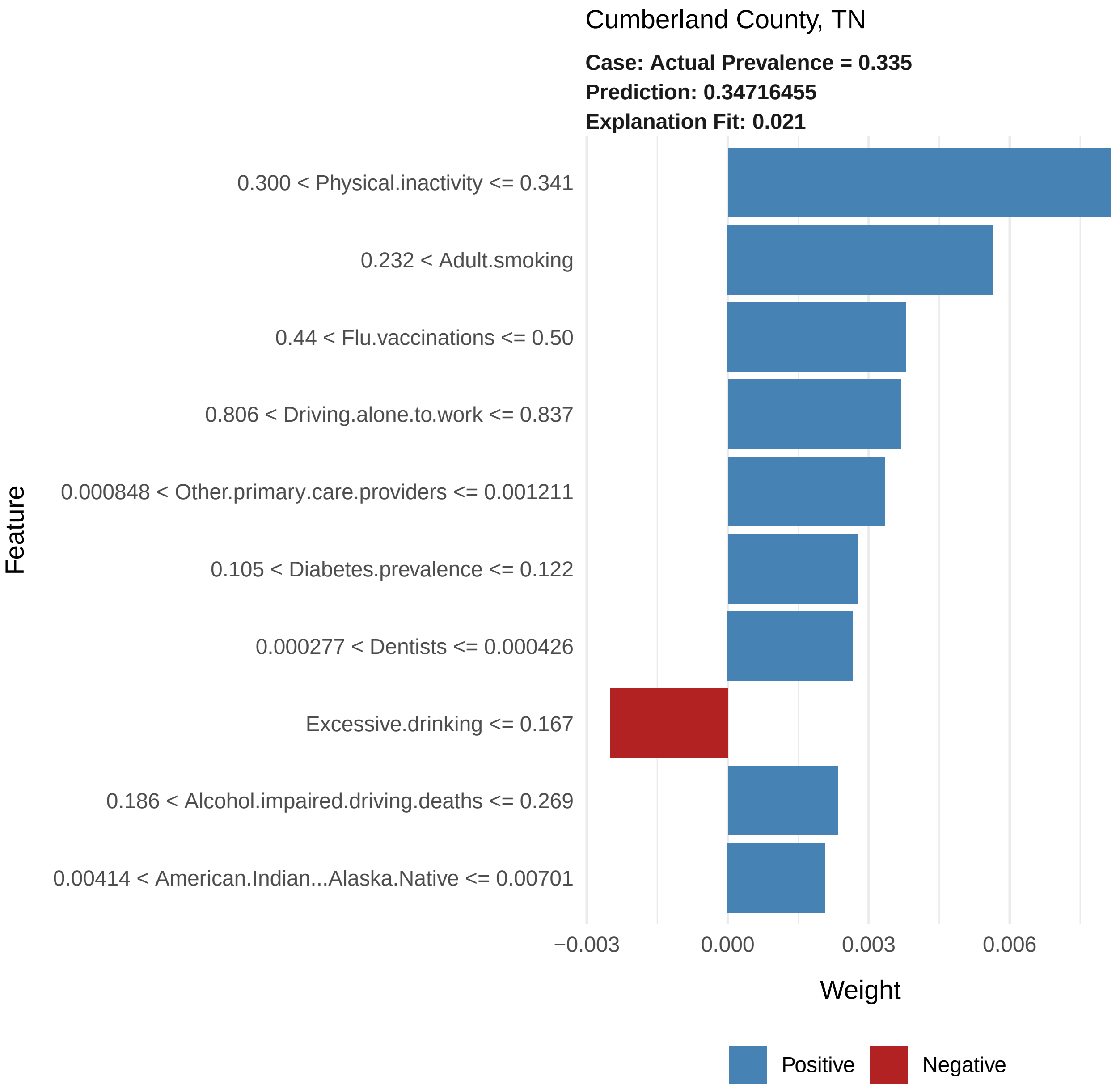

Cumberland County, TN
Case: Actual Prevalence = 0.335
Prediction: 0.34716455
Explanation Fit: 0.021
0.300 < Physical.inactivity <= 0.341
0.232 < Adult.smoking
0.44 < Flu.vaccinations <= 0.50
0.806 < Driving.alone.to.work <= 0.837
0.000848 < Other.primary.care.providers <= 0.001211
0.105 < Diabetes.prevalence <= 0.122
0.000277 < Dentists <= 0.000426
Excessive.drinking <= 0.167
0.186 < Alcohol.impaired.driving.deaths <= 0.269
0.00414 < American.Indian...Alaska.Native <= 0.00701
Feature
−0.003
0.000
0.003
0.006
Weight
Positive
Negative

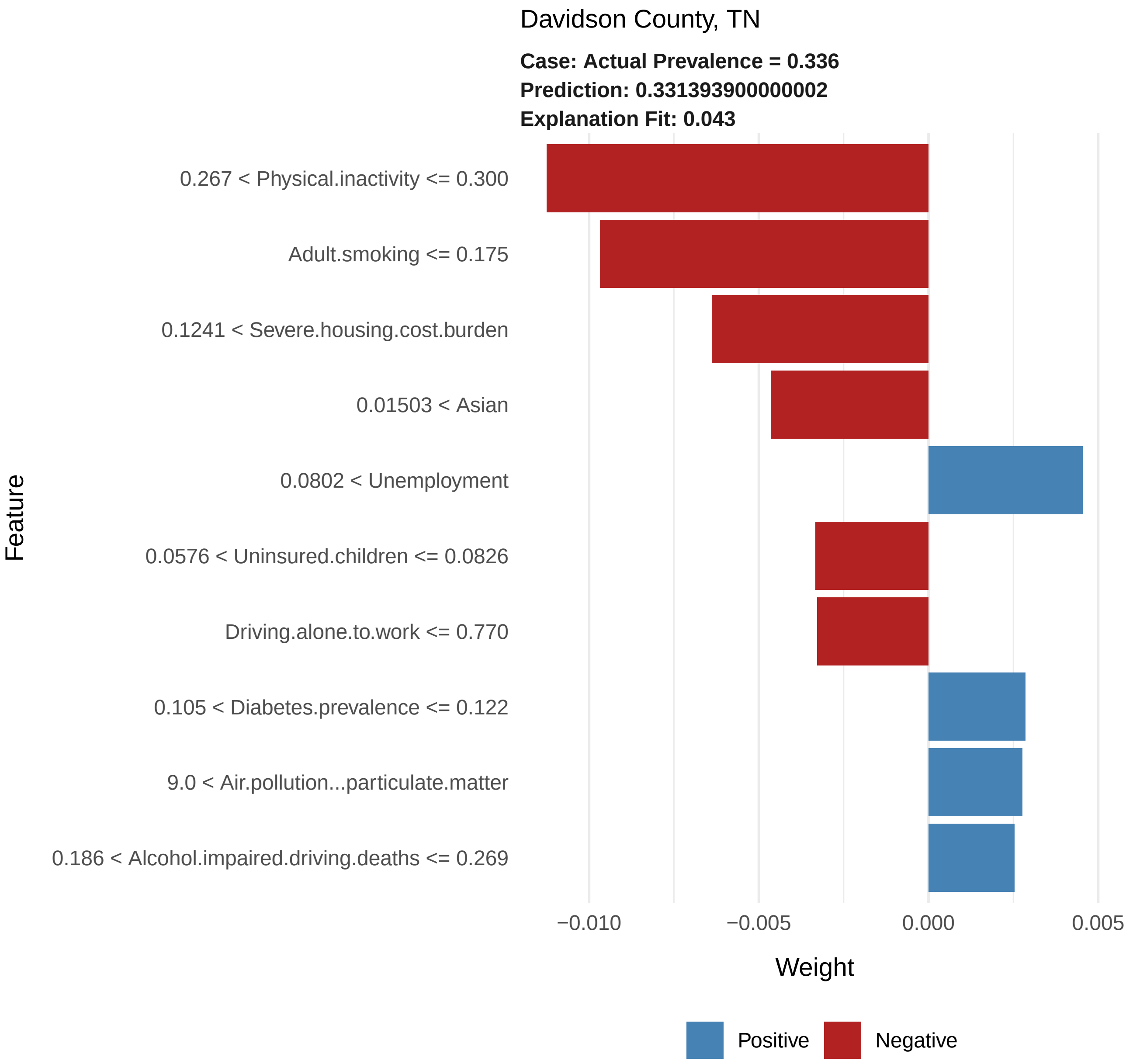
Davidson County, TN
Case: Actual Prevalence = 0.336
Prediction: 0.331393900000002
Explanation Fit: 0.043
0.267 < Physical.inactivity <= 0.300
Adult.smoking <= 0.175
0.1241 < Severe.housing.cost.burden
0.01503 < Asian
0.0802 < Unemployment
0.0576 < Uninsured.children <= 0.0826
Driving.alone.to.work <= 0.770
0.105 < Diabetes.prevalence <= 0.122
9.0 < Air.pollution...particulate.matter
0.186 < Alcohol.impaired.driving.deaths <= 0.269
Feature
−0.010
−0.005
0.000
0.005
Weight
Positive
Negative

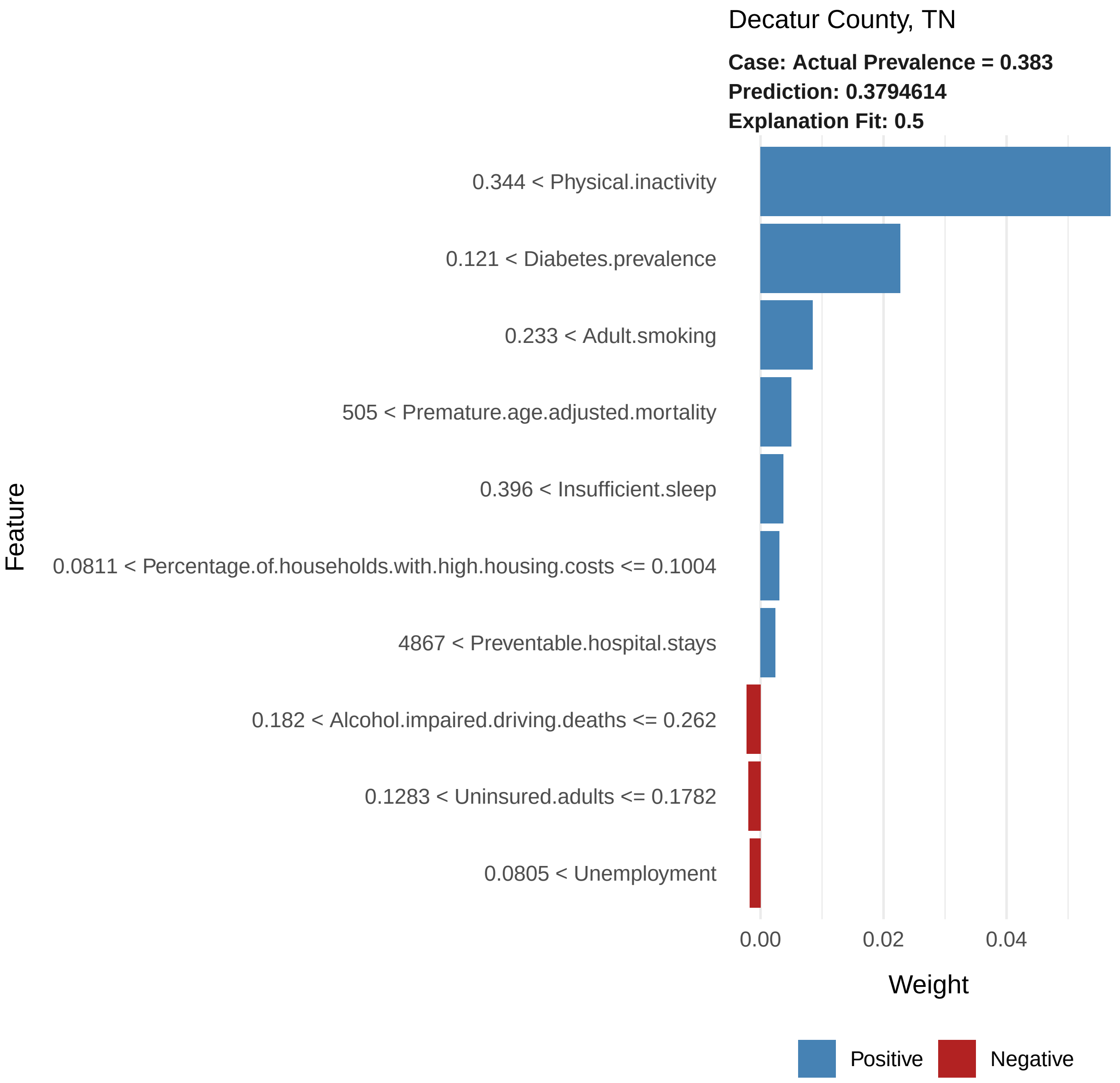
Decatur County, TN
Case: Actual Prevalence = 0.383
Prediction: 0.3794614
Explanation Fit: 0.5
0.344 < Physical.inactivity
0.121 < Diabetes.prevalence
0.233 < Adult.smoking
505 < Premature.age.adjusted.mortality
0.396 < Insufficient.sleep
0.0811 < Percentage.of.households.with.high.housing.costs <= 0.1004
4867 < Preventable.hospital.stays
0.182 < Alcohol.impaired.driving.deaths <= 0.262
0.1283 < Uninsured.adults <= 0.1782
0.0805 < Unemployment
Feature
0.00
0.02
0.04
Weight
Positive
Negative

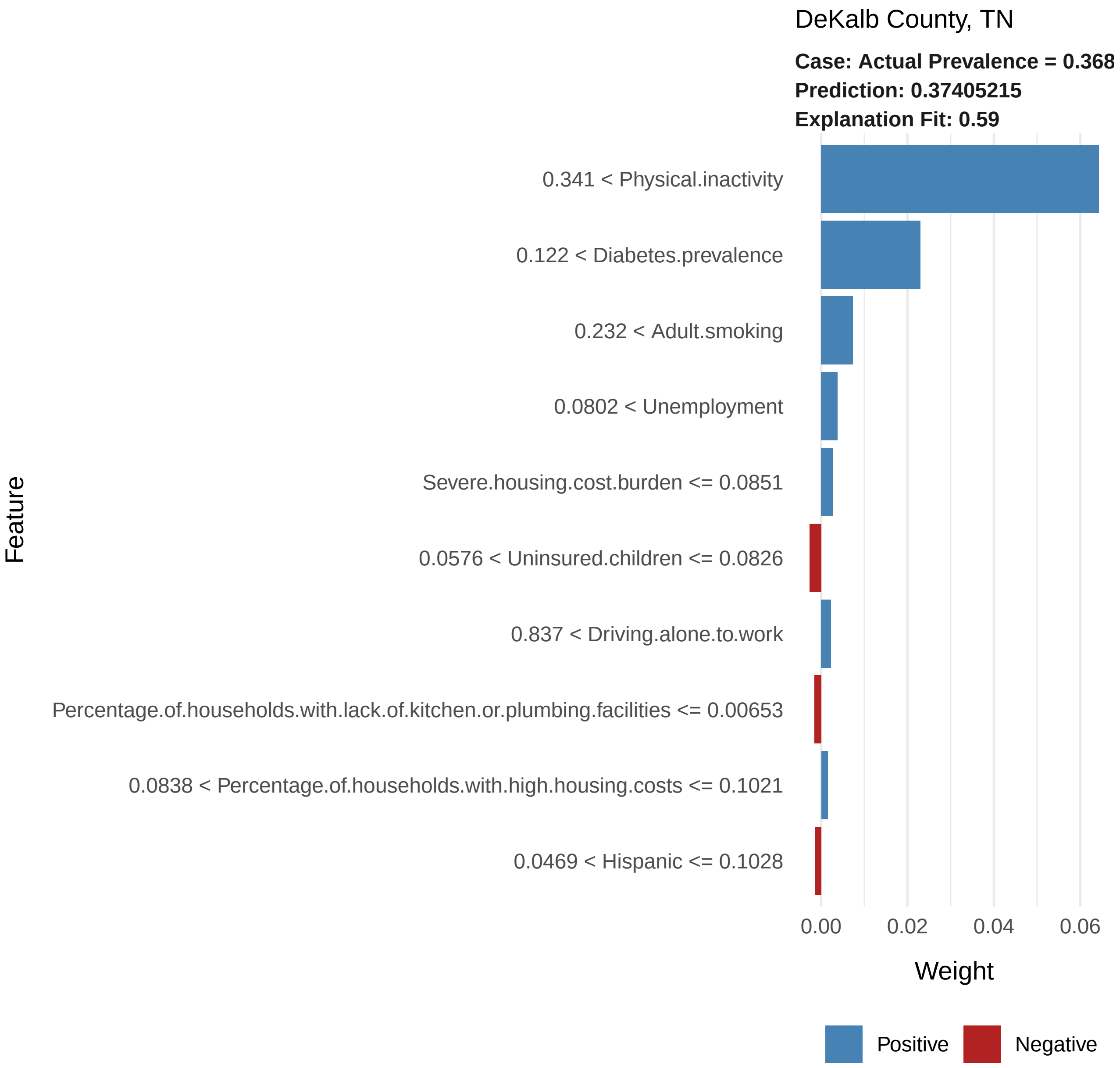
DeKalb County, TN
Case: Actual Prevalence = 0.368
Prediction: 0.37405215
Explanation Fit: 0.59
0.341 < Physical.inactivity
0.122 < Diabetes.prevalence
0.232 < Adult.smoking
0.0802 < Unemployment
Severe.housing.cost.burden <= 0.0851
0.0576 < Uninsured.children <= 0.0826
0.837 < Driving.alone.to.work
Percentage.of.households.with.lack.of.kitchen.or.plumbing.facilities <= 0.00653
0.0838 < Percentage.of.households.with.high.housing.costs <= 0.1021
0.0469 < Hispanic <= 0.1028
Feature
0.00
0.02
0.04
0.06
Weight
Positive
Negative

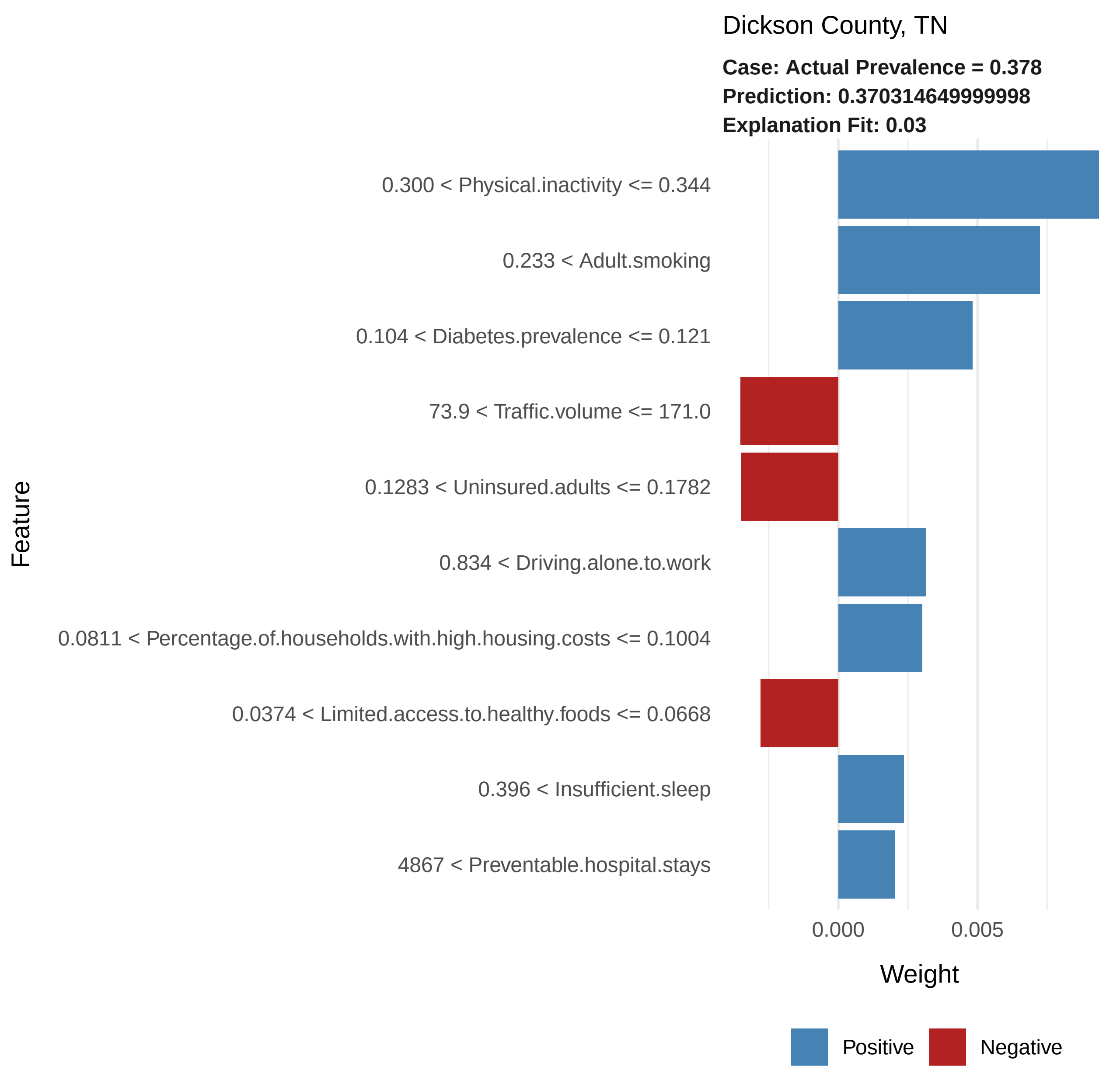
Dickson County, TN
Case: Actual Prevalence = 0.378
Prediction: 0.370314649999998
Explanation Fit: 0.03
0.300 < Physical.inactivity <= 0.344
0.233 < Adult.smoking
0.104 < Diabetes.prevalence <= 0.121
73.9 < Traffic.volume <= 171.0
0.1283 < Uninsured.adults <= 0.1782
0.834 < Driving.alone.to.work
0.0811 < Percentage.of.households.with.high.housing.costs <= 0.1004
0.0374 < Limited.access.to.healthy.foods <= 0.0668
0.396 < Insufficient.sleep
4867 < Preventable.hospital.stays
Feature
0.000
0.005
Weight
Positive
Negative

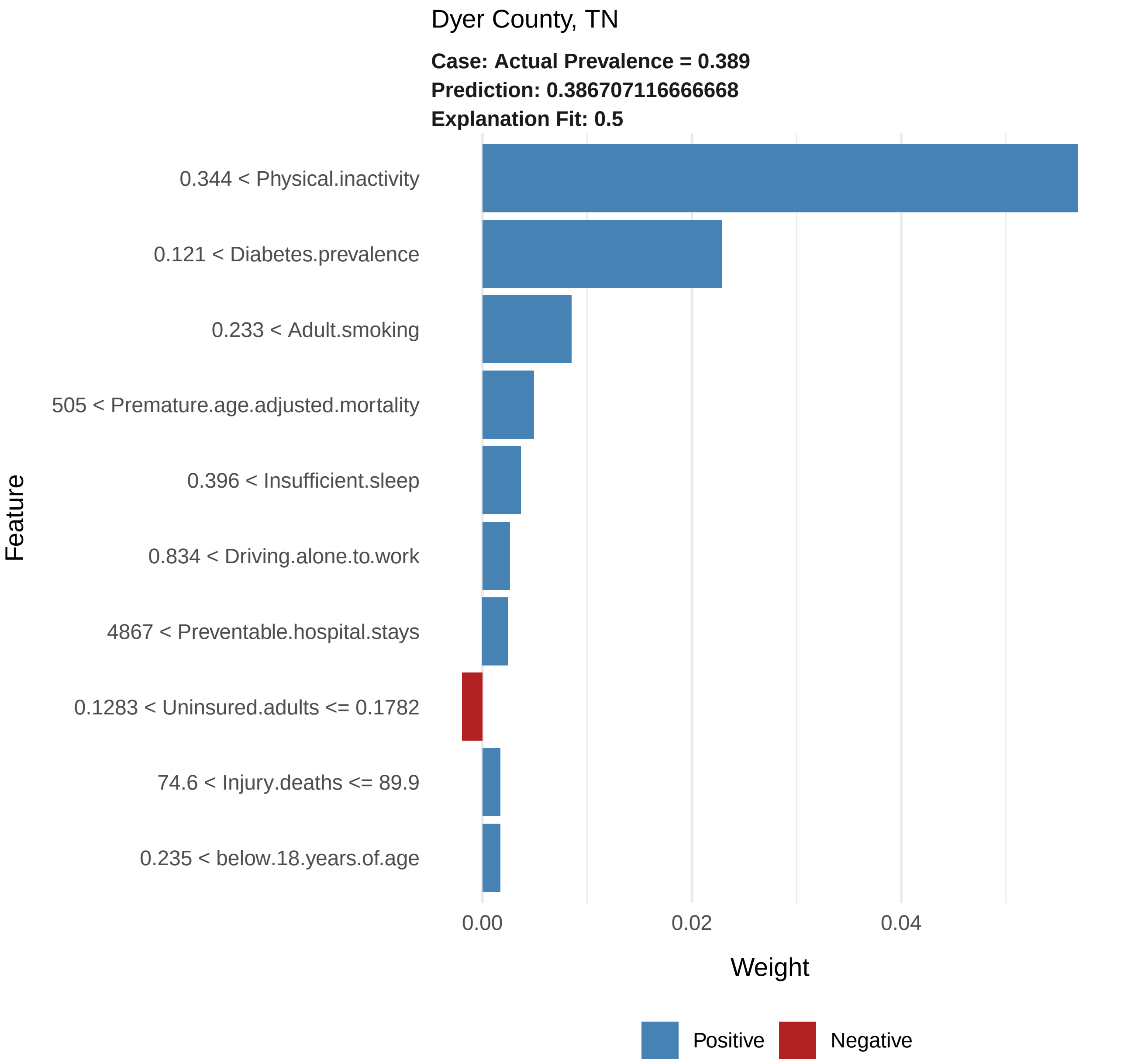

Dyer County, TN
Case: Actual Prevalence = 0.389
Prediction: 0.386707116666668
Explanation Fit: 0.5
0.344 < Physical.inactivity
0.121 < Diabetes.prevalence
0.233 < Adult.smoking
505 < Premature.age.adjusted.mortality
0.396 < Insufficient.sleep
0.834 < Driving.alone.to.work
4867 < Preventable.hospital.stays
0.1283 < Uninsured.adults <= 0.1782
74.6 < Injury.deaths <= 89.9
0.235 < below.18.years.of.age
Feature
0.00
0.02
0.04
Weight
Positive
Negative

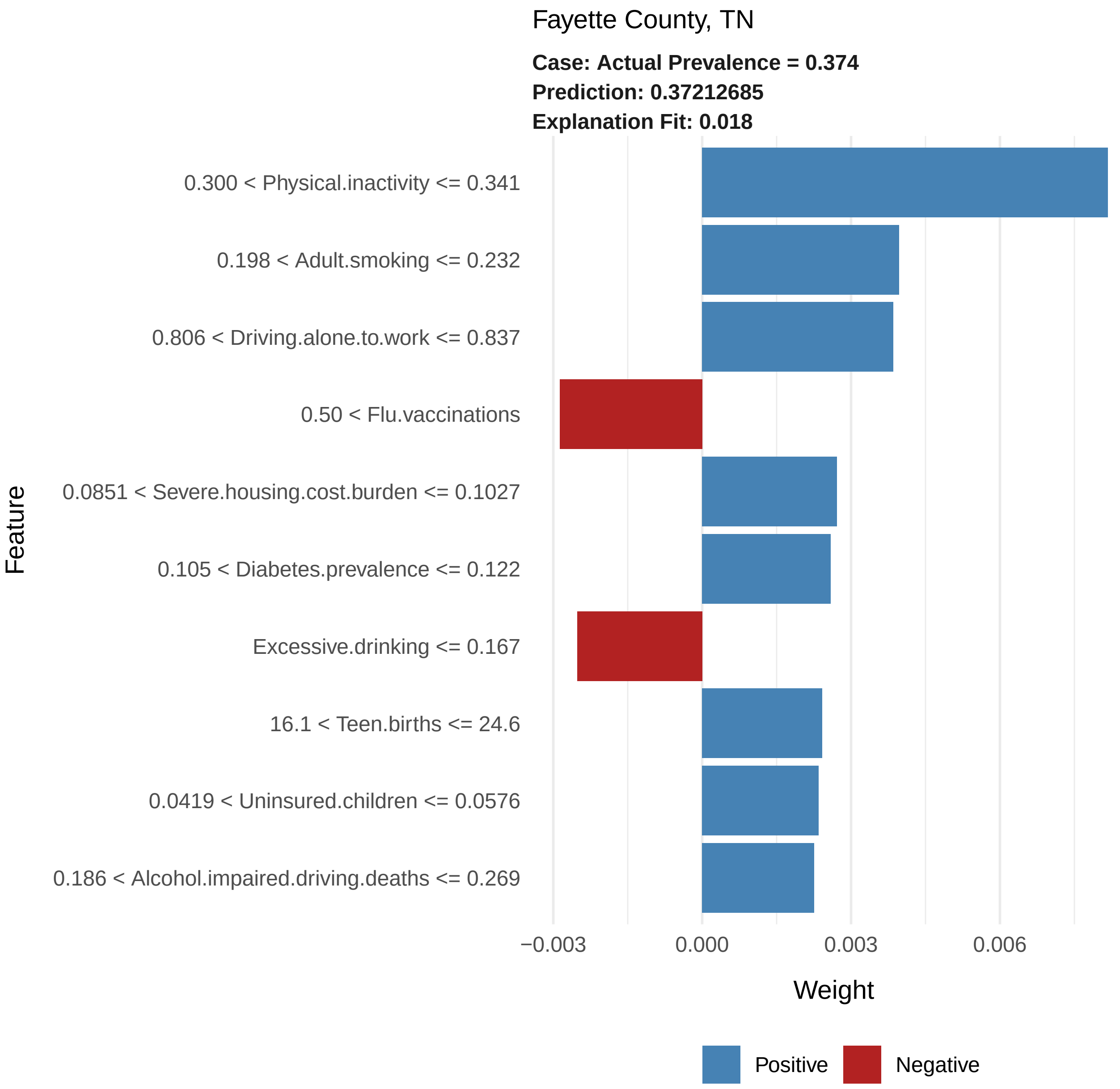

Fayette County, TN
Case: Actual Prevalence = 0.374
Prediction: 0.37212685
Explanation Fit: 0.018
0.300 < Physical.inactivity <= 0.341
0.198 < Adult.smoking <= 0.232
0.806 < Driving.alone.to.work <= 0.837
0.50 < Flu.vaccinations
0.0851 < Severe.housing.cost.burden <= 0.1027
0.105 < Diabetes.prevalence <= 0.122
Excessive.drinking <= 0.167
16.1 < Teen.births <= 24.6
0.0419 < Uninsured.children <= 0.0576
0.186 < Alcohol.impaired.driving.deaths <= 0.269
Feature
−0.003
0.000
0.003
0.006
Weight
Positive
Negative

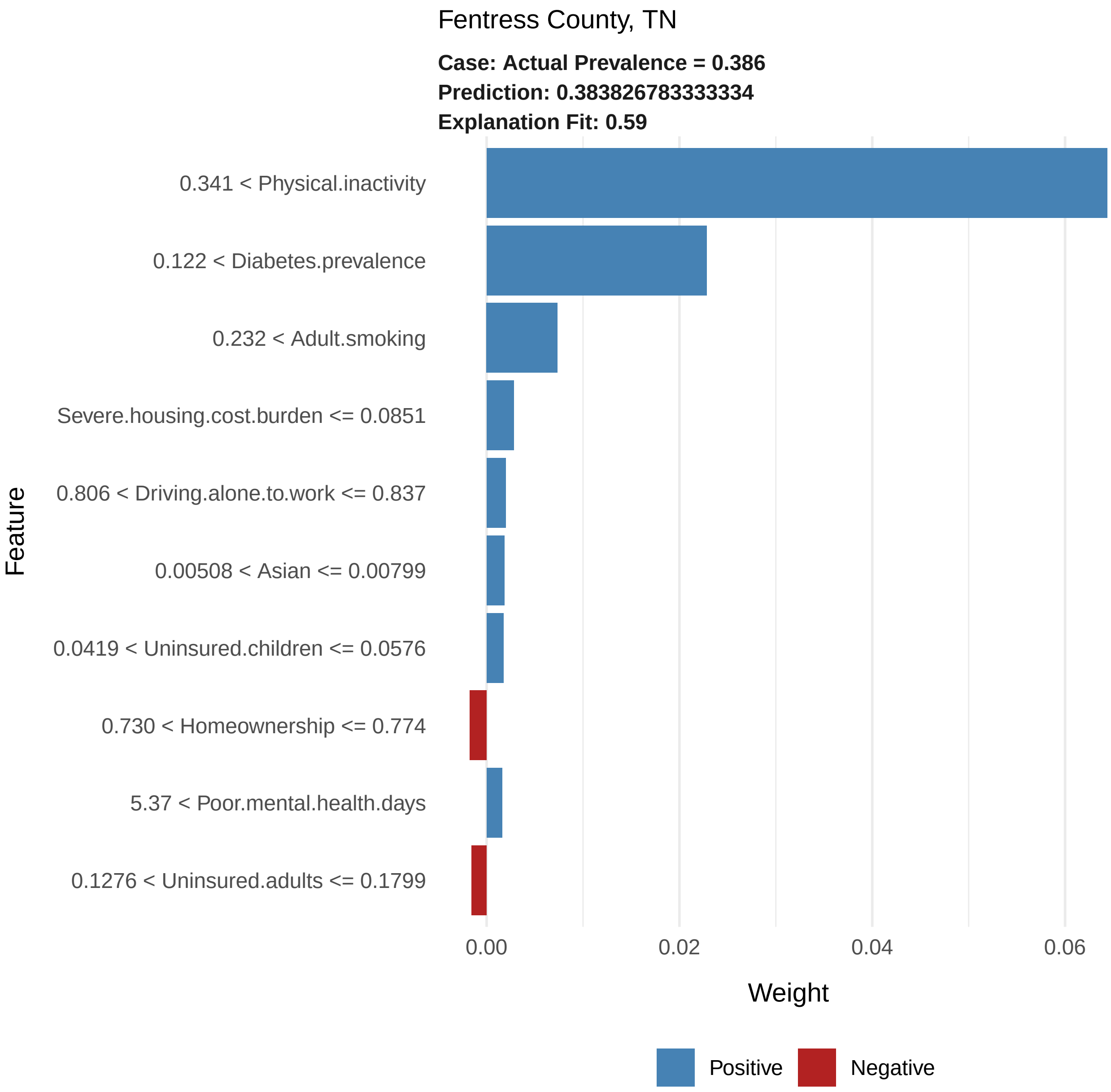
Fentress County, TN
Case: Actual Prevalence = 0.386
Prediction: 0.383826783333334
Explanation Fit: 0.59
Feature
0.341 < Physical.inactivity
0.122 < Diabetes.prevalence
0.232 < Adult.smoking
Severe.housing.cost.burden <= 0.0851
0.806 < Driving.alone.to.work <= 0.837
0.00508 < Asian <= 0.00799
0.0419 < Uninsured.children <= 0.0576
0.730 < Homeownership <= 0.774
5.37 < Poor.mental.health.days
0.1276 < Uninsured.adults <= 0.1799
0.00
0.02
0.04
0.06
Weight
Positive
Negative

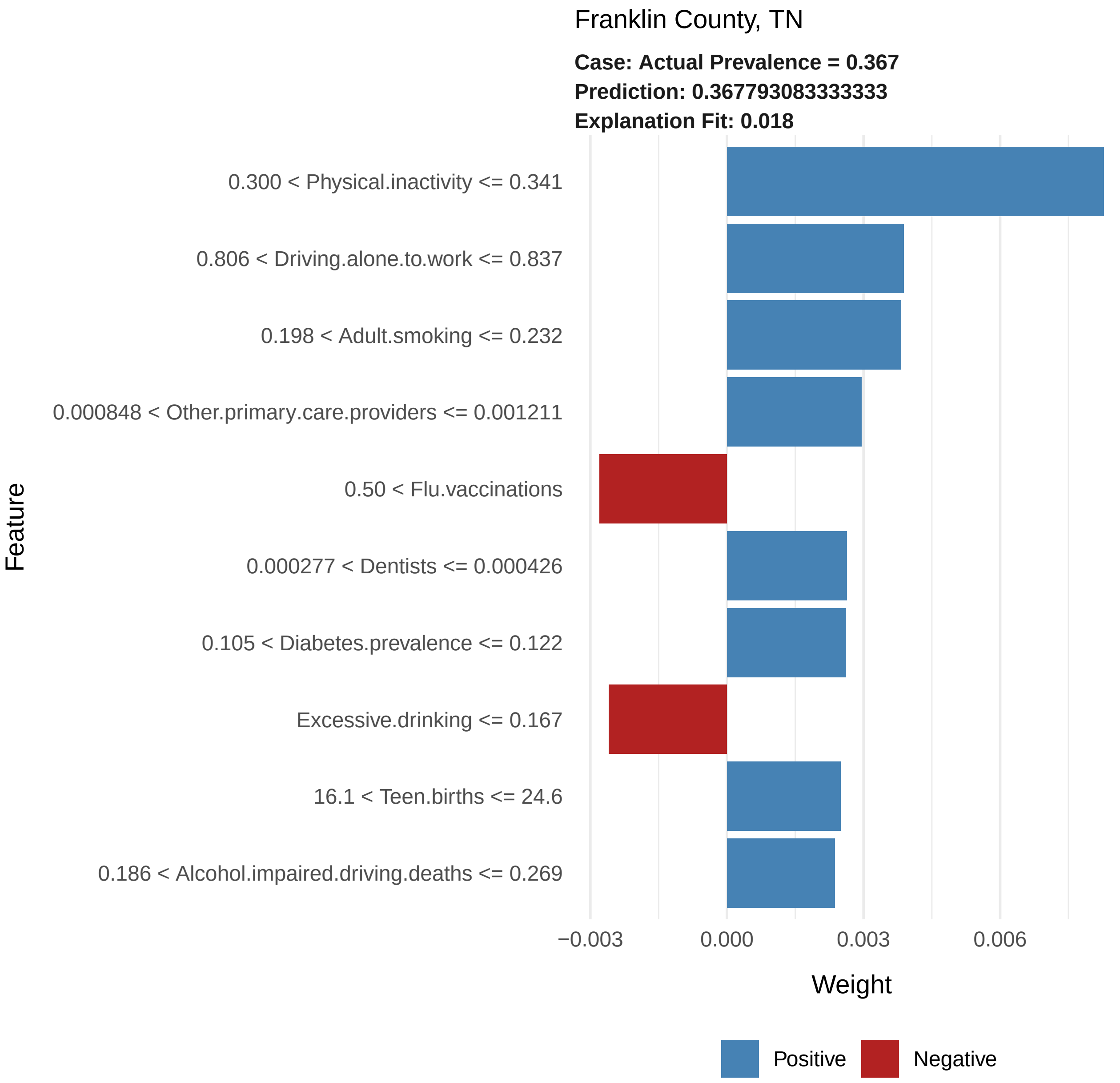

Franklin County, TN
Case: Actual Prevalence = 0.367
Prediction: 0.367793083333333
Explanation Fit: 0.018
0.300 < Physical.inactivity <= 0.341
0.806 < Driving.alone.to.work <= 0.837
0.198 < Adult.smoking <= 0.232
0.000848 < Other.primary.care.providers <= 0.001211
0.50 < Flu.vaccinations
0.000277 < Dentists <= 0.000426
0.105 < Diabetes.prevalence <= 0.122
Excessive.drinking <= 0.167
16.1 < Teen.births <= 24.6
0.186 < Alcohol.impaired.driving.deaths <= 0.269
Feature
−0.003
0.000
0.003
0.006
Weight
Positive
Negative

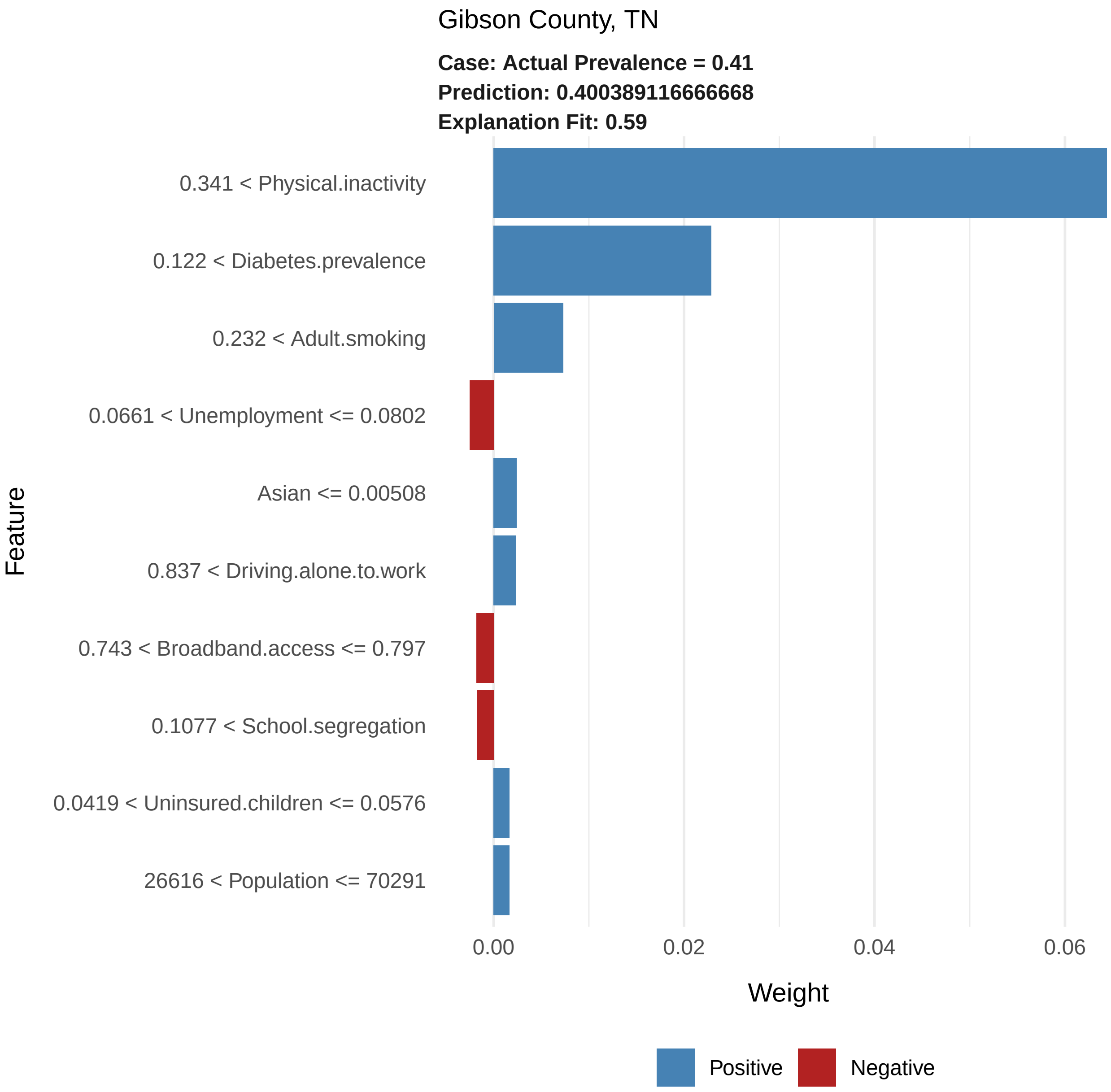

Gibson County, TN
Case: Actual Prevalence = 0.41
Prediction: 0.400389116666668
Explanation Fit: 0.59
0.341 < Physical.inactivity
0.122 < Diabetes.prevalence
0.232 < Adult.smoking
0.0661 < Unemployment <= 0.0802
Asian <= 0.00508
0.837 < Driving.alone.to.work
0.743 < Broadband.access <= 0.797
0.1077 < School.segregation
0.0419 < Uninsured.children <= 0.0576
26616 < Population <= 70291
Feature
0.00
0.02
0.04
0.06
Weight
Positive
Negative

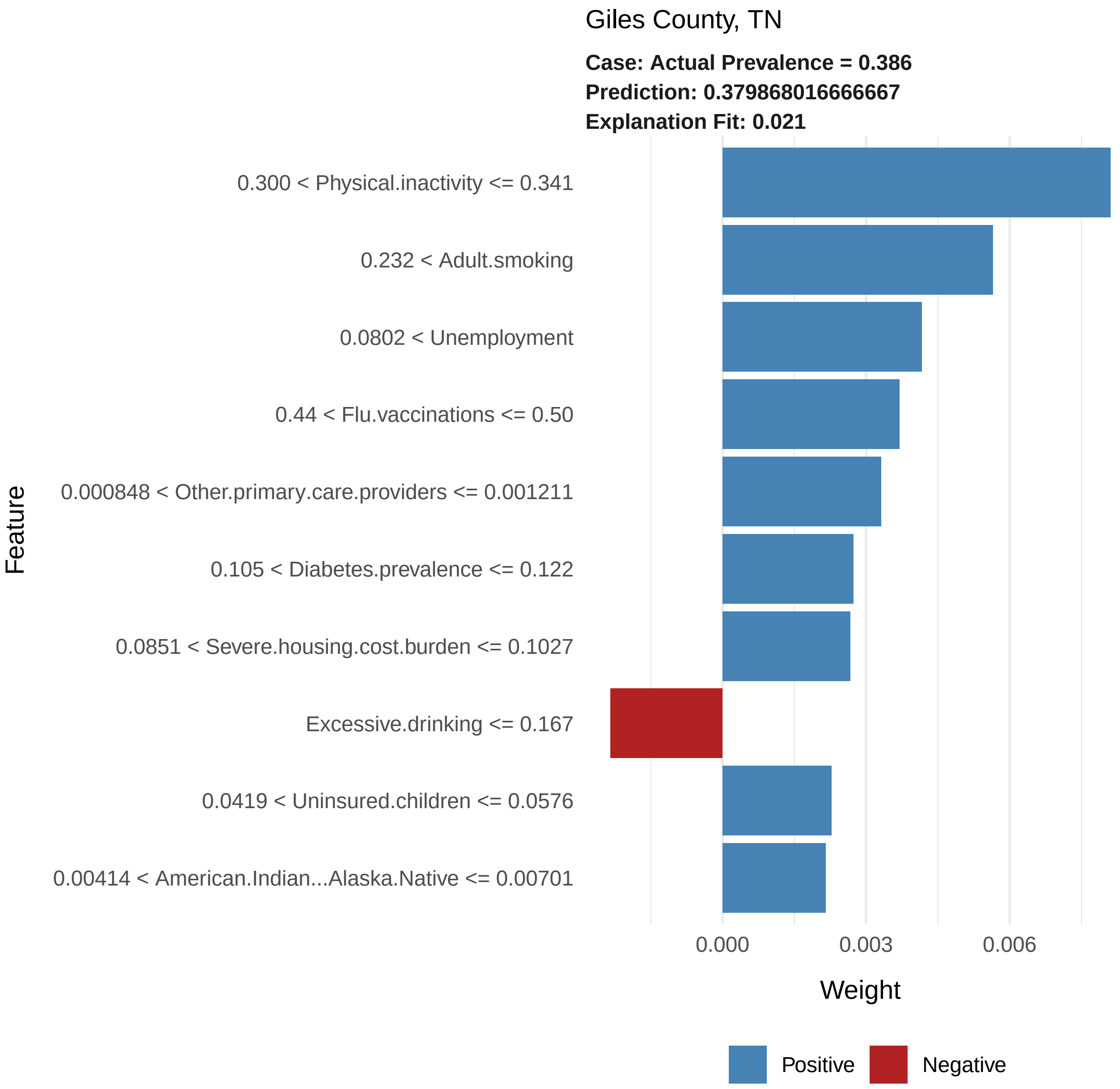

Giles County, TN
Case: Actual Prevalence = 0.386
Prediction: 0.379868016666667
Explanation Fit: 0.021
0.300 < Physical.inactivity <= 0.341
0.232 < Adult.smoking
0.0802 < Unemployment
0.44 < Flu.vaccinations <= 0.50
0.000848 < Other.primary.care.providers <= 0.001211
0.105 < Diabetes.prevalence <= 0.122
0.0851 < Severe.housing.cost.burden <= 0.1027
Excessive.drinking <= 0.167
0.0419 < Uninsured.children <= 0.0576
0.00414 < American.Indian...Alaska.Native <= 0.00701
Feature
0.000
0.003
0.006
Weight
Positive
Negative

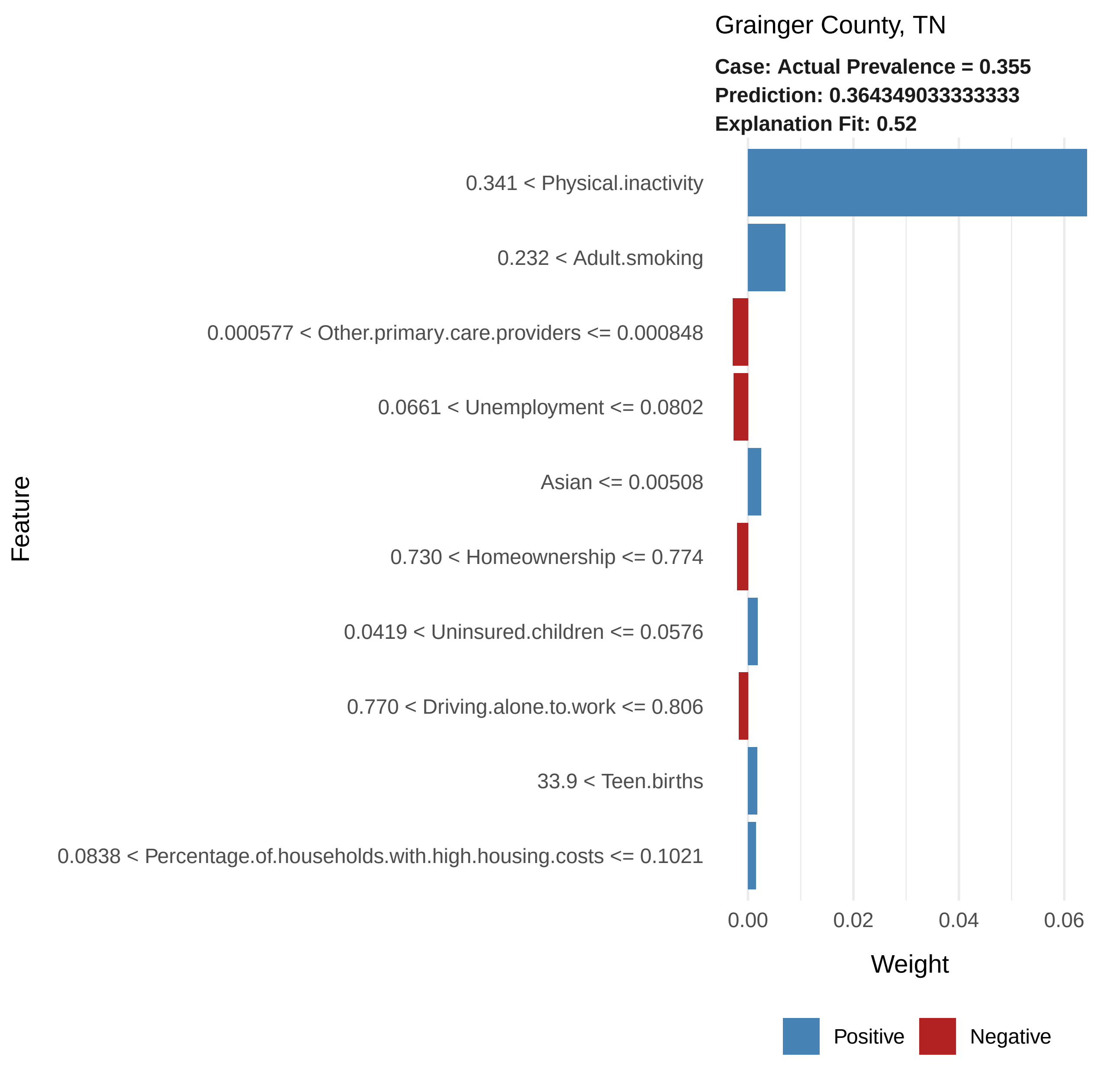

Grainger County, TN
Case: Actual Prevalence = 0.355
Prediction: 0.364349033333333
Explanation Fit: 0.52
0.341 < Physical.inactivity
0.232 < Adult.smoking
0.000577 < Other.primary.care.providers <= 0.000848
0.0661 < Unemployment <= 0.0802
Asian <= 0.00508
0.730 < Homeownership <= 0.774
0.0419 < Uninsured.children <= 0.0576
0.770 < Driving.alone.to.work <= 0.806
33.9 < Teen.births
0.0838 < Percentage.of.households.with.high.housing.costs <= 0.1021
Feature
0.00
0.02
0.04
0.06
Weight
Positive
Negative

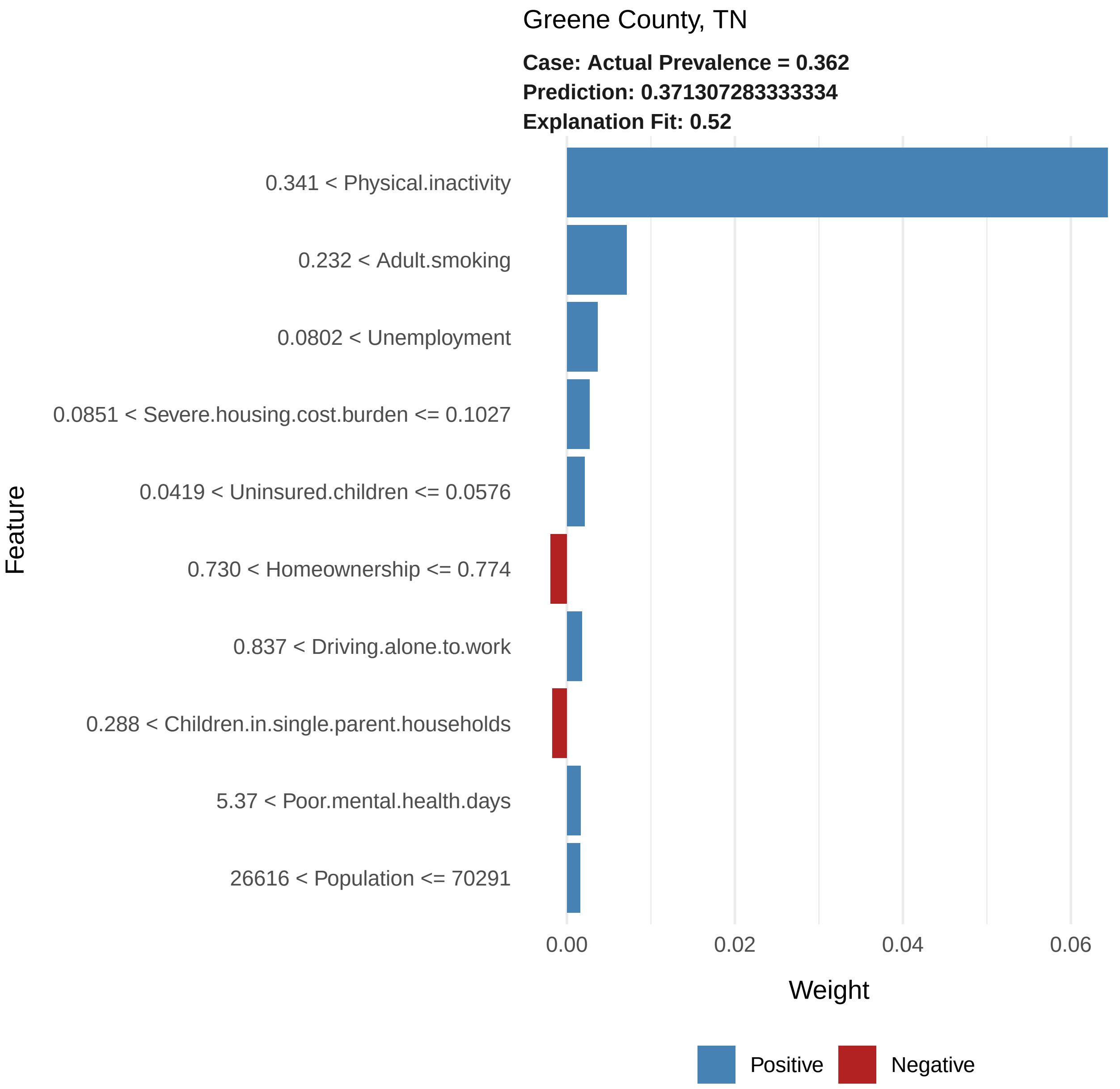

Greene County, TN
Case: Actual Prevalence = 0.362
Prediction: 0.371307283333334
Explanation Fit: 0.52
0.341 < Physical.inactivity
0.232 < Adult.smoking
0.0802 < Unemployment
0.0851 < Severe.housing.cost.burden <= 0.1027
0.0419 < Uninsured.children <= 0.0576
0.730 < Homeownership <= 0.774
0.837 < Driving.alone.to.work
0.288 < Children.in.single.parent.households
5.37 < Poor.mental.health.days
26616 < Population <= 70291
Feature
0.00
0.02
0.04
0.06
Weight
Positive
Negative

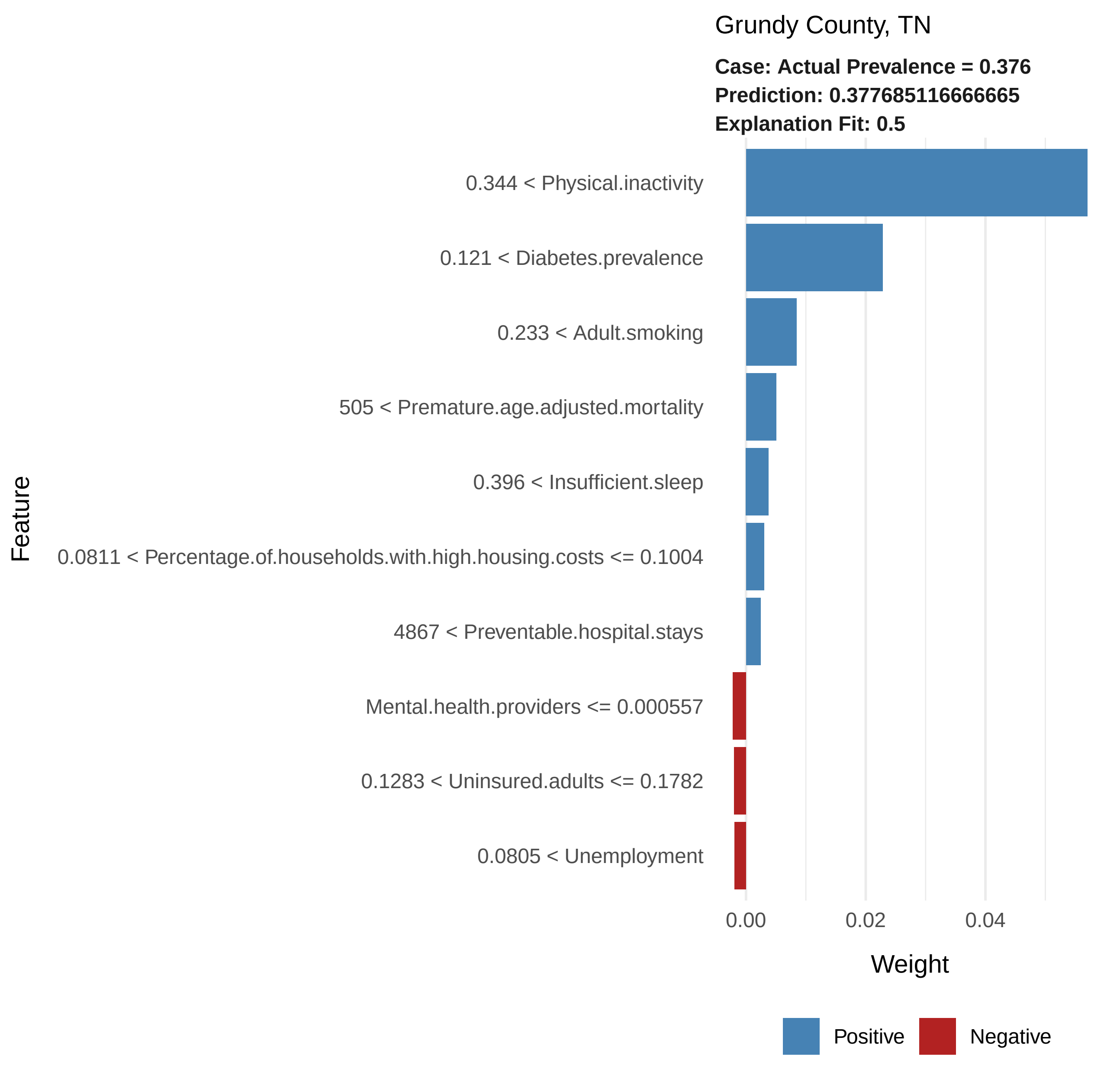

Grundy County, TN
Case: Actual Prevalence = 0.376
Prediction: 0.377685116666665
Explanation Fit: 0.5
0.344 < Physical.inactivity
0.121 < Diabetes.prevalence
0.233 < Adult.smoking
505 < Premature.age.adjusted.mortality
0.396 < Insufficient.sleep
0.0811 < Percentage.of.households.with.high.housing.costs <= 0.1004
4867 < Preventable.hospital.stays
Mental.health.providers <= 0.000557
0.1283 < Uninsured.adults <= 0.1782
0.0805 < Unemployment
Feature
0.00
0.02
0.04
Weight
Positive
Negative

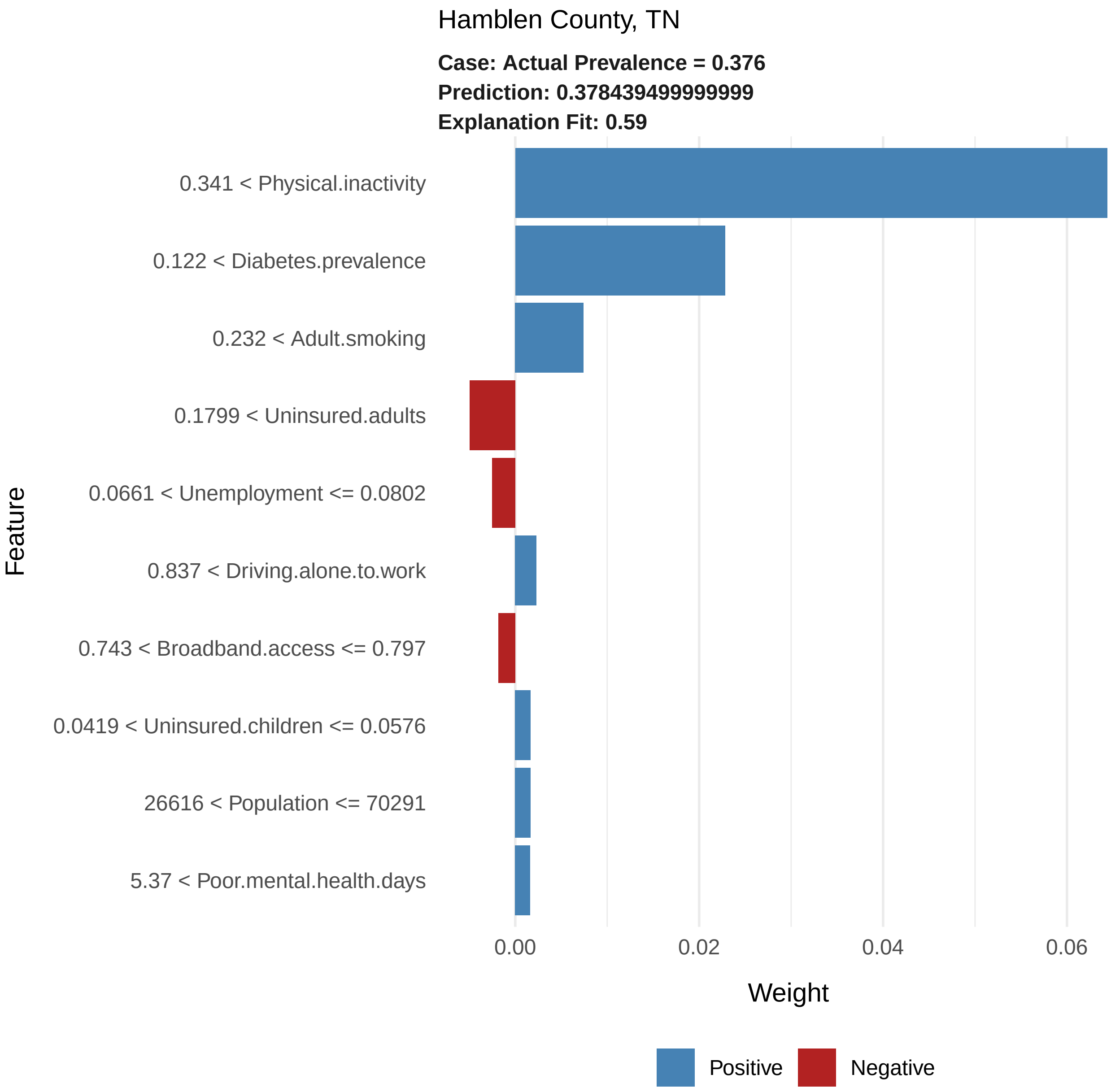
Hamblen County, TN
Case: Actual Prevalence = 0.376
Prediction: 0.378439499999999
Explanation Fit: 0.59
0.341 < Physical.inactivity
0.122 < Diabetes.prevalence
0.232 < Adult.smoking
0.1799 < Uninsured.adults
0.0661 < Unemployment <= 0.0802
0.837 < Driving.alone.to.work
0.743 < Broadband.access <= 0.797
0.0419 < Uninsured.children <= 0.0576
26616 < Population <= 70291
5.37 < Poor.mental.health.days
Feature
0.00
0.02
0.04
0.06
Weight
Positive
Negative

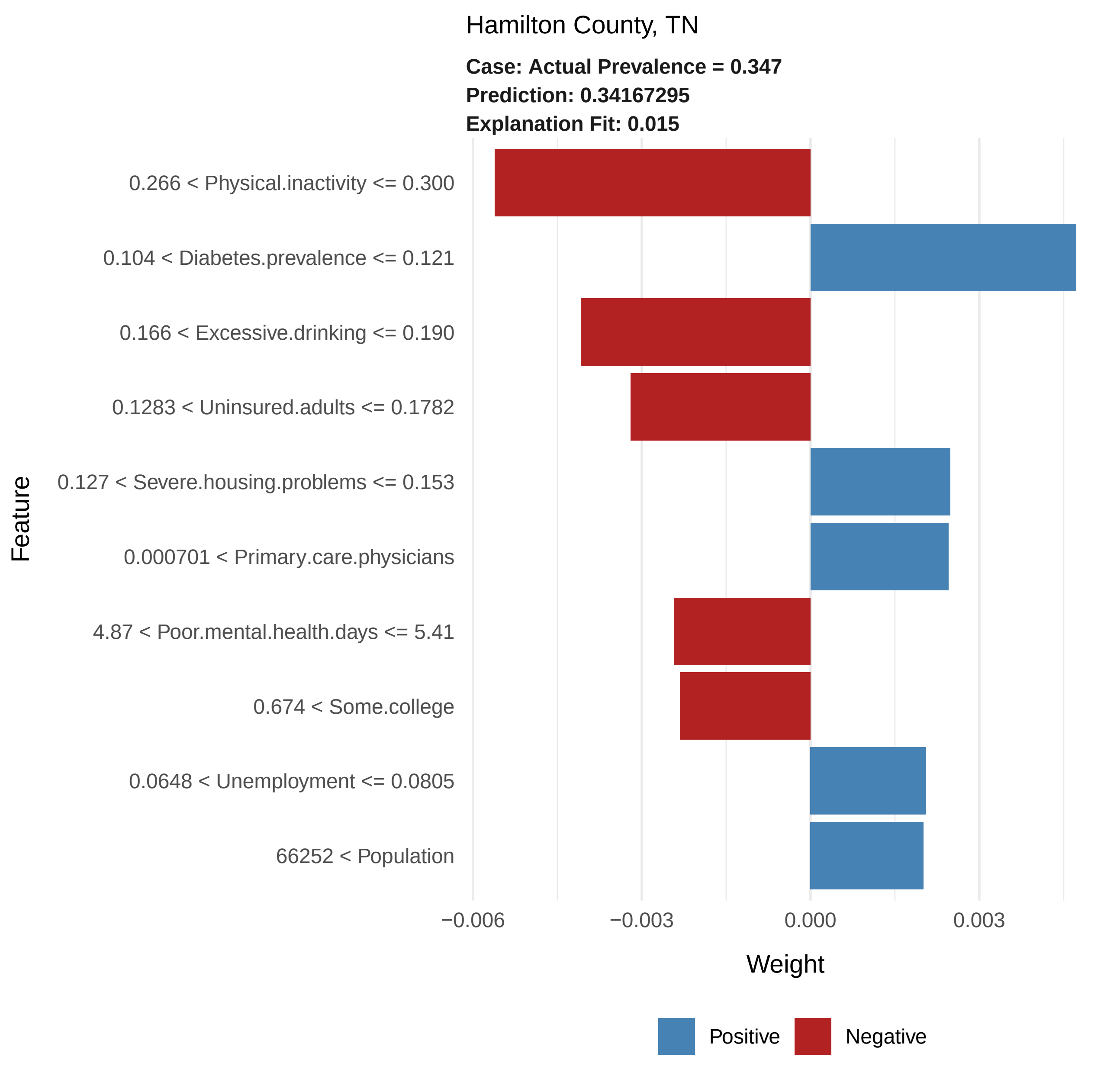
Hamilton County, TN
Case: Actual Prevalence = 0.347
Prediction: 0.34167295
Explanation Fit: 0.015
0.266 < Physical.inactivity <= 0.300
0.104 < Diabetes.prevalence <= 0.121
0.166 < Excessive.drinking <= 0.190
0.1283 < Uninsured.adults <= 0.1782
0.127 < Severe.housing.problems <= 0.153
0.000701 < Primary.care.physicians
4.87 < Poor.mental.health.days <= 5.41
0.674 < Some.college
0.0648 < Unemployment <= 0.0805
66252 < Population
Feature
−0.006
−0.003
0.000
0.003
Weight
Positive
Negative

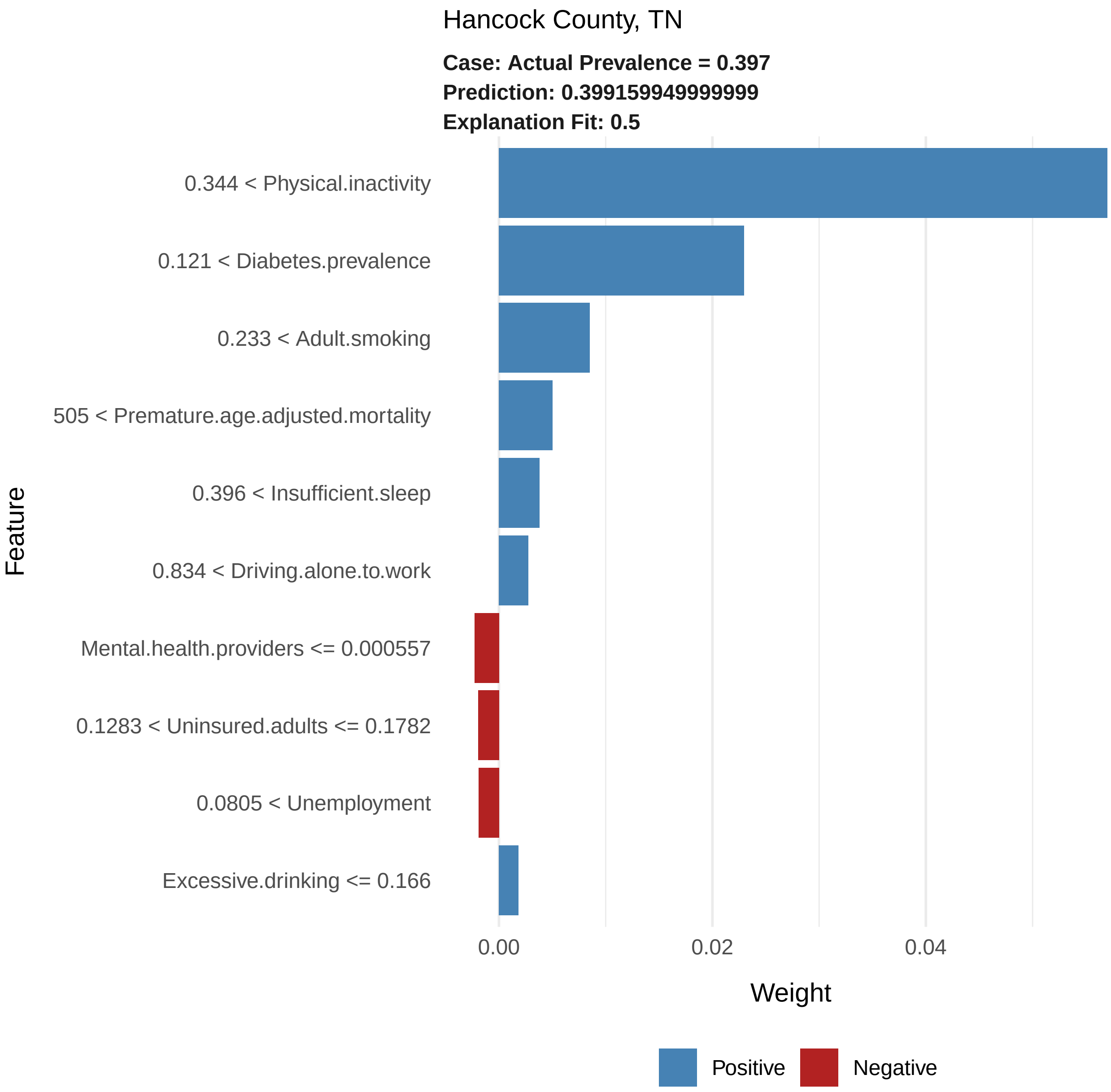

Hancock County, TN
Case: Actual Prevalence = 0.397
Prediction: 0.399159949999999
Explanation Fit: 0.5
0.344 < Physical.inactivity
0.121 < Diabetes.prevalence
0.233 < Adult.smoking
505 < Premature.age.adjusted.mortality
0.396 < Insufficient.sleep
0.834 < Driving.alone.to.work
Mental.health.providers <= 0.000557
0.1283 < Uninsured.adults <= 0.1782
0.0805 < Unemployment
Excessive.drinking <= 0.166
Feature
0.00
0.02
0.04
Weight
Positive
Negative

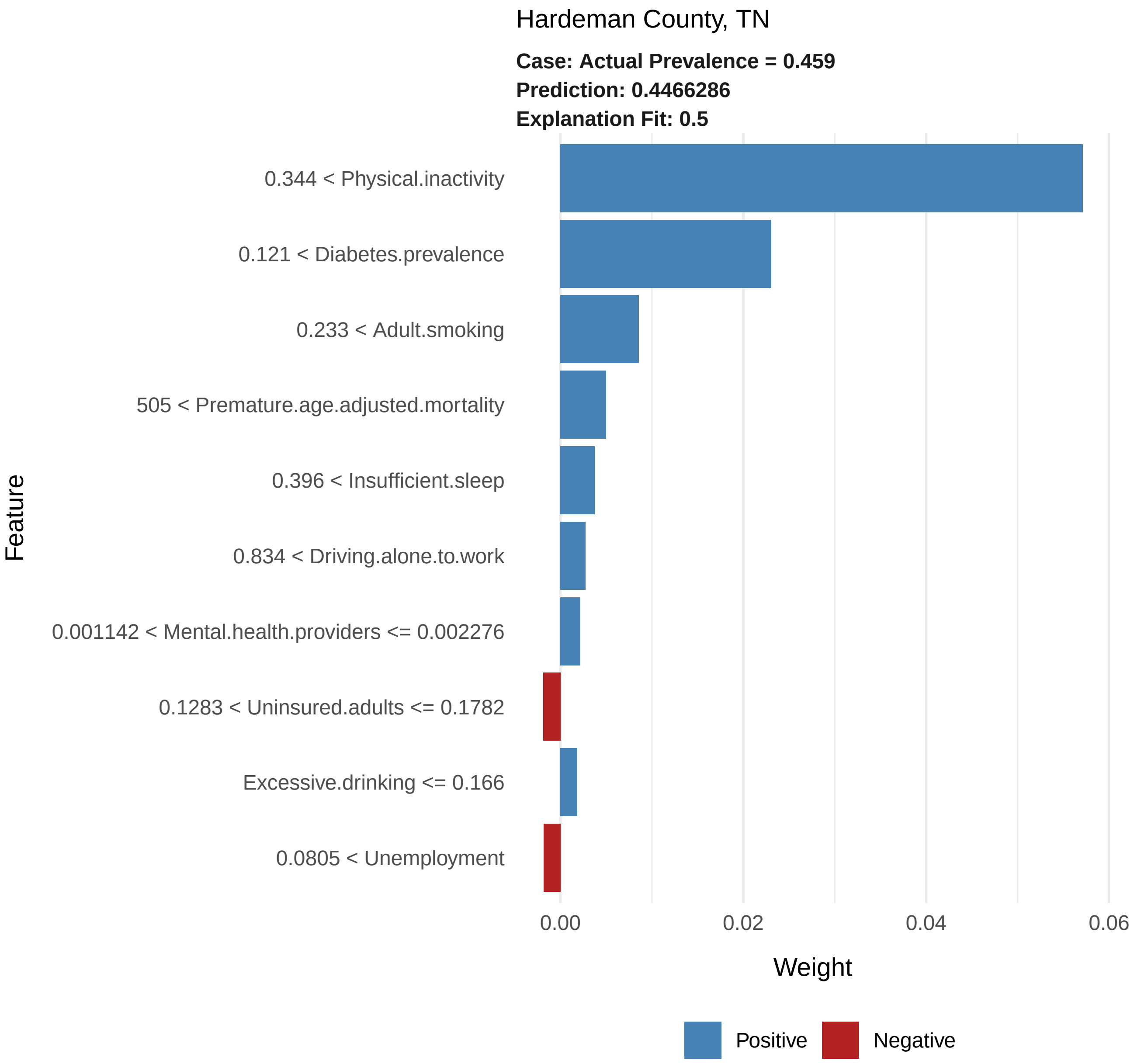

Hardeman County, TN
Case: Actual Prevalence = 0.459
Prediction: 0.4466286
Explanation Fit: 0.5
0.344 < Physical.inactivity
0.121 < Diabetes.prevalence
0.233 < Adult.smoking
505 < Premature.age.adjusted.mortality
0.396 < Insufficient.sleep
0.834 < Driving.alone.to.work
0.001142 < Mental.health.providers <= 0.002276
0.1283 < Uninsured.adults <= 0.1782
Excessive.drinking <= 0.166
0.0805 < Unemployment
Feature
0.00
0.02
0.04
0.06
Weight
Positive
Negative

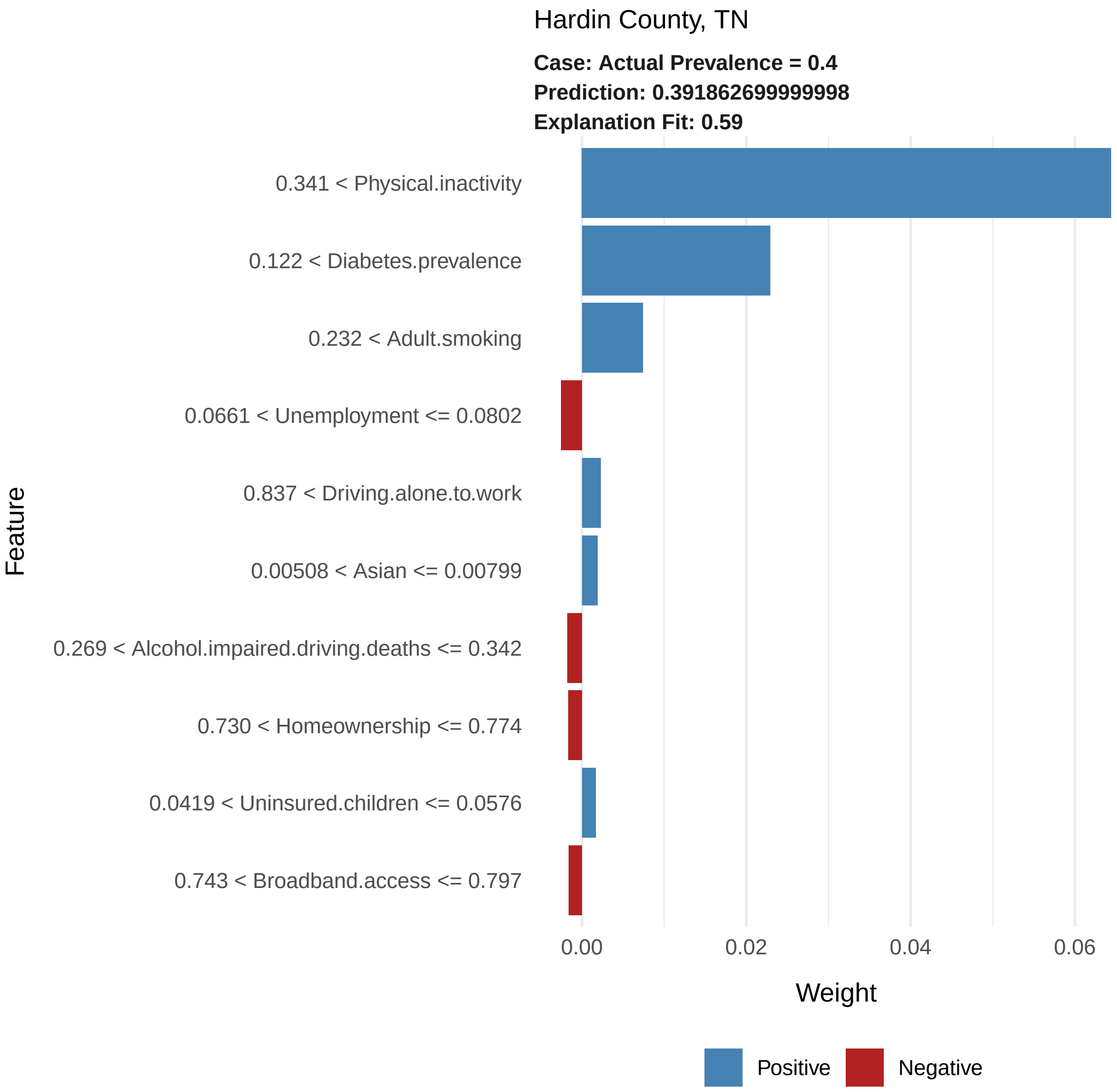

Hardin County, TN
Case: Actual Prevalence = 0.4
Prediction: 0.391862699999998
Explanation Fit: 0.59
0.341 < Physical.inactivity
0.122 < Diabetes.prevalence
0.232 < Adult.smoking
0.0661 < Unemployment <= 0.0802
0.837 < Driving.alone.to.work
0.00508 < Asian <= 0.00799
0.269 < Alcohol.impaired.driving.deaths <= 0.342
0.730 < Homeownership <= 0.774
0.0419 < Uninsured.children <= 0.0576
0.743 < Broadband.access <= 0.797
Feature
0.00
0.02
0.04
0.06
Weight
Positive
Negative

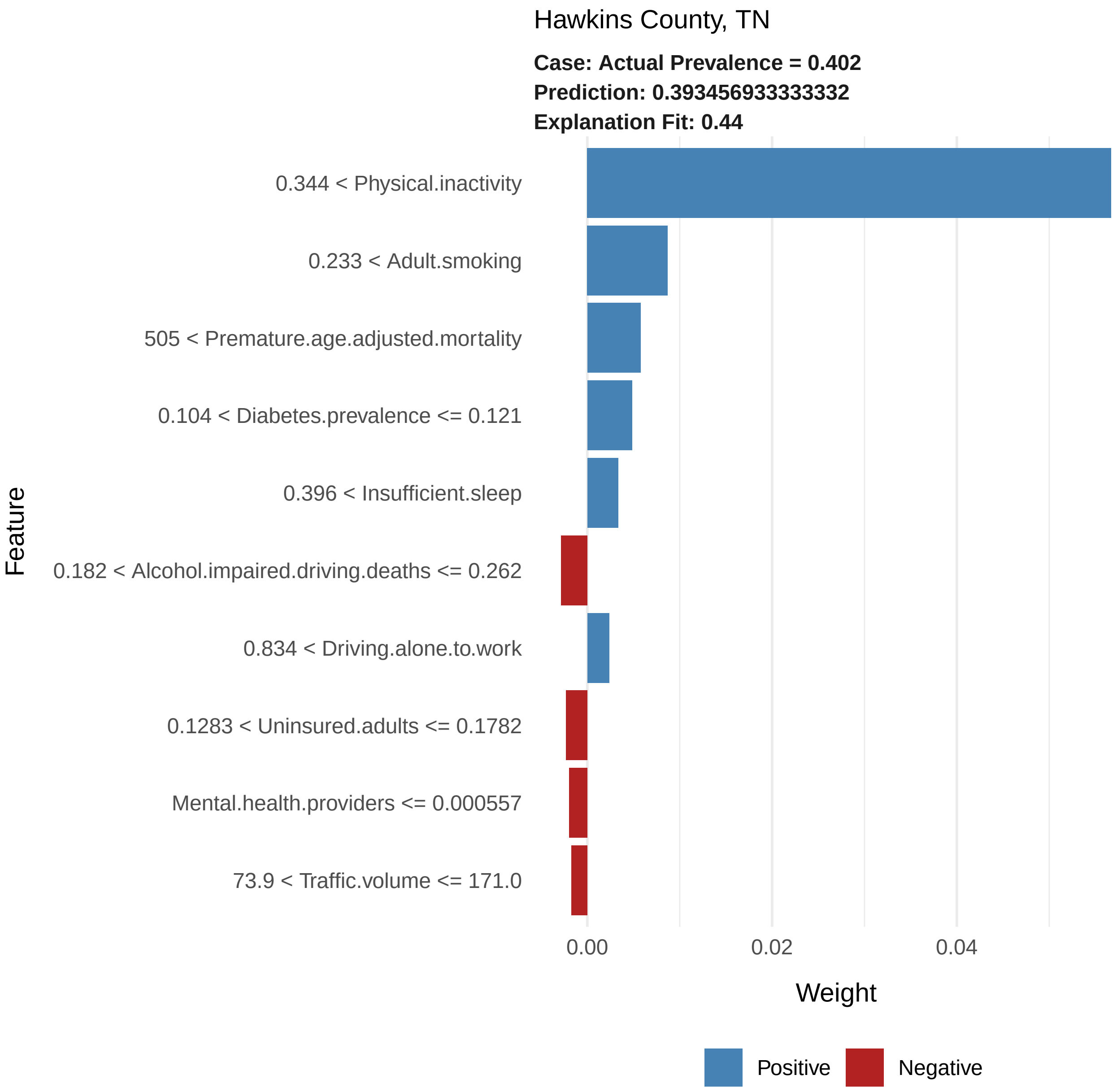
Hawkins County, TN
Case: Actual Prevalence = 0.402
Prediction: 0.393456933333332
Explanation Fit: 0.44
0.344 < Physical.inactivity
0.233 < Adult.smoking
505 < Premature.age.adjusted.mortality
0.104 < Diabetes.prevalence <= 0.121
0.396 < Insufficient.sleep
0.182 < Alcohol.impaired.driving.deaths <= 0.262
0.834 < Driving.alone.to.work
0.1283 < Uninsured.adults <= 0.1782
Mental.health.providers <= 0.000557
73.9 < Traffic.volume <= 171.0
Feature
0.00
0.02
0.04
Weight
Positive
Negative

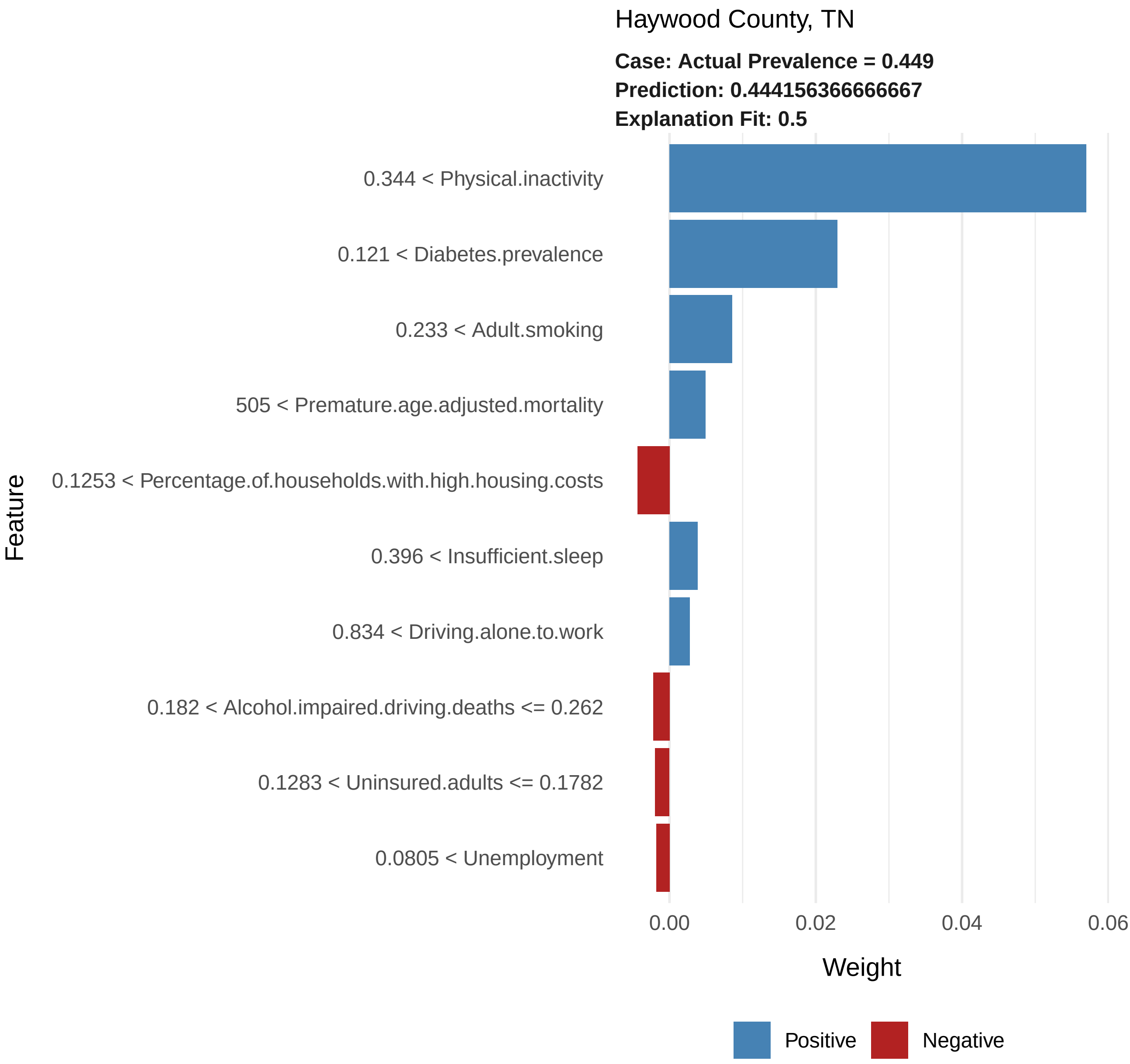
Haywood County, TN
Case: Actual Prevalence = 0.449
Prediction: 0.444156366666667
Explanation Fit: 0.5
0.344 < Physical.inactivity
0.121 < Diabetes.prevalence
0.233 < Adult.smoking
505 < Premature.age.adjusted.mortality
0.1253 < Percentage.of.households.with.high.housing.costs
0.396 < Insufficient.sleep
0.834 < Driving.alone.to.work
0.182 < Alcohol.impaired.driving.deaths <= 0.262
0.1283 < Uninsured.adults <= 0.1782
0.0805 < Unemployment
Feature
0.00
0.02
0.04
0.06
Weight
Positive
Negative

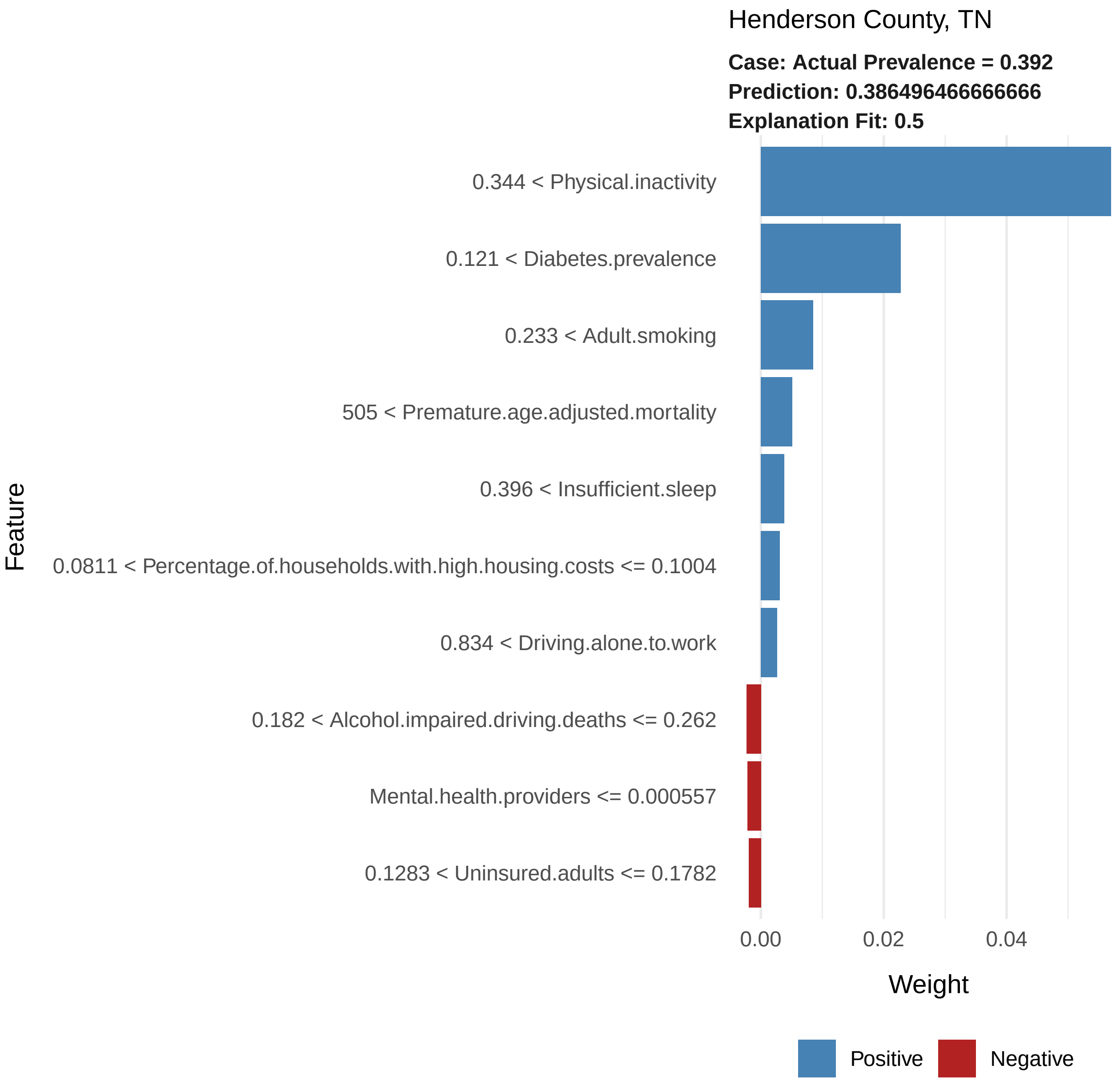

Henderson County, TN
Case: Actual Prevalence = 0.392
Prediction: 0.386496466666666
Explanation Fit: 0.5
0.344 < Physical.inactivity
0.121 < Diabetes.prevalence
0.233 < Adult.smoking
505 < Premature.age.adjusted.mortality
0.396 < Insufficient.sleep
0.0811 < Percentage.of.households.with.high.housing.costs <= 0.1004
0.834 < Driving.alone.to.work
0.182 < Alcohol.impaired.driving.deaths <= 0.262
Mental.health.providers <= 0.000557
0.1283 < Uninsured.adults <= 0.1782
Feature
0.00
0.02
0.04
Weight
Positive
Negative

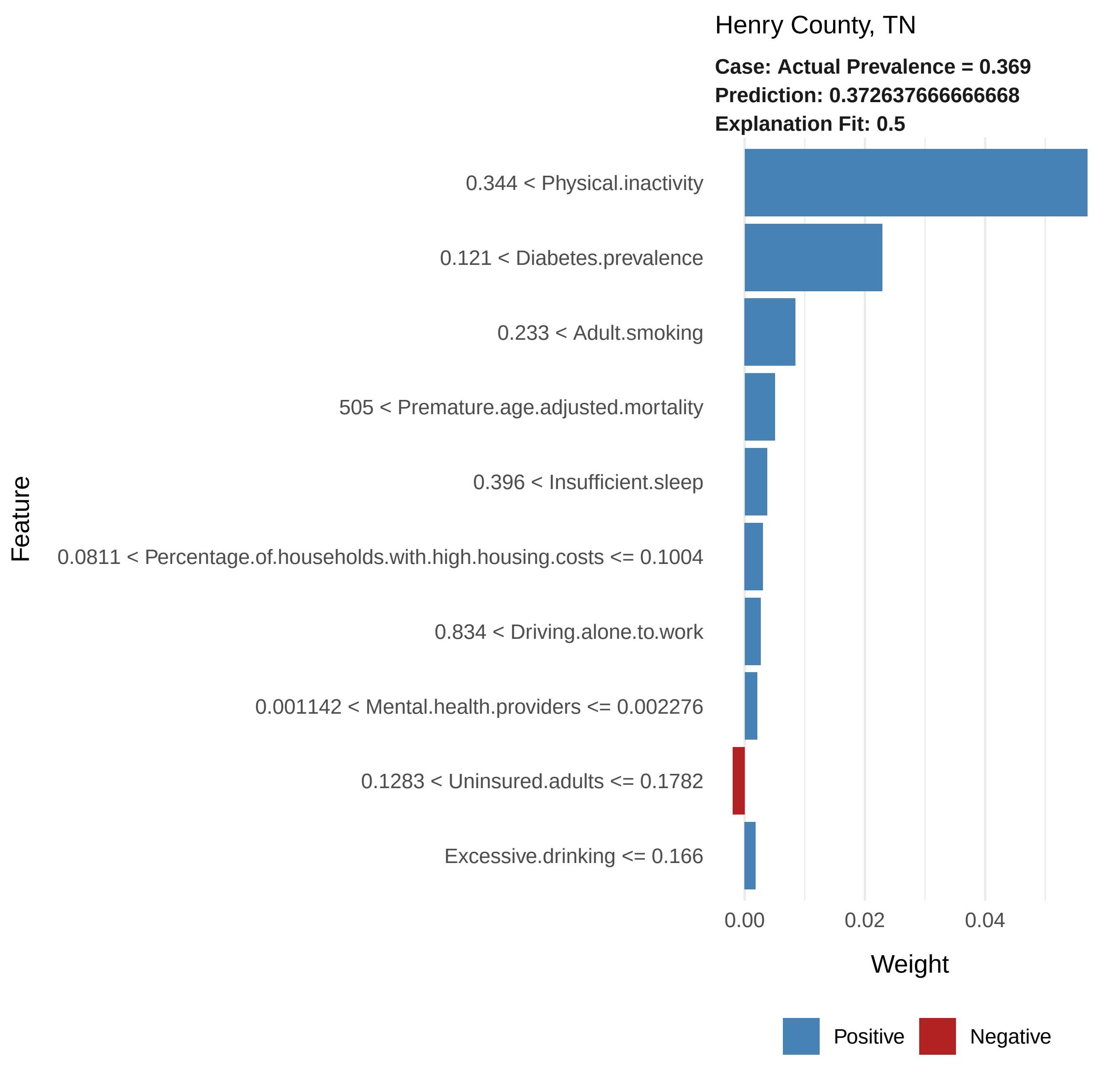

Henry County, TN
Case: Actual Prevalence = 0.369
Prediction: 0.372637666666668
Explanation Fit: 0.5
0.344 < Physical.inactivity
0.121 < Diabetes.prevalence
0.233 < Adult.smoking
505 < Premature.age.adjusted.mortality
0.396 < Insufficient.sleep
0.0811 < Percentage.of.households.with.high.housing.costs <= 0.1004
0.834 < Driving.alone.to.work
0.001142 < Mental.health.providers <= 0.002276
0.1283 < Uninsured.adults <= 0.1782
Excessive.drinking <= 0.166
Feature
0.00
0.02
0.04
Weight
Positive
Negative

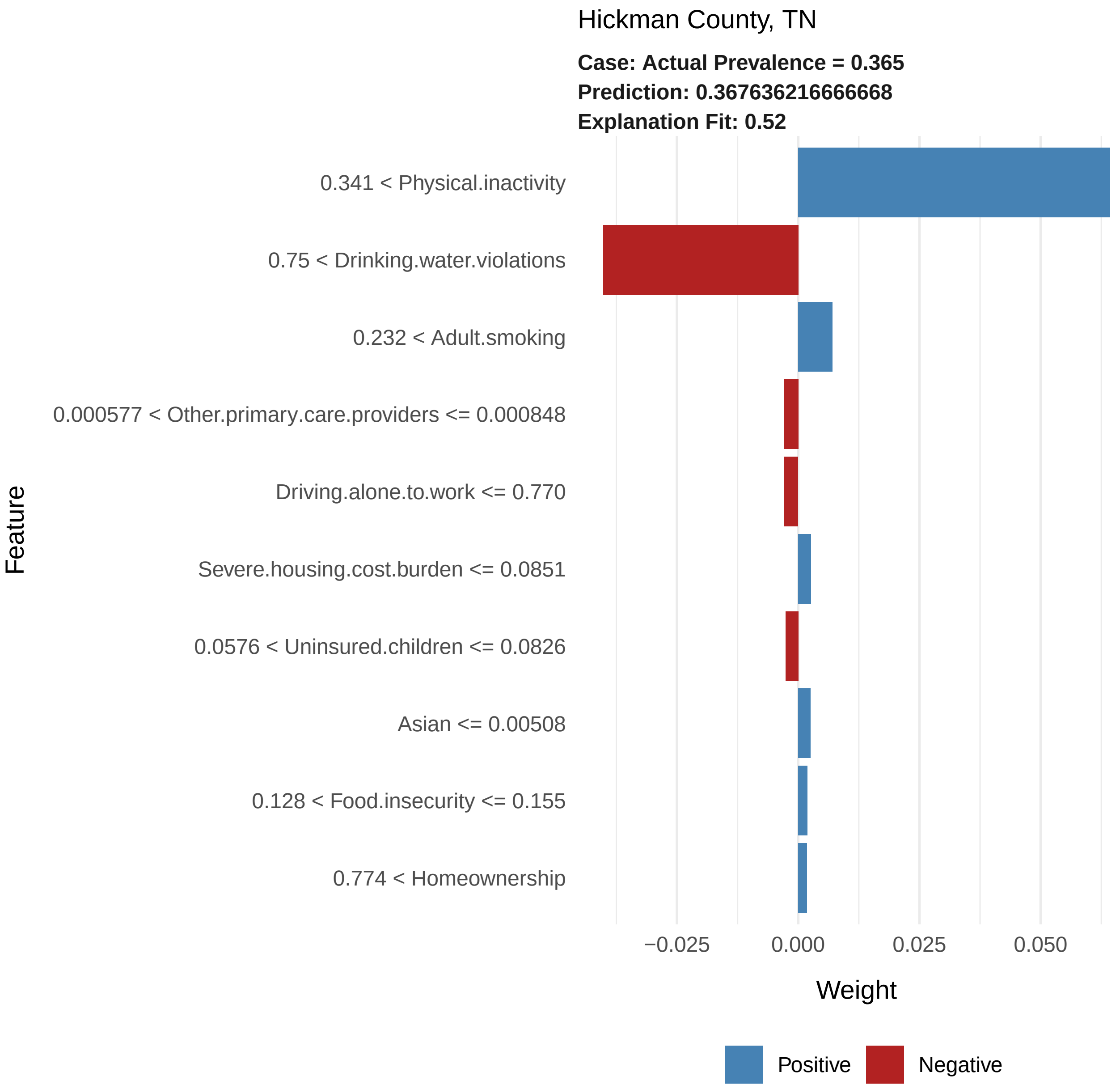

Hickman County, TN
Case: Actual Prevalence = 0.365
Prediction: 0.367636216666668
Explanation Fit: 0.52
0.341 < Physical.inactivity
0.75 < Drinking.water.violations
0.232 < Adult.smoking
0.000577 < Other.primary.care.providers <= 0.000848
Driving.alone.to.work <= 0.770
Severe.housing.cost.burden <= 0.0851
0.0576 < Uninsured.children <= 0.0826
Asian <= 0.00508
0.128 < Food.insecurity <= 0.155
0.774 < Homeownership
Feature
−0.025
0.000
0.025
0.050
Weight
Positive
Negative

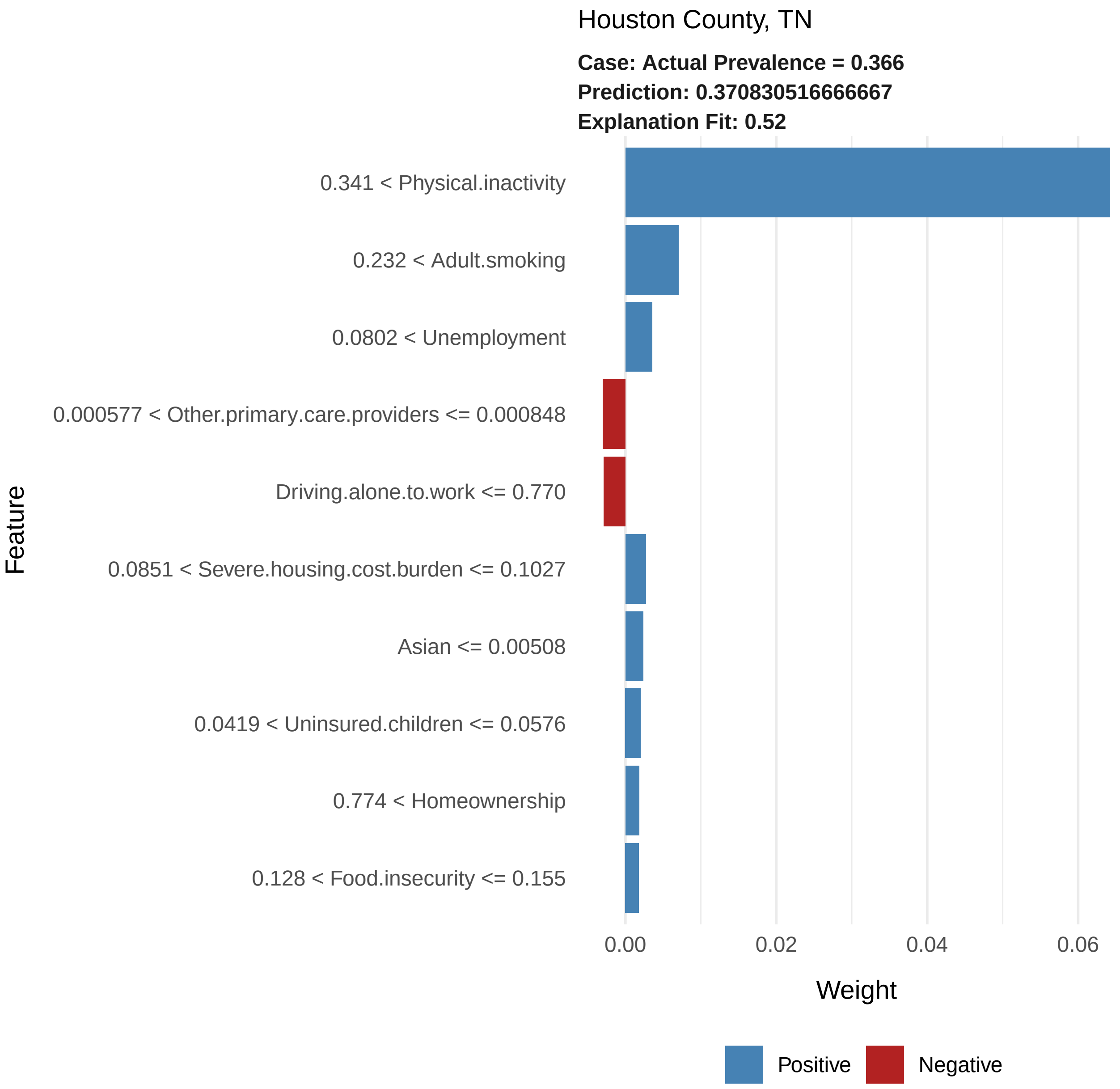
Houston County, TN
Case: Actual Prevalence = 0.366
Prediction: 0.370830516666667
Explanation Fit: 0.52
0.341 < Physical.inactivity
0.232 < Adult.smoking
0.0802 < Unemployment
0.000577 < Other.primary.care.providers <= 0.000848
Driving.alone.to.work <= 0.770
0.0851 < Severe.housing.cost.burden <= 0.1027
Asian <= 0.00508
0.0419 < Uninsured.children <= 0.0576
0.774 < Homeownership
0.128 < Food.insecurity <= 0.155
Feature
0.00
0.02
0.04
0.06
Weight
Positive
Negative

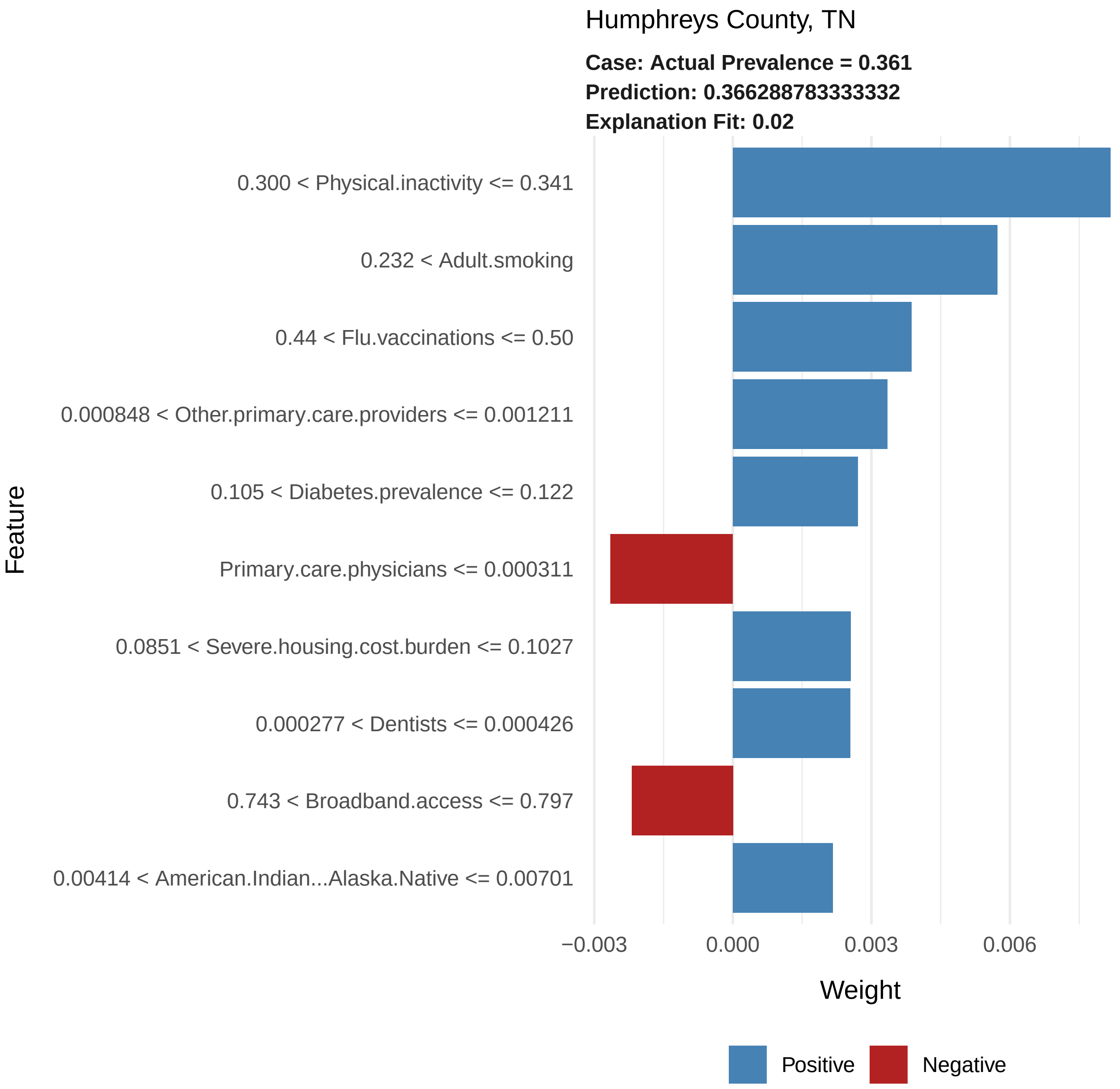

Humphreys County, TN
Case: Actual Prevalence = 0.361
Prediction: 0.366288783333332
Explanation Fit: 0.02
0.300 < Physical.inactivity <= 0.341
0.232 < Adult.smoking
0.44 < Flu.vaccinations <= 0.50
0.000848 < Other.primary.care.providers <= 0.001211
0.105 < Diabetes.prevalence <= 0.122
Primary.care.physicians <= 0.000311
0.0851 < Severe.housing.cost.burden <= 0.1027
0.000277 < Dentists <= 0.000426
0.743 < Broadband.access <= 0.797
0.00414 < American.Indian...Alaska.Native <= 0.00701
Feature
−0.003
0.000
0.003
0.006
Weight
Positive
Negative

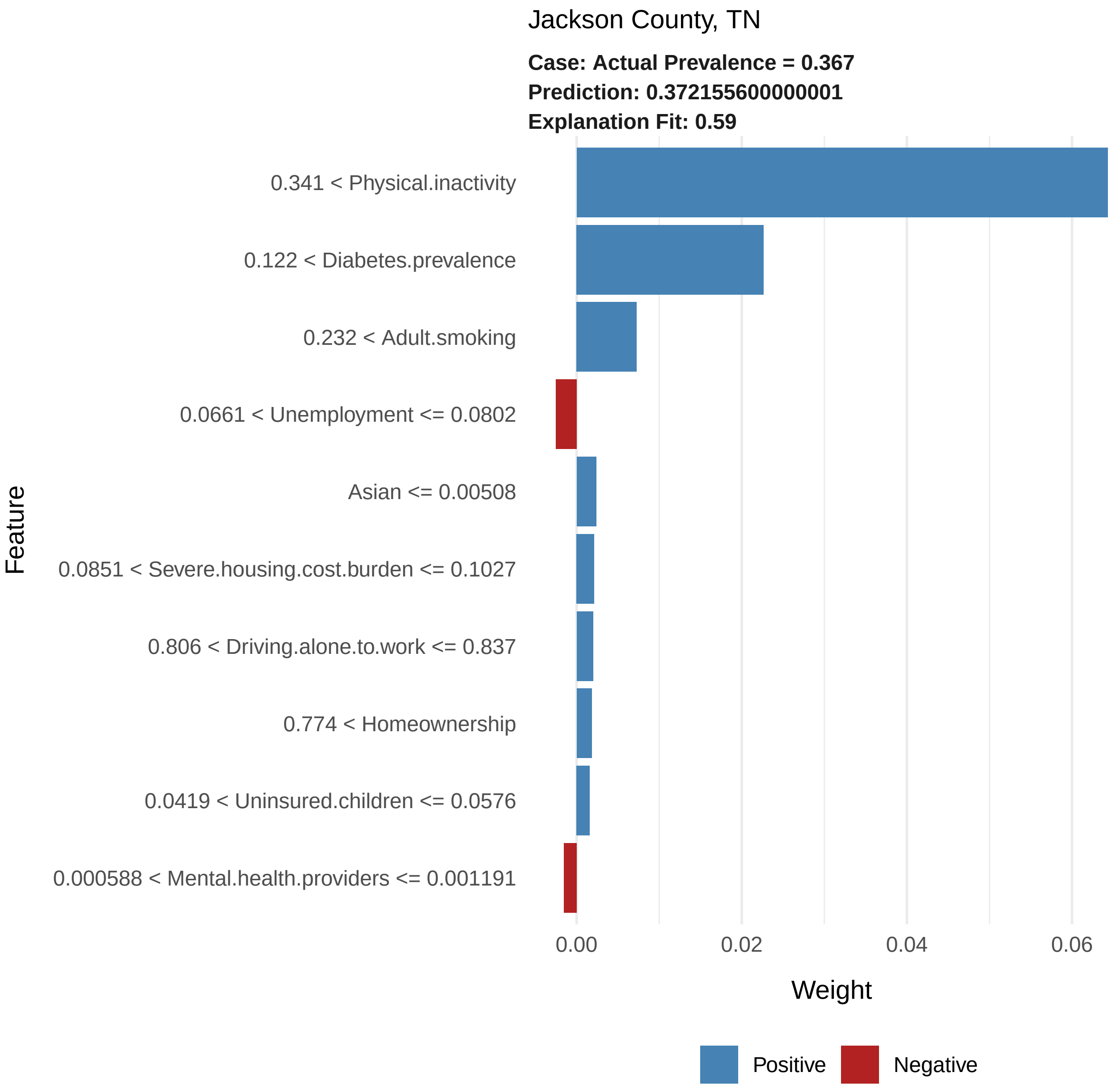

Jackson County, TN
Case: Actual Prevalence = 0.367
Prediction: 0.372155600000001
Explanation Fit: 0.59
0.341 < Physical.inactivity
0.122 < Diabetes.prevalence
0.232 < Adult.smoking
0.0661 < Unemployment <= 0.0802
Asian <= 0.00508
0.0851 < Severe.housing.cost.burden <= 0.1027
0.806 < Driving.alone.to.work <= 0.837
0.774 < Homeownership
0.0419 < Uninsured.children <= 0.0576
0.000588 < Mental.health.providers <= 0.001191
Feature
0.00
0.02
0.04
0.06
Weight
Positive
Negative

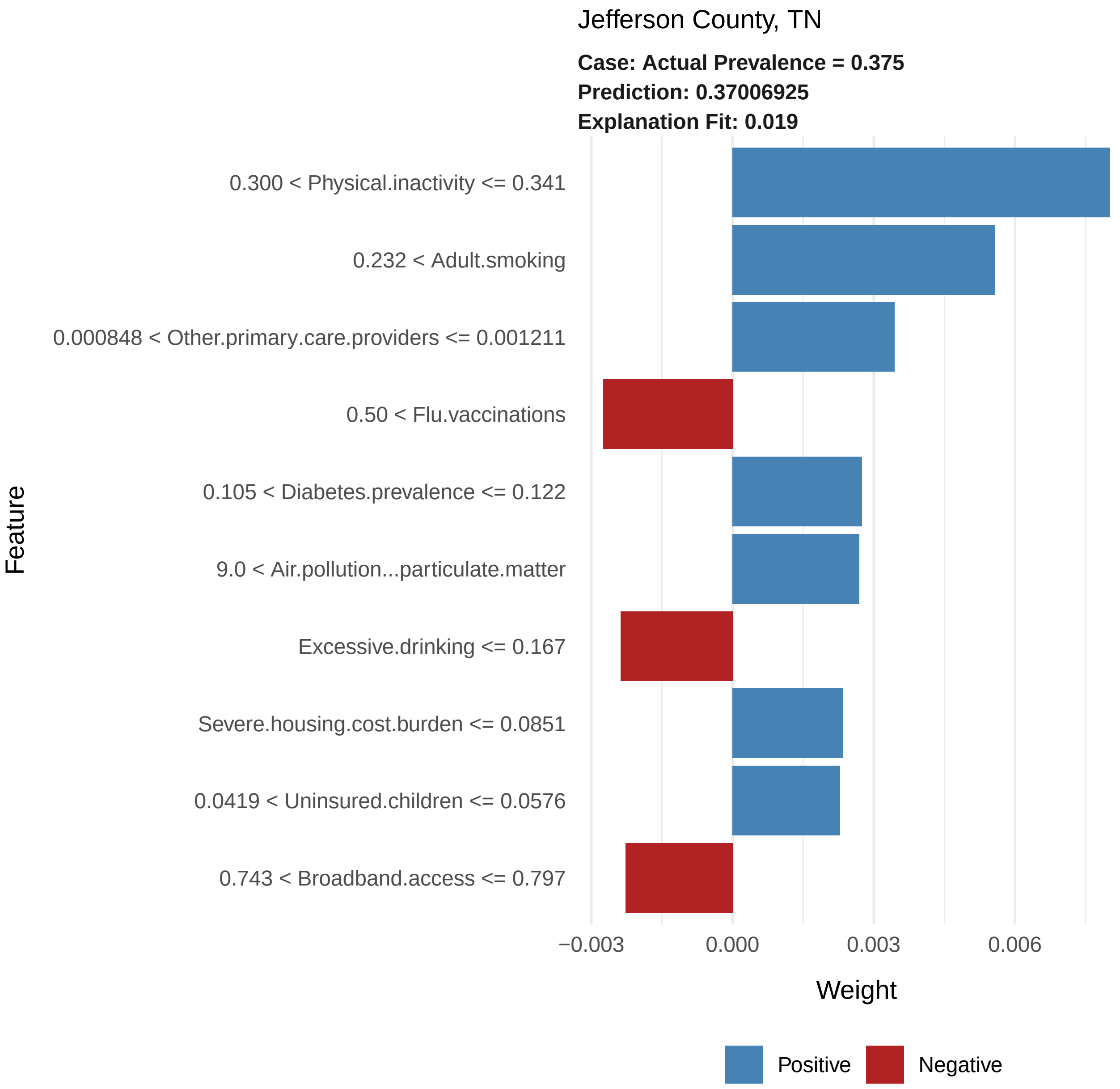

Jefferson County, TN
Case: Actual Prevalence = 0.375
Prediction: 0.37006925
Explanation Fit: 0.019
0.300 < Physical.inactivity <= 0.341
0.232 < Adult.smoking
0.000848 < Other.primary.care.providers <= 0.001211
0.50 < Flu.vaccinations
0.105 < Diabetes.prevalence <= 0.122
9.0 < Air.pollution...particulate.matter
Excessive.drinking <= 0.167
Severe.housing.cost.burden <= 0.0851
0.0419 < Uninsured.children <= 0.0576
0.743 < Broadband.access <= 0.797
Feature
−0.003
0.000
0.003
0.006
Weight
Positive
Negative

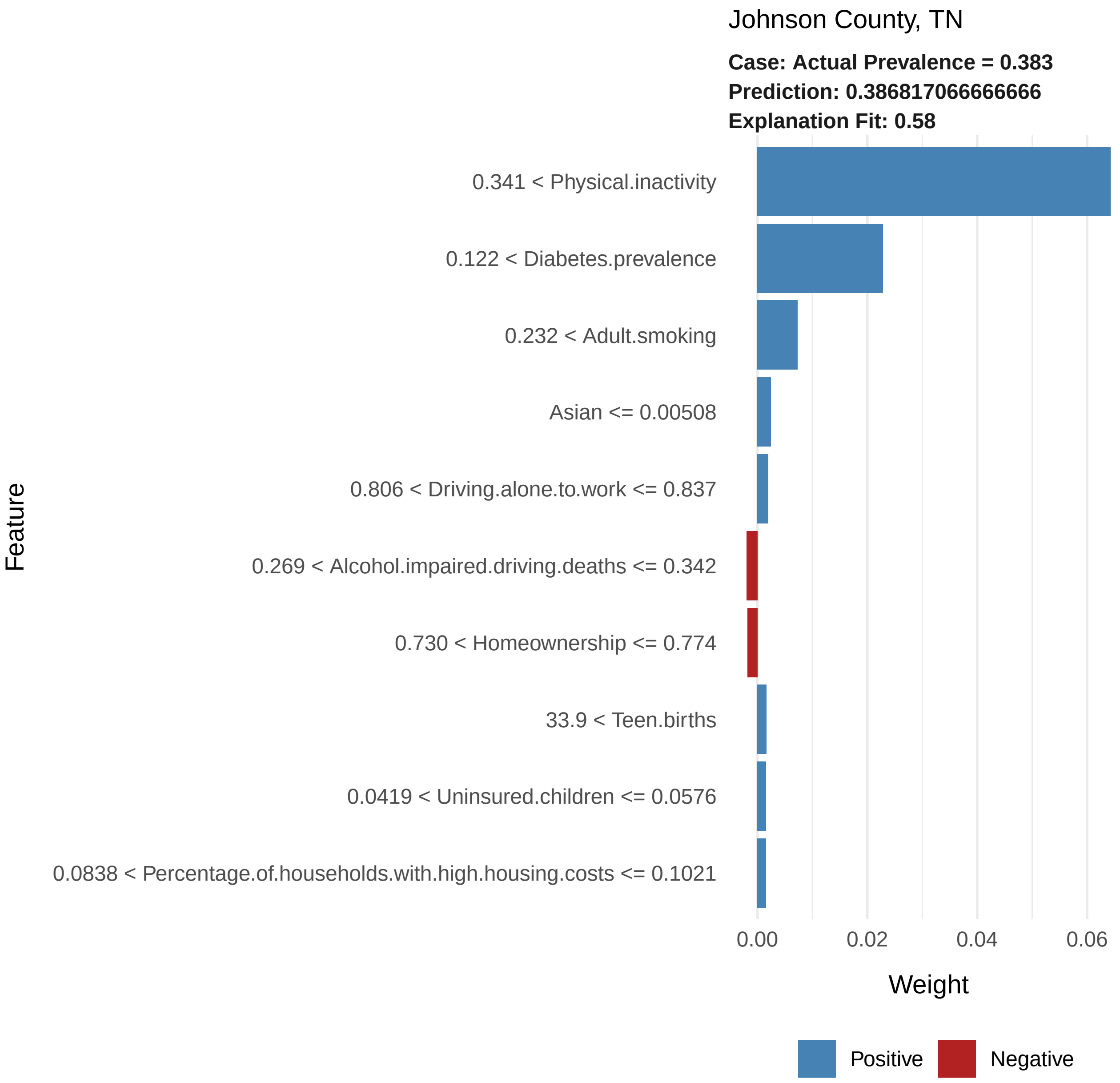

Johnson County, TN
Case: Actual Prevalence = 0.383
Prediction: 0.386817066666666
Explanation Fit: 0.58
0.341 < Physical.inactivity
0.122 < Diabetes.prevalence
0.232 < Adult.smoking
Asian <= 0.00508
0.806 < Driving.alone.to.work <= 0.837
0.269 < Alcohol.impaired.driving.deaths <= 0.342
0.730 < Homeownership <= 0.774
33.9 < Teen.births
0.0419 < Uninsured.children <= 0.0576
0.0838 < Percentage.of.households.with.high.housing.costs <= 0.1021
Feature
0.00
0.02
0.04
0.06
Weight
Positive
Negative

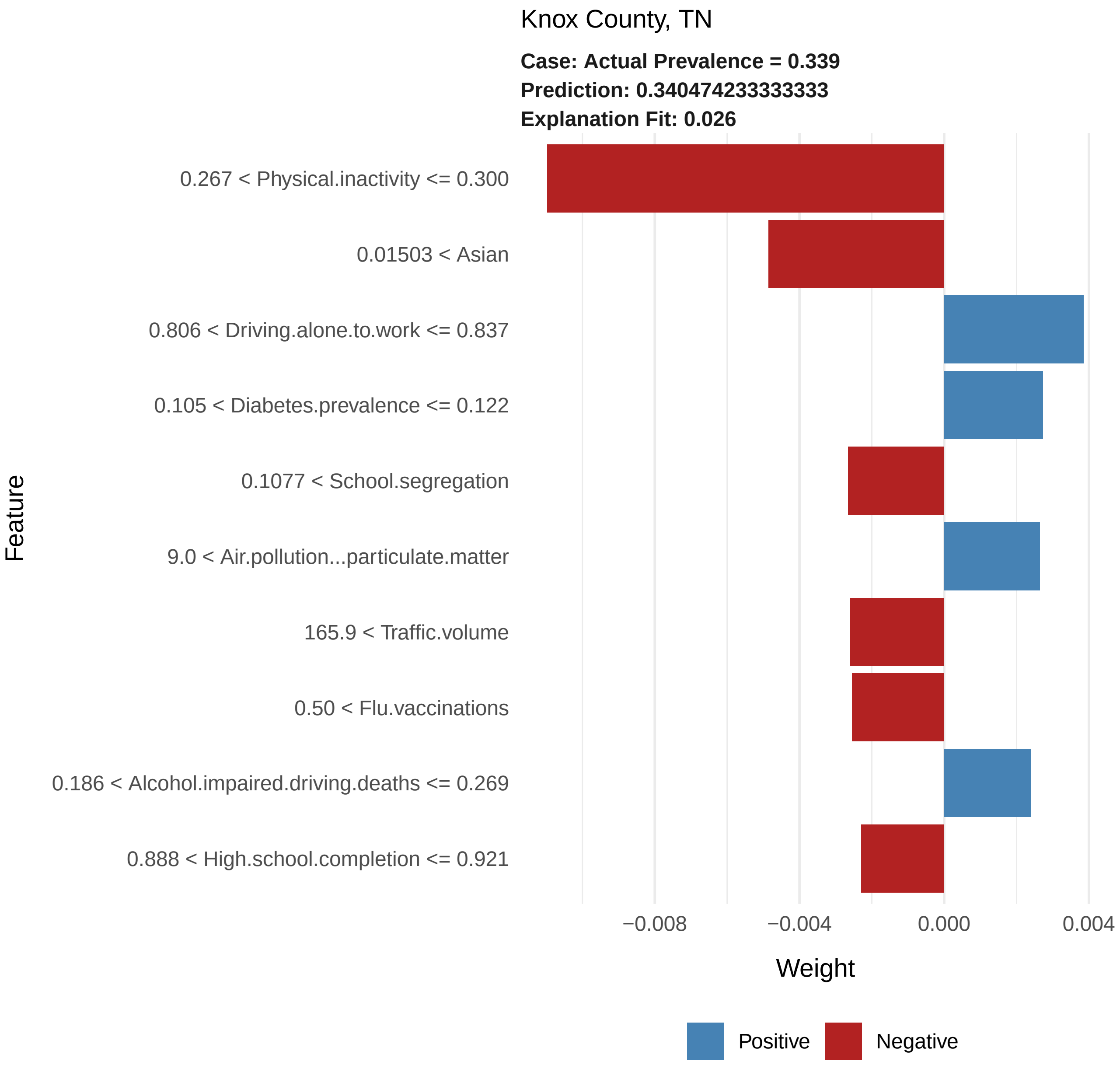
Knox County, TN
Case: Actual Prevalence = 0.339
Prediction: 0.340474233333333
Explanation Fit: 0.026
0.267 < Physical.inactivity <= 0.300
0.01503 < Asian
0.806 < Driving.alone.to.work <= 0.837
0.105 < Diabetes.prevalence <= 0.122
0.1077 < School.segregation
9.0 < Air.pollution...particulate.matter
165.9 < Traffic.volume
0.50 < Flu.vaccinations
0.186 < Alcohol.impaired.driving.deaths <= 0.269
0.888 < High.school.completion <= 0.921
Feature
−0.008
−0.004
0.000
0.004
Weight
Positive
Negative

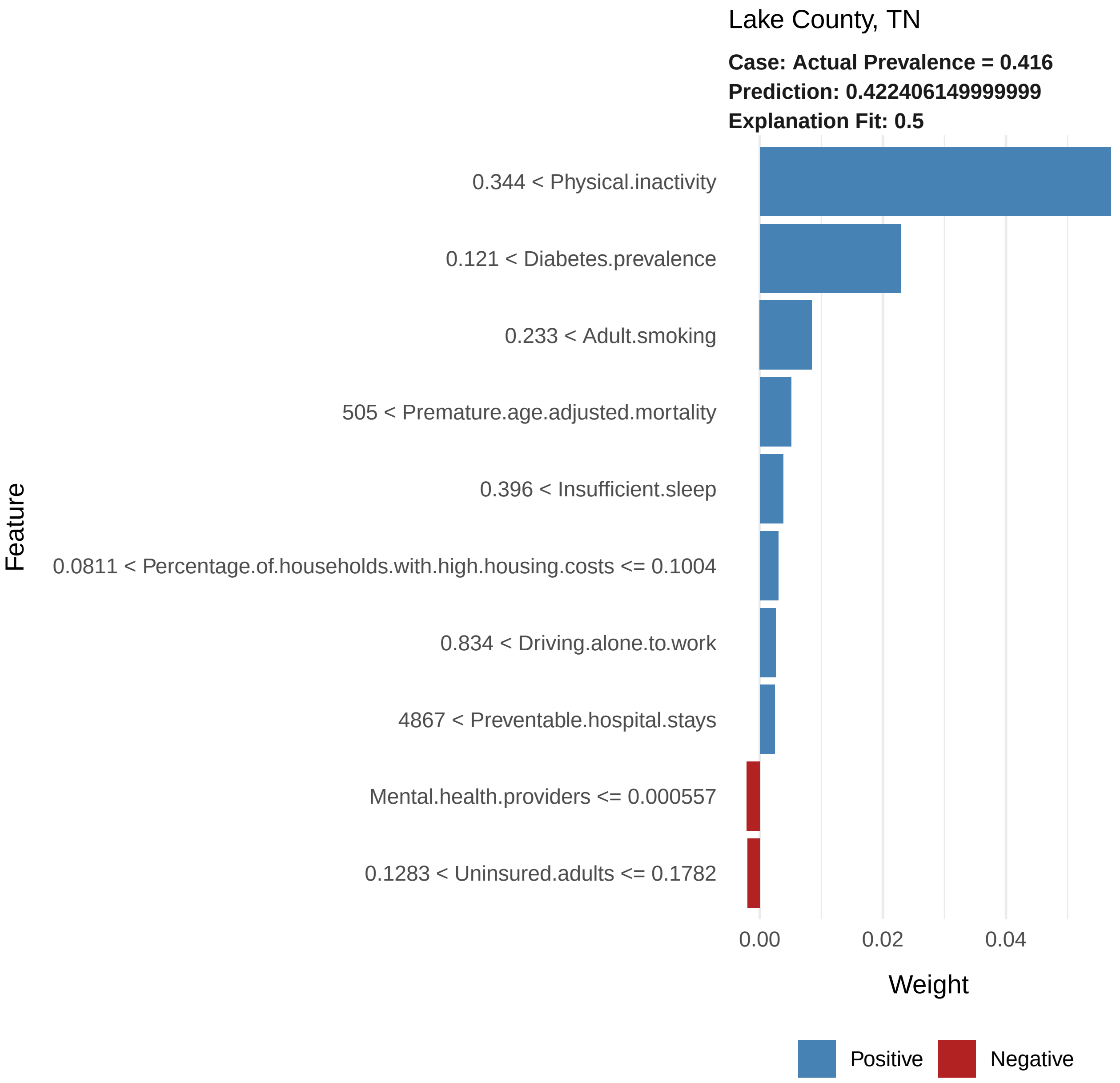
Lake County, TN
Case: Actual Prevalence = 0.416
Prediction: 0.422406149999999
Explanation Fit: 0.5
0.344 < Physical.inactivity
0.121 < Diabetes.prevalence
0.233 < Adult.smoking
505 < Premature.age.adjusted.mortality
0.396 < Insufficient.sleep
0.0811 < Percentage.of.households.with.high.housing.costs <= 0.1004
0.834 < Driving.alone.to.work
4867 < Preventable.hospital.stays
Mental.health.providers <= 0.000557
0.1283 < Uninsured.adults <= 0.1782
Feature
0.00
0.02
0.04
Weight
Positive
Negative

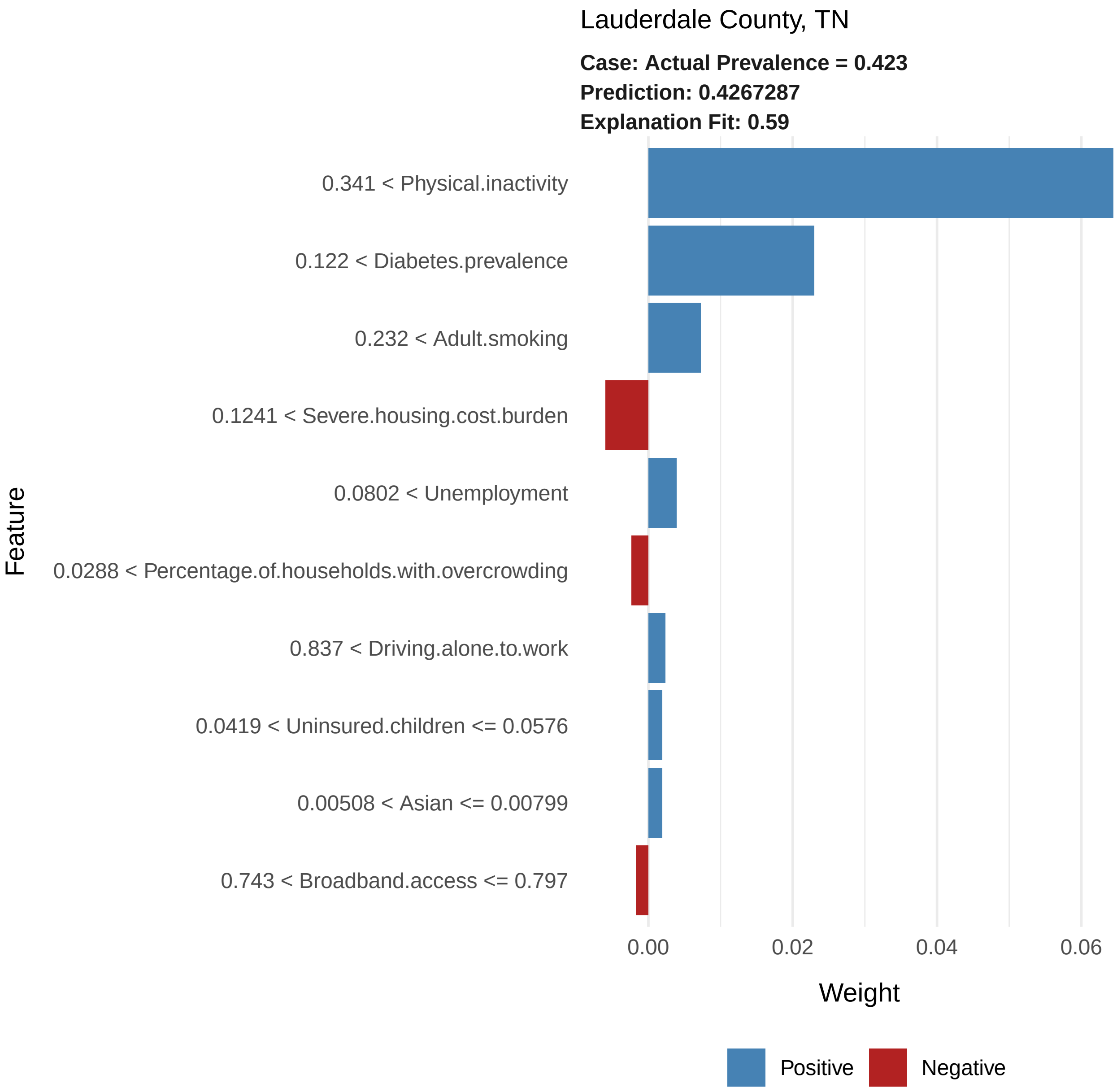
Lauderdale County, TN
Case: Actual Prevalence = 0.423
Prediction: 0.4267287
Explanation Fit: 0.59
0.341 < Physical.inactivity
0.122 < Diabetes.prevalence
0.232 < Adult.smoking
0.1241 < Severe.housing.cost.burden
0.0802 < Unemployment
0.0288 < Percentage.of.households.with.overcrowding
0.837 < Driving.alone.to.work
0.0419 < Uninsured.children <= 0.0576
0.00508 < Asian <= 0.00799
0.743 < Broadband.access <= 0.797
Feature
0.00
0.02
0.04
0.06
Weight
Positive
Negative

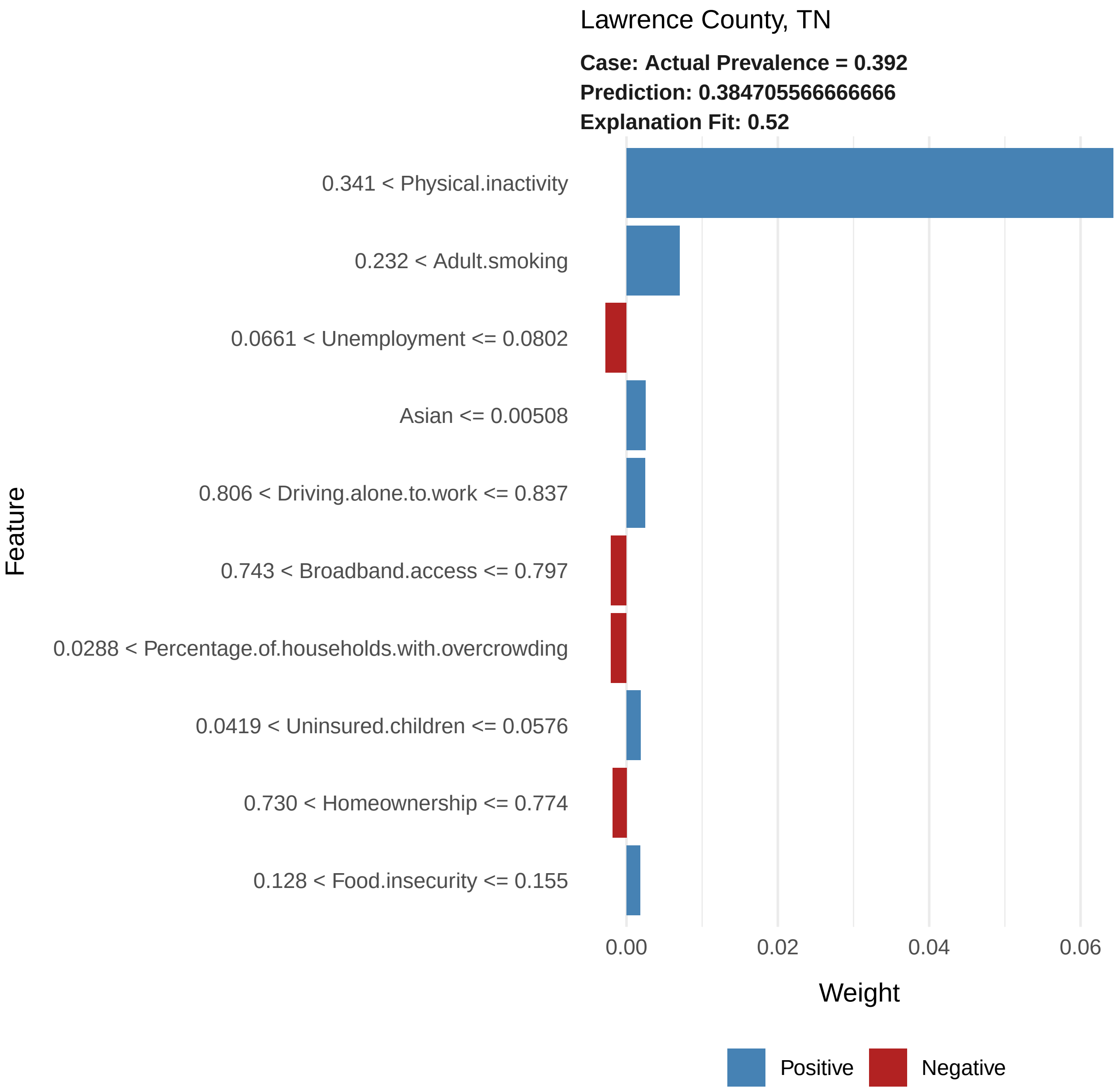
Lawrence County, TN
Case: Actual Prevalence = 0.392
Prediction: 0.384705566666666
Explanation Fit: 0.52
0.341 < Physical.inactivity
0.232 < Adult.smoking
0.0661 < Unemployment <= 0.0802
Asian <= 0.00508
0.806 < Driving.alone.to.work <= 0.837
0.743 < Broadband.access <= 0.797
0.0288 < Percentage.of.households.with.overcrowding
0.0419 < Uninsured.children <= 0.0576
0.730 < Homeownership <= 0.774
0.128 < Food.insecurity <= 0.155
Feature
0.00
0.02
0.04
0.06
Weight
Positive
Negative

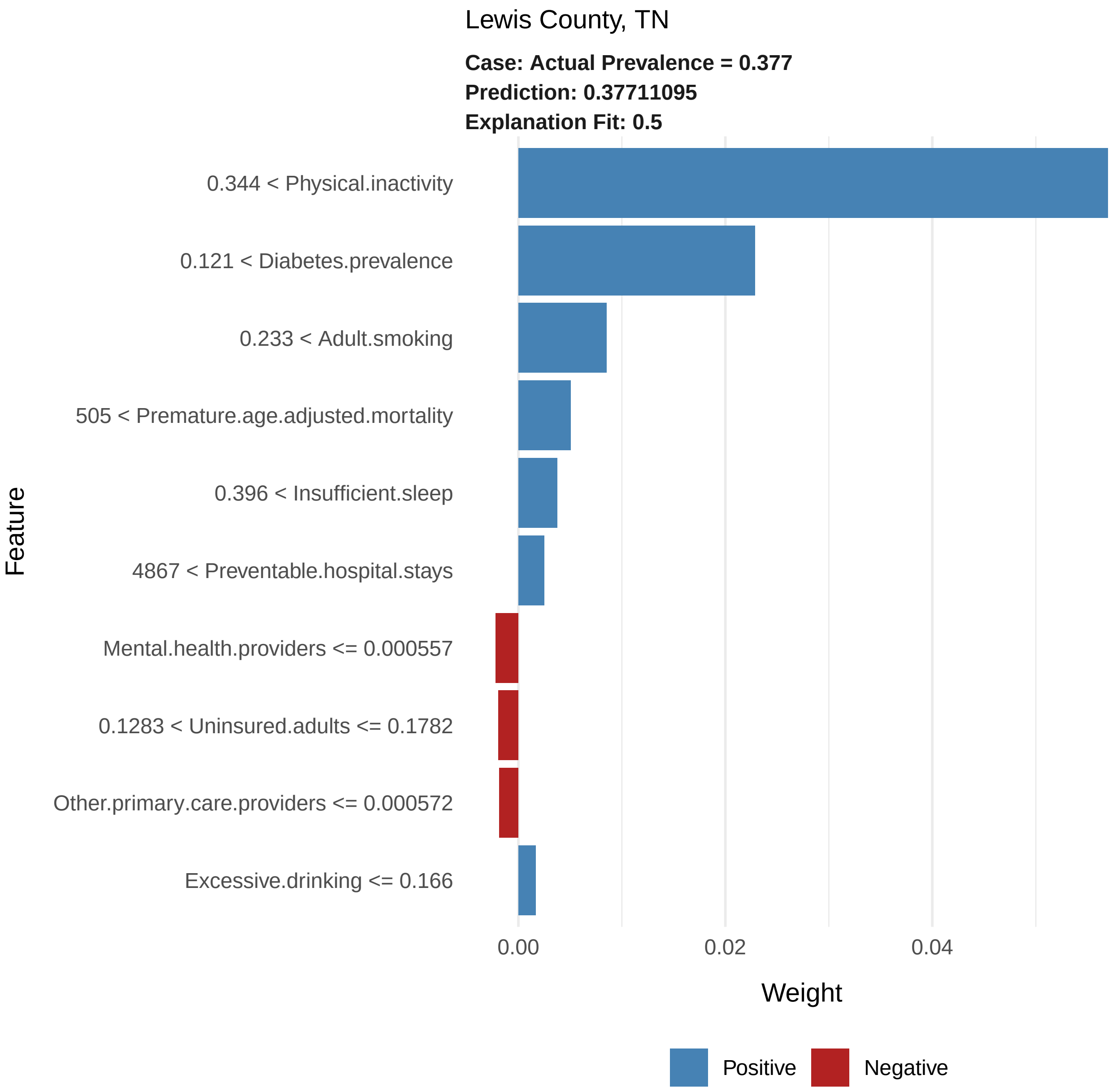

Lewis County, TN
Case: Actual Prevalence = 0.377
Prediction: 0.37711095
Explanation Fit: 0.5
0.344 < Physical.inactivity
0.121 < Diabetes.prevalence
0.233 < Adult.smoking
505 < Premature.age.adjusted.mortality
0.396 < Insufficient.sleep
4867 < Preventable.hospital.stays
Mental.health.providers <= 0.000557
0.1283 < Uninsured.adults <= 0.1782
Other.primary.care.providers <= 0.000572
Excessive.drinking <= 0.166
Feature
0.00
0.02
0.04
Weight
Positive
Negative

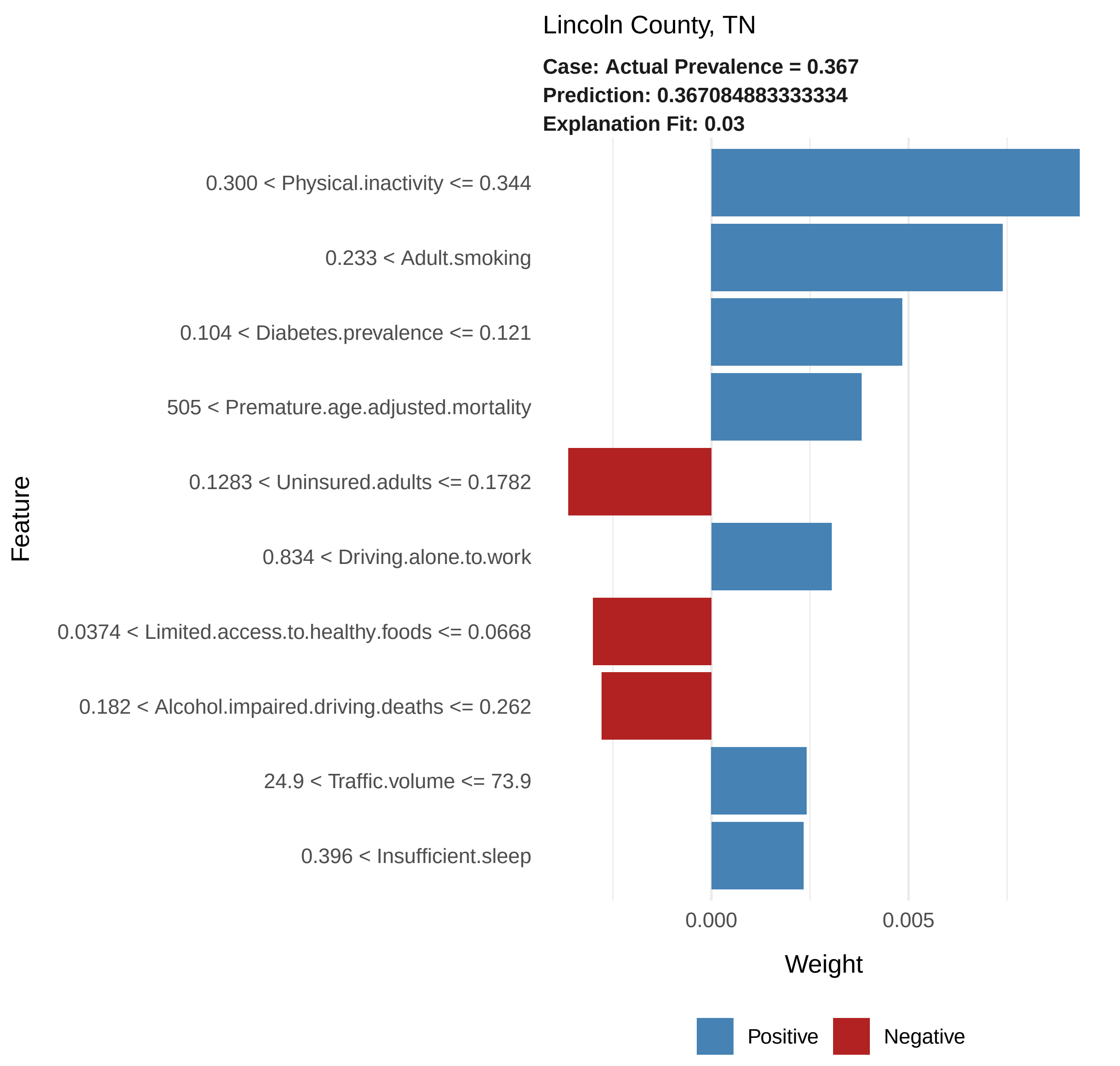

Lincoln County, TN
Case: Actual Prevalence = 0.367
Prediction: 0.367084883333334
Explanation Fit: 0.03
0.300 < Physical.inactivity <= 0.344
0.233 < Adult.smoking
0.104 < Diabetes.prevalence <= 0.121
505 < Premature.age.adjusted.mortality
0.1283 < Uninsured.adults <= 0.1782
0.834 < Driving.alone.to.work
0.0374 < Limited.access.to.healthy.foods <= 0.0668
0.182 < Alcohol.impaired.driving.deaths <= 0.262
24.9 < Traffic.volume <= 73.9
0.396 < Insufficient.sleep
Feature
0.000
0.005
Weight
Positive
Negative

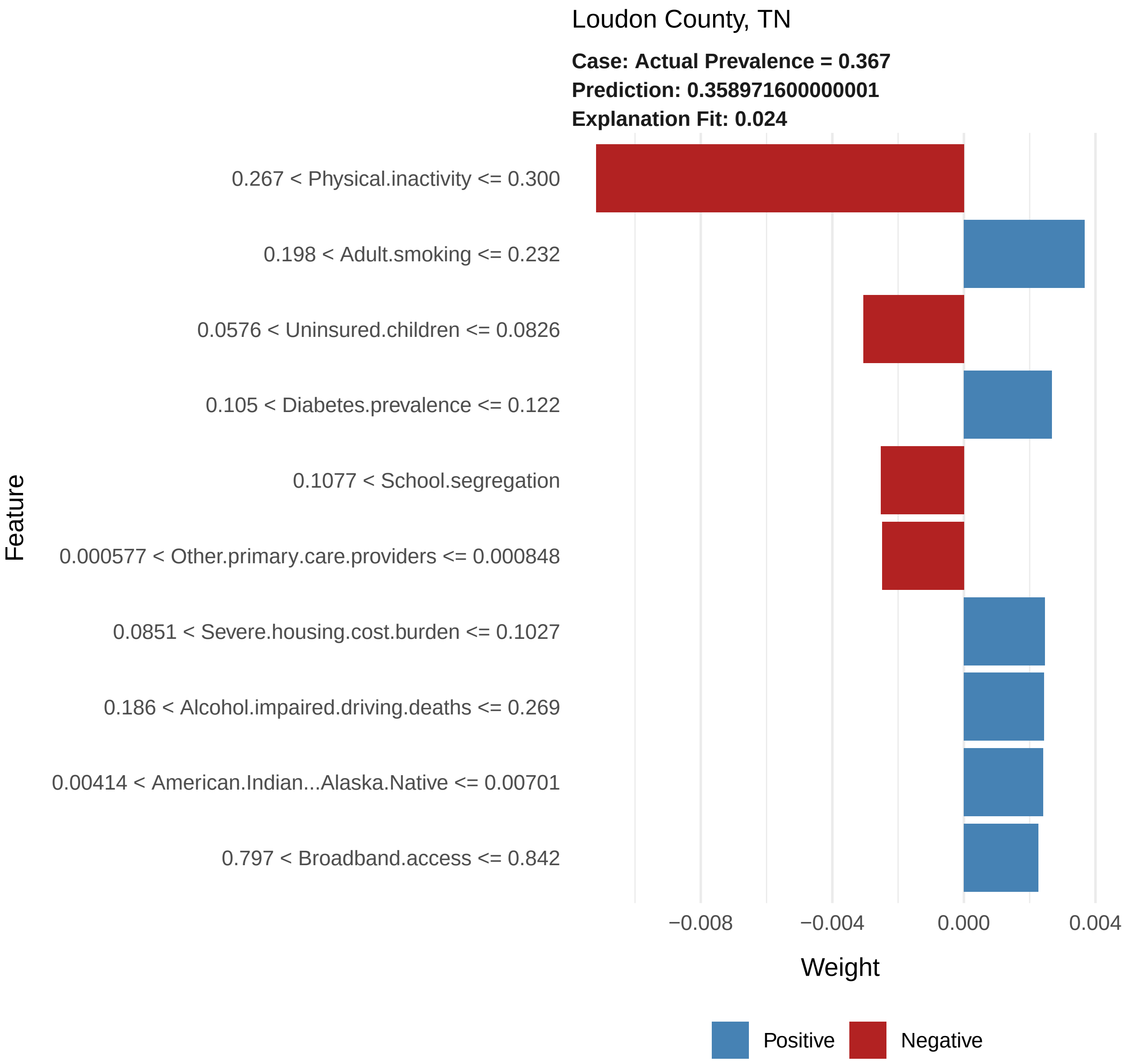
Loudon County, TN
Case: Actual Prevalence = 0.367
Prediction: 0.358971600000001
Explanation Fit: 0.024
0.267 < Physical.inactivity <= 0.300
0.198 < Adult.smoking <= 0.232
0.0576 < Uninsured.children <= 0.0826
0.105 < Diabetes.prevalence <= 0.122
0.1077 < School.segregation
0.000577 < Other.primary.care.providers <= 0.000848
0.0851 < Severe.housing.cost.burden <= 0.1027
0.186 < Alcohol.impaired.driving.deaths <= 0.269
0.00414 < American.Indian...Alaska.Native <= 0.00701
0.797 < Broadband.access <= 0.842
Feature
−0.008
−0.004
0.000
0.004
Weight
Positive
Negative

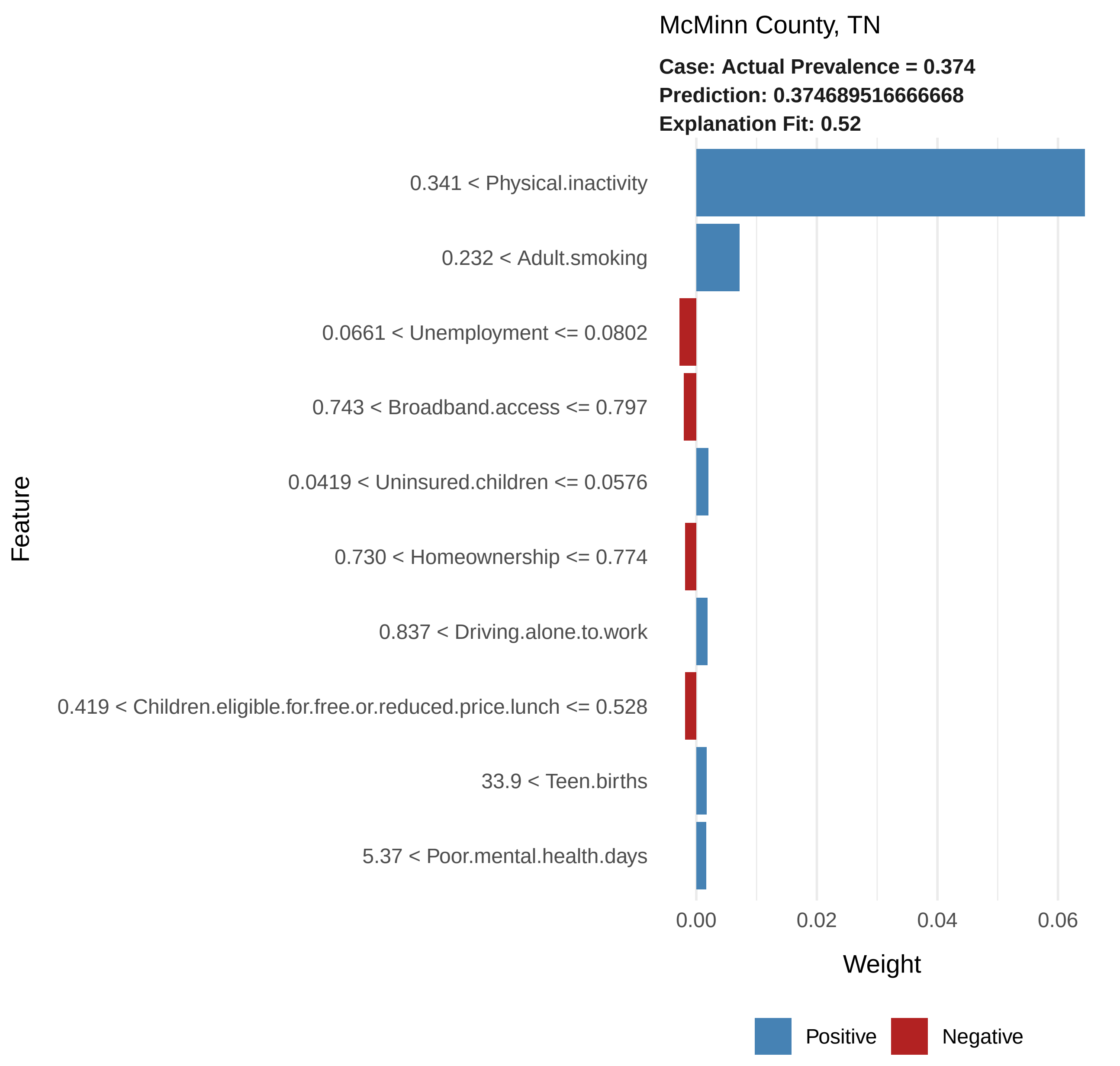

McMinn County, TN
Case: Actual Prevalence = 0.374
Prediction: 0.374689516666668
Explanation Fit: 0.52
0.341 < Physical.inactivity
0.232 < Adult.smoking
0.0661 < Unemployment <= 0.0802
0.743 < Broadband.access <= 0.797
0.0419 < Uninsured.children <= 0.0576
0.730 < Homeownership <= 0.774
0.837 < Driving.alone.to.work
0.419 < Children.eligible.for.free.or.reduced.price.lunch <= 0.528
33.9 < Teen.births
5.37 < Poor.mental.health.days
Feature
0.00
0.02
0.04
0.06
Weight
Positive
Negative

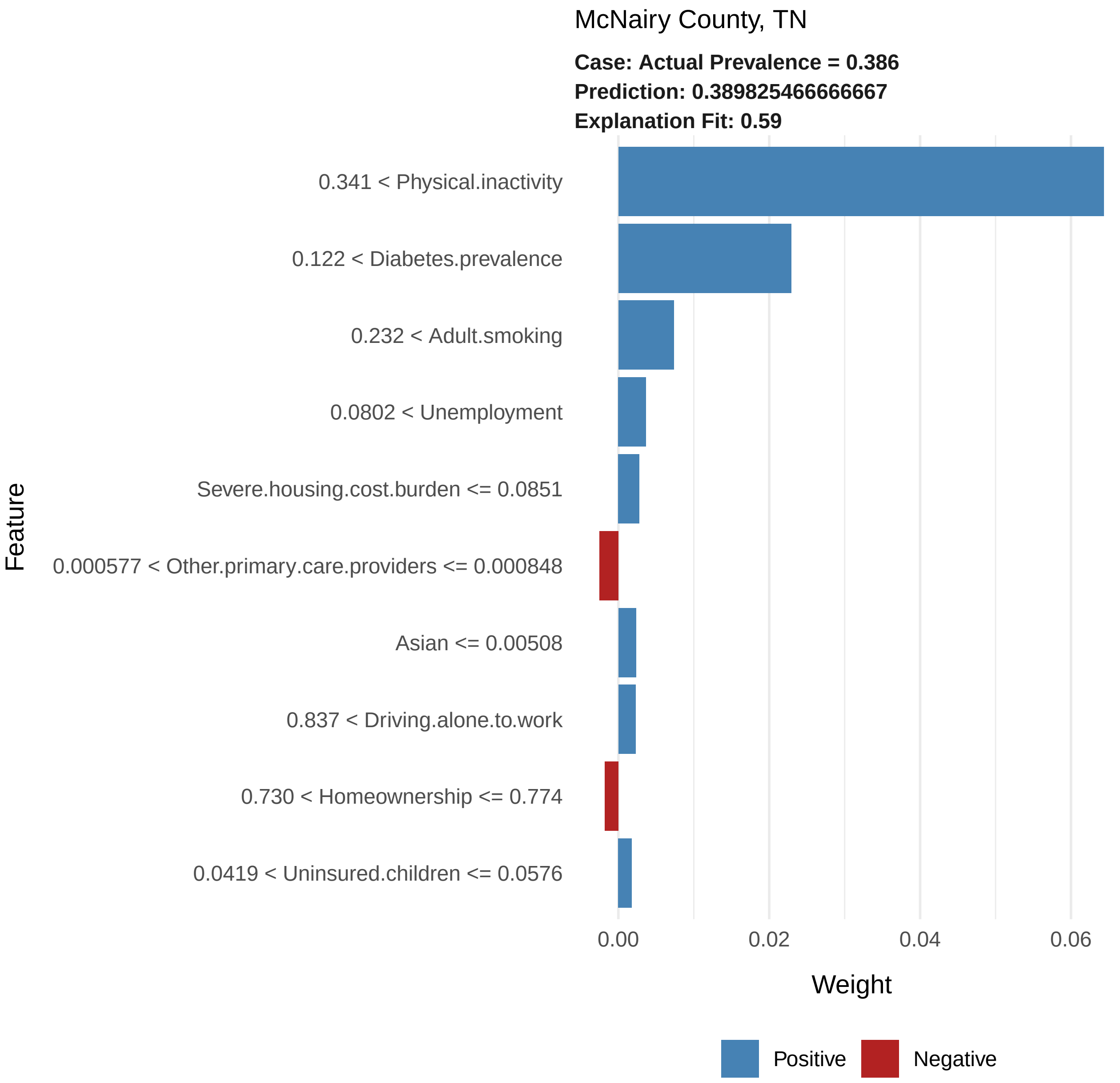
McNairy County, TN
Case: Actual Prevalence = 0.386
Prediction: 0.389825466666667
Explanation Fit: 0.59
0.341 < Physical.inactivity
0.122 < Diabetes.prevalence
0.232 < Adult.smoking
0.0802 < Unemployment
Severe.housing.cost.burden <= 0.0851
0.000577 < Other.primary.care.providers <= 0.000848
Asian <= 0.00508
0.837 < Driving.alone.to.work
0.730 < Homeownership <= 0.774
0.0419 < Uninsured.children <= 0.0576
Feature
0.00
0.02
0.04
0.06
Weight
Positive
Negative

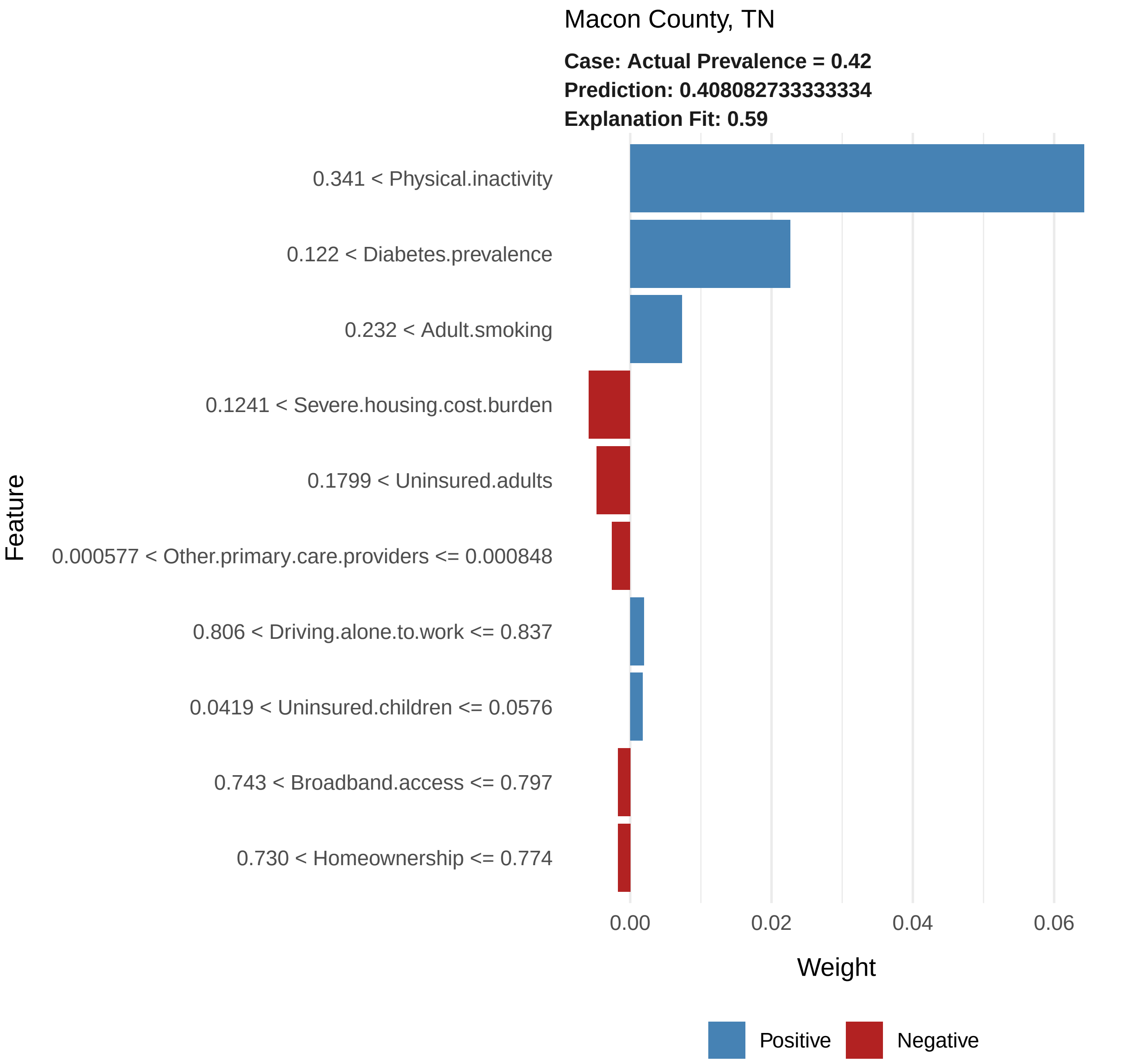

Macon County, TN
Case: Actual Prevalence = 0.42
Prediction: 0.408082733333334
Explanation Fit: 0.59
0.341 < Physical.inactivity
0.122 < Diabetes.prevalence
0.232 < Adult.smoking
0.1241 < Severe.housing.cost.burden
0.1799 < Uninsured.adults
0.000577 < Other.primary.care.providers <= 0.000848
0.806 < Driving.alone.to.work <= 0.837
0.0419 < Uninsured.children <= 0.0576
0.743 < Broadband.access <= 0.797
0.730 < Homeownership <= 0.774
Feature
0.00
0.02
0.04
0.06
Weight
Positive
Negative

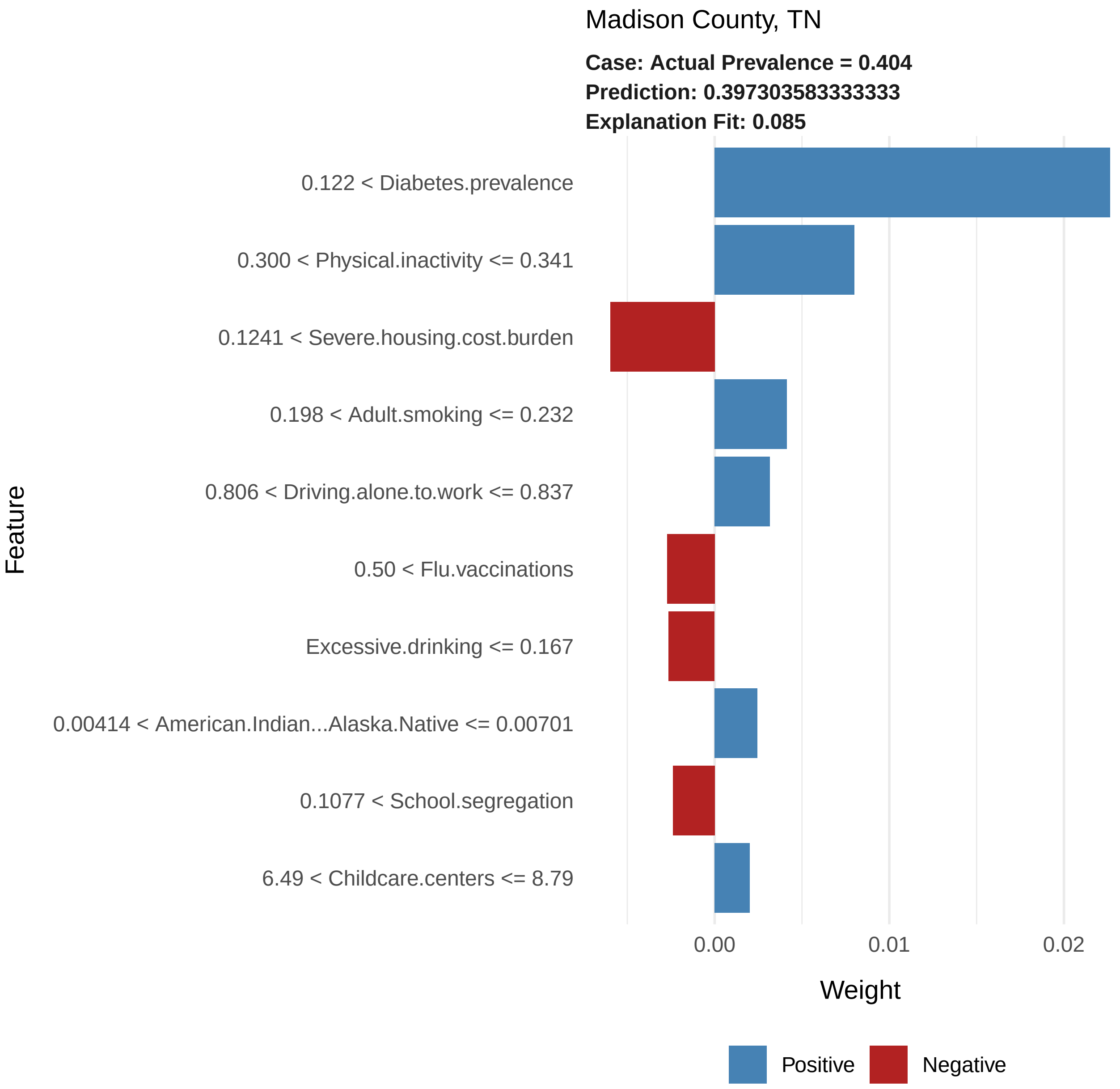

Madison County, TN
Case: Actual Prevalence = 0.404
Prediction: 0.397303583333333
Explanation Fit: 0.085
0.122 < Diabetes.prevalence
0.300 < Physical.inactivity <= 0.341
0.1241 < Severe.housing.cost.burden
0.198 < Adult.smoking <= 0.232
0.806 < Driving.alone.to.work <= 0.837
0.50 < Flu.vaccinations
Excessive.drinking <= 0.167
0.00414 < American.Indian...Alaska.Native <= 0.00701
0.1077 < School.segregation
6.49 < Childcare.centers <= 8.79
Feature
0.00
0.01
0.02
Weight
Positive
Negative

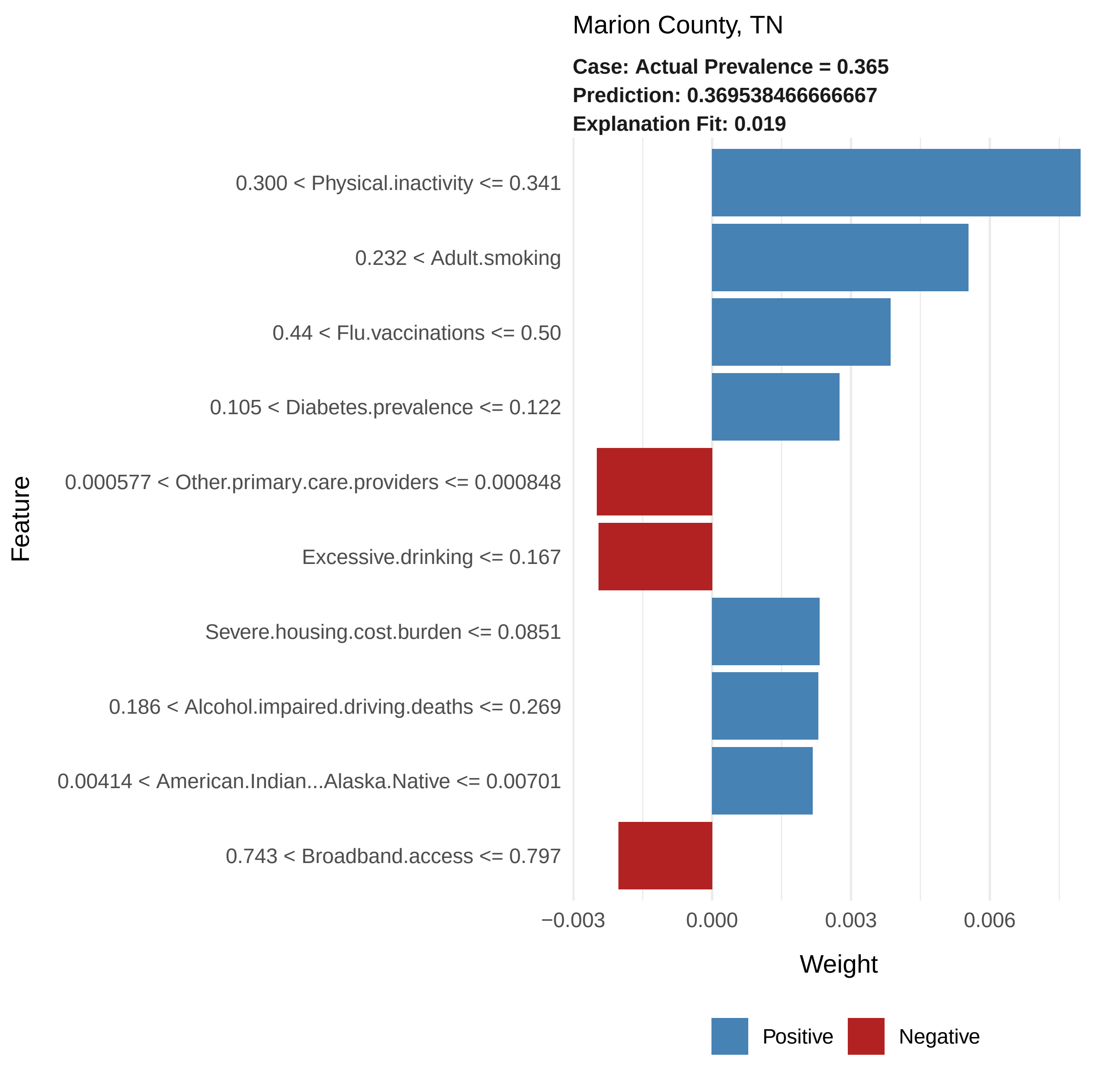
Marion County, TN
Case: Actual Prevalence = 0.365
Prediction: 0.369538466666667
Explanation Fit: 0.019
Feature
0.300 < Physical.inactivity <= 0.341
0.232 < Adult.smoking
0.44 < Flu.vaccinations <= 0.50
0.105 < Diabetes.prevalence <= 0.122
0.000577 < Other.primary.care.providers <= 0.000848
Excessive.drinking <= 0.167
Severe.housing.cost.burden <= 0.0851
0.186 < Alcohol.impaired.driving.deaths <= 0.269
0.00414 < American.Indian...Alaska.Native <= 0.00701
0.743 < Broadband.access <= 0.797
−0.003
0.000
0.003
0.006
Weight
Positive
Negative

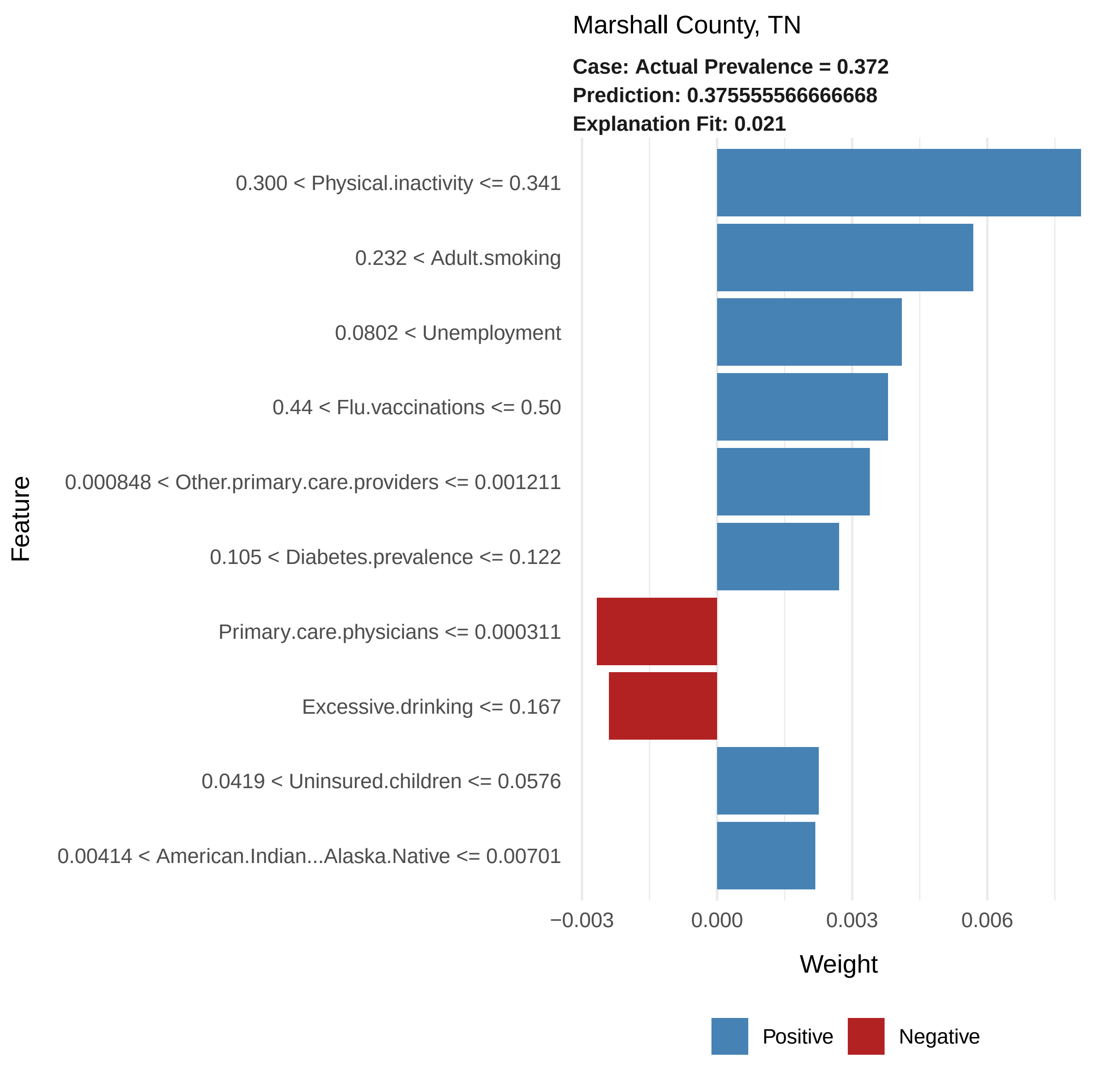

Marshall County, TN
Case: Actual Prevalence = 0.372
Prediction: 0.375555566666668
Explanation Fit: 0.021
0.300 < Physical.inactivity <= 0.341
0.232 < Adult.smoking
0.0802 < Unemployment
0.44 < Flu.vaccinations <= 0.50
0.000848 < Other.primary.care.providers <= 0.001211
0.105 < Diabetes.prevalence <= 0.122
Primary.care.physicians <= 0.000311
Excessive.drinking <= 0.167
0.0419 < Uninsured.children <= 0.0576
0.00414 < American.Indian...Alaska.Native <= 0.00701
Feature
−0.003
0.000
0.003
0.006
Weight
Positive
Negative

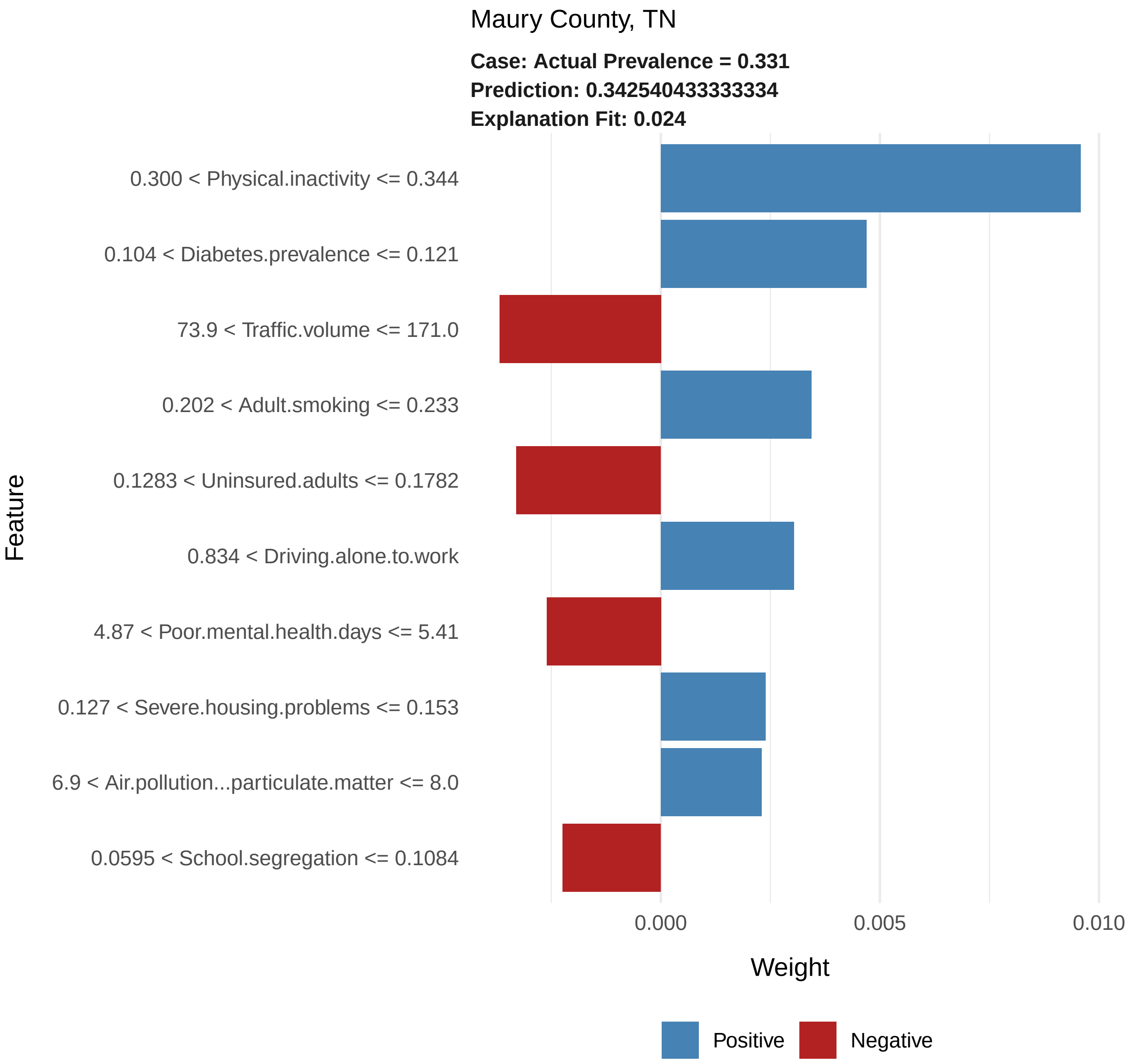
Maury County, TN
Case: Actual Prevalence = 0.331
Prediction: 0.342540433333334
Explanation Fit: 0.024
0.300 < Physical.inactivity <= 0.344
0.104 < Diabetes.prevalence <= 0.121
73.9 < Traffic.volume <= 171.0
0.202 < Adult.smoking <= 0.233
0.1283 < Uninsured.adults <= 0.1782
0.834 < Driving.alone.to.work
4.87 < Poor.mental.health.days <= 5.41
0.127 < Severe.housing.problems <= 0.153
6.9 < Air.pollution...particulate.matter <= 8.0
0.0595 < School.segregation <= 0.1084
Feature
0.000
0.005
0.010
Weight
Positive
Negative

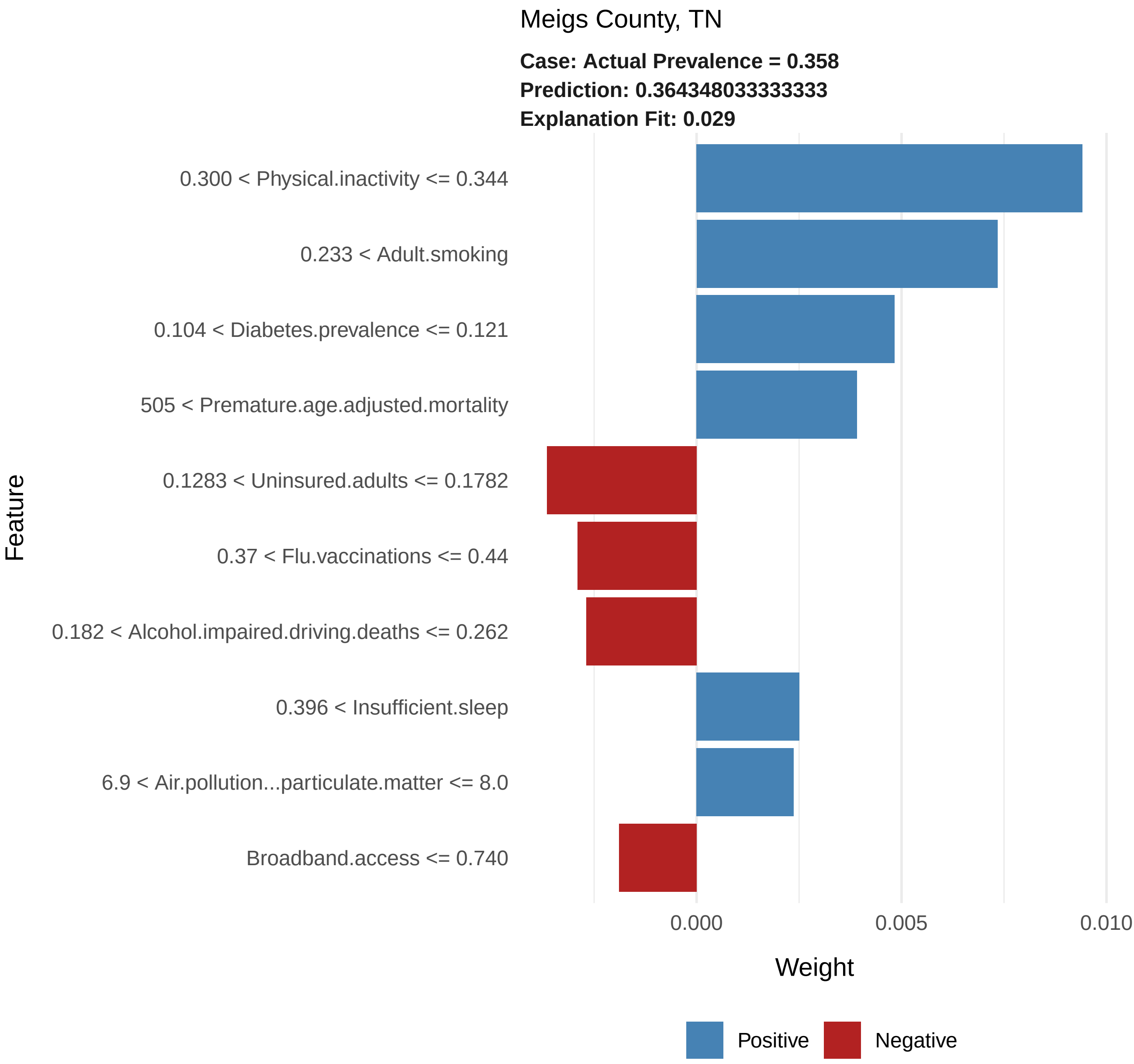

Meigs County, TN
Case: Actual Prevalence = 0.358
Prediction: 0.364348033333333
Explanation Fit: 0.029
0.300 < Physical.inactivity <= 0.344
0.233 < Adult.smoking
0.104 < Diabetes.prevalence <= 0.121
505 < Premature.age.adjusted.mortality
0.1283 < Uninsured.adults <= 0.1782
0.37 < Flu.vaccinations <= 0.44
0.182 < Alcohol.impaired.driving.deaths <= 0.262
0.396 < Insufficient.sleep
6.9 < Air.pollution...particulate.matter <= 8.0
Broadband.access <= 0.740
Feature
0.000
0.005
0.010
Weight
Positive
Negative

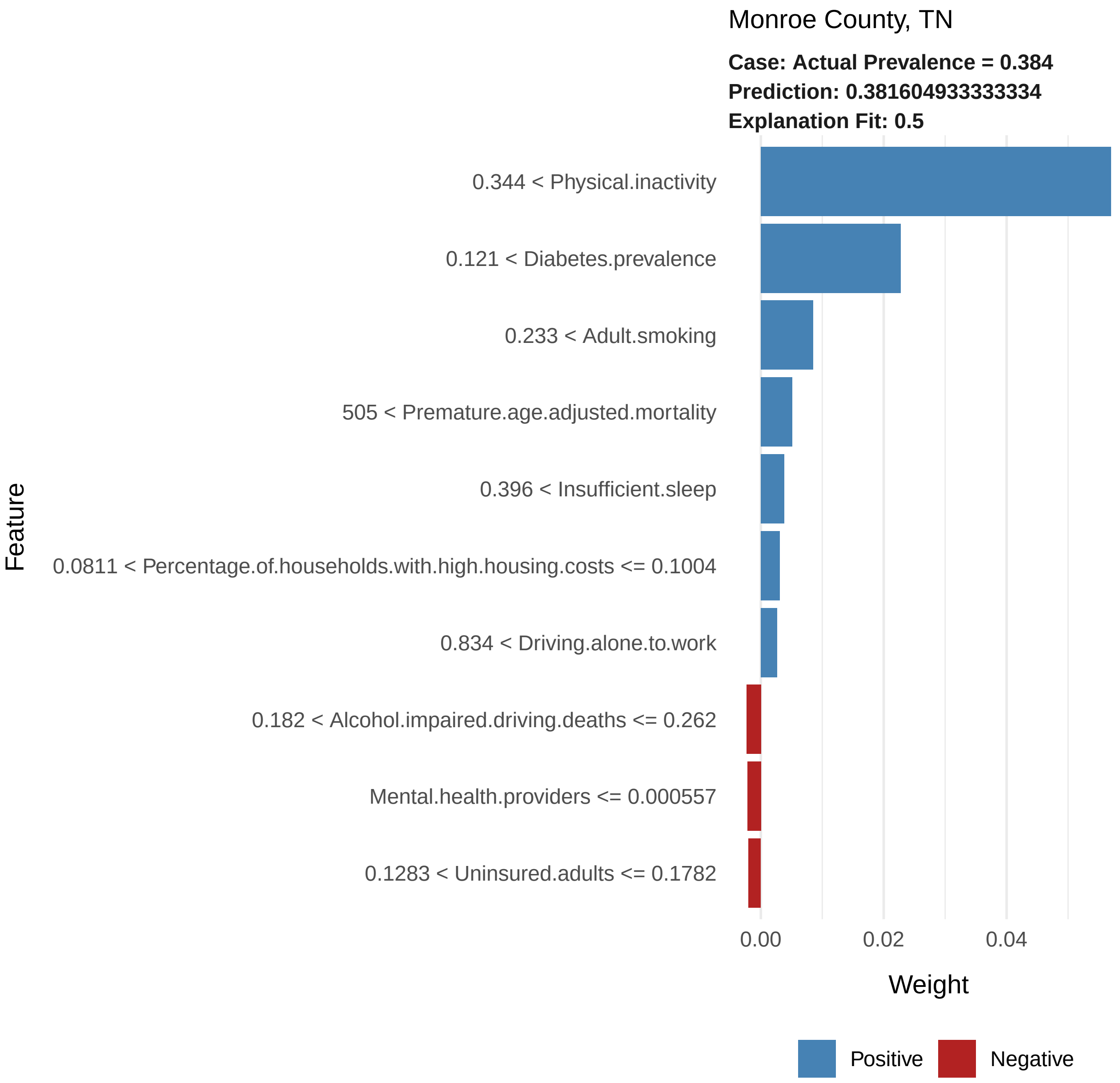
Monroe County, TN
Case: Actual Prevalence = 0.384
Prediction: 0.381604933333334
Explanation Fit: 0.5
0.344 < Physical.inactivity
0.121 < Diabetes.prevalence
0.233 < Adult.smoking
505 < Premature.age.adjusted.mortality
0.396 < Insufficient.sleep
0.0811 < Percentage.of.households.with.high.housing.costs <= 0.1004
0.834 < Driving.alone.to.work
0.182 < Alcohol.impaired.driving.deaths <= 0.262
Mental.health.providers <= 0.000557
0.1283 < Uninsured.adults <= 0.1782
Feature
0.00
0.02
0.04
Weight
Positive
Negative

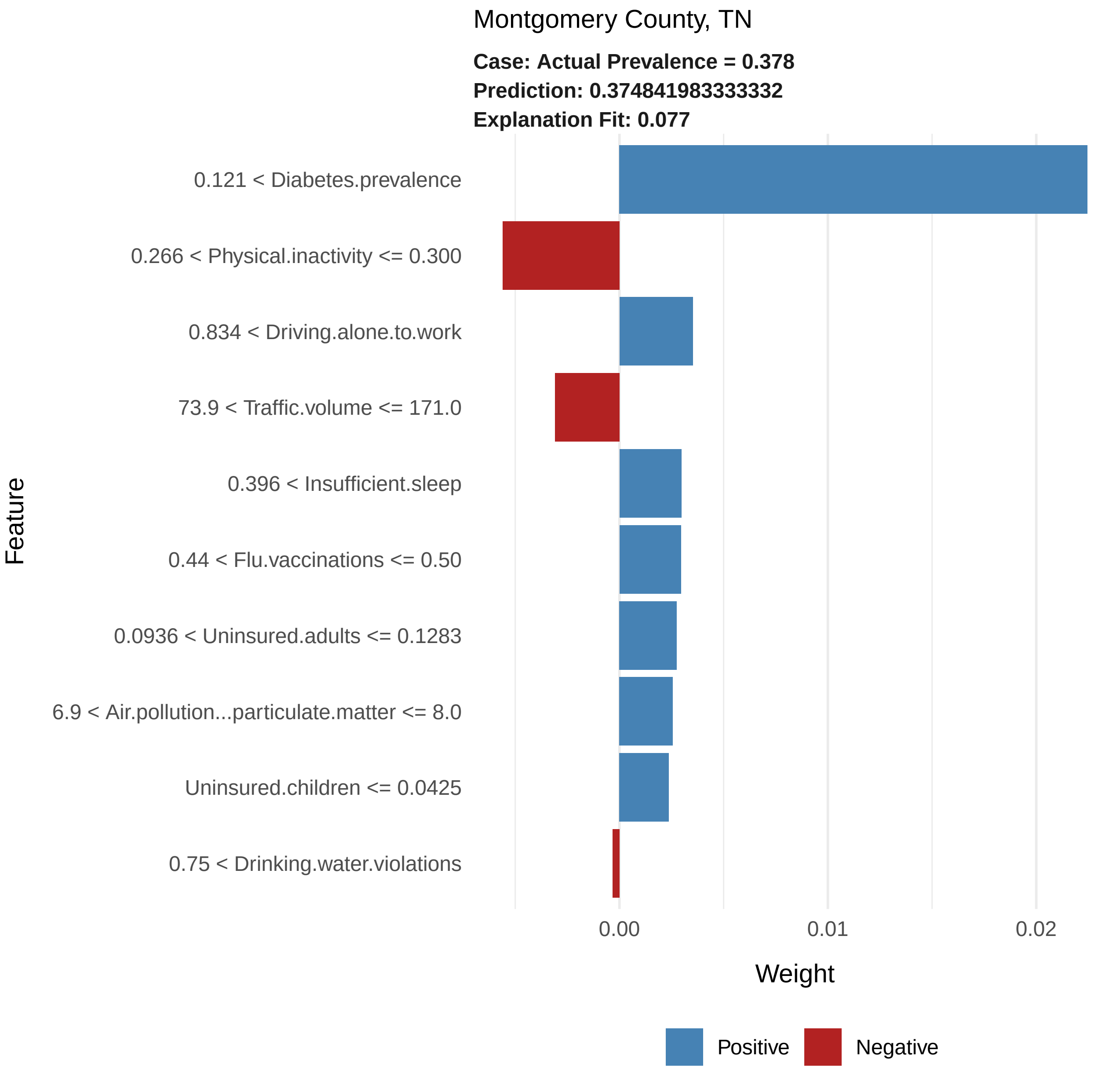

Montgomery County, TN
Case: Actual Prevalence = 0.378
Prediction: 0.374841983333332
Explanation Fit: 0.077
0.121 < Diabetes.prevalence
0.266 < Physical.inactivity <= 0.300
0.834 < Driving.alone.to.work
73.9 < Traffic.volume <= 171.0
0.396 < Insufficient.sleep
0.44 < Flu.vaccinations <= 0.50
0.0936 < Uninsured.adults <= 0.1283
6.9 < Air.pollution...particulate.matter <= 8.0
Uninsured.children <= 0.0425
0.75 < Drinking.water.violations
Feature
0.00
0.01
0.02
Weight
Positive
Negative

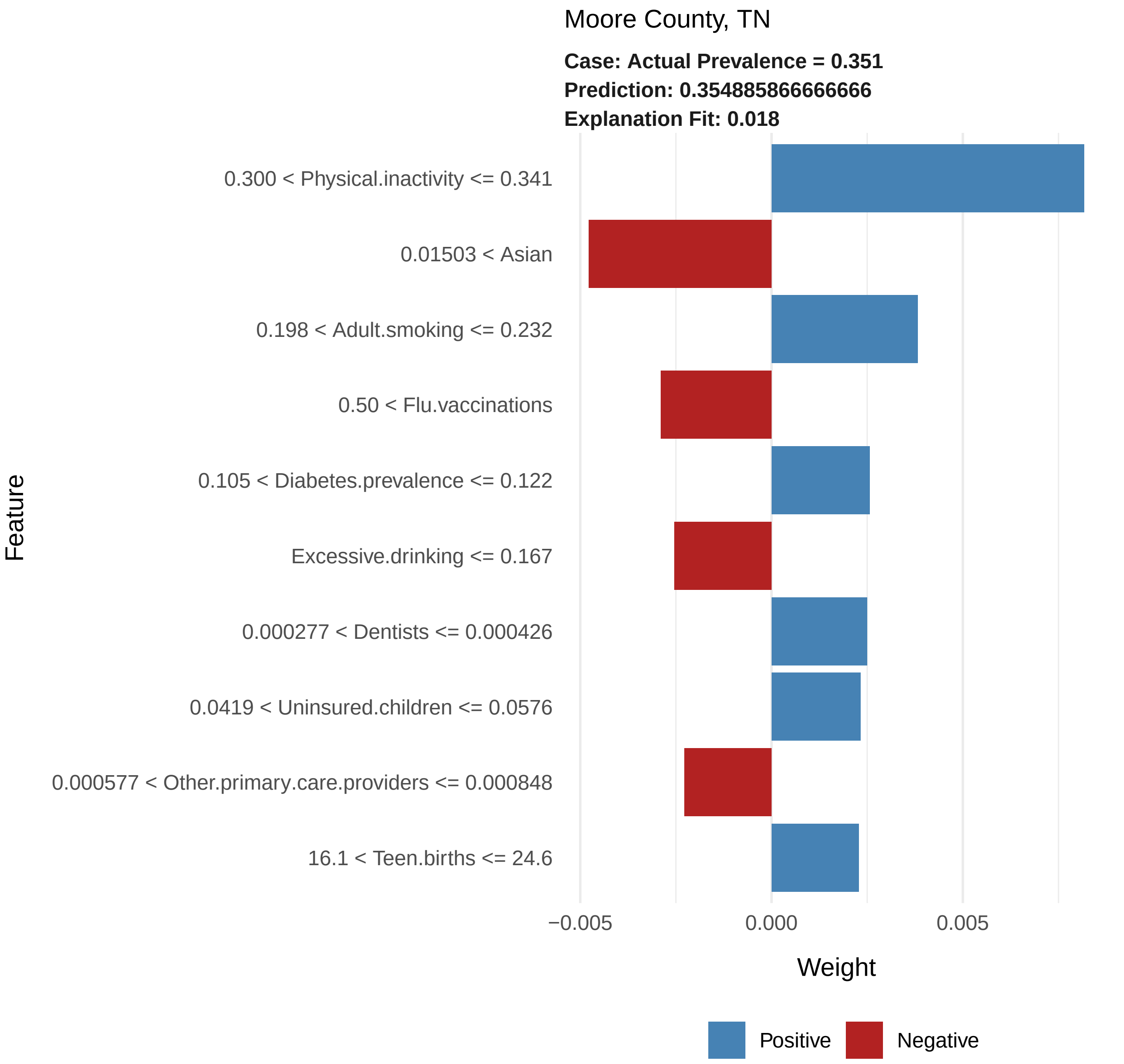
Moore County, TN
Case: Actual Prevalence = 0.351
Prediction: 0.354885866666666
Explanation Fit: 0.018
0.300 < Physical.inactivity <= 0.341
0.01503 < Asian
0.198 < Adult.smoking <= 0.232
0.50 < Flu.vaccinations
0.105 < Diabetes.prevalence <= 0.122
Excessive.drinking <= 0.167
0.000277 < Dentists <= 0.000426
0.0419 < Uninsured.children <= 0.0576
0.000577 < Other.primary.care.providers <= 0.000848
16.1 < Teen.births <= 24.6
Feature
−0.005
0.000
0.005
Weight
Positive
Negative

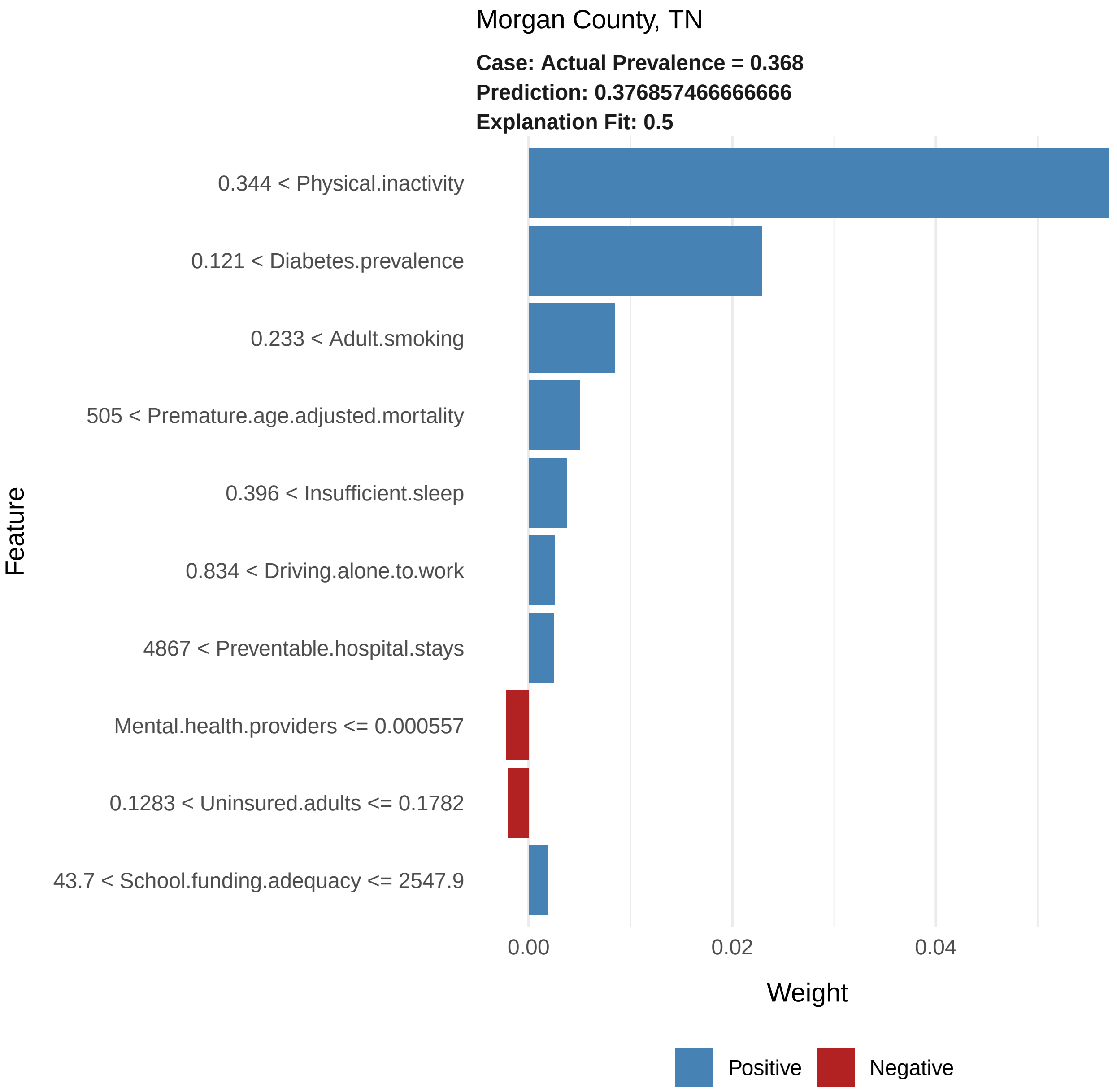

Morgan County, TN
Case: Actual Prevalence = 0.368
Prediction: 0.376857466666666
Explanation Fit: 0.5
0.344 < Physical.inactivity
0.121 < Diabetes.prevalence
0.233 < Adult.smoking
505 < Premature.age.adjusted.mortality
0.396 < Insufficient.sleep
0.834 < Driving.alone.to.work
4867 < Preventable.hospital.stays
Mental.health.providers <= 0.000557
0.1283 < Uninsured.adults <= 0.1782
43.7 < School.funding.adequacy <= 2547.9
Feature
0.00
0.02
0.04
Weight
Positive
Negative

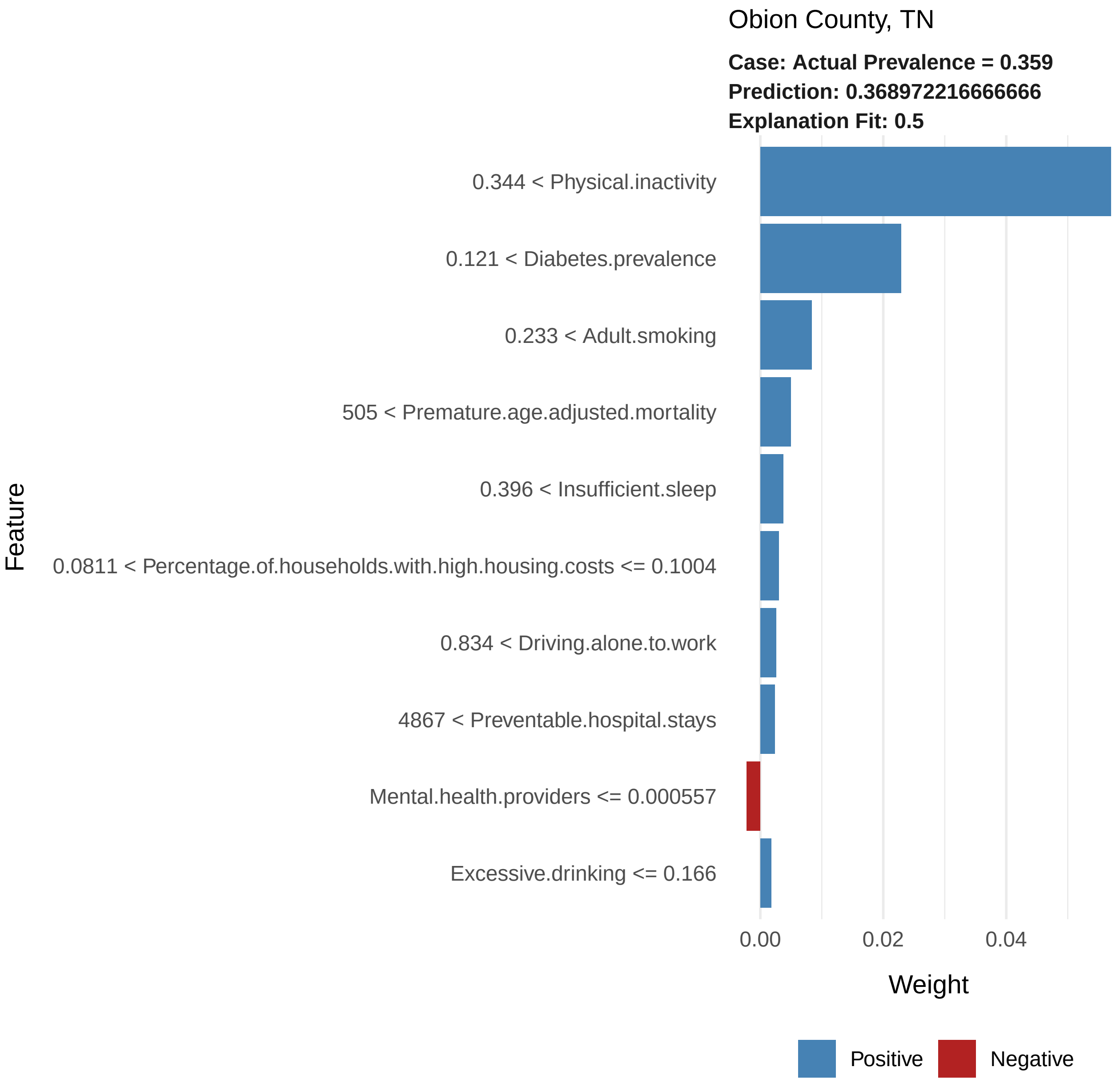
Obion County, TN
Case: Actual Prevalence = 0.359
Prediction: 0.368972216666666
Explanation Fit: 0.5
0.344 < Physical.inactivity
0.121 < Diabetes.prevalence
0.233 < Adult.smoking
505 < Premature.age.adjusted.mortality
0.396 < Insufficient.sleep
0.0811 < Percentage.of.households.with.high.housing.costs <= 0.1004
0.834 < Driving.alone.to.work
4867 < Preventable.hospital.stays
Mental.health.providers <= 0.000557
Excessive.drinking <= 0.166
Feature
0.00
0.02
0.04
Weight
Positive
Negative

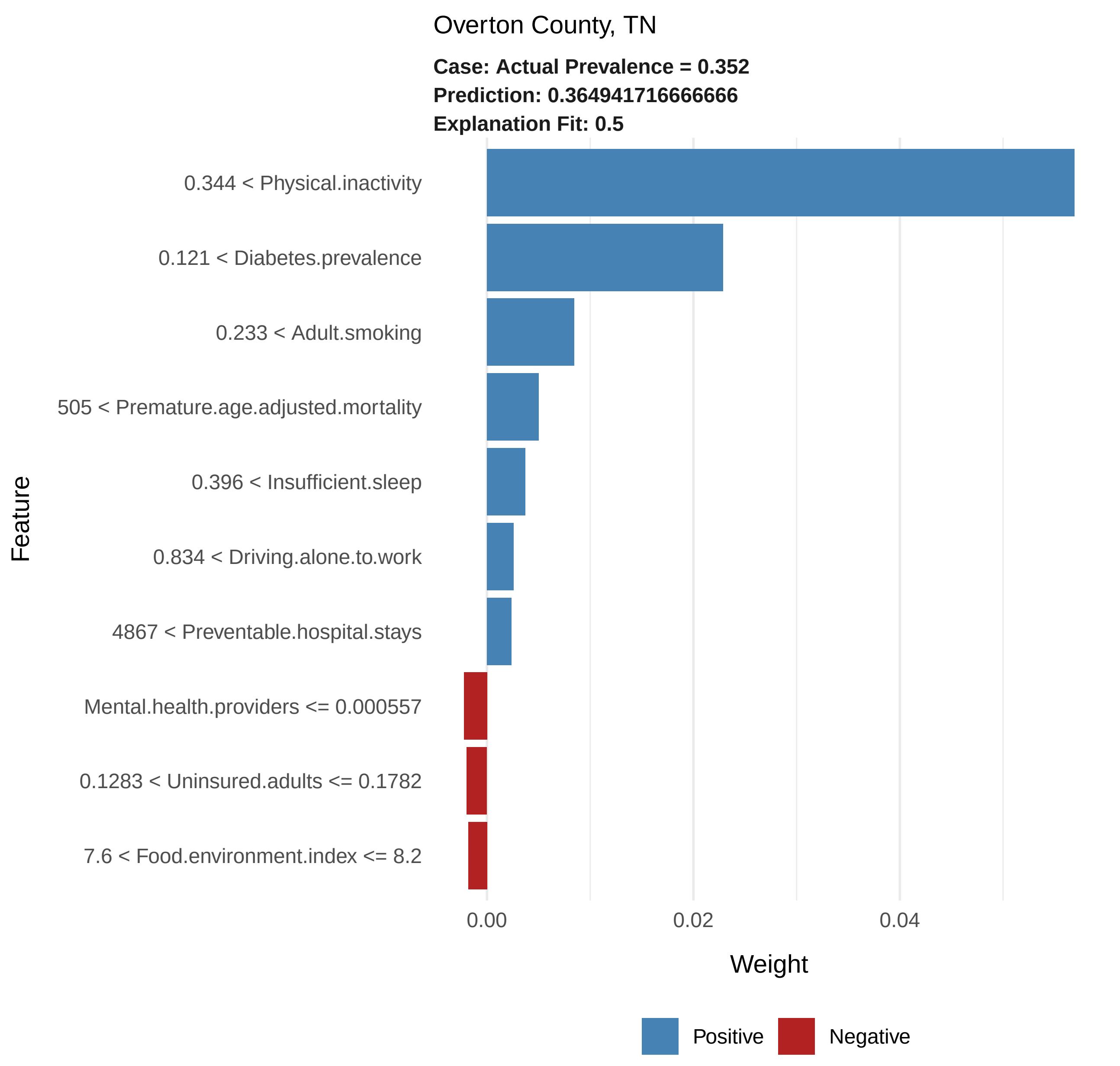

Overton County, TN
Case: Actual Prevalence = 0.352
Prediction: 0.364941716666666
Explanation Fit: 0.5
0.344 < Physical.inactivity
0.121 < Diabetes.prevalence
0.233 < Adult.smoking
505 < Premature.age.adjusted.mortality
0.396 < Insufficient.sleep
0.834 < Driving.alone.to.work
4867 < Preventable.hospital.stays
Mental.health.providers <= 0.000557
0.1283 < Uninsured.adults <= 0.1782
7.6 < Food.environment.index <= 8.2
Feature
0.00
0.02
0.04
Weight
Positive
Negative

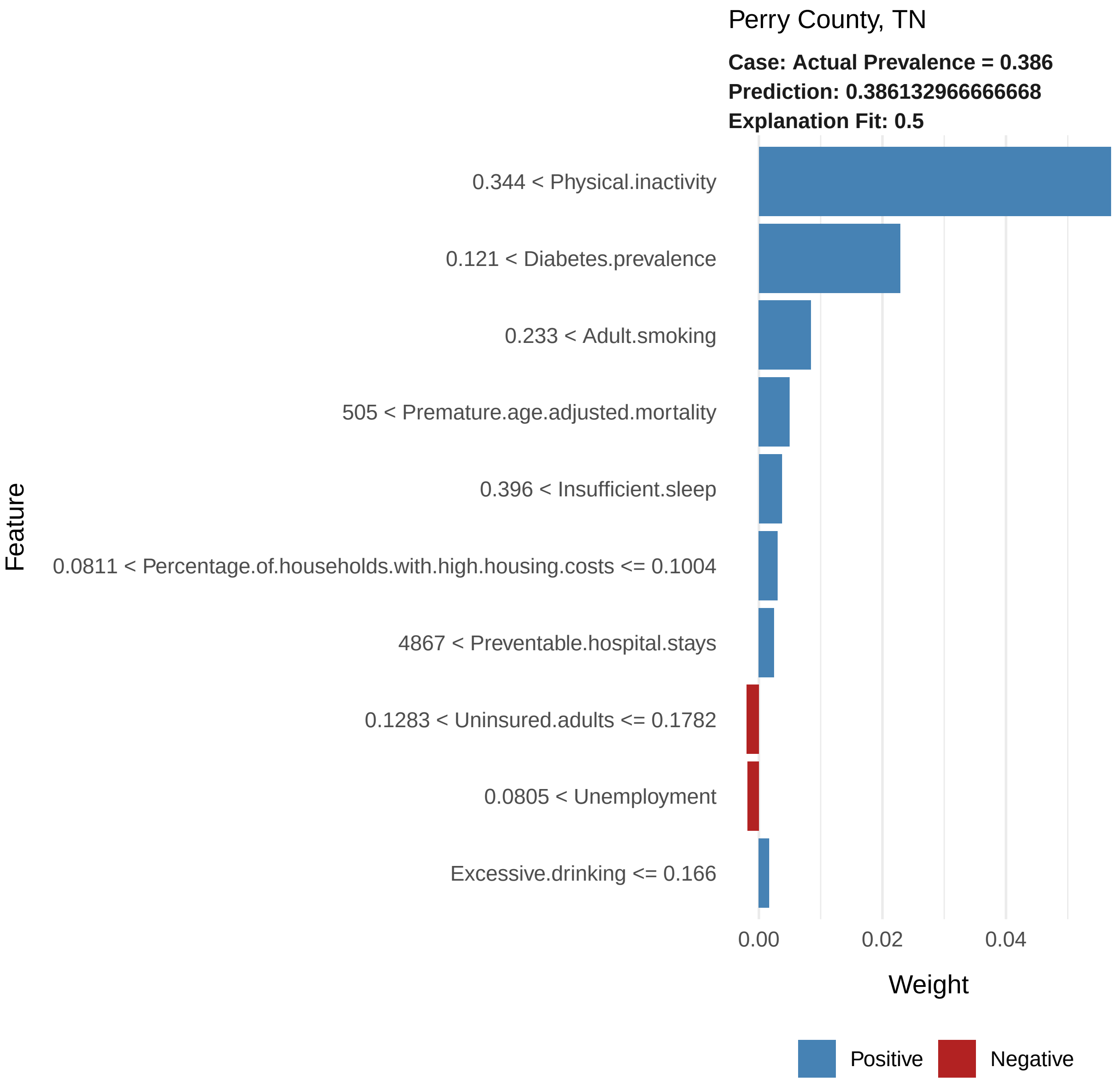

Perry County, TN
Case: Actual Prevalence = 0.386
Prediction: 0.386132966666668
Explanation Fit: 0.5
0.344 < Physical.inactivity
0.121 < Diabetes.prevalence
0.233 < Adult.smoking
505 < Premature.age.adjusted.mortality
0.396 < Insufficient.sleep
0.0811 < Percentage.of.households.with.high.housing.costs <= 0.1004
4867 < Preventable.hospital.stays
0.1283 < Uninsured.adults <= 0.1782
0.0805 < Unemployment
Excessive.drinking <= 0.166
Feature
0.00
0.02
0.04
Weight
Positive
Negative

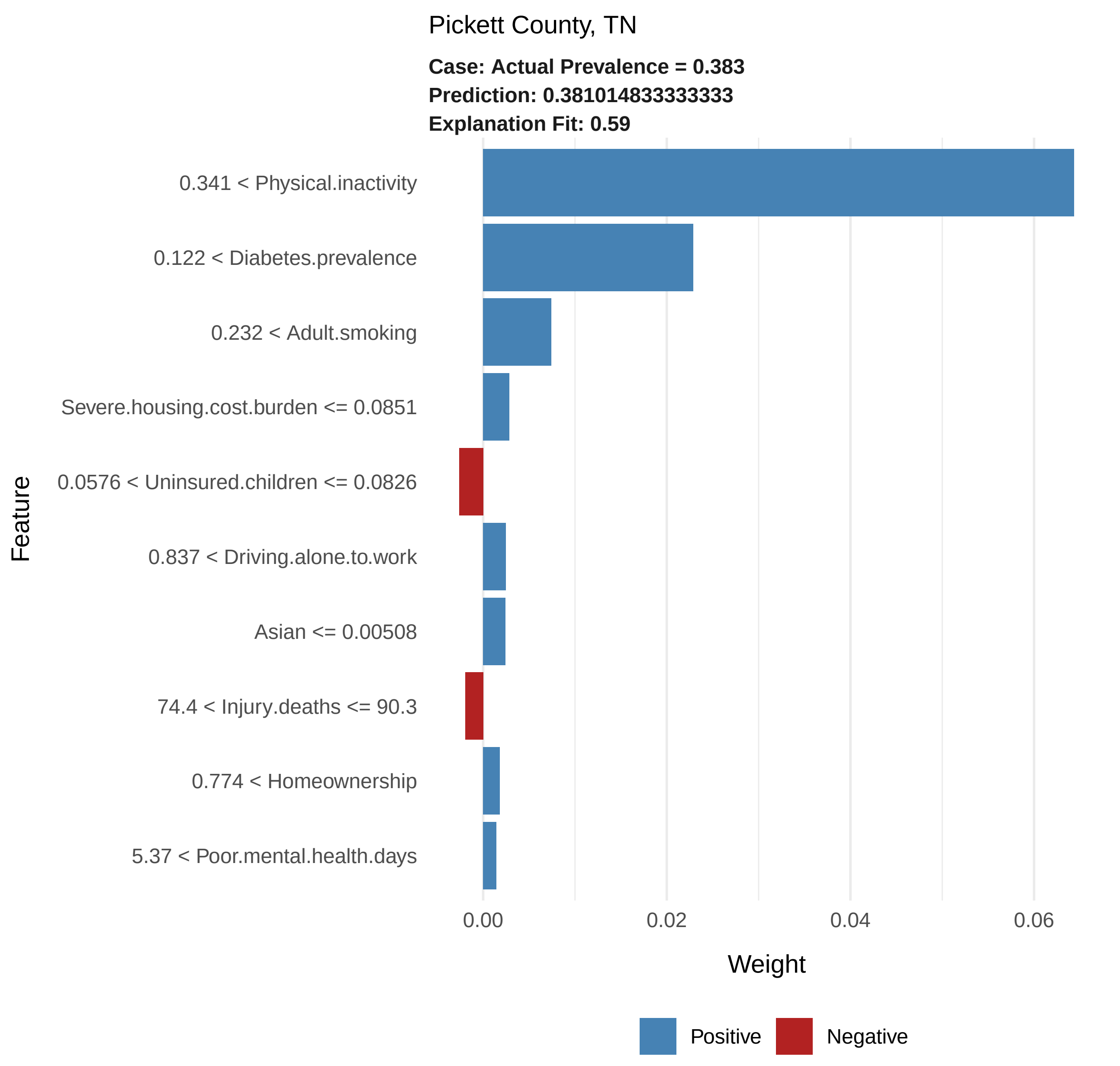
Pickett County, TN
Case: Actual Prevalence = 0.383
Prediction: 0.381014833333333
Explanation Fit: 0.59
Feature
0.341 < Physical.inactivity
0.122 < Diabetes.prevalence
0.232 < Adult.smoking
Severe.housing.cost.burden <= 0.0851
0.0576 < Uninsured.children <= 0.0826
0.837 < Driving.alone.to.work
Asian <= 0.00508
74.4 < Injury.deaths <= 90.3
0.774 < Homeownership
5.37 < Poor.mental.health.days
0.00
0.02
0.04
0.06
Weight
Positive
Negative

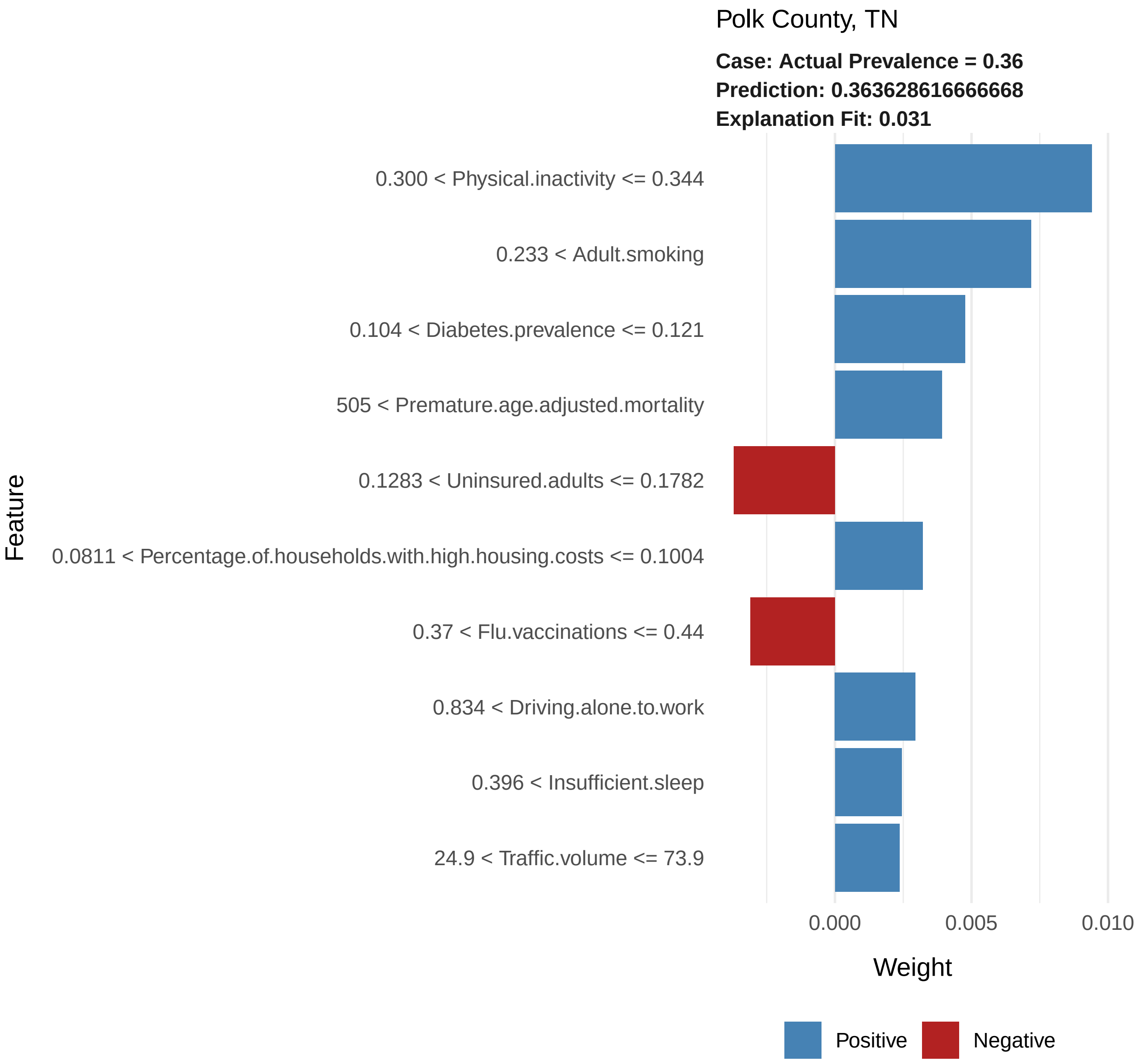

Polk County, TN
Case: Actual Prevalence = 0.36
Prediction: 0.363628616666668
Explanation Fit: 0.031
0.300 < Physical.inactivity <= 0.344
0.233 < Adult.smoking
0.104 < Diabetes.prevalence <= 0.121
505 < Premature.age.adjusted.mortality
0.1283 < Uninsured.adults <= 0.1782
0.0811 < Percentage.of.households.with.high.housing.costs <= 0.1004
0.37 < Flu.vaccinations <= 0.44
0.834 < Driving.alone.to.work
0.396 < Insufficient.sleep
24.9 < Traffic.volume <= 73.9
Feature
0.000
0.005
0.010
Weight
Positive
Negative

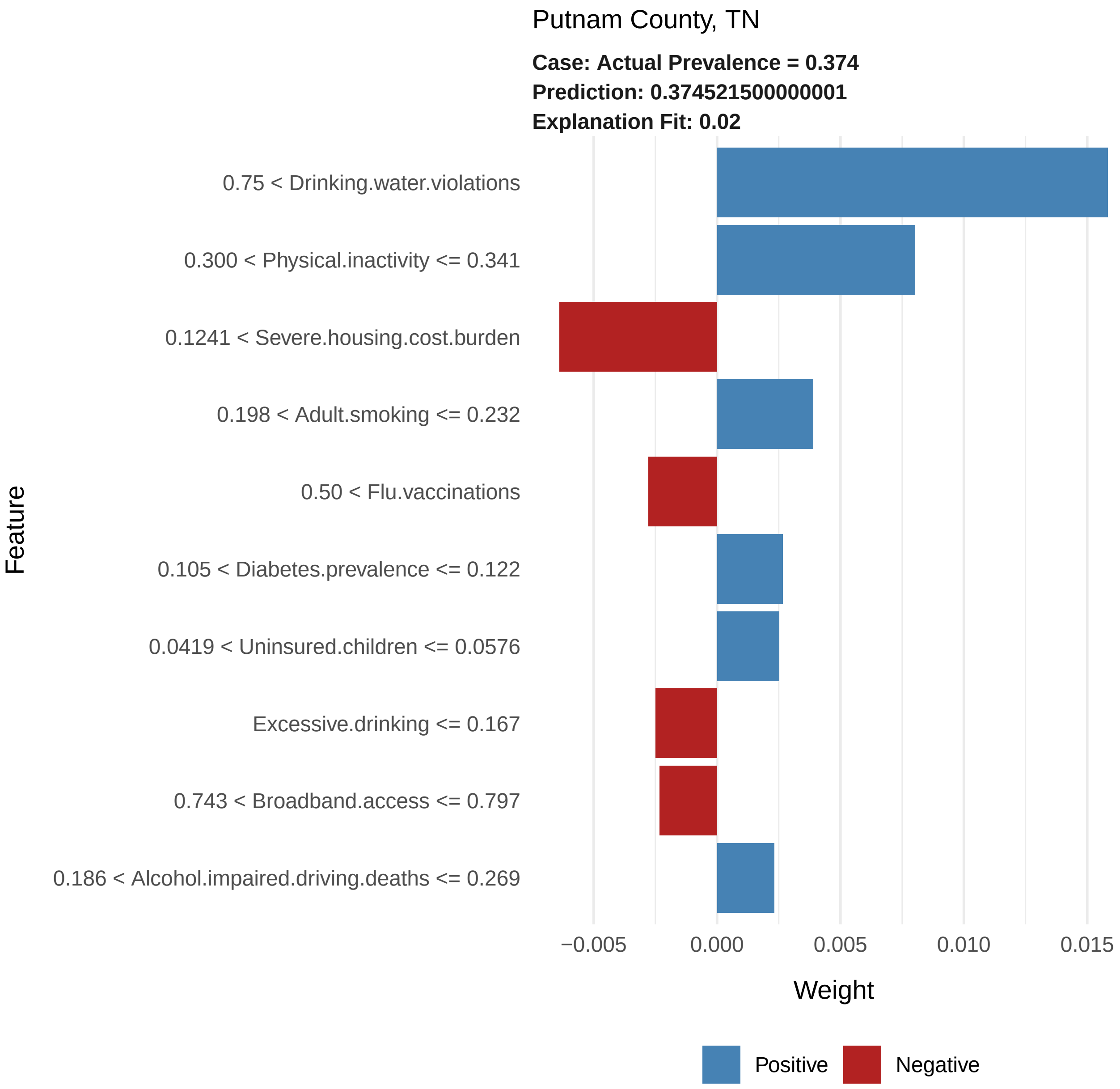

Putnam County, TN
Case: Actual Prevalence = 0.374
Prediction: 0.374521500000001
Explanation Fit: 0.02
0.75 < Drinking.water.violations
0.300 < Physical.inactivity <= 0.341
0.1241 < Severe.housing.cost.burden
0.198 < Adult.smoking <= 0.232
0.50 < Flu.vaccinations
0.105 < Diabetes.prevalence <= 0.122
0.0419 < Uninsured.children <= 0.0576
Excessive.drinking <= 0.167
0.743 < Broadband.access <= 0.797
0.186 < Alcohol.impaired.driving.deaths <= 0.269
Feature
−0.005
0.000
0.005
0.010
0.015
Weight
Positive
Negative

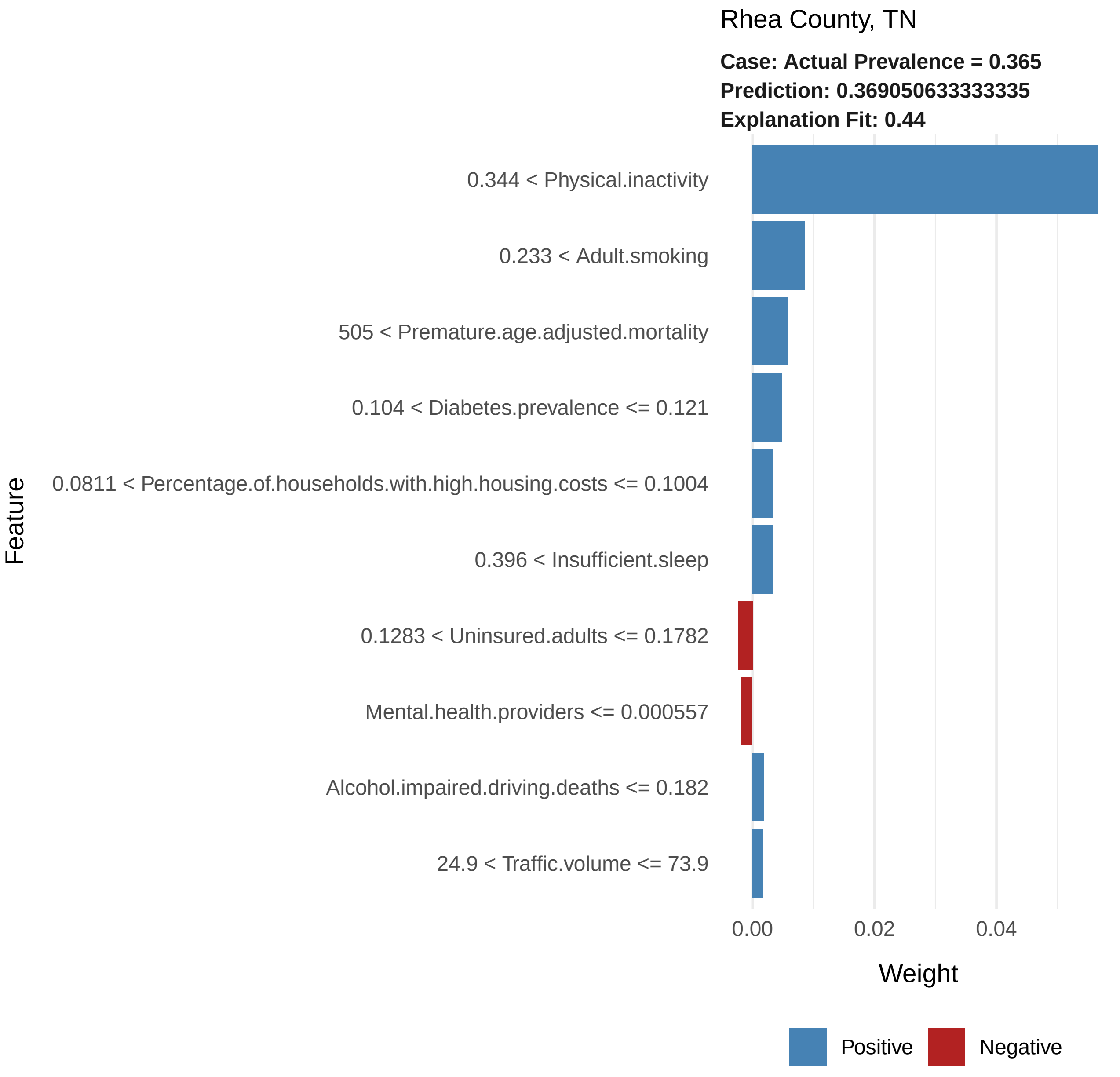

Rhea County, TN
Case: Actual Prevalence = 0.365
Prediction: 0.369050633333335
Explanation Fit: 0.44
0.344 < Physical.inactivity
0.233 < Adult.smoking
505 < Premature.age.adjusted.mortality
0.104 < Diabetes.prevalence <= 0.121
0.0811 < Percentage.of.households.with.high.housing.costs <= 0.1004
0.396 < Insufficient.sleep
0.1283 < Uninsured.adults <= 0.1782
Mental.health.providers <= 0.000557
Alcohol.impaired.driving.deaths <= 0.182
24.9 < Traffic.volume <= 73.9
Feature
0.00
0.02
0.04
Weight
Positive
Negative

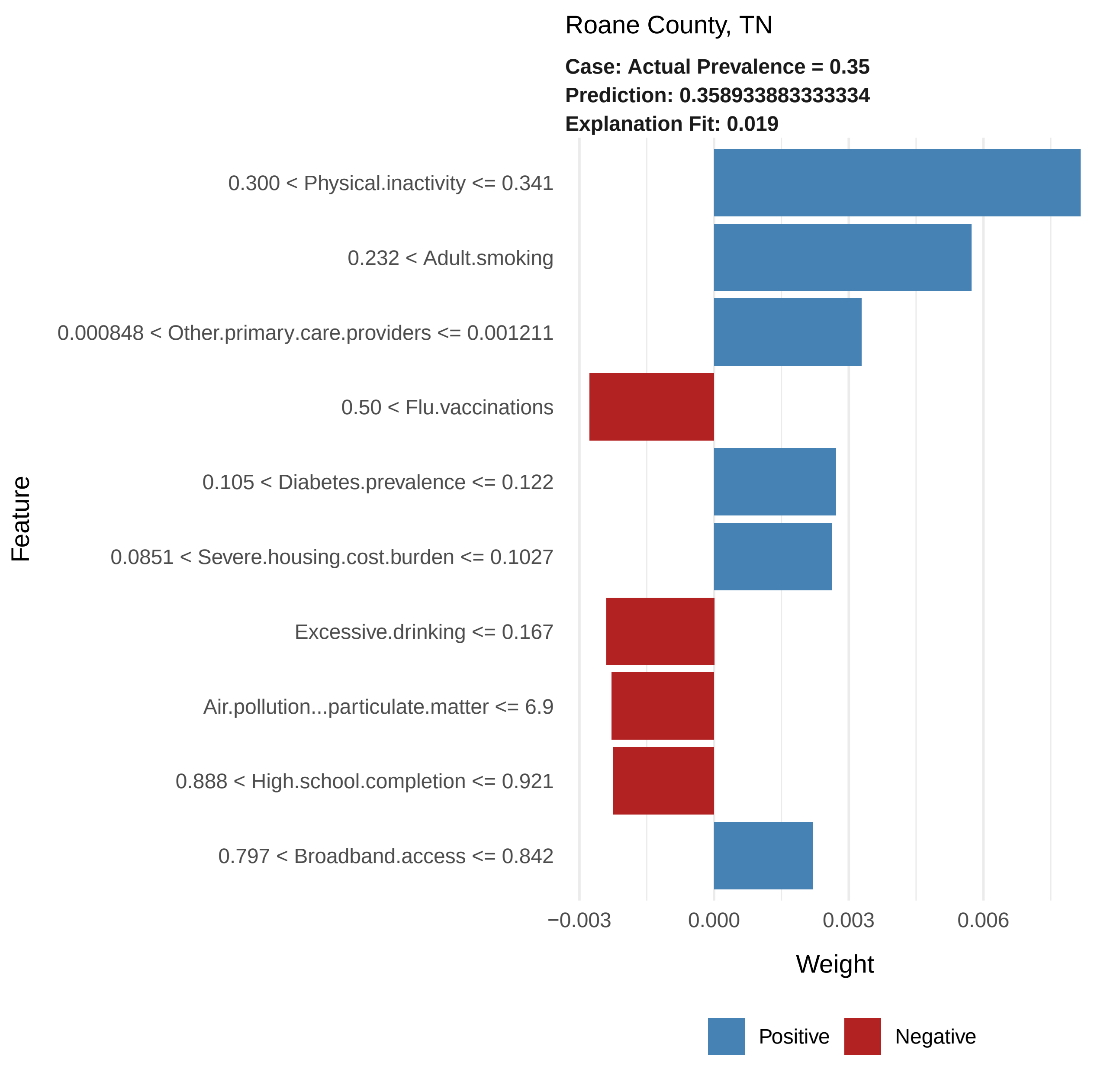

Roane County, TN
Case: Actual Prevalence = 0.35
Prediction: 0.358933883333334
Explanation Fit: 0.019
0.300 < Physical.inactivity <= 0.341
0.232 < Adult.smoking
0.000848 < Other.primary.care.providers <= 0.001211
0.50 < Flu.vaccinations
0.105 < Diabetes.prevalence <= 0.122
0.0851 < Severe.housing.cost.burden <= 0.1027
Excessive.drinking <= 0.167
Air.pollution...particulate.matter <= 6.9
0.888 < High.school.completion <= 0.921
0.797 < Broadband.access <= 0.842
Feature
−0.003
0.000
0.003
0.006
Weight
Positive
Negative

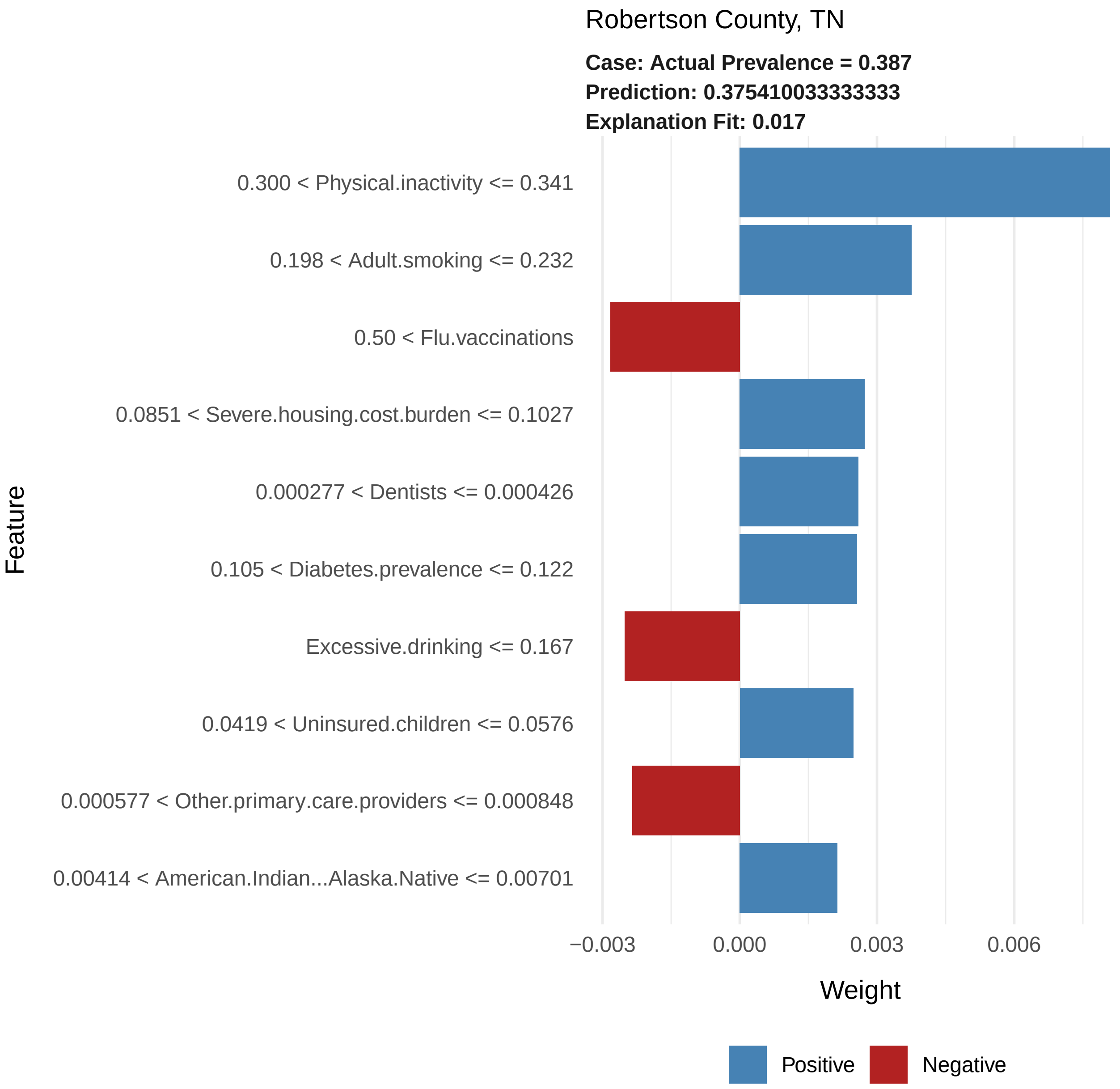

Robertson County, TN
Case: Actual Prevalence = 0.387
Prediction: 0.375410033333333
Explanation Fit: 0.017
0.300 < Physical.inactivity <= 0.341
0.198 < Adult.smoking <= 0.232
0.50 < Flu.vaccinations
0.0851 < Severe.housing.cost.burden <= 0.1027
0.000277 < Dentists <= 0.000426
0.105 < Diabetes.prevalence <= 0.122
Excessive.drinking <= 0.167
0.0419 < Uninsured.children <= 0.0576
0.000577 < Other.primary.care.providers <= 0.000848
0.00414 < American.Indian...Alaska.Native <= 0.00701
Feature
−0.003
0.000
0.003
0.006
Weight
Positive
Negative

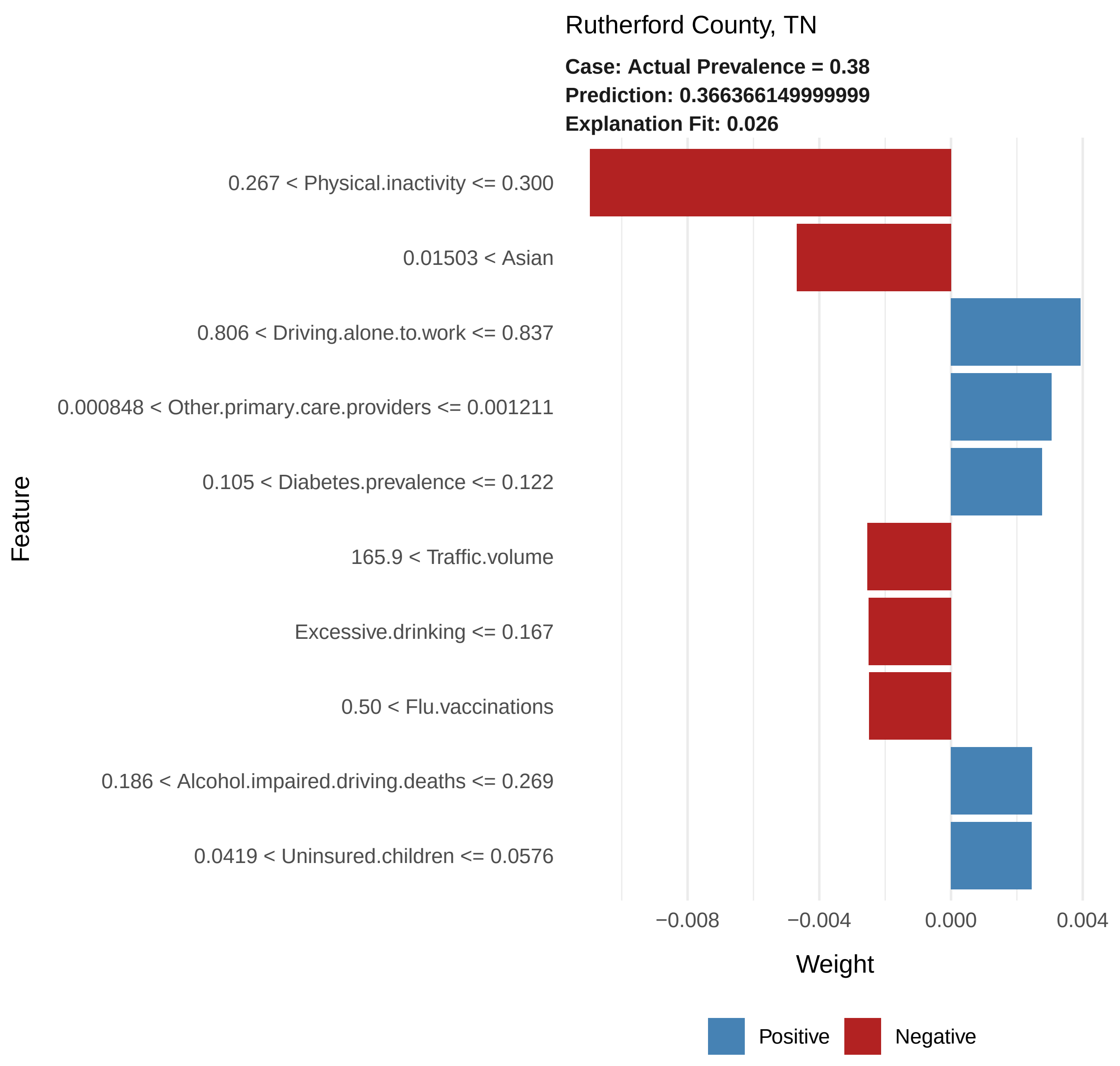
Rutherford County, TN
Case: Actual Prevalence = 0.38
Prediction: 0.366366149999999
Explanation Fit: 0.026
0.267 < Physical.inactivity <= 0.300
0.01503 < Asian
0.806 < Driving.alone.to.work <= 0.837
0.000848 < Other.primary.care.providers <= 0.001211
0.105 < Diabetes.prevalence <= 0.122
165.9 < Traffic.volume
Excessive.drinking <= 0.167
0.50 < Flu.vaccinations
0.186 < Alcohol.impaired.driving.deaths <= 0.269
0.0419 < Uninsured.children <= 0.0576
Feature
−0.008
−0.004
0.000
0.004
Weight
Positive
Negative

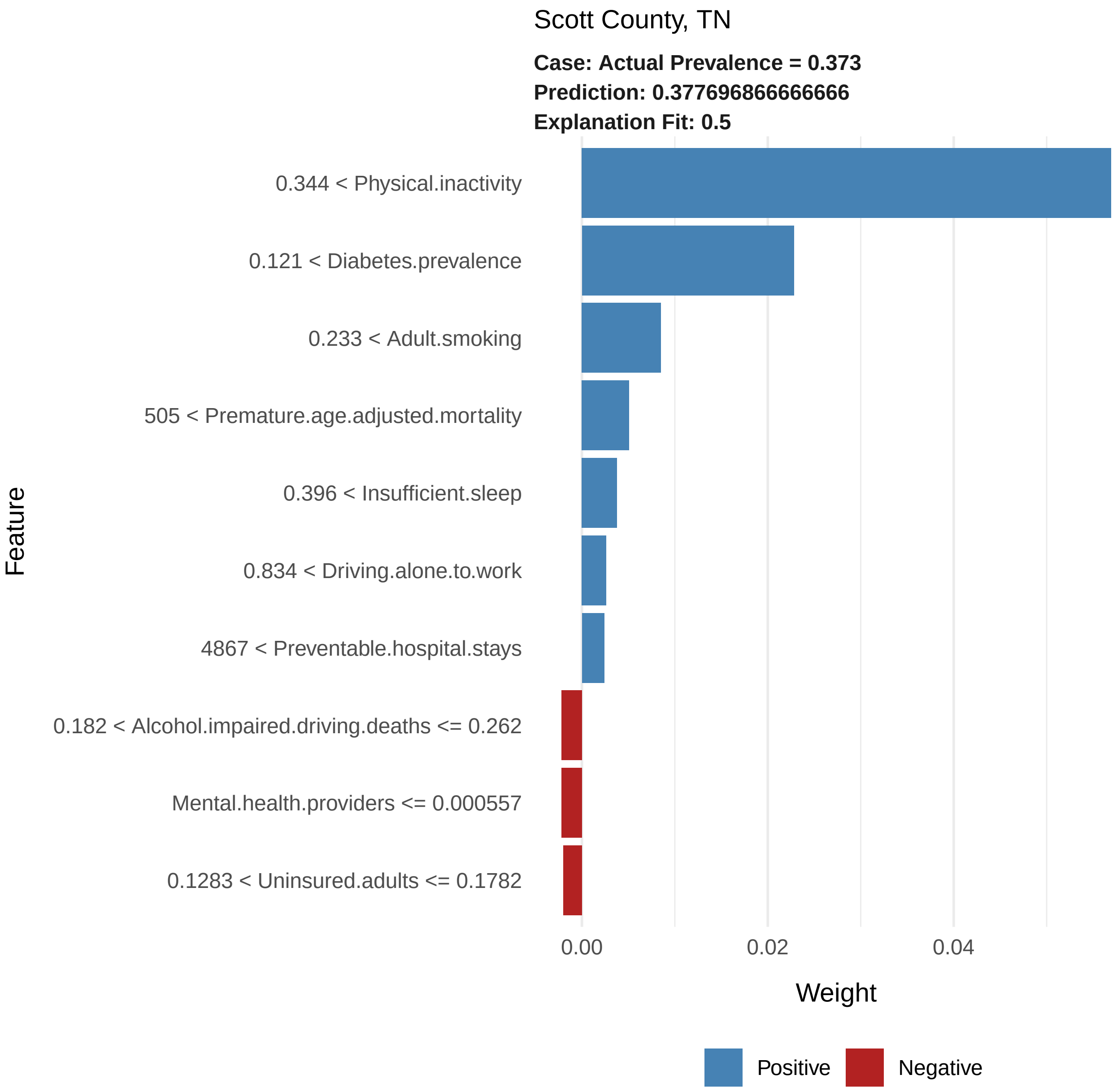

Scott County, TN
Case: Actual Prevalence = 0.373
Prediction: 0.377696866666666
Explanation Fit: 0.5
0.344 < Physical.inactivity
0.121 < Diabetes.prevalence
0.233 < Adult.smoking
505 < Premature.age.adjusted.mortality
0.396 < Insufficient.sleep
0.834 < Driving.alone.to.work
4867 < Preventable.hospital.stays
0.182 < Alcohol.impaired.driving.deaths <= 0.262
Mental.health.providers <= 0.000557
0.1283 < Uninsured.adults <= 0.1782
Feature
0.00
0.02
0.04
Weight
Positive
Negative

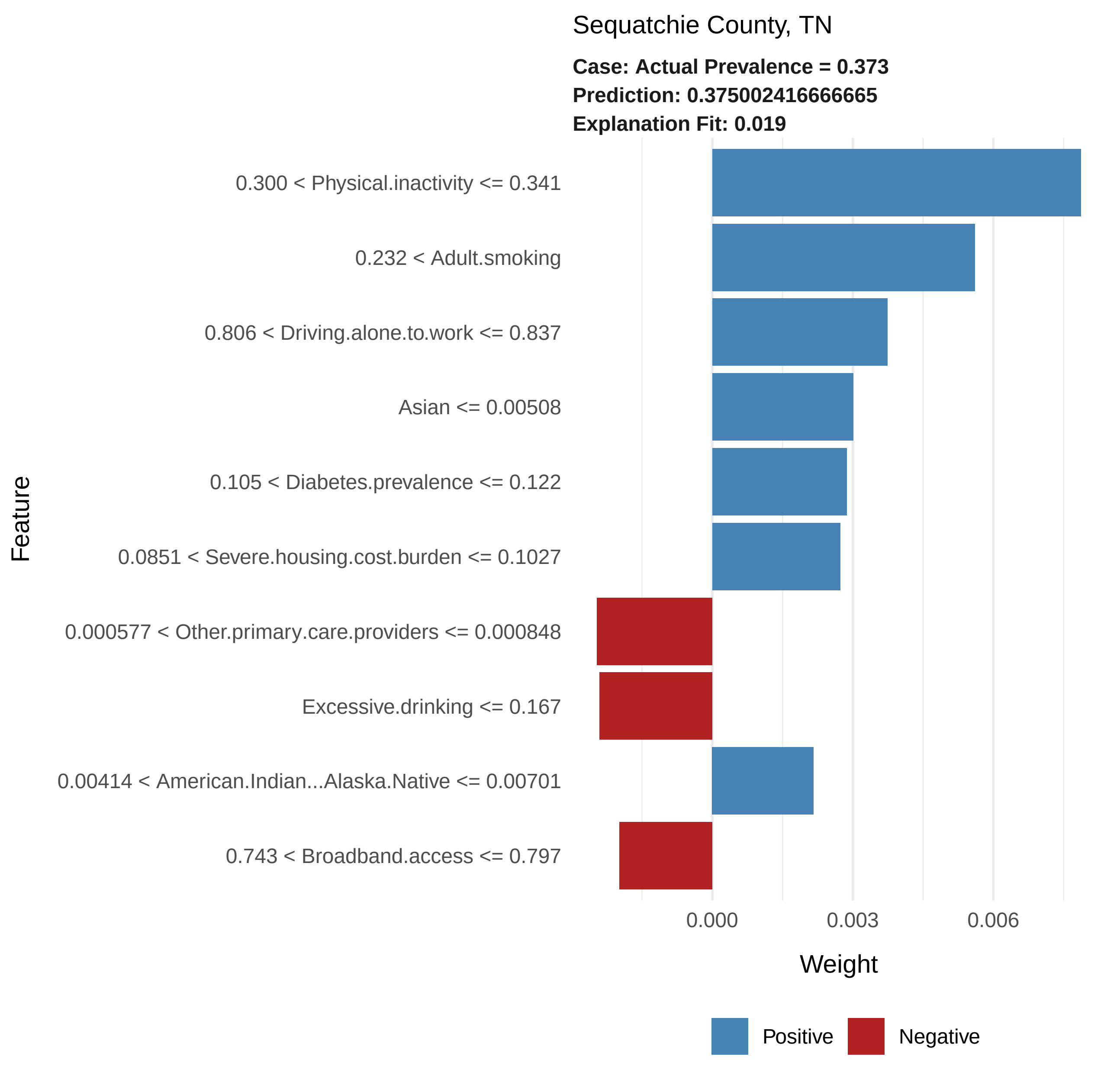

Sequatchie County, TN
Case: Actual Prevalence = 0.373
Prediction: 0.375002416666665
Explanation Fit: 0.019
0.300 < Physical.inactivity <= 0.341
0.232 < Adult.smoking
0.806 < Driving.alone.to.work <= 0.837
Asian <= 0.00508
0.105 < Diabetes.prevalence <= 0.122
0.0851 < Severe.housing.cost.burden <= 0.1027
0.000577 < Other.primary.care.providers <= 0.000848
Excessive.drinking <= 0.167
0.00414 < American.Indian...Alaska.Native <= 0.00701
0.743 < Broadband.access <= 0.797
Feature
0.000
0.003
0.006
Weight
Positive
Negative

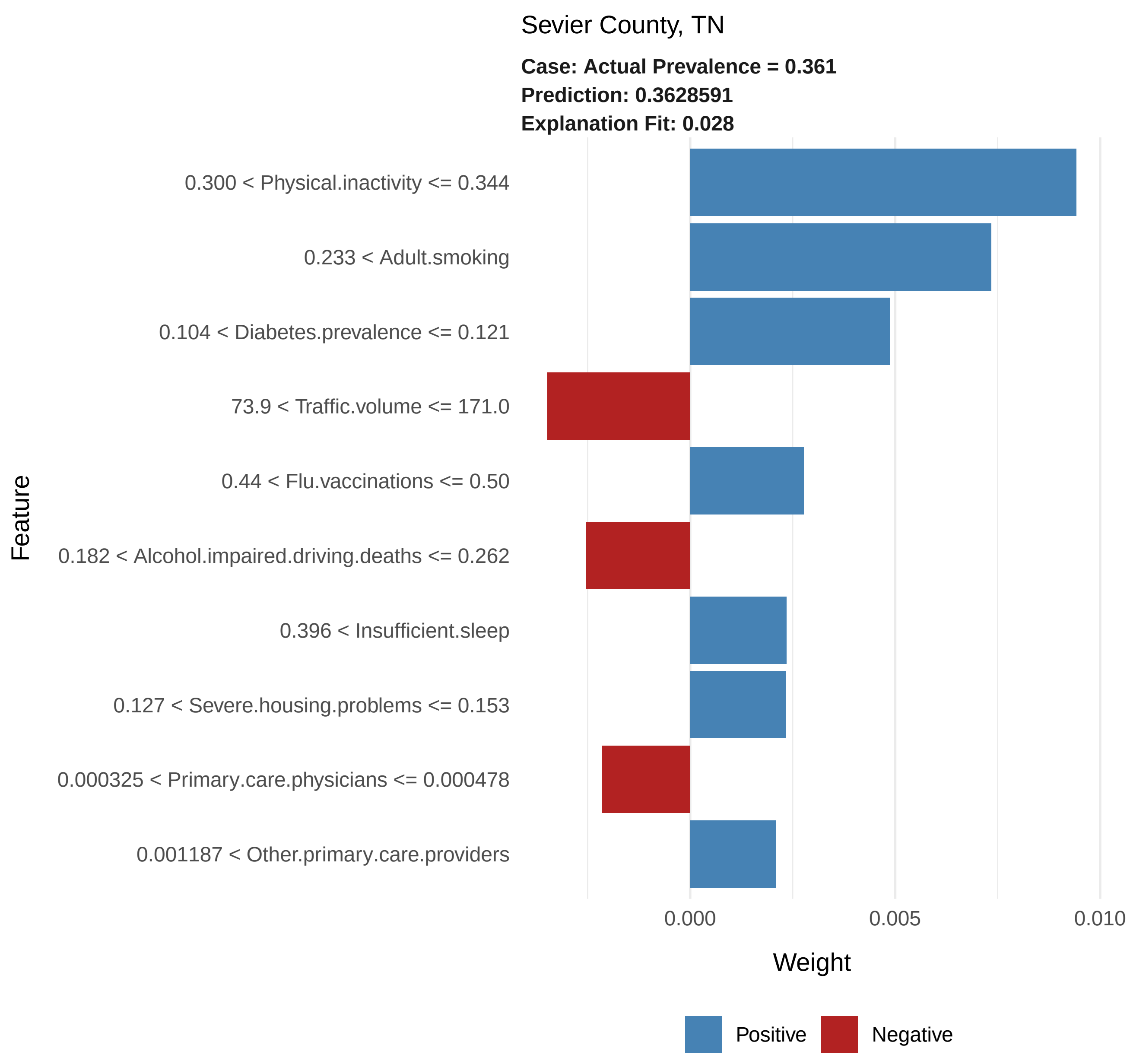
Sevier County, TN
Case: Actual Prevalence = 0.361
Prediction: 0.3628591
Explanation Fit: 0.028
0.300 < Physical.inactivity <= 0.344
0.233 < Adult.smoking
0.104 < Diabetes.prevalence <= 0.121
73.9 < Traffic.volume <= 171.0
0.44 < Flu.vaccinations <= 0.50
0.182 < Alcohol.impaired.driving.deaths <= 0.262
0.396 < Insufficient.sleep
0.127 < Severe.housing.problems <= 0.153
0.000325 < Primary.care.physicians <= 0.000478
0.001187 < Other.primary.care.providers
Feature
0.000
0.005
0.010
Weight
Positive
Negative

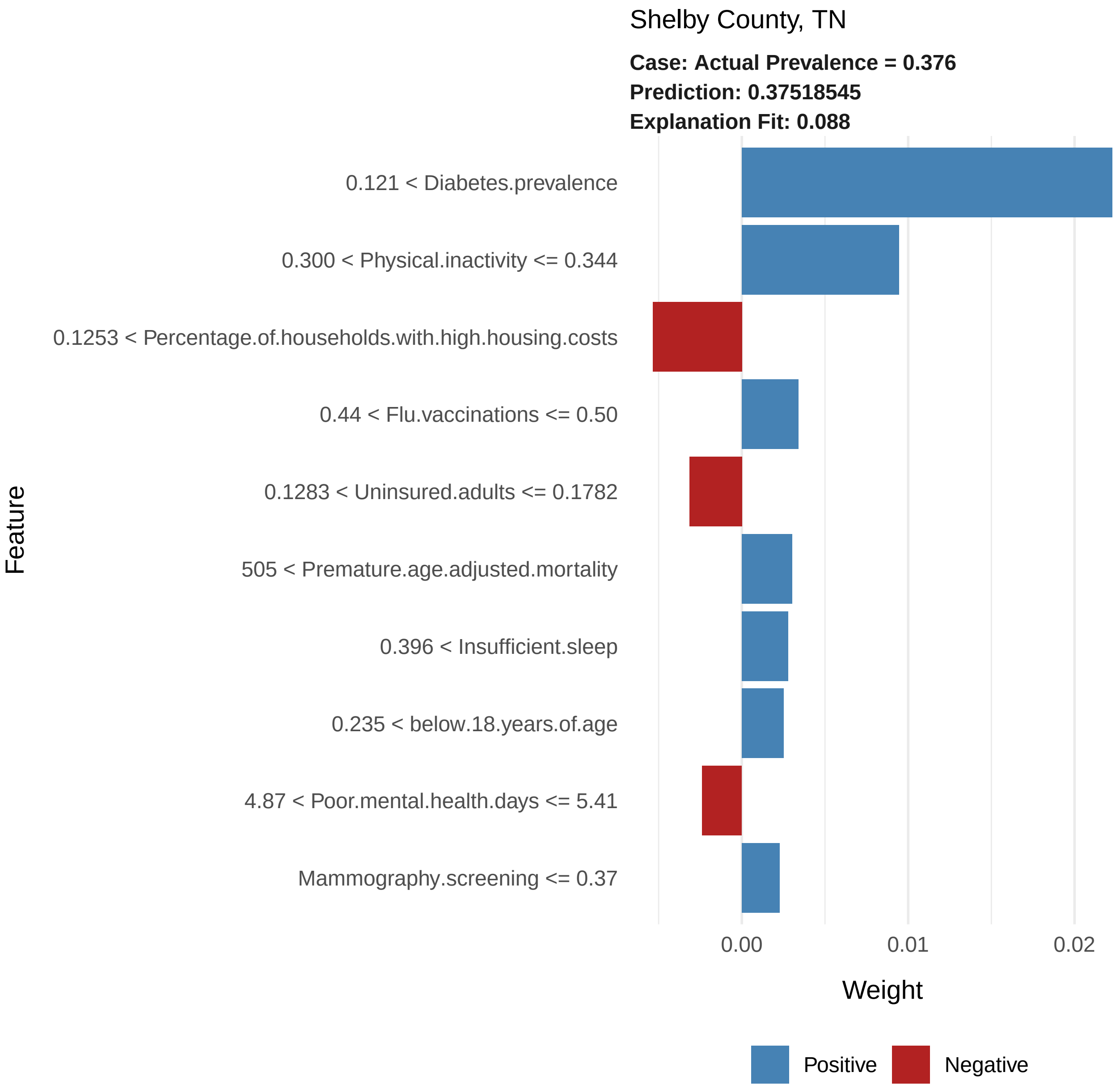
Shelby County, TN
Case: Actual Prevalence = 0.376
Prediction: 0.37518545
Explanation Fit: 0.088
0.121 < Diabetes.prevalence
0.300 < Physical.inactivity <= 0.344
0.1253 < Percentage.of.households.with.high.housing.costs
0.44 < Flu.vaccinations <= 0.50
0.1283 < Uninsured.adults <= 0.1782
505 < Premature.age.adjusted.mortality
0.396 < Insufficient.sleep
0.235 < below.18.years.of.age
4.87 < Poor.mental.health.days <= 5.41
Mammography.screening <= 0.37
Feature
0.00
0.01
0.02
Weight
Positive
Negative

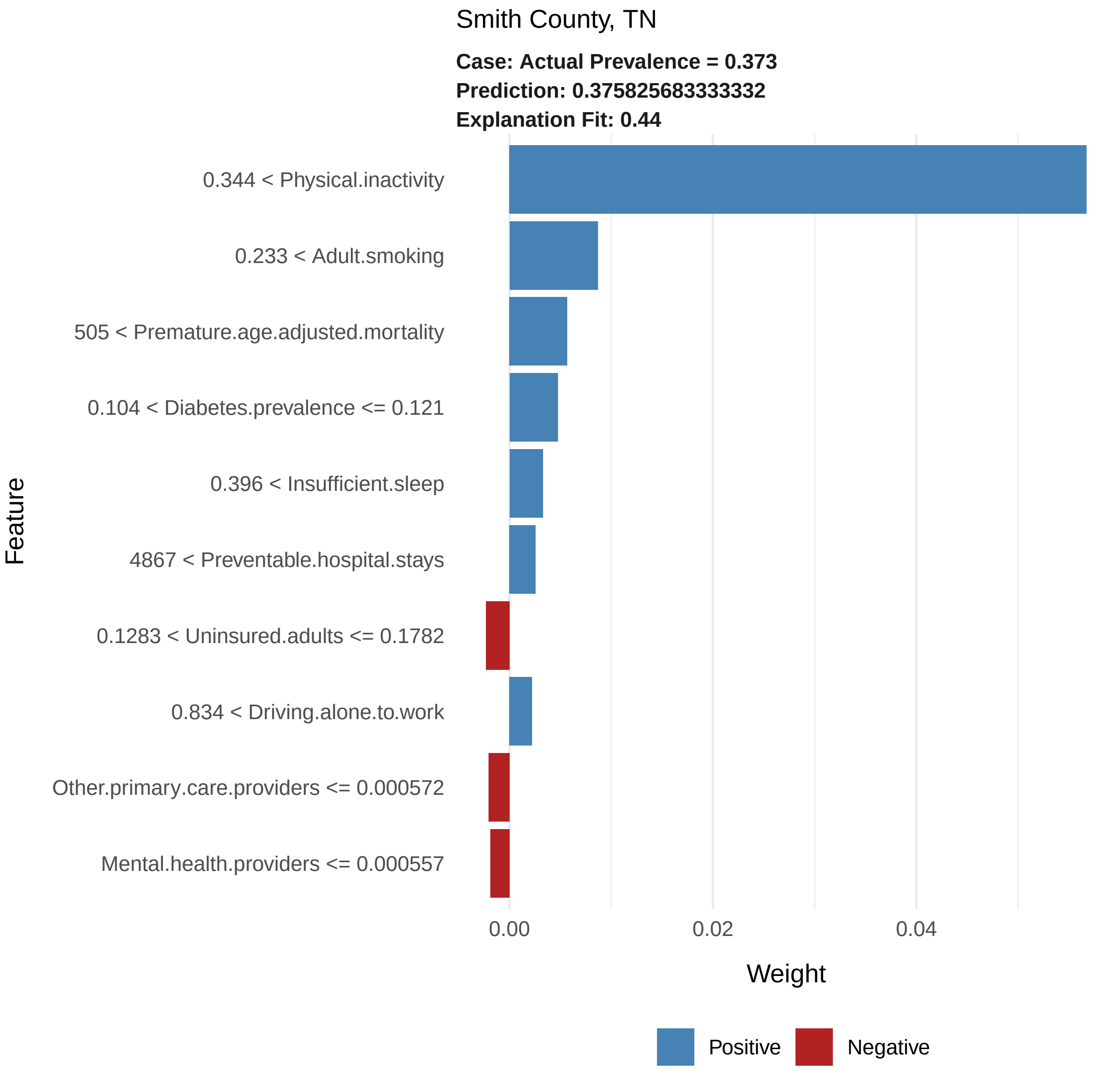
Smith County, TN
Case: Actual Prevalence = 0.373
Prediction: 0.375825683333332
Explanation Fit: 0.44
0.344 < Physical.inactivity
0.233 < Adult.smoking
505 < Premature.age.adjusted.mortality
0.104 < Diabetes.prevalence <= 0.121
0.396 < Insufficient.sleep
4867 < Preventable.hospital.stays
0.1283 < Uninsured.adults <= 0.1782
0.834 < Driving.alone.to.work
Other.primary.care.providers <= 0.000572
Mental.health.providers <= 0.000557
Feature
0.00
0.02
0.04
Weight
Positive
Negative

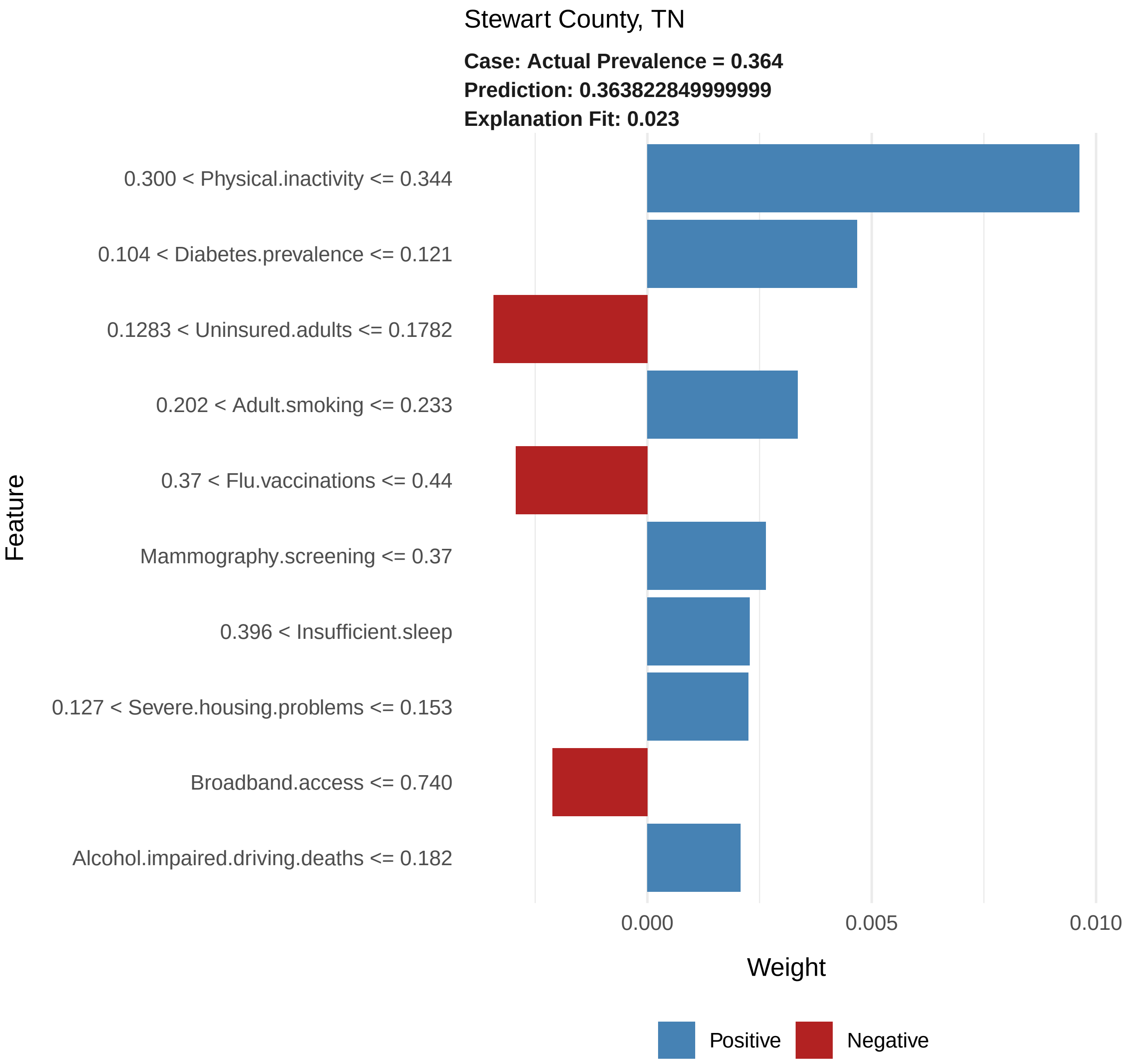

Stewart County, TN
Case: Actual Prevalence = 0.364
Prediction: 0.363822849999999
Explanation Fit: 0.023
0.300 < Physical.inactivity <= 0.344
0.104 < Diabetes.prevalence <= 0.121
0.1283 < Uninsured.adults <= 0.1782
0.202 < Adult.smoking <= 0.233
0.37 < Flu.vaccinations <= 0.44
Mammography.screening <= 0.37
0.396 < Insufficient.sleep
0.127 < Severe.housing.problems <= 0.153
Broadband.access <= 0.740
Alcohol.impaired.driving.deaths <= 0.182
Feature
0.000
0.005
0.010
Weight
Positive
Negative

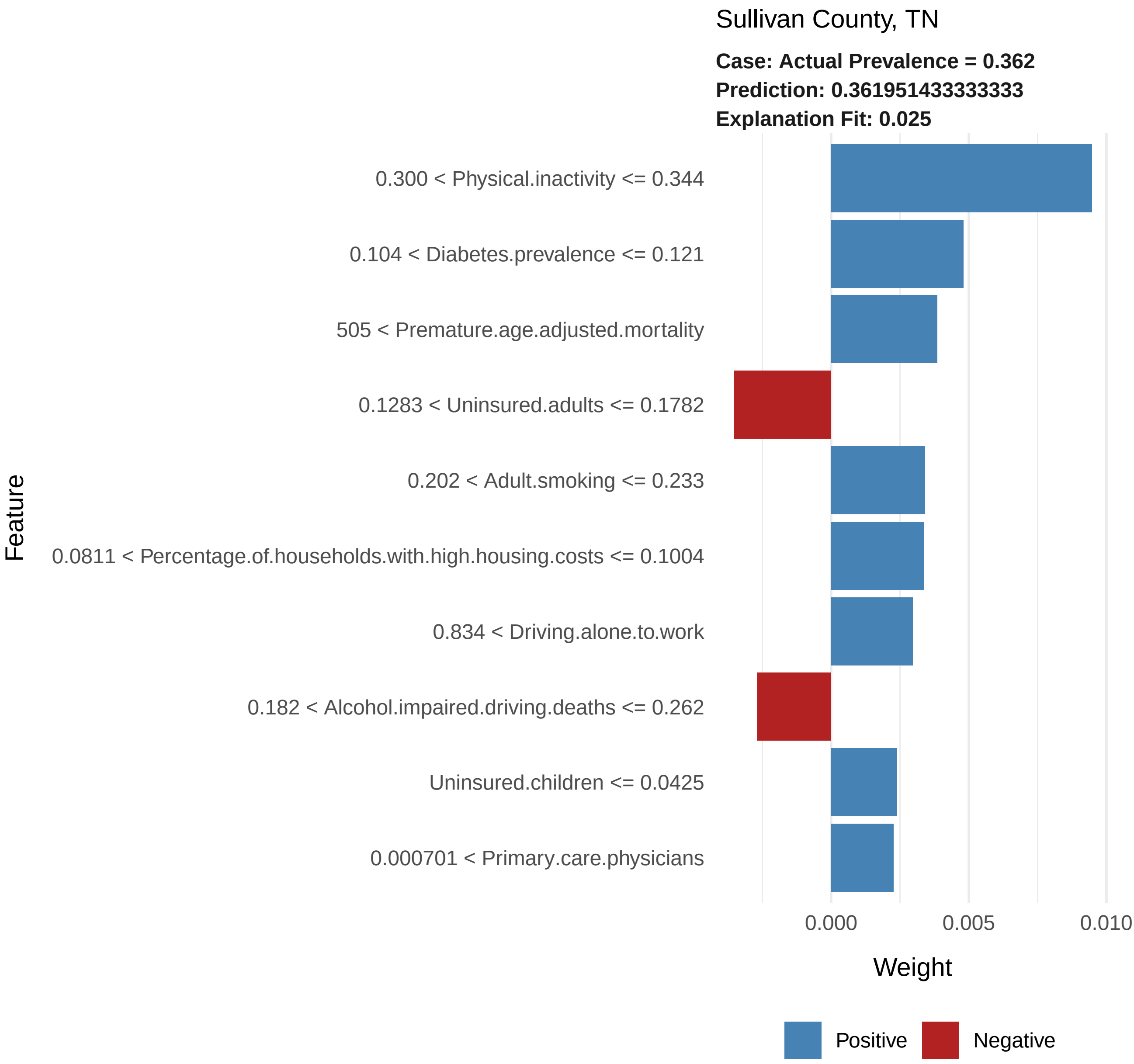

Sullivan County, TN
Case: Actual Prevalence = 0.362
Prediction: 0.361951433333333
Explanation Fit: 0.025
0.300 < Physical.inactivity <= 0.344
0.104 < Diabetes.prevalence <= 0.121
505 < Premature.age.adjusted.mortality
0.1283 < Uninsured.adults <= 0.1782
0.202 < Adult.smoking <= 0.233
0.0811 < Percentage.of.households.with.high.housing.costs <= 0.1004
0.834 < Driving.alone.to.work
0.182 < Alcohol.impaired.driving.deaths <= 0.262
Uninsured.children <= 0.0425
0.000701 < Primary.care.physicians
Feature
0.000
0.005
0.010
Weight
Positive
Negative

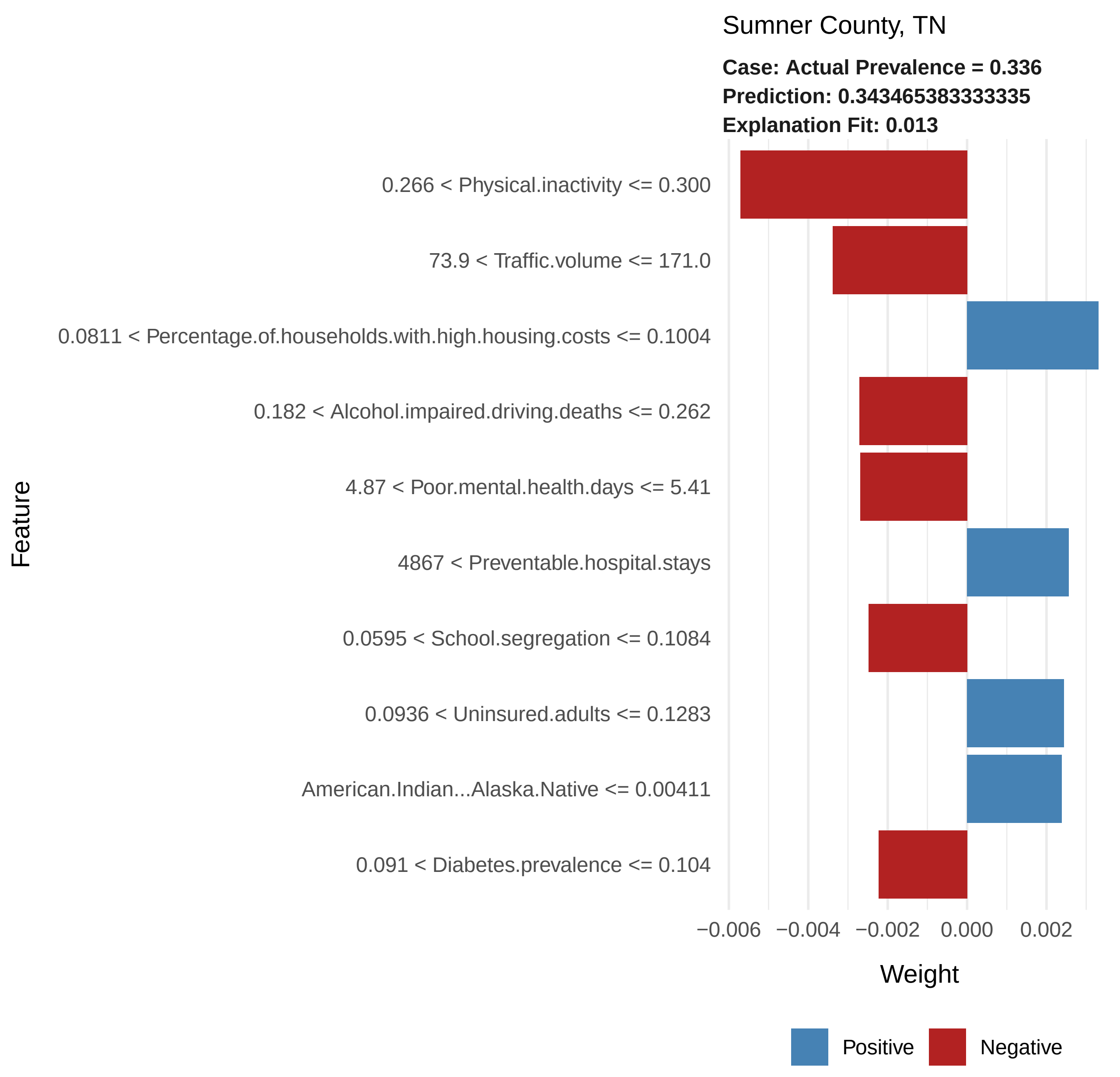

Sumner County, TN
Case: Actual Prevalence = 0.336
Prediction: 0.343465383333335
Explanation Fit: 0.013
0.266 < Physical.inactivity <= 0.300
73.9 < Traffic.volume <= 171.0
0.0811 < Percentage.of.households.with.high.housing.costs <= 0.1004
0.182 < Alcohol.impaired.driving.deaths <= 0.262
4.87 < Poor.mental.health.days <= 5.41
4867 < Preventable.hospital.stays
0.0595 < School.segregation <= 0.1084
0.0936 < Uninsured.adults <= 0.1283
American.Indian...Alaska.Native <= 0.00411
0.091 < Diabetes.prevalence <= 0.104
Feature
−0.006
−0.004
−0.002
0.000
0.002
Weight
Positive
Negative

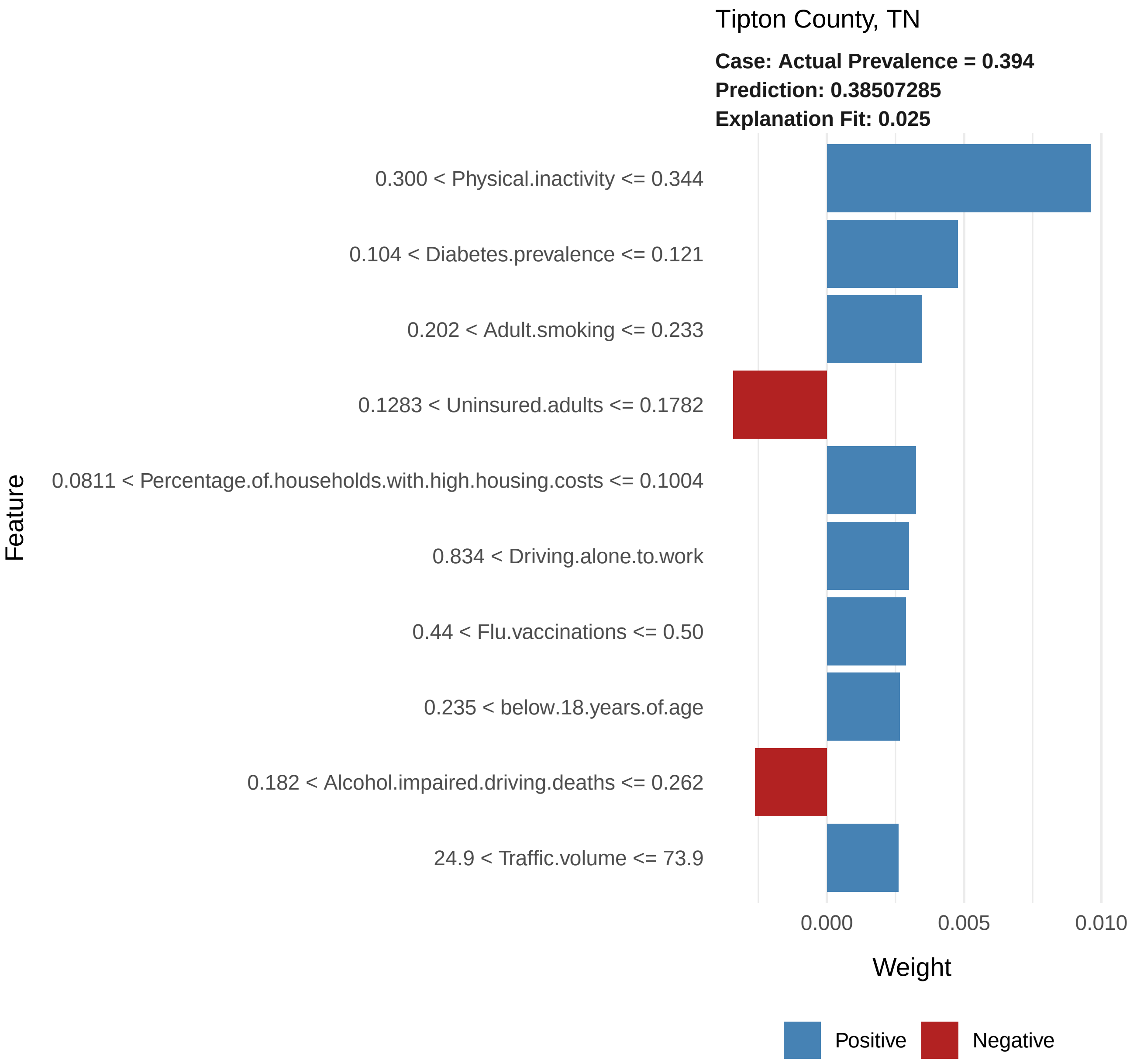

Tipton County, TN
Case: Actual Prevalence = 0.394
Prediction: 0.38507285
Explanation Fit: 0.025
0.300 < Physical.inactivity <= 0.344
0.104 < Diabetes.prevalence <= 0.121
0.202 < Adult.smoking <= 0.233
0.1283 < Uninsured.adults <= 0.1782
0.0811 < Percentage.of.households.with.high.housing.costs <= 0.1004
0.834 < Driving.alone.to.work
0.44 < Flu.vaccinations <= 0.50
0.235 < below.18.years.of.age
0.182 < Alcohol.impaired.driving.deaths <= 0.262
24.9 < Traffic.volume <= 73.9
Feature
0.000
0.005
0.010
Weight
Positive
Negative

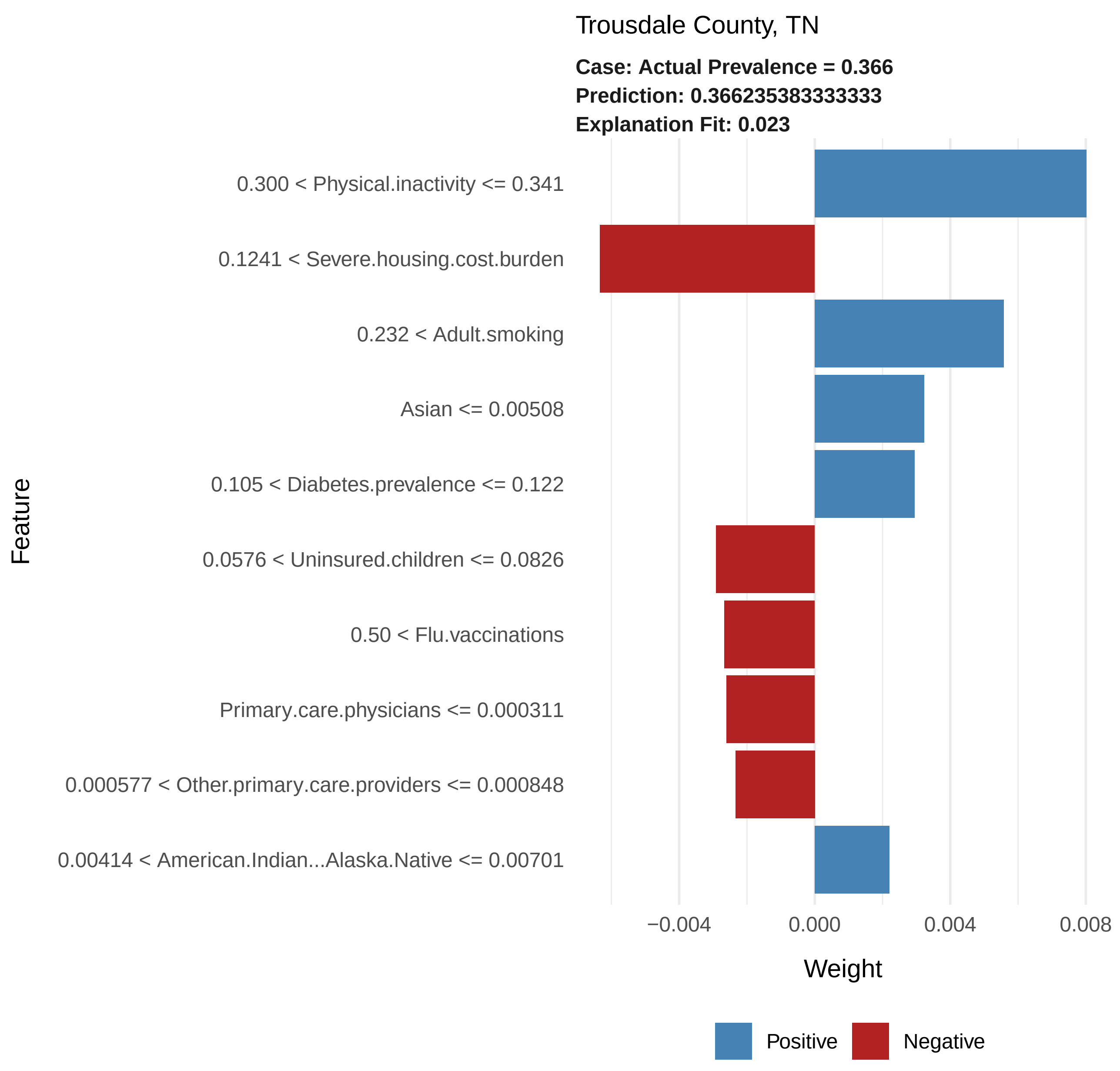

Trousdale County, TN
Case: Actual Prevalence = 0.366
Prediction: 0.366235383333333
Explanation Fit: 0.023
0.300 < Physical.inactivity <= 0.341
0.1241 < Severe.housing.cost.burden
0.232 < Adult.smoking
Asian <= 0.00508
0.105 < Diabetes.prevalence <= 0.122
0.0576 < Uninsured.children <= 0.0826
0.50 < Flu.vaccinations
Primary.care.physicians <= 0.000311
0.000577 < Other.primary.care.providers <= 0.000848
0.00414 < American.Indian...Alaska.Native <= 0.00701
Feature
−0.004
0.000
0.004
0.008
Weight
Positive
Negative

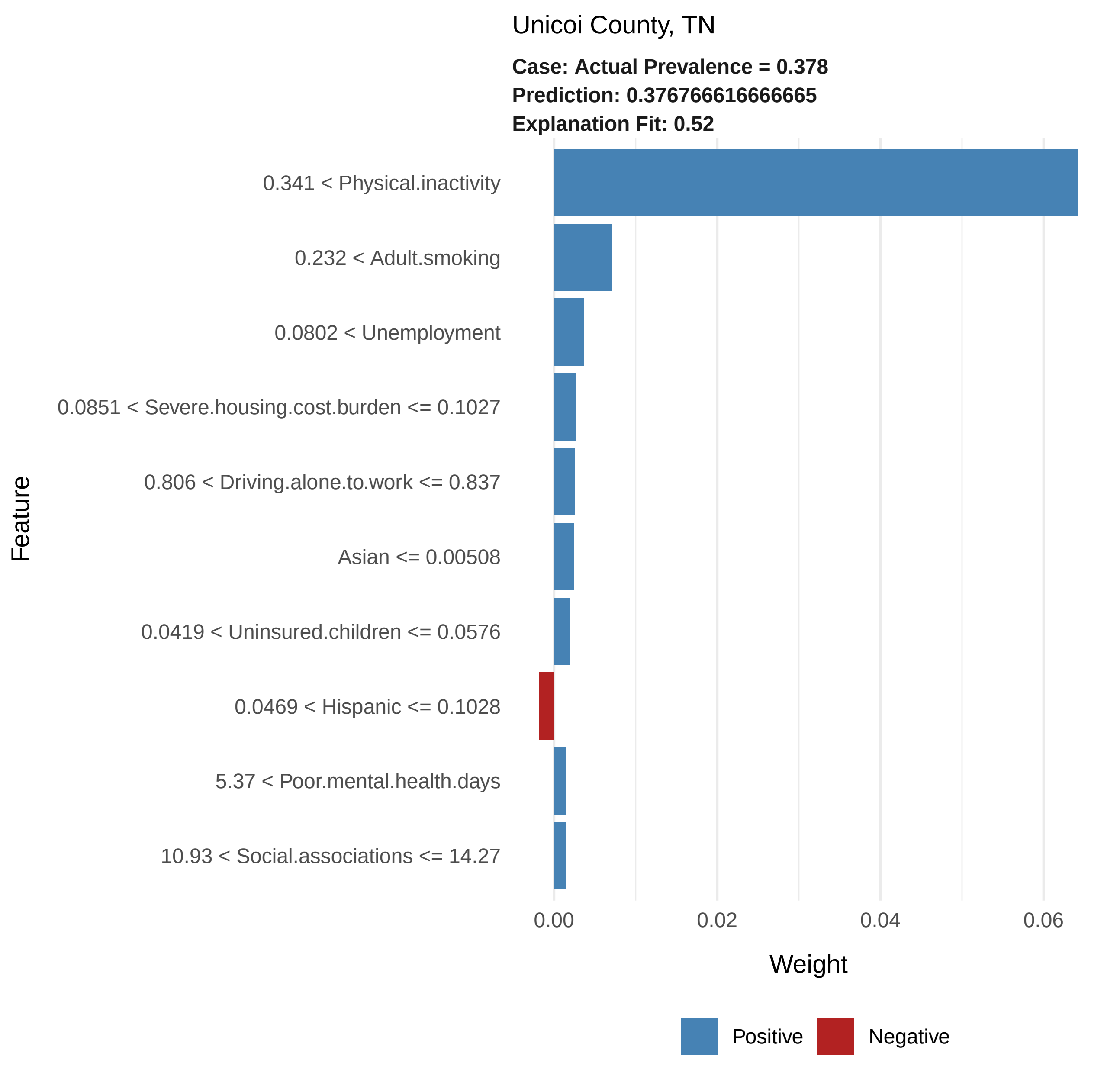

Unicoi County, TN
Case: Actual Prevalence = 0.378
Prediction: 0.376766616666665
Explanation Fit: 0.52
0.341 < Physical.inactivity
0.232 < Adult.smoking
0.0802 < Unemployment
0.0851 < Severe.housing.cost.burden <= 0.1027
0.806 < Driving.alone.to.work <= 0.837
Asian <= 0.00508
0.0419 < Uninsured.children <= 0.0576
0.0469 < Hispanic <= 0.1028
5.37 < Poor.mental.health.days
10.93 < Social.associations <= 14.27
Feature
0.00
0.02
0.04
0.06
Weight
Positive
Negative

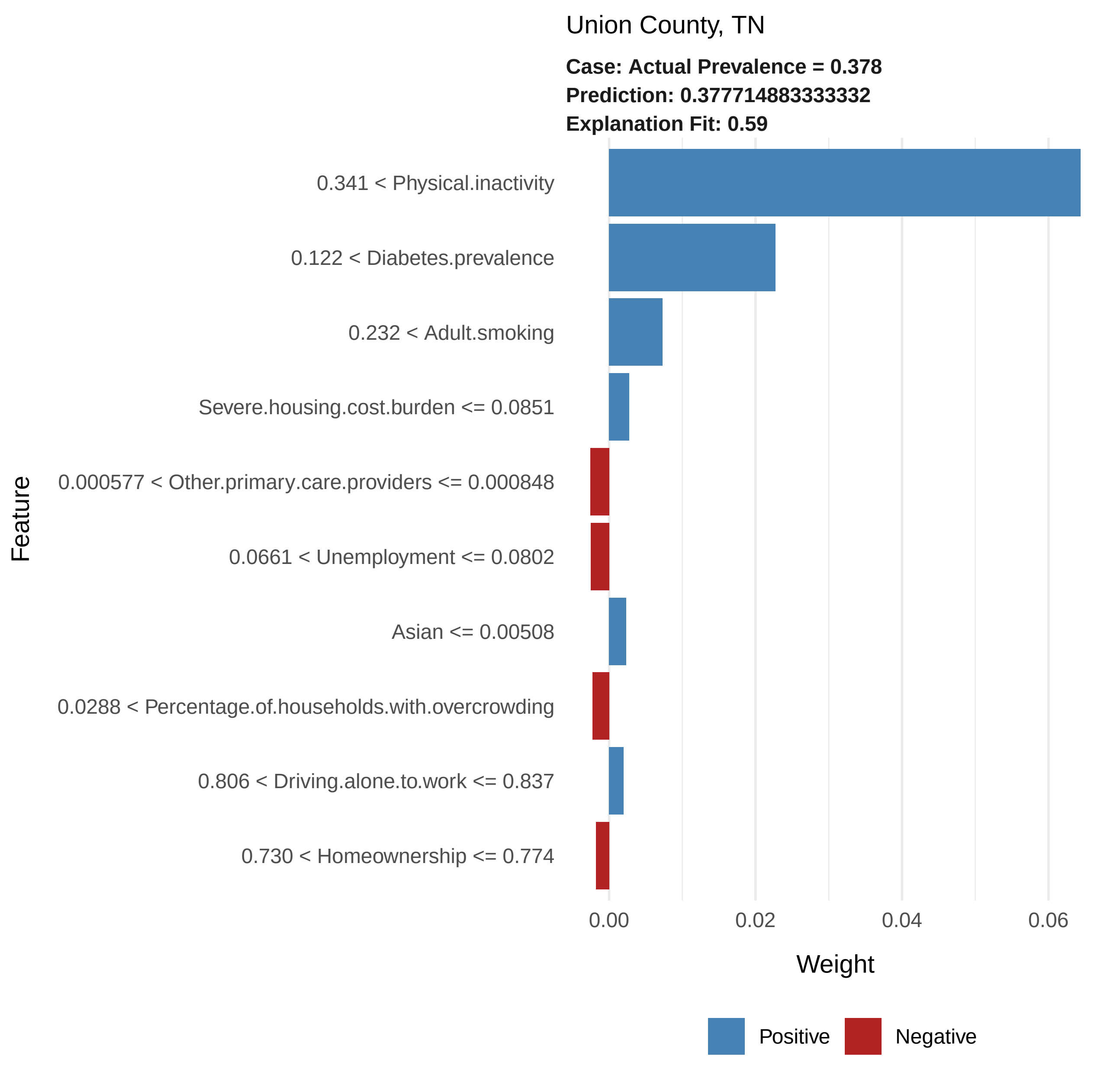

Union County, TN
Case: Actual Prevalence = 0.378
Prediction: 0.377714883333332
Explanation Fit: 0.59
0.341 < Physical.inactivity
0.122 < Diabetes.prevalence
0.232 < Adult.smoking
Severe.housing.cost.burden <= 0.0851
0.000577 < Other.primary.care.providers <= 0.000848
0.0661 < Unemployment <= 0.0802
Asian <= 0.00508
0.0288 < Percentage.of.households.with.overcrowding
0.806 < Driving.alone.to.work <= 0.837
0.730 < Homeownership <= 0.774
Feature
0.00
0.02
0.04
0.06
Weight
Positive
Negative

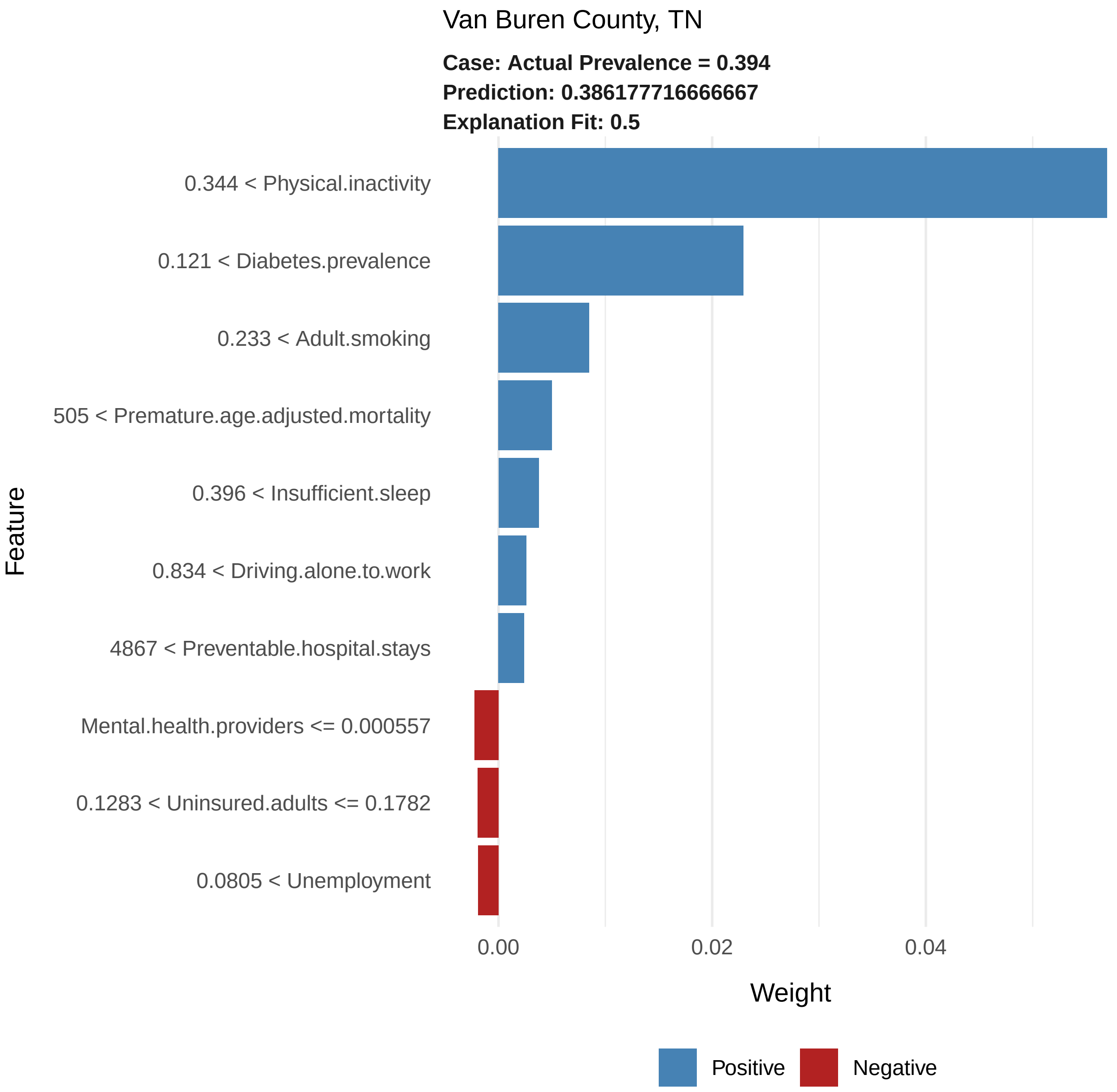
Van Buren County, TN
Case: Actual Prevalence = 0.394
Prediction: 0.386177716666667
Explanation Fit: 0.5
0.344 < Physical.inactivity
0.121 < Diabetes.prevalence
0.233 < Adult.smoking
505 < Premature.age.adjusted.mortality
0.396 < Insufficient.sleep
0.834 < Driving.alone.to.work
4867 < Preventable.hospital.stays
Mental.health.providers <= 0.000557
0.1283 < Uninsured.adults <= 0.1782
0.0805 < Unemployment
Feature
0.00
0.02
0.04
Weight
Positive
Negative

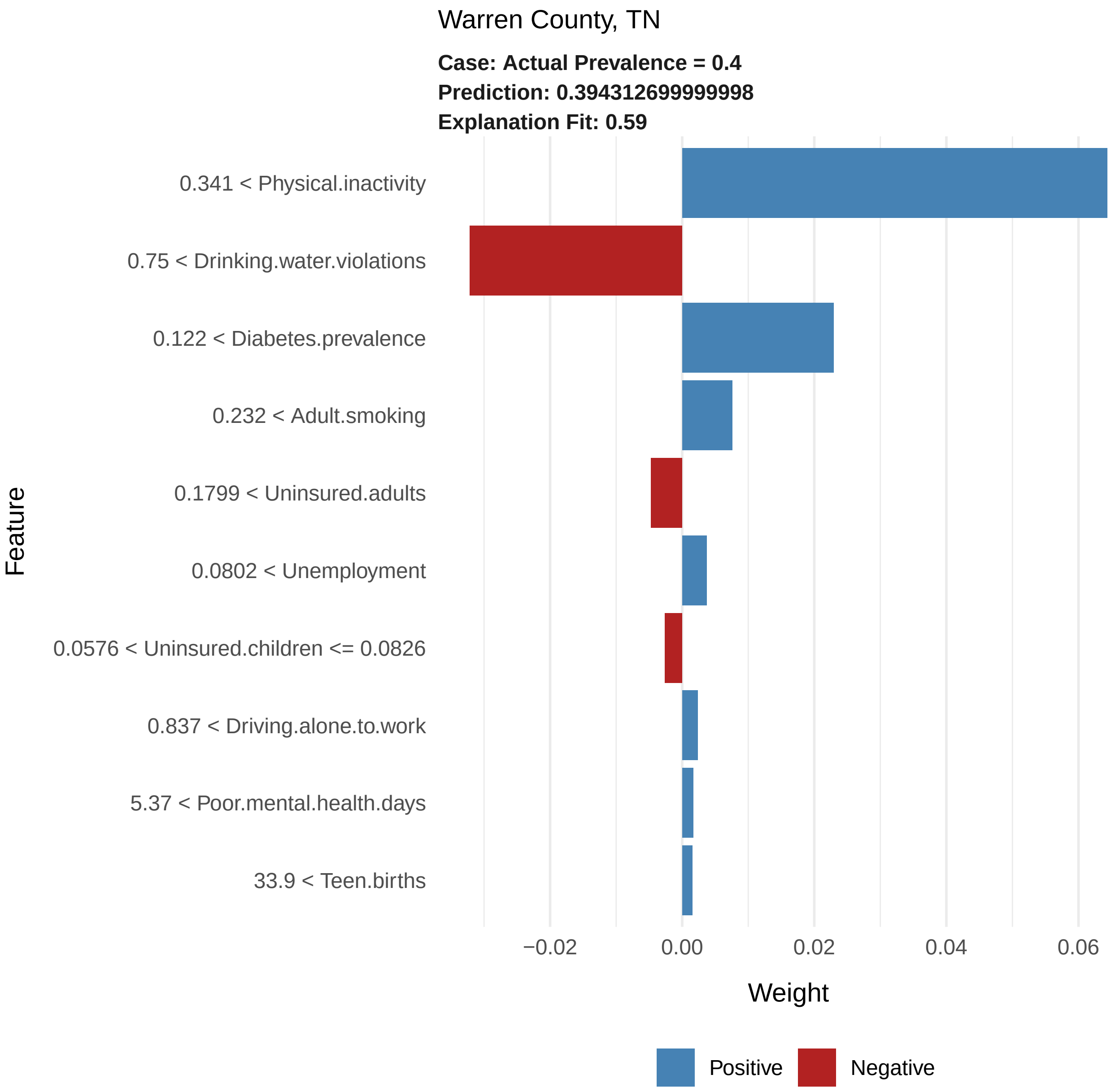

Warren County, TN
Case: Actual Prevalence = 0.4
Prediction: 0.394312699999998
Explanation Fit: 0.59
0.341 < Physical.inactivity
0.75 < Drinking.water.violations
0.122 < Diabetes.prevalence
0.232 < Adult.smoking
0.1799 < Uninsured.adults
0.0802 < Unemployment
0.0576 < Uninsured.children <= 0.0826
0.837 < Driving.alone.to.work
5.37 < Poor.mental.health.days
33.9 < Teen.births
Feature
−0.02
0.00
0.02
0.04
0.06
Weight
Positive
Negative

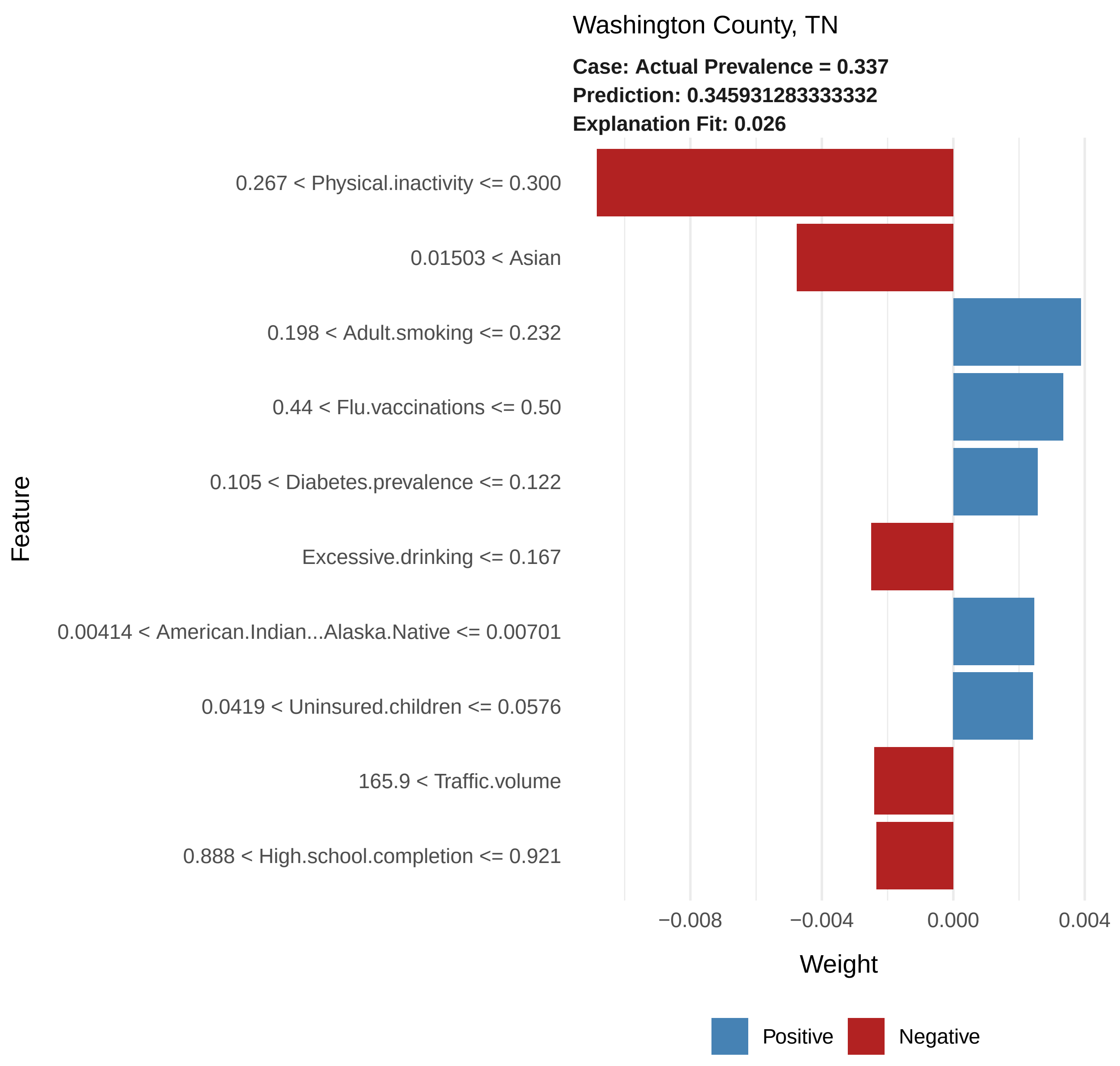

Washington County, TN
Case: Actual Prevalence = 0.337
Prediction: 0.345931283333332
Explanation Fit: 0.026
0.267 < Physical.inactivity <= 0.300
0.01503 < Asian
0.198 < Adult.smoking <= 0.232
0.44 < Flu.vaccinations <= 0.50
0.105 < Diabetes.prevalence <= 0.122
Excessive.drinking <= 0.167
0.00414 < American.Indian...Alaska.Native <= 0.00701
0.0419 < Uninsured.children <= 0.0576
165.9 < Traffic.volume
0.888 < High.school.completion <= 0.921
Feature
−0.008
−0.004
0.000
0.004
Weight
Positive
Negative

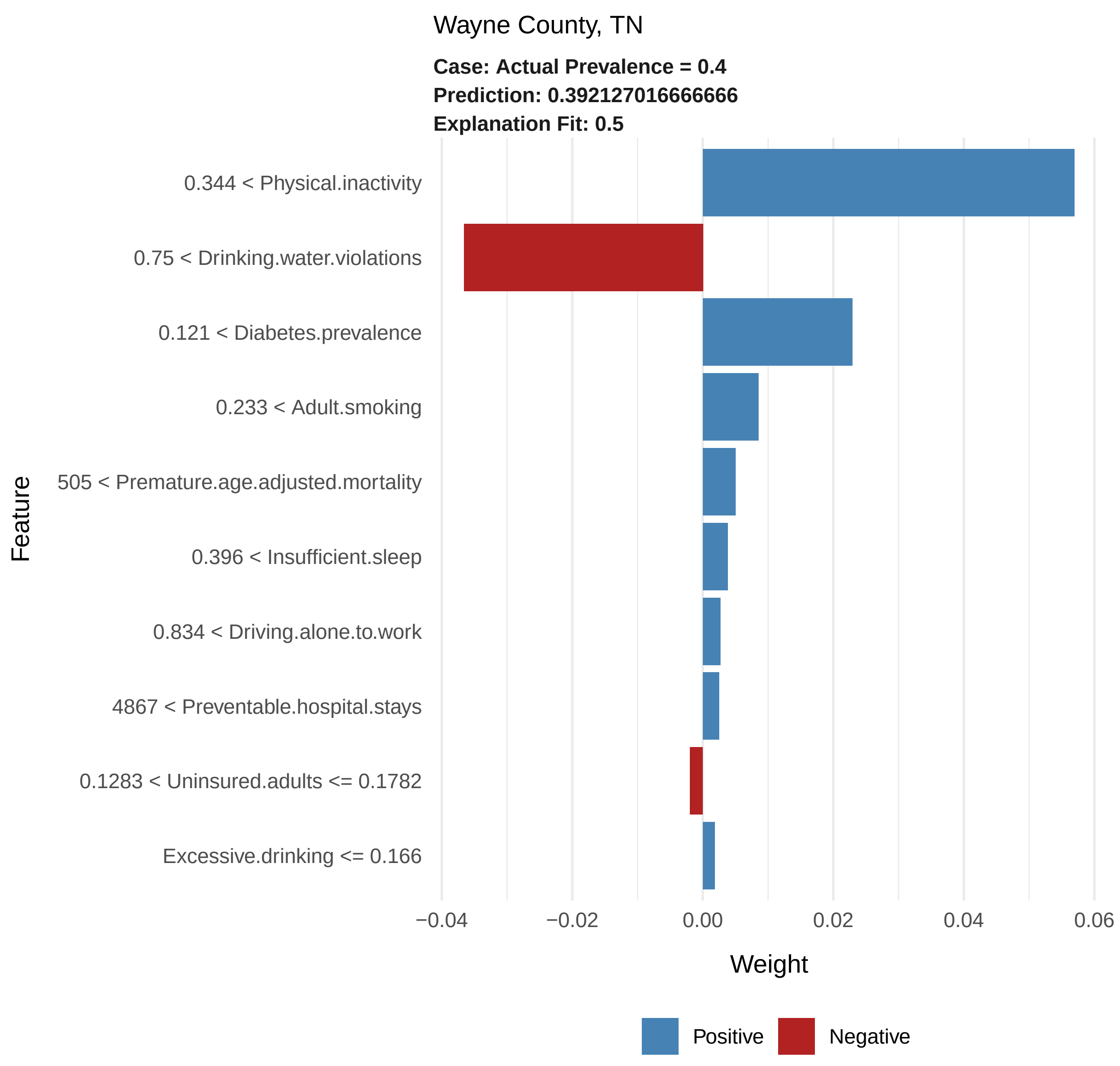
Wayne County, TN
Case: Actual Prevalence = 0.4
Prediction: 0.392127016666666
Explanation Fit: 0.5
0.344 < Physical.inactivity
0.75 < Drinking.water.violations
0.121 < Diabetes.prevalence
0.233 < Adult.smoking
505 < Premature.age.adjusted.mortality
0.396 < Insufficient.sleep
0.834 < Driving.alone.to.work
4867 < Preventable.hospital.stays
0.1283 < Uninsured.adults <= 0.1782
Excessive.drinking <= 0.166
Feature
−0.04
−0.02
0.00
0.02
0.04
0.06
Weight
Positive
Negative

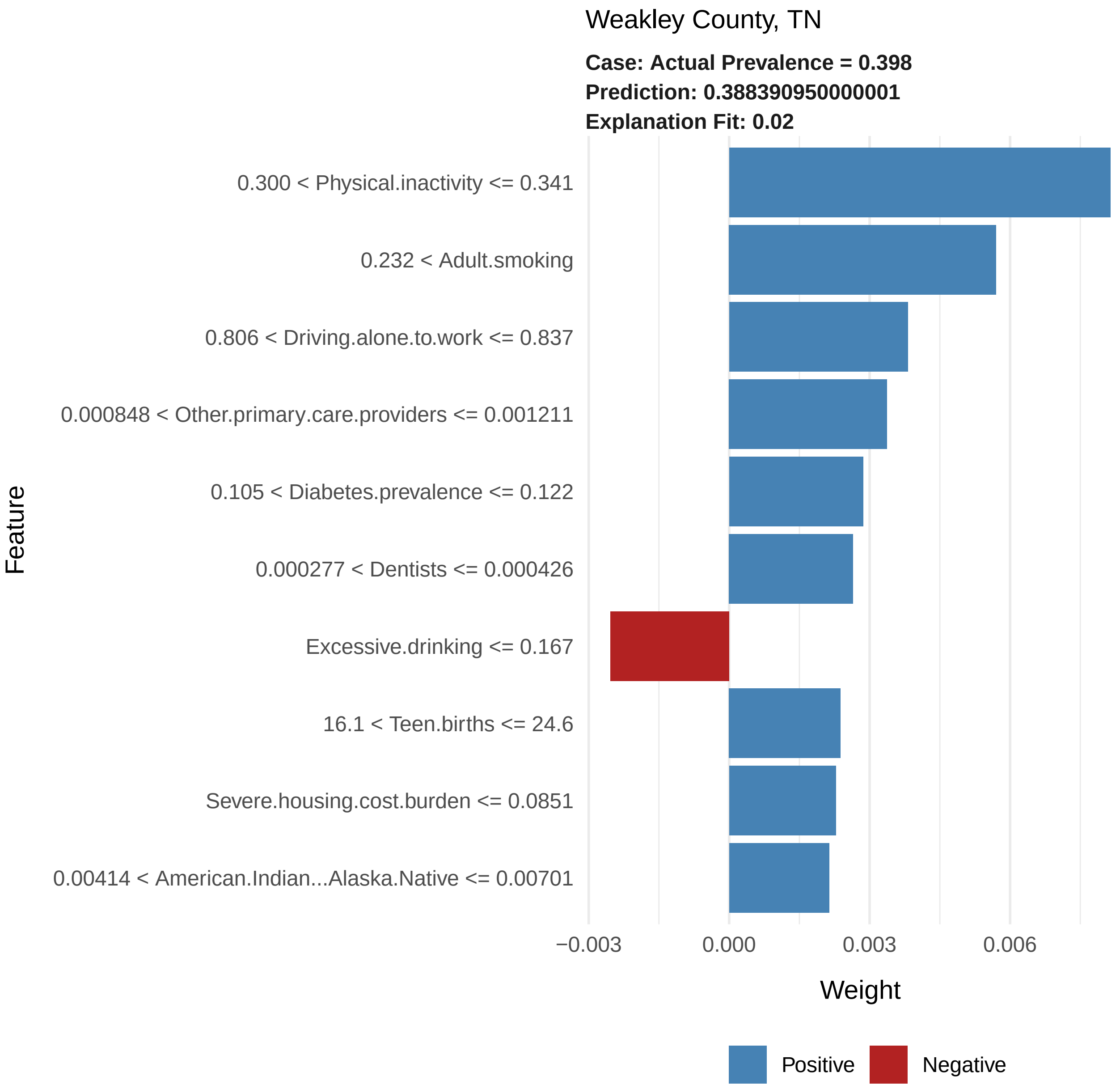

Weakley County, TN
Case: Actual Prevalence = 0.398
Prediction: 0.388390950000001
Explanation Fit: 0.02
0.300 < Physical.inactivity <= 0.341
0.232 < Adult.smoking
0.806 < Driving.alone.to.work <= 0.837
0.000848 < Other.primary.care.providers <= 0.001211
0.105 < Diabetes.prevalence <= 0.122
0.000277 < Dentists <= 0.000426
Excessive.drinking <= 0.167
16.1 < Teen.births <= 24.6
Severe.housing.cost.burden <= 0.0851
0.00414 < American.Indian...Alaska.Native <= 0.00701
Feature
−0.003
0.000
0.003
0.006
Weight
Positive
Negative

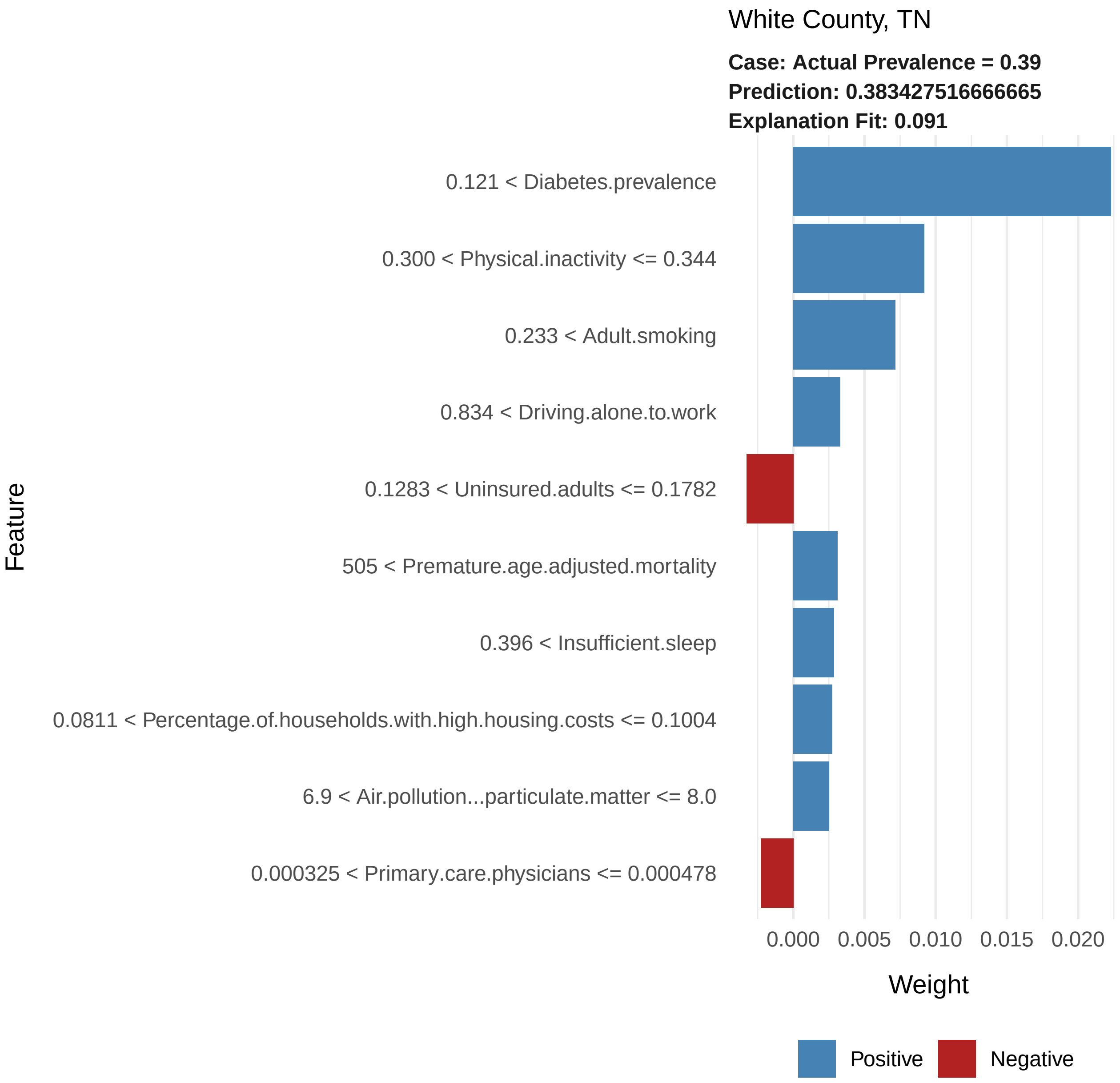
White County, TN
Case: Actual Prevalence = 0.39
Prediction: 0.383427516666665
Explanation Fit: 0.091
0.121 < Diabetes.prevalence
0.300 < Physical.inactivity <= 0.344
0.233 < Adult.smoking
0.834 < Driving.alone.to.work
0.1283 < Uninsured.adults <= 0.1782
505 < Premature.age.adjusted.mortality
0.396 < Insufficient.sleep
0.0811 < Percentage.of.households.with.high.housing.costs <= 0.1004
6.9 < Air.pollution...particulate.matter <= 8.0
0.000325 < Primary.care.physicians <= 0.000478
Feature
0.000
0.005
0.010
0.015
0.020
Weight
Positive
Negative

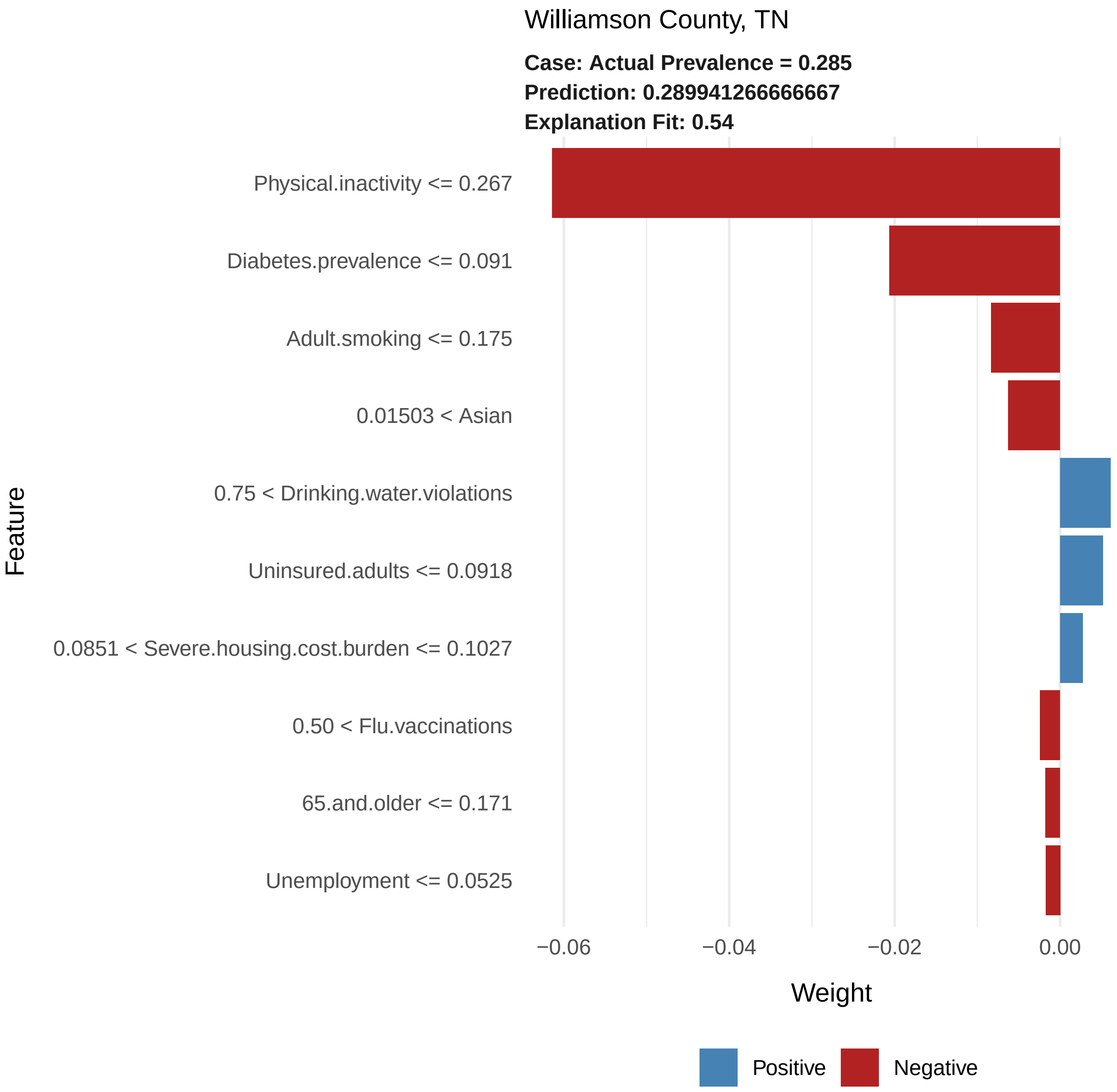
Williamson County, TN
Case: Actual Prevalence = 0.285
Prediction: 0.289941266666667
Explanation Fit: 0.54
Physical.inactivity <= 0.267
Diabetes.prevalence <= 0.091
Adult.smoking <= 0.175
0.01503 < Asian
0.75 < Drinking.water.violations
Uninsured.adults <= 0.0918
0.0851 < Severe.housing.cost.burden <= 0.1027
0.50 < Flu.vaccinations
65.and.older <= 0.171
Unemployment <= 0.0525
Feature
−0.06
−0.04
−0.02
0.00
Weight
Positive
Negative

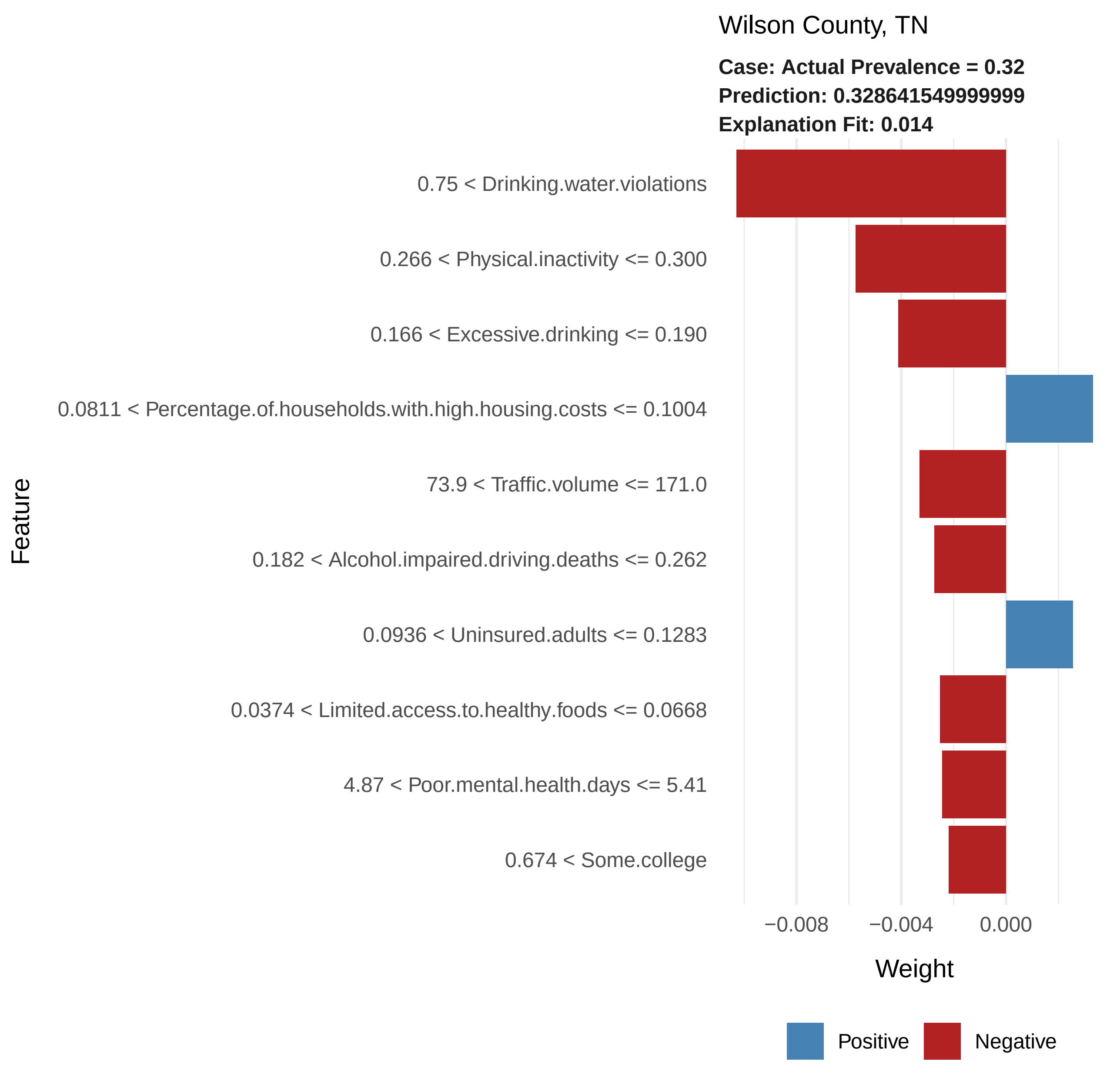
Wilson County, TN
Case: Actual Prevalence = 0.32
Prediction: 0.328641549999999
Explanation Fit: 0.014
0.75 < Drinking.water.violations
0.266 < Physical.inactivity <= 0.300
0.166 < Excessive.drinking <= 0.190
0.0811 < Percentage.of.households.with.high.housing.costs <= 0.1004
73.9 < Traffic.volume <= 171.0
0.182 < Alcohol.impaired.driving.deaths <= 0.262
0.0936 < Uninsured.adults <= 0.1283
0.0374 < Limited.access.to.healthy.foods <= 0.0668
4.87 < Poor.mental.health.days <= 5.41
0.674 < Some.college
Feature
−0.008
−0.004
0.000
Weight
Positive
Negative

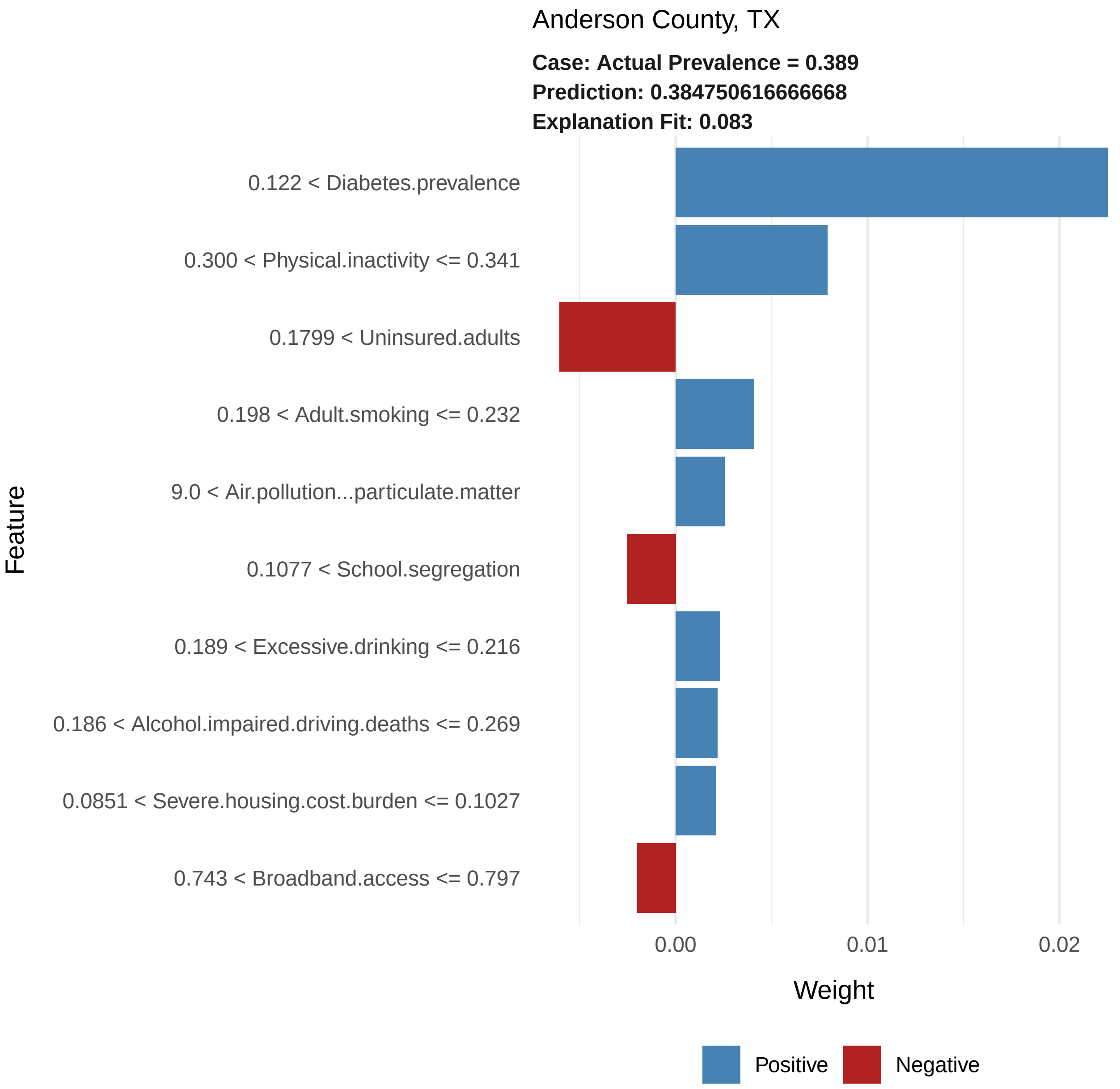

Anderson County, TX
Case: Actual Prevalence = 0.389
Prediction: 0.384750616666668
Explanation Fit: 0.083
0.122 < Diabetes.prevalence
0.300 < Physical.inactivity <= 0.341
0.1799 < Uninsured.adults
0.198 < Adult.smoking <= 0.232
9.0 < Air.pollution...particulate.matter
0.1077 < School.segregation
0.189 < Excessive.drinking <= 0.216
0.186 < Alcohol.impaired.driving.deaths <= 0.269
0.0851 < Severe.housing.cost.burden <= 0.1027
0.743 < Broadband.access <= 0.797
Feature
0.00
0.01
0.02
Weight
Positive
Negative

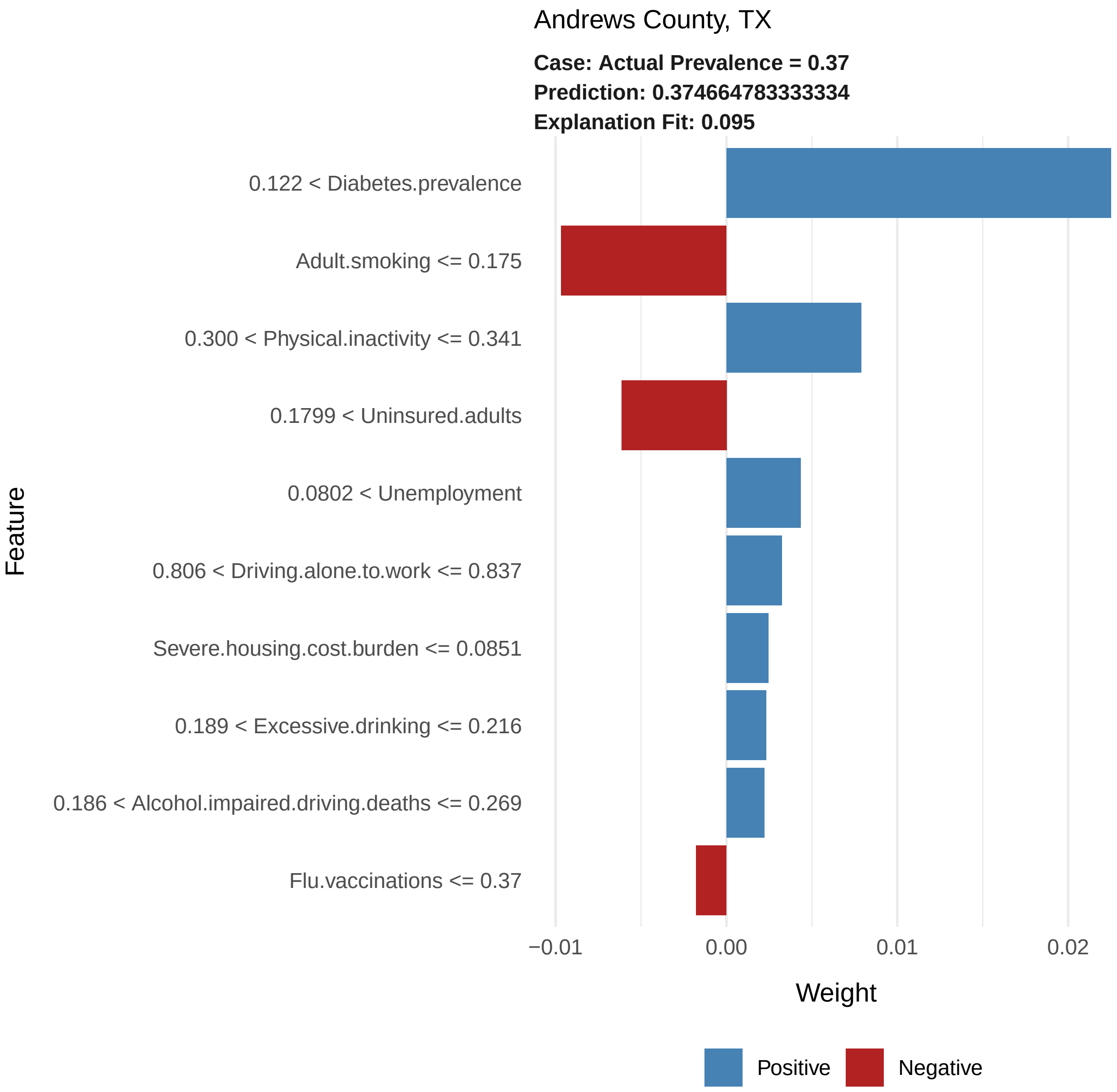

Andrews County, TX
Case: Actual Prevalence = 0.37
Prediction: 0.374664783333334
Explanation Fit: 0.095
0.122 < Diabetes.prevalence
Adult.smoking <= 0.175
0.300 < Physical.inactivity <= 0.341
0.1799 < Uninsured.adults
0.0802 < Unemployment
0.806 < Driving.alone.to.work <= 0.837
Severe.housing.cost.burden <= 0.0851
0.189 < Excessive.drinking <= 0.216
0.186 < Alcohol.impaired.driving.deaths <= 0.269
Flu.vaccinations <= 0.37
Feature
−0.01
0.00
0.01
0.02
Weight
Positive
Negative

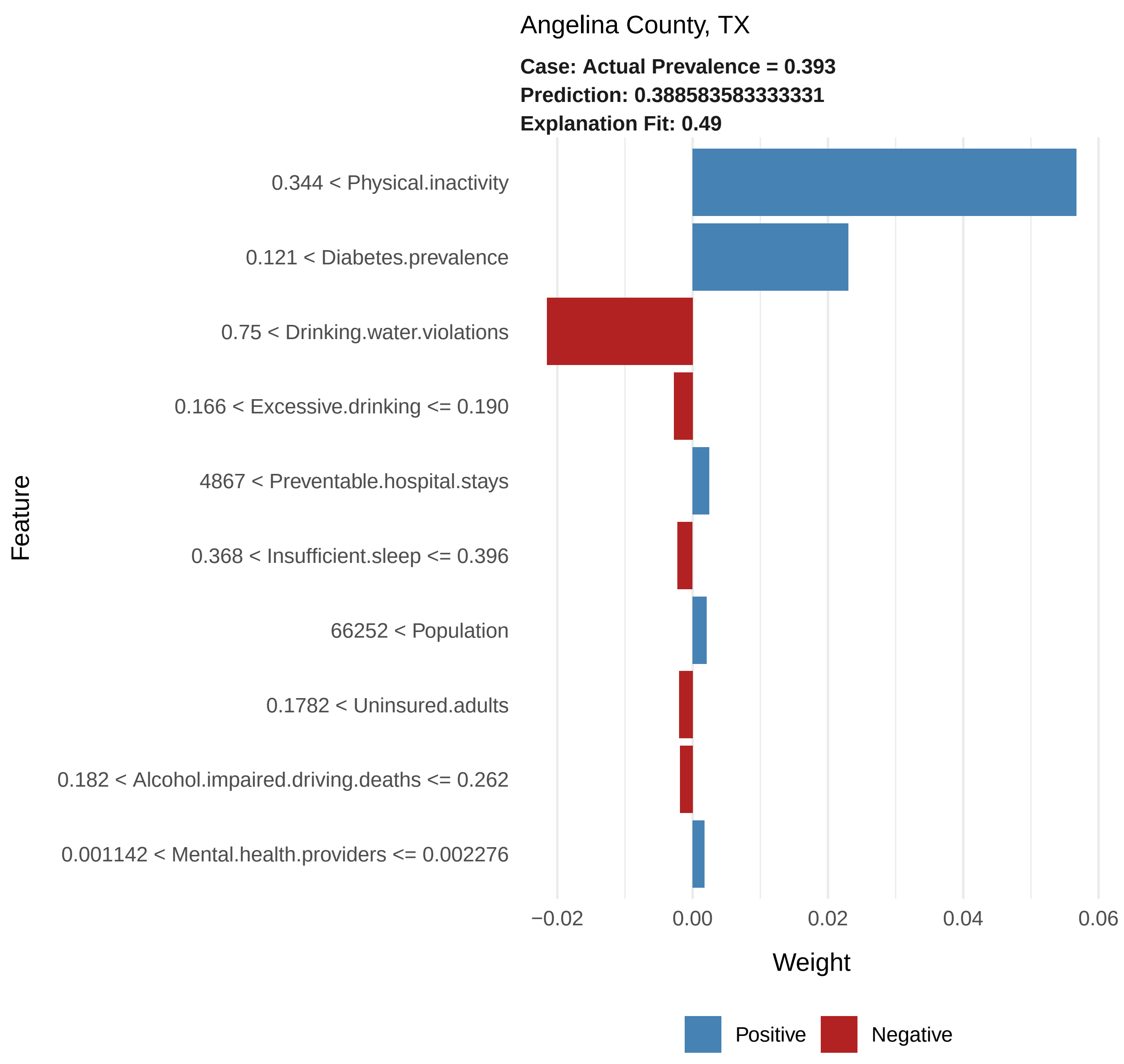
Angelina County, TX
Case: Actual Prevalence = 0.393
Prediction: 0.388583583333331
Explanation Fit: 0.49
0.344 < Physical.inactivity
0.121 < Diabetes.prevalence
0.75 < Drinking.water.violations
0.166 < Excessive.drinking <= 0.190
4867 < Preventable.hospital.stays
0.368 < Insufficient.sleep <= 0.396
66252 < Population
0.1782 < Uninsured.adults
0.182 < Alcohol.impaired.driving.deaths <= 0.262
0.001142 < Mental.health.providers <= 0.002276
Feature
−0.02
0.00
0.02
0.04
0.06
Weight
Positive
Negative

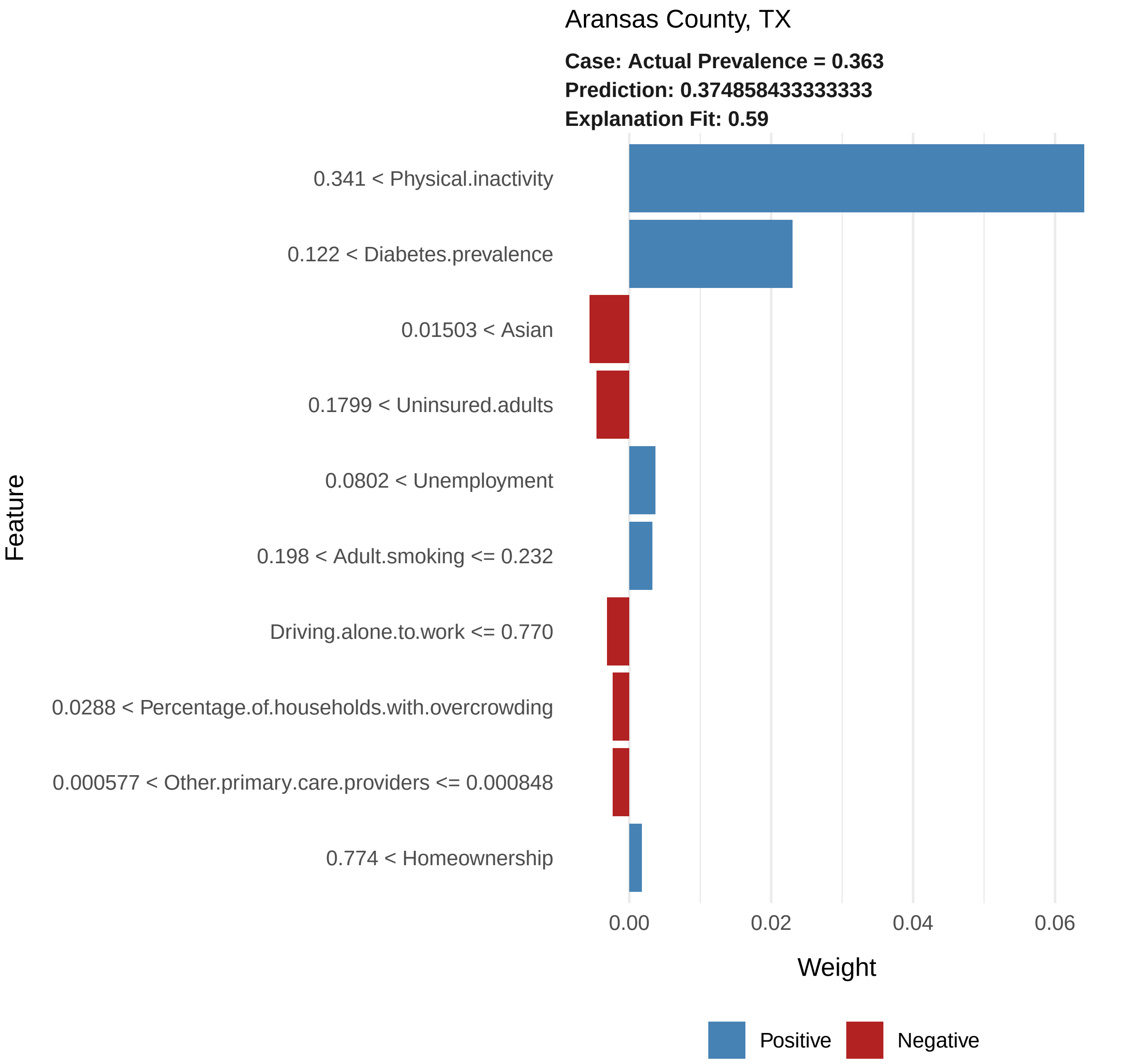

Aransas County, TX
Case: Actual Prevalence = 0.363
Prediction: 0.374858433333333
Explanation Fit: 0.59
0.341 < Physical.inactivity
0.122 < Diabetes.prevalence
0.01503 < Asian
0.1799 < Uninsured.adults
0.0802 < Unemployment
0.198 < Adult.smoking <= 0.232
Driving.alone.to.work <= 0.770
0.0288 < Percentage.of.households.with.overcrowding
0.000577 < Other.primary.care.providers <= 0.000848
0.774 < Homeownership
Feature
0.00
0.02
0.04
0.06
Weight
Positive
Negative

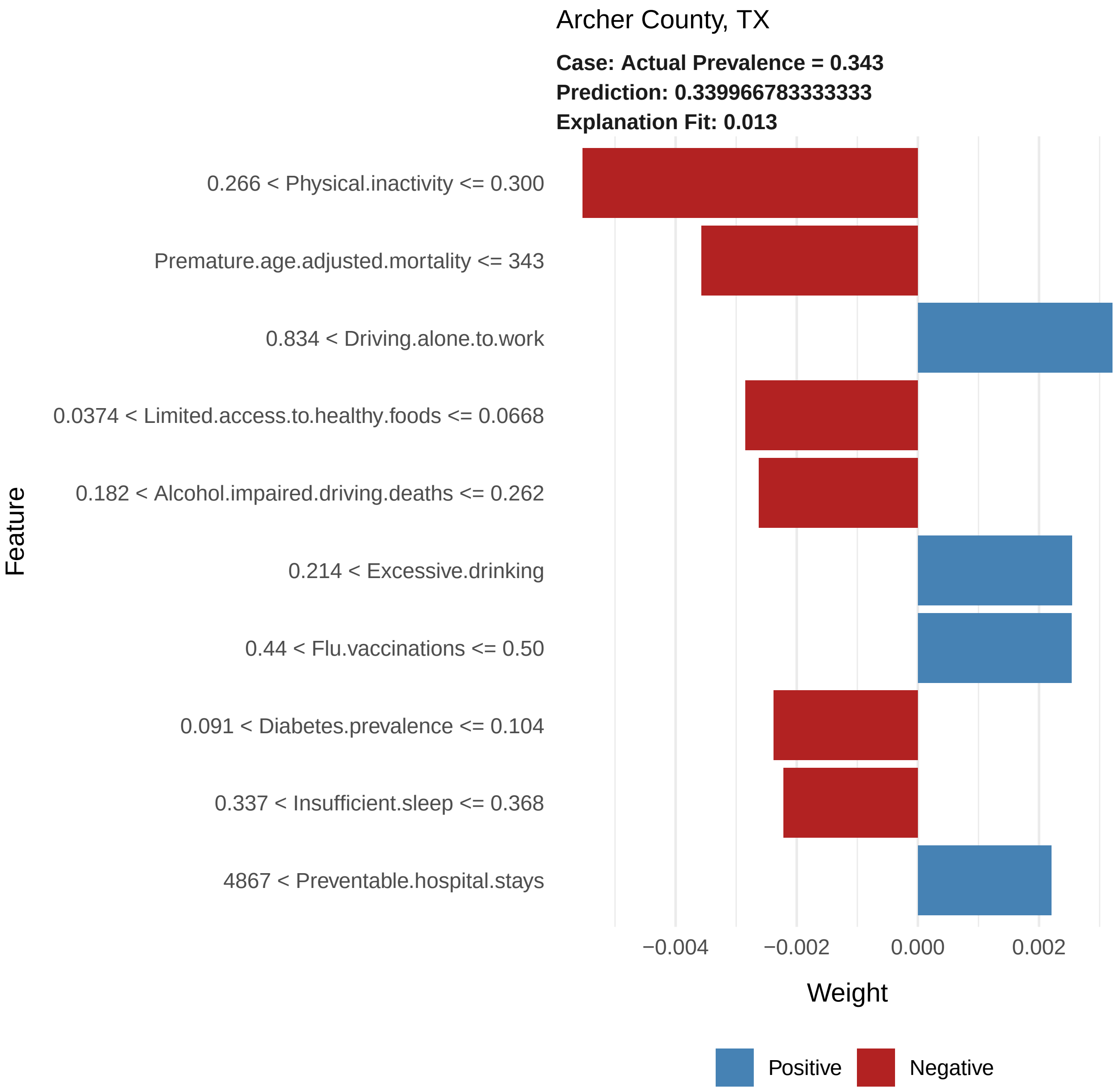
Archer County, TX
Case: Actual Prevalence = 0.343
Prediction: 0.339966783333333
Explanation Fit: 0.013
0.266 < Physical.inactivity <= 0.300
Premature.age.adjusted.mortality <= 343
0.834 < Driving.alone.to.work
0.0374 < Limited.access.to.healthy.foods <= 0.0668
0.182 < Alcohol.impaired.driving.deaths <= 0.262
0.214 < Excessive.drinking
0.44 < Flu.vaccinations <= 0.50
0.091 < Diabetes.prevalence <= 0.104
0.337 < Insufficient.sleep <= 0.368
4867 < Preventable.hospital.stays
Feature
−0.004
−0.002
0.000
0.002
Weight
Positive
Negative

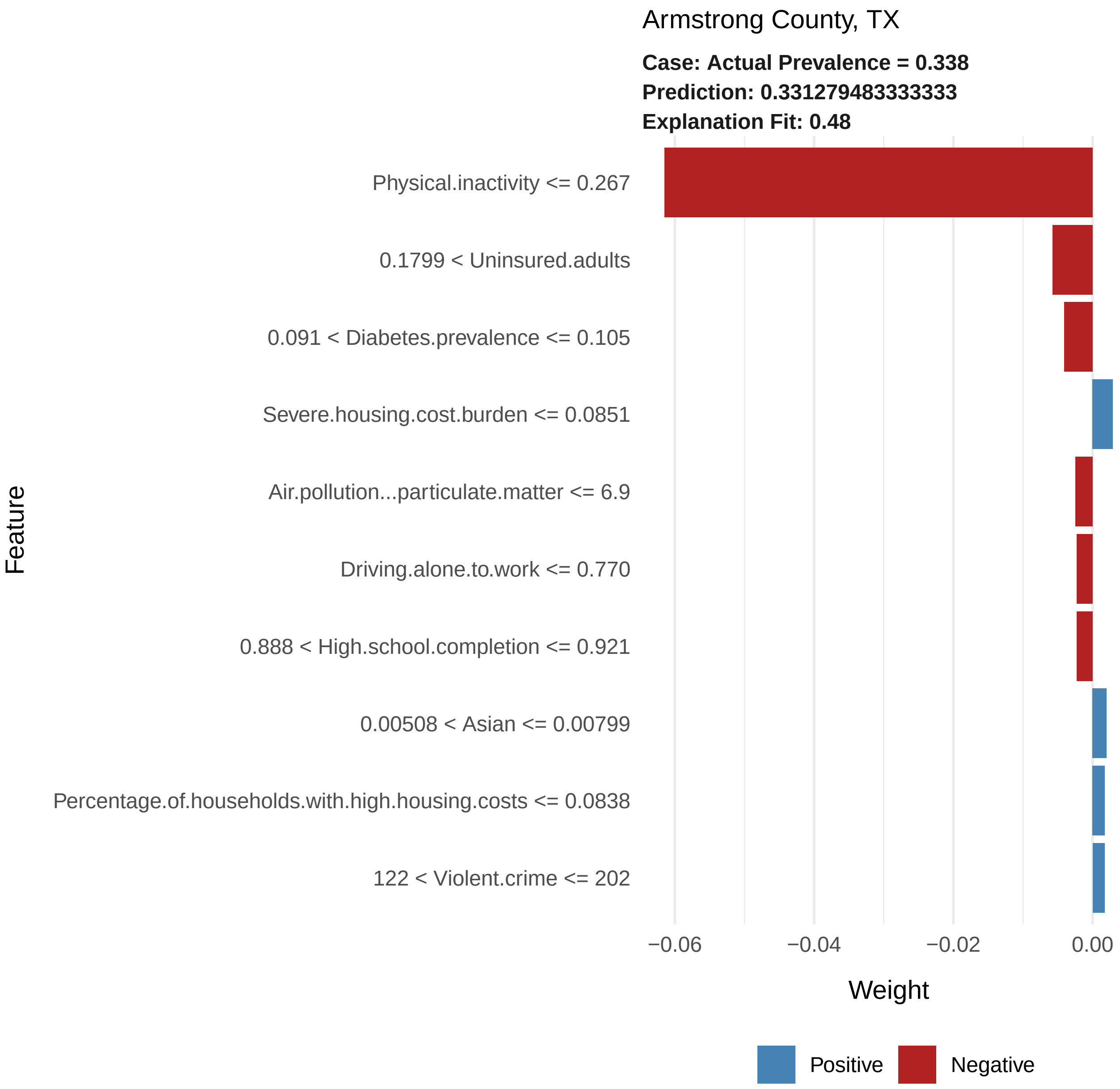
Armstrong County, TX
Case: Actual Prevalence = 0.338
Prediction: 0.331279483333333
Explanation Fit: 0.48
Physical.inactivity <= 0.267
0.1799 < Uninsured.adults
0.091 < Diabetes.prevalence <= 0.105
Severe.housing.cost.burden <= 0.0851
Air.pollution...particulate.matter <= 6.9
Driving.alone.to.work <= 0.770
0.888 < High.school.completion <= 0.921
0.00508 < Asian <= 0.00799
Percentage.of.households.with.high.housing.costs <= 0.0838
122 < Violent.crime <= 202
Feature
−0.06
−0.04
−0.02
0.00
Weight
Positive
Negative

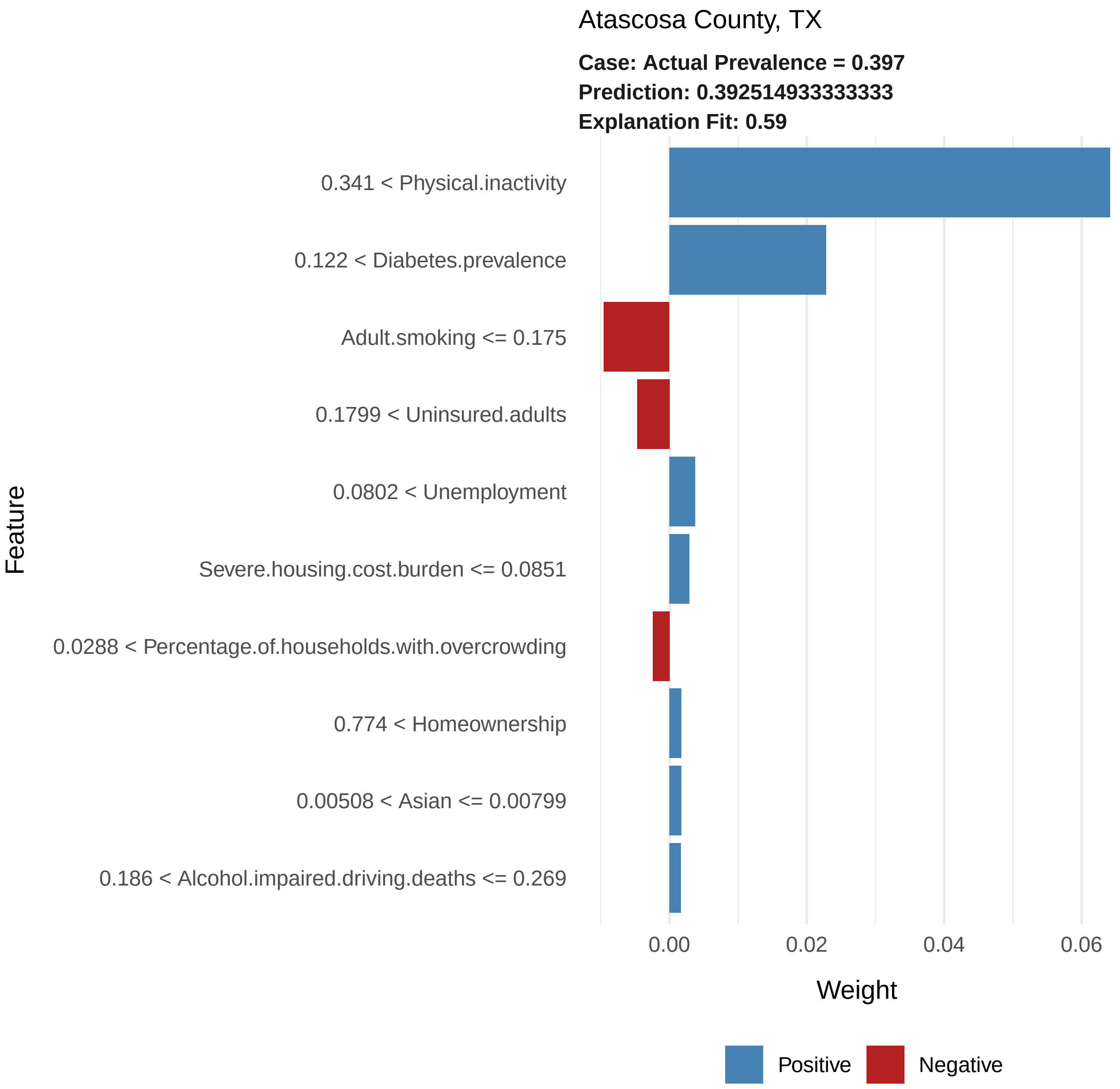
Atascosa County, TX
Case: Actual Prevalence = 0.397
Prediction: 0.392514933333333
Explanation Fit: 0.59
0.341 < Physical.inactivity
0.122 < Diabetes.prevalence
Adult.smoking <= 0.175
0.1799 < Uninsured.adults
0.0802 < Unemployment
Severe.housing.cost.burden <= 0.0851
0.0288 < Percentage.of.households.with.overcrowding
0.774 < Homeownership
0.00508 < Asian <= 0.00799
0.186 < Alcohol.impaired.driving.deaths <= 0.269
Feature
0.00
0.02
0.04
0.06
Weight
Positive
Negative

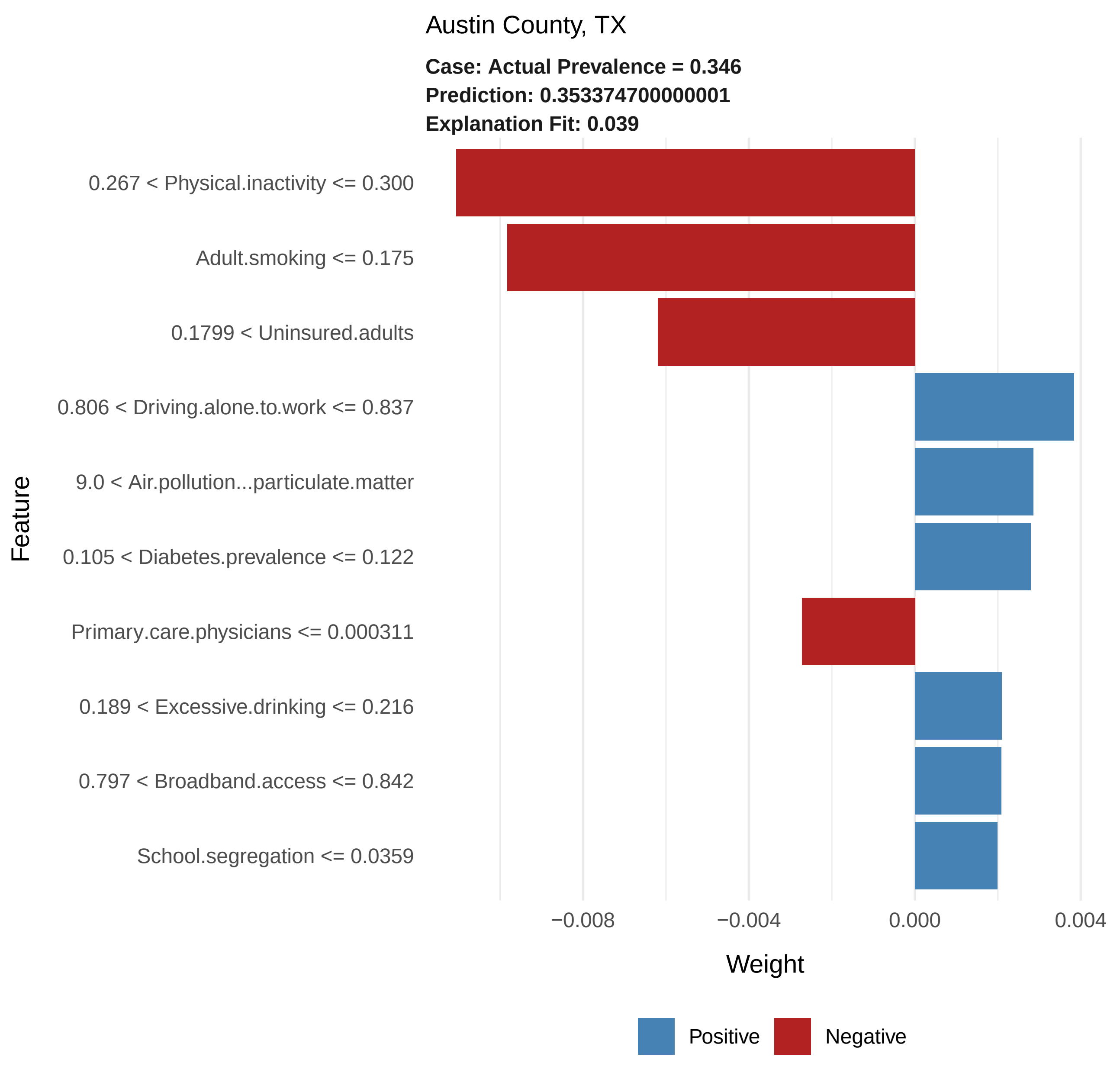
Austin County, TX
Case: Actual Prevalence = 0.346
Prediction: 0.353374700000001
Explanation Fit: 0.039
0.267 < Physical.inactivity <= 0.300
Adult.smoking <= 0.175
0.1799 < Uninsured.adults
0.806 < Driving.alone.to.work <= 0.837
9.0 < Air.pollution...particulate.matter
0.105 < Diabetes.prevalence <= 0.122
Primary.care.physicians <= 0.000311
0.189 < Excessive.drinking <= 0.216
0.797 < Broadband.access <= 0.842
School.segregation <= 0.0359
Feature
−0.008
−0.004
0.000
0.004
Weight
Positive
Negative

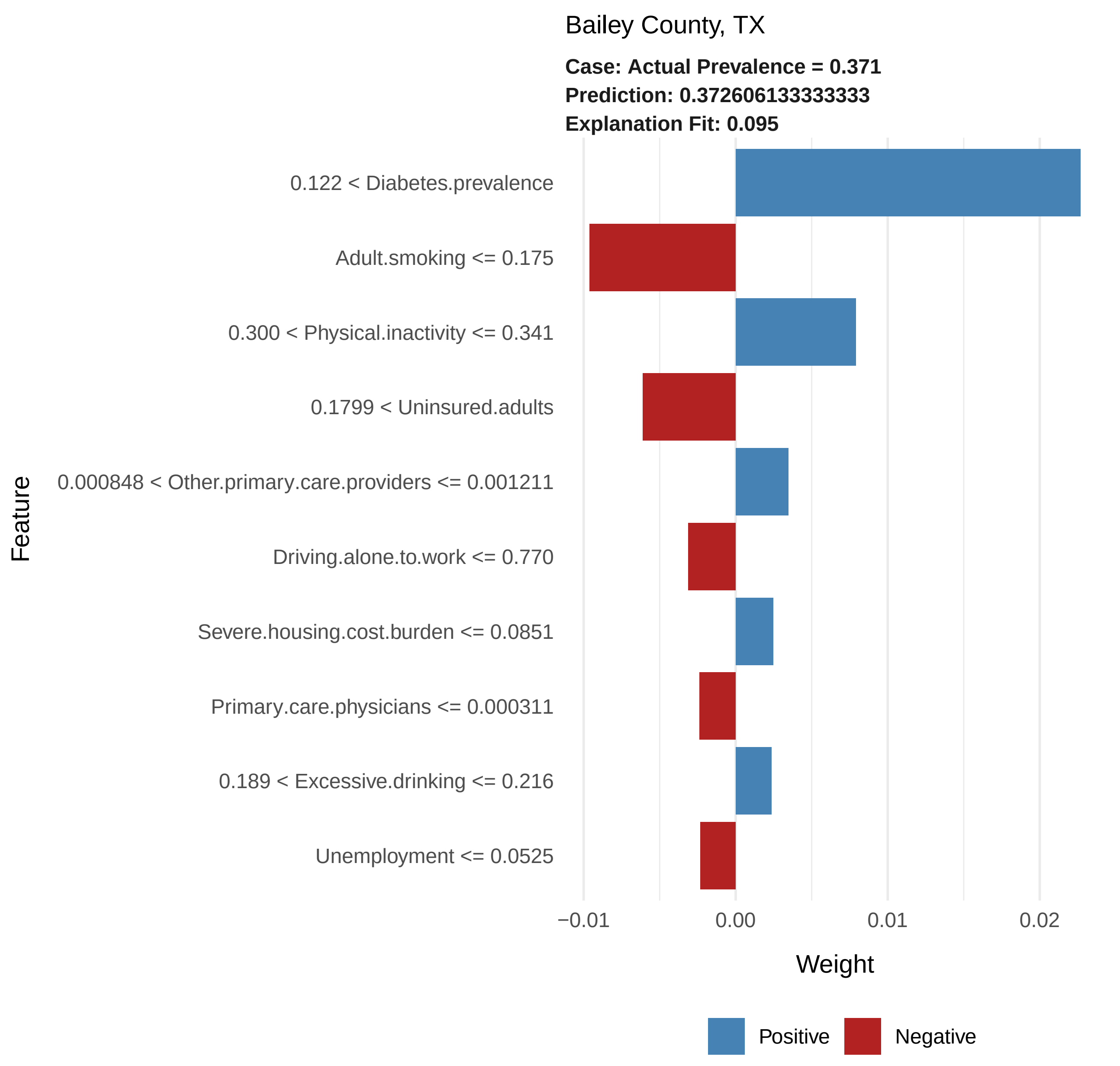
Bailey County, TX
Case: Actual Prevalence = 0.371
Prediction: 0.372606133333333
Explanation Fit: 0.095
0.122 < Diabetes.prevalence
Adult.smoking <= 0.175
0.300 < Physical.inactivity <= 0.341
0.1799 < Uninsured.adults
0.000848 < Other.primary.care.providers <= 0.001211
Driving.alone.to.work <= 0.770
Severe.housing.cost.burden <= 0.0851
Primary.care.physicians <= 0.000311
0.189 < Excessive.drinking <= 0.216
Unemployment <= 0.0525
Feature
−0.01
0.00
0.01
0.02
Weight
Positive
Negative

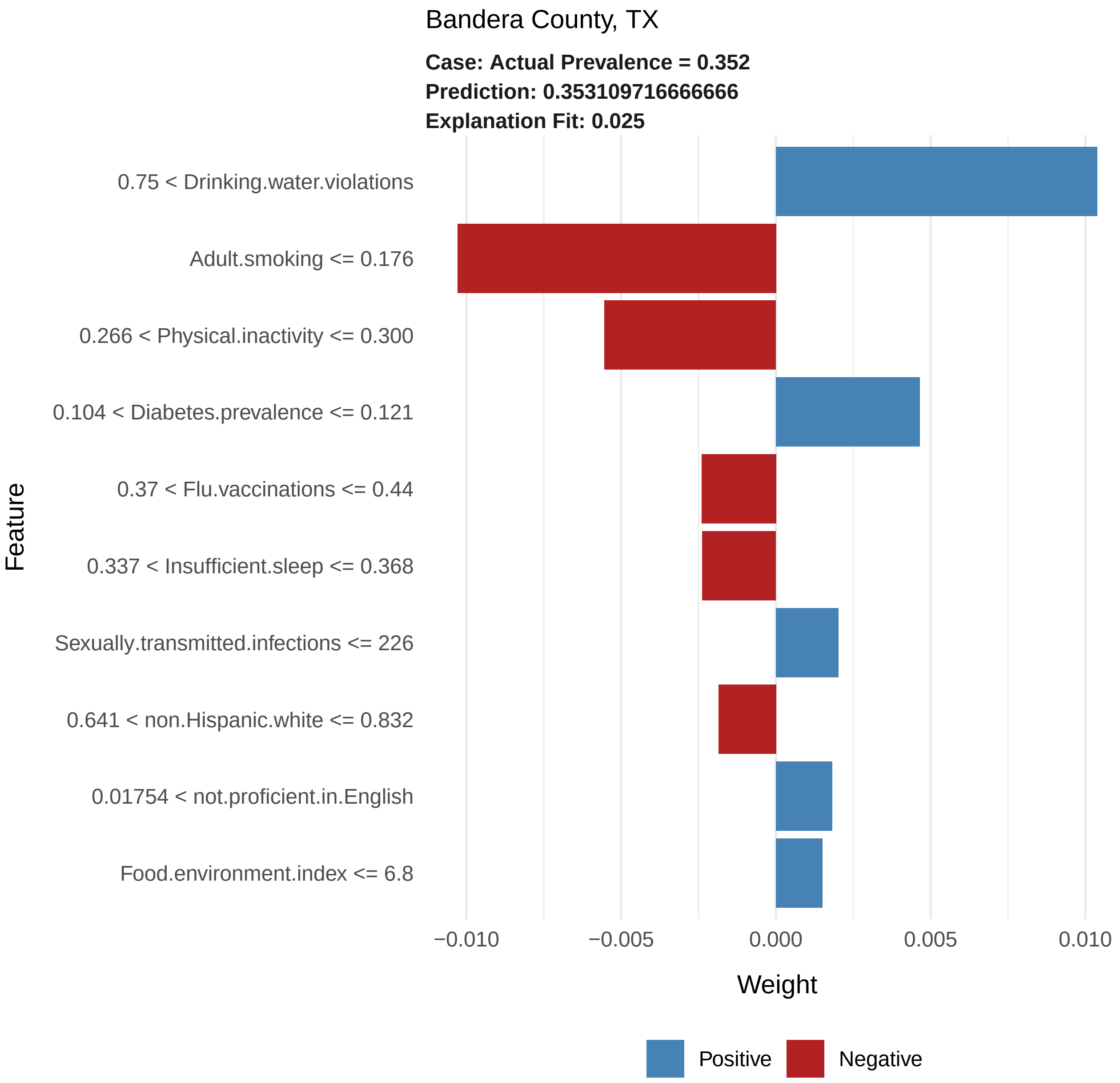
Bandera County, TX
Case: Actual Prevalence = 0.352
Prediction: 0.353109716666666
Explanation Fit: 0.025
0.75 < Drinking.water.violations
Adult.smoking <= 0.176
0.266 < Physical.inactivity <= 0.300
0.104 < Diabetes.prevalence <= 0.121
0.37 < Flu.vaccinations <= 0.44
0.337 < Insufficient.sleep <= 0.368
Sexually.transmitted.infections <= 226
0.641 < non.Hispanic.white <= 0.832
0.01754 < not.proficient.in.English
Food.environment.index <= 6.8
Feature
−0.010
−0.005
0.000
0.005
0.010
Weight
Positive
Negative

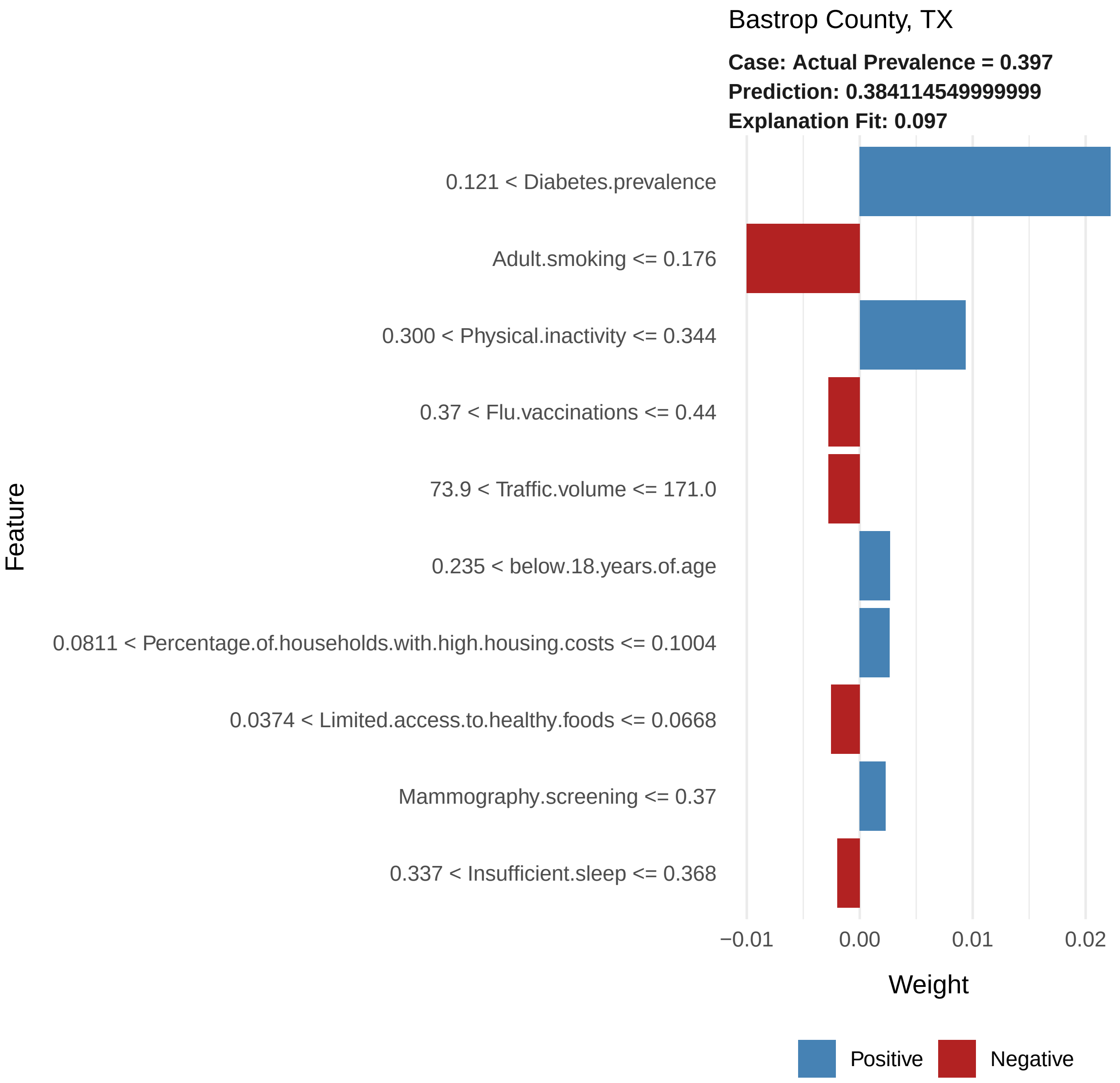

Bastrop County, TX
Case: Actual Prevalence = 0.397
Prediction: 0.384114549999999
Explanation Fit: 0.097
0.121 < Diabetes.prevalence
Adult.smoking <= 0.176
0.300 < Physical.inactivity <= 0.344
0.37 < Flu.vaccinations <= 0.44
73.9 < Traffic.volume <= 171.0
0.235 < below.18.years.of.age
0.0811 < Percentage.of.households.with.high.housing.costs <= 0.1004
0.0374 < Limited.access.to.healthy.foods <= 0.0668
Mammography.screening <= 0.37
0.337 < Insufficient.sleep <= 0.368
Feature
−0.01
0.00
0.01
0.02
Weight
Positive
Negative

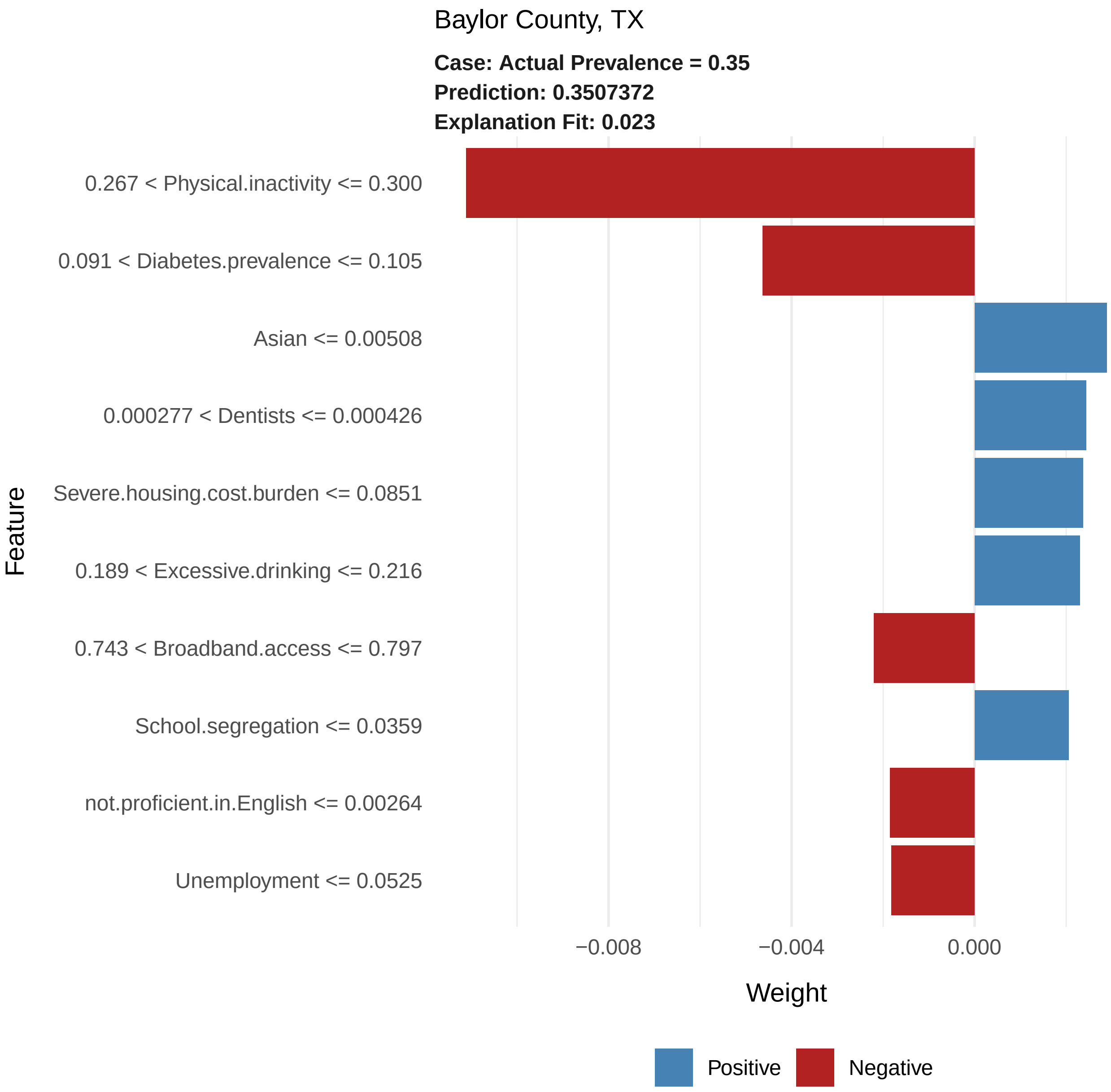

Baylor County, TX
Case: Actual Prevalence = 0.35
Prediction: 0.3507372
Explanation Fit: 0.023
0.267 < Physical.inactivity <= 0.300
0.091 < Diabetes.prevalence <= 0.105
Asian <= 0.00508
0.000277 < Dentists <= 0.000426
Severe.housing.cost.burden <= 0.0851
0.189 < Excessive.drinking <= 0.216
0.743 < Broadband.access <= 0.797
School.segregation <= 0.0359
not.proficient.in.English <= 0.00264
Unemployment <= 0.0525
Feature
−0.008
−0.004
0.000
Weight
Positive
Negative

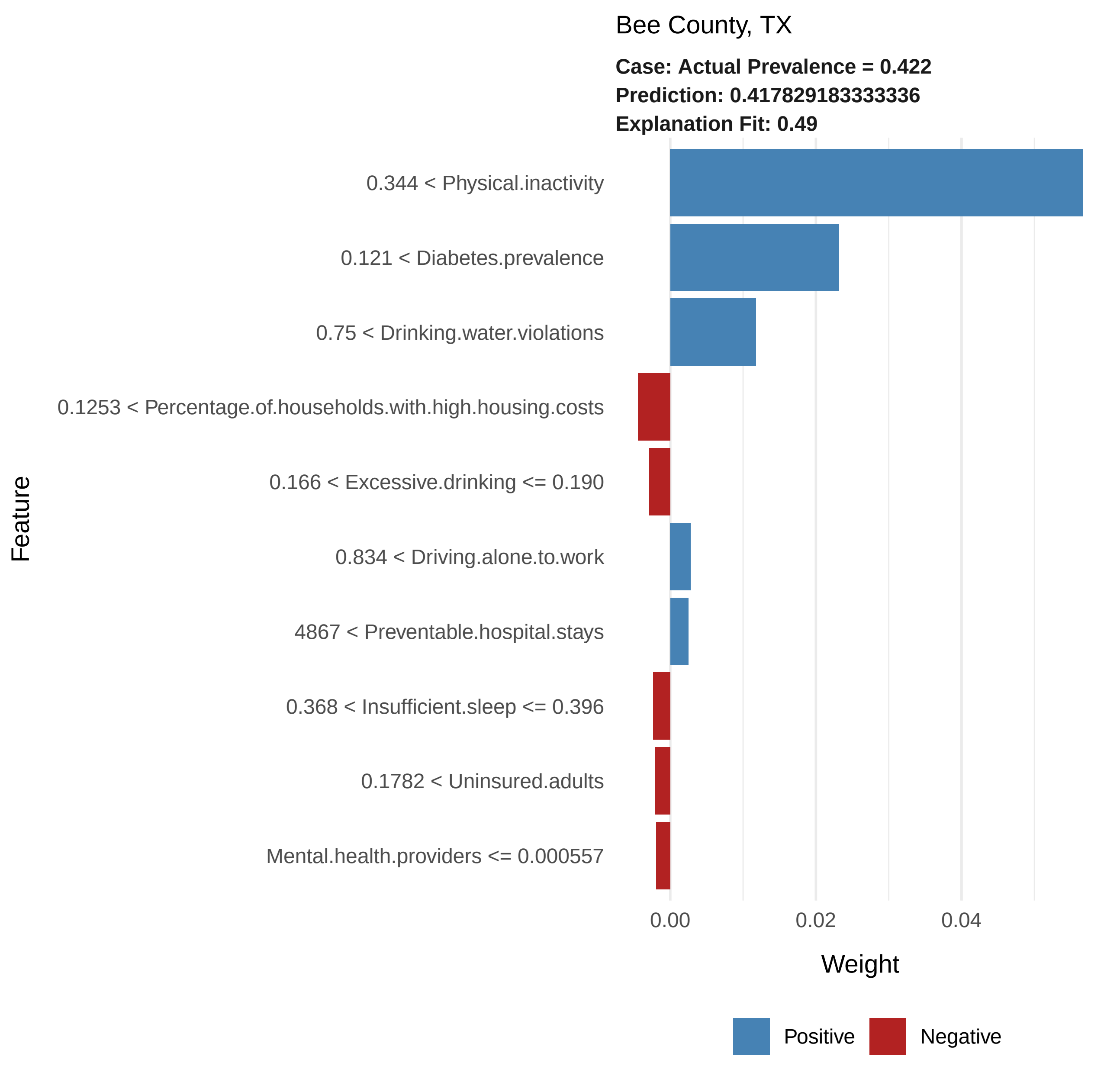
Bee County, TX
Case: Actual Prevalence = 0.422
Prediction: 0.417829183333336
Explanation Fit: 0.49
0.344 < Physical.inactivity
0.121 < Diabetes.prevalence
0.75 < Drinking.water.violations
0.1253 < Percentage.of.households.with.high.housing.costs
0.166 < Excessive.drinking <= 0.190
0.834 < Driving.alone.to.work
4867 < Preventable.hospital.stays
0.368 < Insufficient.sleep <= 0.396
0.1782 < Uninsured.adults
Mental.health.providers <= 0.000557
Feature
0.00
0.02
0.04
Weight
Positive
Negative

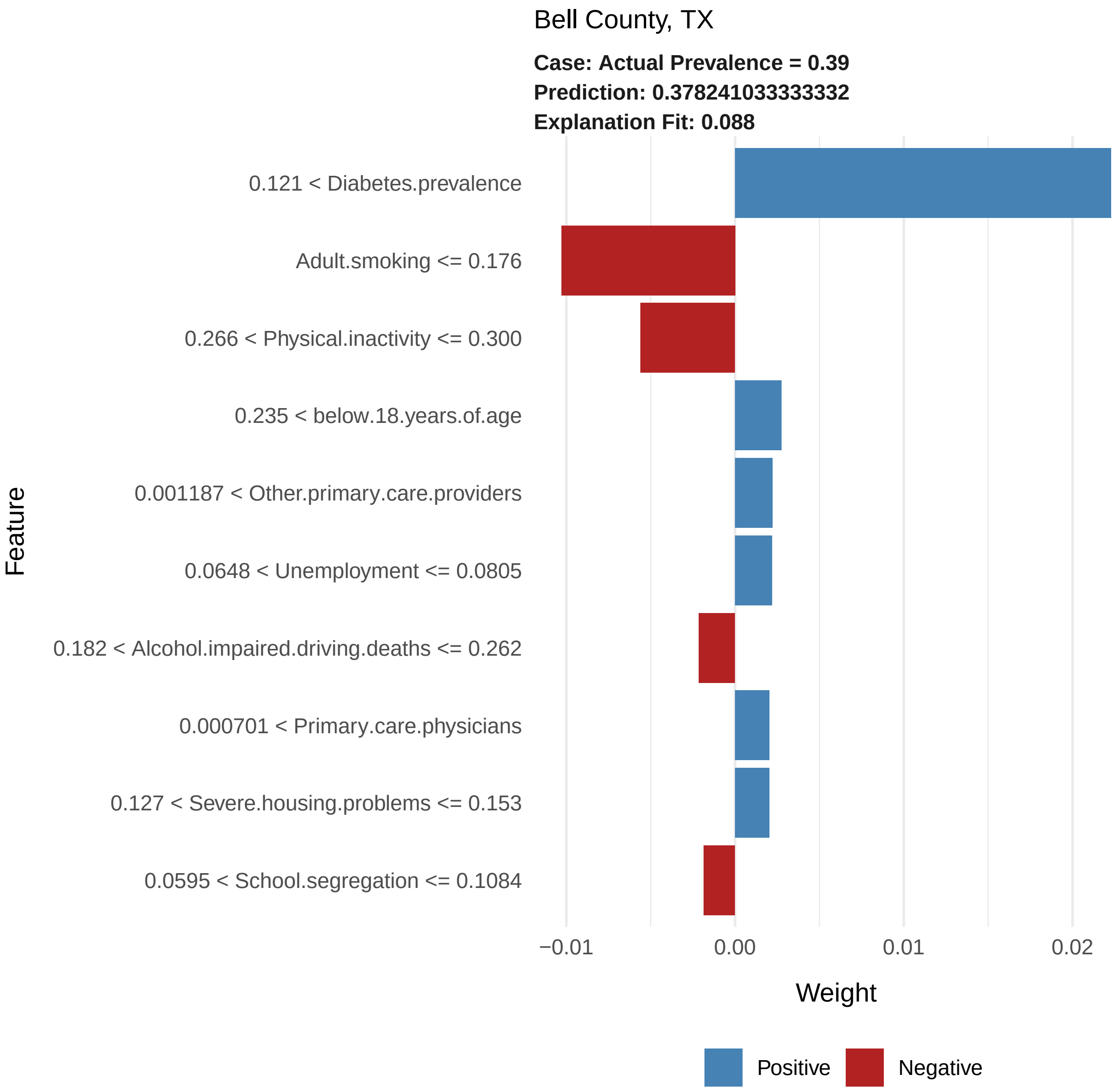
Bell County, TX
Case: Actual Prevalence = 0.39
Prediction: 0.378241033333332
Explanation Fit: 0.088
0.121 < Diabetes.prevalence
Adult.smoking <= 0.176
0.266 < Physical.inactivity <= 0.300
0.235 < below.18.years.of.age
0.001187 < Other.primary.care.providers
0.0648 < Unemployment <= 0.0805
0.182 < Alcohol.impaired.driving.deaths <= 0.262
0.000701 < Primary.care.physicians
0.127 < Severe.housing.problems <= 0.153
0.0595 < School.segregation <= 0.1084
Feature
−0.01
0.00
0.01
0.02
Weight
Positive
Negative

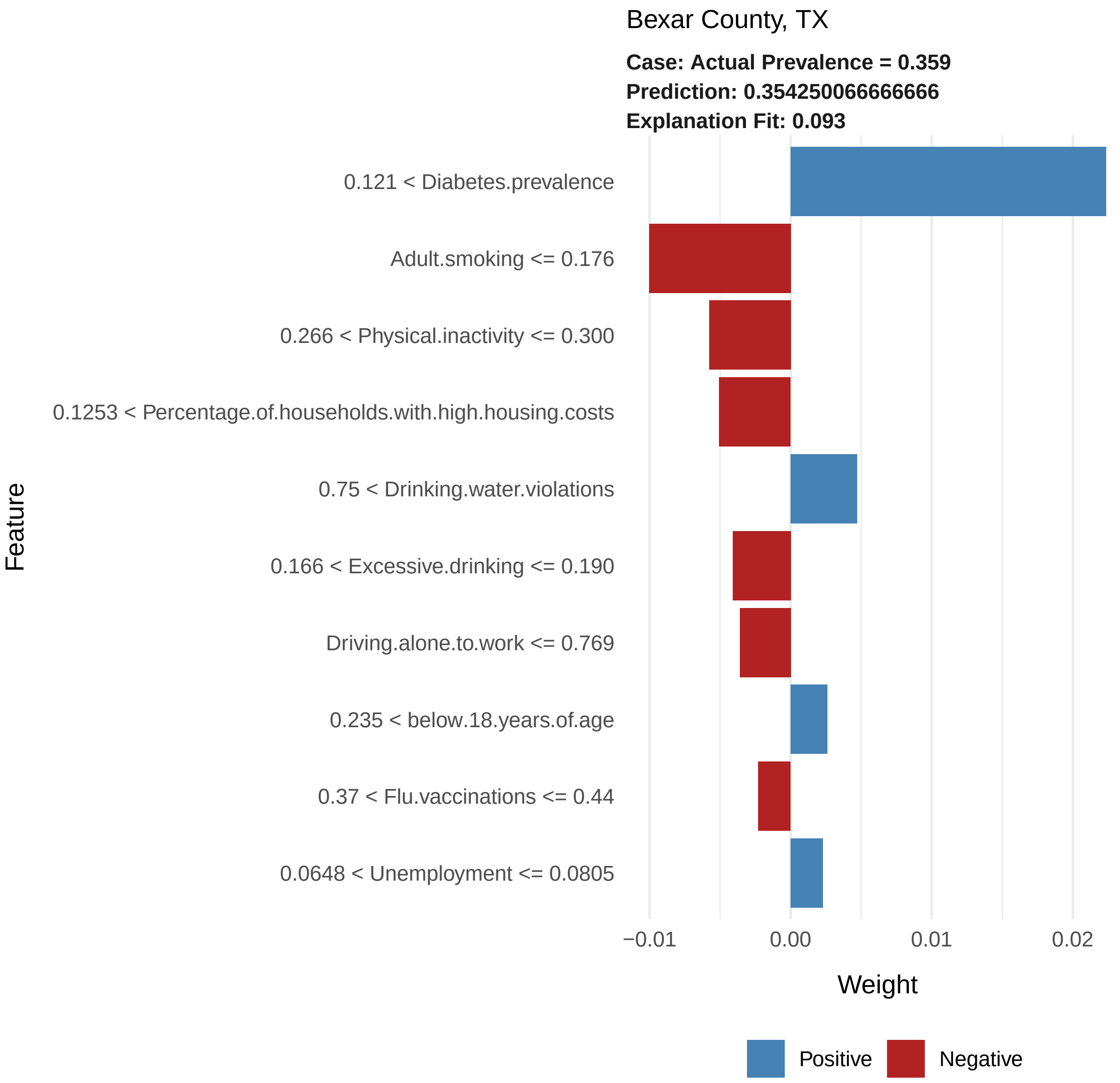
Bexar County, TX
Case: Actual Prevalence = 0.359
Prediction: 0.354250066666666
Explanation Fit: 0.093
0.121 < Diabetes.prevalence
Adult.smoking <= 0.176
0.266 < Physical.inactivity <= 0.300
0.1253 < Percentage.of.households.with.high.housing.costs
0.75 < Drinking.water.violations
0.166 < Excessive.drinking <= 0.190
Driving.alone.to.work <= 0.769
0.235 < below.18.years.of.age
0.37 < Flu.vaccinations <= 0.44
0.0648 < Unemployment <= 0.0805
Feature
−0.01
0.00
0.01
0.02
Weight
Positive
Negative

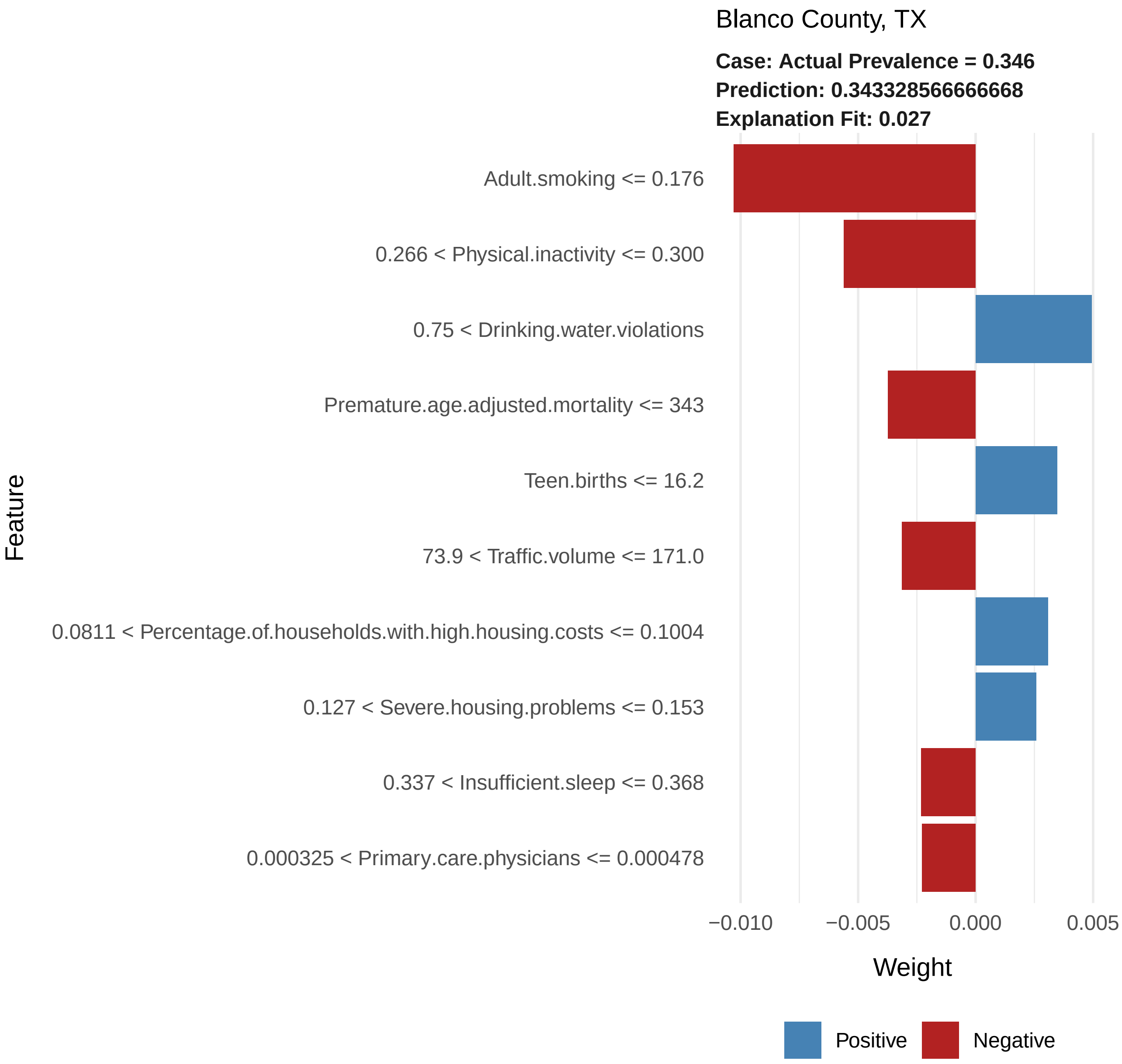

Blanco County, TX
Case: Actual Prevalence = 0.346
Prediction: 0.343328566666668
Explanation Fit: 0.027
Adult.smoking <= 0.176
0.266 < Physical.inactivity <= 0.300
0.75 < Drinking.water.violations
Premature.age.adjusted.mortality <= 343
Teen.births <= 16.2
73.9 < Traffic.volume <= 171.0
0.0811 < Percentage.of.households.with.high.housing.costs <= 0.1004
0.127 < Severe.housing.problems <= 0.153
0.337 < Insufficient.sleep <= 0.368
0.000325 < Primary.care.physicians <= 0.000478
Feature
−0.010
−0.005
0.000
0.005
Weight
Positive
Negative

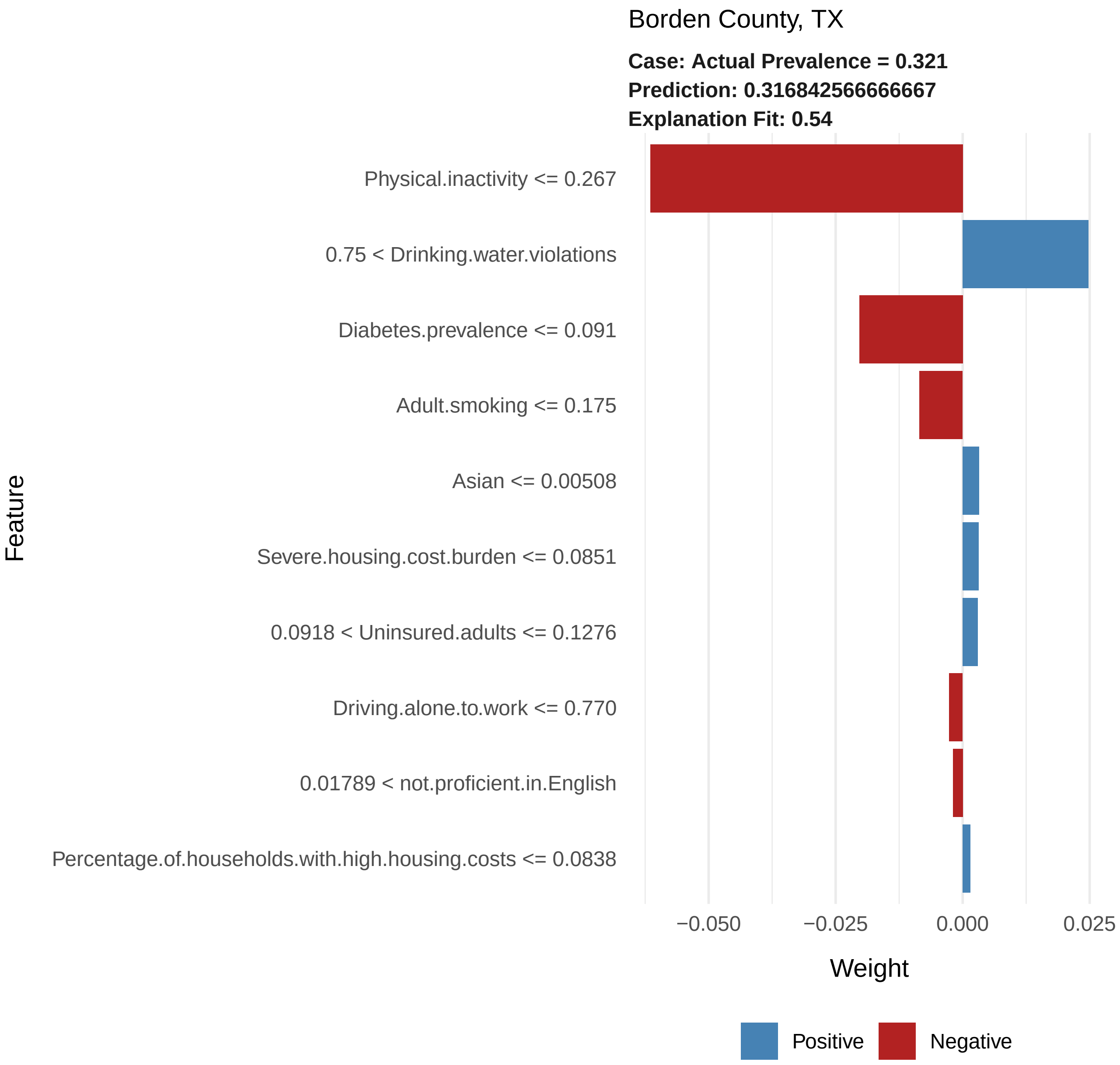
Borden County, TX
Case: Actual Prevalence = 0.321
Prediction: 0.316842566666667
Explanation Fit: 0.54
Physical.inactivity <= 0.267
0.75 < Drinking.water.violations
Diabetes.prevalence <= 0.091
Adult.smoking <= 0.175
Asian <= 0.00508
Severe.housing.cost.burden <= 0.0851
0.0918 < Uninsured.adults <= 0.1276
Driving.alone.to.work <= 0.770
0.01789 < not.proficient.in.English
Percentage.of.households.with.high.housing.costs <= 0.0838
Feature
−0.050
−0.025
0.000
0.025
Weight
Positive
Negative

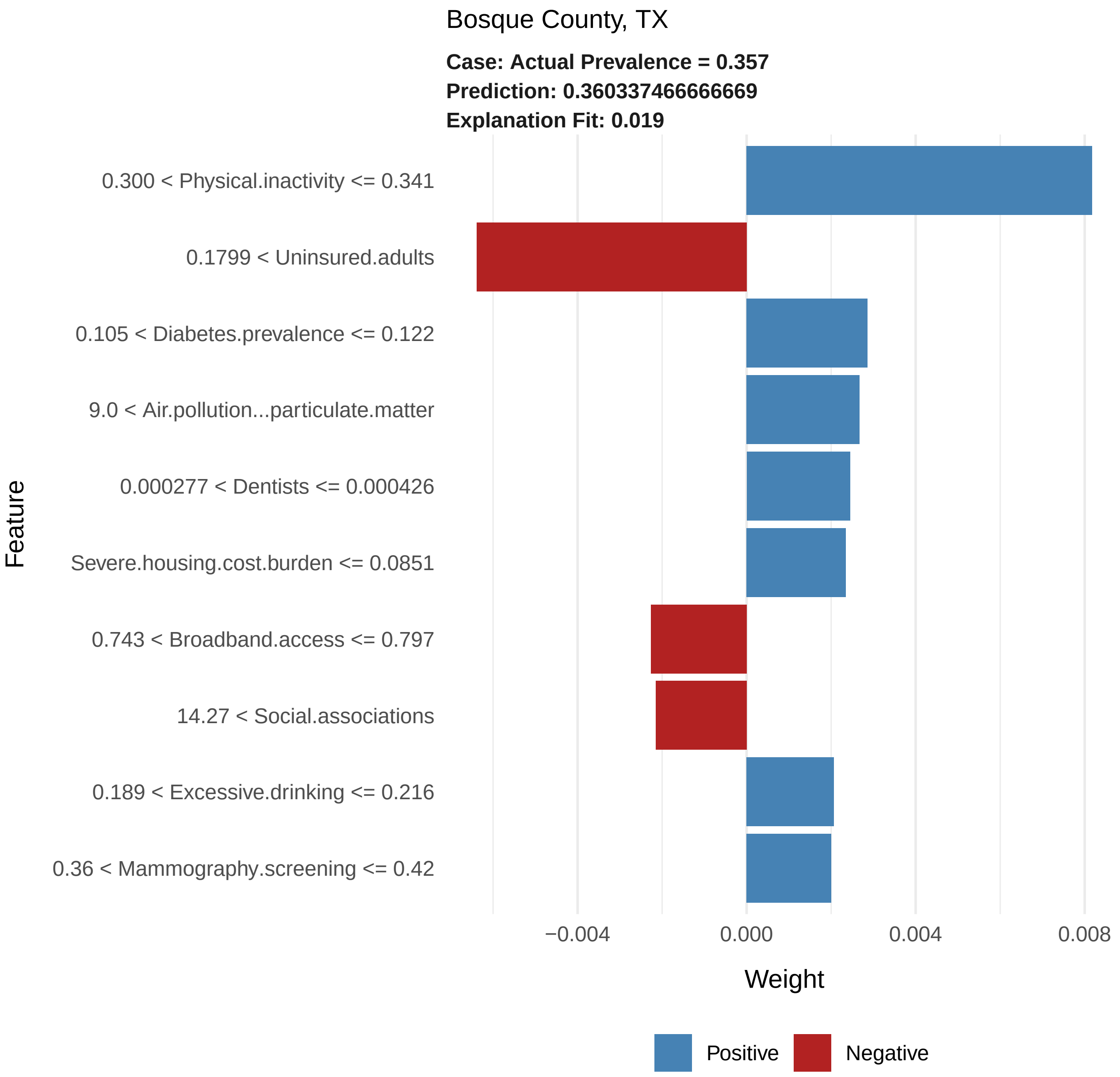

Bosque County, TX
Case: Actual Prevalence = 0.357
Prediction: 0.360337466666669
Explanation Fit: 0.019
0.300 < Physical.inactivity <= 0.341
0.1799 < Uninsured.adults
0.105 < Diabetes.prevalence <= 0.122
9.0 < Air.pollution...particulate.matter
0.000277 < Dentists <= 0.000426
Severe.housing.cost.burden <= 0.0851
0.743 < Broadband.access <= 0.797
14.27 < Social.associations
0.189 < Excessive.drinking <= 0.216
0.36 < Mammography.screening <= 0.42
Feature
−0.004
0.000
0.004
0.008
Weight
Positive
Negative

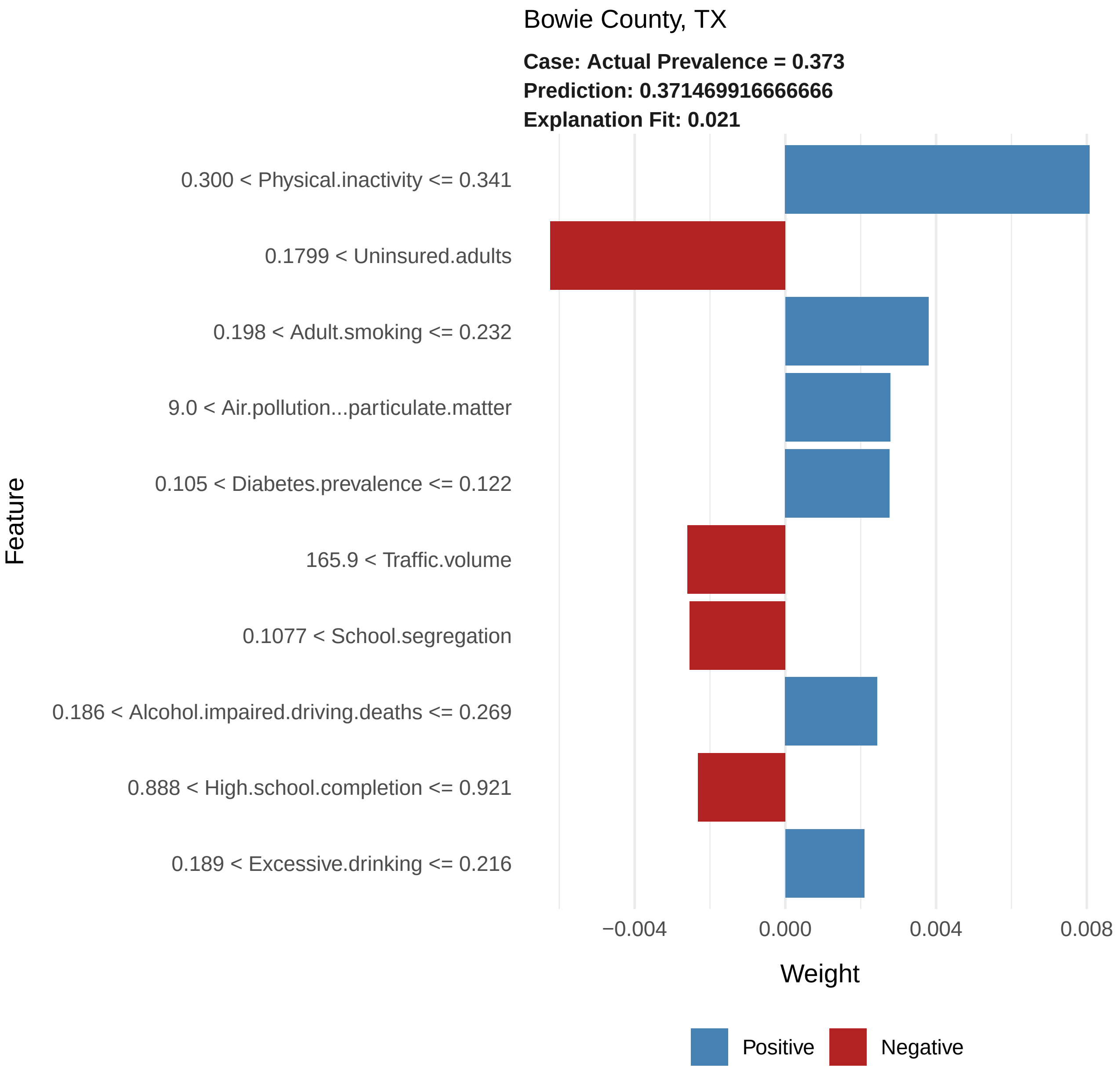
Bowie County, TX
Case: Actual Prevalence = 0.373
Prediction: 0.371469916666666
Explanation Fit: 0.021
0.300 < Physical.inactivity <= 0.341
0.1799 < Uninsured.adults
0.198 < Adult.smoking <= 0.232
9.0 < Air.pollution...particulate.matter
0.105 < Diabetes.prevalence <= 0.122
165.9 < Traffic.volume
0.1077 < School.segregation
0.186 < Alcohol.impaired.driving.deaths <= 0.269
0.888 < High.school.completion <= 0.921
0.189 < Excessive.drinking <= 0.216
Feature
−0.004
0.000
0.004
0.008
Weight
Positive
Negative

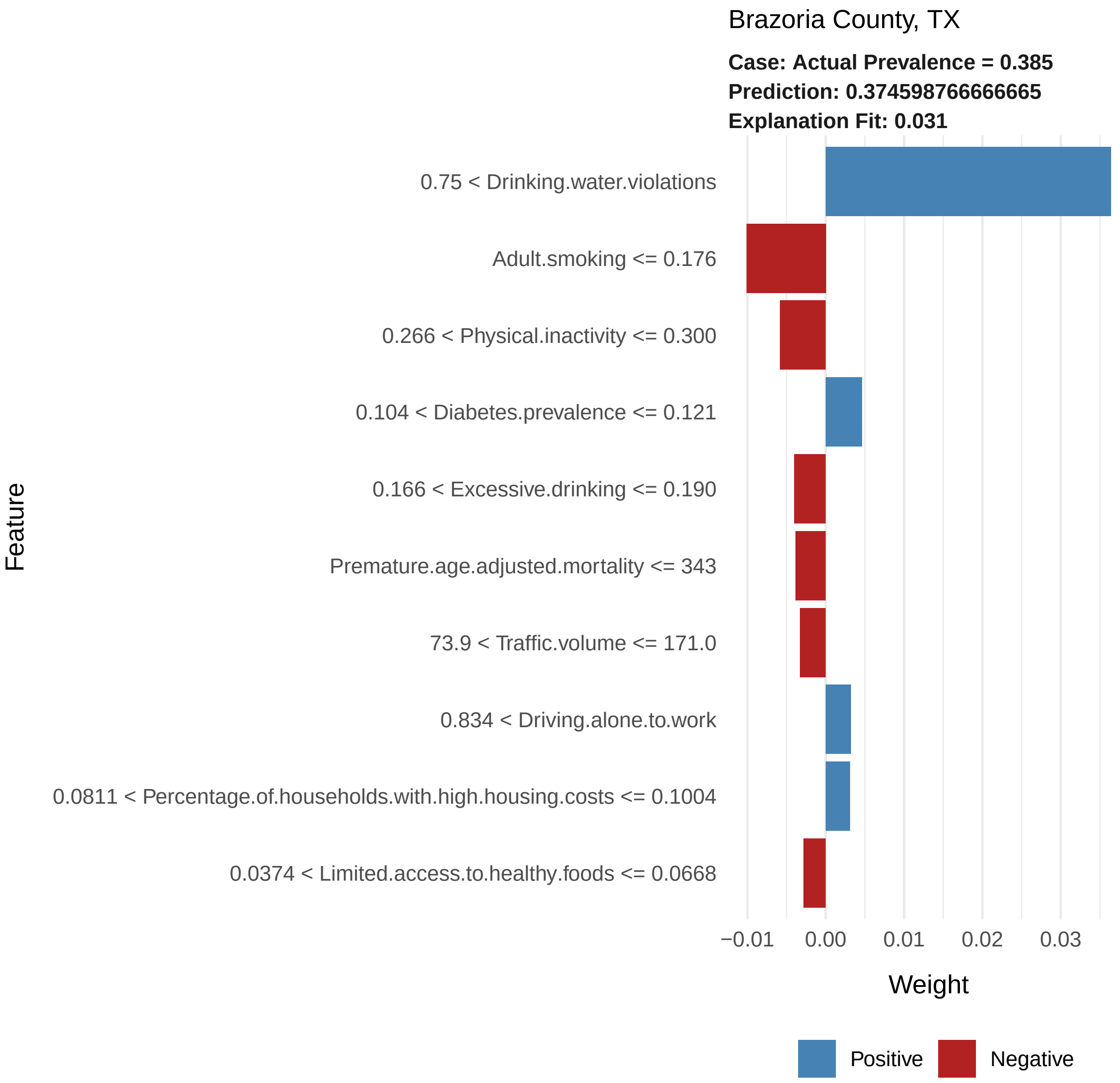

Brazoria County, TX
Case: Actual Prevalence = 0.385
Prediction: 0.374598766666665
Explanation Fit: 0.031
0.75 < Drinking.water.violations
Adult.smoking <= 0.176
0.266 < Physical.inactivity <= 0.300
0.104 < Diabetes.prevalence <= 0.121
0.166 < Excessive.drinking <= 0.190
Premature.age.adjusted.mortality <= 343
73.9 < Traffic.volume <= 171.0
0.834 < Driving.alone.to.work
0.0811 < Percentage.of.households.with.high.housing.costs <= 0.1004
0.0374 < Limited.access.to.healthy.foods <= 0.0668
Feature
−0.01
0.00
0.01
0.02
0.03
Weight
Positive
Negative

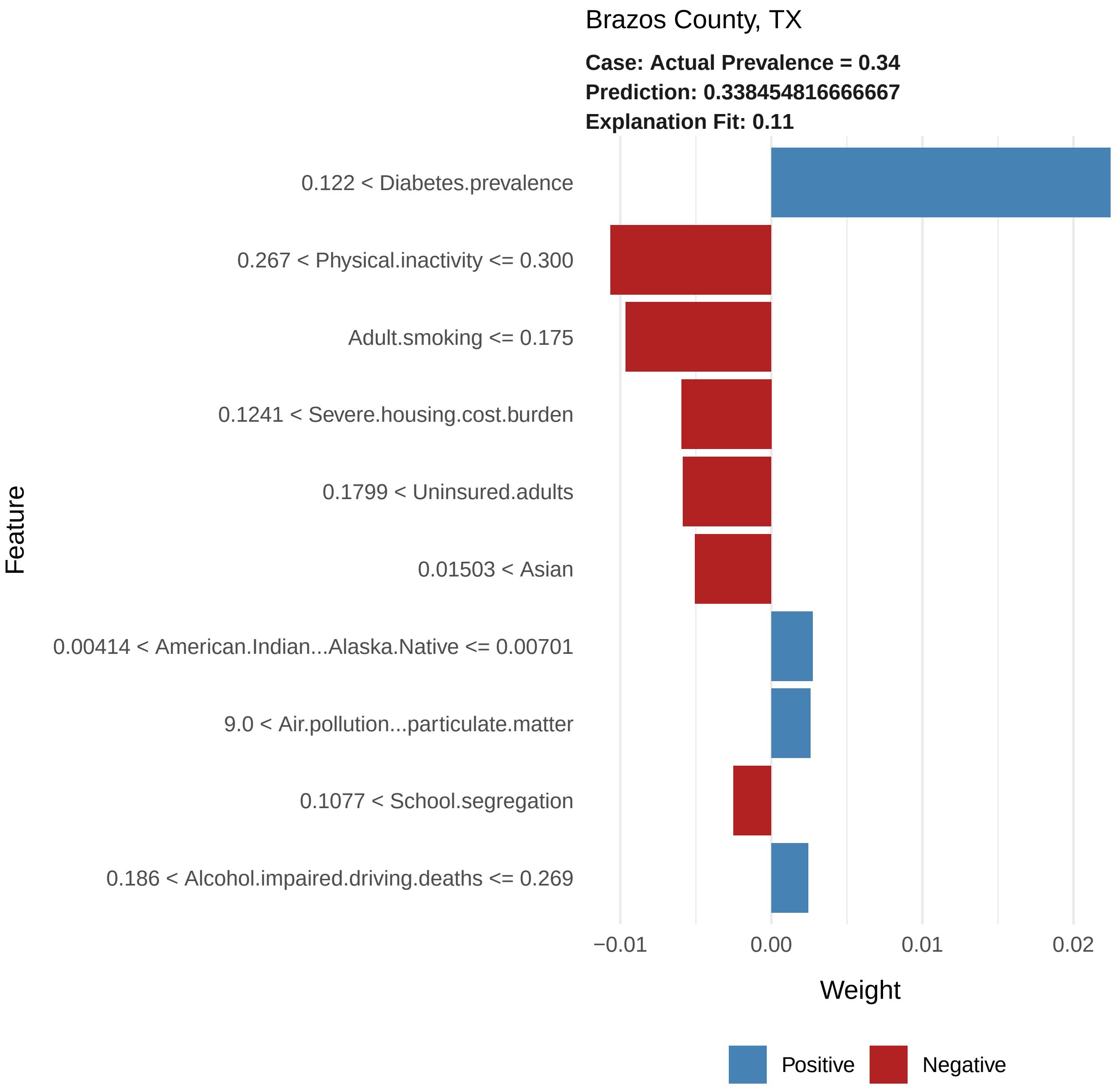

Brazos County, TX
Case: Actual Prevalence = 0.34
Prediction: 0.338454816666667
Explanation Fit: 0.11
Feature
0.122 < Diabetes.prevalence
0.267 < Physical.inactivity <= 0.300
Adult.smoking <= 0.175
0.1241 < Severe.housing.cost.burden
0.1799 < Uninsured.adults
0.01503 < Asian
0.00414 < American.Indian...Alaska.Native <= 0.00701
9.0 < Air.pollution...particulate.matter
0.1077 < School.segregation
0.186 < Alcohol.impaired.driving.deaths <= 0.269
−0.01
0.00
0.01
0.02
Weight
Positive
Negative

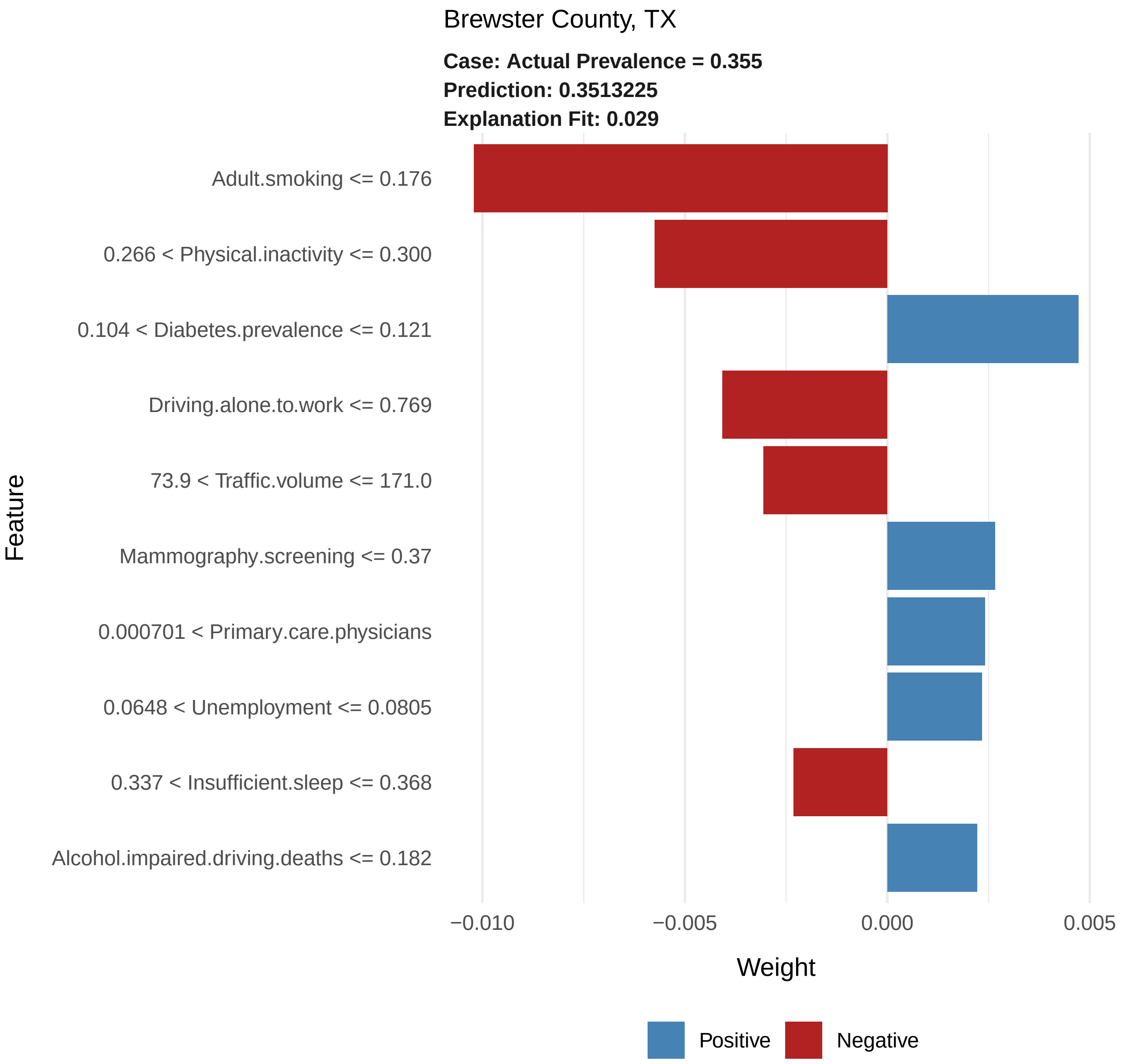

Brewster County, TX
Case: Actual Prevalence = 0.355
Prediction: 0.3513225
Explanation Fit: 0.029
Adult.smoking <= 0.176
0.266 < Physical.inactivity <= 0.300
0.104 < Diabetes.prevalence <= 0.121
Driving.alone.to.work <= 0.769
73.9 < Traffic.volume <= 171.0
Mammography.screening <= 0.37
0.000701 < Primary.care.physicians
0.0648 < Unemployment <= 0.0805
0.337 < Insufficient.sleep <= 0.368
Alcohol.impaired.driving.deaths <= 0.182
Feature
−0.010
−0.005
0.000
0.005
Weight
Positive
Negative

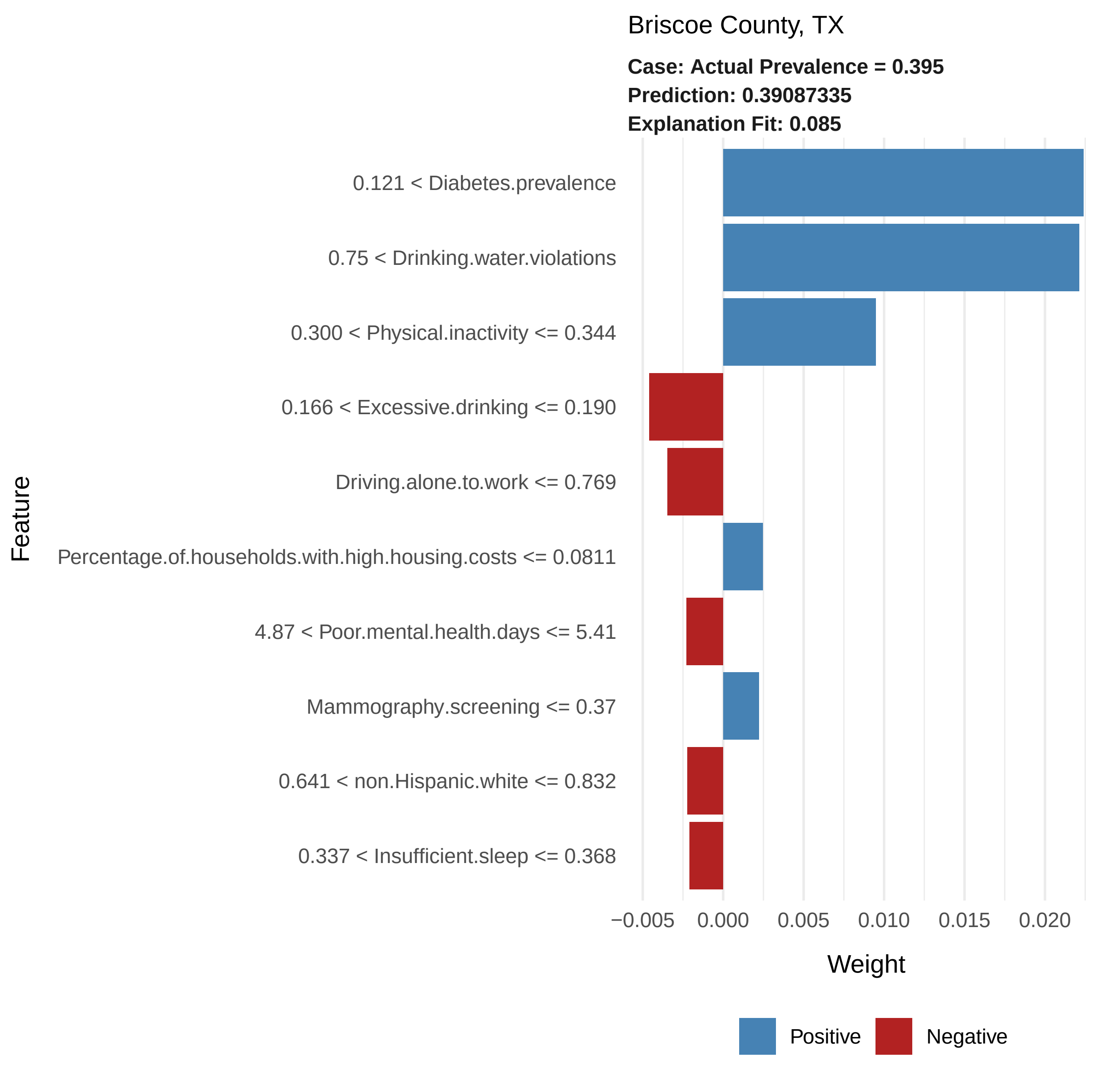

Briscoe County, TX
Case: Actual Prevalence = 0.395
Prediction: 0.39087335
Explanation Fit: 0.085
0.121 < Diabetes.prevalence
0.75 < Drinking.water.violations
0.300 < Physical.inactivity <= 0.344
0.166 < Excessive.drinking <= 0.190
Driving.alone.to.work <= 0.769
Percentage.of.households.with.high.housing.costs <= 0.0811
4.87 < Poor.mental.health.days <= 5.41
Mammography.screening <= 0.37
0.641 < non.Hispanic.white <= 0.832
0.337 < Insufficient.sleep <= 0.368
Feature
−0.005
0.000
0.005
0.010
0.015
0.020
Weight
Positive
Negative

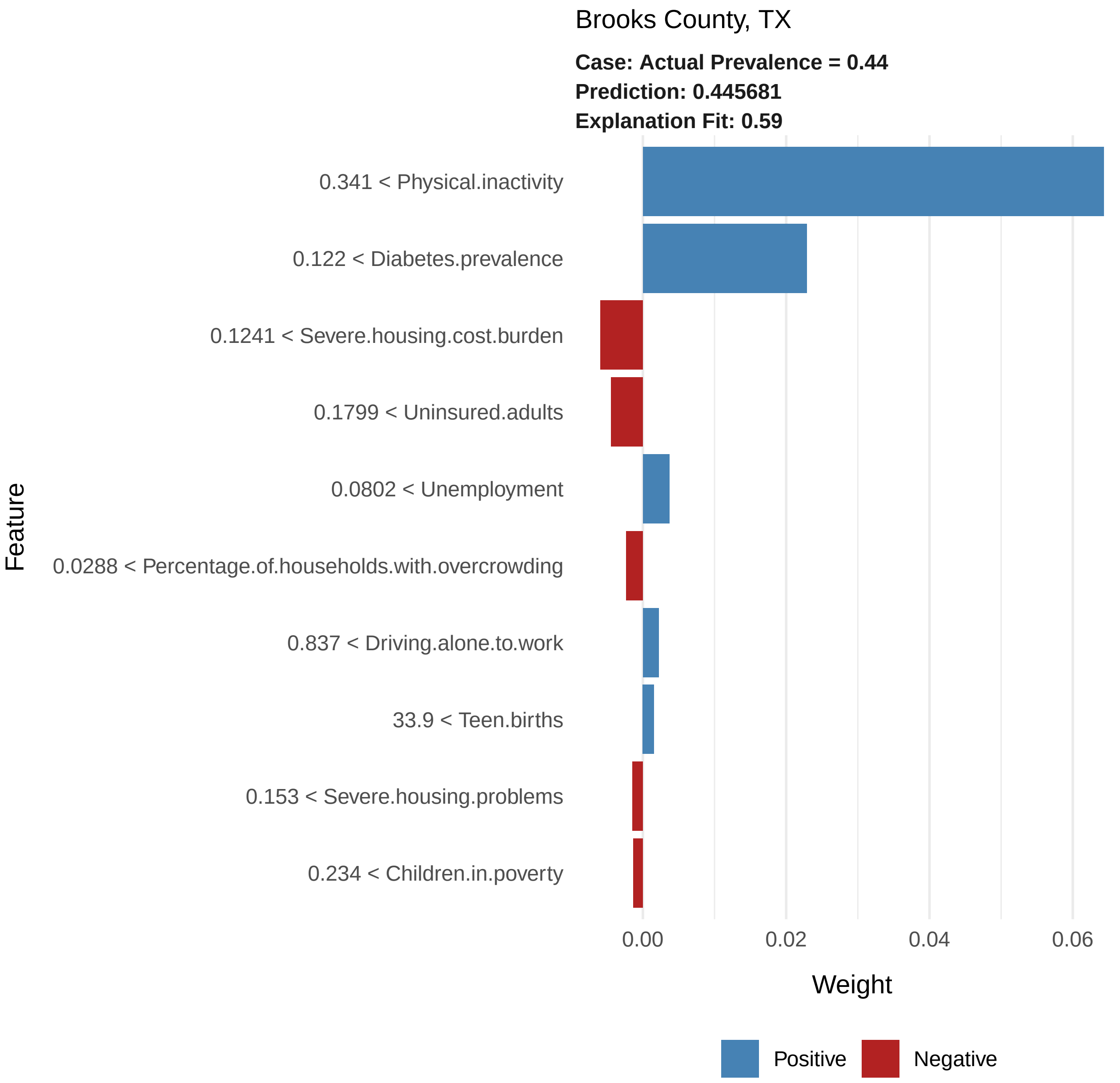
Brooks County, TX
Case: Actual Prevalence = 0.44
Prediction: 0.445681
Explanation Fit: 0.59
0.341 < Physical.inactivity
0.122 < Diabetes.prevalence
0.1241 < Severe.housing.cost.burden
0.1799 < Uninsured.adults
0.0802 < Unemployment
0.0288 < Percentage.of.households.with.overcrowding
0.837 < Driving.alone.to.work
33.9 < Teen.births
0.153 < Severe.housing.problems
0.234 < Children.in.poverty
Feature
0.00
0.02
0.04
0.06
Weight
Positive
Negative

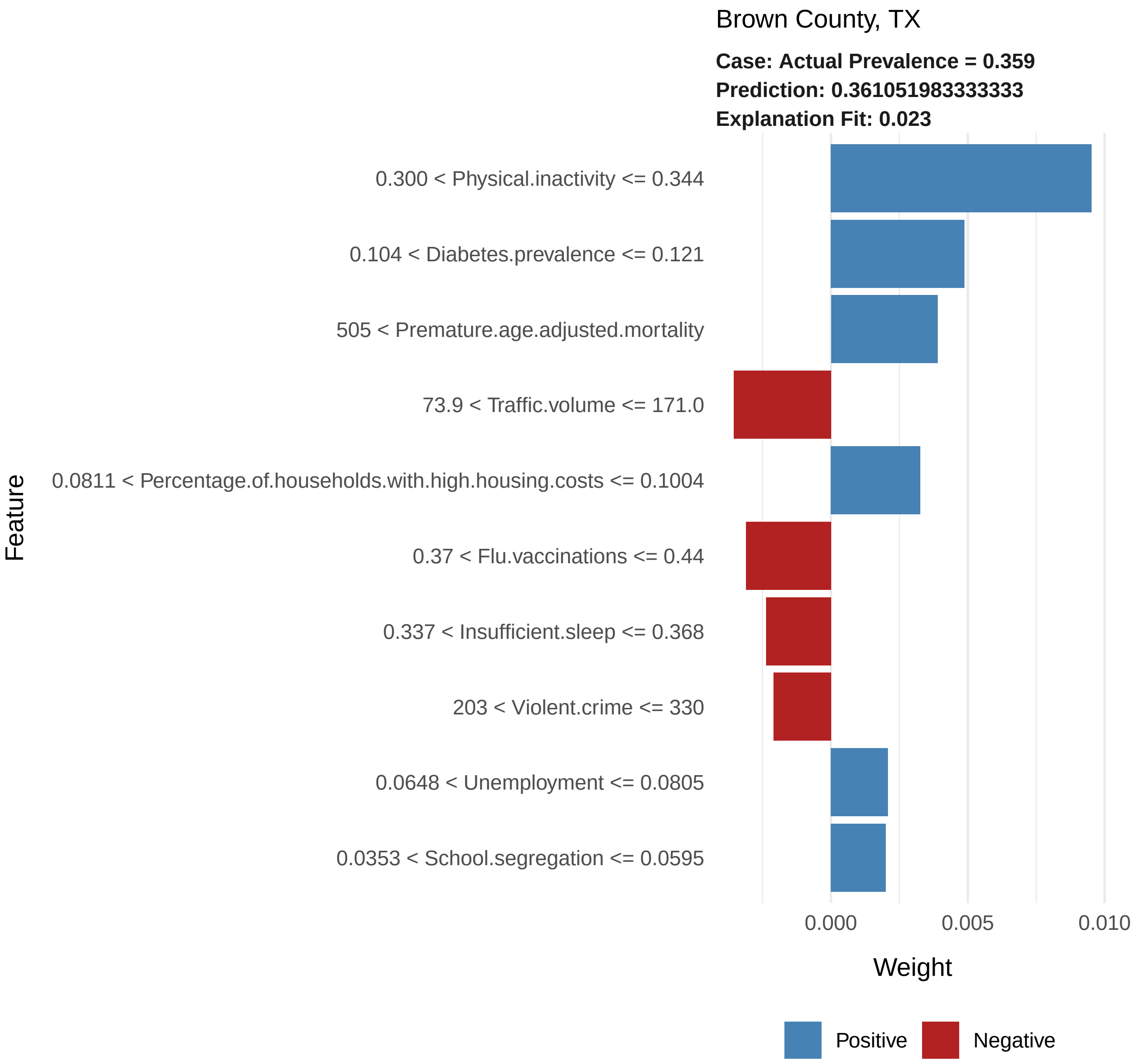

Brown County, TX
Case: Actual Prevalence = 0.359
Prediction: 0.361051983333333
Explanation Fit: 0.023
0.300 < Physical.inactivity <= 0.344
0.104 < Diabetes.prevalence <= 0.121
505 < Premature.age.adjusted.mortality
73.9 < Traffic.volume <= 171.0
0.0811 < Percentage.of.households.with.high.housing.costs <= 0.1004
0.37 < Flu.vaccinations <= 0.44
0.337 < Insufficient.sleep <= 0.368
203 < Violent.crime <= 330
0.0648 < Unemployment <= 0.0805
0.0353 < School.segregation <= 0.0595
Feature
0.000
0.005
0.010
Weight
Positive
Negative

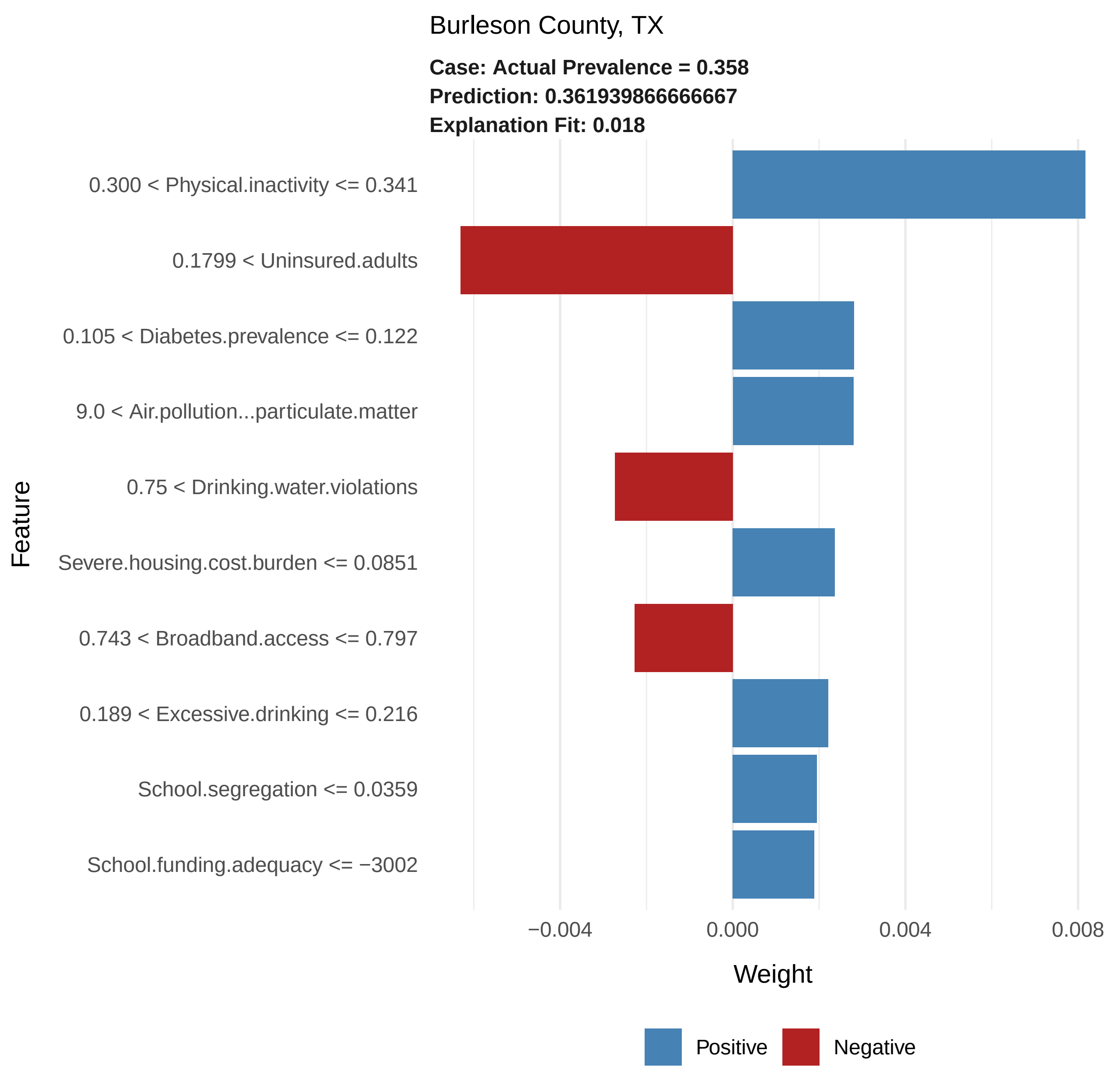

Burleson County, TX
Case: Actual Prevalence = 0.358
Prediction: 0.361939866666667
Explanation Fit: 0.018
0.300 < Physical.inactivity <= 0.341
0.1799 < Uninsured.adults
0.105 < Diabetes.prevalence <= 0.122
9.0 < Air.pollution...particulate.matter
0.75 < Drinking.water.violations
Severe.housing.cost.burden <= 0.0851
0.743 < Broadband.access <= 0.797
0.189 < Excessive.drinking <= 0.216
School.segregation <= 0.0359
School.funding.adequacy <= −3002
Feature
−0.004
0.000
0.004
0.008
Weight
Positive
Negative

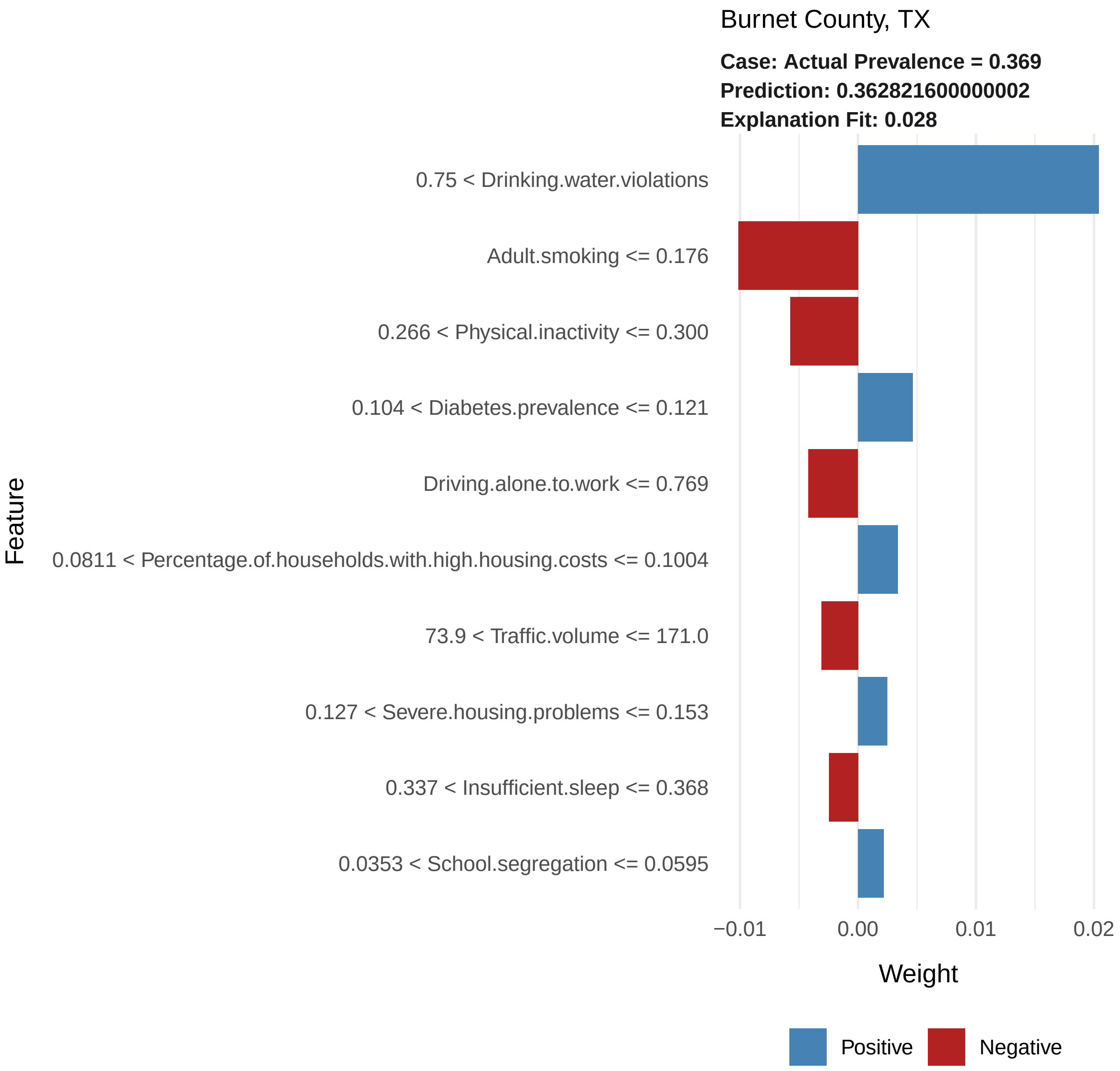
Burnet County, TX
Case: Actual Prevalence = 0.369
Prediction: 0.362821600000002
Explanation Fit: 0.028
0.75 < Drinking.water.violations
Adult.smoking <= 0.176
0.266 < Physical.inactivity <= 0.300
0.104 < Diabetes.prevalence <= 0.121
Driving.alone.to.work <= 0.769
0.0811 < Percentage.of.households.with.high.housing.costs <= 0.1004
73.9 < Traffic.volume <= 171.0
0.127 < Severe.housing.problems <= 0.153
0.337 < Insufficient.sleep <= 0.368
0.0353 < School.segregation <= 0.0595
Feature
−0.01
0.00
0.01
0.02
Weight
Positive
Negative

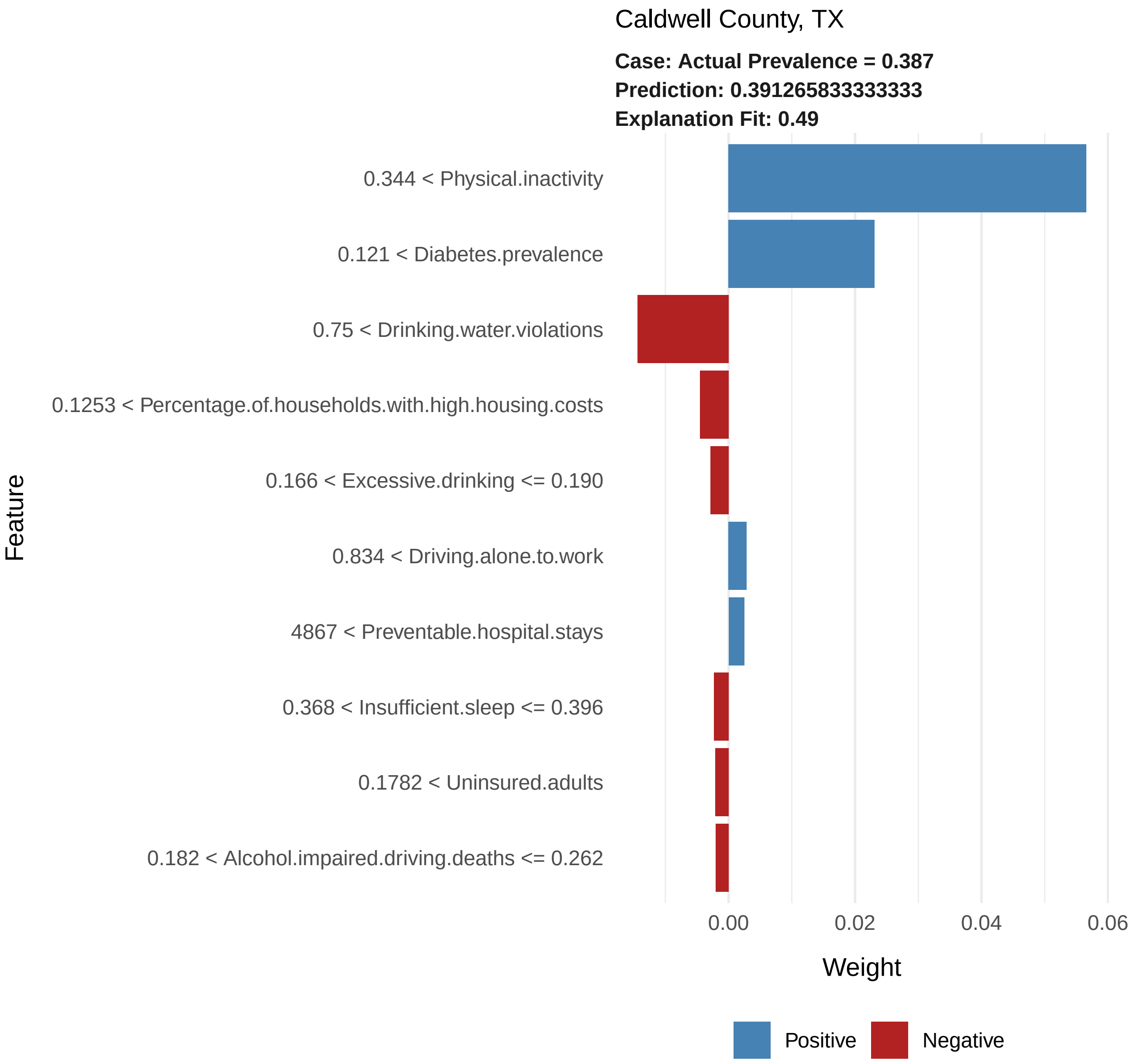
Caldwell County, TX
Case: Actual Prevalence = 0.387
Prediction: 0.391265833333333
Explanation Fit: 0.49
0.344 < Physical.inactivity
0.121 < Diabetes.prevalence
0.75 < Drinking.water.violations
0.1253 < Percentage.of.households.with.high.housing.costs
0.166 < Excessive.drinking <= 0.190
0.834 < Driving.alone.to.work
4867 < Preventable.hospital.stays
0.368 < Insufficient.sleep <= 0.396
0.1782 < Uninsured.adults
0.182 < Alcohol.impaired.driving.deaths <= 0.262
Feature
0.00
0.02
0.04
0.06
Weight
Positive
Negative

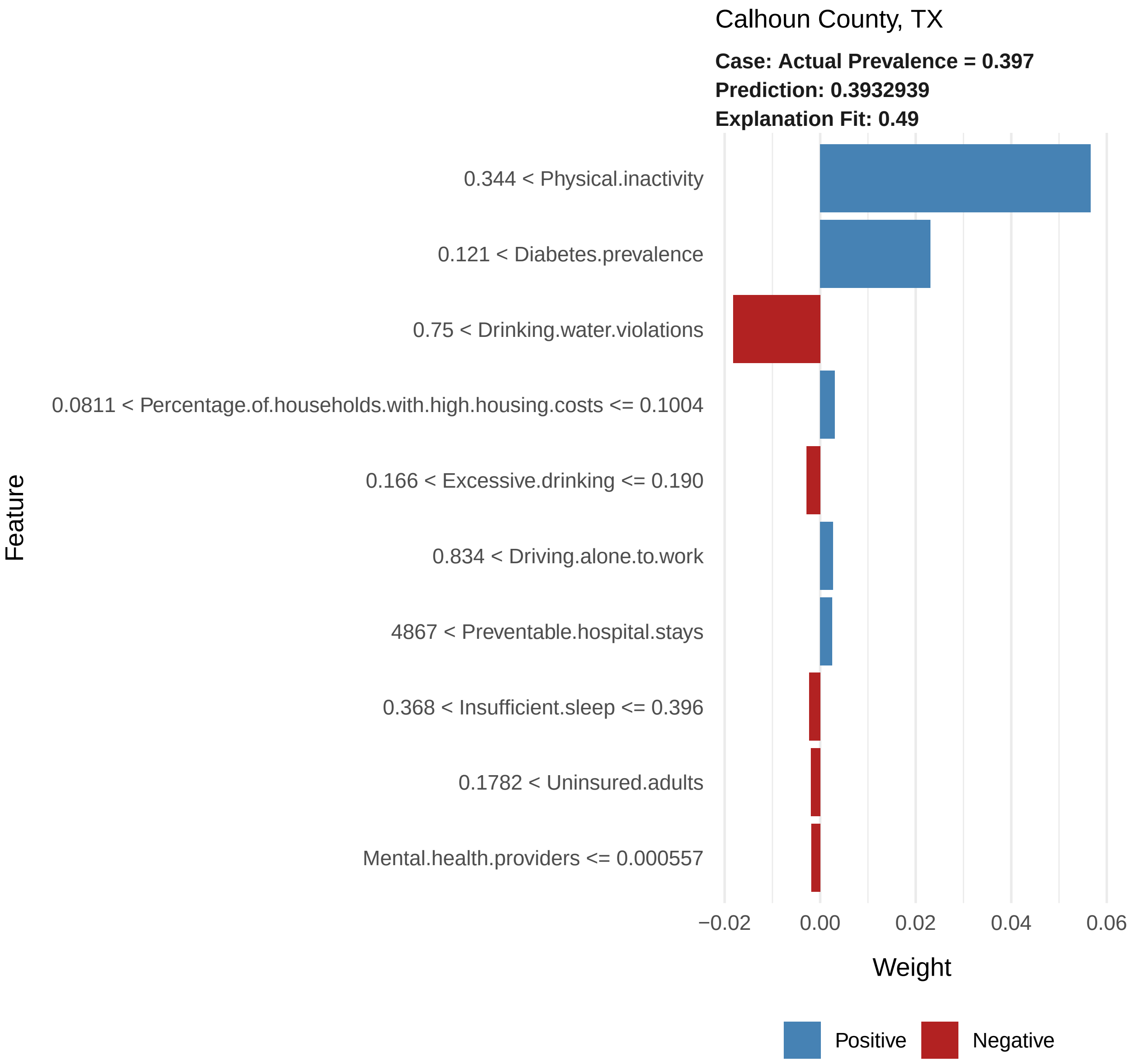
Calhoun County, TX
Case: Actual Prevalence = 0.397
Prediction: 0.3932939
Explanation Fit: 0.49
0.344 < Physical.inactivity
0.121 < Diabetes.prevalence
0.75 < Drinking.water.violations
0.0811 < Percentage.of.households.with.high.housing.costs <= 0.1004
0.166 < Excessive.drinking <= 0.190
0.834 < Driving.alone.to.work
4867 < Preventable.hospital.stays
0.368 < Insufficient.sleep <= 0.396
0.1782 < Uninsured.adults
Mental.health.providers <= 0.000557
Feature
−0.02
0.00
0.02
0.04
0.06
Weight
Positive
Negative

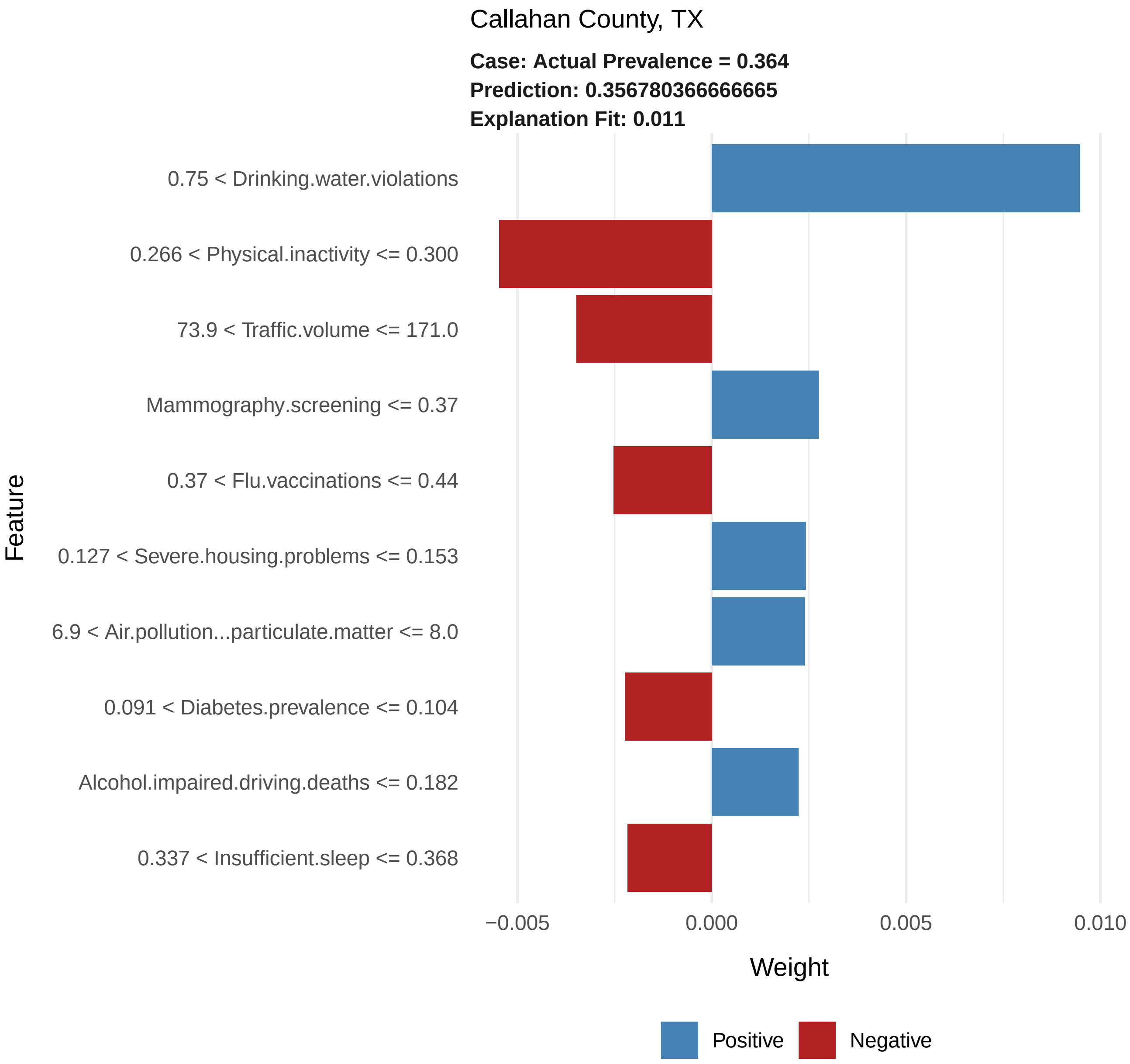
Callahan County, TX
Case: Actual Prevalence = 0.364
Prediction: 0.356780366666665
Explanation Fit: 0.011
0.75 < Drinking.water.violations
0.266 < Physical.inactivity <= 0.300
73.9 < Traffic.volume <= 171.0
Mammography.screening <= 0.37
0.37 < Flu.vaccinations <= 0.44
0.127 < Severe.housing.problems <= 0.153
6.9 < Air.pollution...particulate.matter <= 8.0
0.091 < Diabetes.prevalence <= 0.104
Alcohol.impaired.driving.deaths <= 0.182
0.337 < Insufficient.sleep <= 0.368
Feature
−0.005
0.000
0.005
0.010
Weight
Positive
Negative

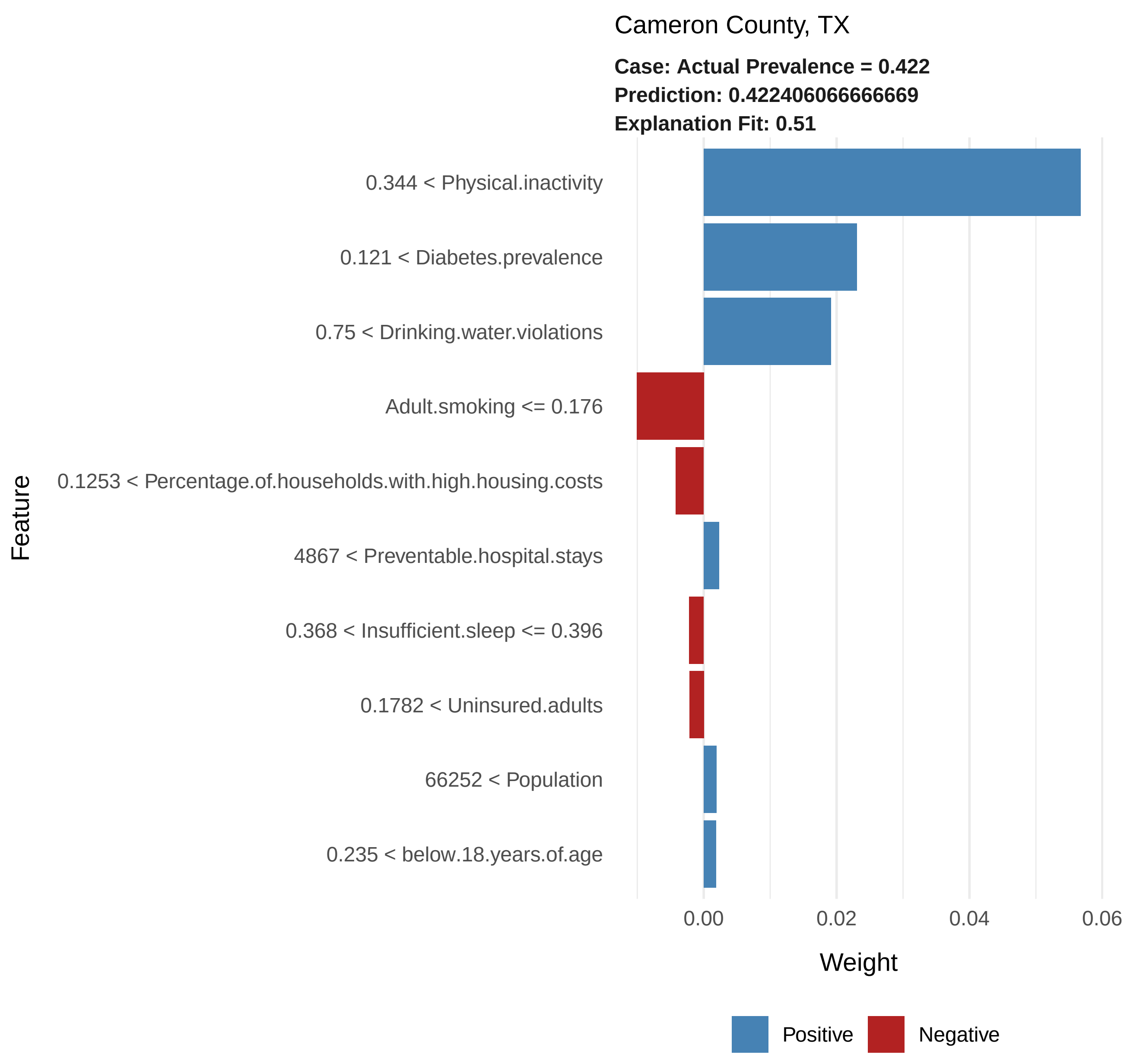

Cameron County, TX
Case: Actual Prevalence = 0.422
Prediction: 0.422406066666669
Explanation Fit: 0.51
0.344 < Physical.inactivity
0.121 < Diabetes.prevalence
0.75 < Drinking.water.violations
Adult.smoking <= 0.176
0.1253 < Percentage.of.households.with.high.housing.costs
4867 < Preventable.hospital.stays
0.368 < Insufficient.sleep <= 0.396
0.1782 < Uninsured.adults
66252 < Population
0.235 < below.18.years.of.age
Feature
0.00
0.02
0.04
0.06
Weight
Positive
Negative

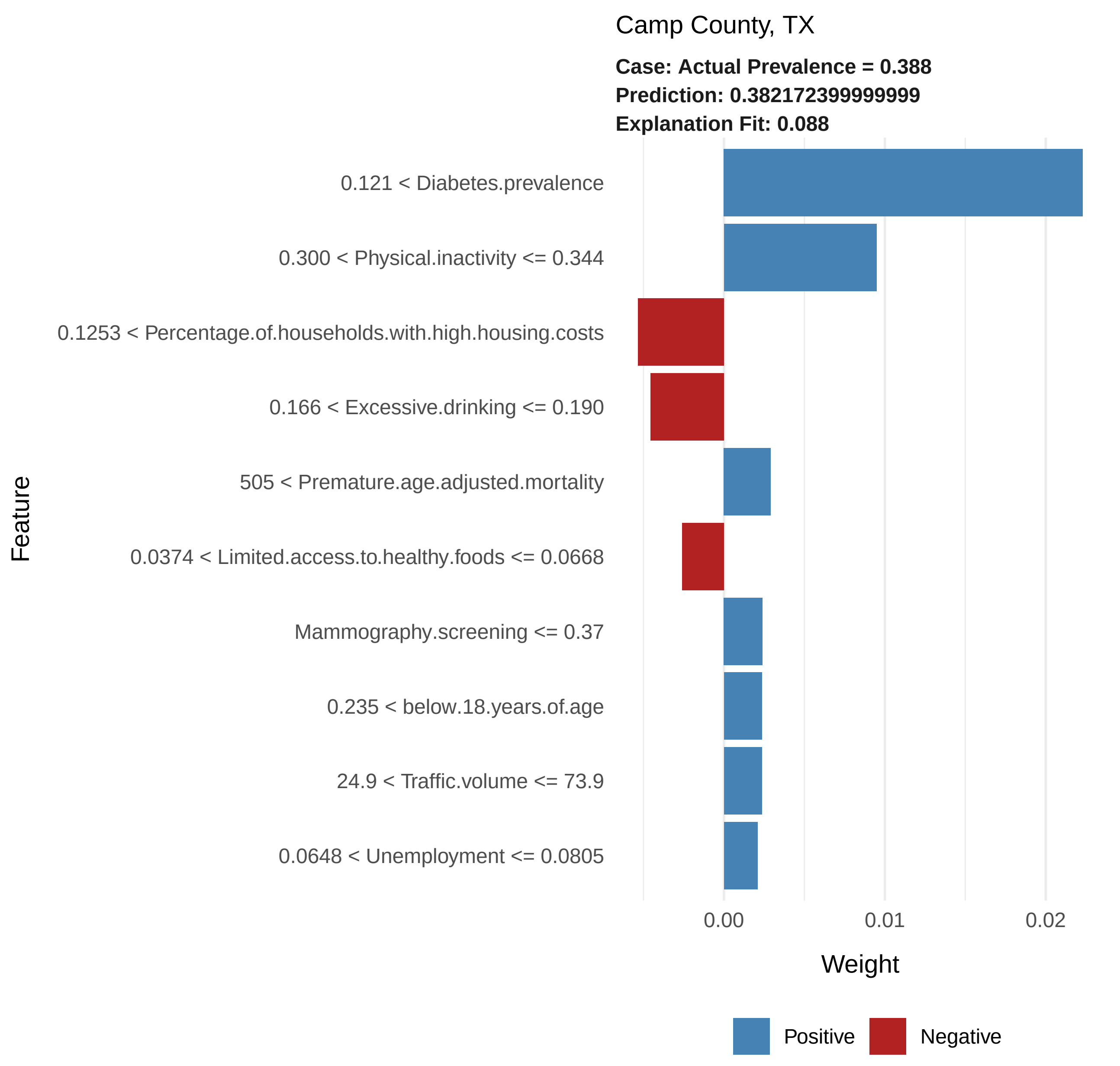

Camp County, TX
Case: Actual Prevalence = 0.388
Prediction: 0.382172399999999
Explanation Fit: 0.088
0.121 < Diabetes.prevalence
0.300 < Physical.inactivity <= 0.344
0.1253 < Percentage.of.households.with.high.housing.costs
0.166 < Excessive.drinking <= 0.190
505 < Premature.age.adjusted.mortality
0.0374 < Limited.access.to.healthy.foods <= 0.0668
Mammography.screening <= 0.37
0.235 < below.18.years.of.age
24.9 < Traffic.volume <= 73.9
0.0648 < Unemployment <= 0.0805
Feature
0.00
0.01
0.02
Weight
Positive
Negative

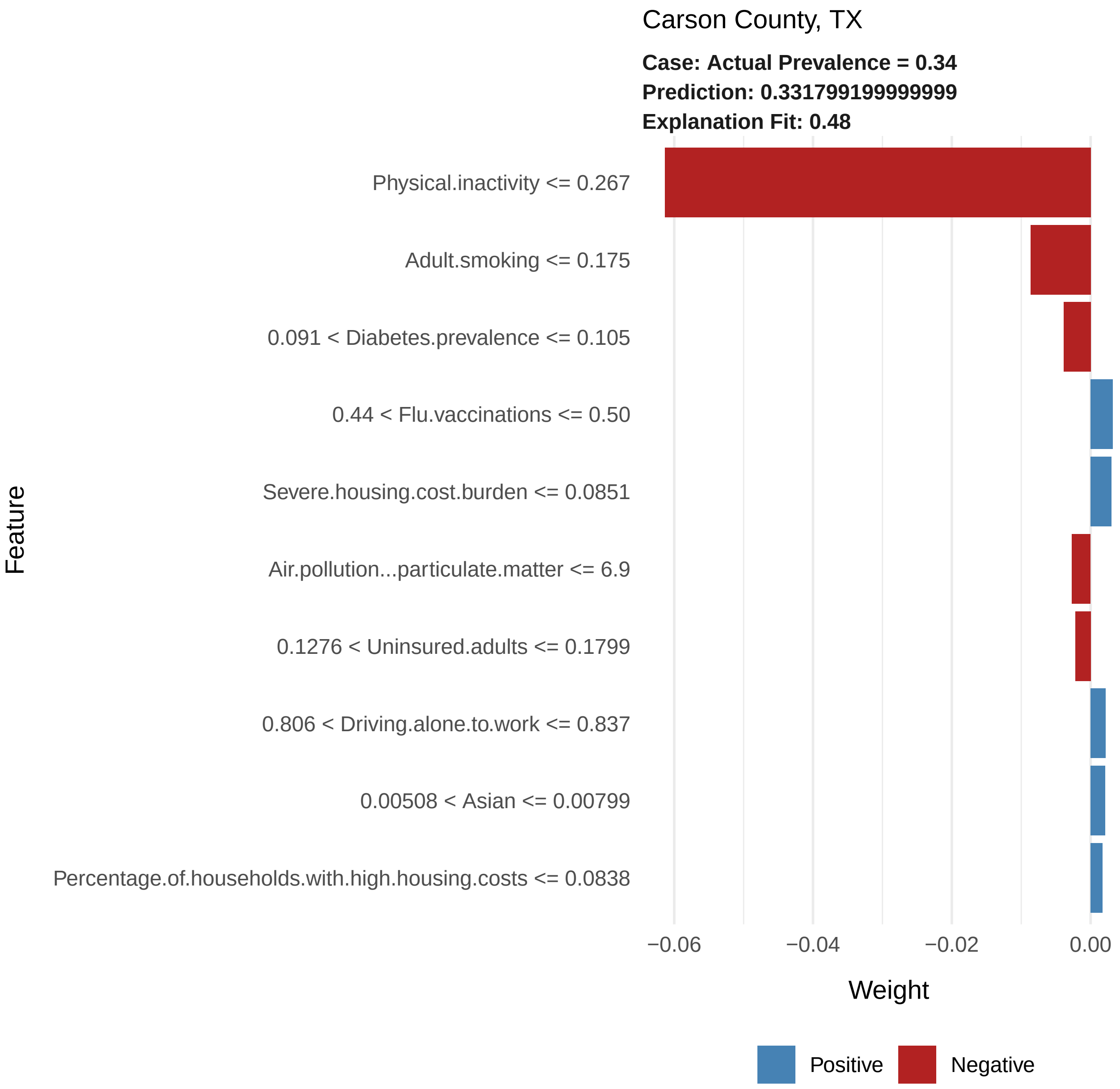

Carson County, TX
Case: Actual Prevalence = 0.34
Prediction: 0.331799199999999
Explanation Fit: 0.48
Physical.inactivity <= 0.267
Adult.smoking <= 0.175
0.091 < Diabetes.prevalence <= 0.105
0.44 < Flu.vaccinations <= 0.50
Severe.housing.cost.burden <= 0.0851
Air.pollution...particulate.matter <= 6.9
0.1276 < Uninsured.adults <= 0.1799
0.806 < Driving.alone.to.work <= 0.837
0.00508 < Asian <= 0.00799
Percentage.of.households.with.high.housing.costs <= 0.0838
Feature
−0.06
−0.04
−0.02
0.00
Weight
Positive
Negative

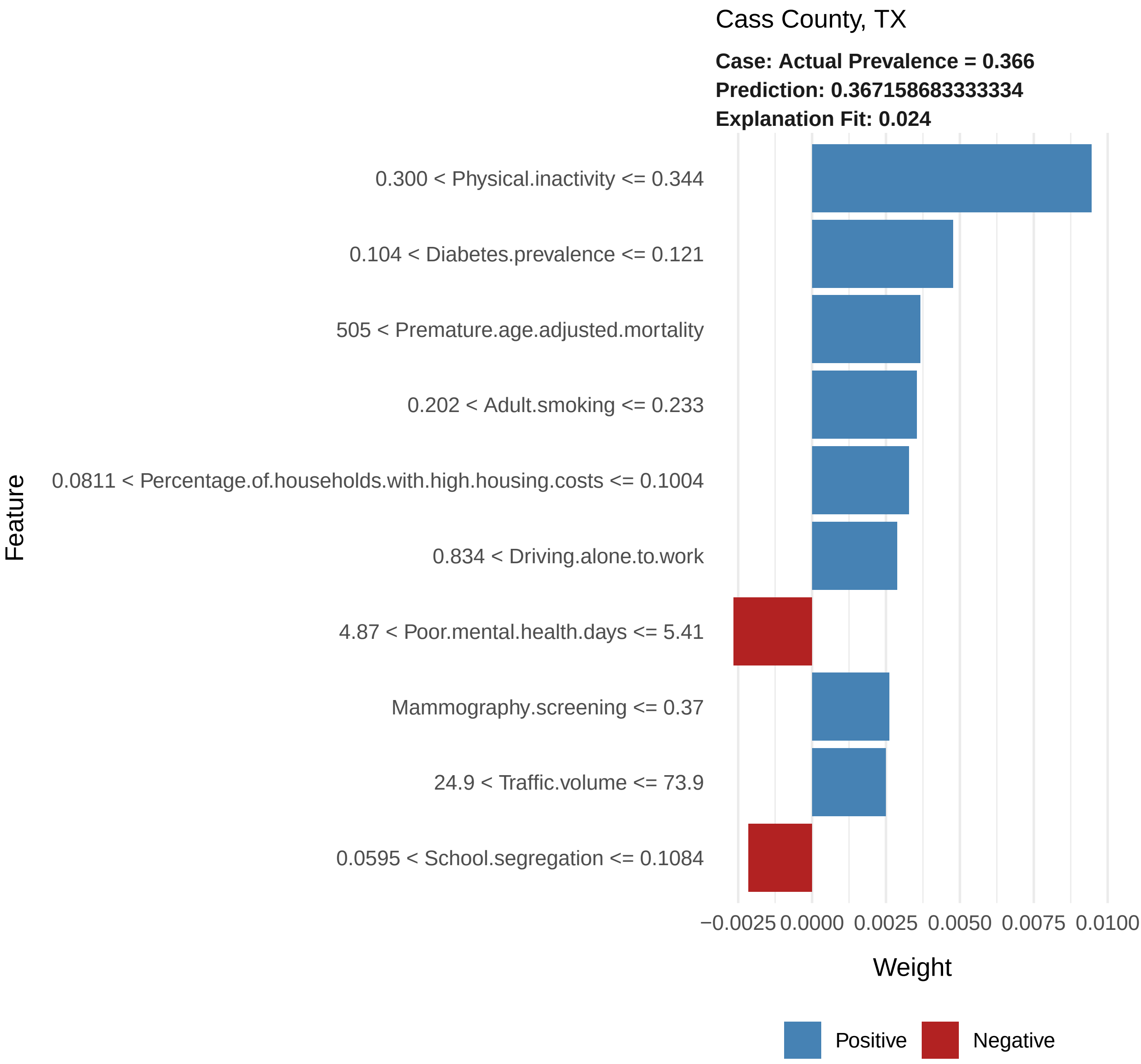
Cass County, TX
Case: Actual Prevalence = 0.366
Prediction: 0.367158683333334
Explanation Fit: 0.024
0.300 < Physical.inactivity <= 0.344
0.104 < Diabetes.prevalence <= 0.121
505 < Premature.age.adjusted.mortality
0.202 < Adult.smoking <= 0.233
0.0811 < Percentage.of.households.with.high.housing.costs <= 0.1004
0.834 < Driving.alone.to.work
4.87 < Poor.mental.health.days <= 5.41
Mammography.screening <= 0.37
24.9 < Traffic.volume <= 73.9
0.0595 < School.segregation <= 0.1084
Feature
−0.0025 0.0000 0.0025 0.0050 0.0075 0.0100
Weight
Positive
Negative

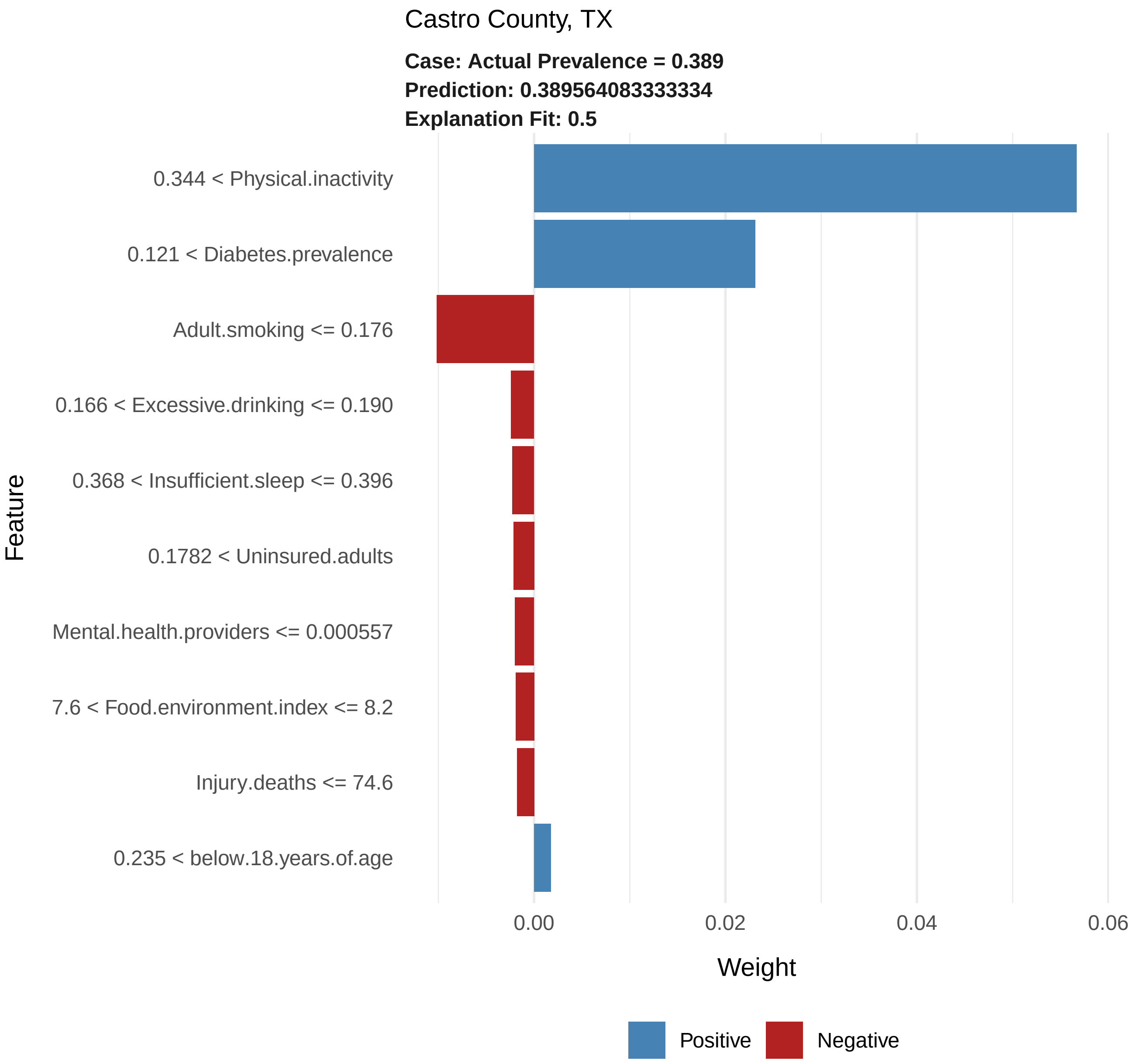
Castro County, TX
Case: Actual Prevalence = 0.389
Prediction: 0.389564083333334
Explanation Fit: 0.5
0.344 < Physical.inactivity
0.121 < Diabetes.prevalence
Adult.smoking <= 0.176
0.166 < Excessive.drinking <= 0.190
0.368 < Insufficient.sleep <= 0.396
0.1782 < Uninsured.adults
Mental.health.providers <= 0.000557
7.6 < Food.environment.index <= 8.2
Injury.deaths <= 74.6
0.235 < below.18.years.of.age
Feature
0.00
0.02
0.04
0.06
Weight
Positive
Negative

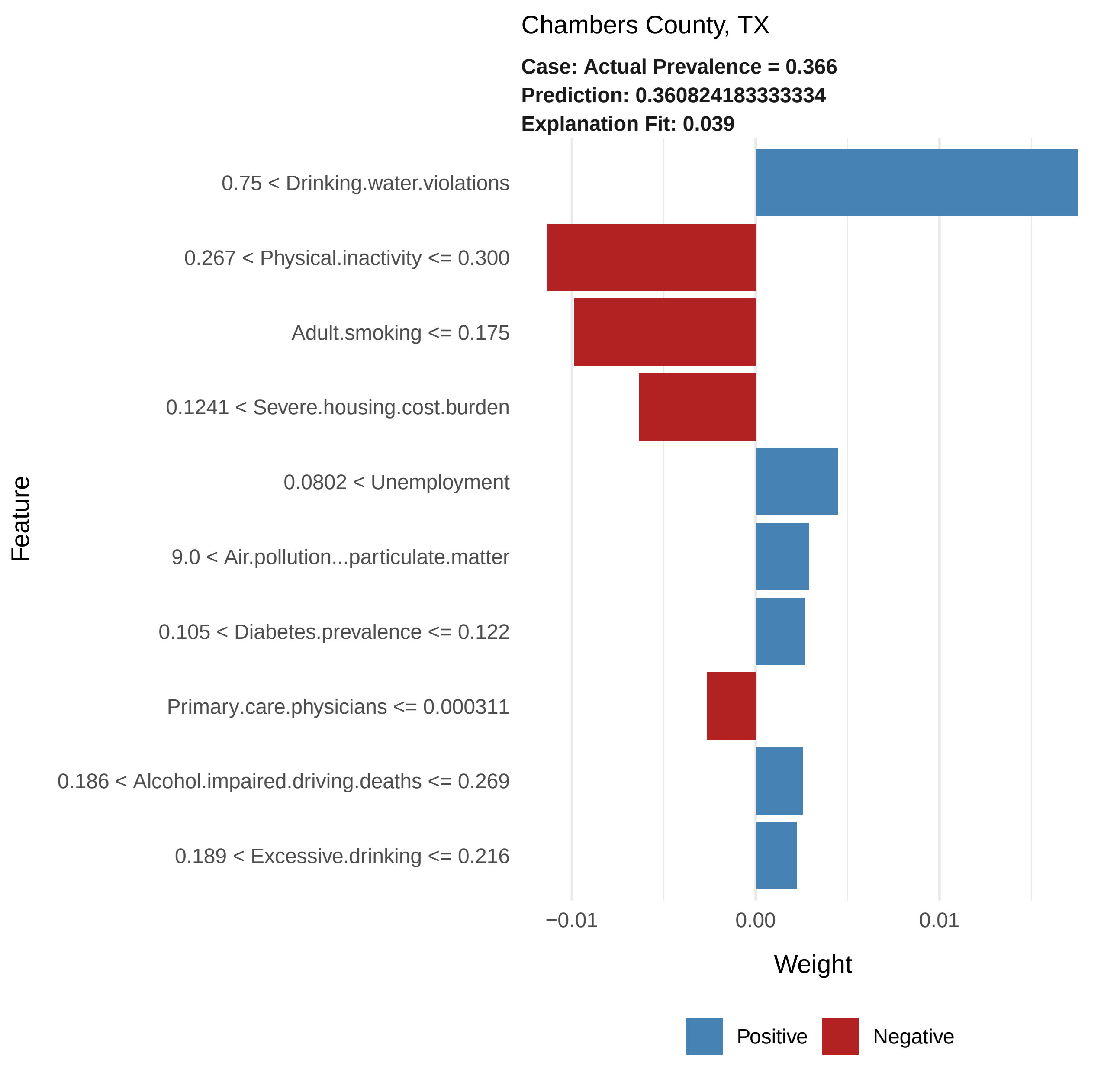
Chambers County, TX
Case: Actual Prevalence = 0.366
Prediction: 0.360824183333334
Explanation Fit: 0.039
0.75 < Drinking.water.violations
0.267 < Physical.inactivity <= 0.300
Adult.smoking <= 0.175
0.1241 < Severe.housing.cost.burden
0.0802 < Unemployment
9.0 < Air.pollution...particulate.matter
0.105 < Diabetes.prevalence <= 0.122
Primary.care.physicians <= 0.000311
0.186 < Alcohol.impaired.driving.deaths <= 0.269
0.189 < Excessive.drinking <= 0.216
Feature
−0.01
0.00
0.01
Weight
Positive
Negative

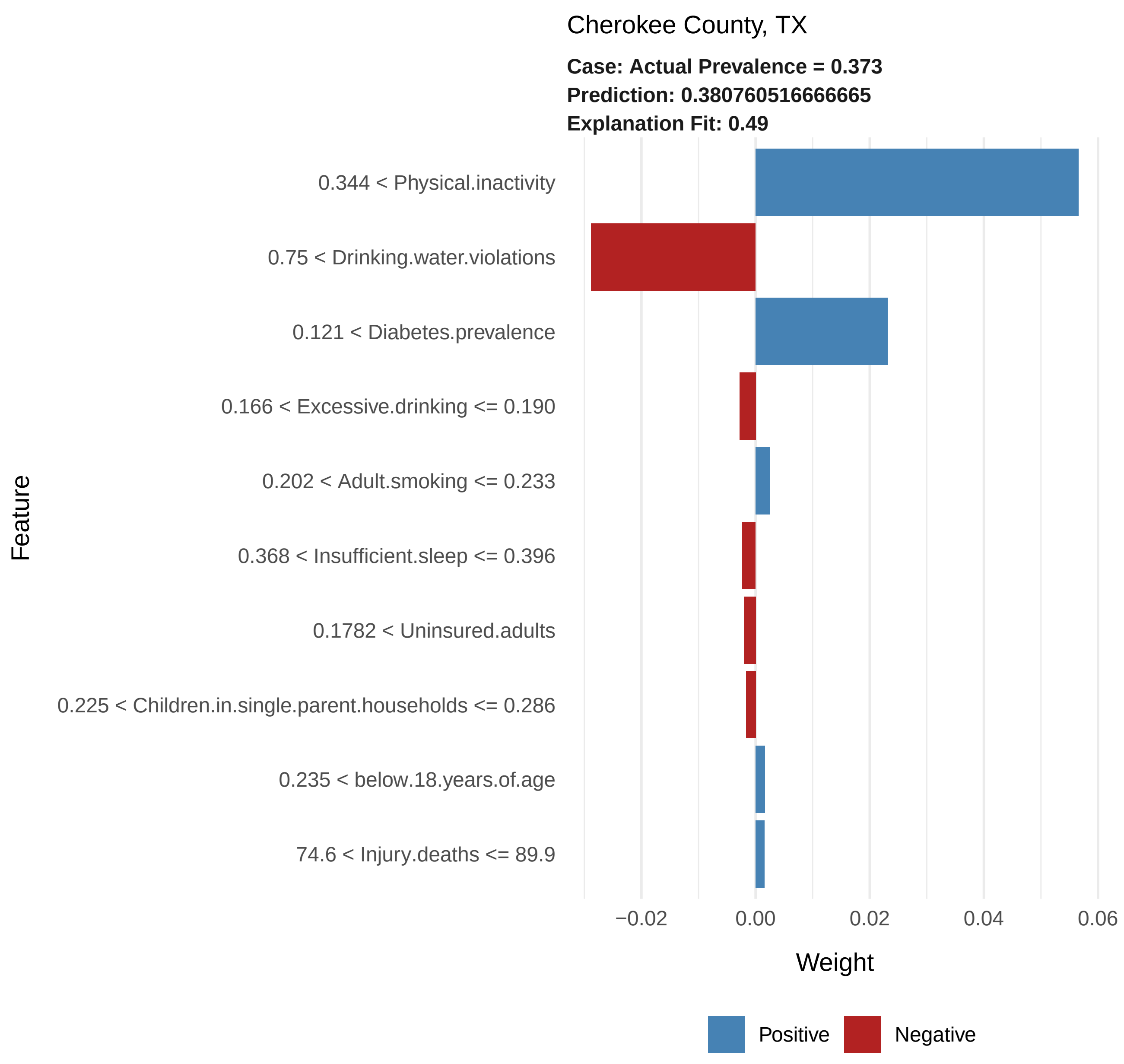
Cherokee County, TX
Case: Actual Prevalence = 0.373
Prediction: 0.380760516666665
Explanation Fit: 0.49
0.344 < Physical.inactivity
0.75 < Drinking.water.violations
0.121 < Diabetes.prevalence
0.166 < Excessive.drinking <= 0.190
0.202 < Adult.smoking <= 0.233
0.368 < Insufficient.sleep <= 0.396
0.1782 < Uninsured.adults
0.225 < Children.in.single.parent.households <= 0.286
0.235 < below.18.years.of.age
74.6 < Injury.deaths <= 89.9
Feature
−0.02
0.00
0.02
0.04
0.06
Weight
Positive
Negative

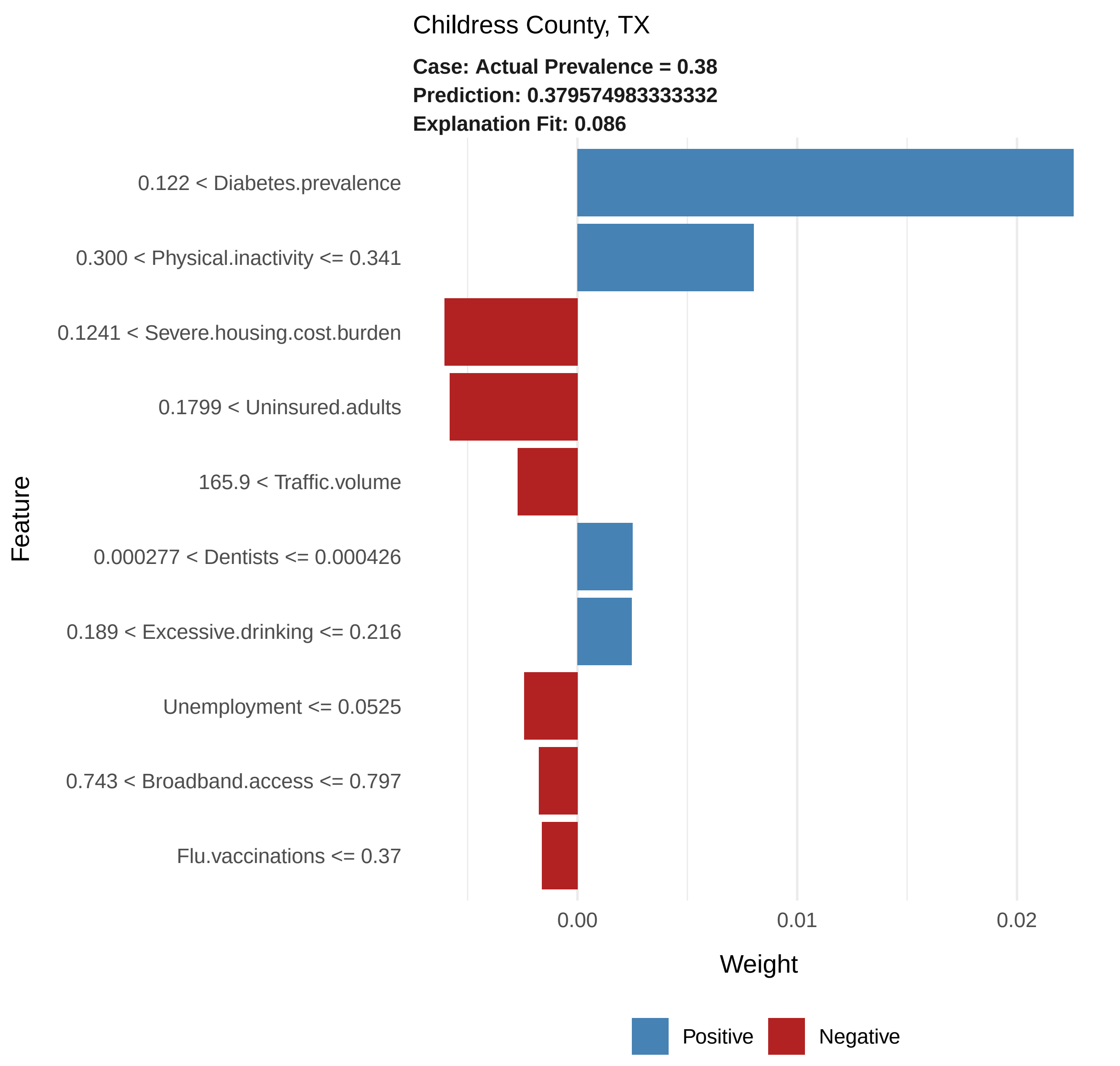

Childress County, TX
Case: Actual Prevalence = 0.38
Prediction: 0.379574983333332
Explanation Fit: 0.086
0.122 < Diabetes.prevalence
0.300 < Physical.inactivity <= 0.341
0.1241 < Severe.housing.cost.burden
0.1799 < Uninsured.adults
165.9 < Traffic.volume
0.000277 < Dentists <= 0.000426
0.189 < Excessive.drinking <= 0.216
Unemployment <= 0.0525
0.743 < Broadband.access <= 0.797
Flu.vaccinations <= 0.37
Feature
0.00
0.01
0.02
Weight
Positive
Negative

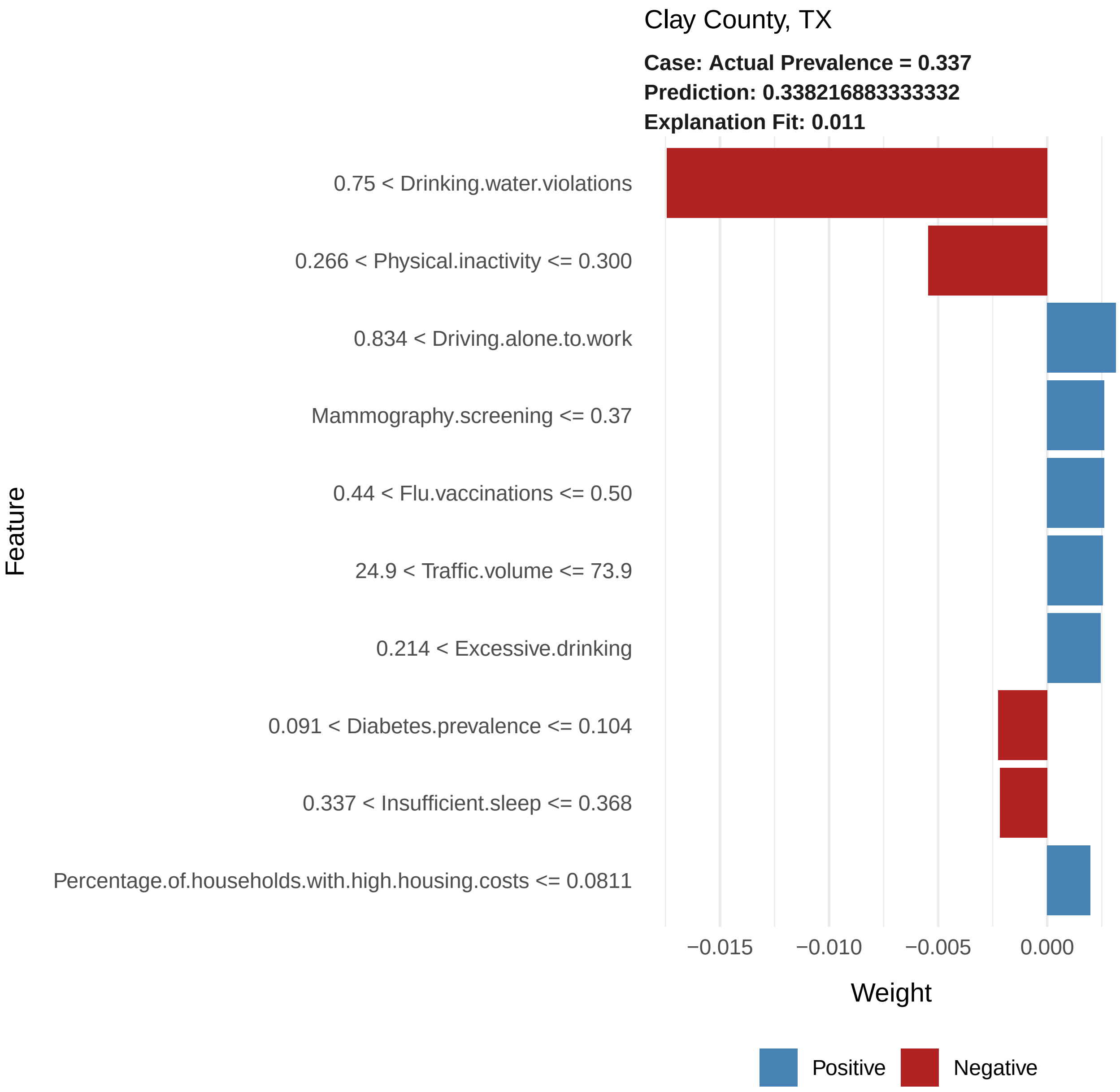

Clay County, TX
Case: Actual Prevalence = 0.337
Prediction: 0.338216883333332
Explanation Fit: 0.011
0.75 < Drinking.water.violations
0.266 < Physical.inactivity <= 0.300
0.834 < Driving.alone.to.work
Mammography.screening <= 0.37
0.44 < Flu.vaccinations <= 0.50
24.9 < Traffic.volume <= 73.9
0.214 < Excessive.drinking
0.091 < Diabetes.prevalence <= 0.104
0.337 < Insufficient.sleep <= 0.368
Percentage.of.households.with.high.housing.costs <= 0.0811
Feature
−0.015
−0.010
−0.005
0.000
Weight
Positive
Negative

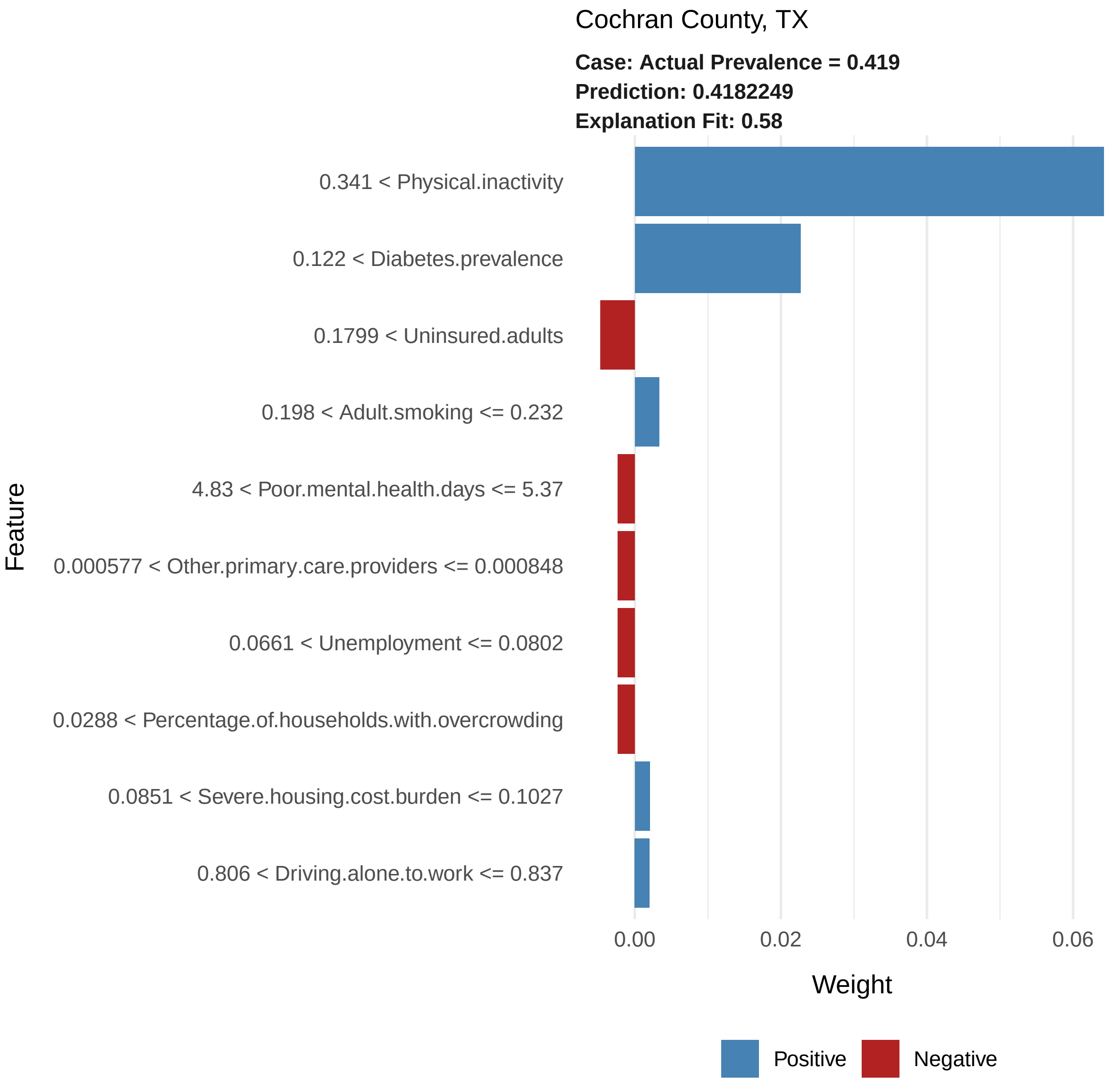

Cochran County, TX
Case: Actual Prevalence = 0.419
Prediction: 0.4182249
Explanation Fit: 0.58
0.341 < Physical.inactivity
0.122 < Diabetes.prevalence
0.1799 < Uninsured.adults
0.198 < Adult.smoking <= 0.232
4.83 < Poor.mental.health.days <= 5.37
0.000577 < Other.primary.care.providers <= 0.000848
0.0661 < Unemployment <= 0.0802
0.0288 < Percentage.of.households.with.overcrowding
0.0851 < Severe.housing.cost.burden <= 0.1027
0.806 < Driving.alone.to.work <= 0.837
Feature
0.00
0.02
0.04
0.06
Weight
Positive
Negative

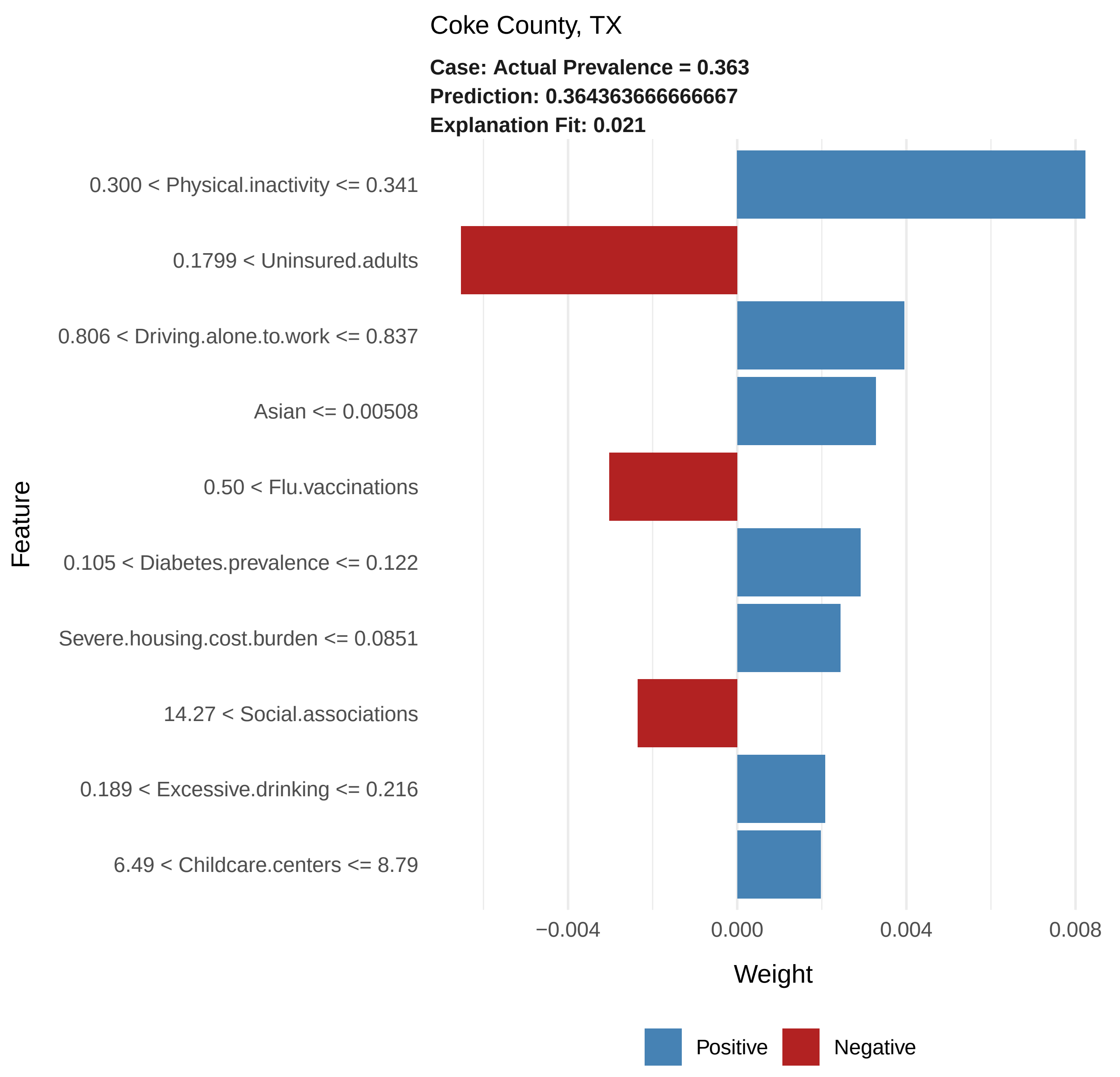
Coke County, TX
Case: Actual Prevalence = 0.363
Prediction: 0.364363666666667
Explanation Fit: 0.021
0.300 < Physical.inactivity <= 0.341
0.1799 < Uninsured.adults
0.806 < Driving.alone.to.work <= 0.837
Asian <= 0.00508
0.50 < Flu.vaccinations
0.105 < Diabetes.prevalence <= 0.122
Severe.housing.cost.burden <= 0.0851
14.27 < Social.associations
0.189 < Excessive.drinking <= 0.216
6.49 < Childcare.centers <= 8.79
Feature
−0.004
0.000
0.004
0.008
Weight
Positive
Negative

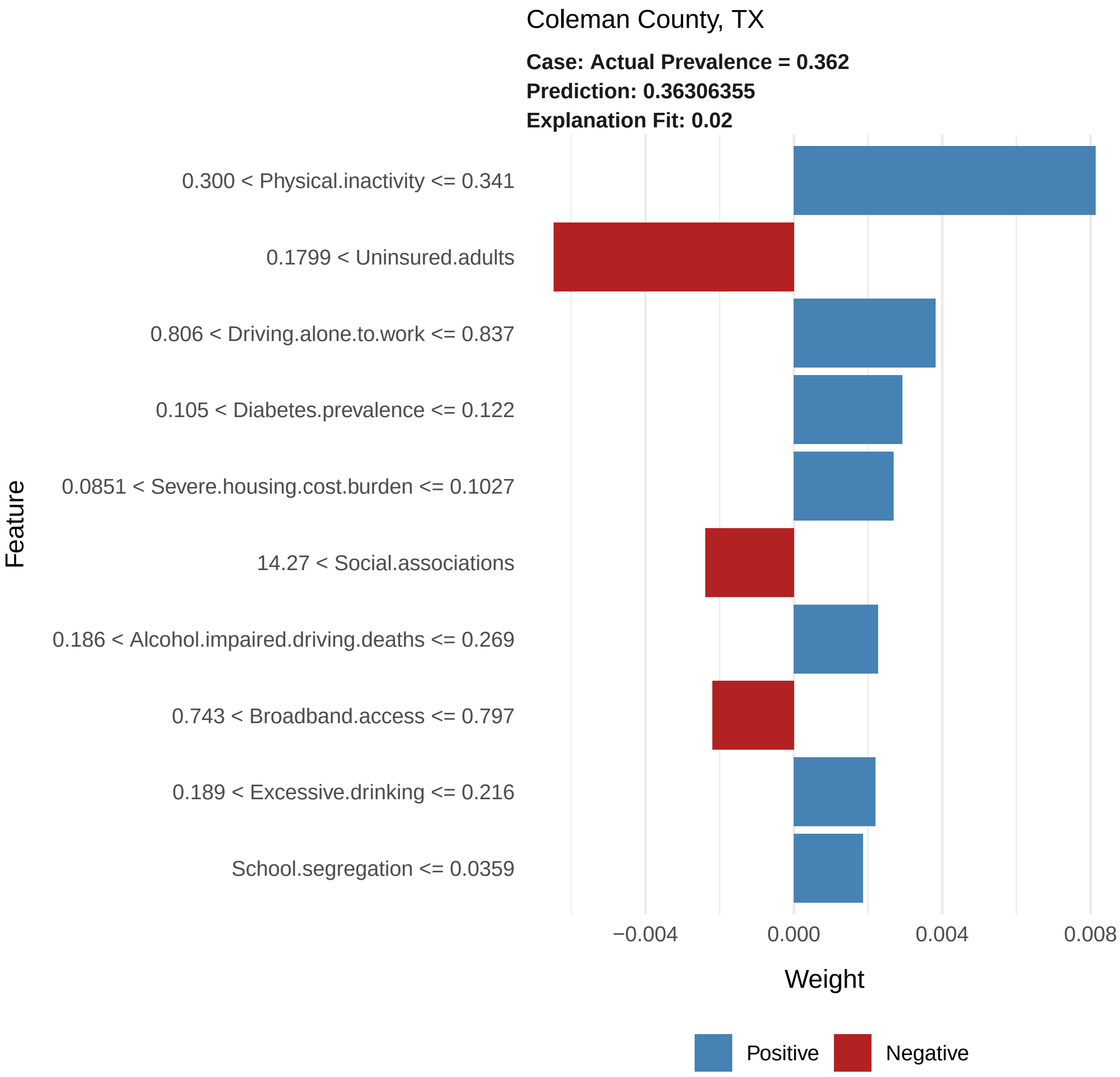
Coleman County, TX
Case: Actual Prevalence = 0.362
Prediction: 0.36306355
Explanation Fit: 0.02
0.300 < Physical.inactivity <= 0.341
0.1799 < Uninsured.adults
0.806 < Driving.alone.to.work <= 0.837
0.105 < Diabetes.prevalence <= 0.122
0.0851 < Severe.housing.cost.burden <= 0.1027
14.27 < Social.associations
0.186 < Alcohol.impaired.driving.deaths <= 0.269
0.743 < Broadband.access <= 0.797
0.189 < Excessive.drinking <= 0.216
School.segregation <= 0.0359
Feature
−0.004
0.000
0.004
0.008
Weight
Positive
Negative

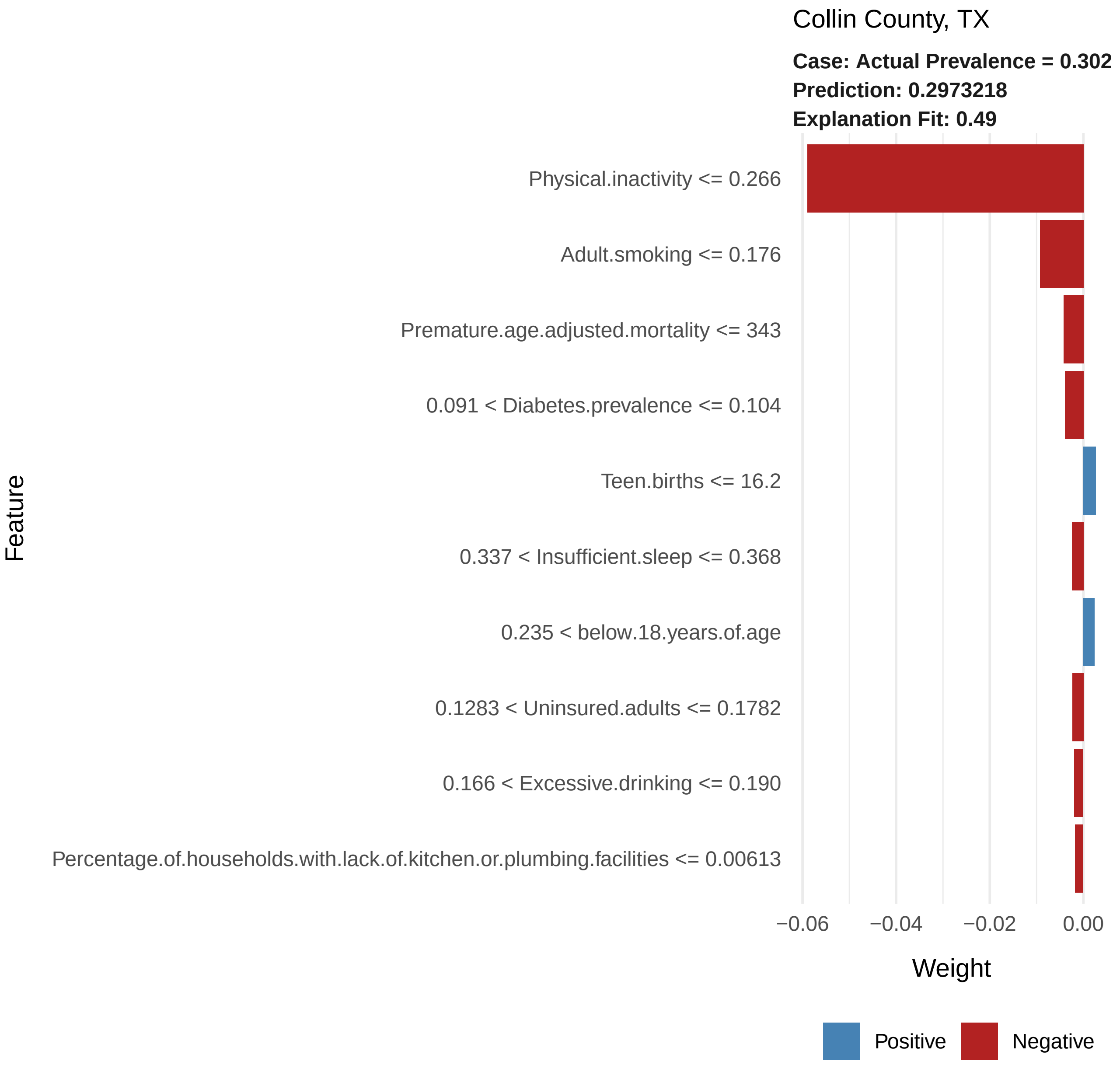

Collin County, TX
Case: Actual Prevalence = 0.302
Prediction: 0.2973218
Explanation Fit: 0.49
Physical.inactivity <= 0.266
Adult.smoking <= 0.176
Premature.age.adjusted.mortality <= 343
0.091 < Diabetes.prevalence <= 0.104
Teen.births <= 16.2
0.337 < Insufficient.sleep <= 0.368
0.235 < below.18.years.of.age
0.1283 < Uninsured.adults <= 0.1782
0.166 < Excessive.drinking <= 0.190
Percentage.of.households.with.lack.of.kitchen.or.plumbing.facilities <= 0.00613
Feature
−0.06
−0.04
−0.02
0.00
Weight
Positive
Negative

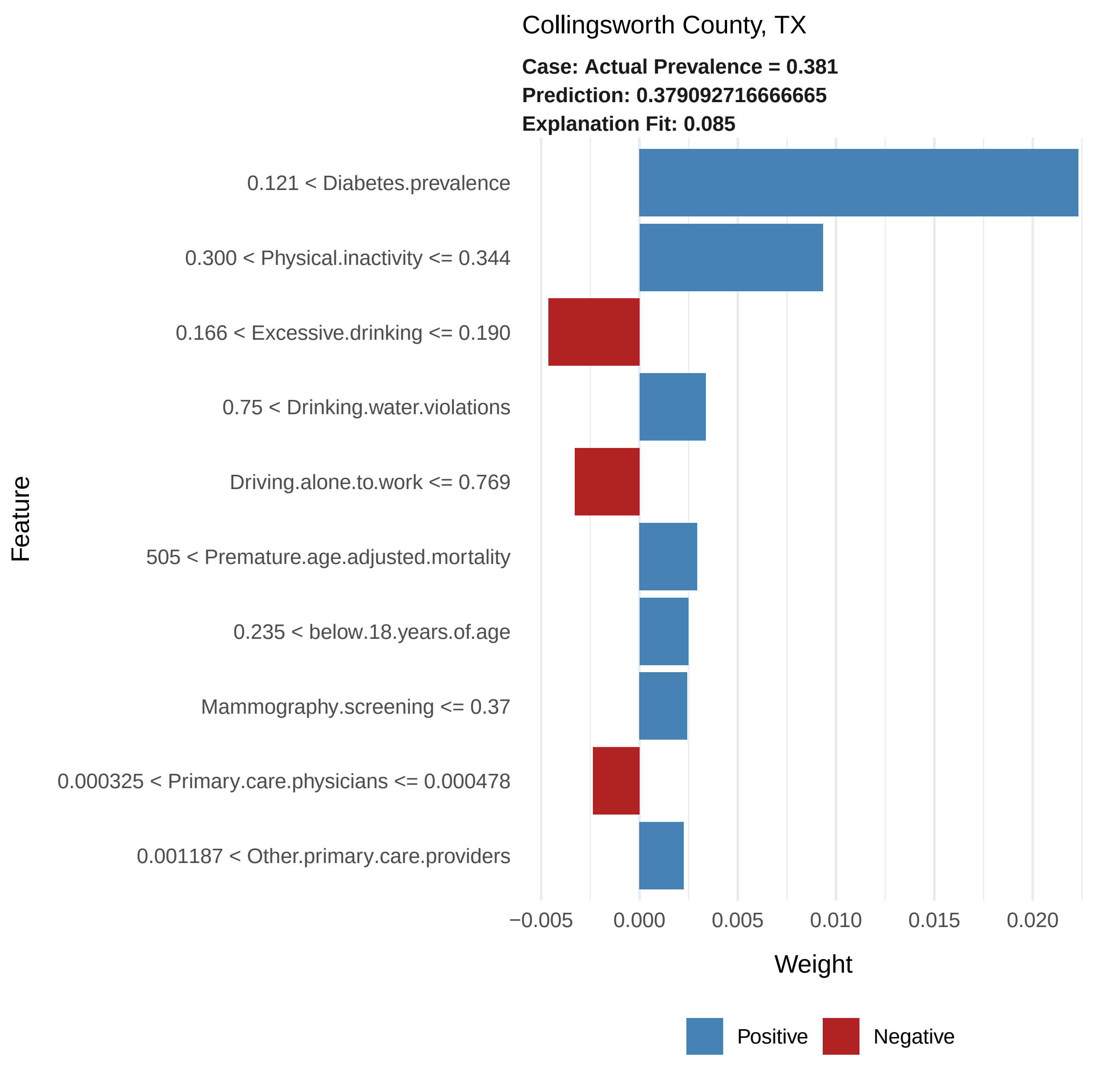
Collingsworth County, TX
Case: Actual Prevalence = 0.381
Prediction: 0.379092716666665
Explanation Fit: 0.085
0.121 < Diabetes.prevalence
0.300 < Physical.inactivity <= 0.344
0.166 < Excessive.drinking <= 0.190
0.75 < Drinking.water.violations
Driving.alone.to.work <= 0.769
505 < Premature.age.adjusted.mortality
0.235 < below.18.years.of.age
Mammography.screening <= 0.37
0.000325 < Primary.care.physicians <= 0.000478
0.001187 < Other.primary.care.providers
Feature
−0.005
0.000
0.005
0.010
0.015
0.020
Weight
Positive
Negative

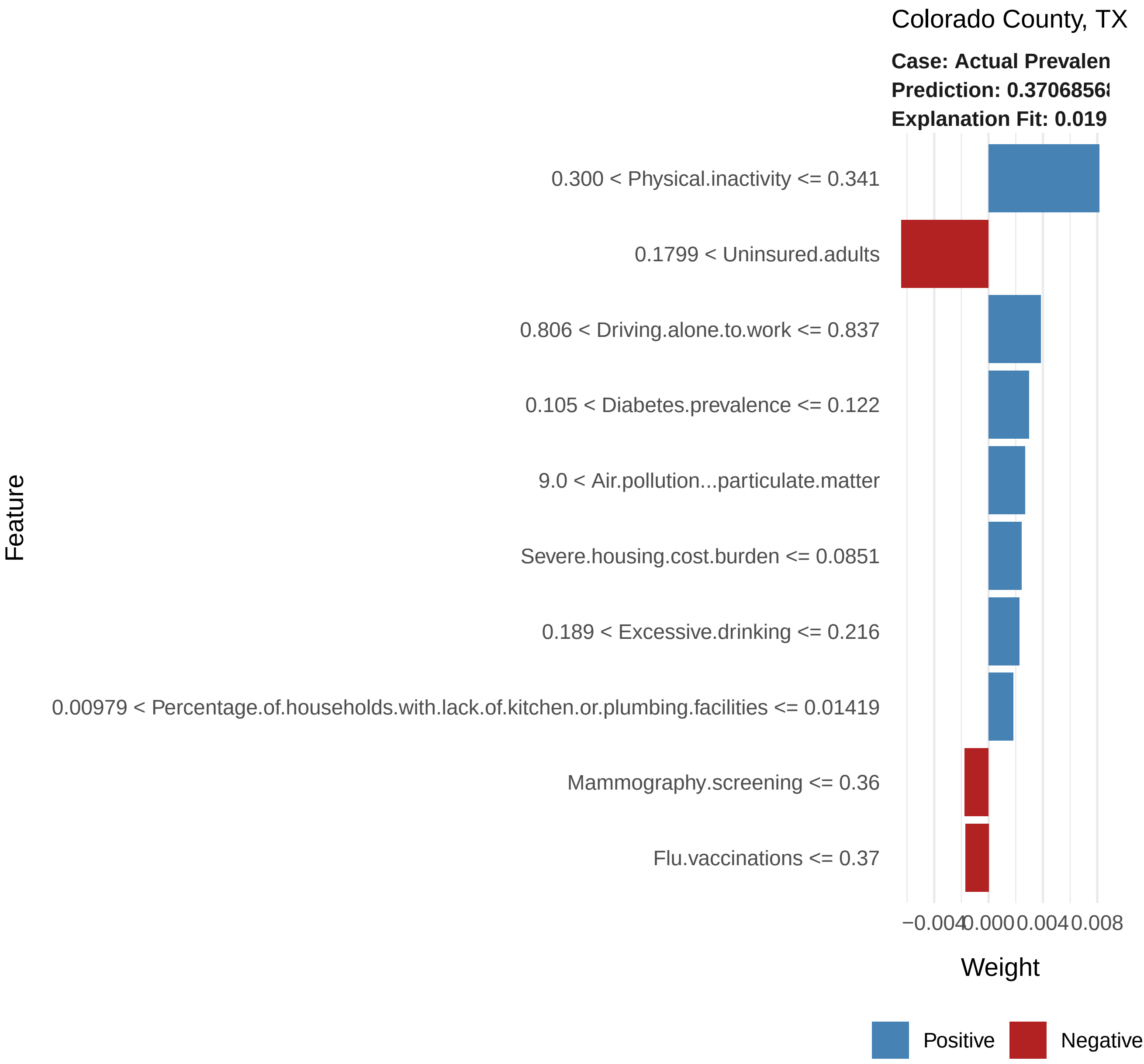
Colorado County, TX
Case: Actual Prevalen
Prediction: 0.37068568
Explanation Fit: 0.019
0.300 < Physical.inactivity <= 0.341
0.1799 < Uninsured.adults
0.806 < Driving.alone.to.work <= 0.837
0.105 < Diabetes.prevalence <= 0.122
9.0 < Air.pollution...particulate.matter
Severe.housing.cost.burden <= 0.0851
0.189 < Excessive.drinking <= 0.216
0.00979 < Percentage.of.households.with.lack.of.kitchen.or.plumbing.facilities <= 0.01419
Mammography.screening <= 0.36
Flu.vaccinations <= 0.37
Feature
−0.004
0.000
0.004
0.008
Weight
Positive
Negative

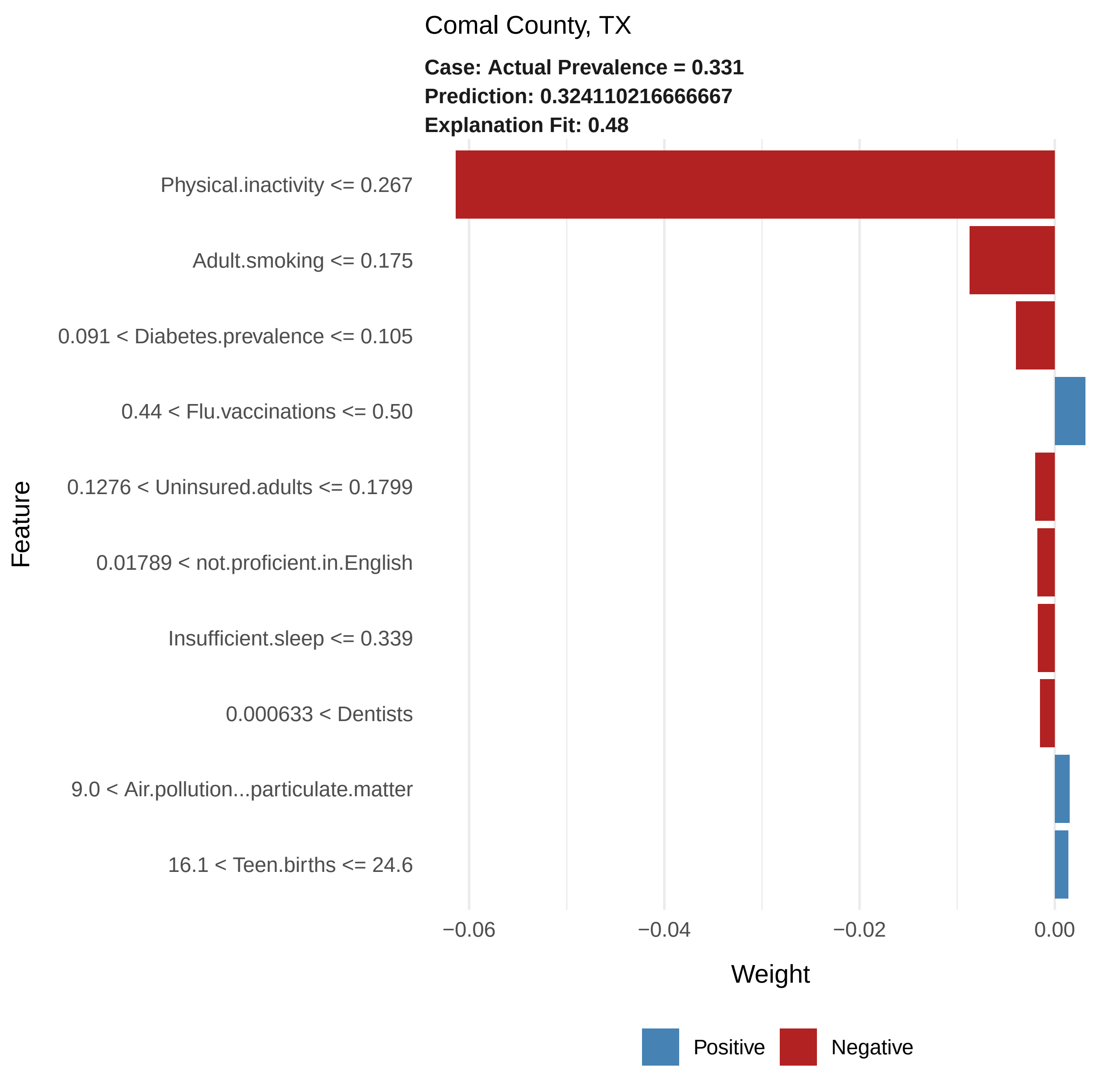
Comal County, TX
Case: Actual Prevalence = 0.331
Prediction: 0.324110216666667
Explanation Fit: 0.48
Physical.inactivity <= 0.267
Adult.smoking <= 0.175
0.091 < Diabetes.prevalence <= 0.105
0.44 < Flu.vaccinations <= 0.50
0.1276 < Uninsured.adults <= 0.1799
0.01789 < not.proficient.in.English
Insufficient.sleep <= 0.339
0.000633 < Dentists
9.0 < Air.pollution...particulate.matter
16.1 < Teen.births <= 24.6
Feature
−0.06
−0.04
−0.02
0.00
Weight
Positive
Negative

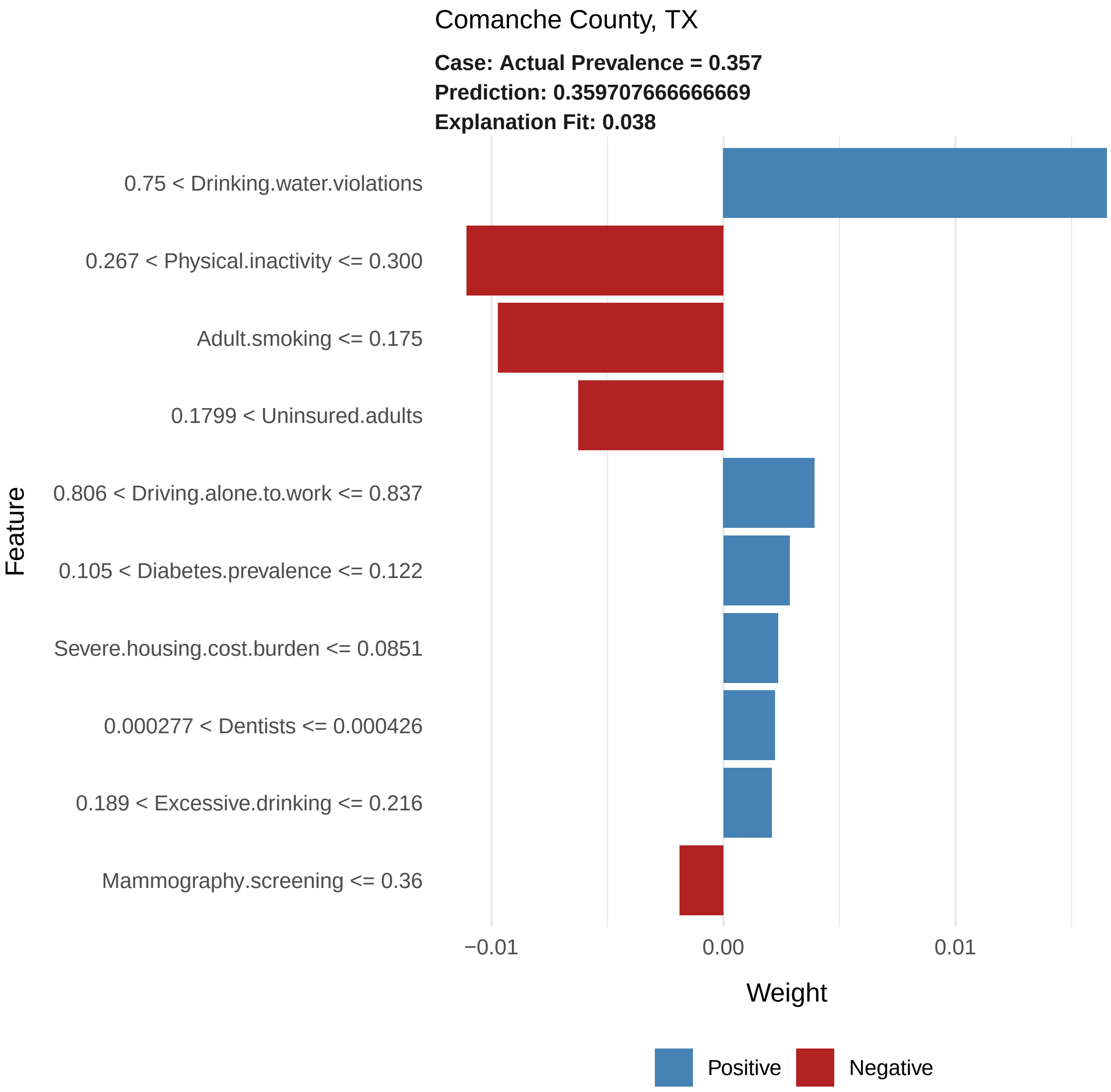

Comanche County, TX
Case: Actual Prevalence = 0.357
Prediction: 0.359707666666669
Explanation Fit: 0.038
0.75 < Drinking.water.violations
0.267 < Physical.inactivity <= 0.300
Adult.smoking <= 0.175
0.1799 < Uninsured.adults
0.806 < Driving.alone.to.work <= 0.837
0.105 < Diabetes.prevalence <= 0.122
Severe.housing.cost.burden <= 0.0851
0.000277 < Dentists <= 0.000426
0.189 < Excessive.drinking <= 0.216
Mammography.screening <= 0.36
Feature
−0.01
0.00
0.01
Weight
Positive
Negative

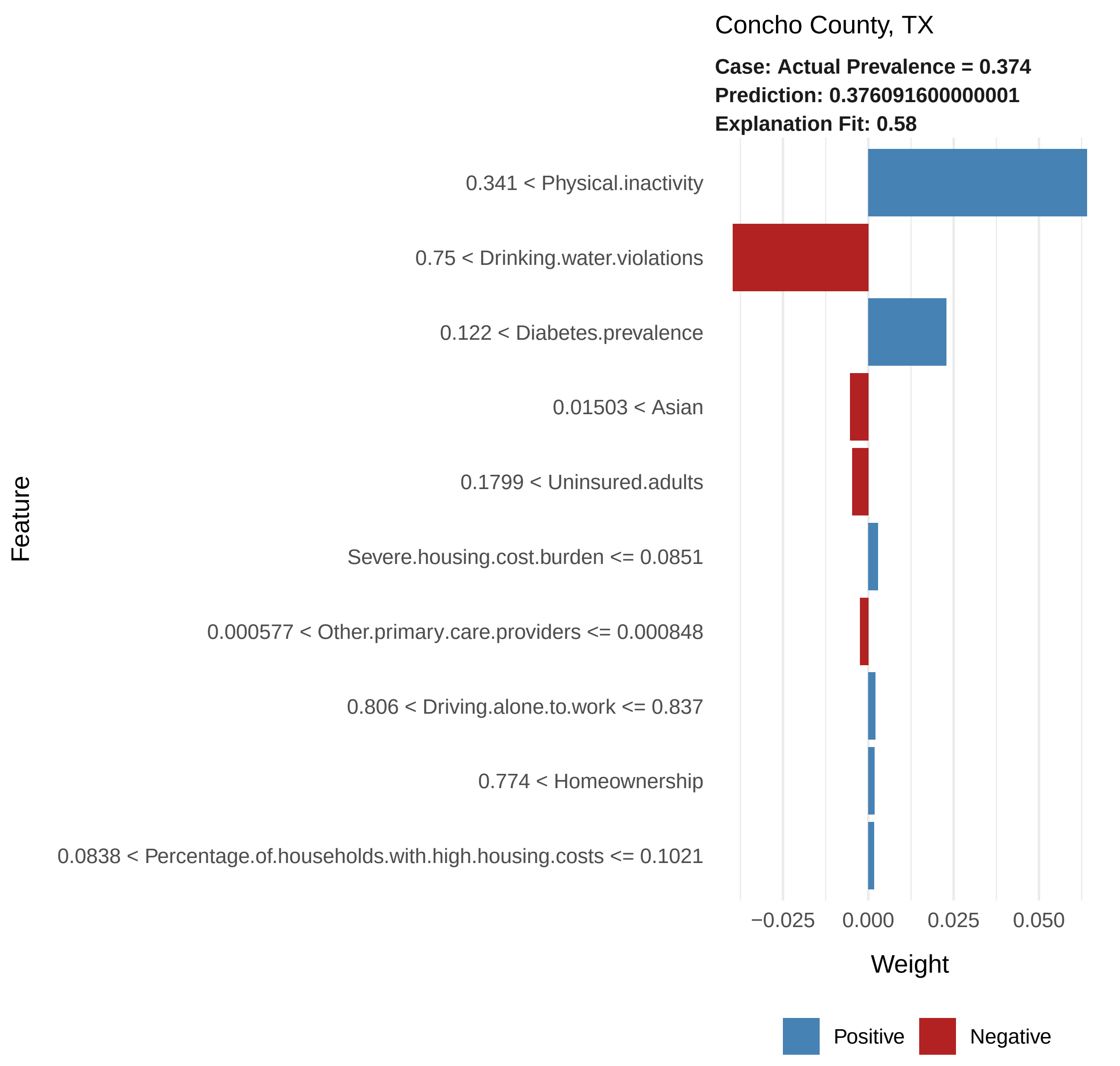
Concho County, TX
Case: Actual Prevalence = 0.374
Prediction: 0.376091600000001
Explanation Fit: 0.58
0.341 < Physical.inactivity
0.75 < Drinking.water.violations
0.122 < Diabetes.prevalence
0.01503 < Asian
0.1799 < Uninsured.adults
Severe.housing.cost.burden <= 0.0851
0.000577 < Other.primary.care.providers <= 0.000848
0.806 < Driving.alone.to.work <= 0.837
0.774 < Homeownership
0.0838 < Percentage.of.households.with.high.housing.costs <= 0.1021
Feature
−0.025
0.000
0.025
0.050
Weight
Positive
Negative

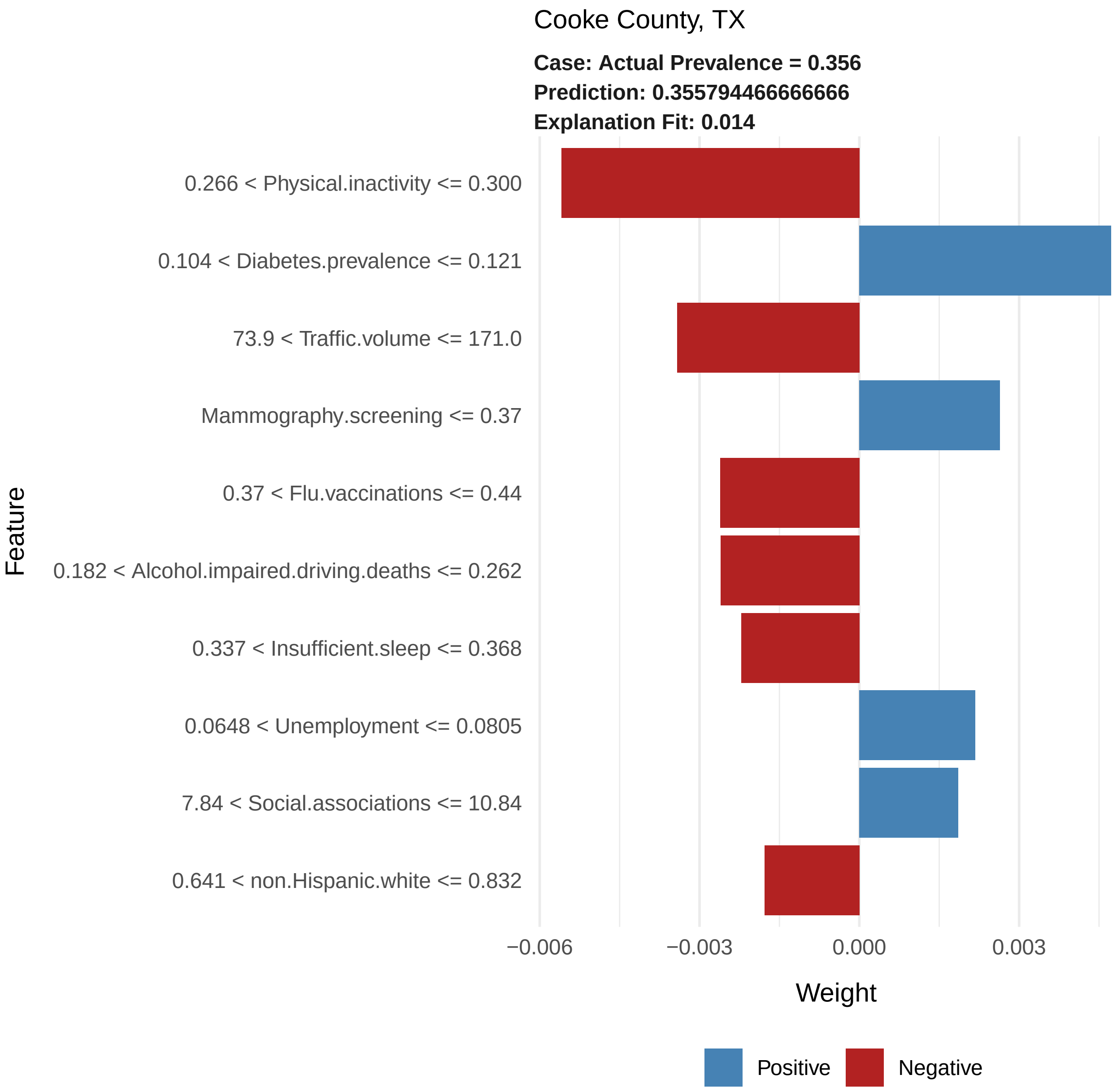

Cooke County, TX
Case: Actual Prevalence = 0.356
Prediction: 0.355794466666666
Explanation Fit: 0.014
0.266 < Physical.inactivity <= 0.300
0.104 < Diabetes.prevalence <= 0.121
73.9 < Traffic.volume <= 171.0
Mammography.screening <= 0.37
0.37 < Flu.vaccinations <= 0.44
0.182 < Alcohol.impaired.driving.deaths <= 0.262
0.337 < Insufficient.sleep <= 0.368
0.0648 < Unemployment <= 0.0805
7.84 < Social.associations <= 10.84
0.641 < non.Hispanic.white <= 0.832
Feature
−0.006
−0.003
0.000
0.003
Weight
Positive
Negative

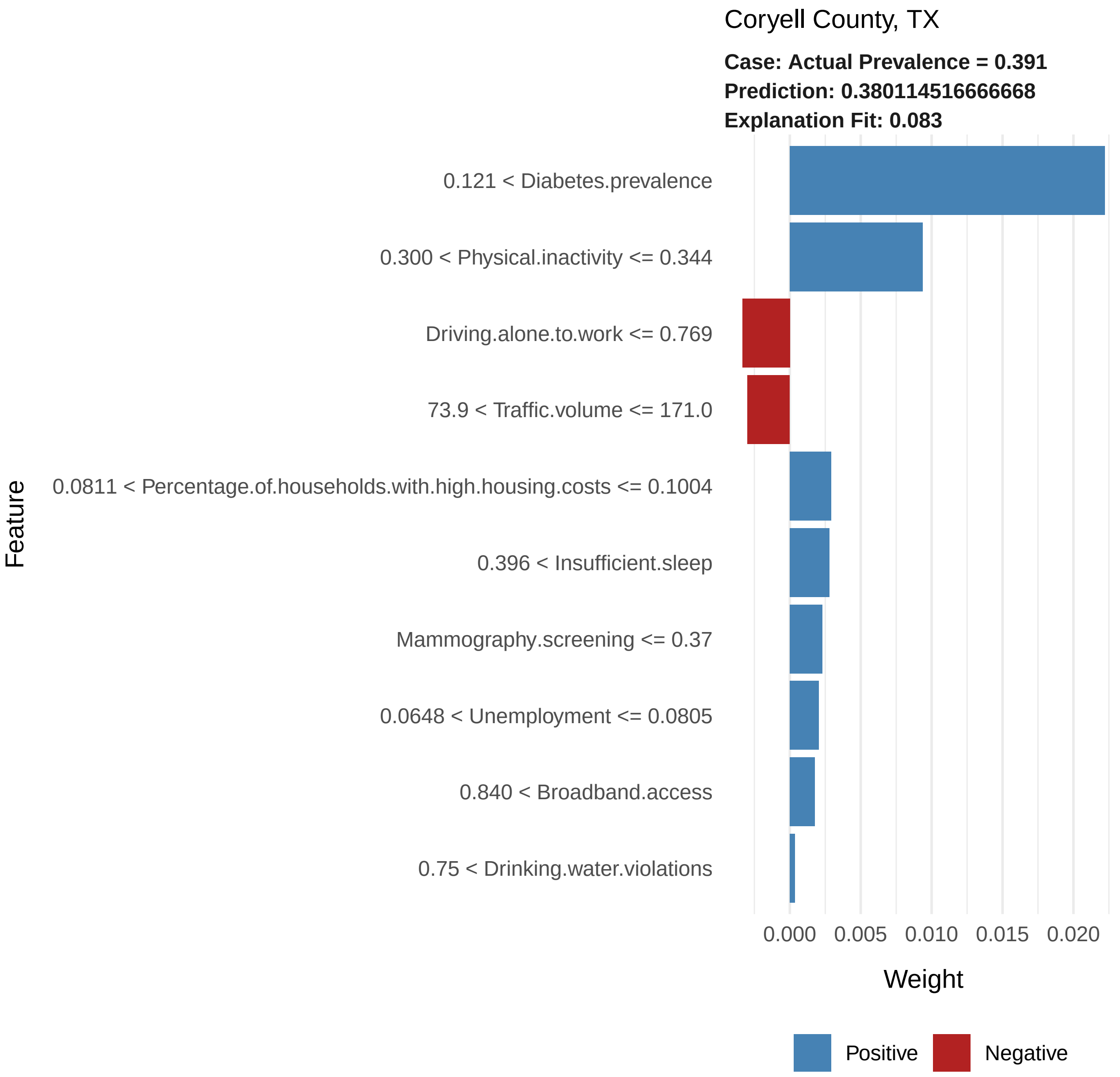

Coryell County, TX
Case: Actual Prevalence = 0.391
Prediction: 0.380114516666668
Explanation Fit: 0.083
0.121 < Diabetes.prevalence
0.300 < Physical.inactivity <= 0.344
Driving.alone.to.work <= 0.769
73.9 < Traffic.volume <= 171.0
0.0811 < Percentage.of.households.with.high.housing.costs <= 0.1004
0.396 < Insufficient.sleep
Mammography.screening <= 0.37
0.0648 < Unemployment <= 0.0805
0.840 < Broadband.access
0.75 < Drinking.water.violations
Feature
0.000
0.005
0.010
0.015
0.020
Weight
Positive
Negative

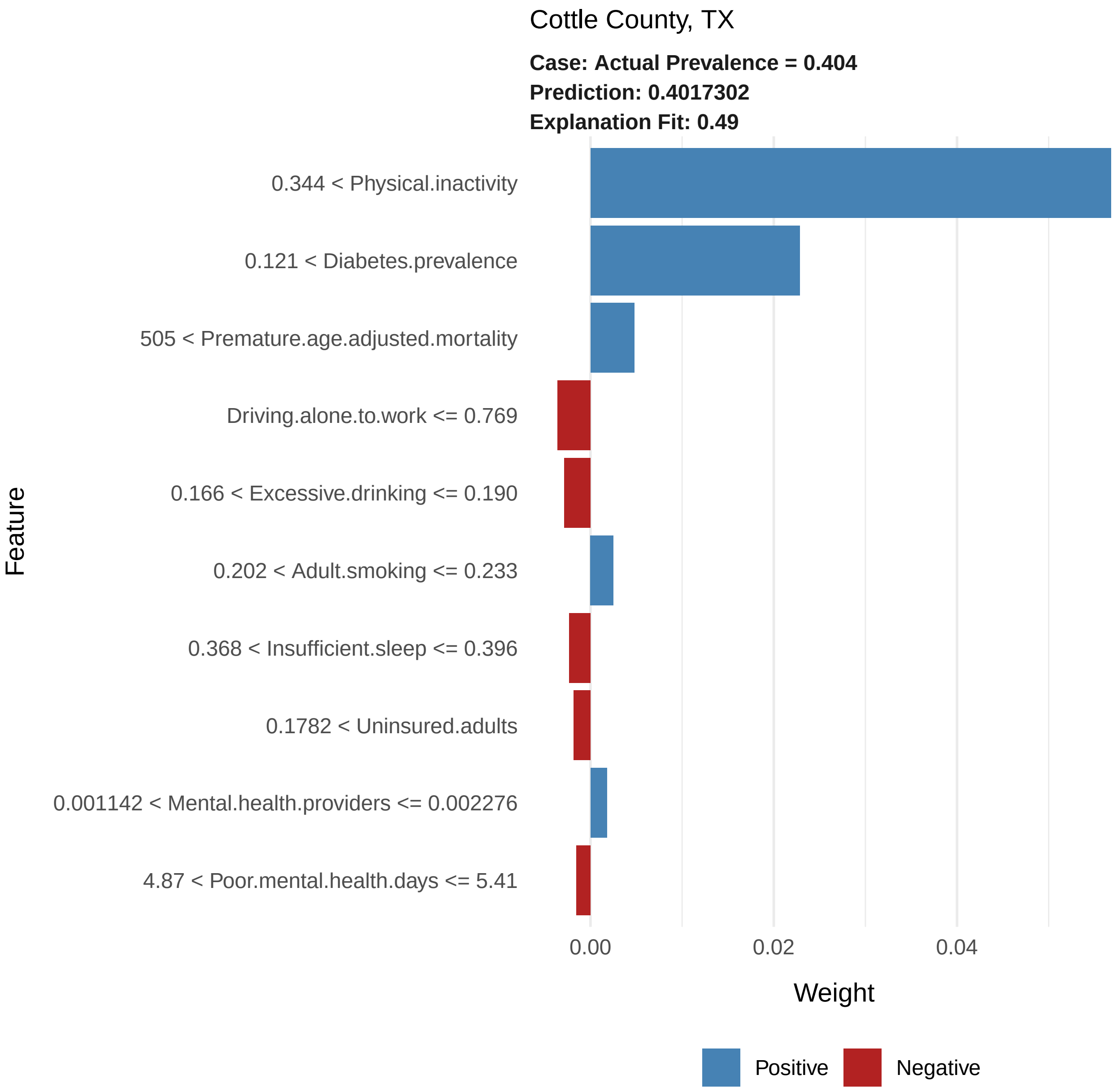

Cottle County, TX
Case: Actual Prevalence = 0.404
Prediction: 0.4017302
Explanation Fit: 0.49
0.344 < Physical.inactivity
0.121 < Diabetes.prevalence
505 < Premature.age.adjusted.mortality
Driving.alone.to.work <= 0.769
0.166 < Excessive.drinking <= 0.190
0.202 < Adult.smoking <= 0.233
0.368 < Insufficient.sleep <= 0.396
0.1782 < Uninsured.adults
0.001142 < Mental.health.providers <= 0.002276
4.87 < Poor.mental.health.days <= 5.41
Feature
0.00
0.02
0.04
Weight
Positive
Negative

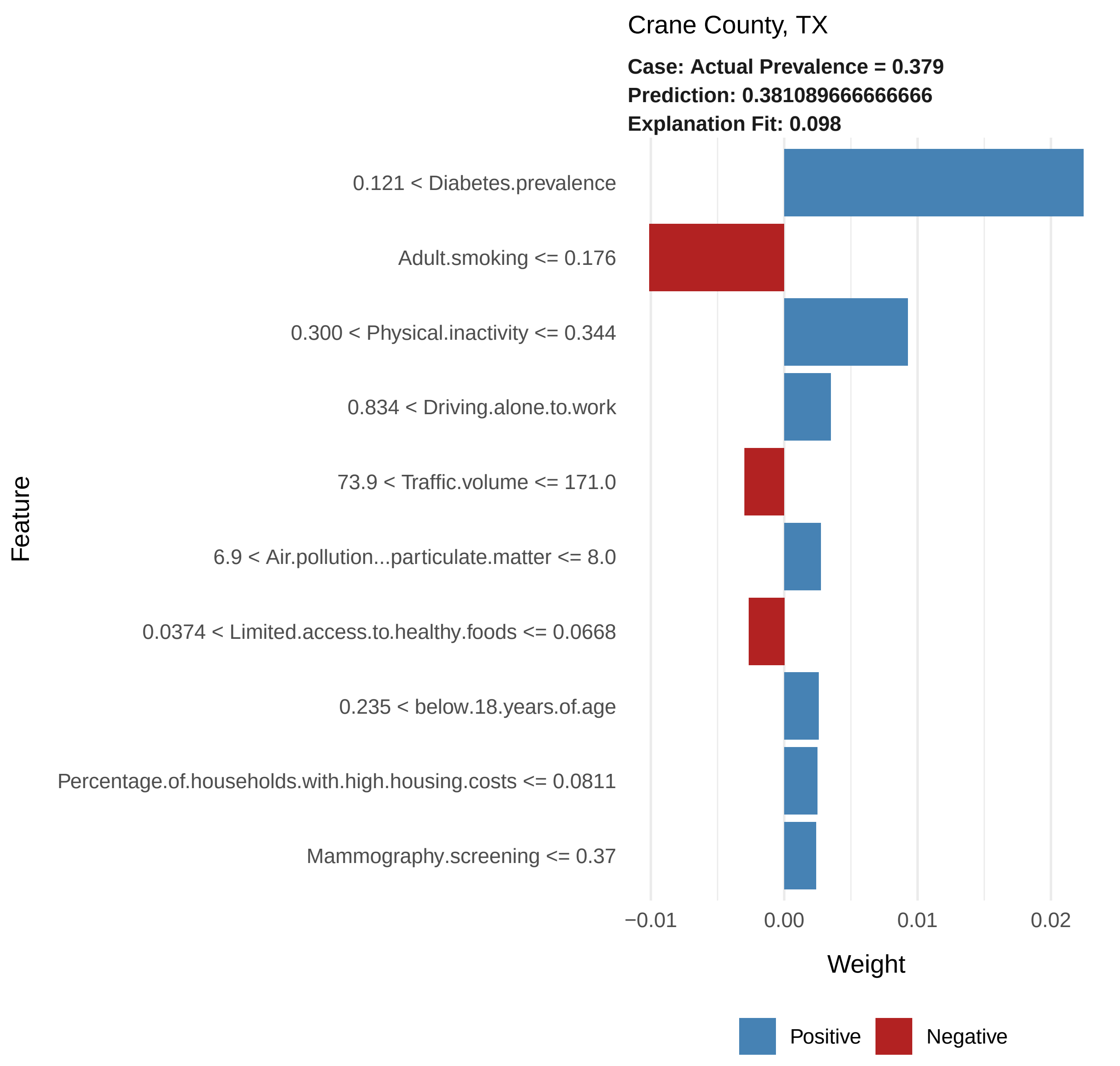
Crane County, TX
Case: Actual Prevalence = 0.379
Prediction: 0.381089666666666
Explanation Fit: 0.098
0.121 < Diabetes.prevalence
Adult.smoking <= 0.176
0.300 < Physical.inactivity <= 0.344
0.834 < Driving.alone.to.work
73.9 < Traffic.volume <= 171.0
6.9 < Air.pollution...particulate.matter <= 8.0
0.0374 < Limited.access.to.healthy.foods <= 0.0668
0.235 < below.18.years.of.age
Percentage.of.households.with.high.housing.costs <= 0.0811
Mammography.screening <= 0.37
Feature
−0.01
0.00
0.01
0.02
Weight
Positive
Negative

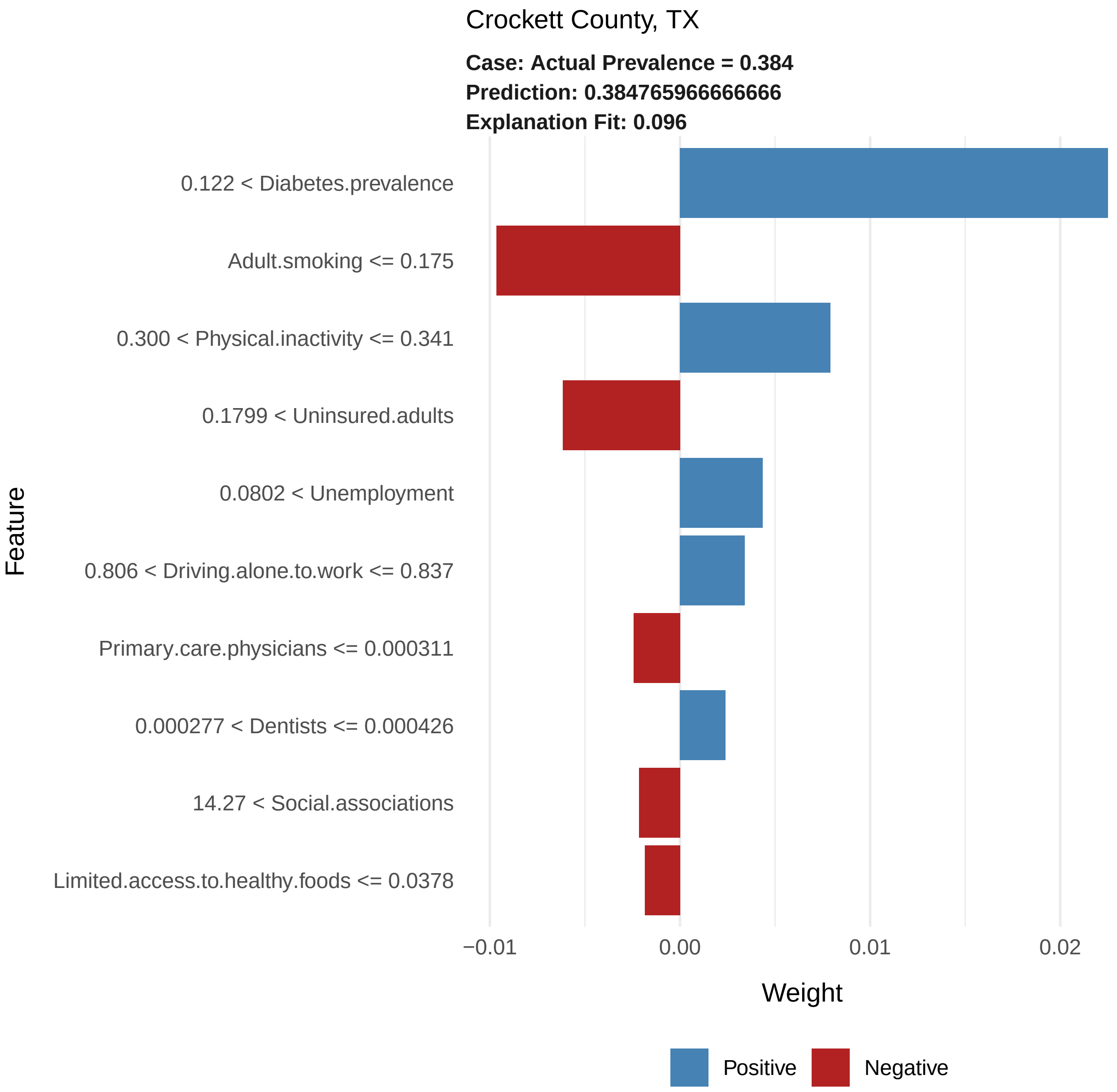

Crockett County, TX
Case: Actual Prevalence = 0.384
Prediction: 0.384765966666666
Explanation Fit: 0.096
0.122 < Diabetes.prevalence
Adult.smoking <= 0.175
0.300 < Physical.inactivity <= 0.341
0.1799 < Uninsured.adults
0.0802 < Unemployment
0.806 < Driving.alone.to.work <= 0.837
Primary.care.physicians <= 0.000311
0.000277 < Dentists <= 0.000426
14.27 < Social.associations
Limited.access.to.healthy.foods <= 0.0378
Feature
−0.01
0.00
0.01
0.02
Weight
Positive
Negative

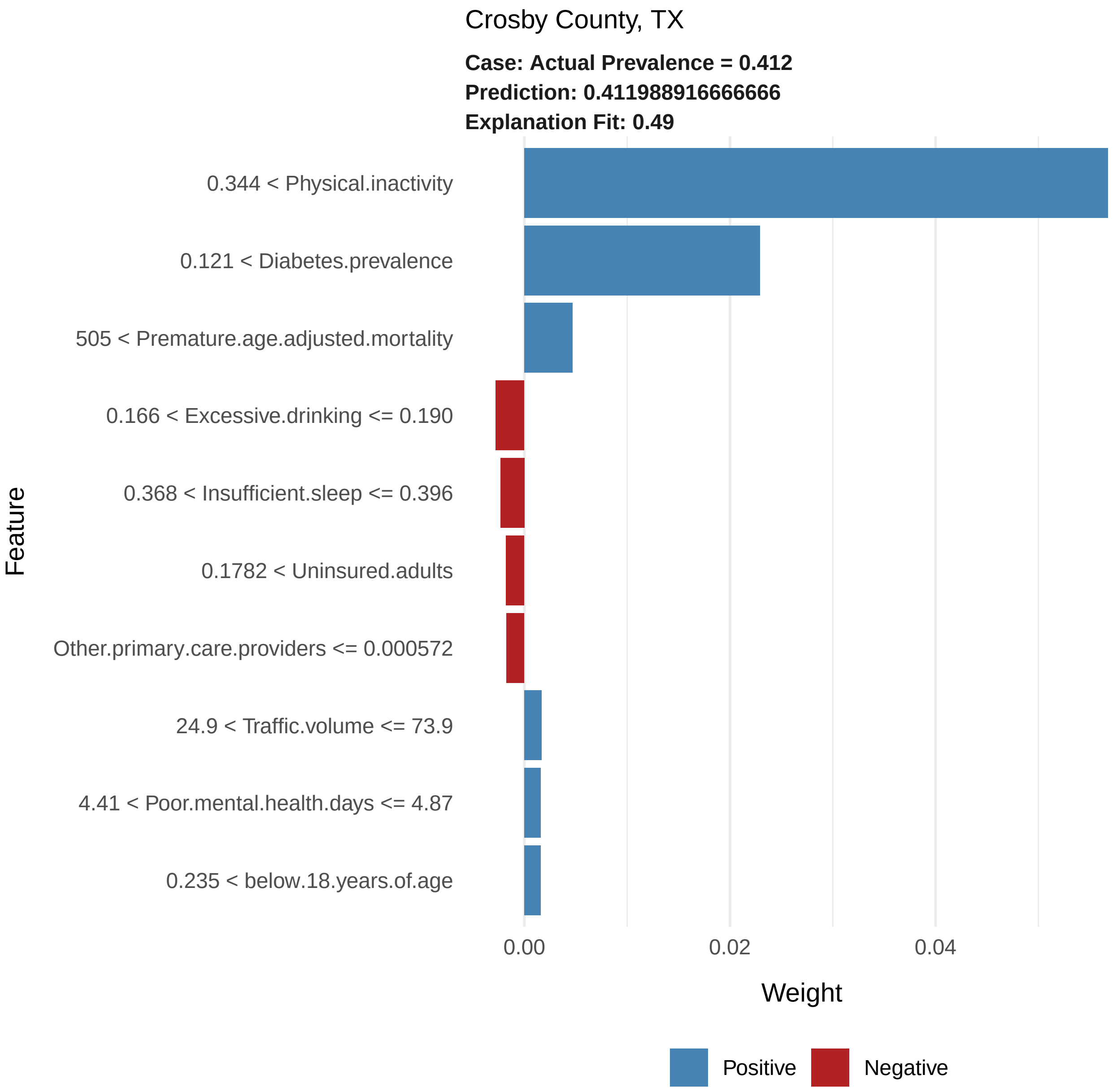

Crosby County, TX
Case: Actual Prevalence = 0.412
Prediction: 0.411988916666666
Explanation Fit: 0.49
0.344 < Physical.inactivity
0.121 < Diabetes.prevalence
505 < Premature.age.adjusted.mortality
0.166 < Excessive.drinking <= 0.190
0.368 < Insufficient.sleep <= 0.396
0.1782 < Uninsured.adults
Other.primary.care.providers <= 0.000572
24.9 < Traffic.volume <= 73.9
4.41 < Poor.mental.health.days <= 4.87
0.235 < below.18.years.of.age
Feature
0.00
0.02
0.04
Weight
Positive
Negative

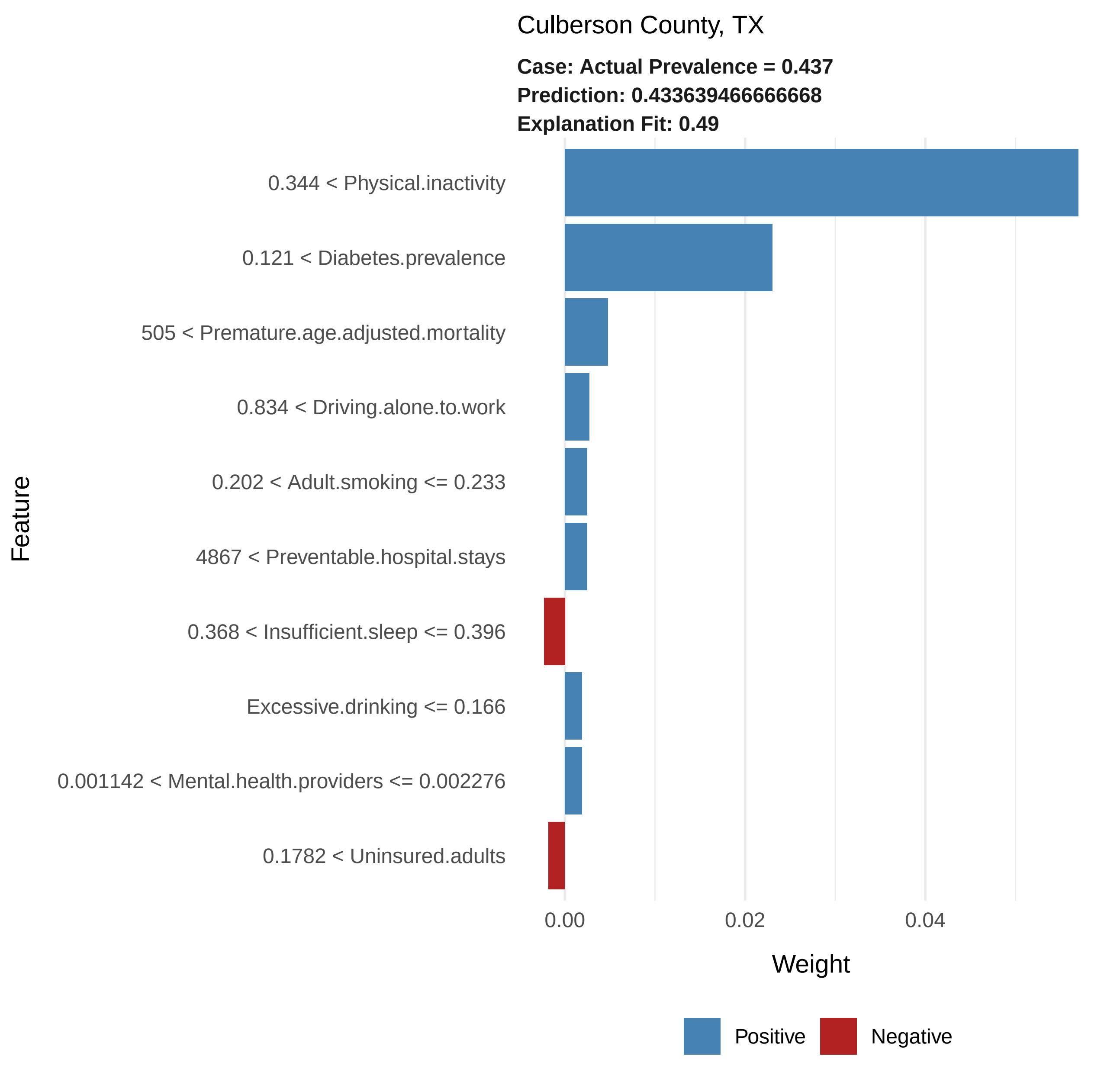

Culberson County, TX
Case: Actual Prevalence = 0.437
Prediction: 0.433639466666668
Explanation Fit: 0.49
0.344 < Physical.inactivity
0.121 < Diabetes.prevalence
505 < Premature.age.adjusted.mortality
0.834 < Driving.alone.to.work
0.202 < Adult.smoking <= 0.233
4867 < Preventable.hospital.stays
0.368 < Insufficient.sleep <= 0.396
Excessive.drinking <= 0.166
0.001142 < Mental.health.providers <= 0.002276
0.1782 < Uninsured.adults
Feature
0.00
0.02
0.04
Weight
Positive
Negative

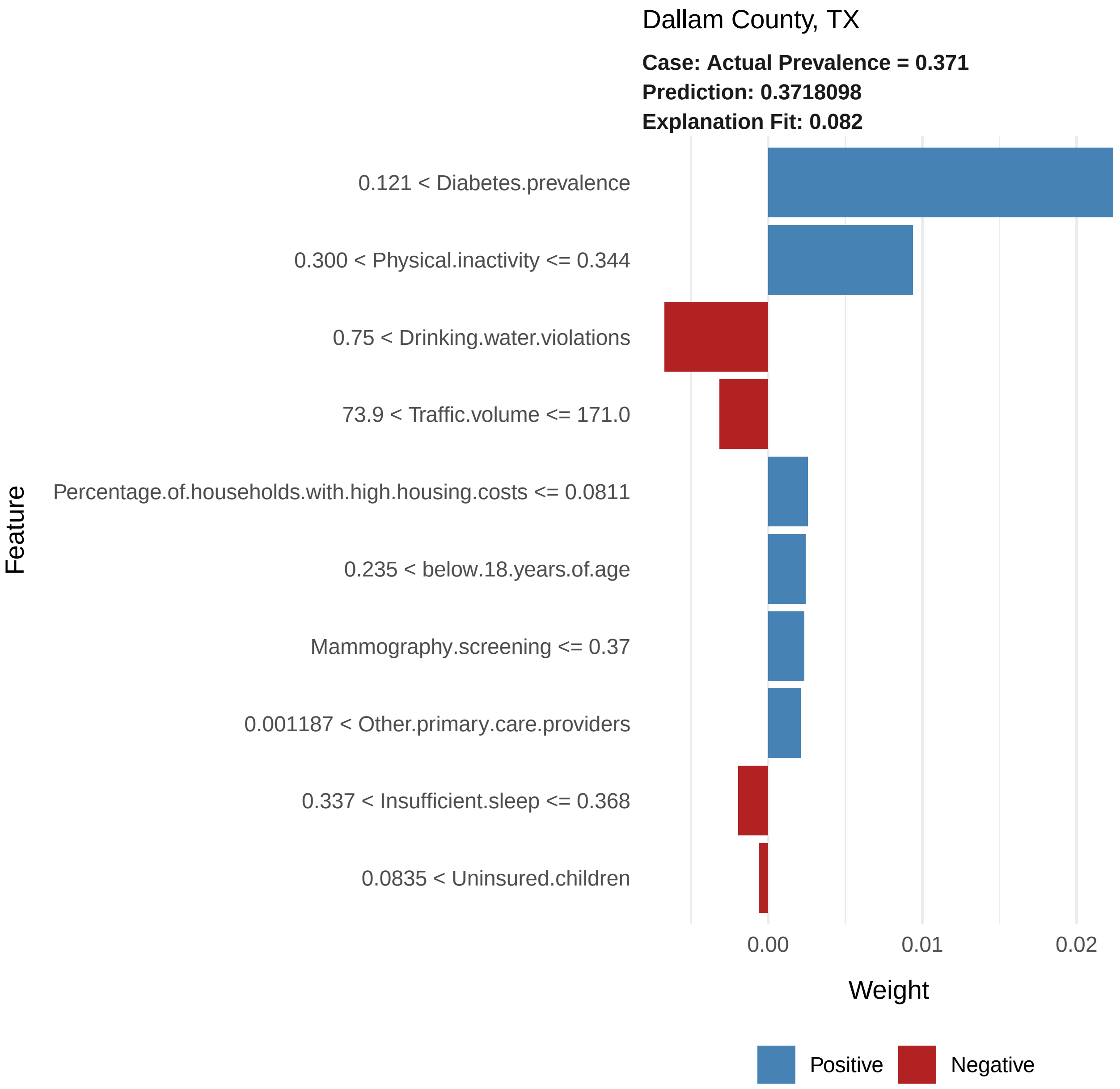
Dallam County, TX
Case: Actual Prevalence = 0.371
Prediction: 0.3718098
Explanation Fit: 0.082
0.121 < Diabetes.prevalence
0.300 < Physical.inactivity <= 0.344
0.75 < Drinking.water.violations
73.9 < Traffic.volume <= 171.0
Percentage.of.households.with.high.housing.costs <= 0.0811
0.235 < below.18.years.of.age
Mammography.screening <= 0.37
0.001187 < Other.primary.care.providers
0.337 < Insufficient.sleep <= 0.368
0.0835 < Uninsured.children
Feature
0.00
0.01
0.02
Weight
Positive
Negative

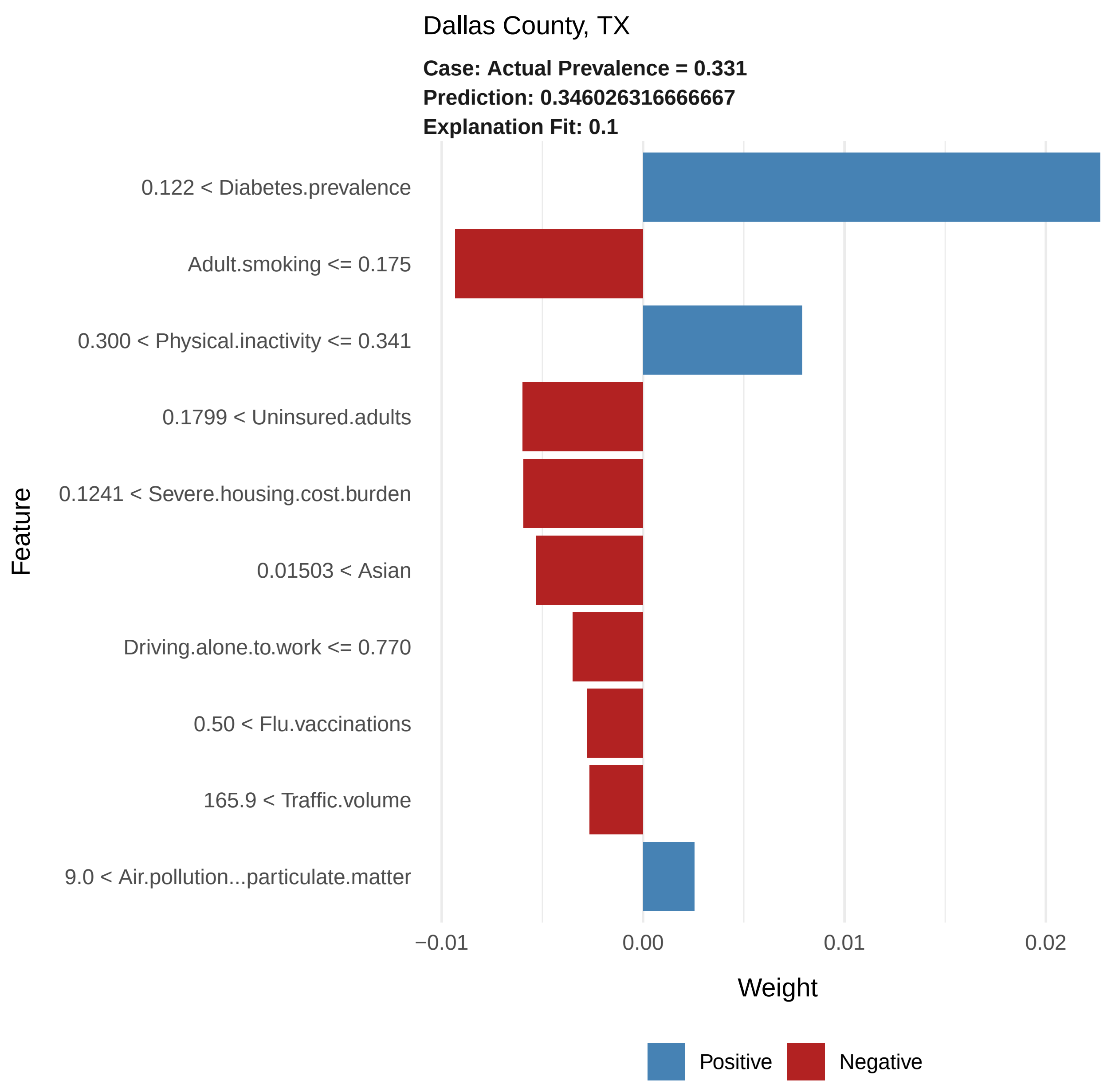

Dallas County, TX
Case: Actual Prevalence = 0.331
Prediction: 0.346026316666667
Explanation Fit: 0.1
0.122 < Diabetes.prevalence
Adult.smoking <= 0.175
0.300 < Physical.inactivity <= 0.341
0.1799 < Uninsured.adults
0.1241 < Severe.housing.cost.burden
0.01503 < Asian
Driving.alone.to.work <= 0.770
0.50 < Flu.vaccinations
165.9 < Traffic.volume
9.0 < Air.pollution...particulate.matter
Feature
−0.01
0.00
0.01
0.02
Weight
Positive
Negative

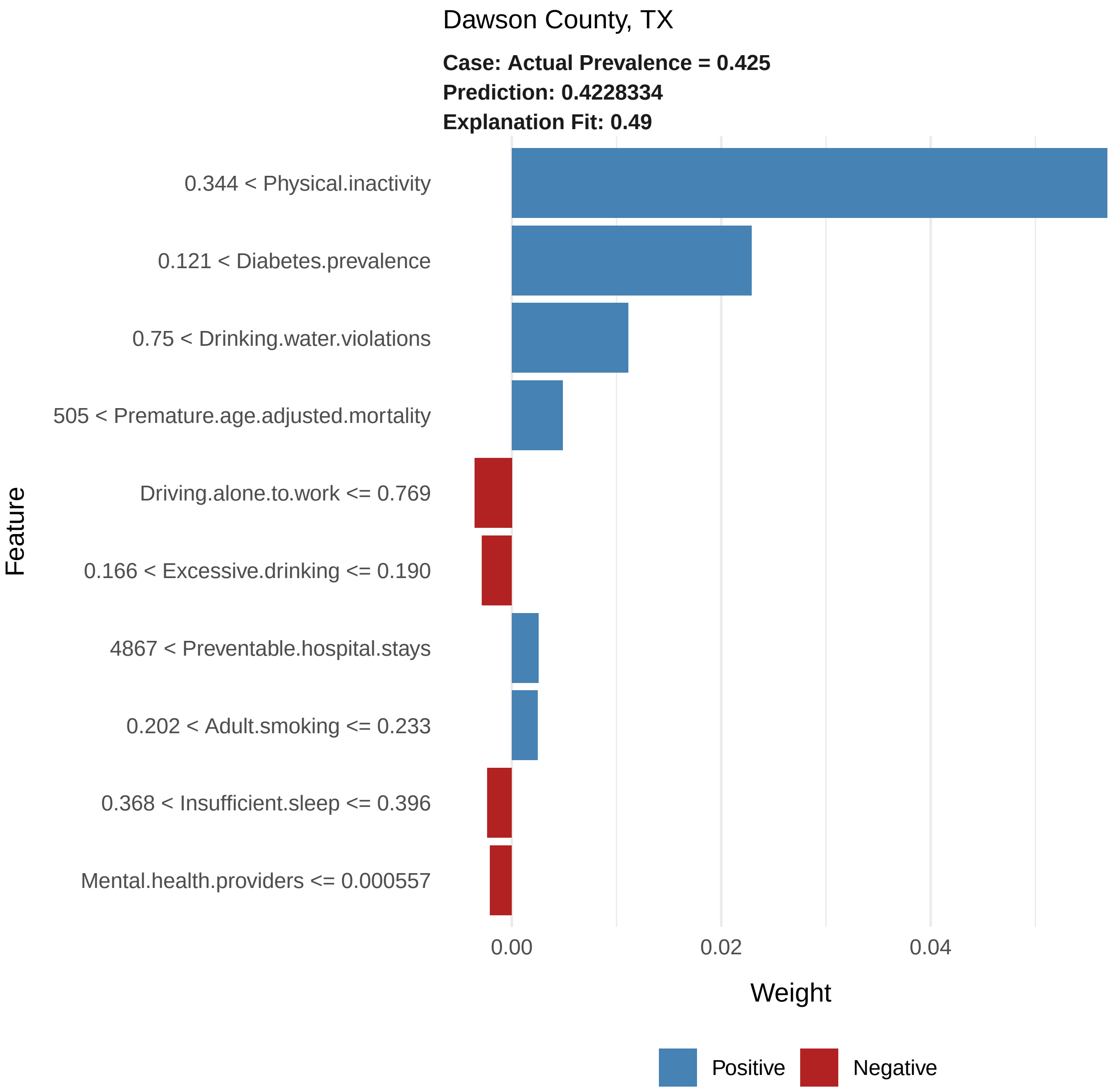

Dawson County, TX
Case: Actual Prevalence = 0.425
Prediction: 0.4228334
Explanation Fit: 0.49
0.344 < Physical.inactivity
0.121 < Diabetes.prevalence
0.75 < Drinking.water.violations
505 < Premature.age.adjusted.mortality
Driving.alone.to.work <= 0.769
0.166 < Excessive.drinking <= 0.190
4867 < Preventable.hospital.stays
0.202 < Adult.smoking <= 0.233
0.368 < Insufficient.sleep <= 0.396
Mental.health.providers <= 0.000557
Feature
0.00
0.02
0.04
Weight
Positive
Negative

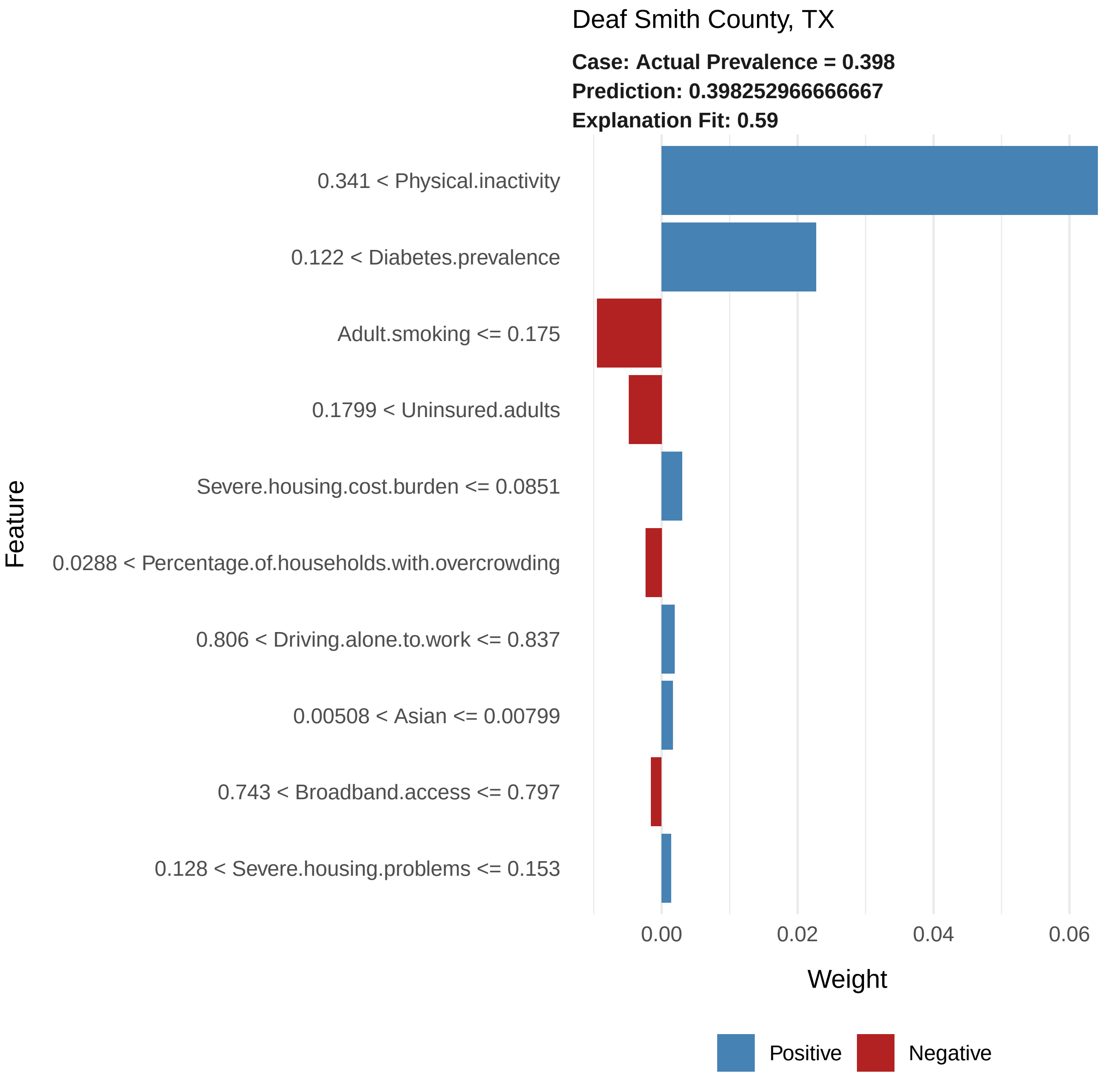

Deaf Smith County, TX
Case: Actual Prevalence = 0.398
Prediction: 0.398252966666667
Explanation Fit: 0.59
0.341 < Physical.inactivity
0.122 < Diabetes.prevalence
Adult.smoking <= 0.175
0.1799 < Uninsured.adults
Severe.housing.cost.burden <= 0.0851
0.0288 < Percentage.of.households.with.overcrowding
0.806 < Driving.alone.to.work <= 0.837
0.00508 < Asian <= 0.00799
0.743 < Broadband.access <= 0.797
0.128 < Severe.housing.problems <= 0.153
Feature
0.00
0.02
0.04
0.06
Weight
Positive
Negative

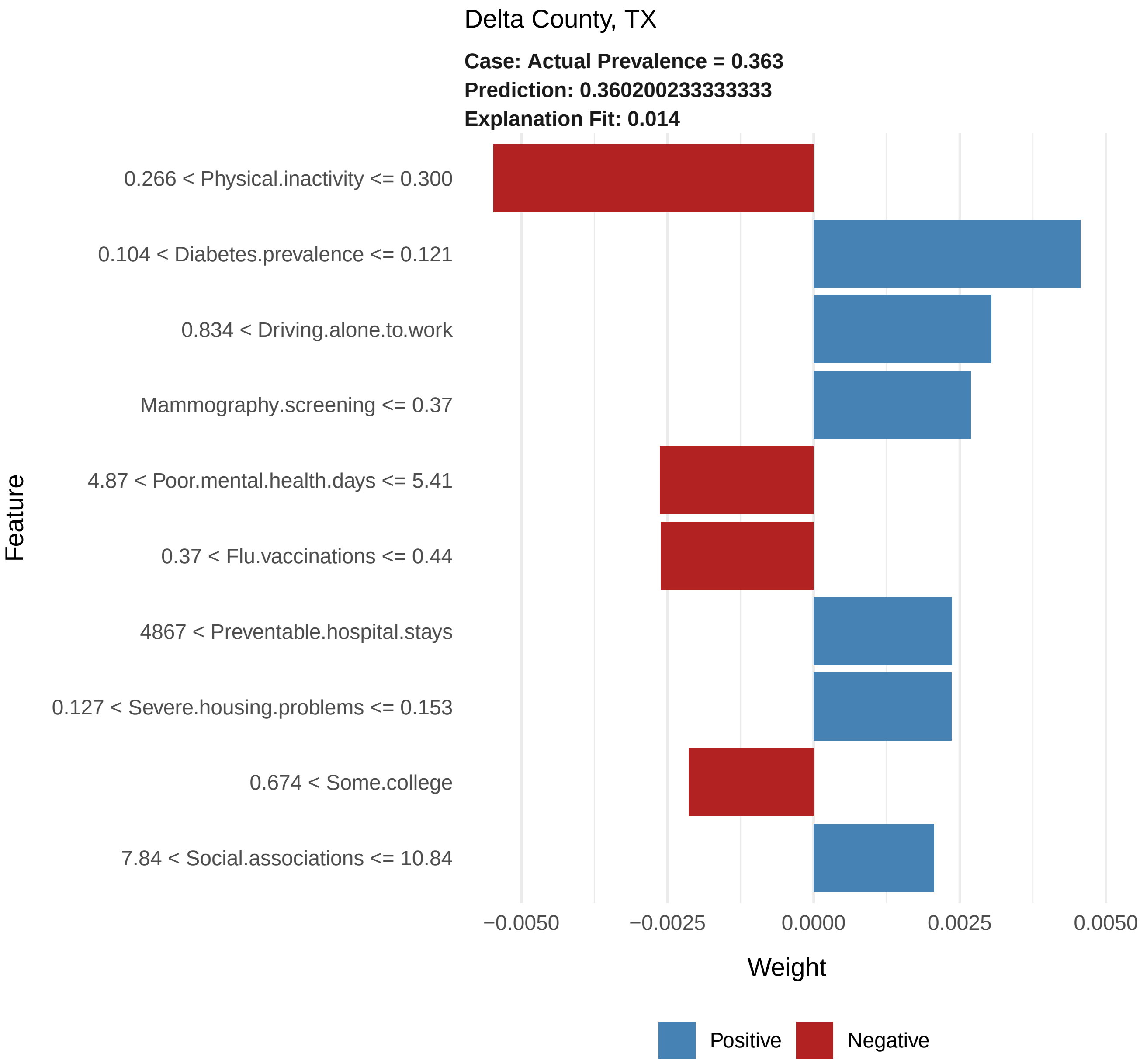
Delta County, TX
Case: Actual Prevalence = 0.363
Prediction: 0.360200233333333
Explanation Fit: 0.014
0.266 < Physical.inactivity <= 0.300
0.104 < Diabetes.prevalence <= 0.121
0.834 < Driving.alone.to.work
Mammography.screening <= 0.37
4.87 < Poor.mental.health.days <= 5.41
0.37 < Flu.vaccinations <= 0.44
4867 < Preventable.hospital.stays
0.127 < Severe.housing.problems <= 0.153
0.674 < Some.college
7.84 < Social.associations <= 10.84
Feature
−0.0050
−0.0025
0.0000
0.0025
0.0050
Weight
Positive
Negative

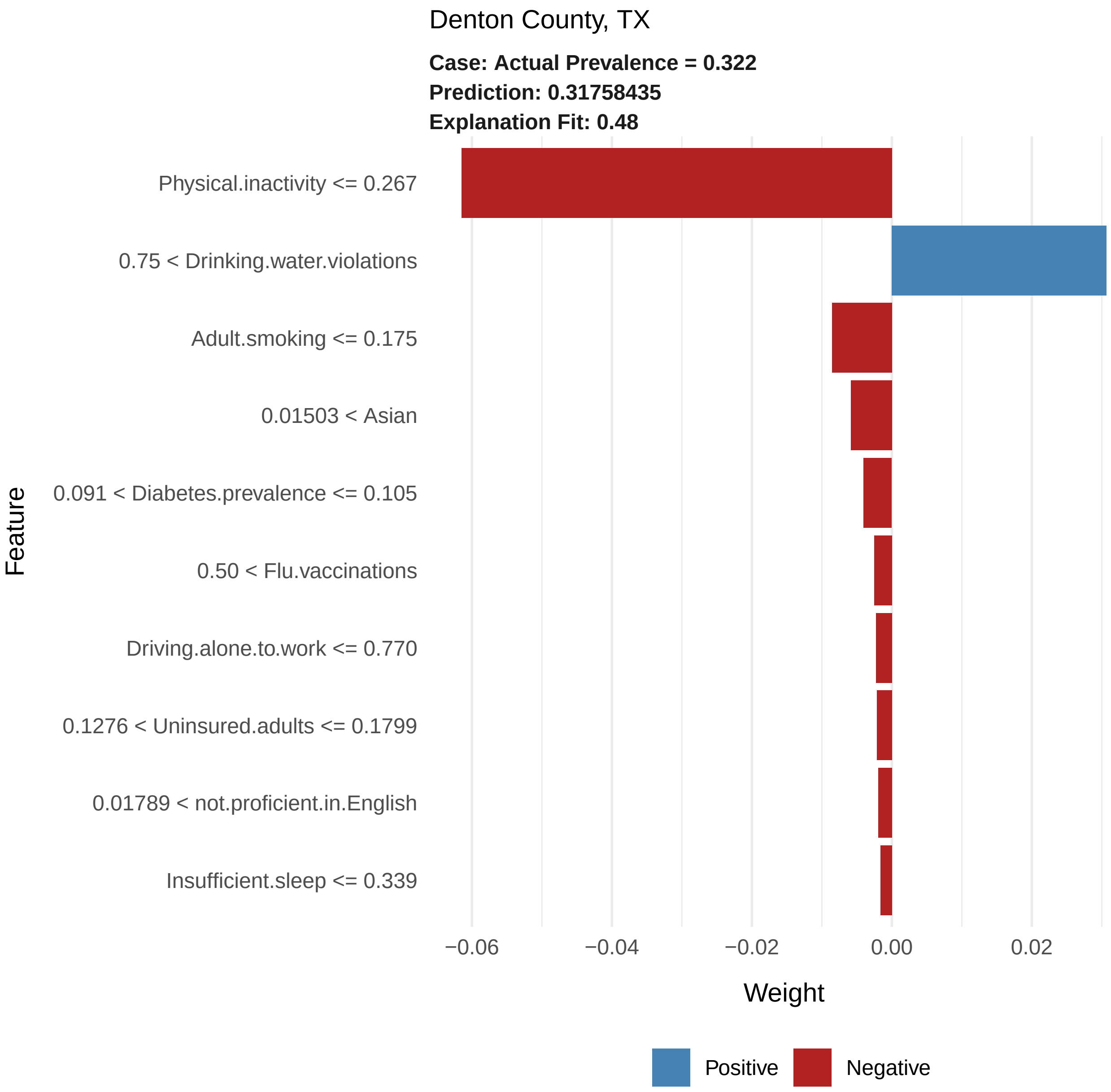
Denton County, TX
Case: Actual Prevalence = 0.322
Prediction: 0.31758435
Explanation Fit: 0.48
Physical.inactivity <= 0.267
0.75 < Drinking.water.violations
Adult.smoking <= 0.175
0.01503 < Asian
0.091 < Diabetes.prevalence <= 0.105
0.50 < Flu.vaccinations
Driving.alone.to.work <= 0.770
0.1276 < Uninsured.adults <= 0.1799
0.01789 < not.proficient.in.English
Insufficient.sleep <= 0.339
Feature
−0.06
−0.04
−0.02
0.00
0.02
Weight
Positive
Negative

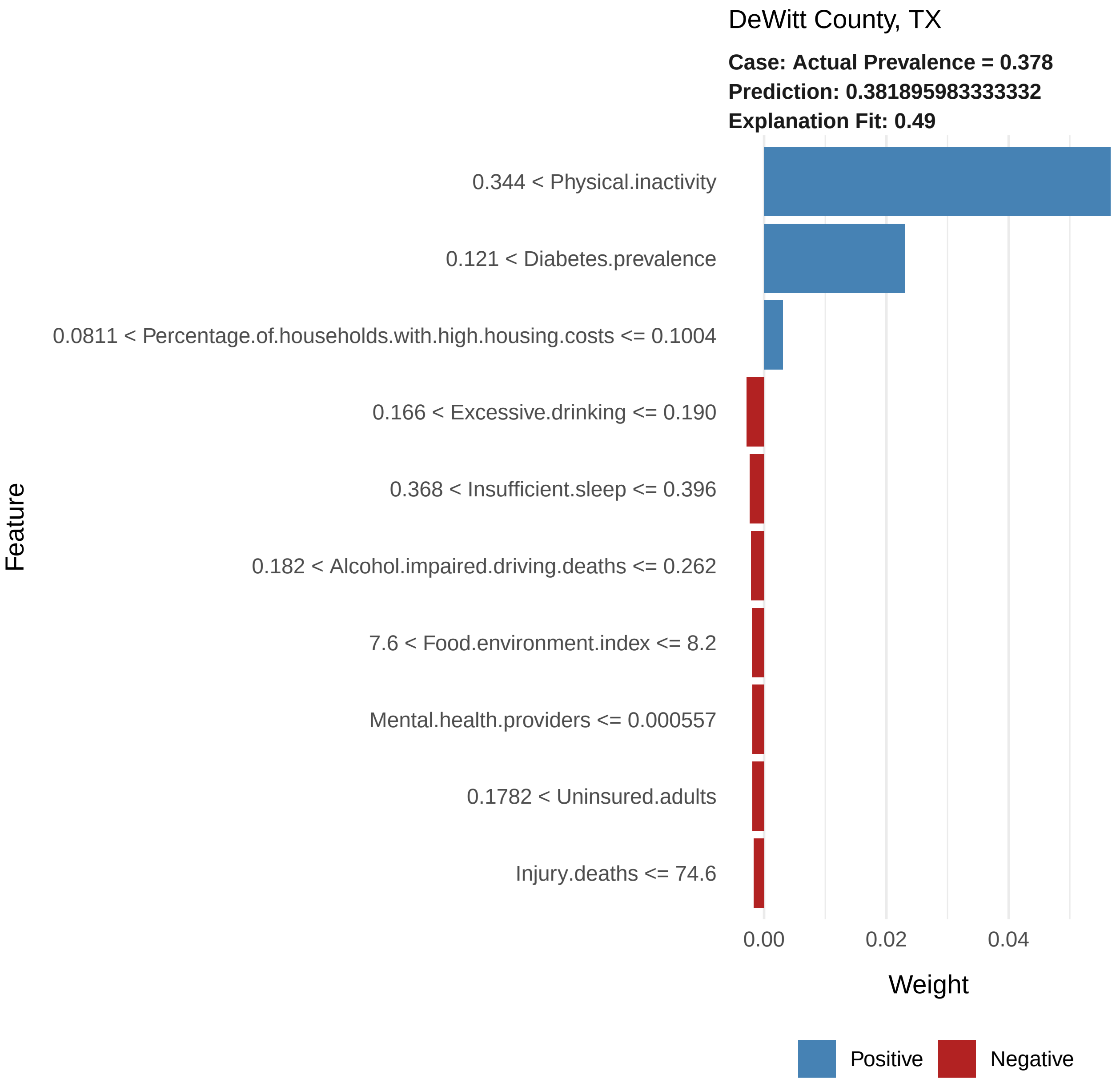
DeWitt County, TX
Case: Actual Prevalence = 0.378
Prediction: 0.381895983333332
Explanation Fit: 0.49
0.344 < Physical.inactivity
0.121 < Diabetes.prevalence
0.0811 < Percentage.of.households.with.high.housing.costs <= 0.1004
0.166 < Excessive.drinking <= 0.190
0.368 < Insufficient.sleep <= 0.396
0.182 < Alcohol.impaired.driving.deaths <= 0.262
7.6 < Food.environment.index <= 8.2
Mental.health.providers <= 0.000557
0.1782 < Uninsured.adults
Injury.deaths <= 74.6
Feature
0.00
0.02
0.04
Weight
Positive
Negative

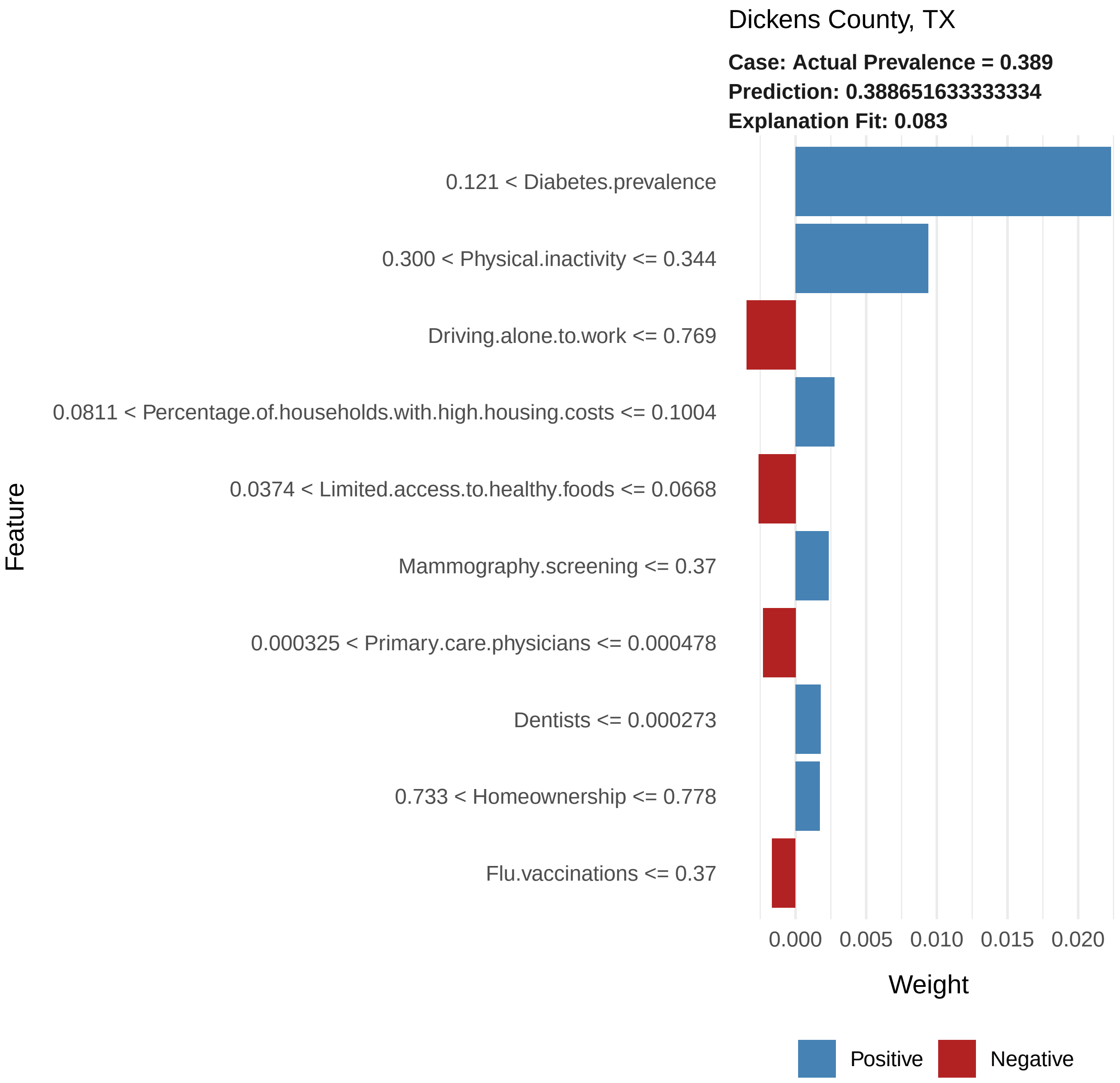

Dickens County, TX
Case: Actual Prevalence = 0.389
Prediction: 0.388651633333334
Explanation Fit: 0.083
0.121 < Diabetes.prevalence
0.300 < Physical.inactivity <= 0.344
Driving.alone.to.work <= 0.769
0.0811 < Percentage.of.households.with.high.housing.costs <= 0.1004
0.0374 < Limited.access.to.healthy.foods <= 0.0668
Mammography.screening <= 0.37
0.000325 < Primary.care.physicians <= 0.000478
Dentists <= 0.000273
0.733 < Homeownership <= 0.778
Flu.vaccinations <= 0.37
Feature
0.000
0.005
0.010
0.015
0.020
Weight
Positive
Negative

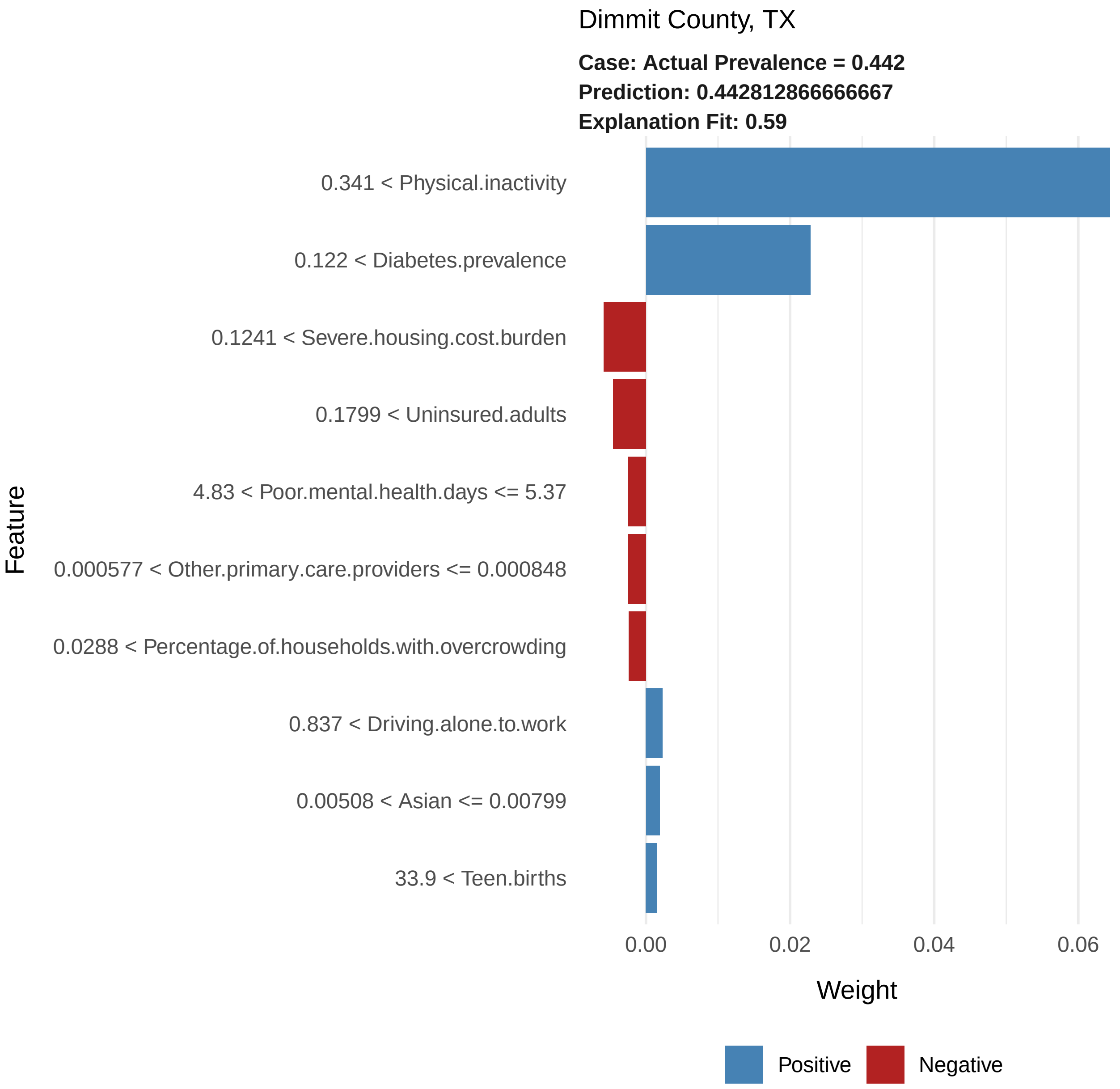

Dimmit County, TX
Case: Actual Prevalence = 0.442
Prediction: 0.442812866666667
Explanation Fit: 0.59
0.341 < Physical.inactivity
0.122 < Diabetes.prevalence
0.1241 < Severe.housing.cost.burden
0.1799 < Uninsured.adults
4.83 < Poor.mental.health.days <= 5.37
0.000577 < Other.primary.care.providers <= 0.000848
0.0288 < Percentage.of.households.with.overcrowding
0.837 < Driving.alone.to.work
0.00508 < Asian <= 0.00799
33.9 < Teen.births
Feature
0.00
0.02
0.04
0.06
Weight
Positive
Negative

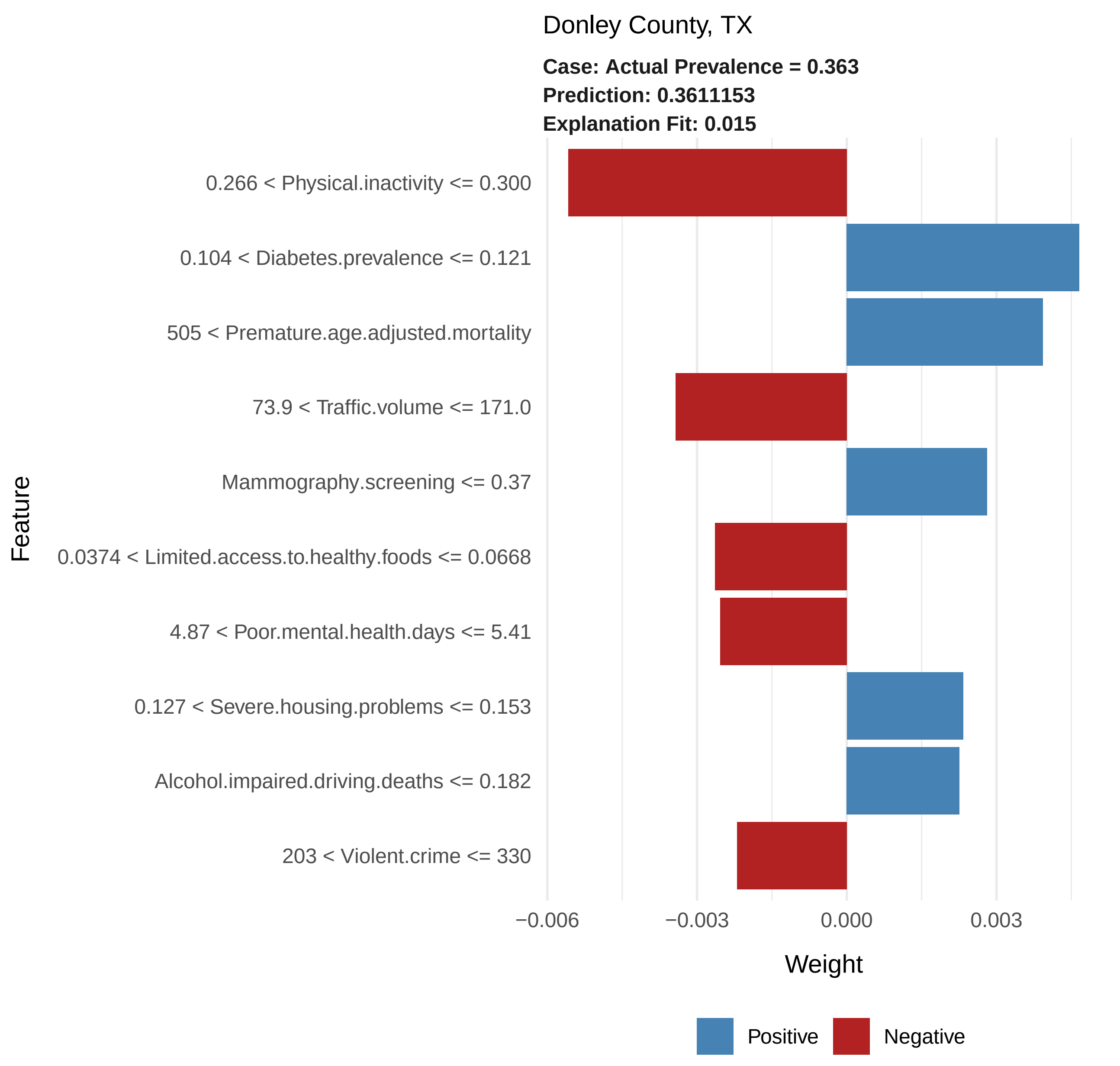
Donley County, TX
Case: Actual Prevalence = 0.363
Prediction: 0.3611153
Explanation Fit: 0.015
Feature
0.266 < Physical.inactivity <= 0.300
0.104 < Diabetes.prevalence <= 0.121
505 < Premature.age.adjusted.mortality
73.9 < Traffic.volume <= 171.0
Mammography.screening <= 0.37
0.0374 < Limited.access.to.healthy.foods <= 0.0668
4.87 < Poor.mental.health.days <= 5.41
0.127 < Severe.housing.problems <= 0.153
Alcohol.impaired.driving.deaths <= 0.182
203 < Violent.crime <= 330
−0.006
−0.003
0.000
0.003
Weight
Positive
Negative

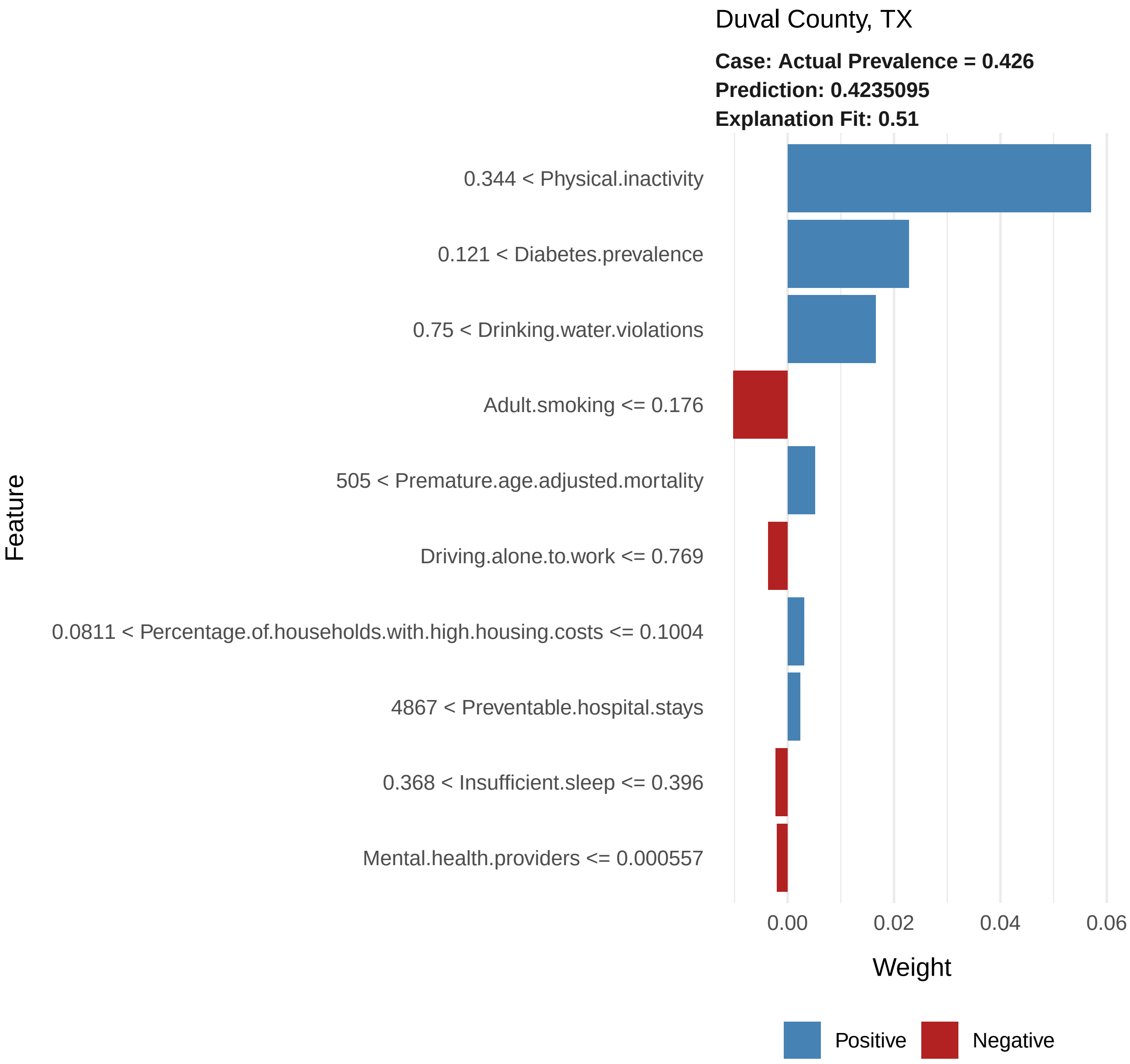
Duval County, TX
Case: Actual Prevalence = 0.426
Prediction: 0.4235095
Explanation Fit: 0.51
0.344 < Physical.inactivity
0.121 < Diabetes.prevalence
0.75 < Drinking.water.violations
Adult.smoking <= 0.176
505 < Premature.age.adjusted.mortality
Driving.alone.to.work <= 0.769
0.0811 < Percentage.of.households.with.high.housing.costs <= 0.1004
4867 < Preventable.hospital.stays
0.368 < Insufficient.sleep <= 0.396
Mental.health.providers <= 0.000557
Feature
0.00
0.02
0.04
0.06
Weight
Positive
Negative

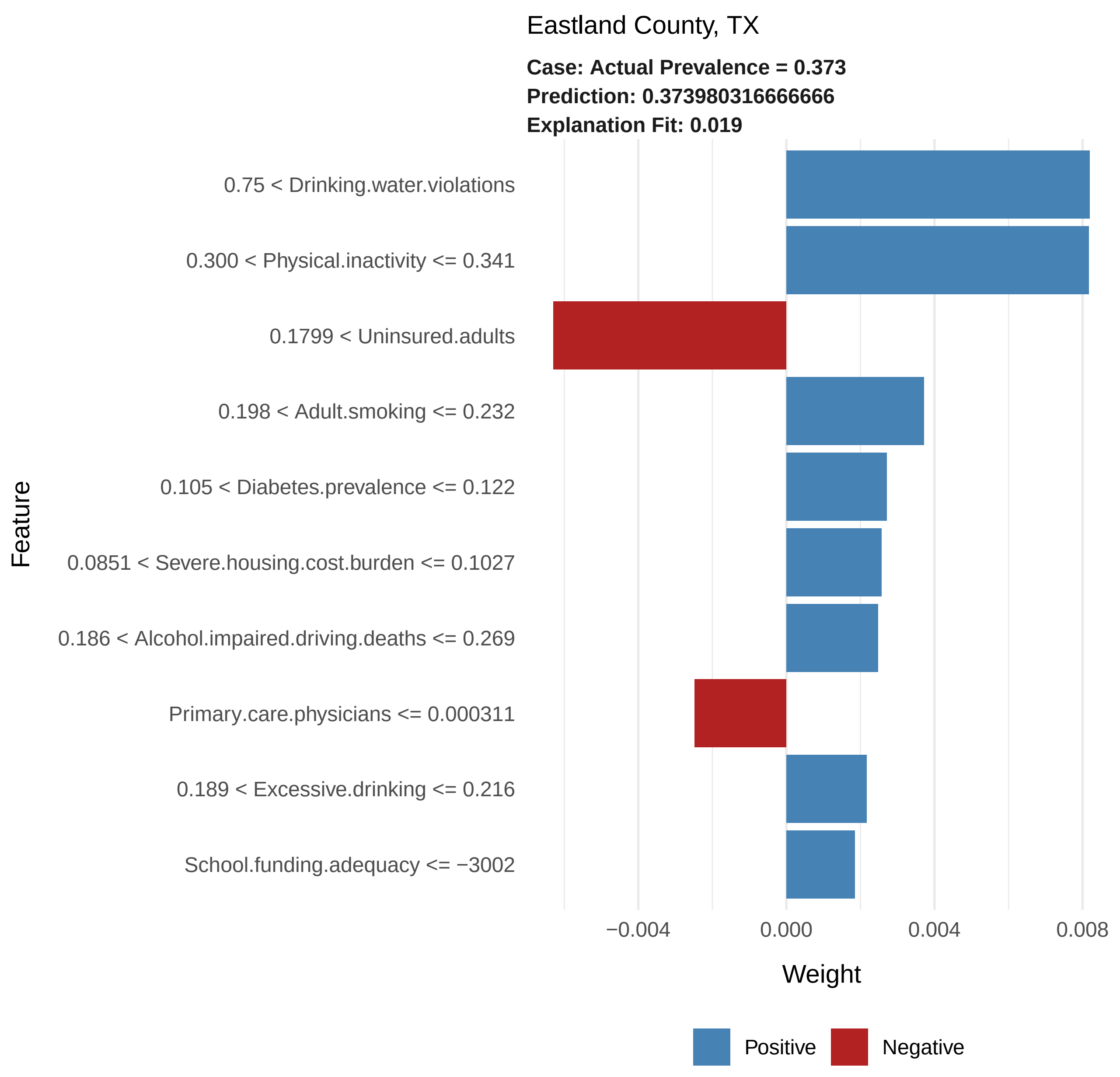
Eastland County, TX
Case: Actual Prevalence = 0.373
Prediction: 0.373980316666666
Explanation Fit: 0.019
0.75 < Drinking.water.violations
0.300 < Physical.inactivity <= 0.341
0.1799 < Uninsured.adults
0.198 < Adult.smoking <= 0.232
0.105 < Diabetes.prevalence <= 0.122
0.0851 < Severe.housing.cost.burden <= 0.1027
0.186 < Alcohol.impaired.driving.deaths <= 0.269
Primary.care.physicians <= 0.000311
0.189 < Excessive.drinking <= 0.216
School.funding.adequacy <= −3002
Feature
−0.004
0.000
0.004
0.008
Weight
Positive
Negative

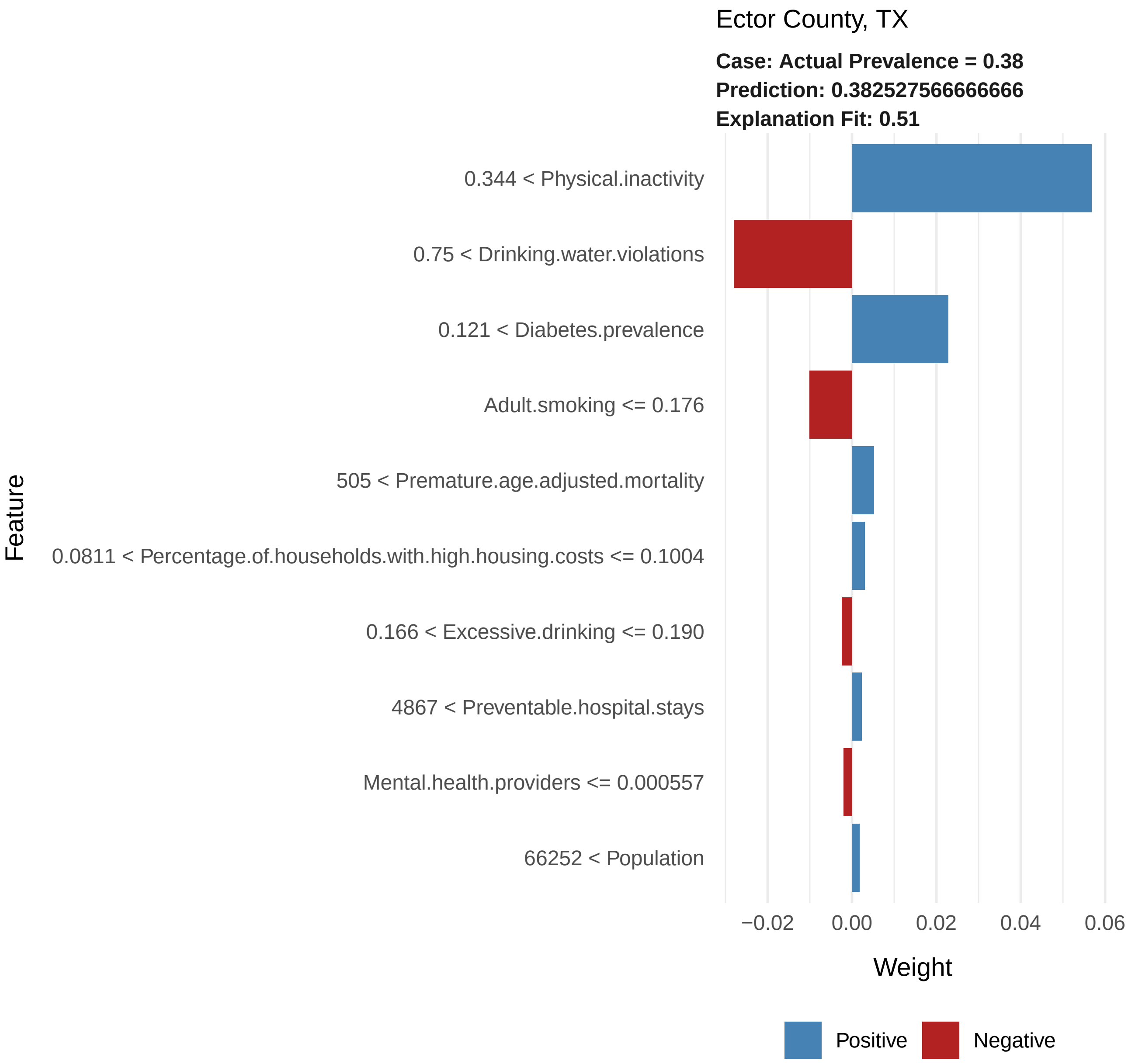

Ector County, TX
Case: Actual Prevalence = 0.38
Prediction: 0.382527566666666
Explanation Fit: 0.51
0.344 < Physical.inactivity
0.75 < Drinking.water.violations
0.121 < Diabetes.prevalence
Adult.smoking <= 0.176
505 < Premature.age.adjusted.mortality
0.0811 < Percentage.of.households.with.high.housing.costs <= 0.1004
0.166 < Excessive.drinking <= 0.190
4867 < Preventable.hospital.stays
Mental.health.providers <= 0.000557
66252 < Population
Feature
−0.02
0.00
0.02
0.04
0.06
Weight
Positive
Negative

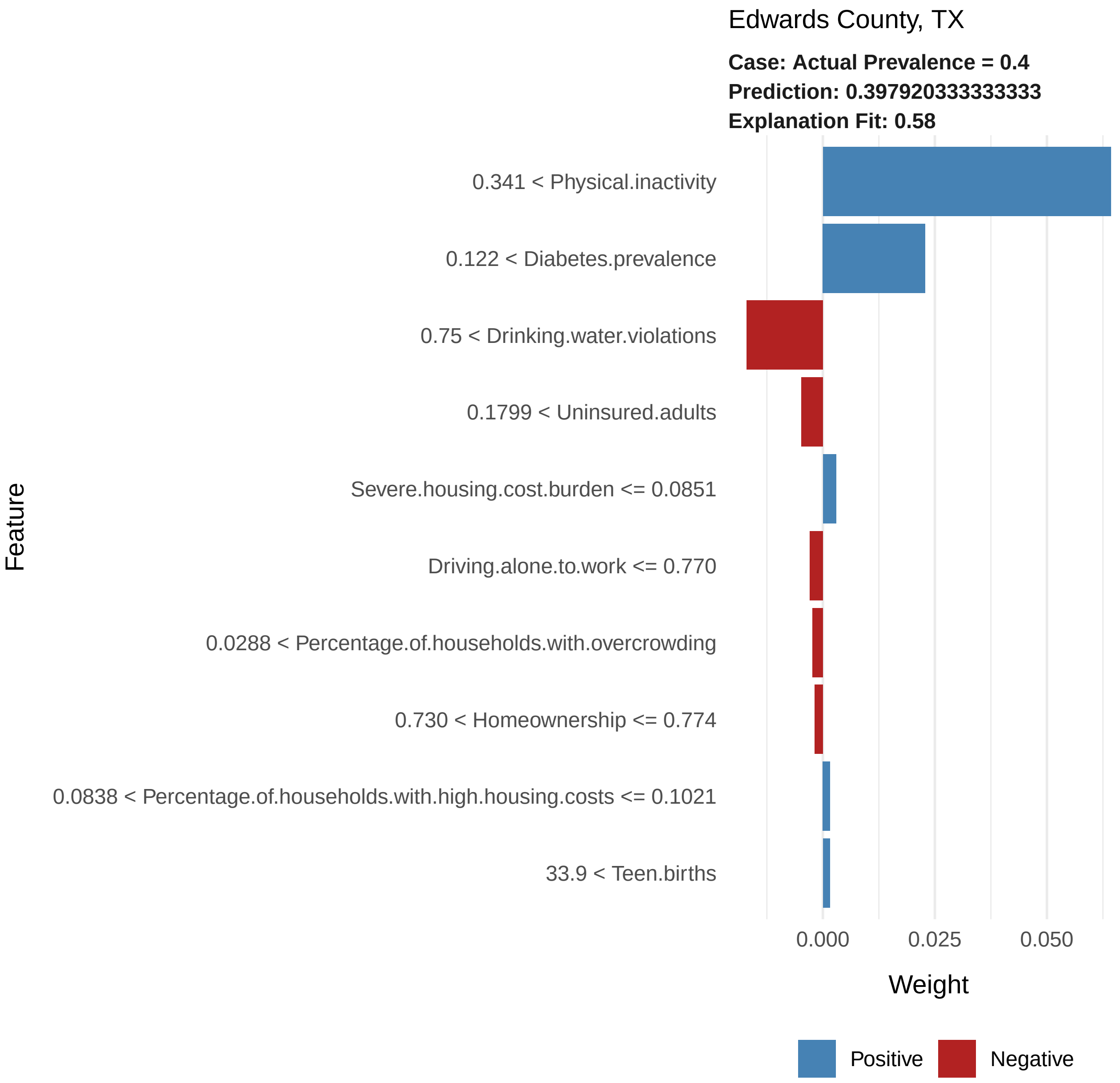
Edwards County, TX
Case: Actual Prevalence = 0.4
Prediction: 0.397920333333333
Explanation Fit: 0.58
0.341 < Physical.inactivity
0.122 < Diabetes.prevalence
0.75 < Drinking.water.violations
0.1799 < Uninsured.adults
Severe.housing.cost.burden <= 0.0851
Driving.alone.to.work <= 0.770
0.0288 < Percentage.of.households.with.overcrowding
0.730 < Homeownership <= 0.774
0.0838 < Percentage.of.households.with.high.housing.costs <= 0.1021
33.9 < Teen.births
Feature
0.000
0.025
0.050
Weight
Positive
Negative

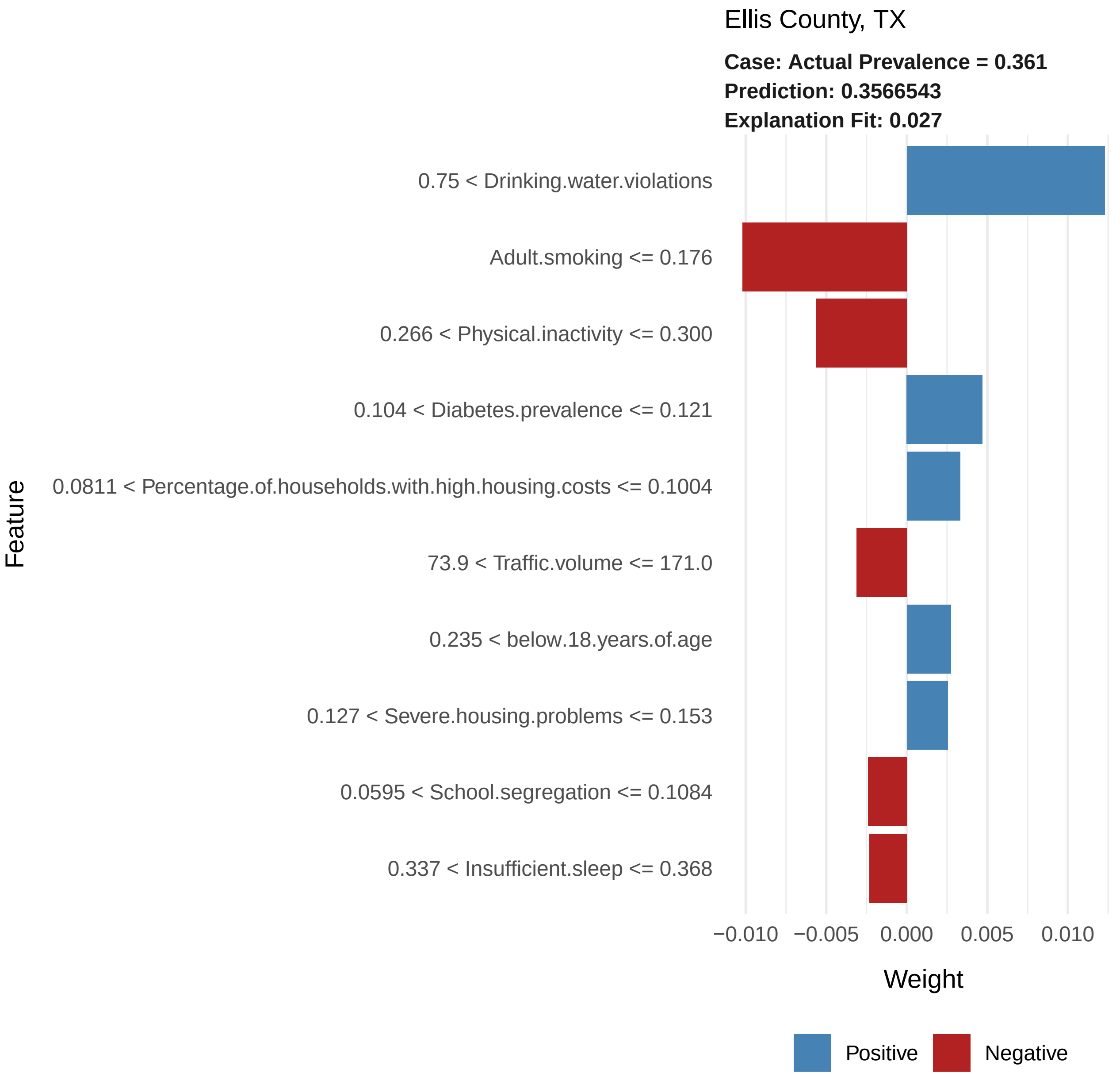

Ellis County, TX
Case: Actual Prevalence = 0.361
Prediction: 0.3566543
Explanation Fit: 0.027
0.75 < Drinking.water.violations
Adult.smoking <= 0.176
0.266 < Physical.inactivity <= 0.300
0.104 < Diabetes.prevalence <= 0.121
0.0811 < Percentage.of.households.with.high.housing.costs <= 0.1004
73.9 < Traffic.volume <= 171.0
0.235 < below.18.years.of.age
0.127 < Severe.housing.problems <= 0.153
0.0595 < School.segregation <= 0.1084
0.337 < Insufficient.sleep <= 0.368
Feature
−0.010
−0.005
0.000
0.005
0.010
Weight
Positive
Negative

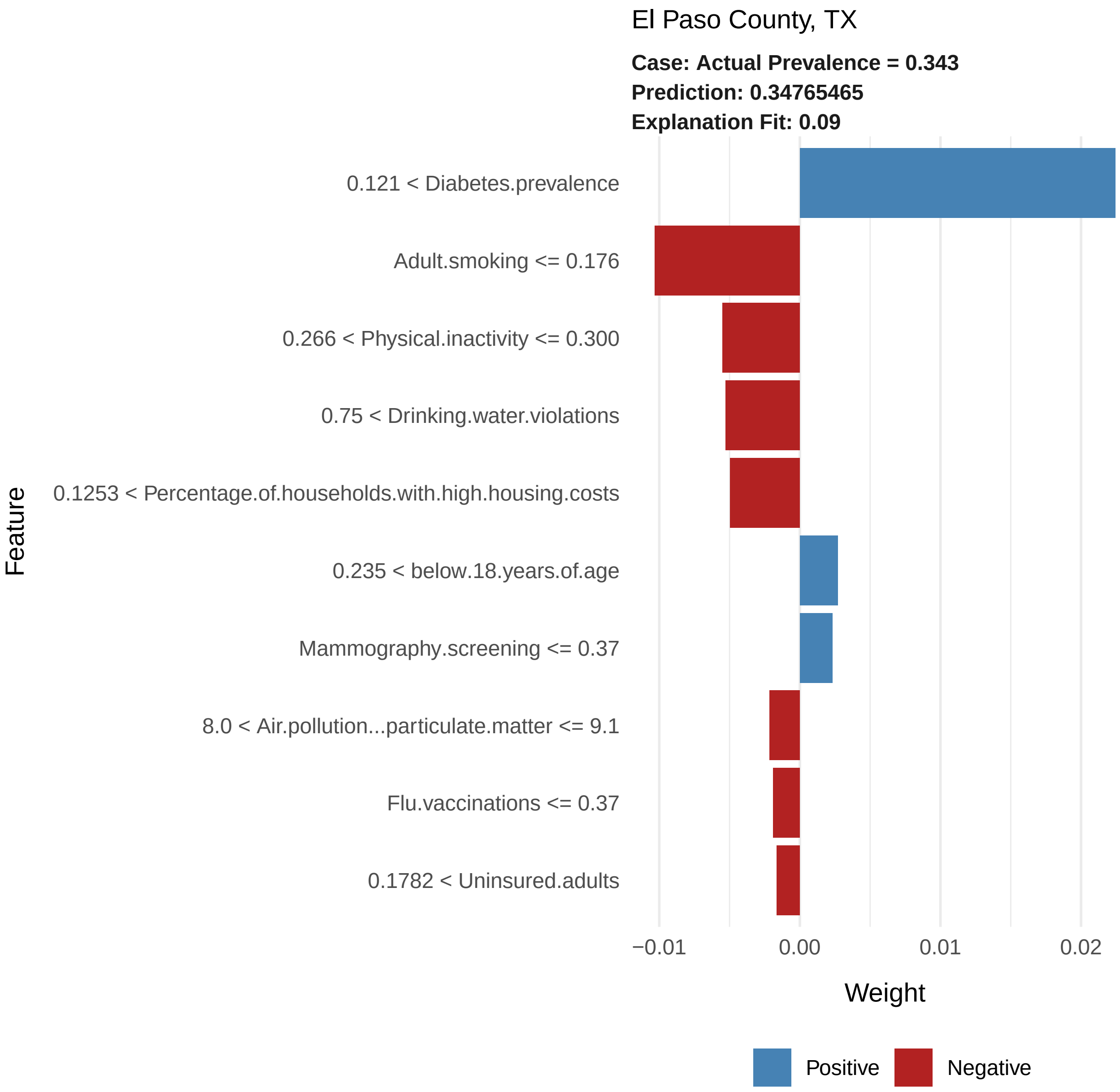
El Paso County, TX
Case: Actual Prevalence = 0.343
Prediction: 0.34765465
Explanation Fit: 0.09
0.121 < Diabetes.prevalence
Adult.smoking <= 0.176
0.266 < Physical.inactivity <= 0.300
0.75 < Drinking.water.violations
0.1253 < Percentage.of.households.with.high.housing.costs
0.235 < below.18.years.of.age
Mammography.screening <= 0.37
8.0 < Air.pollution...particulate.matter <= 9.1
Flu.vaccinations <= 0.37
0.1782 < Uninsured.adults
Feature
−0.01
0.00
0.01
0.02
Weight
Positive
Negative

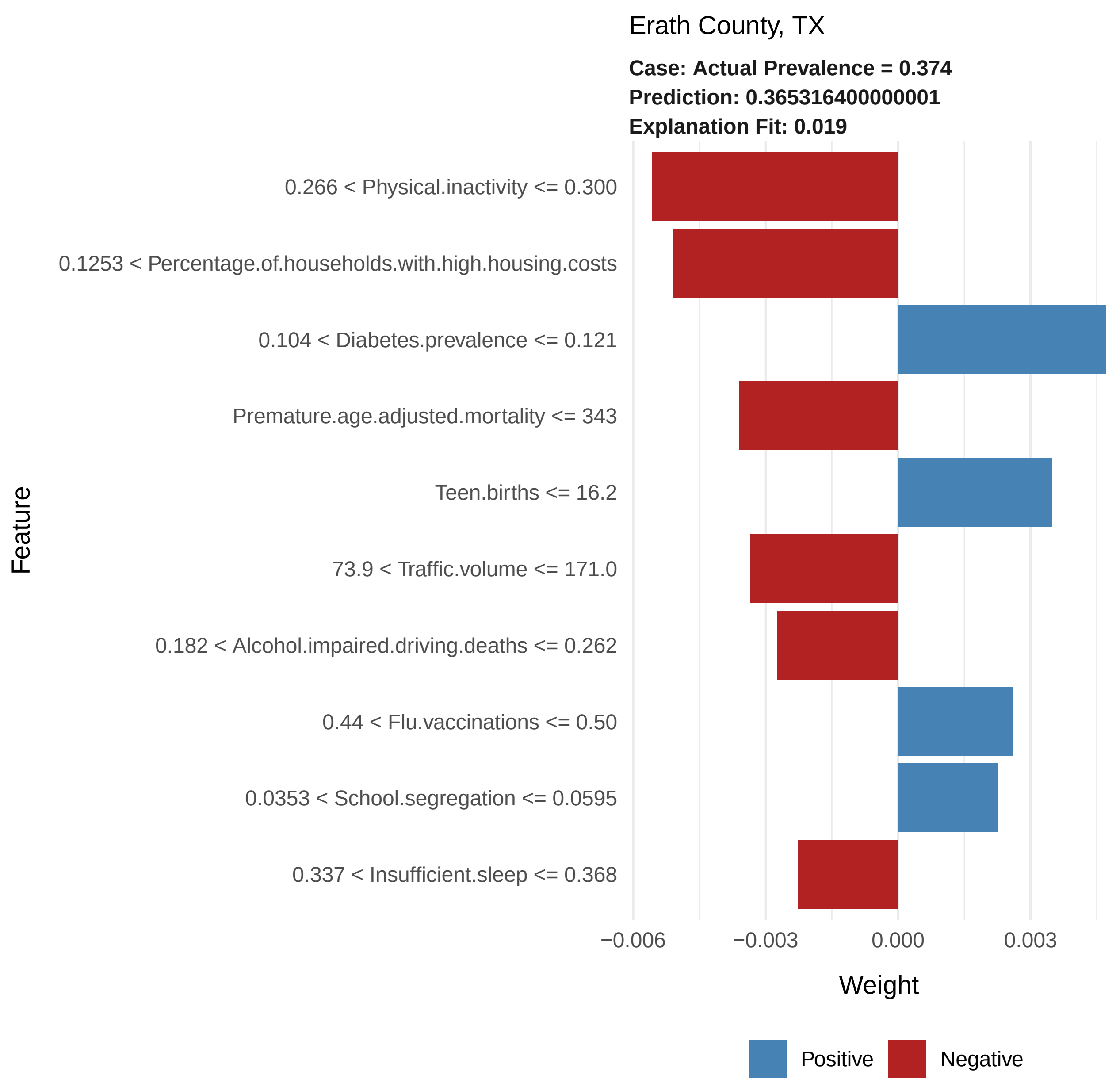
Erath County, TX
Case: Actual Prevalence = 0.374
Prediction: 0.365316400000001
Explanation Fit: 0.019
0.266 < Physical.inactivity <= 0.300
0.1253 < Percentage.of.households.with.high.housing.costs
0.104 < Diabetes.prevalence <= 0.121
Premature.age.adjusted.mortality <= 343
Teen.births <= 16.2
73.9 < Traffic.volume <= 171.0
0.182 < Alcohol.impaired.driving.deaths <= 0.262
0.44 < Flu.vaccinations <= 0.50
0.0353 < School.segregation <= 0.0595
0.337 < Insufficient.sleep <= 0.368
Feature
−0.006
−0.003
0.000
0.003
Weight
Positive
Negative

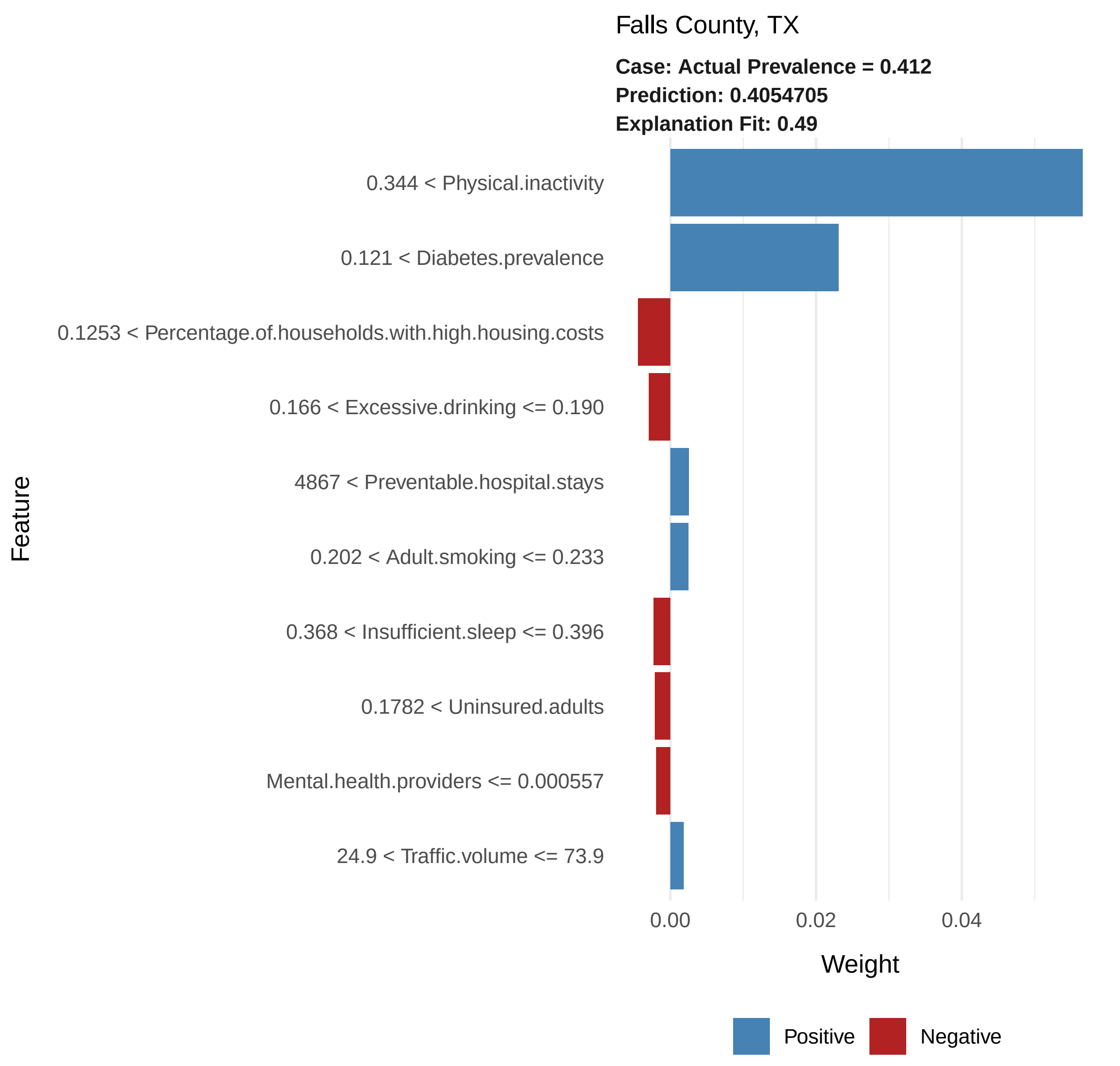
Falls County, TX
Case: Actual Prevalence = 0.412
Prediction: 0.4054705
Explanation Fit: 0.49
0.344 < Physical.inactivity
0.121 < Diabetes.prevalence
0.1253 < Percentage.of.households.with.high.housing.costs
0.166 < Excessive.drinking <= 0.190
4867 < Preventable.hospital.stays
0.202 < Adult.smoking <= 0.233
0.368 < Insufficient.sleep <= 0.396
0.1782 < Uninsured.adults
Mental.health.providers <= 0.000557
24.9 < Traffic.volume <= 73.9
Feature
0.00
0.02
0.04
Weight
Positive
Negative

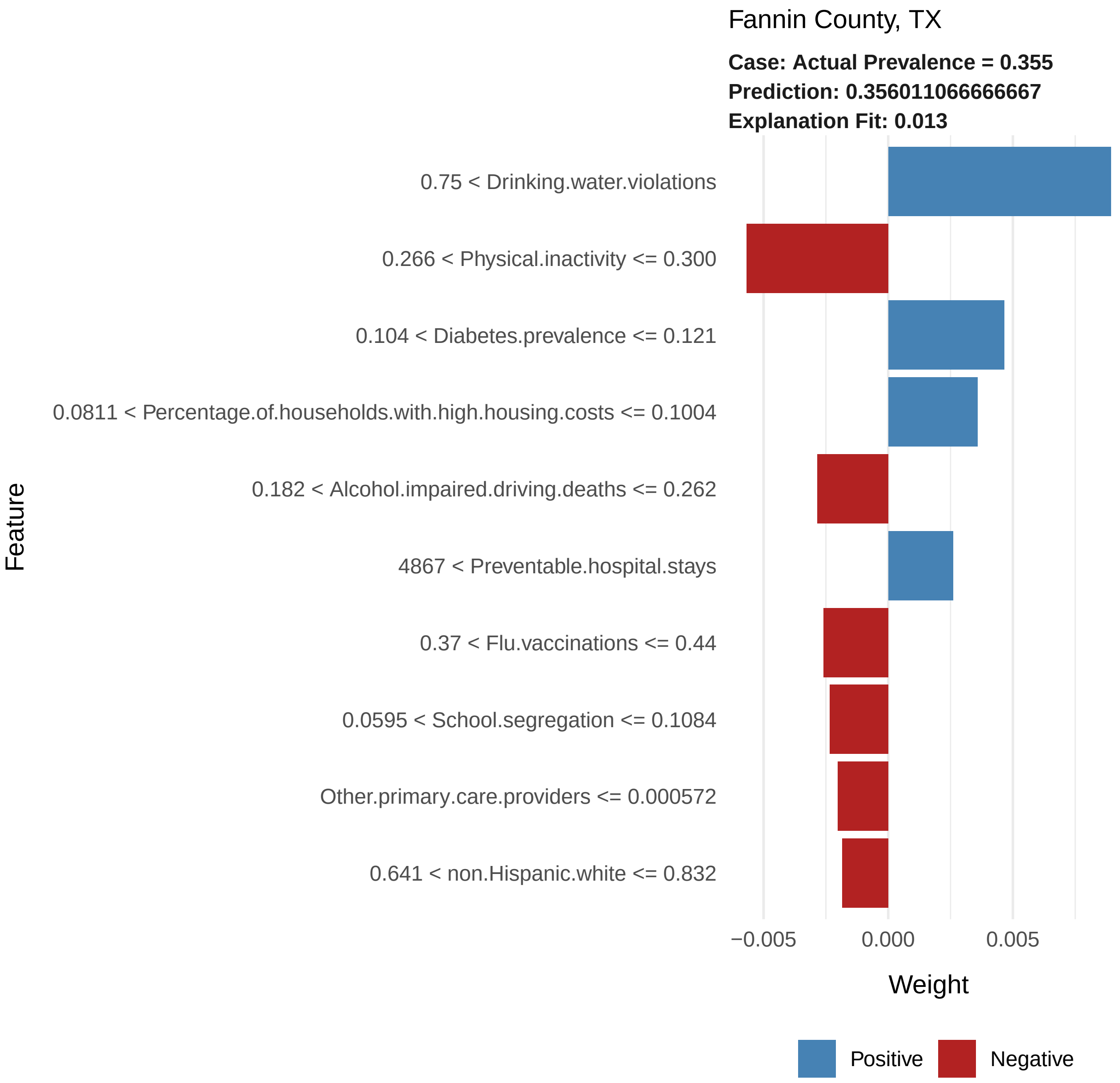

Fannin County, TX
Case: Actual Prevalence = 0.355
Prediction: 0.356011066666667
Explanation Fit: 0.013
0.75 < Drinking.water.violations
0.266 < Physical.inactivity <= 0.300
0.104 < Diabetes.prevalence <= 0.121
0.0811 < Percentage.of.households.with.high.housing.costs <= 0.1004
0.182 < Alcohol.impaired.driving.deaths <= 0.262
4867 < Preventable.hospital.stays
0.37 < Flu.vaccinations <= 0.44
0.0595 < School.segregation <= 0.1084
Other.primary.care.providers <= 0.000572
0.641 < non.Hispanic.white <= 0.832
Feature
−0.005
0.000
0.005
Weight
Positive
Negative

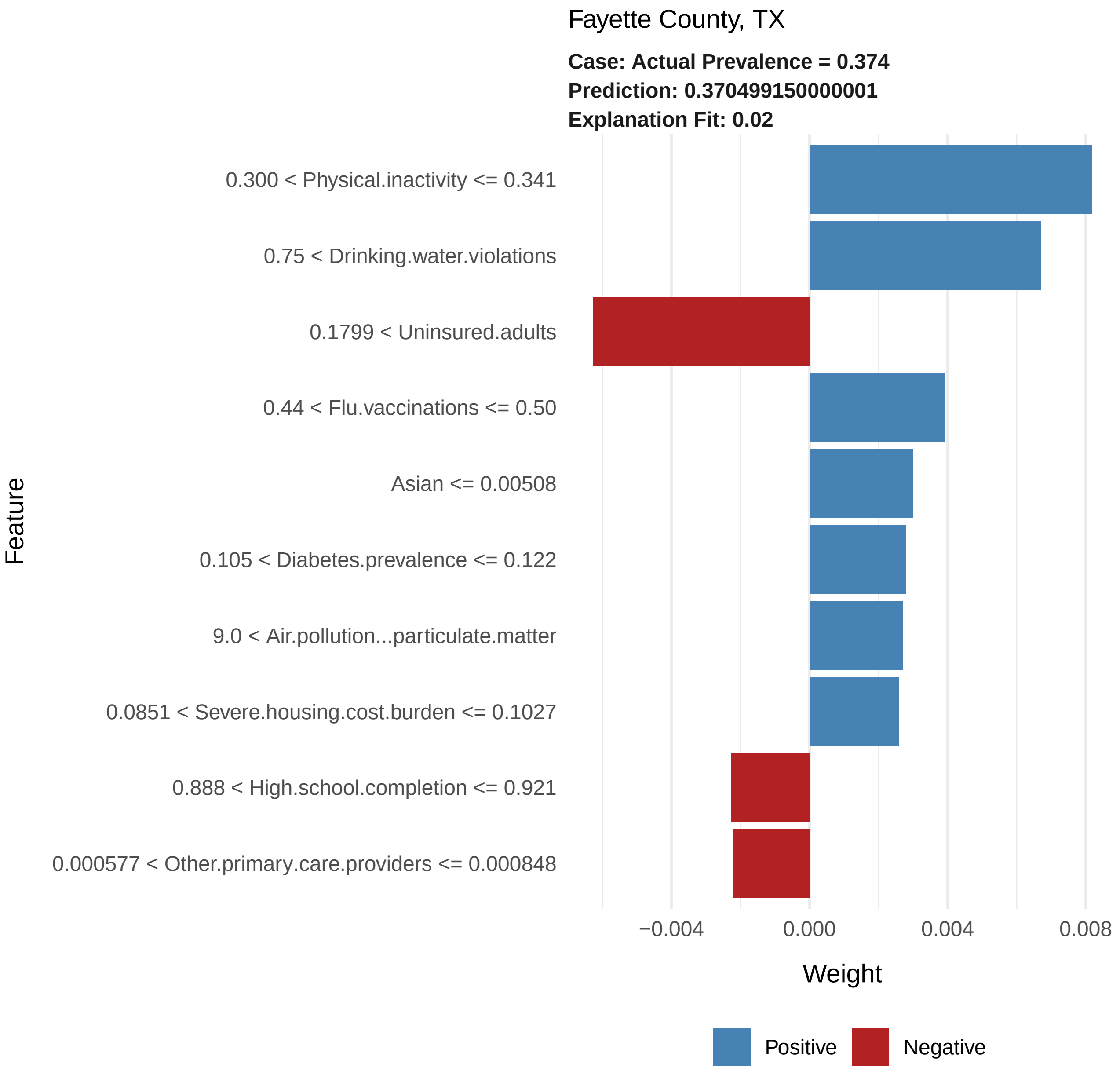

Fayette County, TX
Case: Actual Prevalence = 0.374
Prediction: 0.370499150000001
Explanation Fit: 0.02
0.300 < Physical.inactivity <= 0.341
0.75 < Drinking.water.violations
0.1799 < Uninsured.adults
0.44 < Flu.vaccinations <= 0.50
Asian <= 0.00508
0.105 < Diabetes.prevalence <= 0.122
9.0 < Air.pollution...particulate.matter
0.0851 < Severe.housing.cost.burden <= 0.1027
0.888 < High.school.completion <= 0.921
0.000577 < Other.primary.care.providers <= 0.000848
Feature
−0.004
0.000
0.004
0.008
Weight
Positive
Negative

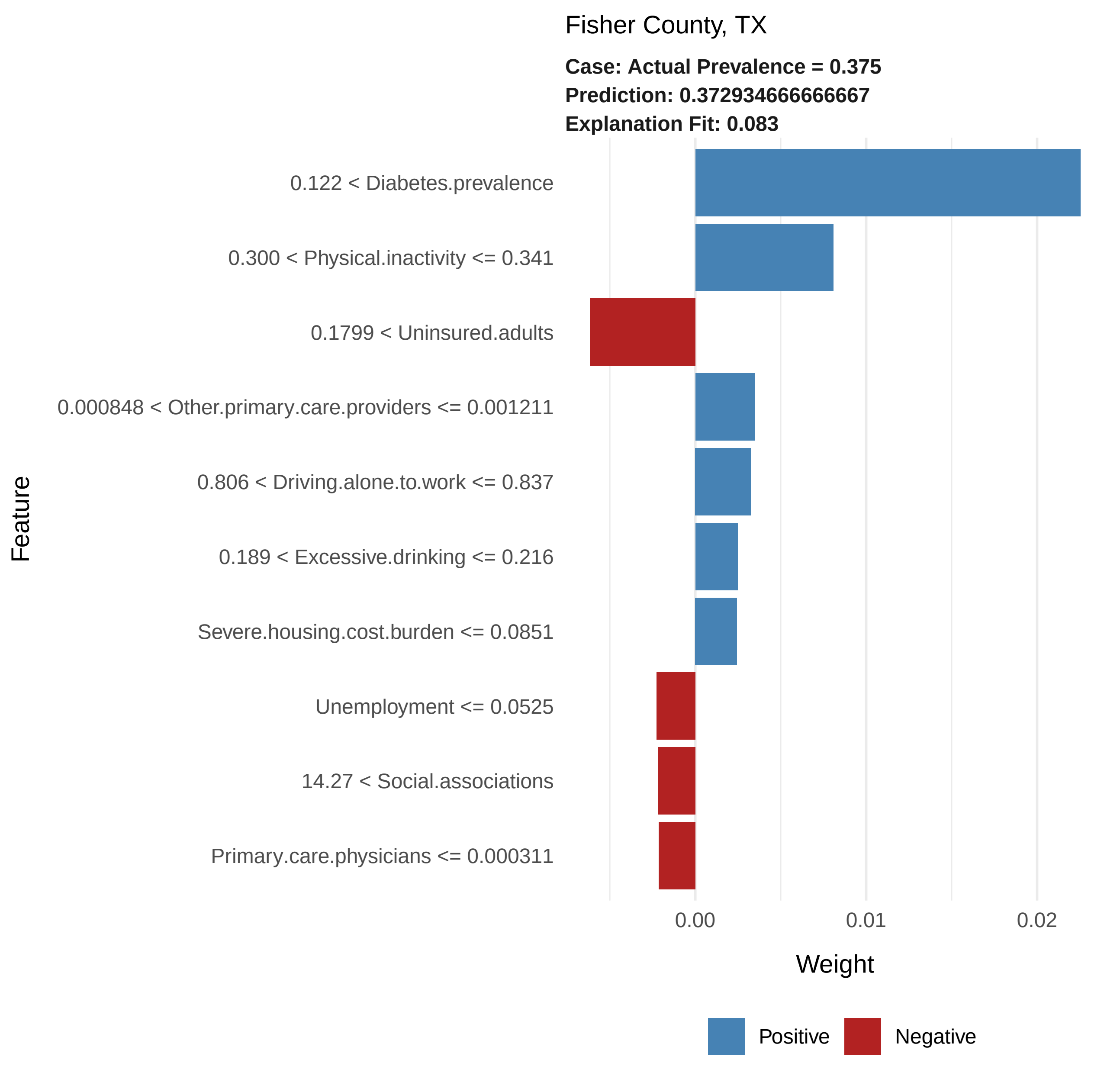

Fisher County, TX
Case: Actual Prevalence = 0.375
Prediction: 0.372934666666667
Explanation Fit: 0.083
0.122 < Diabetes.prevalence
0.300 < Physical.inactivity <= 0.341
0.1799 < Uninsured.adults
0.000848 < Other.primary.care.providers <= 0.001211
0.806 < Driving.alone.to.work <= 0.837
0.189 < Excessive.drinking <= 0.216
Severe.housing.cost.burden <= 0.0851
Unemployment <= 0.0525
14.27 < Social.associations
Primary.care.physicians <= 0.000311
Feature
0.00
0.01
0.02
Weight
Positive
Negative

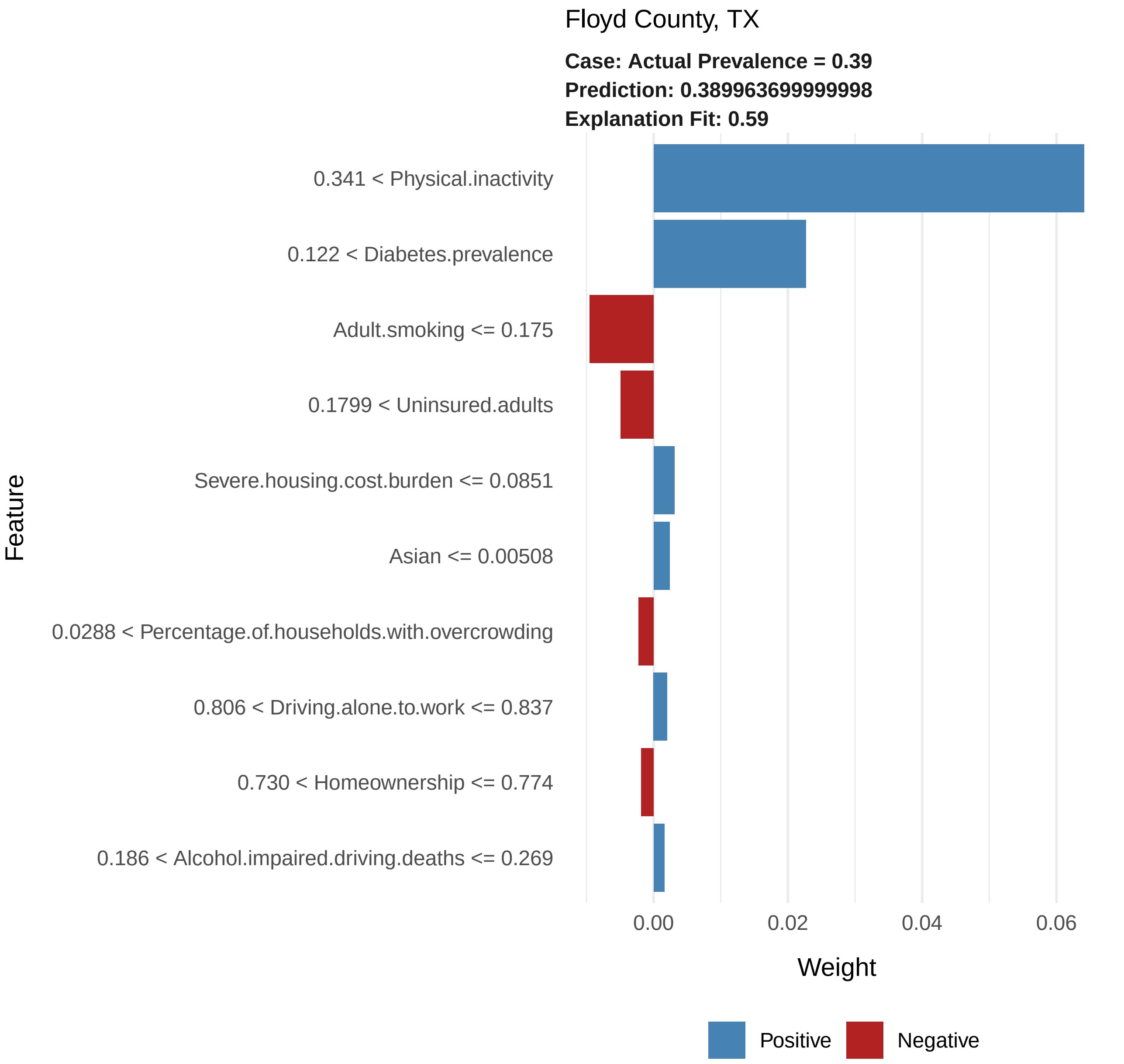

Floyd County, TX
Case: Actual Prevalence = 0.39
Prediction: 0.389963699999998
Explanation Fit: 0.59
0.341 < Physical.inactivity
0.122 < Diabetes.prevalence
Adult.smoking <= 0.175
0.1799 < Uninsured.adults
Severe.housing.cost.burden <= 0.0851
Asian <= 0.00508
0.0288 < Percentage.of.households.with.overcrowding
0.806 < Driving.alone.to.work <= 0.837
0.730 < Homeownership <= 0.774
0.186 < Alcohol.impaired.driving.deaths <= 0.269
Feature
0.00
0.02
0.04
0.06
Weight
Positive
Negative

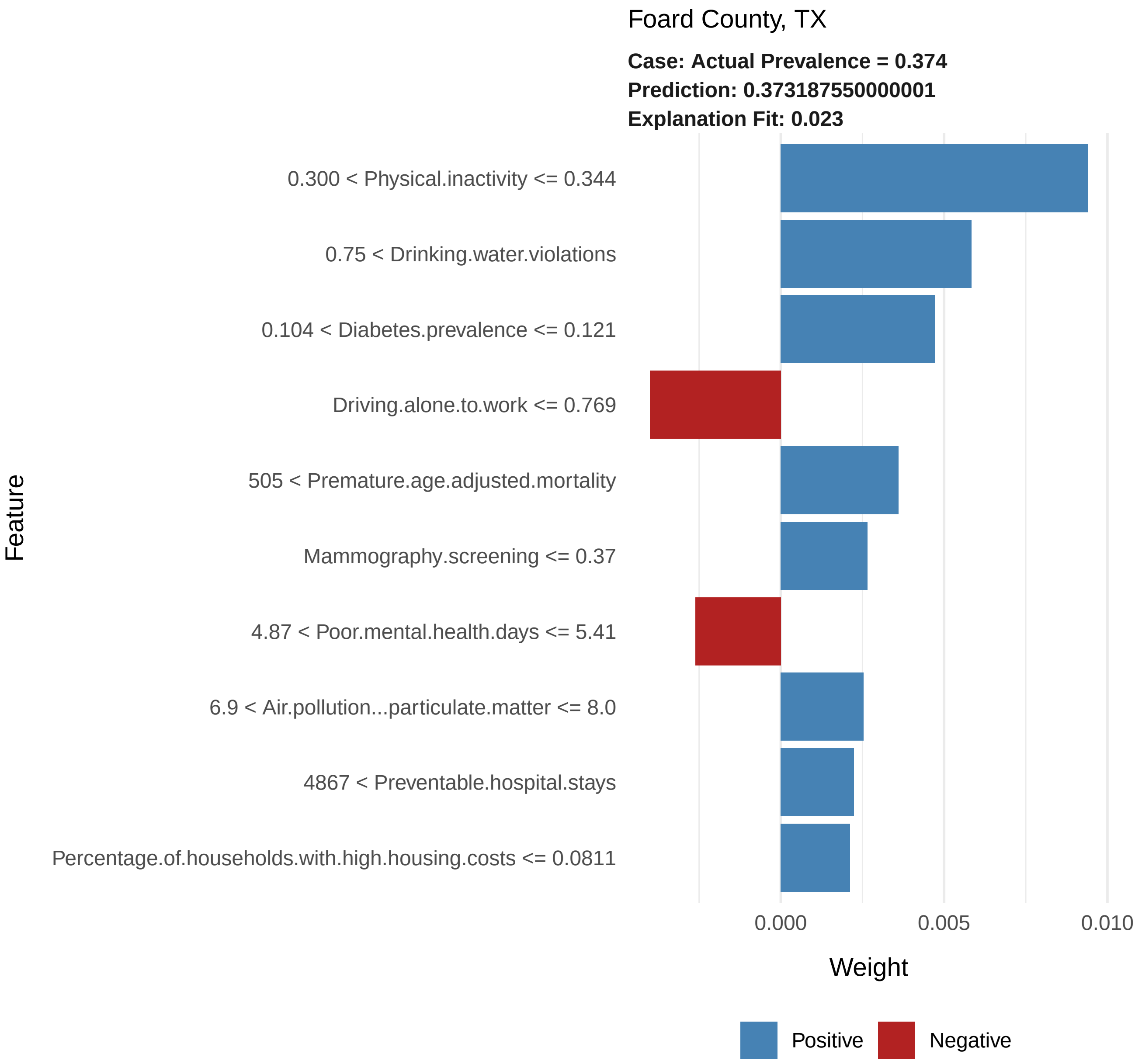

Foard County, TX
Case: Actual Prevalence = 0.374
Prediction: 0.373187550000001
Explanation Fit: 0.023
0.300 < Physical.inactivity <= 0.344
0.75 < Drinking.water.violations
0.104 < Diabetes.prevalence <= 0.121
Driving.alone.to.work <= 0.769
505 < Premature.age.adjusted.mortality
Mammography.screening <= 0.37
4.87 < Poor.mental.health.days <= 5.41
6.9 < Air.pollution...particulate.matter <= 8.0
4867 < Preventable.hospital.stays
Percentage.of.households.with.high.housing.costs <= 0.0811
Feature
0.000
0.005
0.010
Weight
Positive
Negative

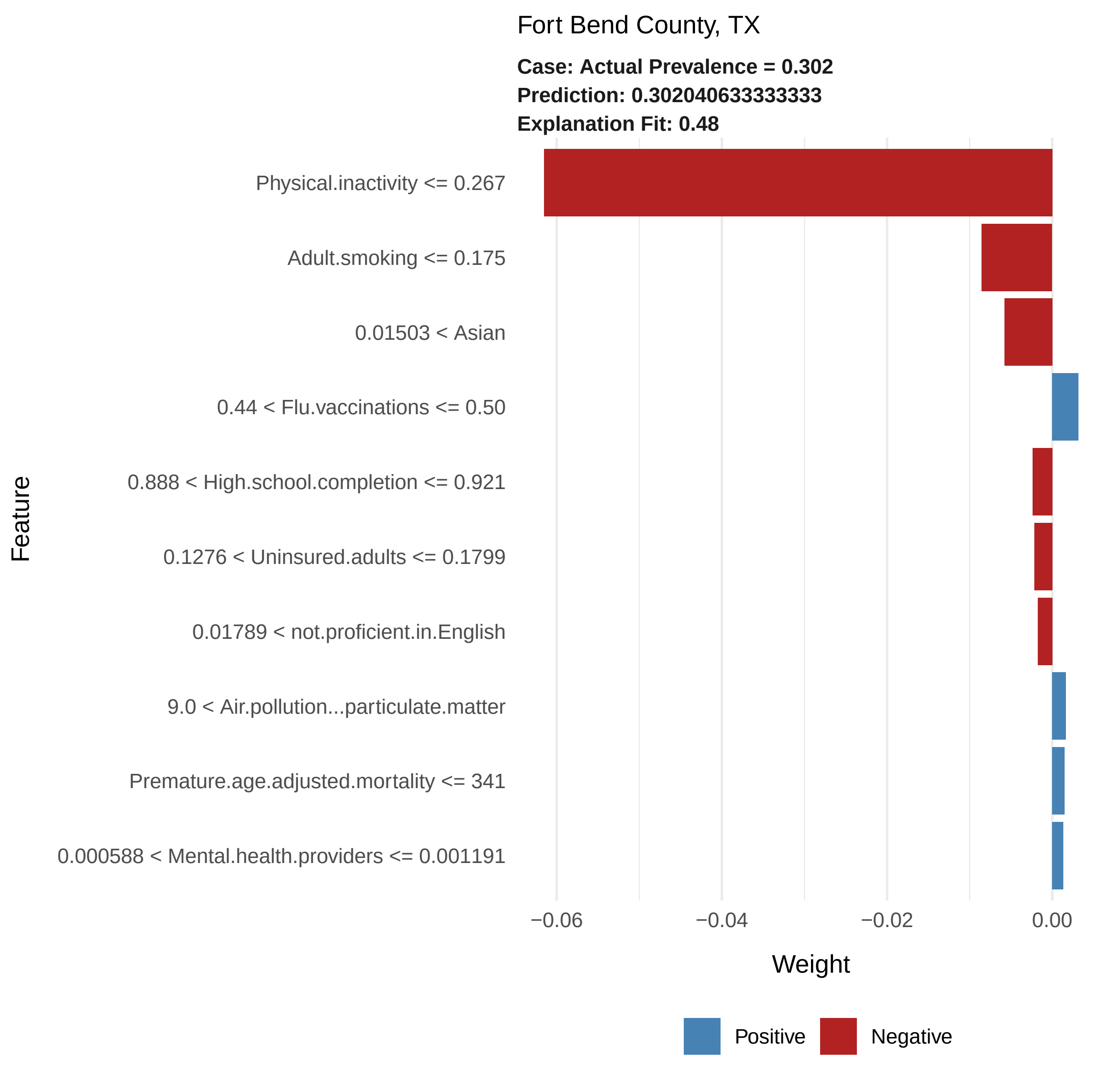
Fort Bend County, TX
Case: Actual Prevalence = 0.302
Prediction: 0.302040633333333
Explanation Fit: 0.48
Physical.inactivity <= 0.267
Adult.smoking <= 0.175
0.01503 < Asian
0.44 < Flu.vaccinations <= 0.50
0.888 < High.school.completion <= 0.921
0.1276 < Uninsured.adults <= 0.1799
0.01789 < not.proficient.in.English
9.0 < Air.pollution...particulate.matter
Premature.age.adjusted.mortality <= 341
0.000588 < Mental.health.providers <= 0.001191
Feature
−0.06
−0.04
−0.02
0.00
Weight
Positive
Negative

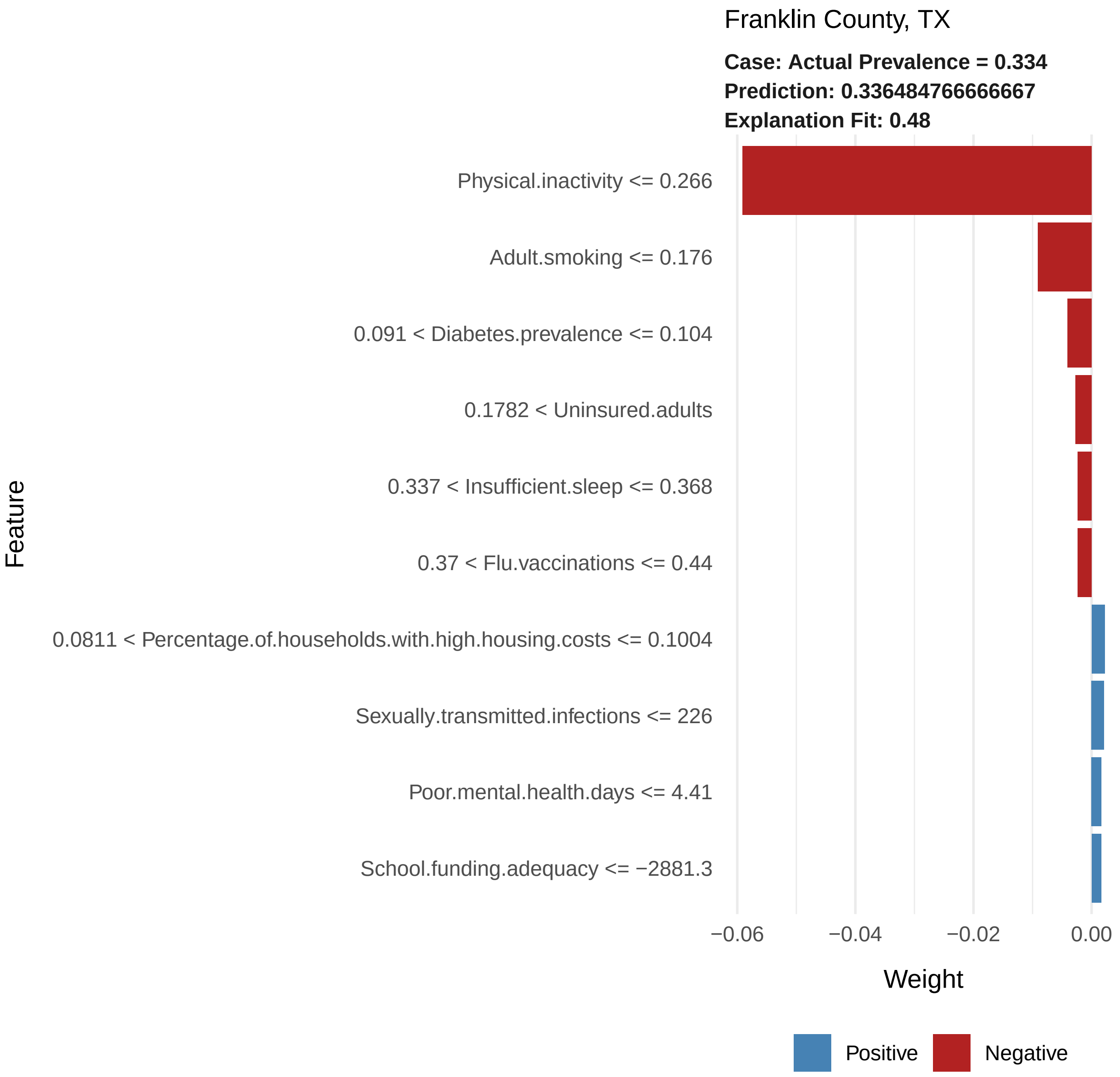
Franklin County, TX
Case: Actual Prevalence = 0.334
Prediction: 0.336484766666667
Explanation Fit: 0.48
Physical.inactivity <= 0.266
Adult.smoking <= 0.176
0.091 < Diabetes.prevalence <= 0.104
0.1782 < Uninsured.adults
0.337 < Insufficient.sleep <= 0.368
0.37 < Flu.vaccinations <= 0.44
0.0811 < Percentage.of.households.with.high.housing.costs <= 0.1004
Sexually.transmitted.infections <= 226
Poor.mental.health.days <= 4.41
School.funding.adequacy <= −2881.3
Feature
−0.06
−0.04
−0.02
0.00
Weight
Positive
Negative

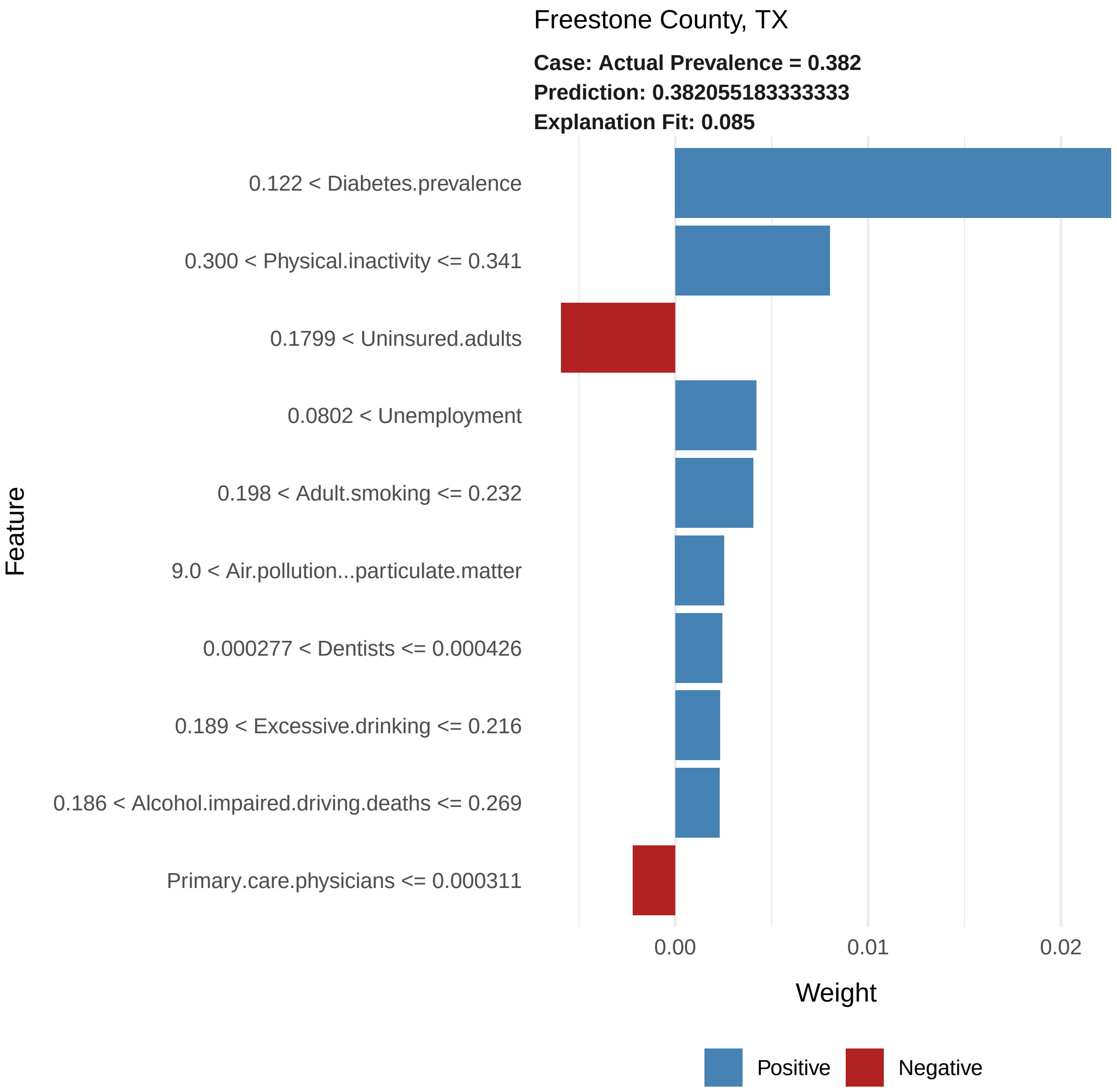
Freestone County, TX
Case: Actual Prevalence = 0.382
Prediction: 0.382055183333333
Explanation Fit: 0.085
Feature
0.122 < Diabetes.prevalence
0.300 < Physical.inactivity <= 0.341
0.1799 < Uninsured.adults
0.0802 < Unemployment
0.198 < Adult.smoking <= 0.232
9.0 < Air.pollution...particulate.matter
0.000277 < Dentists <= 0.000426
0.189 < Excessive.drinking <= 0.216
0.186 < Alcohol.impaired.driving.deaths <= 0.269
Primary.care.physicians <= 0.000311
0.00
0.01
0.02
Weight
Positive
Negative

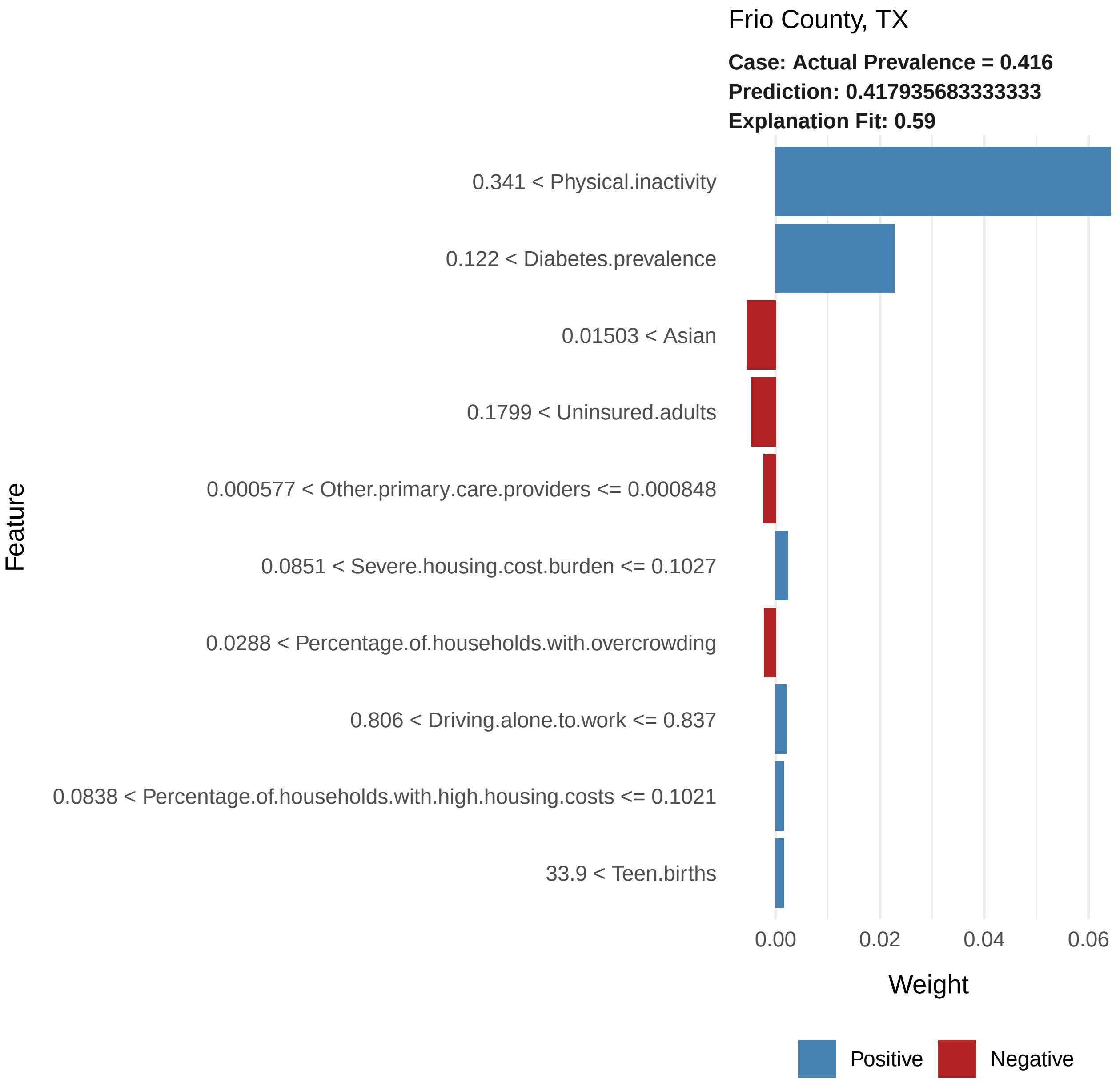

Frio County, TX
Case: Actual Prevalence = 0.416
Prediction: 0.417935683333333
Explanation Fit: 0.59
0.341 < Physical.inactivity
0.122 < Diabetes.prevalence
0.01503 < Asian
0.1799 < Uninsured.adults
0.000577 < Other.primary.care.providers <= 0.000848
0.0851 < Severe.housing.cost.burden <= 0.1027
0.0288 < Percentage.of.households.with.overcrowding
0.806 < Driving.alone.to.work <= 0.837
0.0838 < Percentage.of.households.with.high.housing.costs <= 0.1021
33.9 < Teen.births
Feature
0.00
0.02
0.04
0.06
Weight
Positive
Negative

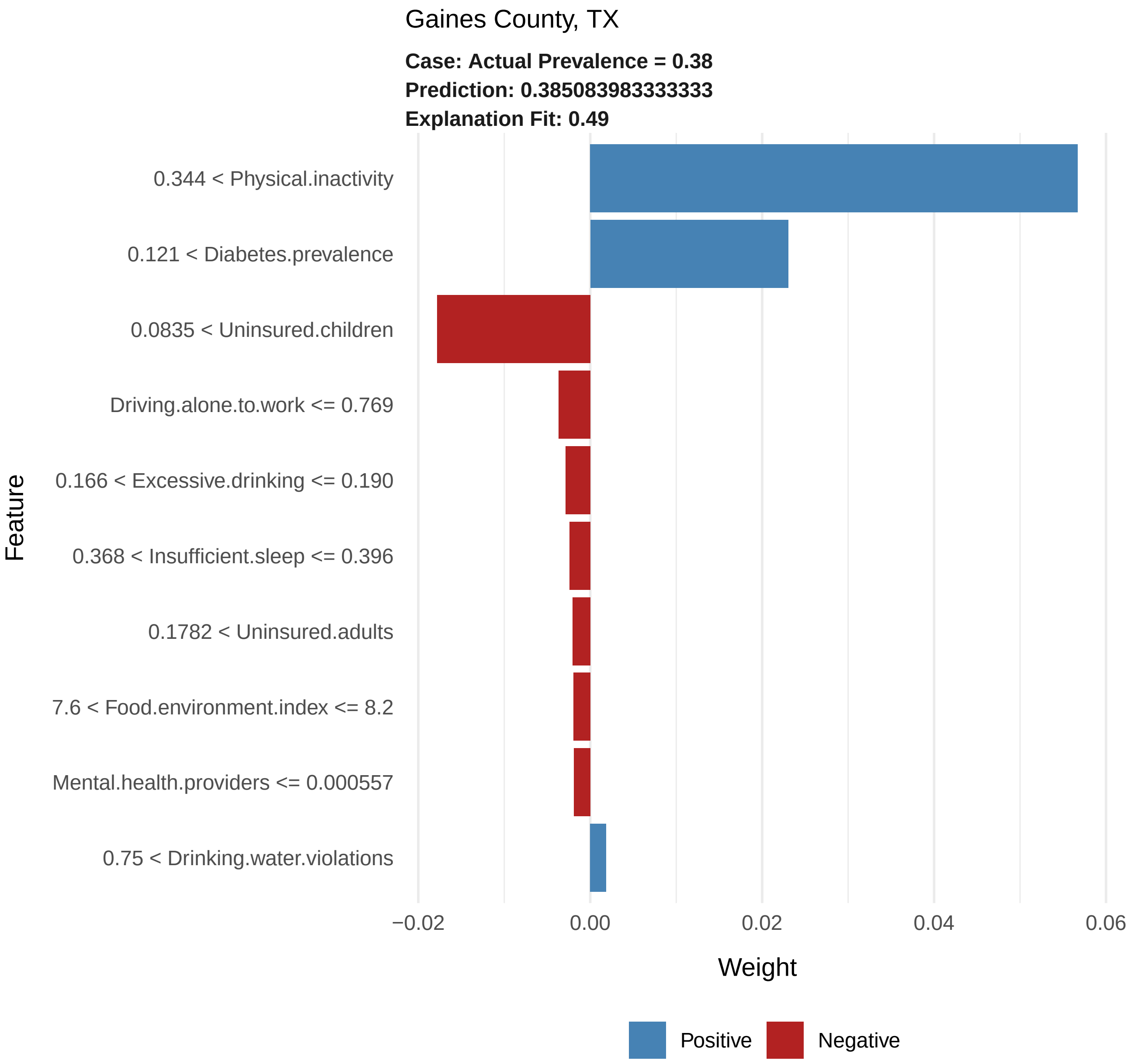
Gaines County, TX
Case: Actual Prevalence = 0.38
Prediction: 0.385083983333333
Explanation Fit: 0.49
0.344 < Physical.inactivity
0.121 < Diabetes.prevalence
0.0835 < Uninsured.children
Driving.alone.to.work <= 0.769
0.166 < Excessive.drinking <= 0.190
0.368 < Insufficient.sleep <= 0.396
0.1782 < Uninsured.adults
7.6 < Food.environment.index <= 8.2
Mental.health.providers <= 0.000557
0.75 < Drinking.water.violations
Feature
−0.02
0.00
0.02
0.04
0.06
Weight
Positive
Negative

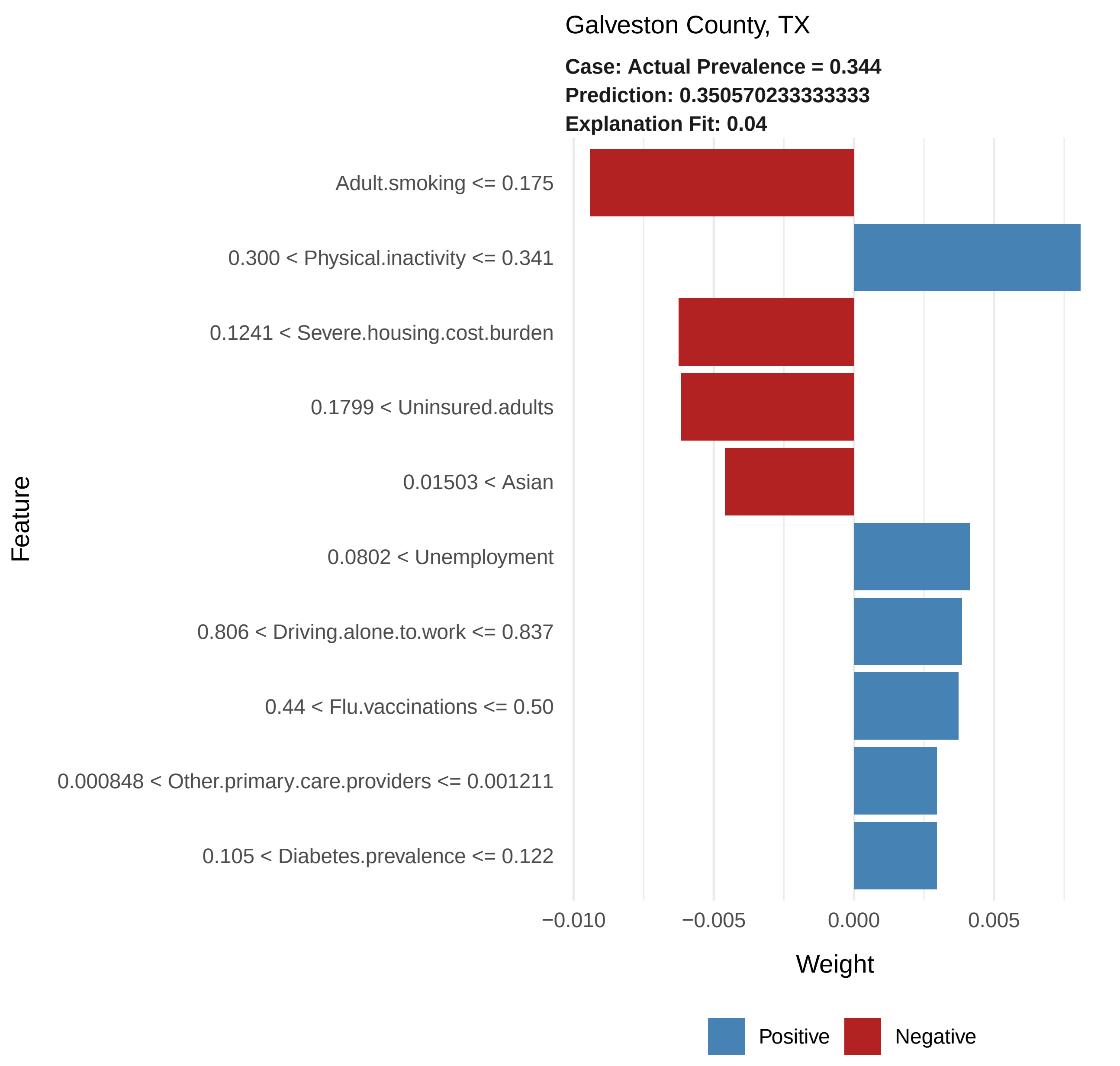

Galveston County, TX
Case: Actual Prevalence = 0.344
Prediction: 0.350570233333333
Explanation Fit: 0.04
Adult.smoking <= 0.175
0.300 < Physical.inactivity <= 0.341
0.1241 < Severe.housing.cost.burden
0.1799 < Uninsured.adults
0.01503 < Asian
0.0802 < Unemployment
0.806 < Driving.alone.to.work <= 0.837
0.44 < Flu.vaccinations <= 0.50
0.000848 < Other.primary.care.providers <= 0.001211
0.105 < Diabetes.prevalence <= 0.122
Feature
−0.010
−0.005
0.000
0.005
Weight
Positive
Negative

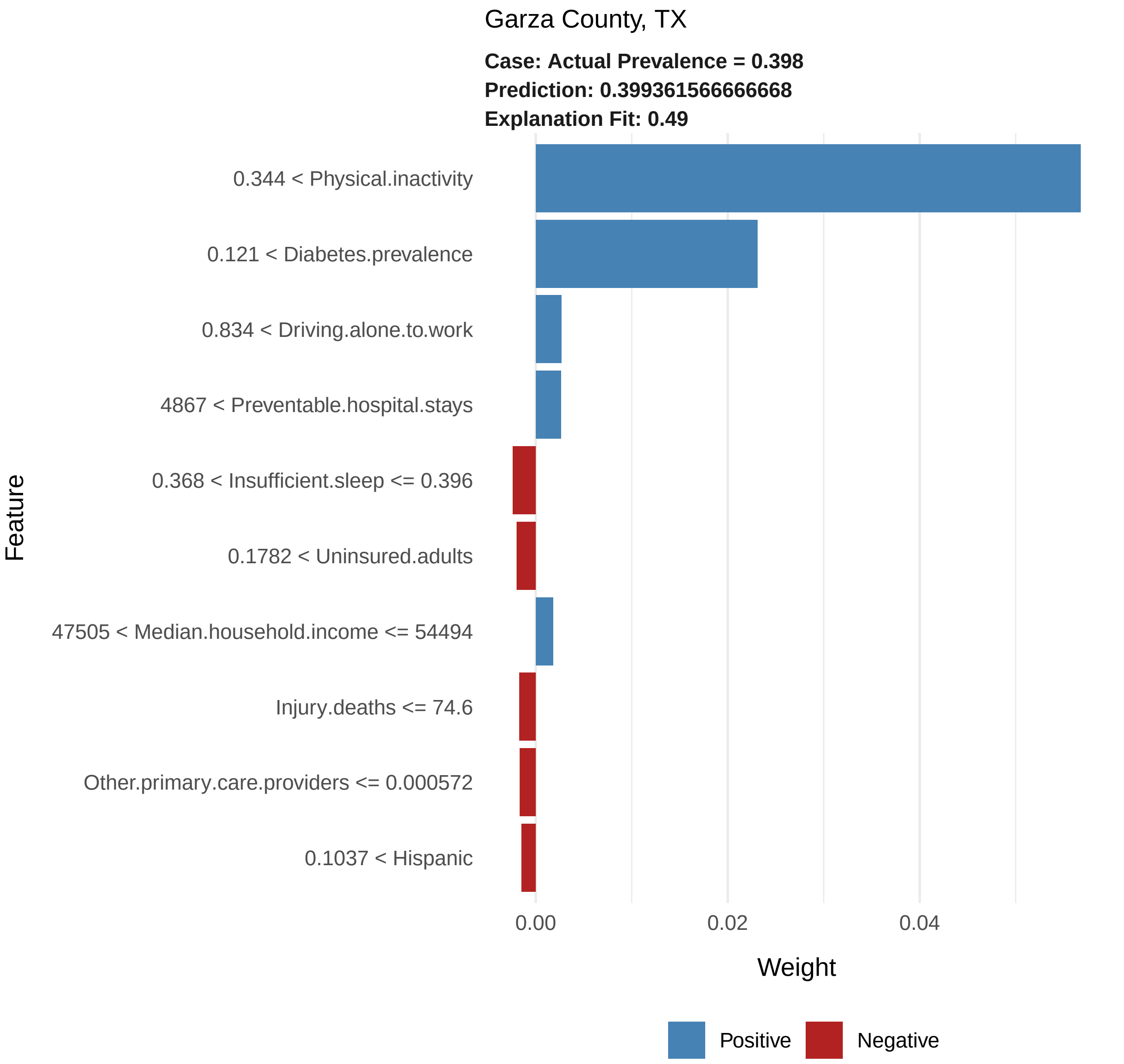
Garza County, TX
Case: Actual Prevalence = 0.398
Prediction: 0.399361566666668
Explanation Fit: 0.49
0.344 < Physical.inactivity
0.121 < Diabetes.prevalence
0.834 < Driving.alone.to.work
4867 < Preventable.hospital.stays
0.368 < Insufficient.sleep <= 0.396
0.1782 < Uninsured.adults
47505 < Median.household.income <= 54494
Injury.deaths <= 74.6
Other.primary.care.providers <= 0.000572
0.1037 < Hispanic
Feature
0.00
0.02
0.04
Weight
Positive
Negative

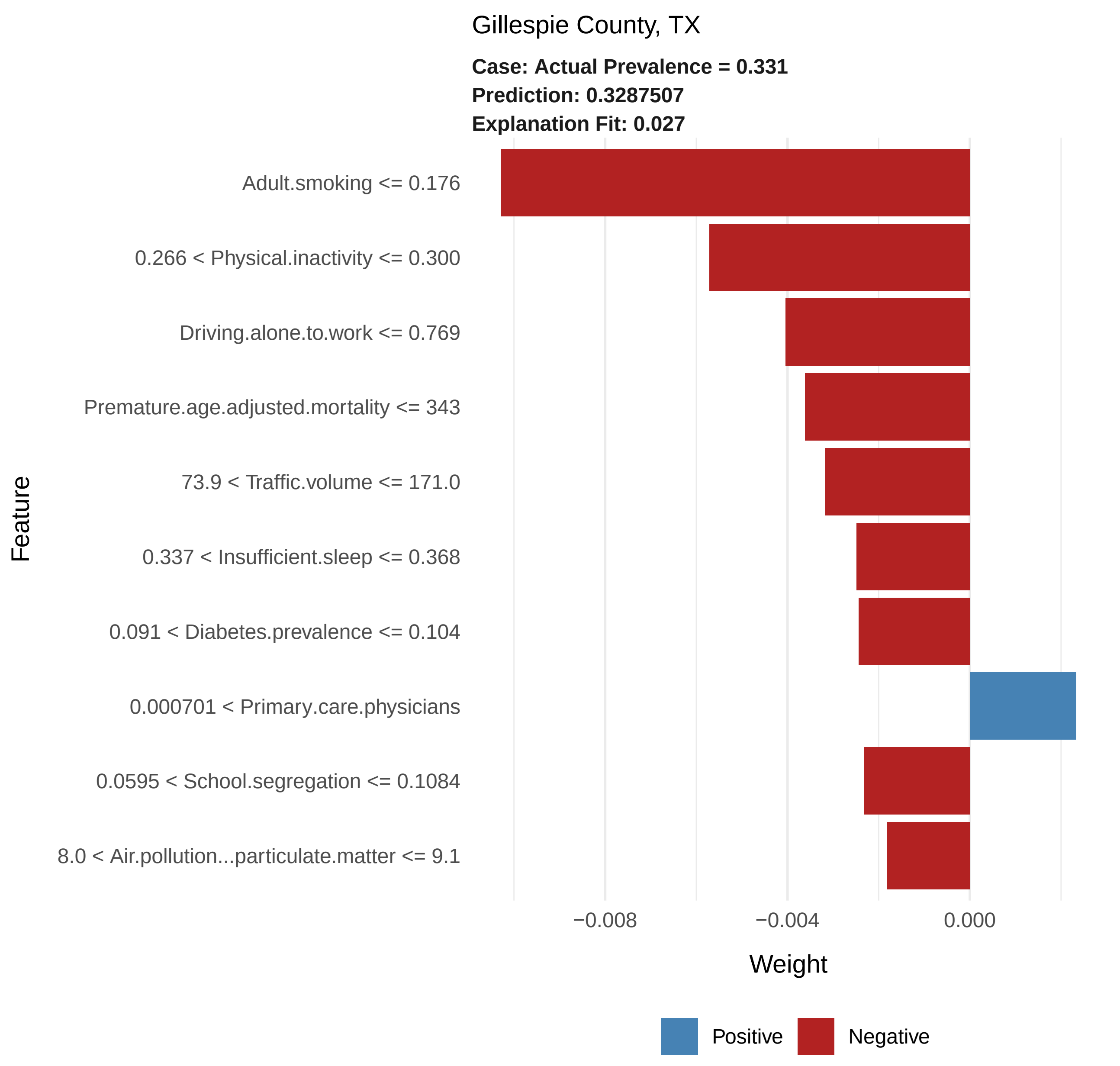

Gillespie County, TX
Case: Actual Prevalence = 0.331
Prediction: 0.3287507
Explanation Fit: 0.027
Adult.smoking <= 0.176
0.266 < Physical.inactivity <= 0.300
Driving.alone.to.work <= 0.769
Premature.age.adjusted.mortality <= 343
73.9 < Traffic.volume <= 171.0
0.337 < Insufficient.sleep <= 0.368
0.091 < Diabetes.prevalence <= 0.104
0.000701 < Primary.care.physicians
0.0595 < School.segregation <= 0.1084
8.0 < Air.pollution...particulate.matter <= 9.1
Feature
−0.008
−0.004
0.000
Weight
Positive
Negative

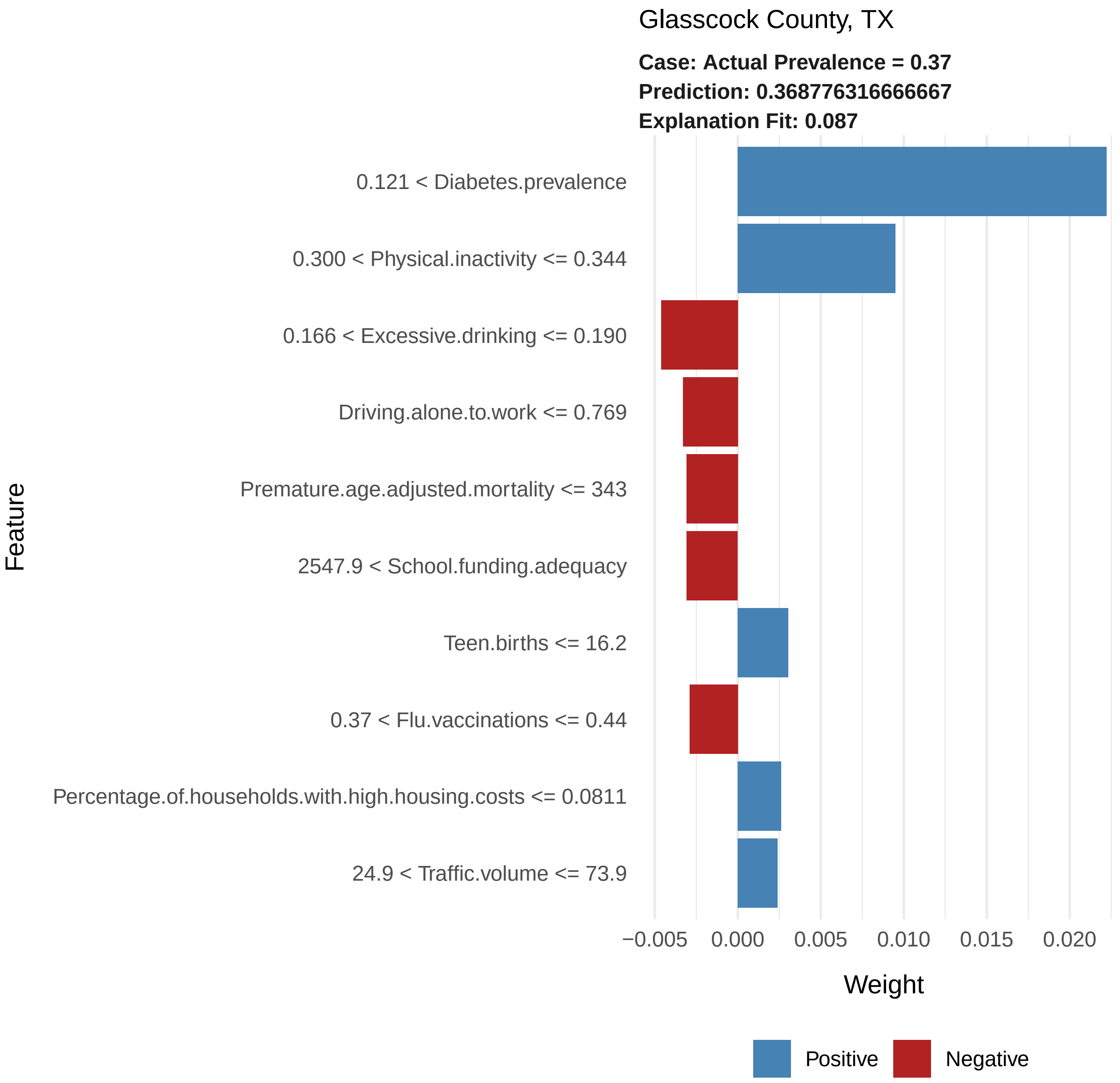

Glasscock County, TX
Case: Actual Prevalence = 0.37
Prediction: 0.368776316666667
Explanation Fit: 0.087
0.121 < Diabetes.prevalence
0.300 < Physical.inactivity <= 0.344
0.166 < Excessive.drinking <= 0.190
Driving.alone.to.work <= 0.769
Premature.age.adjusted.mortality <= 343
2547.9 < School.funding.adequacy
Teen.births <= 16.2
0.37 < Flu.vaccinations <= 0.44
Percentage.of.households.with.high.housing.costs <= 0.0811
24.9 < Traffic.volume <= 73.9
Feature
−0.005
0.000
0.005
0.010
0.015
0.020
Weight
Positive
Negative

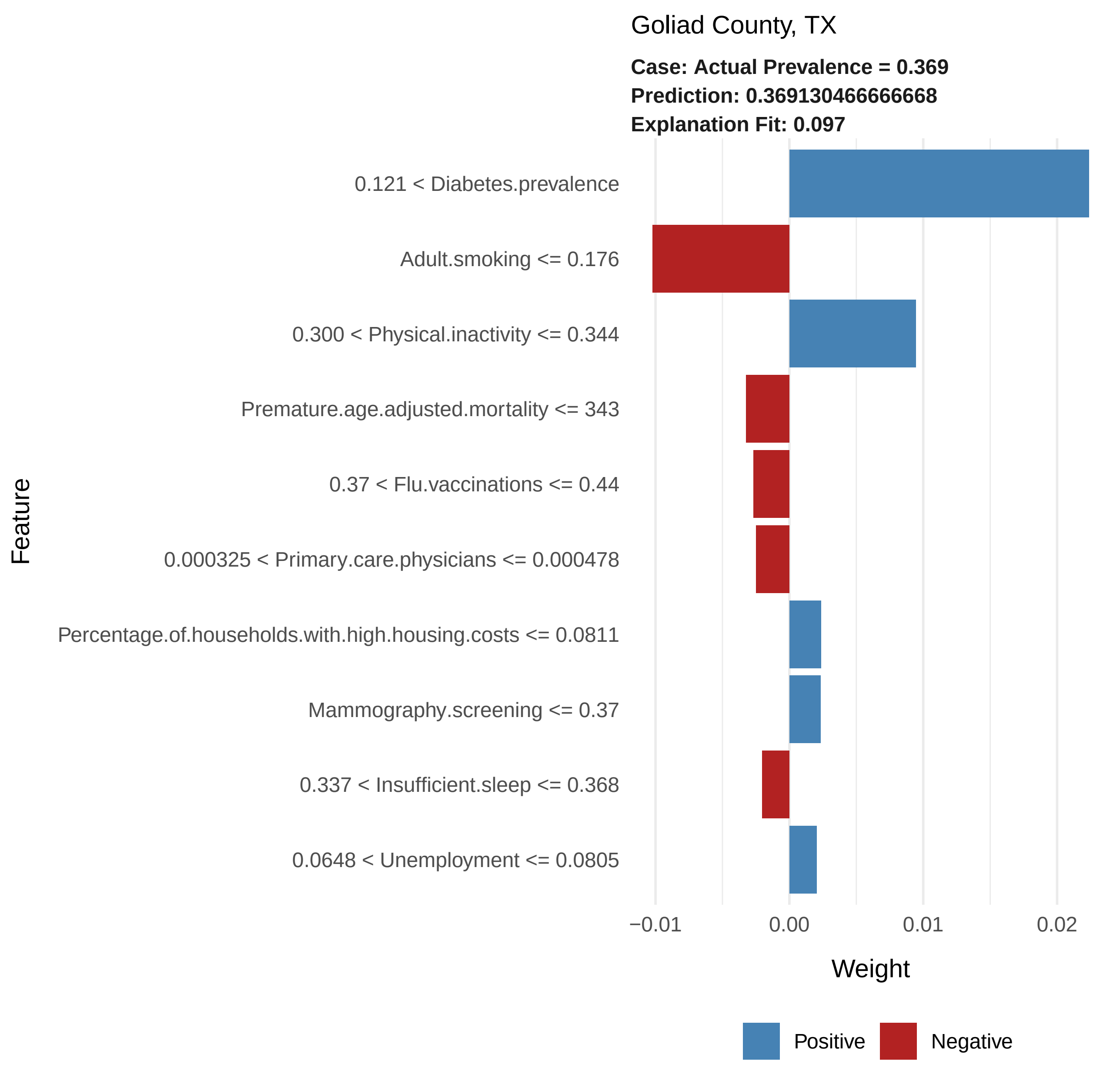

Goliad County, TX
Case: Actual Prevalence = 0.369
Prediction: 0.369130466666668
Explanation Fit: 0.097
0.121 < Diabetes.prevalence
Adult.smoking <= 0.176
0.300 < Physical.inactivity <= 0.344
Premature.age.adjusted.mortality <= 343
0.37 < Flu.vaccinations <= 0.44
0.000325 < Primary.care.physicians <= 0.000478
Percentage.of.households.with.high.housing.costs <= 0.0811
Mammography.screening <= 0.37
0.337 < Insufficient.sleep <= 0.368
0.0648 < Unemployment <= 0.0805
Feature
−0.01
0.00
0.01
0.02
Weight
Positive
Negative

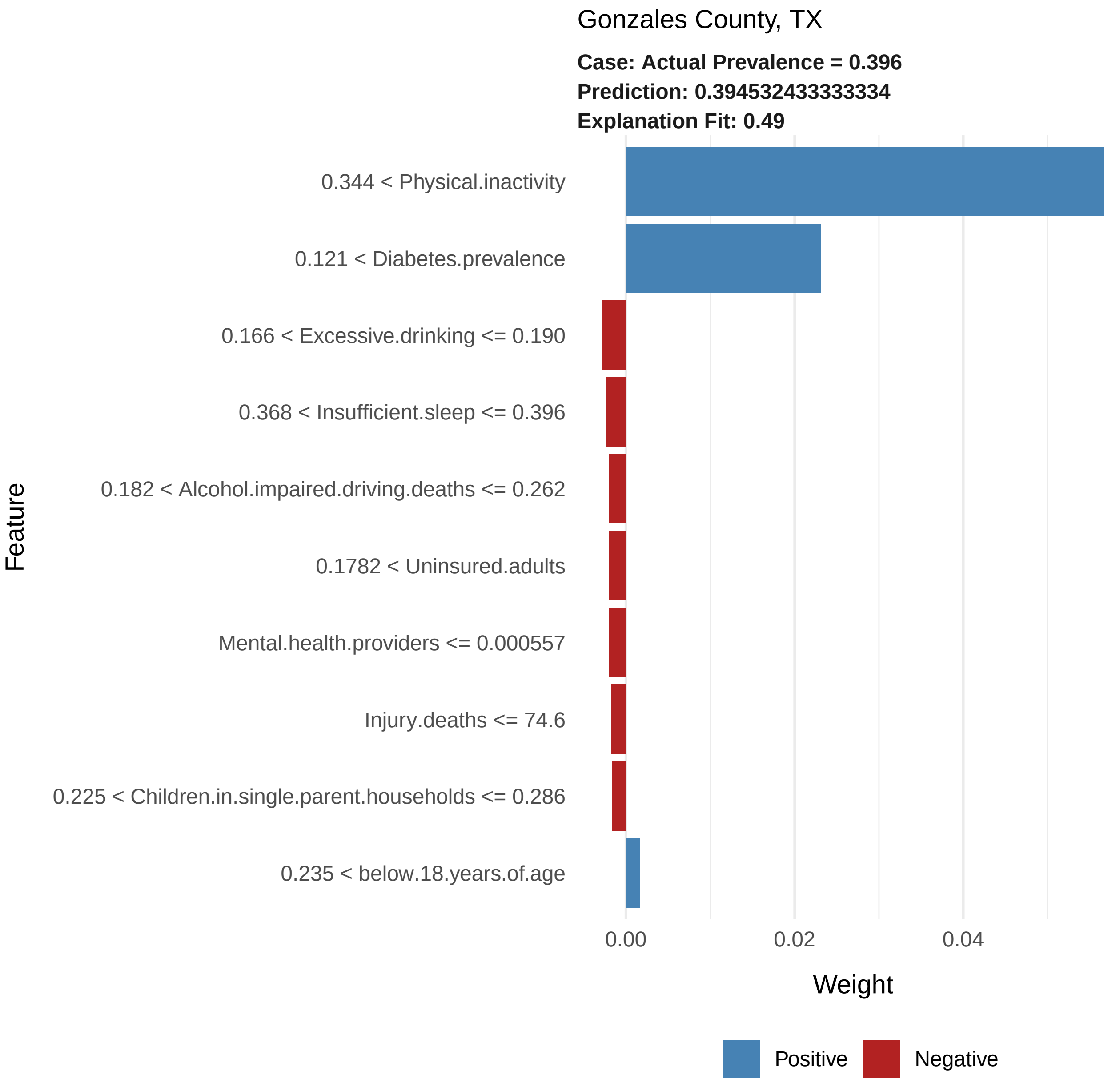
Gonzales County, TX
Case: Actual Prevalence = 0.396
Prediction: 0.394532433333334
Explanation Fit: 0.49
0.344 < Physical.inactivity
0.121 < Diabetes.prevalence
0.166 < Excessive.drinking <= 0.190
0.368 < Insufficient.sleep <= 0.396
0.182 < Alcohol.impaired.driving.deaths <= 0.262
0.1782 < Uninsured.adults
Mental.health.providers <= 0.000557
Injury.deaths <= 74.6
0.225 < Children.in.single.parent.households <= 0.286
0.235 < below.18.years.of.age
Feature
0.00
0.02
0.04
Weight
Positive
Negative

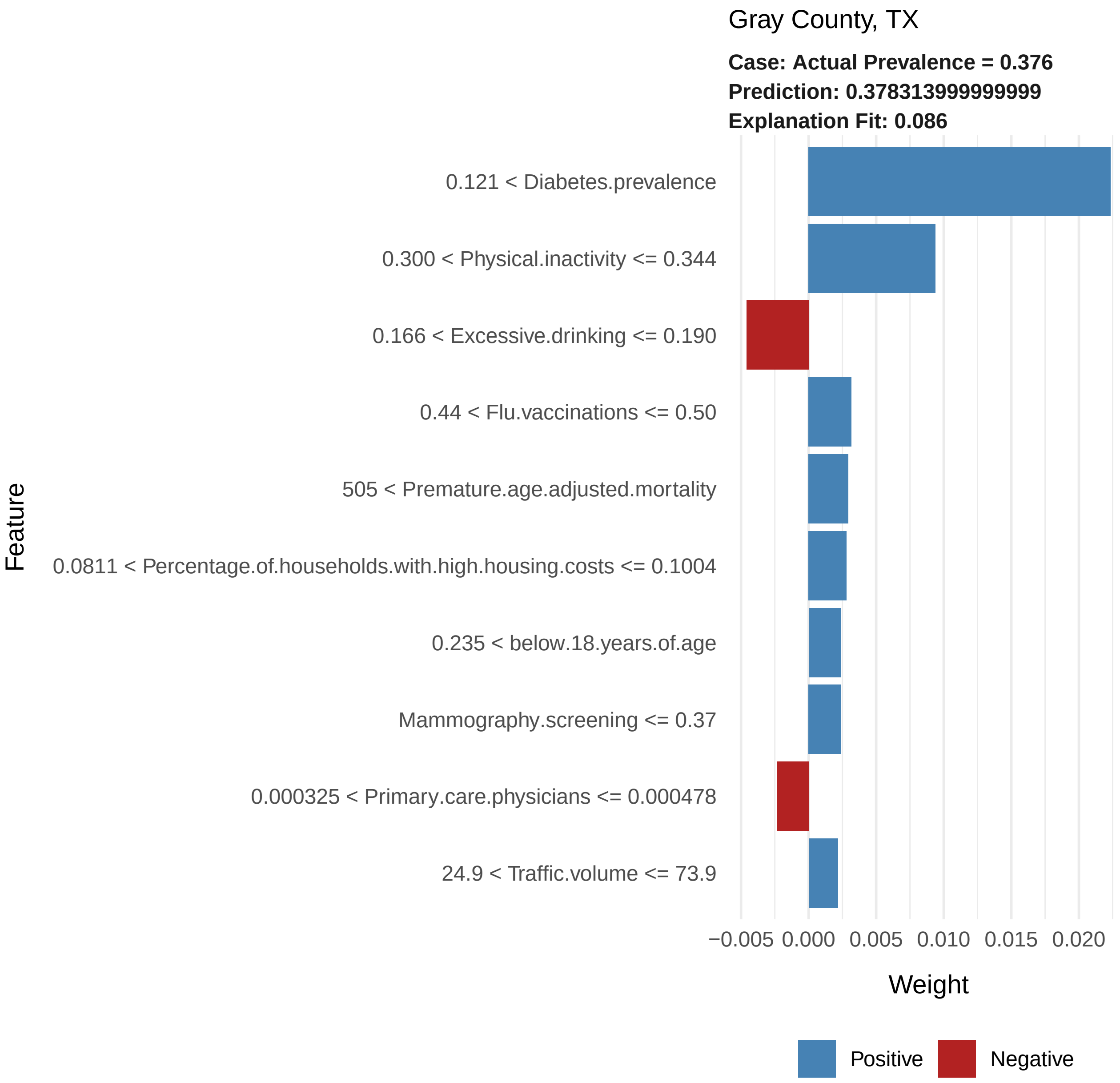
Gray County, TX
Case: Actual Prevalence = 0.376
Prediction: 0.378313999999999
Explanation Fit: 0.086
0.121 < Diabetes.prevalence
0.300 < Physical.inactivity <= 0.344
0.166 < Excessive.drinking <= 0.190
0.44 < Flu.vaccinations <= 0.50
505 < Premature.age.adjusted.mortality
0.0811 < Percentage.of.households.with.high.housing.costs <= 0.1004
0.235 < below.18.years.of.age
Mammography.screening <= 0.37
0.000325 < Primary.care.physicians <= 0.000478
24.9 < Traffic.volume <= 73.9
Feature
−0.005 0.000 0.005 0.010 0.015 0.020
Weight
Positive
Negative

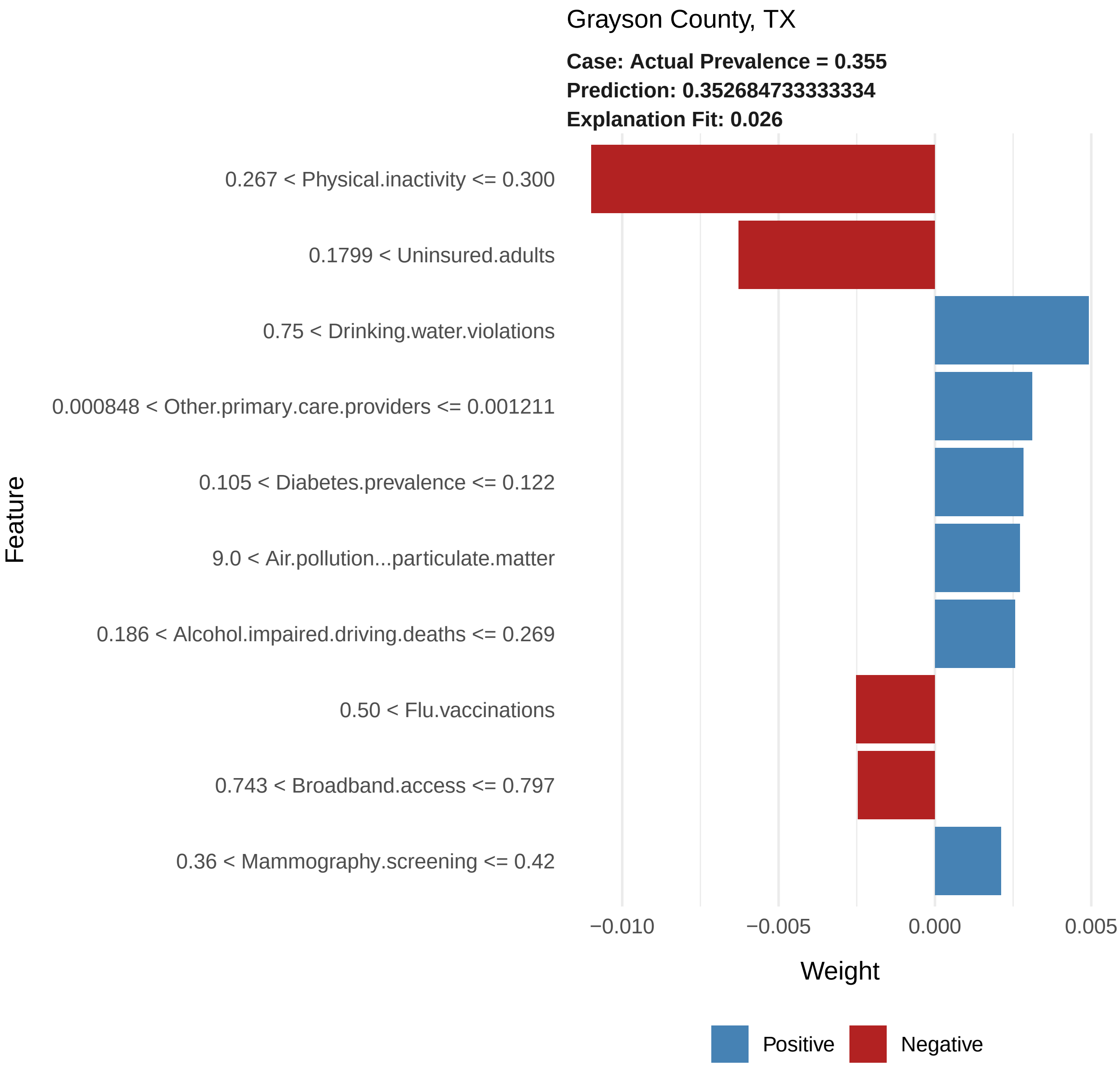

Grayson County, TX
Case: Actual Prevalence = 0.355
Prediction: 0.352684733333334
Explanation Fit: 0.026
0.267 < Physical.inactivity <= 0.300
0.1799 < Uninsured.adults
0.75 < Drinking.water.violations
0.000848 < Other.primary.care.providers <= 0.001211
0.105 < Diabetes.prevalence <= 0.122
9.0 < Air.pollution...particulate.matter
0.186 < Alcohol.impaired.driving.deaths <= 0.269
0.50 < Flu.vaccinations
0.743 < Broadband.access <= 0.797
0.36 < Mammography.screening <= 0.42
Feature
−0.010
−0.005
0.000
0.005
Weight
Positive
Negative

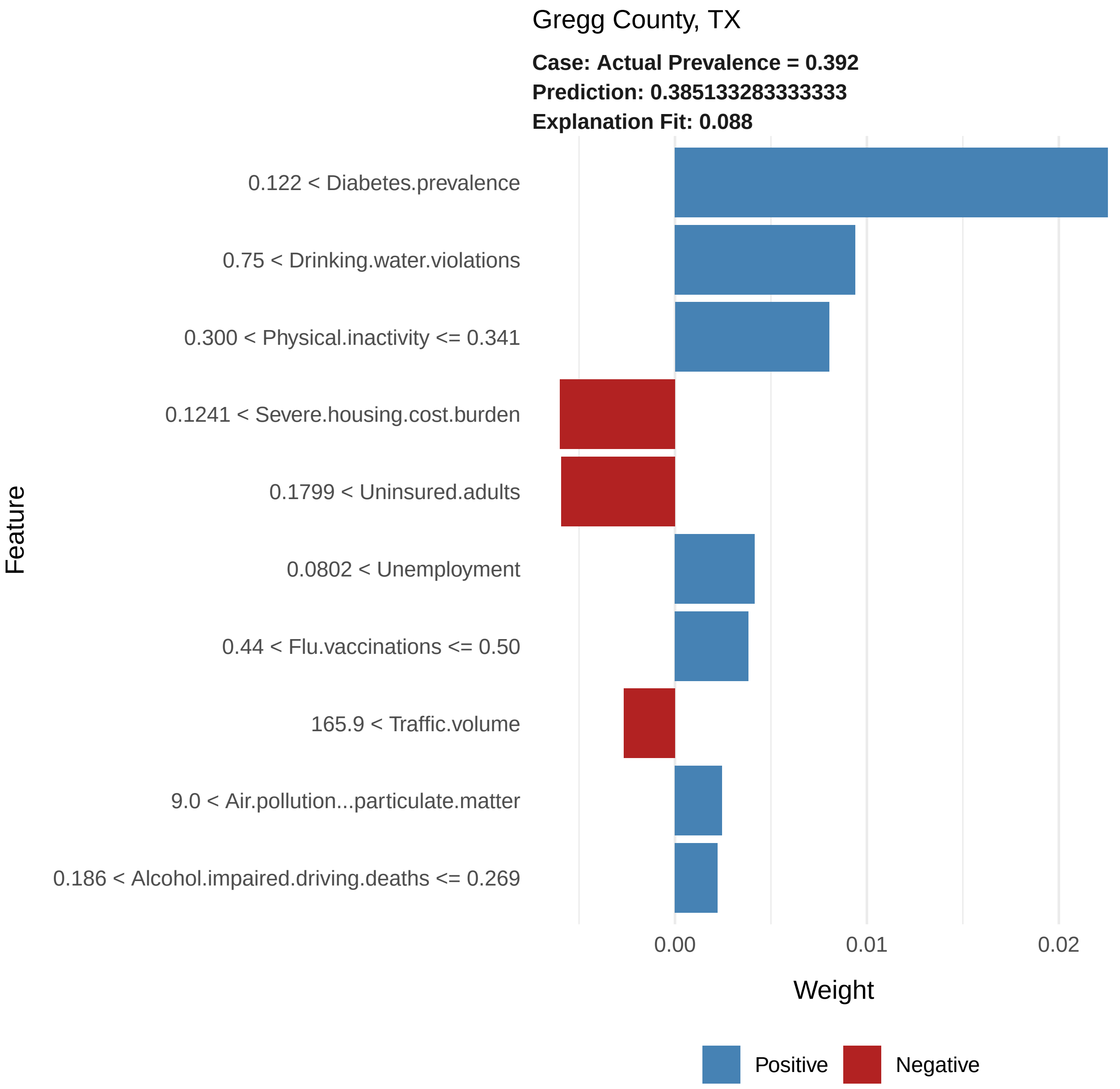

Gregg County, TX
Case: Actual Prevalence = 0.392
Prediction: 0.385133283333333
Explanation Fit: 0.088
0.122 < Diabetes.prevalence
0.75 < Drinking.water.violations
0.300 < Physical.inactivity <= 0.341
0.1241 < Severe.housing.cost.burden
0.1799 < Uninsured.adults
0.0802 < Unemployment
0.44 < Flu.vaccinations <= 0.50
165.9 < Traffic.volume
9.0 < Air.pollution...particulate.matter
0.186 < Alcohol.impaired.driving.deaths <= 0.269
Feature
0.00
0.01
0.02
Weight
Positive
Negative

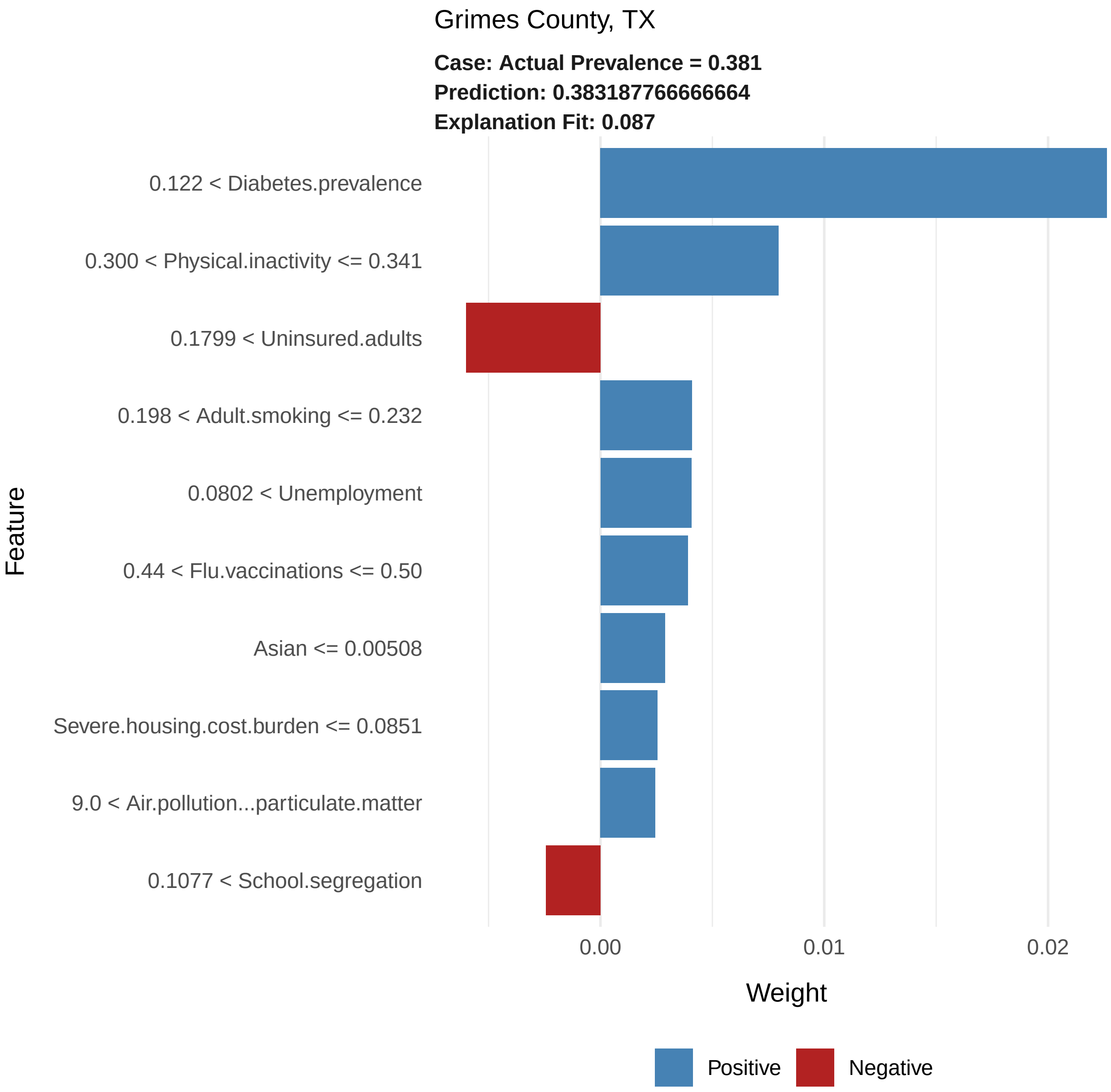

Grimes County, TX
Case: Actual Prevalence = 0.381
Prediction: 0.383187766666664
Explanation Fit: 0.087
0.122 < Diabetes.prevalence
0.300 < Physical.inactivity <= 0.341
0.1799 < Uninsured.adults
0.198 < Adult.smoking <= 0.232
0.0802 < Unemployment
0.44 < Flu.vaccinations <= 0.50
Asian <= 0.00508
Severe.housing.cost.burden <= 0.0851
9.0 < Air.pollution...particulate.matter
0.1077 < School.segregation
Feature
0.00
0.01
0.02
Weight
Positive
Negative

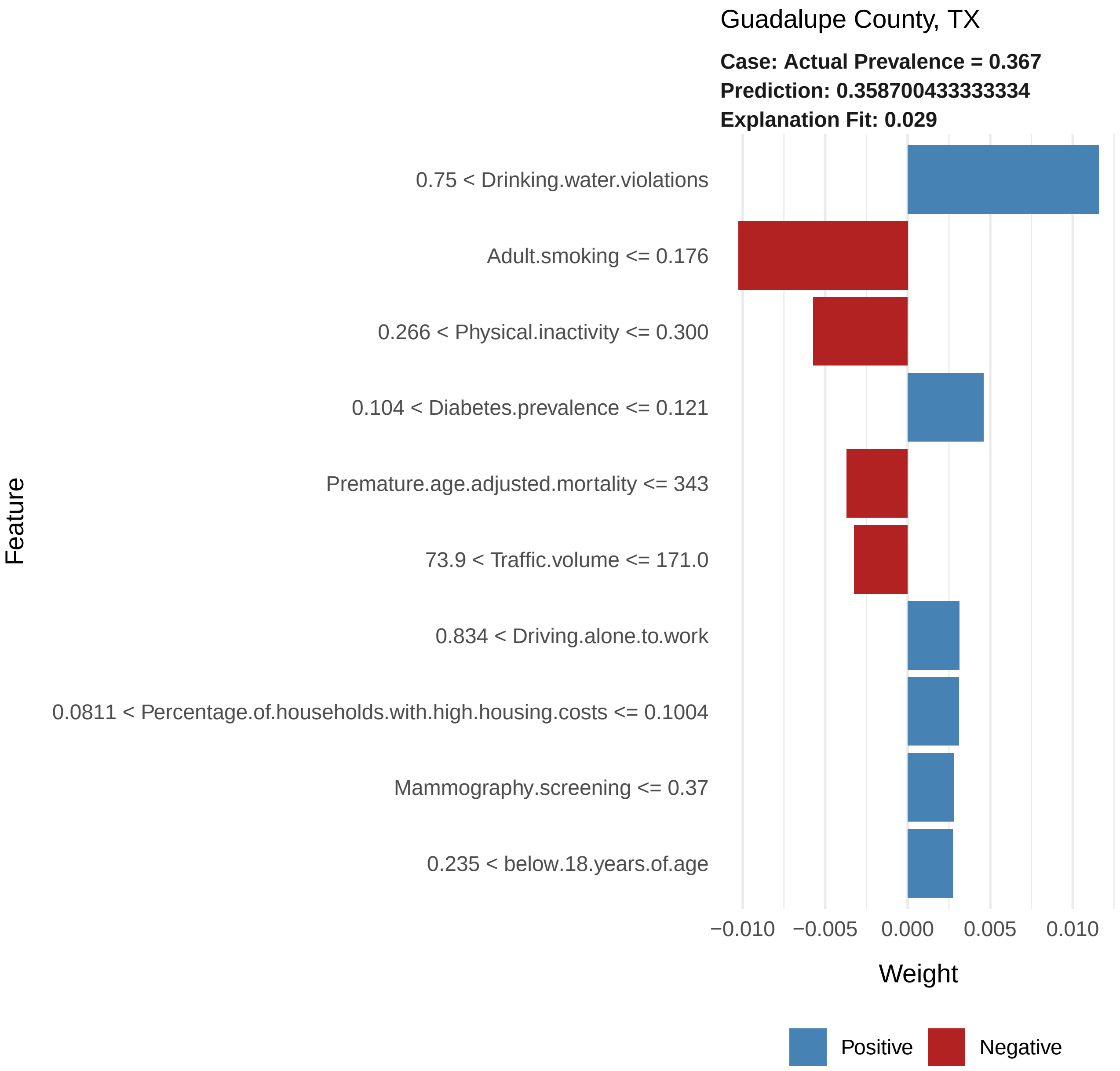
Guadalupe County, TX
Case: Actual Prevalence = 0.367
Prediction: 0.358700433333334
Explanation Fit: 0.029
0.75 < Drinking.water.violations
Adult.smoking <= 0.176
0.266 < Physical.inactivity <= 0.300
0.104 < Diabetes.prevalence <= 0.121
Premature.age.adjusted.mortality <= 343
73.9 < Traffic.volume <= 171.0
0.834 < Driving.alone.to.work
0.0811 < Percentage.of.households.with.high.housing.costs <= 0.1004
Mammography.screening <= 0.37
0.235 < below.18.years.of.age
Feature
−0.010
−0.005
0.000
0.005
0.010
Weight
Positive
Negative

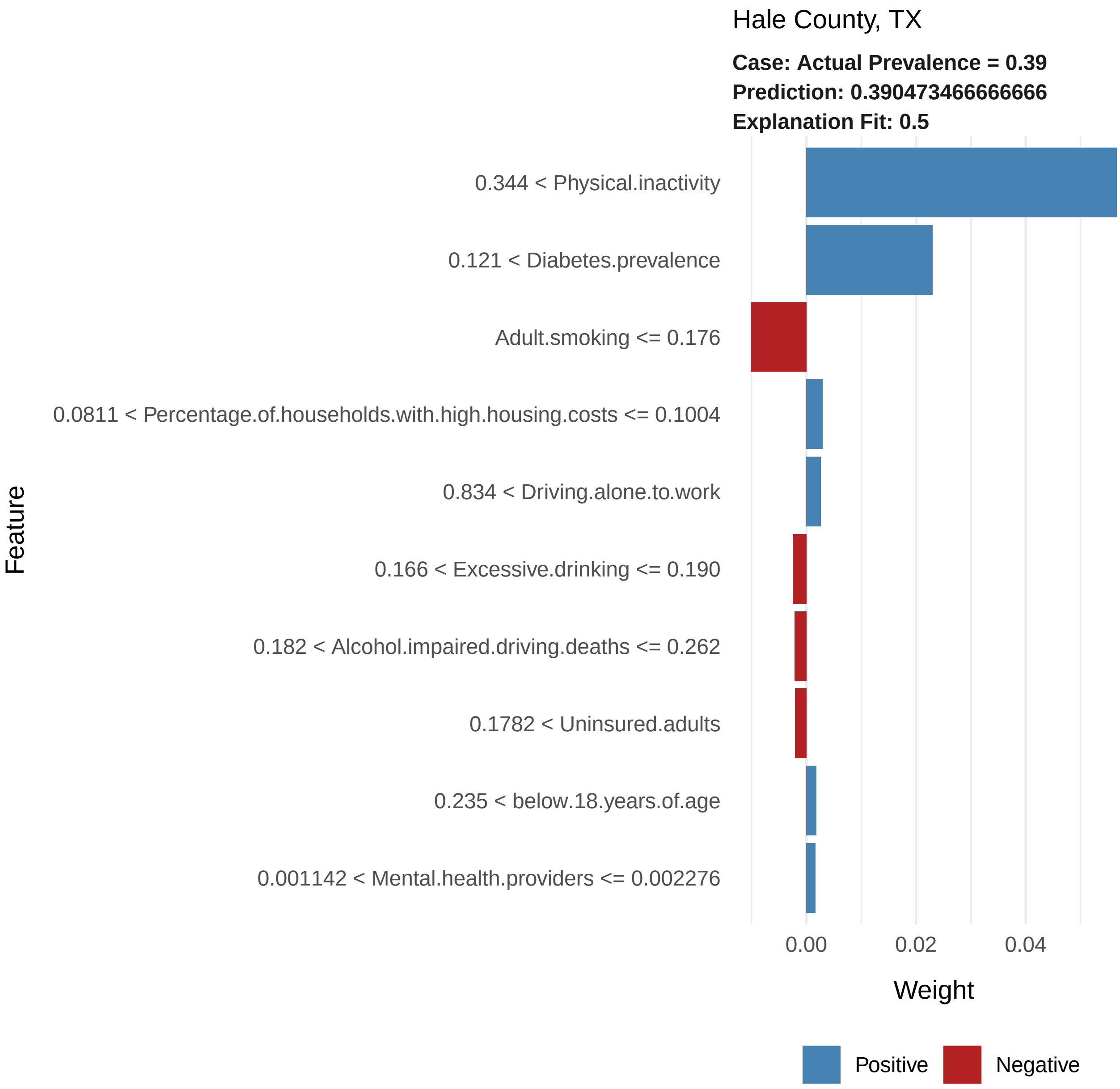
Hale County, TX
Case: Actual Prevalence = 0.39
Prediction: 0.390473466666666
Explanation Fit: 0.5
0.344 < Physical.inactivity
0.121 < Diabetes.prevalence
Adult.smoking <= 0.176
0.0811 < Percentage.of.households.with.high.housing.costs <= 0.1004
0.834 < Driving.alone.to.work
0.166 < Excessive.drinking <= 0.190
0.182 < Alcohol.impaired.driving.deaths <= 0.262
0.1782 < Uninsured.adults
0.235 < below.18.years.of.age
0.001142 < Mental.health.providers <= 0.002276
Feature
0.00
0.02
0.04
Weight
Positive
Negative

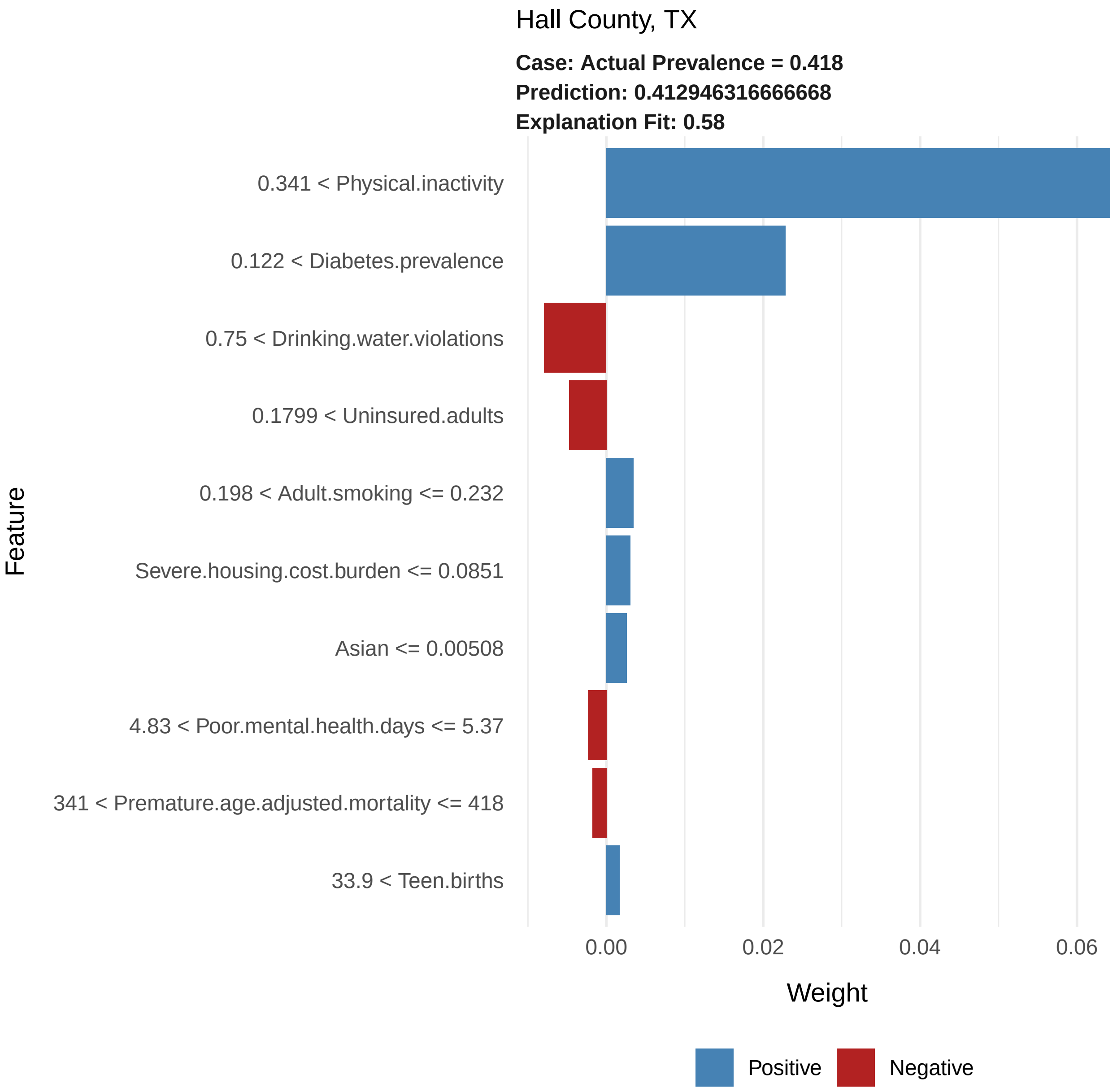

Hall County, TX
Case: Actual Prevalence = 0.418
Prediction: 0.412946316666668
Explanation Fit: 0.58
0.341 < Physical.inactivity
0.122 < Diabetes.prevalence
0.75 < Drinking.water.violations
0.1799 < Uninsured.adults
0.198 < Adult.smoking <= 0.232
Severe.housing.cost.burden <= 0.0851
Asian <= 0.00508
4.83 < Poor.mental.health.days <= 5.37
341 < Premature.age.adjusted.mortality <= 418
33.9 < Teen.births
Feature
0.00
0.02
0.04
0.06
Weight
Positive
Negative

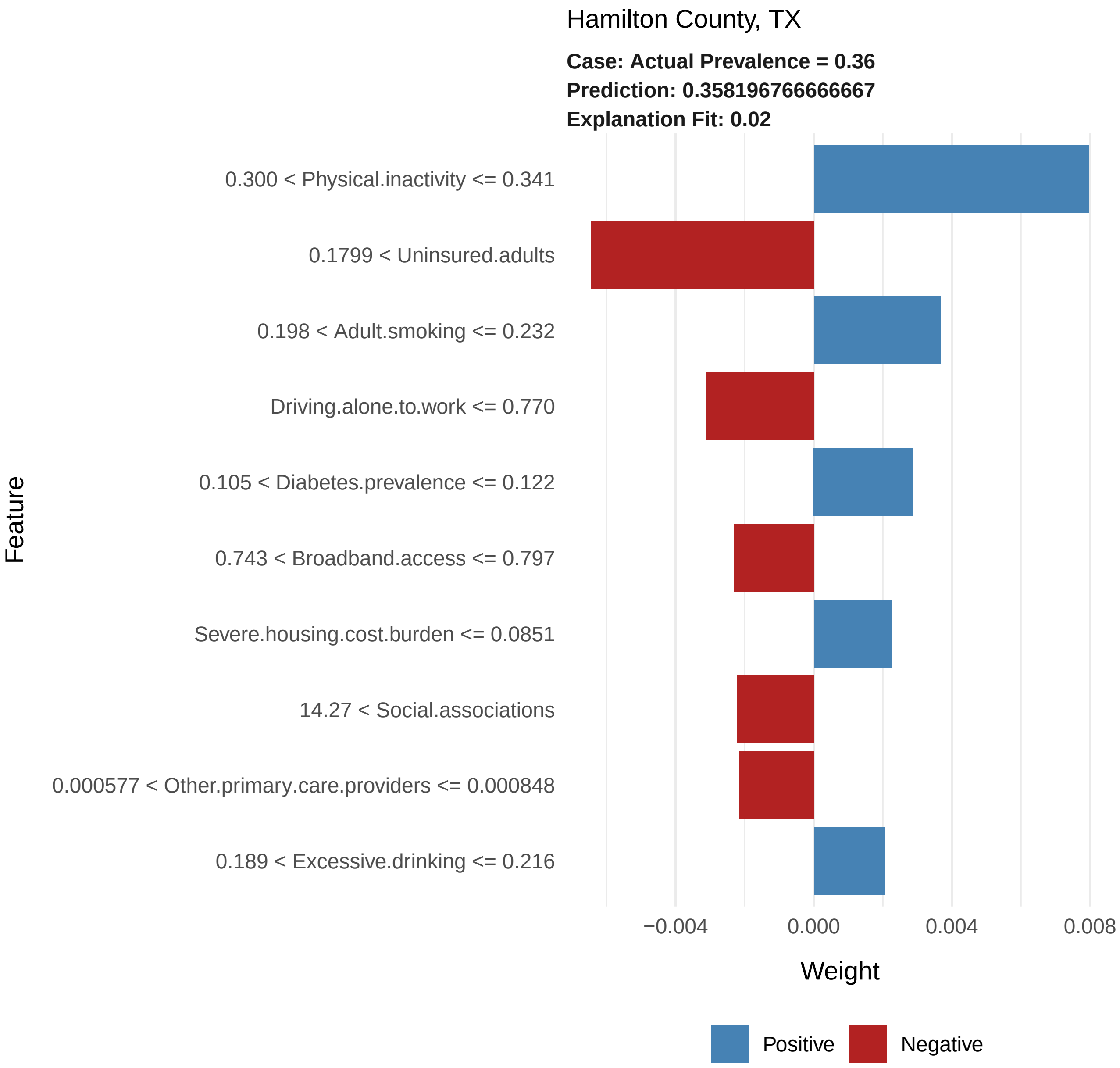

Hamilton County, TX
Case: Actual Prevalence = 0.36
Prediction: 0.358196766666667
Explanation Fit: 0.02
0.300 < Physical.inactivity <= 0.341
0.1799 < Uninsured.adults
0.198 < Adult.smoking <= 0.232
Driving.alone.to.work <= 0.770
0.105 < Diabetes.prevalence <= 0.122
0.743 < Broadband.access <= 0.797
Severe.housing.cost.burden <= 0.0851
14.27 < Social.associations
0.000577 < Other.primary.care.providers <= 0.000848
0.189 < Excessive.drinking <= 0.216
Feature
−0.004
0.000
0.004
0.008
Weight
Positive
Negative

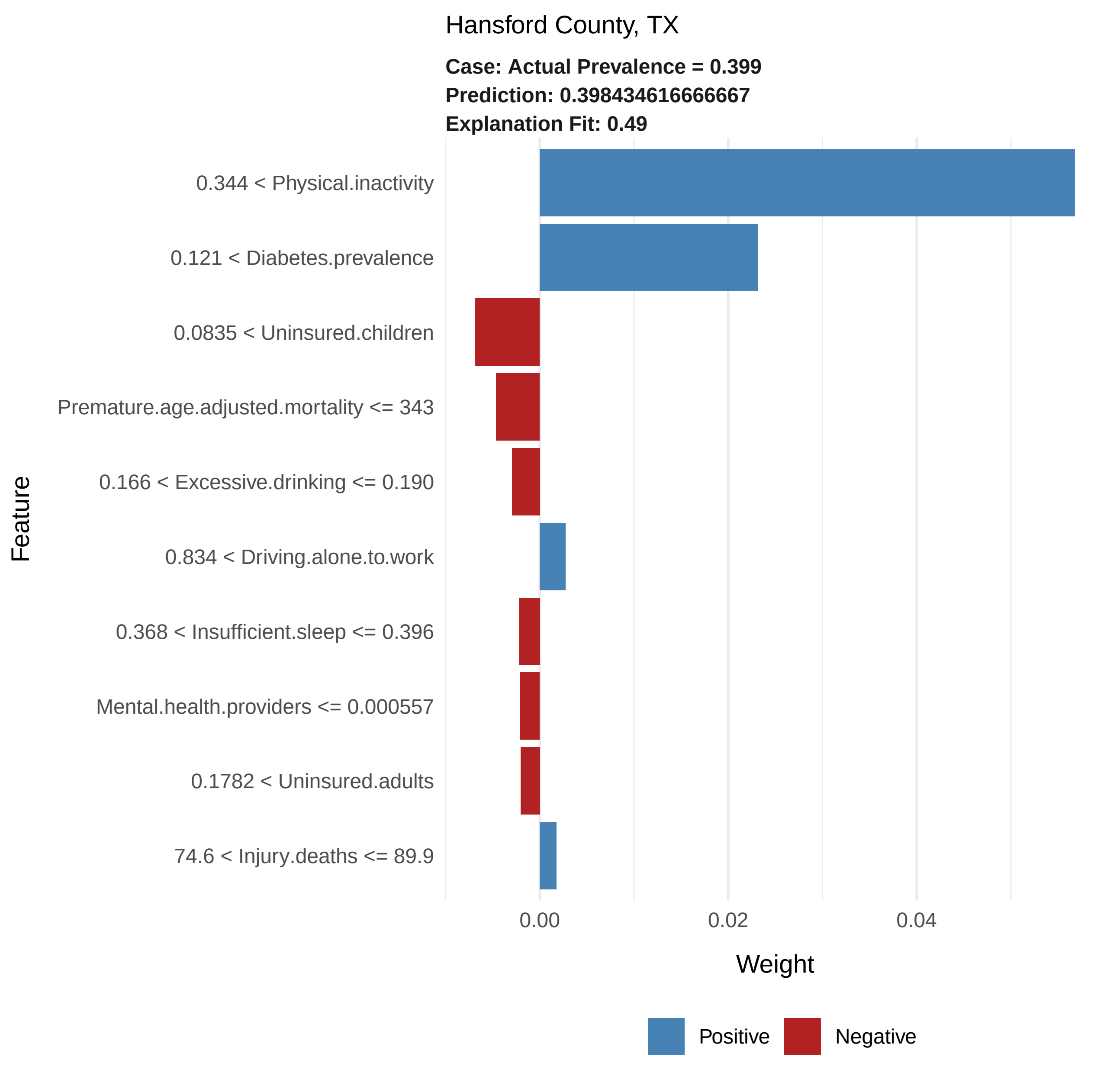

Hansford County, TX
Case: Actual Prevalence = 0.399
Prediction: 0.398434616666667
Explanation Fit: 0.49
0.344 < Physical.inactivity
0.121 < Diabetes.prevalence
0.0835 < Uninsured.children
Premature.age.adjusted.mortality <= 343
0.166 < Excessive.drinking <= 0.190
0.834 < Driving.alone.to.work
0.368 < Insufficient.sleep <= 0.396
Mental.health.providers <= 0.000557
0.1782 < Uninsured.adults
74.6 < Injury.deaths <= 89.9
Feature
0.00
0.02
0.04
Weight
Positive
Negative

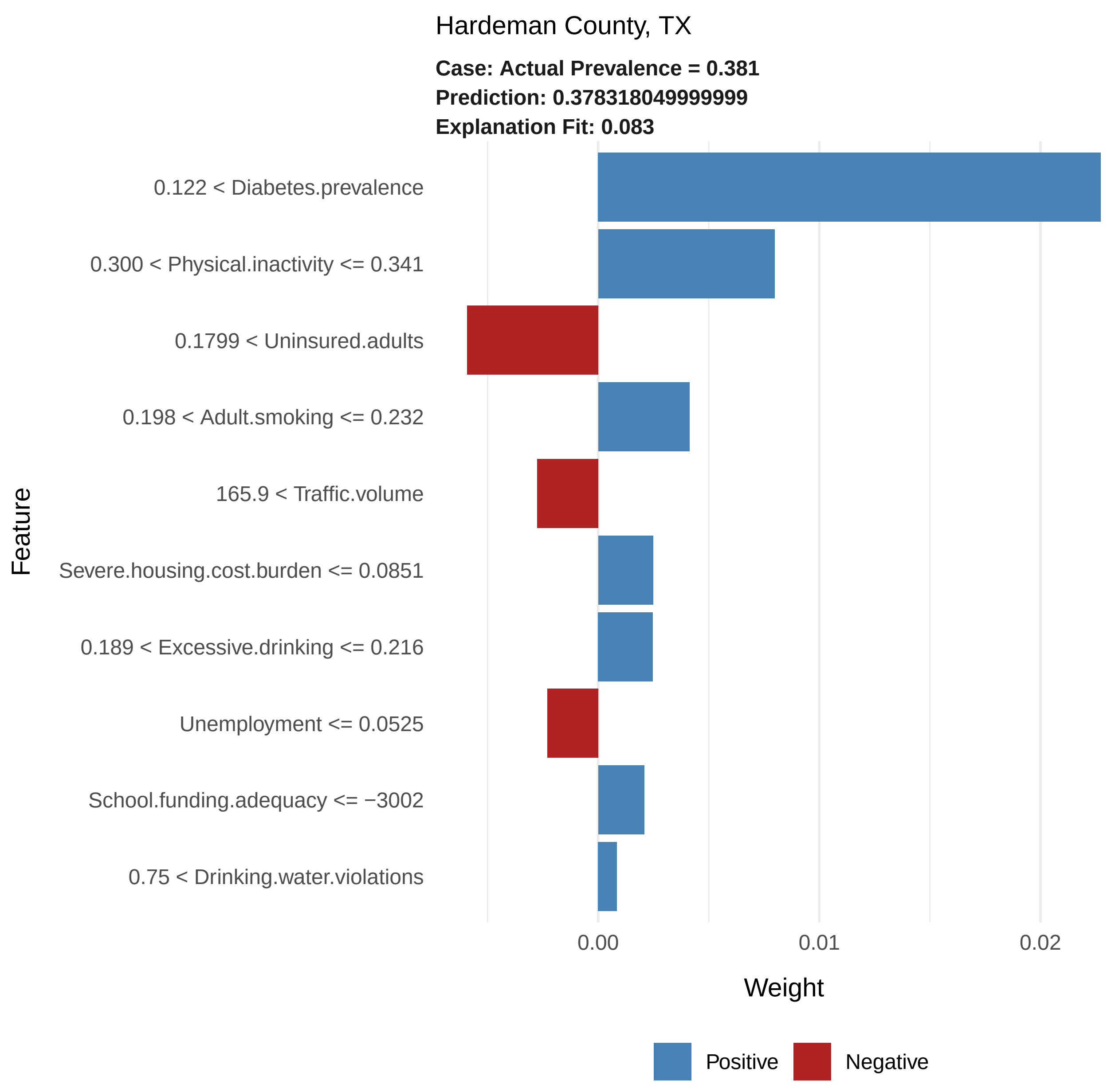

Hardeman County, TX
Case: Actual Prevalence = 0.381
Prediction: 0.378318049999999
Explanation Fit: 0.083
0.122 < Diabetes.prevalence
0.300 < Physical.inactivity <= 0.341
0.1799 < Uninsured.adults
0.198 < Adult.smoking <= 0.232
165.9 < Traffic.volume
Severe.housing.cost.burden <= 0.0851
0.189 < Excessive.drinking <= 0.216
Unemployment <= 0.0525
School.funding.adequacy <= −3002
0.75 < Drinking.water.violations
Feature
0.00
0.01
0.02
Weight
Positive
Negative

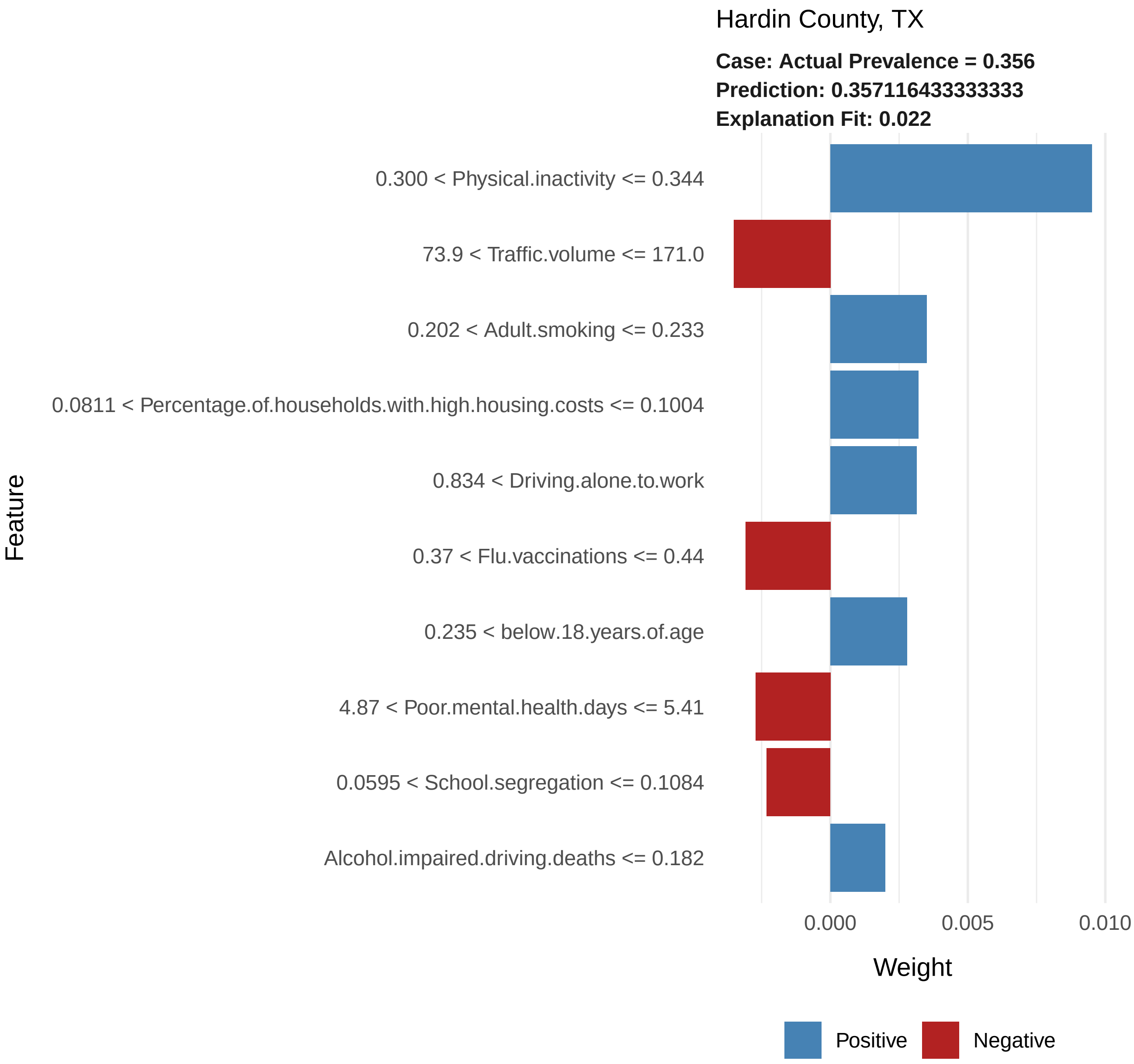
Hardin County, TX
Case: Actual Prevalence = 0.356
Prediction: 0.357116433333333
Explanation Fit: 0.022
0.300 < Physical.inactivity <= 0.344
73.9 < Traffic.volume <= 171.0
0.202 < Adult.smoking <= 0.233
0.0811 < Percentage.of.households.with.high.housing.costs <= 0.1004
0.834 < Driving.alone.to.work
0.37 < Flu.vaccinations <= 0.44
0.235 < below.18.years.of.age
4.87 < Poor.mental.health.days <= 5.41
0.0595 < School.segregation <= 0.1084
Alcohol.impaired.driving.deaths <= 0.182
Feature
0.000
0.005
0.010
Weight
Positive
Negative

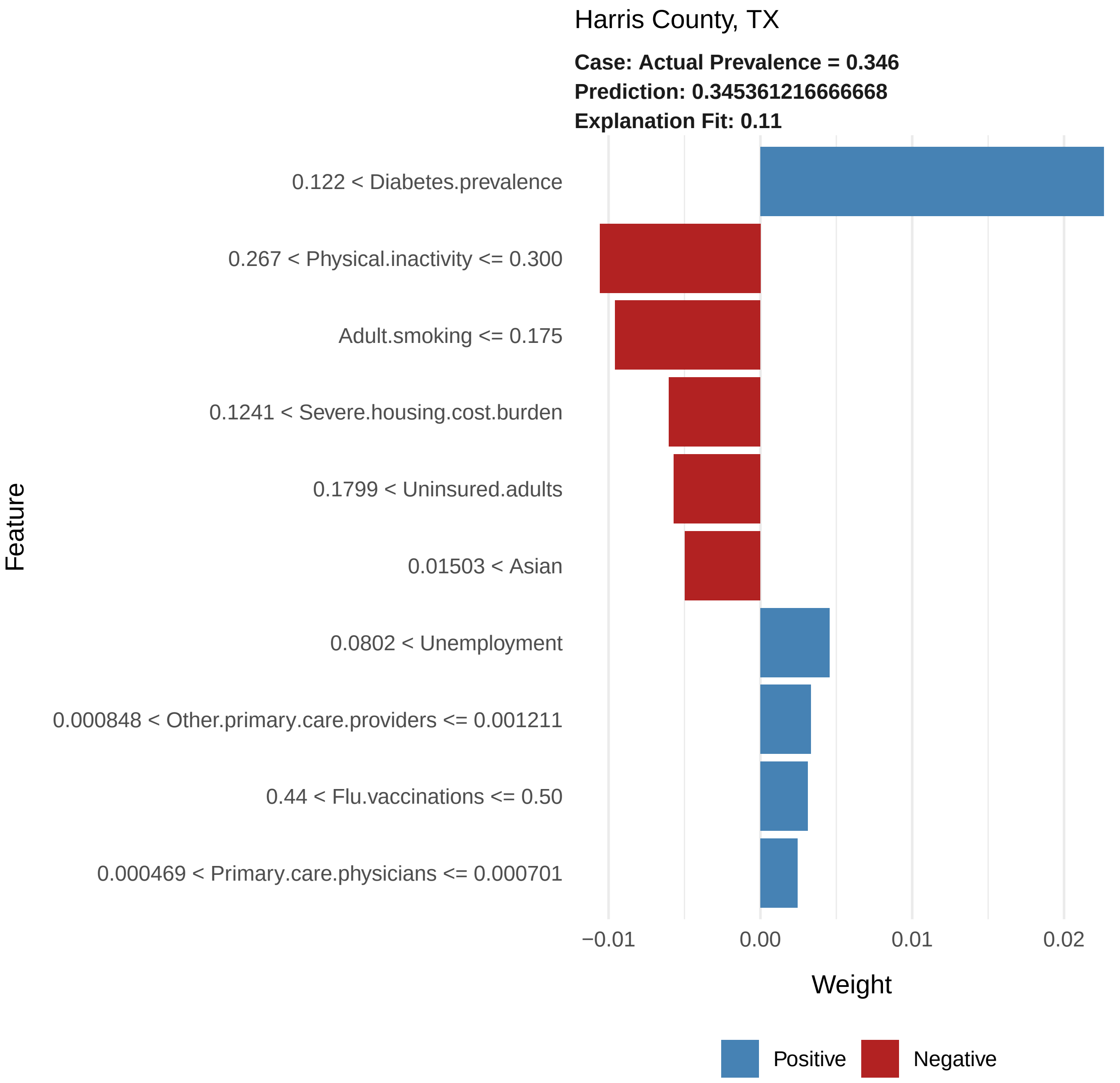

Harris County, TX
Case: Actual Prevalence = 0.346
Prediction: 0.345361216666668
Explanation Fit: 0.11
0.122 < Diabetes.prevalence
0.267 < Physical.inactivity <= 0.300
Adult.smoking <= 0.175
0.1241 < Severe.housing.cost.burden
0.1799 < Uninsured.adults
0.01503 < Asian
0.0802 < Unemployment
0.000848 < Other.primary.care.providers <= 0.001211
0.44 < Flu.vaccinations <= 0.50
0.000469 < Primary.care.physicians <= 0.000701
Feature
−0.01
0.00
0.01
0.02
Weight
Positive
Negative

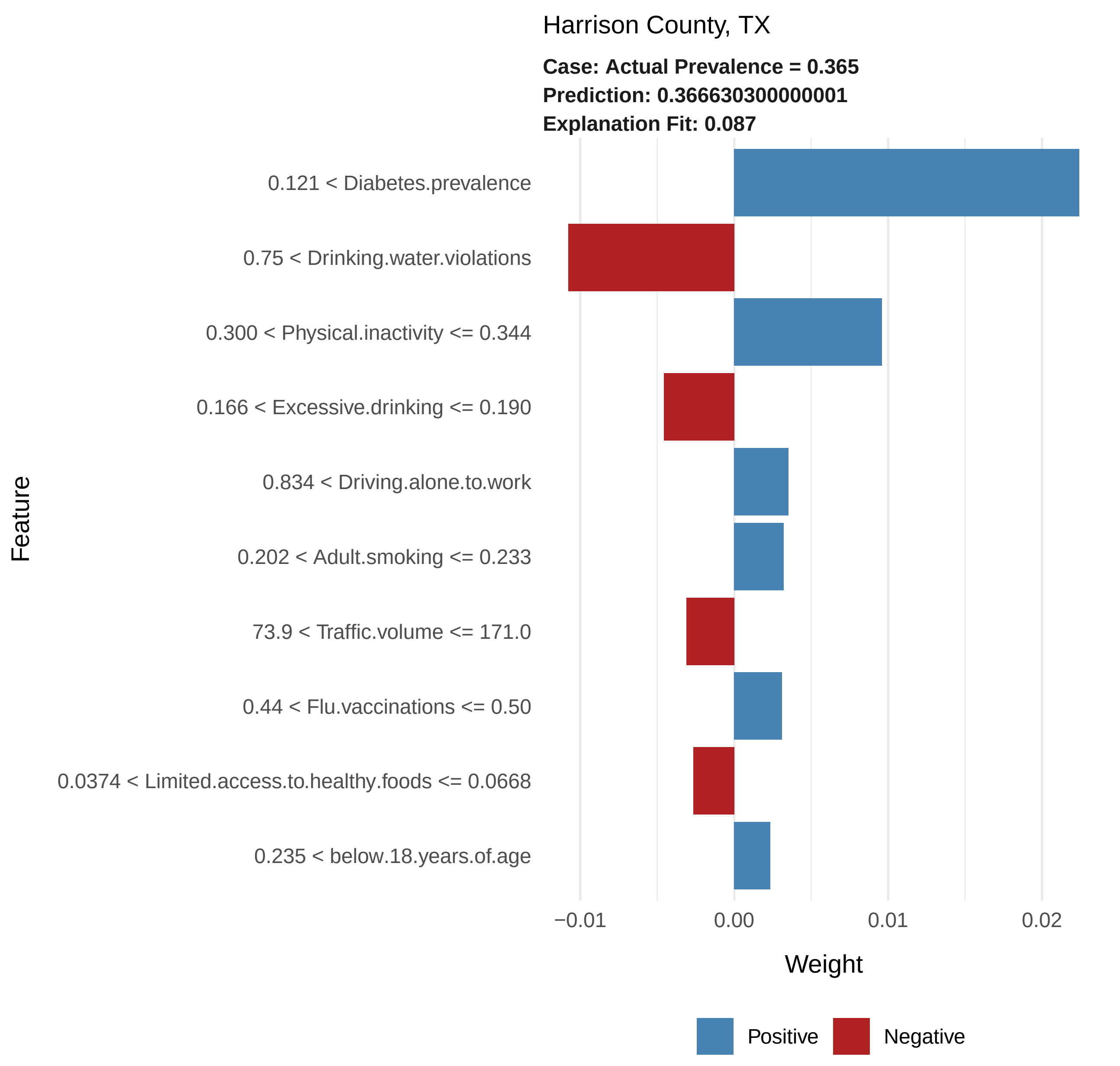

Harrison County, TX
Case: Actual Prevalence = 0.365
Prediction: 0.366630300000001
Explanation Fit: 0.087
Feature
0.121 < Diabetes.prevalence
0.75 < Drinking.water.violations
0.300 < Physical.inactivity <= 0.344
0.166 < Excessive.drinking <= 0.190
0.834 < Driving.alone.to.work
0.202 < Adult.smoking <= 0.233
73.9 < Traffic.volume <= 171.0
0.44 < Flu.vaccinations <= 0.50
0.0374 < Limited.access.to.healthy.foods <= 0.0668
0.235 < below.18.years.of.age
−0.01
0.00
0.01
0.02
Weight
Positive
Negative

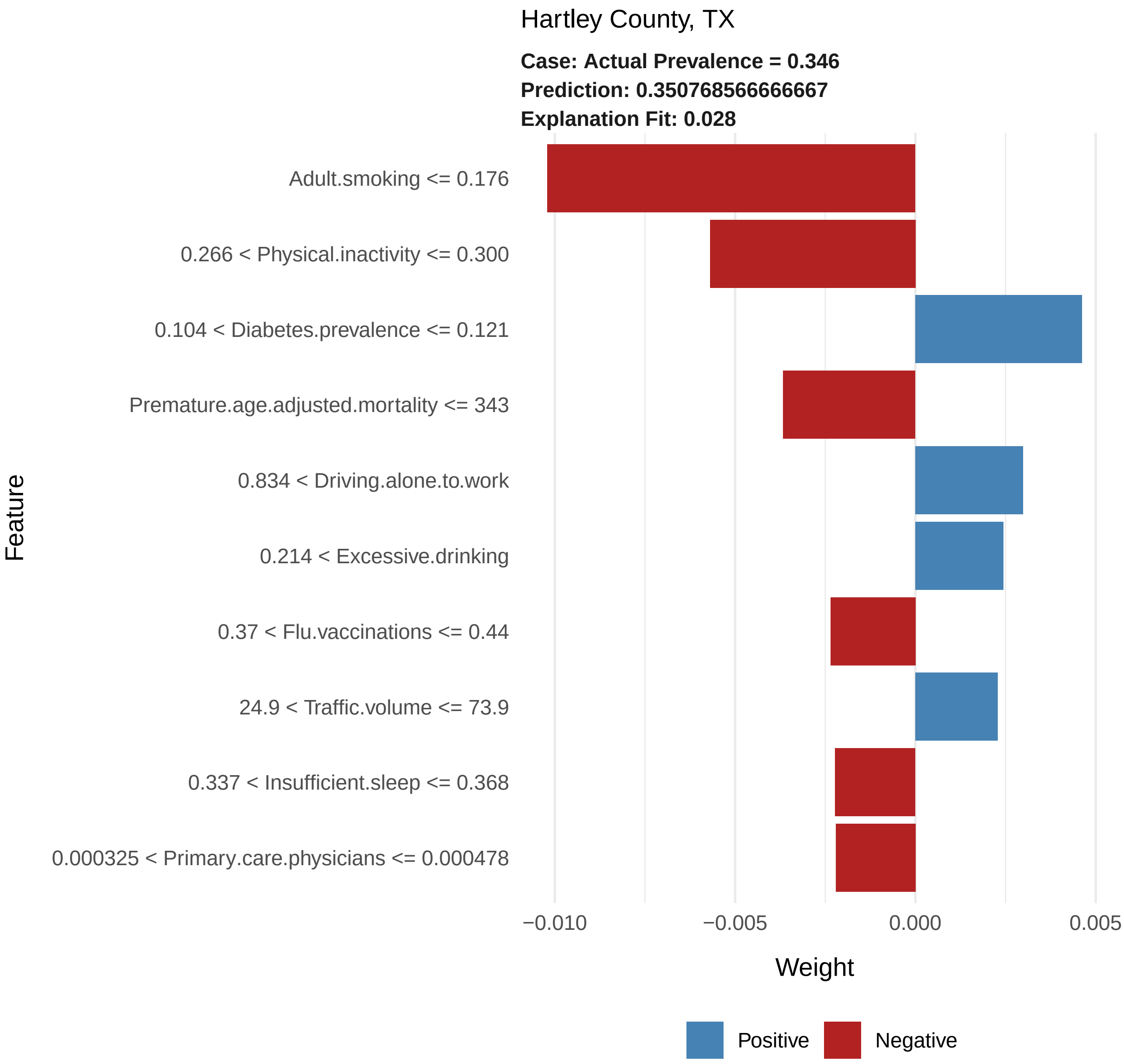

Hartley County, TX
Case: Actual Prevalence = 0.346
Prediction: 0.350768566666667
Explanation Fit: 0.028
Adult.smoking <= 0.176
0.266 < Physical.inactivity <= 0.300
0.104 < Diabetes.prevalence <= 0.121
Premature.age.adjusted.mortality <= 343
0.834 < Driving.alone.to.work
0.214 < Excessive.drinking
0.37 < Flu.vaccinations <= 0.44
24.9 < Traffic.volume <= 73.9
0.337 < Insufficient.sleep <= 0.368
0.000325 < Primary.care.physicians <= 0.000478
Feature
−0.010
−0.005
0.000
0.005
Weight
Positive
Negative

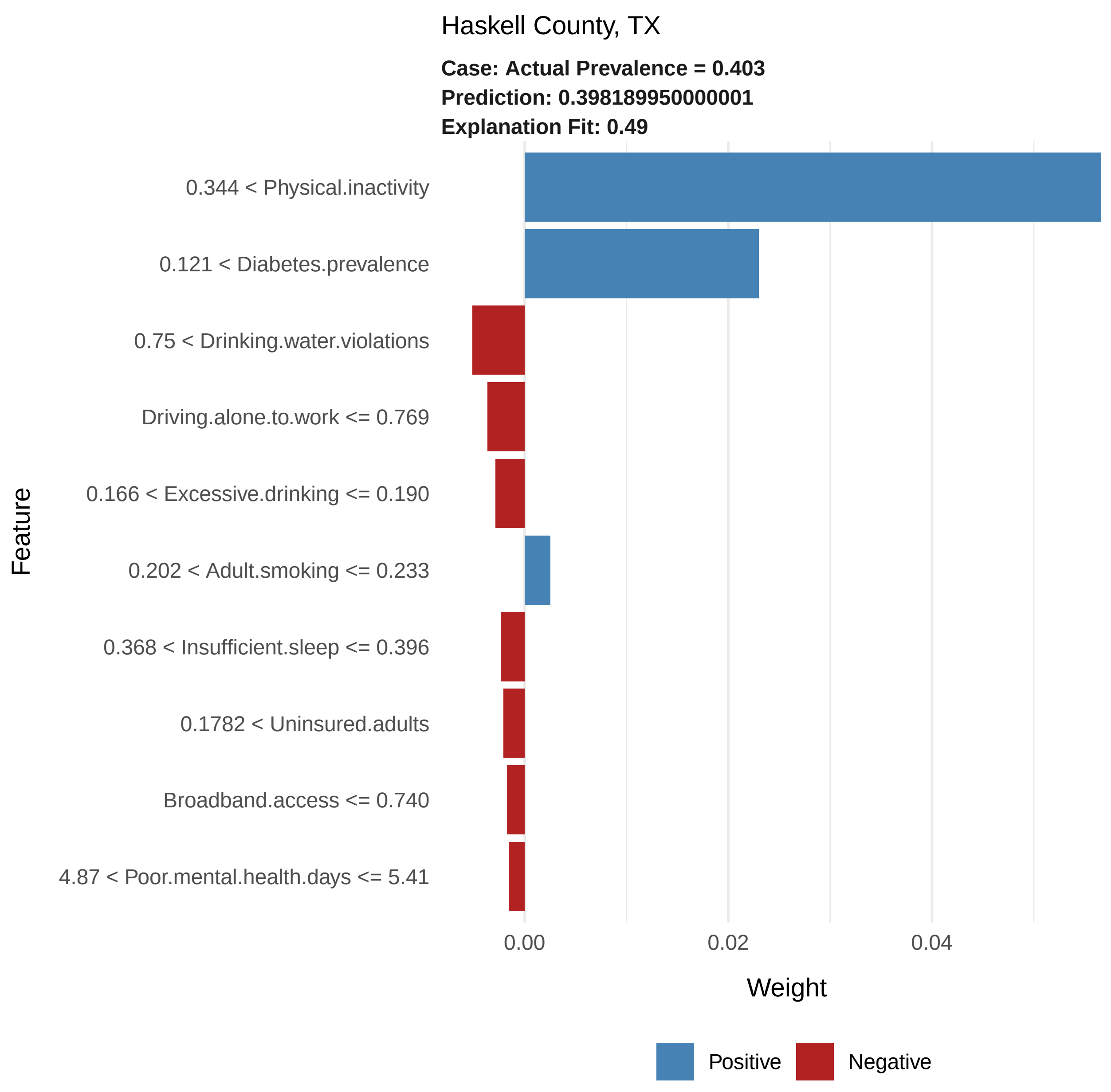

Haskell County, TX
Case: Actual Prevalence = 0.403
Prediction: 0.398189950000001
Explanation Fit: 0.49
0.344 < Physical.inactivity
0.121 < Diabetes.prevalence
0.75 < Drinking.water.violations
Driving.alone.to.work <= 0.769
0.166 < Excessive.drinking <= 0.190
0.202 < Adult.smoking <= 0.233
0.368 < Insufficient.sleep <= 0.396
0.1782 < Uninsured.adults
Broadband.access <= 0.740
4.87 < Poor.mental.health.days <= 5.41
Feature
0.00
0.02
0.04
Weight
Positive
Negative

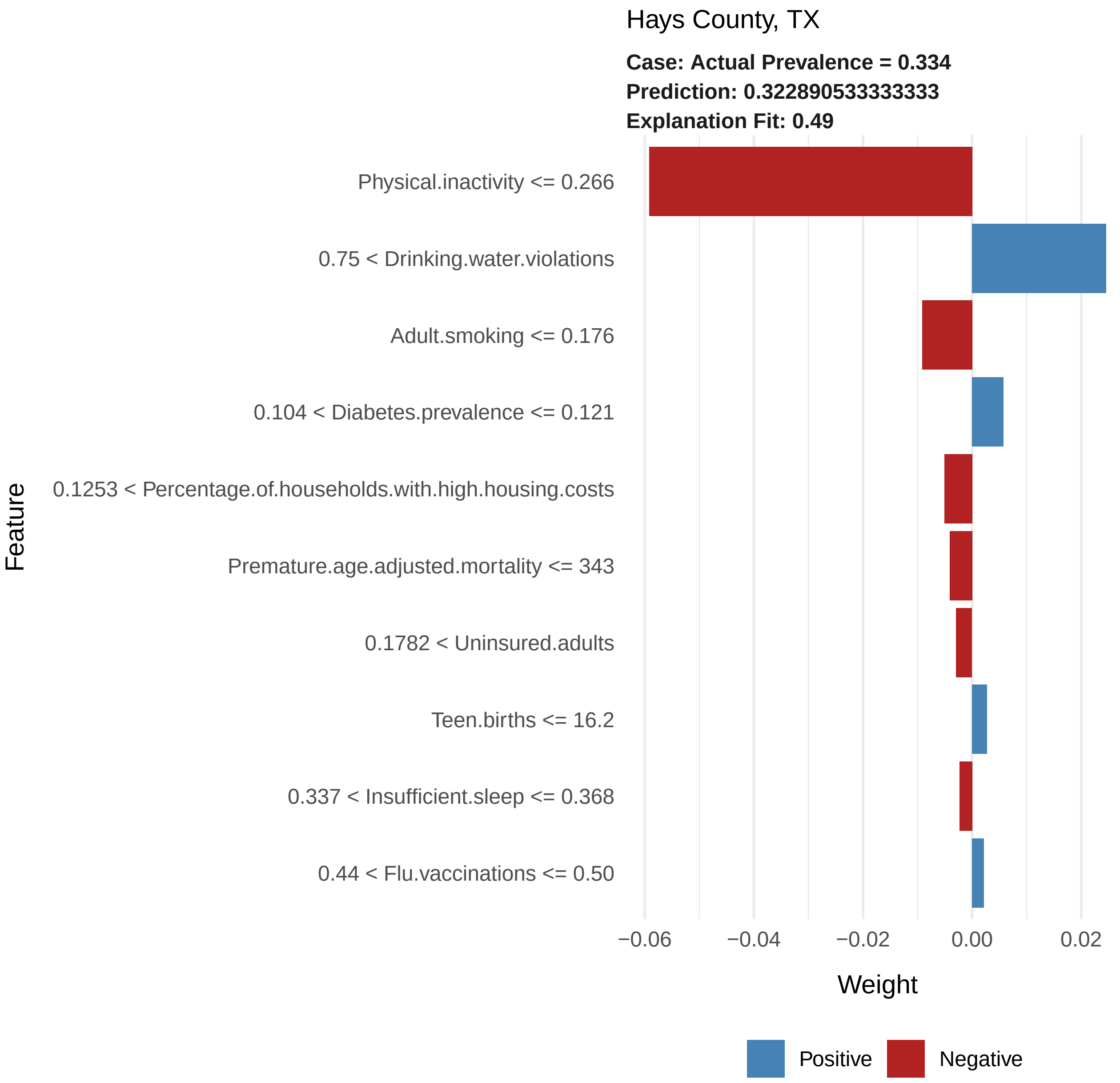
Hays County, TX
Case: Actual Prevalence = 0.334
Prediction: 0.322890533333333
Explanation Fit: 0.49
Physical.inactivity <= 0.266
0.75 < Drinking.water.violations
Adult.smoking <= 0.176
0.104 < Diabetes.prevalence <= 0.121
0.1253 < Percentage.of.households.with.high.housing.costs
Premature.age.adjusted.mortality <= 343
0.1782 < Uninsured.adults
Teen.births <= 16.2
0.337 < Insufficient.sleep <= 0.368
0.44 < Flu.vaccinations <= 0.50
Feature
−0.06
−0.04
−0.02
0.00
0.02
Weight
Positive
Negative

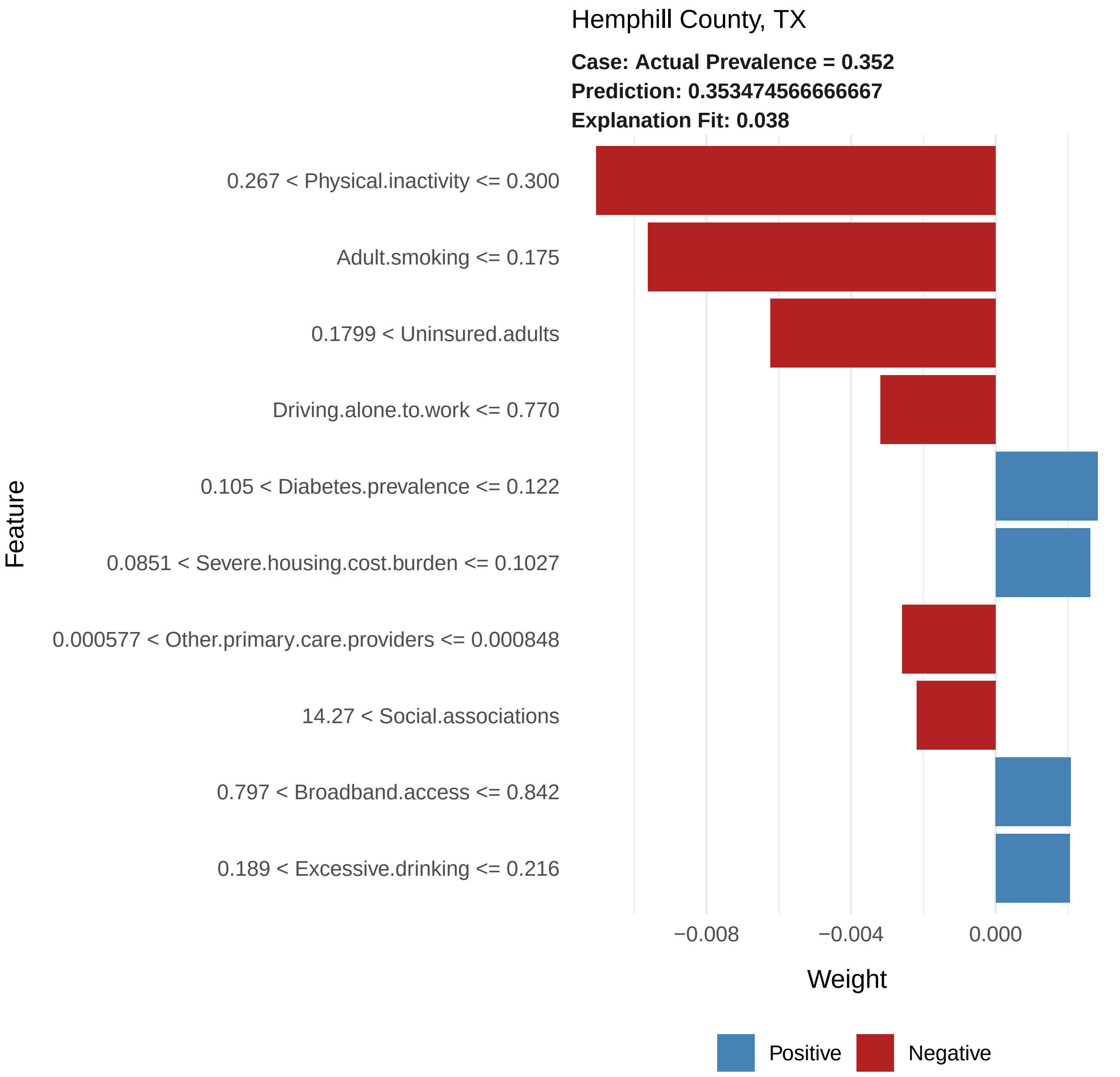
Hemphill County, TX
Case: Actual Prevalence = 0.352
Prediction: 0.353474566666667
Explanation Fit: 0.038
0.267 < Physical.inactivity <= 0.300
Adult.smoking <= 0.175
0.1799 < Uninsured.adults
Driving.alone.to.work <= 0.770
0.105 < Diabetes.prevalence <= 0.122
0.0851 < Severe.housing.cost.burden <= 0.1027
0.000577 < Other.primary.care.providers <= 0.000848
14.27 < Social.associations
0.797 < Broadband.access <= 0.842
0.189 < Excessive.drinking <= 0.216
Feature
−0.008
−0.004
0.000
Weight
Positive
Negative

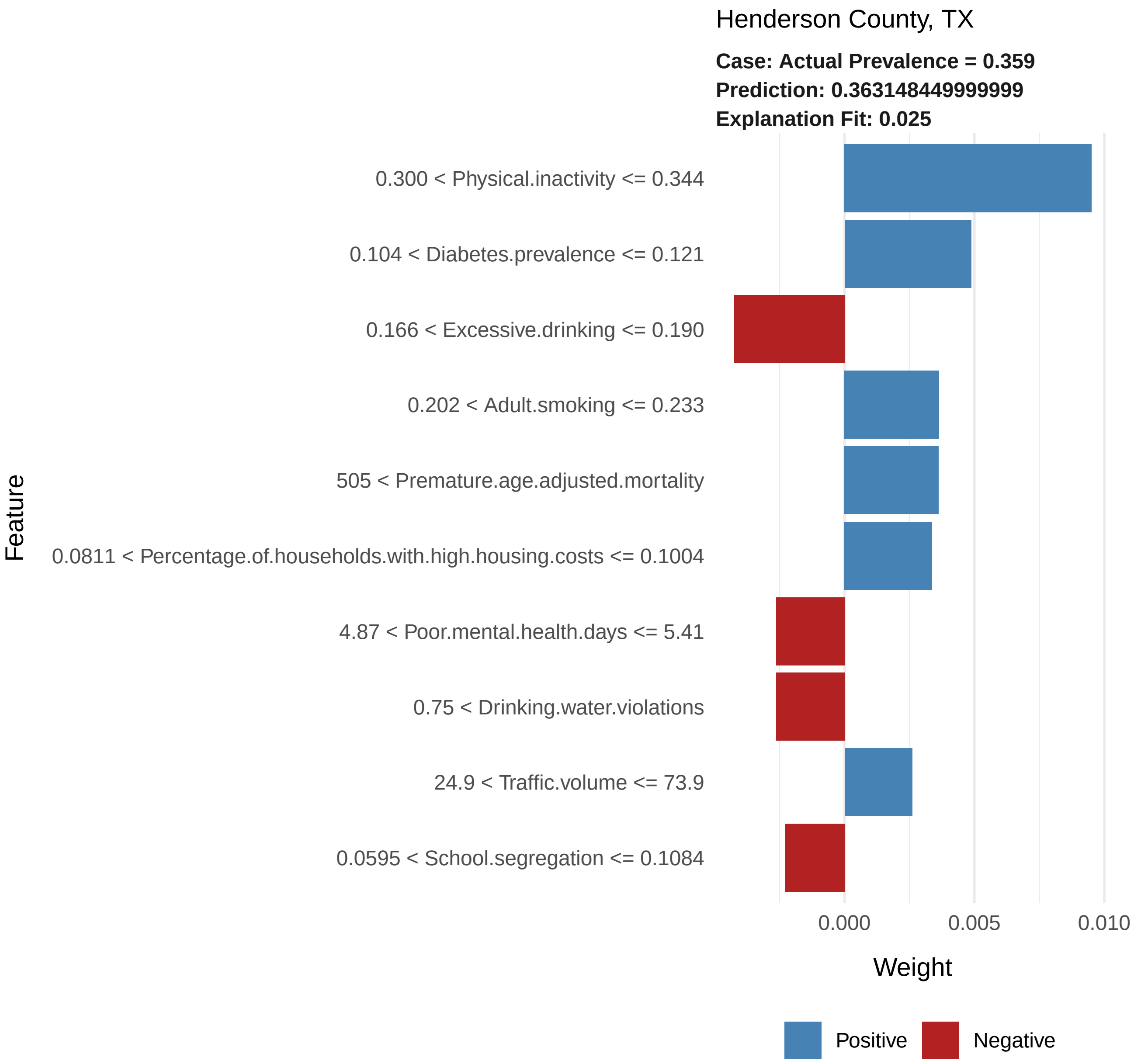

Henderson County, TX
Case: Actual Prevalence = 0.359
Prediction: 0.363148449999999
Explanation Fit: 0.025
0.300 < Physical.inactivity <= 0.344
0.104 < Diabetes.prevalence <= 0.121
0.166 < Excessive.drinking <= 0.190
0.202 < Adult.smoking <= 0.233
505 < Premature.age.adjusted.mortality
0.0811 < Percentage.of.households.with.high.housing.costs <= 0.1004
4.87 < Poor.mental.health.days <= 5.41
0.75 < Drinking.water.violations
24.9 < Traffic.volume <= 73.9
0.0595 < School.segregation <= 0.1084
Feature
0.000
0.005
0.010
Weight
Positive
Negative

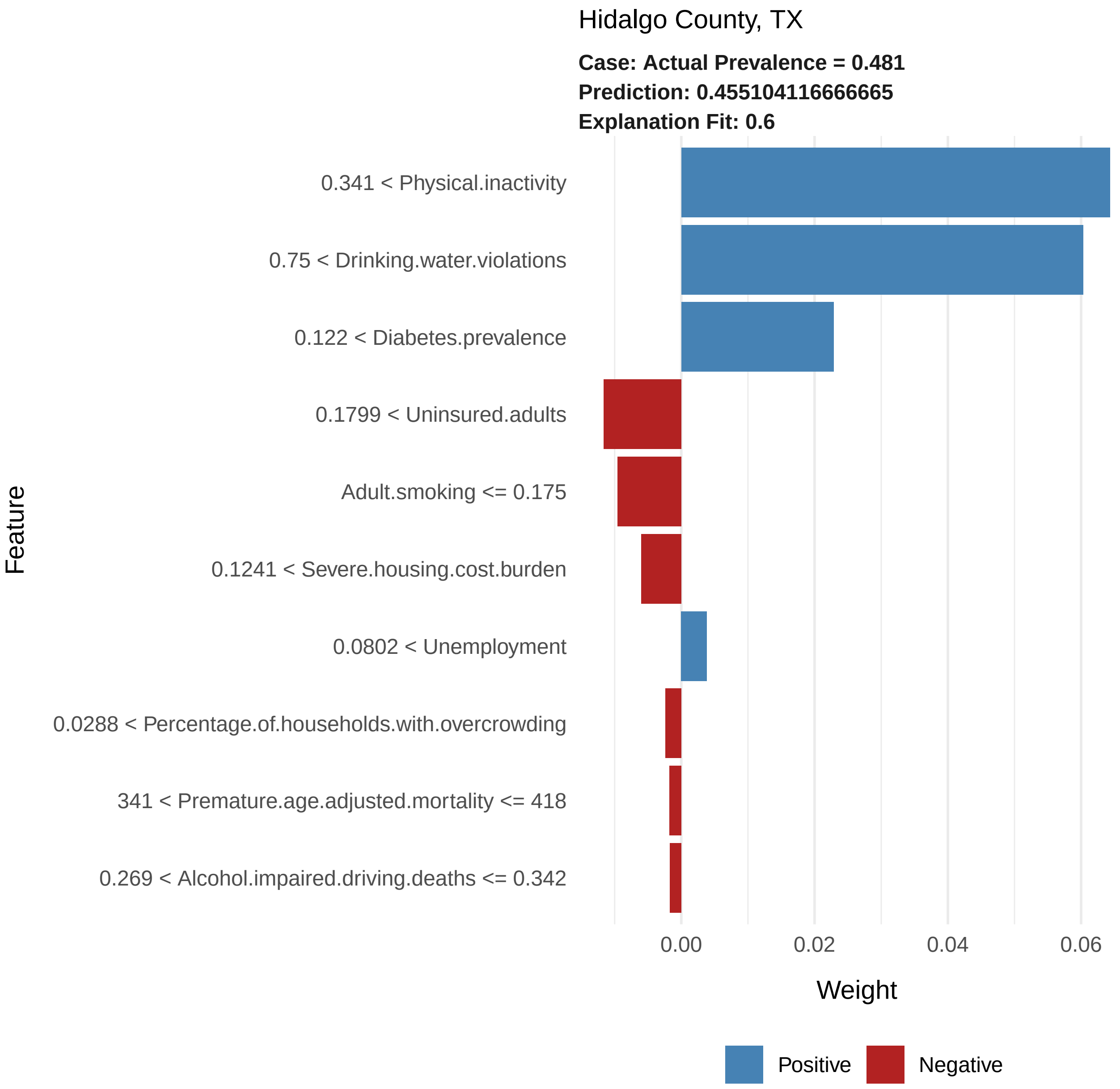
Hidalgo County, TX
Case: Actual Prevalence = 0.481
Prediction: 0.455104116666665
Explanation Fit: 0.6
0.341 < Physical.inactivity
0.75 < Drinking.water.violations
0.122 < Diabetes.prevalence
0.1799 < Uninsured.adults
Adult.smoking <= 0.175
0.1241 < Severe.housing.cost.burden
0.0802 < Unemployment
0.0288 < Percentage.of.households.with.overcrowding
341 < Premature.age.adjusted.mortality <= 418
0.269 < Alcohol.impaired.driving.deaths <= 0.342
Feature
0.00
0.02
0.04
0.06
Weight
Positive
Negative

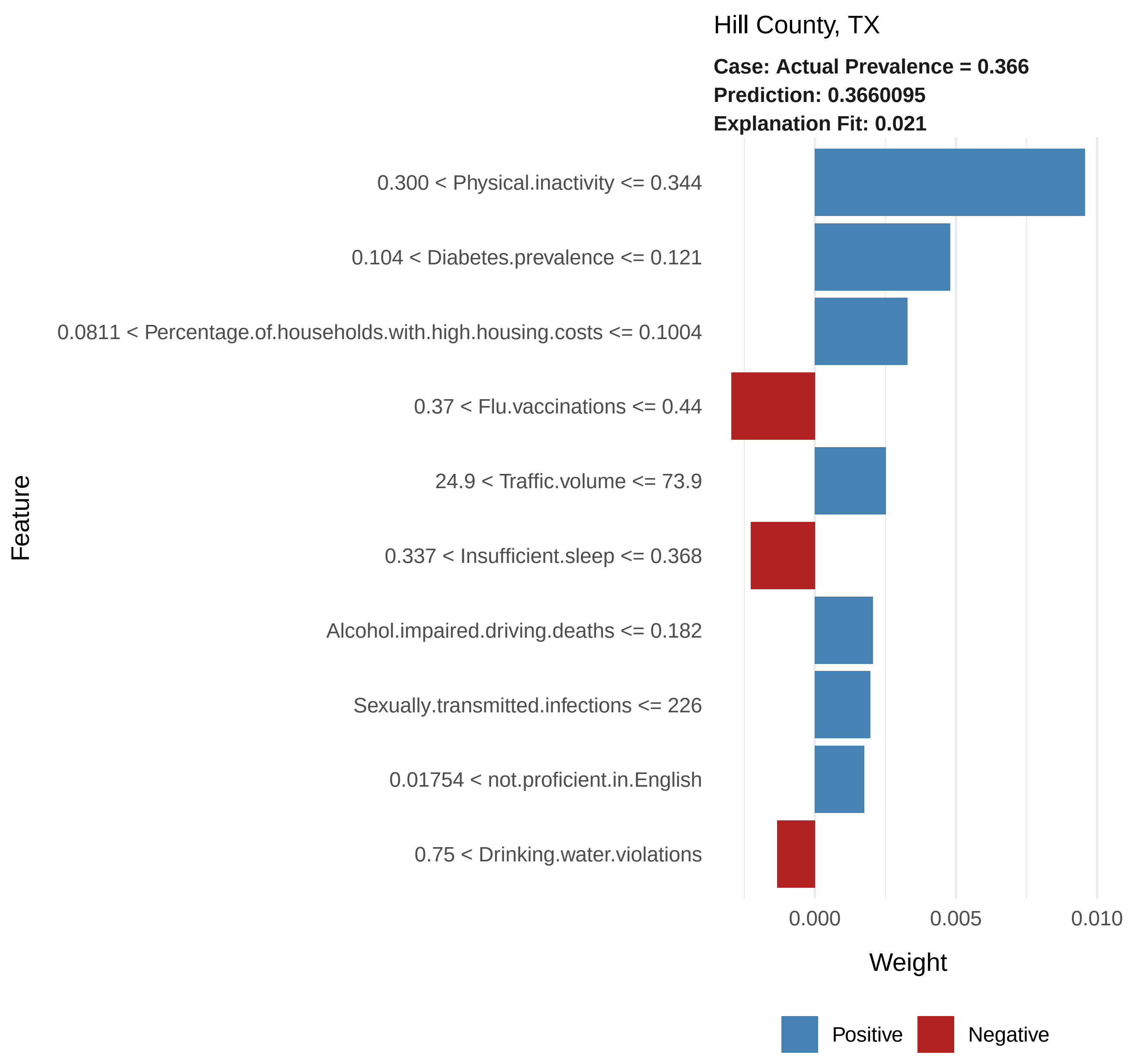
Hill County, TX
Case: Actual Prevalence = 0.366
Prediction: 0.3660095
Explanation Fit: 0.021
0.300 < Physical.inactivity <= 0.344
0.104 < Diabetes.prevalence <= 0.121
0.0811 < Percentage.of.households.with.high.housing.costs <= 0.1004
0.37 < Flu.vaccinations <= 0.44
24.9 < Traffic.volume <= 73.9
0.337 < Insufficient.sleep <= 0.368
Alcohol.impaired.driving.deaths <= 0.182
Sexually.transmitted.infections <= 226
0.01754 < not.proficient.in.English
0.75 < Drinking.water.violations
Feature
0.000
0.005
0.010
Weight
Positive
Negative

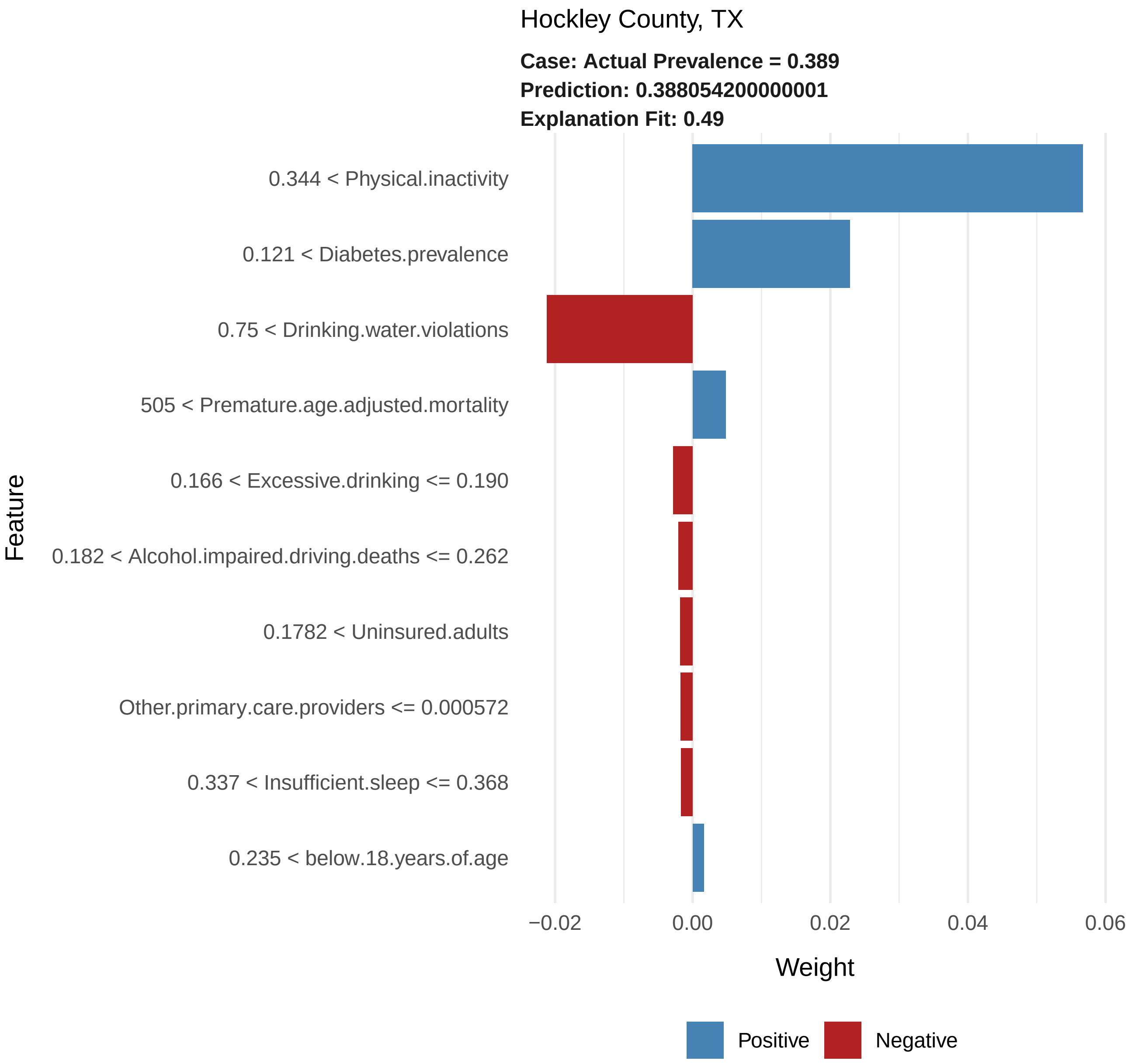
Hockley County, TX
Case: Actual Prevalence = 0.389
Prediction: 0.388054200000001
Explanation Fit: 0.49
0.344 < Physical.inactivity
0.121 < Diabetes.prevalence
0.75 < Drinking.water.violations
505 < Premature.age.adjusted.mortality
0.166 < Excessive.drinking <= 0.190
0.182 < Alcohol.impaired.driving.deaths <= 0.262
0.1782 < Uninsured.adults
Other.primary.care.providers <= 0.000572
0.337 < Insufficient.sleep <= 0.368
0.235 < below.18.years.of.age
Feature
−0.02
0.00
0.02
0.04
0.06
Weight
Positive
Negative

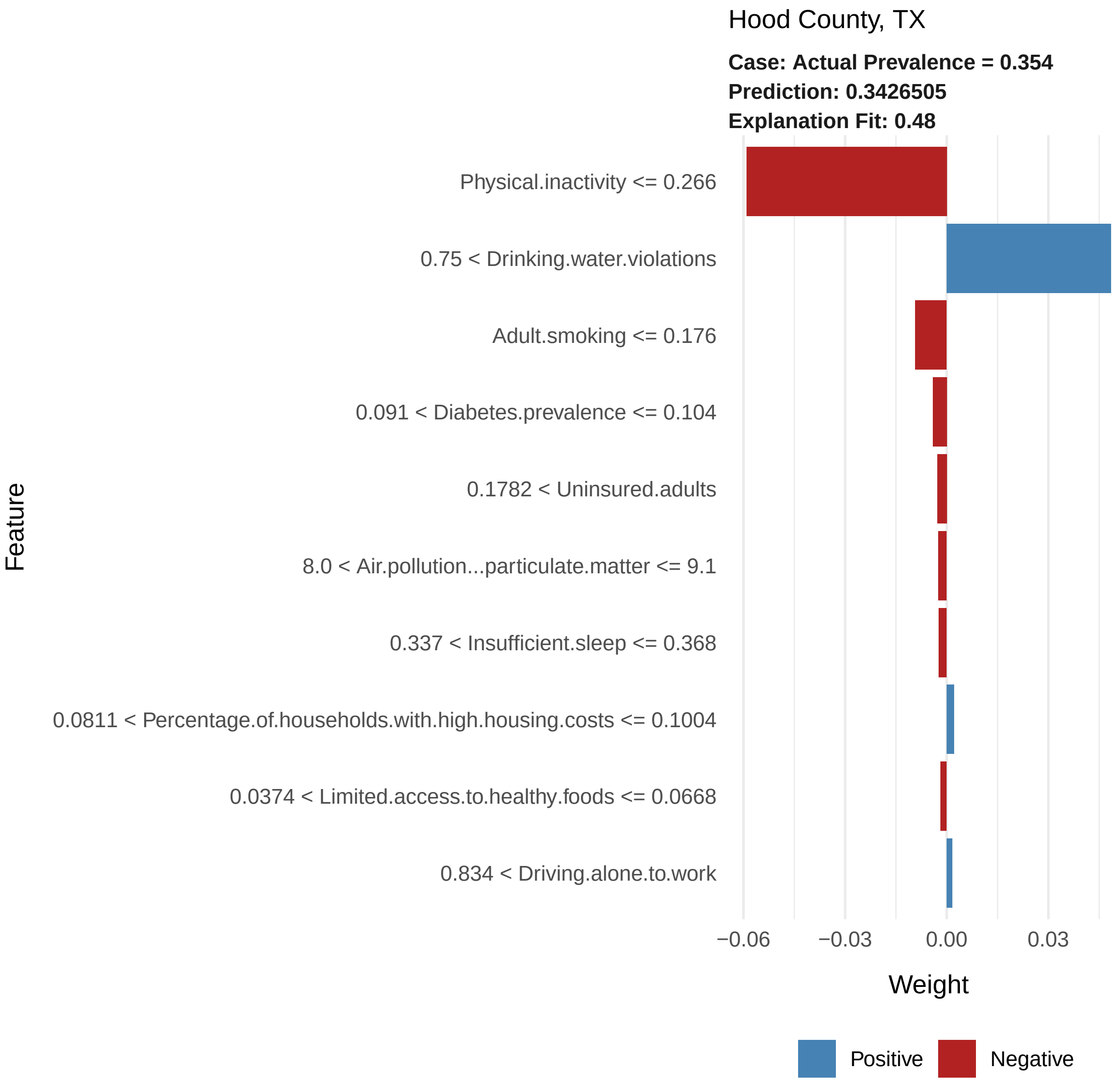
Hood County, TX
Case: Actual Prevalence = 0.354
Prediction: 0.3426505
Explanation Fit: 0.48
Physical.inactivity <= 0.266
0.75 < Drinking.water.violations
Adult.smoking <= 0.176
0.091 < Diabetes.prevalence <= 0.104
0.1782 < Uninsured.adults
8.0 < Air.pollution...particulate.matter <= 9.1
0.337 < Insufficient.sleep <= 0.368
0.0811 < Percentage.of.households.with.high.housing.costs <= 0.1004
0.0374 < Limited.access.to.healthy.foods <= 0.0668
0.834 < Driving.alone.to.work
Feature
−0.06
−0.03
0.00
0.03
Weight
Positive
Negative

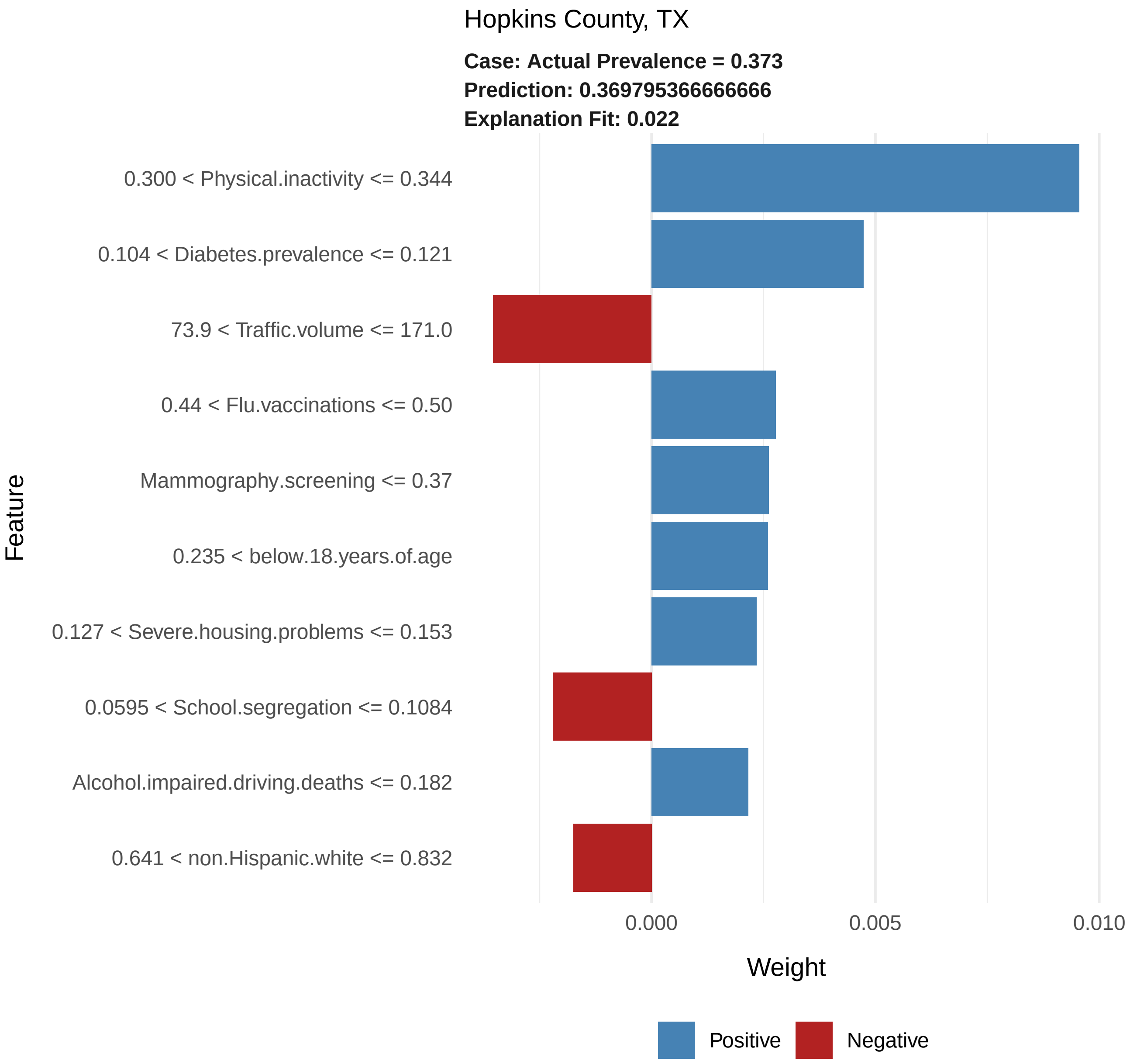
Hopkins County, TX
Case: Actual Prevalence = 0.373
Prediction: 0.369795366666666
Explanation Fit: 0.022
0.300 < Physical.inactivity <= 0.344
0.104 < Diabetes.prevalence <= 0.121
73.9 < Traffic.volume <= 171.0
0.44 < Flu.vaccinations <= 0.50
Mammography.screening <= 0.37
0.235 < below.18.years.of.age
0.127 < Severe.housing.problems <= 0.153
0.0595 < School.segregation <= 0.1084
Alcohol.impaired.driving.deaths <= 0.182
0.641 < non.Hispanic.white <= 0.832
Feature
0.000
0.005
0.010
Weight
Positive
Negative

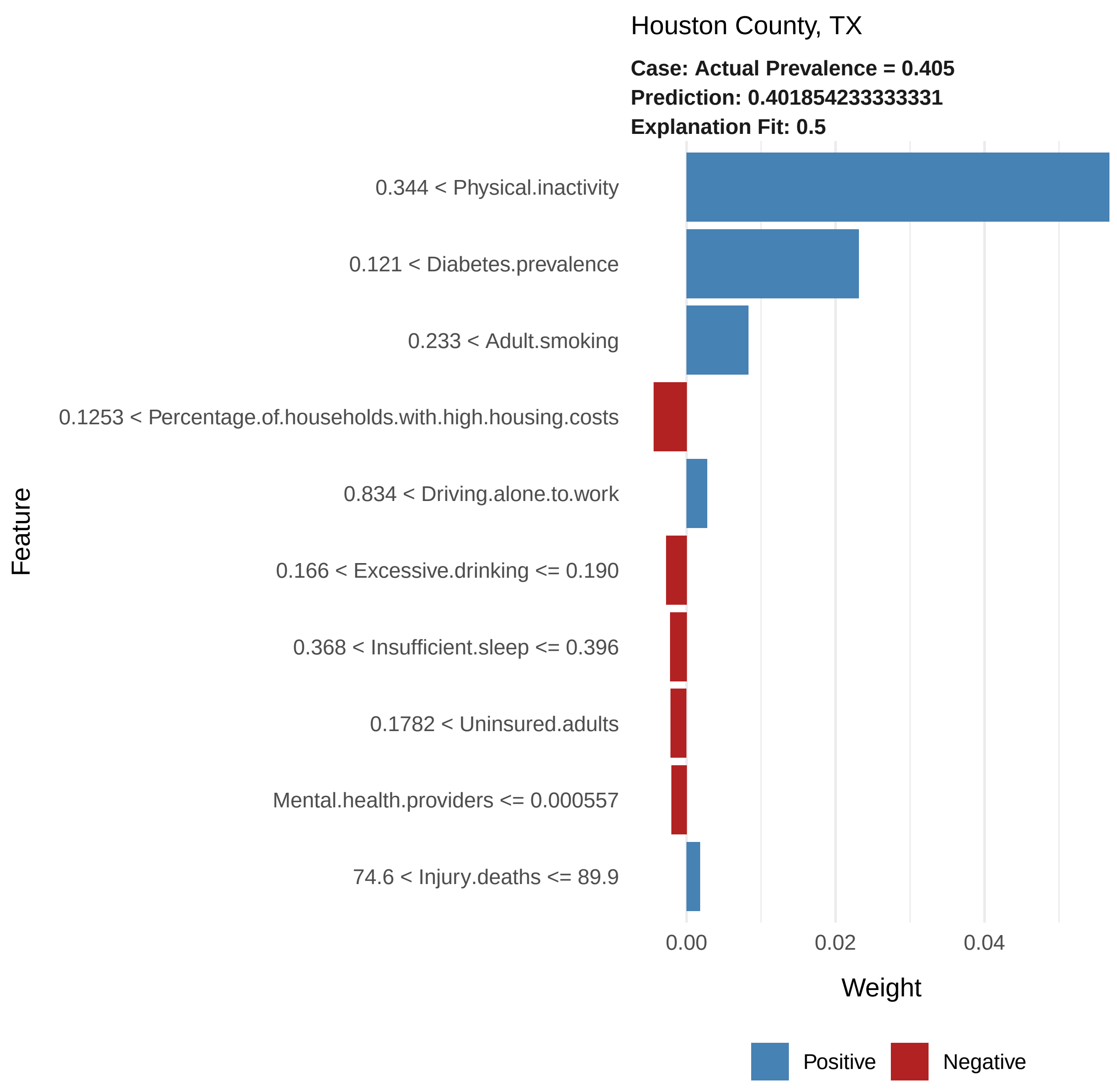

Houston County, TX
Case: Actual Prevalence = 0.405
Prediction: 0.401854233333331
Explanation Fit: 0.5
0.344 < Physical.inactivity
0.121 < Diabetes.prevalence
0.233 < Adult.smoking
0.1253 < Percentage.of.households.with.high.housing.costs
0.834 < Driving.alone.to.work
0.166 < Excessive.drinking <= 0.190
0.368 < Insufficient.sleep <= 0.396
0.1782 < Uninsured.adults
Mental.health.providers <= 0.000557
74.6 < Injury.deaths <= 89.9
Feature
0.00
0.02
0.04
Weight
Positive
Negative

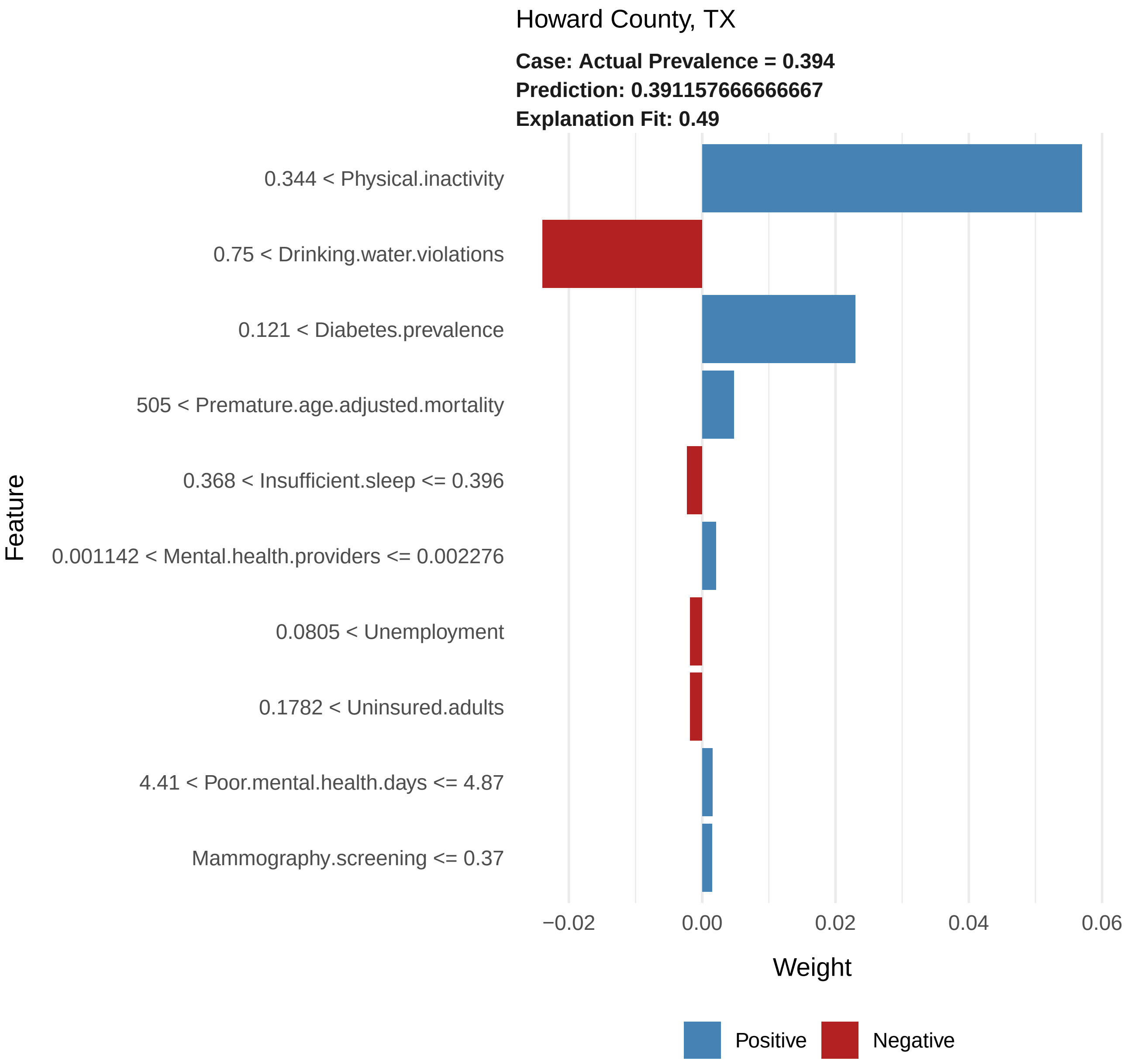

Howard County, TX
Case: Actual Prevalence = 0.394
Prediction: 0.391157666666667
Explanation Fit: 0.49
0.344 < Physical.inactivity
0.75 < Drinking.water.violations
0.121 < Diabetes.prevalence
505 < Premature.age.adjusted.mortality
0.368 < Insufficient.sleep <= 0.396
0.001142 < Mental.health.providers <= 0.002276
0.0805 < Unemployment
0.1782 < Uninsured.adults
4.41 < Poor.mental.health.days <= 4.87
Mammography.screening <= 0.37
Feature
−0.02
0.00
0.02
0.04
0.06
Weight
Positive
Negative

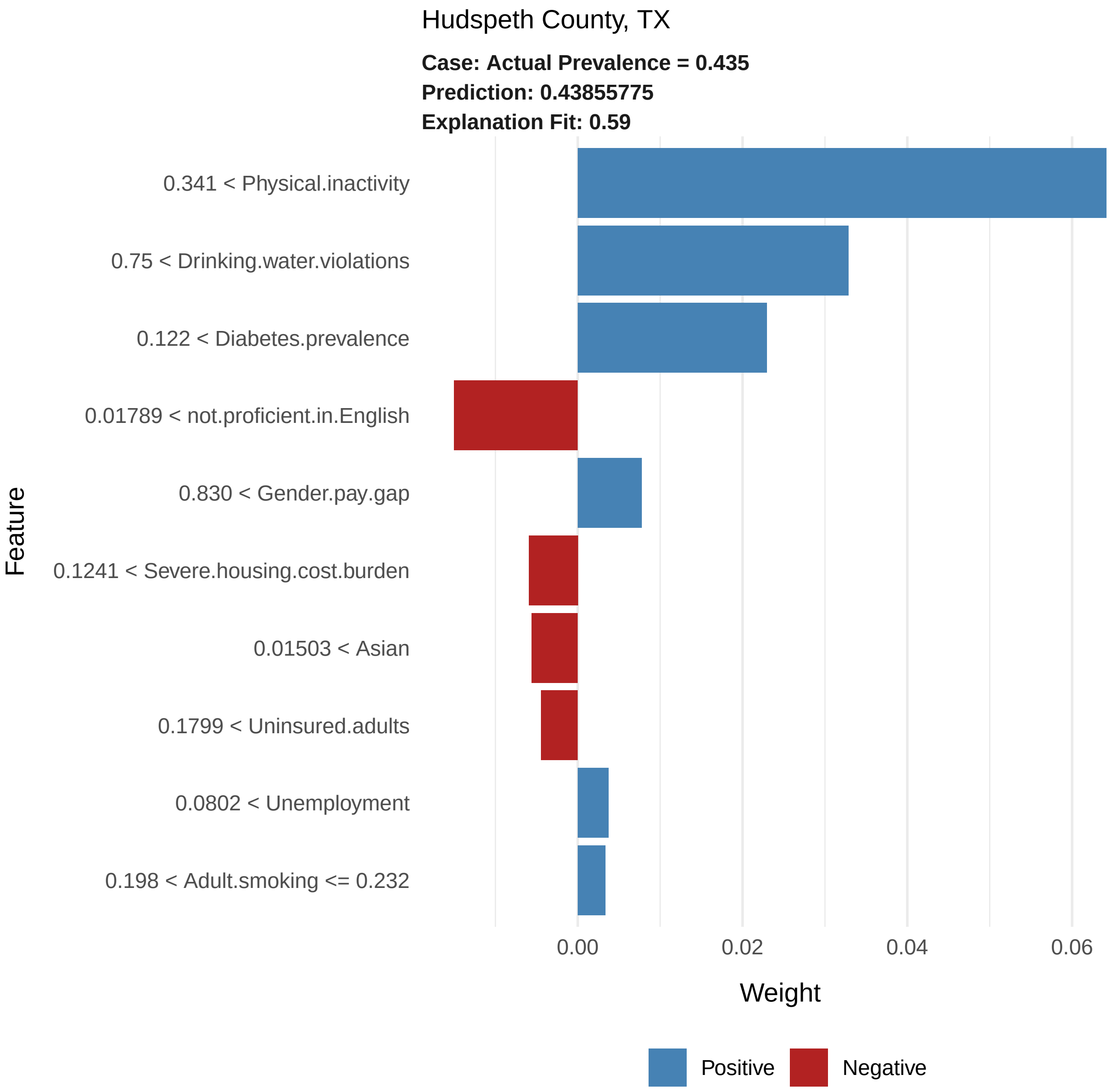

Hudspeth County, TX
Case: Actual Prevalence = 0.435
Prediction: 0.43855775
Explanation Fit: 0.59
0.341 < Physical.inactivity
0.75 < Drinking.water.violations
0.122 < Diabetes.prevalence
0.01789 < not.proficient.in.English
0.830 < Gender.pay.gap
0.1241 < Severe.housing.cost.burden
0.01503 < Asian
0.1799 < Uninsured.adults
0.0802 < Unemployment
0.198 < Adult.smoking <= 0.232
Feature
0.00
0.02
0.04
0.06
Weight
Positive
Negative

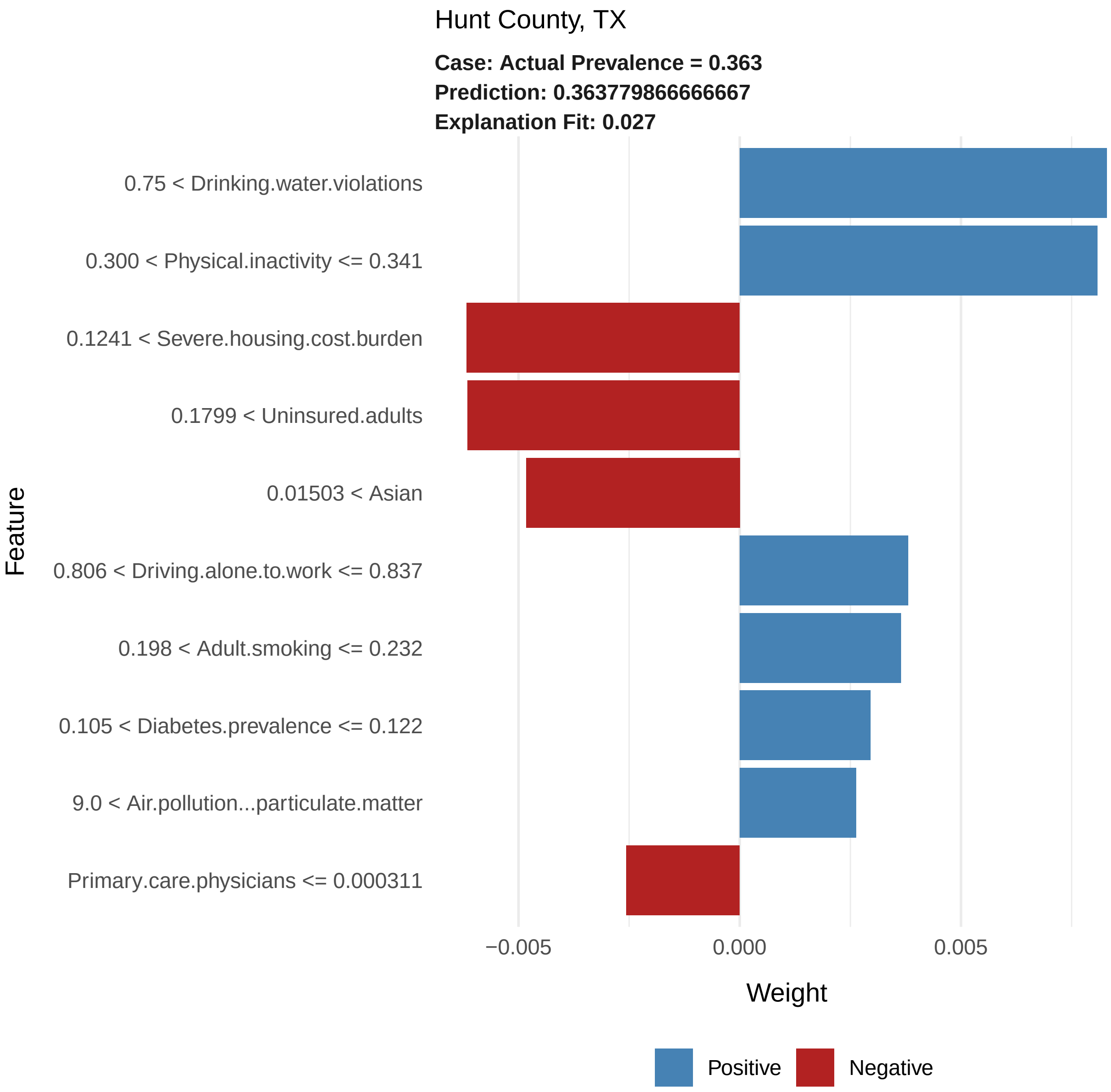

Hunt County, TX
Case: Actual Prevalence = 0.363
Prediction: 0.363779866666667
Explanation Fit: 0.027
0.75 < Drinking.water.violations
0.300 < Physical.inactivity <= 0.341
0.1241 < Severe.housing.cost.burden
0.1799 < Uninsured.adults
0.01503 < Asian
0.806 < Driving.alone.to.work <= 0.837
0.198 < Adult.smoking <= 0.232
0.105 < Diabetes.prevalence <= 0.122
9.0 < Air.pollution...particulate.matter
Primary.care.physicians <= 0.000311
Feature
−0.005
0.000
0.005
Weight
Positive
Negative

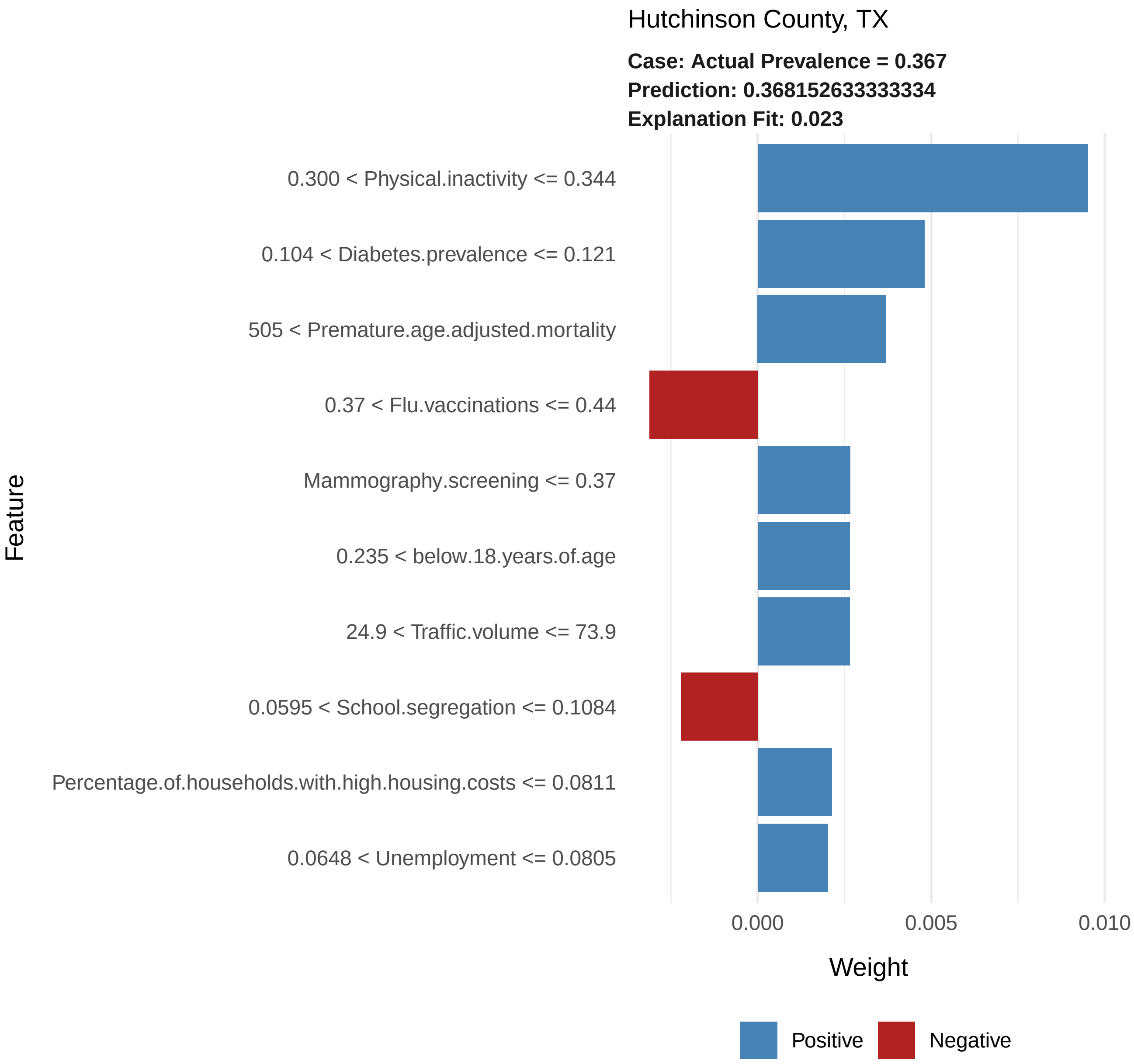
Hutchinson County, TX
Case: Actual Prevalence = 0.367
Prediction: 0.368152633333334
Explanation Fit: 0.023
0.300 < Physical.inactivity <= 0.344
0.104 < Diabetes.prevalence <= 0.121
505 < Premature.age.adjusted.mortality
0.37 < Flu.vaccinations <= 0.44
Mammography.screening <= 0.37
0.235 < below.18.years.of.age
24.9 < Traffic.volume <= 73.9
0.0595 < School.segregation <= 0.1084
Percentage.of.households.with.high.housing.costs <= 0.0811
0.0648 < Unemployment <= 0.0805
Feature
0.000
0.005
0.010
Weight
Positive
Negative

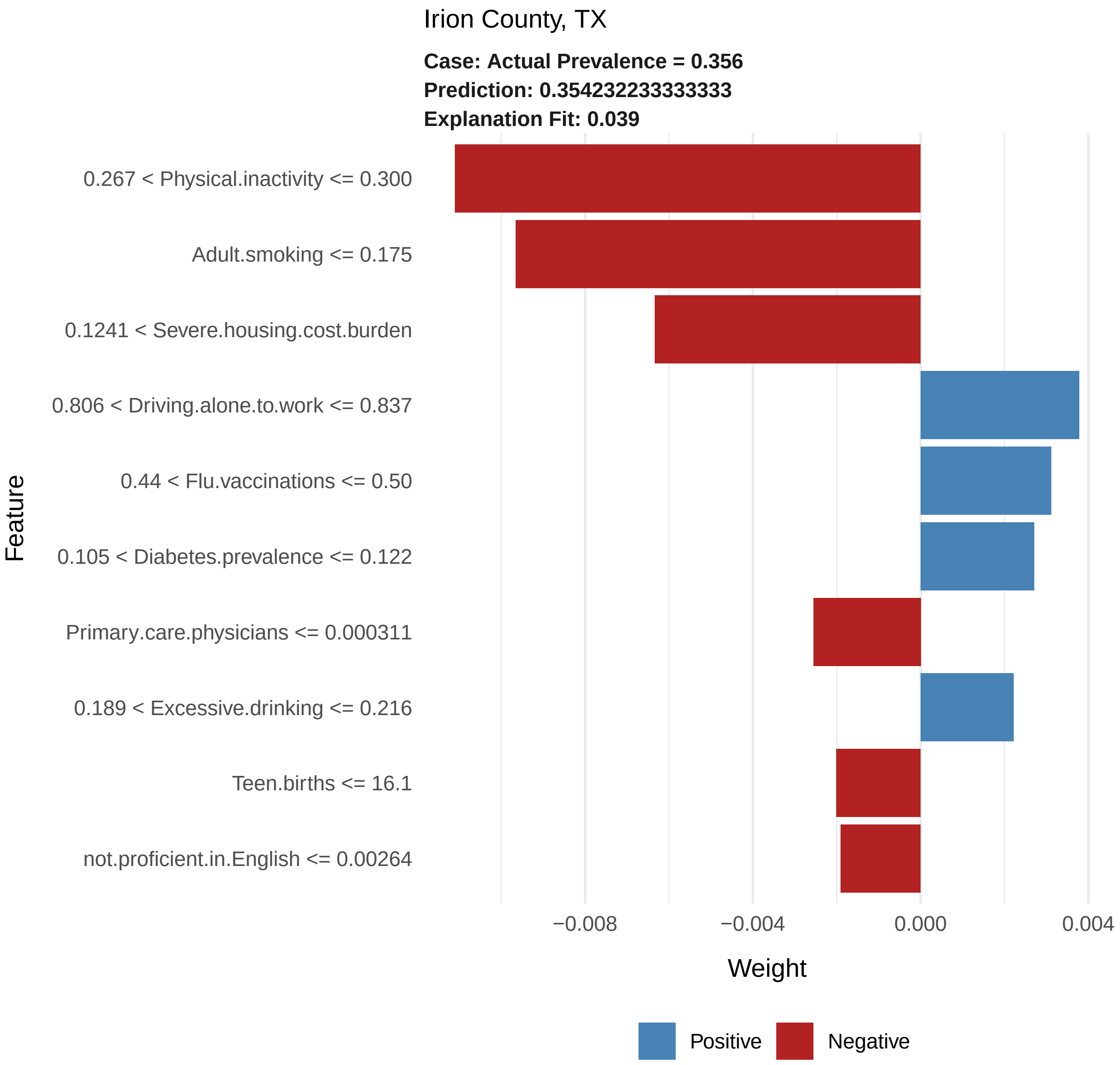

Irion County, TX
Case: Actual Prevalence = 0.356
Prediction: 0.354232233333333
Explanation Fit: 0.039
0.267 < Physical.inactivity <= 0.300
Adult.smoking <= 0.175
0.1241 < Severe.housing.cost.burden
0.806 < Driving.alone.to.work <= 0.837
0.44 < Flu.vaccinations <= 0.50
0.105 < Diabetes.prevalence <= 0.122
Primary.care.physicians <= 0.000311
0.189 < Excessive.drinking <= 0.216
Teen.births <= 16.1
not.proficient.in.English <= 0.00264
Feature
−0.008
−0.004
0.000
0.004
Weight
Positive
Negative

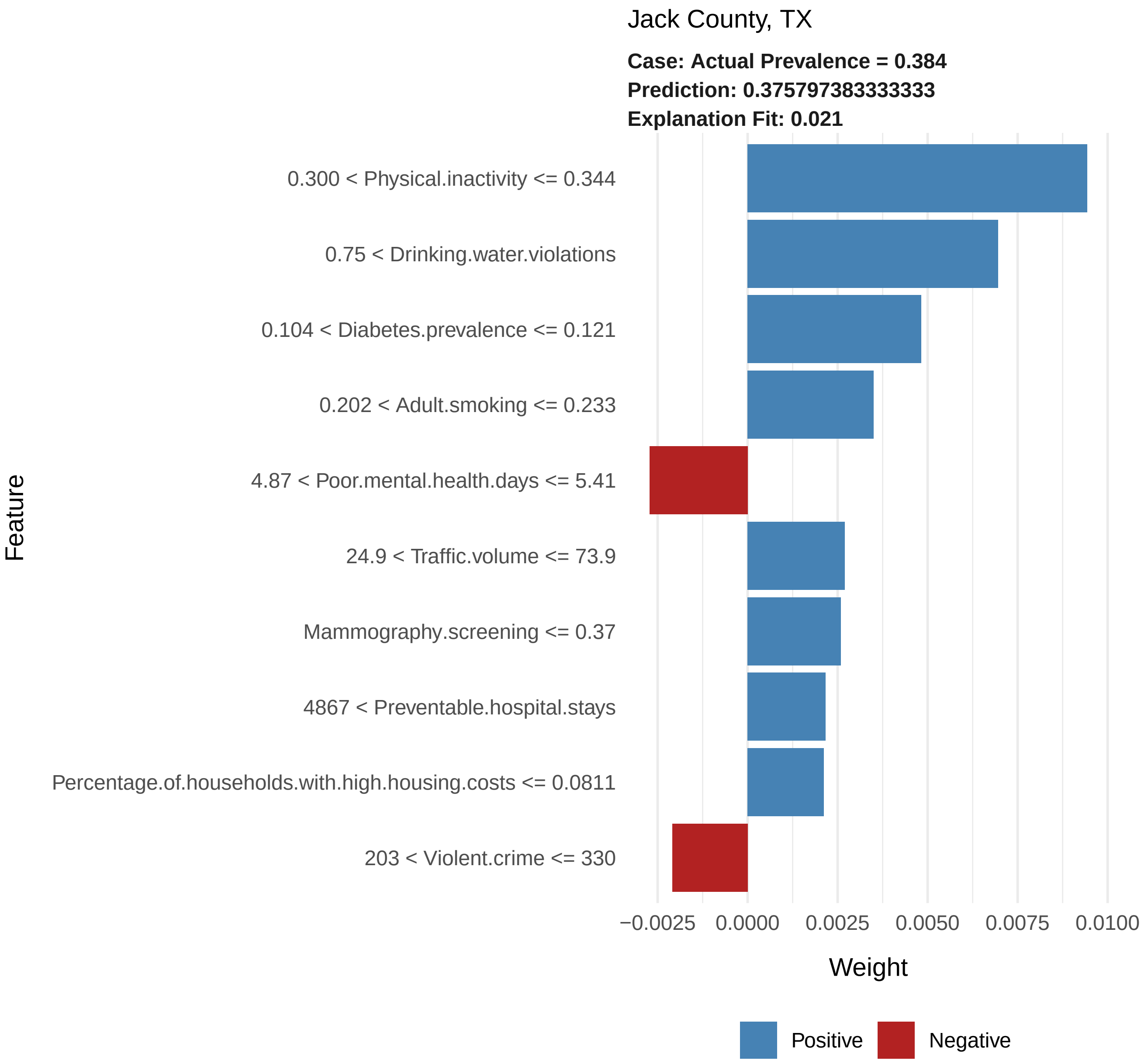
Jack County, TX
Case: Actual Prevalence = 0.384
Prediction: 0.375797383333333
Explanation Fit: 0.021
0.300 < Physical.inactivity <= 0.344
0.75 < Drinking.water.violations
0.104 < Diabetes.prevalence <= 0.121
0.202 < Adult.smoking <= 0.233
4.87 < Poor.mental.health.days <= 5.41
24.9 < Traffic.volume <= 73.9
Mammography.screening <= 0.37
4867 < Preventable.hospital.stays
Percentage.of.households.with.high.housing.costs <= 0.0811
203 < Violent.crime <= 330
Feature
−0.0025
0.0000
0.0025
0.0050
0.0075
0.0100
Weight
Positive
Negative

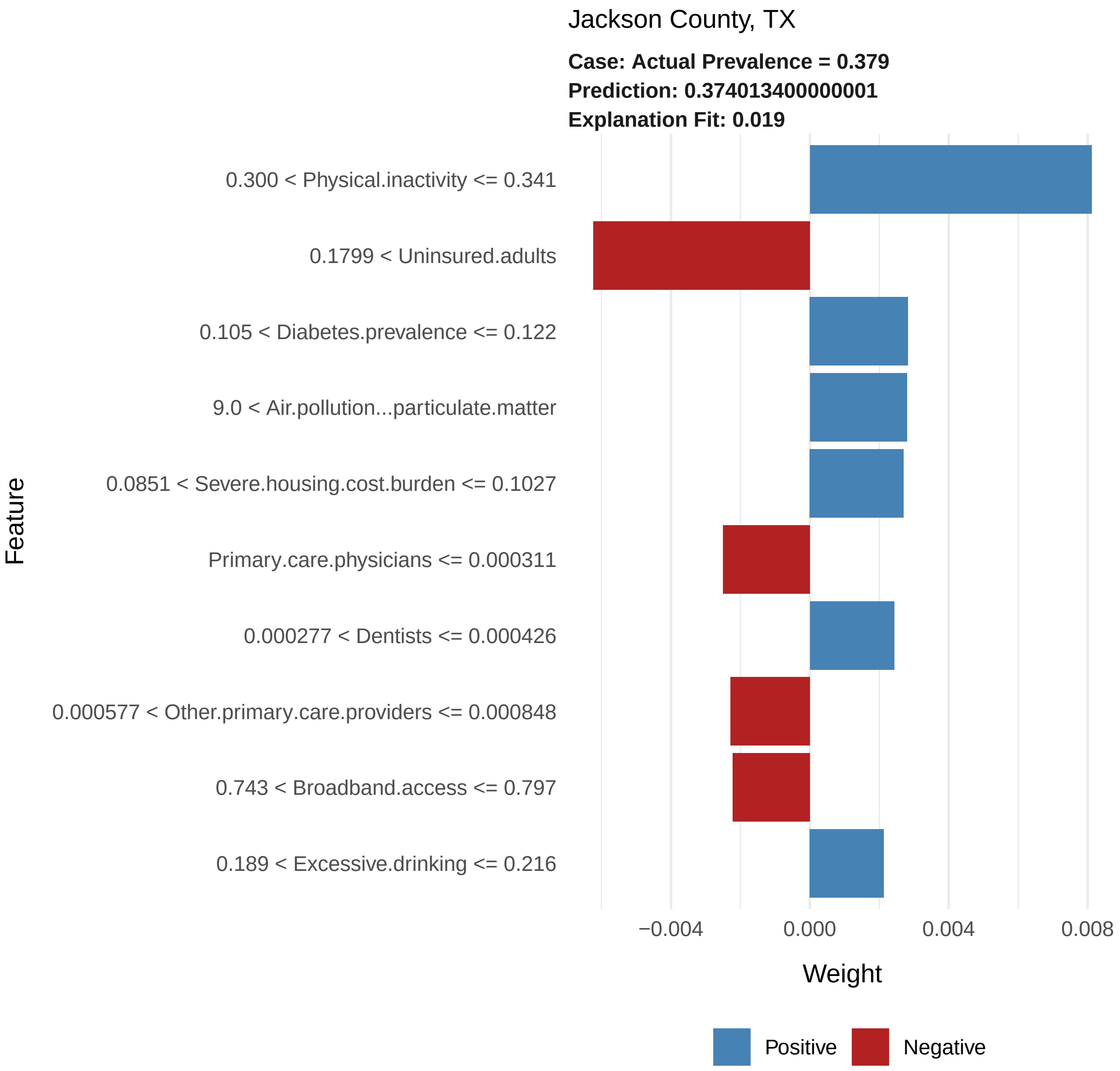

Jackson County, TX
Case: Actual Prevalence = 0.379
Prediction: 0.374013400000001
Explanation Fit: 0.019
0.300 < Physical.inactivity <= 0.341
0.1799 < Uninsured.adults
0.105 < Diabetes.prevalence <= 0.122
9.0 < Air.pollution...particulate.matter
0.0851 < Severe.housing.cost.burden <= 0.1027
Primary.care.physicians <= 0.000311
0.000277 < Dentists <= 0.000426
0.000577 < Other.primary.care.providers <= 0.000848
0.743 < Broadband.access <= 0.797
0.189 < Excessive.drinking <= 0.216
Feature
−0.004
0.000
0.004
0.008
Weight
Positive
Negative

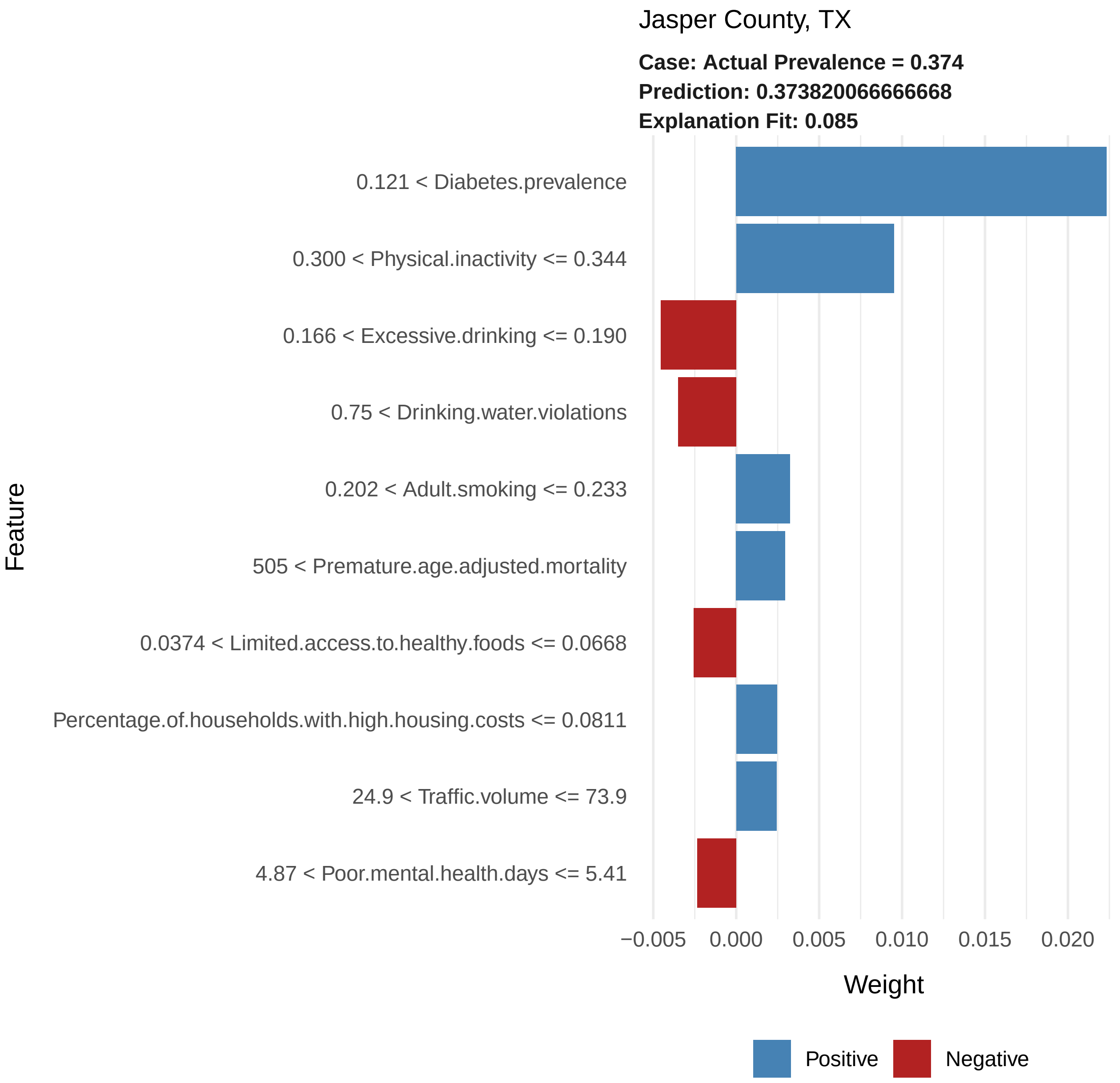

Jasper County, TX
Case: Actual Prevalence = 0.374
Prediction: 0.373820066666668
Explanation Fit: 0.085
0.121 < Diabetes.prevalence
0.300 < Physical.inactivity <= 0.344
0.166 < Excessive.drinking <= 0.190
0.75 < Drinking.water.violations
0.202 < Adult.smoking <= 0.233
505 < Premature.age.adjusted.mortality
0.0374 < Limited.access.to.healthy.foods <= 0.0668
Percentage.of.households.with.high.housing.costs <= 0.0811
24.9 < Traffic.volume <= 73.9
4.87 < Poor.mental.health.days <= 5.41
Feature
−0.005
0.000
0.005
0.010
0.015
0.020
Weight
Positive
Negative

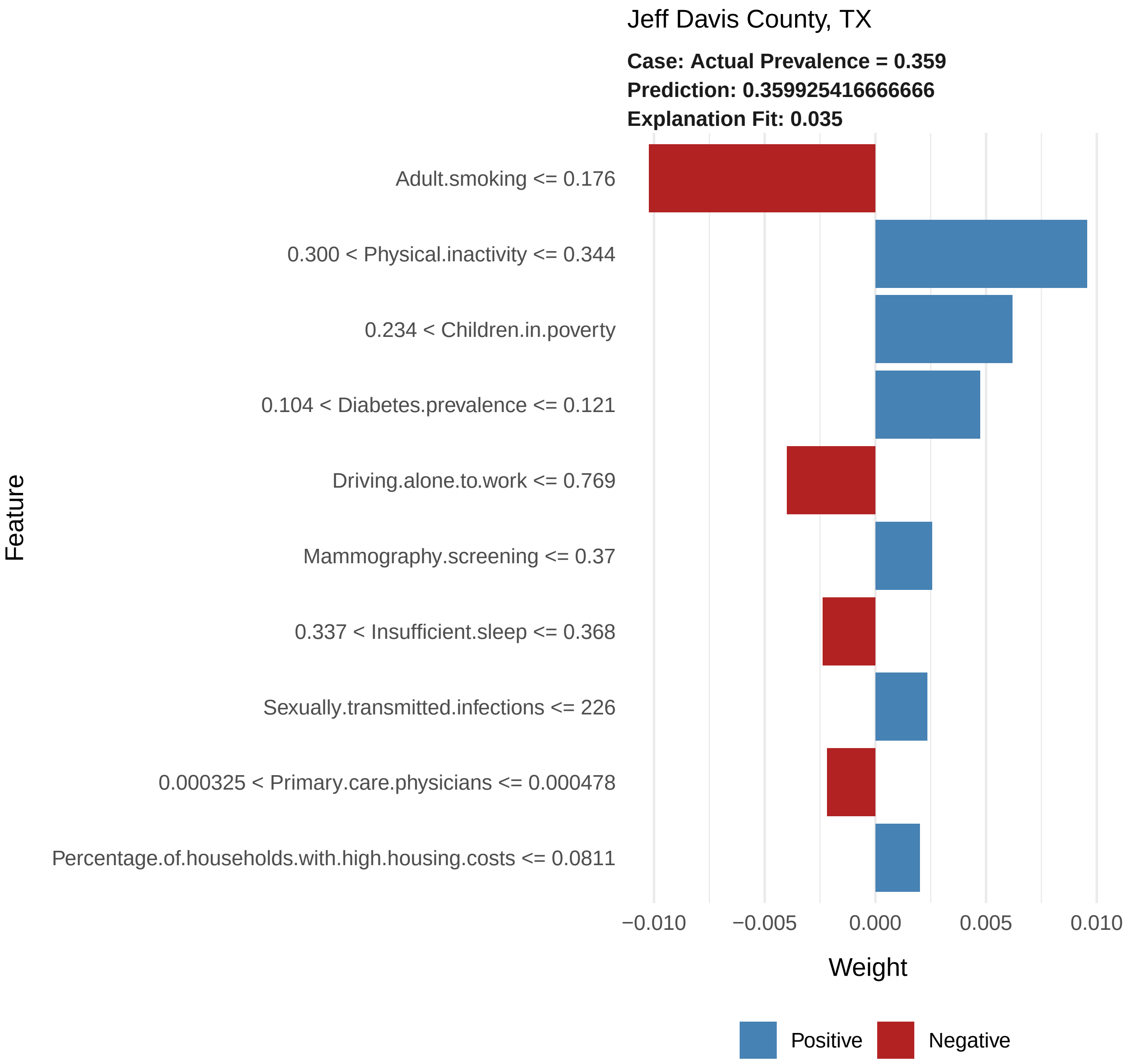

Jeff Davis County, TX
Case: Actual Prevalence = 0.359
Prediction: 0.359925416666666
Explanation Fit: 0.035
Adult.smoking <= 0.176
0.300 < Physical.inactivity <= 0.344
0.234 < Children.in.poverty
0.104 < Diabetes.prevalence <= 0.121
Driving.alone.to.work <= 0.769
Mammography.screening <= 0.37
0.337 < Insufficient.sleep <= 0.368
Sexually.transmitted.infections <= 226
0.000325 < Primary.care.physicians <= 0.000478
Percentage.of.households.with.high.housing.costs <= 0.0811
Feature
−0.010
−0.005
0.000
0.005
0.010
Weight
Positive
Negative

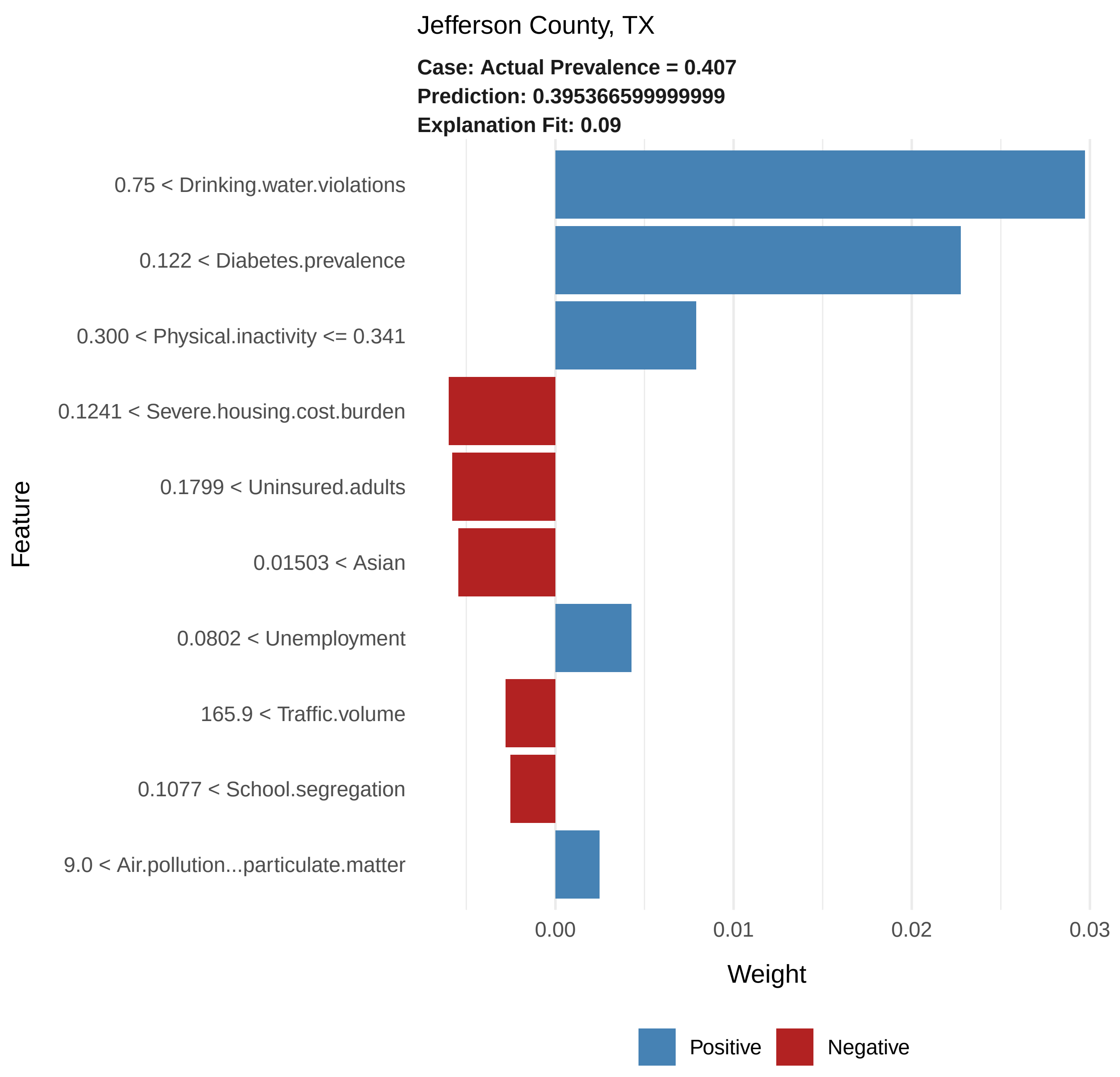
Jefferson County, TX
Case: Actual Prevalence = 0.407
Prediction: 0.395366599999999
Explanation Fit: 0.09
0.75 < Drinking.water.violations
0.122 < Diabetes.prevalence
0.300 < Physical.inactivity <= 0.341
0.1241 < Severe.housing.cost.burden
0.1799 < Uninsured.adults
0.01503 < Asian
0.0802 < Unemployment
165.9 < Traffic.volume
0.1077 < School.segregation
9.0 < Air.pollution...particulate.matter
Feature
0.00
0.01
0.02
0.03
Weight
Positive
Negative

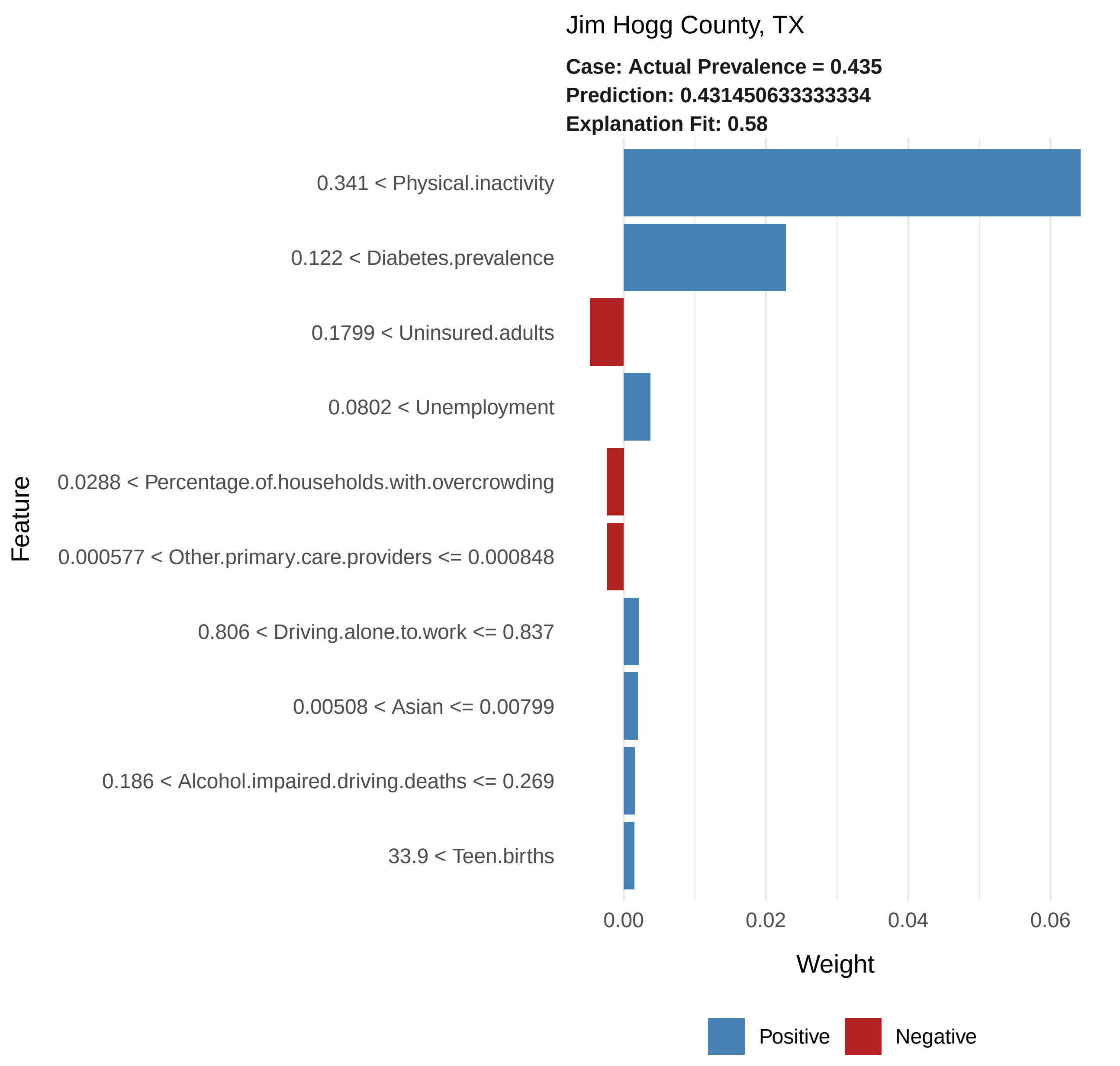
Jim Hogg County, TX
Case: Actual Prevalence = 0.435
Prediction: 0.431450633333334
Explanation Fit: 0.58
0.341 < Physical.inactivity
0.122 < Diabetes.prevalence
0.1799 < Uninsured.adults
0.0802 < Unemployment
0.0288 < Percentage.of.households.with.overcrowding
0.000577 < Other.primary.care.providers <= 0.000848
0.806 < Driving.alone.to.work <= 0.837
0.00508 < Asian <= 0.00799
0.186 < Alcohol.impaired.driving.deaths <= 0.269
33.9 < Teen.births
Feature
0.00
0.02
0.04
0.06
Weight
Positive
Negative

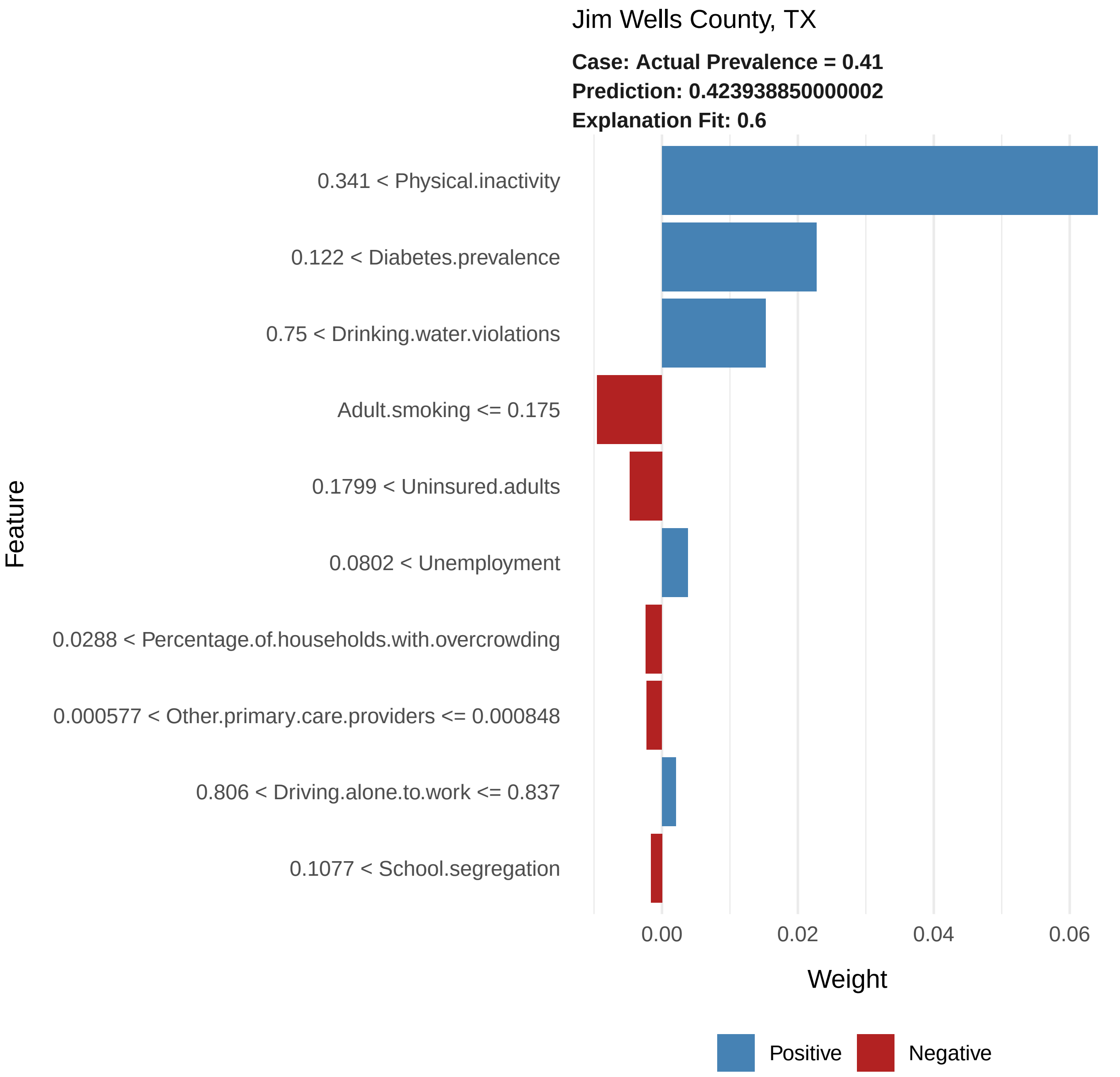
Jim Wells County, TX
Case: Actual Prevalence = 0.41
Prediction: 0.423938850000002
Explanation Fit: 0.6
0.341 < Physical.inactivity
0.122 < Diabetes.prevalence
0.75 < Drinking.water.violations
Adult.smoking <= 0.175
0.1799 < Uninsured.adults
0.0802 < Unemployment
0.0288 < Percentage.of.households.with.overcrowding
0.000577 < Other.primary.care.providers <= 0.000848
0.806 < Driving.alone.to.work <= 0.837
0.1077 < School.segregation
Feature
0.00
0.02
0.04
0.06
Weight
Positive
Negative

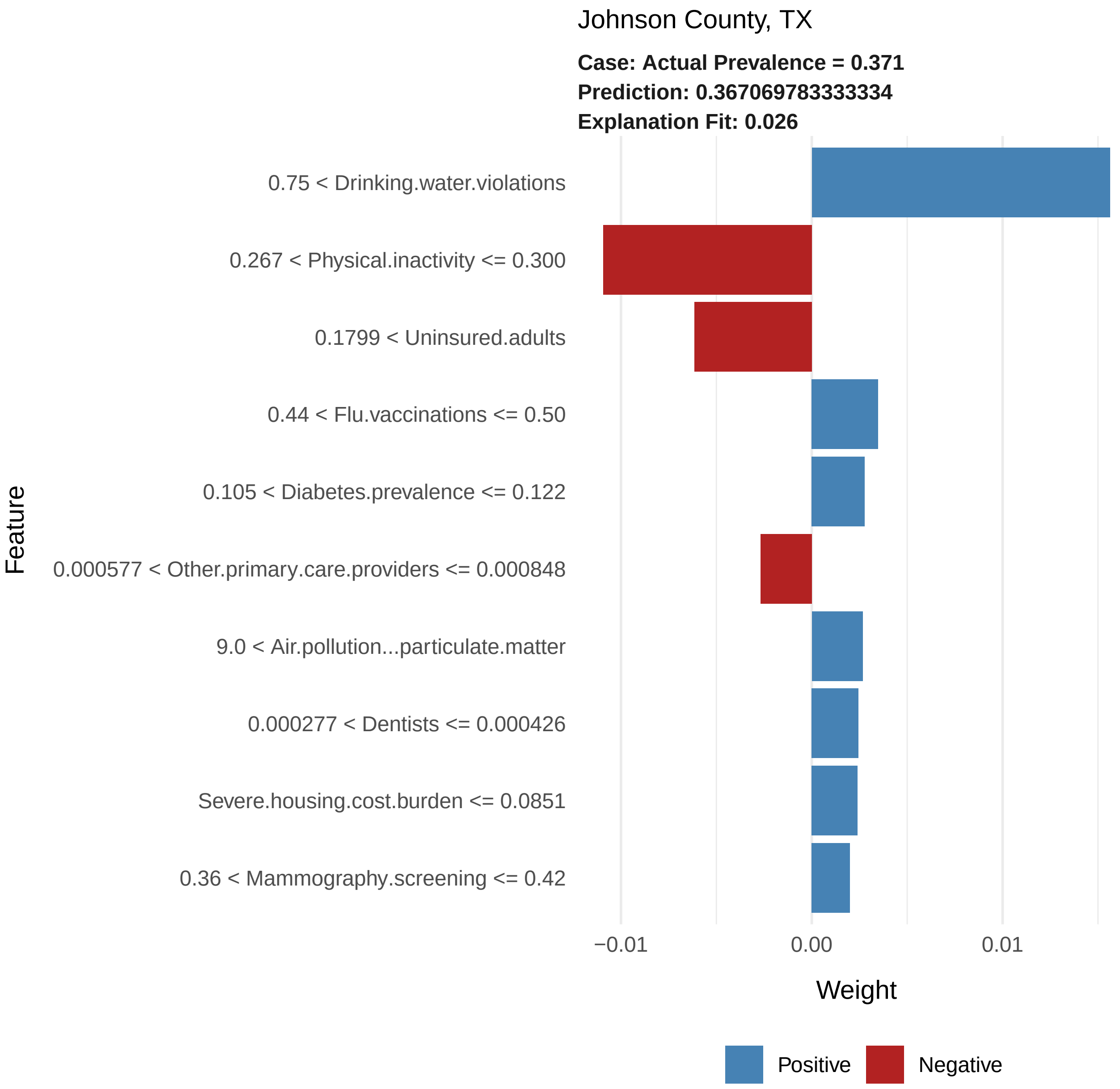

Johnson County, TX
Case: Actual Prevalence = 0.371
Prediction: 0.367069783333334
Explanation Fit: 0.026
0.75 < Drinking.water.violations
0.267 < Physical.inactivity <= 0.300
0.1799 < Uninsured.adults
0.44 < Flu.vaccinations <= 0.50
0.105 < Diabetes.prevalence <= 0.122
0.000577 < Other.primary.care.providers <= 0.000848
9.0 < Air.pollution...particulate.matter
0.000277 < Dentists <= 0.000426
Severe.housing.cost.burden <= 0.0851
0.36 < Mammography.screening <= 0.42
Feature
−0.01
0.00
0.01
Weight
Positive
Negative

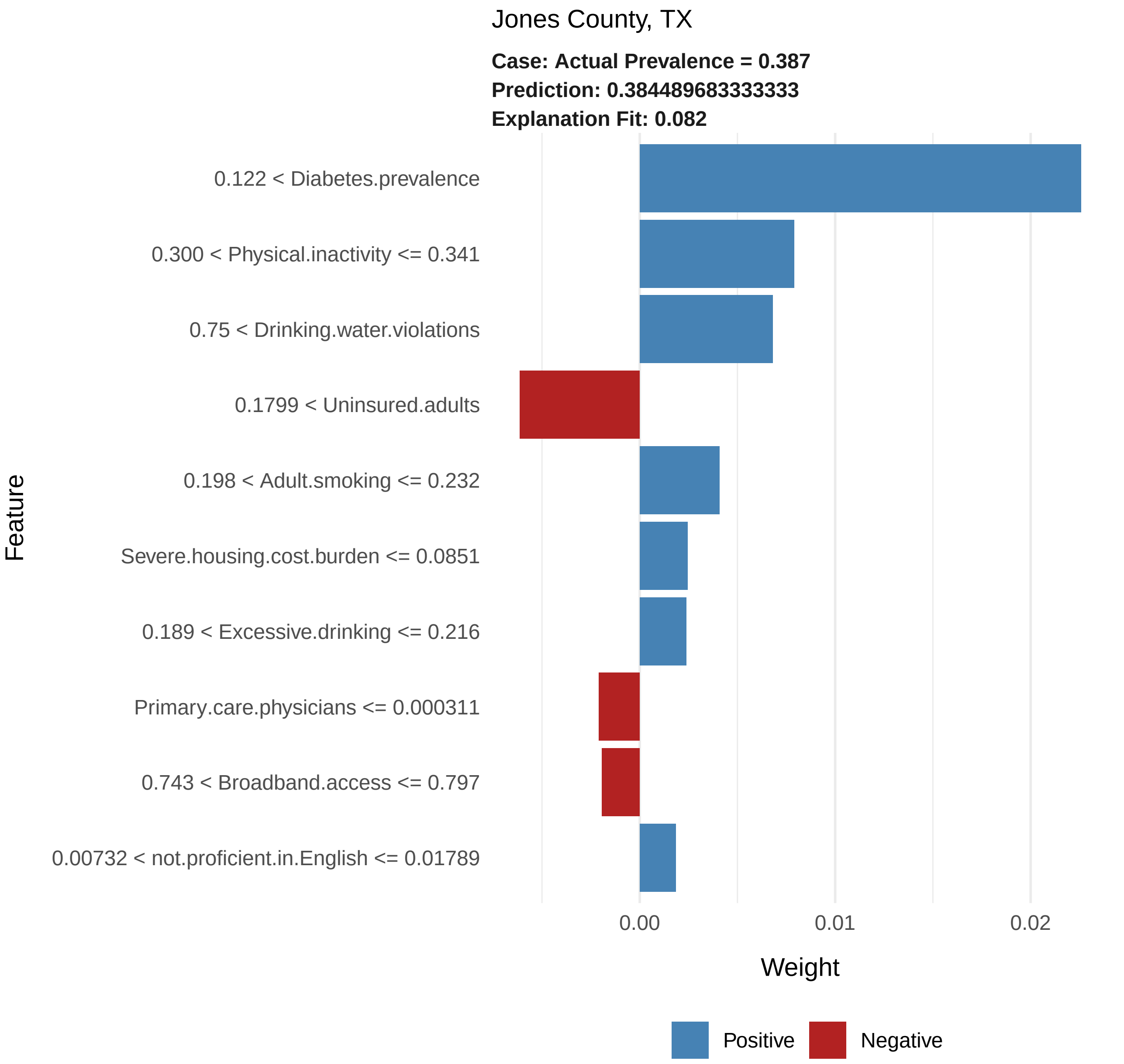
Jones County, TX
Case: Actual Prevalence = 0.387
Prediction: 0.384489683333333
Explanation Fit: 0.082
0.122 < Diabetes.prevalence
0.300 < Physical.inactivity <= 0.341
0.75 < Drinking.water.violations
0.1799 < Uninsured.adults
0.198 < Adult.smoking <= 0.232
Severe.housing.cost.burden <= 0.0851
0.189 < Excessive.drinking <= 0.216
Primary.care.physicians <= 0.000311
0.743 < Broadband.access <= 0.797
0.00732 < not.proficient.in.English <= 0.01789
Feature
0.00
0.01
0.02
Weight
Positive
Negative

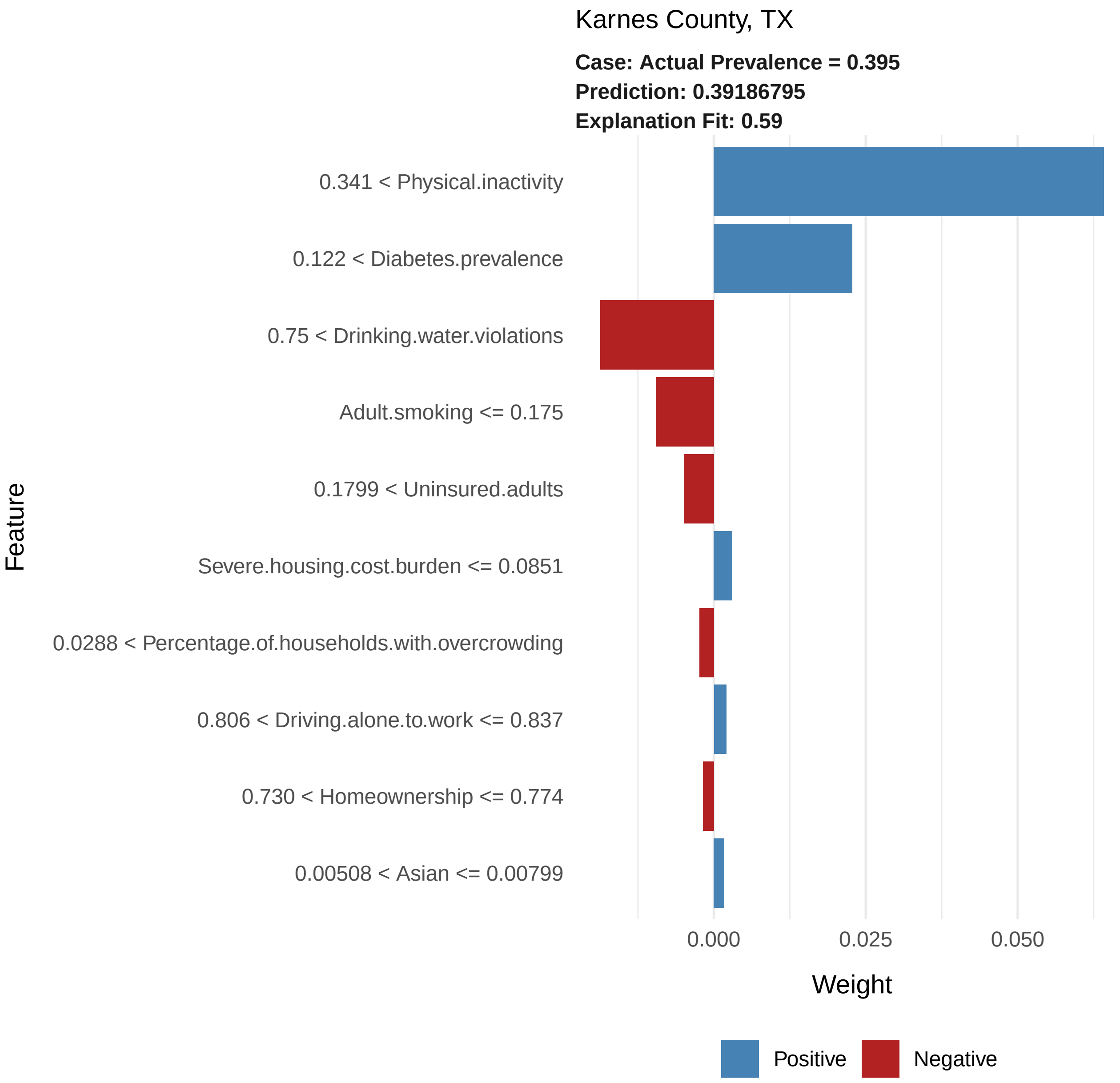
Karnes County, TX
Case: Actual Prevalence = 0.395
Prediction: 0.39186795
Explanation Fit: 0.59
0.341 < Physical.inactivity
0.122 < Diabetes.prevalence
0.75 < Drinking.water.violations
Adult.smoking <= 0.175
0.1799 < Uninsured.adults
Severe.housing.cost.burden <= 0.0851
0.0288 < Percentage.of.households.with.overcrowding
0.806 < Driving.alone.to.work <= 0.837
0.730 < Homeownership <= 0.774
0.00508 < Asian <= 0.00799
Feature
0.000
0.025
0.050
Weight
Positive
Negative

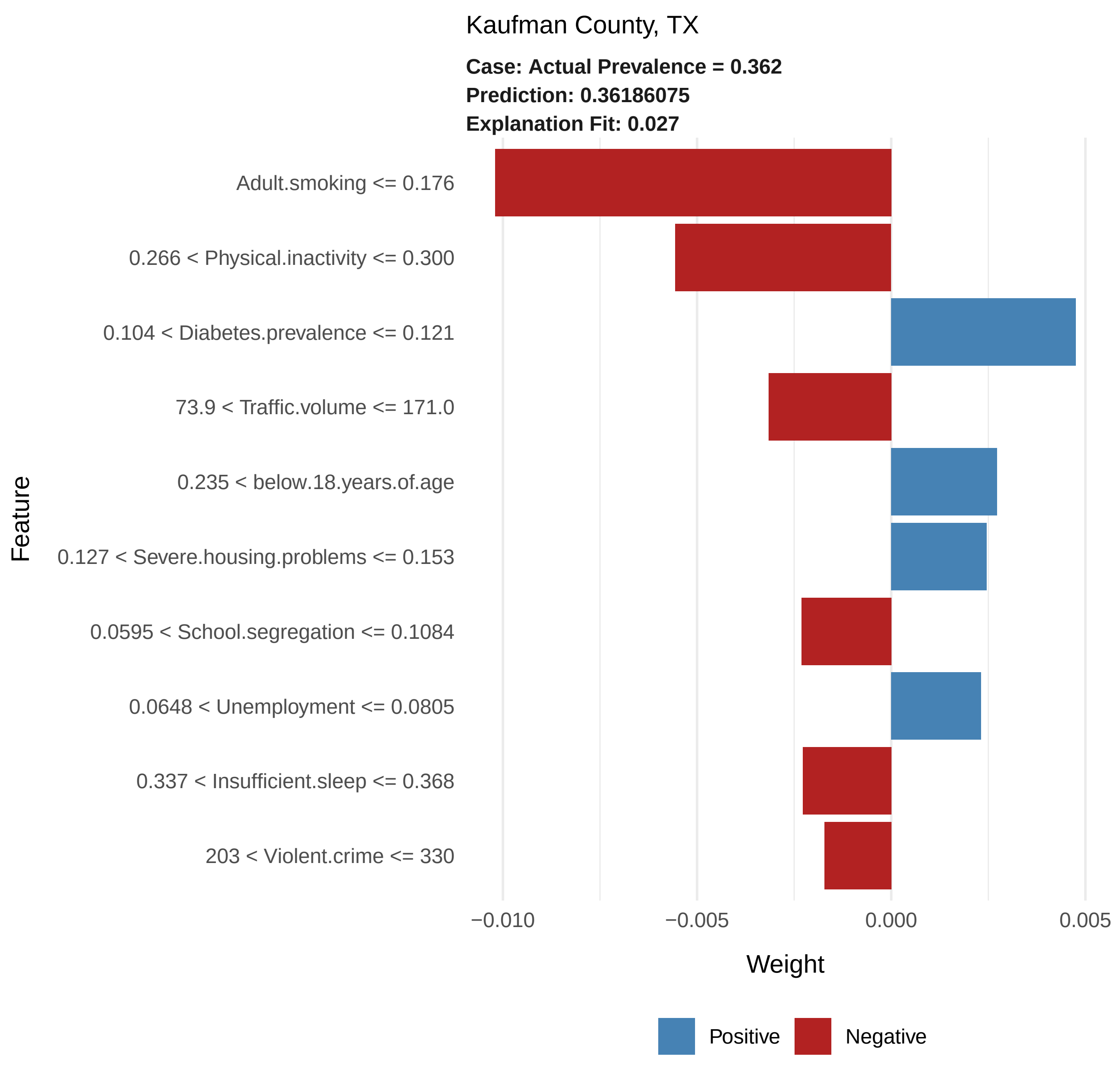

Kaufman County, TX
Case: Actual Prevalence = 0.362
Prediction: 0.36186075
Explanation Fit: 0.027
Adult.smoking <= 0.176
0.266 < Physical.inactivity <= 0.300
0.104 < Diabetes.prevalence <= 0.121
73.9 < Traffic.volume <= 171.0
0.235 < below.18.years.of.age
0.127 < Severe.housing.problems <= 0.153
0.0595 < School.segregation <= 0.1084
0.0648 < Unemployment <= 0.0805
0.337 < Insufficient.sleep <= 0.368
203 < Violent.crime <= 330
Feature
−0.010
−0.005
0.000
0.005
Weight
Positive
Negative

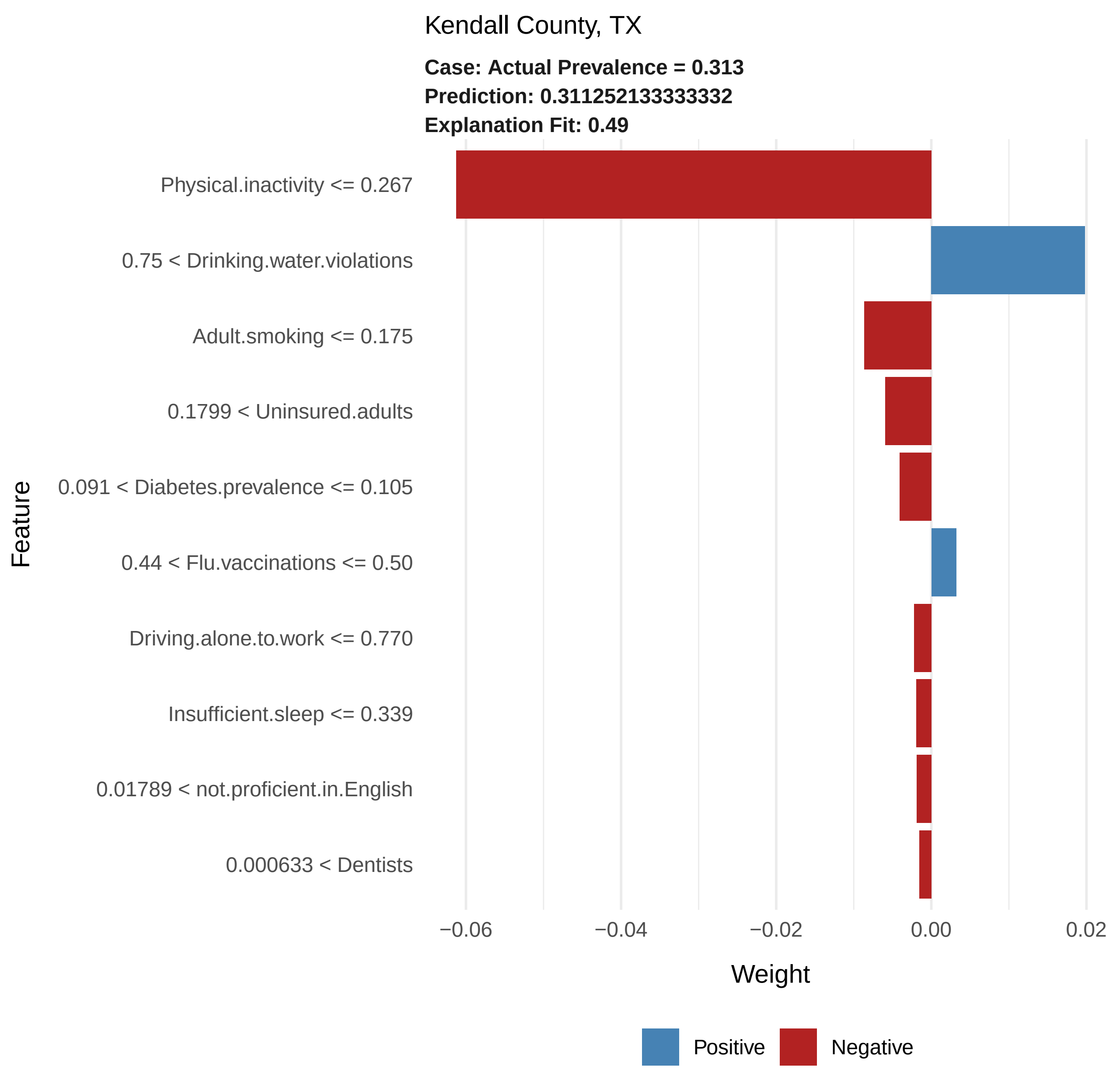
Kendall County, TX
Case: Actual Prevalence = 0.313
Prediction: 0.311252133333332
Explanation Fit: 0.49
Physical.inactivity <= 0.267
0.75 < Drinking.water.violations
Adult.smoking <= 0.175
0.1799 < Uninsured.adults
0.091 < Diabetes.prevalence <= 0.105
0.44 < Flu.vaccinations <= 0.50
Driving.alone.to.work <= 0.770
Insufficient.sleep <= 0.339
0.01789 < not.proficient.in.English
0.000633 < Dentists
Feature
−0.06
−0.04
−0.02
0.00
0.02
Weight
Positive
Negative

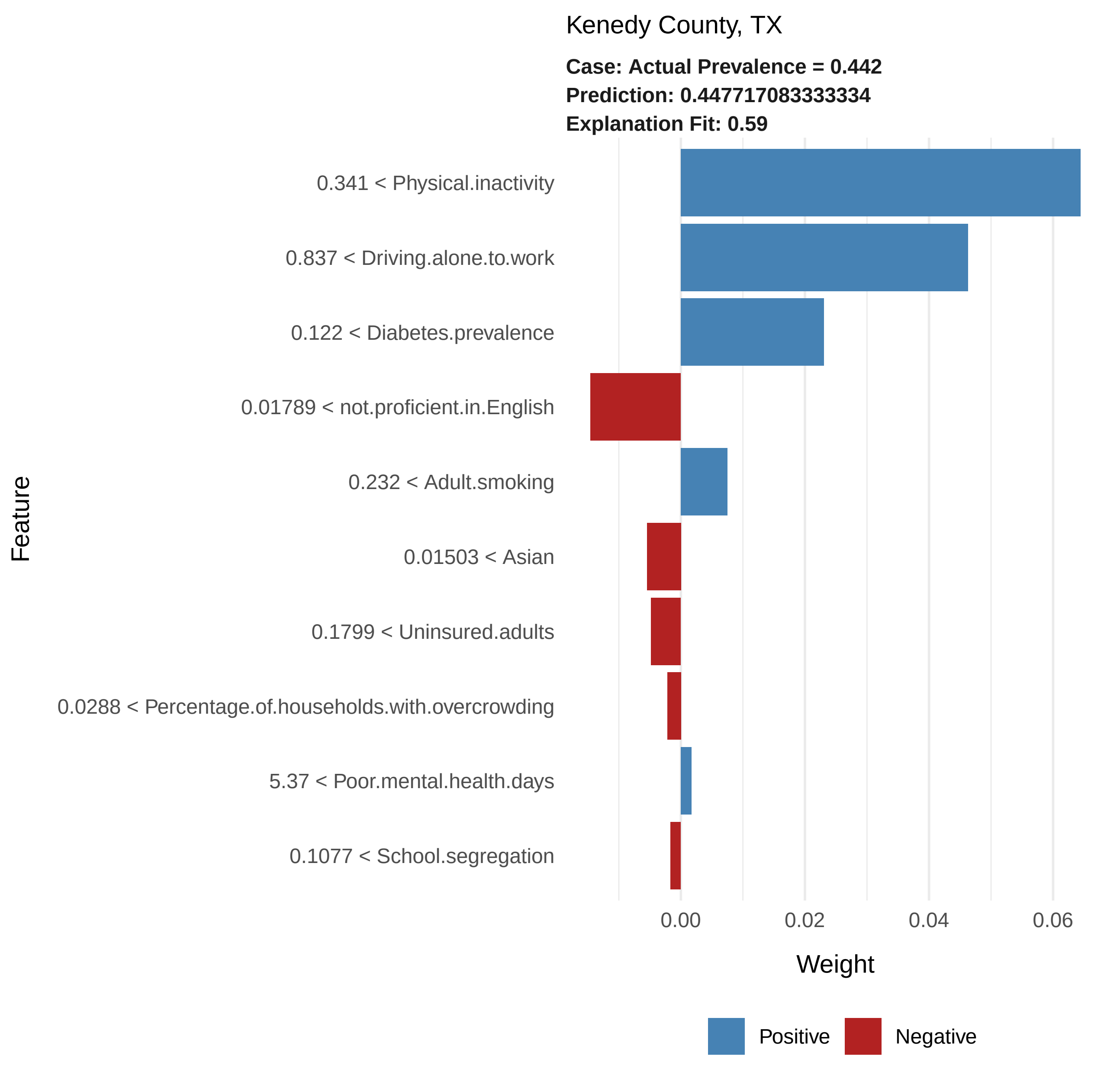
Kenedy County, TX
Case: Actual Prevalence = 0.442
Prediction: 0.447717083333334
Explanation Fit: 0.59
0.341 < Physical.inactivity
0.837 < Driving.alone.to.work
0.122 < Diabetes.prevalence
0.01789 < not.proficient.in.English
0.232 < Adult.smoking
0.01503 < Asian
0.1799 < Uninsured.adults
0.0288 < Percentage.of.households.with.overcrowding
5.37 < Poor.mental.health.days
0.1077 < School.segregation
Feature
0.00
0.02
0.04
0.06
Weight
Positive
Negative

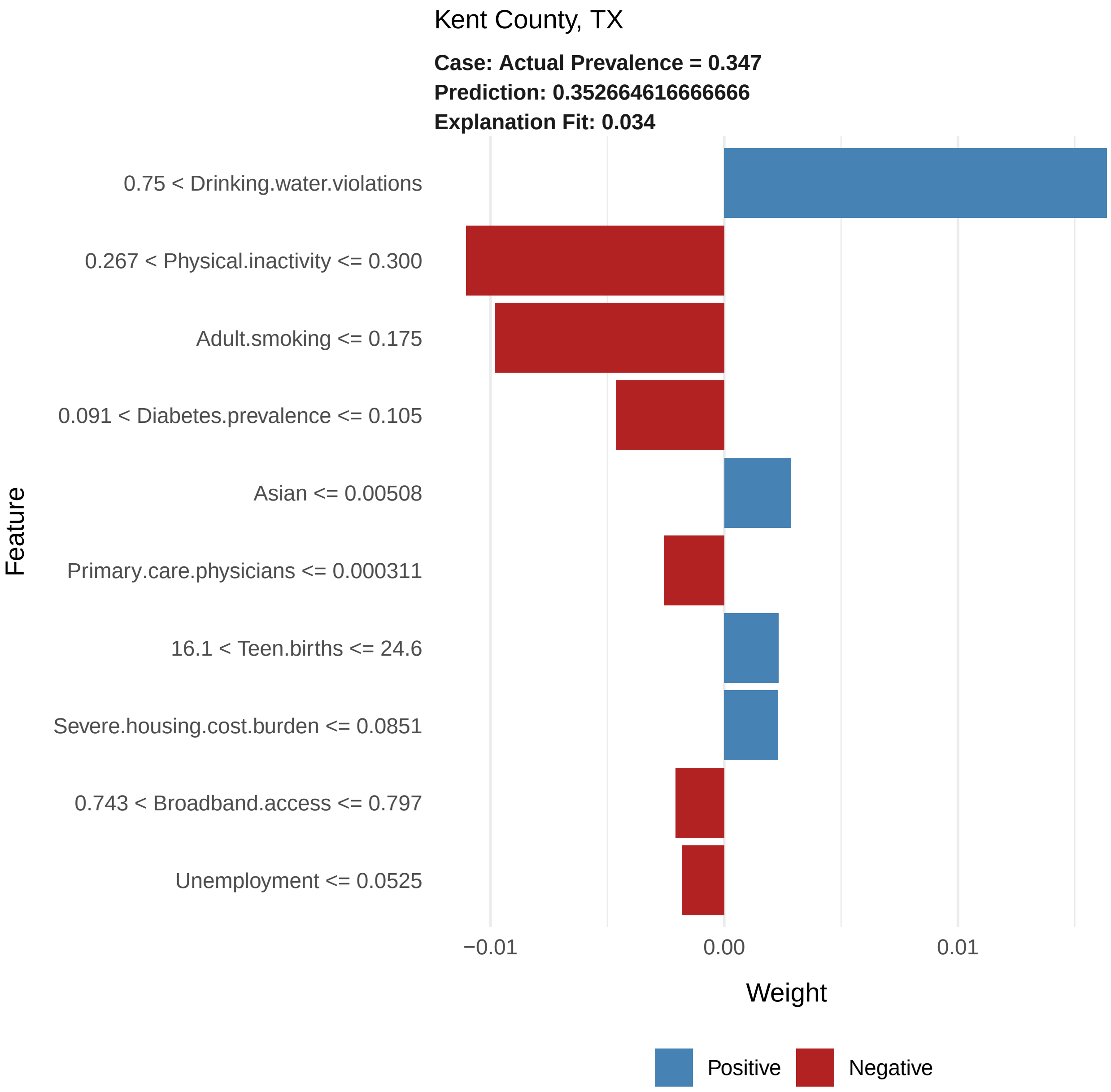

Kent County, TX
Case: Actual Prevalence = 0.347
Prediction: 0.352664616666666
Explanation Fit: 0.034
0.75 < Drinking.water.violations
0.267 < Physical.inactivity <= 0.300
Adult.smoking <= 0.175
0.091 < Diabetes.prevalence <= 0.105
Asian <= 0.00508
Primary.care.physicians <= 0.000311
16.1 < Teen.births <= 24.6
Severe.housing.cost.burden <= 0.0851
0.743 < Broadband.access <= 0.797
Unemployment <= 0.0525
Feature
−0.01
0.00
0.01
Weight
Positive
Negative

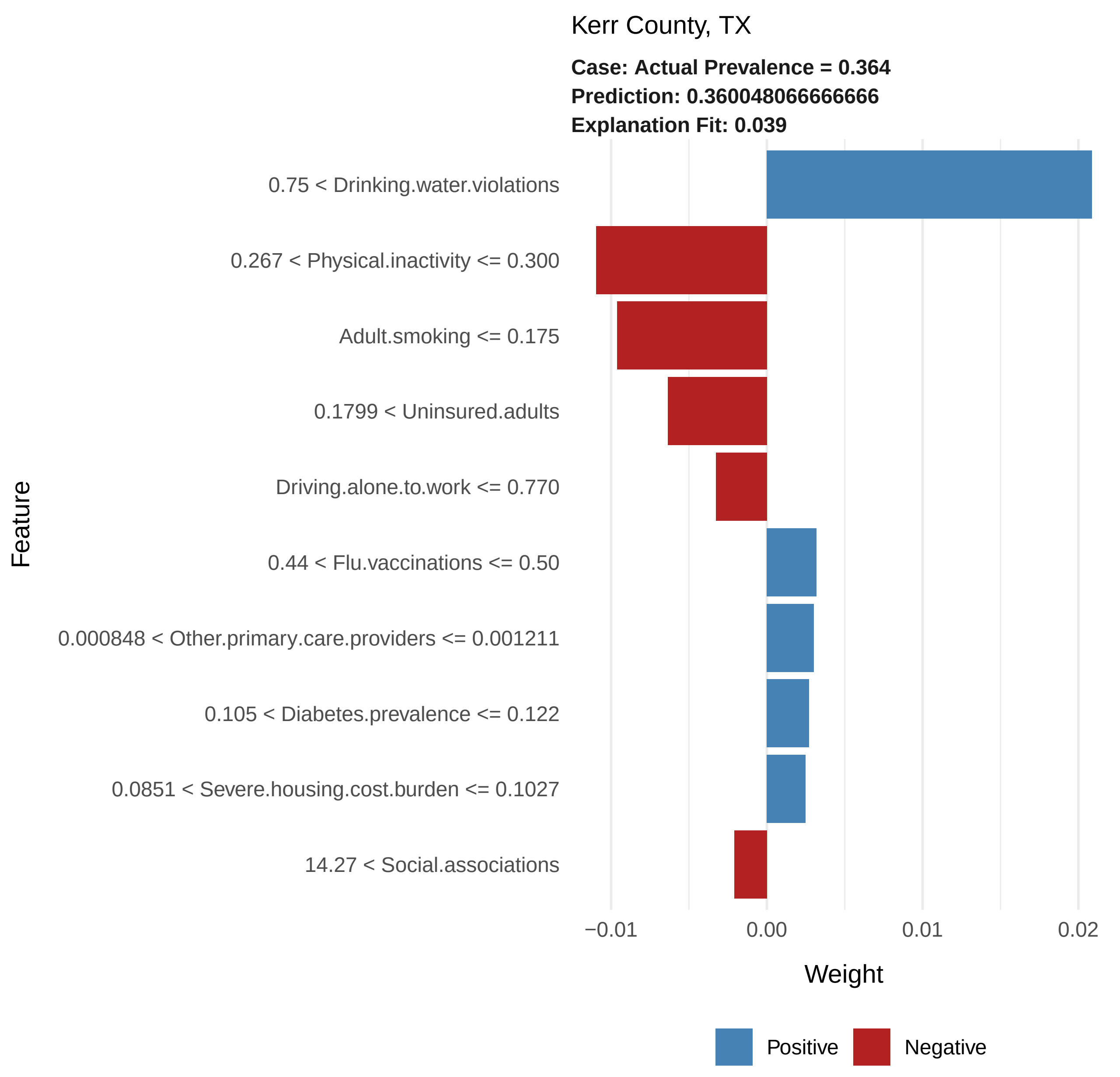

Kerr County, TX
Case: Actual Prevalence = 0.364
Prediction: 0.360048066666666
Explanation Fit: 0.039
0.75 < Drinking.water.violations
0.267 < Physical.inactivity <= 0.300
Adult.smoking <= 0.175
0.1799 < Uninsured.adults
Driving.alone.to.work <= 0.770
0.44 < Flu.vaccinations <= 0.50
0.000848 < Other.primary.care.providers <= 0.001211
0.105 < Diabetes.prevalence <= 0.122
0.0851 < Severe.housing.cost.burden <= 0.1027
14.27 < Social.associations
Feature
−0.01
0.00
0.01
0.02
Weight
Positive
Negative

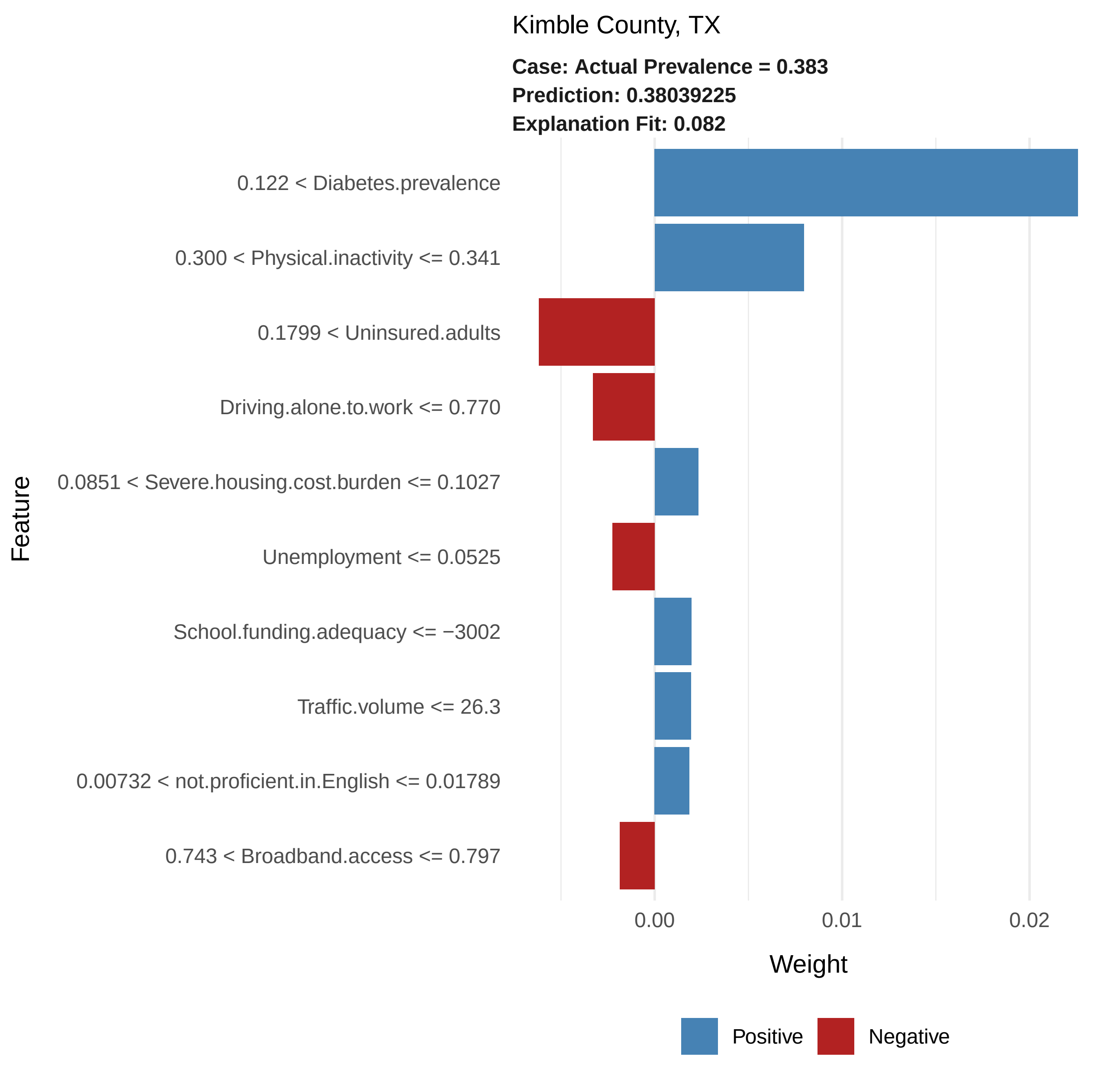
Kimble County, TX
Case: Actual Prevalence = 0.383
Prediction: 0.38039225
Explanation Fit: 0.082
0.122 < Diabetes.prevalence
0.300 < Physical.inactivity <= 0.341
0.1799 < Uninsured.adults
Driving.alone.to.work <= 0.770
0.0851 < Severe.housing.cost.burden <= 0.1027
Unemployment <= 0.0525
School.funding.adequacy <= −3002
Traffic.volume <= 26.3
0.00732 < not.proficient.in.English <= 0.01789
0.743 < Broadband.access <= 0.797
Feature
0.00
0.01
0.02
Weight
Positive
Negative

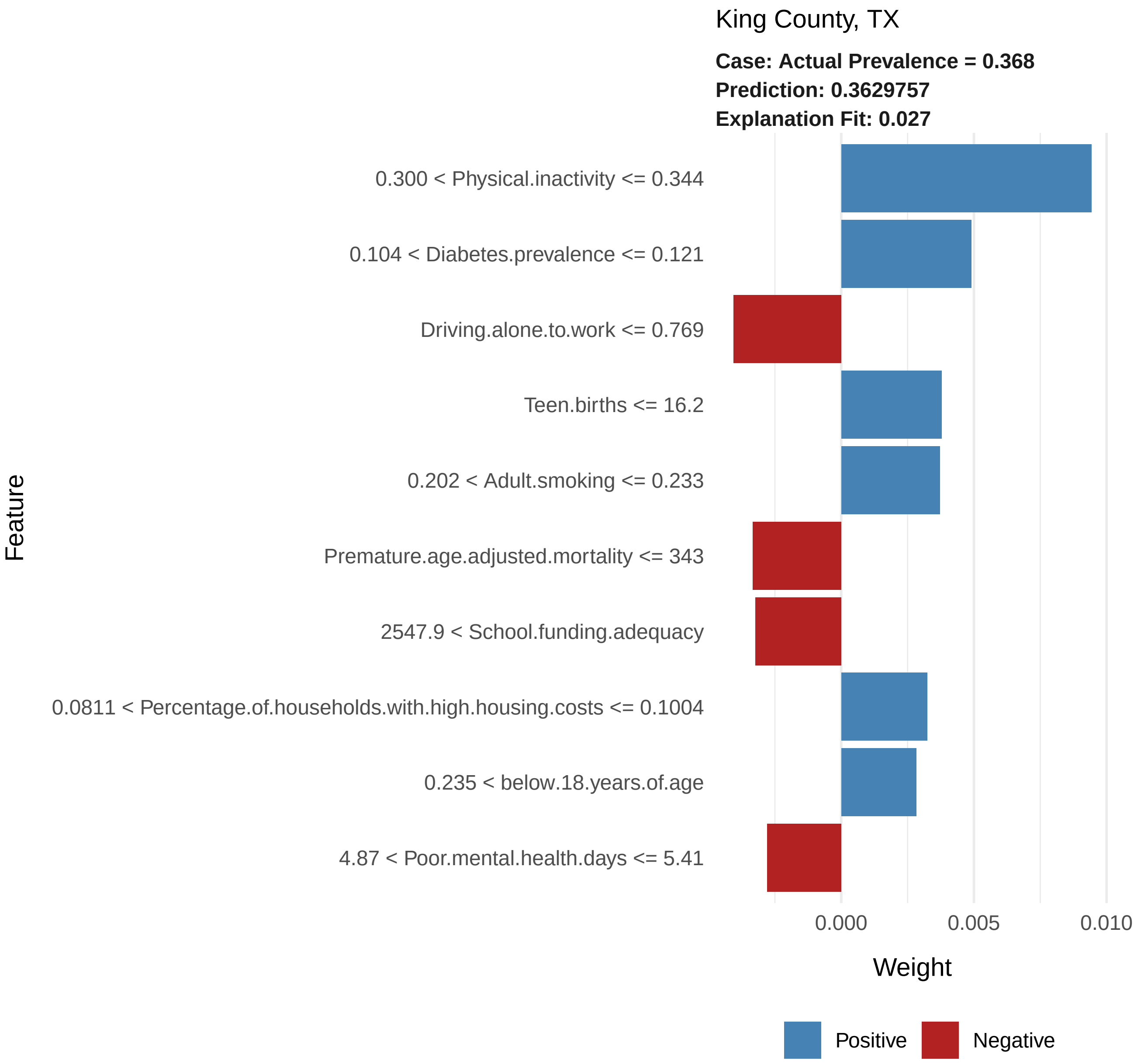
King County, TX
Case: Actual Prevalence = 0.368
Prediction: 0.3629757
Explanation Fit: 0.027
0.300 < Physical.inactivity <= 0.344
0.104 < Diabetes.prevalence <= 0.121
Driving.alone.to.work <= 0.769
Teen.births <= 16.2
0.202 < Adult.smoking <= 0.233
Premature.age.adjusted.mortality <= 343
2547.9 < School.funding.adequacy
0.0811 < Percentage.of.households.with.high.housing.costs <= 0.1004
0.235 < below.18.years.of.age
4.87 < Poor.mental.health.days <= 5.41
Feature
0.000
0.005
0.010
Weight
Positive
Negative

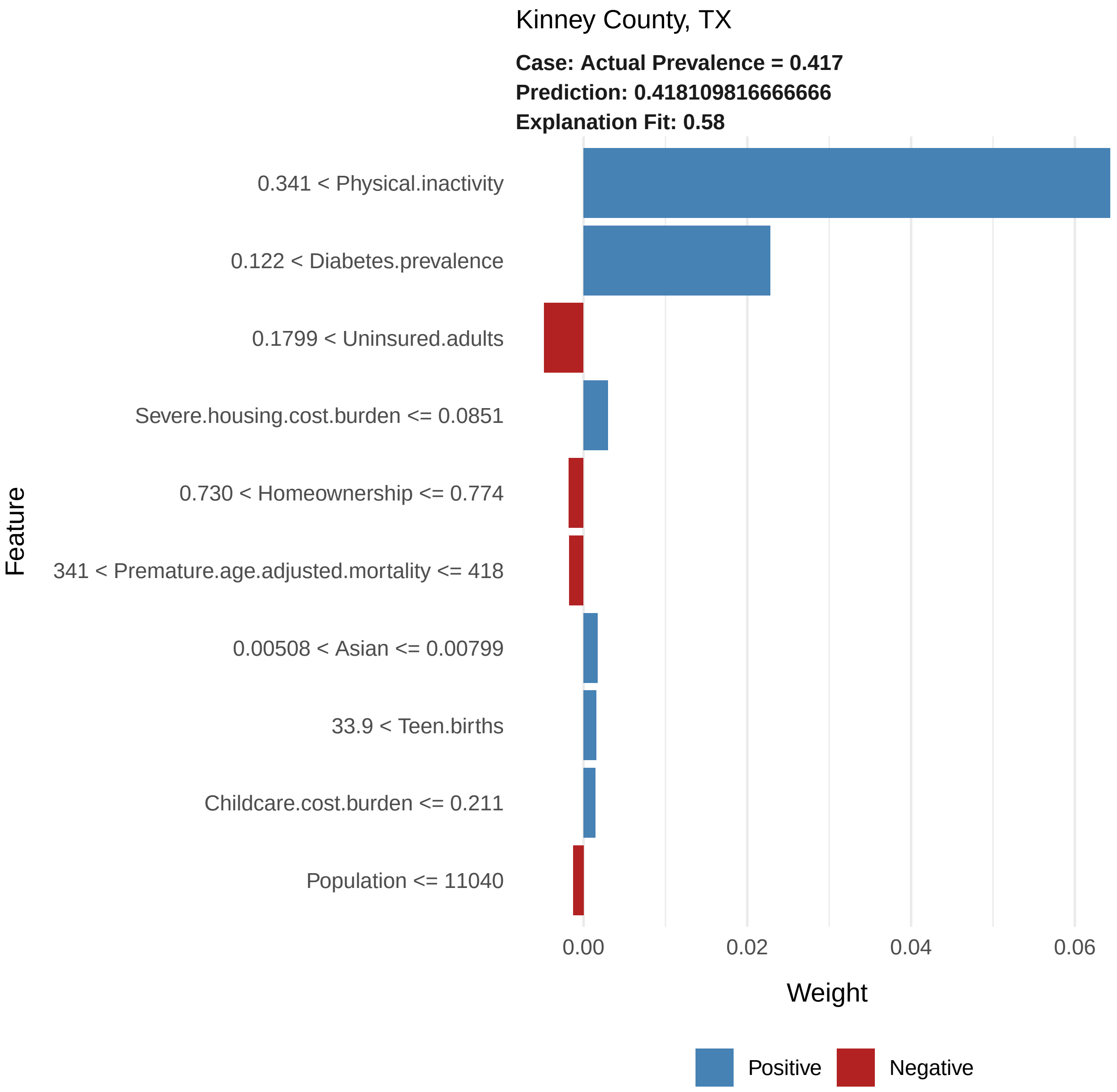

Kinney County, TX
Case: Actual Prevalence = 0.417
Prediction: 0.418109816666666
Explanation Fit: 0.58
0.341 < Physical.inactivity
0.122 < Diabetes.prevalence
0.1799 < Uninsured.adults
Severe.housing.cost.burden <= 0.0851
0.730 < Homeownership <= 0.774
341 < Premature.age.adjusted.mortality <= 418
0.00508 < Asian <= 0.00799
33.9 < Teen.births
Childcare.cost.burden <= 0.211
Population <= 11040
Feature
0.00
0.02
0.04
0.06
Weight
Positive
Negative

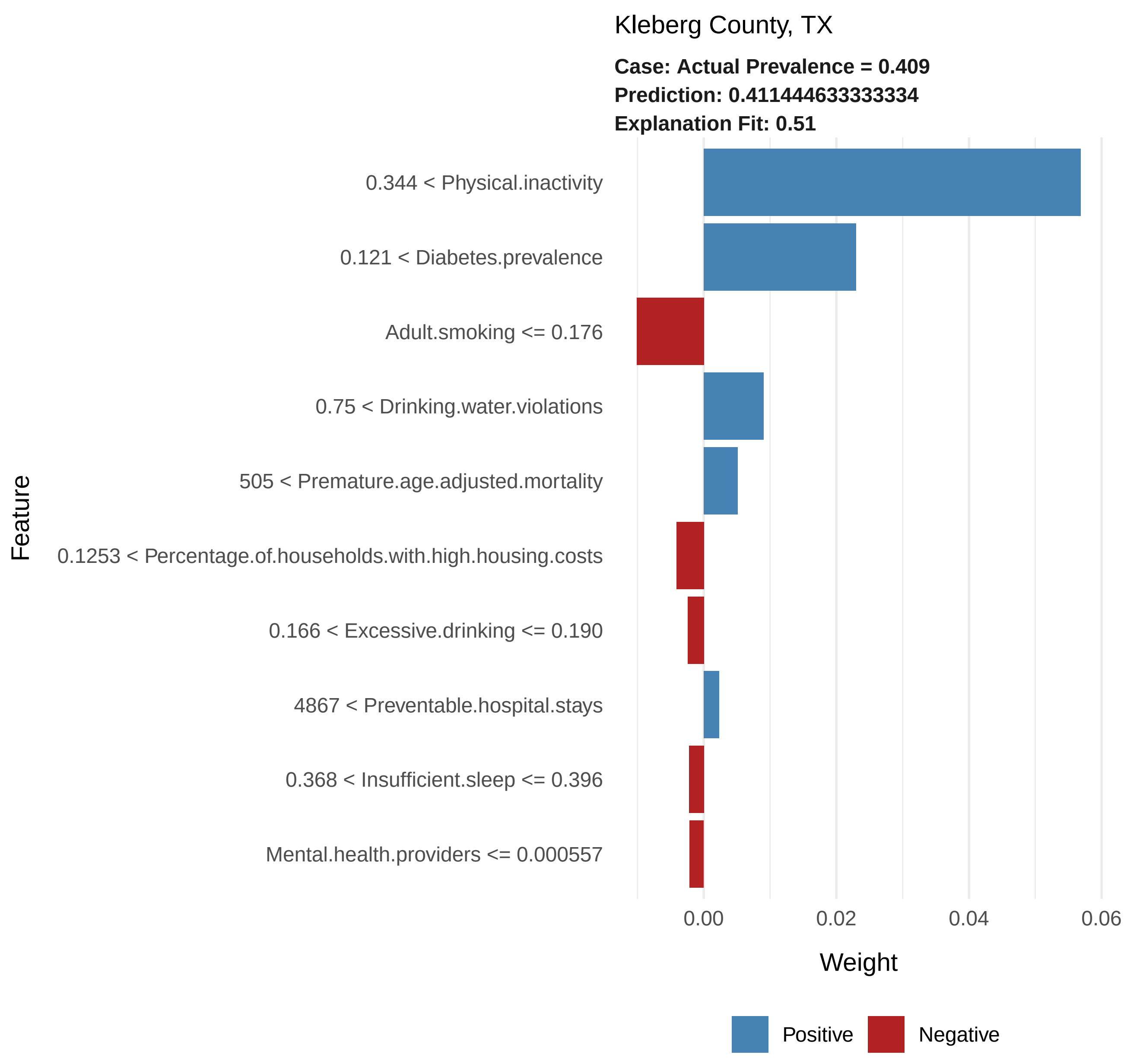

Kleberg County, TX
Case: Actual Prevalence = 0.409
Prediction: 0.411444633333334
Explanation Fit: 0.51
0.344 < Physical.inactivity
0.121 < Diabetes.prevalence
Adult.smoking <= 0.176
0.75 < Drinking.water.violations
505 < Premature.age.adjusted.mortality
0.1253 < Percentage.of.households.with.high.housing.costs
0.166 < Excessive.drinking <= 0.190
4867 < Preventable.hospital.stays
0.368 < Insufficient.sleep <= 0.396
Mental.health.providers <= 0.000557
Feature
0.00
0.02
0.04
0.06
Weight
Positive
Negative

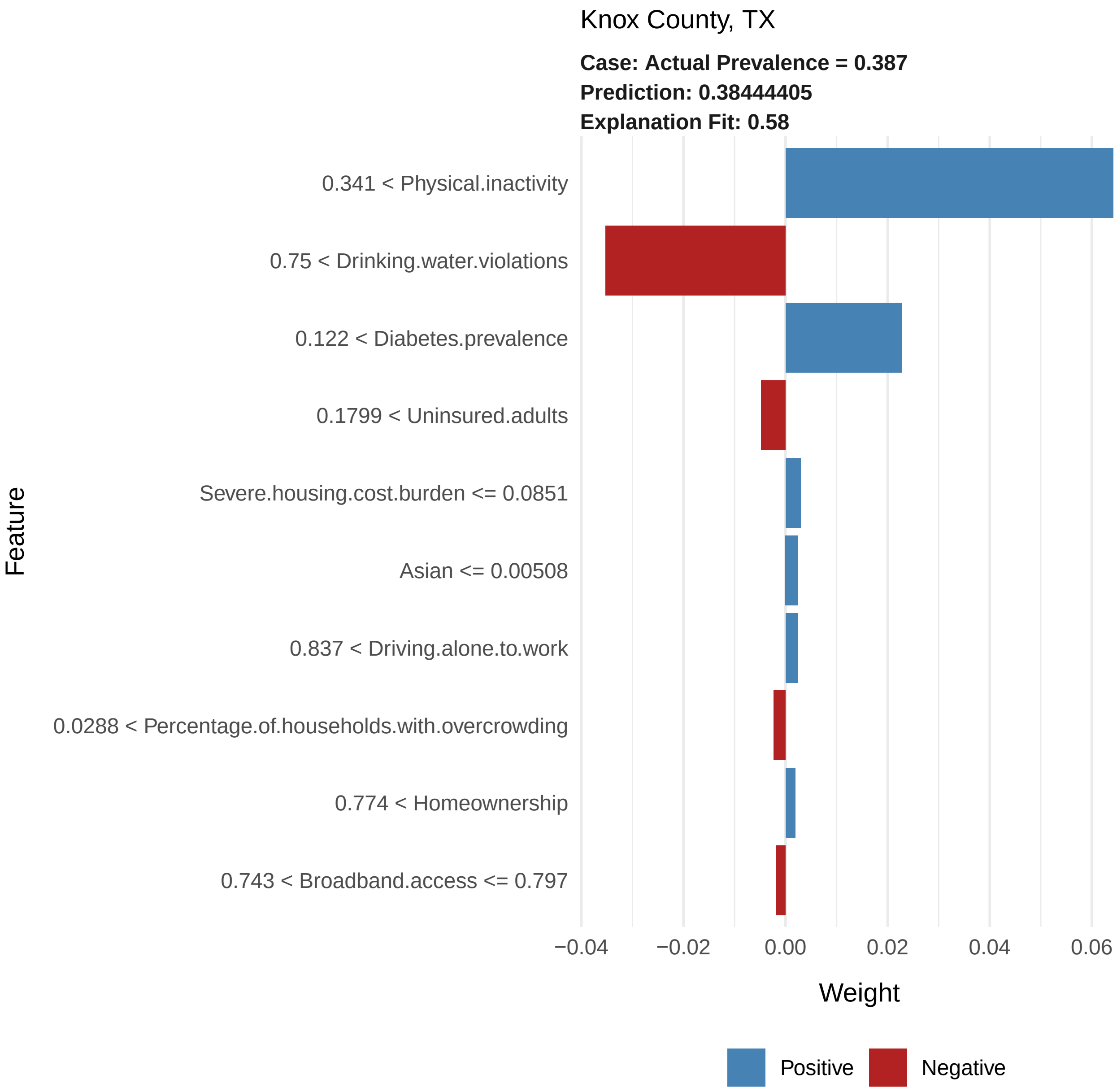

Knox County, TX
Case: Actual Prevalence = 0.387
Prediction: 0.38444405
Explanation Fit: 0.58
0.341 < Physical.inactivity
0.75 < Drinking.water.violations
0.122 < Diabetes.prevalence
0.1799 < Uninsured.adults
Severe.housing.cost.burden <= 0.0851
Asian <= 0.00508
0.837 < Driving.alone.to.work
0.0288 < Percentage.of.households.with.overcrowding
0.774 < Homeownership
0.743 < Broadband.access <= 0.797
Feature
−0.04
−0.02
0.00
0.02
0.04
0.06
Weight
Positive
Negative

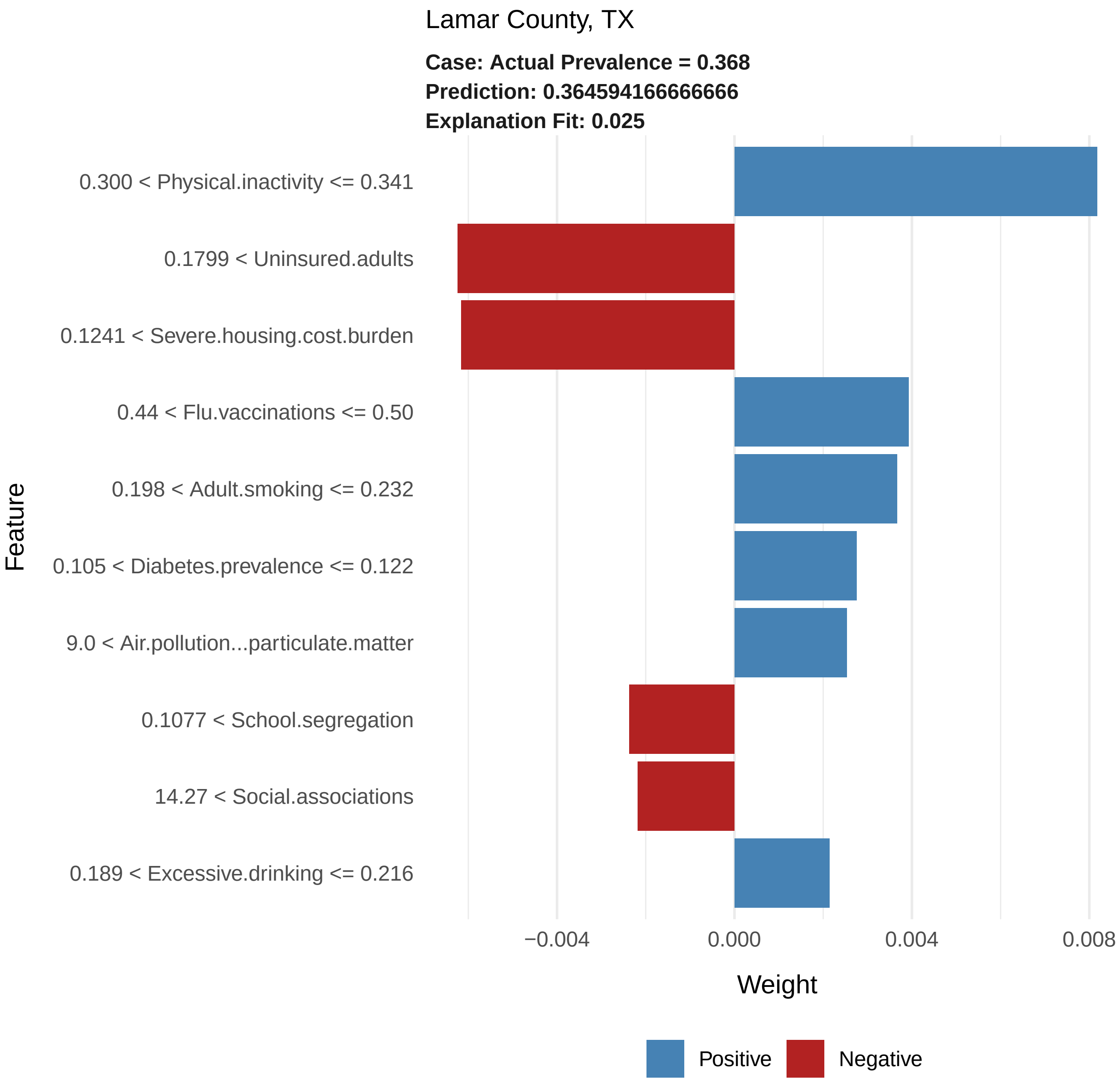
Lamar County, TX
Case: Actual Prevalence = 0.368
Prediction: 0.364594166666666
Explanation Fit: 0.025
0.300 < Physical.inactivity <= 0.341
0.1799 < Uninsured.adults
0.1241 < Severe.housing.cost.burden
0.44 < Flu.vaccinations <= 0.50
0.198 < Adult.smoking <= 0.232
0.105 < Diabetes.prevalence <= 0.122
9.0 < Air.pollution...particulate.matter
0.1077 < School.segregation
14.27 < Social.associations
0.189 < Excessive.drinking <= 0.216
Feature
−0.004
0.000
0.004
0.008
Weight
Positive
Negative

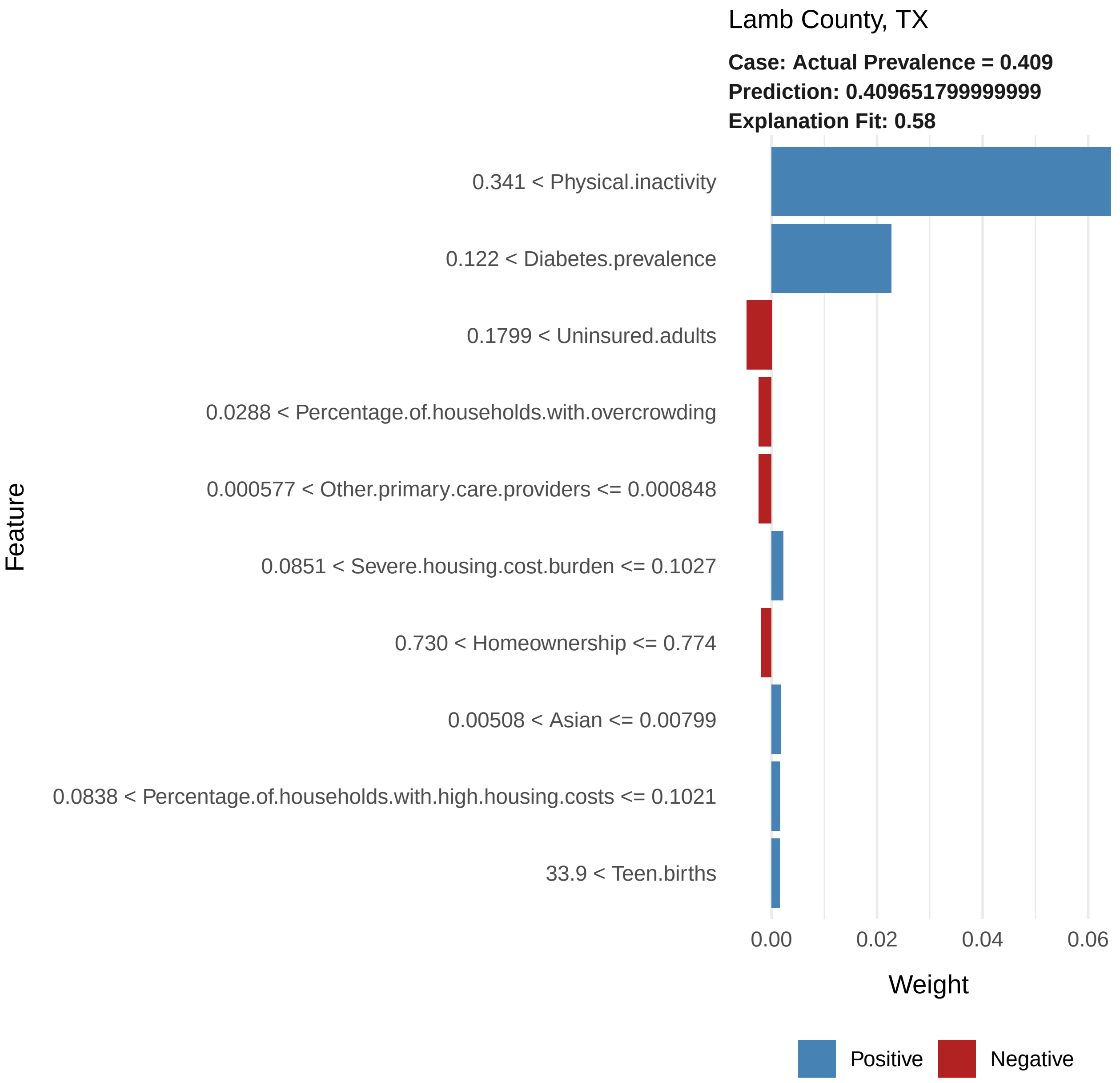
Lamb County, TX
Case: Actual Prevalence = 0.409
Prediction: 0.409651799999999
Explanation Fit: 0.58
0.341 < Physical.inactivity
0.122 < Diabetes.prevalence
0.1799 < Uninsured.adults
0.0288 < Percentage.of.households.with.overcrowding
0.000577 < Other.primary.care.providers <= 0.000848
0.0851 < Severe.housing.cost.burden <= 0.1027
0.730 < Homeownership <= 0.774
0.00508 < Asian <= 0.00799
0.0838 < Percentage.of.households.with.high.housing.costs <= 0.1021
33.9 < Teen.births
Feature
0.00
0.02
0.04
0.06
Weight
Positive
Negative

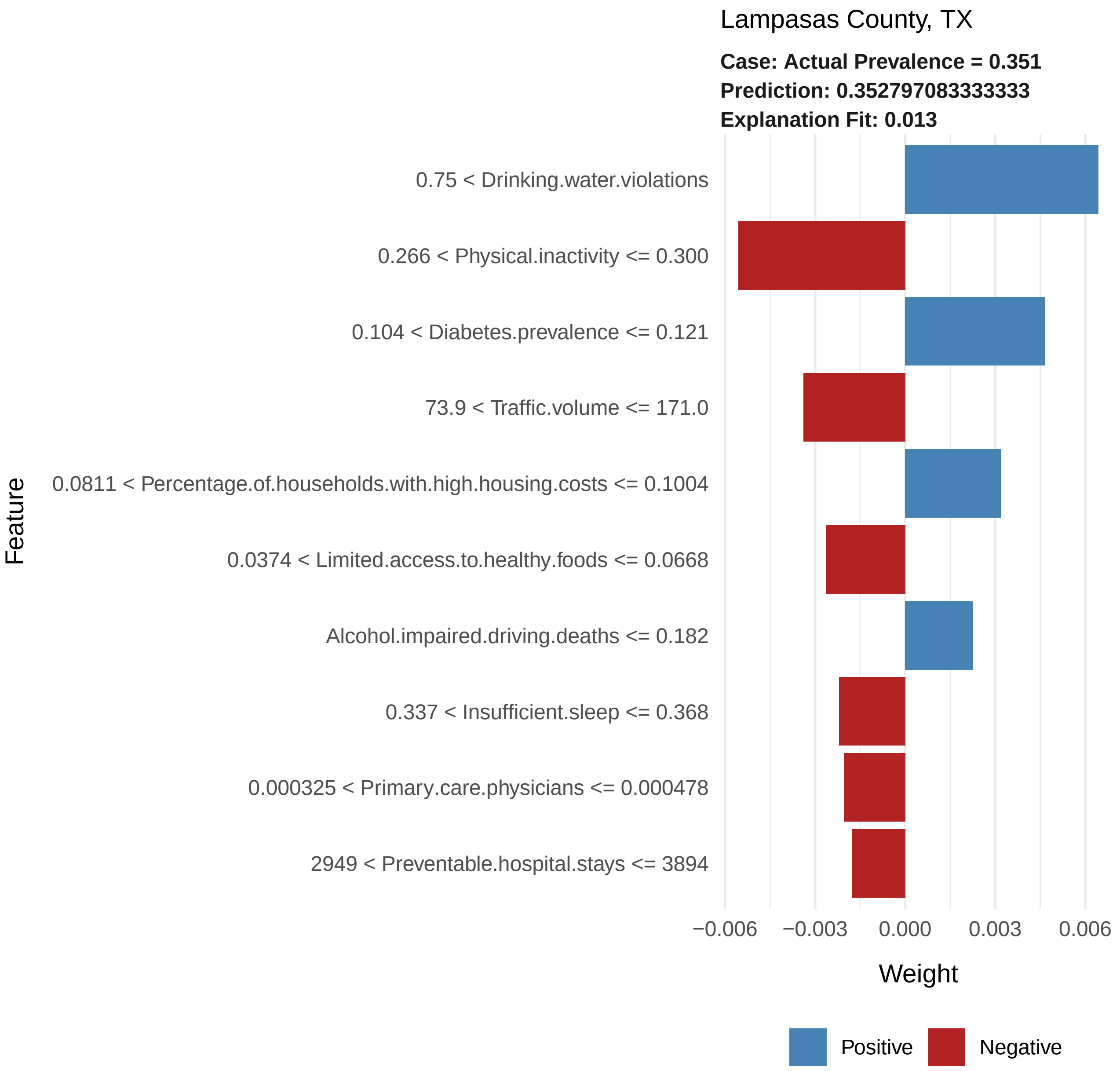
Lampasas County, TX
Case: Actual Prevalence = 0.351
Prediction: 0.352797083333333
Explanation Fit: 0.013
0.75 < Drinking.water.violations
0.266 < Physical.inactivity <= 0.300
0.104 < Diabetes.prevalence <= 0.121
73.9 < Traffic.volume <= 171.0
0.0811 < Percentage.of.households.with.high.housing.costs <= 0.1004
0.0374 < Limited.access.to.healthy.foods <= 0.0668
Alcohol.impaired.driving.deaths <= 0.182
0.337 < Insufficient.sleep <= 0.368
0.000325 < Primary.care.physicians <= 0.000478
2949 < Preventable.hospital.stays <= 3894
Feature
−0.006
−0.003
0.000
0.003
0.006
Weight
Positive
Negative

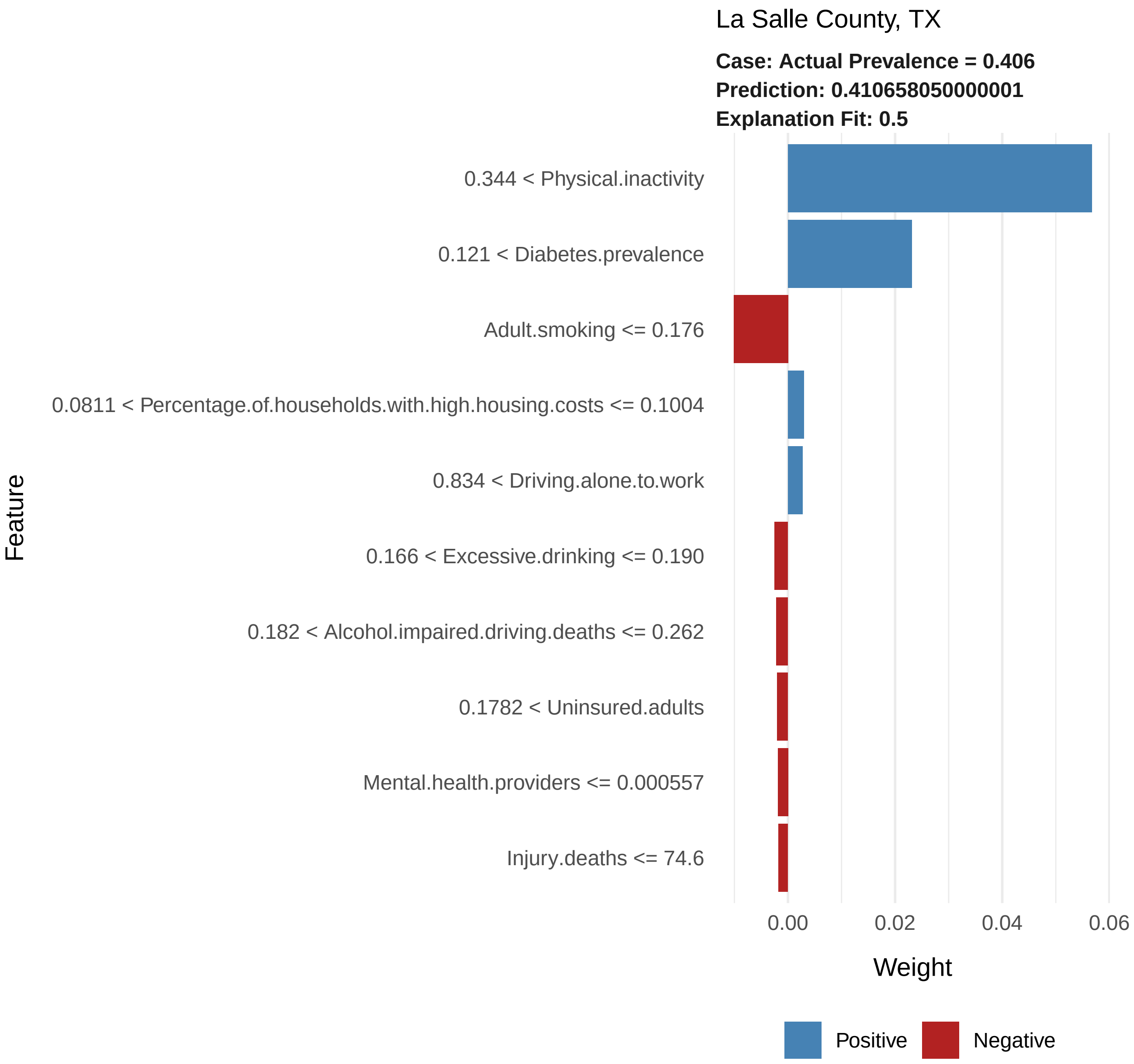
La Salle County, TX
Case: Actual Prevalence = 0.406
Prediction: 0.410658050000001
Explanation Fit: 0.5
0.344 < Physical.inactivity
0.121 < Diabetes.prevalence
Adult.smoking <= 0.176
0.0811 < Percentage.of.households.with.high.housing.costs <= 0.1004
0.834 < Driving.alone.to.work
0.166 < Excessive.drinking <= 0.190
0.182 < Alcohol.impaired.driving.deaths <= 0.262
0.1782 < Uninsured.adults
Mental.health.providers <= 0.000557
Injury.deaths <= 74.6
Feature
0.00
0.02
0.04
0.06
Weight
Positive
Negative

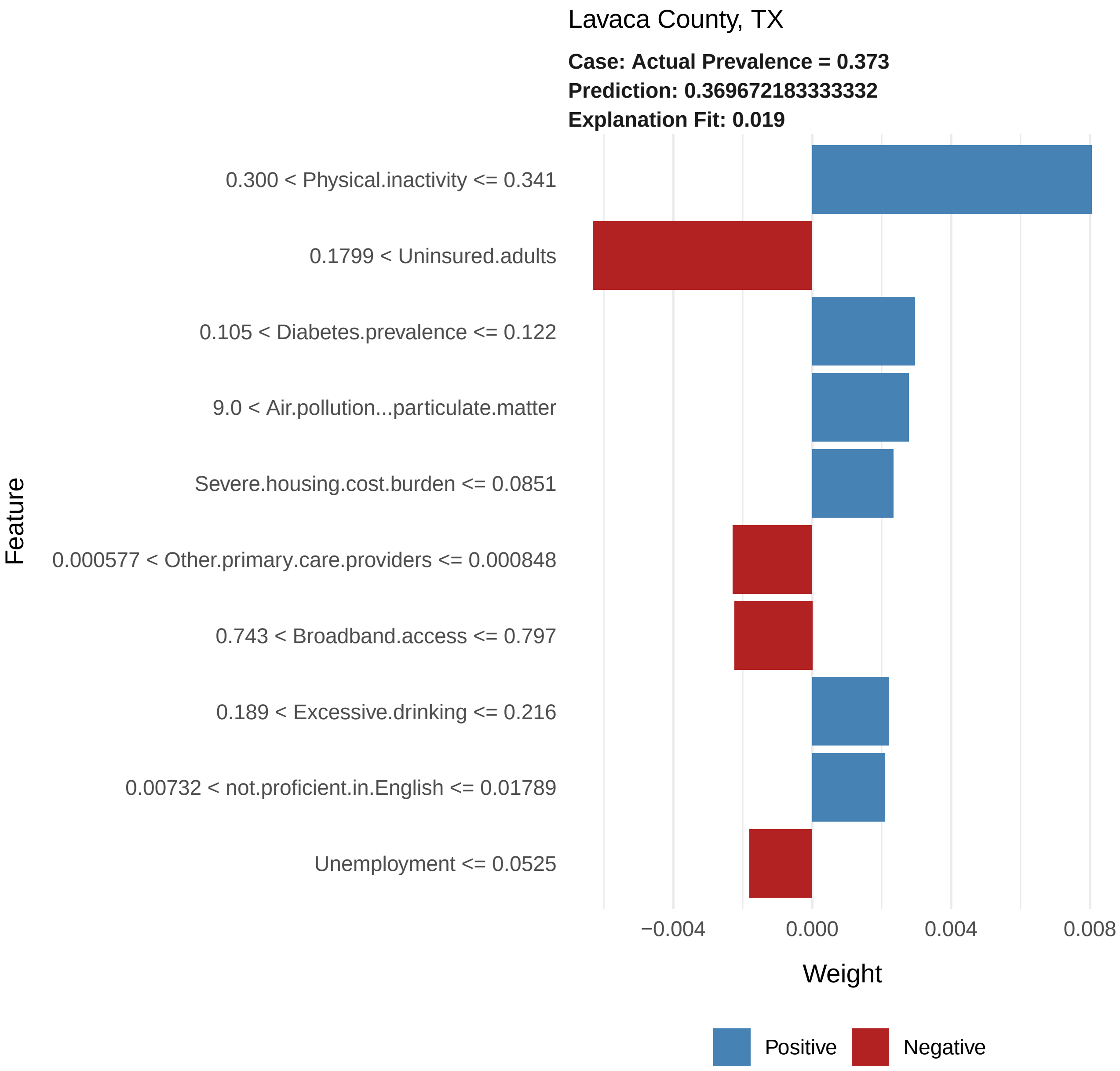

Lavaca County, TX
Case: Actual Prevalence = 0.373
Prediction: 0.369672183333332
Explanation Fit: 0.019
0.300 < Physical.inactivity <= 0.341
0.1799 < Uninsured.adults
0.105 < Diabetes.prevalence <= 0.122
9.0 < Air.pollution...particulate.matter
Severe.housing.cost.burden <= 0.0851
0.000577 < Other.primary.care.providers <= 0.000848
0.743 < Broadband.access <= 0.797
0.189 < Excessive.drinking <= 0.216
0.00732 < not.proficient.in.English <= 0.01789
Unemployment <= 0.0525
Feature
−0.004
0.000
0.004
0.008
Weight
Positive
Negative

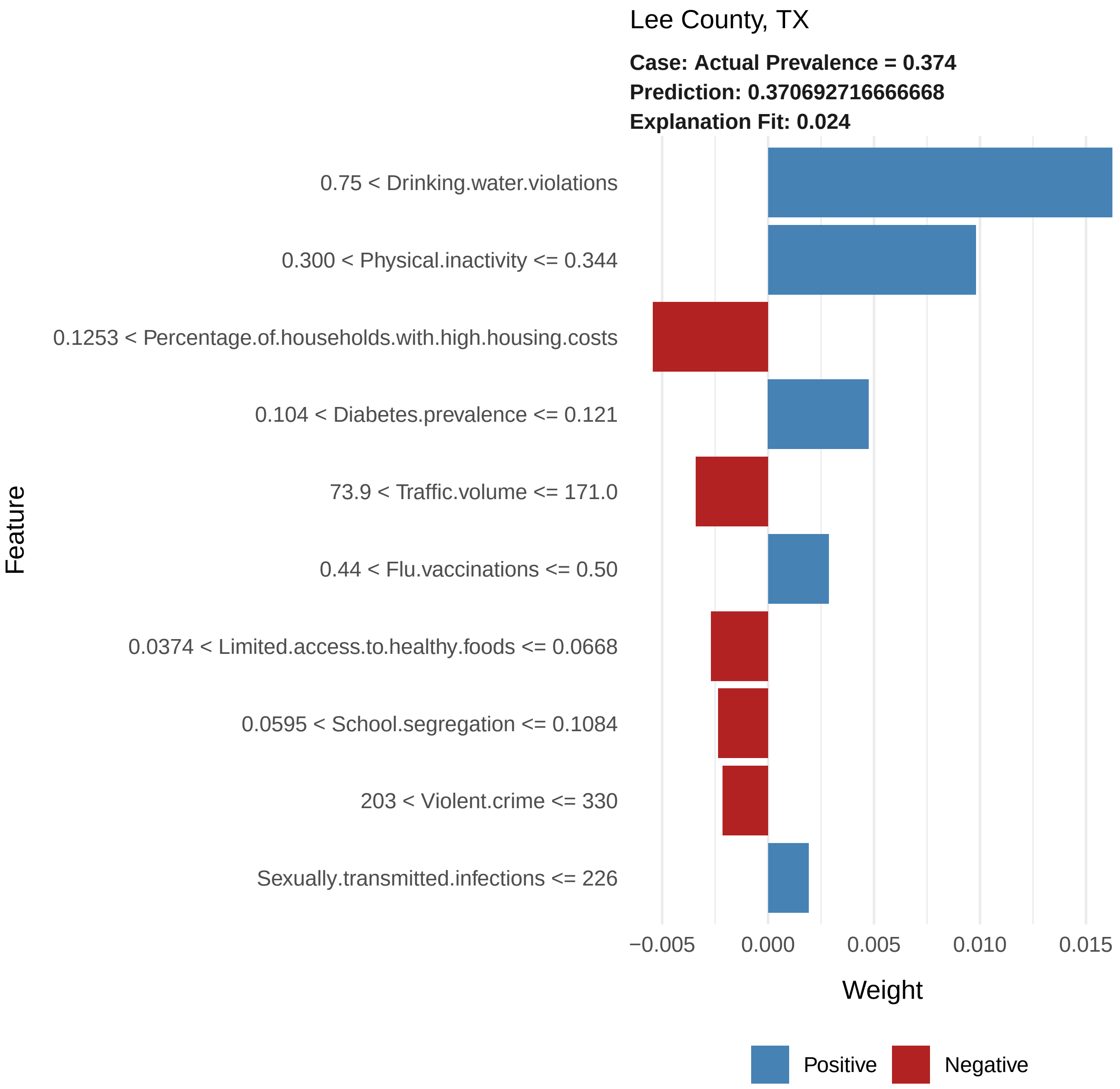

Lee County, TX
Case: Actual Prevalence = 0.374
Prediction: 0.370692716666668
Explanation Fit: 0.024
0.75 < Drinking.water.violations
0.300 < Physical.inactivity <= 0.344
0.1253 < Percentage.of.households.with.high.housing.costs
0.104 < Diabetes.prevalence <= 0.121
73.9 < Traffic.volume <= 171.0
0.44 < Flu.vaccinations <= 0.50
0.0374 < Limited.access.to.healthy.foods <= 0.0668
0.0595 < School.segregation <= 0.1084
203 < Violent.crime <= 330
Sexually.transmitted.infections <= 226
Feature
−0.005
0.000
0.005
0.010
0.015
Weight
Positive
Negative

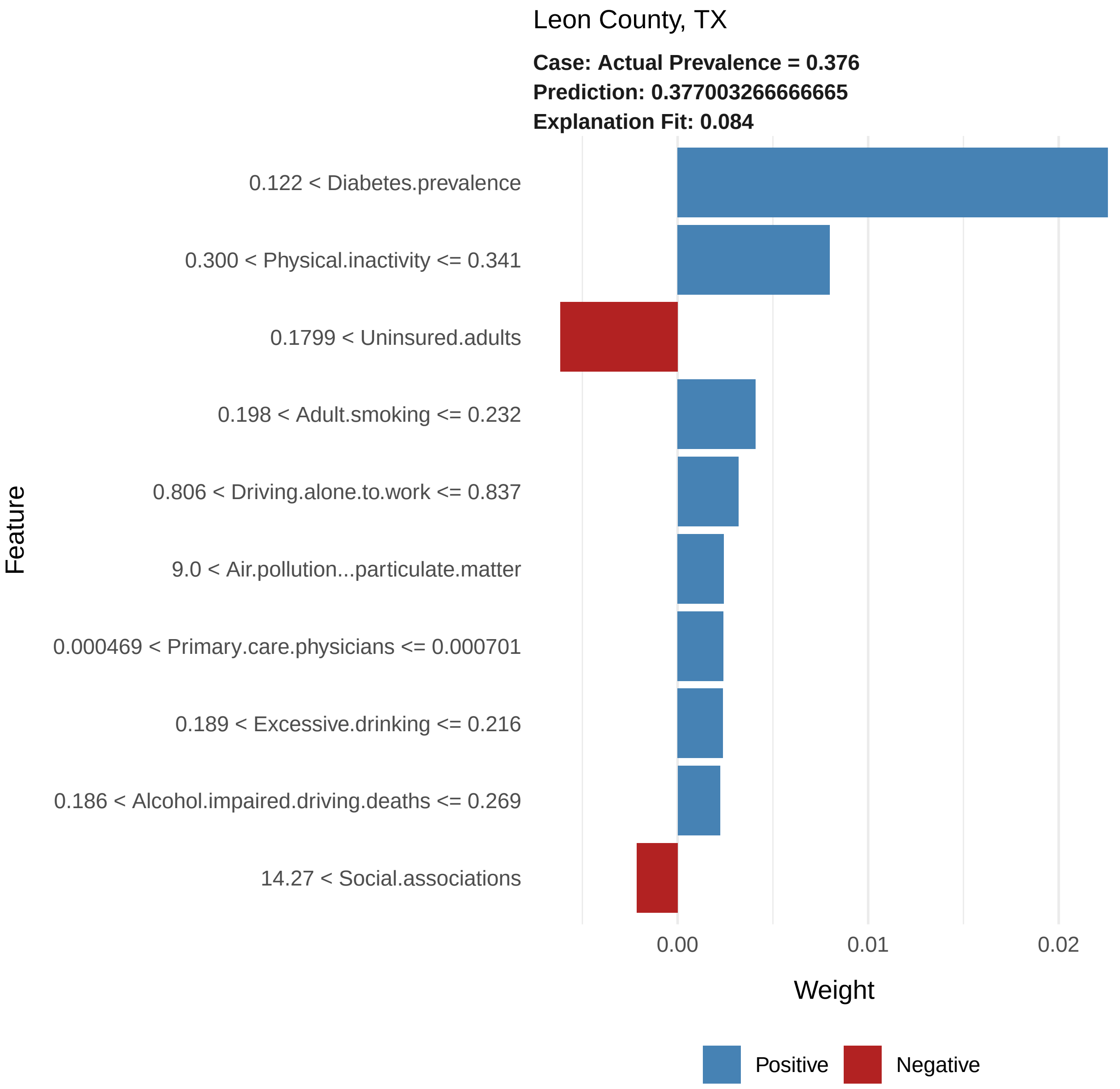
Leon County, TX
Case: Actual Prevalence = 0.376
Prediction: 0.377003266666665
Explanation Fit: 0.084
0.122 < Diabetes.prevalence
0.300 < Physical.inactivity <= 0.341
0.1799 < Uninsured.adults
0.198 < Adult.smoking <= 0.232
0.806 < Driving.alone.to.work <= 0.837
9.0 < Air.pollution...particulate.matter
0.000469 < Primary.care.physicians <= 0.000701
0.189 < Excessive.drinking <= 0.216
0.186 < Alcohol.impaired.driving.deaths <= 0.269
14.27 < Social.associations
Feature
0.00
0.01
0.02
Weight
Positive
Negative

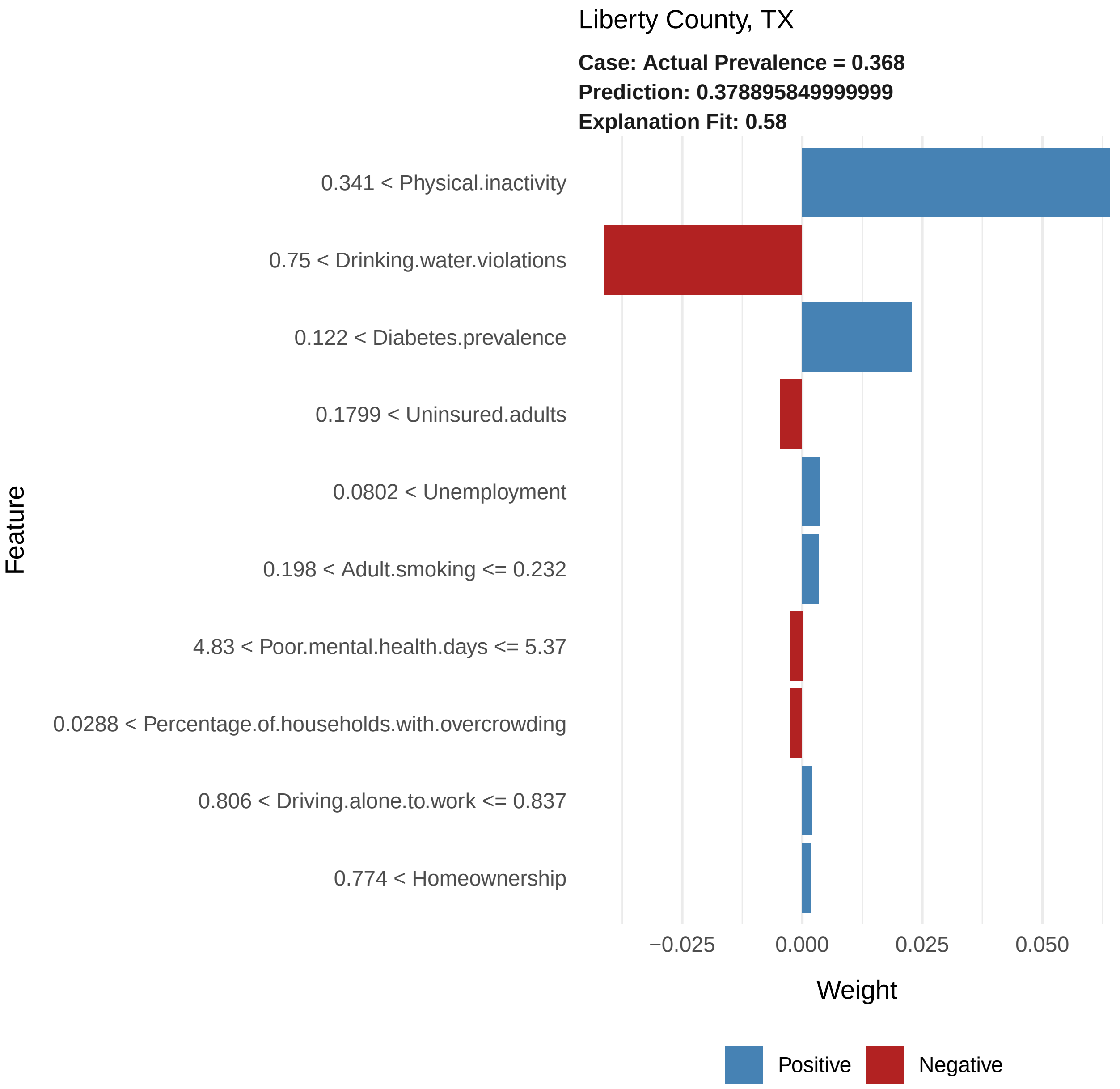
Liberty County, TX
Case: Actual Prevalence = 0.368
Prediction: 0.378895849999999
Explanation Fit: 0.58
0.341 < Physical.inactivity
0.75 < Drinking.water.violations
0.122 < Diabetes.prevalence
0.1799 < Uninsured.adults
0.0802 < Unemployment
0.198 < Adult.smoking <= 0.232
4.83 < Poor.mental.health.days <= 5.37
0.0288 < Percentage.of.households.with.overcrowding
0.806 < Driving.alone.to.work <= 0.837
0.774 < Homeownership
Feature
−0.025
0.000
0.025
0.050
Weight
Positive
Negative

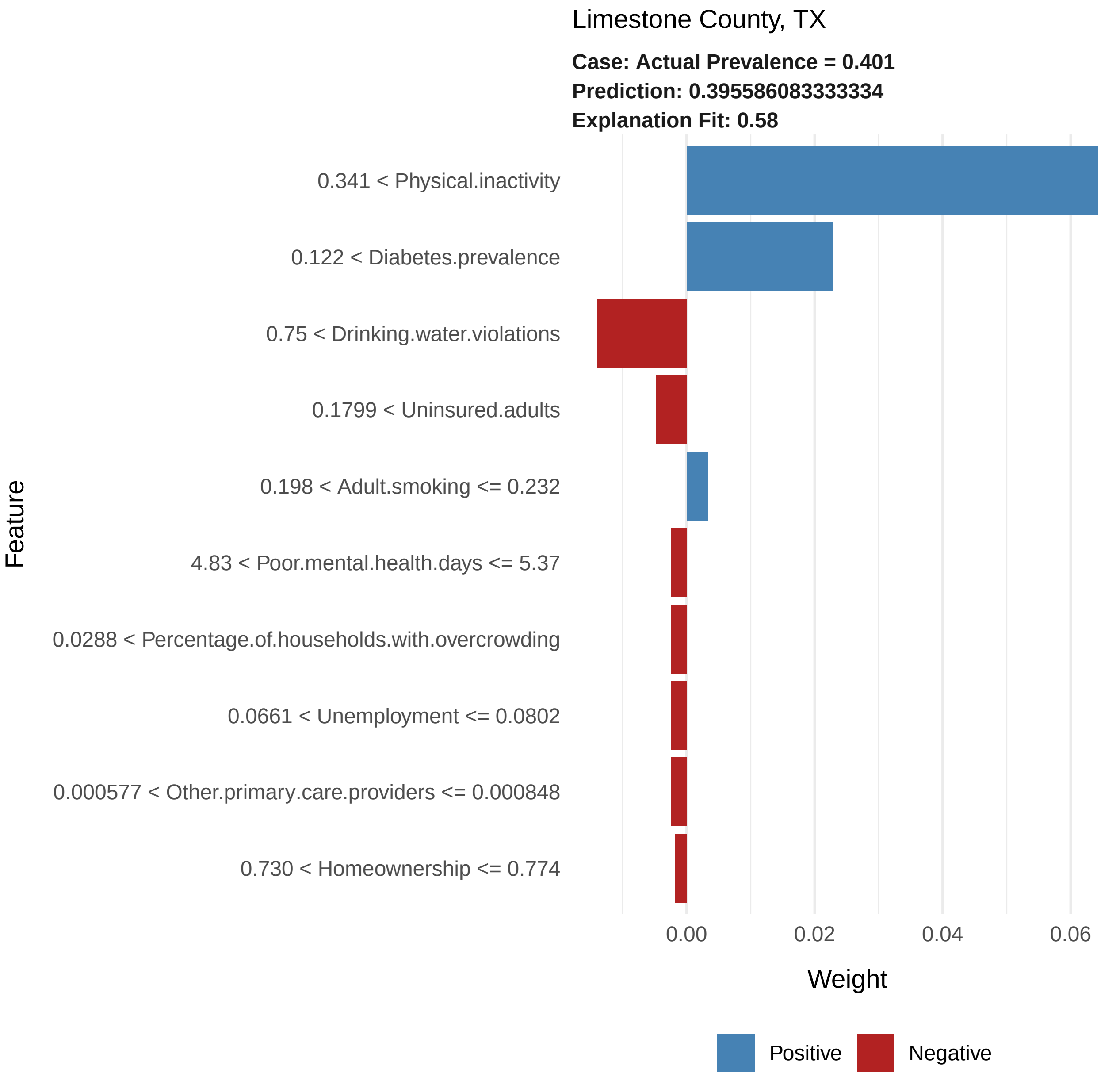

Limestone County, TX
Case: Actual Prevalence = 0.401
Prediction: 0.395586083333334
Explanation Fit: 0.58
0.341 < Physical.inactivity
0.122 < Diabetes.prevalence
0.75 < Drinking.water.violations
0.1799 < Uninsured.adults
0.198 < Adult.smoking <= 0.232
4.83 < Poor.mental.health.days <= 5.37
0.0288 < Percentage.of.households.with.overcrowding
0.0661 < Unemployment <= 0.0802
0.000577 < Other.primary.care.providers <= 0.000848
0.730 < Homeownership <= 0.774
Feature
0.00
0.02
0.04
0.06
Weight
Positive
Negative

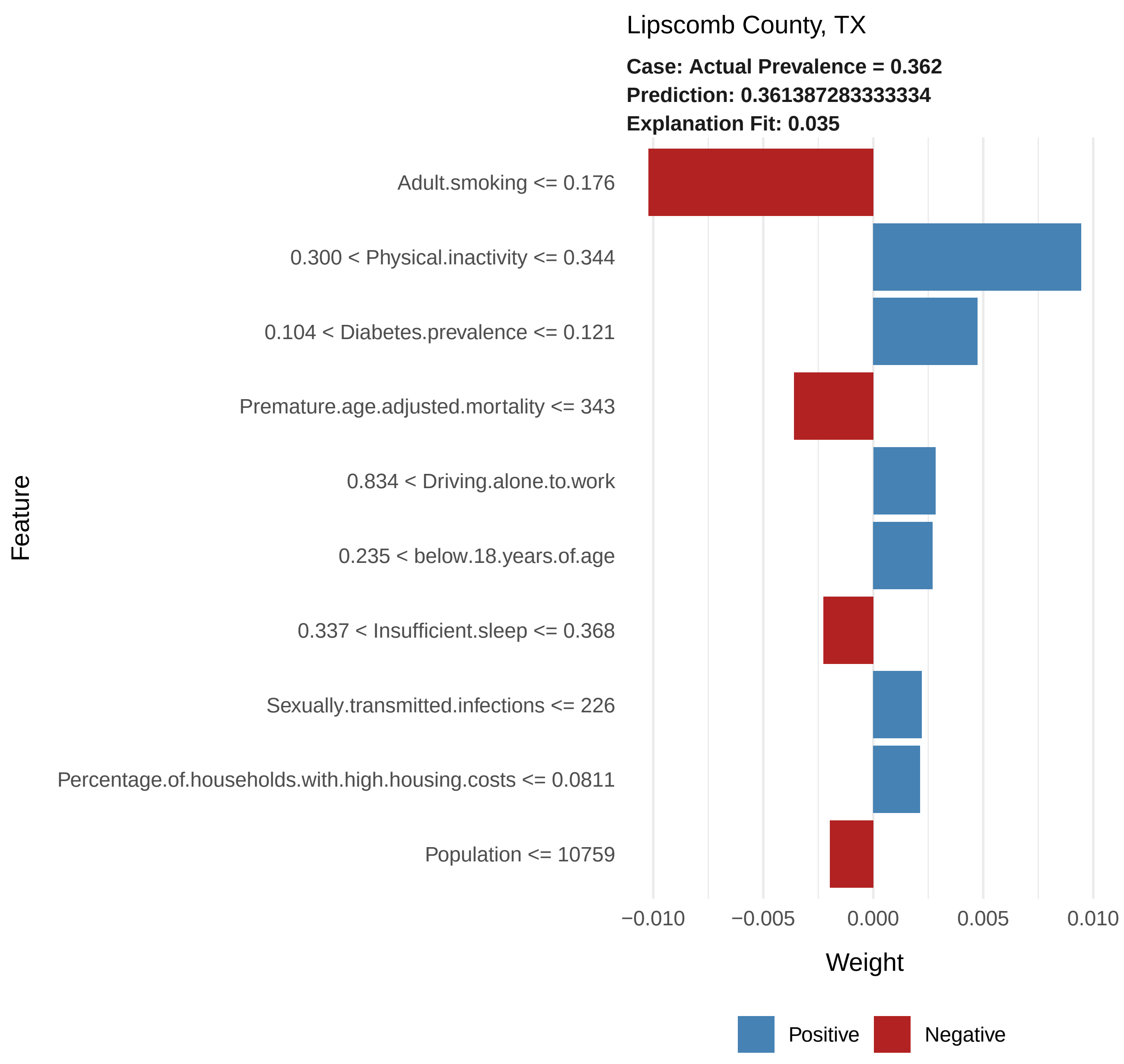

Lipscomb County, TX
Case: Actual Prevalence = 0.362
Prediction: 0.361387283333334
Explanation Fit: 0.035
Adult.smoking <= 0.176
0.300 < Physical.inactivity <= 0.344
0.104 < Diabetes.prevalence <= 0.121
Premature.age.adjusted.mortality <= 343
0.834 < Driving.alone.to.work
0.235 < below.18.years.of.age
0.337 < Insufficient.sleep <= 0.368
Sexually.transmitted.infections <= 226
Percentage.of.households.with.high.housing.costs <= 0.0811
Population <= 10759
Feature
−0.010
−0.005
0.000
0.005
0.010
Weight
Positive
Negative

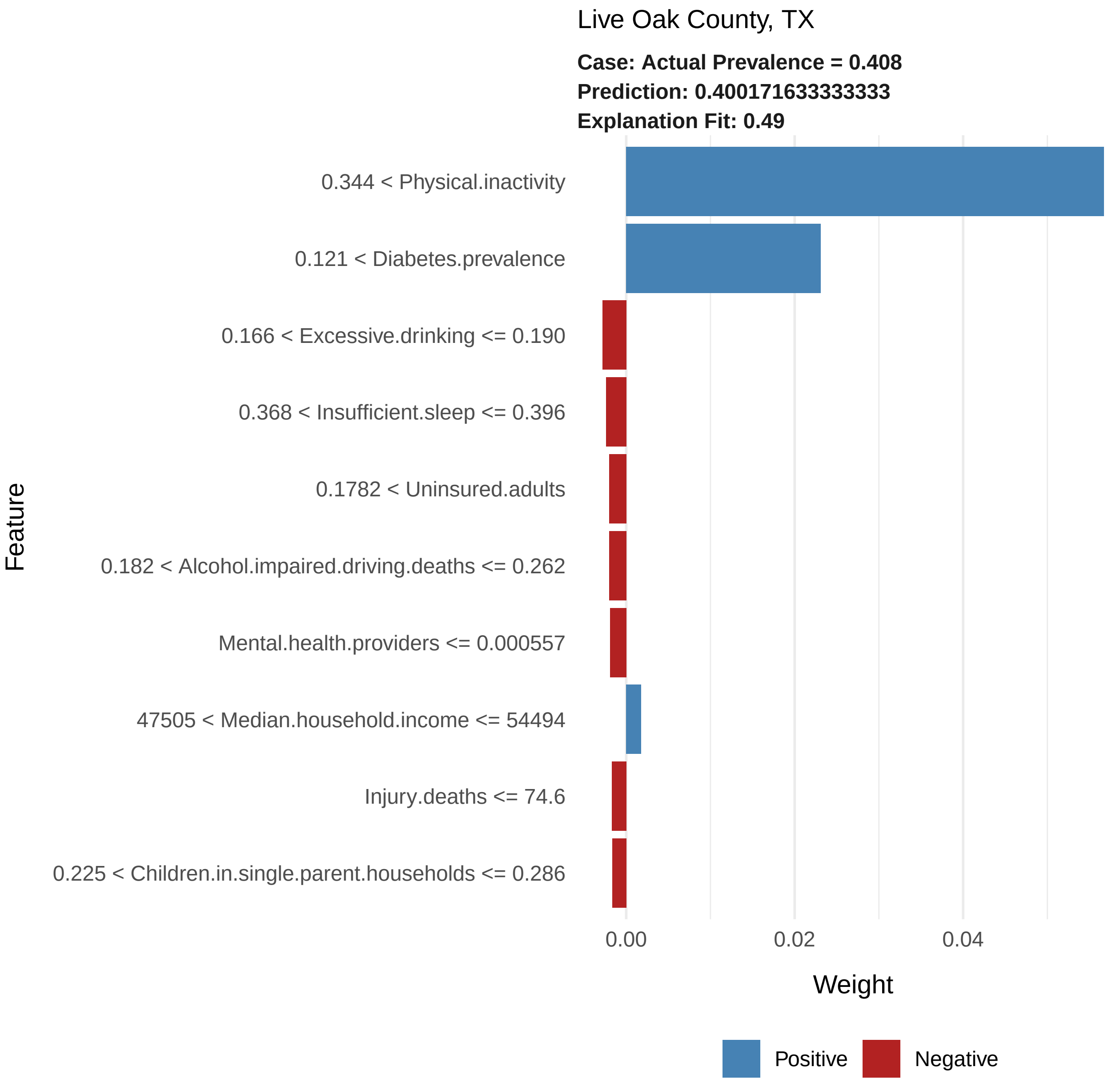
Live Oak County, TX
Case: Actual Prevalence = 0.408
Prediction: 0.400171633333333
Explanation Fit: 0.49
0.344 < Physical.inactivity
0.121 < Diabetes.prevalence
0.166 < Excessive.drinking <= 0.190
0.368 < Insufficient.sleep <= 0.396
0.1782 < Uninsured.adults
0.182 < Alcohol.impaired.driving.deaths <= 0.262
Mental.health.providers <= 0.000557
47505 < Median.household.income <= 54494
Injury.deaths <= 74.6
0.225 < Children.in.single.parent.households <= 0.286
Feature
0.00
0.02
0.04
Weight
Positive
Negative

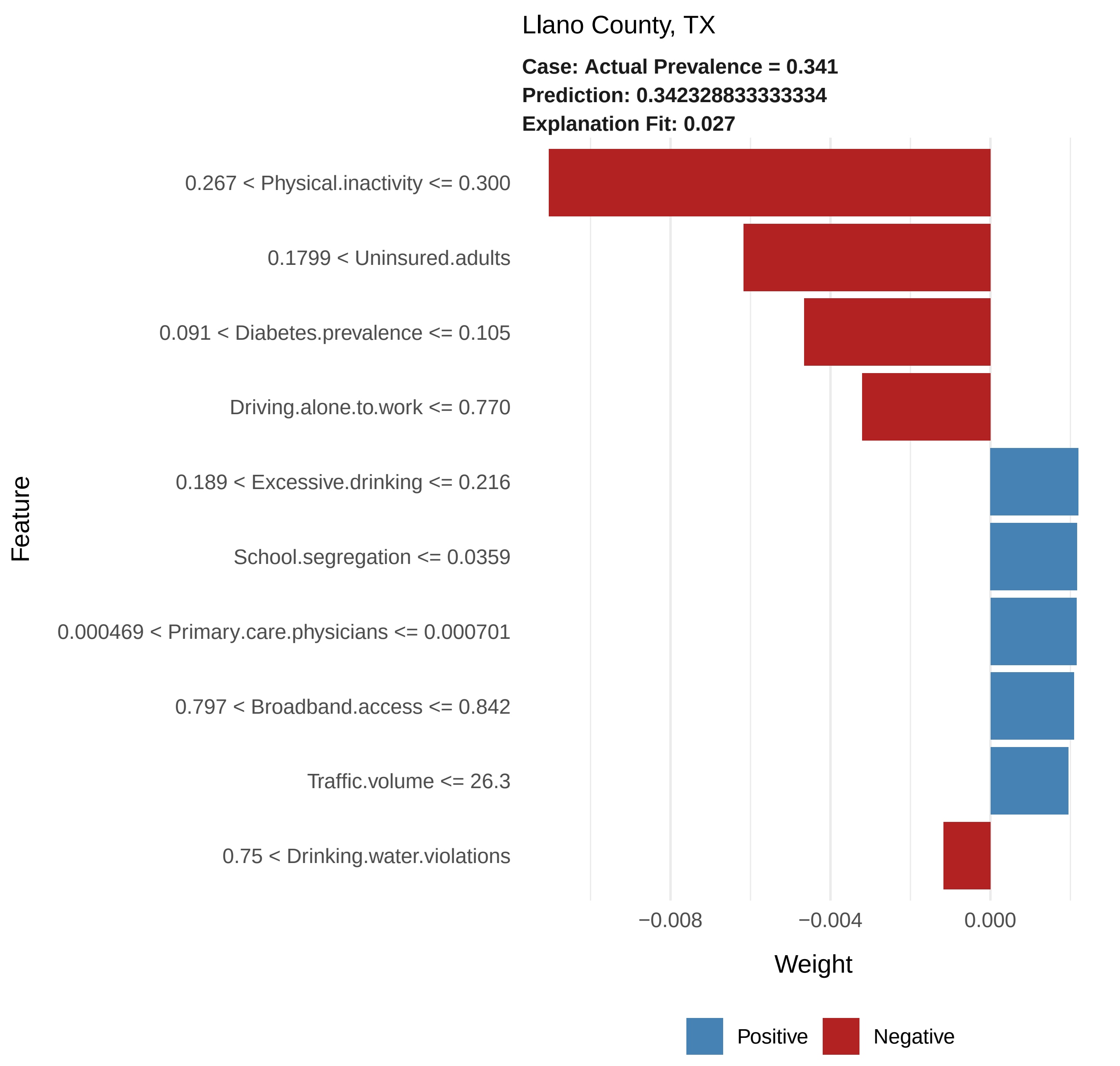
Llano County, TX
Case: Actual Prevalence = 0.341
Prediction: 0.342328833333334
Explanation Fit: 0.027
0.267 < Physical.inactivity <= 0.300
0.1799 < Uninsured.adults
0.091 < Diabetes.prevalence <= 0.105
Driving.alone.to.work <= 0.770
0.189 < Excessive.drinking <= 0.216
School.segregation <= 0.0359
0.000469 < Primary.care.physicians <= 0.000701
0.797 < Broadband.access <= 0.842
Traffic.volume <= 26.3
0.75 < Drinking.water.violations
Feature
−0.008
−0.004
0.000
Weight
Positive
Negative

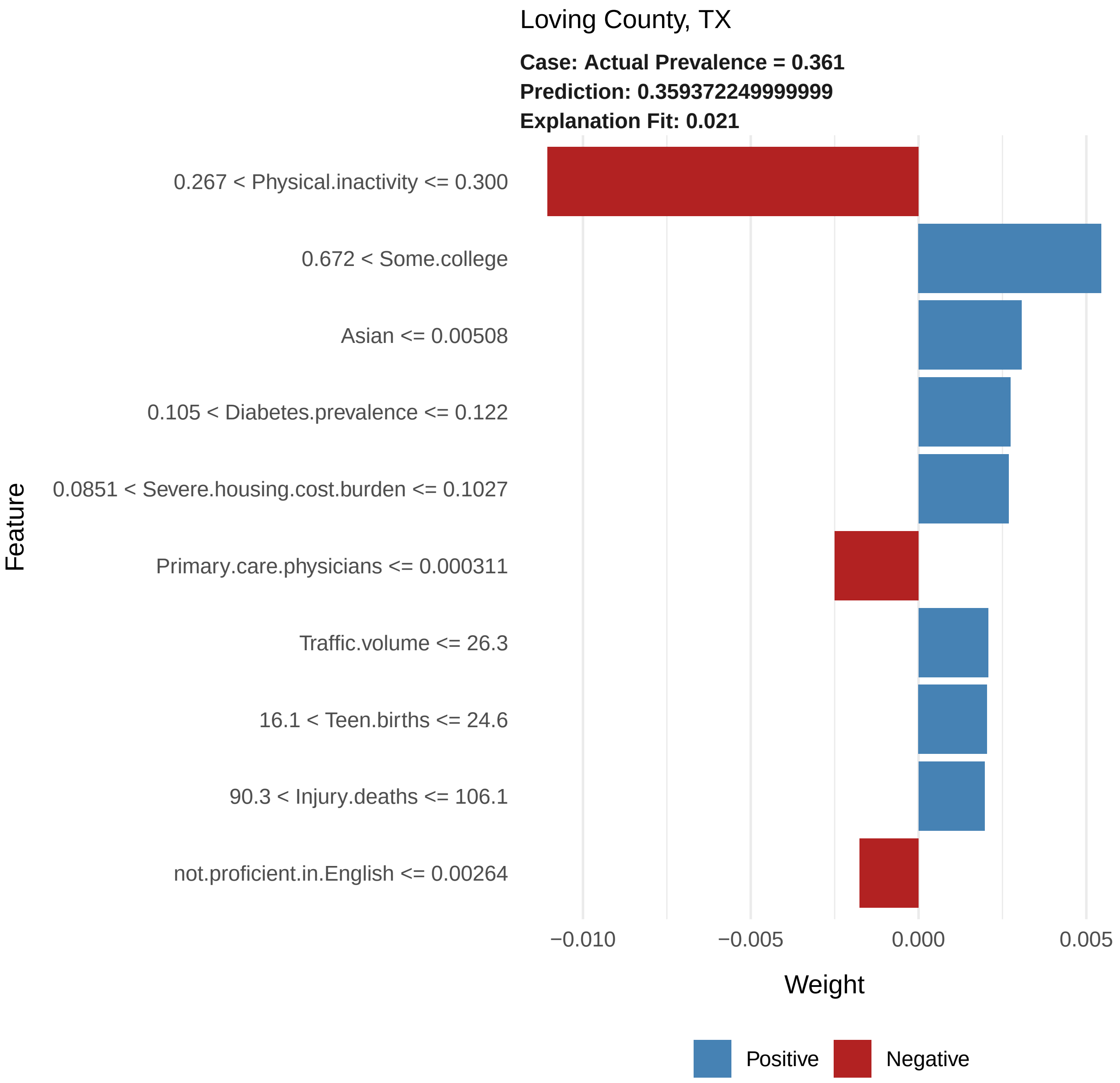

Loving County, TX
Case: Actual Prevalence = 0.361
Prediction: 0.359372249999999
Explanation Fit: 0.021
0.267 < Physical.inactivity <= 0.300
0.672 < Some.college
Asian <= 0.00508
0.105 < Diabetes.prevalence <= 0.122
0.0851 < Severe.housing.cost.burden <= 0.1027
Primary.care.physicians <= 0.000311
Traffic.volume <= 26.3
16.1 < Teen.births <= 24.6
90.3 < Injury.deaths <= 106.1
not.proficient.in.English <= 0.00264
Feature
−0.010
−0.005
0.000
0.005
Weight
Positive
Negative

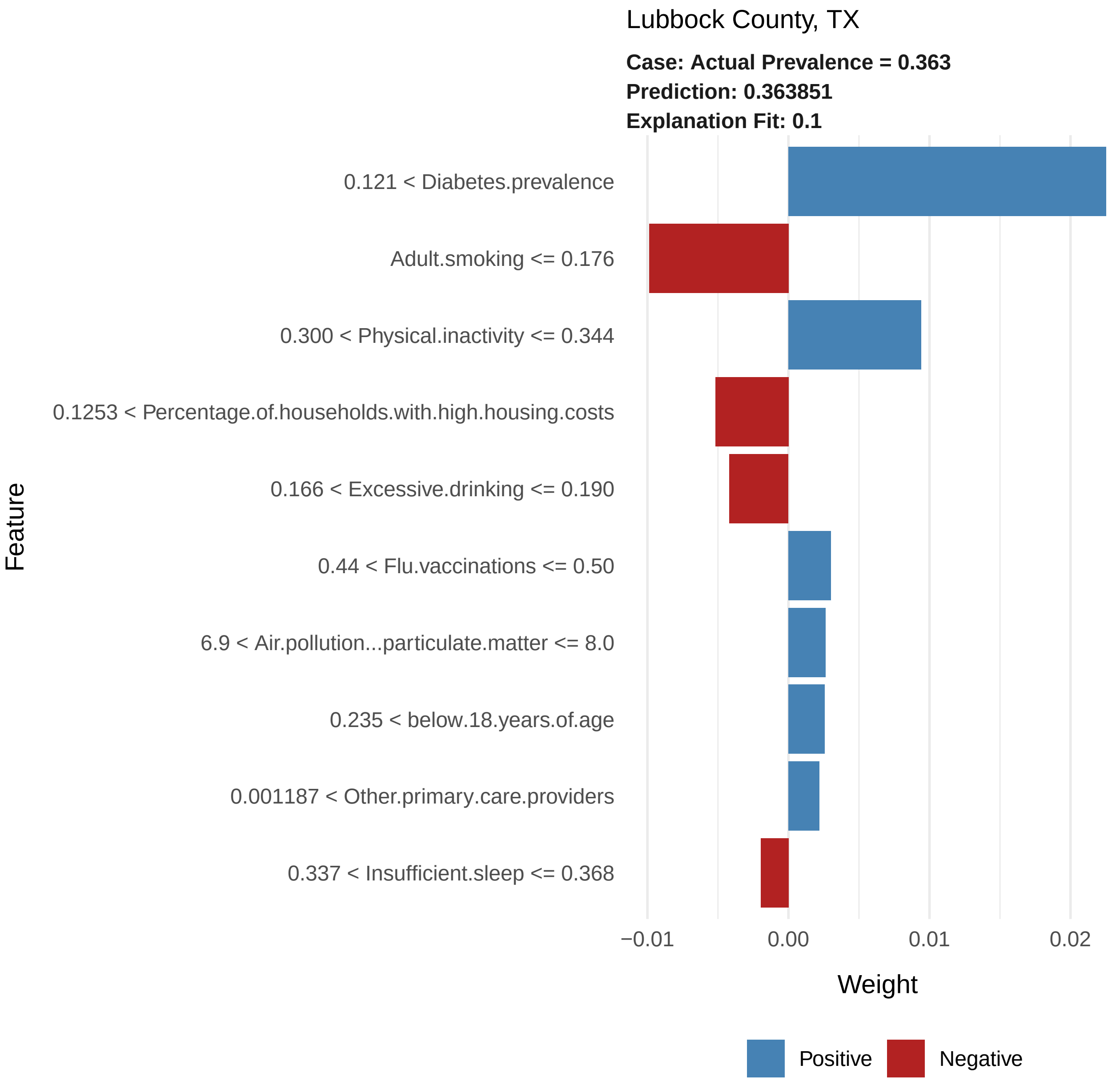

Lubbock County, TX
Case: Actual Prevalence = 0.363
Prediction: 0.363851
Explanation Fit: 0.1
0.121 < Diabetes.prevalence
Adult.smoking <= 0.176
0.300 < Physical.inactivity <= 0.344
0.1253 < Percentage.of.households.with.high.housing.costs
0.166 < Excessive.drinking <= 0.190
0.44 < Flu.vaccinations <= 0.50
6.9 < Air.pollution...particulate.matter <= 8.0
0.235 < below.18.years.of.age
0.001187 < Other.primary.care.providers
0.337 < Insufficient.sleep <= 0.368
Feature
−0.01
0.00
0.01
0.02
Weight
Positive
Negative

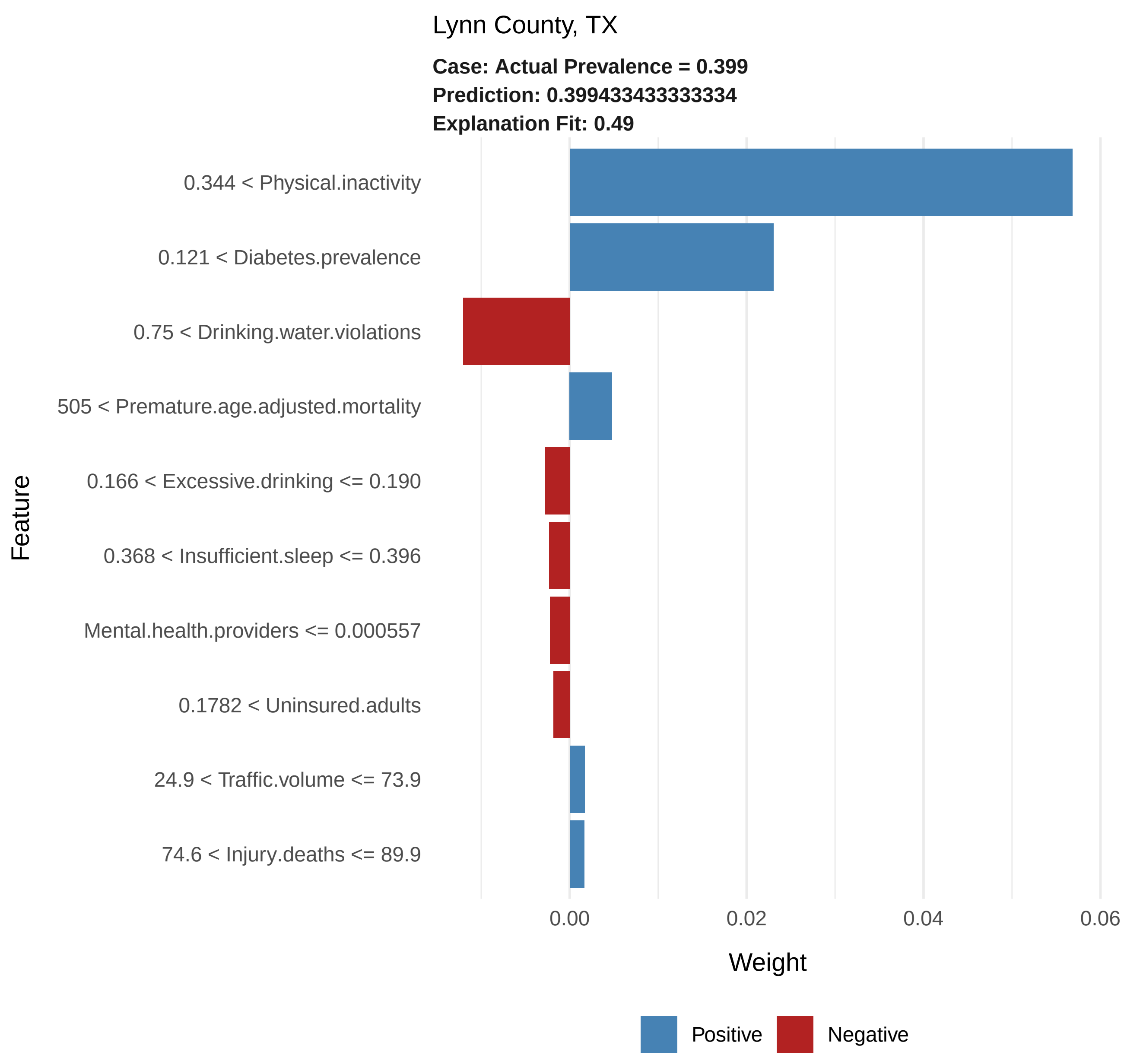
Lynn County, TX
Case: Actual Prevalence = 0.399
Prediction: 0.399433433333334
Explanation Fit: 0.49
0.344 < Physical.inactivity
0.121 < Diabetes.prevalence
0.75 < Drinking.water.violations
505 < Premature.age.adjusted.mortality
0.166 < Excessive.drinking <= 0.190
0.368 < Insufficient.sleep <= 0.396
Mental.health.providers <= 0.000557
0.1782 < Uninsured.adults
24.9 < Traffic.volume <= 73.9
74.6 < Injury.deaths <= 89.9
Feature
0.00
0.02
0.04
0.06
Weight
Positive
Negative

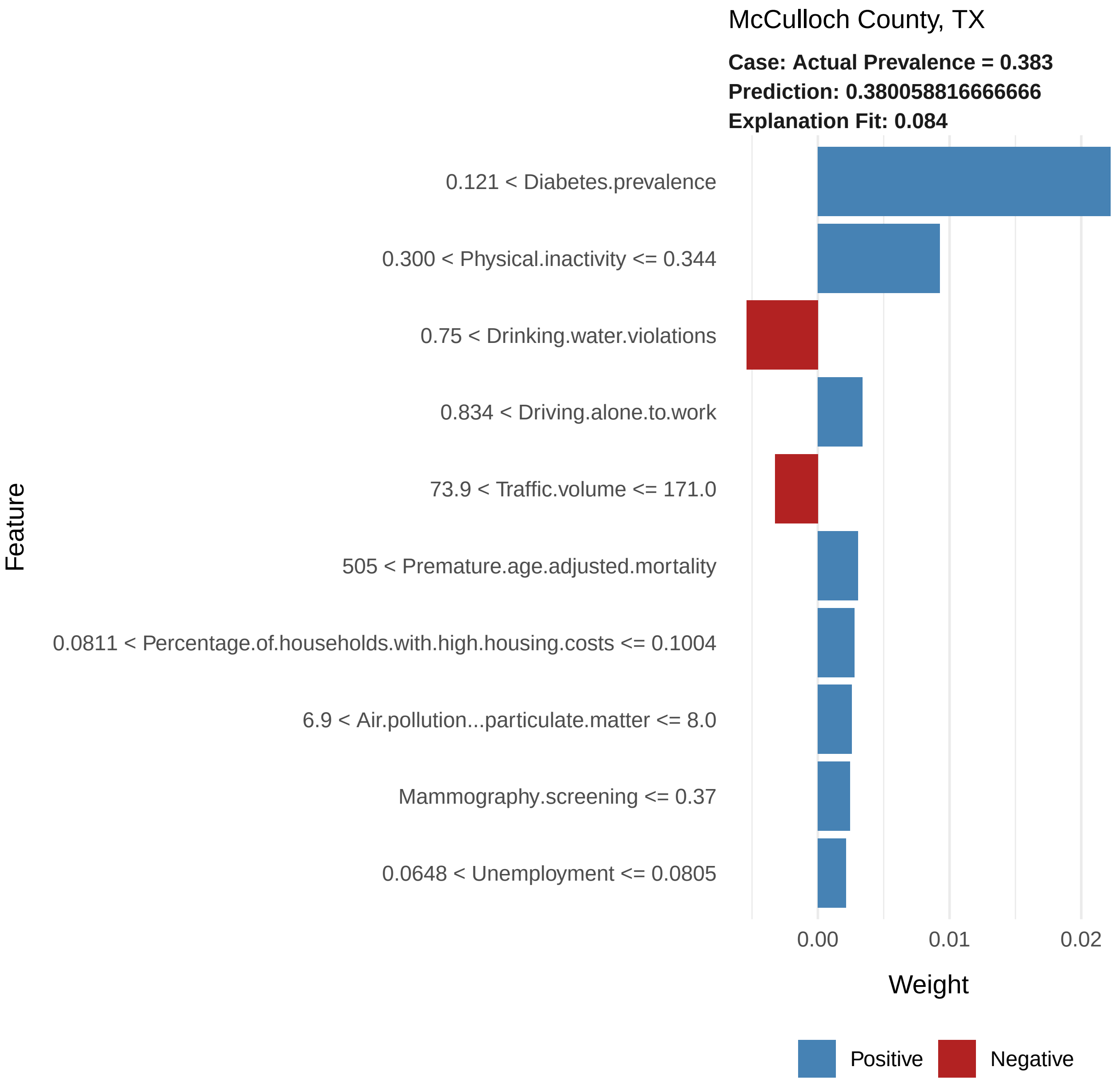
McCulloch County, TX
Case: Actual Prevalence = 0.383
Prediction: 0.380058816666666
Explanation Fit: 0.084
0.121 < Diabetes.prevalence
0.300 < Physical.inactivity <= 0.344
0.75 < Drinking.water.violations
0.834 < Driving.alone.to.work
73.9 < Traffic.volume <= 171.0
505 < Premature.age.adjusted.mortality
0.0811 < Percentage.of.households.with.high.housing.costs <= 0.1004
6.9 < Air.pollution...particulate.matter <= 8.0
Mammography.screening <= 0.37
0.0648 < Unemployment <= 0.0805
Feature
0.00
0.01
0.02
Weight
Positive
Negative

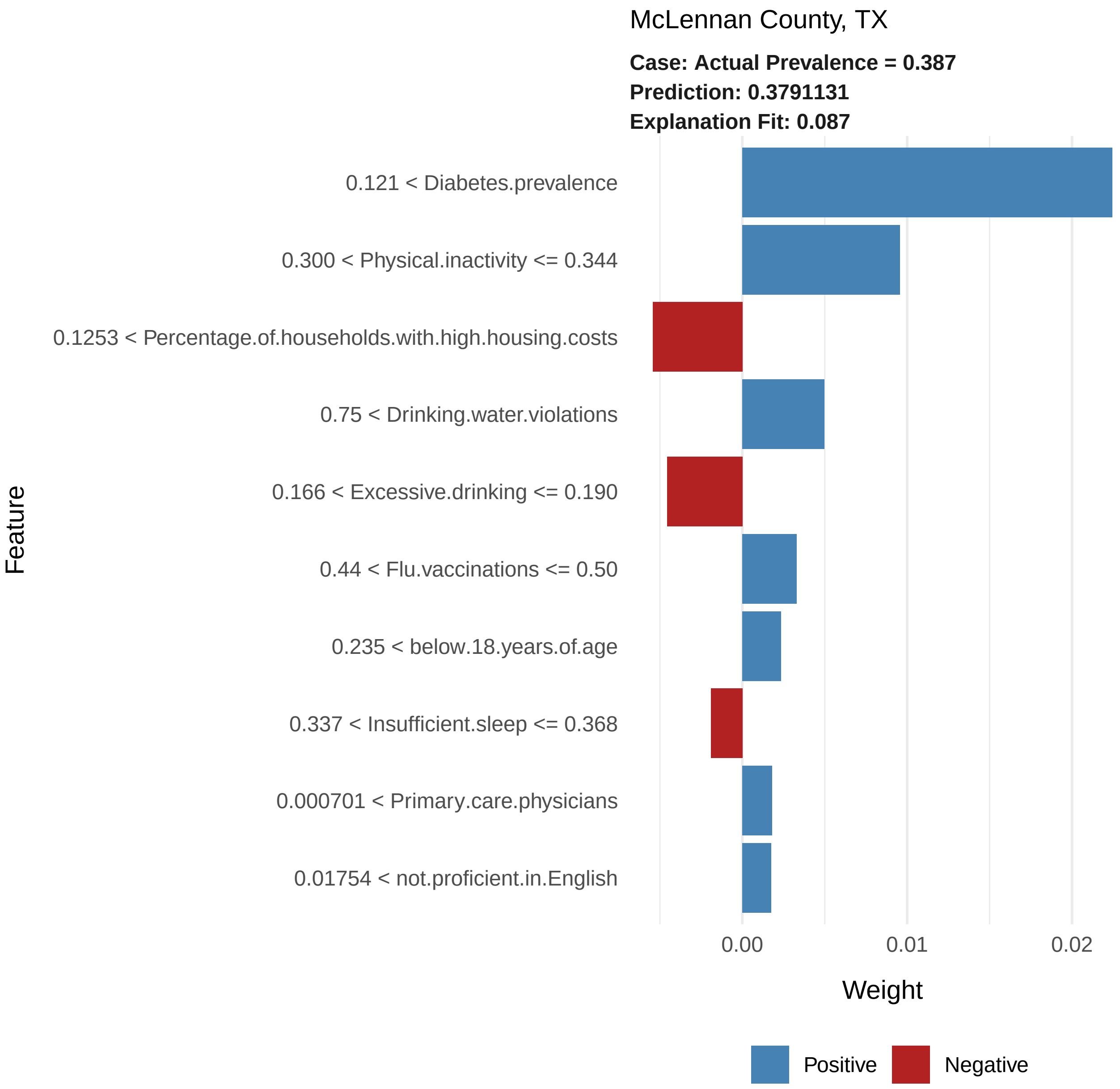
McLennan County, TX
Case: Actual Prevalence = 0.387
Prediction: 0.3791131
Explanation Fit: 0.087
0.121 < Diabetes.prevalence
0.300 < Physical.inactivity <= 0.344
0.1253 < Percentage.of.households.with.high.housing.costs
0.75 < Drinking.water.violations
0.166 < Excessive.drinking <= 0.190
0.44 < Flu.vaccinations <= 0.50
0.235 < below.18.years.of.age
0.337 < Insufficient.sleep <= 0.368
0.000701 < Primary.care.physicians
0.01754 < not.proficient.in.English
Feature
0.00
0.01
0.02
Weight
Positive
Negative

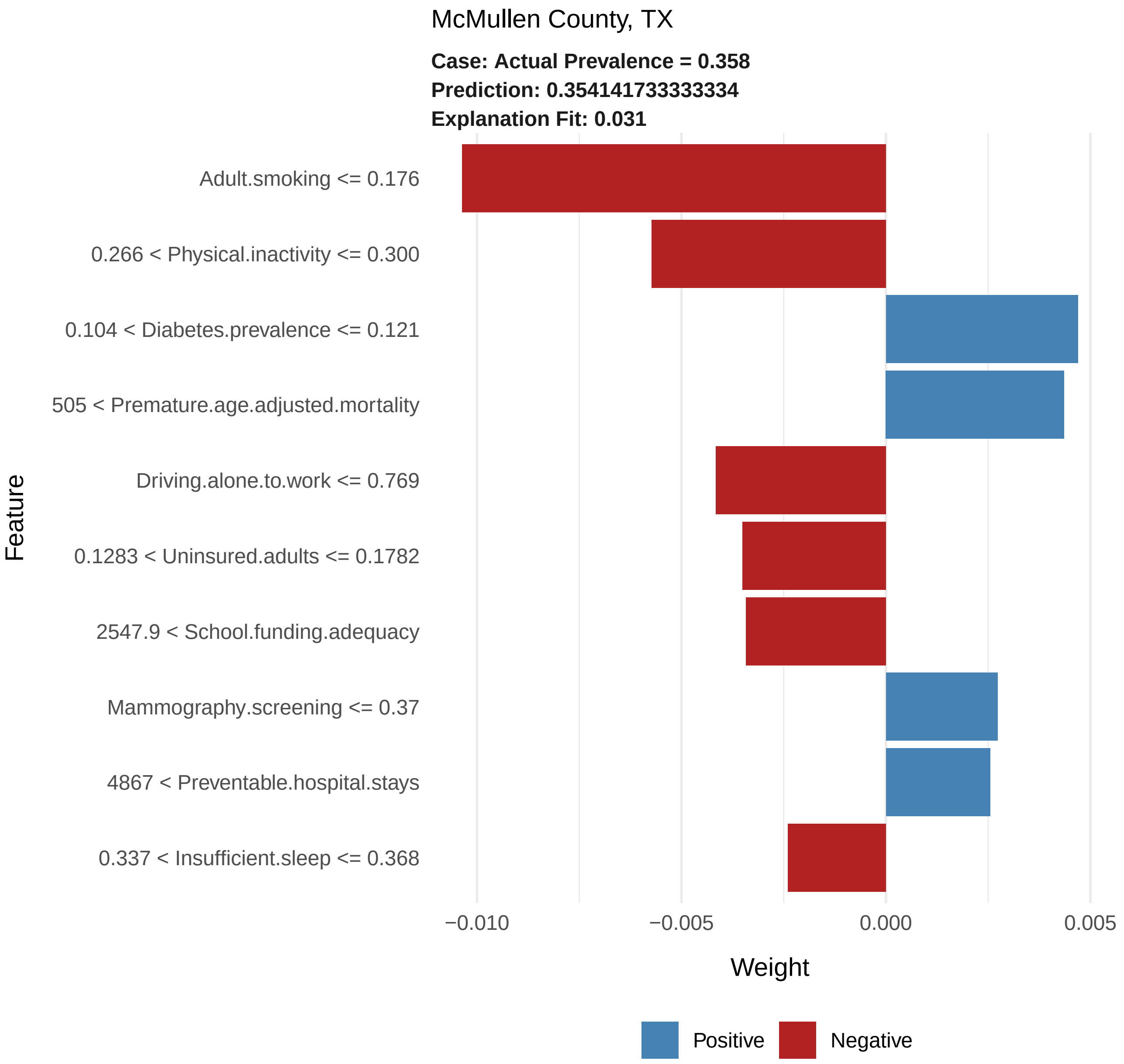

McMullen County, TX
Case: Actual Prevalence = 0.358
Prediction: 0.354141733333334
Explanation Fit: 0.031
Adult.smoking <= 0.176
0.266 < Physical.inactivity <= 0.300
0.104 < Diabetes.prevalence <= 0.121
505 < Premature.age.adjusted.mortality
Driving.alone.to.work <= 0.769
0.1283 < Uninsured.adults <= 0.1782
2547.9 < School.funding.adequacy
Mammography.screening <= 0.37
4867 < Preventable.hospital.stays
0.337 < Insufficient.sleep <= 0.368
Feature
−0.010
−0.005
0.000
0.005
Weight
Positive
Negative

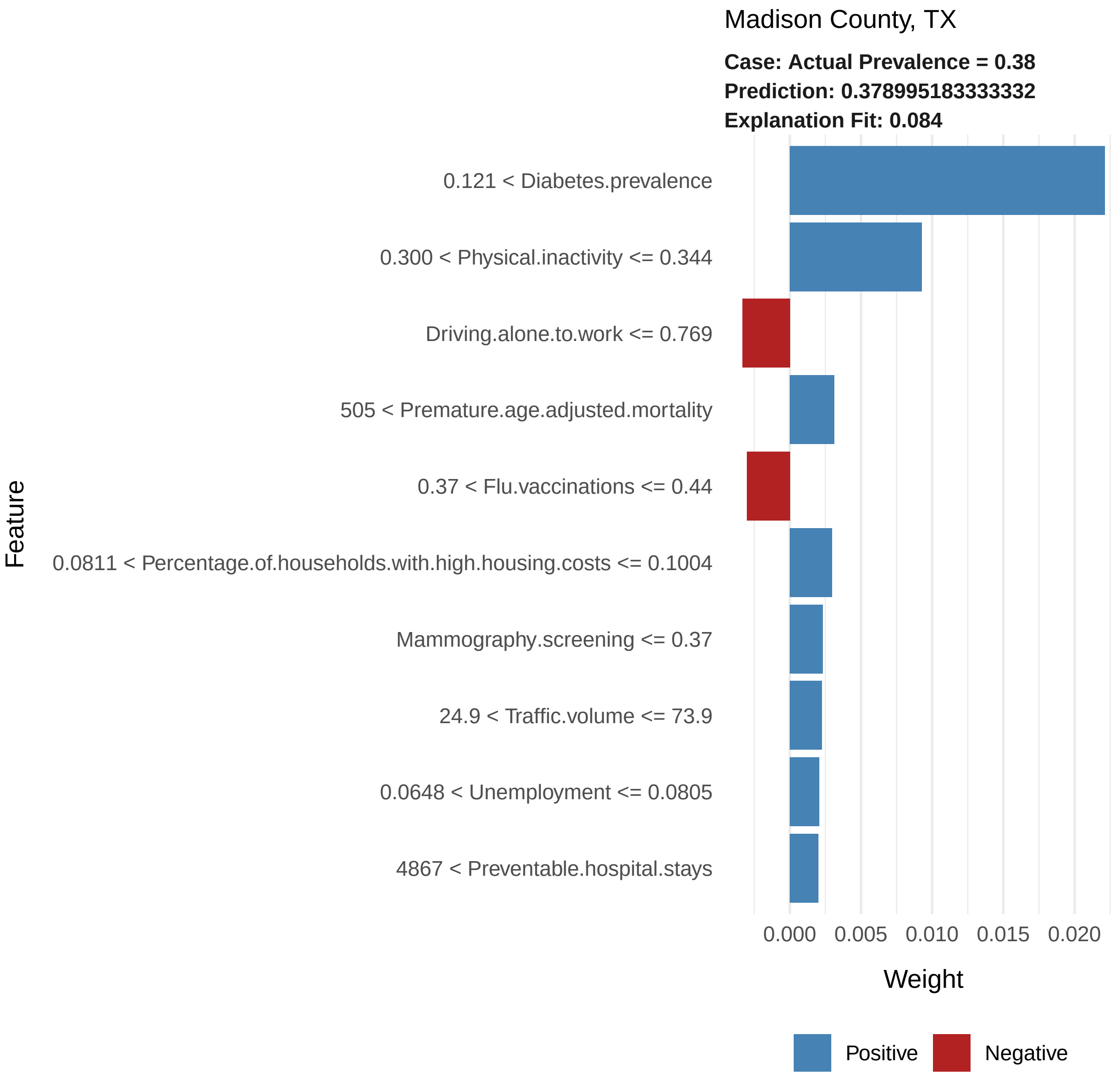

Madison County, TX
Case: Actual Prevalence = 0.38
Prediction: 0.378995183333332
Explanation Fit: 0.084
0.121 < Diabetes.prevalence
0.300 < Physical.inactivity <= 0.344
Driving.alone.to.work <= 0.769
505 < Premature.age.adjusted.mortality
0.37 < Flu.vaccinations <= 0.44
0.0811 < Percentage.of.households.with.high.housing.costs <= 0.1004
Mammography.screening <= 0.37
24.9 < Traffic.volume <= 73.9
0.0648 < Unemployment <= 0.0805
4867 < Preventable.hospital.stays
Feature
0.000
0.005
0.010
0.015
0.020
Weight
Positive
Negative

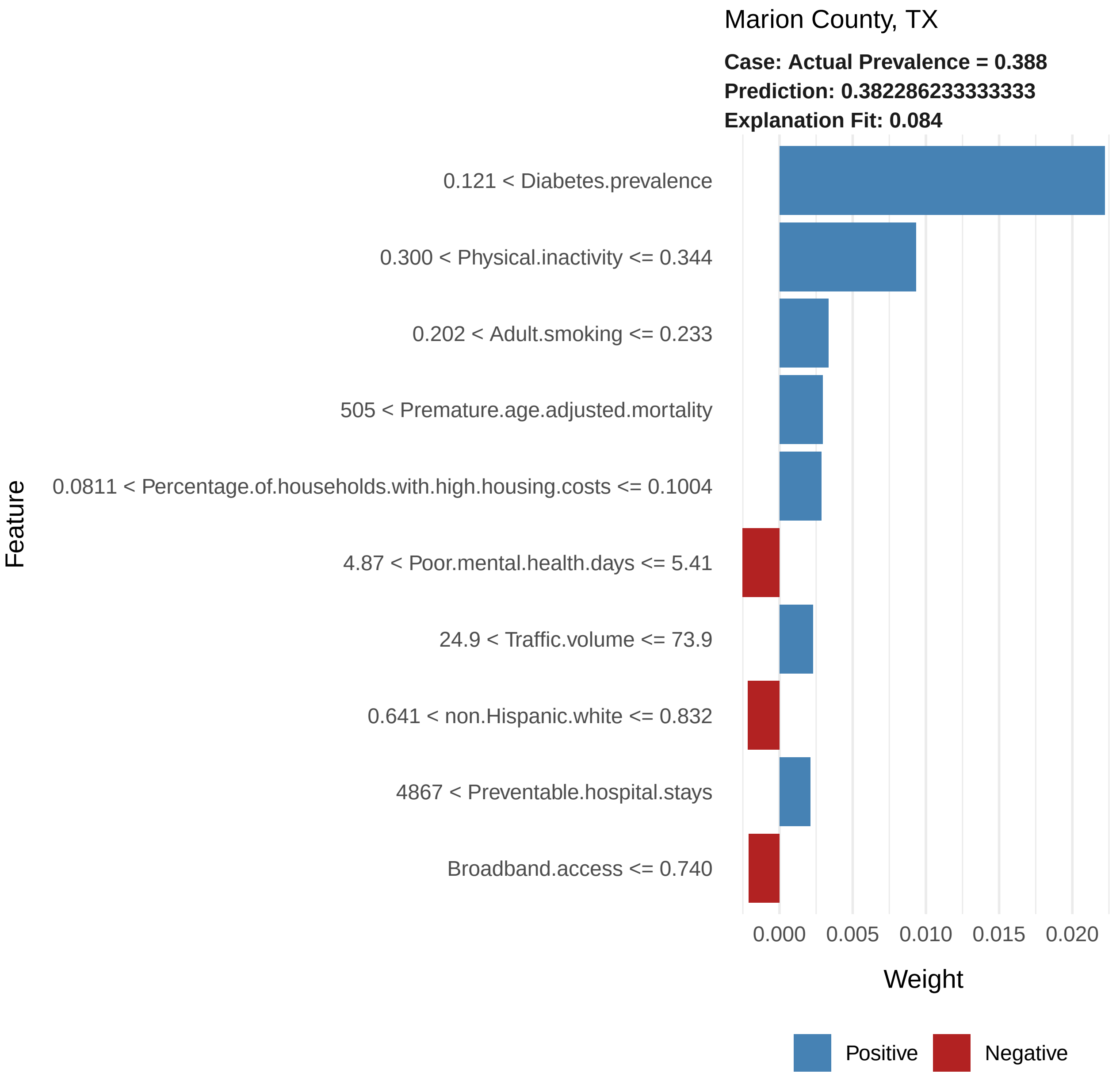
Marion County, TX
Case: Actual Prevalence = 0.388
Prediction: 0.382286233333333
Explanation Fit: 0.084
Feature
0.121 < Diabetes.prevalence
0.300 < Physical.inactivity <= 0.344
0.202 < Adult.smoking <= 0.233
505 < Premature.age.adjusted.mortality
0.0811 < Percentage.of.households.with.high.housing.costs <= 0.1004
4.87 < Poor.mental.health.days <= 5.41
24.9 < Traffic.volume <= 73.9
0.641 < non.Hispanic.white <= 0.832
4867 < Preventable.hospital.stays
Broadband.access <= 0.740
0.000
0.005
0.010
0.015
0.020
Weight
Positive
Negative

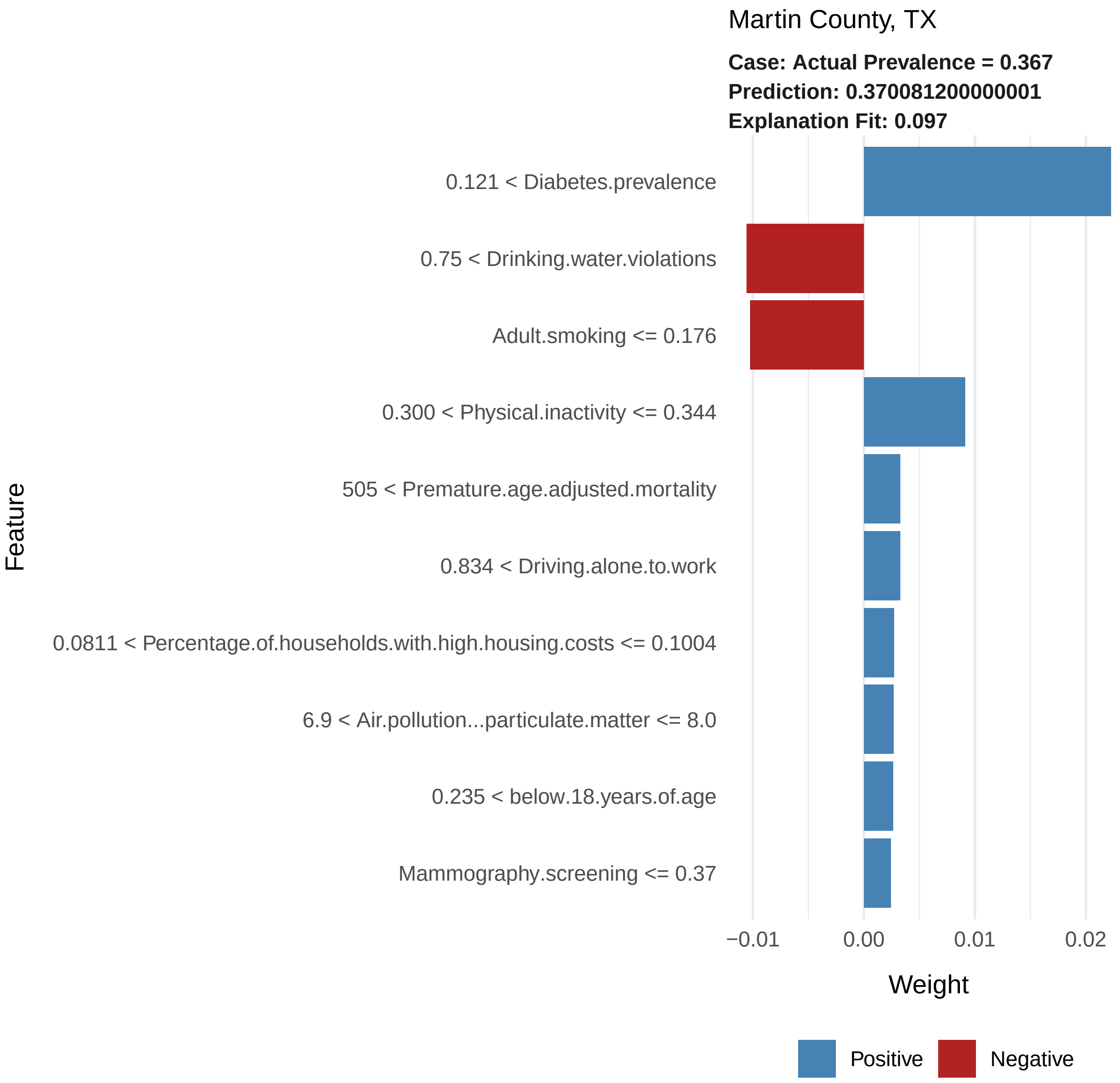

Martin County, TX
Case: Actual Prevalence = 0.367
Prediction: 0.370081200000001
Explanation Fit: 0.097
0.121 < Diabetes.prevalence
0.75 < Drinking.water.violations
Adult.smoking <= 0.176
0.300 < Physical.inactivity <= 0.344
505 < Premature.age.adjusted.mortality
0.834 < Driving.alone.to.work
0.0811 < Percentage.of.households.with.high.housing.costs <= 0.1004
6.9 < Air.pollution...particulate.matter <= 8.0
0.235 < below.18.years.of.age
Mammography.screening <= 0.37
Feature
−0.01
0.00
0.01
0.02
Weight
Positive
Negative

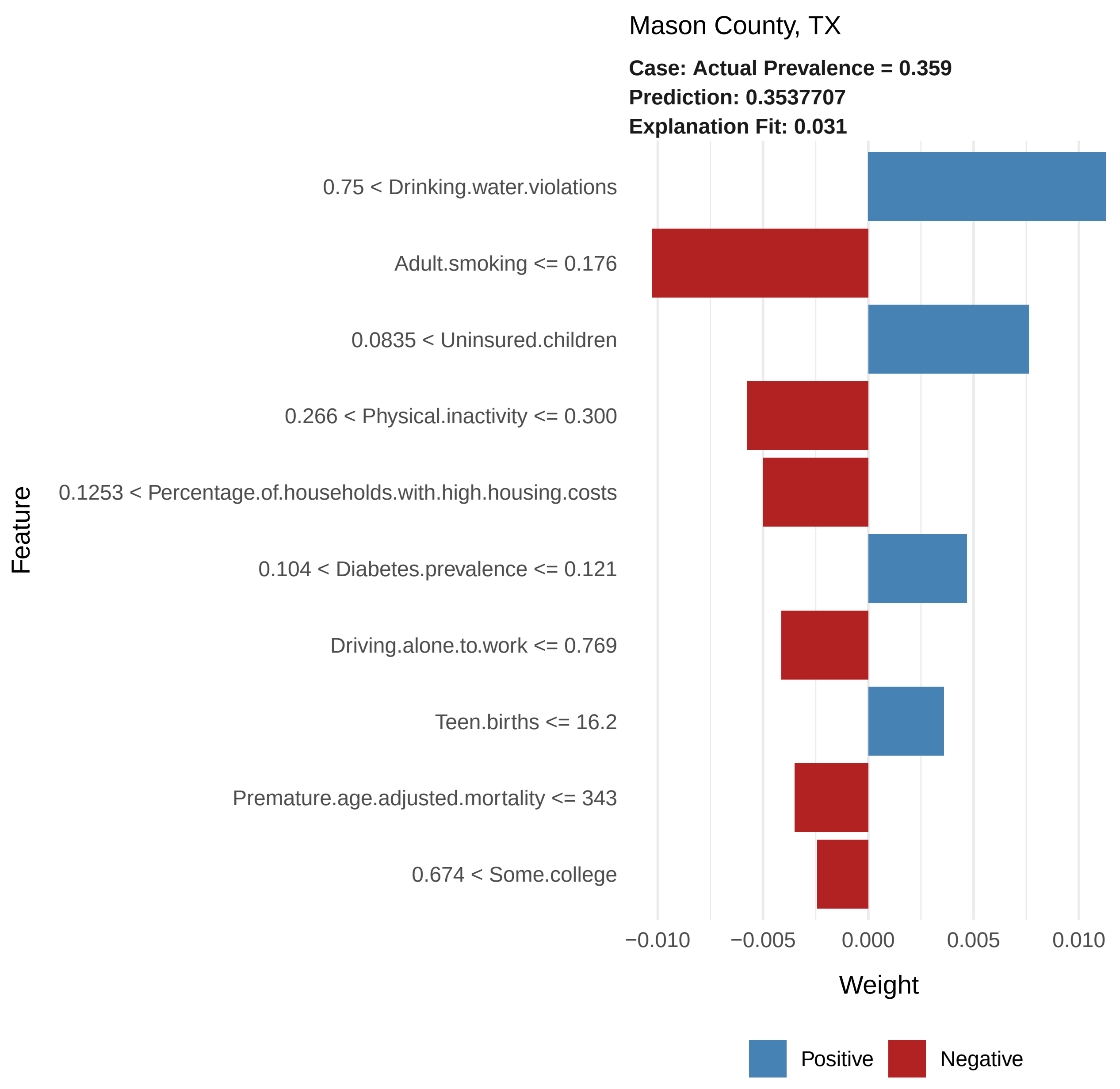

Mason County, TX
Case: Actual Prevalence = 0.359
Prediction: 0.3537707
Explanation Fit: 0.031
0.75 < Drinking.water.violations
Adult.smoking <= 0.176
0.0835 < Uninsured.children
0.266 < Physical.inactivity <= 0.300
0.1253 < Percentage.of.households.with.high.housing.costs
0.104 < Diabetes.prevalence <= 0.121
Driving.alone.to.work <= 0.769
Teen.births <= 16.2
Premature.age.adjusted.mortality <= 343
0.674 < Some.college
Feature
−0.010
−0.005
0.000
0.005
0.010
Weight
Positive
Negative

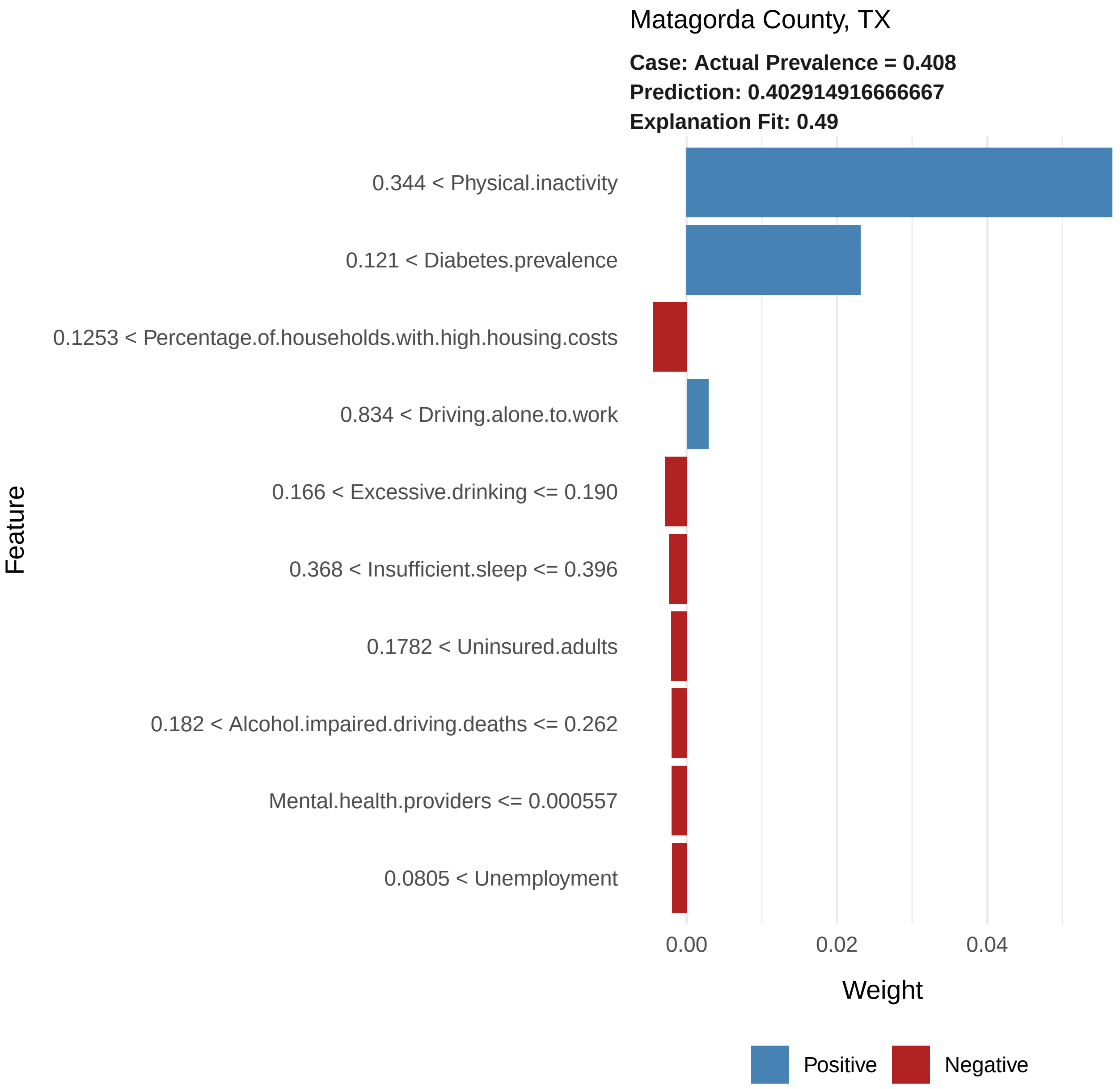
Matagorda County, TX
Case: Actual Prevalence = 0.408
Prediction: 0.402914916666667
Explanation Fit: 0.49
0.344 < Physical.inactivity
0.121 < Diabetes.prevalence
0.1253 < Percentage.of.households.with.high.housing.costs
0.834 < Driving.alone.to.work
0.166 < Excessive.drinking <= 0.190
0.368 < Insufficient.sleep <= 0.396
0.1782 < Uninsured.adults
0.182 < Alcohol.impaired.driving.deaths <= 0.262
Mental.health.providers <= 0.000557
0.0805 < Unemployment
Feature
0.00
0.02
0.04
Weight
Positive
Negative

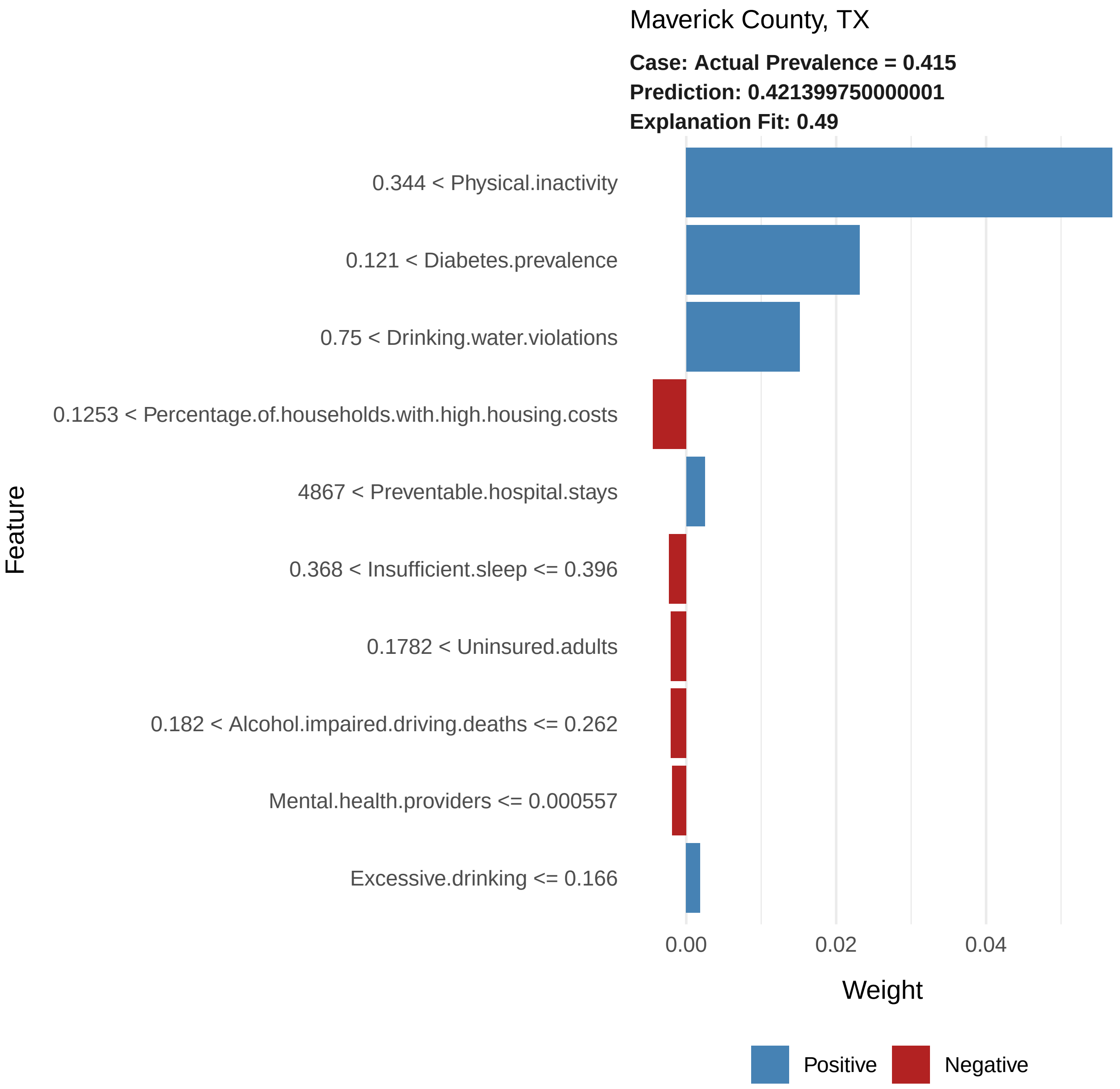
Maverick County, TX
Case: Actual Prevalence = 0.415
Prediction: 0.421399750000001
Explanation Fit: 0.49
0.344 < Physical.inactivity
0.121 < Diabetes.prevalence
0.75 < Drinking.water.violations
0.1253 < Percentage.of.households.with.high.housing.costs
4867 < Preventable.hospital.stays
0.368 < Insufficient.sleep <= 0.396
0.1782 < Uninsured.adults
0.182 < Alcohol.impaired.driving.deaths <= 0.262
Mental.health.providers <= 0.000557
Excessive.drinking <= 0.166
Feature
0.00
0.02
0.04
Weight
Positive
Negative

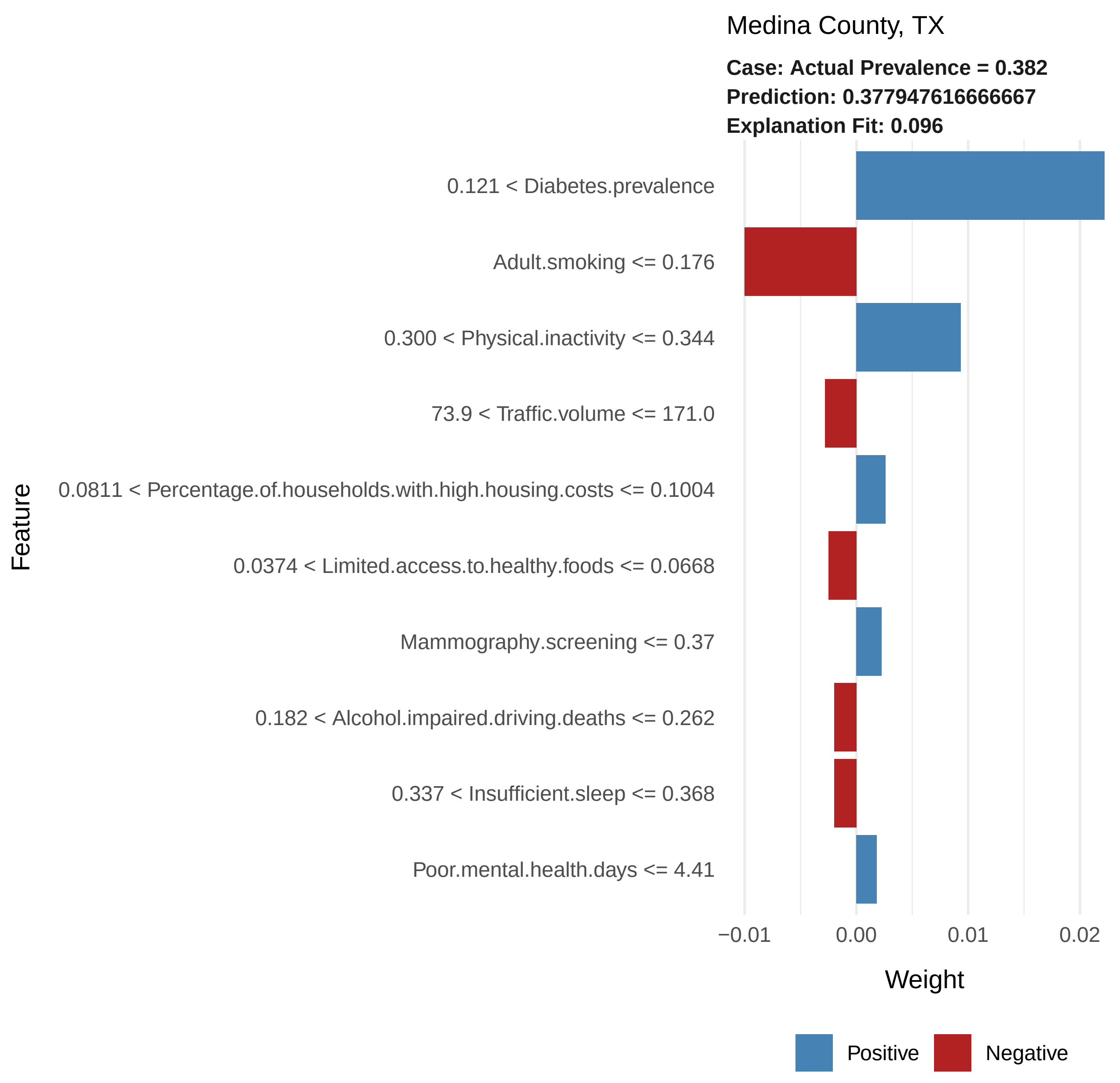
Medina County, TX
Case: Actual Prevalence = 0.382
Prediction: 0.377947616666667
Explanation Fit: 0.096
0.121 < Diabetes.prevalence
Adult.smoking <= 0.176
0.300 < Physical.inactivity <= 0.344
73.9 < Traffic.volume <= 171.0
0.0811 < Percentage.of.households.with.high.housing.costs <= 0.1004
0.0374 < Limited.access.to.healthy.foods <= 0.0668
Mammography.screening <= 0.37
0.182 < Alcohol.impaired.driving.deaths <= 0.262
0.337 < Insufficient.sleep <= 0.368
Poor.mental.health.days <= 4.41
Feature
−0.01
0.00
0.01
0.02
Weight
Positive
Negative

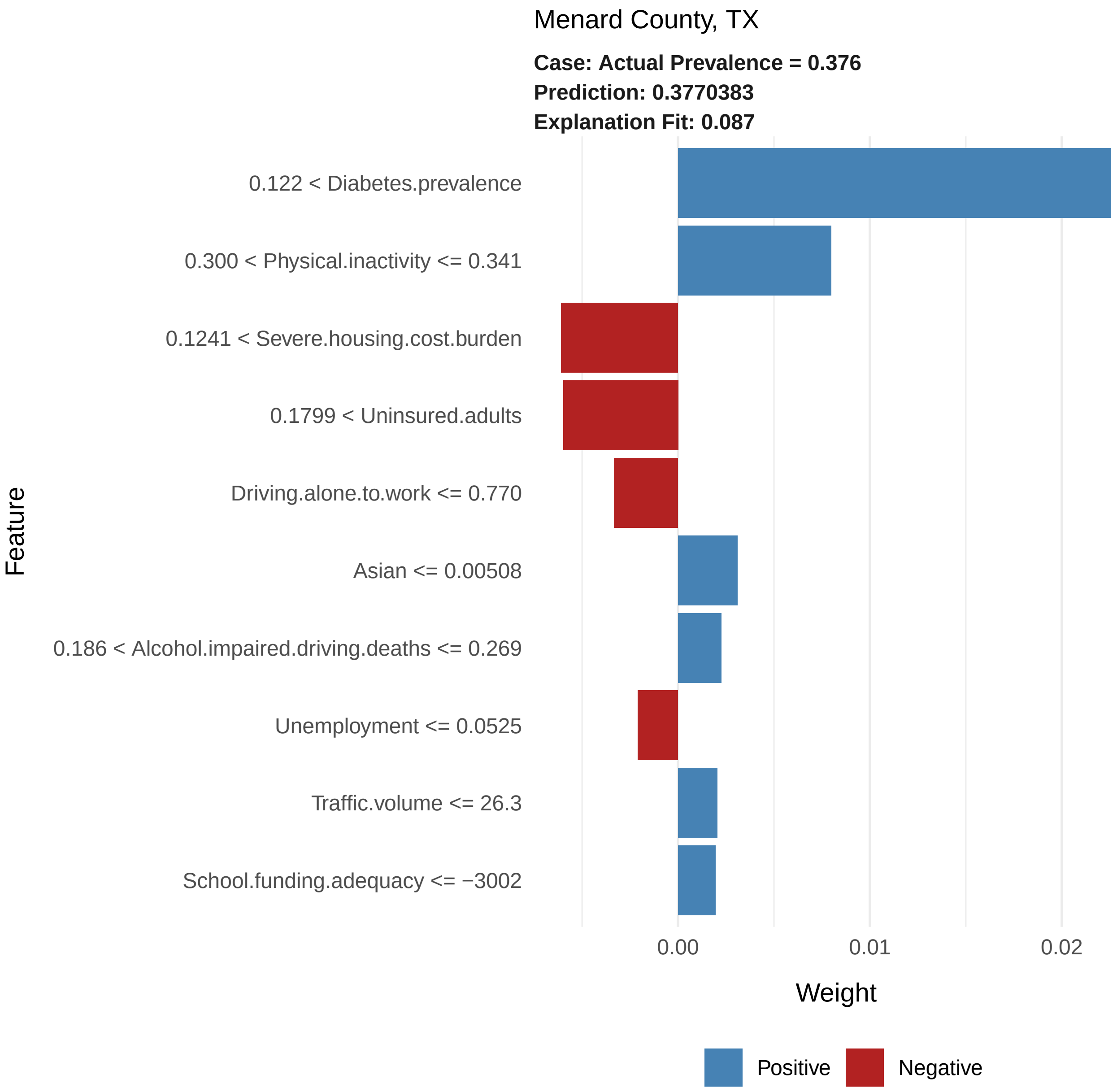

Menard County, TX
Case: Actual Prevalence = 0.376
Prediction: 0.3770383
Explanation Fit: 0.087
0.122 < Diabetes.prevalence
0.300 < Physical.inactivity <= 0.341
0.1241 < Severe.housing.cost.burden
0.1799 < Uninsured.adults
Driving.alone.to.work <= 0.770
Asian <= 0.00508
0.186 < Alcohol.impaired.driving.deaths <= 0.269
Unemployment <= 0.0525
Traffic.volume <= 26.3
School.funding.adequacy <= −3002
Feature
0.00
0.01
0.02
Weight
Positive
Negative

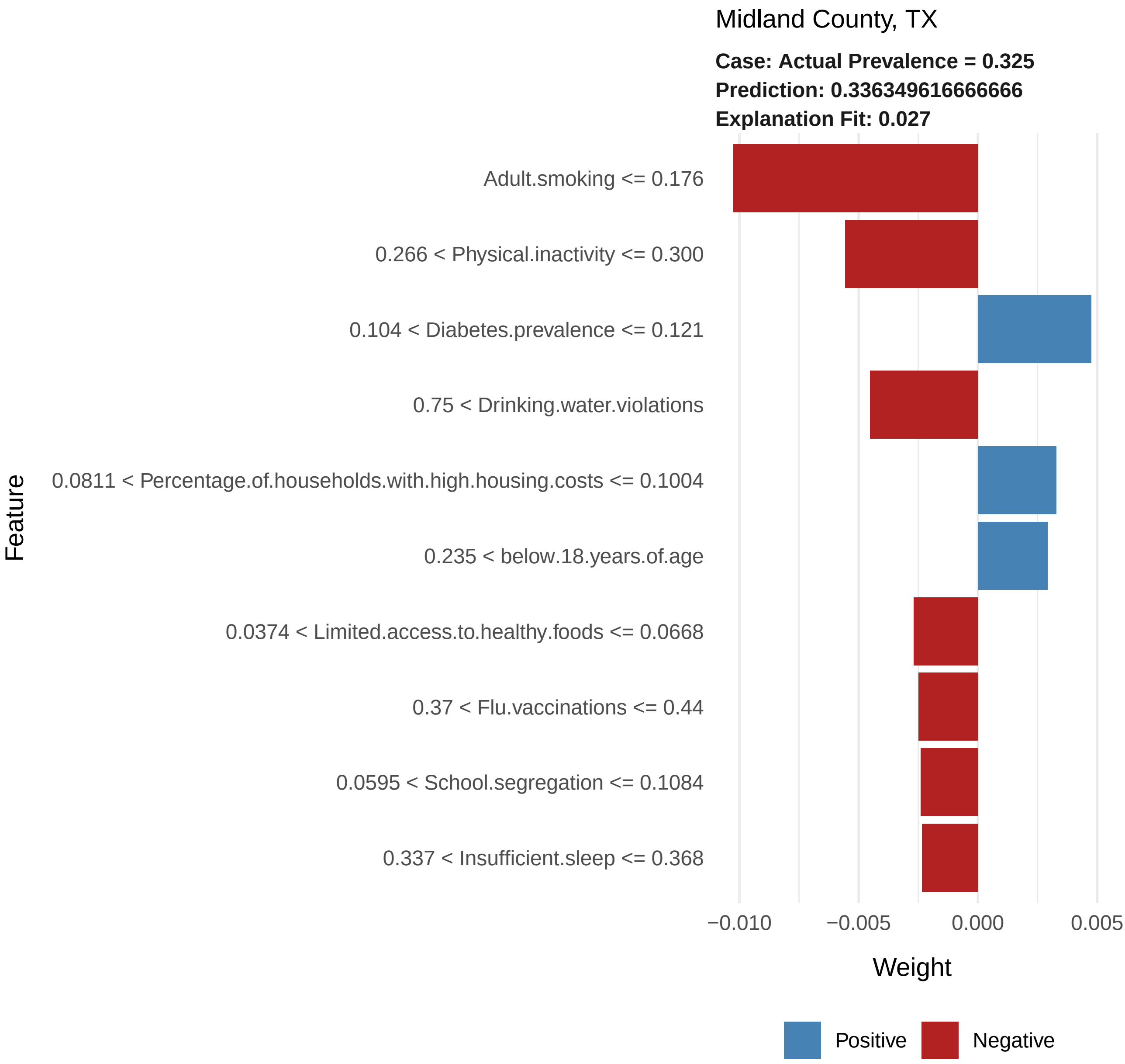
Midland County, TX
Case: Actual Prevalence = 0.325
Prediction: 0.336349616666666
Explanation Fit: 0.027
Adult.smoking <= 0.176
0.266 < Physical.inactivity <= 0.300
0.104 < Diabetes.prevalence <= 0.121
0.75 < Drinking.water.violations
0.0811 < Percentage.of.households.with.high.housing.costs <= 0.1004
0.235 < below.18.years.of.age
0.0374 < Limited.access.to.healthy.foods <= 0.0668
0.37 < Flu.vaccinations <= 0.44
0.0595 < School.segregation <= 0.1084
0.337 < Insufficient.sleep <= 0.368
Feature
−0.010
−0.005
0.000
0.005
Weight
Positive
Negative

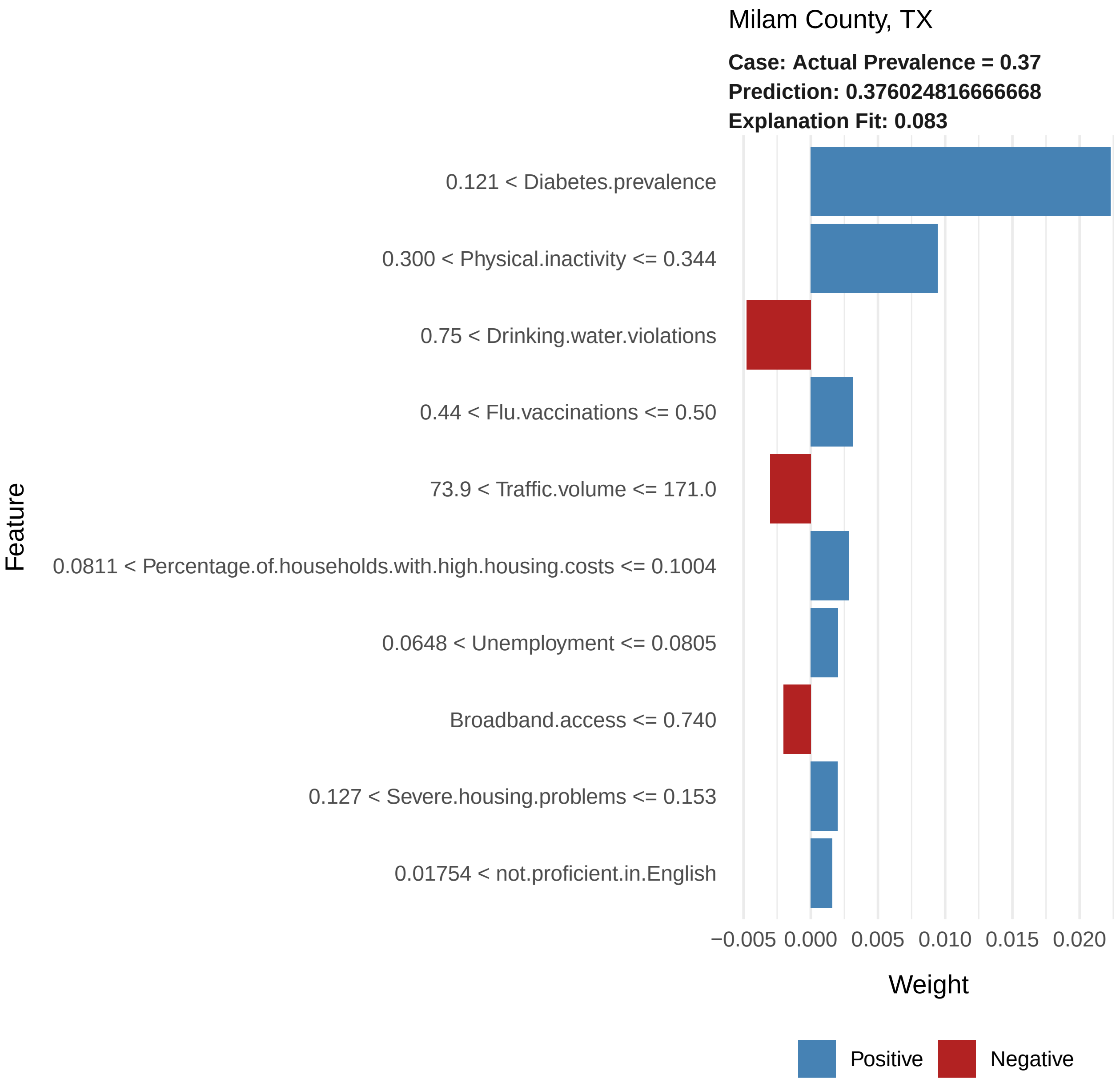

Milam County, TX
Case: Actual Prevalence = 0.37
Prediction: 0.376024816666668
Explanation Fit: 0.083
0.121 < Diabetes.prevalence
0.300 < Physical.inactivity <= 0.344
0.75 < Drinking.water.violations
0.44 < Flu.vaccinations <= 0.50
73.9 < Traffic.volume <= 171.0
0.0811 < Percentage.of.households.with.high.housing.costs <= 0.1004
0.0648 < Unemployment <= 0.0805
Broadband.access <= 0.740
0.127 < Severe.housing.problems <= 0.153
0.01754 < not.proficient.in.English
Feature
−0.005
0.000
0.005
0.010
0.015
0.020
Weight
Positive
Negative

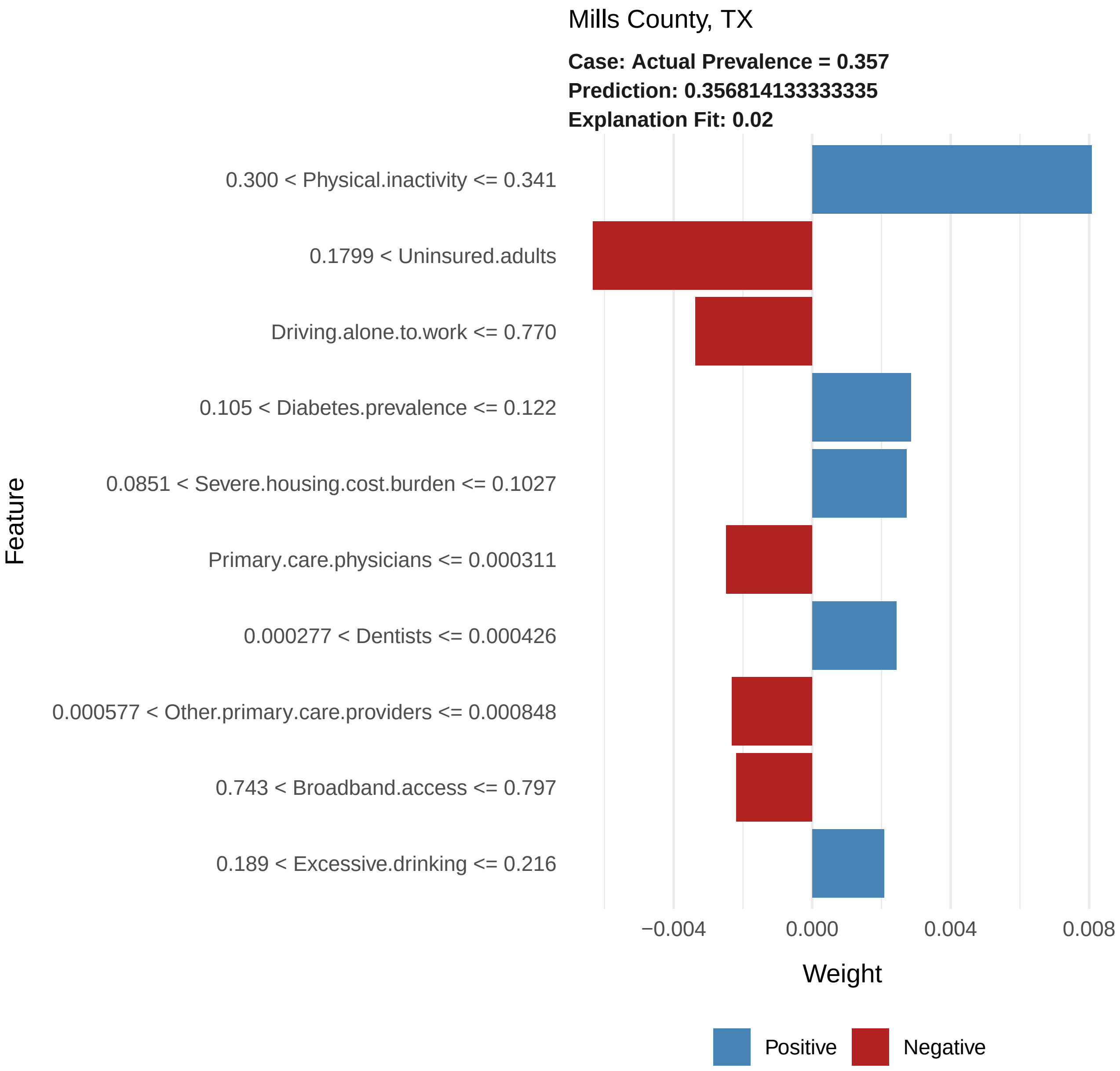

Mills County, TX
Case: Actual Prevalence = 0.357
Prediction: 0.356814133333335
Explanation Fit: 0.02
0.300 < Physical.inactivity <= 0.341
0.1799 < Uninsured.adults
Driving.alone.to.work <= 0.770
0.105 < Diabetes.prevalence <= 0.122
0.0851 < Severe.housing.cost.burden <= 0.1027
Primary.care.physicians <= 0.000311
0.000277 < Dentists <= 0.000426
0.000577 < Other.primary.care.providers <= 0.000848
0.743 < Broadband.access <= 0.797
0.189 < Excessive.drinking <= 0.216
Feature
−0.004
0.000
0.004
0.008
Weight
Positive
Negative

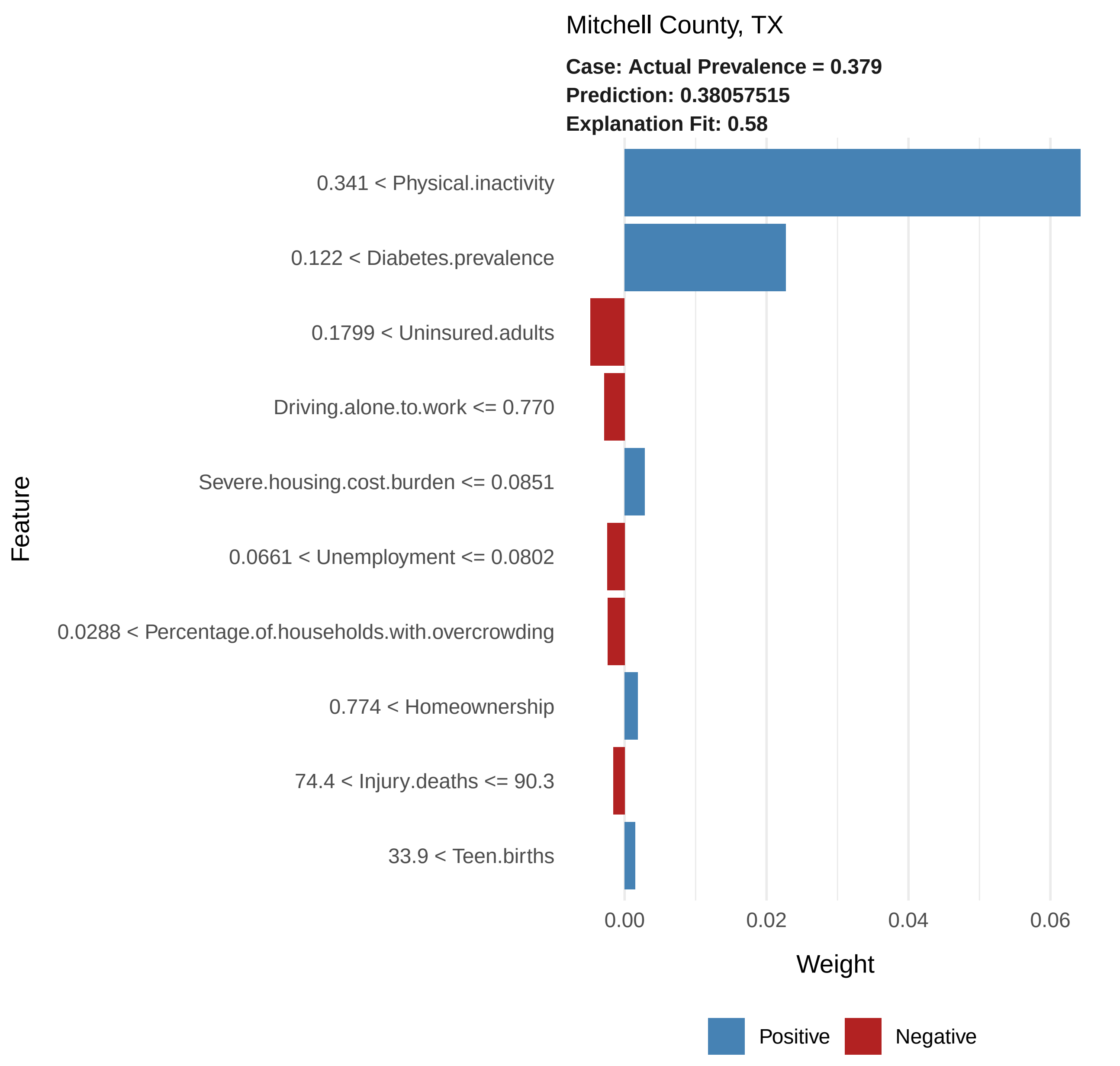

Mitchell County, TX
Case: Actual Prevalence = 0.379
Prediction: 0.38057515
Explanation Fit: 0.58
0.341 < Physical.inactivity
0.122 < Diabetes.prevalence
0.1799 < Uninsured.adults
Driving.alone.to.work <= 0.770
Severe.housing.cost.burden <= 0.0851
0.0661 < Unemployment <= 0.0802
0.0288 < Percentage.of.households.with.overcrowding
0.774 < Homeownership
74.4 < Injury.deaths <= 90.3
33.9 < Teen.births
Feature
0.00
0.02
0.04
0.06
Weight
Positive
Negative

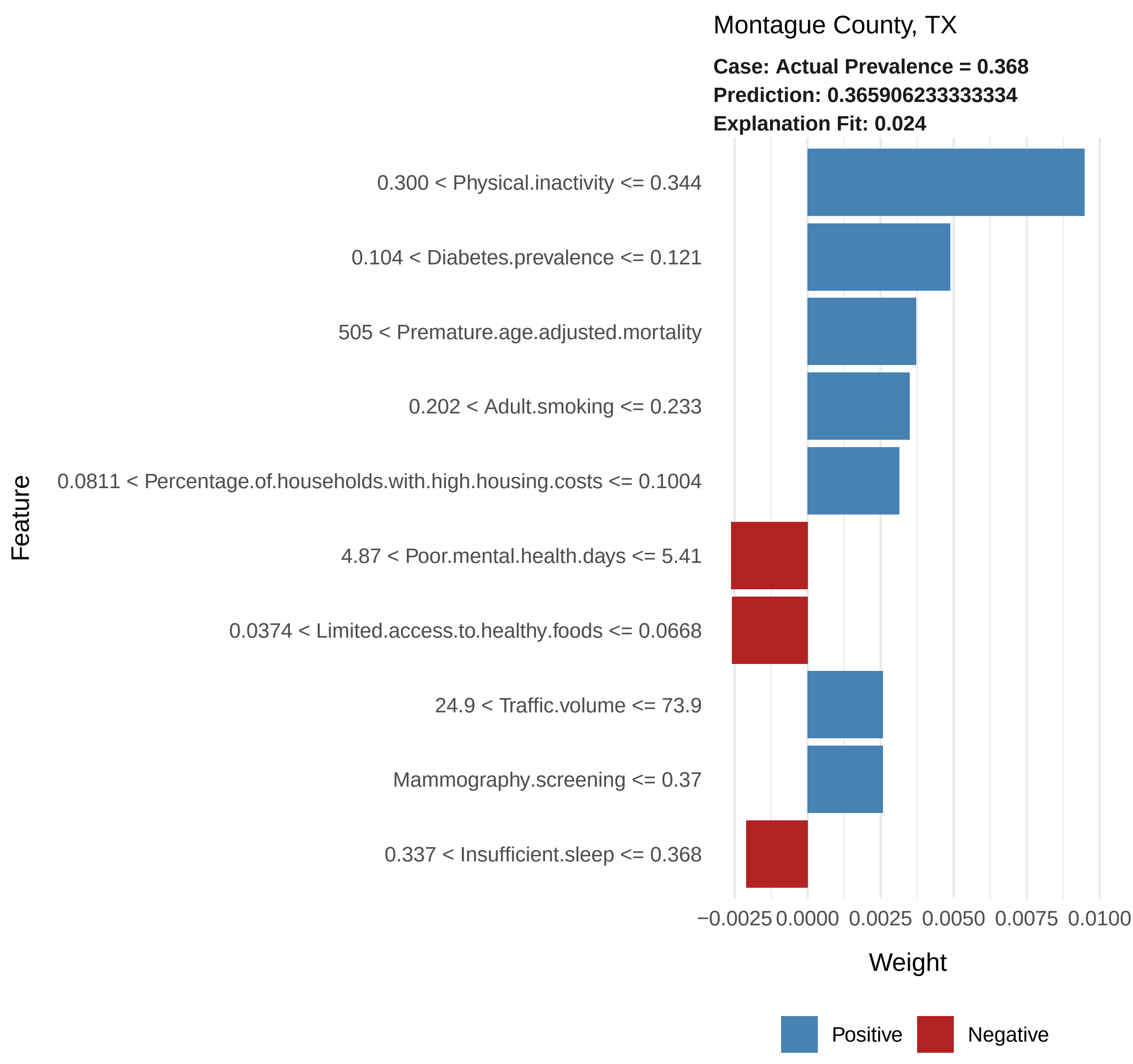
Montague County, TX
Case: Actual Prevalence = 0.368
Prediction: 0.365906233333334
Explanation Fit: 0.024
0.300 < Physical.inactivity <= 0.344
0.104 < Diabetes.prevalence <= 0.121
505 < Premature.age.adjusted.mortality
0.202 < Adult.smoking <= 0.233
0.0811 < Percentage.of.households.with.high.housing.costs <= 0.1004
4.87 < Poor.mental.health.days <= 5.41
0.0374 < Limited.access.to.healthy.foods <= 0.0668
24.9 < Traffic.volume <= 73.9
Mammography.screening <= 0.37
0.337 < Insufficient.sleep <= 0.368
Feature
−0.0025
0.0000
0.0025
0.0050
0.0075
0.0100
Weight
Positive
Negative

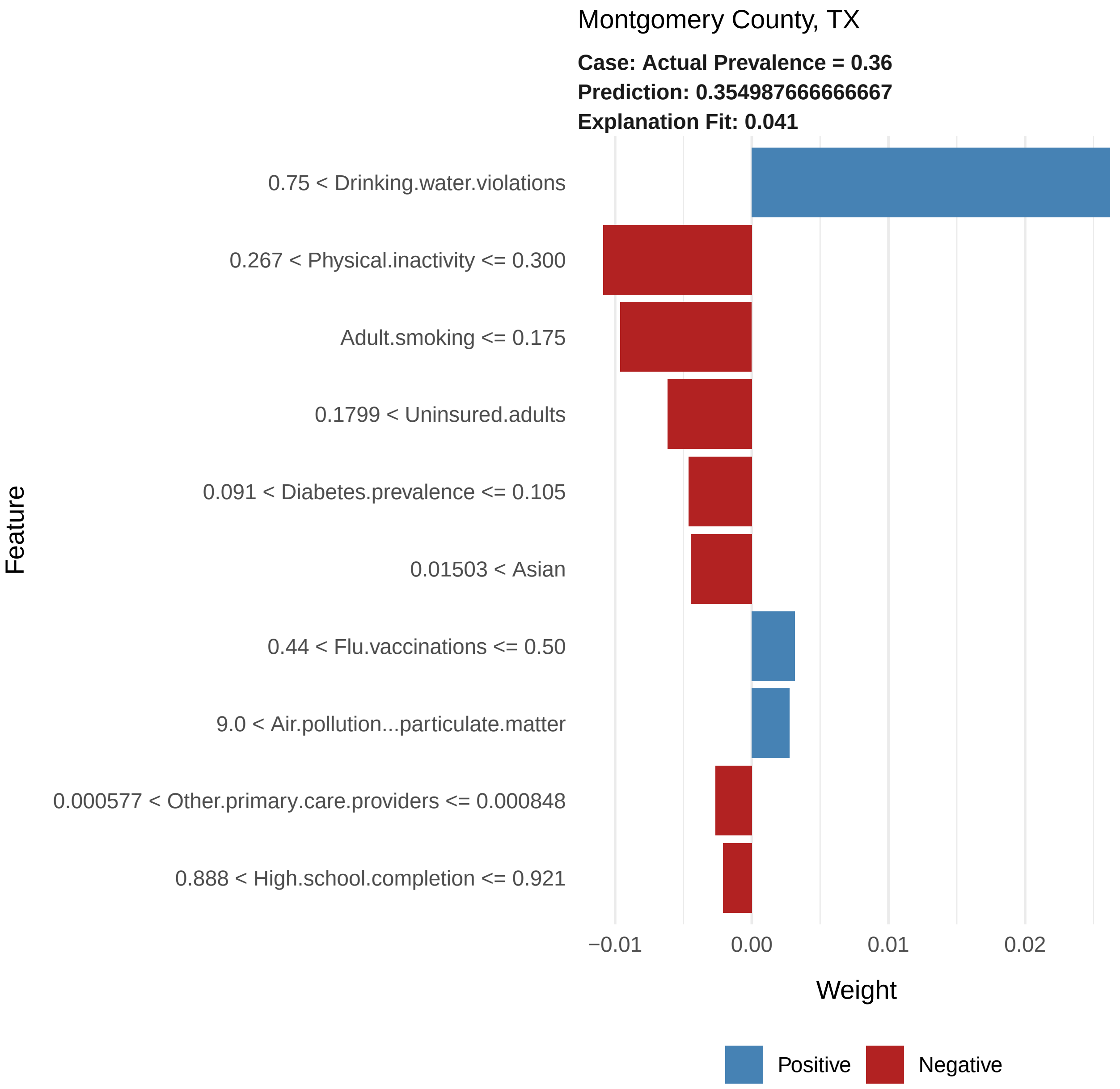
Montgomery County, TX
Case: Actual Prevalence = 0.36
Prediction: 0.354987666666667
Explanation Fit: 0.041
0.75 < Drinking.water.violations
0.267 < Physical.inactivity <= 0.300
Adult.smoking <= 0.175
0.1799 < Uninsured.adults
0.091 < Diabetes.prevalence <= 0.105
0.01503 < Asian
0.44 < Flu.vaccinations <= 0.50
9.0 < Air.pollution...particulate.matter
0.000577 < Other.primary.care.providers <= 0.000848
0.888 < High.school.completion <= 0.921
Feature
−0.01
0.00
0.01
0.02
Weight
Positive
Negative

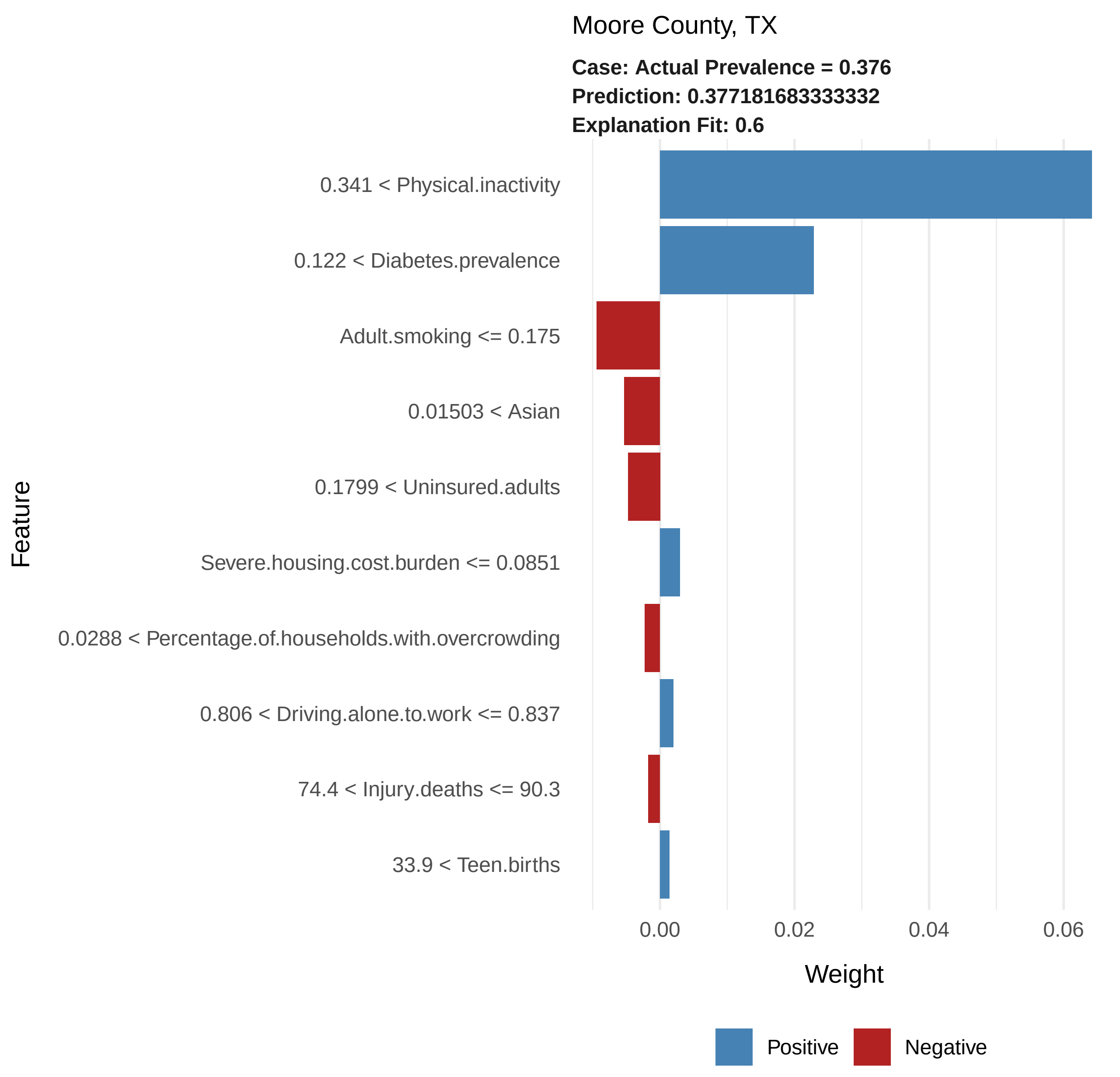

Moore County, TX
Case: Actual Prevalence = 0.376
Prediction: 0.377181683333332
Explanation Fit: 0.6
0.341 < Physical.inactivity
0.122 < Diabetes.prevalence
Adult.smoking <= 0.175
0.01503 < Asian
0.1799 < Uninsured.adults
Severe.housing.cost.burden <= 0.0851
0.0288 < Percentage.of.households.with.overcrowding
0.806 < Driving.alone.to.work <= 0.837
74.4 < Injury.deaths <= 90.3
33.9 < Teen.births
Feature
0.00
0.02
0.04
0.06
Weight
Positive
Negative

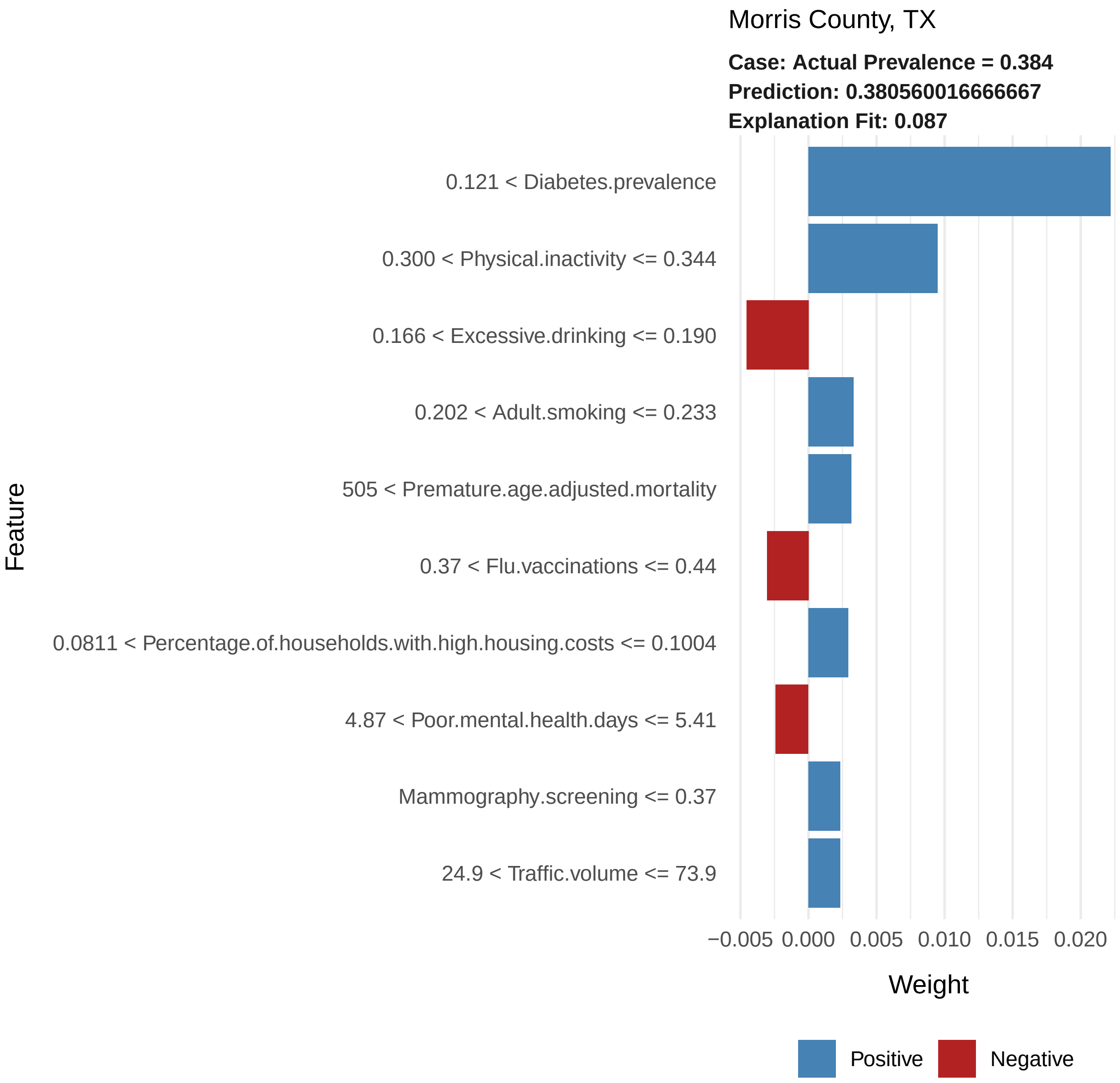
Morris County, TX
Case: Actual Prevalence = 0.384
Prediction: 0.380560016666667
Explanation Fit: 0.087
0.121 < Diabetes.prevalence
0.300 < Physical.inactivity <= 0.344
0.166 < Excessive.drinking <= 0.190
0.202 < Adult.smoking <= 0.233
505 < Premature.age.adjusted.mortality
0.37 < Flu.vaccinations <= 0.44
0.0811 < Percentage.of.households.with.high.housing.costs <= 0.1004
4.87 < Poor.mental.health.days <= 5.41
Mammography.screening <= 0.37
24.9 < Traffic.volume <= 73.9
Feature
−0.005
0.000
0.005
0.010
0.015
0.020
Weight
Positive
Negative

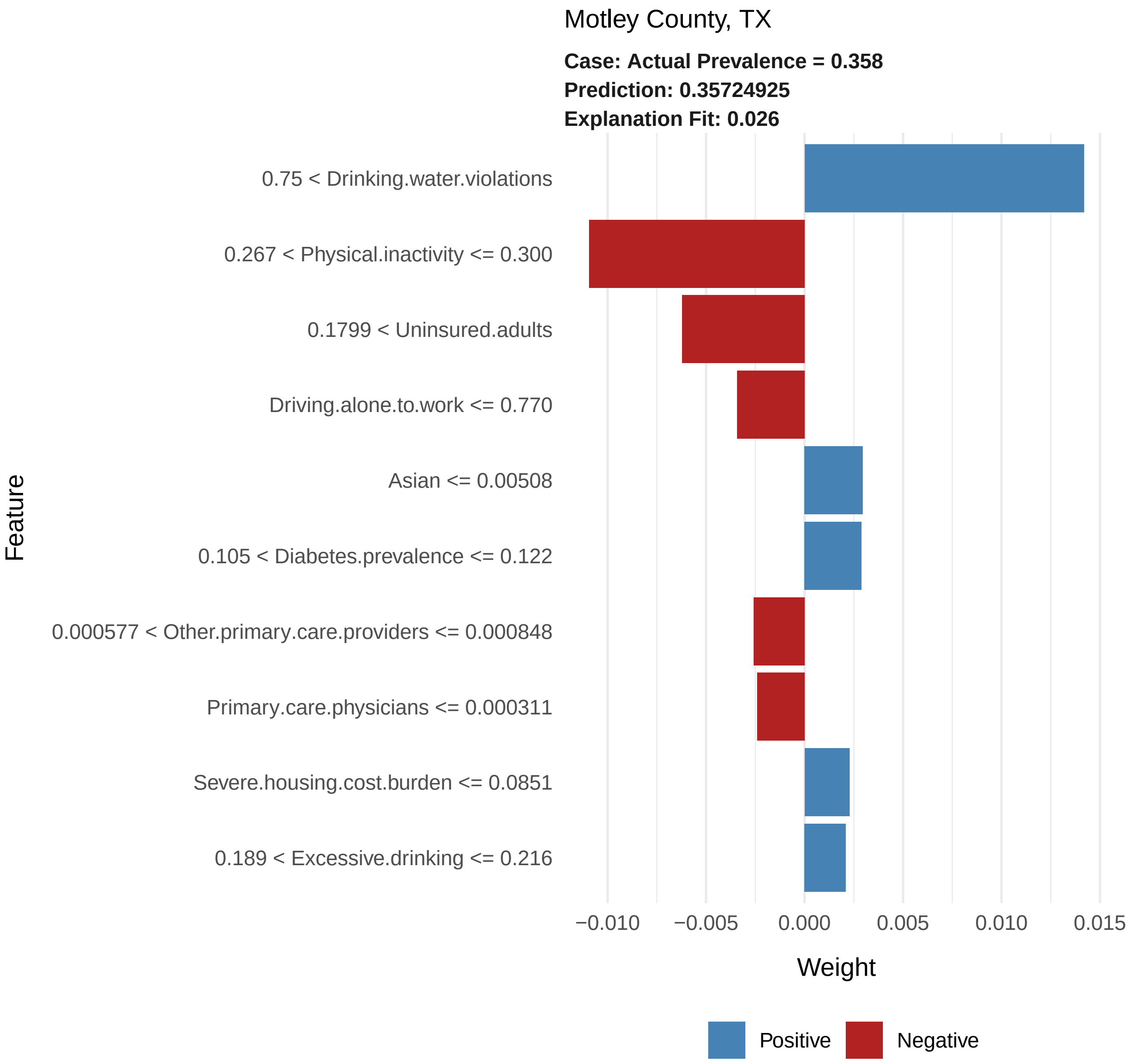
Motley County, TX
Case: Actual Prevalence = 0.358
Prediction: 0.35724925
Explanation Fit: 0.026
0.75 < Drinking.water.violations
0.267 < Physical.inactivity <= 0.300
0.1799 < Uninsured.adults
Driving.alone.to.work <= 0.770
Asian <= 0.00508
0.105 < Diabetes.prevalence <= 0.122
0.000577 < Other.primary.care.providers <= 0.000848
Primary.care.physicians <= 0.000311
Severe.housing.cost.burden <= 0.0851
0.189 < Excessive.drinking <= 0.216
Feature
−0.010
−0.005
0.000
0.005
0.010
0.015
Weight
Positive
Negative

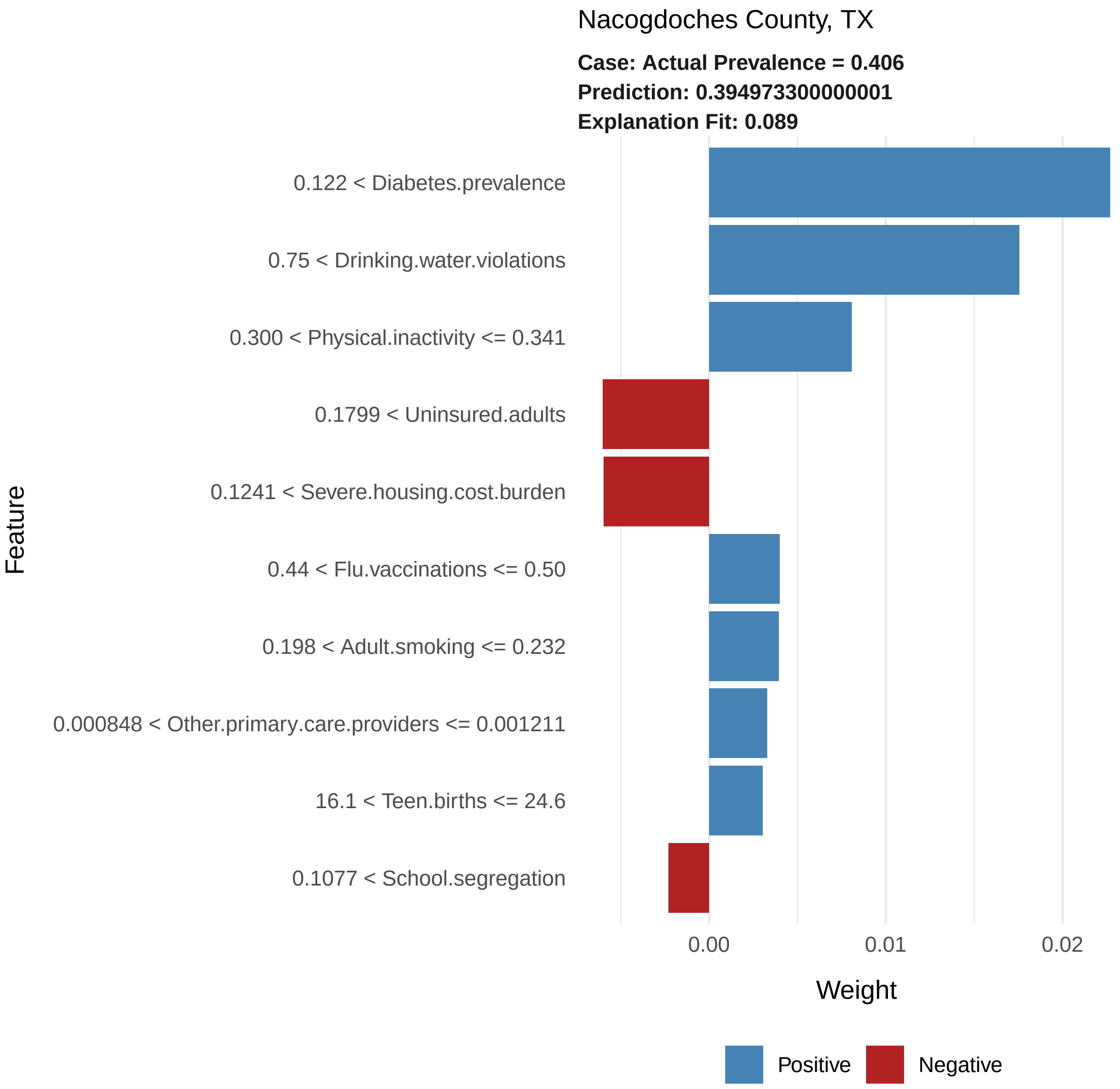

Nacogdoches County, TX
Case: Actual Prevalence = 0.406
Prediction: 0.394973300000001
Explanation Fit: 0.089
0.122 < Diabetes.prevalence
0.75 < Drinking.water.violations
0.300 < Physical.inactivity <= 0.341
0.1799 < Uninsured.adults
0.1241 < Severe.housing.cost.burden
0.44 < Flu.vaccinations <= 0.50
0.198 < Adult.smoking <= 0.232
0.000848 < Other.primary.care.providers <= 0.001211
16.1 < Teen.births <= 24.6
0.1077 < School.segregation
Feature
0.00
0.01
0.02
Weight
Positive
Negative

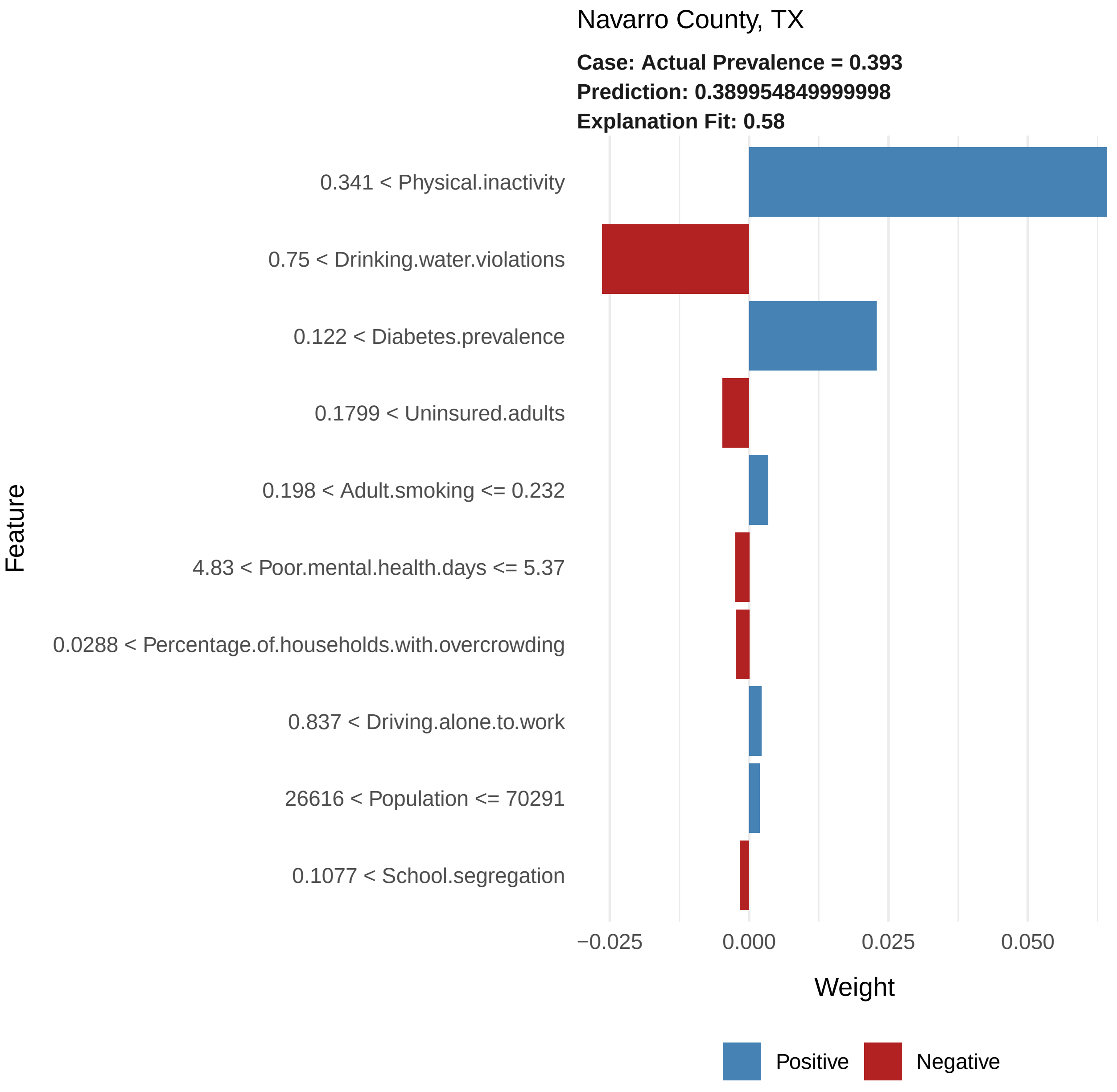
Navarro County, TX
Case: Actual Prevalence = 0.393
Prediction: 0.389954849999998
Explanation Fit: 0.58
0.341 < Physical.inactivity
0.75 < Drinking.water.violations
0.122 < Diabetes.prevalence
0.1799 < Uninsured.adults
0.198 < Adult.smoking <= 0.232
4.83 < Poor.mental.health.days <= 5.37
0.0288 < Percentage.of.households.with.overcrowding
0.837 < Driving.alone.to.work
26616 < Population <= 70291
0.1077 < School.segregation
Feature
−0.025
0.000
0.025
0.050
Weight
Positive
Negative

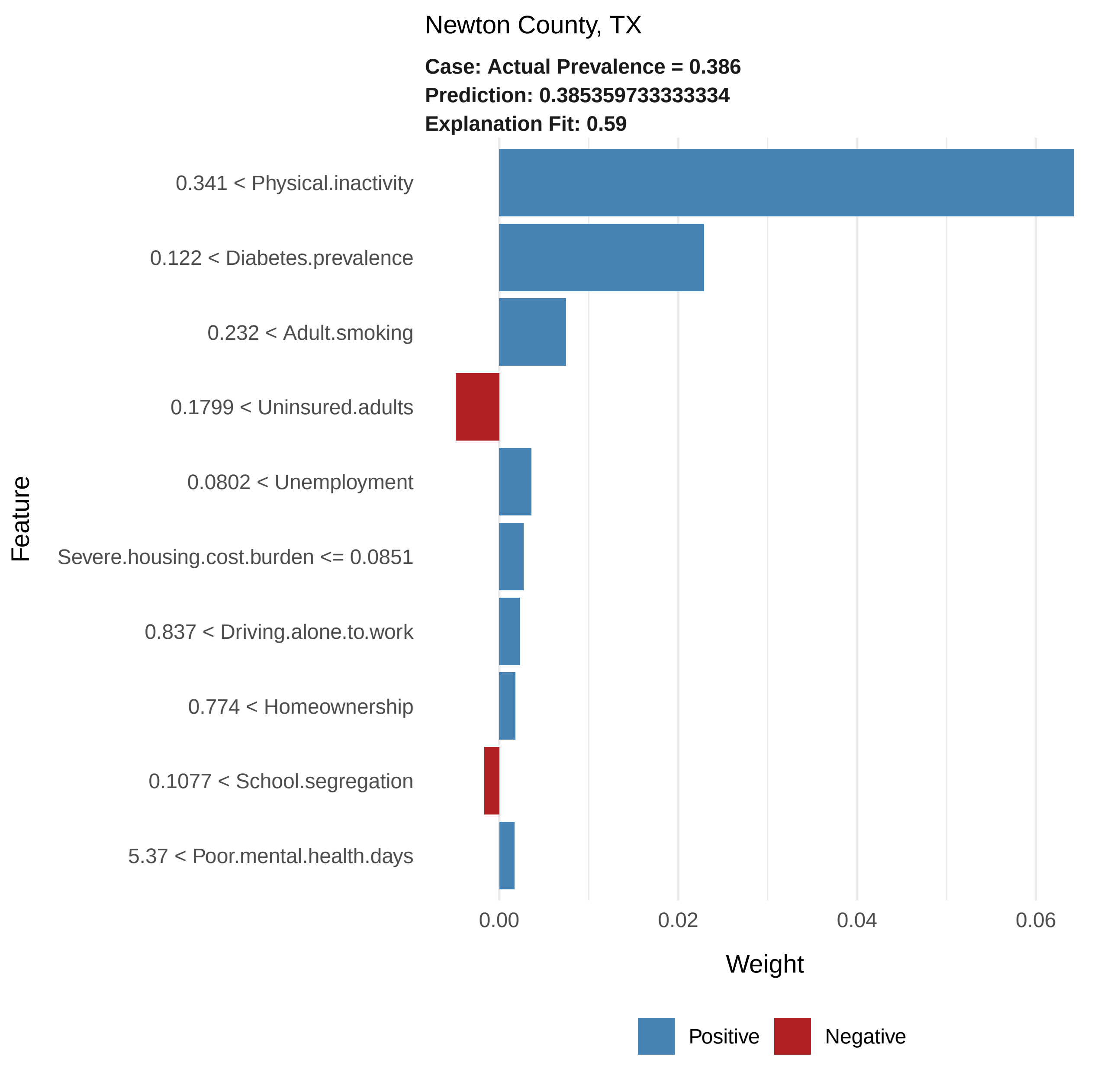
Newton County, TX
Case: Actual Prevalence = 0.386
Prediction: 0.385359733333334
Explanation Fit: 0.59
0.341 < Physical.inactivity
0.122 < Diabetes.prevalence
0.232 < Adult.smoking
0.1799 < Uninsured.adults
0.0802 < Unemployment
Severe.housing.cost.burden <= 0.0851
0.837 < Driving.alone.to.work
0.774 < Homeownership
0.1077 < School.segregation
5.37 < Poor.mental.health.days
Feature
0.00
0.02
0.04
0.06
Weight
Positive
Negative

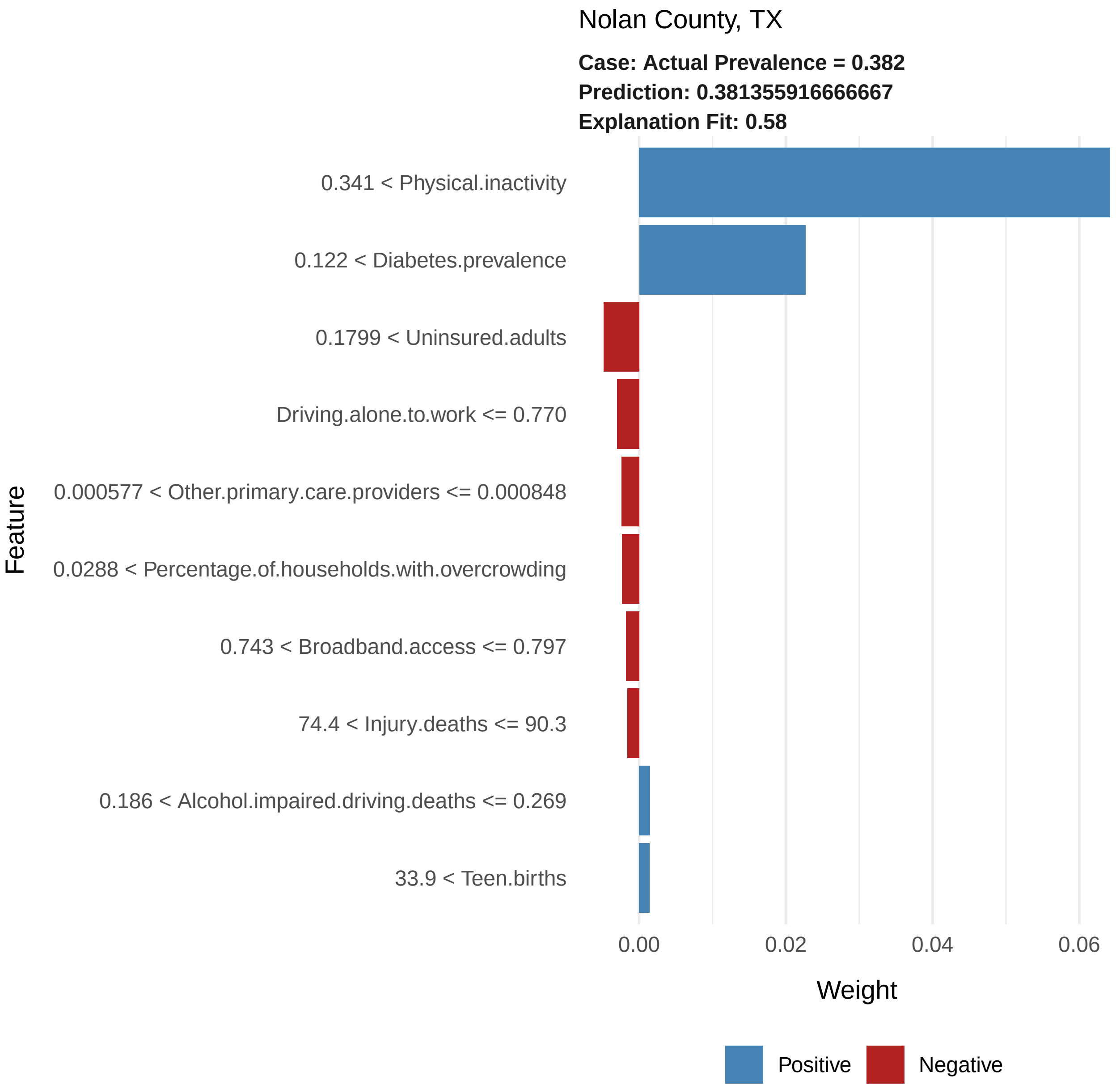
Nolan County, TX
Case: Actual Prevalence = 0.382
Prediction: 0.381355916666667
Explanation Fit: 0.58
0.341 < Physical.inactivity
0.122 < Diabetes.prevalence
0.1799 < Uninsured.adults
Driving.alone.to.work <= 0.770
0.000577 < Other.primary.care.providers <= 0.000848
0.0288 < Percentage.of.households.with.overcrowding
0.743 < Broadband.access <= 0.797
74.4 < Injury.deaths <= 90.3
0.186 < Alcohol.impaired.driving.deaths <= 0.269
33.9 < Teen.births
Feature
0.00
0.02
0.04
0.06
Weight
Positive
Negative

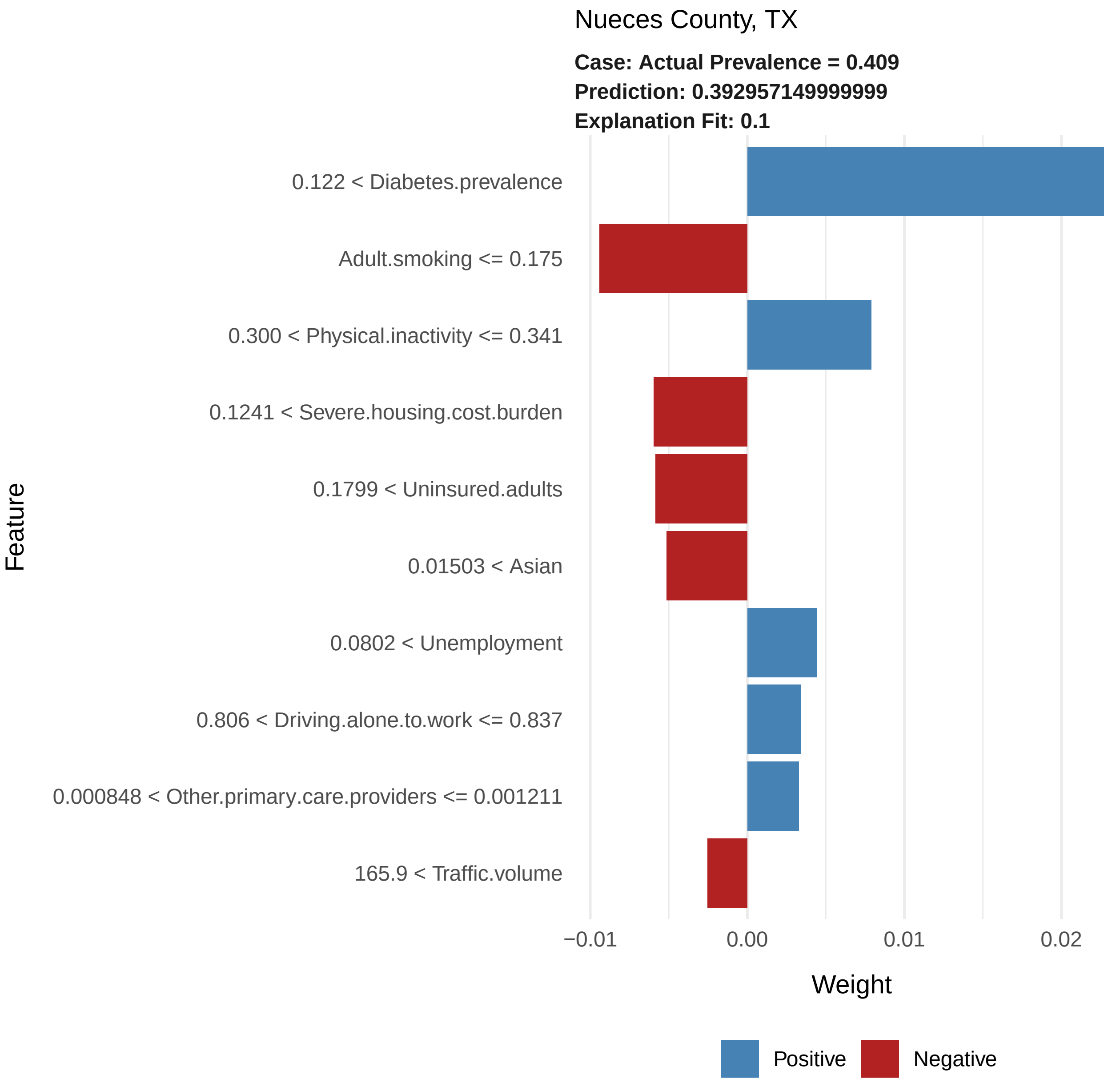

Nueces County, TX
Case: Actual Prevalence = 0.409
Prediction: 0.392957149999999
Explanation Fit: 0.1
0.122 < Diabetes.prevalence
Adult.smoking <= 0.175
0.300 < Physical.inactivity <= 0.341
0.1241 < Severe.housing.cost.burden
0.1799 < Uninsured.adults
0.01503 < Asian
0.0802 < Unemployment
0.806 < Driving.alone.to.work <= 0.837
0.000848 < Other.primary.care.providers <= 0.001211
165.9 < Traffic.volume
Feature
−0.01
0.00
0.01
0.02
Weight
Positive
Negative

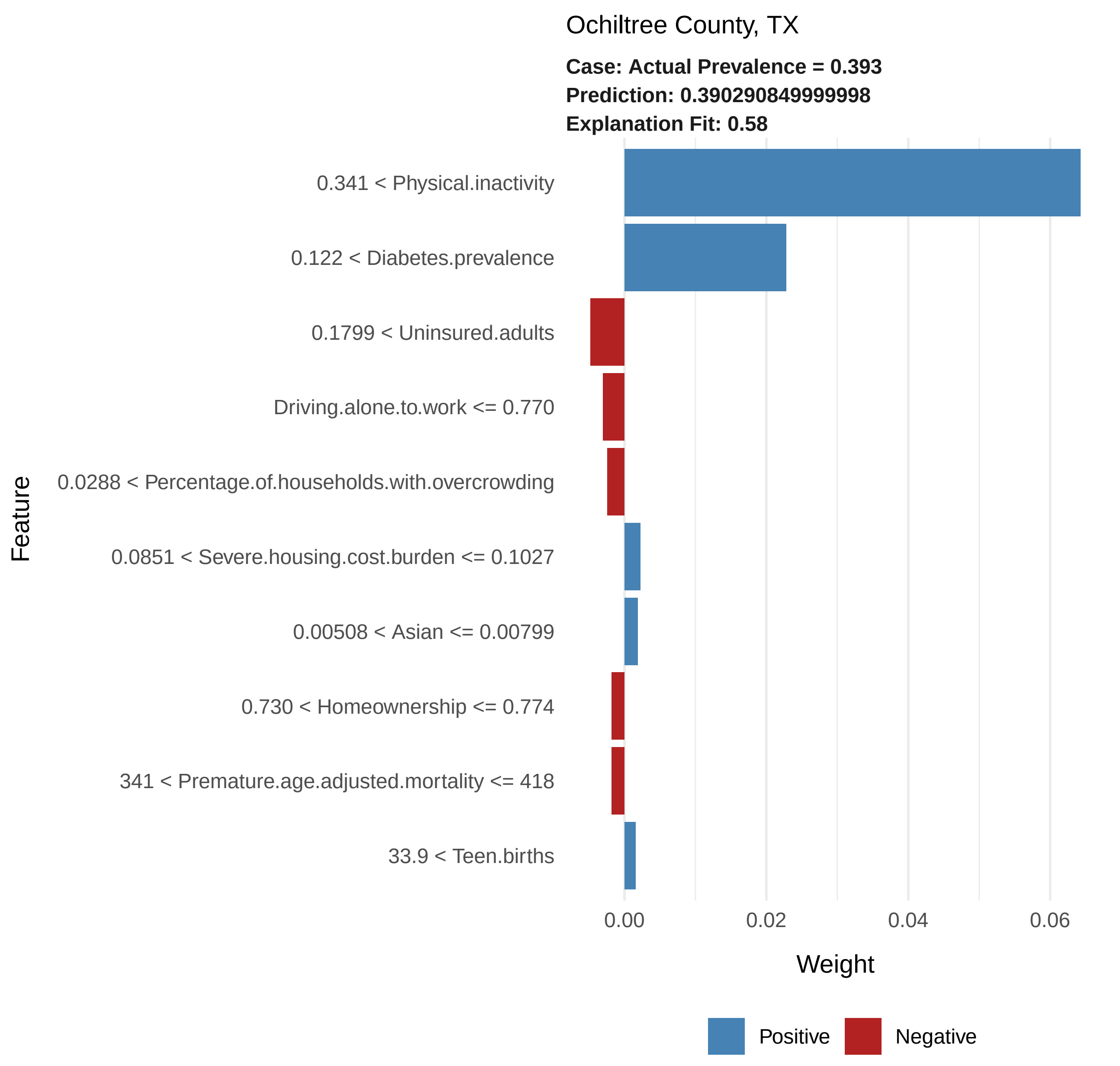

Ochiltree County, TX
Case: Actual Prevalence = 0.393
Prediction: 0.390290849999998
Explanation Fit: 0.58
0.341 < Physical.inactivity
0.122 < Diabetes.prevalence
0.1799 < Uninsured.adults
Driving.alone.to.work <= 0.770
0.0288 < Percentage.of.households.with.overcrowding
0.0851 < Severe.housing.cost.burden <= 0.1027
0.00508 < Asian <= 0.00799
0.730 < Homeownership <= 0.774
341 < Premature.age.adjusted.mortality <= 418
33.9 < Teen.births
Feature
0.00
0.02
0.04
0.06
Weight
Positive
Negative

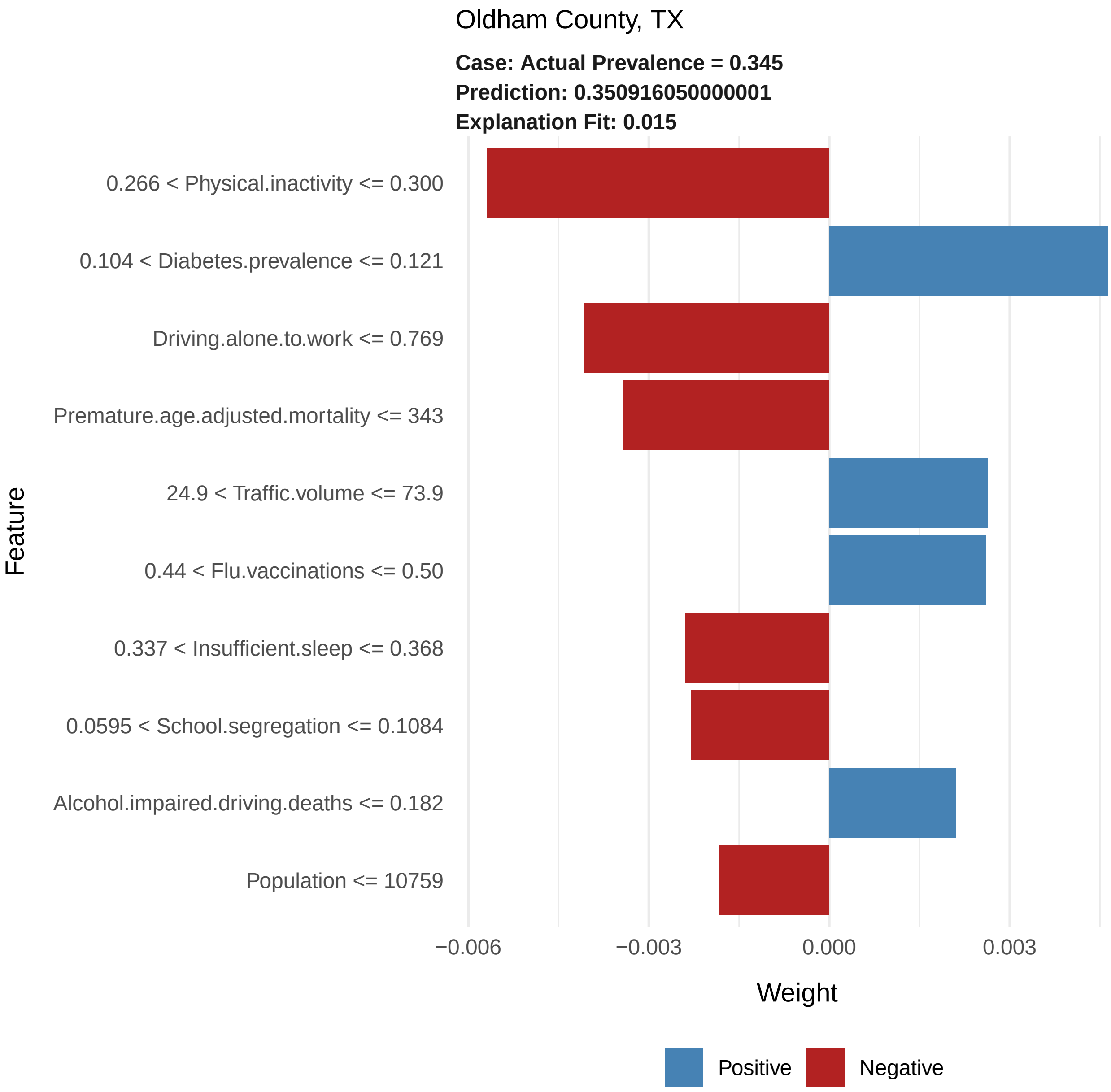

Oldham County, TX
Case: Actual Prevalence = 0.345
Prediction: 0.350916050000001
Explanation Fit: 0.015
0.266 < Physical.inactivity <= 0.300
0.104 < Diabetes.prevalence <= 0.121
Driving.alone.to.work <= 0.769
Premature.age.adjusted.mortality <= 343
24.9 < Traffic.volume <= 73.9
0.44 < Flu.vaccinations <= 0.50
0.337 < Insufficient.sleep <= 0.368
0.0595 < School.segregation <= 0.1084
Alcohol.impaired.driving.deaths <= 0.182
Population <= 10759
Feature
−0.006
−0.003
0.000
0.003
Weight
Positive
Negative

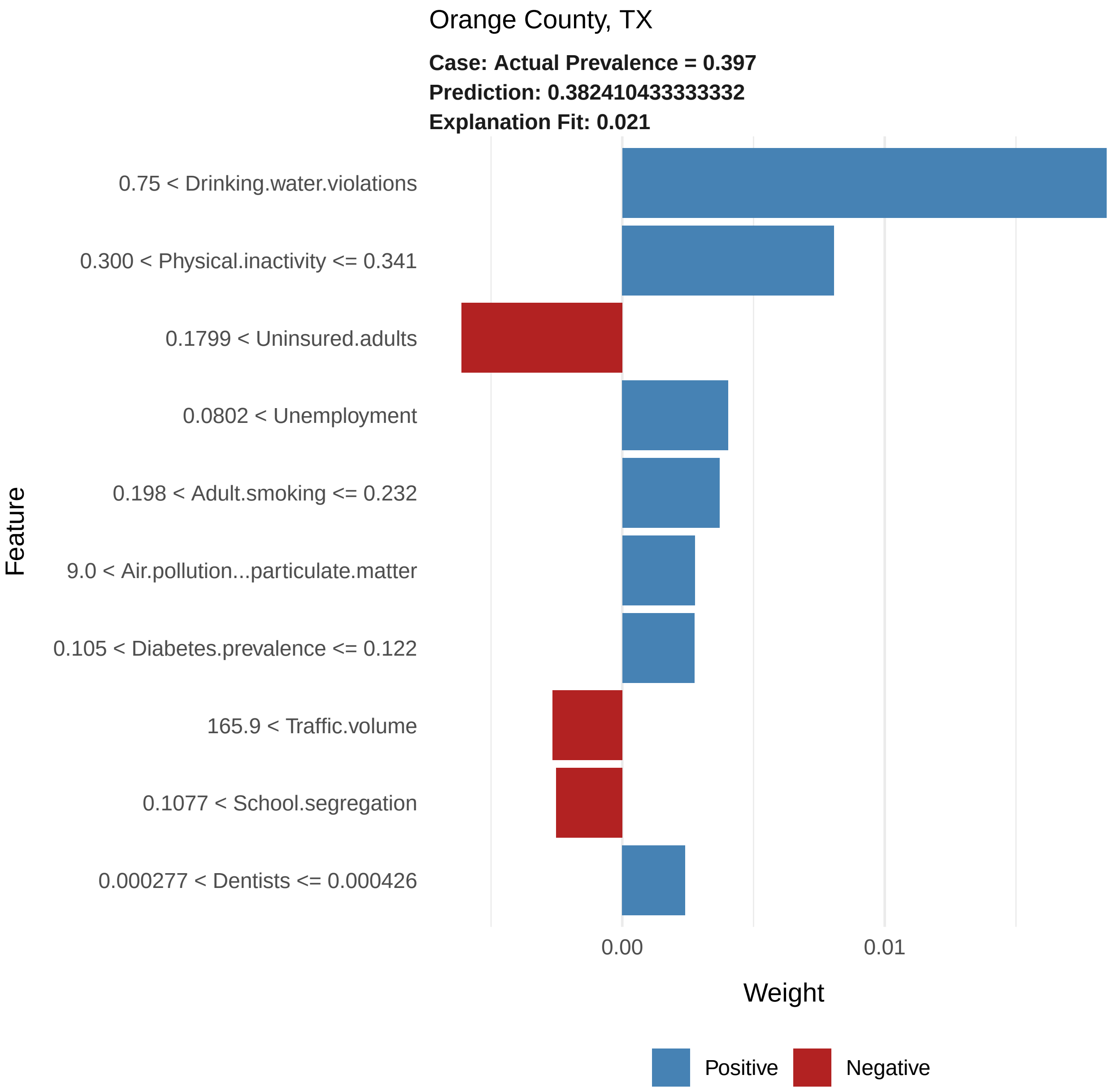

Orange County, TX
Case: Actual Prevalence = 0.397
Prediction: 0.382410433333332
Explanation Fit: 0.021
0.75 < Drinking.water.violations
0.300 < Physical.inactivity <= 0.341
0.1799 < Uninsured.adults
0.0802 < Unemployment
0.198 < Adult.smoking <= 0.232
9.0 < Air.pollution...particulate.matter
0.105 < Diabetes.prevalence <= 0.122
165.9 < Traffic.volume
0.1077 < School.segregation
0.000277 < Dentists <= 0.000426
Feature
0.00
0.01
Weight
Positive
Negative

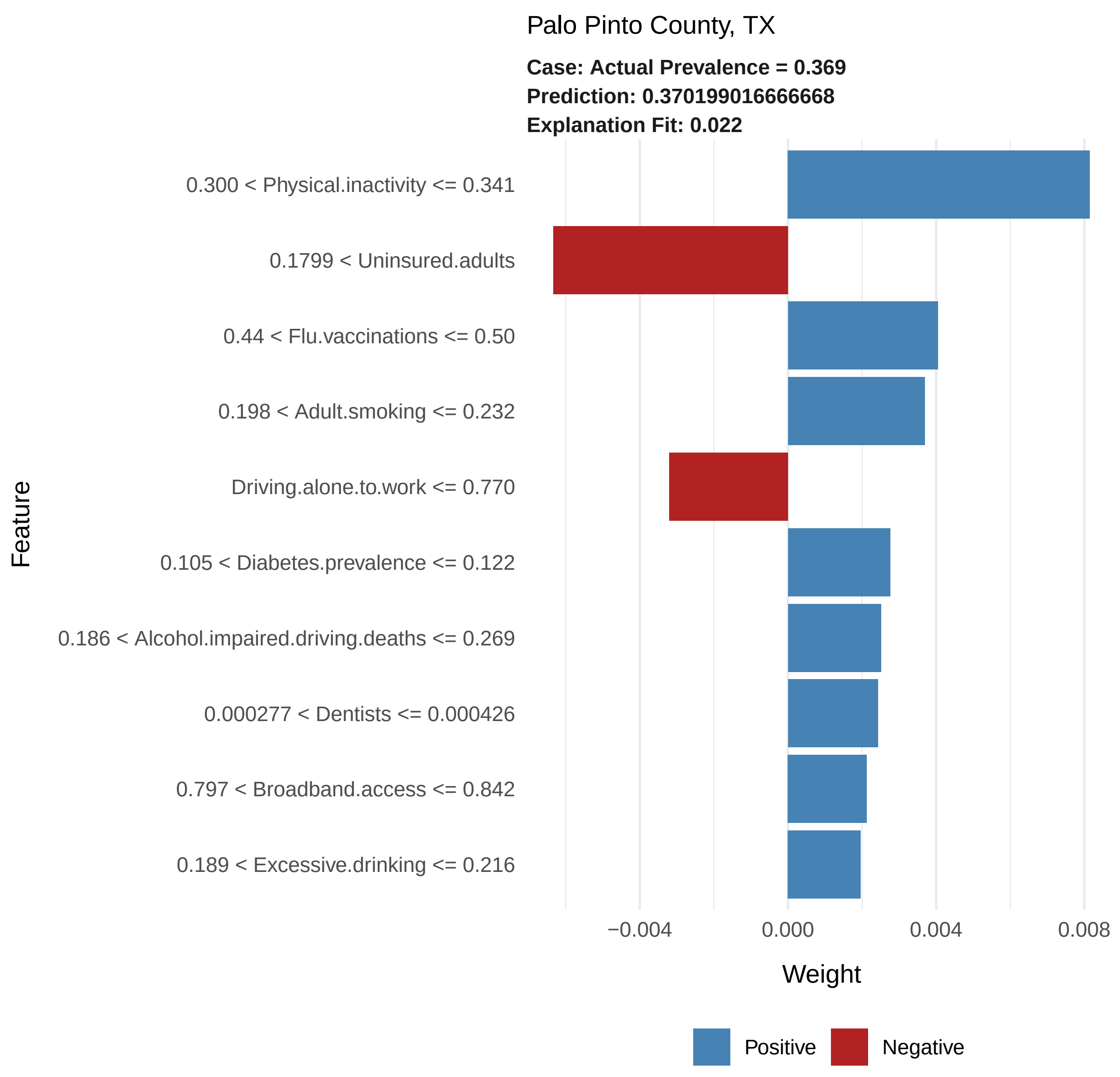
Palo Pinto County, TX
Case: Actual Prevalence = 0.369
Prediction: 0.370199016666668
Explanation Fit: 0.022
0.300 < Physical.inactivity <= 0.341
0.1799 < Uninsured.adults
0.44 < Flu.vaccinations <= 0.50
0.198 < Adult.smoking <= 0.232
Driving.alone.to.work <= 0.770
0.105 < Diabetes.prevalence <= 0.122
0.186 < Alcohol.impaired.driving.deaths <= 0.269
0.000277 < Dentists <= 0.000426
0.797 < Broadband.access <= 0.842
0.189 < Excessive.drinking <= 0.216
Feature
−0.004
0.000
0.004
0.008
Weight
Positive
Negative

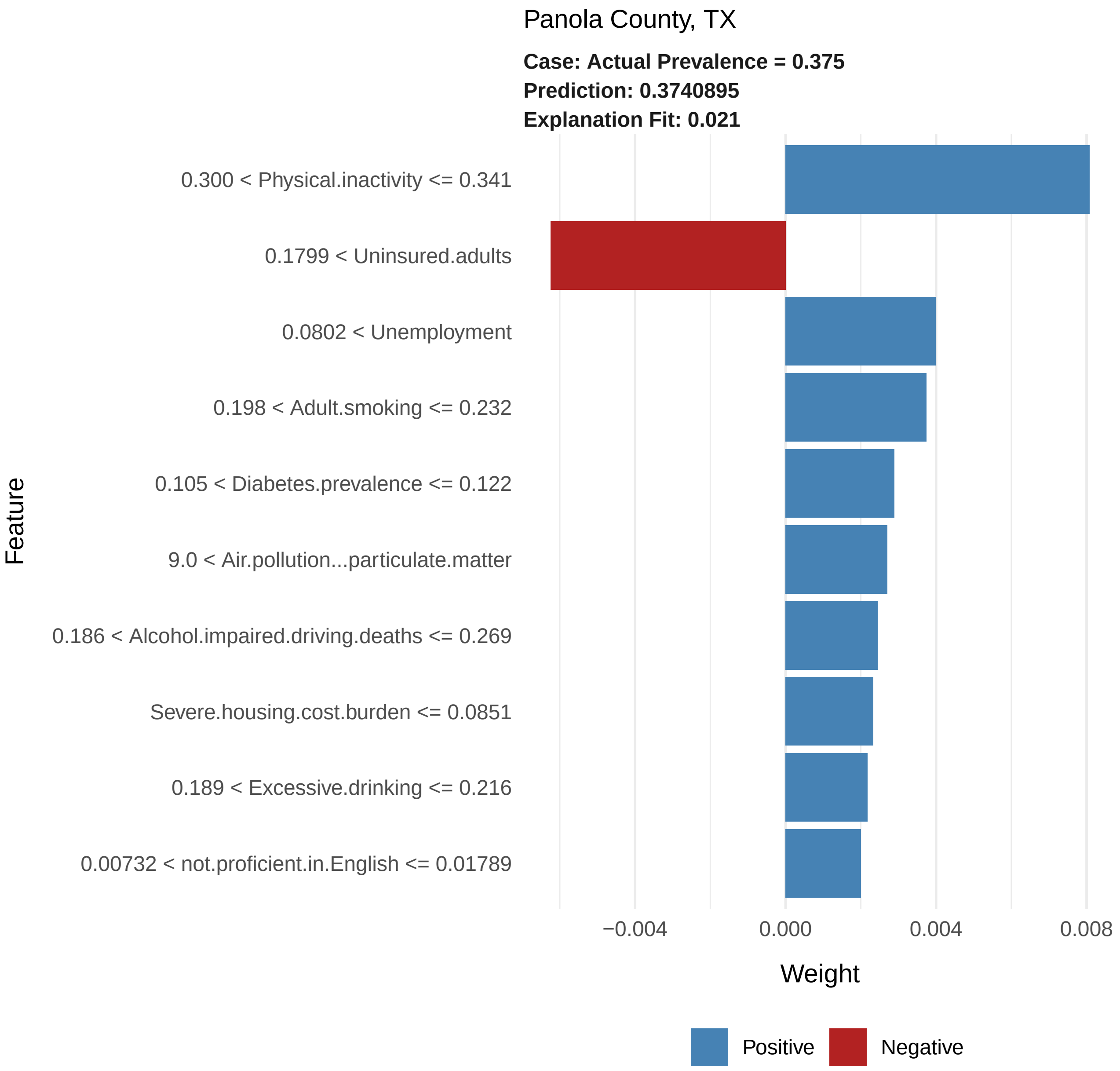

Panola County, TX
Case: Actual Prevalence = 0.375
Prediction: 0.3740895
Explanation Fit: 0.021
0.300 < Physical.inactivity <= 0.341
0.1799 < Uninsured.adults
0.0802 < Unemployment
0.198 < Adult.smoking <= 0.232
0.105 < Diabetes.prevalence <= 0.122
9.0 < Air.pollution...particulate.matter
0.186 < Alcohol.impaired.driving.deaths <= 0.269
Severe.housing.cost.burden <= 0.0851
0.189 < Excessive.drinking <= 0.216
0.00732 < not.proficient.in.English <= 0.01789
Feature
−0.004
0.000
0.004
0.008
Weight
Positive
Negative

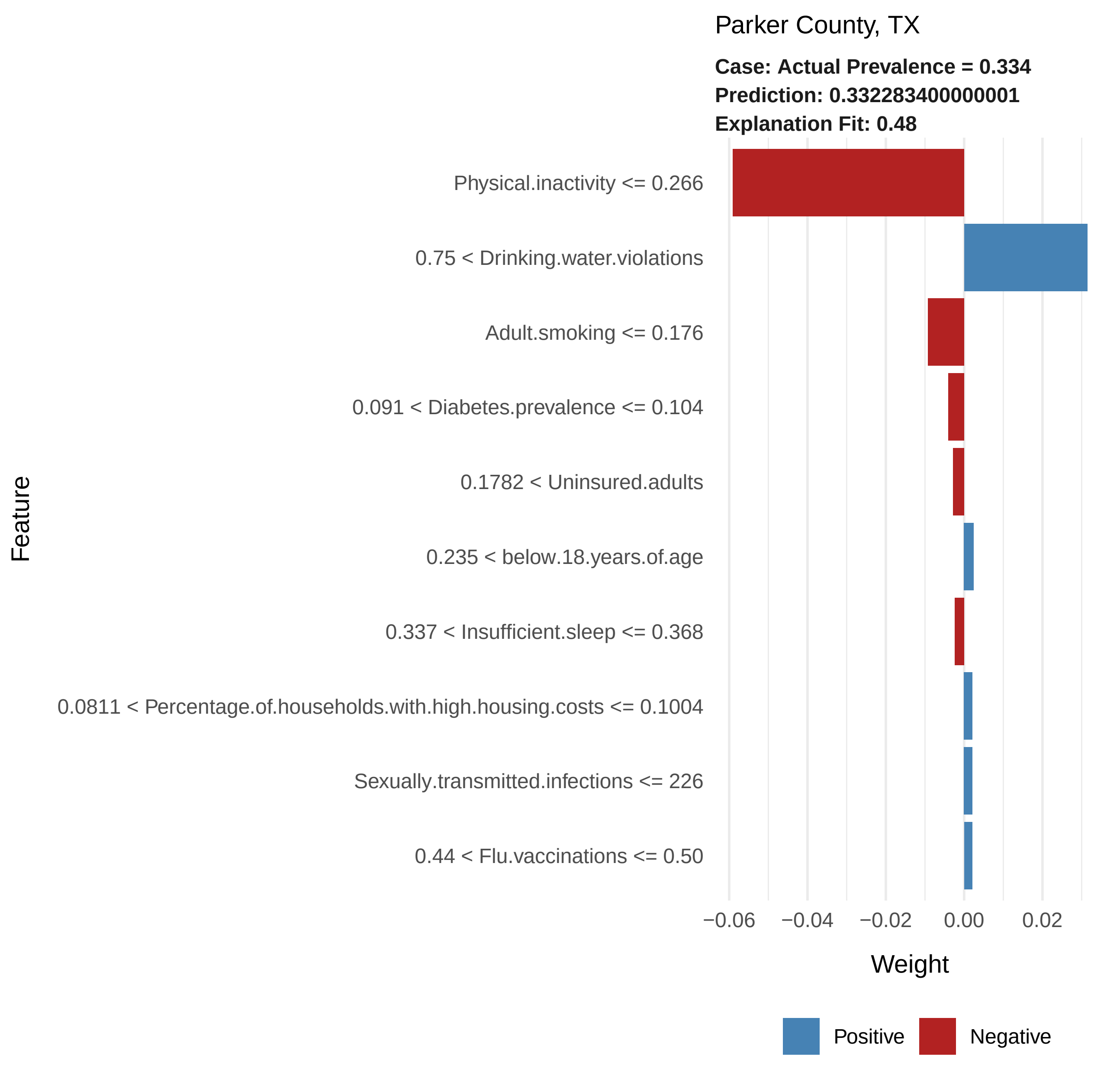
Parker County, TX
Case: Actual Prevalence = 0.334
Prediction: 0.332283400000001
Explanation Fit: 0.48
Physical.inactivity <= 0.266
0.75 < Drinking.water.violations
Adult.smoking <= 0.176
0.091 < Diabetes.prevalence <= 0.104
0.1782 < Uninsured.adults
0.235 < below.18.years.of.age
0.337 < Insufficient.sleep <= 0.368
0.0811 < Percentage.of.households.with.high.housing.costs <= 0.1004
Sexually.transmitted.infections <= 226
0.44 < Flu.vaccinations <= 0.50
Feature
−0.06
−0.04
−0.02
0.00
0.02
Weight
Positive
Negative

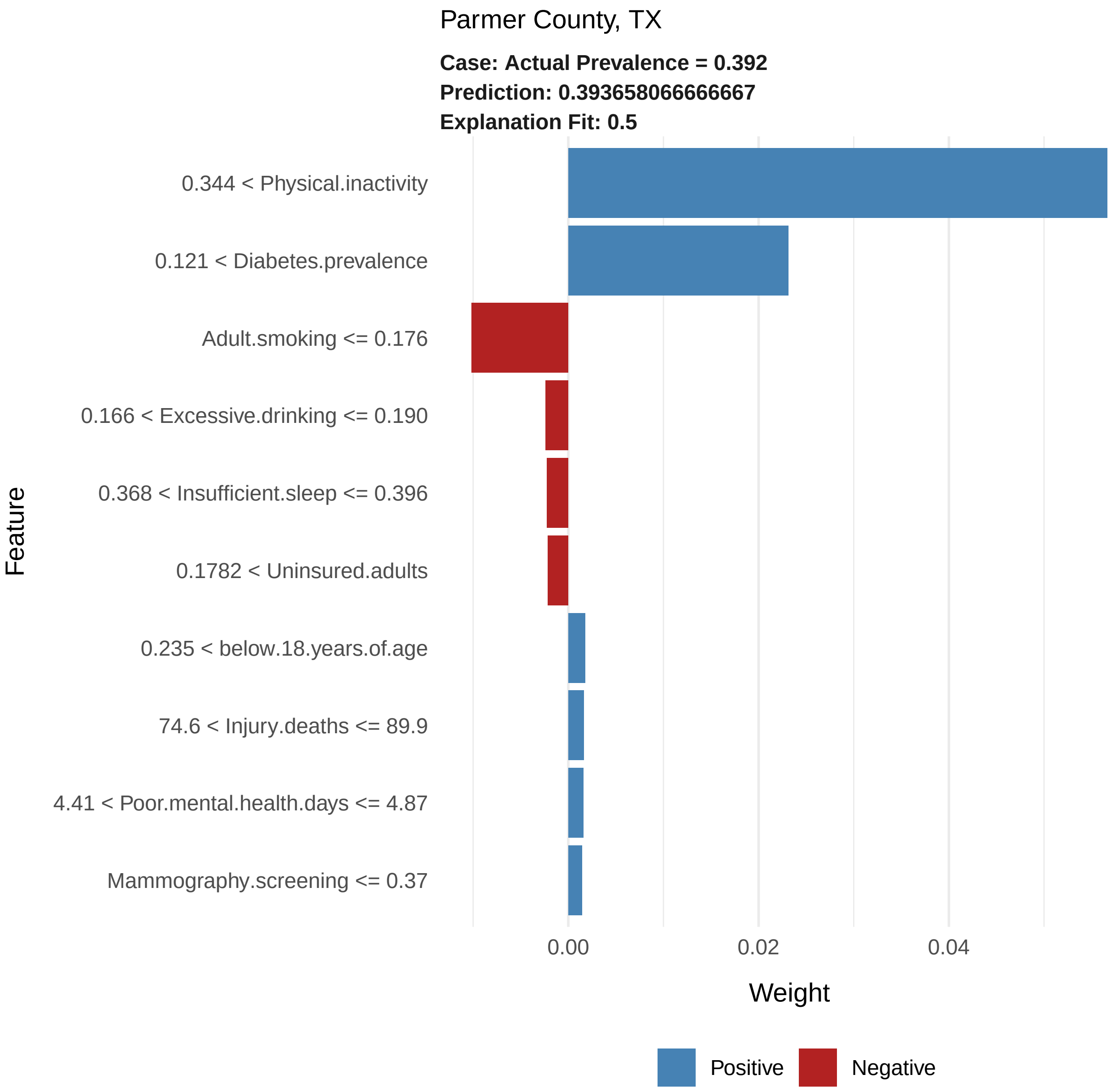
Parmer County, TX
Case: Actual Prevalence = 0.392
Prediction: 0.393658066666667
Explanation Fit: 0.5
0.344 < Physical.inactivity
0.121 < Diabetes.prevalence
Adult.smoking <= 0.176
0.166 < Excessive.drinking <= 0.190
0.368 < Insufficient.sleep <= 0.396
0.1782 < Uninsured.adults
0.235 < below.18.years.of.age
74.6 < Injury.deaths <= 89.9
4.41 < Poor.mental.health.days <= 4.87
Mammography.screening <= 0.37
Feature
0.00
0.02
0.04
Weight
Positive
Negative

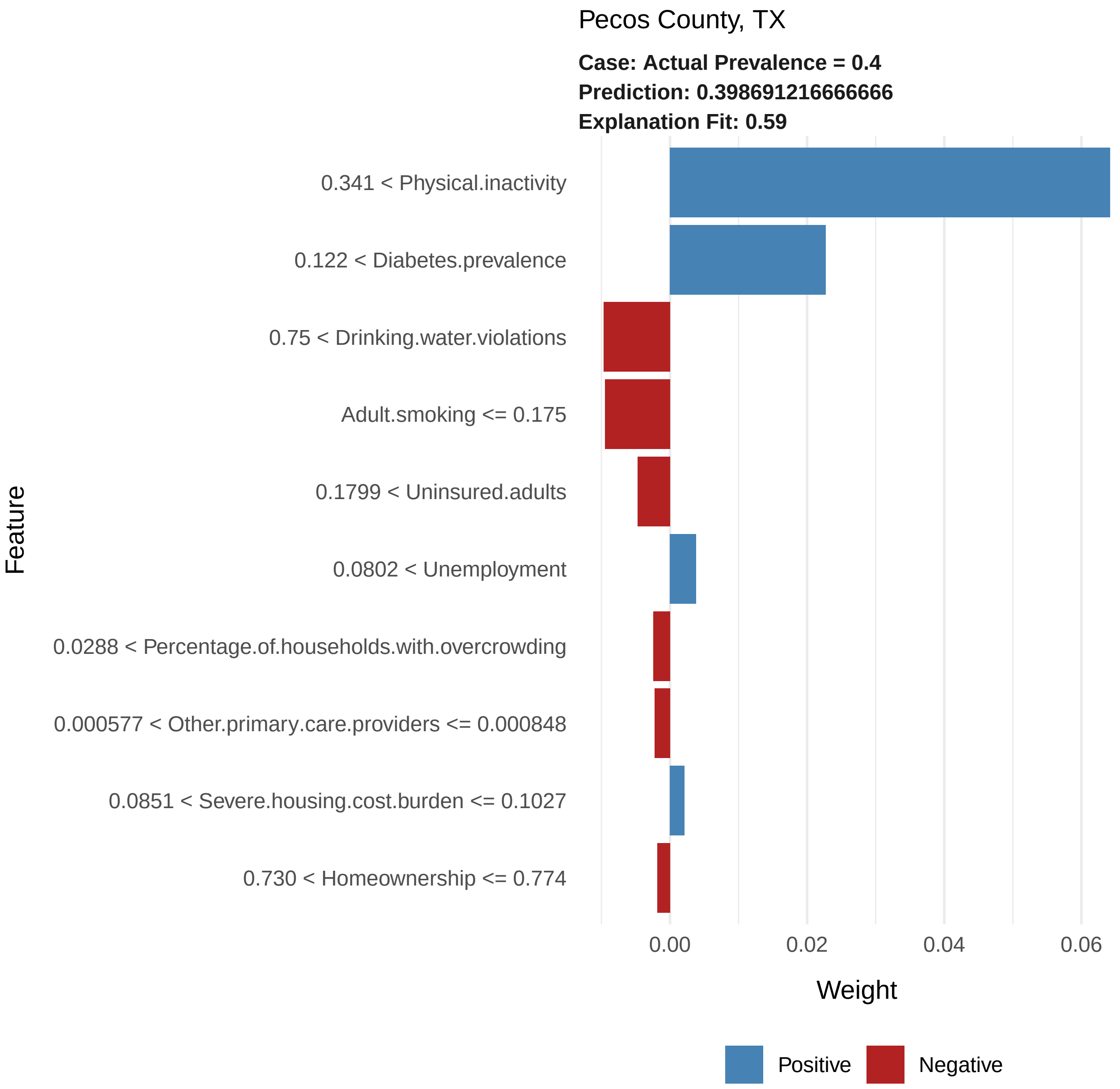

Pecos County, TX
Case: Actual Prevalence = 0.4
Prediction: 0.398691216666666
Explanation Fit: 0.59
0.341 < Physical.inactivity
0.122 < Diabetes.prevalence
0.75 < Drinking.water.violations
Adult.smoking <= 0.175
0.1799 < Uninsured.adults
0.0802 < Unemployment
0.0288 < Percentage.of.households.with.overcrowding
0.000577 < Other.primary.care.providers <= 0.000848
0.0851 < Severe.housing.cost.burden <= 0.1027
0.730 < Homeownership <= 0.774
Feature
0.00
0.02
0.04
0.06
Weight
Positive
Negative

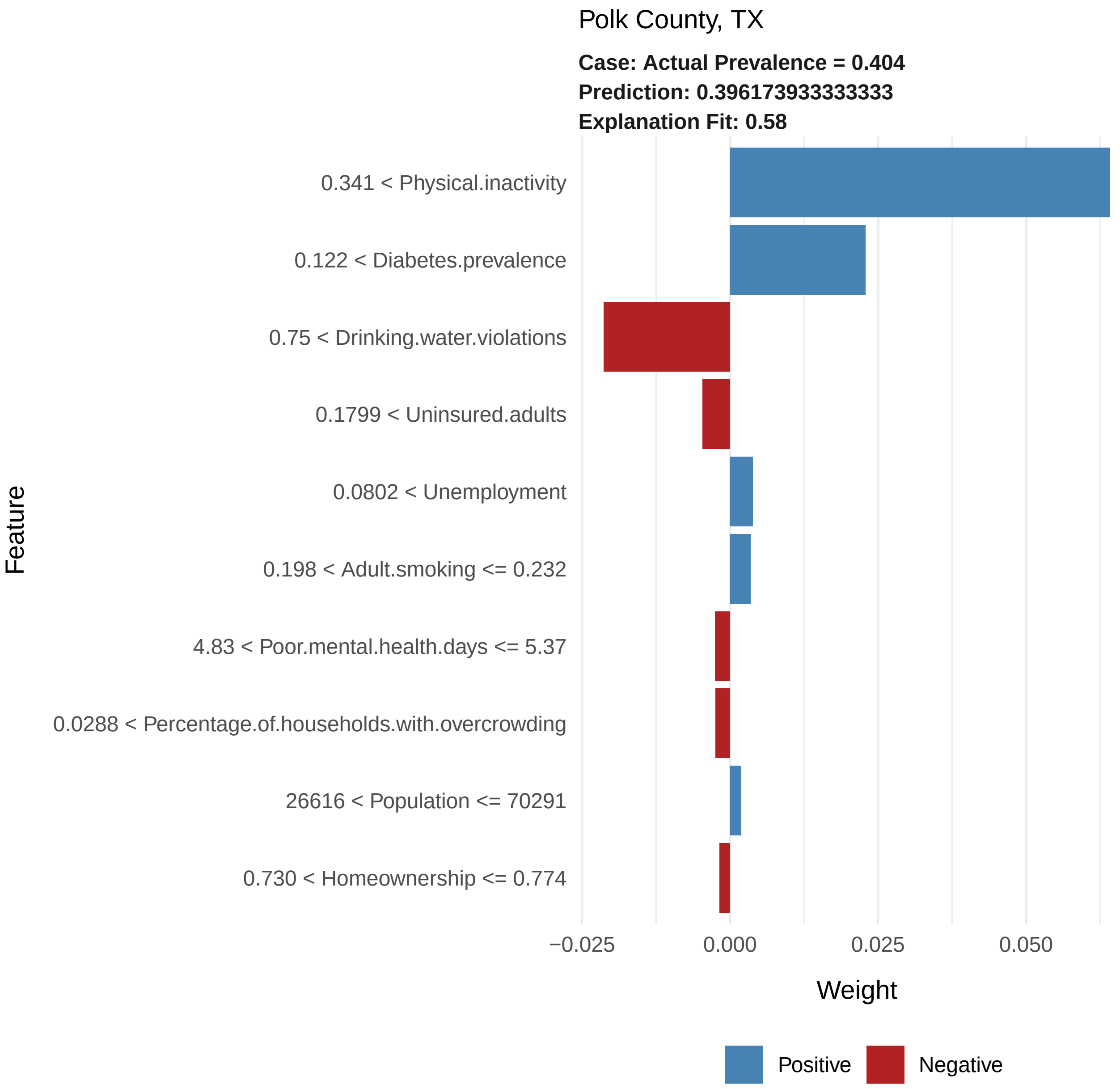
Polk County, TX
Case: Actual Prevalence = 0.404
Prediction: 0.396173933333333
Explanation Fit: 0.58
0.341 < Physical.inactivity
0.122 < Diabetes.prevalence
0.75 < Drinking.water.violations
0.1799 < Uninsured.adults
0.0802 < Unemployment
0.198 < Adult.smoking <= 0.232
4.83 < Poor.mental.health.days <= 5.37
0.0288 < Percentage.of.households.with.overcrowding
26616 < Population <= 70291
0.730 < Homeownership <= 0.774
Feature
−0.025
0.000
0.025
0.050
Weight
Positive
Negative

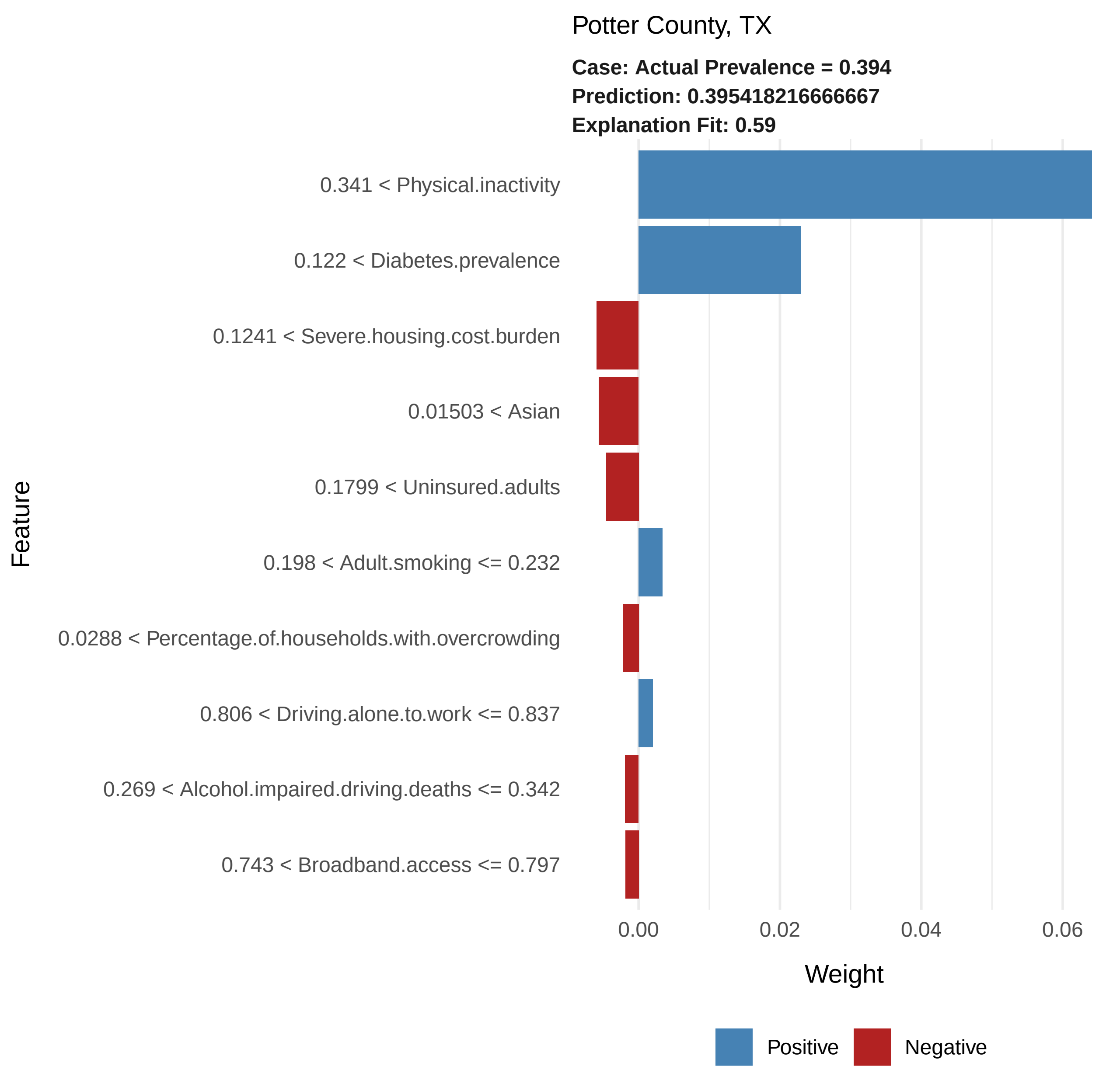

Potter County, TX
Case: Actual Prevalence = 0.394
Prediction: 0.395418216666667
Explanation Fit: 0.59
0.341 < Physical.inactivity
0.122 < Diabetes.prevalence
0.1241 < Severe.housing.cost.burden
0.01503 < Asian
0.1799 < Uninsured.adults
0.198 < Adult.smoking <= 0.232
0.0288 < Percentage.of.households.with.overcrowding
0.806 < Driving.alone.to.work <= 0.837
0.269 < Alcohol.impaired.driving.deaths <= 0.342
0.743 < Broadband.access <= 0.797
Feature
0.00
0.02
0.04
0.06
Weight
Positive
Negative

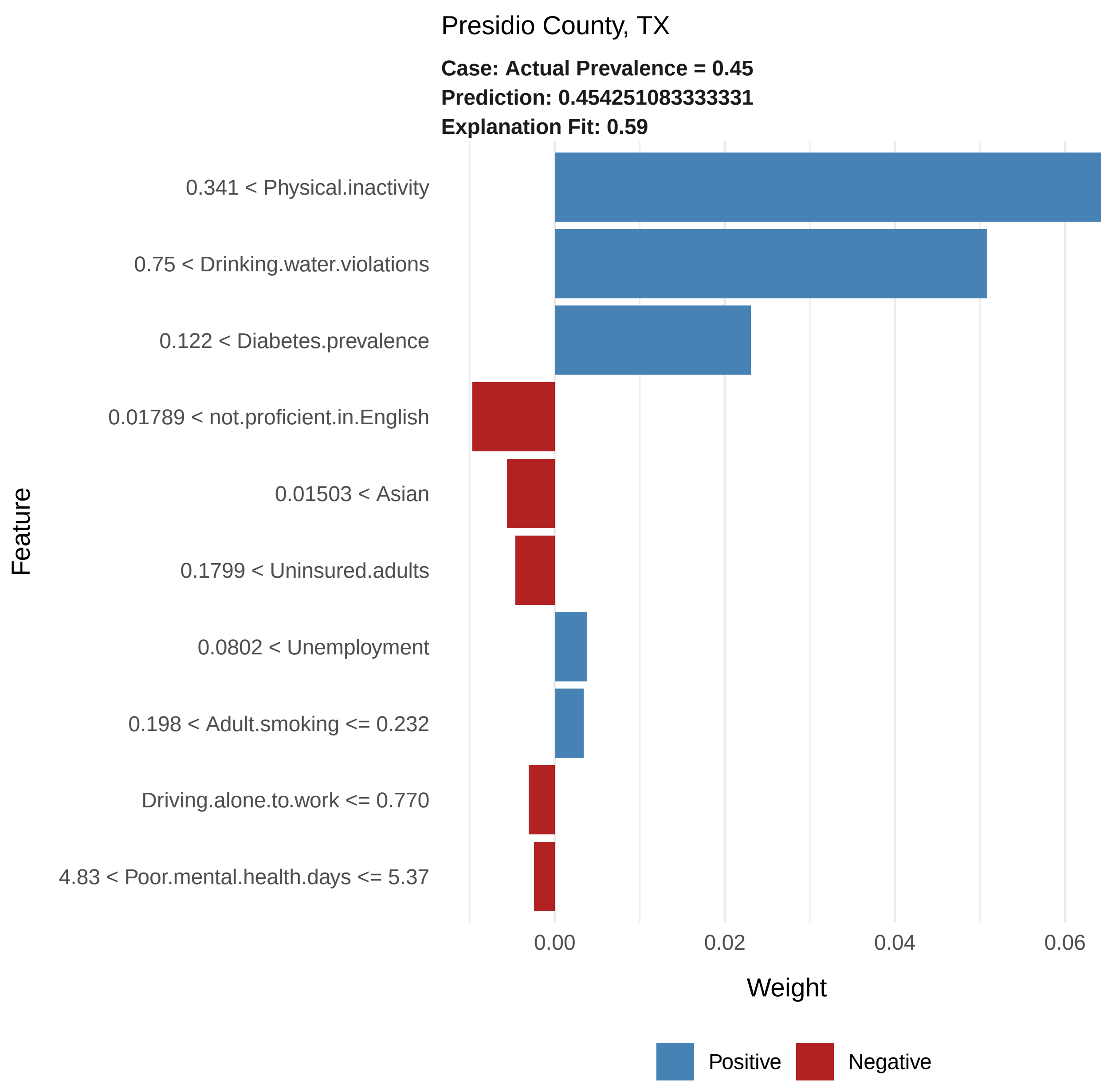
Presidio County, TX
Case: Actual Prevalence = 0.45
Prediction: 0.454251083333331
Explanation Fit: 0.59
0.341 < Physical.inactivity
0.75 < Drinking.water.violations
0.122 < Diabetes.prevalence
0.01789 < not.proficient.in.English
0.01503 < Asian
0.1799 < Uninsured.adults
0.0802 < Unemployment
0.198 < Adult.smoking <= 0.232
Driving.alone.to.work <= 0.770
4.83 < Poor.mental.health.days <= 5.37
Feature
0.00
0.02
0.04
0.06
Weight
Positive
Negative

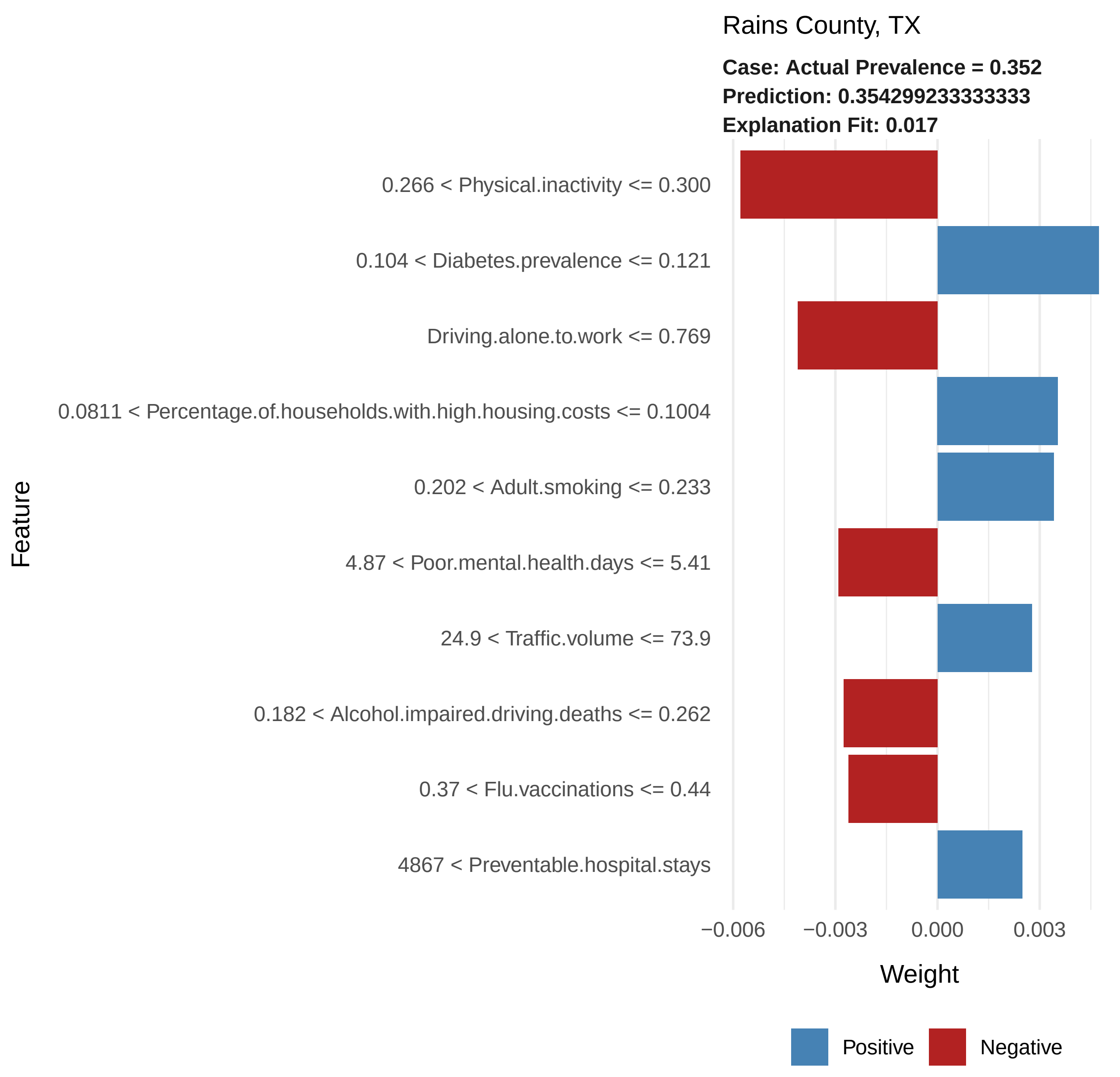
Rains County, TX
Case: Actual Prevalence = 0.352
Prediction: 0.354299233333333
Explanation Fit: 0.017
0.266 < Physical.inactivity <= 0.300
0.104 < Diabetes.prevalence <= 0.121
Driving.alone.to.work <= 0.769
0.0811 < Percentage.of.households.with.high.housing.costs <= 0.1004
0.202 < Adult.smoking <= 0.233
4.87 < Poor.mental.health.days <= 5.41
24.9 < Traffic.volume <= 73.9
0.182 < Alcohol.impaired.driving.deaths <= 0.262
0.37 < Flu.vaccinations <= 0.44
4867 < Preventable.hospital.stays
Feature
−0.006
−0.003
0.000
0.003
Weight
Positive
Negative

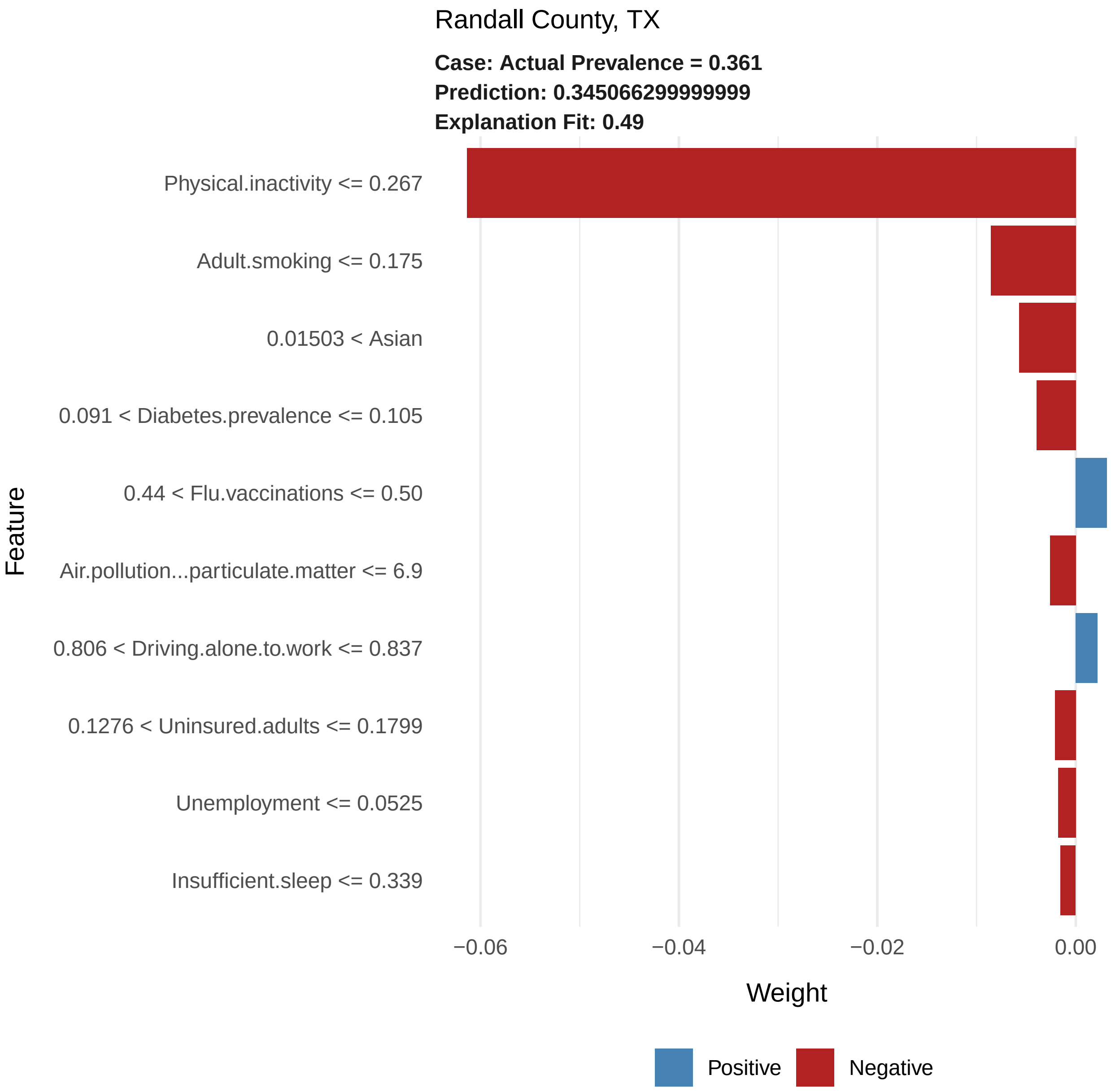

Randall County, TX
Case: Actual Prevalence = 0.361
Prediction: 0.345066299999999
Explanation Fit: 0.49
Physical.inactivity <= 0.267
Adult.smoking <= 0.175
0.01503 < Asian
0.091 < Diabetes.prevalence <= 0.105
0.44 < Flu.vaccinations <= 0.50
Air.pollution...particulate.matter <= 6.9
0.806 < Driving.alone.to.work <= 0.837
0.1276 < Uninsured.adults <= 0.1799
Unemployment <= 0.0525
Insufficient.sleep <= 0.339
Feature
−0.06
−0.04
−0.02
0.00
Weight
Positive
Negative

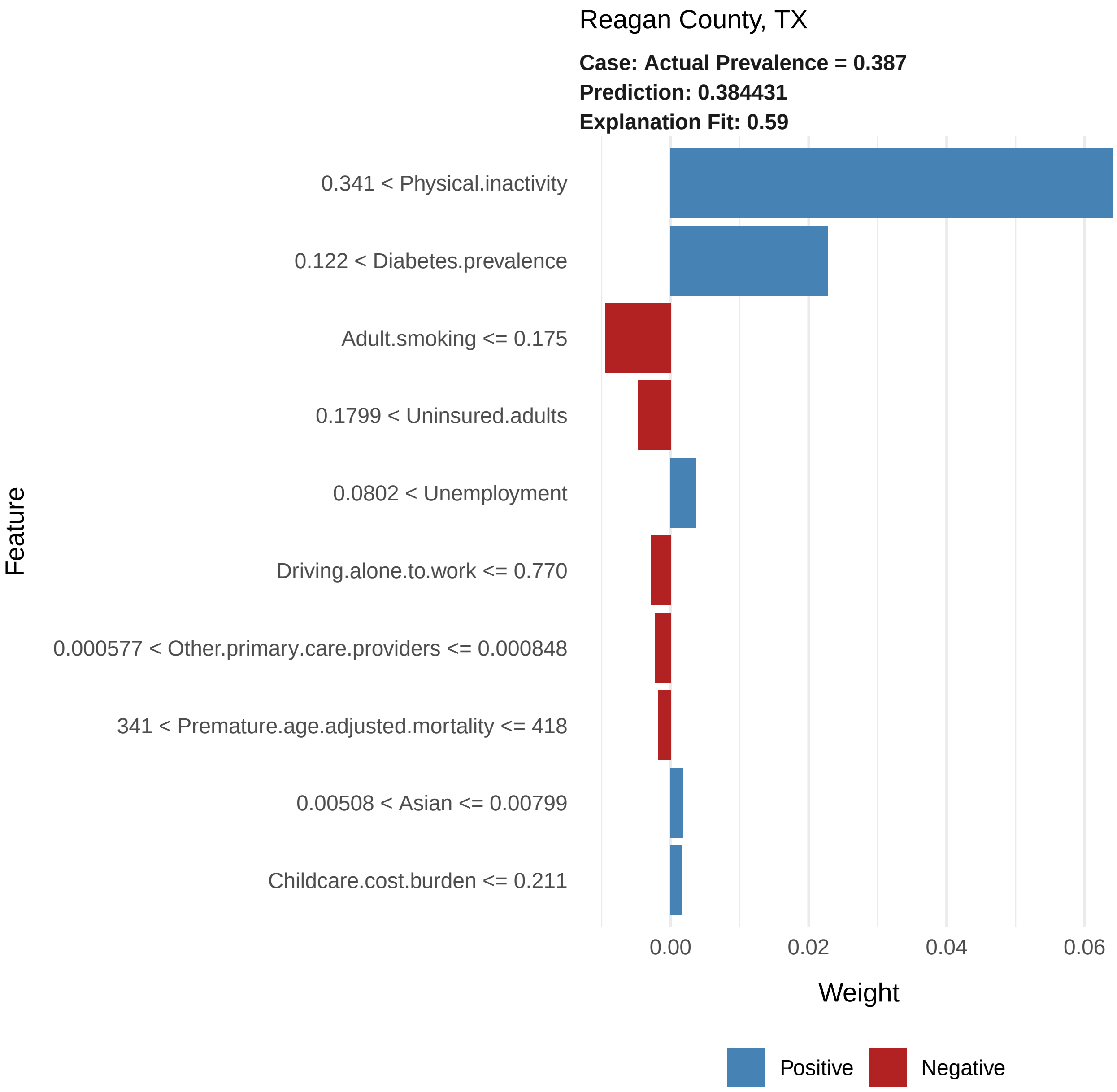

Reagan County, TX
Case: Actual Prevalence = 0.387
Prediction: 0.384431
Explanation Fit: 0.59
0.341 < Physical.inactivity
0.122 < Diabetes.prevalence
Adult.smoking <= 0.175
0.1799 < Uninsured.adults
0.0802 < Unemployment
Driving.alone.to.work <= 0.770
0.000577 < Other.primary.care.providers <= 0.000848
341 < Premature.age.adjusted.mortality <= 418
0.00508 < Asian <= 0.00799
Childcare.cost.burden <= 0.211
Feature
0.00
0.02
0.04
0.06
Weight
Positive
Negative

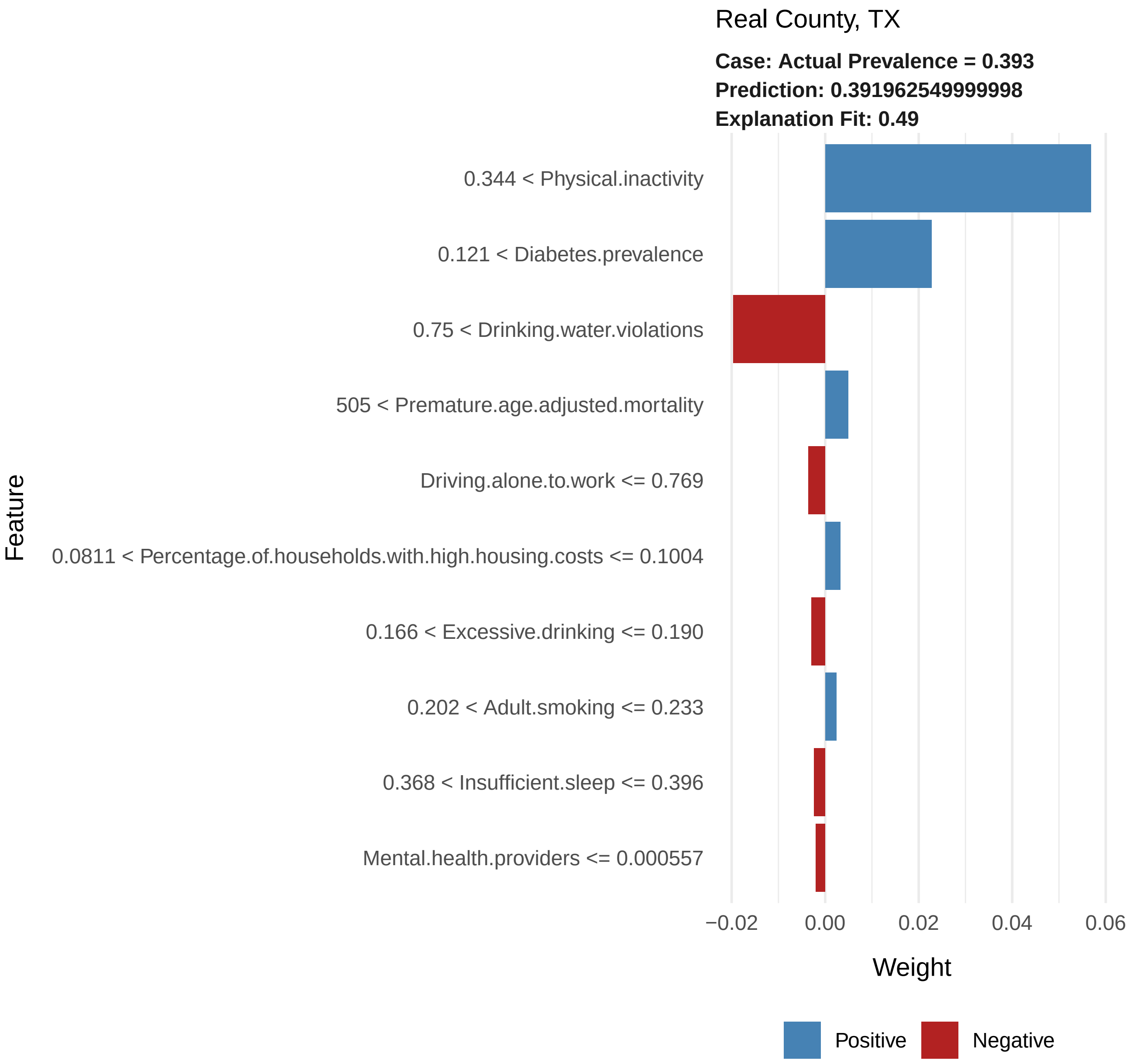

Real County, TX
Case: Actual Prevalence = 0.393
Prediction: 0.391962549999998
Explanation Fit: 0.49
0.344 < Physical.inactivity
0.121 < Diabetes.prevalence
0.75 < Drinking.water.violations
505 < Premature.age.adjusted.mortality
Driving.alone.to.work <= 0.769
0.0811 < Percentage.of.households.with.high.housing.costs <= 0.1004
0.166 < Excessive.drinking <= 0.190
0.202 < Adult.smoking <= 0.233
0.368 < Insufficient.sleep <= 0.396
Mental.health.providers <= 0.000557
Feature
−0.02
0.00
0.02
0.04
0.06
Weight
Positive
Negative

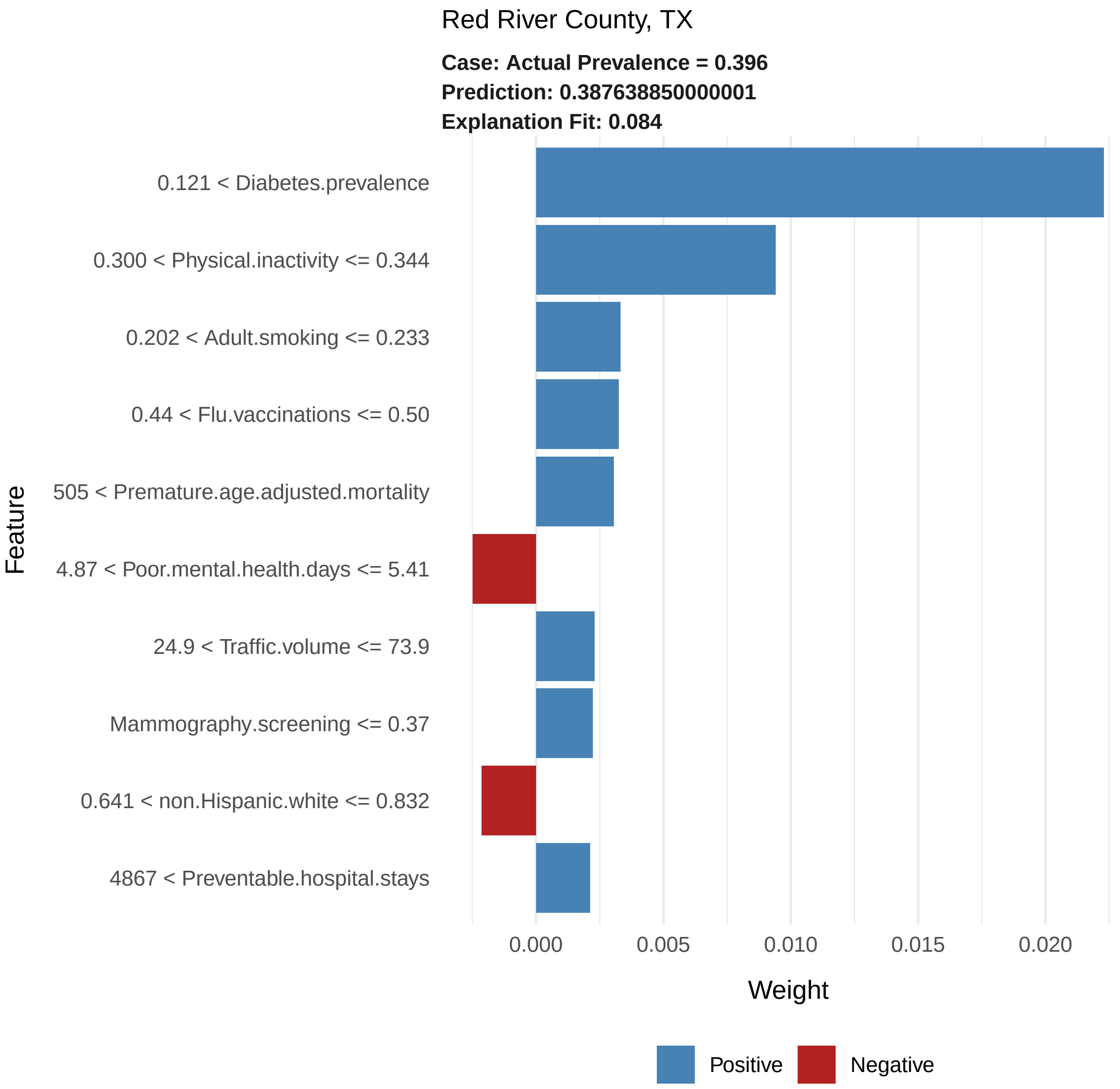

Red River County, TX
Case: Actual Prevalence = 0.396
Prediction: 0.387638850000001
Explanation Fit: 0.084
0.121 < Diabetes.prevalence
0.300 < Physical.inactivity <= 0.344
0.202 < Adult.smoking <= 0.233
0.44 < Flu.vaccinations <= 0.50
505 < Premature.age.adjusted.mortality
4.87 < Poor.mental.health.days <= 5.41
24.9 < Traffic.volume <= 73.9
Mammography.screening <= 0.37
0.641 < non.Hispanic.white <= 0.832
4867 < Preventable.hospital.stays
Feature
0.000
0.005
0.010
0.015
0.020
Weight
Positive
Negative

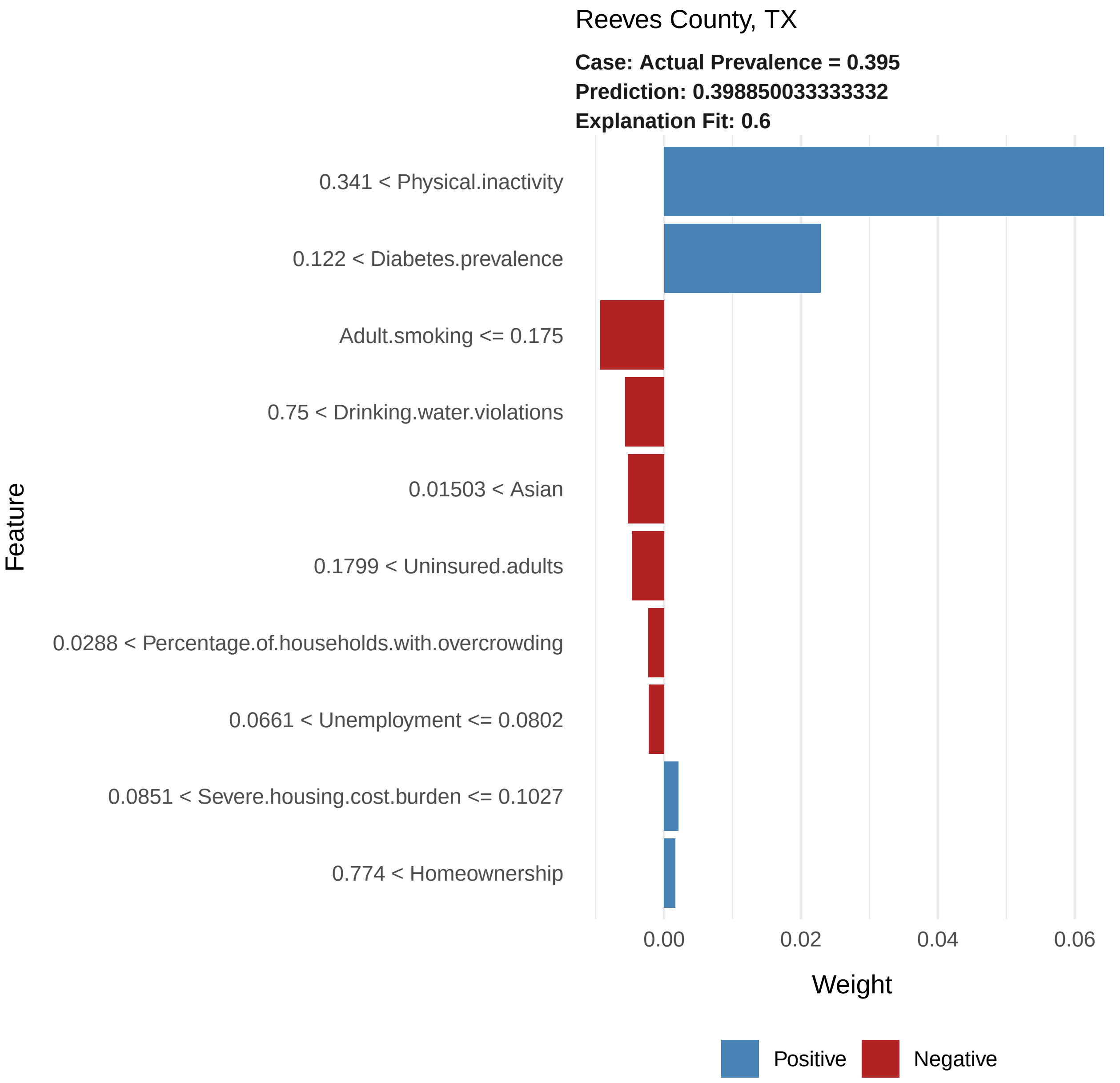

Reeves County, TX
Case: Actual Prevalence = 0.395
Prediction: 0.398850033333332
Explanation Fit: 0.6
0.341 < Physical.inactivity
0.122 < Diabetes.prevalence
Adult.smoking <= 0.175
0.75 < Drinking.water.violations
0.01503 < Asian
0.1799 < Uninsured.adults
0.0288 < Percentage.of.households.with.overcrowding
0.0661 < Unemployment <= 0.0802
0.0851 < Severe.housing.cost.burden <= 0.1027
0.774 < Homeownership
Feature
0.00
0.02
0.04
0.06
Weight
Positive
Negative

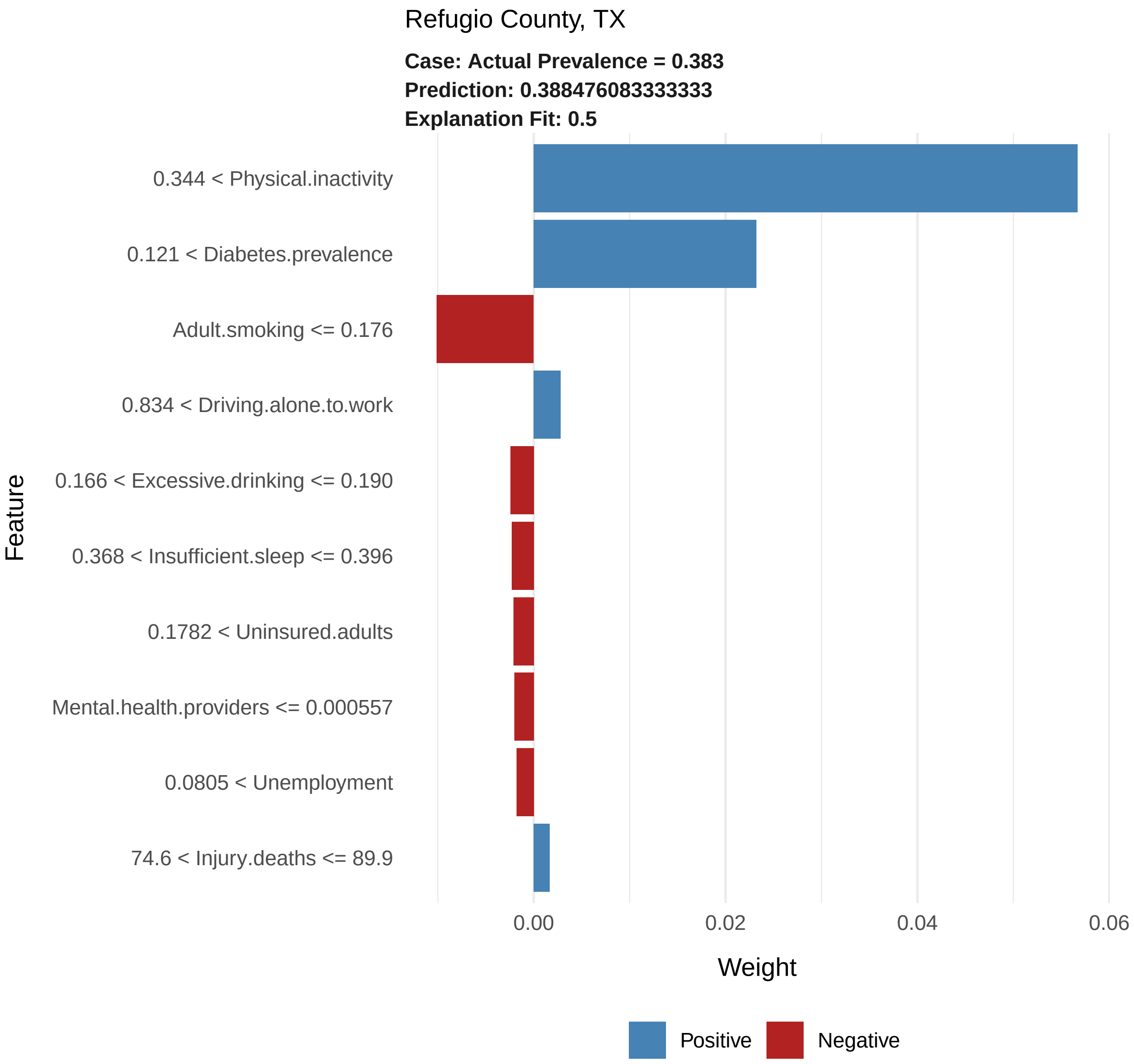

Refugio County, TX
Case: Actual Prevalence = 0.383
Prediction: 0.388476083333333
Explanation Fit: 0.5
0.344 < Physical.inactivity
0.121 < Diabetes.prevalence
Adult.smoking <= 0.176
0.834 < Driving.alone.to.work
0.166 < Excessive.drinking <= 0.190
0.368 < Insufficient.sleep <= 0.396
0.1782 < Uninsured.adults
Mental.health.providers <= 0.000557
0.0805 < Unemployment
74.6 < Injury.deaths <= 89.9
Feature
0.00
0.02
0.04
0.06
Weight
Positive
Negative

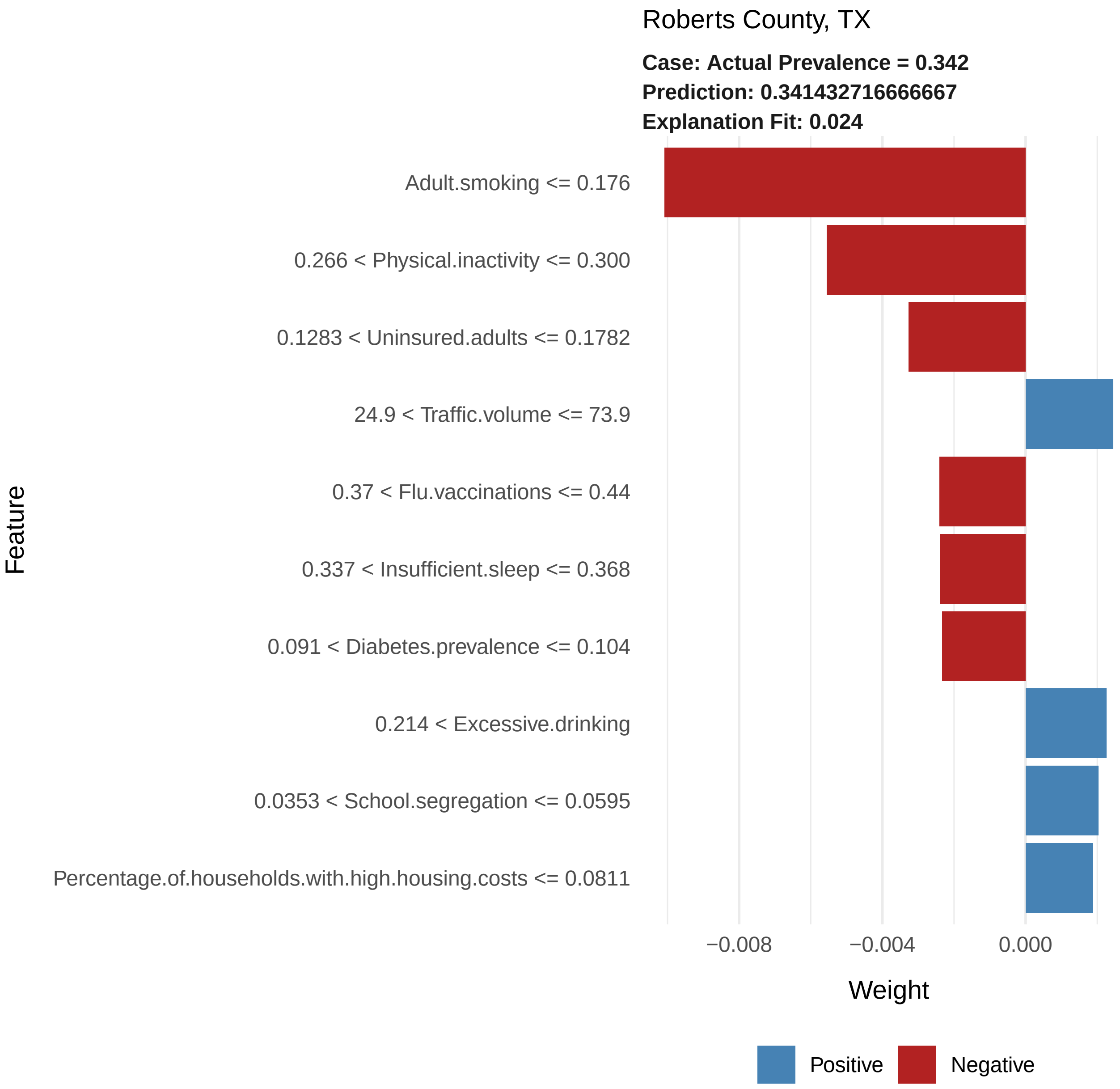
Roberts County, TX
Case: Actual Prevalence = 0.342
Prediction: 0.341432716666667
Explanation Fit: 0.024
Adult.smoking <= 0.176
0.266 < Physical.inactivity <= 0.300
0.1283 < Uninsured.adults <= 0.1782
24.9 < Traffic.volume <= 73.9
0.37 < Flu.vaccinations <= 0.44
0.337 < Insufficient.sleep <= 0.368
0.091 < Diabetes.prevalence <= 0.104
0.214 < Excessive.drinking
0.0353 < School.segregation <= 0.0595
Percentage.of.households.with.high.housing.costs <= 0.0811
Feature
−0.008
−0.004
0.000
Weight
Positive
Negative

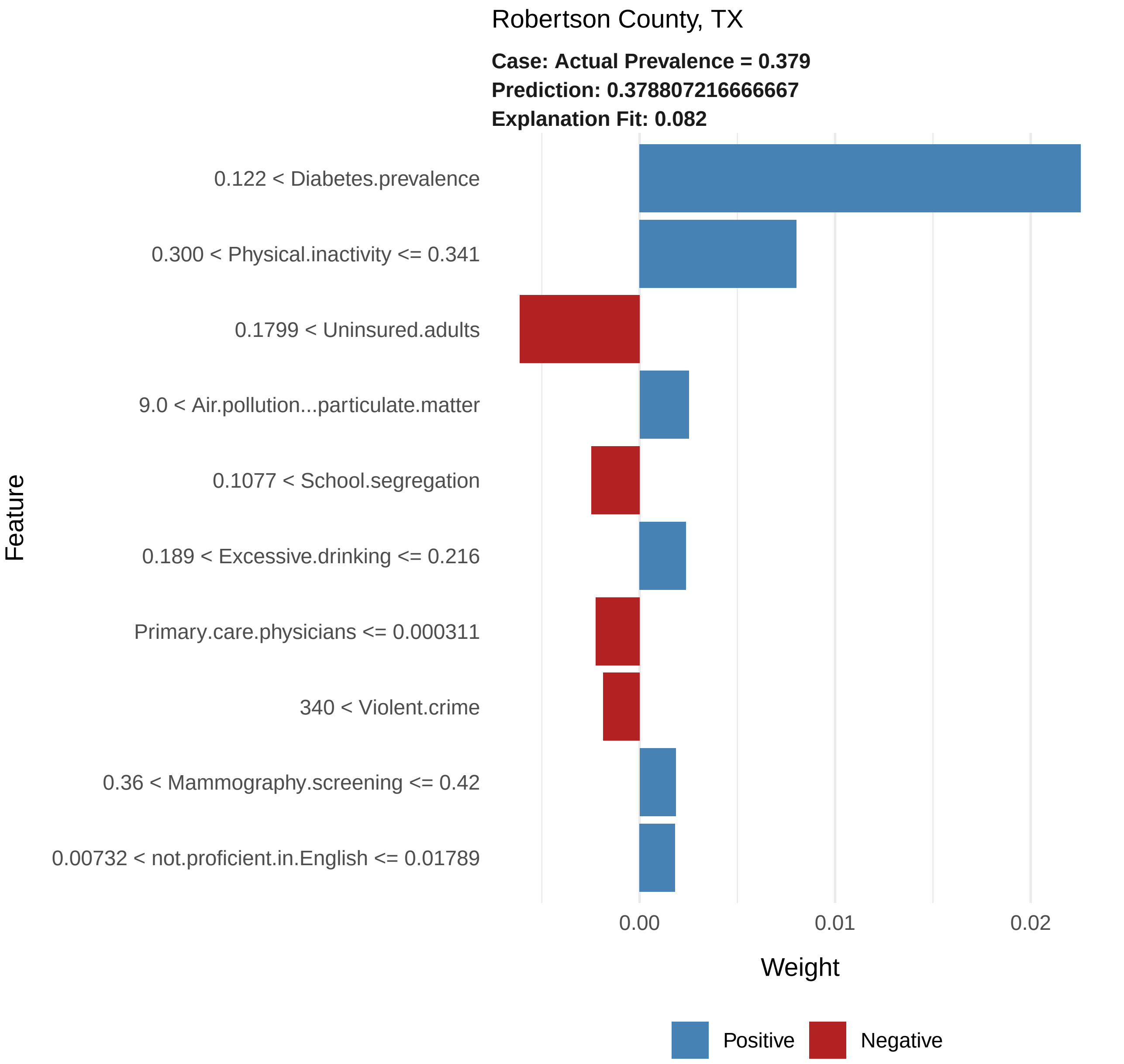
Robertson County, TX
Case: Actual Prevalence = 0.379
Prediction: 0.378807216666667
Explanation Fit: 0.082
Feature
0.122 < Diabetes.prevalence
0.300 < Physical.inactivity <= 0.341
0.1799 < Uninsured.adults
9.0 < Air.pollution...particulate.matter
0.1077 < School.segregation
0.189 < Excessive.drinking <= 0.216
Primary.care.physicians <= 0.000311
340 < Violent.crime
0.36 < Mammography.screening <= 0.42
0.00732 < not.proficient.in.English <= 0.01789
0.00
0.01
0.02
Weight
Positive
Negative

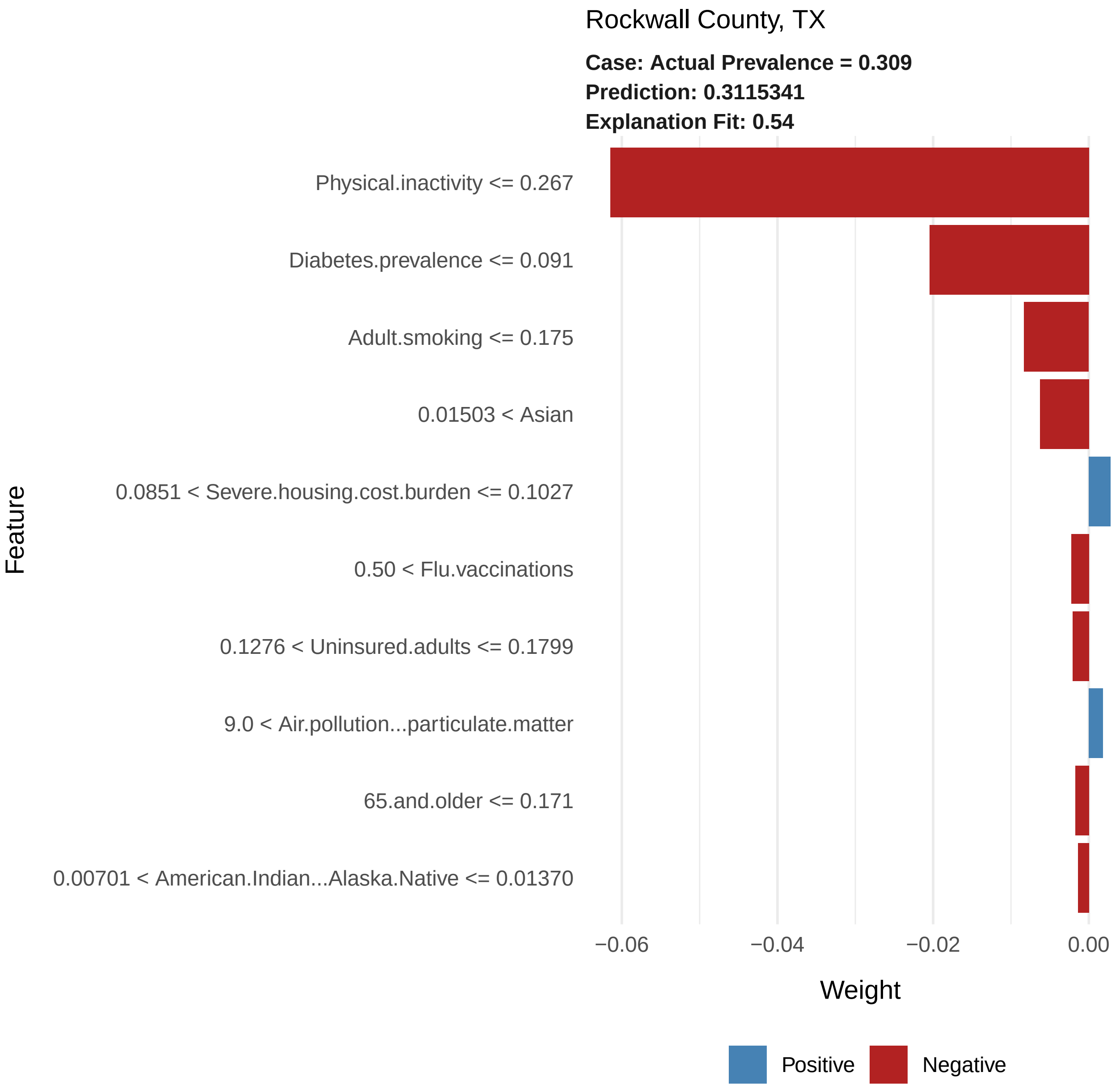
Rockwall County, TX
Case: Actual Prevalence = 0.309
Prediction: 0.3115341
Explanation Fit: 0.54
Physical.inactivity <= 0.267
Diabetes.prevalence <= 0.091
Adult.smoking <= 0.175
0.01503 < Asian
0.0851 < Severe.housing.cost.burden <= 0.1027
0.50 < Flu.vaccinations
0.1276 < Uninsured.adults <= 0.1799
9.0 < Air.pollution...particulate.matter
65.and.older <= 0.171
0.00701 < American.Indian...Alaska.Native <= 0.01370
Feature
−0.06
−0.04
−0.02
0.00
Weight
Positive
Negative

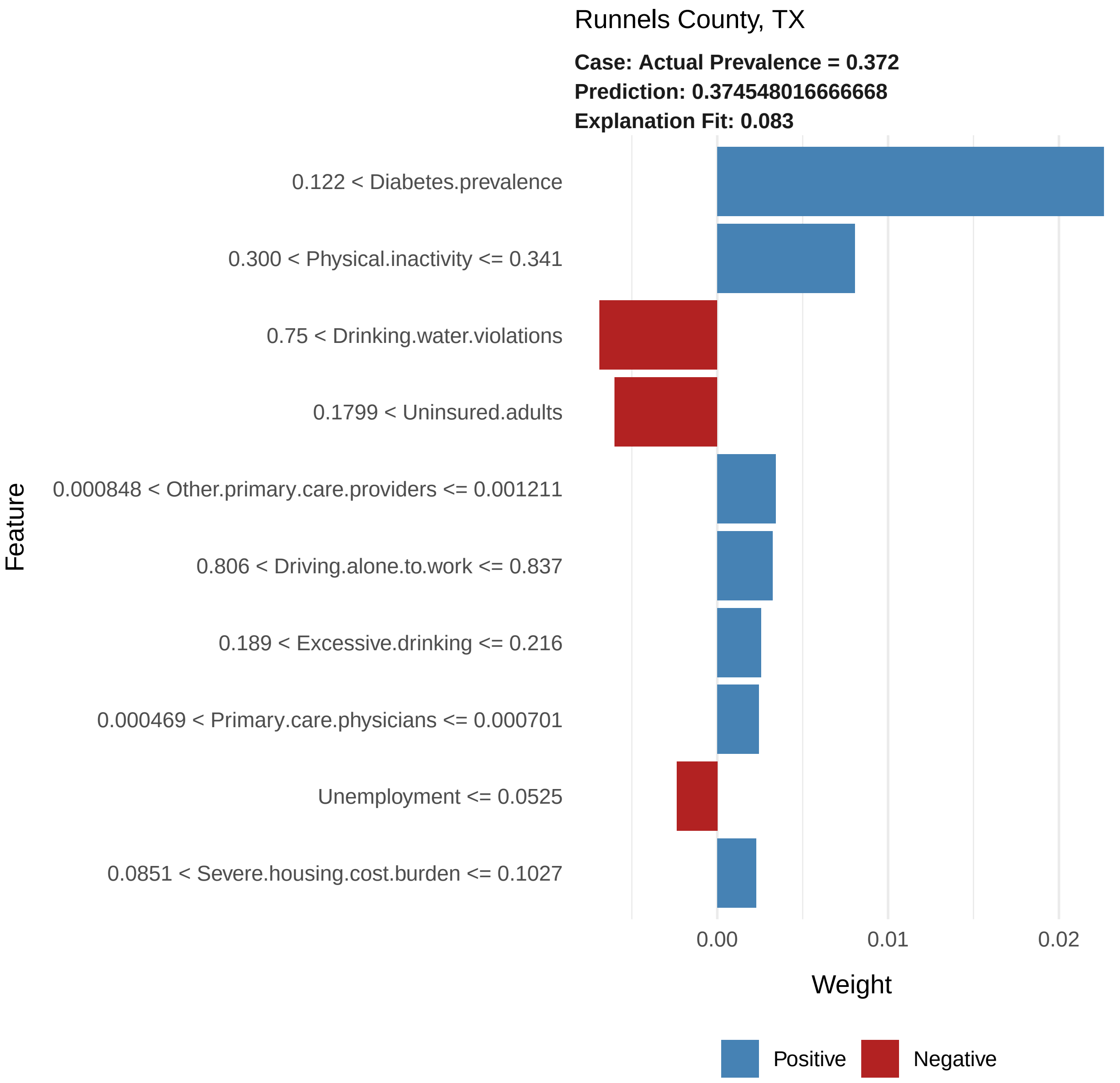

Runnels County, TX
Case: Actual Prevalence = 0.372
Prediction: 0.374548016666668
Explanation Fit: 0.083
0.122 < Diabetes.prevalence
0.300 < Physical.inactivity <= 0.341
0.75 < Drinking.water.violations
0.1799 < Uninsured.adults
0.000848 < Other.primary.care.providers <= 0.001211
0.806 < Driving.alone.to.work <= 0.837
0.189 < Excessive.drinking <= 0.216
0.000469 < Primary.care.physicians <= 0.000701
Unemployment <= 0.0525
0.0851 < Severe.housing.cost.burden <= 0.1027
Feature
0.00
0.01
0.02
Weight
Positive
Negative

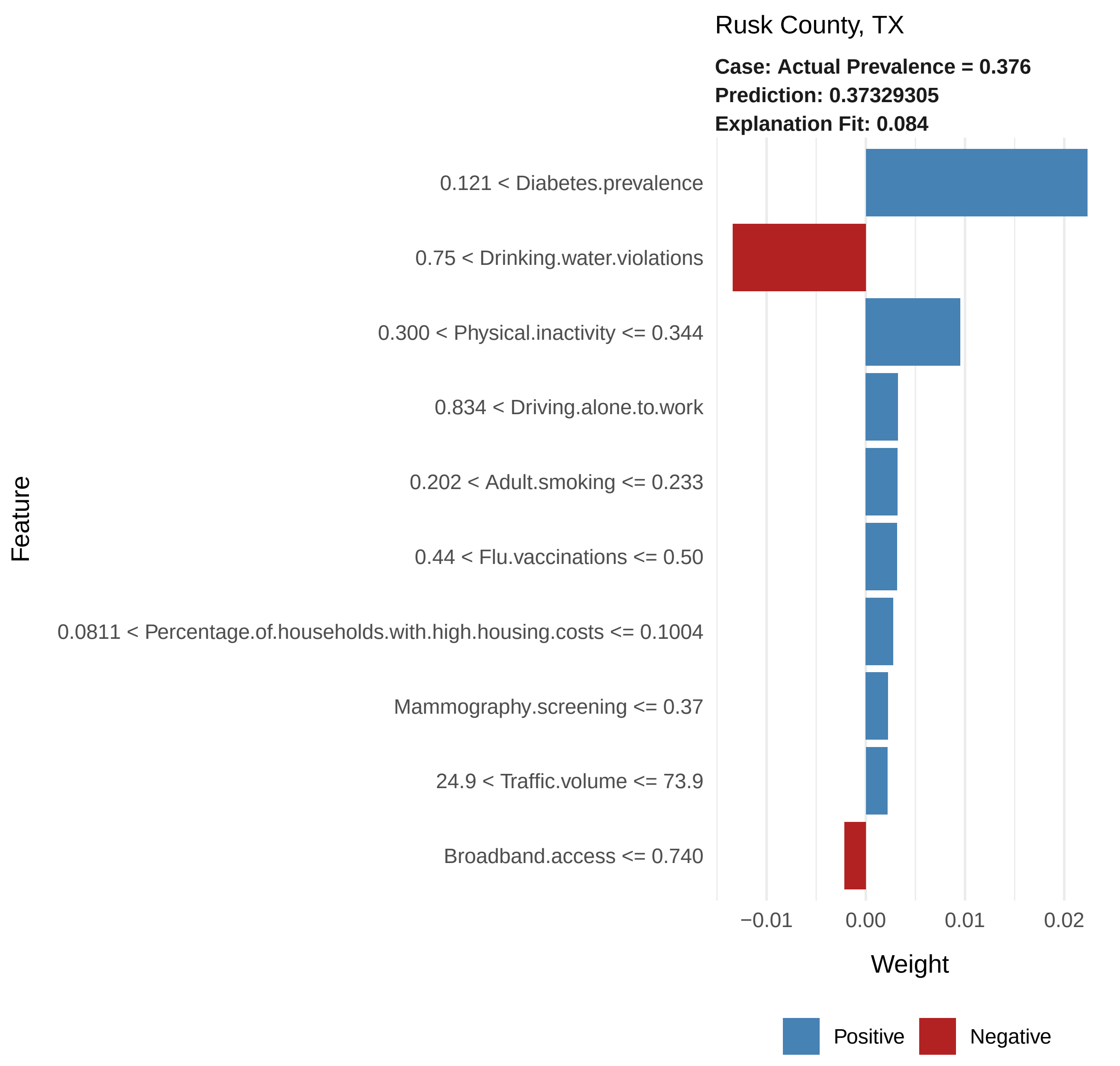

Rusk County, TX
Case: Actual Prevalence = 0.376
Prediction: 0.37329305
Explanation Fit: 0.084
Feature
0.121 < Diabetes.prevalence
0.75 < Drinking.water.violations
0.300 < Physical.inactivity <= 0.344
0.834 < Driving.alone.to.work
0.202 < Adult.smoking <= 0.233
0.44 < Flu.vaccinations <= 0.50
0.0811 < Percentage.of.households.with.high.housing.costs <= 0.1004
Mammography.screening <= 0.37
24.9 < Traffic.volume <= 73.9
Broadband.access <= 0.740
−0.01
0.00
0.01
0.02
Weight
Positive
Negative

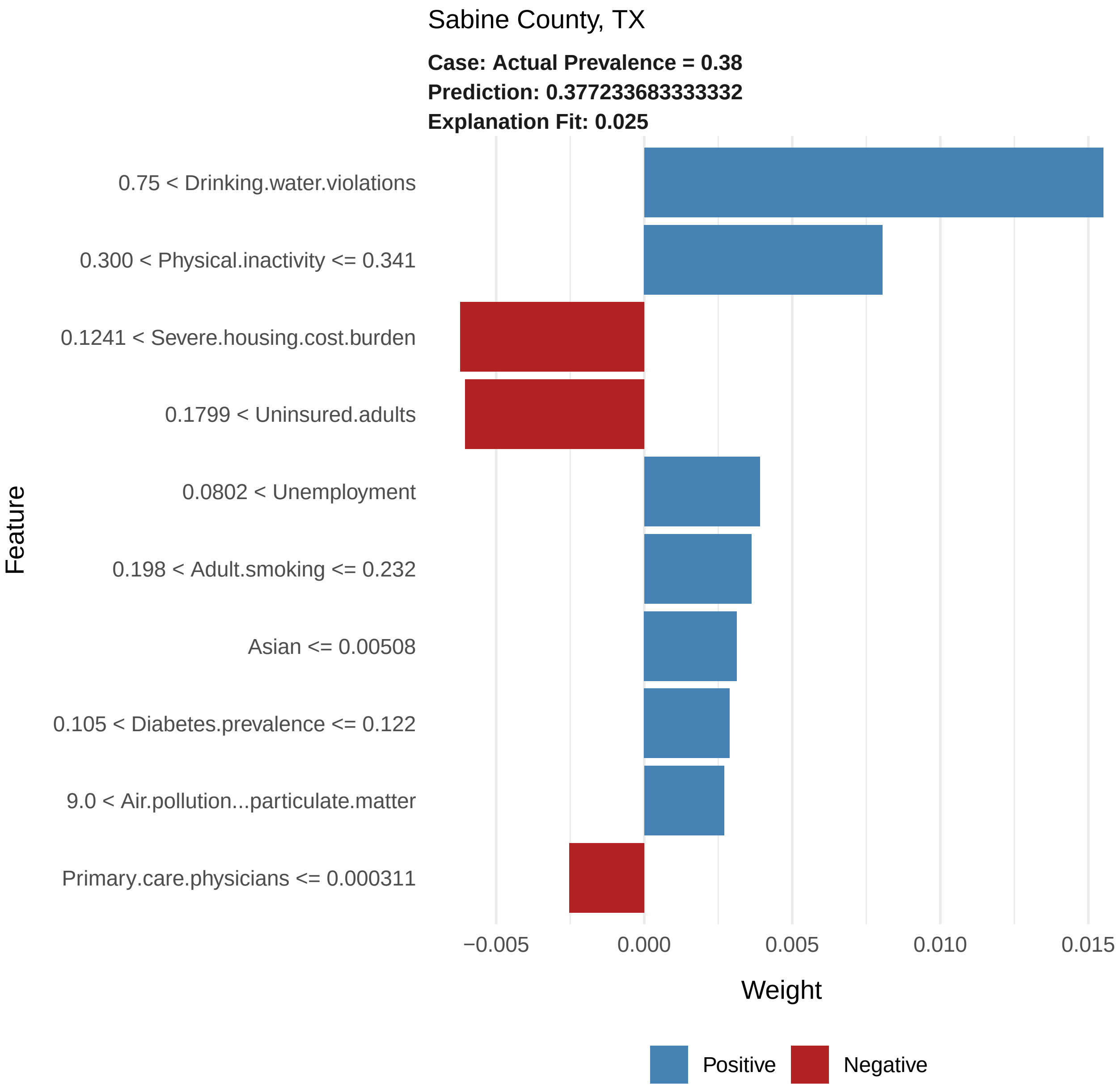
Sabine County, TX
Case: Actual Prevalence = 0.38
Prediction: 0.377233683333332
Explanation Fit: 0.025
0.75 < Drinking.water.violations
0.300 < Physical.inactivity <= 0.341
0.1241 < Severe.housing.cost.burden
0.1799 < Uninsured.adults
0.0802 < Unemployment
0.198 < Adult.smoking <= 0.232
Asian <= 0.00508
0.105 < Diabetes.prevalence <= 0.122
9.0 < Air.pollution...particulate.matter
Primary.care.physicians <= 0.000311
Feature
−0.005
0.000
0.005
0.010
0.015
Weight
Positive
Negative

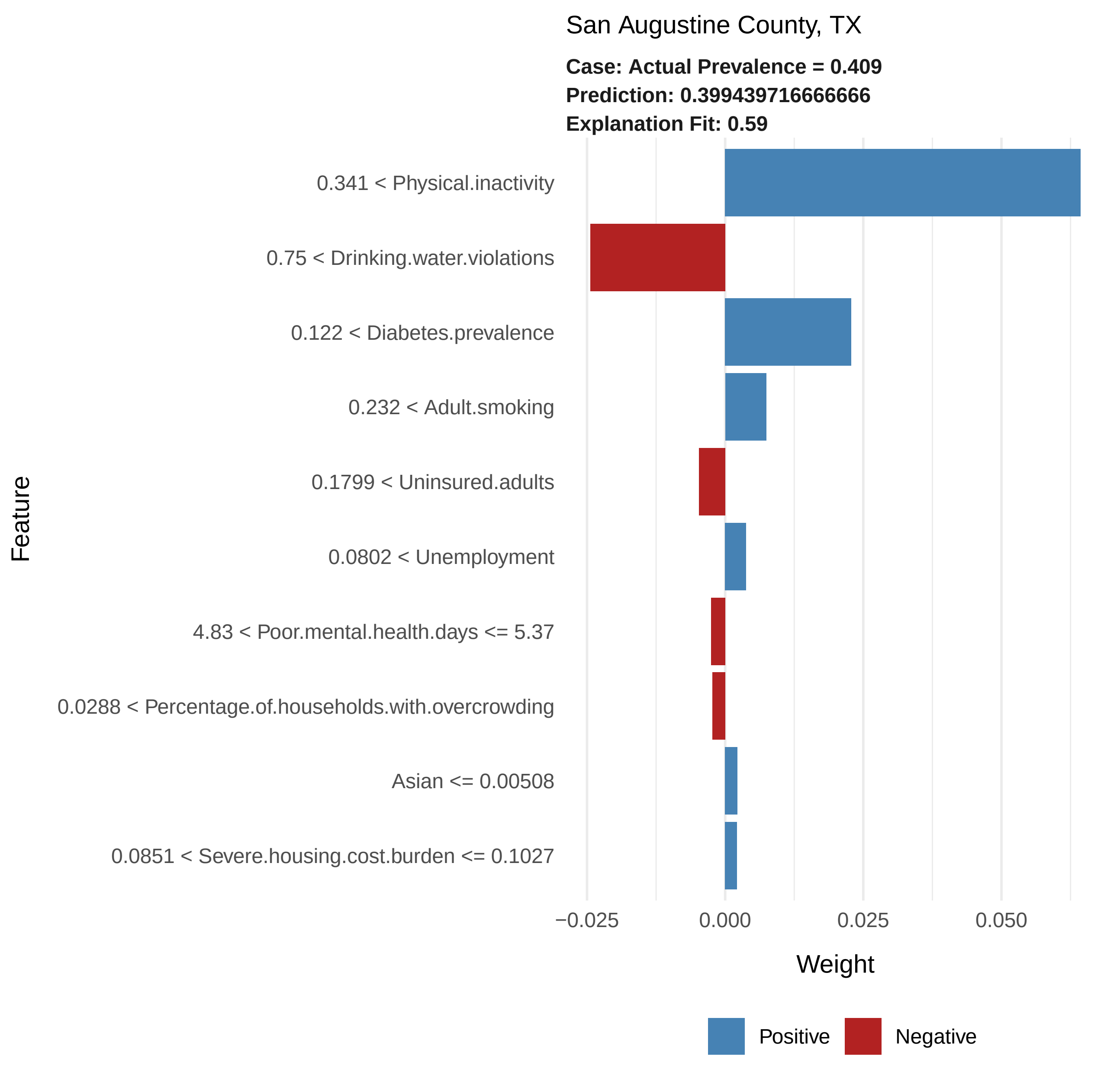

San Augustine County, TX
Case: Actual Prevalence = 0.409
Prediction: 0.399439716666666
Explanation Fit: 0.59
0.341 < Physical.inactivity
0.75 < Drinking.water.violations
0.122 < Diabetes.prevalence
0.232 < Adult.smoking
0.1799 < Uninsured.adults
0.0802 < Unemployment
4.83 < Poor.mental.health.days <= 5.37
0.0288 < Percentage.of.households.with.overcrowding
Asian <= 0.00508
0.0851 < Severe.housing.cost.burden <= 0.1027
Feature
−0.025
0.000
0.025
0.050
Weight
Positive
Negative

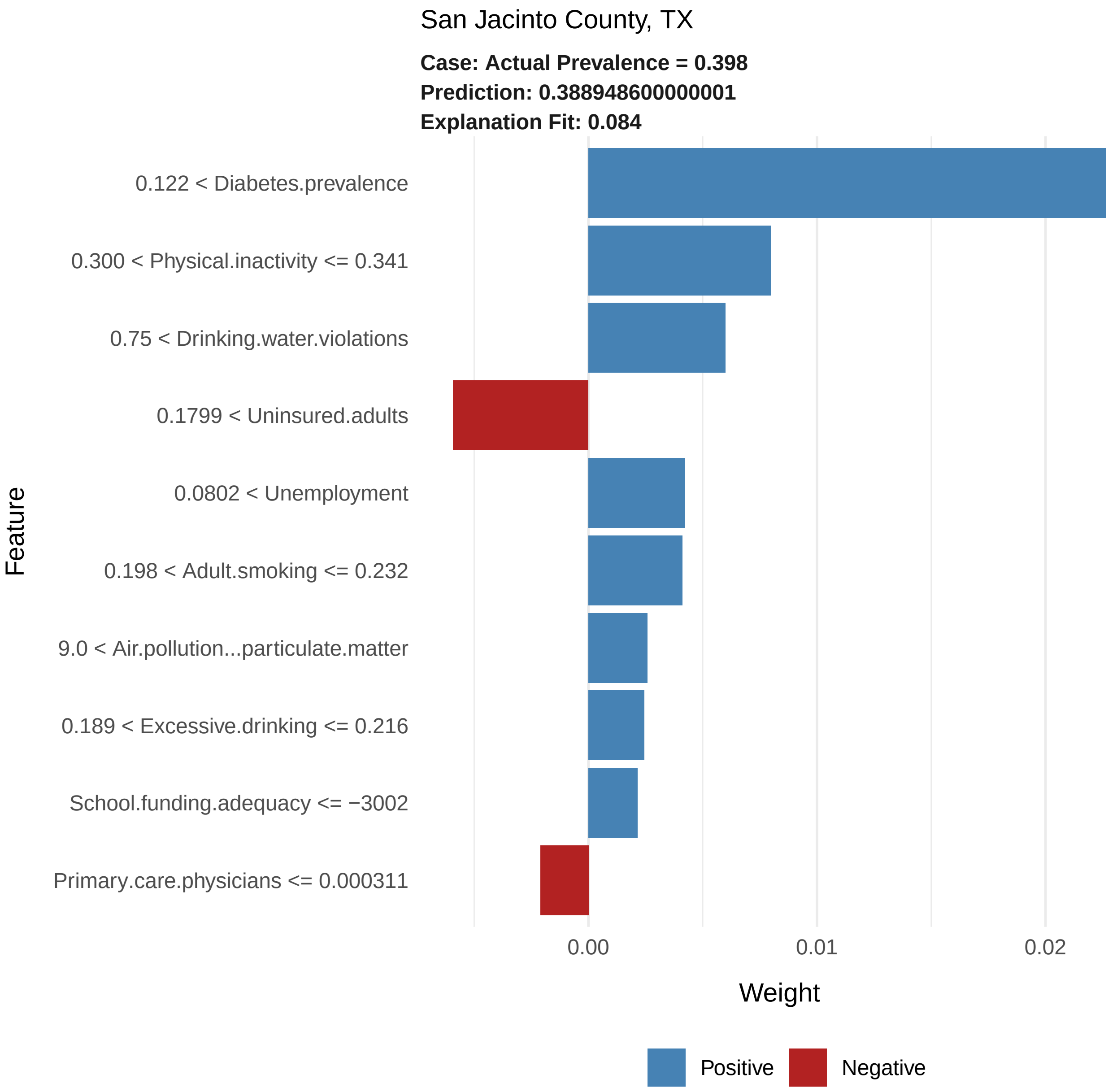
San Jacinto County, TX
Case: Actual Prevalence = 0.398
Prediction: 0.388948600000001
Explanation Fit: 0.084
0.122 < Diabetes.prevalence
0.300 < Physical.inactivity <= 0.341
0.75 < Drinking.water.violations
0.1799 < Uninsured.adults
0.0802 < Unemployment
0.198 < Adult.smoking <= 0.232
9.0 < Air.pollution...particulate.matter
0.189 < Excessive.drinking <= 0.216
School.funding.adequacy <= −3002
Primary.care.physicians <= 0.000311
Feature
0.00
0.01
0.02
Weight
Positive
Negative

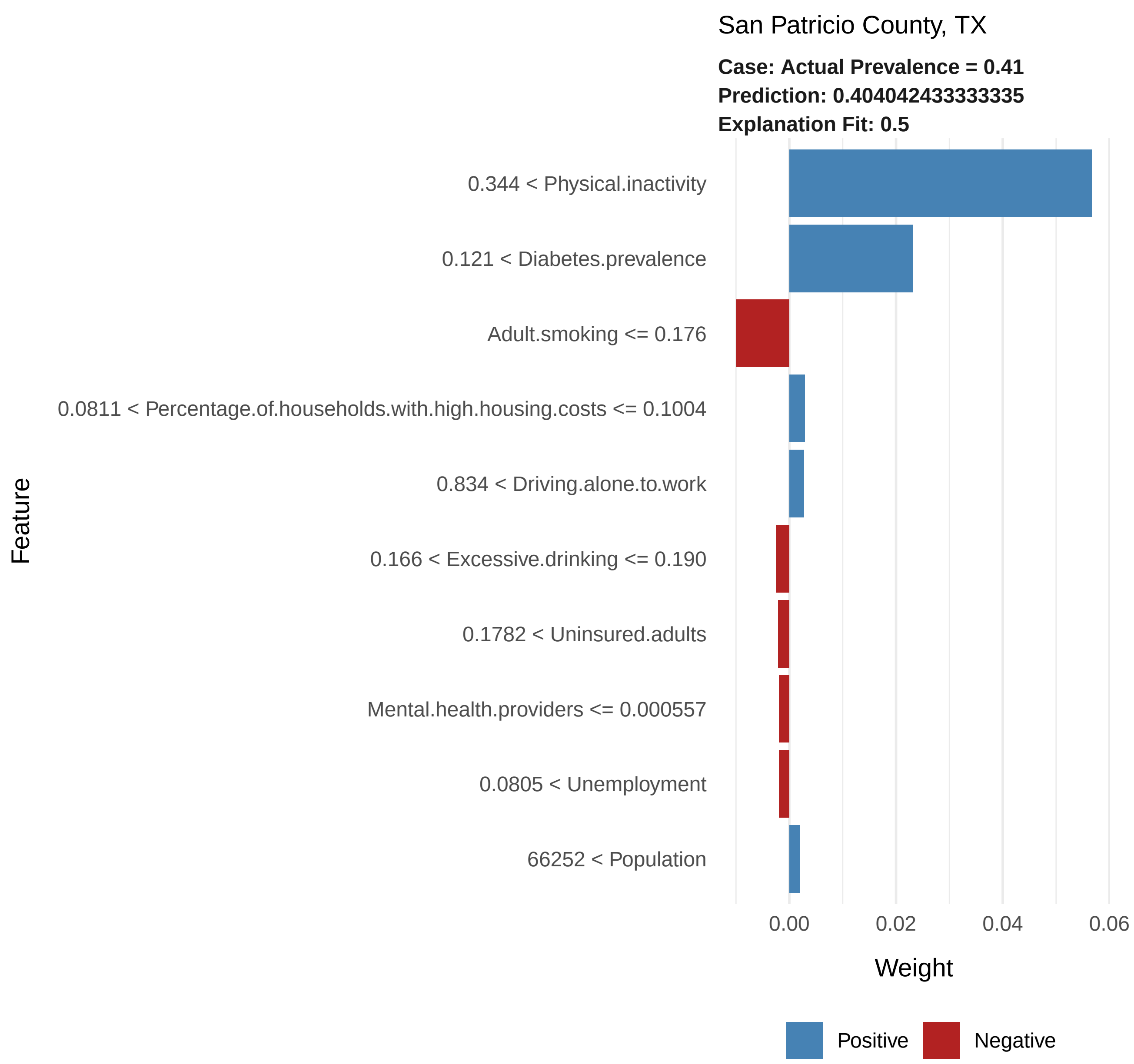
San Patricio County, TX
Case: Actual Prevalence = 0.41
Prediction: 0.404042433333335
Explanation Fit: 0.5
0.344 < Physical.inactivity
0.121 < Diabetes.prevalence
Adult.smoking <= 0.176
0.0811 < Percentage.of.households.with.high.housing.costs <= 0.1004
0.834 < Driving.alone.to.work
0.166 < Excessive.drinking <= 0.190
0.1782 < Uninsured.adults
Mental.health.providers <= 0.000557
0.0805 < Unemployment
66252 < Population
Feature
0.00
0.02
0.04
0.06
Weight
Positive
Negative

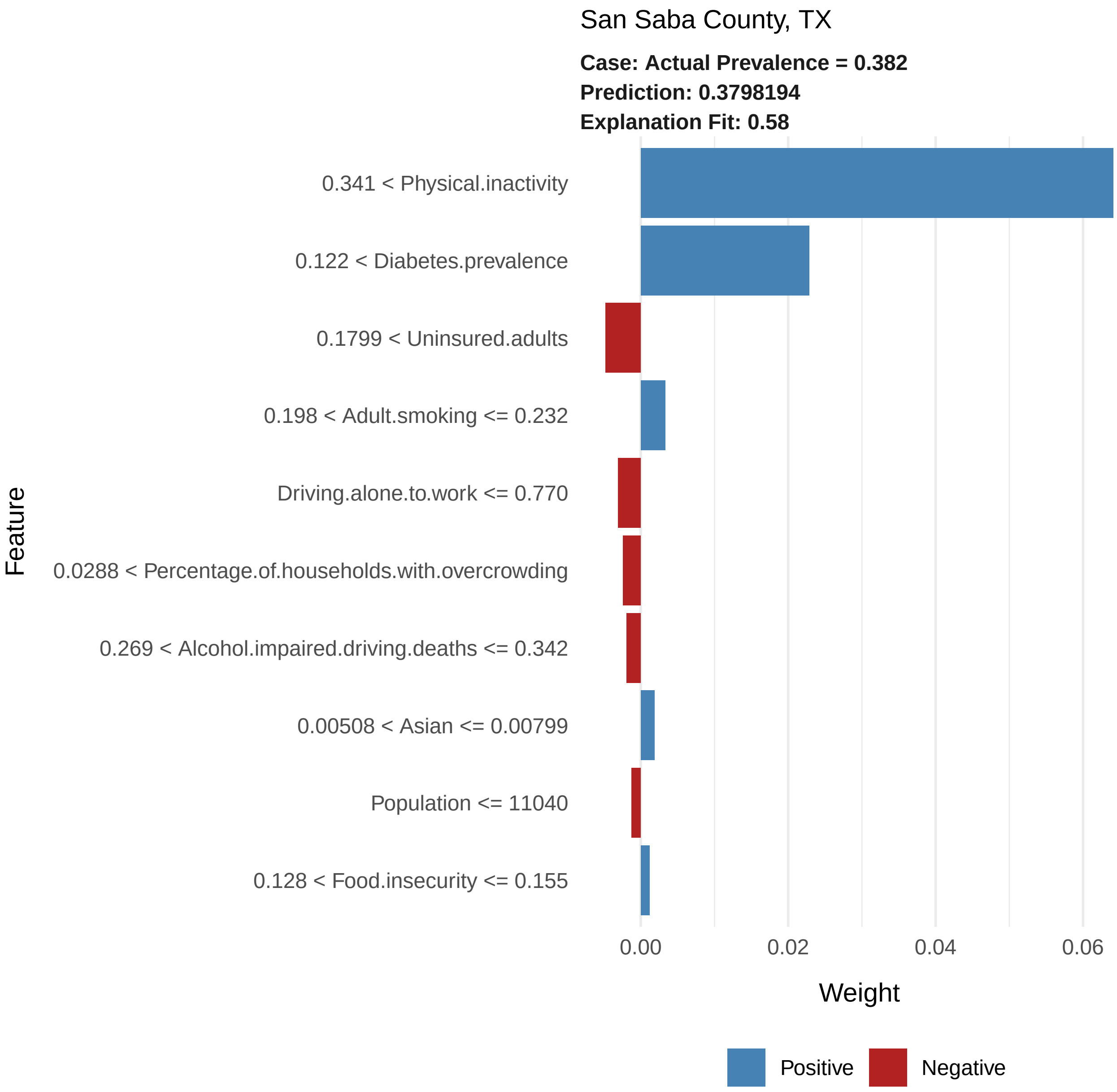
San Saba County, TX
Case: Actual Prevalence = 0.382
Prediction: 0.3798194
Explanation Fit: 0.58
0.341 < Physical.inactivity
0.122 < Diabetes.prevalence
0.1799 < Uninsured.adults
0.198 < Adult.smoking <= 0.232
Driving.alone.to.work <= 0.770
0.0288 < Percentage.of.households.with.overcrowding
0.269 < Alcohol.impaired.driving.deaths <= 0.342
0.00508 < Asian <= 0.00799
Population <= 11040
0.128 < Food.insecurity <= 0.155
Feature
0.00
0.02
0.04
0.06
Weight
Positive
Negative

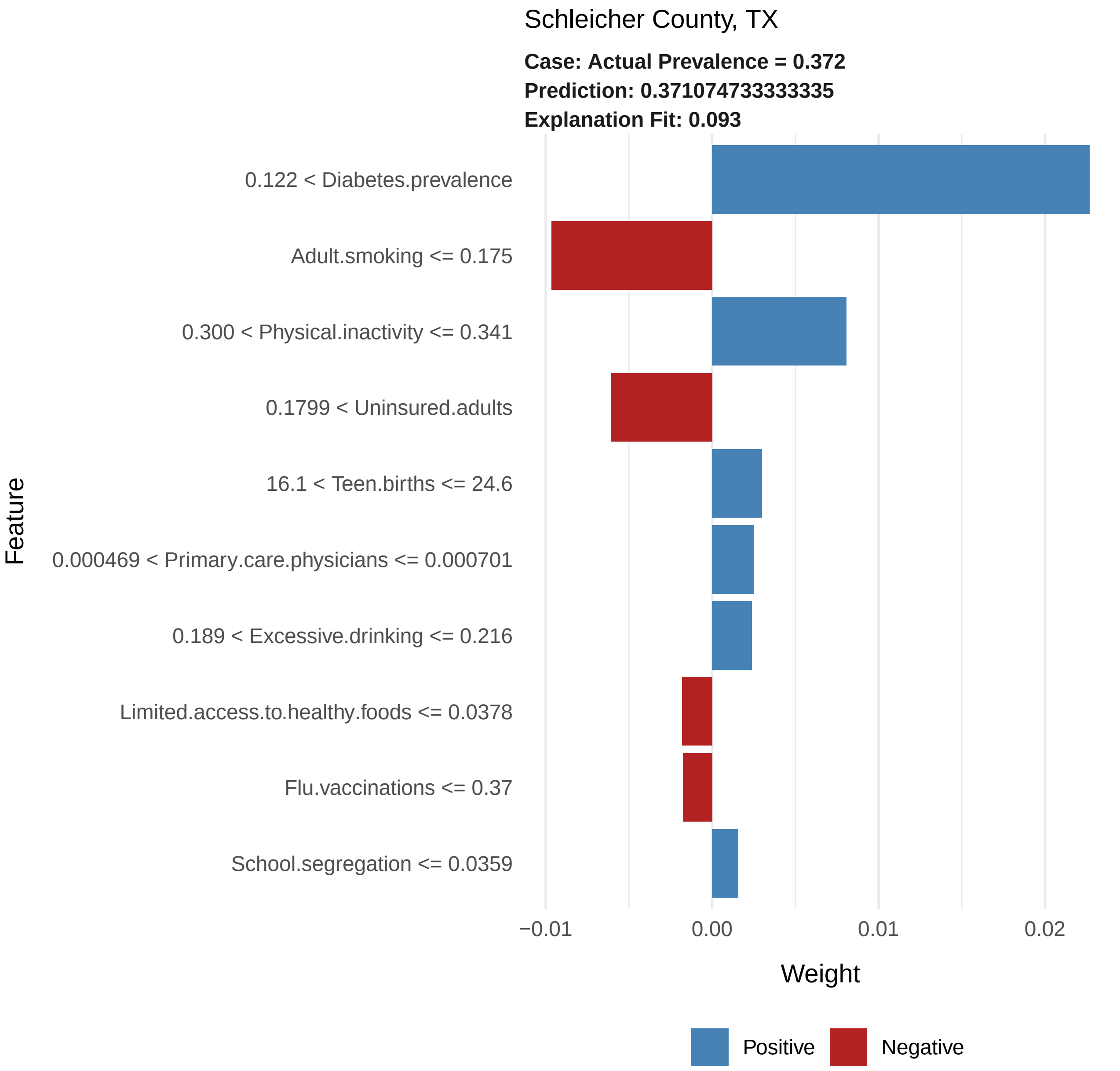

Schleicher County, TX
Case: Actual Prevalence = 0.372
Prediction: 0.371074733333335
Explanation Fit: 0.093
0.122 < Diabetes.prevalence
Adult.smoking <= 0.175
0.300 < Physical.inactivity <= 0.341
0.1799 < Uninsured.adults
16.1 < Teen.births <= 24.6
0.000469 < Primary.care.physicians <= 0.000701
0.189 < Excessive.drinking <= 0.216
Limited.access.to.healthy.foods <= 0.0378
Flu.vaccinations <= 0.37
School.segregation <= 0.0359
Feature
−0.01
0.00
0.01
0.02
Weight
Positive
Negative

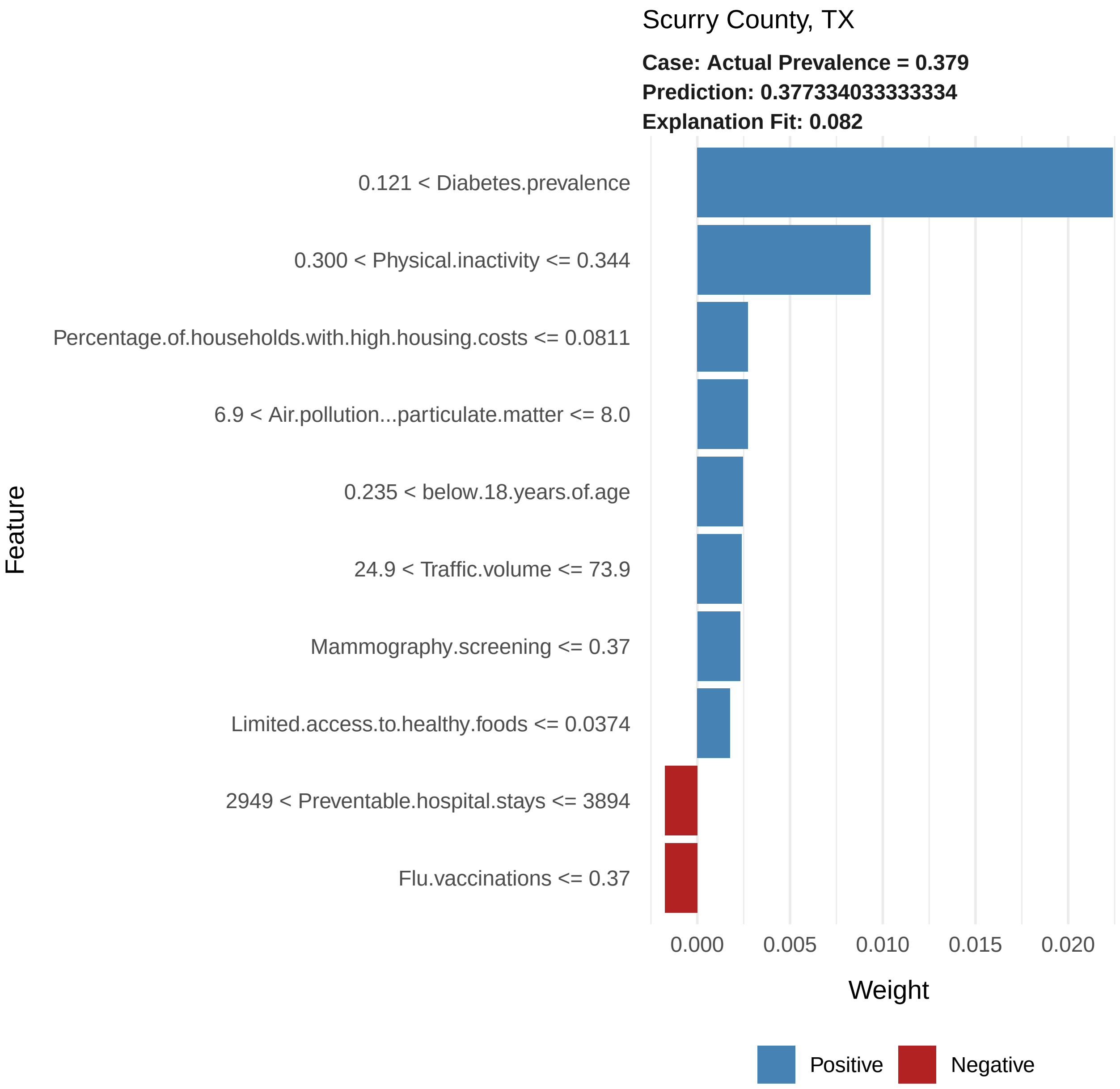

Scurry County, TX
Case: Actual Prevalence = 0.379
Prediction: 0.377334033333334
Explanation Fit: 0.082
0.121 < Diabetes.prevalence
0.300 < Physical.inactivity <= 0.344
Percentage.of.households.with.high.housing.costs <= 0.0811
6.9 < Air.pollution...particulate.matter <= 8.0
0.235 < below.18.years.of.age
24.9 < Traffic.volume <= 73.9
Mammography.screening <= 0.37
Limited.access.to.healthy.foods <= 0.0374
2949 < Preventable.hospital.stays <= 3894
Flu.vaccinations <= 0.37
Feature
0.000
0.005
0.010
0.015
0.020
Weight
Positive
Negative

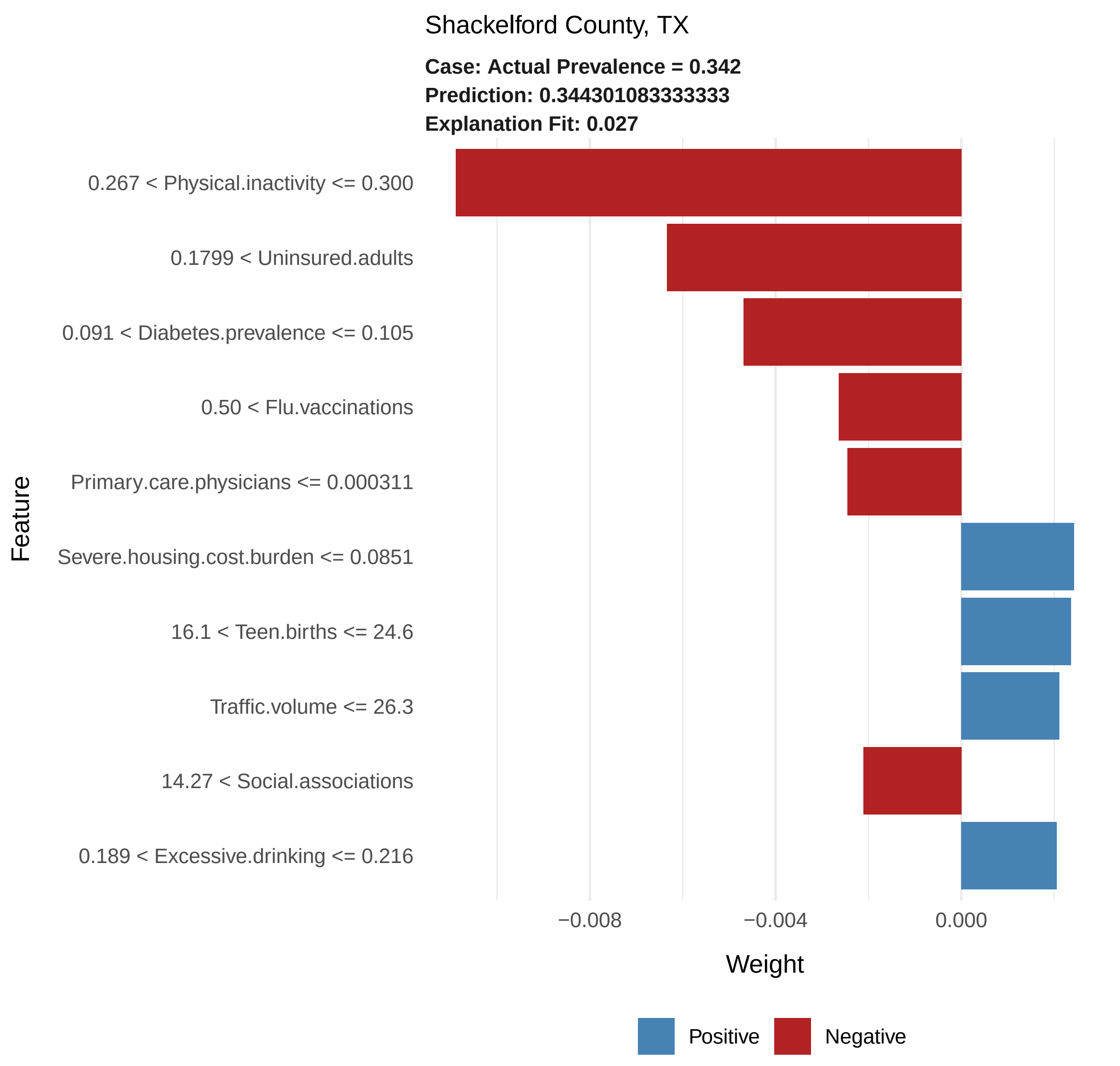

Shackelford County, TX
Case: Actual Prevalence = 0.342
Prediction: 0.344301083333333
Explanation Fit: 0.027
0.267 < Physical.inactivity <= 0.300
0.1799 < Uninsured.adults
0.091 < Diabetes.prevalence <= 0.105
0.50 < Flu.vaccinations
Primary.care.physicians <= 0.000311
Severe.housing.cost.burden <= 0.0851
16.1 < Teen.births <= 24.6
Traffic.volume <= 26.3
14.27 < Social.associations
0.189 < Excessive.drinking <= 0.216
Feature
−0.008
−0.004
0.000
Weight
Positive
Negative

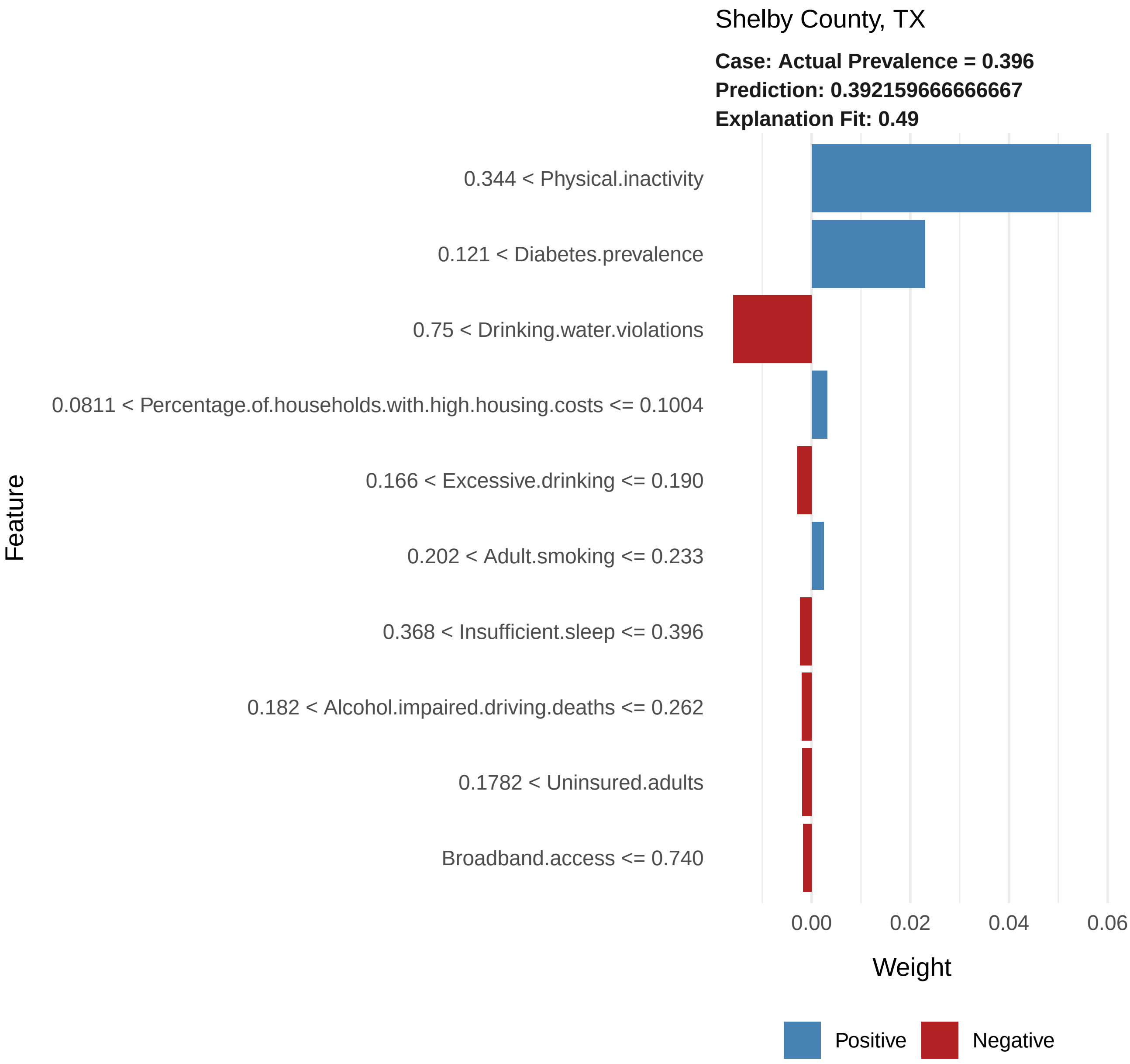

Shelby County, TX
Case: Actual Prevalence = 0.396
Prediction: 0.392159666666667
Explanation Fit: 0.49
0.344 < Physical.inactivity
0.121 < Diabetes.prevalence
0.75 < Drinking.water.violations
0.0811 < Percentage.of.households.with.high.housing.costs <= 0.1004
0.166 < Excessive.drinking <= 0.190
0.202 < Adult.smoking <= 0.233
0.368 < Insufficient.sleep <= 0.396
0.182 < Alcohol.impaired.driving.deaths <= 0.262
0.1782 < Uninsured.adults
Broadband.access <= 0.740
Feature
0.00
0.02
0.04
0.06
Weight
Positive
Negative

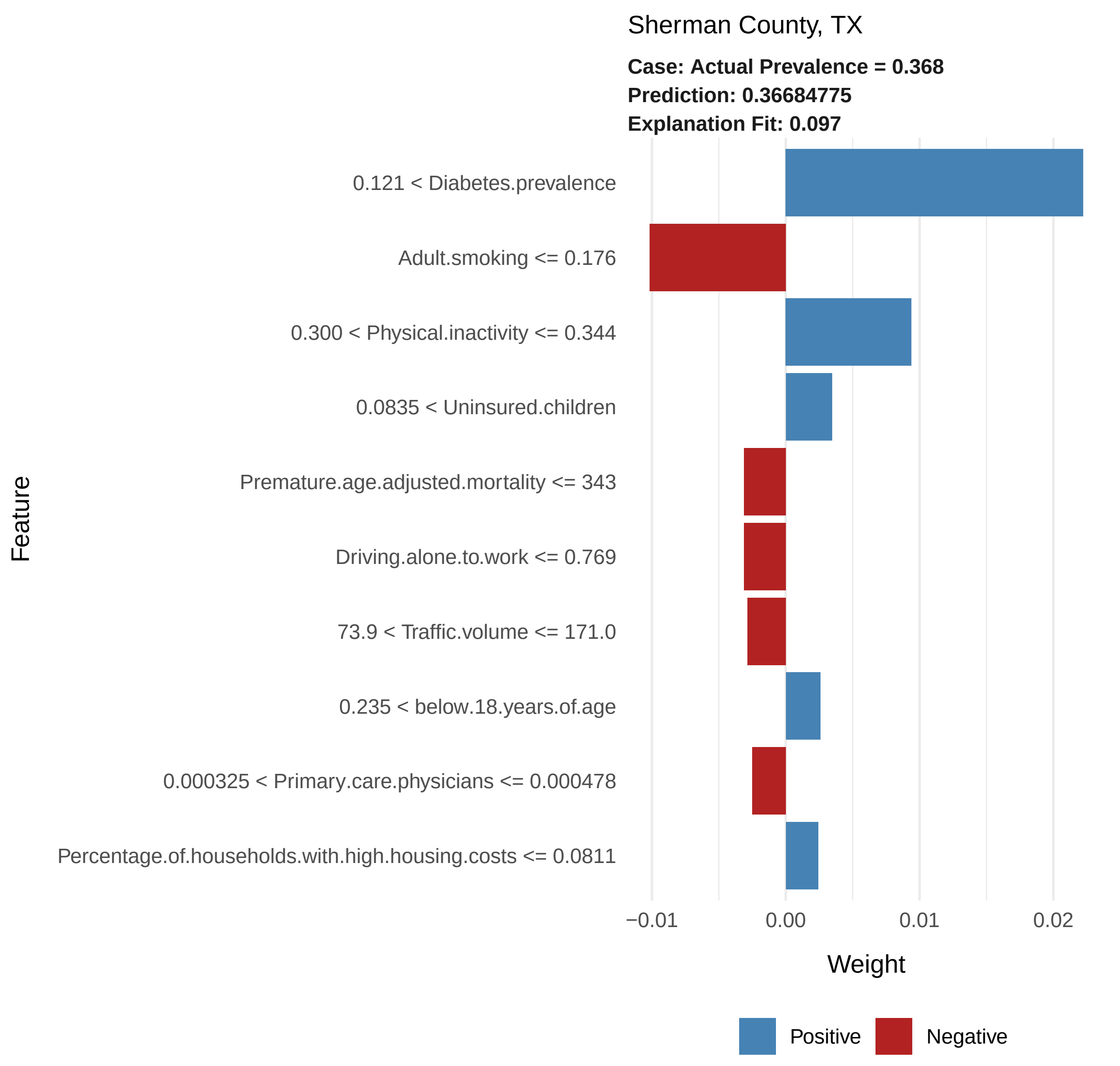

Sherman County, TX
Case: Actual Prevalence = 0.368
Prediction: 0.36684775
Explanation Fit: 0.097
0.121 < Diabetes.prevalence
Adult.smoking <= 0.176
0.300 < Physical.inactivity <= 0.344
0.0835 < Uninsured.children
Premature.age.adjusted.mortality <= 343
Driving.alone.to.work <= 0.769
73.9 < Traffic.volume <= 171.0
0.235 < below.18.years.of.age
0.000325 < Primary.care.physicians <= 0.000478
Percentage.of.households.with.high.housing.costs <= 0.0811
Feature
−0.01
0.00
0.01
0.02
Weight
Positive
Negative

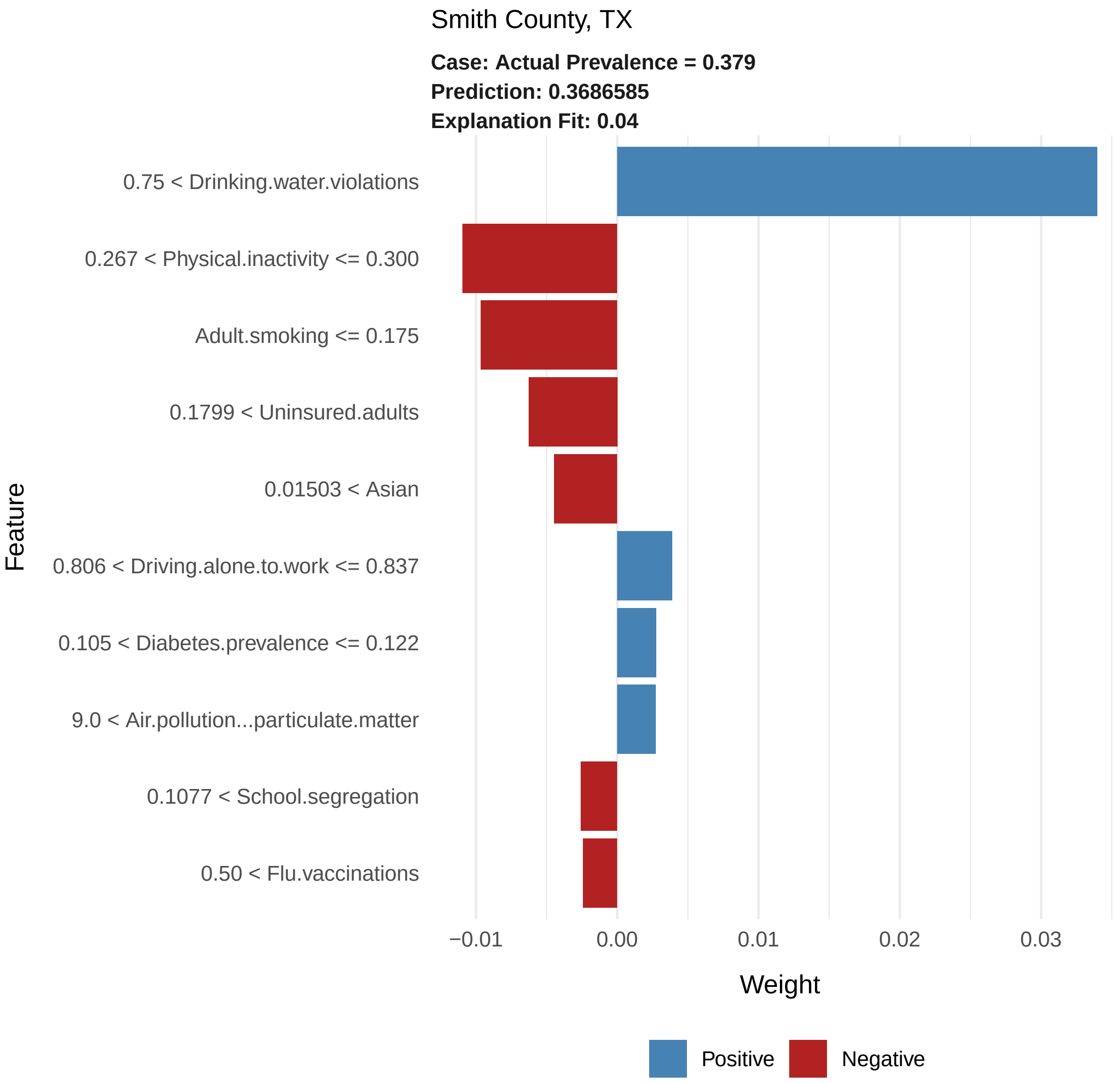
Smith County, TX
Case: Actual Prevalence = 0.379
Prediction: 0.3686585
Explanation Fit: 0.04
0.75 < Drinking.water.violations
0.267 < Physical.inactivity <= 0.300
Adult.smoking <= 0.175
0.1799 < Uninsured.adults
0.01503 < Asian
0.806 < Driving.alone.to.work <= 0.837
0.105 < Diabetes.prevalence <= 0.122
9.0 < Air.pollution...particulate.matter
0.1077 < School.segregation
0.50 < Flu.vaccinations
Feature
−0.01
0.00
0.01
0.02
0.03
Weight
Positive
Negative

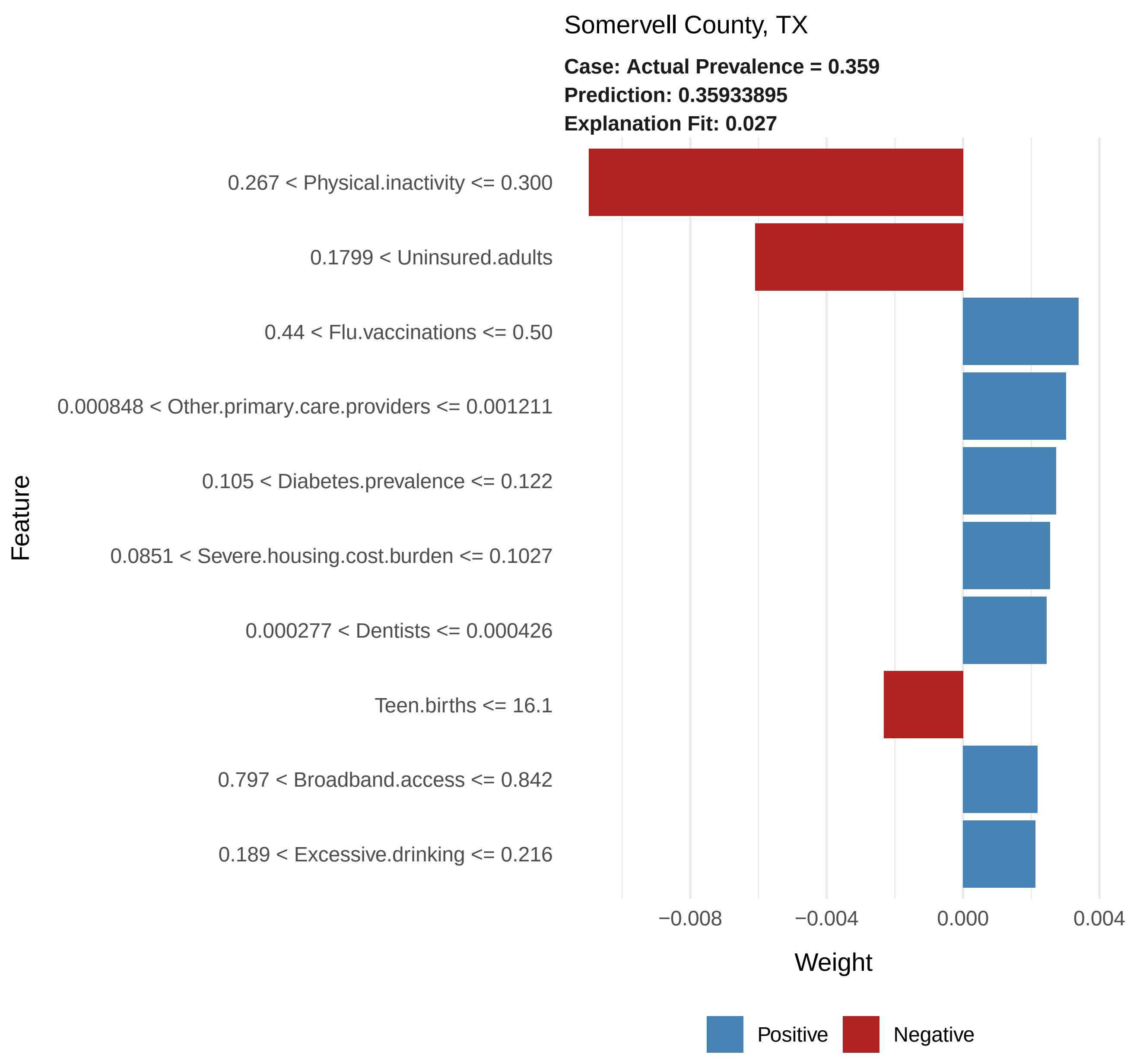

Somervell County, TX
Case: Actual Prevalence = 0.359
Prediction: 0.35933895
Explanation Fit: 0.027
0.267 < Physical.inactivity <= 0.300
0.1799 < Uninsured.adults
0.44 < Flu.vaccinations <= 0.50
0.000848 < Other.primary.care.providers <= 0.001211
0.105 < Diabetes.prevalence <= 0.122
0.0851 < Severe.housing.cost.burden <= 0.1027
0.000277 < Dentists <= 0.000426
Teen.births <= 16.1
0.797 < Broadband.access <= 0.842
0.189 < Excessive.drinking <= 0.216
Feature
−0.008
−0.004
0.000
0.004
Weight
Positive
Negative

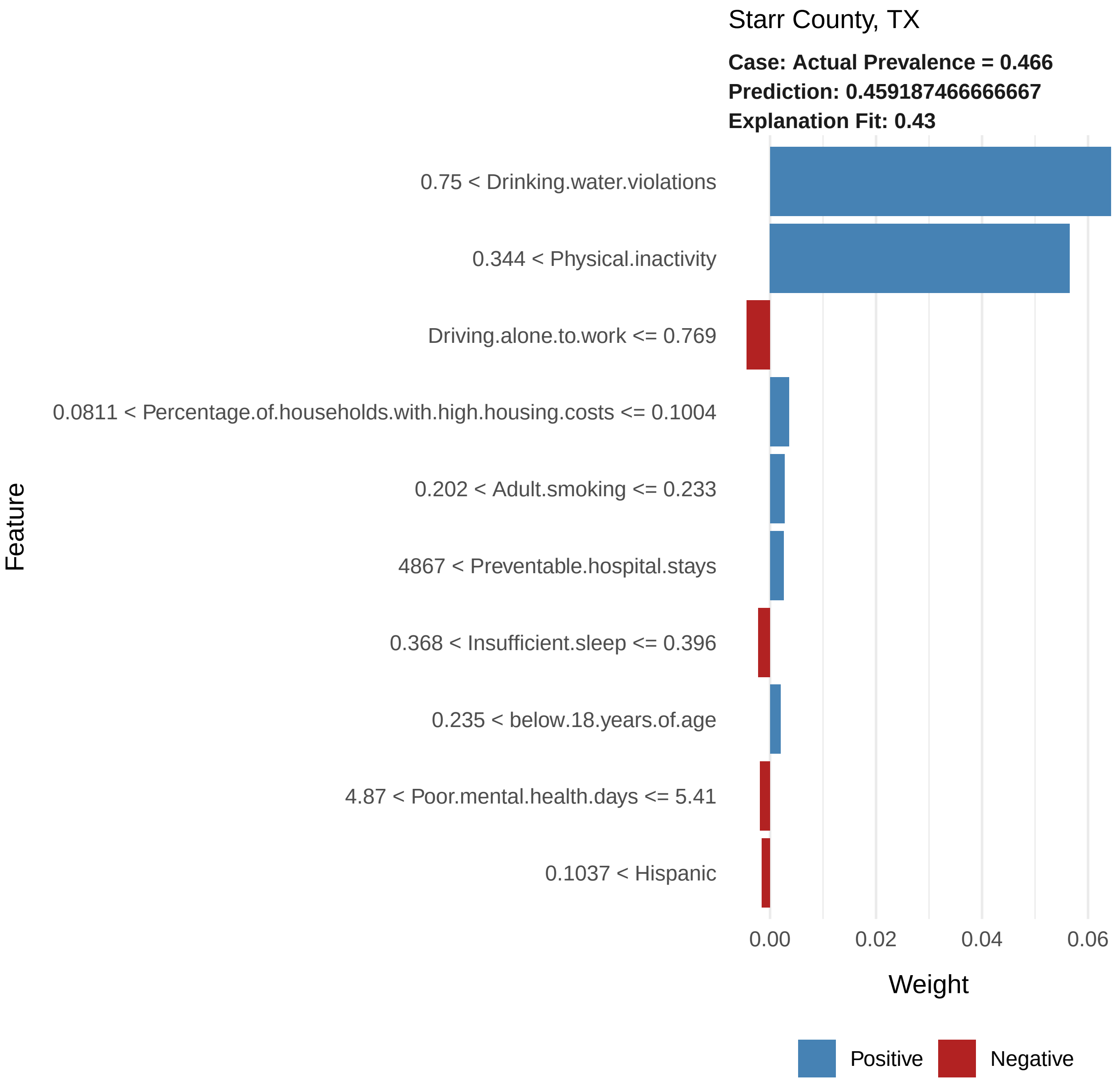
Starr County, TX
Case: Actual Prevalence = 0.466
Prediction: 0.459187466666667
Explanation Fit: 0.43
0.75 < Drinking.water.violations
0.344 < Physical.inactivity
Driving.alone.to.work <= 0.769
0.0811 < Percentage.of.households.with.high.housing.costs <= 0.1004
0.202 < Adult.smoking <= 0.233
4867 < Preventable.hospital.stays
0.368 < Insufficient.sleep <= 0.396
0.235 < below.18.years.of.age
4.87 < Poor.mental.health.days <= 5.41
0.1037 < Hispanic
Feature
0.00
0.02
0.04
0.06
Weight
Positive
Negative

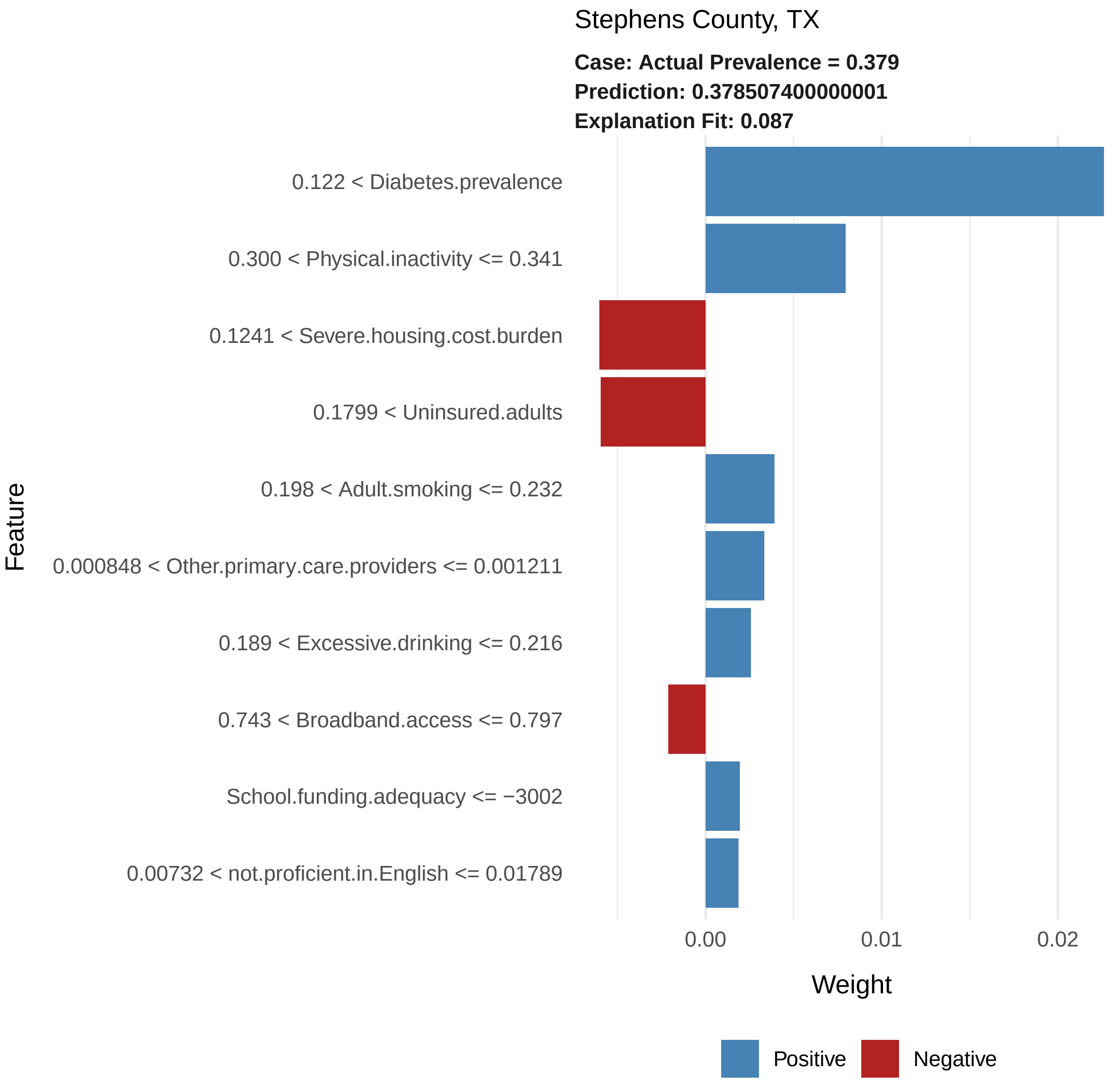

Stephens County, TX
Case: Actual Prevalence = 0.379
Prediction: 0.378507400000001
Explanation Fit: 0.087
0.122 < Diabetes.prevalence
0.300 < Physical.inactivity <= 0.341
0.1241 < Severe.housing.cost.burden
0.1799 < Uninsured.adults
0.198 < Adult.smoking <= 0.232
0.000848 < Other.primary.care.providers <= 0.001211
0.189 < Excessive.drinking <= 0.216
0.743 < Broadband.access <= 0.797
School.funding.adequacy <= −3002
0.00732 < not.proficient.in.English <= 0.01789
Feature
0.00
0.01
0.02
Weight
Positive
Negative

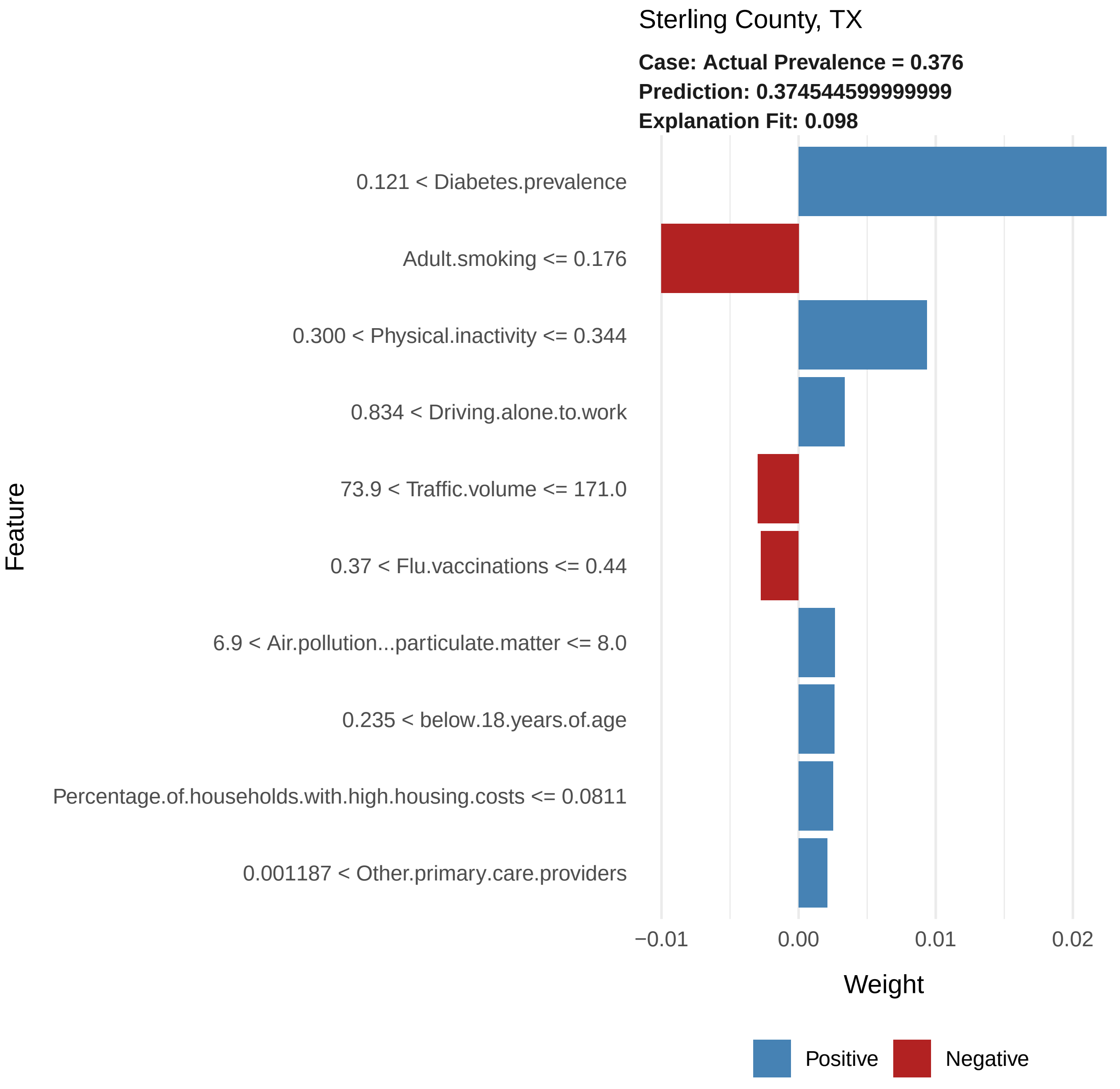

Sterling County, TX
Case: Actual Prevalence = 0.376
Prediction: 0.374544599999999
Explanation Fit: 0.098
0.121 < Diabetes.prevalence
Adult.smoking <= 0.176
0.300 < Physical.inactivity <= 0.344
0.834 < Driving.alone.to.work
73.9 < Traffic.volume <= 171.0
0.37 < Flu.vaccinations <= 0.44
6.9 < Air.pollution...particulate.matter <= 8.0
0.235 < below.18.years.of.age
Percentage.of.households.with.high.housing.costs <= 0.0811
0.001187 < Other.primary.care.providers
Feature
−0.01
0.00
0.01
0.02
Weight
Positive
Negative

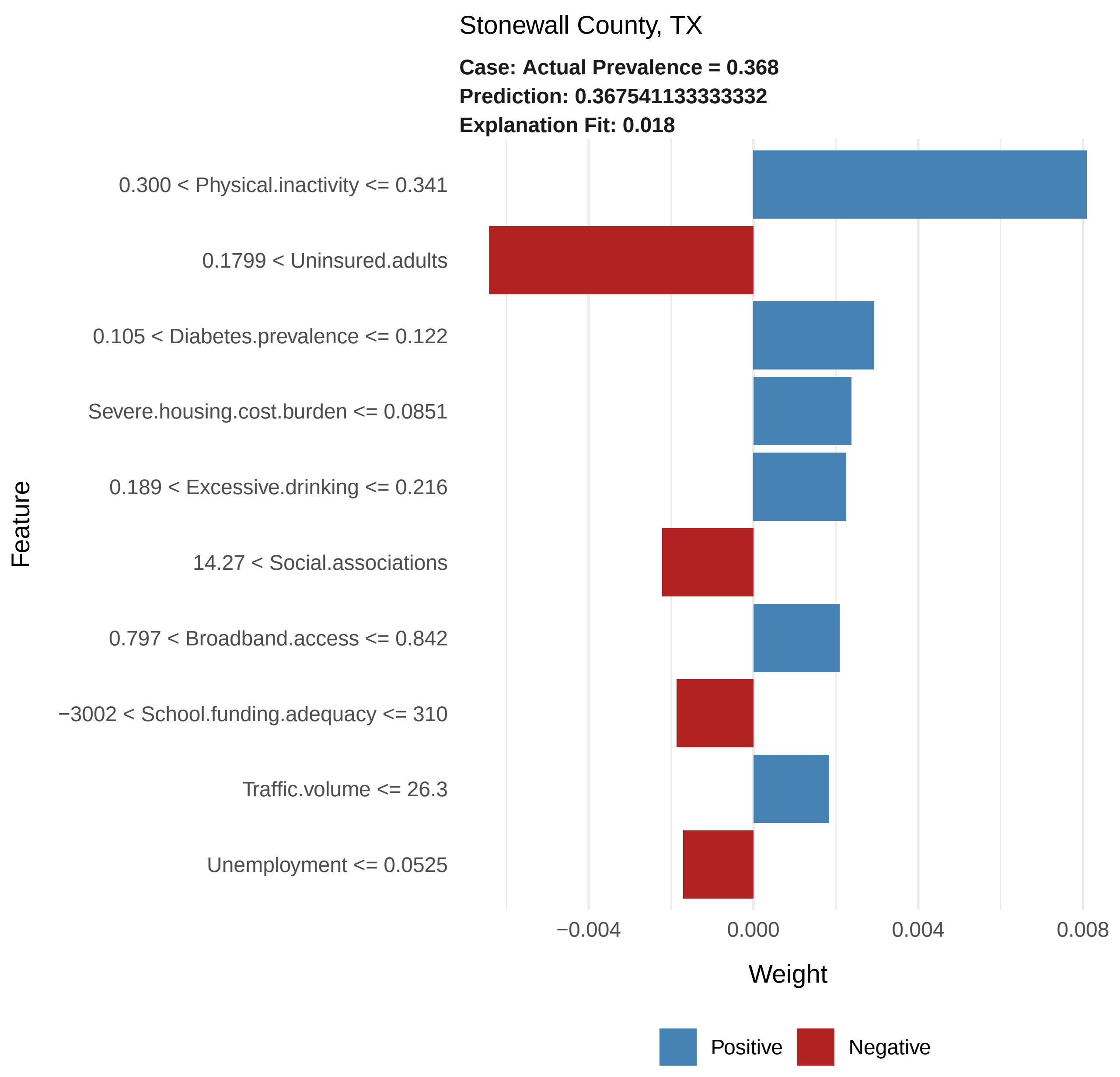
Stonewall County, TX
Case: Actual Prevalence = 0.368
Prediction: 0.367541133333332
Explanation Fit: 0.018
0.300 < Physical.inactivity <= 0.341
0.1799 < Uninsured.adults
0.105 < Diabetes.prevalence <= 0.122
Severe.housing.cost.burden <= 0.0851
0.189 < Excessive.drinking <= 0.216
14.27 < Social.associations
0.797 < Broadband.access <= 0.842
−3002 < School.funding.adequacy <= 310
Traffic.volume <= 26.3
Unemployment <= 0.0525
Feature
−0.004
0.000
0.004
0.008
Weight
Positive
Negative

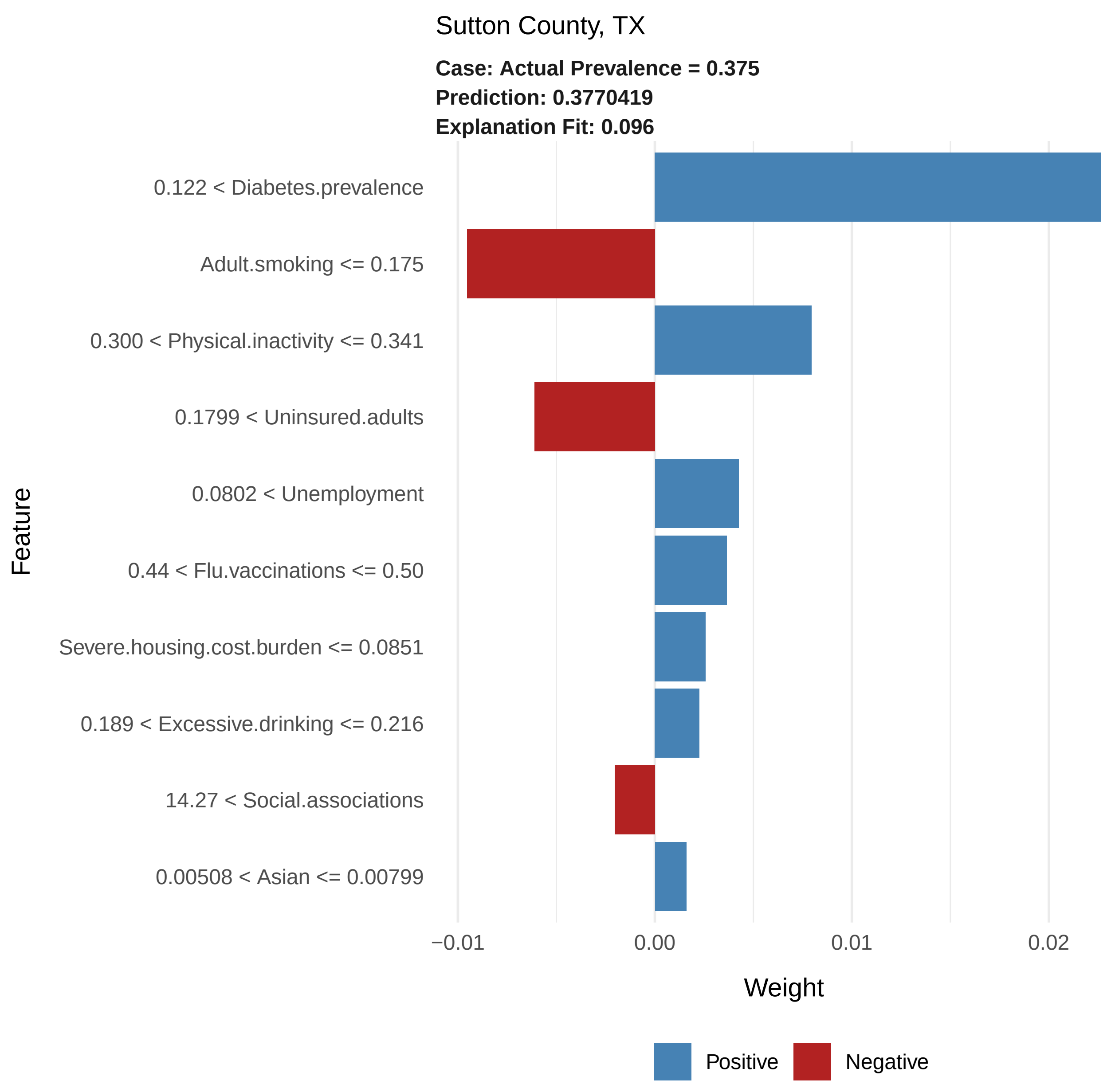

Sutton County, TX
Case: Actual Prevalence = 0.375
Prediction: 0.3770419
Explanation Fit: 0.096
0.122 < Diabetes.prevalence
Adult.smoking <= 0.175
0.300 < Physical.inactivity <= 0.341
0.1799 < Uninsured.adults
0.0802 < Unemployment
0.44 < Flu.vaccinations <= 0.50
Severe.housing.cost.burden <= 0.0851
0.189 < Excessive.drinking <= 0.216
14.27 < Social.associations
0.00508 < Asian <= 0.00799
Feature
−0.01
0.00
0.01
0.02
Weight
Positive
Negative

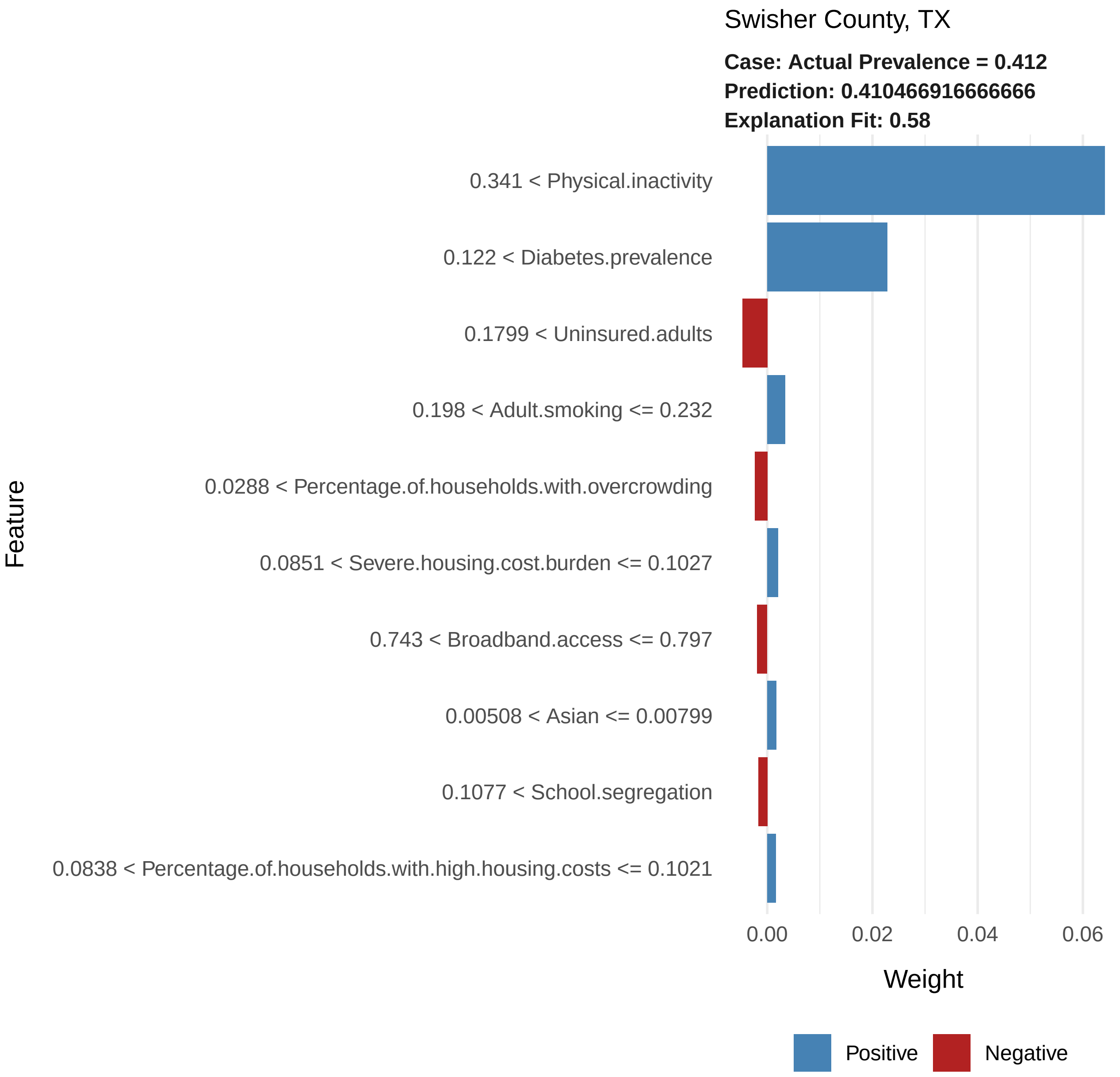

Swisher County, TX
Case: Actual Prevalence = 0.412
Prediction: 0.410466916666666
Explanation Fit: 0.58
0.341 < Physical.inactivity
0.122 < Diabetes.prevalence
0.1799 < Uninsured.adults
0.198 < Adult.smoking <= 0.232
0.0288 < Percentage.of.households.with.overcrowding
0.0851 < Severe.housing.cost.burden <= 0.1027
0.743 < Broadband.access <= 0.797
0.00508 < Asian <= 0.00799
0.1077 < School.segregation
0.0838 < Percentage.of.households.with.high.housing.costs <= 0.1021
Feature
0.00
0.02
0.04
0.06
Weight
Positive
Negative

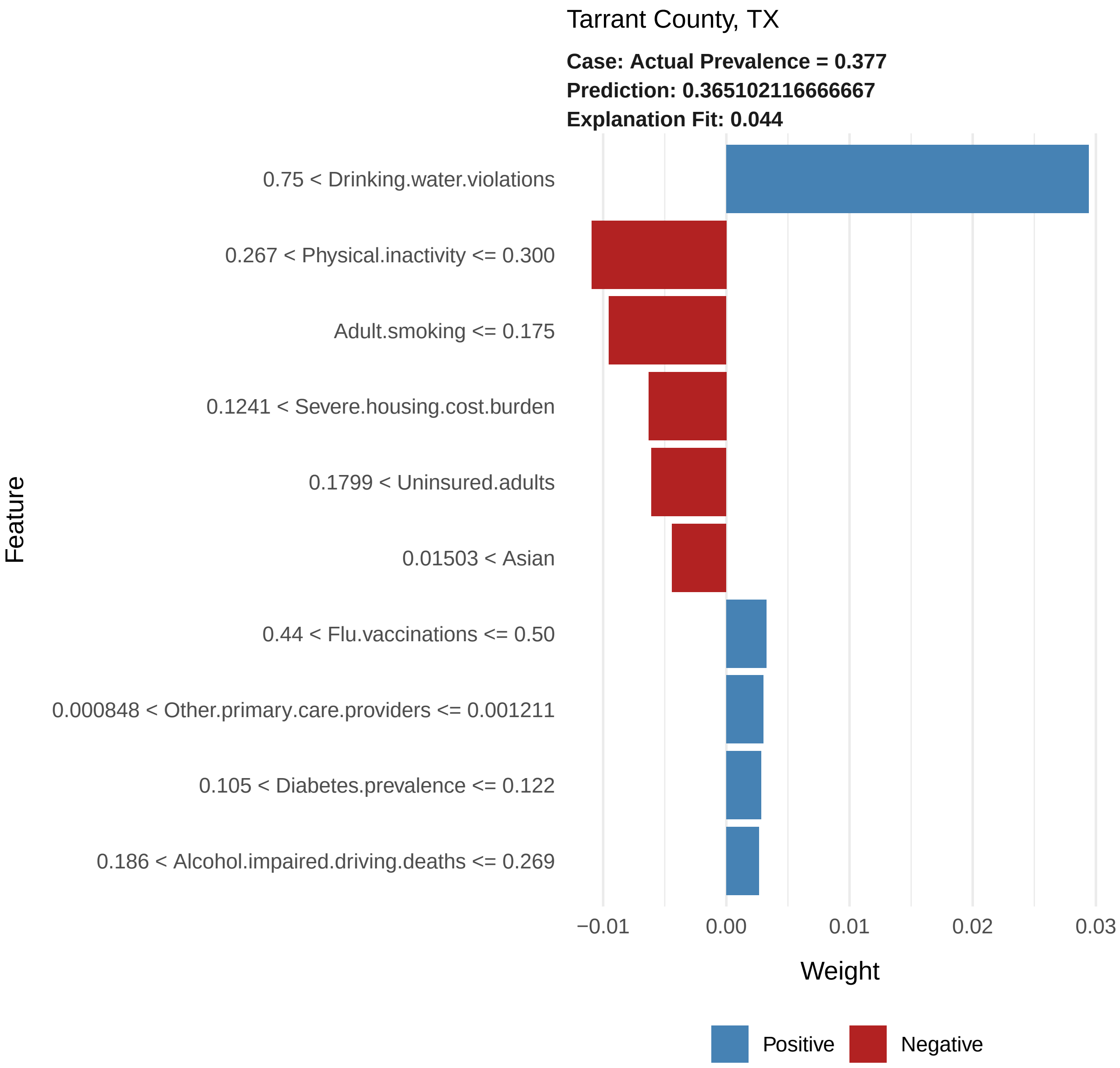

Tarrant County, TX
Case: Actual Prevalence = 0.377
Prediction: 0.365102116666667
Explanation Fit: 0.044
0.75 < Drinking.water.violations
0.267 < Physical.inactivity <= 0.300
Adult.smoking <= 0.175
0.1241 < Severe.housing.cost.burden
0.1799 < Uninsured.adults
0.01503 < Asian
0.44 < Flu.vaccinations <= 0.50
0.000848 < Other.primary.care.providers <= 0.001211
0.105 < Diabetes.prevalence <= 0.122
0.186 < Alcohol.impaired.driving.deaths <= 0.269
Feature
−0.01
0.00
0.01
0.02
0.03
Weight
Positive
Negative

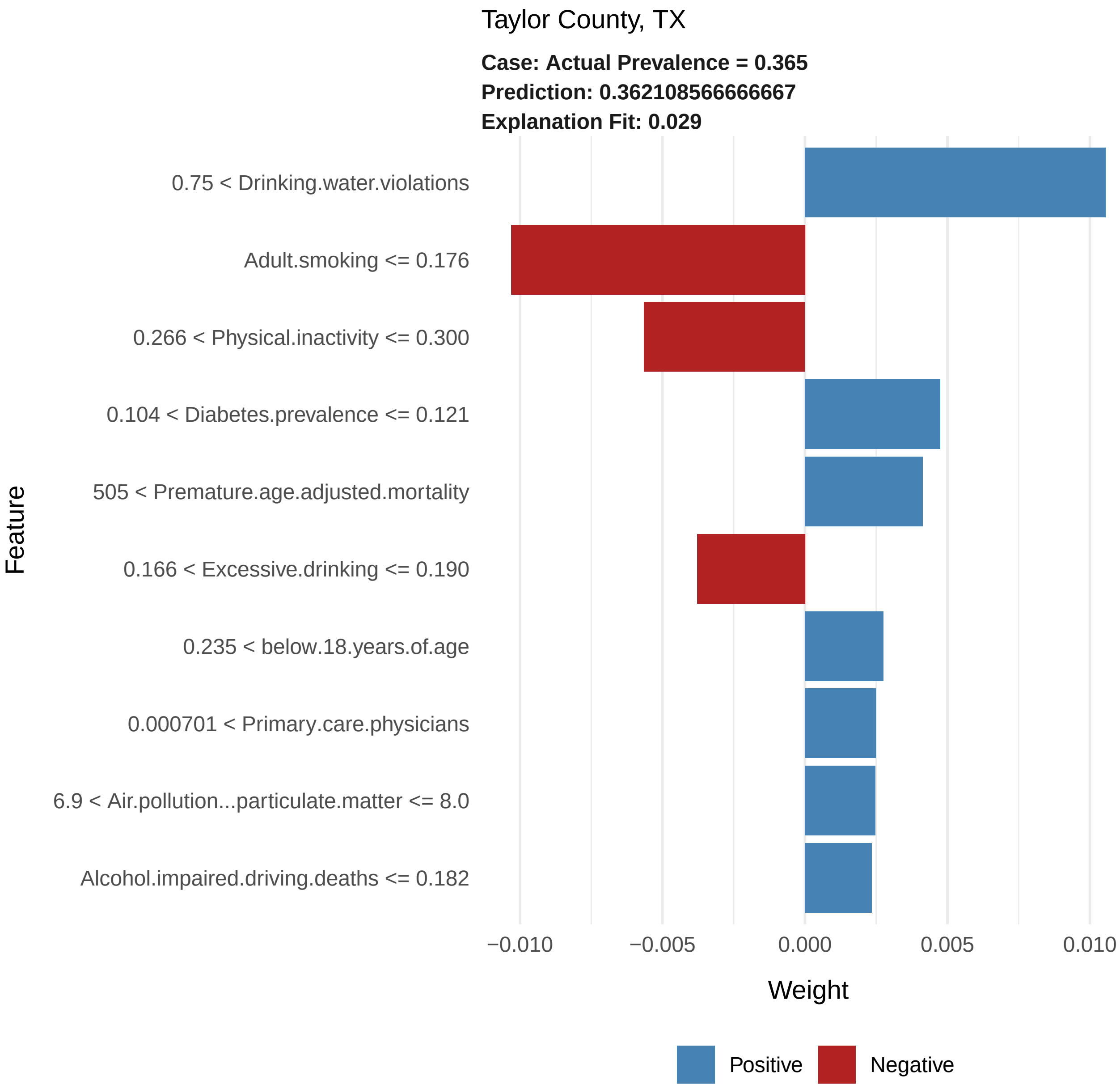

Taylor County, TX
Case: Actual Prevalence = 0.365
Prediction: 0.362108566666667
Explanation Fit: 0.029
0.75 < Drinking.water.violations
Adult.smoking <= 0.176
0.266 < Physical.inactivity <= 0.300
0.104 < Diabetes.prevalence <= 0.121
505 < Premature.age.adjusted.mortality
0.166 < Excessive.drinking <= 0.190
0.235 < below.18.years.of.age
0.000701 < Primary.care.physicians
6.9 < Air.pollution...particulate.matter <= 8.0
Alcohol.impaired.driving.deaths <= 0.182
Feature
−0.010
−0.005
0.000
0.005
0.010
Weight
Positive
Negative

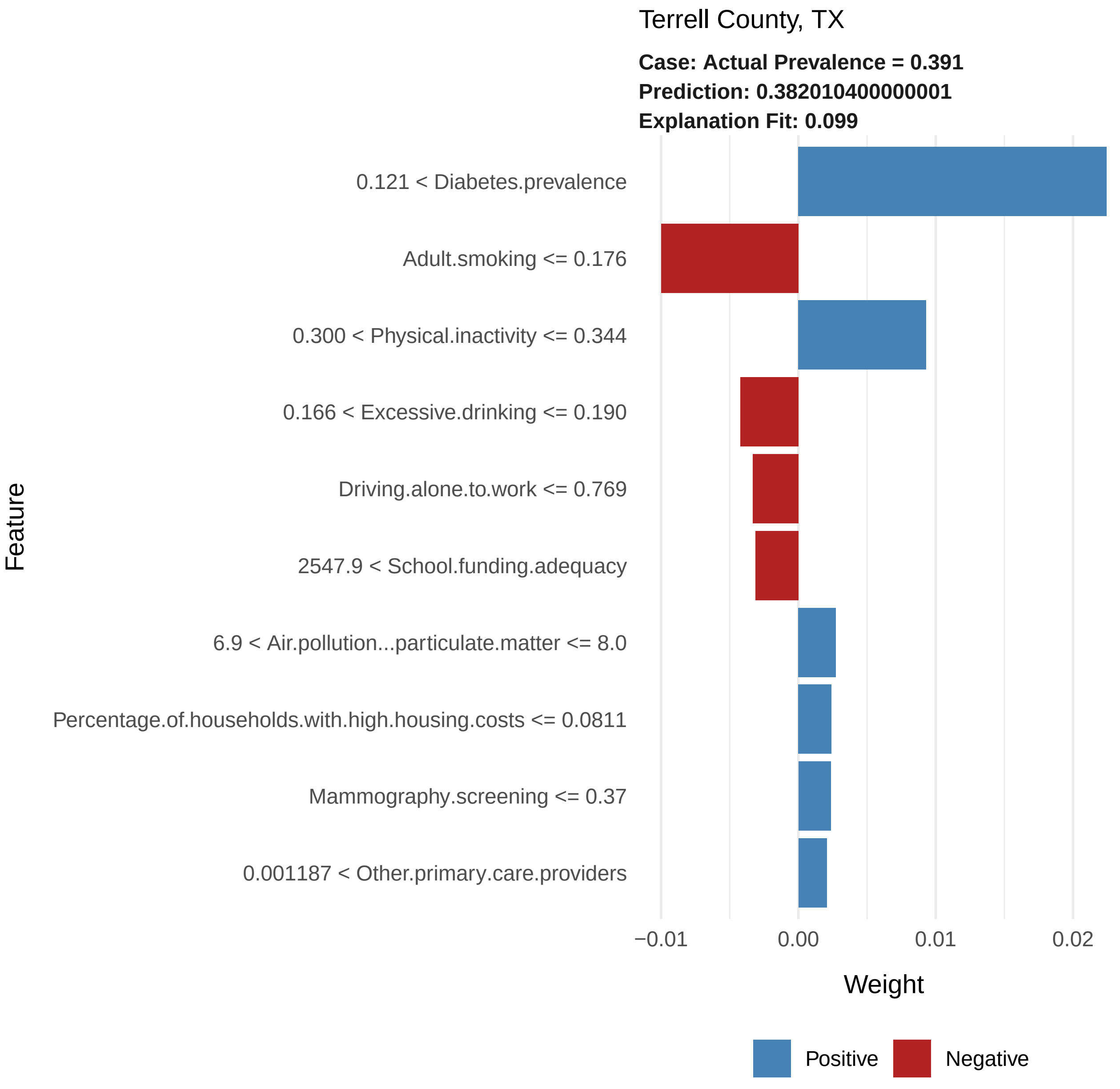

Terrell County, TX
Case: Actual Prevalence = 0.391
Prediction: 0.382010400000001
Explanation Fit: 0.099
Feature
0.121 < Diabetes.prevalence
Adult.smoking <= 0.176
0.300 < Physical.inactivity <= 0.344
0.166 < Excessive.drinking <= 0.190
Driving.alone.to.work <= 0.769
2547.9 < School.funding.adequacy
6.9 < Air.pollution...particulate.matter <= 8.0
Percentage.of.households.with.high.housing.costs <= 0.0811
Mammography.screening <= 0.37
0.001187 < Other.primary.care.providers
−0.01
0.00
0.01
0.02
Weight
Positive
Negative

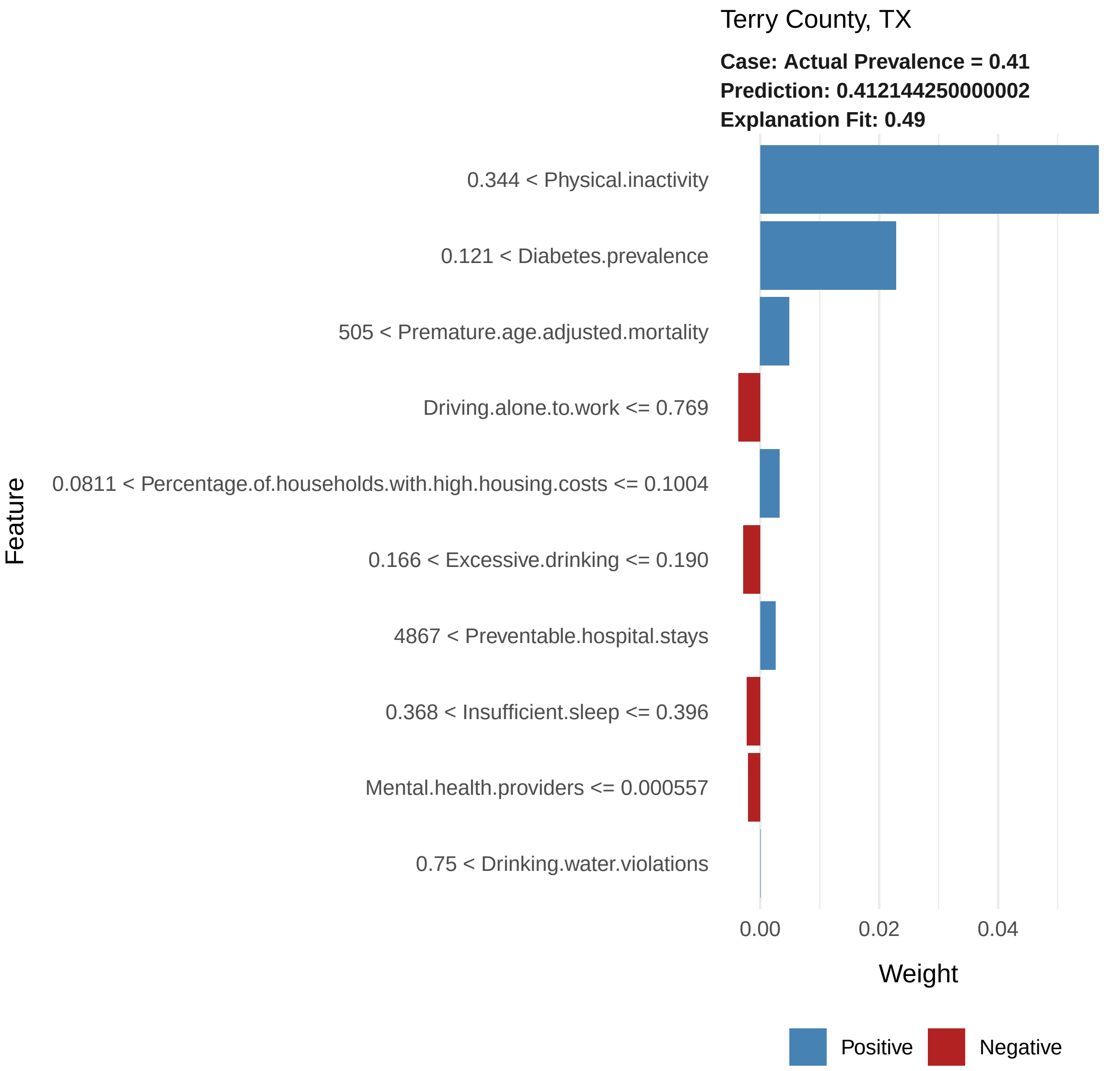
Terry County, TX
Case: Actual Prevalence = 0.41
Prediction: 0.412144250000002
Explanation Fit: 0.49
0.344 < Physical.inactivity
0.121 < Diabetes.prevalence
505 < Premature.age.adjusted.mortality
Driving.alone.to.work <= 0.769
0.0811 < Percentage.of.households.with.high.housing.costs <= 0.1004
0.166 < Excessive.drinking <= 0.190
4867 < Preventable.hospital.stays
0.368 < Insufficient.sleep <= 0.396
Mental.health.providers <= 0.000557
0.75 < Drinking.water.violations
Feature
0.00
0.02
0.04
Weight
Positive
Negative

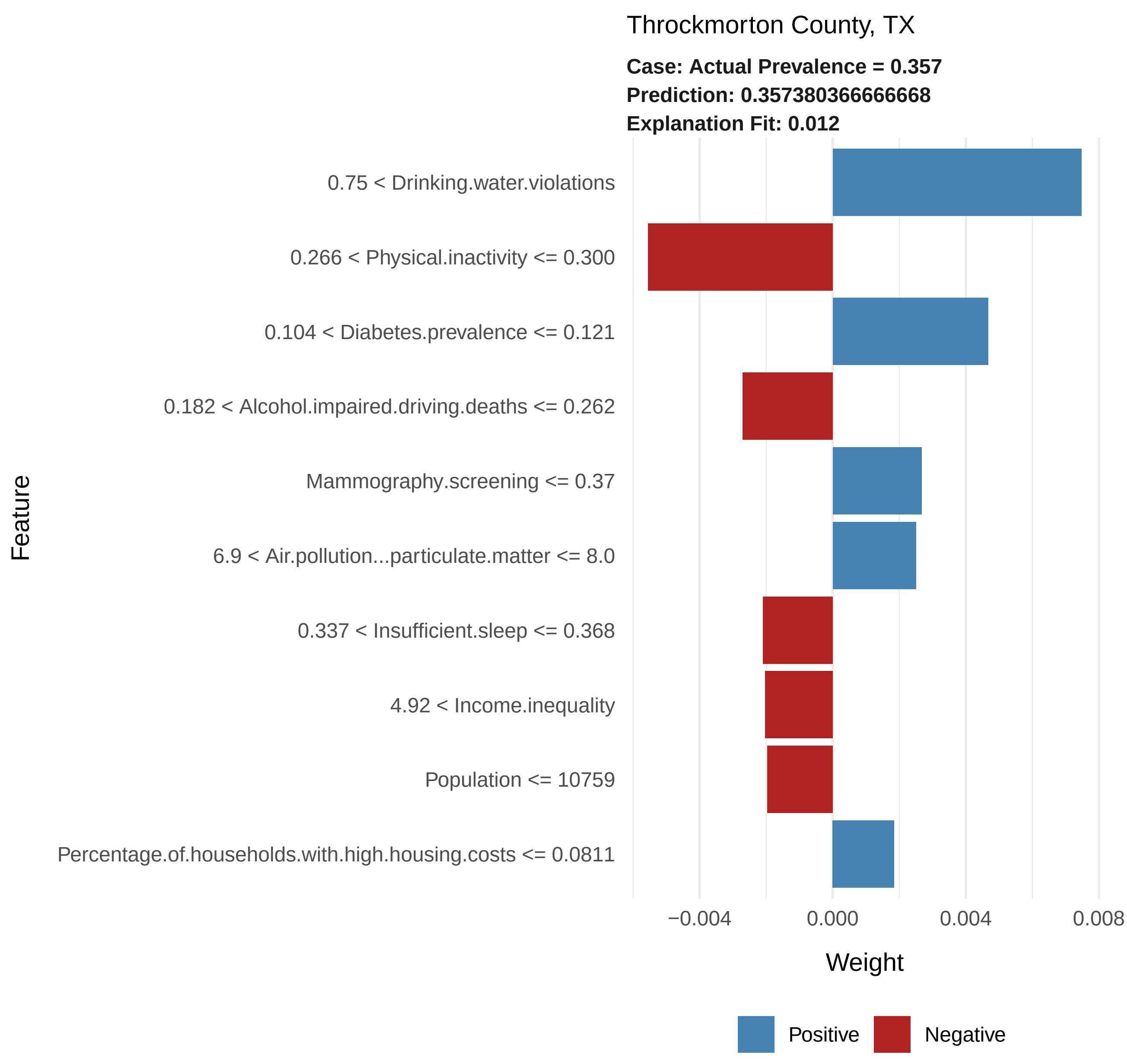

Throckmorton County, TX
Case: Actual Prevalence = 0.357
Prediction: 0.357380366666668
Explanation Fit: 0.012
0.75 < Drinking.water.violations
0.266 < Physical.inactivity <= 0.300
0.104 < Diabetes.prevalence <= 0.121
0.182 < Alcohol.impaired.driving.deaths <= 0.262
Mammography.screening <= 0.37
6.9 < Air.pollution...particulate.matter <= 8.0
0.337 < Insufficient.sleep <= 0.368
4.92 < Income.inequality
Population <= 10759
Percentage.of.households.with.high.housing.costs <= 0.0811
Feature
−0.004
0.000
0.004
0.008
Weight
Positive
Negative

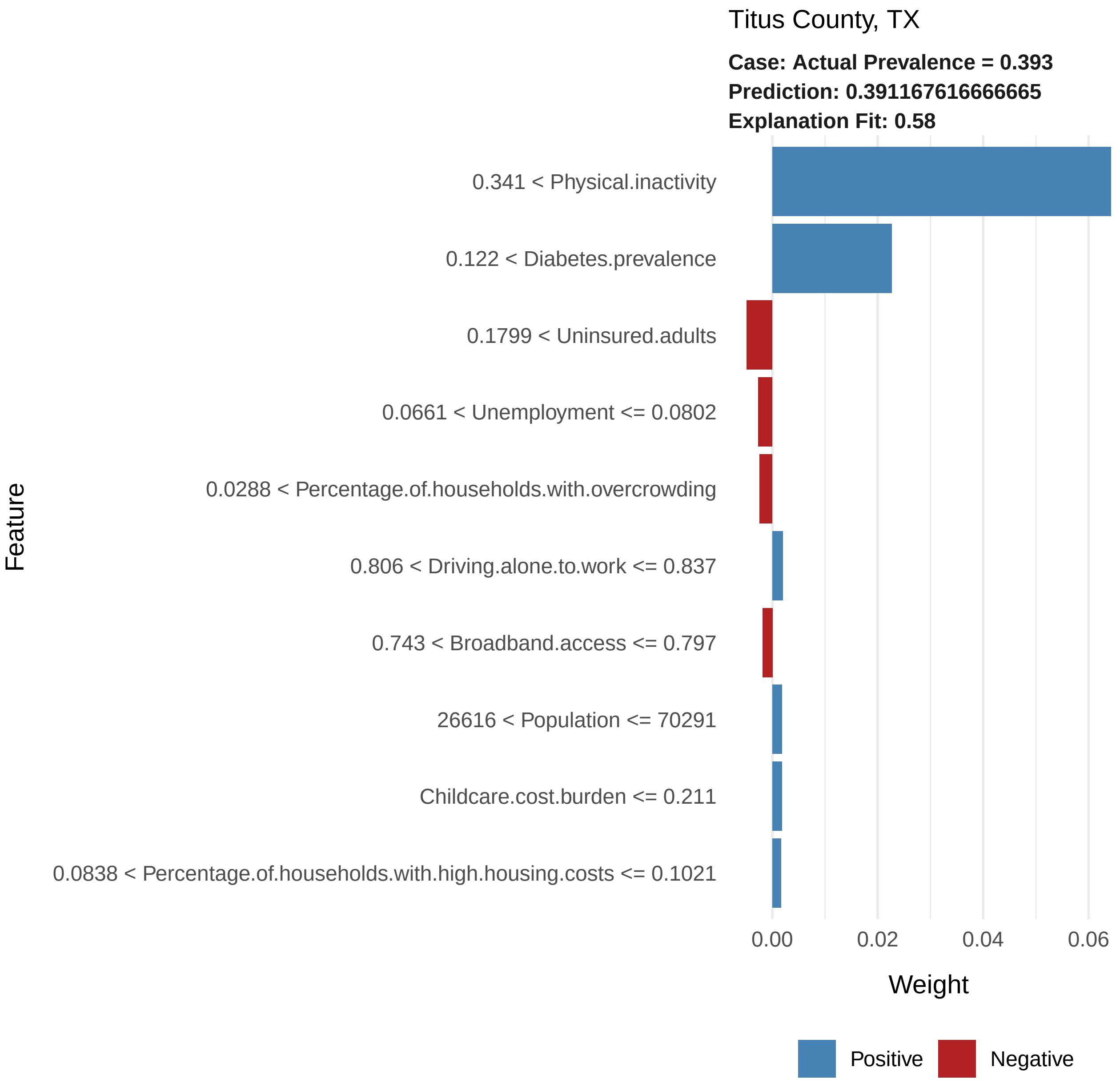

Titus County, TX
Case: Actual Prevalence = 0.393
Prediction: 0.391167616666665
Explanation Fit: 0.58
0.341 < Physical.inactivity
0.122 < Diabetes.prevalence
0.1799 < Uninsured.adults
0.0661 < Unemployment <= 0.0802
0.0288 < Percentage.of.households.with.overcrowding
0.806 < Driving.alone.to.work <= 0.837
0.743 < Broadband.access <= 0.797
26616 < Population <= 70291
Childcare.cost.burden <= 0.211
0.0838 < Percentage.of.households.with.high.housing.costs <= 0.1021
Feature
0.00
0.02
0.04
0.06
Weight
Positive
Negative

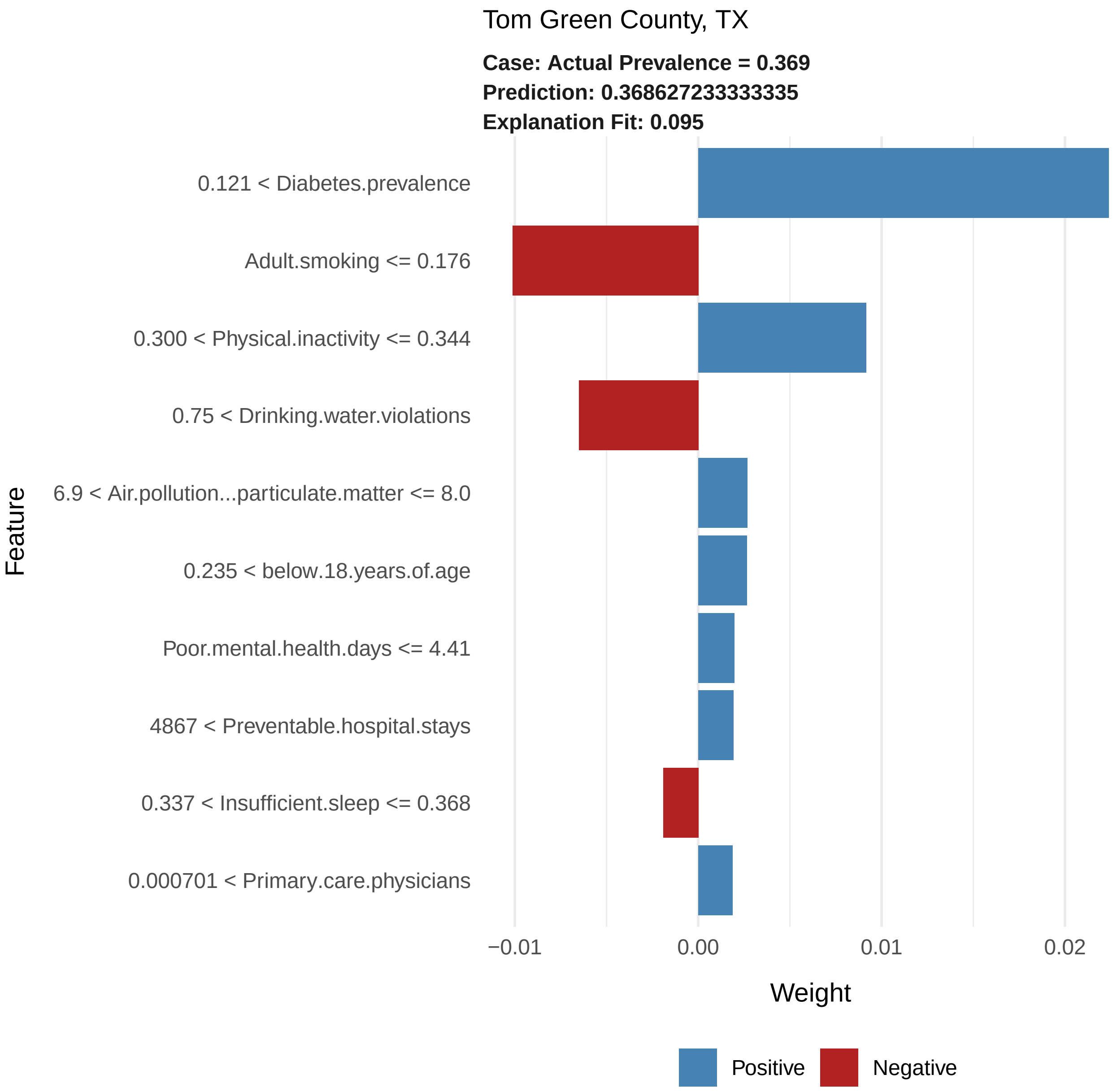

Tom Green County, TX
Case: Actual Prevalence = 0.369
Prediction: 0.368627233333335
Explanation Fit: 0.095
0.121 < Diabetes.prevalence
Adult.smoking <= 0.176
0.300 < Physical.inactivity <= 0.344
0.75 < Drinking.water.violations
6.9 < Air.pollution...particulate.matter <= 8.0
0.235 < below.18.years.of.age
Poor.mental.health.days <= 4.41
4867 < Preventable.hospital.stays
0.337 < Insufficient.sleep <= 0.368
0.000701 < Primary.care.physicians
Feature
−0.01
0.00
0.01
0.02
Weight
Positive
Negative

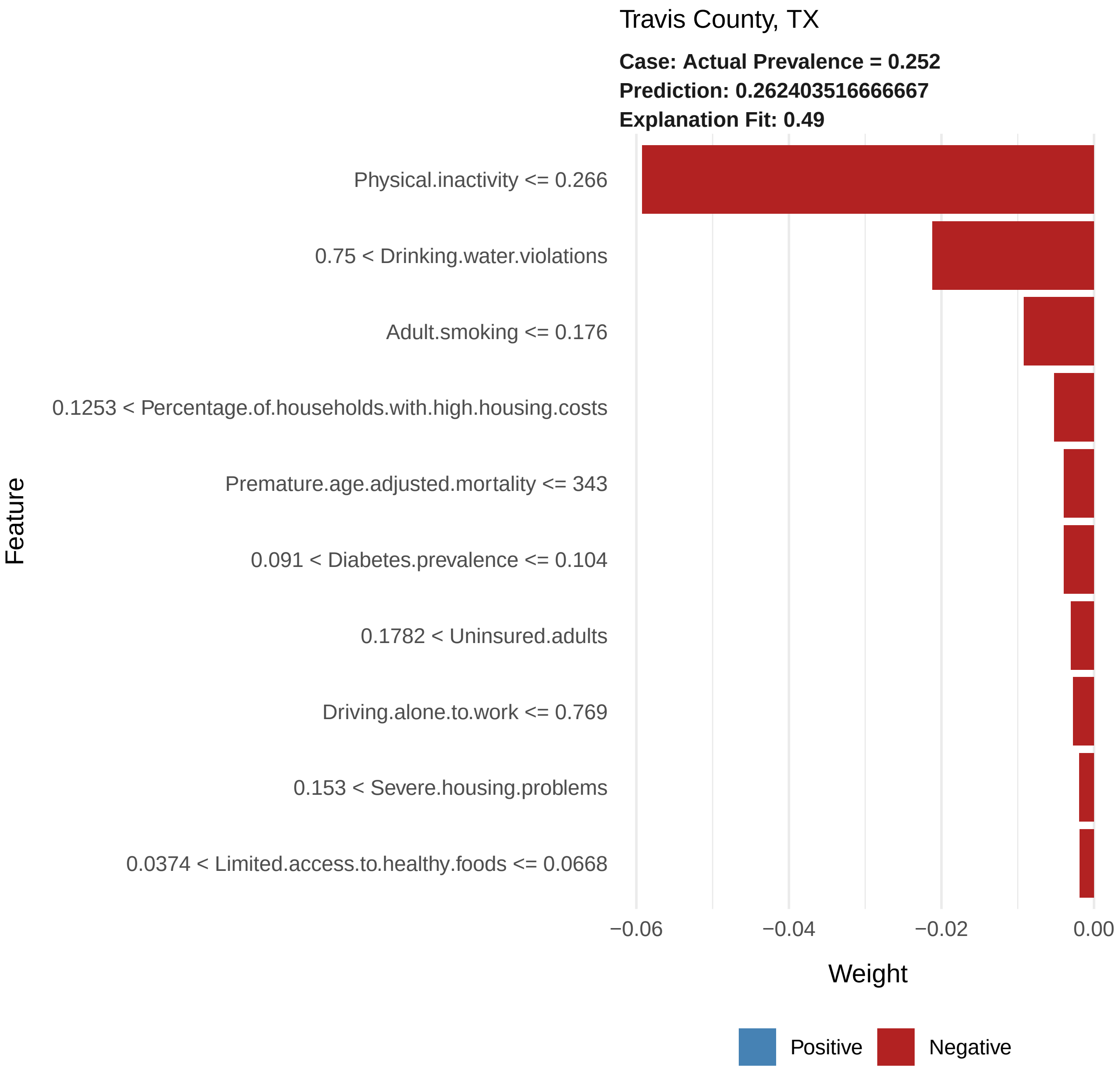
Travis County, TX
Case: Actual Prevalence = 0.252
Prediction: 0.262403516666667
Explanation Fit: 0.49
Physical.inactivity <= 0.266
0.75 < Drinking.water.violations
Adult.smoking <= 0.176
0.1253 < Percentage.of.households.with.high.housing.costs
Premature.age.adjusted.mortality <= 343
0.091 < Diabetes.prevalence <= 0.104
0.1782 < Uninsured.adults
Driving.alone.to.work <= 0.769
0.153 < Severe.housing.problems
0.0374 < Limited.access.to.healthy.foods <= 0.0668
Feature
−0.06
−0.04
−0.02
0.00
Weight
Positive
Negative

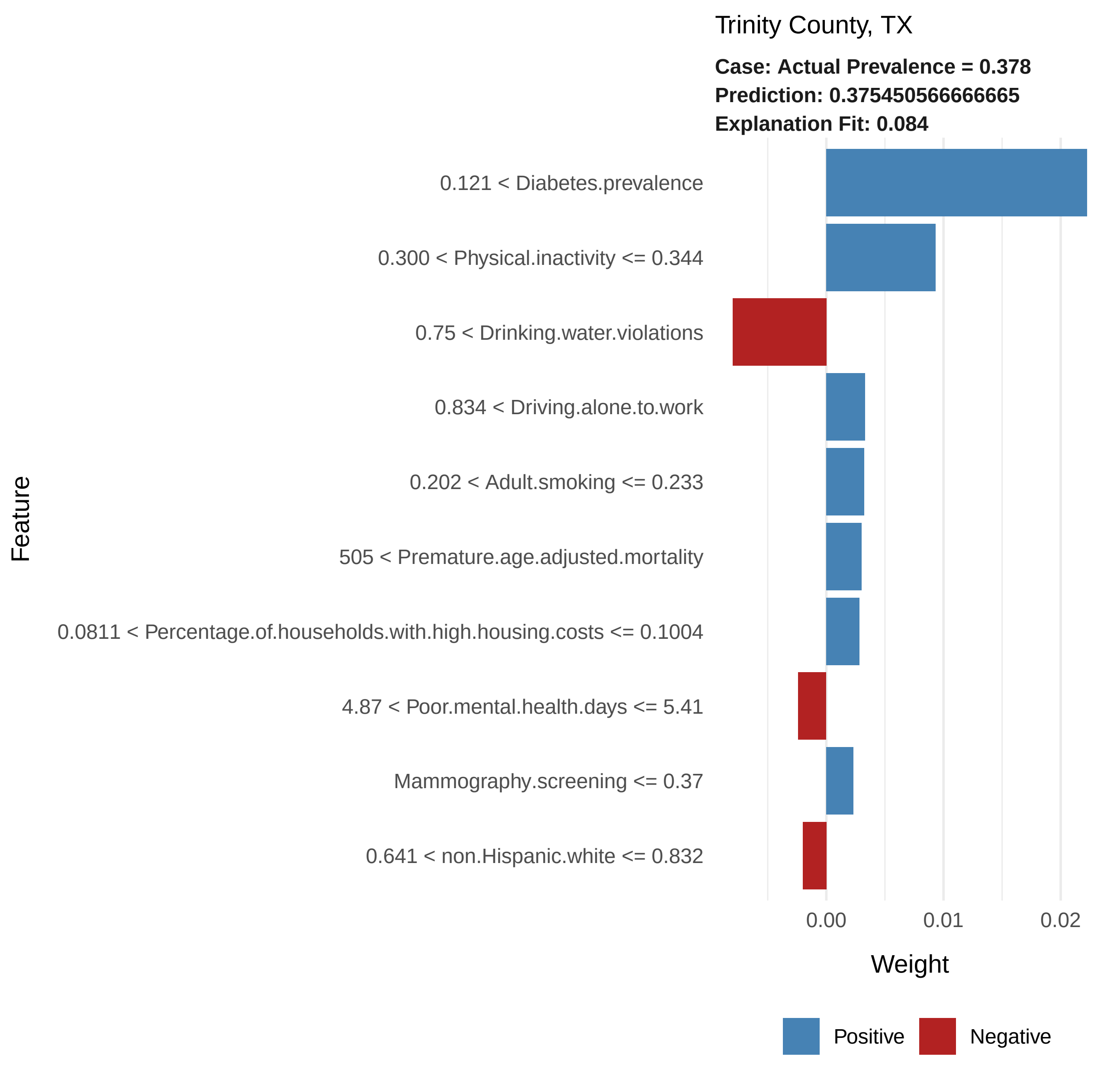
Trinity County, TX
Case: Actual Prevalence = 0.378
Prediction: 0.375450566666665
Explanation Fit: 0.084
0.121 < Diabetes.prevalence
0.300 < Physical.inactivity <= 0.344
0.75 < Drinking.water.violations
0.834 < Driving.alone.to.work
0.202 < Adult.smoking <= 0.233
505 < Premature.age.adjusted.mortality
0.0811 < Percentage.of.households.with.high.housing.costs <= 0.1004
4.87 < Poor.mental.health.days <= 5.41
Mammography.screening <= 0.37
0.641 < non.Hispanic.white <= 0.832
Feature
0.00
0.01
0.02
Weight
Positive
Negative

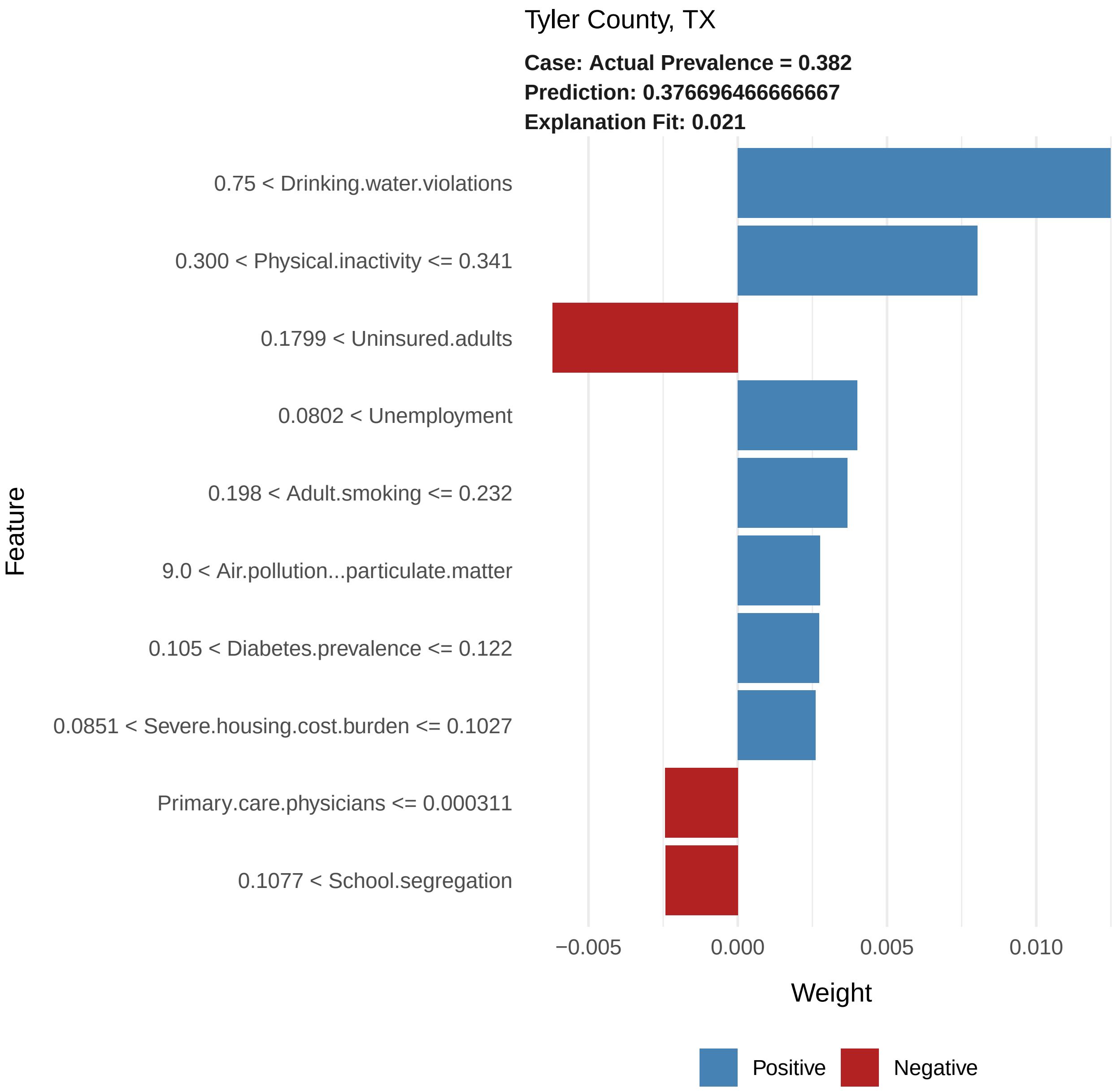

Tyler County, TX
Case: Actual Prevalence = 0.382
Prediction: 0.376696466666667
Explanation Fit: 0.021
0.75 < Drinking.water.violations
0.300 < Physical.inactivity <= 0.341
0.1799 < Uninsured.adults
0.0802 < Unemployment
0.198 < Adult.smoking <= 0.232
9.0 < Air.pollution...particulate.matter
0.105 < Diabetes.prevalence <= 0.122
0.0851 < Severe.housing.cost.burden <= 0.1027
Primary.care.physicians <= 0.000311
0.1077 < School.segregation
Feature
−0.005
0.000
0.005
0.010
Weight
Positive
Negative

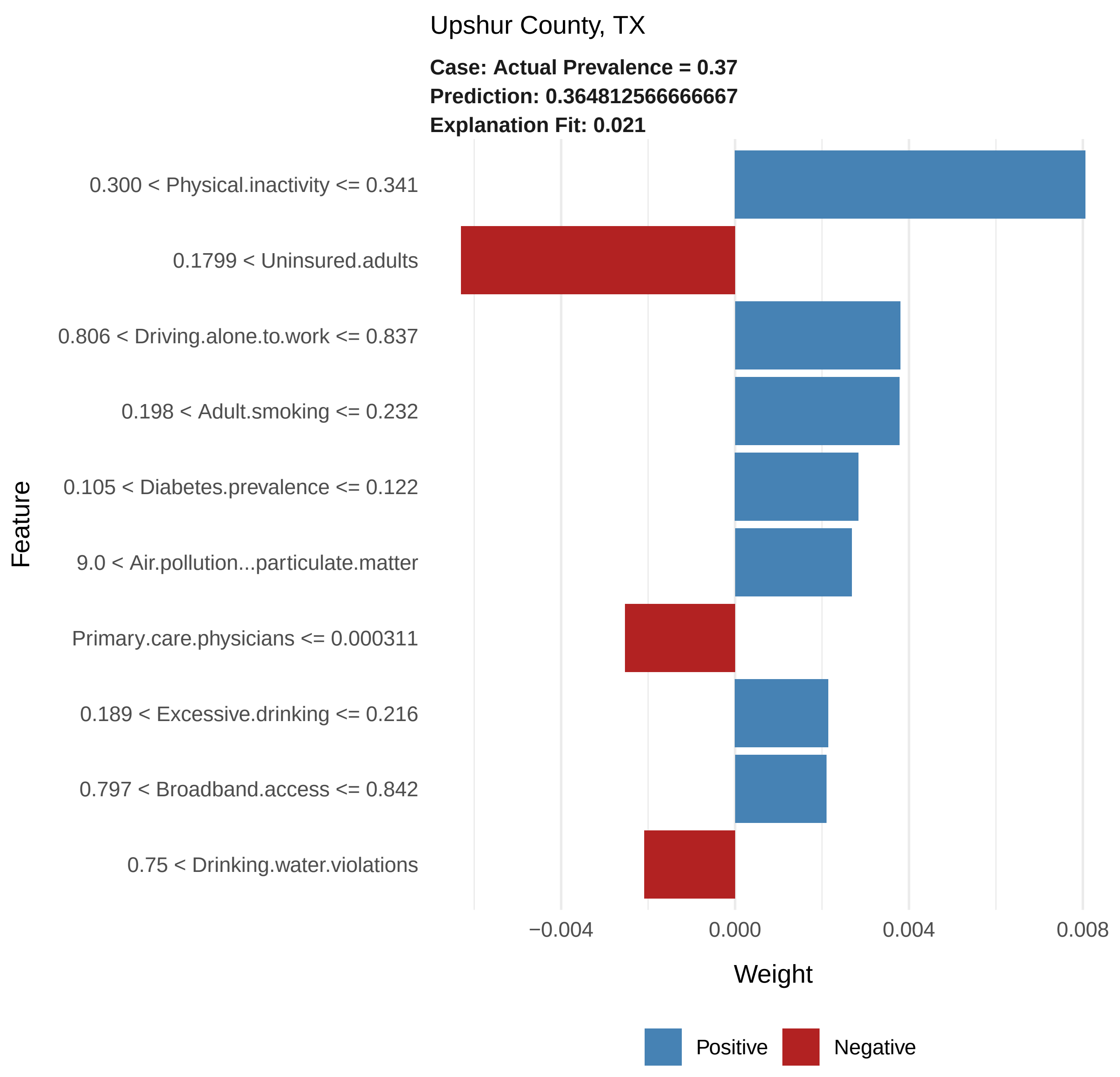
Upshur County, TX
Case: Actual Prevalence = 0.37
Prediction: 0.364812566666667
Explanation Fit: 0.021
0.300 < Physical.inactivity <= 0.341
0.1799 < Uninsured.adults
0.806 < Driving.alone.to.work <= 0.837
0.198 < Adult.smoking <= 0.232
0.105 < Diabetes.prevalence <= 0.122
9.0 < Air.pollution...particulate.matter
Primary.care.physicians <= 0.000311
0.189 < Excessive.drinking <= 0.216
0.797 < Broadband.access <= 0.842
0.75 < Drinking.water.violations
Feature
−0.004
0.000
0.004
0.008
Weight
Positive
Negative

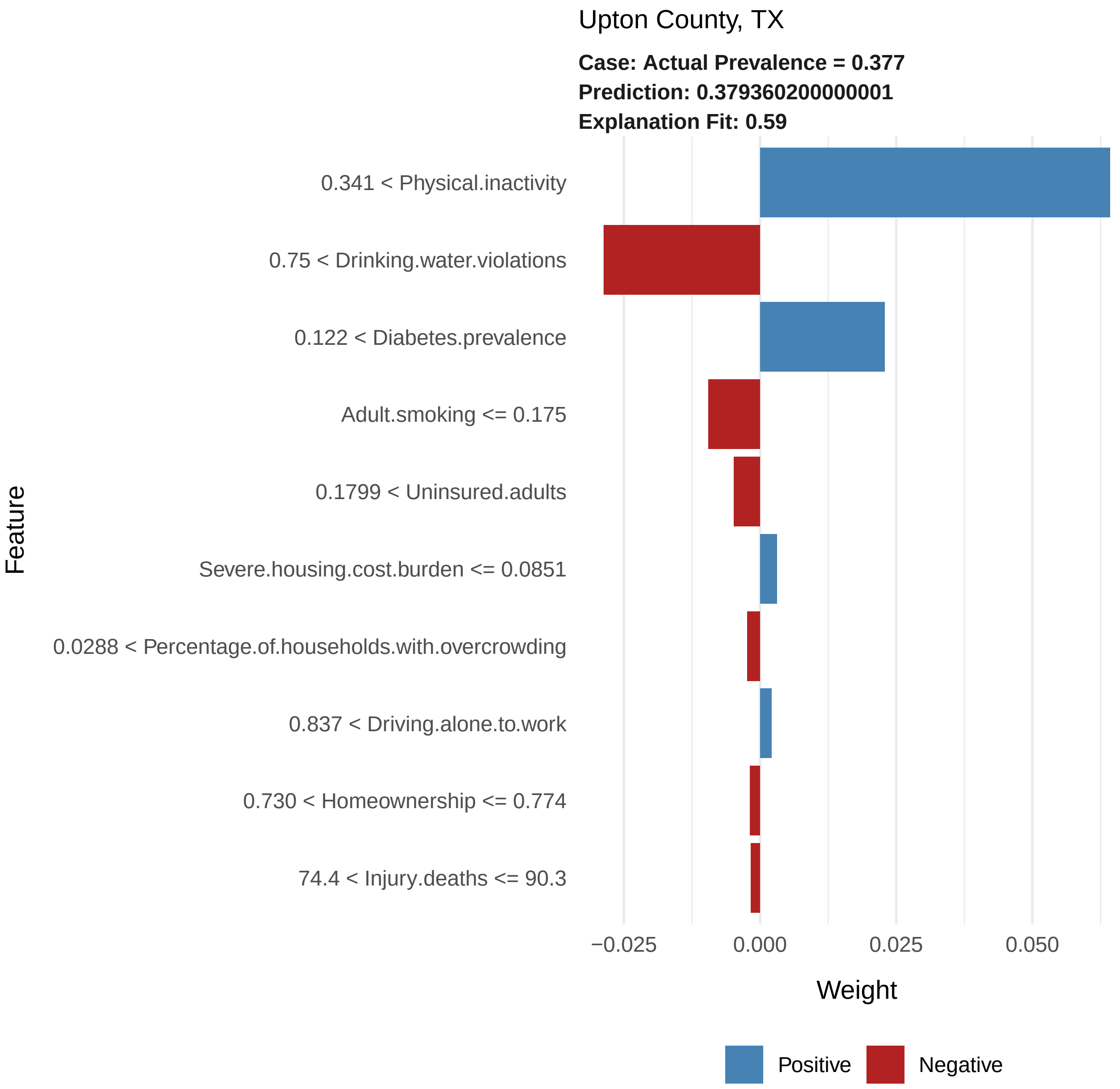

Upton County, TX
Case: Actual Prevalence = 0.377
Prediction: 0.379360200000001
Explanation Fit: 0.59
0.341 < Physical.inactivity
0.75 < Drinking.water.violations
0.122 < Diabetes.prevalence
Adult.smoking <= 0.175
0.1799 < Uninsured.adults
Severe.housing.cost.burden <= 0.0851
0.0288 < Percentage.of.households.with.overcrowding
0.837 < Driving.alone.to.work
0.730 < Homeownership <= 0.774
74.4 < Injury.deaths <= 90.3
Feature
−0.025
0.000
0.025
0.050
Weight
Positive
Negative

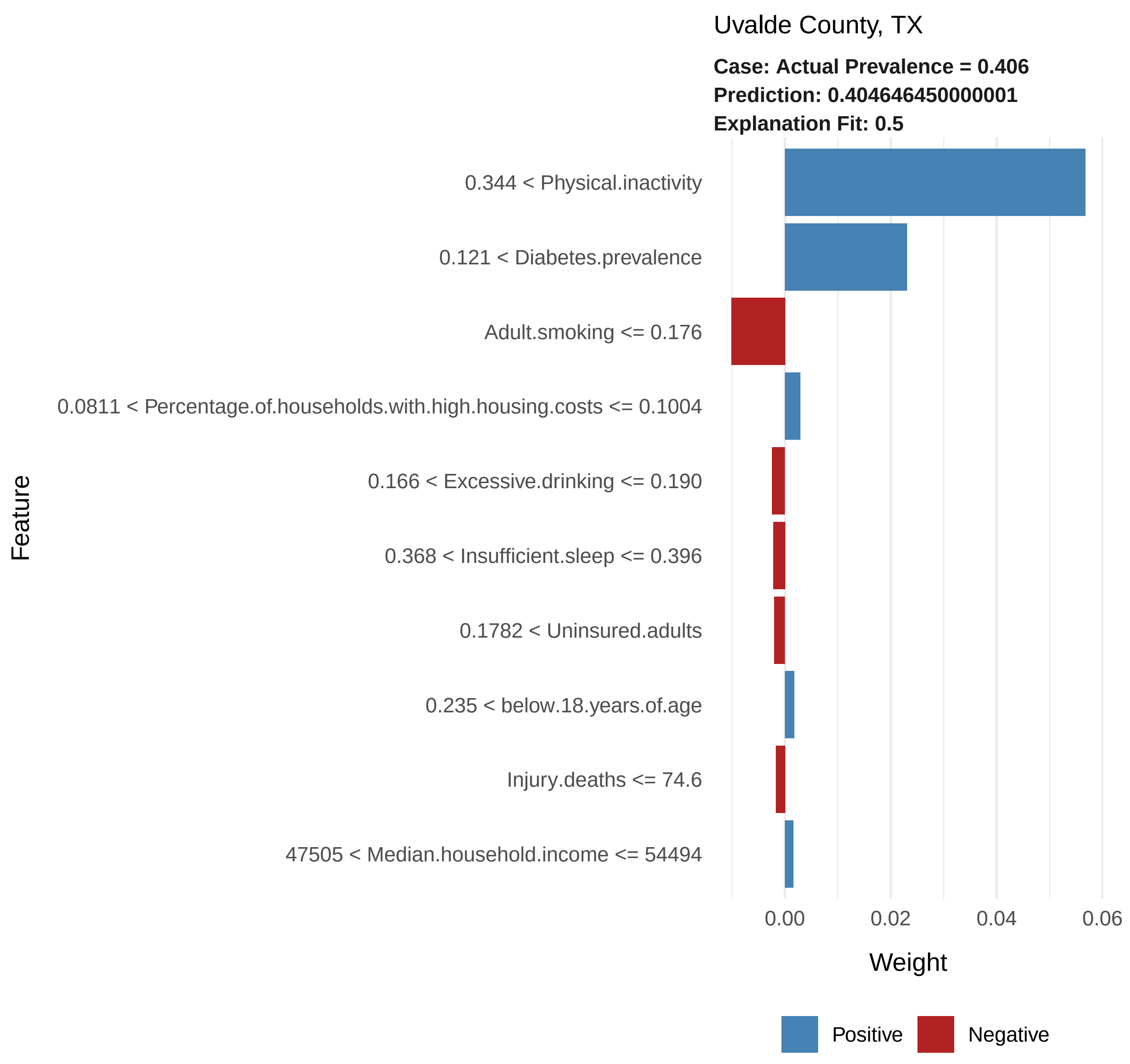
Uvalde County, TX
Case: Actual Prevalence = 0.406
Prediction: 0.404646450000001
Explanation Fit: 0.5
0.344 < Physical.inactivity
0.121 < Diabetes.prevalence
Adult.smoking <= 0.176
0.0811 < Percentage.of.households.with.high.housing.costs <= 0.1004
0.166 < Excessive.drinking <= 0.190
0.368 < Insufficient.sleep <= 0.396
0.1782 < Uninsured.adults
0.235 < below.18.years.of.age
Injury.deaths <= 74.6
47505 < Median.household.income <= 54494
Feature
0.00
0.02
0.04
0.06
Weight
Positive
Negative

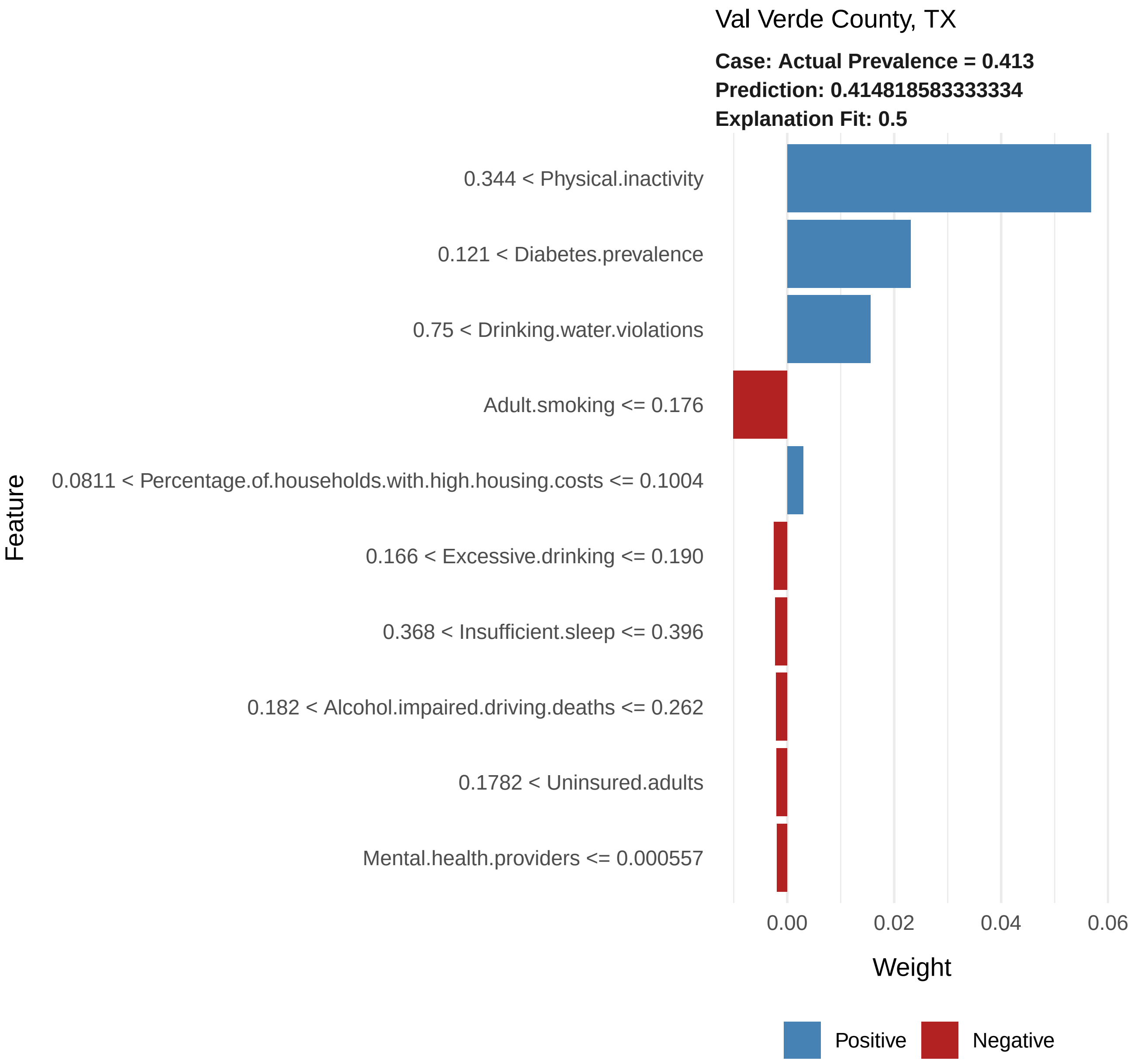

Val Verde County, TX
Case: Actual Prevalence = 0.413
Prediction: 0.414818583333334
Explanation Fit: 0.5
0.344 < Physical.inactivity
0.121 < Diabetes.prevalence
0.75 < Drinking.water.violations
Adult.smoking <= 0.176
0.0811 < Percentage.of.households.with.high.housing.costs <= 0.1004
0.166 < Excessive.drinking <= 0.190
0.368 < Insufficient.sleep <= 0.396
0.182 < Alcohol.impaired.driving.deaths <= 0.262
0.1782 < Uninsured.adults
Mental.health.providers <= 0.000557
Feature
0.00
0.02
0.04
0.06
Weight
Positive
Negative

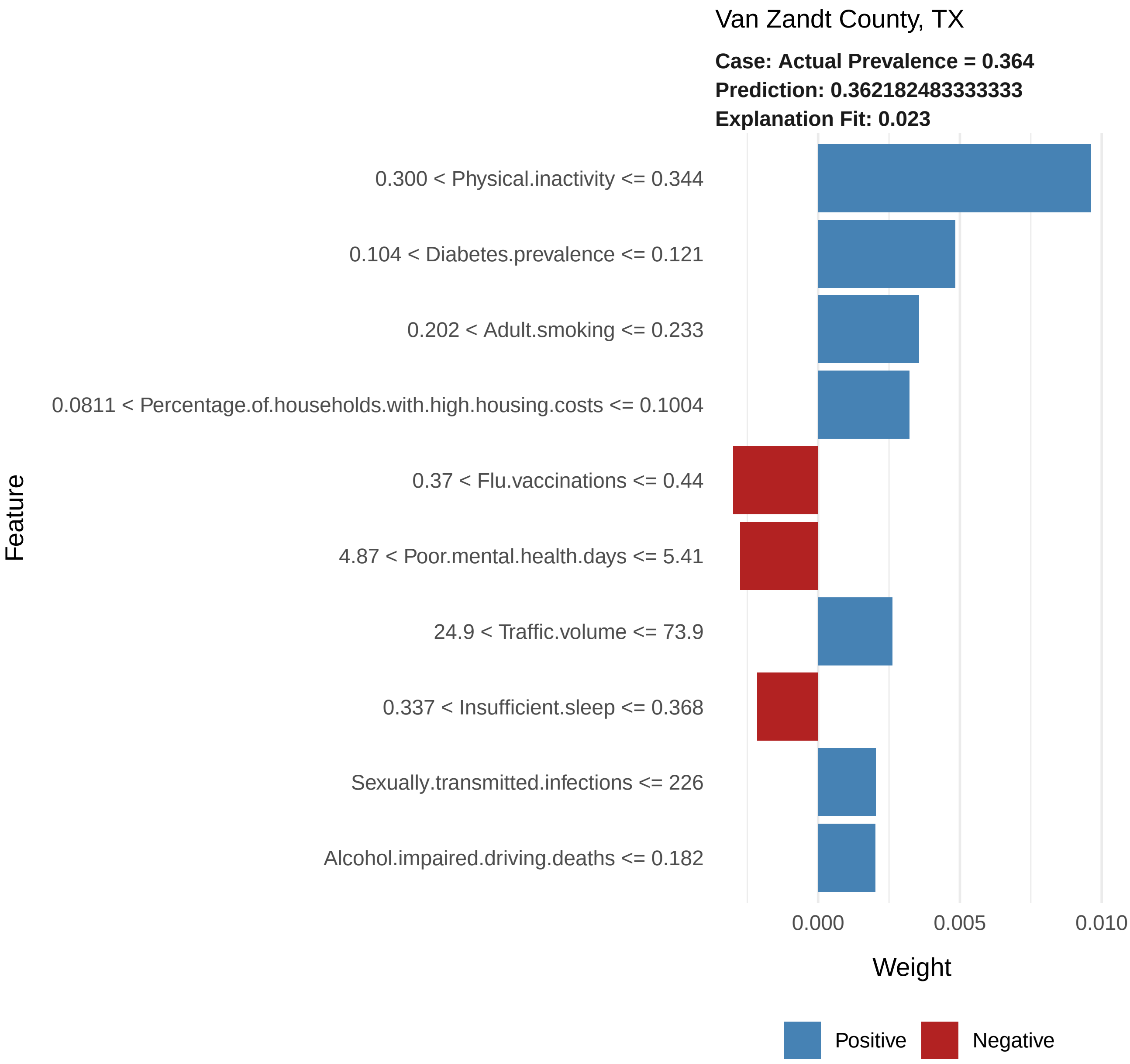

Van Zandt County, TX
Case: Actual Prevalence = 0.364
Prediction: 0.362182483333333
Explanation Fit: 0.023
0.300 < Physical.inactivity <= 0.344
0.104 < Diabetes.prevalence <= 0.121
0.202 < Adult.smoking <= 0.233
0.0811 < Percentage.of.households.with.high.housing.costs <= 0.1004
0.37 < Flu.vaccinations <= 0.44
4.87 < Poor.mental.health.days <= 5.41
24.9 < Traffic.volume <= 73.9
0.337 < Insufficient.sleep <= 0.368
Sexually.transmitted.infections <= 226
Alcohol.impaired.driving.deaths <= 0.182
Feature
0.000
0.005
0.010
Weight
Positive
Negative

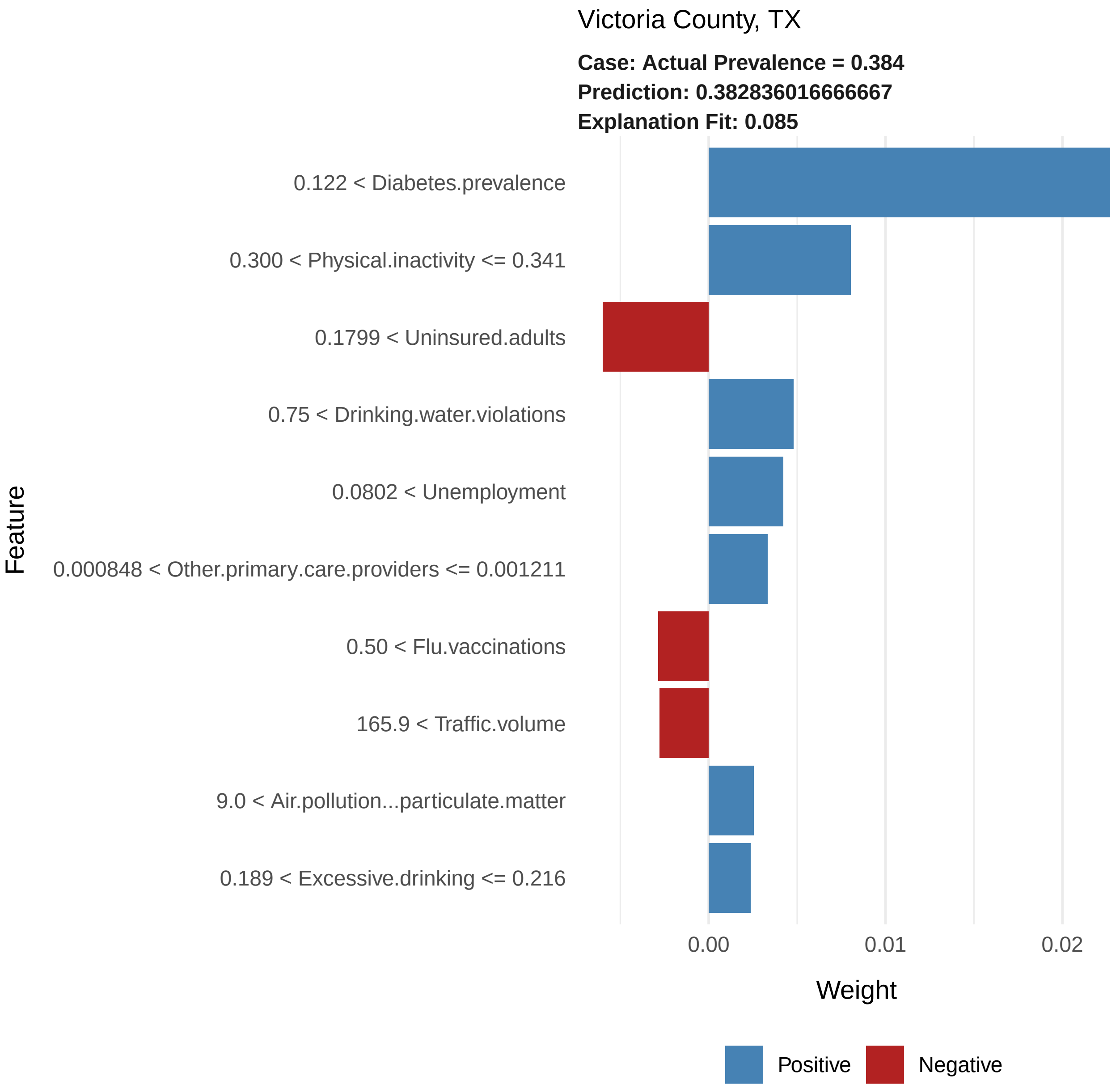

Victoria County, TX
Case: Actual Prevalence = 0.384
Prediction: 0.382836016666667
Explanation Fit: 0.085
0.122 < Diabetes.prevalence
0.300 < Physical.inactivity <= 0.341
0.1799 < Uninsured.adults
0.75 < Drinking.water.violations
0.0802 < Unemployment
0.000848 < Other.primary.care.providers <= 0.001211
0.50 < Flu.vaccinations
165.9 < Traffic.volume
9.0 < Air.pollution...particulate.matter
0.189 < Excessive.drinking <= 0.216
Feature
0.00
0.01
0.02
Weight
Positive
Negative

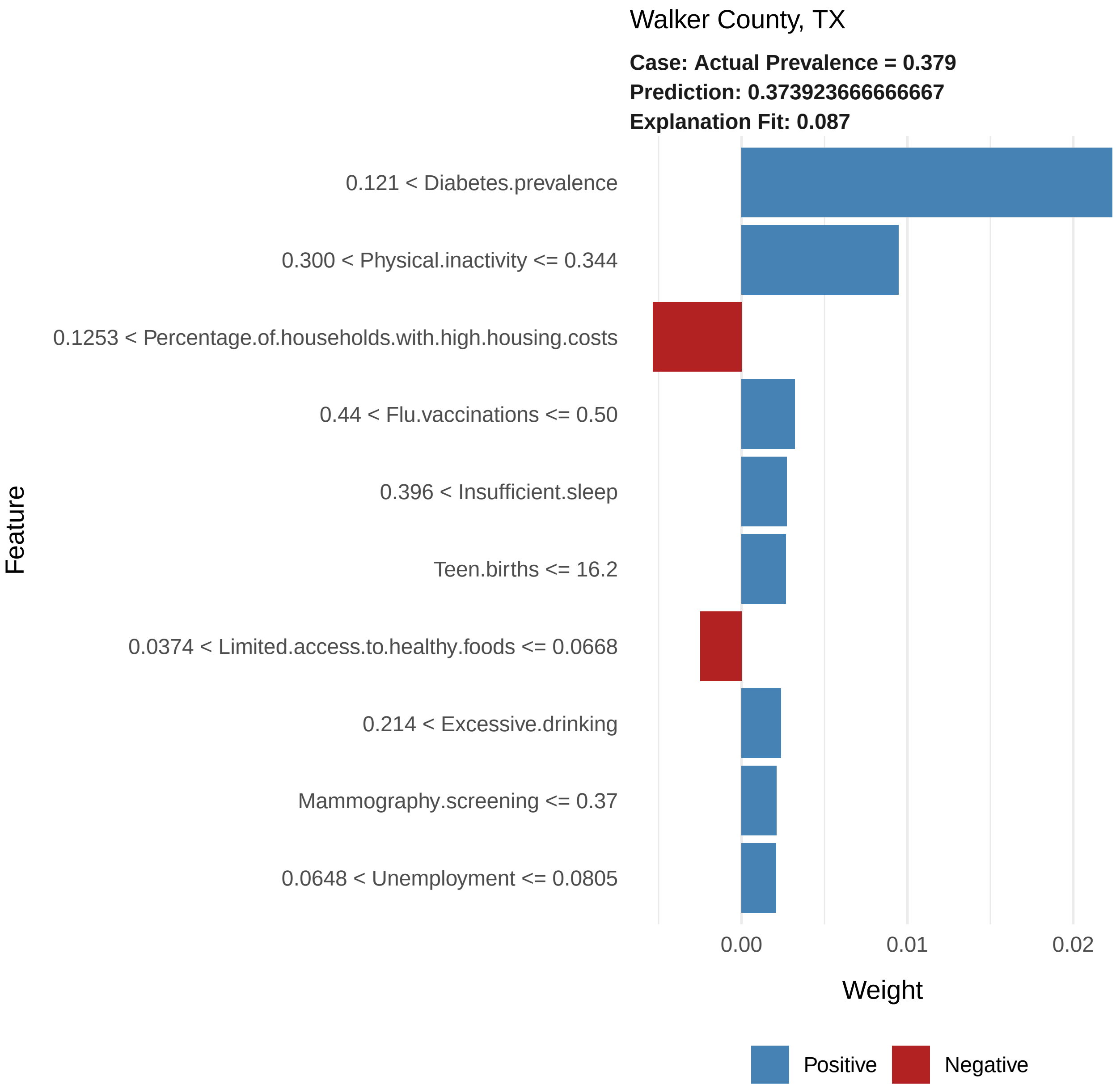

Walker County, TX
Case: Actual Prevalence = 0.379
Prediction: 0.373923666666667
Explanation Fit: 0.087
0.121 < Diabetes.prevalence
0.300 < Physical.inactivity <= 0.344
0.1253 < Percentage.of.households.with.high.housing.costs
0.44 < Flu.vaccinations <= 0.50
0.396 < Insufficient.sleep
Teen.births <= 16.2
0.0374 < Limited.access.to.healthy.foods <= 0.0668
0.214 < Excessive.drinking
Mammography.screening <= 0.37
0.0648 < Unemployment <= 0.0805
Feature
0.00
0.01
0.02
Weight
Positive
Negative

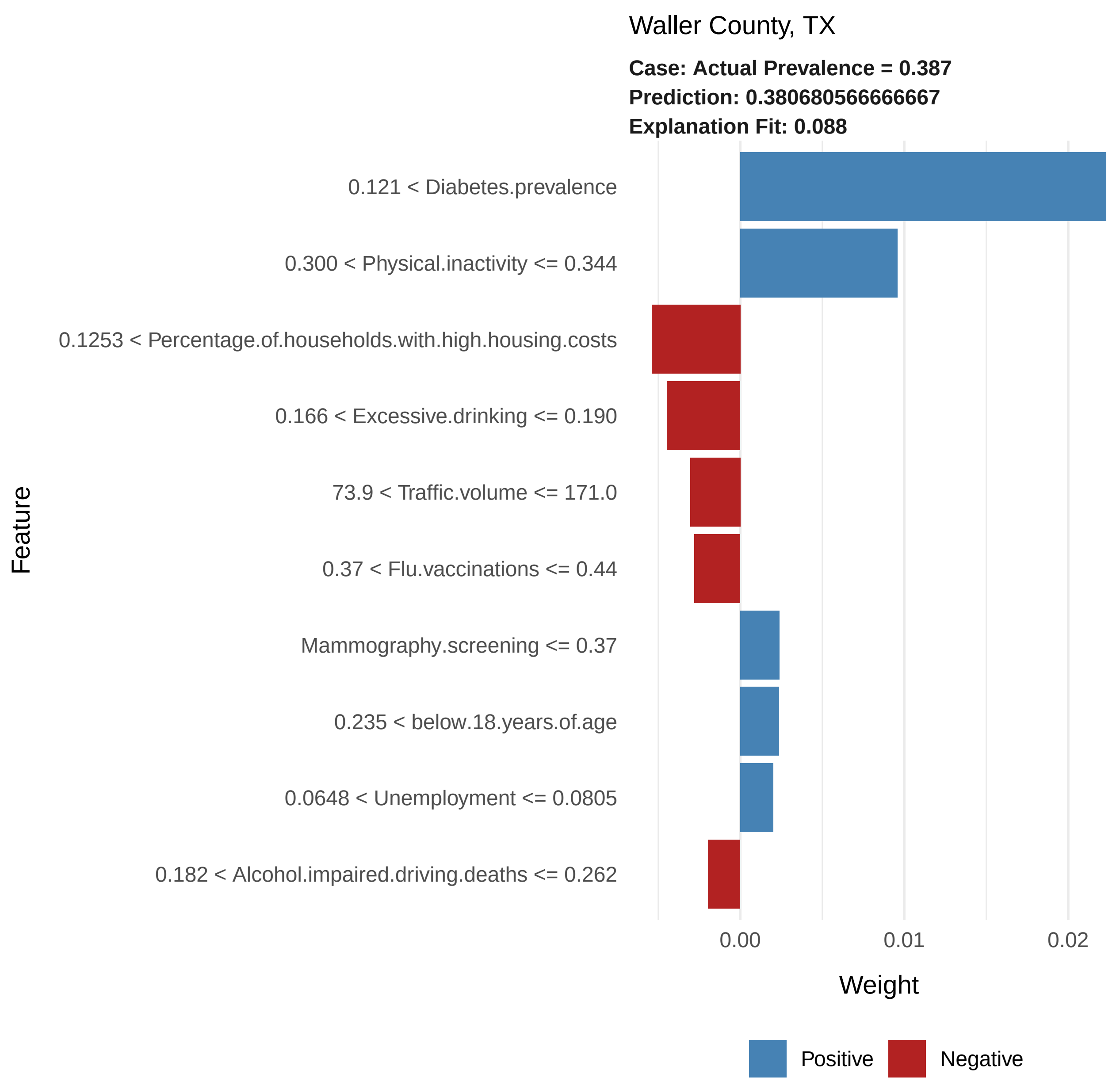
Waller County, TX
Case: Actual Prevalence = 0.387
Prediction: 0.380680566666667
Explanation Fit: 0.088
0.121 < Diabetes.prevalence
0.300 < Physical.inactivity <= 0.344
0.1253 < Percentage.of.households.with.high.housing.costs
0.166 < Excessive.drinking <= 0.190
73.9 < Traffic.volume <= 171.0
0.37 < Flu.vaccinations <= 0.44
Mammography.screening <= 0.37
0.235 < below.18.years.of.age
0.0648 < Unemployment <= 0.0805
0.182 < Alcohol.impaired.driving.deaths <= 0.262
Feature
0.00
0.01
0.02
Weight
Positive
Negative

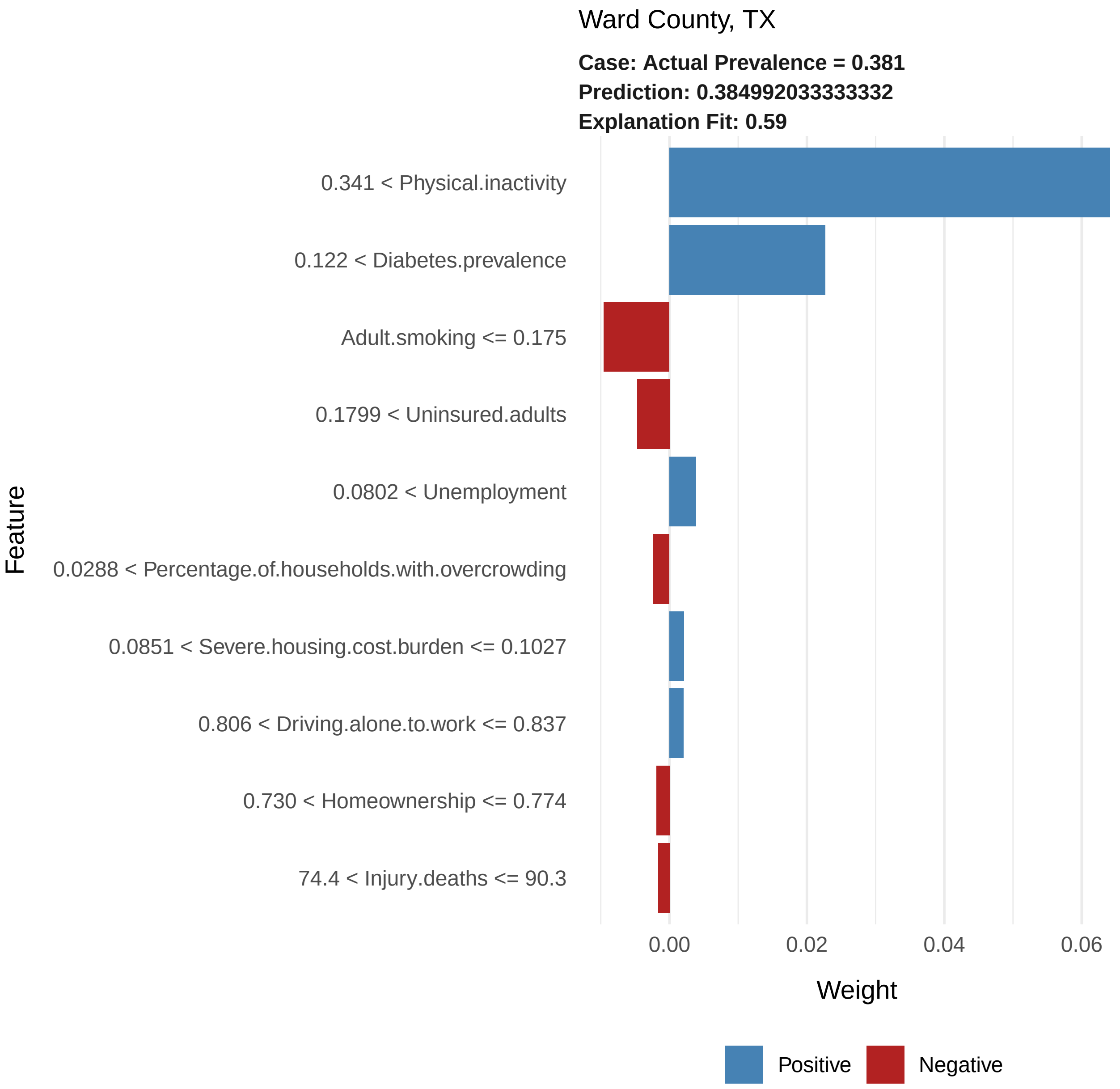

Ward County, TX
Case: Actual Prevalence = 0.381
Prediction: 0.384992033333332
Explanation Fit: 0.59
0.341 < Physical.inactivity
0.122 < Diabetes.prevalence
Adult.smoking <= 0.175
0.1799 < Uninsured.adults
0.0802 < Unemployment
0.0288 < Percentage.of.households.with.overcrowding
0.0851 < Severe.housing.cost.burden <= 0.1027
0.806 < Driving.alone.to.work <= 0.837
0.730 < Homeownership <= 0.774
74.4 < Injury.deaths <= 90.3
Feature
0.00
0.02
0.04
0.06
Weight
Positive
Negative

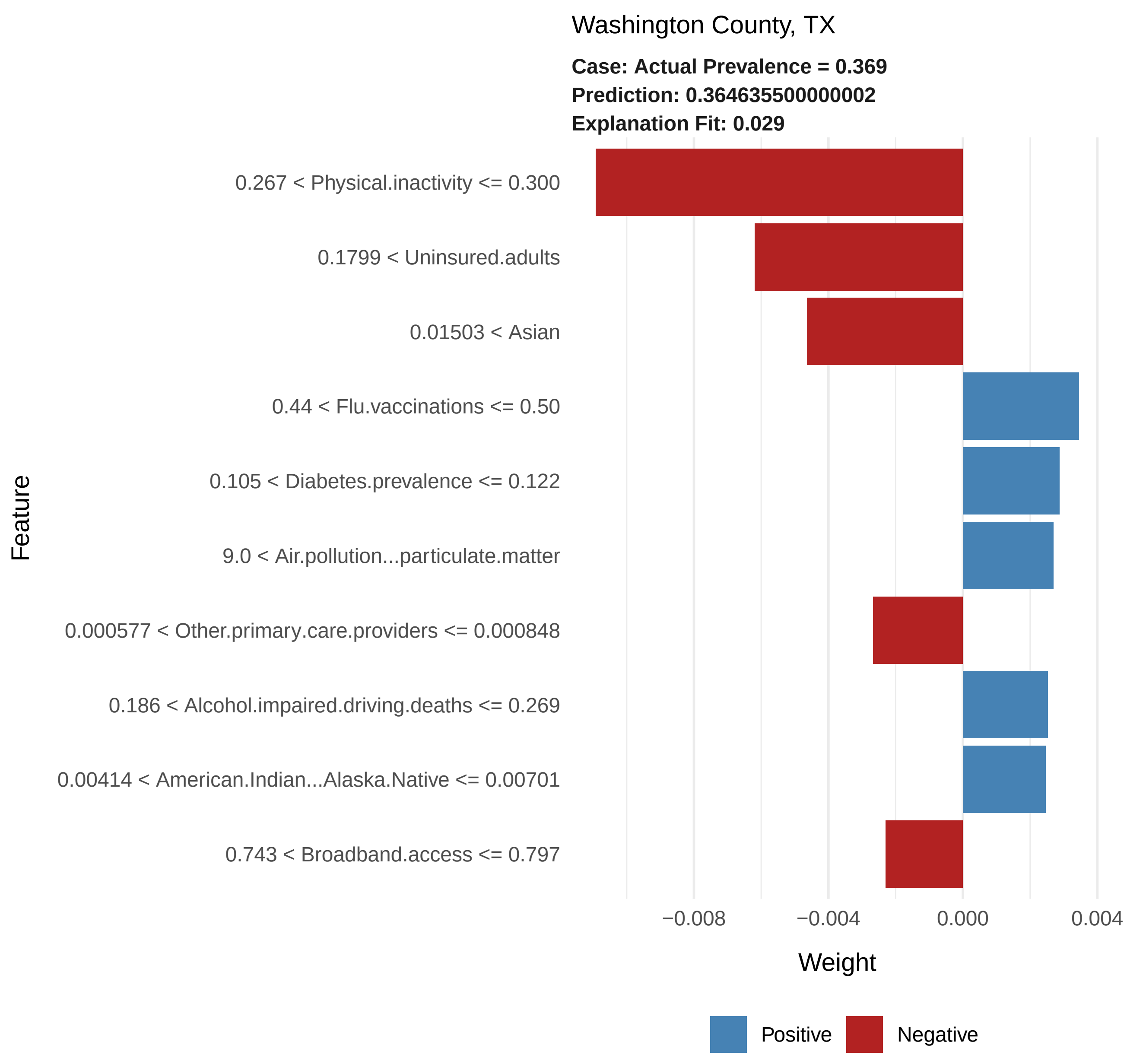
Washington County, TX
Case: Actual Prevalence = 0.369
Prediction: 0.364635500000002
Explanation Fit: 0.029
0.267 < Physical.inactivity <= 0.300
0.1799 < Uninsured.adults
0.01503 < Asian
0.44 < Flu.vaccinations <= 0.50
0.105 < Diabetes.prevalence <= 0.122
9.0 < Air.pollution...particulate.matter
0.000577 < Other.primary.care.providers <= 0.000848
0.186 < Alcohol.impaired.driving.deaths <= 0.269
0.00414 < American.Indian...Alaska.Native <= 0.00701
0.743 < Broadband.access <= 0.797
Feature
−0.008
−0.004
0.000
0.004
Weight
Positive
Negative

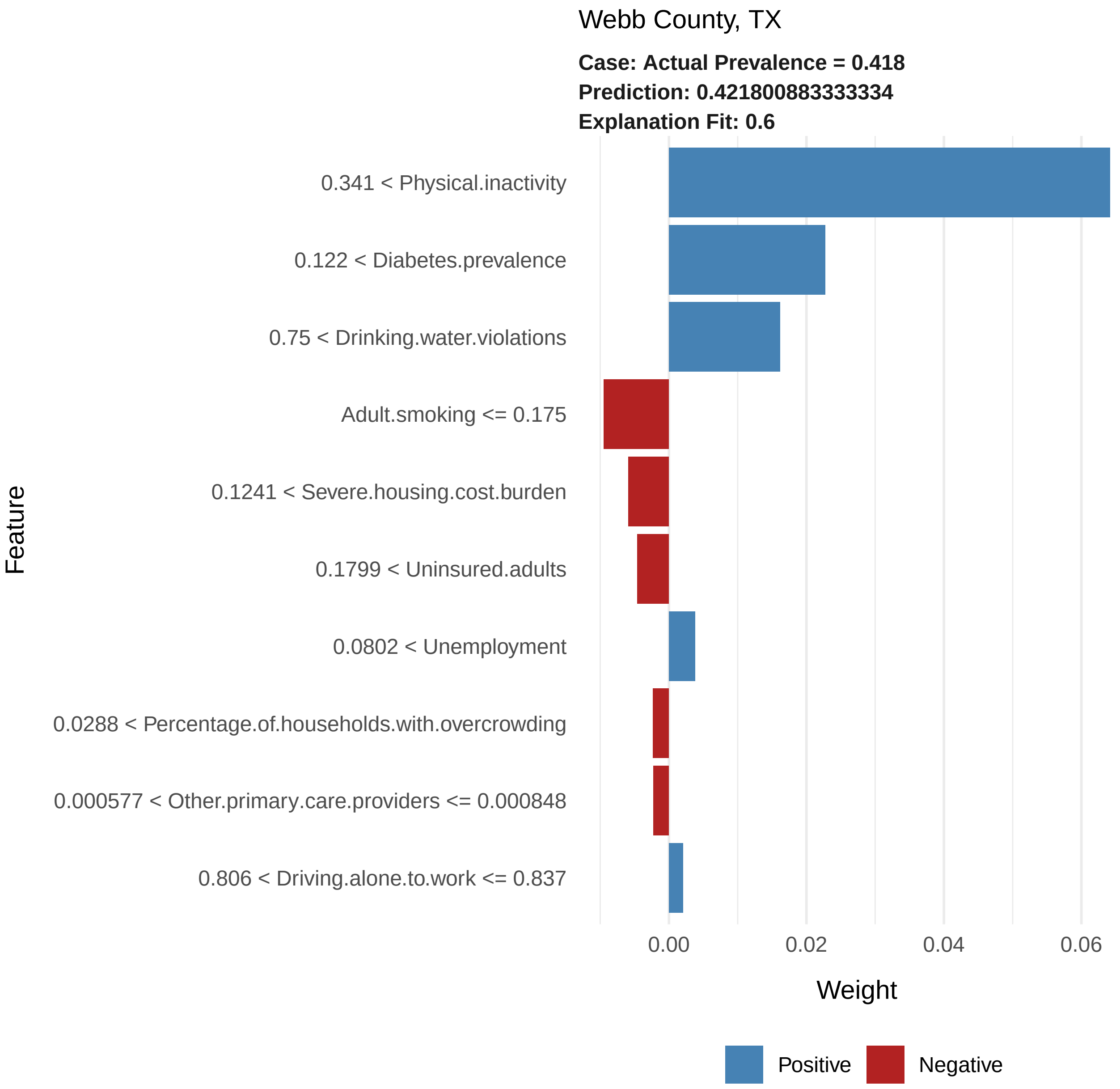
Webb County, TX
Case: Actual Prevalence = 0.418
Prediction: 0.421800883333334
Explanation Fit: 0.6
0.341 < Physical.inactivity
0.122 < Diabetes.prevalence
0.75 < Drinking.water.violations
Adult.smoking <= 0.175
0.1241 < Severe.housing.cost.burden
0.1799 < Uninsured.adults
0.0802 < Unemployment
0.0288 < Percentage.of.households.with.overcrowding
0.000577 < Other.primary.care.providers <= 0.000848
0.806 < Driving.alone.to.work <= 0.837
Feature
0.00
0.02
0.04
0.06
Weight
Positive
Negative

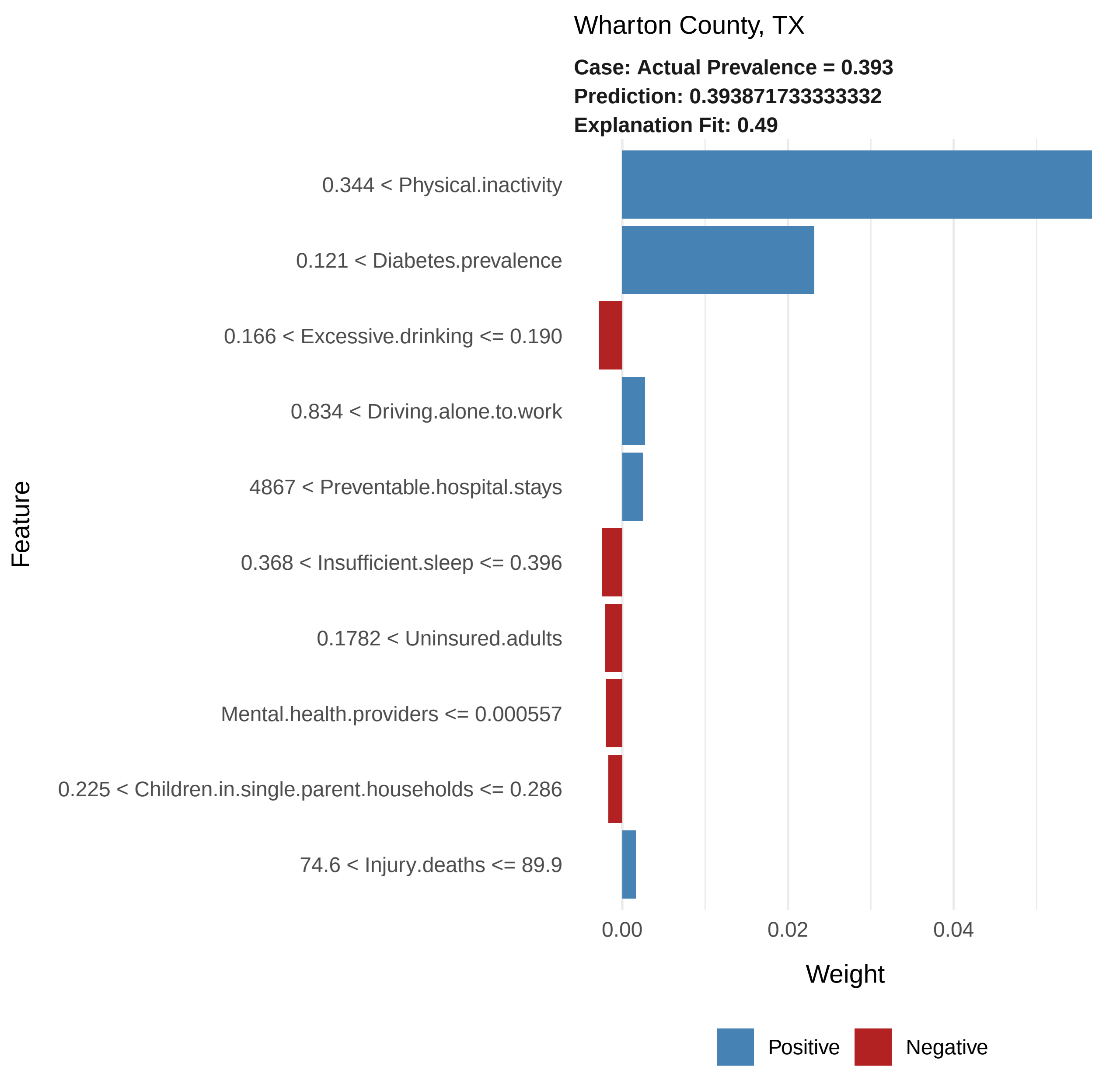

Wharton County, TX
Case: Actual Prevalence = 0.393
Prediction: 0.393871733333332
Explanation Fit: 0.49
0.344 < Physical.inactivity
0.121 < Diabetes.prevalence
0.166 < Excessive.drinking <= 0.190
0.834 < Driving.alone.to.work
4867 < Preventable.hospital.stays
0.368 < Insufficient.sleep <= 0.396
0.1782 < Uninsured.adults
Mental.health.providers <= 0.000557
0.225 < Children.in.single.parent.households <= 0.286
74.6 < Injury.deaths <= 89.9
Feature
0.00
0.02
0.04
Weight
Positive
Negative

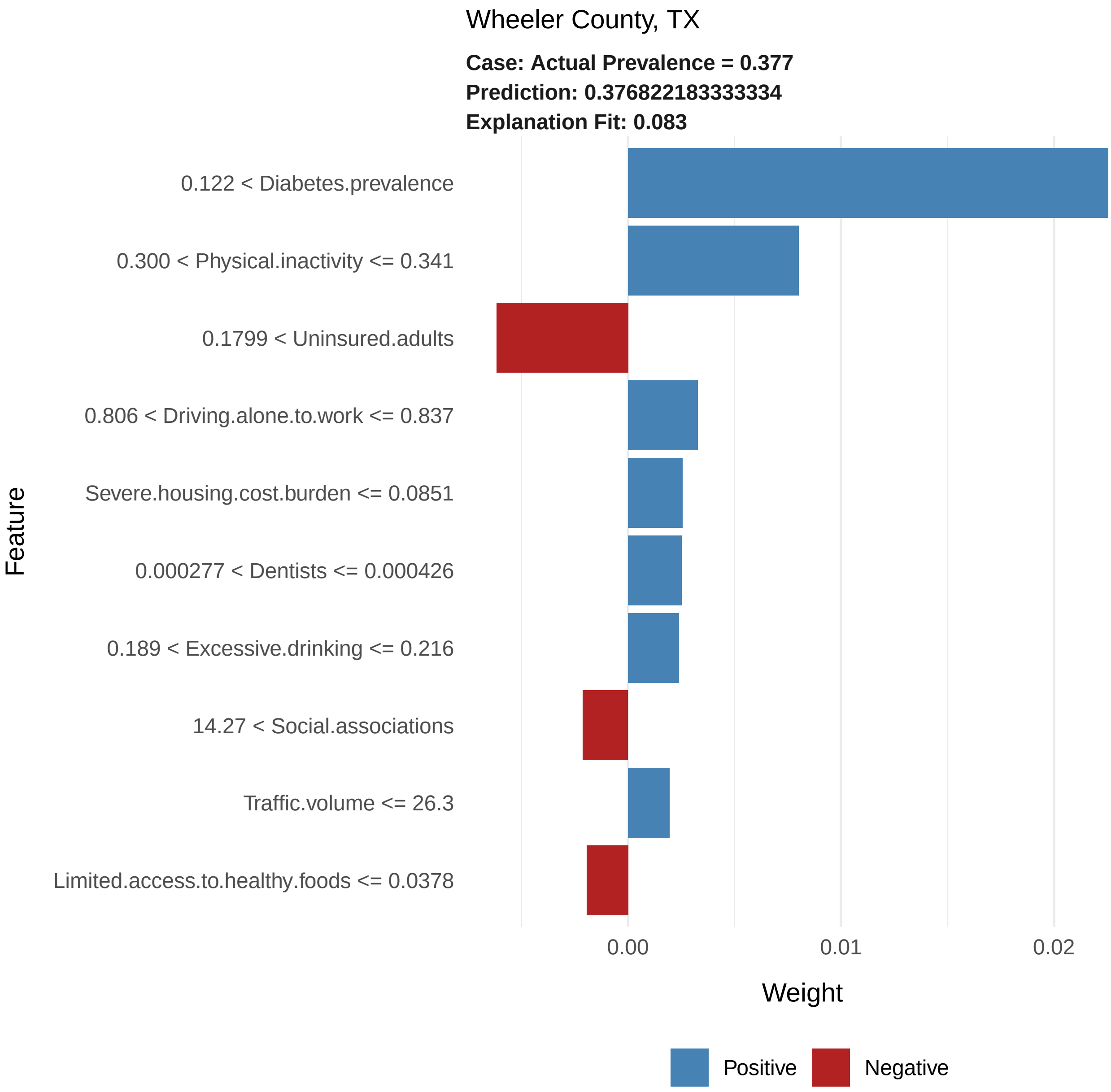

Wheeler County, TX
Case: Actual Prevalence = 0.377
Prediction: 0.376822183333334
Explanation Fit: 0.083
0.122 < Diabetes.prevalence
0.300 < Physical.inactivity <= 0.341
0.1799 < Uninsured.adults
0.806 < Driving.alone.to.work <= 0.837
Severe.housing.cost.burden <= 0.0851
0.000277 < Dentists <= 0.000426
0.189 < Excessive.drinking <= 0.216
14.27 < Social.associations
Traffic.volume <= 26.3
Limited.access.to.healthy.foods <= 0.0378
Feature
0.00
0.01
0.02
Weight
Positive
Negative

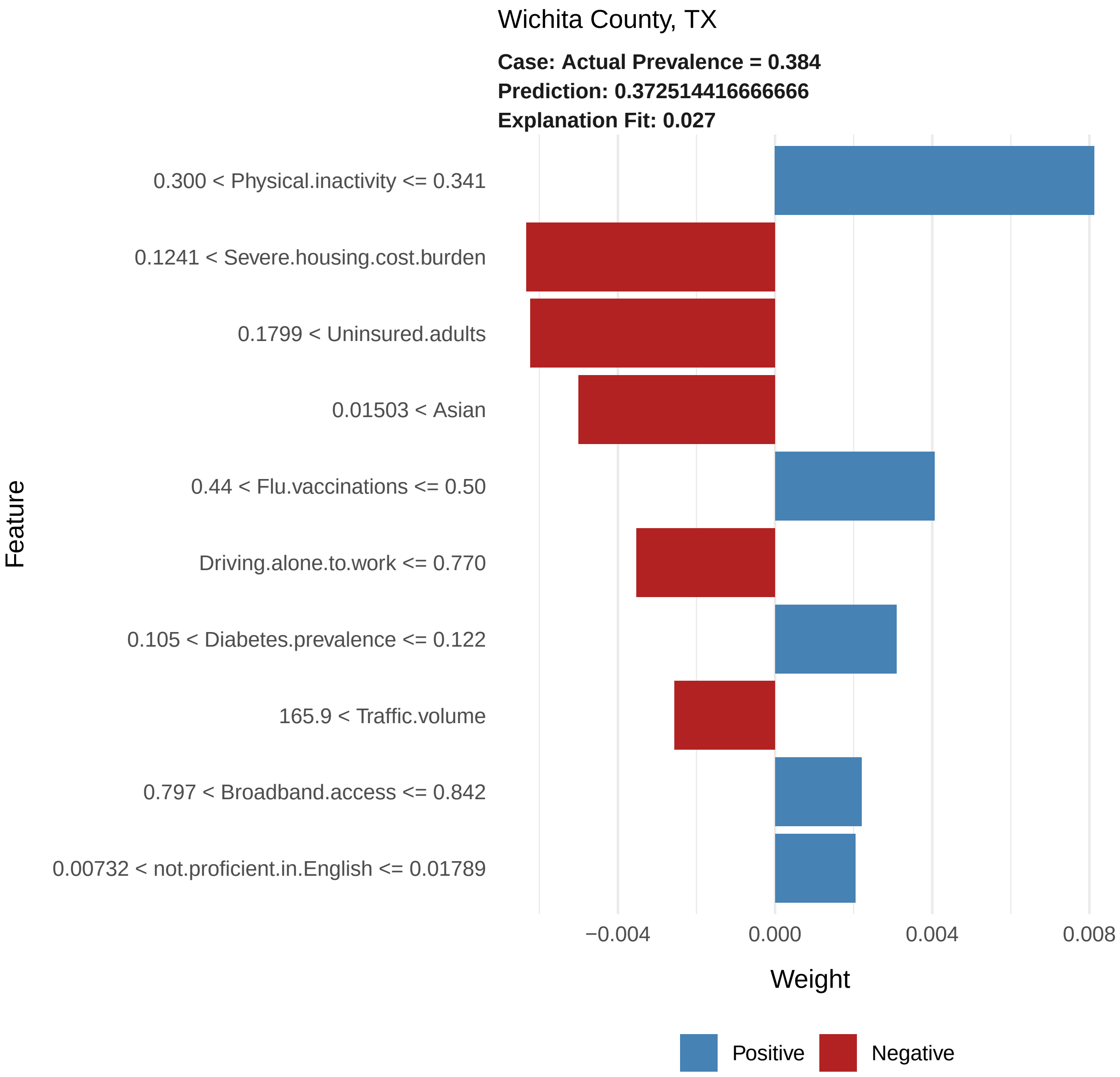

Wichita County, TX
Case: Actual Prevalence = 0.384
Prediction: 0.372514416666666
Explanation Fit: 0.027
0.300 < Physical.inactivity <= 0.341
0.1241 < Severe.housing.cost.burden
0.1799 < Uninsured.adults
0.01503 < Asian
0.44 < Flu.vaccinations <= 0.50
Driving.alone.to.work <= 0.770
0.105 < Diabetes.prevalence <= 0.122
165.9 < Traffic.volume
0.797 < Broadband.access <= 0.842
0.00732 < not.proficient.in.English <= 0.01789
Feature
−0.004
0.000
0.004
0.008
Weight
Positive
Negative

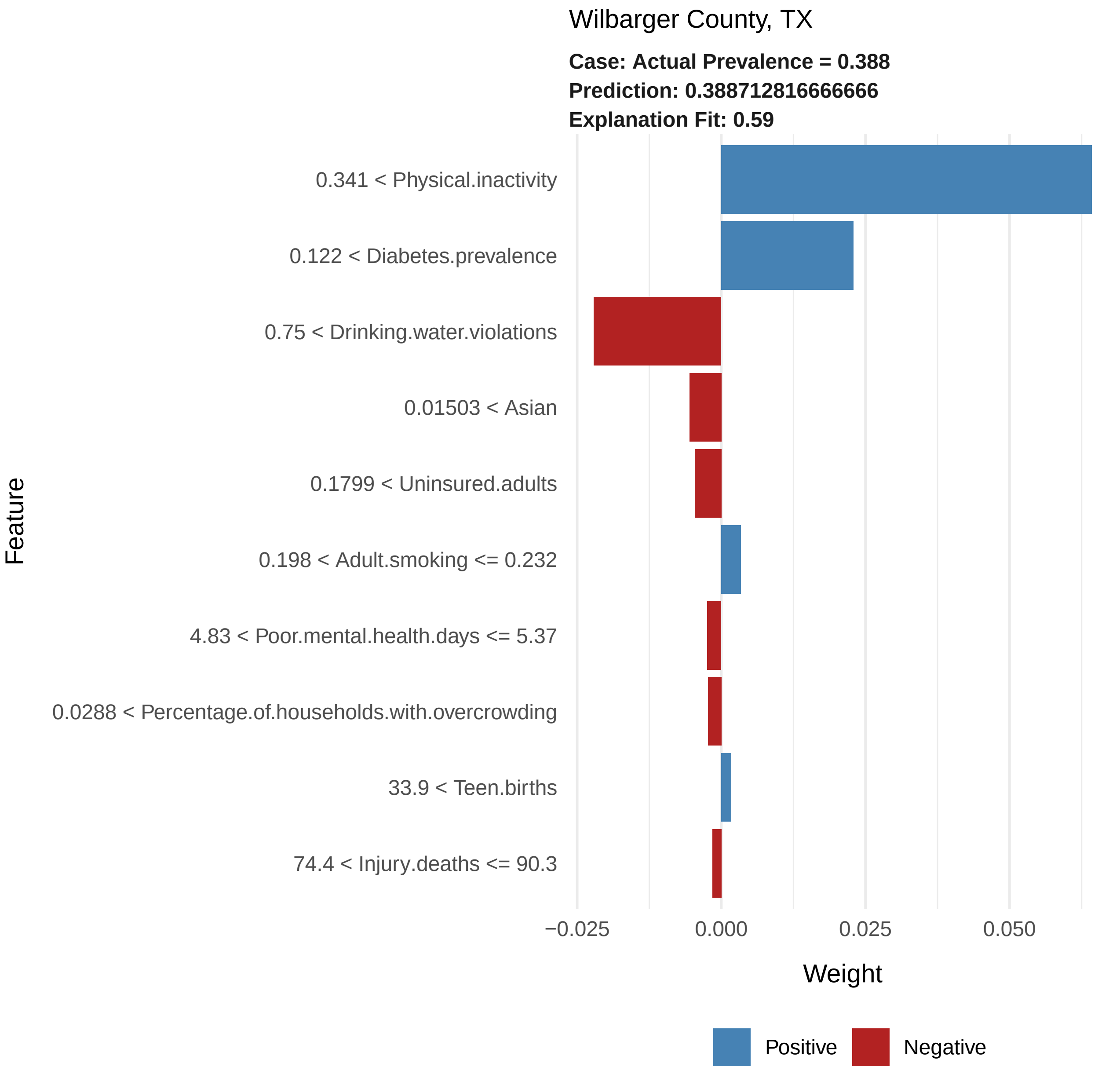

Wilbarger County, TX
Case: Actual Prevalence = 0.388
Prediction: 0.388712816666666
Explanation Fit: 0.59
0.341 < Physical.inactivity
0.122 < Diabetes.prevalence
0.75 < Drinking.water.violations
0.01503 < Asian
0.1799 < Uninsured.adults
0.198 < Adult.smoking <= 0.232
4.83 < Poor.mental.health.days <= 5.37
0.0288 < Percentage.of.households.with.overcrowding
33.9 < Teen.births
74.4 < Injury.deaths <= 90.3
Feature
−0.025
0.000
0.025
0.050
Weight
Positive
Negative

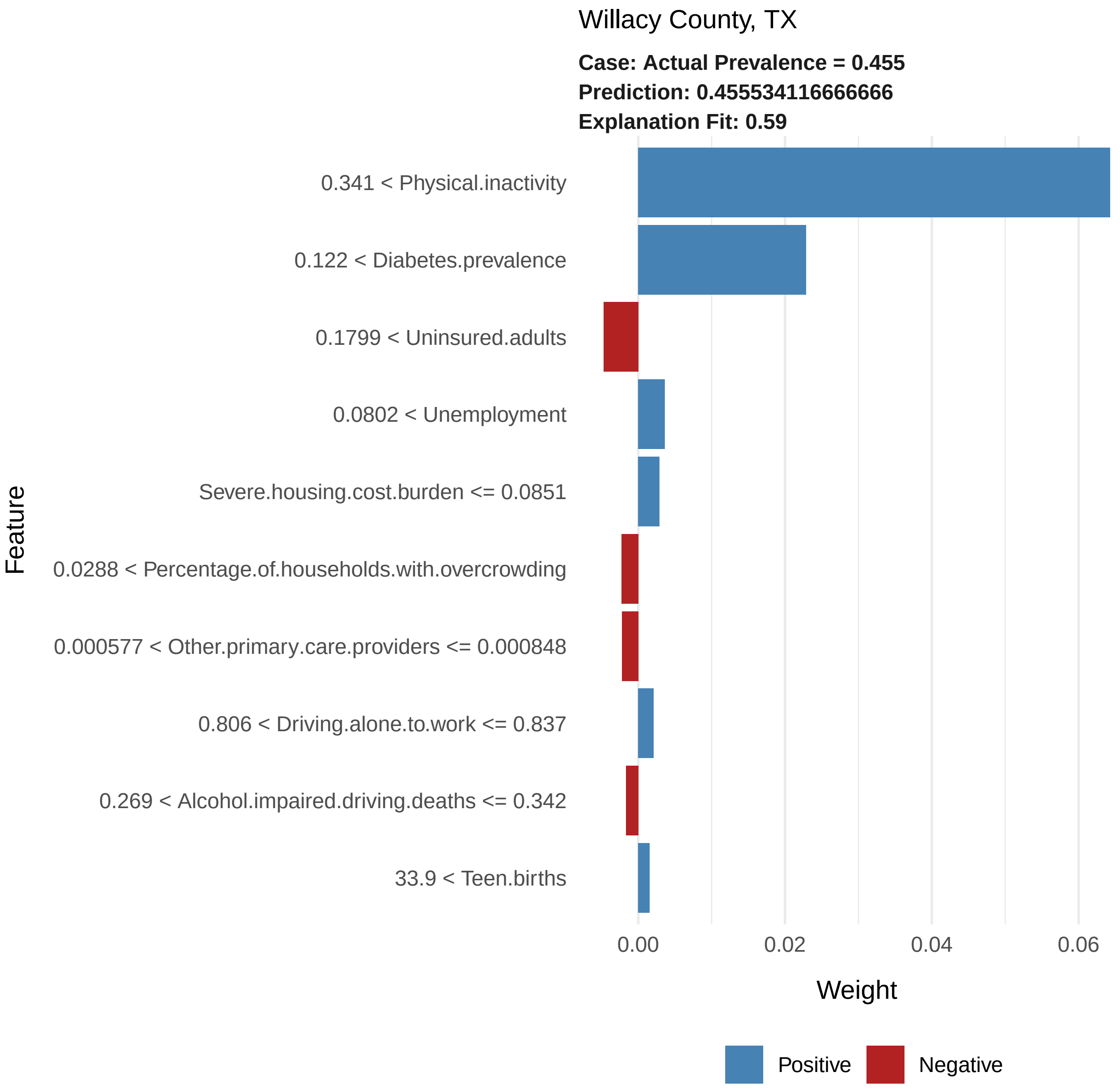

Willacy County, TX
Case: Actual Prevalence = 0.455
Prediction: 0.455534116666666
Explanation Fit: 0.59
0.341 < Physical.inactivity
0.122 < Diabetes.prevalence
0.1799 < Uninsured.adults
0.0802 < Unemployment
Severe.housing.cost.burden <= 0.0851
0.0288 < Percentage.of.households.with.overcrowding
0.000577 < Other.primary.care.providers <= 0.000848
0.806 < Driving.alone.to.work <= 0.837
0.269 < Alcohol.impaired.driving.deaths <= 0.342
33.9 < Teen.births
Feature
0.00
0.02
0.04
0.06
Weight
Positive
Negative

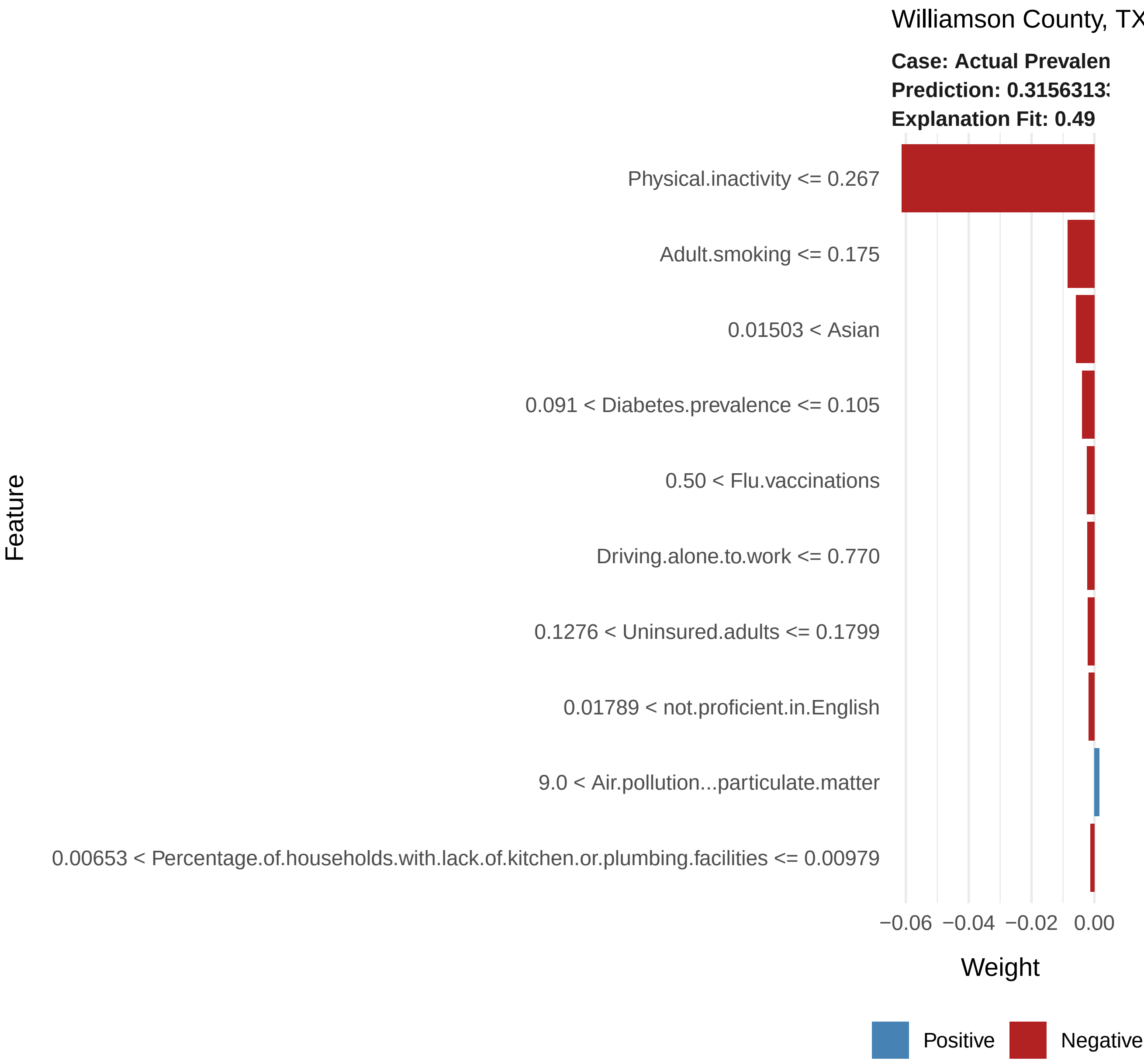

Williamson County, TX
Case: Actual Prevalen
Prediction: 0.3156313
Explanation Fit: 0.49
Physical.inactivity <= 0.267
Adult.smoking <= 0.175
0.01503 < Asian
0.091 < Diabetes.prevalence <= 0.105
0.50 < Flu.vaccinations
Driving.alone.to.work <= 0.770
0.1276 < Uninsured.adults <= 0.1799
0.01789 < not.proficient.in.English
9.0 < Air.pollution...particulate.matter
0.00653 < Percentage.of.households.with.lack.of.kitchen.or.plumbing.facilities <= 0.00979
Feature
−0.06
−0.04
−0.02
0.00
Weight
Positive
Negative

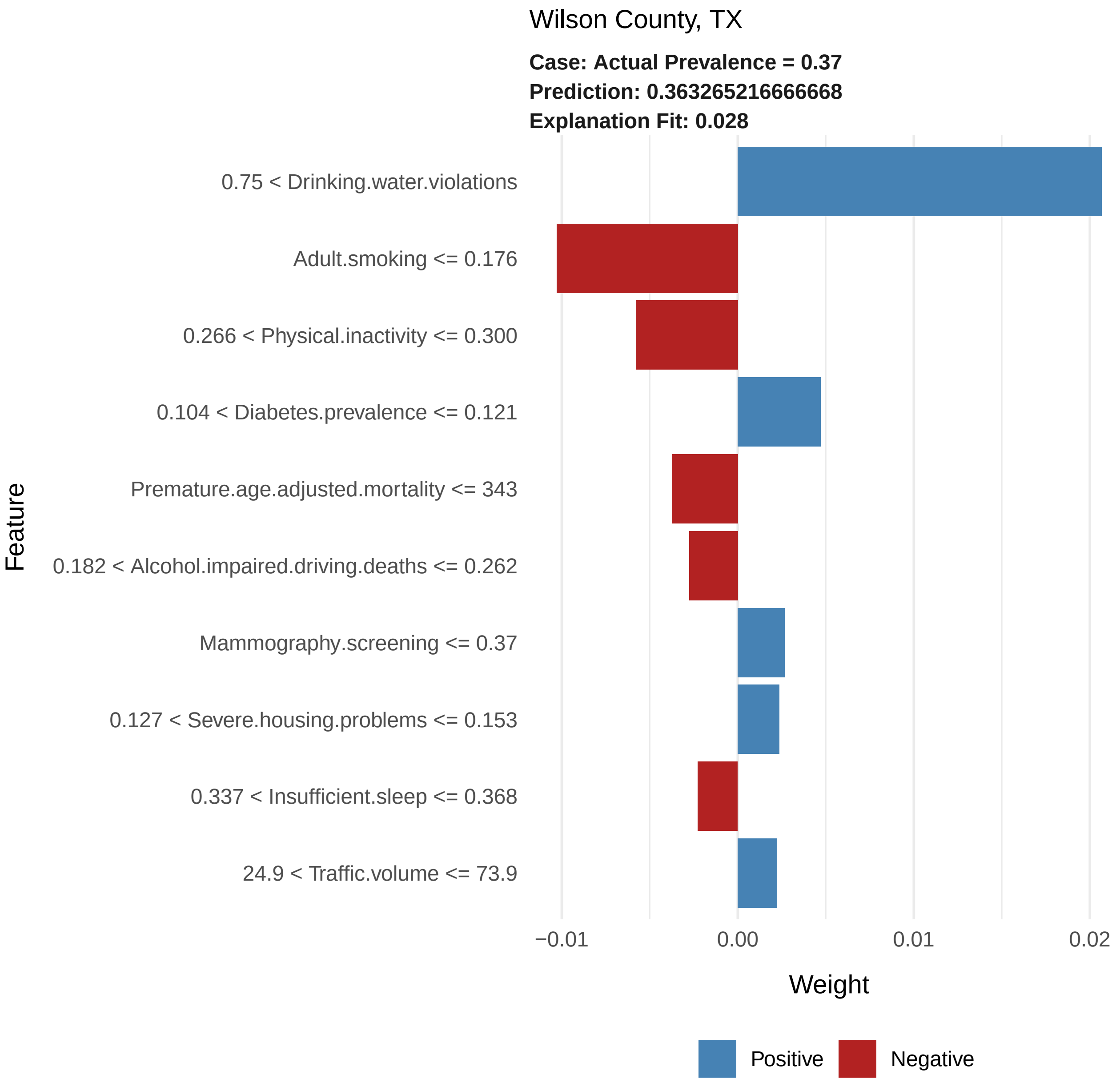

Wilson County, TX
Case: Actual Prevalence = 0.37
Prediction: 0.363265216666668
Explanation Fit: 0.028
0.75 < Drinking.water.violations
Adult.smoking <= 0.176
0.266 < Physical.inactivity <= 0.300
0.104 < Diabetes.prevalence <= 0.121
Premature.age.adjusted.mortality <= 343
0.182 < Alcohol.impaired.driving.deaths <= 0.262
Mammography.screening <= 0.37
0.127 < Severe.housing.problems <= 0.153
0.337 < Insufficient.sleep <= 0.368
24.9 < Traffic.volume <= 73.9
Feature
−0.01
0.00
0.01
0.02
Weight
Positive
Negative

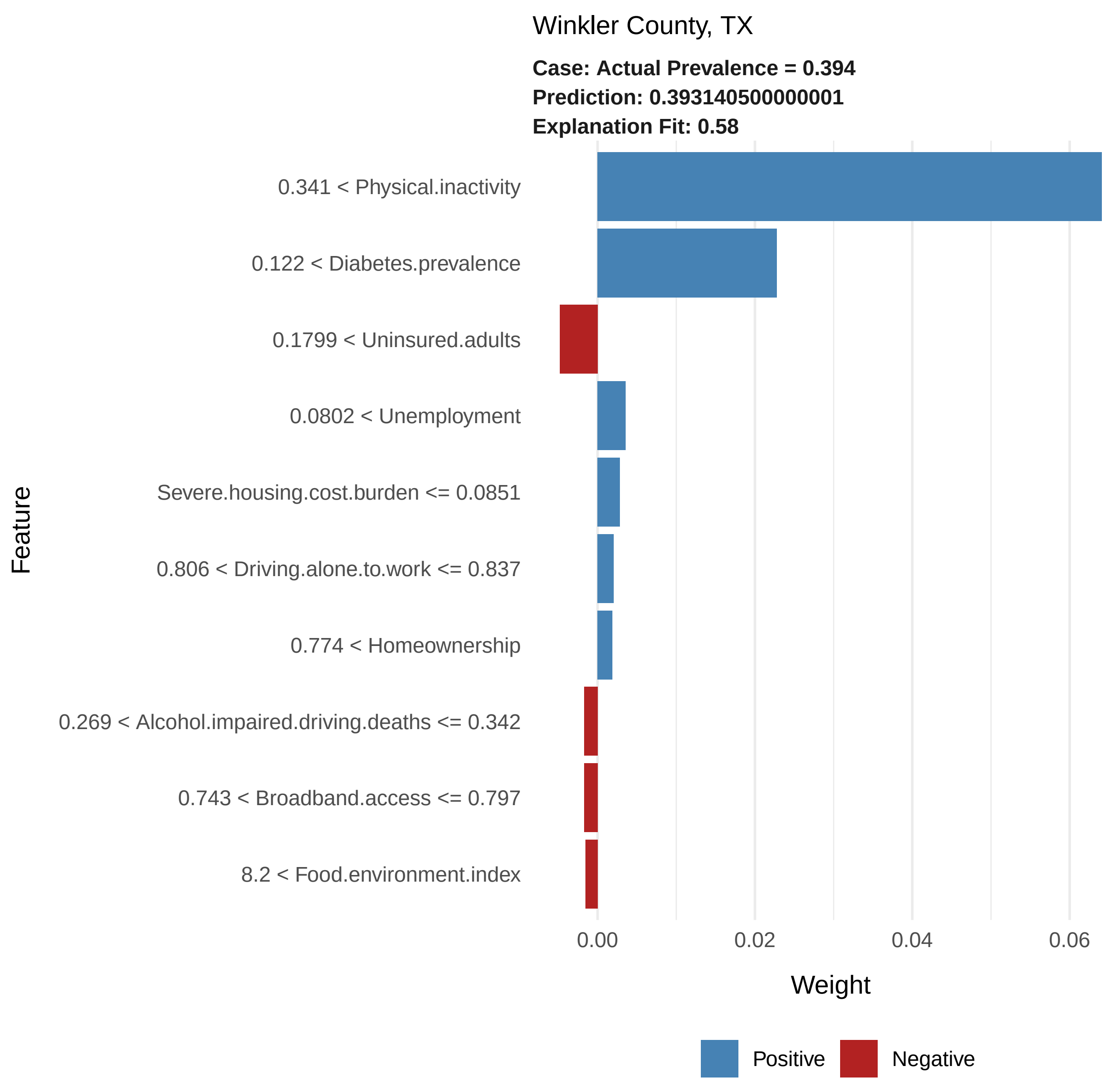
Winkler County, TX
Case: Actual Prevalence = 0.394
Prediction: 0.393140500000001
Explanation Fit: 0.58
0.341 < Physical.inactivity
0.122 < Diabetes.prevalence
0.1799 < Uninsured.adults
0.0802 < Unemployment
Severe.housing.cost.burden <= 0.0851
0.806 < Driving.alone.to.work <= 0.837
0.774 < Homeownership
0.269 < Alcohol.impaired.driving.deaths <= 0.342
0.743 < Broadband.access <= 0.797
8.2 < Food.environment.index
Feature
0.00
0.02
0.04
0.06
Weight
Positive
Negative

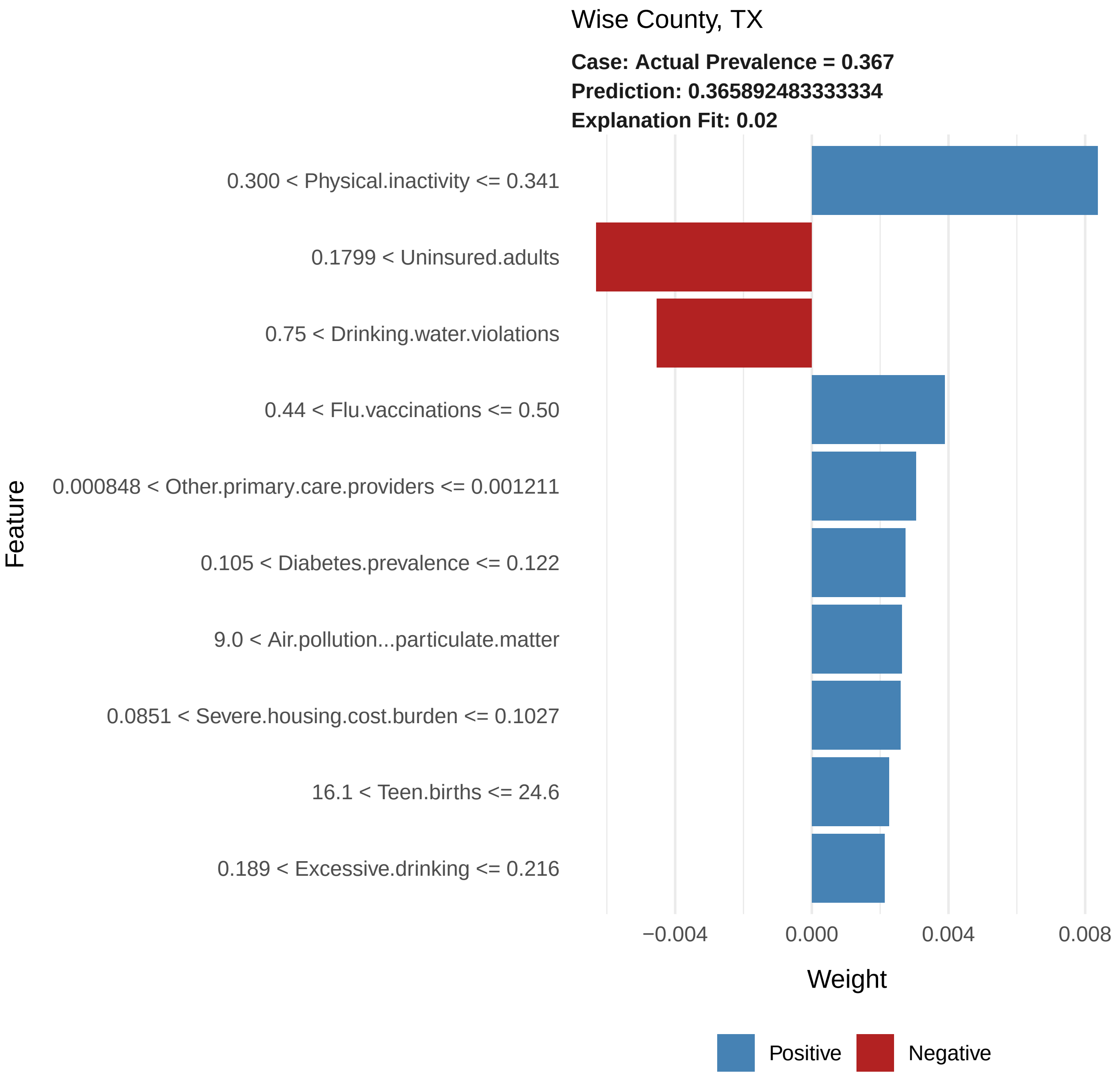
Wise County, TX
Case: Actual Prevalence = 0.367
Prediction: 0.365892483333334
Explanation Fit: 0.02
0.300 < Physical.inactivity <= 0.341
0.1799 < Uninsured.adults
0.75 < Drinking.water.violations
0.44 < Flu.vaccinations <= 0.50
0.000848 < Other.primary.care.providers <= 0.001211
0.105 < Diabetes.prevalence <= 0.122
9.0 < Air.pollution...particulate.matter
0.0851 < Severe.housing.cost.burden <= 0.1027
16.1 < Teen.births <= 24.6
0.189 < Excessive.drinking <= 0.216
Feature
−0.004
0.000
0.004
0.008
Weight
Positive
Negative

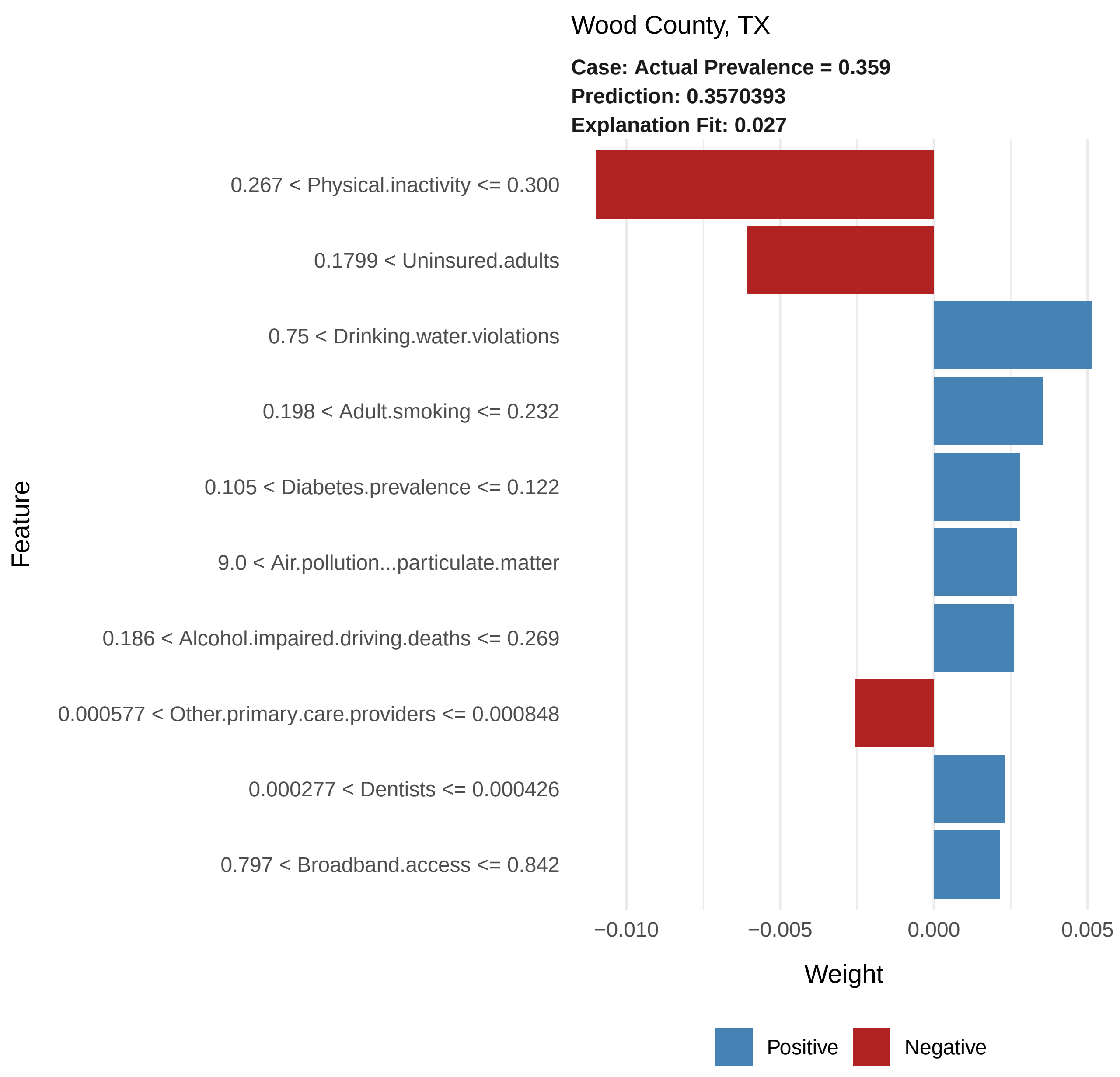
Wood County, TX
Case: Actual Prevalence = 0.359
Prediction: 0.3570393
Explanation Fit: 0.027
0.267 < Physical.inactivity <= 0.300
0.1799 < Uninsured.adults
0.75 < Drinking.water.violations
0.198 < Adult.smoking <= 0.232
0.105 < Diabetes.prevalence <= 0.122
9.0 < Air.pollution...particulate.matter
0.186 < Alcohol.impaired.driving.deaths <= 0.269
0.000577 < Other.primary.care.providers <= 0.000848
0.000277 < Dentists <= 0.000426
0.797 < Broadband.access <= 0.842
Feature
−0.010
−0.005
0.000
0.005
Weight
Positive
Negative

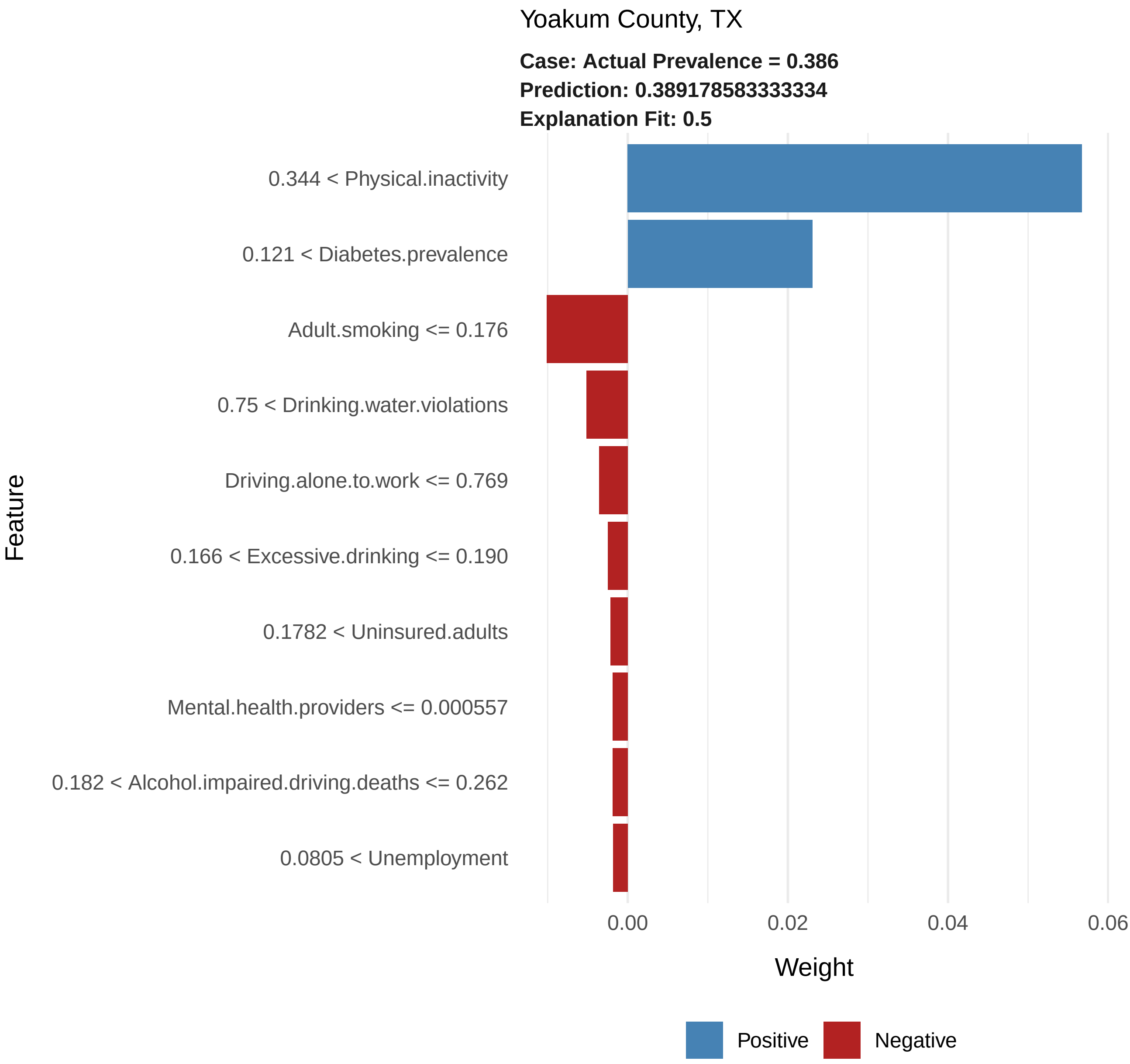
Yoakum County, TX
Case: Actual Prevalence = 0.386
Prediction: 0.389178583333334
Explanation Fit: 0.5
0.344 < Physical.inactivity
0.121 < Diabetes.prevalence
Adult.smoking <= 0.176
0.75 < Drinking.water.violations
Driving.alone.to.work <= 0.769
0.166 < Excessive.drinking <= 0.190
0.1782 < Uninsured.adults
Mental.health.providers <= 0.000557
0.182 < Alcohol.impaired.driving.deaths <= 0.262
0.0805 < Unemployment
Feature
0.00
0.02
0.04
0.06
Weight
Positive
Negative

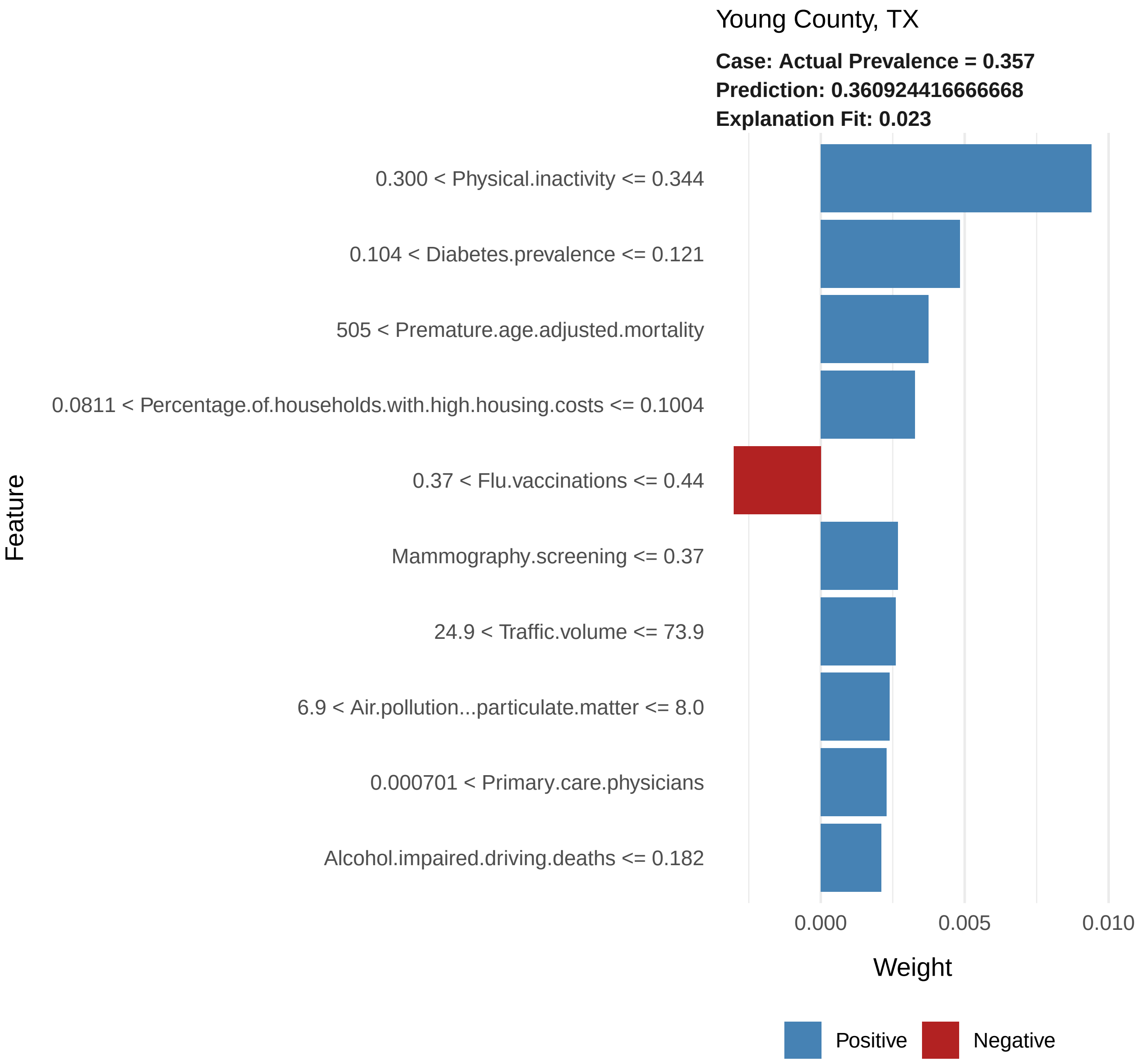
Young County, TX
Case: Actual Prevalence = 0.357
Prediction: 0.360924416666668
Explanation Fit: 0.023
0.300 < Physical.inactivity <= 0.344
0.104 < Diabetes.prevalence <= 0.121
505 < Premature.age.adjusted.mortality
0.0811 < Percentage.of.households.with.high.housing.costs <= 0.1004
0.37 < Flu.vaccinations <= 0.44
Mammography.screening <= 0.37
24.9 < Traffic.volume <= 73.9
6.9 < Air.pollution...particulate.matter <= 8.0
0.000701 < Primary.care.physicians
Alcohol.impaired.driving.deaths <= 0.182
Feature
0.000
0.005
0.010
Weight
Positive
Negative

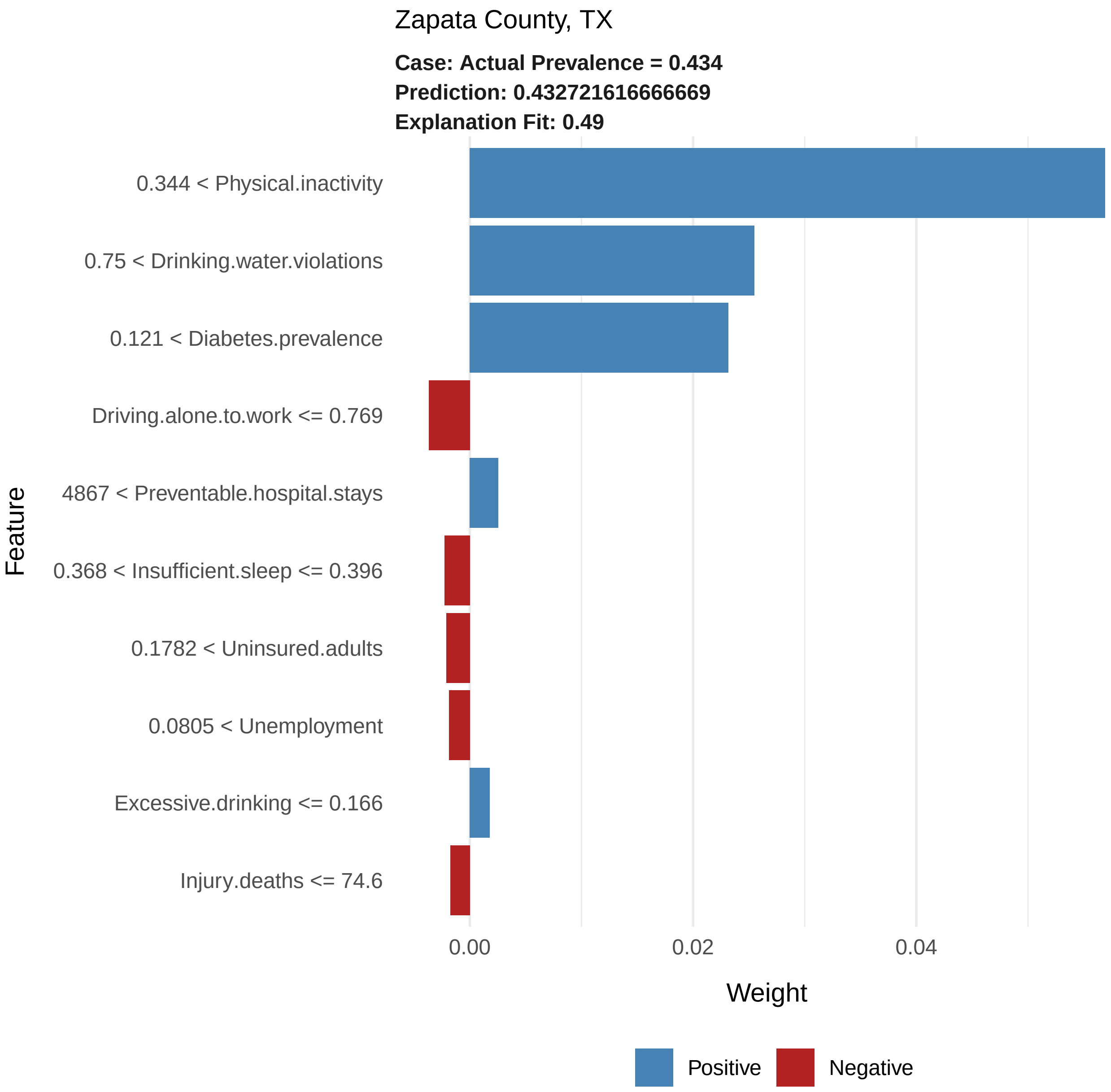
Zapata County, TX
Case: Actual Prevalence = 0.434
Prediction: 0.432721616666669
Explanation Fit: 0.49
0.344 < Physical.inactivity
0.75 < Drinking.water.violations
0.121 < Diabetes.prevalence
Driving.alone.to.work <= 0.769
4867 < Preventable.hospital.stays
0.368 < Insufficient.sleep <= 0.396
0.1782 < Uninsured.adults
0.0805 < Unemployment
Excessive.drinking <= 0.166
Injury.deaths <= 74.6
Feature
0.00
0.02
0.04
Weight
Positive
Negative

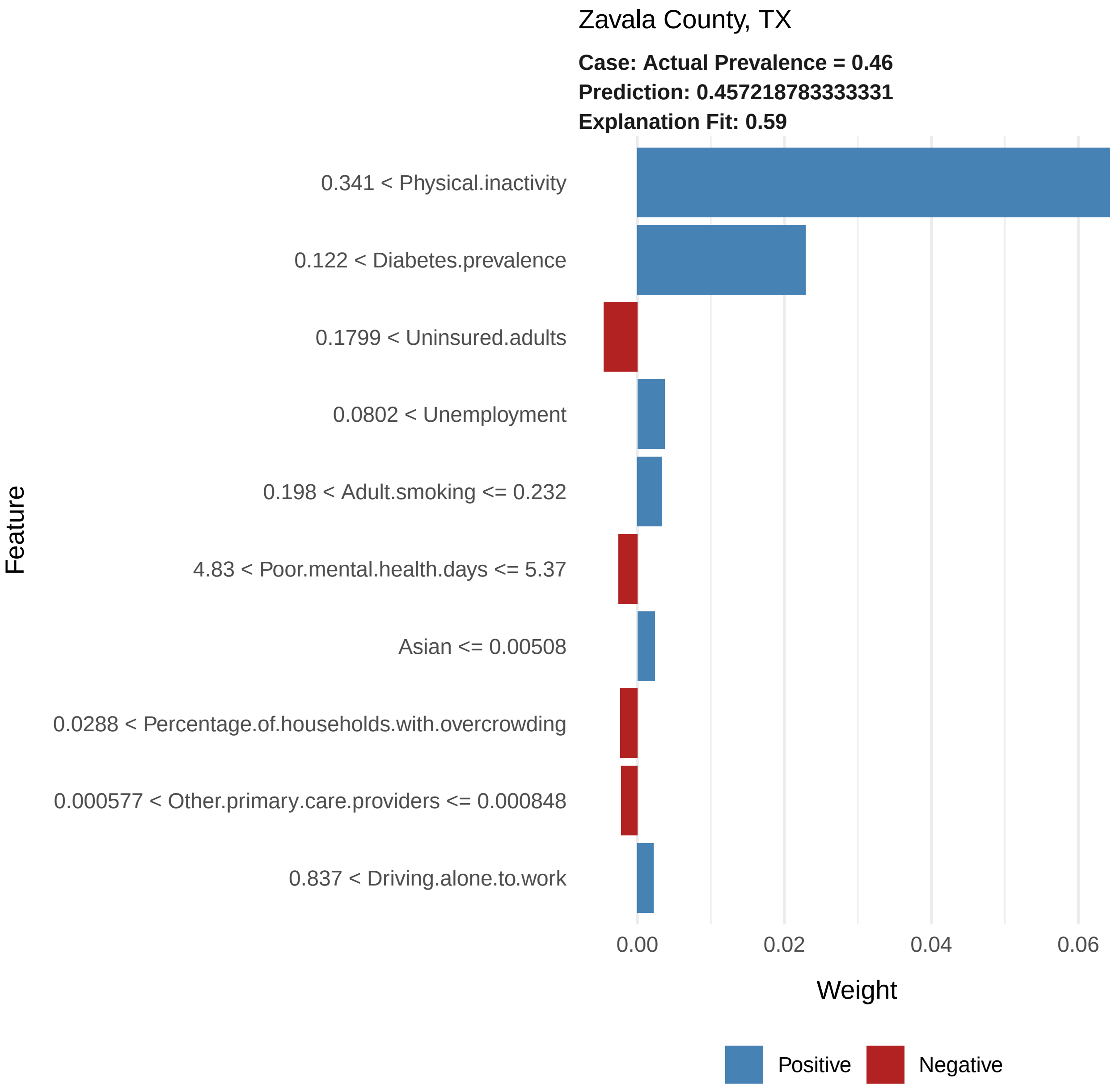
Zavala County, TX
Case: Actual Prevalence = 0.46
Prediction: 0.457218783333331
Explanation Fit: 0.59
0.341 < Physical.inactivity
0.122 < Diabetes.prevalence
0.1799 < Uninsured.adults
0.0802 < Unemployment
0.198 < Adult.smoking <= 0.232
4.83 < Poor.mental.health.days <= 5.37
Asian <= 0.00508
0.0288 < Percentage.of.households.with.overcrowding
0.000577 < Other.primary.care.providers <= 0.000848
0.837 < Driving.alone.to.work
Feature
0.00
0.02
0.04
0.06
Weight
Positive
Negative

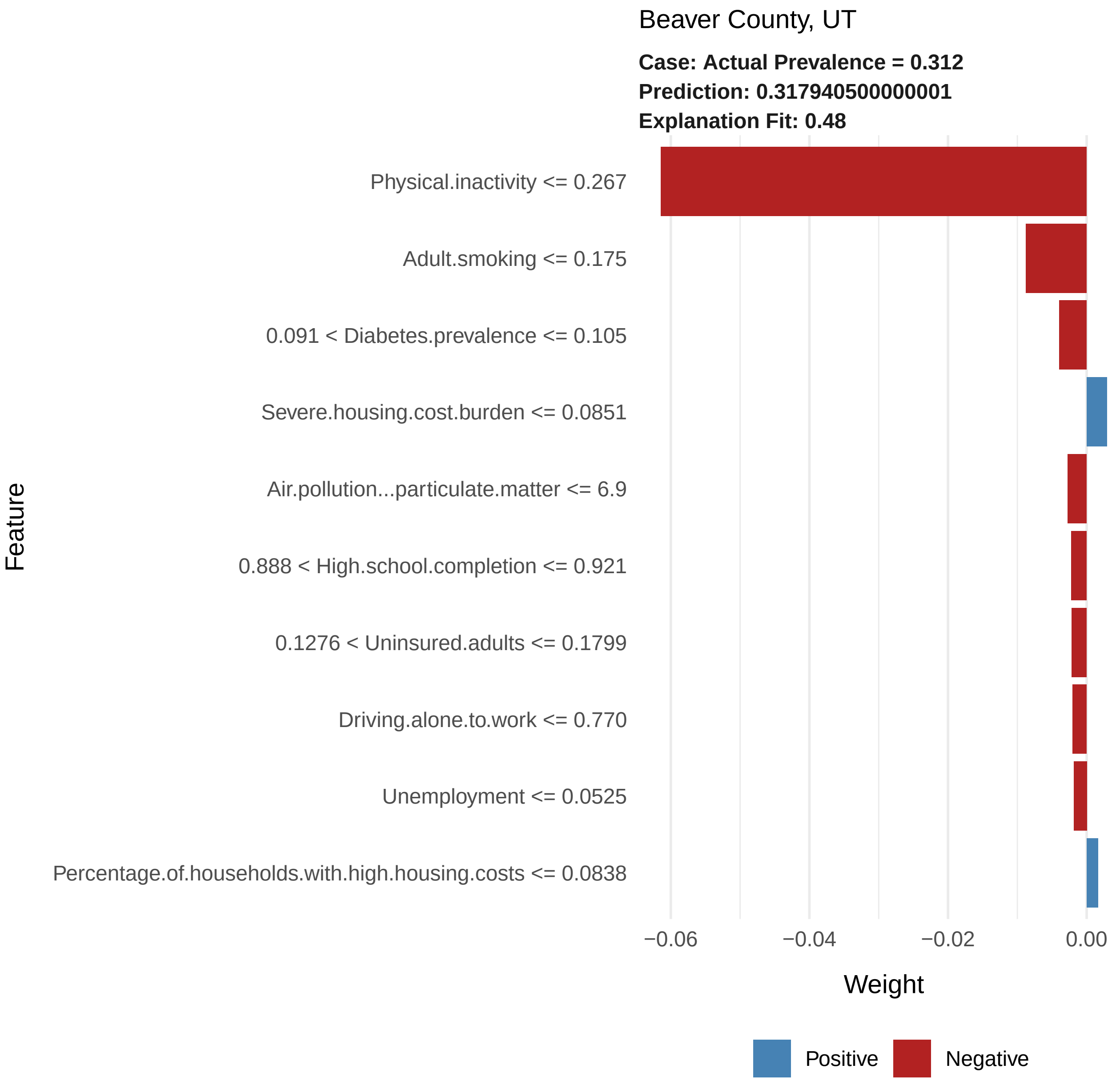

Beaver County, UT
Case: Actual Prevalence = 0.312
Prediction: 0.317940500000001
Explanation Fit: 0.48
Feature
Physical.inactivity <= 0.267
Adult.smoking <= 0.175
0.091 < Diabetes.prevalence <= 0.105
Severe.housing.cost.burden <= 0.0851
Air.pollution...particulate.matter <= 6.9
0.888 < High.school.completion <= 0.921
0.1276 < Uninsured.adults <= 0.1799
Driving.alone.to.work <= 0.770
Unemployment <= 0.0525
Percentage.of.households.with.high.housing.costs <= 0.0838
−0.06
−0.04
−0.02
0.00
Weight
Positive
Negative

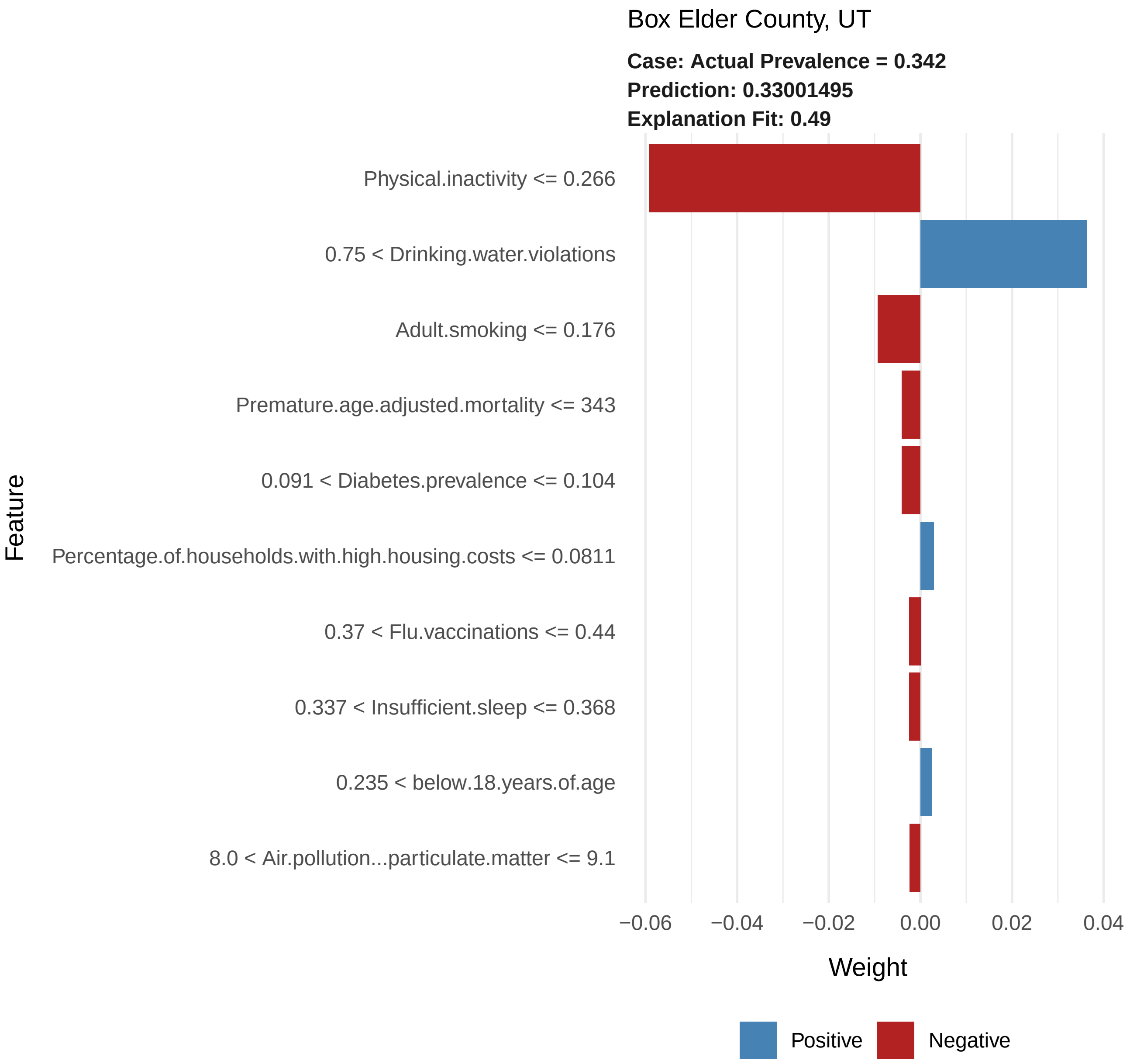

Box Elder County, UT
Case: Actual Prevalence = 0.342
Prediction: 0.33001495
Explanation Fit: 0.49
Physical.inactivity <= 0.266
0.75 < Drinking.water.violations
Adult.smoking <= 0.176
Premature.age.adjusted.mortality <= 343
0.091 < Diabetes.prevalence <= 0.104
Percentage.of.households.with.high.housing.costs <= 0.0811
0.37 < Flu.vaccinations <= 0.44
0.337 < Insufficient.sleep <= 0.368
0.235 < below.18.years.of.age
8.0 < Air.pollution...particulate.matter <= 9.1
Feature
−0.06
−0.04
−0.02
0.00
0.02
0.04
Weight
Positive
Negative

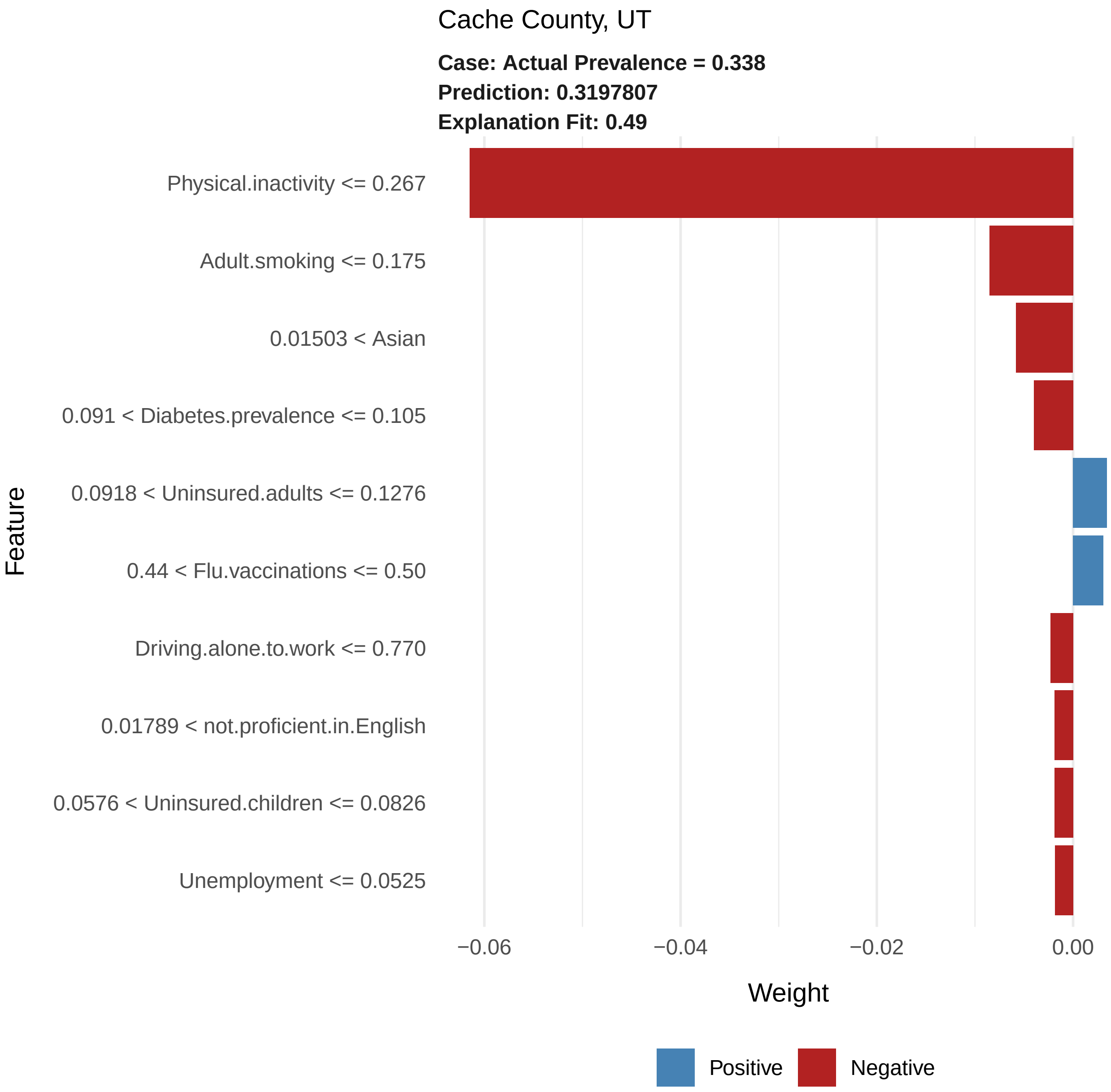

Cache County, UT
Case: Actual Prevalence = 0.338
Prediction: 0.3197807
Explanation Fit: 0.49
Physical.inactivity <= 0.267
Adult.smoking <= 0.175
0.01503 < Asian
0.091 < Diabetes.prevalence <= 0.105
0.0918 < Uninsured.adults <= 0.1276
0.44 < Flu.vaccinations <= 0.50
Driving.alone.to.work <= 0.770
0.01789 < not.proficient.in.English
0.0576 < Uninsured.children <= 0.0826
Unemployment <= 0.0525
Feature
−0.06
−0.04
−0.02
0.00
Weight
Positive
Negative

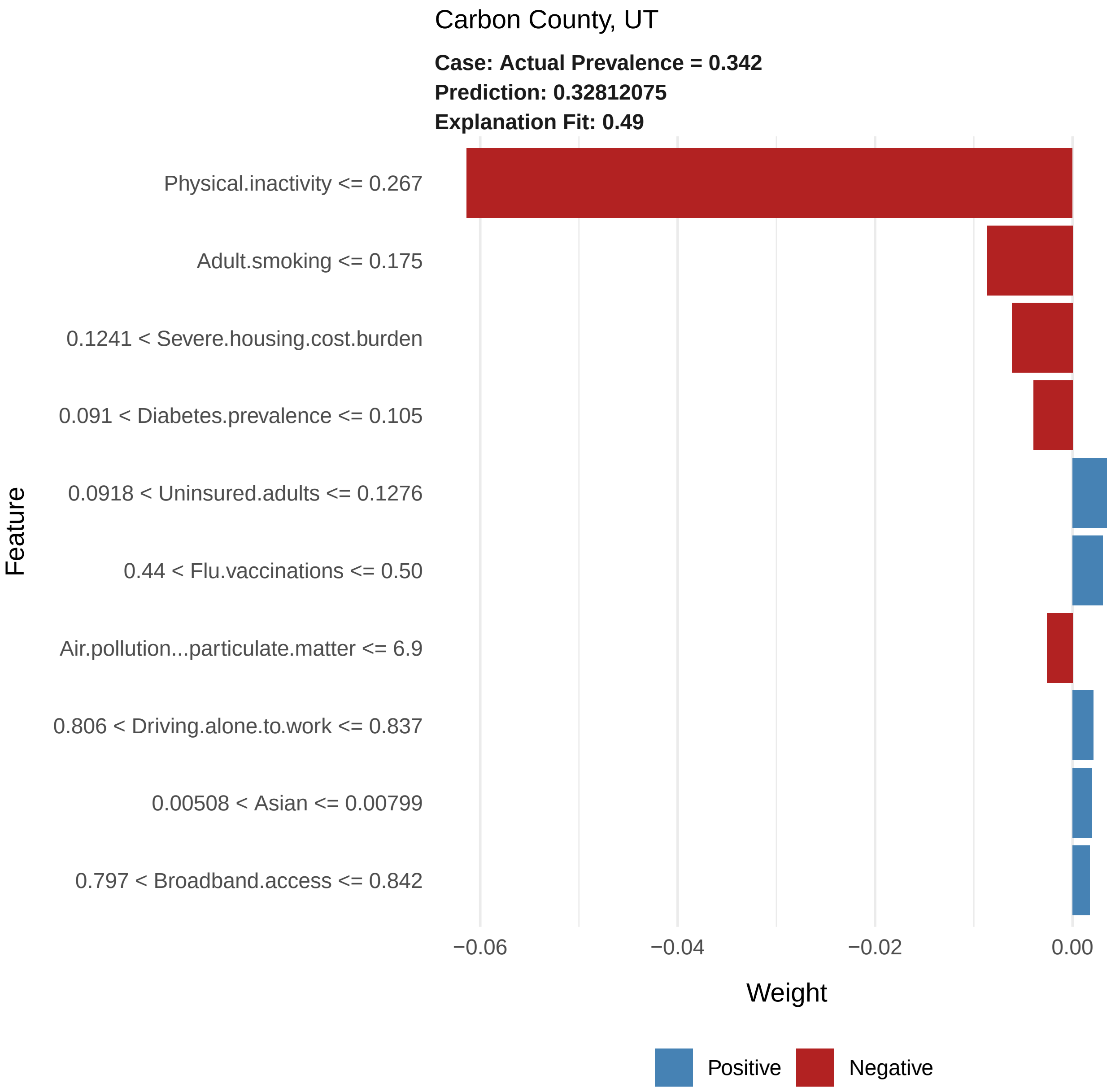
Carbon County, UT
Case: Actual Prevalence = 0.342
Prediction: 0.32812075
Explanation Fit: 0.49
Feature
Physical.inactivity <= 0.267
Adult.smoking <= 0.175
0.1241 < Severe.housing.cost.burden
0.091 < Diabetes.prevalence <= 0.105
0.0918 < Uninsured.adults <= 0.1276
0.44 < Flu.vaccinations <= 0.50
Air.pollution...particulate.matter <= 6.9
0.806 < Driving.alone.to.work <= 0.837
0.00508 < Asian <= 0.00799
0.797 < Broadband.access <= 0.842
−0.06
−0.04
−0.02
0.00
Weight
Positive
Negative

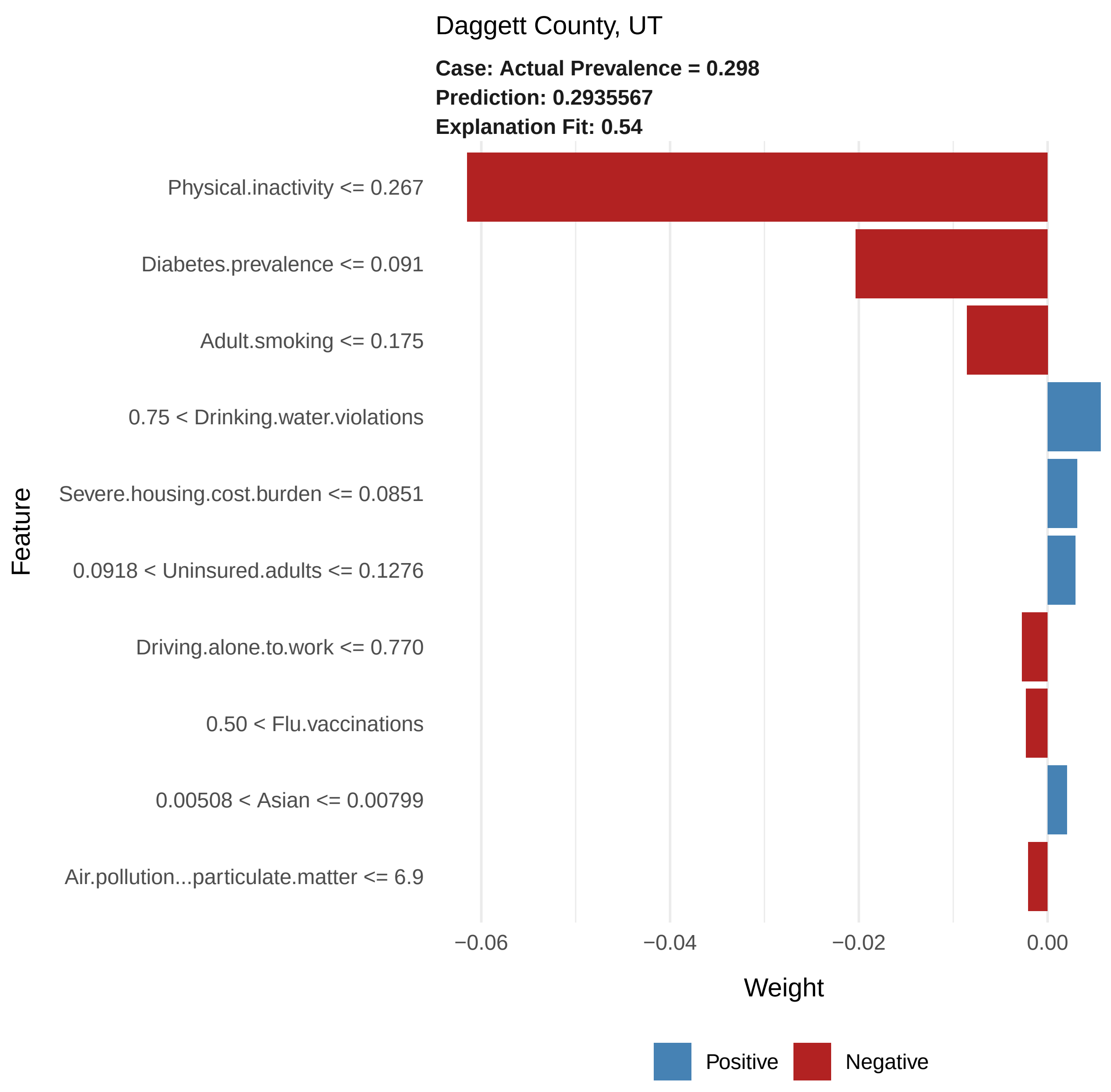

Daggett County, UT
Case: Actual Prevalence = 0.298
Prediction: 0.2935567
Explanation Fit: 0.54
Physical.inactivity <= 0.267
Diabetes.prevalence <= 0.091
Adult.smoking <= 0.175
0.75 < Drinking.water.violations
Severe.housing.cost.burden <= 0.0851
0.0918 < Uninsured.adults <= 0.1276
Driving.alone.to.work <= 0.770
0.50 < Flu.vaccinations
0.00508 < Asian <= 0.00799
Air.pollution...particulate.matter <= 6.9
Feature
−0.06
−0.04
−0.02
0.00
Weight
Positive
Negative

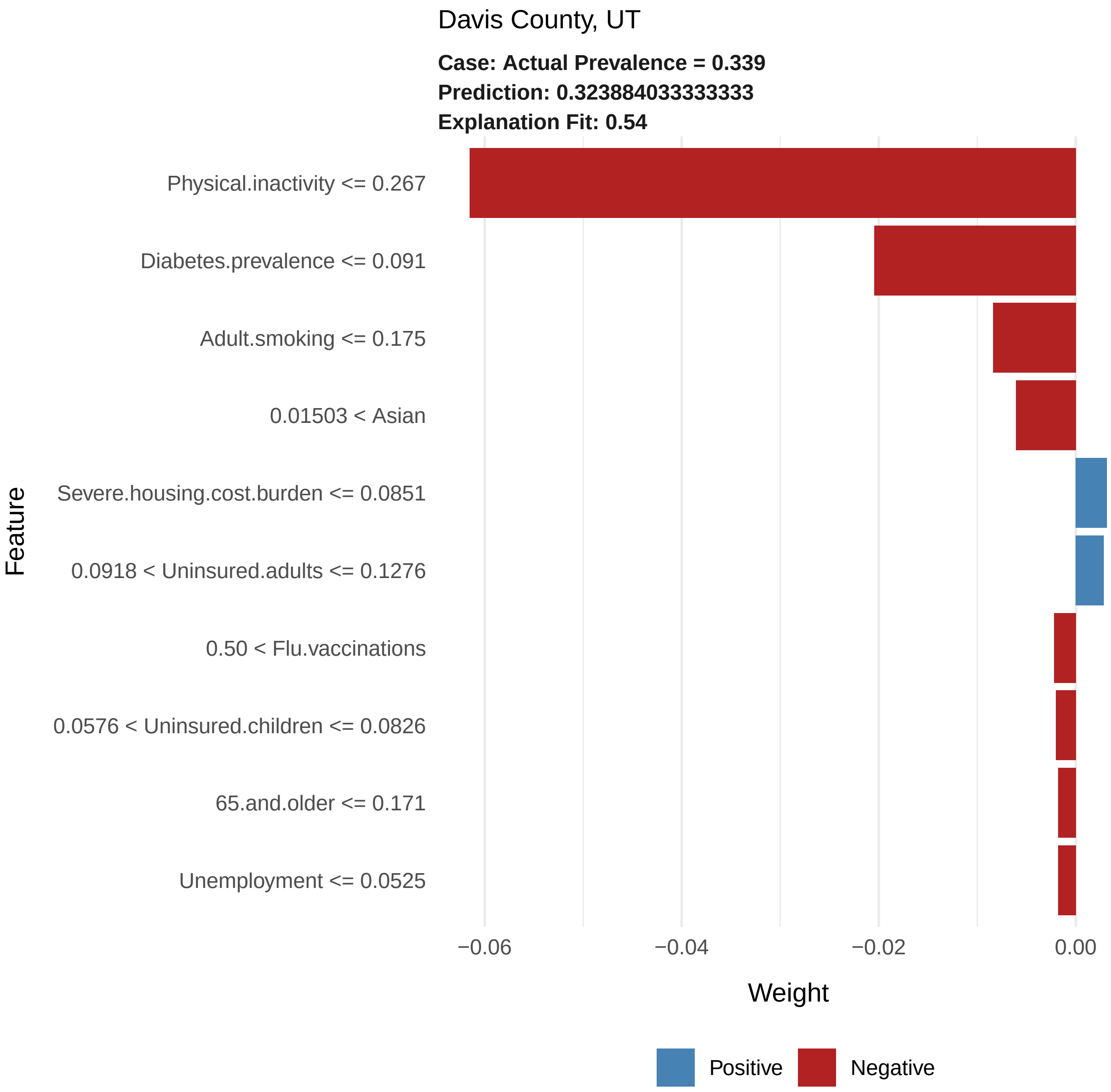
Davis County, UT
Case: Actual Prevalence = 0.339
Prediction: 0.323884033333333
Explanation Fit: 0.54
Physical.inactivity <= 0.267
Diabetes.prevalence <= 0.091
Adult.smoking <= 0.175
0.01503 < Asian
Severe.housing.cost.burden <= 0.0851
0.0918 < Uninsured.adults <= 0.1276
0.50 < Flu.vaccinations
0.0576 < Uninsured.children <= 0.0826
65.and.older <= 0.171
Unemployment <= 0.0525
Feature
−0.06
−0.04
−0.02
0.00
Weight
Positive
Negative

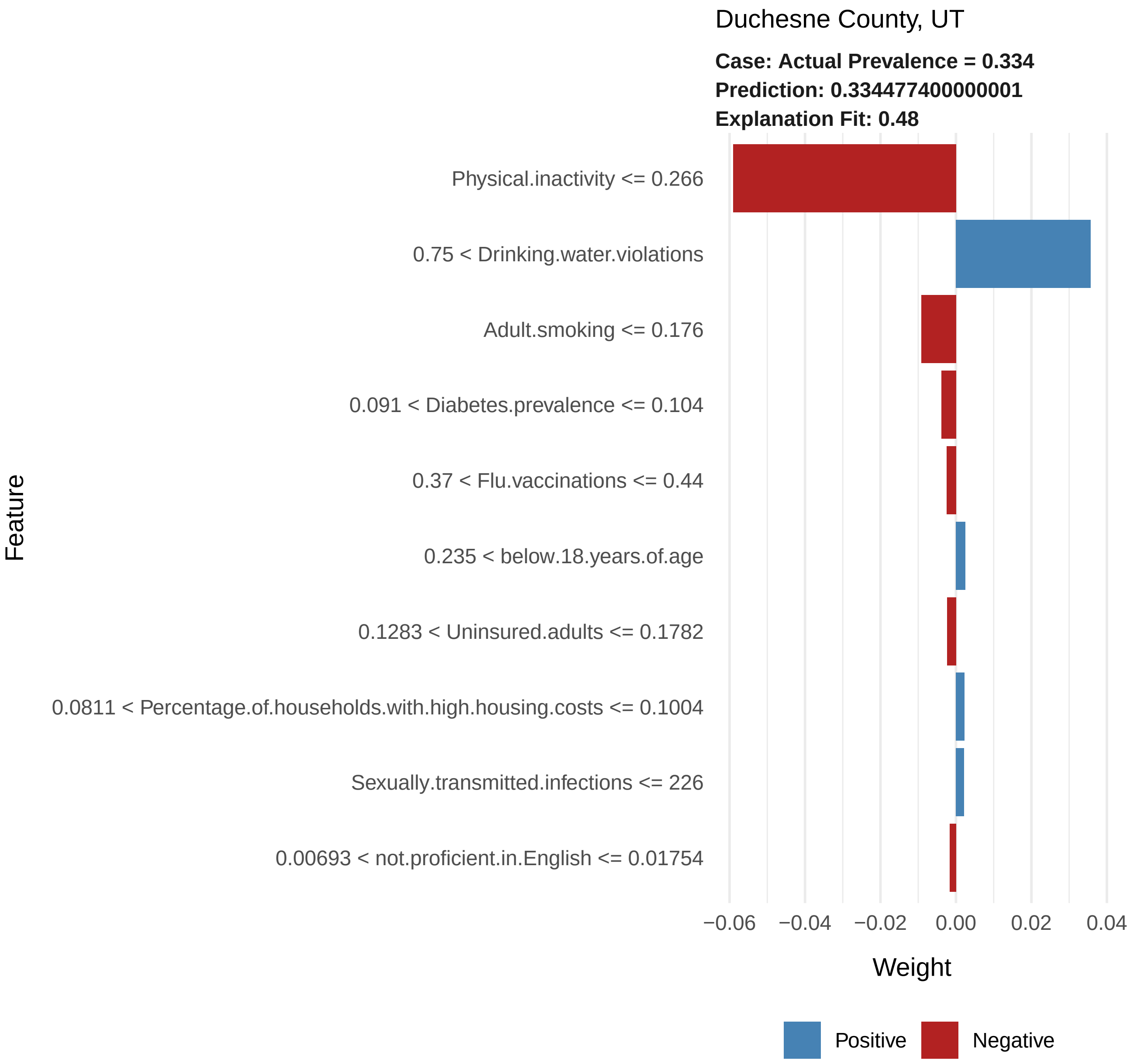
Duchesne County, UT
Case: Actual Prevalence = 0.334
Prediction: 0.334477400000001
Explanation Fit: 0.48
Physical.inactivity <= 0.266
0.75 < Drinking.water.violations
Adult.smoking <= 0.176
0.091 < Diabetes.prevalence <= 0.104
0.37 < Flu.vaccinations <= 0.44
0.235 < below.18.years.of.age
0.1283 < Uninsured.adults <= 0.1782
0.0811 < Percentage.of.households.with.high.housing.costs <= 0.1004
Sexually.transmitted.infections <= 226
0.00693 < not.proficient.in.English <= 0.01754
Feature
−0.06
−0.04
−0.02
0.00
0.02
0.04
Weight
Positive
Negative

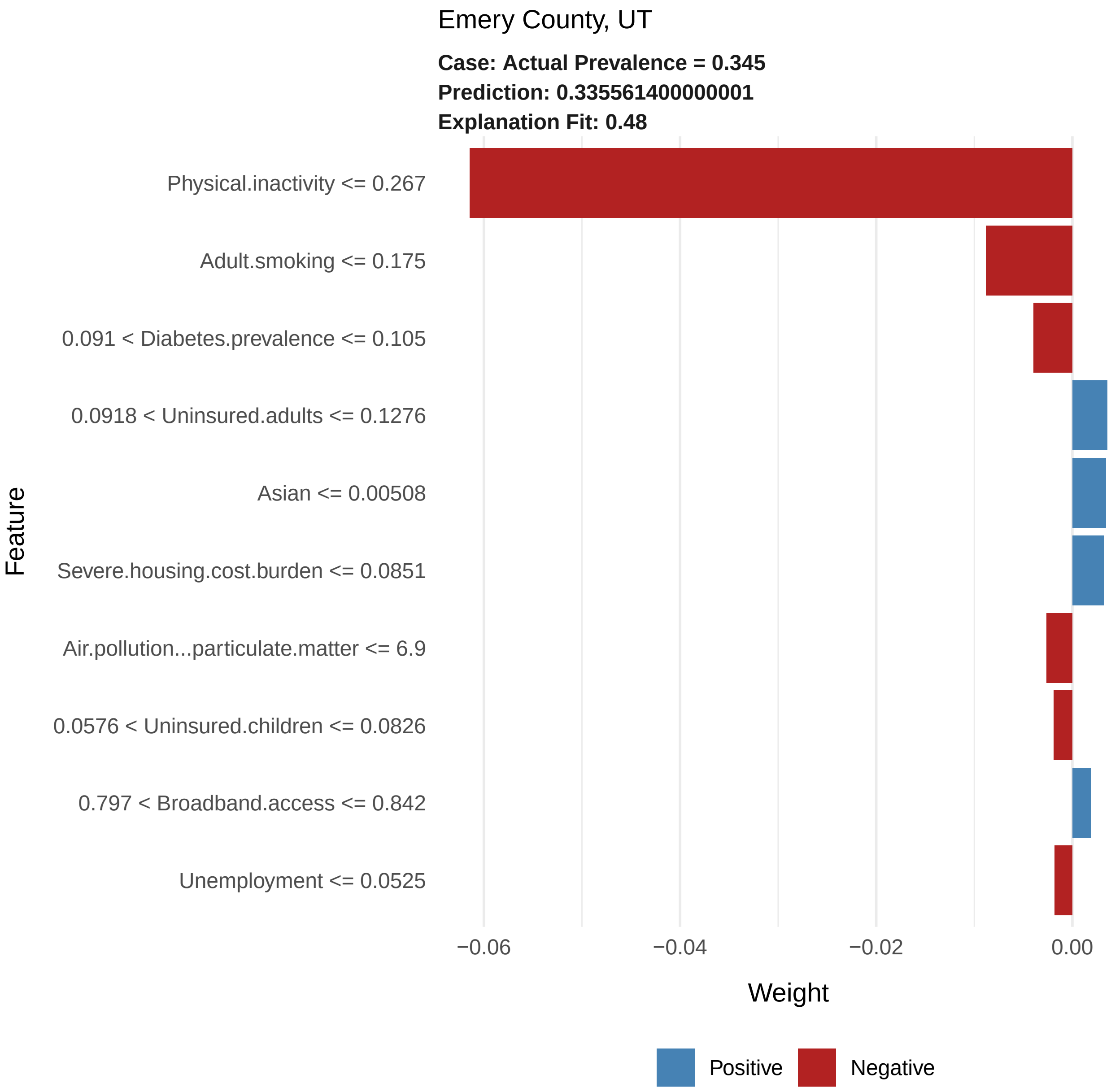

Emery County, UT
Case: Actual Prevalence = 0.345
Prediction: 0.335561400000001
Explanation Fit: 0.48
Physical.inactivity <= 0.267
Adult.smoking <= 0.175
0.091 < Diabetes.prevalence <= 0.105
0.0918 < Uninsured.adults <= 0.1276
Asian <= 0.00508
Severe.housing.cost.burden <= 0.0851
Air.pollution...particulate.matter <= 6.9
0.0576 < Uninsured.children <= 0.0826
0.797 < Broadband.access <= 0.842
Unemployment <= 0.0525
Feature
−0.06
−0.04
−0.02
0.00
Weight
Positive
Negative

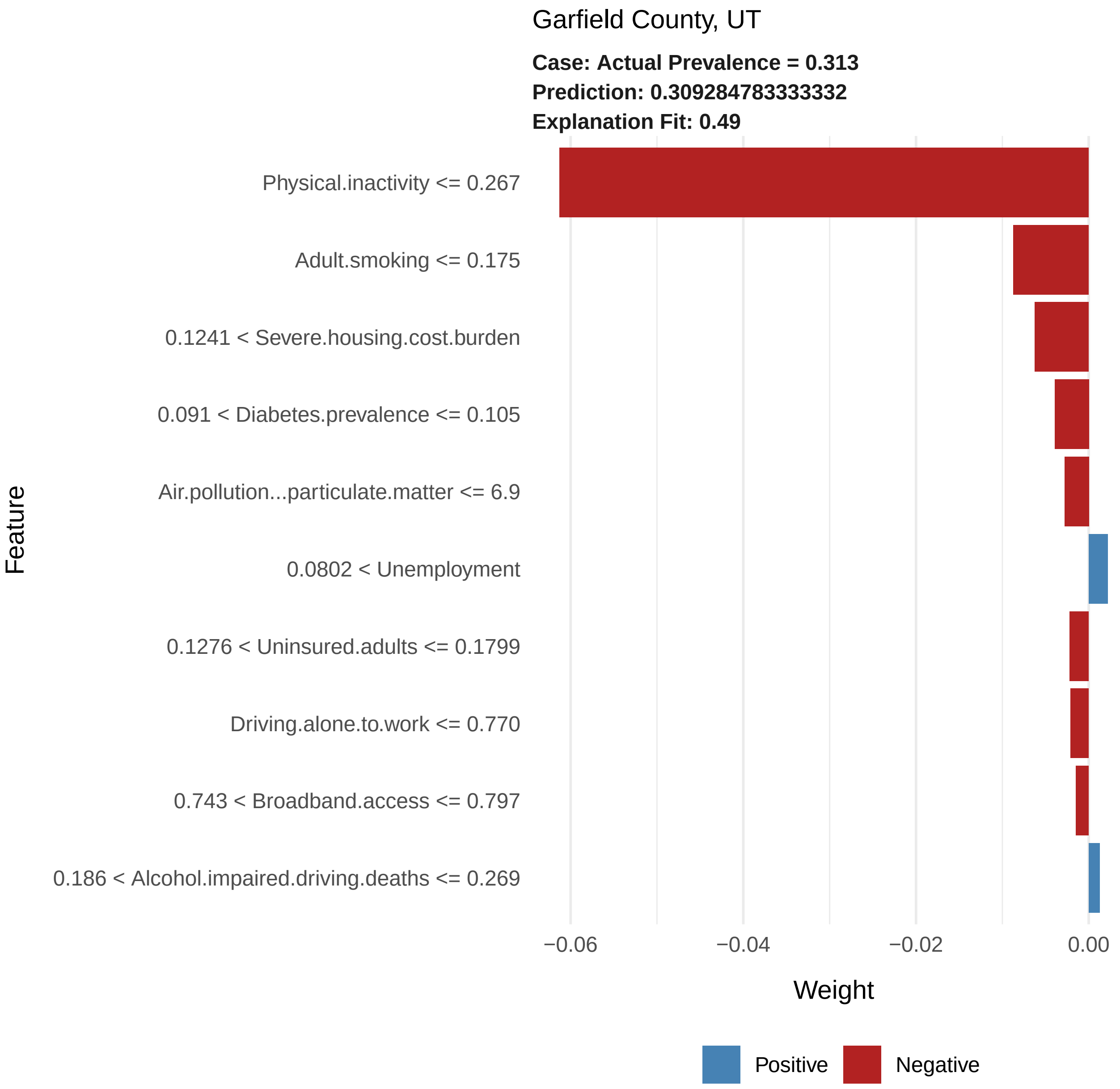

Garfield County, UT
Case: Actual Prevalence = 0.313
Prediction: 0.309284783333332
Explanation Fit: 0.49
Physical.inactivity <= 0.267
Adult.smoking <= 0.175
0.1241 < Severe.housing.cost.burden
0.091 < Diabetes.prevalence <= 0.105
Air.pollution...particulate.matter <= 6.9
0.0802 < Unemployment
0.1276 < Uninsured.adults <= 0.1799
Driving.alone.to.work <= 0.770
0.743 < Broadband.access <= 0.797
0.186 < Alcohol.impaired.driving.deaths <= 0.269
Feature
−0.06
−0.04
−0.02
0.00
Weight
Positive
Negative

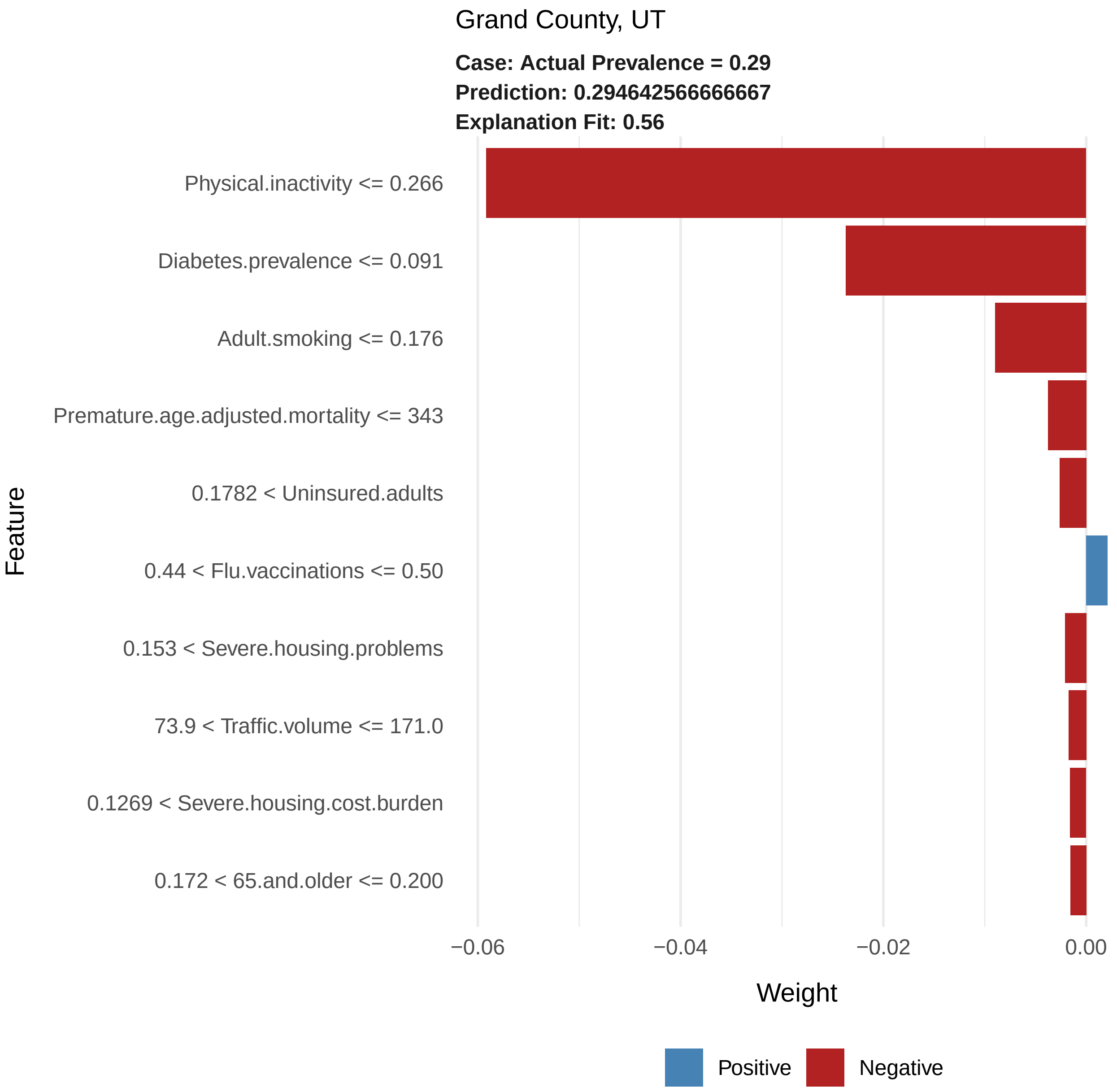
Grand County, UT
Case: Actual Prevalence = 0.29
Prediction: 0.294642566666667
Explanation Fit: 0.56
Feature
Physical.inactivity <= 0.266
Diabetes.prevalence <= 0.091
Adult.smoking <= 0.176
Premature.age.adjusted.mortality <= 343
0.1782 < Uninsured.adults
0.44 < Flu.vaccinations <= 0.50
0.153 < Severe.housing.problems
73.9 < Traffic.volume <= 171.0
0.1269 < Severe.housing.cost.burden
0.172 < 65.and.older <= 0.200
−0.06
−0.04
−0.02
0.00
Weight
Positive
Negative

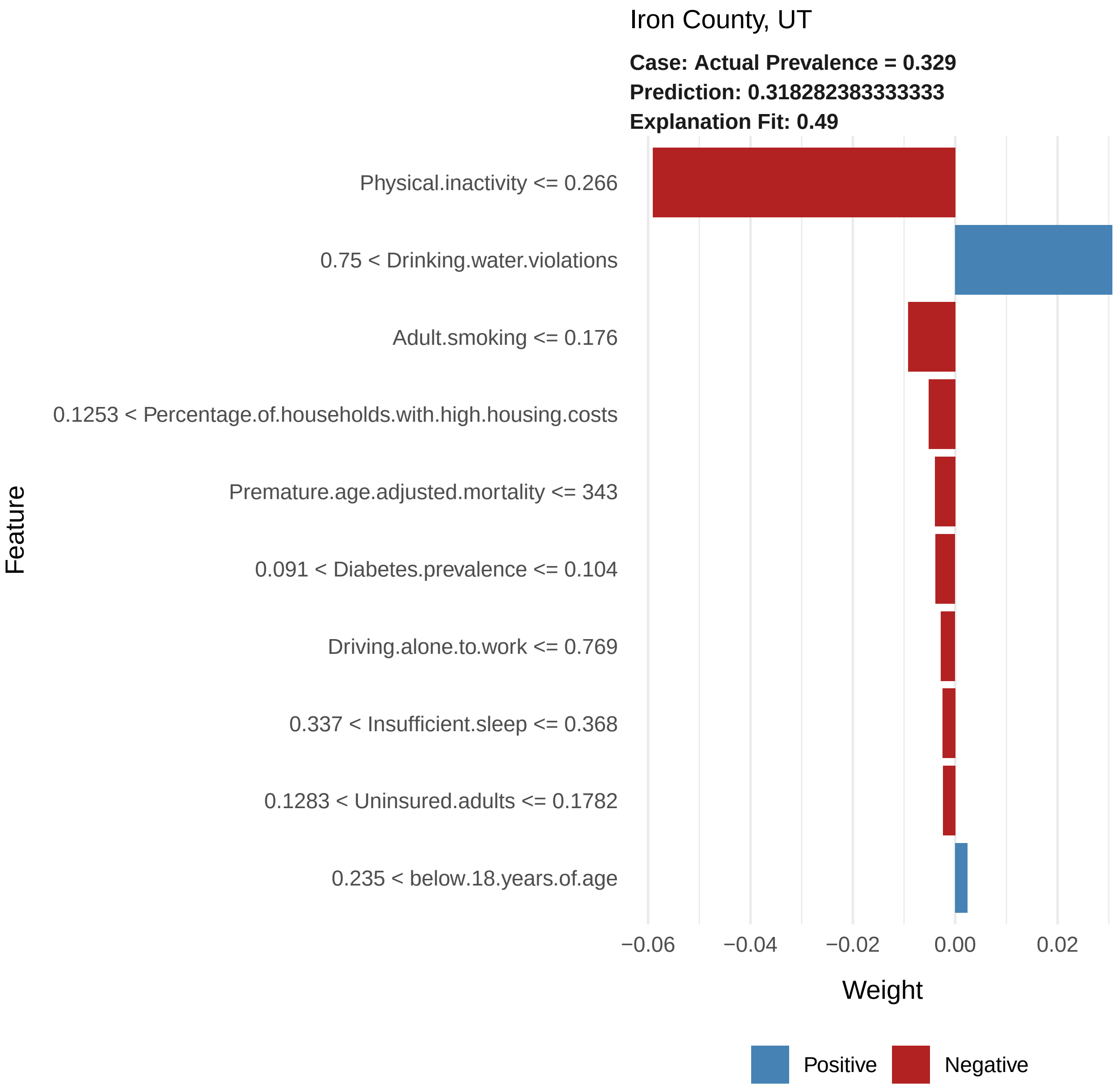

Iron County, UT
Case: Actual Prevalence = 0.329
Prediction: 0.318282383333333
Explanation Fit: 0.49
Physical.inactivity <= 0.266
0.75 < Drinking.water.violations
Adult.smoking <= 0.176
0.1253 < Percentage.of.households.with.high.housing.costs
Premature.age.adjusted.mortality <= 343
0.091 < Diabetes.prevalence <= 0.104
Driving.alone.to.work <= 0.769
0.337 < Insufficient.sleep <= 0.368
0.1283 < Uninsured.adults <= 0.1782
0.235 < below.18.years.of.age
Feature
−0.06
−0.04
−0.02
0.00
0.02
Weight
Positive
Negative

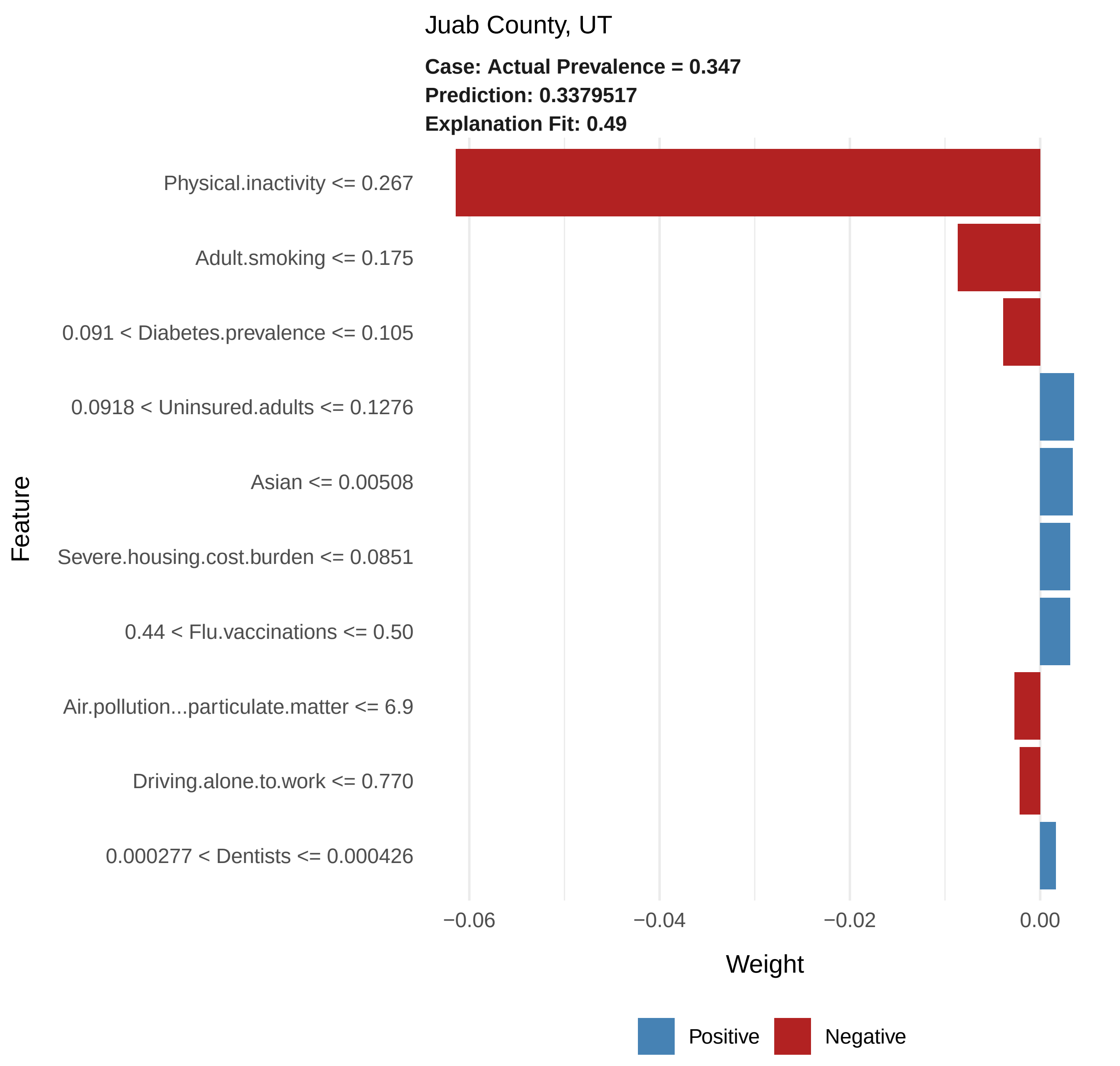
Juab County, UT
Case: Actual Prevalence = 0.347
Prediction: 0.3379517
Explanation Fit: 0.49
Physical.inactivity <= 0.267
Adult.smoking <= 0.175
0.091 < Diabetes.prevalence <= 0.105
0.0918 < Uninsured.adults <= 0.1276
Asian <= 0.00508
Severe.housing.cost.burden <= 0.0851
0.44 < Flu.vaccinations <= 0.50
Air.pollution...particulate.matter <= 6.9
Driving.alone.to.work <= 0.770
0.000277 < Dentists <= 0.000426
Feature
−0.06
−0.04
−0.02
0.00
Weight
Positive
Negative

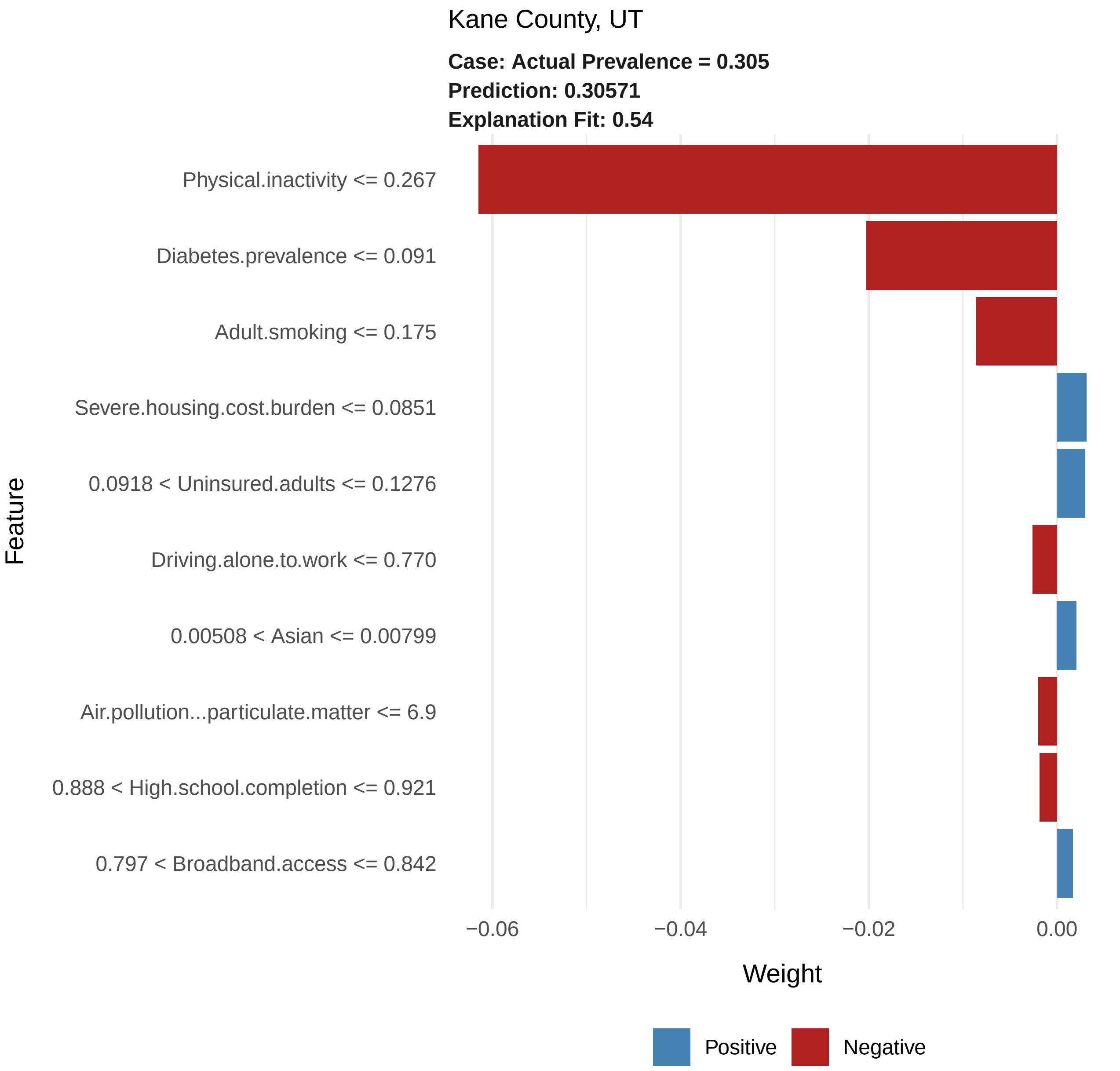

Kane County, UT
Case: Actual Prevalence = 0.305
Prediction: 0.30571
Explanation Fit: 0.54
Physical.inactivity <= 0.267
Diabetes.prevalence <= 0.091
Adult.smoking <= 0.175
Severe.housing.cost.burden <= 0.0851
0.0918 < Uninsured.adults <= 0.1276
Driving.alone.to.work <= 0.770
0.00508 < Asian <= 0.00799
Air.pollution...particulate.matter <= 6.9
0.888 < High.school.completion <= 0.921
0.797 < Broadband.access <= 0.842
Feature
−0.06
−0.04
−0.02
0.00
Weight
Positive
Negative

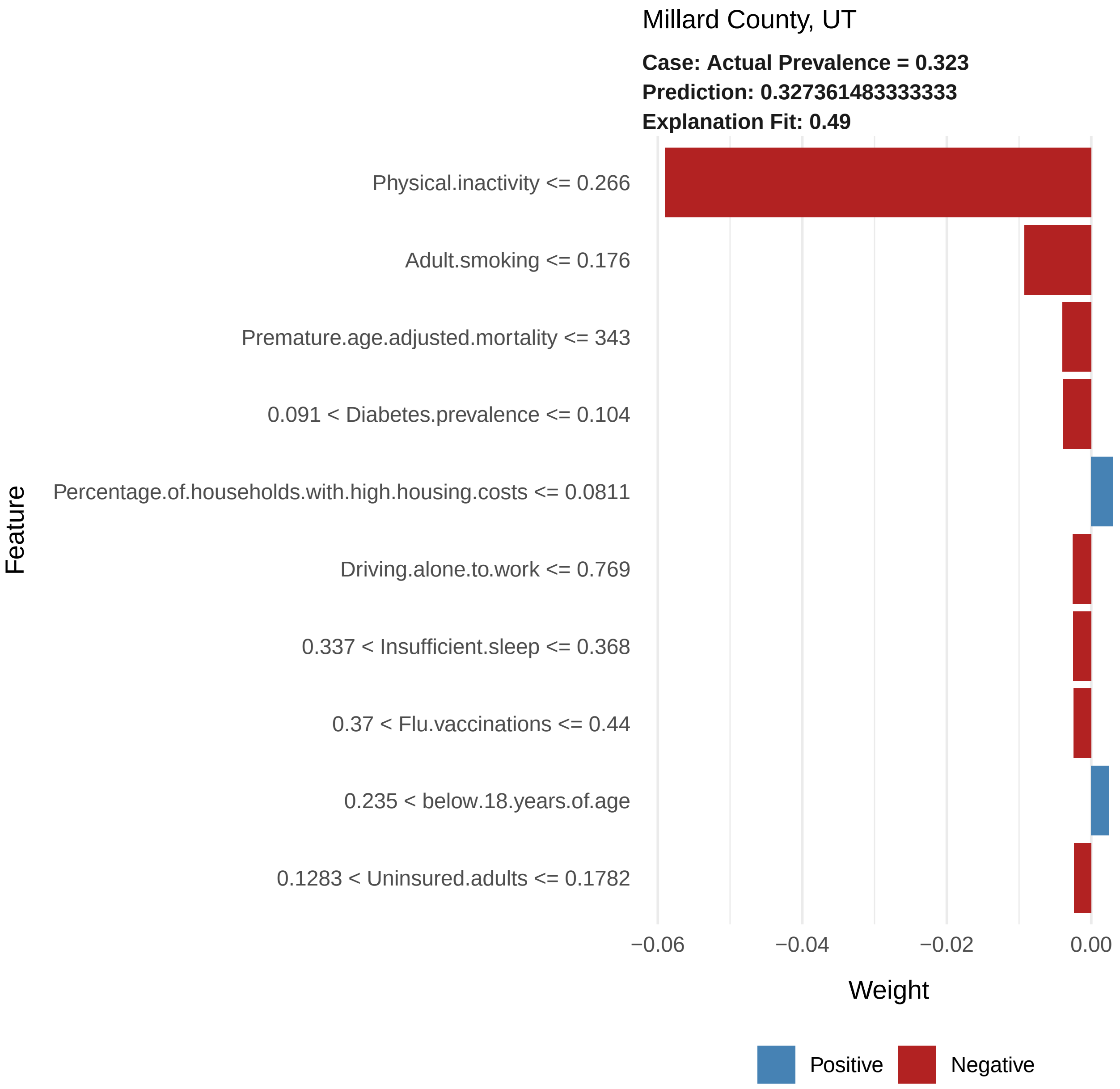
Millard County, UT
Case: Actual Prevalence = 0.323
Prediction: 0.327361483333333
Explanation Fit: 0.49
Physical.inactivity <= 0.266
Adult.smoking <= 0.176
Premature.age.adjusted.mortality <= 343
0.091 < Diabetes.prevalence <= 0.104
Percentage.of.households.with.high.housing.costs <= 0.0811
Driving.alone.to.work <= 0.769
0.337 < Insufficient.sleep <= 0.368
0.37 < Flu.vaccinations <= 0.44
0.235 < below.18.years.of.age
0.1283 < Uninsured.adults <= 0.1782
Feature
−0.06
−0.04
−0.02
0.00
Weight
Positive
Negative

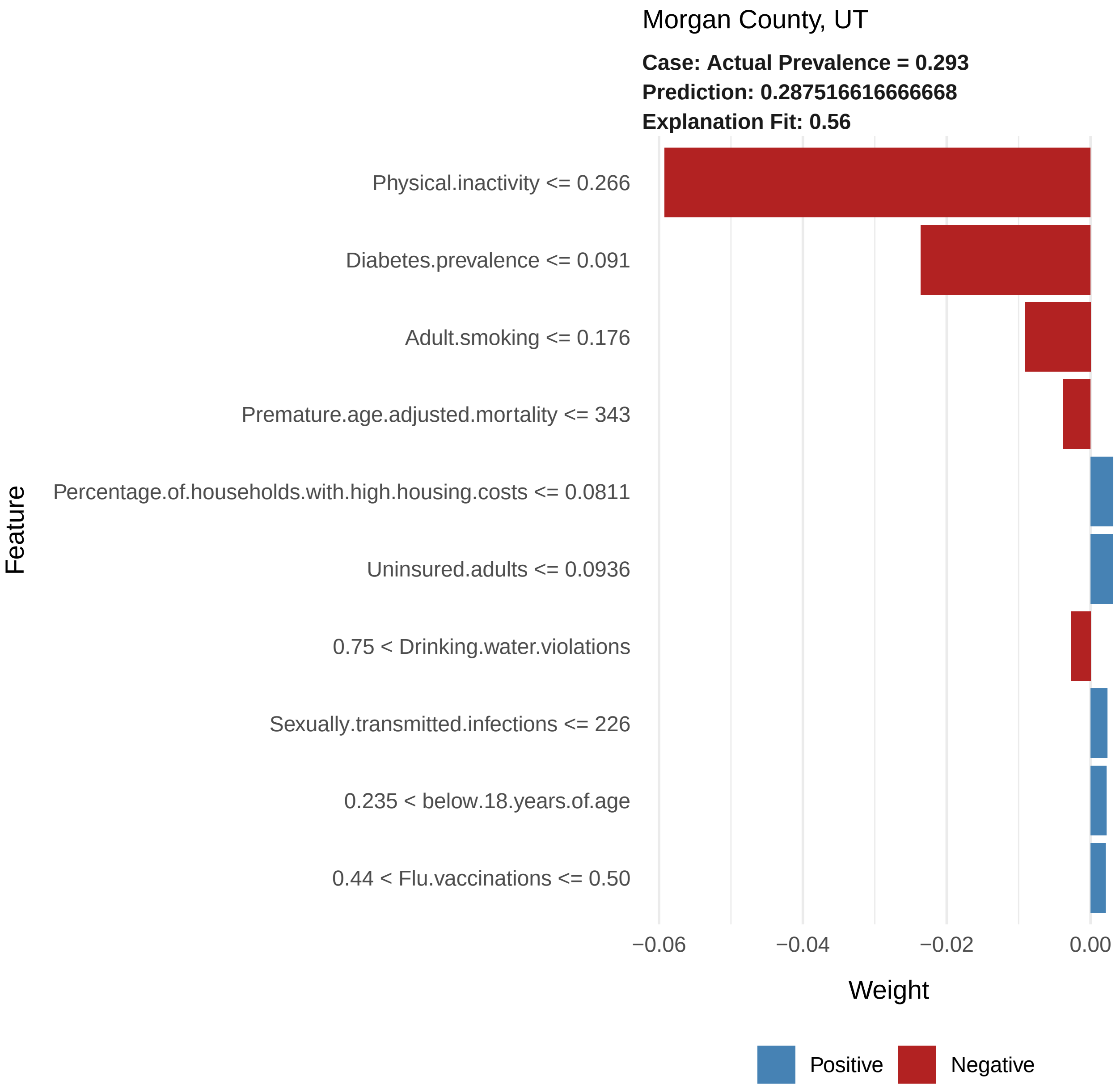

Morgan County, UT
Case: Actual Prevalence = 0.293
Prediction: 0.287516616666668
Explanation Fit: 0.56
Physical.inactivity <= 0.266
Diabetes.prevalence <= 0.091
Adult.smoking <= 0.176
Premature.age.adjusted.mortality <= 343
Percentage.of.households.with.high.housing.costs <= 0.0811
Uninsured.adults <= 0.0936
0.75 < Drinking.water.violations
Sexually.transmitted.infections <= 226
0.235 < below.18.years.of.age
0.44 < Flu.vaccinations <= 0.50
Feature
−0.06
−0.04
−0.02
0.00
Weight
Positive
Negative

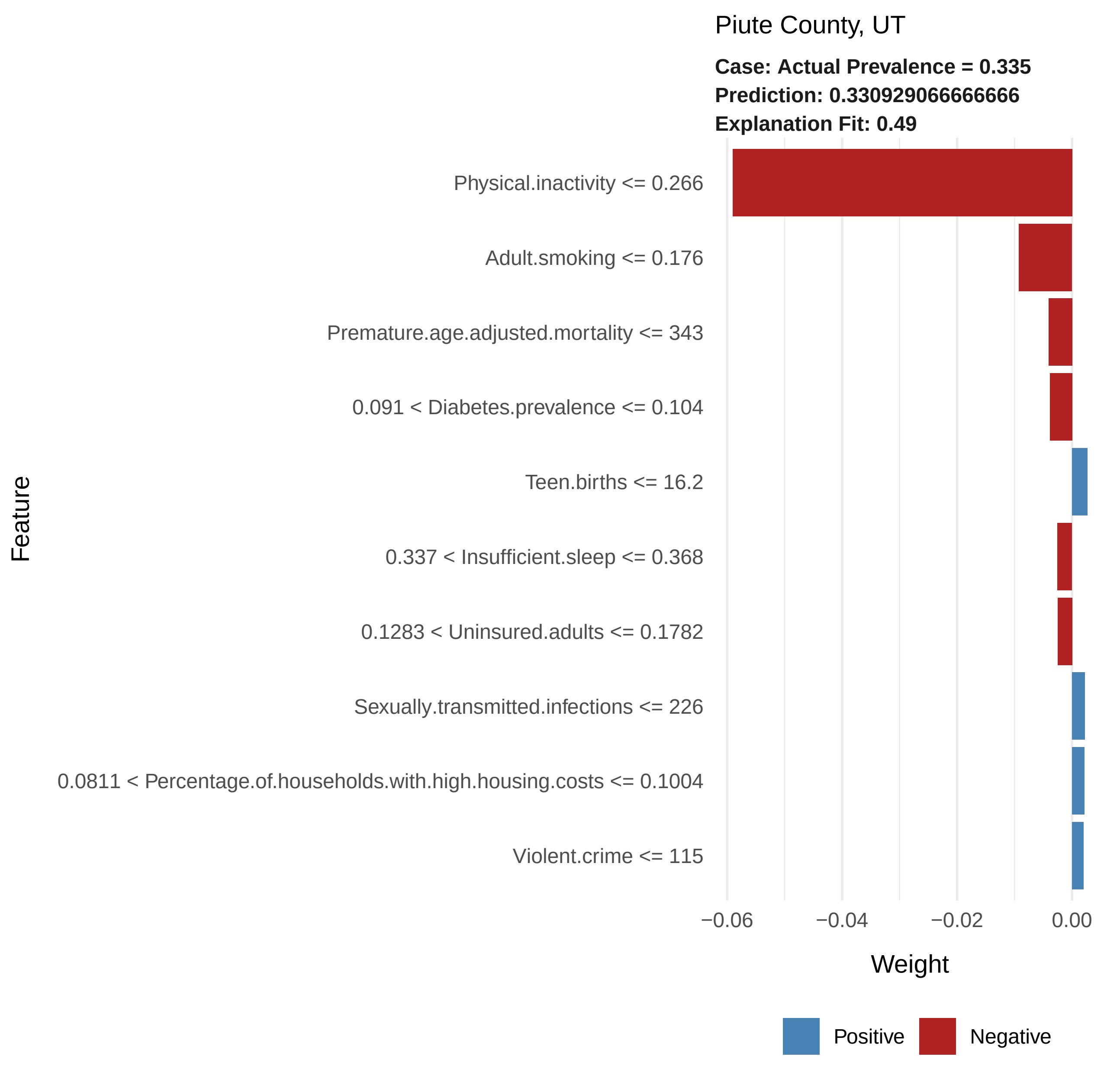

Piute County, UT
Case: Actual Prevalence = 0.335
Prediction: 0.330929066666666
Explanation Fit: 0.49
Physical.inactivity <= 0.266
Adult.smoking <= 0.176
Premature.age.adjusted.mortality <= 343
0.091 < Diabetes.prevalence <= 0.104
Teen.births <= 16.2
0.337 < Insufficient.sleep <= 0.368
0.1283 < Uninsured.adults <= 0.1782
Sexually.transmitted.infections <= 226
0.0811 < Percentage.of.households.with.high.housing.costs <= 0.1004
Violent.crime <= 115
Feature
−0.06
−0.04
−0.02
0.00
Weight
Positive
Negative

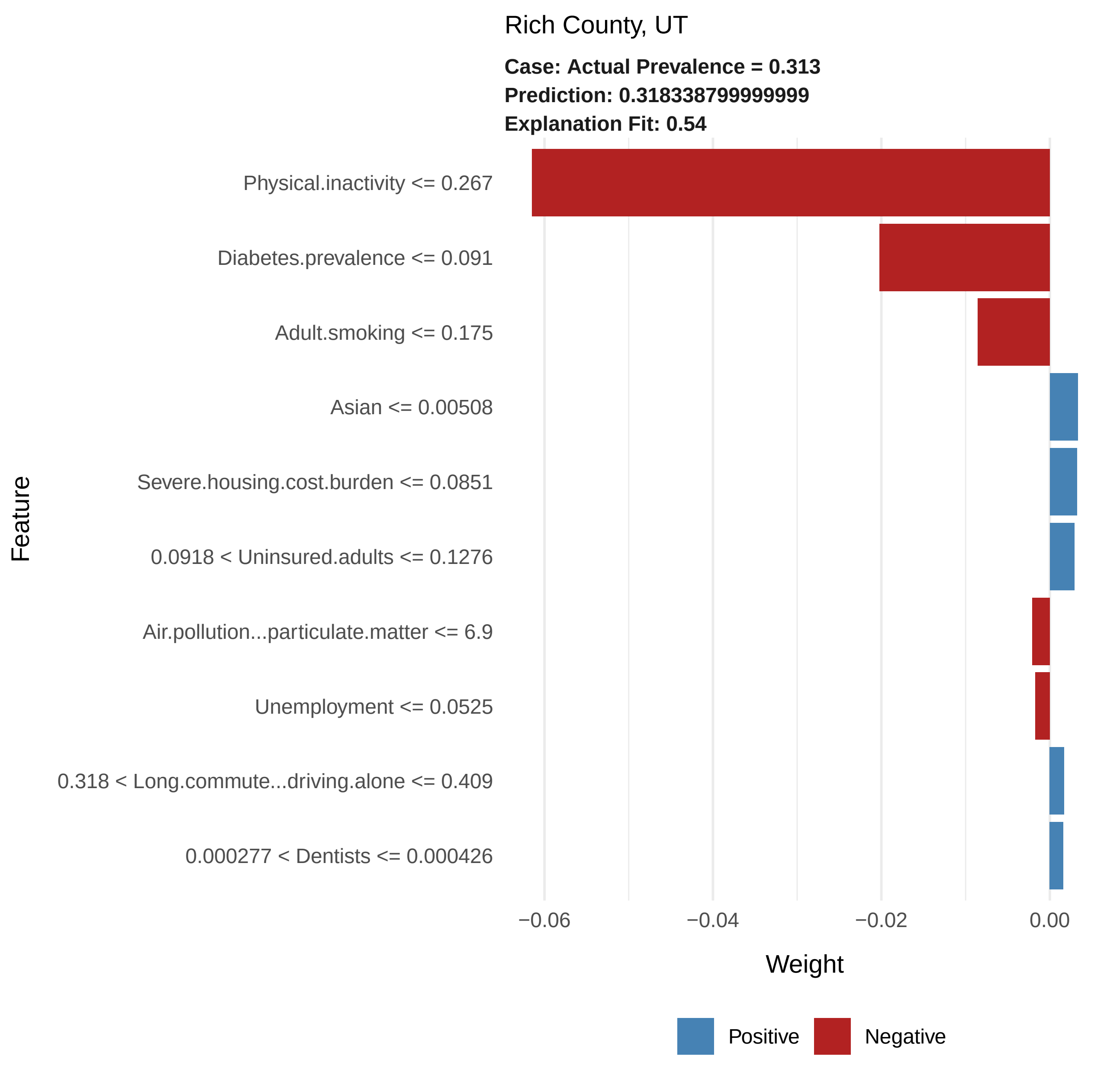

Rich County, UT
Case: Actual Prevalence = 0.313
Prediction: 0.318338799999999
Explanation Fit: 0.54
Physical.inactivity <= 0.267
Diabetes.prevalence <= 0.091
Adult.smoking <= 0.175
Asian <= 0.00508
Severe.housing.cost.burden <= 0.0851
0.0918 < Uninsured.adults <= 0.1276
Air.pollution...particulate.matter <= 6.9
Unemployment <= 0.0525
0.318 < Long.commute...driving.alone <= 0.409
0.000277 < Dentists <= 0.000426
Feature
−0.06
−0.04
−0.02
0.00
Weight
Positive
Negative

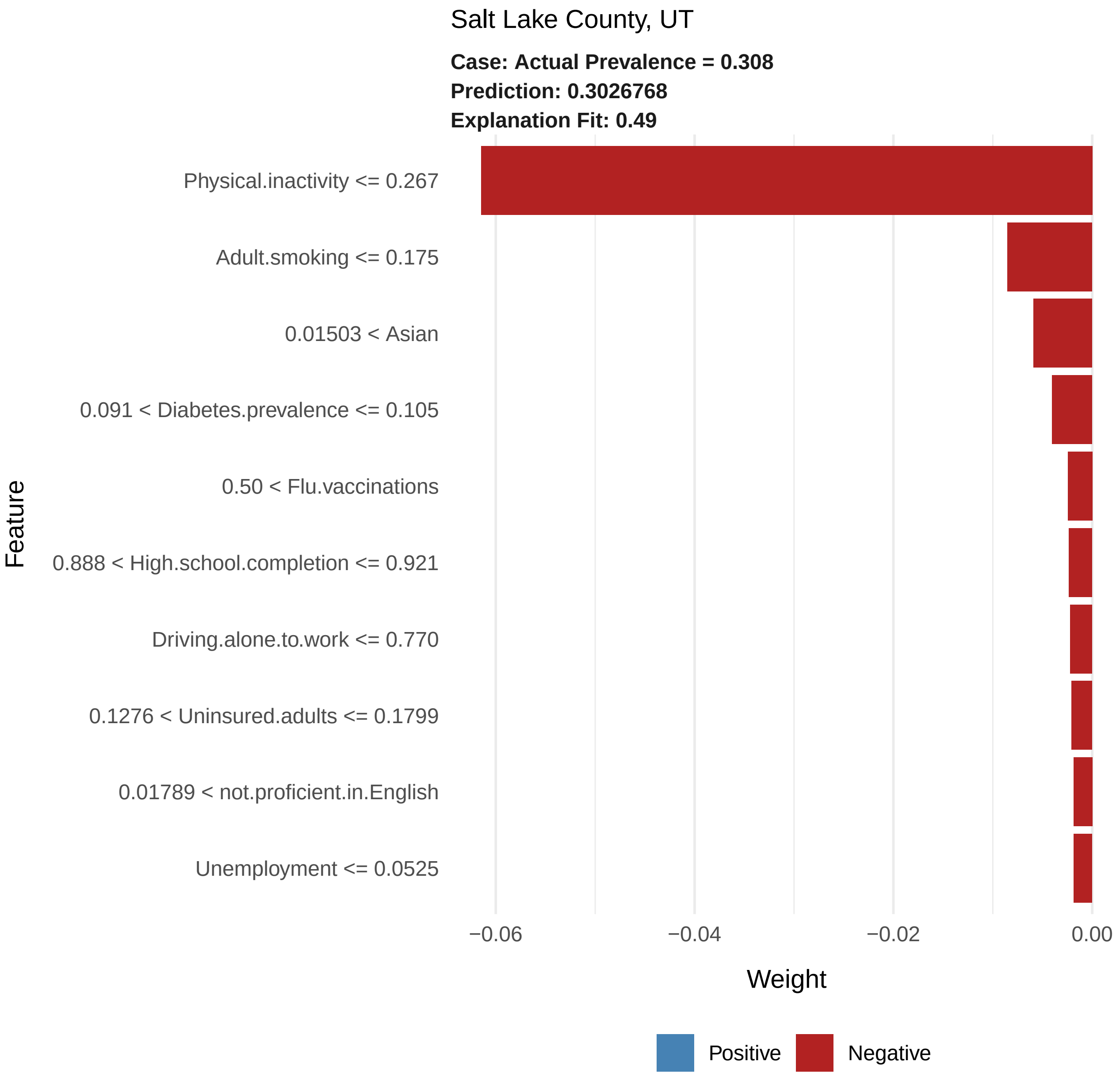

Salt Lake County, UT
Case: Actual Prevalence = 0.308
Prediction: 0.3026768
Explanation Fit: 0.49
Physical.inactivity <= 0.267
Adult.smoking <= 0.175
0.01503 < Asian
0.091 < Diabetes.prevalence <= 0.105
0.50 < Flu.vaccinations
0.888 < High.school.completion <= 0.921
Driving.alone.to.work <= 0.770
0.1276 < Uninsured.adults <= 0.1799
0.01789 < not.proficient.in.English
Unemployment <= 0.0525
Feature
−0.06
−0.04
−0.02
0.00
Weight
Positive
Negative

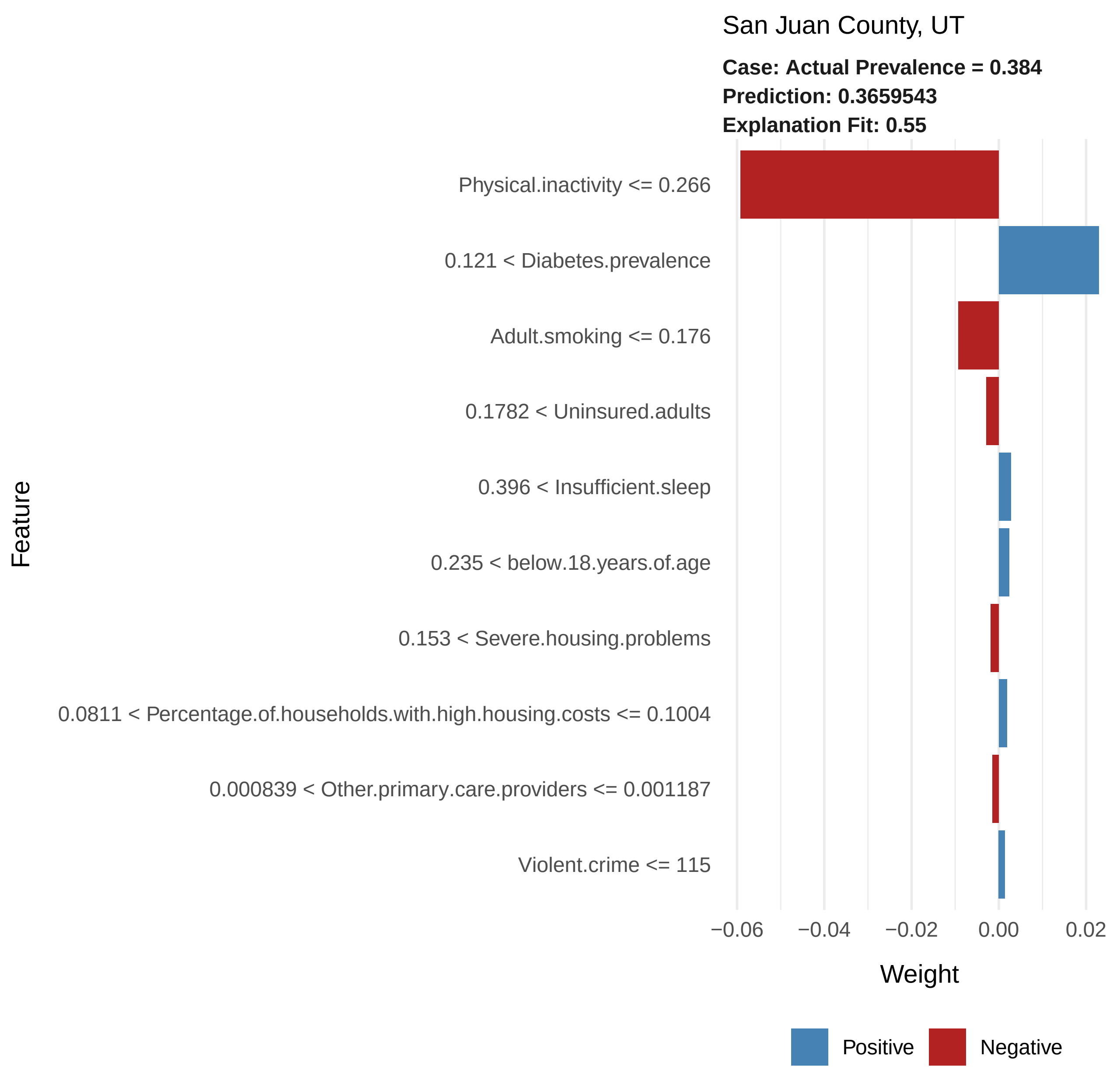
San Juan County, UT
Case: Actual Prevalence = 0.384
Prediction: 0.3659543
Explanation Fit: 0.55
Feature
Physical.inactivity <= 0.266
0.121 < Diabetes.prevalence
Adult.smoking <= 0.176
0.1782 < Uninsured.adults
0.396 < Insufficient.sleep
0.235 < below.18.years.of.age
0.153 < Severe.housing.problems
0.0811 < Percentage.of.households.with.high.housing.costs <= 0.1004
0.000839 < Other.primary.care.providers <= 0.001187
Violent.crime <= 115
−0.06
−0.04
−0.02
0.00
0.02
Weight
Positive
Negative

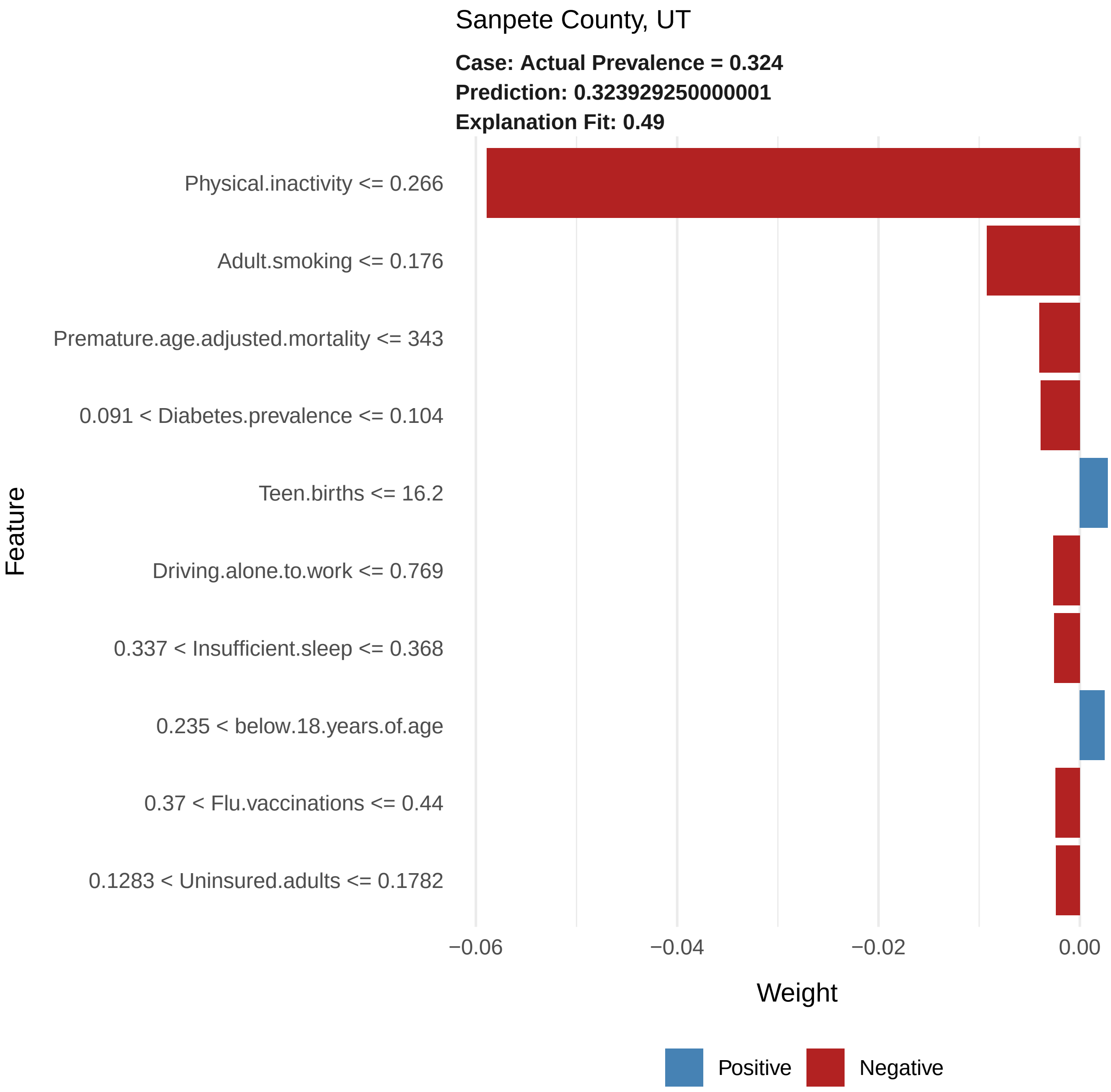

Sanpete County, UT
Case: Actual Prevalence = 0.324
Prediction: 0.323929250000001
Explanation Fit: 0.49
Physical.inactivity <= 0.266
Adult.smoking <= 0.176
Premature.age.adjusted.mortality <= 343
0.091 < Diabetes.prevalence <= 0.104
Teen.births <= 16.2
Driving.alone.to.work <= 0.769
0.337 < Insufficient.sleep <= 0.368
0.235 < below.18.years.of.age
0.37 < Flu.vaccinations <= 0.44
0.1283 < Uninsured.adults <= 0.1782
Feature
−0.06
−0.04
−0.02
0.00
Weight
Positive
Negative

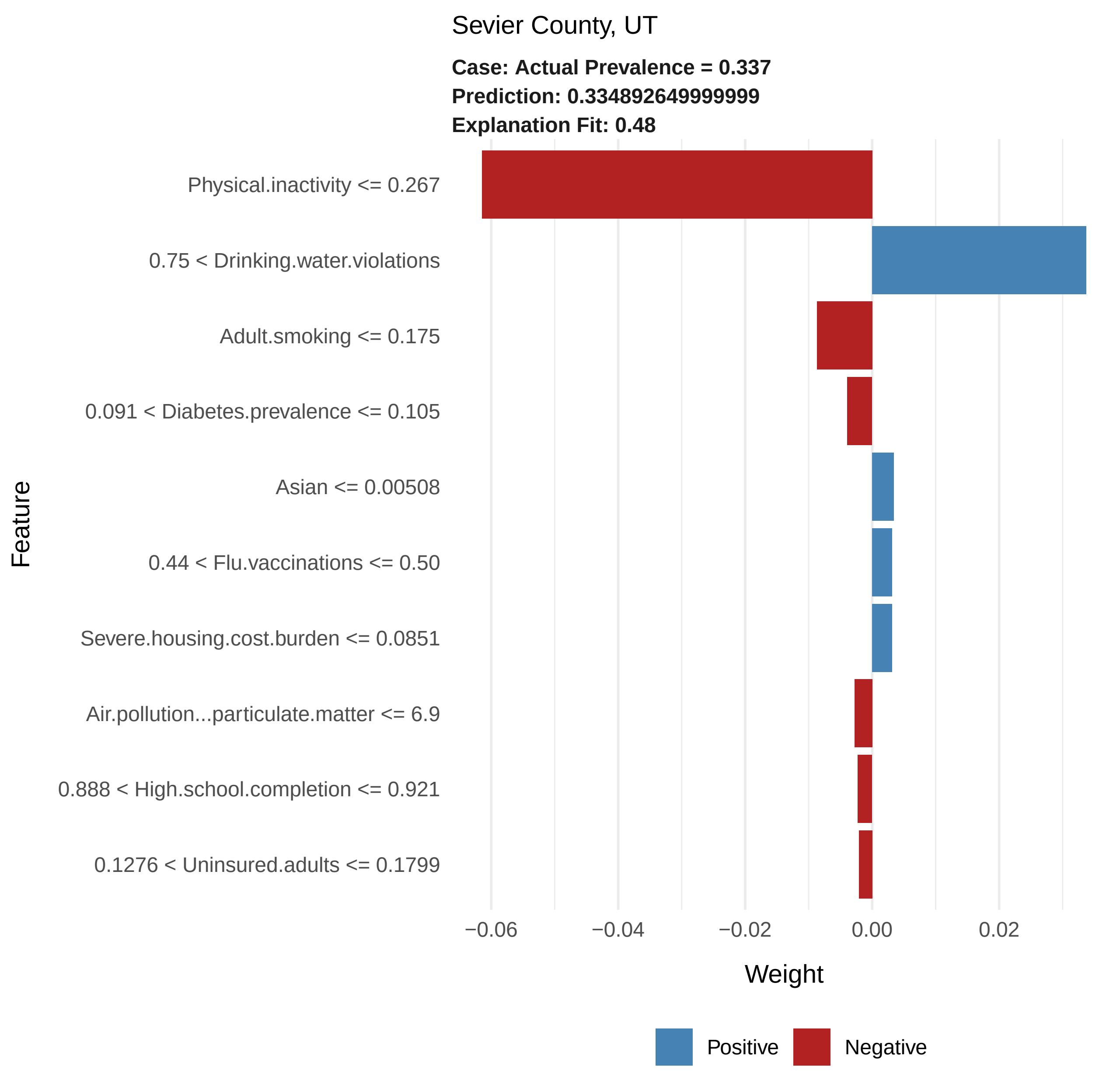
Sevier County, UT
Case: Actual Prevalence = 0.337
Prediction: 0.334892649999999
Explanation Fit: 0.48
Physical.inactivity <= 0.267
0.75 < Drinking.water.violations
Adult.smoking <= 0.175
0.091 < Diabetes.prevalence <= 0.105
Asian <= 0.00508
0.44 < Flu.vaccinations <= 0.50
Severe.housing.cost.burden <= 0.0851
Air.pollution...particulate.matter <= 6.9
0.888 < High.school.completion <= 0.921
0.1276 < Uninsured.adults <= 0.1799
Feature
−0.06
−0.04
−0.02
0.00
0.02
Weight
Positive
Negative

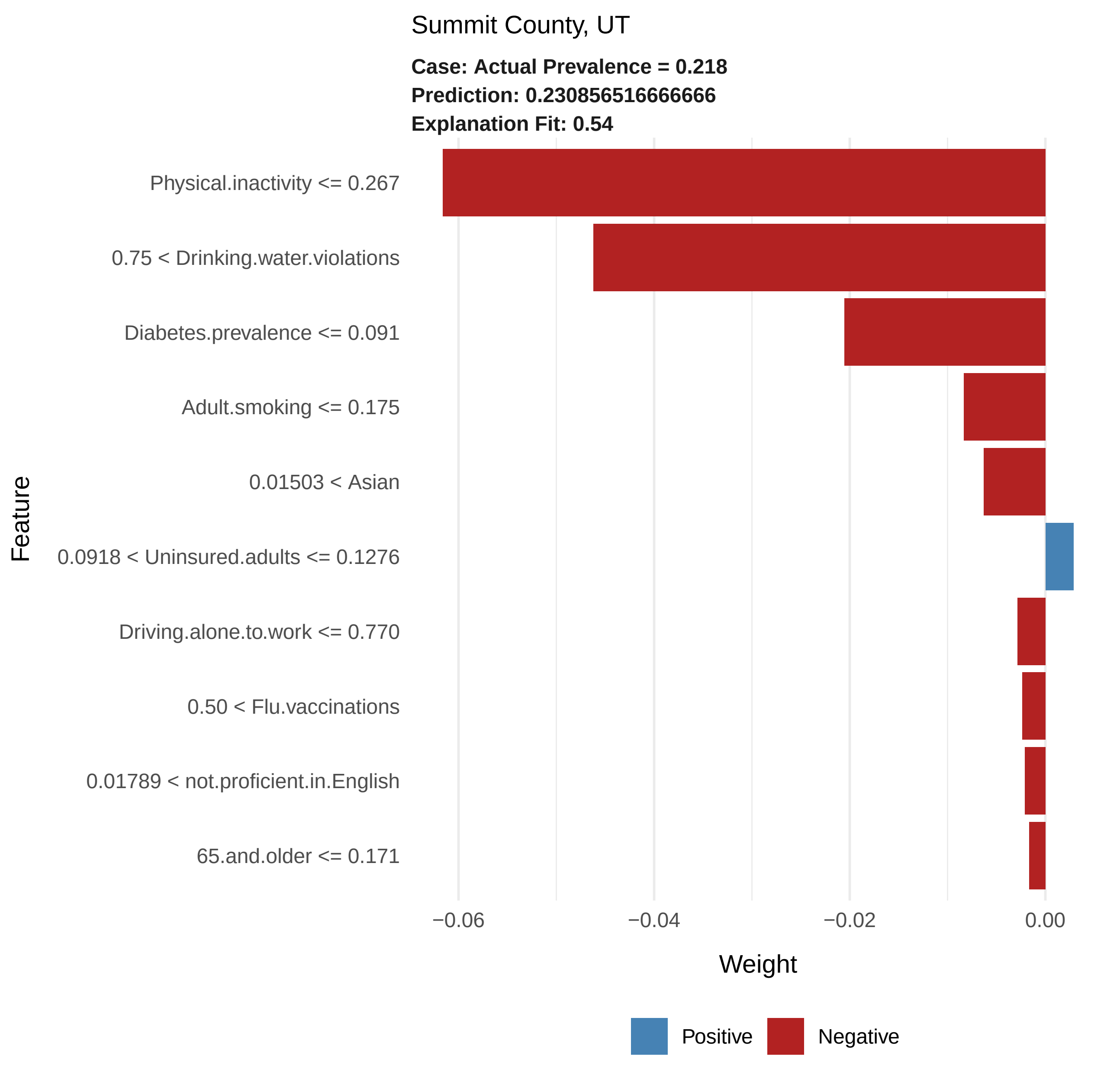
Summit County, UT
Case: Actual Prevalence = 0.218
Prediction: 0.230856516666666
Explanation Fit: 0.54
Physical.inactivity <= 0.267
0.75 < Drinking.water.violations
Diabetes.prevalence <= 0.091
Adult.smoking <= 0.175
0.01503 < Asian
0.0918 < Uninsured.adults <= 0.1276
Driving.alone.to.work <= 0.770
0.50 < Flu.vaccinations
0.01789 < not.proficient.in.English
65.and.older <= 0.171
Feature
−0.06
−0.04
−0.02
0.00
Weight
Positive
Negative

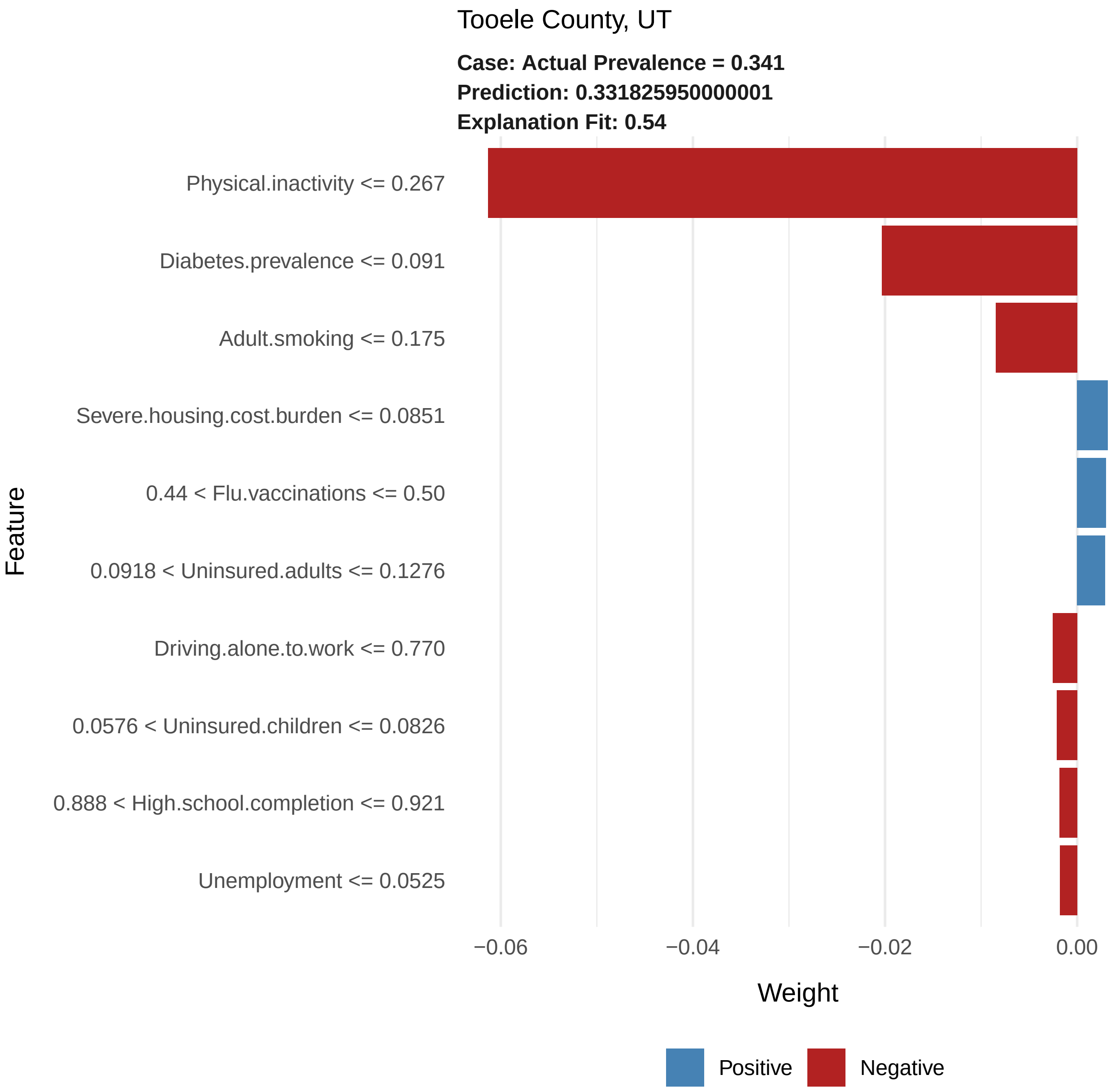

Tooele County, UT
Case: Actual Prevalence = 0.341
Prediction: 0.331825950000001
Explanation Fit: 0.54
Physical.inactivity <= 0.267
Diabetes.prevalence <= 0.091
Adult.smoking <= 0.175
Severe.housing.cost.burden <= 0.0851
0.44 < Flu.vaccinations <= 0.50
0.0918 < Uninsured.adults <= 0.1276
Driving.alone.to.work <= 0.770
0.0576 < Uninsured.children <= 0.0826
0.888 < High.school.completion <= 0.921
Unemployment <= 0.0525
Feature
−0.06
−0.04
−0.02
0.00
Weight
Positive
Negative

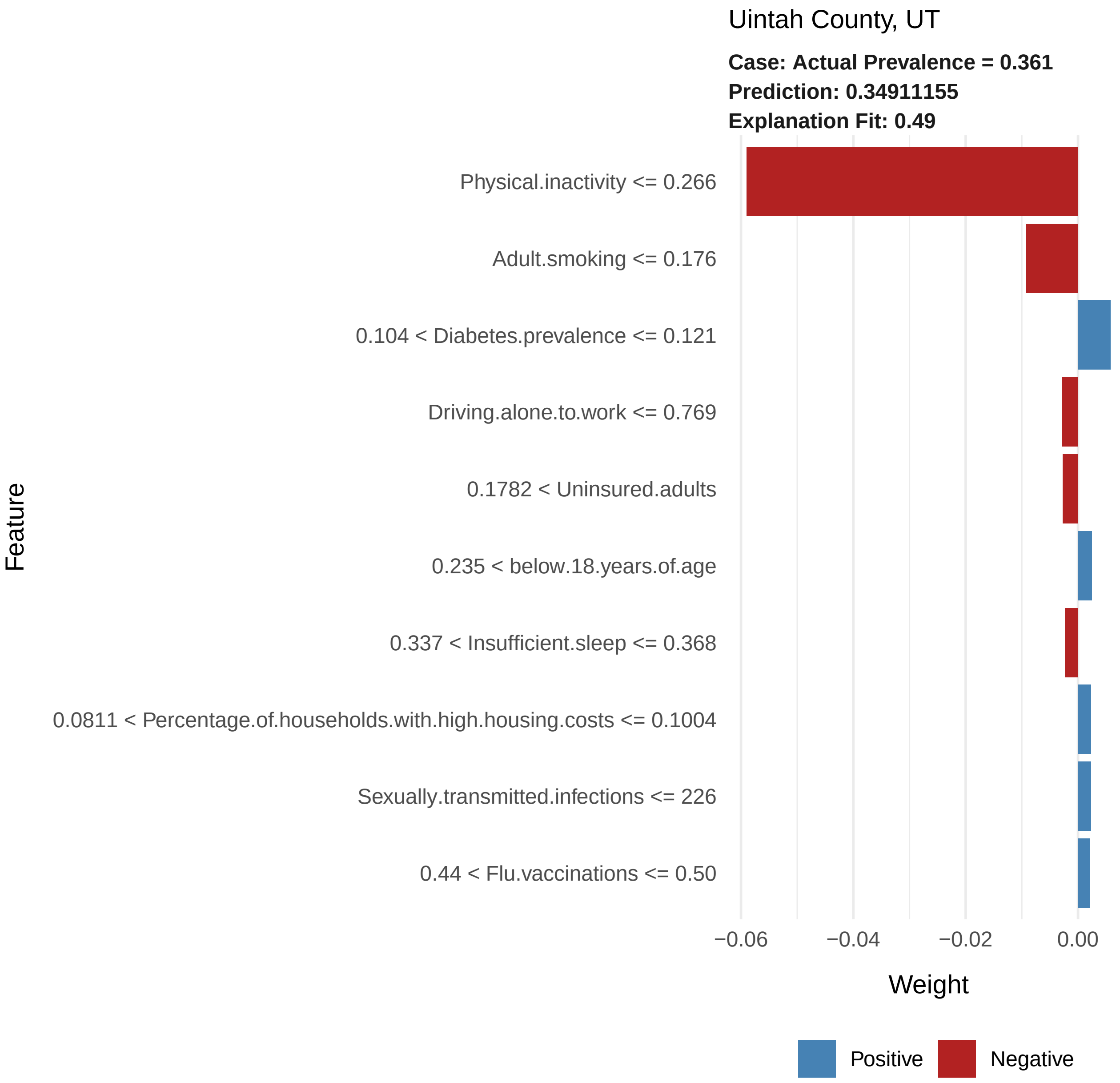

Uintah County, UT
Case: Actual Prevalence = 0.361
Prediction: 0.34911155
Explanation Fit: 0.49
Physical.inactivity <= 0.266
Adult.smoking <= 0.176
0.104 < Diabetes.prevalence <= 0.121
Driving.alone.to.work <= 0.769
0.1782 < Uninsured.adults
0.235 < below.18.years.of.age
0.337 < Insufficient.sleep <= 0.368
0.0811 < Percentage.of.households.with.high.housing.costs <= 0.1004
Sexually.transmitted.infections <= 226
0.44 < Flu.vaccinations <= 0.50
Feature
−0.06
−0.04
−0.02
0.00
Weight
Positive
Negative

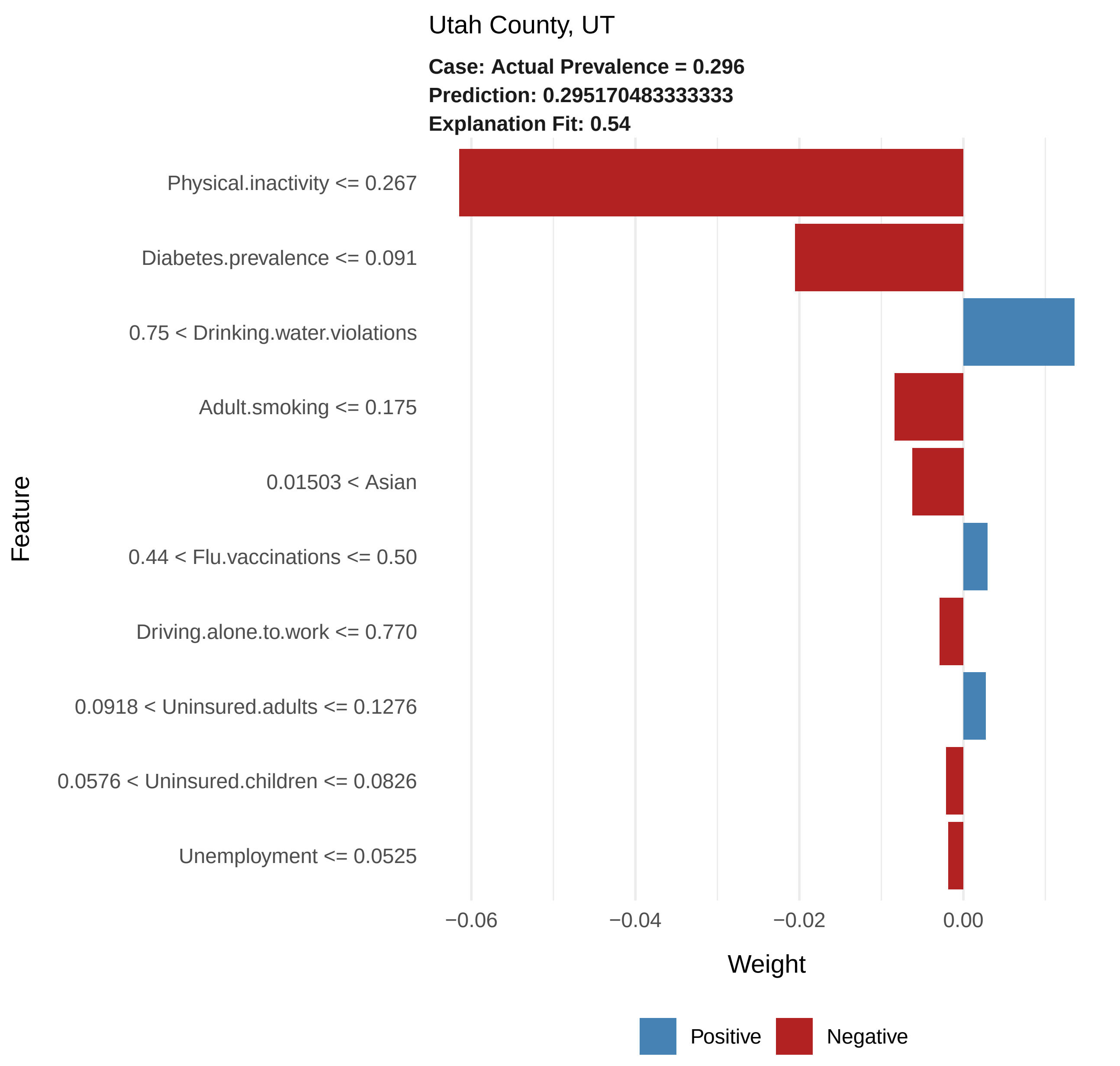

Utah County, UT
Case: Actual Prevalence = 0.296
Prediction: 0.295170483333333
Explanation Fit: 0.54
Physical.inactivity <= 0.267
Diabetes.prevalence <= 0.091
0.75 < Drinking.water.violations
Adult.smoking <= 0.175
0.01503 < Asian
0.44 < Flu.vaccinations <= 0.50
Driving.alone.to.work <= 0.770
0.0918 < Uninsured.adults <= 0.1276
0.0576 < Uninsured.children <= 0.0826
Unemployment <= 0.0525
Feature
−0.06
−0.04
−0.02
0.00
Weight
Positive
Negative

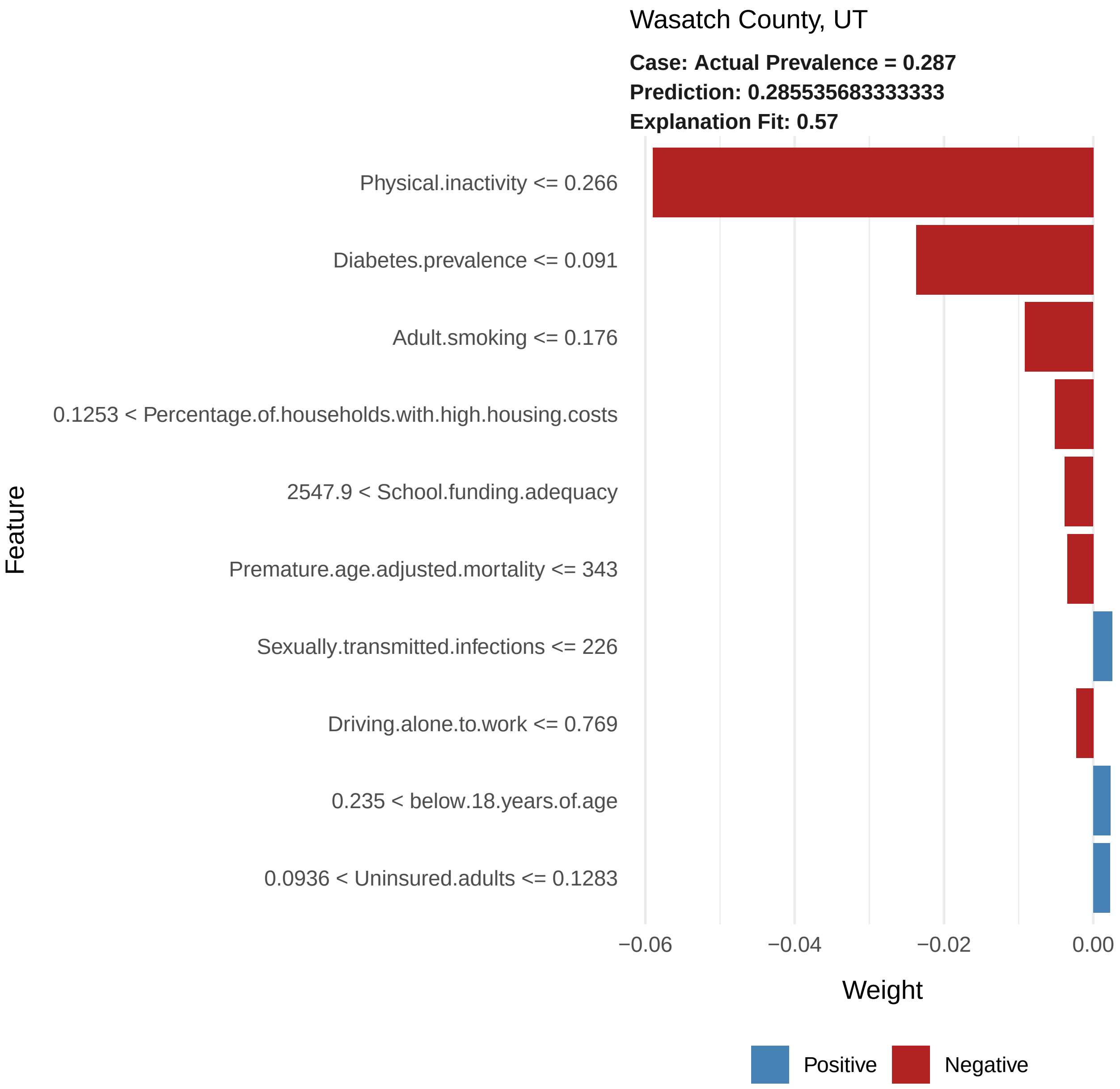
Wasatch County, UT
Case: Actual Prevalence = 0.287
Prediction: 0.285535683333333
Explanation Fit: 0.57
Physical.inactivity <= 0.266
Diabetes.prevalence <= 0.091
Adult.smoking <= 0.176
0.1253 < Percentage.of.households.with.high.housing.costs
2547.9 < School.funding.adequacy
Premature.age.adjusted.mortality <= 343
Sexually.transmitted.infections <= 226
Driving.alone.to.work <= 0.769
0.235 < below.18.years.of.age
0.0936 < Uninsured.adults <= 0.1283
Feature
−0.06
−0.04
−0.02
0.00
Weight
Positive
Negative

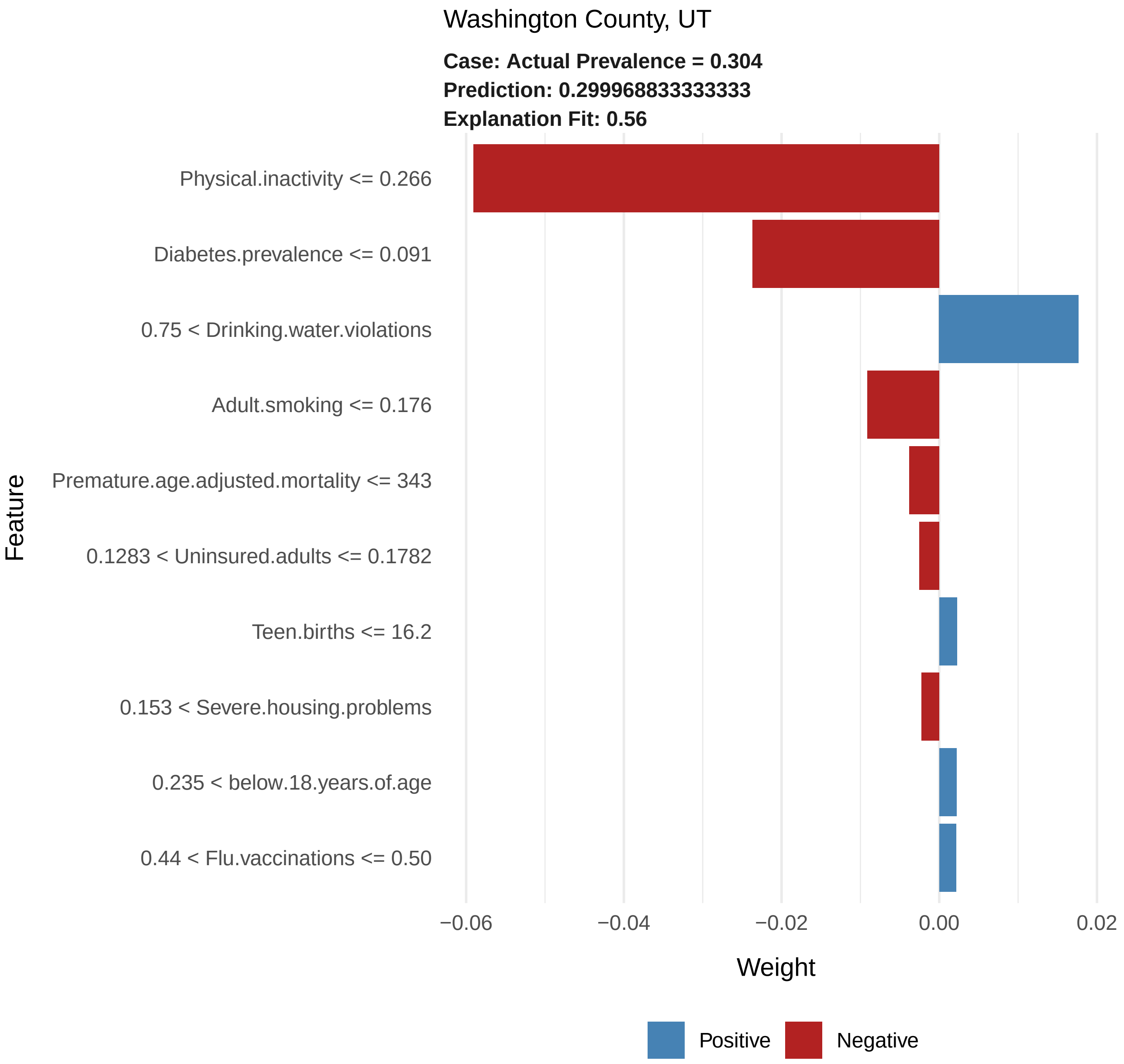
Washington County, UT
Case: Actual Prevalence = 0.304
Prediction: 0.299968833333333
Explanation Fit: 0.56
Physical.inactivity <= 0.266
Diabetes.prevalence <= 0.091
0.75 < Drinking.water.violations
Adult.smoking <= 0.176
Premature.age.adjusted.mortality <= 343
0.1283 < Uninsured.adults <= 0.1782
Teen.births <= 16.2
0.153 < Severe.housing.problems
0.235 < below.18.years.of.age
0.44 < Flu.vaccinations <= 0.50
Feature
−0.06
−0.04
−0.02
0.00
0.02
Weight
Positive
Negative

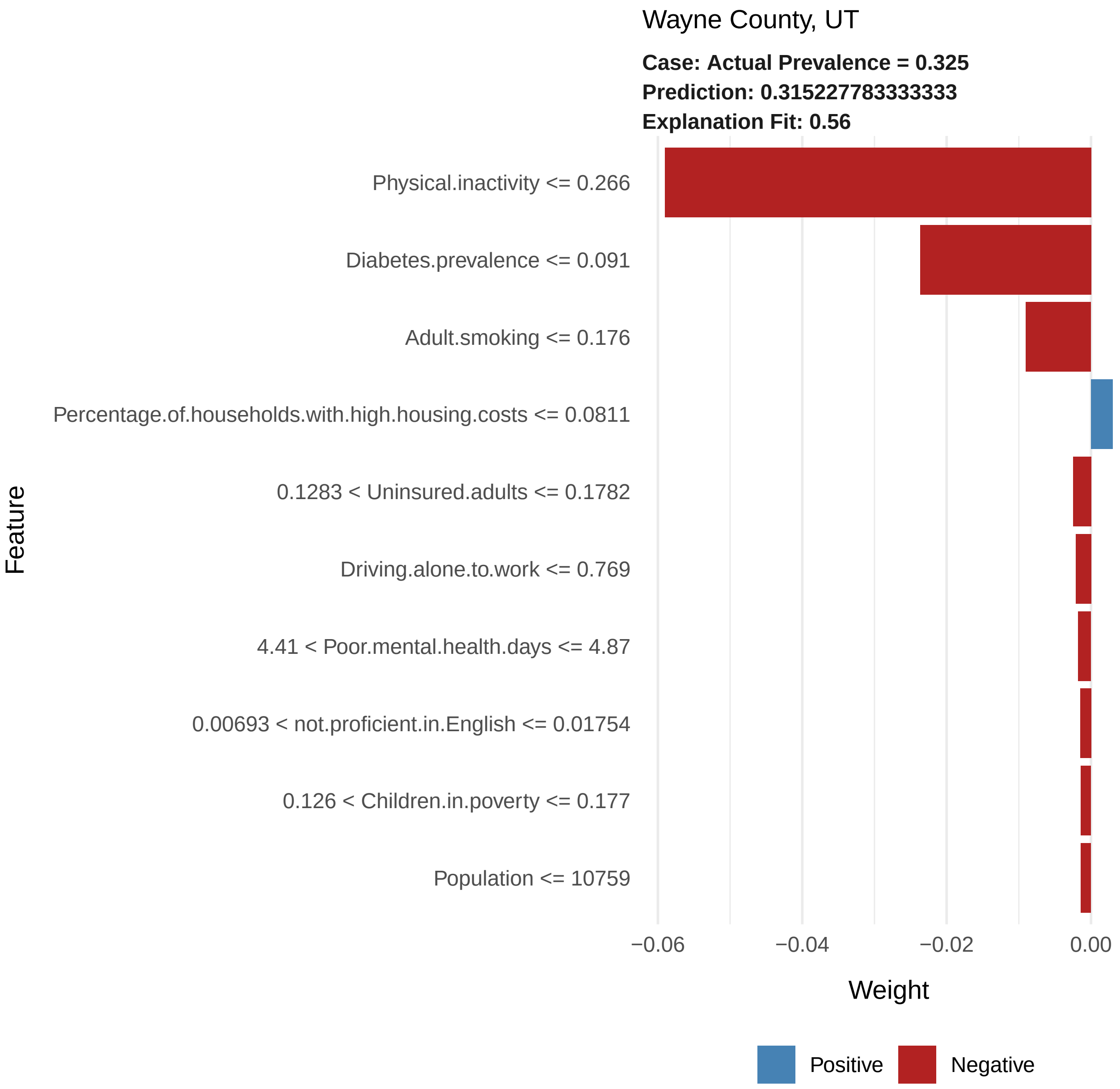
Wayne County, UT
Case: Actual Prevalence = 0.325
Prediction: 0.315227783333333
Explanation Fit: 0.56
Physical.inactivity <= 0.266
Diabetes.prevalence <= 0.091
Adult.smoking <= 0.176
Percentage.of.households.with.high.housing.costs <= 0.0811
0.1283 < Uninsured.adults <= 0.1782
Driving.alone.to.work <= 0.769
4.41 < Poor.mental.health.days <= 4.87
0.00693 < not.proficient.in.English <= 0.01754
0.126 < Children.in.poverty <= 0.177
Population <= 10759
Feature
−0.06
−0.04
−0.02
0.00
Weight
Positive
Negative

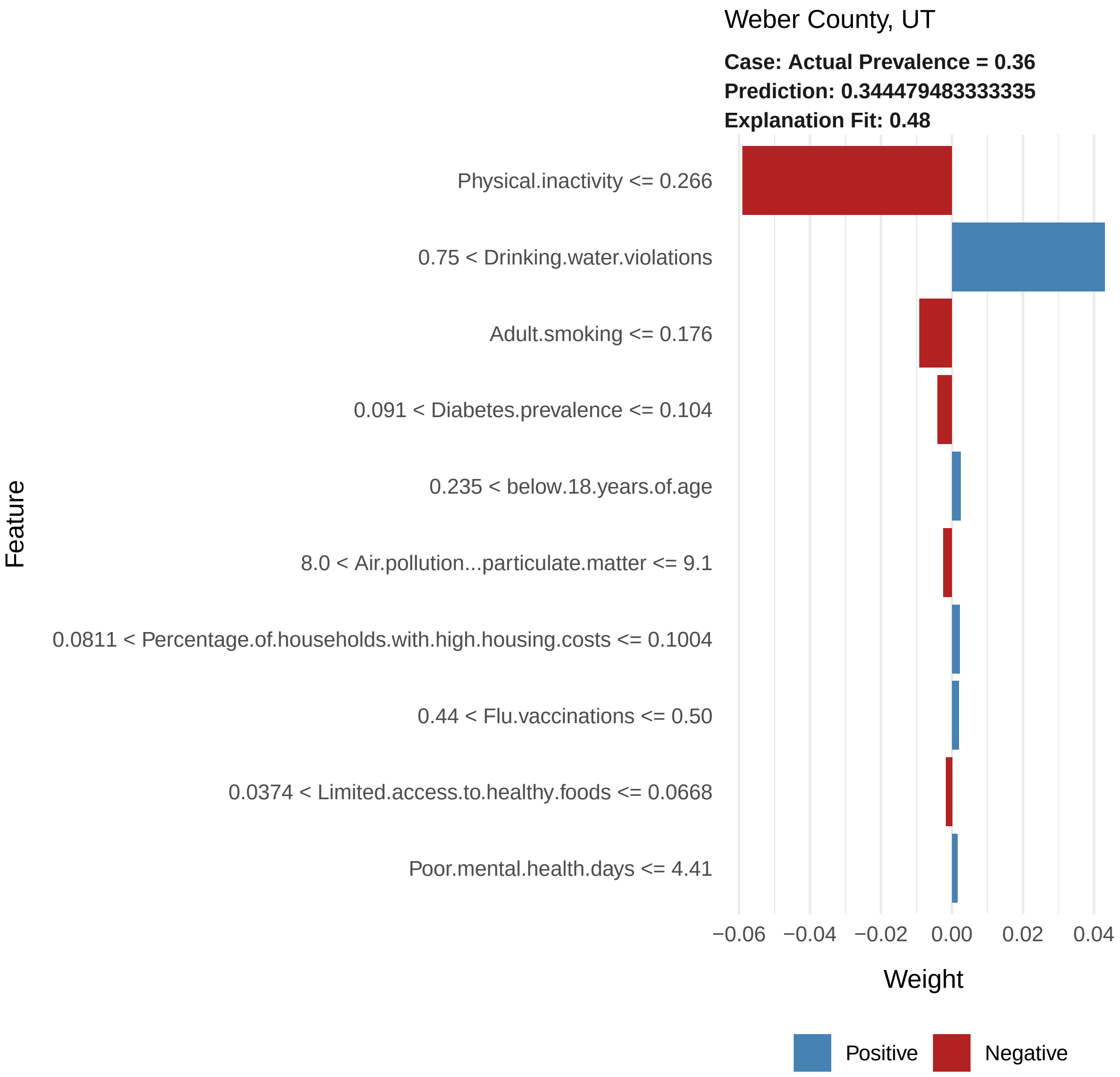

Weber County, UT
Case: Actual Prevalence = 0.36
Prediction: 0.344479483333335
Explanation Fit: 0.48
Physical.inactivity <= 0.266
0.75 < Drinking.water.violations
Adult.smoking <= 0.176
0.091 < Diabetes.prevalence <= 0.104
0.235 < below.18.years.of.age
8.0 < Air.pollution...particulate.matter <= 9.1
0.0811 < Percentage.of.households.with.high.housing.costs <= 0.1004
0.44 < Flu.vaccinations <= 0.50
0.0374 < Limited.access.to.healthy.foods <= 0.0668
Poor.mental.health.days <= 4.41
Feature
−0.06
−0.04
−0.02
0.00
0.02
0.04
Weight
Positive
Negative

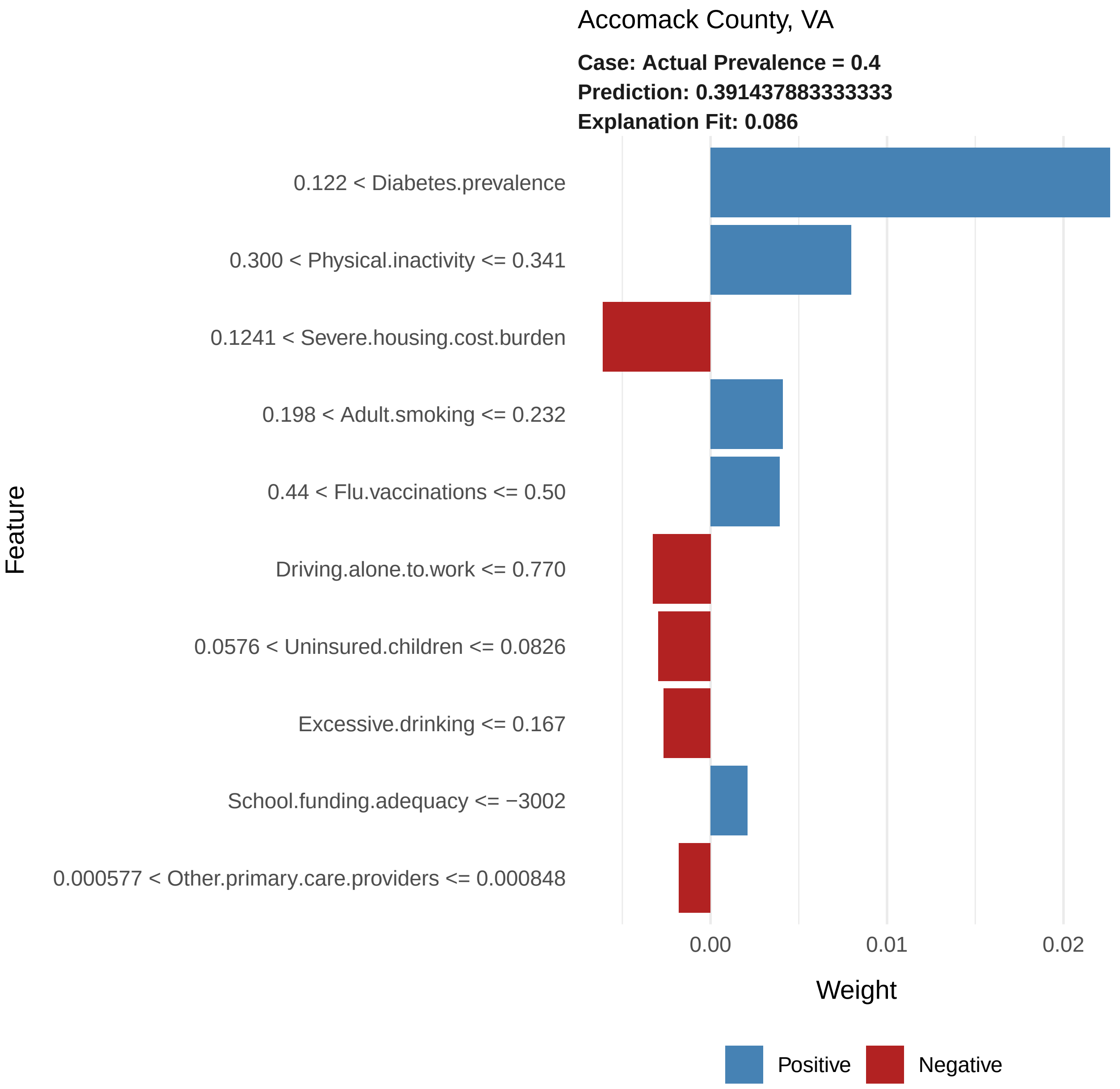

Accomack County, VA
Case: Actual Prevalence = 0.4
Prediction: 0.391437883333333
Explanation Fit: 0.086
0.122 < Diabetes.prevalence
0.300 < Physical.inactivity <= 0.341
0.1241 < Severe.housing.cost.burden
0.198 < Adult.smoking <= 0.232
0.44 < Flu.vaccinations <= 0.50
Driving.alone.to.work <= 0.770
0.0576 < Uninsured.children <= 0.0826
Excessive.drinking <= 0.167
School.funding.adequacy <= −3002
0.000577 < Other.primary.care.providers <= 0.000848
Feature
0.00
0.01
0.02
Weight
Positive
Negative

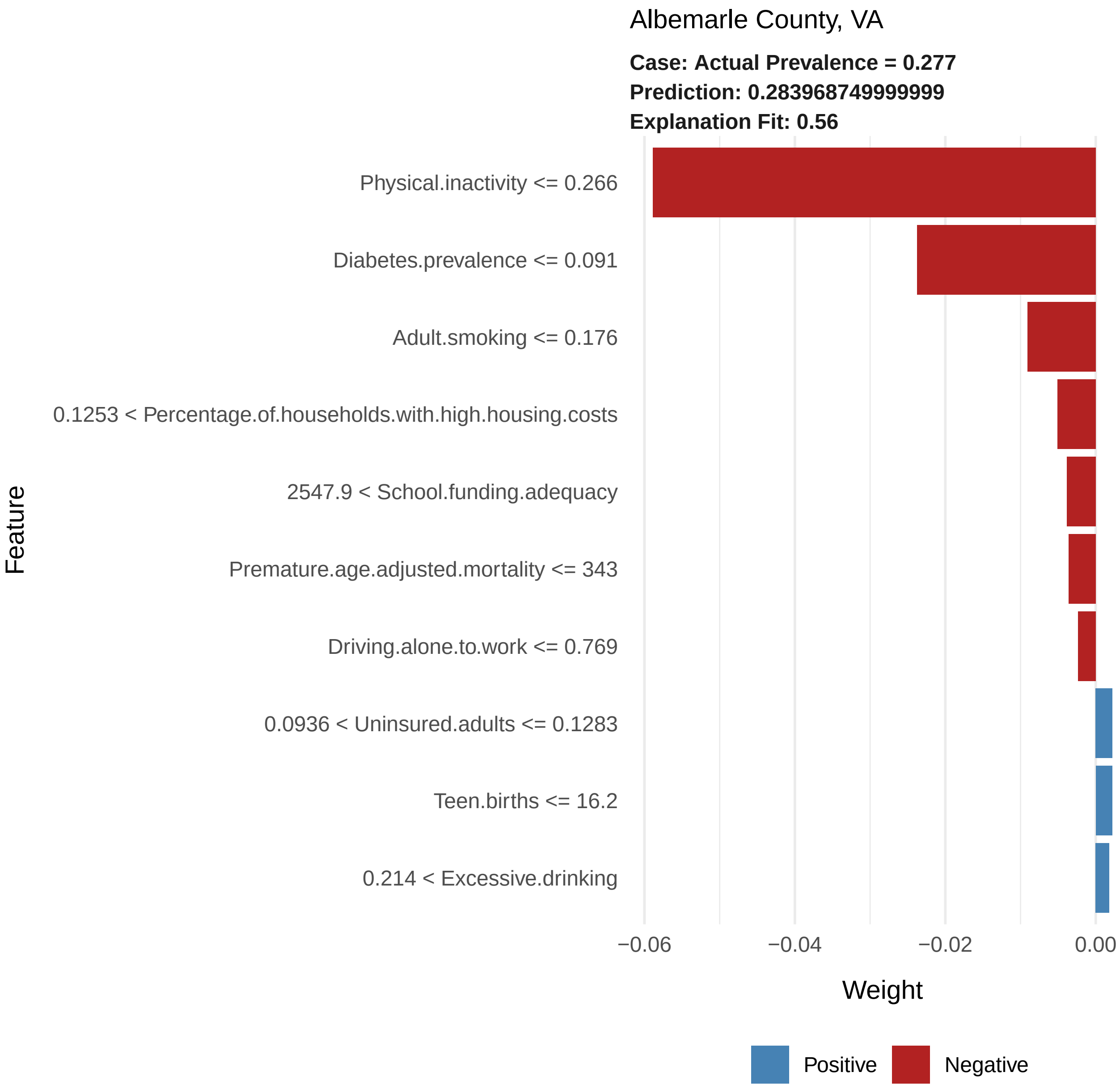

Albemarle County, VA
Case: Actual Prevalence = 0.277
Prediction: 0.283968749999999
Explanation Fit: 0.56
Physical.inactivity <= 0.266
Diabetes.prevalence <= 0.091
Adult.smoking <= 0.176
0.1253 < Percentage.of.households.with.high.housing.costs
2547.9 < School.funding.adequacy
Premature.age.adjusted.mortality <= 343
Driving.alone.to.work <= 0.769
0.0936 < Uninsured.adults <= 0.1283
Teen.births <= 16.2
0.214 < Excessive.drinking
Feature
−0.06
−0.04
−0.02
0.00
Weight
Positive
Negative

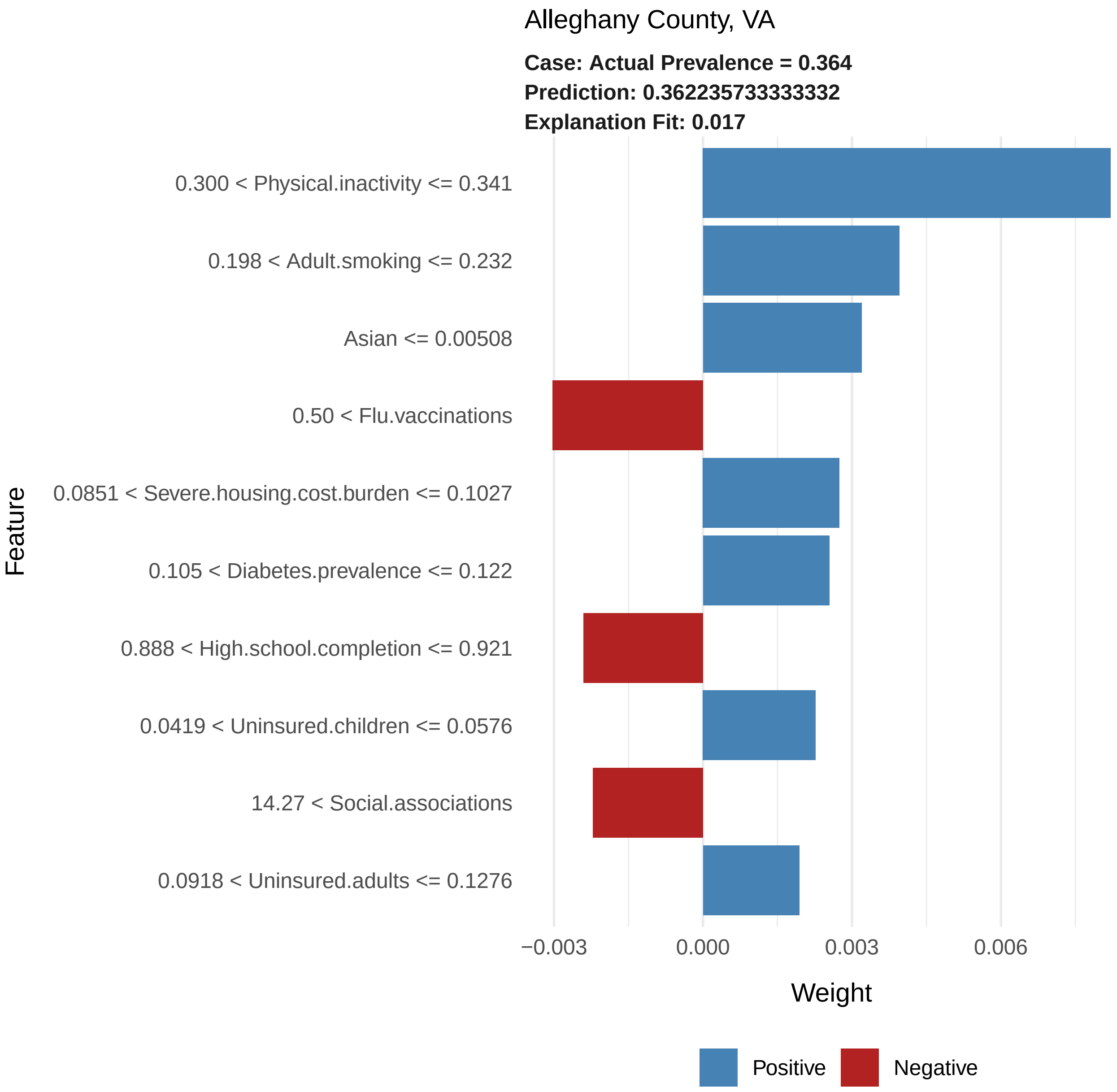
Alleghany County, VA
Case: Actual Prevalence = 0.364
Prediction: 0.362235733333332
Explanation Fit: 0.017
0.300 < Physical.inactivity <= 0.341
0.198 < Adult.smoking <= 0.232
Asian <= 0.00508
0.50 < Flu.vaccinations
0.0851 < Severe.housing.cost.burden <= 0.1027
0.105 < Diabetes.prevalence <= 0.122
0.888 < High.school.completion <= 0.921
0.0419 < Uninsured.children <= 0.0576
14.27 < Social.associations
0.0918 < Uninsured.adults <= 0.1276
Feature
−0.003
0.000
0.003
0.006
Weight
Positive
Negative

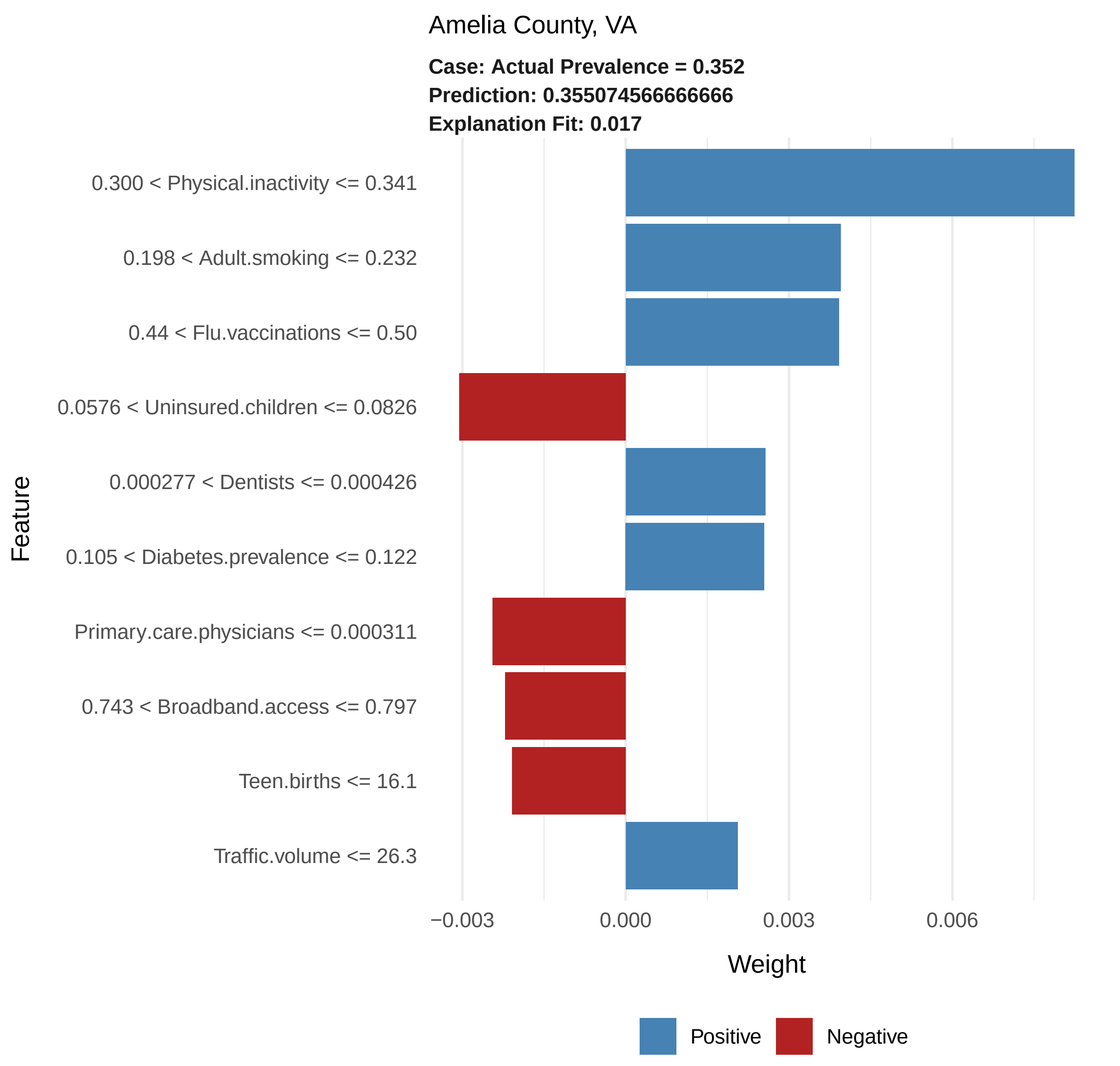
Amelia County, VA
Case: Actual Prevalence = 0.352
Prediction: 0.355074566666666
Explanation Fit: 0.017
0.300 < Physical.inactivity <= 0.341
0.198 < Adult.smoking <= 0.232
0.44 < Flu.vaccinations <= 0.50
0.0576 < Uninsured.children <= 0.0826
0.000277 < Dentists <= 0.000426
0.105 < Diabetes.prevalence <= 0.122
Primary.care.physicians <= 0.000311
0.743 < Broadband.access <= 0.797
Teen.births <= 16.1
Traffic.volume <= 26.3
Feature
−0.003
0.000
0.003
0.006
Weight
Positive
Negative

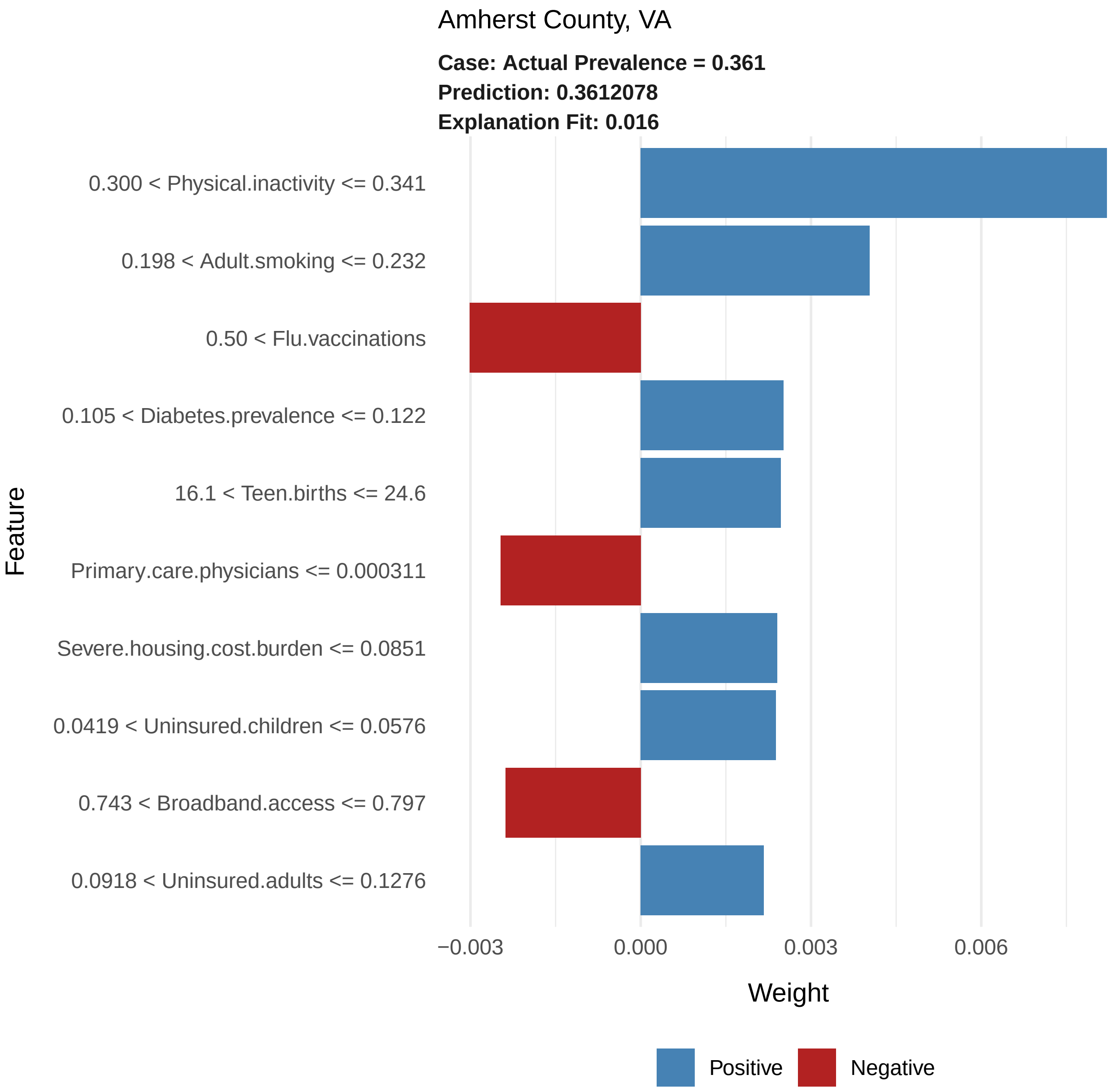
Amherst County, VA
Case: Actual Prevalence = 0.361
Prediction: 0.3612078
Explanation Fit: 0.016
0.300 < Physical.inactivity <= 0.341
0.198 < Adult.smoking <= 0.232
0.50 < Flu.vaccinations
0.105 < Diabetes.prevalence <= 0.122
16.1 < Teen.births <= 24.6
Primary.care.physicians <= 0.000311
Severe.housing.cost.burden <= 0.0851
0.0419 < Uninsured.children <= 0.0576
0.743 < Broadband.access <= 0.797
0.0918 < Uninsured.adults <= 0.1276
Feature
−0.003
0.000
0.003
0.006
Weight
Positive
Negative

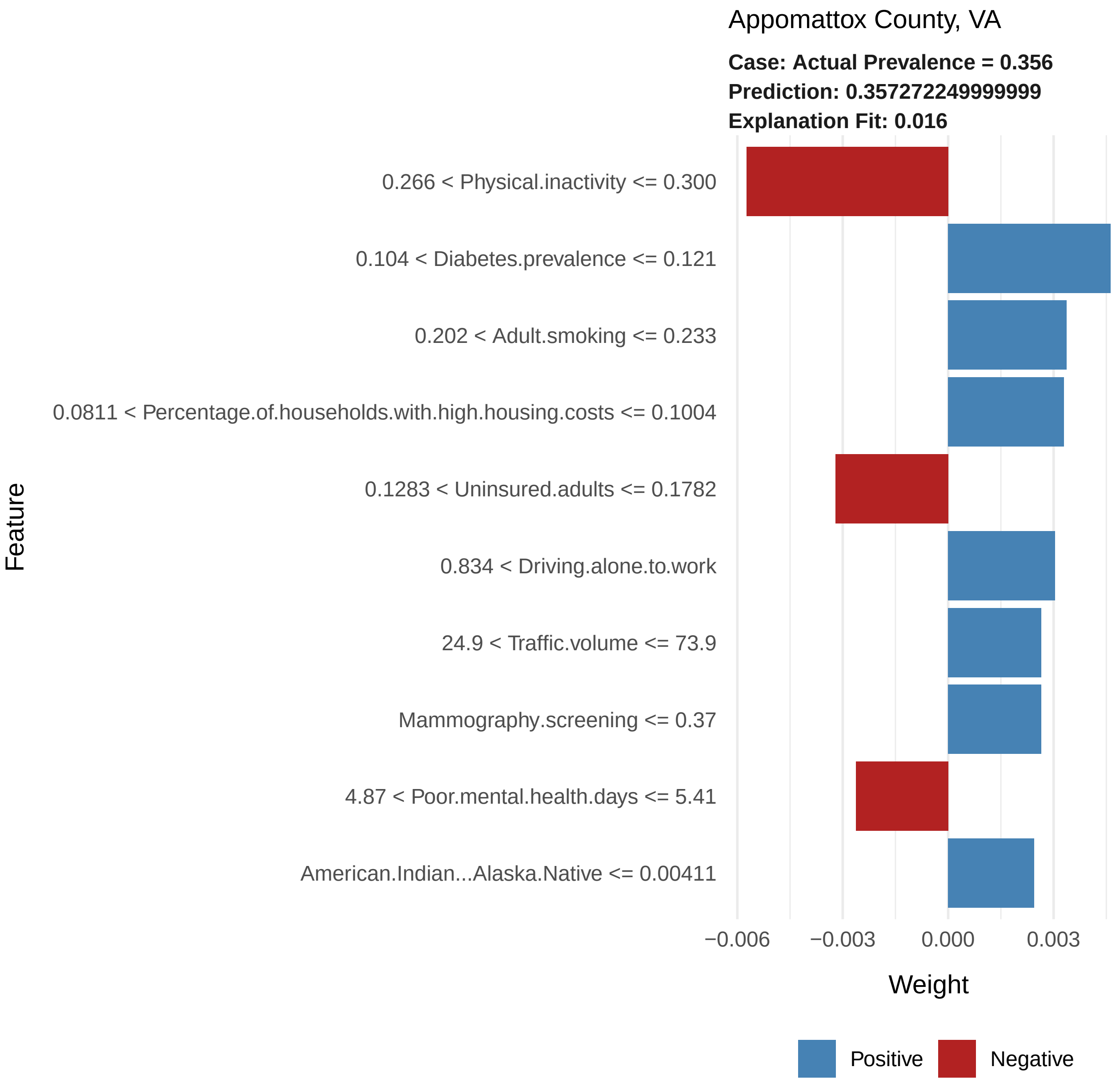

Appomattox County, VA
Case: Actual Prevalence = 0.356
Prediction: 0.357272249999999
Explanation Fit: 0.016
0.266 < Physical.inactivity <= 0.300
0.104 < Diabetes.prevalence <= 0.121
0.202 < Adult.smoking <= 0.233
0.0811 < Percentage.of.households.with.high.housing.costs <= 0.1004
0.1283 < Uninsured.adults <= 0.1782
0.834 < Driving.alone.to.work
24.9 < Traffic.volume <= 73.9
Mammography.screening <= 0.37
4.87 < Poor.mental.health.days <= 5.41
American.Indian...Alaska.Native <= 0.00411
Feature
−0.006
−0.003
0.000
0.003
Weight
Positive
Negative

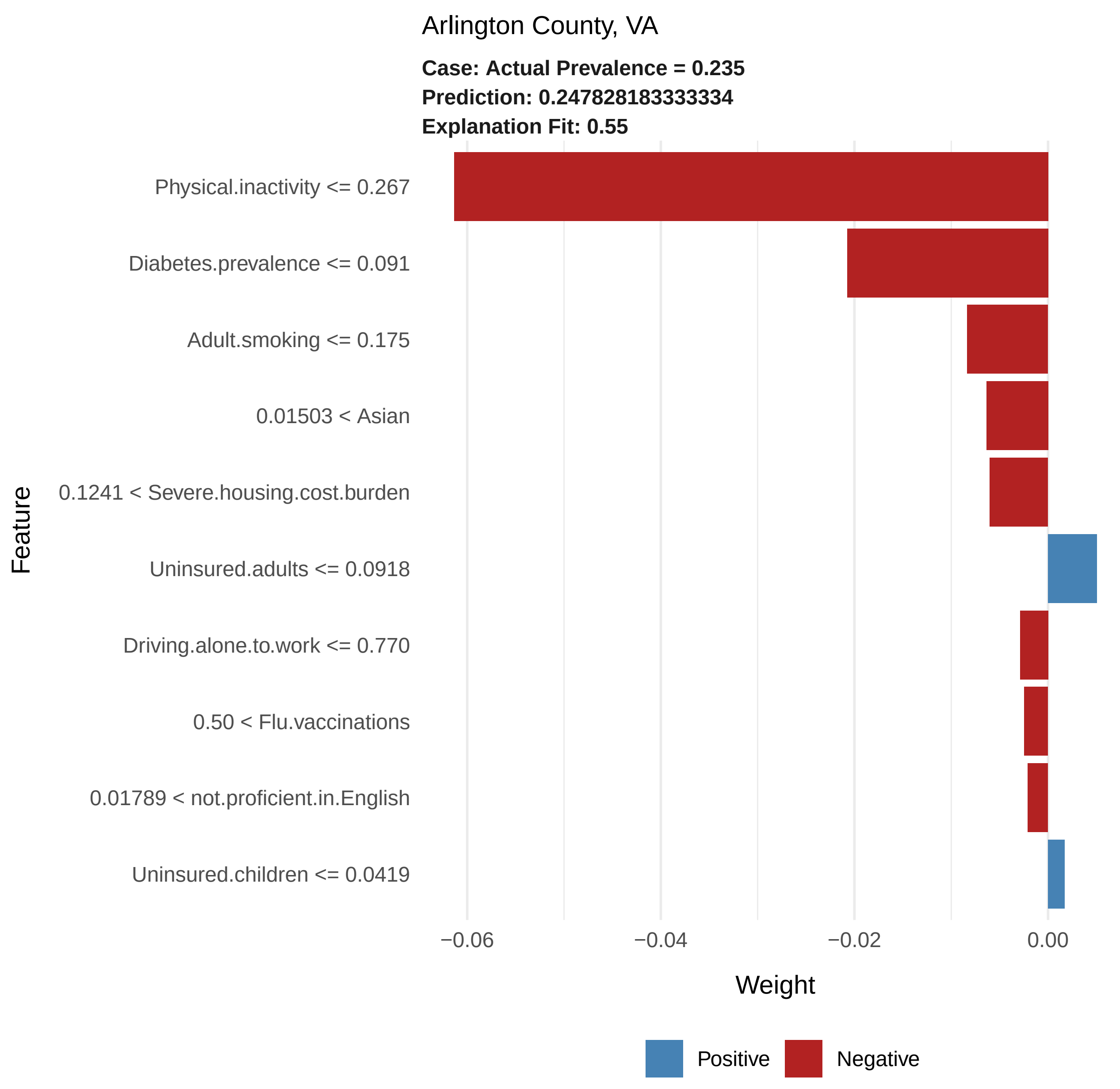
Arlington County, VA
Case: Actual Prevalence = 0.235
Prediction: 0.247828183333334
Explanation Fit: 0.55
Physical.inactivity <= 0.267
Diabetes.prevalence <= 0.091
Adult.smoking <= 0.175
0.01503 < Asian
0.1241 < Severe.housing.cost.burden
Uninsured.adults <= 0.0918
Driving.alone.to.work <= 0.770
0.50 < Flu.vaccinations
0.01789 < not.proficient.in.English
Uninsured.children <= 0.0419
Feature
−0.06
−0.04
−0.02
0.00
Weight
Positive
Negative

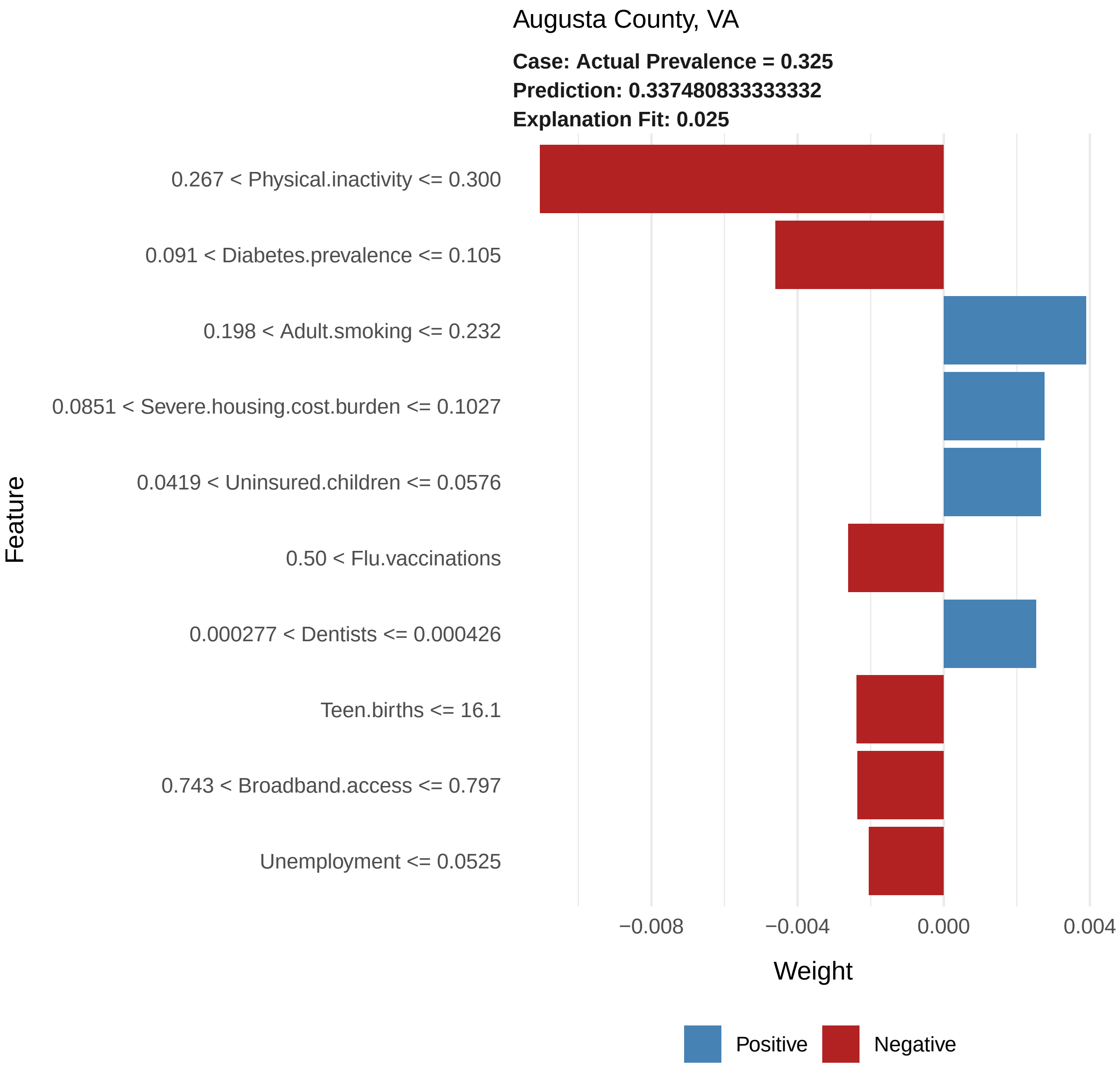

Augusta County, VA
Case: Actual Prevalence = 0.325
Prediction: 0.337480833333332
Explanation Fit: 0.025
0.267 < Physical.inactivity <= 0.300
0.091 < Diabetes.prevalence <= 0.105
0.198 < Adult.smoking <= 0.232
0.0851 < Severe.housing.cost.burden <= 0.1027
0.0419 < Uninsured.children <= 0.0576
0.50 < Flu.vaccinations
0.000277 < Dentists <= 0.000426
Teen.births <= 16.1
0.743 < Broadband.access <= 0.797
Unemployment <= 0.0525
Feature
−0.008
−0.004
0.000
0.004
Weight
Positive
Negative

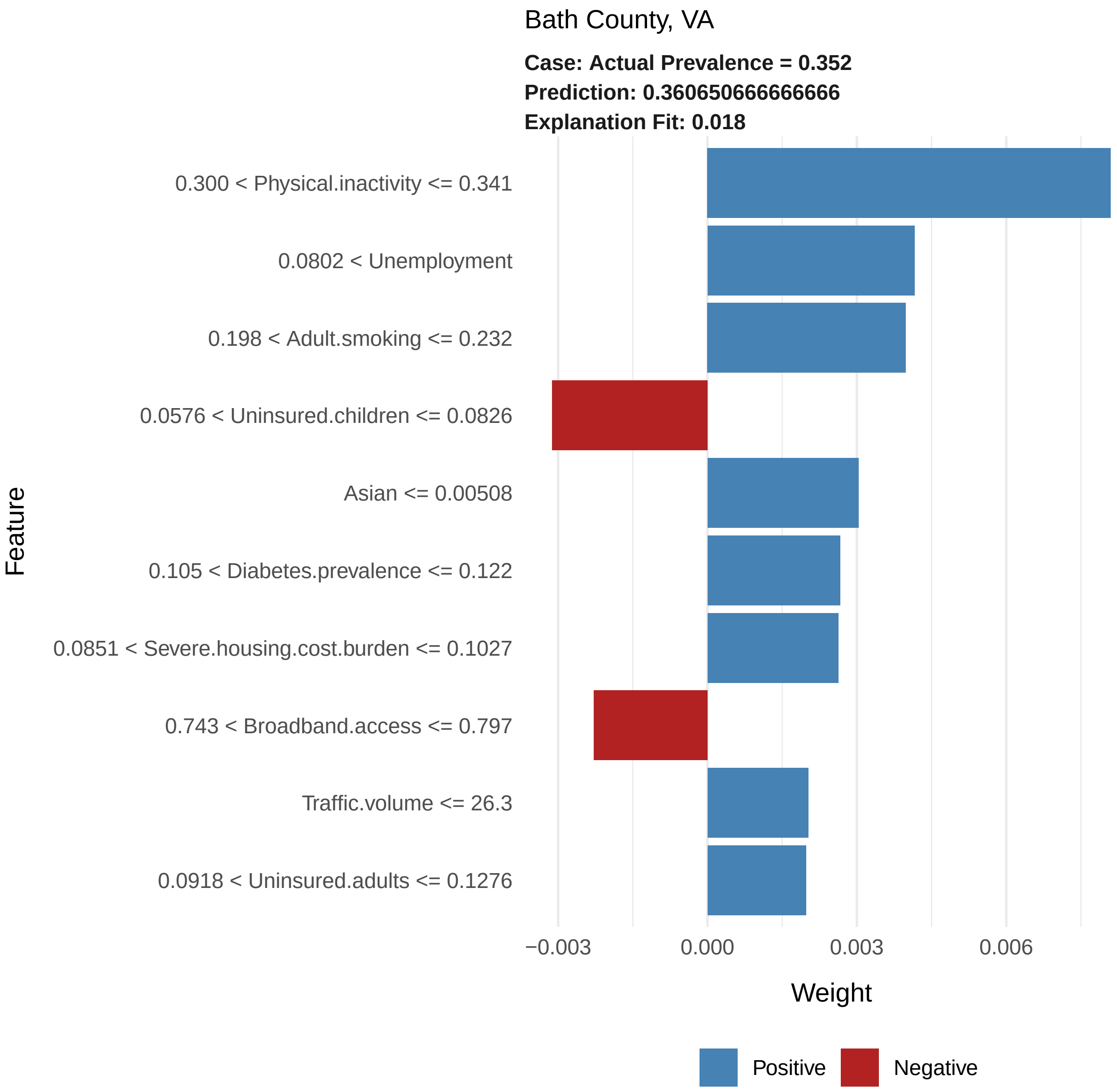

Bath County, VA
Case: Actual Prevalence = 0.352
Prediction: 0.360650666666666
Explanation Fit: 0.018
0.300 < Physical.inactivity <= 0.341
0.0802 < Unemployment
0.198 < Adult.smoking <= 0.232
0.0576 < Uninsured.children <= 0.0826
Asian <= 0.00508
0.105 < Diabetes.prevalence <= 0.122
0.0851 < Severe.housing.cost.burden <= 0.1027
0.743 < Broadband.access <= 0.797
Traffic.volume <= 26.3
0.0918 < Uninsured.adults <= 0.1276
Feature
−0.003
0.000
0.003
0.006
Weight
Positive
Negative

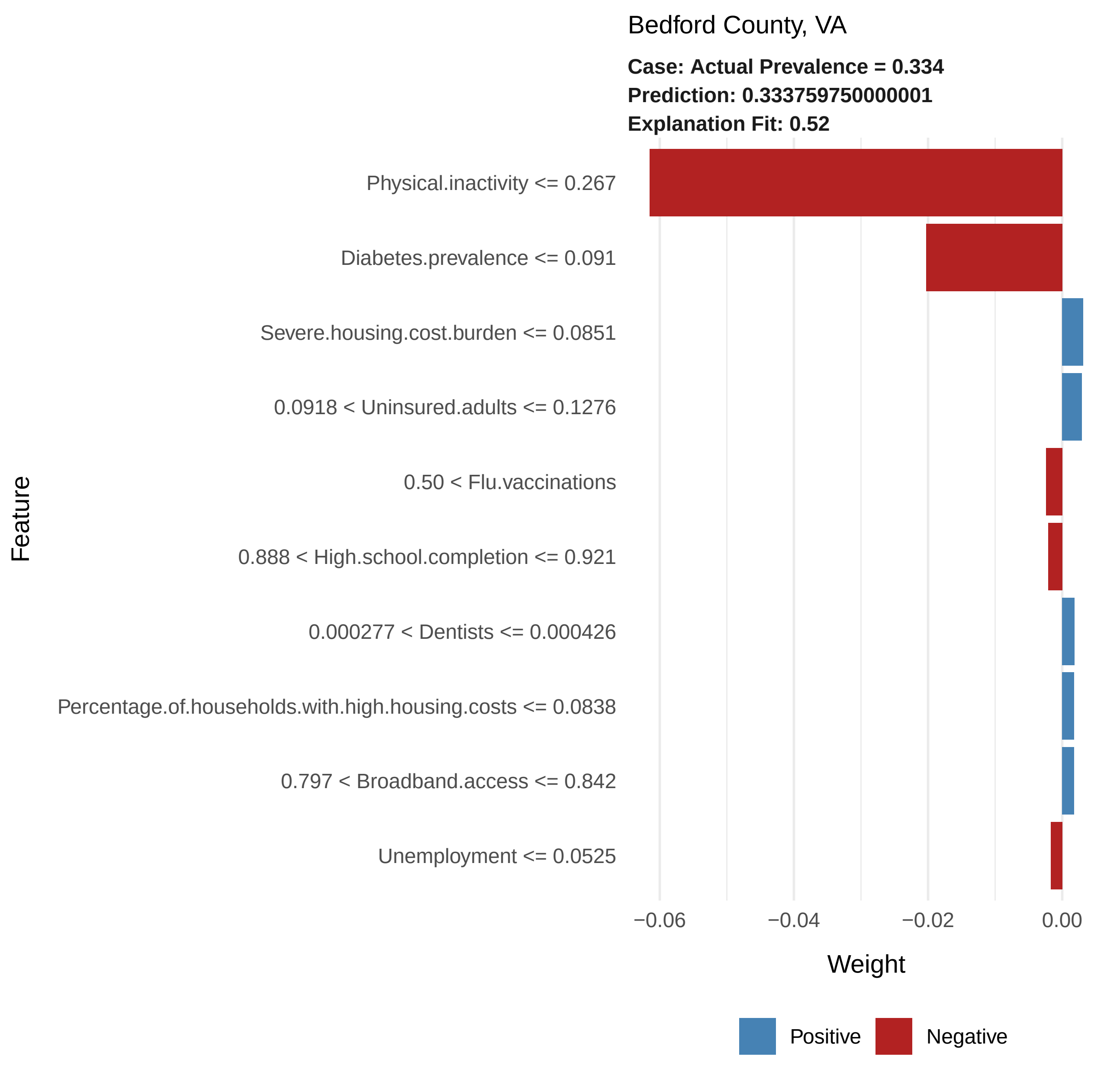
Bedford County, VA
Case: Actual Prevalence = 0.334
Prediction: 0.333759750000001
Explanation Fit: 0.52
Physical.inactivity <= 0.267
Diabetes.prevalence <= 0.091
Severe.housing.cost.burden <= 0.0851
0.0918 < Uninsured.adults <= 0.1276
0.50 < Flu.vaccinations
0.888 < High.school.completion <= 0.921
0.000277 < Dentists <= 0.000426
Percentage.of.households.with.high.housing.costs <= 0.0838
0.797 < Broadband.access <= 0.842
Unemployment <= 0.0525
Feature
−0.06
−0.04
−0.02
0.00
Weight
Positive
Negative

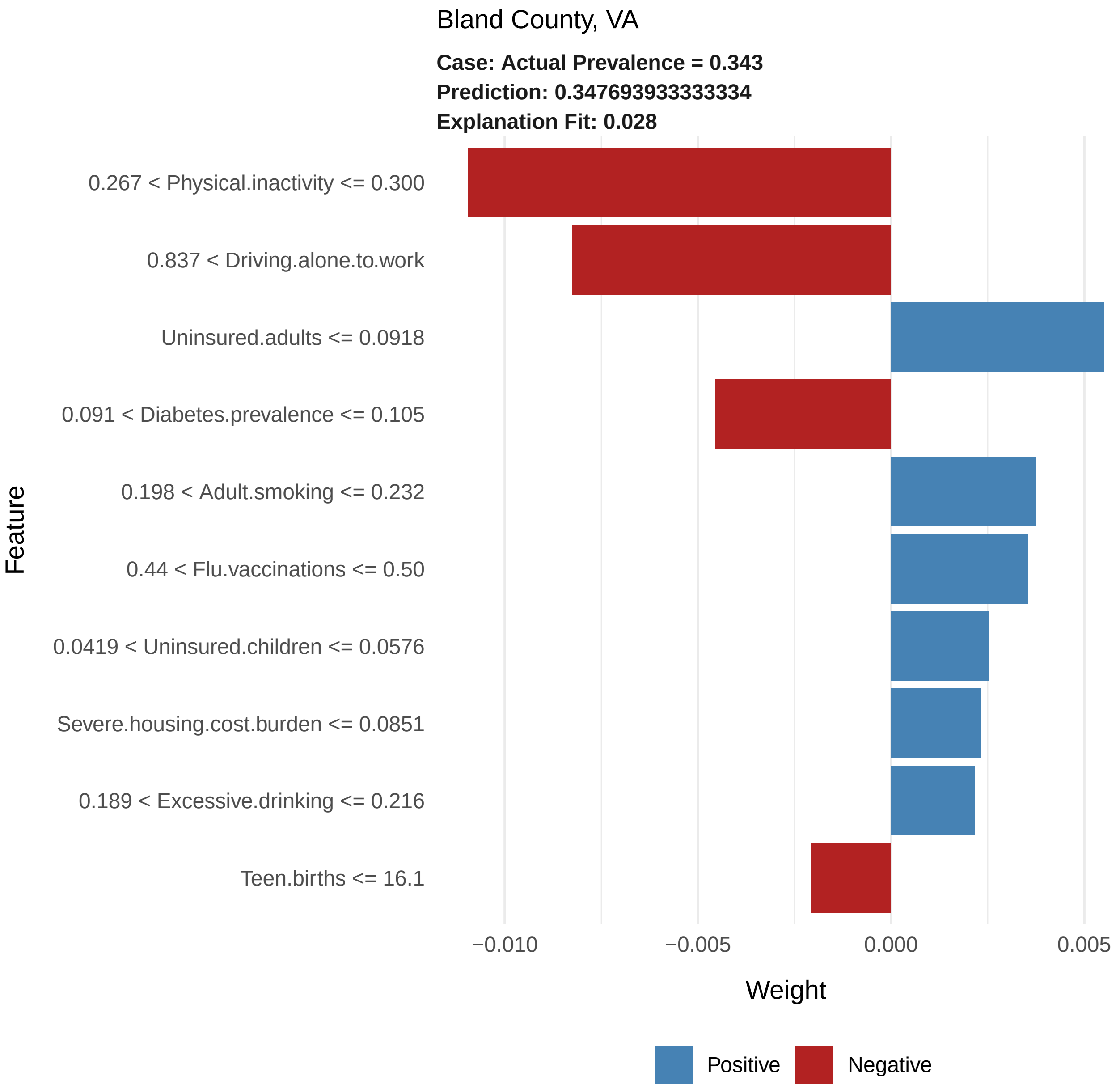
Bland County, VA
Case: Actual Prevalence = 0.343
Prediction: 0.347693933333334
Explanation Fit: 0.028
0.267 < Physical.inactivity <= 0.300
0.837 < Driving.alone.to.work
Uninsured.adults <= 0.0918
0.091 < Diabetes.prevalence <= 0.105
0.198 < Adult.smoking <= 0.232
0.44 < Flu.vaccinations <= 0.50
0.0419 < Uninsured.children <= 0.0576
Severe.housing.cost.burden <= 0.0851
0.189 < Excessive.drinking <= 0.216
Teen.births <= 16.1
Feature
−0.010
−0.005
0.000
0.005
Weight
Positive
Negative

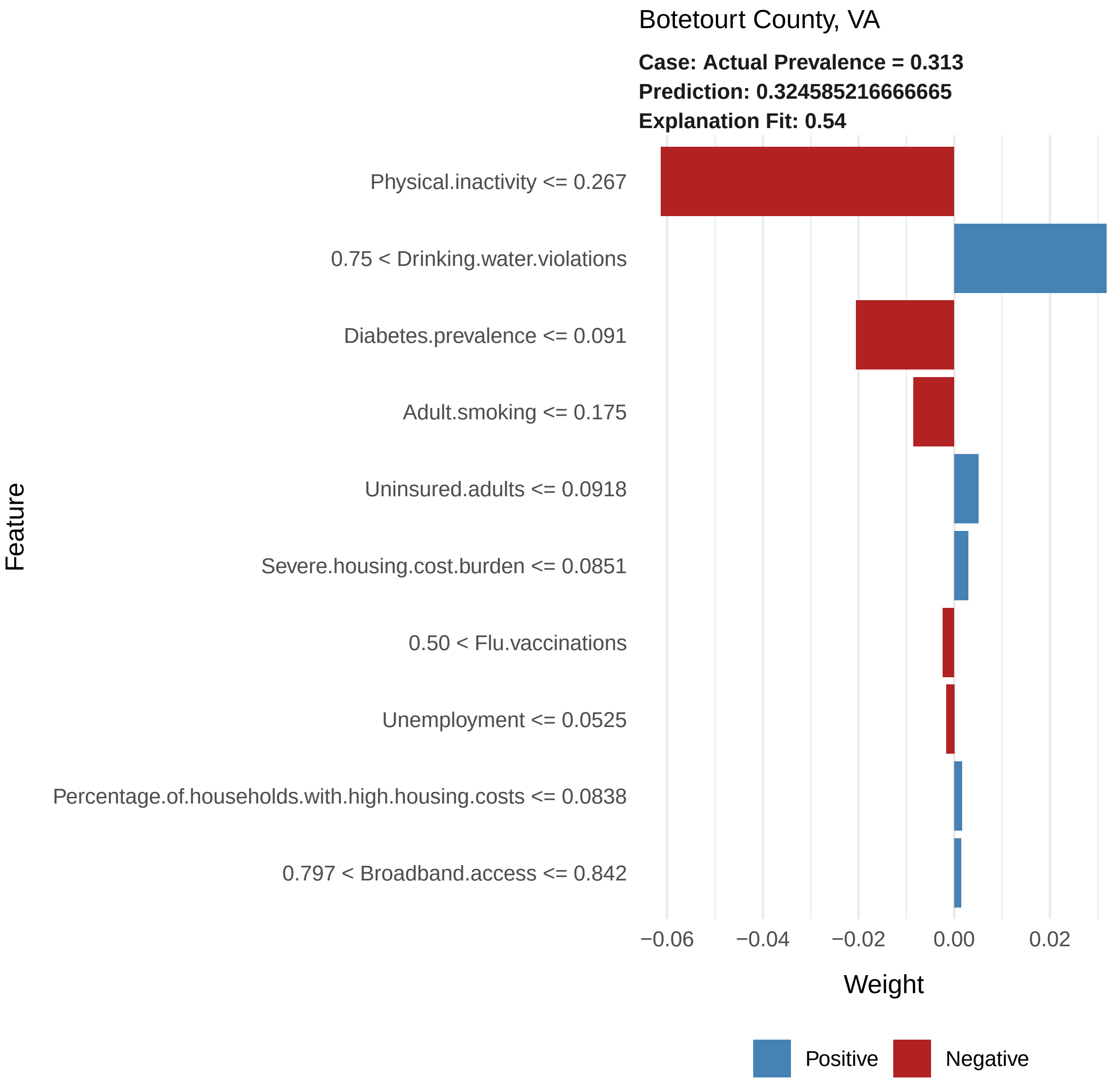
Botetourt County, VA
Case: Actual Prevalence = 0.313
Prediction: 0.324585216666665
Explanation Fit: 0.54
Physical.inactivity <= 0.267
0.75 < Drinking.water.violations
Diabetes.prevalence <= 0.091
Adult.smoking <= 0.175
Uninsured.adults <= 0.0918
Severe.housing.cost.burden <= 0.0851
0.50 < Flu.vaccinations
Unemployment <= 0.0525
Percentage.of.households.with.high.housing.costs <= 0.0838
0.797 < Broadband.access <= 0.842
Feature
−0.06
−0.04
−0.02
0.00
0.02
Weight
Positive
Negative

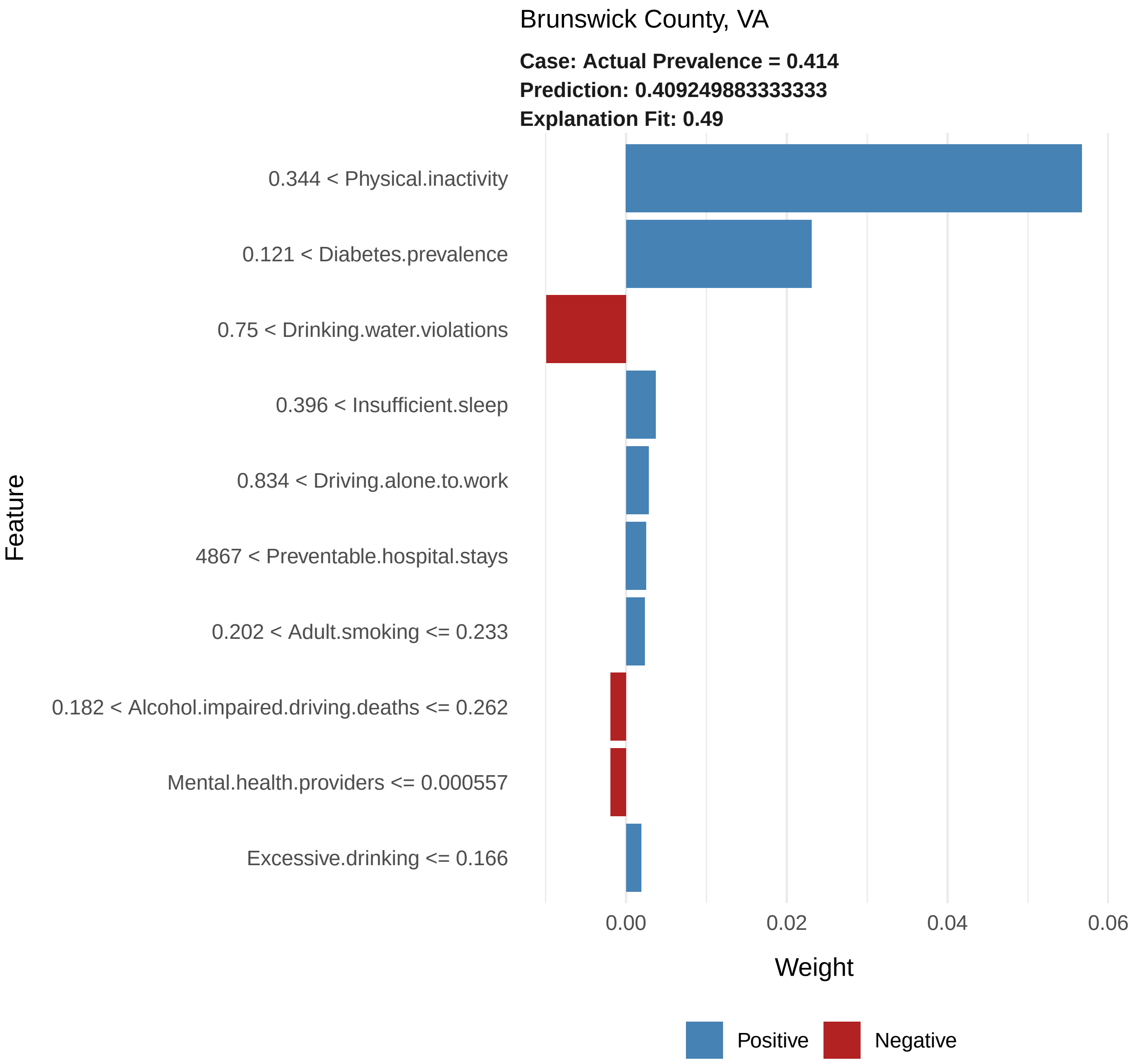

Brunswick County, VA
Case: Actual Prevalence = 0.414
Prediction: 0.409249883333333
Explanation Fit: 0.49
0.344 < Physical.inactivity
0.121 < Diabetes.prevalence
0.75 < Drinking.water.violations
0.396 < Insufficient.sleep
0.834 < Driving.alone.to.work
4867 < Preventable.hospital.stays
0.202 < Adult.smoking <= 0.233
0.182 < Alcohol.impaired.driving.deaths <= 0.262
Mental.health.providers <= 0.000557
Excessive.drinking <= 0.166
Feature
0.00
0.02
0.04
0.06
Weight
Positive
Negative

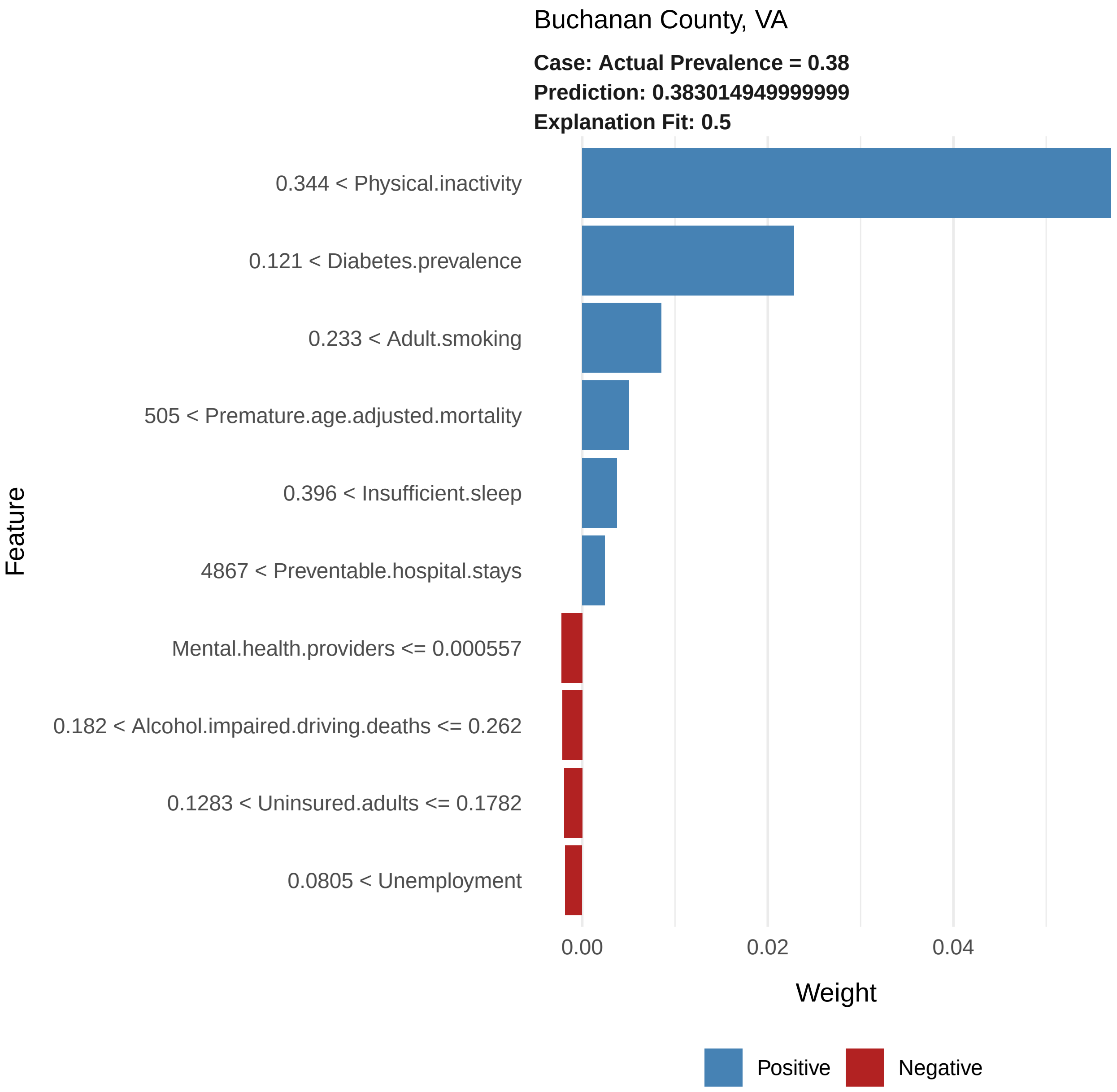

Buchanan County, VA
Case: Actual Prevalence = 0.38
Prediction: 0.383014949999999
Explanation Fit: 0.5
0.344 < Physical.inactivity
0.121 < Diabetes.prevalence
0.233 < Adult.smoking
505 < Premature.age.adjusted.mortality
0.396 < Insufficient.sleep
4867 < Preventable.hospital.stays
Mental.health.providers <= 0.000557
0.182 < Alcohol.impaired.driving.deaths <= 0.262
0.1283 < Uninsured.adults <= 0.1782
0.0805 < Unemployment
Feature
0.00
0.02
0.04
Weight
Positive
Negative

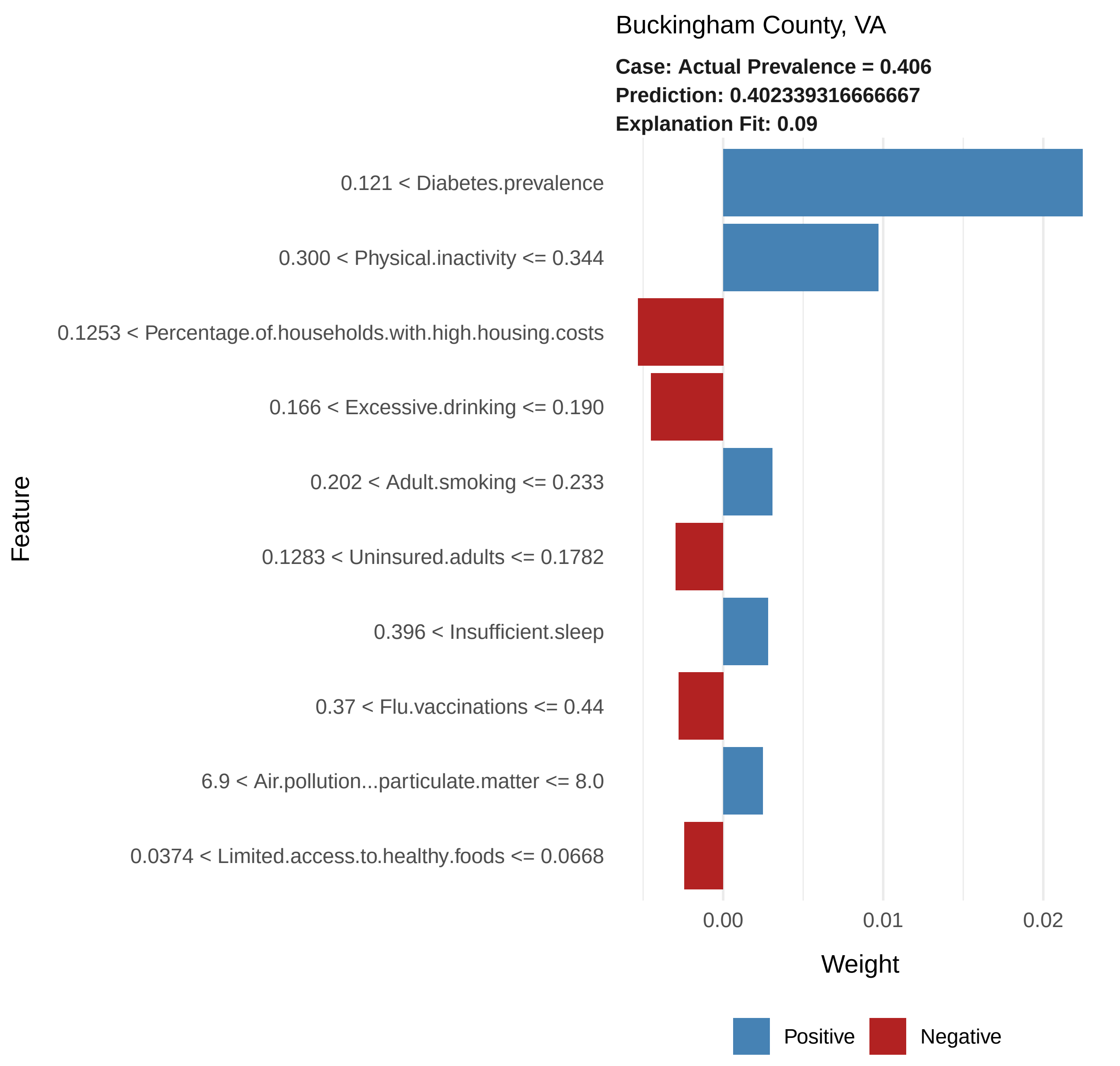
Buckingham County, VA
Case: Actual Prevalence = 0.406
Prediction: 0.402339316666667
Explanation Fit: 0.09
0.121 < Diabetes.prevalence
0.300 < Physical.inactivity <= 0.344
0.1253 < Percentage.of.households.with.high.housing.costs
0.166 < Excessive.drinking <= 0.190
0.202 < Adult.smoking <= 0.233
0.1283 < Uninsured.adults <= 0.1782
0.396 < Insufficient.sleep
0.37 < Flu.vaccinations <= 0.44
6.9 < Air.pollution...particulate.matter <= 8.0
0.0374 < Limited.access.to.healthy.foods <= 0.0668
Feature
0.00
0.01
0.02
Weight
Positive
Negative

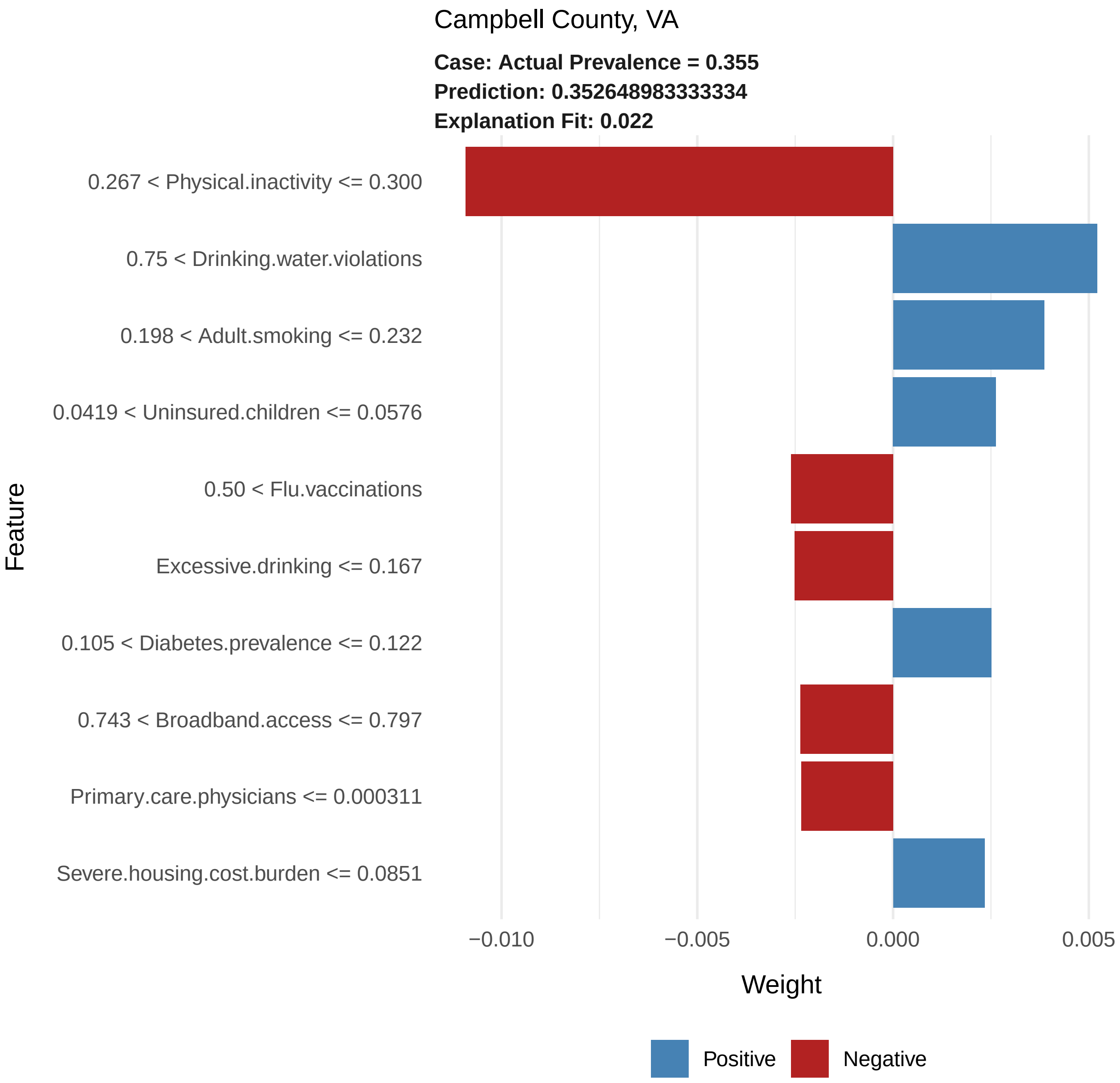

Campbell County, VA
Case: Actual Prevalence = 0.355
Prediction: 0.352648983333334
Explanation Fit: 0.022
0.267 < Physical.inactivity <= 0.300
0.75 < Drinking.water.violations
0.198 < Adult.smoking <= 0.232
0.0419 < Uninsured.children <= 0.0576
0.50 < Flu.vaccinations
Excessive.drinking <= 0.167
0.105 < Diabetes.prevalence <= 0.122
0.743 < Broadband.access <= 0.797
Primary.care.physicians <= 0.000311
Severe.housing.cost.burden <= 0.0851
Feature
−0.010
−0.005
0.000
0.005
Weight
Positive
Negative

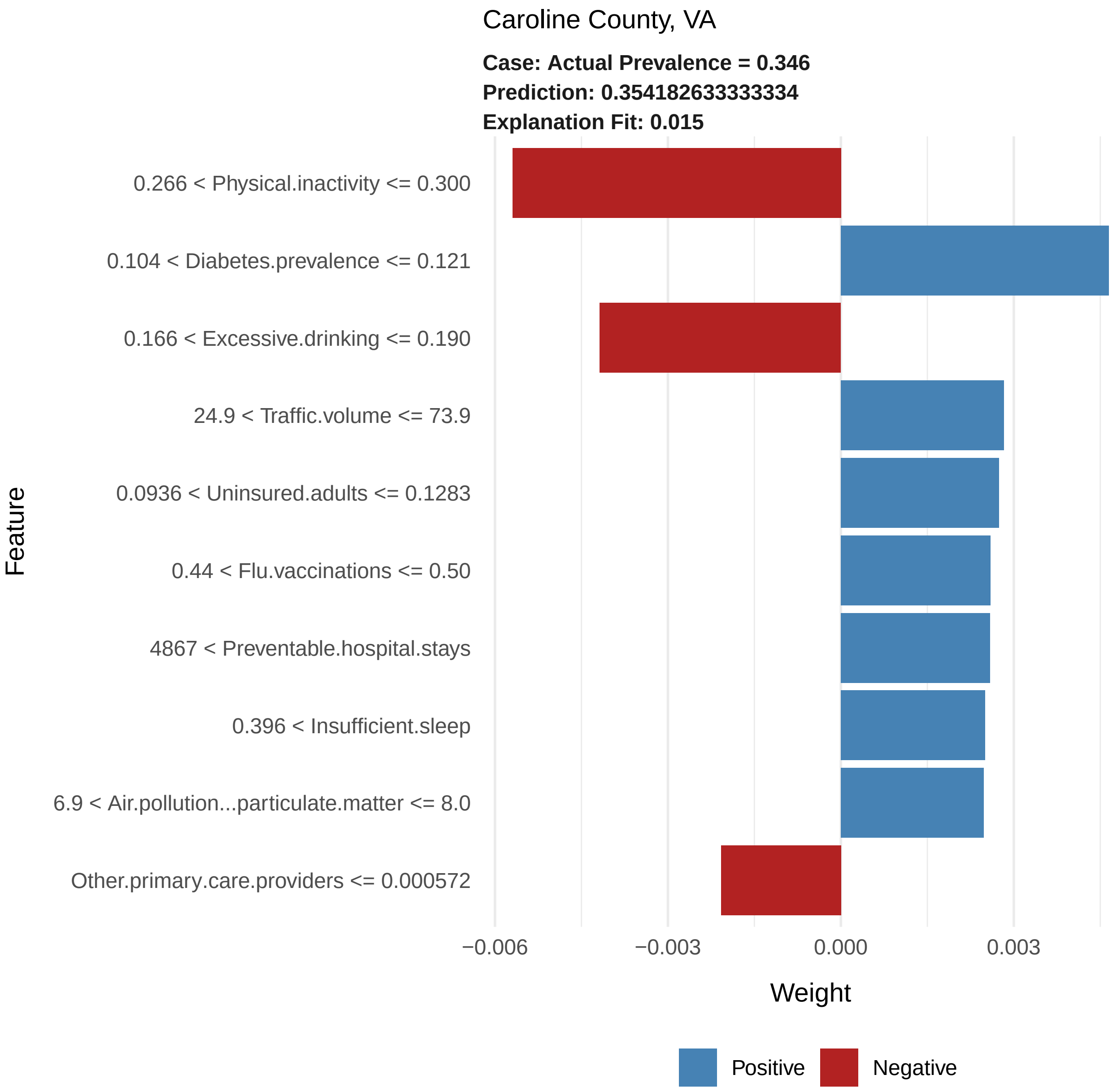

Caroline County, VA
Case: Actual Prevalence = 0.346
Prediction: 0.354182633333334
Explanation Fit: 0.015
0.266 < Physical.inactivity <= 0.300
0.104 < Diabetes.prevalence <= 0.121
0.166 < Excessive.drinking <= 0.190
24.9 < Traffic.volume <= 73.9
0.0936 < Uninsured.adults <= 0.1283
0.44 < Flu.vaccinations <= 0.50
4867 < Preventable.hospital.stays
0.396 < Insufficient.sleep
6.9 < Air.pollution...particulate.matter <= 8.0
Other.primary.care.providers <= 0.000572
Feature
−0.006
−0.003
0.000
0.003
Weight
Positive
Negative

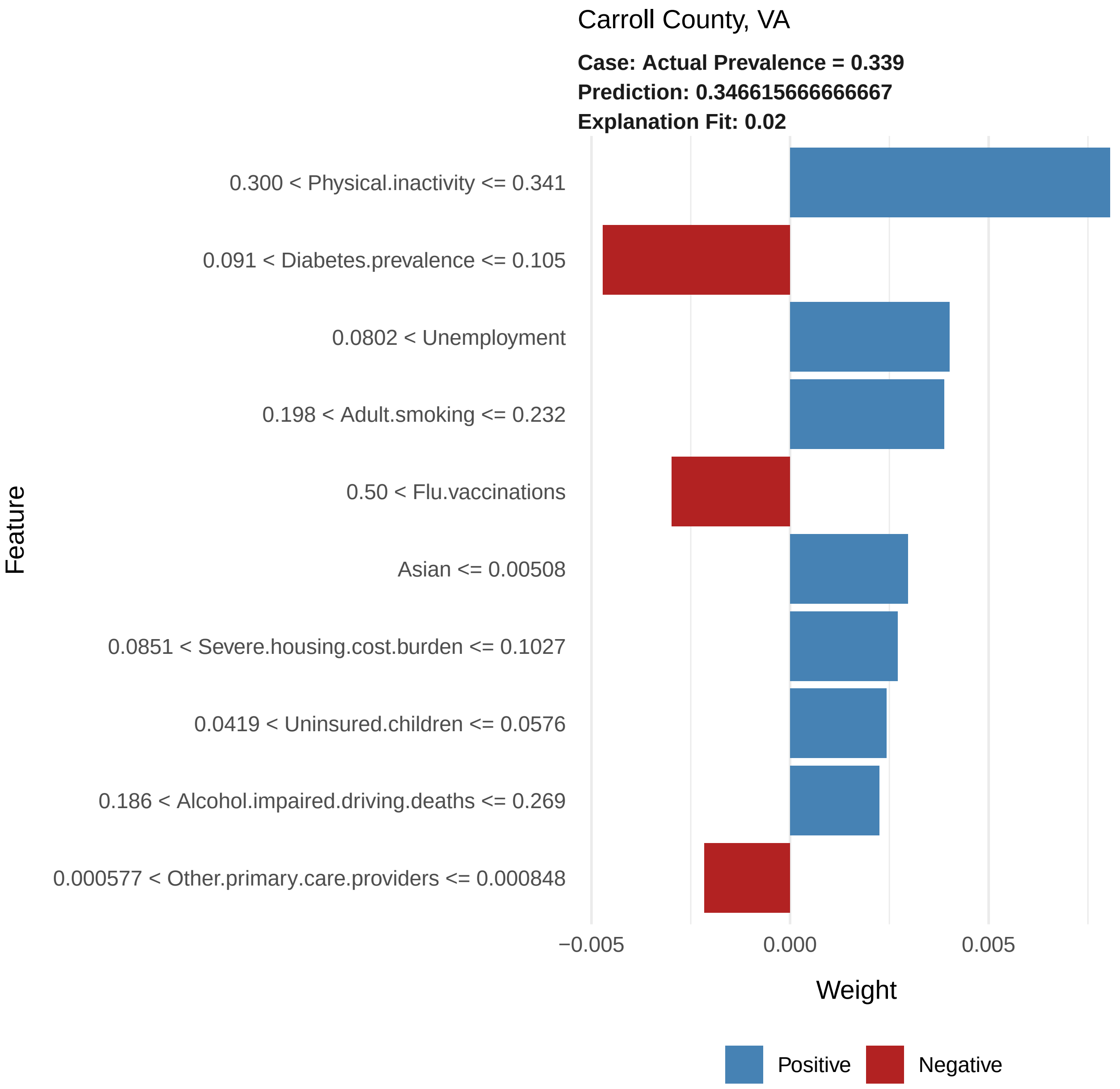
Carroll County, VA
Case: Actual Prevalence = 0.339
Prediction: 0.346615666666667
Explanation Fit: 0.02
0.300 < Physical.inactivity <= 0.341
0.091 < Diabetes.prevalence <= 0.105
0.0802 < Unemployment
0.198 < Adult.smoking <= 0.232
0.50 < Flu.vaccinations
Asian <= 0.00508
0.0851 < Severe.housing.cost.burden <= 0.1027
0.0419 < Uninsured.children <= 0.0576
0.186 < Alcohol.impaired.driving.deaths <= 0.269
0.000577 < Other.primary.care.providers <= 0.000848
Feature
−0.005
0.000
0.005
Weight
Positive
Negative

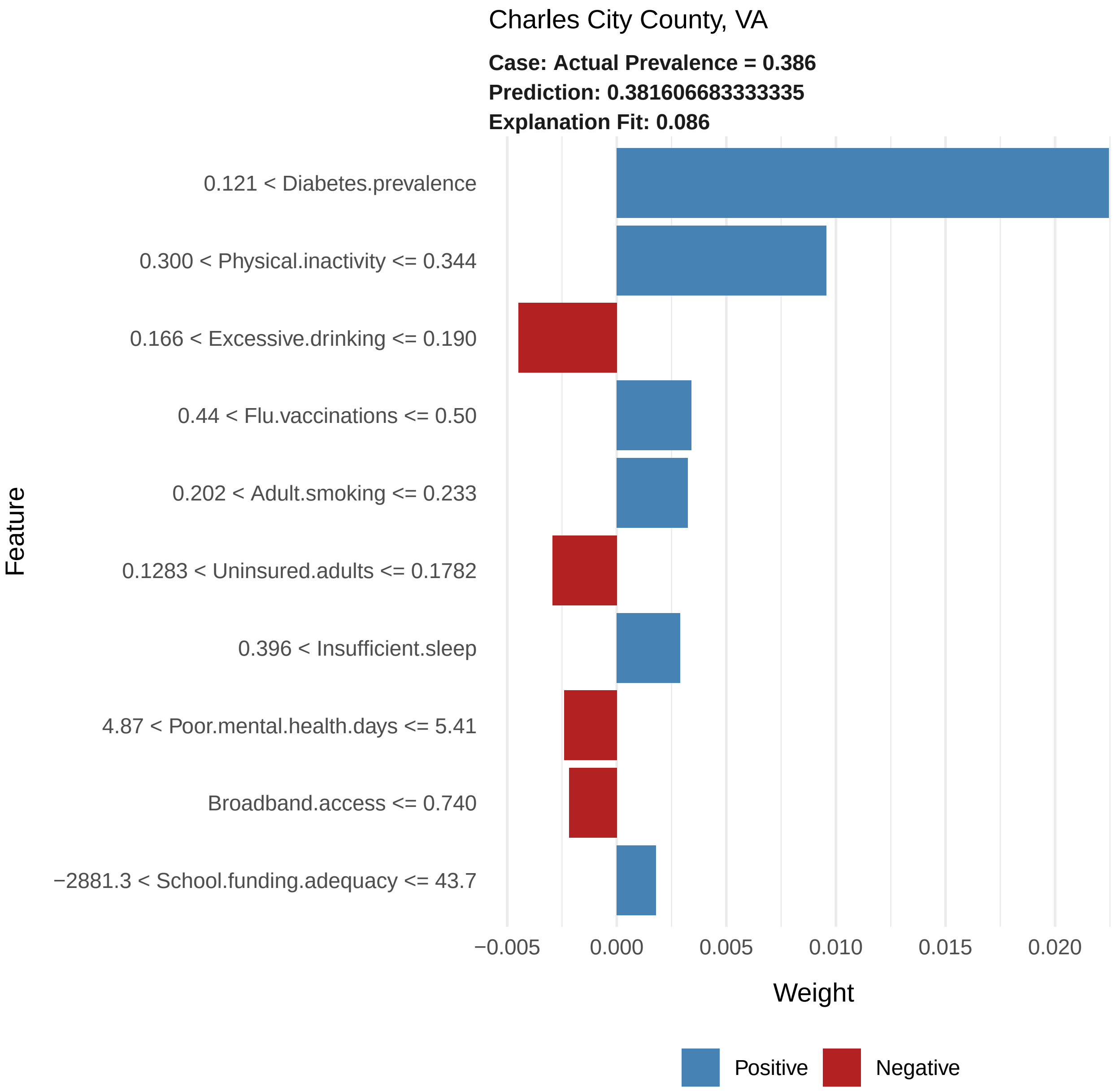

Charles City County, VA
Case: Actual Prevalence = 0.386
Prediction: 0.381606683333335
Explanation Fit: 0.086
0.121 < Diabetes.prevalence
0.300 < Physical.inactivity <= 0.344
0.166 < Excessive.drinking <= 0.190
0.44 < Flu.vaccinations <= 0.50
0.202 < Adult.smoking <= 0.233
0.1283 < Uninsured.adults <= 0.1782
0.396 < Insufficient.sleep
4.87 < Poor.mental.health.days <= 5.41
Broadband.access <= 0.740
−2881.3 < School.funding.adequacy <= 43.7
Feature
−0.005
0.000
0.005
0.010
0.015
0.020
Weight
Positive
Negative

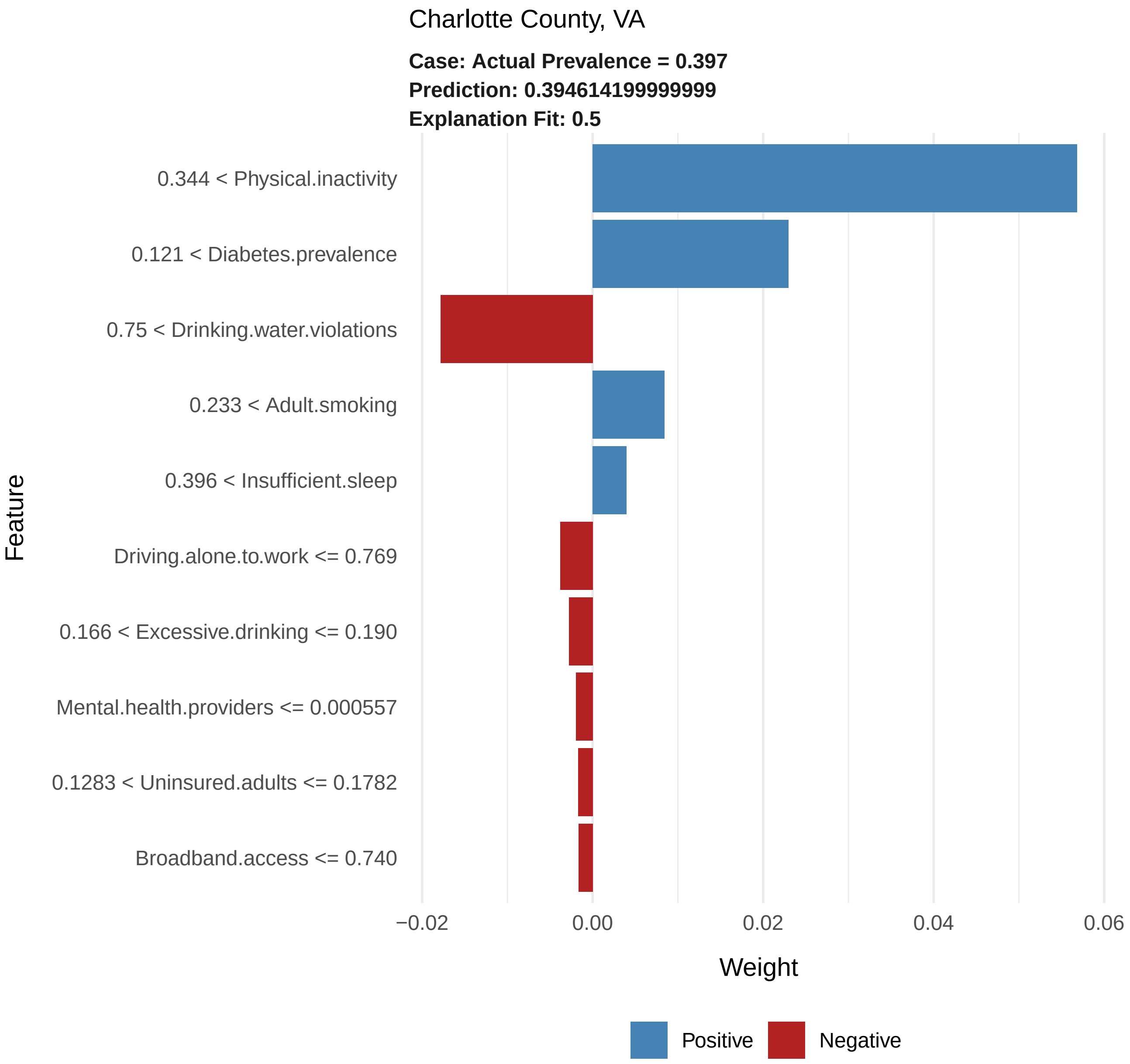

Charlotte County, VA
Case: Actual Prevalence = 0.397
Prediction: 0.394614199999999
Explanation Fit: 0.5
0.344 < Physical.inactivity
0.121 < Diabetes.prevalence
0.75 < Drinking.water.violations
0.233 < Adult.smoking
0.396 < Insufficient.sleep
Driving.alone.to.work <= 0.769
0.166 < Excessive.drinking <= 0.190
Mental.health.providers <= 0.000557
0.1283 < Uninsured.adults <= 0.1782
Broadband.access <= 0.740
Feature
−0.02
0.00
0.02
0.04
0.06
Weight
Positive
Negative

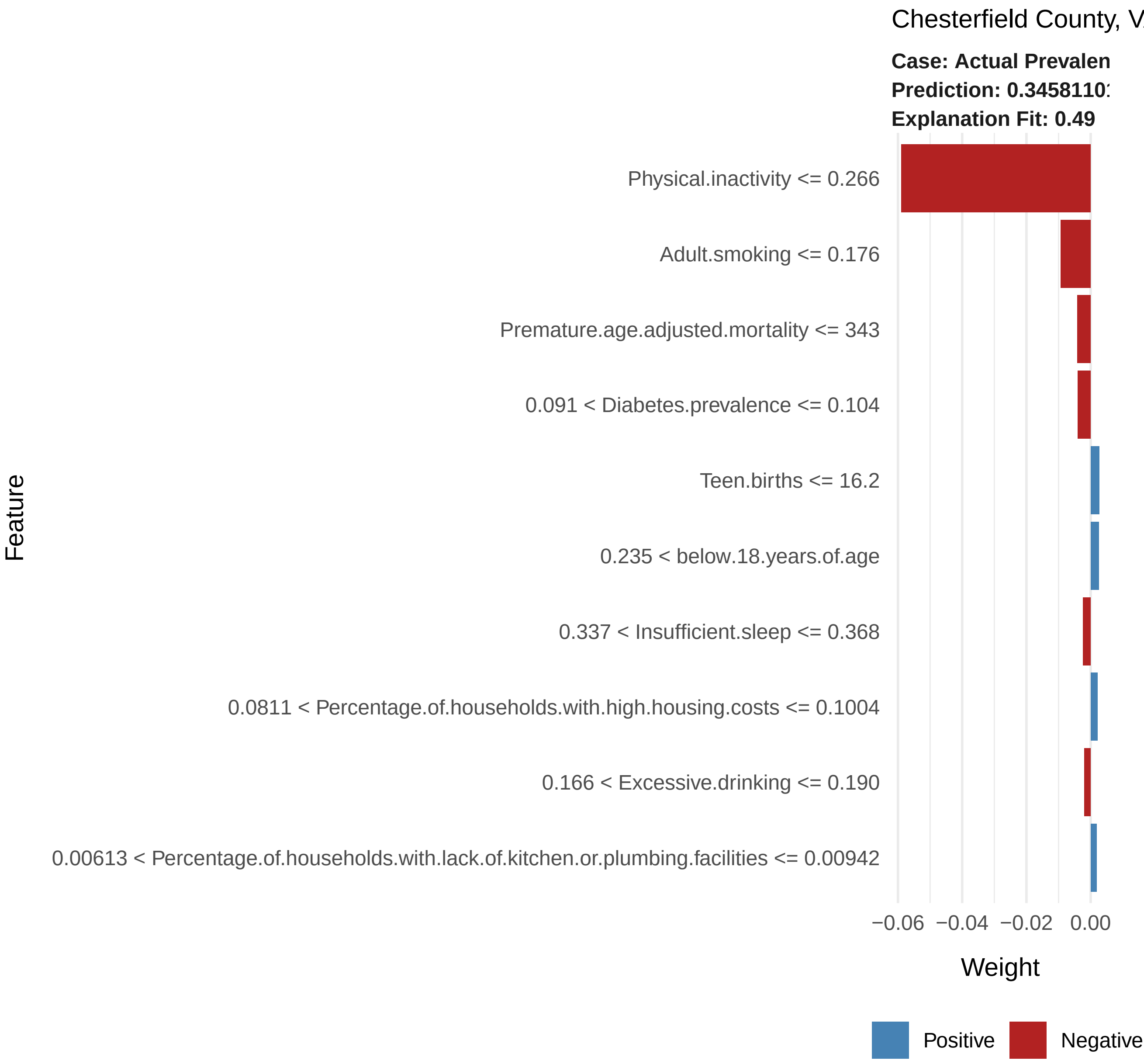

Chesterfield County, V
Case: Actual Prevalen
Prediction: 0.3458110
Explanation Fit: 0.49
Physical.inactivity <= 0.266
Adult.smoking <= 0.176
Premature.age.adjusted.mortality <= 343
0.091 < Diabetes.prevalence <= 0.104
Teen.births <= 16.2
0.235 < below.18.years.of.age
0.337 < Insufficient.sleep <= 0.368
0.0811 < Percentage.of.households.with.high.housing.costs <= 0.1004
0.166 < Excessive.drinking <= 0.190
0.00613 < Percentage.of.households.with.lack.of.kitchen.or.plumbing.facilities <= 0.00942
Feature
−0.06
−0.04
−0.02
0.00
Weight
Positive
Negative

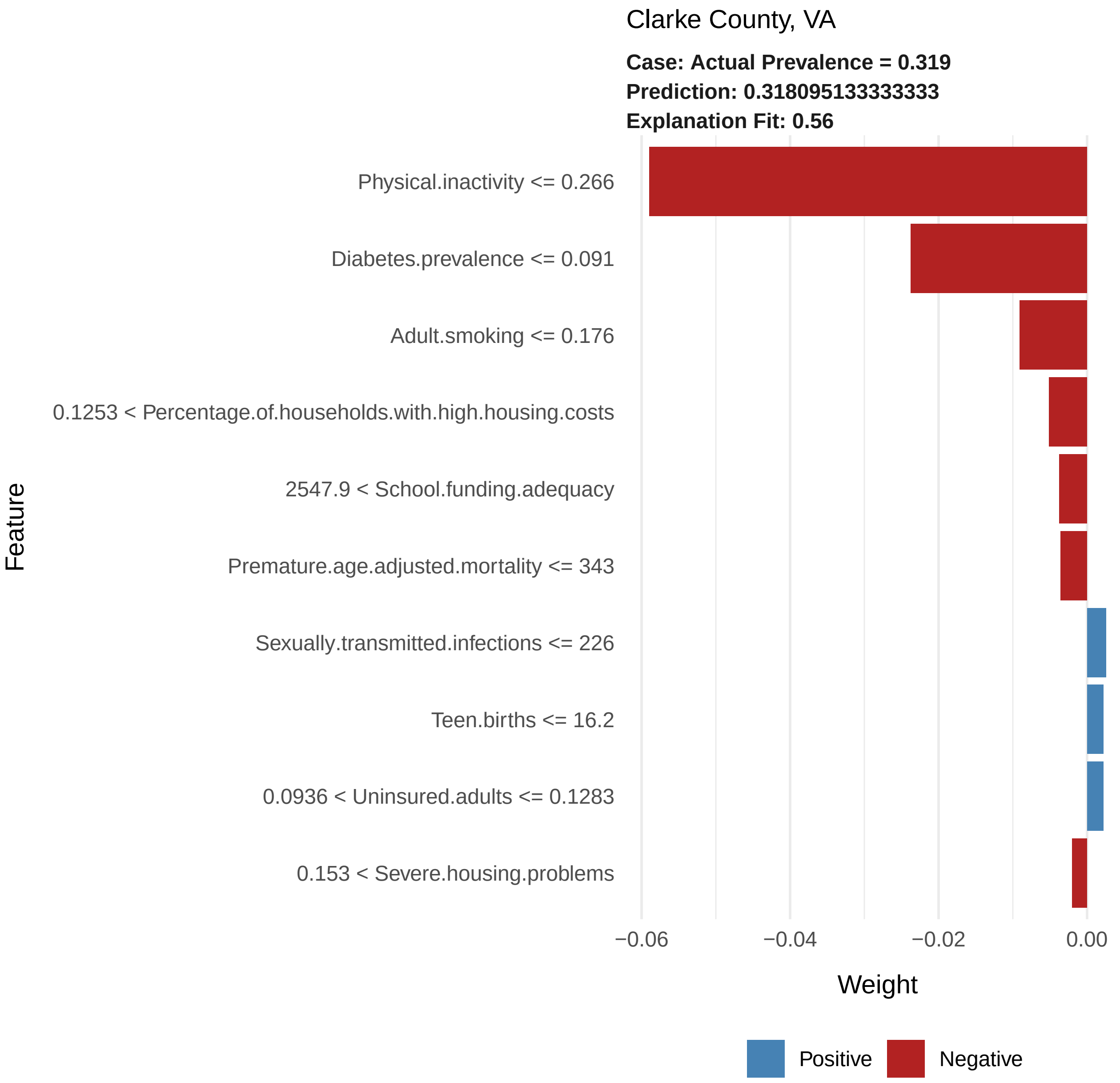
Clarke County, VA
Case: Actual Prevalence = 0.319
Prediction: 0.318095133333333
Explanation Fit: 0.56
Physical.inactivity <= 0.266
Diabetes.prevalence <= 0.091
Adult.smoking <= 0.176
0.1253 < Percentage.of.households.with.high.housing.costs
2547.9 < School.funding.adequacy
Premature.age.adjusted.mortality <= 343
Sexually.transmitted.infections <= 226
Teen.births <= 16.2
0.0936 < Uninsured.adults <= 0.1283
0.153 < Severe.housing.problems
Feature
−0.06
−0.04
−0.02
0.00
Weight
Positive
Negative

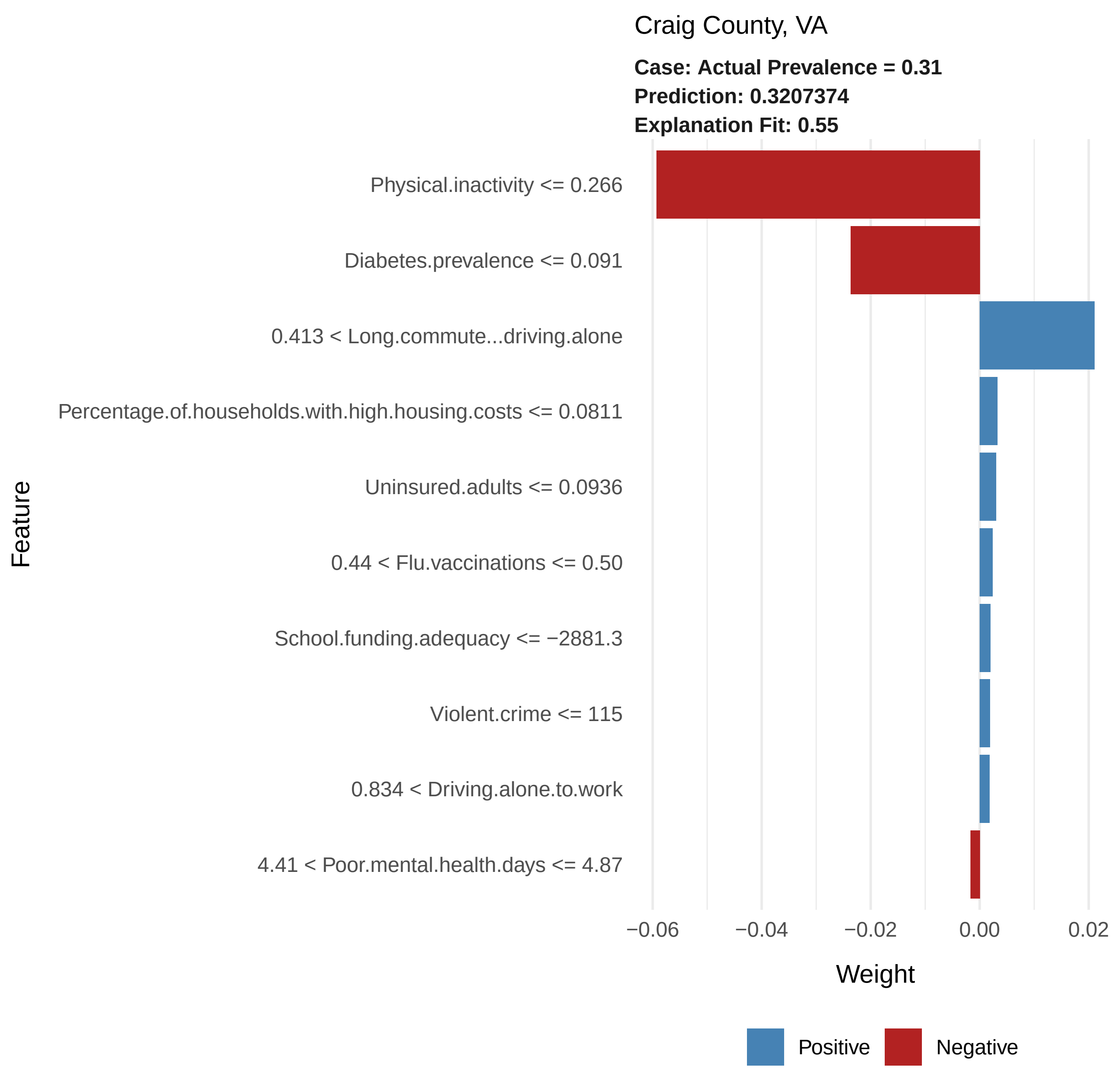

Craig County, VA
Case: Actual Prevalence = 0.31
Prediction: 0.3207374
Explanation Fit: 0.55
Physical.inactivity <= 0.266
Diabetes.prevalence <= 0.091
0.413 < Long.commute...driving.alone
Percentage.of.households.with.high.housing.costs <= 0.0811
Uninsured.adults <= 0.0936
0.44 < Flu.vaccinations <= 0.50
School.funding.adequacy <= −2881.3
Violent.crime <= 115
0.834 < Driving.alone.to.work
4.41 < Poor.mental.health.days <= 4.87
Feature
−0.06
−0.04
−0.02
0.00
0.02
Weight
Positive
Negative

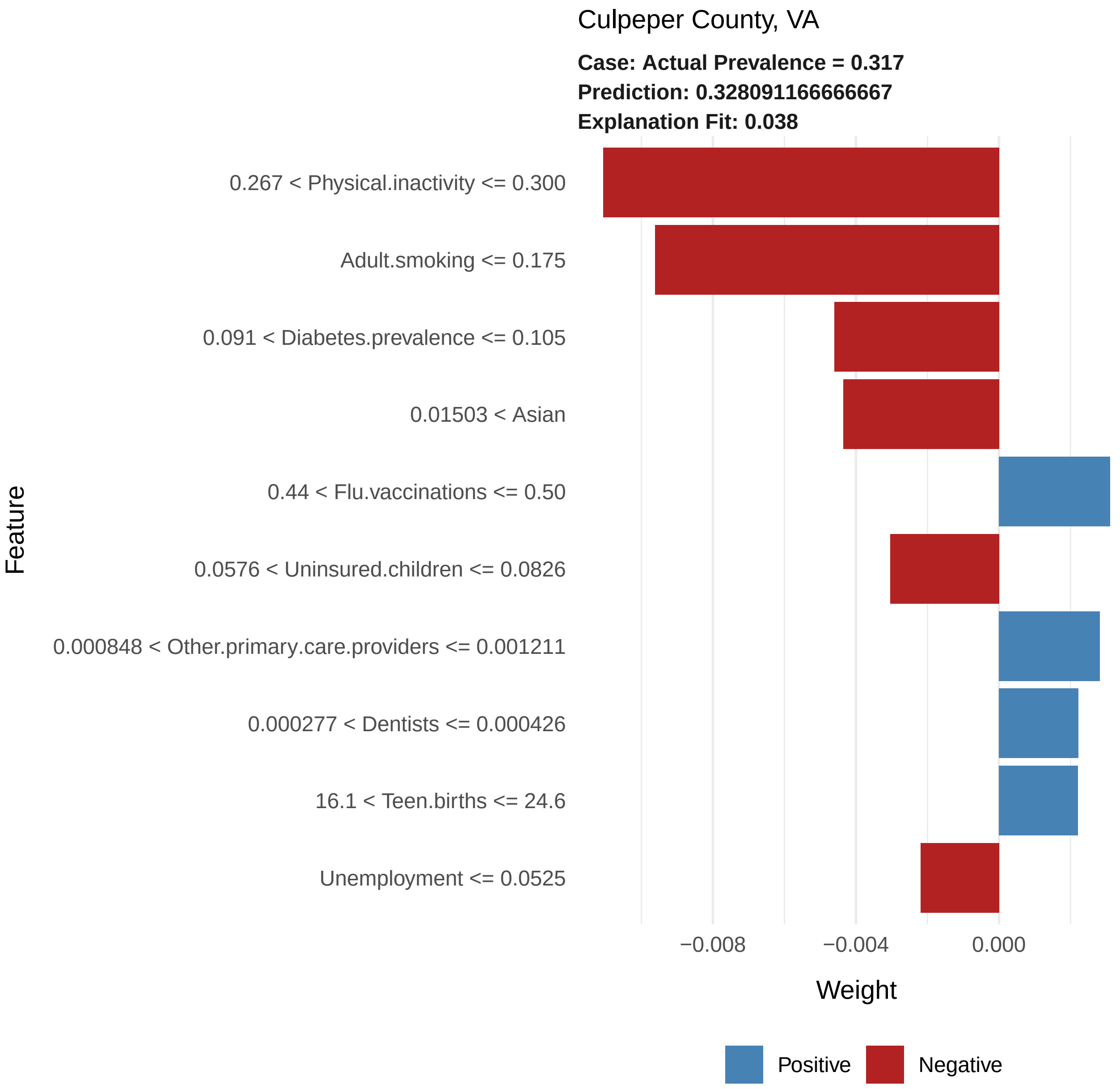

Culpeper County, VA
Case: Actual Prevalence = 0.317
Prediction: 0.328091166666667
Explanation Fit: 0.038
0.267 < Physical.inactivity <= 0.300
Adult.smoking <= 0.175
0.091 < Diabetes.prevalence <= 0.105
0.01503 < Asian
0.44 < Flu.vaccinations <= 0.50
0.0576 < Uninsured.children <= 0.0826
0.000848 < Other.primary.care.providers <= 0.001211
0.000277 < Dentists <= 0.000426
16.1 < Teen.births <= 24.6
Unemployment <= 0.0525
Feature
−0.008
−0.004
0.000
Weight
Positive
Negative

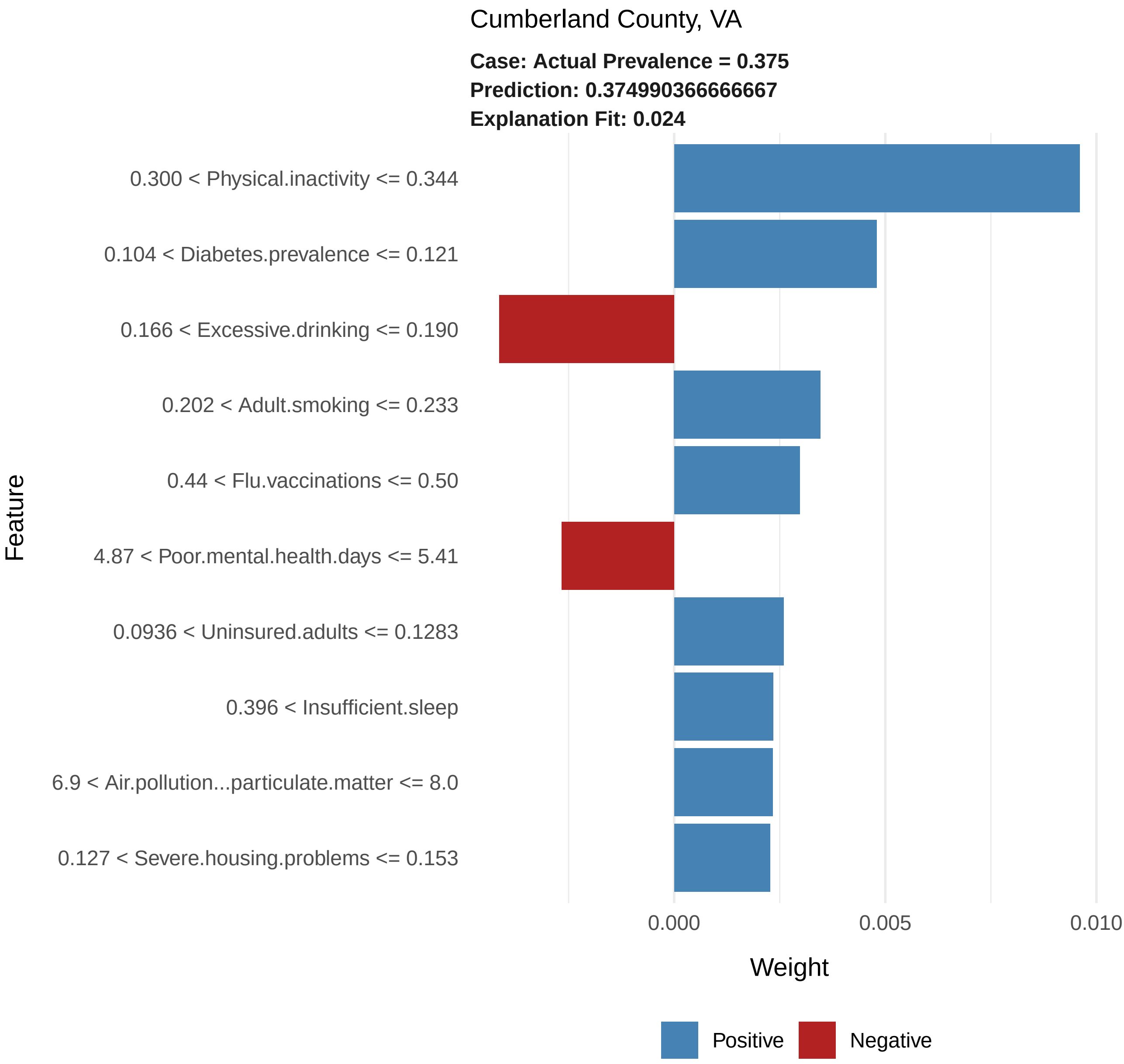
Cumberland County, VA
Case: Actual Prevalence = 0.375
Prediction: 0.374990366666667
Explanation Fit: 0.024
0.300 < Physical.inactivity <= 0.344
0.104 < Diabetes.prevalence <= 0.121
0.166 < Excessive.drinking <= 0.190
0.202 < Adult.smoking <= 0.233
0.44 < Flu.vaccinations <= 0.50
4.87 < Poor.mental.health.days <= 5.41
0.0936 < Uninsured.adults <= 0.1283
0.396 < Insufficient.sleep
6.9 < Air.pollution...particulate.matter <= 8.0
0.127 < Severe.housing.problems <= 0.153
Feature
0.000
0.005
0.010
Weight
Positive
Negative

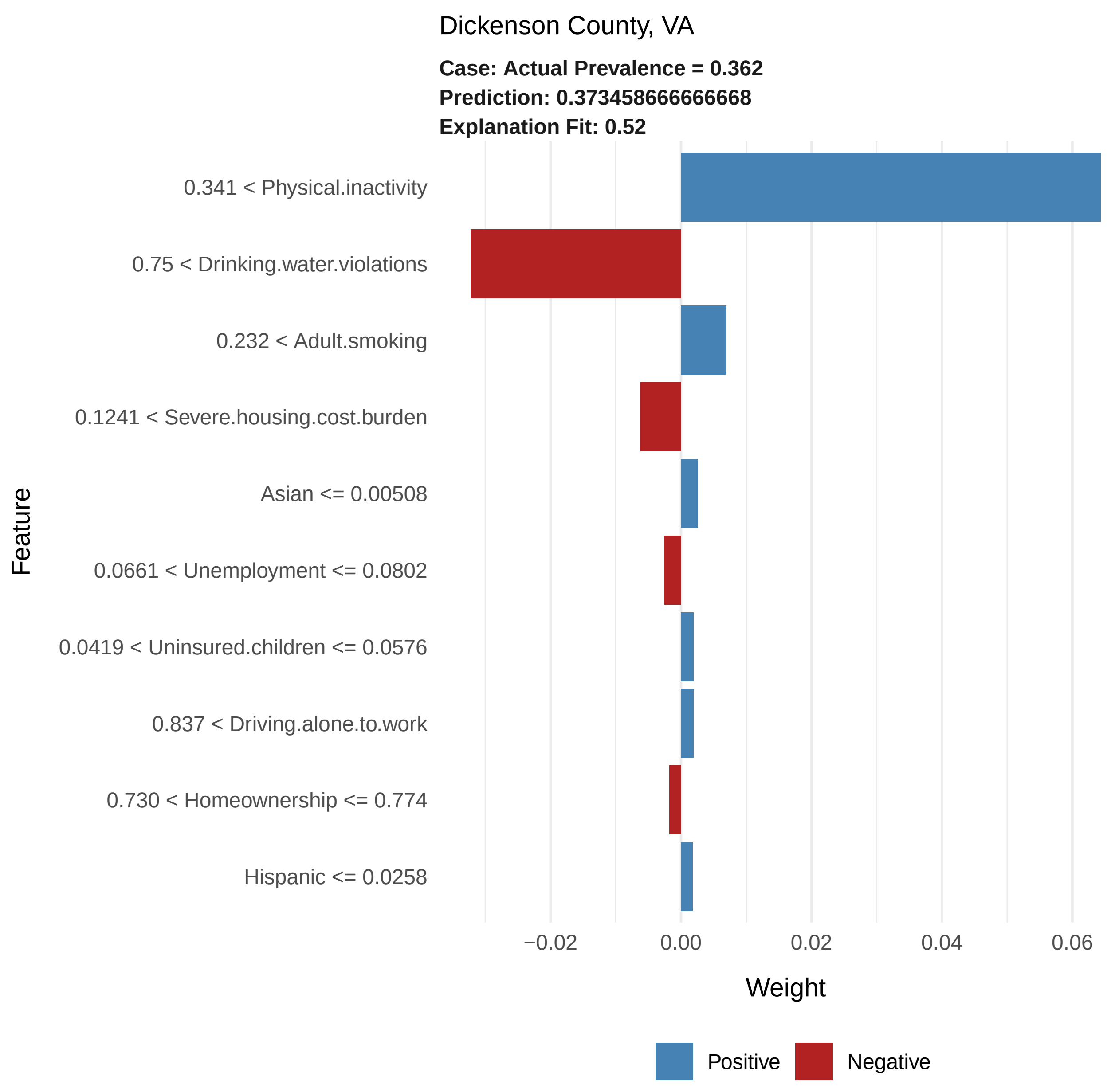

Dickenson County, VA
Case: Actual Prevalence = 0.362
Prediction: 0.373458666666668
Explanation Fit: 0.52
0.341 < Physical.inactivity
0.75 < Drinking.water.violations
0.232 < Adult.smoking
0.1241 < Severe.housing.cost.burden
Asian <= 0.00508
0.0661 < Unemployment <= 0.0802
0.0419 < Uninsured.children <= 0.0576
0.837 < Driving.alone.to.work
0.730 < Homeownership <= 0.774
Hispanic <= 0.0258
Feature
−0.02
0.00
0.02
0.04
0.06
Weight
Positive
Negative

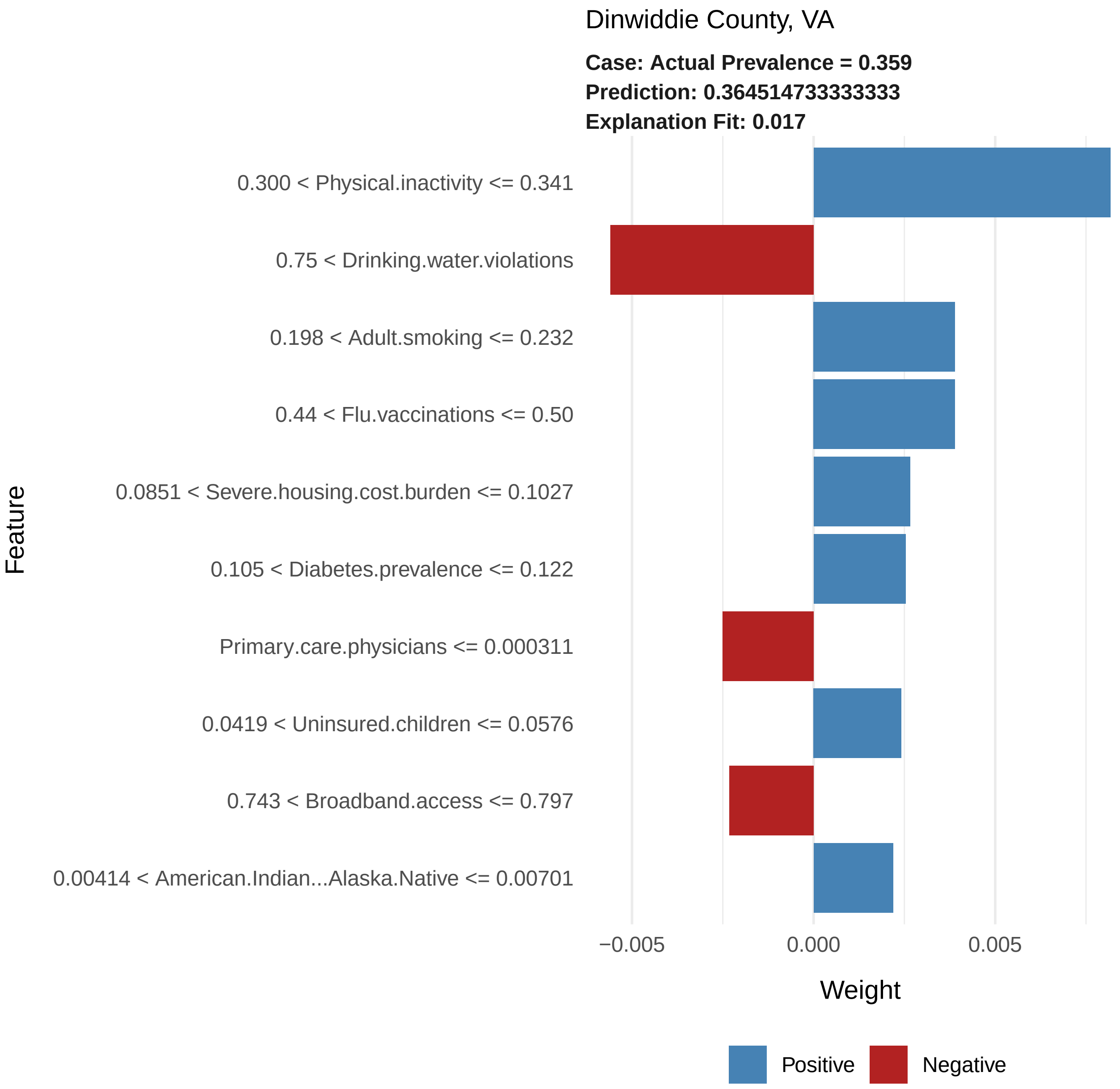

Dinwiddie County, VA
Case: Actual Prevalence = 0.359
Prediction: 0.364514733333333
Explanation Fit: 0.017
0.300 < Physical.inactivity <= 0.341
0.75 < Drinking.water.violations
0.198 < Adult.smoking <= 0.232
0.44 < Flu.vaccinations <= 0.50
0.0851 < Severe.housing.cost.burden <= 0.1027
0.105 < Diabetes.prevalence <= 0.122
Primary.care.physicians <= 0.000311
0.0419 < Uninsured.children <= 0.0576
0.743 < Broadband.access <= 0.797
0.00414 < American.Indian...Alaska.Native <= 0.00701
Feature
−0.005
0.000
0.005
Weight
Positive
Negative

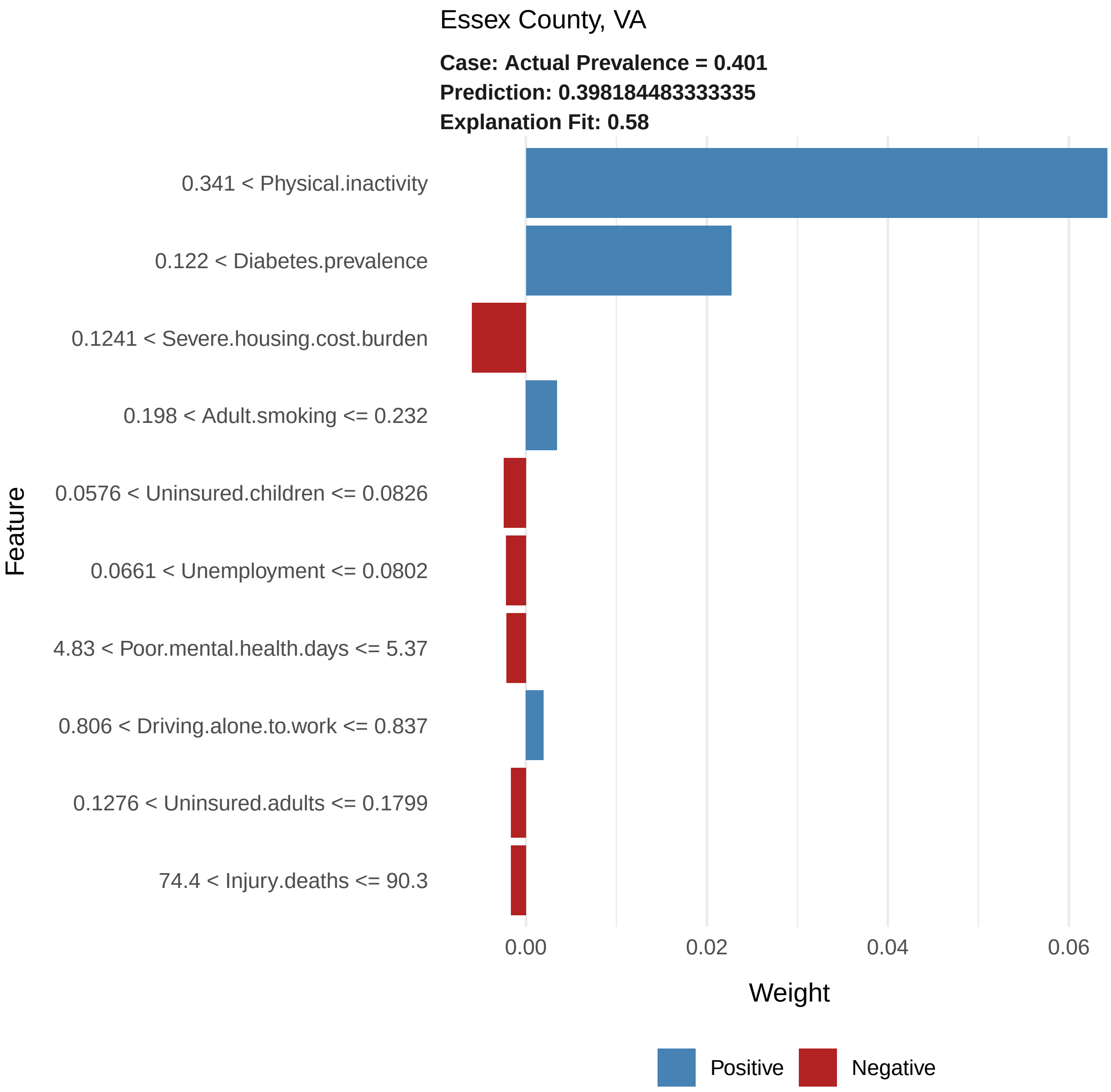

Essex County, VA
Case: Actual Prevalence = 0.401
Prediction: 0.398184483333335
Explanation Fit: 0.58
0.341 < Physical.inactivity
0.122 < Diabetes.prevalence
0.1241 < Severe.housing.cost.burden
0.198 < Adult.smoking <= 0.232
0.0576 < Uninsured.children <= 0.0826
0.0661 < Unemployment <= 0.0802
4.83 < Poor.mental.health.days <= 5.37
0.806 < Driving.alone.to.work <= 0.837
0.1276 < Uninsured.adults <= 0.1799
74.4 < Injury.deaths <= 90.3
Feature
0.00
0.02
0.04
0.06
Weight
Positive
Negative

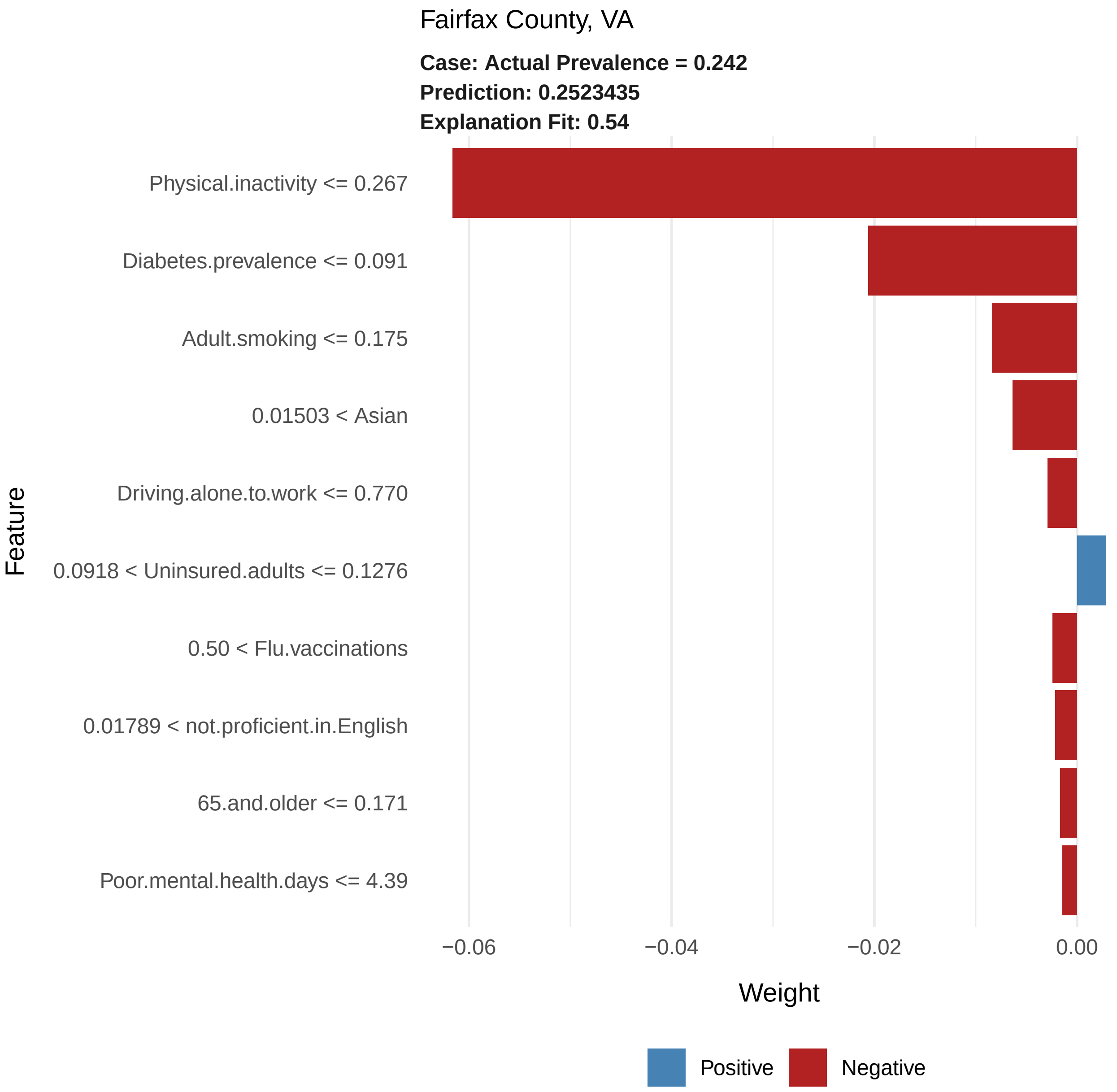

Fairfax County, VA
Case: Actual Prevalence = 0.242
Prediction: 0.2523435
Explanation Fit: 0.54
Physical.inactivity <= 0.267
Diabetes.prevalence <= 0.091
Adult.smoking <= 0.175
0.01503 < Asian
Driving.alone.to.work <= 0.770
0.0918 < Uninsured.adults <= 0.1276
0.50 < Flu.vaccinations
0.01789 < not.proficient.in.English
65.and.older <= 0.171
Poor.mental.health.days <= 4.39
Feature
−0.06
−0.04
−0.02
0.00
Weight
Positive
Negative

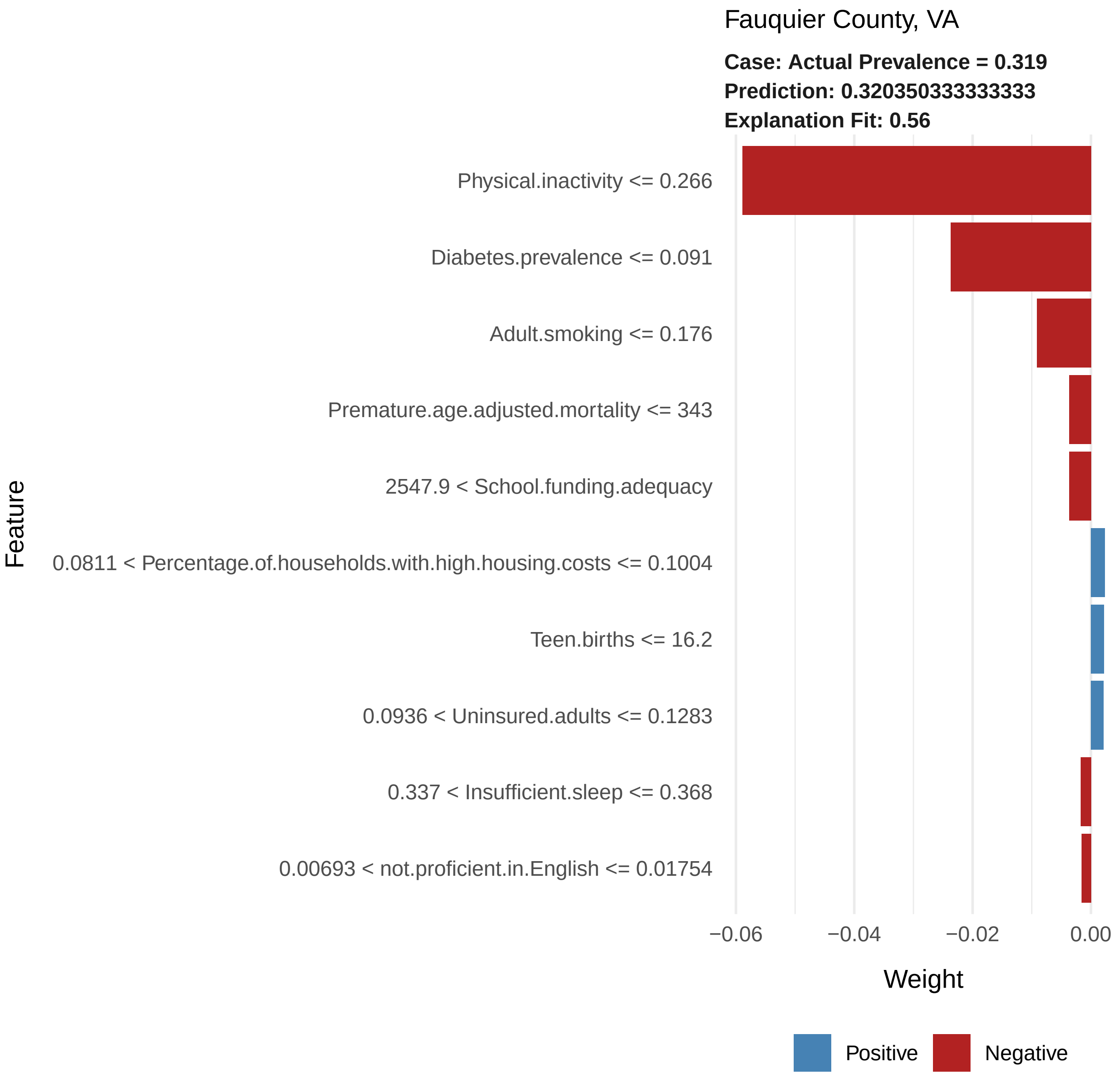

Fauquier County, VA
Case: Actual Prevalence = 0.319
Prediction: 0.320350333333333
Explanation Fit: 0.56
Physical.inactivity <= 0.266
Diabetes.prevalence <= 0.091
Adult.smoking <= 0.176
Premature.age.adjusted.mortality <= 343
2547.9 < School.funding.adequacy
0.0811 < Percentage.of.households.with.high.housing.costs <= 0.1004
Teen.births <= 16.2
0.0936 < Uninsured.adults <= 0.1283
0.337 < Insufficient.sleep <= 0.368
0.00693 < not.proficient.in.English <= 0.01754
Feature
−0.06
−0.04
−0.02
0.00
Weight
Positive
Negative

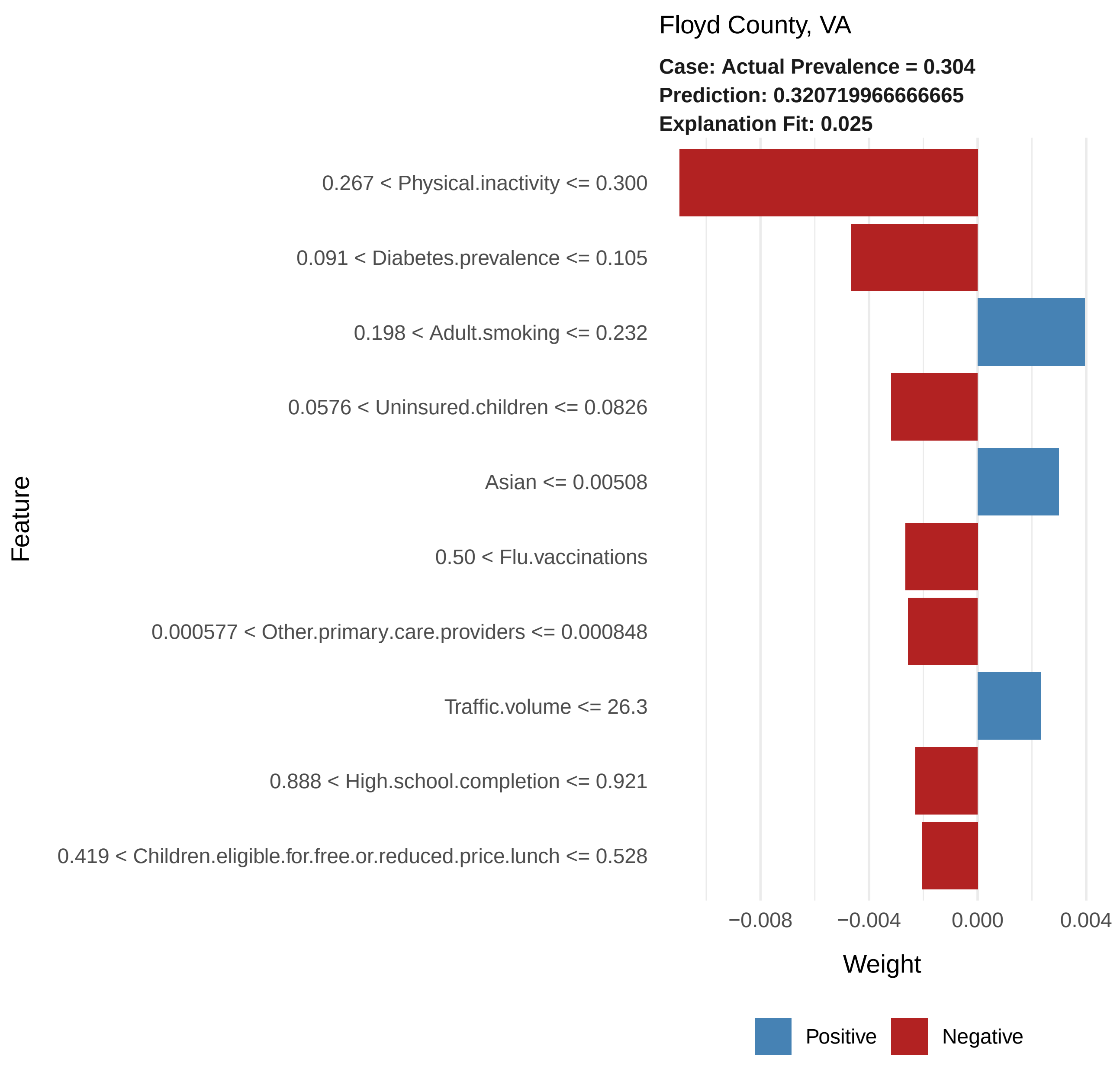
Floyd County, VA
Case: Actual Prevalence = 0.304
Prediction: 0.320719966666665
Explanation Fit: 0.025
0.267 < Physical.inactivity <= 0.300
0.091 < Diabetes.prevalence <= 0.105
0.198 < Adult.smoking <= 0.232
0.0576 < Uninsured.children <= 0.0826
Asian <= 0.00508
0.50 < Flu.vaccinations
0.000577 < Other.primary.care.providers <= 0.000848
Traffic.volume <= 26.3
0.888 < High.school.completion <= 0.921
0.419 < Children.eligible.for.free.or.reduced.price.lunch <= 0.528
Feature
−0.008
−0.004
0.000
0.004
Weight
Positive
Negative

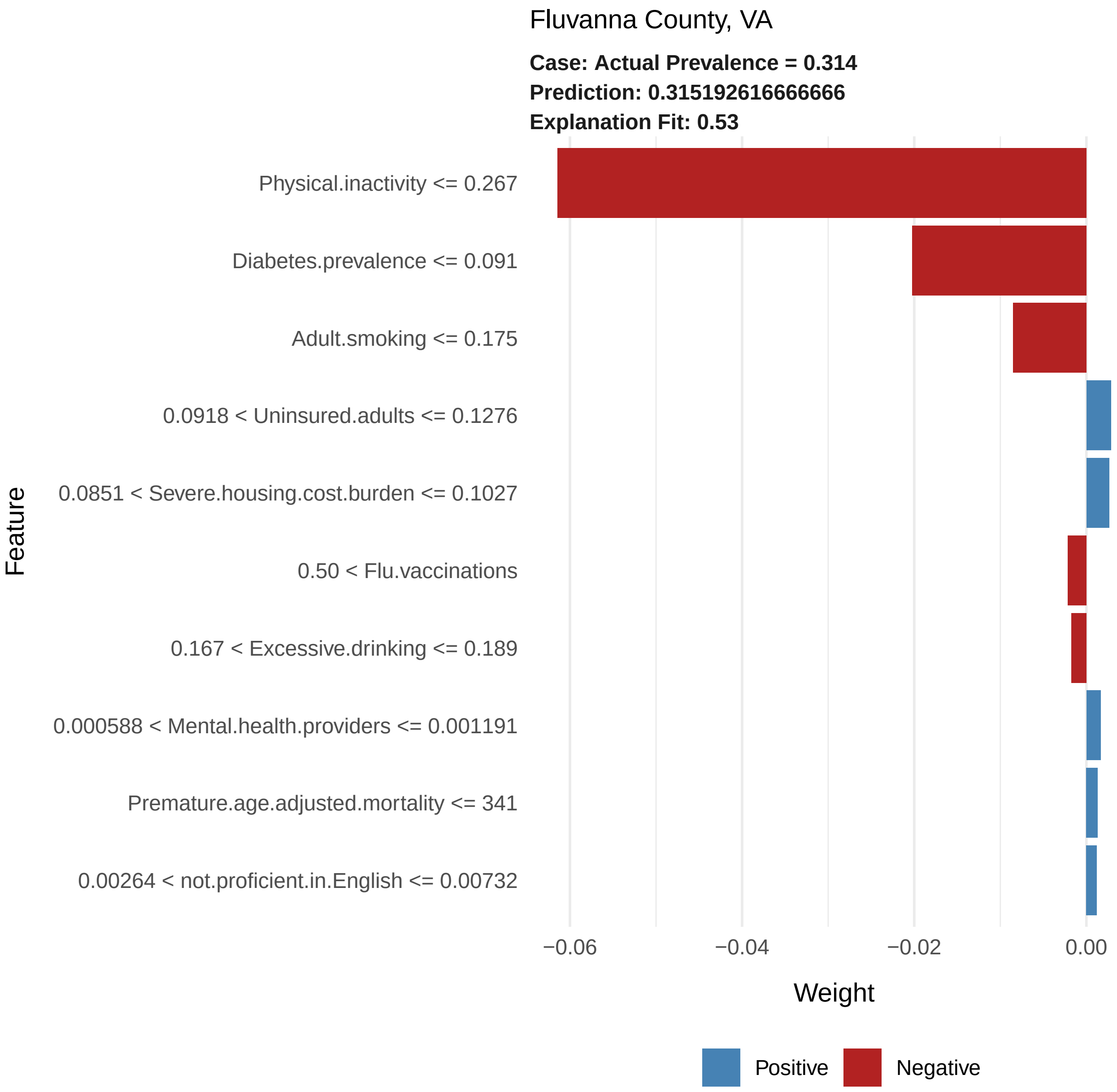

Fluvanna County, VA
Case: Actual Prevalence = 0.314
Prediction: 0.315192616666666
Explanation Fit: 0.53
Physical.inactivity <= 0.267
Diabetes.prevalence <= 0.091
Adult.smoking <= 0.175
0.0918 < Uninsured.adults <= 0.1276
0.0851 < Severe.housing.cost.burden <= 0.1027
0.50 < Flu.vaccinations
0.167 < Excessive.drinking <= 0.189
0.000588 < Mental.health.providers <= 0.001191
Premature.age.adjusted.mortality <= 341
0.00264 < not.proficient.in.English <= 0.00732
Feature
−0.06
−0.04
−0.02
0.00
Weight
Positive
Negative

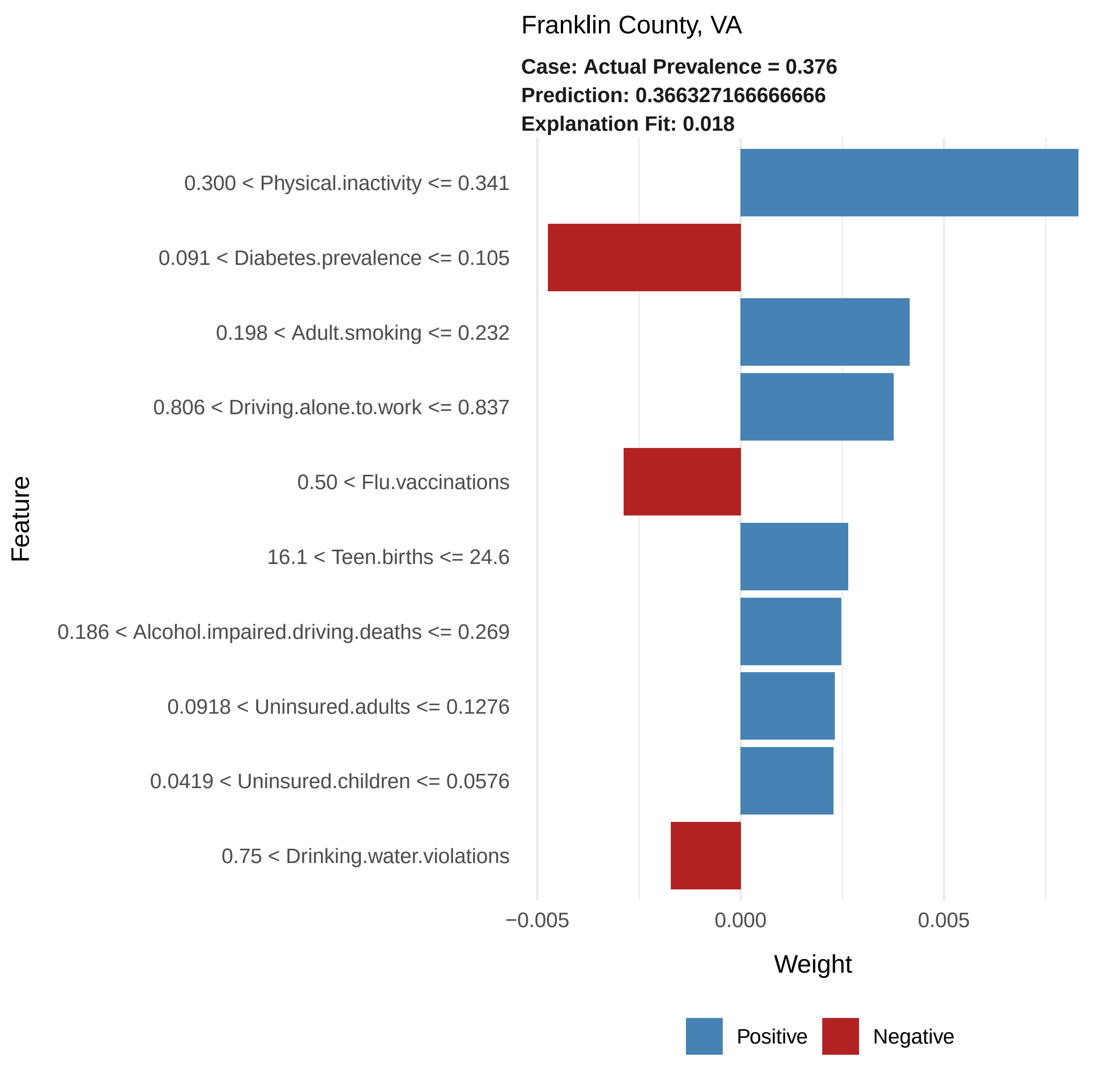

Franklin County, VA
Case: Actual Prevalence = 0.376
Prediction: 0.366327166666666
Explanation Fit: 0.018
0.300 < Physical.inactivity <= 0.341
0.091 < Diabetes.prevalence <= 0.105
0.198 < Adult.smoking <= 0.232
0.806 < Driving.alone.to.work <= 0.837
0.50 < Flu.vaccinations
16.1 < Teen.births <= 24.6
0.186 < Alcohol.impaired.driving.deaths <= 0.269
0.0918 < Uninsured.adults <= 0.1276
0.0419 < Uninsured.children <= 0.0576
0.75 < Drinking.water.violations
Feature
−0.005
0.000
0.005
Weight
Positive
Negative

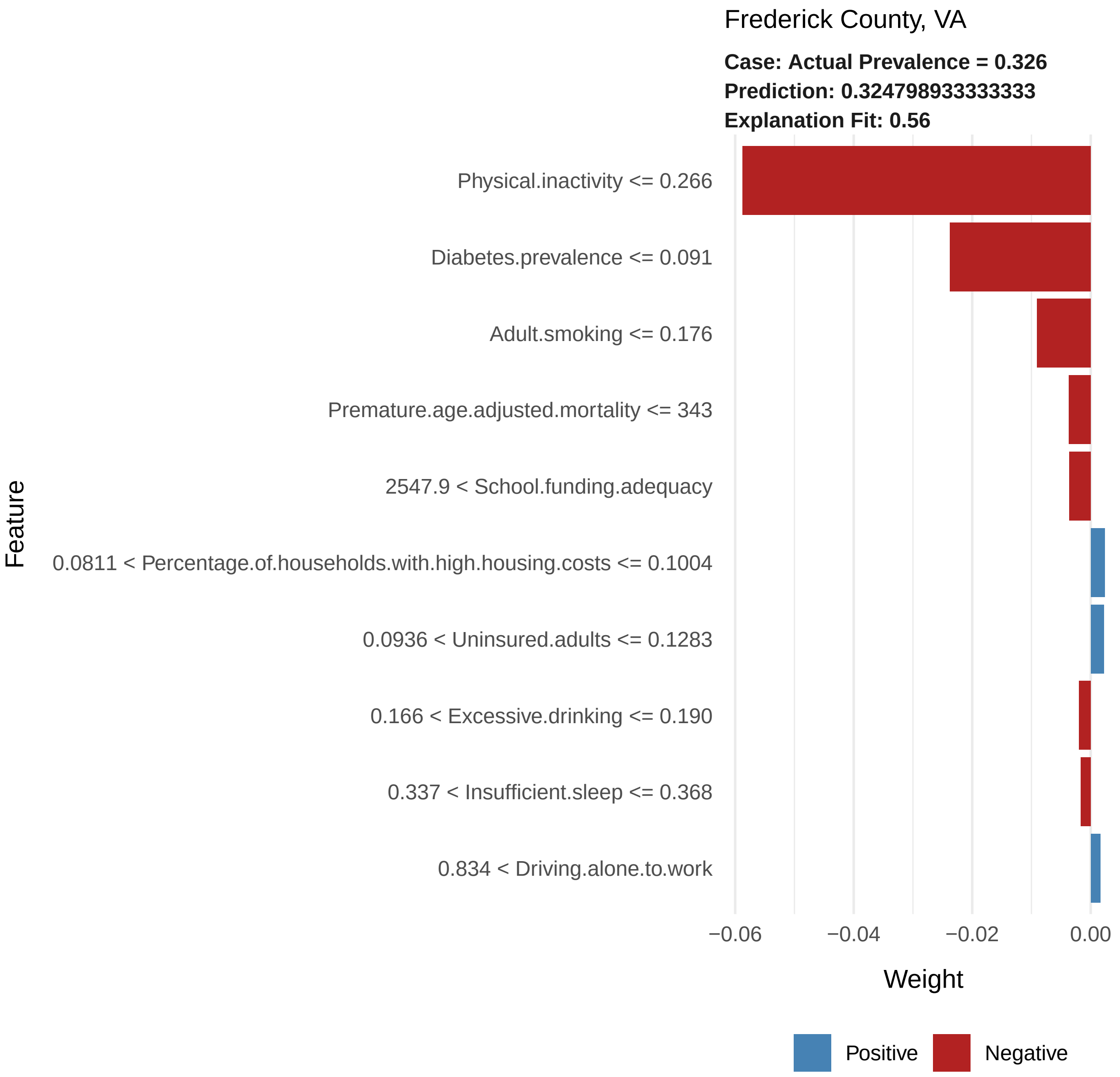

Frederick County, VA
Case: Actual Prevalence = 0.326
Prediction: 0.324798933333333
Explanation Fit: 0.56
Physical.inactivity <= 0.266
Diabetes.prevalence <= 0.091
Adult.smoking <= 0.176
Premature.age.adjusted.mortality <= 343
2547.9 < School.funding.adequacy
0.0811 < Percentage.of.households.with.high.housing.costs <= 0.1004
0.0936 < Uninsured.adults <= 0.1283
0.166 < Excessive.drinking <= 0.190
0.337 < Insufficient.sleep <= 0.368
0.834 < Driving.alone.to.work
Feature
−0.06
−0.04
−0.02
0.00
Weight
Positive
Negative

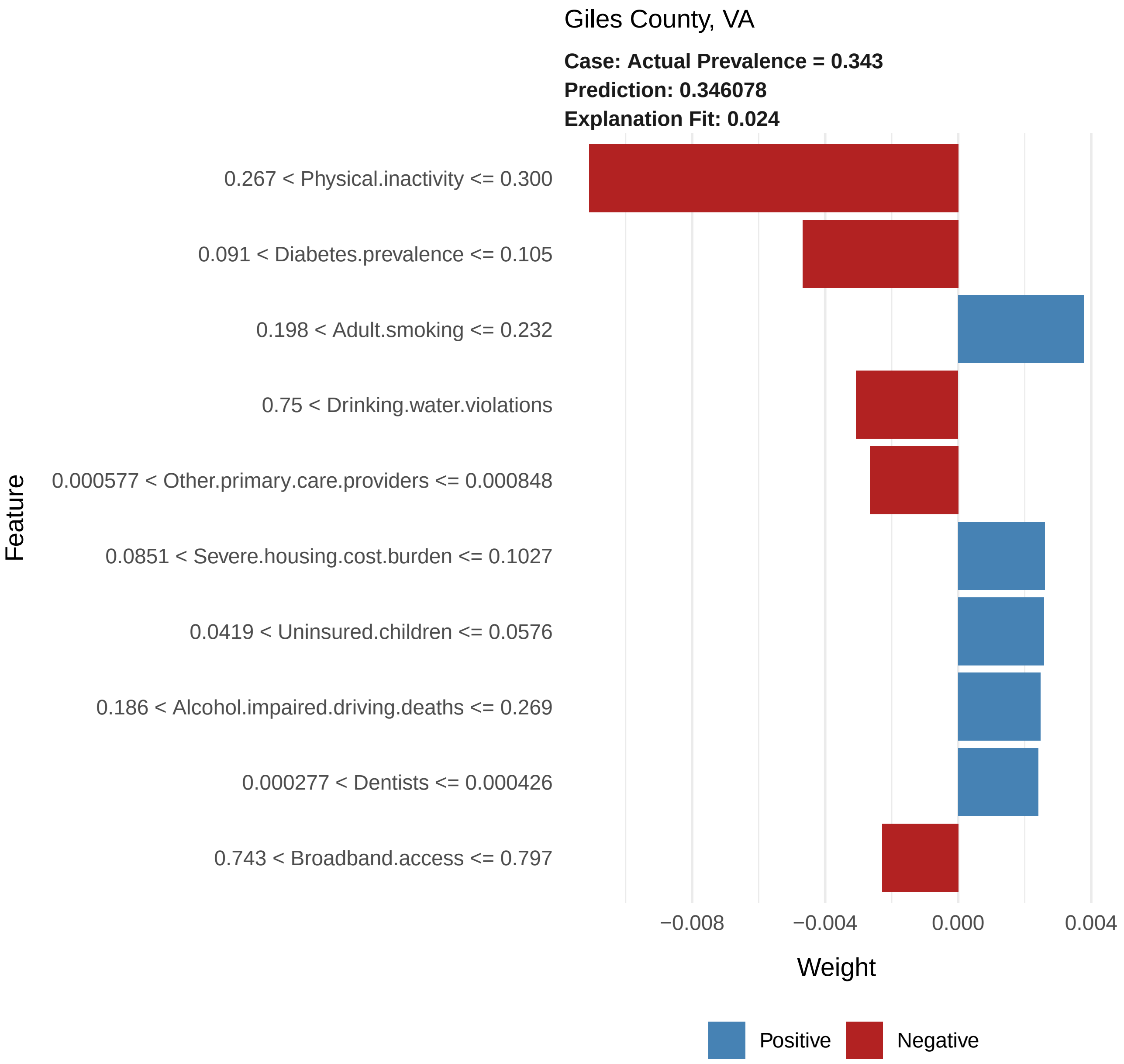

Giles County, VA
Case: Actual Prevalence = 0.343
Prediction: 0.346078
Explanation Fit: 0.024
0.267 < Physical.inactivity <= 0.300
0.091 < Diabetes.prevalence <= 0.105
0.198 < Adult.smoking <= 0.232
0.75 < Drinking.water.violations
0.000577 < Other.primary.care.providers <= 0.000848
0.0851 < Severe.housing.cost.burden <= 0.1027
0.0419 < Uninsured.children <= 0.0576
0.186 < Alcohol.impaired.driving.deaths <= 0.269
0.000277 < Dentists <= 0.000426
0.743 < Broadband.access <= 0.797
Feature
−0.008
−0.004
0.000
0.004
Weight
Positive
Negative

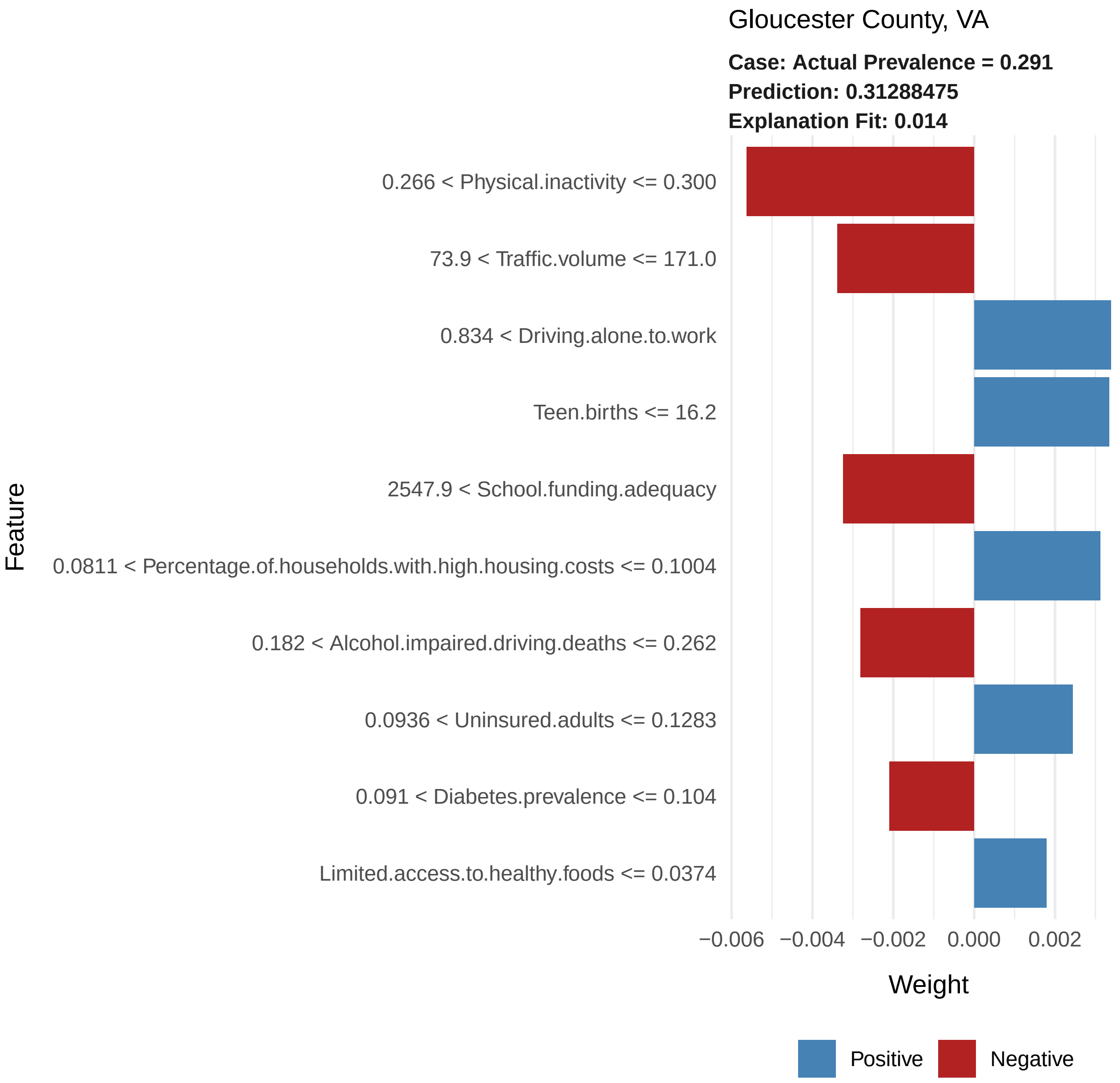
Gloucester County, VA
Case: Actual Prevalence = 0.291
Prediction: 0.31288475
Explanation Fit: 0.014
Feature
0.266 < Physical.inactivity <= 0.300
73.9 < Traffic.volume <= 171.0
0.834 < Driving.alone.to.work
Teen.births <= 16.2
2547.9 < School.funding.adequacy
0.0811 < Percentage.of.households.with.high.housing.costs <= 0.1004
0.182 < Alcohol.impaired.driving.deaths <= 0.262
0.0936 < Uninsured.adults <= 0.1283
0.091 < Diabetes.prevalence <= 0.104
Limited.access.to.healthy.foods <= 0.0374
−0.006
−0.004
−0.002
0.000
0.002
Weight
Positive
Negative

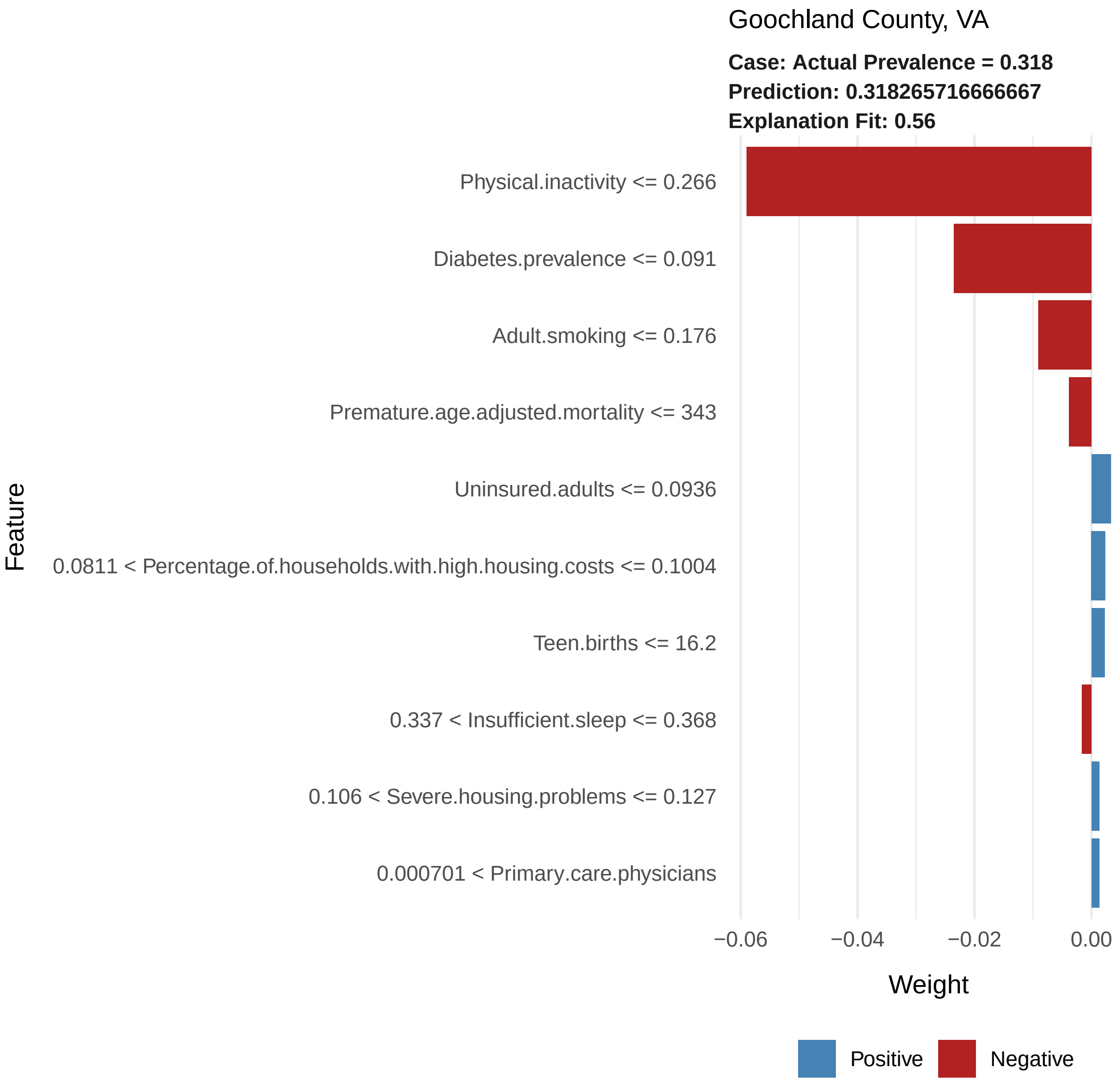
Goochland County, VA
Case: Actual Prevalence = 0.318
Prediction: 0.318265716666667
Explanation Fit: 0.56
Physical.inactivity <= 0.266
Diabetes.prevalence <= 0.091
Adult.smoking <= 0.176
Premature.age.adjusted.mortality <= 343
Uninsured.adults <= 0.0936
0.0811 < Percentage.of.households.with.high.housing.costs <= 0.1004
Teen.births <= 16.2
0.337 < Insufficient.sleep <= 0.368
0.106 < Severe.housing.problems <= 0.127
0.000701 < Primary.care.physicians
Feature
−0.06
−0.04
−0.02
0.00
Weight
Positive
Negative

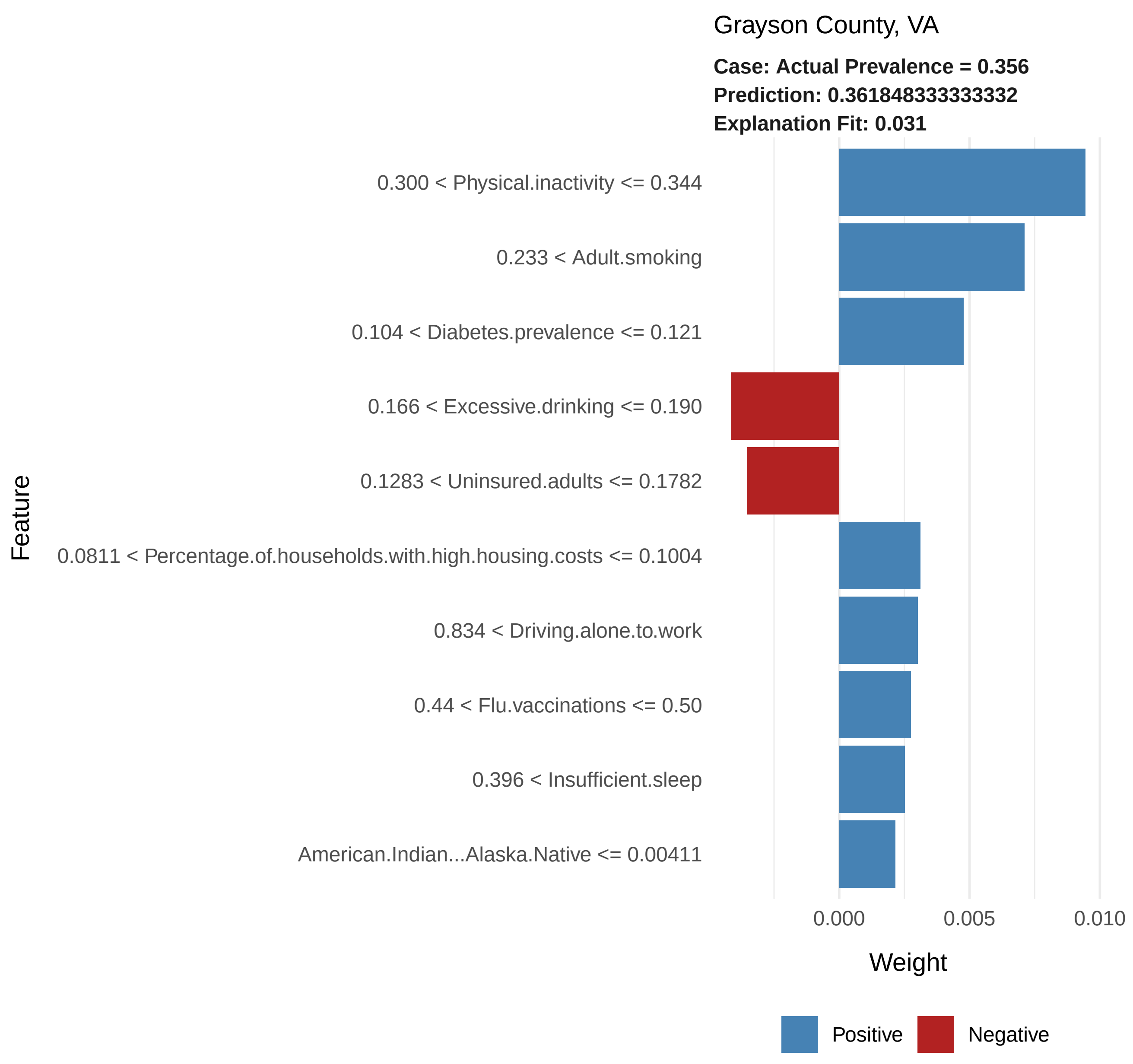

Grayson County, VA
Case: Actual Prevalence = 0.356
Prediction: 0.361848333333332
Explanation Fit: 0.031
0.300 < Physical.inactivity <= 0.344
0.233 < Adult.smoking
0.104 < Diabetes.prevalence <= 0.121
0.166 < Excessive.drinking <= 0.190
0.1283 < Uninsured.adults <= 0.1782
0.0811 < Percentage.of.households.with.high.housing.costs <= 0.1004
0.834 < Driving.alone.to.work
0.44 < Flu.vaccinations <= 0.50
0.396 < Insufficient.sleep
American.Indian...Alaska.Native <= 0.00411
Feature
0.000
0.005
0.010
Weight
Positive
Negative

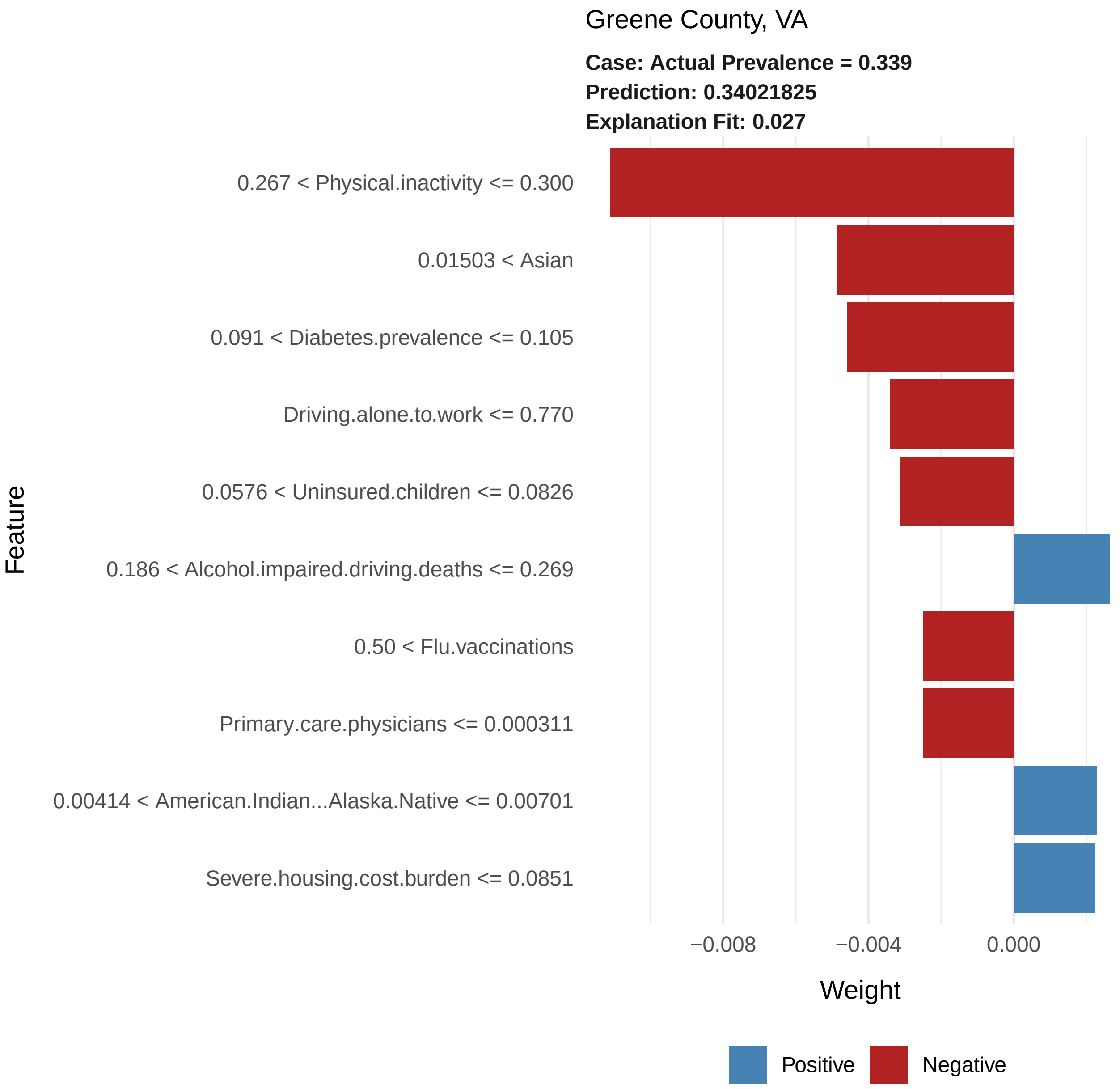

Greene County, VA
Case: Actual Prevalence = 0.339
Prediction: 0.34021825
Explanation Fit: 0.027
0.267 < Physical.inactivity <= 0.300
0.01503 < Asian
0.091 < Diabetes.prevalence <= 0.105
Driving.alone.to.work <= 0.770
0.0576 < Uninsured.children <= 0.0826
0.186 < Alcohol.impaired.driving.deaths <= 0.269
0.50 < Flu.vaccinations
Primary.care.physicians <= 0.000311
0.00414 < American.Indian...Alaska.Native <= 0.00701
Severe.housing.cost.burden <= 0.0851
Feature
−0.008
−0.004
0.000
Weight
Positive
Negative

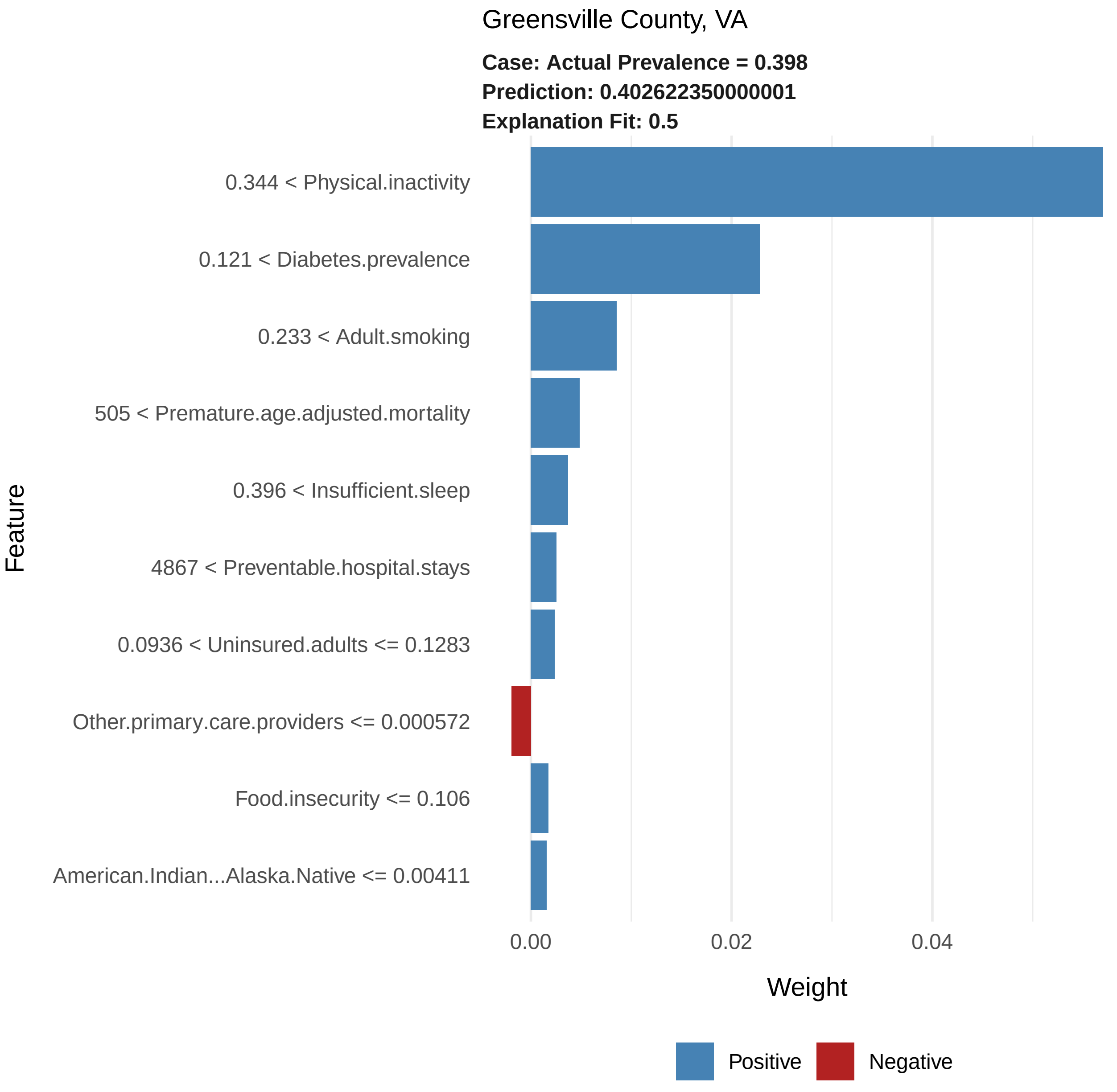
Greensville County, VA
Case: Actual Prevalence = 0.398
Prediction: 0.402622350000001
Explanation Fit: 0.5
0.344 < Physical.inactivity
0.121 < Diabetes.prevalence
0.233 < Adult.smoking
505 < Premature.age.adjusted.mortality
0.396 < Insufficient.sleep
4867 < Preventable.hospital.stays
0.0936 < Uninsured.adults <= 0.1283
Other.primary.care.providers <= 0.000572
Food.insecurity <= 0.106
American.Indian...Alaska.Native <= 0.00411
Feature
0.00
0.02
0.04
Weight
Positive
Negative

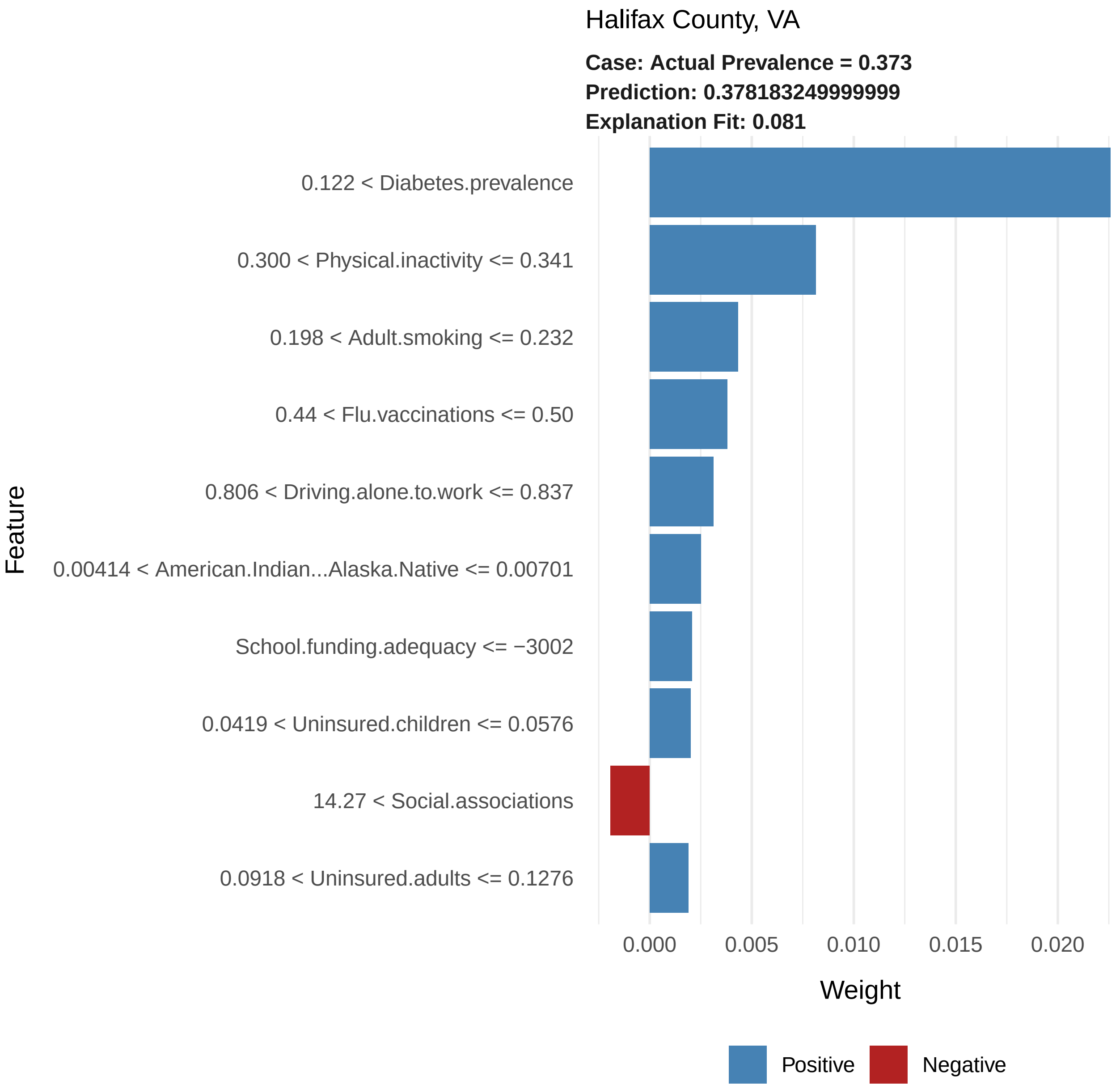
Halifax County, VA
Case: Actual Prevalence = 0.373
Prediction: 0.378183249999999
Explanation Fit: 0.081
0.122 < Diabetes.prevalence
0.300 < Physical.inactivity <= 0.341
0.198 < Adult.smoking <= 0.232
0.44 < Flu.vaccinations <= 0.50
0.806 < Driving.alone.to.work <= 0.837
0.00414 < American.Indian...Alaska.Native <= 0.00701
School.funding.adequacy <= −3002
0.0419 < Uninsured.children <= 0.0576
14.27 < Social.associations
0.0918 < Uninsured.adults <= 0.1276
Feature
0.000
0.005
0.010
0.015
0.020
Weight
Positive
Negative

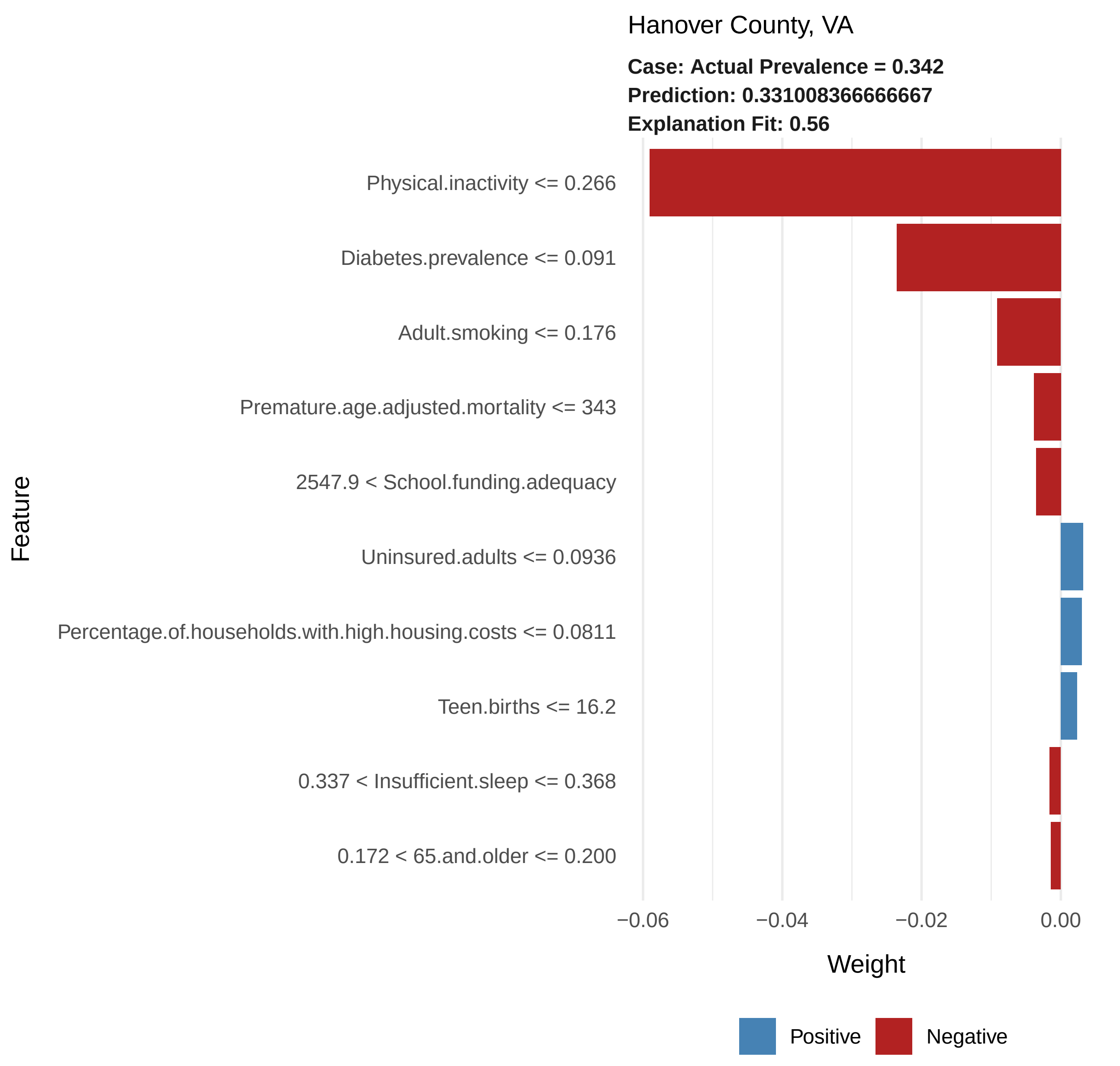

Hanover County, VA
Case: Actual Prevalence = 0.342
Prediction: 0.331008366666667
Explanation Fit: 0.56
Physical.inactivity <= 0.266
Diabetes.prevalence <= 0.091
Adult.smoking <= 0.176
Premature.age.adjusted.mortality <= 343
2547.9 < School.funding.adequacy
Uninsured.adults <= 0.0936
Percentage.of.households.with.high.housing.costs <= 0.0811
Teen.births <= 16.2
0.337 < Insufficient.sleep <= 0.368
0.172 < 65.and.older <= 0.200
Feature
−0.06
−0.04
−0.02
0.00
Weight
Positive
Negative

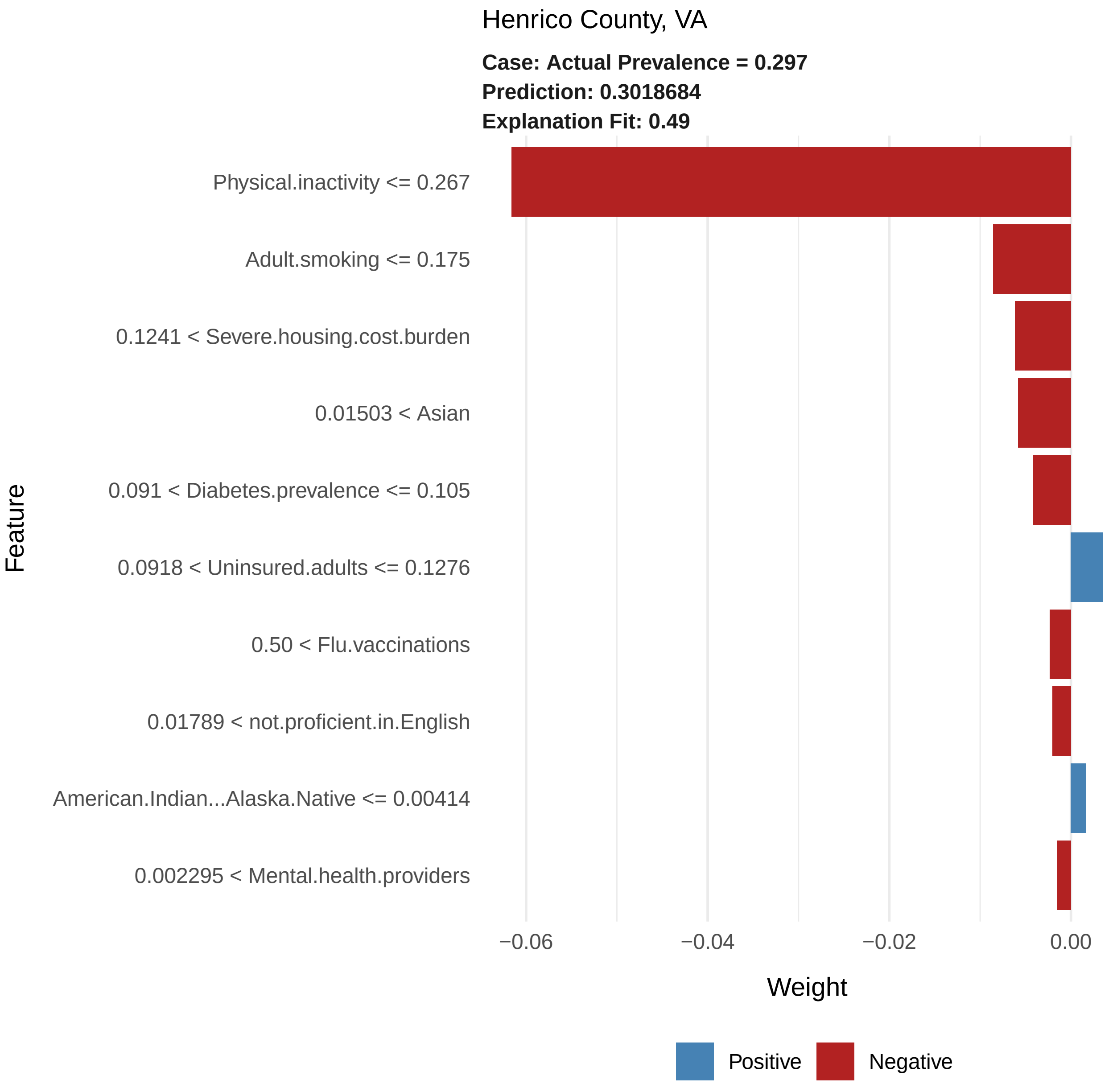

Henrico County, VA
Case: Actual Prevalence = 0.297
Prediction: 0.3018684
Explanation Fit: 0.49
Physical.inactivity <= 0.267
Adult.smoking <= 0.175
0.1241 < Severe.housing.cost.burden
0.01503 < Asian
0.091 < Diabetes.prevalence <= 0.105
0.0918 < Uninsured.adults <= 0.1276
0.50 < Flu.vaccinations
0.01789 < not.proficient.in.English
American.Indian...Alaska.Native <= 0.00414
0.002295 < Mental.health.providers
Feature
−0.06
−0.04
−0.02
0.00
Weight
Positive
Negative

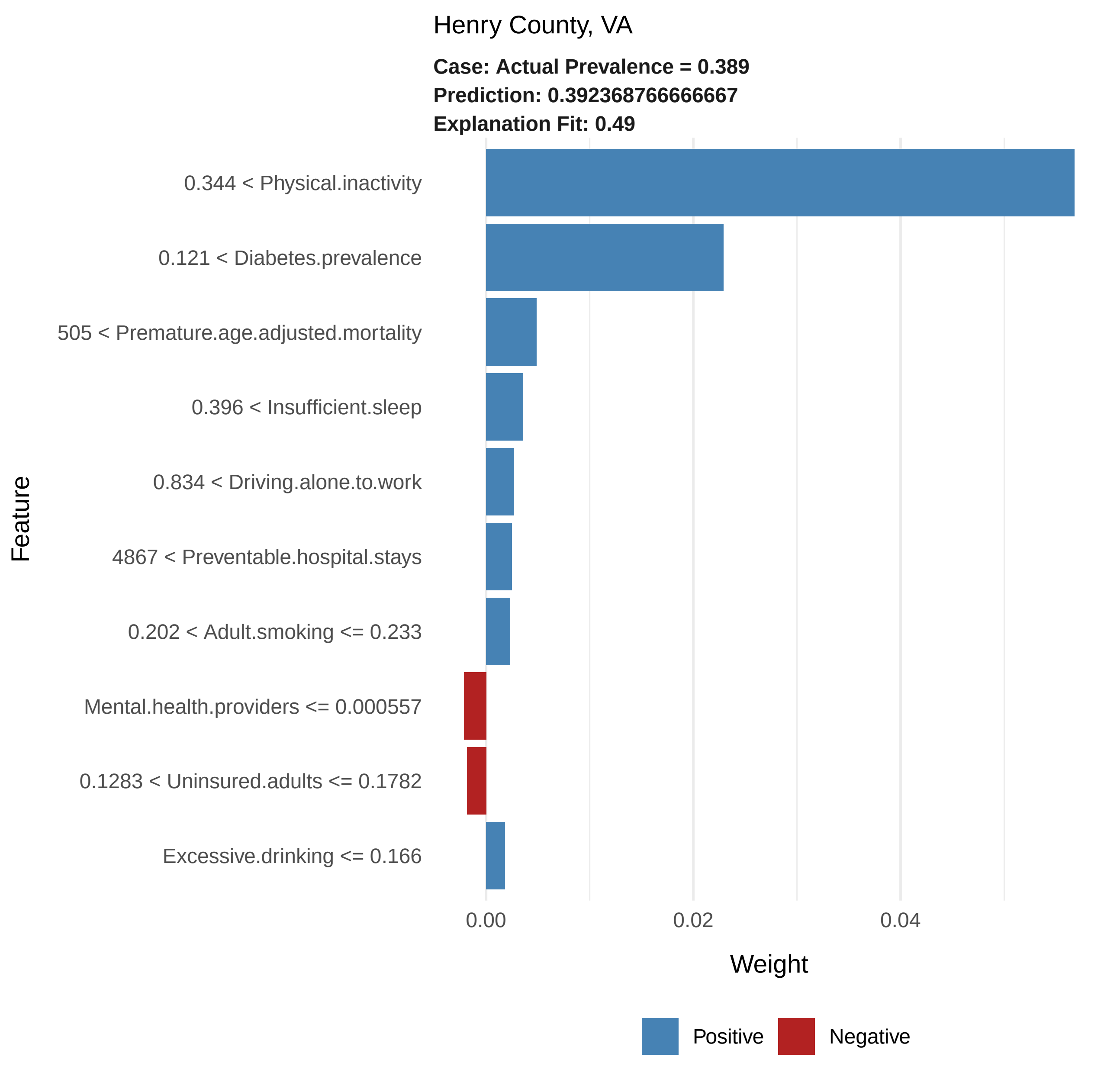

Henry County, VA
Case: Actual Prevalence = 0.389
Prediction: 0.392368766666667
Explanation Fit: 0.49
0.344 < Physical.inactivity
0.121 < Diabetes.prevalence
505 < Premature.age.adjusted.mortality
0.396 < Insufficient.sleep
0.834 < Driving.alone.to.work
4867 < Preventable.hospital.stays
0.202 < Adult.smoking <= 0.233
Mental.health.providers <= 0.000557
0.1283 < Uninsured.adults <= 0.1782
Excessive.drinking <= 0.166
Feature
0.00
0.02
0.04
Weight
Positive
Negative

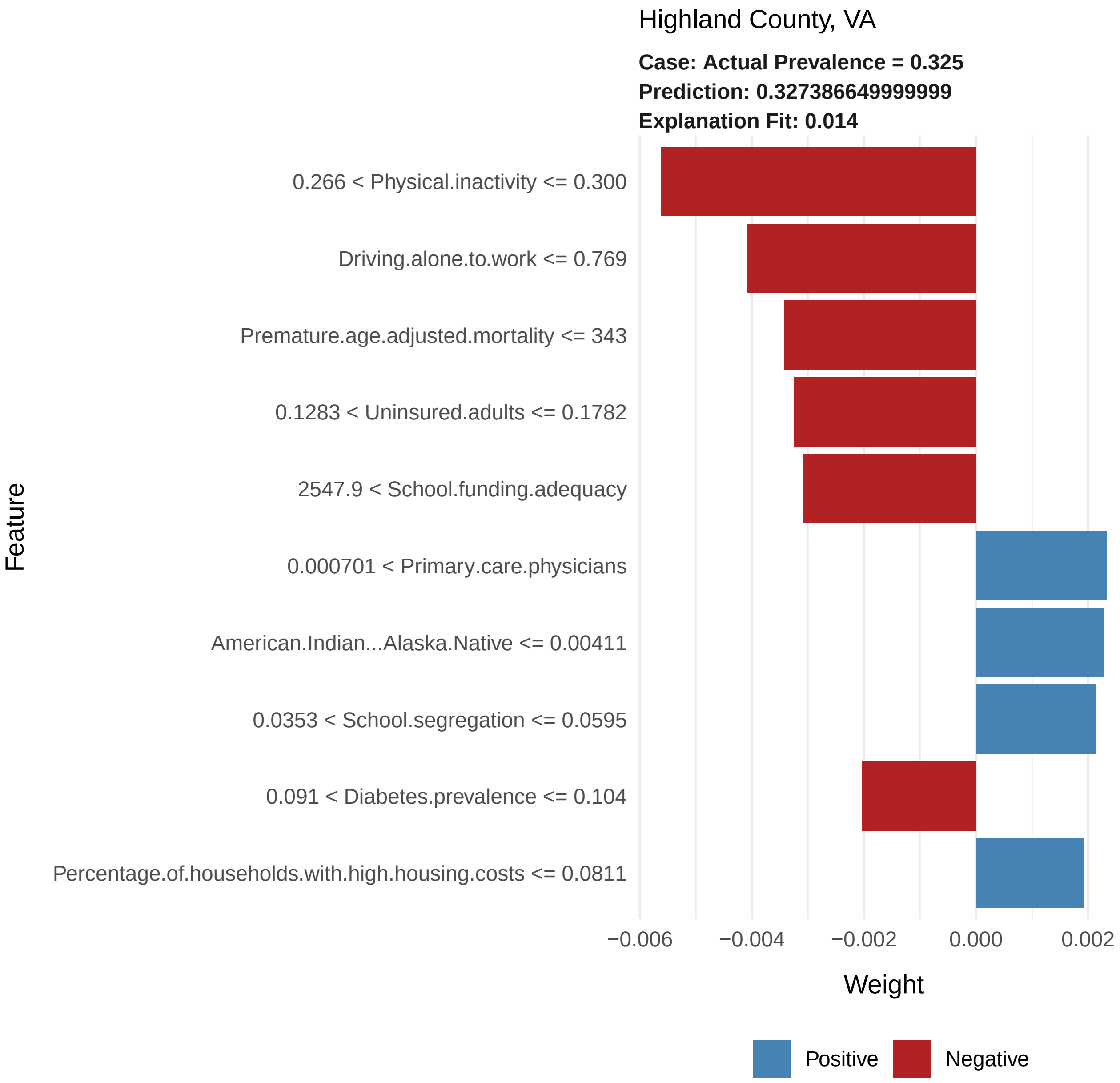

Highland County, VA
Case: Actual Prevalence = 0.325
Prediction: 0.327386649999999
Explanation Fit: 0.014
0.266 < Physical.inactivity <= 0.300
Driving.alone.to.work <= 0.769
Premature.age.adjusted.mortality <= 343
0.1283 < Uninsured.adults <= 0.1782
2547.9 < School.funding.adequacy
0.000701 < Primary.care.physicians
American.Indian...Alaska.Native <= 0.00411
0.0353 < School.segregation <= 0.0595
0.091 < Diabetes.prevalence <= 0.104
Percentage.of.households.with.high.housing.costs <= 0.0811
Feature
−0.006
−0.004
−0.002
0.000
0.002
Weight
Positive
Negative

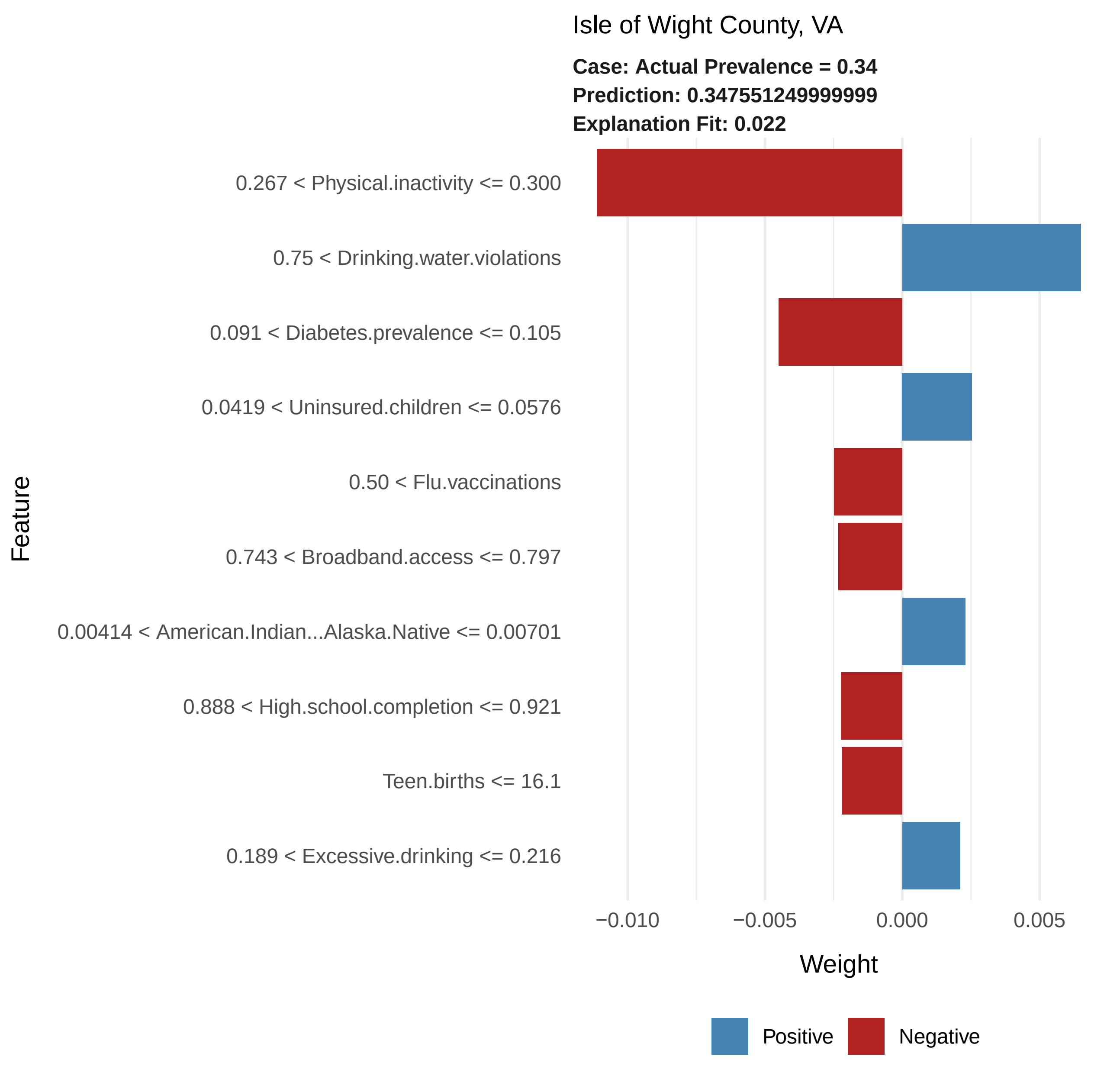

Isle of Wight County, VA
Case: Actual Prevalence = 0.34
Prediction: 0.347551249999999
Explanation Fit: 0.022
0.267 < Physical.inactivity <= 0.300
0.75 < Drinking.water.violations
0.091 < Diabetes.prevalence <= 0.105
0.0419 < Uninsured.children <= 0.0576
0.50 < Flu.vaccinations
0.743 < Broadband.access <= 0.797
0.00414 < American.Indian...Alaska.Native <= 0.00701
0.888 < High.school.completion <= 0.921
Teen.births <= 16.1
0.189 < Excessive.drinking <= 0.216
Feature
−0.010
−0.005
0.000
0.005
Weight
Positive
Negative

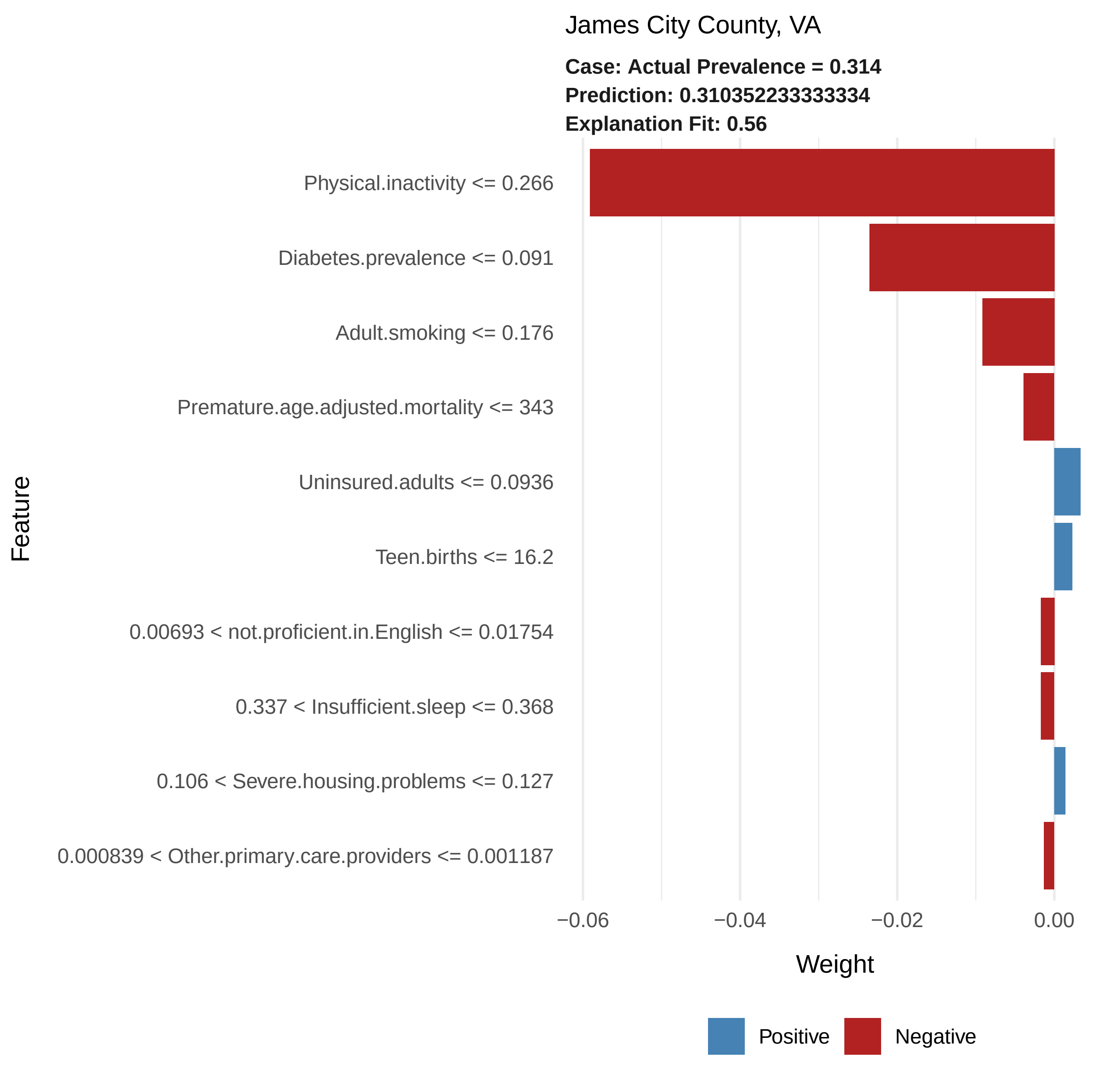
James City County, VA
Case: Actual Prevalence = 0.314
Prediction: 0.310352233333334
Explanation Fit: 0.56
Physical.inactivity <= 0.266
Diabetes.prevalence <= 0.091
Adult.smoking <= 0.176
Premature.age.adjusted.mortality <= 343
Uninsured.adults <= 0.0936
Teen.births <= 16.2
0.00693 < not.proficient.in.English <= 0.01754
0.337 < Insufficient.sleep <= 0.368
0.106 < Severe.housing.problems <= 0.127
0.000839 < Other.primary.care.providers <= 0.001187
Feature
−0.06
−0.04
−0.02
0.00
Weight
Positive
Negative

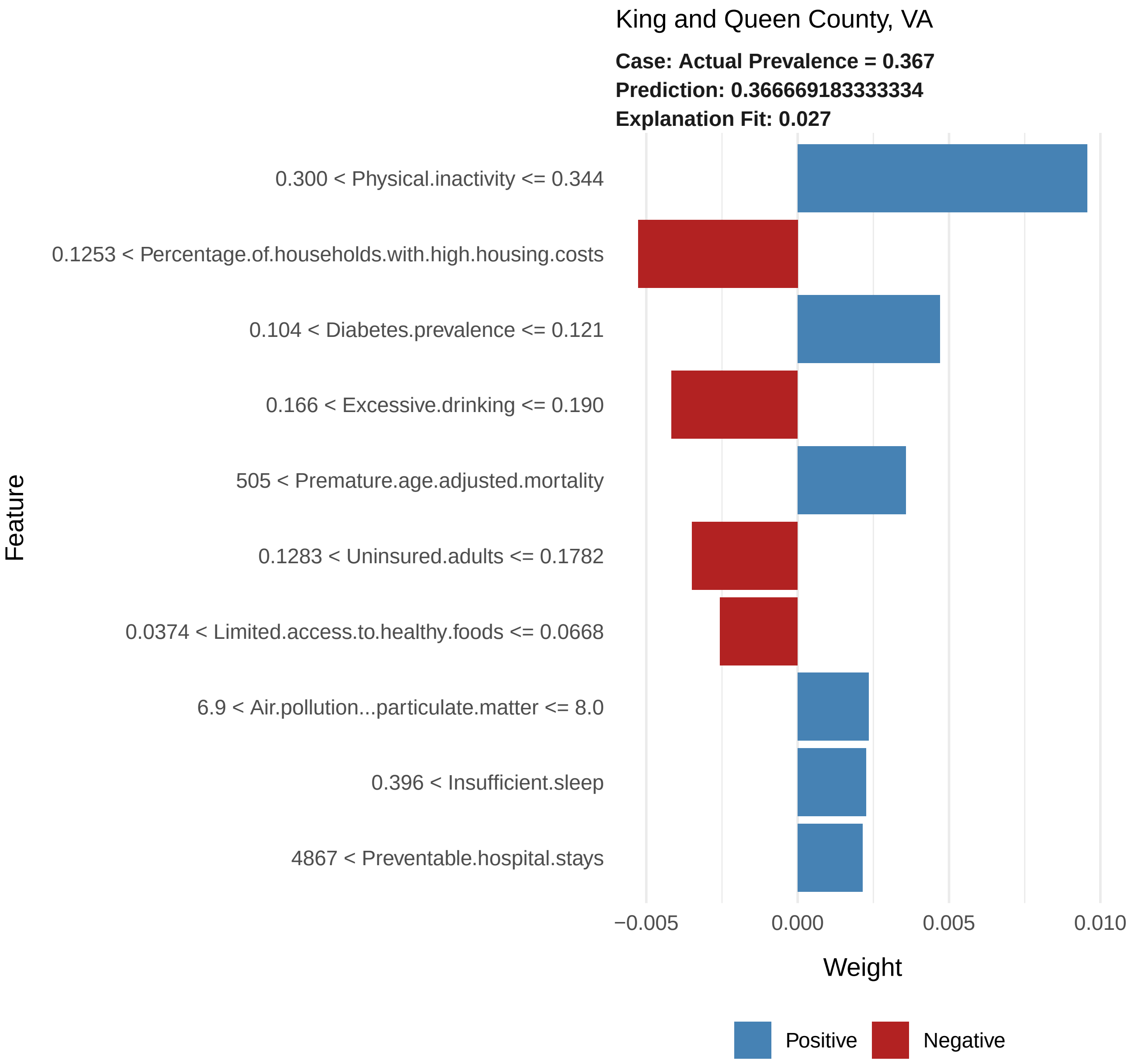

King and Queen County, VA
Case: Actual Prevalence = 0.367
Prediction: 0.366669183333334
Explanation Fit: 0.027
0.300 < Physical.inactivity <= 0.344
0.1253 < Percentage.of.households.with.high.housing.costs
0.104 < Diabetes.prevalence <= 0.121
0.166 < Excessive.drinking <= 0.190
505 < Premature.age.adjusted.mortality
0.1283 < Uninsured.adults <= 0.1782
0.0374 < Limited.access.to.healthy.foods <= 0.0668
6.9 < Air.pollution...particulate.matter <= 8.0
0.396 < Insufficient.sleep
4867 < Preventable.hospital.stays
Feature
−0.005
0.000
0.005
0.010
Weight
Positive
Negative

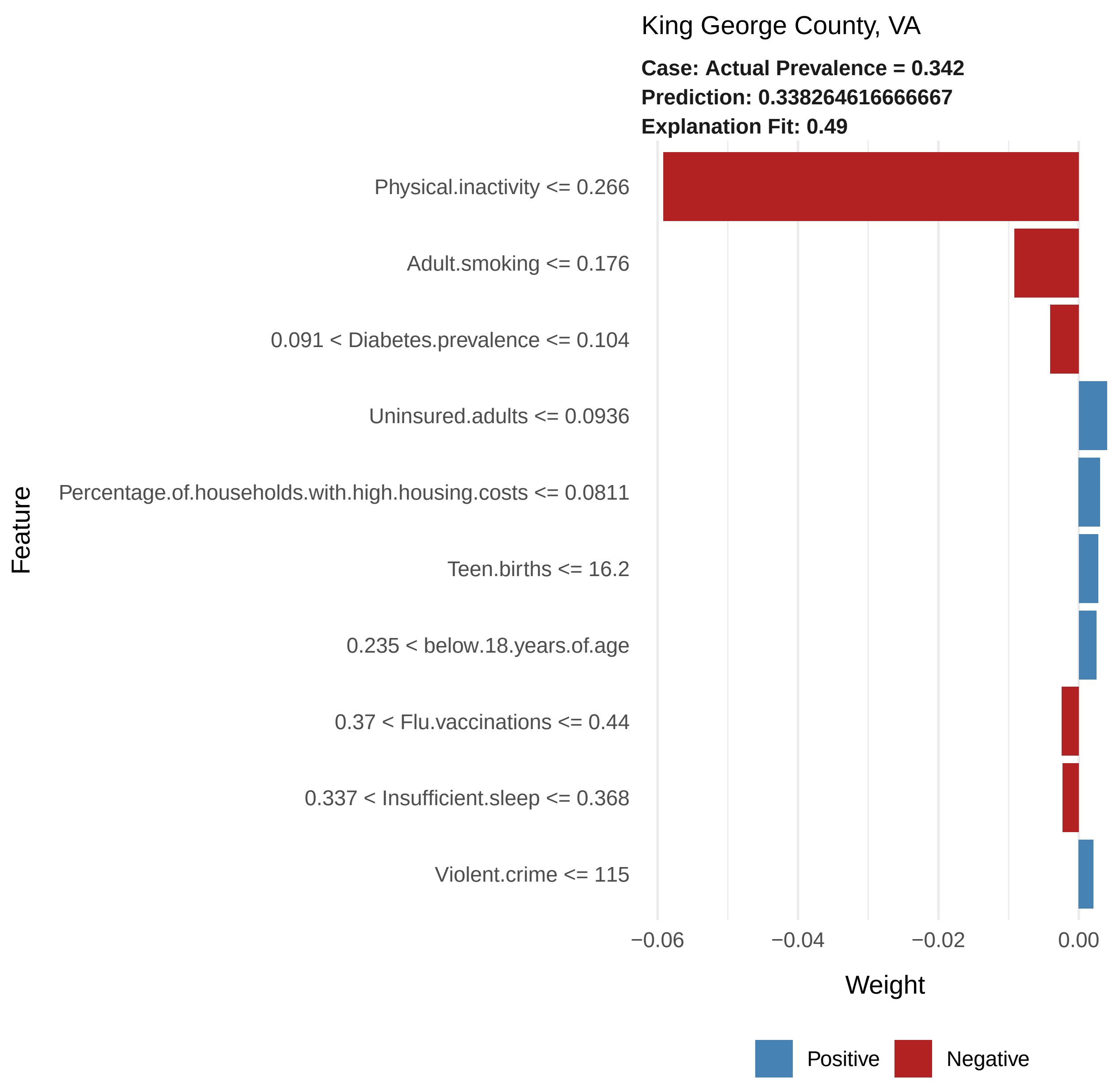

King George County, VA
Case: Actual Prevalence = 0.342
Prediction: 0.338264616666667
Explanation Fit: 0.49
Physical.inactivity <= 0.266
Adult.smoking <= 0.176
0.091 < Diabetes.prevalence <= 0.104
Uninsured.adults <= 0.0936
Percentage.of.households.with.high.housing.costs <= 0.0811
Teen.births <= 16.2
0.235 < below.18.years.of.age
0.37 < Flu.vaccinations <= 0.44
0.337 < Insufficient.sleep <= 0.368
Violent.crime <= 115
Feature
−0.06
−0.04
−0.02
0.00
Weight
Positive
Negative

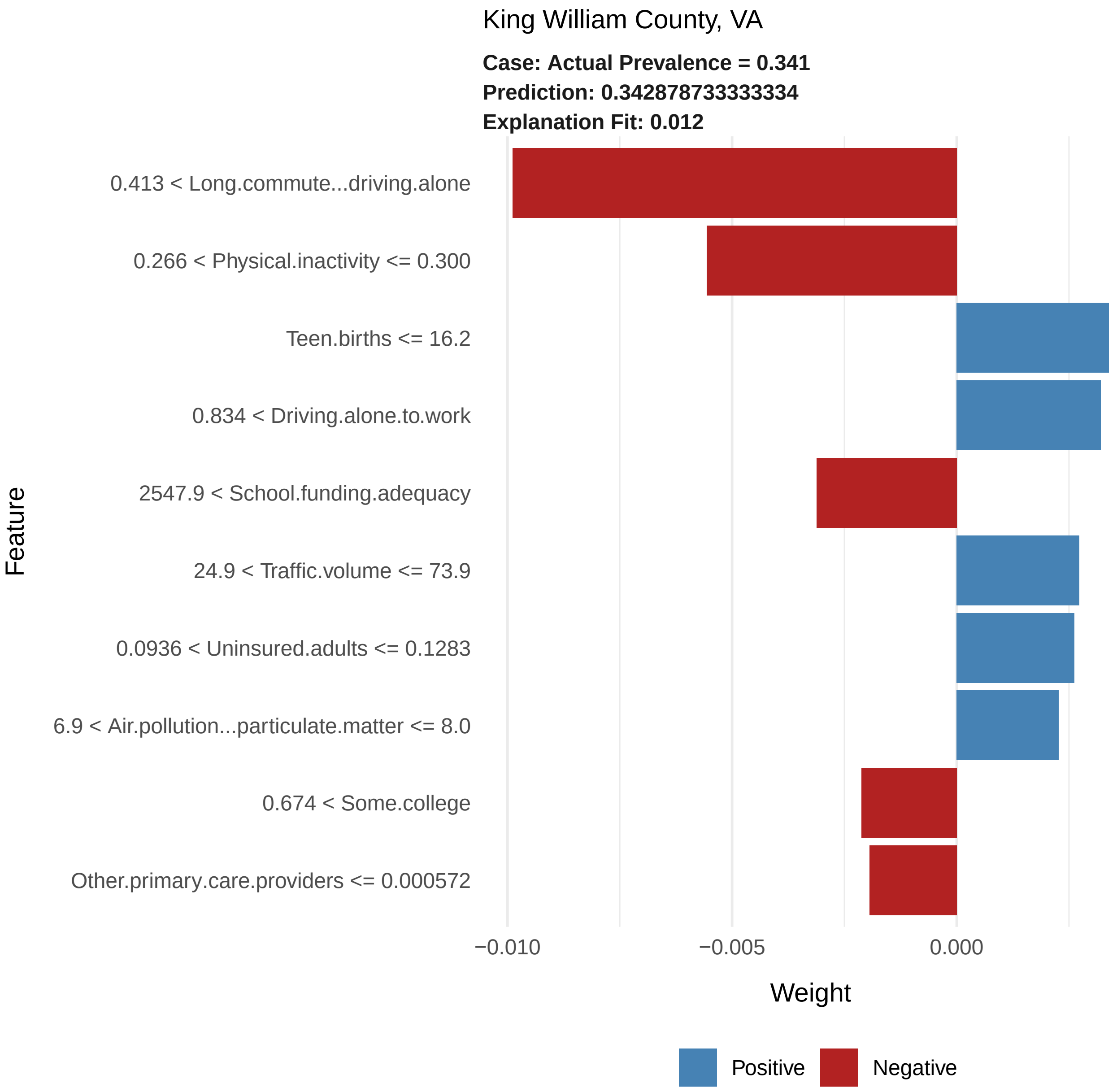

King William County, VA
Case: Actual Prevalence = 0.341
Prediction: 0.342878733333334
Explanation Fit: 0.012
0.413 < Long.commute...driving.alone
0.266 < Physical.inactivity <= 0.300
Teen.births <= 16.2
0.834 < Driving.alone.to.work
2547.9 < School.funding.adequacy
24.9 < Traffic.volume <= 73.9
0.0936 < Uninsured.adults <= 0.1283
6.9 < Air.pollution...particulate.matter <= 8.0
0.674 < Some.college
Other.primary.care.providers <= 0.000572
Feature
−0.010
−0.005
0.000
Weight
Positive
Negative

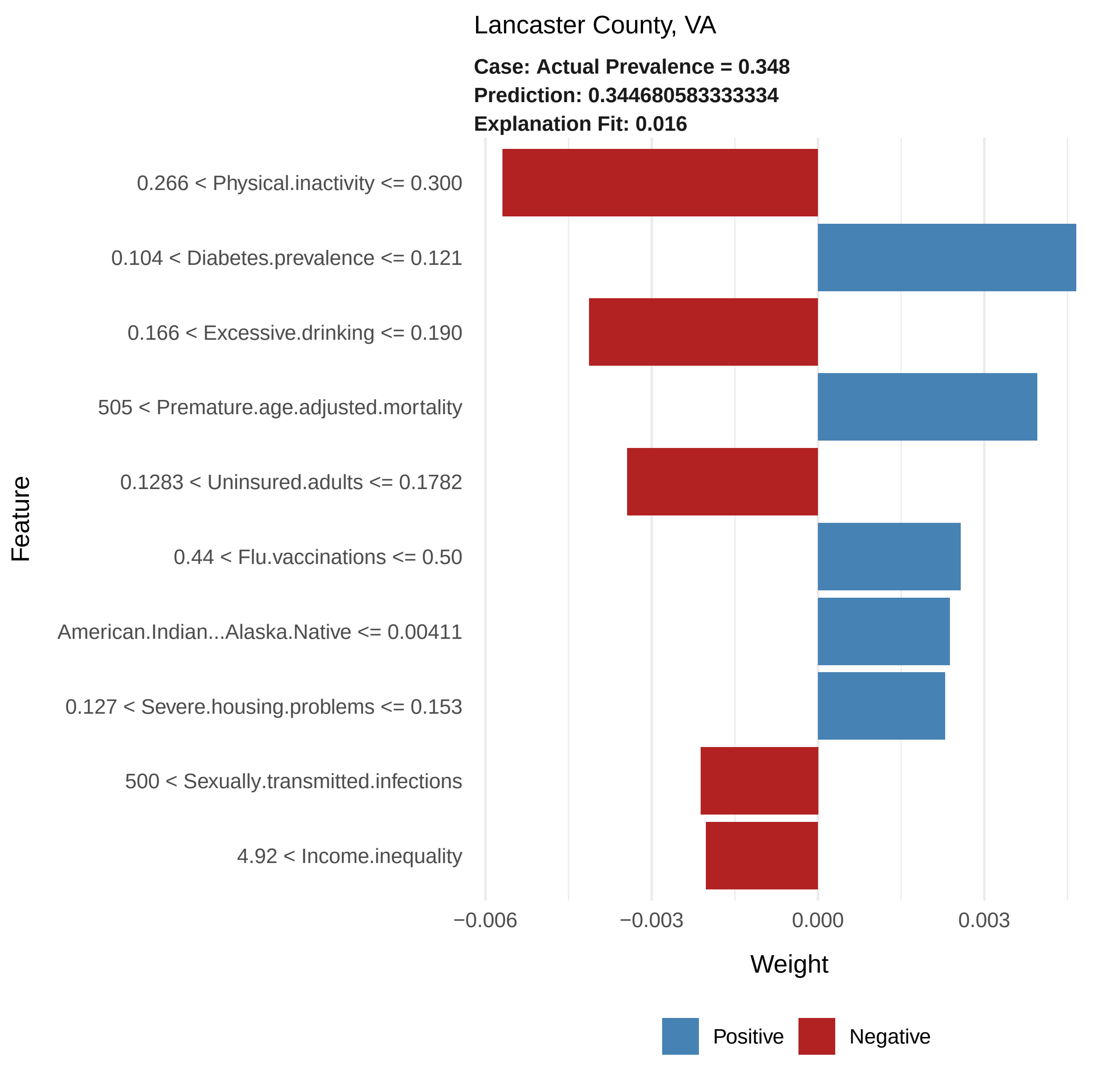
Lancaster County, VA
Case: Actual Prevalence = 0.348
Prediction: 0.344680583333334
Explanation Fit: 0.016
0.266 < Physical.inactivity <= 0.300
0.104 < Diabetes.prevalence <= 0.121
0.166 < Excessive.drinking <= 0.190
505 < Premature.age.adjusted.mortality
0.1283 < Uninsured.adults <= 0.1782
0.44 < Flu.vaccinations <= 0.50
American.Indian...Alaska.Native <= 0.00411
0.127 < Severe.housing.problems <= 0.153
500 < Sexually.transmitted.infections
4.92 < Income.inequality
Feature
−0.006
−0.003
0.000
0.003
Weight
Positive
Negative

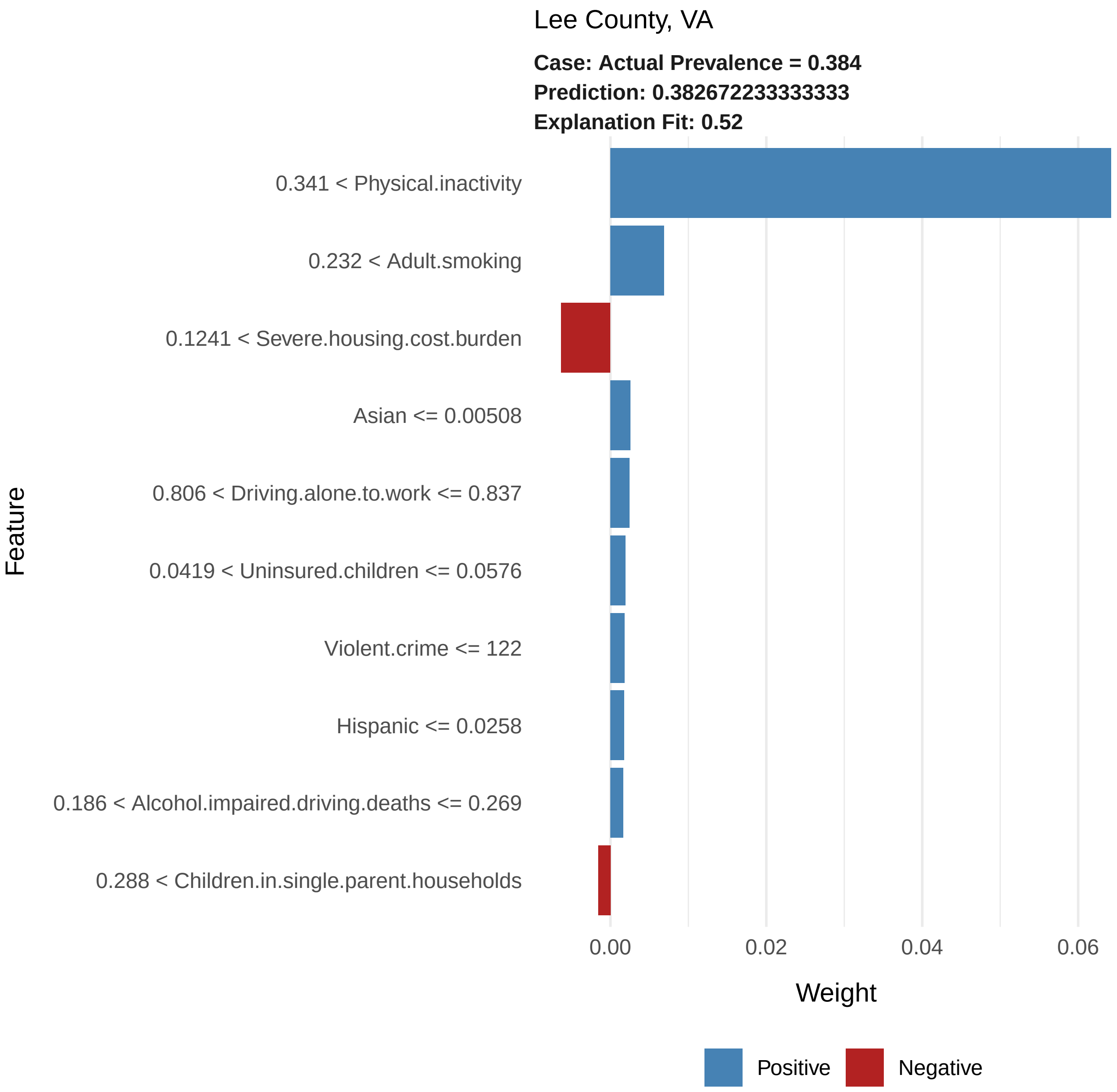
Lee County, VA
Case: Actual Prevalence = 0.384
Prediction: 0.382672233333333
Explanation Fit: 0.52
0.341 < Physical.inactivity
0.232 < Adult.smoking
0.1241 < Severe.housing.cost.burden
Asian <= 0.00508
0.806 < Driving.alone.to.work <= 0.837
0.0419 < Uninsured.children <= 0.0576
Violent.crime <= 122
Hispanic <= 0.0258
0.186 < Alcohol.impaired.driving.deaths <= 0.269
0.288 < Children.in.single.parent.households
Feature
0.00
0.02
0.04
0.06
Weight
Positive
Negative

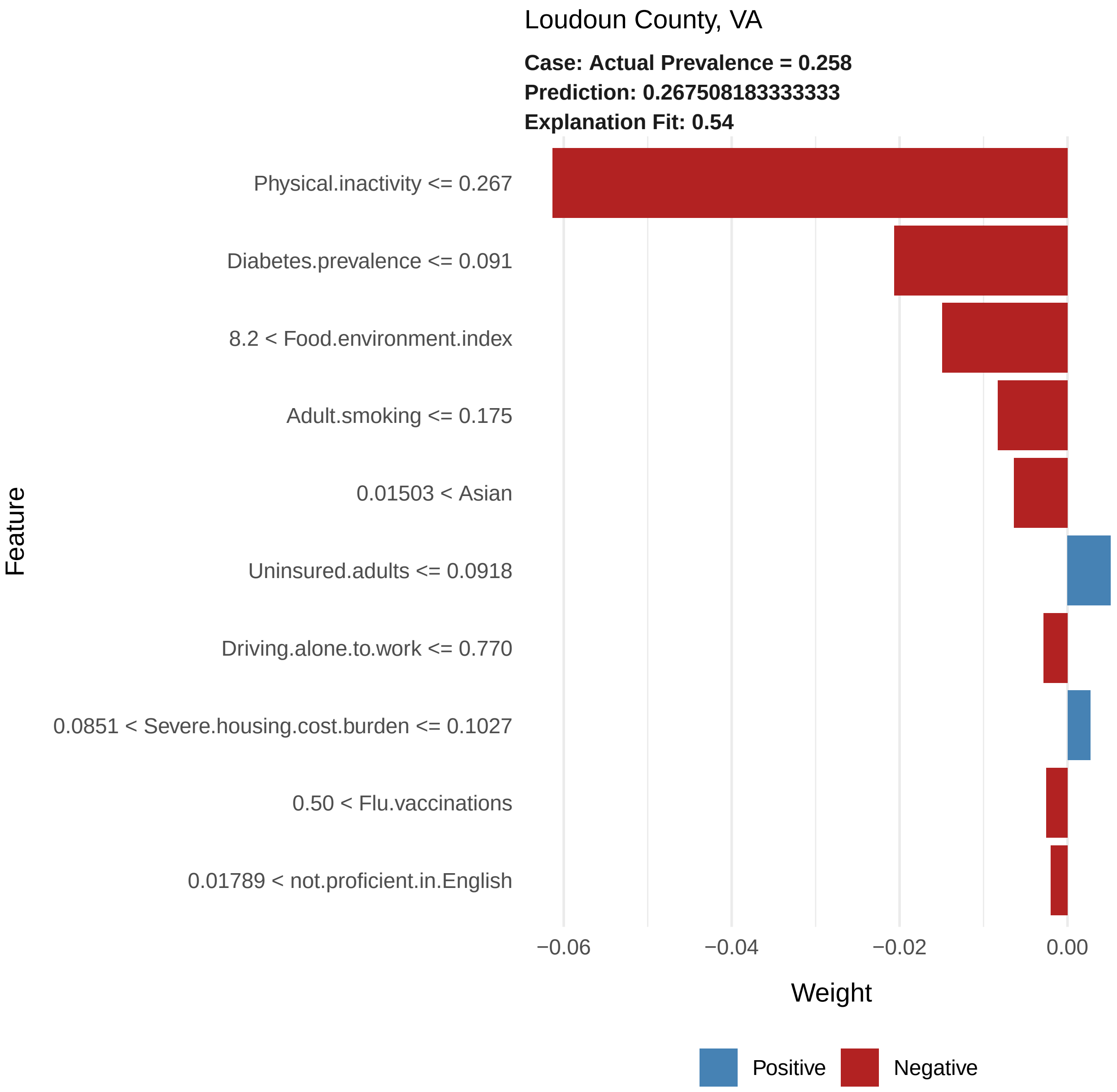

Loudoun County, VA
Case: Actual Prevalence = 0.258
Prediction: 0.267508183333333
Explanation Fit: 0.54
Physical.inactivity <= 0.267
Diabetes.prevalence <= 0.091
8.2 < Food.environment.index
Adult.smoking <= 0.175
0.01503 < Asian
Uninsured.adults <= 0.0918
Driving.alone.to.work <= 0.770
0.0851 < Severe.housing.cost.burden <= 0.1027
0.50 < Flu.vaccinations
0.01789 < not.proficient.in.English
Feature
−0.06
−0.04
−0.02
0.00
Weight
Positive
Negative

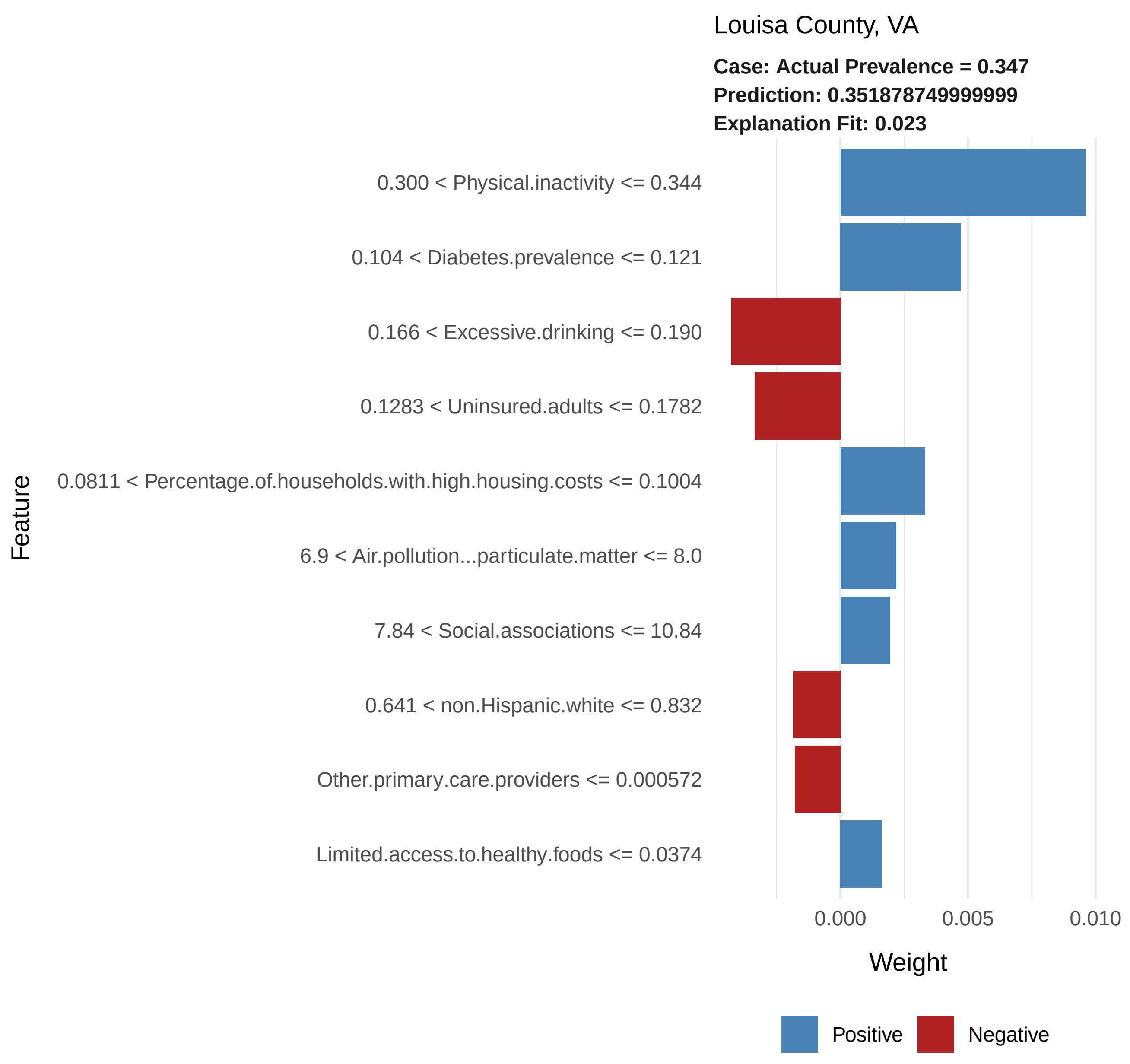

Louisa County, VA
Case: Actual Prevalence = 0.347
Prediction: 0.351878749999999
Explanation Fit: 0.023
0.300 < Physical.inactivity <= 0.344
0.104 < Diabetes.prevalence <= 0.121
0.166 < Excessive.drinking <= 0.190
0.1283 < Uninsured.adults <= 0.1782
0.0811 < Percentage.of.households.with.high.housing.costs <= 0.1004
6.9 < Air.pollution...particulate.matter <= 8.0
7.84 < Social.associations <= 10.84
0.641 < non.Hispanic.white <= 0.832
Other.primary.care.providers <= 0.000572
Limited.access.to.healthy.foods <= 0.0374
Feature
0.000
0.005
0.010
Weight
Positive
Negative

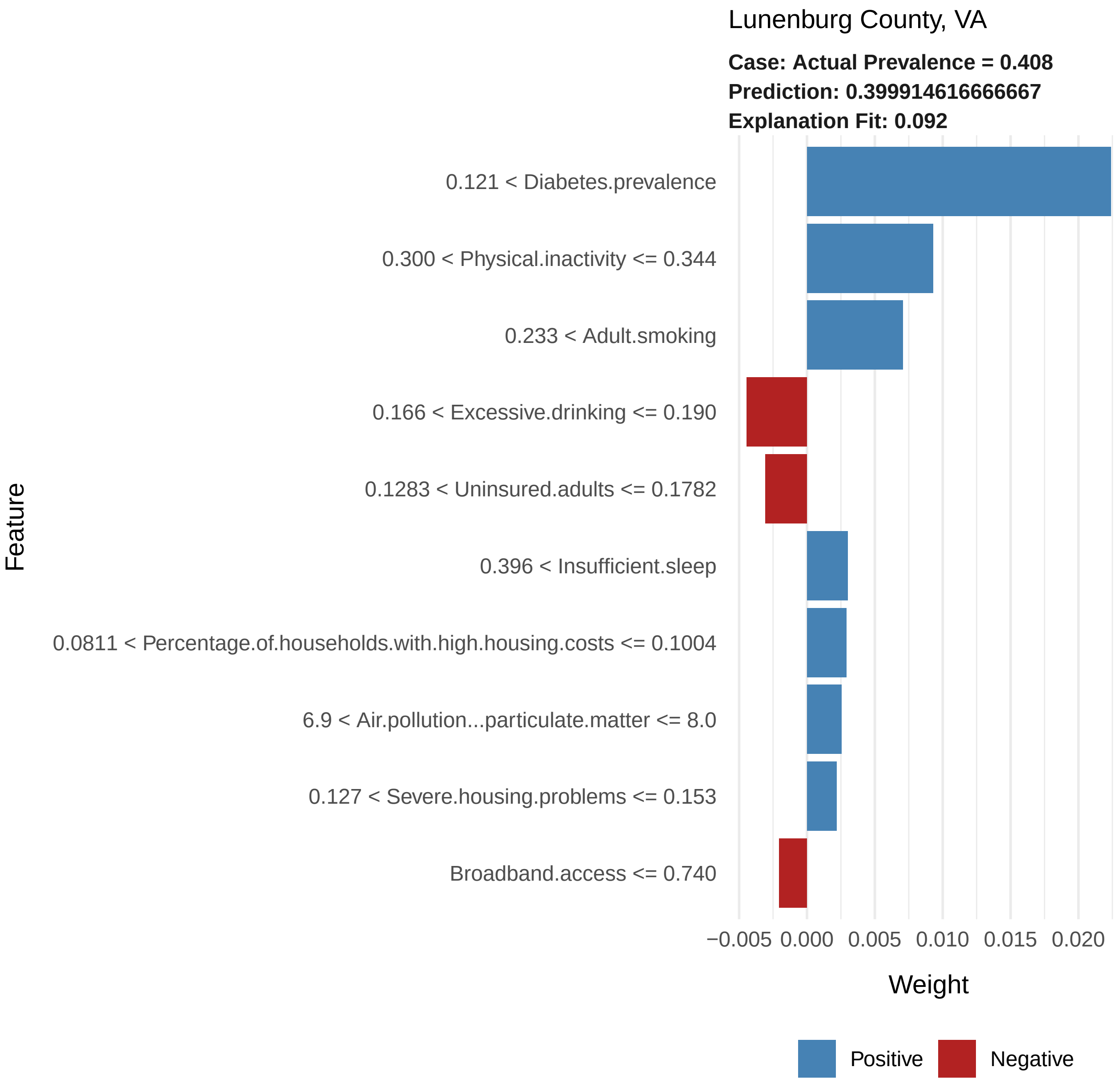

Lunenburg County, VA
Case: Actual Prevalence = 0.408
Prediction: 0.399914616666667
Explanation Fit: 0.092
0.121 < Diabetes.prevalence
0.300 < Physical.inactivity <= 0.344
0.233 < Adult.smoking
0.166 < Excessive.drinking <= 0.190
0.1283 < Uninsured.adults <= 0.1782
0.396 < Insufficient.sleep
0.0811 < Percentage.of.households.with.high.housing.costs <= 0.1004
6.9 < Air.pollution...particulate.matter <= 8.0
0.127 < Severe.housing.problems <= 0.153
Broadband.access <= 0.740
Feature
−0.005
0.000
0.005
0.010
0.015
0.020
Weight
Positive
Negative

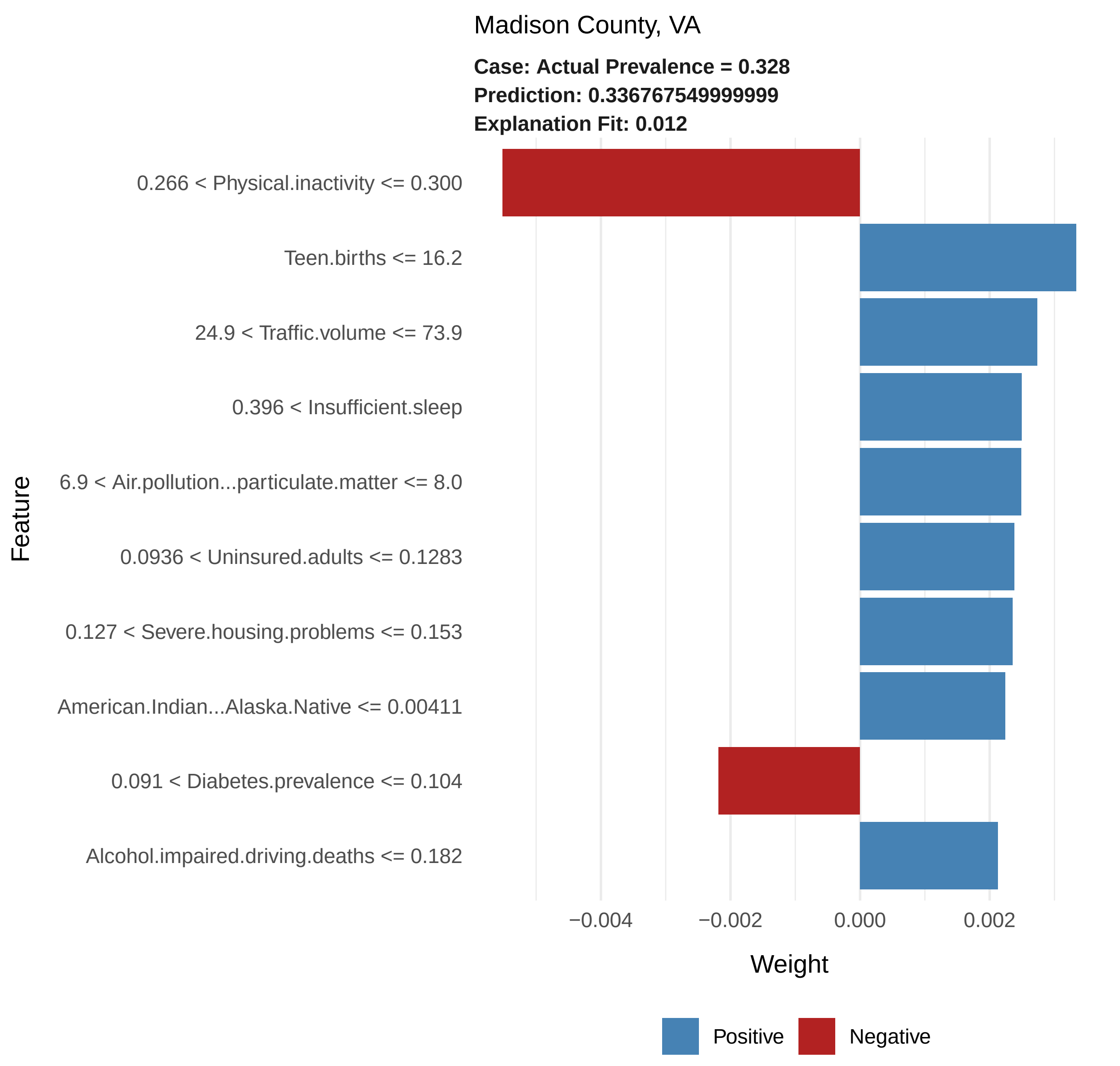

Madison County, VA
Case: Actual Prevalence = 0.328
Prediction: 0.336767549999999
Explanation Fit: 0.012
0.266 < Physical.inactivity <= 0.300
Teen.births <= 16.2
24.9 < Traffic.volume <= 73.9
0.396 < Insufficient.sleep
6.9 < Air.pollution...particulate.matter <= 8.0
0.0936 < Uninsured.adults <= 0.1283
0.127 < Severe.housing.problems <= 0.153
American.Indian...Alaska.Native <= 0.00411
0.091 < Diabetes.prevalence <= 0.104
Alcohol.impaired.driving.deaths <= 0.182
Feature
−0.004
−0.002
0.000
0.002
Weight
Positive
Negative

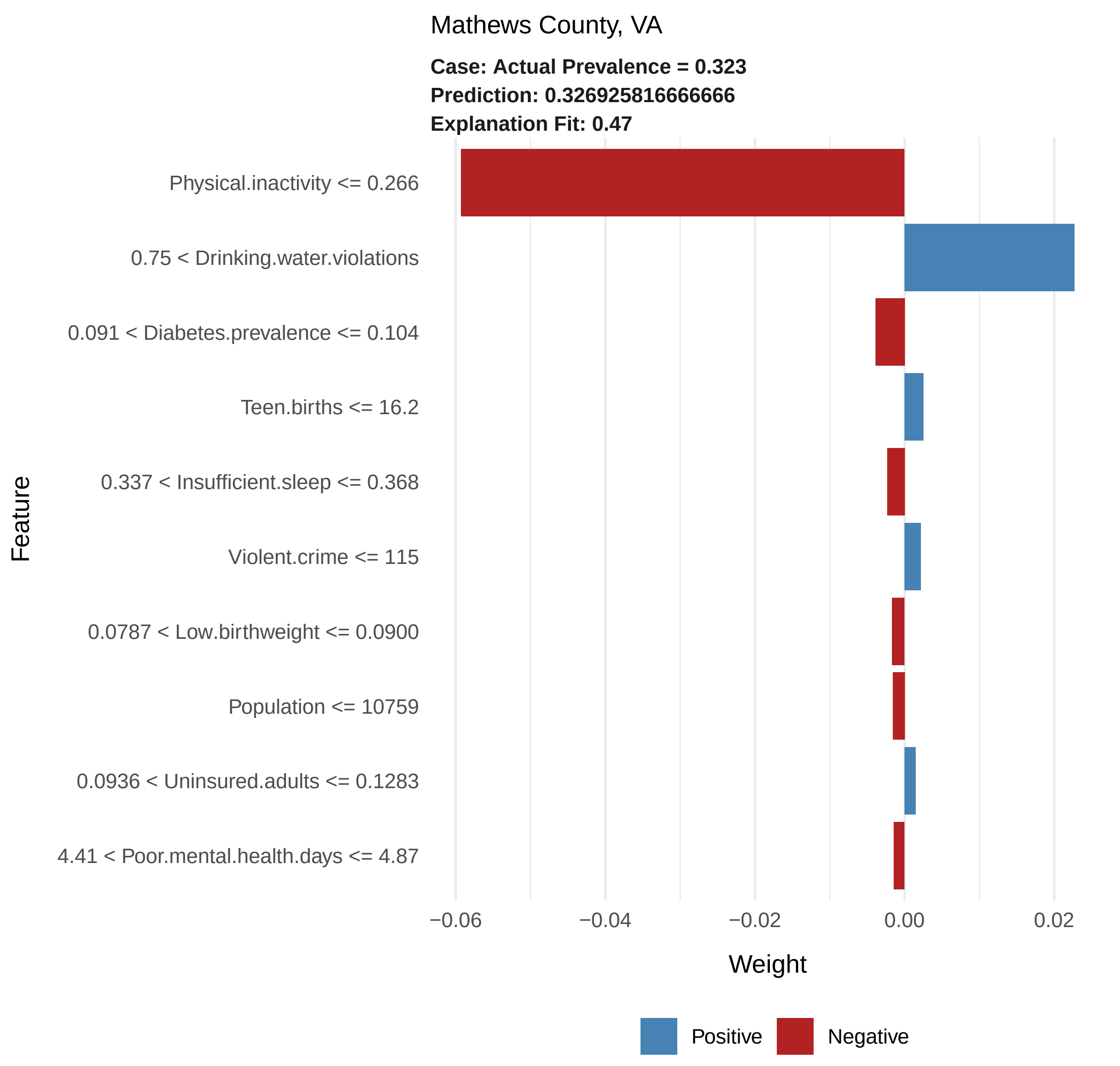
Mathews County, VA
Case: Actual Prevalence = 0.323
Prediction: 0.326925816666666
Explanation Fit: 0.47
Physical.inactivity <= 0.266
0.75 < Drinking.water.violations
0.091 < Diabetes.prevalence <= 0.104
Teen.births <= 16.2
0.337 < Insufficient.sleep <= 0.368
Violent.crime <= 115
0.0787 < Low.birthweight <= 0.0900
Population <= 10759
0.0936 < Uninsured.adults <= 0.1283
4.41 < Poor.mental.health.days <= 4.87
Feature
−0.06
−0.04
−0.02
0.00
0.02
Weight
Positive
Negative

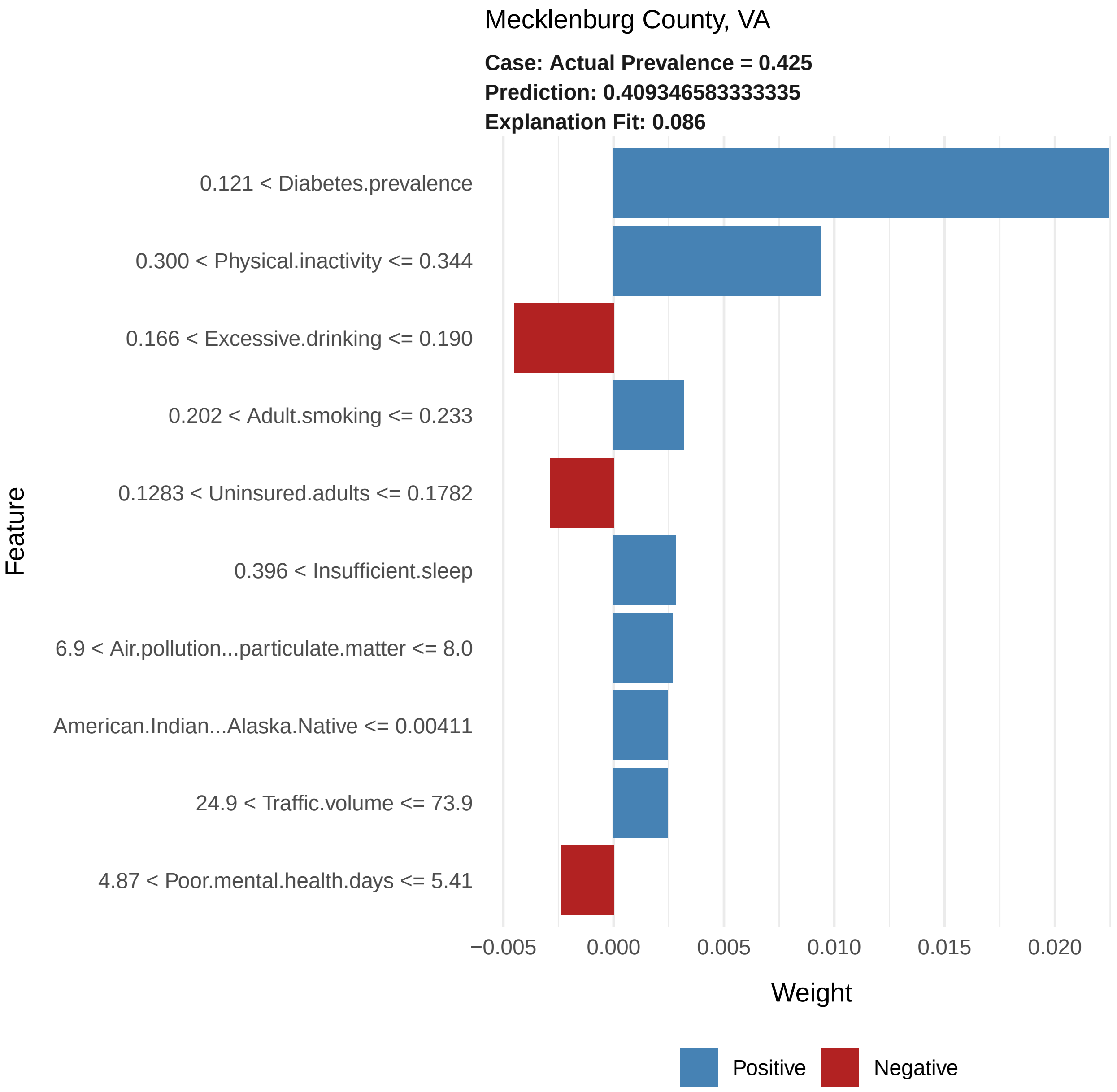
Mecklenburg County, VA
Case: Actual Prevalence = 0.425
Prediction: 0.409346583333335
Explanation Fit: 0.086
0.121 < Diabetes.prevalence
0.300 < Physical.inactivity <= 0.344
0.166 < Excessive.drinking <= 0.190
0.202 < Adult.smoking <= 0.233
0.1283 < Uninsured.adults <= 0.1782
0.396 < Insufficient.sleep
6.9 < Air.pollution...particulate.matter <= 8.0
American.Indian...Alaska.Native <= 0.00411
24.9 < Traffic.volume <= 73.9
4.87 < Poor.mental.health.days <= 5.41
Feature
−0.005
0.000
0.005
0.010
0.015
0.020
Weight
Positive
Negative

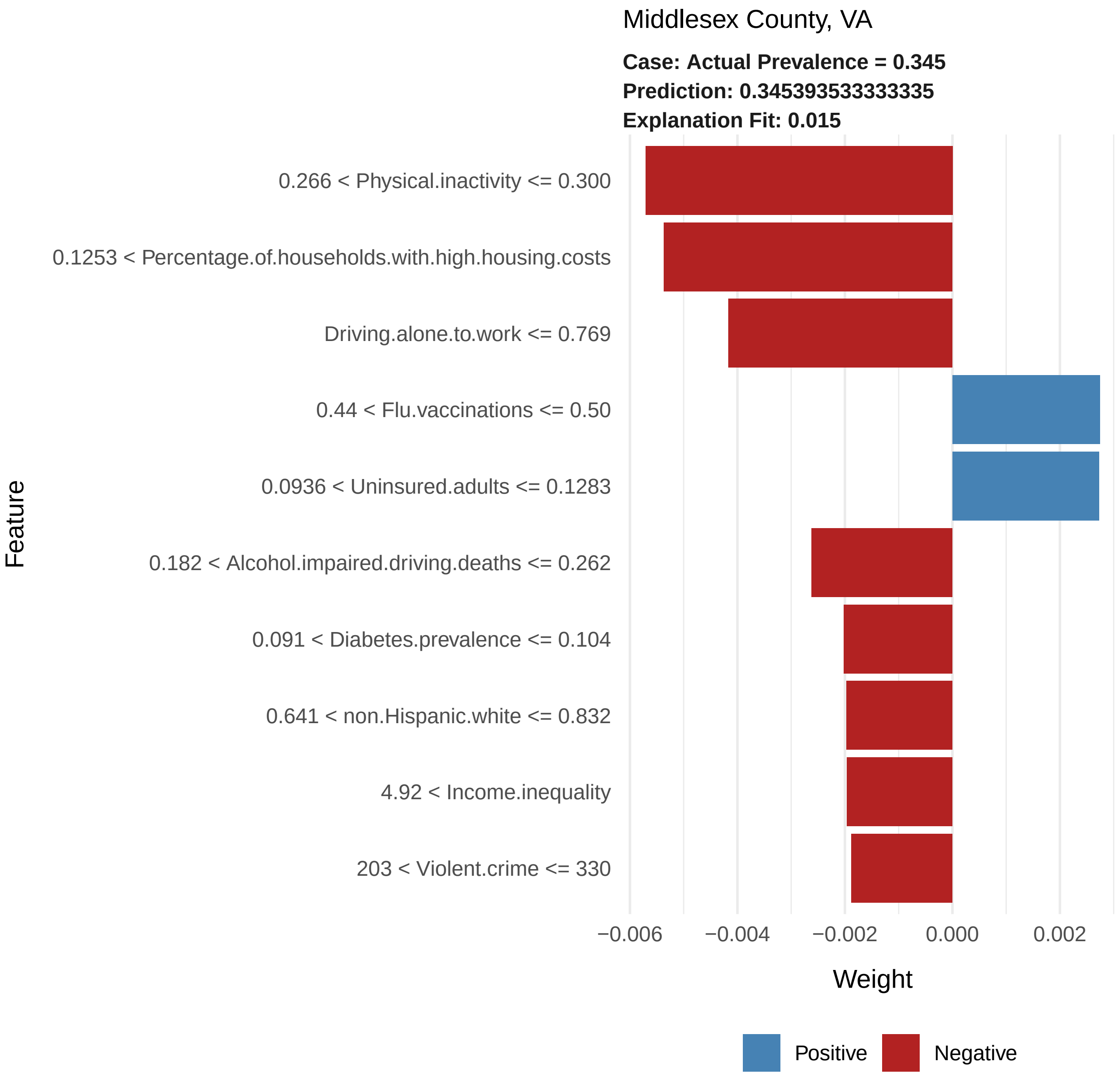

Middlesex County, VA
Case: Actual Prevalence = 0.345
Prediction: 0.345393533333335
Explanation Fit: 0.015
0.266 < Physical.inactivity <= 0.300
0.1253 < Percentage.of.households.with.high.housing.costs
Driving.alone.to.work <= 0.769
0.44 < Flu.vaccinations <= 0.50
0.0936 < Uninsured.adults <= 0.1283
0.182 < Alcohol.impaired.driving.deaths <= 0.262
0.091 < Diabetes.prevalence <= 0.104
0.641 < non.Hispanic.white <= 0.832
4.92 < Income.inequality
203 < Violent.crime <= 330
Feature
−0.006
−0.004
−0.002
0.000
0.002
Weight
Positive
Negative

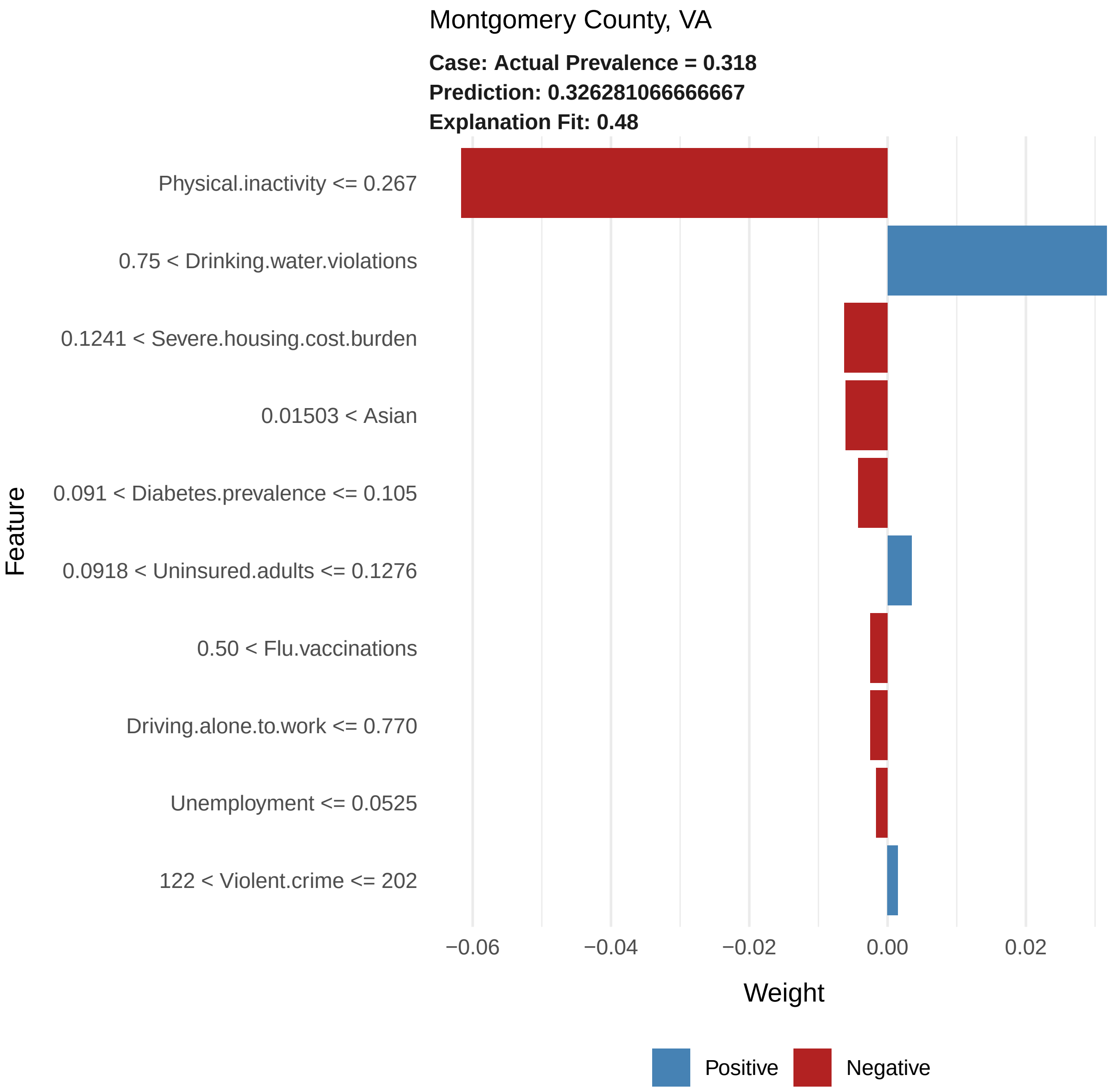

Montgomery County, VA
Case: Actual Prevalence = 0.318
Prediction: 0.326281066666667
Explanation Fit: 0.48
Physical.inactivity <= 0.267
0.75 < Drinking.water.violations
0.1241 < Severe.housing.cost.burden
0.01503 < Asian
0.091 < Diabetes.prevalence <= 0.105
0.0918 < Uninsured.adults <= 0.1276
0.50 < Flu.vaccinations
Driving.alone.to.work <= 0.770
Unemployment <= 0.0525
122 < Violent.crime <= 202
Feature
−0.06
−0.04
−0.02
0.00
0.02
Weight
Positive
Negative

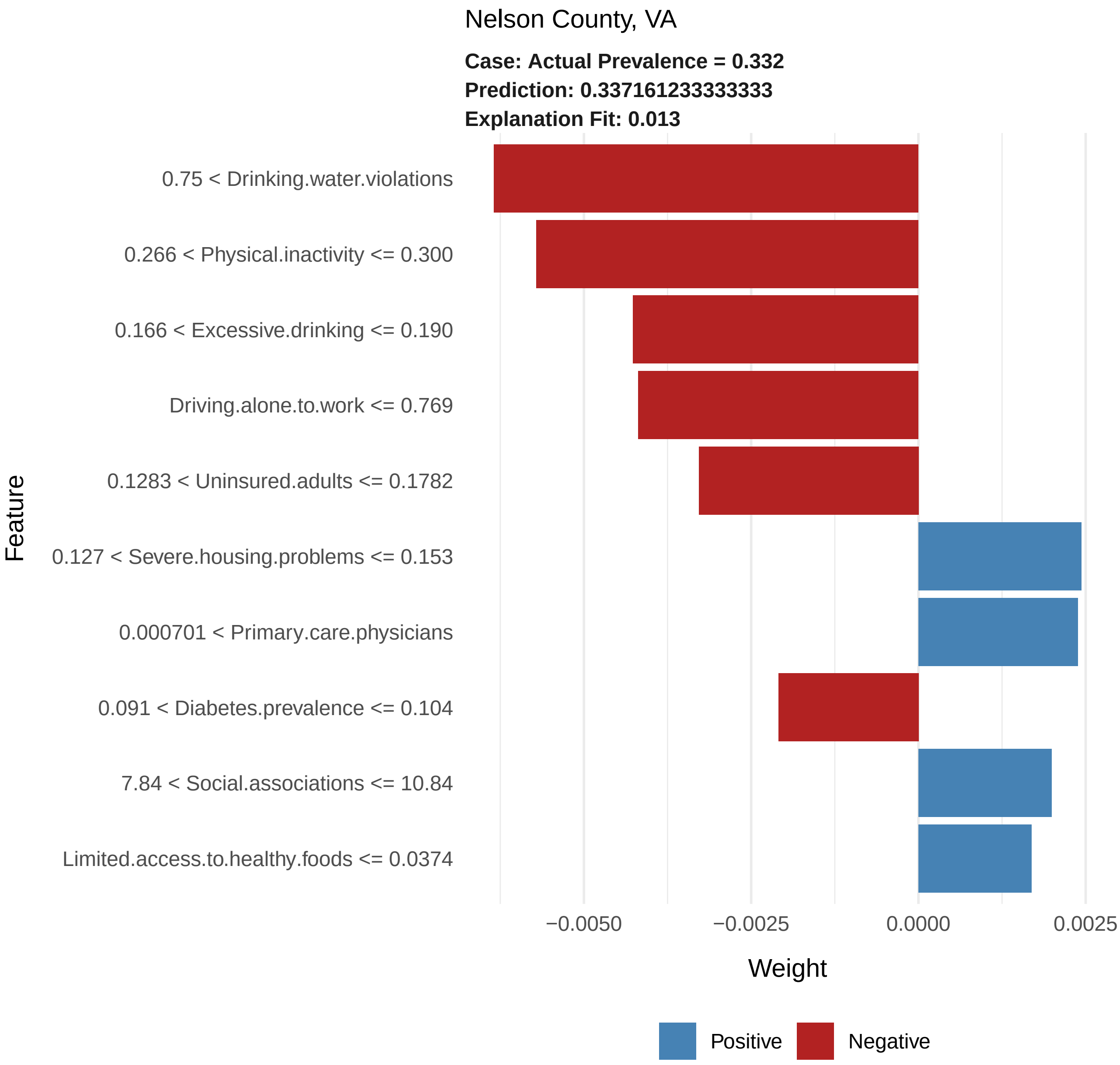

Nelson County, VA
Case: Actual Prevalence = 0.332
Prediction: 0.337161233333333
Explanation Fit: 0.013
0.75 < Drinking.water.violations
0.266 < Physical.inactivity <= 0.300
0.166 < Excessive.drinking <= 0.190
Driving.alone.to.work <= 0.769
0.1283 < Uninsured.adults <= 0.1782
0.127 < Severe.housing.problems <= 0.153
0.000701 < Primary.care.physicians
0.091 < Diabetes.prevalence <= 0.104
7.84 < Social.associations <= 10.84
Limited.access.to.healthy.foods <= 0.0374
Feature
−0.0050
−0.0025
0.0000
0.0025
Weight
Positive
Negative

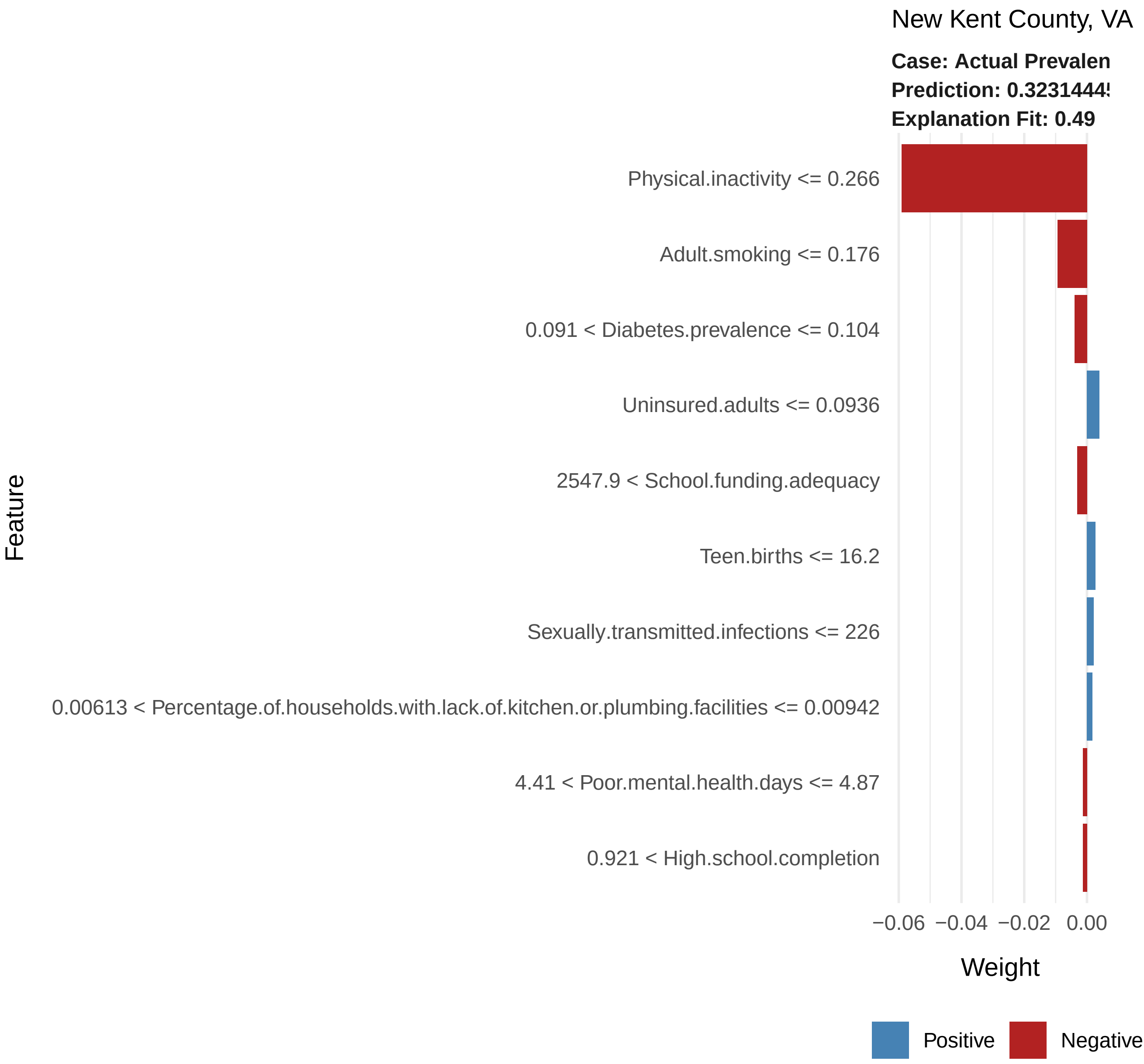

New Kent County, VA
Case: Actual Prevalen
Prediction: 0.3231444
Explanation Fit: 0.49
Physical.inactivity <= 0.266
Adult.smoking <= 0.176
0.091 < Diabetes.prevalence <= 0.104
Uninsured.adults <= 0.0936
2547.9 < School.funding.adequacy
Teen.births <= 16.2
Sexually.transmitted.infections <= 226
0.00613 < Percentage.of.households.with.lack.of.kitchen.or.plumbing.facilities <= 0.00942
4.41 < Poor.mental.health.days <= 4.87
0.921 < High.school.completion
Feature
−0.06 −0.04 −0.02 0.00
Weight
Positive
Negative

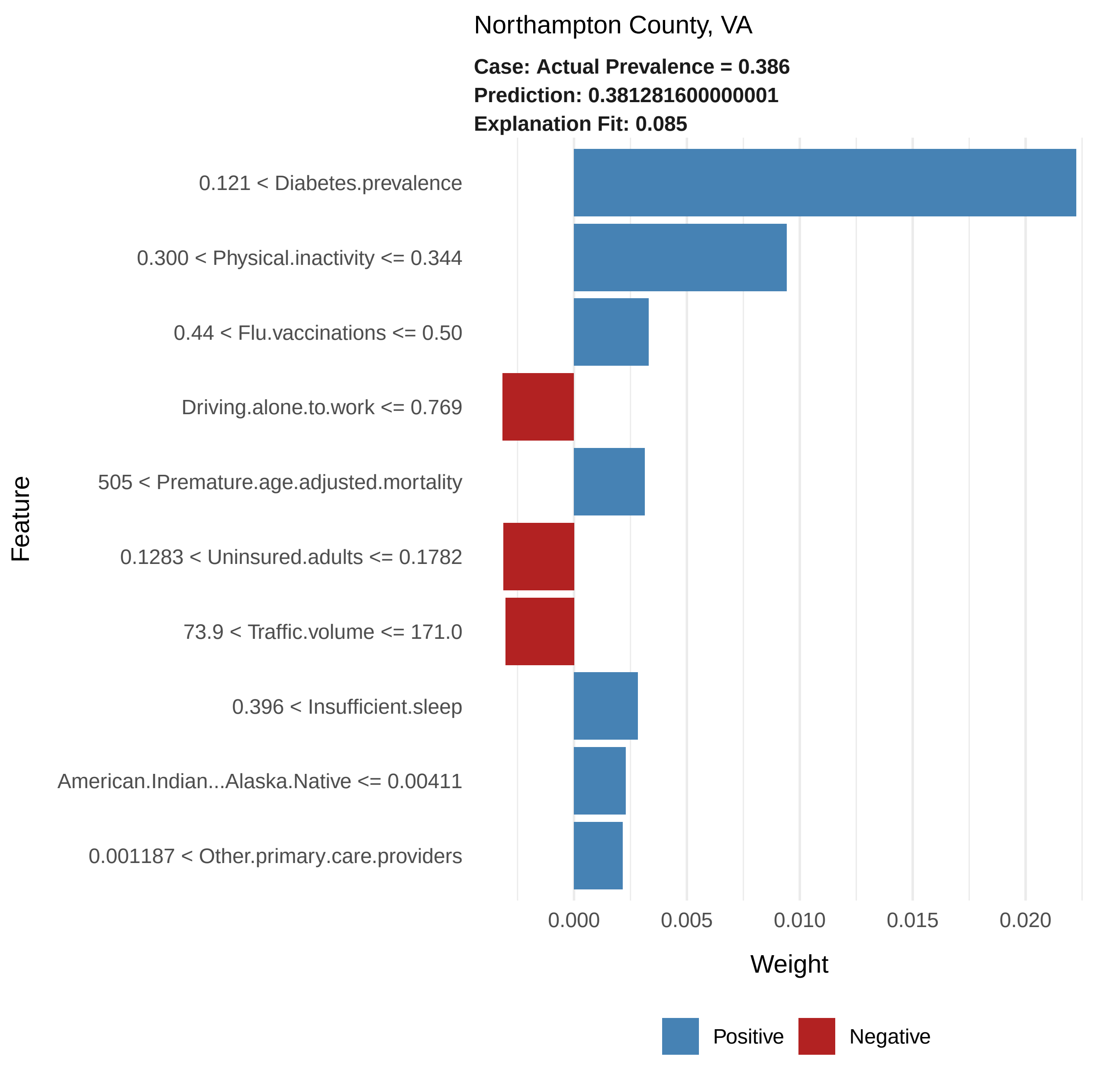

Northampton County, VA
Case: Actual Prevalence = 0.386
Prediction: 0.381281600000001
Explanation Fit: 0.085
0.121 < Diabetes.prevalence
0.300 < Physical.inactivity <= 0.344
0.44 < Flu.vaccinations <= 0.50
Driving.alone.to.work <= 0.769
505 < Premature.age.adjusted.mortality
0.1283 < Uninsured.adults <= 0.1782
73.9 < Traffic.volume <= 171.0
0.396 < Insufficient.sleep
American.Indian...Alaska.Native <= 0.00411
0.001187 < Other.primary.care.providers
Feature
0.000
0.005
0.010
0.015
0.020
Weight
Positive
Negative

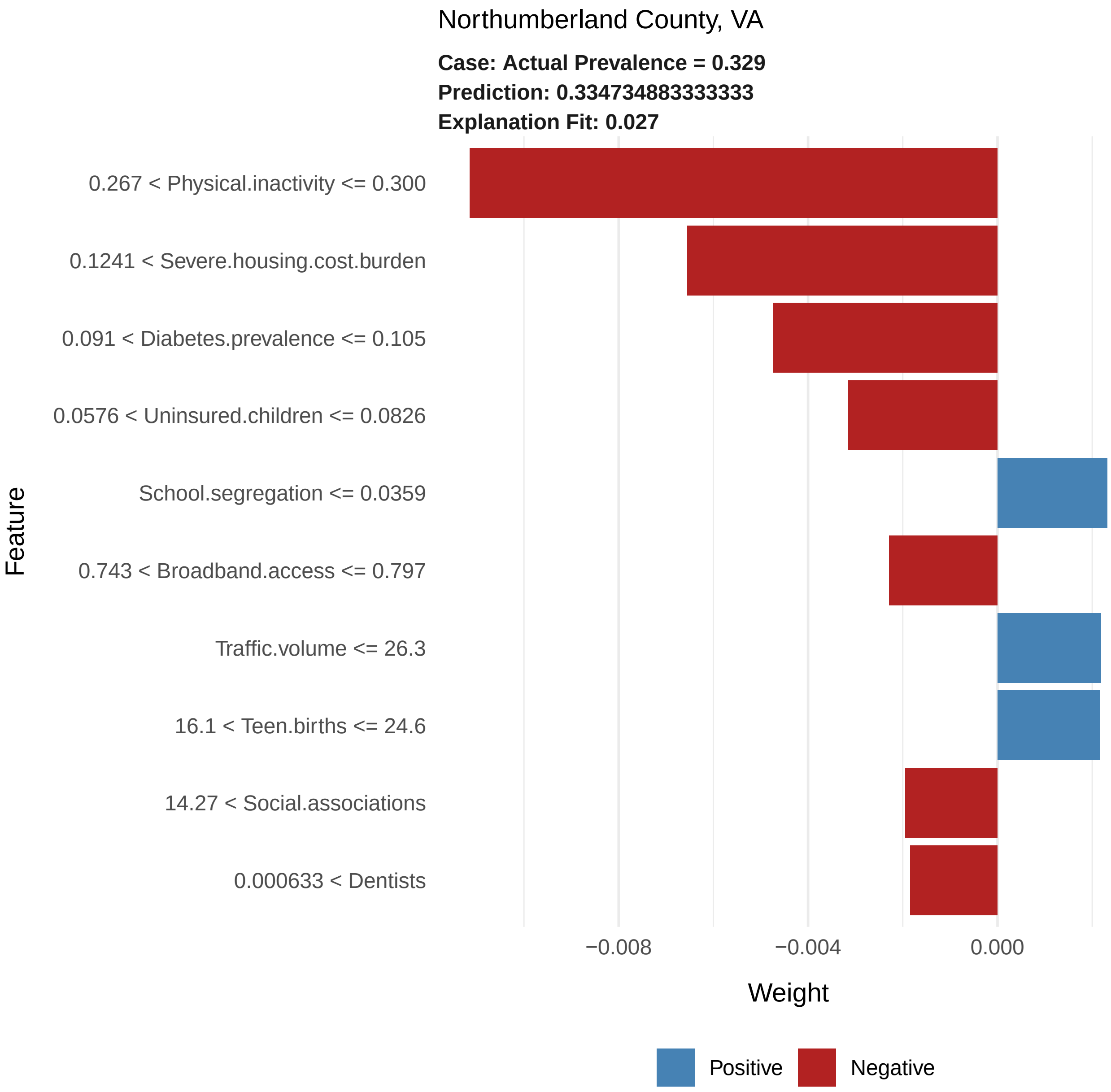

Northumberland County, VA
Case: Actual Prevalence = 0.329
Prediction: 0.334734883333333
Explanation Fit: 0.027
0.267 < Physical.inactivity <= 0.300
0.1241 < Severe.housing.cost.burden
0.091 < Diabetes.prevalence <= 0.105
0.0576 < Uninsured.children <= 0.0826
School.segregation <= 0.0359
0.743 < Broadband.access <= 0.797
Traffic.volume <= 26.3
16.1 < Teen.births <= 24.6
14.27 < Social.associations
0.000633 < Dentists
Feature
−0.008
−0.004
0.000
Weight
Positive
Negative

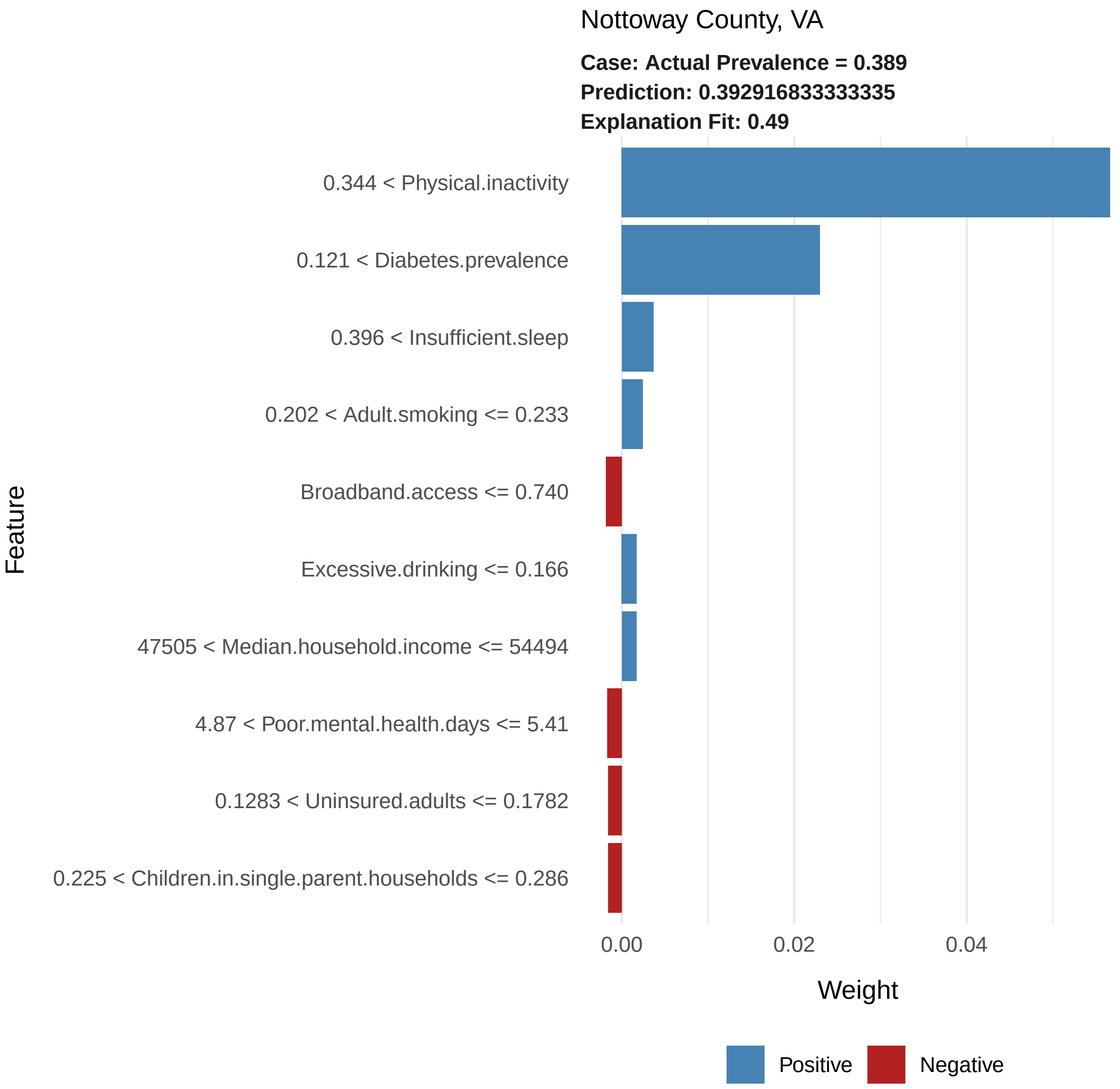

Nottoway County, VA
Case: Actual Prevalence = 0.389
Prediction: 0.392916833333335
Explanation Fit: 0.49
0.344 < Physical.inactivity
0.121 < Diabetes.prevalence
0.396 < Insufficient.sleep
0.202 < Adult.smoking <= 0.233
Broadband.access <= 0.740
Excessive.drinking <= 0.166
47505 < Median.household.income <= 54494
4.87 < Poor.mental.health.days <= 5.41
0.1283 < Uninsured.adults <= 0.1782
0.225 < Children.in.single.parent.households <= 0.286
Feature
0.00
0.02
0.04
Weight
Positive
Negative

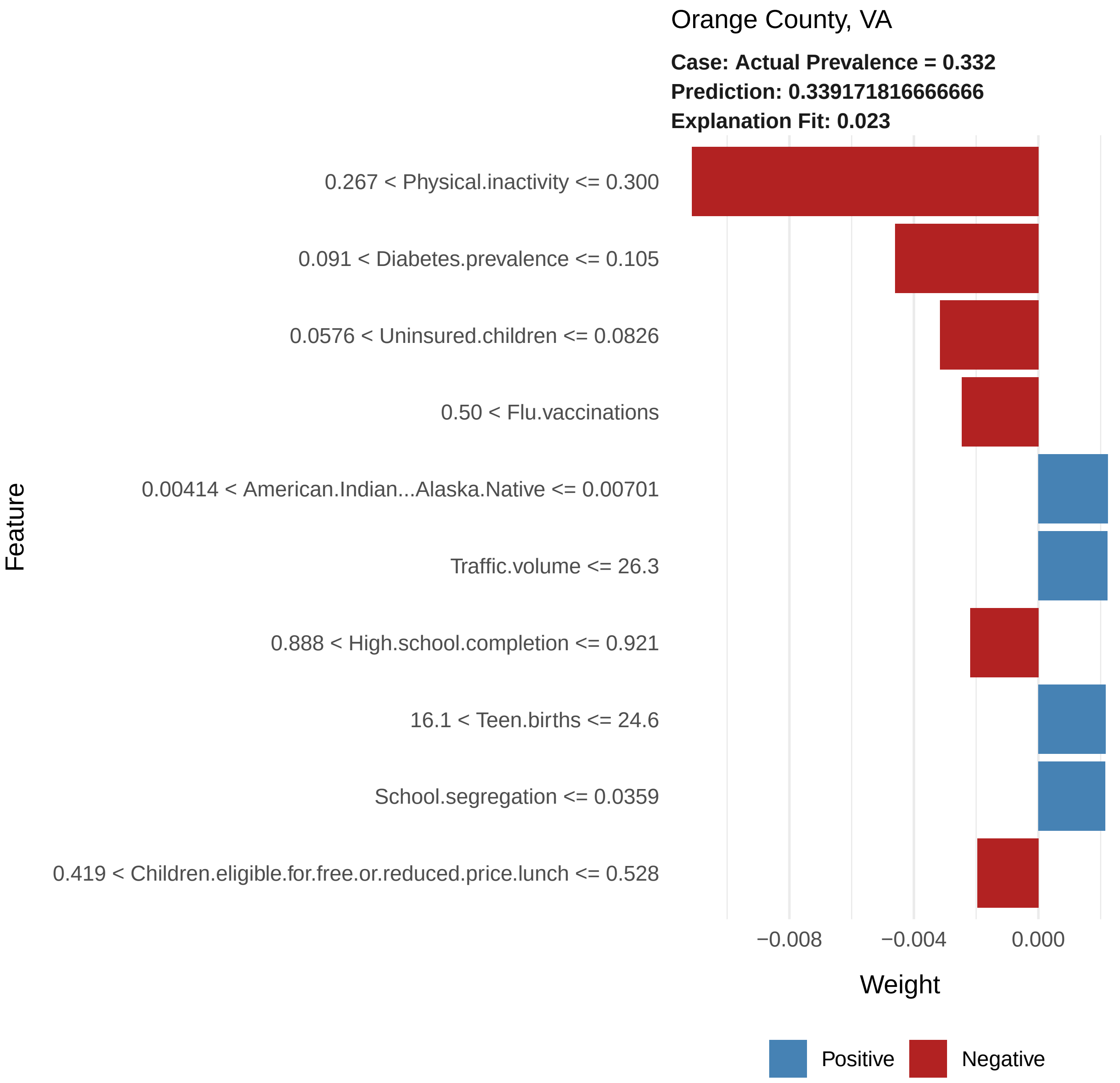

Orange County, VA
Case: Actual Prevalence = 0.332
Prediction: 0.339171816666666
Explanation Fit: 0.023
0.267 < Physical.inactivity <= 0.300
0.091 < Diabetes.prevalence <= 0.105
0.0576 < Uninsured.children <= 0.0826
0.50 < Flu.vaccinations
0.00414 < American.Indian...Alaska.Native <= 0.00701
Traffic.volume <= 26.3
0.888 < High.school.completion <= 0.921
16.1 < Teen.births <= 24.6
School.segregation <= 0.0359
0.419 < Children.eligible.for.free.or.reduced.price.lunch <= 0.528
Feature
−0.008
−0.004
0.000
Weight
Positive
Negative

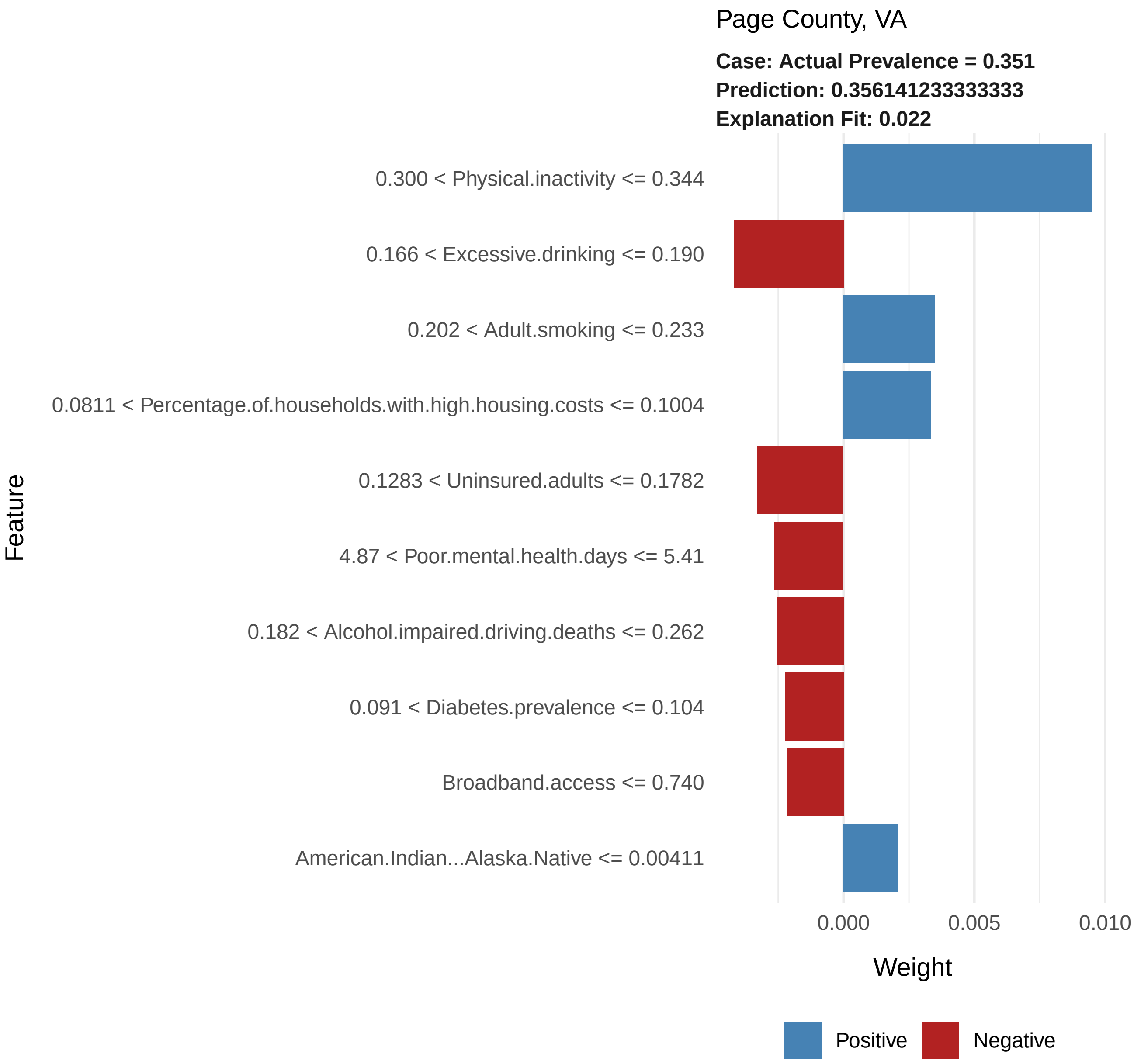

Page County, VA
Case: Actual Prevalence = 0.351
Prediction: 0.356141233333333
Explanation Fit: 0.022
0.300 < Physical.inactivity <= 0.344
0.166 < Excessive.drinking <= 0.190
0.202 < Adult.smoking <= 0.233
0.0811 < Percentage.of.households.with.high.housing.costs <= 0.1004
0.1283 < Uninsured.adults <= 0.1782
4.87 < Poor.mental.health.days <= 5.41
0.182 < Alcohol.impaired.driving.deaths <= 0.262
0.091 < Diabetes.prevalence <= 0.104
Broadband.access <= 0.740
American.Indian...Alaska.Native <= 0.00411
Feature
0.000
0.005
0.010
Weight
Positive
Negative

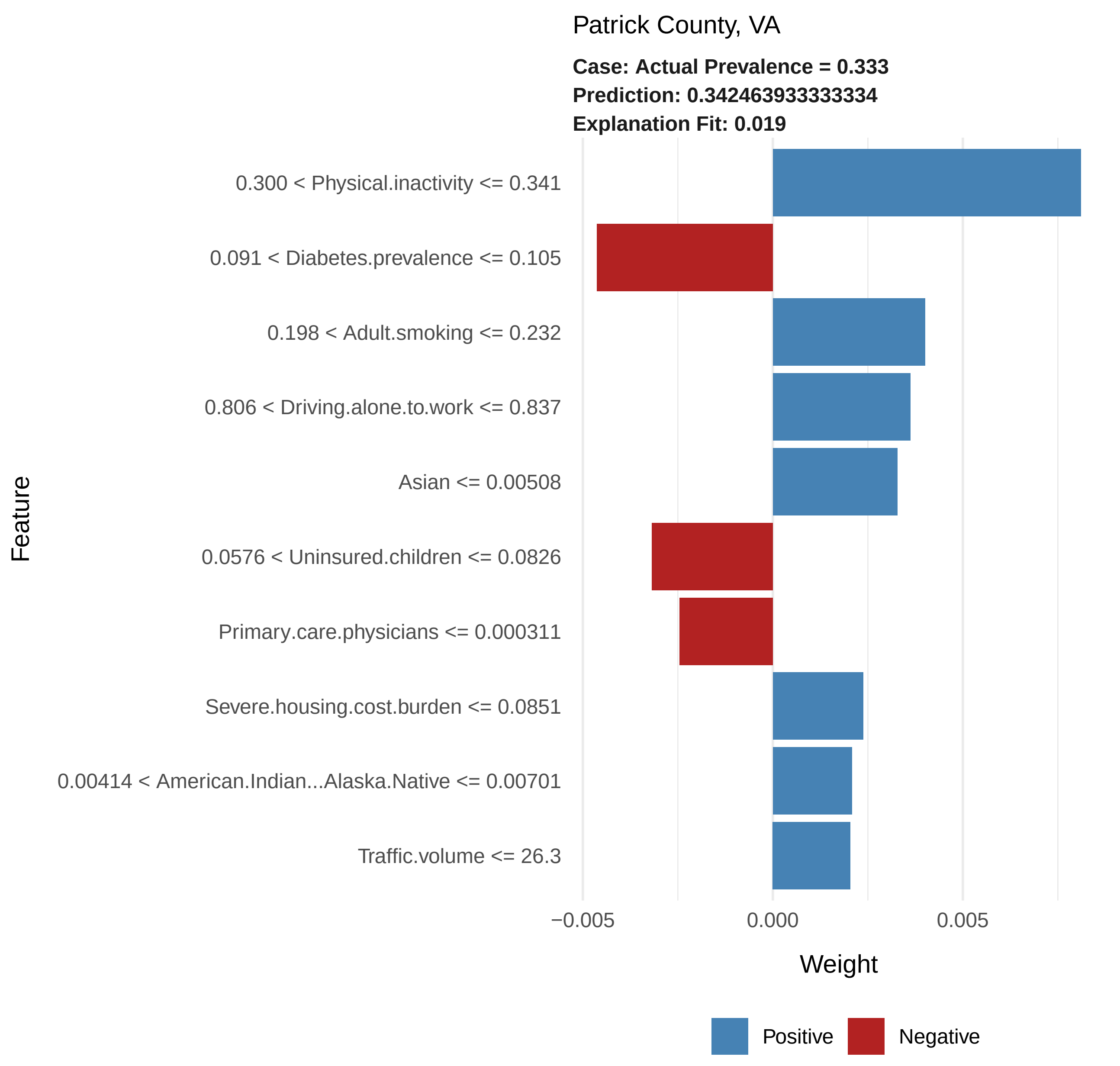
Patrick County, VA
Case: Actual Prevalence = 0.333
Prediction: 0.342463933333334
Explanation Fit: 0.019
0.300 < Physical.inactivity <= 0.341
0.091 < Diabetes.prevalence <= 0.105
0.198 < Adult.smoking <= 0.232
0.806 < Driving.alone.to.work <= 0.837
Asian <= 0.00508
0.0576 < Uninsured.children <= 0.0826
Primary.care.physicians <= 0.000311
Severe.housing.cost.burden <= 0.0851
0.00414 < American.Indian...Alaska.Native <= 0.00701
Traffic.volume <= 26.3
Feature
−0.005
0.000
0.005
Weight
Positive
Negative

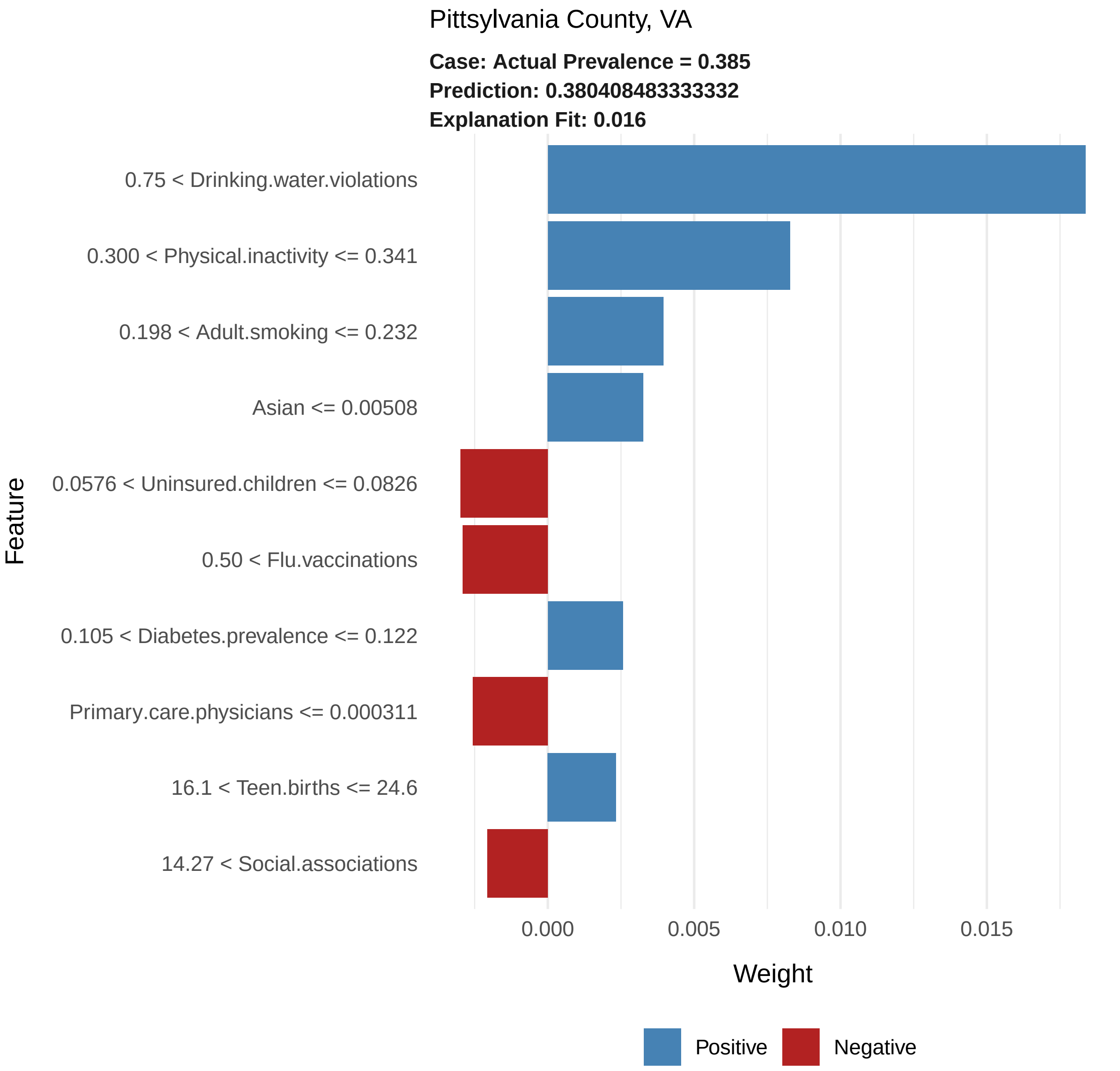
Pittsylvania County, VA
Case: Actual Prevalence = 0.385
Prediction: 0.380408483333332
Explanation Fit: 0.016
0.75 < Drinking.water.violations
0.300 < Physical.inactivity <= 0.341
0.198 < Adult.smoking <= 0.232
Asian <= 0.00508
0.0576 < Uninsured.children <= 0.0826
0.50 < Flu.vaccinations
0.105 < Diabetes.prevalence <= 0.122
Primary.care.physicians <= 0.000311
16.1 < Teen.births <= 24.6
14.27 < Social.associations
Feature
0.000
0.005
0.010
0.015
Weight
Positive
Negative

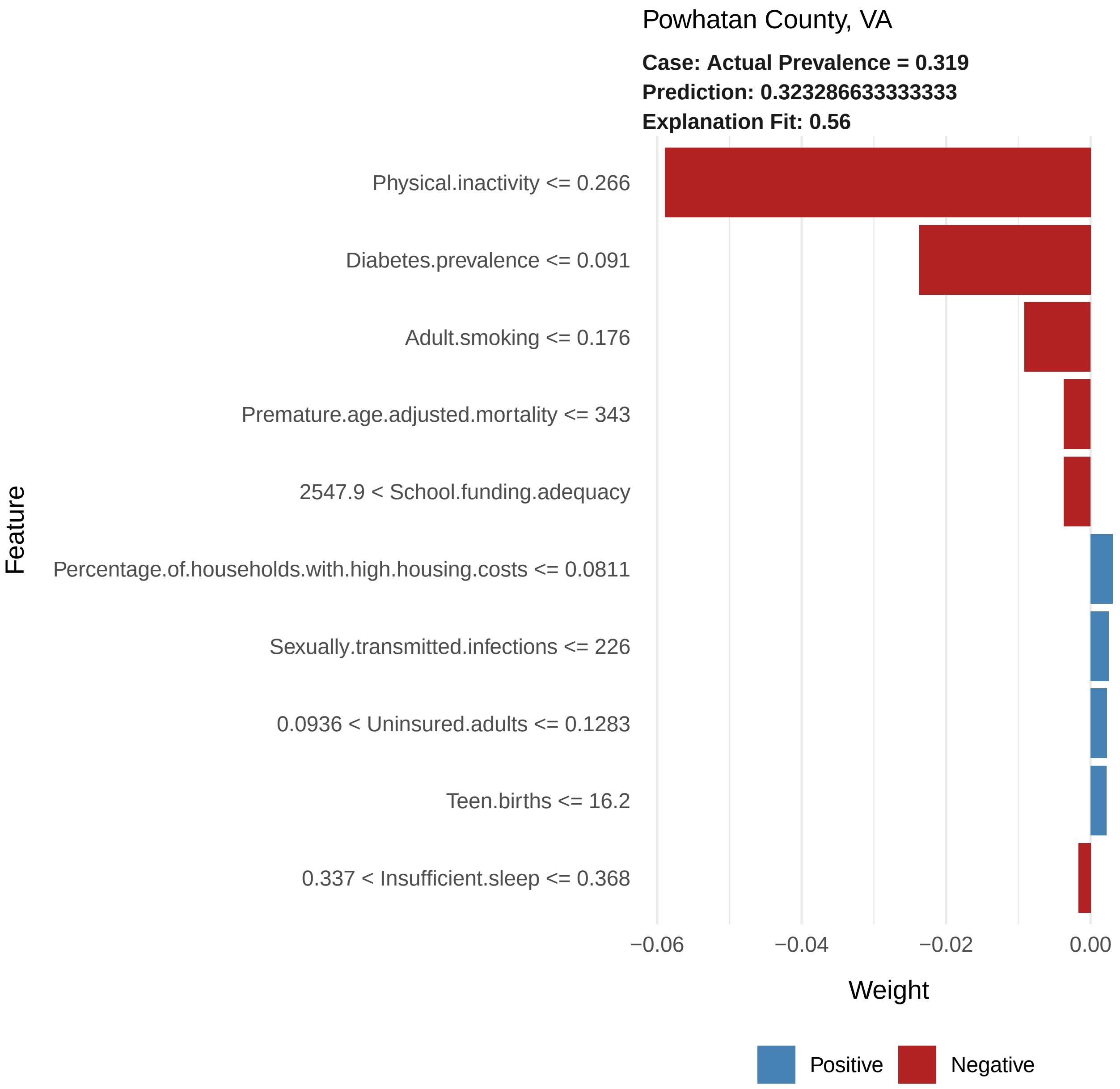

Powhatan County, VA
Case: Actual Prevalence = 0.319
Prediction: 0.323286633333333
Explanation Fit: 0.56
Physical.inactivity <= 0.266
Diabetes.prevalence <= 0.091
Adult.smoking <= 0.176
Premature.age.adjusted.mortality <= 343
2547.9 < School.funding.adequacy
Percentage.of.households.with.high.housing.costs <= 0.0811
Sexually.transmitted.infections <= 226
0.0936 < Uninsured.adults <= 0.1283
Teen.births <= 16.2
0.337 < Insufficient.sleep <= 0.368
Feature
−0.06
−0.04
−0.02
0.00
Weight
Positive
Negative

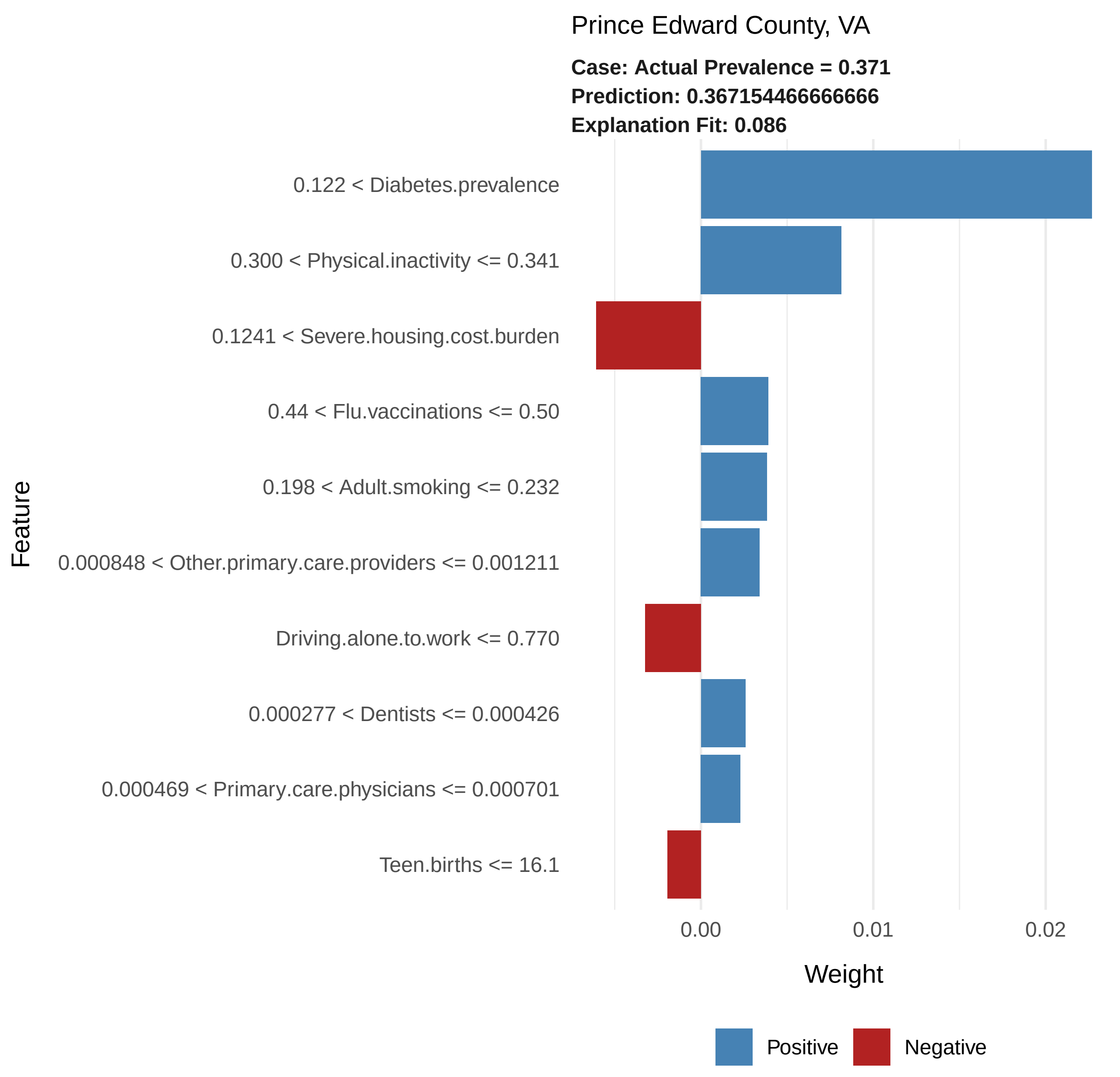
Prince Edward County, VA
Case: Actual Prevalence = 0.371
Prediction: 0.367154466666666
Explanation Fit: 0.086
0.122 < Diabetes.prevalence
0.300 < Physical.inactivity <= 0.341
0.1241 < Severe.housing.cost.burden
0.44 < Flu.vaccinations <= 0.50
0.198 < Adult.smoking <= 0.232
0.000848 < Other.primary.care.providers <= 0.001211
Driving.alone.to.work <= 0.770
0.000277 < Dentists <= 0.000426
0.000469 < Primary.care.physicians <= 0.000701
Teen.births <= 16.1
Feature
0.00
0.01
0.02
Weight
Positive
Negative

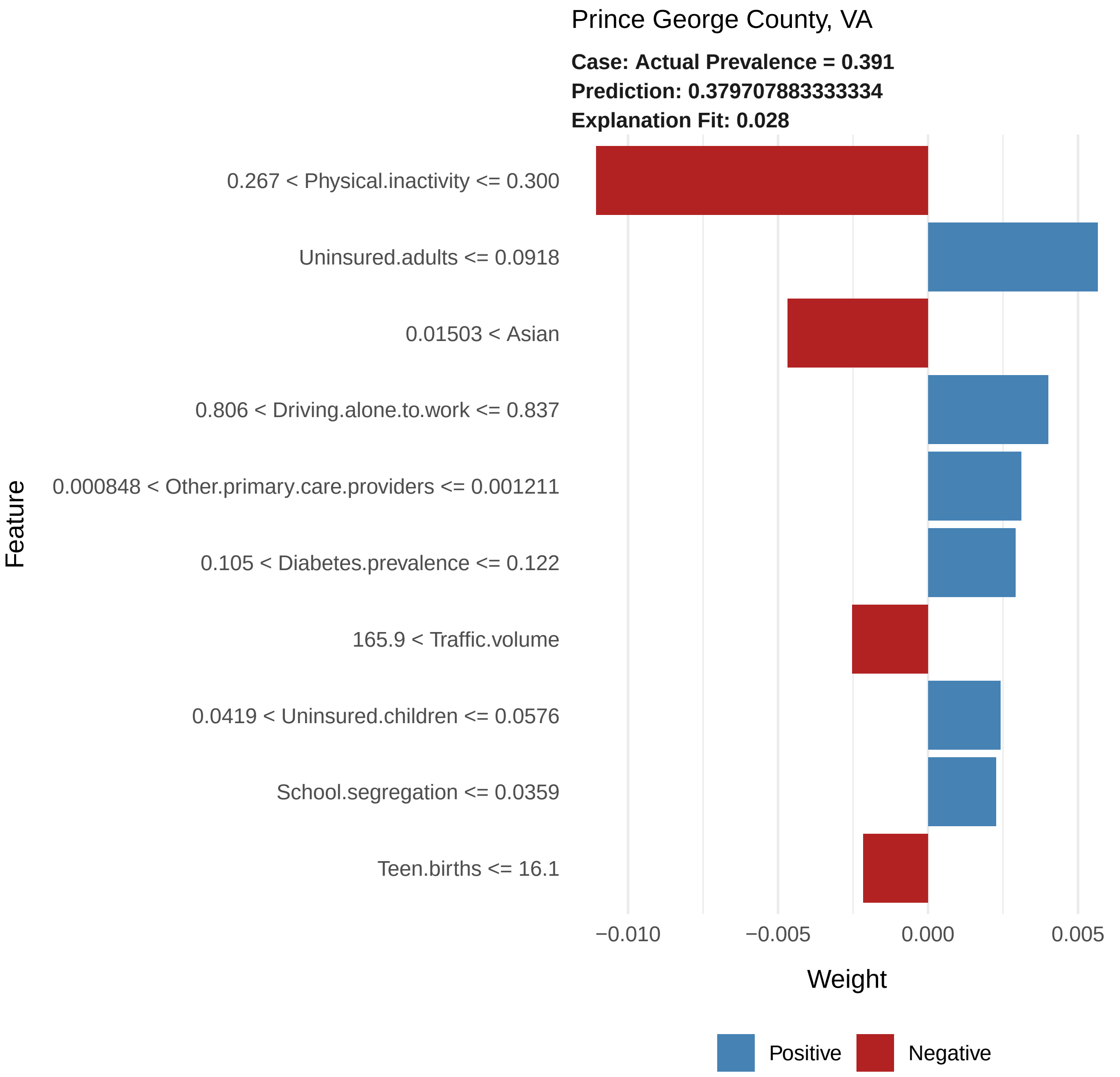

Prince George County, VA
Case: Actual Prevalence = 0.391
Prediction: 0.379707883333334
Explanation Fit: 0.028
0.267 < Physical.inactivity <= 0.300
Uninsured.adults <= 0.0918
0.01503 < Asian
0.806 < Driving.alone.to.work <= 0.837
0.000848 < Other.primary.care.providers <= 0.001211
0.105 < Diabetes.prevalence <= 0.122
165.9 < Traffic.volume
0.0419 < Uninsured.children <= 0.0576
School.segregation <= 0.0359
Teen.births <= 16.1
Feature
−0.010
−0.005
0.000
0.005
Weight
Positive
Negative

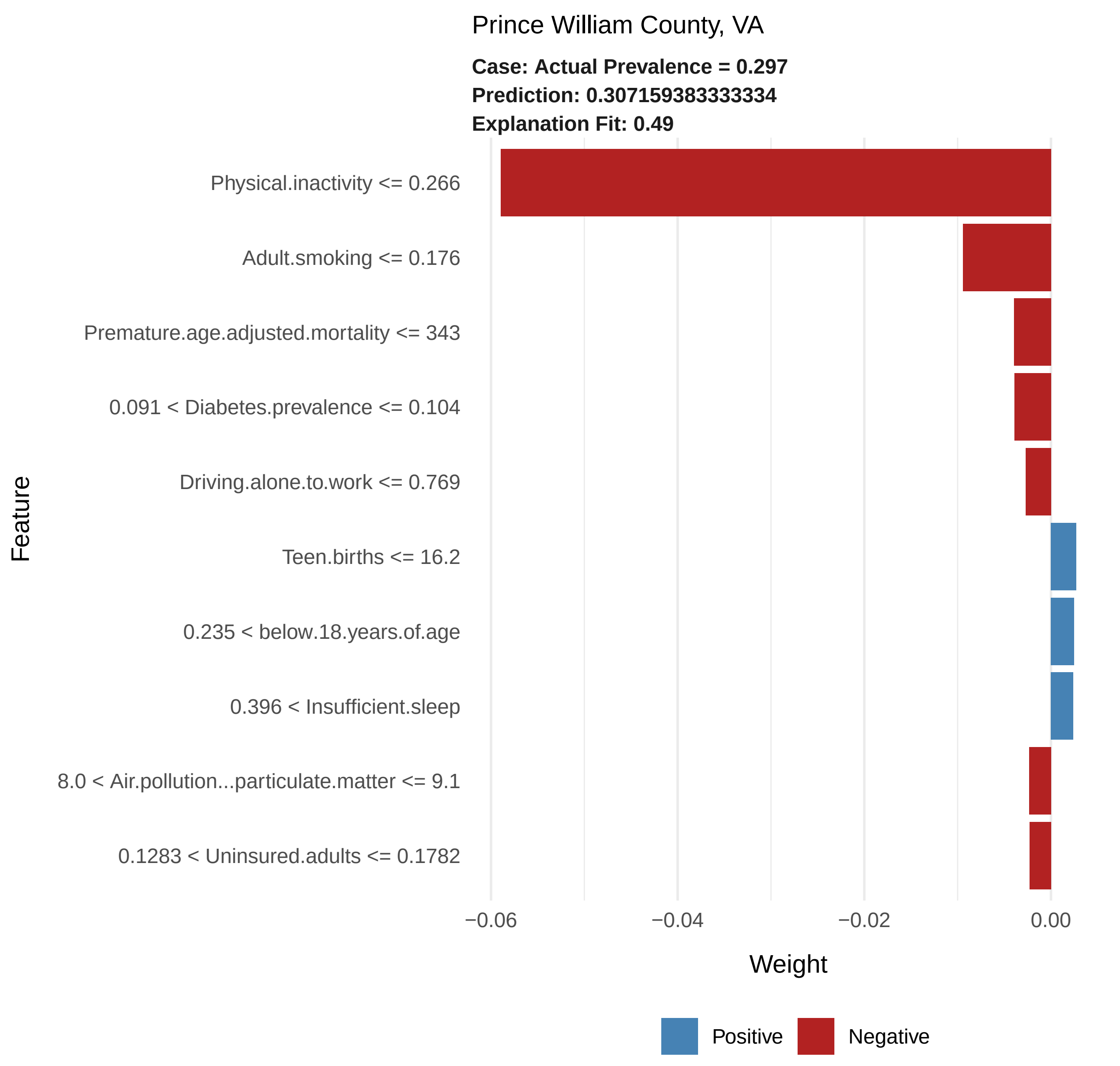
Prince William County, VA
Case: Actual Prevalence = 0.297
Prediction: 0.307159383333334
Explanation Fit: 0.49
Physical.inactivity <= 0.266
Adult.smoking <= 0.176
Premature.age.adjusted.mortality <= 343
0.091 < Diabetes.prevalence <= 0.104
Driving.alone.to.work <= 0.769
Teen.births <= 16.2
0.235 < below.18.years.of.age
0.396 < Insufficient.sleep
8.0 < Air.pollution...particulate.matter <= 9.1
0.1283 < Uninsured.adults <= 0.1782
Feature
−0.06
−0.04
−0.02
0.00
Weight
Positive
Negative

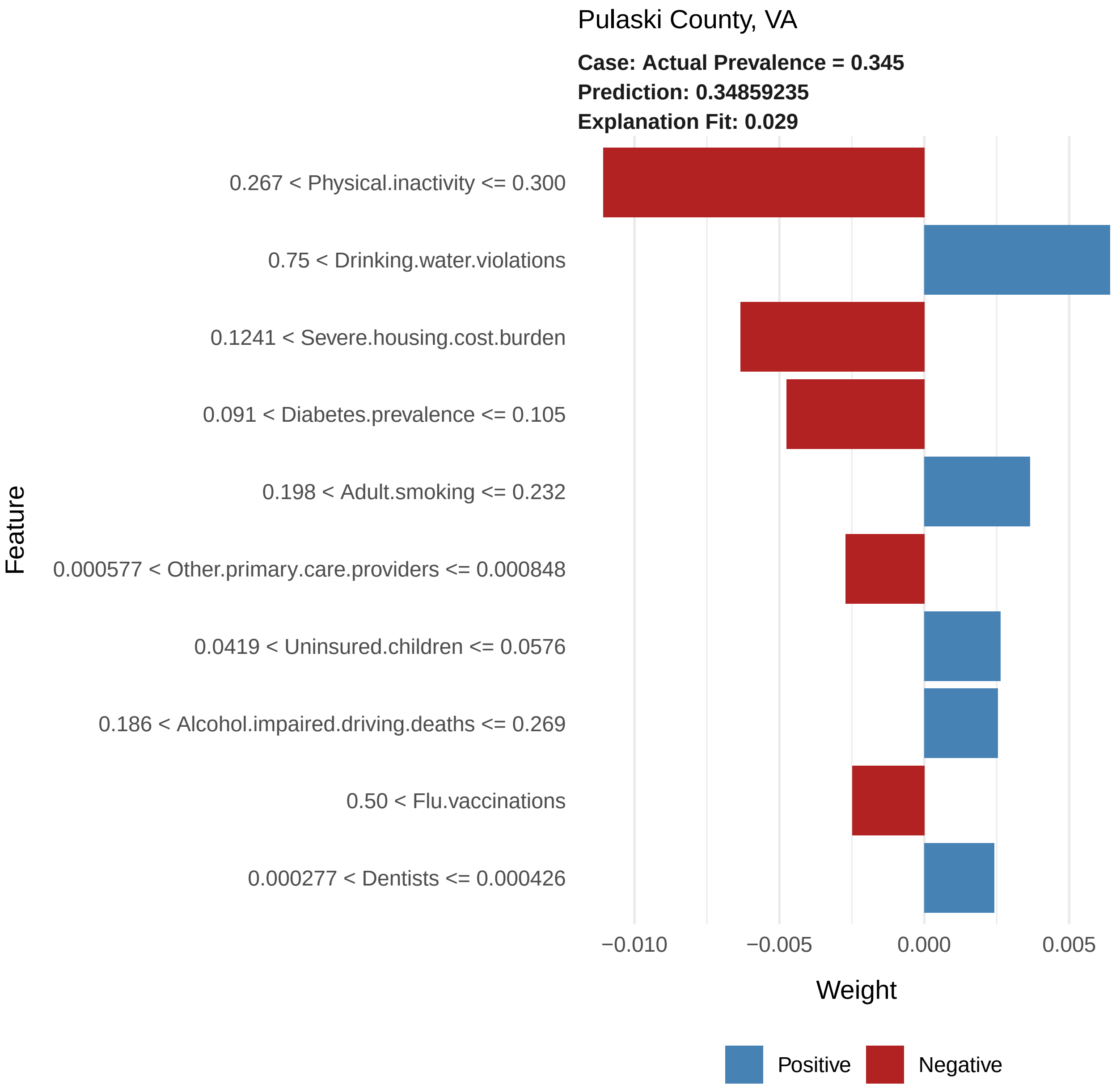
Pulaski County, VA
Case: Actual Prevalence = 0.345
Prediction: 0.34859235
Explanation Fit: 0.029
0.267 < Physical.inactivity <= 0.300
0.75 < Drinking.water.violations
0.1241 < Severe.housing.cost.burden
0.091 < Diabetes.prevalence <= 0.105
0.198 < Adult.smoking <= 0.232
0.000577 < Other.primary.care.providers <= 0.000848
0.0419 < Uninsured.children <= 0.0576
0.186 < Alcohol.impaired.driving.deaths <= 0.269
0.50 < Flu.vaccinations
0.000277 < Dentists <= 0.000426
Feature
−0.010
−0.005
0.000
0.005
Weight
Positive
Negative

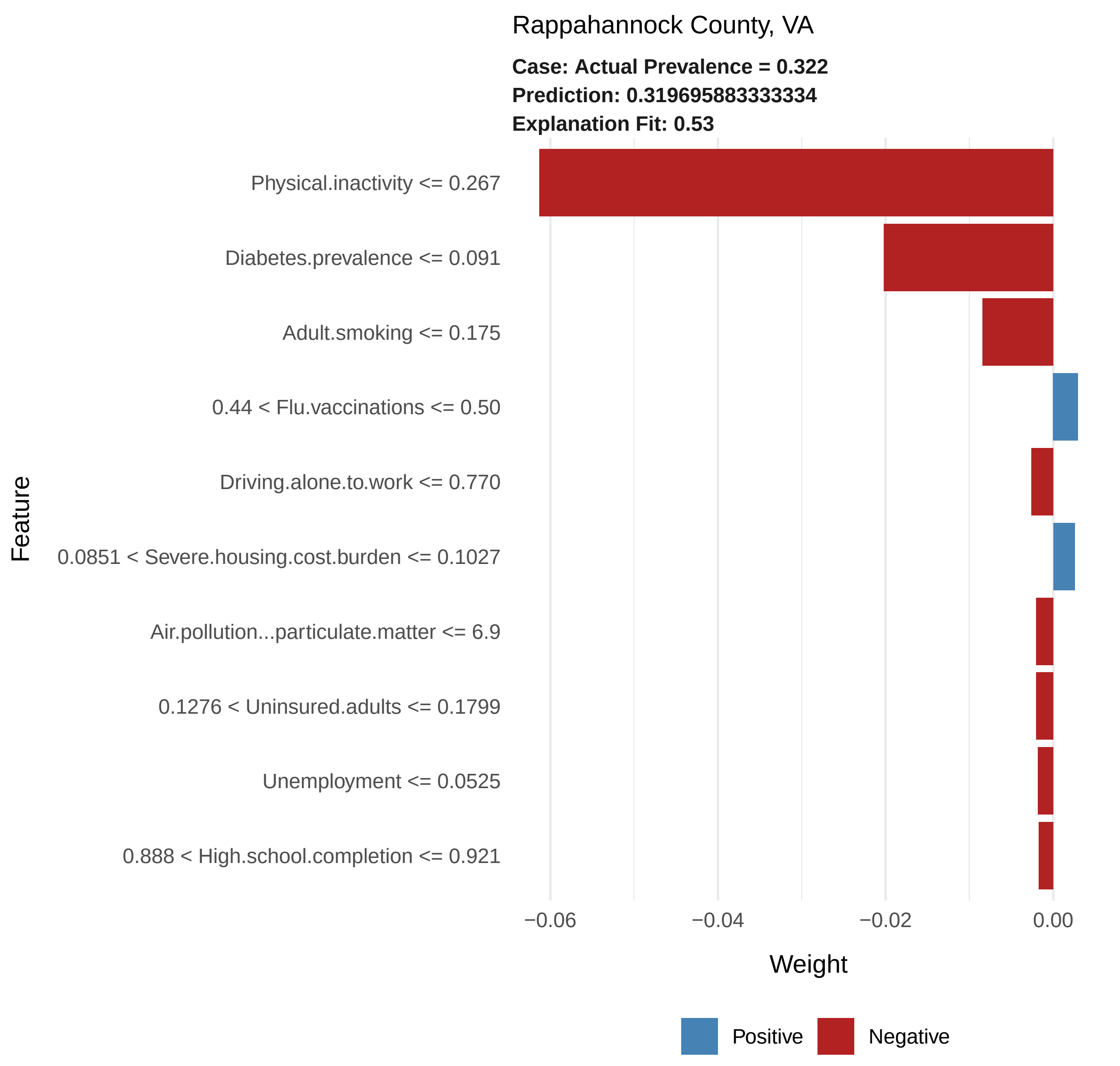
Rappahannock County, VA
Case: Actual Prevalence = 0.322
Prediction: 0.319695883333334
Explanation Fit: 0.53
Physical.inactivity <= 0.267
Diabetes.prevalence <= 0.091
Adult.smoking <= 0.175
0.44 < Flu.vaccinations <= 0.50
Driving.alone.to.work <= 0.770
0.0851 < Severe.housing.cost.burden <= 0.1027
Air.pollution...particulate.matter <= 6.9
0.1276 < Uninsured.adults <= 0.1799
Unemployment <= 0.0525
0.888 < High.school.completion <= 0.921
Feature
−0.06
−0.04
−0.02
0.00
Weight
Positive
Negative

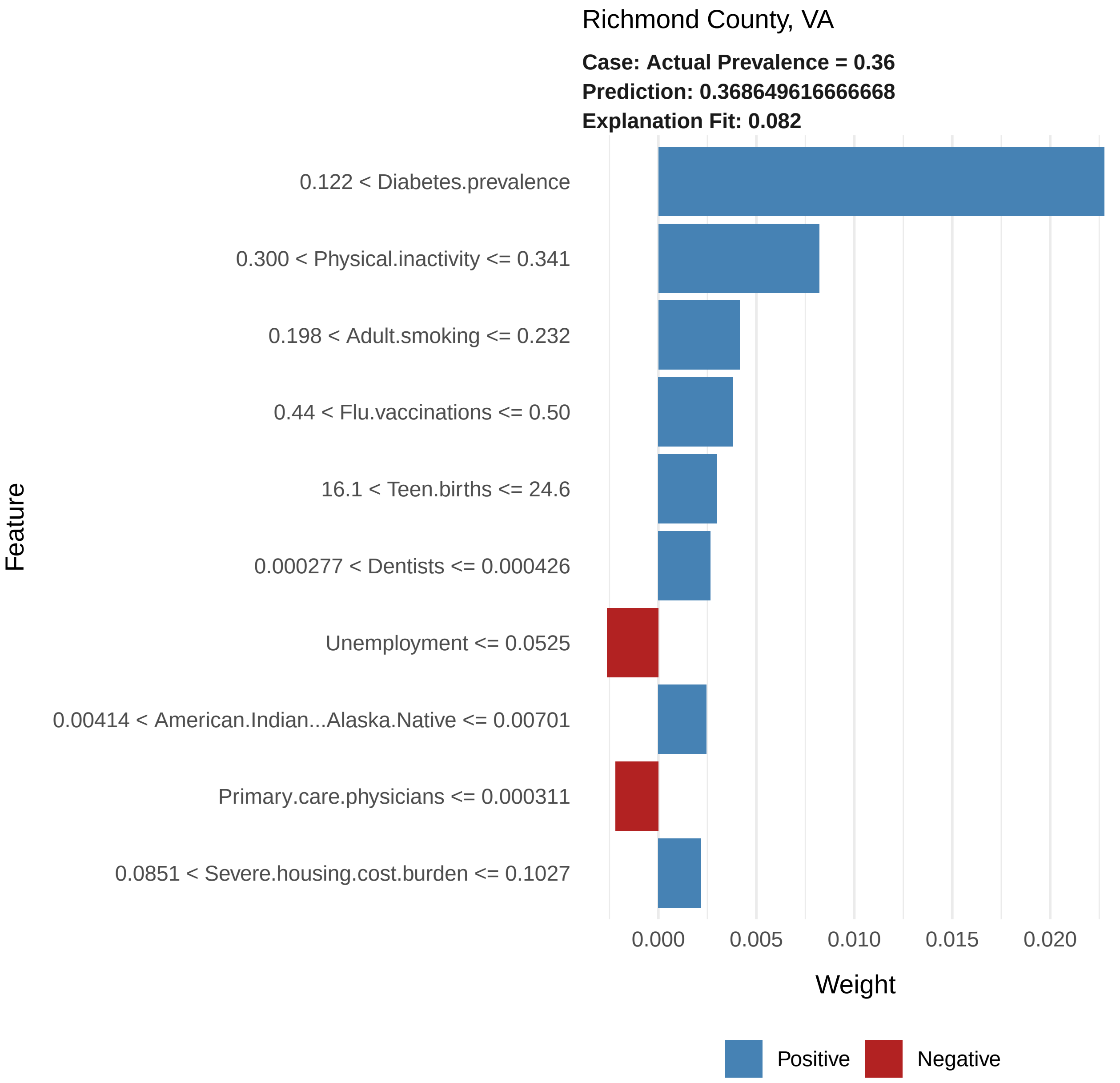

Richmond County, VA
Case: Actual Prevalence = 0.36
Prediction: 0.368649616666668
Explanation Fit: 0.082
0.122 < Diabetes.prevalence
0.300 < Physical.inactivity <= 0.341
0.198 < Adult.smoking <= 0.232
0.44 < Flu.vaccinations <= 0.50
16.1 < Teen.births <= 24.6
0.000277 < Dentists <= 0.000426
Unemployment <= 0.0525
0.00414 < American.Indian...Alaska.Native <= 0.00701
Primary.care.physicians <= 0.000311
0.0851 < Severe.housing.cost.burden <= 0.1027
Feature
0.000
0.005
0.010
0.015
0.020
Weight
Positive
Negative

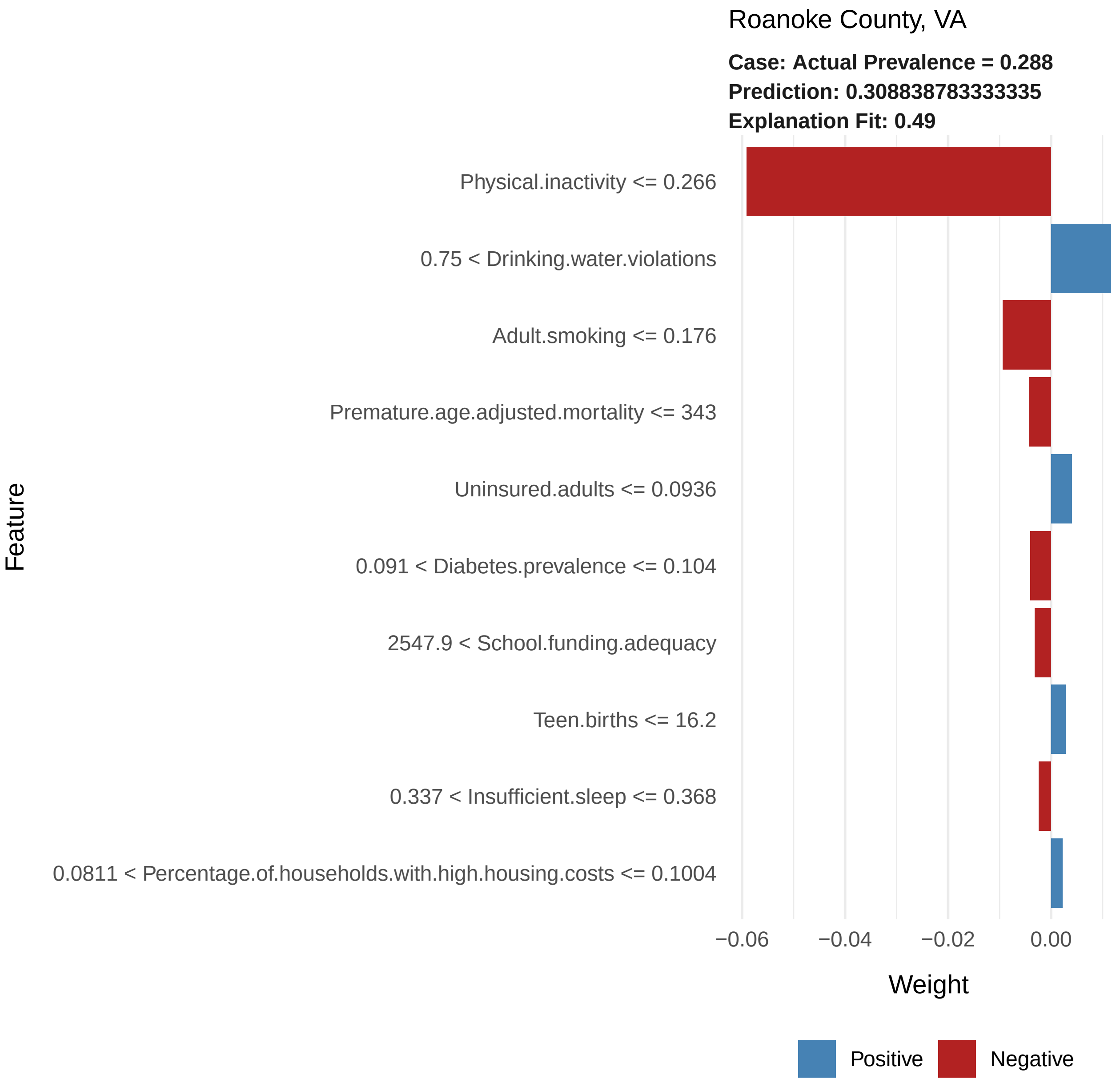
Roanoke County, VA
Case: Actual Prevalence = 0.288
Prediction: 0.308838783333335
Explanation Fit: 0.49
Physical.inactivity <= 0.266
0.75 < Drinking.water.violations
Adult.smoking <= 0.176
Premature.age.adjusted.mortality <= 343
Uninsured.adults <= 0.0936
0.091 < Diabetes.prevalence <= 0.104
2547.9 < School.funding.adequacy
Teen.births <= 16.2
0.337 < Insufficient.sleep <= 0.368
0.0811 < Percentage.of.households.with.high.housing.costs <= 0.1004
Feature
−0.06
−0.04
−0.02
0.00
Weight
Positive
Negative

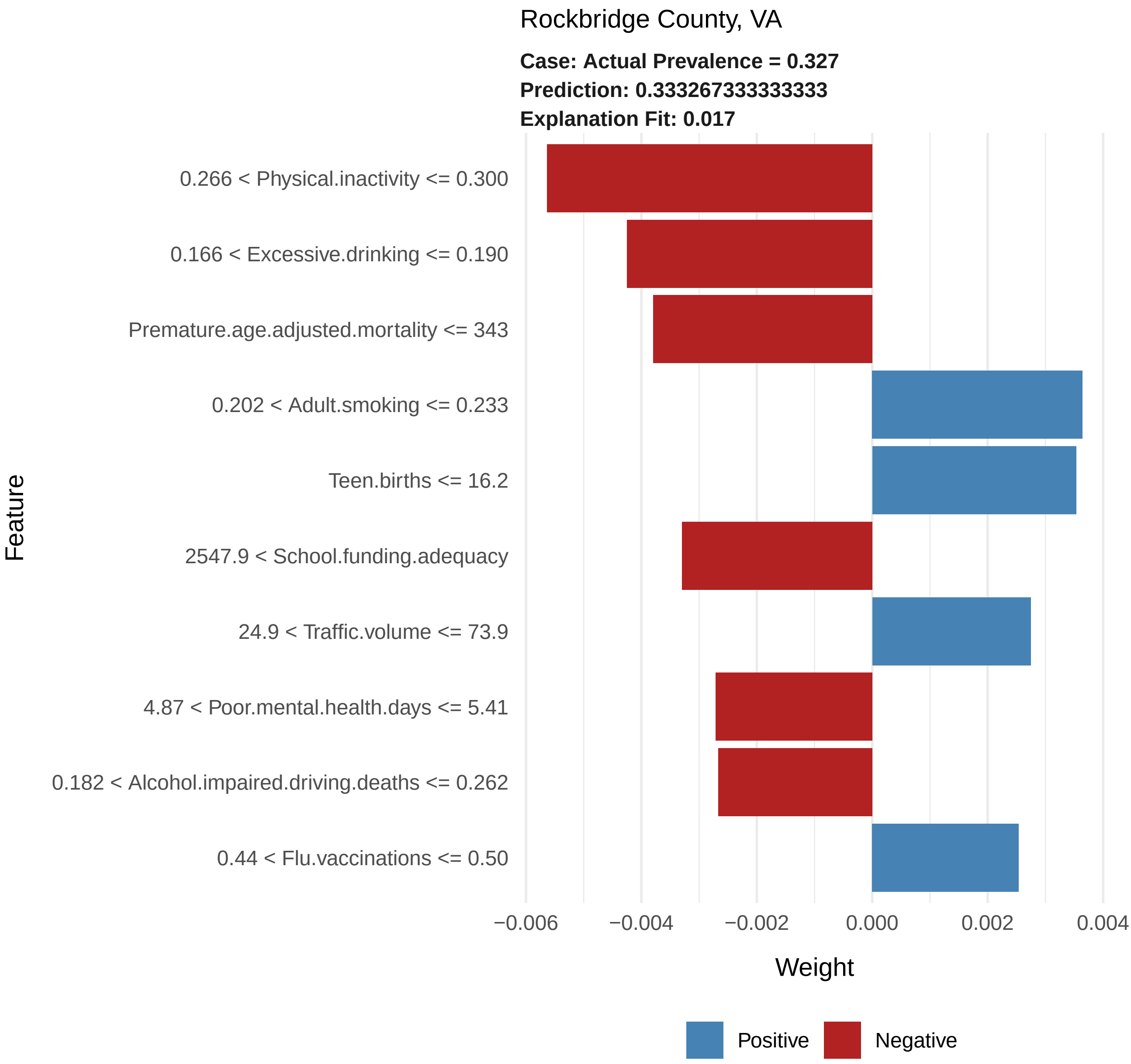

Rockbridge County, VA
Case: Actual Prevalence = 0.327
Prediction: 0.333267333333333
Explanation Fit: 0.017
0.266 < Physical.inactivity <= 0.300
0.166 < Excessive.drinking <= 0.190
Premature.age.adjusted.mortality <= 343
0.202 < Adult.smoking <= 0.233
Teen.births <= 16.2
2547.9 < School.funding.adequacy
24.9 < Traffic.volume <= 73.9
4.87 < Poor.mental.health.days <= 5.41
0.182 < Alcohol.impaired.driving.deaths <= 0.262
0.44 < Flu.vaccinations <= 0.50
Feature
−0.006
−0.004
−0.002
0.000
0.002
0.004
Weight
Positive
Negative

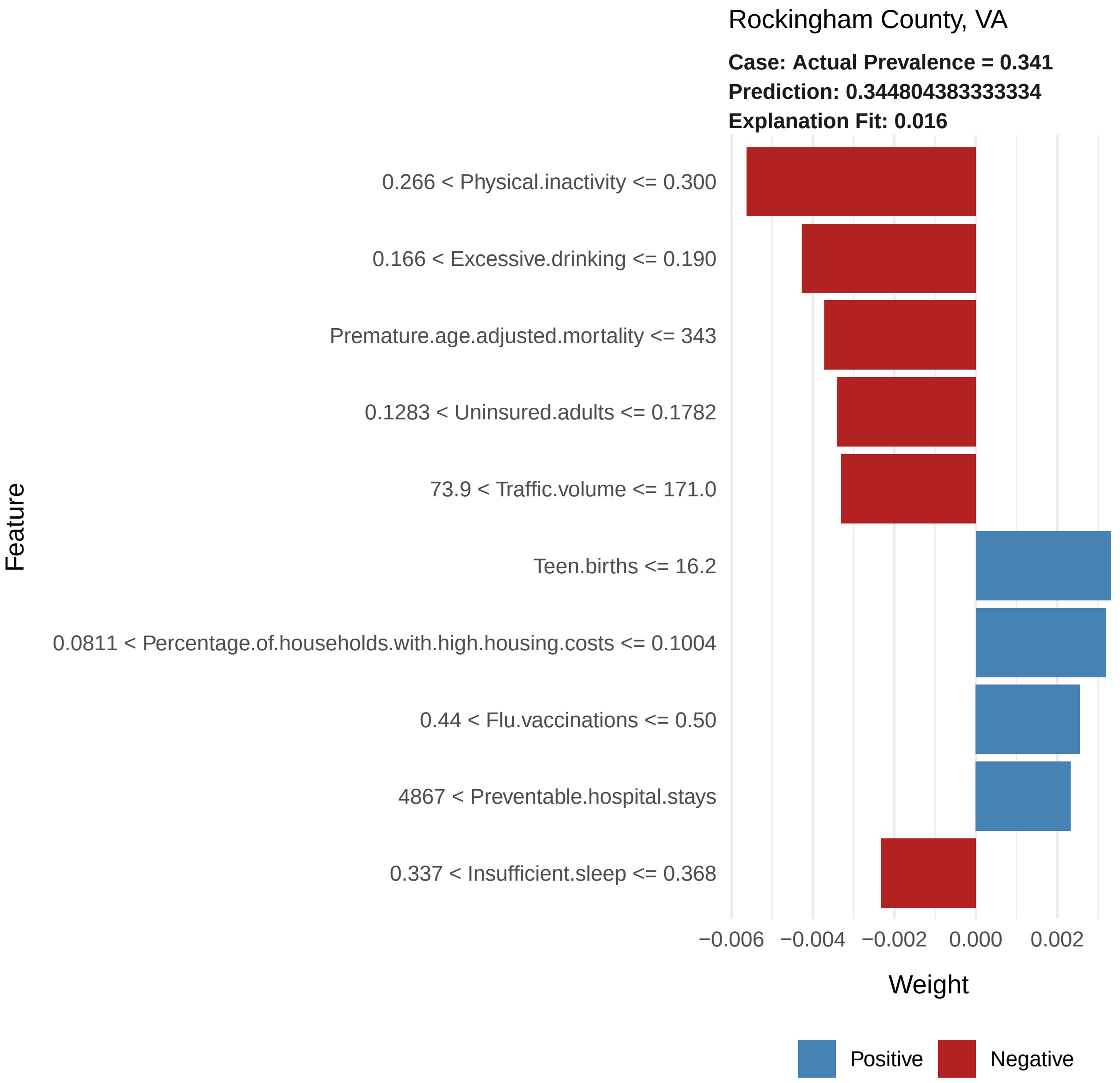

Rockingham County, VA
Case: Actual Prevalence = 0.341
Prediction: 0.344804383333334
Explanation Fit: 0.016
Feature
0.266 < Physical.inactivity <= 0.300
0.166 < Excessive.drinking <= 0.190
Premature.age.adjusted.mortality <= 343
0.1283 < Uninsured.adults <= 0.1782
73.9 < Traffic.volume <= 171.0
Teen.births <= 16.2
0.0811 < Percentage.of.households.with.high.housing.costs <= 0.1004
0.44 < Flu.vaccinations <= 0.50
4867 < Preventable.hospital.stays
0.337 < Insufficient.sleep <= 0.368
−0.006
−0.004
−0.002
0.000
0.002
Weight
Positive
Negative

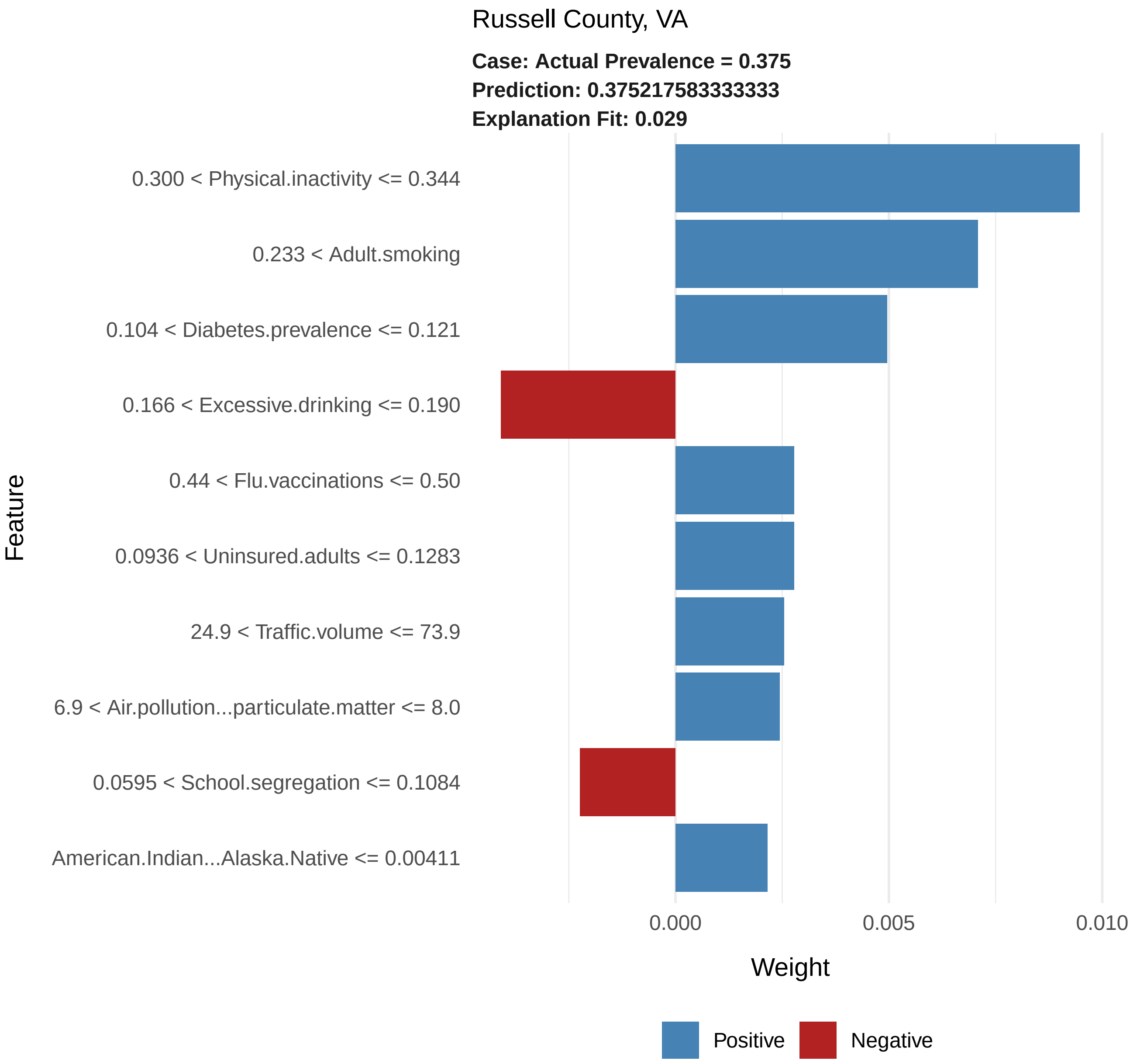

Russell County, VA
Case: Actual Prevalence = 0.375
Prediction: 0.375217583333333
Explanation Fit: 0.029
0.300 < Physical.inactivity <= 0.344
0.233 < Adult.smoking
0.104 < Diabetes.prevalence <= 0.121
0.166 < Excessive.drinking <= 0.190
0.44 < Flu.vaccinations <= 0.50
0.0936 < Uninsured.adults <= 0.1283
24.9 < Traffic.volume <= 73.9
6.9 < Air.pollution...particulate.matter <= 8.0
0.0595 < School.segregation <= 0.1084
American.Indian...Alaska.Native <= 0.00411
Feature
0.000
0.005
0.010
Weight
Positive
Negative

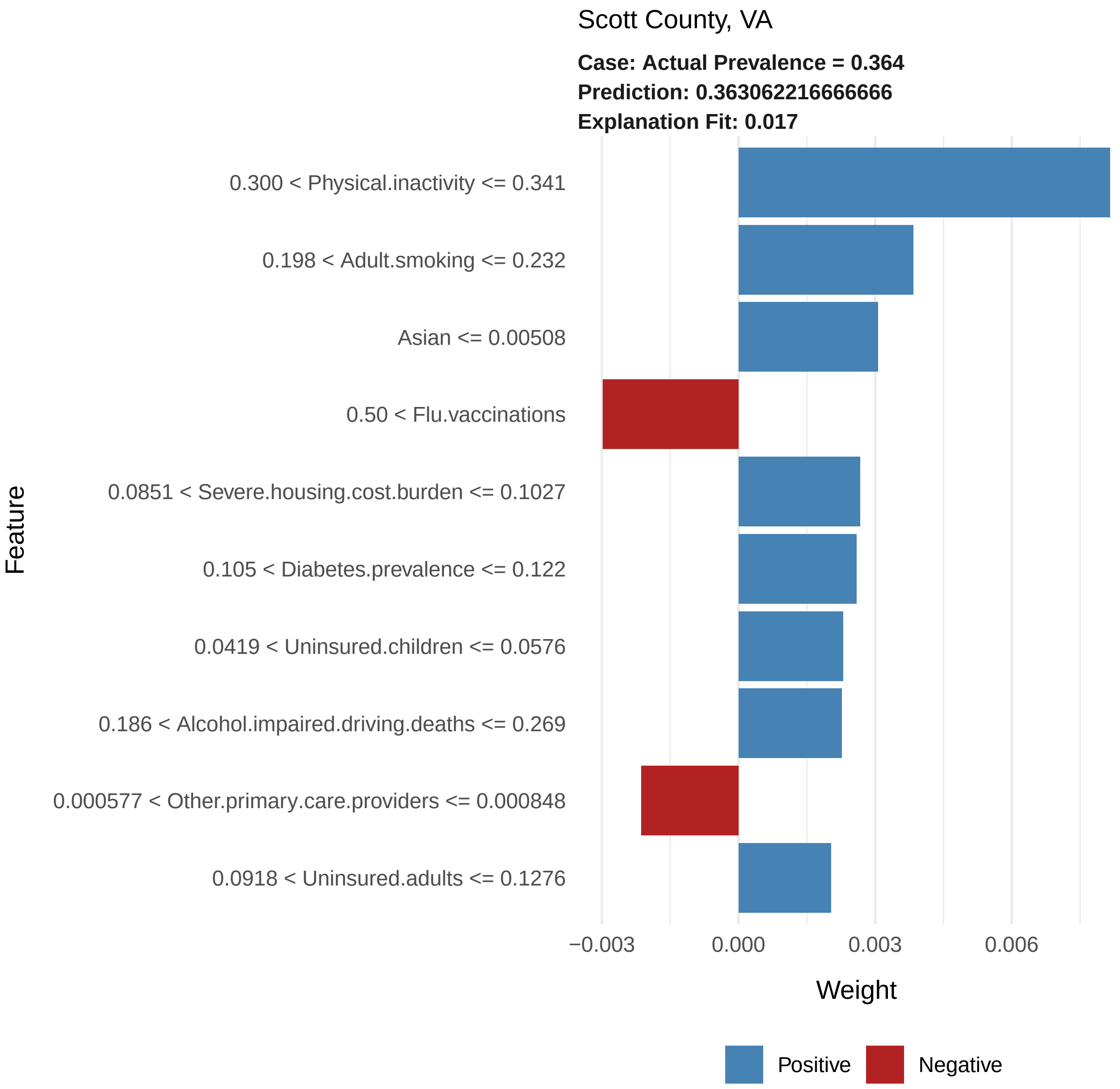

Scott County, VA
Case: Actual Prevalence = 0.364
Prediction: 0.363062216666666
Explanation Fit: 0.017
0.300 < Physical.inactivity <= 0.341
0.198 < Adult.smoking <= 0.232
Asian <= 0.00508
0.50 < Flu.vaccinations
0.0851 < Severe.housing.cost.burden <= 0.1027
0.105 < Diabetes.prevalence <= 0.122
0.0419 < Uninsured.children <= 0.0576
0.186 < Alcohol.impaired.driving.deaths <= 0.269
0.000577 < Other.primary.care.providers <= 0.000848
0.0918 < Uninsured.adults <= 0.1276
Feature
−0.003
0.000
0.003
0.006
Weight
Positive
Negative

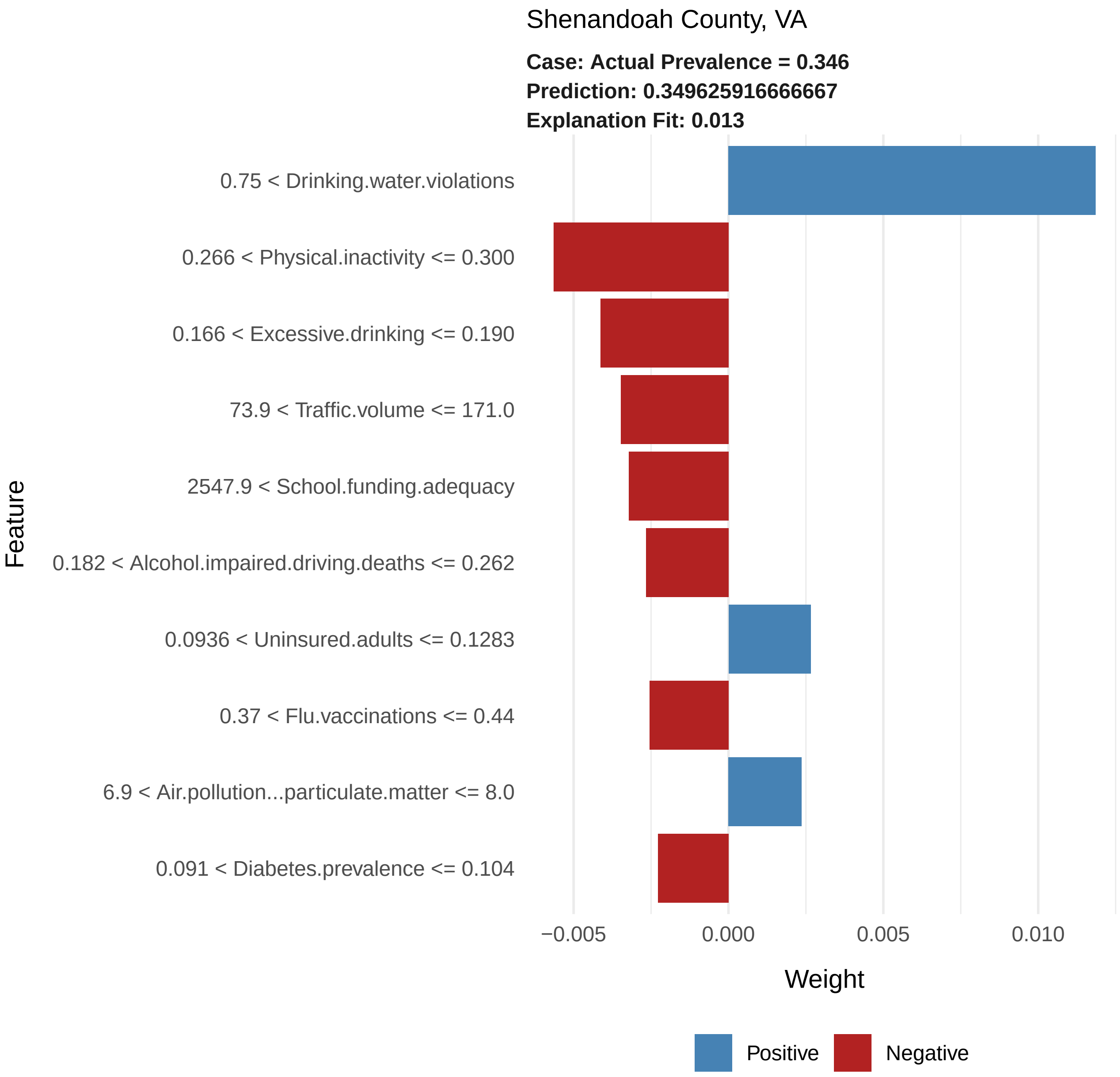
Shenandoah County, VA
Case: Actual Prevalence = 0.346
Prediction: 0.349625916666667
Explanation Fit: 0.013
Feature
0.75 < Drinking.water.violations
0.266 < Physical.inactivity <= 0.300
0.166 < Excessive.drinking <= 0.190
73.9 < Traffic.volume <= 171.0
2547.9 < School.funding.adequacy
0.182 < Alcohol.impaired.driving.deaths <= 0.262
0.0936 < Uninsured.adults <= 0.1283
0.37 < Flu.vaccinations <= 0.44
6.9 < Air.pollution...particulate.matter <= 8.0
0.091 < Diabetes.prevalence <= 0.104
−0.005
0.000
0.005
0.010
Weight
Positive
Negative

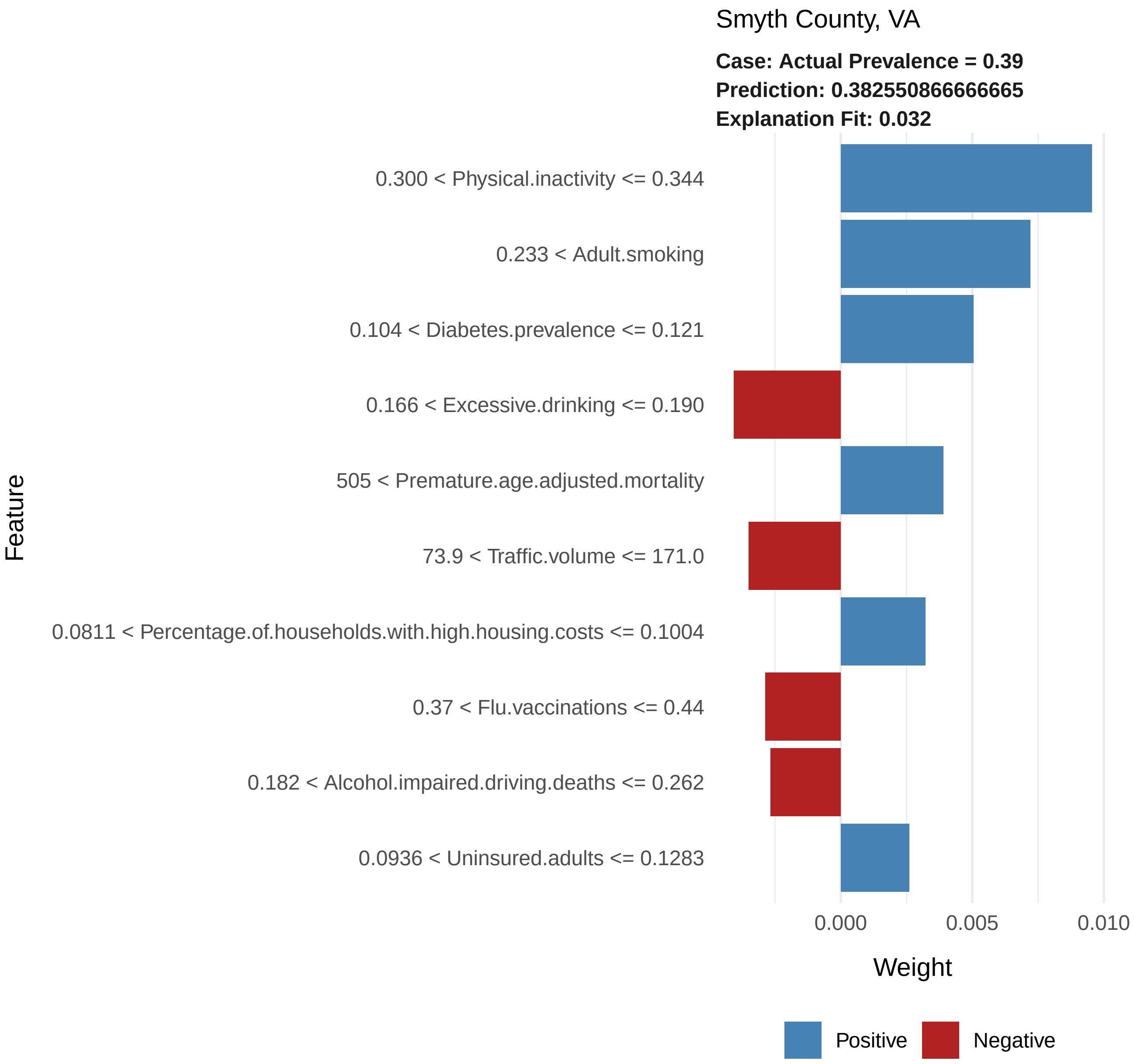

Smyth County, VA
Case: Actual Prevalence = 0.39
Prediction: 0.382550866666665
Explanation Fit: 0.032
Feature
0.300 < Physical.inactivity <= 0.344
0.233 < Adult.smoking
0.104 < Diabetes.prevalence <= 0.121
0.166 < Excessive.drinking <= 0.190
505 < Premature.age.adjusted.mortality
73.9 < Traffic.volume <= 171.0
0.0811 < Percentage.of.households.with.high.housing.costs <= 0.1004
0.37 < Flu.vaccinations <= 0.44
0.182 < Alcohol.impaired.driving.deaths <= 0.262
0.0936 < Uninsured.adults <= 0.1283
0.000
0.005
0.010
Weight
Positive
Negative

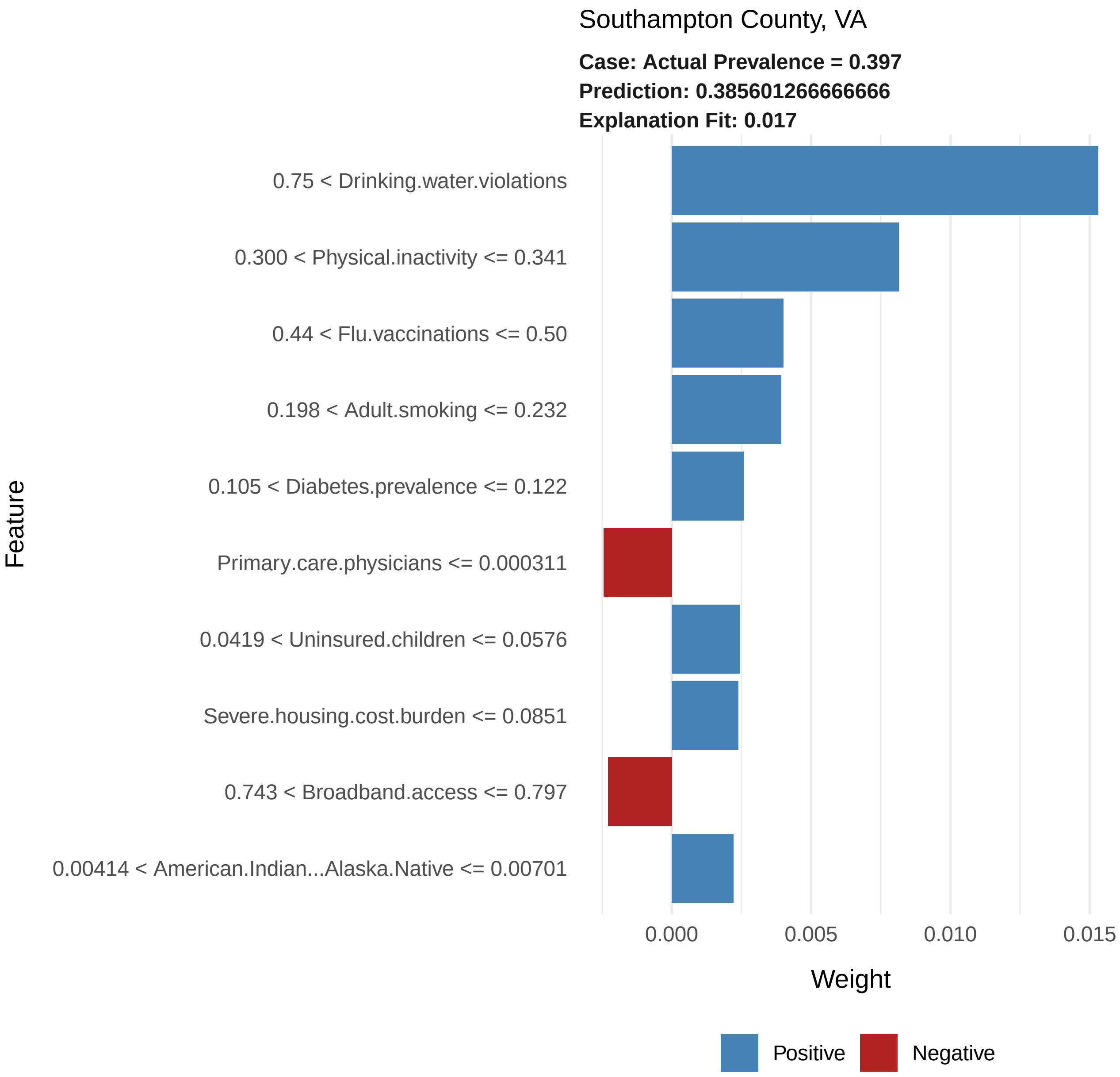
Southampton County, VA
Case: Actual Prevalence = 0.397
Prediction: 0.385601266666666
Explanation Fit: 0.017
0.75 < Drinking.water.violations
0.300 < Physical.inactivity <= 0.341
0.44 < Flu.vaccinations <= 0.50
0.198 < Adult.smoking <= 0.232
0.105 < Diabetes.prevalence <= 0.122
Primary.care.physicians <= 0.000311
0.0419 < Uninsured.children <= 0.0576
Severe.housing.cost.burden <= 0.0851
0.743 < Broadband.access <= 0.797
0.00414 < American.Indian...Alaska.Native <= 0.00701
Feature
0.000
0.005
0.010
0.015
Weight
Positive
Negative

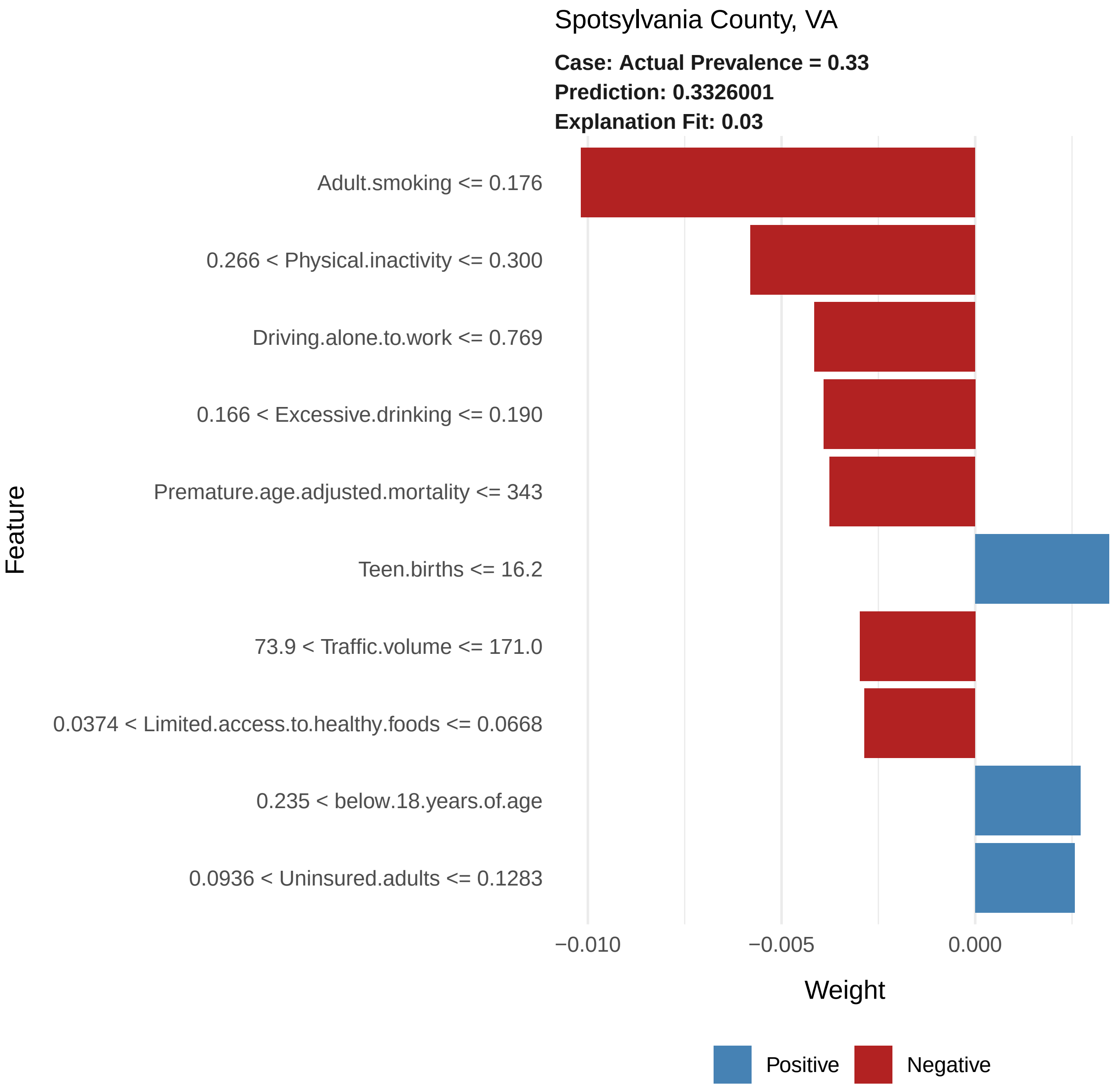
Spotsylvania County, VA
Case: Actual Prevalence = 0.33
Prediction: 0.3326001
Explanation Fit: 0.03
Adult.smoking <= 0.176
0.266 < Physical.inactivity <= 0.300
Driving.alone.to.work <= 0.769
0.166 < Excessive.drinking <= 0.190
Premature.age.adjusted.mortality <= 343
Teen.births <= 16.2
73.9 < Traffic.volume <= 171.0
0.0374 < Limited.access.to.healthy.foods <= 0.0668
0.235 < below.18.years.of.age
0.0936 < Uninsured.adults <= 0.1283
Feature
−0.010
−0.005
0.000
Weight
Positive
Negative

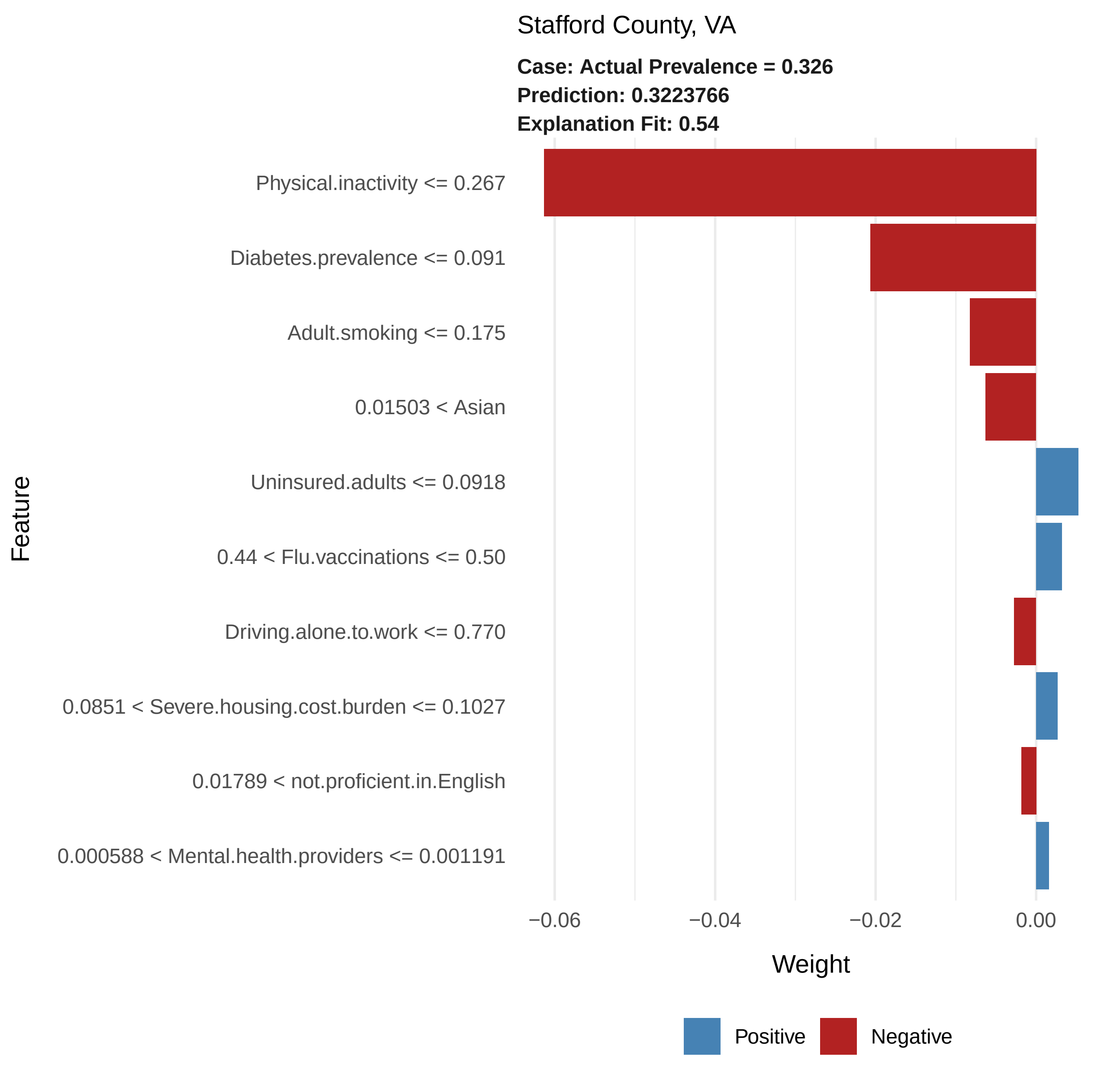

Stafford County, VA
Case: Actual Prevalence = 0.326
Prediction: 0.3223766
Explanation Fit: 0.54
Physical.inactivity <= 0.267
Diabetes.prevalence <= 0.091
Adult.smoking <= 0.175
0.01503 < Asian
Uninsured.adults <= 0.0918
0.44 < Flu.vaccinations <= 0.50
Driving.alone.to.work <= 0.770
0.0851 < Severe.housing.cost.burden <= 0.1027
0.01789 < not.proficient.in.English
0.000588 < Mental.health.providers <= 0.001191
Feature
−0.06
−0.04
−0.02
0.00
Weight
Positive
Negative

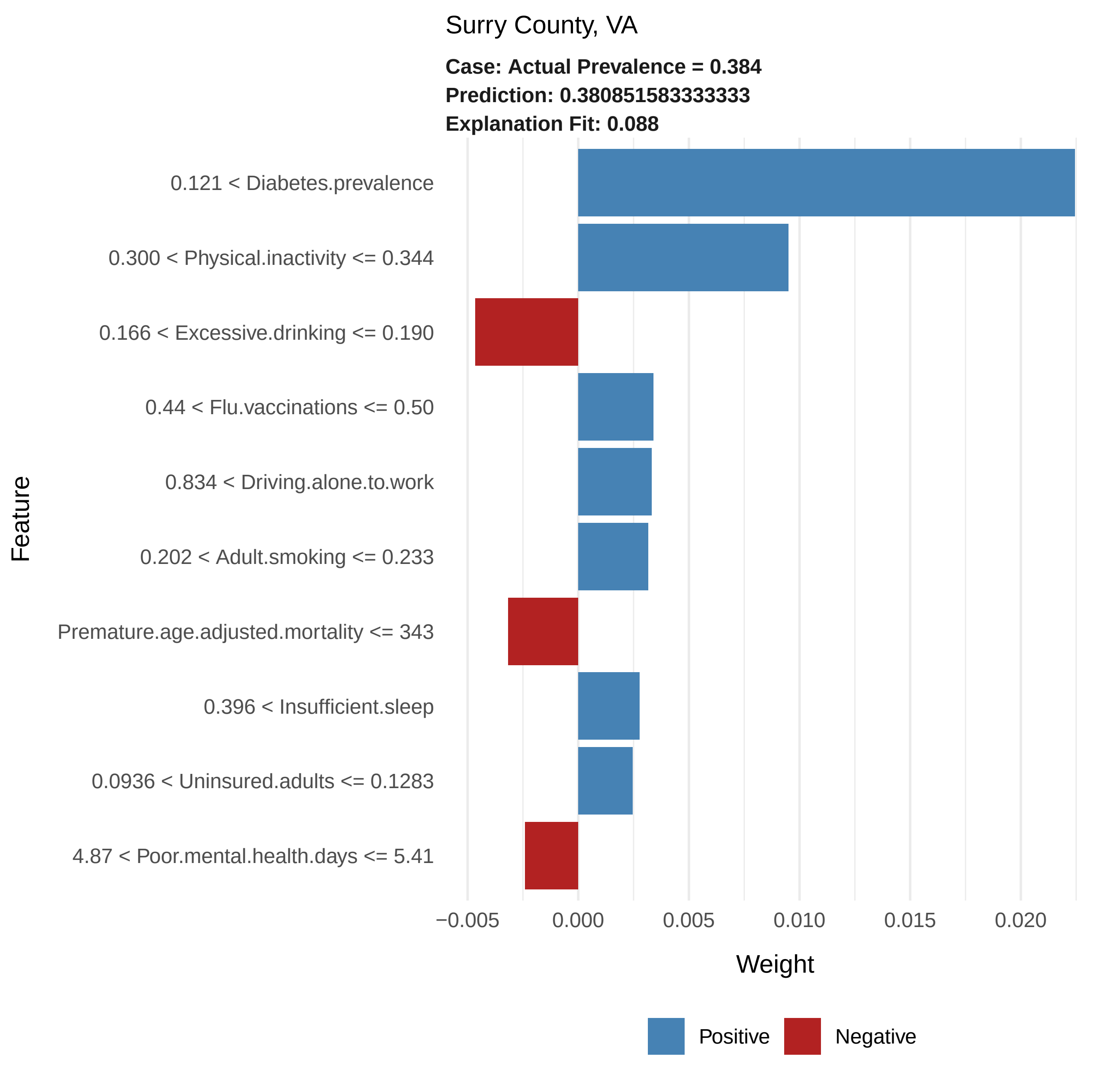
Surry County, VA
Case: Actual Prevalence = 0.384
Prediction: 0.380851583333333
Explanation Fit: 0.088
0.121 < Diabetes.prevalence
0.300 < Physical.inactivity <= 0.344
0.166 < Excessive.drinking <= 0.190
0.44 < Flu.vaccinations <= 0.50
0.834 < Driving.alone.to.work
0.202 < Adult.smoking <= 0.233
Premature.age.adjusted.mortality <= 343
0.396 < Insufficient.sleep
0.0936 < Uninsured.adults <= 0.1283
4.87 < Poor.mental.health.days <= 5.41
Feature
−0.005
0.000
0.005
0.010
0.015
0.020
Weight
Positive
Negative

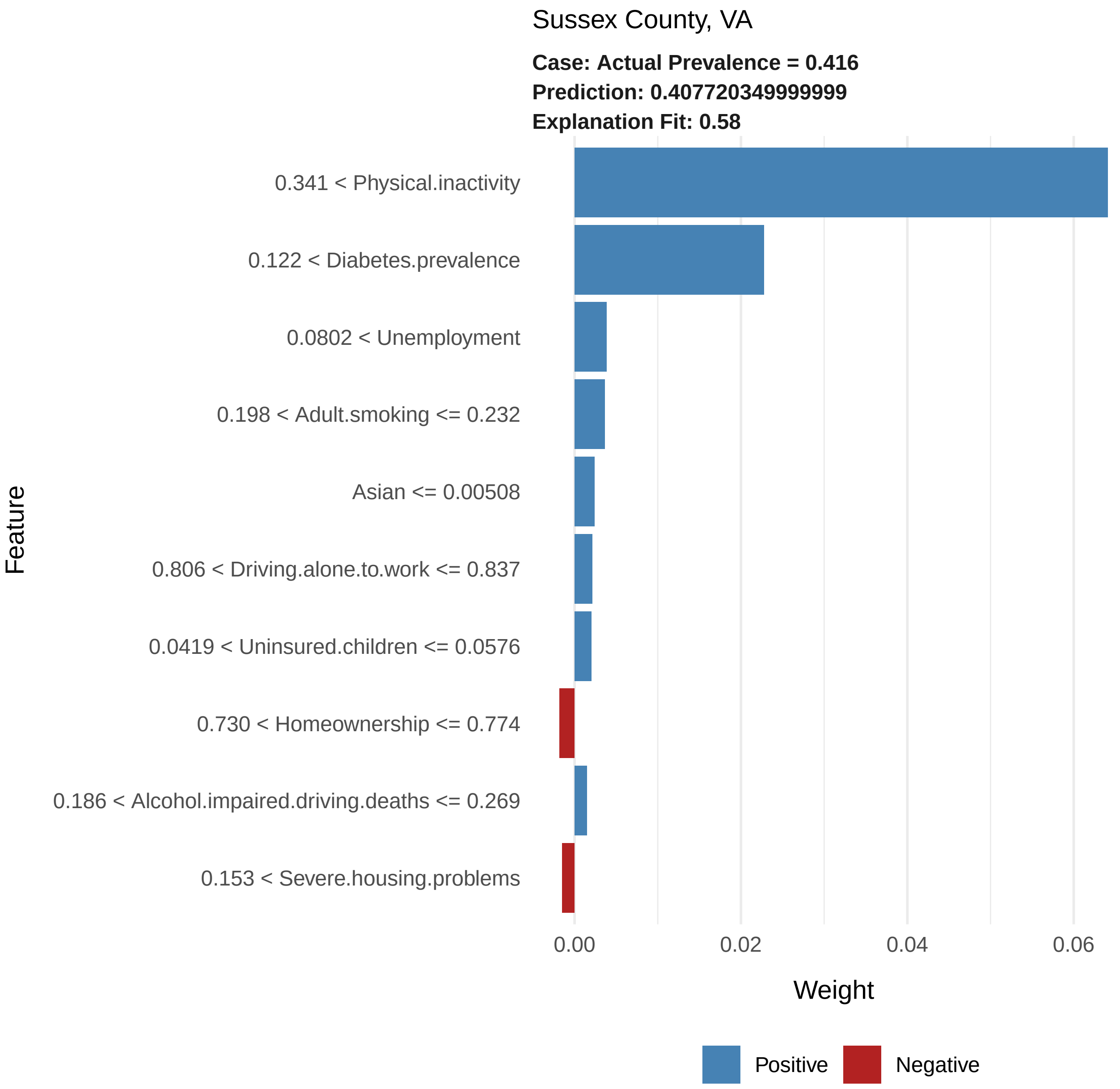
Sussex County, VA
Case: Actual Prevalence = 0.416
Prediction: 0.407720349999999
Explanation Fit: 0.58
0.341 < Physical.inactivity
0.122 < Diabetes.prevalence
0.0802 < Unemployment
0.198 < Adult.smoking <= 0.232
Asian <= 0.00508
0.806 < Driving.alone.to.work <= 0.837
0.0419 < Uninsured.children <= 0.0576
0.730 < Homeownership <= 0.774
0.186 < Alcohol.impaired.driving.deaths <= 0.269
0.153 < Severe.housing.problems
Feature
0.00
0.02
0.04
0.06
Weight
Positive
Negative

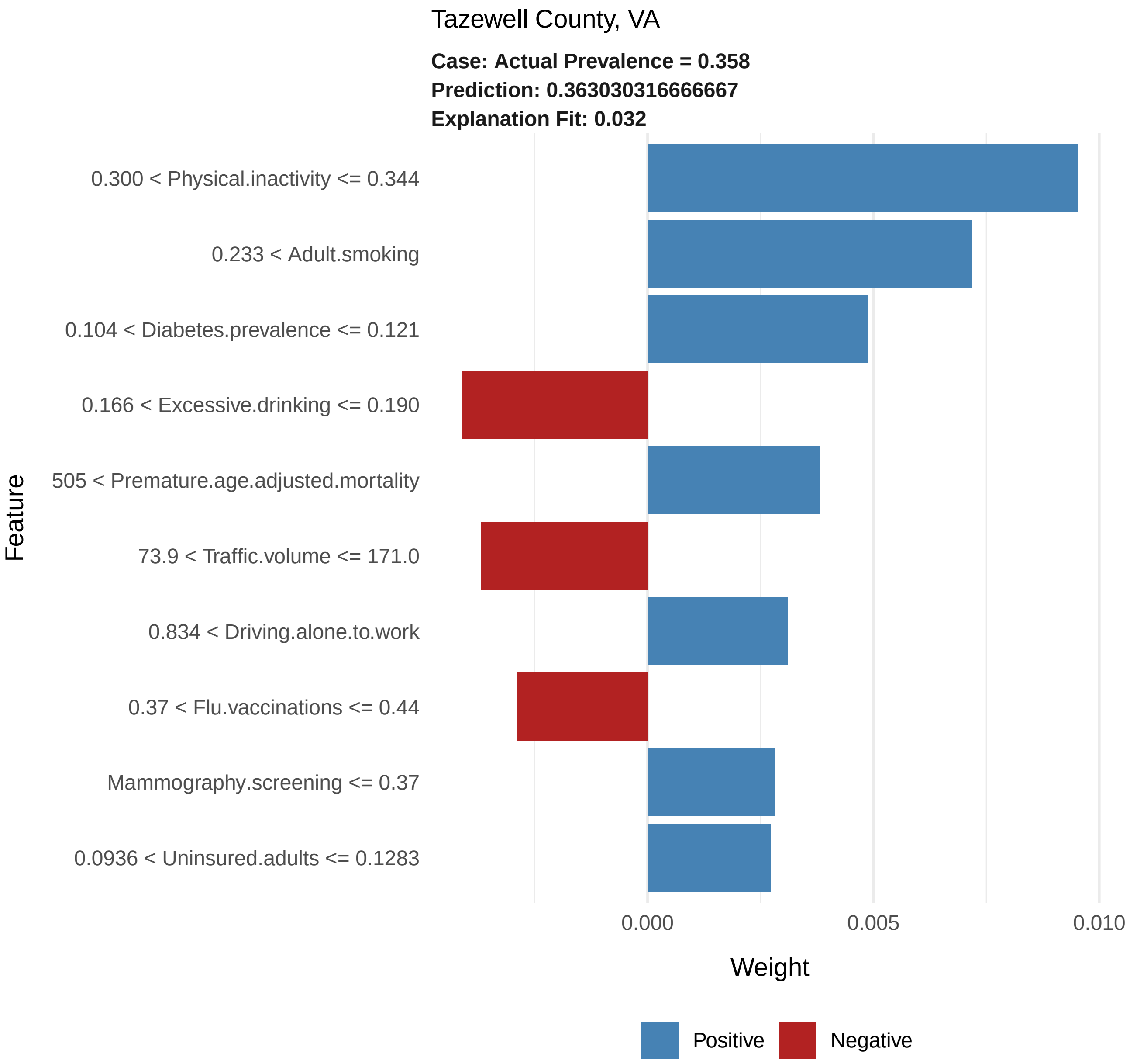

Tazewell County, VA
Case: Actual Prevalence = 0.358
Prediction: 0.363030316666667
Explanation Fit: 0.032
0.300 < Physical.inactivity <= 0.344
0.233 < Adult.smoking
0.104 < Diabetes.prevalence <= 0.121
0.166 < Excessive.drinking <= 0.190
505 < Premature.age.adjusted.mortality
73.9 < Traffic.volume <= 171.0
0.834 < Driving.alone.to.work
0.37 < Flu.vaccinations <= 0.44
Mammography.screening <= 0.37
0.0936 < Uninsured.adults <= 0.1283
Feature
0.000
0.005
0.010
Weight
Positive
Negative

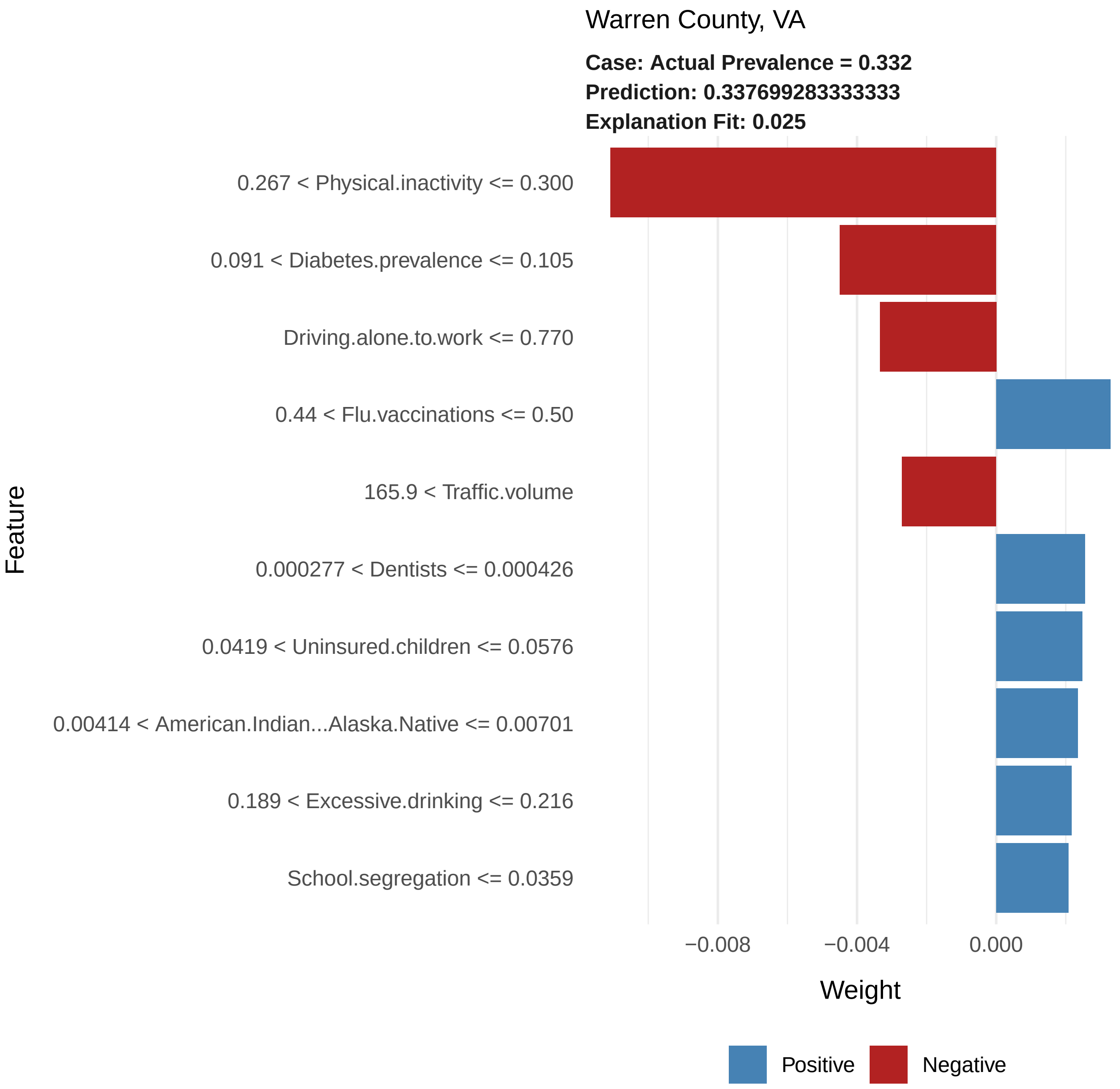

Warren County, VA
Case: Actual Prevalence = 0.332
Prediction: 0.337699283333333
Explanation Fit: 0.025
0.267 < Physical.inactivity <= 0.300
0.091 < Diabetes.prevalence <= 0.105
Driving.alone.to.work <= 0.770
0.44 < Flu.vaccinations <= 0.50
165.9 < Traffic.volume
0.000277 < Dentists <= 0.000426
0.0419 < Uninsured.children <= 0.0576
0.00414 < American.Indian...Alaska.Native <= 0.00701
0.189 < Excessive.drinking <= 0.216
School.segregation <= 0.0359
Feature
−0.008
−0.004
0.000
Weight
Positive
Negative

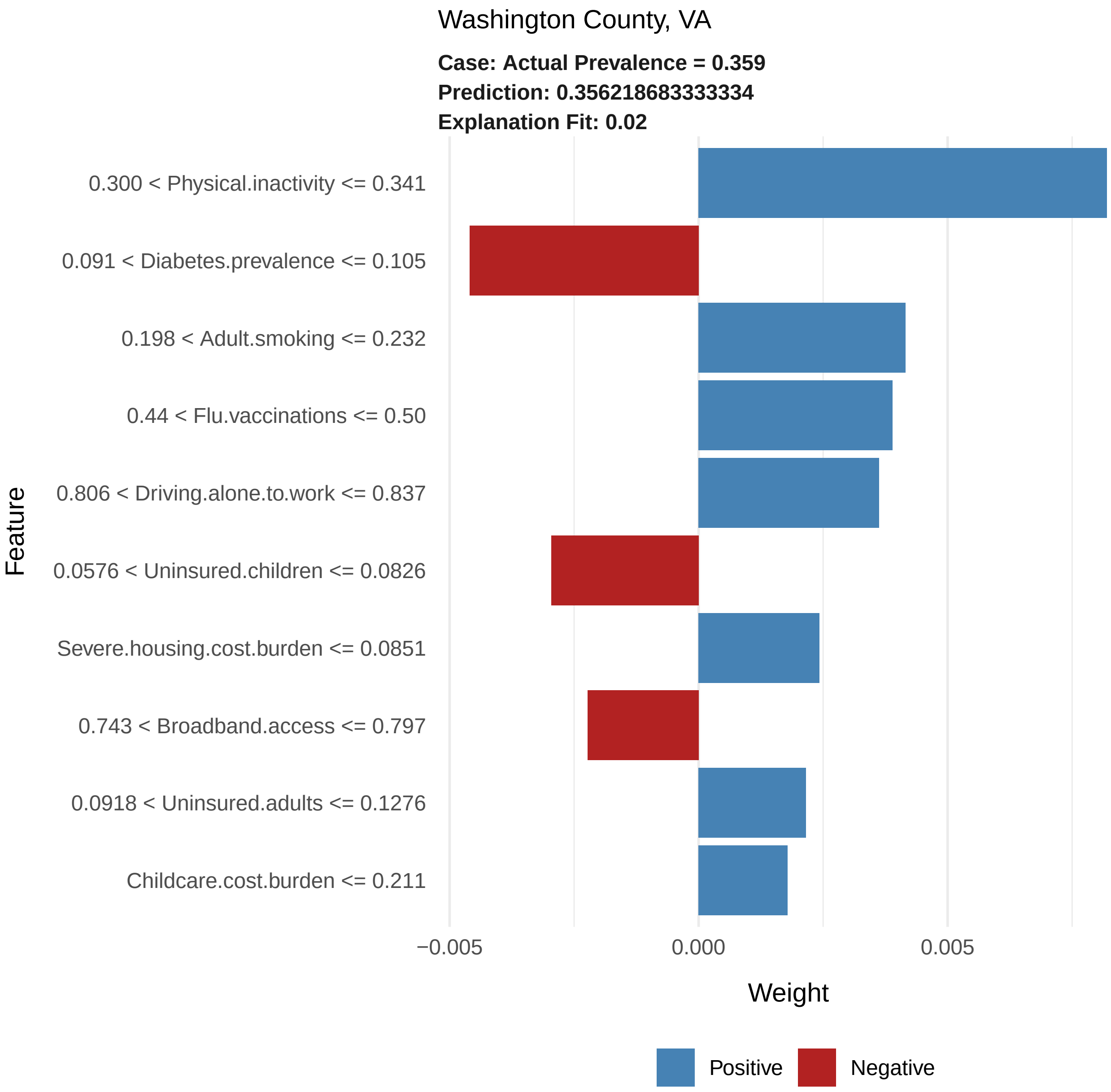
Washington County, VA
Case: Actual Prevalence = 0.359
Prediction: 0.356218683333334
Explanation Fit: 0.02
0.300 < Physical.inactivity <= 0.341
0.091 < Diabetes.prevalence <= 0.105
0.198 < Adult.smoking <= 0.232
0.44 < Flu.vaccinations <= 0.50
0.806 < Driving.alone.to.work <= 0.837
0.0576 < Uninsured.children <= 0.0826
Severe.housing.cost.burden <= 0.0851
0.743 < Broadband.access <= 0.797
0.0918 < Uninsured.adults <= 0.1276
Childcare.cost.burden <= 0.211
Feature
−0.005
0.000
0.005
Weight
Positive
Negative

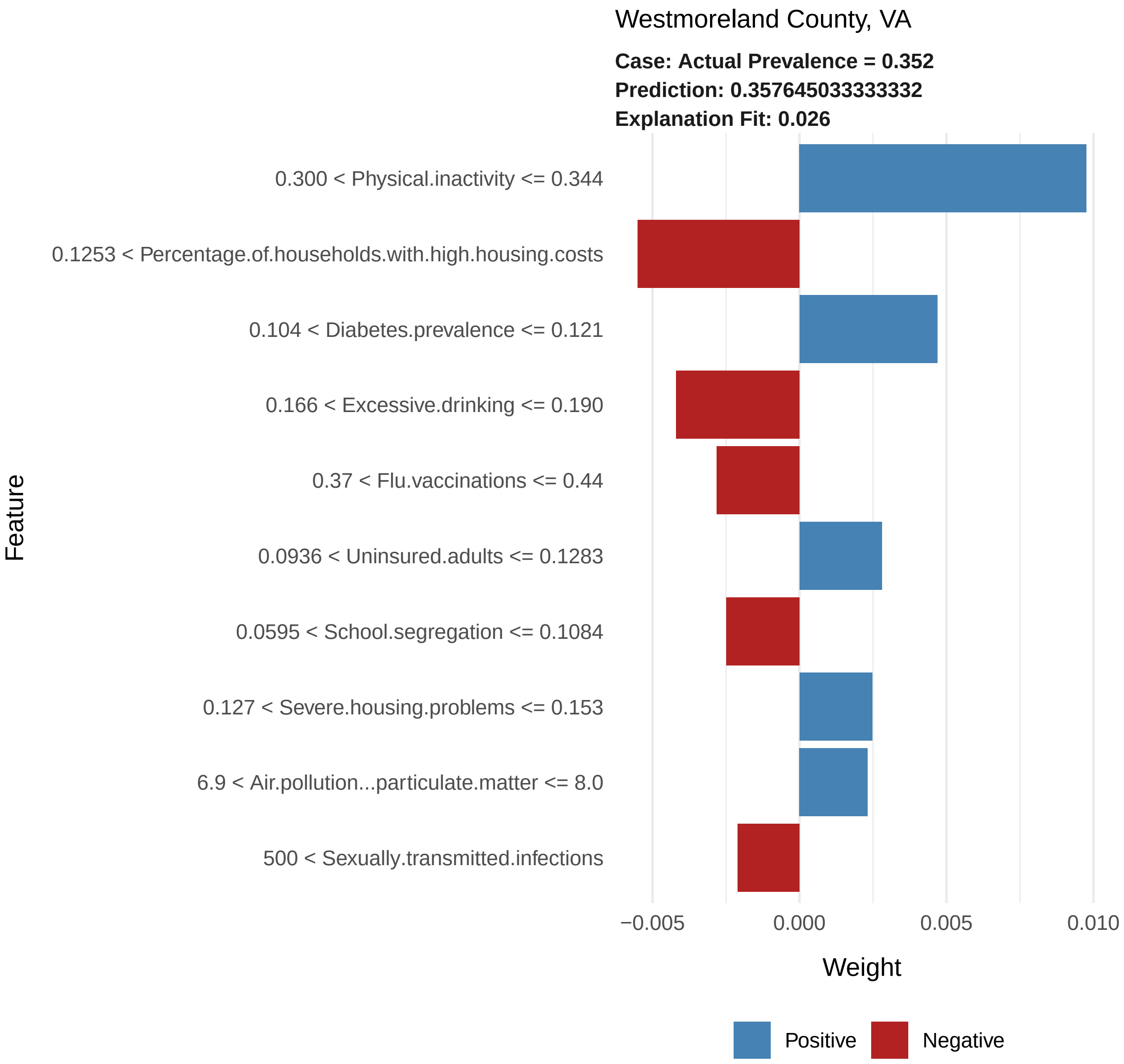
Westmoreland County, VA
Case: Actual Prevalence = 0.352
Prediction: 0.357645033333332
Explanation Fit: 0.026
0.300 < Physical.inactivity <= 0.344
0.1253 < Percentage.of.households.with.high.housing.costs
0.104 < Diabetes.prevalence <= 0.121
0.166 < Excessive.drinking <= 0.190
0.37 < Flu.vaccinations <= 0.44
0.0936 < Uninsured.adults <= 0.1283
0.0595 < School.segregation <= 0.1084
0.127 < Severe.housing.problems <= 0.153
6.9 < Air.pollution...particulate.matter <= 8.0
500 < Sexually.transmitted.infections
Feature
−0.005
0.000
0.005
0.010
Weight
Positive
Negative

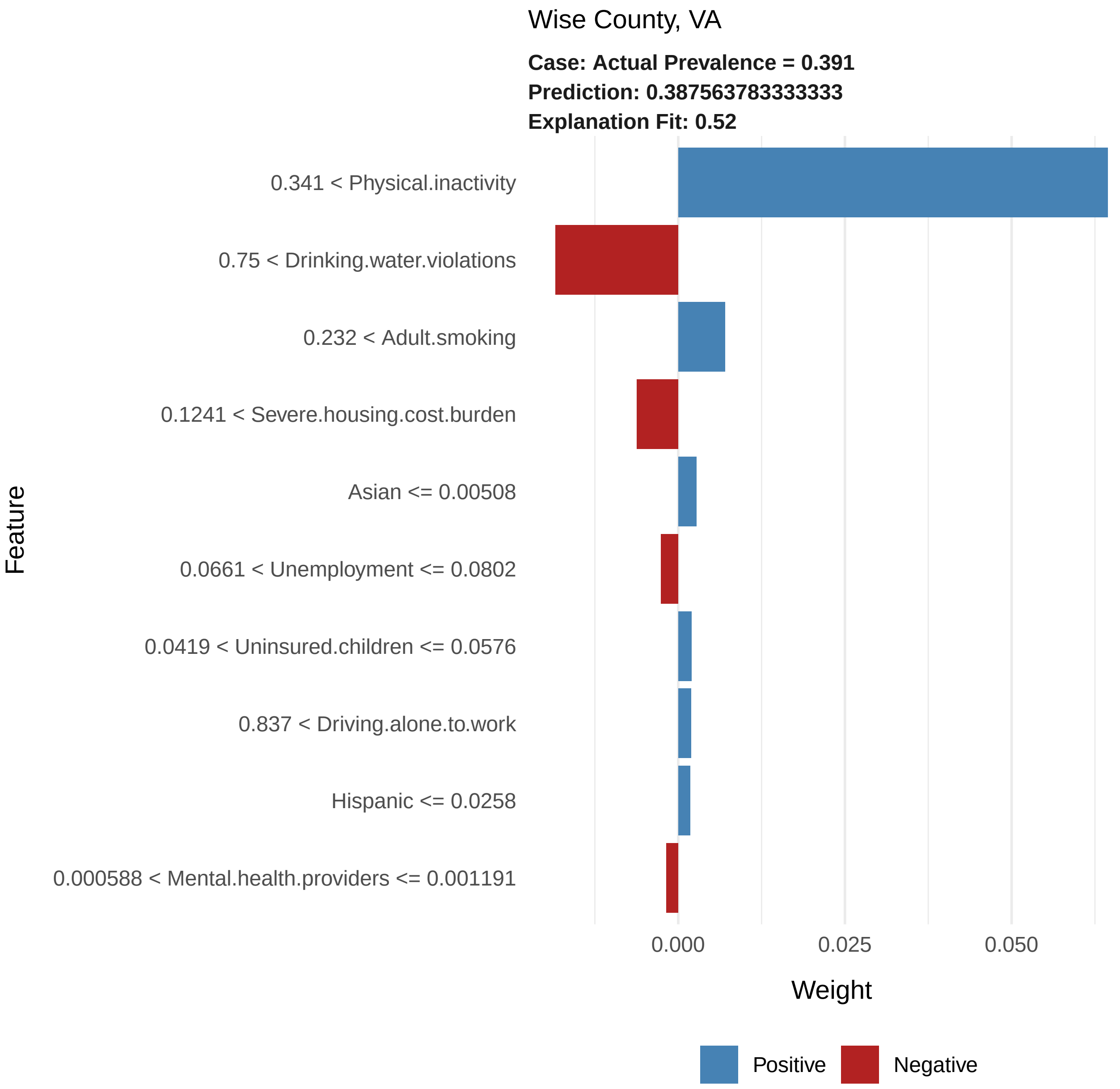
Wise County, VA
Case: Actual Prevalence = 0.391
Prediction: 0.387563783333333
Explanation Fit: 0.52
0.341 < Physical.inactivity
0.75 < Drinking.water.violations
0.232 < Adult.smoking
0.1241 < Severe.housing.cost.burden
Asian <= 0.00508
0.0661 < Unemployment <= 0.0802
0.0419 < Uninsured.children <= 0.0576
0.837 < Driving.alone.to.work
Hispanic <= 0.0258
0.000588 < Mental.health.providers <= 0.001191
Feature
0.000
0.025
0.050
Weight
Positive
Negative

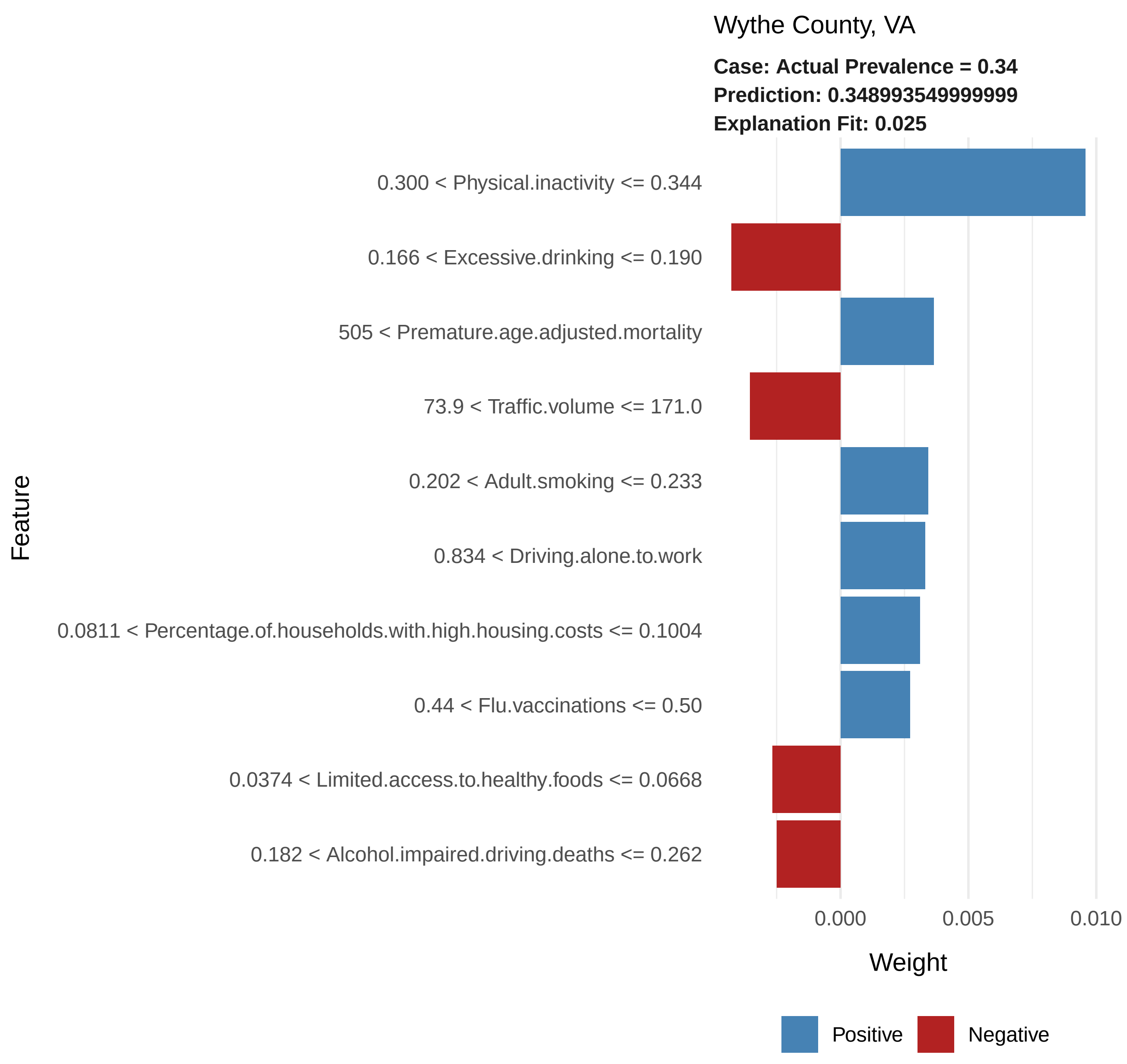

Wythe County, VA
Case: Actual Prevalence = 0.34
Prediction: 0.348993549999999
Explanation Fit: 0.025
0.300 < Physical.inactivity <= 0.344
0.166 < Excessive.drinking <= 0.190
505 < Premature.age.adjusted.mortality
73.9 < Traffic.volume <= 171.0
0.202 < Adult.smoking <= 0.233
0.834 < Driving.alone.to.work
0.0811 < Percentage.of.households.with.high.housing.costs <= 0.1004
0.44 < Flu.vaccinations <= 0.50
0.0374 < Limited.access.to.healthy.foods <= 0.0668
0.182 < Alcohol.impaired.driving.deaths <= 0.262
Feature
0.000
0.005
0.010
Weight
Positive
Negative

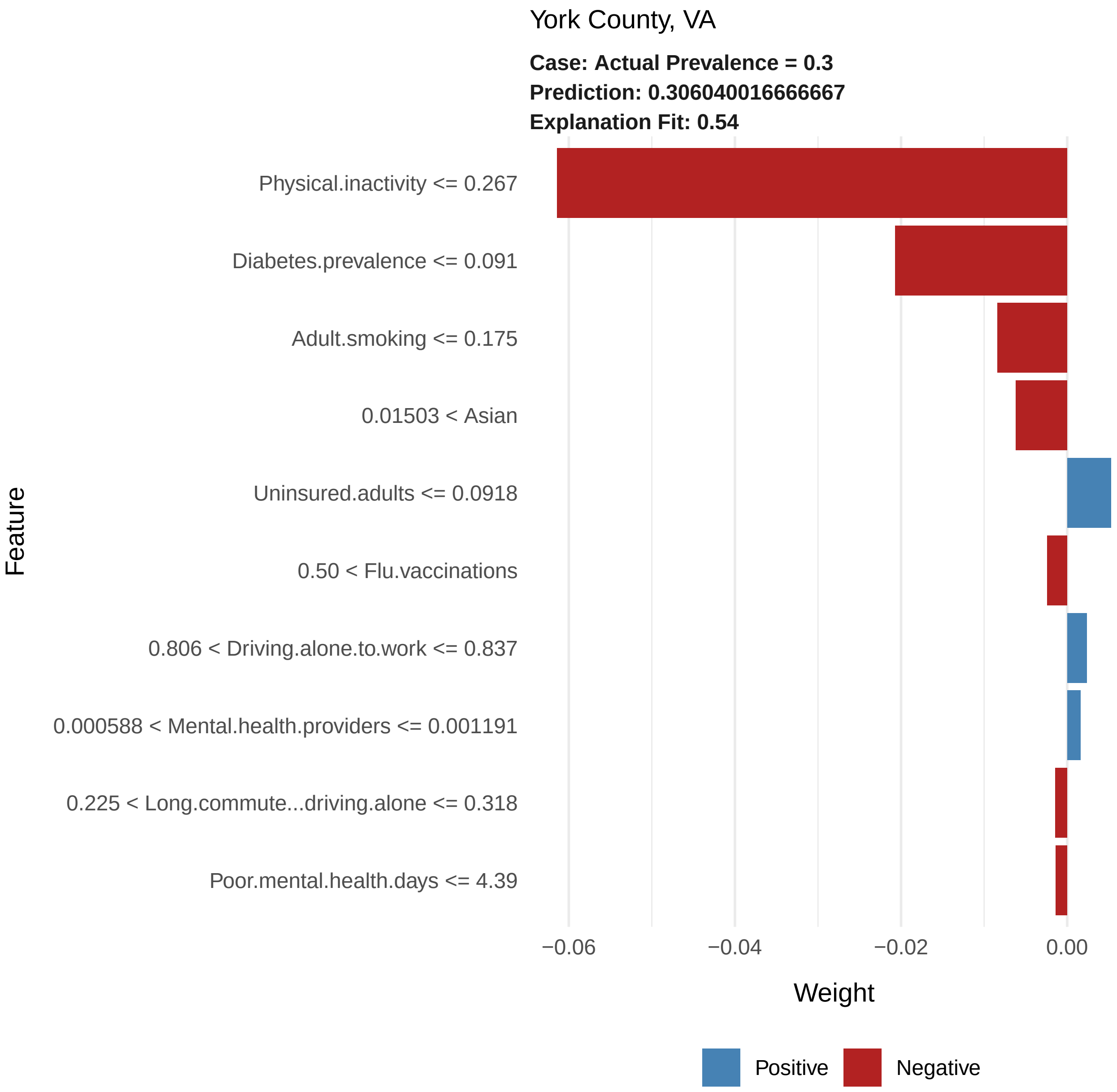
York County, VA
Case: Actual Prevalence = 0.3
Prediction: 0.306040016666667
Explanation Fit: 0.54
Physical.inactivity <= 0.267
Diabetes.prevalence <= 0.091
Adult.smoking <= 0.175
0.01503 < Asian
Uninsured.adults <= 0.0918
0.50 < Flu.vaccinations
0.806 < Driving.alone.to.work <= 0.837
0.000588 < Mental.health.providers <= 0.001191
0.225 < Long.commute...driving.alone <= 0.318
Poor.mental.health.days <= 4.39
Feature
−0.06
−0.04
−0.02
0.00
Weight
Positive
Negative

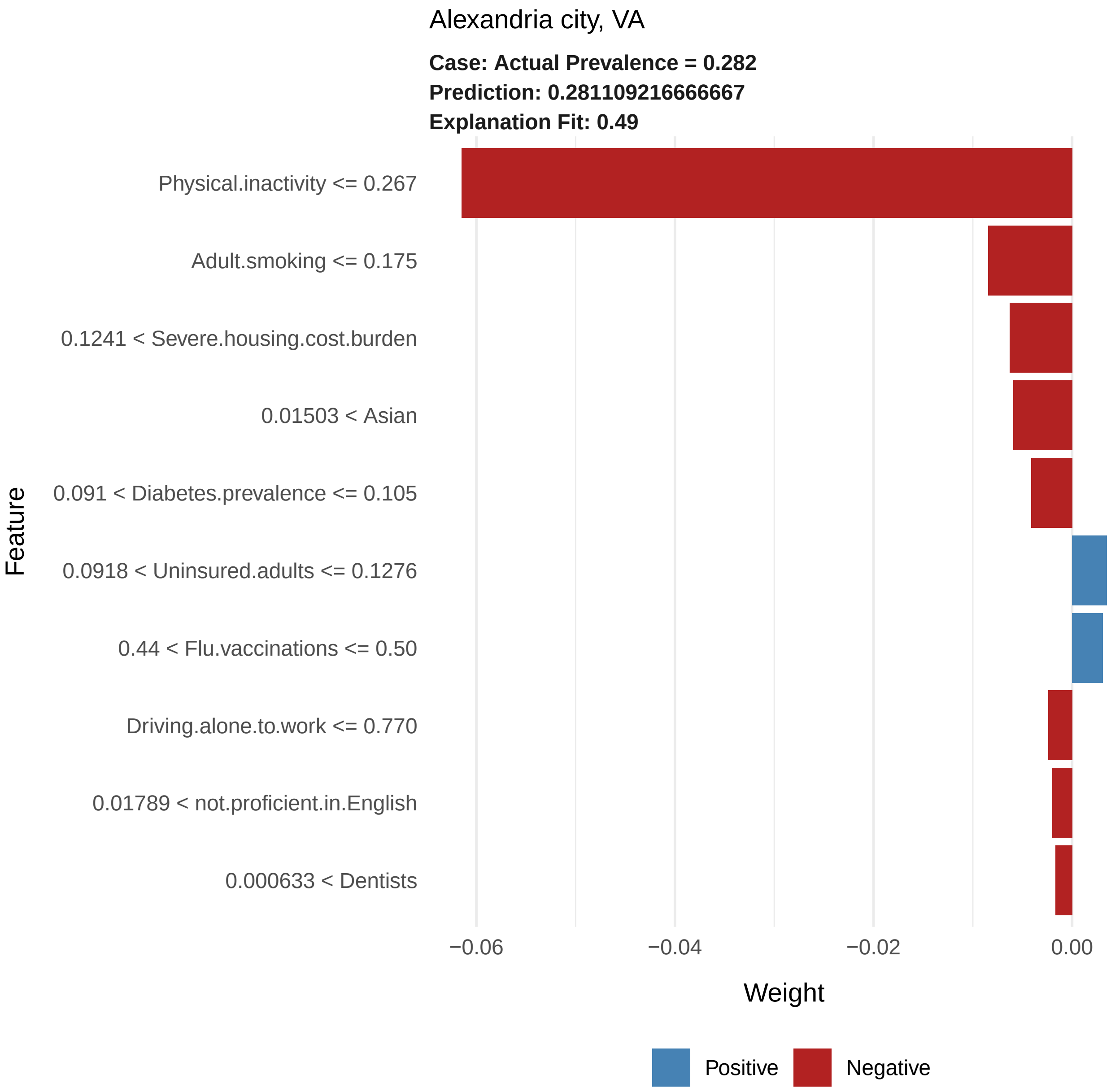

Alexandria city, VA
Case: Actual Prevalence = 0.282
Prediction: 0.281109216666667
Explanation Fit: 0.49
Feature
Physical.inactivity <= 0.267
Adult.smoking <= 0.175
0.1241 < Severe.housing.cost.burden
0.01503 < Asian
0.091 < Diabetes.prevalence <= 0.105
0.0918 < Uninsured.adults <= 0.1276
0.44 < Flu.vaccinations <= 0.50
Driving.alone.to.work <= 0.770
0.01789 < not.proficient.in.English
0.000633 < Dentists
−0.06
−0.04
−0.02
0.00
Weight
Positive
Negative

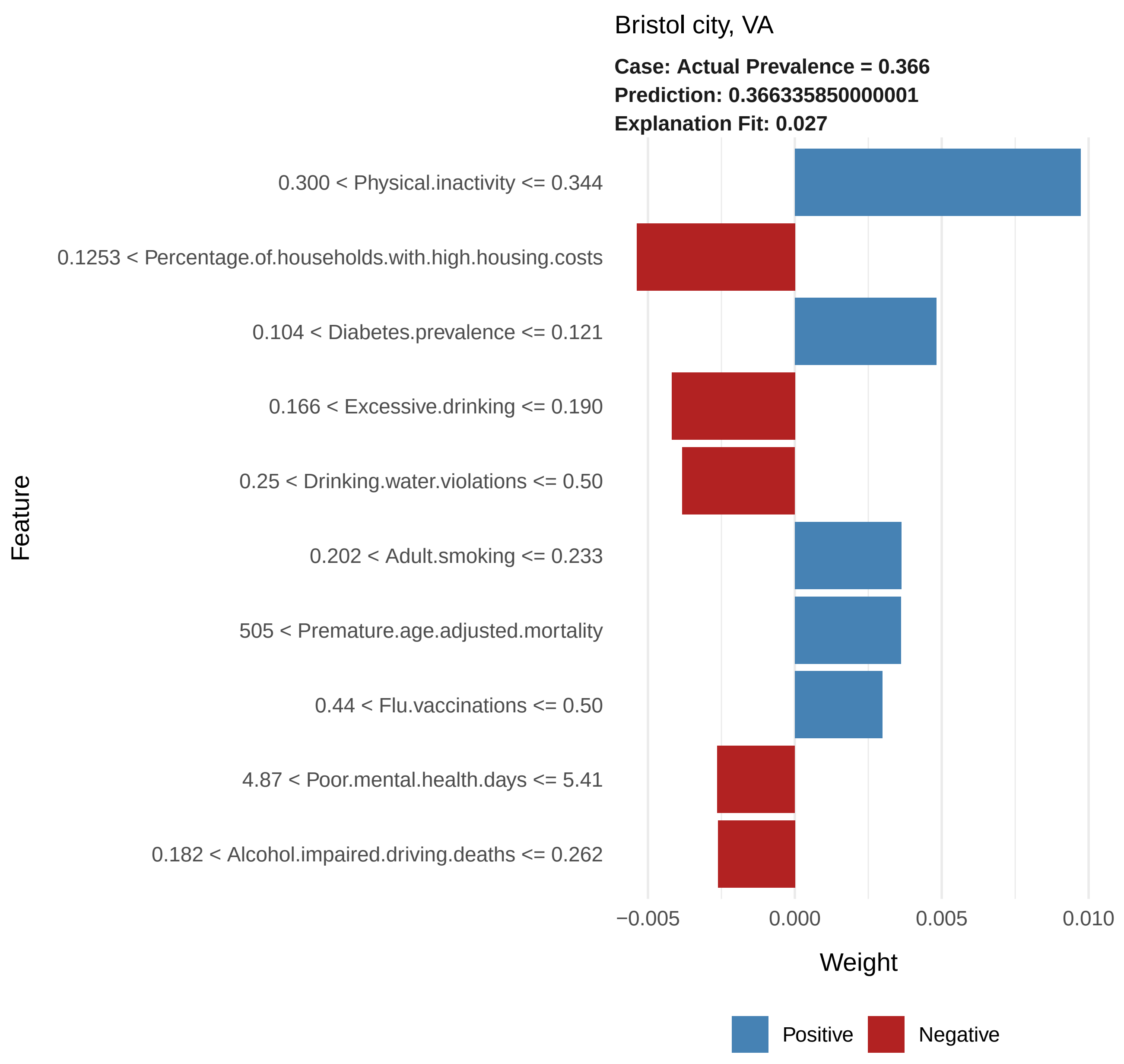
Bristol city, VA
Case: Actual Prevalence = 0.366
Prediction: 0.366335850000001
Explanation Fit: 0.027
0.300 < Physical.inactivity <= 0.344
0.1253 < Percentage.of.households.with.high.housing.costs
0.104 < Diabetes.prevalence <= 0.121
0.166 < Excessive.drinking <= 0.190
0.25 < Drinking.water.violations <= 0.50
0.202 < Adult.smoking <= 0.233
505 < Premature.age.adjusted.mortality
0.44 < Flu.vaccinations <= 0.50
4.87 < Poor.mental.health.days <= 5.41
0.182 < Alcohol.impaired.driving.deaths <= 0.262
Feature
−0.005
0.000
0.005
0.010
Weight
Positive
Negative

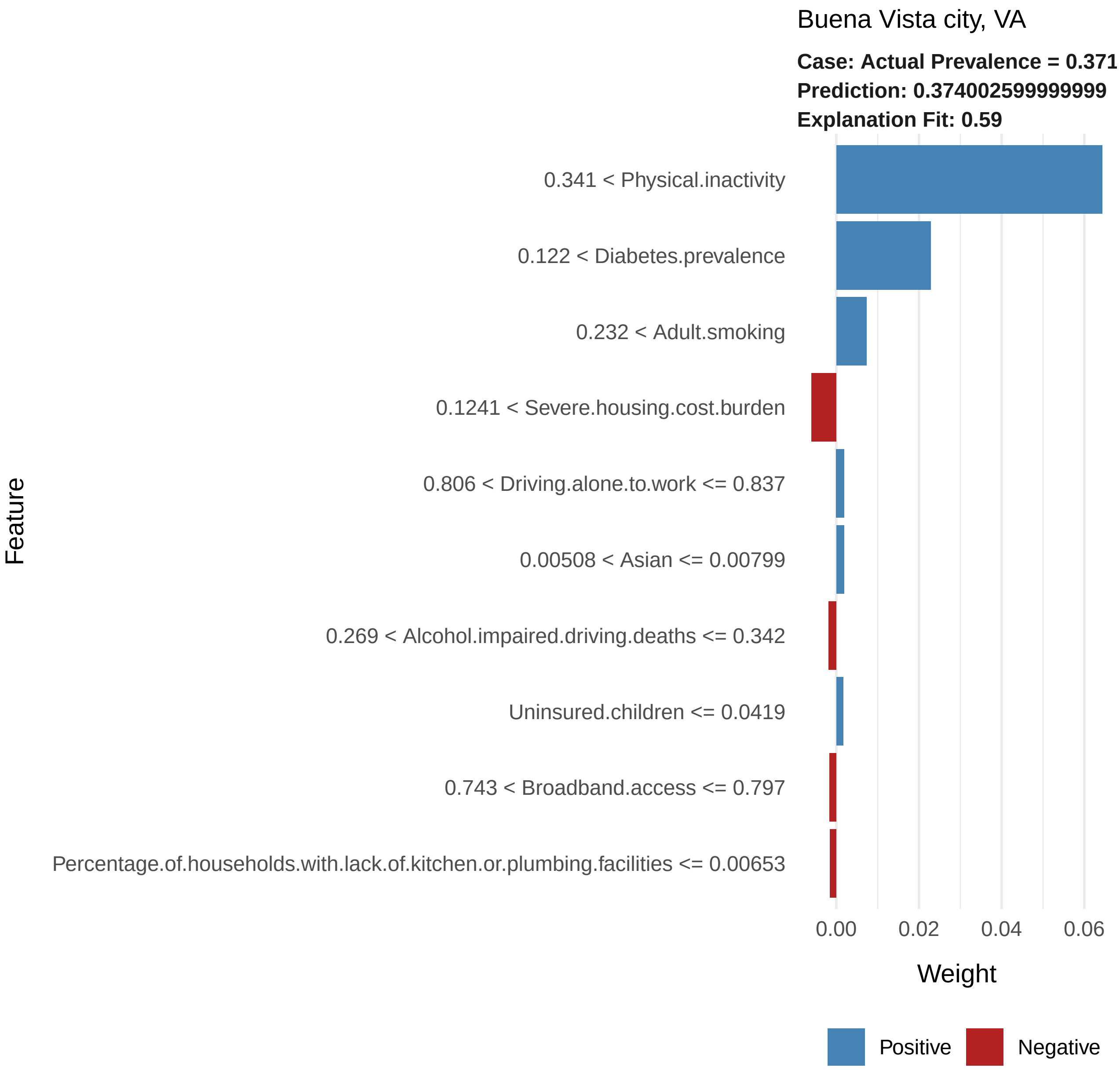
Buena Vista city, VA
Case: Actual Prevalence = 0.371
Prediction: 0.374002599999999
Explanation Fit: 0.59
Feature
0.341 < Physical.inactivity
0.122 < Diabetes.prevalence
0.232 < Adult.smoking
0.1241 < Severe.housing.cost.burden
0.806 < Driving.alone.to.work <= 0.837
0.00508 < Asian <= 0.00799
0.269 < Alcohol.impaired.driving.deaths <= 0.342
Uninsured.children <= 0.0419
0.743 < Broadband.access <= 0.797
Percentage.of.households.with.lack.of.kitchen.or.plumbing.facilities <= 0.00653
0.00
0.02
0.04
0.06
Weight
Positive
Negative

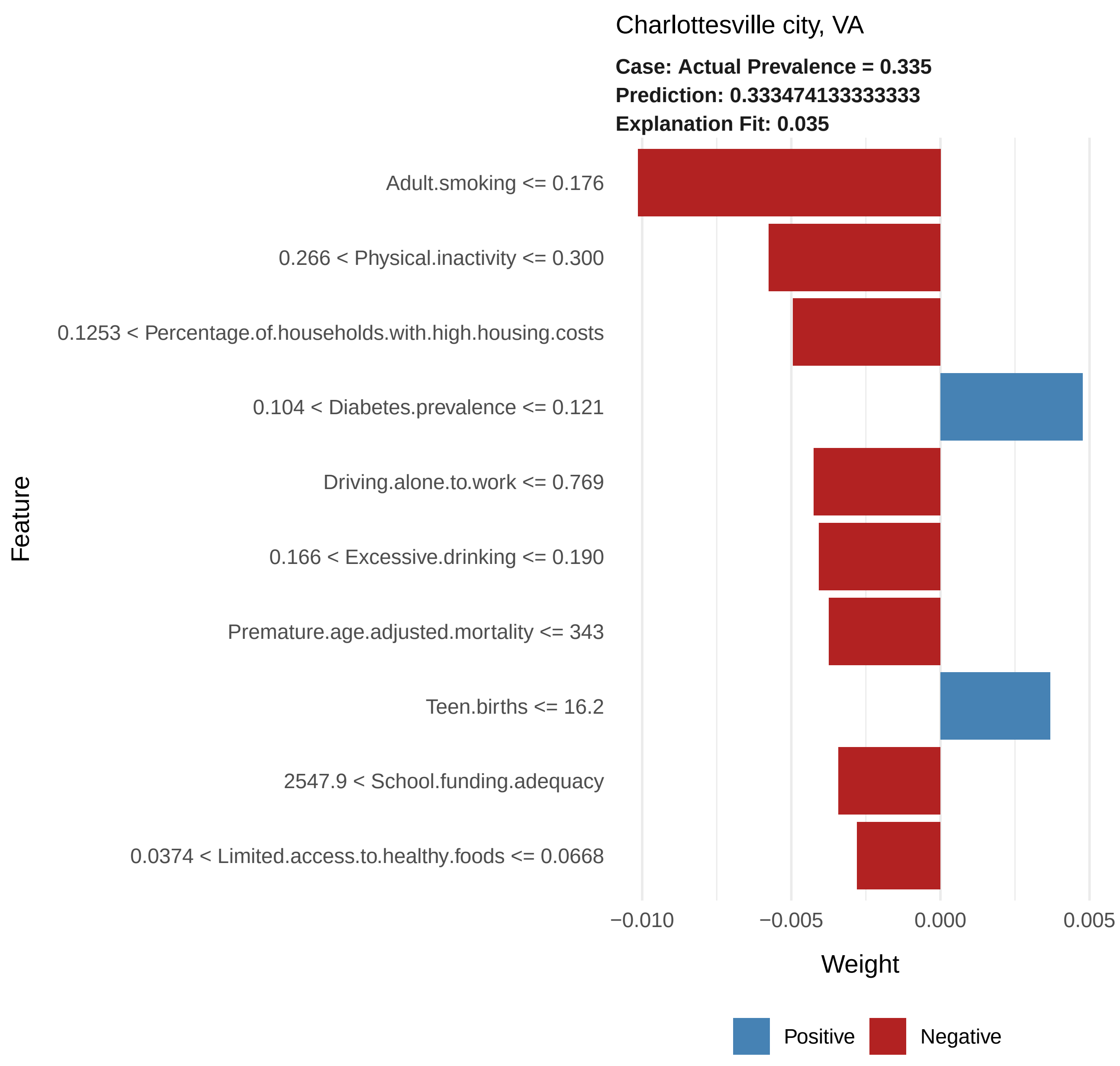

Charlottesville city, VA
Case: Actual Prevalence = 0.335
Prediction: 0.333474133333333
Explanation Fit: 0.035
Adult.smoking <= 0.176
0.266 < Physical.inactivity <= 0.300
0.1253 < Percentage.of.households.with.high.housing.costs
0.104 < Diabetes.prevalence <= 0.121
Driving.alone.to.work <= 0.769
0.166 < Excessive.drinking <= 0.190
Premature.age.adjusted.mortality <= 343
Teen.births <= 16.2
2547.9 < School.funding.adequacy
0.0374 < Limited.access.to.healthy.foods <= 0.0668
Feature
−0.010
−0.005
0.000
0.005
Weight
Positive
Negative

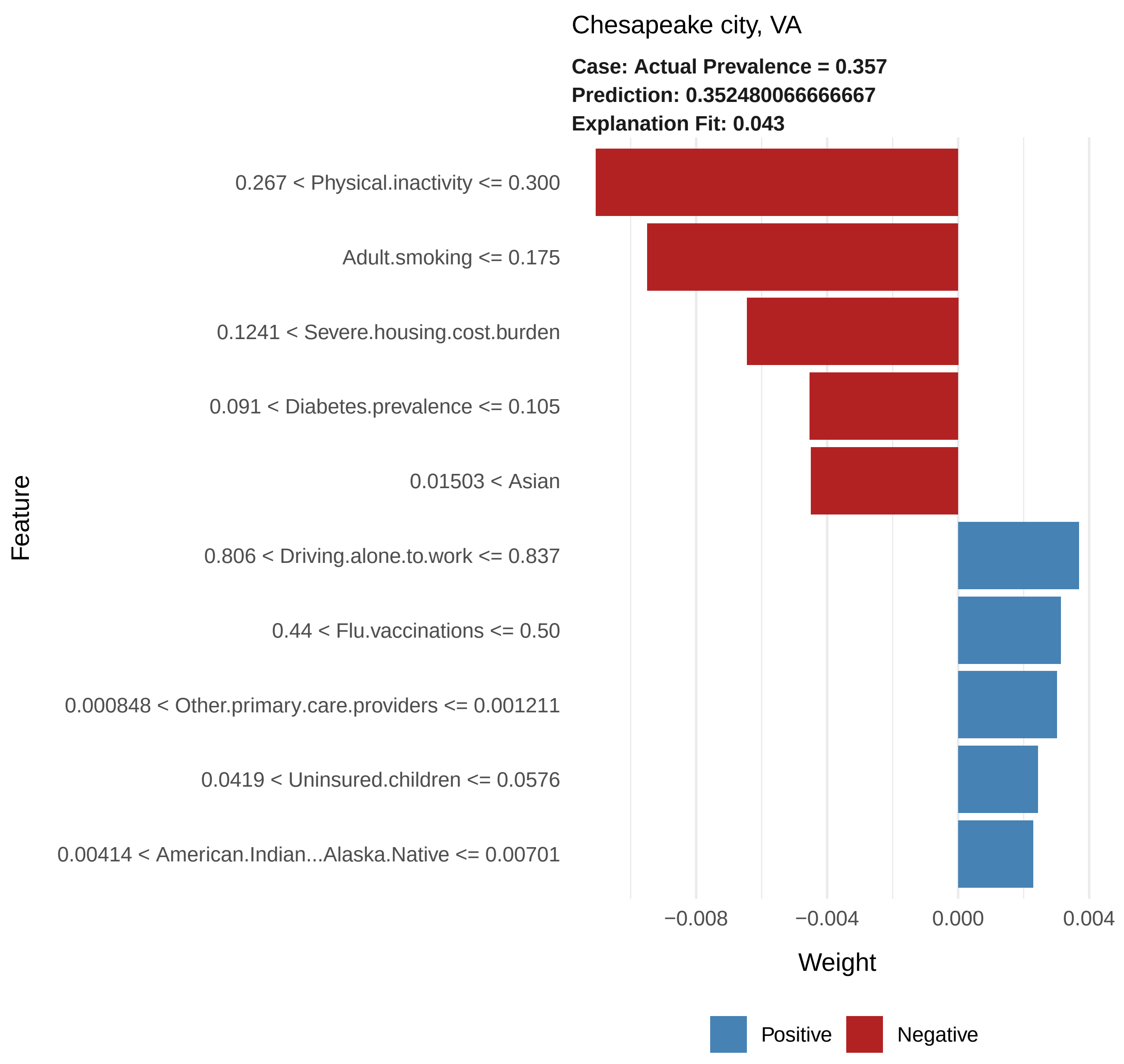
Chesapeake city, VA
Case: Actual Prevalence = 0.357
Prediction: 0.352480066666667
Explanation Fit: 0.043
0.267 < Physical.inactivity <= 0.300
Adult.smoking <= 0.175
0.1241 < Severe.housing.cost.burden
0.091 < Diabetes.prevalence <= 0.105
0.01503 < Asian
0.806 < Driving.alone.to.work <= 0.837
0.44 < Flu.vaccinations <= 0.50
0.000848 < Other.primary.care.providers <= 0.001211
0.0419 < Uninsured.children <= 0.0576
0.00414 < American.Indian...Alaska.Native <= 0.00701
Feature
−0.008
−0.004
0.000
0.004
Weight
Positive
Negative

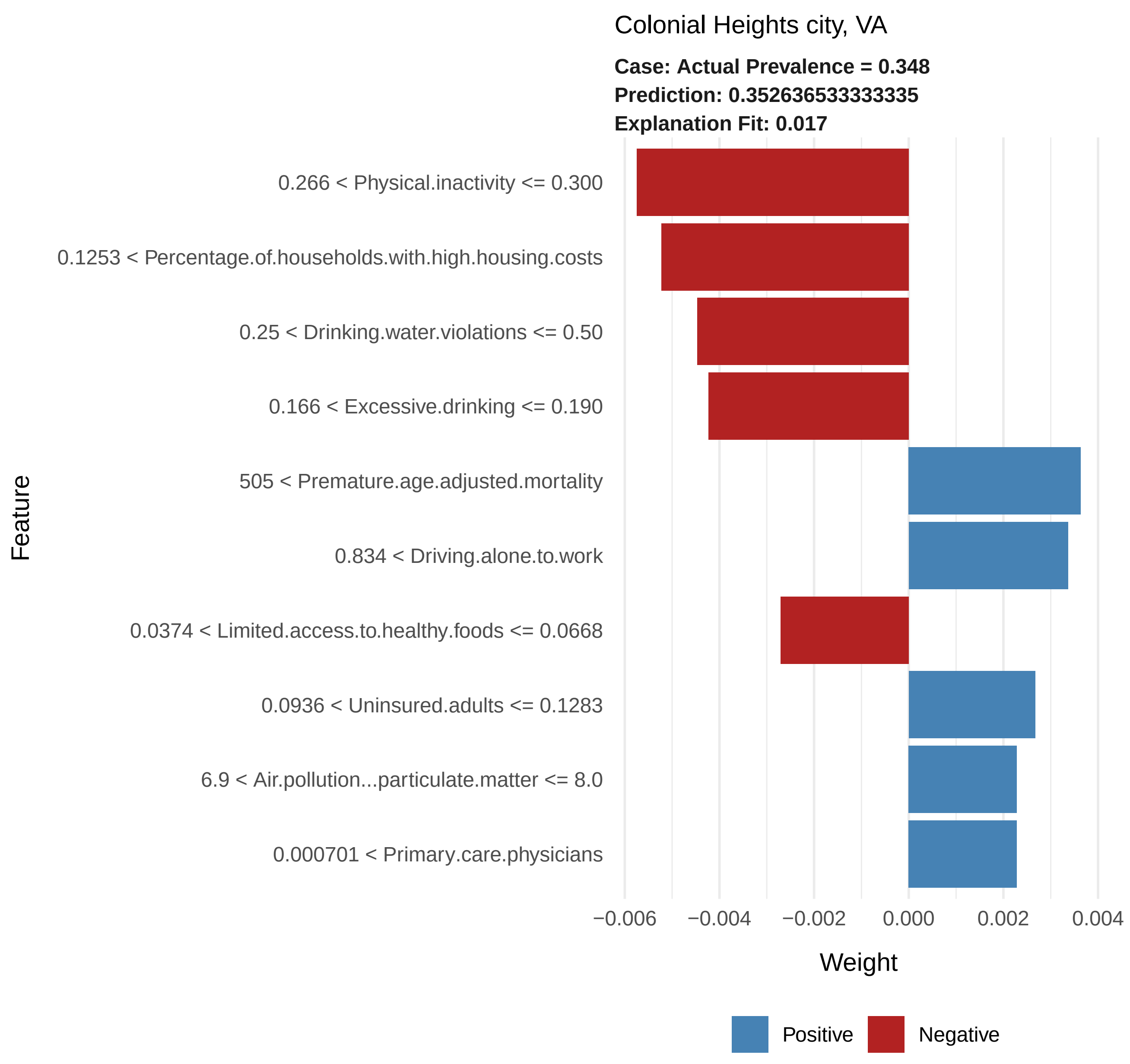

Colonial Heights city, VA
Case: Actual Prevalence = 0.348
Prediction: 0.352636533333335
Explanation Fit: 0.017
0.266 < Physical.inactivity <= 0.300
0.1253 < Percentage.of.households.with.high.housing.costs
0.25 < Drinking.water.violations <= 0.50
0.166 < Excessive.drinking <= 0.190
505 < Premature.age.adjusted.mortality
0.834 < Driving.alone.to.work
0.0374 < Limited.access.to.healthy.foods <= 0.0668
0.0936 < Uninsured.adults <= 0.1283
6.9 < Air.pollution...particulate.matter <= 8.0
0.000701 < Primary.care.physicians
Feature
−0.006
−0.004
−0.002
0.000
0.002
0.004
Weight
Positive
Negative

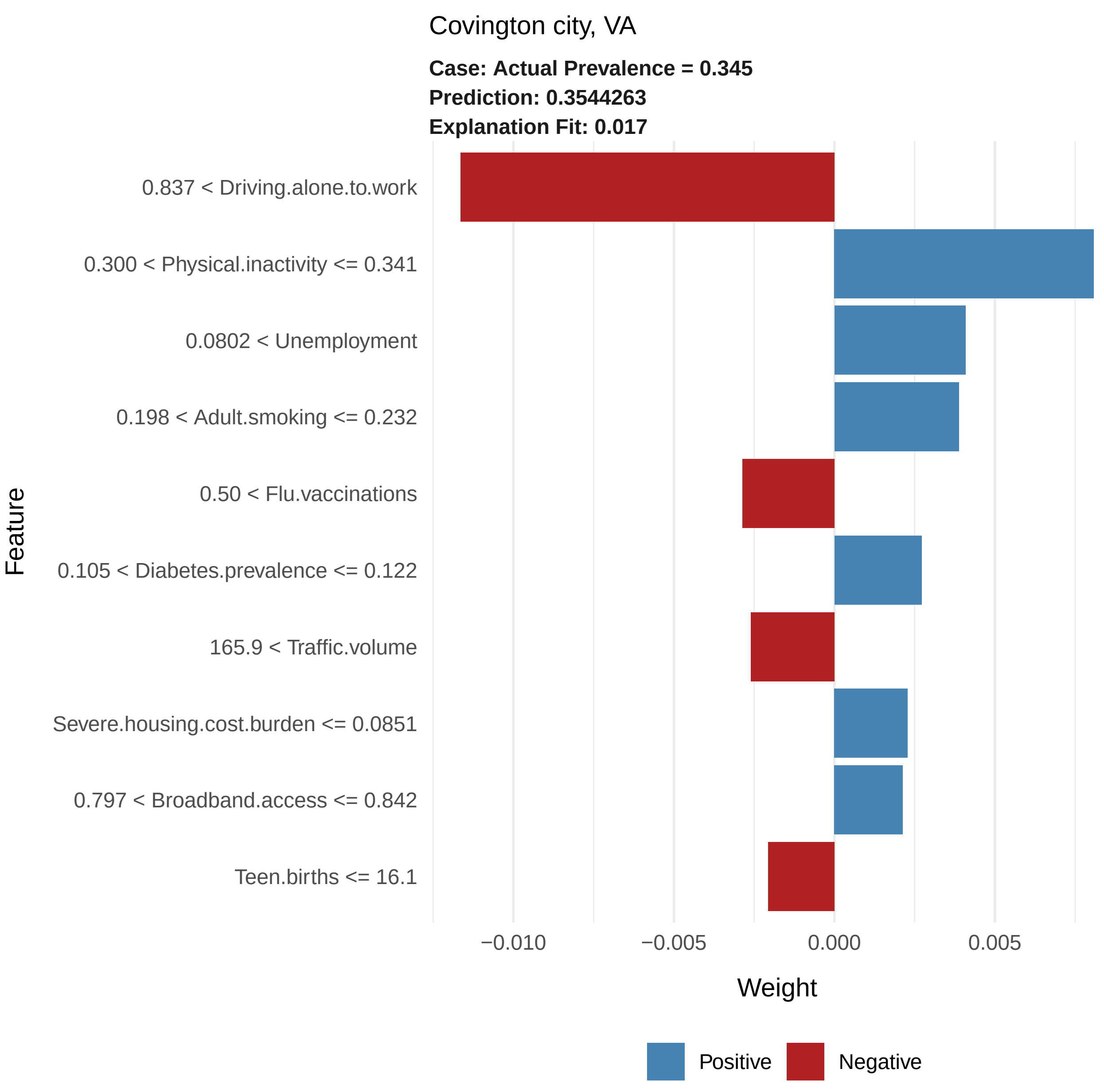
Covington city, VA
Case: Actual Prevalence = 0.345
Prediction: 0.3544263
Explanation Fit: 0.017
0.837 < Driving.alone.to.work
0.300 < Physical.inactivity <= 0.341
0.0802 < Unemployment
0.198 < Adult.smoking <= 0.232
0.50 < Flu.vaccinations
0.105 < Diabetes.prevalence <= 0.122
165.9 < Traffic.volume
Severe.housing.cost.burden <= 0.0851
0.797 < Broadband.access <= 0.842
Teen.births <= 16.1
Feature
−0.010
−0.005
0.000
0.005
Weight
Positive
Negative

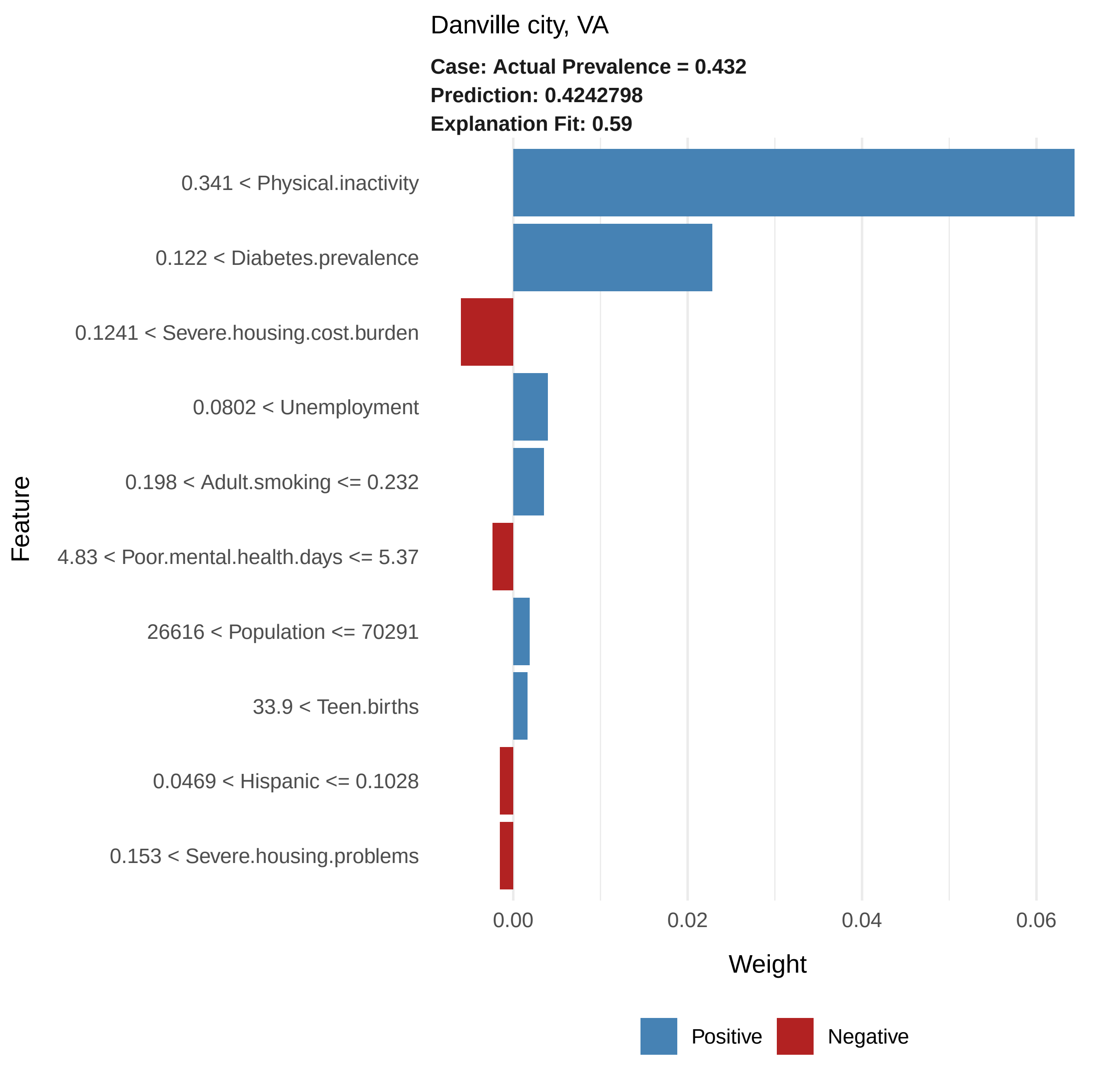
Danville city, VA
Case: Actual Prevalence = 0.432
Prediction: 0.4242798
Explanation Fit: 0.59
0.341 < Physical.inactivity
0.122 < Diabetes.prevalence
0.1241 < Severe.housing.cost.burden
0.0802 < Unemployment
0.198 < Adult.smoking <= 0.232
4.83 < Poor.mental.health.days <= 5.37
26616 < Population <= 70291
33.9 < Teen.births
0.0469 < Hispanic <= 0.1028
0.153 < Severe.housing.problems
Feature
0.00
0.02
0.04
0.06
Weight
Positive
Negative

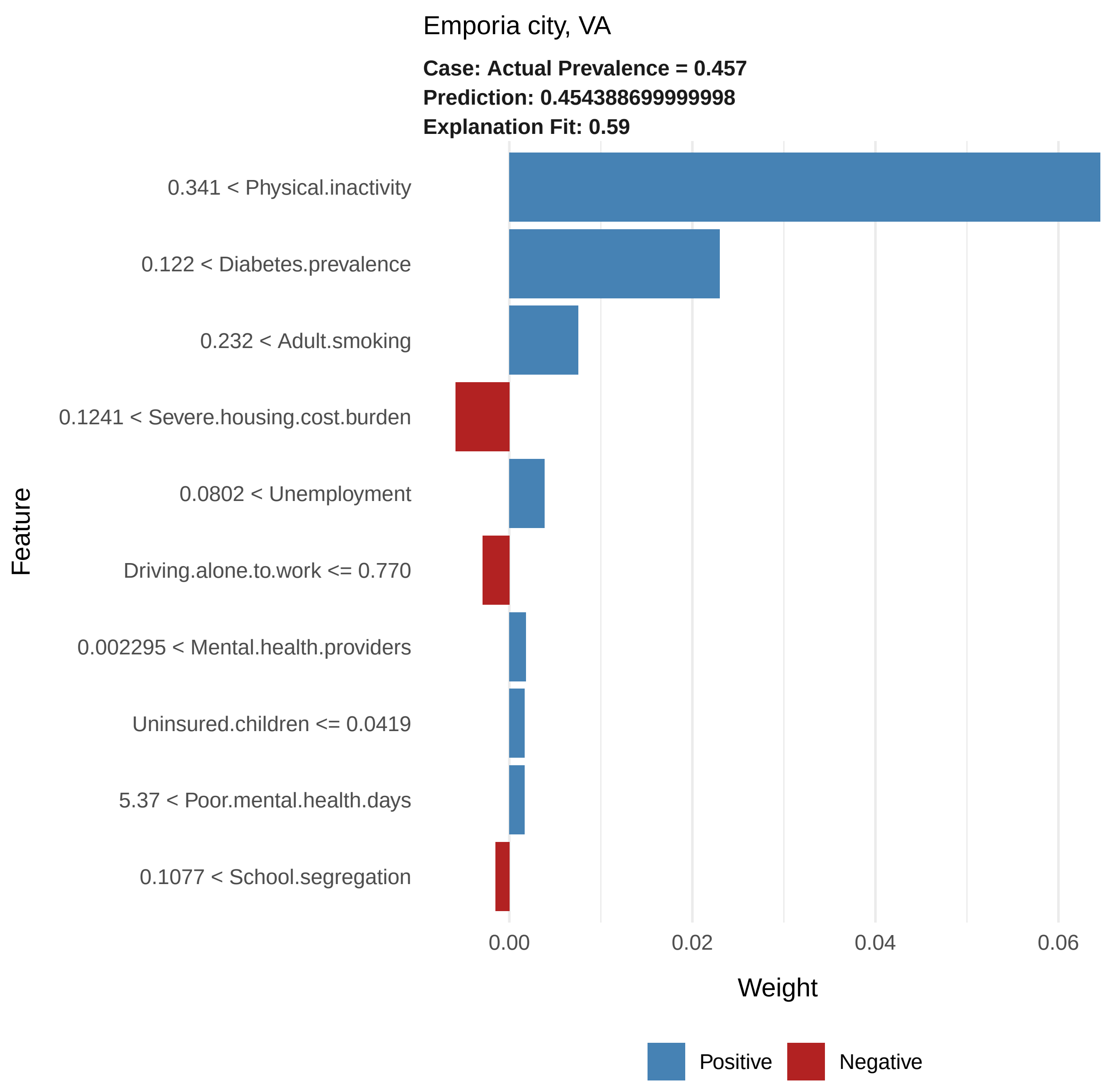
Emporia city, VA
Case: Actual Prevalence = 0.457
Prediction: 0.454388699999998
Explanation Fit: 0.59
0.341 < Physical.inactivity
0.122 < Diabetes.prevalence
0.232 < Adult.smoking
0.1241 < Severe.housing.cost.burden
0.0802 < Unemployment
Driving.alone.to.work <= 0.770
0.002295 < Mental.health.providers
Uninsured.children <= 0.0419
5.37 < Poor.mental.health.days
0.1077 < School.segregation
Feature
0.00
0.02
0.04
0.06
Weight
Positive
Negative

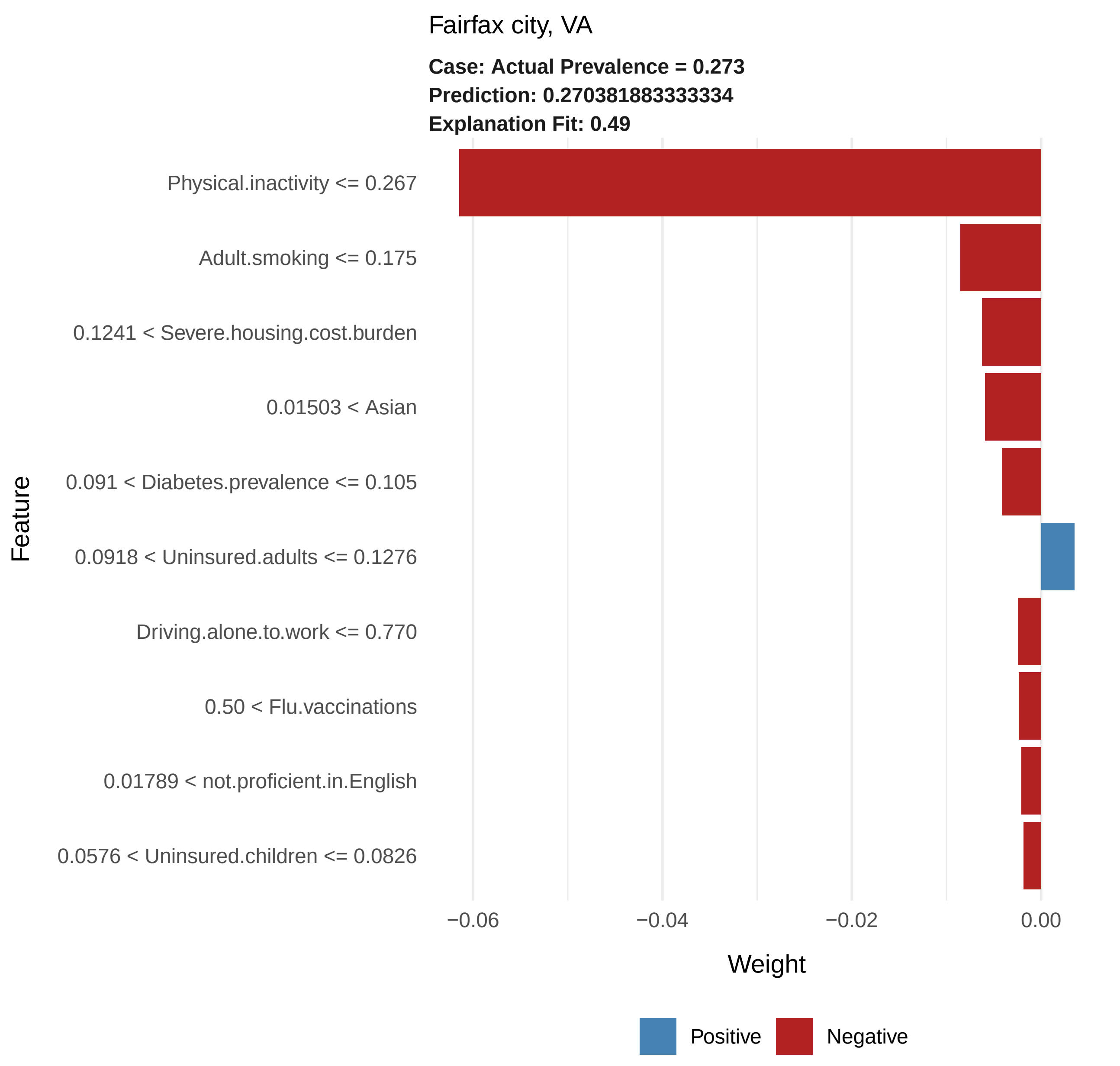

Fairfax city, VA
Case: Actual Prevalence = 0.273
Prediction: 0.270381883333334
Explanation Fit: 0.49
Physical.inactivity <= 0.267
Adult.smoking <= 0.175
0.1241 < Severe.housing.cost.burden
0.01503 < Asian
0.091 < Diabetes.prevalence <= 0.105
0.0918 < Uninsured.adults <= 0.1276
Driving.alone.to.work <= 0.770
0.50 < Flu.vaccinations
0.01789 < not.proficient.in.English
0.0576 < Uninsured.children <= 0.0826
Feature
−0.06
−0.04
−0.02
0.00
Weight
Positive
Negative

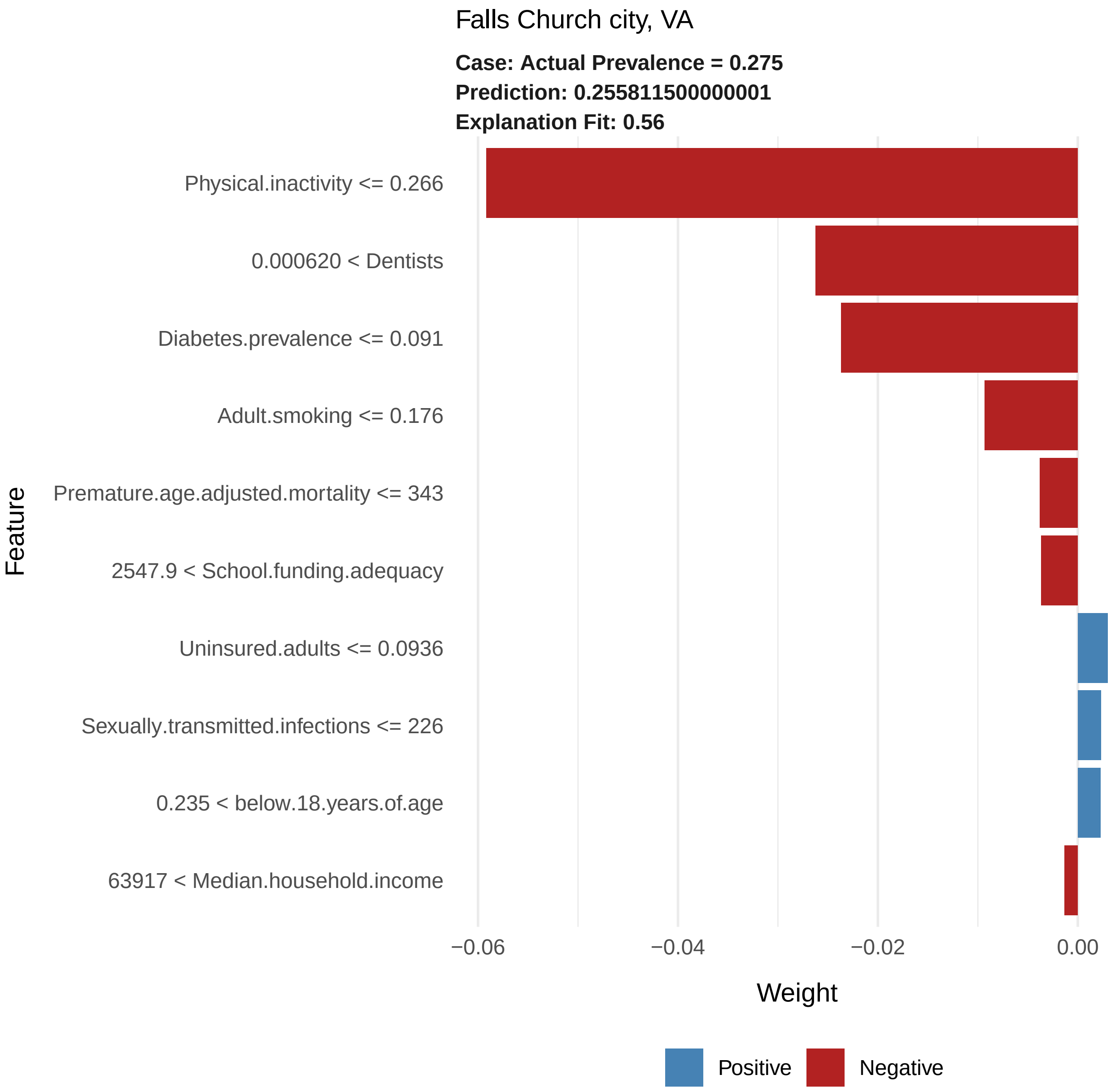

Falls Church city, VA
Case: Actual Prevalence = 0.275
Prediction: 0.255811500000001
Explanation Fit: 0.56
Physical.inactivity <= 0.266
0.000620 < Dentists
Diabetes.prevalence <= 0.091
Adult.smoking <= 0.176
Premature.age.adjusted.mortality <= 343
2547.9 < School.funding.adequacy
Uninsured.adults <= 0.0936
Sexually.transmitted.infections <= 226
0.235 < below.18.years.of.age
63917 < Median.household.income
Feature
−0.06
−0.04
−0.02
0.00
Weight
Positive
Negative

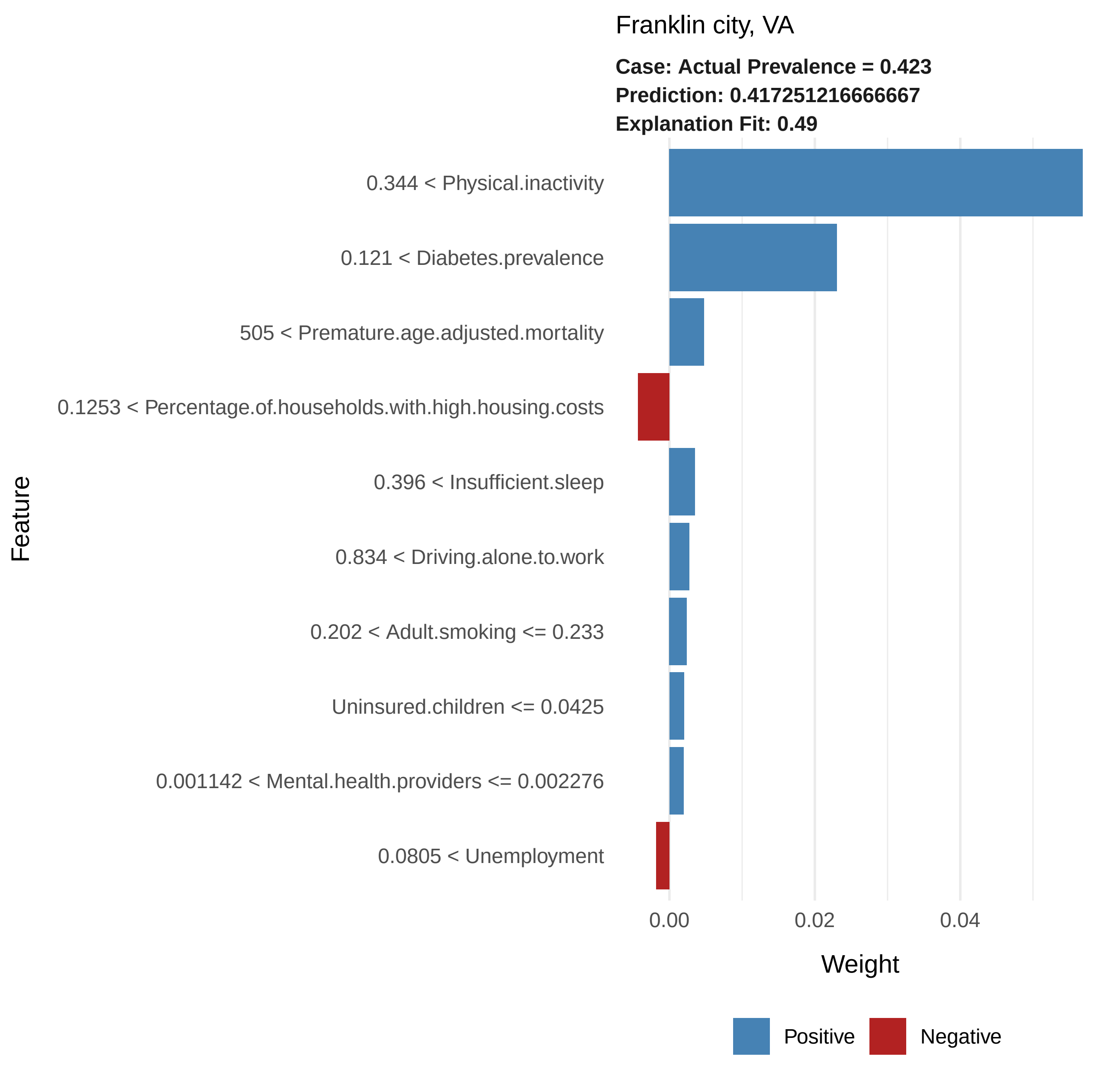

Franklin city, VA
Case: Actual Prevalence = 0.423
Prediction: 0.417251216666667
Explanation Fit: 0.49
0.344 < Physical.inactivity
0.121 < Diabetes.prevalence
505 < Premature.age.adjusted.mortality
0.1253 < Percentage.of.households.with.high.housing.costs
0.396 < Insufficient.sleep
0.834 < Driving.alone.to.work
0.202 < Adult.smoking <= 0.233
Uninsured.children <= 0.0425
0.001142 < Mental.health.providers <= 0.002276
0.0805 < Unemployment
Feature
0.00
0.02
0.04
Weight
Positive
Negative

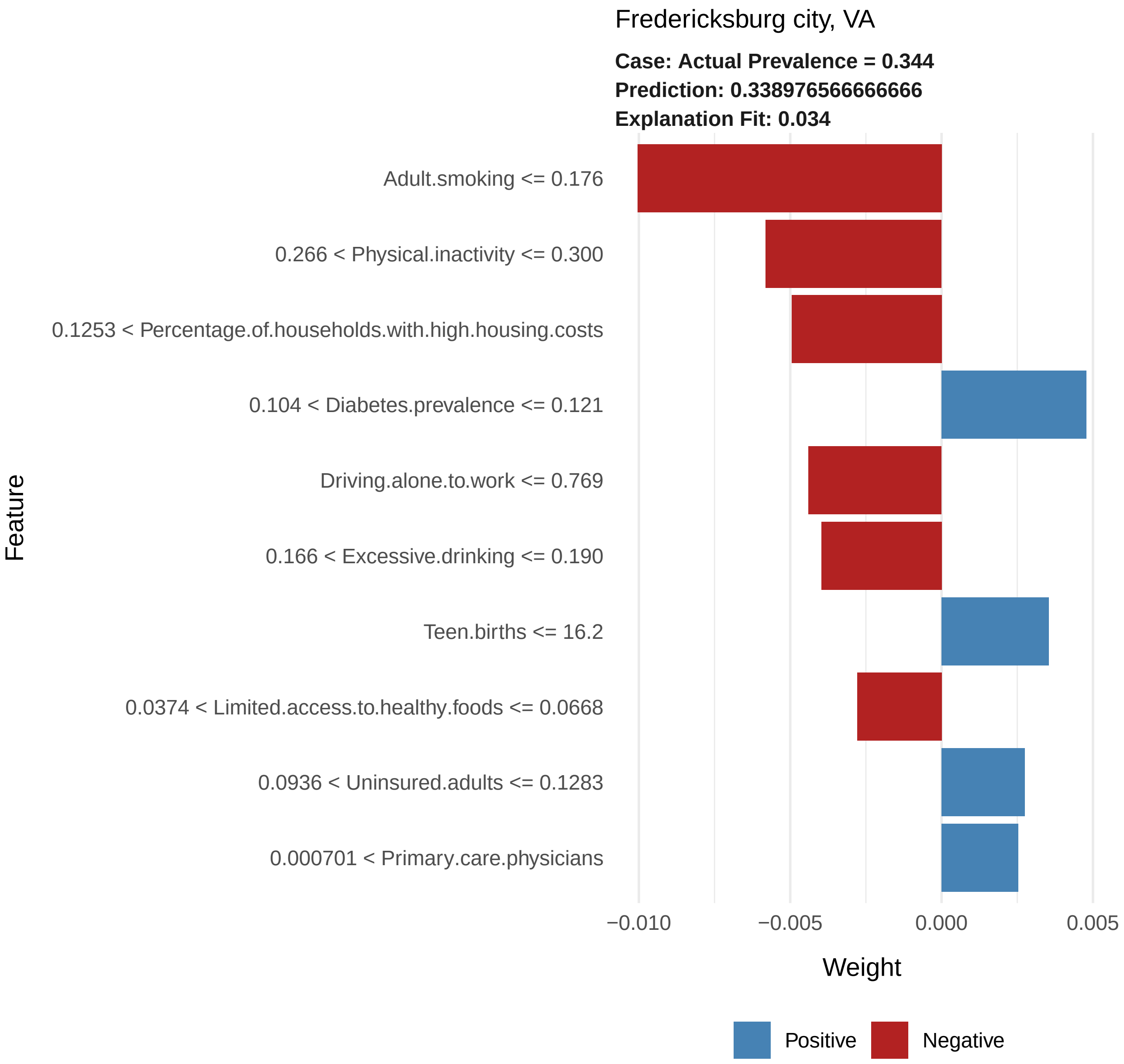

Fredericksburg city, VA
Case: Actual Prevalence = 0.344
Prediction: 0.338976566666666
Explanation Fit: 0.034
Adult.smoking <= 0.176
0.266 < Physical.inactivity <= 0.300
0.1253 < Percentage.of.households.with.high.housing.costs
0.104 < Diabetes.prevalence <= 0.121
Driving.alone.to.work <= 0.769
0.166 < Excessive.drinking <= 0.190
Teen.births <= 16.2
0.0374 < Limited.access.to.healthy.foods <= 0.0668
0.0936 < Uninsured.adults <= 0.1283
0.000701 < Primary.care.physicians
Feature
−0.010
−0.005
0.000
0.005
Weight
Positive
Negative

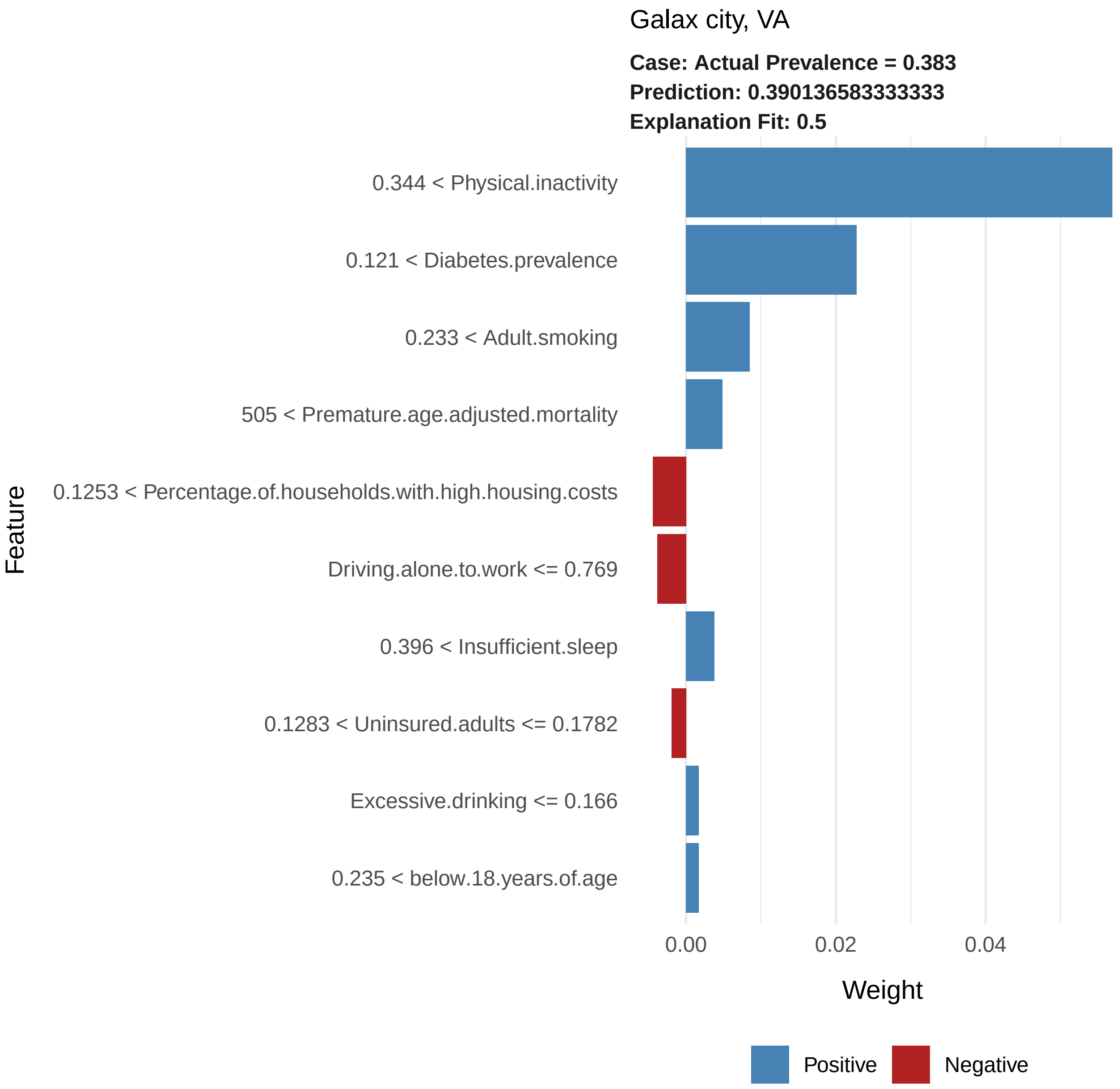

Galax city, VA
Case: Actual Prevalence = 0.383
Prediction: 0.390136583333333
Explanation Fit: 0.5
0.344 < Physical.inactivity
0.121 < Diabetes.prevalence
0.233 < Adult.smoking
505 < Premature.age.adjusted.mortality
0.1253 < Percentage.of.households.with.high.housing.costs
Driving.alone.to.work <= 0.769
0.396 < Insufficient.sleep
0.1283 < Uninsured.adults <= 0.1782
Excessive.drinking <= 0.166
0.235 < below.18.years.of.age
Feature
0.00
0.02
0.04
Weight
Positive
Negative

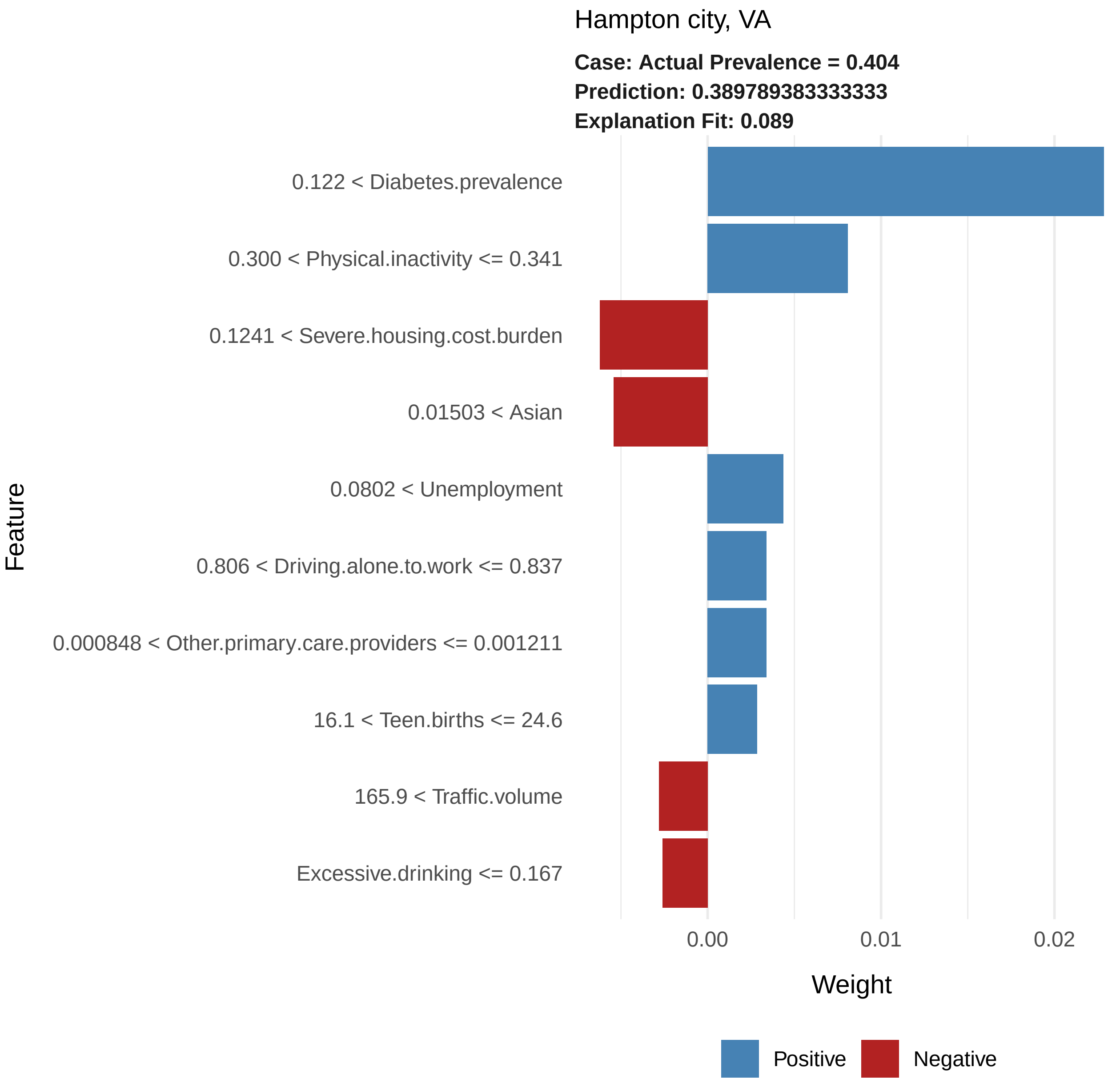

Hampton city, VA
Case: Actual Prevalence = 0.404
Prediction: 0.389789383333333
Explanation Fit: 0.089
0.122 < Diabetes.prevalence
0.300 < Physical.inactivity <= 0.341
0.1241 < Severe.housing.cost.burden
0.01503 < Asian
0.0802 < Unemployment
0.806 < Driving.alone.to.work <= 0.837
0.000848 < Other.primary.care.providers <= 0.001211
16.1 < Teen.births <= 24.6
165.9 < Traffic.volume
Excessive.drinking <= 0.167
Feature
0.00
0.01
0.02
Weight
Positive
Negative

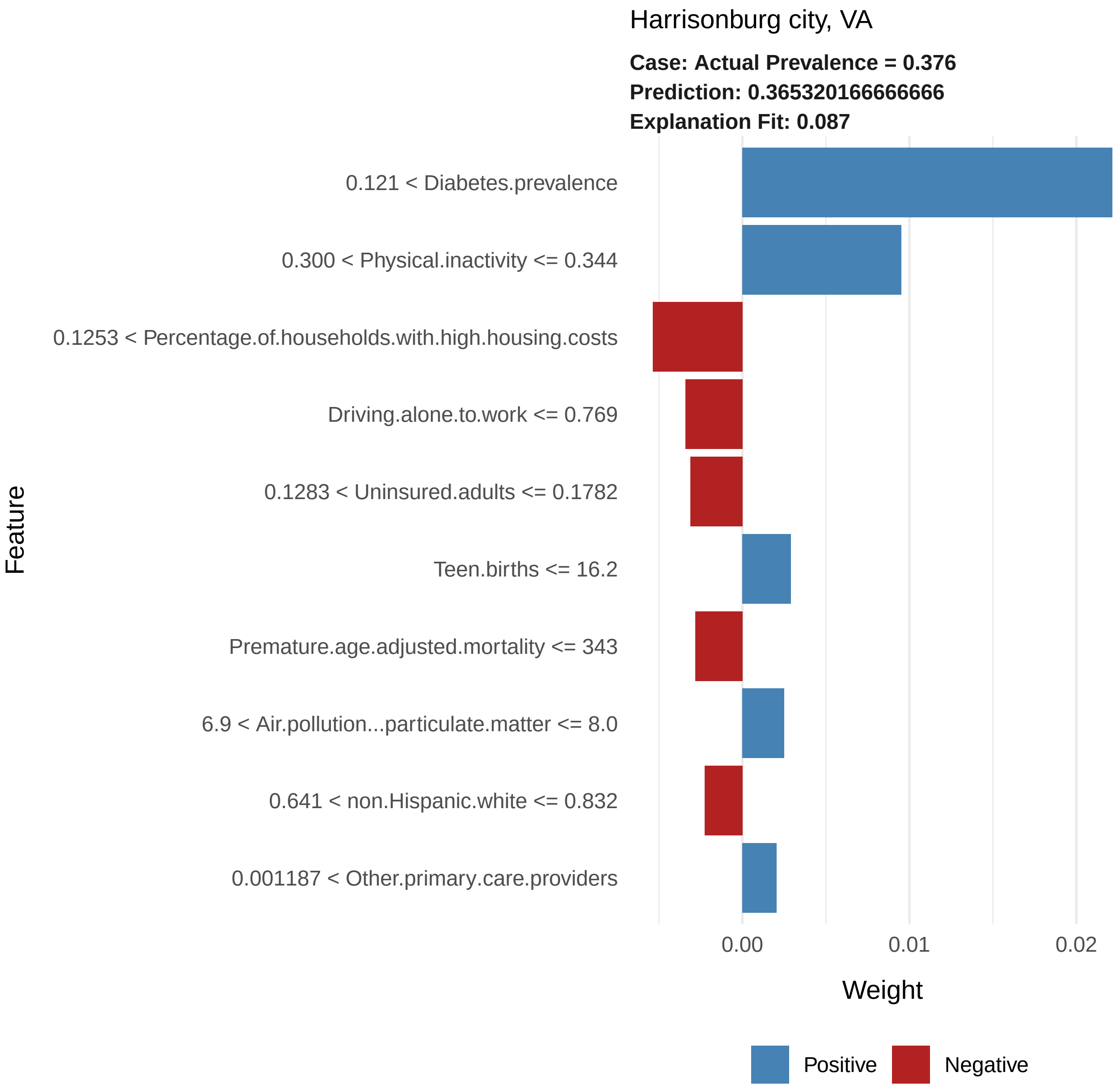

Harrisonburg city, VA
Case: Actual Prevalence = 0.376
Prediction: 0.365320166666666
Explanation Fit: 0.087
0.121 < Diabetes.prevalence
0.300 < Physical.inactivity <= 0.344
0.1253 < Percentage.of.households.with.high.housing.costs
Driving.alone.to.work <= 0.769
0.1283 < Uninsured.adults <= 0.1782
Teen.births <= 16.2
Premature.age.adjusted.mortality <= 343
6.9 < Air.pollution...particulate.matter <= 8.0
0.641 < non.Hispanic.white <= 0.832
0.001187 < Other.primary.care.providers
Feature
0.00
0.01
0.02
Weight
Positive
Negative

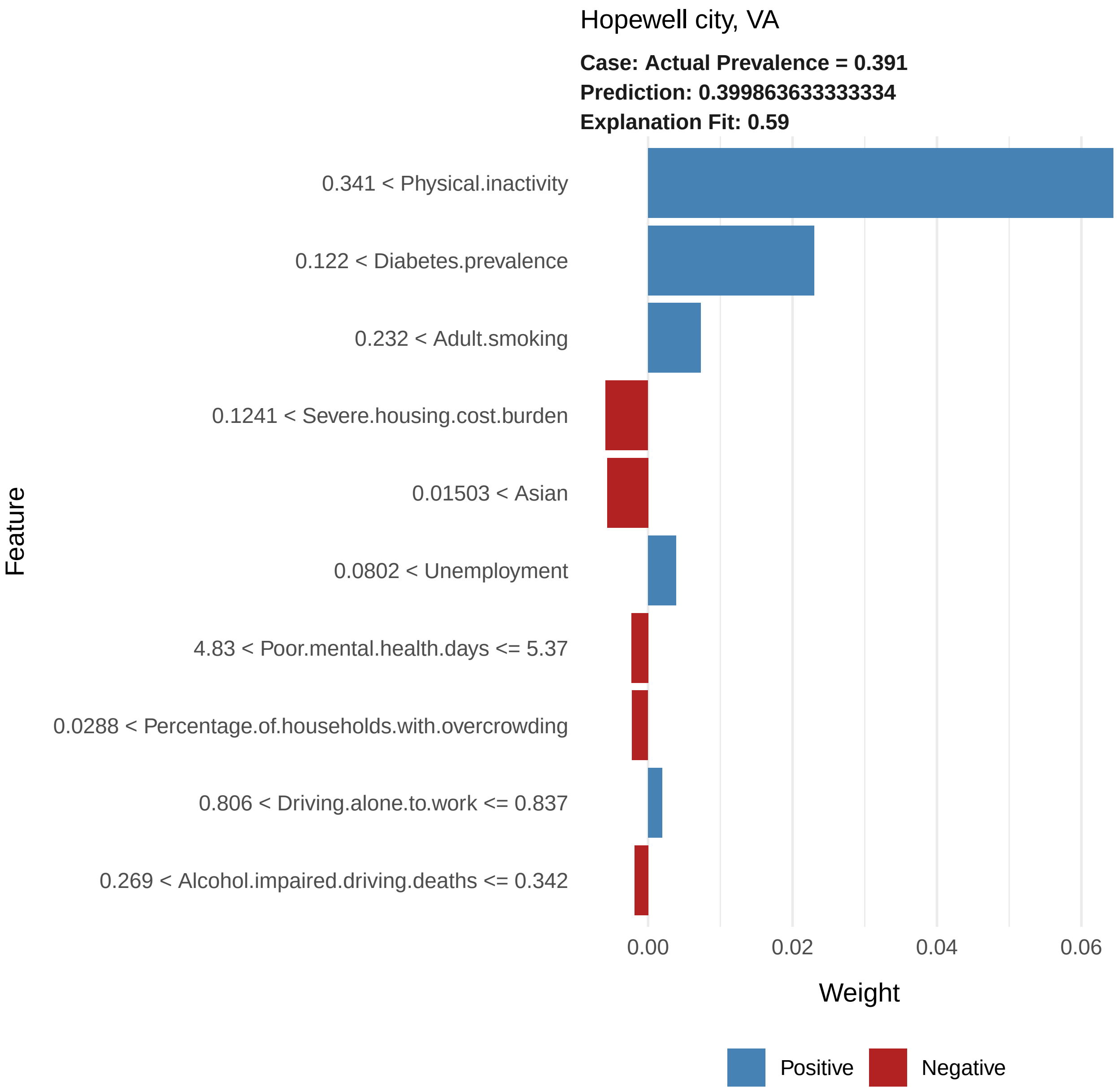

Hopewell city, VA
Case: Actual Prevalence = 0.391
Prediction: 0.399863633333334
Explanation Fit: 0.59
Feature
0.341 < Physical.inactivity
0.122 < Diabetes.prevalence
0.232 < Adult.smoking
0.1241 < Severe.housing.cost.burden
0.01503 < Asian
0.0802 < Unemployment
4.83 < Poor.mental.health.days <= 5.37
0.0288 < Percentage.of.households.with.overcrowding
0.806 < Driving.alone.to.work <= 0.837
0.269 < Alcohol.impaired.driving.deaths <= 0.342
0.00
0.02
0.04
0.06
Weight
Positive
Negative

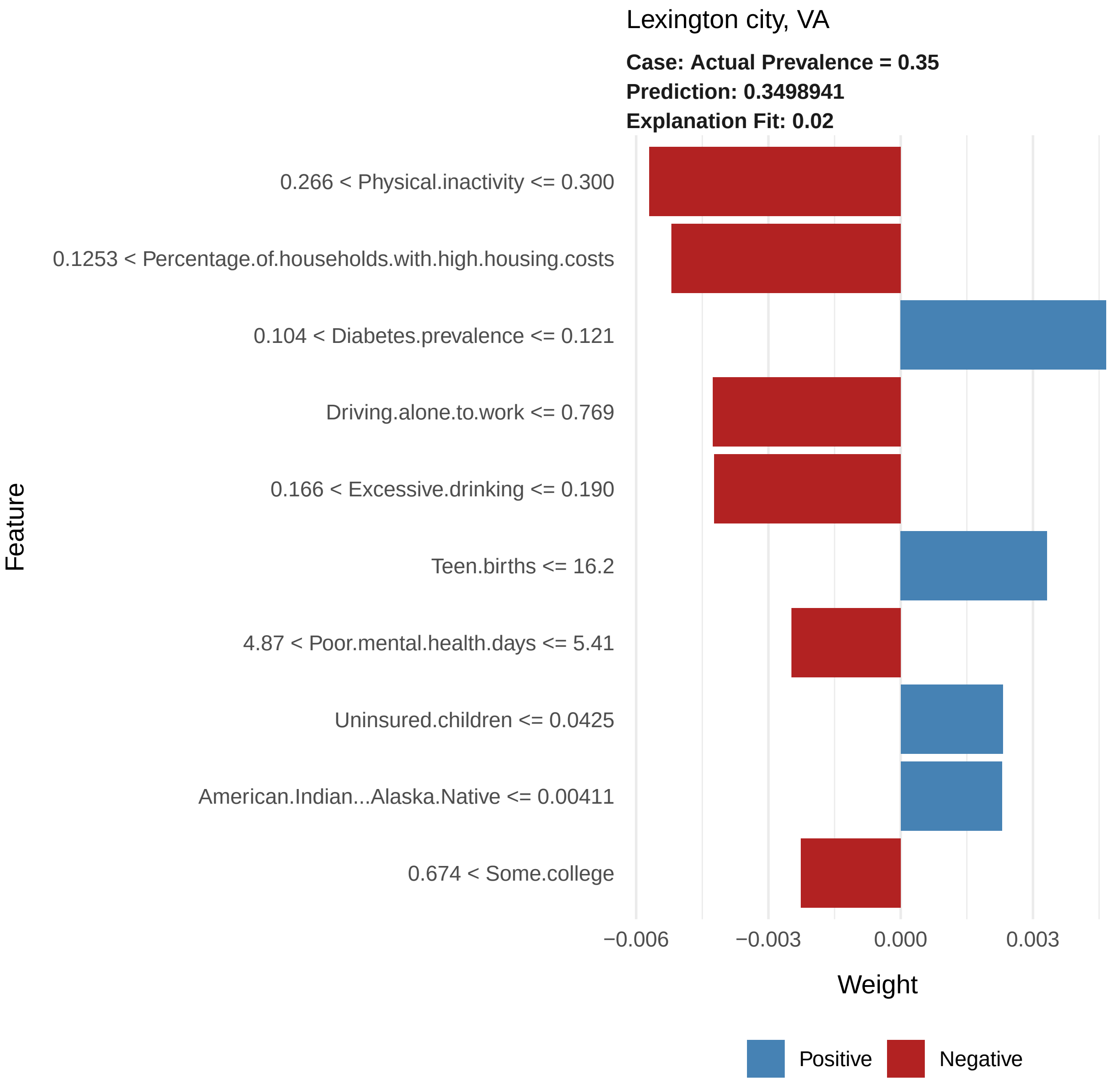
Lexington city, VA
Case: Actual Prevalence = 0.35
Prediction: 0.3498941
Explanation Fit: 0.02
0.266 < Physical.inactivity <= 0.300
0.1253 < Percentage.of.households.with.high.housing.costs
0.104 < Diabetes.prevalence <= 0.121
Driving.alone.to.work <= 0.769
0.166 < Excessive.drinking <= 0.190
Teen.births <= 16.2
4.87 < Poor.mental.health.days <= 5.41
Uninsured.children <= 0.0425
American.Indian...Alaska.Native <= 0.00411
0.674 < Some.college
Feature
−0.006
−0.003
0.000
0.003
Weight
Positive
Negative

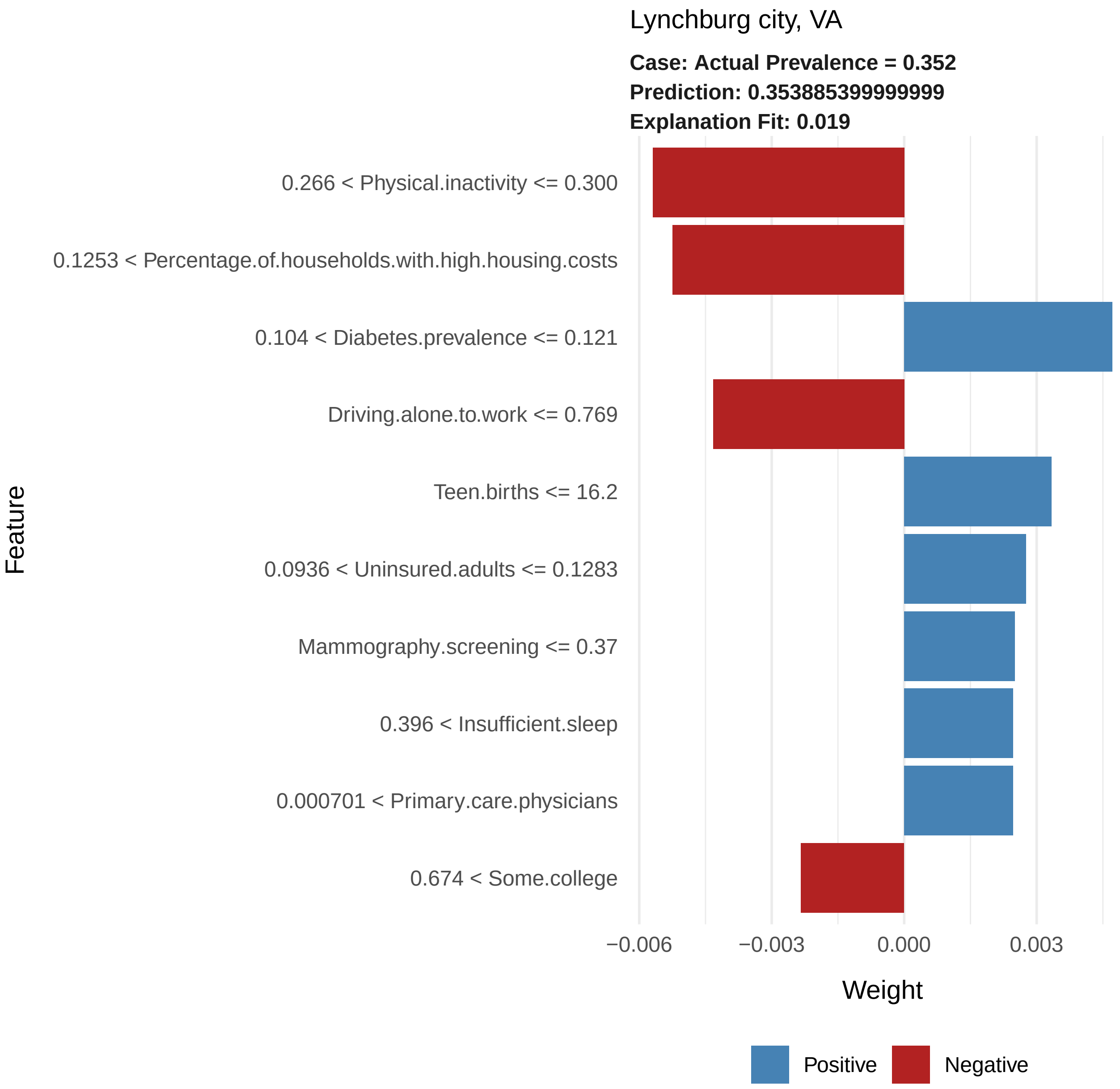

Lynchburg city, VA
Case: Actual Prevalence = 0.352
Prediction: 0.353885399999999
Explanation Fit: 0.019
0.266 < Physical.inactivity <= 0.300
0.1253 < Percentage.of.households.with.high.housing.costs
0.104 < Diabetes.prevalence <= 0.121
Driving.alone.to.work <= 0.769
Teen.births <= 16.2
0.0936 < Uninsured.adults <= 0.1283
Mammography.screening <= 0.37
0.396 < Insufficient.sleep
0.000701 < Primary.care.physicians
0.674 < Some.college
Feature
−0.006
−0.003
0.000
0.003
Weight
Positive
Negative

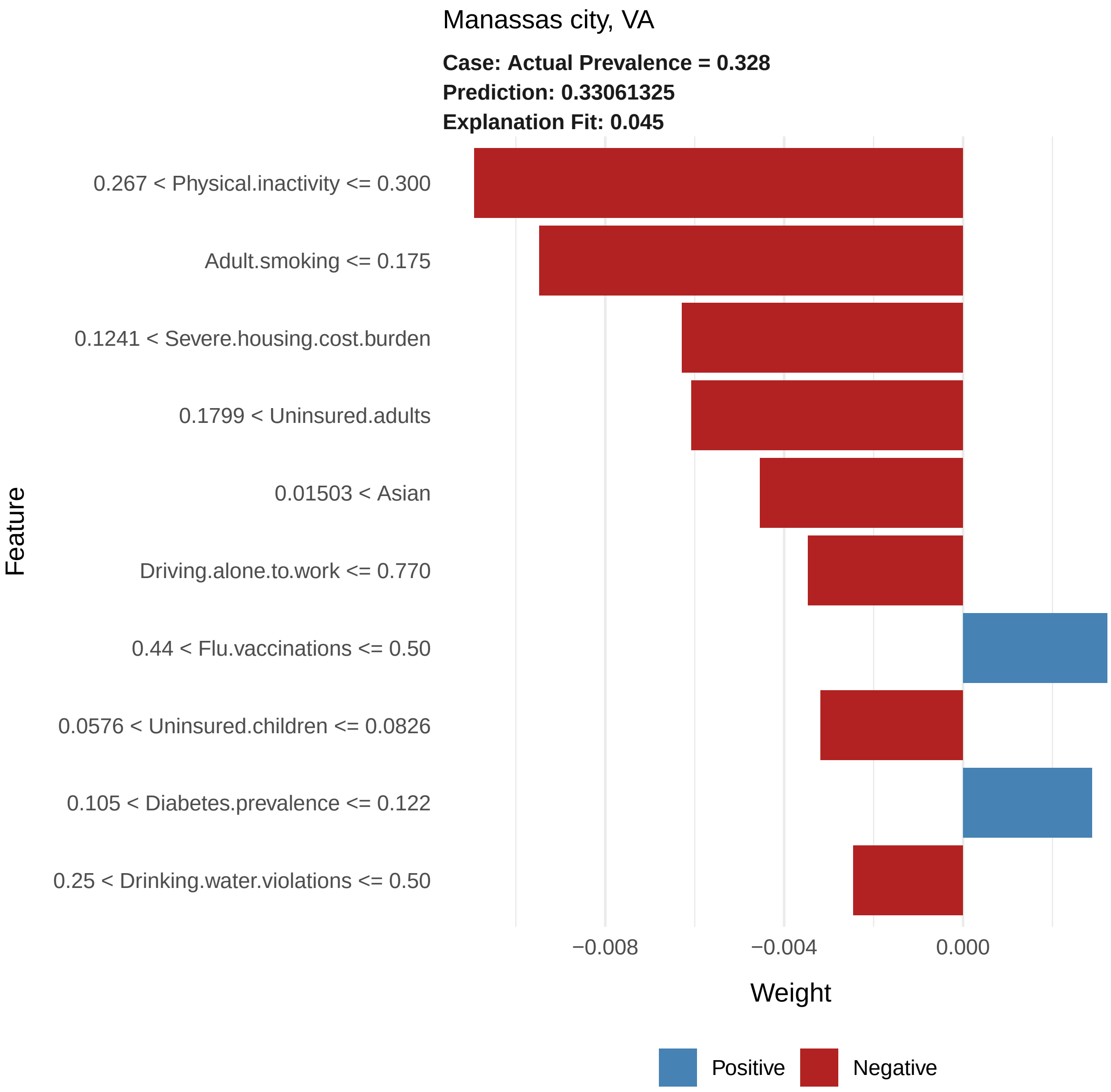
Manassas city, VA
Case: Actual Prevalence = 0.328
Prediction: 0.33061325
Explanation Fit: 0.045
0.267 < Physical.inactivity <= 0.300
Adult.smoking <= 0.175
0.1241 < Severe.housing.cost.burden
0.1799 < Uninsured.adults
0.01503 < Asian
Driving.alone.to.work <= 0.770
0.44 < Flu.vaccinations <= 0.50
0.0576 < Uninsured.children <= 0.0826
0.105 < Diabetes.prevalence <= 0.122
0.25 < Drinking.water.violations <= 0.50
Feature
−0.008
−0.004
0.000
Weight
Positive
Negative

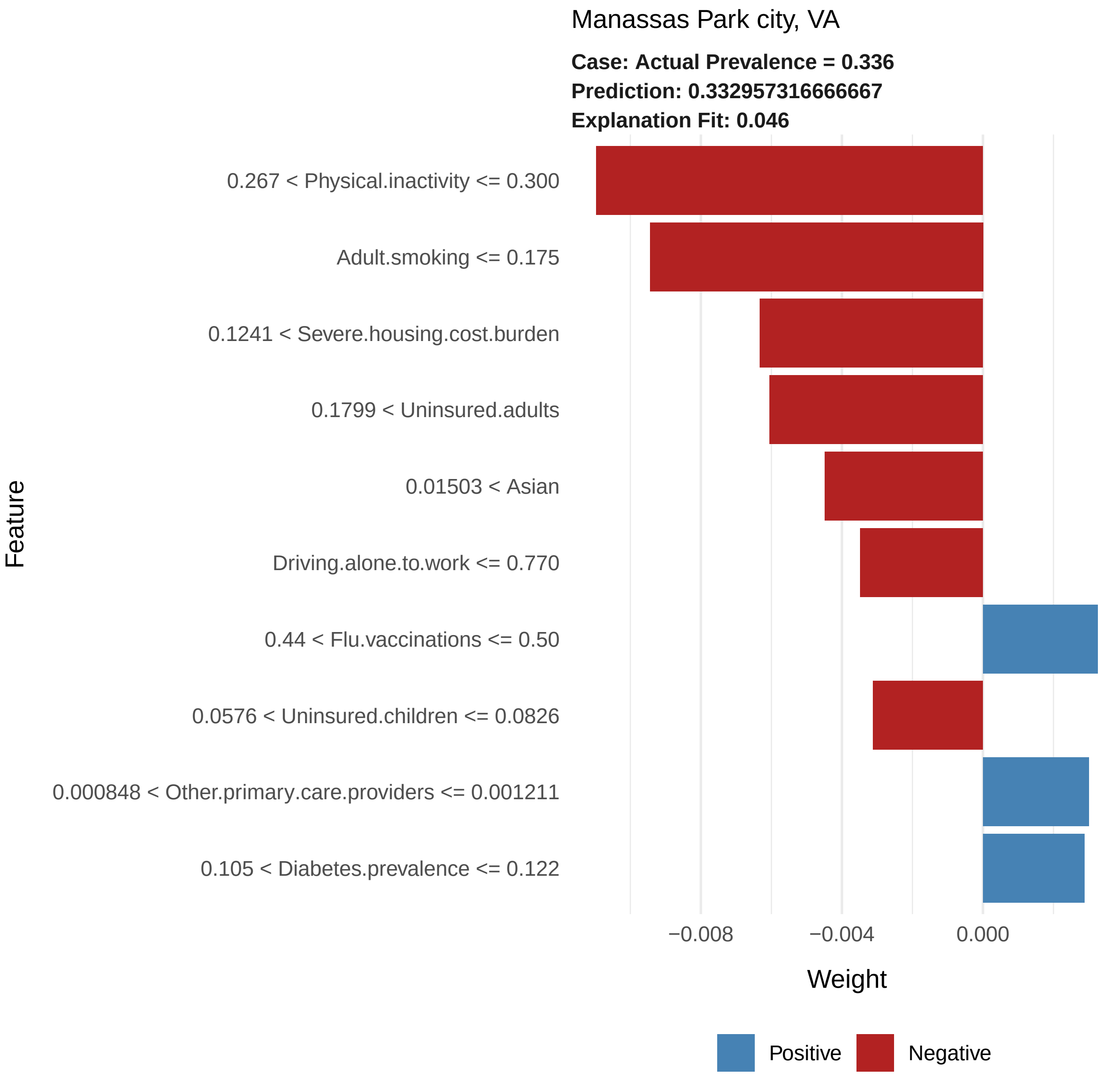
Manassas Park city, VA
Case: Actual Prevalence = 0.336
Prediction: 0.332957316666667
Explanation Fit: 0.046
0.267 < Physical.inactivity <= 0.300
Adult.smoking <= 0.175
0.1241 < Severe.housing.cost.burden
0.1799 < Uninsured.adults
0.01503 < Asian
Driving.alone.to.work <= 0.770
0.44 < Flu.vaccinations <= 0.50
0.0576 < Uninsured.children <= 0.0826
0.000848 < Other.primary.care.providers <= 0.001211
0.105 < Diabetes.prevalence <= 0.122
Feature
−0.008
−0.004
0.000
Weight
Positive
Negative

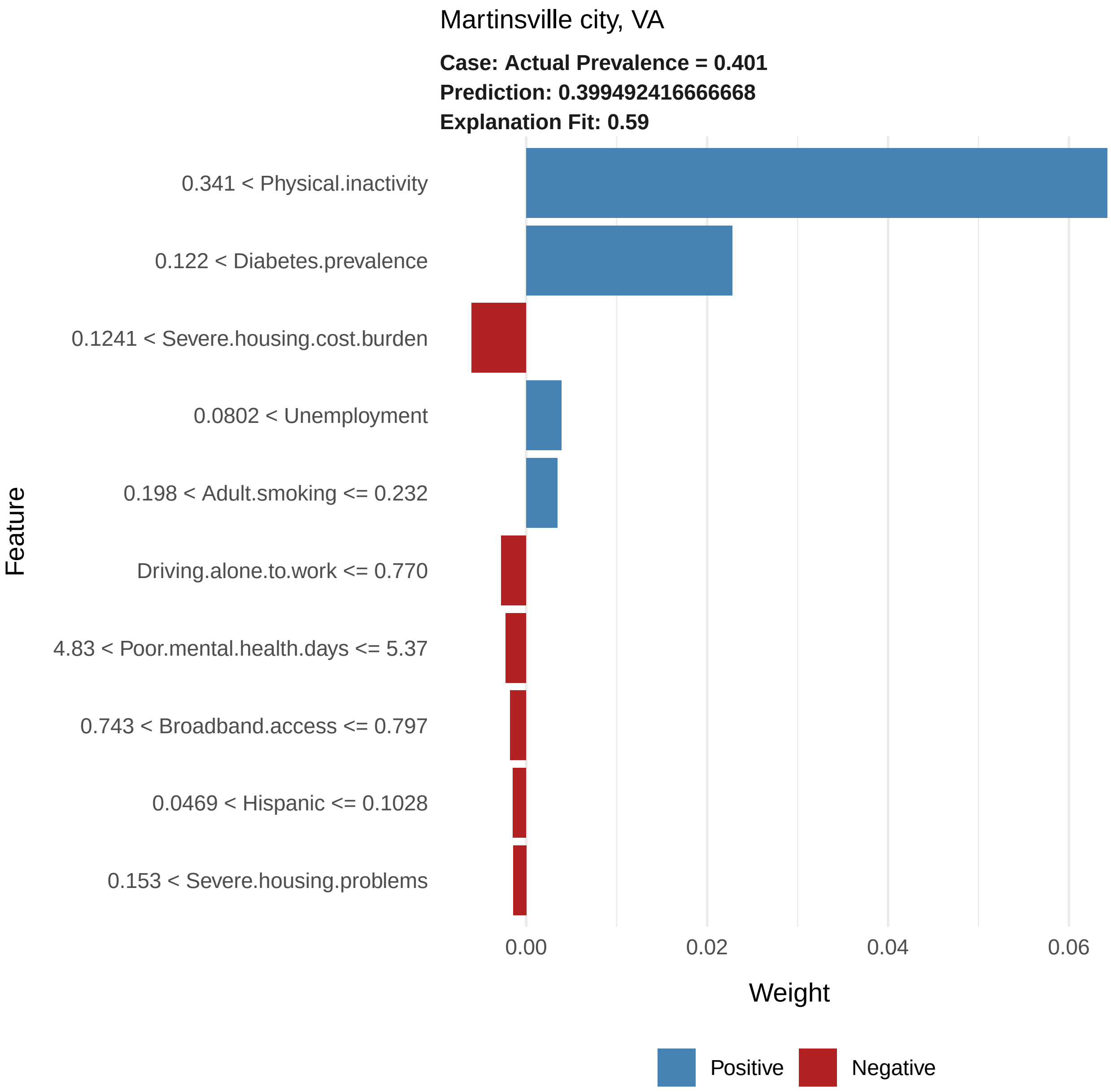
Martinsville city, VA
Case: Actual Prevalence = 0.401
Prediction: 0.399492416666668
Explanation Fit: 0.59
0.341 < Physical.inactivity
0.122 < Diabetes.prevalence
0.1241 < Severe.housing.cost.burden
0.0802 < Unemployment
0.198 < Adult.smoking <= 0.232
Driving.alone.to.work <= 0.770
4.83 < Poor.mental.health.days <= 5.37
0.743 < Broadband.access <= 0.797
0.0469 < Hispanic <= 0.1028
0.153 < Severe.housing.problems
Feature
0.00
0.02
0.04
0.06
Weight
Positive
Negative

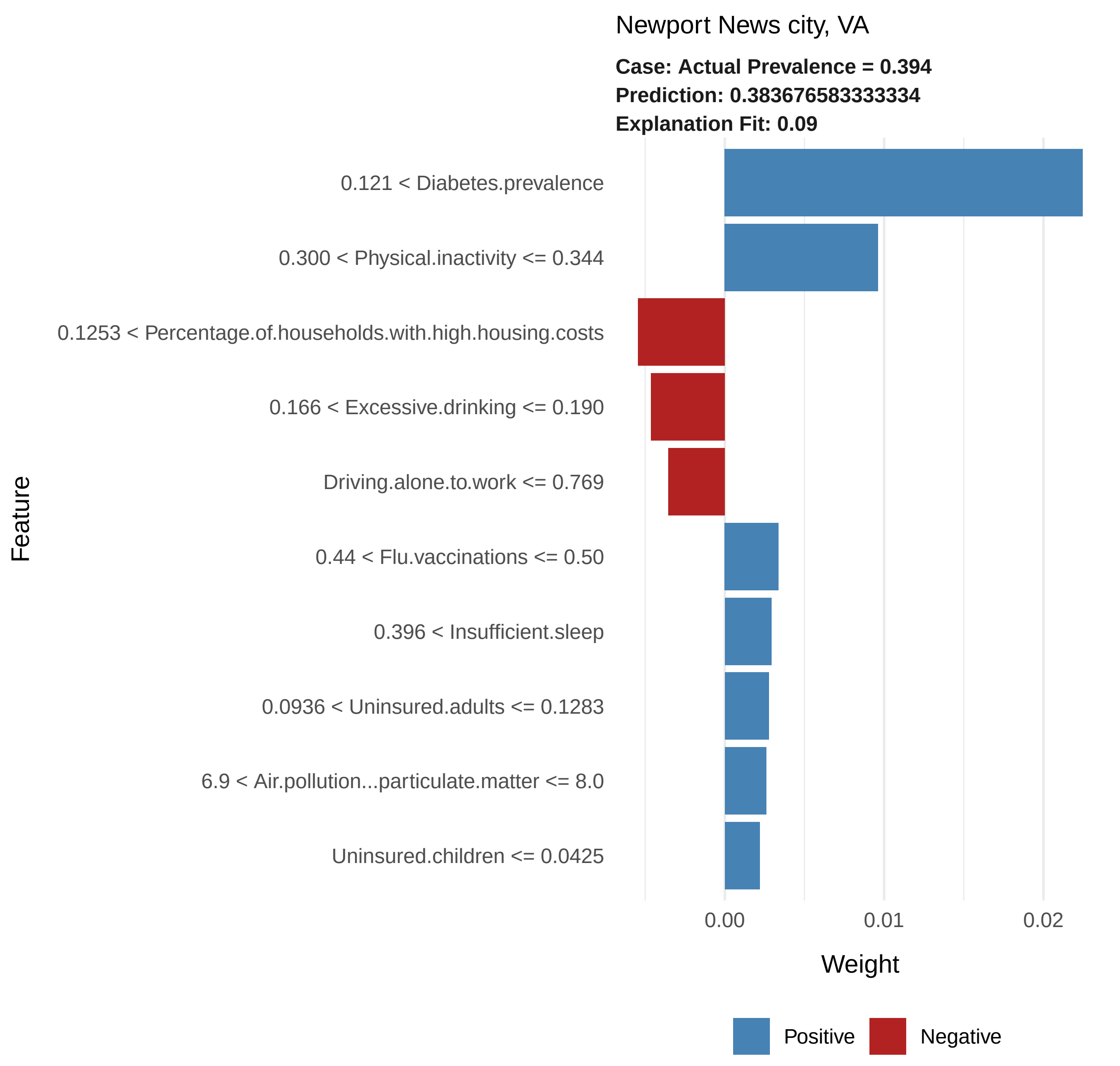

Newport News city, VA
Case: Actual Prevalence = 0.394
Prediction: 0.383676583333334
Explanation Fit: 0.09
0.121 < Diabetes.prevalence
0.300 < Physical.inactivity <= 0.344
0.1253 < Percentage.of.households.with.high.housing.costs
0.166 < Excessive.drinking <= 0.190
Driving.alone.to.work <= 0.769
0.44 < Flu.vaccinations <= 0.50
0.396 < Insufficient.sleep
0.0936 < Uninsured.adults <= 0.1283
6.9 < Air.pollution...particulate.matter <= 8.0
Uninsured.children <= 0.0425
Feature
0.00
0.01
0.02
Weight
Positive
Negative

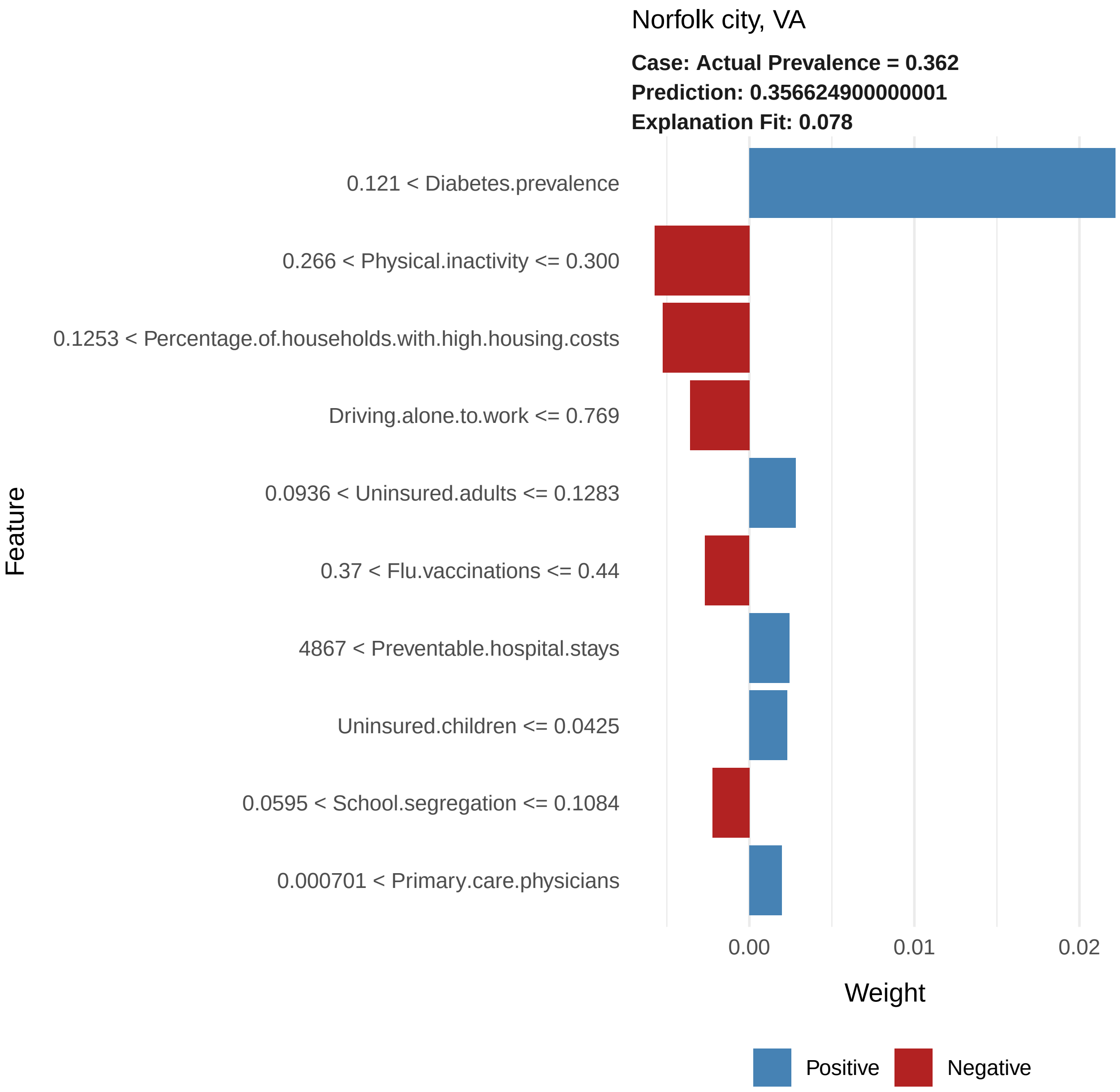

Norfolk city, VA
Case: Actual Prevalence = 0.362
Prediction: 0.356624900000001
Explanation Fit: 0.078
0.121 < Diabetes.prevalence
0.266 < Physical.inactivity <= 0.300
0.1253 < Percentage.of.households.with.high.housing.costs
Driving.alone.to.work <= 0.769
0.0936 < Uninsured.adults <= 0.1283
0.37 < Flu.vaccinations <= 0.44
4867 < Preventable.hospital.stays
Uninsured.children <= 0.0425
0.0595 < School.segregation <= 0.1084
0.000701 < Primary.care.physicians
Feature
0.00
0.01
0.02
Weight
Positive
Negative

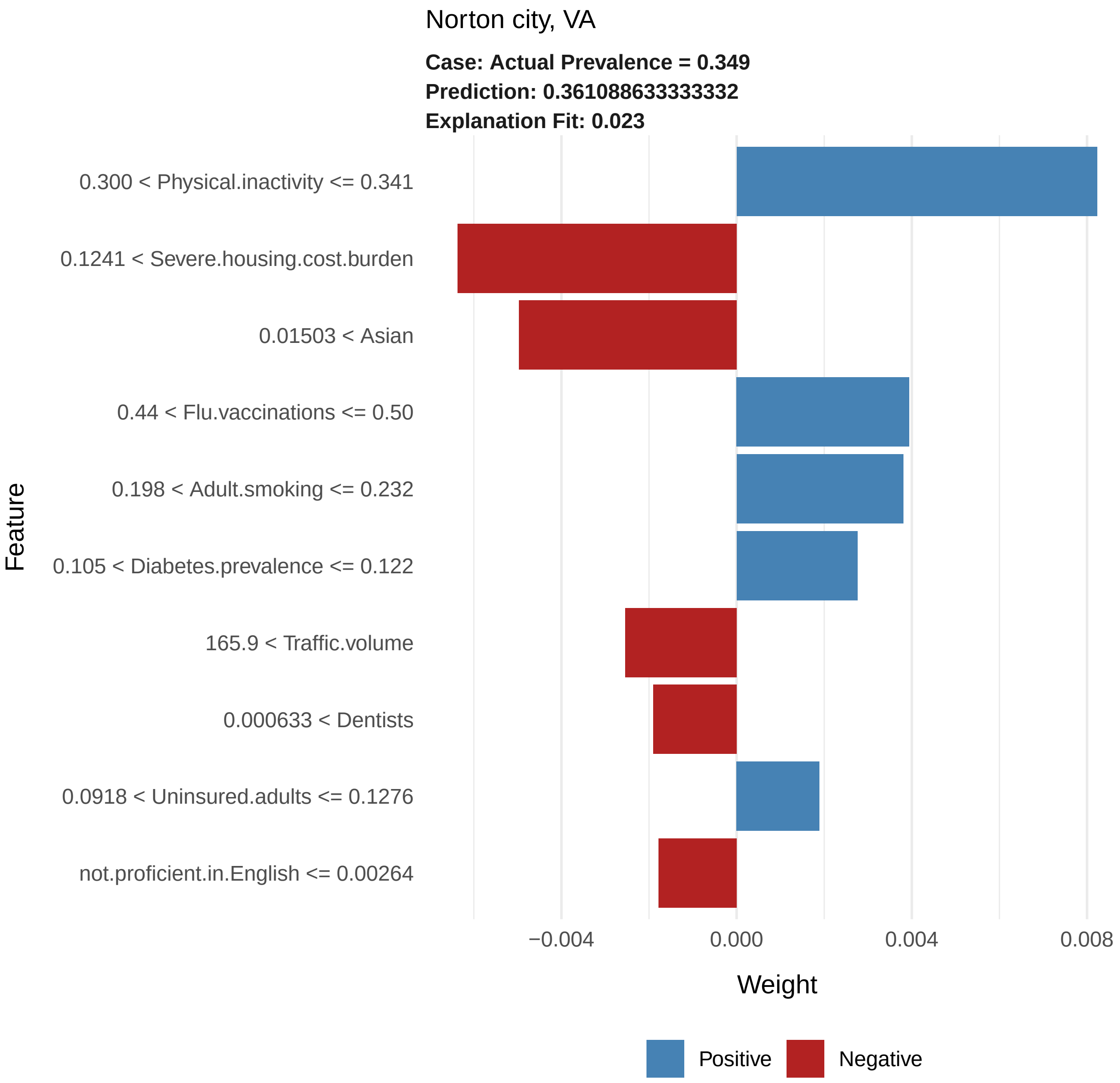

Norton city, VA
Case: Actual Prevalence = 0.349
Prediction: 0.361088633333332
Explanation Fit: 0.023
0.300 < Physical.inactivity <= 0.341
0.1241 < Severe.housing.cost.burden
0.01503 < Asian
0.44 < Flu.vaccinations <= 0.50
0.198 < Adult.smoking <= 0.232
0.105 < Diabetes.prevalence <= 0.122
165.9 < Traffic.volume
0.000633 < Dentists
0.0918 < Uninsured.adults <= 0.1276
not.proficient.in.English <= 0.00264
Feature
−0.004
0.000
0.004
0.008
Weight
Positive
Negative

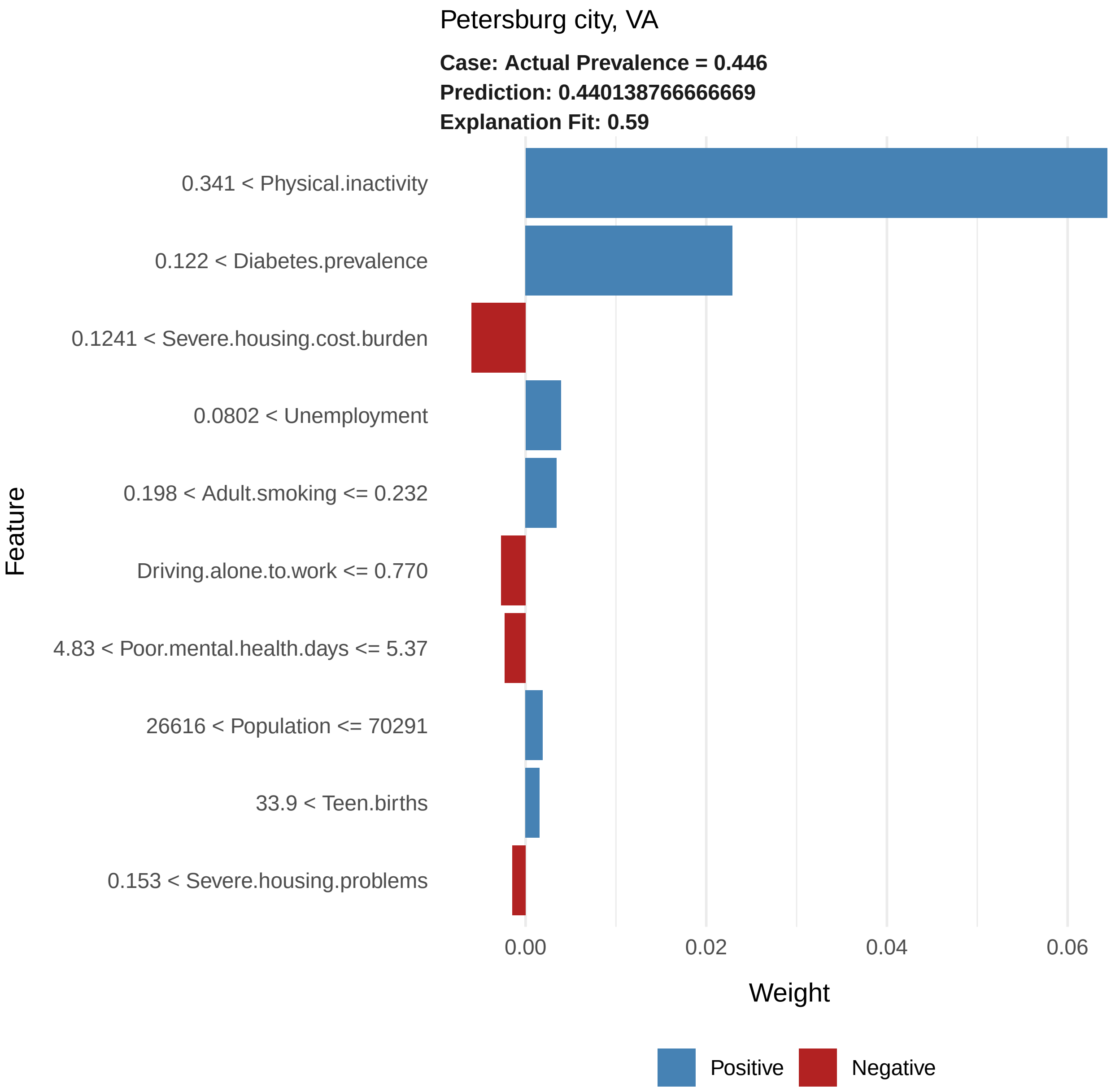
Petersburg city, VA
Case: Actual Prevalence = 0.446
Prediction: 0.440138766666669
Explanation Fit: 0.59
0.341 < Physical.inactivity
0.122 < Diabetes.prevalence
0.1241 < Severe.housing.cost.burden
0.0802 < Unemployment
0.198 < Adult.smoking <= 0.232
Driving.alone.to.work <= 0.770
4.83 < Poor.mental.health.days <= 5.37
26616 < Population <= 70291
33.9 < Teen.births
0.153 < Severe.housing.problems
Feature
0.00
0.02
0.04
0.06
Weight
Positive
Negative

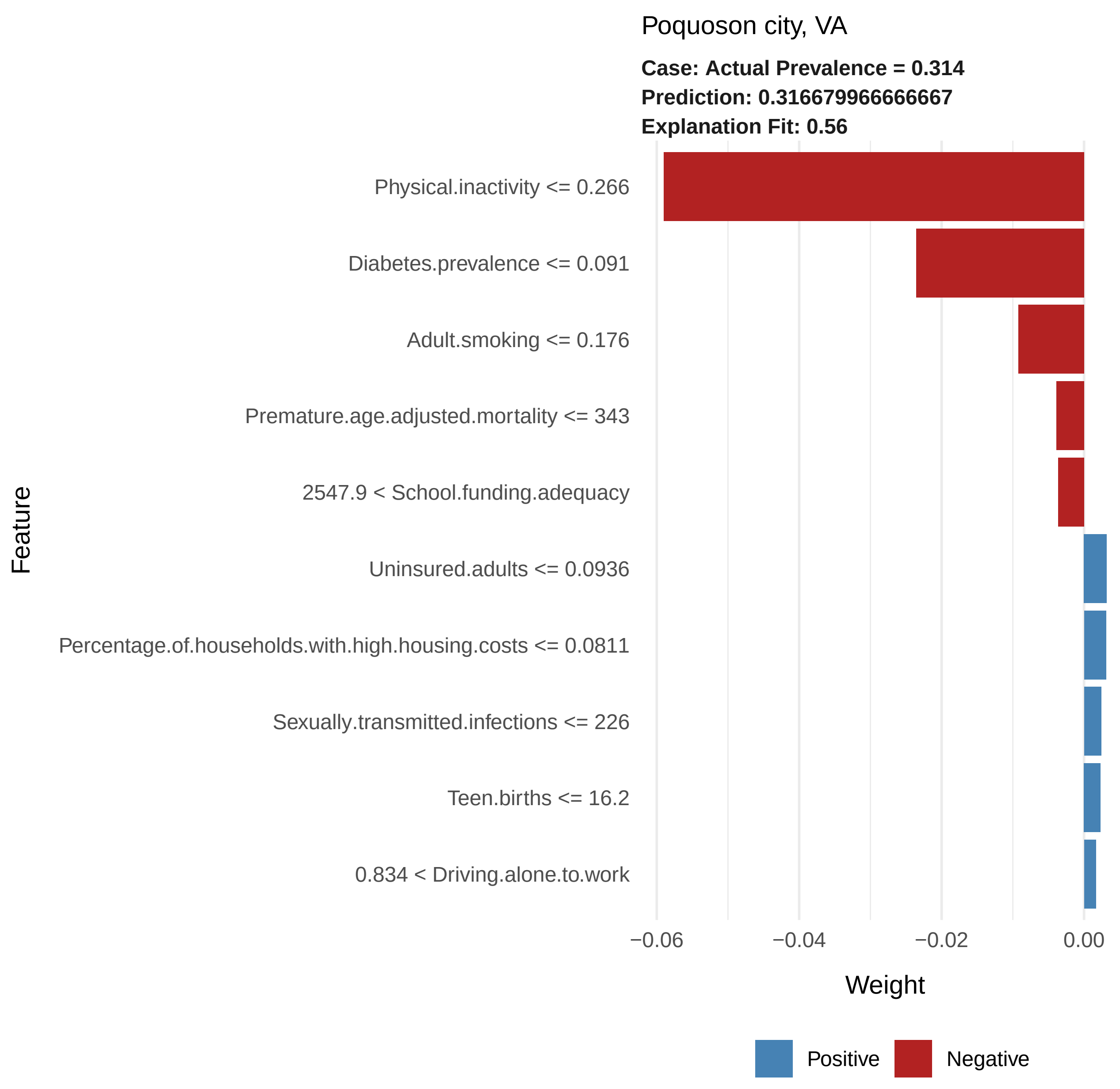

Poquoson city, VA
Case: Actual Prevalence = 0.314
Prediction: 0.316679966666667
Explanation Fit: 0.56
Physical.inactivity <= 0.266
Diabetes.prevalence <= 0.091
Adult.smoking <= 0.176
Premature.age.adjusted.mortality <= 343
2547.9 < School.funding.adequacy
Uninsured.adults <= 0.0936
Percentage.of.households.with.high.housing.costs <= 0.0811
Sexually.transmitted.infections <= 226
Teen.births <= 16.2
0.834 < Driving.alone.to.work
Feature
−0.06
−0.04
−0.02
0.00
Weight
Positive
Negative

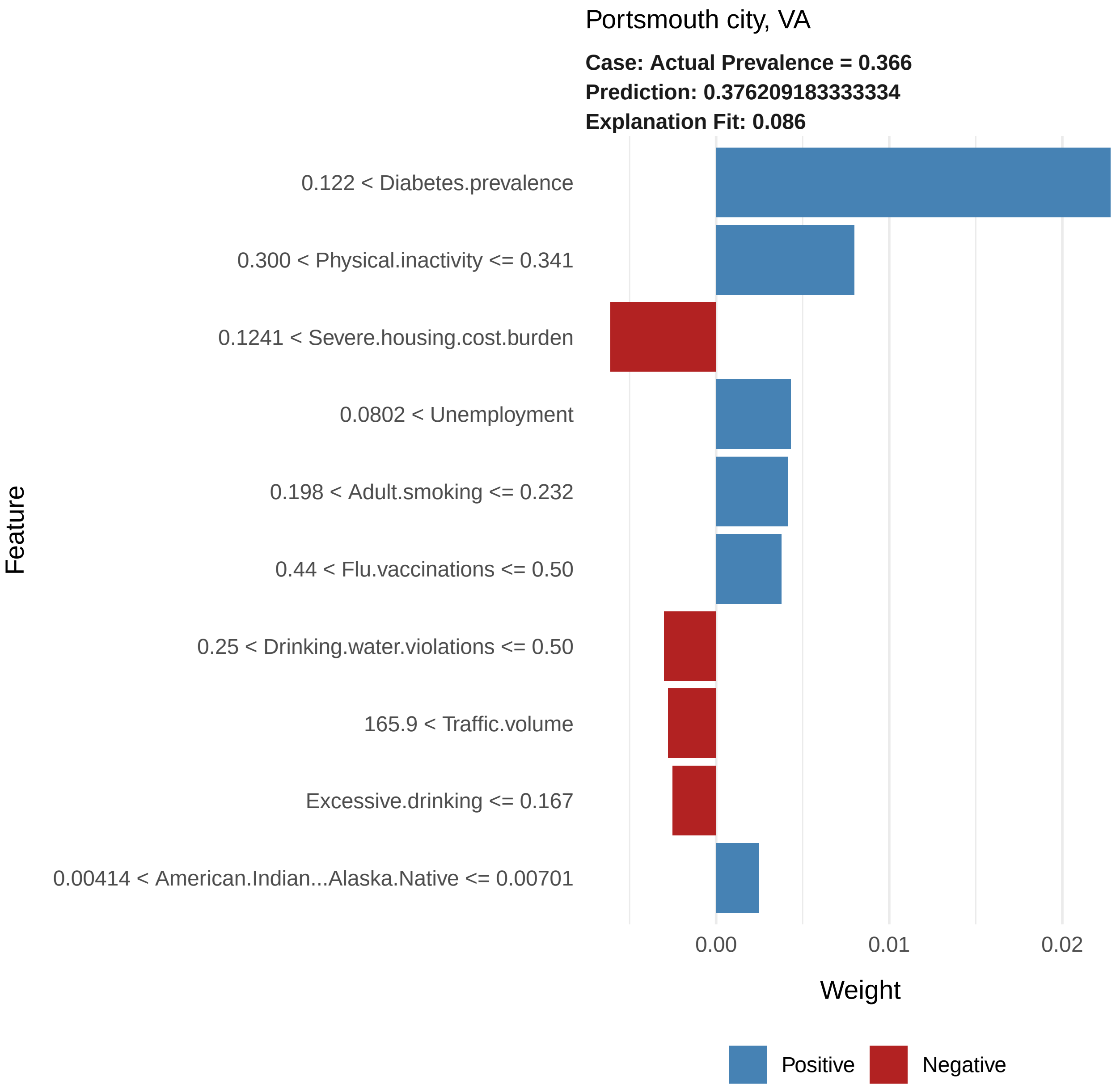
Portsmouth city, VA
Case: Actual Prevalence = 0.366
Prediction: 0.376209183333334
Explanation Fit: 0.086
0.122 < Diabetes.prevalence
0.300 < Physical.inactivity <= 0.341
0.1241 < Severe.housing.cost.burden
0.0802 < Unemployment
0.198 < Adult.smoking <= 0.232
0.44 < Flu.vaccinations <= 0.50
0.25 < Drinking.water.violations <= 0.50
165.9 < Traffic.volume
Excessive.drinking <= 0.167
0.00414 < American.Indian...Alaska.Native <= 0.00701
Feature
0.00
0.01
0.02
Weight
Positive
Negative

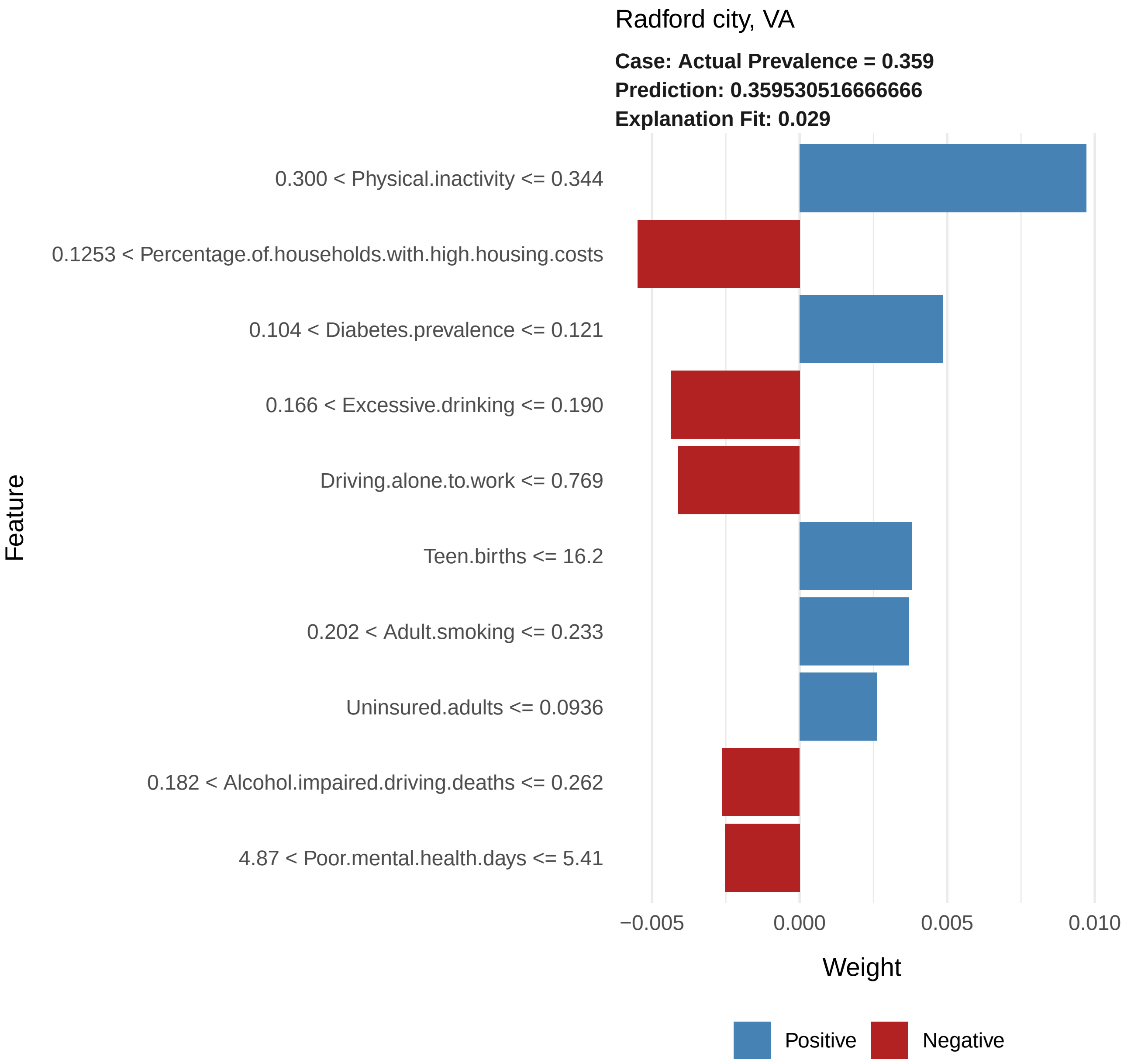
Radford city, VA
Case: Actual Prevalence = 0.359
Prediction: 0.359530516666666
Explanation Fit: 0.029
0.300 < Physical.inactivity <= 0.344
0.1253 < Percentage.of.households.with.high.housing.costs
0.104 < Diabetes.prevalence <= 0.121
0.166 < Excessive.drinking <= 0.190
Driving.alone.to.work <= 0.769
Teen.births <= 16.2
0.202 < Adult.smoking <= 0.233
Uninsured.adults <= 0.0936
0.182 < Alcohol.impaired.driving.deaths <= 0.262
4.87 < Poor.mental.health.days <= 5.41
Feature
−0.005
0.000
0.005
0.010
Weight
Positive
Negative

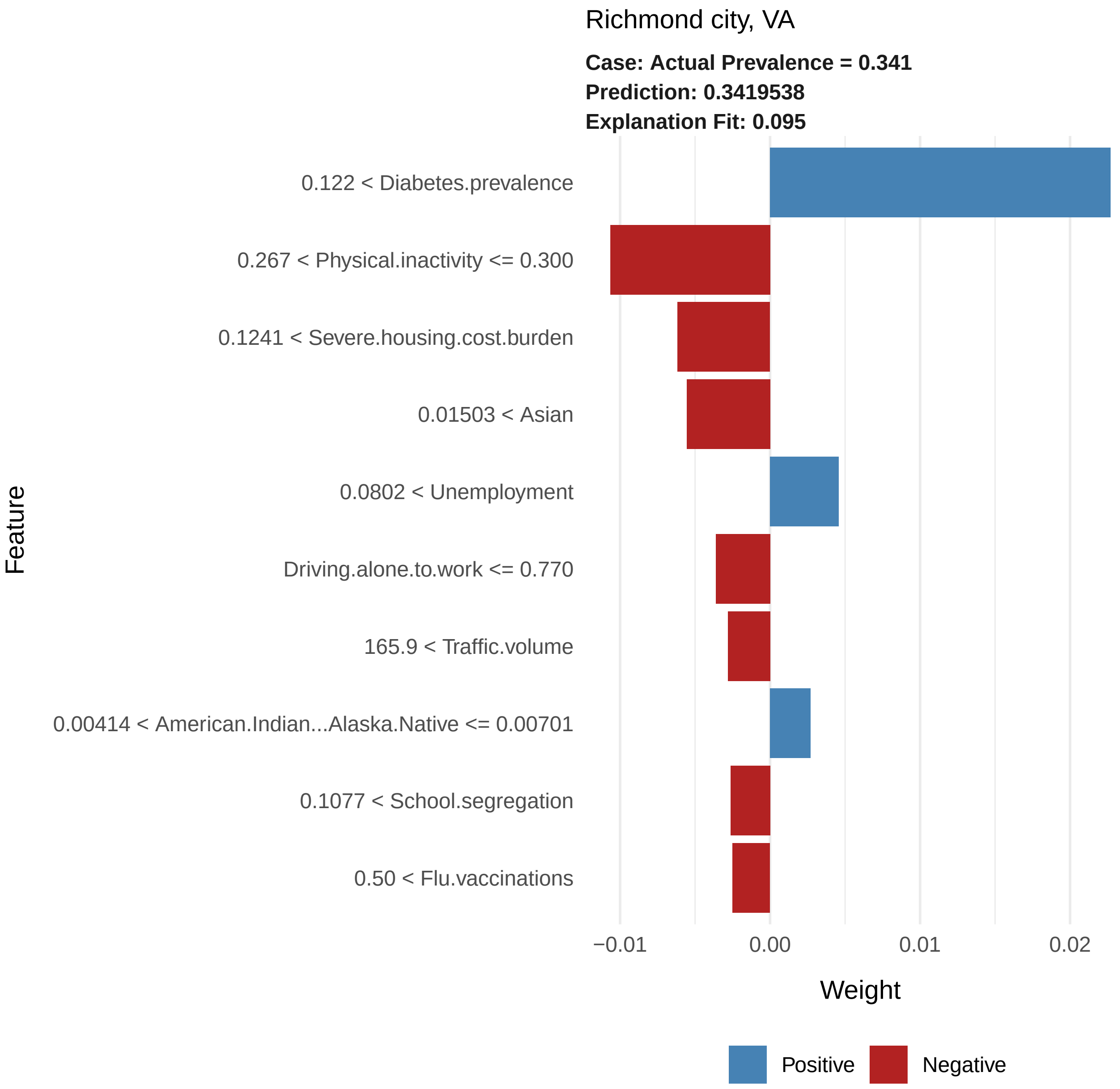

Richmond city, VA
Case: Actual Prevalence = 0.341
Prediction: 0.3419538
Explanation Fit: 0.095
0.122 < Diabetes.prevalence
0.267 < Physical.inactivity <= 0.300
0.1241 < Severe.housing.cost.burden
0.01503 < Asian
0.0802 < Unemployment
Driving.alone.to.work <= 0.770
165.9 < Traffic.volume
0.00414 < American.Indian...Alaska.Native <= 0.00701
0.1077 < School.segregation
0.50 < Flu.vaccinations
Feature
−0.01
0.00
0.01
0.02
Weight
Positive
Negative

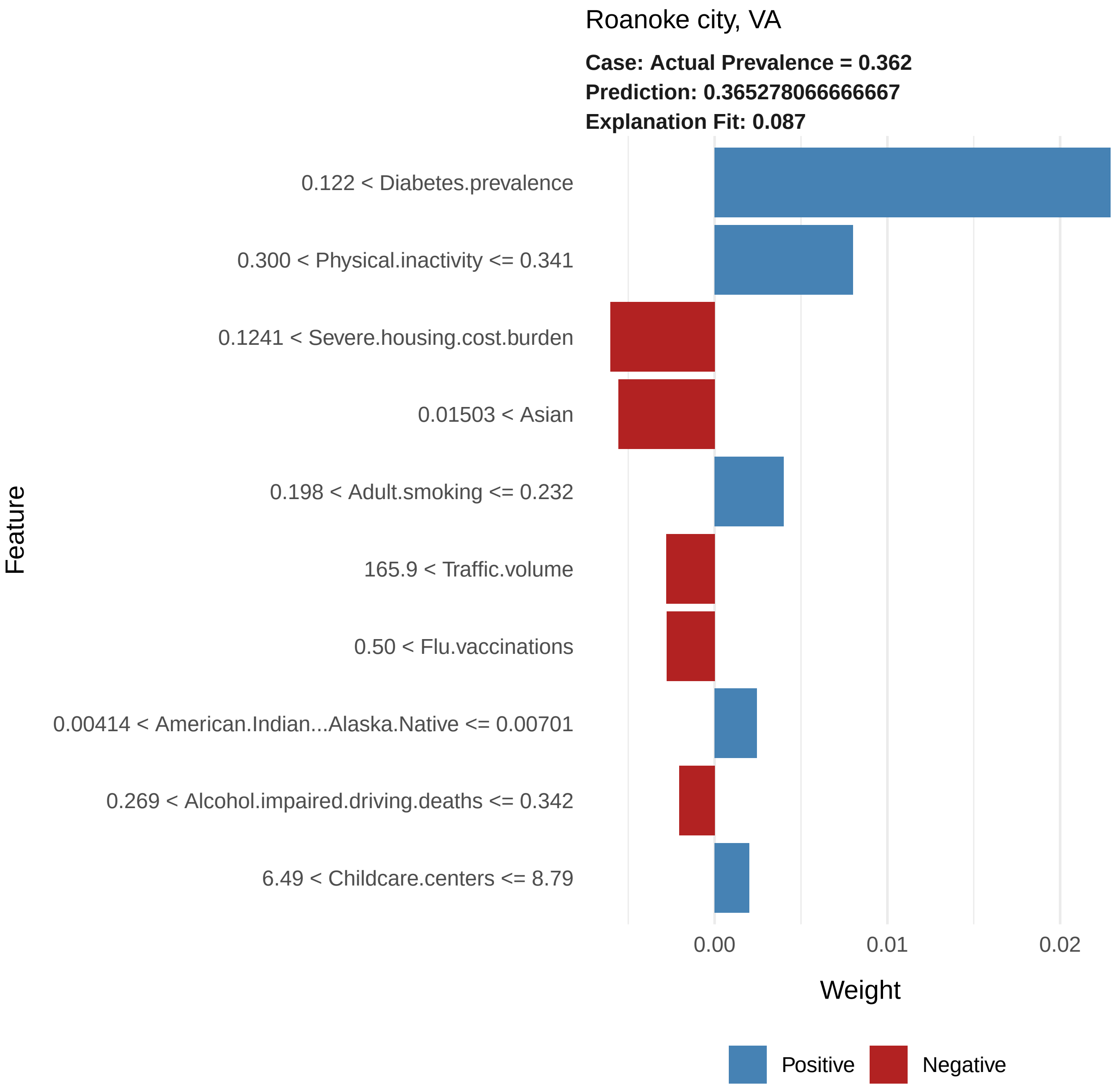

Roanoke city, VA
Case: Actual Prevalence = 0.362
Prediction: 0.365278066666667
Explanation Fit: 0.087
0.122 < Diabetes.prevalence
0.300 < Physical.inactivity <= 0.341
0.1241 < Severe.housing.cost.burden
0.01503 < Asian
0.198 < Adult.smoking <= 0.232
165.9 < Traffic.volume
0.50 < Flu.vaccinations
0.00414 < American.Indian...Alaska.Native <= 0.00701
0.269 < Alcohol.impaired.driving.deaths <= 0.342
6.49 < Childcare.centers <= 8.79
Feature
0.00
0.01
0.02
Weight
Positive
Negative

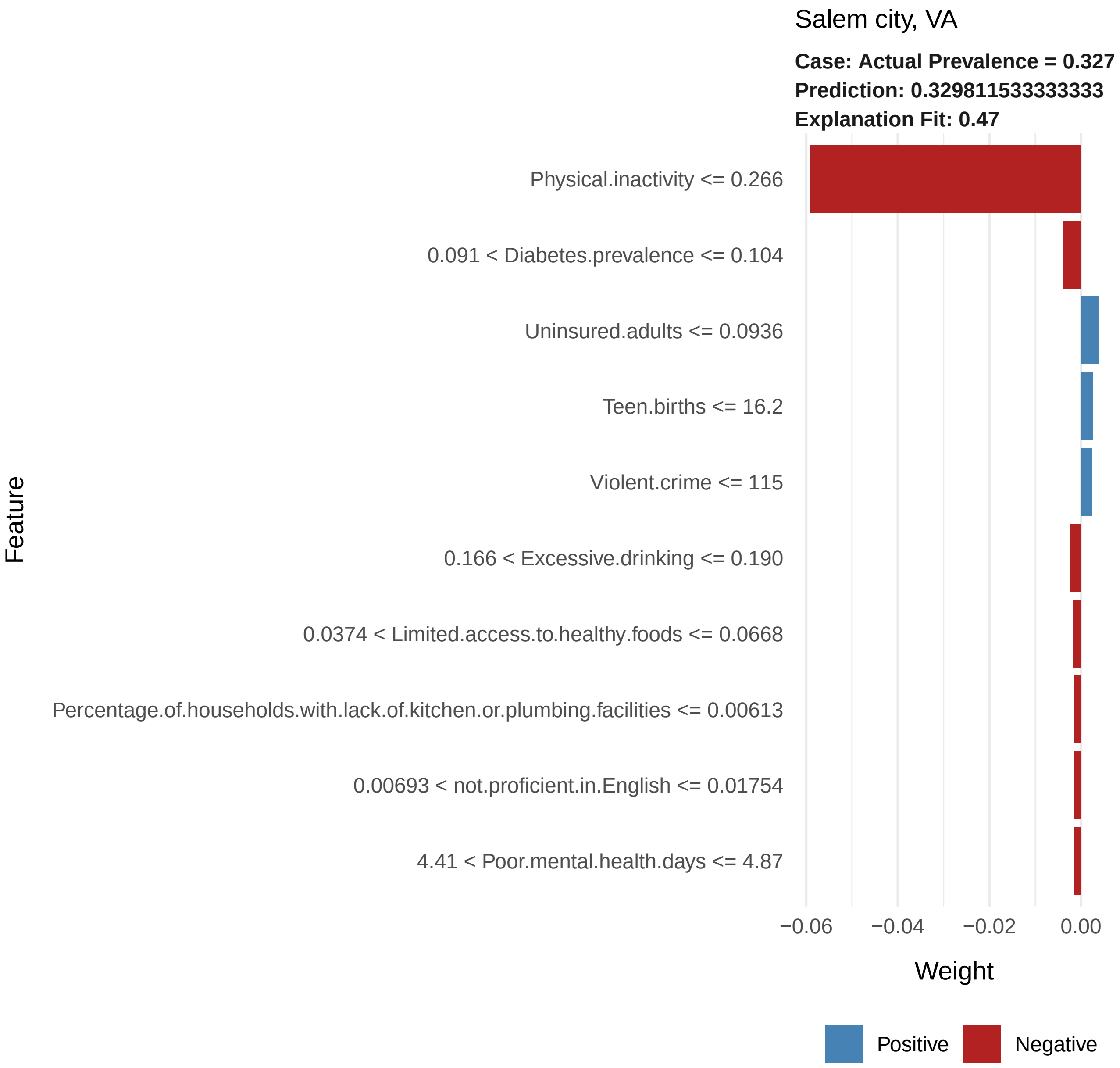

Salem city, VA
Case: Actual Prevalence = 0.327
Prediction: 0.329811533333333
Explanation Fit: 0.47
Physical.inactivity <= 0.266
0.091 < Diabetes.prevalence <= 0.104
Uninsured.adults <= 0.0936
Teen.births <= 16.2
Violent.crime <= 115
0.166 < Excessive.drinking <= 0.190
0.0374 < Limited.access.to.healthy.foods <= 0.0668
Percentage.of.households.with.lack.of.kitchen.or.plumbing.facilities <= 0.00613
0.00693 < not.proficient.in.English <= 0.01754
4.41 < Poor.mental.health.days <= 4.87
Feature
−0.06
−0.04
−0.02
0.00
Weight
Positive
Negative

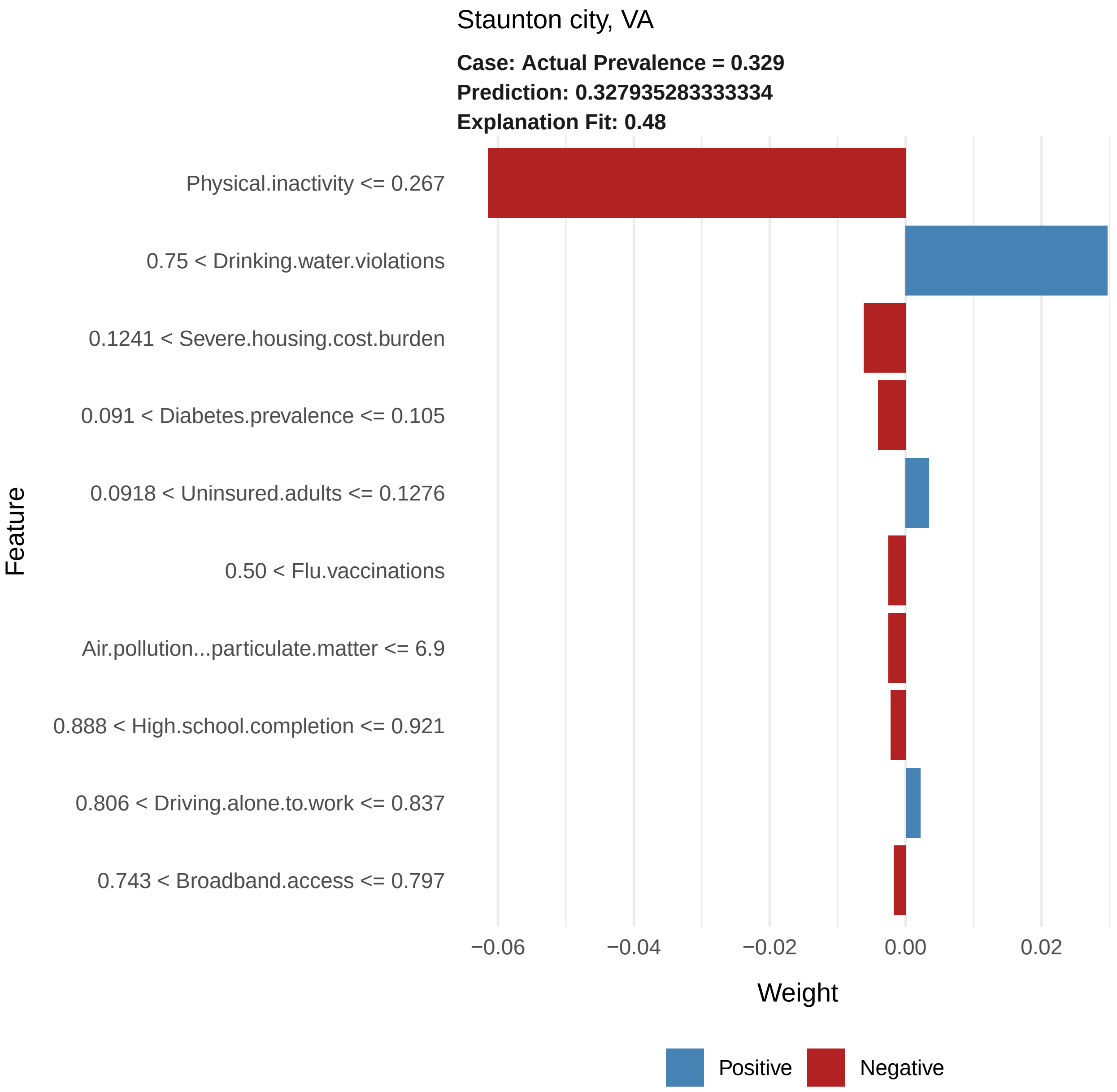
Staunton city, VA
Case: Actual Prevalence = 0.329
Prediction: 0.327935283333334
Explanation Fit: 0.48
Physical.inactivity <= 0.267
0.75 < Drinking.water.violations
0.1241 < Severe.housing.cost.burden
0.091 < Diabetes.prevalence <= 0.105
0.0918 < Uninsured.adults <= 0.1276
0.50 < Flu.vaccinations
Air.pollution...particulate.matter <= 6.9
0.888 < High.school.completion <= 0.921
0.806 < Driving.alone.to.work <= 0.837
0.743 < Broadband.access <= 0.797
Feature
−0.06
−0.04
−0.02
0.00
0.02
Weight
Positive
Negative

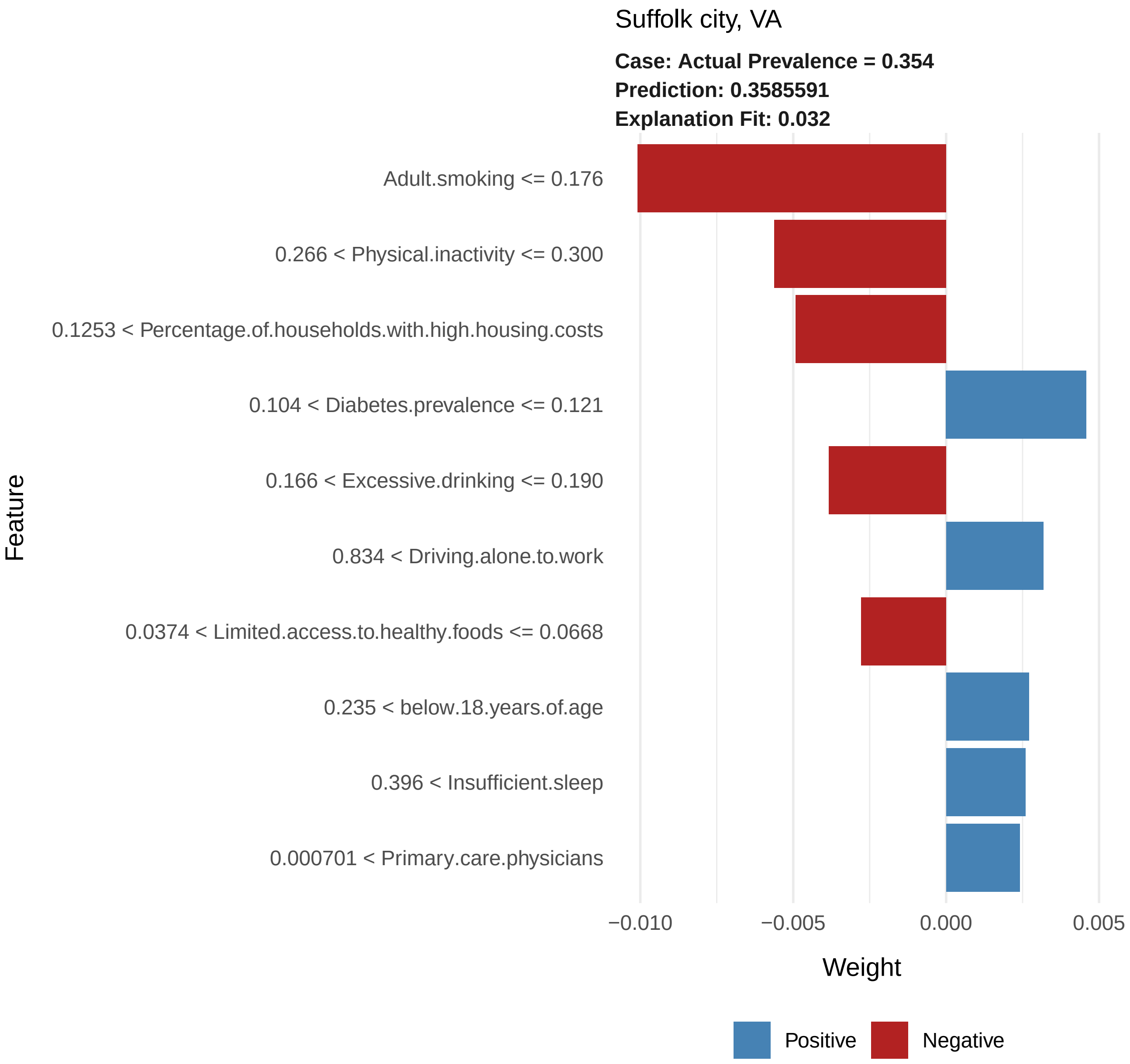
Suffolk city, VA
Case: Actual Prevalence = 0.354
Prediction: 0.3585591
Explanation Fit: 0.032
Adult.smoking <= 0.176
0.266 < Physical.inactivity <= 0.300
0.1253 < Percentage.of.households.with.high.housing.costs
0.104 < Diabetes.prevalence <= 0.121
0.166 < Excessive.drinking <= 0.190
0.834 < Driving.alone.to.work
0.0374 < Limited.access.to.healthy.foods <= 0.0668
0.235 < below.18.years.of.age
0.396 < Insufficient.sleep
0.000701 < Primary.care.physicians
Feature
−0.010
−0.005
0.000
0.005
Weight
Positive
Negative

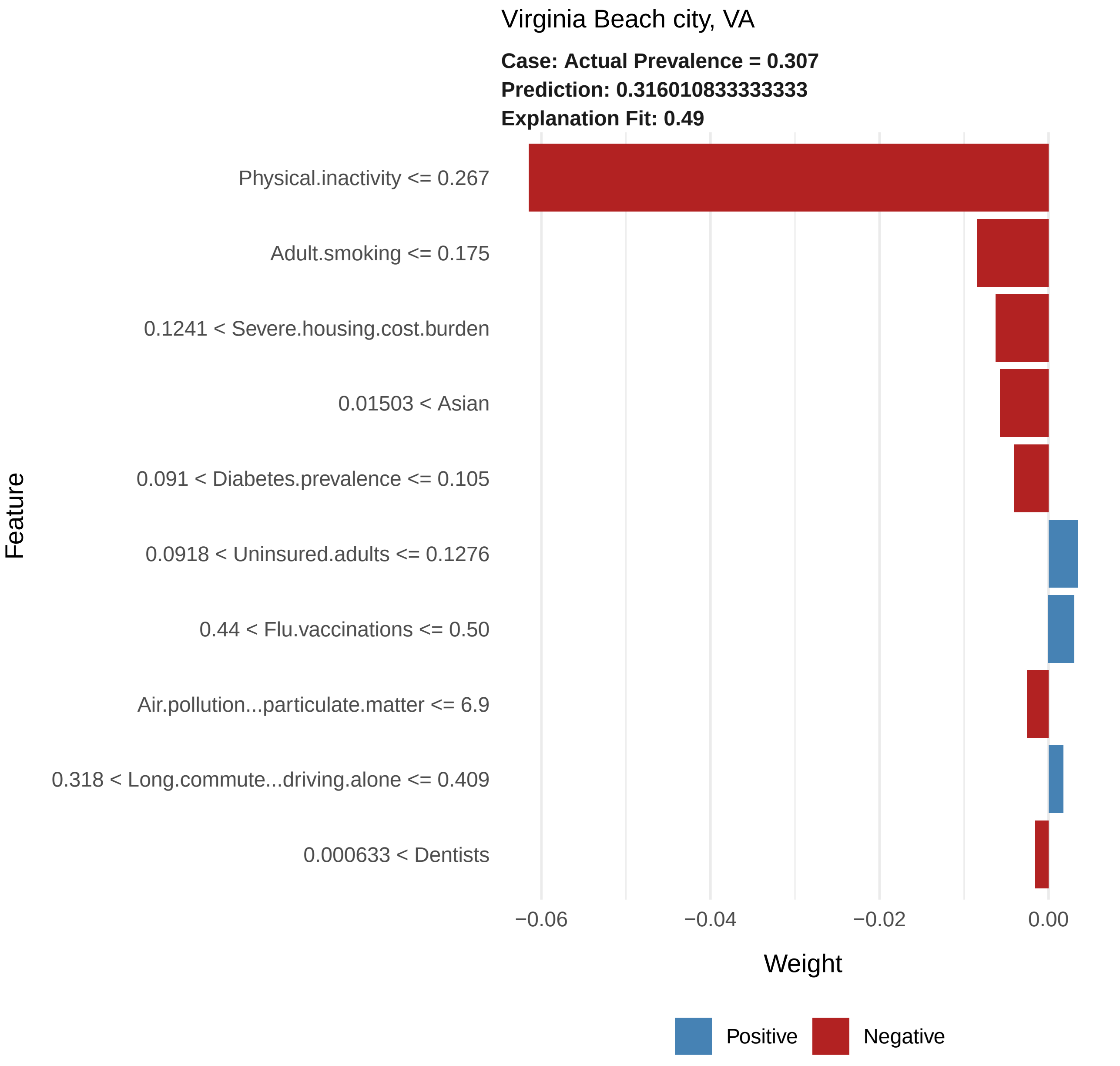

Virginia Beach city, VA
Case: Actual Prevalence = 0.307
Prediction: 0.316010833333333
Explanation Fit: 0.49
Physical.inactivity <= 0.267
Adult.smoking <= 0.175
0.1241 < Severe.housing.cost.burden
0.01503 < Asian
0.091 < Diabetes.prevalence <= 0.105
0.0918 < Uninsured.adults <= 0.1276
0.44 < Flu.vaccinations <= 0.50
Air.pollution...particulate.matter <= 6.9
0.318 < Long.commute...driving.alone <= 0.409
0.000633 < Dentists
Feature
−0.06
−0.04
−0.02
0.00
Weight
Positive
Negative

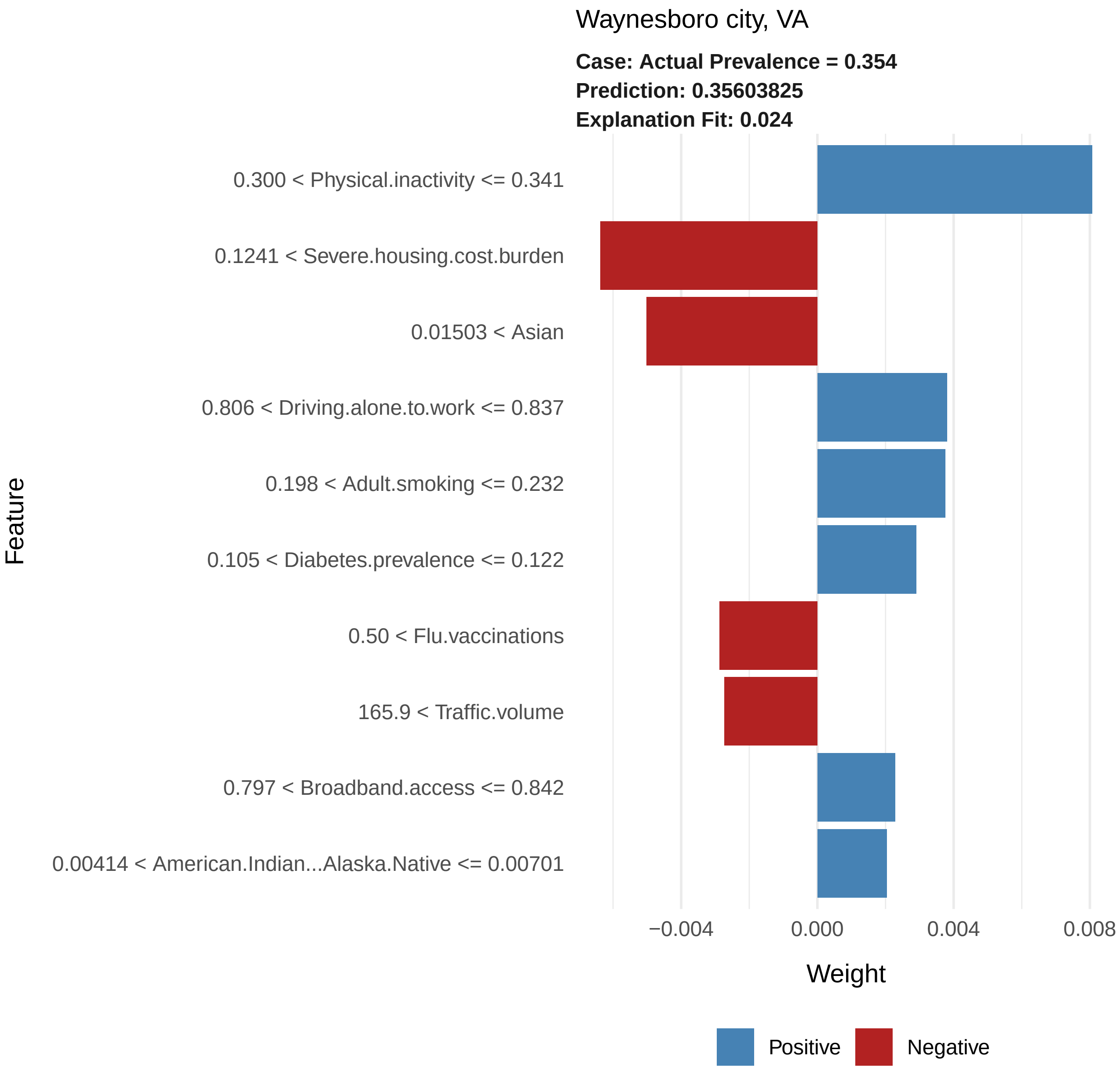

Waynesboro city, VA
Case: Actual Prevalence = 0.354
Prediction: 0.35603825
Explanation Fit: 0.024
Feature
0.300 < Physical.inactivity <= 0.341
0.1241 < Severe.housing.cost.burden
0.01503 < Asian
0.806 < Driving.alone.to.work <= 0.837
0.198 < Adult.smoking <= 0.232
0.105 < Diabetes.prevalence <= 0.122
0.50 < Flu.vaccinations
165.9 < Traffic.volume
0.797 < Broadband.access <= 0.842
0.00414 < American.Indian...Alaska.Native <= 0.00701
−0.004
0.000
0.004
0.008
Weight
Positive
Negative

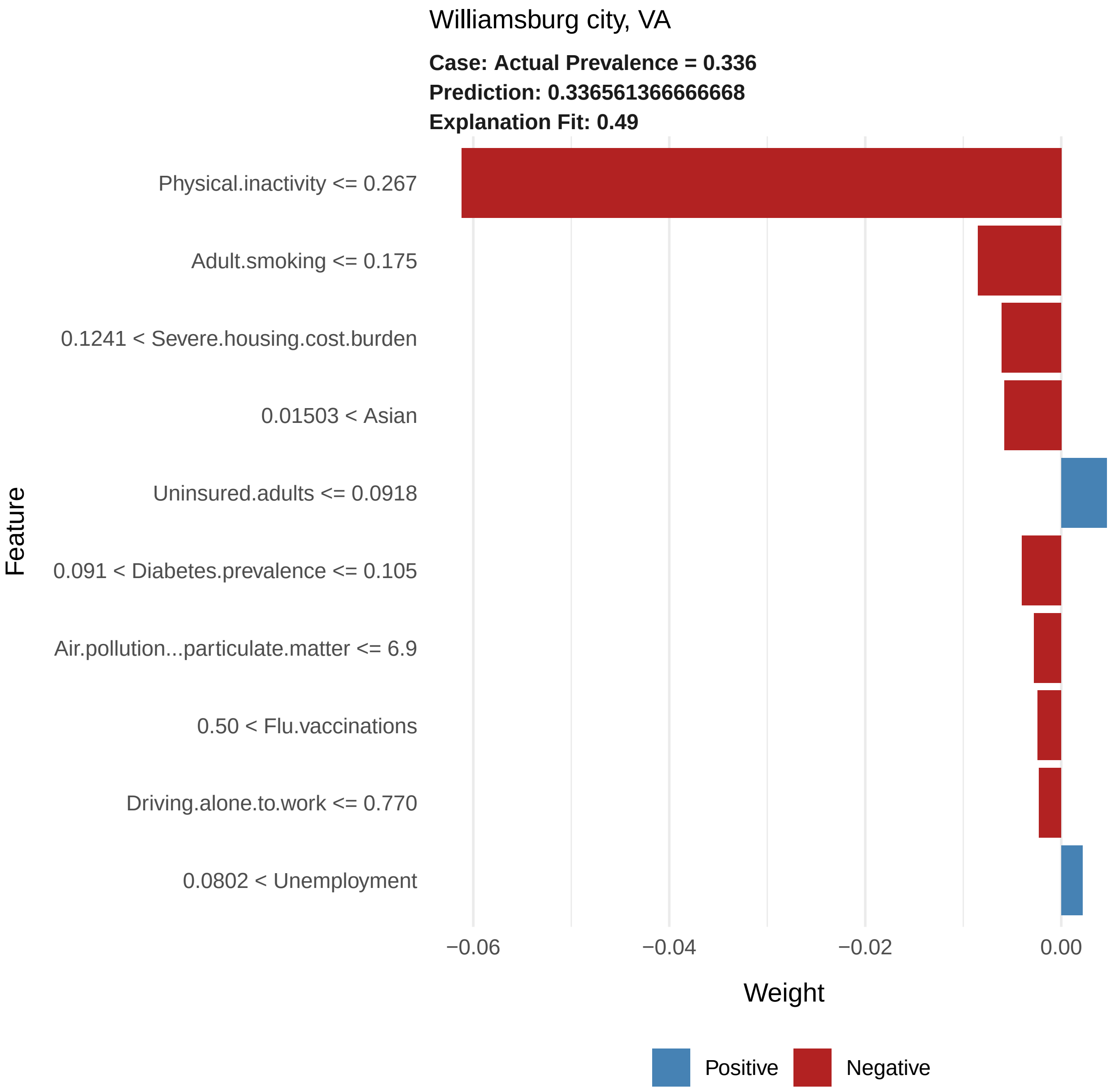

Williamsburg city, VA
Case: Actual Prevalence = 0.336
Prediction: 0.336561366666668
Explanation Fit: 0.49
Physical.inactivity <= 0.267
Adult.smoking <= 0.175
0.1241 < Severe.housing.cost.burden
0.01503 < Asian
Uninsured.adults <= 0.0918
0.091 < Diabetes.prevalence <= 0.105
Air.pollution...particulate.matter <= 6.9
0.50 < Flu.vaccinations
Driving.alone.to.work <= 0.770
0.0802 < Unemployment
Feature
−0.06
−0.04
−0.02
0.00
Weight
Positive
Negative

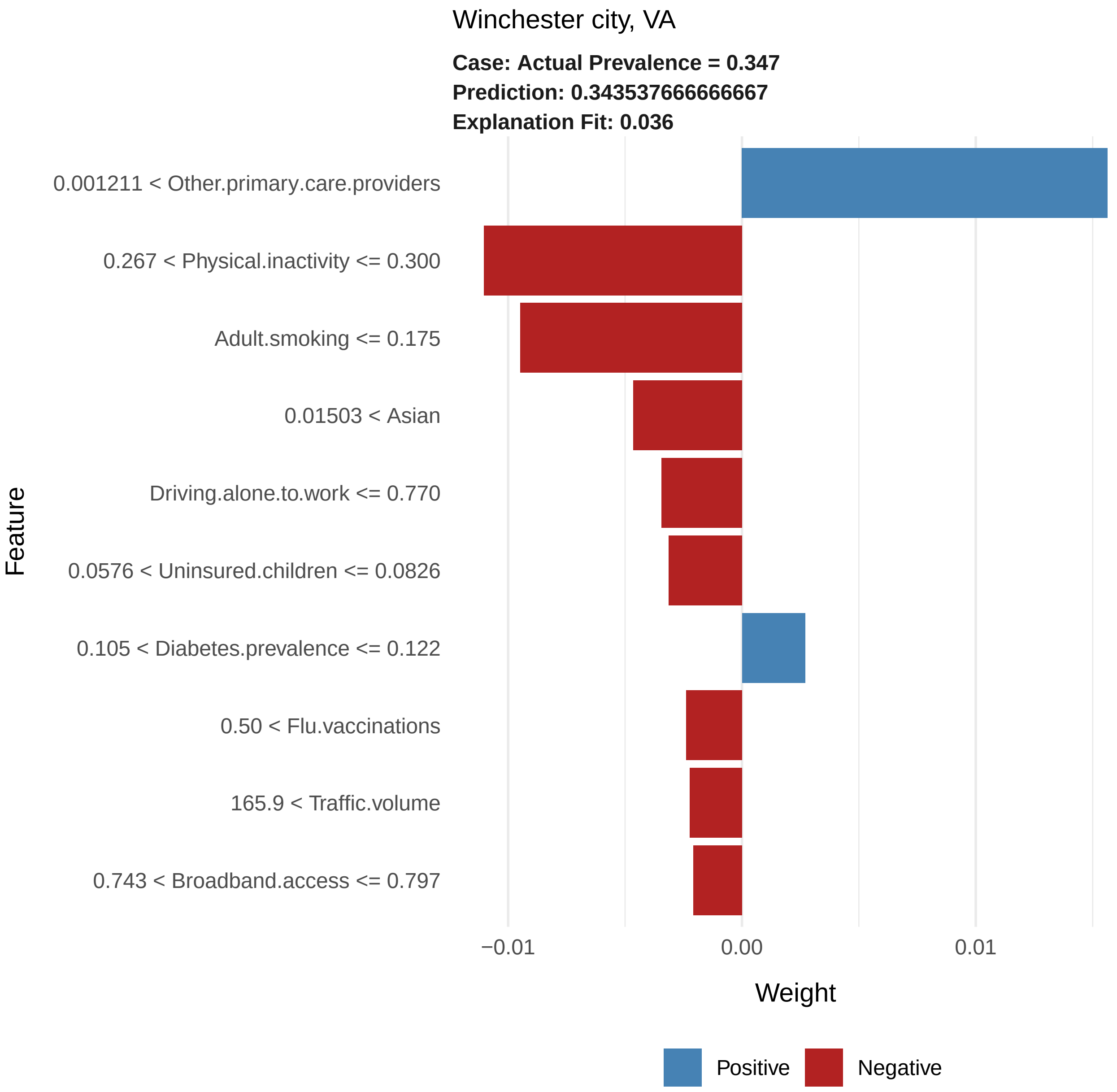

Winchester city, VA
Case: Actual Prevalence = 0.347
Prediction: 0.343537666666667
Explanation Fit: 0.036
0.001211 < Other.primary.care.providers
0.267 < Physical.inactivity <= 0.300
Adult.smoking <= 0.175
0.01503 < Asian
Driving.alone.to.work <= 0.770
0.0576 < Uninsured.children <= 0.0826
0.105 < Diabetes.prevalence <= 0.122
0.50 < Flu.vaccinations
165.9 < Traffic.volume
0.743 < Broadband.access <= 0.797
Feature
−0.01
0.00
0.01
Weight
Positive
Negative

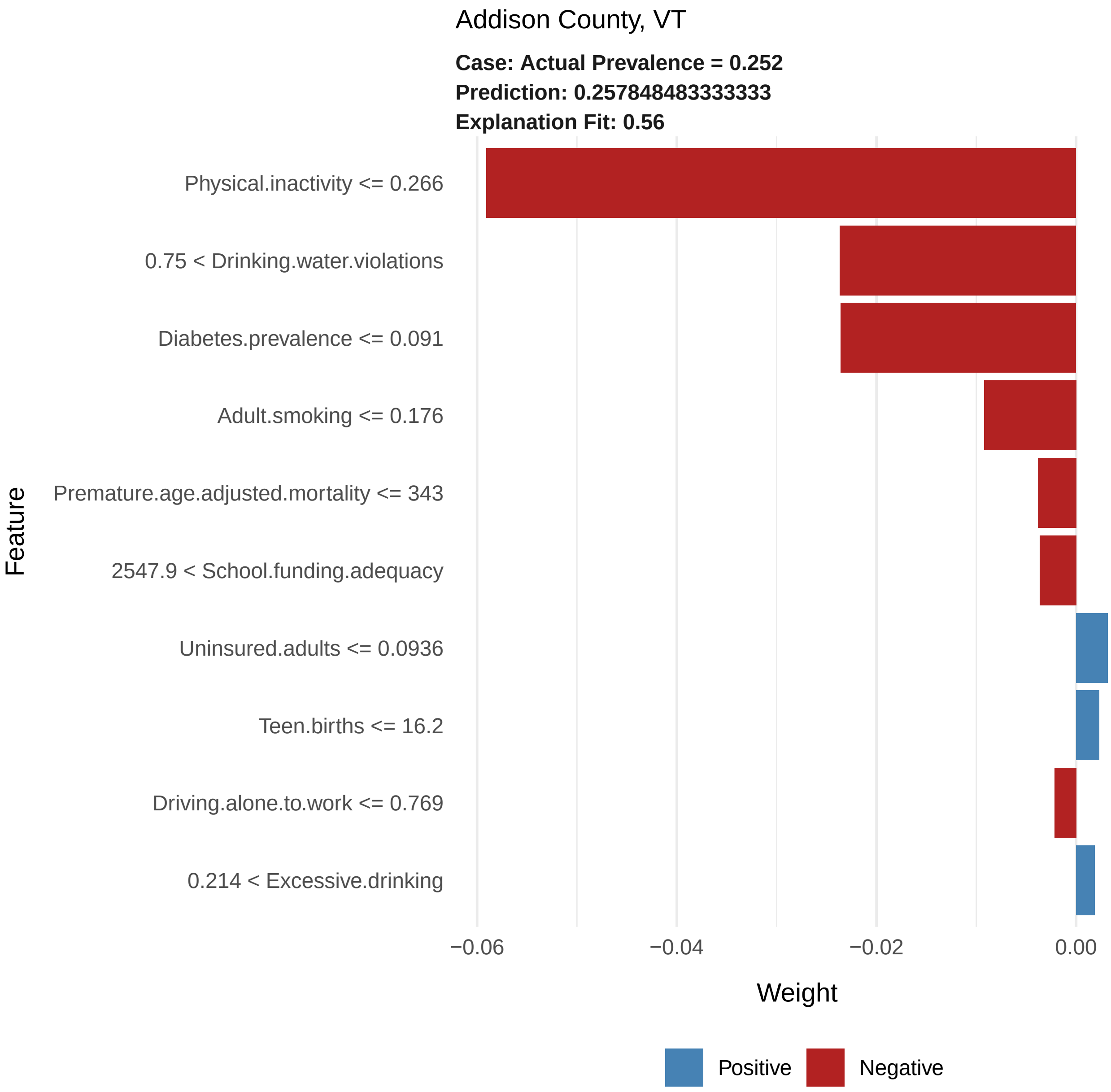
Addison County, VT
Case: Actual Prevalence = 0.252
Prediction: 0.257848483333333
Explanation Fit: 0.56
Physical.inactivity <= 0.266
0.75 < Drinking.water.violations
Diabetes.prevalence <= 0.091
Adult.smoking <= 0.176
Premature.age.adjusted.mortality <= 343
2547.9 < School.funding.adequacy
Uninsured.adults <= 0.0936
Teen.births <= 16.2
Driving.alone.to.work <= 0.769
0.214 < Excessive.drinking
Feature
−0.06
−0.04
−0.02
0.00
Weight
Positive
Negative

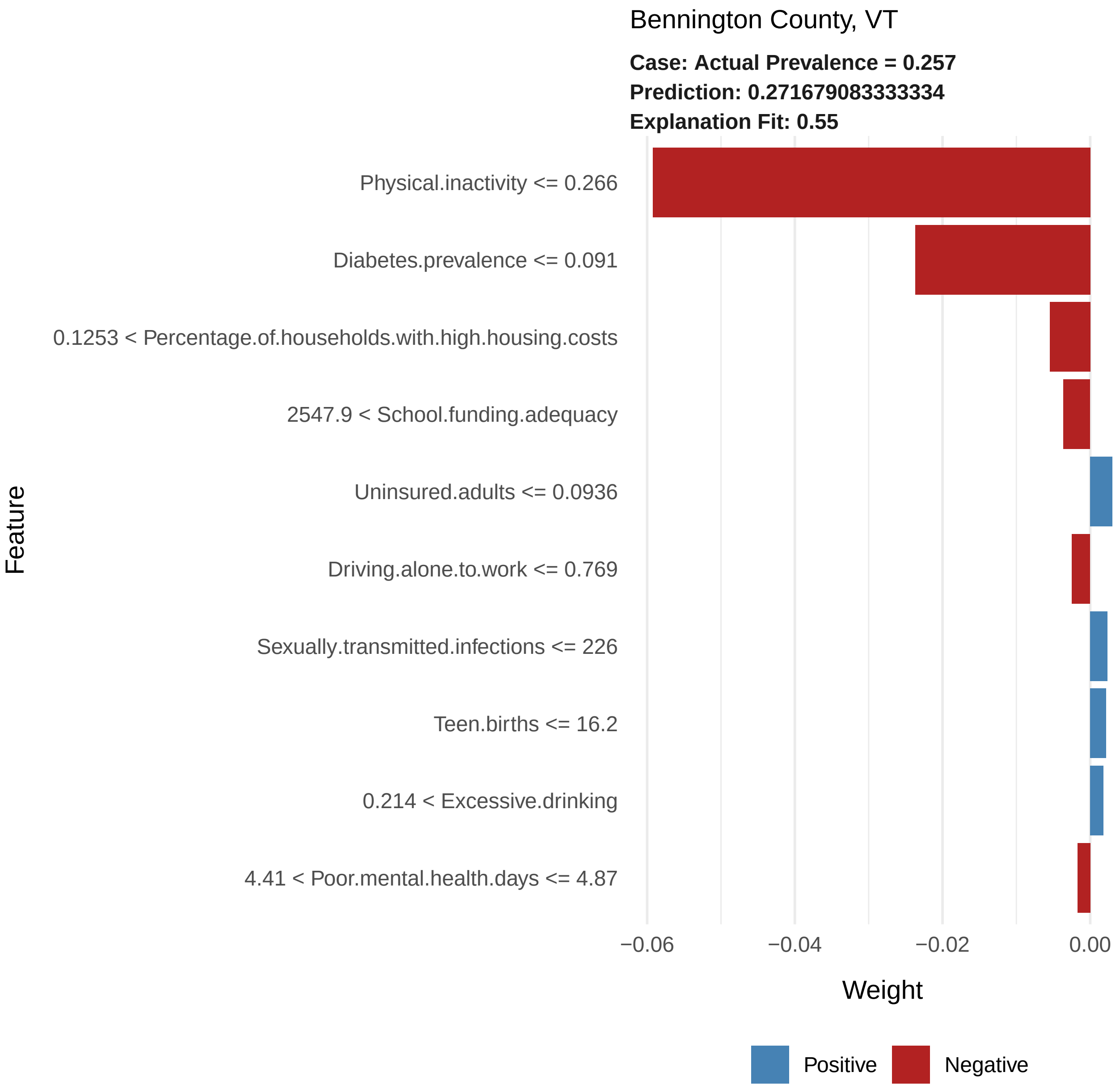

Bennington County, VT
Case: Actual Prevalence = 0.257
Prediction: 0.271679083333334
Explanation Fit: 0.55
Physical.inactivity <= 0.266
Diabetes.prevalence <= 0.091
0.1253 < Percentage.of.households.with.high.housing.costs
2547.9 < School.funding.adequacy
Uninsured.adults <= 0.0936
Driving.alone.to.work <= 0.769
Sexually.transmitted.infections <= 226
Teen.births <= 16.2
0.214 < Excessive.drinking
4.41 < Poor.mental.health.days <= 4.87
Feature
−0.06
−0.04
−0.02
0.00
Weight
Positive
Negative

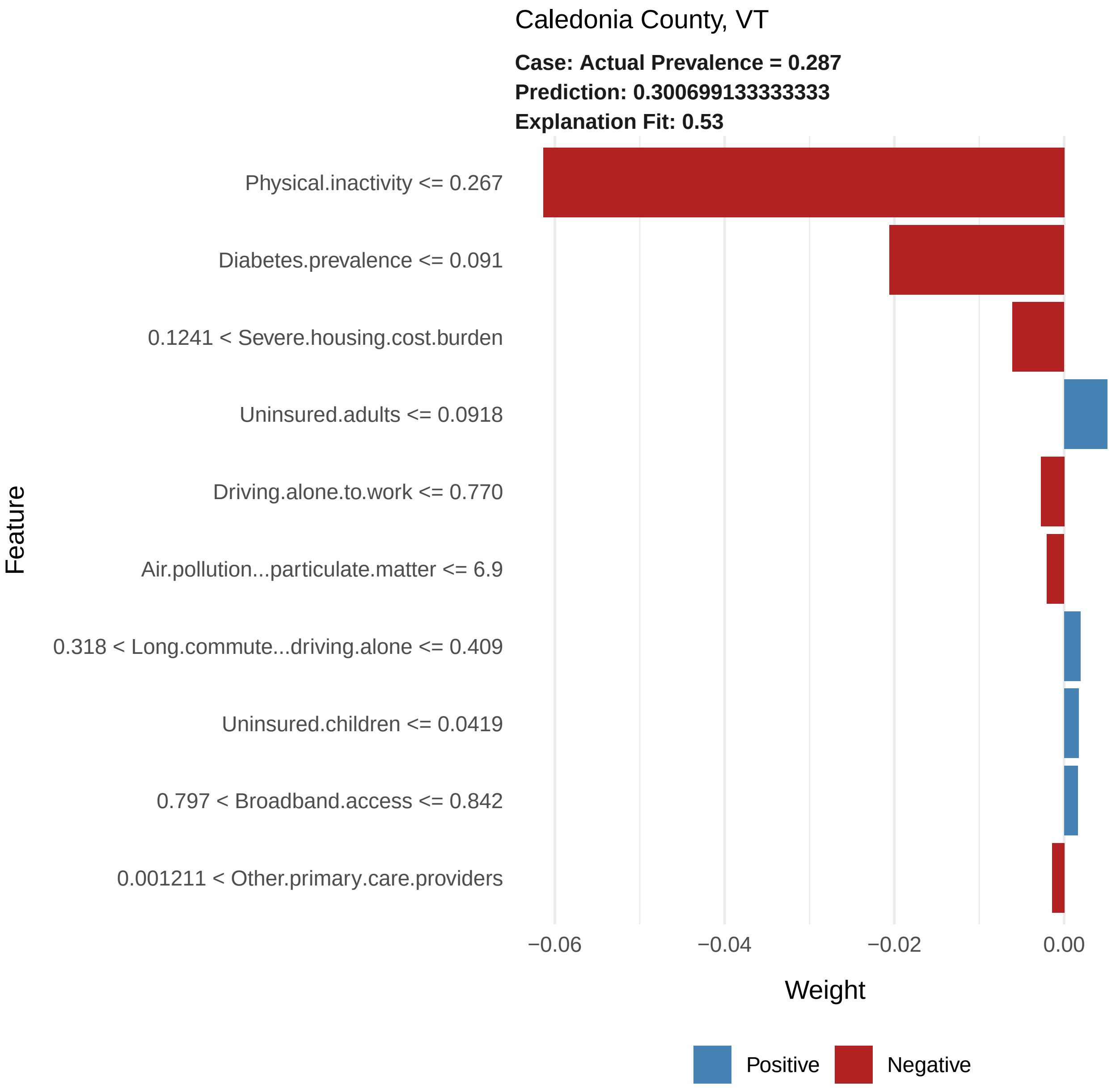
Caledonia County, VT
Case: Actual Prevalence = 0.287
Prediction: 0.300699133333333
Explanation Fit: 0.53
Physical.inactivity <= 0.267
Diabetes.prevalence <= 0.091
0.1241 < Severe.housing.cost.burden
Uninsured.adults <= 0.0918
Driving.alone.to.work <= 0.770
Air.pollution...particulate.matter <= 6.9
0.318 < Long.commute...driving.alone <= 0.409
Uninsured.children <= 0.0419
0.797 < Broadband.access <= 0.842
0.001211 < Other.primary.care.providers
Feature
−0.06
−0.04
−0.02
0.00
Weight
Positive
Negative

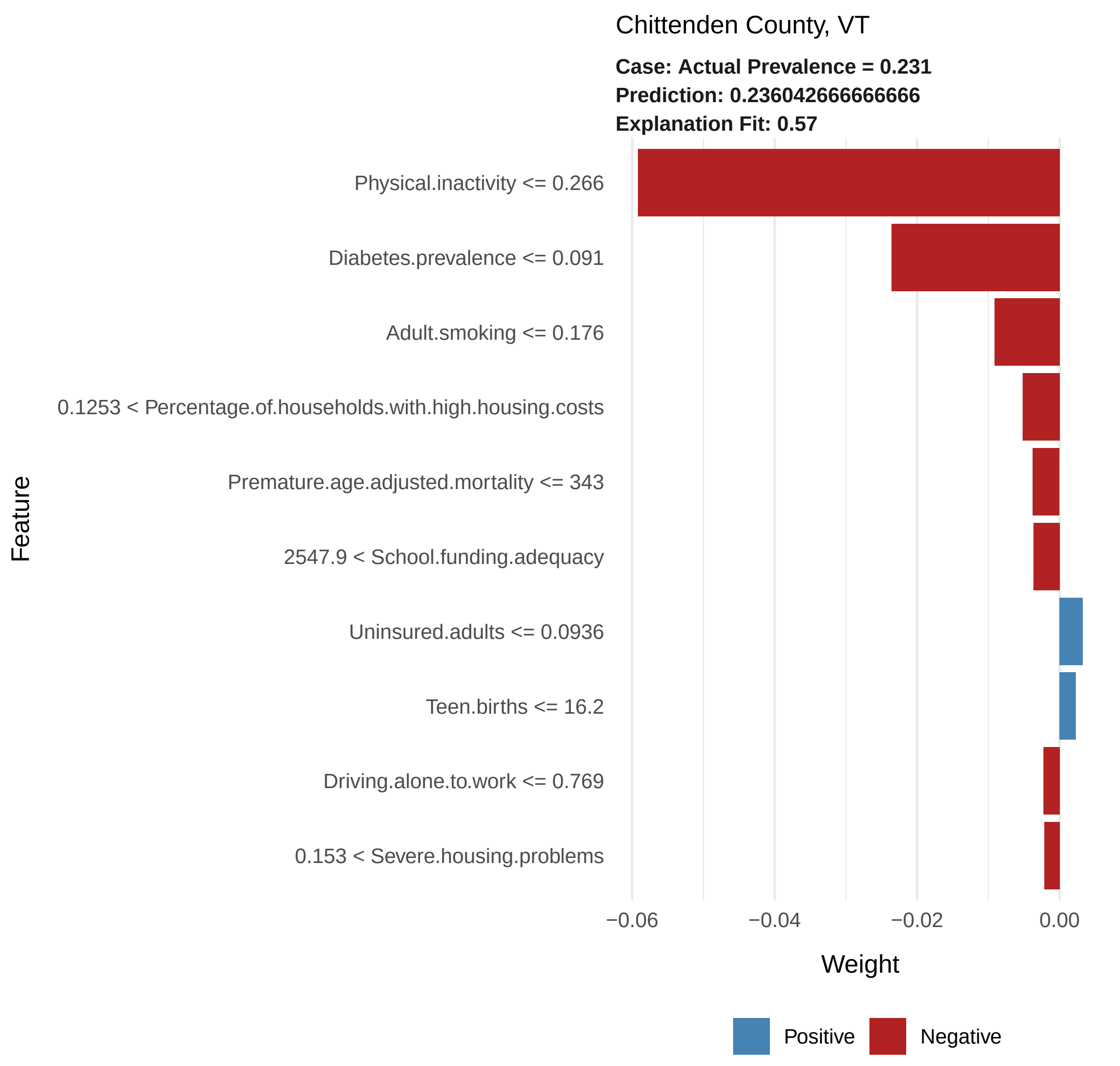

Chittenden County, VT
Case: Actual Prevalence = 0.231
Prediction: 0.236042666666666
Explanation Fit: 0.57
Physical.inactivity <= 0.266
Diabetes.prevalence <= 0.091
Adult.smoking <= 0.176
0.1253 < Percentage.of.households.with.high.housing.costs
Premature.age.adjusted.mortality <= 343
2547.9 < School.funding.adequacy
Uninsured.adults <= 0.0936
Teen.births <= 16.2
Driving.alone.to.work <= 0.769
0.153 < Severe.housing.problems
Feature
−0.06
−0.04
−0.02
0.00
Weight
Positive
Negative

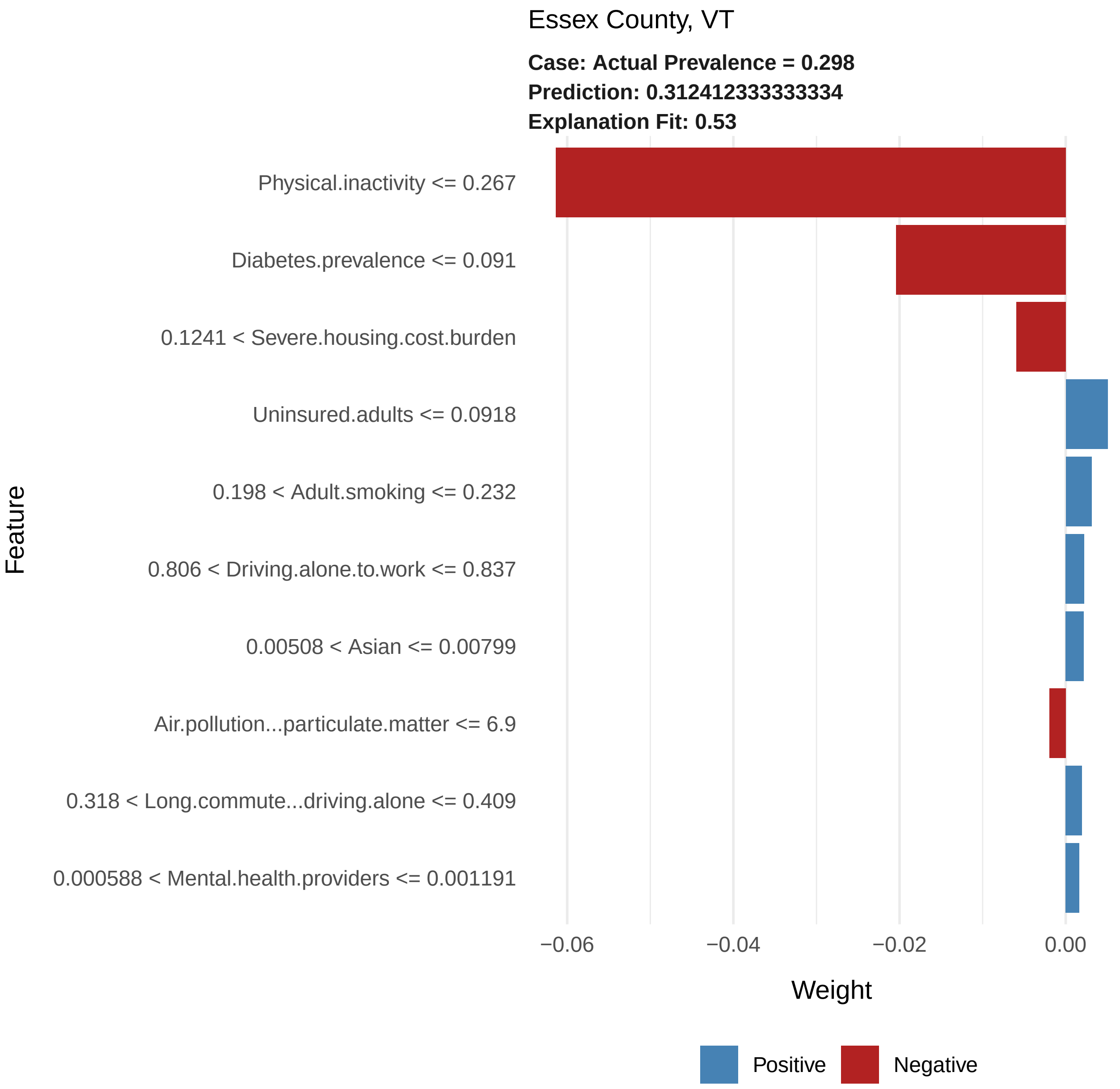
Essex County, VT
Case: Actual Prevalence = 0.298
Prediction: 0.312412333333334
Explanation Fit: 0.53
Physical.inactivity <= 0.267
Diabetes.prevalence <= 0.091
0.1241 < Severe.housing.cost.burden
Uninsured.adults <= 0.0918
0.198 < Adult.smoking <= 0.232
0.806 < Driving.alone.to.work <= 0.837
0.00508 < Asian <= 0.00799
Air.pollution...particulate.matter <= 6.9
0.318 < Long.commute...driving.alone <= 0.409
0.000588 < Mental.health.providers <= 0.001191
Feature
−0.06
−0.04
−0.02
0.00
Weight
Positive
Negative

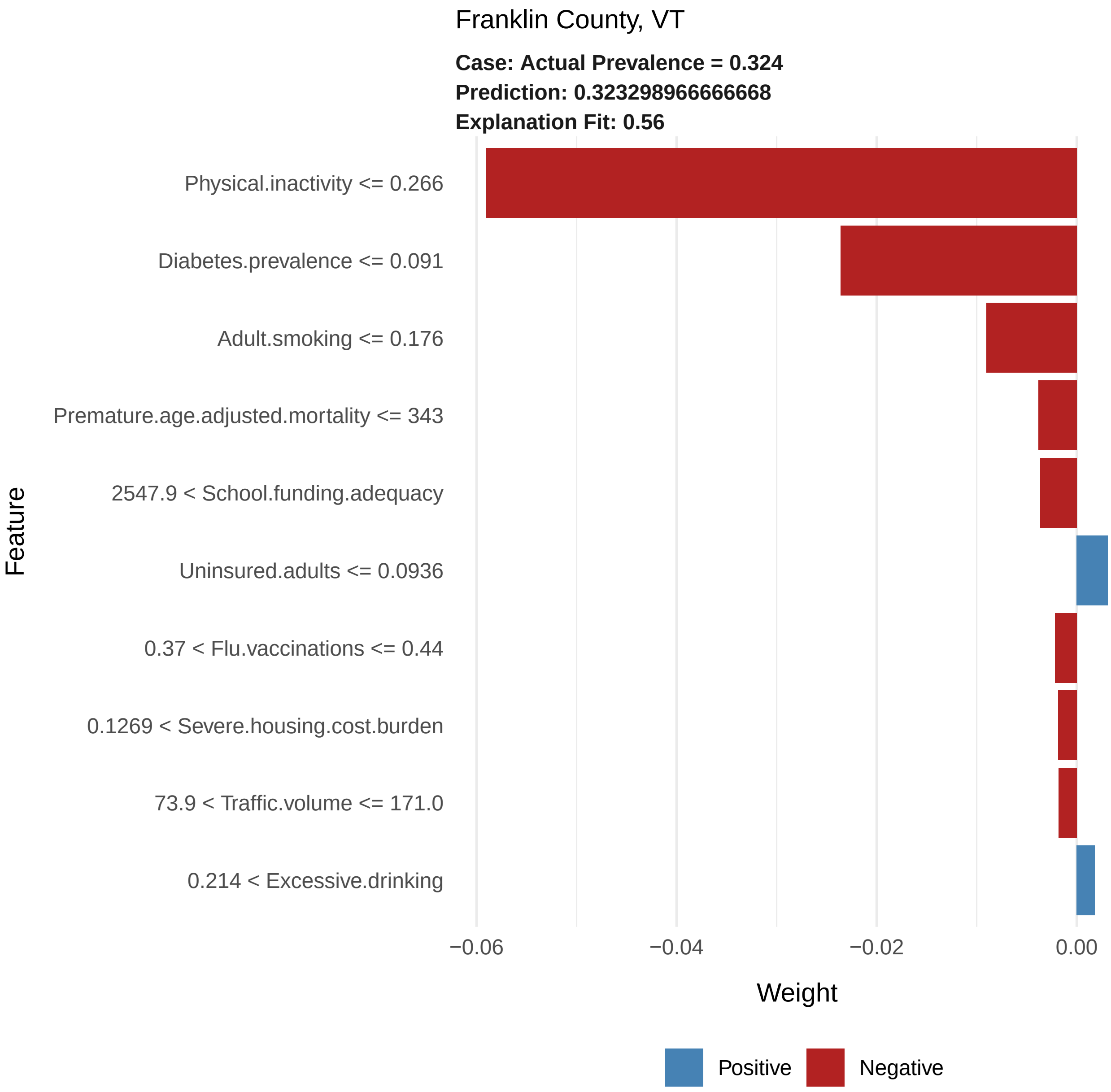
Franklin County, VT
Case: Actual Prevalence = 0.324
Prediction: 0.323298966666668
Explanation Fit: 0.56
Physical.inactivity <= 0.266
Diabetes.prevalence <= 0.091
Adult.smoking <= 0.176
Premature.age.adjusted.mortality <= 343
2547.9 < School.funding.adequacy
Uninsured.adults <= 0.0936
0.37 < Flu.vaccinations <= 0.44
0.1269 < Severe.housing.cost.burden
73.9 < Traffic.volume <= 171.0
0.214 < Excessive.drinking
Feature
−0.06
−0.04
−0.02
0.00
Weight
Positive
Negative

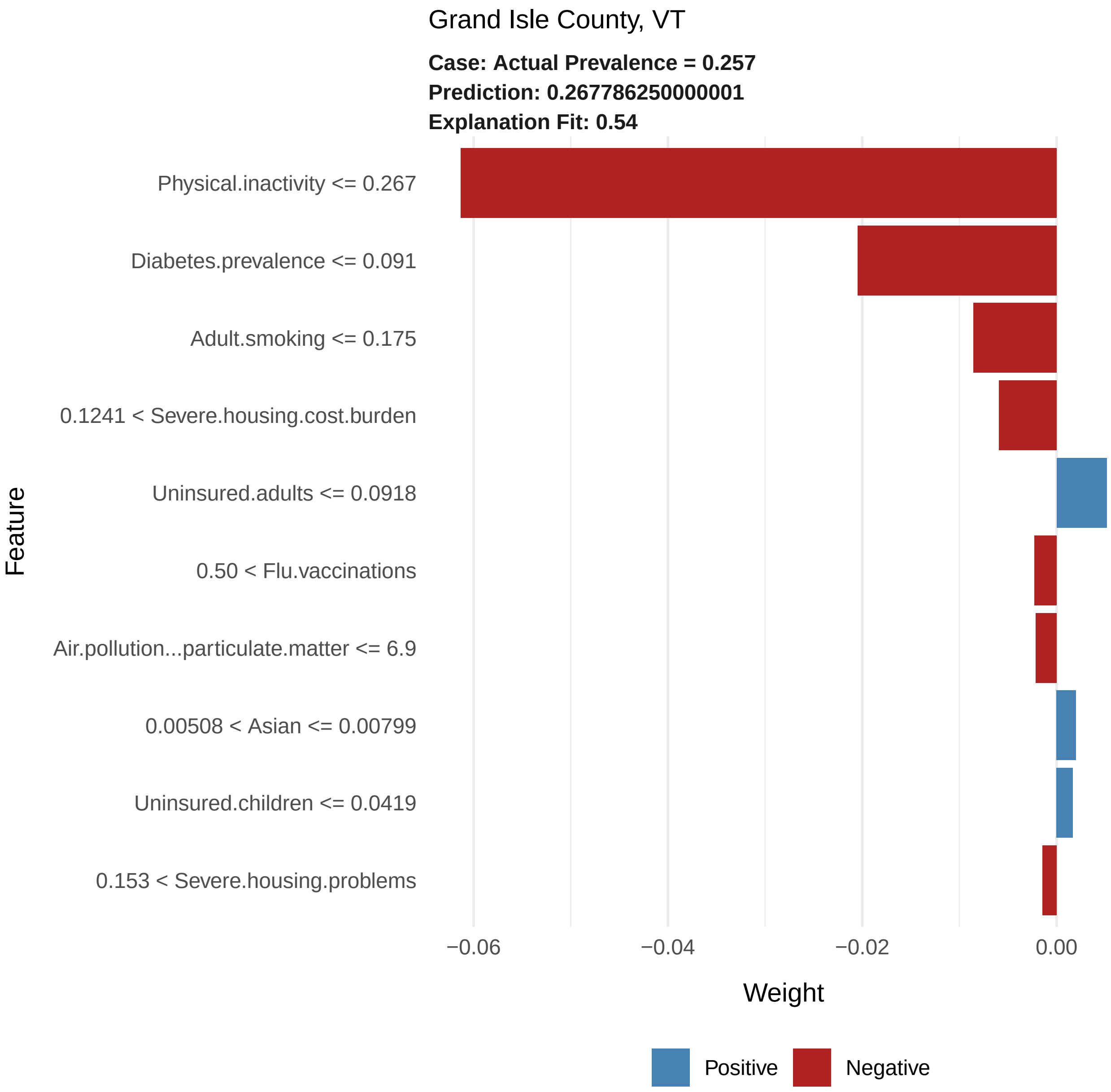

Grand Isle County, VT
Case: Actual Prevalence = 0.257
Prediction: 0.267786250000001
Explanation Fit: 0.54
Physical.inactivity <= 0.267
Diabetes.prevalence <= 0.091
Adult.smoking <= 0.175
0.1241 < Severe.housing.cost.burden
Uninsured.adults <= 0.0918
0.50 < Flu.vaccinations
Air.pollution...particulate.matter <= 6.9
0.00508 < Asian <= 0.00799
Uninsured.children <= 0.0419
0.153 < Severe.housing.problems
Feature
−0.06
−0.04
−0.02
0.00
Weight
Positive
Negative

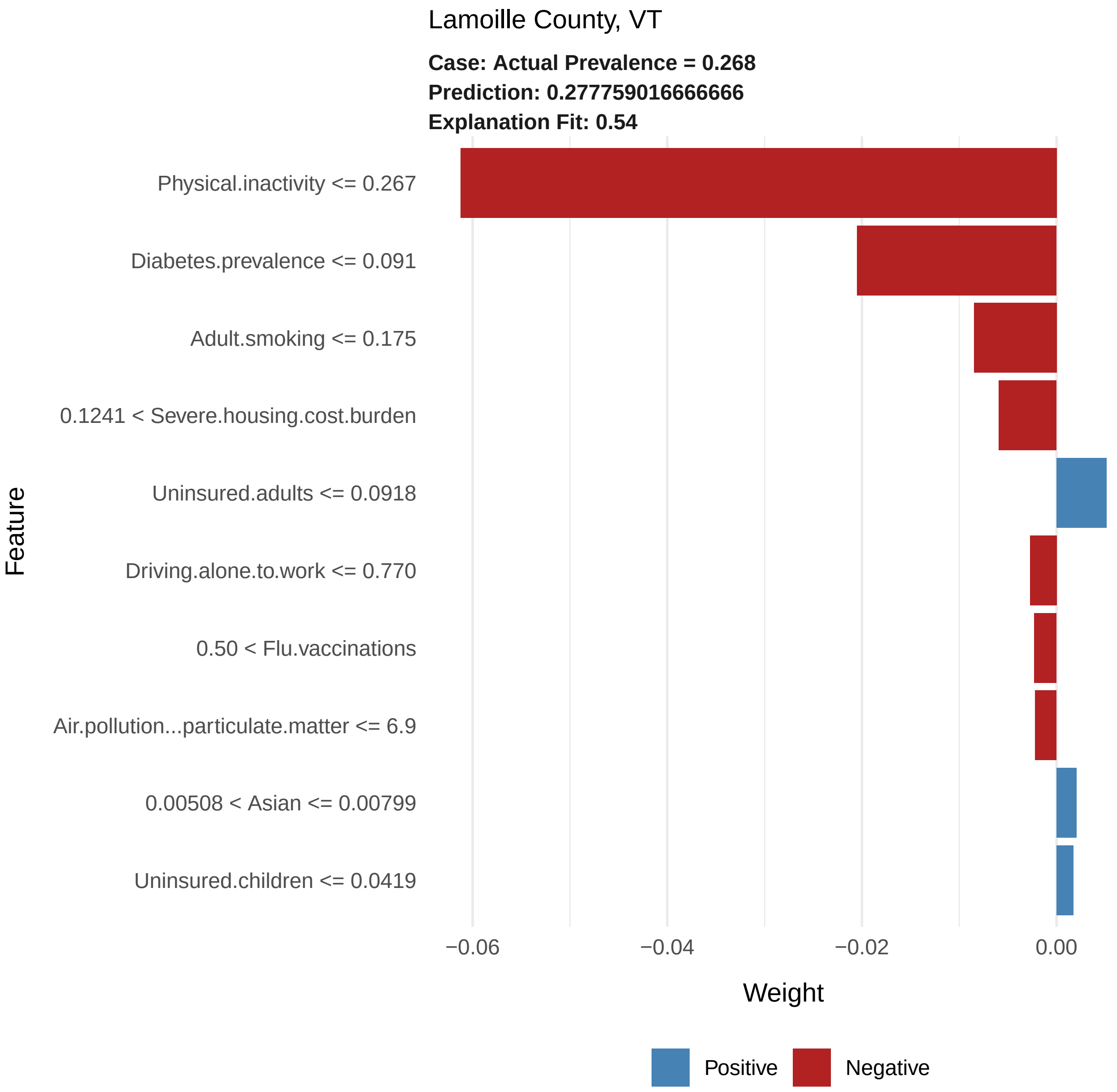

Lamoille County, VT
Case: Actual Prevalence = 0.268
Prediction: 0.277759016666666
Explanation Fit: 0.54
Feature
Physical.inactivity <= 0.267
Diabetes.prevalence <= 0.091
Adult.smoking <= 0.175
0.1241 < Severe.housing.cost.burden
Uninsured.adults <= 0.0918
Driving.alone.to.work <= 0.770
0.50 < Flu.vaccinations
Air.pollution...particulate.matter <= 6.9
0.00508 < Asian <= 0.00799
Uninsured.children <= 0.0419
−0.06
−0.04
−0.02
0.00
Weight
Positive
Negative

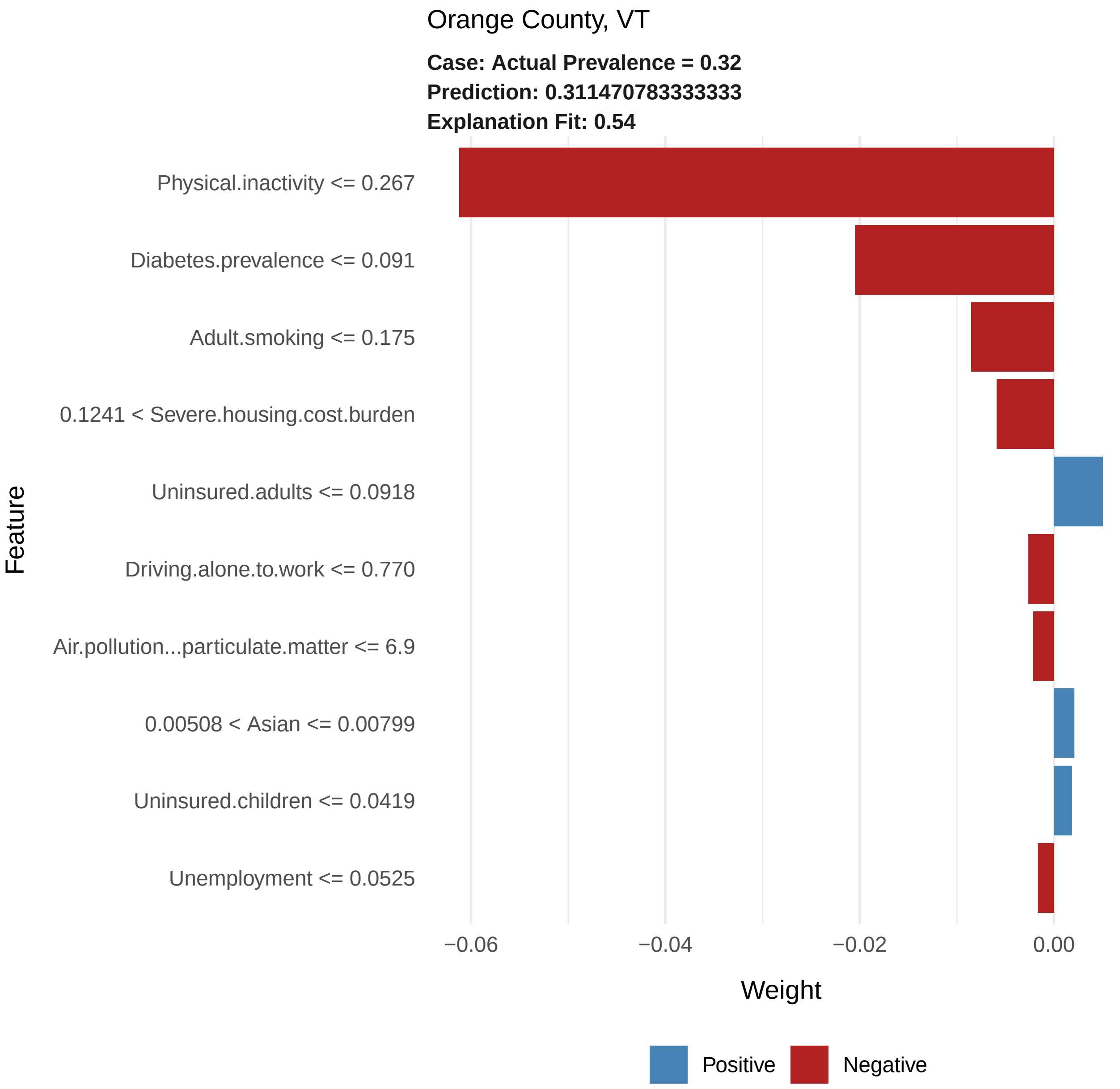
Orange County, VT
Case: Actual Prevalence = 0.32
Prediction: 0.311470783333333
Explanation Fit: 0.54
Feature
Physical.inactivity <= 0.267
Diabetes.prevalence <= 0.091
Adult.smoking <= 0.175
0.1241 < Severe.housing.cost.burden
Uninsured.adults <= 0.0918
Driving.alone.to.work <= 0.770
Air.pollution...particulate.matter <= 6.9
0.00508 < Asian <= 0.00799
Uninsured.children <= 0.0419
Unemployment <= 0.0525
−0.06
−0.04
−0.02
0.00
Weight
Positive
Negative

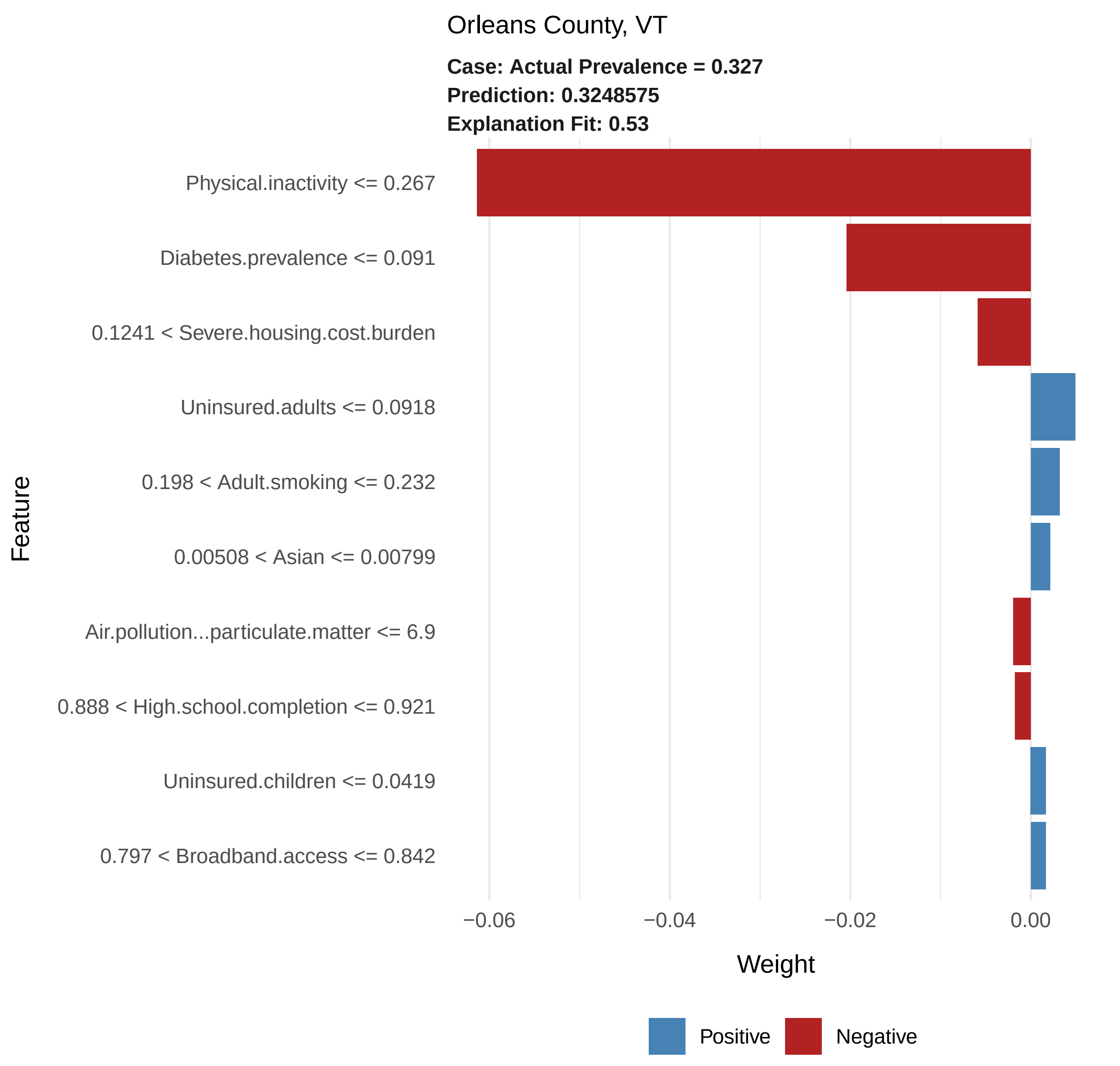
Orleans County, VT
Case: Actual Prevalence = 0.327
Prediction: 0.3248575
Explanation Fit: 0.53
Physical.inactivity <= 0.267
Diabetes.prevalence <= 0.091
0.1241 < Severe.housing.cost.burden
Uninsured.adults <= 0.0918
0.198 < Adult.smoking <= 0.232
0.00508 < Asian <= 0.00799
Air.pollution...particulate.matter <= 6.9
0.888 < High.school.completion <= 0.921
Uninsured.children <= 0.0419
0.797 < Broadband.access <= 0.842
Feature
−0.06
−0.04
−0.02
0.00
Weight
Positive
Negative

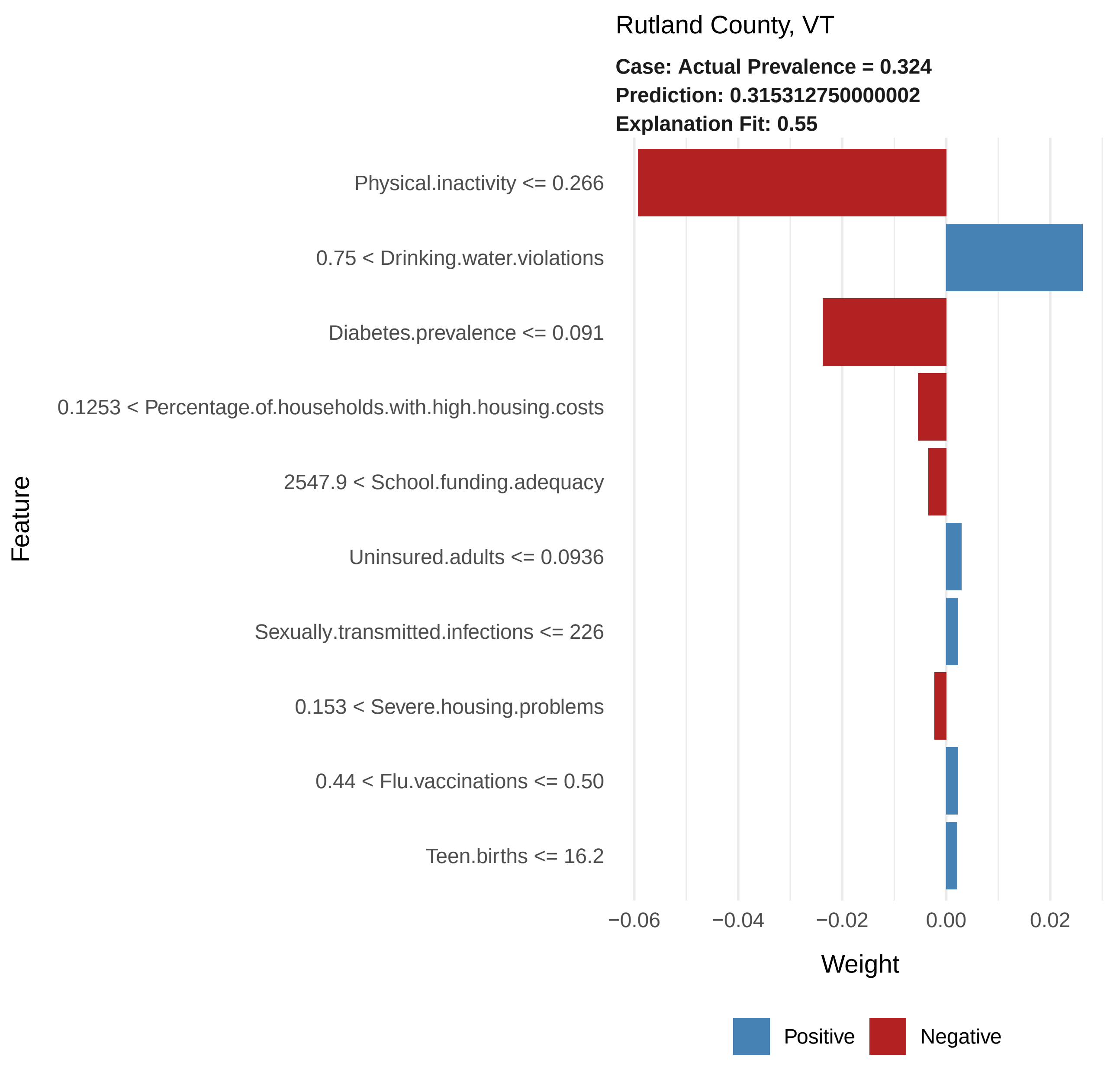
Rutland County, VT
Case: Actual Prevalence = 0.324
Prediction: 0.315312750000002
Explanation Fit: 0.55
Physical.inactivity <= 0.266
0.75 < Drinking.water.violations
Diabetes.prevalence <= 0.091
0.1253 < Percentage.of.households.with.high.housing.costs
2547.9 < School.funding.adequacy
Uninsured.adults <= 0.0936
Sexually.transmitted.infections <= 226
0.153 < Severe.housing.problems
0.44 < Flu.vaccinations <= 0.50
Teen.births <= 16.2
Feature
−0.06
−0.04
−0.02
0.00
0.02
Weight
Positive
Negative

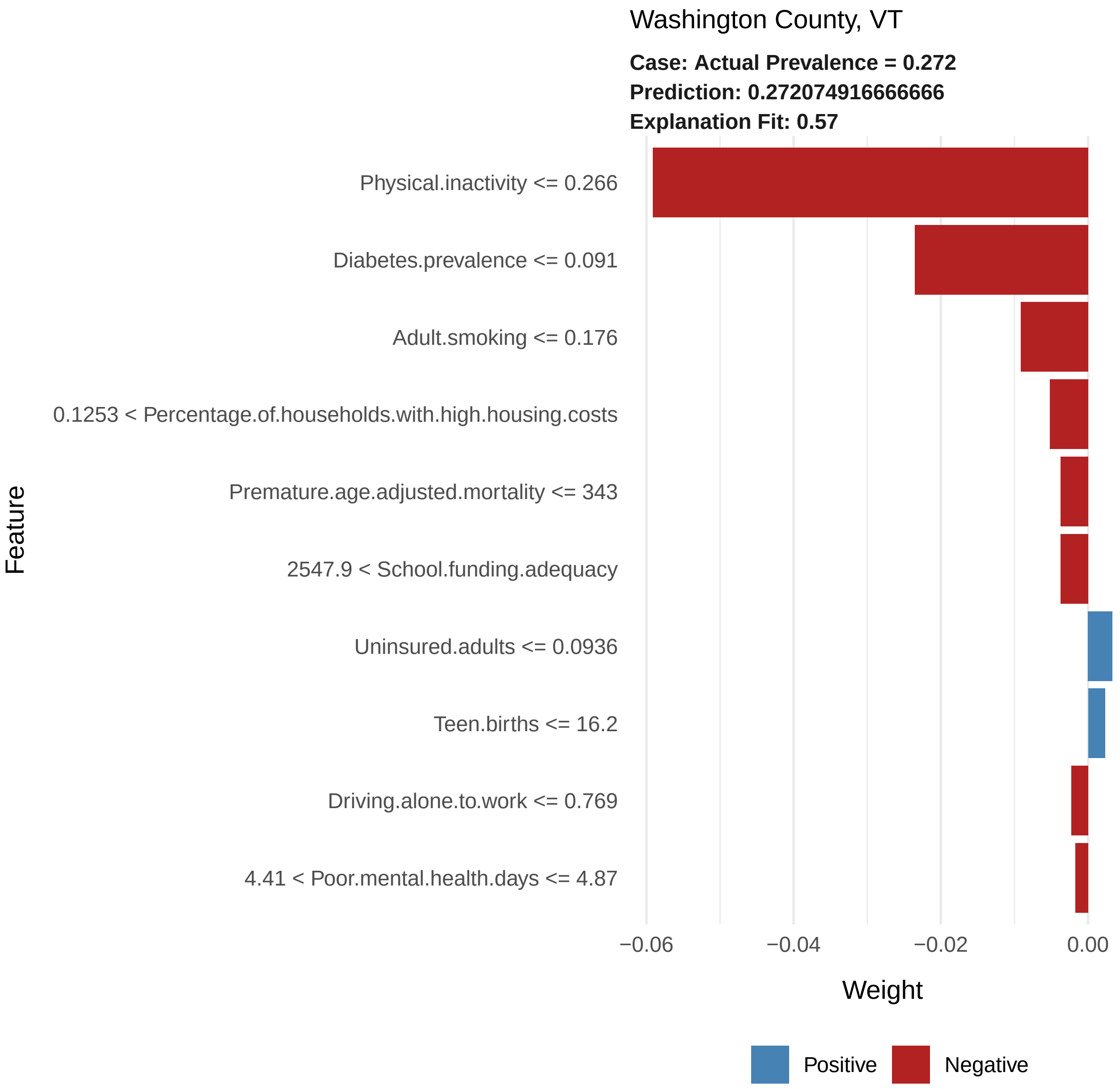
Washington County, VT
Case: Actual Prevalence = 0.272
Prediction: 0.272074916666666
Explanation Fit: 0.57
Physical.inactivity <= 0.266
Diabetes.prevalence <= 0.091
Adult.smoking <= 0.176
0.1253 < Percentage.of.households.with.high.housing.costs
Premature.age.adjusted.mortality <= 343
2547.9 < School.funding.adequacy
Uninsured.adults <= 0.0936
Teen.births <= 16.2
Driving.alone.to.work <= 0.769
4.41 < Poor.mental.health.days <= 4.87
Feature
−0.06
−0.04
−0.02
0.00
Weight
Positive
Negative

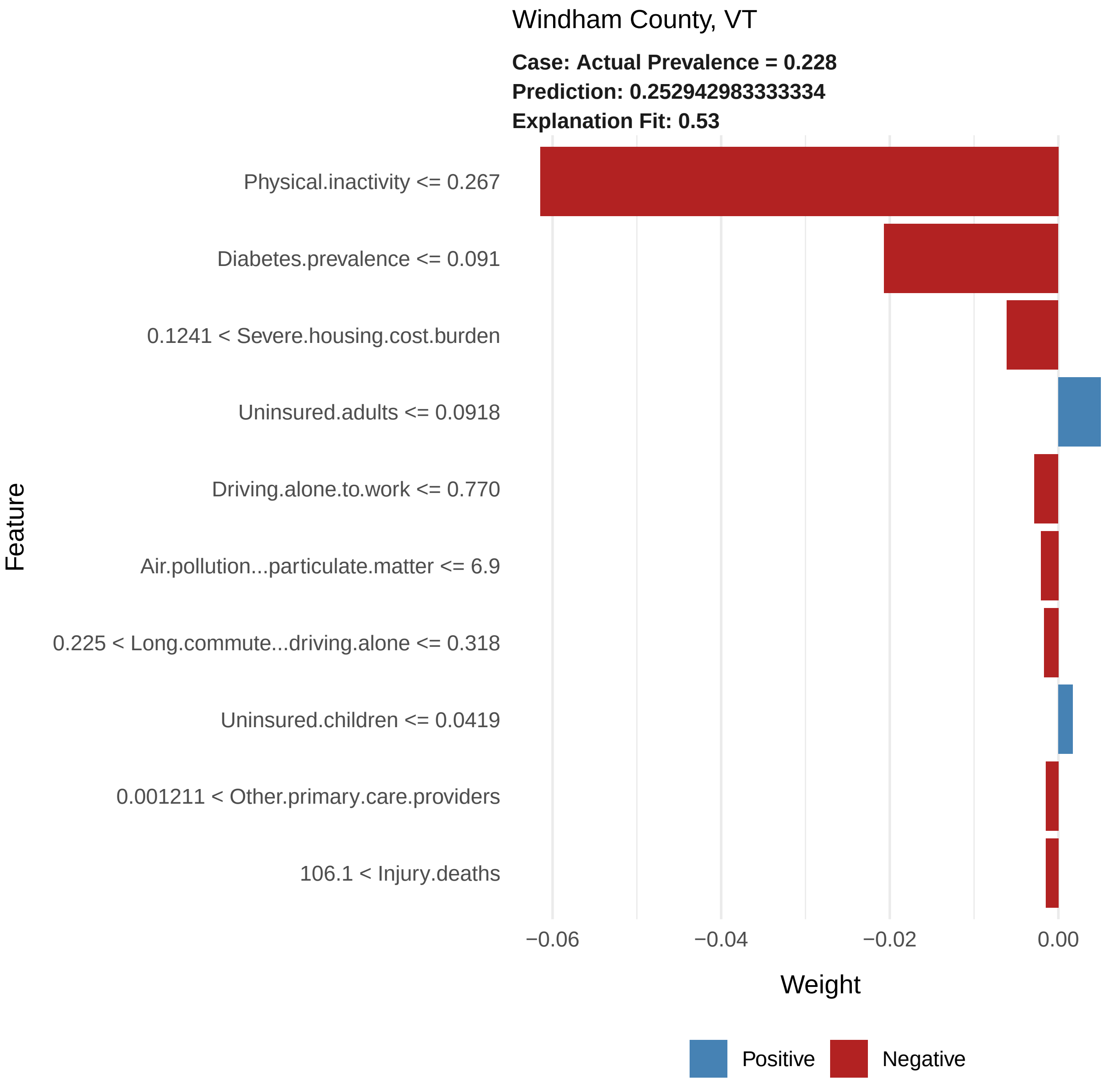
Windham County, VT
Case: Actual Prevalence = 0.228
Prediction: 0.252942983333334
Explanation Fit: 0.53
Physical.inactivity <= 0.267
Diabetes.prevalence <= 0.091
0.1241 < Severe.housing.cost.burden
Uninsured.adults <= 0.0918
Driving.alone.to.work <= 0.770
Air.pollution...particulate.matter <= 6.9
0.225 < Long.commute...driving.alone <= 0.318
Uninsured.children <= 0.0419
0.001211 < Other.primary.care.providers
106.1 < Injury.deaths
Feature
−0.06
−0.04
−0.02
0.00
Weight
Positive
Negative

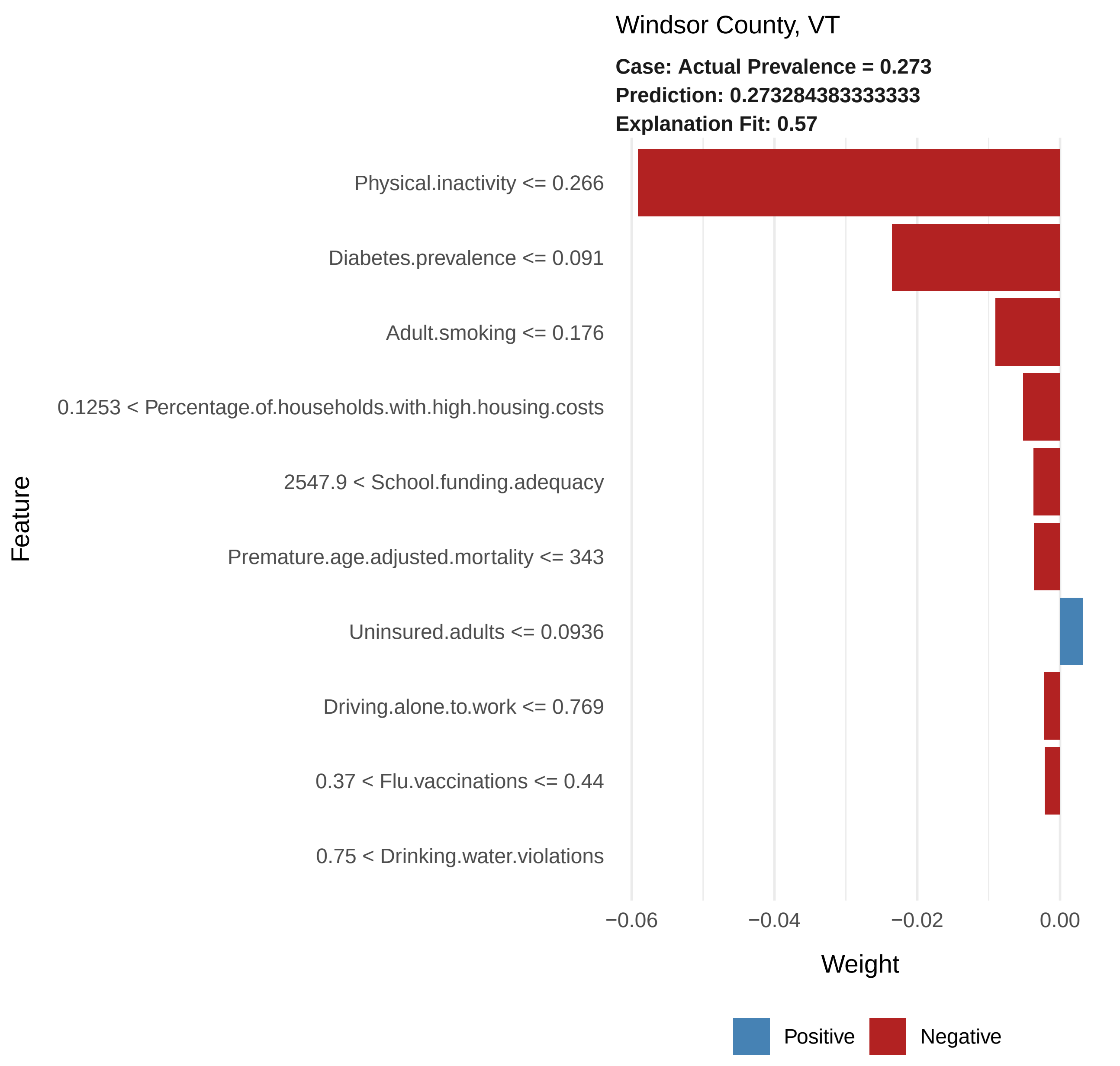

Windsor County, VT
Case: Actual Prevalence = 0.273
Prediction: 0.273284383333333
Explanation Fit: 0.57
Feature
Physical.inactivity <= 0.266
Diabetes.prevalence <= 0.091
Adult.smoking <= 0.176
0.1253 < Percentage.of.households.with.high.housing.costs
2547.9 < School.funding.adequacy
Premature.age.adjusted.mortality <= 343
Uninsured.adults <= 0.0936
Driving.alone.to.work <= 0.769
0.37 < Flu.vaccinations <= 0.44
0.75 < Drinking.water.violations
−0.06
−0.04
−0.02
0.00
Weight
Positive
Negative

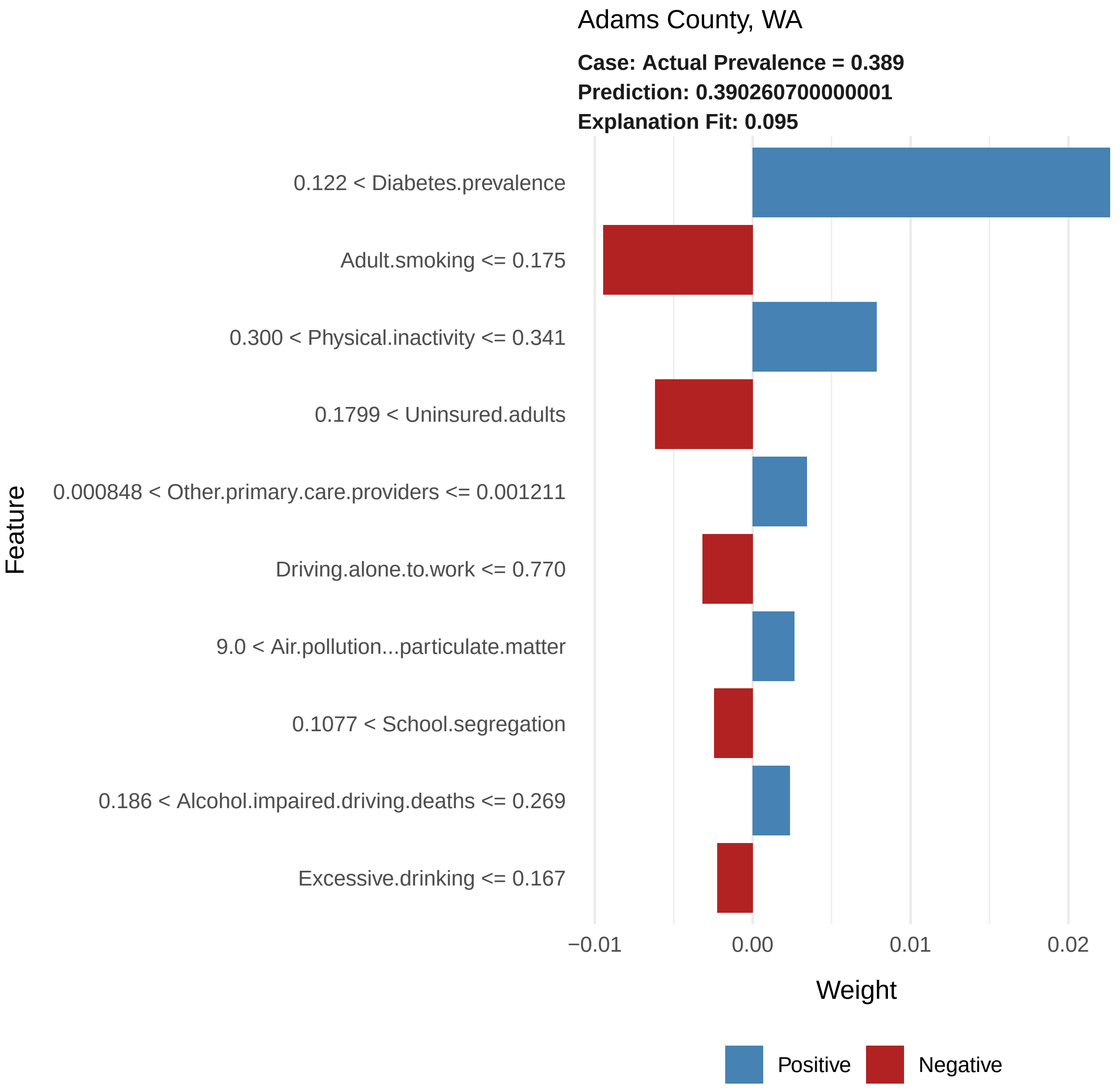

Adams County, WA
Case: Actual Prevalence = 0.389
Prediction: 0.390260700000001
Explanation Fit: 0.095
0.122 < Diabetes.prevalence
Adult.smoking <= 0.175
0.300 < Physical.inactivity <= 0.341
0.1799 < Uninsured.adults
0.000848 < Other.primary.care.providers <= 0.001211
Driving.alone.to.work <= 0.770
9.0 < Air.pollution...particulate.matter
0.1077 < School.segregation
0.186 < Alcohol.impaired.driving.deaths <= 0.269
Excessive.drinking <= 0.167
Feature
−0.01
0.00
0.01
0.02
Weight
Positive
Negative

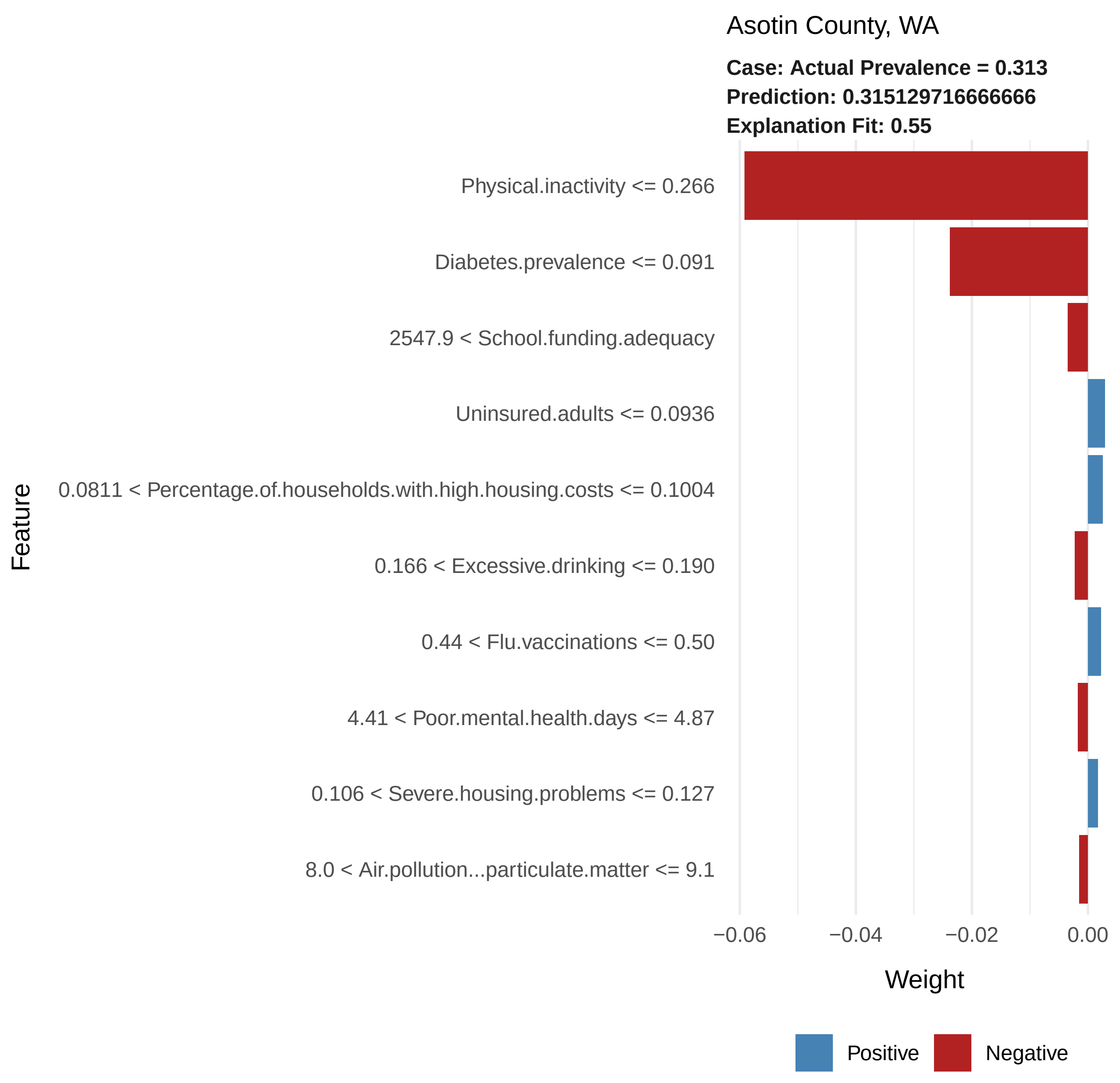

Asotin County, WA
Case: Actual Prevalence = 0.313
Prediction: 0.315129716666666
Explanation Fit: 0.55
Physical.inactivity <= 0.266
Diabetes.prevalence <= 0.091
2547.9 < School.funding.adequacy
Uninsured.adults <= 0.0936
0.0811 < Percentage.of.households.with.high.housing.costs <= 0.1004
0.166 < Excessive.drinking <= 0.190
0.44 < Flu.vaccinations <= 0.50
4.41 < Poor.mental.health.days <= 4.87
0.106 < Severe.housing.problems <= 0.127
8.0 < Air.pollution...particulate.matter <= 9.1
Feature
−0.06
−0.04
−0.02
0.00
Weight
Positive
Negative

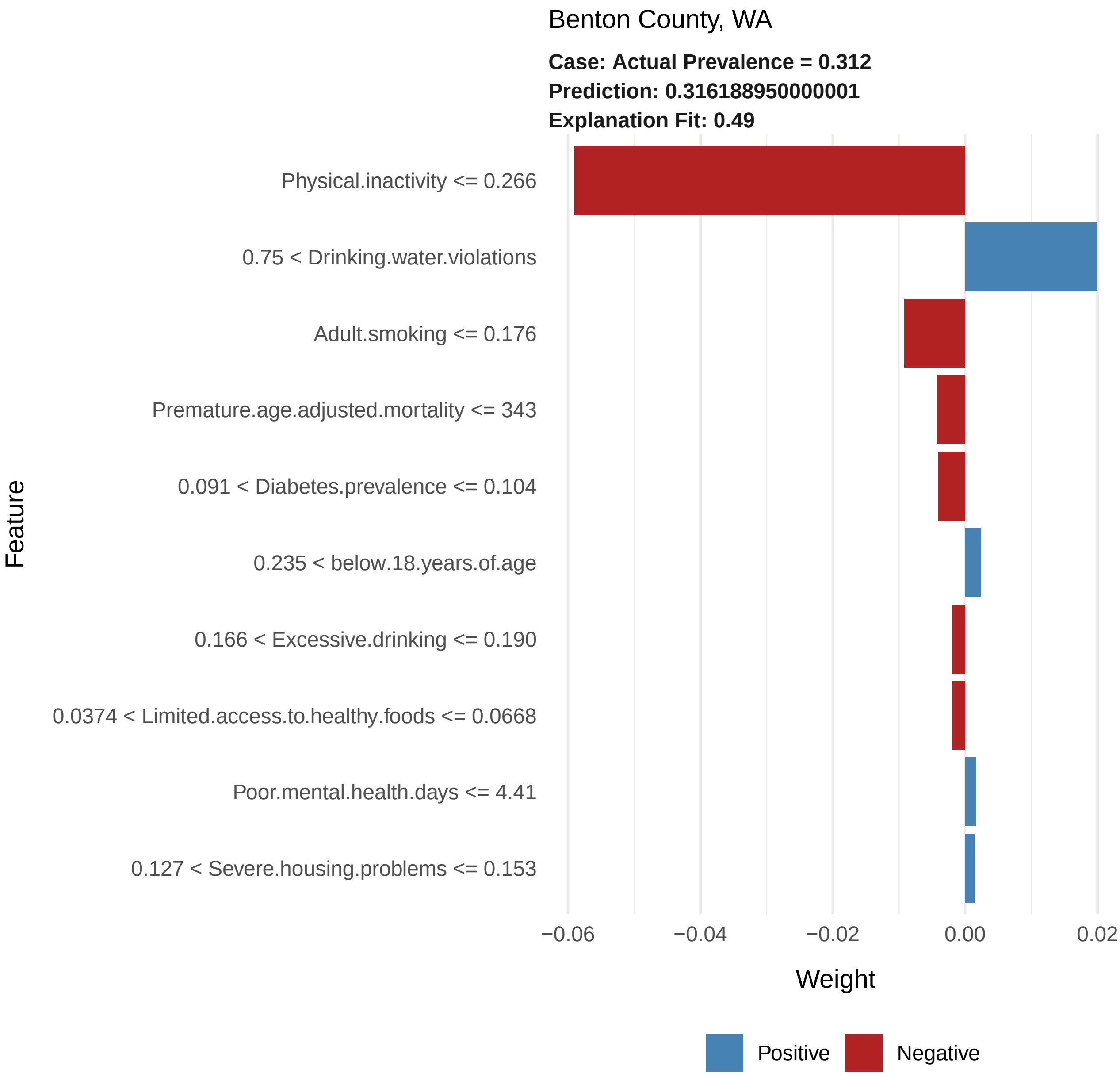
Benton County, WA
Case: Actual Prevalence = 0.312
Prediction: 0.316188950000001
Explanation Fit: 0.49
Physical.inactivity <= 0.266
0.75 < Drinking.water.violations
Adult.smoking <= 0.176
Premature.age.adjusted.mortality <= 343
0.091 < Diabetes.prevalence <= 0.104
0.235 < below.18.years.of.age
0.166 < Excessive.drinking <= 0.190
0.0374 < Limited.access.to.healthy.foods <= 0.0668
Poor.mental.health.days <= 4.41
0.127 < Severe.housing.problems <= 0.153
Feature
−0.06
−0.04
−0.02
0.00
0.02
Weight
Positive
Negative

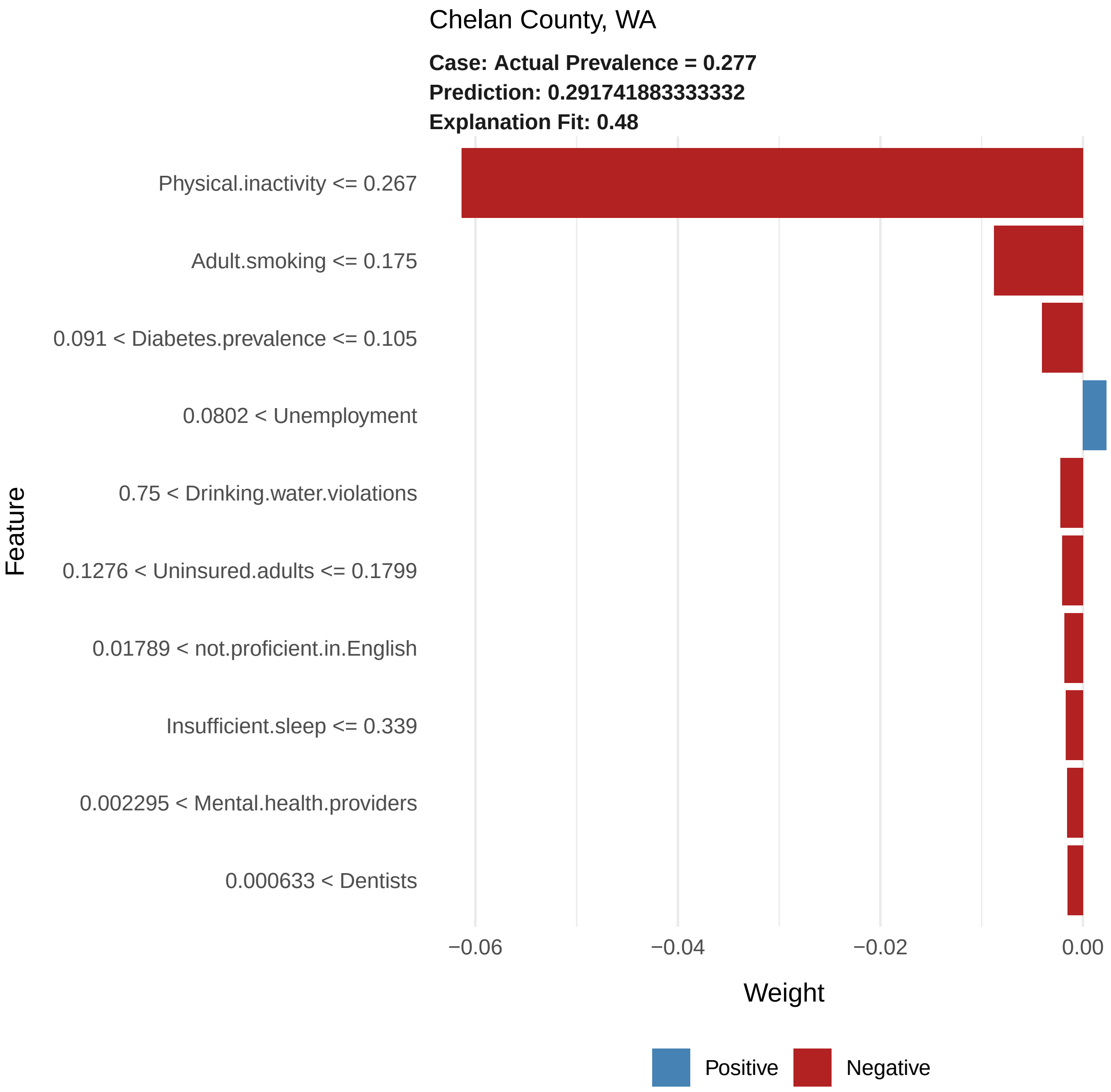
Chelan County, WA
Case: Actual Prevalence = 0.277
Prediction: 0.291741883333332
Explanation Fit: 0.48
Physical.inactivity <= 0.267
Adult.smoking <= 0.175
0.091 < Diabetes.prevalence <= 0.105
0.0802 < Unemployment
0.75 < Drinking.water.violations
0.1276 < Uninsured.adults <= 0.1799
0.01789 < not.proficient.in.English
Insufficient.sleep <= 0.339
0.002295 < Mental.health.providers
0.000633 < Dentists
Feature
−0.06
−0.04
−0.02
0.00
Weight
Positive
Negative

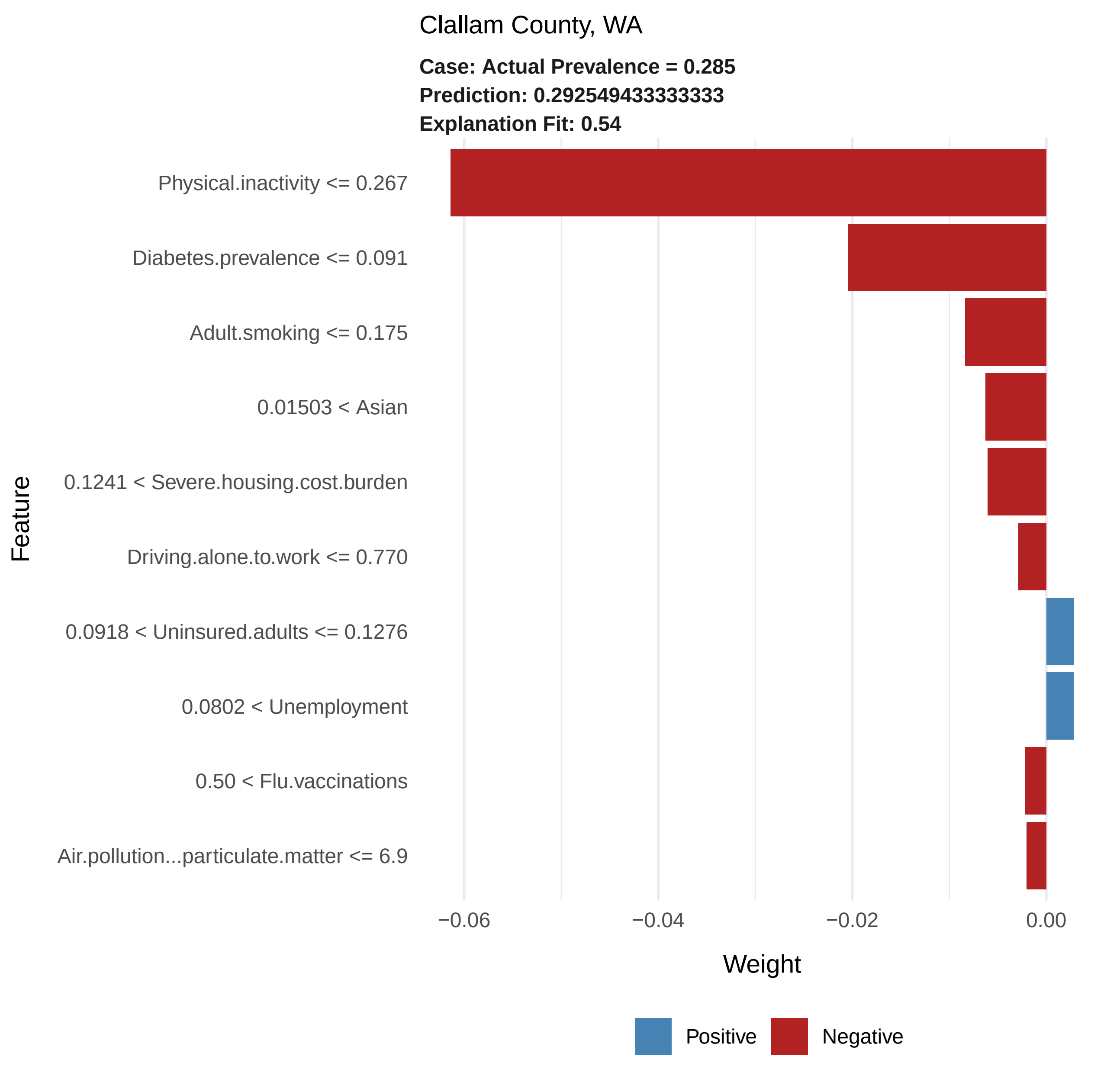
Clallam County, WA
Case: Actual Prevalence = 0.285
Prediction: 0.292549433333333
Explanation Fit: 0.54
Physical.inactivity <= 0.267
Diabetes.prevalence <= 0.091
Adult.smoking <= 0.175
0.01503 < Asian
0.1241 < Severe.housing.cost.burden
Driving.alone.to.work <= 0.770
0.0918 < Uninsured.adults <= 0.1276
0.0802 < Unemployment
0.50 < Flu.vaccinations
Air.pollution...particulate.matter <= 6.9
Feature
−0.06
−0.04
−0.02
0.00
Weight
Positive
Negative

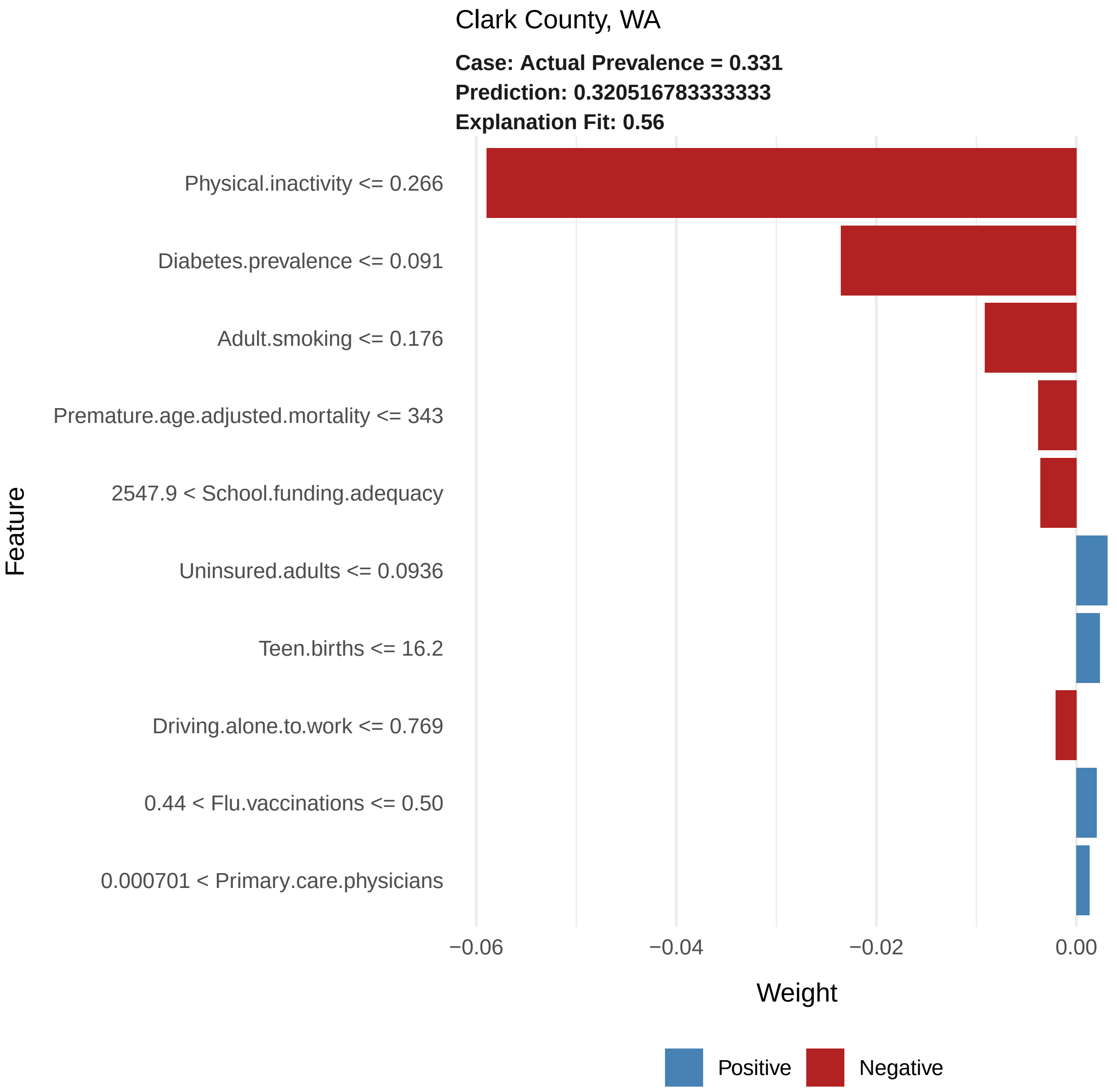
Clark County, WA
Case: Actual Prevalence = 0.331
Prediction: 0.320516783333333
Explanation Fit: 0.56
Physical.inactivity <= 0.266
Diabetes.prevalence <= 0.091
Adult.smoking <= 0.176
Premature.age.adjusted.mortality <= 343
2547.9 < School.funding.adequacy
Uninsured.adults <= 0.0936
Teen.births <= 16.2
Driving.alone.to.work <= 0.769
0.44 < Flu.vaccinations <= 0.50
0.000701 < Primary.care.physicians
Feature
−0.06
−0.04
−0.02
0.00
Weight
Positive
Negative

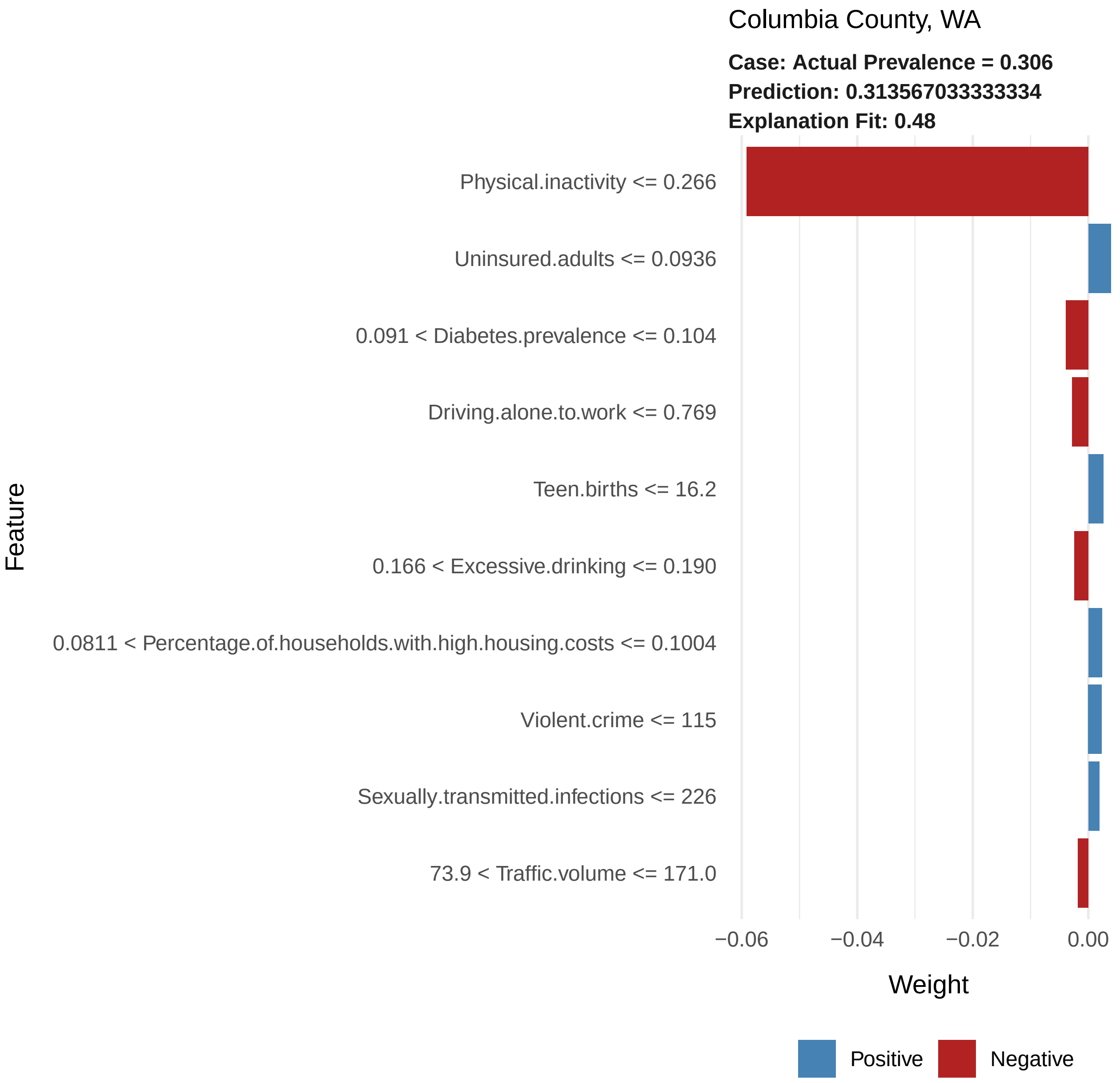
Columbia County, WA
Case: Actual Prevalence = 0.306
Prediction: 0.313567033333334
Explanation Fit: 0.48
Physical.inactivity <= 0.266
Uninsured.adults <= 0.0936
0.091 < Diabetes.prevalence <= 0.104
Driving.alone.to.work <= 0.769
Teen.births <= 16.2
0.166 < Excessive.drinking <= 0.190
0.0811 < Percentage.of.households.with.high.housing.costs <= 0.1004
Violent.crime <= 115
Sexually.transmitted.infections <= 226
73.9 < Traffic.volume <= 171.0
Feature
−0.06
−0.04
−0.02
0.00
Weight
Positive
Negative

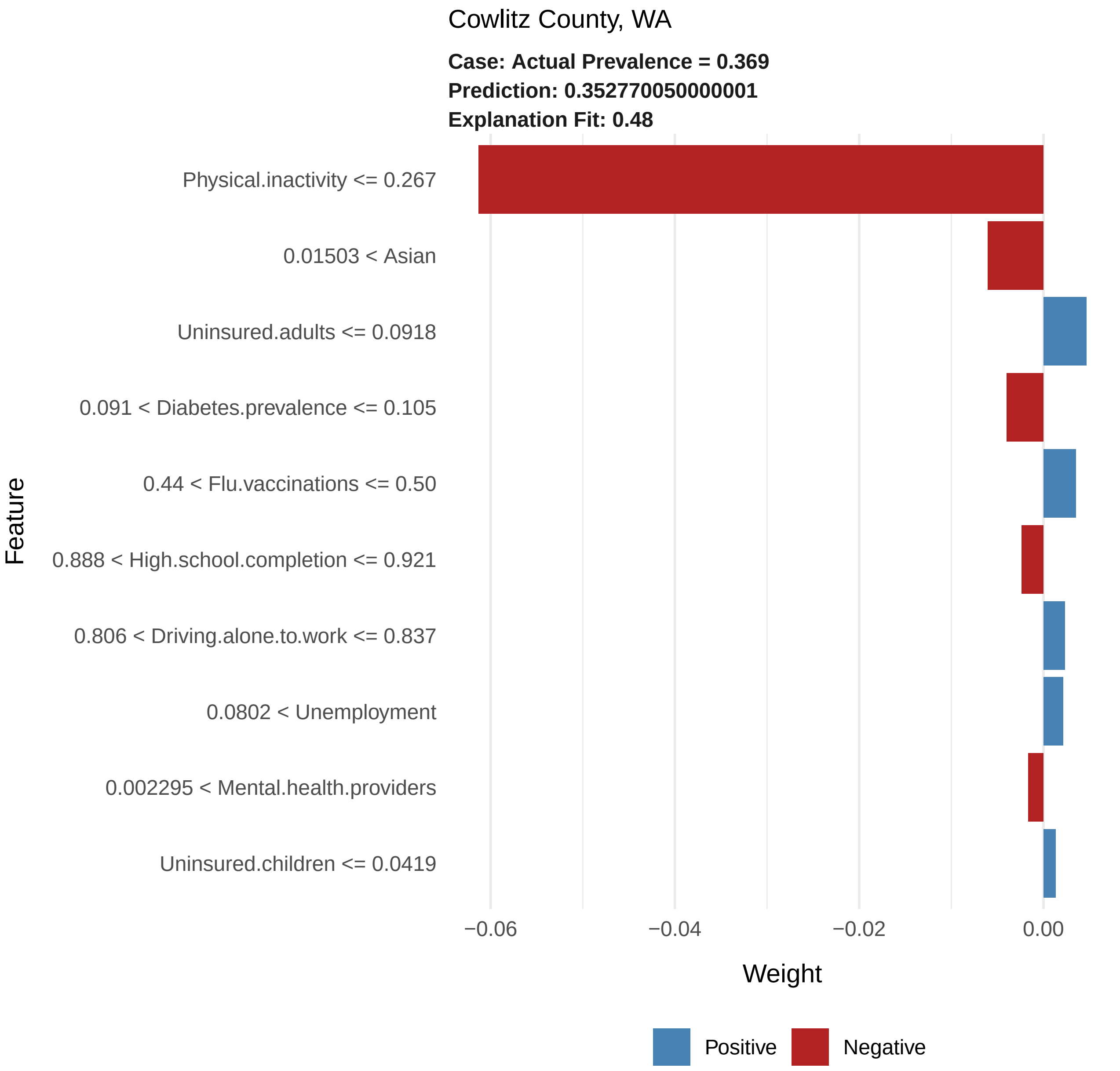
Cowlitz County, WA
Case: Actual Prevalence = 0.369
Prediction: 0.352770050000001
Explanation Fit: 0.48
Physical.inactivity <= 0.267
0.01503 < Asian
Uninsured.adults <= 0.0918
0.091 < Diabetes.prevalence <= 0.105
0.44 < Flu.vaccinations <= 0.50
0.888 < High.school.completion <= 0.921
0.806 < Driving.alone.to.work <= 0.837
0.0802 < Unemployment
0.002295 < Mental.health.providers
Uninsured.children <= 0.0419
Feature
−0.06
−0.04
−0.02
0.00
Weight
Positive
Negative

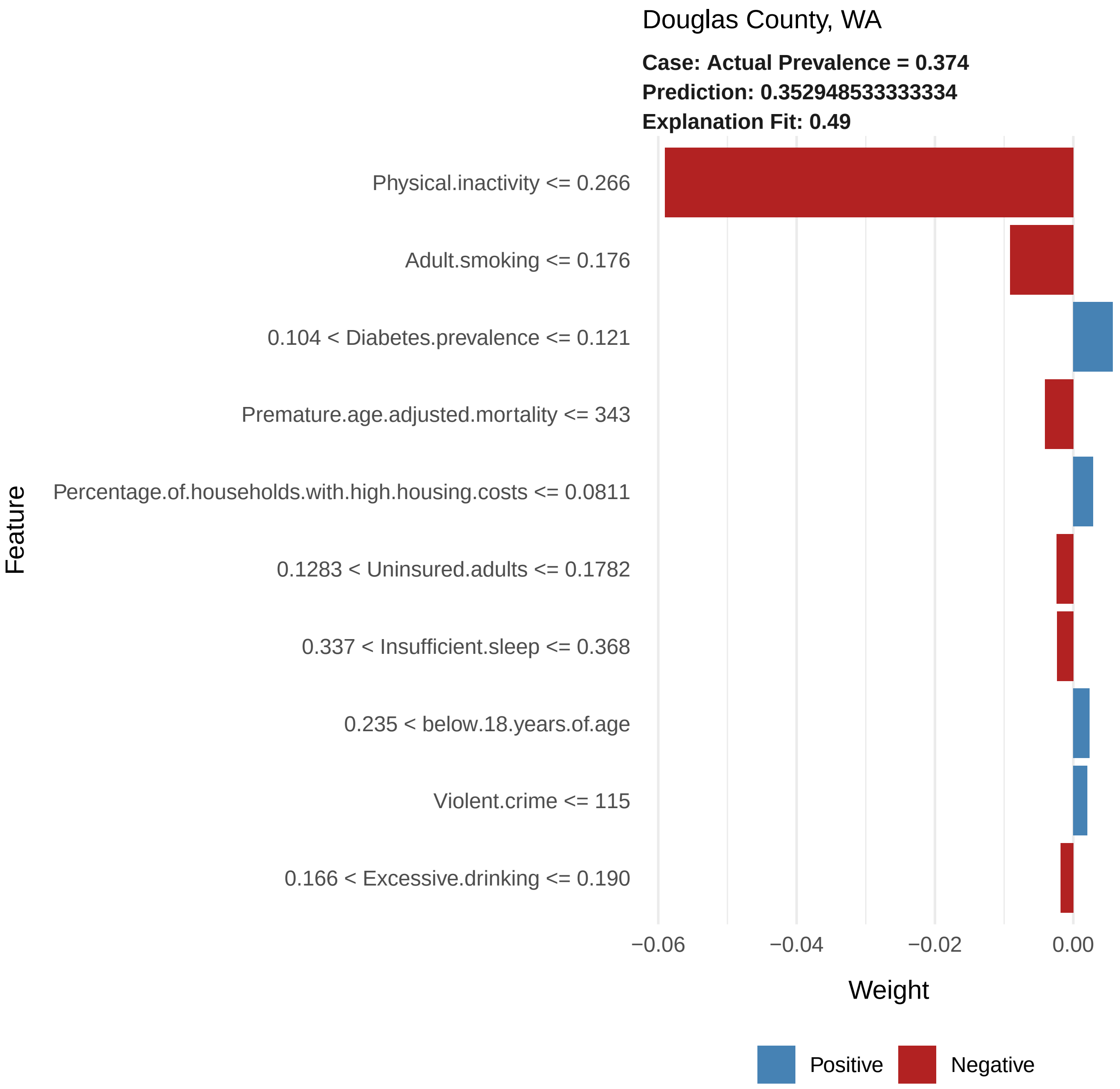
Douglas County, WA
Case: Actual Prevalence = 0.374
Prediction: 0.352948533333334
Explanation Fit: 0.49
Physical.inactivity <= 0.266
Adult.smoking <= 0.176
0.104 < Diabetes.prevalence <= 0.121
Premature.age.adjusted.mortality <= 343
Percentage.of.households.with.high.housing.costs <= 0.0811
0.1283 < Uninsured.adults <= 0.1782
0.337 < Insufficient.sleep <= 0.368
0.235 < below.18.years.of.age
Violent.crime <= 115
0.166 < Excessive.drinking <= 0.190
Feature
−0.06
−0.04
−0.02
0.00
Weight
Positive
Negative

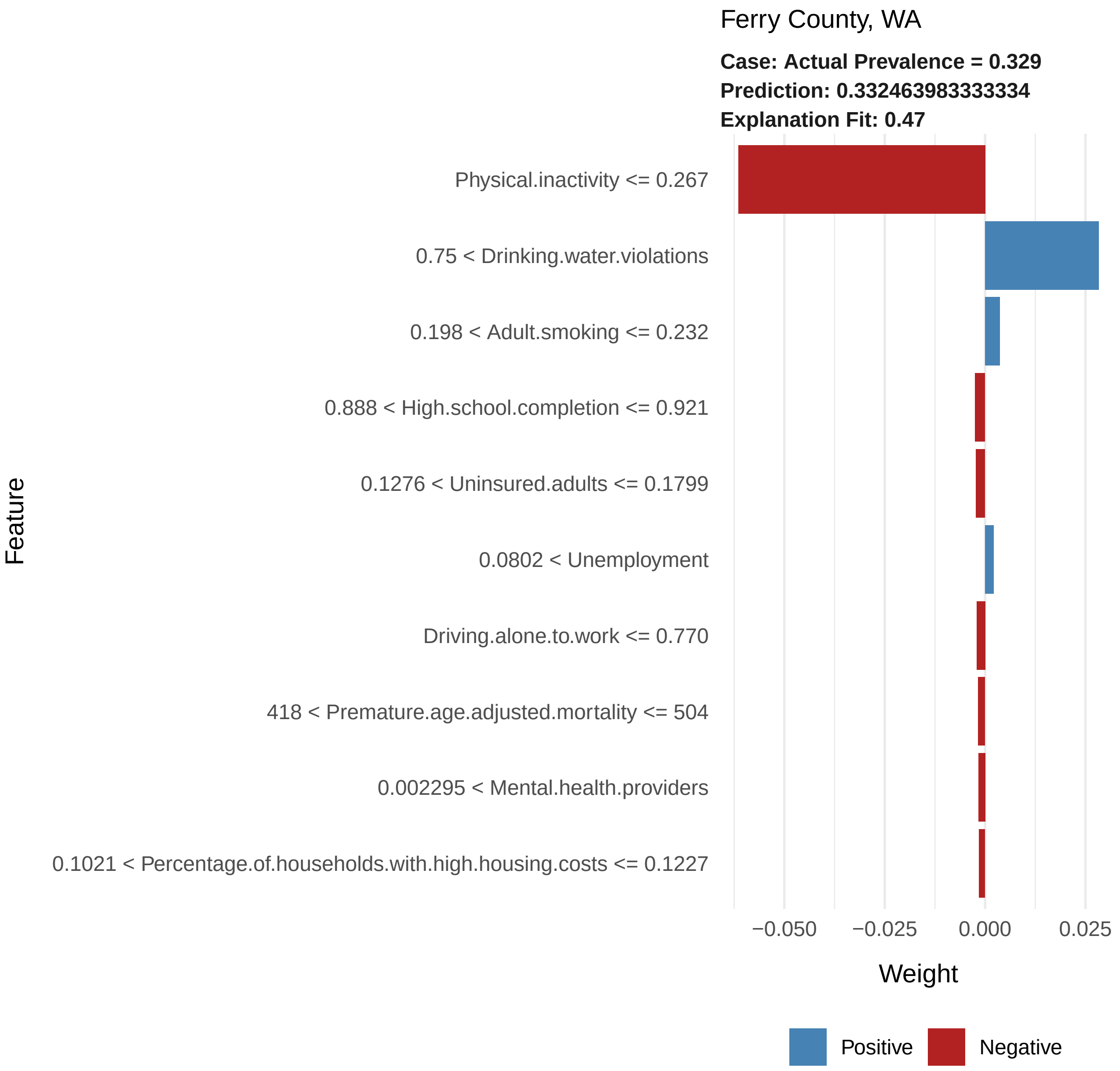

Ferry County, WA
Case: Actual Prevalence = 0.329
Prediction: 0.332463983333334
Explanation Fit: 0.47
Physical.inactivity <= 0.267
0.75 < Drinking.water.violations
0.198 < Adult.smoking <= 0.232
0.888 < High.school.completion <= 0.921
0.1276 < Uninsured.adults <= 0.1799
0.0802 < Unemployment
Driving.alone.to.work <= 0.770
418 < Premature.age.adjusted.mortality <= 504
0.002295 < Mental.health.providers
0.1021 < Percentage.of.households.with.high.housing.costs <= 0.1227
Feature
−0.050
−0.025
0.000
0.025
Weight
Positive
Negative

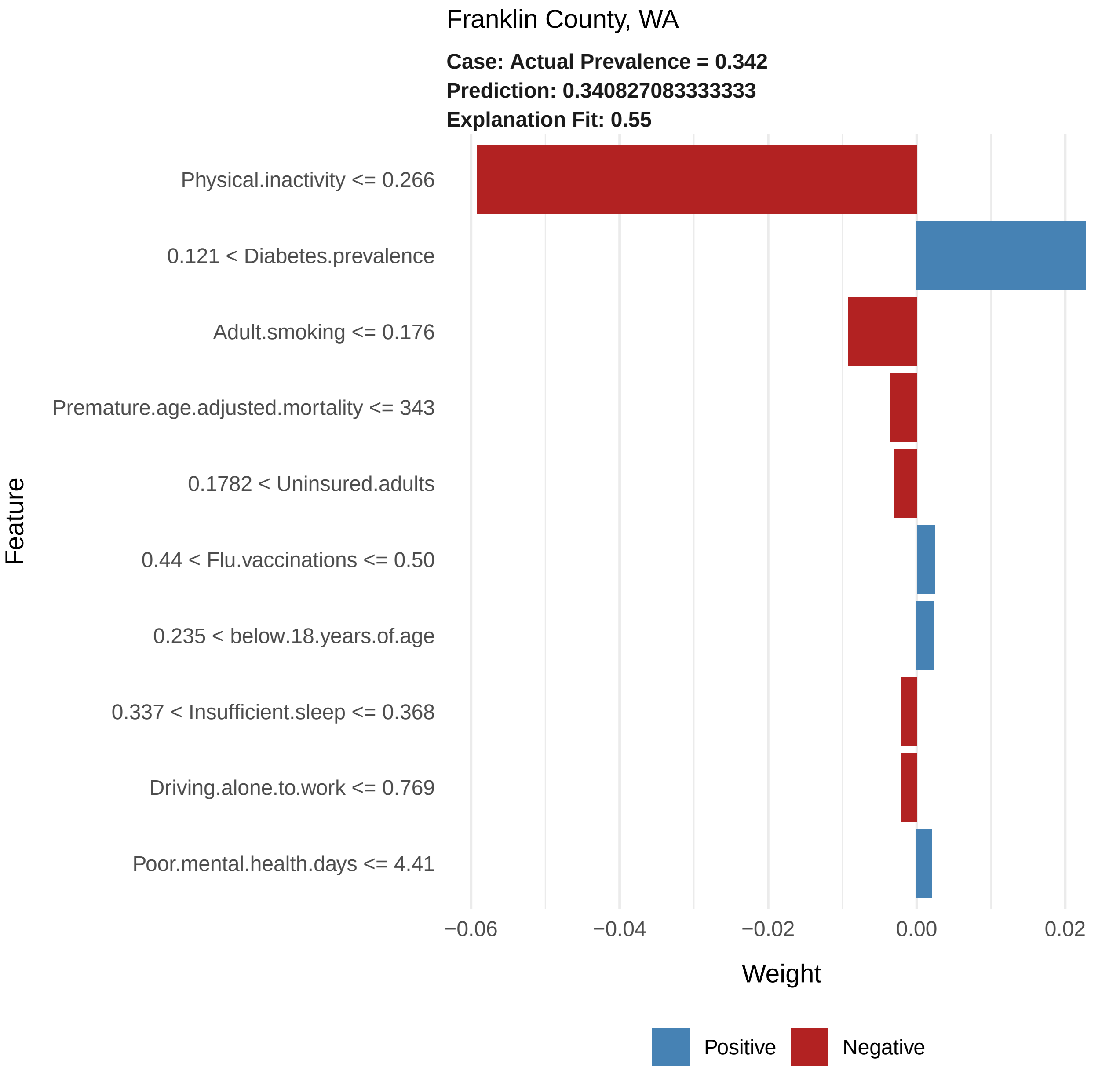

Franklin County, WA
Case: Actual Prevalence = 0.342
Prediction: 0.340827083333333
Explanation Fit: 0.55
Physical.inactivity <= 0.266
0.121 < Diabetes.prevalence
Adult.smoking <= 0.176
Premature.age.adjusted.mortality <= 343
0.1782 < Uninsured.adults
0.44 < Flu.vaccinations <= 0.50
0.235 < below.18.years.of.age
0.337 < Insufficient.sleep <= 0.368
Driving.alone.to.work <= 0.769
Poor.mental.health.days <= 4.41
Feature
−0.06
−0.04
−0.02
0.00
0.02
Weight
Positive
Negative

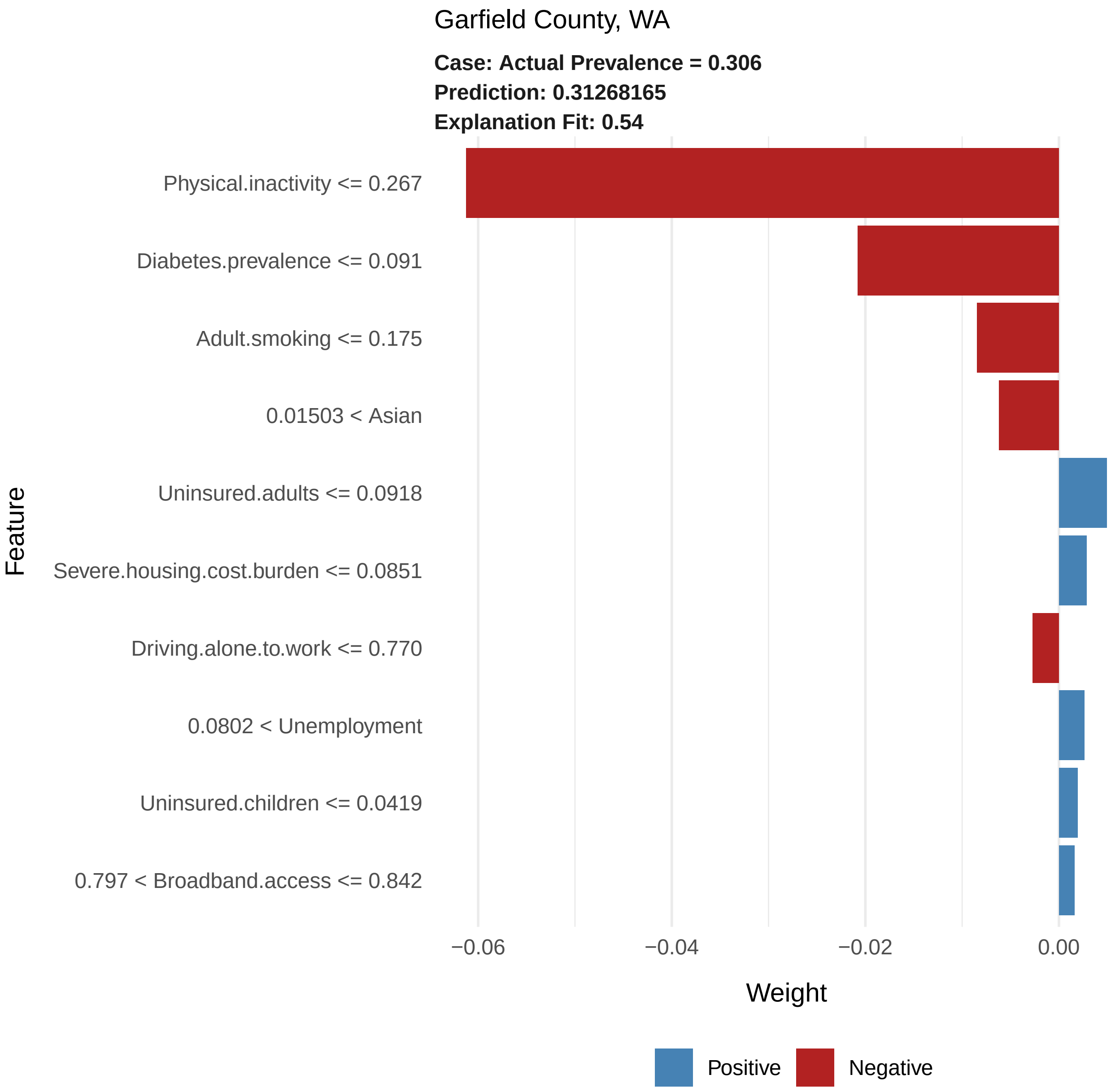

Garfield County, WA
Case: Actual Prevalence = 0.306
Prediction: 0.31268165
Explanation Fit: 0.54
Physical.inactivity <= 0.267
Diabetes.prevalence <= 0.091
Adult.smoking <= 0.175
0.01503 < Asian
Uninsured.adults <= 0.0918
Severe.housing.cost.burden <= 0.0851
Driving.alone.to.work <= 0.770
0.0802 < Unemployment
Uninsured.children <= 0.0419
0.797 < Broadband.access <= 0.842
Feature
−0.06
−0.04
−0.02
0.00
Weight
Positive
Negative

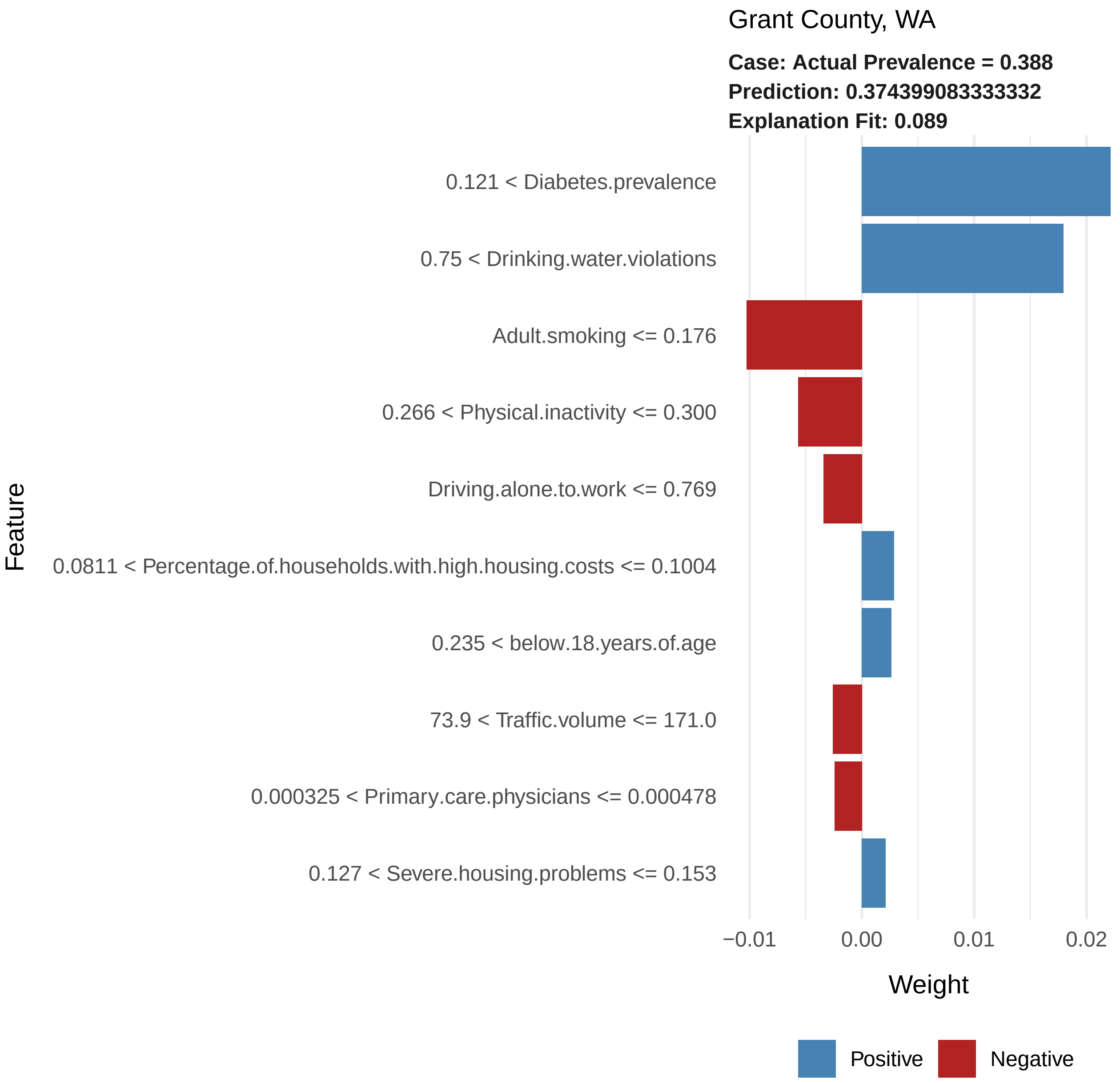
Grant County, WA
Case: Actual Prevalence = 0.388
Prediction: 0.374399083333332
Explanation Fit: 0.089
0.121 < Diabetes.prevalence
0.75 < Drinking.water.violations
Adult.smoking <= 0.176
0.266 < Physical.inactivity <= 0.300
Driving.alone.to.work <= 0.769
0.0811 < Percentage.of.households.with.high.housing.costs <= 0.1004
0.235 < below.18.years.of.age
73.9 < Traffic.volume <= 171.0
0.000325 < Primary.care.physicians <= 0.000478
0.127 < Severe.housing.problems <= 0.153
Feature
−0.01
0.00
0.01
0.02
Weight
Positive
Negative

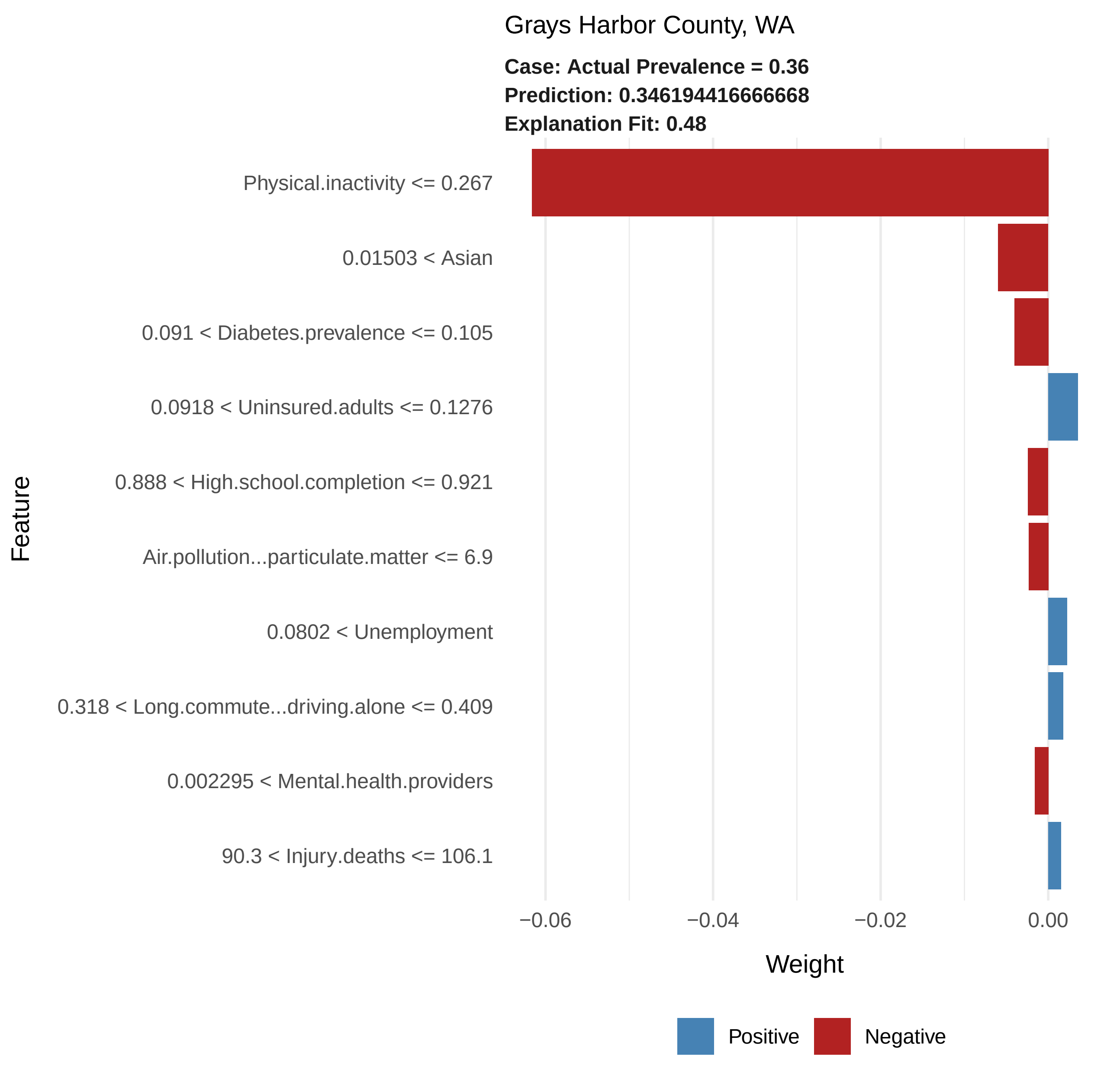

Grays Harbor County, WA
Case: Actual Prevalence = 0.36
Prediction: 0.346194416666668
Explanation Fit: 0.48
Feature
Physical.inactivity <= 0.267
0.01503 < Asian
0.091 < Diabetes.prevalence <= 0.105
0.0918 < Uninsured.adults <= 0.1276
0.888 < High.school.completion <= 0.921
Air.pollution...particulate.matter <= 6.9
0.0802 < Unemployment
0.318 < Long.commute...driving.alone <= 0.409
0.002295 < Mental.health.providers
90.3 < Injury.deaths <= 106.1
−0.06
−0.04
−0.02
0.00
Weight
Positive
Negative

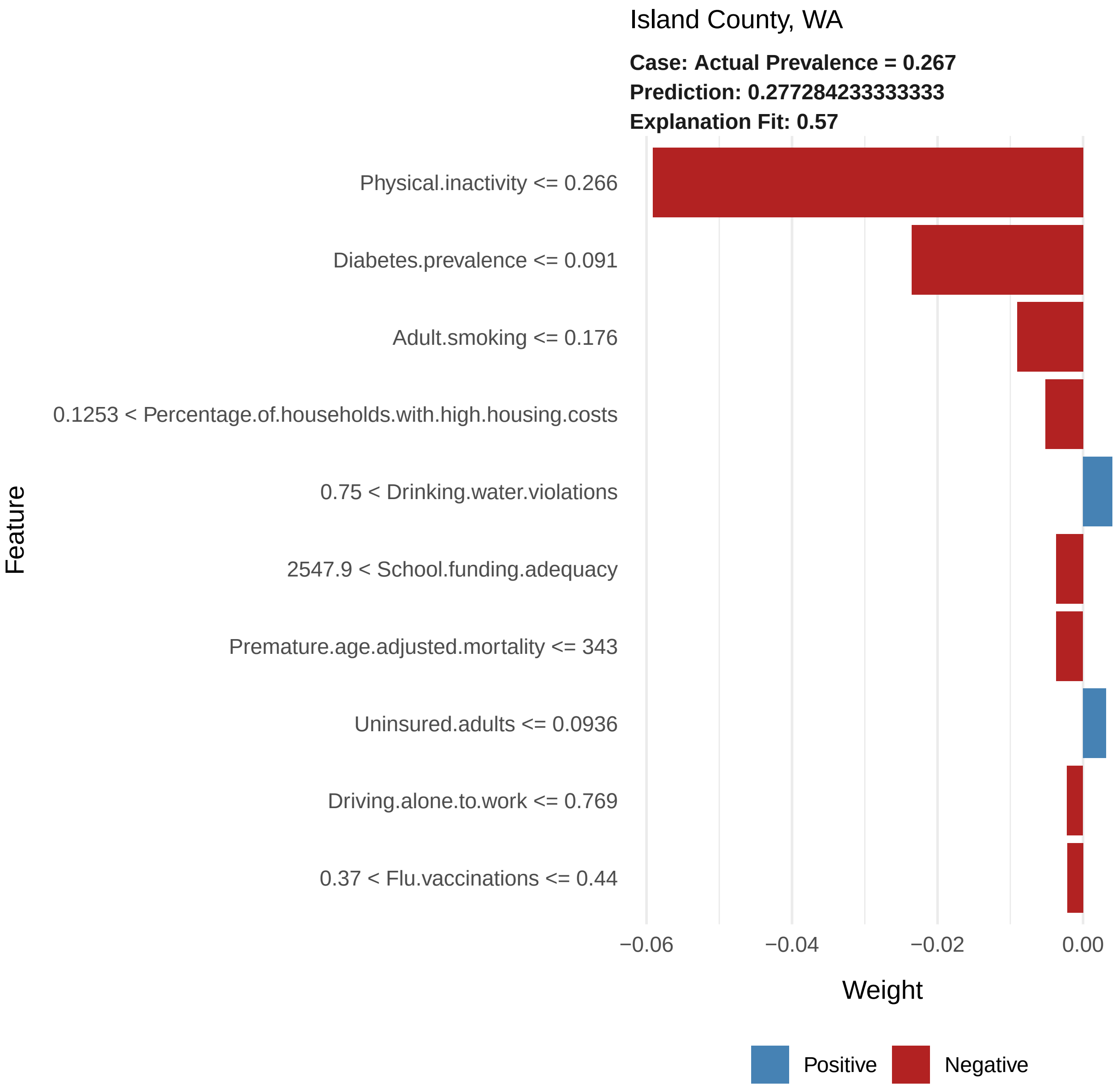
Island County, WA
Case: Actual Prevalence = 0.267
Prediction: 0.277284233333333
Explanation Fit: 0.57
Physical.inactivity <= 0.266
Diabetes.prevalence <= 0.091
Adult.smoking <= 0.176
0.1253 < Percentage.of.households.with.high.housing.costs
0.75 < Drinking.water.violations
2547.9 < School.funding.adequacy
Premature.age.adjusted.mortality <= 343
Uninsured.adults <= 0.0936
Driving.alone.to.work <= 0.769
0.37 < Flu.vaccinations <= 0.44
Feature
−0.06
−0.04
−0.02
0.00
Weight
Positive
Negative

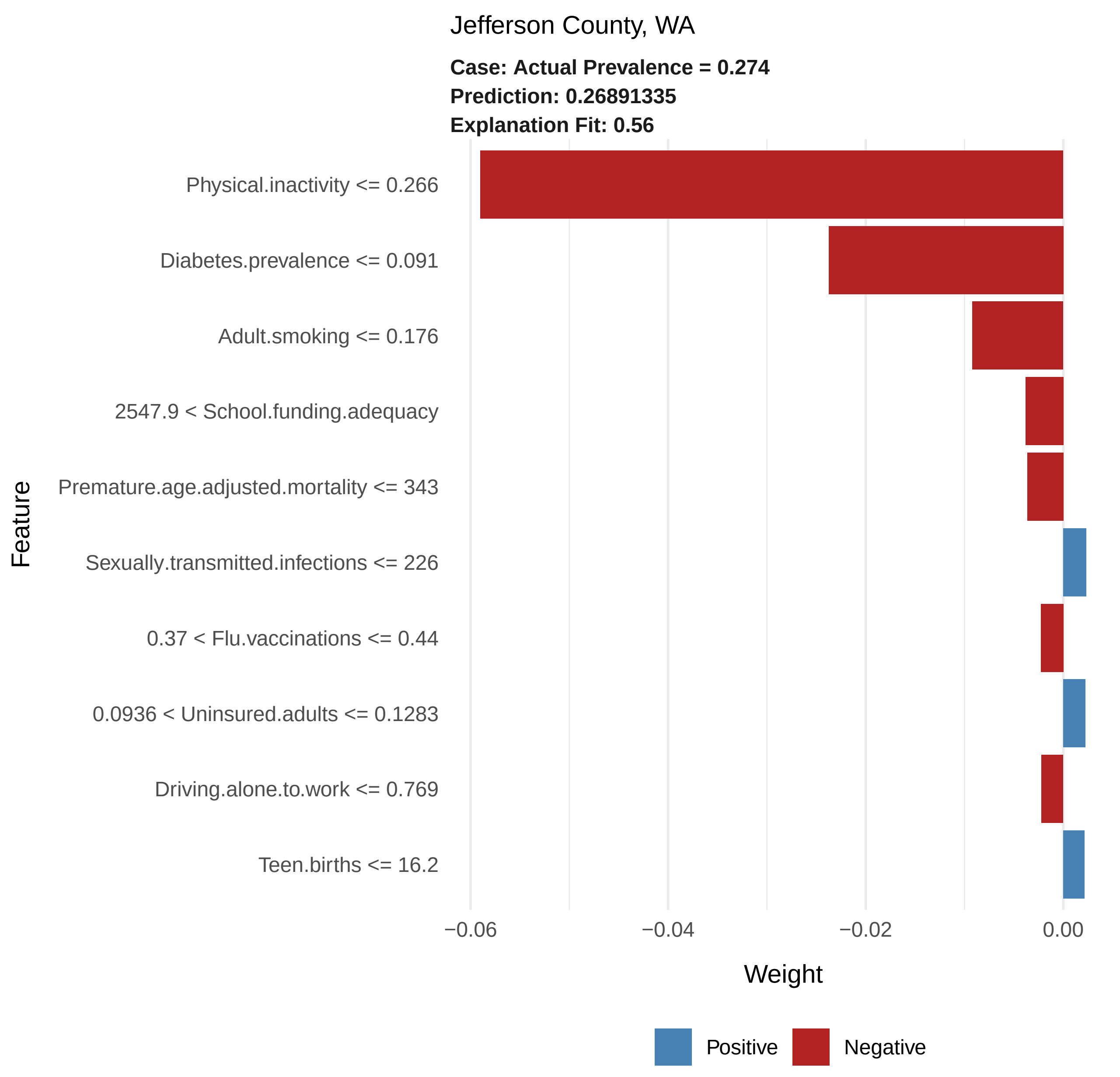

Jefferson County, WA
Case: Actual Prevalence = 0.274
Prediction: 0.26891335
Explanation Fit: 0.56
Physical.inactivity <= 0.266
Diabetes.prevalence <= 0.091
Adult.smoking <= 0.176
2547.9 < School.funding.adequacy
Premature.age.adjusted.mortality <= 343
Sexually.transmitted.infections <= 226
0.37 < Flu.vaccinations <= 0.44
0.0936 < Uninsured.adults <= 0.1283
Driving.alone.to.work <= 0.769
Teen.births <= 16.2
Feature
−0.06
−0.04
−0.02
0.00
Weight
Positive
Negative

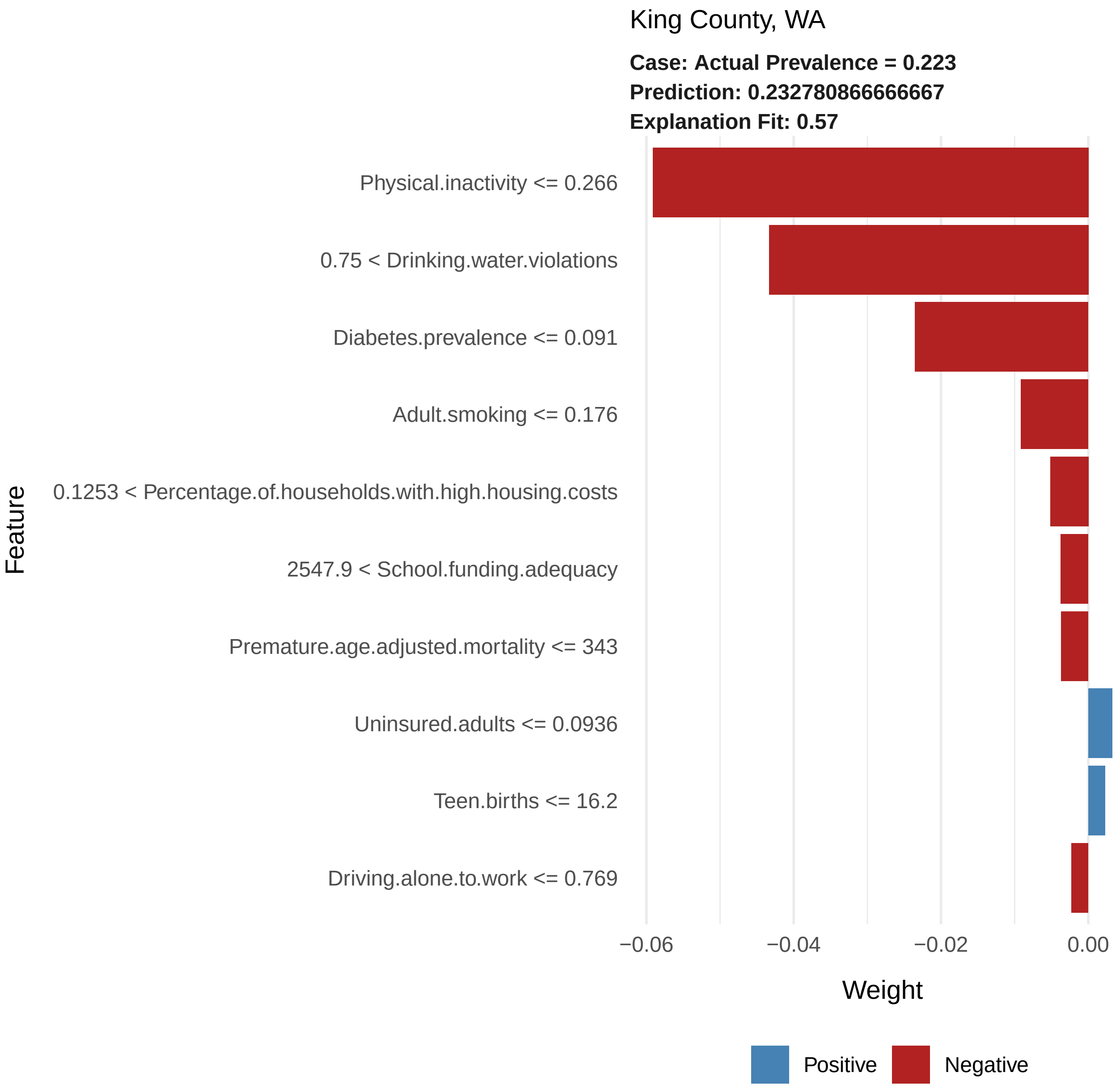
King County, WA
Case: Actual Prevalence = 0.223
Prediction: 0.232780866666667
Explanation Fit: 0.57
Physical.inactivity <= 0.266
0.75 < Drinking.water.violations
Diabetes.prevalence <= 0.091
Adult.smoking <= 0.176
0.1253 < Percentage.of.households.with.high.housing.costs
2547.9 < School.funding.adequacy
Premature.age.adjusted.mortality <= 343
Uninsured.adults <= 0.0936
Teen.births <= 16.2
Driving.alone.to.work <= 0.769
Feature
−0.06
−0.04
−0.02
0.00
Weight
Positive
Negative

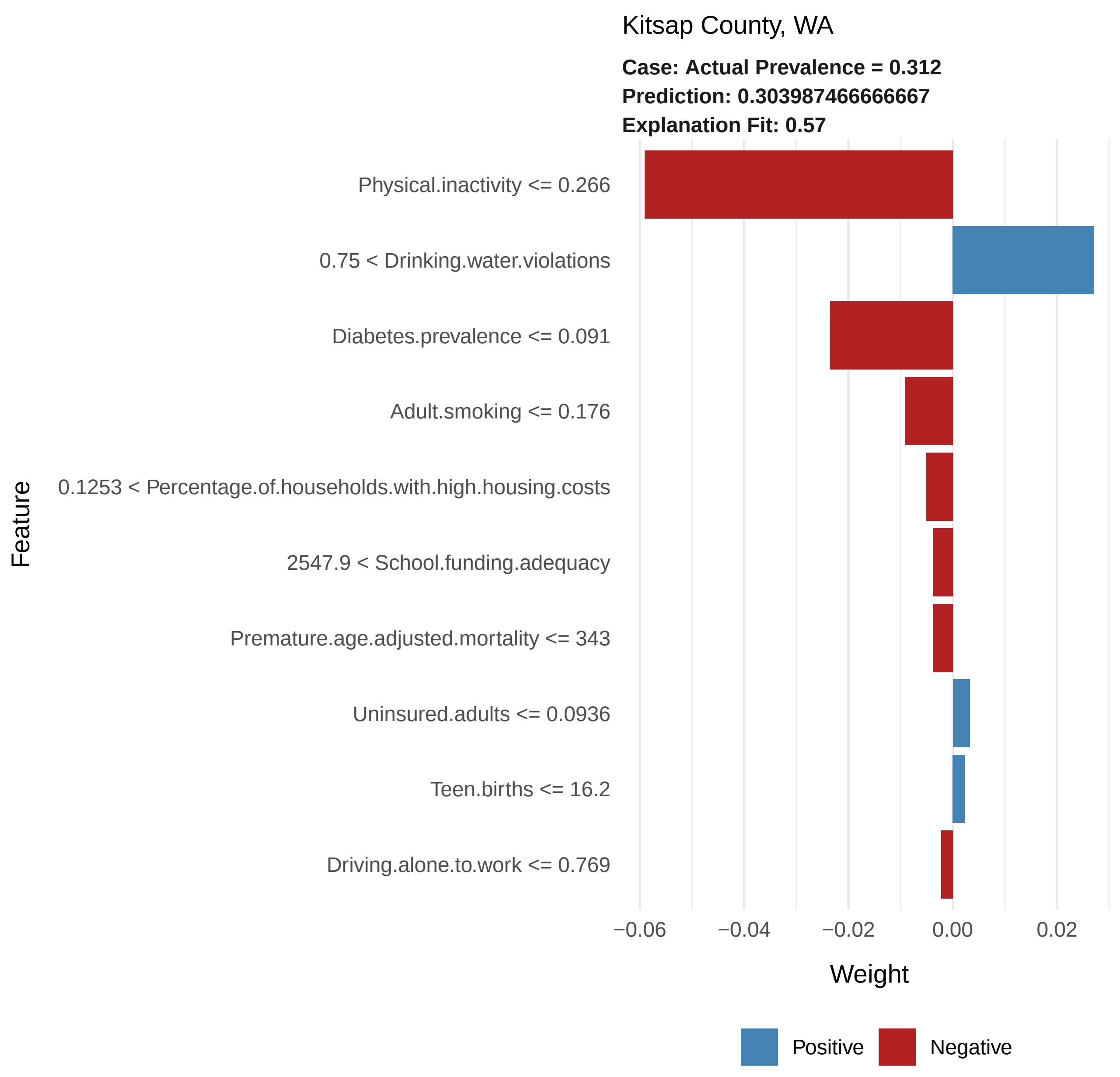
Kitsap County, WA
Case: Actual Prevalence = 0.312
Prediction: 0.303987466666667
Explanation Fit: 0.57
Physical.inactivity <= 0.266
0.75 < Drinking.water.violations
Diabetes.prevalence <= 0.091
Adult.smoking <= 0.176
0.1253 < Percentage.of.households.with.high.housing.costs
2547.9 < School.funding.adequacy
Premature.age.adjusted.mortality <= 343
Uninsured.adults <= 0.0936
Teen.births <= 16.2
Driving.alone.to.work <= 0.769
Feature
−0.06
−0.04
−0.02
0.00
0.02
Weight
Positive
Negative

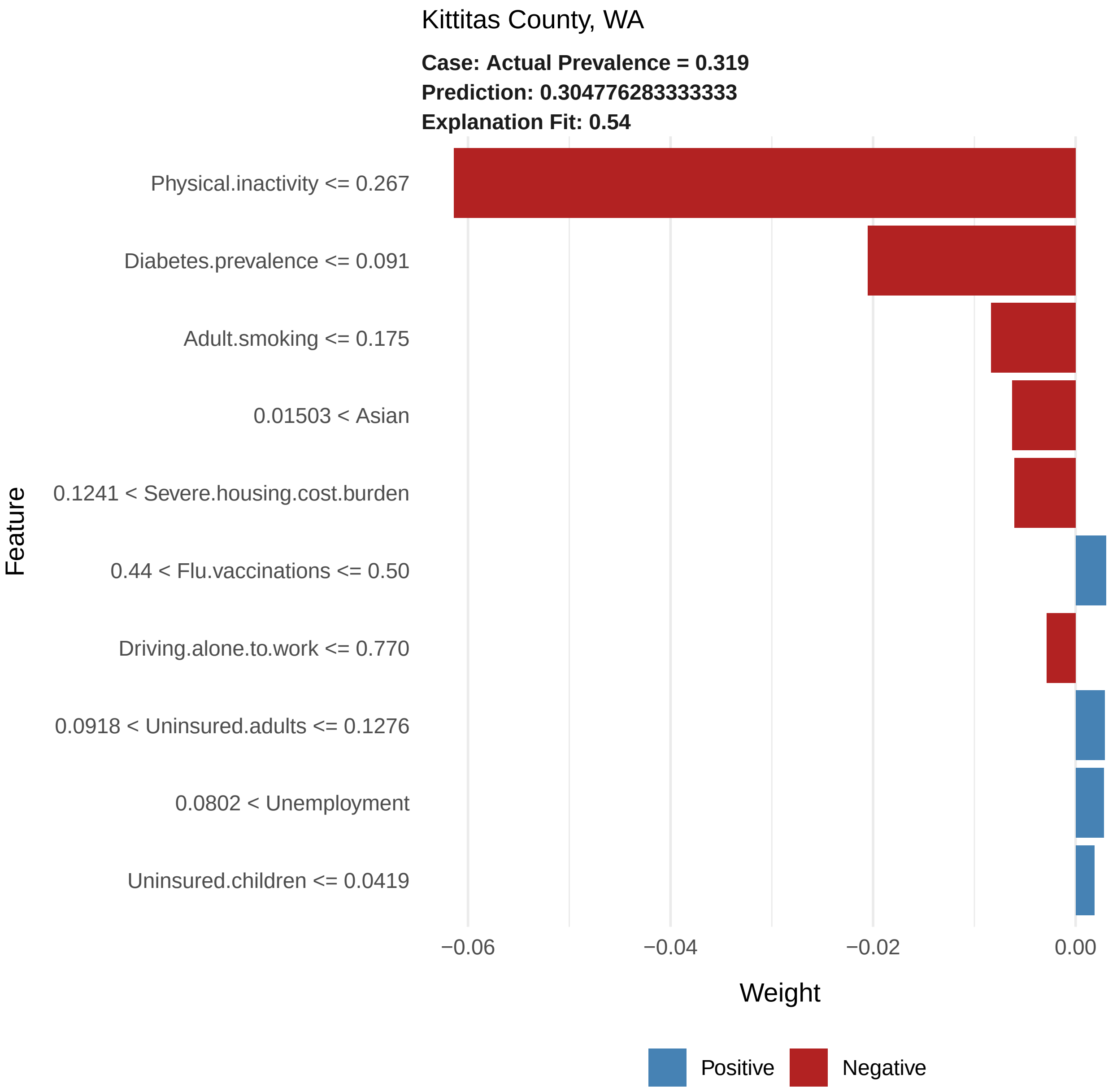

Kittitas County, WA
Case: Actual Prevalence = 0.319
Prediction: 0.304776283333333
Explanation Fit: 0.54
Physical.inactivity <= 0.267
Diabetes.prevalence <= 0.091
Adult.smoking <= 0.175
0.01503 < Asian
0.1241 < Severe.housing.cost.burden
0.44 < Flu.vaccinations <= 0.50
Driving.alone.to.work <= 0.770
0.0918 < Uninsured.adults <= 0.1276
0.0802 < Unemployment
Uninsured.children <= 0.0419
Feature
−0.06
−0.04
−0.02
0.00
Weight
Positive
Negative

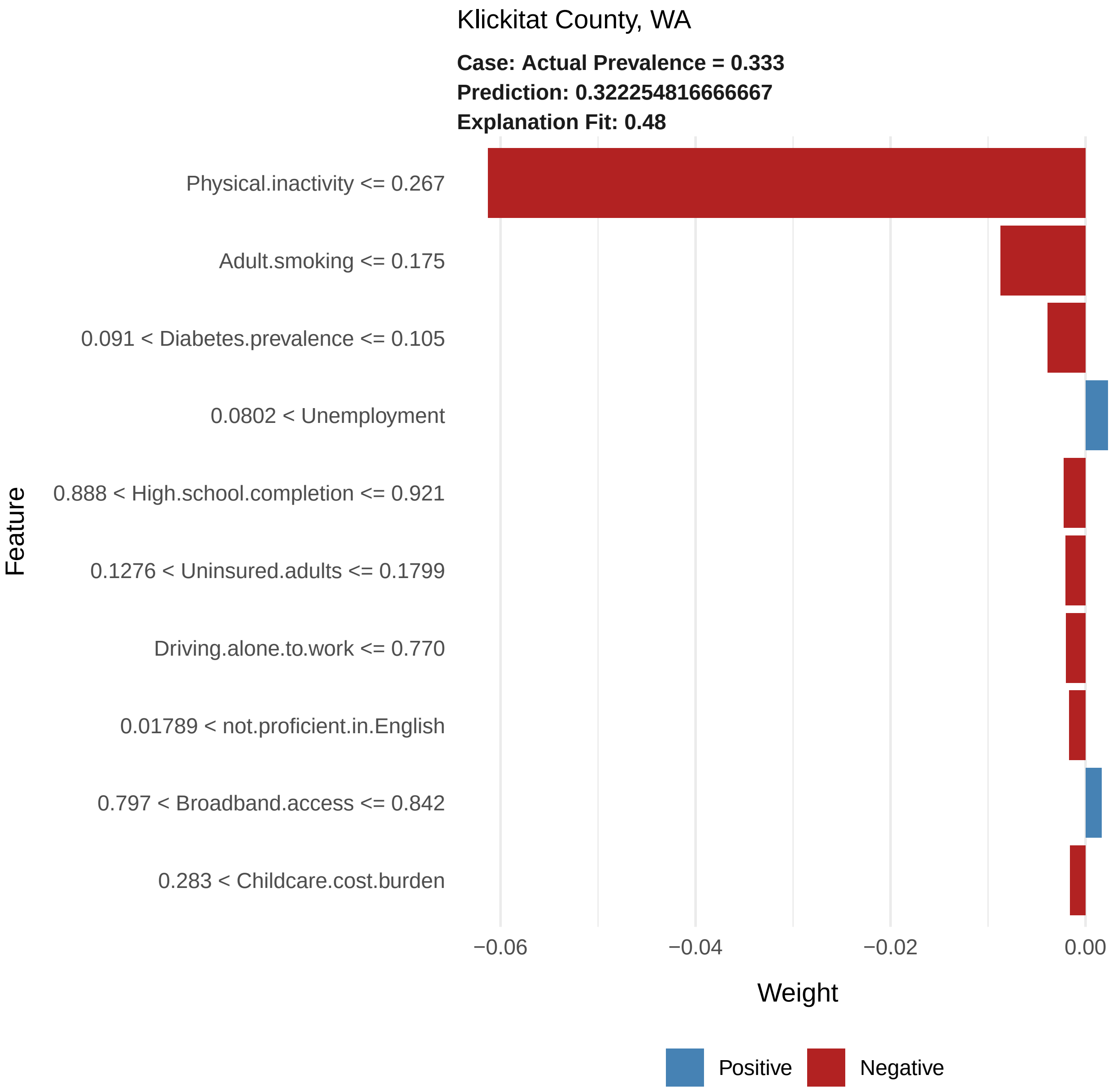

Klickitat County, WA
Case: Actual Prevalence = 0.333
Prediction: 0.322254816666667
Explanation Fit: 0.48
Physical.inactivity <= 0.267
Adult.smoking <= 0.175
0.091 < Diabetes.prevalence <= 0.105
0.0802 < Unemployment
0.888 < High.school.completion <= 0.921
0.1276 < Uninsured.adults <= 0.1799
Driving.alone.to.work <= 0.770
0.01789 < not.proficient.in.English
0.797 < Broadband.access <= 0.842
0.283 < Childcare.cost.burden
Feature
−0.06
−0.04
−0.02
0.00
Weight
Positive
Negative

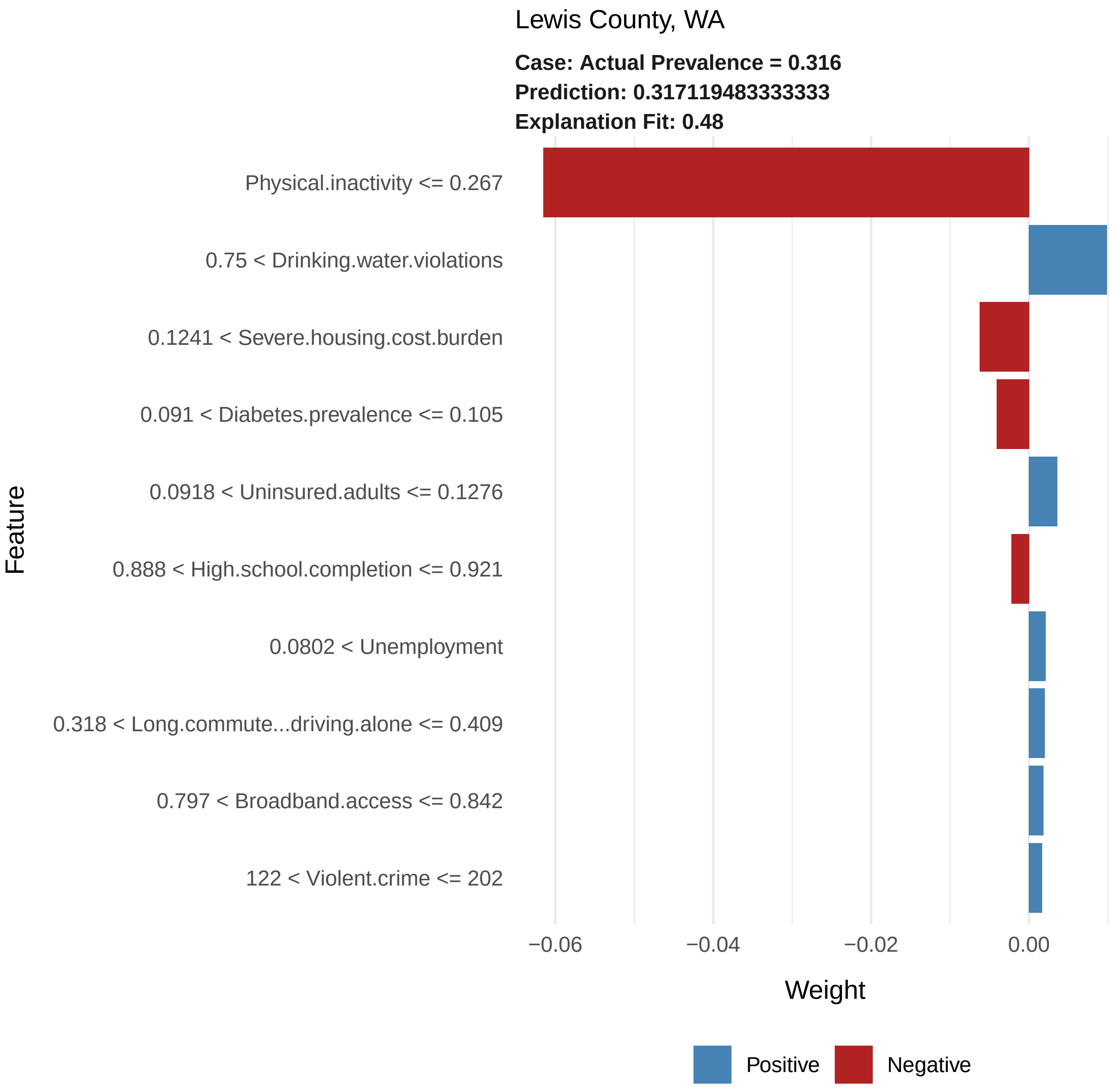
Lewis County, WA
Case: Actual Prevalence = 0.316
Prediction: 0.317119483333333
Explanation Fit: 0.48
Physical.inactivity <= 0.267
0.75 < Drinking.water.violations
0.1241 < Severe.housing.cost.burden
0.091 < Diabetes.prevalence <= 0.105
0.0918 < Uninsured.adults <= 0.1276
0.888 < High.school.completion <= 0.921
0.0802 < Unemployment
0.318 < Long.commute...driving.alone <= 0.409
0.797 < Broadband.access <= 0.842
122 < Violent.crime <= 202
Feature
−0.06
−0.04
−0.02
0.00
Weight
Positive
Negative

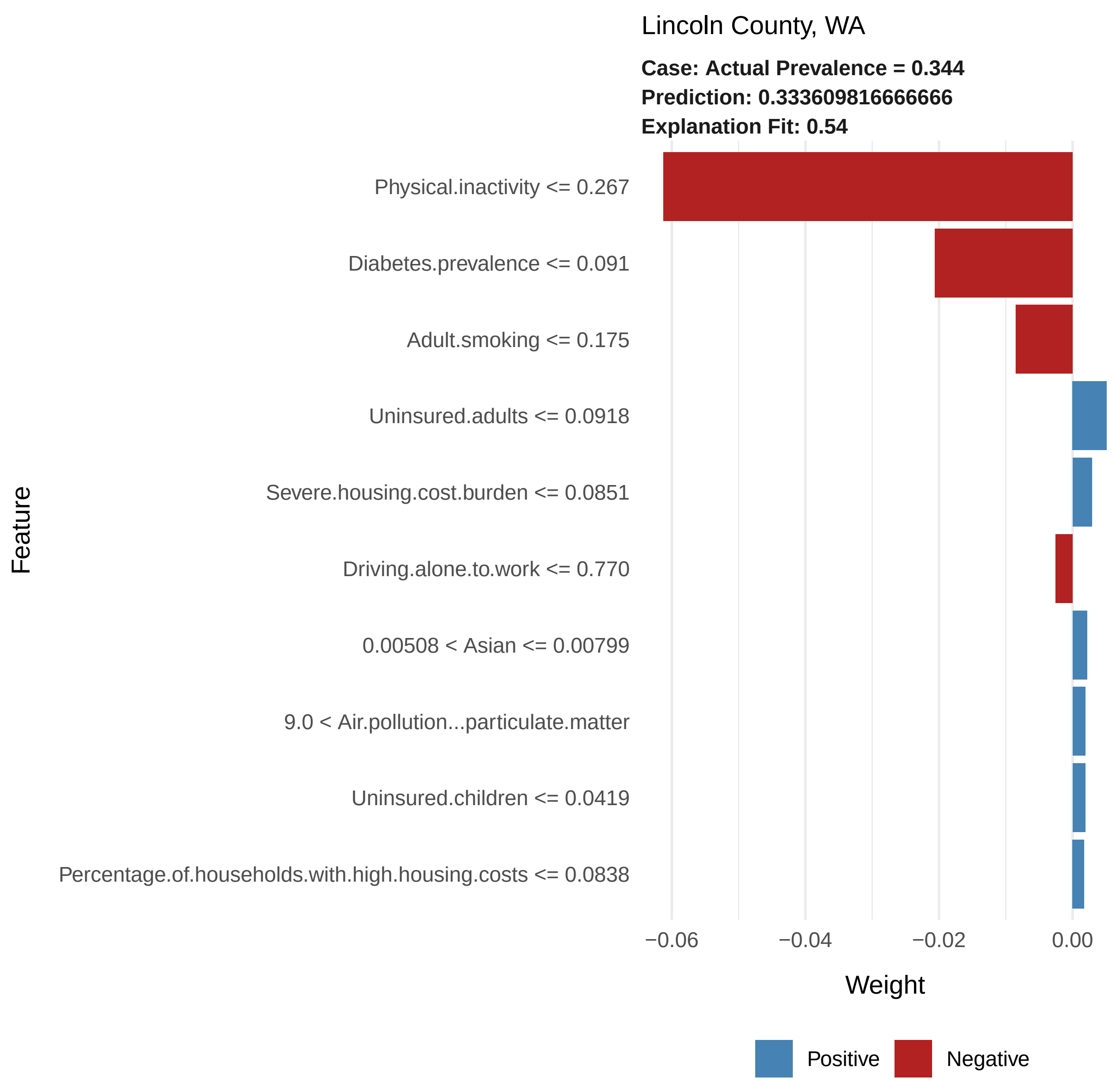

Lincoln County, WA
Case: Actual Prevalence = 0.344
Prediction: 0.333609816666666
Explanation Fit: 0.54
Physical.inactivity <= 0.267
Diabetes.prevalence <= 0.091
Adult.smoking <= 0.175
Uninsured.adults <= 0.0918
Severe.housing.cost.burden <= 0.0851
Driving.alone.to.work <= 0.770
0.00508 < Asian <= 0.00799
9.0 < Air.pollution...particulate.matter
Uninsured.children <= 0.0419
Percentage.of.households.with.high.housing.costs <= 0.0838
Feature
−0.06
−0.04
−0.02
0.00
Weight
Positive
Negative

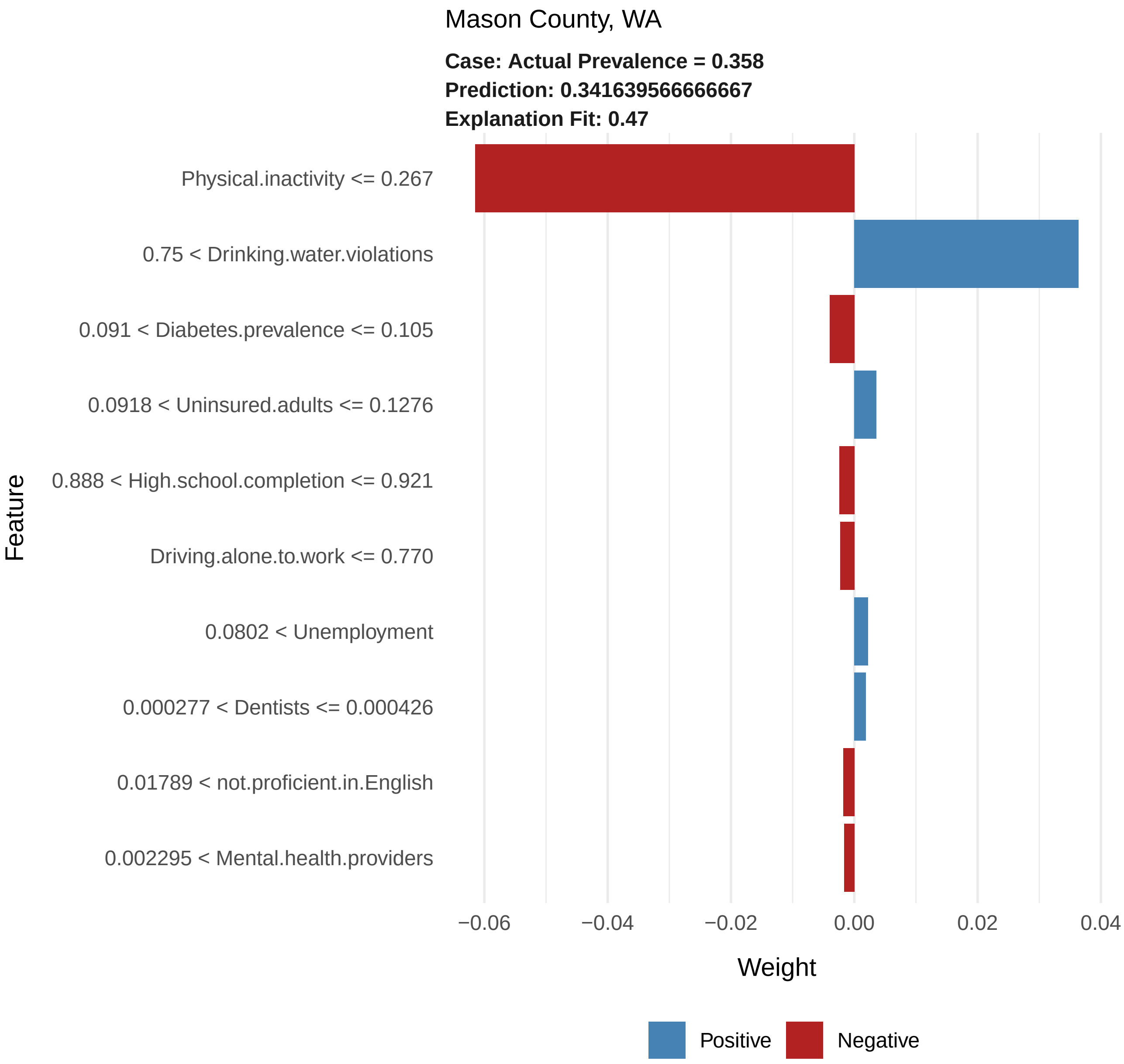

Mason County, WA
Case: Actual Prevalence = 0.358
Prediction: 0.341639566666667
Explanation Fit: 0.47
Physical.inactivity <= 0.267
0.75 < Drinking.water.violations
0.091 < Diabetes.prevalence <= 0.105
0.0918 < Uninsured.adults <= 0.1276
0.888 < High.school.completion <= 0.921
Driving.alone.to.work <= 0.770
0.0802 < Unemployment
0.000277 < Dentists <= 0.000426
0.01789 < not.proficient.in.English
0.002295 < Mental.health.providers
Feature
−0.06
−0.04
−0.02
0.00
0.02
0.04
Weight
Positive
Negative

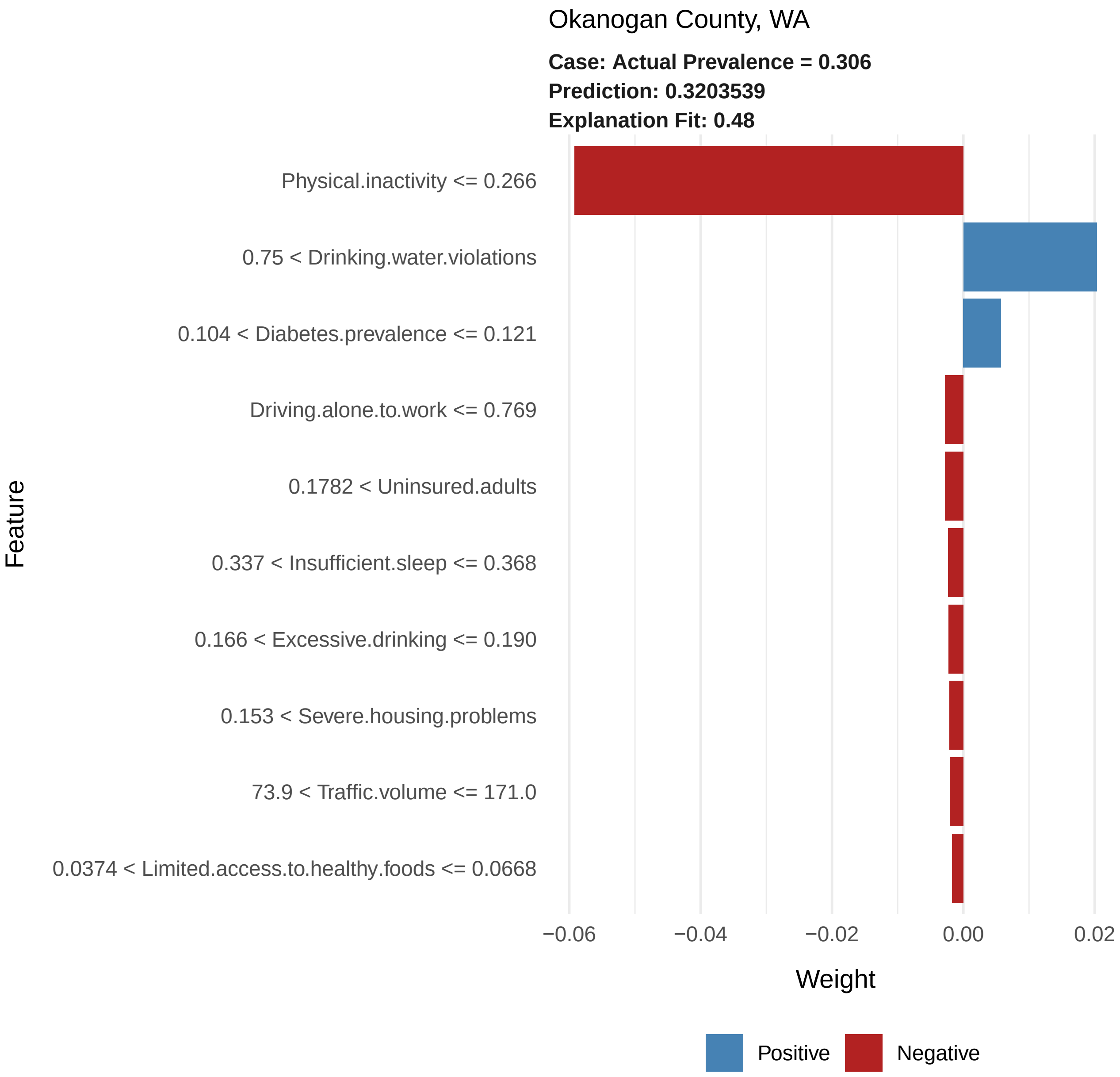

Okanogan County, WA
Case: Actual Prevalence = 0.306
Prediction: 0.3203539
Explanation Fit: 0.48
Physical.inactivity <= 0.266
0.75 < Drinking.water.violations
0.104 < Diabetes.prevalence <= 0.121
Driving.alone.to.work <= 0.769
0.1782 < Uninsured.adults
0.337 < Insufficient.sleep <= 0.368
0.166 < Excessive.drinking <= 0.190
0.153 < Severe.housing.problems
73.9 < Traffic.volume <= 171.0
0.0374 < Limited.access.to.healthy.foods <= 0.0668
Feature
−0.06
−0.04
−0.02
0.00
0.02
Weight
Positive
Negative

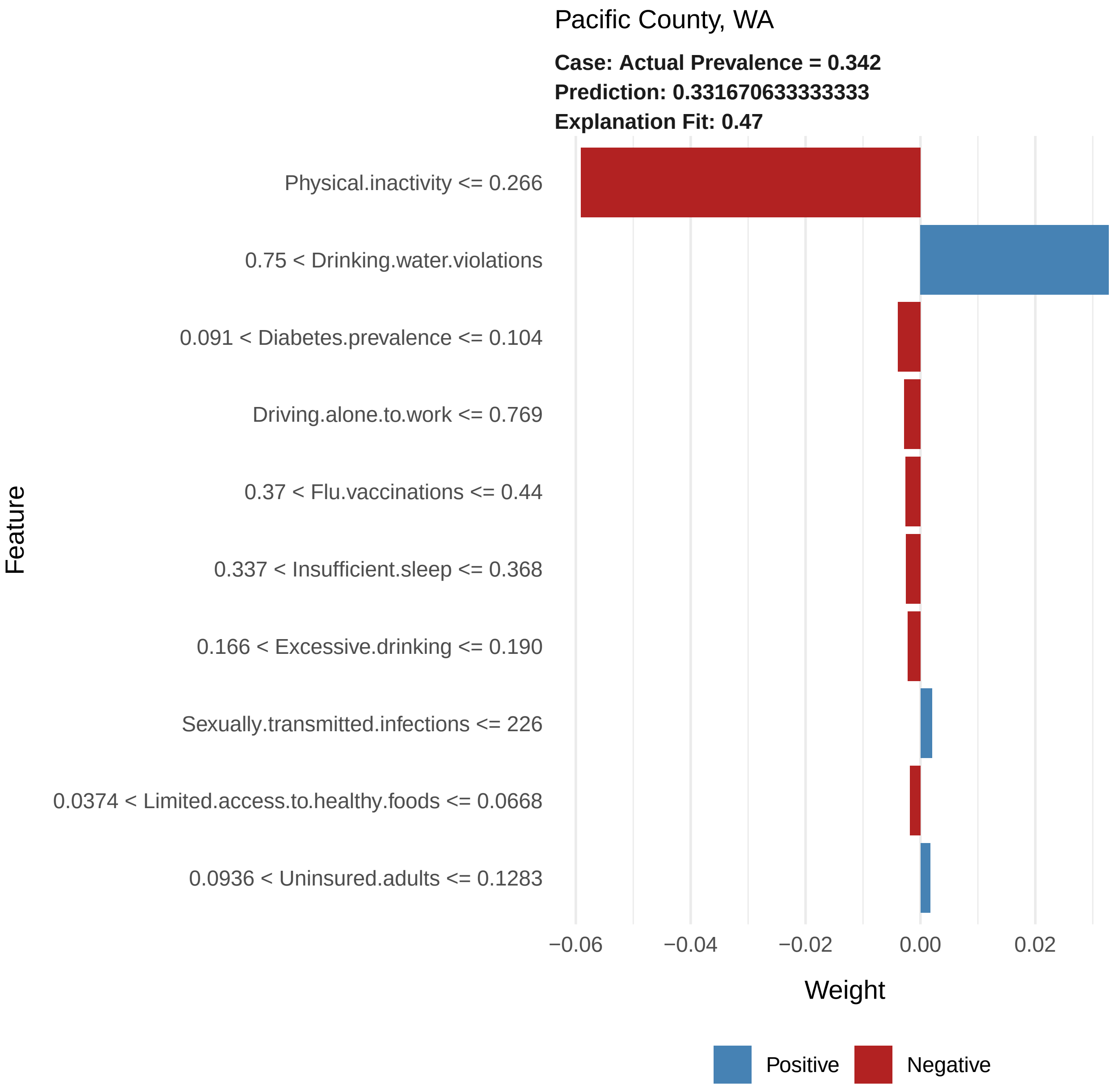
Pacific County, WA
Case: Actual Prevalence = 0.342
Prediction: 0.331670633333333
Explanation Fit: 0.47
Physical.inactivity <= 0.266
0.75 < Drinking.water.violations
0.091 < Diabetes.prevalence <= 0.104
Driving.alone.to.work <= 0.769
0.37 < Flu.vaccinations <= 0.44
0.337 < Insufficient.sleep <= 0.368
0.166 < Excessive.drinking <= 0.190
Sexually.transmitted.infections <= 226
0.0374 < Limited.access.to.healthy.foods <= 0.0668
0.0936 < Uninsured.adults <= 0.1283
Feature
−0.06
−0.04
−0.02
0.00
0.02
Weight
Positive
Negative

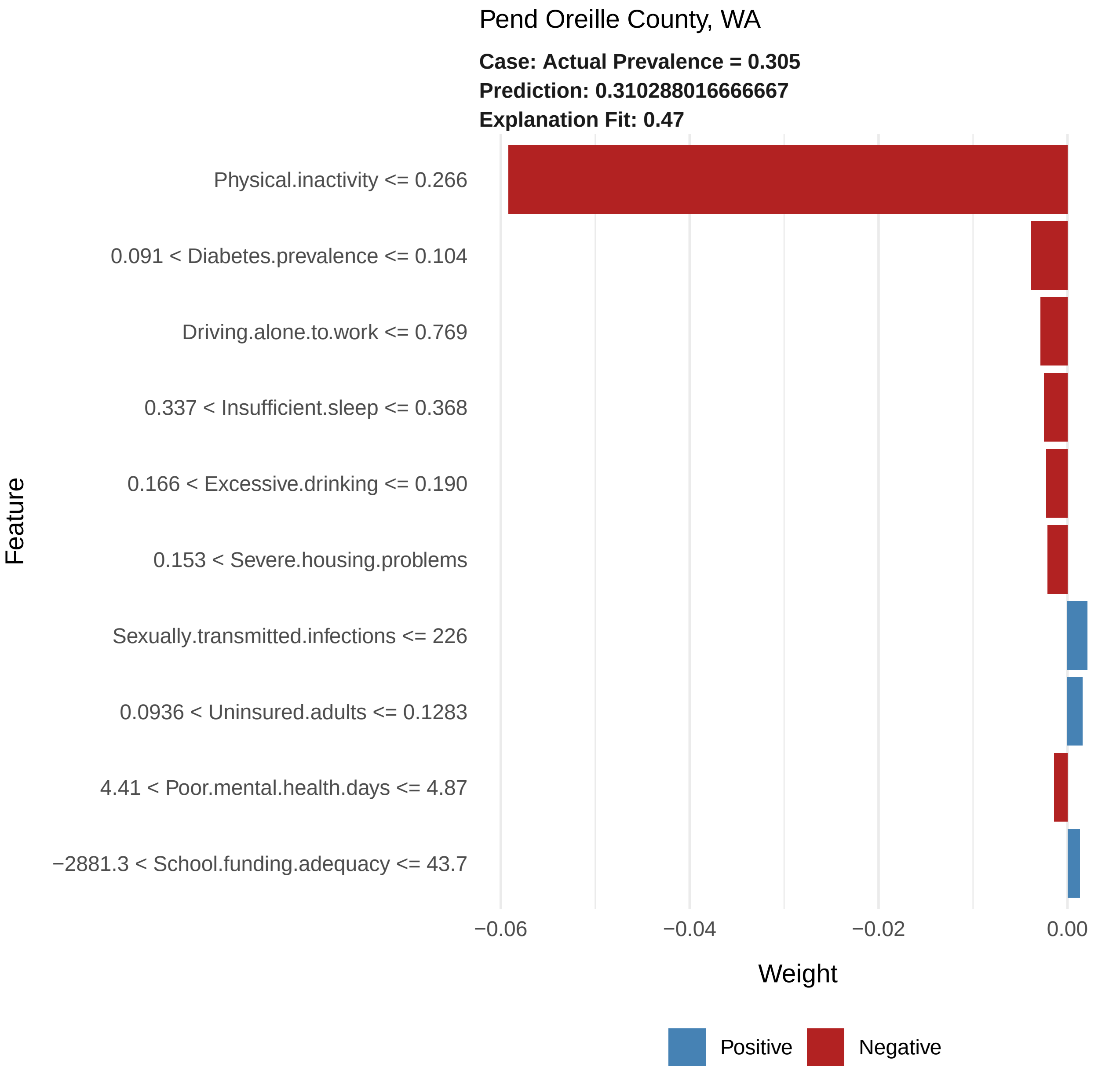

Pend Oreille County, WA
Case: Actual Prevalence = 0.305
Prediction: 0.310288016666667
Explanation Fit: 0.47
Physical.inactivity <= 0.266
0.091 < Diabetes.prevalence <= 0.104
Driving.alone.to.work <= 0.769
0.337 < Insufficient.sleep <= 0.368
0.166 < Excessive.drinking <= 0.190
0.153 < Severe.housing.problems
Sexually.transmitted.infections <= 226
0.0936 < Uninsured.adults <= 0.1283
4.41 < Poor.mental.health.days <= 4.87
−2881.3 < School.funding.adequacy <= 43.7
Feature
−0.06
−0.04
−0.02
0.00
Weight
Positive
Negative

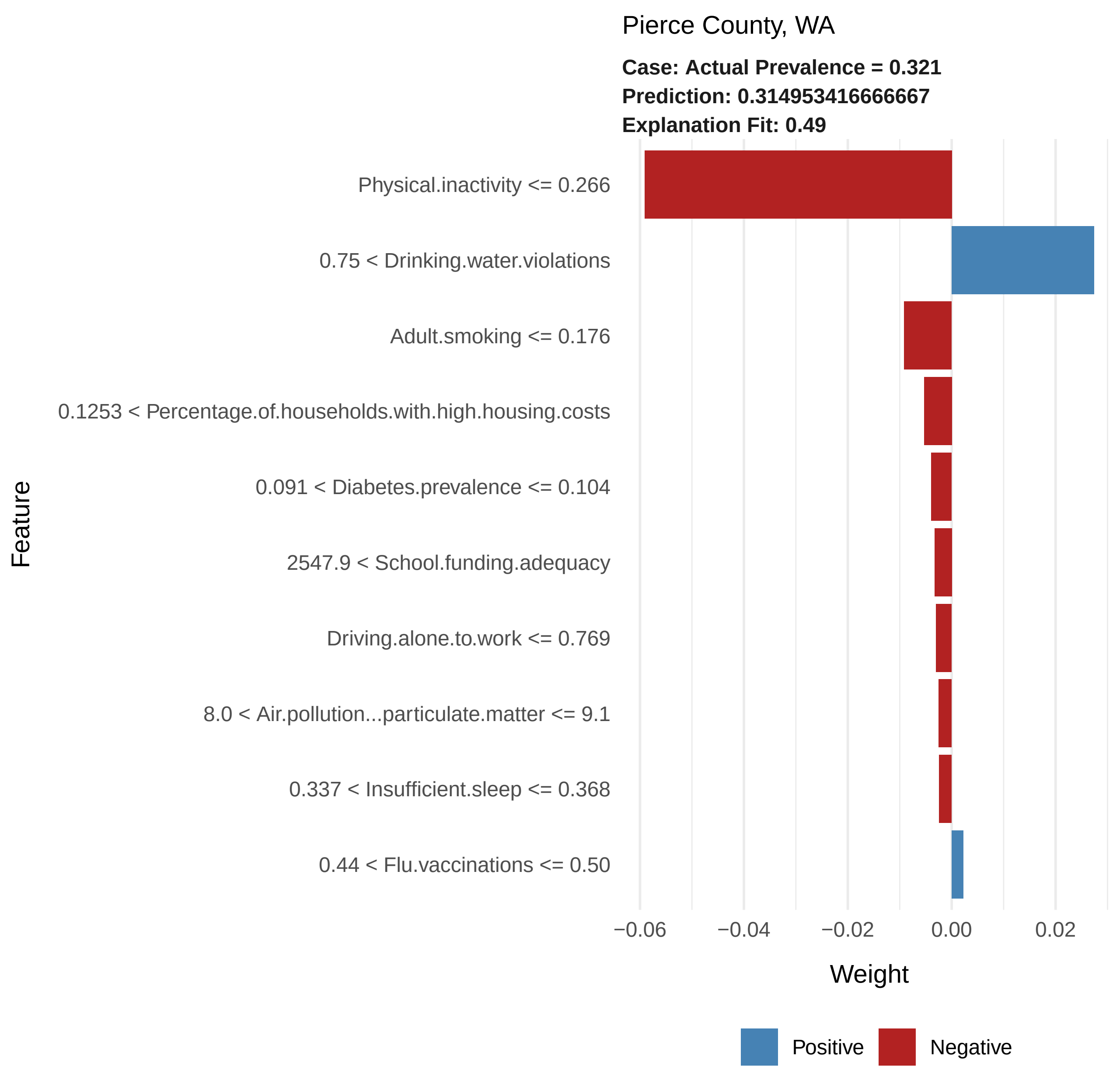

Pierce County, WA
Case: Actual Prevalence = 0.321
Prediction: 0.314953416666667
Explanation Fit: 0.49
Physical.inactivity <= 0.266
0.75 < Drinking.water.violations
Adult.smoking <= 0.176
0.1253 < Percentage.of.households.with.high.housing.costs
0.091 < Diabetes.prevalence <= 0.104
2547.9 < School.funding.adequacy
Driving.alone.to.work <= 0.769
8.0 < Air.pollution...particulate.matter <= 9.1
0.337 < Insufficient.sleep <= 0.368
0.44 < Flu.vaccinations <= 0.50
Feature
−0.06
−0.04
−0.02
0.00
0.02
Weight
Positive
Negative

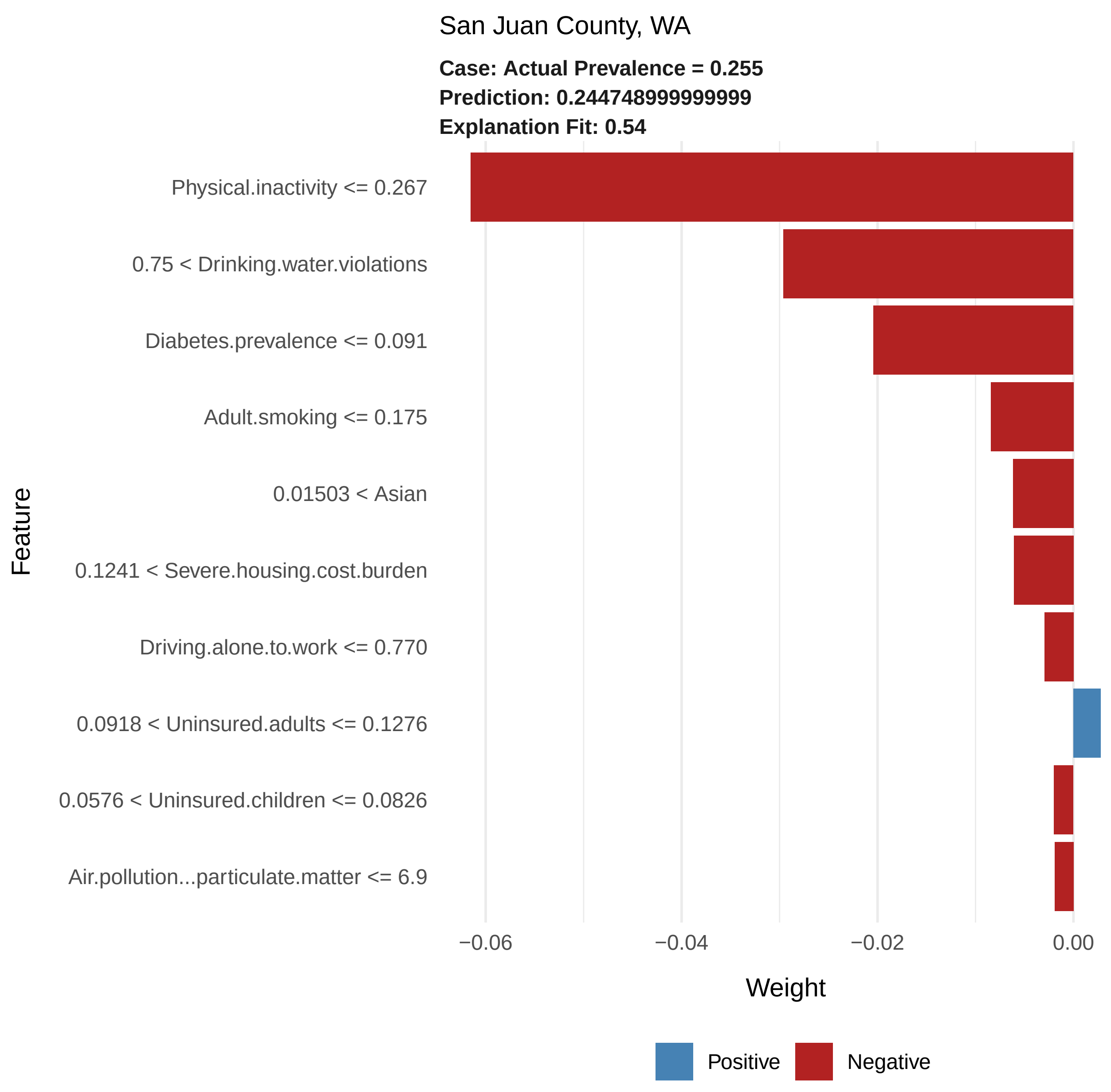

San Juan County, WA
Case: Actual Prevalence = 0.255
Prediction: 0.244748999999999
Explanation Fit: 0.54
Physical.inactivity <= 0.267
0.75 < Drinking.water.violations
Diabetes.prevalence <= 0.091
Adult.smoking <= 0.175
0.01503 < Asian
0.1241 < Severe.housing.cost.burden
Driving.alone.to.work <= 0.770
0.0918 < Uninsured.adults <= 0.1276
0.0576 < Uninsured.children <= 0.0826
Air.pollution...particulate.matter <= 6.9
Feature
−0.06
−0.04
−0.02
0.00
Weight
Positive
Negative

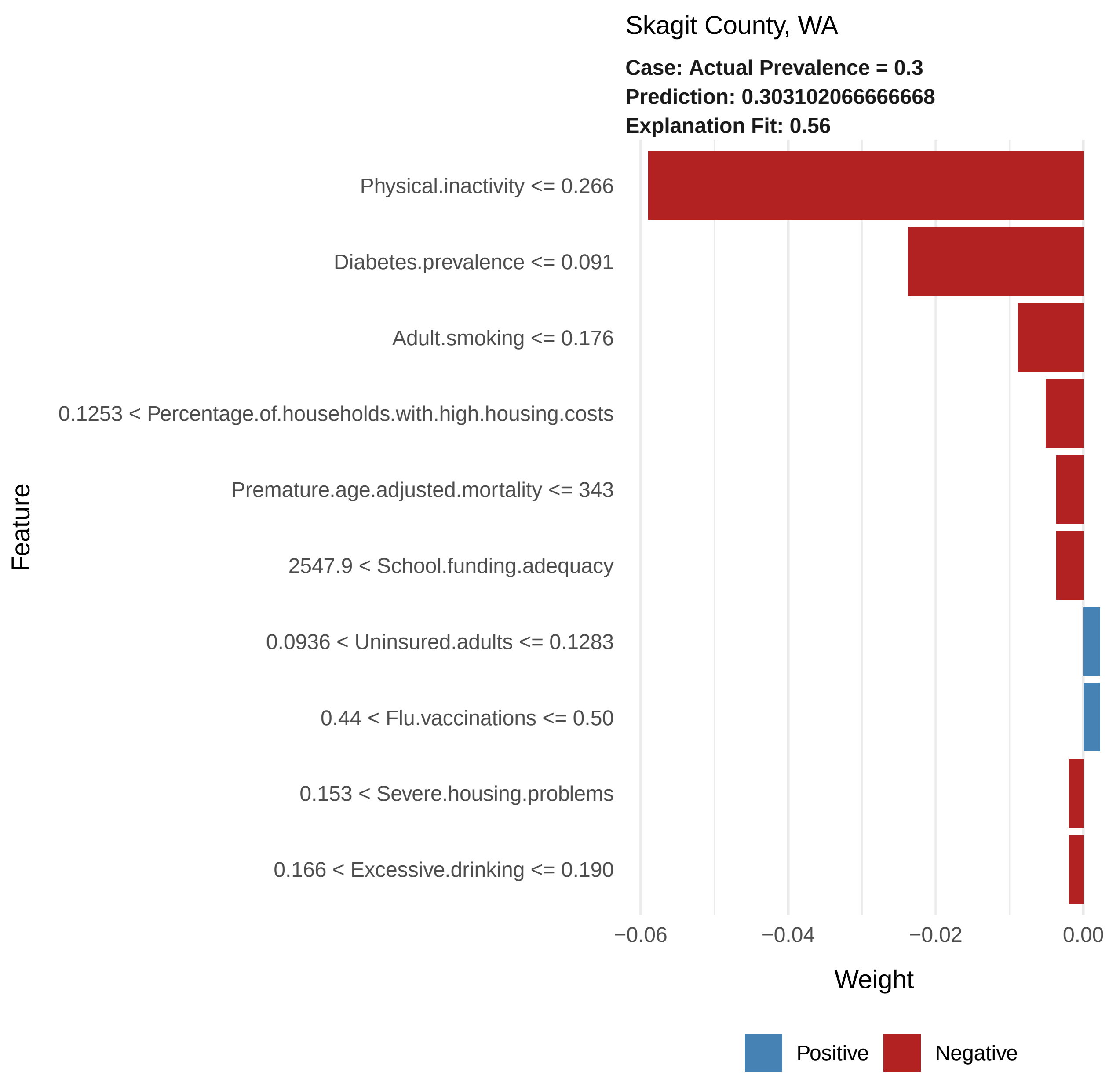
Skagit County, WA
Case: Actual Prevalence = 0.3
Prediction: 0.303102066666668
Explanation Fit: 0.56
Physical.inactivity <= 0.266
Diabetes.prevalence <= 0.091
Adult.smoking <= 0.176
0.1253 < Percentage.of.households.with.high.housing.costs
Premature.age.adjusted.mortality <= 343
2547.9 < School.funding.adequacy
0.0936 < Uninsured.adults <= 0.1283
0.44 < Flu.vaccinations <= 0.50
0.153 < Severe.housing.problems
0.166 < Excessive.drinking <= 0.190
Feature
−0.06
−0.04
−0.02
0.00
Weight
Positive
Negative

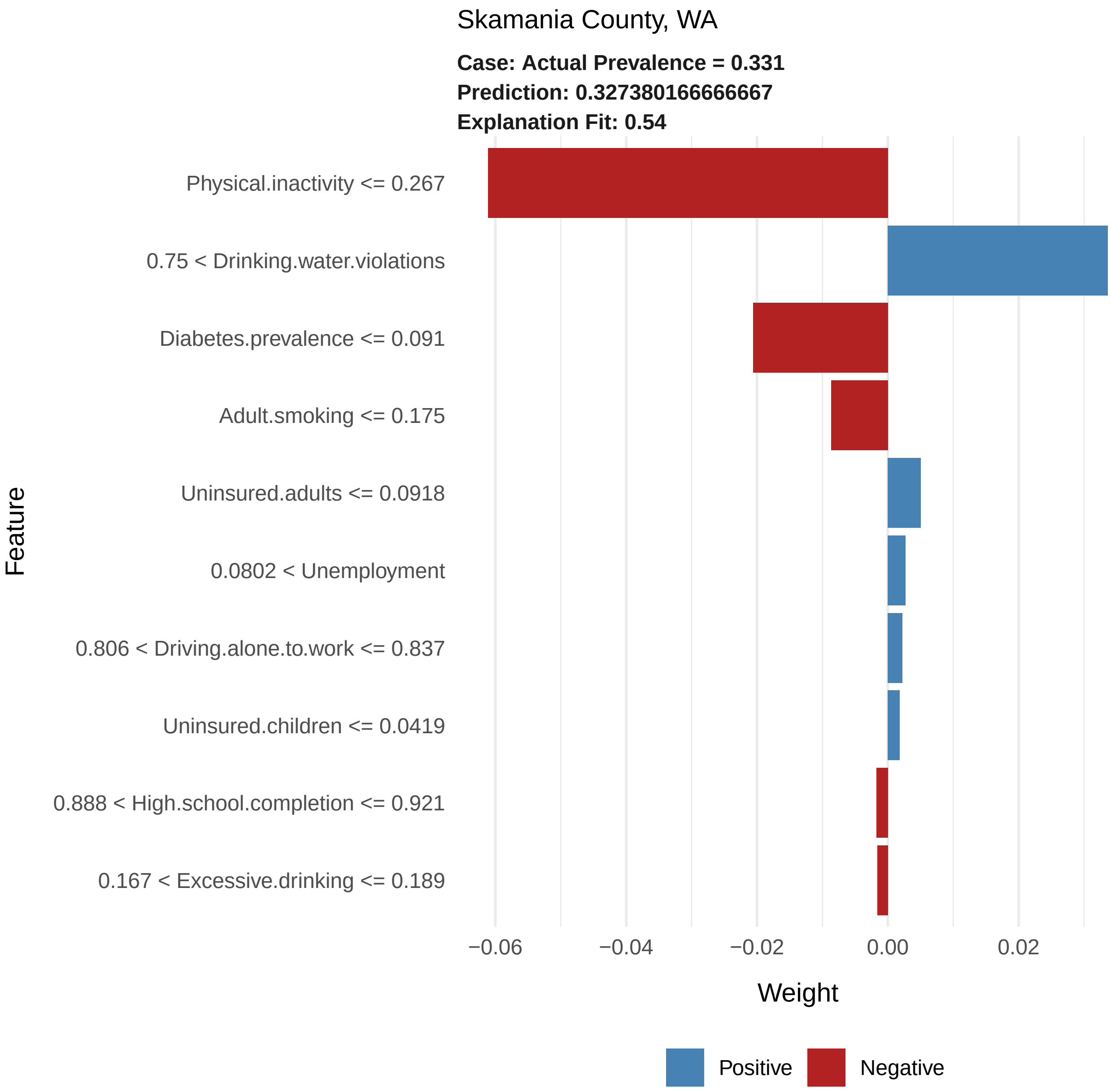

Skamania County, WA
Case: Actual Prevalence = 0.331
Prediction: 0.327380166666667
Explanation Fit: 0.54
Physical.inactivity <= 0.267
0.75 < Drinking.water.violations
Diabetes.prevalence <= 0.091
Adult.smoking <= 0.175
Uninsured.adults <= 0.0918
0.0802 < Unemployment
0.806 < Driving.alone.to.work <= 0.837
Uninsured.children <= 0.0419
0.888 < High.school.completion <= 0.921
0.167 < Excessive.drinking <= 0.189
Feature
−0.06
−0.04
−0.02
0.00
0.02
Weight
Positive
Negative

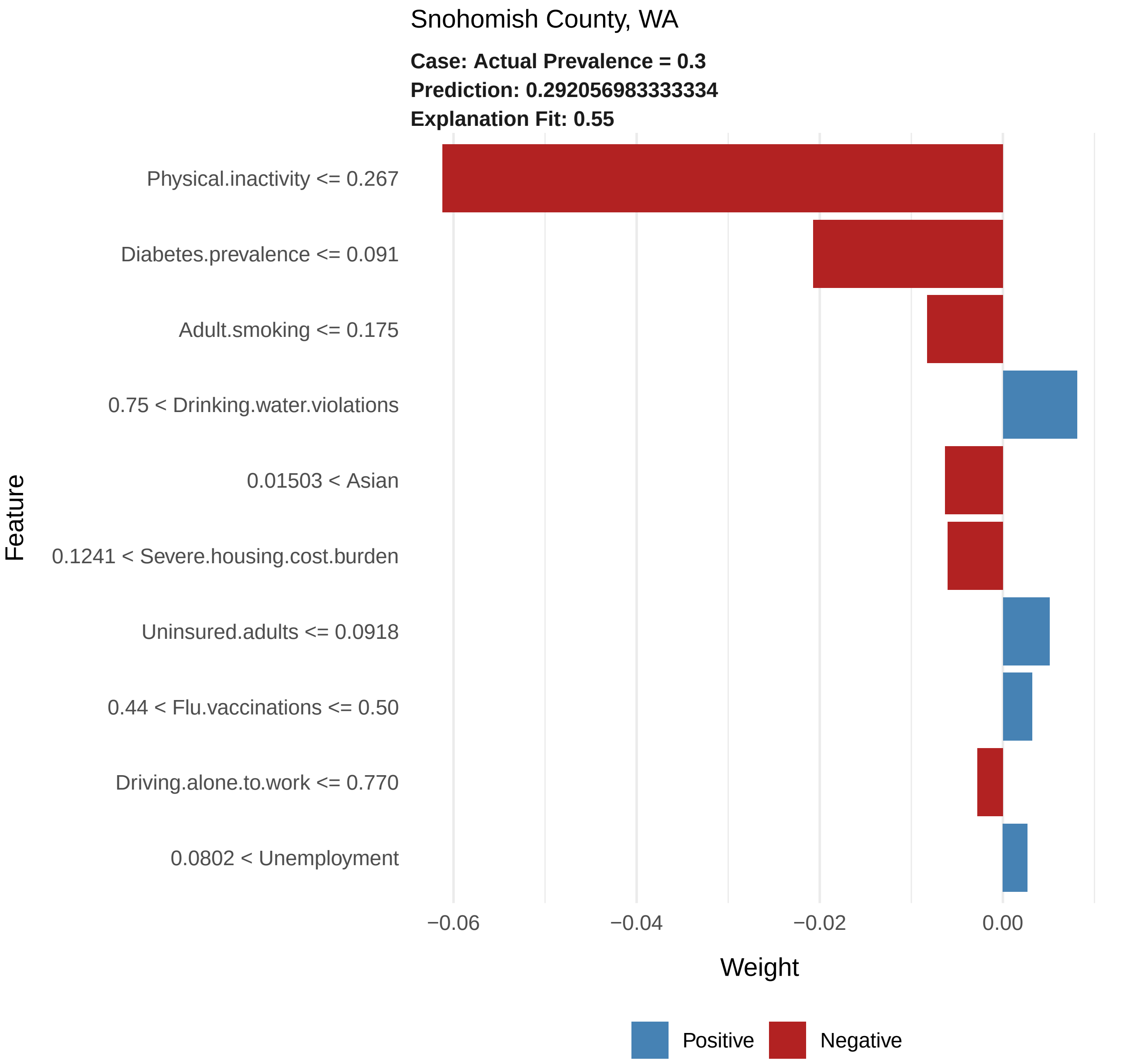

Snohomish County, WA
Case: Actual Prevalence = 0.3
Prediction: 0.292056983333334
Explanation Fit: 0.55
Physical.inactivity <= 0.267
Diabetes.prevalence <= 0.091
Adult.smoking <= 0.175
0.75 < Drinking.water.violations
0.01503 < Asian
0.1241 < Severe.housing.cost.burden
Uninsured.adults <= 0.0918
0.44 < Flu.vaccinations <= 0.50
Driving.alone.to.work <= 0.770
0.0802 < Unemployment
Feature
−0.06
−0.04
−0.02
0.00
Weight
Positive
Negative

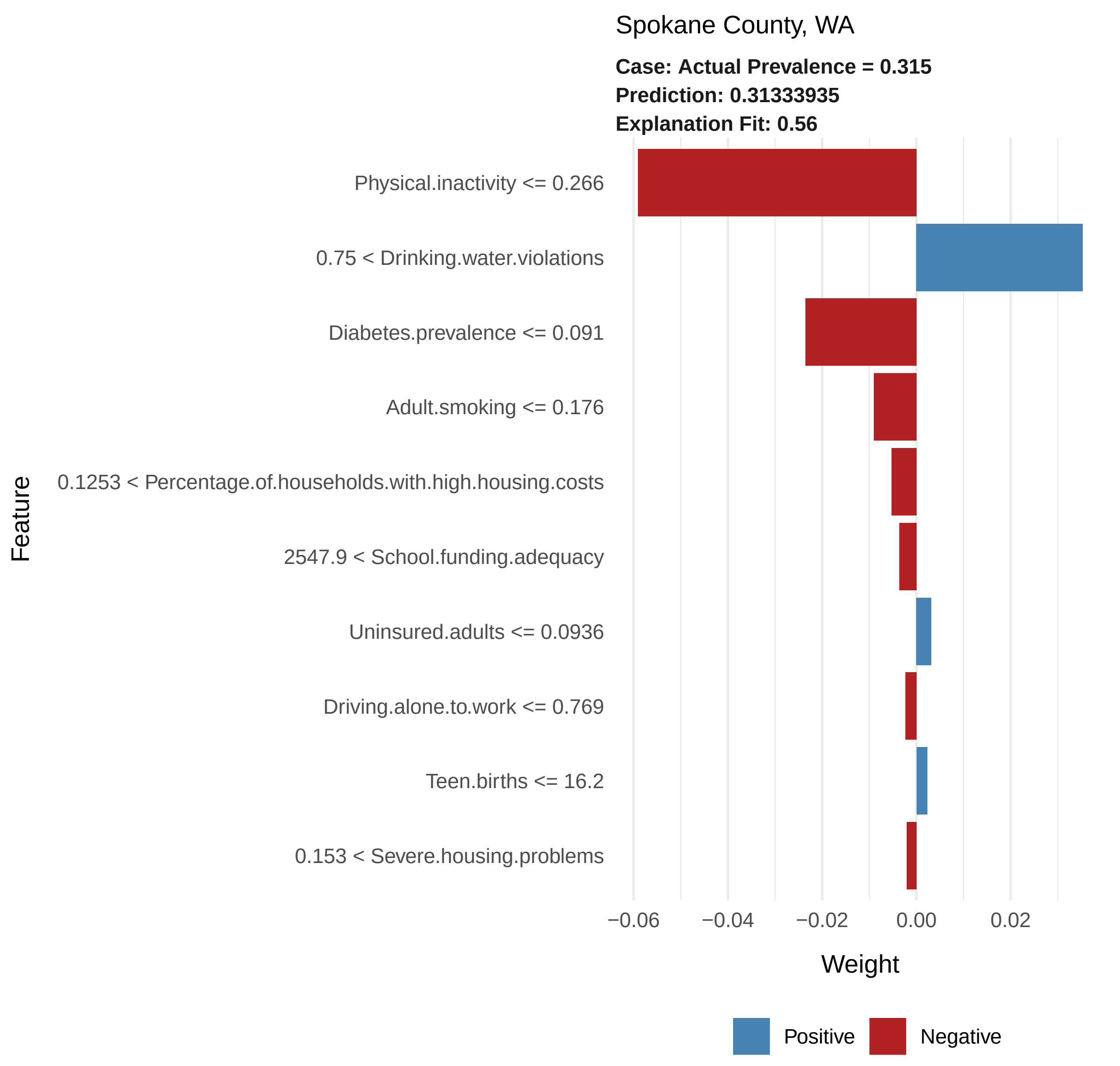

Spokane County, WA
Case: Actual Prevalence = 0.315
Prediction: 0.31333935
Explanation Fit: 0.56
Physical.inactivity <= 0.266
0.75 < Drinking.water.violations
Diabetes.prevalence <= 0.091
Adult.smoking <= 0.176
0.1253 < Percentage.of.households.with.high.housing.costs
2547.9 < School.funding.adequacy
Uninsured.adults <= 0.0936
Driving.alone.to.work <= 0.769
Teen.births <= 16.2
0.153 < Severe.housing.problems
Feature
−0.06
−0.04
−0.02
0.00
0.02
Weight
Positive
Negative

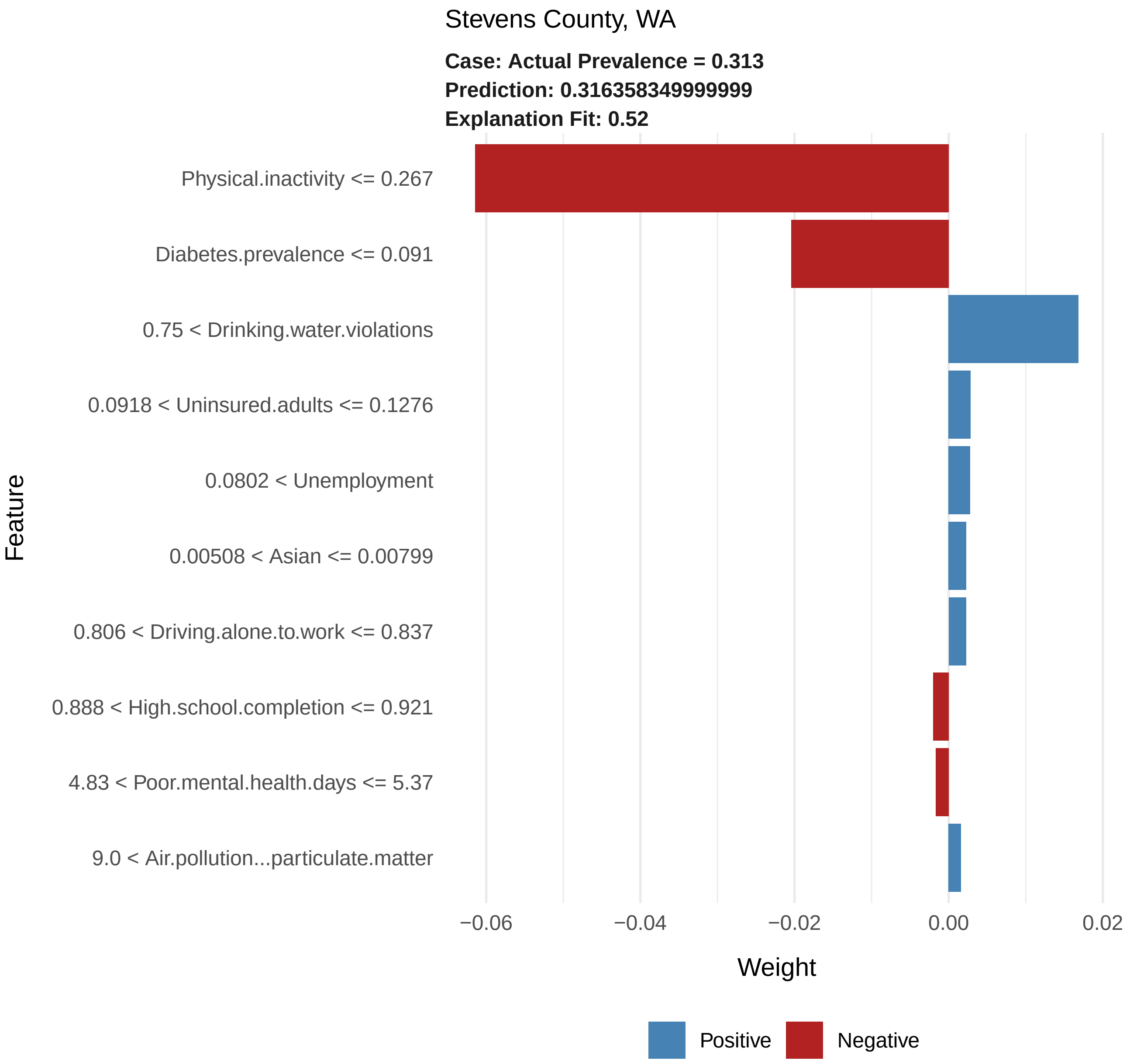
Stevens County, WA
Case: Actual Prevalence = 0.313
Prediction: 0.316358349999999
Explanation Fit: 0.52
Physical.inactivity <= 0.267
Diabetes.prevalence <= 0.091
0.75 < Drinking.water.violations
0.0918 < Uninsured.adults <= 0.1276
0.0802 < Unemployment
0.00508 < Asian <= 0.00799
0.806 < Driving.alone.to.work <= 0.837
0.888 < High.school.completion <= 0.921
4.83 < Poor.mental.health.days <= 5.37
9.0 < Air.pollution...particulate.matter
Feature
−0.06
−0.04
−0.02
0.00
0.02
Weight
Positive
Negative

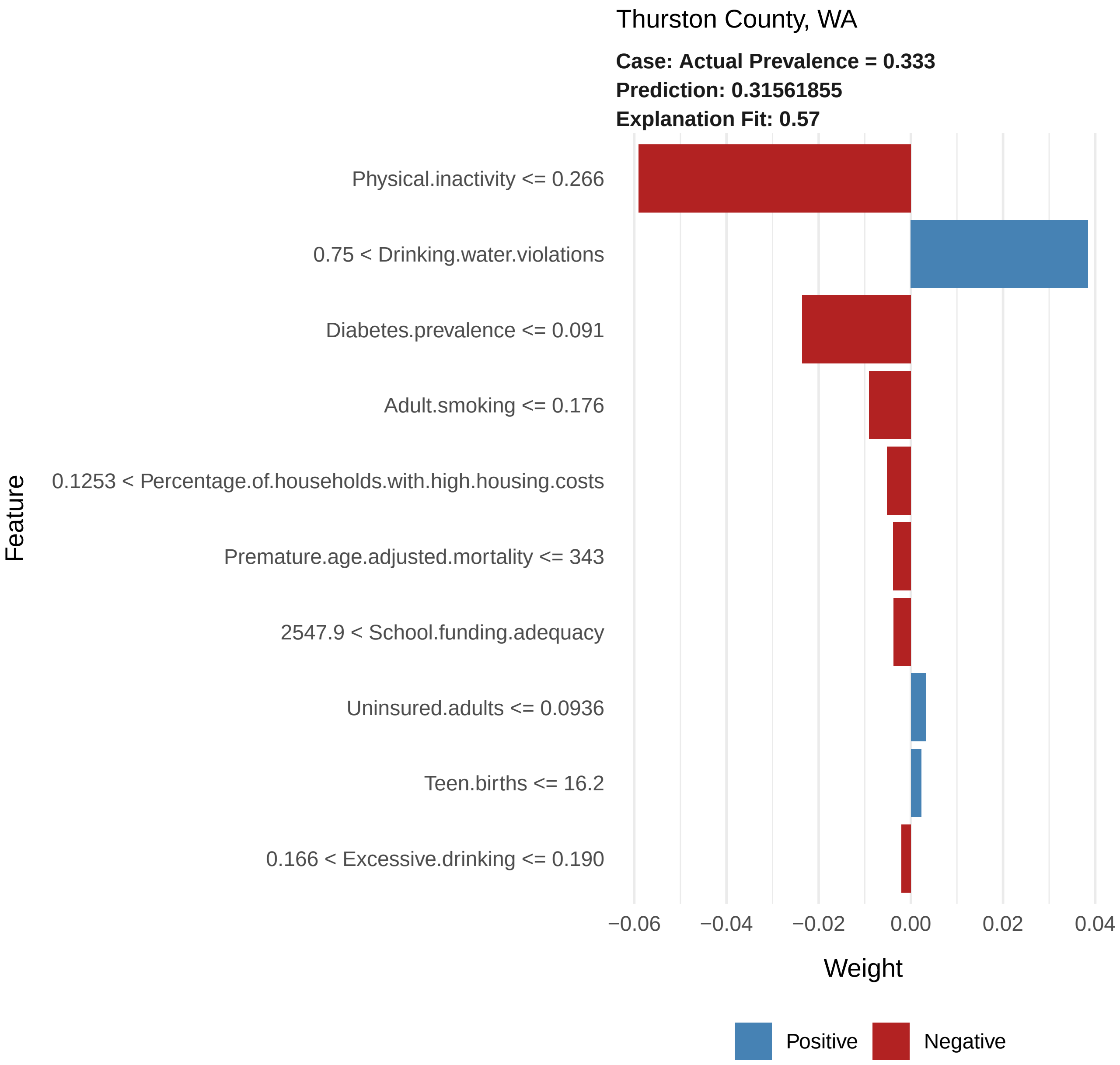
Thurston County, WA
Case: Actual Prevalence = 0.333
Prediction: 0.31561855
Explanation Fit: 0.57
Physical.inactivity <= 0.266
0.75 < Drinking.water.violations
Diabetes.prevalence <= 0.091
Adult.smoking <= 0.176
0.1253 < Percentage.of.households.with.high.housing.costs
Premature.age.adjusted.mortality <= 343
2547.9 < School.funding.adequacy
Uninsured.adults <= 0.0936
Teen.births <= 16.2
0.166 < Excessive.drinking <= 0.190
Feature
−0.06
−0.04
−0.02
0.00
0.02
0.04
Weight
Positive
Negative

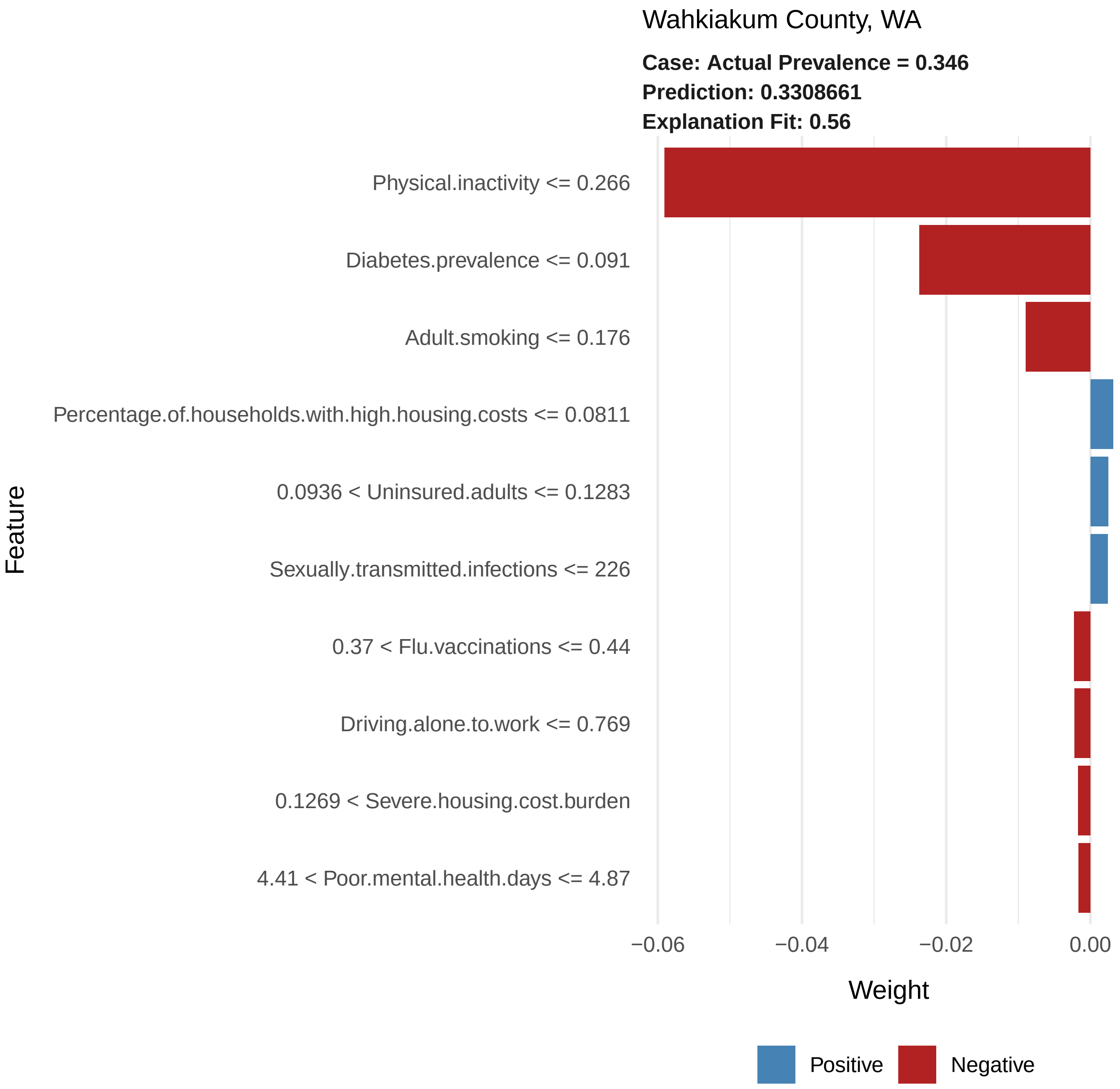
Wahkiakum County, WA
Case: Actual Prevalence = 0.346
Prediction: 0.3308661
Explanation Fit: 0.56
Physical.inactivity <= 0.266
Diabetes.prevalence <= 0.091
Adult.smoking <= 0.176
Percentage.of.households.with.high.housing.costs <= 0.0811
0.0936 < Uninsured.adults <= 0.1283
Sexually.transmitted.infections <= 226
0.37 < Flu.vaccinations <= 0.44
Driving.alone.to.work <= 0.769
0.1269 < Severe.housing.cost.burden
4.41 < Poor.mental.health.days <= 4.87
Feature
−0.06
−0.04
−0.02
0.00
Weight
Positive
Negative

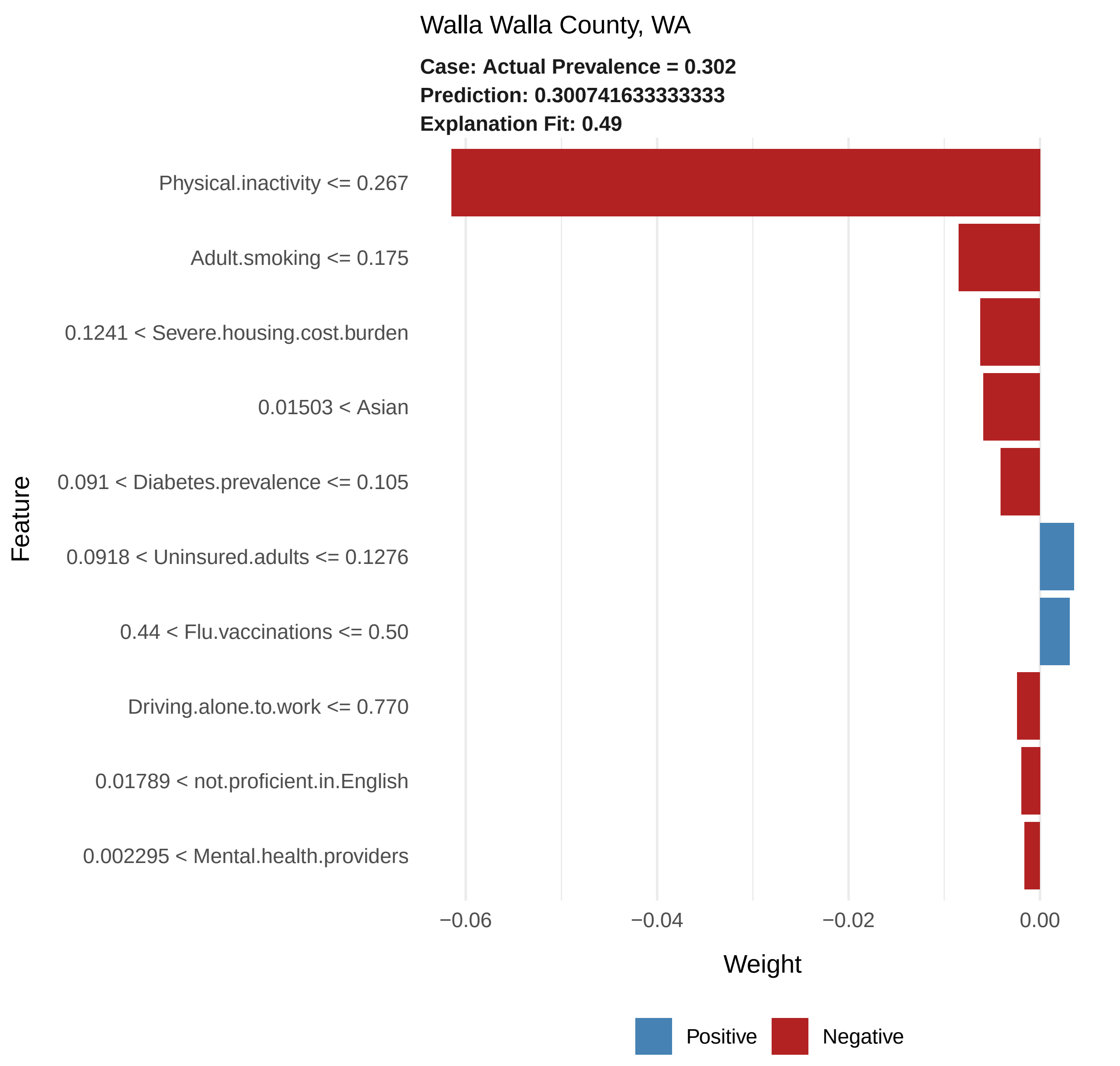

Walla Walla County, WA
Case: Actual Prevalence = 0.302
Prediction: 0.300741633333333
Explanation Fit: 0.49
Physical.inactivity <= 0.267
Adult.smoking <= 0.175
0.1241 < Severe.housing.cost.burden
0.01503 < Asian
0.091 < Diabetes.prevalence <= 0.105
0.0918 < Uninsured.adults <= 0.1276
0.44 < Flu.vaccinations <= 0.50
Driving.alone.to.work <= 0.770
0.01789 < not.proficient.in.English
0.002295 < Mental.health.providers
Feature
−0.06
−0.04
−0.02
0.00
Weight
Positive
Negative

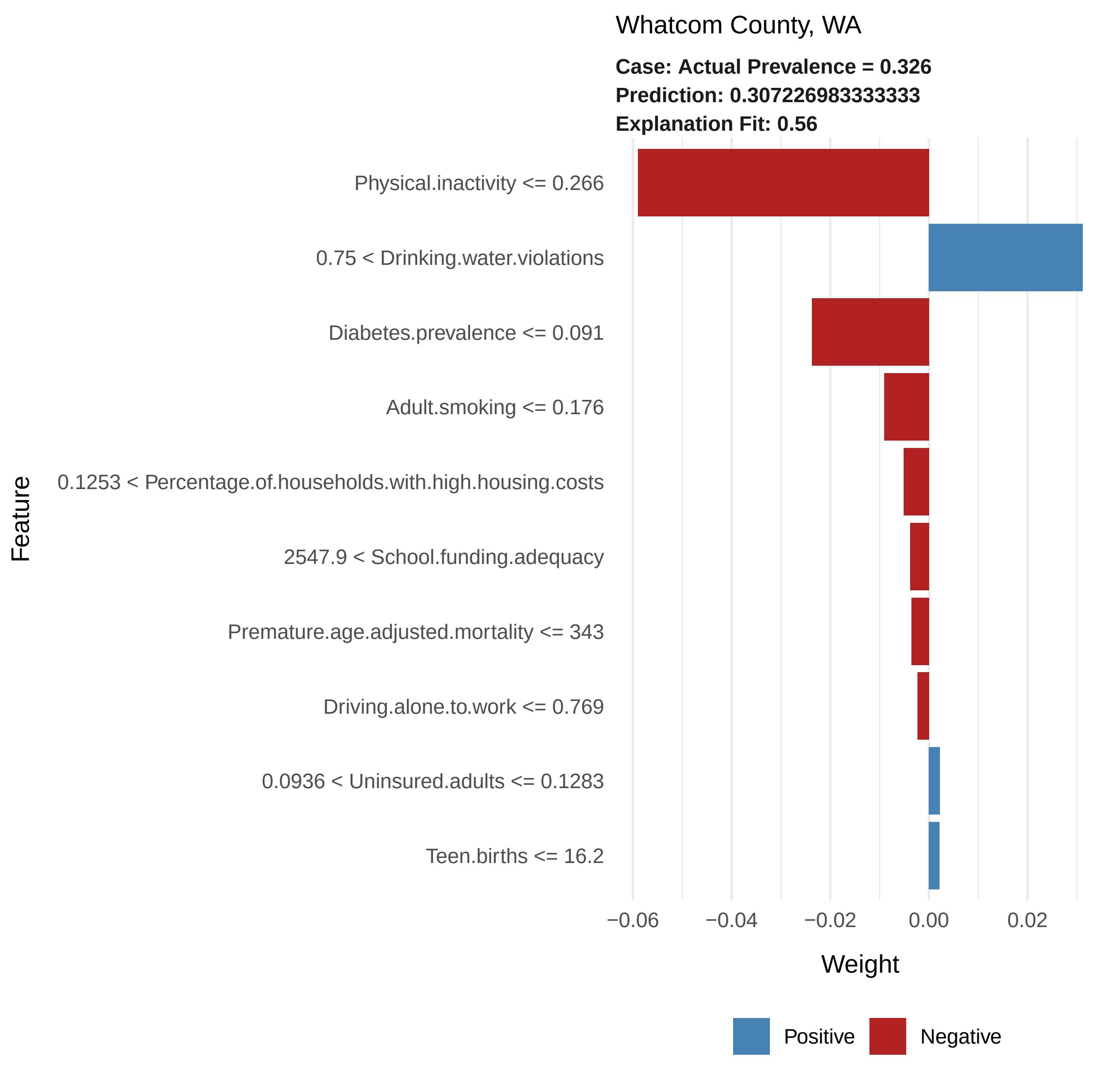
Whatcom County, WA
Case: Actual Prevalence = 0.326
Prediction: 0.307226983333333
Explanation Fit: 0.56
Physical.inactivity <= 0.266
0.75 < Drinking.water.violations
Diabetes.prevalence <= 0.091
Adult.smoking <= 0.176
0.1253 < Percentage.of.households.with.high.housing.costs
2547.9 < School.funding.adequacy
Premature.age.adjusted.mortality <= 343
Driving.alone.to.work <= 0.769
0.0936 < Uninsured.adults <= 0.1283
Teen.births <= 16.2
Feature
−0.06
−0.04
−0.02
0.00
0.02
Weight
Positive
Negative

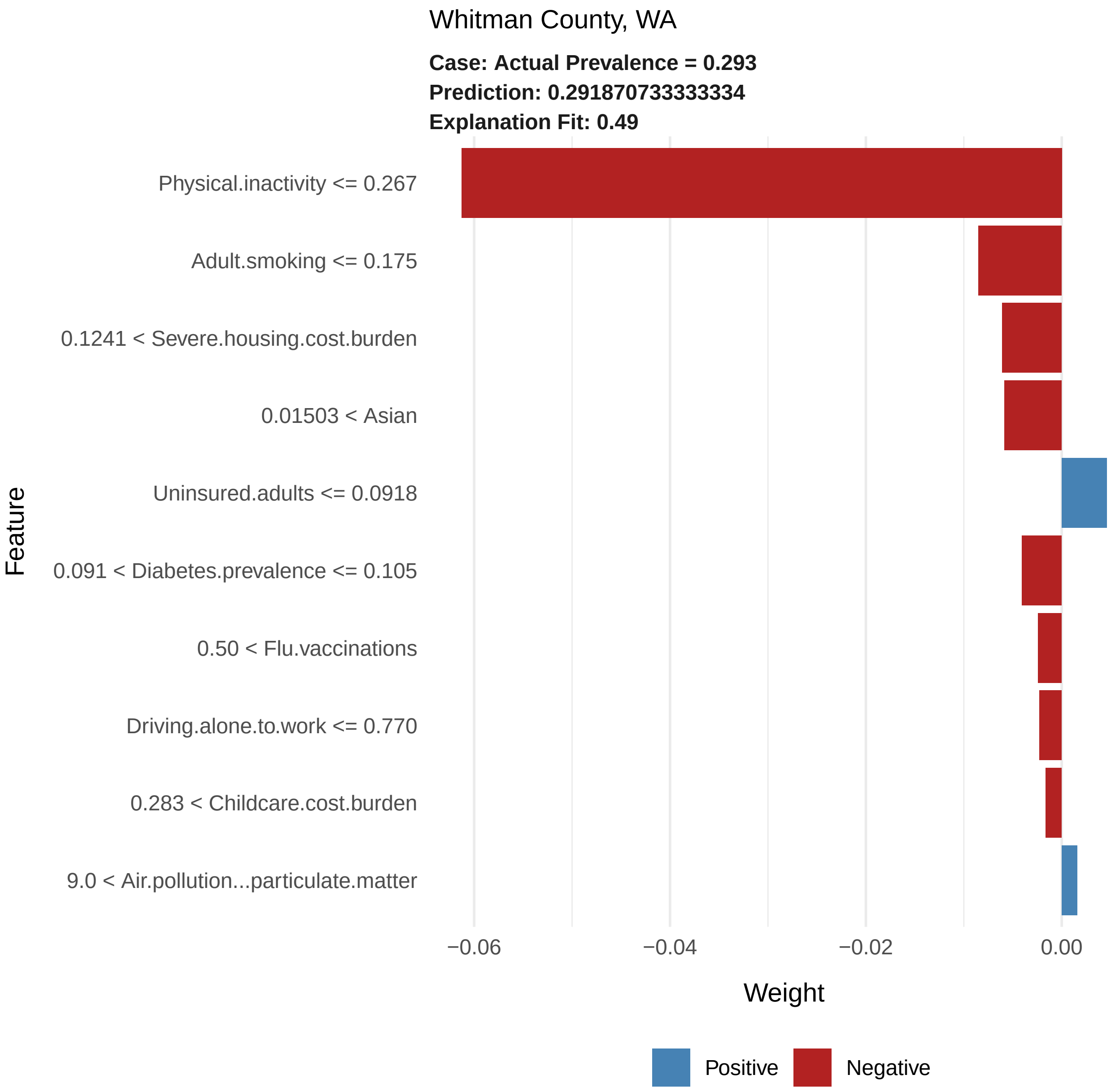

Whitman County, WA
Case: Actual Prevalence = 0.293
Prediction: 0.291870733333334
Explanation Fit: 0.49
Physical.inactivity <= 0.267
Adult.smoking <= 0.175
0.1241 < Severe.housing.cost.burden
0.01503 < Asian
Uninsured.adults <= 0.0918
0.091 < Diabetes.prevalence <= 0.105
0.50 < Flu.vaccinations
Driving.alone.to.work <= 0.770
0.283 < Childcare.cost.burden
9.0 < Air.pollution...particulate.matter
Feature
−0.06
−0.04
−0.02
0.00
Weight
Positive
Negative

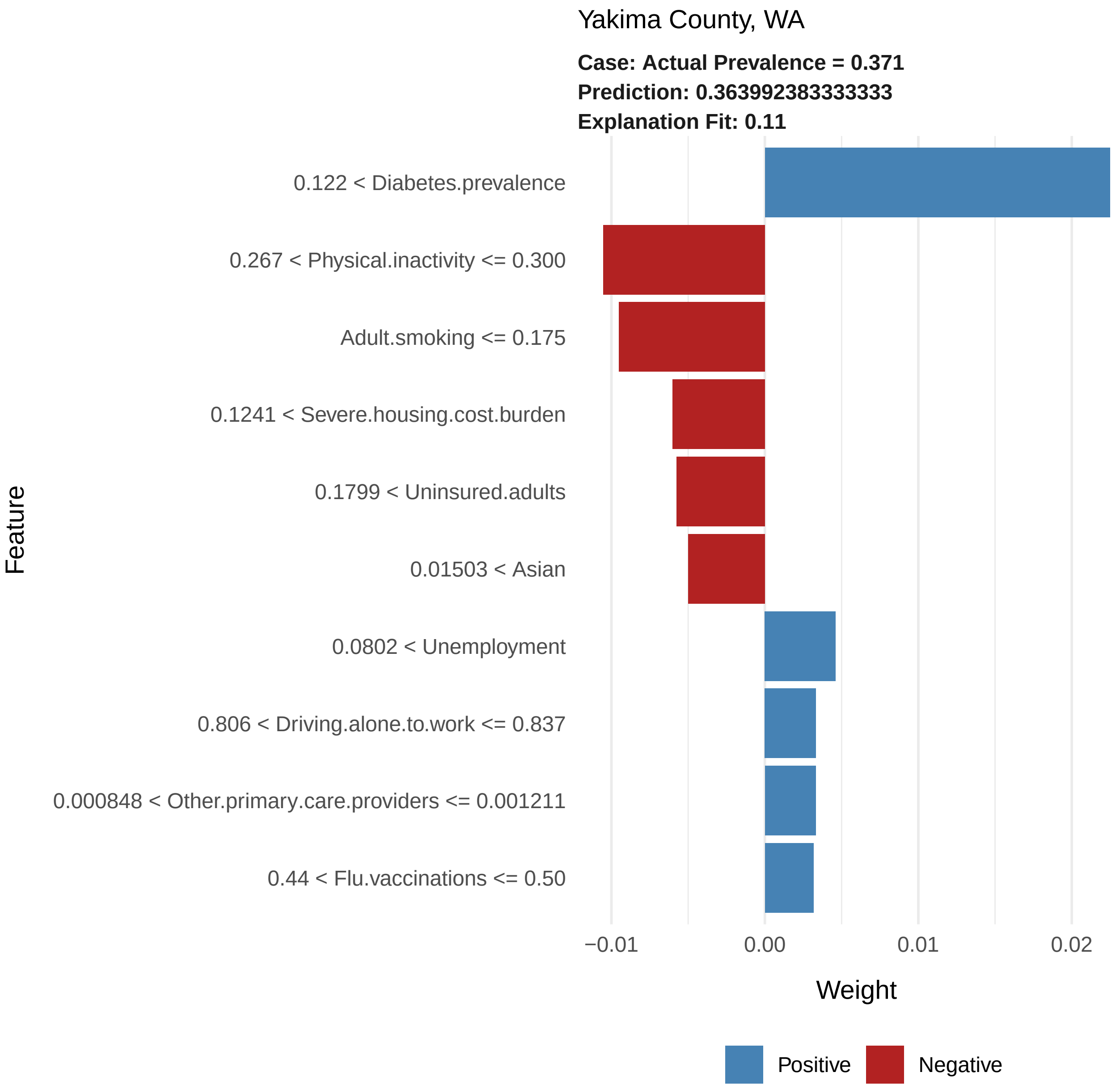
Yakima County, WA
Case: Actual Prevalence = 0.371
Prediction: 0.363992383333333
Explanation Fit: 0.11
Feature
0.122 < Diabetes.prevalence
0.267 < Physical.inactivity <= 0.300
Adult.smoking <= 0.175
0.1241 < Severe.housing.cost.burden
0.1799 < Uninsured.adults
0.01503 < Asian
0.0802 < Unemployment
0.806 < Driving.alone.to.work <= 0.837
0.000848 < Other.primary.care.providers <= 0.001211
0.44 < Flu.vaccinations <= 0.50
−0.01
0.00
0.01
0.02
Weight
Positive
Negative

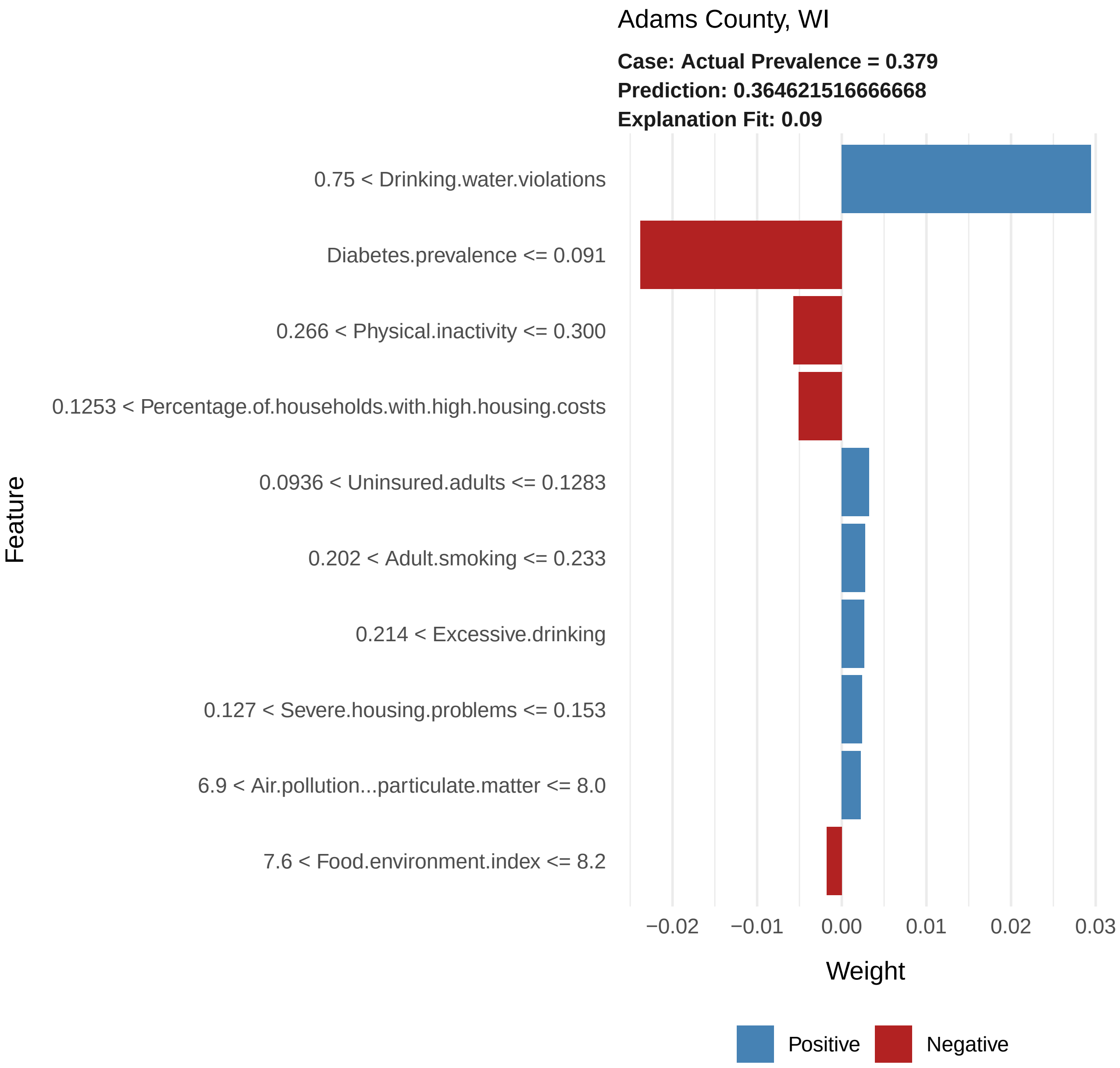
Adams County, WI
Case: Actual Prevalence = 0.379
Prediction: 0.364621516666668
Explanation Fit: 0.09
0.75 < Drinking.water.violations
Diabetes.prevalence <= 0.091
0.266 < Physical.inactivity <= 0.300
0.1253 < Percentage.of.households.with.high.housing.costs
0.0936 < Uninsured.adults <= 0.1283
0.202 < Adult.smoking <= 0.233
0.214 < Excessive.drinking
0.127 < Severe.housing.problems <= 0.153
6.9 < Air.pollution...particulate.matter <= 8.0
7.6 < Food.environment.index <= 8.2
Feature
−0.02
−0.01
0.00
0.01
0.02
0.03
Weight
Positive
Negative

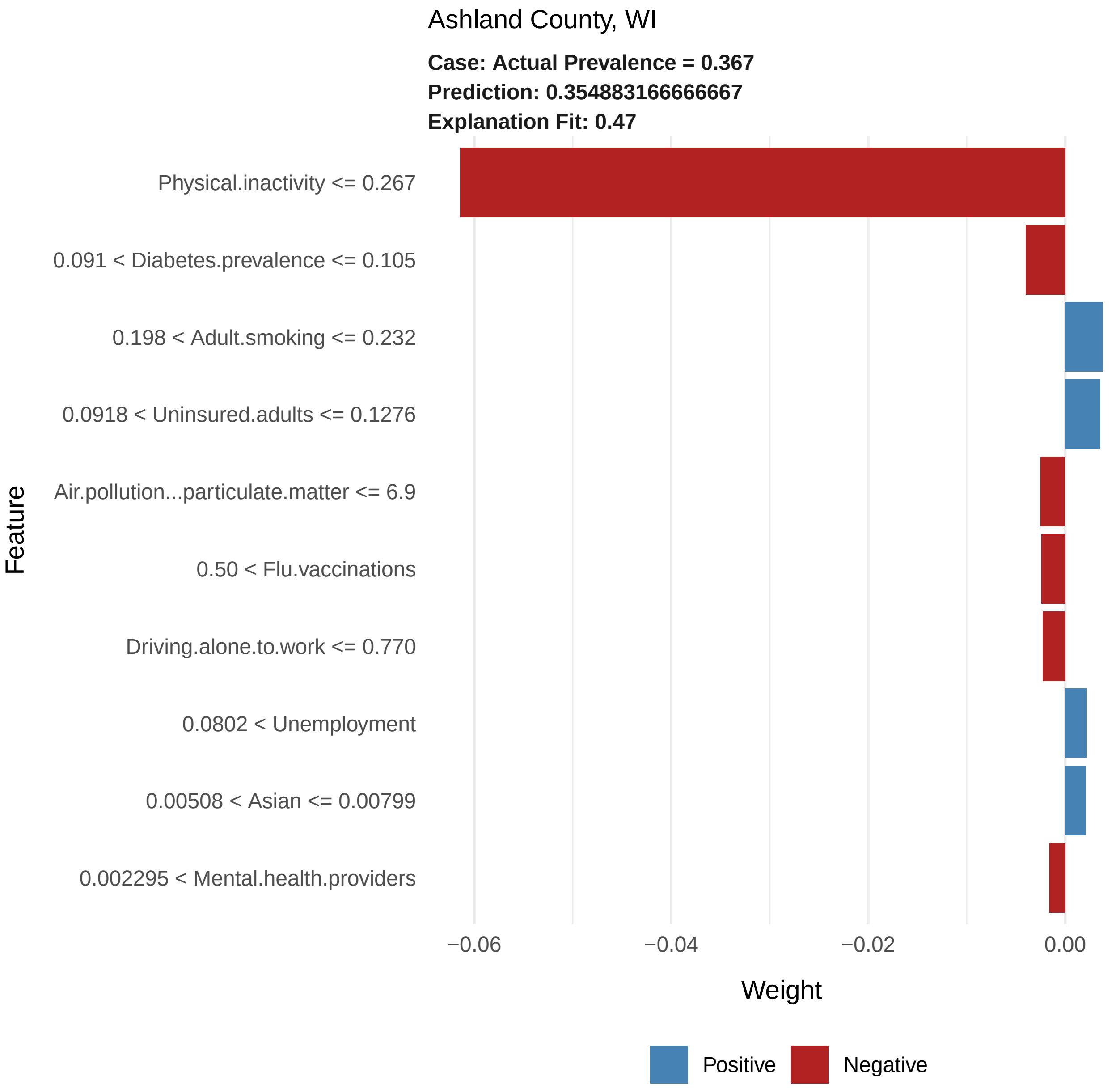
Ashland County, WI
Case: Actual Prevalence = 0.367
Prediction: 0.354883166666667
Explanation Fit: 0.47
Physical.inactivity <= 0.267
0.091 < Diabetes.prevalence <= 0.105
0.198 < Adult.smoking <= 0.232
0.0918 < Uninsured.adults <= 0.1276
Air.pollution...particulate.matter <= 6.9
0.50 < Flu.vaccinations
Driving.alone.to.work <= 0.770
0.0802 < Unemployment
0.00508 < Asian <= 0.00799
0.002295 < Mental.health.providers
Feature
−0.06
−0.04
−0.02
0.00
Weight
Positive
Negative

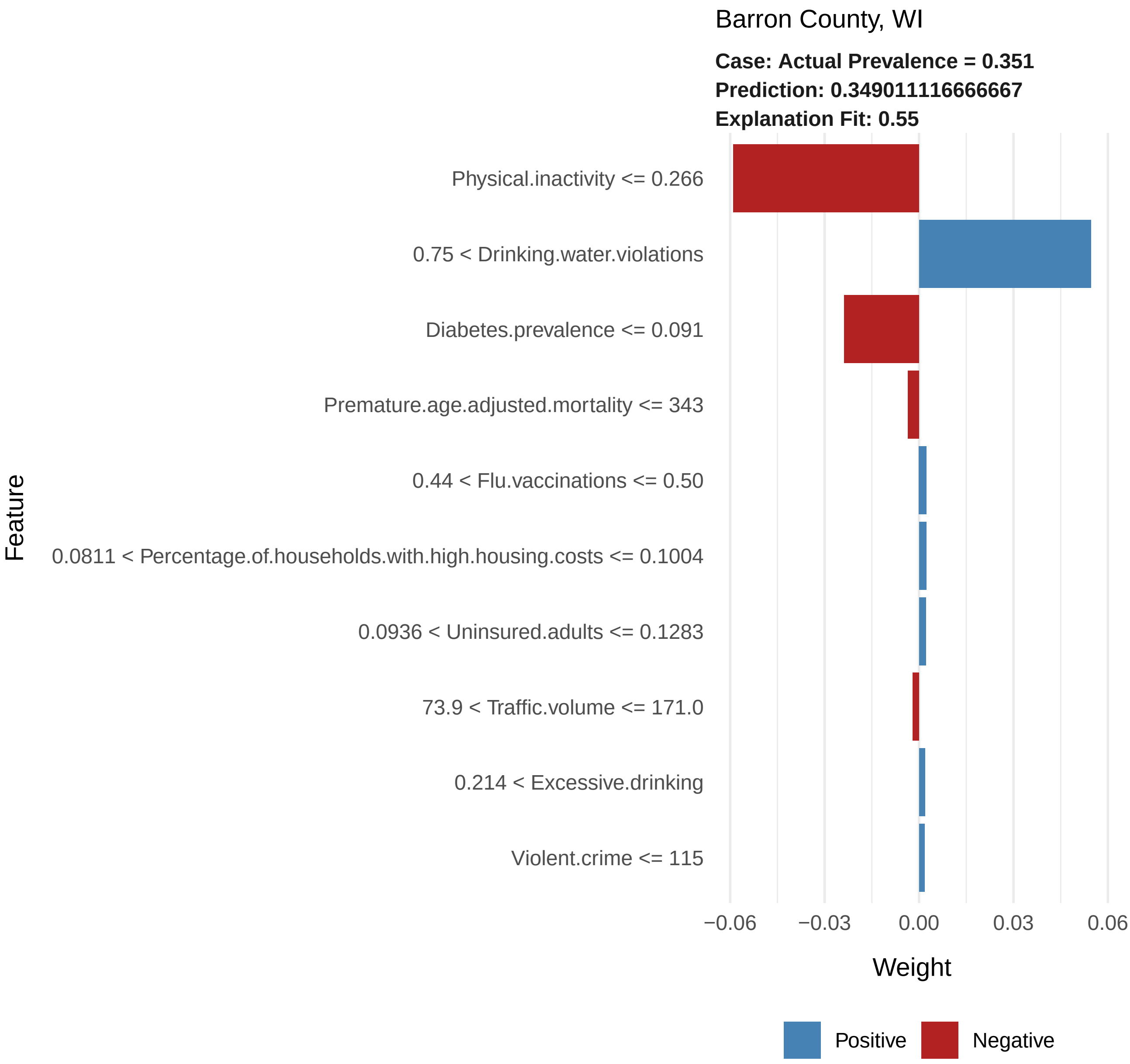
Barron County, WI
Case: Actual Prevalence = 0.351
Prediction: 0.349011116666667
Explanation Fit: 0.55
Physical.inactivity <= 0.266
0.75 < Drinking.water.violations
Diabetes.prevalence <= 0.091
Premature.age.adjusted.mortality <= 343
0.44 < Flu.vaccinations <= 0.50
0.0811 < Percentage.of.households.with.high.housing.costs <= 0.1004
0.0936 < Uninsured.adults <= 0.1283
73.9 < Traffic.volume <= 171.0
0.214 < Excessive.drinking
Violent.crime <= 115
Feature
−0.06
−0.03
0.00
0.03
0.06
Weight
Positive
Negative

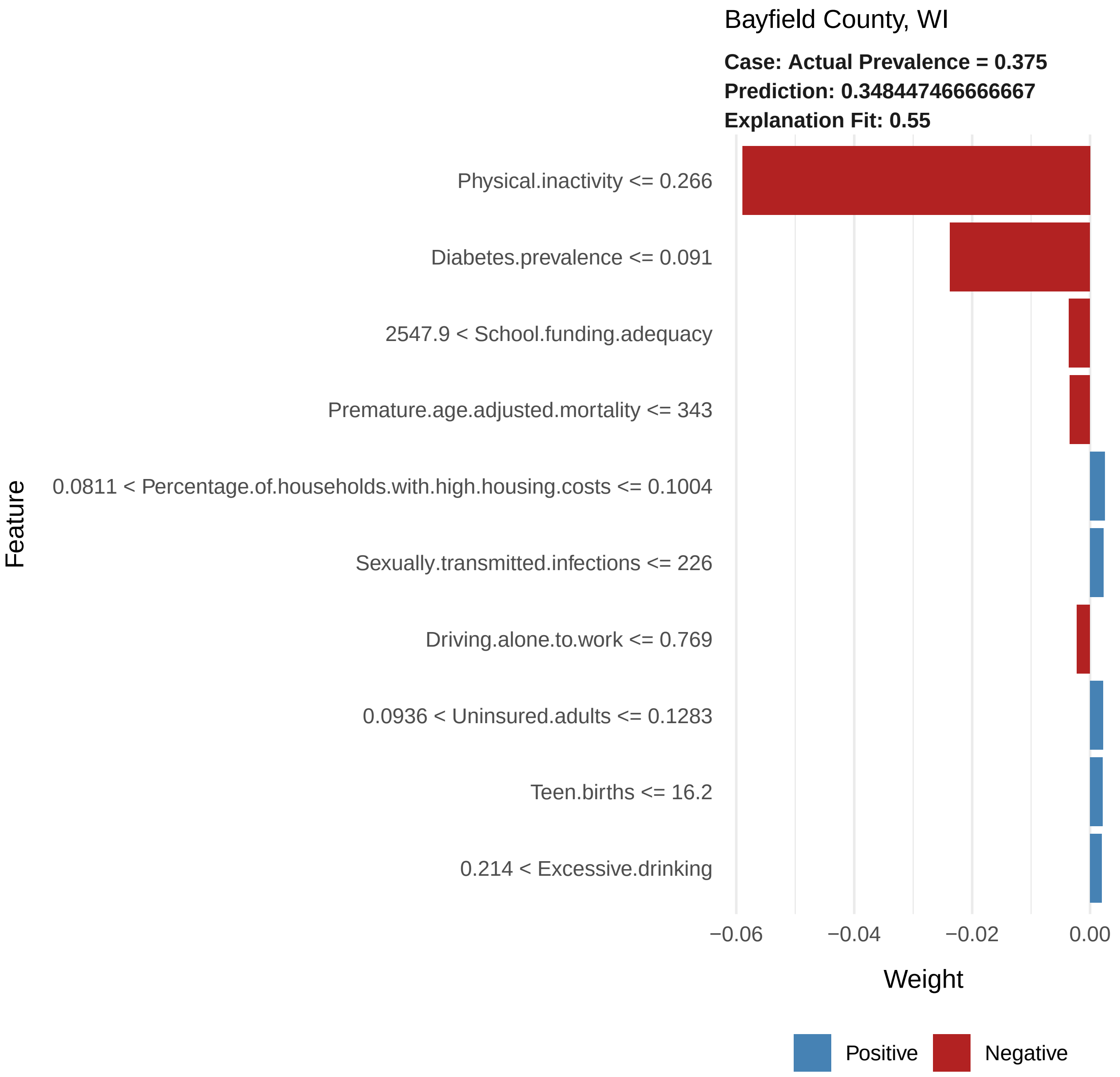

Bayfield County, WI
Case: Actual Prevalence = 0.375
Prediction: 0.348447466666667
Explanation Fit: 0.55
Physical.inactivity <= 0.266
Diabetes.prevalence <= 0.091
2547.9 < School.funding.adequacy
Premature.age.adjusted.mortality <= 343
0.0811 < Percentage.of.households.with.high.housing.costs <= 0.1004
Sexually.transmitted.infections <= 226
Driving.alone.to.work <= 0.769
0.0936 < Uninsured.adults <= 0.1283
Teen.births <= 16.2
0.214 < Excessive.drinking
Feature
−0.06
−0.04
−0.02
0.00
Weight
Positive
Negative

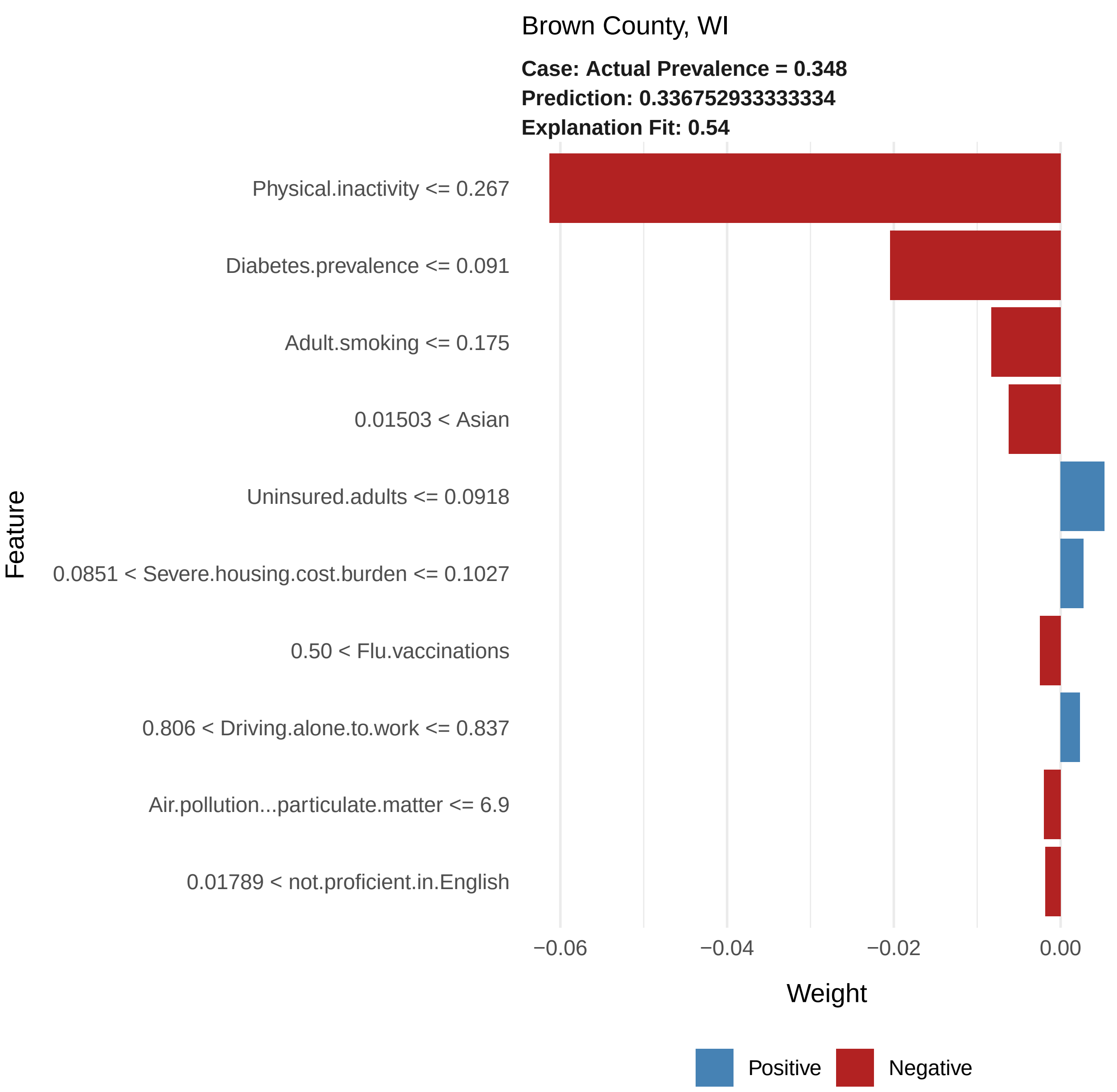

Brown County, WI
Case: Actual Prevalence = 0.348
Prediction: 0.336752933333334
Explanation Fit: 0.54
Physical.inactivity <= 0.267
Diabetes.prevalence <= 0.091
Adult.smoking <= 0.175
0.01503 < Asian
Uninsured.adults <= 0.0918
0.0851 < Severe.housing.cost.burden <= 0.1027
0.50 < Flu.vaccinations
0.806 < Driving.alone.to.work <= 0.837
Air.pollution...particulate.matter <= 6.9
0.01789 < not.proficient.in.English
Feature
−0.06
−0.04
−0.02
0.00
Weight
Positive
Negative

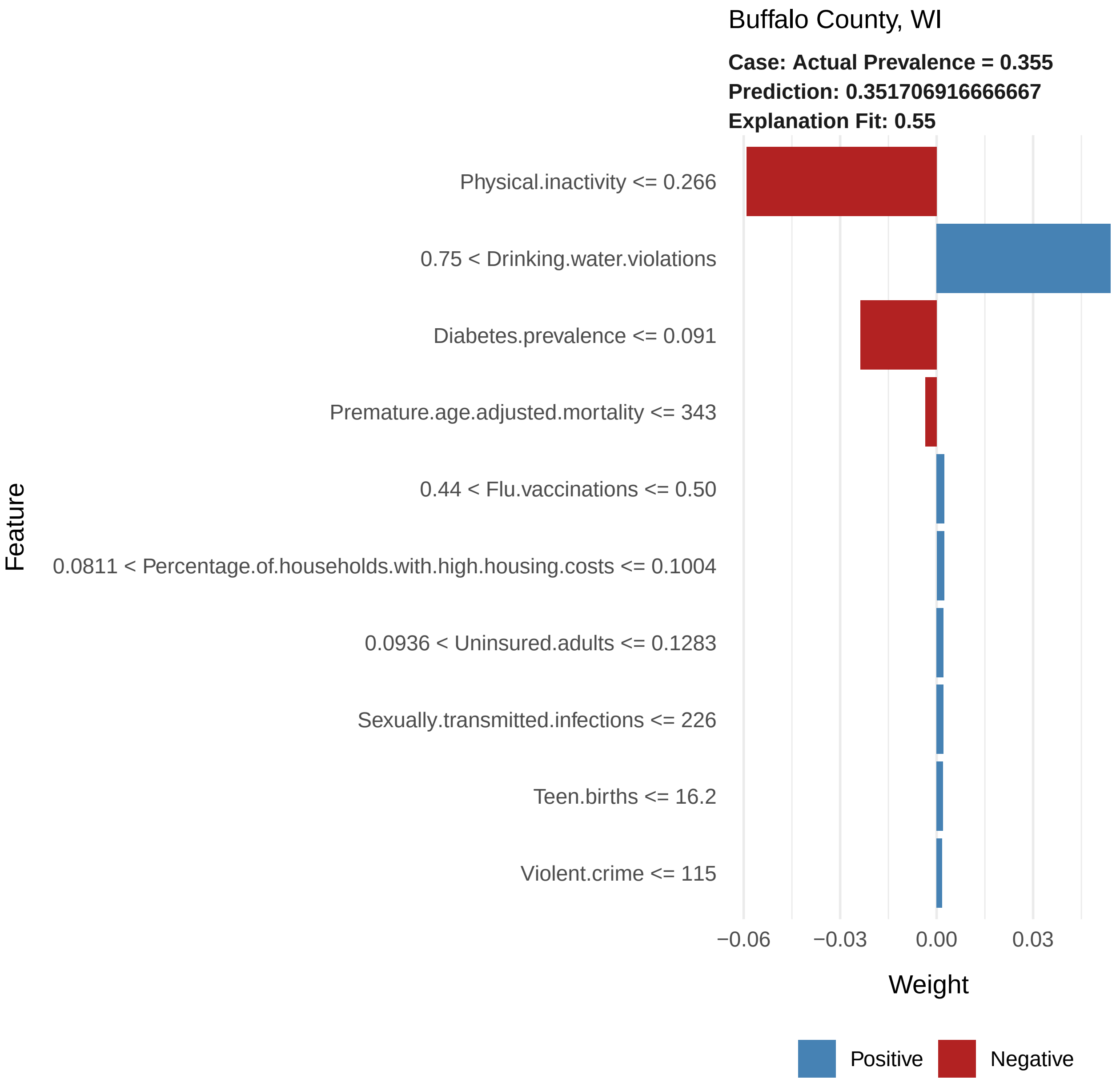

Buffalo County, WI
Case: Actual Prevalence = 0.355
Prediction: 0.351706916666667
Explanation Fit: 0.55
Physical.inactivity <= 0.266
0.75 < Drinking.water.violations
Diabetes.prevalence <= 0.091
Premature.age.adjusted.mortality <= 343
0.44 < Flu.vaccinations <= 0.50
0.0811 < Percentage.of.households.with.high.housing.costs <= 0.1004
0.0936 < Uninsured.adults <= 0.1283
Sexually.transmitted.infections <= 226
Teen.births <= 16.2
Violent.crime <= 115
Feature
−0.06
−0.03
0.00
0.03
Weight
Positive
Negative

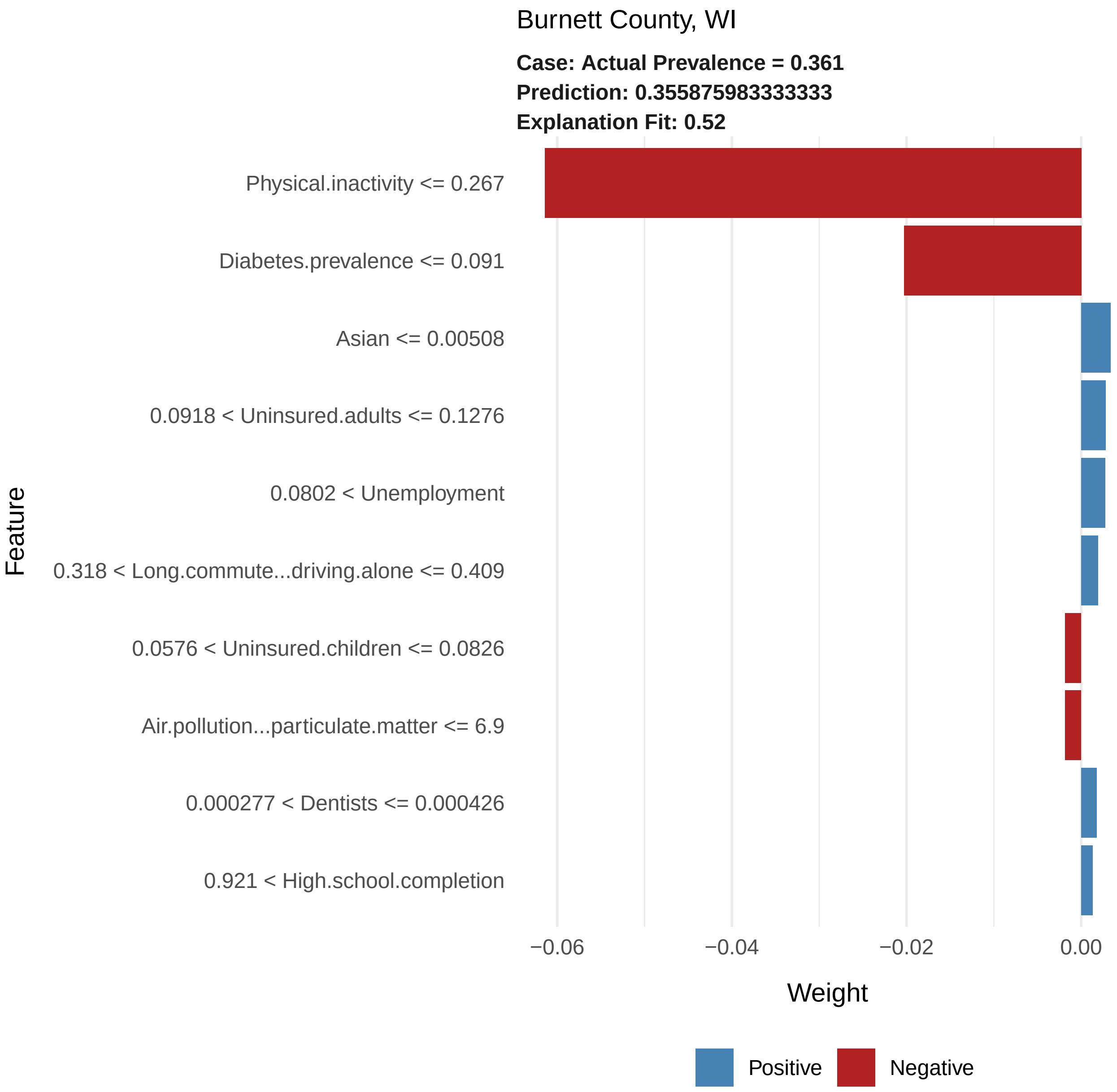

Burnett County, WI
Case: Actual Prevalence = 0.361
Prediction: 0.355875983333333
Explanation Fit: 0.52
Physical.inactivity <= 0.267
Diabetes.prevalence <= 0.091
Asian <= 0.00508
0.0918 < Uninsured.adults <= 0.1276
0.0802 < Unemployment
0.318 < Long.commute...driving.alone <= 0.409
0.0576 < Uninsured.children <= 0.0826
Air.pollution...particulate.matter <= 6.9
0.000277 < Dentists <= 0.000426
0.921 < High.school.completion
Feature
−0.06
−0.04
−0.02
0.00
Weight
Positive
Negative

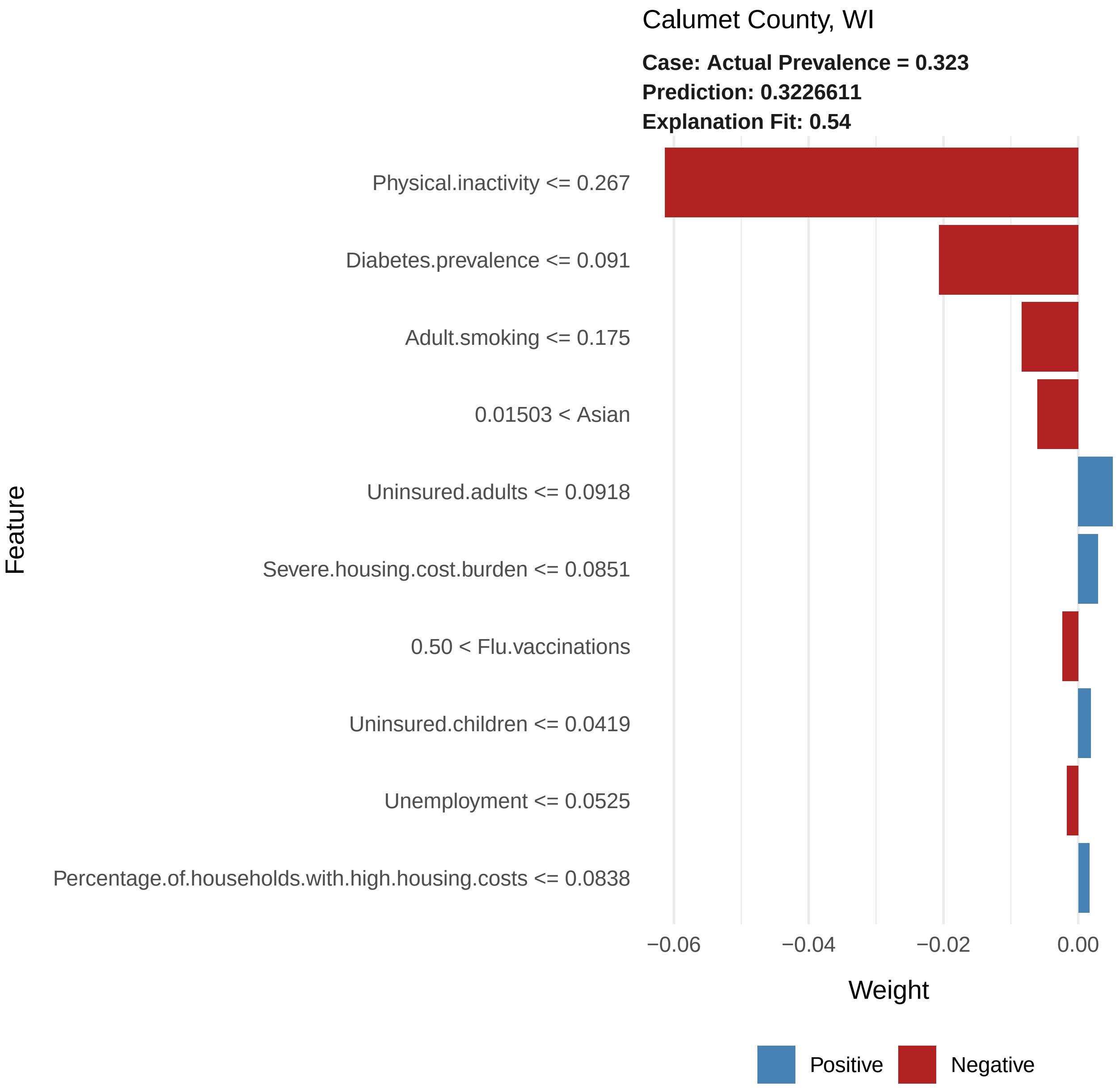
Calumet County, WI
Case: Actual Prevalence = 0.323
Prediction: 0.3226611
Explanation Fit: 0.54
Physical.inactivity <= 0.267
Diabetes.prevalence <= 0.091
Adult.smoking <= 0.175
0.01503 < Asian
Uninsured.adults <= 0.0918
Severe.housing.cost.burden <= 0.0851
0.50 < Flu.vaccinations
Uninsured.children <= 0.0419
Unemployment <= 0.0525
Percentage.of.households.with.high.housing.costs <= 0.0838
Feature
−0.06
−0.04
−0.02
0.00
Weight
Positive
Negative

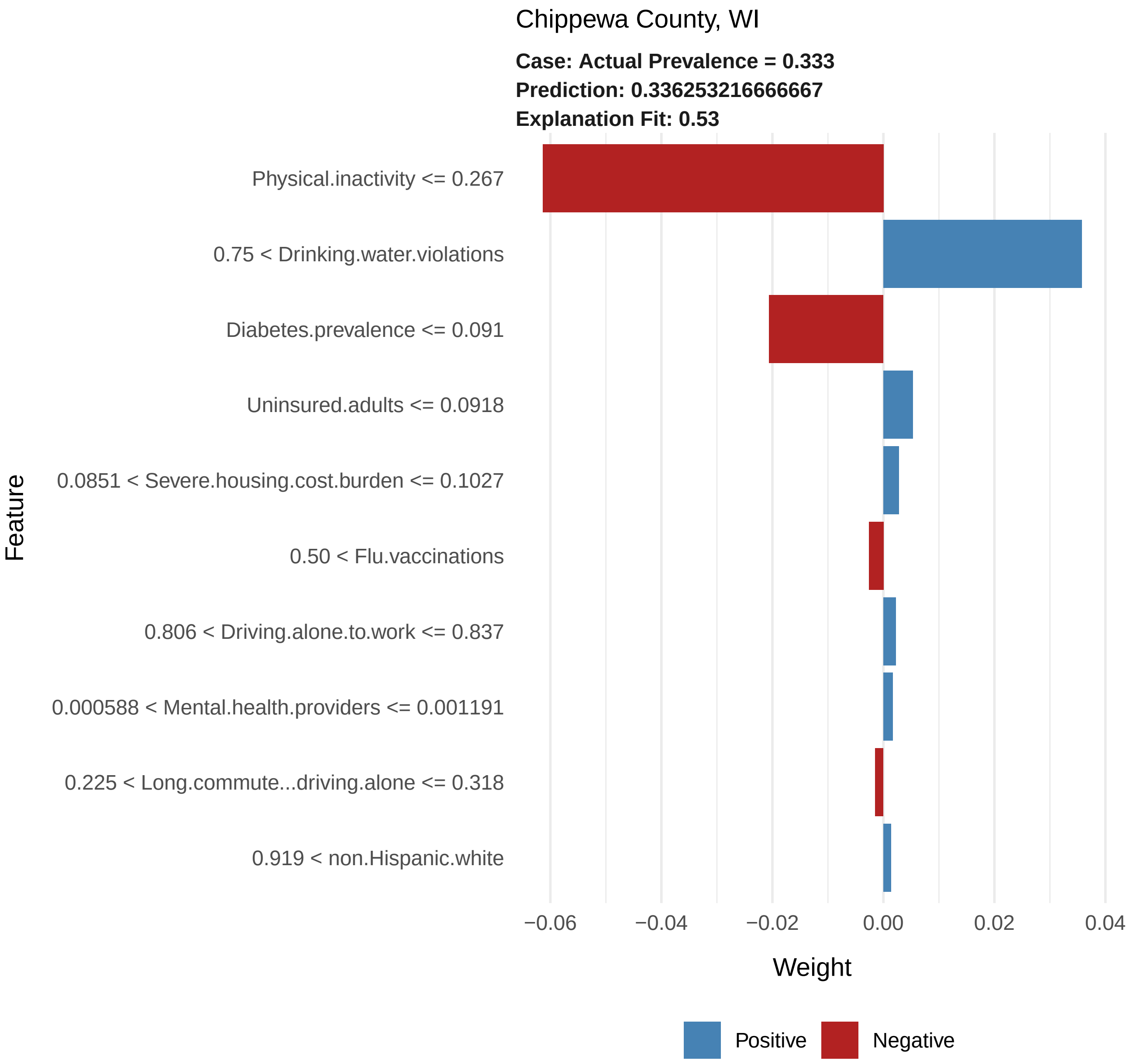
Chippewa County, WI
Case: Actual Prevalence = 0.333
Prediction: 0.336253216666667
Explanation Fit: 0.53
Physical.inactivity <= 0.267
0.75 < Drinking.water.violations
Diabetes.prevalence <= 0.091
Uninsured.adults <= 0.0918
0.0851 < Severe.housing.cost.burden <= 0.1027
0.50 < Flu.vaccinations
0.806 < Driving.alone.to.work <= 0.837
0.000588 < Mental.health.providers <= 0.001191
0.225 < Long.commute...driving.alone <= 0.318
0.919 < non.Hispanic.white
Feature
−0.06
−0.04
−0.02
0.00
0.02
0.04
Weight
Positive
Negative

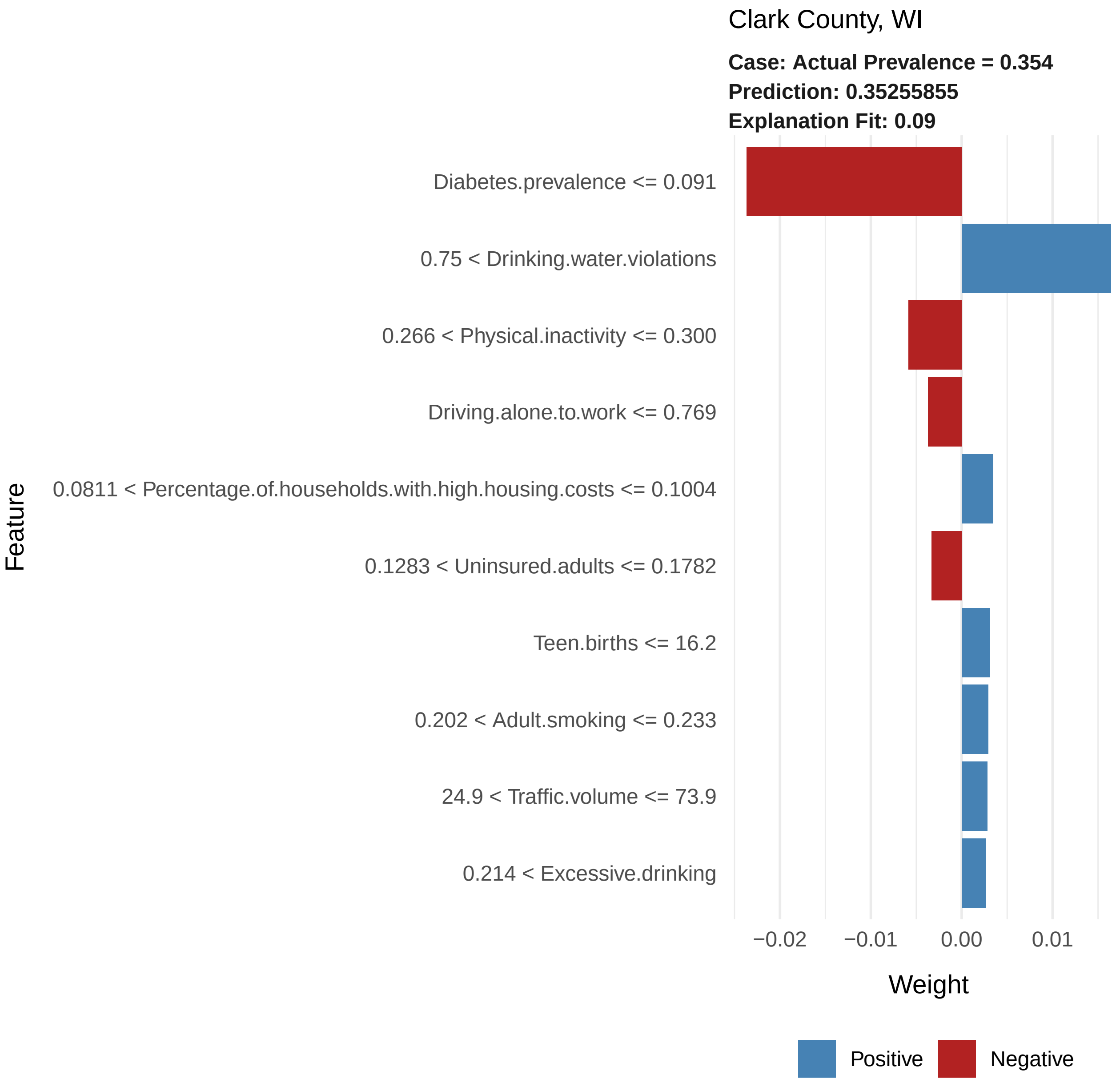

Clark County, WI
Case: Actual Prevalence = 0.354
Prediction: 0.35255855
Explanation Fit: 0.09
Feature
Diabetes.prevalence <= 0.091
0.75 < Drinking.water.violations
0.266 < Physical.inactivity <= 0.300
Driving.alone.to.work <= 0.769
0.0811 < Percentage.of.households.with.high.housing.costs <= 0.1004
0.1283 < Uninsured.adults <= 0.1782
Teen.births <= 16.2
0.202 < Adult.smoking <= 0.233
24.9 < Traffic.volume <= 73.9
0.214 < Excessive.drinking
−0.02
−0.01
0.00
0.01
Weight
Positive
Negative

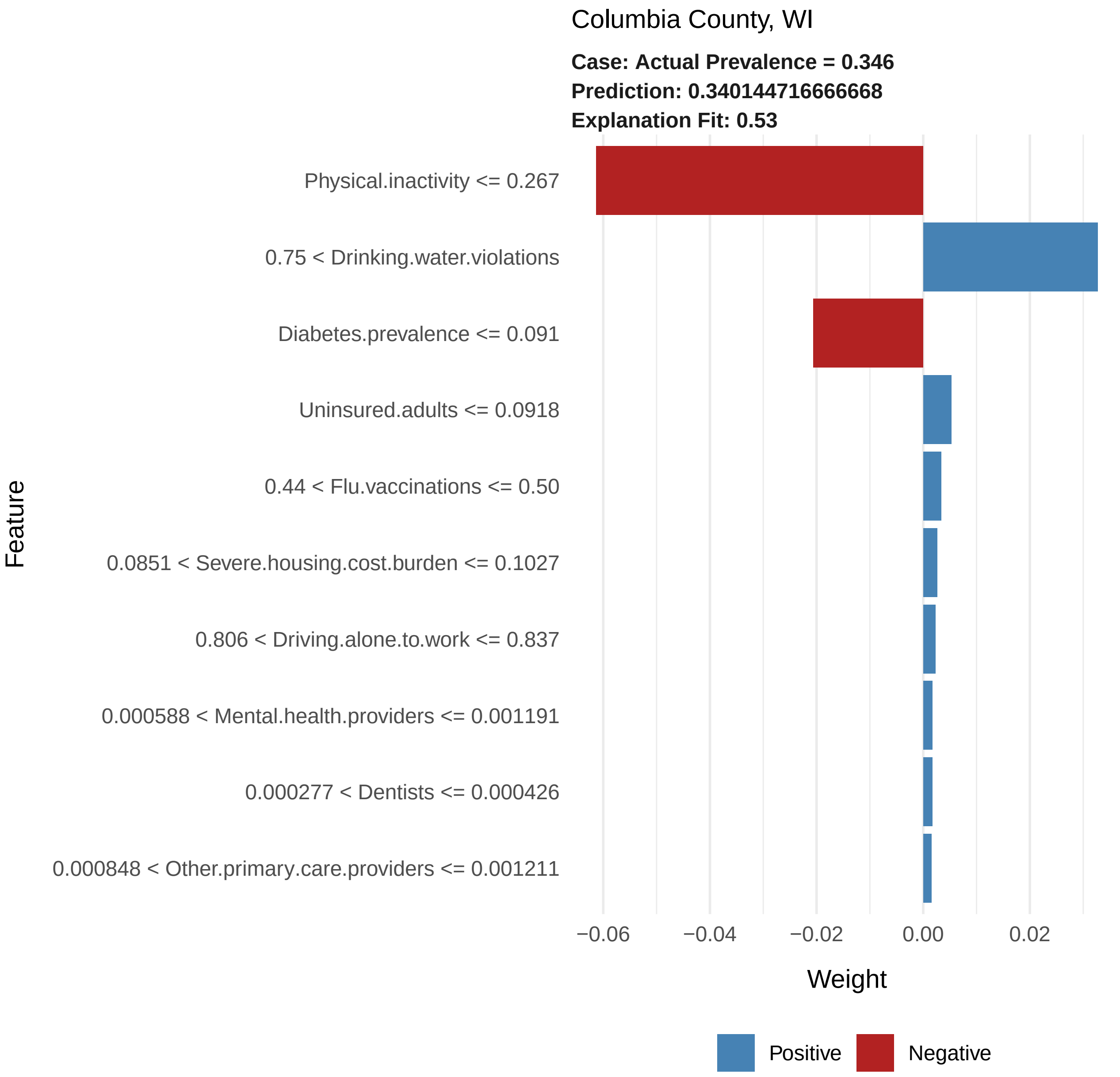
Columbia County, WI
Case: Actual Prevalence = 0.346
Prediction: 0.340144716666668
Explanation Fit: 0.53
Physical.inactivity <= 0.267
0.75 < Drinking.water.violations
Diabetes.prevalence <= 0.091
Uninsured.adults <= 0.0918
0.44 < Flu.vaccinations <= 0.50
0.0851 < Severe.housing.cost.burden <= 0.1027
0.806 < Driving.alone.to.work <= 0.837
0.000588 < Mental.health.providers <= 0.001191
0.000277 < Dentists <= 0.000426
0.000848 < Other.primary.care.providers <= 0.001211
Feature
−0.06
−0.04
−0.02
0.00
0.02
Weight
Positive
Negative

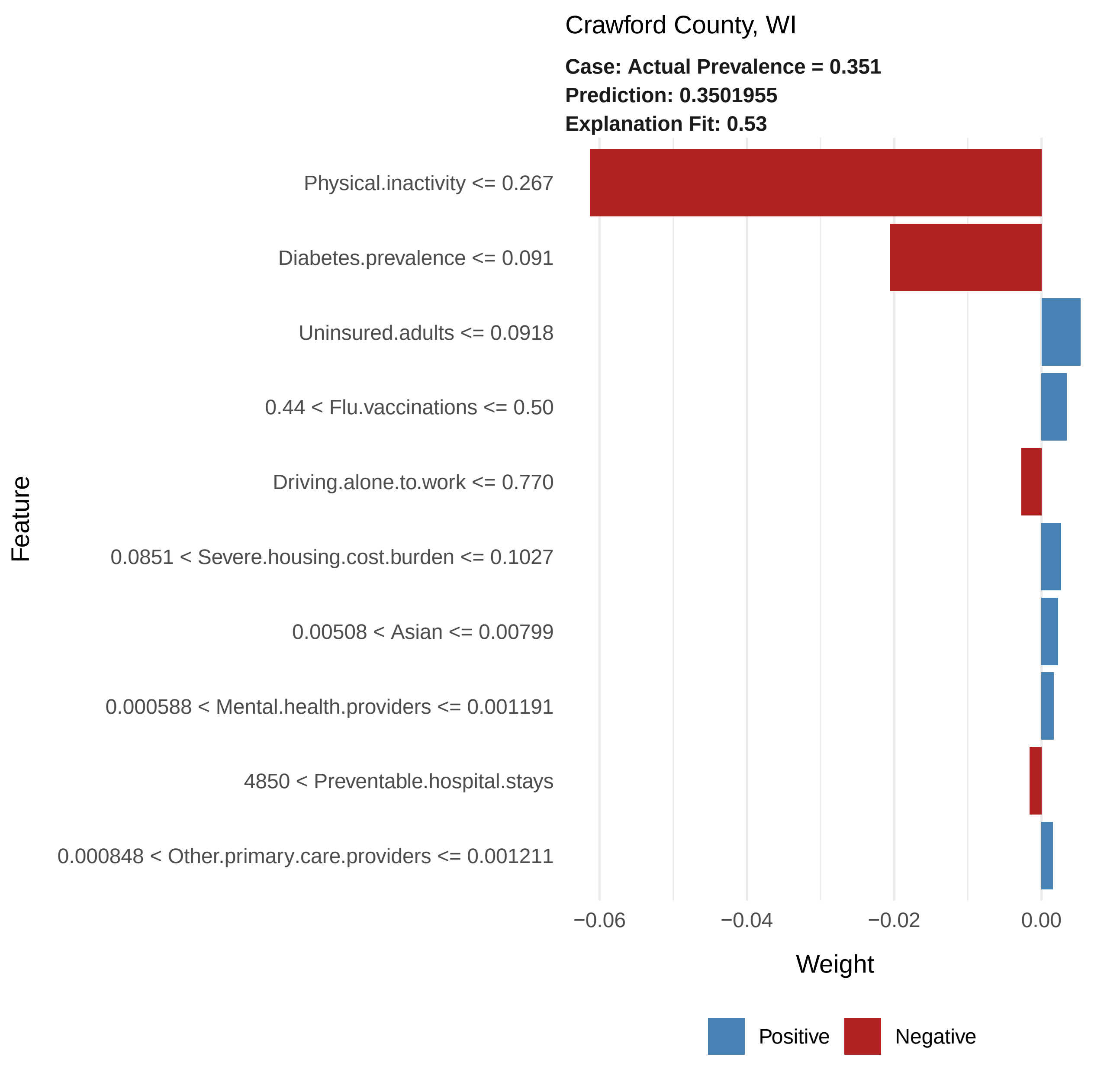

Crawford County, WI
Case: Actual Prevalence = 0.351
Prediction: 0.3501955
Explanation Fit: 0.53
Physical.inactivity <= 0.267
Diabetes.prevalence <= 0.091
Uninsured.adults <= 0.0918
0.44 < Flu.vaccinations <= 0.50
Driving.alone.to.work <= 0.770
0.0851 < Severe.housing.cost.burden <= 0.1027
0.00508 < Asian <= 0.00799
0.000588 < Mental.health.providers <= 0.001191
4850 < Preventable.hospital.stays
0.000848 < Other.primary.care.providers <= 0.001211
Feature
−0.06
−0.04
−0.02
0.00
Weight
Positive
Negative

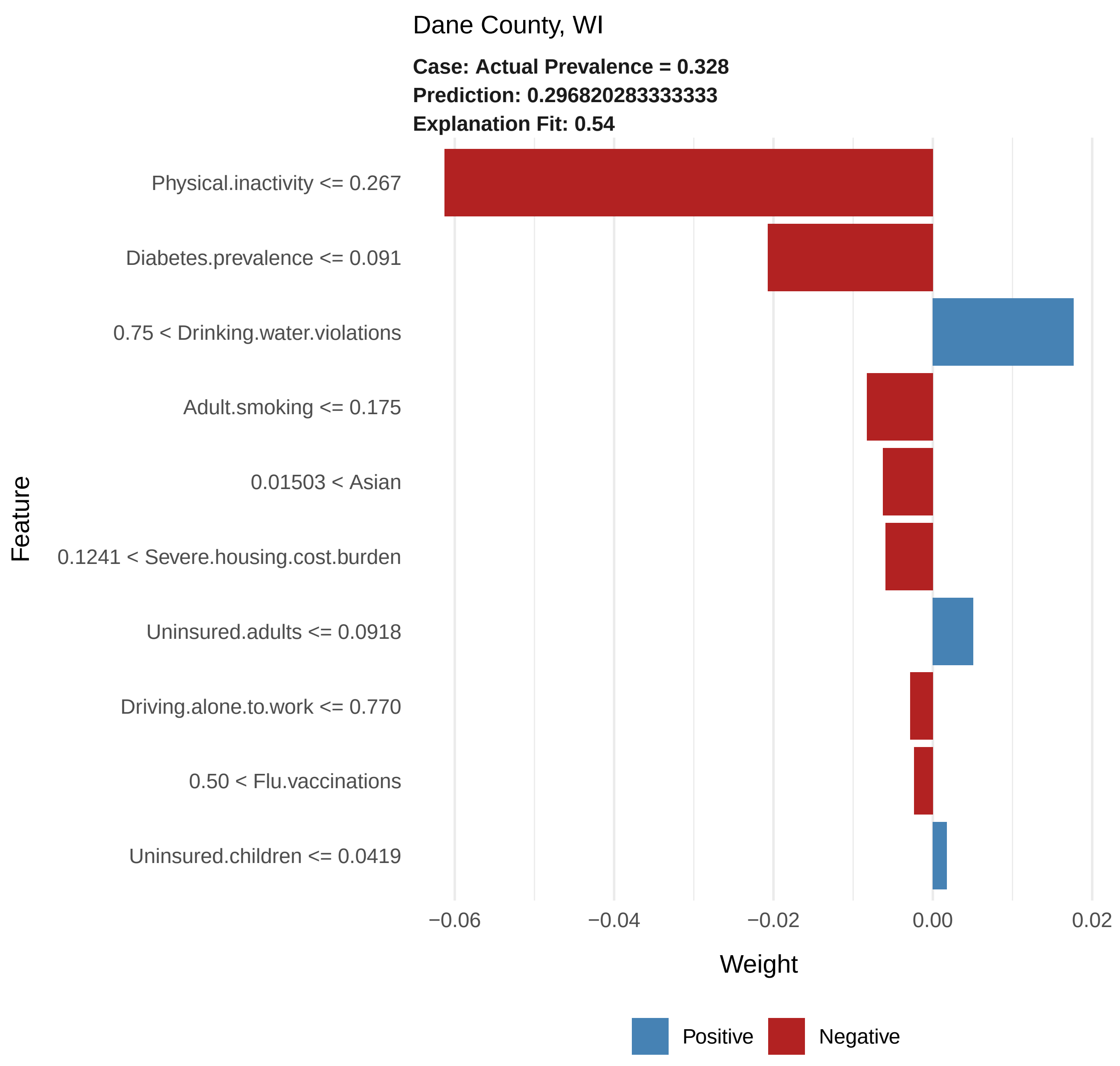
Dane County, WI
Case: Actual Prevalence = 0.328
Prediction: 0.296820283333333
Explanation Fit: 0.54
Physical.inactivity <= 0.267
Diabetes.prevalence <= 0.091
0.75 < Drinking.water.violations
Adult.smoking <= 0.175
0.01503 < Asian
0.1241 < Severe.housing.cost.burden
Uninsured.adults <= 0.0918
Driving.alone.to.work <= 0.770
0.50 < Flu.vaccinations
Uninsured.children <= 0.0419
Feature
−0.06
−0.04
−0.02
0.00
0.02
Weight
Positive
Negative

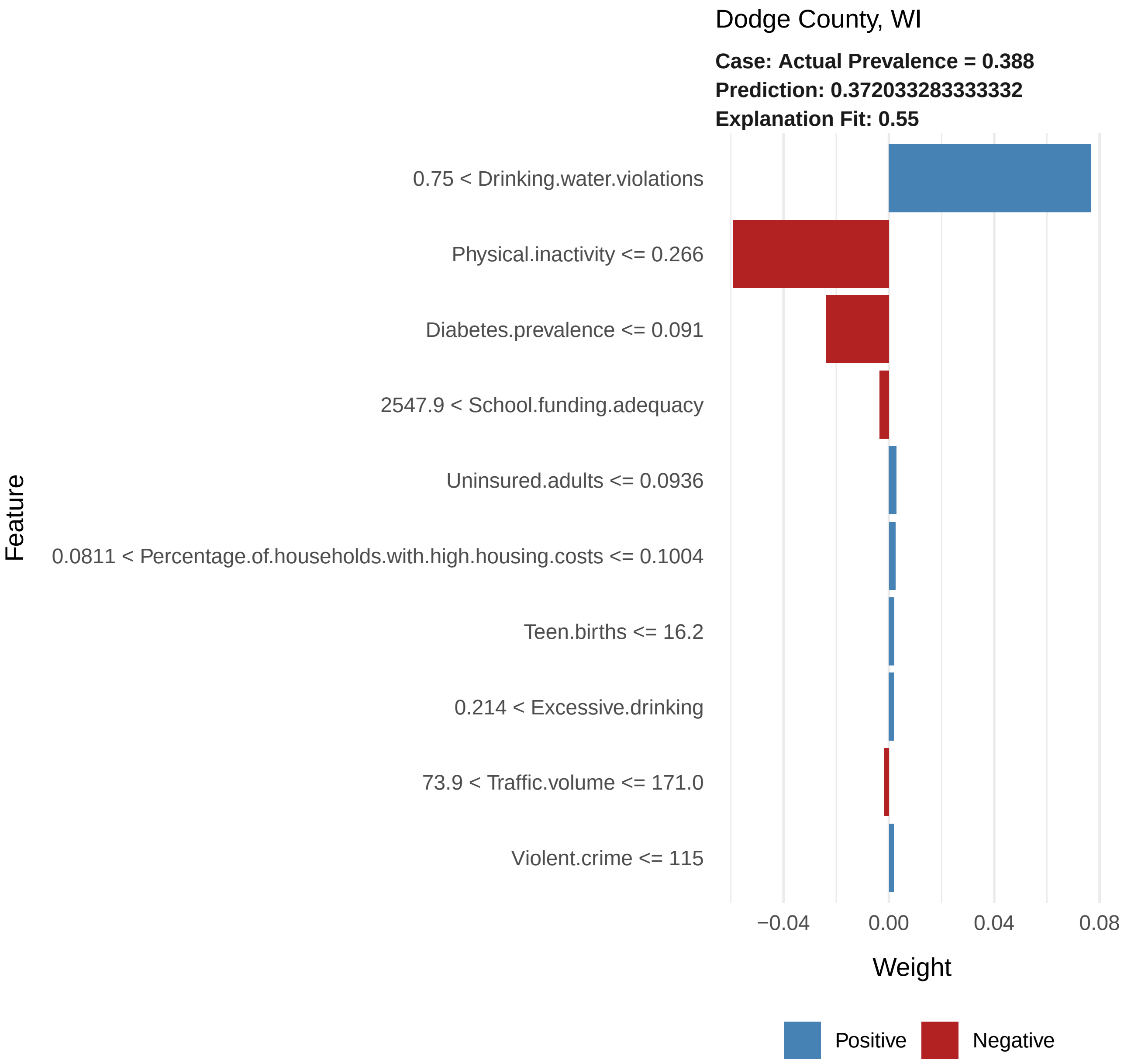

Dodge County, WI
Case: Actual Prevalence = 0.388
Prediction: 0.372033283333332
Explanation Fit: 0.55
0.75 < Drinking.water.violations
Physical.inactivity <= 0.266
Diabetes.prevalence <= 0.091
2547.9 < School.funding.adequacy
Uninsured.adults <= 0.0936
0.0811 < Percentage.of.households.with.high.housing.costs <= 0.1004
Teen.births <= 16.2
0.214 < Excessive.drinking
73.9 < Traffic.volume <= 171.0
Violent.crime <= 115
Feature
−0.04
0.00
0.04
0.08
Weight
Positive
Negative

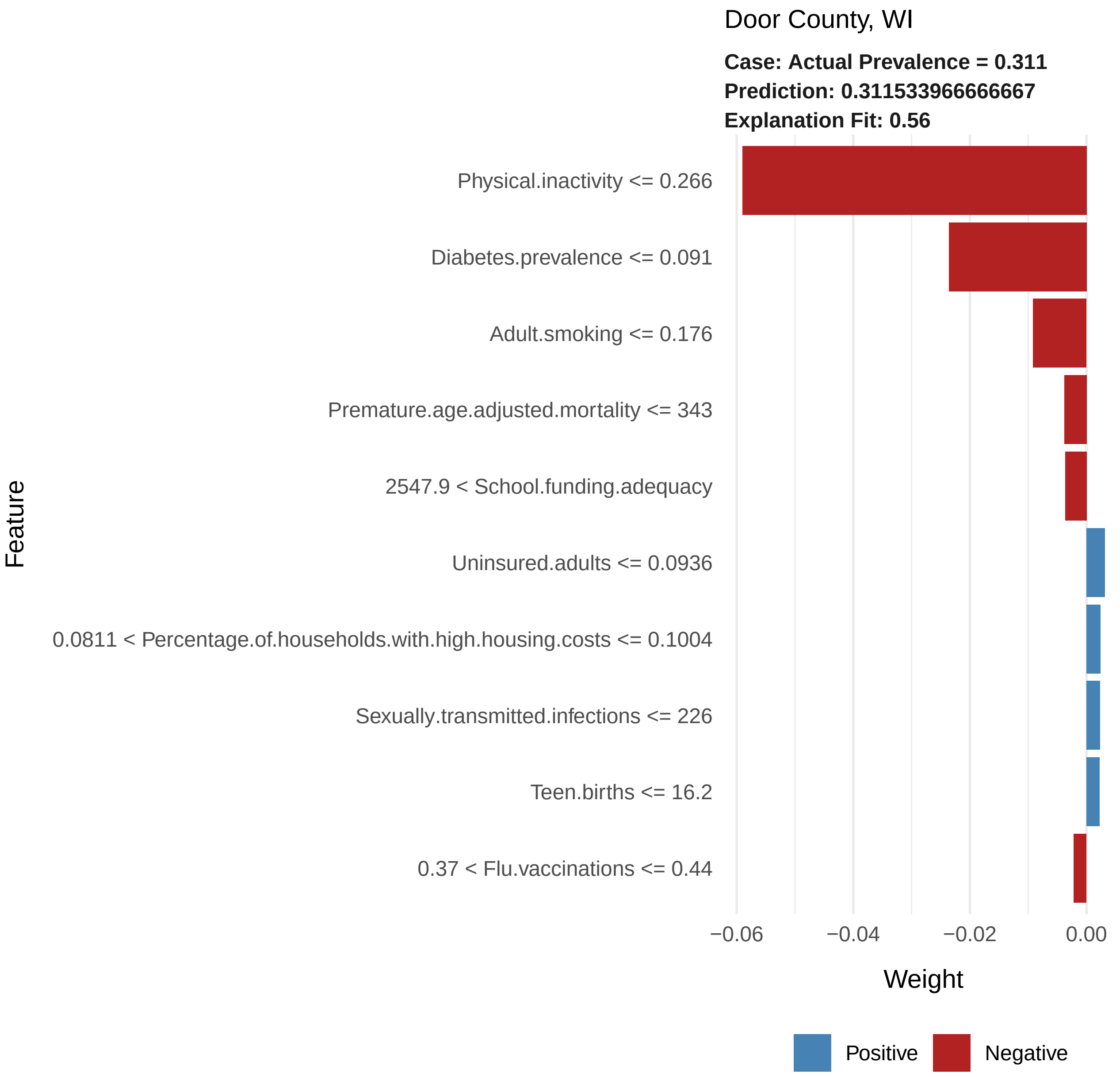

Door County, WI
Case: Actual Prevalence = 0.311
Prediction: 0.311533966666667
Explanation Fit: 0.56
Physical.inactivity <= 0.266
Diabetes.prevalence <= 0.091
Adult.smoking <= 0.176
Premature.age.adjusted.mortality <= 343
2547.9 < School.funding.adequacy
Uninsured.adults <= 0.0936
0.0811 < Percentage.of.households.with.high.housing.costs <= 0.1004
Sexually.transmitted.infections <= 226
Teen.births <= 16.2
0.37 < Flu.vaccinations <= 0.44
Feature
−0.06
−0.04
−0.02
0.00
Weight
Positive
Negative

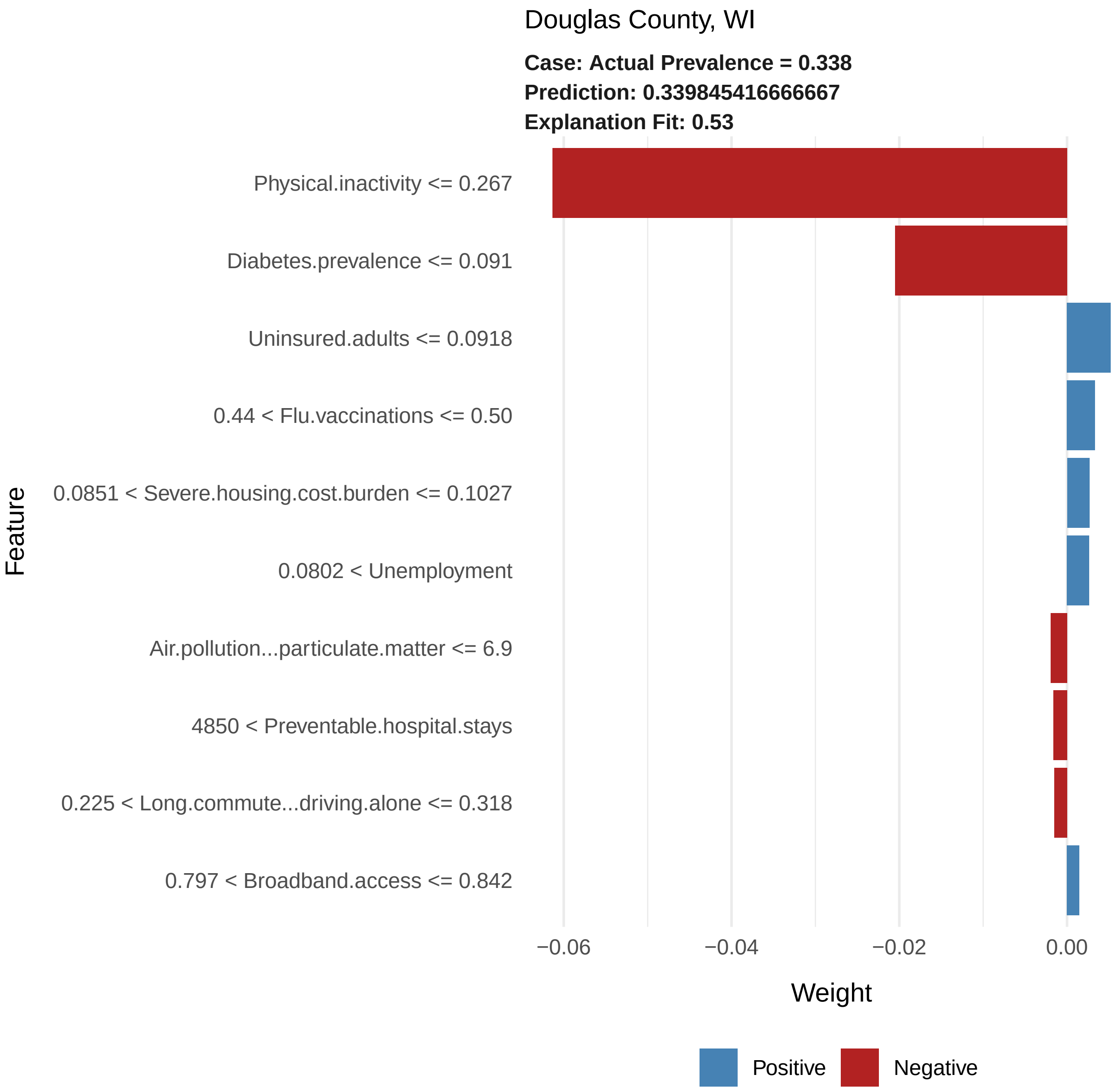
Douglas County, WI
Case: Actual Prevalence = 0.338
Prediction: 0.339845416666667
Explanation Fit: 0.53
Physical.inactivity <= 0.267
Diabetes.prevalence <= 0.091
Uninsured.adults <= 0.0918
0.44 < Flu.vaccinations <= 0.50
0.0851 < Severe.housing.cost.burden <= 0.1027
0.0802 < Unemployment
Air.pollution...particulate.matter <= 6.9
4850 < Preventable.hospital.stays
0.225 < Long.commute...driving.alone <= 0.318
0.797 < Broadband.access <= 0.842
Feature
−0.06
−0.04
−0.02
0.00
Weight
Positive
Negative

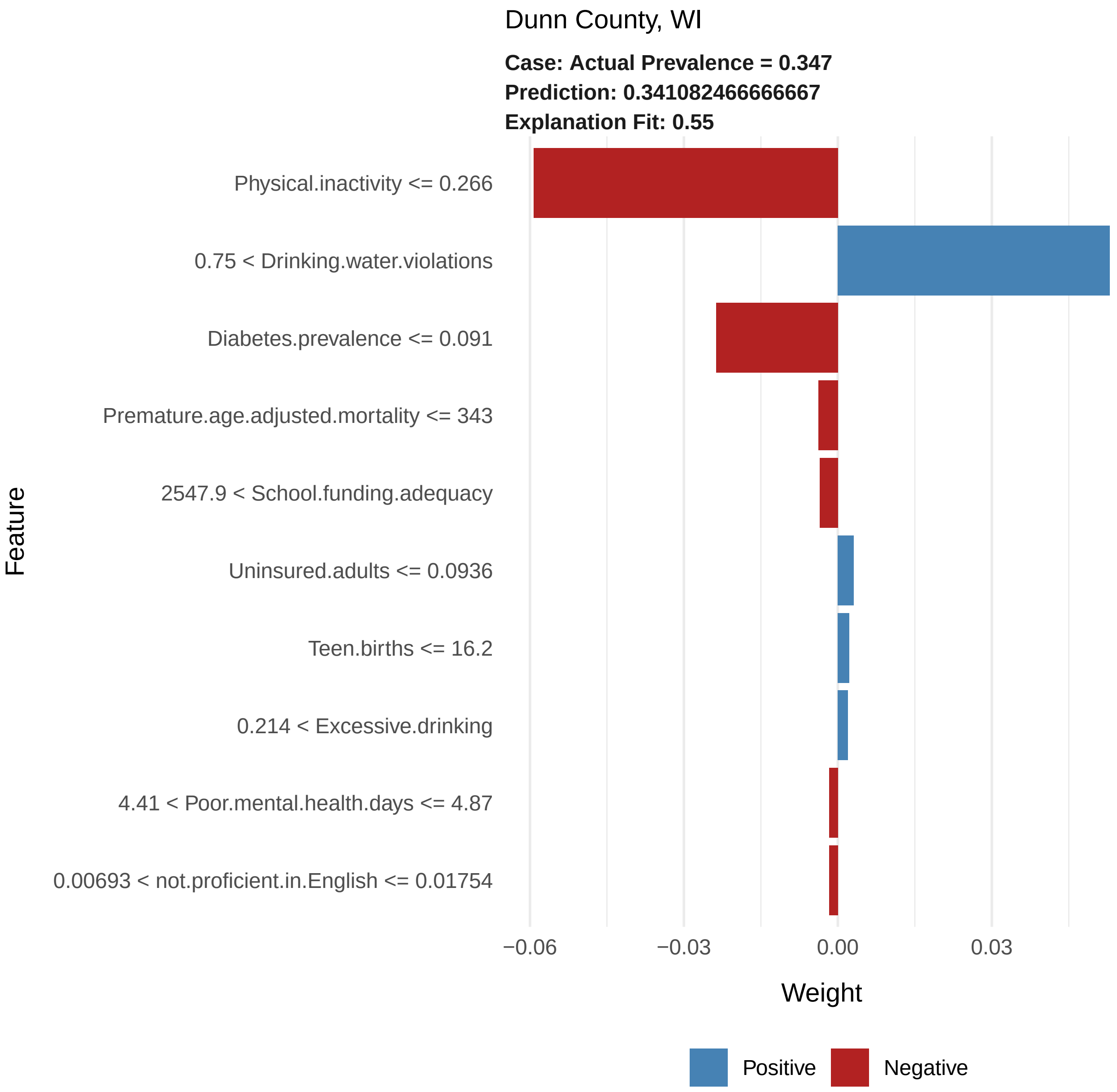

Dunn County, WI
Case: Actual Prevalence = 0.347
Prediction: 0.341082466666667
Explanation Fit: 0.55
Physical.inactivity <= 0.266
0.75 < Drinking.water.violations
Diabetes.prevalence <= 0.091
Premature.age.adjusted.mortality <= 343
2547.9 < School.funding.adequacy
Uninsured.adults <= 0.0936
Teen.births <= 16.2
0.214 < Excessive.drinking
4.41 < Poor.mental.health.days <= 4.87
0.00693 < not.proficient.in.English <= 0.01754
Feature
−0.06
−0.03
0.00
0.03
Weight
Positive
Negative

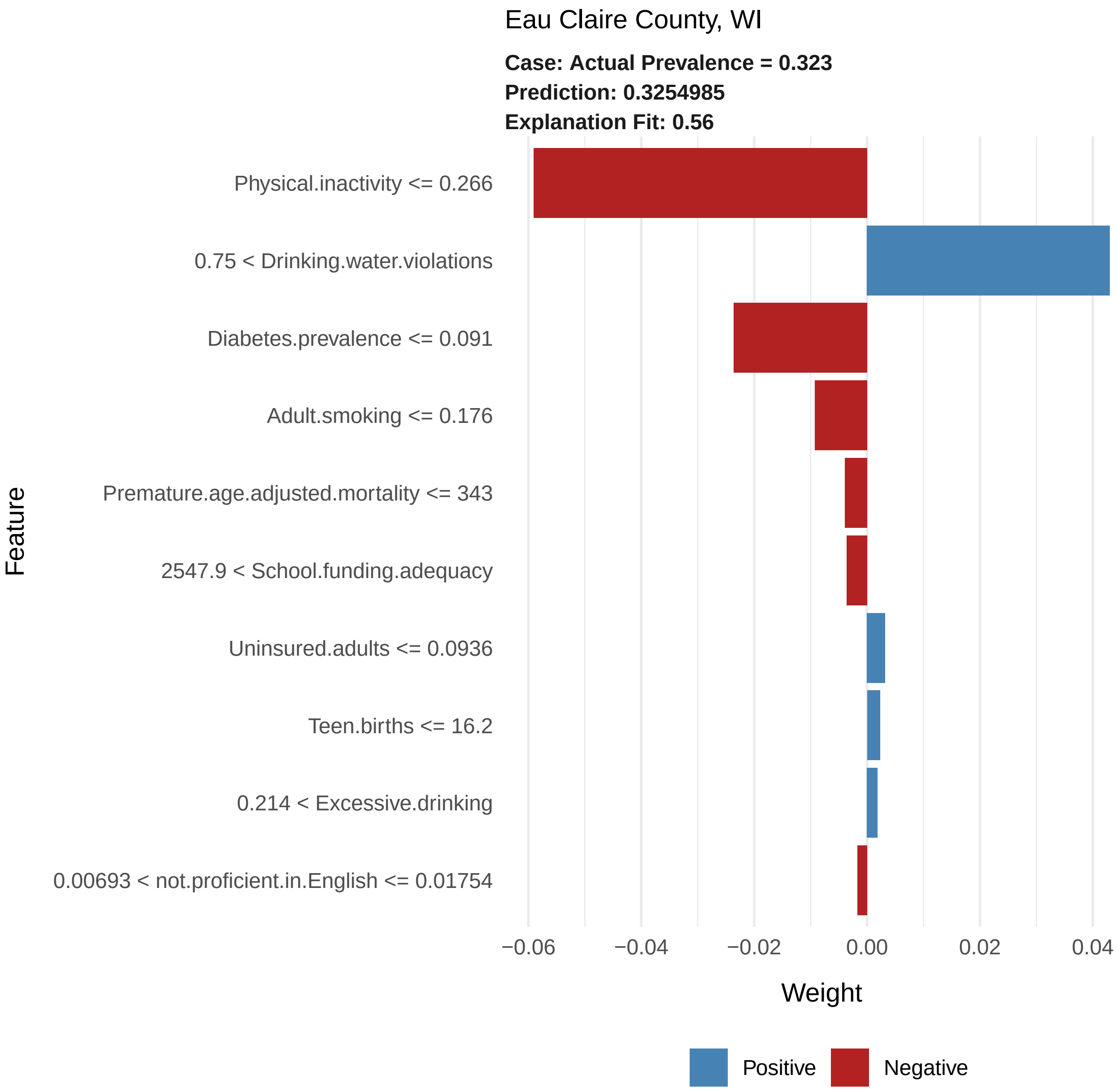
Eau Claire County, WI
Case: Actual Prevalence = 0.323
Prediction: 0.3254985
Explanation Fit: 0.56
Physical.inactivity <= 0.266
0.75 < Drinking.water.violations
Diabetes.prevalence <= 0.091
Adult.smoking <= 0.176
Premature.age.adjusted.mortality <= 343
2547.9 < School.funding.adequacy
Uninsured.adults <= 0.0936
Teen.births <= 16.2
0.214 < Excessive.drinking
0.00693 < not.proficient.in.English <= 0.01754
Feature
−0.06
−0.04
−0.02
0.00
0.02
0.04
Weight
Positive
Negative

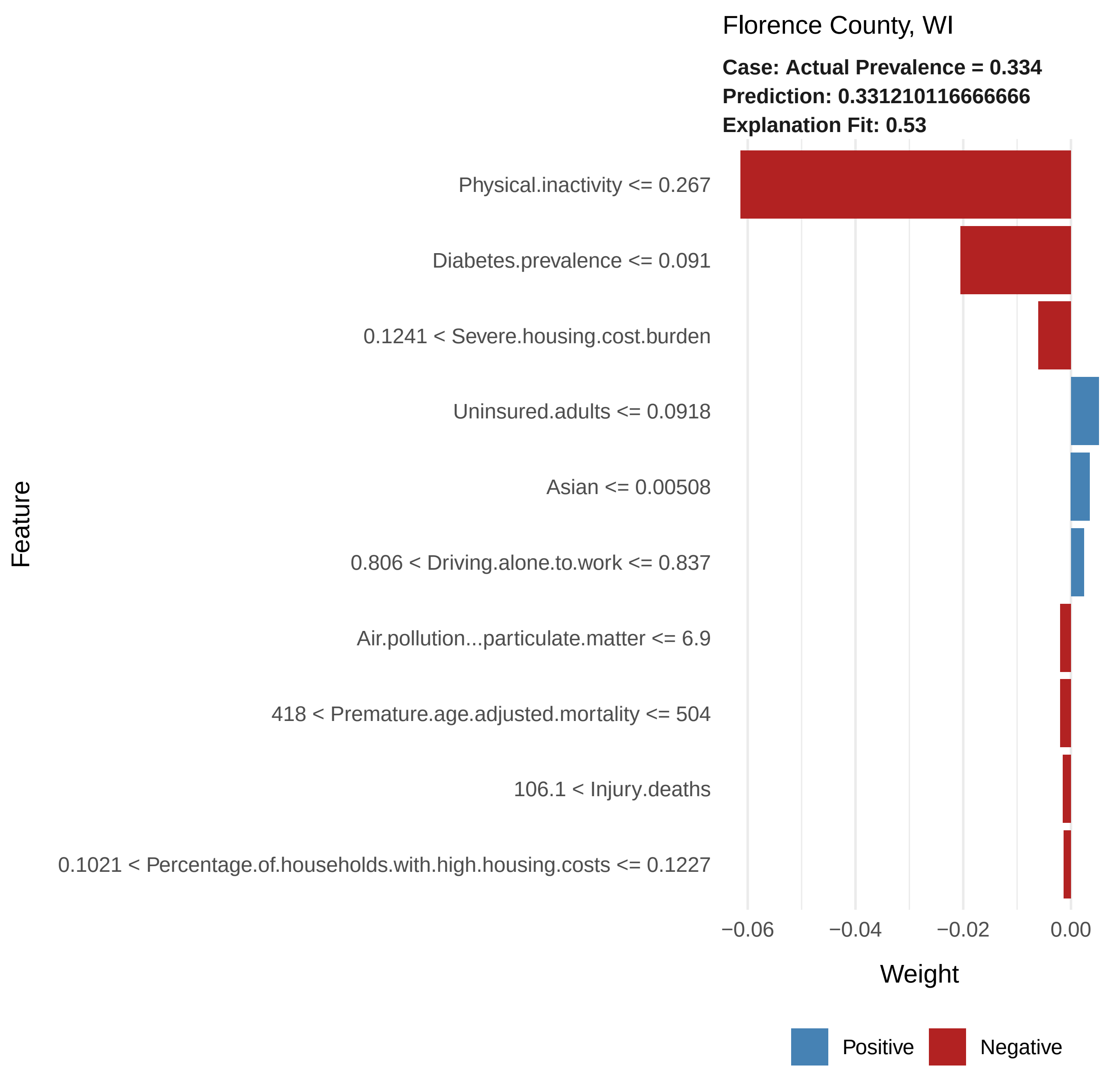

Florence County, WI
Case: Actual Prevalence = 0.334
Prediction: 0.331210116666666
Explanation Fit: 0.53
Feature
Physical.inactivity <= 0.267
Diabetes.prevalence <= 0.091
0.1241 < Severe.housing.cost.burden
Uninsured.adults <= 0.0918
Asian <= 0.00508
0.806 < Driving.alone.to.work <= 0.837
Air.pollution...particulate.matter <= 6.9
418 < Premature.age.adjusted.mortality <= 504
106.1 < Injury.deaths
0.1021 < Percentage.of.households.with.high.housing.costs <= 0.1227
−0.06
−0.04
−0.02
0.00
Weight
Positive
Negative

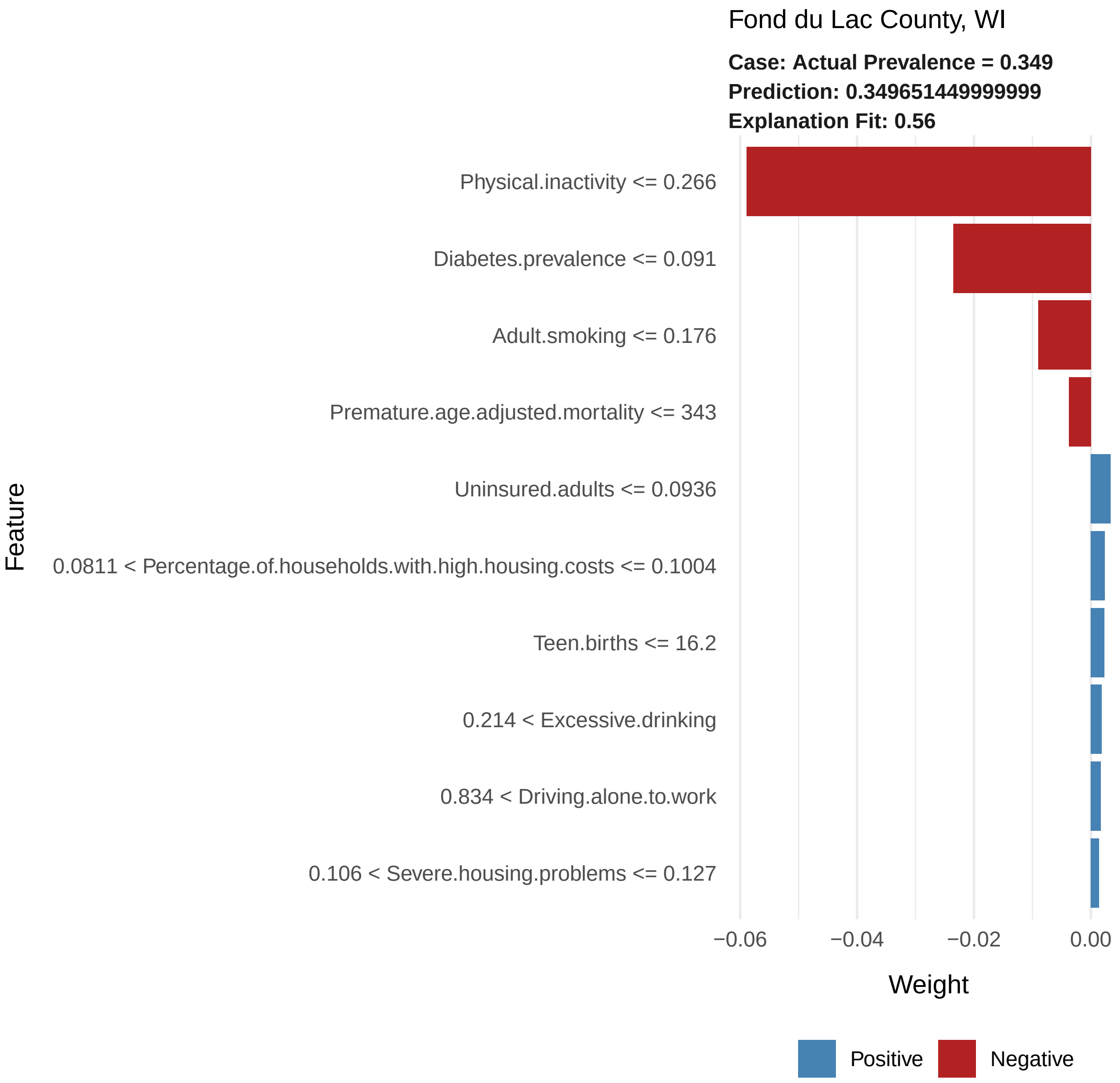
Fond du Lac County, WI
Case: Actual Prevalence = 0.349
Prediction: 0.349651449999999
Explanation Fit: 0.56
Physical.inactivity <= 0.266
Diabetes.prevalence <= 0.091
Adult.smoking <= 0.176
Premature.age.adjusted.mortality <= 343
Uninsured.adults <= 0.0936
0.0811 < Percentage.of.households.with.high.housing.costs <= 0.1004
Teen.births <= 16.2
0.214 < Excessive.drinking
0.834 < Driving.alone.to.work
0.106 < Severe.housing.problems <= 0.127
Feature
−0.06
−0.04
−0.02
0.00
Weight
Positive
Negative

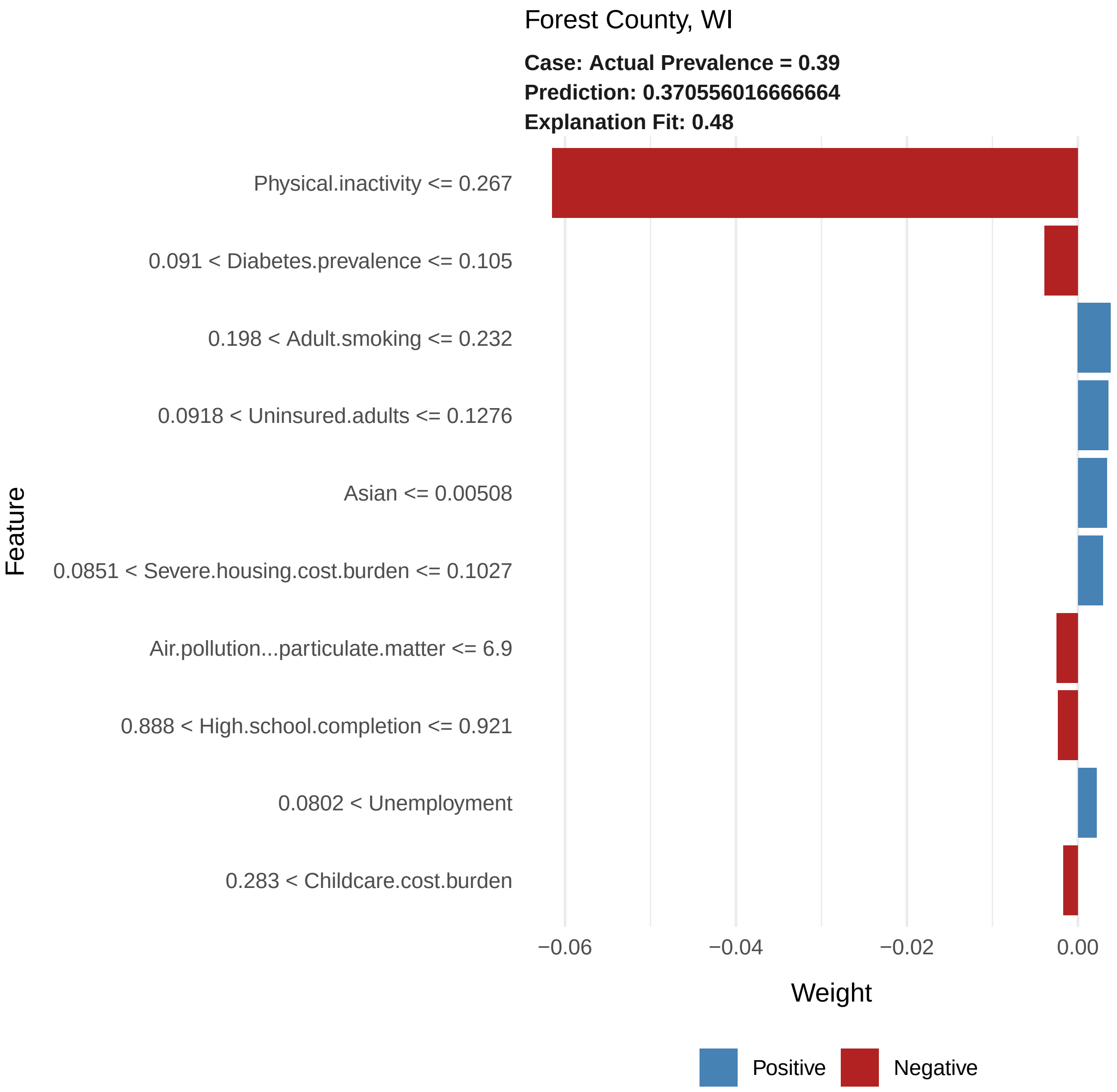

Forest County, WI
Case: Actual Prevalence = 0.39
Prediction: 0.370556016666664
Explanation Fit: 0.48
Physical.inactivity <= 0.267
0.091 < Diabetes.prevalence <= 0.105
0.198 < Adult.smoking <= 0.232
0.0918 < Uninsured.adults <= 0.1276
Asian <= 0.00508
0.0851 < Severe.housing.cost.burden <= 0.1027
Air.pollution...particulate.matter <= 6.9
0.888 < High.school.completion <= 0.921
0.0802 < Unemployment
0.283 < Childcare.cost.burden
Feature
−0.06
−0.04
−0.02
0.00
Weight
Positive
Negative

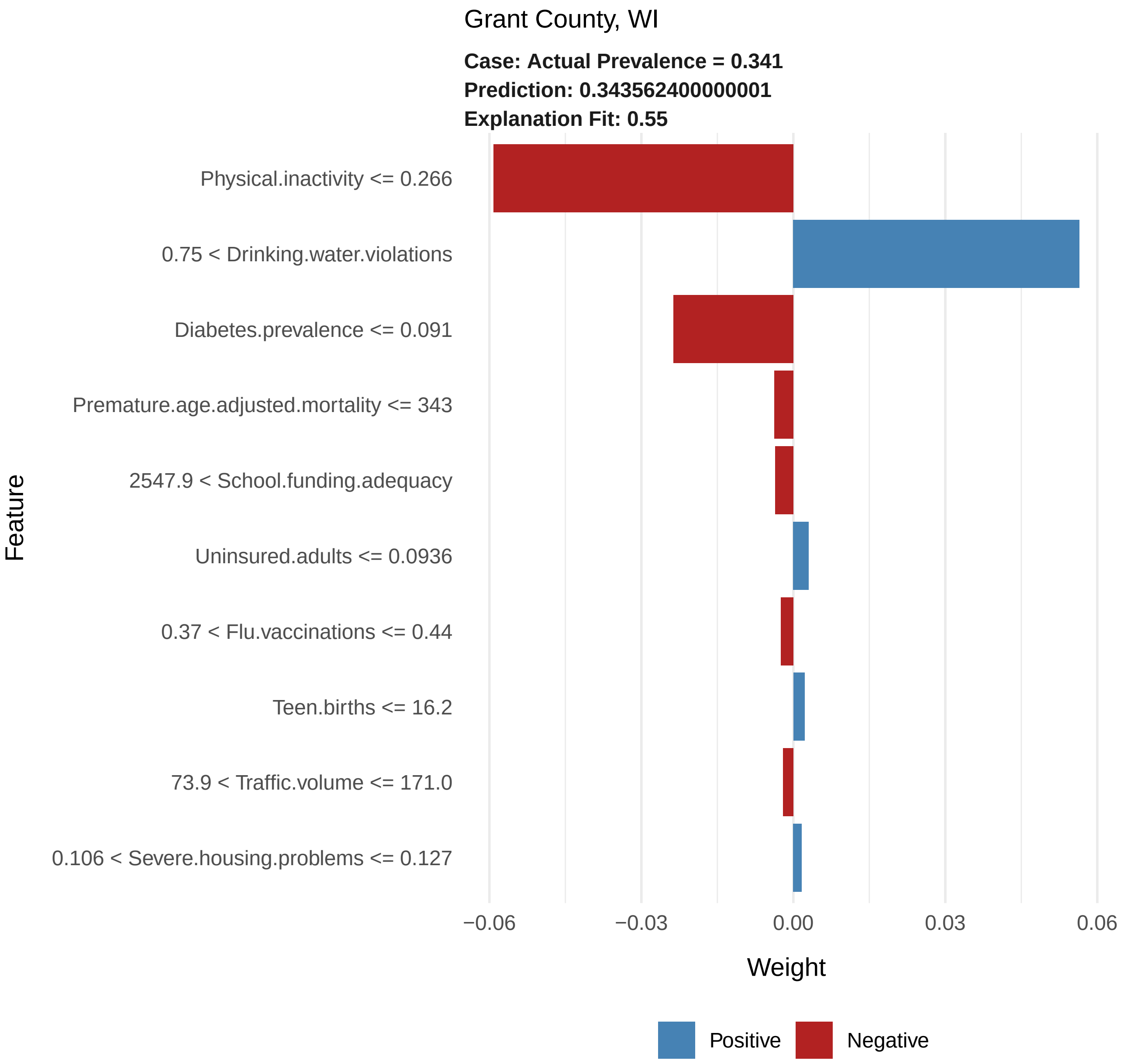
Grant County, WI
Case: Actual Prevalence = 0.341
Prediction: 0.343562400000001
Explanation Fit: 0.55
Physical.inactivity <= 0.266
0.75 < Drinking.water.violations
Diabetes.prevalence <= 0.091
Premature.age.adjusted.mortality <= 343
2547.9 < School.funding.adequacy
Uninsured.adults <= 0.0936
0.37 < Flu.vaccinations <= 0.44
Teen.births <= 16.2
73.9 < Traffic.volume <= 171.0
0.106 < Severe.housing.problems <= 0.127
Feature
−0.06
−0.03
0.00
0.03
0.06
Weight
Positive
Negative

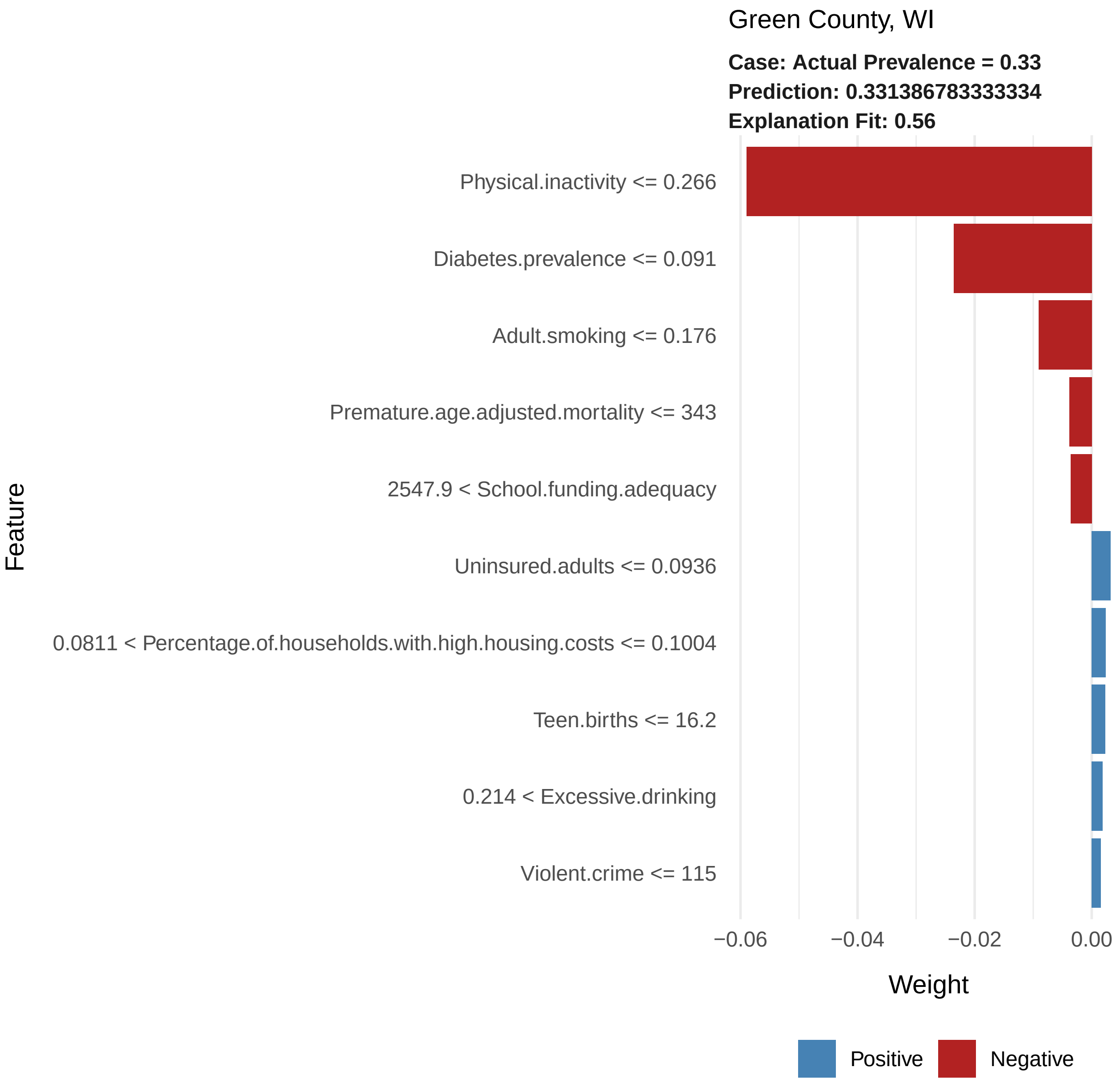

Green County, WI
Case: Actual Prevalence = 0.33
Prediction: 0.331386783333334
Explanation Fit: 0.56
Physical.inactivity <= 0.266
Diabetes.prevalence <= 0.091
Adult.smoking <= 0.176
Premature.age.adjusted.mortality <= 343
2547.9 < School.funding.adequacy
Uninsured.adults <= 0.0936
0.0811 < Percentage.of.households.with.high.housing.costs <= 0.1004
Teen.births <= 16.2
0.214 < Excessive.drinking
Violent.crime <= 115
Feature
−0.06
−0.04
−0.02
0.00
Weight
Positive
Negative

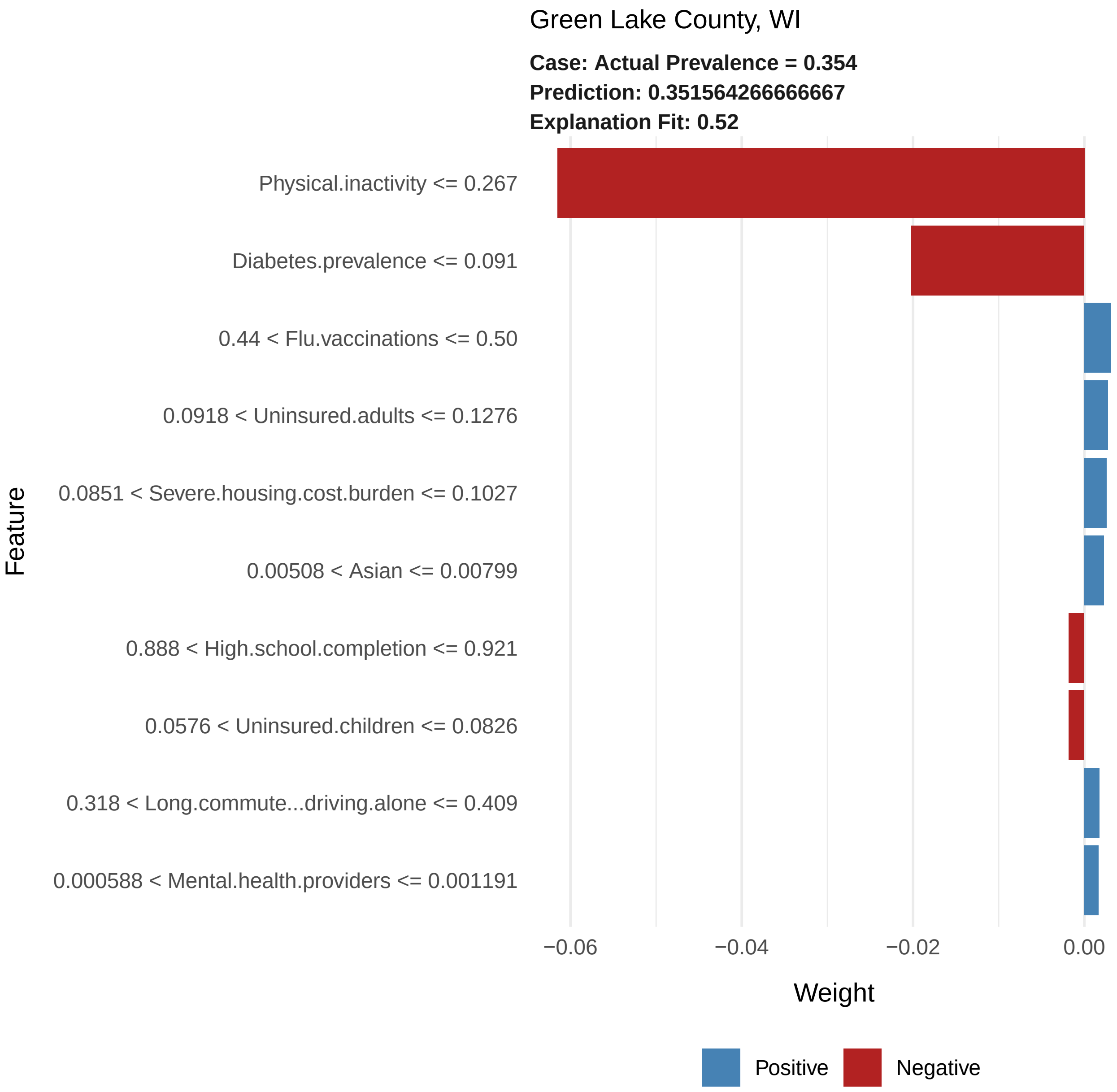

Green Lake County, WI
Case: Actual Prevalence = 0.354
Prediction: 0.351564266666667
Explanation Fit: 0.52
Physical.inactivity <= 0.267
Diabetes.prevalence <= 0.091
0.44 < Flu.vaccinations <= 0.50
0.0918 < Uninsured.adults <= 0.1276
0.0851 < Severe.housing.cost.burden <= 0.1027
0.00508 < Asian <= 0.00799
0.888 < High.school.completion <= 0.921
0.0576 < Uninsured.children <= 0.0826
0.318 < Long.commute...driving.alone <= 0.409
0.000588 < Mental.health.providers <= 0.001191
Feature
−0.06
−0.04
−0.02
0.00
Weight
Positive
Negative

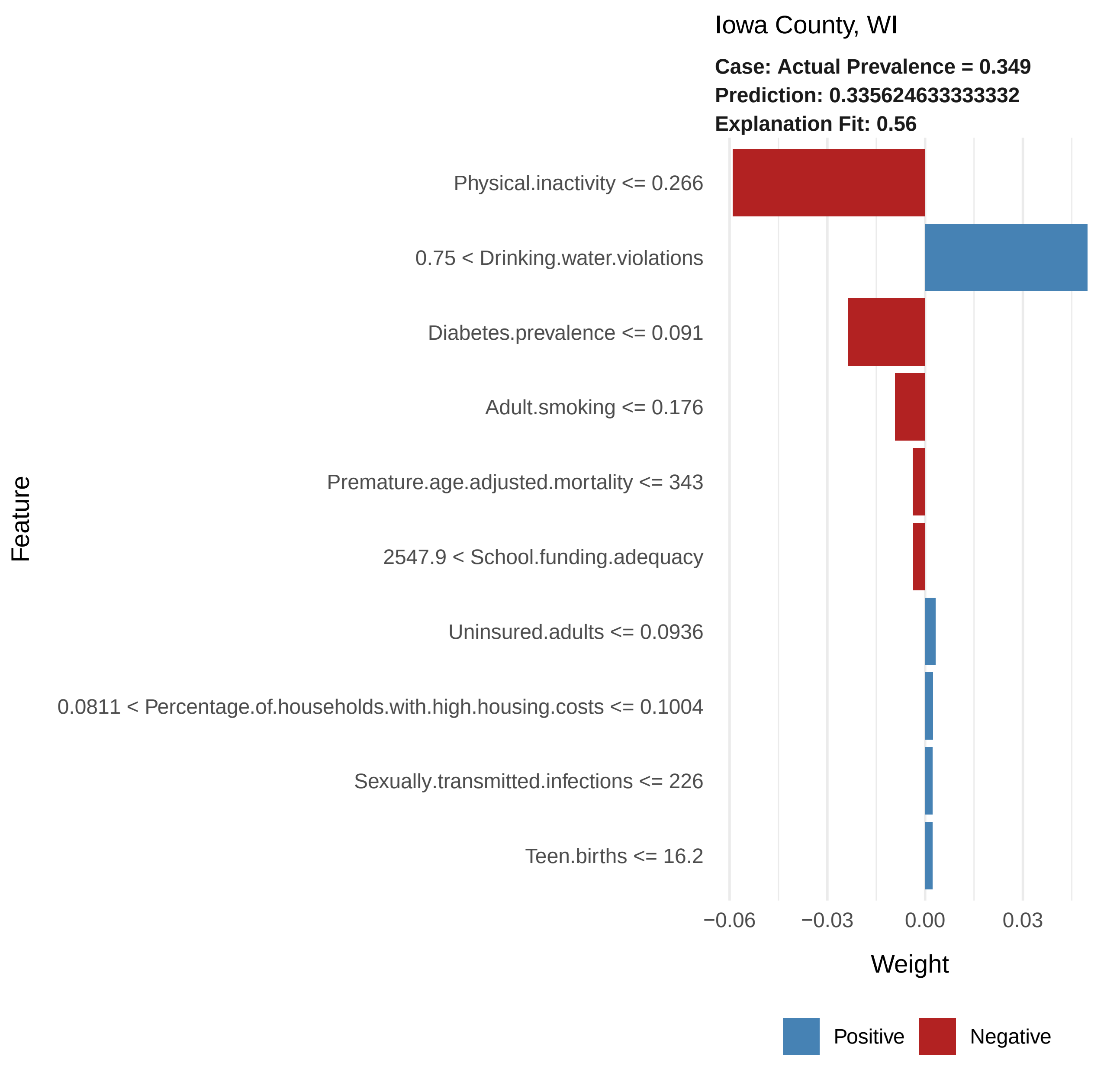
Iowa County, WI
Case: Actual Prevalence = 0.349
Prediction: 0.335624633333332
Explanation Fit: 0.56
Physical.inactivity <= 0.266
0.75 < Drinking.water.violations
Diabetes.prevalence <= 0.091
Adult.smoking <= 0.176
Premature.age.adjusted.mortality <= 343
2547.9 < School.funding.adequacy
Uninsured.adults <= 0.0936
0.0811 < Percentage.of.households.with.high.housing.costs <= 0.1004
Sexually.transmitted.infections <= 226
Teen.births <= 16.2
Feature
−0.06
−0.03
0.00
0.03
Weight
Positive
Negative

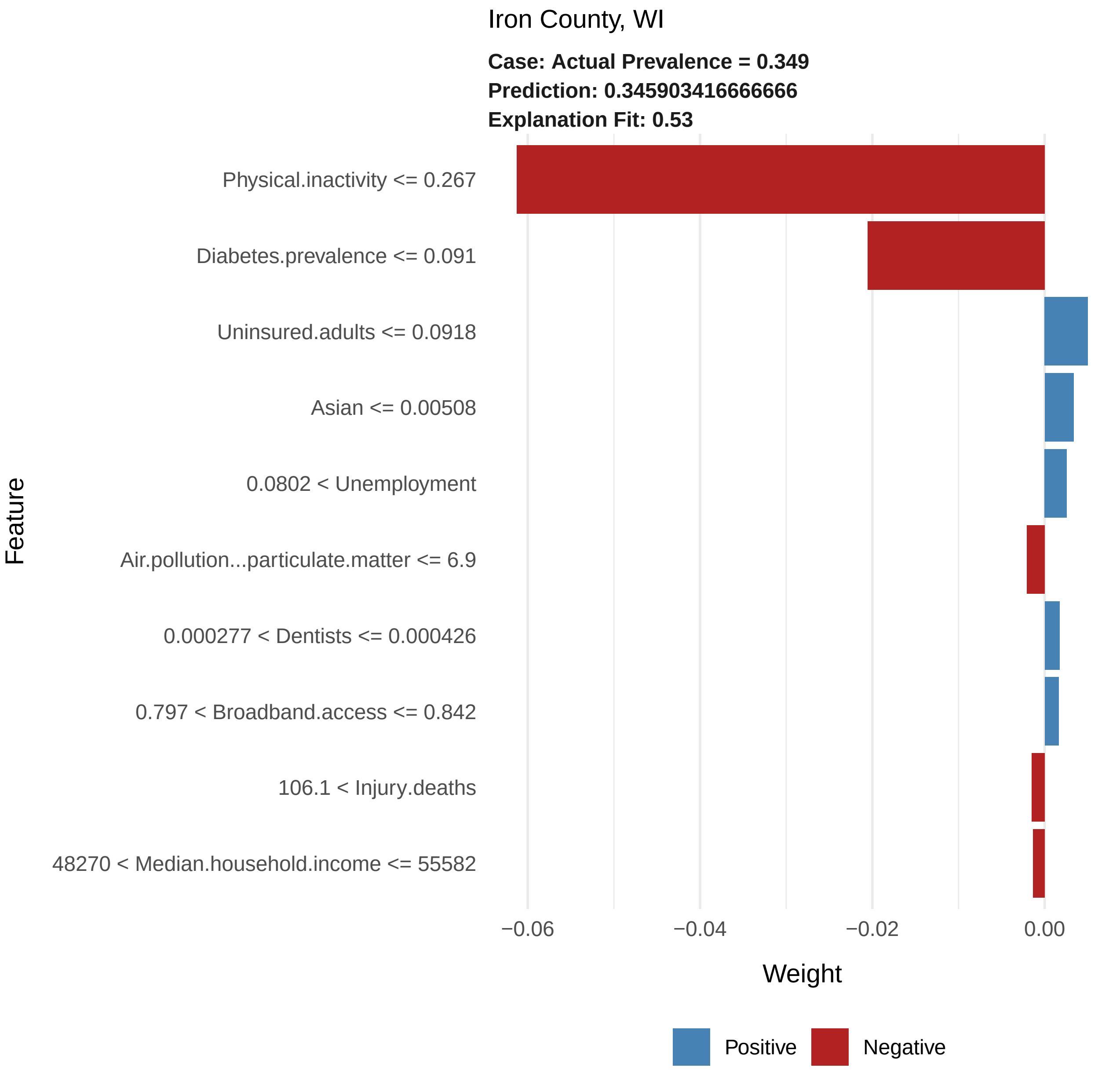

Iron County, WI
Case: Actual Prevalence = 0.349
Prediction: 0.345903416666666
Explanation Fit: 0.53
Physical.inactivity <= 0.267
Diabetes.prevalence <= 0.091
Uninsured.adults <= 0.0918
Asian <= 0.00508
0.0802 < Unemployment
Air.pollution...particulate.matter <= 6.9
0.000277 < Dentists <= 0.000426
0.797 < Broadband.access <= 0.842
106.1 < Injury.deaths
48270 < Median.household.income <= 55582
Feature
−0.06
−0.04
−0.02
0.00
Weight
Positive
Negative

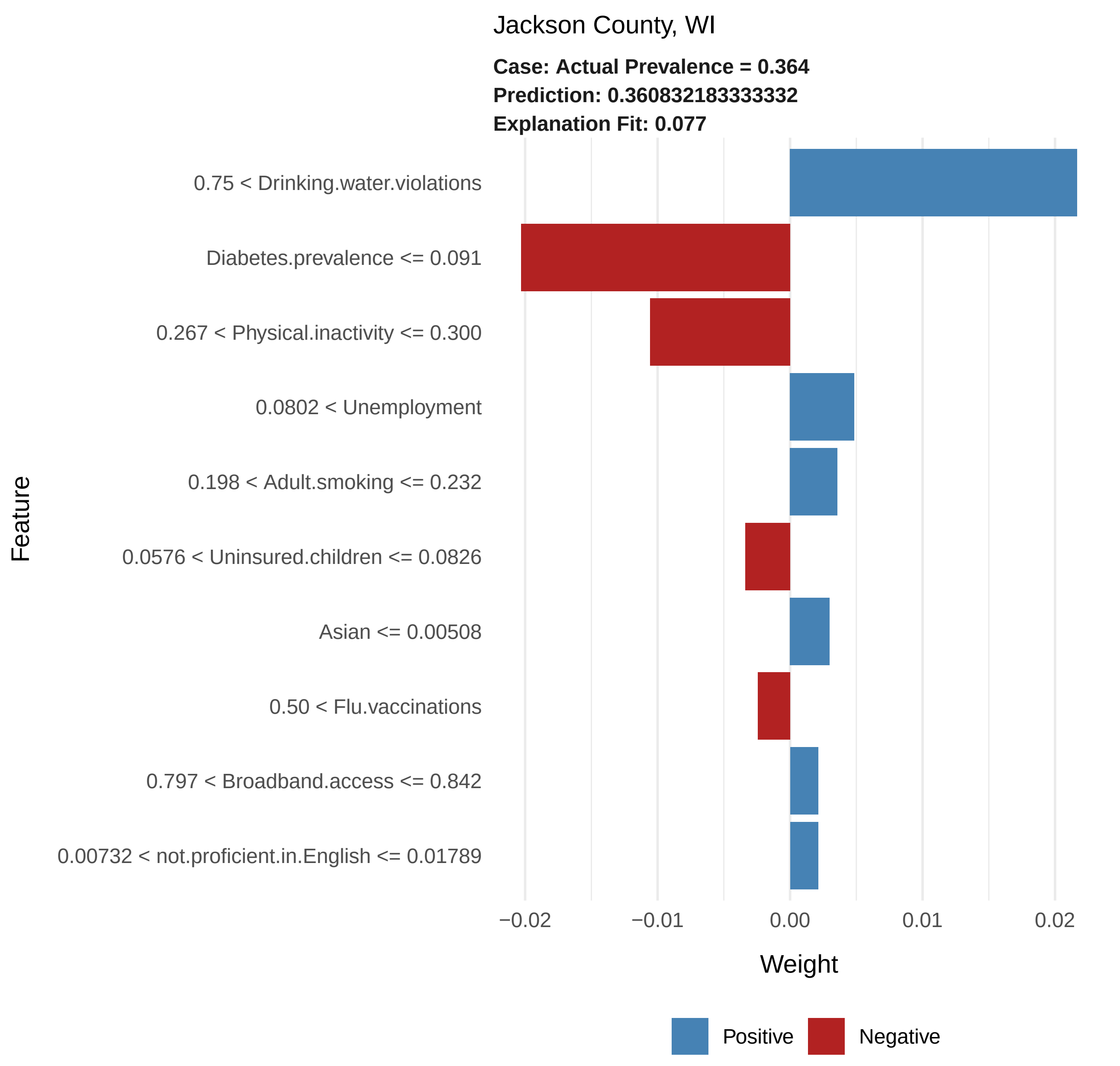
Jackson County, WI
Case: Actual Prevalence = 0.364
Prediction: 0.360832183333332
Explanation Fit: 0.077
0.75 < Drinking.water.violations
Diabetes.prevalence <= 0.091
0.267 < Physical.inactivity <= 0.300
0.0802 < Unemployment
0.198 < Adult.smoking <= 0.232
0.0576 < Uninsured.children <= 0.0826
Asian <= 0.00508
0.50 < Flu.vaccinations
0.797 < Broadband.access <= 0.842
0.00732 < not.proficient.in.English <= 0.01789
Feature
−0.02
−0.01
0.00
0.01
0.02
Weight
Positive
Negative

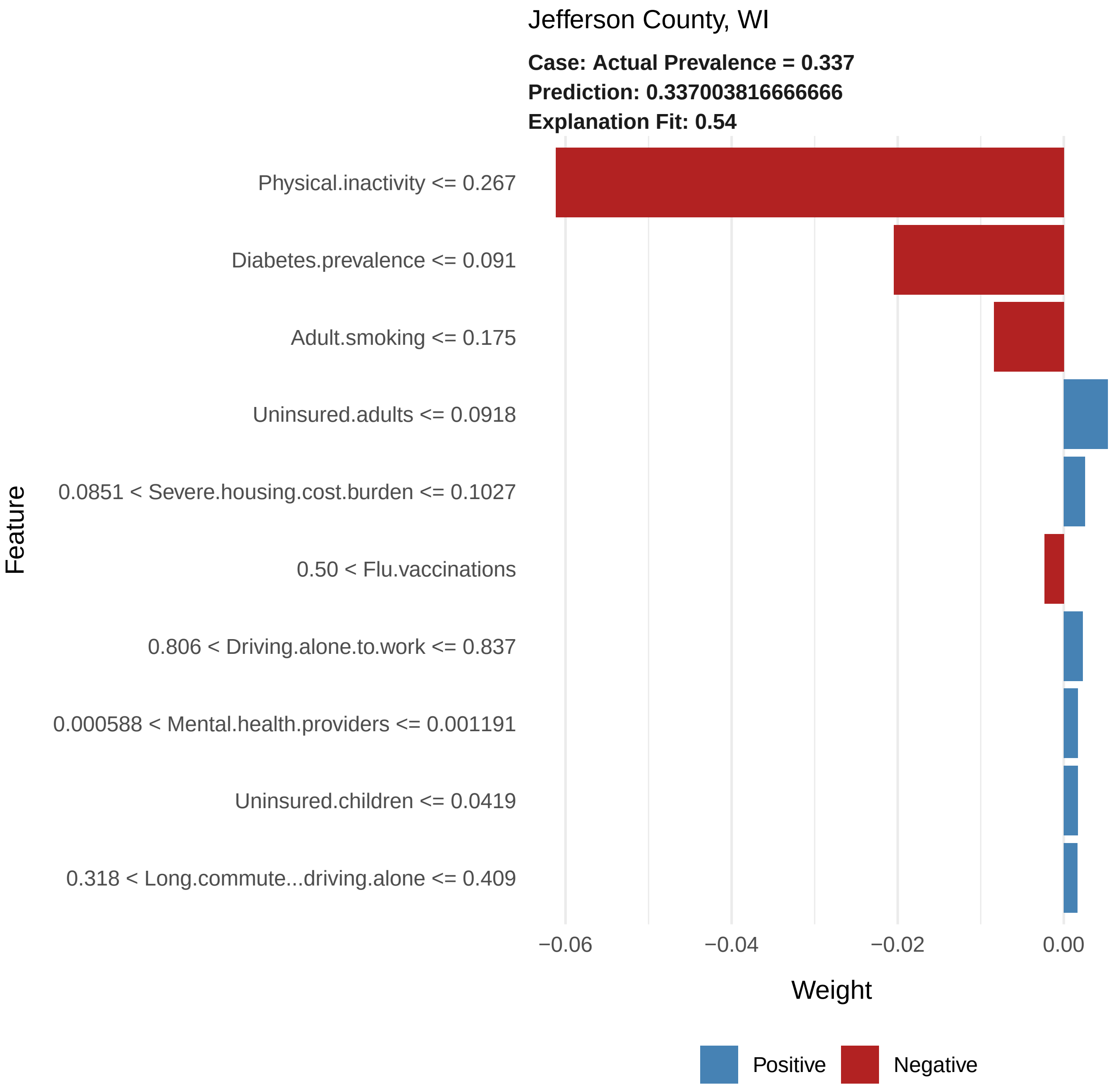

Jefferson County, WI
Case: Actual Prevalence = 0.337
Prediction: 0.337003816666666
Explanation Fit: 0.54
Physical.inactivity <= 0.267
Diabetes.prevalence <= 0.091
Adult.smoking <= 0.175
Uninsured.adults <= 0.0918
0.0851 < Severe.housing.cost.burden <= 0.1027
0.50 < Flu.vaccinations
0.806 < Driving.alone.to.work <= 0.837
0.000588 < Mental.health.providers <= 0.001191
Uninsured.children <= 0.0419
0.318 < Long.commute...driving.alone <= 0.409
Feature
−0.06
−0.04
−0.02
0.00
Weight
Positive
Negative

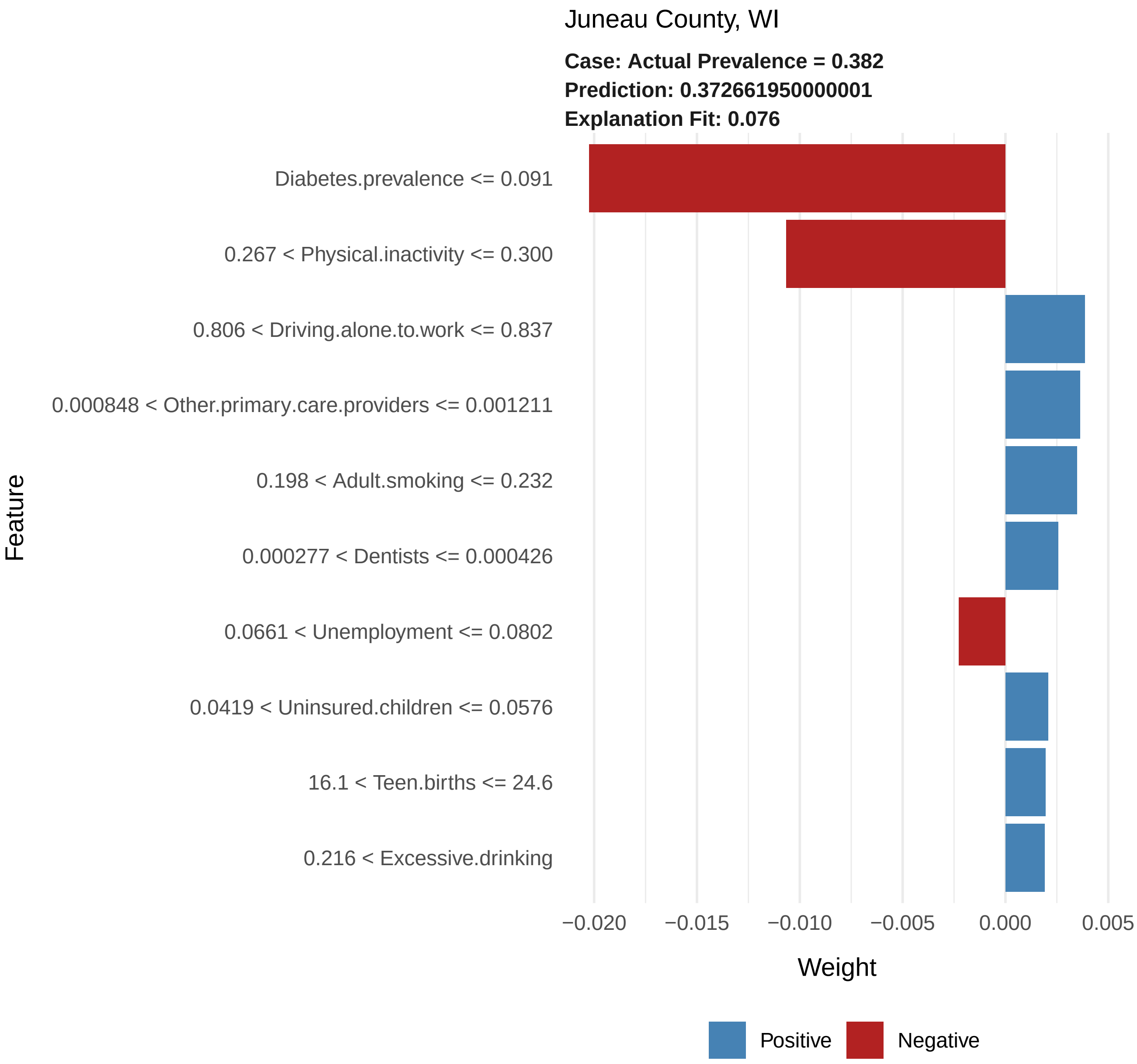

Juneau County, WI
Case: Actual Prevalence = 0.382
Prediction: 0.372661950000001
Explanation Fit: 0.076
Diabetes.prevalence <= 0.091
0.267 < Physical.inactivity <= 0.300
0.806 < Driving.alone.to.work <= 0.837
0.000848 < Other.primary.care.providers <= 0.001211
0.198 < Adult.smoking <= 0.232
0.000277 < Dentists <= 0.000426
0.0661 < Unemployment <= 0.0802
0.0419 < Uninsured.children <= 0.0576
16.1 < Teen.births <= 24.6
0.216 < Excessive.drinking
Feature
−0.020
−0.015
−0.010
−0.005
0.000
0.005
Weight
Positive
Negative

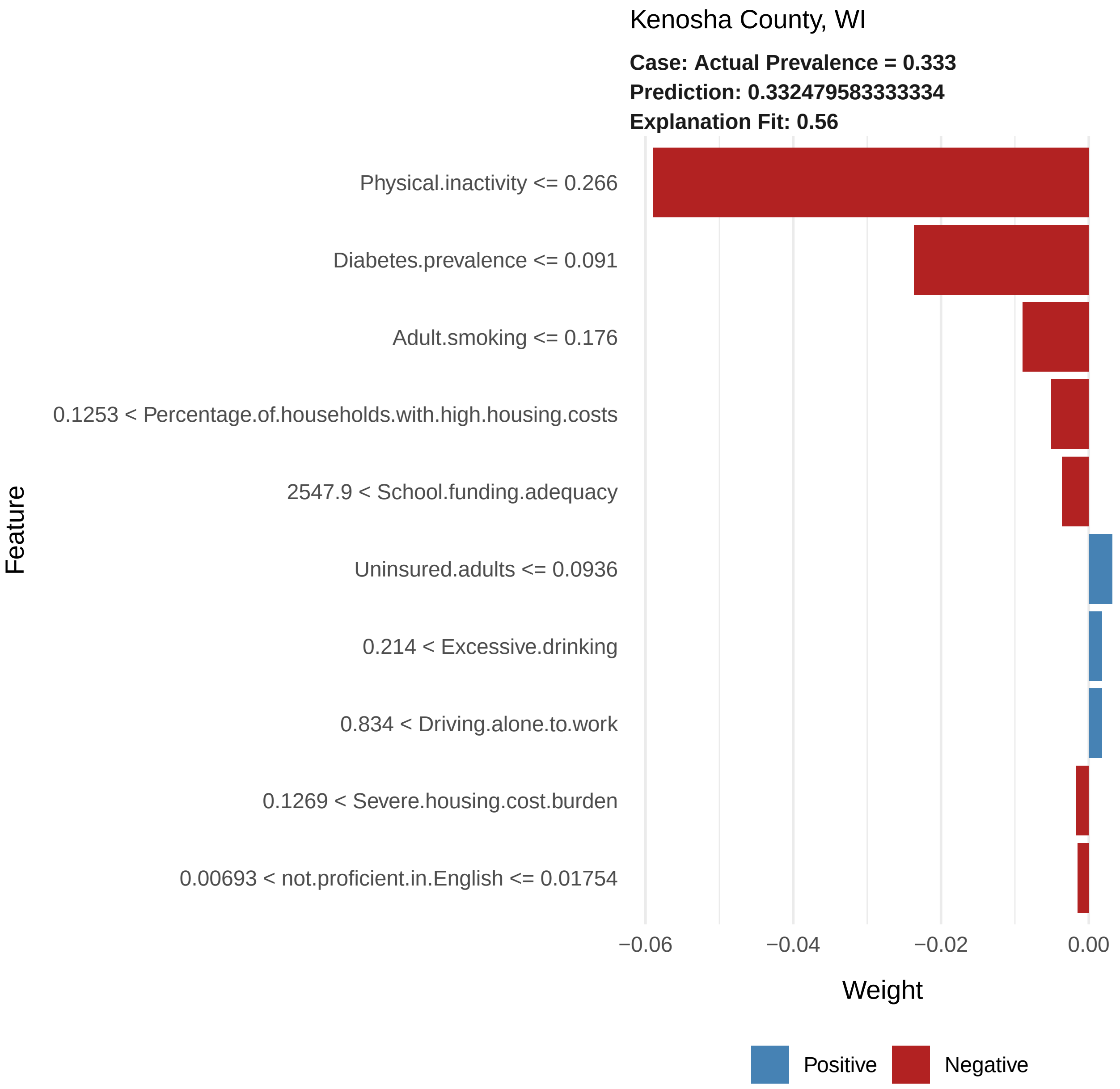

Kenosha County, WI
Case: Actual Prevalence = 0.333
Prediction: 0.332479583333334
Explanation Fit: 0.56
Physical.inactivity <= 0.266
Diabetes.prevalence <= 0.091
Adult.smoking <= 0.176
0.1253 < Percentage.of.households.with.high.housing.costs
2547.9 < School.funding.adequacy
Uninsured.adults <= 0.0936
0.214 < Excessive.drinking
0.834 < Driving.alone.to.work
0.1269 < Severe.housing.cost.burden
0.00693 < not.proficient.in.English <= 0.01754
Feature
−0.06
−0.04
−0.02
0.00
Weight
Positive
Negative

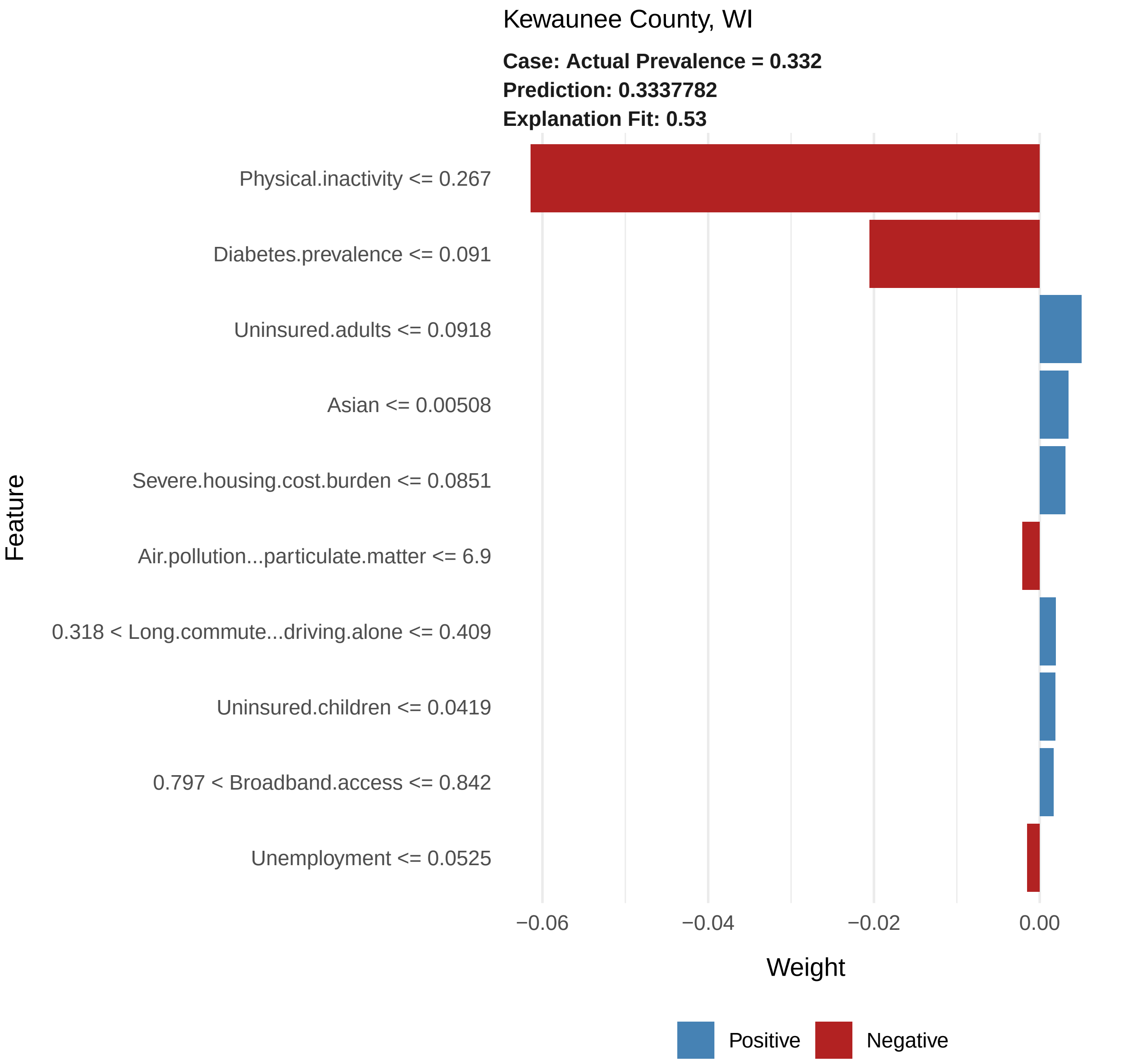
Kewaunee County, WI
Case: Actual Prevalence = 0.332
Prediction: 0.3337782
Explanation Fit: 0.53
Physical.inactivity <= 0.267
Diabetes.prevalence <= 0.091
Uninsured.adults <= 0.0918
Asian <= 0.00508
Severe.housing.cost.burden <= 0.0851
Air.pollution...particulate.matter <= 6.9
0.318 < Long.commute...driving.alone <= 0.409
Uninsured.children <= 0.0419
0.797 < Broadband.access <= 0.842
Unemployment <= 0.0525
Feature
−0.06
−0.04
−0.02
0.00
Weight
Positive
Negative

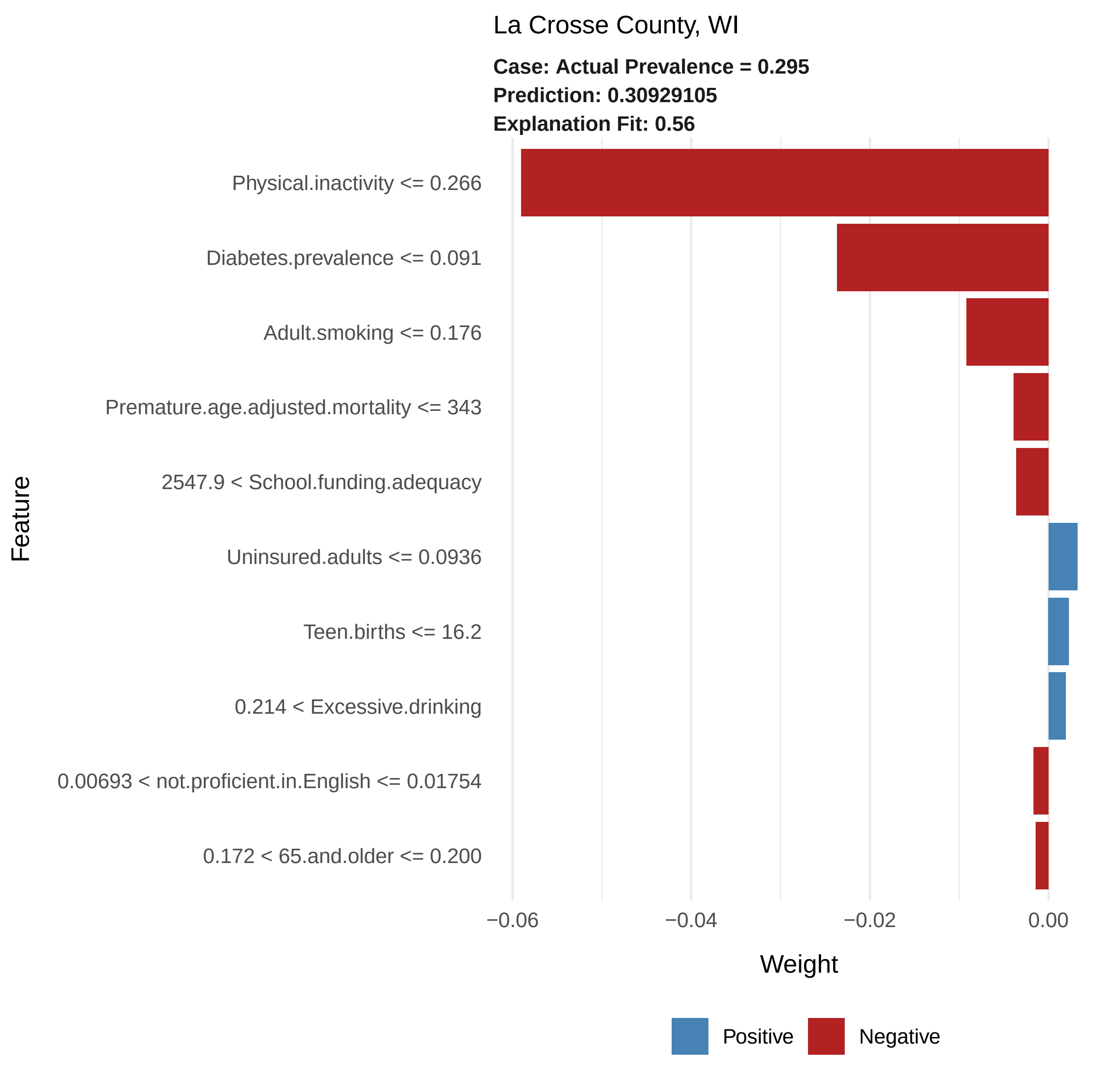
La Crosse County, WI
Case: Actual Prevalence = 0.295
Prediction: 0.30929105
Explanation Fit: 0.56
Physical.inactivity <= 0.266
Diabetes.prevalence <= 0.091
Adult.smoking <= 0.176
Premature.age.adjusted.mortality <= 343
2547.9 < School.funding.adequacy
Uninsured.adults <= 0.0936
Teen.births <= 16.2
0.214 < Excessive.drinking
0.00693 < not.proficient.in.English <= 0.01754
0.172 < 65.and.older <= 0.200
Feature
−0.06
−0.04
−0.02
0.00
Weight
Positive
Negative

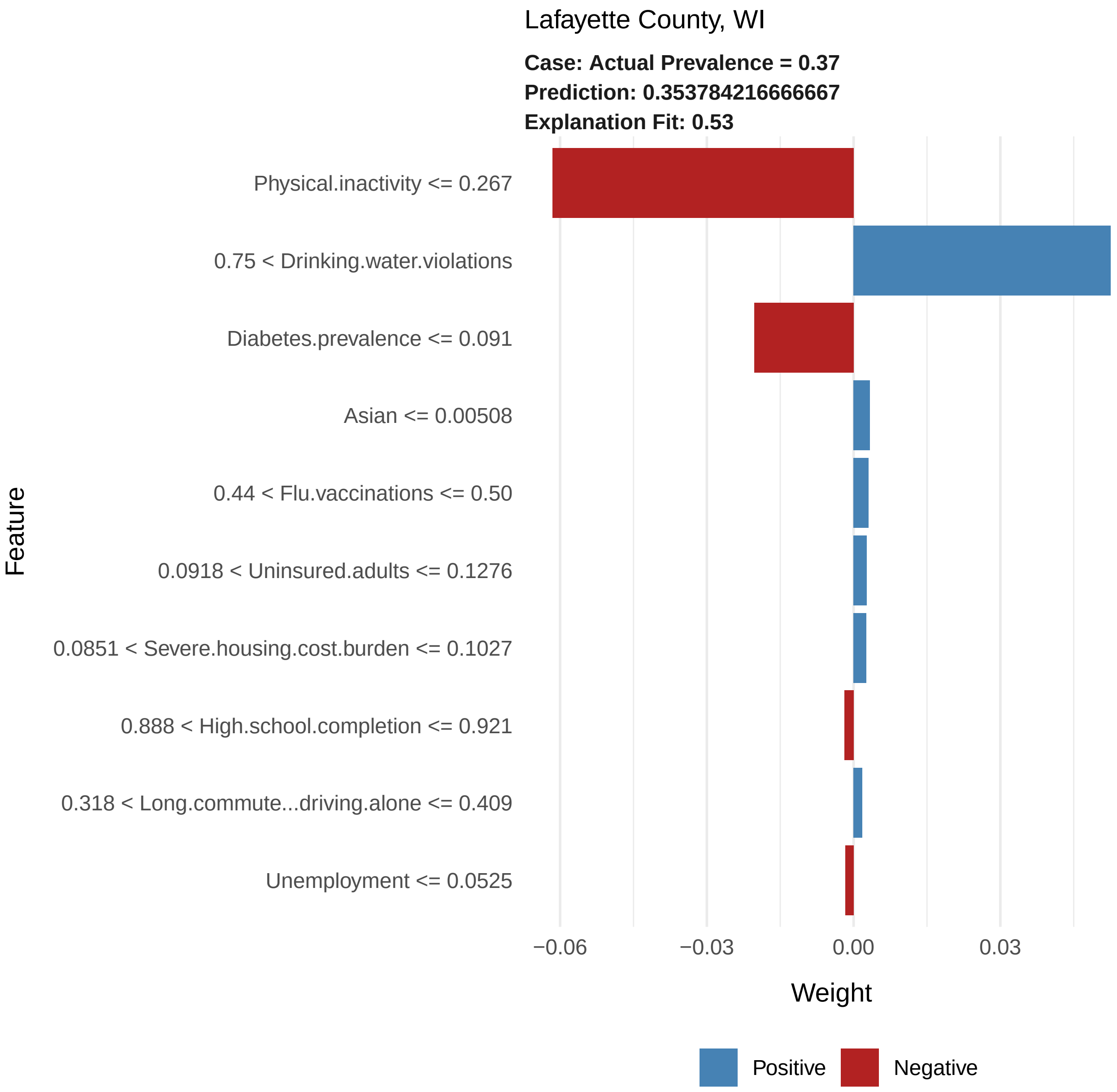

Lafayette County, WI
Case: Actual Prevalence = 0.37
Prediction: 0.353784216666667
Explanation Fit: 0.53
Physical.inactivity <= 0.267
0.75 < Drinking.water.violations
Diabetes.prevalence <= 0.091
Asian <= 0.00508
0.44 < Flu.vaccinations <= 0.50
0.0918 < Uninsured.adults <= 0.1276
0.0851 < Severe.housing.cost.burden <= 0.1027
0.888 < High.school.completion <= 0.921
0.318 < Long.commute...driving.alone <= 0.409
Unemployment <= 0.0525
Feature
−0.06
−0.03
0.00
0.03
Weight
Positive
Negative

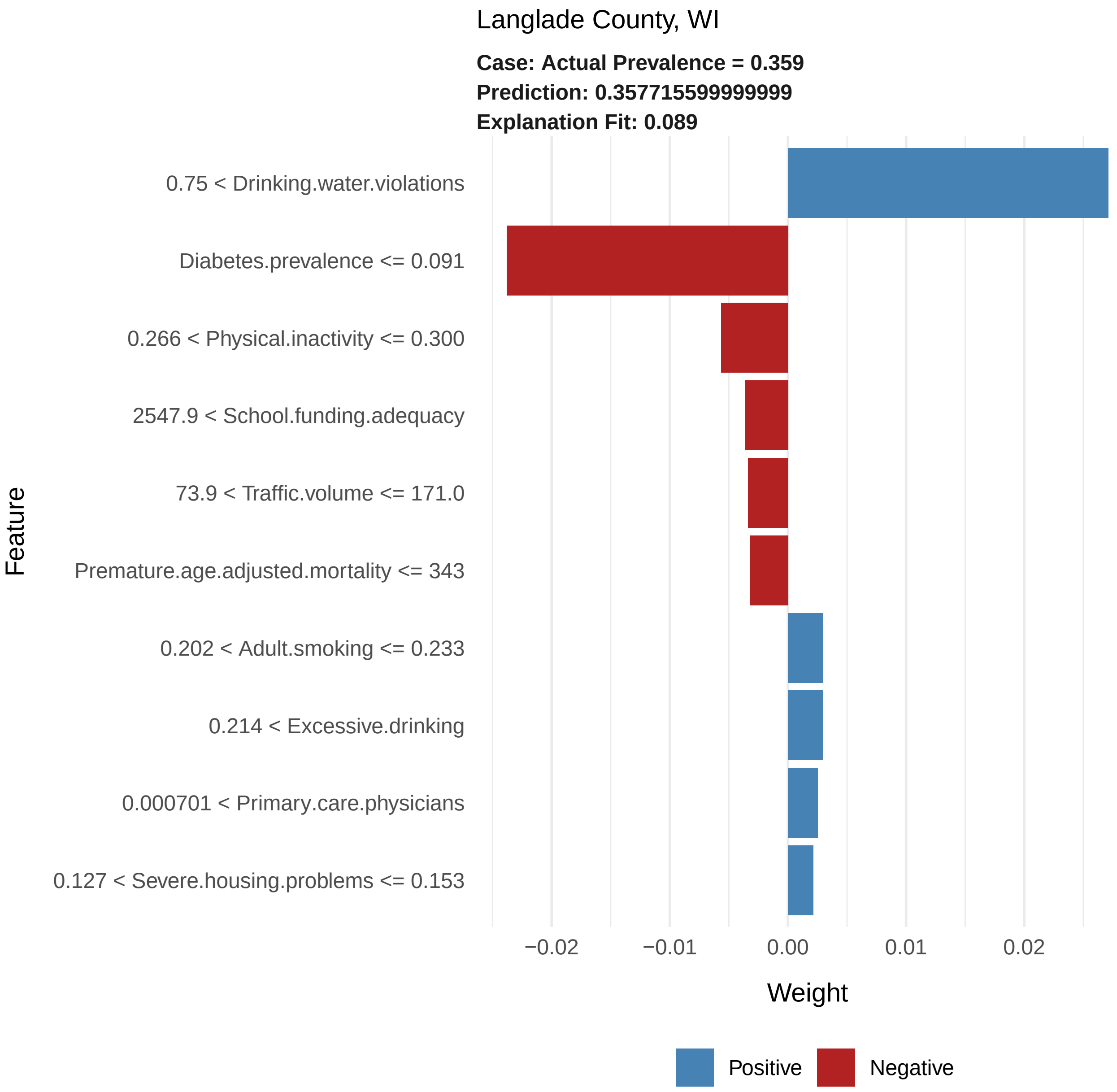
Langlade County, WI
Case: Actual Prevalence = 0.359
Prediction: 0.357715599999999
Explanation Fit: 0.089
0.75 < Drinking.water.violations
Diabetes.prevalence <= 0.091
0.266 < Physical.inactivity <= 0.300
2547.9 < School.funding.adequacy
73.9 < Traffic.volume <= 171.0
Premature.age.adjusted.mortality <= 343
0.202 < Adult.smoking <= 0.233
0.214 < Excessive.drinking
0.000701 < Primary.care.physicians
0.127 < Severe.housing.problems <= 0.153
Feature
−0.02
−0.01
0.00
0.01
0.02
Weight
Positive
Negative

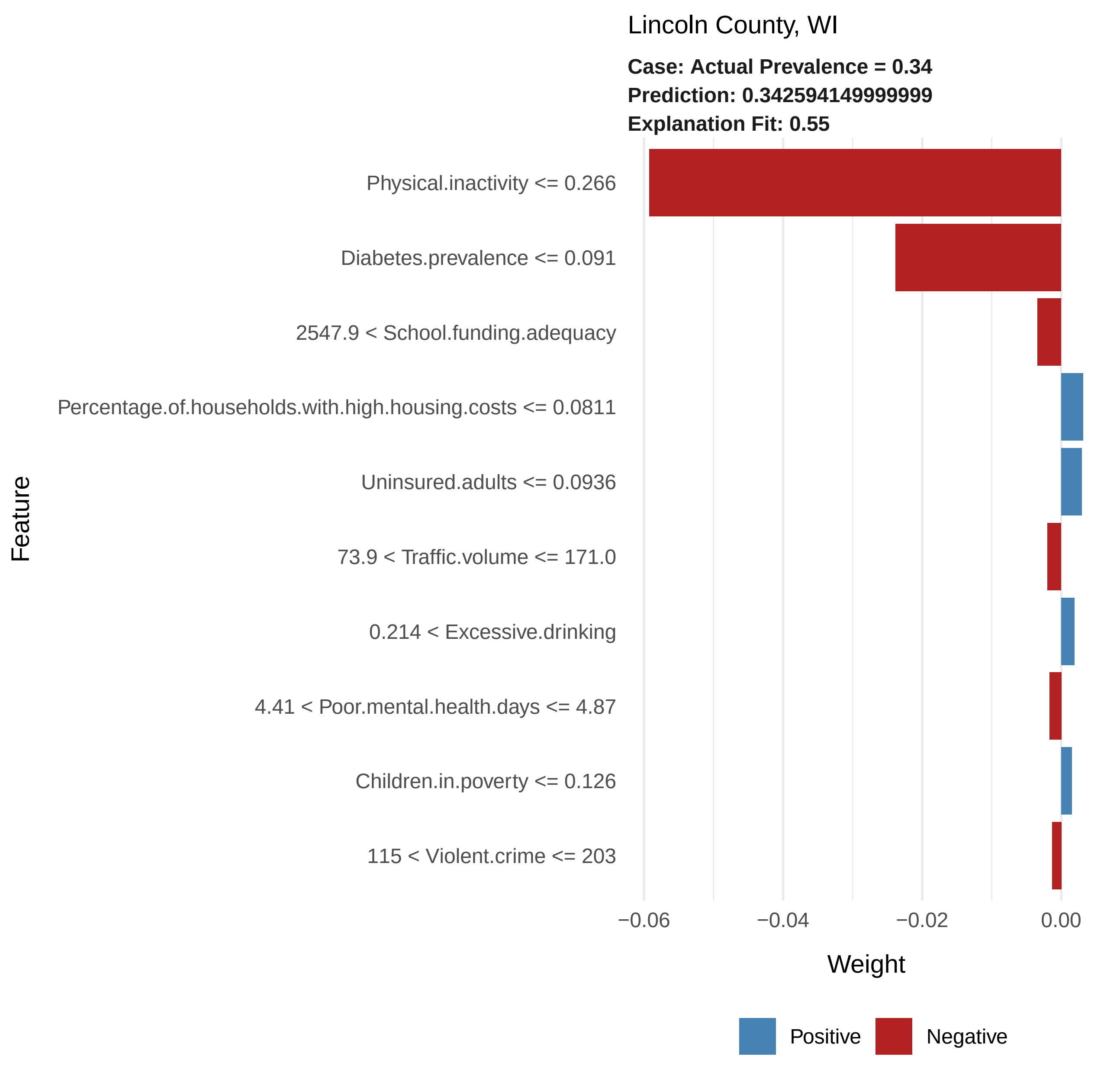
Lincoln County, WI
Case: Actual Prevalence = 0.34
Prediction: 0.342594149999999
Explanation Fit: 0.55
Physical.inactivity <= 0.266
Diabetes.prevalence <= 0.091
2547.9 < School.funding.adequacy
Percentage.of.households.with.high.housing.costs <= 0.0811
Uninsured.adults <= 0.0936
73.9 < Traffic.volume <= 171.0
0.214 < Excessive.drinking
4.41 < Poor.mental.health.days <= 4.87
Children.in.poverty <= 0.126
115 < Violent.crime <= 203
Feature
−0.06
−0.04
−0.02
0.00
Weight
Positive
Negative

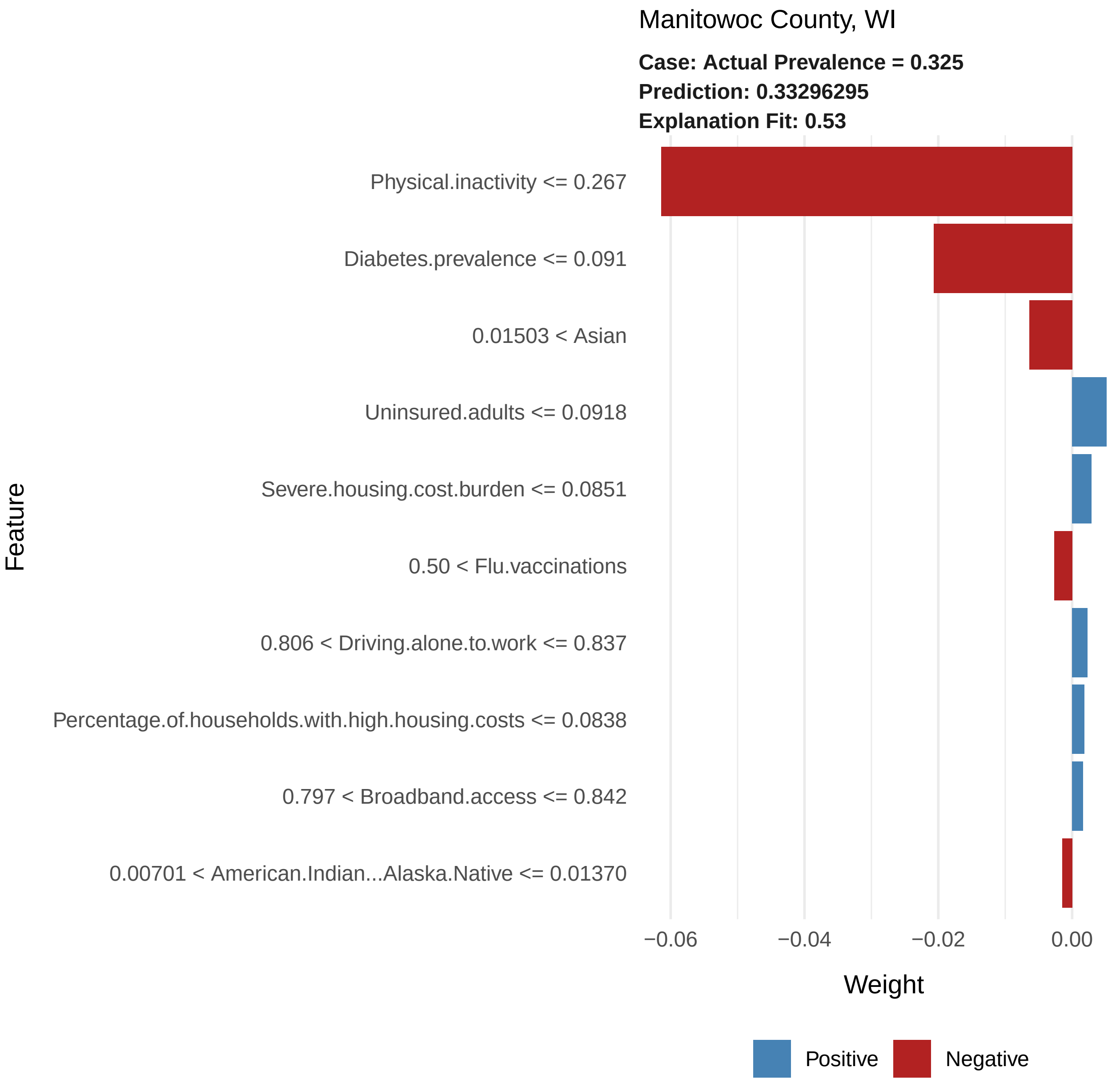
Manitowoc County, WI
Case: Actual Prevalence = 0.325
Prediction: 0.33296295
Explanation Fit: 0.53
Physical.inactivity <= 0.267
Diabetes.prevalence <= 0.091
0.01503 < Asian
Uninsured.adults <= 0.0918
Severe.housing.cost.burden <= 0.0851
0.50 < Flu.vaccinations
0.806 < Driving.alone.to.work <= 0.837
Percentage.of.households.with.high.housing.costs <= 0.0838
0.797 < Broadband.access <= 0.842
0.00701 < American.Indian...Alaska.Native <= 0.01370
Feature
−0.06
−0.04
−0.02
0.00
Weight
Positive
Negative

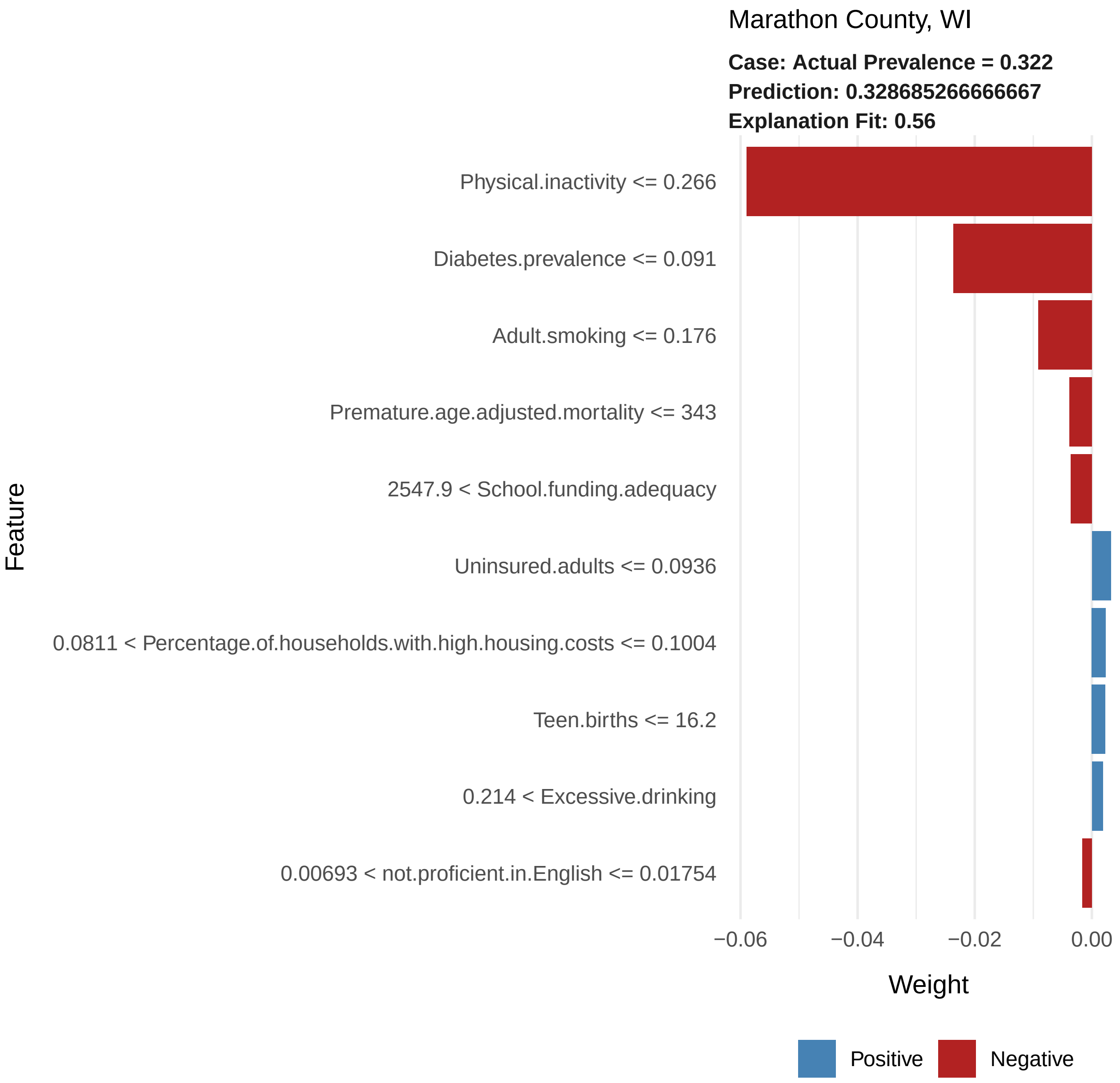
Marathon County, WI
Case: Actual Prevalence = 0.322
Prediction: 0.328685266666667
Explanation Fit: 0.56
Physical.inactivity <= 0.266
Diabetes.prevalence <= 0.091
Adult.smoking <= 0.176
Premature.age.adjusted.mortality <= 343
2547.9 < School.funding.adequacy
Uninsured.adults <= 0.0936
0.0811 < Percentage.of.households.with.high.housing.costs <= 0.1004
Teen.births <= 16.2
0.214 < Excessive.drinking
0.00693 < not.proficient.in.English <= 0.01754
Feature
−0.06
−0.04
−0.02
0.00
Weight
Positive
Negative

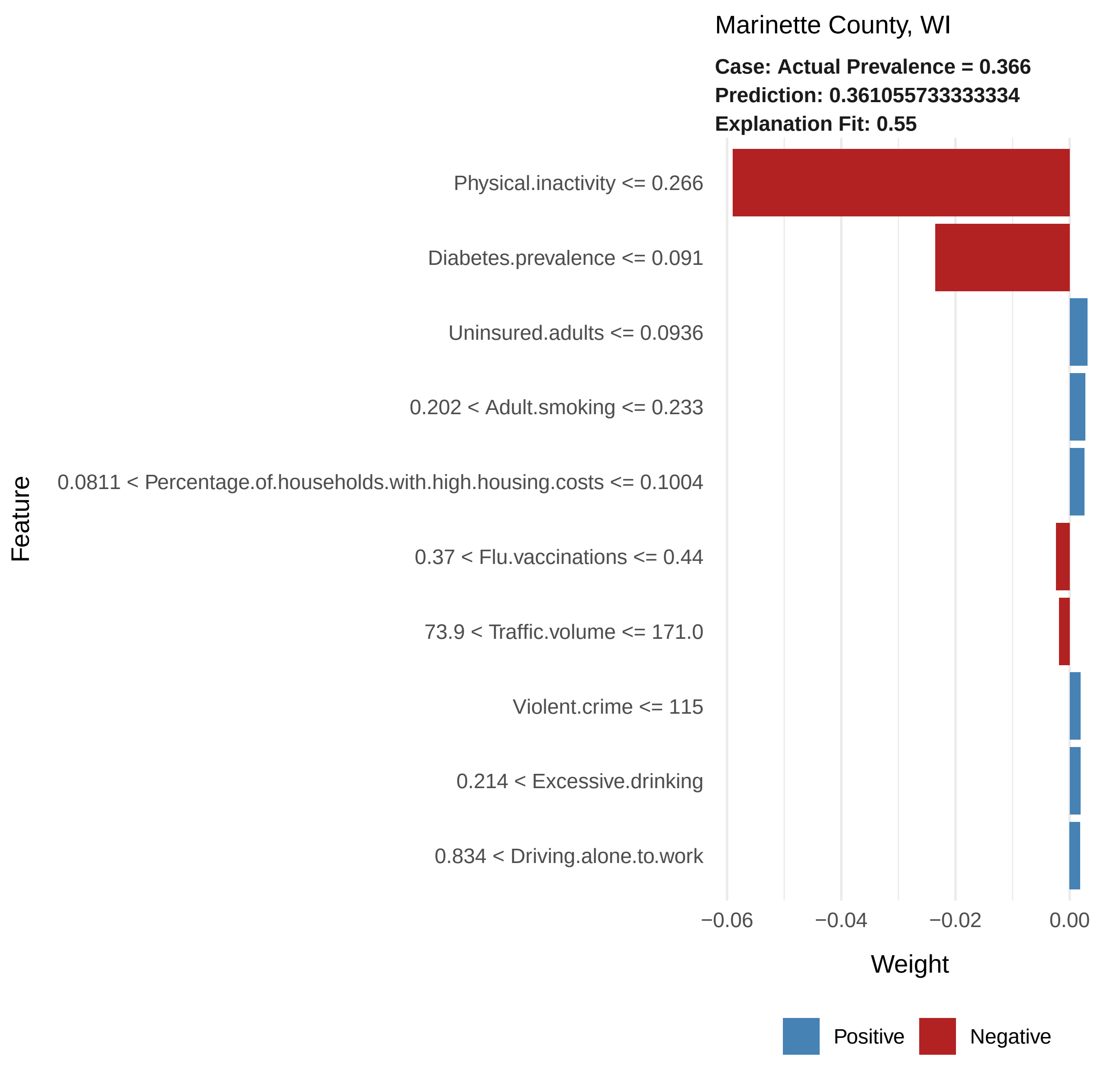

Marinette County, WI
Case: Actual Prevalence = 0.366
Prediction: 0.361055733333334
Explanation Fit: 0.55
Physical.inactivity <= 0.266
Diabetes.prevalence <= 0.091
Uninsured.adults <= 0.0936
0.202 < Adult.smoking <= 0.233
0.0811 < Percentage.of.households.with.high.housing.costs <= 0.1004
0.37 < Flu.vaccinations <= 0.44
73.9 < Traffic.volume <= 171.0
Violent.crime <= 115
0.214 < Excessive.drinking
0.834 < Driving.alone.to.work
Feature
−0.06
−0.04
−0.02
0.00
Weight
Positive
Negative

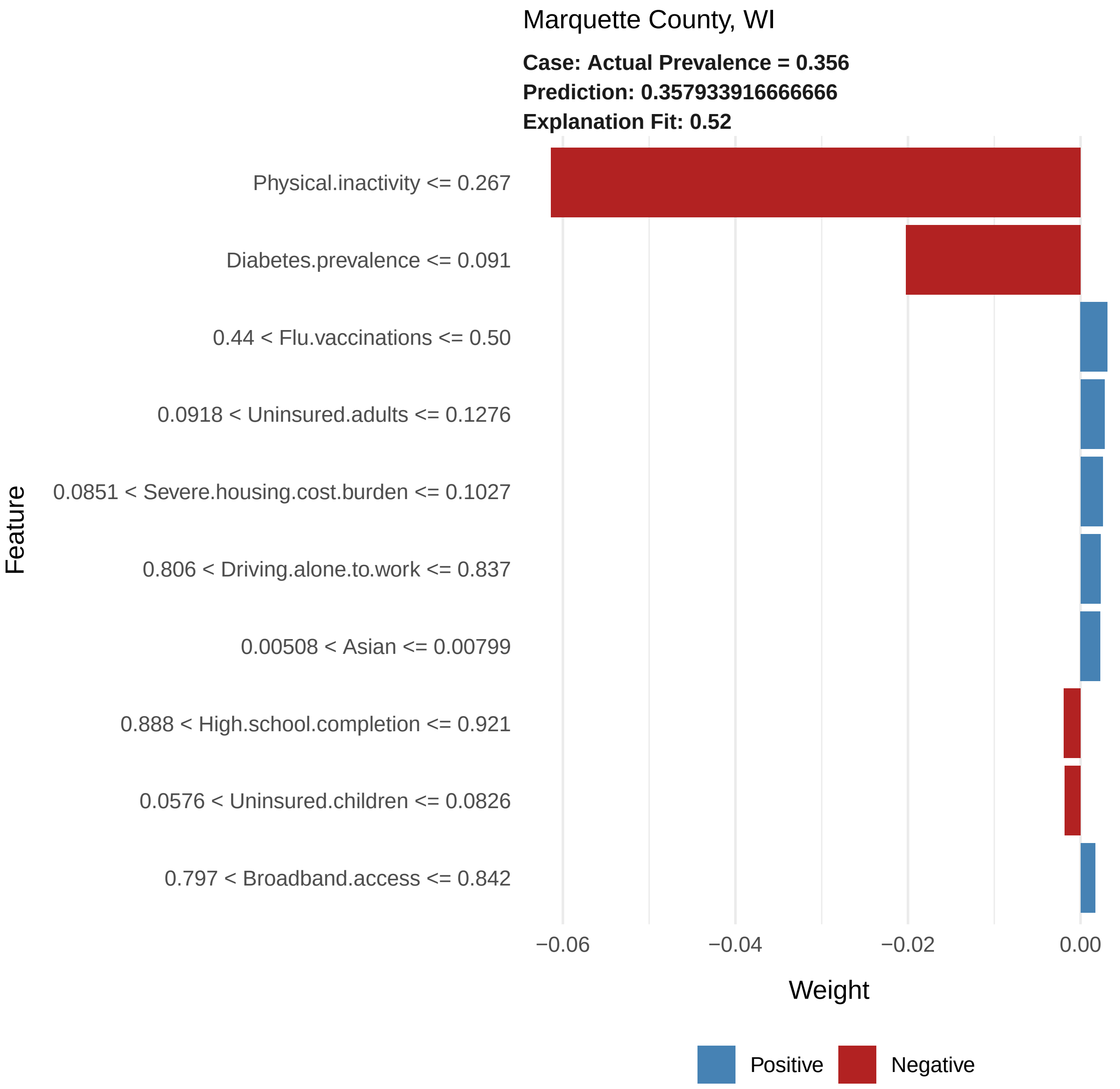
Marquette County, WI
Case: Actual Prevalence = 0.356
Prediction: 0.357933916666666
Explanation Fit: 0.52
Physical.inactivity <= 0.267
Diabetes.prevalence <= 0.091
0.44 < Flu.vaccinations <= 0.50
0.0918 < Uninsured.adults <= 0.1276
0.0851 < Severe.housing.cost.burden <= 0.1027
0.806 < Driving.alone.to.work <= 0.837
0.00508 < Asian <= 0.00799
0.888 < High.school.completion <= 0.921
0.0576 < Uninsured.children <= 0.0826
0.797 < Broadband.access <= 0.842
Feature
−0.06
−0.04
−0.02
0.00
Weight
Positive
Negative

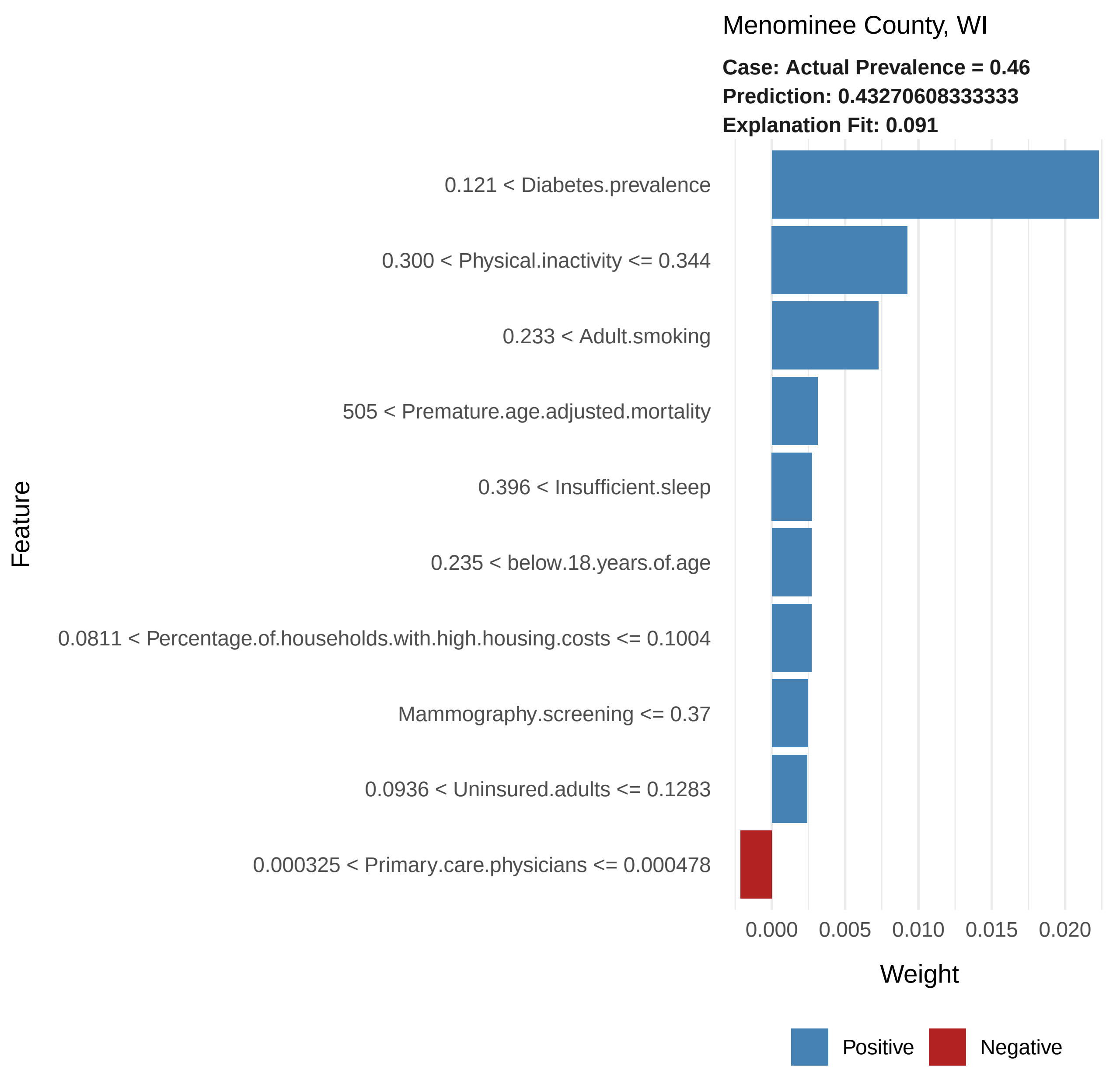
Menominee County, WI
Case: Actual Prevalence = 0.46
Prediction: 0.432706083333333
Explanation Fit: 0.091
0.121 < Diabetes.prevalence
0.300 < Physical.inactivity <= 0.344
0.233 < Adult.smoking
505 < Premature.age.adjusted.mortality
0.396 < Insufficient.sleep
0.235 < below.18.years.of.age
0.0811 < Percentage.of.households.with.high.housing.costs <= 0.1004
Mammography.screening <= 0.37
0.0936 < Uninsured.adults <= 0.1283
0.000325 < Primary.care.physicians <= 0.000478
Feature
0.000
0.005
0.010
0.015
0.020
Weight
Positive
Negative

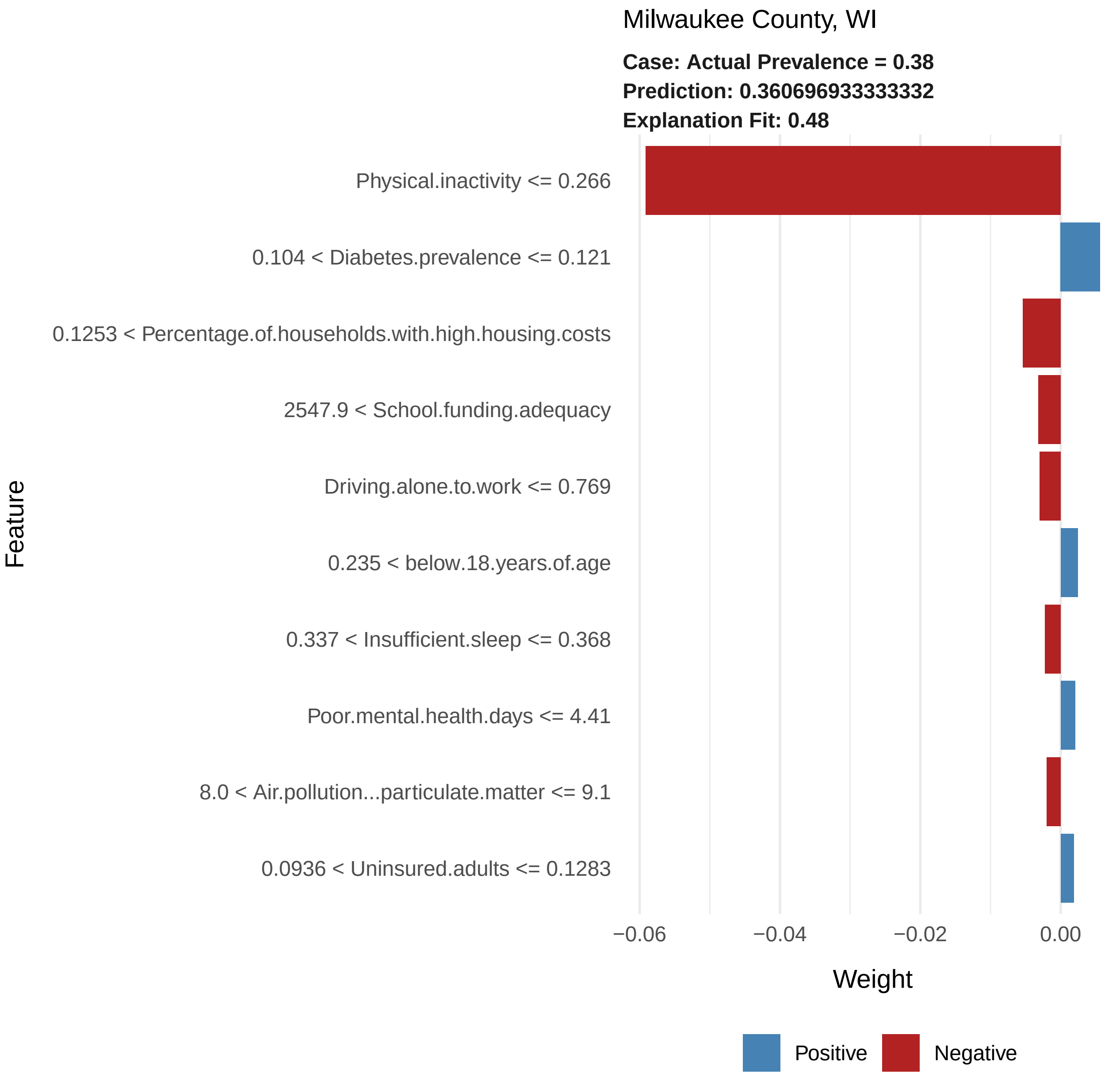

Milwaukee County, WI
Case: Actual Prevalence = 0.38
Prediction: 0.360696933333332
Explanation Fit: 0.48
Physical.inactivity <= 0.266
0.104 < Diabetes.prevalence <= 0.121
0.1253 < Percentage.of.households.with.high.housing.costs
2547.9 < School.funding.adequacy
Driving.alone.to.work <= 0.769
0.235 < below.18.years.of.age
0.337 < Insufficient.sleep <= 0.368
Poor.mental.health.days <= 4.41
8.0 < Air.pollution...particulate.matter <= 9.1
0.0936 < Uninsured.adults <= 0.1283
Feature
−0.06
−0.04
−0.02
0.00
Weight
Positive
Negative

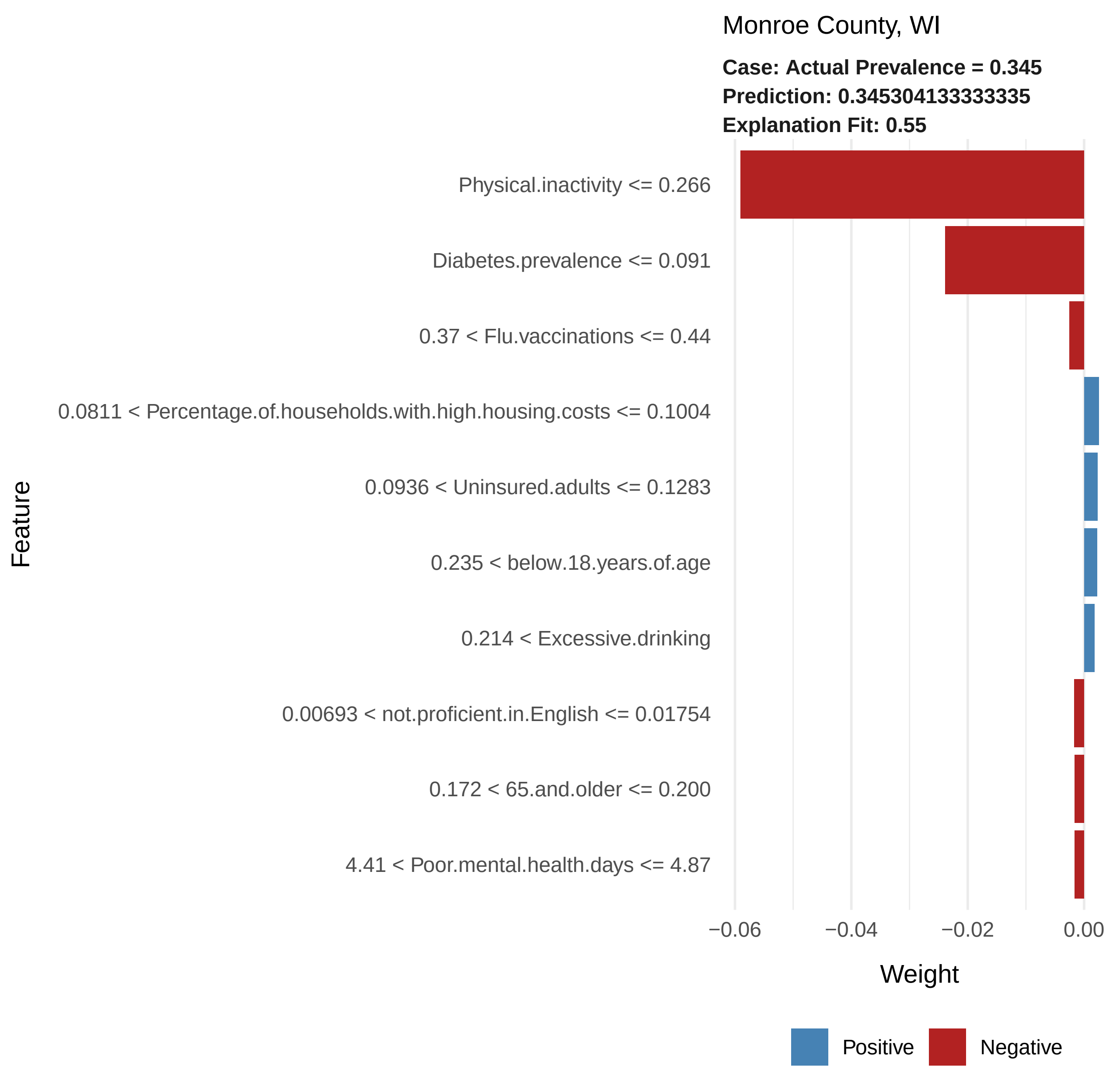
Monroe County, WI
Case: Actual Prevalence = 0.345
Prediction: 0.345304133333335
Explanation Fit: 0.55
Physical.inactivity <= 0.266
Diabetes.prevalence <= 0.091
0.37 < Flu.vaccinations <= 0.44
0.0811 < Percentage.of.households.with.high.housing.costs <= 0.1004
0.0936 < Uninsured.adults <= 0.1283
0.235 < below.18.years.of.age
0.214 < Excessive.drinking
0.00693 < not.proficient.in.English <= 0.01754
0.172 < 65.and.older <= 0.200
4.41 < Poor.mental.health.days <= 4.87
Feature
−0.06
−0.04
−0.02
0.00
Weight
Positive
Negative

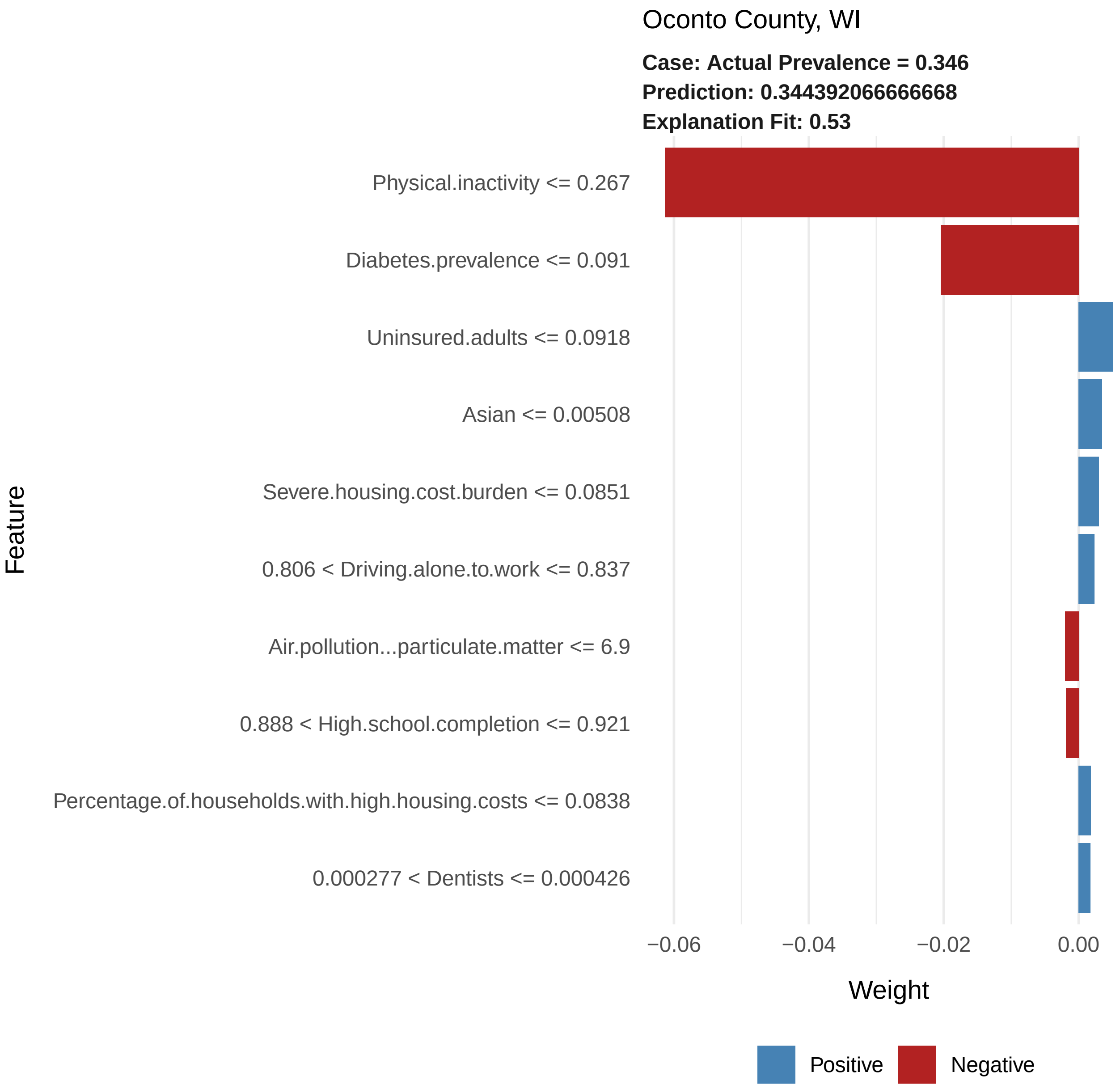
Oconto County, WI
Case: Actual Prevalence = 0.346
Prediction: 0.344392066666668
Explanation Fit: 0.53
Feature
Physical.inactivity <= 0.267
Diabetes.prevalence <= 0.091
Uninsured.adults <= 0.0918
Asian <= 0.00508
Severe.housing.cost.burden <= 0.0851
0.806 < Driving.alone.to.work <= 0.837
Air.pollution...particulate.matter <= 6.9
0.888 < High.school.completion <= 0.921
Percentage.of.households.with.high.housing.costs <= 0.0838
0.000277 < Dentists <= 0.000426
−0.06
−0.04
−0.02
0.00
Weight
Positive
Negative

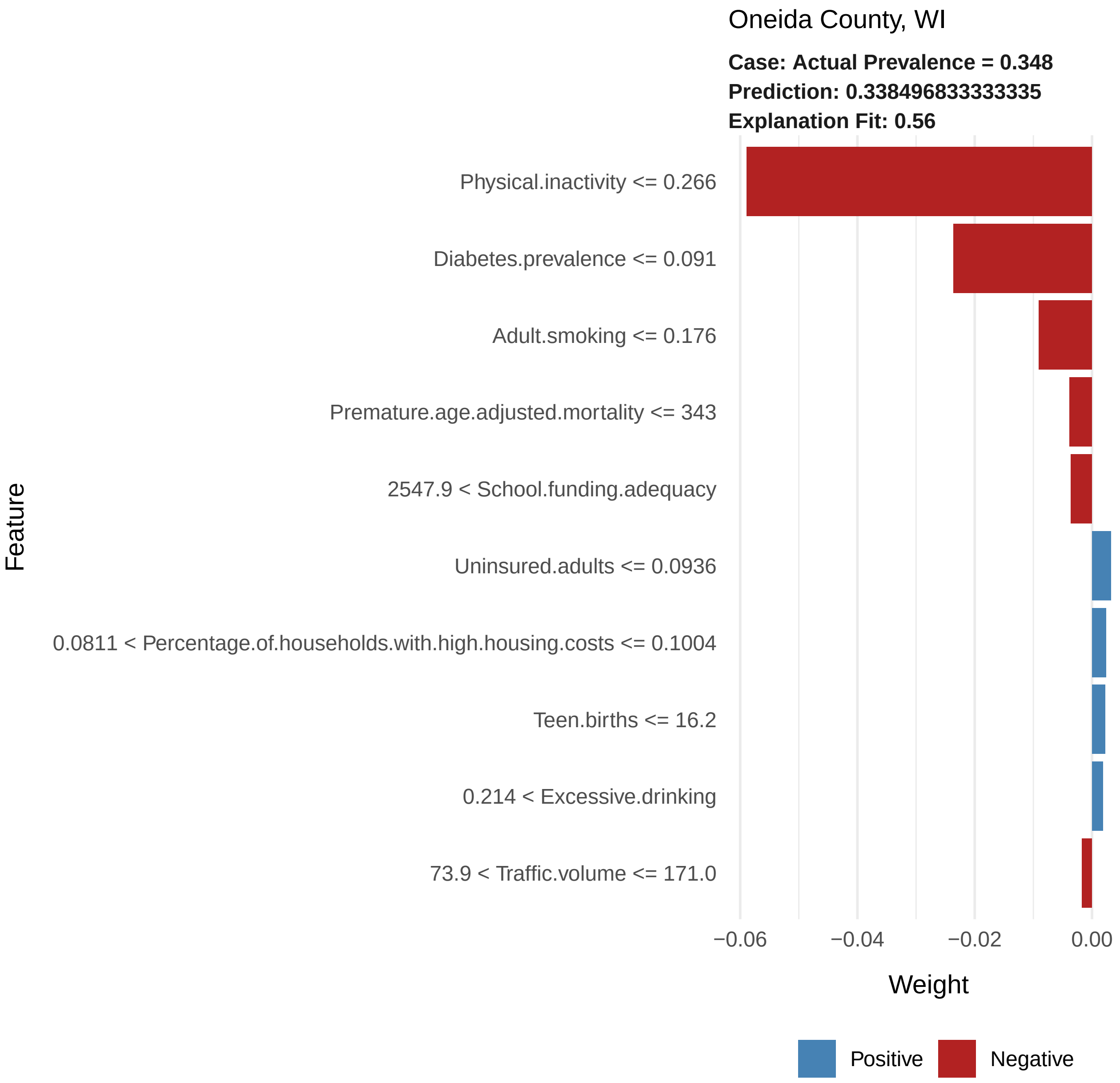
Oneida County, WI
Case: Actual Prevalence = 0.348
Prediction: 0.338496833333335
Explanation Fit: 0.56
Physical.inactivity <= 0.266
Diabetes.prevalence <= 0.091
Adult.smoking <= 0.176
Premature.age.adjusted.mortality <= 343
2547.9 < School.funding.adequacy
Uninsured.adults <= 0.0936
0.0811 < Percentage.of.households.with.high.housing.costs <= 0.1004
Teen.births <= 16.2
0.214 < Excessive.drinking
73.9 < Traffic.volume <= 171.0
Feature
−0.06
−0.04
−0.02
0.00
Weight
Positive
Negative

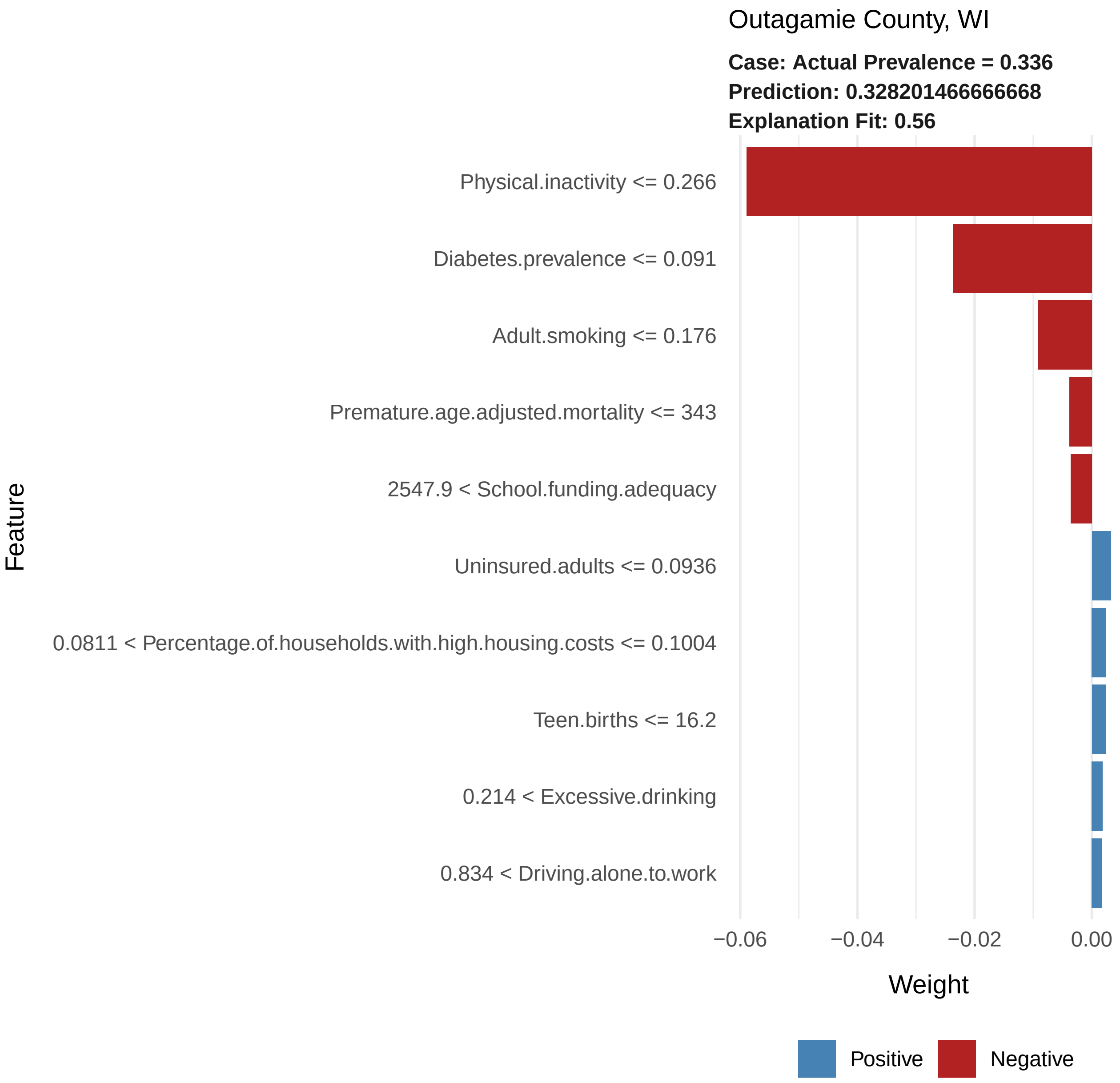
Outagamie County, WI
Case: Actual Prevalence = 0.336
Prediction: 0.328201466666668
Explanation Fit: 0.56
Physical.inactivity <= 0.266
Diabetes.prevalence <= 0.091
Adult.smoking <= 0.176
Premature.age.adjusted.mortality <= 343
2547.9 < School.funding.adequacy
Uninsured.adults <= 0.0936
0.0811 < Percentage.of.households.with.high.housing.costs <= 0.1004
Teen.births <= 16.2
0.214 < Excessive.drinking
0.834 < Driving.alone.to.work
Feature
−0.06
−0.04
−0.02
0.00
Weight
Positive
Negative

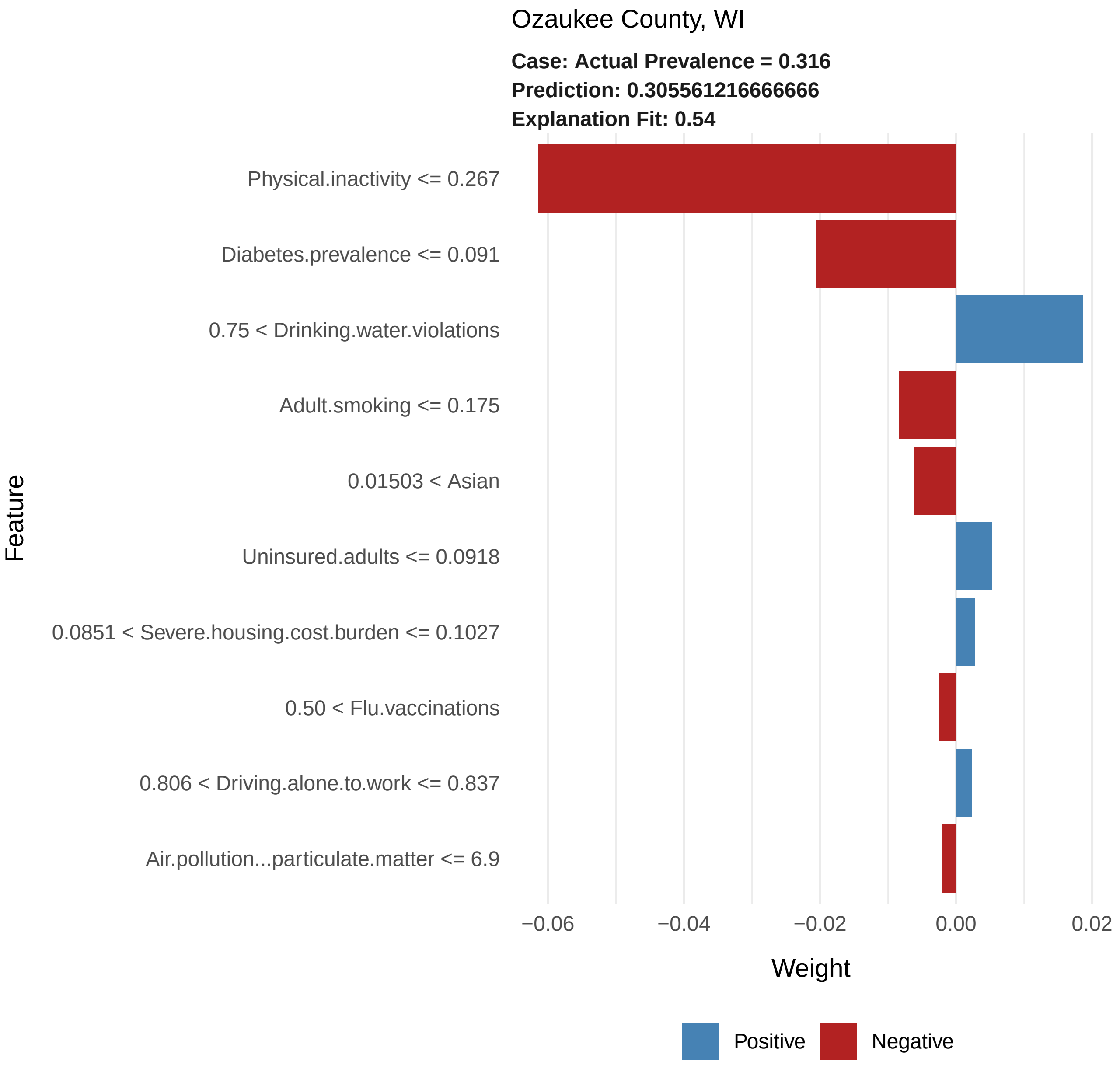
Ozaukee County, WI
Case: Actual Prevalence = 0.316
Prediction: 0.305561216666666
Explanation Fit: 0.54
Physical.inactivity <= 0.267
Diabetes.prevalence <= 0.091
0.75 < Drinking.water.violations
Adult.smoking <= 0.175
0.01503 < Asian
Uninsured.adults <= 0.0918
0.0851 < Severe.housing.cost.burden <= 0.1027
0.50 < Flu.vaccinations
0.806 < Driving.alone.to.work <= 0.837
Air.pollution...particulate.matter <= 6.9
Feature
−0.06
−0.04
−0.02
0.00
0.02
Weight
Positive
Negative

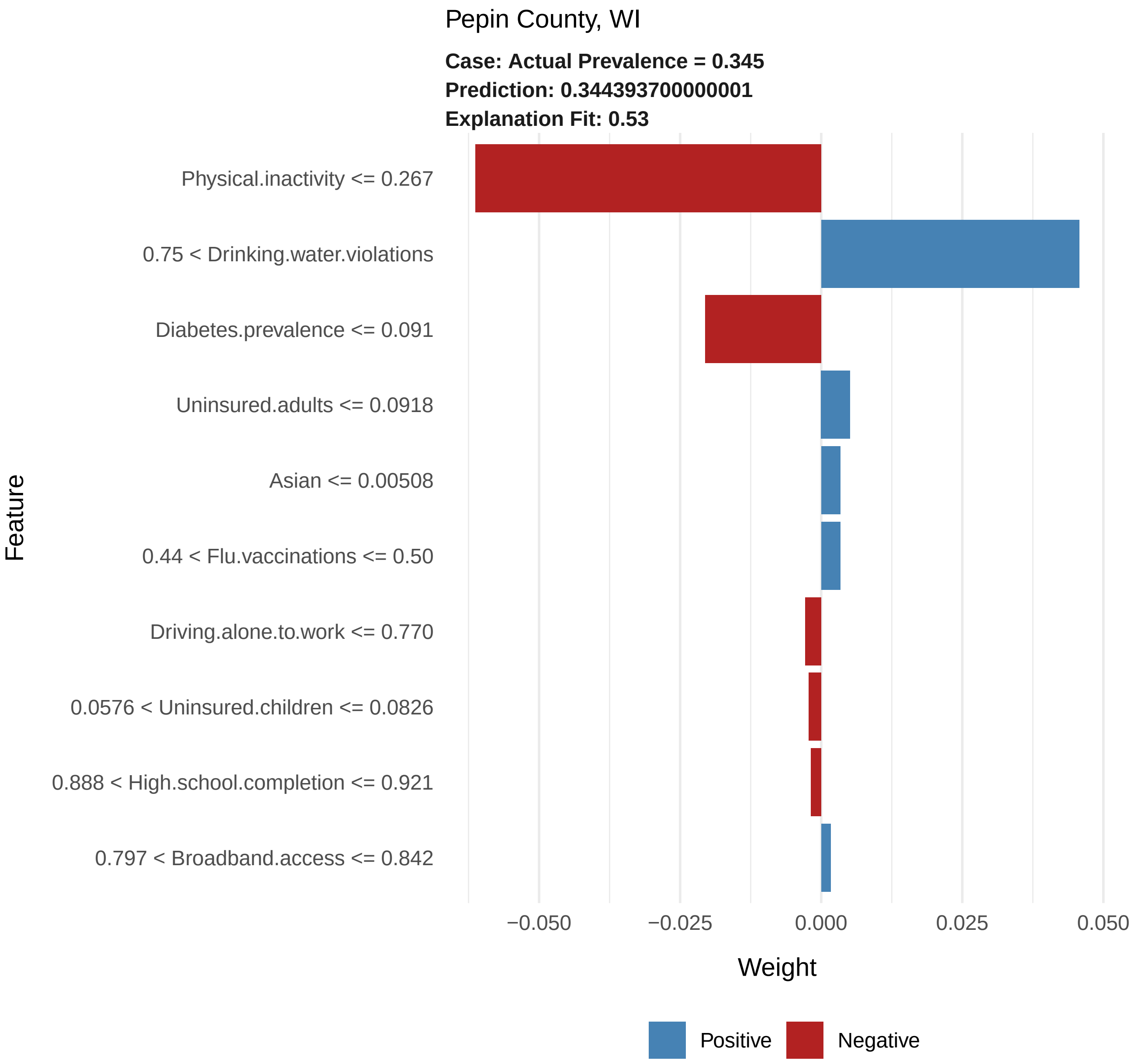
Pepin County, WI
Case: Actual Prevalence = 0.345
Prediction: 0.344393700000001
Explanation Fit: 0.53
Physical.inactivity <= 0.267
0.75 < Drinking.water.violations
Diabetes.prevalence <= 0.091
Uninsured.adults <= 0.0918
Asian <= 0.00508
0.44 < Flu.vaccinations <= 0.50
Driving.alone.to.work <= 0.770
0.0576 < Uninsured.children <= 0.0826
0.888 < High.school.completion <= 0.921
0.797 < Broadband.access <= 0.842
Feature
−0.050
−0.025
0.000
0.025
0.050
Weight
Positive
Negative

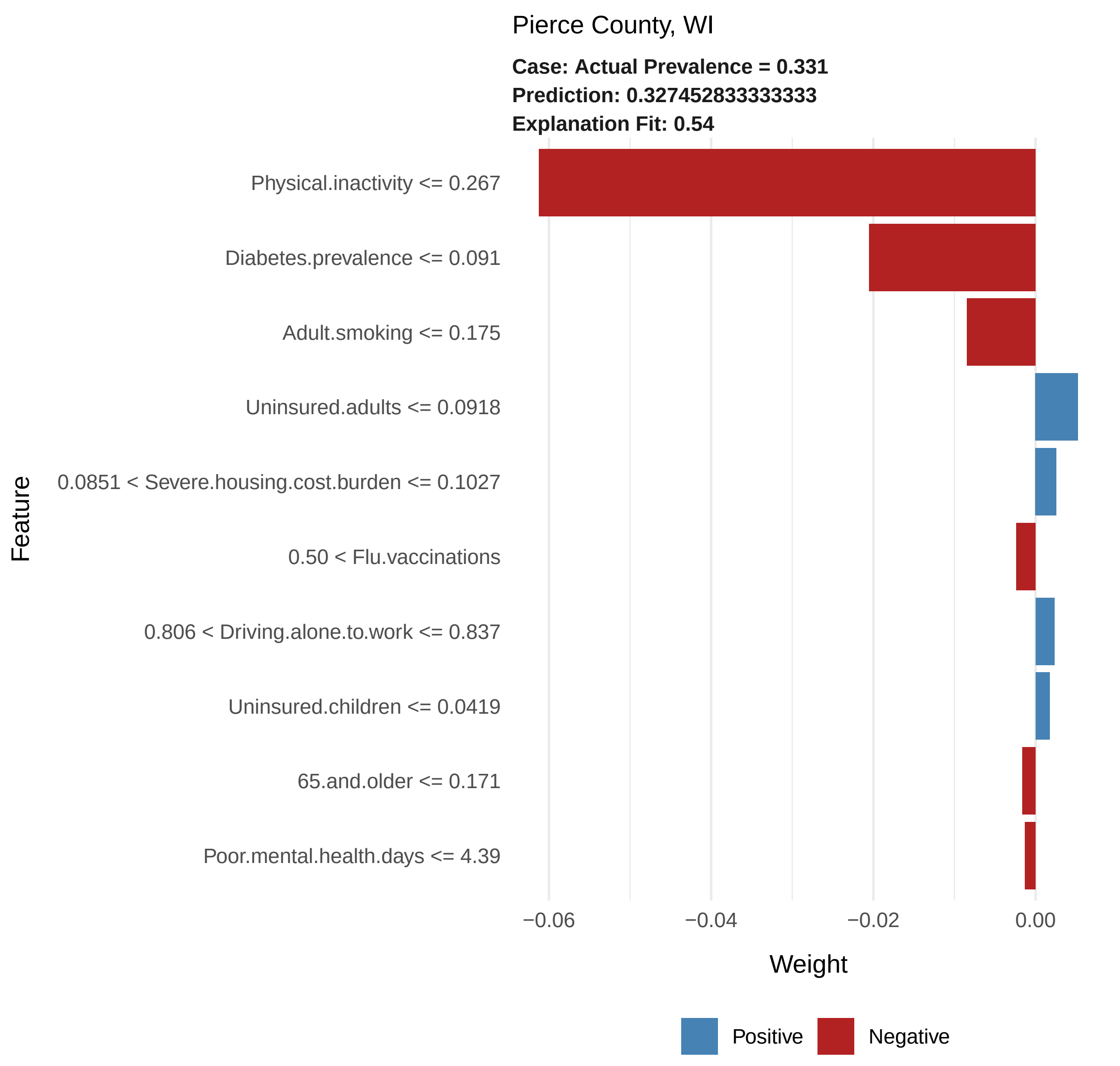

Pierce County, WI
Case: Actual Prevalence = 0.331
Prediction: 0.327452833333333
Explanation Fit: 0.54
Physical.inactivity <= 0.267
Diabetes.prevalence <= 0.091
Adult.smoking <= 0.175
Uninsured.adults <= 0.0918
0.0851 < Severe.housing.cost.burden <= 0.1027
0.50 < Flu.vaccinations
0.806 < Driving.alone.to.work <= 0.837
Uninsured.children <= 0.0419
65.and.older <= 0.171
Poor.mental.health.days <= 4.39
Feature
−0.06
−0.04
−0.02
0.00
Weight
Positive
Negative

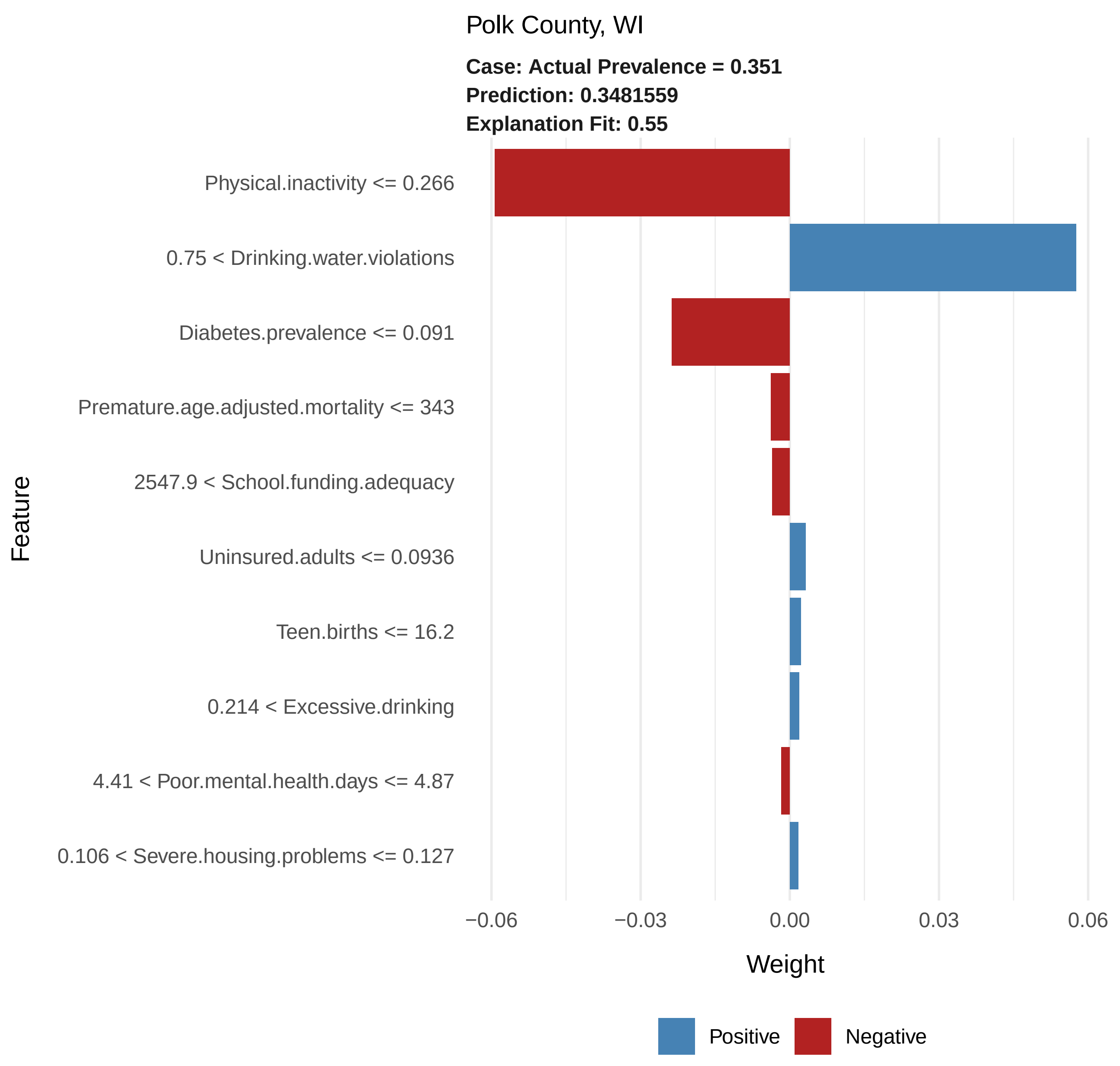
Polk County, WI
Case: Actual Prevalence = 0.351
Prediction: 0.3481559
Explanation Fit: 0.55
Physical.inactivity <= 0.266
0.75 < Drinking.water.violations
Diabetes.prevalence <= 0.091
Premature.age.adjusted.mortality <= 343
2547.9 < School.funding.adequacy
Uninsured.adults <= 0.0936
Teen.births <= 16.2
0.214 < Excessive.drinking
4.41 < Poor.mental.health.days <= 4.87
0.106 < Severe.housing.problems <= 0.127
Feature
−0.06
−0.03
0.00
0.03
0.06
Weight
Positive
Negative

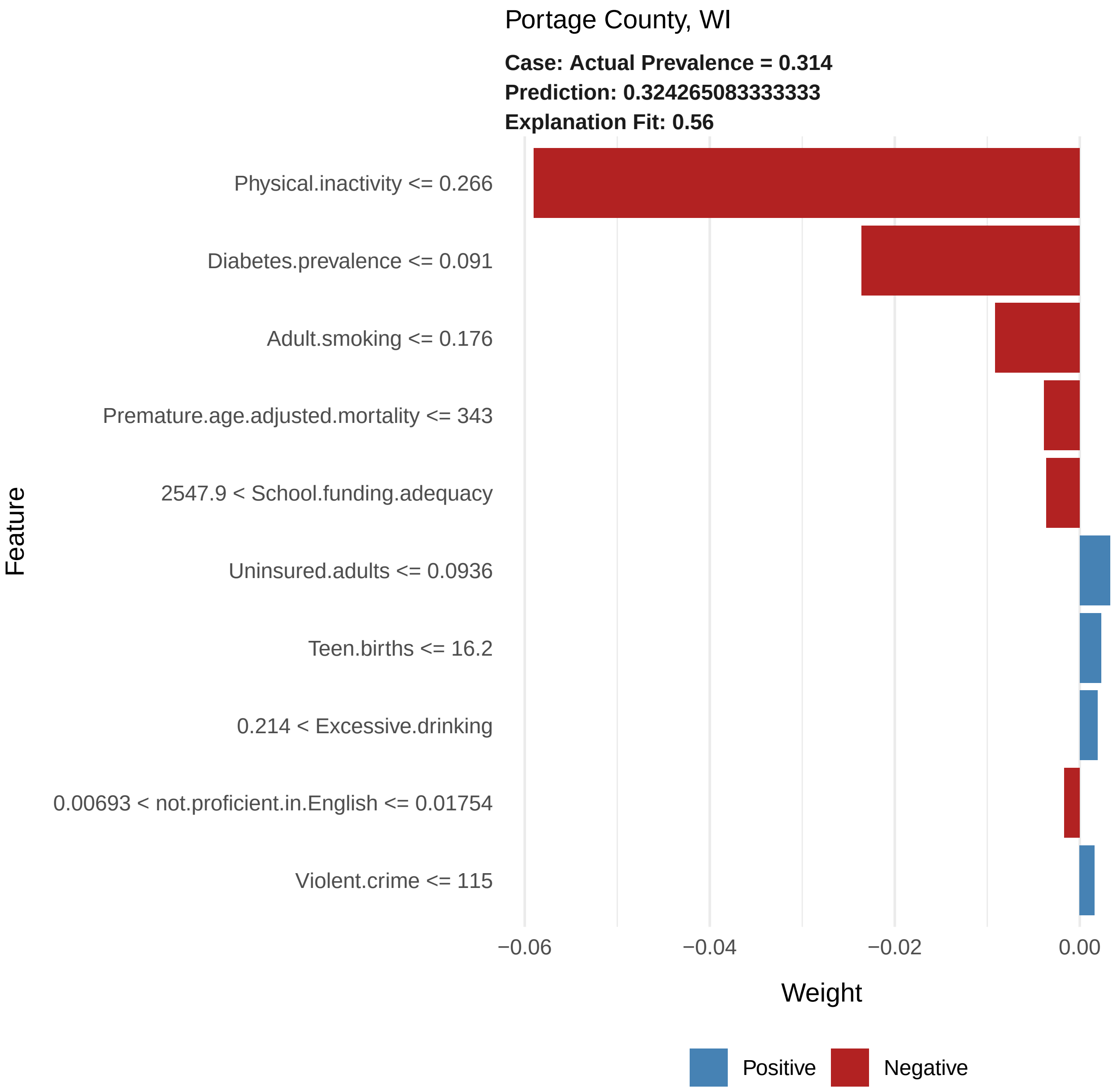

Portage County, WI
Case: Actual Prevalence = 0.314
Prediction: 0.324265083333333
Explanation Fit: 0.56
Physical.inactivity <= 0.266
Diabetes.prevalence <= 0.091
Adult.smoking <= 0.176
Premature.age.adjusted.mortality <= 343
2547.9 < School.funding.adequacy
Uninsured.adults <= 0.0936
Teen.births <= 16.2
0.214 < Excessive.drinking
0.00693 < not.proficient.in.English <= 0.01754
Violent.crime <= 115
Feature
−0.06
−0.04
−0.02
0.00
Weight
Positive
Negative

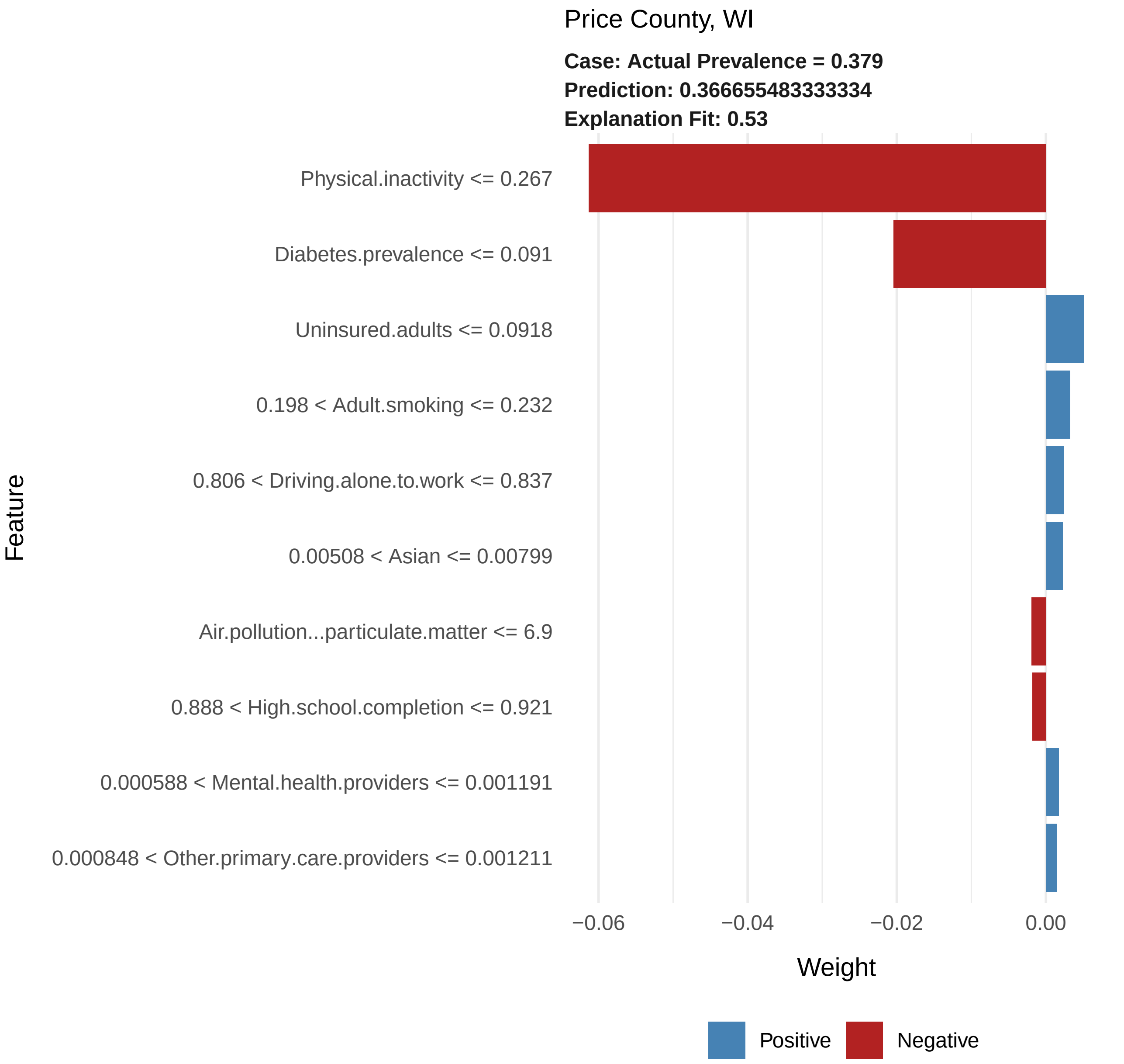

Price County, WI
Case: Actual Prevalence = 0.379
Prediction: 0.366655483333334
Explanation Fit: 0.53
Physical.inactivity <= 0.267
Diabetes.prevalence <= 0.091
Uninsured.adults <= 0.0918
0.198 < Adult.smoking <= 0.232
0.806 < Driving.alone.to.work <= 0.837
0.00508 < Asian <= 0.00799
Air.pollution...particulate.matter <= 6.9
0.888 < High.school.completion <= 0.921
0.000588 < Mental.health.providers <= 0.001191
0.000848 < Other.primary.care.providers <= 0.001211
Feature
−0.06
−0.04
−0.02
0.00
Weight
Positive
Negative

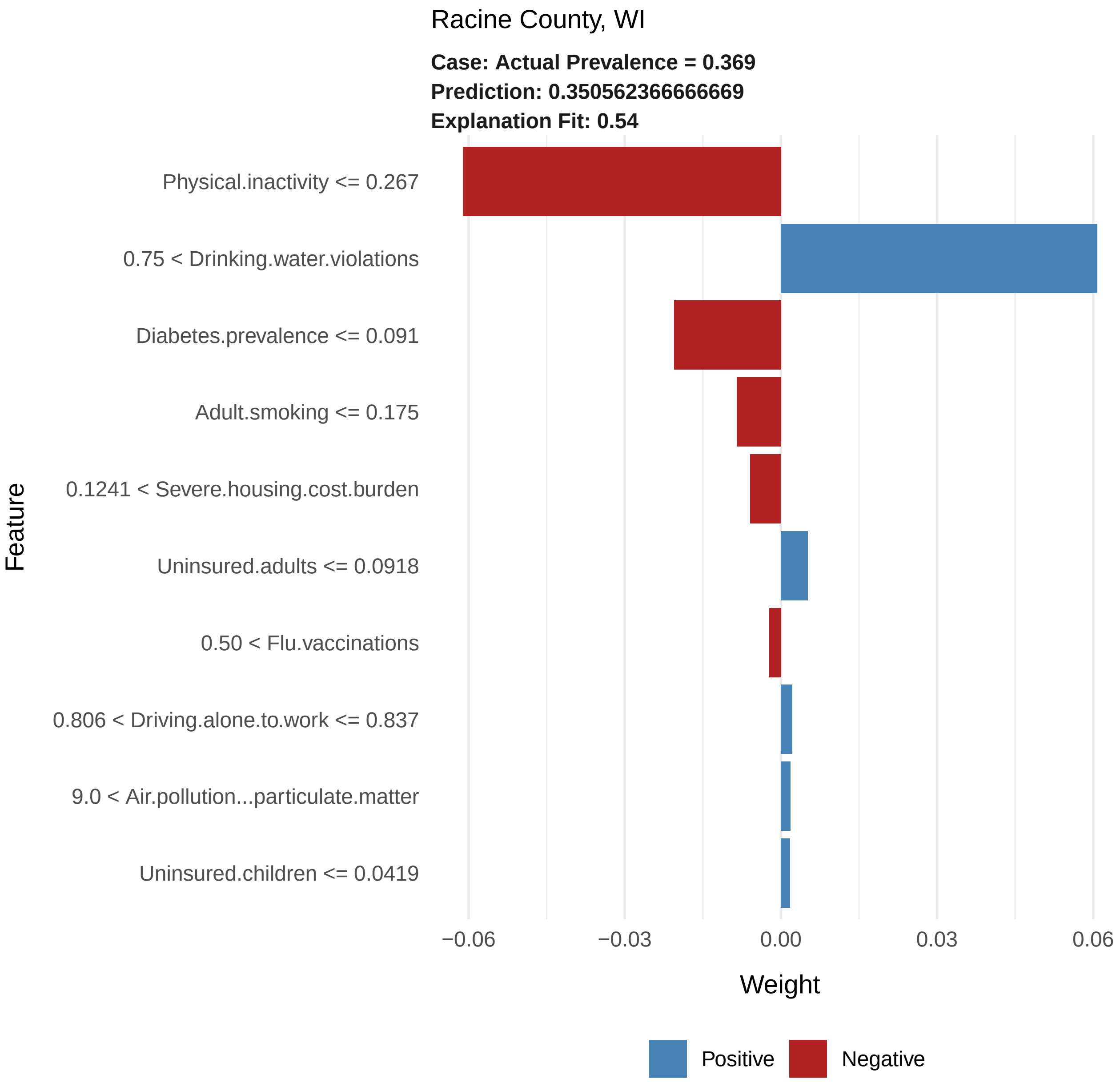

Racine County, WI
Case: Actual Prevalence = 0.369
Prediction: 0.350562366666669
Explanation Fit: 0.54
Physical.inactivity <= 0.267
0.75 < Drinking.water.violations
Diabetes.prevalence <= 0.091
Adult.smoking <= 0.175
0.1241 < Severe.housing.cost.burden
Uninsured.adults <= 0.0918
0.50 < Flu.vaccinations
0.806 < Driving.alone.to.work <= 0.837
9.0 < Air.pollution...particulate.matter
Uninsured.children <= 0.0419
Feature
−0.06
−0.03
0.00
0.03
0.06
Weight
Positive
Negative

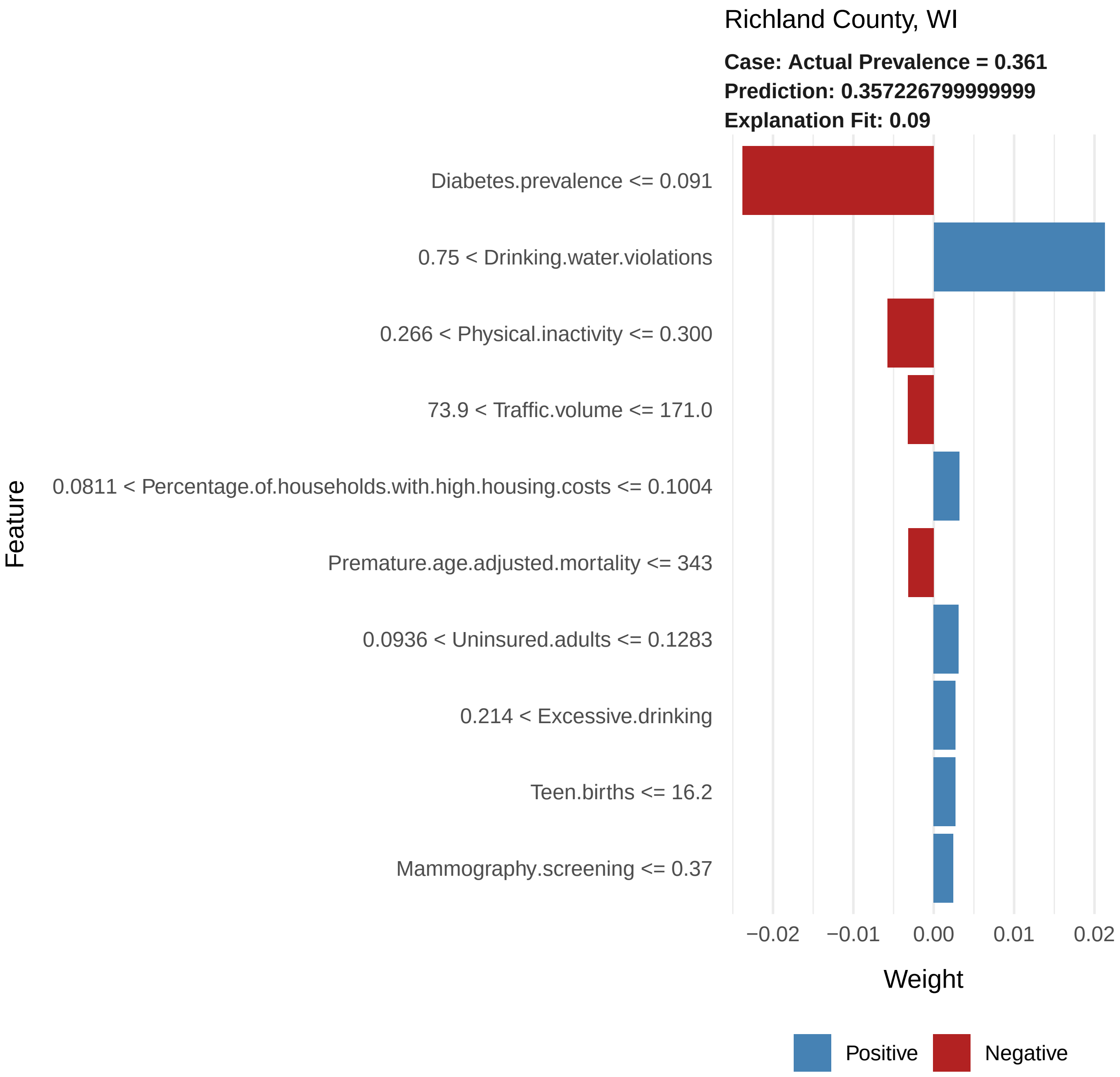

Richland County, WI
Case: Actual Prevalence = 0.361
Prediction: 0.357226799999999
Explanation Fit: 0.09
Diabetes.prevalence <= 0.091
0.75 < Drinking.water.violations
0.266 < Physical.inactivity <= 0.300
73.9 < Traffic.volume <= 171.0
0.0811 < Percentage.of.households.with.high.housing.costs <= 0.1004
Premature.age.adjusted.mortality <= 343
0.0936 < Uninsured.adults <= 0.1283
0.214 < Excessive.drinking
Teen.births <= 16.2
Mammography.screening <= 0.37
Feature
−0.02
−0.01
0.00
0.01
0.02
Weight
Positive
Negative

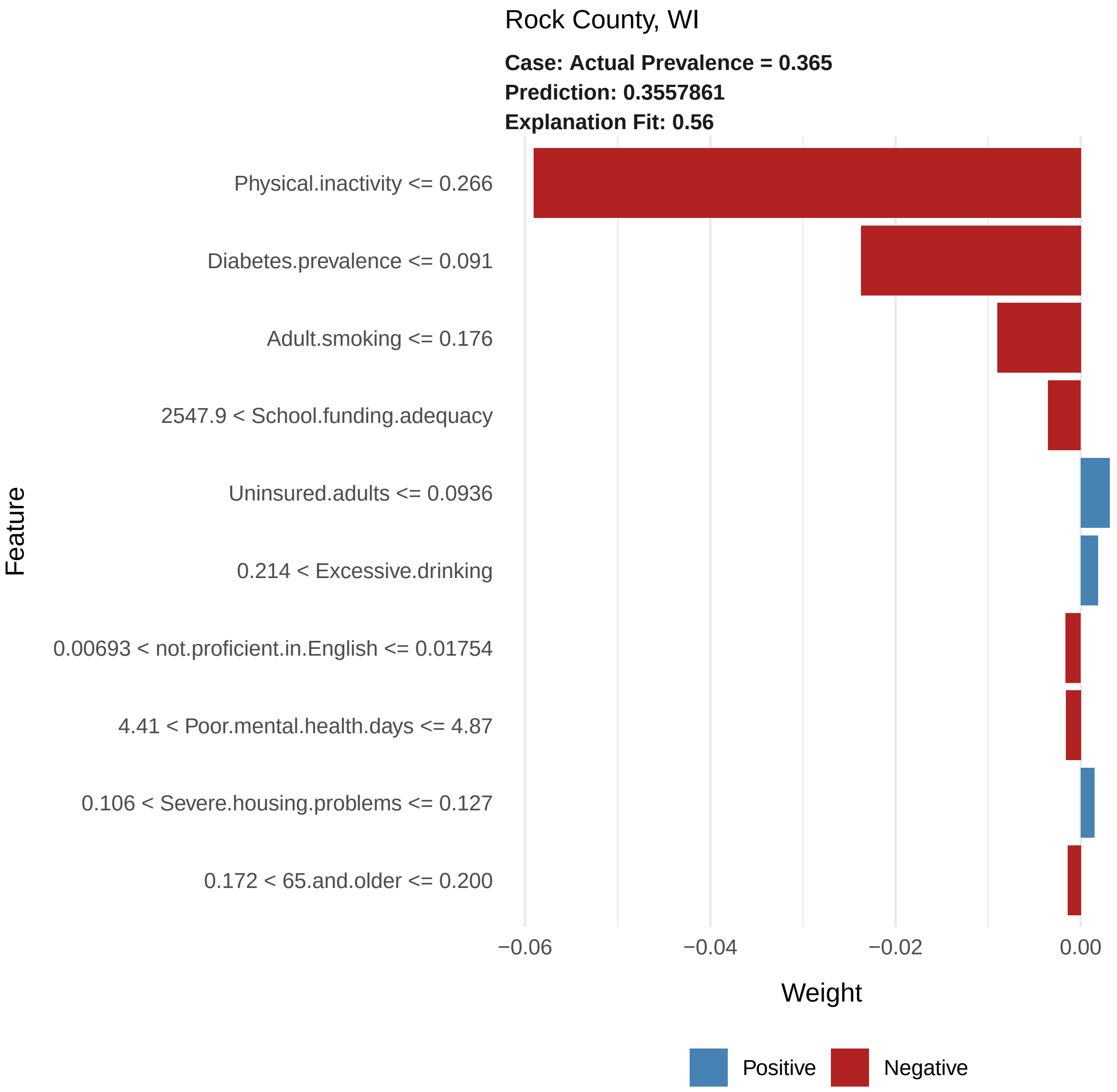

Rock County, WI
Case: Actual Prevalence = 0.365
Prediction: 0.3557861
Explanation Fit: 0.56
Physical.inactivity <= 0.266
Diabetes.prevalence <= 0.091
Adult.smoking <= 0.176
2547.9 < School.funding.adequacy
Uninsured.adults <= 0.0936
0.214 < Excessive.drinking
0.00693 < not.proficient.in.English <= 0.01754
4.41 < Poor.mental.health.days <= 4.87
0.106 < Severe.housing.problems <= 0.127
0.172 < 65.and.older <= 0.200
Feature
−0.06
−0.04
−0.02
0.00
Weight
Positive
Negative

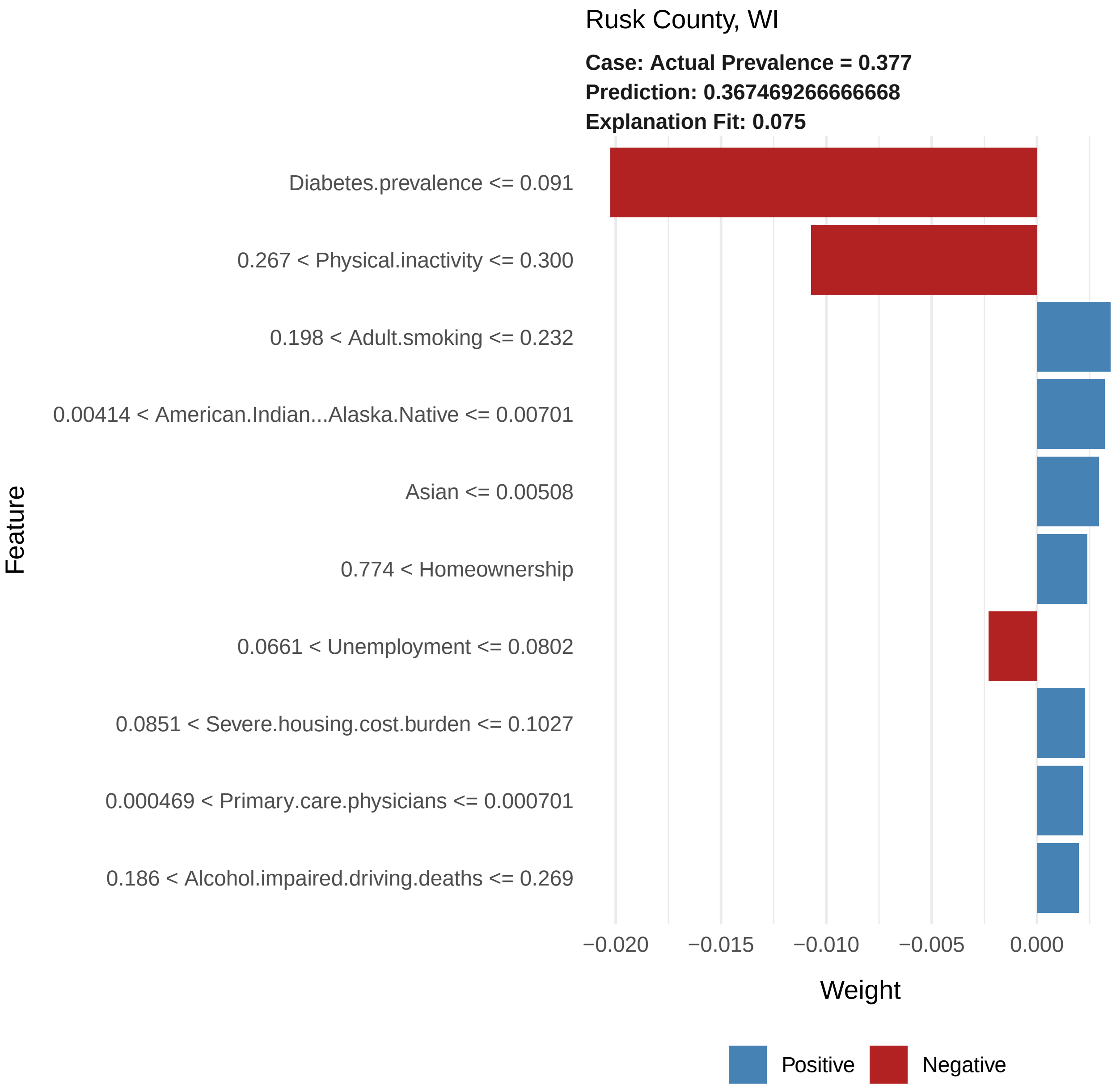

Rusk County, WI
Case: Actual Prevalence = 0.377
Prediction: 0.367469266666668
Explanation Fit: 0.075
Diabetes.prevalence <= 0.091
0.267 < Physical.inactivity <= 0.300
0.198 < Adult.smoking <= 0.232
0.00414 < American.Indian...Alaska.Native <= 0.00701
Asian <= 0.00508
0.774 < Homeownership
0.0661 < Unemployment <= 0.0802
0.0851 < Severe.housing.cost.burden <= 0.1027
0.000469 < Primary.care.physicians <= 0.000701
0.186 < Alcohol.impaired.driving.deaths <= 0.269
Feature
−0.020
−0.015
−0.010
−0.005
0.000
Weight
Positive
Negative

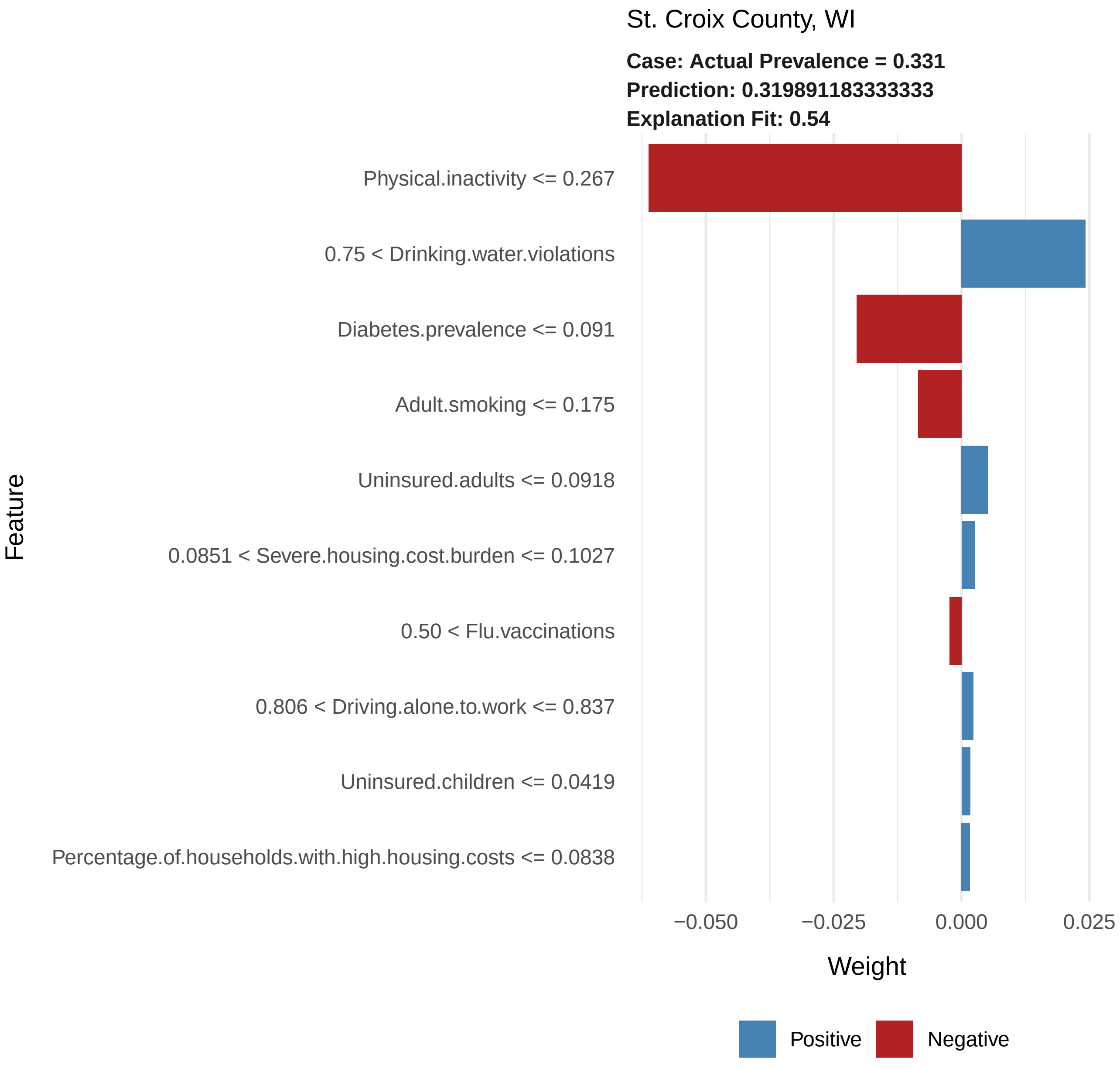
St. Croix County, WI
Case: Actual Prevalence = 0.331
Prediction: 0.319891183333333
Explanation Fit: 0.54
Physical.inactivity <= 0.267
0.75 < Drinking.water.violations
Diabetes.prevalence <= 0.091
Adult.smoking <= 0.175
Uninsured.adults <= 0.0918
0.0851 < Severe.housing.cost.burden <= 0.1027
0.50 < Flu.vaccinations
0.806 < Driving.alone.to.work <= 0.837
Uninsured.children <= 0.0419
Percentage.of.households.with.high.housing.costs <= 0.0838
Feature
−0.050
−0.025
0.000
0.025
Weight
Positive
Negative

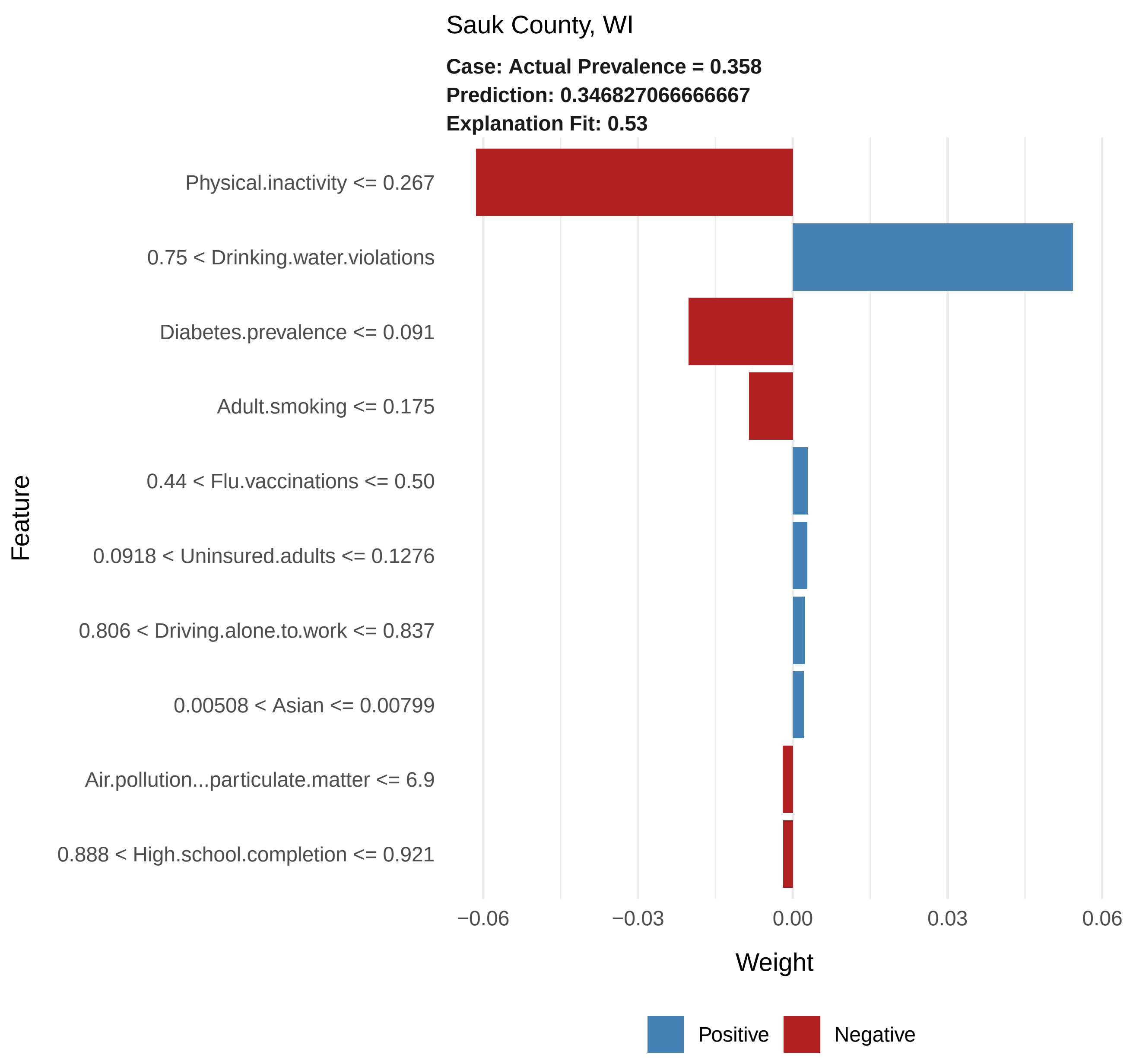
Sauk County, WI
Case: Actual Prevalence = 0.358
Prediction: 0.346827066666667
Explanation Fit: 0.53
Physical.inactivity <= 0.267
0.75 < Drinking.water.violations
Diabetes.prevalence <= 0.091
Adult.smoking <= 0.175
0.44 < Flu.vaccinations <= 0.50
0.0918 < Uninsured.adults <= 0.1276
0.806 < Driving.alone.to.work <= 0.837
0.00508 < Asian <= 0.00799
Air.pollution...particulate.matter <= 6.9
0.888 < High.school.completion <= 0.921
Feature
−0.06
−0.03
0.00
0.03
0.06
Weight
Positive
Negative

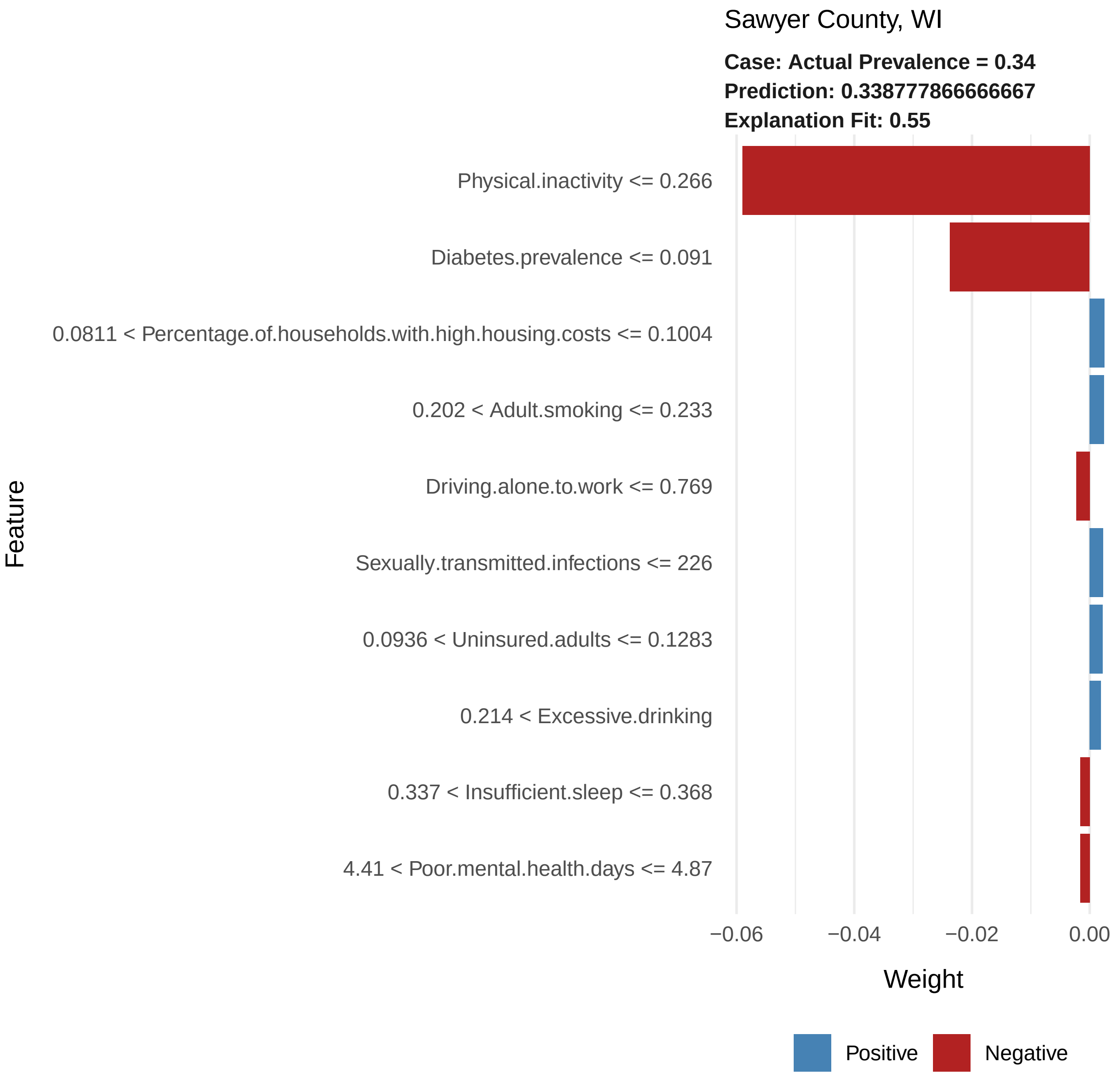

Sawyer County, WI
Case: Actual Prevalence = 0.34
Prediction: 0.338777866666667
Explanation Fit: 0.55
Physical.inactivity <= 0.266
Diabetes.prevalence <= 0.091
0.0811 < Percentage.of.households.with.high.housing.costs <= 0.1004
0.202 < Adult.smoking <= 0.233
Driving.alone.to.work <= 0.769
Sexually.transmitted.infections <= 226
0.0936 < Uninsured.adults <= 0.1283
0.214 < Excessive.drinking
0.337 < Insufficient.sleep <= 0.368
4.41 < Poor.mental.health.days <= 4.87
Feature
−0.06
−0.04
−0.02
0.00
Weight
Positive
Negative

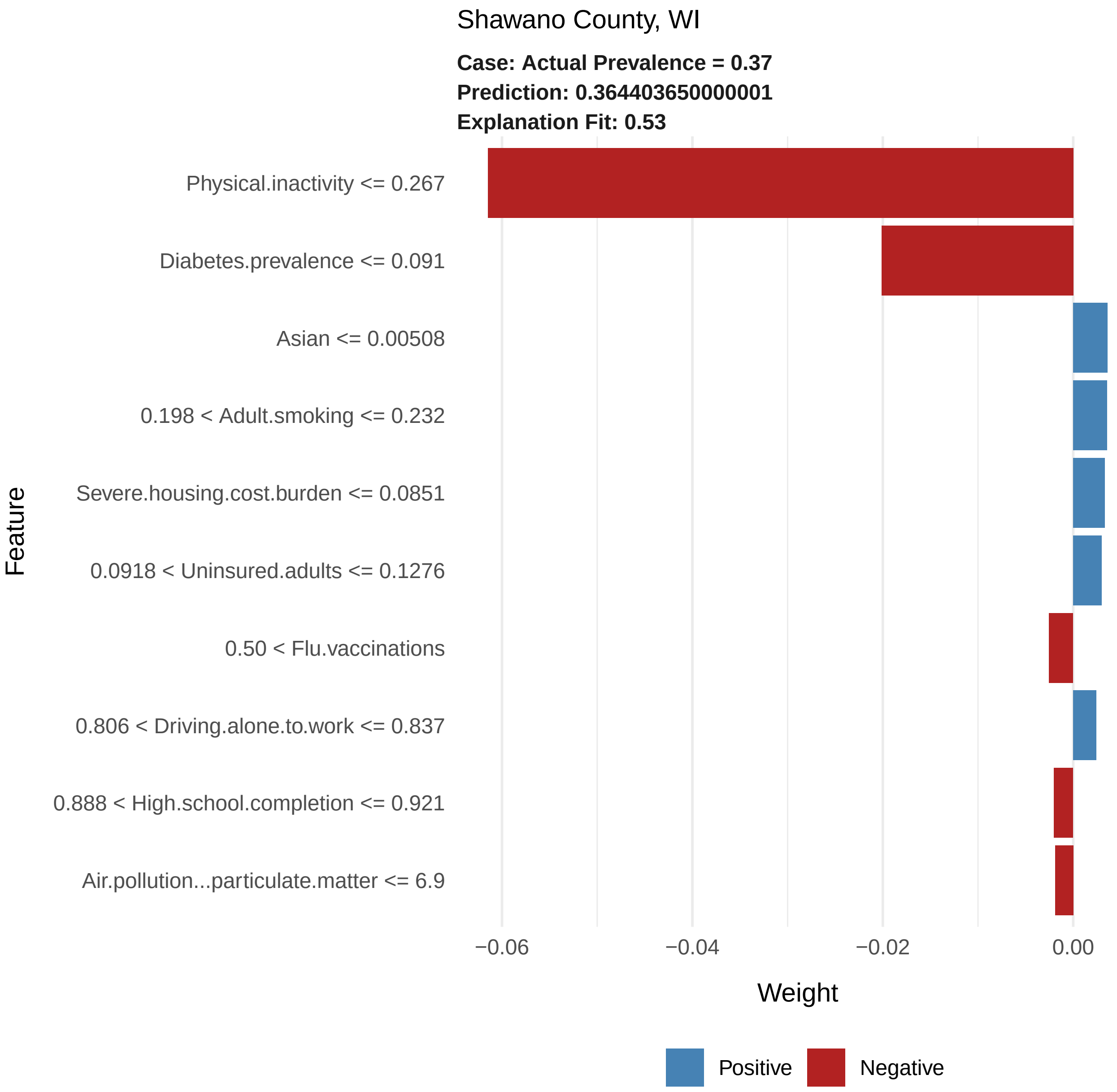

Shawano County, WI
Case: Actual Prevalence = 0.37
Prediction: 0.364403650000001
Explanation Fit: 0.53
Physical.inactivity <= 0.267
Diabetes.prevalence <= 0.091
Asian <= 0.00508
0.198 < Adult.smoking <= 0.232
Severe.housing.cost.burden <= 0.0851
0.0918 < Uninsured.adults <= 0.1276
0.50 < Flu.vaccinations
0.806 < Driving.alone.to.work <= 0.837
0.888 < High.school.completion <= 0.921
Air.pollution...particulate.matter <= 6.9
Feature
−0.06
−0.04
−0.02
0.00
Weight
Positive
Negative

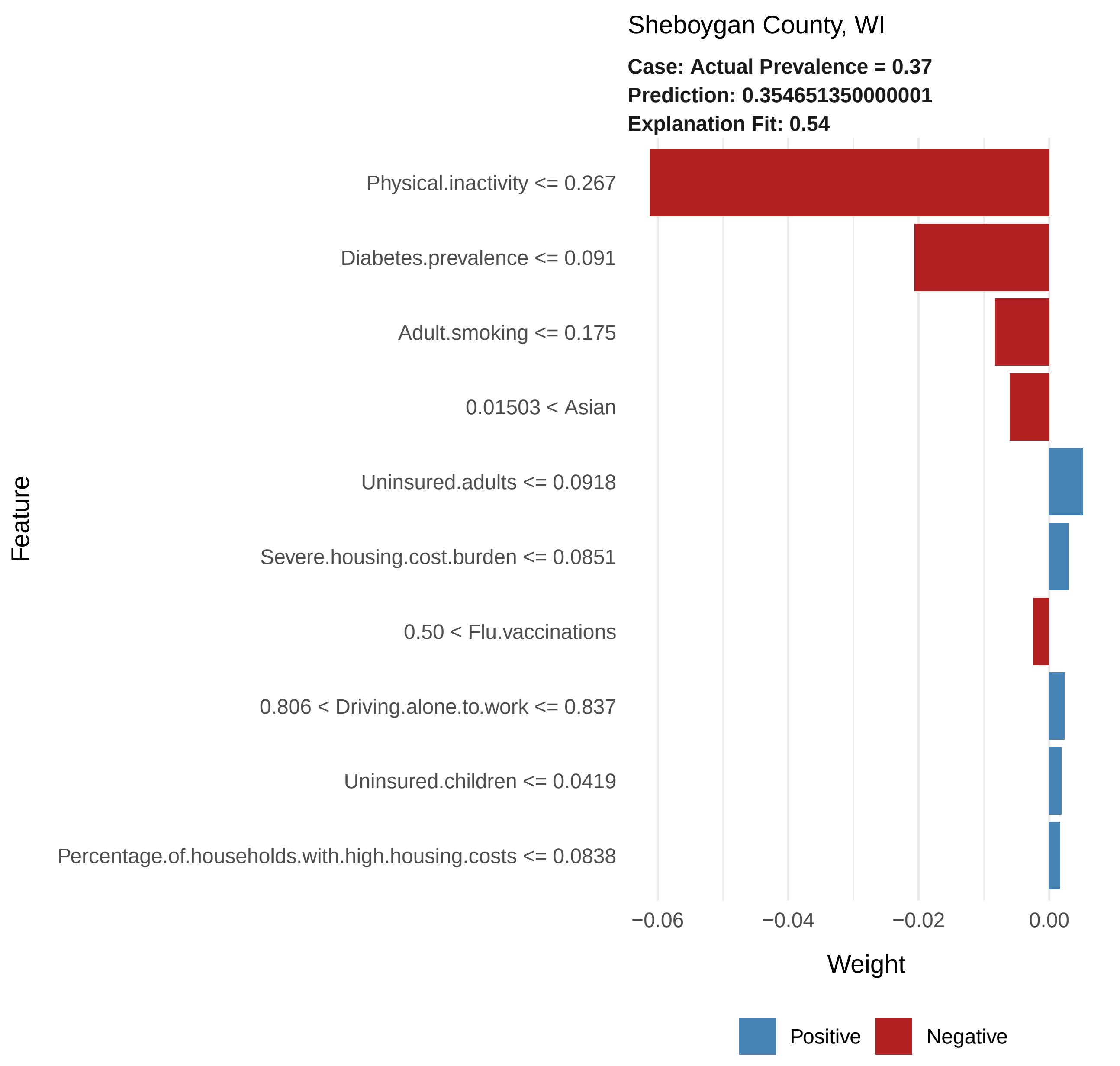

Sheboygan County, WI
Case: Actual Prevalence = 0.37
Prediction: 0.354651350000001
Explanation Fit: 0.54
Physical.inactivity <= 0.267
Diabetes.prevalence <= 0.091
Adult.smoking <= 0.175
0.01503 < Asian
Uninsured.adults <= 0.0918
Severe.housing.cost.burden <= 0.0851
0.50 < Flu.vaccinations
0.806 < Driving.alone.to.work <= 0.837
Uninsured.children <= 0.0419
Percentage.of.households.with.high.housing.costs <= 0.0838
Feature
−0.06
−0.04
−0.02
0.00
Weight
Positive
Negative

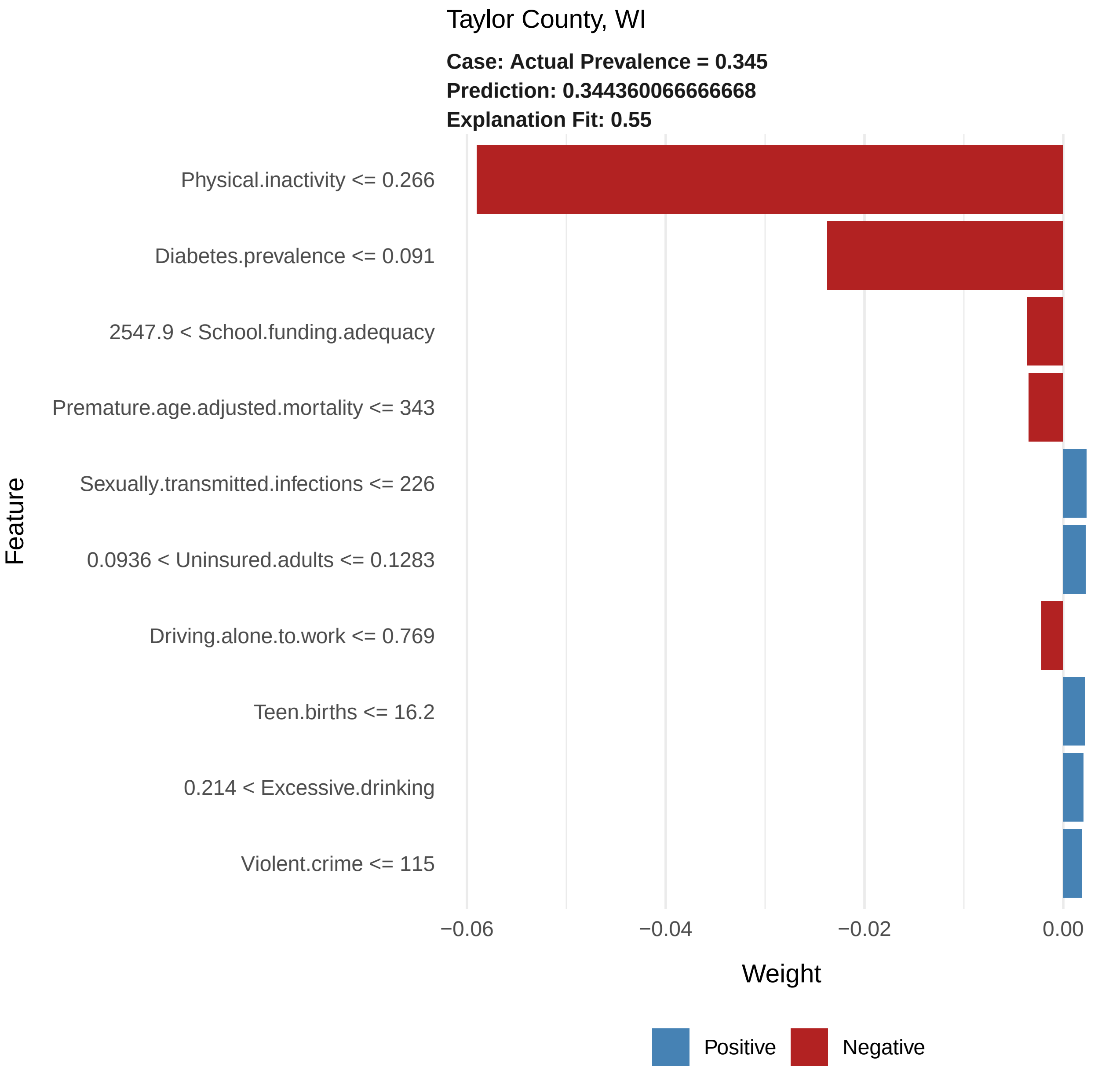

Taylor County, WI
Case: Actual Prevalence = 0.345
Prediction: 0.344360066666668
Explanation Fit: 0.55
Physical.inactivity <= 0.266
Diabetes.prevalence <= 0.091
2547.9 < School.funding.adequacy
Premature.age.adjusted.mortality <= 343
Sexually.transmitted.infections <= 226
0.0936 < Uninsured.adults <= 0.1283
Driving.alone.to.work <= 0.769
Teen.births <= 16.2
0.214 < Excessive.drinking
Violent.crime <= 115
Feature
−0.06
−0.04
−0.02
0.00
Weight
Positive
Negative

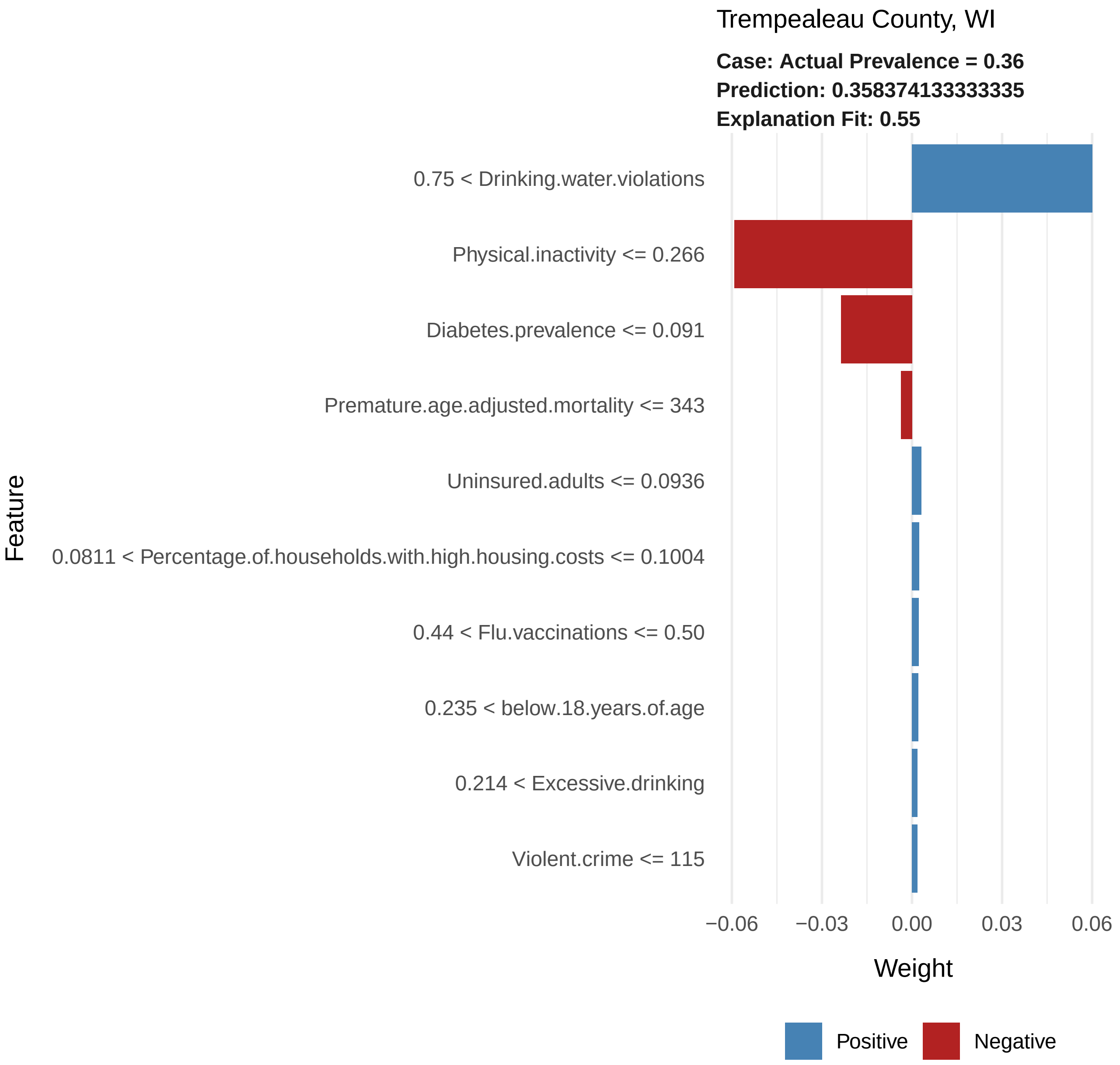

Trempealeau County, WI
Case: Actual Prevalence = 0.36
Prediction: 0.358374133333335
Explanation Fit: 0.55
0.75 < Drinking.water.violations
Physical.inactivity <= 0.266
Diabetes.prevalence <= 0.091
Premature.age.adjusted.mortality <= 343
Uninsured.adults <= 0.0936
0.0811 < Percentage.of.households.with.high.housing.costs <= 0.1004
0.44 < Flu.vaccinations <= 0.50
0.235 < below.18.years.of.age
0.214 < Excessive.drinking
Violent.crime <= 115
Feature
−0.06
−0.03
0.00
0.03
0.06
Weight
Positive
Negative

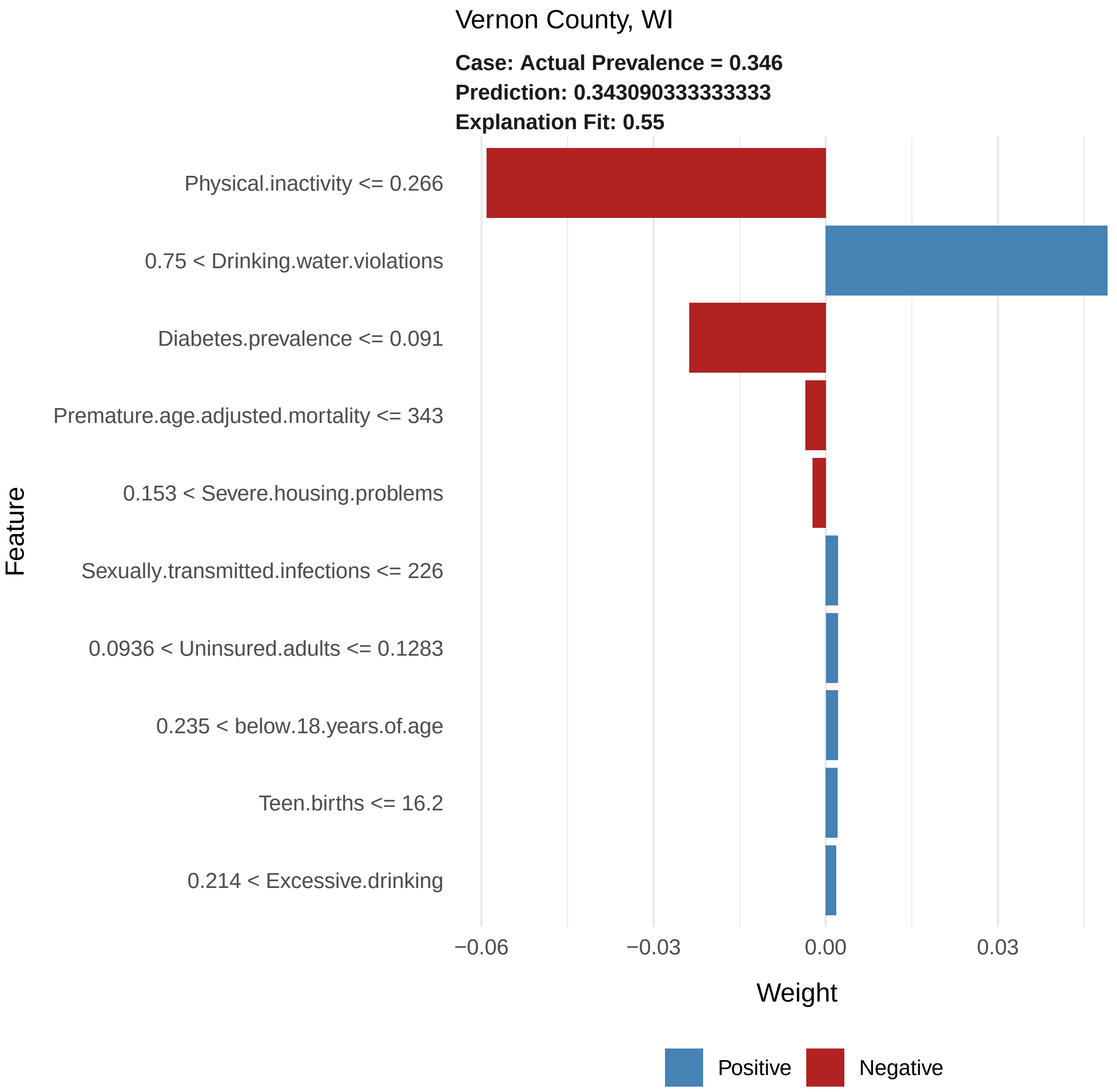

Vernon County, WI
Case: Actual Prevalence = 0.346
Prediction: 0.343090333333333
Explanation Fit: 0.55
Physical.inactivity <= 0.266
0.75 < Drinking.water.violations
Diabetes.prevalence <= 0.091
Premature.age.adjusted.mortality <= 343
0.153 < Severe.housing.problems
Sexually.transmitted.infections <= 226
0.0936 < Uninsured.adults <= 0.1283
0.235 < below.18.years.of.age
Teen.births <= 16.2
0.214 < Excessive.drinking
Feature
−0.06
−0.03
0.00
0.03
Weight
Positive
Negative

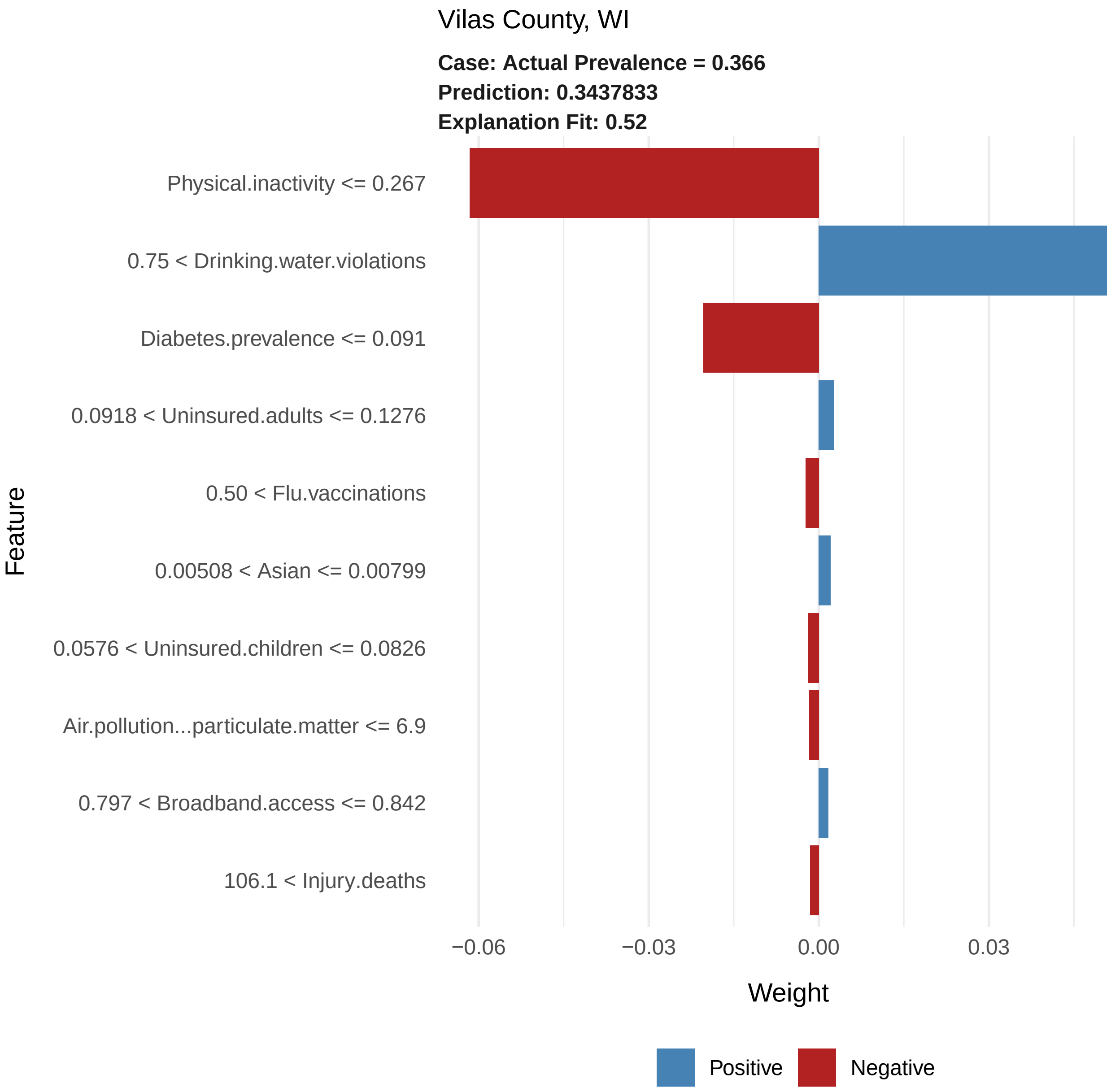

Vilas County, WI
Case: Actual Prevalence = 0.366
Prediction: 0.3437833
Explanation Fit: 0.52
Physical.inactivity <= 0.267
0.75 < Drinking.water.violations
Diabetes.prevalence <= 0.091
0.0918 < Uninsured.adults <= 0.1276
0.50 < Flu.vaccinations
0.00508 < Asian <= 0.00799
0.0576 < Uninsured.children <= 0.0826
Air.pollution...particulate.matter <= 6.9
0.797 < Broadband.access <= 0.842
106.1 < Injury.deaths
Feature
−0.06
−0.03
0.00
0.03
Weight
Positive
Negative

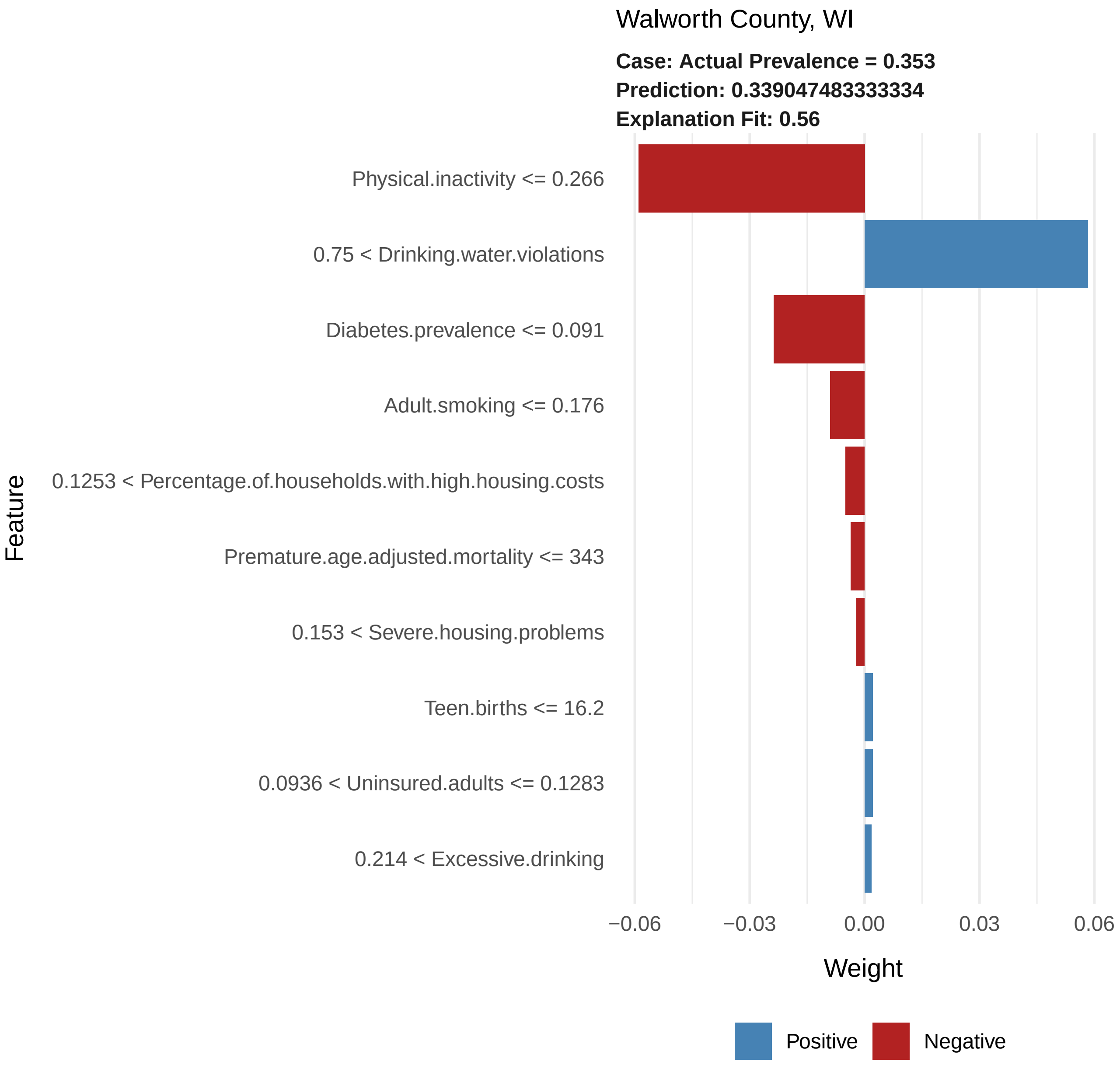

Walworth County, WI
Case: Actual Prevalence = 0.353
Prediction: 0.339047483333334
Explanation Fit: 0.56
Physical.inactivity <= 0.266
0.75 < Drinking.water.violations
Diabetes.prevalence <= 0.091
Adult.smoking <= 0.176
0.1253 < Percentage.of.households.with.high.housing.costs
Premature.age.adjusted.mortality <= 343
0.153 < Severe.housing.problems
Teen.births <= 16.2
0.0936 < Uninsured.adults <= 0.1283
0.214 < Excessive.drinking
Feature
−0.06
−0.03
0.00
0.03
0.06
Weight
Positive
Negative

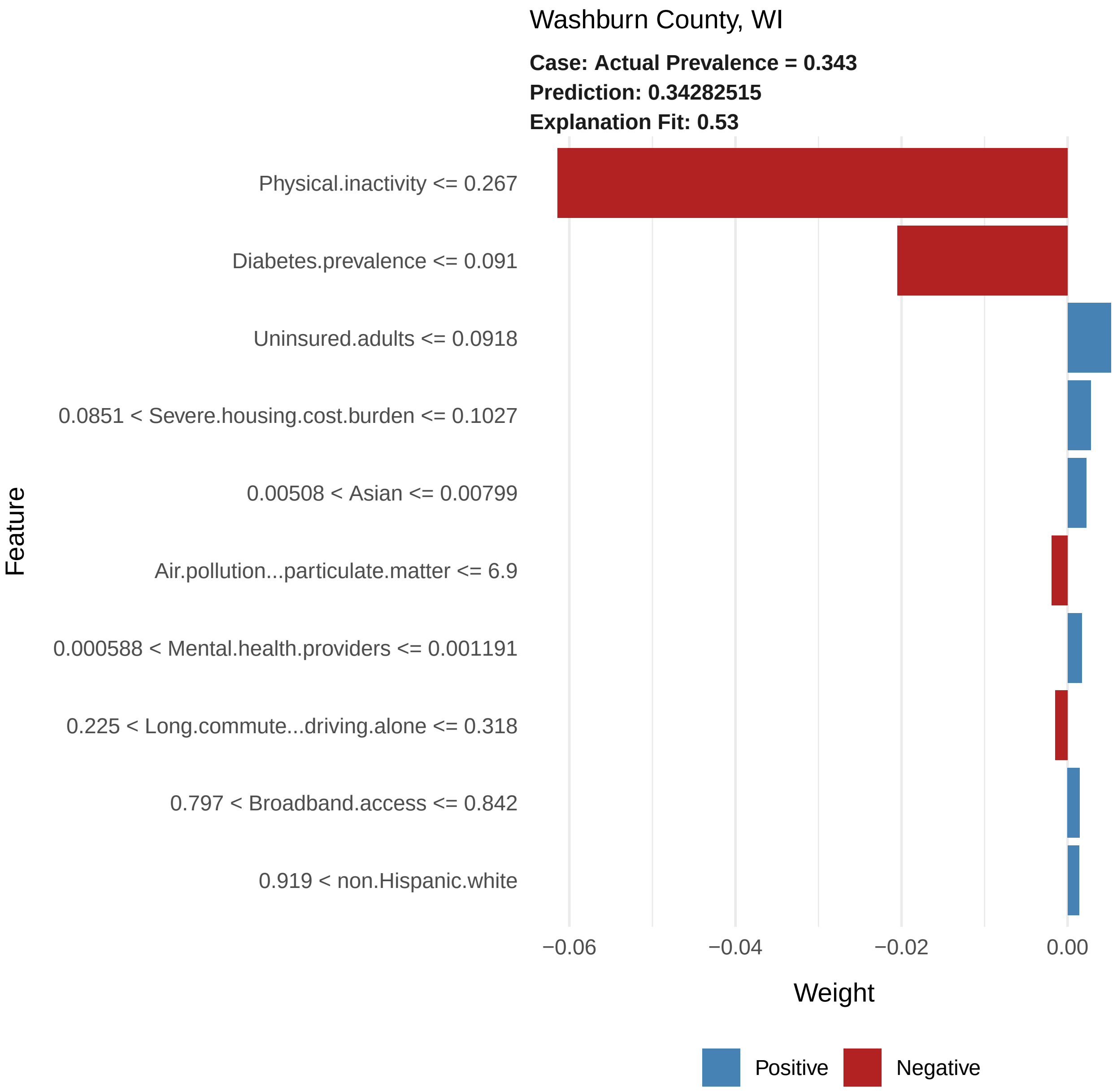
Washburn County, WI
Case: Actual Prevalence = 0.343
Prediction: 0.34282515
Explanation Fit: 0.53
Physical.inactivity <= 0.267
Diabetes.prevalence <= 0.091
Uninsured.adults <= 0.0918
0.0851 < Severe.housing.cost.burden <= 0.1027
0.00508 < Asian <= 0.00799
Air.pollution...particulate.matter <= 6.9
0.000588 < Mental.health.providers <= 0.001191
0.225 < Long.commute...driving.alone <= 0.318
0.797 < Broadband.access <= 0.842
0.919 < non.Hispanic.white
Feature
−0.06
−0.04
−0.02
0.00
Weight
Positive
Negative

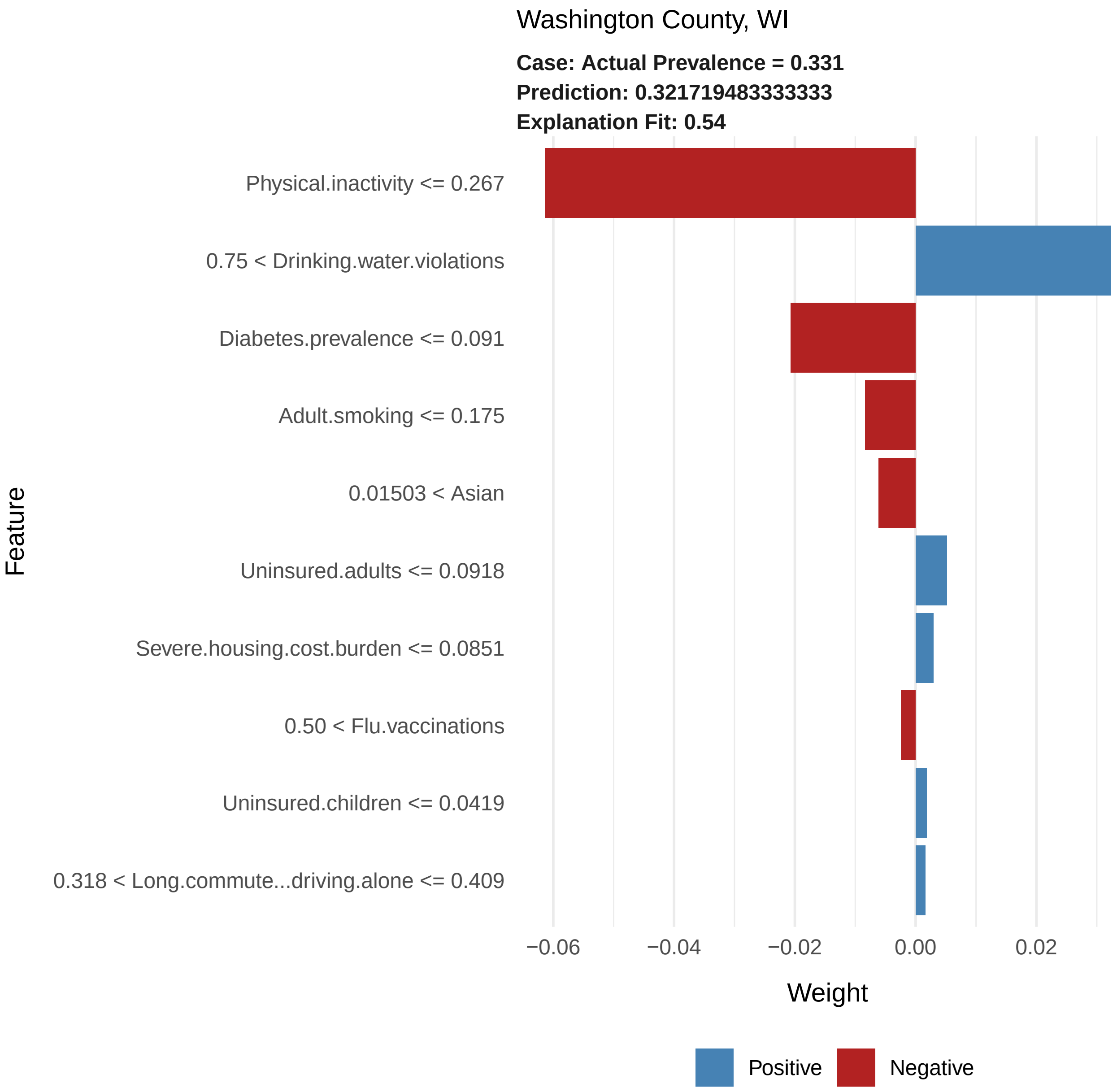

Washington County, WI
Case: Actual Prevalence = 0.331
Prediction: 0.321719483333333
Explanation Fit: 0.54
Physical.inactivity <= 0.267
0.75 < Drinking.water.violations
Diabetes.prevalence <= 0.091
Adult.smoking <= 0.175
0.01503 < Asian
Uninsured.adults <= 0.0918
Severe.housing.cost.burden <= 0.0851
0.50 < Flu.vaccinations
Uninsured.children <= 0.0419
0.318 < Long.commute...driving.alone <= 0.409
Feature
−0.06
−0.04
−0.02
0.00
0.02
Weight
Positive
Negative

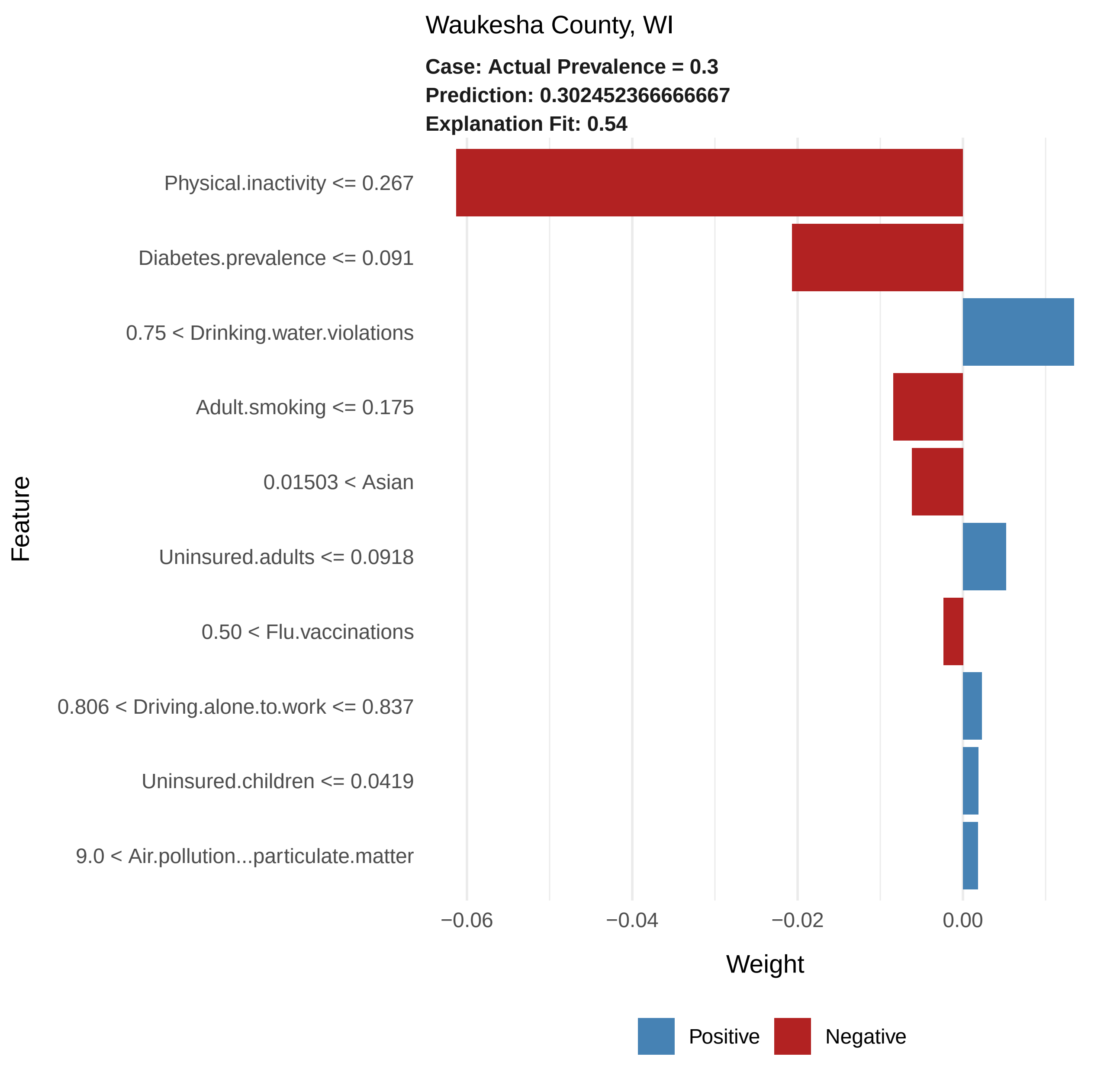
Waukesha County, WI
Case: Actual Prevalence = 0.3
Prediction: 0.302452366666667
Explanation Fit: 0.54
Physical.inactivity <= 0.267
Diabetes.prevalence <= 0.091
0.75 < Drinking.water.violations
Adult.smoking <= 0.175
0.01503 < Asian
Uninsured.adults <= 0.0918
0.50 < Flu.vaccinations
0.806 < Driving.alone.to.work <= 0.837
Uninsured.children <= 0.0419
9.0 < Air.pollution...particulate.matter
Feature
−0.06
−0.04
−0.02
0.00
Weight
Positive
Negative

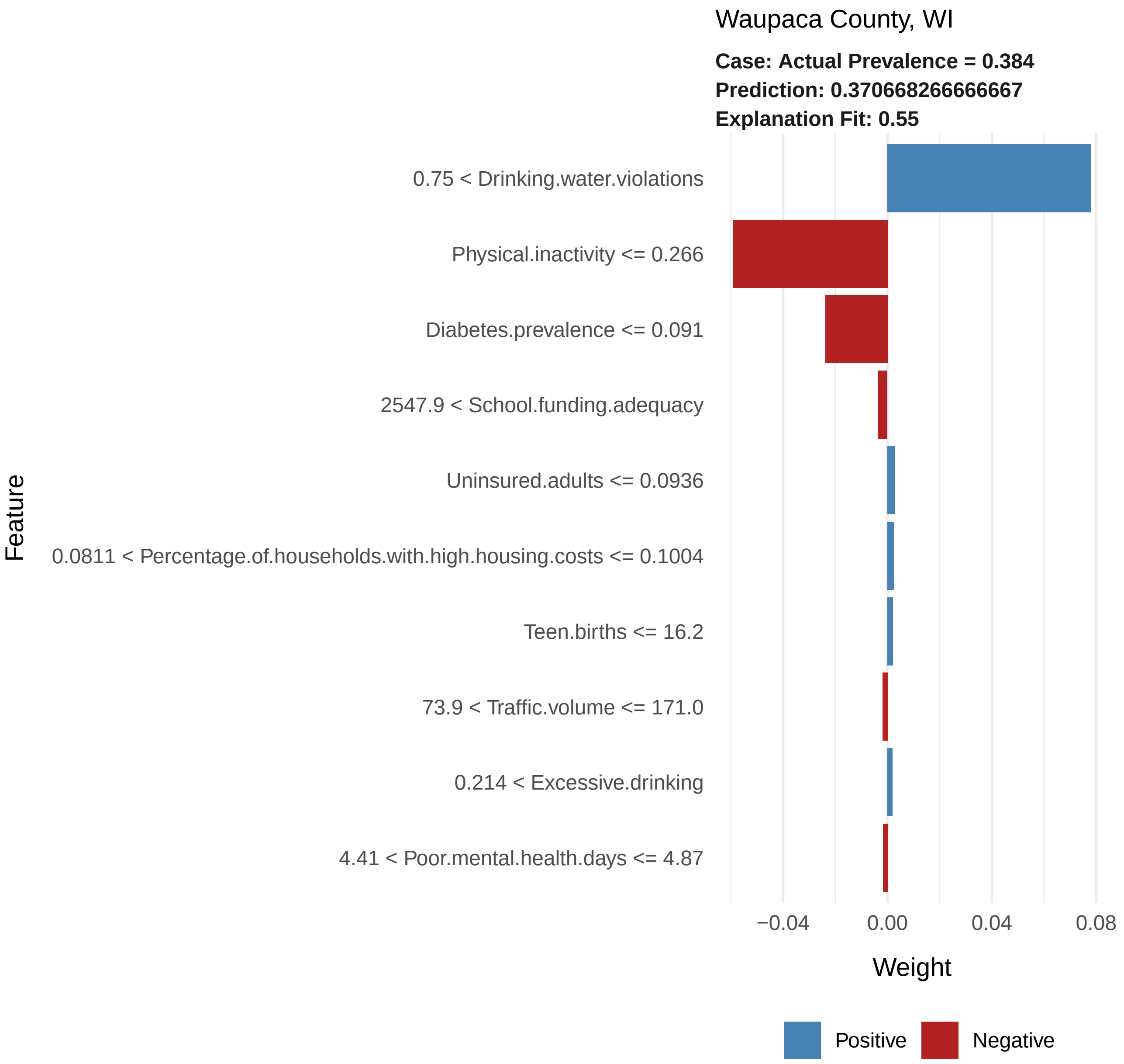
Waupaca County, WI
Case: Actual Prevalence = 0.384
Prediction: 0.370668266666667
Explanation Fit: 0.55
0.75 < Drinking.water.violations
Physical.inactivity <= 0.266
Diabetes.prevalence <= 0.091
2547.9 < School.funding.adequacy
Uninsured.adults <= 0.0936
0.0811 < Percentage.of.households.with.high.housing.costs <= 0.1004
Teen.births <= 16.2
73.9 < Traffic.volume <= 171.0
0.214 < Excessive.drinking
4.41 < Poor.mental.health.days <= 4.87
Feature
−0.04
0.00
0.04
0.08
Weight
Positive
Negative

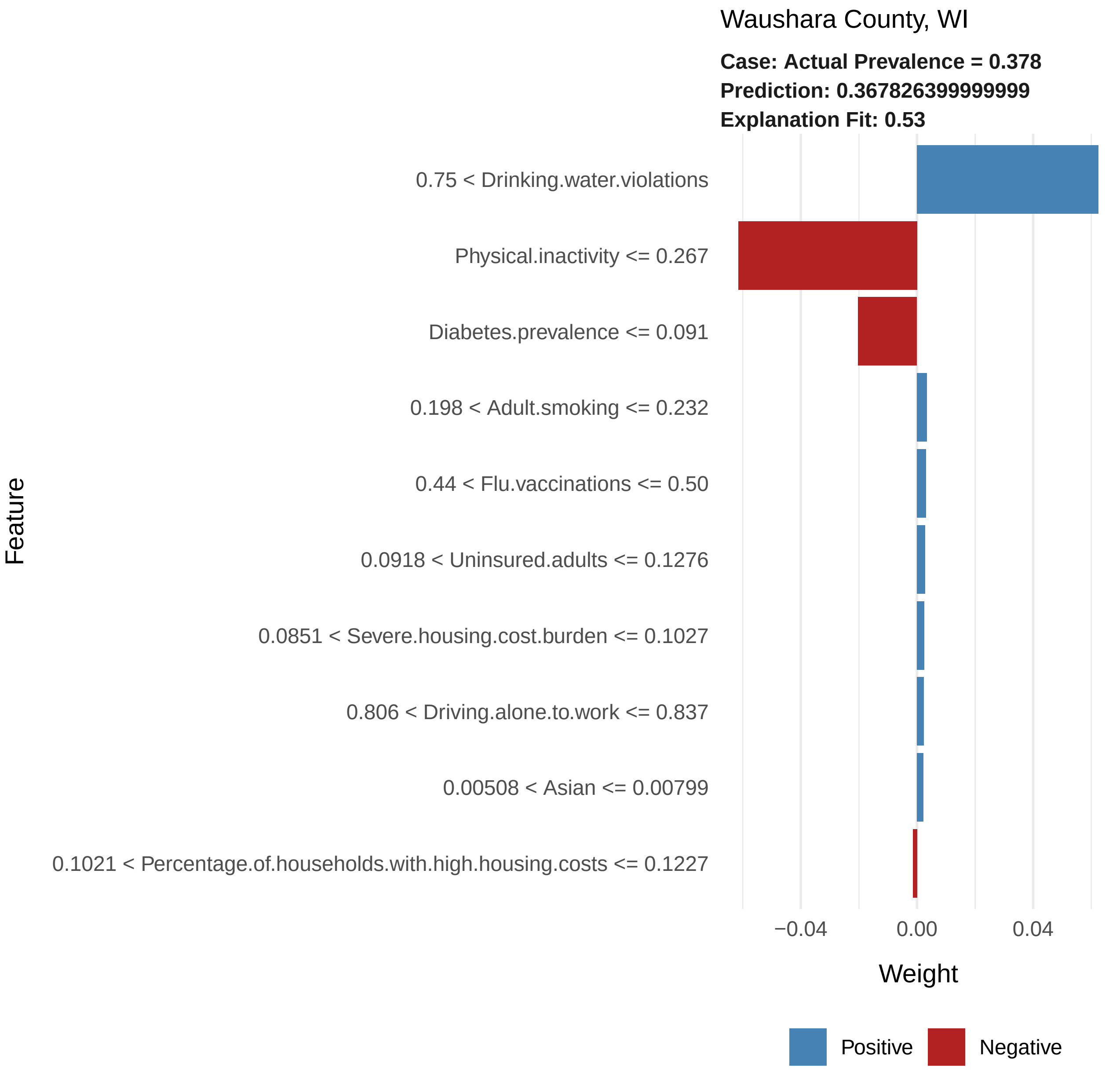
Waushara County, WI
Case: Actual Prevalence = 0.378
Prediction: 0.367826399999999
Explanation Fit: 0.53
0.75 < Drinking.water.violations
Physical.inactivity <= 0.267
Diabetes.prevalence <= 0.091
0.198 < Adult.smoking <= 0.232
0.44 < Flu.vaccinations <= 0.50
0.0918 < Uninsured.adults <= 0.1276
0.0851 < Severe.housing.cost.burden <= 0.1027
0.806 < Driving.alone.to.work <= 0.837
0.00508 < Asian <= 0.00799
0.1021 < Percentage.of.households.with.high.housing.costs <= 0.1227
Feature
−0.04
0.00
0.04
Weight
Positive
Negative

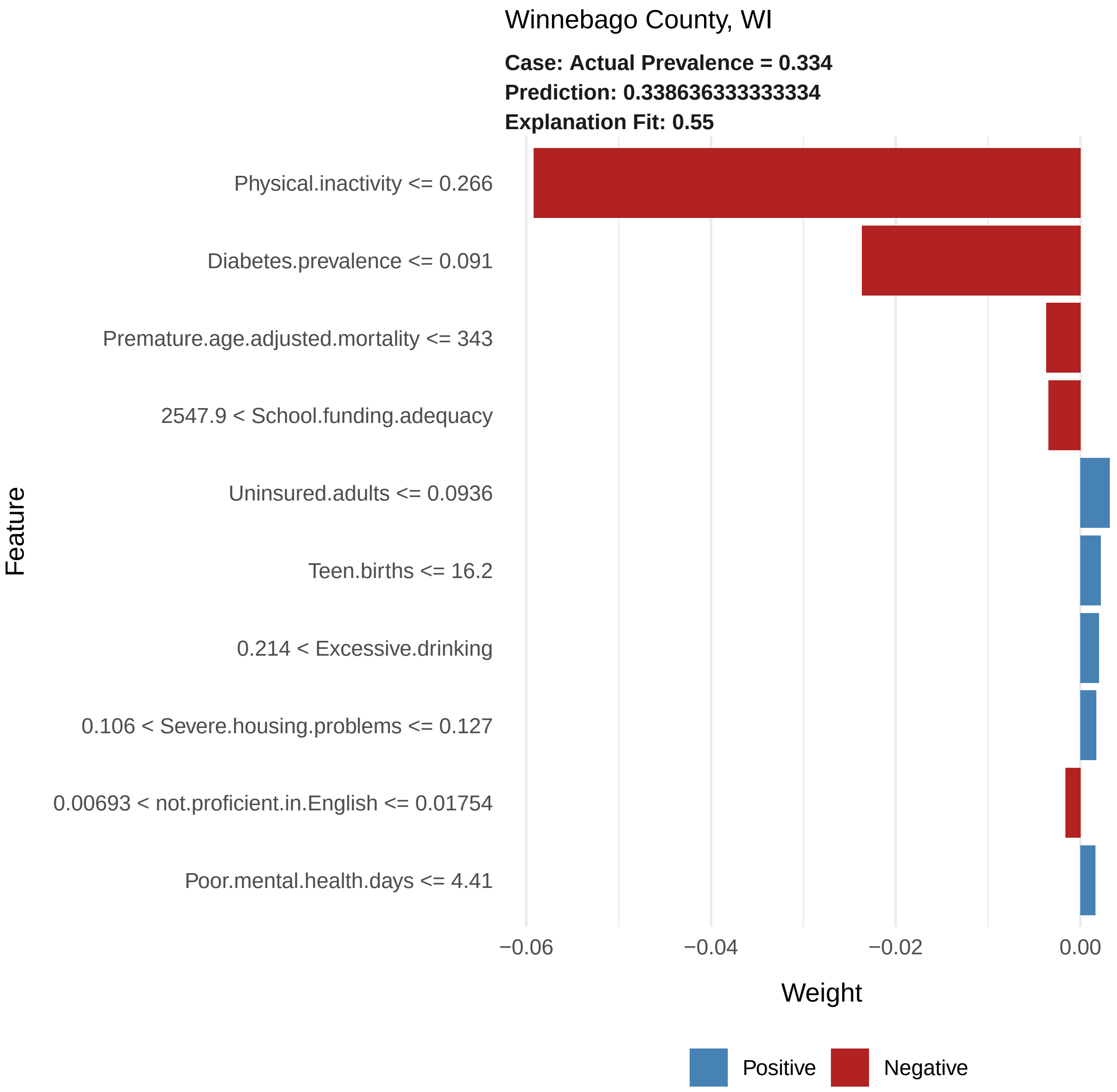

Winnebago County, WI
Case: Actual Prevalence = 0.334
Prediction: 0.338636333333334
Explanation Fit: 0.55
Physical.inactivity <= 0.266
Diabetes.prevalence <= 0.091
Premature.age.adjusted.mortality <= 343
2547.9 < School.funding.adequacy
Uninsured.adults <= 0.0936
Teen.births <= 16.2
0.214 < Excessive.drinking
0.106 < Severe.housing.problems <= 0.127
0.00693 < not.proficient.in.English <= 0.01754
Poor.mental.health.days <= 4.41
Feature
−0.06
−0.04
−0.02
0.00
Weight
Positive
Negative

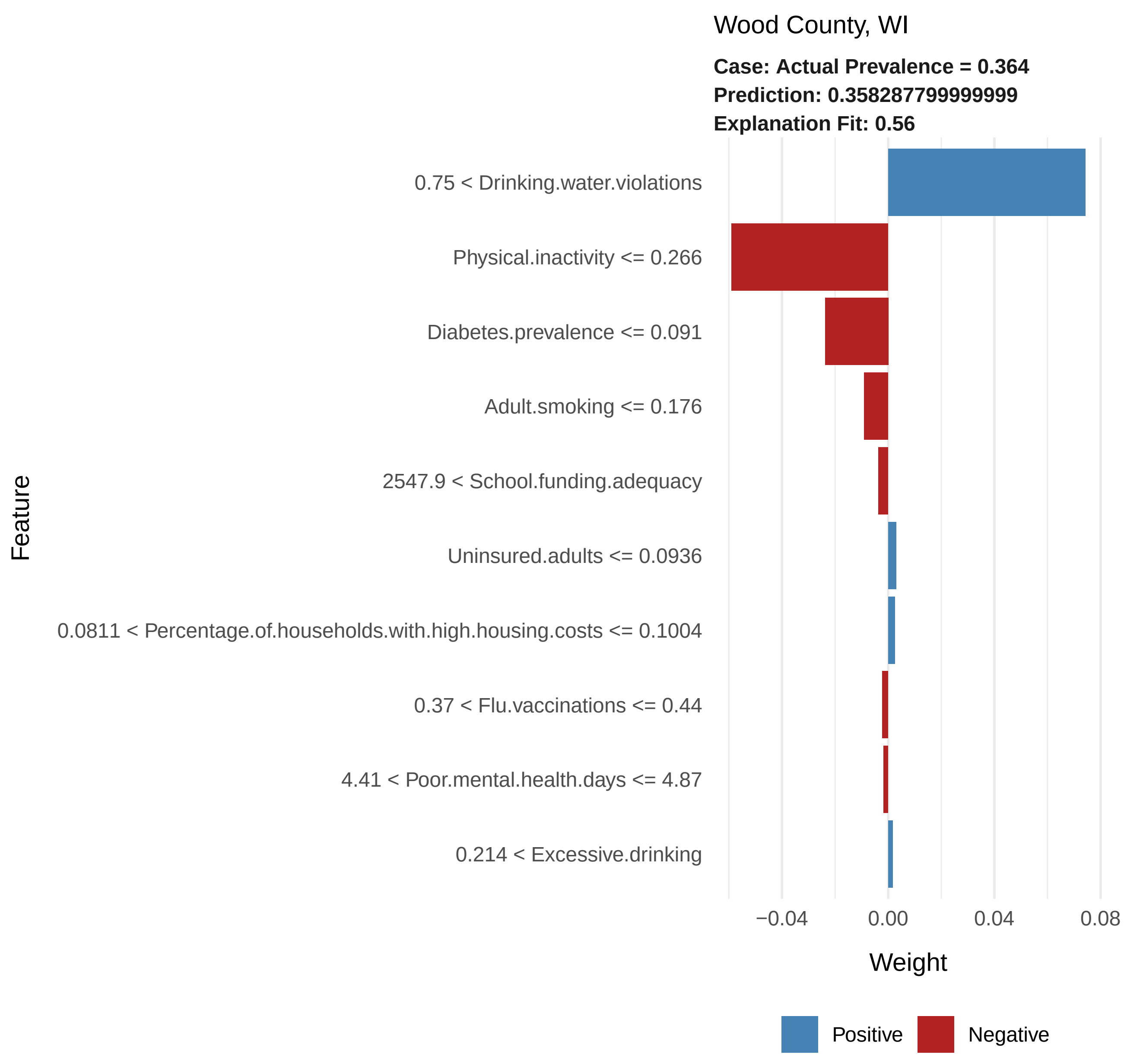
Wood County, WI
Case: Actual Prevalence = 0.364
Prediction: 0.358287799999999
Explanation Fit: 0.56
0.75 < Drinking.water.violations
Physical.inactivity <= 0.266
Diabetes.prevalence <= 0.091
Adult.smoking <= 0.176
2547.9 < School.funding.adequacy
Uninsured.adults <= 0.0936
0.0811 < Percentage.of.households.with.high.housing.costs <= 0.1004
0.37 < Flu.vaccinations <= 0.44
4.41 < Poor.mental.health.days <= 4.87
0.214 < Excessive.drinking
Feature
−0.04
0.00
0.04
0.08
Weight
Positive
Negative

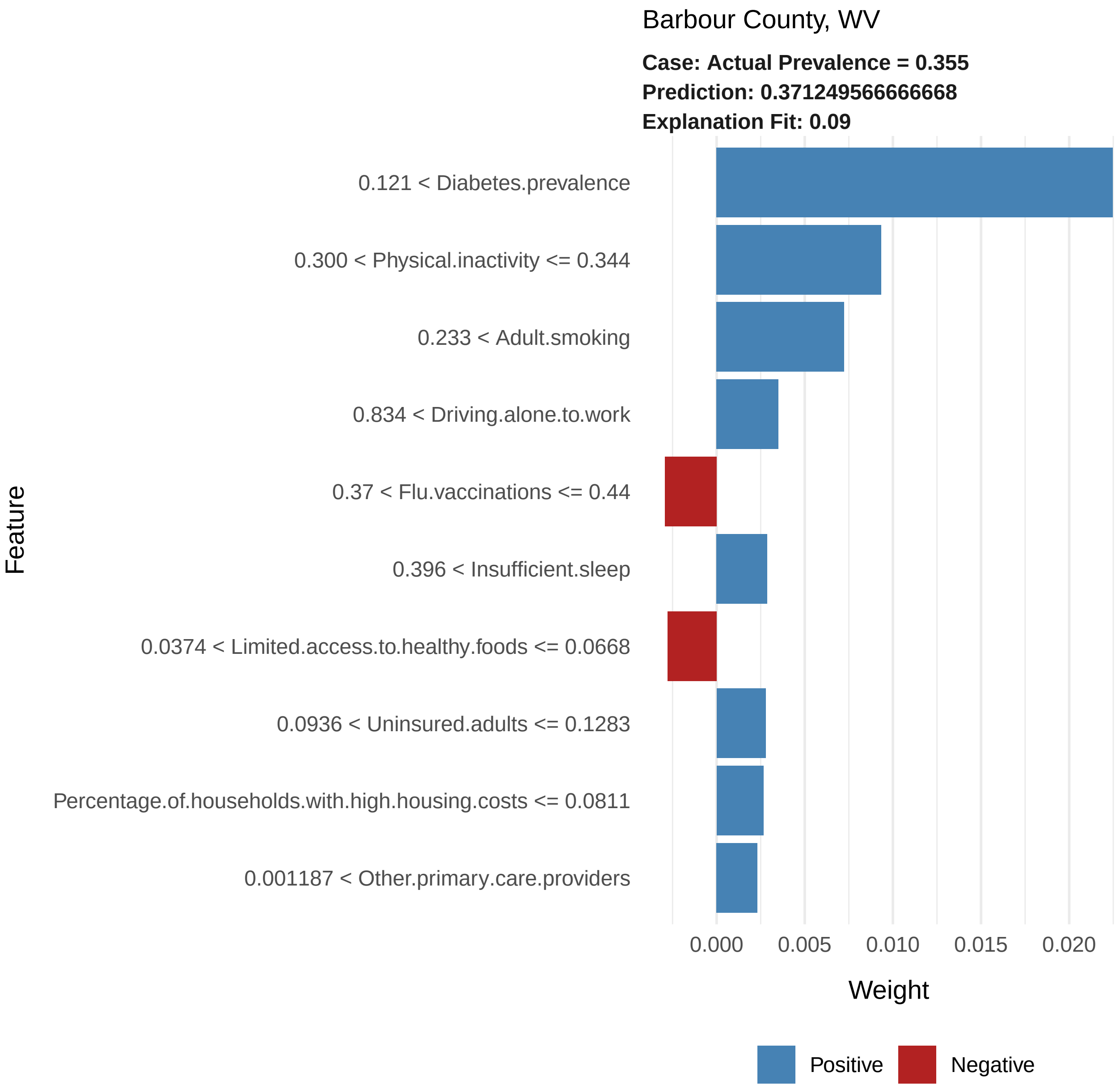

Barbour County, WV
Case: Actual Prevalence = 0.355
Prediction: 0.371249566666668
Explanation Fit: 0.09
0.121 < Diabetes.prevalence
0.300 < Physical.inactivity <= 0.344
0.233 < Adult.smoking
0.834 < Driving.alone.to.work
0.37 < Flu.vaccinations <= 0.44
0.396 < Insufficient.sleep
0.0374 < Limited.access.to.healthy.foods <= 0.0668
0.0936 < Uninsured.adults <= 0.1283
Percentage.of.households.with.high.housing.costs <= 0.0811
0.001187 < Other.primary.care.providers
Feature
0.000
0.005
0.010
0.015
0.020
Weight
Positive
Negative

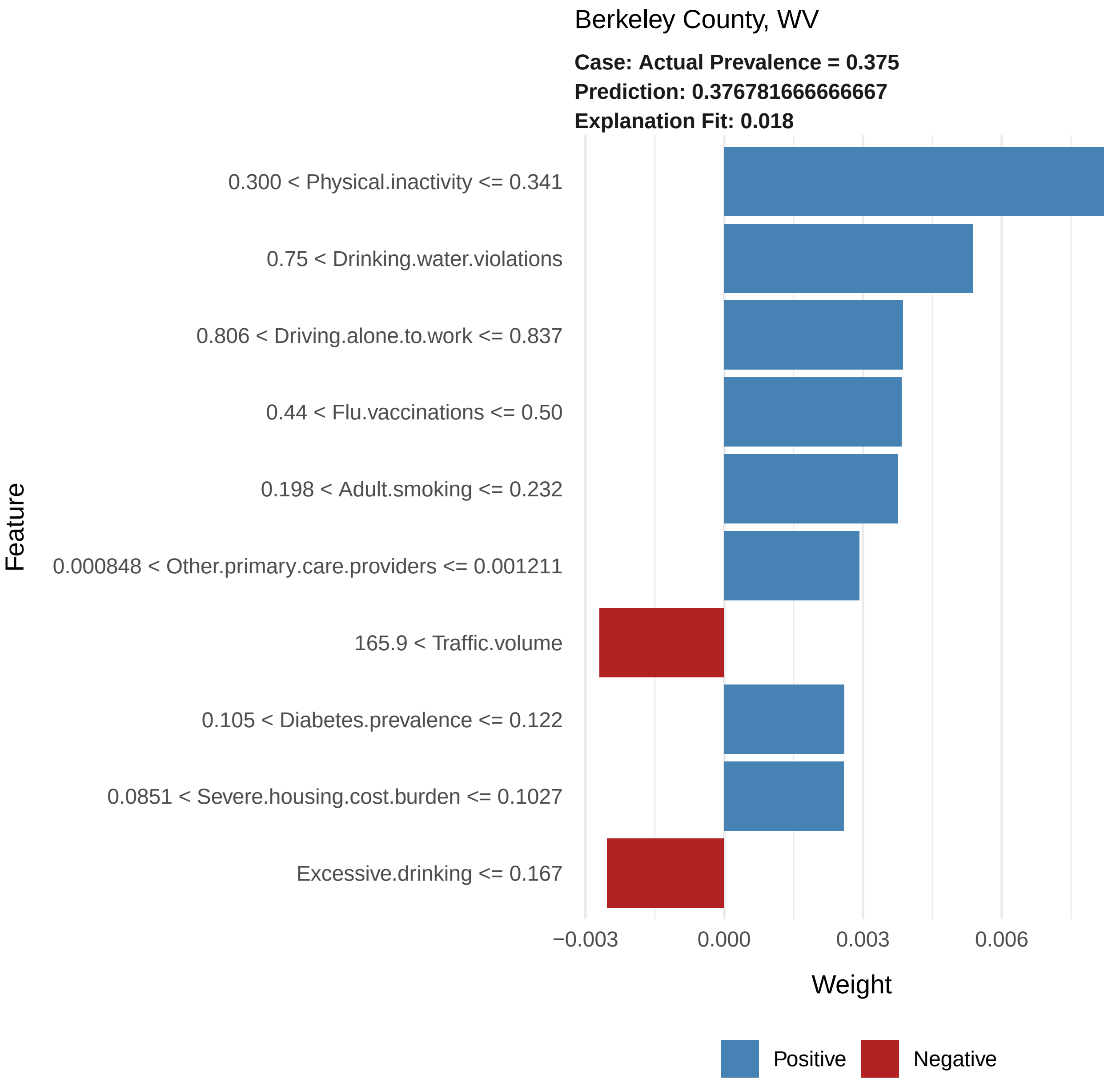
Berkeley County, WV
Case: Actual Prevalence = 0.375
Prediction: 0.376781666666667
Explanation Fit: 0.018
0.300 < Physical.inactivity <= 0.341
0.75 < Drinking.water.violations
0.806 < Driving.alone.to.work <= 0.837
0.44 < Flu.vaccinations <= 0.50
0.198 < Adult.smoking <= 0.232
0.000848 < Other.primary.care.providers <= 0.001211
165.9 < Traffic.volume
0.105 < Diabetes.prevalence <= 0.122
0.0851 < Severe.housing.cost.burden <= 0.1027
Excessive.drinking <= 0.167
Feature
−0.003
0.000
0.003
0.006
Weight
Positive
Negative

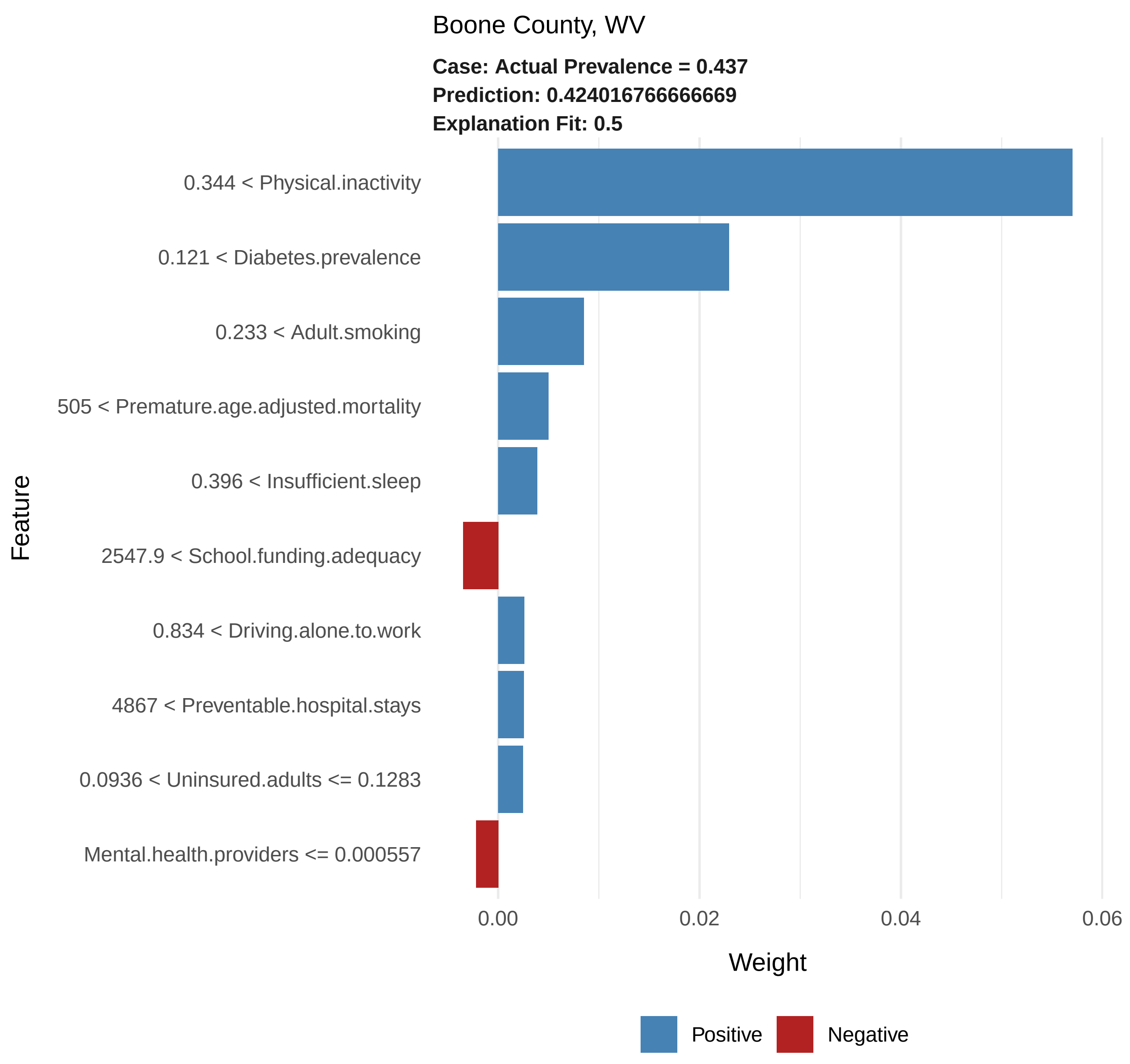
Boone County, WV
Case: Actual Prevalence = 0.437
Prediction: 0.424016766666669
Explanation Fit: 0.5
0.344 < Physical.inactivity
0.121 < Diabetes.prevalence
0.233 < Adult.smoking
505 < Premature.age.adjusted.mortality
0.396 < Insufficient.sleep
2547.9 < School.funding.adequacy
0.834 < Driving.alone.to.work
4867 < Preventable.hospital.stays
0.0936 < Uninsured.adults <= 0.1283
Mental.health.providers <= 0.000557
Feature
0.00
0.02
0.04
0.06
Weight
Positive
Negative

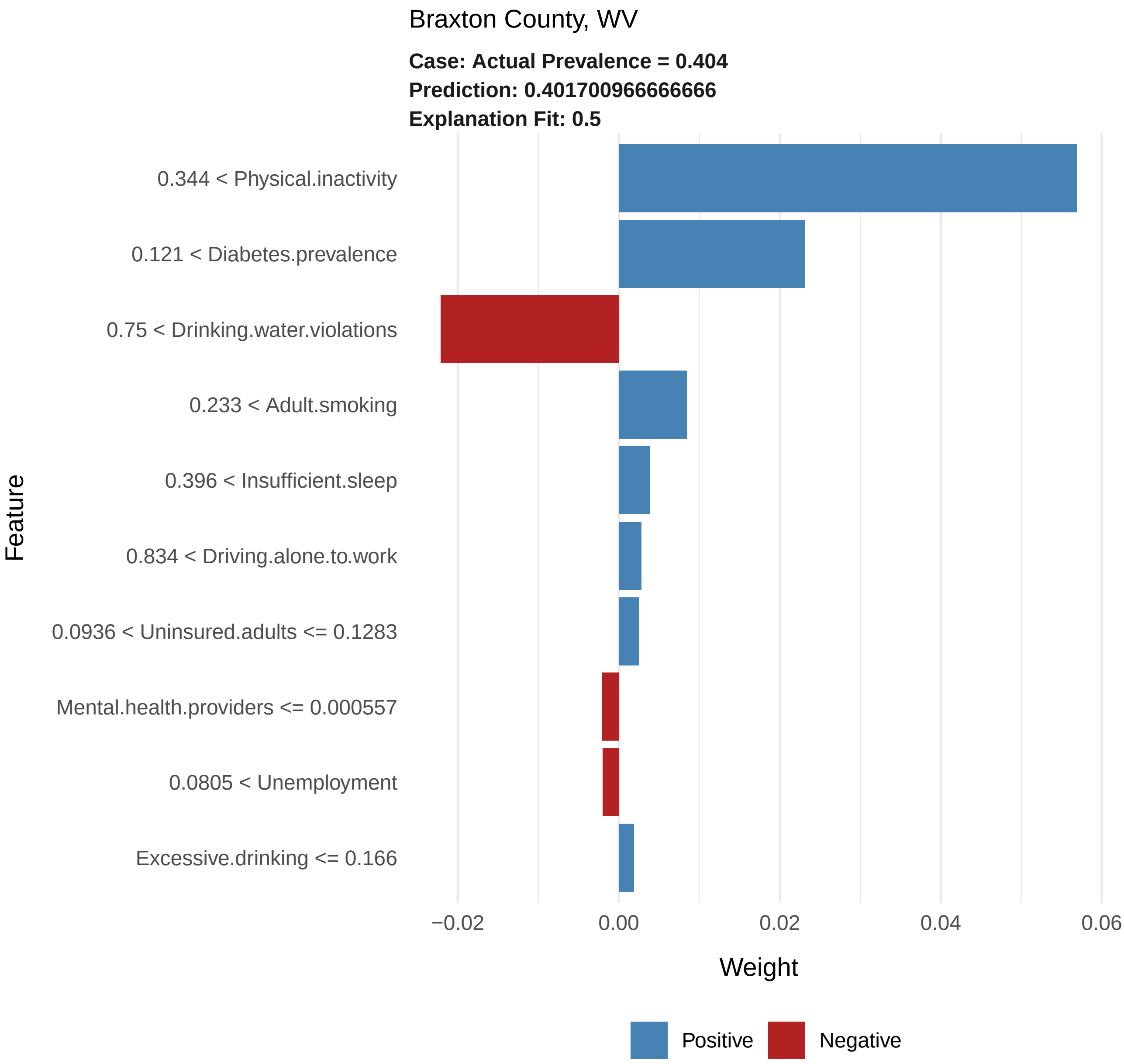

Braxton County, WV
Case: Actual Prevalence = 0.404
Prediction: 0.401700966666666
Explanation Fit: 0.5
0.344 < Physical.inactivity
0.121 < Diabetes.prevalence
0.75 < Drinking.water.violations
0.233 < Adult.smoking
0.396 < Insufficient.sleep
0.834 < Driving.alone.to.work
0.0936 < Uninsured.adults <= 0.1283
Mental.health.providers <= 0.000557
0.0805 < Unemployment
Excessive.drinking <= 0.166
Feature
−0.02
0.00
0.02
0.04
0.06
Weight
Positive
Negative

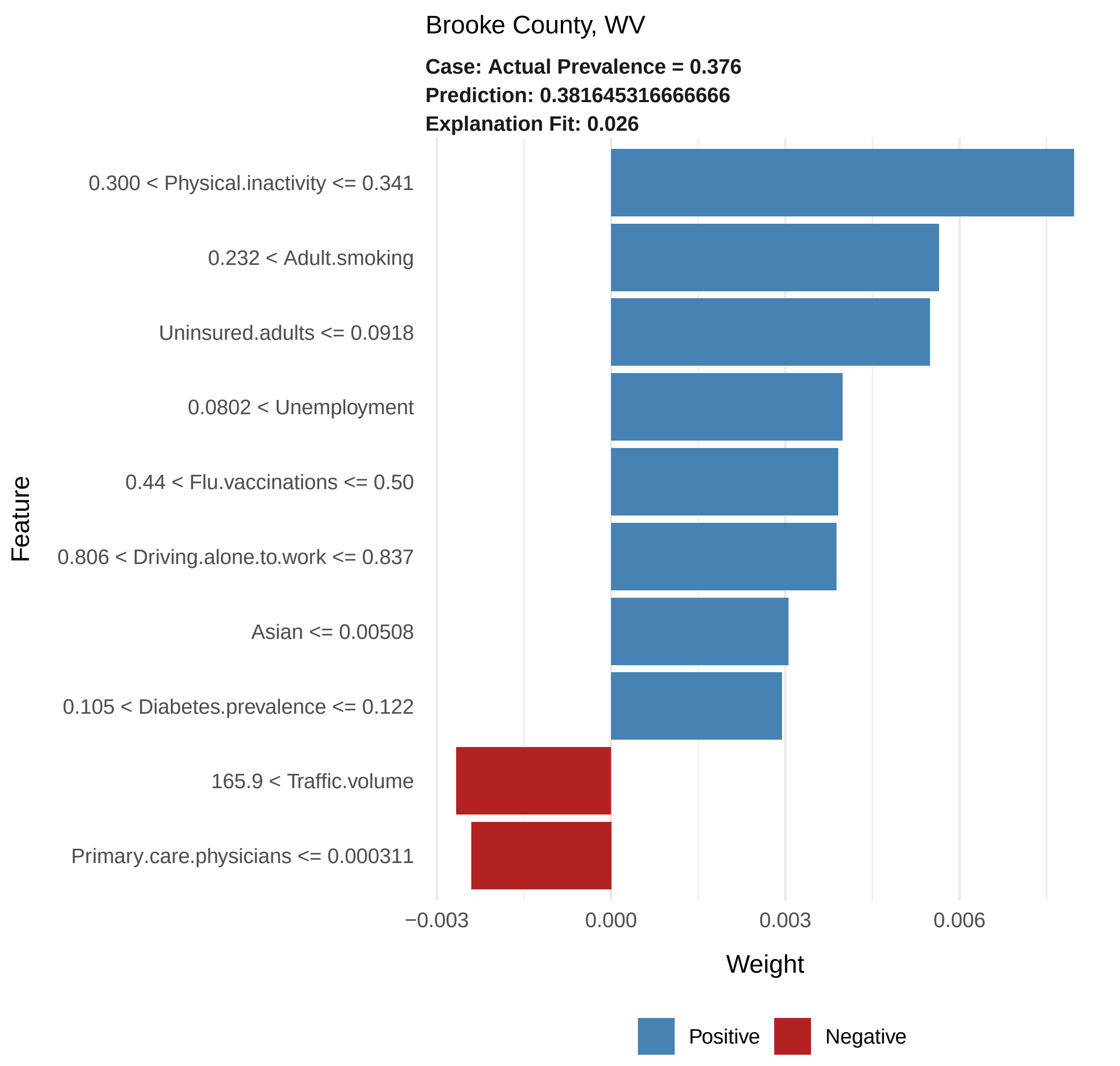
Brooke County, WV
Case: Actual Prevalence = 0.376
Prediction: 0.381645316666666
Explanation Fit: 0.026
0.300 < Physical.inactivity <= 0.341
0.232 < Adult.smoking
Uninsured.adults <= 0.0918
0.0802 < Unemployment
0.44 < Flu.vaccinations <= 0.50
0.806 < Driving.alone.to.work <= 0.837
Asian <= 0.00508
0.105 < Diabetes.prevalence <= 0.122
165.9 < Traffic.volume
Primary.care.physicians <= 0.000311
Feature
−0.003
0.000
0.003
0.006
Weight
Positive
Negative

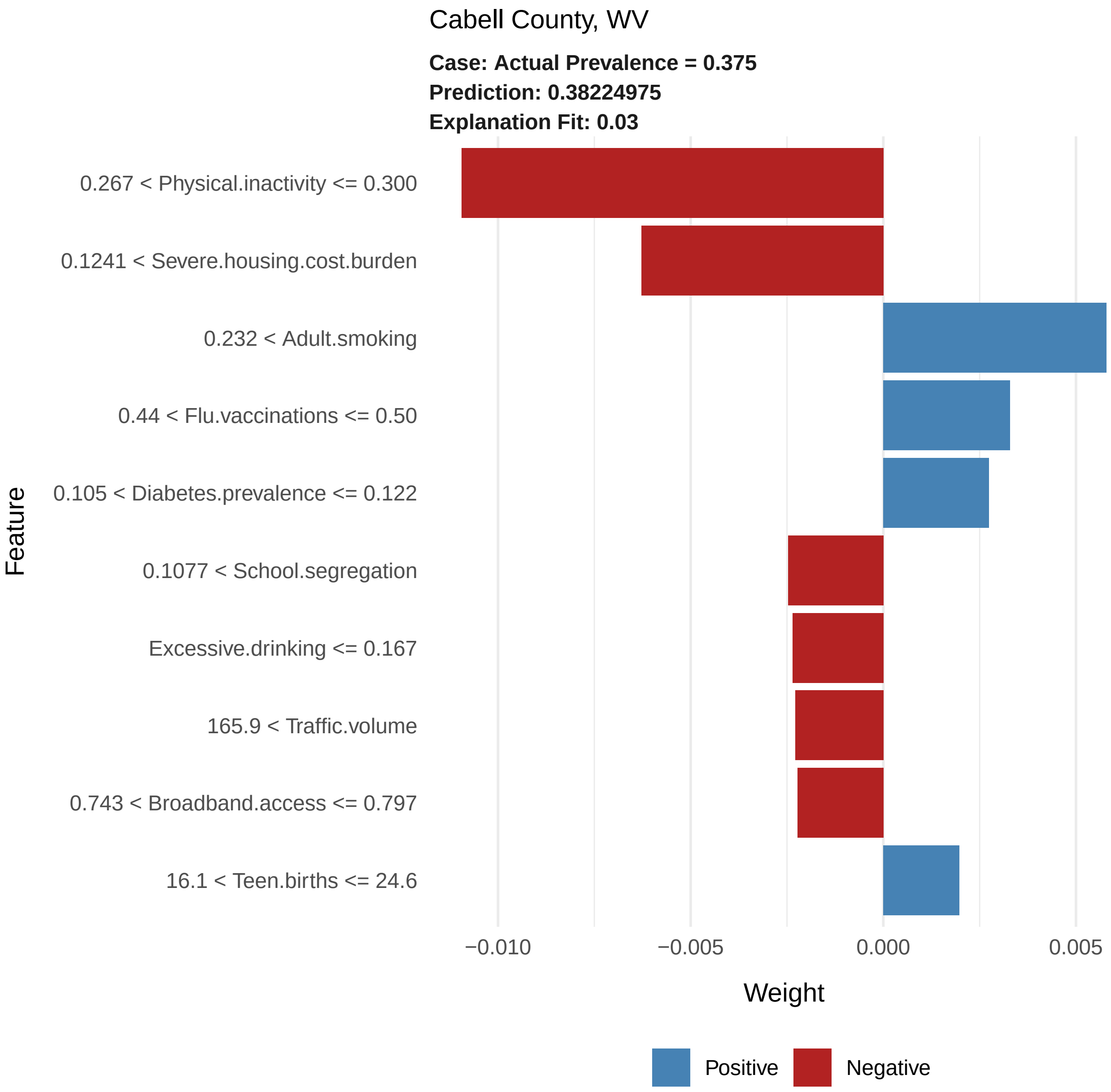

Cabell County, WV
Case: Actual Prevalence = 0.375
Prediction: 0.38224975
Explanation Fit: 0.03
Feature
0.267 < Physical.inactivity <= 0.300
0.1241 < Severe.housing.cost.burden
0.232 < Adult.smoking
0.44 < Flu.vaccinations <= 0.50
0.105 < Diabetes.prevalence <= 0.122
0.1077 < School.segregation
Excessive.drinking <= 0.167
165.9 < Traffic.volume
0.743 < Broadband.access <= 0.797
16.1 < Teen.births <= 24.6
−0.010
−0.005
0.000
0.005
Weight
Positive
Negative

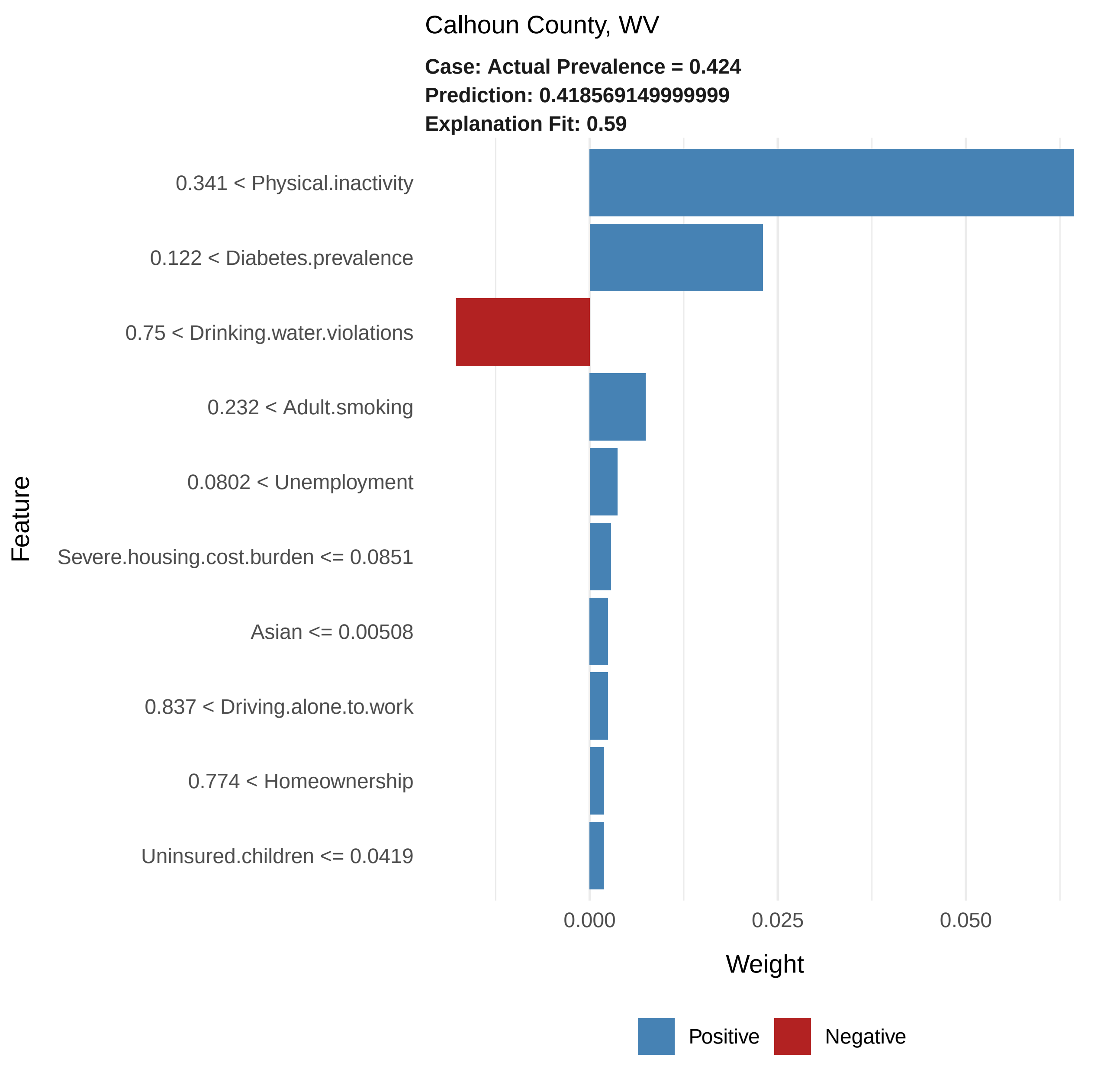
Calhoun County, WV
Case: Actual Prevalence = 0.424
Prediction: 0.418569149999999
Explanation Fit: 0.59
0.341 < Physical.inactivity
0.122 < Diabetes.prevalence
0.75 < Drinking.water.violations
0.232 < Adult.smoking
0.0802 < Unemployment
Severe.housing.cost.burden <= 0.0851
Asian <= 0.00508
0.837 < Driving.alone.to.work
0.774 < Homeownership
Uninsured.children <= 0.0419
Feature
0.000
0.025
0.050
Weight
Positive
Negative

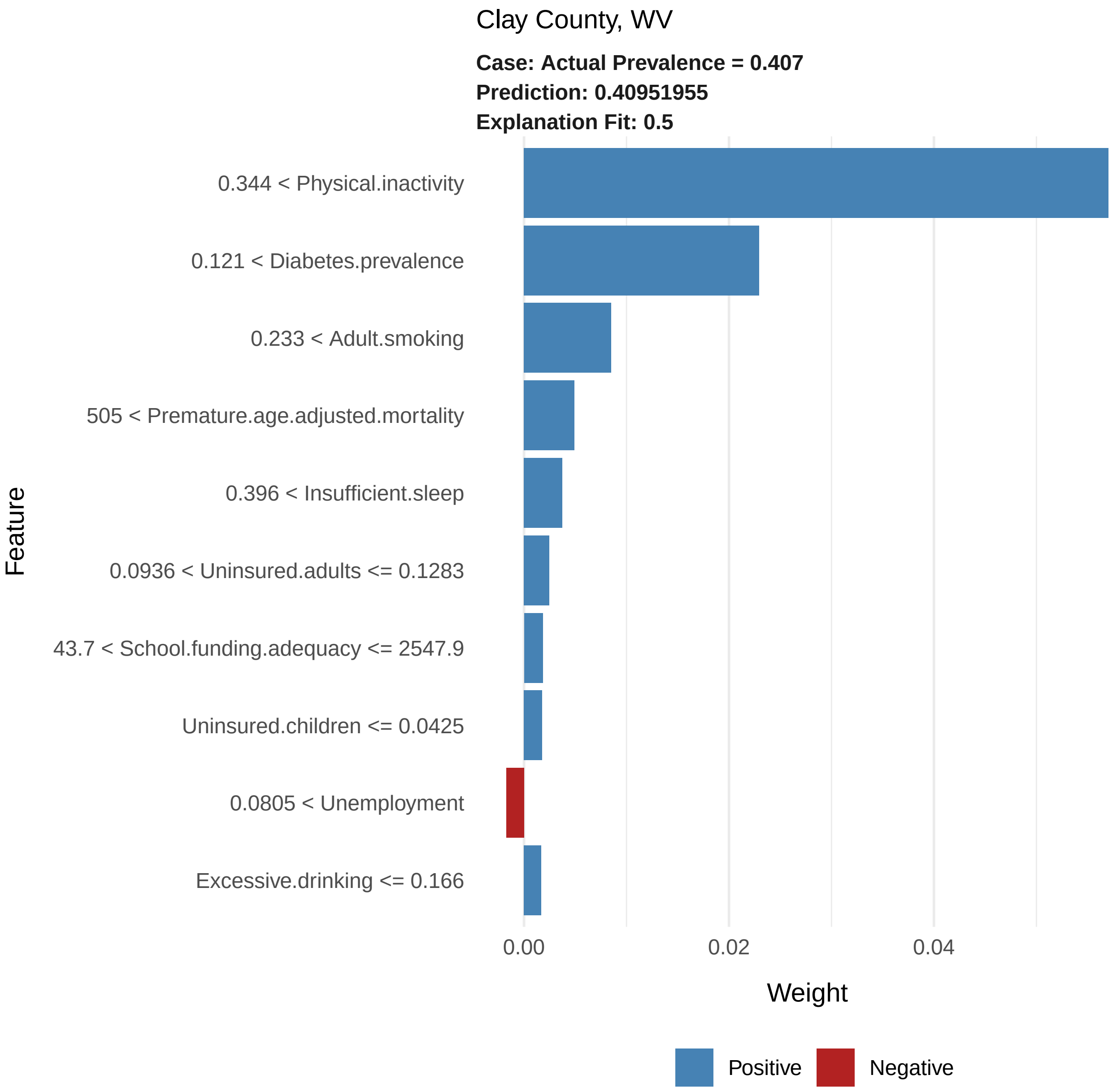
Clay County, WV
Case: Actual Prevalence = 0.407
Prediction: 0.40951955
Explanation Fit: 0.5
0.344 < Physical.inactivity
0.121 < Diabetes.prevalence
0.233 < Adult.smoking
505 < Premature.age.adjusted.mortality
0.396 < Insufficient.sleep
0.0936 < Uninsured.adults <= 0.1283
43.7 < School.funding.adequacy <= 2547.9
Uninsured.children <= 0.0425
0.0805 < Unemployment
Excessive.drinking <= 0.166
Feature
0.00
0.02
0.04
Weight
Positive
Negative

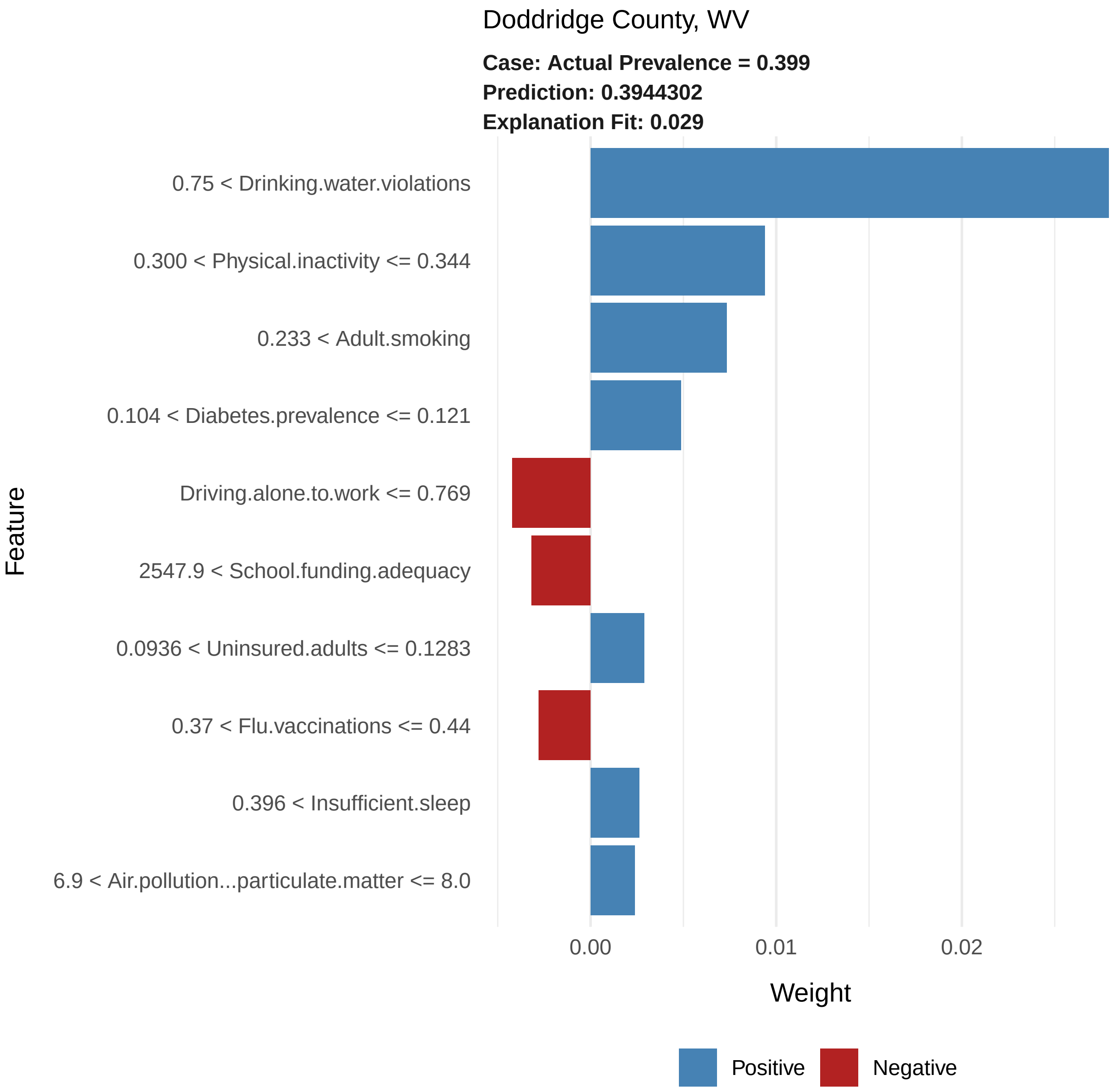

Doddridge County, WV
Case: Actual Prevalence = 0.399
Prediction: 0.3944302
Explanation Fit: 0.029
0.75 < Drinking.water.violations
0.300 < Physical.inactivity <= 0.344
0.233 < Adult.smoking
0.104 < Diabetes.prevalence <= 0.121
Driving.alone.to.work <= 0.769
2547.9 < School.funding.adequacy
0.0936 < Uninsured.adults <= 0.1283
0.37 < Flu.vaccinations <= 0.44
0.396 < Insufficient.sleep
6.9 < Air.pollution...particulate.matter <= 8.0
Feature
0.00
0.01
0.02
Weight
Positive
Negative

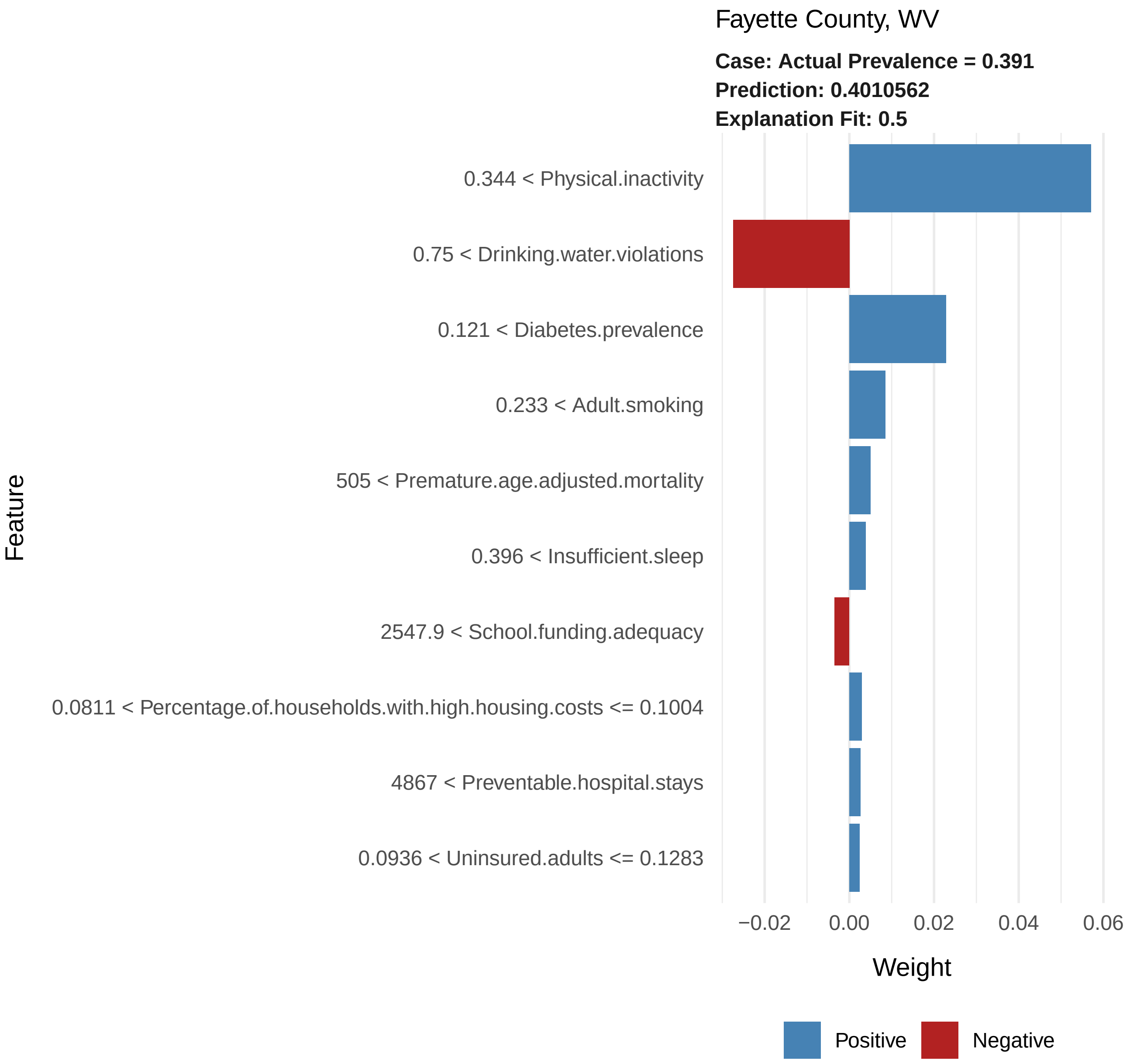
Fayette County, WV
Case: Actual Prevalence = 0.391
Prediction: 0.4010562
Explanation Fit: 0.5
0.344 < Physical.inactivity
0.75 < Drinking.water.violations
0.121 < Diabetes.prevalence
0.233 < Adult.smoking
505 < Premature.age.adjusted.mortality
0.396 < Insufficient.sleep
2547.9 < School.funding.adequacy
0.0811 < Percentage.of.households.with.high.housing.costs <= 0.1004
4867 < Preventable.hospital.stays
0.0936 < Uninsured.adults <= 0.1283
Feature
−0.02
0.00
0.02
0.04
0.06
Weight
Positive
Negative

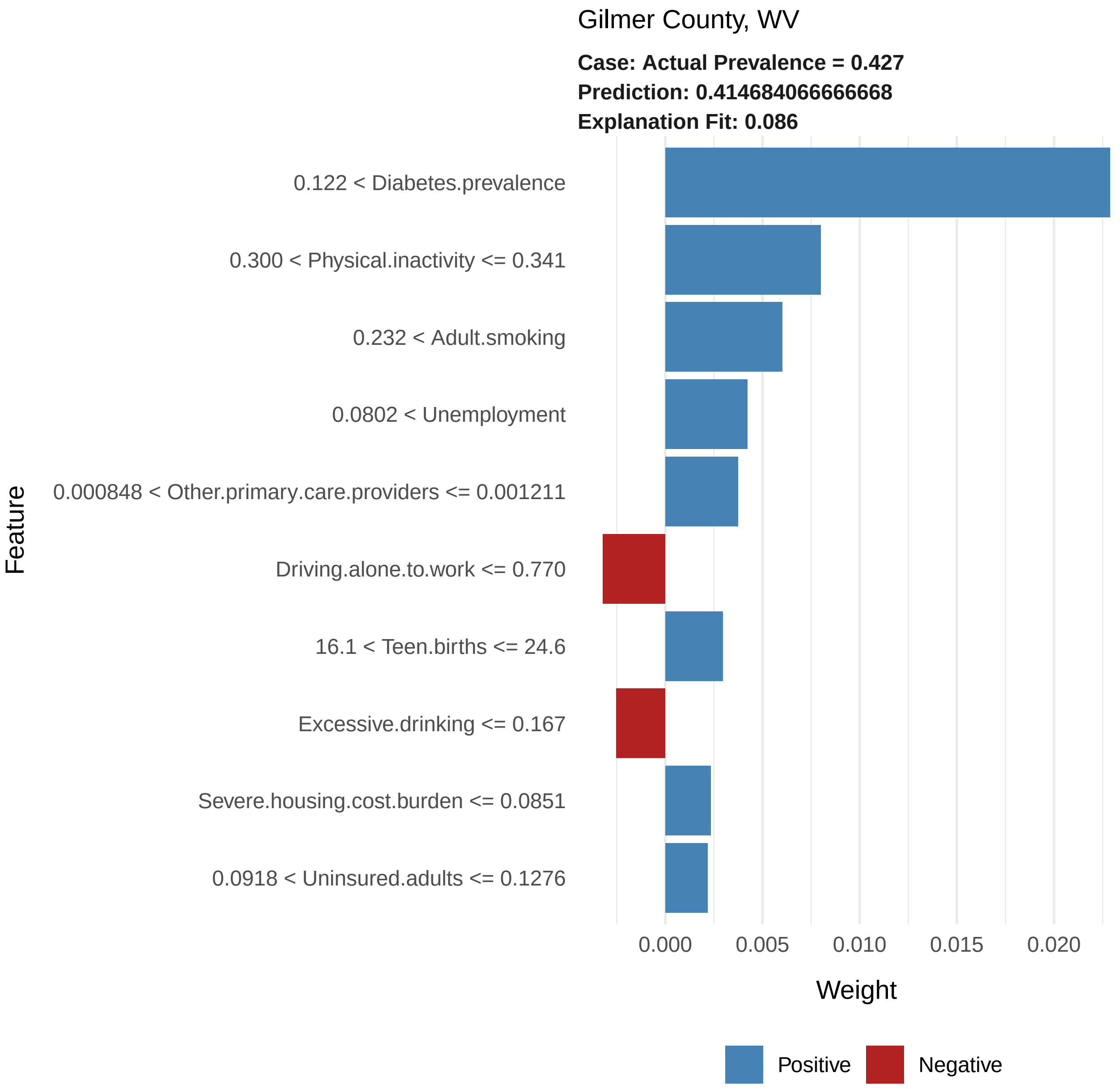

Gilmer County, WV
Case: Actual Prevalence = 0.427
Prediction: 0.414684066666668
Explanation Fit: 0.086
0.122 < Diabetes.prevalence
0.300 < Physical.inactivity <= 0.341
0.232 < Adult.smoking
0.0802 < Unemployment
0.000848 < Other.primary.care.providers <= 0.001211
Driving.alone.to.work <= 0.770
16.1 < Teen.births <= 24.6
Excessive.drinking <= 0.167
Severe.housing.cost.burden <= 0.0851
0.0918 < Uninsured.adults <= 0.1276
Feature
0.000
0.005
0.010
0.015
0.020
Weight
Positive
Negative

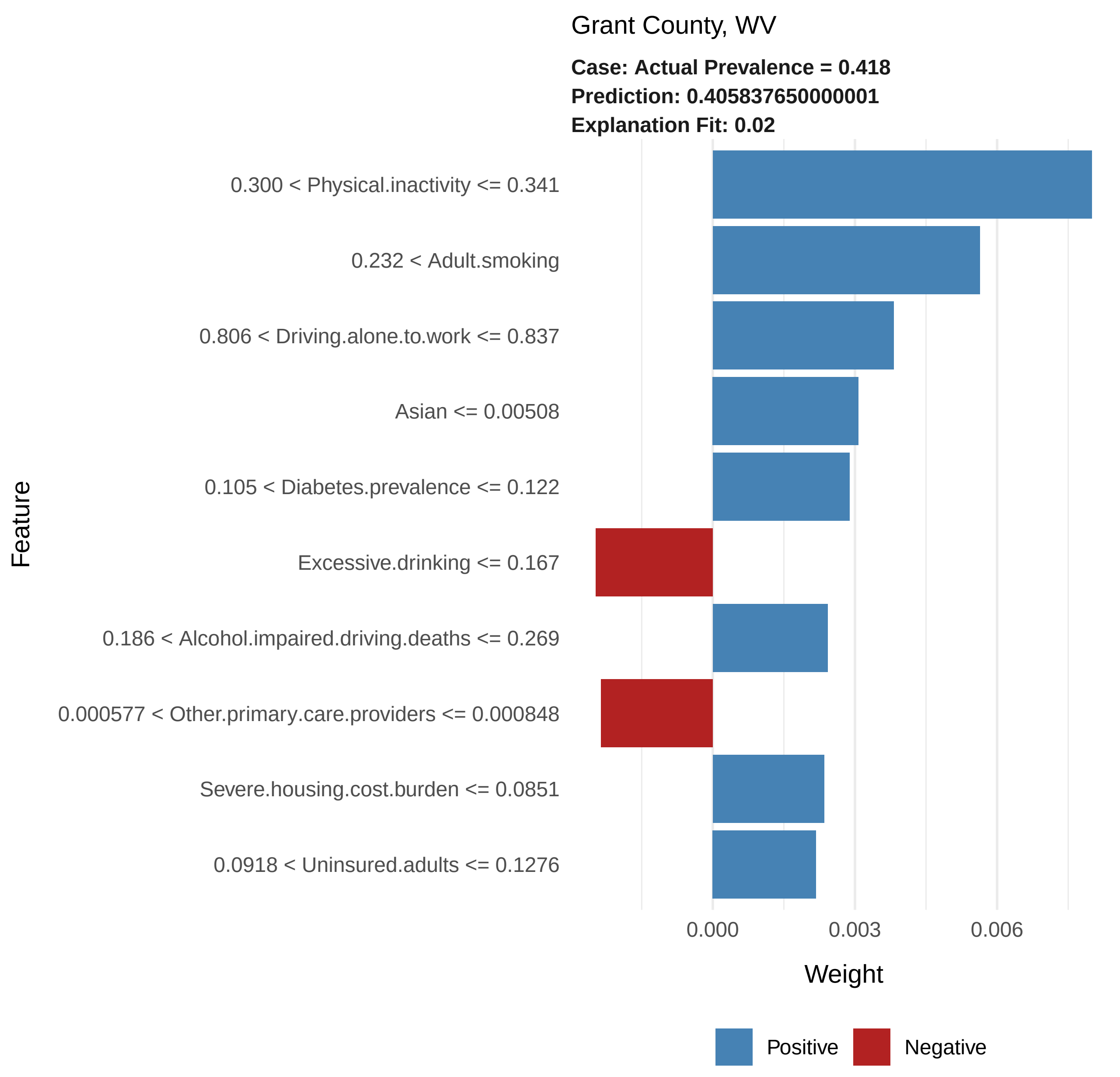

Grant County, WV
Case: Actual Prevalence = 0.418
Prediction: 0.405837650000001
Explanation Fit: 0.02
Feature
0.300 < Physical.inactivity <= 0.341
0.232 < Adult.smoking
0.806 < Driving.alone.to.work <= 0.837
Asian <= 0.00508
0.105 < Diabetes.prevalence <= 0.122
Excessive.drinking <= 0.167
0.186 < Alcohol.impaired.driving.deaths <= 0.269
0.000577 < Other.primary.care.providers <= 0.000848
Severe.housing.cost.burden <= 0.0851
0.0918 < Uninsured.adults <= 0.1276
0.000
0.003
0.006
Weight
Positive
Negative

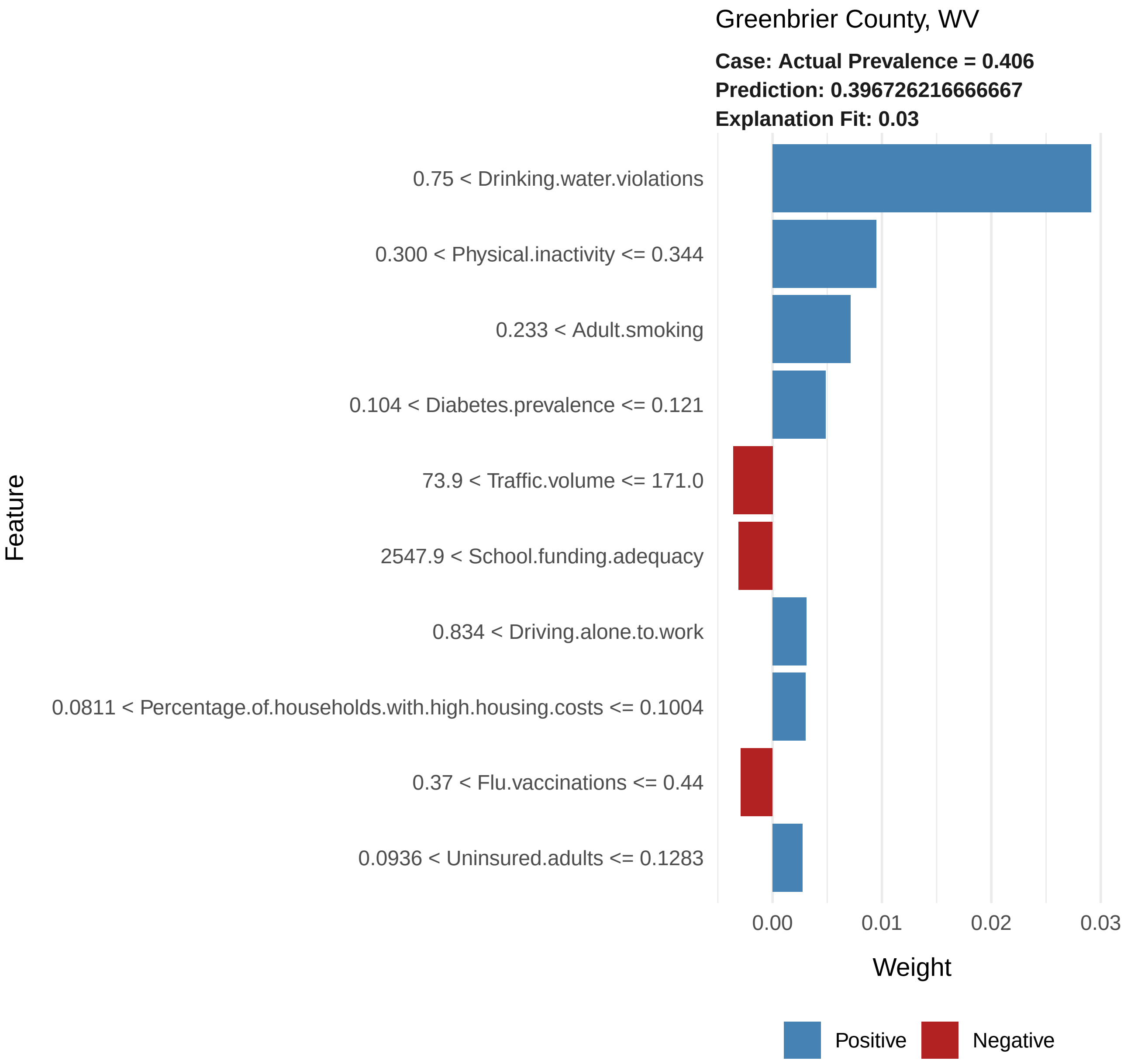
Greenbrier County, WV
Case: Actual Prevalence = 0.406
Prediction: 0.396726216666667
Explanation Fit: 0.03
0.75 < Drinking.water.violations
0.300 < Physical.inactivity <= 0.344
0.233 < Adult.smoking
0.104 < Diabetes.prevalence <= 0.121
73.9 < Traffic.volume <= 171.0
2547.9 < School.funding.adequacy
0.834 < Driving.alone.to.work
0.0811 < Percentage.of.households.with.high.housing.costs <= 0.1004
0.37 < Flu.vaccinations <= 0.44
0.0936 < Uninsured.adults <= 0.1283
Feature
0.00
0.01
0.02
0.03
Weight
Positive
Negative

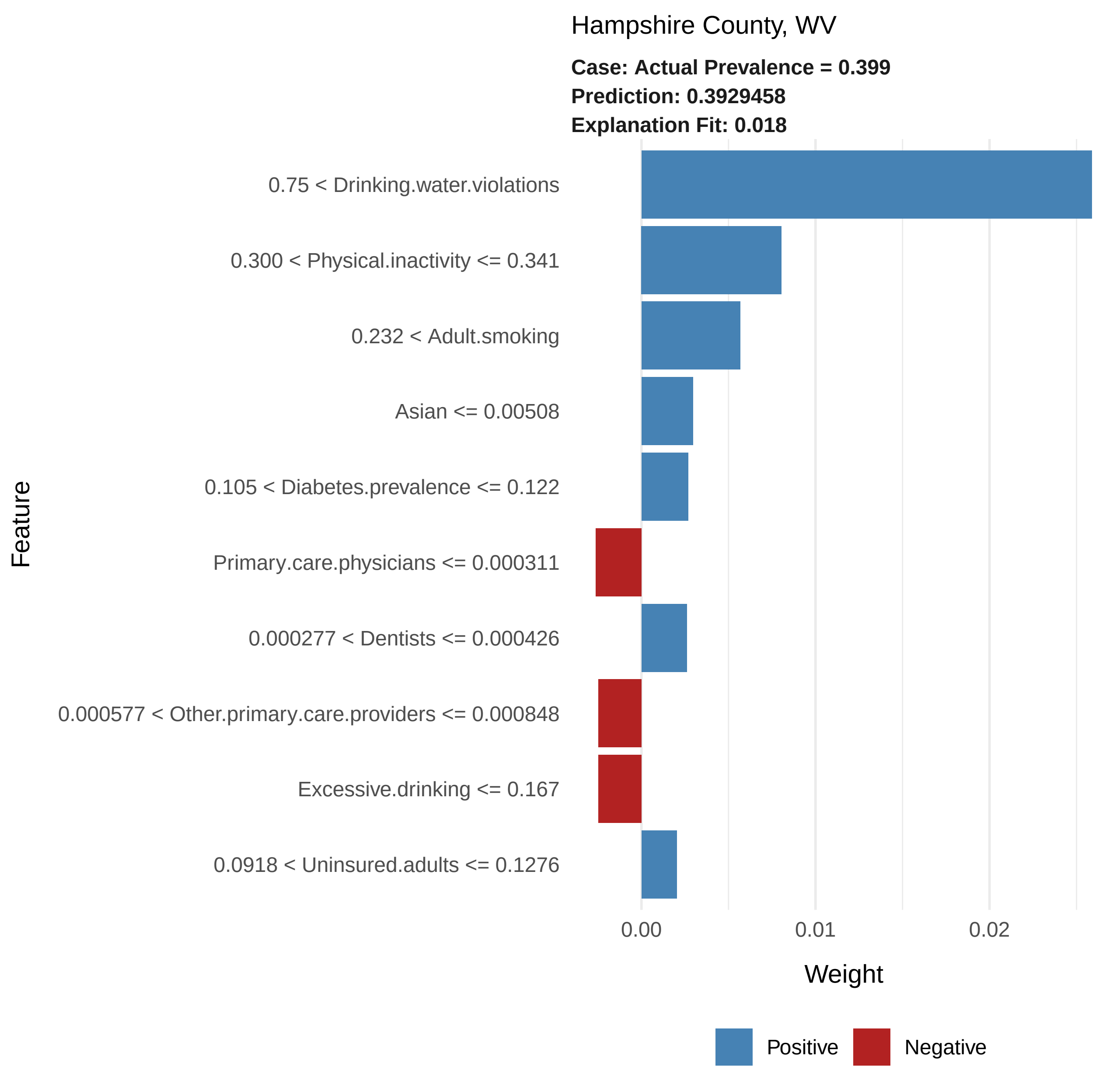

Hampshire County, WV
Case: Actual Prevalence = 0.399
Prediction: 0.3929458
Explanation Fit: 0.018
0.75 < Drinking.water.violations
0.300 < Physical.inactivity <= 0.341
0.232 < Adult.smoking
Asian <= 0.00508
0.105 < Diabetes.prevalence <= 0.122
Primary.care.physicians <= 0.000311
0.000277 < Dentists <= 0.000426
0.000577 < Other.primary.care.providers <= 0.000848
Excessive.drinking <= 0.167
0.0918 < Uninsured.adults <= 0.1276
Feature
0.00
0.01
0.02
Weight
Positive
Negative

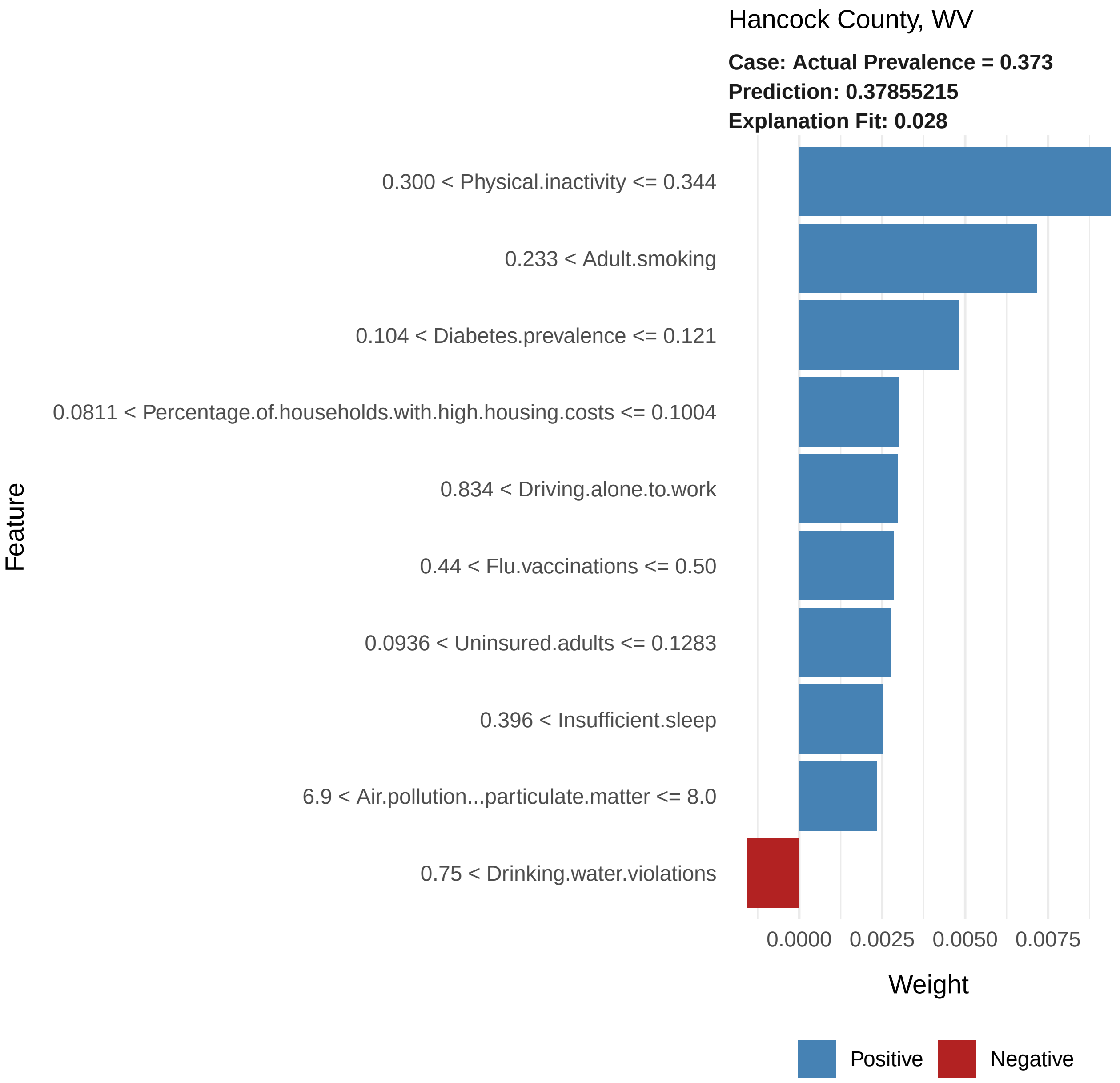

Hancock County, WV
Case: Actual Prevalence = 0.373
Prediction: 0.37855215
Explanation Fit: 0.028
0.300 < Physical.inactivity <= 0.344
0.233 < Adult.smoking
0.104 < Diabetes.prevalence <= 0.121
0.0811 < Percentage.of.households.with.high.housing.costs <= 0.1004
0.834 < Driving.alone.to.work
0.44 < Flu.vaccinations <= 0.50
0.0936 < Uninsured.adults <= 0.1283
0.396 < Insufficient.sleep
6.9 < Air.pollution...particulate.matter <= 8.0
0.75 < Drinking.water.violations
Feature
0.0000
0.0025
0.0050
0.0075
Weight
Positive
Negative

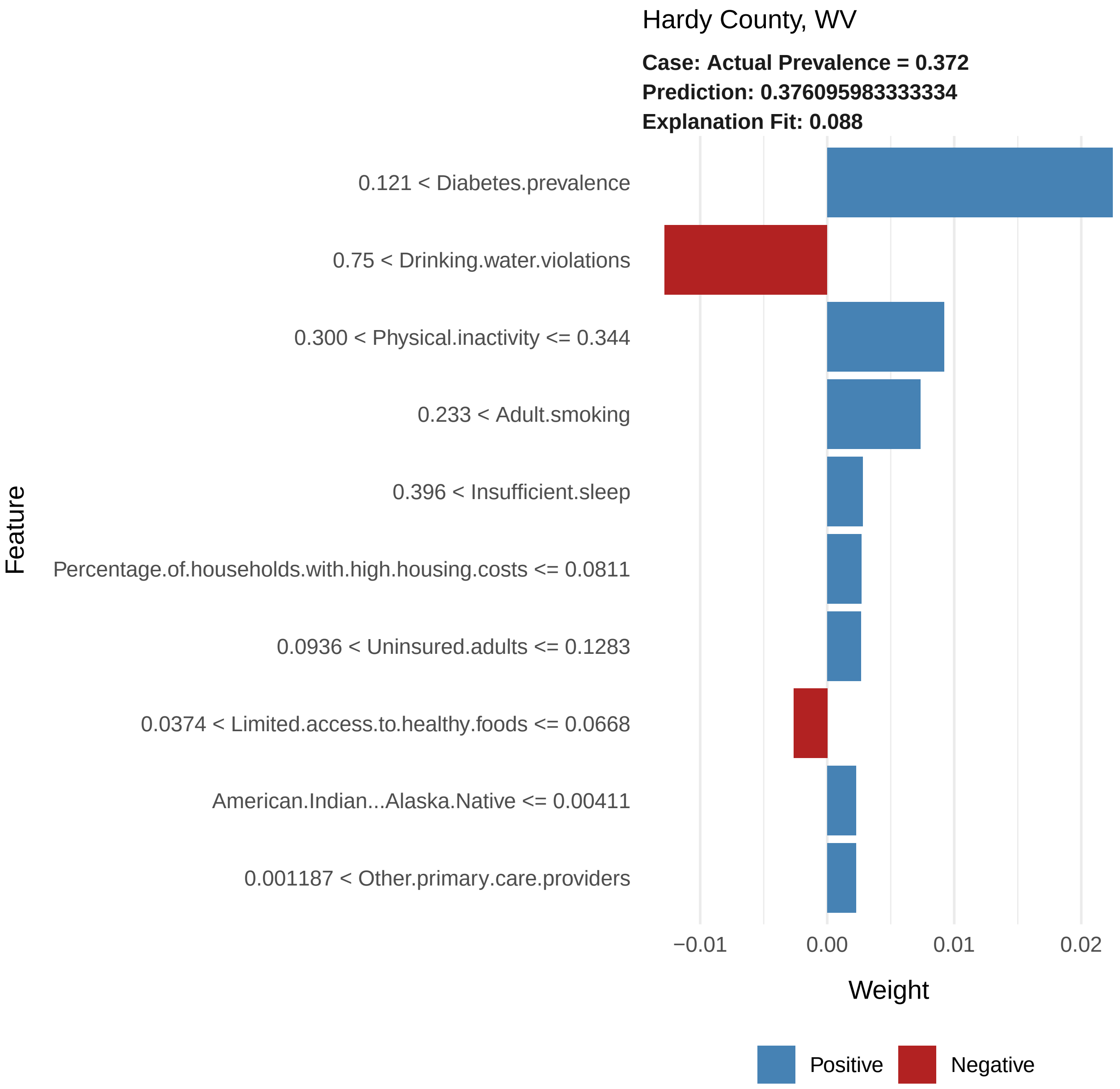
Hardy County, WV
Case: Actual Prevalence = 0.372
Prediction: 0.376095983333334
Explanation Fit: 0.088
Feature
0.121 < Diabetes.prevalence
0.75 < Drinking.water.violations
0.300 < Physical.inactivity <= 0.344
0.233 < Adult.smoking
0.396 < Insufficient.sleep
Percentage.of.households.with.high.housing.costs <= 0.0811
0.0936 < Uninsured.adults <= 0.1283
0.0374 < Limited.access.to.healthy.foods <= 0.0668
American.Indian...Alaska.Native <= 0.00411
0.001187 < Other.primary.care.providers
−0.01
0.00
0.01
0.02
Weight
Positive
Negative

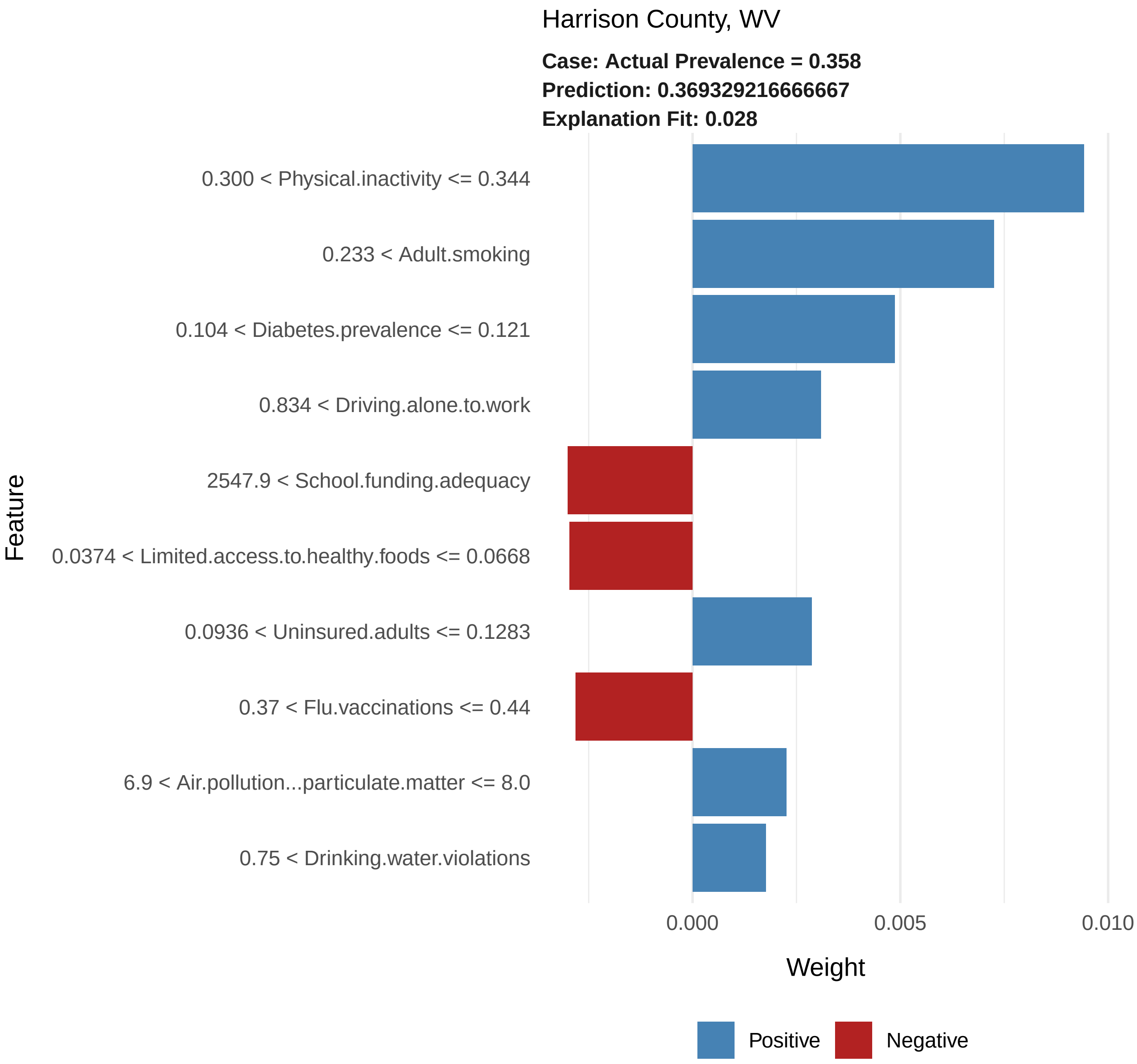

Harrison County, WV
Case: Actual Prevalence = 0.358
Prediction: 0.369329216666667
Explanation Fit: 0.028
0.300 < Physical.inactivity <= 0.344
0.233 < Adult.smoking
0.104 < Diabetes.prevalence <= 0.121
0.834 < Driving.alone.to.work
2547.9 < School.funding.adequacy
0.0374 < Limited.access.to.healthy.foods <= 0.0668
0.0936 < Uninsured.adults <= 0.1283
0.37 < Flu.vaccinations <= 0.44
6.9 < Air.pollution...particulate.matter <= 8.0
0.75 < Drinking.water.violations
Feature
0.000
0.005
0.010
Weight
Positive
Negative

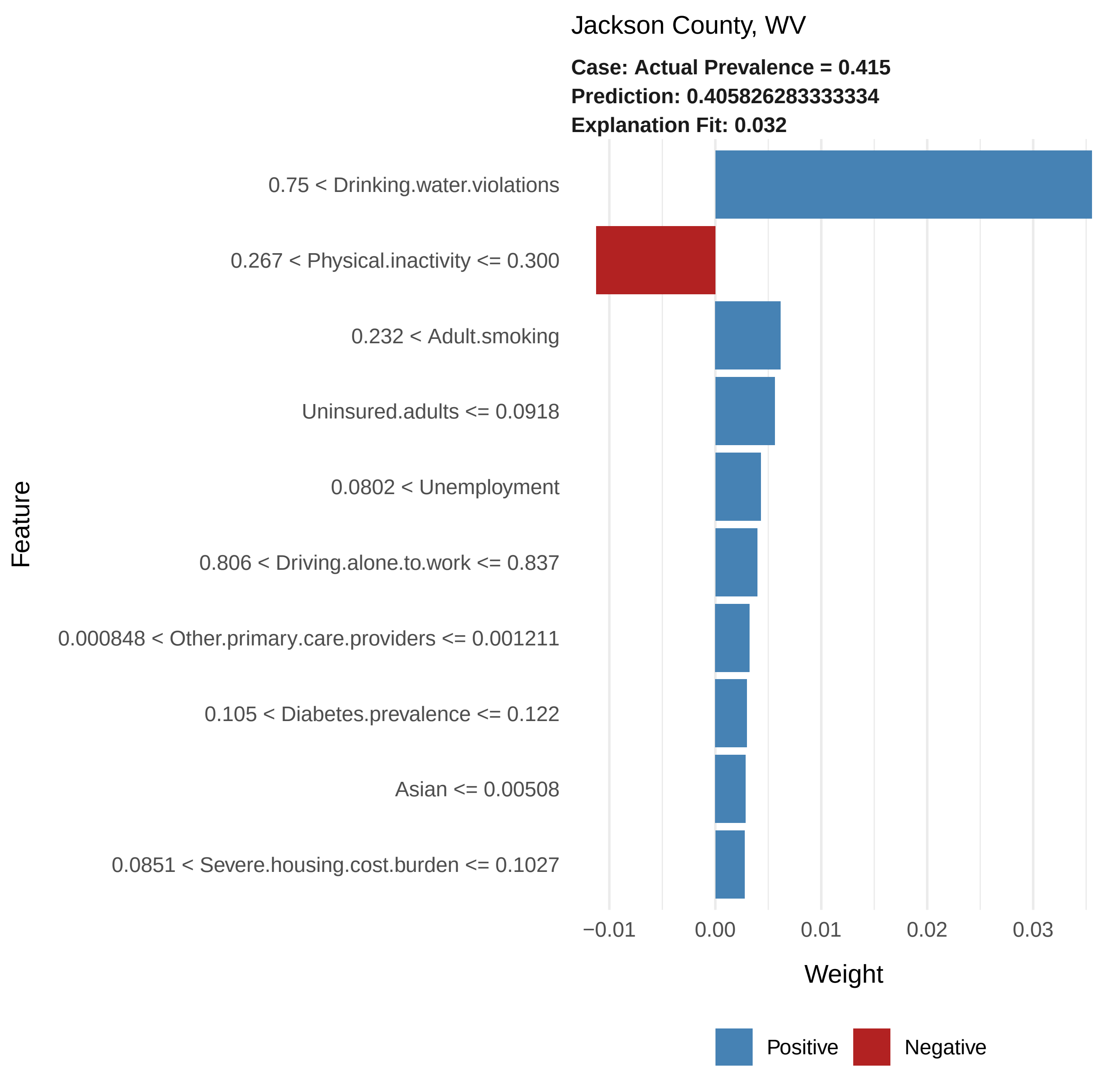
Jackson County, WV
Case: Actual Prevalence = 0.415
Prediction: 0.405826283333334
Explanation Fit: 0.032
0.75 < Drinking.water.violations
0.267 < Physical.inactivity <= 0.300
0.232 < Adult.smoking
Uninsured.adults <= 0.0918
0.0802 < Unemployment
0.806 < Driving.alone.to.work <= 0.837
0.000848 < Other.primary.care.providers <= 0.001211
0.105 < Diabetes.prevalence <= 0.122
Asian <= 0.00508
0.0851 < Severe.housing.cost.burden <= 0.1027
Feature
−0.01
0.00
0.01
0.02
0.03
Weight
Positive
Negative

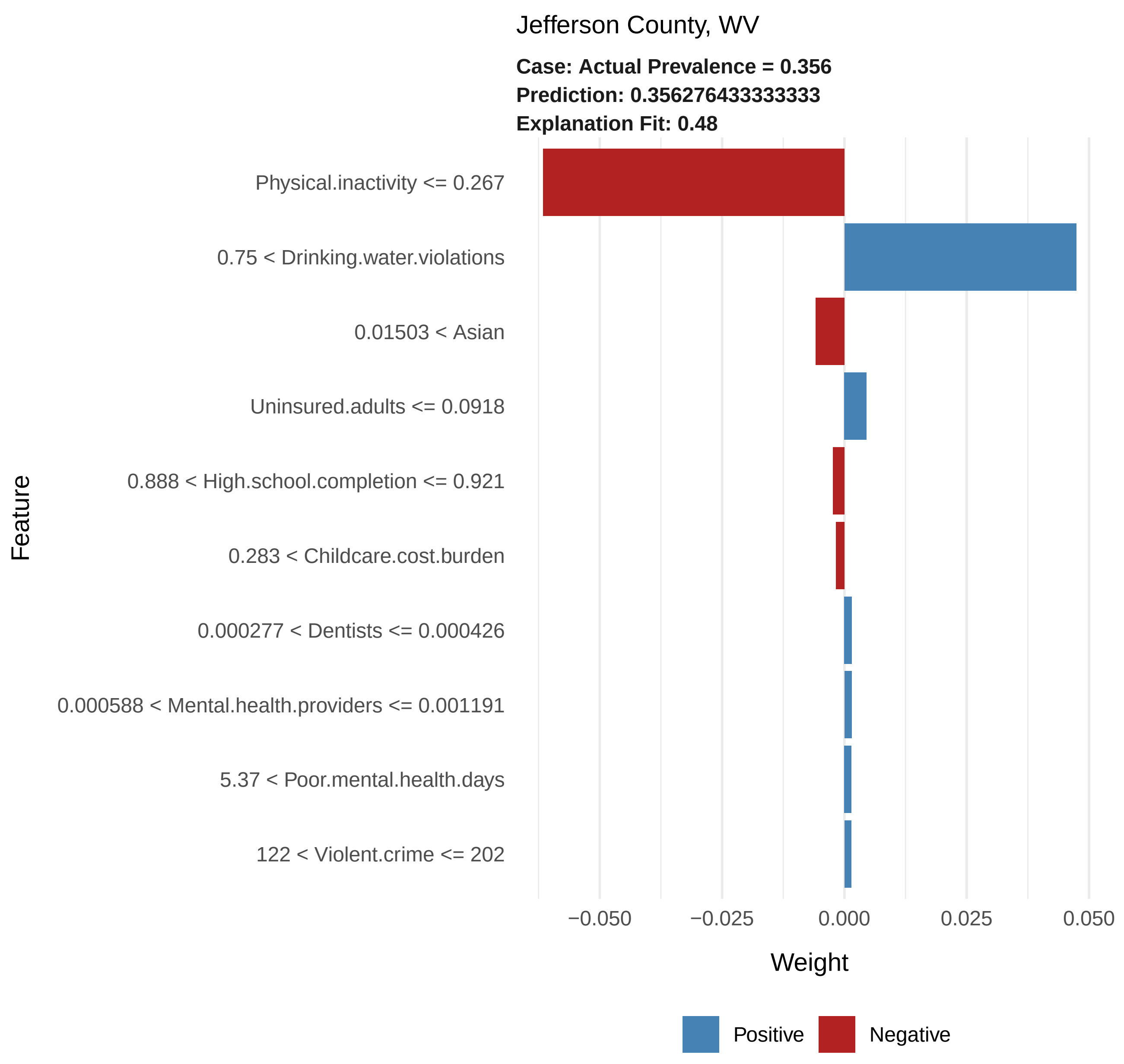
Jefferson County, WV
Case: Actual Prevalence = 0.356
Prediction: 0.356276433333333
Explanation Fit: 0.48
Physical.inactivity <= 0.267
0.75 < Drinking.water.violations
0.01503 < Asian
Uninsured.adults <= 0.0918
0.888 < High.school.completion <= 0.921
0.283 < Childcare.cost.burden
0.000277 < Dentists <= 0.000426
0.000588 < Mental.health.providers <= 0.001191
5.37 < Poor.mental.health.days
122 < Violent.crime <= 202
Feature
−0.050
−0.025
0.000
0.025
0.050
Weight
Positive
Negative

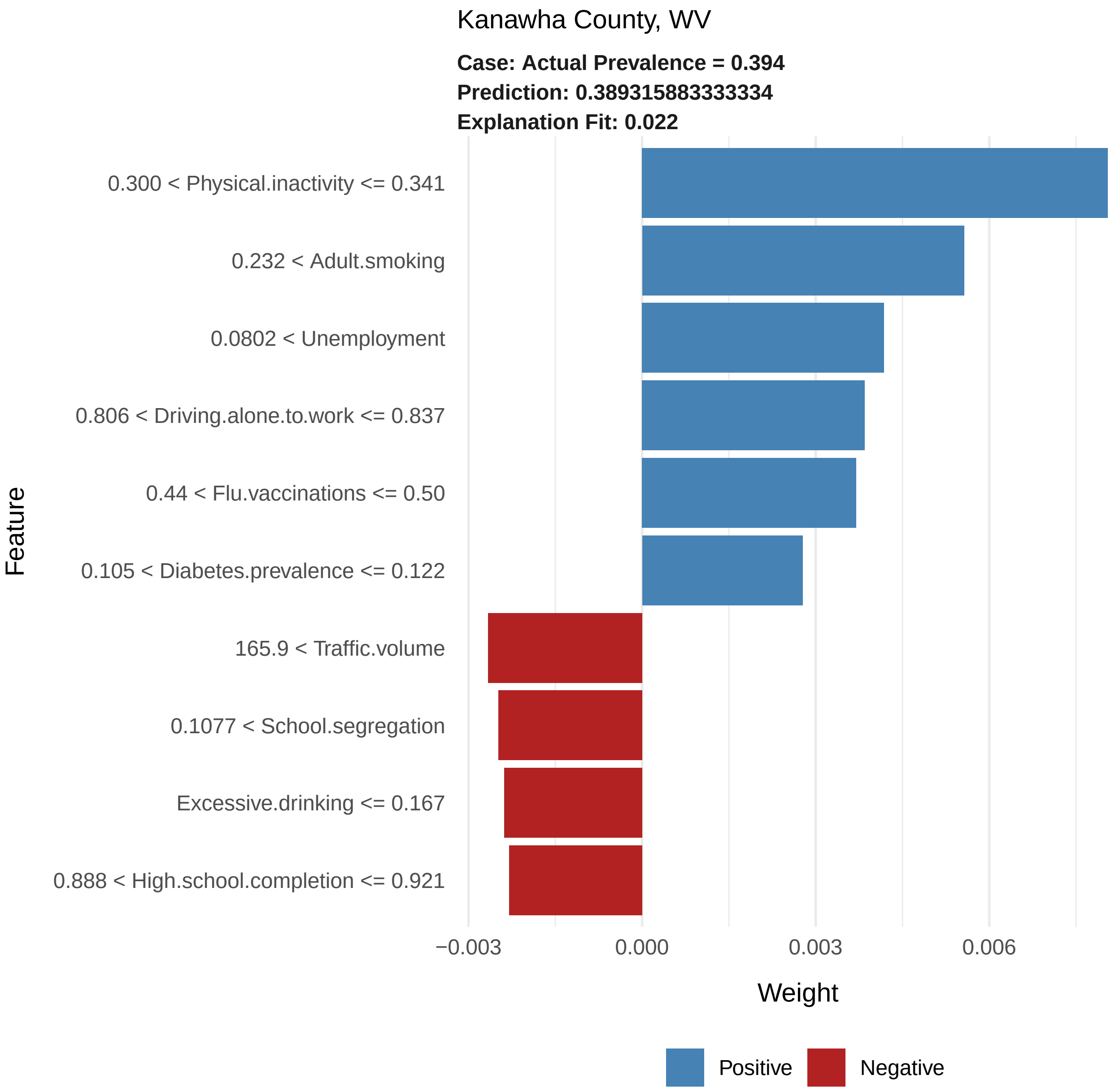
Kanawha County, WV
Case: Actual Prevalence = 0.394
Prediction: 0.389315883333334
Explanation Fit: 0.022
0.300 < Physical.inactivity <= 0.341
0.232 < Adult.smoking
0.0802 < Unemployment
0.806 < Driving.alone.to.work <= 0.837
0.44 < Flu.vaccinations <= 0.50
0.105 < Diabetes.prevalence <= 0.122
165.9 < Traffic.volume
0.1077 < School.segregation
Excessive.drinking <= 0.167
0.888 < High.school.completion <= 0.921
Feature
−0.003
0.000
0.003
0.006
Weight
Positive
Negative

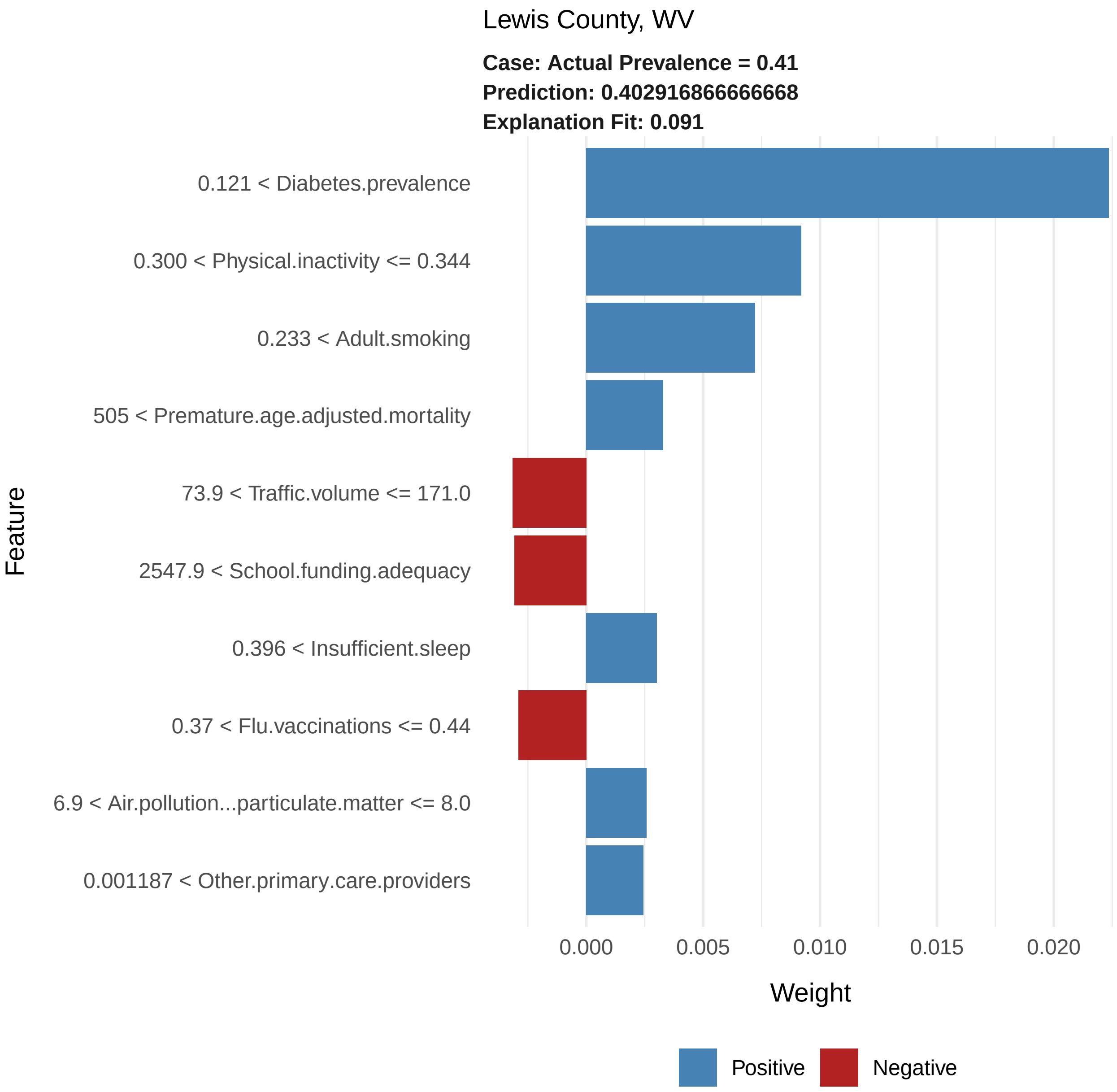

Lewis County, WV
Case: Actual Prevalence = 0.41
Prediction: 0.402916866666668
Explanation Fit: 0.091
0.121 < Diabetes.prevalence
0.300 < Physical.inactivity <= 0.344
0.233 < Adult.smoking
505 < Premature.age.adjusted.mortality
73.9 < Traffic.volume <= 171.0
2547.9 < School.funding.adequacy
0.396 < Insufficient.sleep
0.37 < Flu.vaccinations <= 0.44
6.9 < Air.pollution...particulate.matter <= 8.0
0.001187 < Other.primary.care.providers
Feature
0.000
0.005
0.010
0.015
0.020
Weight
Positive
Negative

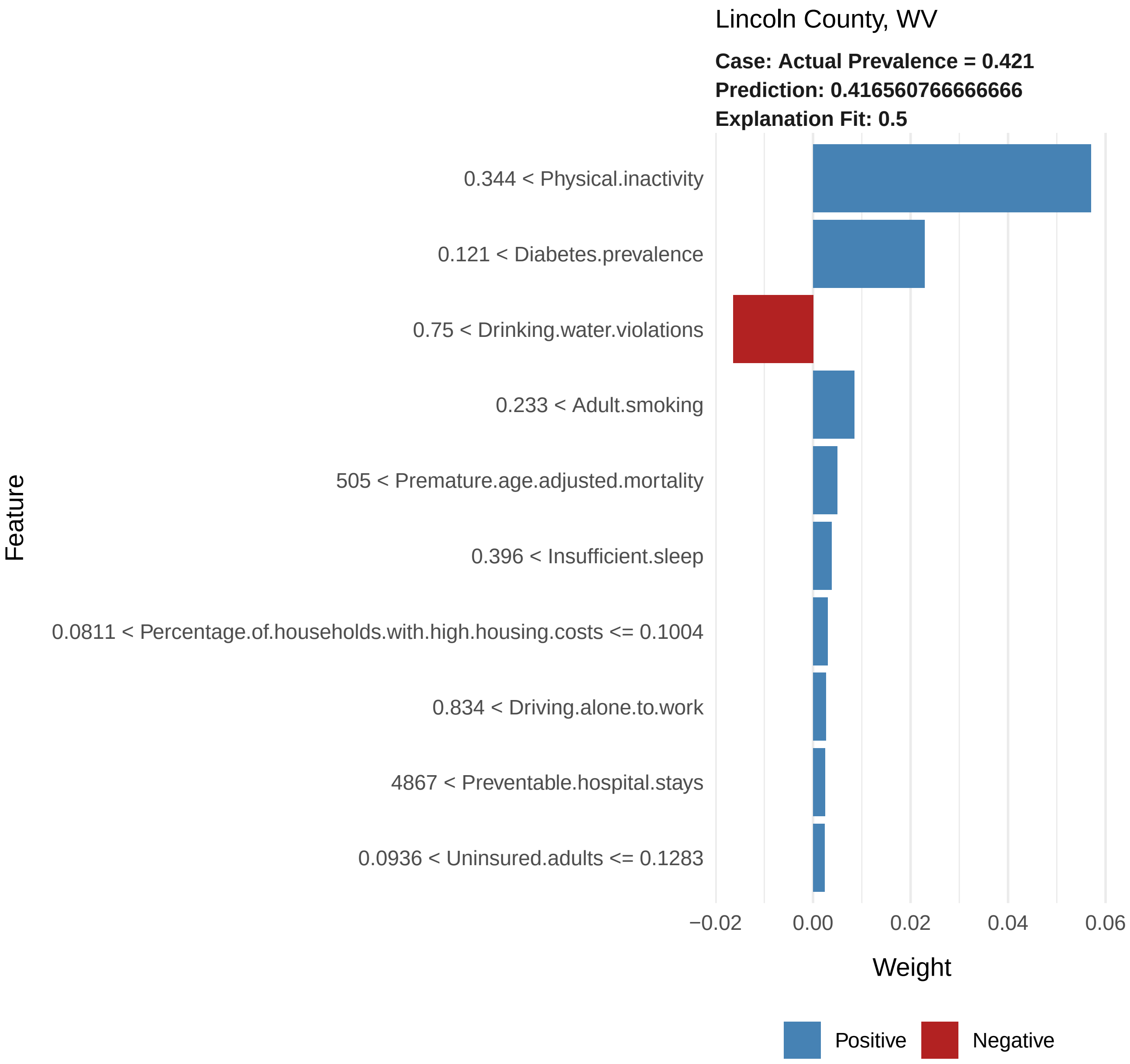
Lincoln County, WV
Case: Actual Prevalence = 0.421
Prediction: 0.416560766666666
Explanation Fit: 0.5
0.344 < Physical.inactivity
0.121 < Diabetes.prevalence
0.75 < Drinking.water.violations
0.233 < Adult.smoking
505 < Premature.age.adjusted.mortality
0.396 < Insufficient.sleep
0.0811 < Percentage.of.households.with.high.housing.costs <= 0.1004
0.834 < Driving.alone.to.work
4867 < Preventable.hospital.stays
0.0936 < Uninsured.adults <= 0.1283
Feature
−0.02
0.00
0.02
0.04
0.06
Weight
Positive
Negative

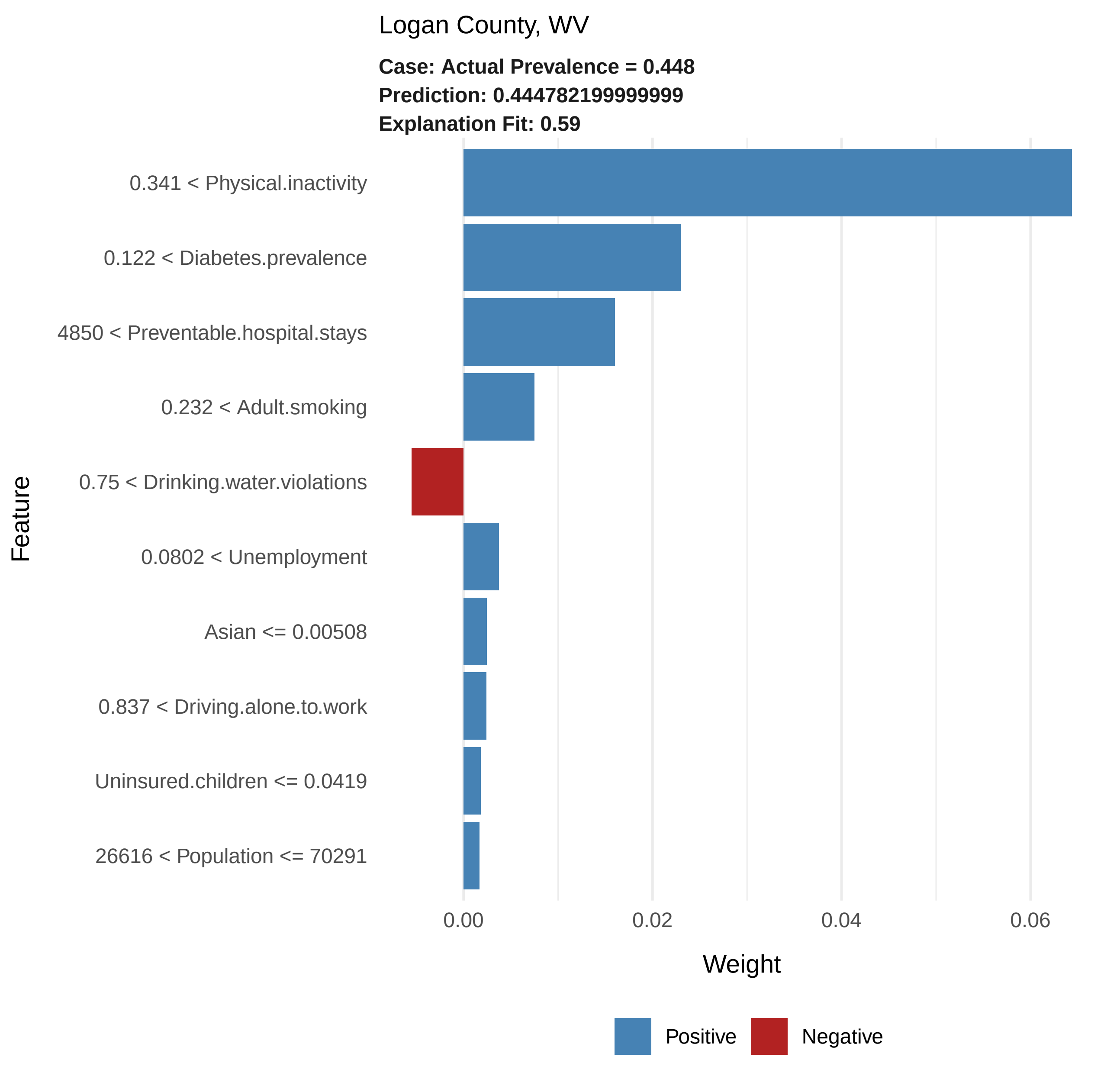

Logan County, WV
Case: Actual Prevalence = 0.448
Prediction: 0.447821999999999
Explanation Fit: 0.59
0.341 < Physical.inactivity
0.122 < Diabetes.prevalence
4850 < Preventable.hospital.stays
0.232 < Adult.smoking
0.75 < Drinking.water.violations
0.0802 < Unemployment
Asian <= 0.00508
0.837 < Driving.alone.to.work
Uninsured.children <= 0.0419
26616 < Population <= 70291
Feature
0.00
0.02
0.04
0.06
Weight
Positive
Negative

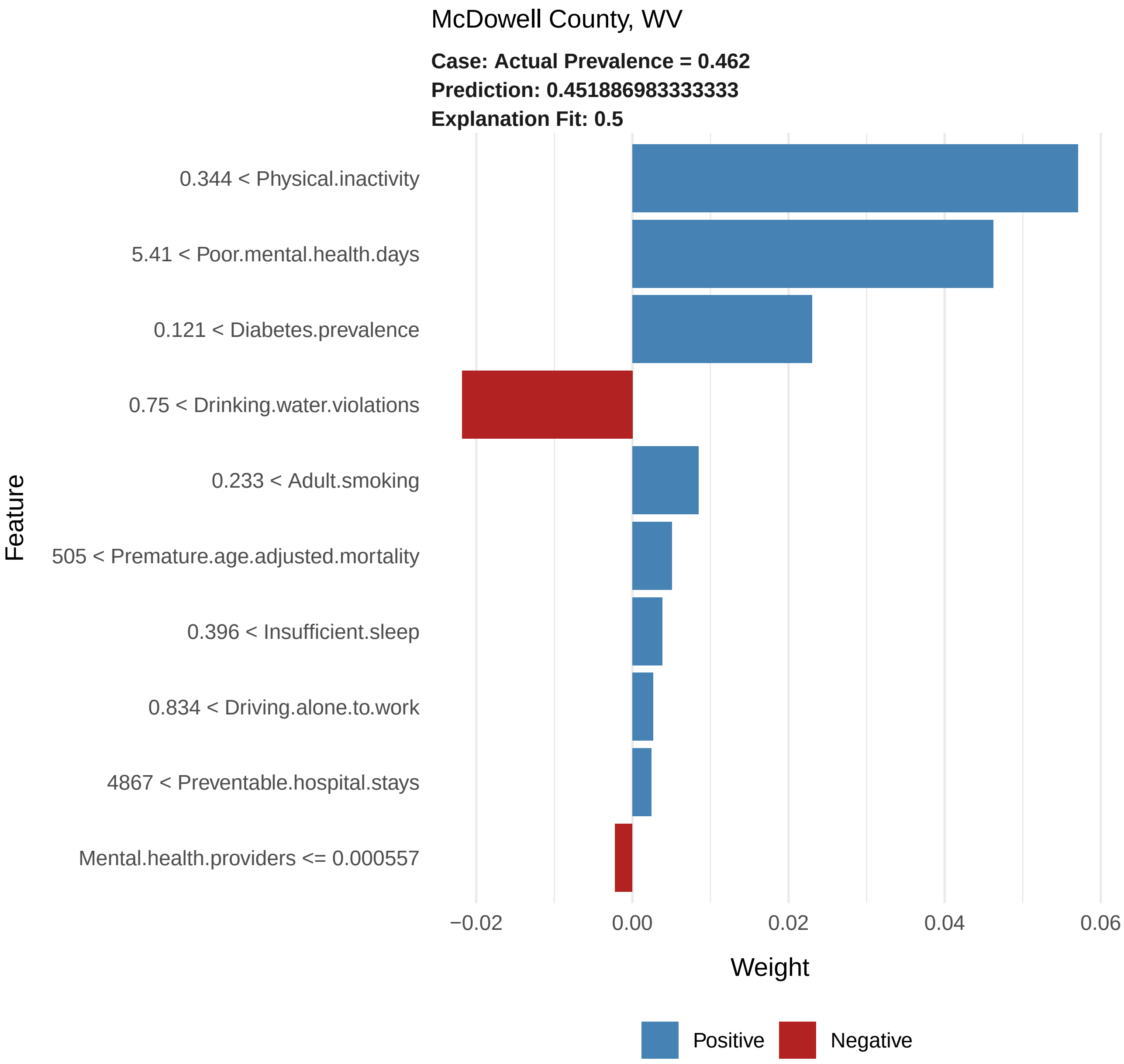
McDowell County, WV
Case: Actual Prevalence = 0.462
Prediction: 0.451886983333333
Explanation Fit: 0.5
0.344 < Physical.inactivity
5.41 < Poor.mental.health.days
0.121 < Diabetes.prevalence
0.75 < Drinking.water.violations
0.233 < Adult.smoking
505 < Premature.age.adjusted.mortality
0.396 < Insufficient.sleep
0.834 < Driving.alone.to.work
4867 < Preventable.hospital.stays
Mental.health.providers <= 0.000557
Feature
−0.02
0.00
0.02
0.04
0.06
Weight
Positive
Negative

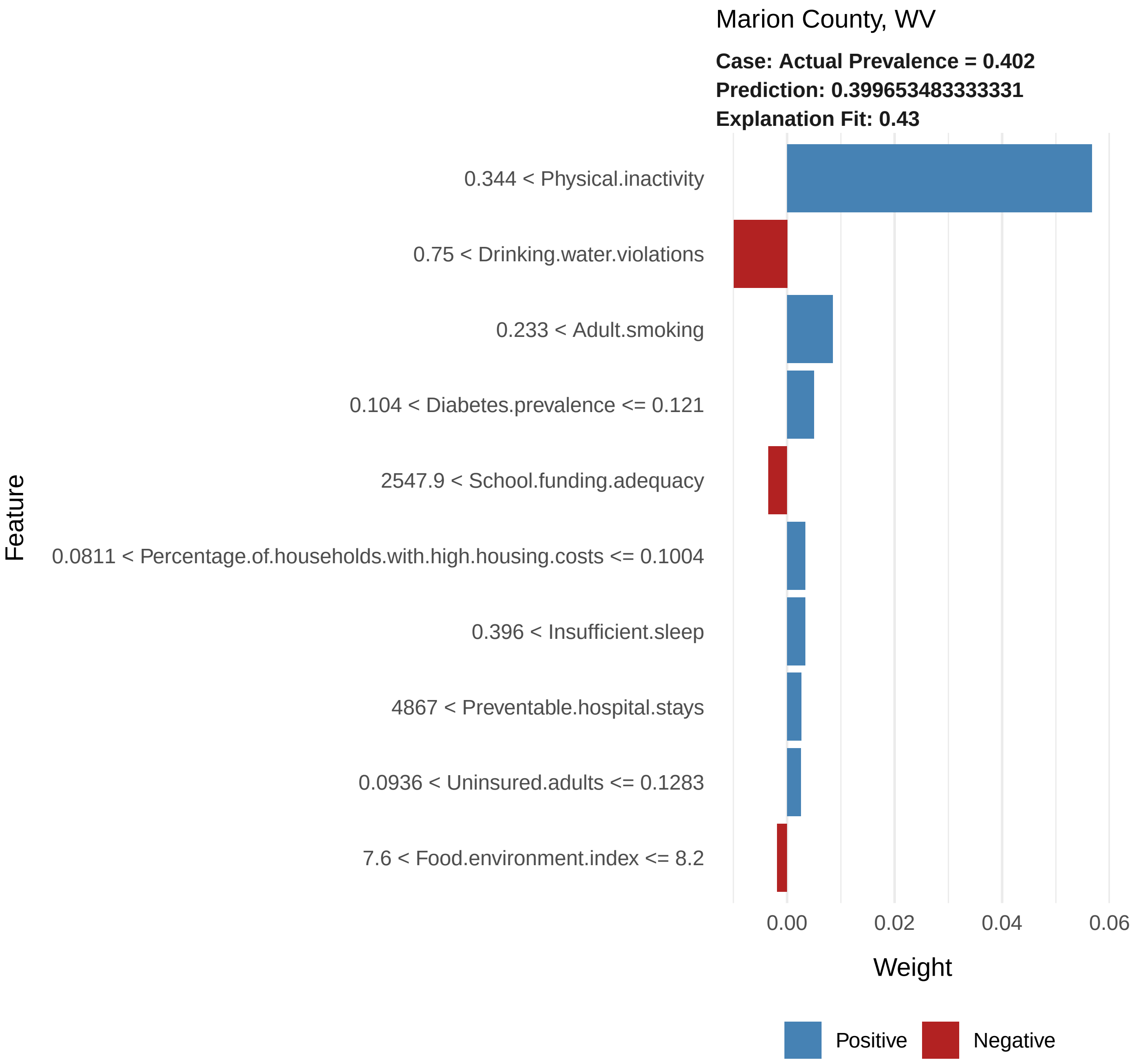
Marion County, WV
Case: Actual Prevalence = 0.402
Prediction: 0.399653483333331
Explanation Fit: 0.43
0.344 < Physical.inactivity
0.75 < Drinking.water.violations
0.233 < Adult.smoking
0.104 < Diabetes.prevalence <= 0.121
2547.9 < School.funding.adequacy
0.0811 < Percentage.of.households.with.high.housing.costs <= 0.1004
0.396 < Insufficient.sleep
4867 < Preventable.hospital.stays
0.0936 < Uninsured.adults <= 0.1283
7.6 < Food.environment.index <= 8.2
Feature
0.00
0.02
0.04
0.06
Weight
Positive
Negative

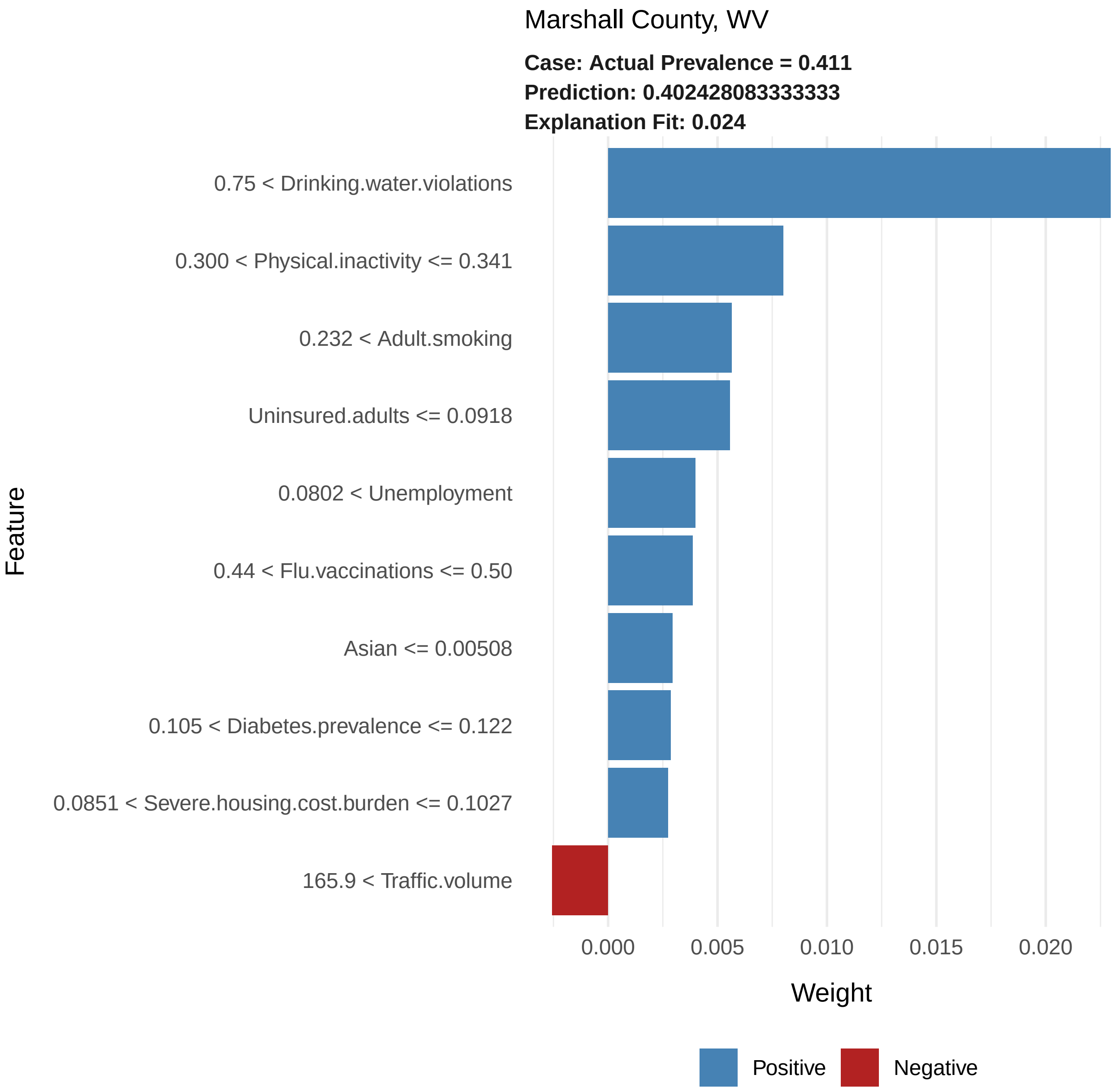

Marshall County, WV
Case: Actual Prevalence = 0.411
Prediction: 0.402428083333333
Explanation Fit: 0.024
0.75 < Drinking.water.violations
0.300 < Physical.inactivity <= 0.341
0.232 < Adult.smoking
Uninsured.adults <= 0.0918
0.0802 < Unemployment
0.44 < Flu.vaccinations <= 0.50
Asian <= 0.00508
0.105 < Diabetes.prevalence <= 0.122
0.0851 < Severe.housing.cost.burden <= 0.1027
165.9 < Traffic.volume
Feature
0.000
0.005
0.010
0.015
0.020
Weight
Positive
Negative

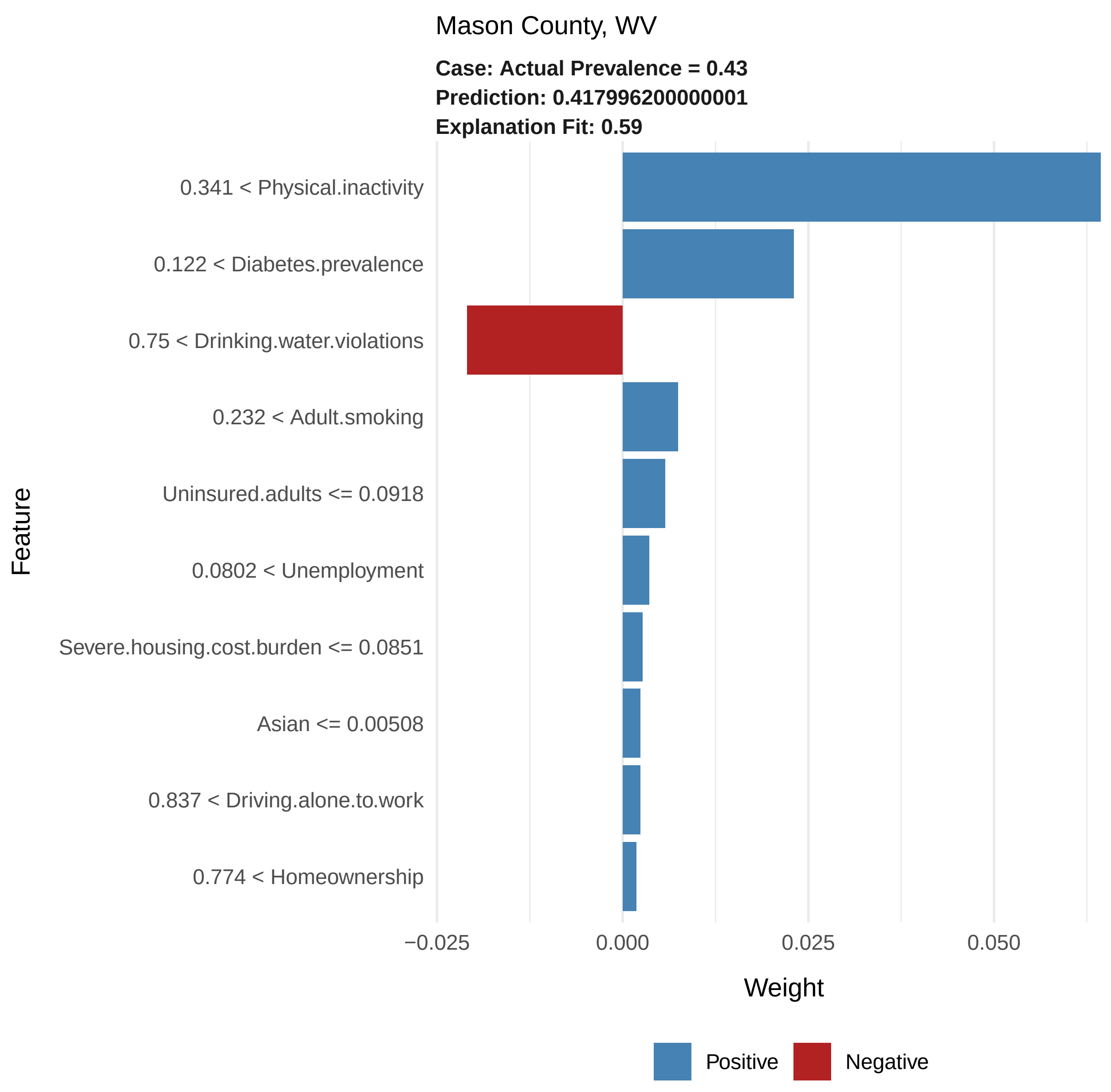

Mason County, WV
Case: Actual Prevalence = 0.43
Prediction: 0.4179962000000001
Explanation Fit: 0.59
0.341 < Physical.inactivity
0.122 < Diabetes.prevalence
0.75 < Drinking.water.violations
0.232 < Adult.smoking
Uninsured.adults <= 0.0918
0.0802 < Unemployment
Severe.housing.cost.burden <= 0.0851
Asian <= 0.00508
0.837 < Driving.alone.to.work
0.774 < Homeownership
Feature
−0.025
0.000
0.025
0.050
Weight
Positive
Negative

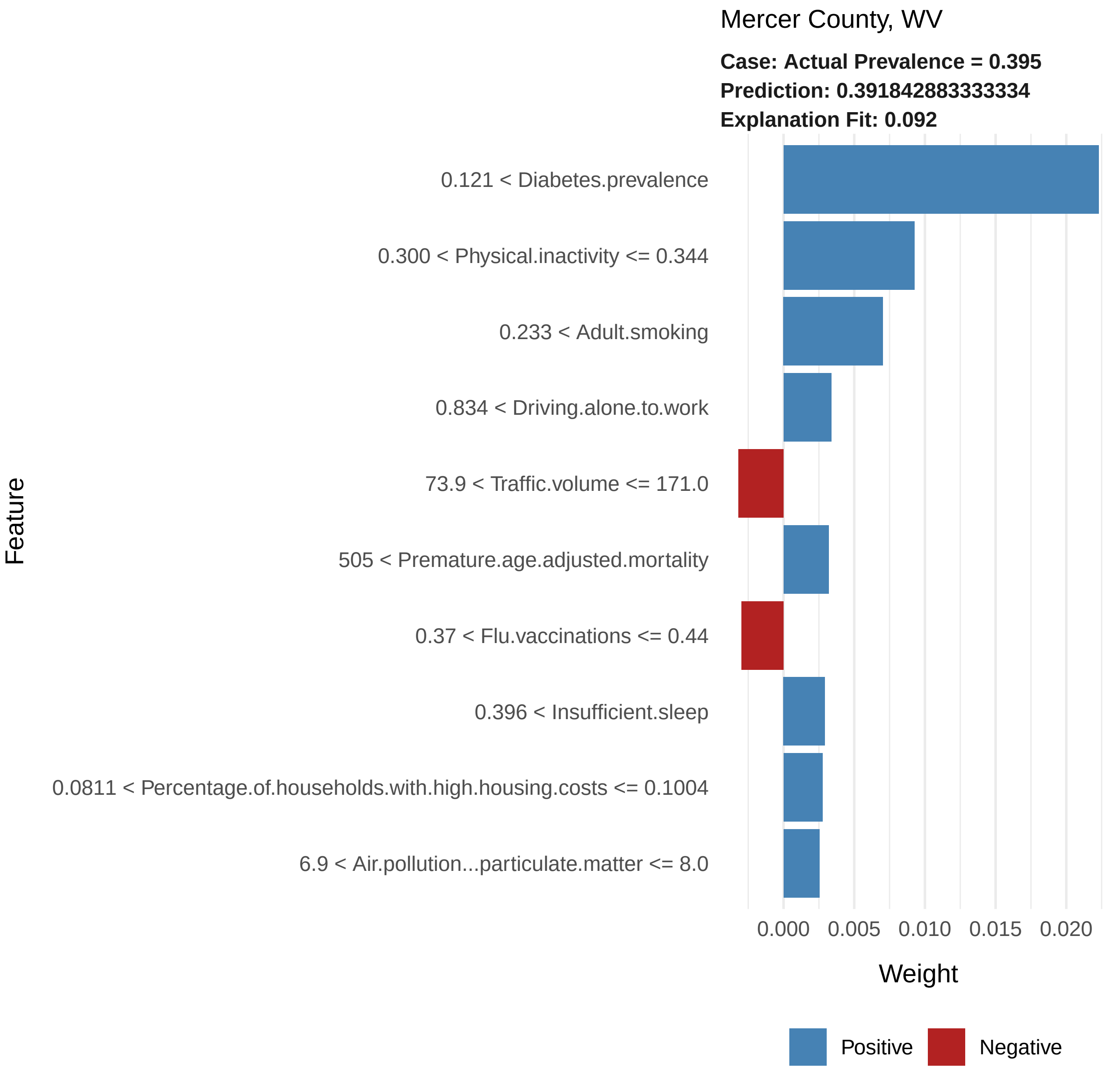

Mercer County, WV
Case: Actual Prevalence = 0.395
Prediction: 0.391842883333334
Explanation Fit: 0.092
0.121 < Diabetes.prevalence
0.300 < Physical.inactivity <= 0.344
0.233 < Adult.smoking
0.834 < Driving.alone.to.work
73.9 < Traffic.volume <= 171.0
505 < Premature.age.adjusted.mortality
0.37 < Flu.vaccinations <= 0.44
0.396 < Insufficient.sleep
0.0811 < Percentage.of.households.with.high.housing.costs <= 0.1004
6.9 < Air.pollution...particulate.matter <= 8.0
Feature
0.000
0.005
0.010
0.015
0.020
Weight
Positive
Negative

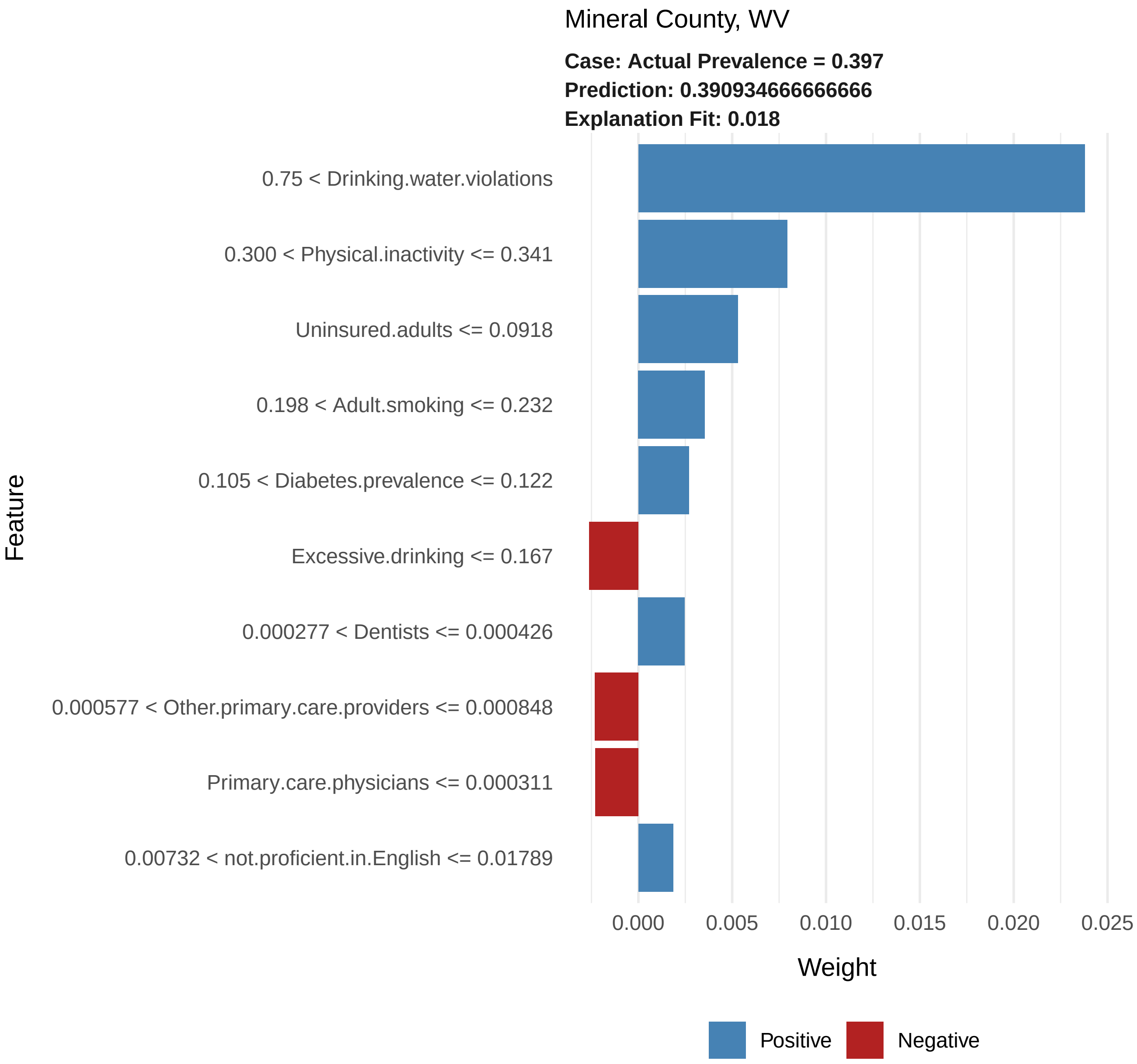

Mineral County, WV
Case: Actual Prevalence = 0.397
Prediction: 0.390934666666666
Explanation Fit: 0.018
0.75 < Drinking.water.violations
0.300 < Physical.inactivity <= 0.341
Uninsured.adults <= 0.0918
0.198 < Adult.smoking <= 0.232
0.105 < Diabetes.prevalence <= 0.122
Excessive.drinking <= 0.167
0.000277 < Dentists <= 0.000426
0.000577 < Other.primary.care.providers <= 0.000848
Primary.care.physicians <= 0.000311
0.00732 < not.proficient.in.English <= 0.01789
Feature
0.000
0.005
0.010
0.015
0.020
0.025
Weight
Positive
Negative

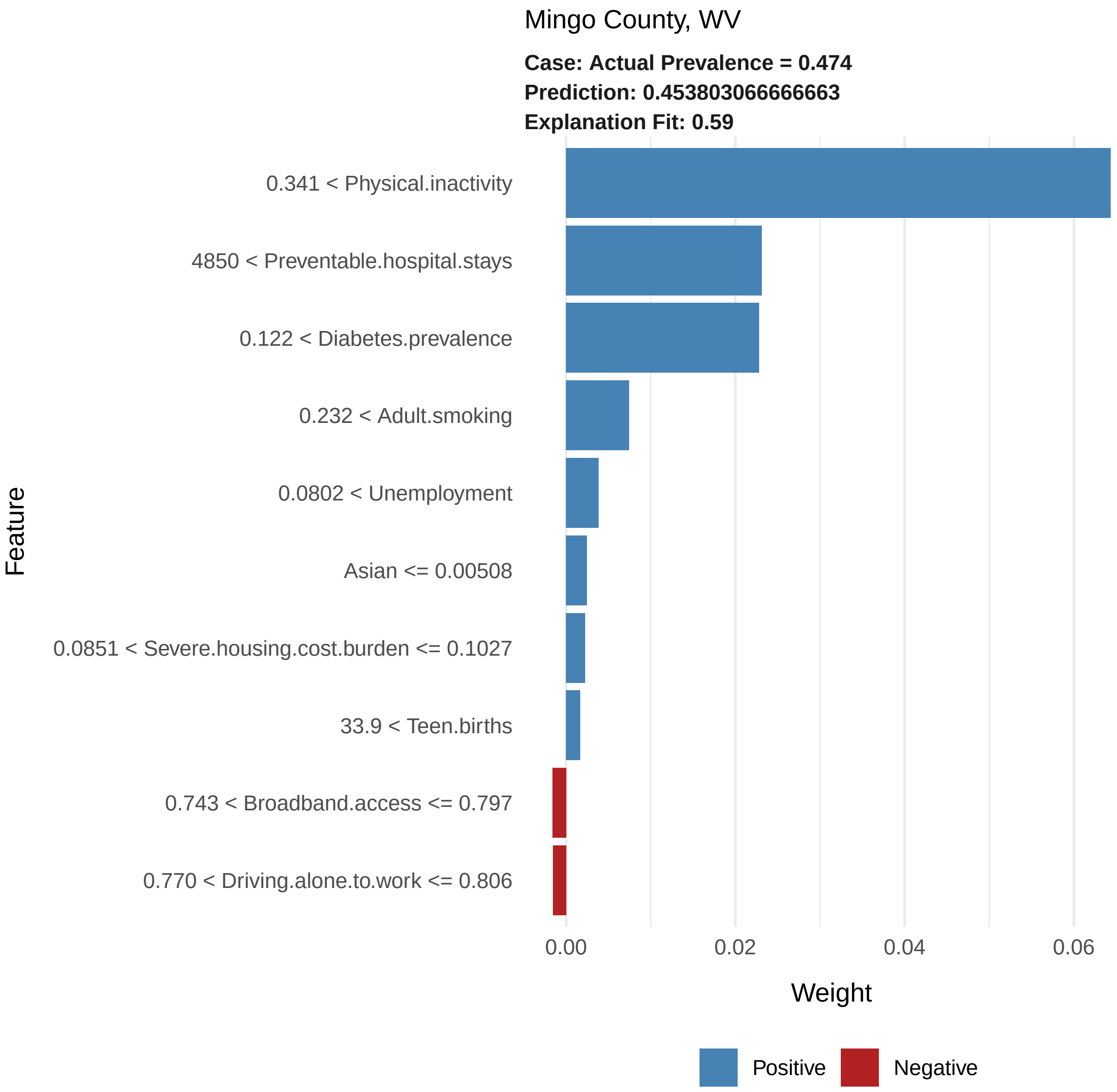
Mingo County, WV
Case: Actual Prevalence = 0.474
Prediction: 0.453803066666663
Explanation Fit: 0.59
0.341 < Physical.inactivity
4850 < Preventable.hospital.stays
0.122 < Diabetes.prevalence
0.232 < Adult.smoking
0.0802 < Unemployment
Asian <= 0.00508
0.0851 < Severe.housing.cost.burden <= 0.1027
33.9 < Teen.births
0.743 < Broadband.access <= 0.797
0.770 < Driving.alone.to.work <= 0.806
Feature
0.00
0.02
0.04
0.06
Weight
Positive
Negative

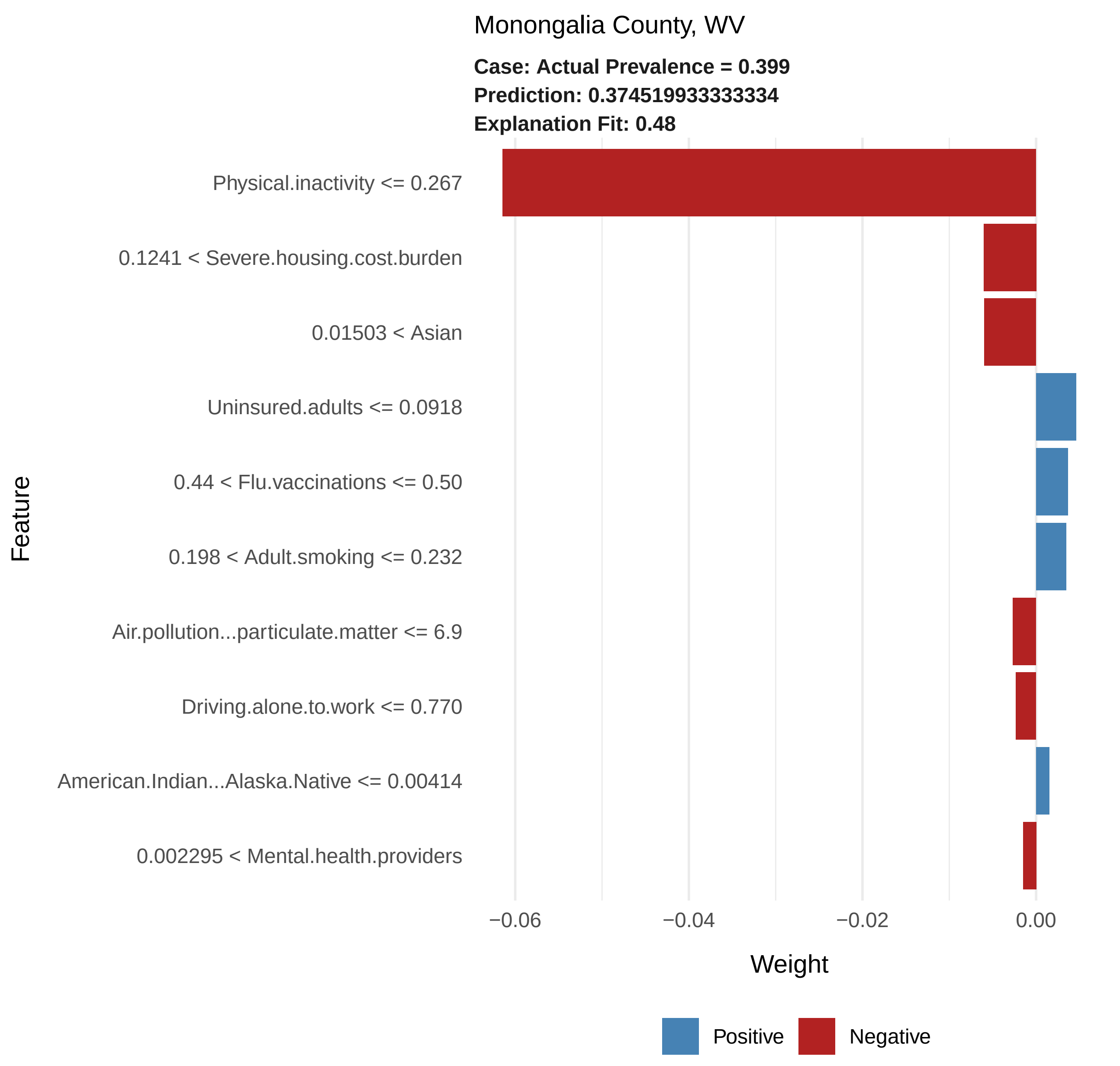
Monongalia County, WV
Case: Actual Prevalence = 0.399
Prediction: 0.374519933333334
Explanation Fit: 0.48
Physical.inactivity <= 0.267
0.1241 < Severe.housing.cost.burden
0.01503 < Asian
Uninsured.adults <= 0.0918
0.44 < Flu.vaccinations <= 0.50
0.198 < Adult.smoking <= 0.232
Air.pollution...particulate.matter <= 6.9
Driving.alone.to.work <= 0.770
American.Indian...Alaska.Native <= 0.00414
0.002295 < Mental.health.providers
Feature
−0.06
−0.04
−0.02
0.00
Weight
Positive
Negative

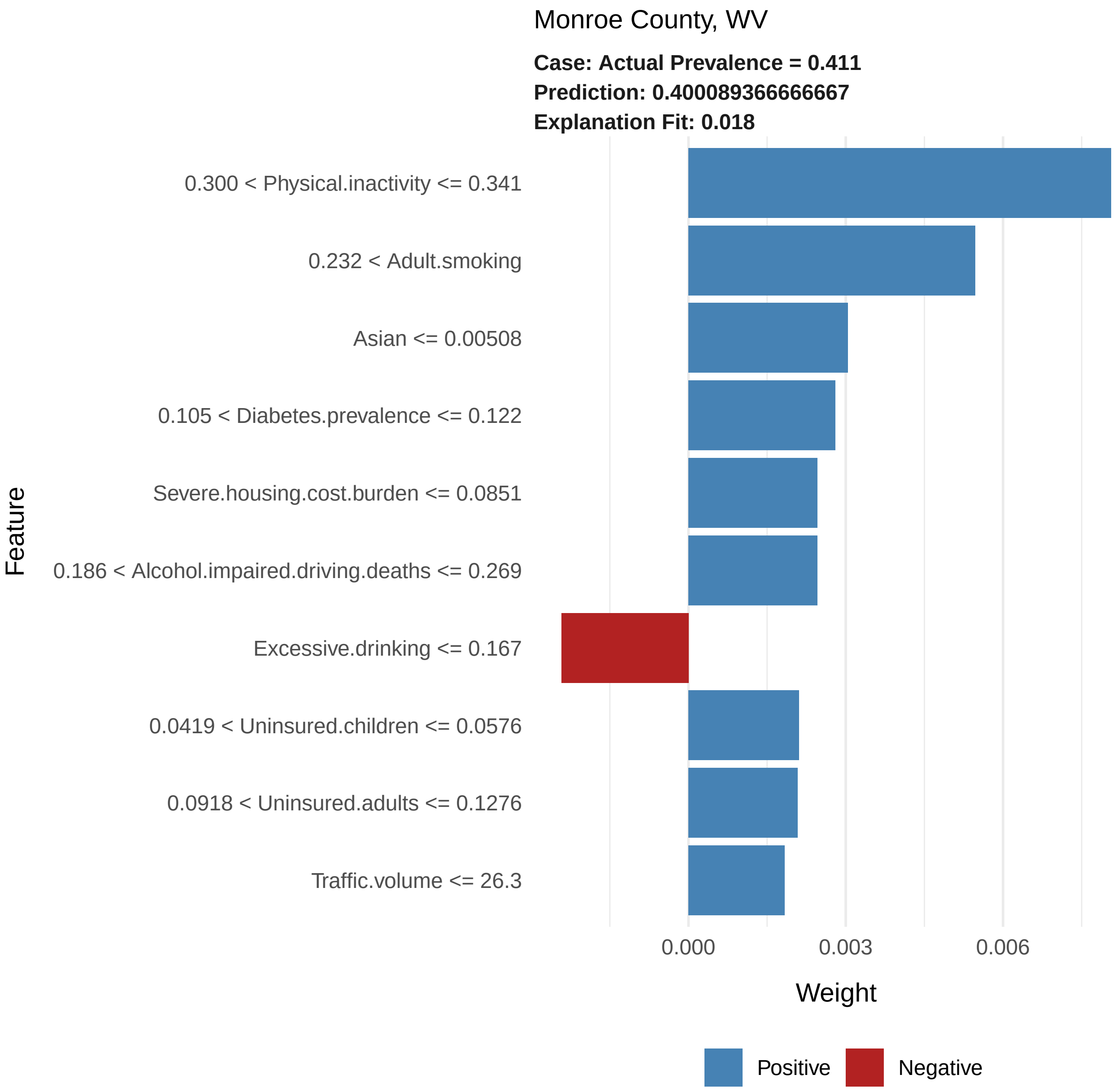

Monroe County, WV
Case: Actual Prevalence = 0.411
Prediction: 0.400089366666667
Explanation Fit: 0.018
0.300 < Physical.inactivity <= 0.341
0.232 < Adult.smoking
Asian <= 0.00508
0.105 < Diabetes.prevalence <= 0.122
Severe.housing.cost.burden <= 0.0851
0.186 < Alcohol.impaired.driving.deaths <= 0.269
Excessive.drinking <= 0.167
0.0419 < Uninsured.children <= 0.0576
0.0918 < Uninsured.adults <= 0.1276
Traffic.volume <= 26.3
Feature
0.000
0.003
0.006
Weight
Positive
Negative

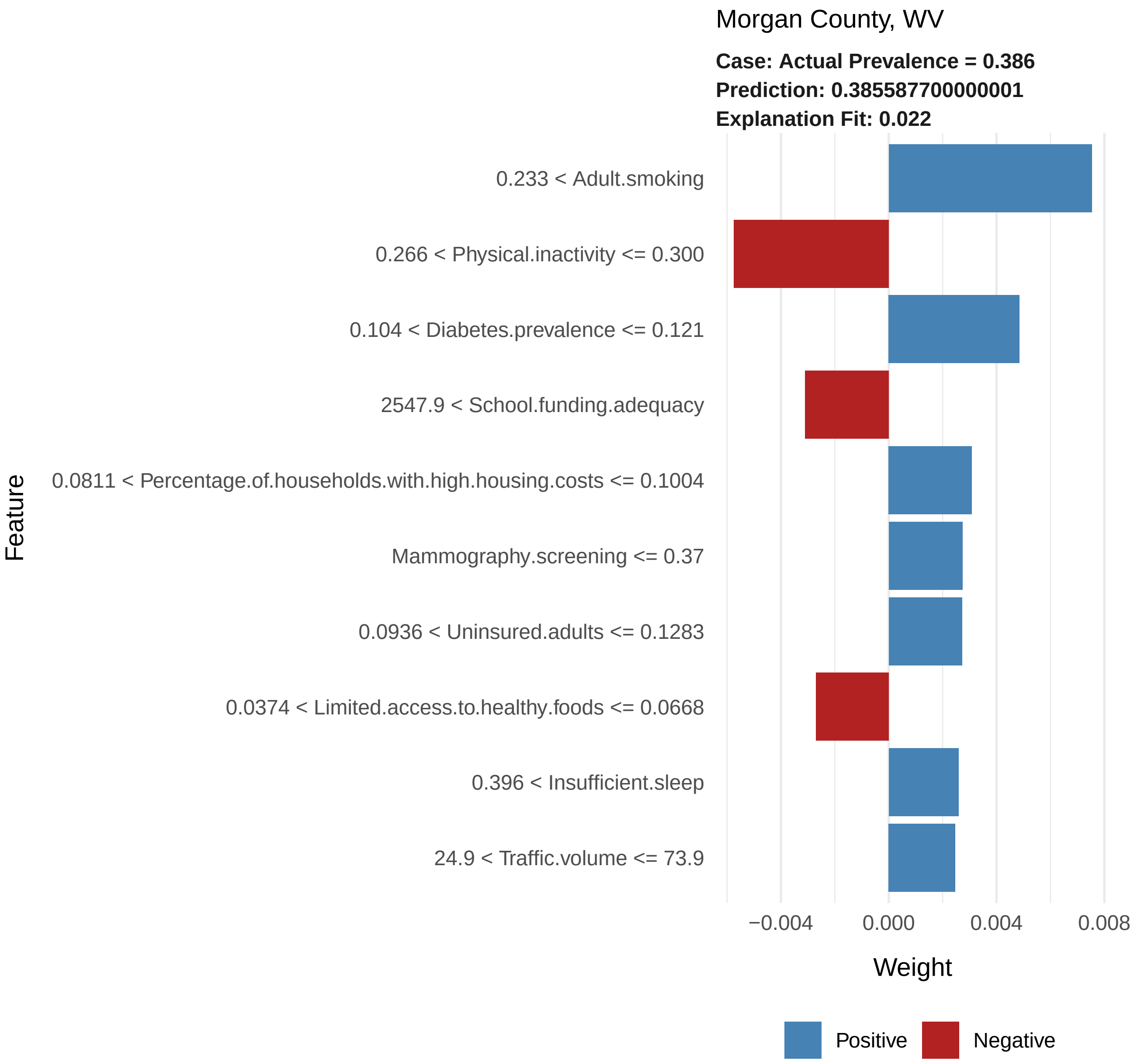

Morgan County, WV
Case: Actual Prevalence = 0.386
Prediction: 0.385587700000001
Explanation Fit: 0.022
0.233 < Adult.smoking
0.266 < Physical.inactivity <= 0.300
0.104 < Diabetes.prevalence <= 0.121
2547.9 < School.funding.adequacy
0.0811 < Percentage.of.households.with.high.housing.costs <= 0.1004
Mammography.screening <= 0.37
0.0936 < Uninsured.adults <= 0.1283
0.0374 < Limited.access.to.healthy.foods <= 0.0668
0.396 < Insufficient.sleep
24.9 < Traffic.volume <= 73.9
Feature
−0.004
0.000
0.004
0.008
Weight
Positive
Negative

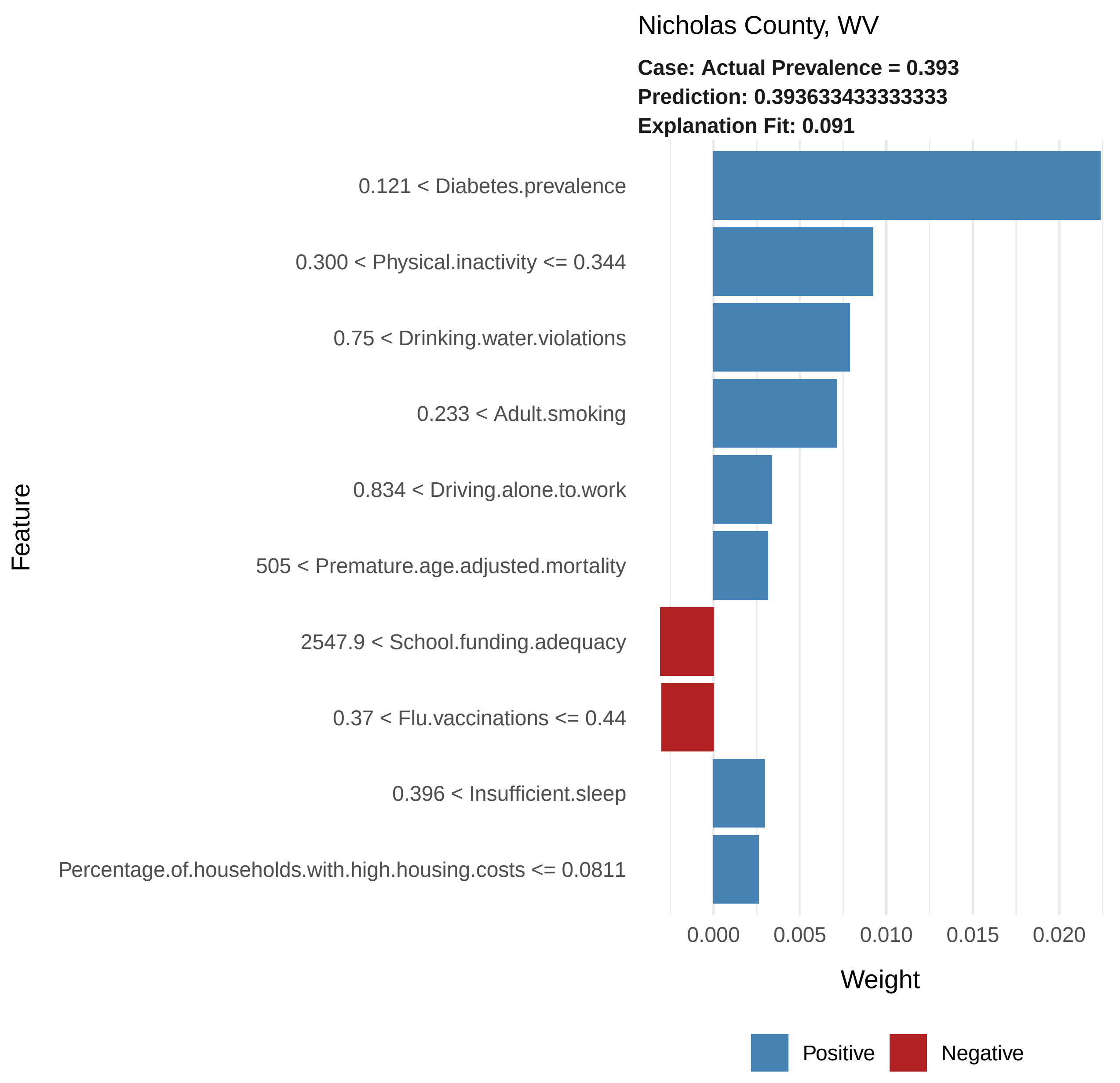
Nicholas County, WV
Case: Actual Prevalence = 0.393
Prediction: 0.393633433333333
Explanation Fit: 0.091
0.121 < Diabetes.prevalence
0.300 < Physical.inactivity <= 0.344
0.75 < Drinking.water.violations
0.233 < Adult.smoking
0.834 < Driving.alone.to.work
505 < Premature.age.adjusted.mortality
2547.9 < School.funding.adequacy
0.37 < Flu.vaccinations <= 0.44
0.396 < Insufficient.sleep
Percentage.of.households.with.high.housing.costs <= 0.0811
Feature
0.000
0.005
0.010
0.015
0.020
Weight
Positive
Negative

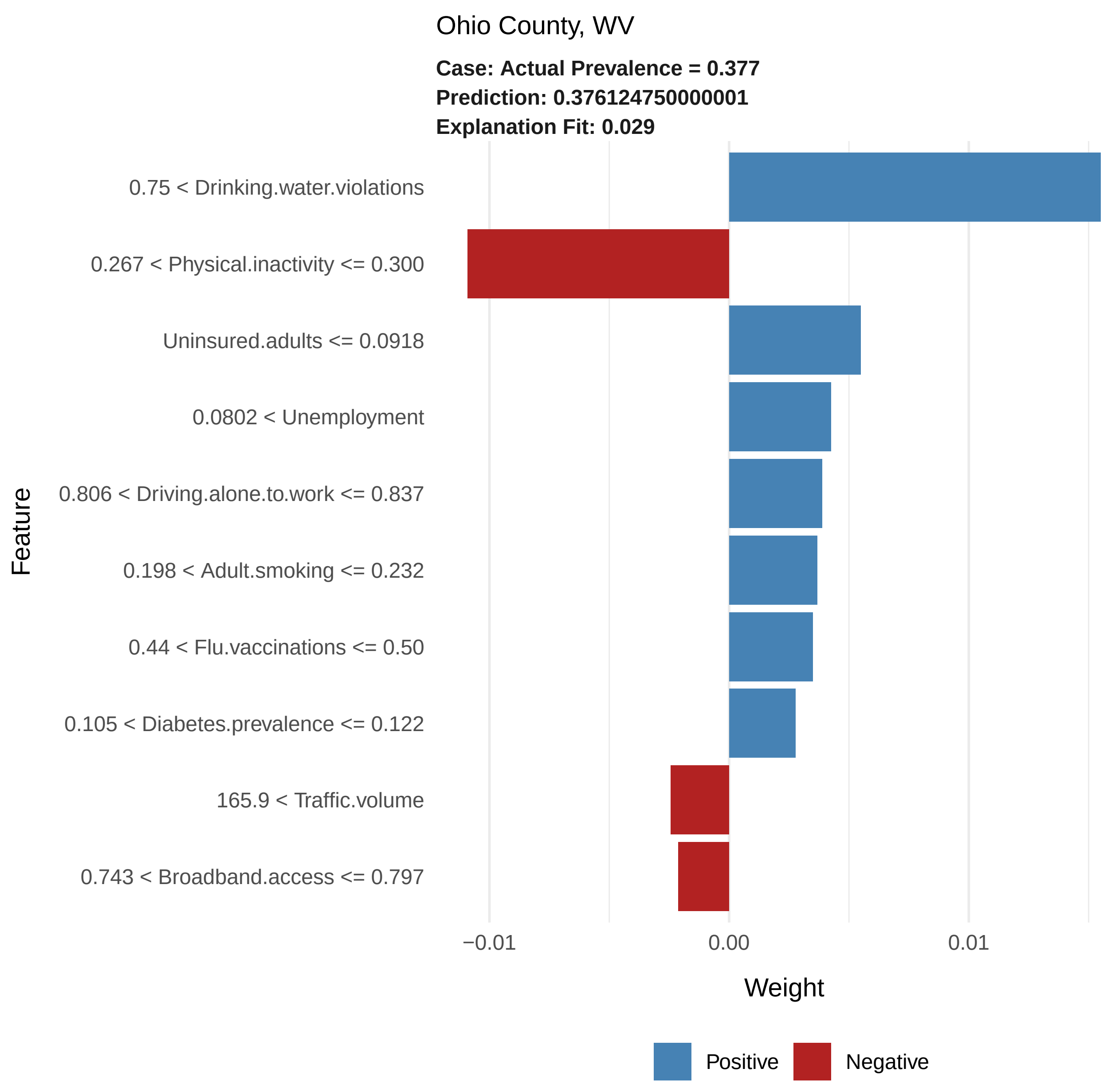
Ohio County, WV
Case: Actual Prevalence = 0.377
Prediction: 0.376124750000001
Explanation Fit: 0.029
0.75 < Drinking.water.violations
0.267 < Physical.inactivity <= 0.300
Uninsured.adults <= 0.0918
0.0802 < Unemployment
0.806 < Driving.alone.to.work <= 0.837
0.198 < Adult.smoking <= 0.232
0.44 < Flu.vaccinations <= 0.50
0.105 < Diabetes.prevalence <= 0.122
165.9 < Traffic.volume
0.743 < Broadband.access <= 0.797
Feature
−0.01
0.00
0.01
Weight
Positive
Negative

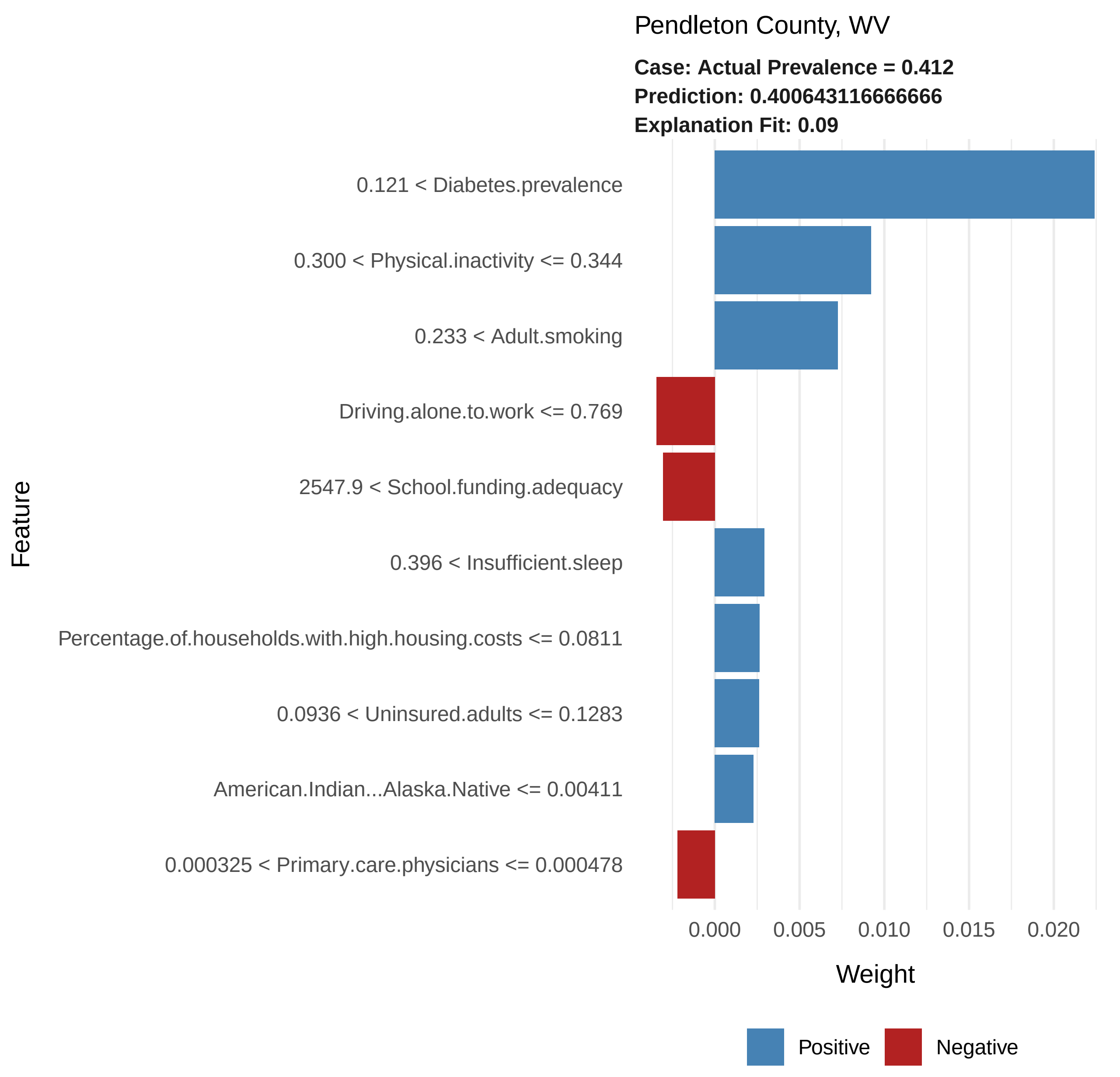
Pendleton County, WV
Case: Actual Prevalence = 0.412
Prediction: 0.400643116666666
Explanation Fit: 0.09
0.121 < Diabetes.prevalence
0.300 < Physical.inactivity <= 0.344
0.233 < Adult.smoking
Driving.alone.to.work <= 0.769
2547.9 < School.funding.adequacy
0.396 < Insufficient.sleep
Percentage.of.households.with.high.housing.costs <= 0.0811
0.0936 < Uninsured.adults <= 0.1283
American.Indian...Alaska.Native <= 0.00411
0.000325 < Primary.care.physicians <= 0.000478
Feature
0.000
0.005
0.010
0.015
0.020
Weight
Positive
Negative

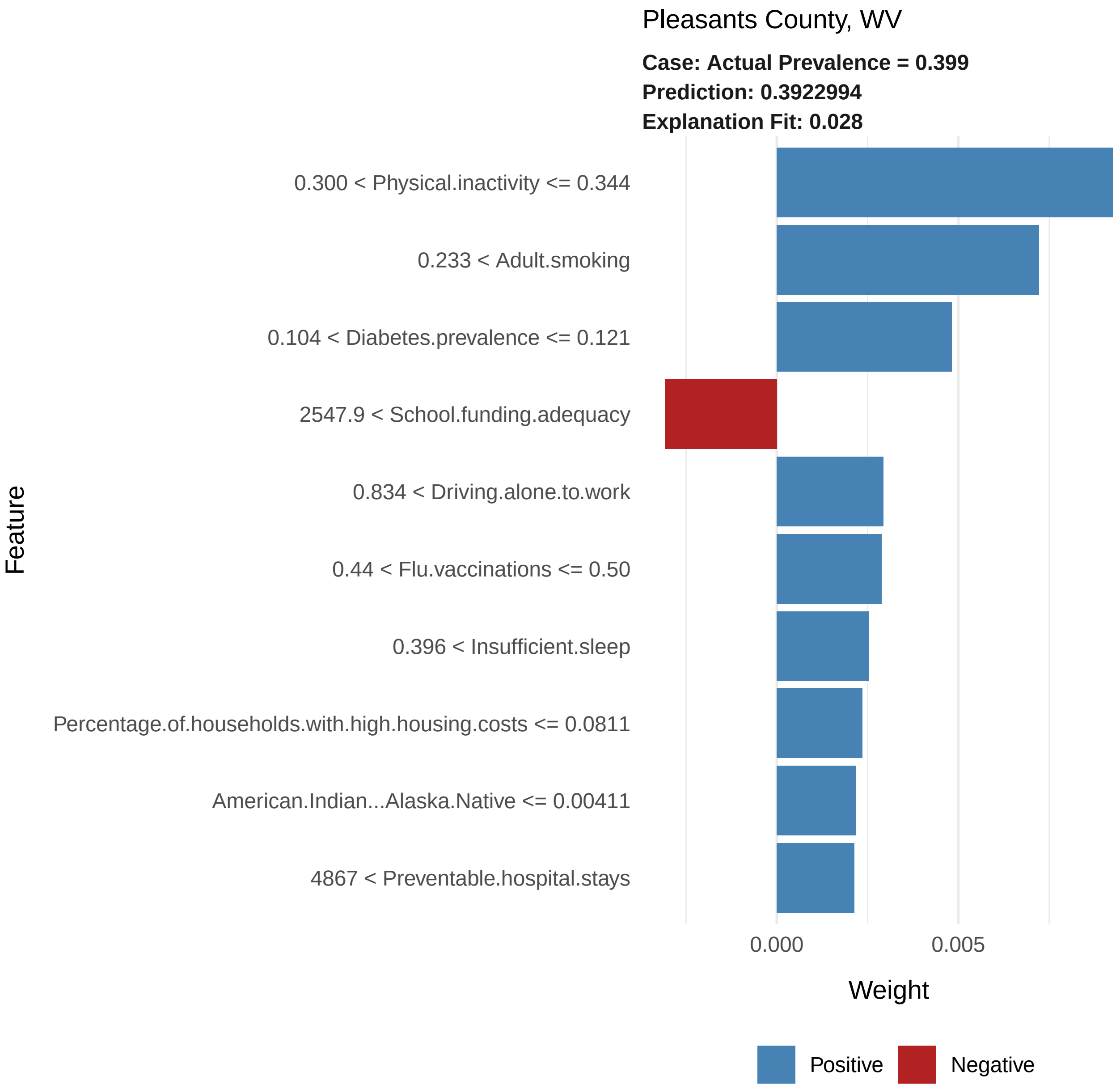
Pleasants County, WV
Case: Actual Prevalence = 0.399
Prediction: 0.3922994
Explanation Fit: 0.028
0.300 < Physical.inactivity <= 0.344
0.233 < Adult.smoking
0.104 < Diabetes.prevalence <= 0.121
2547.9 < School.funding.adequacy
0.834 < Driving.alone.to.work
0.44 < Flu.vaccinations <= 0.50
0.396 < Insufficient.sleep
Percentage.of.households.with.high.housing.costs <= 0.0811
American.Indian...Alaska.Native <= 0.00411
4867 < Preventable.hospital.stays
Feature
0.000
0.005
Weight
Positive
Negative

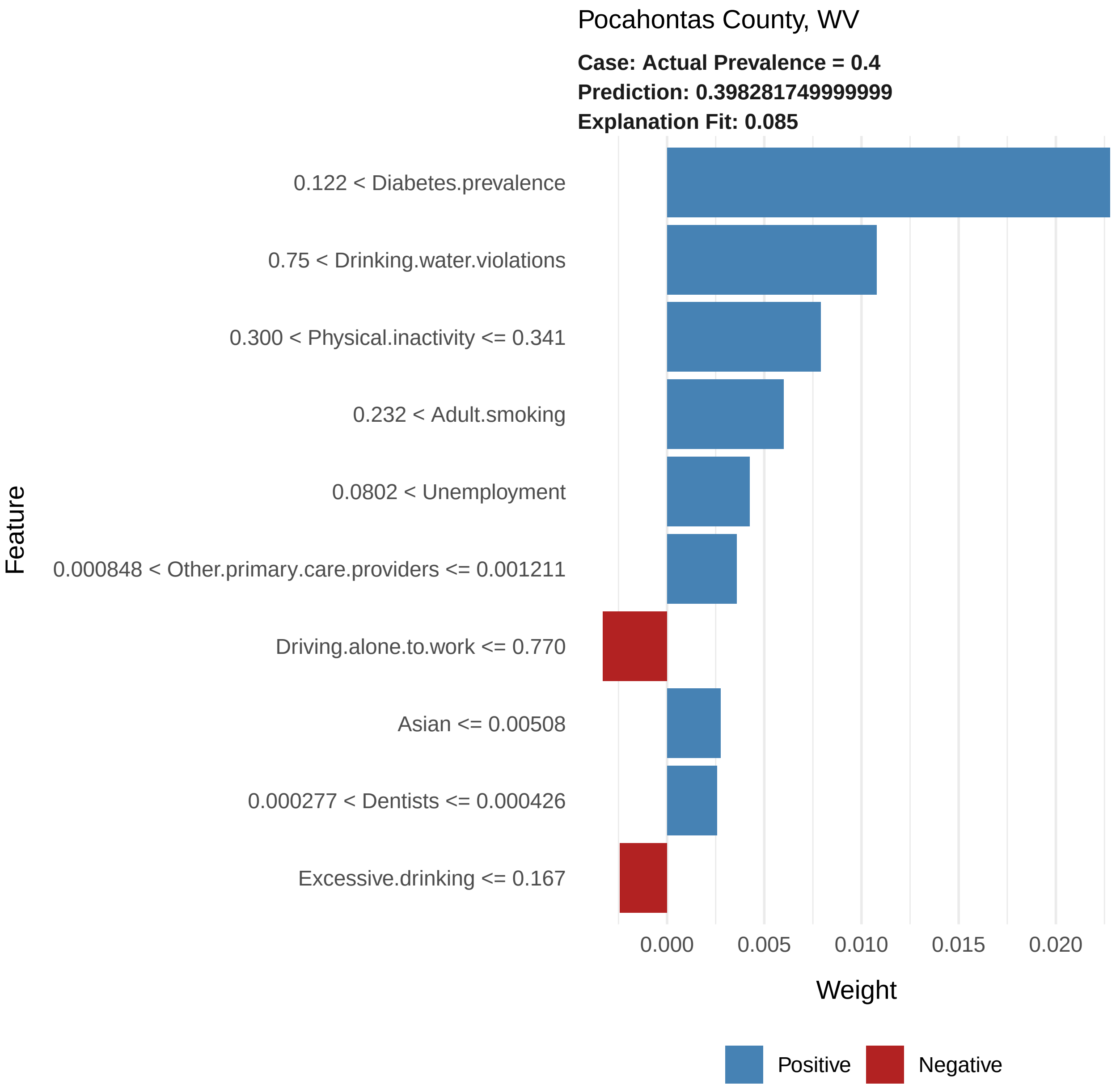

Pocahontas County, WV
Case: Actual Prevalence = 0.4
Prediction: 0.398281749999999
Explanation Fit: 0.085
0.122 < Diabetes.prevalence
0.75 < Drinking.water.violations
0.300 < Physical.inactivity <= 0.341
0.232 < Adult.smoking
0.0802 < Unemployment
0.000848 < Other.primary.care.providers <= 0.001211
Driving.alone.to.work <= 0.770
Asian <= 0.00508
0.000277 < Dentists <= 0.000426
Excessive.drinking <= 0.167
Feature
0.000
0.005
0.010
0.015
0.020
Weight
Positive
Negative

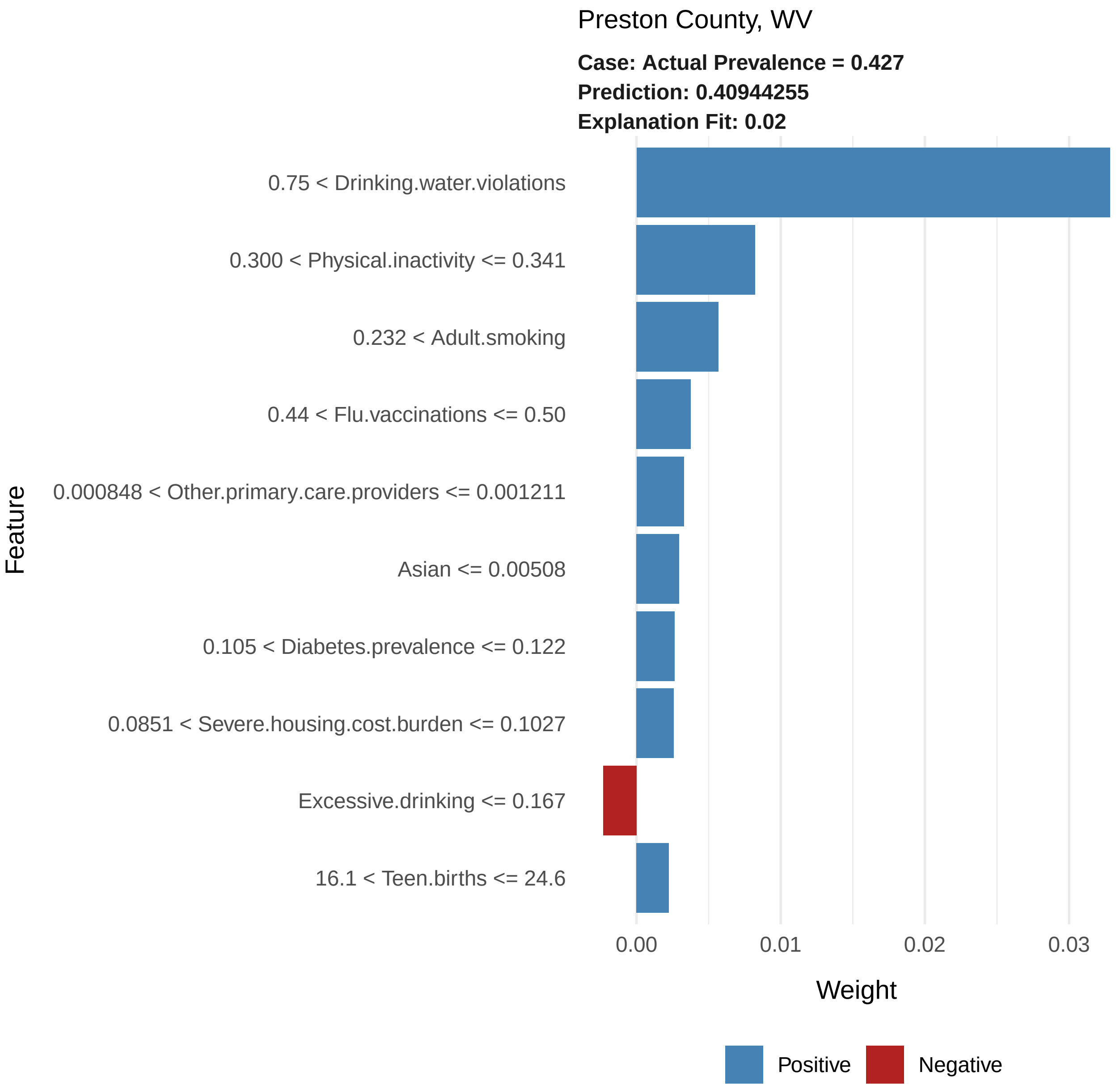
Preston County, WV
Case: Actual Prevalence = 0.427
Prediction: 0.40944255
Explanation Fit: 0.02
0.75 < Drinking.water.violations
0.300 < Physical.inactivity <= 0.341
0.232 < Adult.smoking
0.44 < Flu.vaccinations <= 0.50
0.000848 < Other.primary.care.providers <= 0.001211
Asian <= 0.00508
0.105 < Diabetes.prevalence <= 0.122
0.0851 < Severe.housing.cost.burden <= 0.1027
Excessive.drinking <= 0.167
16.1 < Teen.births <= 24.6
Feature
0.00
0.01
0.02
0.03
Weight
Positive
Negative

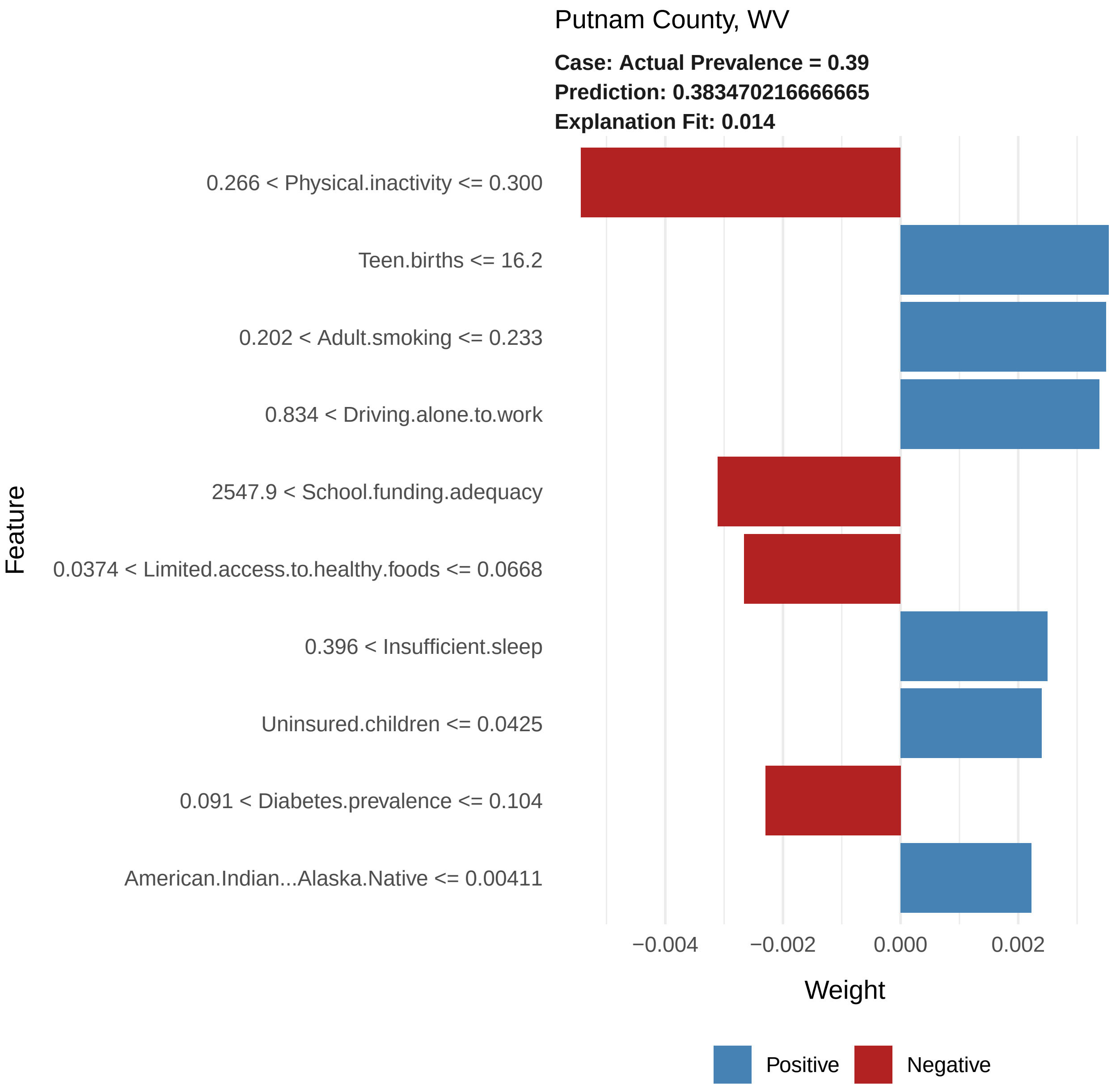
Putnam County, WV
Case: Actual Prevalence = 0.39
Prediction: 0.383470216666665
Explanation Fit: 0.014
0.266 < Physical.inactivity <= 0.300
Teen.births <= 16.2
0.202 < Adult.smoking <= 0.233
0.834 < Driving.alone.to.work
2547.9 < School.funding.adequacy
0.0374 < Limited.access.to.healthy.foods <= 0.0668
0.396 < Insufficient.sleep
Uninsured.children <= 0.0425
0.091 < Diabetes.prevalence <= 0.104
American.Indian...Alaska.Native <= 0.00411
Feature
−0.004
−0.002
0.000
0.002
Weight
Positive
Negative

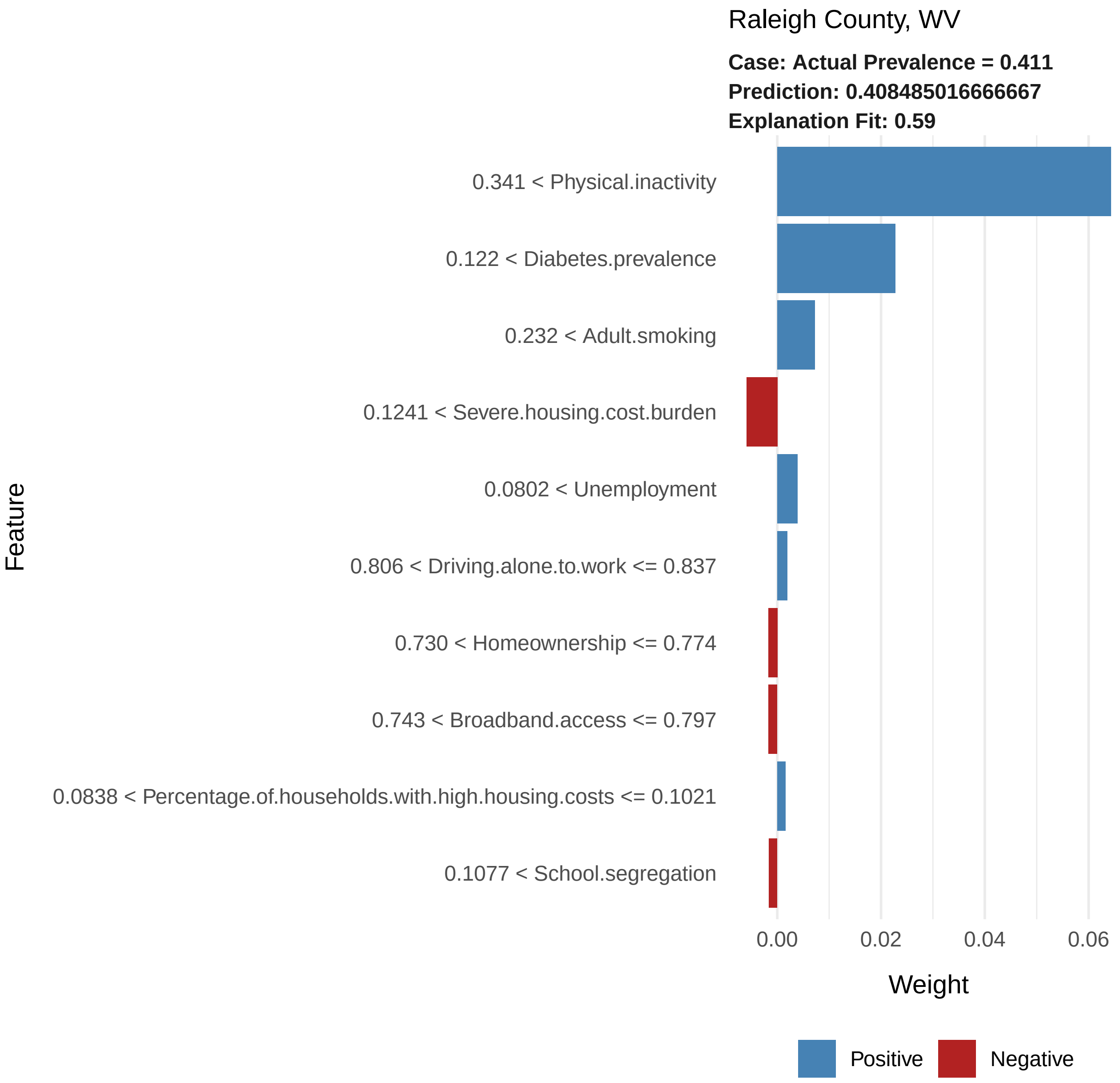

Raleigh County, WV
Case: Actual Prevalence = 0.411
Prediction: 0.408485016666667
Explanation Fit: 0.59
0.341 < Physical.inactivity
0.122 < Diabetes.prevalence
0.232 < Adult.smoking
0.1241 < Severe.housing.cost.burden
0.0802 < Unemployment
0.806 < Driving.alone.to.work <= 0.837
0.730 < Homeownership <= 0.774
0.743 < Broadband.access <= 0.797
0.0838 < Percentage.of.households.with.high.housing.costs <= 0.1021
0.1077 < School.segregation
Feature
0.00
0.02
0.04
0.06
Weight
Positive
Negative

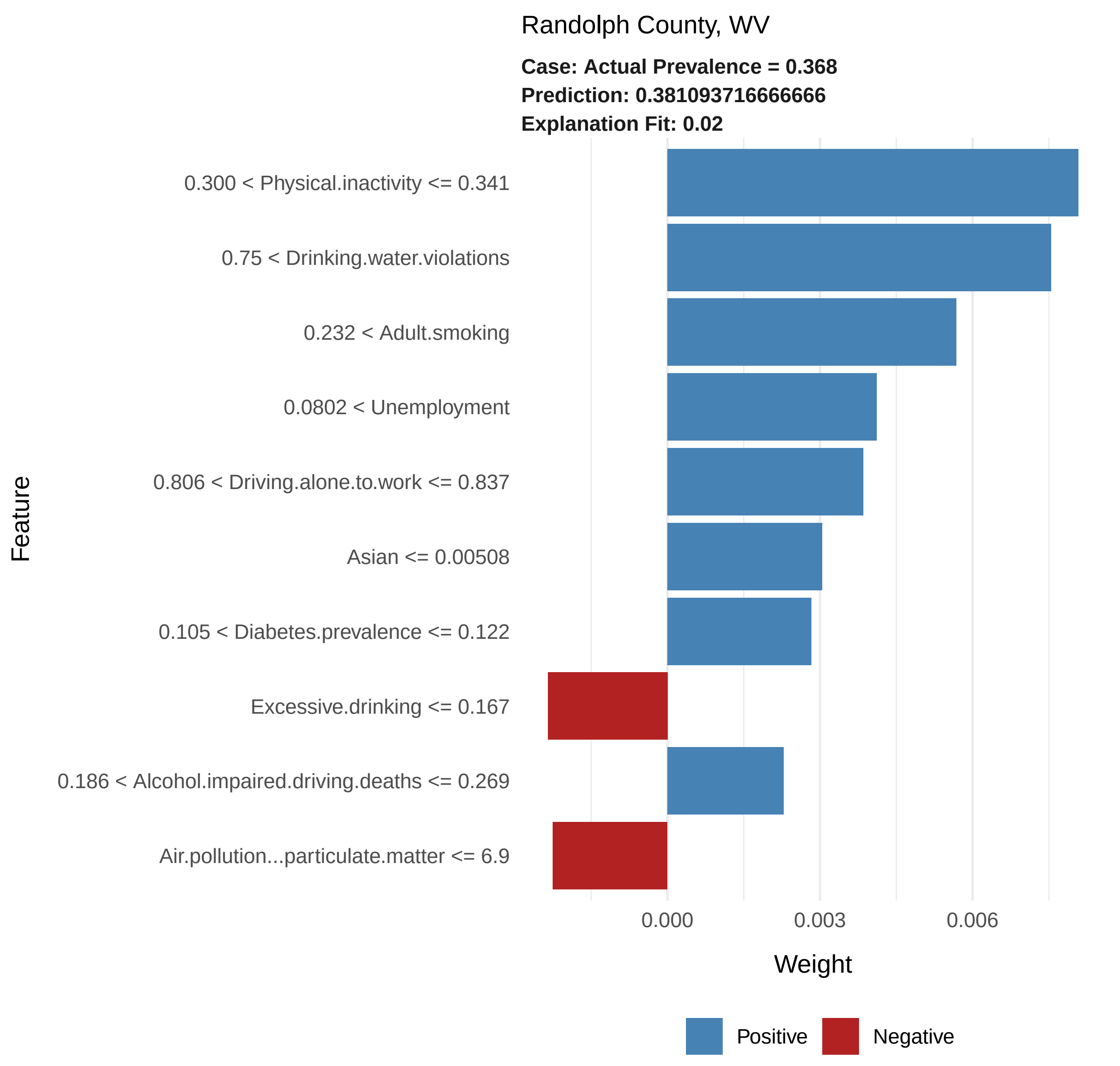

Randolph County, WV
Case: Actual Prevalence = 0.368
Prediction: 0.381093716666666
Explanation Fit: 0.02
0.300 < Physical.inactivity <= 0.341
0.75 < Drinking.water.violations
0.232 < Adult.smoking
0.0802 < Unemployment
0.806 < Driving.alone.to.work <= 0.837
Asian <= 0.00508
0.105 < Diabetes.prevalence <= 0.122
Excessive.drinking <= 0.167
0.186 < Alcohol.impaired.driving.deaths <= 0.269
Air.pollution...particulate.matter <= 6.9
Feature
0.000
0.003
0.006
Weight
Positive
Negative

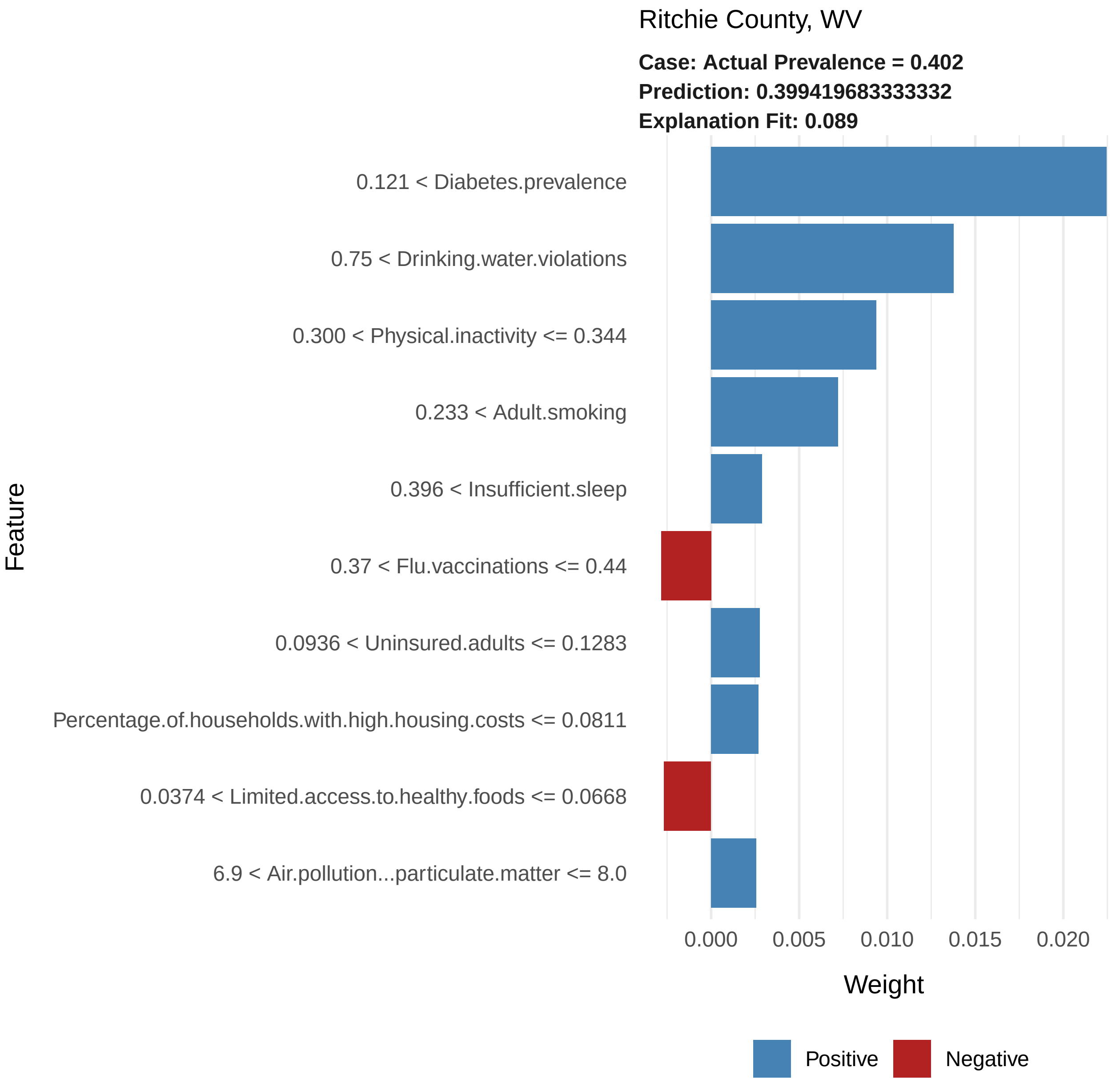

Ritchie County, WV
Case: Actual Prevalence = 0.402
Prediction: 0.399419683333332
Explanation Fit: 0.089
0.121 < Diabetes.prevalence
0.75 < Drinking.water.violations
0.300 < Physical.inactivity <= 0.344
0.233 < Adult.smoking
0.396 < Insufficient.sleep
0.37 < Flu.vaccinations <= 0.44
0.0936 < Uninsured.adults <= 0.1283
Percentage.of.households.with.high.housing.costs <= 0.0811
0.0374 < Limited.access.to.healthy.foods <= 0.0668
6.9 < Air.pollution...particulate.matter <= 8.0
Feature
0.000
0.005
0.010
0.015
0.020
Weight
Positive
Negative

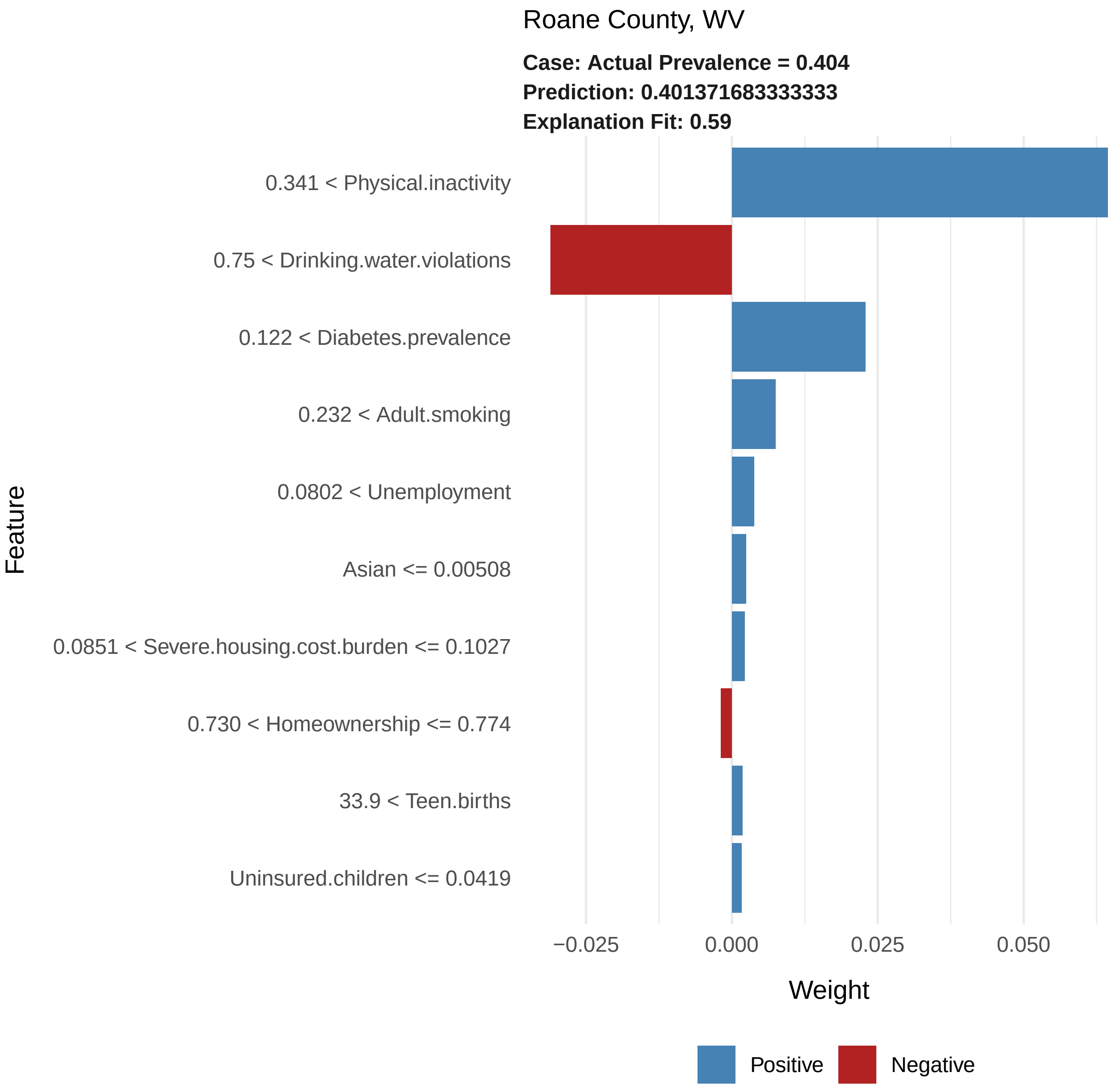

Roane County, WV
Case: Actual Prevalence = 0.404
Prediction: 0.401371683333333
Explanation Fit: 0.59
0.341 < Physical.inactivity
0.75 < Drinking.water.violations
0.122 < Diabetes.prevalence
0.232 < Adult.smoking
0.0802 < Unemployment
Asian <= 0.00508
0.0851 < Severe.housing.cost.burden <= 0.1027
0.730 < Homeownership <= 0.774
33.9 < Teen.births
Uninsured.children <= 0.0419
Feature
−0.025
0.000
0.025
0.050
Weight
Positive
Negative

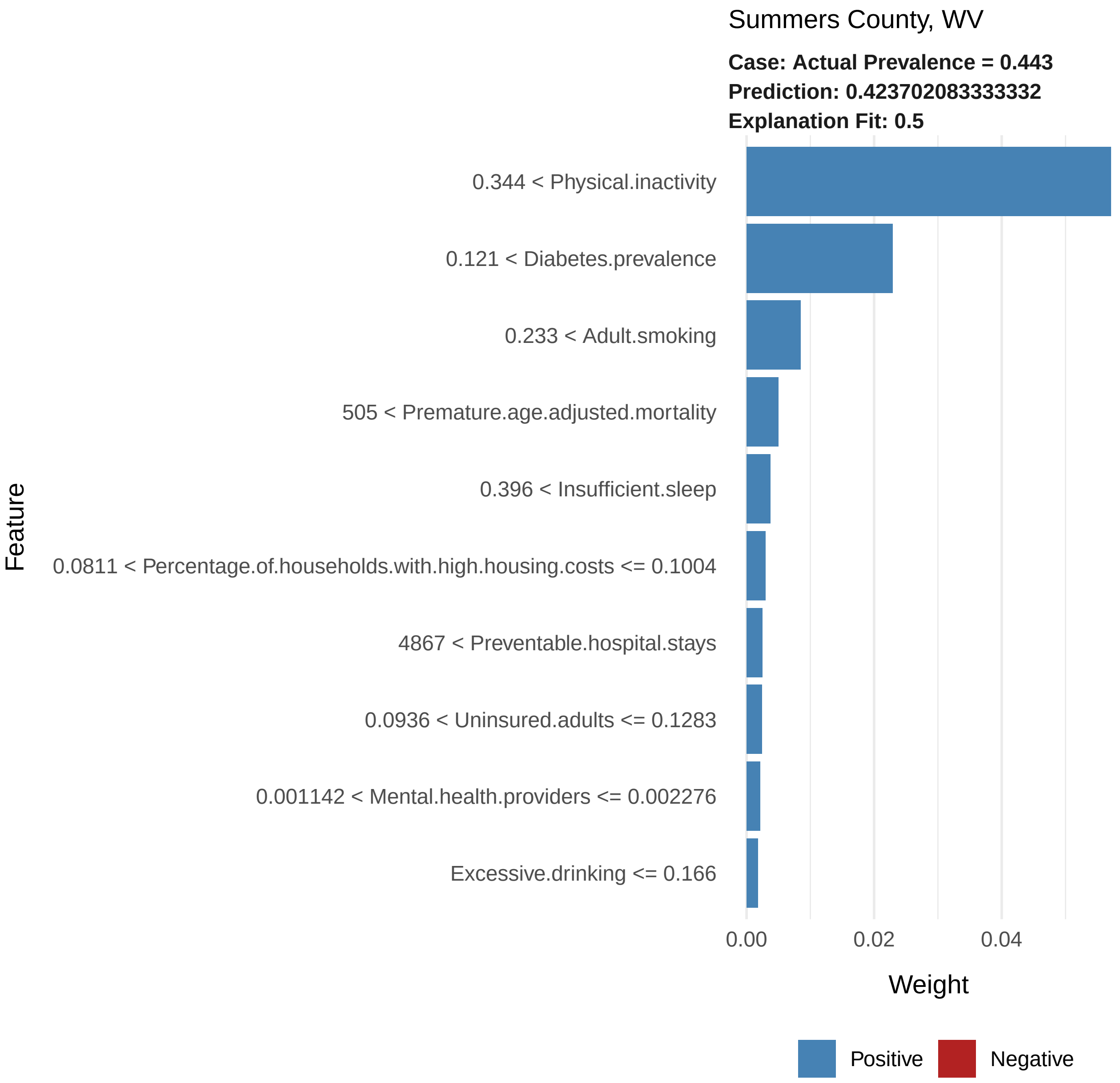
Summers County, WV
Case: Actual Prevalence = 0.443
Prediction: 0.423702083333332
Explanation Fit: 0.5
0.344 < Physical.inactivity
0.121 < Diabetes.prevalence
0.233 < Adult.smoking
505 < Premature.age.adjusted.mortality
0.396 < Insufficient.sleep
0.0811 < Percentage.of.households.with.high.housing.costs <= 0.1004
4867 < Preventable.hospital.stays
0.0936 < Uninsured.adults <= 0.1283
0.001142 < Mental.health.providers <= 0.002276
Excessive.drinking <= 0.166
Feature
0.00
0.02
0.04
Weight
Positive
Negative

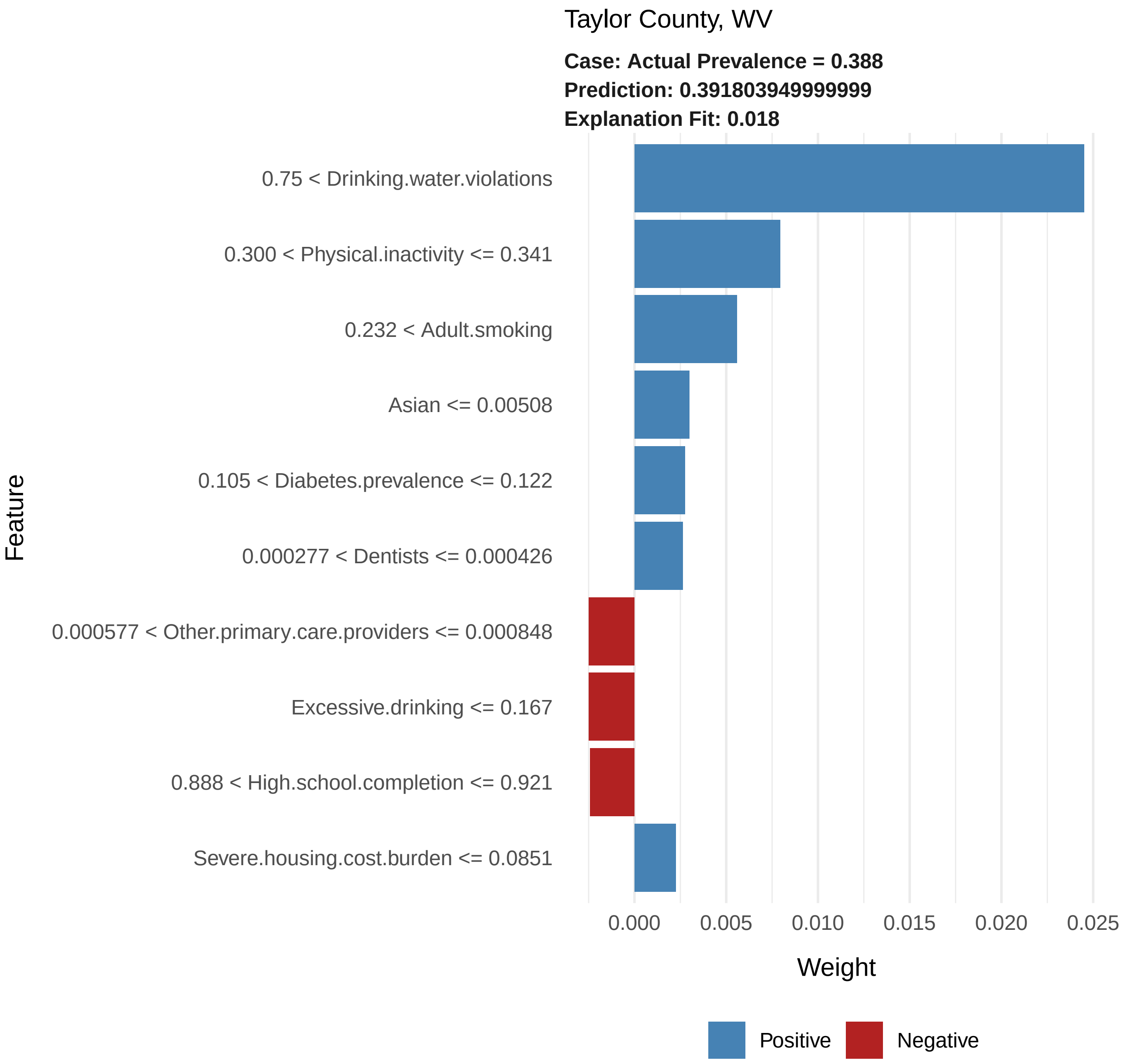

Taylor County, WV
Case: Actual Prevalence = 0.388
Prediction: 0.391803949999999
Explanation Fit: 0.018
0.75 < Drinking.water.violations
0.300 < Physical.inactivity <= 0.341
0.232 < Adult.smoking
Asian <= 0.00508
0.105 < Diabetes.prevalence <= 0.122
0.000277 < Dentists <= 0.000426
0.000577 < Other.primary.care.providers <= 0.000848
Excessive.drinking <= 0.167
0.888 < High.school.completion <= 0.921
Severe.housing.cost.burden <= 0.0851
Feature
0.000
0.005
0.010
0.015
0.020
0.025
Weight
Positive
Negative

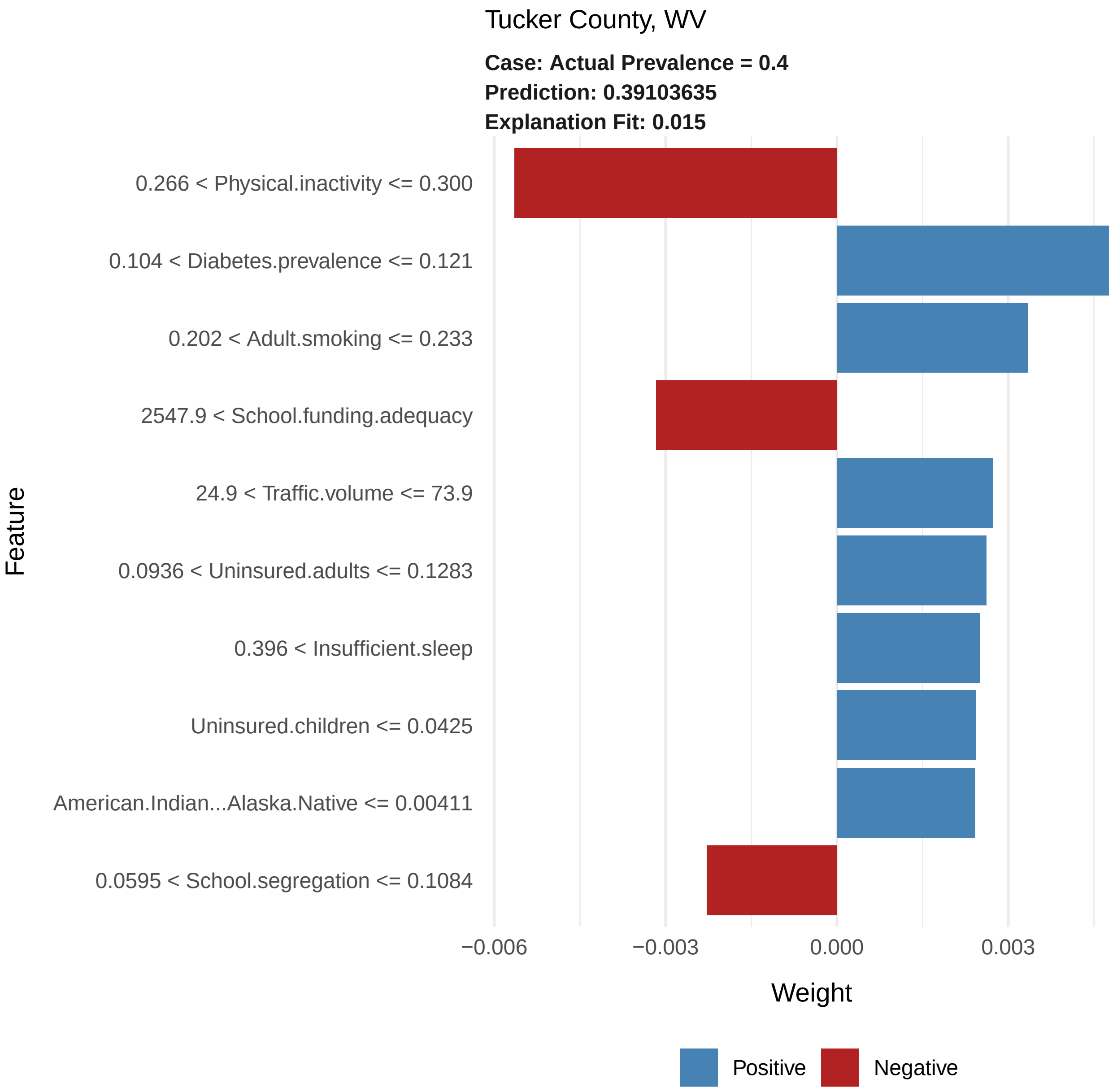
Tucker County, WV
Case: Actual Prevalence = 0.4
Prediction: 0.39103635
Explanation Fit: 0.015
0.266 < Physical.inactivity <= 0.300
0.104 < Diabetes.prevalence <= 0.121
0.202 < Adult.smoking <= 0.233
2547.9 < School.funding.adequacy
24.9 < Traffic.volume <= 73.9
0.0936 < Uninsured.adults <= 0.1283
0.396 < Insufficient.sleep
Uninsured.children <= 0.0425
American.Indian...Alaska.Native <= 0.00411
0.0595 < School.segregation <= 0.1084
Feature
−0.006
−0.003
0.000
0.003
Weight
Positive
Negative

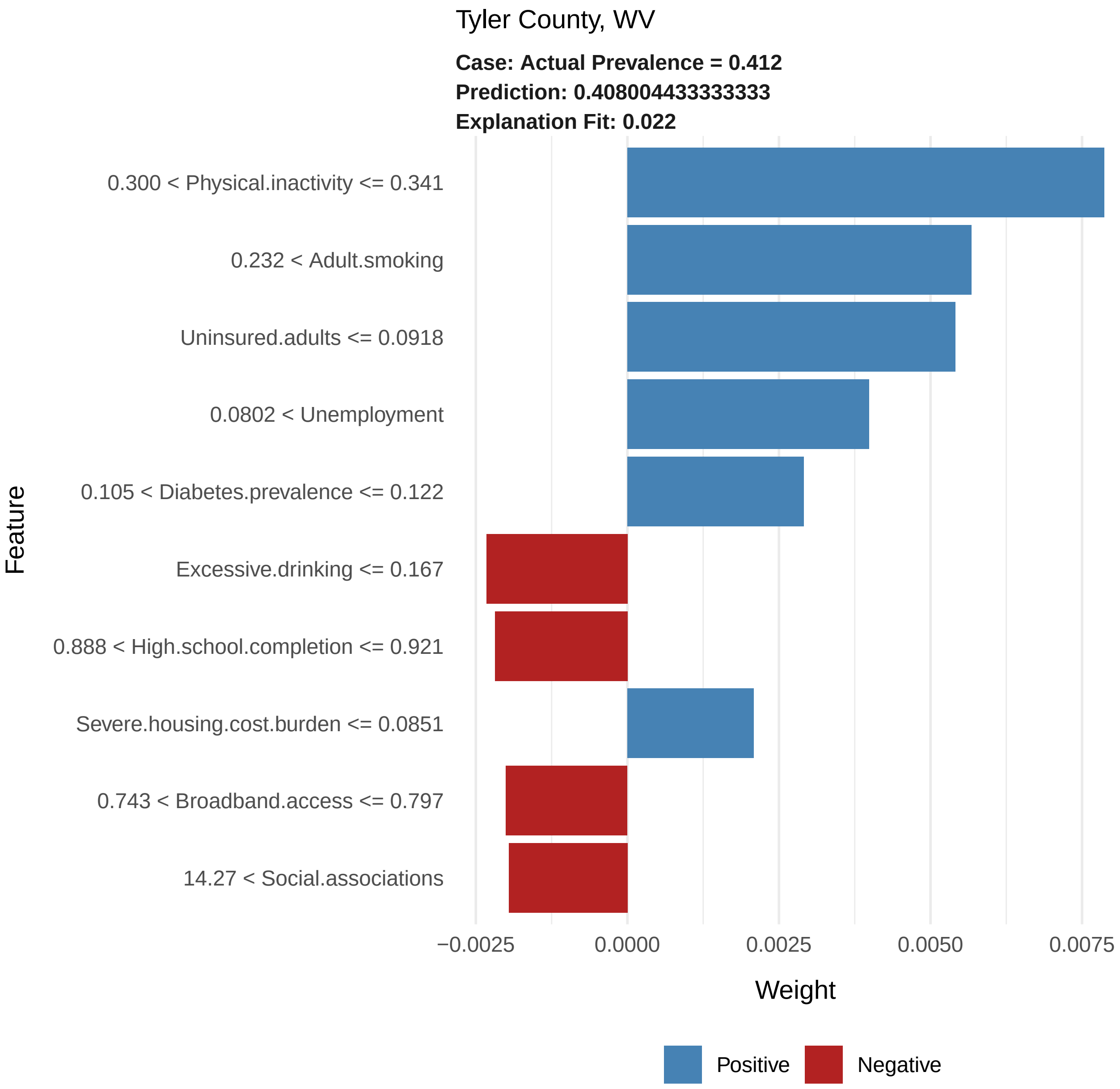

Tyler County, WV
Case: Actual Prevalence = 0.412
Prediction: 0.408004433333333
Explanation Fit: 0.022
0.300 < Physical.inactivity <= 0.341
0.232 < Adult.smoking
Uninsured.adults <= 0.0918
0.0802 < Unemployment
0.105 < Diabetes.prevalence <= 0.122
Excessive.drinking <= 0.167
0.888 < High.school.completion <= 0.921
Severe.housing.cost.burden <= 0.0851
0.743 < Broadband.access <= 0.797
14.27 < Social.associations
Feature
−0.0025
0.0000
0.0025
0.0050
0.0075
Weight
Positive
Negative

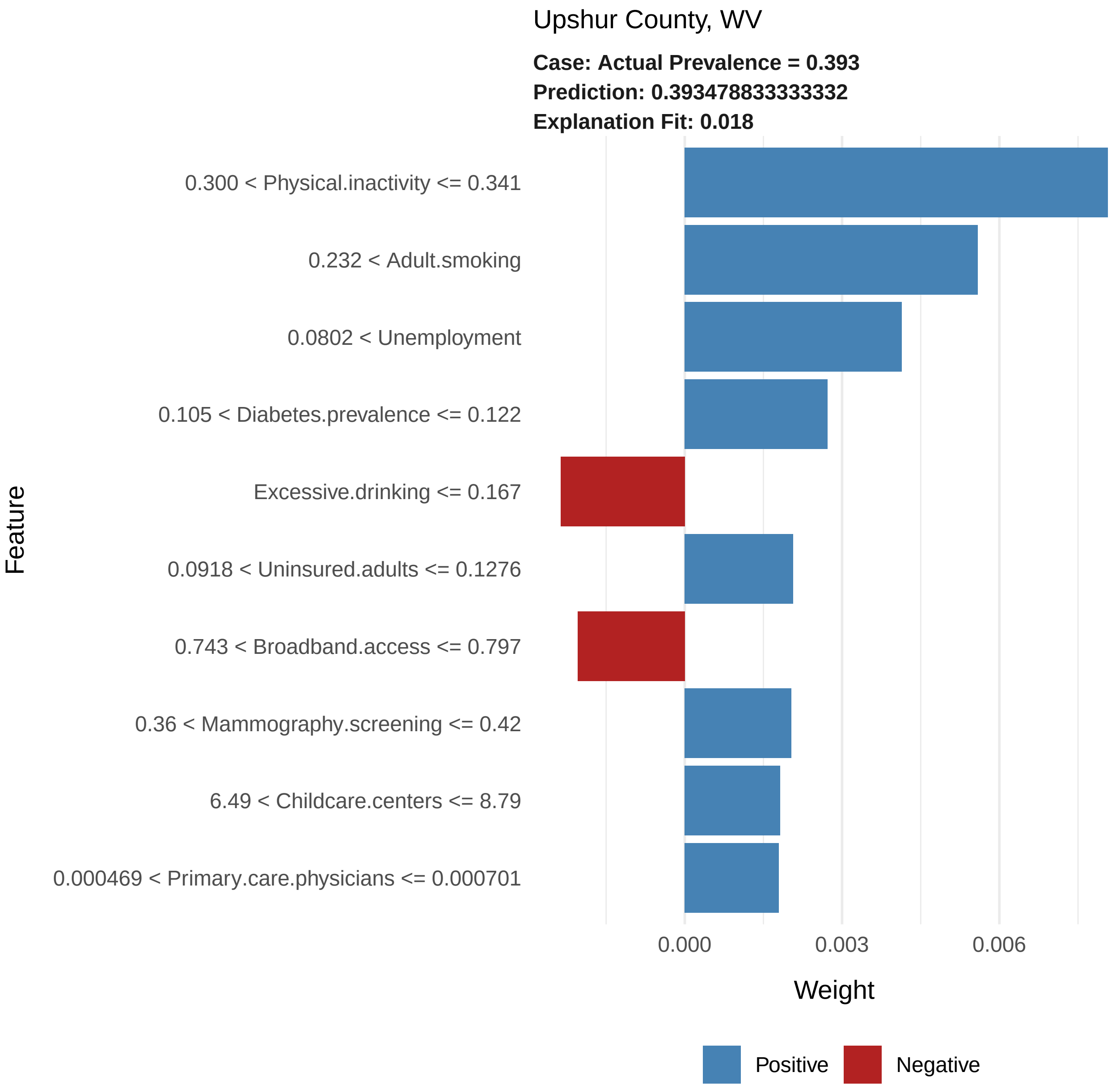
Upshur County, WV
Case: Actual Prevalence = 0.393
Prediction: 0.393478833333332
Explanation Fit: 0.018
Feature
0.300 < Physical.inactivity <= 0.341
0.232 < Adult.smoking
0.0802 < Unemployment
0.105 < Diabetes.prevalence <= 0.122
Excessive.drinking <= 0.167
0.0918 < Uninsured.adults <= 0.1276
0.743 < Broadband.access <= 0.797
0.36 < Mammography.screening <= 0.42
6.49 < Childcare.centers <= 8.79
0.000469 < Primary.care.physicians <= 0.000701
0.000
0.003
0.006
Weight
Positive
Negative

Wayne County, WV

Case: Actual Prevalence = 0.396
Prediction: 0.40523075
Explanation Fit: 0.5

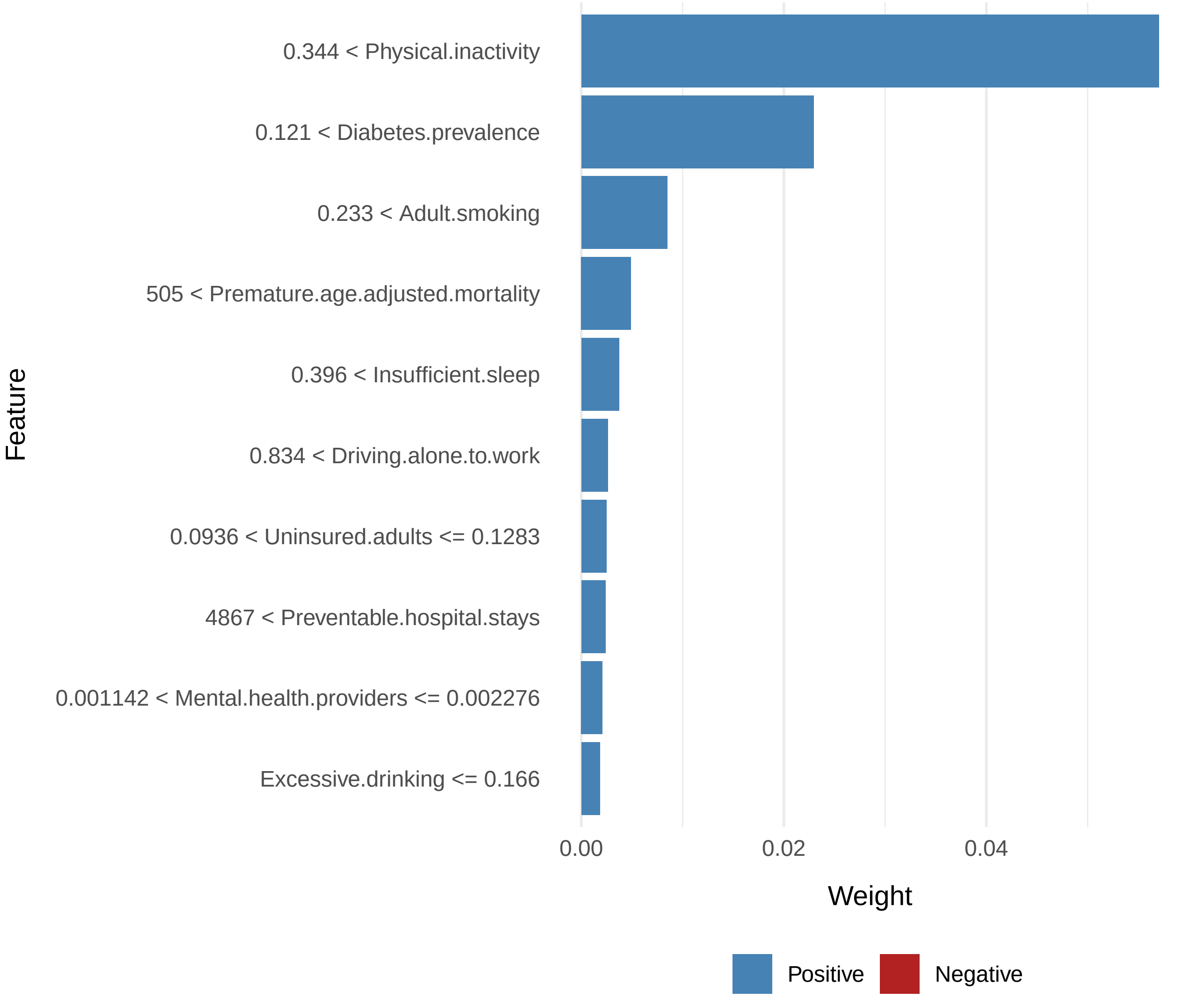

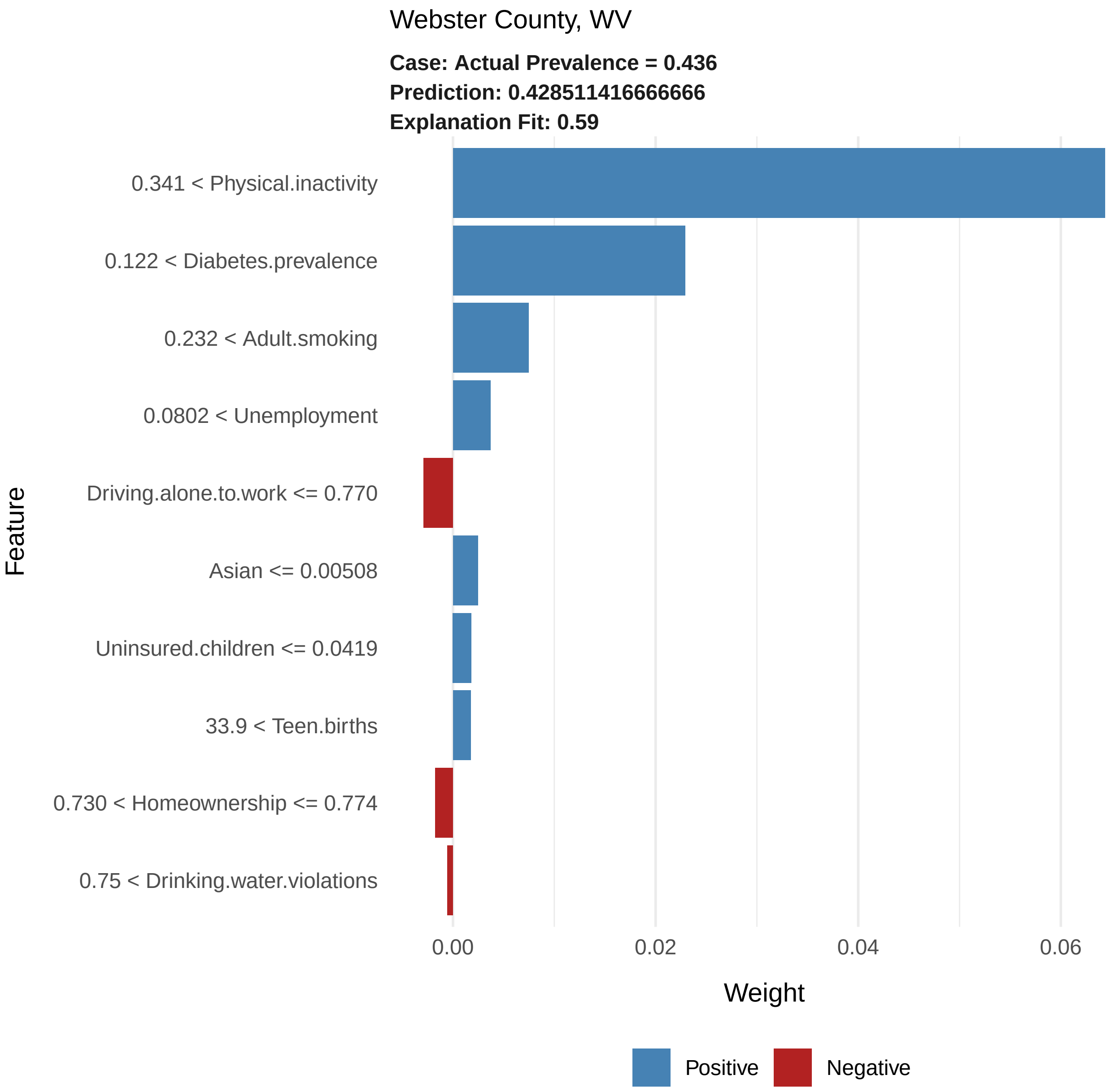

Webster County, WV
Case: Actual Prevalence = 0.436
Prediction: 0.428511416666666
Explanation Fit: 0.59
0.341 < Physical.inactivity
0.122 < Diabetes.prevalence
0.232 < Adult.smoking
0.0802 < Unemployment
Driving.alone.to.work <= 0.770
Asian <= 0.00508
Uninsured.children <= 0.0419
33.9 < Teen.births
0.730 < Homeownership <= 0.774
0.75 < Drinking.water.violations
Feature
0.00
0.02
0.04
0.06
Weight
Positive
Negative

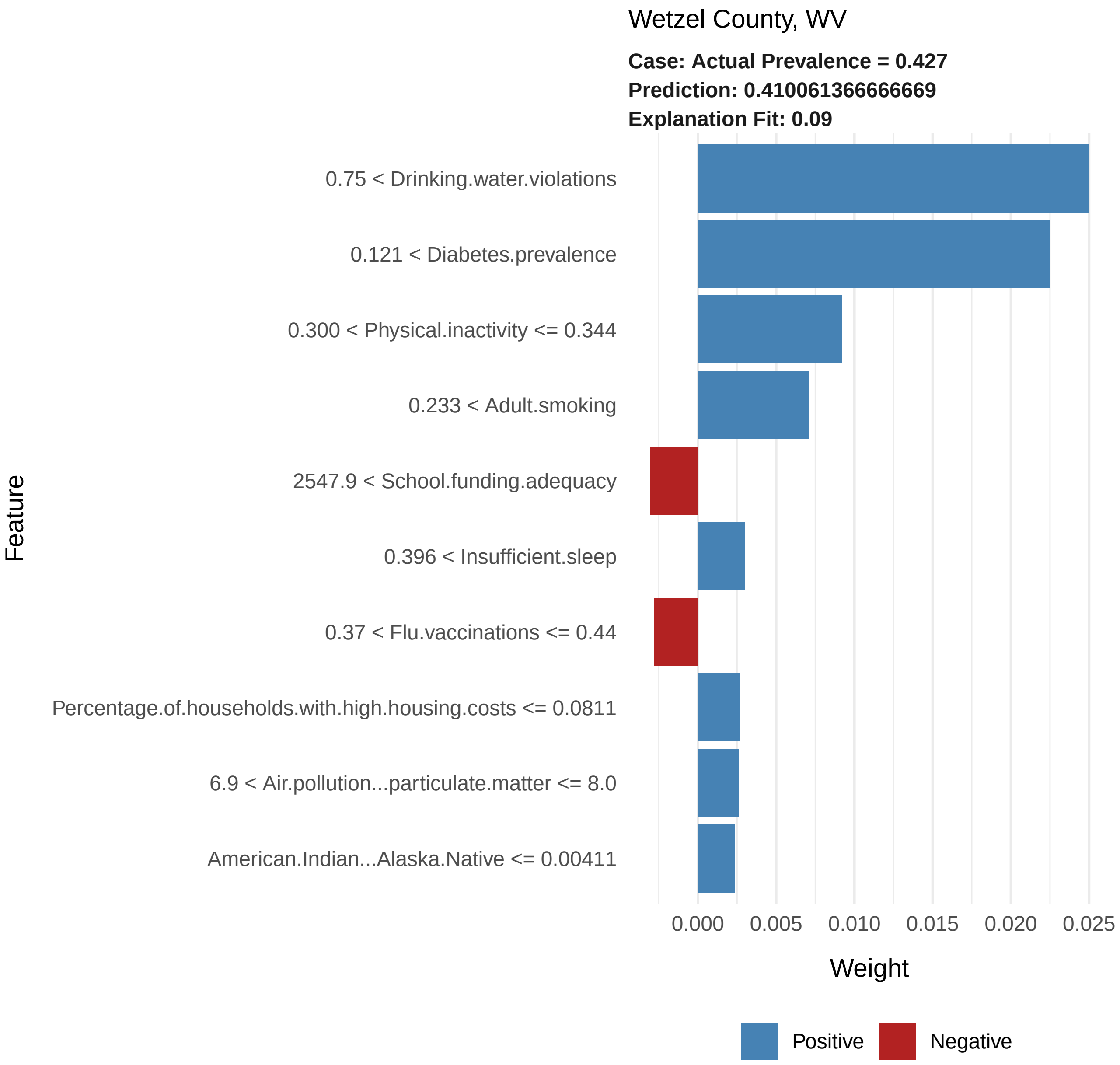

Wetzel County, WV
Case: Actual Prevalence = 0.427
Prediction: 0.410061366666669
Explanation Fit: 0.09
0.75 < Drinking.water.violations
0.121 < Diabetes.prevalence
0.300 < Physical.inactivity <= 0.344
0.233 < Adult.smoking
2547.9 < School.funding.adequacy
0.396 < Insufficient.sleep
0.37 < Flu.vaccinations <= 0.44
Percentage.of.households.with.high.housing.costs <= 0.0811
6.9 < Air.pollution...particulate.matter <= 8.0
American.Indian...Alaska.Native <= 0.00411
Feature
0.000
0.005
0.010
0.015
0.020
0.025
Weight
Positive
Negative

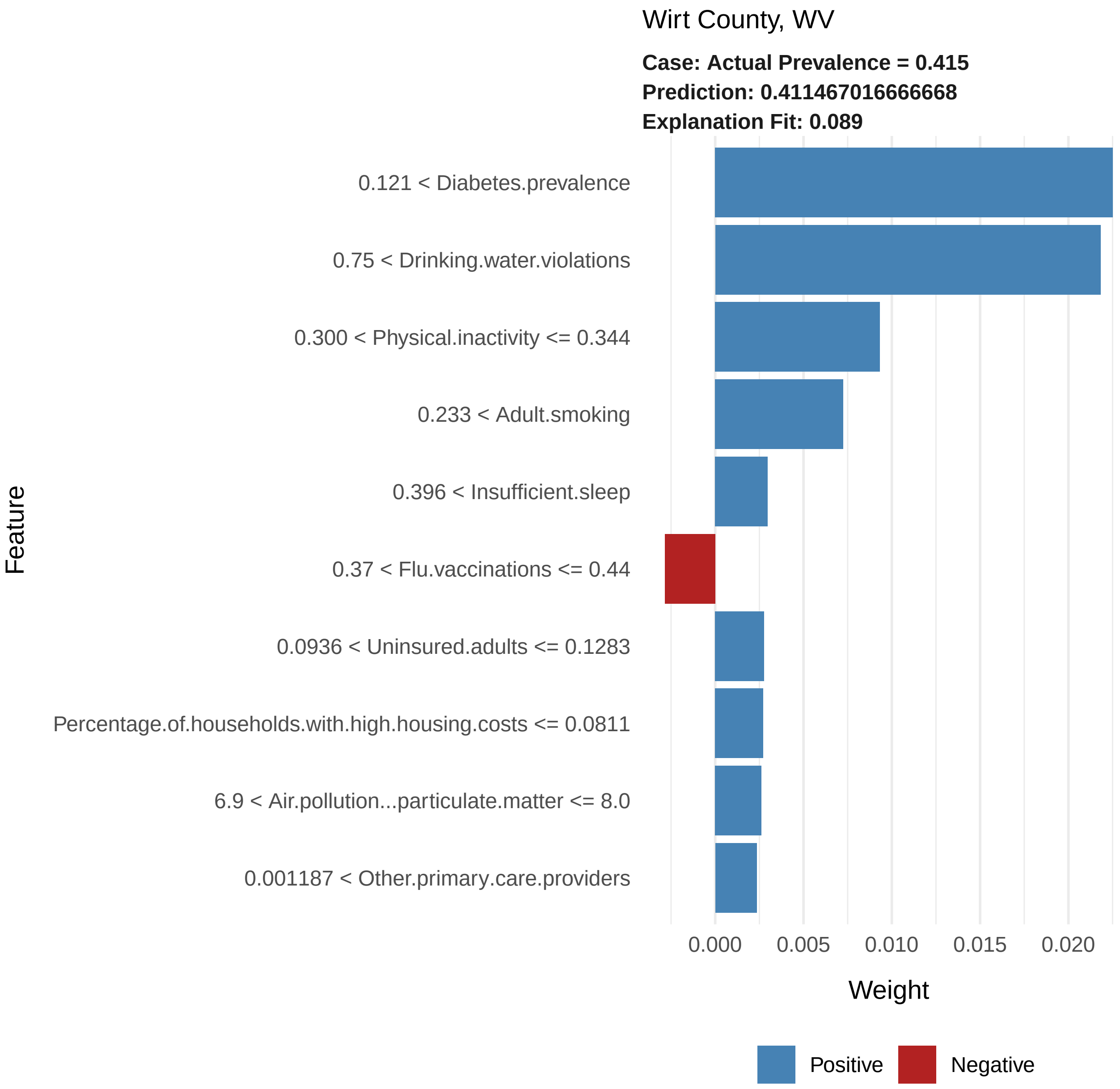
Wirt County, WV
Case: Actual Prevalence = 0.415
Prediction: 0.411467016666668
Explanation Fit: 0.089
0.121 < Diabetes.prevalence
0.75 < Drinking.water.violations
0.300 < Physical.inactivity <= 0.344
0.233 < Adult.smoking
0.396 < Insufficient.sleep
0.37 < Flu.vaccinations <= 0.44
0.0936 < Uninsured.adults <= 0.1283
Percentage.of.households.with.high.housing.costs <= 0.0811
6.9 < Air.pollution...particulate.matter <= 8.0
0.001187 < Other.primary.care.providers
Feature
0.000
0.005
0.010
0.015
0.020
Weight
Positive
Negative

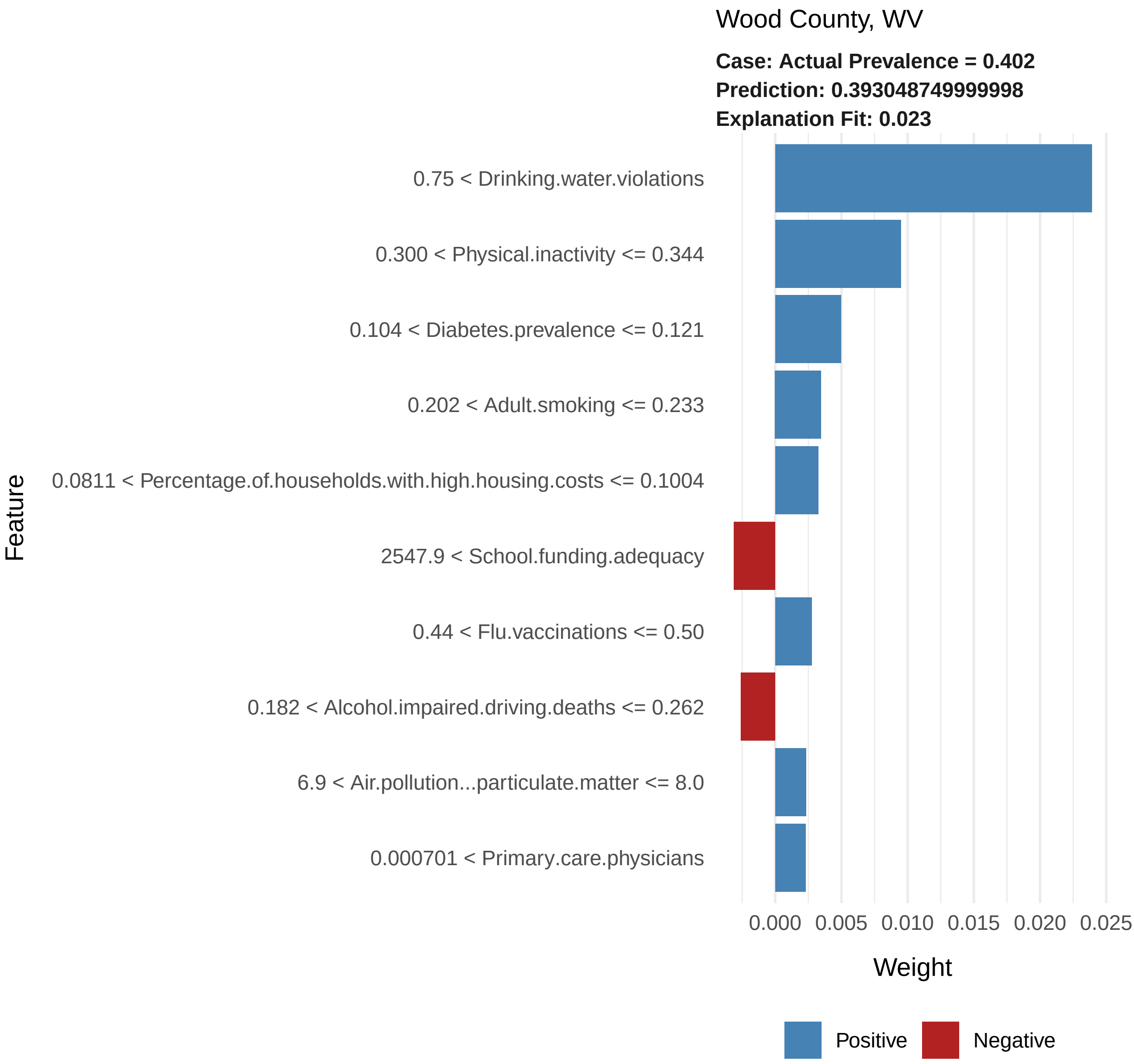

Wood County, WV
Case: Actual Prevalence = 0.402
Prediction: 0.393048749999998
Explanation Fit: 0.023
0.75 < Drinking.water.violations
0.300 < Physical.inactivity <= 0.344
0.104 < Diabetes.prevalence <= 0.121
0.202 < Adult.smoking <= 0.233
0.0811 < Percentage.of.households.with.high.housing.costs <= 0.1004
2547.9 < School.funding.adequacy
0.44 < Flu.vaccinations <= 0.50
0.182 < Alcohol.impaired.driving.deaths <= 0.262
6.9 < Air.pollution...particulate.matter <= 8.0
0.000701 < Primary.care.physicians
Feature
0.000
0.005
0.010
0.015
0.020
0.025
Weight
Positive
Negative

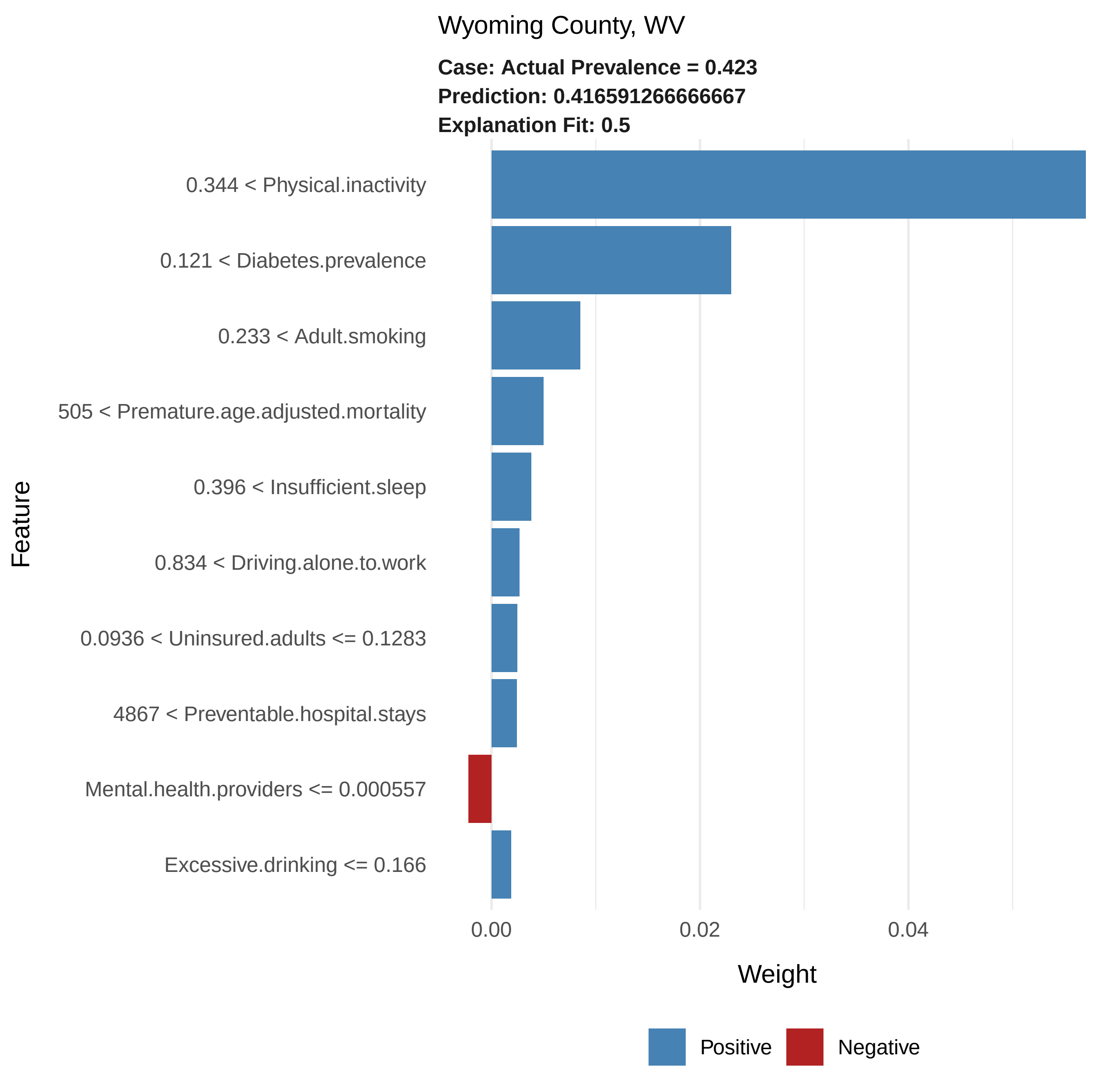
Wyoming County, WV
Case: Actual Prevalence = 0.423
Prediction: 0.416591266666667
Explanation Fit: 0.5
0.344 < Physical.inactivity
0.121 < Diabetes.prevalence
0.233 < Adult.smoking
505 < Premature.age.adjusted.mortality
0.396 < Insufficient.sleep
0.834 < Driving.alone.to.work
0.0936 < Uninsured.adults <= 0.1283
4867 < Preventable.hospital.stays
Mental.health.providers <= 0.000557
Excessive.drinking <= 0.166
Feature
0.00
0.02
0.04
Weight
Positive
Negative

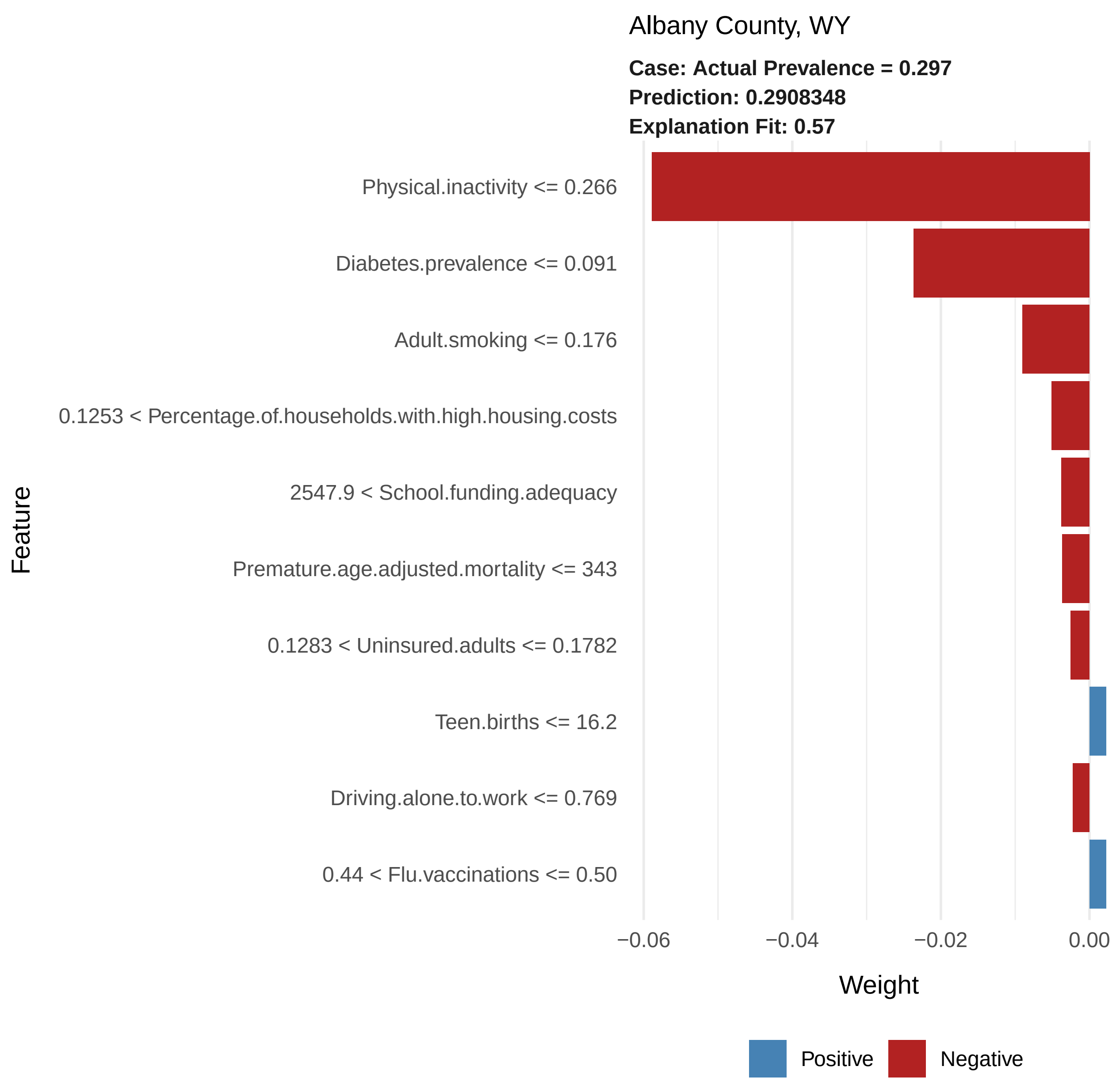
Albany County, WY
Case: Actual Prevalence = 0.297
Prediction: 0.2908348
Explanation Fit: 0.57
Physical.inactivity <= 0.266
Diabetes.prevalence <= 0.091
Adult.smoking <= 0.176
0.1253 < Percentage.of.households.with.high.housing.costs
2547.9 < School.funding.adequacy
Premature.age.adjusted.mortality <= 343
0.1283 < Uninsured.adults <= 0.1782
Teen.births <= 16.2
Driving.alone.to.work <= 0.769
0.44 < Flu.vaccinations <= 0.50
Feature
−0.06
−0.04
−0.02
0.00
Weight
Positive
Negative

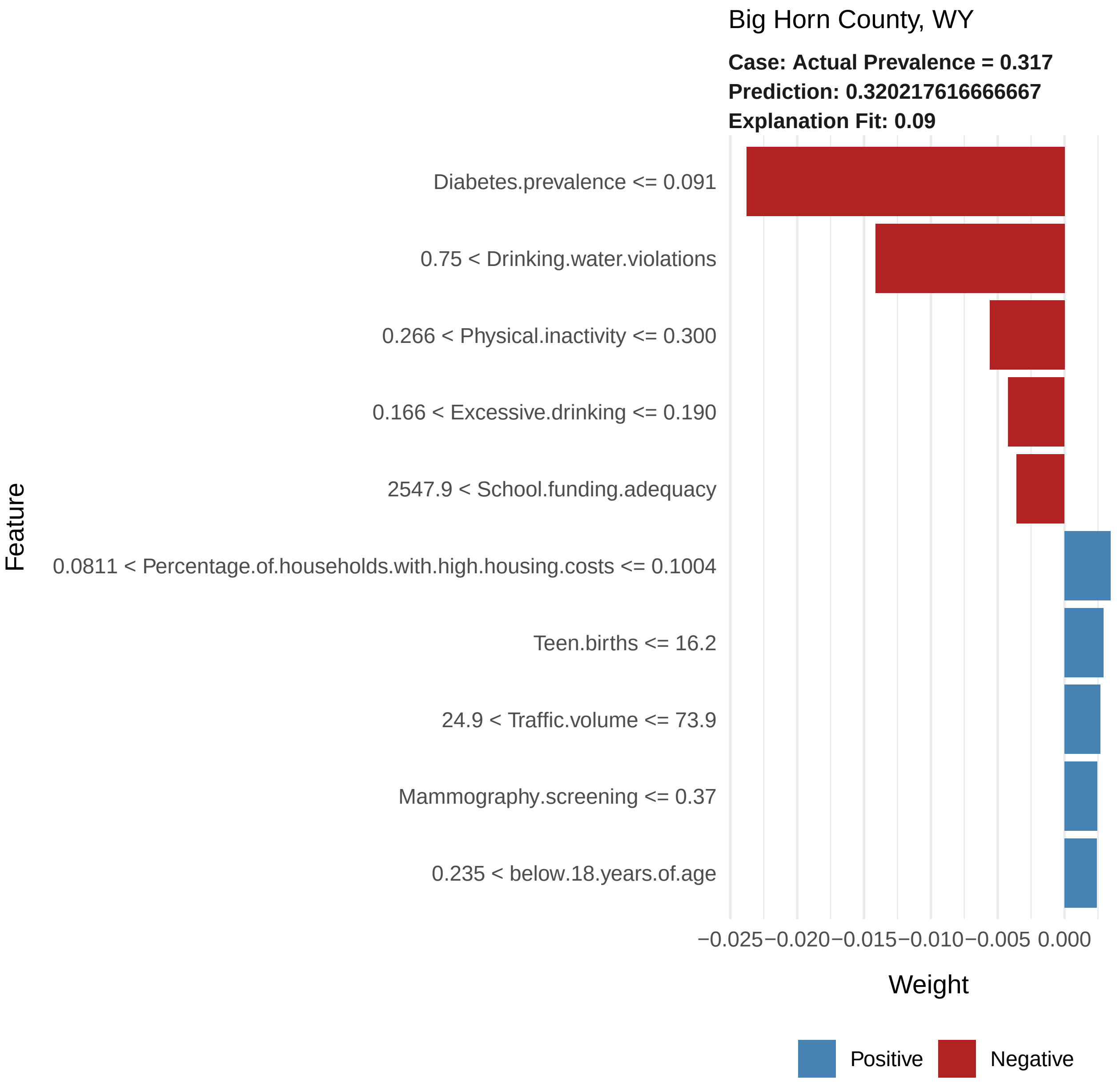

Big Horn County, WY
Case: Actual Prevalence = 0.317
Prediction: 0.320217616666667
Explanation Fit: 0.09
Diabetes.prevalence <= 0.091
0.75 < Drinking.water.violations
0.266 < Physical.inactivity <= 0.300
0.166 < Excessive.drinking <= 0.190
2547.9 < School.funding.adequacy
0.0811 < Percentage.of.households.with.high.housing.costs <= 0.1004
Teen.births <= 16.2
24.9 < Traffic.volume <= 73.9
Mammography.screening <= 0.37
0.235 < below.18.years.of.age
Feature
−0.025
−0.020
−0.015
−0.010
−0.005
0.000
Weight
Positive
Negative

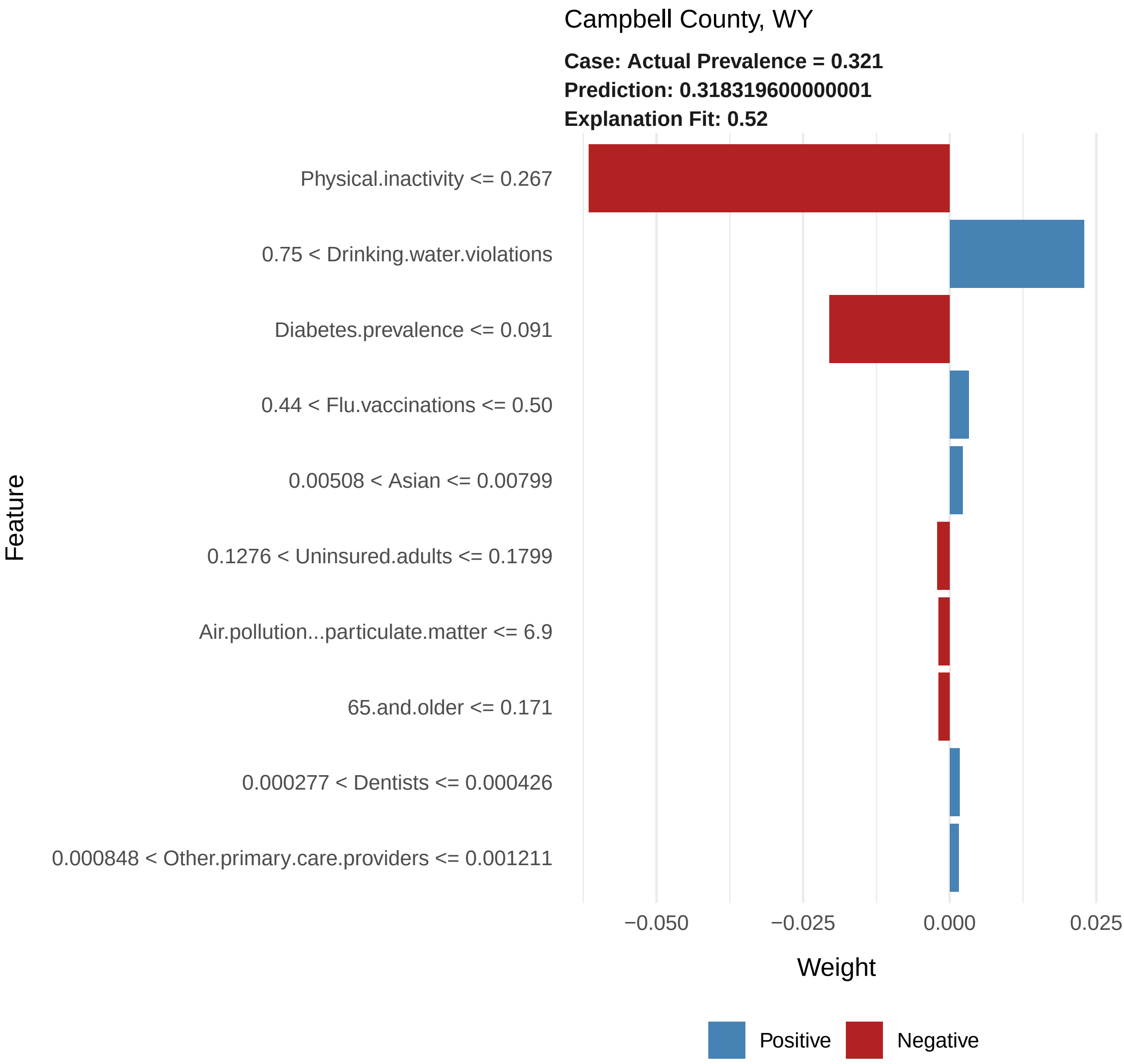
Campbell County, WY
Case: Actual Prevalence = 0.321
Prediction: 0.318319600000001
Explanation Fit: 0.52
Physical.inactivity <= 0.267
0.75 < Drinking.water.violations
Diabetes.prevalence <= 0.091
0.44 < Flu.vaccinations <= 0.50
0.00508 < Asian <= 0.00799
0.1276 < Uninsured.adults <= 0.1799
Air.pollution...particulate.matter <= 6.9
65.and.older <= 0.171
0.000277 < Dentists <= 0.000426
0.000848 < Other.primary.care.providers <= 0.001211
Feature
−0.050
−0.025
0.000
0.025
Weight
Positive
Negative

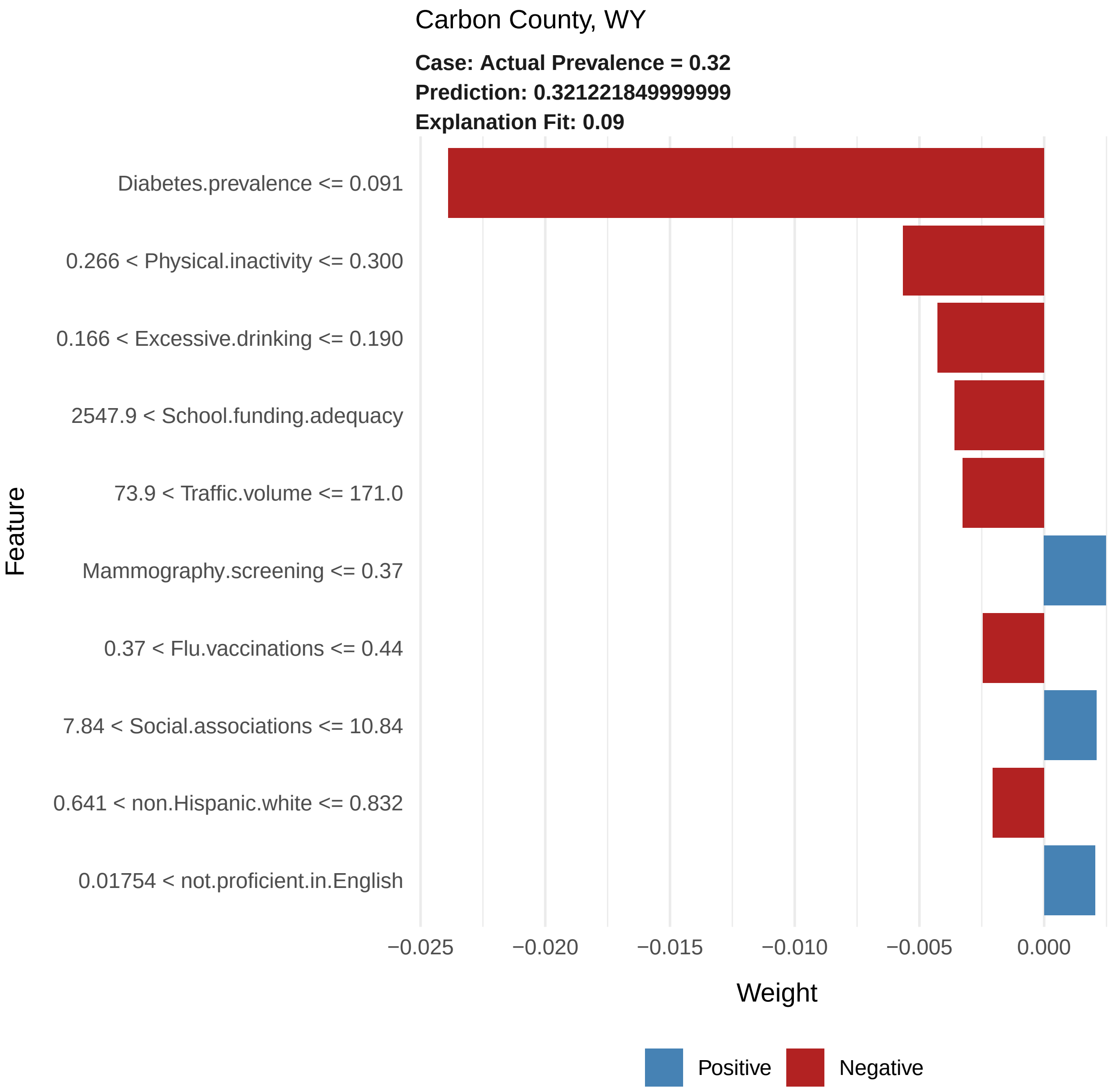

Carbon County, WY
Case: Actual Prevalence = 0.32
Prediction: 0.321221849999999
Explanation Fit: 0.09
Feature
Diabetes.prevalence <= 0.091
0.266 < Physical.inactivity <= 0.300
0.166 < Excessive.drinking <= 0.190
2547.9 < School.funding.adequacy
73.9 < Traffic.volume <= 171.0
Mammography.screening <= 0.37
0.37 < Flu.vaccinations <= 0.44
7.84 < Social.associations <= 10.84
0.641 < non.Hispanic.white <= 0.832
0.01754 < not.proficient.in.English
−0.025
−0.020
−0.015
−0.010
−0.005
0.000
Weight
Positive
Negative

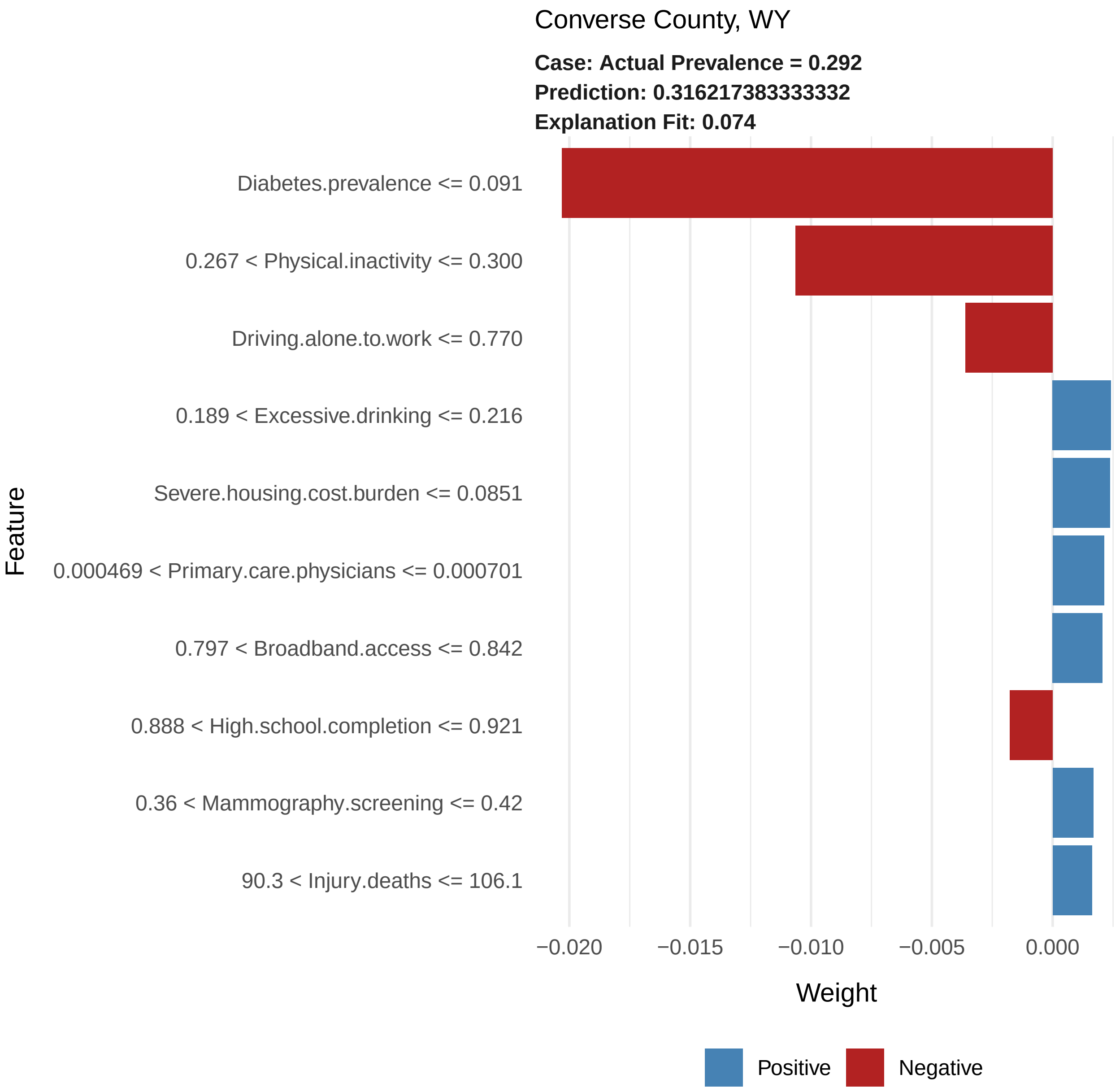

Converse County, WY
Case: Actual Prevalence = 0.292
Prediction: 0.316217383333332
Explanation Fit: 0.074
Diabetes.prevalence <= 0.091
0.267 < Physical.inactivity <= 0.300
Driving.alone.to.work <= 0.770
0.189 < Excessive.drinking <= 0.216
Severe.housing.cost.burden <= 0.0851
0.000469 < Primary.care.physicians <= 0.000701
0.797 < Broadband.access <= 0.842
0.888 < High.school.completion <= 0.921
0.36 < Mammography.screening <= 0.42
90.3 < Injury.deaths <= 106.1
Feature
−0.020
−0.015
−0.010
−0.005
0.000
Weight
Positive
Negative

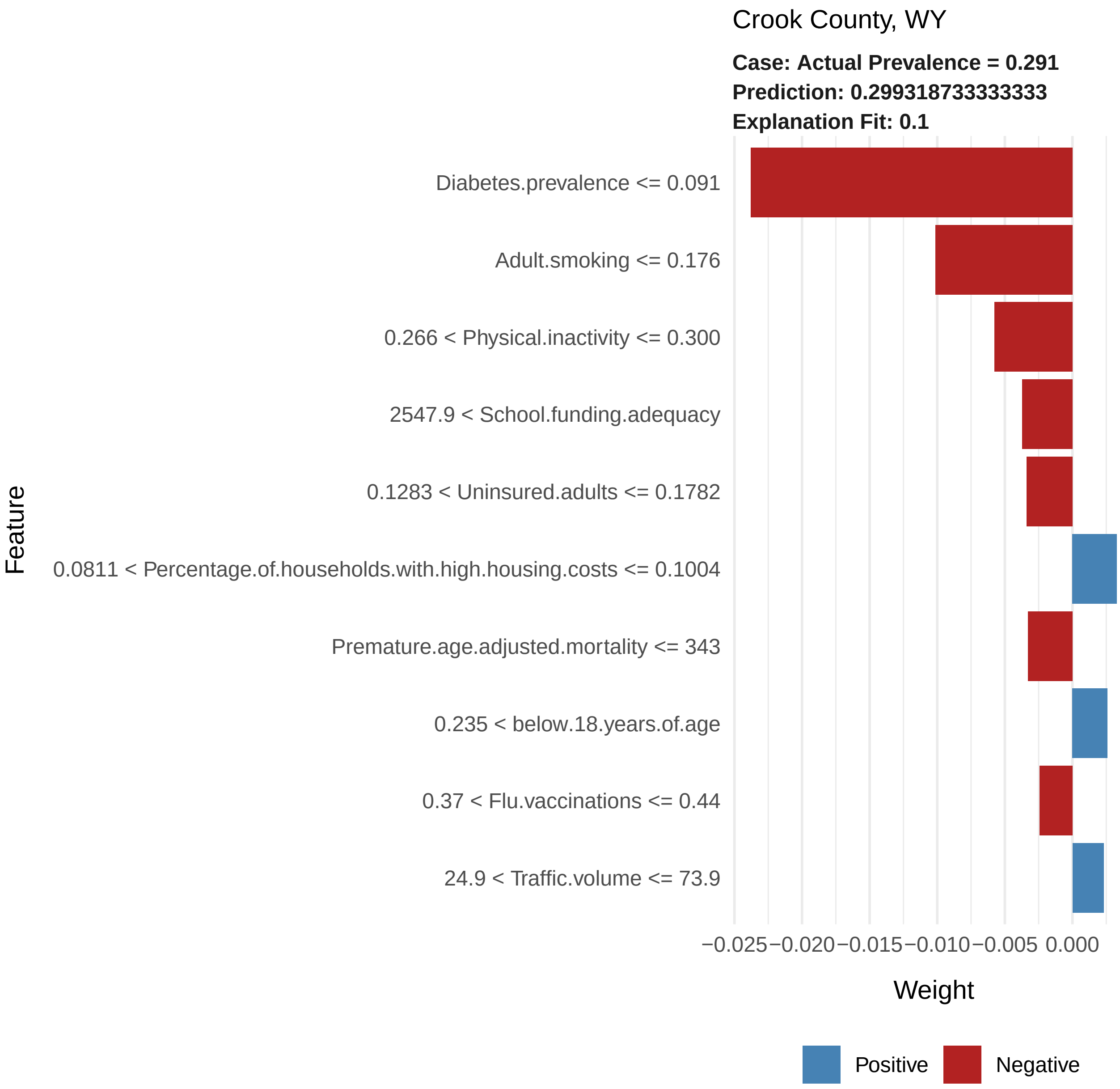

Crook County, WY
Case: Actual Prevalence = 0.291
Prediction: 0.299318733333333
Explanation Fit: 0.1
Diabetes.prevalence <= 0.091
Adult.smoking <= 0.176
0.266 < Physical.inactivity <= 0.300
2547.9 < School.funding.adequacy
0.1283 < Uninsured.adults <= 0.1782
0.0811 < Percentage.of.households.with.high.housing.costs <= 0.1004
Premature.age.adjusted.mortality <= 343
0.235 < below.18.years.of.age
0.37 < Flu.vaccinations <= 0.44
24.9 < Traffic.volume <= 73.9
Feature
−0.025
−0.020
−0.015
−0.010
−0.005
0.000
Weight
Positive
Negative

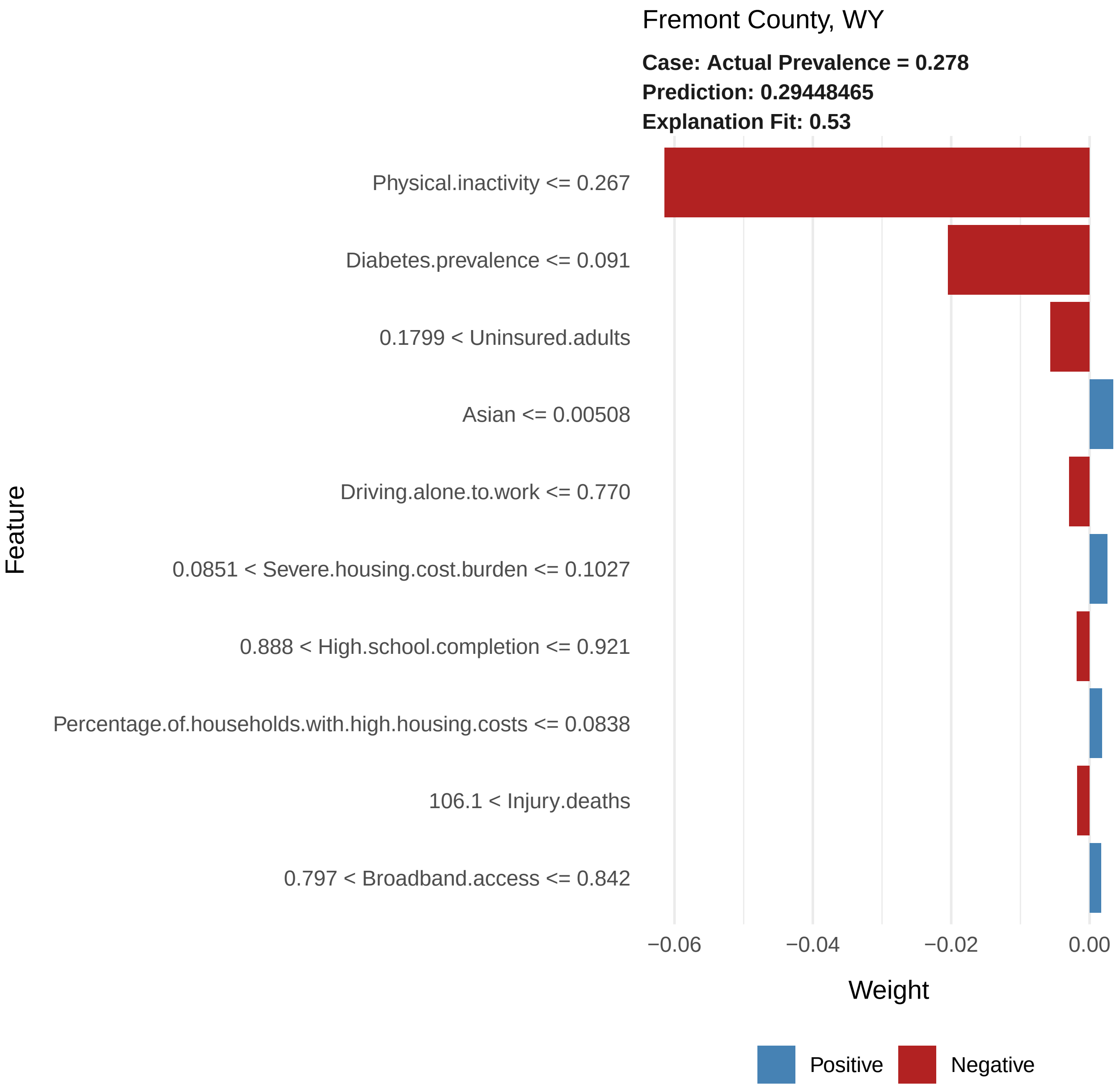
Fremont County, WY
Case: Actual Prevalence = 0.278
Prediction: 0.29448465
Explanation Fit: 0.53
Physical.inactivity <= 0.267
Diabetes.prevalence <= 0.091
0.1799 < Uninsured.adults
Asian <= 0.00508
Driving.alone.to.work <= 0.770
0.0851 < Severe.housing.cost.burden <= 0.1027
0.888 < High.school.completion <= 0.921
Percentage.of.households.with.high.housing.costs <= 0.0838
106.1 < Injury.deaths
0.797 < Broadband.access <= 0.842
Feature
−0.06
−0.04
−0.02
0.00
Weight
Positive
Negative

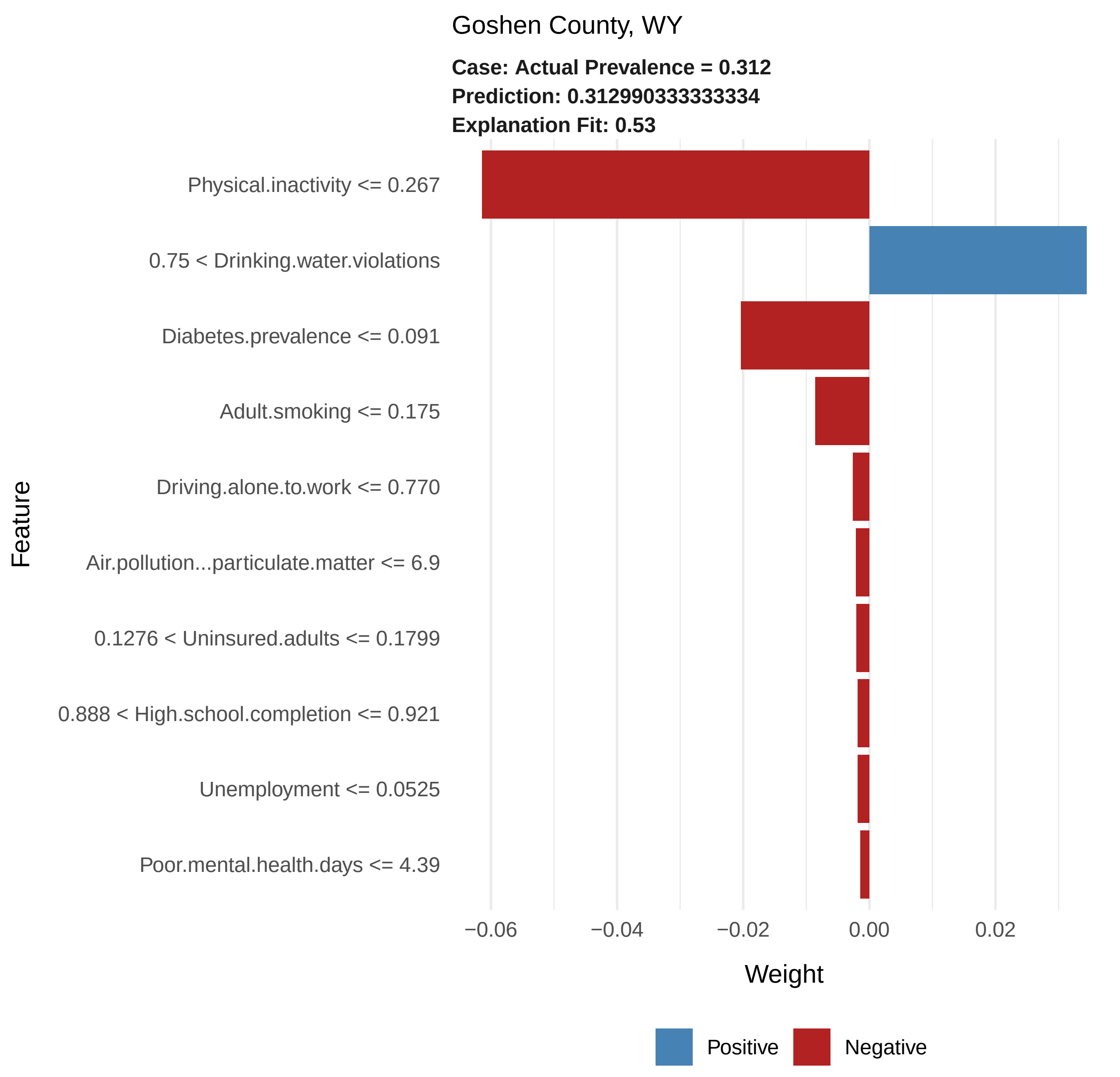
Goshen County, WY
Case: Actual Prevalence = 0.312
Prediction: 0.312990333333334
Explanation Fit: 0.53
Physical.inactivity <= 0.267
0.75 < Drinking.water.violations
Diabetes.prevalence <= 0.091
Adult.smoking <= 0.175
Driving.alone.to.work <= 0.770
Air.pollution...particulate.matter <= 6.9
0.1276 < Uninsured.adults <= 0.1799
0.888 < High.school.completion <= 0.921
Unemployment <= 0.0525
Poor.mental.health.days <= 4.39
Feature
−0.06
−0.04
−0.02
0.00
0.02
Weight
Positive
Negative

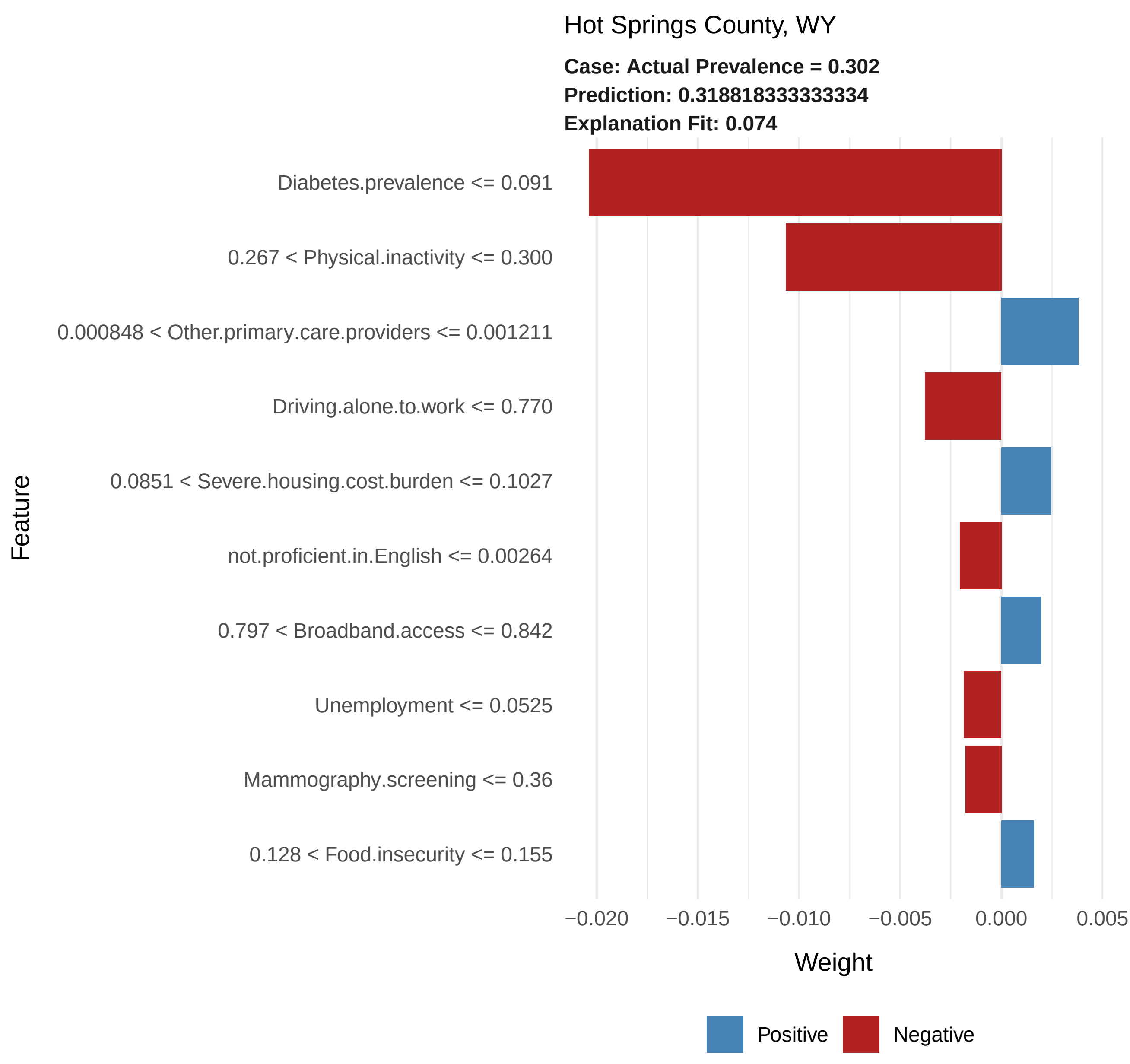
Hot Springs County, WY
Case: Actual Prevalence = 0.302
Prediction: 0.318818333333334
Explanation Fit: 0.074
Feature
Diabetes.prevalence <= 0.091
0.267 < Physical.inactivity <= 0.300
0.000848 < Other.primary.care.providers <= 0.001211
Driving.alone.to.work <= 0.770
0.0851 < Severe.housing.cost.burden <= 0.1027
not.proficient.in.English <= 0.00264
0.797 < Broadband.access <= 0.842
Unemployment <= 0.0525
Mammography.screening <= 0.36
0.128 < Food.insecurity <= 0.155
−0.020
−0.015
−0.010
−0.005
0.000
0.005
Weight
Positive
Negative

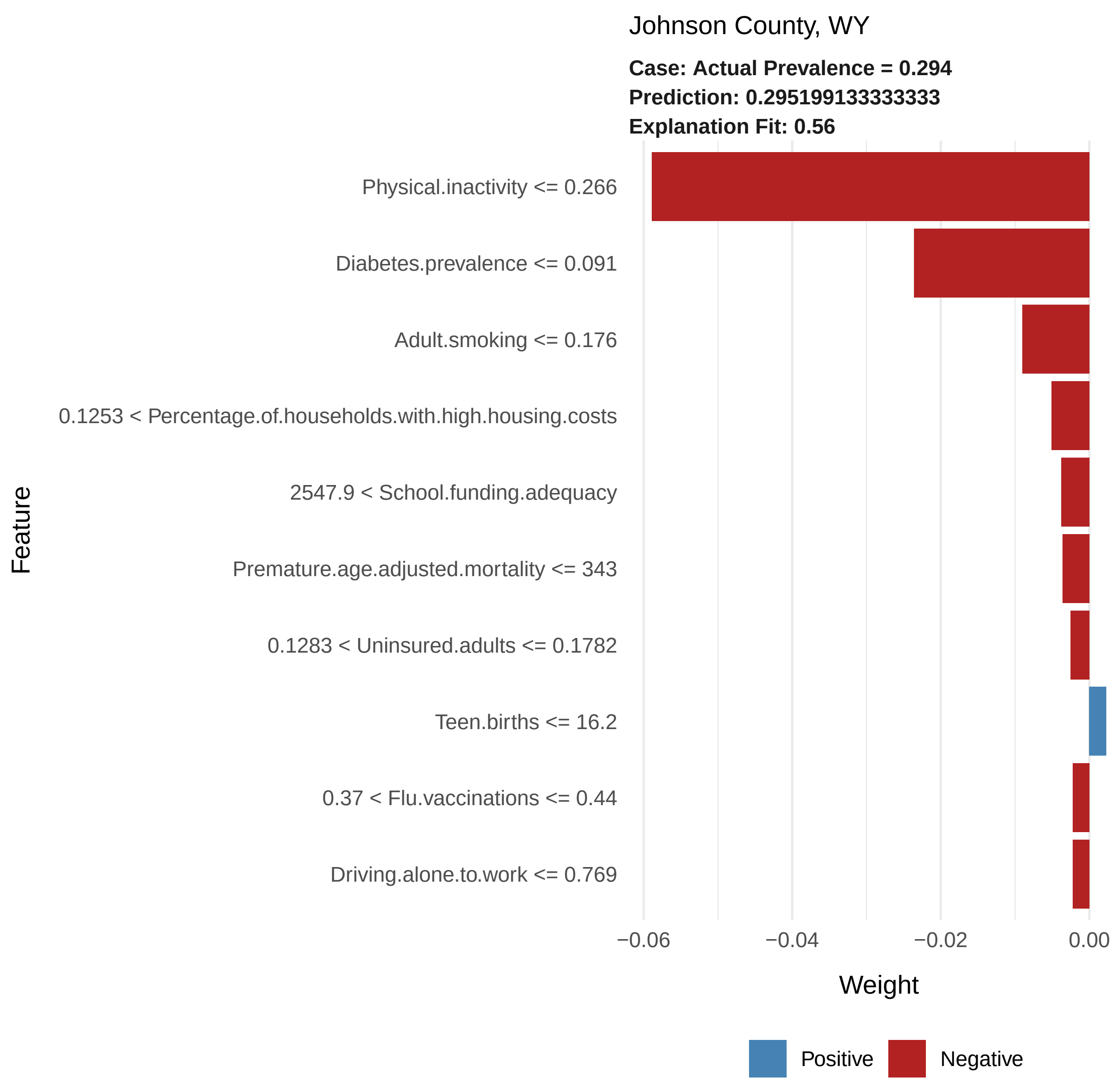

Johnson County, WY
Case: Actual Prevalence = 0.294
Prediction: 0.295199133333333
Explanation Fit: 0.56
Physical.inactivity <= 0.266
Diabetes.prevalence <= 0.091
Adult.smoking <= 0.176
0.1253 < Percentage.of.households.with.high.housing.costs
2547.9 < School.funding.adequacy
Premature.age.adjusted.mortality <= 343
0.1283 < Uninsured.adults <= 0.1782
Teen.births <= 16.2
0.37 < Flu.vaccinations <= 0.44
Driving.alone.to.work <= 0.769
Feature
−0.06
−0.04
−0.02
0.00
Weight
Positive
Negative

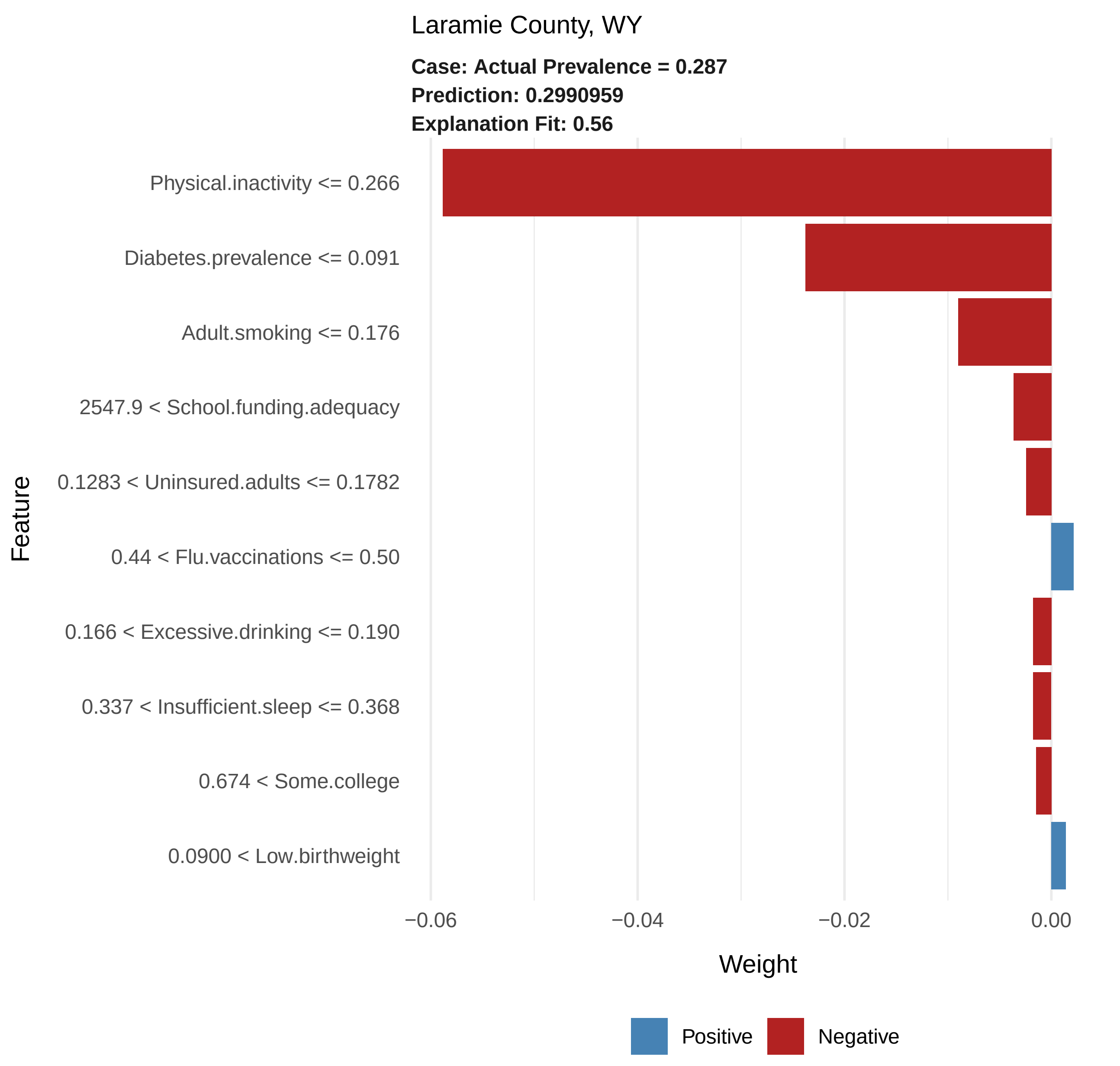
Laramie County, WY
Case: Actual Prevalence = 0.287
Prediction: 0.2990959
Explanation Fit: 0.56
Physical.inactivity <= 0.266
Diabetes.prevalence <= 0.091
Adult.smoking <= 0.176
2547.9 < School.funding.adequacy
0.1283 < Uninsured.adults <= 0.1782
0.44 < Flu.vaccinations <= 0.50
0.166 < Excessive.drinking <= 0.190
0.337 < Insufficient.sleep <= 0.368
0.674 < Some.college
0.0900 < Low.birthweight
Feature
−0.06
−0.04
−0.02
0.00
Weight
Positive
Negative

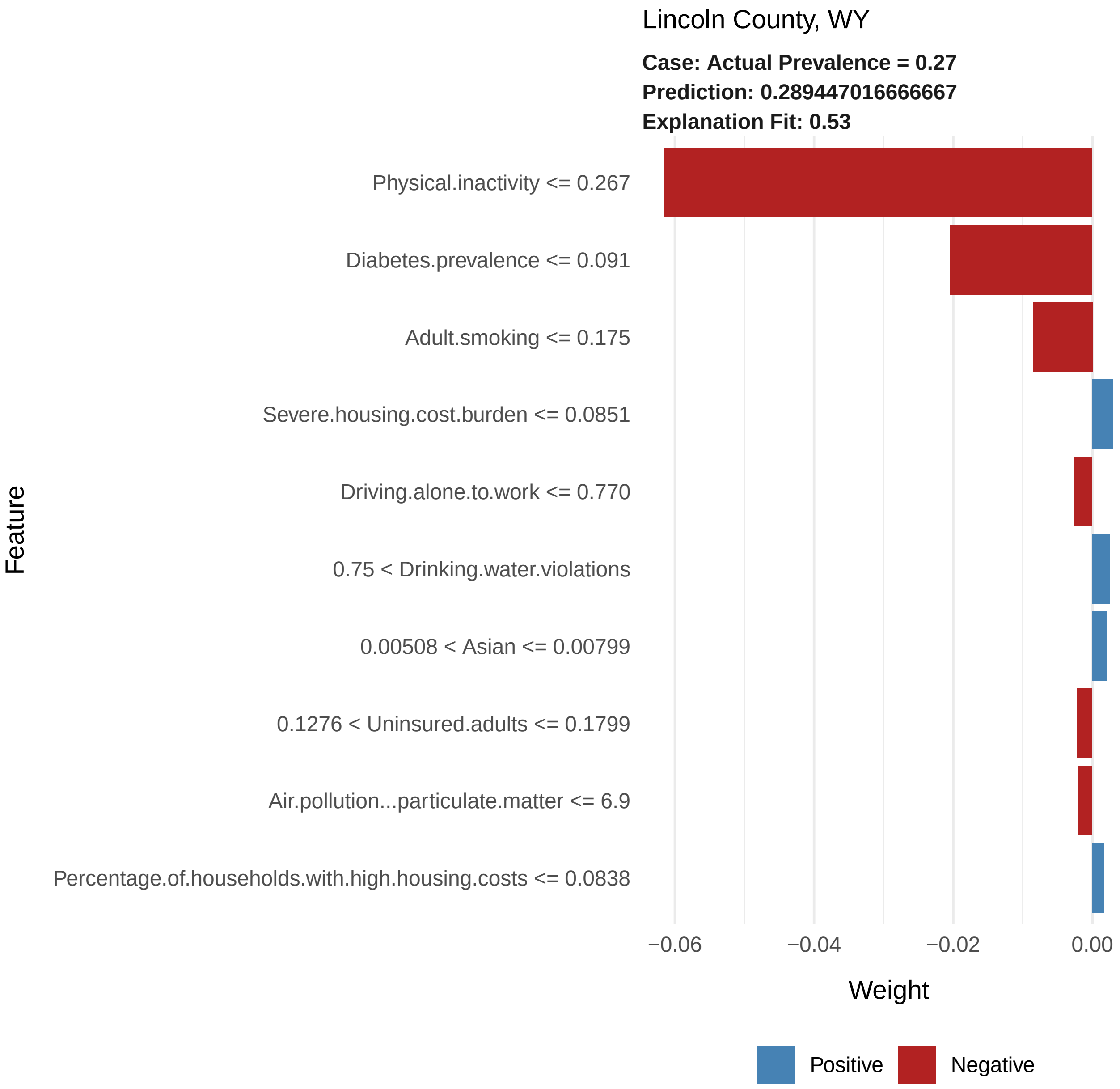
Lincoln County, WY
Case: Actual Prevalence = 0.27
Prediction: 0.289447016666667
Explanation Fit: 0.53
Physical.inactivity <= 0.267
Diabetes.prevalence <= 0.091
Adult.smoking <= 0.175
Severe.housing.cost.burden <= 0.0851
Driving.alone.to.work <= 0.770
0.75 < Drinking.water.violations
0.00508 < Asian <= 0.00799
0.1276 < Uninsured.adults <= 0.1799
Air.pollution...particulate.matter <= 6.9
Percentage.of.households.with.high.housing.costs <= 0.0838
Feature
−0.06
−0.04
−0.02
0.00
Weight
Positive
Negative

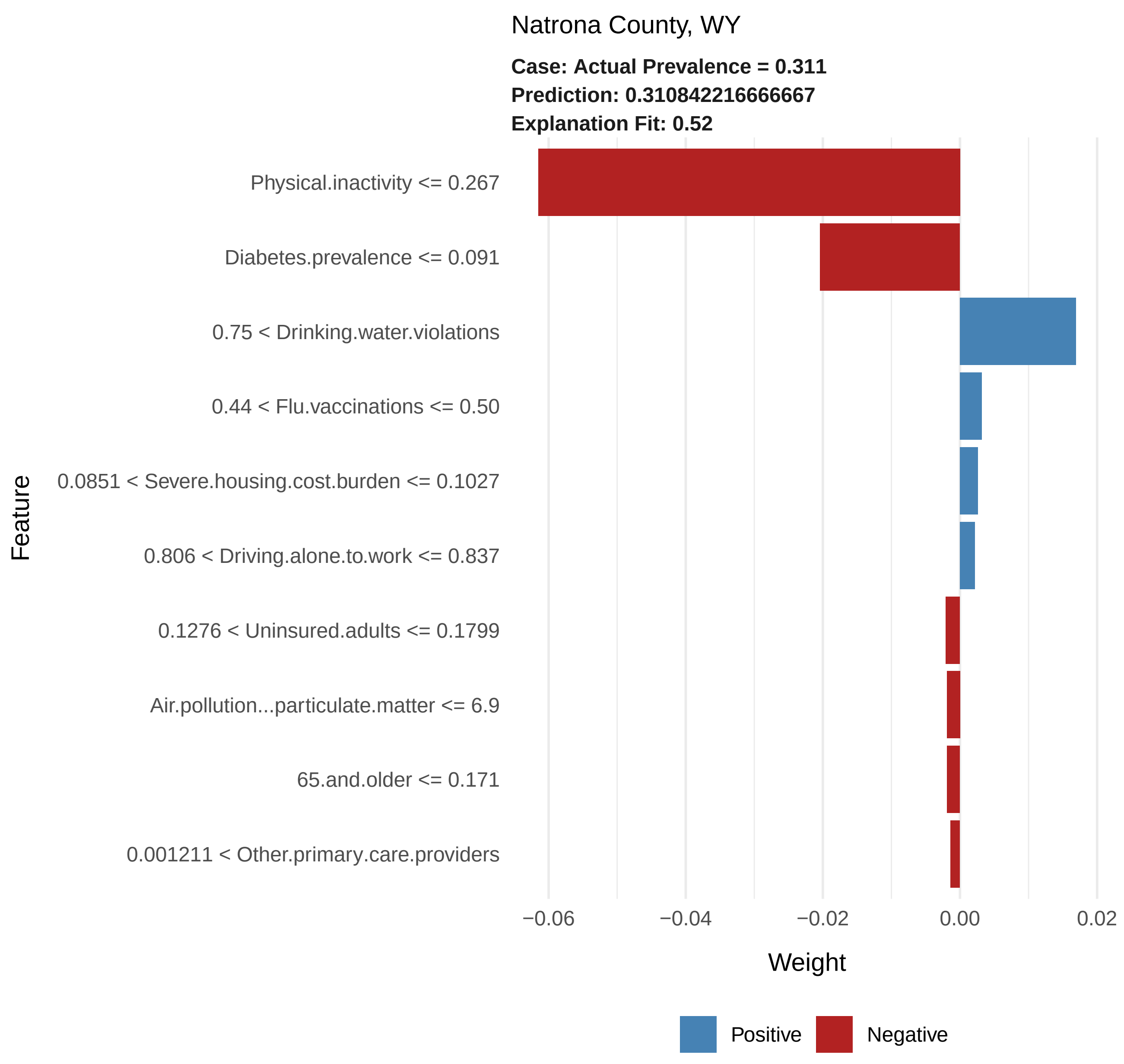

Natrona County, WY
Case: Actual Prevalence = 0.311
Prediction: 0.310842216666667
Explanation Fit: 0.52
Physical.inactivity <= 0.267
Diabetes.prevalence <= 0.091
0.75 < Drinking.water.violations
0.44 < Flu.vaccinations <= 0.50
0.0851 < Severe.housing.cost.burden <= 0.1027
0.806 < Driving.alone.to.work <= 0.837
0.1276 < Uninsured.adults <= 0.1799
Air.pollution...particulate.matter <= 6.9
65.and.older <= 0.171
0.001211 < Other.primary.care.providers
Feature
−0.06
−0.04
−0.02
0.00
0.02
Weight
Positive
Negative

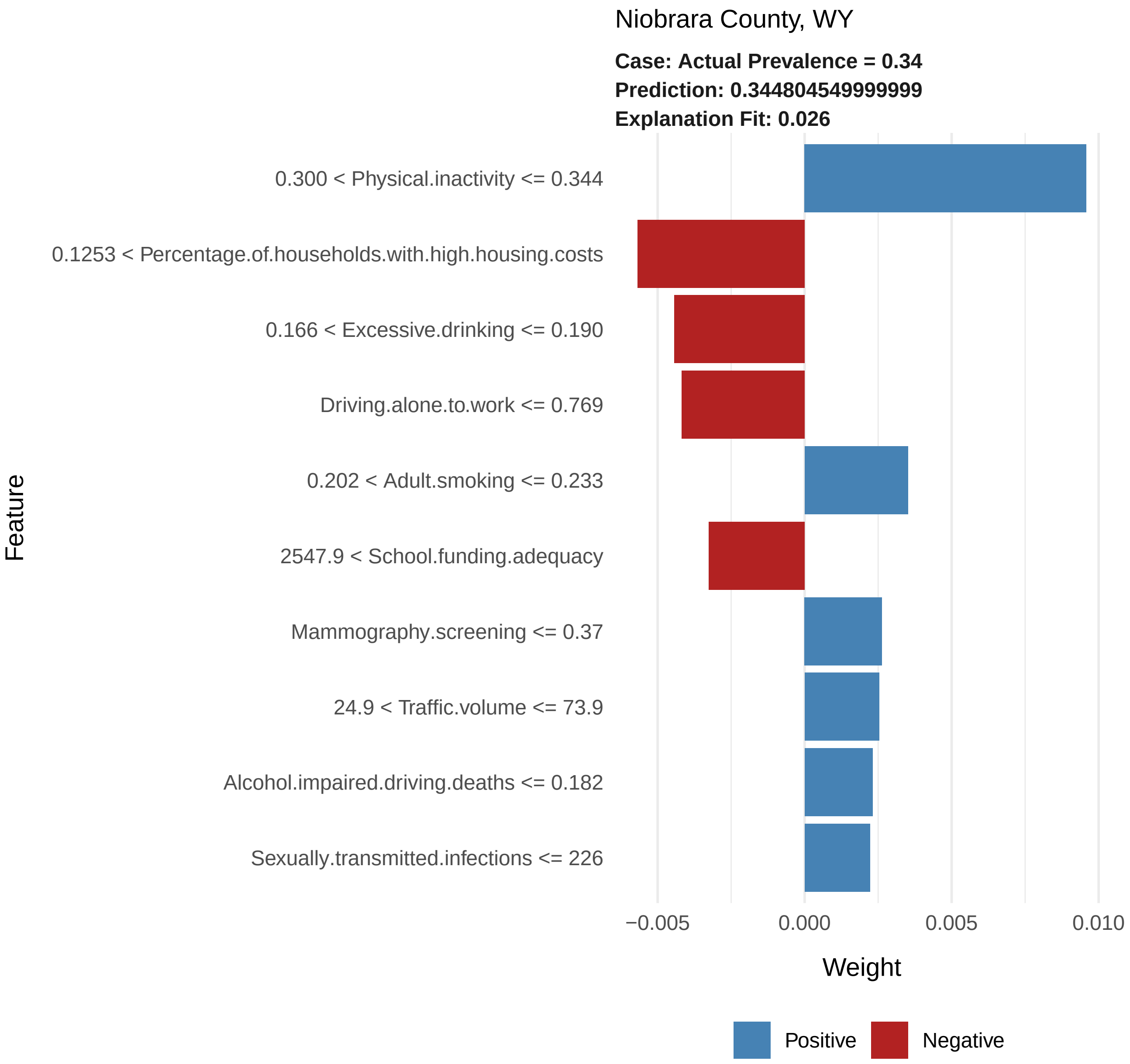
Niobrara County, WY
Case: Actual Prevalence = 0.34
Prediction: 0.344804549999999
Explanation Fit: 0.026
0.300 < Physical.inactivity <= 0.344
0.1253 < Percentage.of.households.with.high.housing.costs
0.166 < Excessive.drinking <= 0.190
Driving.alone.to.work <= 0.769
0.202 < Adult.smoking <= 0.233
2547.9 < School.funding.adequacy
Mammography.screening <= 0.37
24.9 < Traffic.volume <= 73.9
Alcohol.impaired.driving.deaths <= 0.182
Sexually.transmitted.infections <= 226
Feature
−0.005
0.000
0.005
0.010
Weight
Positive
Negative

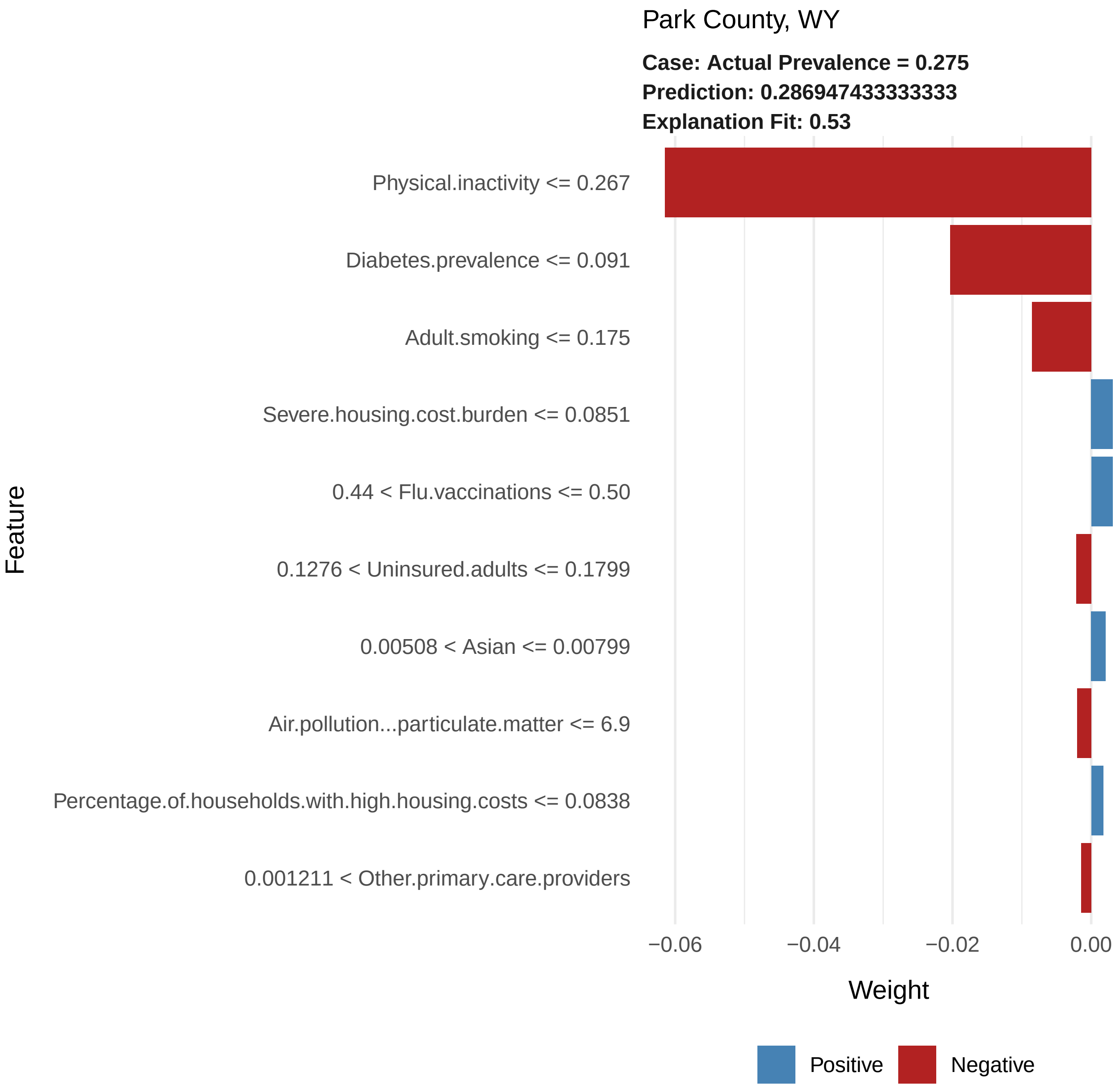
Park County, WY
Case: Actual Prevalence = 0.275
Prediction: 0.286947433333333
Explanation Fit: 0.53
Physical.inactivity <= 0.267
Diabetes.prevalence <= 0.091
Adult.smoking <= 0.175
Severe.housing.cost.burden <= 0.0851
0.44 < Flu.vaccinations <= 0.50
0.1276 < Uninsured.adults <= 0.1799
0.00508 < Asian <= 0.00799
Air.pollution...particulate.matter <= 6.9
Percentage.of.households.with.high.housing.costs <= 0.0838
0.001211 < Other.primary.care.providers
Feature
−0.06
−0.04
−0.02
0.00
Weight
Positive
Negative

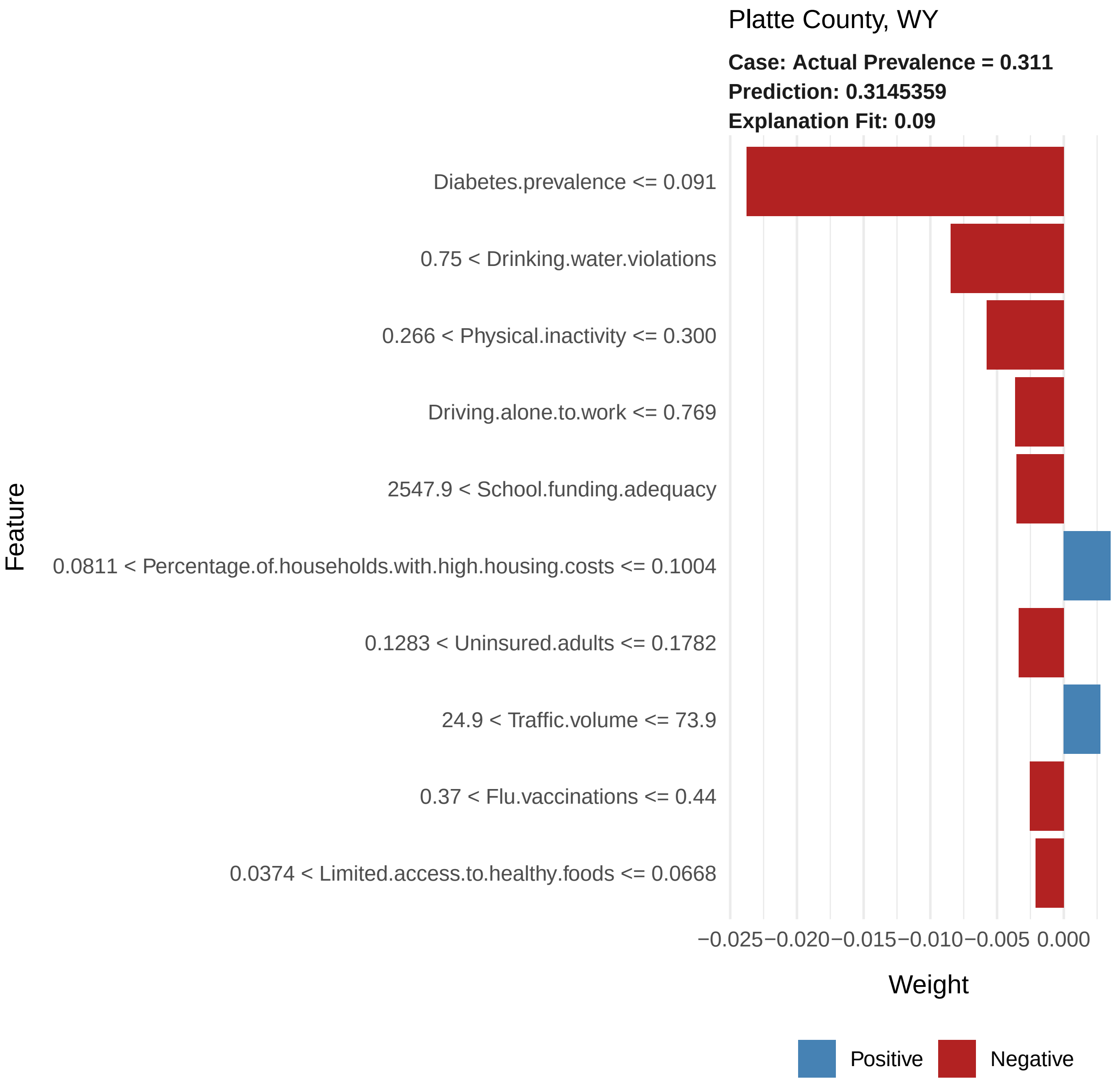
Platte County, WY
Case: Actual Prevalence = 0.311
Prediction: 0.3145359
Explanation Fit: 0.09
Diabetes.prevalence <= 0.091
0.75 < Drinking.water.violations
0.266 < Physical.inactivity <= 0.300
Driving.alone.to.work <= 0.769
2547.9 < School.funding.adequacy
0.0811 < Percentage.of.households.with.high.housing.costs <= 0.1004
0.1283 < Uninsured.adults <= 0.1782
24.9 < Traffic.volume <= 73.9
0.37 < Flu.vaccinations <= 0.44
0.0374 < Limited.access.to.healthy.foods <= 0.0668
Feature
−0.025 −0.020 −0.015 −0.010 −0.005 0.000
Weight
Positive
Negative

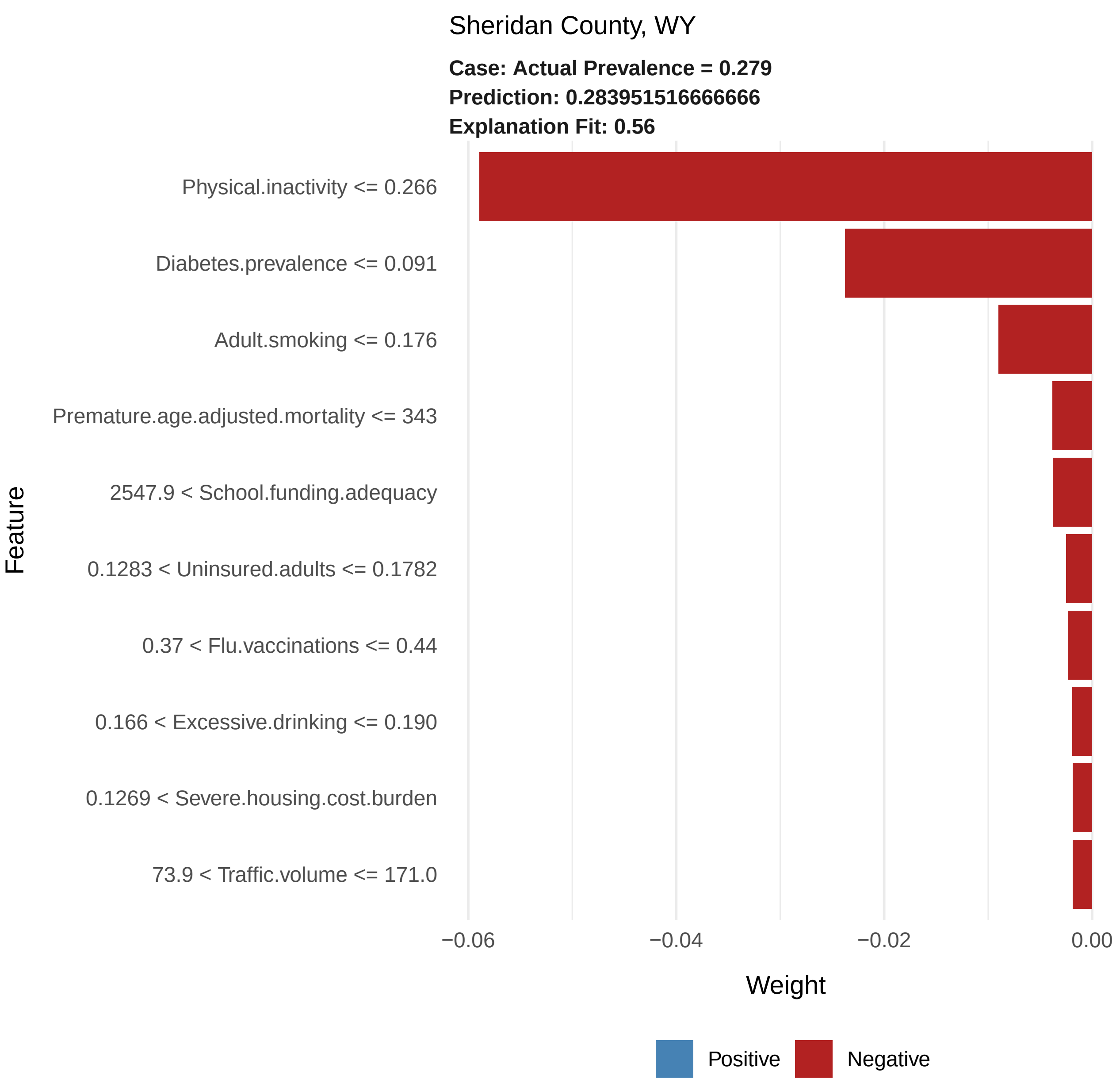

Sheridan County, WY
Case: Actual Prevalence = 0.279
Prediction: 0.283951516666666
Explanation Fit: 0.56
Physical.inactivity <= 0.266
Diabetes.prevalence <= 0.091
Adult.smoking <= 0.176
Premature.age.adjusted.mortality <= 343
2547.9 < School.funding.adequacy
0.1283 < Uninsured.adults <= 0.1782
0.37 < Flu.vaccinations <= 0.44
0.166 < Excessive.drinking <= 0.190
0.1269 < Severe.housing.cost.burden
73.9 < Traffic.volume <= 171.0
Feature
−0.06
−0.04
−0.02
0.00
Weight
Positive
Negative

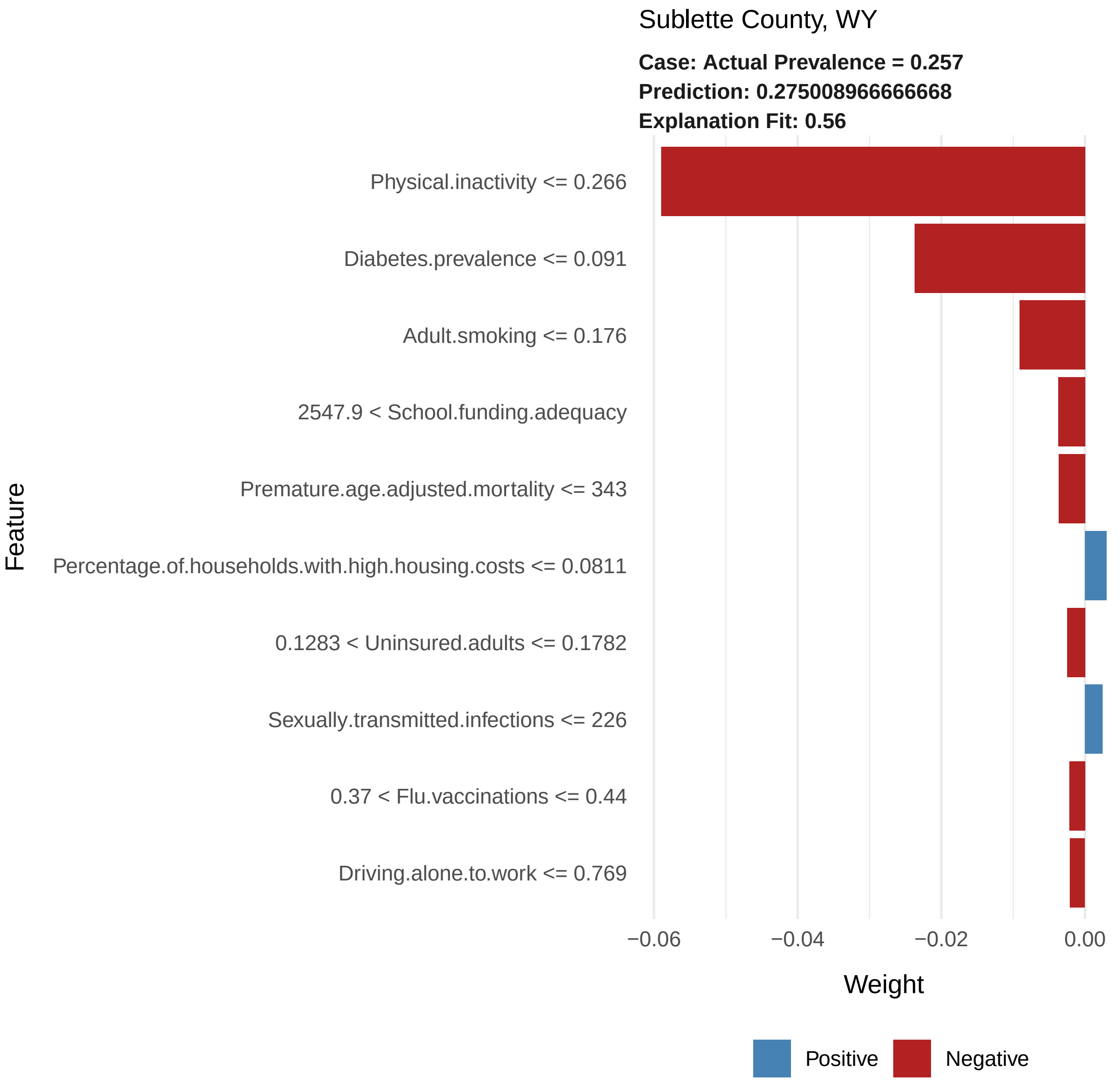
Sublette County, WY
Case: Actual Prevalence = 0.257
Prediction: 0.275008966666668
Explanation Fit: 0.56
Physical.inactivity <= 0.266
Diabetes.prevalence <= 0.091
Adult.smoking <= 0.176
2547.9 < School.funding.adequacy
Premature.age.adjusted.mortality <= 343
Percentage.of.households.with.high.housing.costs <= 0.0811
0.1283 < Uninsured.adults <= 0.1782
Sexually.transmitted.infections <= 226
0.37 < Flu.vaccinations <= 0.44
Driving.alone.to.work <= 0.769
Feature
−0.06
−0.04
−0.02
0.00
Weight
Positive
Negative

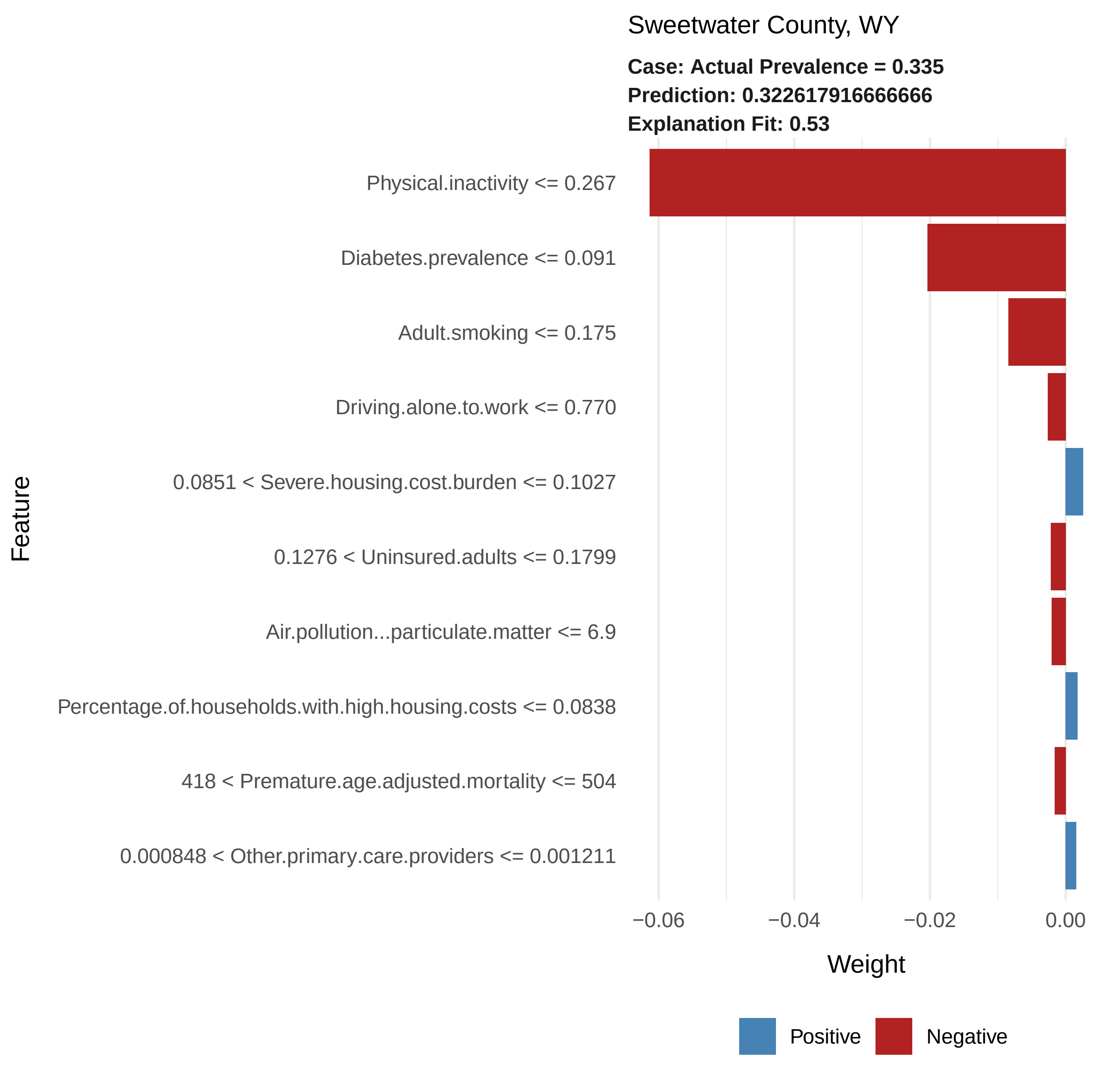
Sweetwater County, WY
Case: Actual Prevalence = 0.335
Prediction: 0.322617916666666
Explanation Fit: 0.53
Physical.inactivity <= 0.267
Diabetes.prevalence <= 0.091
Adult.smoking <= 0.175
Driving.alone.to.work <= 0.770
0.0851 < Severe.housing.cost.burden <= 0.1027
0.1276 < Uninsured.adults <= 0.1799
Air.pollution...particulate.matter <= 6.9
Percentage.of.households.with.high.housing.costs <= 0.0838
418 < Premature.age.adjusted.mortality <= 504
0.000848 < Other.primary.care.providers <= 0.001211
Feature
−0.06
−0.04
−0.02
0.00
Weight
Positive
Negative

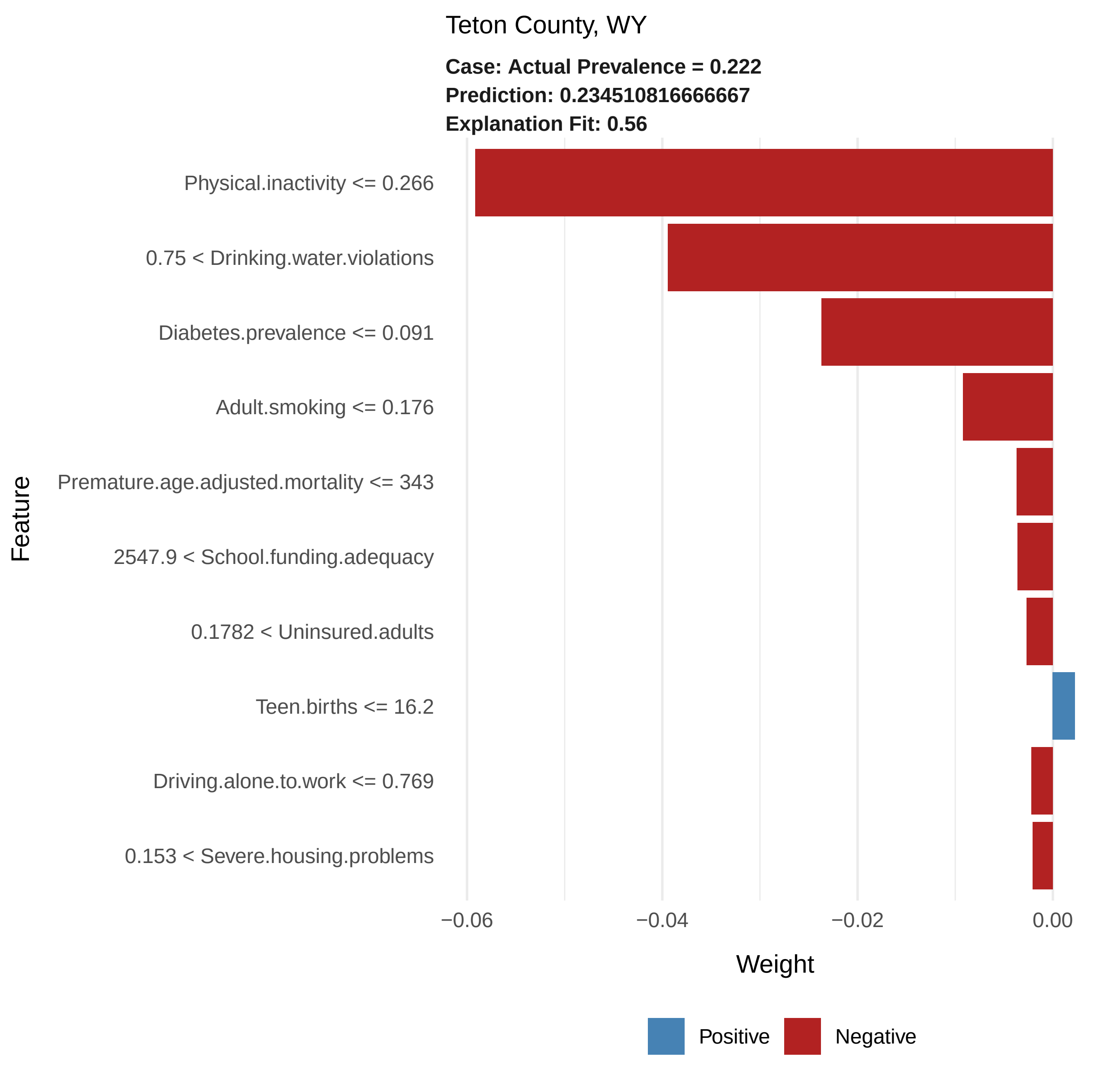
Teton County, WY
Case: Actual Prevalence = 0.222
Prediction: 0.234510816666667
Explanation Fit: 0.56
Physical.inactivity <= 0.266
0.75 < Drinking.water.violations
Diabetes.prevalence <= 0.091
Adult.smoking <= 0.176
Premature.age.adjusted.mortality <= 343
2547.9 < School.funding.adequacy
0.1782 < Uninsured.adults
Teen.births <= 16.2
Driving.alone.to.work <= 0.769
0.153 < Severe.housing.problems
Feature
−0.06
−0.04
−0.02
0.00
Weight
Positive
Negative

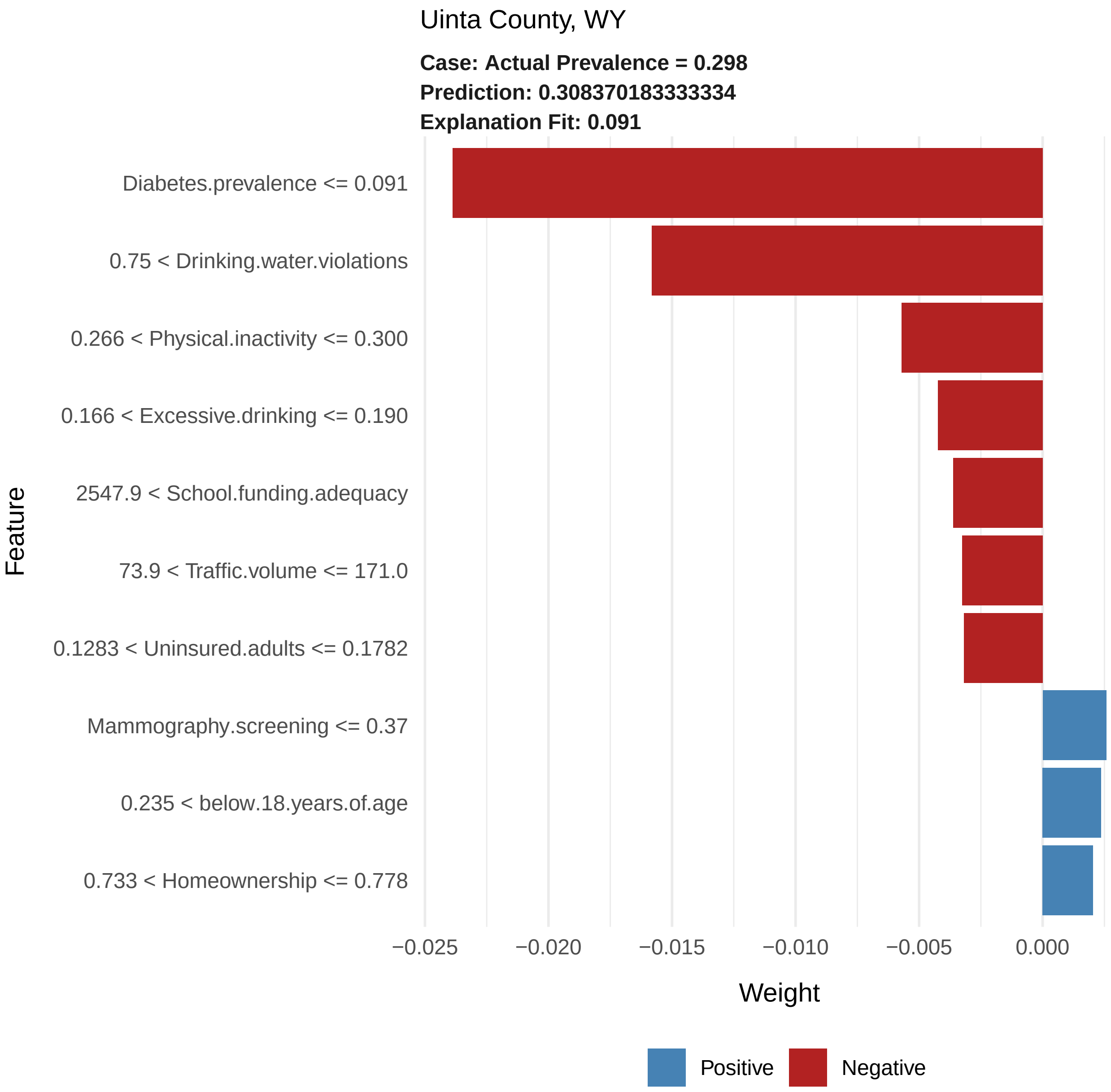

Uinta County, WY
Case: Actual Prevalence = 0.298
Prediction: 0.308370183333334
Explanation Fit: 0.091
Diabetes.prevalence <= 0.091
0.75 < Drinking.water.violations
0.266 < Physical.inactivity <= 0.300
0.166 < Excessive.drinking <= 0.190
2547.9 < School.funding.adequacy
73.9 < Traffic.volume <= 171.0
0.1283 < Uninsured.adults <= 0.1782
Mammography.screening <= 0.37
0.235 < below.18.years.of.age
0.733 < Homeownership <= 0.778
Feature
−0.025
−0.020
−0.015
−0.010
−0.005
0.000
Weight
Positive
Negative

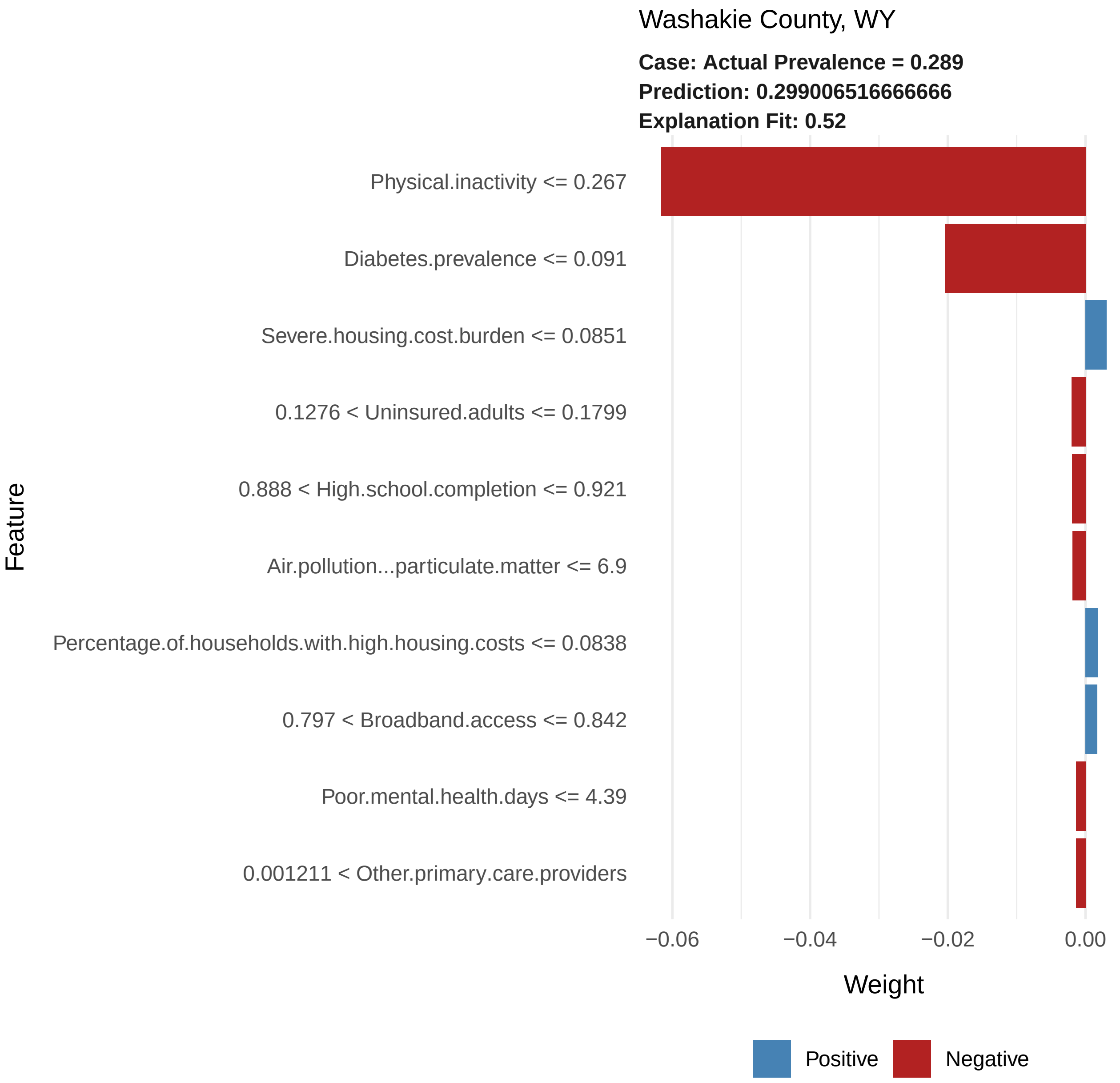
Washakie County, WY
Case: Actual Prevalence = 0.289
Prediction: 0.299006516666666
Explanation Fit: 0.52
Physical.inactivity <= 0.267
Diabetes.prevalence <= 0.091
Severe.housing.cost.burden <= 0.0851
0.1276 < Uninsured.adults <= 0.1799
0.888 < High.school.completion <= 0.921
Air.pollution...particulate.matter <= 6.9
Percentage.of.households.with.high.housing.costs <= 0.0838
0.797 < Broadband.access <= 0.842
Poor.mental.health.days <= 4.39
0.001211 < Other.primary.care.providers
Feature
−0.06
−0.04
−0.02
0.00
Weight
Positive
Negative

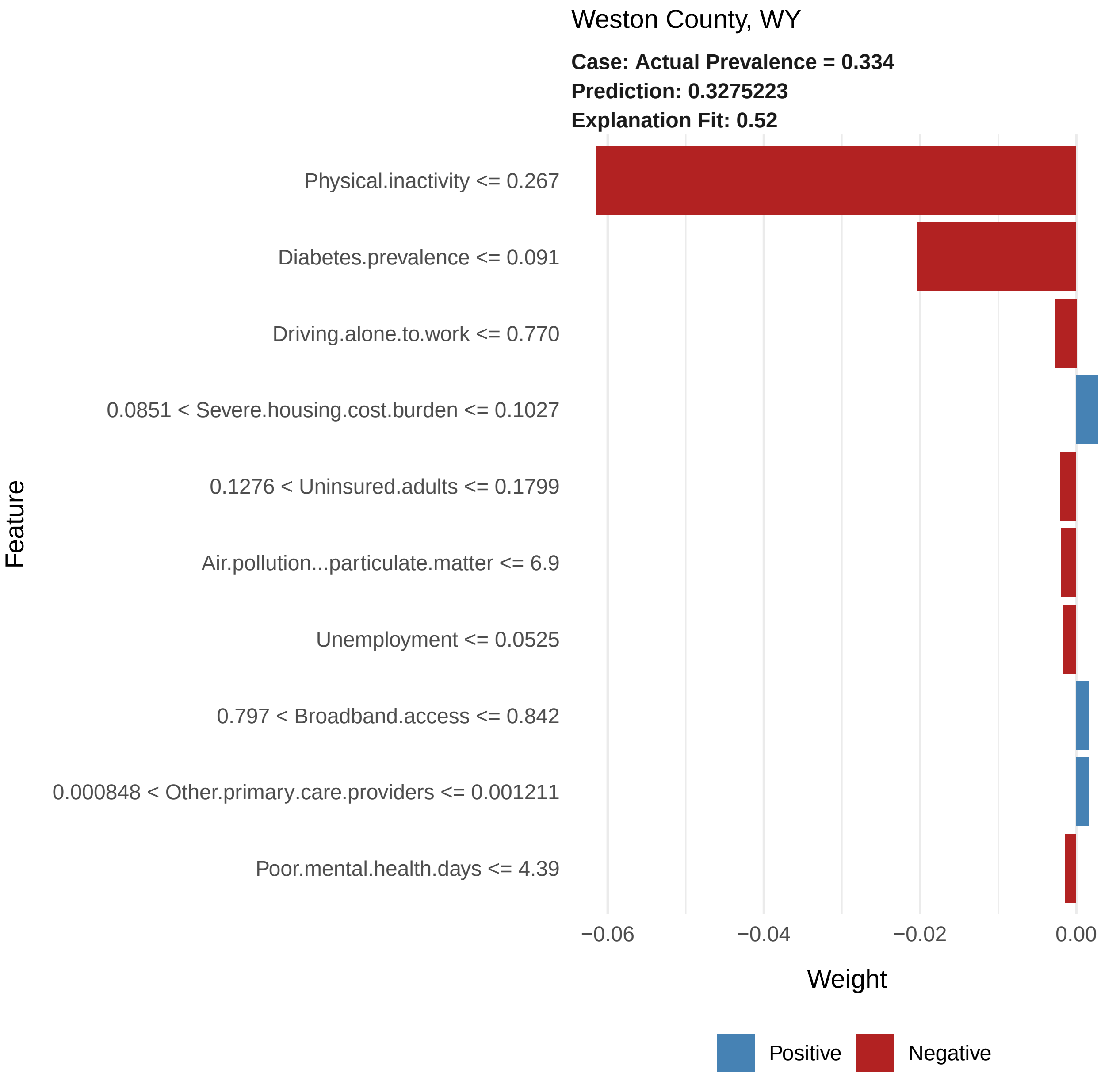

Weston County, WY
Case: Actual Prevalence = 0.334
Prediction: 0.3275223
Explanation Fit: 0.52
Physical.inactivity <= 0.267
Diabetes.prevalence <= 0.091
Driving.alone.to.work <= 0.770
0.0851 < Severe.housing.cost.burden <= 0.1027
0.1276 < Uninsured.adults <= 0.1799
Air.pollution...particulate.matter <= 6.9
Unemployment <= 0.0525
0.797 < Broadband.access <= 0.842
0.000848 < Other.primary.care.providers <= 0.001211
Poor.mental.health.days <= 4.39
Feature
−0.06
−0.04
−0.02
0.00
Weight
Positive
Negative
