# Supplementary material for: An interpretable machine learning model of cross-sectional U.S. county-level obesity prevalence using explainable artificial intelligence
Source: PLoS One. 2023 Oct 5;18(10):e0292341. doi: 10.1371/journal.pone.0292341 (PMC10553328; doi:10.1371/journal.pone.0292341)
Supplement: Attachment — Submitted filename: Rebuttal_to_reviewers_comments_07072023.pdf [file pone.0292341.s006.pdf]

Thank you for the opportunity to submit a revised manuscript.

Below are my responses to journal requirements and reviewer comments.

Response to Journal Requirements:

1. Please ensure that your manuscript meets PLOS ONE's style requirements, including those for file naming.

The manuscript should now meet PLOS ONE's style requirements, including those for file naming.

2. We note that Figure 1 in your submission contain [map/satellite] images which may be copyrighted.

Figure 1 was created for this manuscript by the author using R studio. The data presented in the figures was derived from the models reported in the manuscript as well. I have never published this elsewhere.

3. Please include captions for your Supporting Information files at the end of your manuscript, and update any in-text citations to match accordingly.

Captions for supporting information are now at the end of the manuscript, and in-text citations are updated.

Response to Reviewers' comments:

Reviewer #1: • Statistical tests for hypothesis testing and their assumptions should be specified in the study's statistical analysis in the Materials and Methods section.

The submitted manuscript does not follow a traditional "hypothesis testing" approach. Hypothesis testing requires defining an a priori null hypothesis and an alternative hypothesis. That is not the goal of the submitted paper. Instead, the paper reports the use of machine learning to form a predictive model that performs well on unseen data, that is then probed for knowledge discovery. This process is already described in the Methods section.

• The details (version, license number, etc.) of the statistical package(s) or program(s) should be given in the section of "Data Analysis or Statistical Analysis".

These were already provided in the manuscript. Section *2.2 Data Pre-Processing* shows the version of R used (R version 4.2.1 (2022-06-23)), and the version of each statistical package (i.e., the groupdata2 R package (version 2.0.2)).

• It should be explained how the qualitative and quantitative data are summarized under the sub-heading of Statistical Analyses in the Materials and Methods section of the study.

The paper reports no qualitative data. The only summary statistics reported for quantitative data are means, standard deviations, minimum and maximum values, which require no further explanation.

• Data analysis or Statistical analysis sub-section title should be added to the Materials and Methods.

The paper already has a sub-section titled: *2.3 Statistical Analysis*

• The exact P values should be added to the table(s) (e.g., p=0.25; p=0.03).

The manuscript does not report any p-values in a table, so adding exact p-values is impossible.

• Which methods are used to model relationships between variables?

The manuscript reports in section *2.2 Data Pre-Processing* that Spearman correlations were used to examine relationships between variables. But this was only part of pre-processing the data, not a part of the main analyses.

• The descriptions and other descriptive values/data should be defined on the tables and shapes.

The only tables in the manuscript show the data sources and the decision rules, no descriptive values, making this comment difficult to understand. All figures have descriptive captions.

• Are the data subjected to pre-processing?

The pre-processing of the data is described in section *2.2 Data Pre-Processing*

• How were extreme/outlier values in the data determined and resolved?

All of the reported data is county-level averages from public data sets that have already been heavily cleaned. Any extreme values in the data are indicative of extreme reality, and were not 'resolved'. Nonetheless, the random forest approach utilized in the manuscript is robust to outliers since they get averaged out by the aggregation of multiple tree output.

• What approaches were used to test the validity of the models?

The main approach used to test validity was cross-validation described in section *2.2 Data Pre-Processing*. Moreover, the validity of the machine learning models were assessed based on their ability to accurately predict obesity values in counties not used in training the model.

• Which metrics were used in the performance evaluation of the estimates of models/algorithms?

Model performance was quantified using variance explained in the evaluation data (i.e., fold not used in training), as well as the mean absolute difference between the predicted and actual prevalence rate. This is now explicity stated in section *2.3 Statistical Analysis.*

• How were the predictive models selected in this study?

Iterative random forest models were selected in this study based on a previous report [8] comparing different machine learning algorithms and showing that tree-based models perform well when predicting county-level obesity prevalence. The iterative approach to tree-based models employed in the submitted manuscript has also shown better performance than traditional random forest models, but also lends itself to explainable methods as well. [9] Both of these papers are cited in the manuscript.

8. Scheinker D, Valencia A, Rodriguez F. Identification of Factors Associated With Variation in US County-Level Obesity Prevalence Rates Using Epidemiologic vs Machine Learning Models. *JAMA Netw Open*. 2019;2(4):e192884. doi:10.1001/jamanetworkopen.2019.2884

9. Iterative random forests to discover predictive and stable high-order interactions | PNAS. Accessed March 30, 2023. https://www.pnas.org/doi/abs/10.1073/pnas.1711236115

• Which method(s) was/were used to optimize the hyperparameters of models/algorithms?

Hyperparamters were not optimized for the iterative random forest models. The creators of the iterative random forest approach have demonstrated that prediction accuracy is fairly robust to hyperparameter choices.[9]

9. Iterative random forests to discover predictive and stable high-order interactions | PNAS. Accessed March 30, 2023. https://www.pnas.org/doi/abs/10.1073/pnas.1711236115

• How was the most suitable cut-off point determined using the receiver operator characteristic (ROC) curve analysis?

The paper does not include a receiver operator characteristic (ROC) curve analysis. That analysis is used primarily for evaluating the performance of binary classifiers, where the outcome variable is categorical with two classes. The outcome variable in the submitted manuscript is continuous, making the ROC inappropriate.

• The number of current references on the subject of the study should be increased.

The number of references has been increased based on specific references mentioned by Reviewer 2.

Reviewer #2: The authors propose an Explainable AI model to predict the Cross-Sectional U.S. county-level obesity prevalence rates. The data was split into 2 folds for training and testing almost

50-50. The dataset has 64 variables from 7 categories. The AI model is built based-on random forest. The manuscript is well-writen and the idea is interesting. However, I have minor concerns/suggestions:

- I lightly surfed the literature and found anothe applications for Explainable AI models in health in general e.g. Covid-19 PMID: 36738712 and for obesity in particular PMID: 35954804. The authors may highlight these types of studies.

Thank you for the suggested citations. These papers are now cited in the introduction.

- Why the square root of 64. The authors may highlight why decide that or refer to the reference who initially explained this choice.

This is simply the default parameter for random forest models with a continuous outcome. This is now noted in the method section.

- How this model avoided over fitting

The main strategy used to avoid over-fitting was cross-validation. Model performance was based on the accuracy of predictions on data not used in training. A second strategy was to use a large number of trees (i.e., 1000) in the random forest models. Increasing the number of trees in the forest allows for a more robust aggregation of predictions and reduces the impact of individual noisy trees.

- AUCROc curve could be added to show the performance of the model.

The AUC-ROC (Area Under the Receiver Operating Characteristic) curve is a commonly used metric for evaluating the performance of binary classification models. However, the submitted manuscript employs a prediction model of a continuous outcome, making the AUC-ROC inappropriate.

- the importance of the findings from bio/medical side could be added in the conclusion (2-3 statements).

The conclusion has been revised to better reflect the importance of the findings and implications for biomedicine research.
