# Supplementary material for: An interpretable machine learning model of cross-sectional U.S. county-level obesity prevalence using explainable artificial intelligence
Source: PLoS One. 2023 Oct 5;18(10):e0292341. doi: 10.1371/journal.pone.0292341 (PMC10553328; doi:10.1371/journal.pone.0292341)
Supplement: Attachment — Submitted filename: response to reviewers.docx [file pone.0292341.s007.docx]

**Comments to the Author**

1. If the authors have adequately addressed your comments raised in a previous round of review and you feel that this manuscript is now acceptable for publication, you may indicate that here to bypass the “Comments to the Author” section, enter your conflict of interest statement in the “Confidential to Editor” section, and submit your "Accept" recommendation.

Reviewer #2: All comments have been addressed

Reviewer #3: (No Response)

2. Is the manuscript technically sound, and do the data support the conclusions?

The manuscript must describe a technically sound piece of scientific research with data that supports the conclusions. Experiments must have been conducted rigorously, with appropriate controls, replication, and sample sizes. The conclusions must be drawn appropriately based on the data presented.

Reviewer #2: Yes

Reviewer #3: Partly

3. Has the statistical analysis been performed appropriately and rigorously?

Reviewer #2: Yes

Reviewer #3: N/A

4. Have the authors made all data underlying the findings in their manuscript fully available?

The [PLOS Data policy](https://nam10.safelinks.protection.outlook.com/?url=http%3A%2F%2Fwww.plosone.org%2Fstatic%2Fpolicies.action%23sharing&data=05%7C01%7Cbenallen%40ku.edu%7Ced1f8e3ac1db4b21c40c08dbb003207b%7C3c176536afe643f5b96636feabbe3c1a%7C0%7C0%7C638297299736429878%7CUnknown%7CTWFpbGZsb3d8eyJWIjoiMC4wLjAwMDAiLCJQIjoiV2luMzIiLCJBTiI6Ik1haWwiLCJXVCI6Mn0%3D%7C3000%7C%7C%7C&sdata=YCS8JZX5ilyQDIpNynYx3OKeRq7FLtKNGFtfC01A1%2B8%3D&reserved=0) requires authors to make all data underlying the findings described in their manuscript fully available without restriction, with rare exception (please refer to the Data Availability Statement in the manuscript PDF file). The data should be provided as part of the manuscript or its supporting information, or deposited to a public repository. For example, in addition to summary statistics, the data points behind means, medians and variance measures should be available. If there are restrictions on publicly sharing data—e.g. participant privacy or use of data from a third party—those must be specified.

Reviewer #2: Yes

Reviewer #3: Yes

5. Is the manuscript presented in an intelligible fashion and written in standard English?

PLOS ONE does not copyedit accepted manuscripts, so the language in submitted articles must be clear, correct, and unambiguous. Any typographical or grammatical errors should be corrected at revision, so please note any specific errors here.

Reviewer #2: Yes

Reviewer #3: Yes

6. Review Comments to the Author

Please use the space provided to explain your answers to the questions above. You may also include additional comments for the author, including concerns about dual publication, research ethics, or publication ethics. (Please upload your review as an attachment if it exceeds 20,000 characters)

Reviewer #2: Te authors have done fair efforts to address the comments. The manuscript is in a very good shape now.

- Thank you for the review

Reviewer #3: This is a revised version of a manuscript that proposes applying iterative RF to predict, in an interpretable fashion, obesity prevalence rates at county level. The study is sound overall, and the prior critique for the most part has been addressed satisfactorily, notably improving the manuscript. Minor issues remain:

1. Why the iterative RF? Ref 8 suggests decision tree-based approaches in general (and gradient boosting in particular), not iterative RF. Regardless, iterative RF is a relatively novel method, and it is not clear to this reviewer that it will perform better than, say, well-established RF + SHAP in this context.

- Thank you for emphasizing the lack of explanation for the iterative vs. regular RF. Ref 8 does indeed argue for tree-based solutions. But, it should be noted that the main paper describing the iterative RF approach was published at roughly the same time as Ref 8, which is the main reason ref 8 doesn't utilize iterative RF. The main reason we used iterative random forests is because of research in the past 5 years showing that the iterative approach provides more stable estimates of variable importance and the final random forest model generally performs better than the first iteration. The manuscript now includes justification for the iterative RF approach in the beginning of section 2.3 Statistical Analysis.

2. Section 2.5 is laconic to the point of incomprehensibility. The opening sentence feels orphaned, the closing sentence is difficult to parse --- what parameters? Hyperparameters? Branching order? Threshold values?

- Section 2.5 is now revised and provides a rich description of the decision tree surrogate.
  The surrogate decision tree was completely rerun based on the reviewer's next comment.

3. Relatedly, just how robust are the splits at the bottom of the tree in Figure 4? Is there any point in growing the tree that deep?

- We thank the reviewer for raising the issue of how deep the surrogate decision tree should grow and the robustness of the splits at the bottom. The goal of the surrogate decision tree is to create an interpretable model that approximates the behavior of the random forest model. We agree that some of the splits at the bottom of the decision tree reflected splits of features with low importance. The main issue, as pointed out by the reviewer, is that the surrogate decision tree was not pruned. The revised manuscript addresses this issue by following the practice of pruning decision trees recommended by Breiman et al., (1984) to prune decision trees.
- Breiman, L., Friedman, J., Olshen, R., and Stone, C. (1984). Classification and regression trees. Statistics/Probability Series. Wadsworth & Brooks/Cole Advanced Books & Software.
- In the revised manuscript, the surrogate decision tree is grown unrestricted and then pruned based on the complexity parameter and 10-fold cross-validation estimates of the error of each split (Breiman et al., 1984). The tree was pruned using the highest complexity parameter (shortest decision tree) within 1 standard error of the complexity parameter with the smallest error during cross-validation.
- Unfortunately, this recommended practice resulted in an even deeper tree than the one in the previous manuscript. The highest complexity parameter (shortest decision tree) within 1 standard error of the complexity parameter with the smallest error during cross-validation was 0.001, meaning any split that explained 0.1% of the variance was included. To address this issue, we pruned the original decision tree with a more conservative complexity (0.01), which resulted in a short tree that included the major features of the random forest model and only splits that explained at least 1% of the variance in the outcome. This revision makes the surrogate decision tree do a better job of summarizing the model without including less robust features.

  4. In general, overfitting concerns were raised by the prior reviewer(s). This reviewer agrees that growing a large forest of trees addresses the issue somewhat (although higher robustness does not necessarily mean less overfitting/bias); however, the unpruned surrogate tree (Figure 4) and the local explainers (Figure 5) look overfit. Likewise, in Figure 3, it is difficult to quantify just how significant the lower-ranking features are. Yes, this is not a "conventional statistical analysis with p-values" study --- but this is no excuse. How does the reader know whether, for example, the effect in Figure 3 I is significant or not? Why top ten variables in the first place, what was the motivation behind the cutoff?
- We appreciate the reviewer raising the issue of overfitting. The concern with overfitting is that a machine learning model may perform well on training data but not generalize to unseen data because the model learned noise or other patterns specific to the training data. Cross-validation is one of the main approaches we used to mitigate overfitting, where the random forest model is evaluated based on unseen data withheld from training. One of the ways we tested for overfitting was looking at the variation in mean variable importance during cross-validation (as shown in Figure 2). Fig. 2 shows the standard error bars are quite small, suggesting the models learned similar patterns across data partitions.
- The reason 10 features are plotted is because the tenth most important feature has a mean variable importance that overlaps with zero when considering the standard error, meaning all remaining variables had an average importance of zero, so they aren't plotted. Likewise, the remaining plots focus on the top ten features. However, the local explainers show that the top ten differ across counties, in that the local explainer may not have the same top ten variables as the global model.
- As for overfitting with respect to the interpretable models, the purpose of the explainable methods used in the submitted manuscript is not to predict unseen data but to describe the machine learning model in an interpretable way, to see what is inside. The simpler models (i.e., surrogate decision tree, accumulated local effects, local interpretable model-agnostic explanations) are trained on the predictions of the machine learning model rather than the training data itself. The general goal of these models is to fit the predictions as closely as possible. But there is also the issue of interpretabilty, which came into play with the surrogate decision tree. The surrogate tree was pruned to highlight how the most important features make predictions.

7. PLOS authors have the option to publish the peer review history of their article ([what does this mean?](https://nam10.safelinks.protection.outlook.com/?url=https%3A%2F%2Fjournals.plos.org%2Fplosone%2Fs%2Feditorial-and-peer-review-process%23loc-peer-review-history&data=05%7C01%7Cbenallen%40ku.edu%7Ced1f8e3ac1db4b21c40c08dbb003207b%7C3c176536afe643f5b96636feabbe3c1a%7C0%7C0%7C638297299736429878%7CUnknown%7CTWFpbGZsb3d8eyJWIjoiMC4wLjAwMDAiLCJQIjoiV2luMzIiLCJBTiI6Ik1haWwiLCJXVCI6Mn0%3D%7C3000%7C%7C%7C&sdata=ZuXeIlrpPYAZz162xHU6vWQf1VmfvgfCUpduFtDhFHk%3D&reserved=0)). If published, this will include your full peer review and any attached files.


If you choose “no”, your identity will remain anonymous but your review may still be made public.


**Do you want your identity to be public for this peer review?** For information about this choice, including consent withdrawal, please see our [Privacy Policy](https://nam10.safelinks.protection.outlook.com/?url=https%3A%2F%2Fwww.plos.org%2Fprivacy-policy&data=05%7C01%7Cbenallen%40ku.edu%7Ced1f8e3ac1db4b21c40c08dbb003207b%7C3c176536afe643f5b96636feabbe3c1a%7C0%7C0%7C638297299736429878%7CUnknown%7CTWFpbGZsb3d8eyJWIjoiMC4wLjAwMDAiLCJQIjoiV2luMzIiLCJBTiI6Ik1haWwiLCJXVCI6Mn0%3D%7C3000%7C%7C%7C&sdata=%2B57KfdeAvvJnif60%2FU9P1BXN3Piuy1RwmNjgSSfQ%2B10%3D&reserved=0).

Reviewer #2: **Yes:** Abedalrhman Alkhateeb

Reviewer #3: No
